# Monocyte human leukocyte antigen-DR-mediated diabetic nephropathy progression is a promising therapeutic target

**DOI:** 10.3389/fendo.2025.1733139

**Published:** 2025-12-09

**Authors:** Yue Wang, Shaojie Fu, Sensen Su, Zhonggao Xu

**Affiliations:** Department of Nephrology, The First Hospital of Jilin University, Changchun, China

**Keywords:** causal inference, diabetic nephropathy, immunocyte, Mendelian randomization analysis, clinical verification, therapeutic target

## Abstract

**Objective:**

Increasing evidence has suggested that immune cells are known to play a pivotal role in the progression of diabetic nephropathy (DN). However, the specific causal role of monocyte surface markers, particularly human leukocyte antigen-DR (HLA-DR), remains unclear. This study aims to investigate the causal relationship between monocyte HLA-DR expression and DN risk and to validate its clinical relevance.

**Methods:**

We first employed a detailed two-sample Mendelian randomization (MR) analysis to investigate the potential causal relationships involving immune cell signatures particularly monocytes and DN risk. Then, to clinically validate these findings, we used flow cytometry to detect the median fluorescence intensity (MFI) of HLA-DR on monocyte in peripheral blood from DN patients and normal controls and analyzed its correlation with key renal function indicators of DN patients.

**Results:**

Two monocyte-related immune cell signatures were identified significantly associated with DN risk via MR analyses after false discovery rate (FDR) correction: *HLA-DR on CD14+ CD16− monocyte* (OR = 1.189, 95% CI: 1.113–1.270, P_FDR_ = 1.83 × 10^−4^) and *HLA-DR on CD14+ monocyte* (OR = 1.188, 95% CI: 1.107–1.276, P_FDR_ = 6.69 × 10^−4^), as determined by the inverse variance weighting method. The MFI of *HLA-DR on CD14+ CD16−* (*p* <0.001) and *CD14+ monocyte* (*p* <0.05) was higher in DN patients compared with normal controls, respectively. Clinical validation confirmed that the elevated HLA-DR MFI on these monocyte subsets was significantly correlated with worsening renal function, showing positive correlations with serum creatinine levels and negative correlations with estimated glomerular filtration rate.

**Conclusions:**

Our findings demonstrate that the elevated MFI of HLA-DR *on CD14+* and *CD14+ CD16− monocyte* is associated with DN and is significantly associated with worsened renal function, highlighting monocyte HLA-DR as a key immune mediator in DN progression and a promising therapeutic target for intervention.

## 1 Introduction

Diabetic nephropathy (DN) is a major complication of diabetes mellitus and has become the leading cause of end-stage renal disease worldwide (1). Its management poses a substantial economic burden and is frequently complicated by comorbidities such as hypertension, stroke, and cardiovascular disease (2). At present, there are no specific treatments for DN, except symptomatic treatment by actively controlling blood pressure and glucose and lipid levels as well as improving lifestyle (3, 4). Once the disease progresses to the middle or late stages, it cannot be reversed (5). Therefore, understanding the pathogenesis of DN is crucial for developing new interventions.

Traditionally, the pathogenesis of DN primarily includes metabolic disturbance, hemodynamic changes, oxidative stress, inflammation, and immune dysregulation (6, 7). Among diverse immune cells implicated in DN, such as mast cells, neutrophils, T cells, B cells, natural killer (NK) cells, and dendritic cells, monocytes and macrophages have emerged as pivotal mediators in the initiation and progression of renal injury (8–11). Abnormal DNA methylation in peripheral blood mononuclear cells can promote the inflammatory activation of immune cells in DN (12). Among various immune cells, monocytes also play an important role in DN. A study found that elevated growth hormone in patients with type 1 diabetes stimulates an increase in tumor necrosis factor-α signaling derived from podocytes. The elevated tumor necrosis factor-α further induces the differentiation of monocytes into macrophages, leading to an increase in macrophage recruitment, podocyte damage, and proteinuria, thereby promoting inflammation and fibrosis in the kidneys (13). Another study found that in multiple diabetic mouse models accompanied by dyslipidemia and elevated triglyceride-rich lipoproteins, diabetic dyslipidemia can induce lipid accumulation in monocytes and promote the increase of foam monocytes in the circulation, thereby increasing lipid uptake in the circulation and promoting the progression of atherosclerosis and DN (14). Cagney Cristancho et al. found in their research that in patients with type 2 diabetes, circulating monocytes and serum albumin are significantly associated with albuminuria; their results support the potential role of the innate immune system in microvascular terminal organ damage and urine protein loss in diabetes, suggesting that it may be transformed into clinical markers and incorporated into the risk assessment model for diabetes prognosis (15). Monocytes are key mediators of the innate immune response, and their functional state is often defined by the expression of surface markers. Human leukocyte antigen DR (HLA-DR), a major histocompatibility complex class II molecule, is of particular importance. It is a critical marker of monocyte activation and immunocompetence, essential for antigen presentation and the subsequent activation of adaptive immunity (16). However, the specific role and clinical significance of HLA-DR expression on monocyte in DN remain poorly defined. Elucidating this link is essential for a comprehensive understanding of DN immunopathogenesis and could reveal novel therapeutic targets for slowing disease progression.

While observational studies have implicated immune cell features such as monocyte HLA-DR expression in DN (17, 18), these findings are susceptible to bias and cannot establish causality. Mendelian randomization (MR) addresses this limitation by serving as a powerful causal inference method that leverages instrumental variables to minimize confounding (19). In this study, we undertook a comprehensive two-sample MR assessment to explore the causal link involving immune cell signatures particularly monocyte as well as DN. To further validate these genetic findings and assess their clinical relevance, we collected peripheral blood from patients with DN and normal controls to examine the correlation between HLA-DR expression on monocyte and key indicators of renal function.

## 2 Materials and methods

### 2.1 Study design

In this study, we examined the causal link between 731 immune cell signatures, categorized into seven groups, and DN using a two-sample MR approach. MR leverages genetic variations as proxies for risk factors. To ensure valid instrumental variable (IV) selection in causal inference, three critical conditions must be met (20): (1) the genetic variation is associated with the exposure; (2) it is not linked with confounders affecting the outcome; (3) it influences the outcome solely through the exposure pathway. Ethical approval was obtained from relevant institutional review boards, and informed consent was secured from all participants.

### 2.2 Data source description

The study utilized summary statistics for each immunophenotype, accessible from the GWAS catalog (accession numbers GCST90001391 to GCST90002121) (21). The analysis encompassed 731 immunophenotypes, including 118 absolute counts (AC), 389 median fluorescence intensities (MFI) indicating surface antigen levels, 32 morphological parameters (MP), and 192 relative counts (RC); for specific information, refer to **Supplementary Table 1**. The MFI, AC, and RC data encompassed various cell types like B cells, conventional dendritic cells, mature T cells, monocytes, myeloid cells, TBNK, and Treg panels, whereas MP data included conventional dendritic cell and TBNK panels. The original genome-wide association study (GWAS) for immune traits involved 3,757 European individuals from the central-eastern coast of Sardinia, Italy, with no overlapping cohorts. Around 22 million genotyped single-nucleotide polymorphisms (SNPs), imputed using the Sardinian sequence-based reference panel, were assessed with adjustments for covariates like sex, age, and age squared (22). The datasets presented in this study come from IEU Open GWAS (https://gwas.mrcieu.ac.uk/datasets/), and the GWAS data related to DN were obtained from the FinnGen study (accession numbers finn-bDM_NEPHROPATHY_EXMORE). FinnGen is a large-scale genomic initiative analyzing over 500,000 samples from Finnish biobanks to link genetic variations with health data, aiming to elucidate disease mechanisms and susceptibility. This project represents a collaboration between research institutions and biobanks across Finland and international industry partners (23). The dataset included 3,283 cases and 181,704 controls of European ancestry. After quality control and imputation, approximately 16 million variants were assessed. The participants in the selected exposure and outcome datasets were all of European descent, encompassing both male and female subjects, which minimizes the risk of population stratification. Moreover, the exposure and outcome datasets were from two different geographical locations, Italy and Finland, with no overlapping sample. This separation effectively mitigates the risk of weak instrument bias caused by sample overlap (24).

### 2.3 Selection of instrumental variables

IV significance levels for each immunophenotype were set at 1 × 10^−5^ based on a recent MR study of immune traits (25). The 1000 Genomes Project linkage disequilibrium structure (r^2^< 0.1 with any other associated SNP within 10 Mb) was tested with the initially selected SNPs, ensuring that selected IVs could independently predict exposure. For each IV, we calculated the percentage of phenotypic variation it explained, along with the F-statistic, to gauge its efficacy and mitigate weak instrumental bias. SNPs with F-statistic< 10 were considered weak instruments and excluded from IVs (26). The F-statistic was calculated using the following formula:


$$F=\frac{R^{2}}{1-R^{2}}\times \frac{N-K-1}{K}$$

where *R*^2^ represents the proportion of exposure variance explained by the IVs, *N* denotes the sample size of the exposure GWAS, and *K* indicates the number of SNPs.

### 2.4 Sample collection and flow cytometry

Peripheral venous blood samples were collected from 24 patients diagnosed with DN by renal biopsy and 24 healthy volunteers. The inclusion criteria for DN patients were the following: (1) age ≥18 years at diagnosis of DN; (2) the patients had relatively complete clinical data; (3) the patients were diagnosed as DN by renal biopsy. The exclusion criteria were as follows: (1) patients with infection, viral hepatitis type B, viral hepatitis type C, tuberculosis, tumors, hematological diseases, autoimmune diseases (such as antinuclear antibody with obvious abnormalities), and other systemic diseases; (2) the pathological results of renal biopsy were associated with membranous nephropathy, IgA nephropathy, ischemic renal injury, mesangial proliferative glomerulonephritis, obesity-related nephropathy, podocytosis, tubular interstitial nephropathy, hepatitis B virus antigen positive deposition, and other renal pathologic types. The inclusion criteria for the healthy control group were as follows: physically healthy adults (age ≥18 years), with age and gender matched to the DN group. Peripheral blood monocyte cells were isolated from control and DN patients according to Ficoll. Then, 5×10^5^ PBMC were resuspended with 100 μL phosphate buffered saline in the flow sample tube. PBMC were stained with fluorochrome-coupled antibodies against Brilliant Violet 421™ anti-human CD14 (BioLegend, # 325627), CD16-FITC (Beckman, # B49215), and PE anti-human HLA-DR (BioLegend, # 307605) and incubated at 4 °C for 30 min in the dark; eBioscience™ Fixable Viability Dye eFluor™ 780 (Invitrogen, # 65-0865-14) antibody staining was used to exclude dead cells. After staining, the cells were washed with 1 mL phosphate buffered saline and centrifuged at 450×g for 7 min; the supernatant was discarded, and then the stained cells were suspended with 100 μL phosphate-buffered saline for flow cytometry. All samples were acquired on a flow cytometer (Beckman) and analyzed with FlowJo software (v10.8.1, BD Biosciences). This study was conducted with the approval of the Ethics Committee of the First Hospital of Jilin University, under approval number 2022-417.

### 2.5 Statistical analysis

All of the MR analyses were performed using R software (version 4.3.1). To determine the causal relationship between the 731 immunophenotypes and DN, we primarily employed inverse variance weighted (IVW), weighted median (WM), and Mendelian randomization–Egger (MR-Egger) methodologies, utilizing the “Mendelian Randomization” package (V 0.4.3) (27). Heterogeneity among the selected IVs was assessed using Cochran’s Q statistic and corresponding p-values, and a random effects model was applied to depict this heterogeneity (19). The MR-Egger method was instrumental in examining the potential asymmetry due to horizontal pleiotropy of multiple genetic variants (28). Additionally, the MR pleiotropy residual sum and outlier (MR-PRESSO) method was implemented to identify and exclude significant horizontal pleiotropic outliers that could skew the results (29). Scatter and funnel plots were generated to confirm the absence of outliers in the scatter plots and demonstrate the robustness and homogeneity of the correlations in the funnel plots (30). Finally, we conducted a reverse Mendelian randomization analysis by swapping the exposure and outcome datasets, using the same parameters as in the forward analysis. Statistical analysis was performed using GraphPad Prism 8.0 (San Diego, CA). Continuous variables with normal distribution were presented as mean ± standard deviation; non-normal variables reported as median (interquartile range) were used to describe the mean value of the data subject to non-normal distribution. Student’s *t*-test and Mann–Whitney U test were used to compare between two groups. The chi-square test was used to compare the categorical data between the two groups. Pearson or Spearman correlation was used for correlation analysis. For the two continuous variables that met the normality assumption, we applied Pearson correlation analysis, and for those that did not, we applied Spearman correlation analysis. A value of *p<*0.05 was considered as statistically significant.

## 3 Results

### 3.1 Development of IVs used to genetically predict each immunophenotype

Across different batches of analysis, between 3 and 724 independent, non-palindromic, and genome-wide significant SNP loci were selected as IVs to investigate 731 immune phenotypes. These generated IVs explained between 0.005% and 5.199% of the variance in their respective immune phenotypes. All genetic instruments exhibited F-statistics greater than 10, indicating satisfactory robustness (**Supplementary Table 2**).

### 3.2 Exploration of causal effect of immunophenotypes on DN

**Figure 1:**
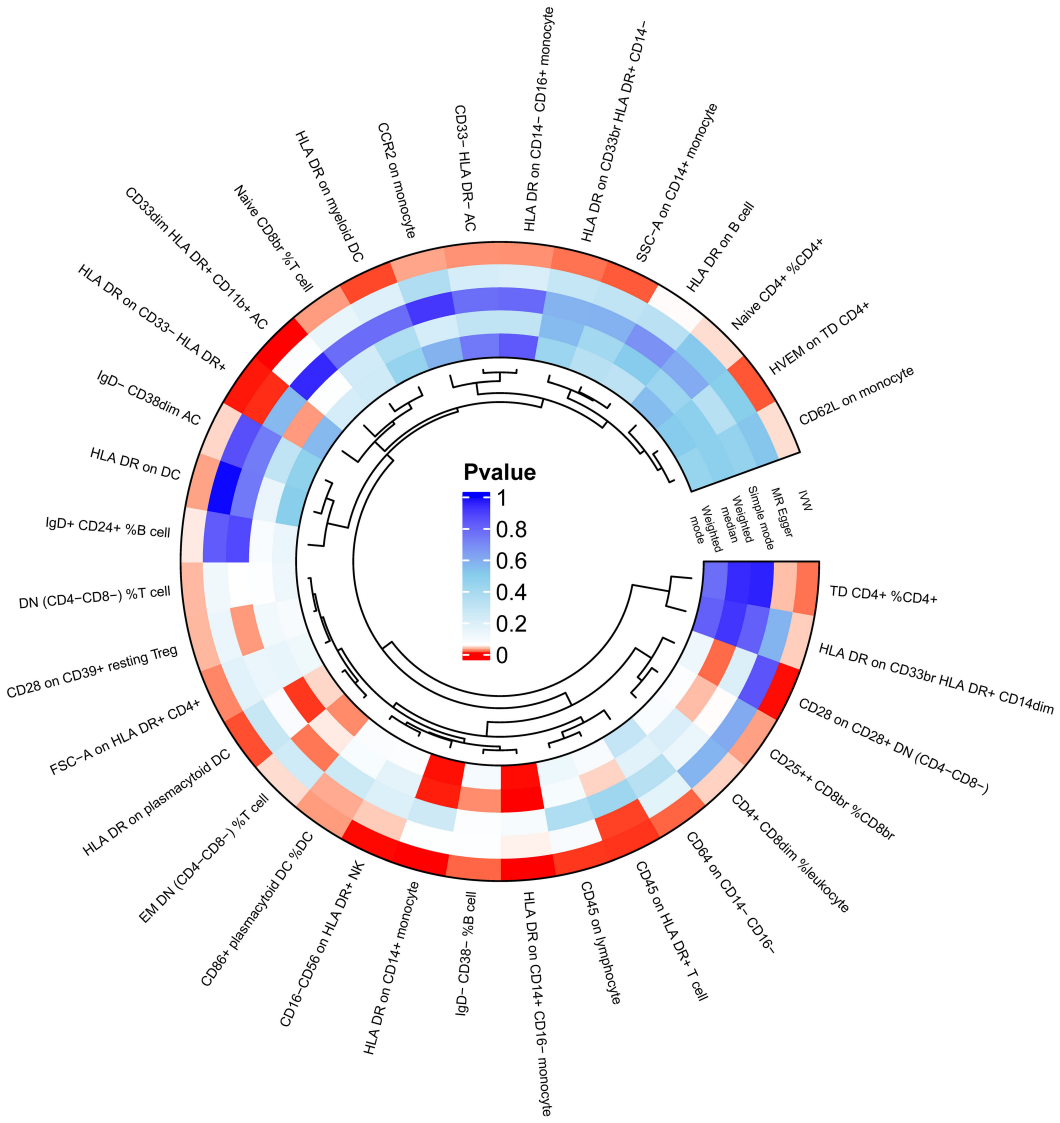
MR results with a p-value of less than 0.05 for the IVW method without FDR correction were visualized in circle plots. MR, mendelian randomization; IVW, inverse variance weighting; FDR, false discovery rate.

A two-sample MR analysis was conducted to assess the causal effects of immunophenotypes upon DN, utilizing the IVW method. A total of 34 MR results for immunophenotypes with a P-value of less than 0.05 for the IVW method without correction for the false discovery rate (FDR) method were visualized in the circle graph (**Figure 1**). Following adjusting for multiple tests using the FDR method, four immunophenotypes were identified at the 0.05 significance level, exhibiting harmful effects on DN. These included *CD16− CD56 on HLA DR+ NK* (TBNK panel), *CD33dim HLA DR+ CD11b+ Activated Cells* (myeloid cell panel), *HLA DR on CD14+ CD16− monocyte* (monocyte panel), and *HLA DR on CD14+ monocyte* (monocyte panel).

**Figure 2:**
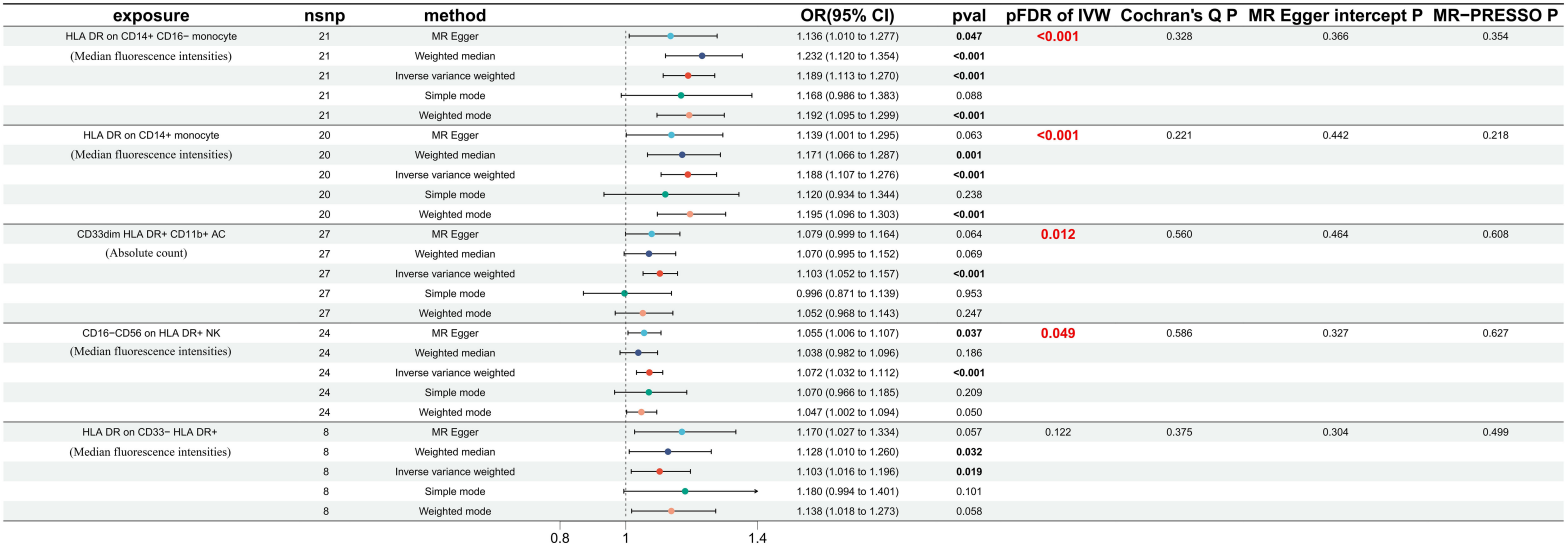
Positive MR results of 731 immunophenotypes on DN with FDR correction (pFDR< 0.2). MR, mendelian randomization; DN, diabetic nephropathy; FDR, false discovery rate.

**Figure 3:**
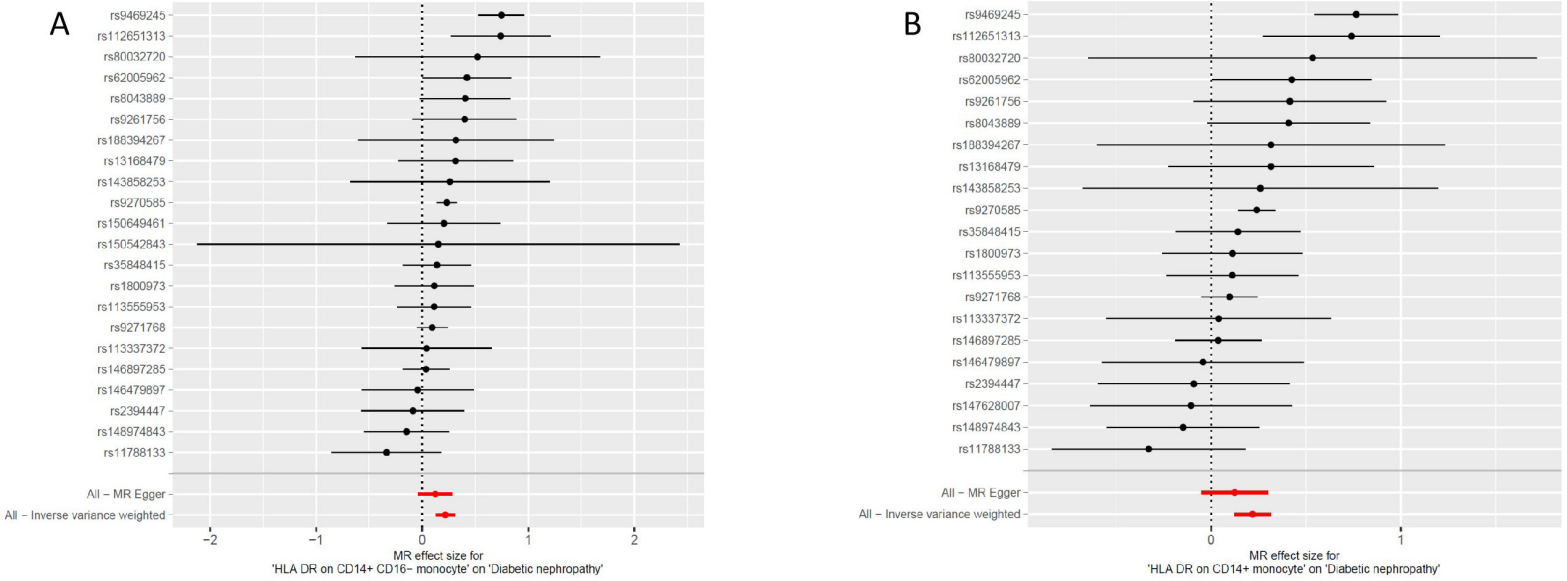
Detailed forest plots with the estimated MR effect of each IV in IVW models. **(A)***HLA DR on CD14+ CD16− monocyte*; **(B)***HLA DR on CD14+monocyte*. MR, mendelian randomization; IV, instrumental variable; IVW, inverse variance weighting; HLA, human leukocyte antigen.

**Figure 4:**
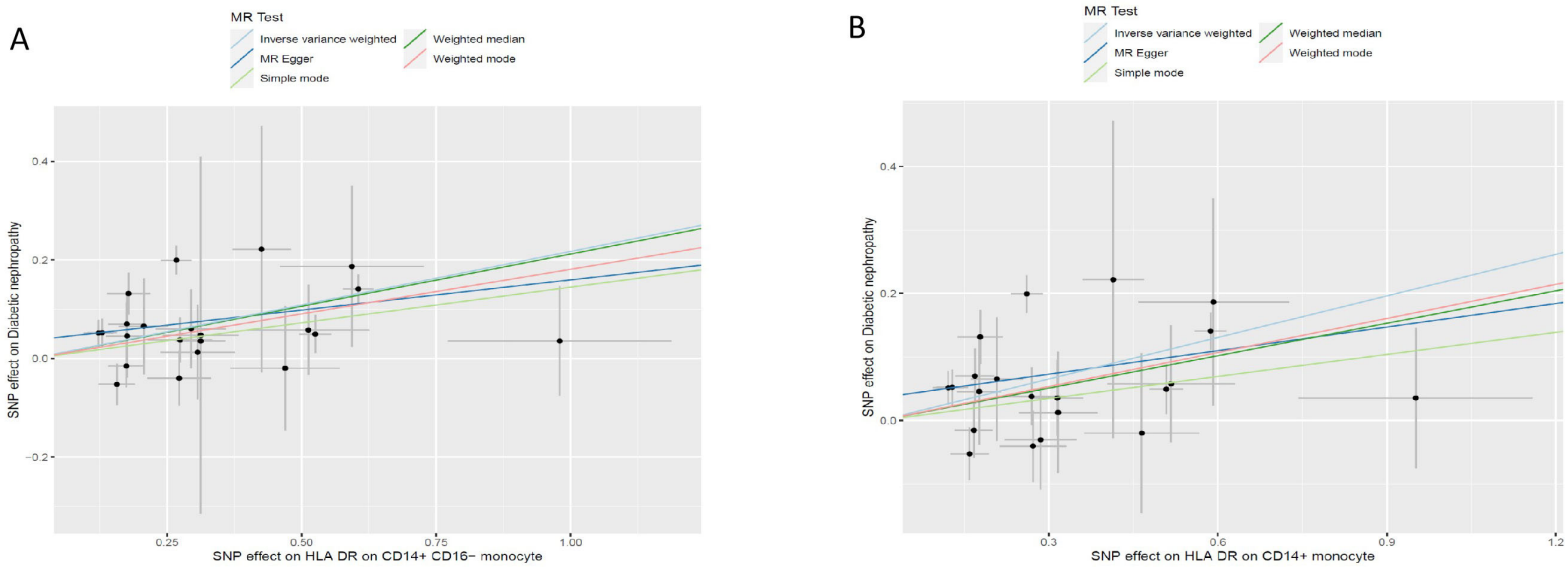
Scatter plots of causality. The slope of each line corresponding to the estimated MR effect in different models. **(A)***HLA DR on CD14+ CD16− monocyte*; **(B)***HLA DR on CD14+monocyte*. MR, mendelian randomization; HLA, human leukocyte antigen.

**Figure 5:**
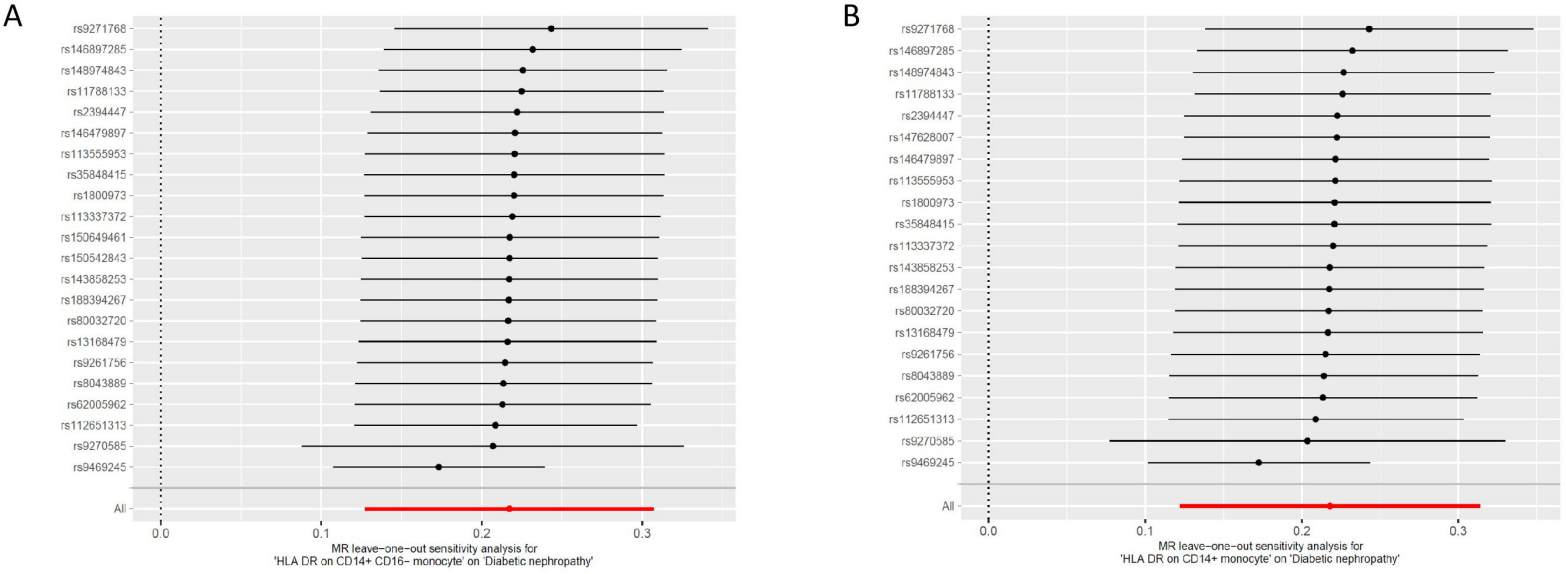
Leave one out of sensitivity tests. Calculate the MR results of the remaining IVs after removing the IVs one by one. **(A)***HLA DR on CD14+ CD16− monocyte*; **(B)***HLA DR on CD14+monocyte*. MR, mendelian randomization; IV, instrumental variable; HLA, human leukocyte antigen.

The odds ratio (OR) of *HLA DR on CD14+ CD16− monocyte* on DN risk was ~1.189 (95% confidence interval (CI) = 1.113–1.270, *p* = 2.51 × 10^−7^, *P_FDR_* = 1.83 × 10^−4^). The MR-Egger and WM method as complementary tests also obtained a similar result: MR-Egger (OR = 1.136, 95% CI = 1.010–1.277, *p* = 0.047) and WM (OR = 1.232, 95% CI = 1.120–1.354, *p* = 1.58 × 10^−5^). OR of *HLA DR on CD14+ monocyte* for DN risk was ~1.188 (95% CI = 1.107–1.276, *p* = 1.83 × 10^−6^, *P_FDR_* = 6.69 × 10^−4^) using the IVW method. WM also produced a similar result: (OR = 1.171, 95% CI = 1.066–1.287, *p* = 0.001); however, the association estimate using MR-Egger was not statistically significant (OR = 1.139, 95% CI = 1.001–1.295, *p* = 0.063). OR of *CD33dim HLA DR+ CD11b+ AC* for DN risk was ~1.103 (95% CI = 1.052–1.157, *p* = 4.8 × 10^−5^, *P_FDR_* = 0.012) using the IVW method. The association estimates calculated by WM and MR-Egger were not statistically significant: WM (OR = 1.070, 95% CI = 0.995–1.152, *p* = 0.069) and MR-Egger (OR = 1.079, 95% CI = 0.999–1.164, *p* = 0.064). OR of *CD16− CD56 on HLA DR+ NK* for DN risk was ~1.072 (95% CI = 1.032–1.112, *p* = 2.75 × 10^−4^, *P_FDR_* = 0.049) using the IVW method. The MR-Egger method also yielded a similar result: (OR = 1.055, 95% CI = 1.006–1.107, *p* = 0.037); however, the association estimates using WM were not statistically significant (OR = 1.038, 95% CI = 0.982–1.096, *p* = 0.186). Regardless of the statistical significance of the association estimates using WM and MR-Egger as the complementary tests, the direction was consistent with the IVW analysis (**Figure 2**), further strengthening our results. Concerning causality, the p-value of Cochran’s Q > 0.05 indicated no heterogeneity in the results. Additionally, MR-Egger detected no evidence of pleiotropy because the p-value for the intercept was >0.05. Neither MR-PRESSSO nor leave-one-out plots detected outliers (**Supplementary Figure 1**). Furthermore, with pFDR< 0.2 as a screening criterion, one immunophenotype exhibiting a correlation with DN was *HLA DR on CD33− HLA DR+*, belonging to the myeloid cell panel and is at risk for DN. OR on DN risk was ~1.1023 (95% CI = 1.016–1.196, *p* = 0.019, *P_FDR_* = 0.1223), and the direction of the results based on WM and MR-Egger methods was also consistent with IVW analysis (**Figure 2**). The forest plot and scatter plot of causal relationships between genetically predicted HLR-DR-associated immunophenotypes and the risk of DN are shown in **Figures 3** and **4**, and the details of sensitivity analyses are shown in **Figure 5**. Furthermore, detailed MR results for all 731 immunophenotypes are shown in **Supplementary Table 3**, whereas the scatter, forest, and funnel plots were presented in **Supplementary Figures 2**–**4**.

### 3.3 Reverse Mendelian randomization analysis

**Figure 6:**
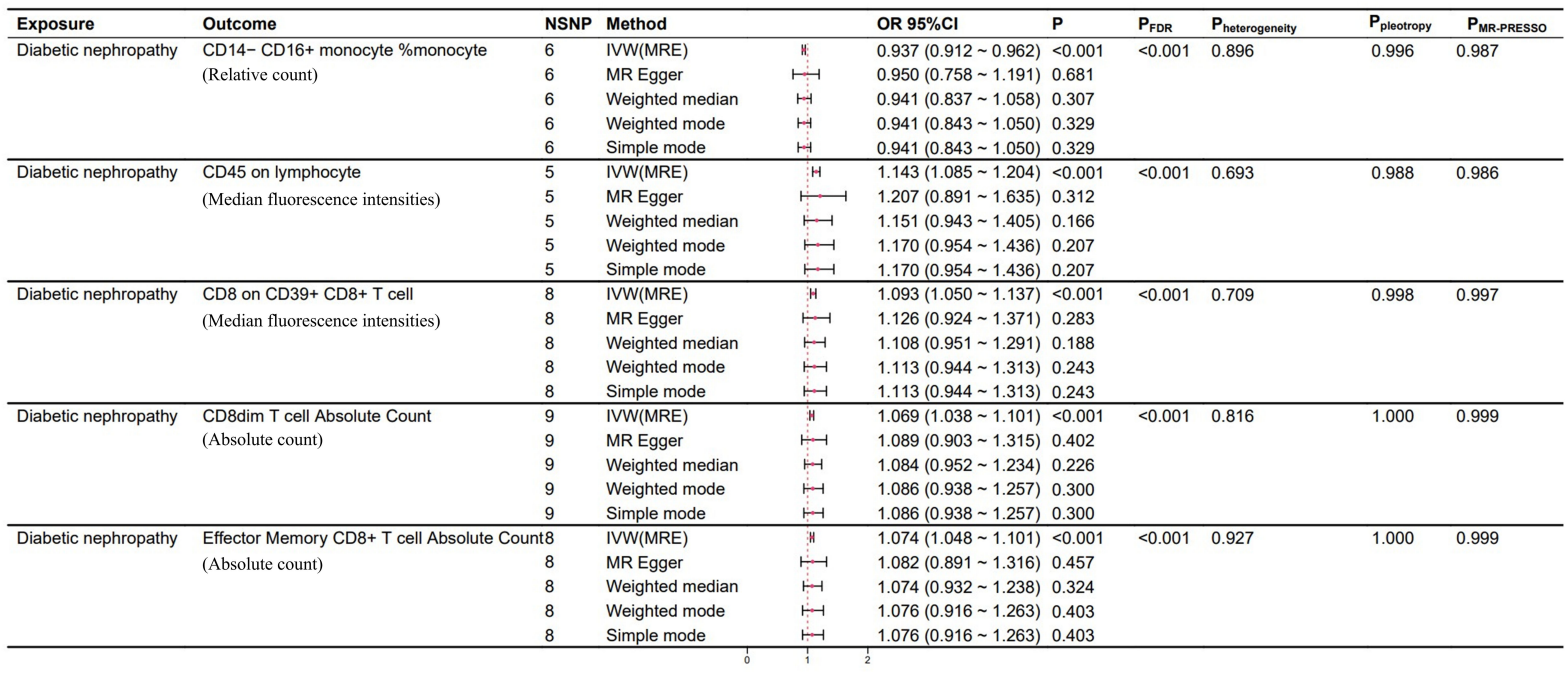
Reverse Mendelian randomization effects of diabetic nephropathy on immune cell phenotypes (top five most significant).

To further investigate the impact of DN on immune cell phenotypes, a reverse MR analysis was conducted by swapping the exposure and outcome. After performing FDR correction for multiple comparisons, it was observed that the onset of DN led to alterations in 152 immune cell phenotypes (**Supplementary Table 4**). Among these, the five most significant immune cell phenotype changes were as follows: DN was associated with a reduced percentage of *CD14− CD16+ monocyte* (OR 95% CI: 0.937 [0.912–0.962], *p* < 0.001). In contrast, DN significantly increased the expression of *CD45 on lymphocytes* (OR 95% CI: 1.143 [1.085–1.204], *p* < 0.001), *CD8 on CD39+ CD8+ T cells* (OR 95% CI: 1.093 [1.050–1.137], *p* < 0.001), *CD8dim T cell* absolute count (OR 95% CI: 1.069 [1.038–1.101], p<0.001), and effector memory CD8+ T cell absolute count (OR 95% CI: 1.074 [1.048–1.101], *p* < 0.001), as detailed in **Figure 6**.

### 3.4 Clinical validation of immunophenotypes in DN patients

Through MR analysis, four immunophenotypes, including *HLA DR on CD14+ CD16− monocyte* and *HLA DR on CD14+ monocyte*, were found to be causally associated with the development of DN. In order to further understand their expression in the blood of DN patients versus normal controls, we performed flow cytometry analysis. Flow cytometry was used to analyze the peripheral blood of 24 patients with DN and 24 normal controls. All patients with DN and normal controls met the inclusion and exclusion criteria. The clinical information of patients with DN and normal controls are shown in **Table 1**.

**Table 1:** General clinical and pathological data of normal controls and diabetic nephropathy patients.

Variables	Normal controls	Diabetic nephropathy patients	*p* value
Male.no. (%)	13(54.17)	14(58.33)	0.771
Age (years), mean ± SD	47.04 ± 16.73	47.00 ± 11.42	0.992
Diabetes course (years), mean ± SD	–	8.97 ± 5.54	–
Body mass index, mean ± SD	24.91 ± 4.12	27.54 ± 3.48	0.021
Hypertension, no. (%) ^a^	4 (16.67)	20 (83.33)	<0.0001
Fasting blood glucose (mmol/L), mean ± SD	5.00 ± 0.53	8.36 ± 3.33	<0.0001
Blood urea nitrogen (mmol/L), mean ± SD/median (IQR)	6.23± 1.75	8.44 (6.13, 10.24)	0.0046
Serum creatinine (μmol/L),mean ± SD/median (IQR)	68.83 ± 12.64	118.1 (52.30, 150.20)	<0.0001
eGFR (ml/min/1.73m^2^), mean ± SD	102.00 ± 18.13	59.33 ± 27.41	<0.0001
Serum albumin (g/L), mean ± SD	42.98 ± 4.61	31.03 ± 6.04	<0.0001
Urinary protein excretion (g/24h), mean ± SD	–	5.96 ± 2.43	–

mean ± SD, mean± standard deviation; IQR, interquartile range; eGFR, estimate glomerular filtration rate. a: Hypertension: blood pressure > 140/90 mmHg or the use of anti-hypertensive agents.

**Figure 7:**
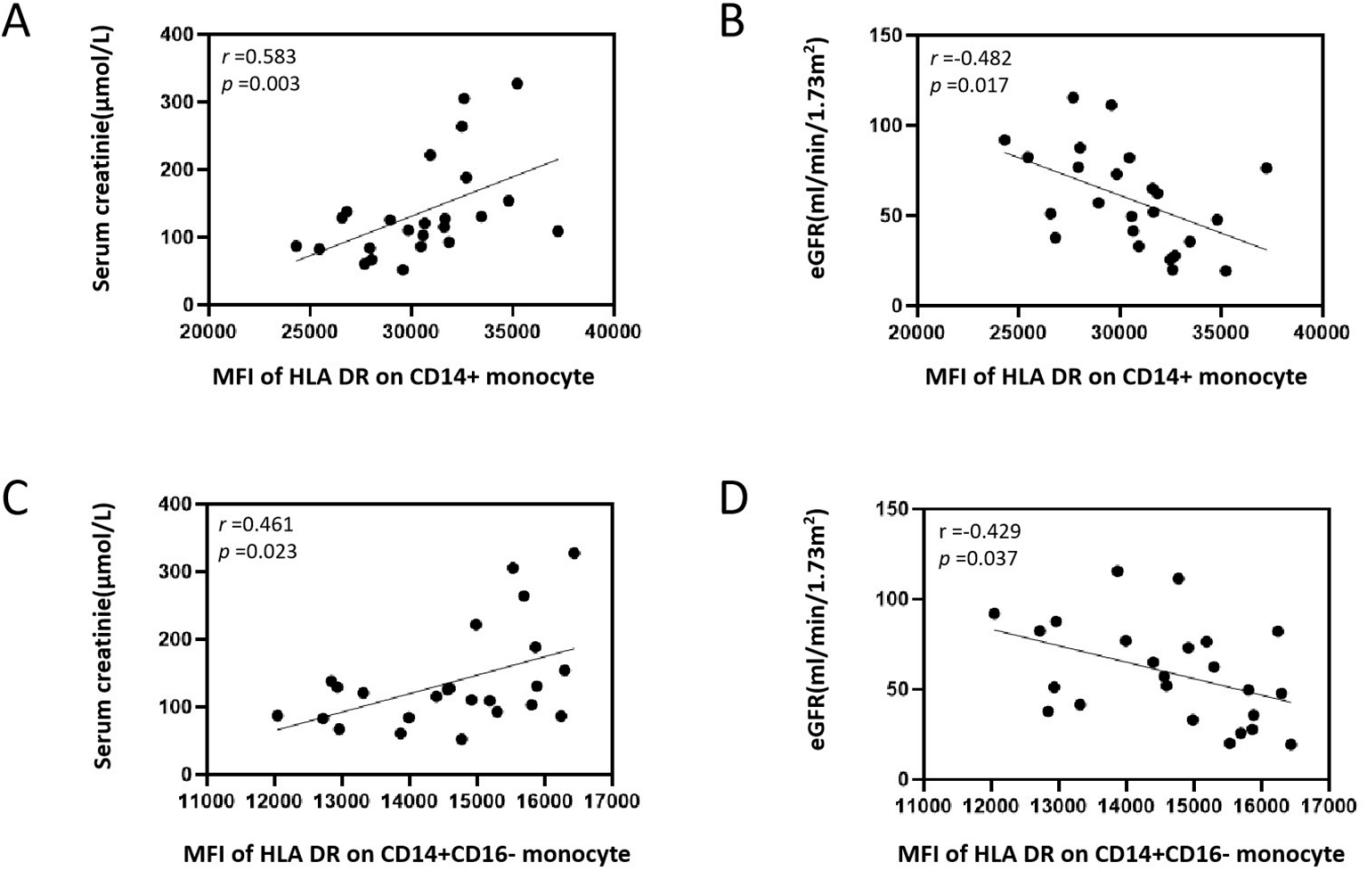
Correlation analysis between MFI of *HLA DR on CD14+ and CD14+ CD16− monocyte* with serum creatinine and eGFR in patients with DN. **(A)** Correlation analysis between MFI of *HLA DR on CD14+ monocyte* and serum creatinine. **(B)** Correlation analysis between MFI of *HLA DR on CD14+ monocyte* and eGFR. **(C)** Correlation analysis between MFI of *HLA DR on CD14+ CD16− monocyte* and serum creatinine. **(D)** Correlation analysis between MFI of *HLA DR on CD14+ CD16− monocyte* and eGFR. MFI, median fluorescence intensities; HLA, human leukocyte antigen; eGFR, estimate glomerular filtration rate; DN, diabetic nephropathy.

Correlation analysis showed that MFI of *HLA DR on CD14+ monocyte* was positively correlated with serum creatinine (Scr) level (*r* = 0.583, *p* = 0.003) and negatively correlated with estimate glomerular filtration rate (eGFR) level (*r* = -0.482, *p* = 0.017) (**Figures 7A, B**). MFI of *HLA DR on CD14+ CD16− monocyte* was positively correlated with Scr level (*r* = 0.461, *p* = 0.023) and negatively correlated with eGFR level (*r* = −0.429, *p* = 0.037) (**Figures 7C, D**). Correlation analysis showed that MFI of *HLA DR on CD14+* and MFI of *HLA DR on CD14+ CD16− monocyte* had no correlation with blood urea nitrogen, urinary protein excretion, fasting blood glucose, and serum albumin in DN patients (**Supplementary Figure 5**). Correlation analysis showed that in the normal control group, the MFI of *HLA DR on CD14+* and the MFI of *HLA DR on CD14+ CD16− monocyte* were not correlated with blood urea nitrogen, Scr, eGFR, fasting blood glucose, and serum albumin (**Supplementary Figure 6**).

**Figure 8:**
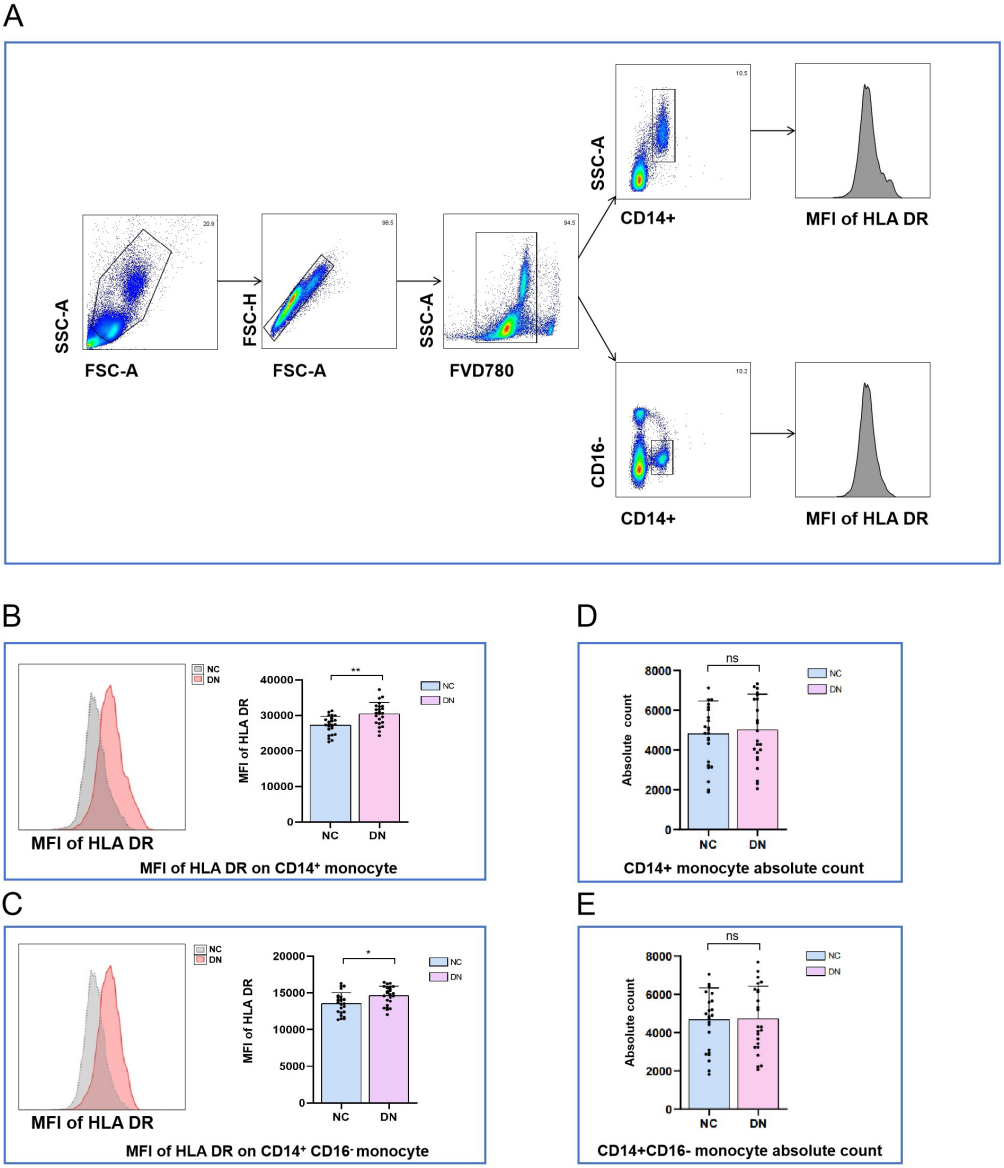
Immunophenotypes in peripheral blood monocyte cells between DN patients and normal controls. **(A)** Gating strategy of *CD14+ monocyte* and *CD14+ CD16− monocyte*. **(B)** MFI of *HLA DR on CD14+ monocyte*. **(C)** MFI of *HLA DR on CD14+ CD16− monocyte*. **(D)***CD14+ monocyte* absolute count. **(E)***CD14+ CD16− monocyte* absolute count. DN, diabetic nephropathy; MFI, median fluorescence intensities; HLA, human leukocyte antigen; NC, normal controls. The values are expressed as mean ± standard deviation. NC group, n=24; DN group, n=24. *, *p* <0.05 vs. NC group; **, *p* <0.001 vs. NC group.

We gated *CD14+ monocyte* and *CD14+ CD16− monocyte* according to the gating strategy in **Figure 8A**. The mean value of MFI of *HLA DR on CD14+ monocyte* in the normal control group was 27,266 ± 2,499, and that of MFI of *HLA DR on CD14+ monocyte* in the DN group was 30,482 ± 3159. As shown in **Figure 8B**, the MFI of *HLA DR on CD14+ monocyte* in the peripheral blood of DN patients was higher than that of the normal control group, and the difference was statistically significant (*p* <0.001). The mean value of the MFI of *HLA DR on CD14+ CD16− monocyte* in the normal control group was 13,541 ± 1,487, and that of MFI of *HLA DR on CD14+ CD16− monocyte* in the DN group was 14,629 ± 1,285. As shown in **Figure 8C**, the MFI of *HLA DR on CD14+ CD16− monocyte* in the peripheral blood of DN patients was higher than that of the normal control group, and the difference was statistically significant (*p* <0.05). The average *CD14+ monocyte* count in the normal control group was 4,841 ± 1,694, and that in the DN group was 5,025 ± 1,787. As shown in **Figure 8D**, there was no statistical difference between the two groups (*p* = 0.679). The average count of *CD14+ CD16− monocyte* in the normal control group was 4,686 ± 1656, and that in the DN group was 4,727 ± 1704. As shown in **Figure 8E**, there was no statistical difference between the two groups (*p* = 0.934). We further divided 24 DN patients into two groups, grade II and grades III-IV, based on the degree of glomerular pathological lesions in renal puncture tissues, with 12 patients in each group, to further explore the MFI of HLA DR on monocyte in different pathological grades of DN patients. The mean value of the MFI of *HLA DR on CD14+ monocyte* in the DN glomerular pathological grade II group was 30,183 ± 3,013, and that of the MFI of *HLA DR on CD14+ monocyte* in the DN glomerular pathological grade III-IV group was 30,782 ± 3,405. As shown in **Supplementary Figure 7A**, there was no statistical difference between the two groups (*p* = 0.653). The mean value of the MFI of *HLA DR on CD14+ CD16− monocyte* in the DN glomerular pathological grade II group was 14,635 ± 1,105, and that of the MFI of *HLA DR on CD14+ CD16− monocyte* in the DN glomerular pathological grade III-IV group was 14,623 ± 1,494. As shown in **Supplementary Figure 7B**, there was no statistical difference between the two groups (*p* = 0.983).

## 4 Discussion

DN is the major cause of chronic kidney disease that leads to renal fibrosis worldwide (1). The pathogenesis of DN is complex (6, 31–33). Currently, emerging evidence highlights that oxidative stress and inflammation play a central role in DN and underscores the critical contribution of inflammation and immune dysregulation to DN progression (34–37). Although current treatment strategies have reduced the incidence of DN and slowed its progression (38, 39), new therapies are still needed for the prevention and treatment of DN (40, 41).

Many studies have indicated a role for the immune system and cells in DN occurrence and development (42, 43); however, the specific causal relationships involving diverse immunophenotypes and DN development have remained unclear. The role of immune disorders in the occurrence of DN has attracted more and more attention. Recently, some scholars have used MR to conduct the causal relationship between immunophenotype and DN at the gene level (44–47). Different from previous studies, we not only used publicly available genetic datasets to establish the causal relationship between immunophenotypes and DN but also verified the causal immunophenotypes of patients using flow cytometry and conducted correlation analyses on the clinical laboratory indicators of patients. Of the various immune traits analyzed (MFI, RC, AC, and MP), four immunophenotypes (*HLA DR* on *CD14+ CD16− monocyte*, *HLA DR* on *CD14+ monocyte*, *CD16− CD56* on *HLA DR+ NK*, and *CD33dim HLA DR+ CD11b+ Activated Cells*) showed significant causal effects on DN (*P_FDR_* < 0.05). Particularly, we conducted clinical validation for two HLA-DR-related monocyte immunophenotypes. The MFI of *HLA-DR on CD14+ CD16−* and *CD14+ monocyte* was higher in DN patients compared with normal controls, respectively. However, we did not find the difference of MFI of *HLA DR* on *CD14+* and *CD14+ CD16−* monocyte among different grades of renal pathology in DN patients. Correlation analysis showed that MFI of *HLA DR* on *CD14+* and *HLA DR* on *CD14+ CD16− monocyte* had a positive correlation with Scr and negative correlation with eGFR in DN patients, but had no correlation with blood urea nitrogen, urinary protein excretion, fasting blood glucose, and serum albumin. In addition, to explore the effect of DN on immune cell phenotype, this study additionally performed reverse MR analysis by exchanging exposure and outcome. The onset of DN led to alterations in many immune cell phenotypes. Among them, the five most significant immune cell phenotype changes were the percentage of *CD14− CD16+ monocyte*, the expression of *CD45 on lymphocytes* and *CD8* on *CD39+ CD8+ T cells*, *CD8dim T cell* absolute count, and effector memory CD8+ T-cell absolute count. This suggests that the occurrence of DN also leads to the disorder of the immune system. However, the reverse MR analysis did not reveal any significant effect of DN on *HLA DR* on *CD14+ monocyte* and *CD14+ CD16− monocyte*, further indicating the directionality of the causal relationship between HLA DR expression on monocytes and DN.

CD14 is a marker for mature monocytes and an important receptor for lipopolysaccharide in the immune system. HLA-DR is a universally recognized marker of immune status. *HLA DR* on *CD14+* monocyte is associated with several diseases, including sepsis and coronary atherosclerosis (48, 49). Studies have shown that *CD14+ CD16+* monocyte levels are elevated in patients with DN (50), and the ratio of monocyte to high-density lipoprotein cholesterol was elevated in patients with DN (51). However, studies on the correlation between HLA-DR on monocytes and DN have not been established. In the present study, we found that *HLA DR on CD14+ monocyte* and *HLA DR on CD14+ CD16− monocyte* are associated with harmful effects on DN. Monocytes may contribute to the establishment and resolution of local inflammatory reactions and participate in the innate immune surveillance of the organism (52). Monocyte heterogeneity has long been recognized; however, in recent years, three functional subsets of human monocytes, classical (*CD14++CD16−*), intermediate (*CD14++CD16+*), and nonclassical (*CD14+CD16+*) monocytes, have been identified (53). Classical monocytes synthesize monocyte chemoattractant protein-1 (MCP-1) and express C–C motif chemokine receptor 2 (54). MCP-1 influences the whole cascade of monocyte-associated inflammation and has a key role in mediating the migration of monocytes and macrophages into kidney tissue (55). MCP-1/C–C motif chemokine receptor 2 is associated with inflammation and morphological changes, such as tubular atrophy and glomerular injury (56). Even in the early DN stages, an increase in MCP-1 urinary excretion can be detected, highlighting its relevance (57). Critically, the role of monocytes extends beyond chemokine production. The functional competence of these cells, as indicated by the expression of HLA-DR, serves as a pivotal upstream regulator in the DN inflammatory cascade. As a key molecule for antigen presentation, the level of HLA-DR expression directly determines the monocyte’s ability to orchestrate adaptive immune responses. In addition, non-classical monocytes are closely associated with DN development, primarily through Toll-like receptors (TLRs) and nuclear factor-kappa B (NF-κB) interaction (58). TLRs are a unique germinal line of pattern recognition receptors that participate in the recognition and activation of innate immunity, leading to a cascade in the presence of inflammation and the release of proinflammatory cytokines (59). Emerging evidence indicates a pivotal role for TLR2 and TLR4 in the perpetuation of inflammation in DN (60, 61). TLR2 may be the predominant long-term mediator of NF-κB activation in transducing inflammation during DN. TLR4 sends signals through its downstream partner myeloid differentiation primary response protein 88, activating NF-κB and resulting in the production of cytokines and reactive oxygen species (62). All of the above have emphasized the significant role of monocytes in the upstream of the DN inflammatory cascade reaction. This supports our findings that immune activation of *CD14+ monocyte*, particularly *CD14+ CD16− monocyte*, may exert harmful effects on DN patients.

DN is characterized by albuminuria, loss of renal function, renal fibrosis, and infiltration of macrophages originating from peripheral monocytes inside kidneys. The infiltration of monocytes/macrophages and the macrophage–myofibroblast transformation in the glomerulus during DN are significant in renal dysfunction and glomerulosclerosis (63, 64). In DN, damaged intrinsic renal cells recruit monocytes/macrophages to the tissue damage area to defend against and clear cell damage; however, the polarization of macrophages aggravates the inflammatory response, extracellular matrix secretion, fibrosis, and necroptosis of intrinsic kidney cells (65). Tissue-infiltrating *CD14+* monocytes/macrophages are regarded as the producers of the extracellular matrix (66). The fluorescence intensity of *CD14+ CD16+* and the expression of TLR4 in patients with uremia complicated with type 2 diabetes mellitus were both higher than those in the normal control group; this immune dysfunction may be related to the activation of the TLR4/NF-κB and signal transducers and activators of transcription 5 signaling pathways (58). The expression level of TLR4 and the proportion of *CD14+ CD16+* monocyte in the peripheral blood of patients with early-stage DN are elevated (50). Furthermore, the high expression of *HLA-DR* on *CD14− CD16−* immune cells may indicate a lower risk of diabetic microvascular complications, such as diabetic retinopathy and DN (47). Our research found that the MFI of HLA DR on *CD14+ monocyte* and *CD14+ CD16− monocyte* were associated with the risk of DN, which was consistent with the previous research results (47, 67).

Our two-sample MR analysis drew upon data from extensive GWAS cohorts, encompassing approximately 185,000 subjects, to ensure robust statistical validity. The conclusions of this study are grounded in genetic instrumental variables, with causal inferences derived from multiple MR methodologies. The findings are resilient, showing no significant distortion from horizontal pleiotropy or other external influences. However, there were still some limitations to our MR study. Despite thorough sensitivity analyses, assessments of horizontal pleiotropy remain somewhat incomplete. Additionally, the absence of individual participant data precluded further stratified analysis. The study’s reliance on a European-centric database also limits the generalizability of the findings to remaining ethnic groups. A broader threshold for evaluating results was employed, which, while potentially increasing the rate of false positives, also facilitated a more comprehensive exploration of the strong associations involving immunophenotypes as well as DN. We collected peripheral blood samples from healthy controls and clinical DN patients and performed flow cytometry analysis on the positive results of HLA DR on monocytes screened by MR. This is where our research has an advantage over previous similar studies. At present, the research results on the expression of immune cell phenotypes in DN are not consistent. This may be related to the different stages of DN, the severity of the disease, whether there are complications, the selection of clinical samples and the size of the sample, etc. Our research still lacks studies on the mechanism and pathophysiology of HLA DR on monocyte in DN. Therefore, when necessary, further *in vitro* cell experiments, animal experiments, clinical studies with larger sample sizes, and the combined application of databases are also required to conduct more in-depth exploration of the role of HLA DR on monocytes in DN.

## 5 Conclusions

By integrating systematic MR with clinical correlation analyses, this study demonstrates that elevated HLA DR expression on *CD14+* and *CD14+ CD16− monocyte* is associated with DN and is significantly associated with worsened renal function. These findings establish that activated monocytes may serve as a key driver of DN pathogenesis and HLA DR may serve as a translatable biomarker for prognostic and therapeutic development.

## Data Availability

The original contributions presented in the study are included in the article/**Supplementary Material**. Further inquiries can be directed to the corresponding author.

## Supplementary material

The tary Material for this article can be found online at: https://www.frontiersin.org/articles/10.3389/fendo.2025.1733139/full#supplementary-material

Supplementary Figure 1 Leave one out analyses of all MR analyses of 731 immunophenotypes on DN. MR, mendelian randomization; DN, diabetic nephropathy.

Supplementary Figure 2 Scatter plots of all MR analyses of 731 immunophenotypes on DN. MR, mendelian randomization; DN, diabetic nephropathy.

Supplementary Figure 3 Forest plots of all MR analyses of 731 immunophenotypes on DN. MR, mendelian randomization; DN, diabetic nephropathy.

Supplementary Figure 4 Funnel plots of all MR analyses of 731 immunophenotypes on DN. MR, mendelian randomization; DN, diabetic nephropathy.

Supplementary Figure 5 Correlation analysis between MFI of *HLA DR on CD14+ and CD14+ CD16− monocyte* with blood urea nitrogen, urinary protein excretion, fasting blood glucose and serum albumin in patients with DN. MFI, median fluorescence intensities; HLA, human leukocyte antigen; DN, diabetic nephropathy.

Supplementary Figure 6 Correlation analysis between MFI of *HLA-DR on CD14+ and CD14+ CD16- monocyte* with clinical indicators in normal controls. MFI, median fluorescence intensities; HLA, human leukocyte antigen; eGFR, estimate glomerular filtration rate.

Supplementary Figure 7 MFI of HLA DR on monocyte of different grades in renal pathology of DN patients. MFI, median fluorescence intensities; HLA, human leukocyte antigen; DN, diabetic nephropathy.

Supplementary Table 1 The specific immune signatures corresponding to each immunophenotype.

Supplementary Table 2 The instrumental variables used to genetically predict each immune trait.

Supplementary Table 3 IVW results of the causal effect of immune cells on Diabetic nephropathy.

Supplementary Table 4 Results of the causal effect of Diabetic nephropathy on immune cells.
