# Supplementary material for: Monocyte human leukocyte antigen-DR-mediated diabetic nephropathy progression is a promising therapeutic target
Source: Front Endocrinol (Lausanne). 2025 Dec 9;16:1733139. doi: 10.3389/fendo.2025.1733139 (PMC12722863; doi:10.3389/fendo.2025.1733139)
Supplement: Supplementary Figure 2 — Scatter plots of all MR analyses of 731 immunophenotypes on DN. MR, mendelian randomization; DN, diabetic nephropathy. [file DataSheet2.pdf]

Supplementary Figure 2: Scatter plots of all MR analyses of 731 immunophenotypes on diabetic nephropathy

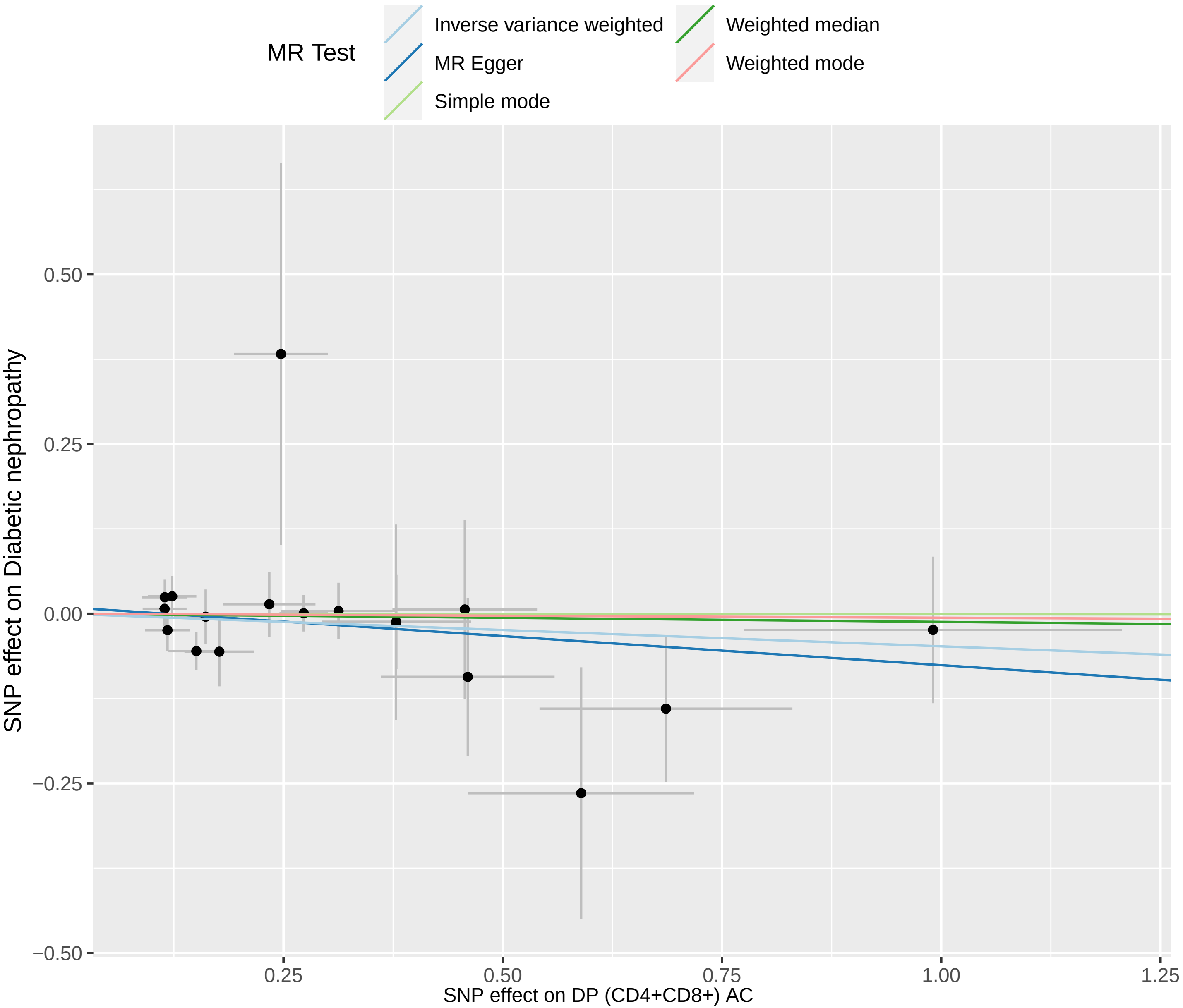

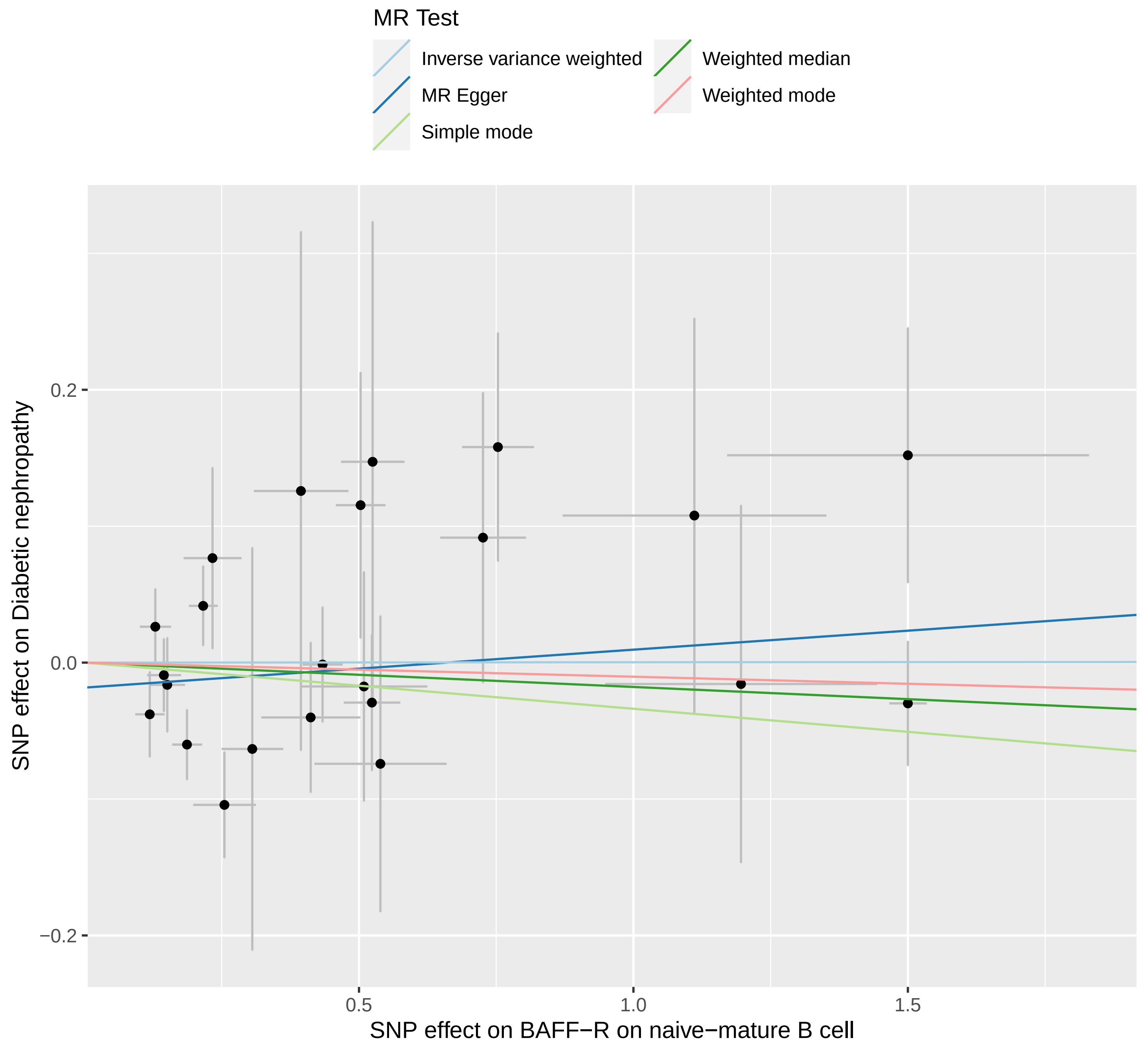
MR Test
Inverse variance weighted
MR Egger
Simple mode
Weighted median
Weighted mode
0.2
0.0
−0.2
0.5
1.0
1.5
SNP effect on Diabetic nephropathy
SNP effect on BAFF−R on naive−mature B cell

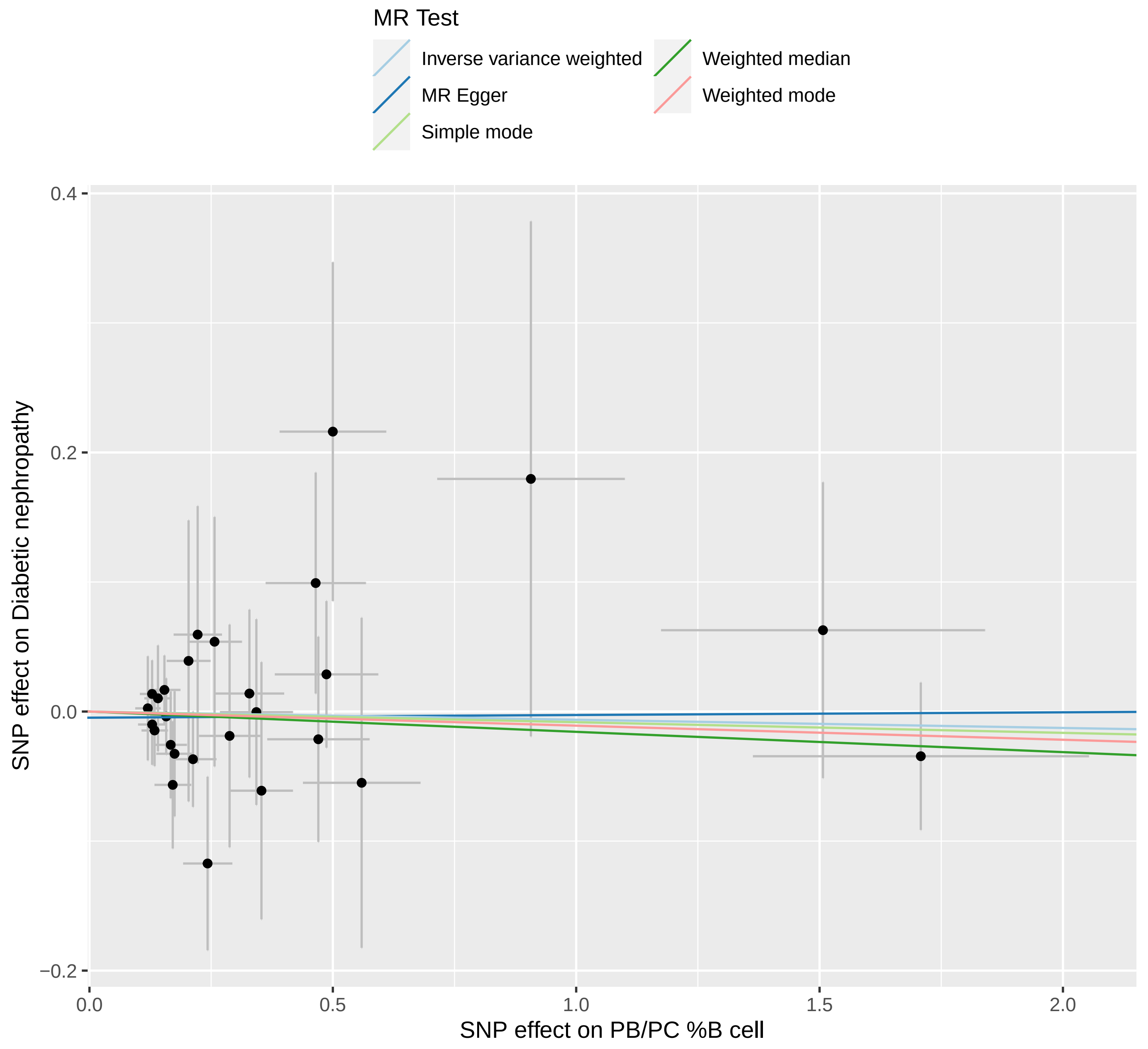
MR Test
Inverse variance weighted
MR Egger
Simple mode
Weighted median
Weighted mode
SNP effect on Diabetic nephropathy
SNP effect on PB/PC %B cell
0.4
0.2
0.0
−0.2
0.0
0.5
1.0
1.5
2.0

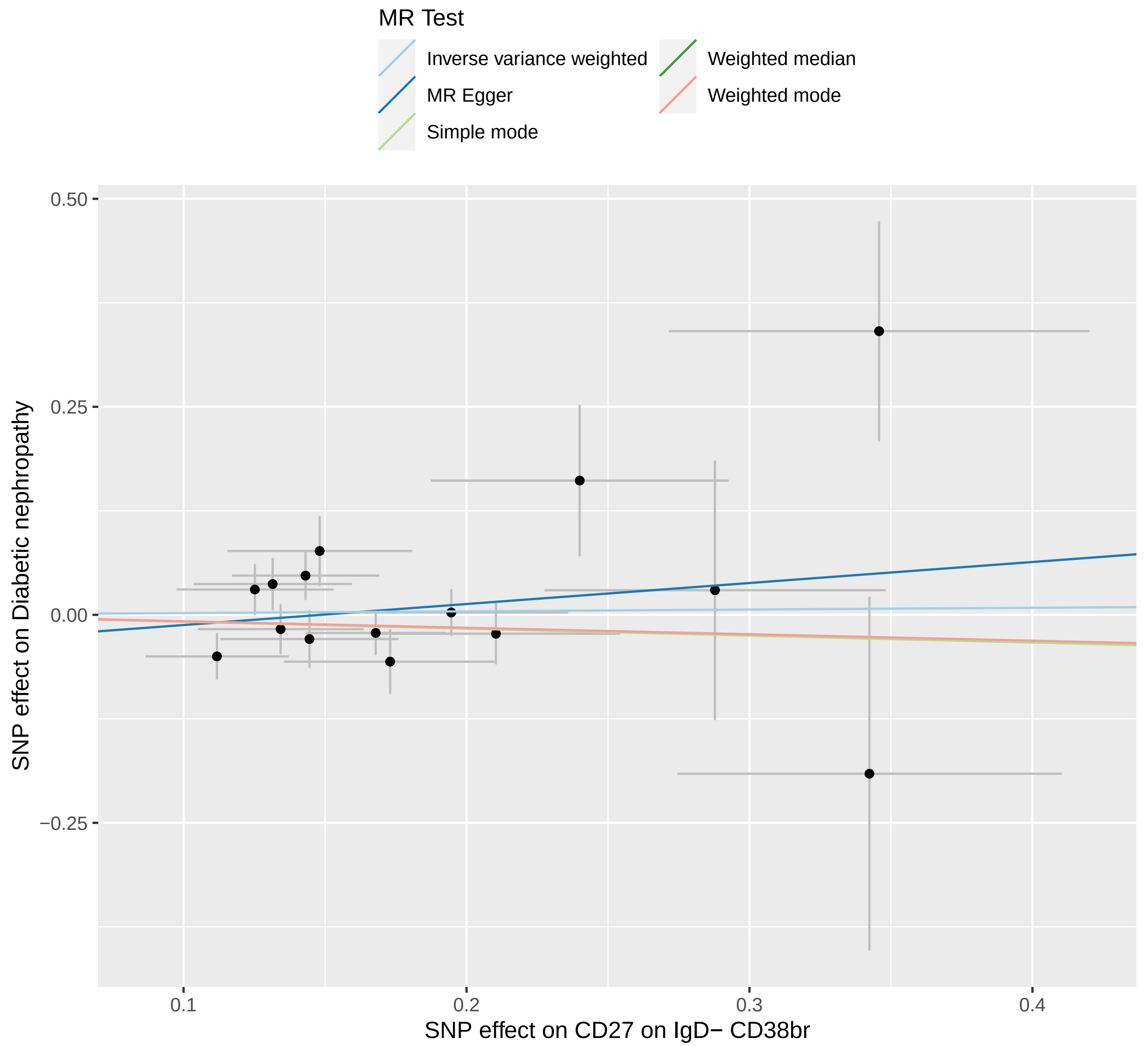
MR Test
Inverse variance weighted
MR Egger
Simple mode
Weighted median
Weighted mode
0.50
0.25
0.00
−0.25
SNP effect on Diabetic nephropathy
0.1
0.2
0.3
0.4
SNP effect on CD27 on IgD− CD38br

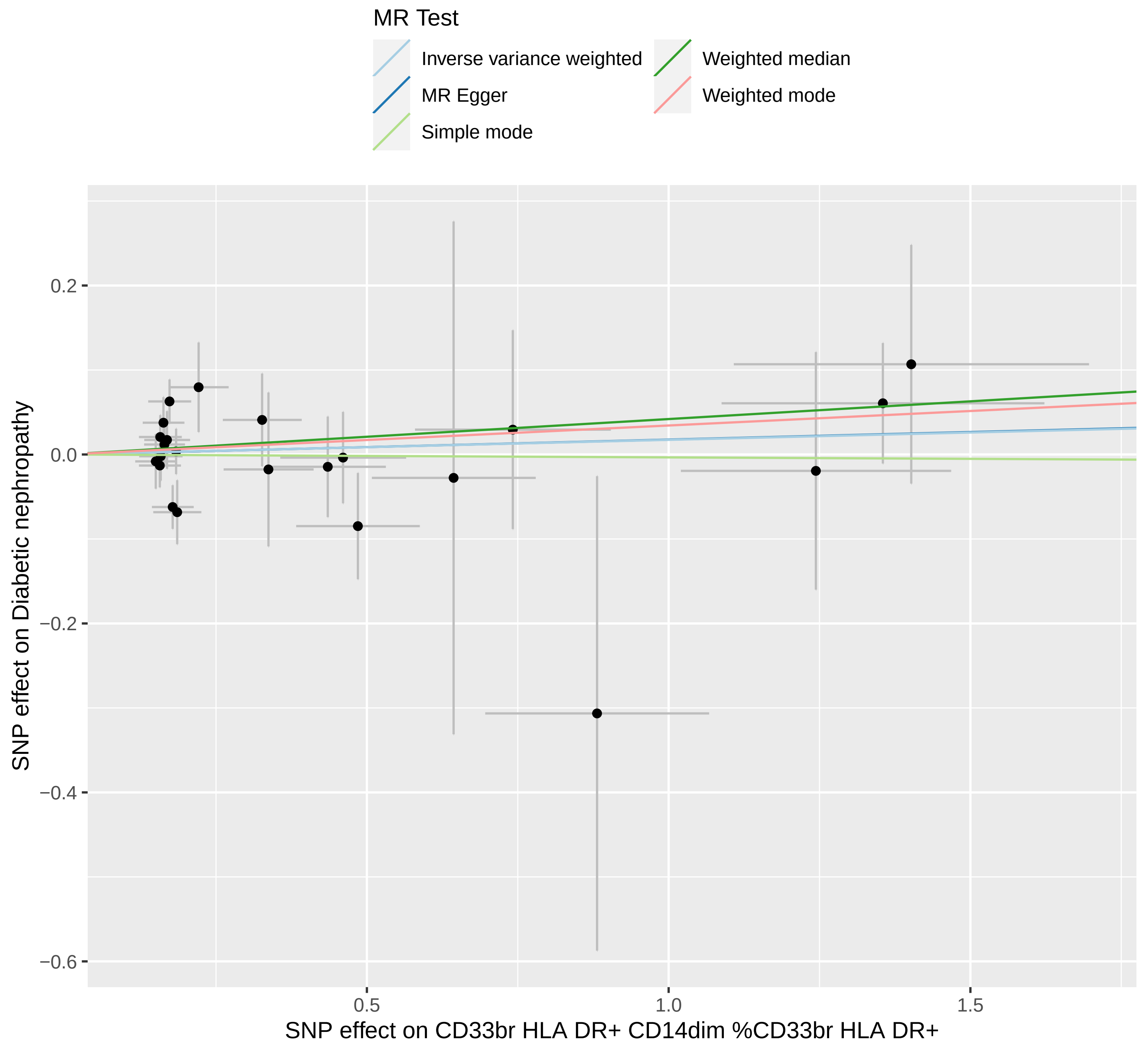
MR Test
Inverse variance weighted
MR Egger
Simple mode
Weighted median
Weighted mode
0.2
0.0
−0.2
−0.4
−0.6
SNP effect on Diabetic nephropathy
0.5
1.0
1.5
SNP effect on CD33br HLA DR+ CD14dim %CD33br HLA DR+

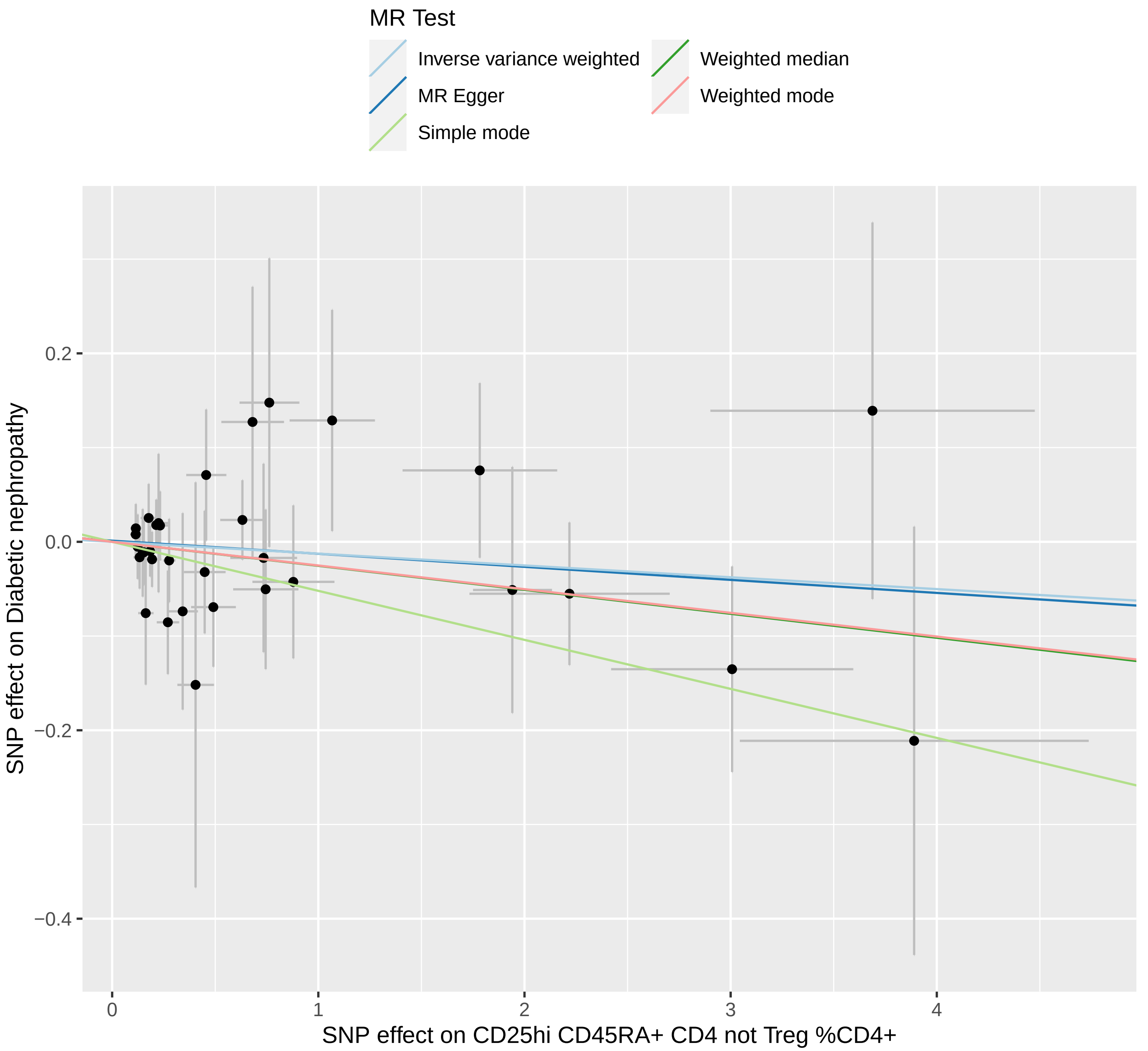

MR Test
Inverse variance weighted
MR Egger
Simple mode
Weighted median
Weighted mode
0.2
0.0
−0.2
−0.4
0
1
2
3
4
SNP effect on Diabetic nephropathy
SNP effect on CD25hi CD45RA+ CD4 not Treg %CD4+

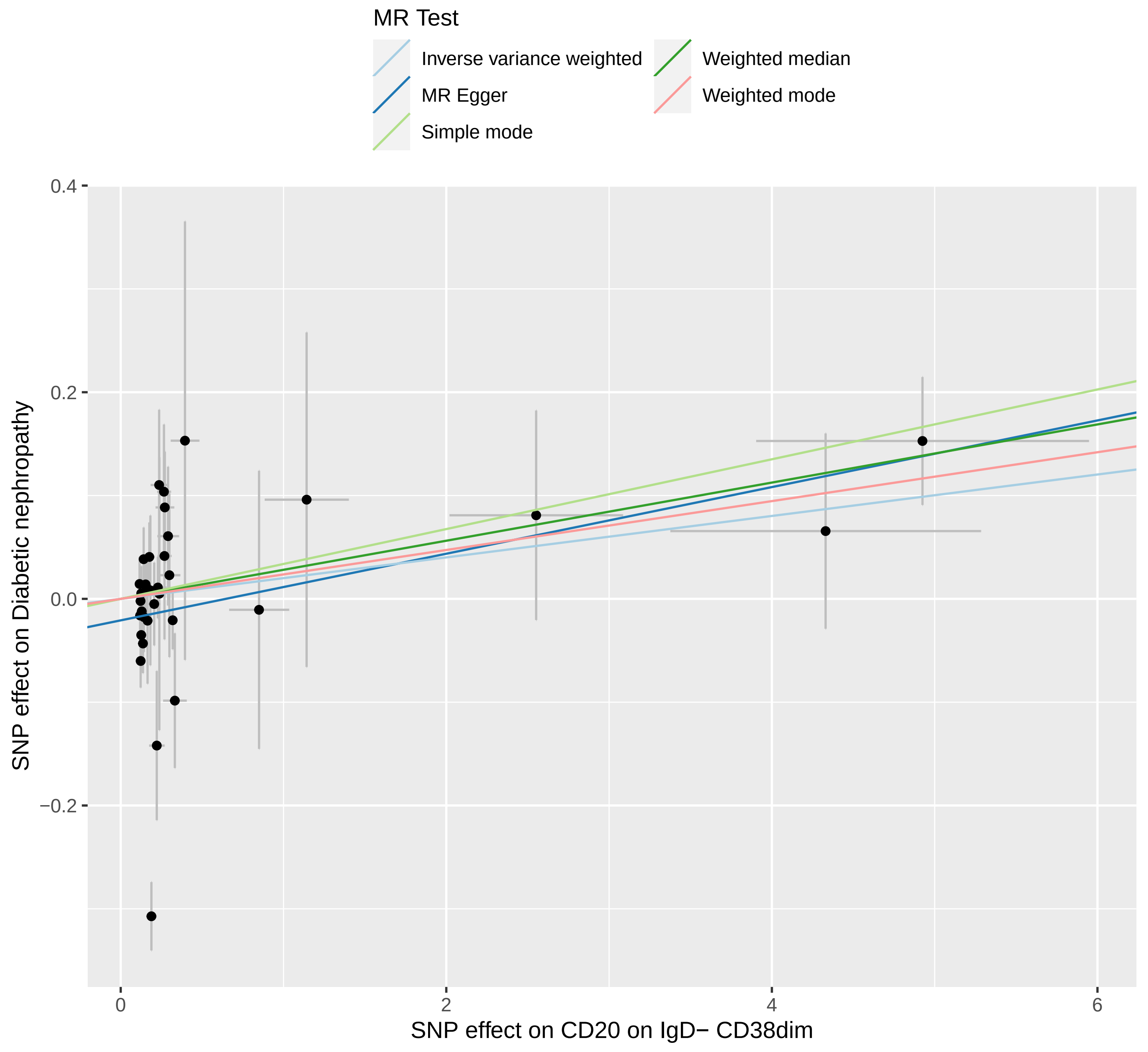
MR Test
Inverse variance weighted
MR Egger
Simple mode
Weighted median
Weighted mode
0.4
0.2
0.0
−0.2
SNP effect on Diabetic nephropathy
0
2
4
6
SNP effect on CD20 on IgD− CD38dim

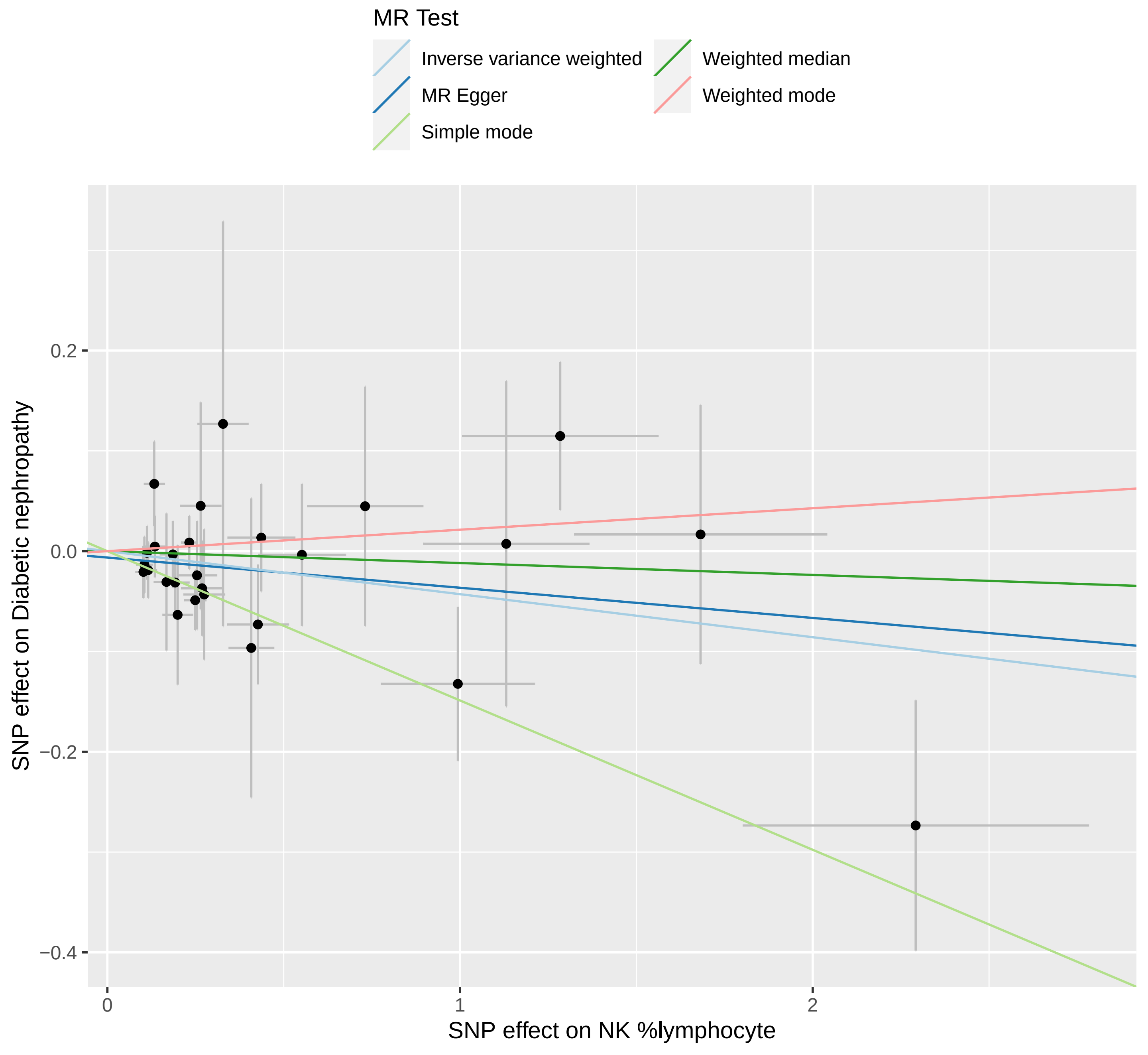

MR Test
Inverse variance weighted
MR Egger
Simple mode
Weighted median
Weighted mode
SNP effect on Diabetic nephropathy
0.2
0.0
−0.2
−0.4
0
1
2
SNP effect on NK %lymphocyte

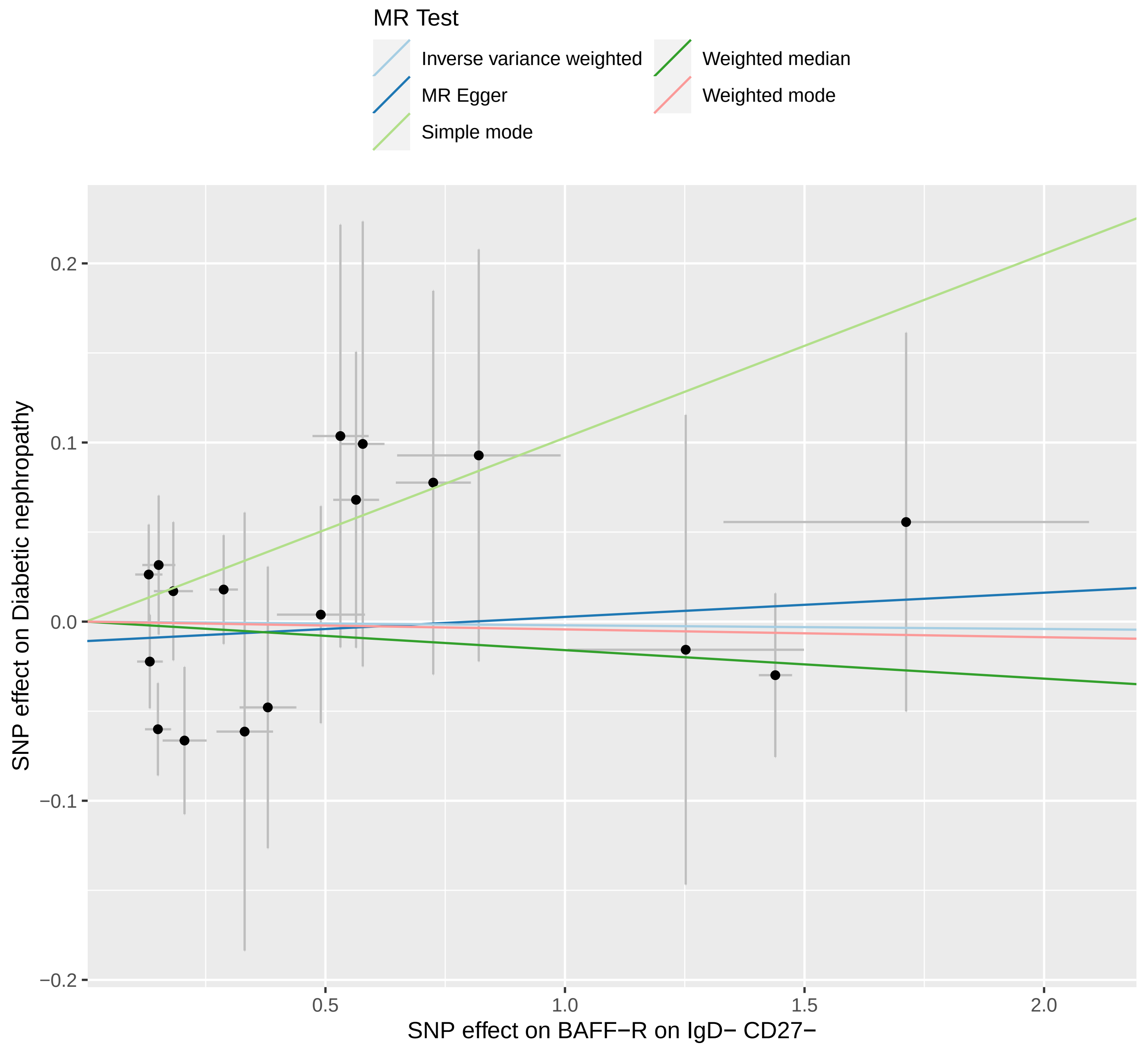

MR Test
Inverse variance weighted
MR Egger
Simple mode
Weighted median
Weighted mode
SNP effect on Diabetic nephropathy
0.2
0.1
0.0
−0.1
−0.2
0.5
1.0
1.5
2.0
SNP effect on BAFF−R on IgD− CD27−

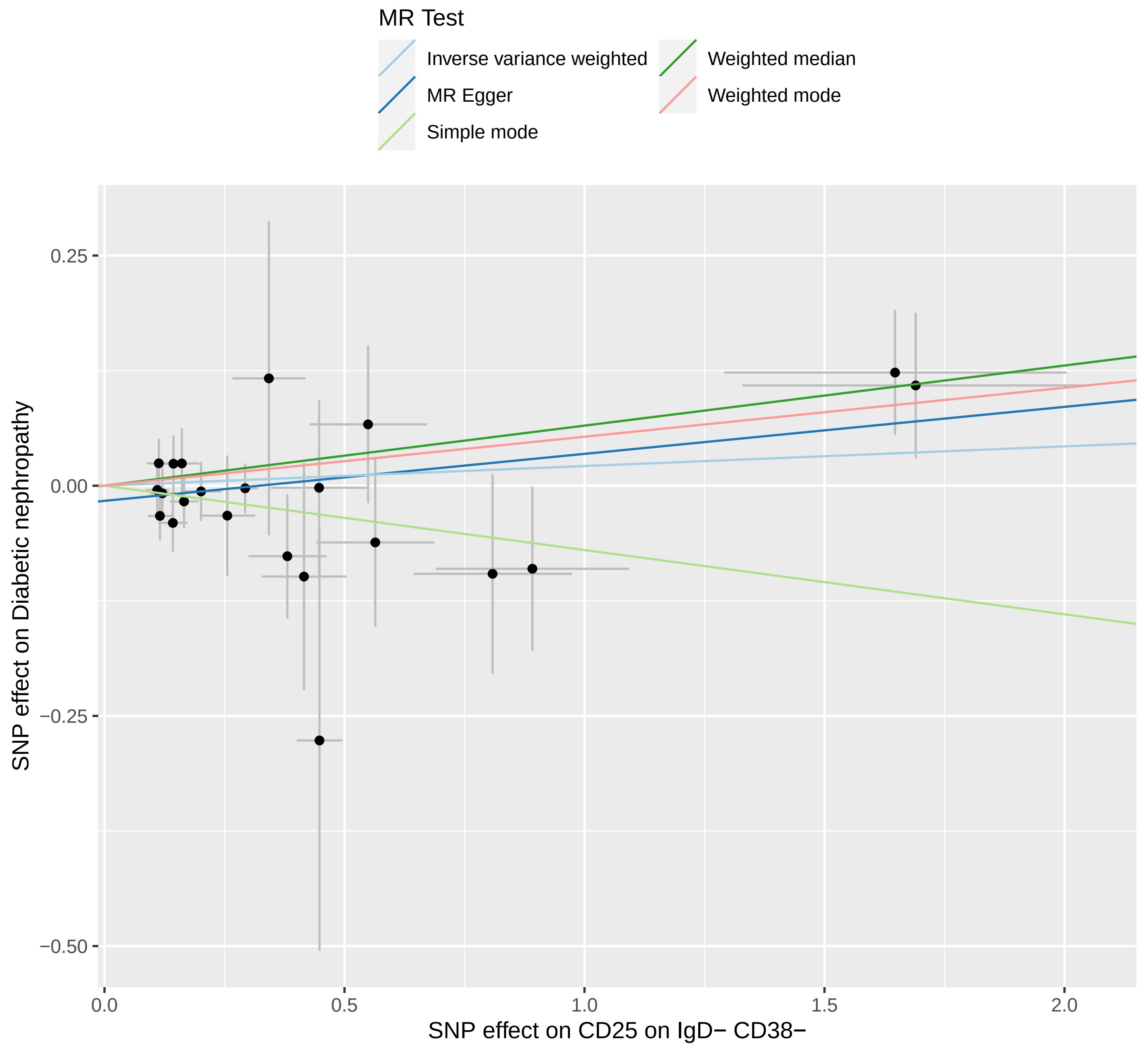
MR Test
Inverse variance weighted
MR Egger
Simple mode
Weighted median
Weighted mode
SNP effect on Diabetic nephropathy
0.25
0.00
−0.25
−0.50
0.0
0.5
1.0
1.5
2.0
SNP effect on CD25 on IgD− CD38−

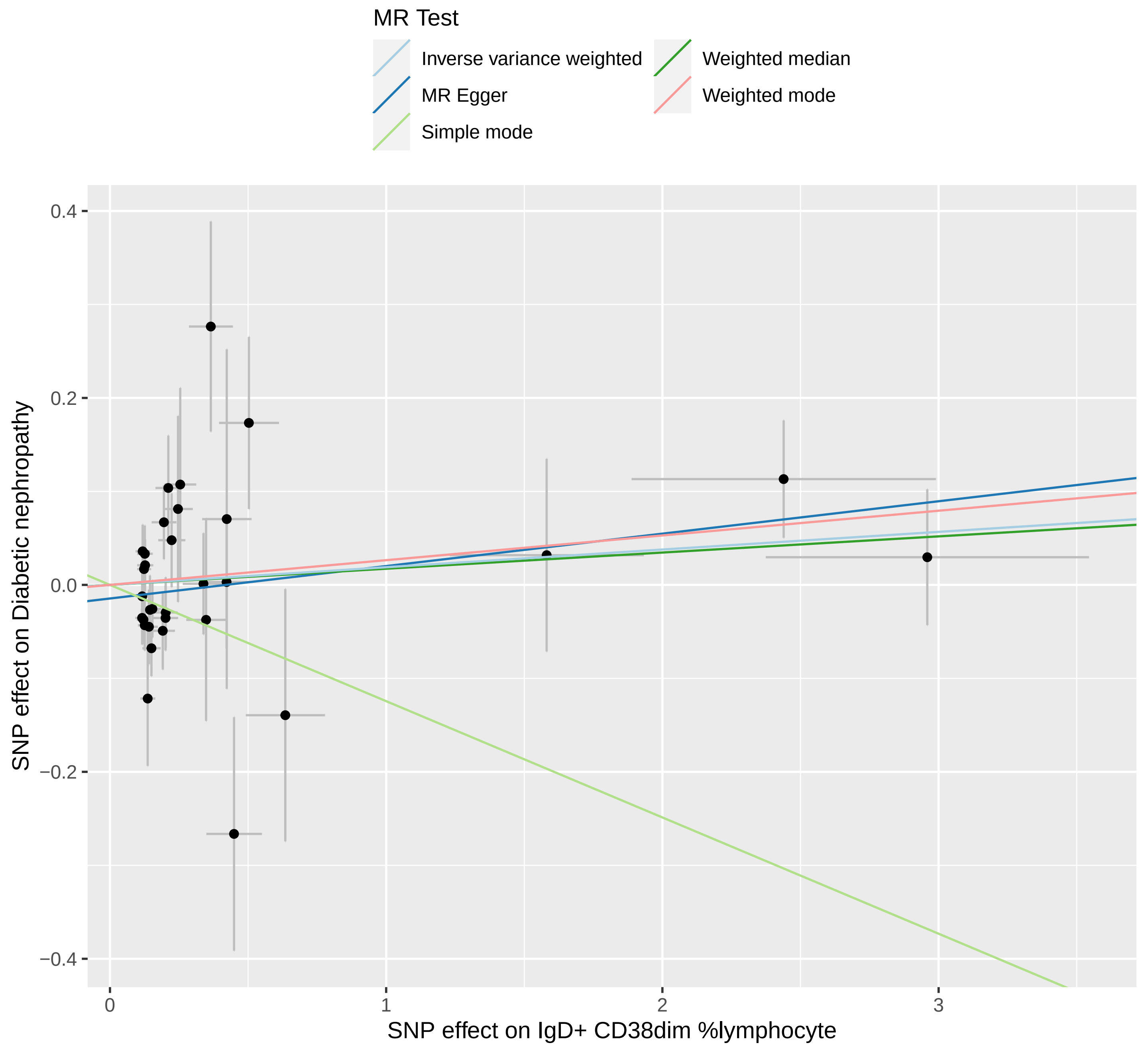

MR Test
Inverse variance weighted
MR Egger
Simple mode
Weighted median
Weighted mode
0.4
0.2
0.0
−0.2
−0.4
0
1
2
3
SNP effect on Diabetic nephropathy
SNP effect on IgD+ CD38dim %lymphocyte

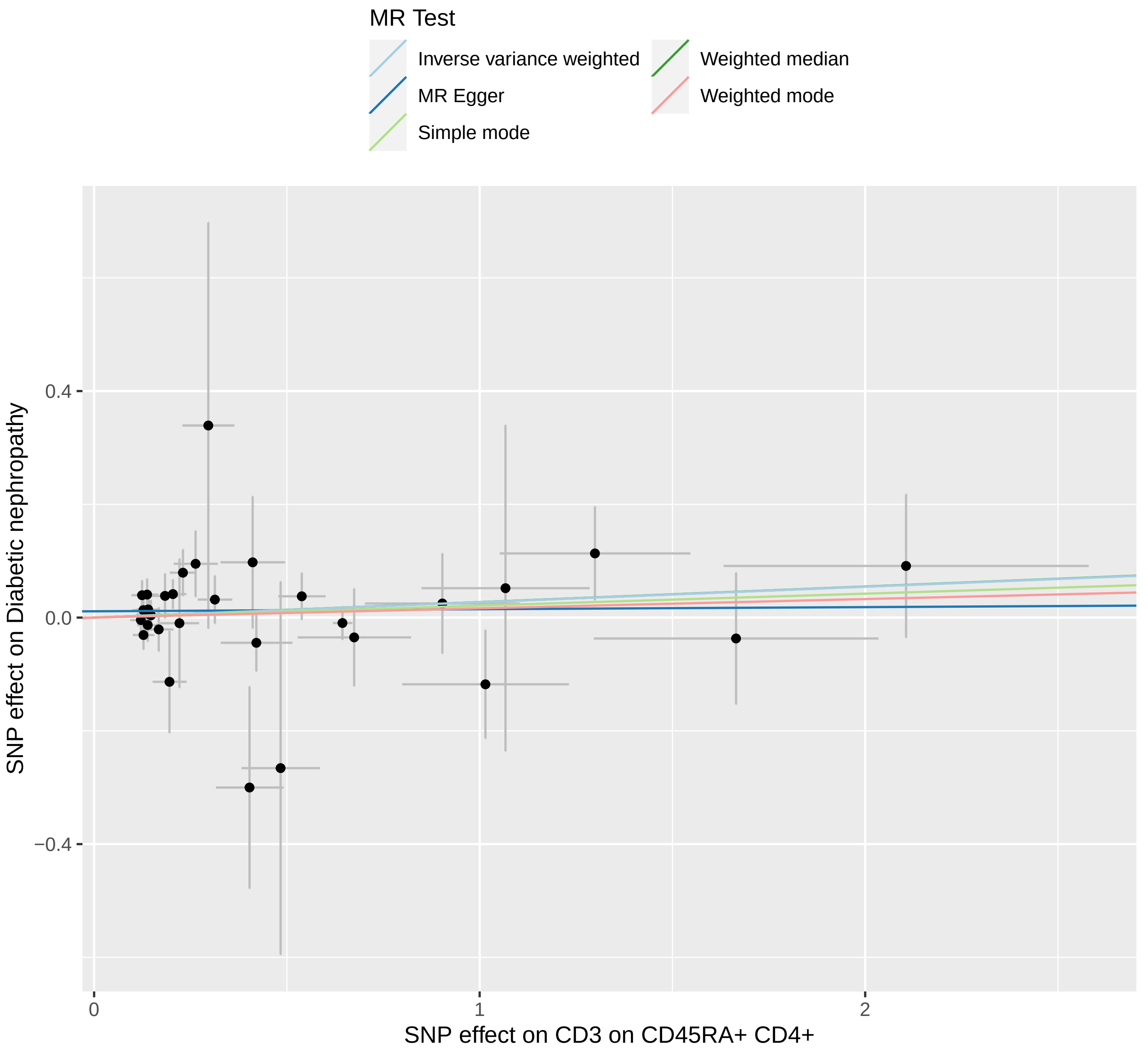

MR Test
Inverse variance weighted
MR Egger
Simple mode
Weighted median
Weighted mode
0.4
0.0
−0.4
0
1
2
SNP effect on Diabetic nephropathy
SNP effect on CD3 on CD45RA+ CD4+

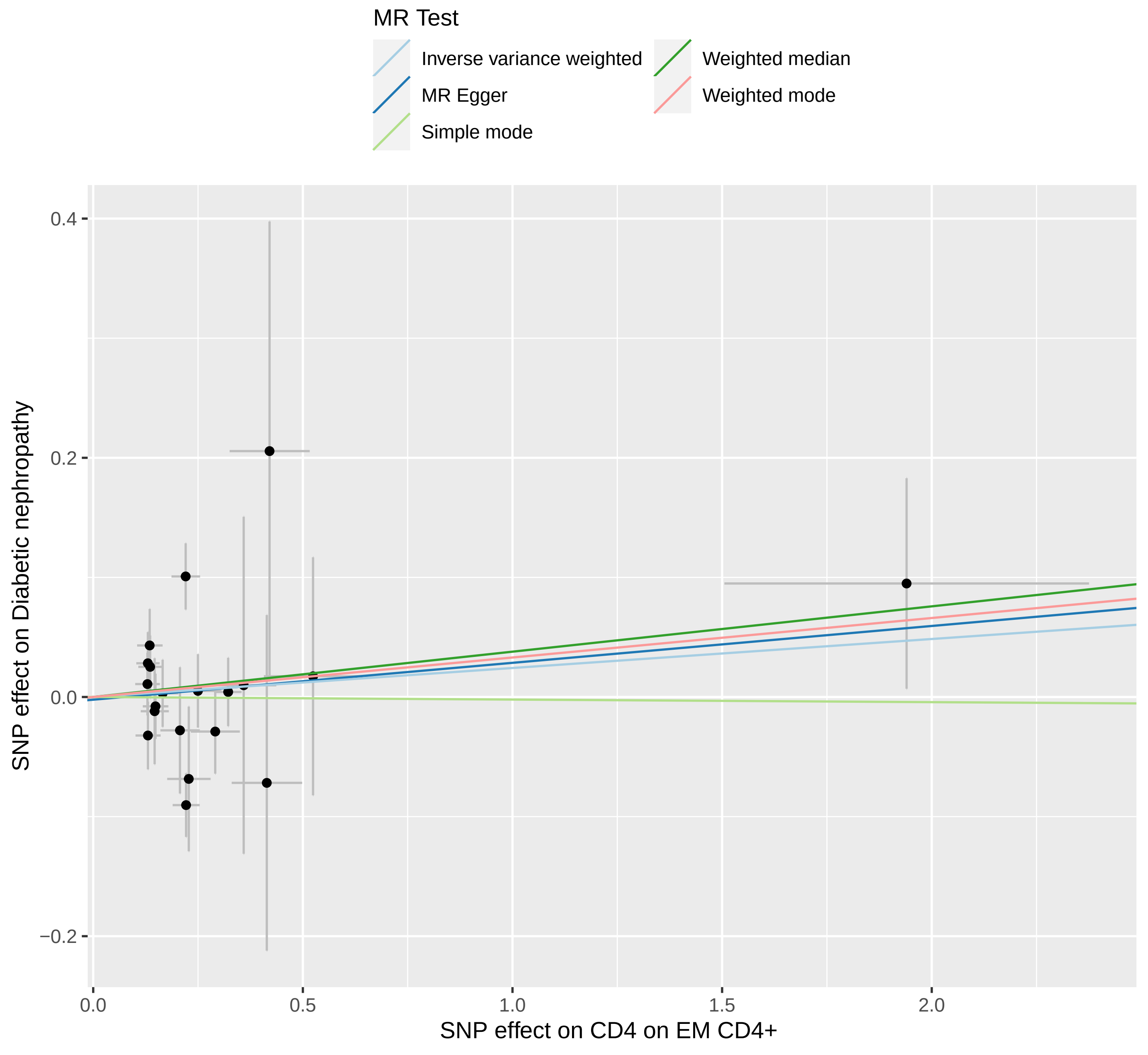

MR Test
Inverse variance weighted
MR Egger
Simple mode
Weighted median
Weighted mode
0.4
0.2
0.0
−0.2
0.0
0.5
1.0
1.5
2.0
SNP effect on Diabetic nephropathy
SNP effect on CD4 on EM CD4+

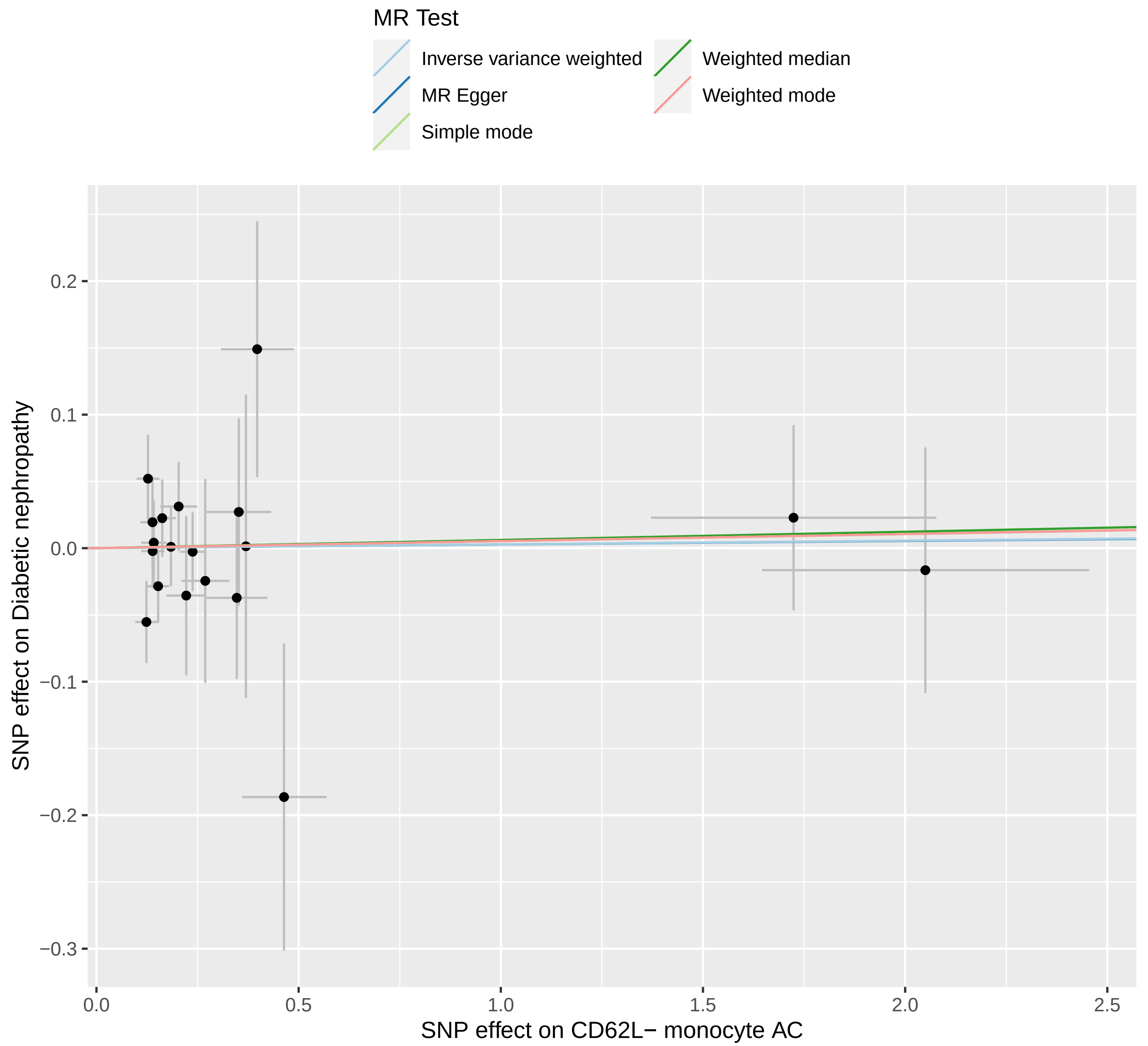
MR Test
Inverse variance weighted
MR Egger
Simple mode
Weighted median
Weighted mode
SNP effect on Diabetic nephropathy
SNP effect on CD62L− monocyte AC
0.2
0.1
0.0
−0.1
−0.2
−0.3
0.0
0.5
1.0
1.5
2.0
2.5

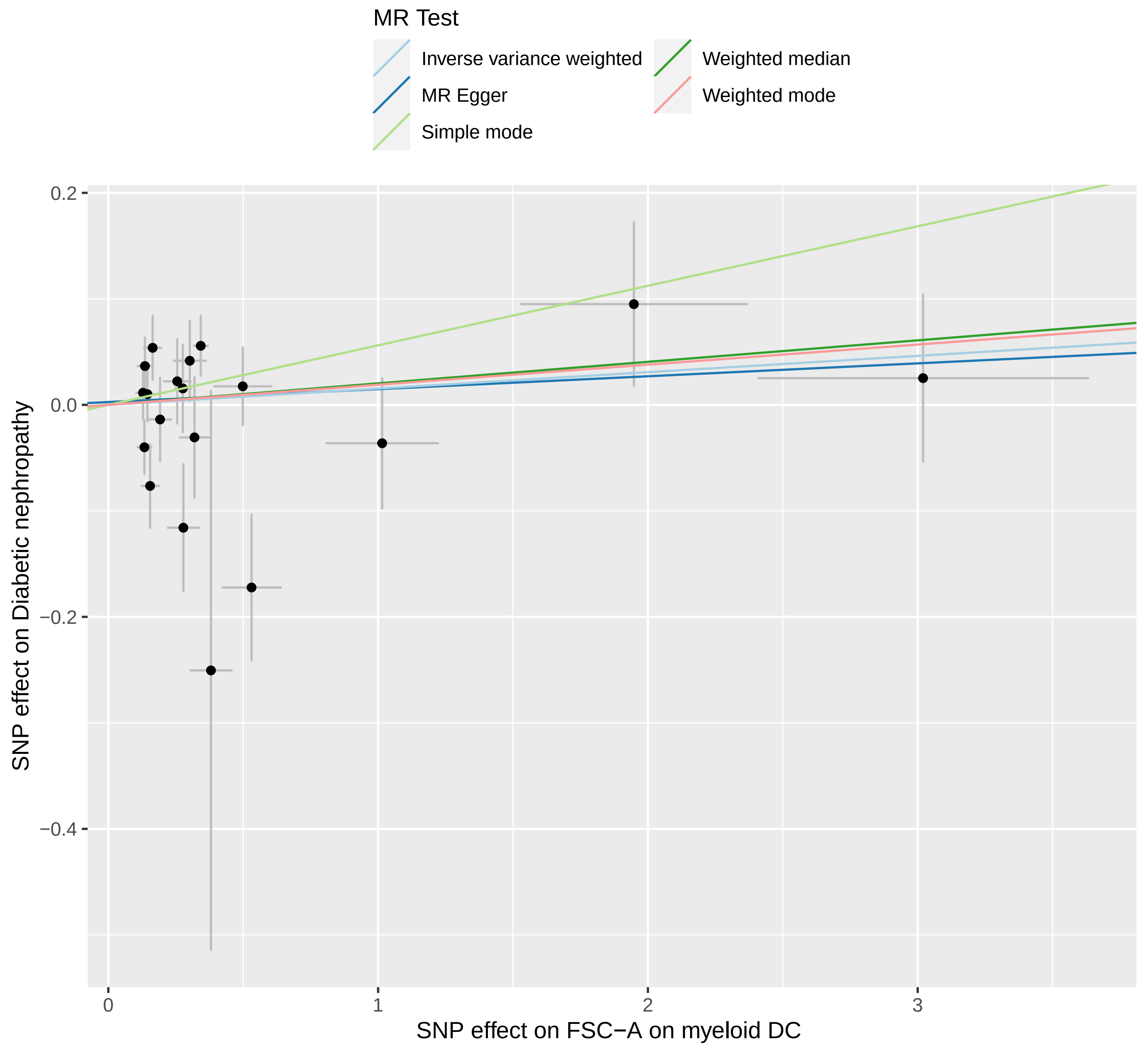

MR Test
Inverse variance weighted
MR Egger
Simple mode
Weighted median
Weighted mode
0.2
0.0
−0.2
−0.4
0
1
2
3
SNP effect on Diabetic nephropathy
SNP effect on FSC−A on myeloid DC

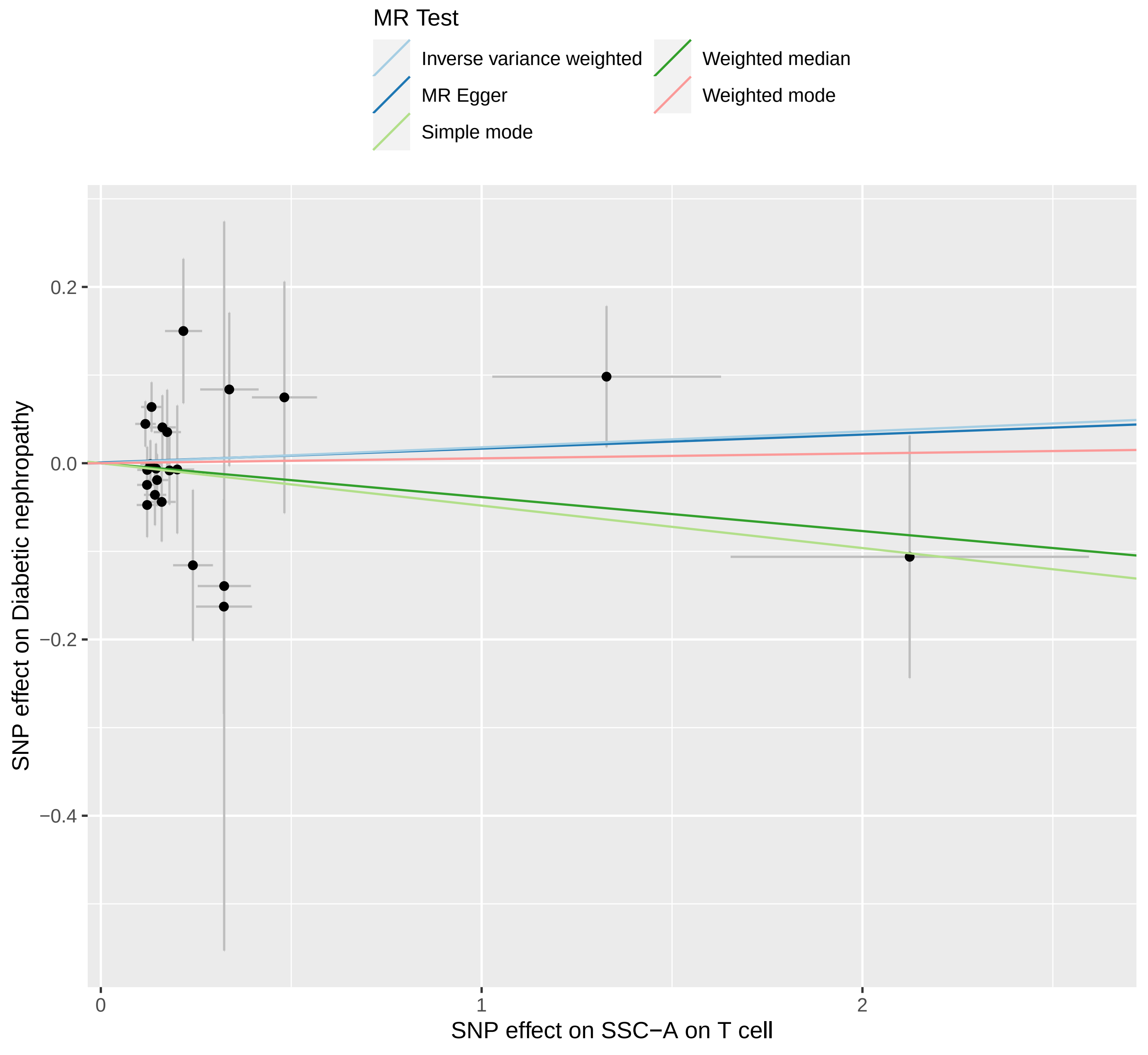

MR Test
Inverse variance weighted
MR Egger
Simple mode
Weighted median
Weighted mode
0.2
0.0
−0.2
−0.4
SNP effect on Diabetic nephropathy
0
1
2
SNP effect on SSC−A on T cell

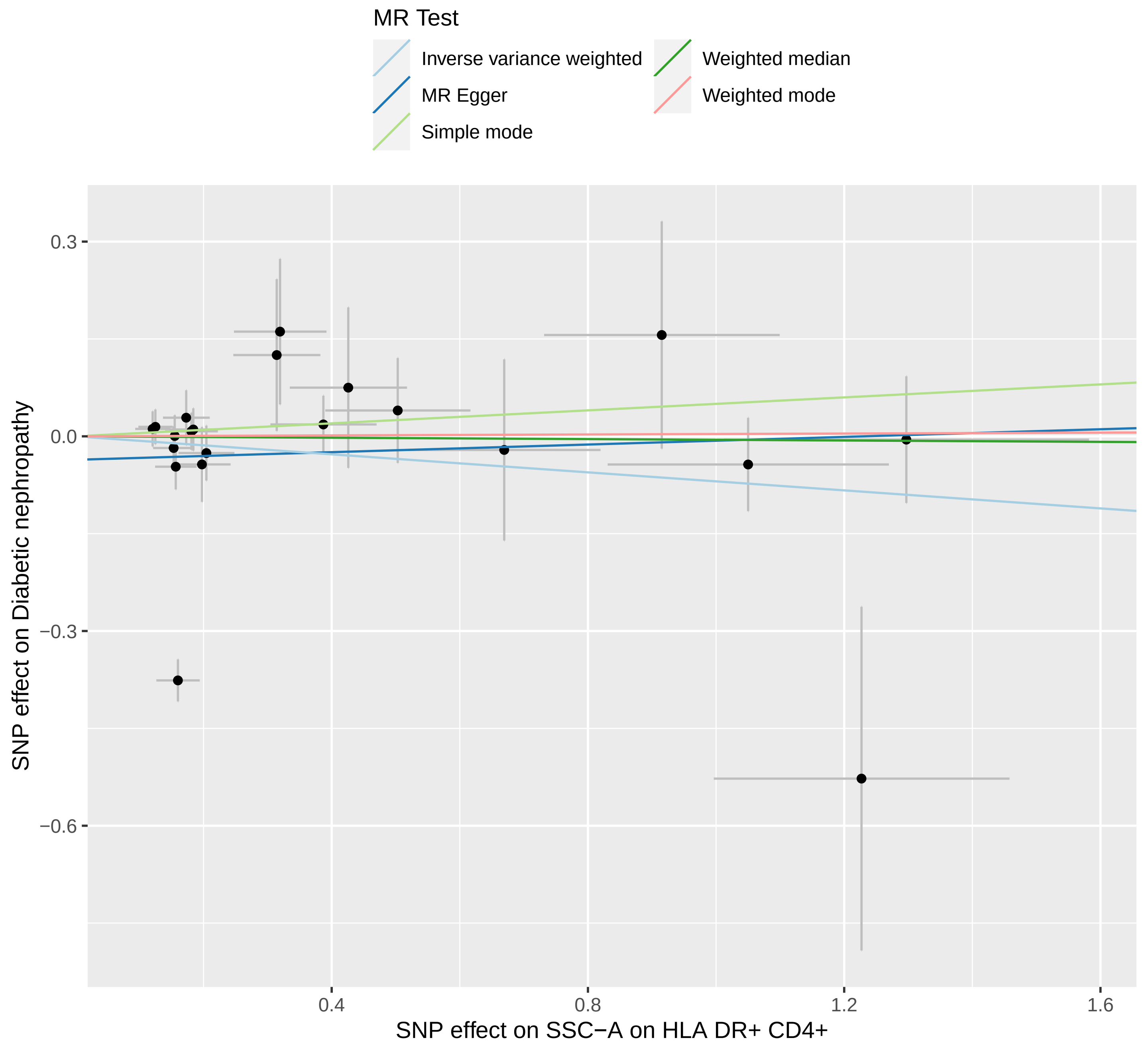
MR Test
Inverse variance weighted
MR Egger
Simple mode
Weighted median
Weighted mode
0.3
0.0
−0.3
−0.6
0.4
0.8
1.2
1.6
SNP effect on Diabetic nephropathy
SNP effect on SSC−A on HLA DR+ CD4+

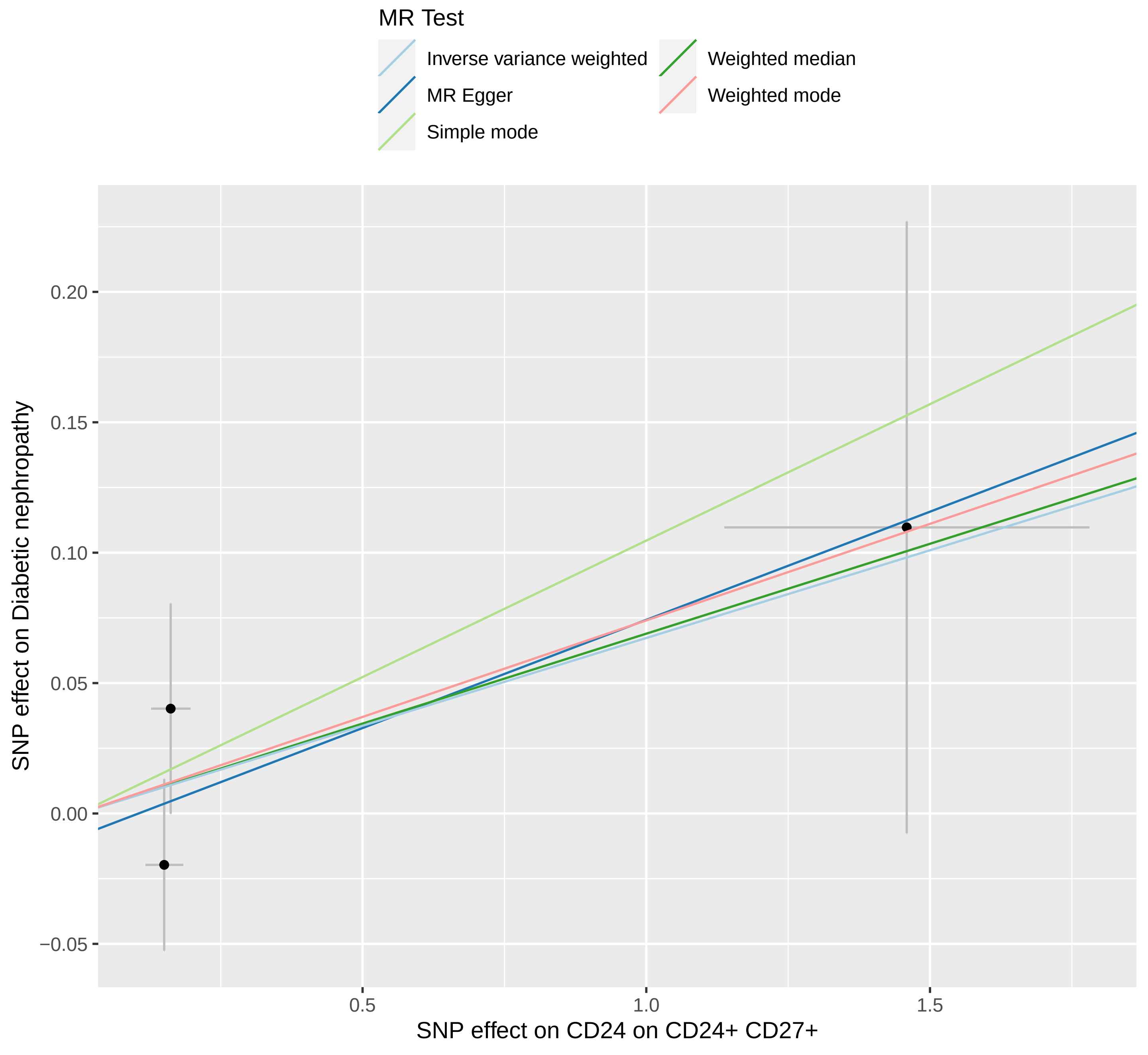

MR Test
Inverse variance weighted
MR Egger
Simple mode
Weighted median
Weighted mode
0.20
0.15
0.10
0.05
0.00
−0.05
0.5
1.0
1.5
SNP effect on Diabetic nephropathy
SNP effect on CD24 on CD24+ CD27+

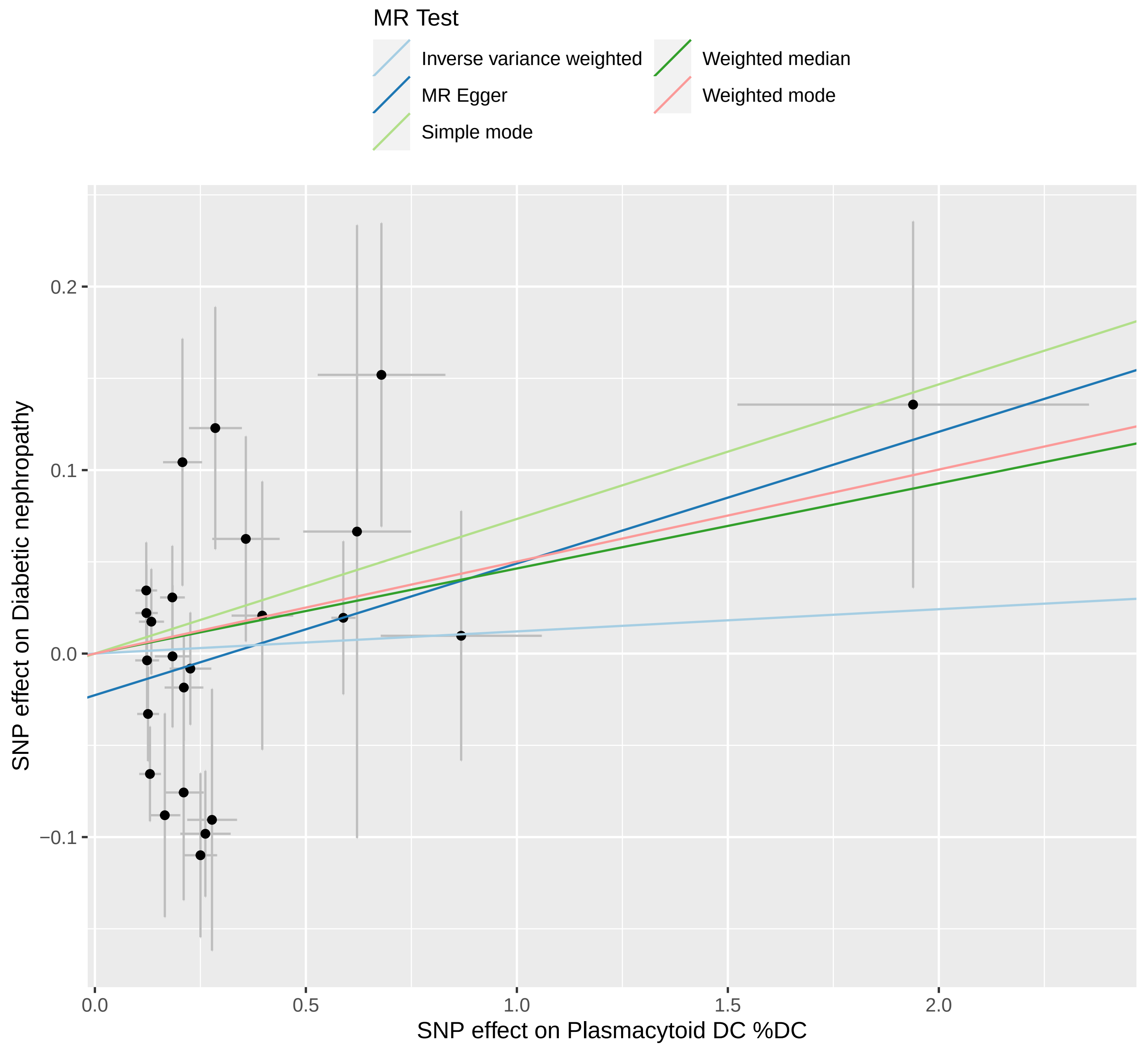
MR Test
Inverse variance weighted
MR Egger
Simple mode
Weighted median
Weighted mode
0.2
0.1
0.0
−0.1
0.0
0.5
1.0
1.5
2.0
SNP effect on Diabetic nephropathy
SNP effect on Plasmacytoid DC %DC

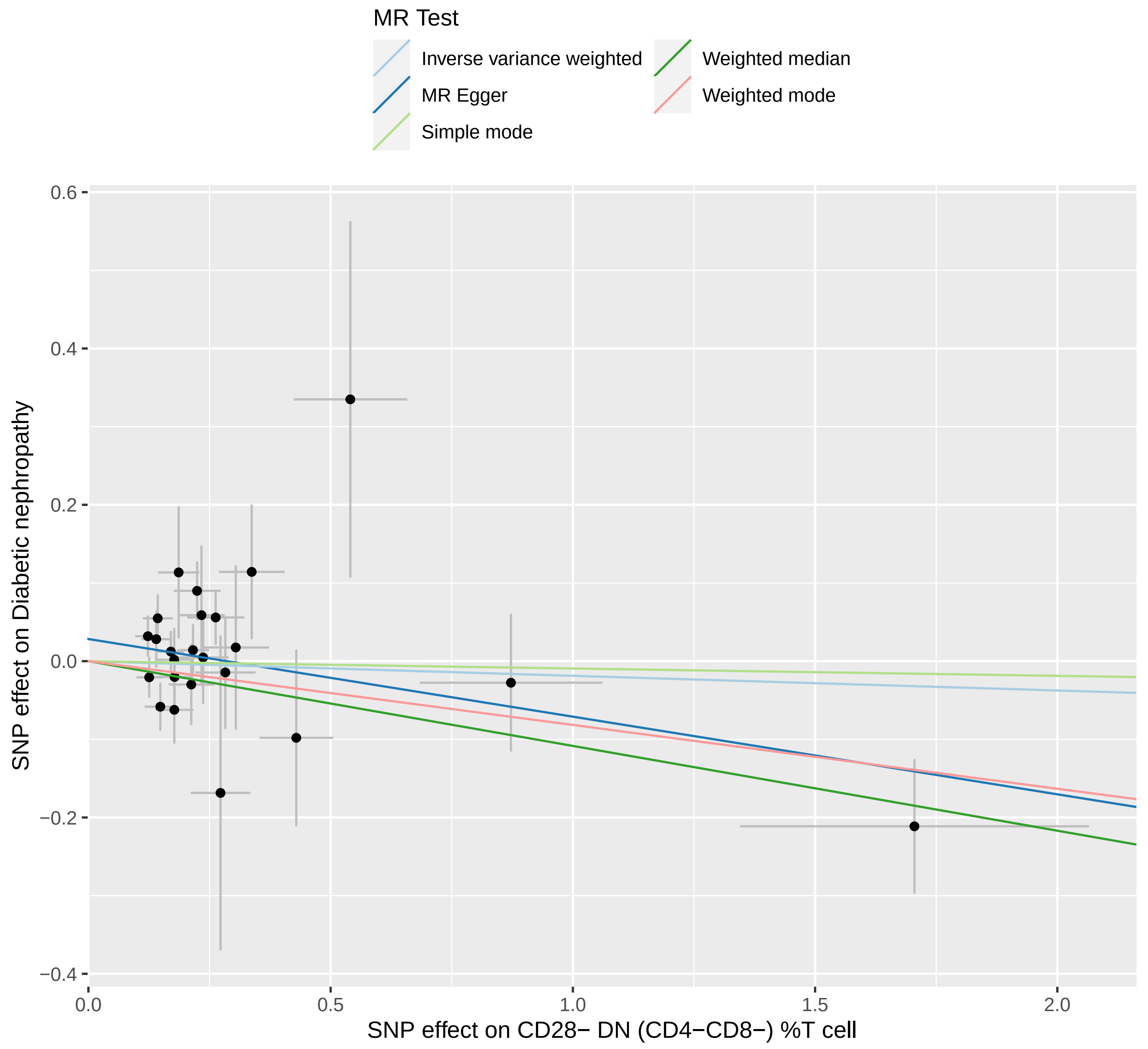

MR Test
Inverse variance weighted
MR Egger
Simple mode
Weighted median
Weighted mode
0.6
0.4
0.2
0.0
−0.2
−0.4
SNP effect on Diabetic nephropathy
0.0
0.5
1.0
1.5
2.0
SNP effect on CD28− DN (CD4−CD8−) %T cell

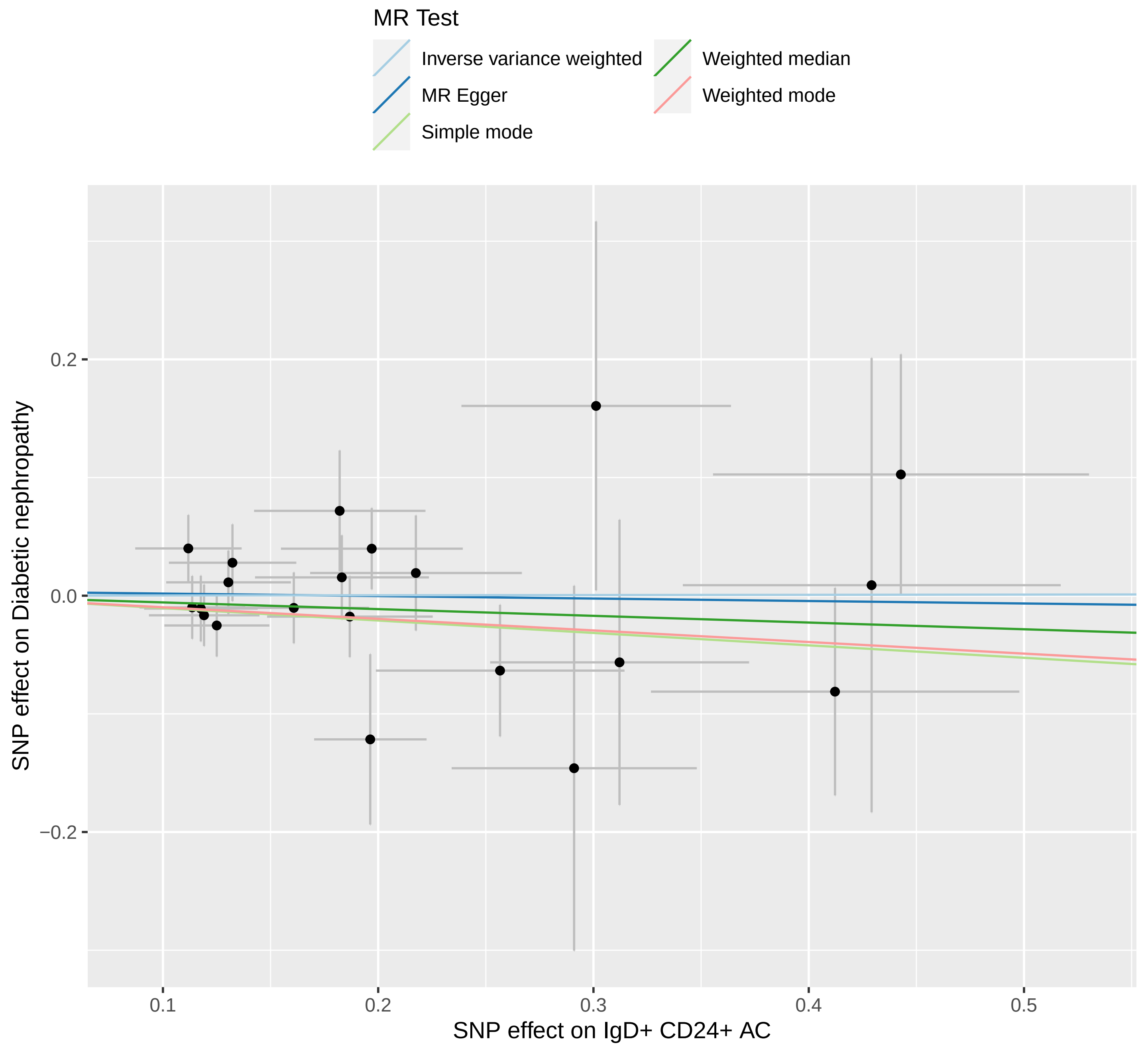
MR Test
Inverse variance weighted
MR Egger
Simple mode
Weighted median
Weighted mode
SNP effect on Diabetic nephropathy
0.2
0.0
−0.2
0.1
0.2
0.3
0.4
0.5
SNP effect on IgD+ CD24+ AC

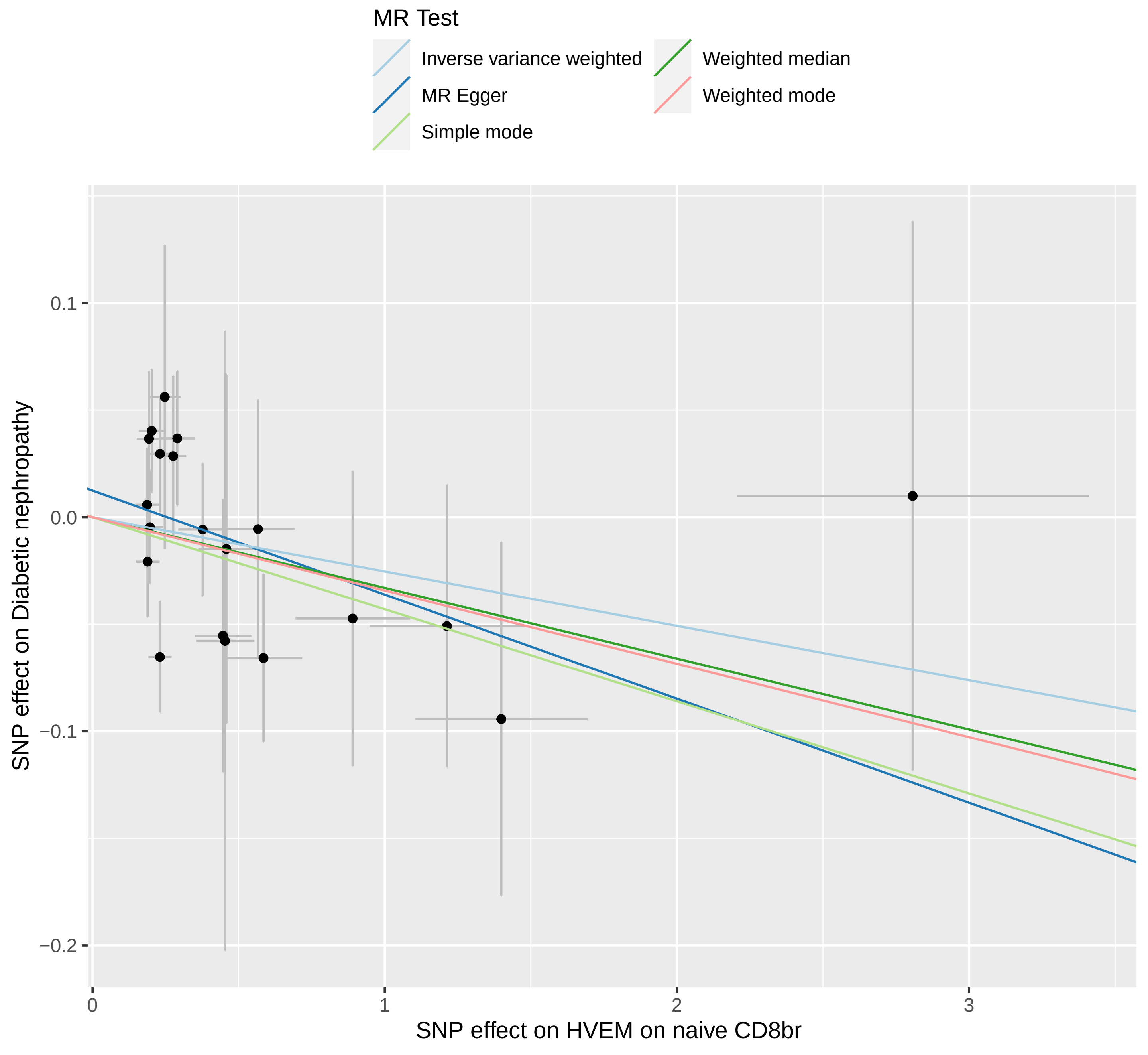

MR Test
Inverse variance weighted
MR Egger
Simple mode
Weighted median
Weighted mode
0.1
0.0
−0.1
−0.2
0
1
2
3
SNP effect on Diabetic nephropathy
SNP effect on HVEM on naive CD8br

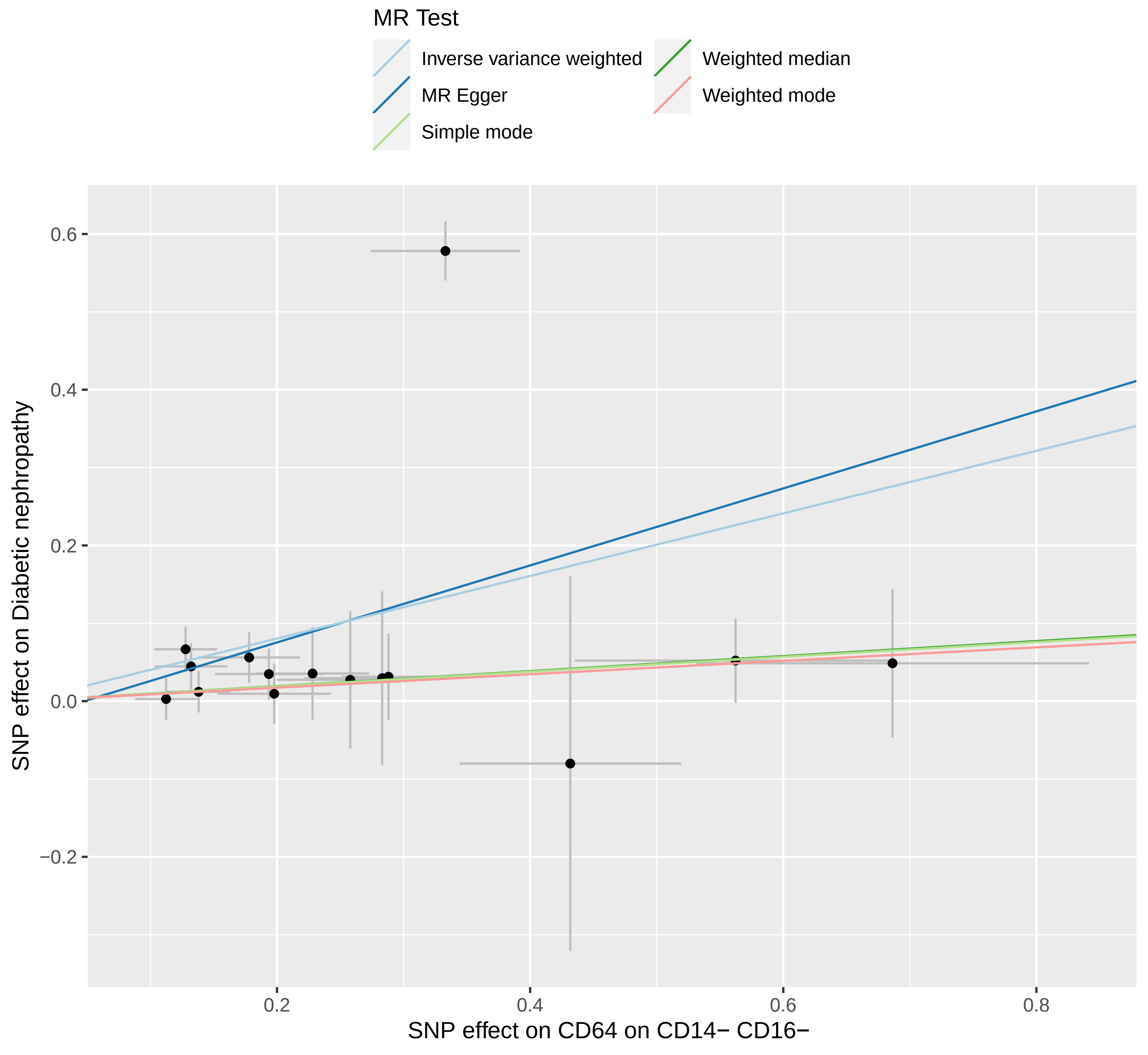
MR Test
Inverse variance weighted
MR Egger
Simple mode
Weighted median
Weighted mode
0.6
0.4
0.2
0.0
−0.2
SNP effect on Diabetic nephropathy
0.2
0.4
0.6
0.8
SNP effect on CD64 on CD14− CD16−

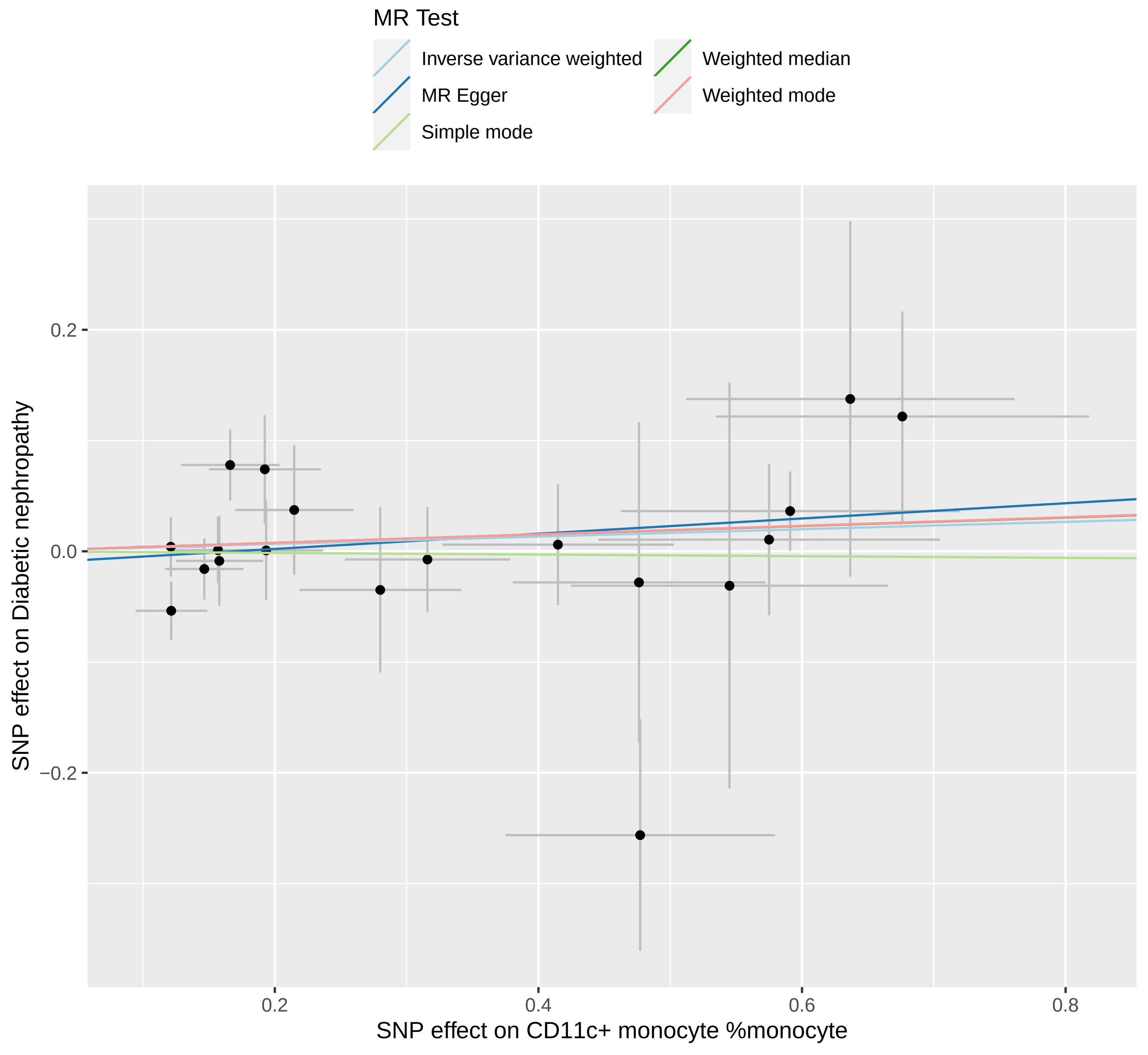
MR Test
Inverse variance weighted
MR Egger
Simple mode
Weighted median
Weighted mode
0.2
0.0
−0.2
0.2
0.4
0.6
0.8
SNP effect on Diabetic nephropathy
SNP effect on CD11c+ monocyte %monocyte

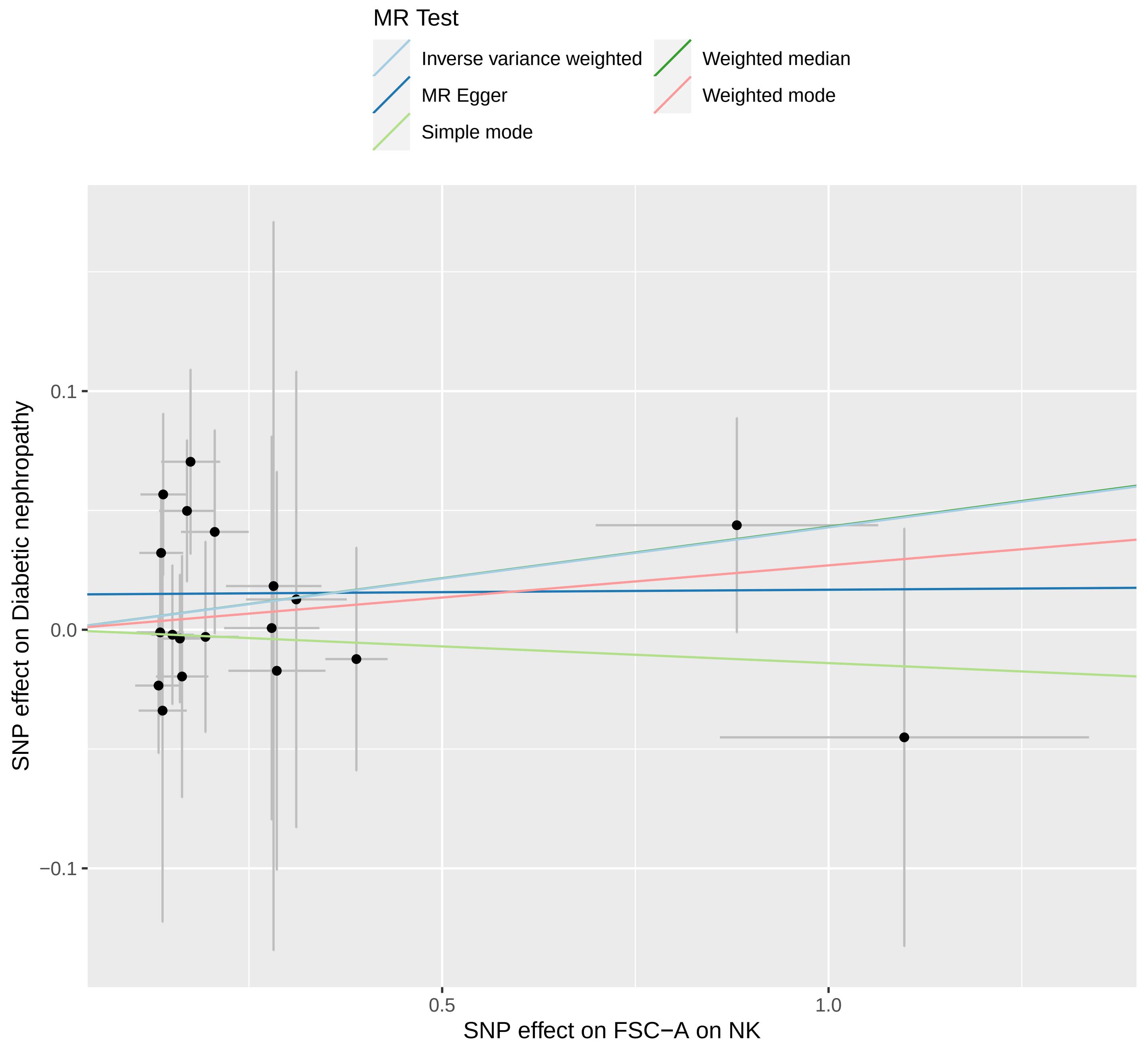

MR Test
Inverse variance weighted
MR Egger
Simple mode
Weighted median
Weighted mode
0.1
0.0
−0.1
0.5
1.0
SNP effect on Diabetic nephropathy
SNP effect on FSC−A on NK

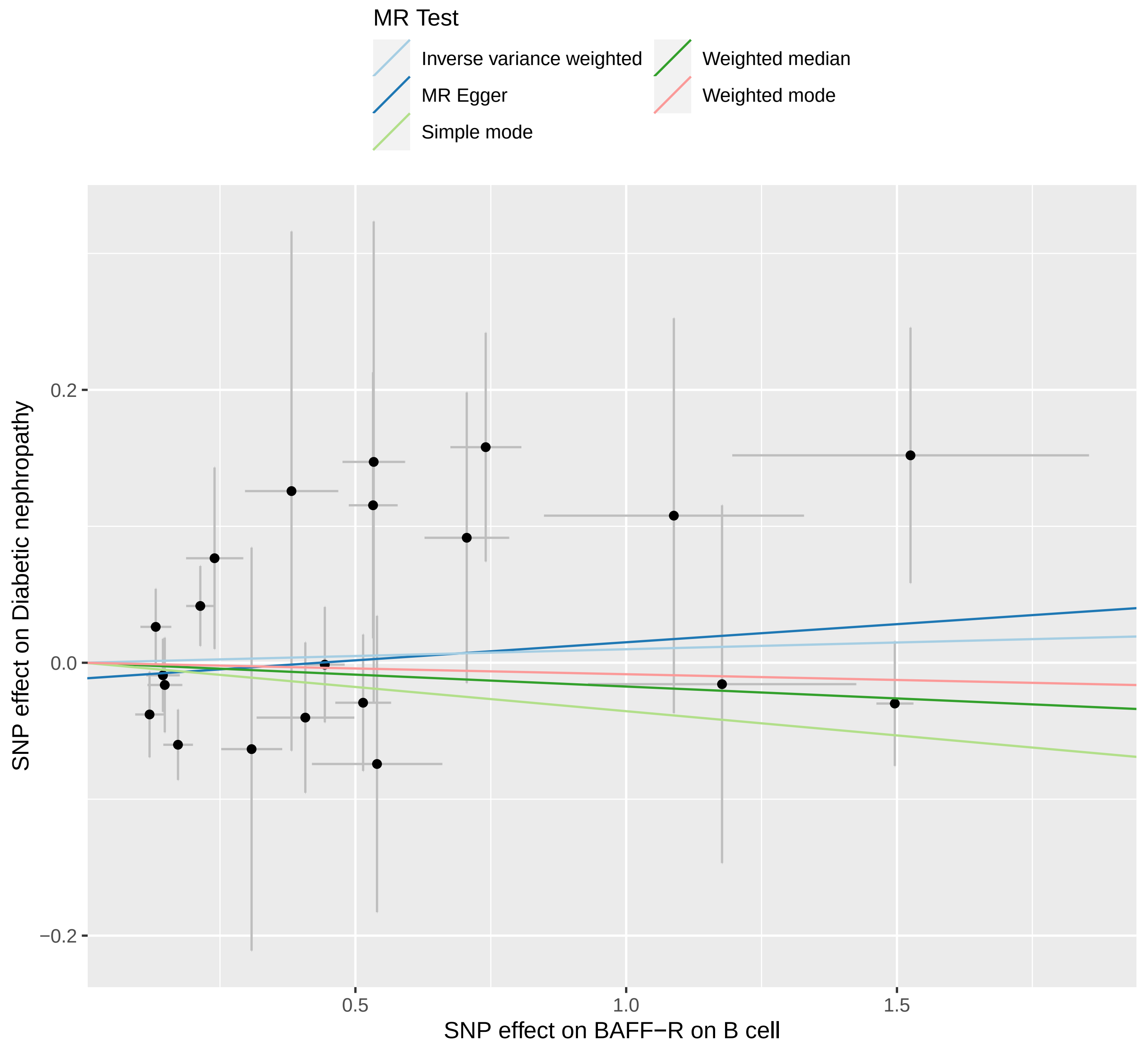

MR Test
Inverse variance weighted
MR Egger
Simple mode
Weighted median
Weighted mode
0.2
0.0
−0.2
0.5
1.0
1.5
SNP effect on Diabetic nephropathy
SNP effect on BAFF−R on B cell

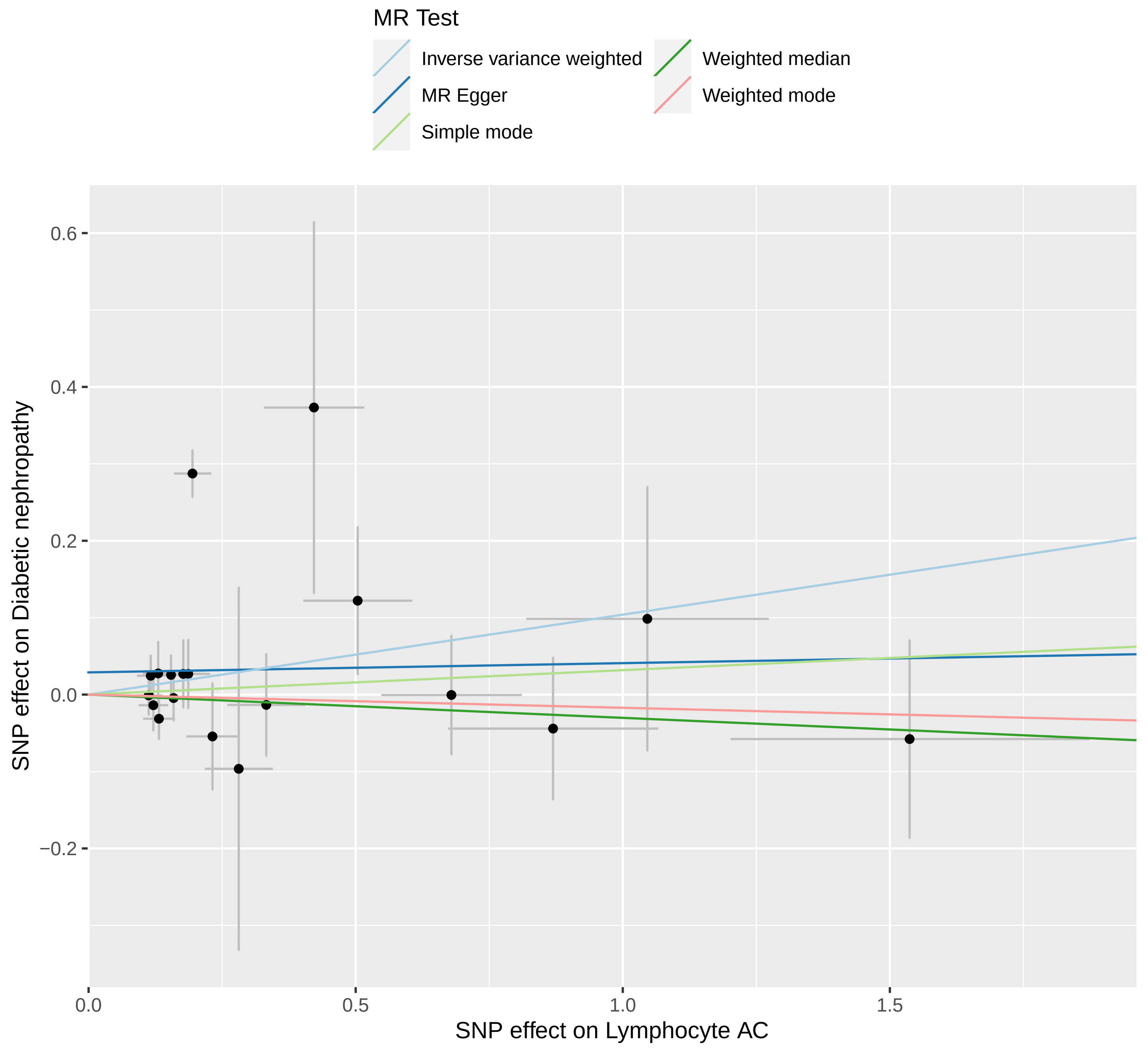
MR Test
Inverse variance weighted
MR Egger
Simple mode
Weighted median
Weighted mode
0.6
0.4
0.2
0.0
−0.2
SNP effect on Diabetic nephropathy
0.0
0.5
1.0
1.5
SNP effect on Lymphocyte AC

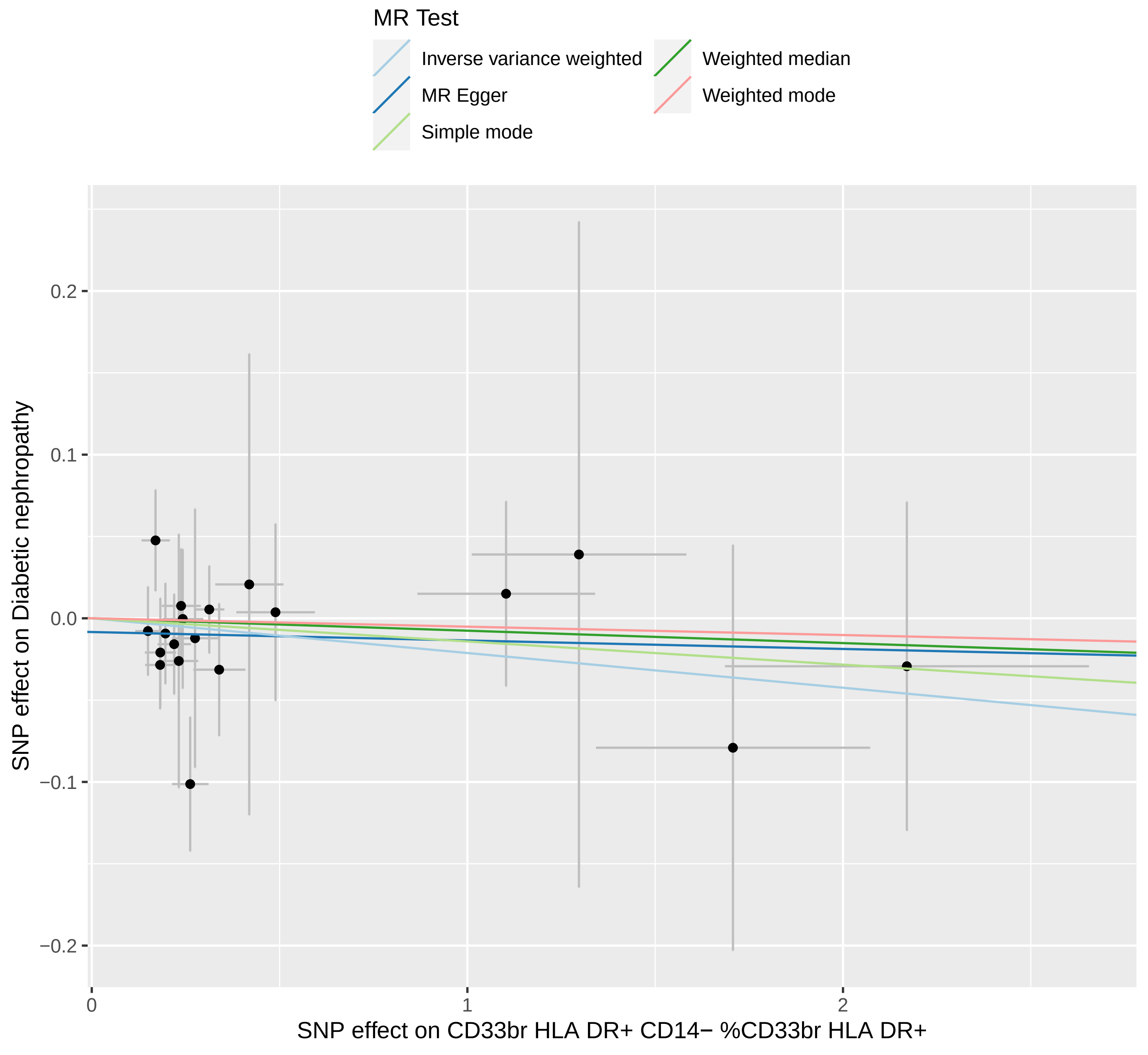

MR Test
Inverse variance weighted
MR Egger
Simple mode
Weighted median
Weighted mode
SNP effect on Diabetic nephropathy
0.2
0.1
0.0
−0.1
−0.2
0
1
2
SNP effect on CD33br HLA DR+ CD14− %CD33br HLA DR+

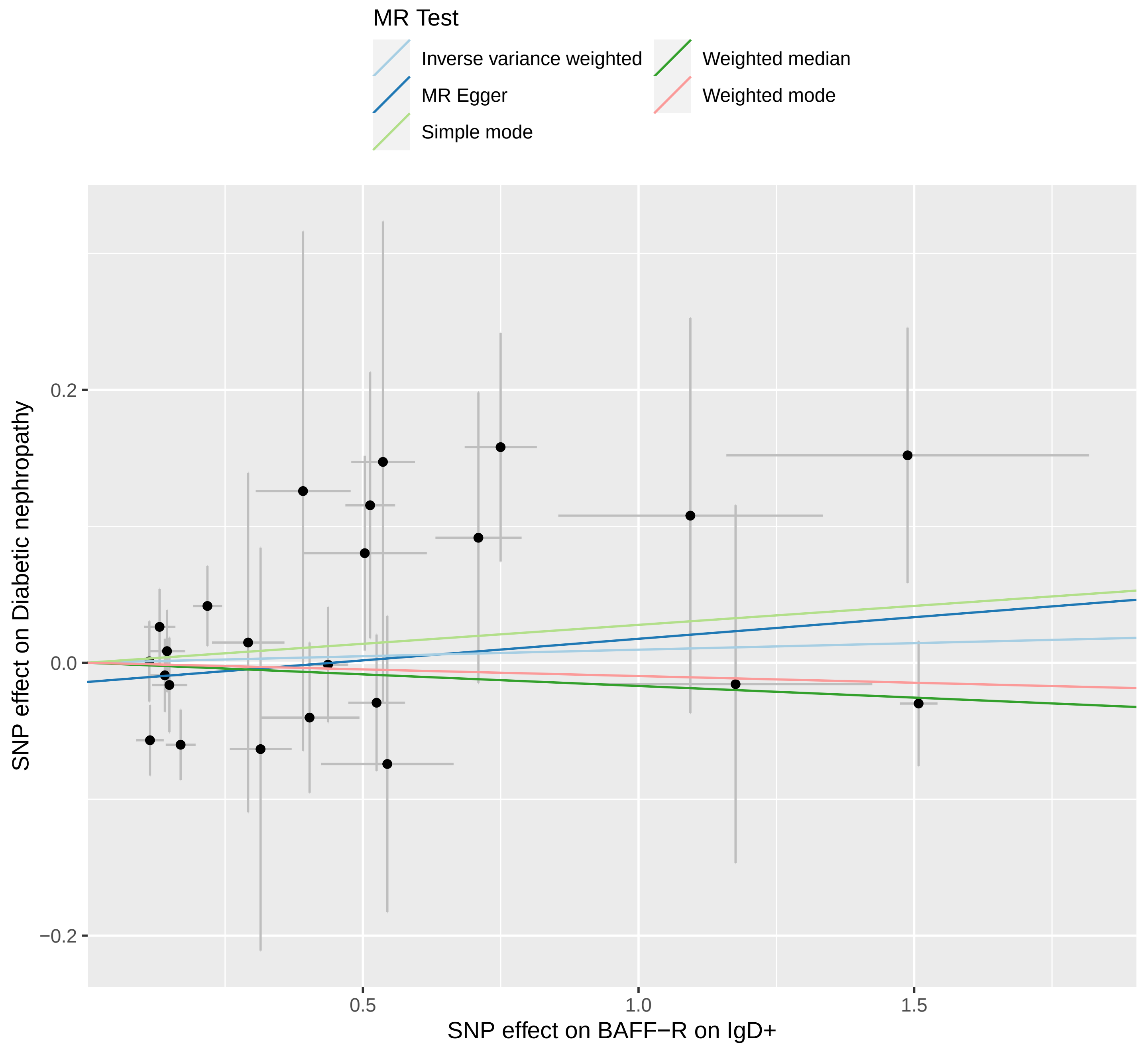
MR Test
Inverse variance weighted
MR Egger
Simple mode
Weighted median
Weighted mode
0.2
0.0
−0.2
0.5
1.0
1.5
SNP effect on Diabetic nephropathy
SNP effect on BAFF−R on IgD+

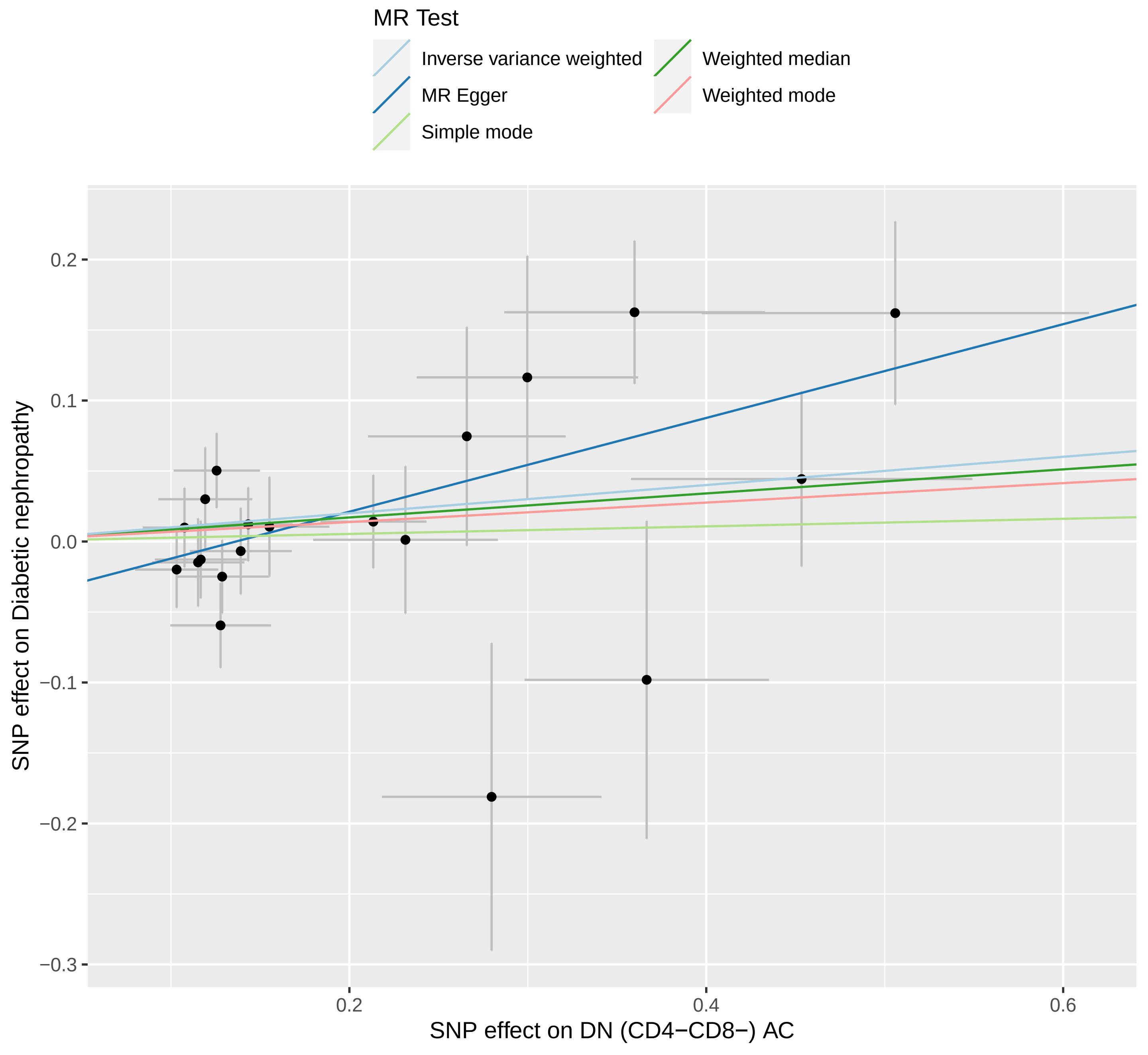

MR Test
Inverse variance weighted
MR Egger
Simple mode
Weighted median
Weighted mode
0.2
0.1
0.0
−0.1
−0.2
−0.3
SNP effect on Diabetic nephropathy
0.2
0.4
0.6
SNP effect on DN (CD4−CD8−) AC

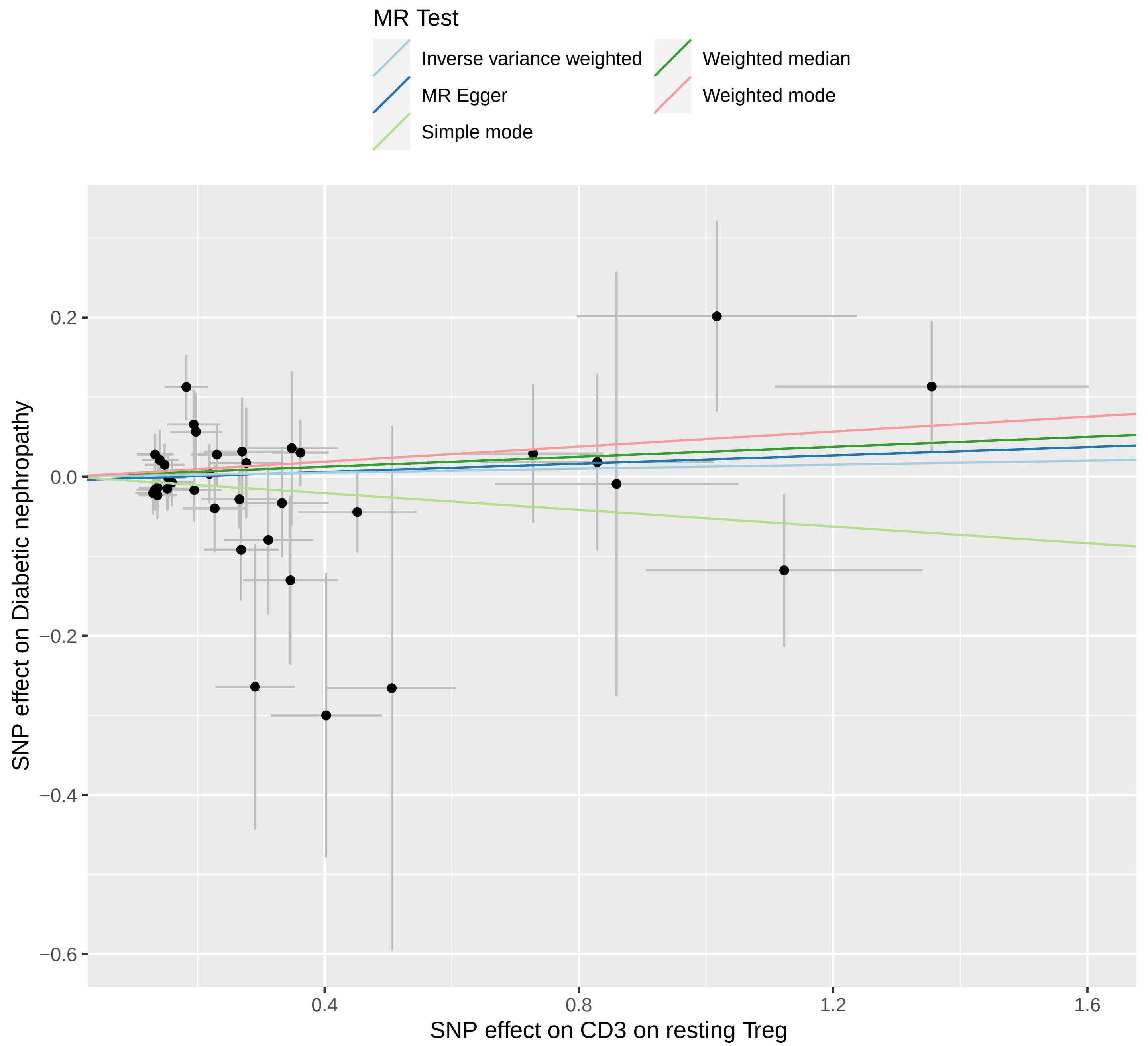
MR Test
Inverse variance weighted
MR Egger
Simple mode
Weighted median
Weighted mode
0.2
0.0
−0.2
−0.4
−0.6
0.4
0.8
1.2
1.6
SNP effect on Diabetic nephropathy
SNP effect on CD3 on resting Treg

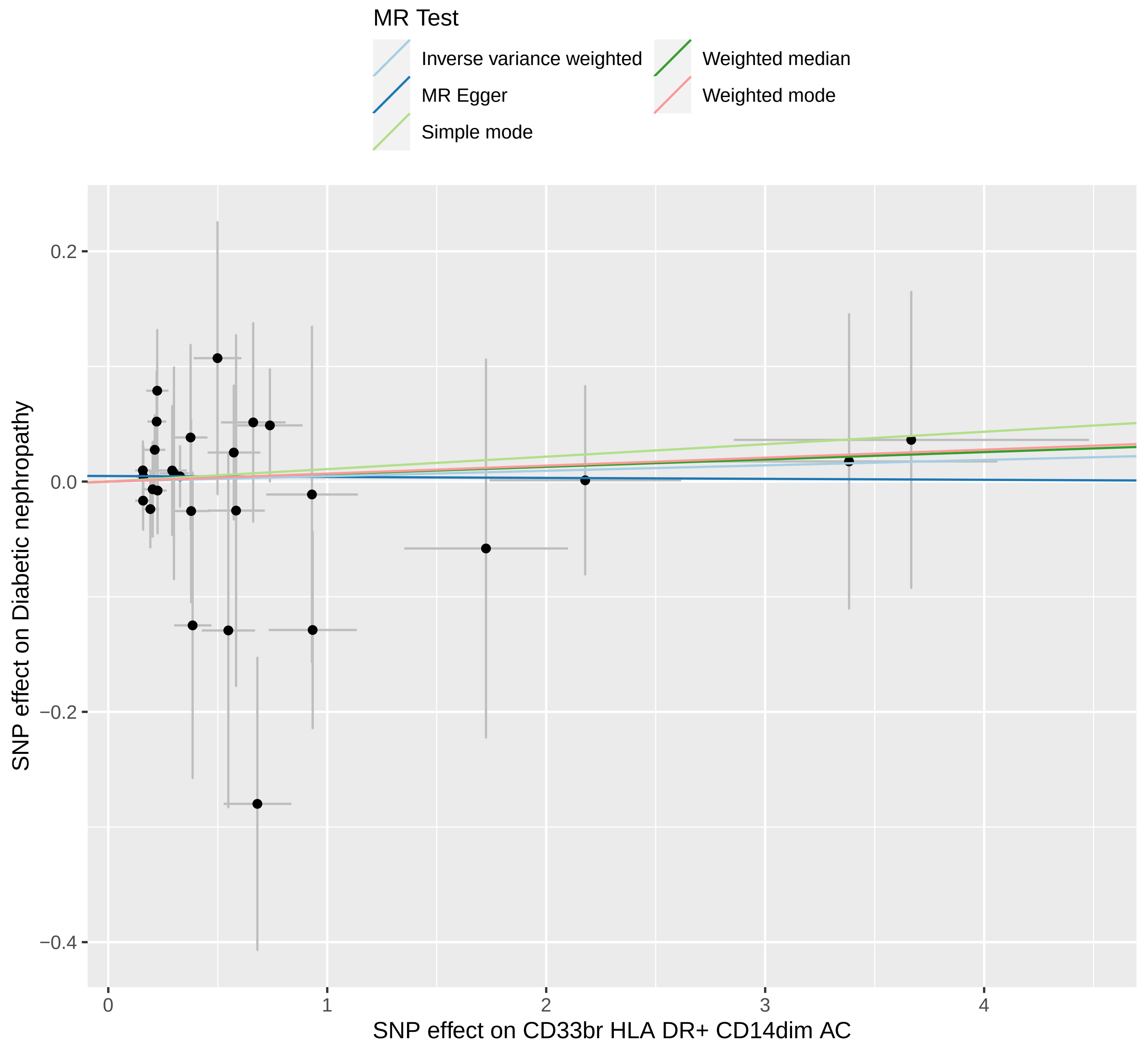
MR Test
Inverse variance weighted
MR Egger
Simple mode
Weighted median
Weighted mode
SNP effect on Diabetic nephropathy
0.2
0.0
−0.2
−0.4
0
1
2
3
4
SNP effect on CD33br HLA DR+ CD14dim AC

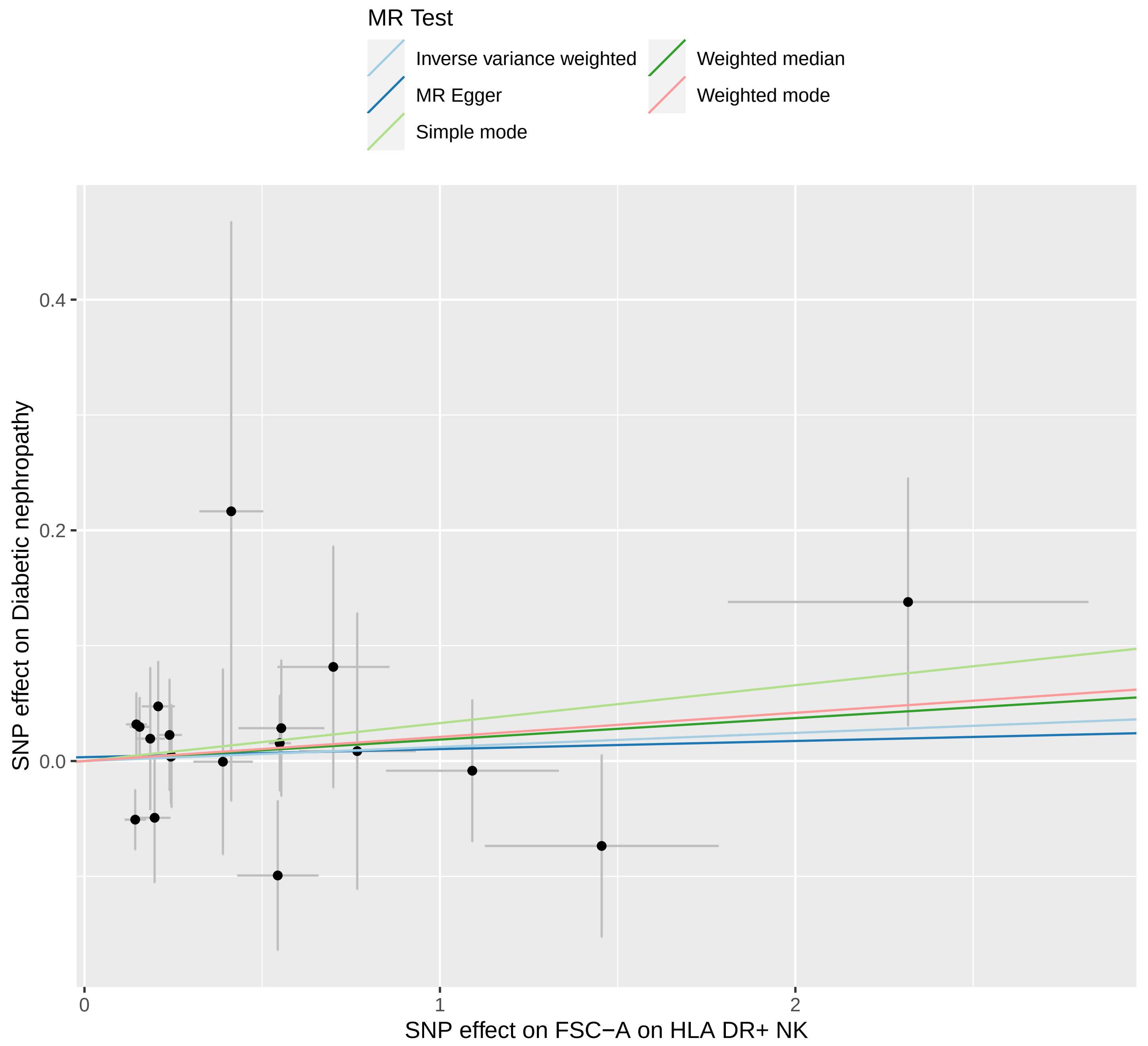
MR Test
Inverse variance weighted
MR Egger
Simple mode
Weighted median
Weighted mode
0.4
0.2
0.0
0
1
2
SNP effect on Diabetic nephropathy
SNP effect on FSC−A on HLA DR+ NK

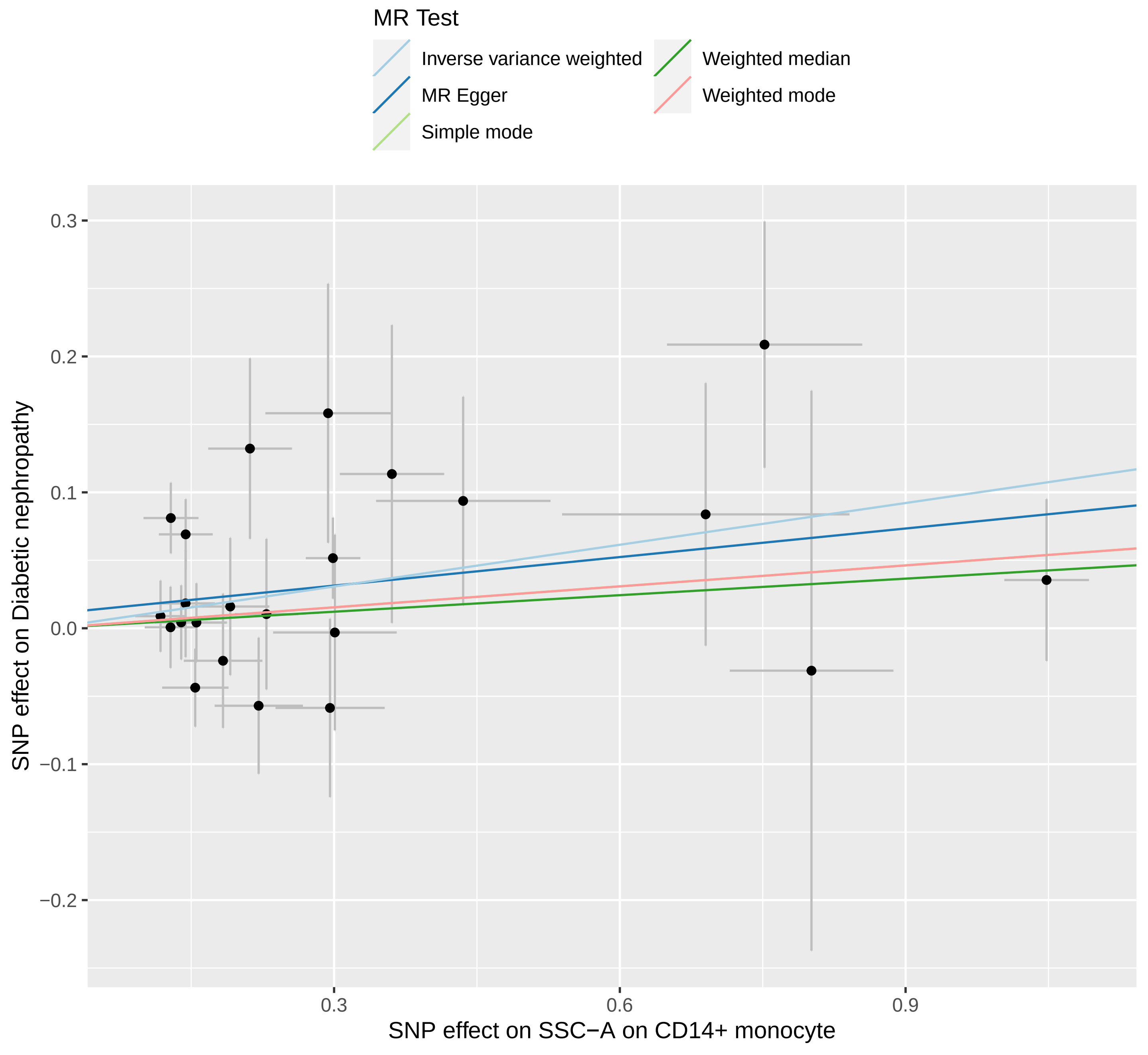

MR Test
Inverse variance weighted
MR Egger
Simple mode
Weighted median
Weighted mode
0.3
0.2
0.1
0.0
−0.1
−0.2
SNP effect on Diabetic nephropathy
0.3
0.6
0.9
SNP effect on SSC−A on CD14+ monocyte

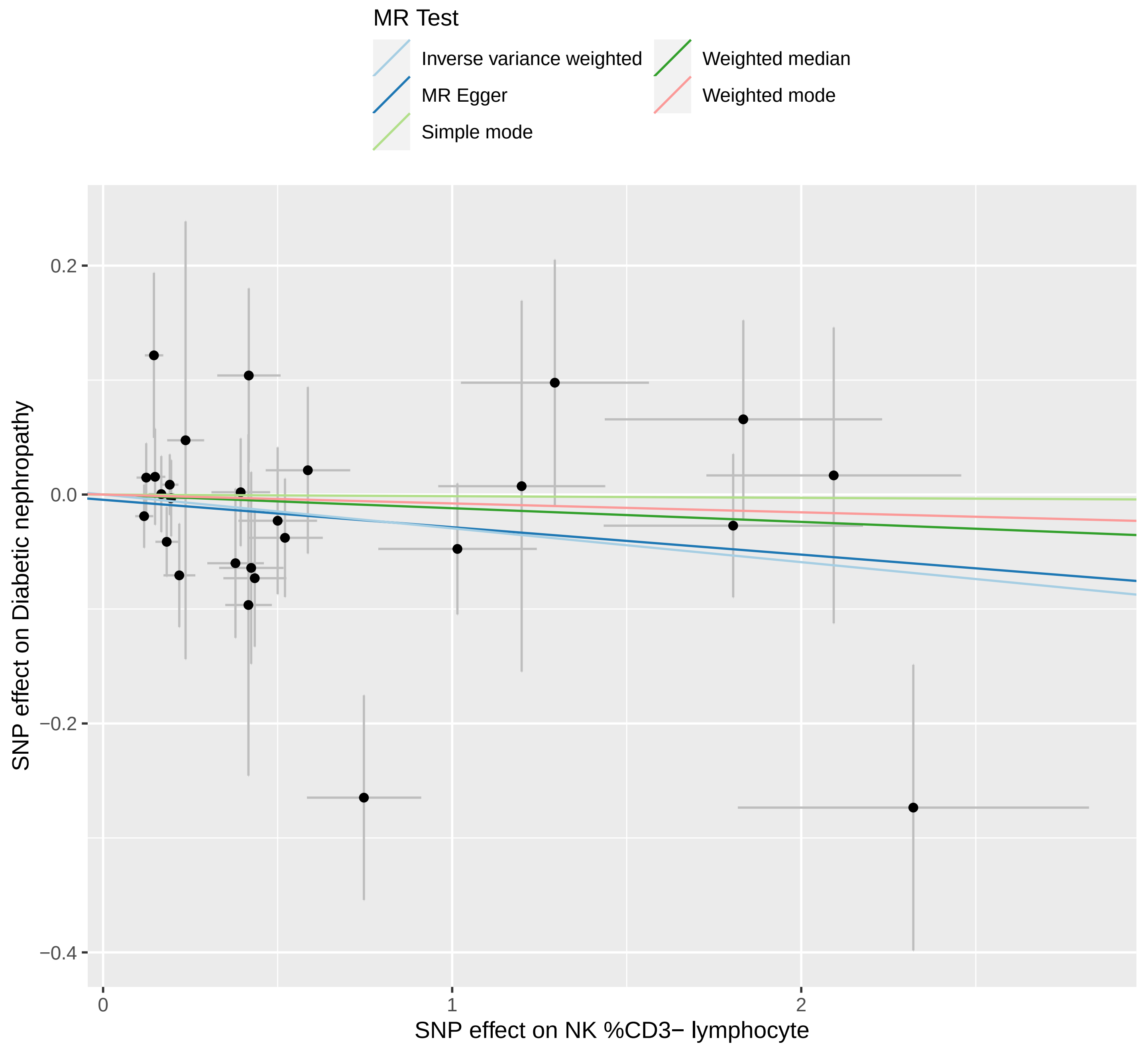
MR Test
Inverse variance weighted
MR Egger
Simple mode
Weighted median
Weighted mode
0.2
0.0
−0.2
−0.4
0
1
2
SNP effect on Diabetic nephropathy
SNP effect on NK %CD3− lymphocyte

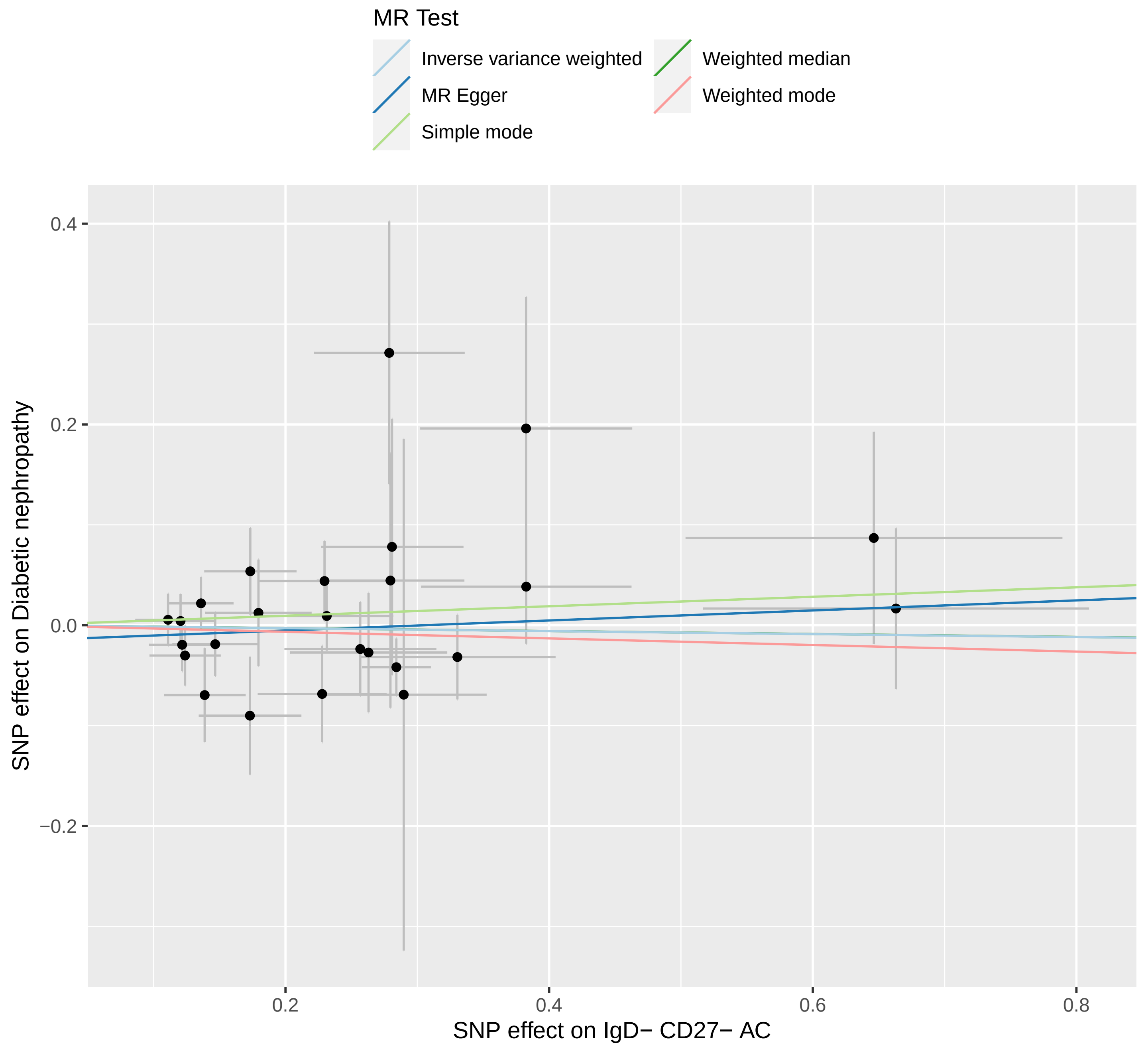
MR Test
Inverse variance weighted
MR Egger
Simple mode
Weighted median
Weighted mode
SNP effect on Diabetic nephropathy
0.4
0.2
0.0
−0.2
0.2
0.4
0.6
0.8
SNP effect on IgD− CD27− AC

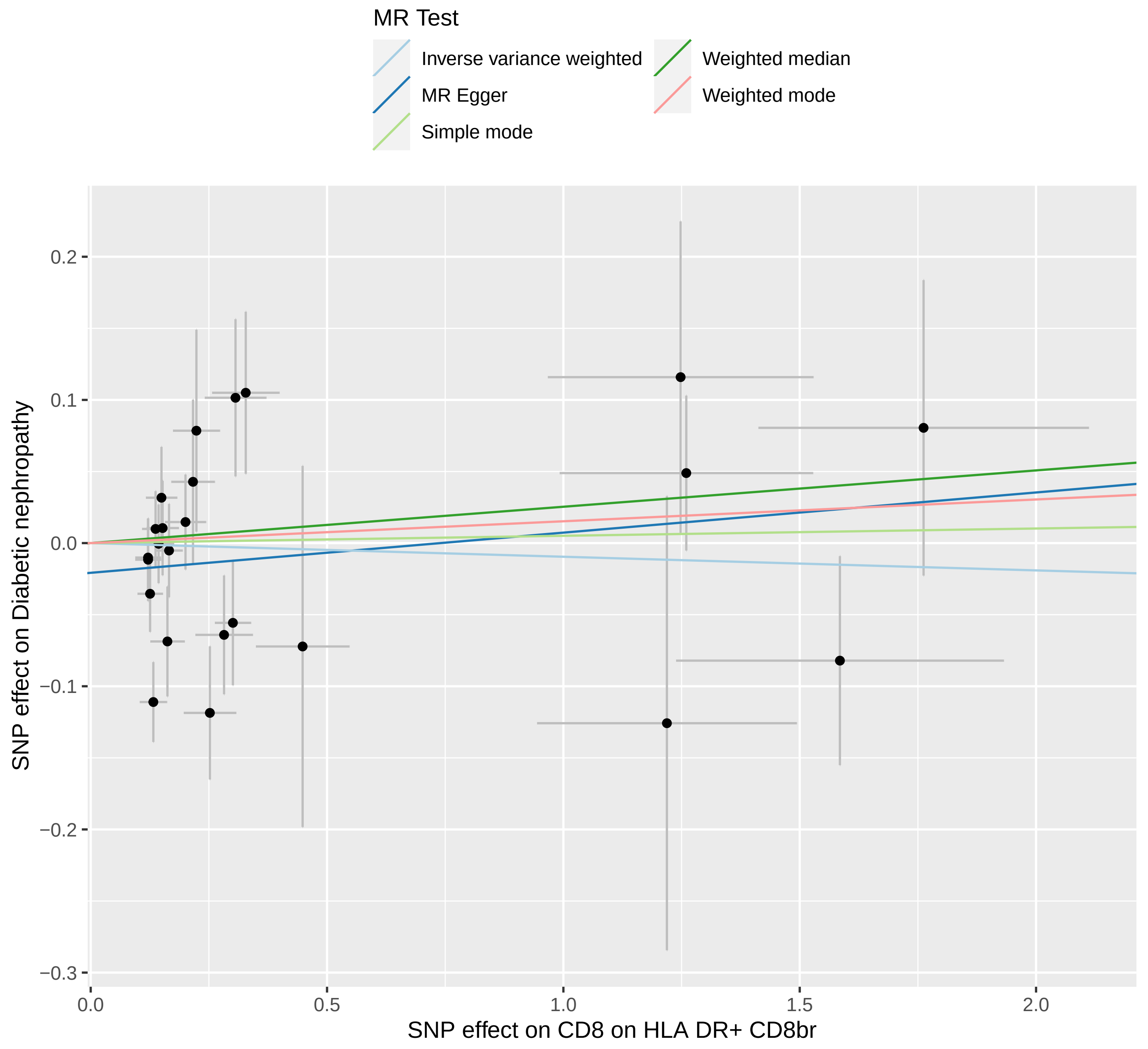
MR Test
Inverse variance weighted
MR Egger
Simple mode
Weighted median
Weighted mode
SNP effect on Diabetic nephropathy
0.2
0.1
0.0
−0.1
−0.2
−0.3
0.0
0.5
1.0
1.5
2.0
SNP effect on CD8 on HLA DR+ CD8br

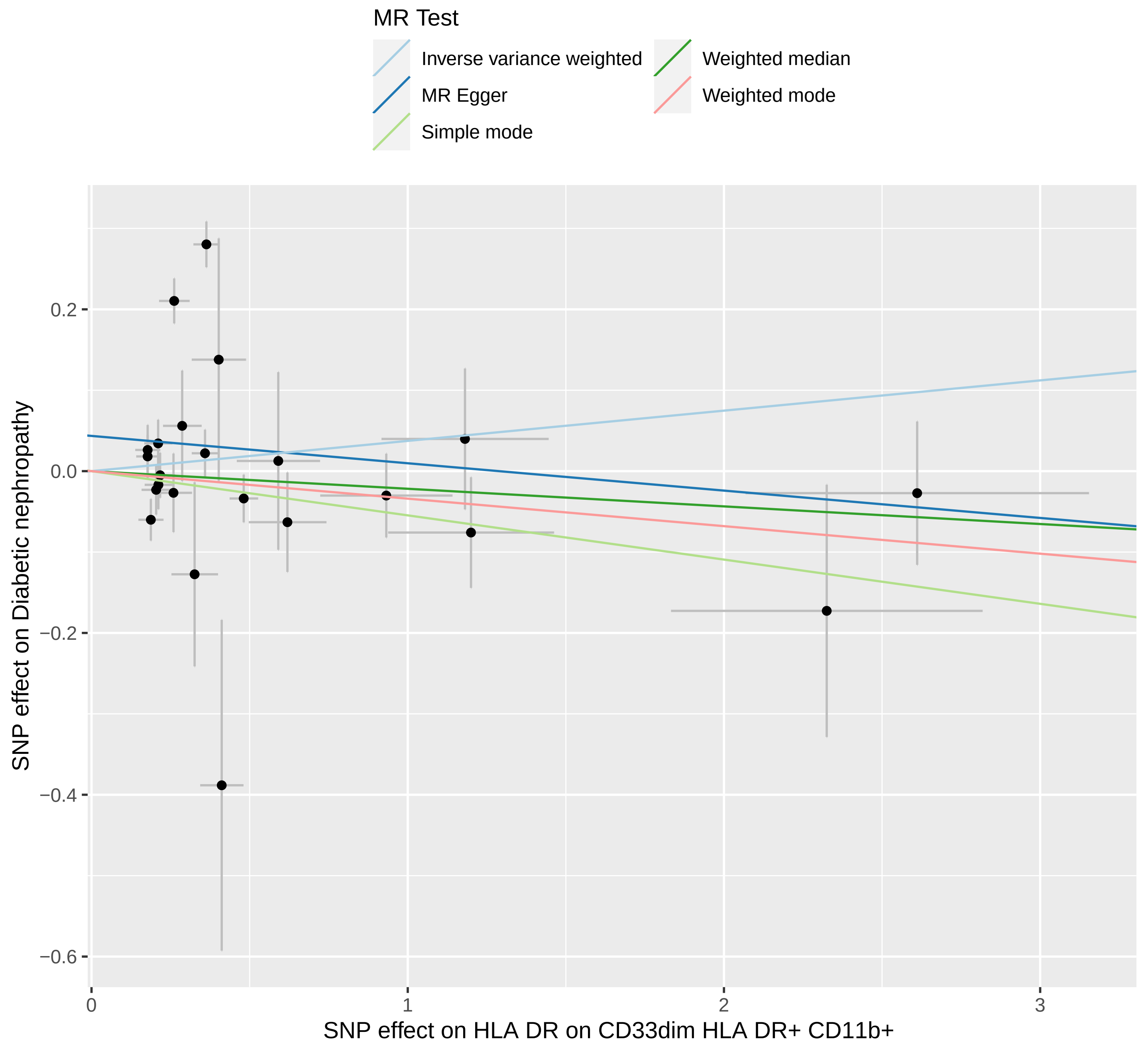
MR Test
Inverse variance weighted
MR Egger
Simple mode
Weighted median
Weighted mode
0.2
0.0
−0.2
−0.4
−0.6
0
1
2
3
SNP effect on Diabetic nephropathy
SNP effect on HLA DR on CD33dim HLA DR+ CD11b+

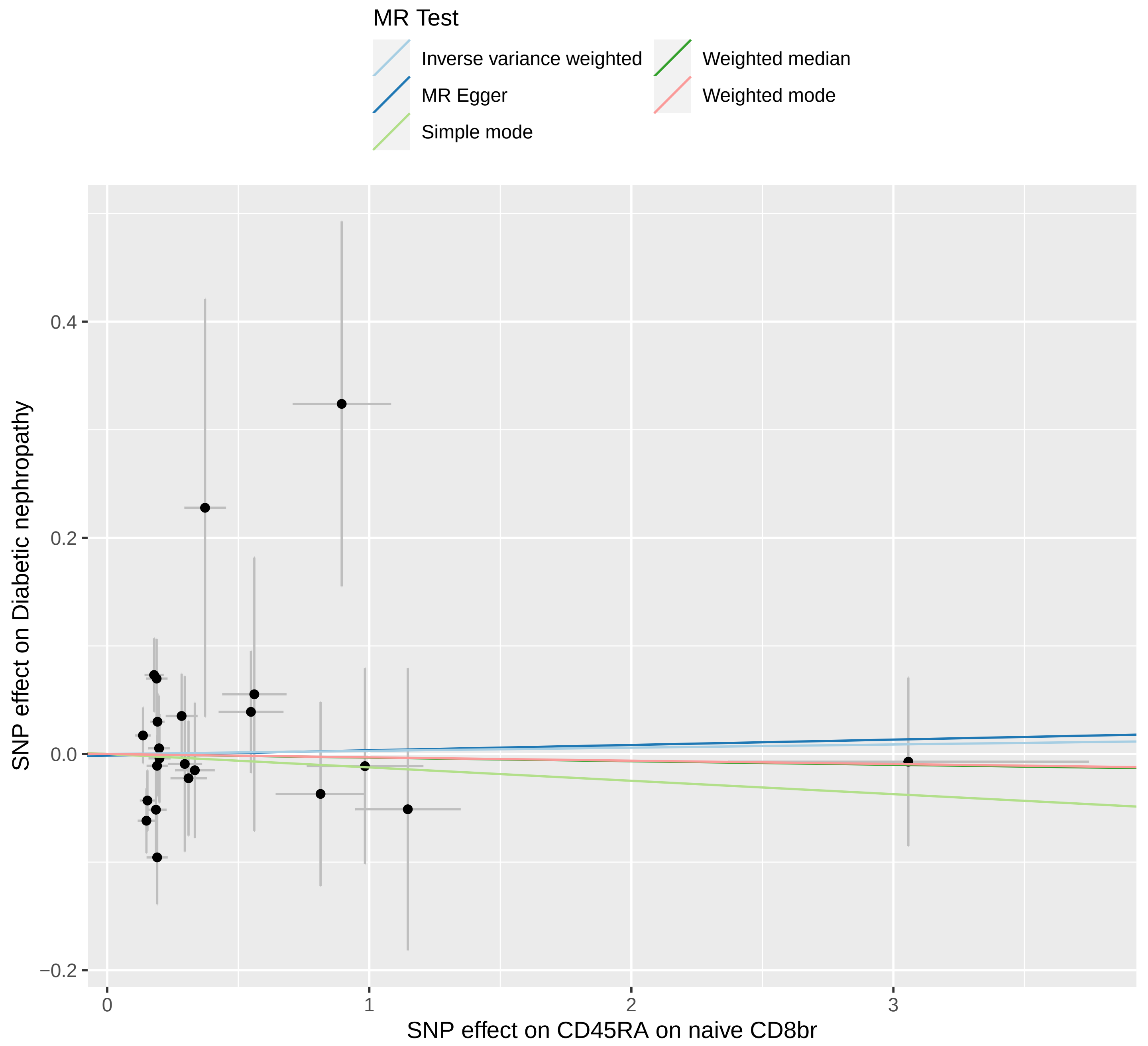

MR Test
Inverse variance weighted
MR Egger
Simple mode
Weighted median
Weighted mode
SNP effect on Diabetic nephropathy
0.4
0.2
0.0
−0.2
0
1
2
3
SNP effect on CD45RA on naive CD8br

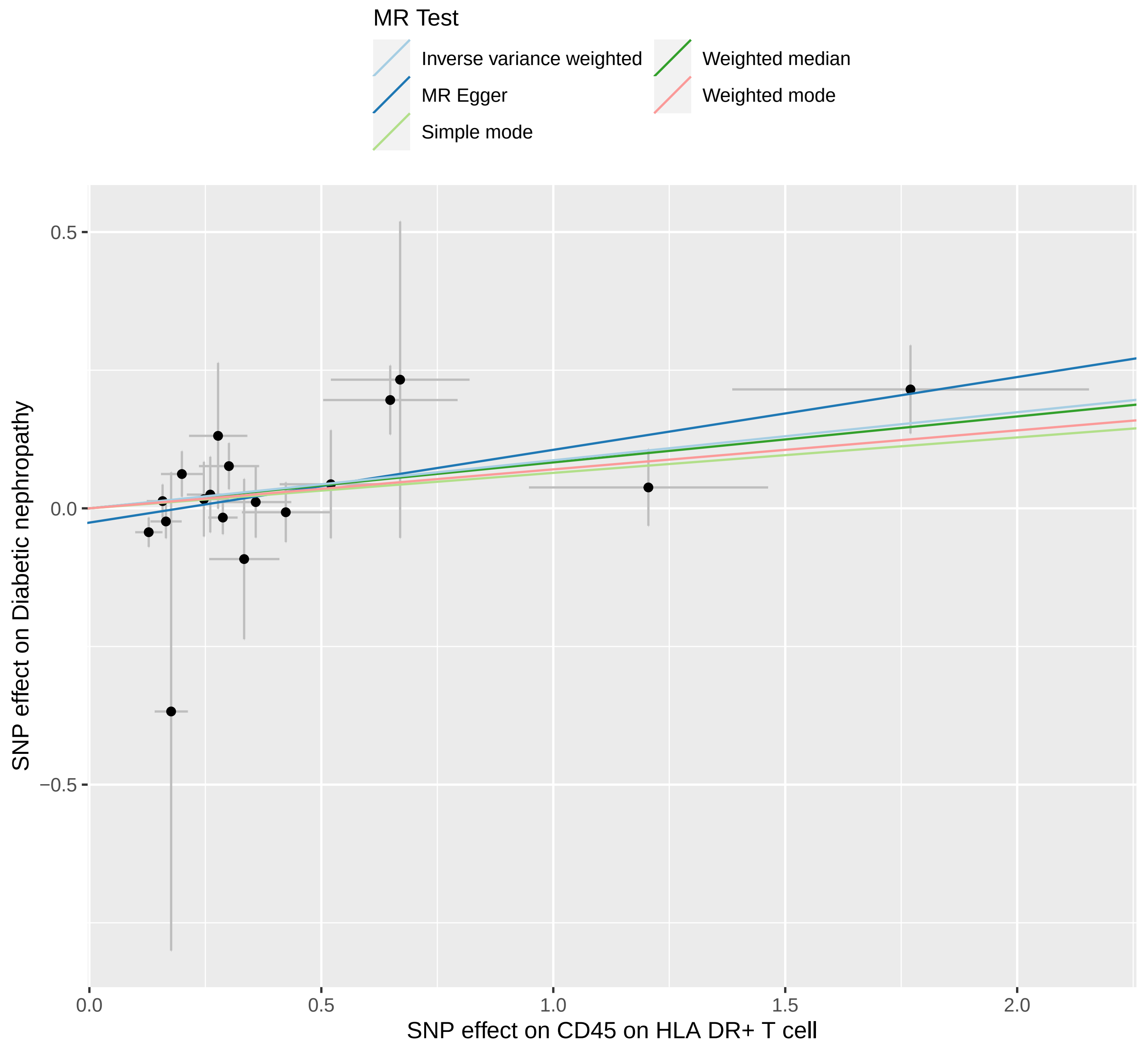
MR Test
Inverse variance weighted
MR Egger
Simple mode
Weighted median
Weighted mode
0.5
0.0
−0.5
SNP effect on Diabetic nephropathy
0.0
0.5
1.0
1.5
2.0
SNP effect on CD45 on HLA DR+ T cell

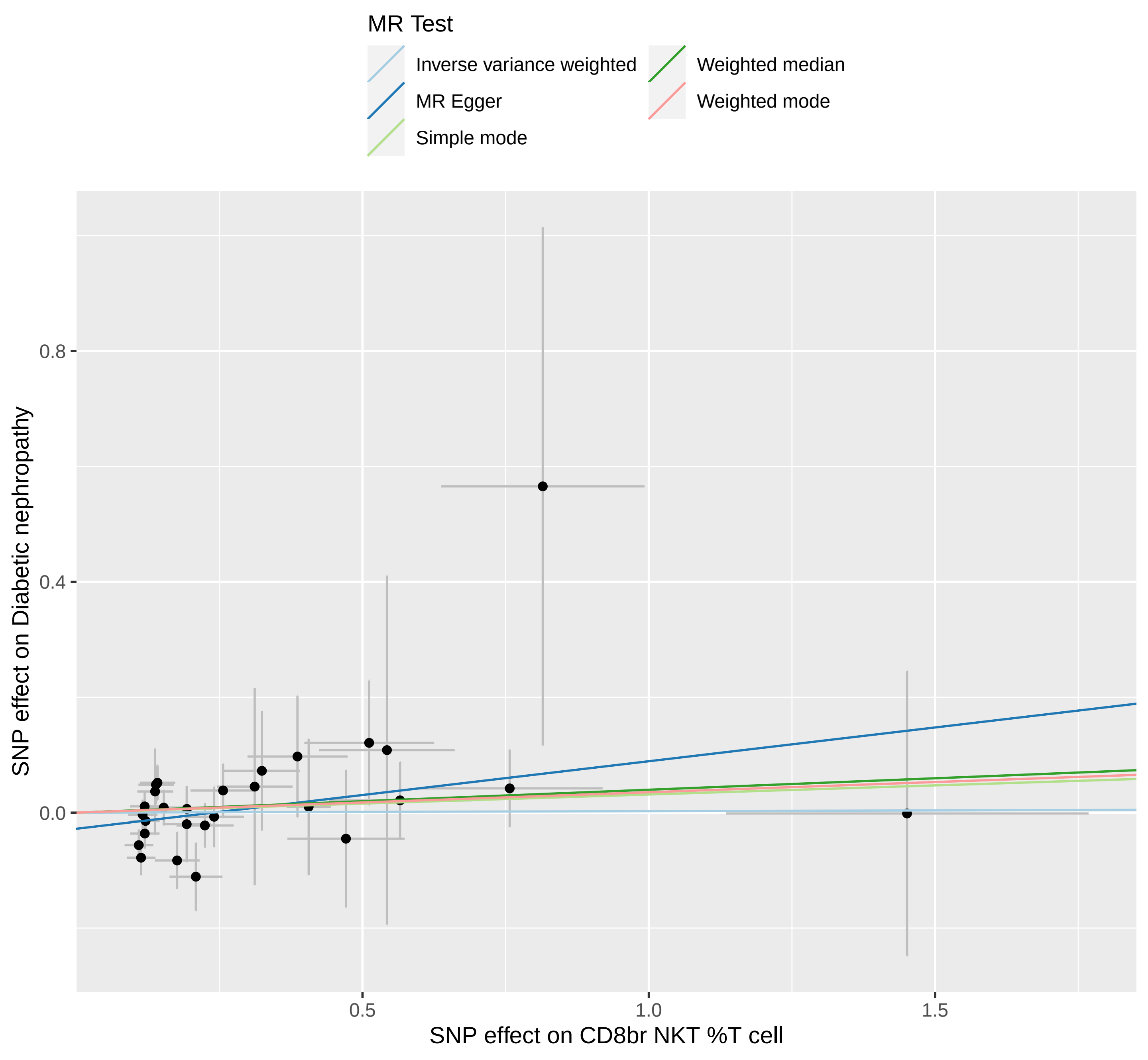
MR Test
Inverse variance weighted
MR Egger
Simple mode
Weighted median
Weighted mode
0.8
0.4
0.0
0.5
1.0
1.5
SNP effect on Diabetic nephropathy
SNP effect on CD8br NKT %T cell

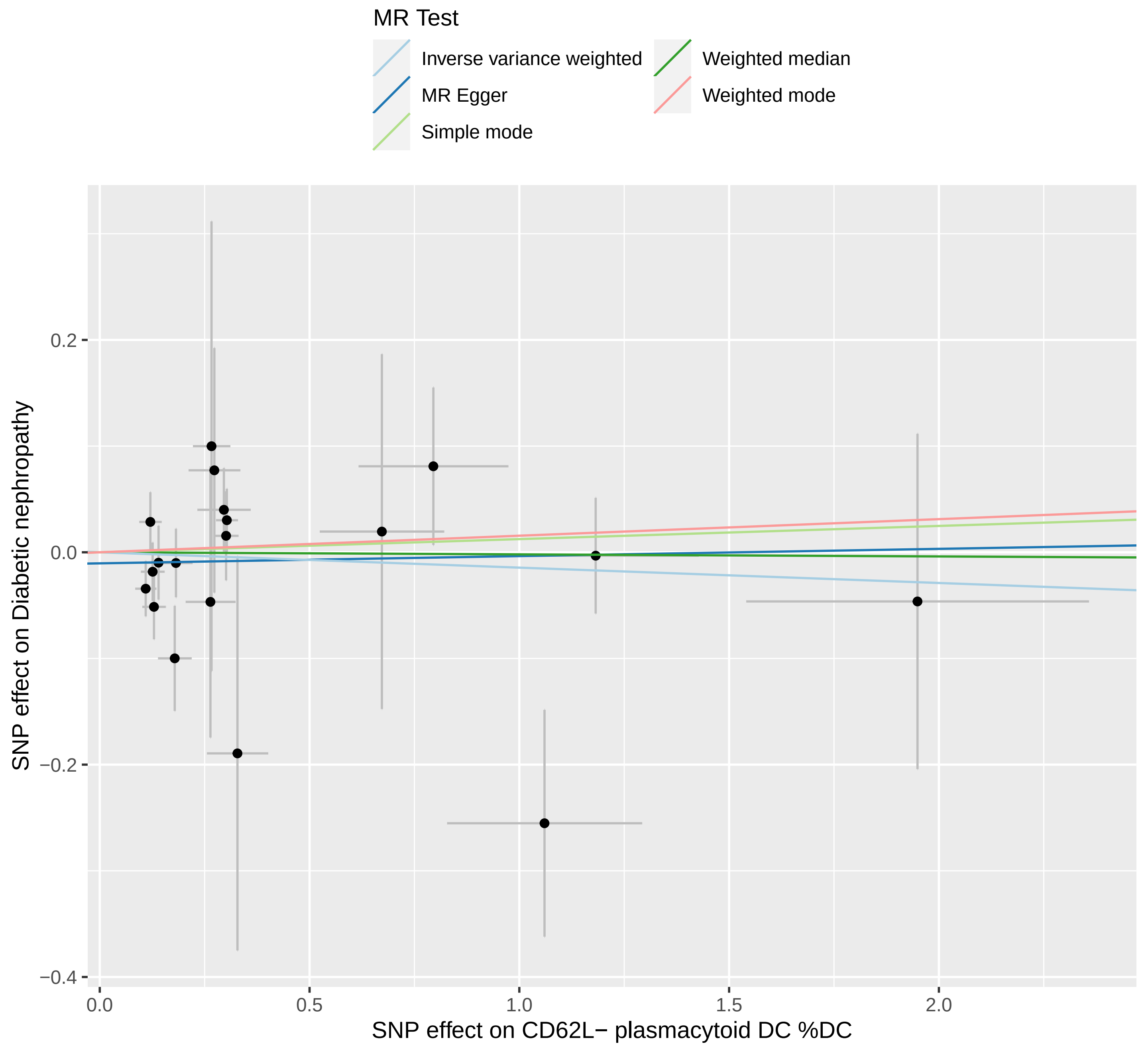
MR Test
Inverse variance weighted
MR Egger
Simple mode
Weighted median
Weighted mode
0.2
0.0
−0.2
−0.4
0.0
0.5
1.0
1.5
2.0
SNP effect on Diabetic nephropathy
SNP effect on CD62L− plasmacytoid DC %DC

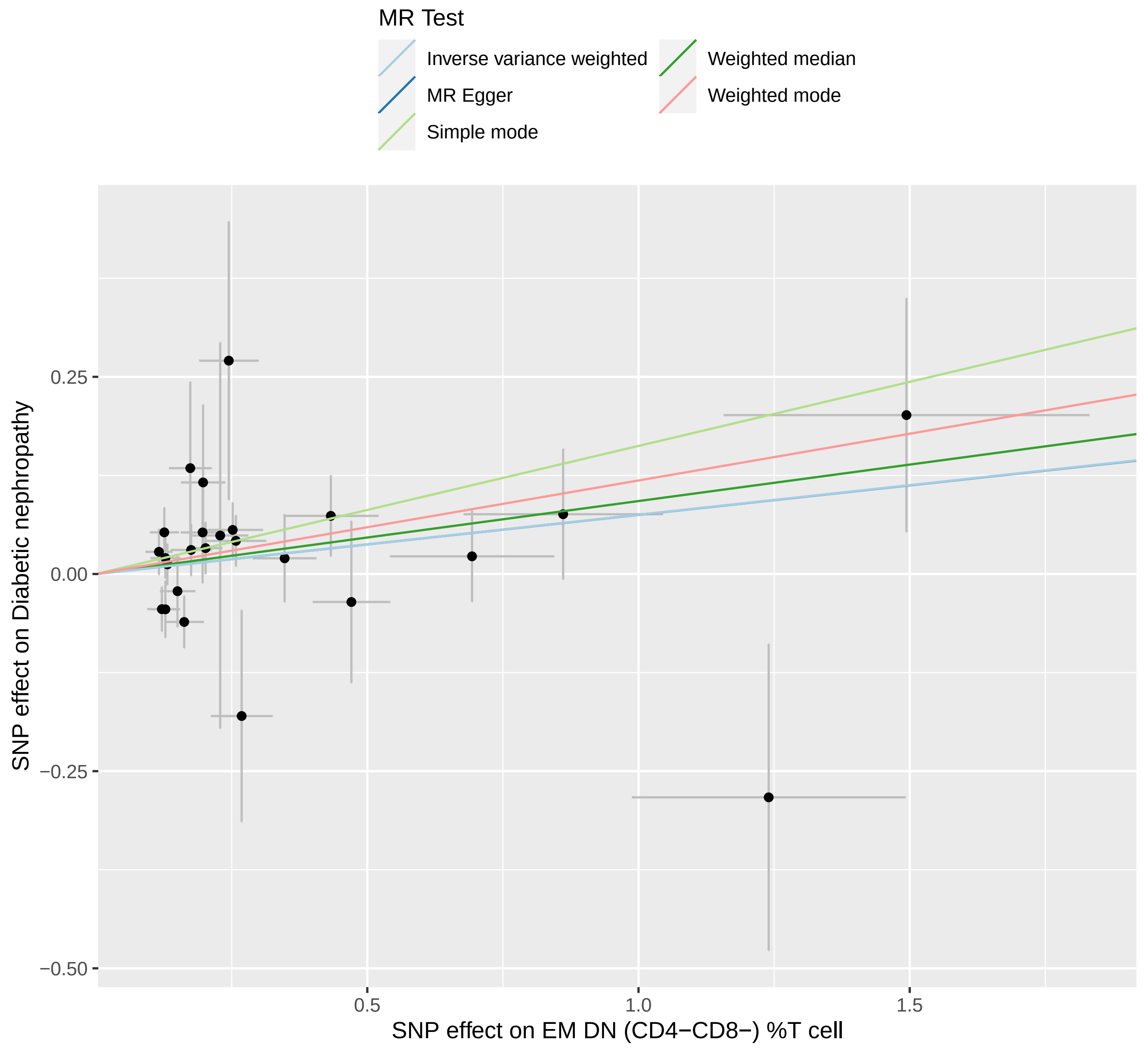

MR Test
Inverse variance weighted
MR Egger
Simple mode
Weighted median
Weighted mode
0.25
0.00
−0.25
−0.50
0.5
1.0
1.5
SNP effect on Diabetic nephropathy
SNP effect on EM DN (CD4−CD8−) %T cell

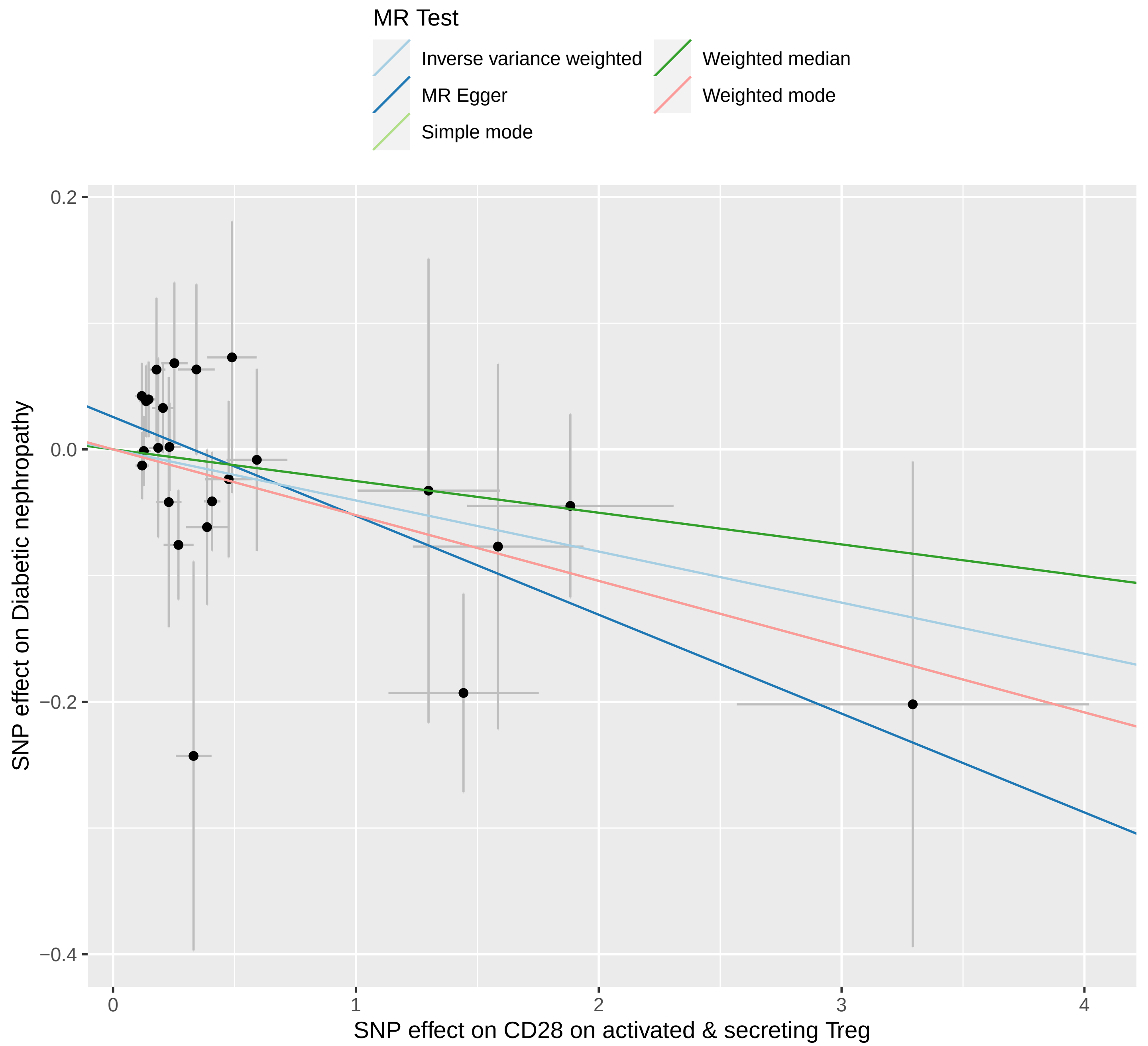

MR Test
Inverse variance weighted
MR Egger
Simple mode
Weighted median
Weighted mode
0.2
0.0
−0.2
−0.4
0
1
2
3
4
SNP effect on Diabetic nephropathy
SNP effect on CD28 on activated & secreting Treg

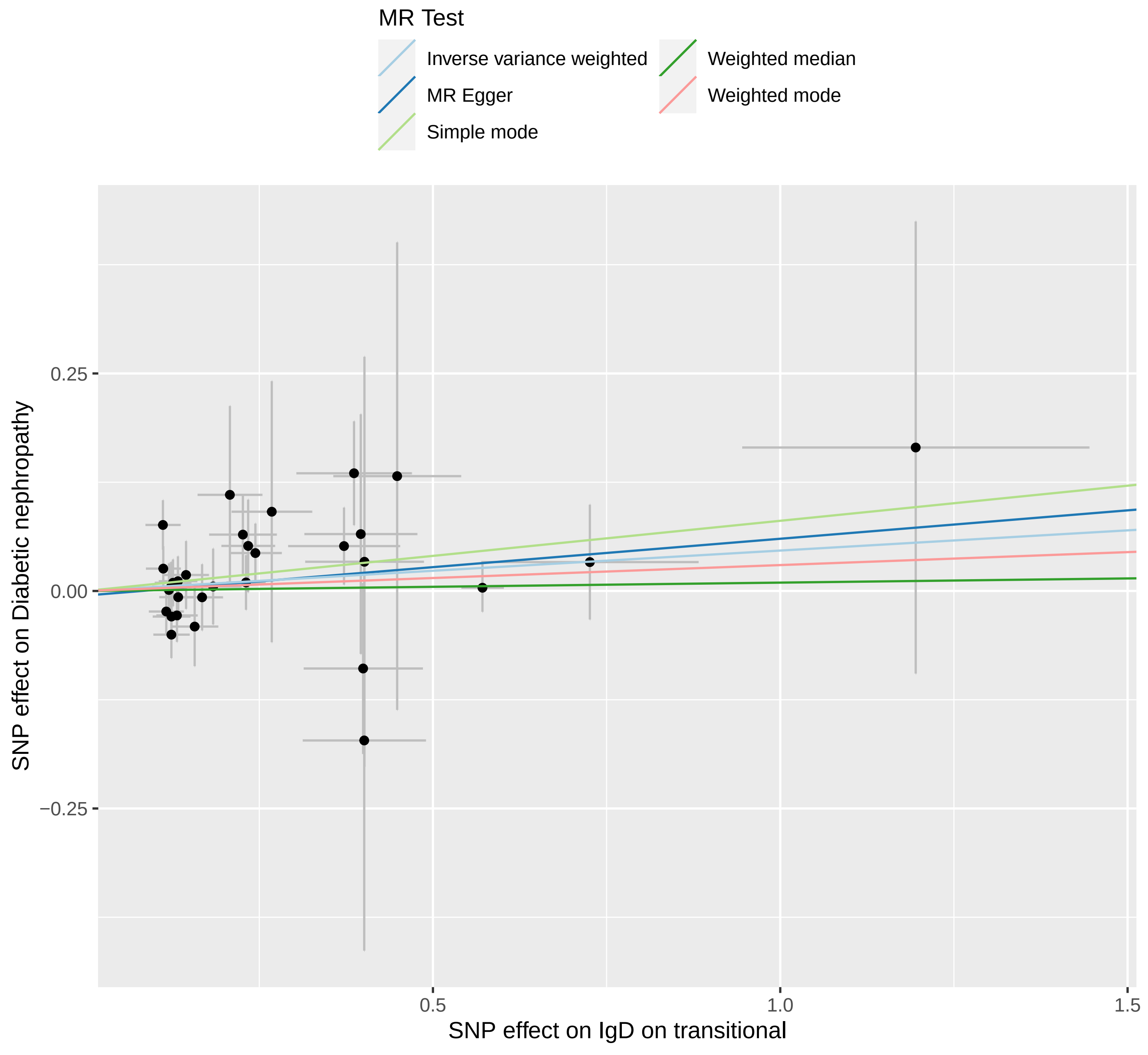
MR Test
Inverse variance weighted
MR Egger
Simple mode
Weighted median
Weighted mode
0.25
0.00
−0.25
SNP effect on Diabetic nephropathy
0.5
1.0
1.5
SNP effect on IgD on transitional

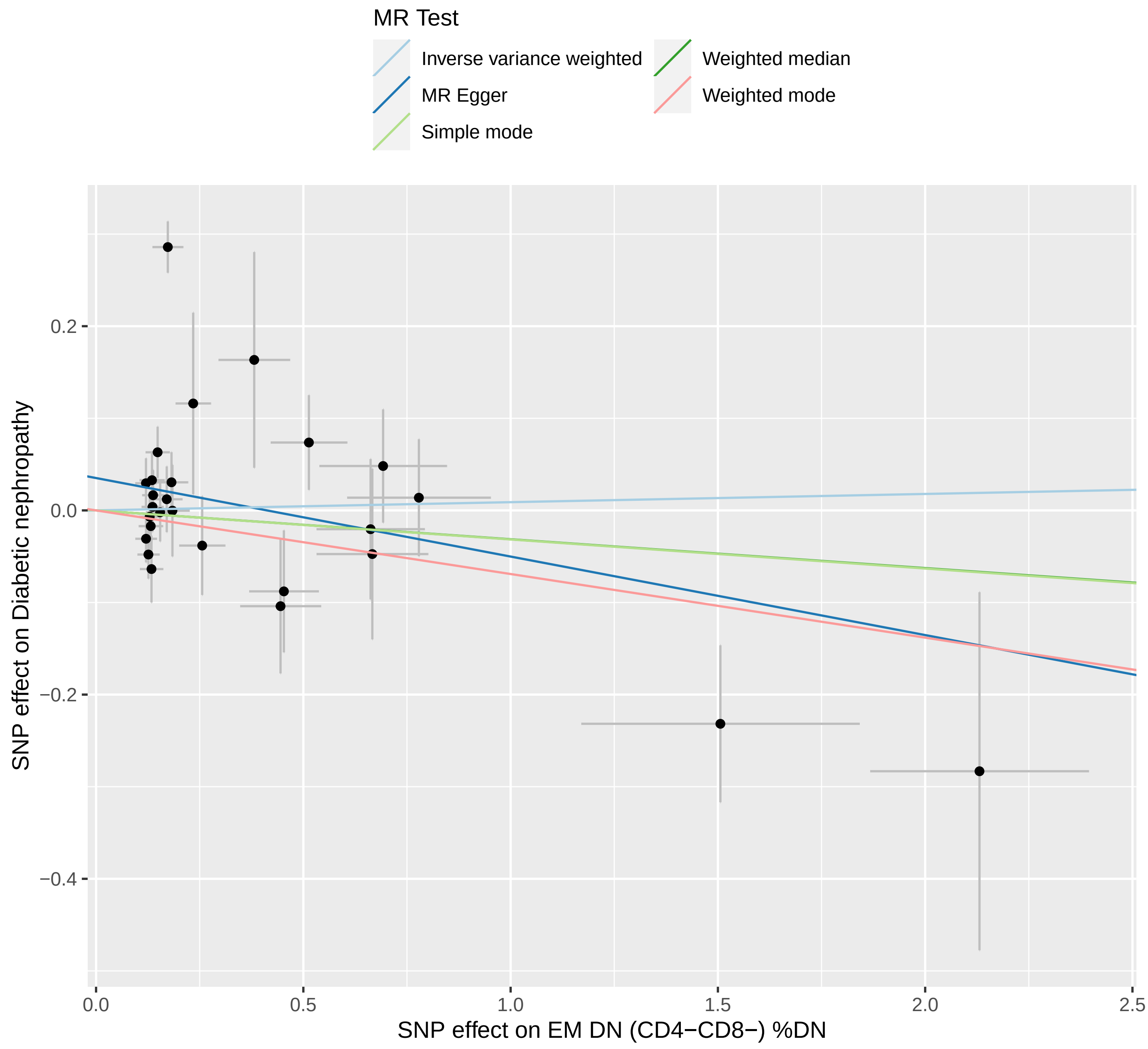
MR Test
Inverse variance weighted
MR Egger
Simple mode
Weighted median
Weighted mode
SNP effect on Diabetic nephropathy
0.2
0.0
−0.2
−0.4
0.0
0.5
1.0
1.5
2.0
2.5
SNP effect on EM DN (CD4−CD8−) %DN

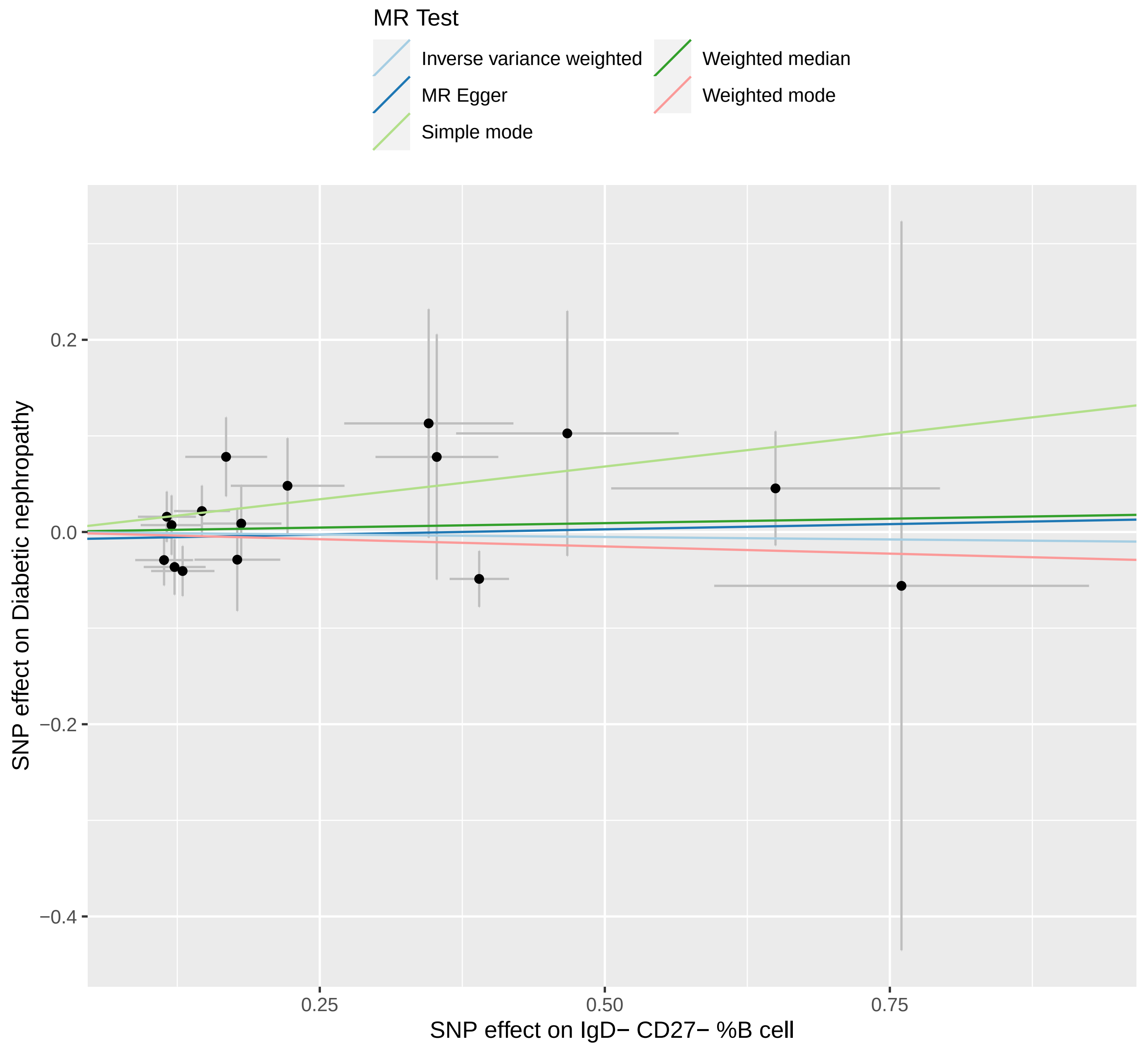

MR Test
Inverse variance weighted
MR Egger
Simple mode
Weighted median
Weighted mode
0.2
0.0
−0.2
−0.4
SNP effect on Diabetic nephropathy
0.25
0.50
0.75
SNP effect on IgD− CD27− %B cell

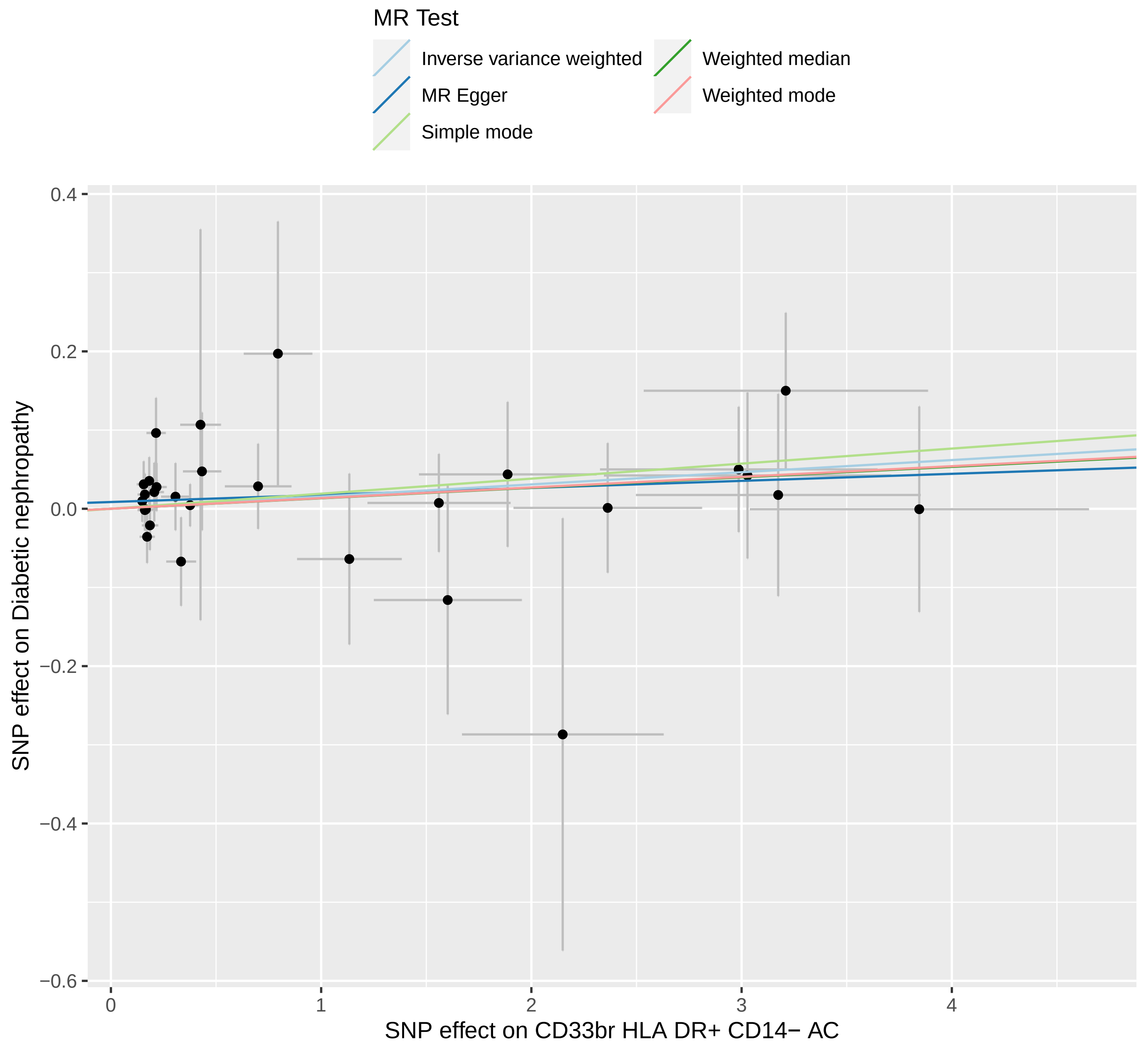

MR Test
Inverse variance weighted
MR Egger
Simple mode
Weighted median
Weighted mode
SNP effect on Diabetic nephropathy
SNP effect on CD33br HLA DR+ CD14− AC
0.4
0.2
0.0
−0.2
−0.4
−0.6
0
1
2
3
4

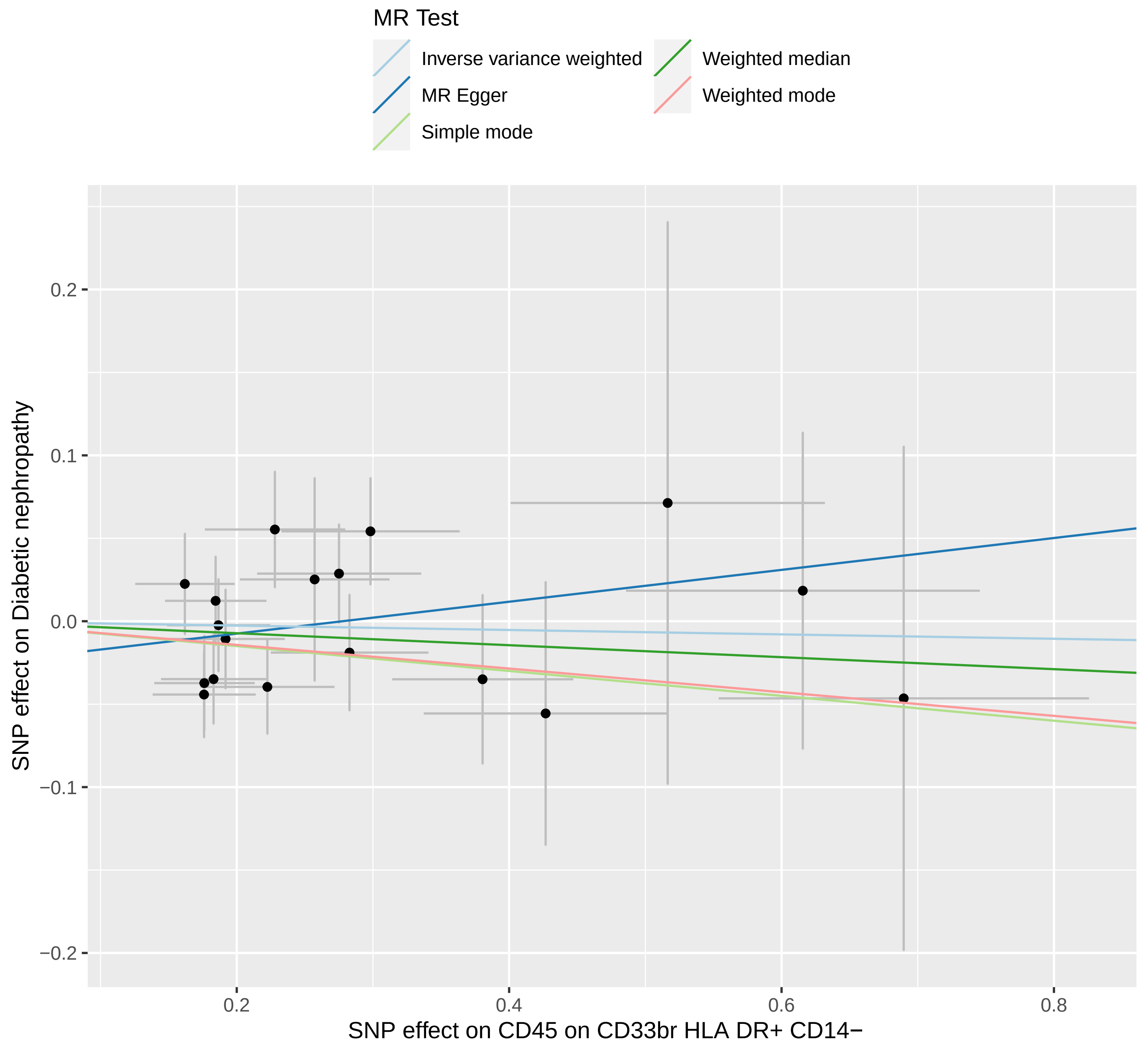

MR Test
Inverse variance weighted
Weighted median
MR Egger
Weighted mode
Simple mode
0.2
0.1
0.0
−0.1
−0.2
SNP effect on Diabetic nephropathy
0.2
0.4
0.6
0.8
SNP effect on CD45 on CD33br HLA DR+ CD14−

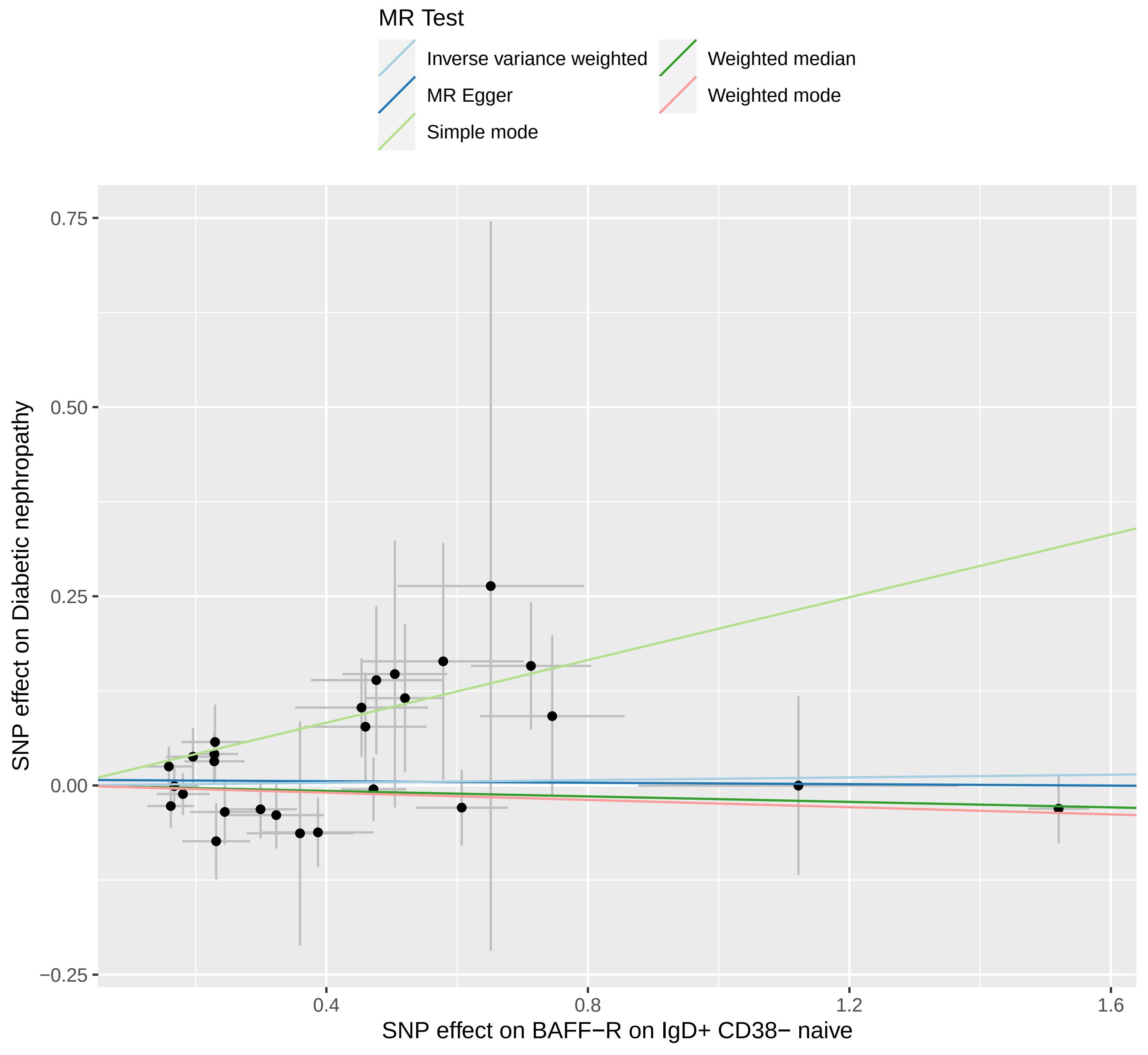

MR Test
Inverse variance weighted
MR Egger
Simple mode
Weighted median
Weighted mode
0.75
0.50
0.25
0.00
−0.25
0.4
0.8
1.2
1.6
SNP effect on Diabetic nephropathy
SNP effect on BAFF−R on IgD+ CD38− naive

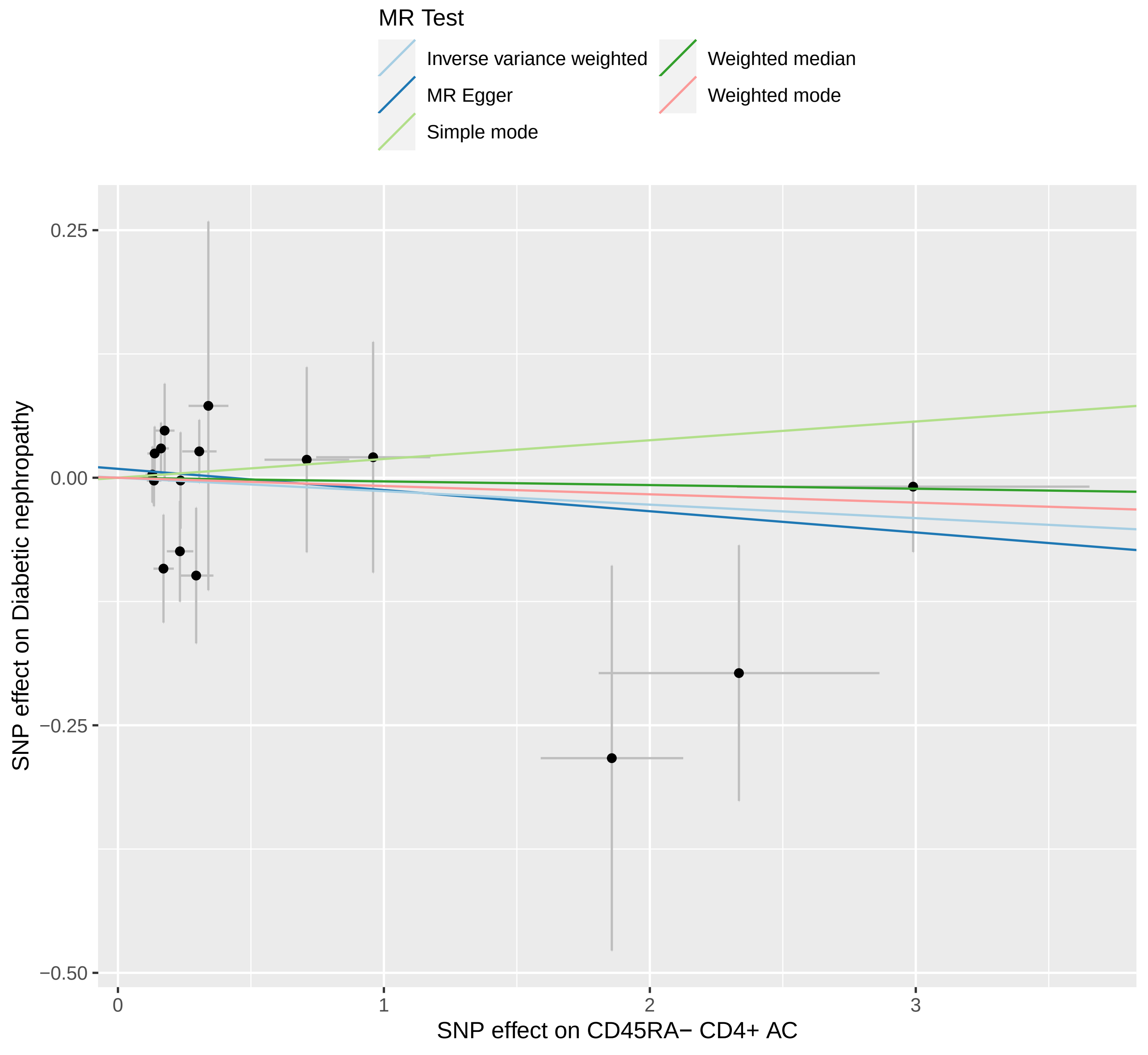

MR Test
Inverse variance weighted
MR Egger
Simple mode
Weighted median
Weighted mode
0.25
0.00
−0.25
−0.50
0
1
2
3
SNP effect on Diabetic nephropathy
SNP effect on CD45RA− CD4+ AC

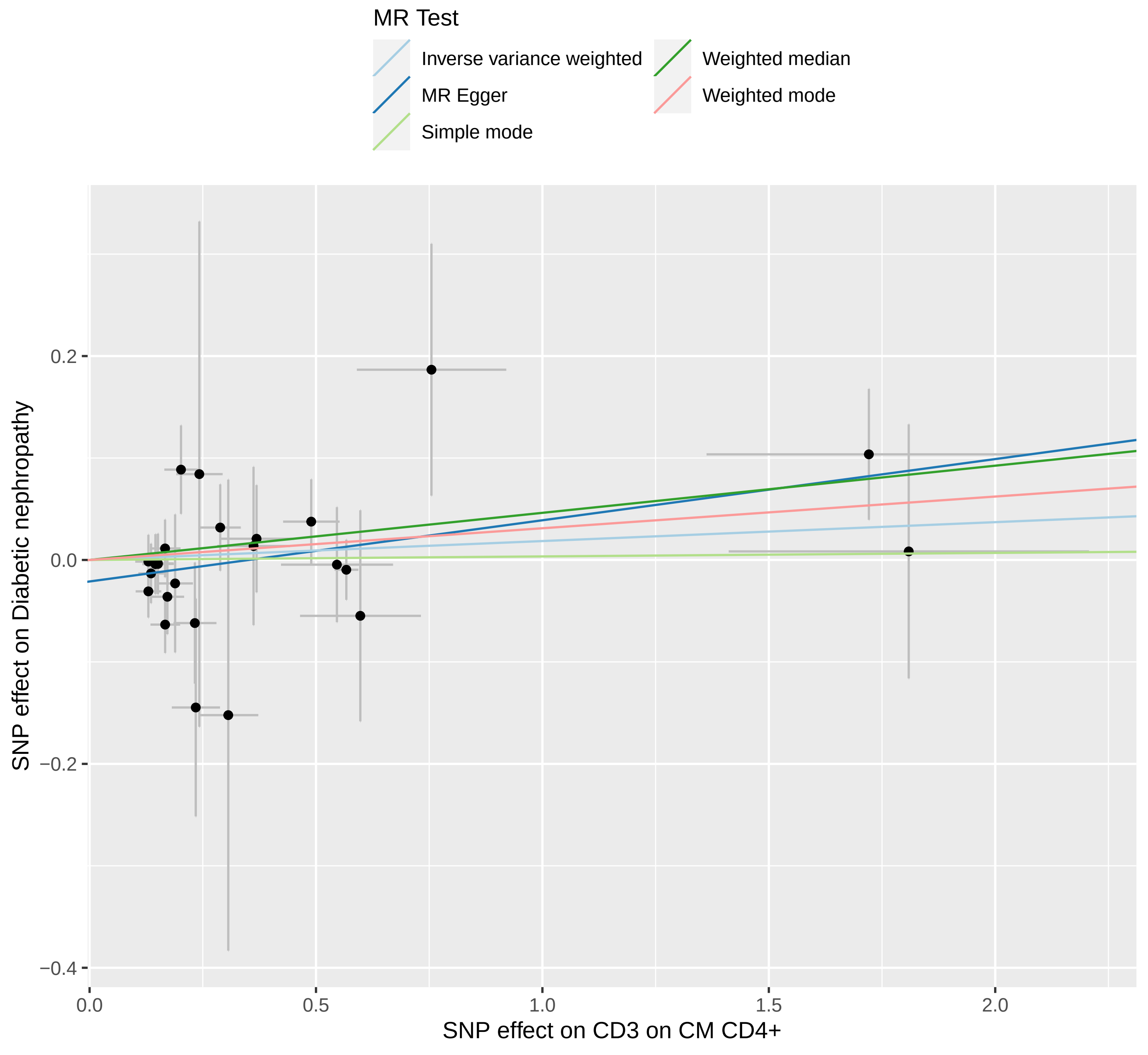

MR Test
Inverse variance weighted
MR Egger
Simple mode
Weighted median
Weighted mode
0.2
0.0
−0.2
−0.4
0.0
0.5
1.0
1.5
2.0
SNP effect on Diabetic nephropathy
SNP effect on CD3 on CM CD4+

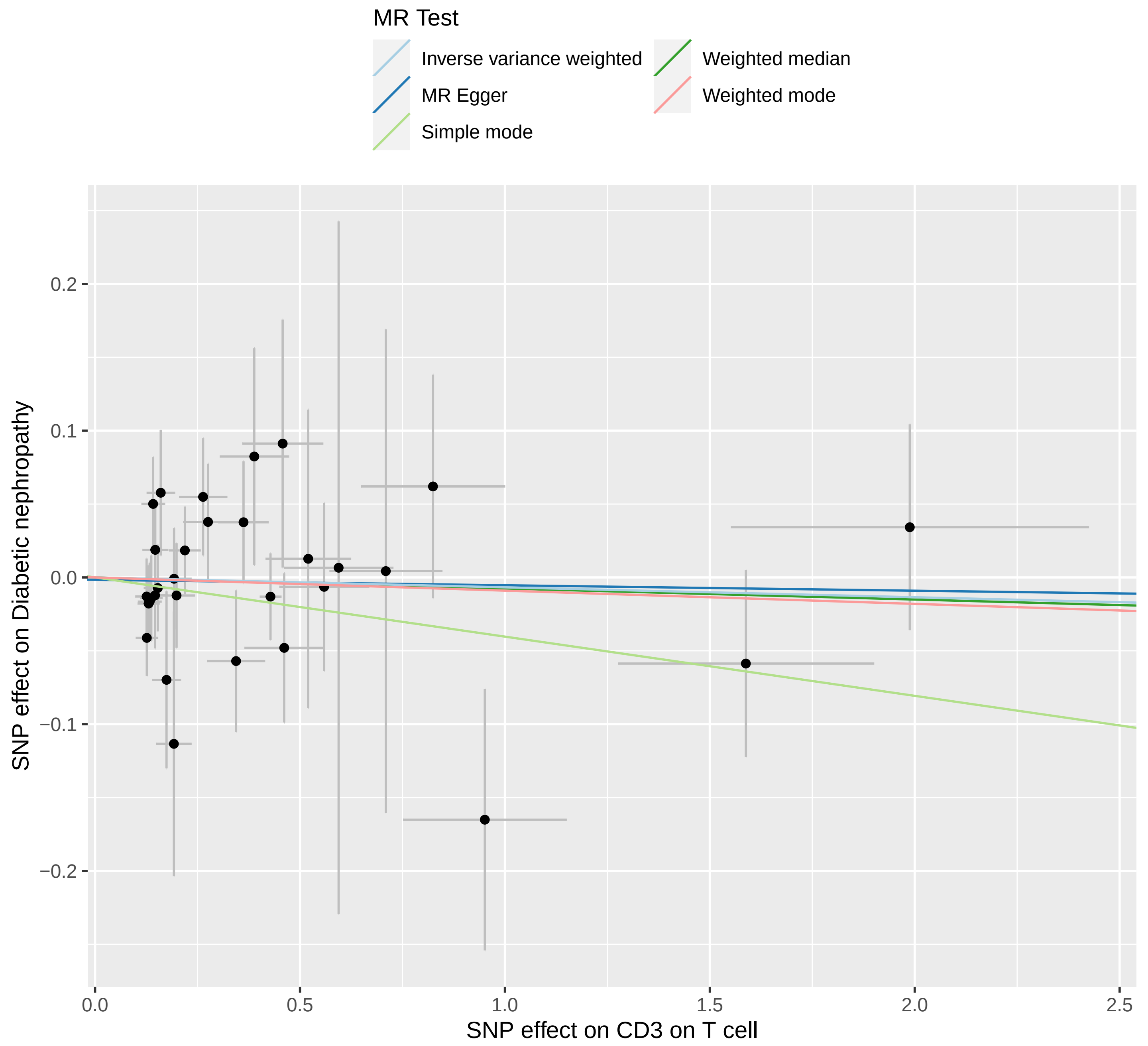

MR Test
Inverse variance weighted
MR Egger
Simple mode
Weighted median
Weighted mode
0.2
0.1
0.0
−0.1
−0.2
SNP effect on Diabetic nephropathy
0.0
0.5
1.0
1.5
2.0
2.5
SNP effect on CD3 on T cell

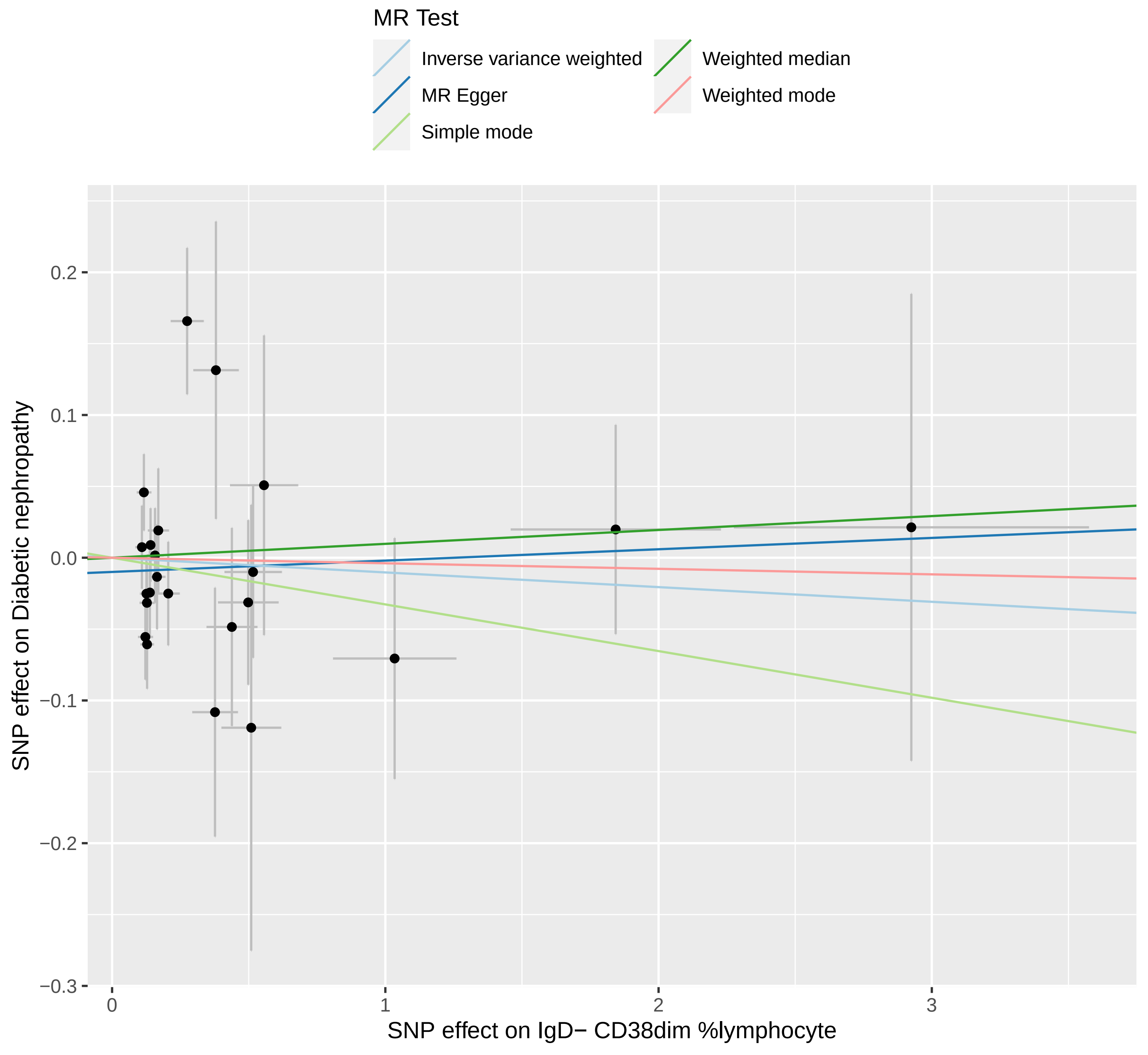
MR Test
Inverse variance weighted
Weighted median
MR Egger
Weighted mode
Simple mode
0.2
0.1
0.0
−0.1
−0.2
−0.3
SNP effect on Diabetic nephropathy
0
1
2
3
SNP effect on IgD− CD38dim %lymphocyte

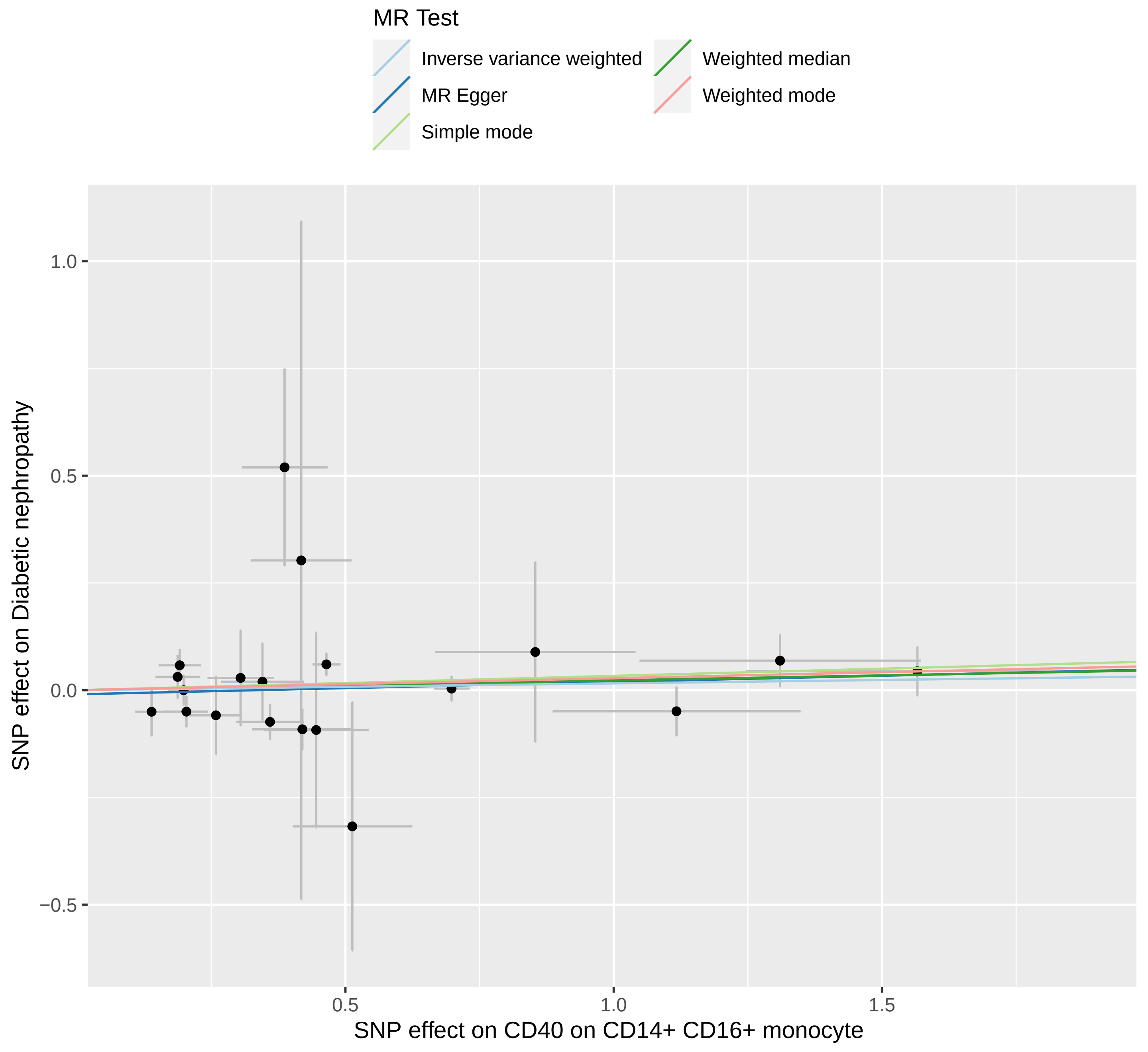

MR Test
Inverse variance weighted
MR Egger
Simple mode
Weighted median
Weighted mode
1.0
0.5
0.0
−0.5
0.5
1.0
1.5
SNP effect on Diabetic nephropathy
SNP effect on CD40 on CD14+ CD16+ monocyte

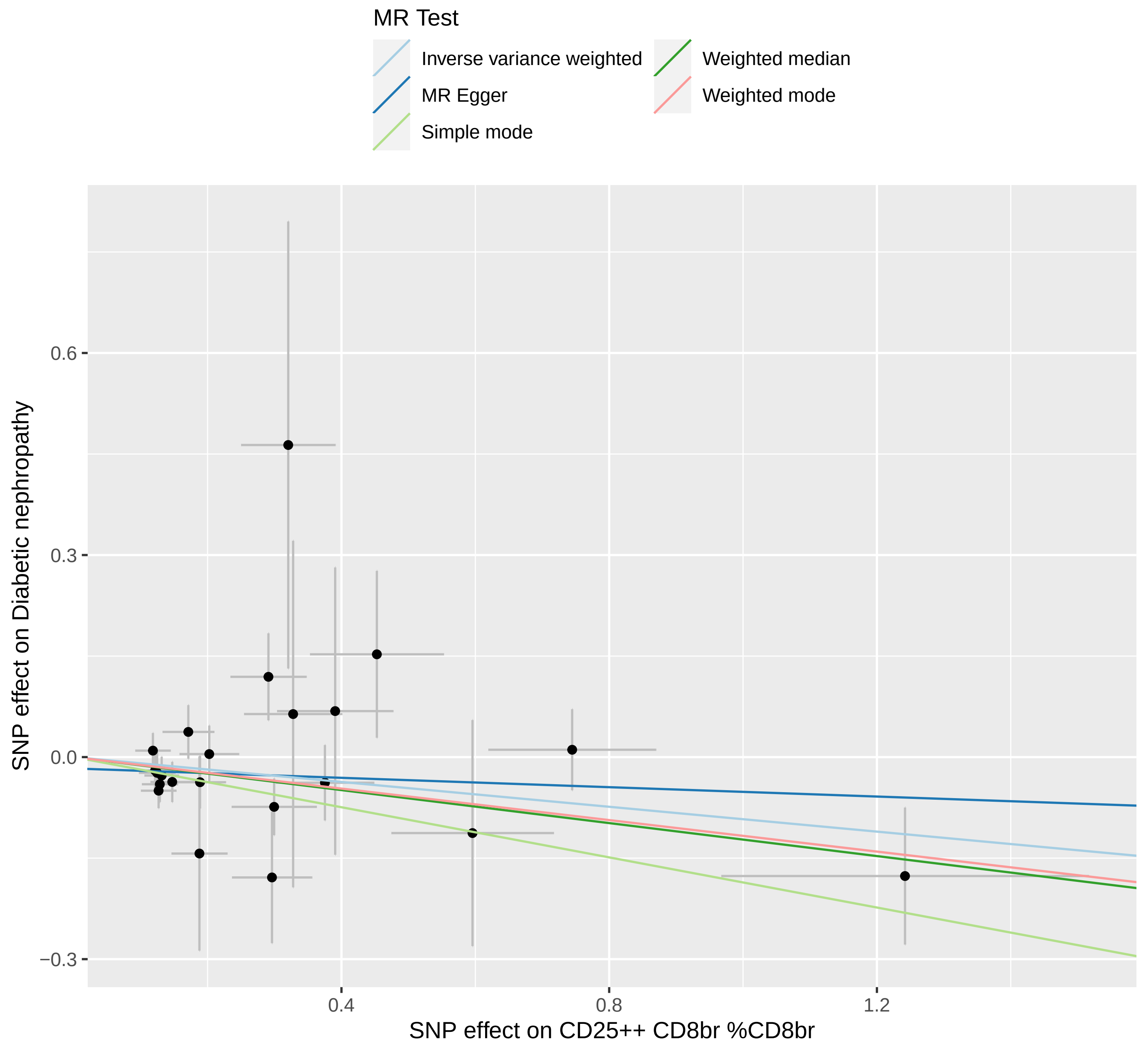

MR Test
Inverse variance weighted
MR Egger
Simple mode
Weighted median
Weighted mode
0.6
0.3
0.0
−0.3
0.4
0.8
1.2
SNP effect on Diabetic nephropathy
SNP effect on CD25++ CD8br %CD8br

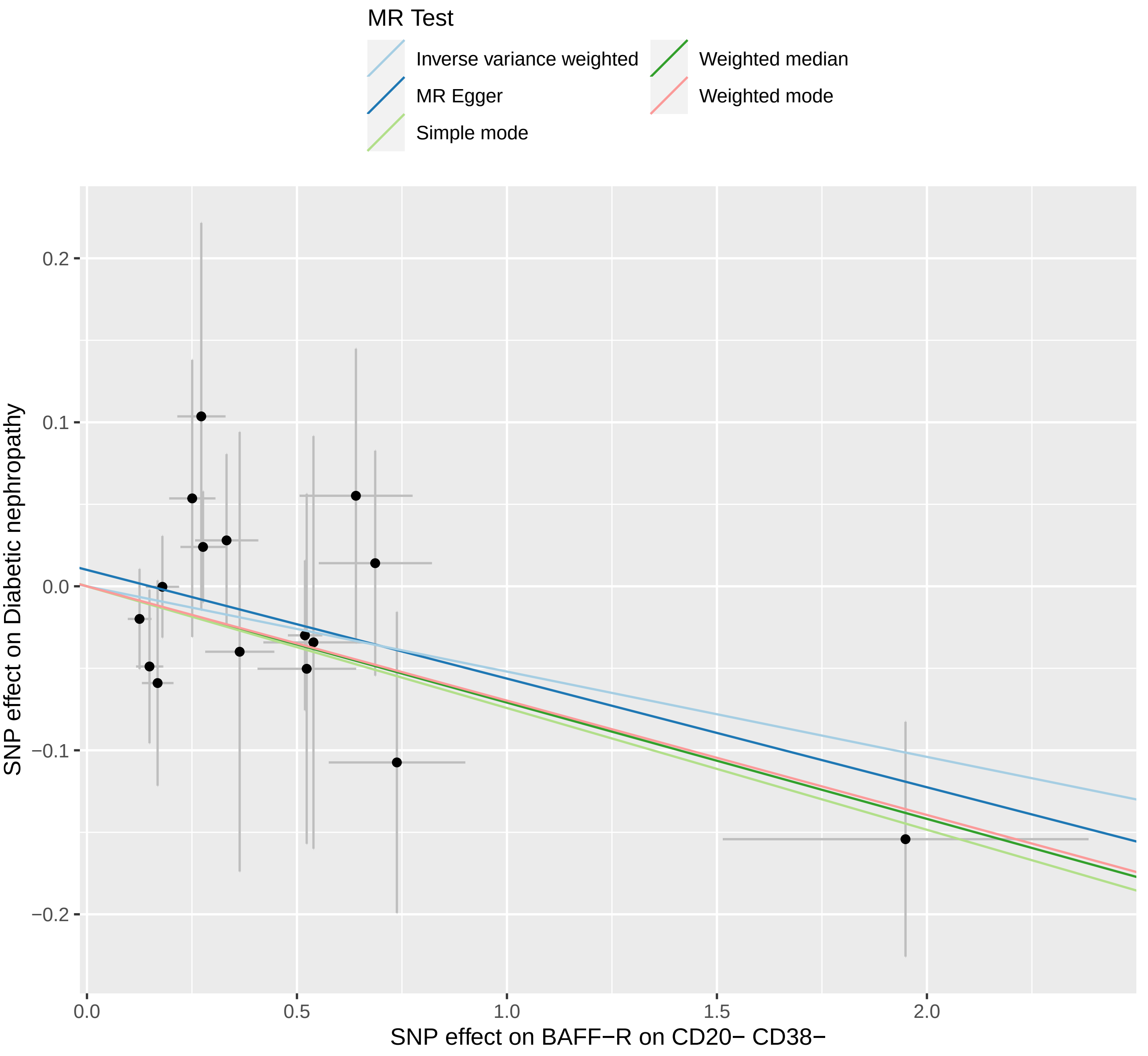
MR Test
Inverse variance weighted
MR Egger
Simple mode
Weighted median
Weighted mode
SNP effect on Diabetic nephropathy
0.2
0.1
0.0
−0.1
−0.2
0.0
0.5
1.0
1.5
2.0
SNP effect on BAFF−R on CD20− CD38−

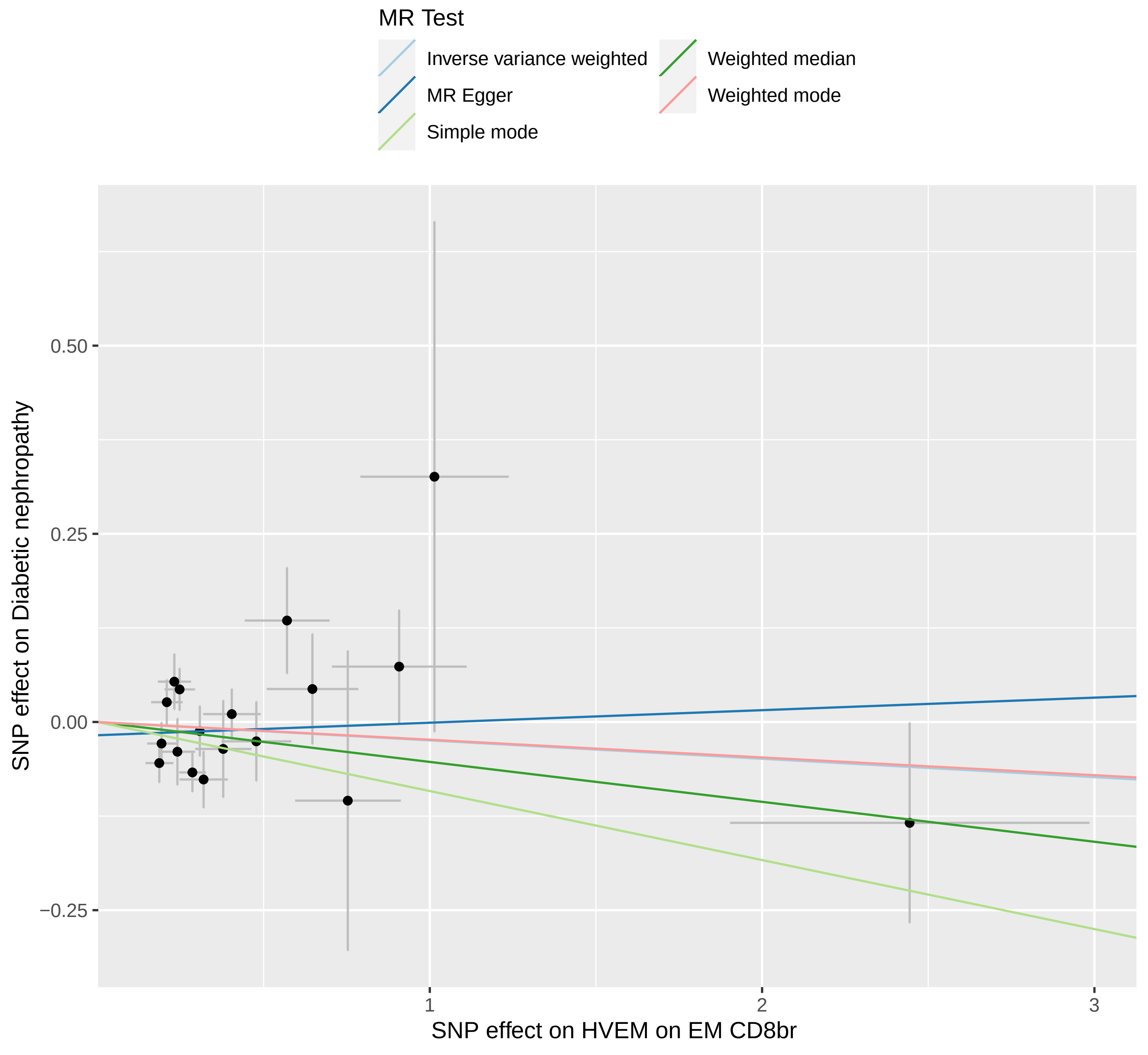

MR Test
Inverse variance weighted
MR Egger
Simple mode
Weighted median
Weighted mode
0.50
0.25
0.00
−0.25
1
2
3
SNP effect on Diabetic nephropathy
SNP effect on HVEM on EM CD8br

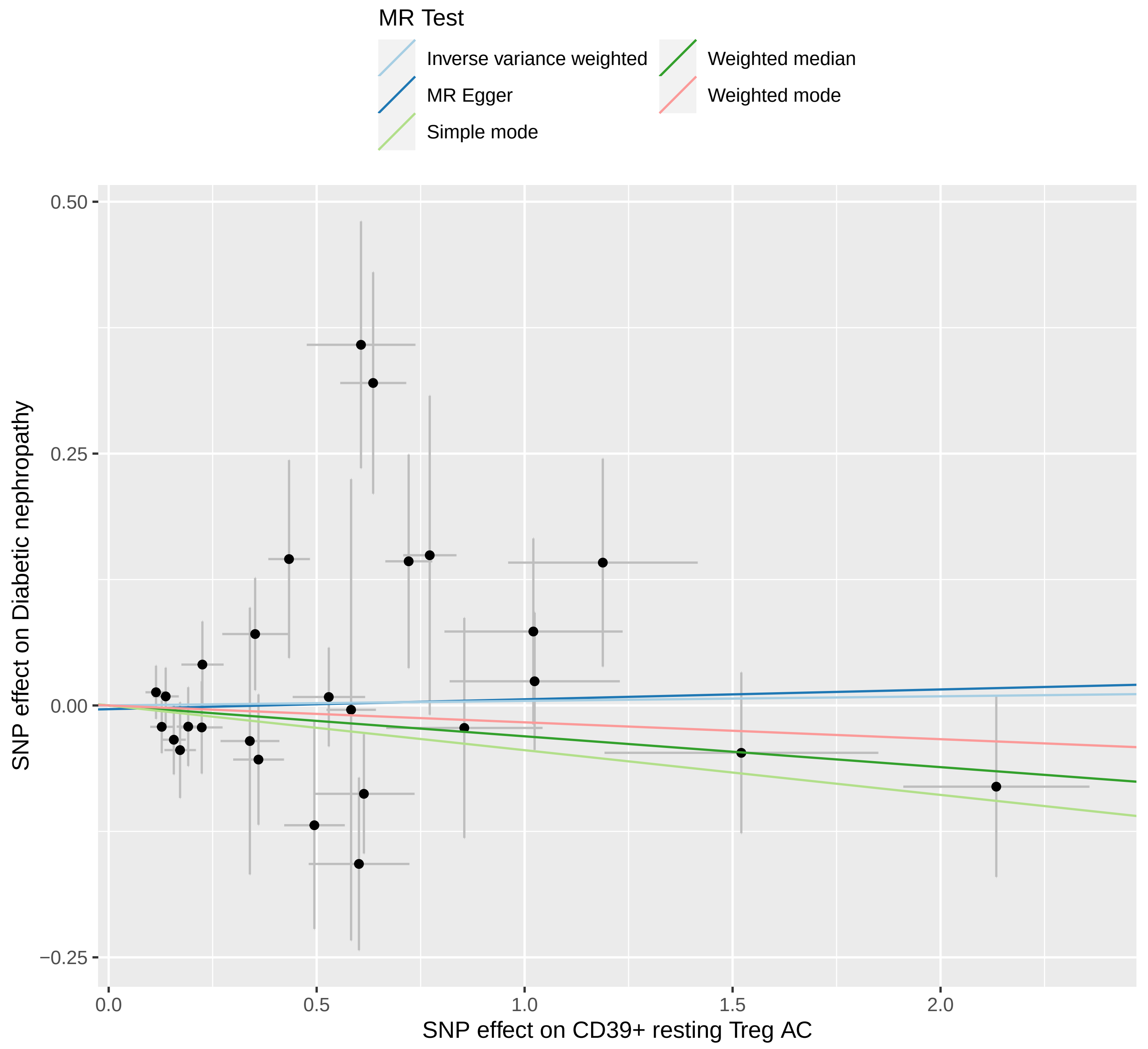
MR Test
Inverse variance weighted
MR Egger
Simple mode
Weighted median
Weighted mode
0.50
0.25
0.00
−0.25
0.0
0.5
1.0
1.5
2.0
SNP effect on Diabetic nephropathy
SNP effect on CD39+ resting Treg AC

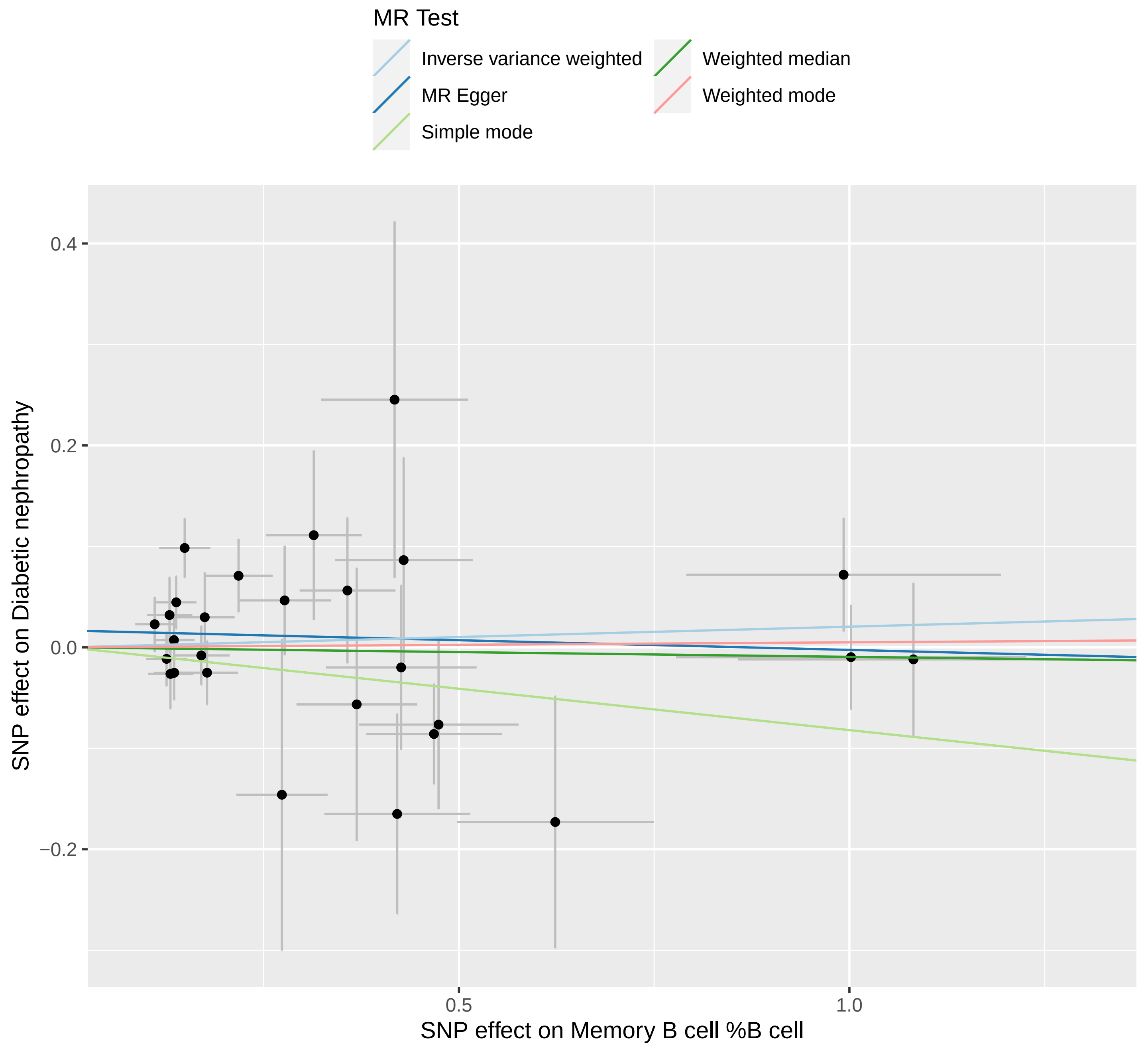
MR Test
Inverse variance weighted
MR Egger
Simple mode
Weighted median
Weighted mode
0.4
0.2
0.0
−0.2
0.5
1.0
SNP effect on Diabetic nephropathy
SNP effect on Memory B cell %B cell

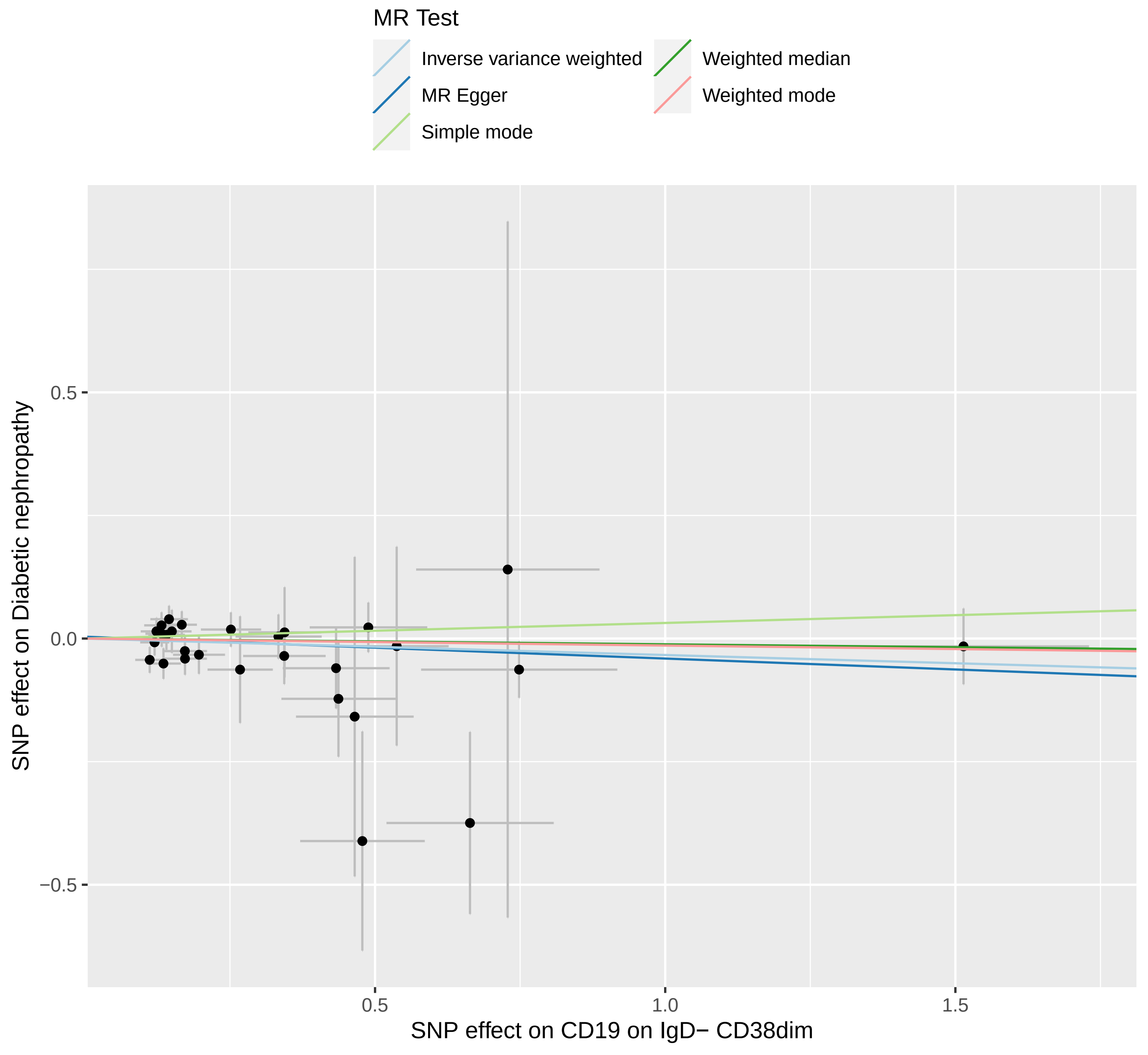
MR Test
Inverse variance weighted
MR Egger
Simple mode
Weighted median
Weighted mode
0.5
0.0
−0.5
0.5
1.0
1.5
SNP effect on Diabetic nephropathy
SNP effect on CD19 on IgD− CD38dim

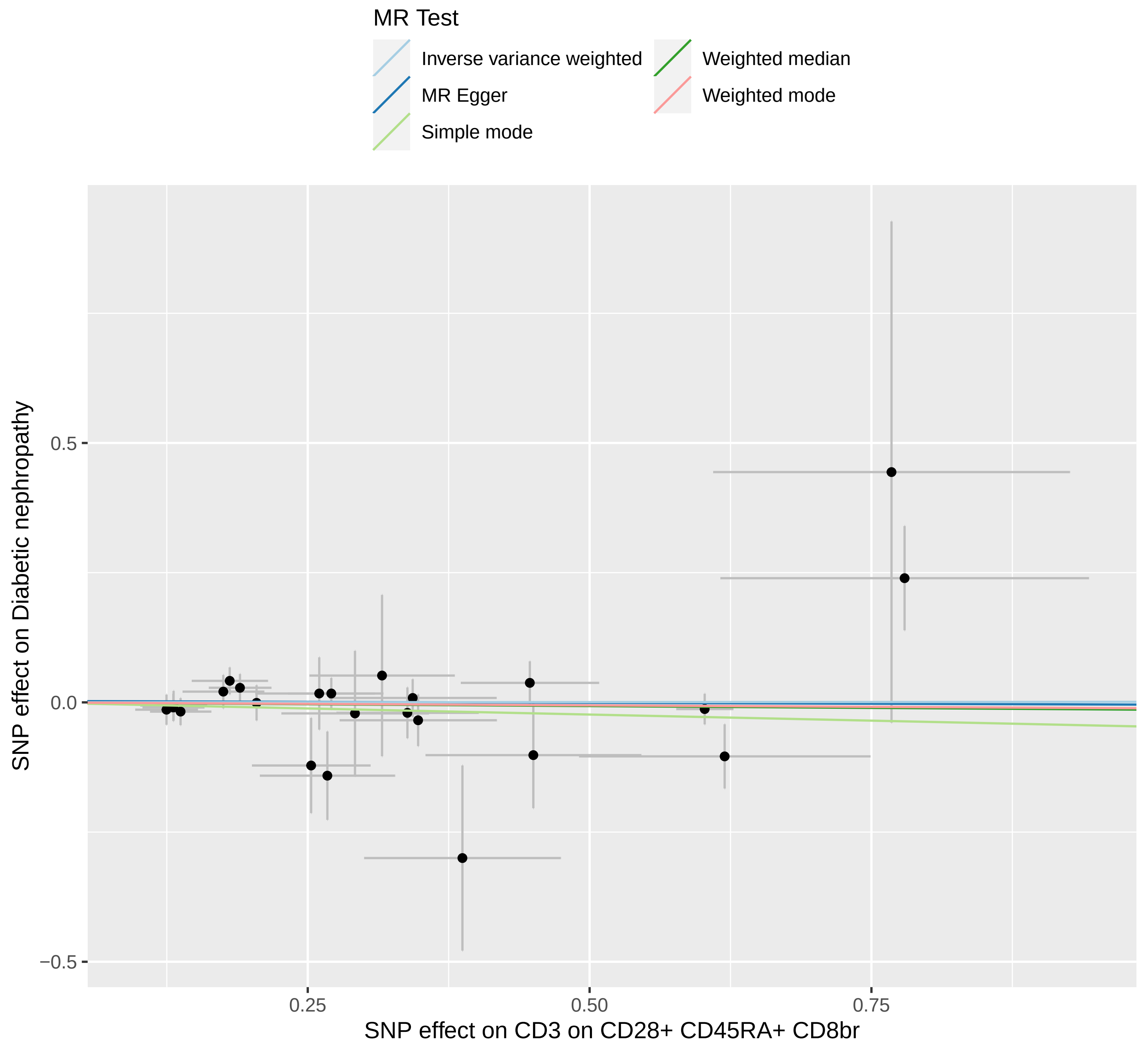
MR Test
Inverse variance weighted
MR Egger
Simple mode
Weighted median
Weighted mode
SNP effect on Diabetic nephropathy
0.5
0.0
−0.5
0.25
0.50
0.75
SNP effect on CD3 on CD28+ CD45RA+ CD8br

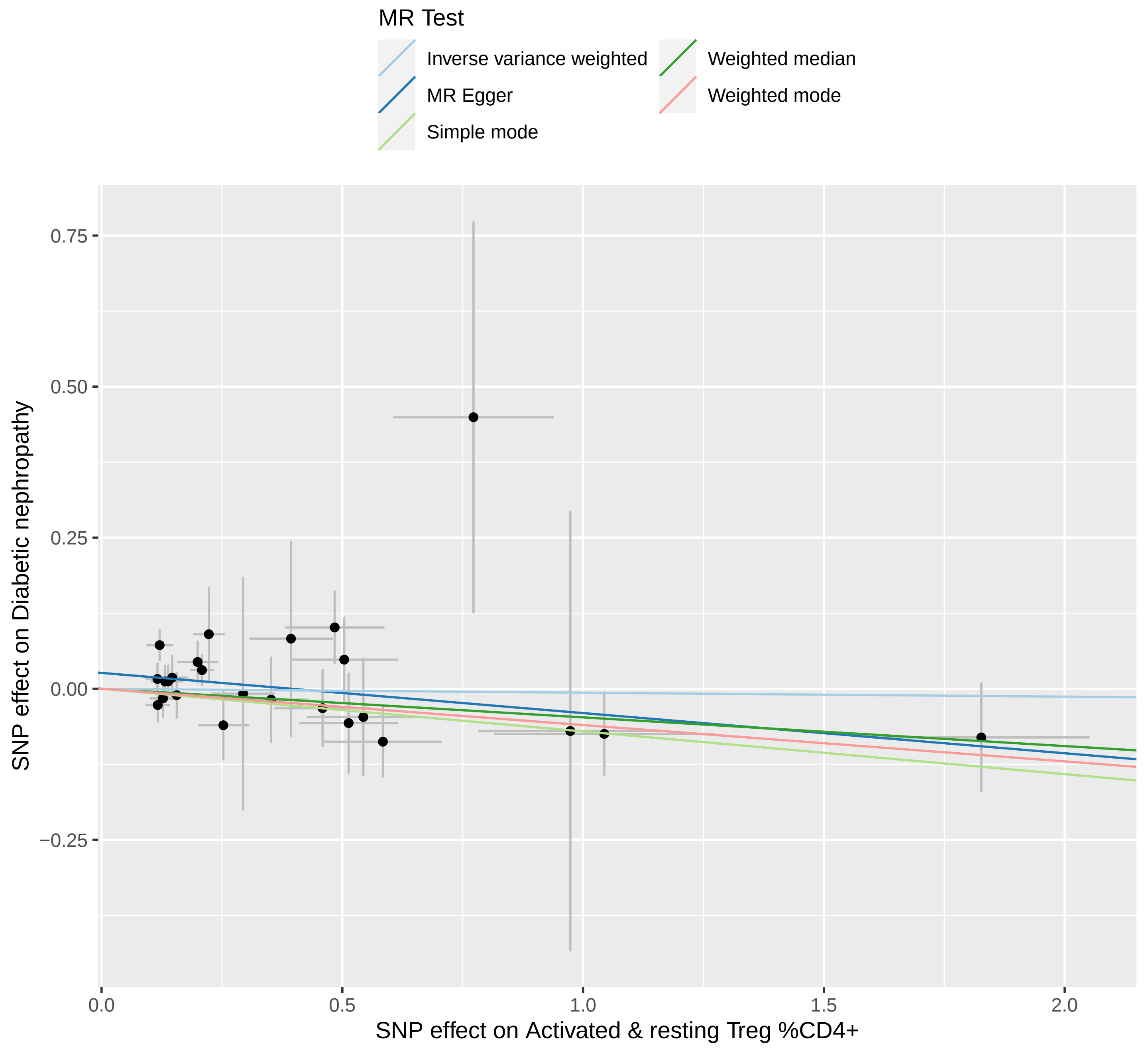

MR Test
Inverse variance weighted
MR Egger
Simple mode
Weighted median
Weighted mode
SNP effect on Diabetic nephropathy
SNP effect on Activated & resting Treg %CD4+
0.75
0.50
0.25
0.00
−0.25
0.0
0.5
1.0
1.5
2.0

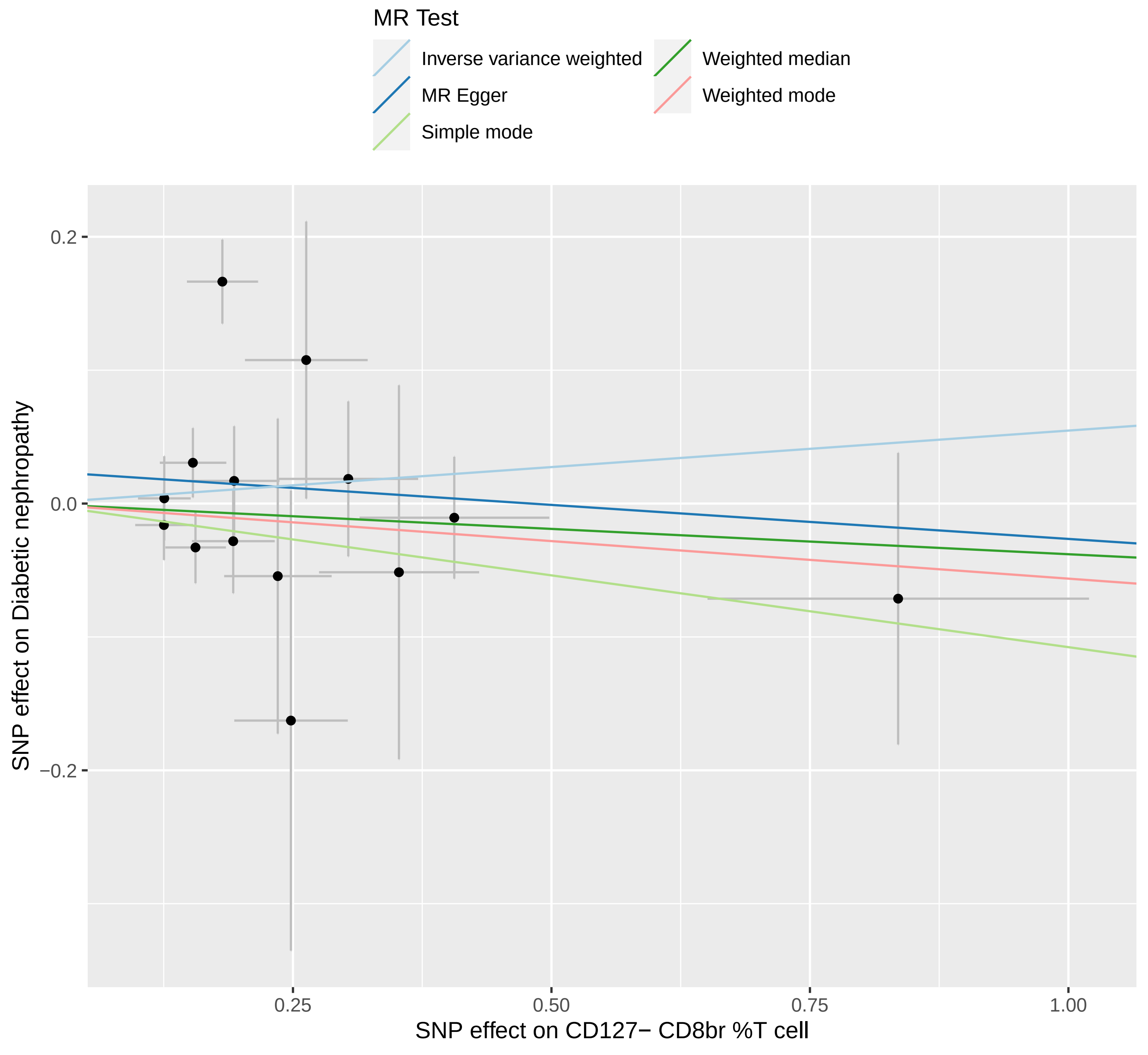
MR Test
Inverse variance weighted
MR Egger
Simple mode
Weighted median
Weighted mode
0.2
0.0
−0.2
0.25
0.50
0.75
1.00
SNP effect on Diabetic nephropathy
SNP effect on CD127− CD8br %T cell

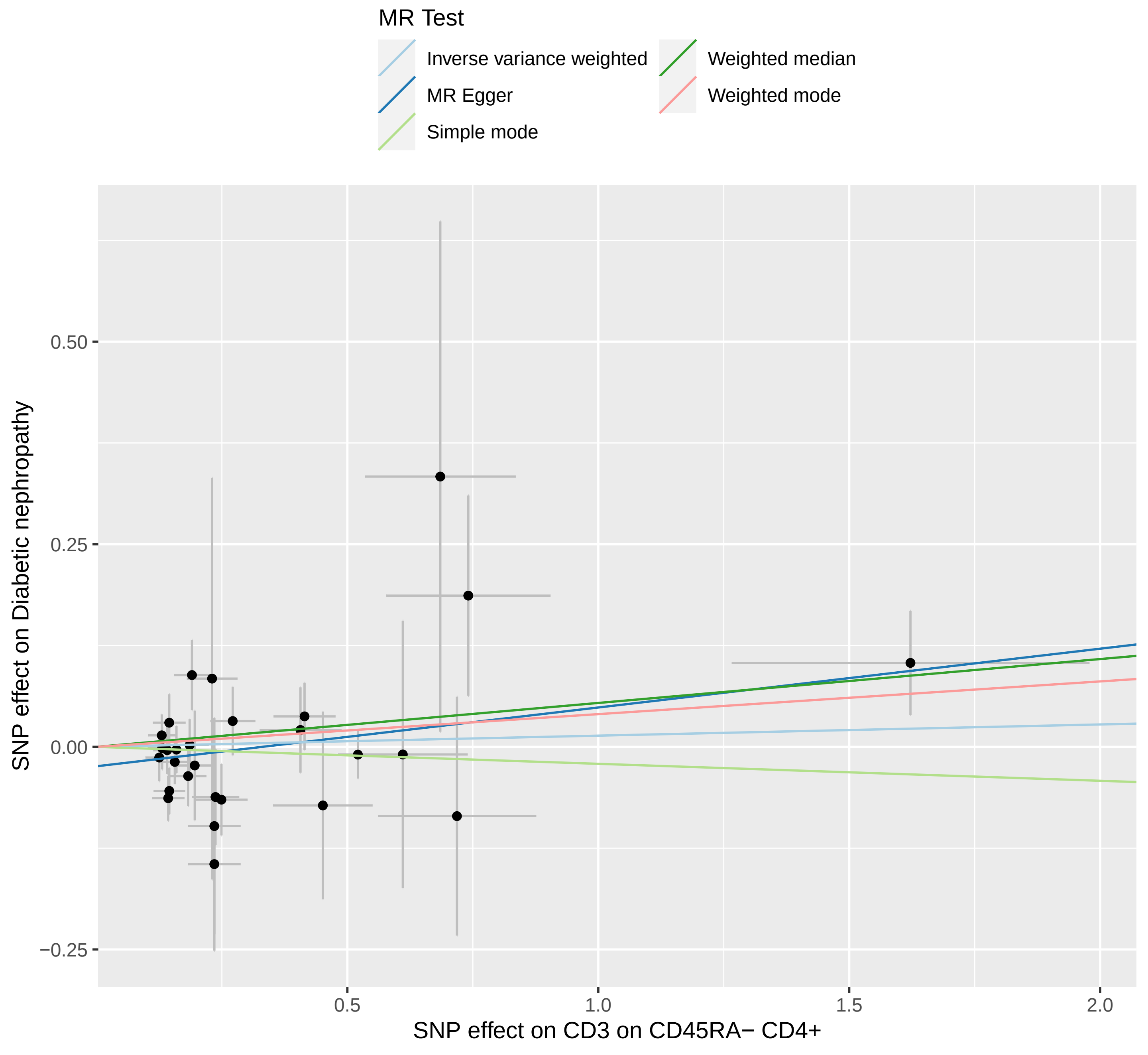

MR Test
Inverse variance weighted
MR Egger
Simple mode
Weighted median
Weighted mode
SNP effect on Diabetic nephropathy
0.50
0.25
0.00
−0.25
0.5
1.0
1.5
2.0
SNP effect on CD3 on CD45RA− CD4+

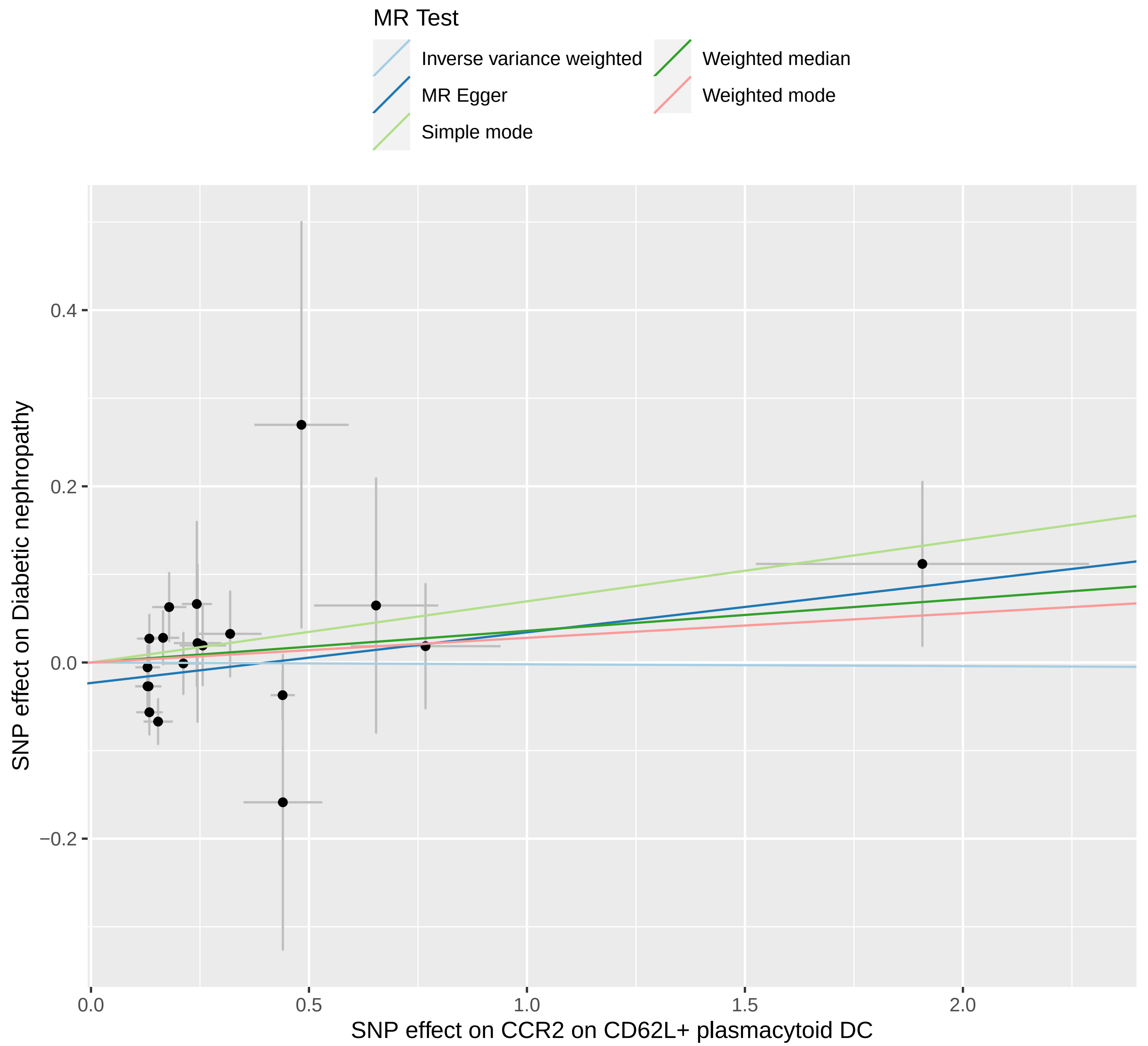
MR Test
Inverse variance weighted
MR Egger
Simple mode
Weighted median
Weighted mode
SNP effect on Diabetic nephropathy
0.4
0.2
0.0
−0.2
0.0
0.5
1.0
1.5
2.0
SNP effect on CCR2 on CD62L+ plasmacytoid DC

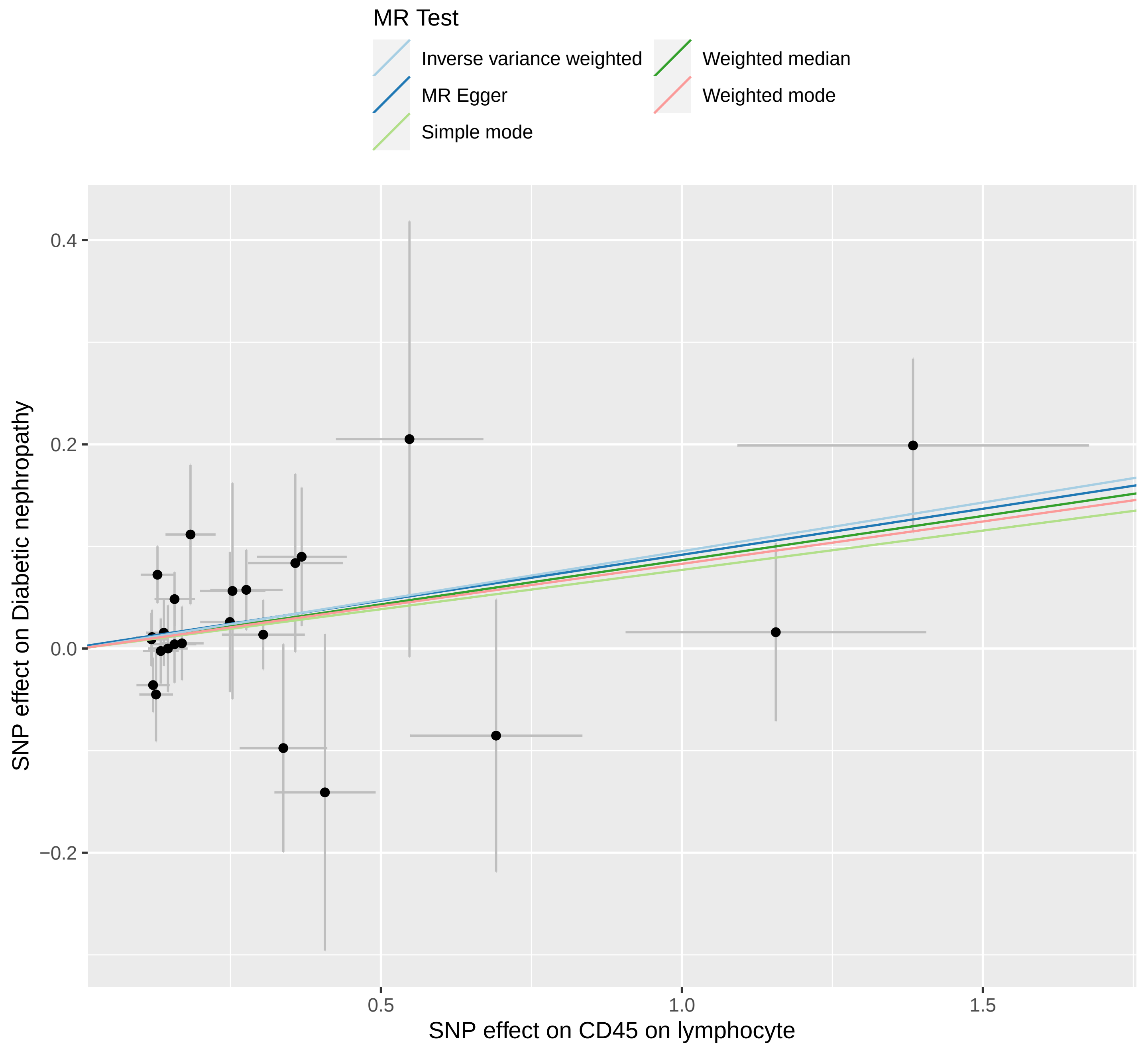

MR Test
Inverse variance weighted
MR Egger
Simple mode
Weighted median
Weighted mode
SNP effect on Diabetic nephropathy
0.4
0.2
0.0
−0.2
0.5
1.0
1.5
SNP effect on CD45 on lymphocyte

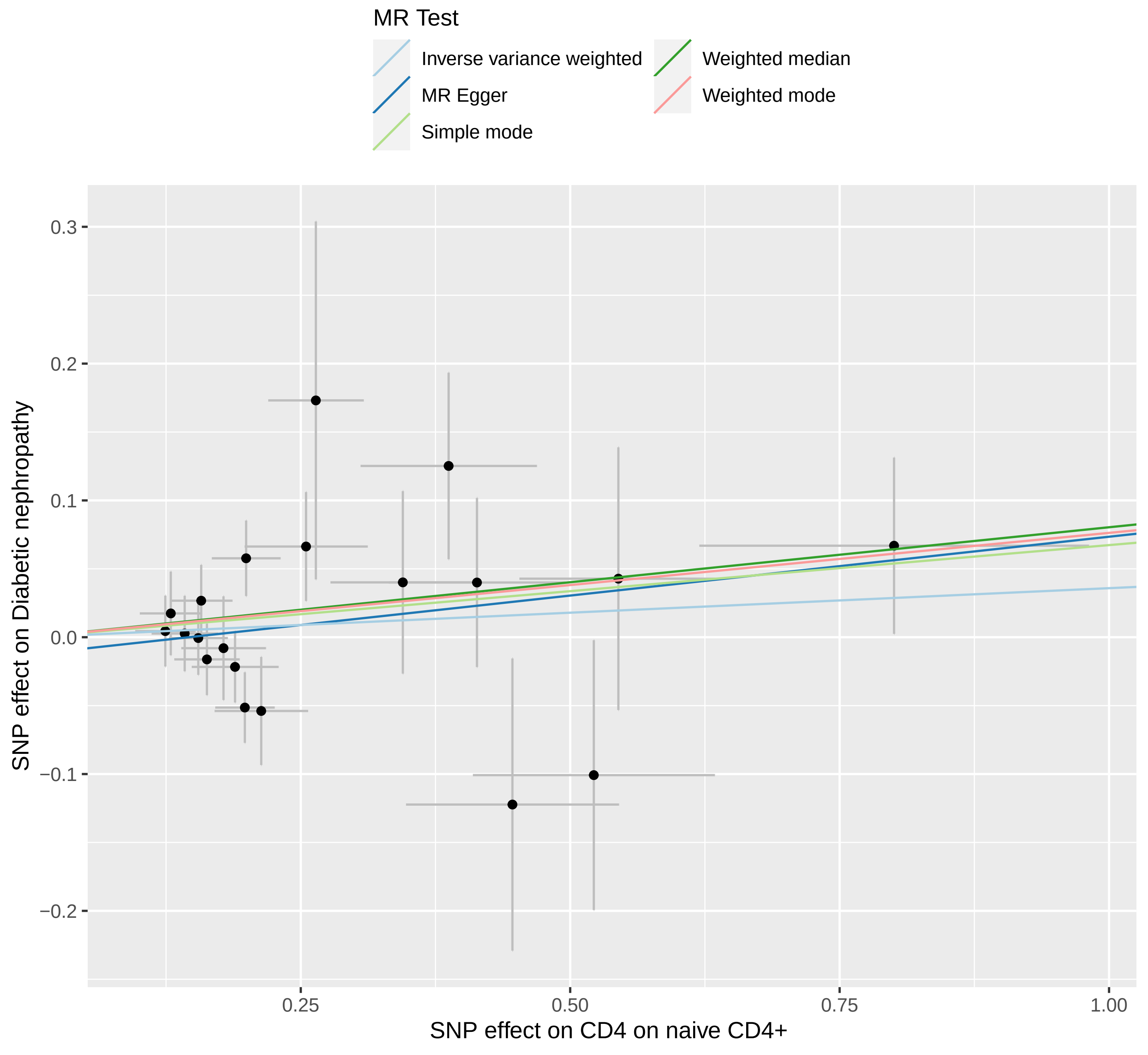

MR Test
Inverse variance weighted
MR Egger
Simple mode
Weighted median
Weighted mode
0.3
0.2
0.1
0.0
−0.1
−0.2
0.25
0.50
0.75
1.00
SNP effect on Diabetic nephropathy
SNP effect on CD4 on naive CD4+

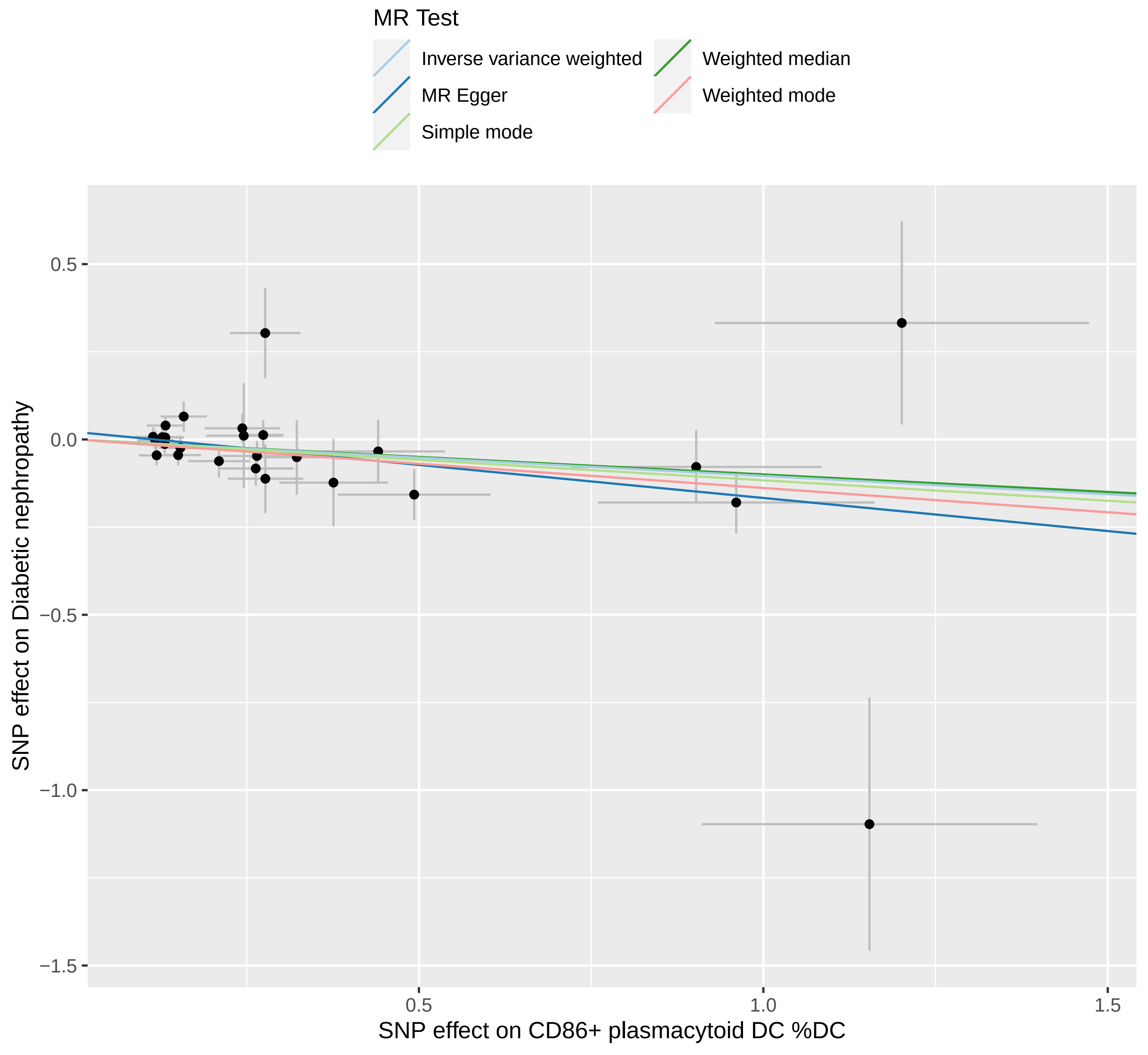
MR Test
Inverse variance weighted
MR Egger
Simple mode
Weighted median
Weighted mode
SNP effect on Diabetic nephropathy
0.5
0.0
−0.5
−1.0
−1.5
0.5
1.0
1.5
SNP effect on CD86+ plasmacytoid DC %DC

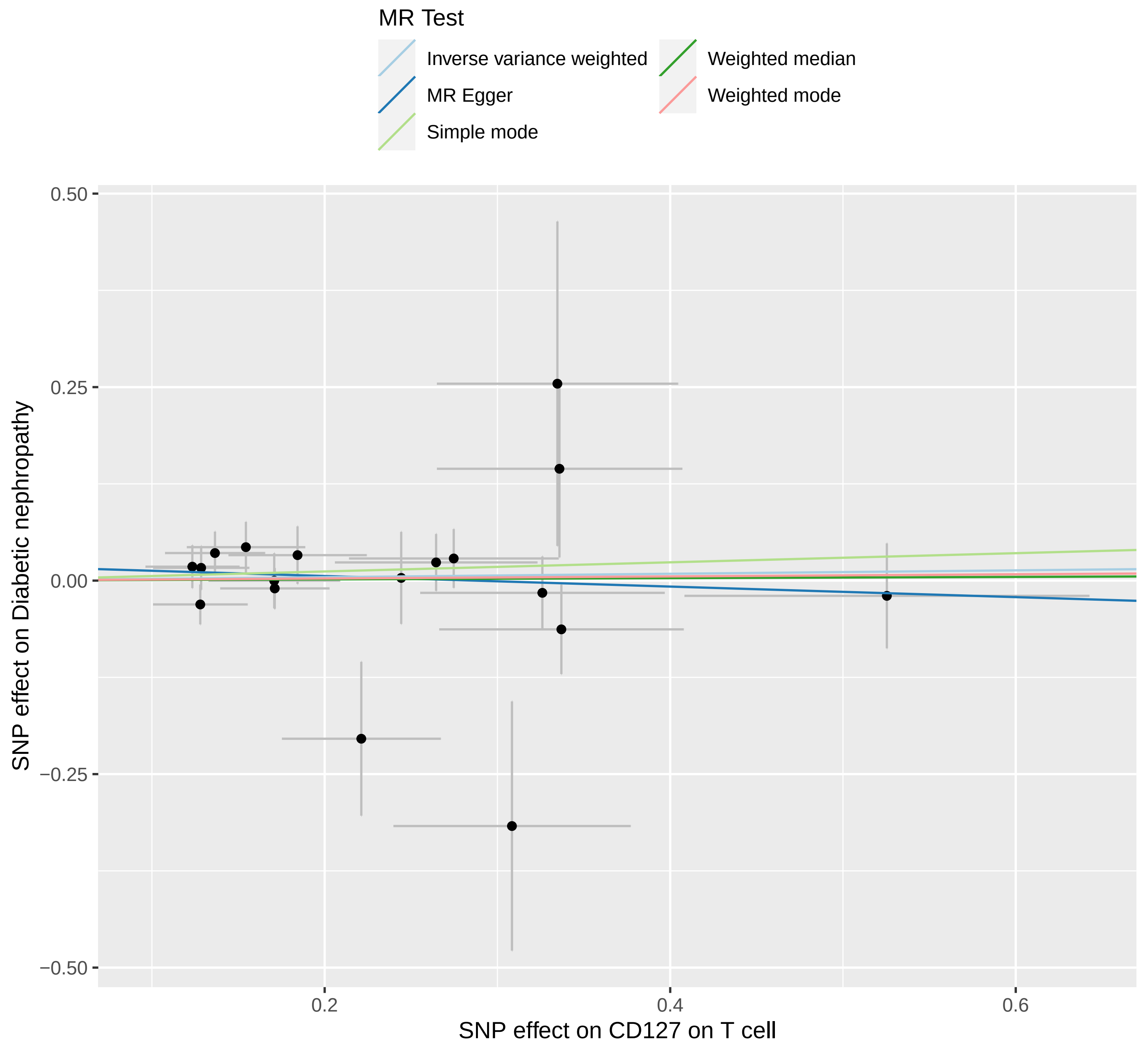
MR Test
Inverse variance weighted
MR Egger
Simple mode
Weighted median
Weighted mode
0.50
0.25
0.00
−0.25
−0.50
0.2
0.4
0.6
SNP effect on Diabetic nephropathy
SNP effect on CD127 on T cell

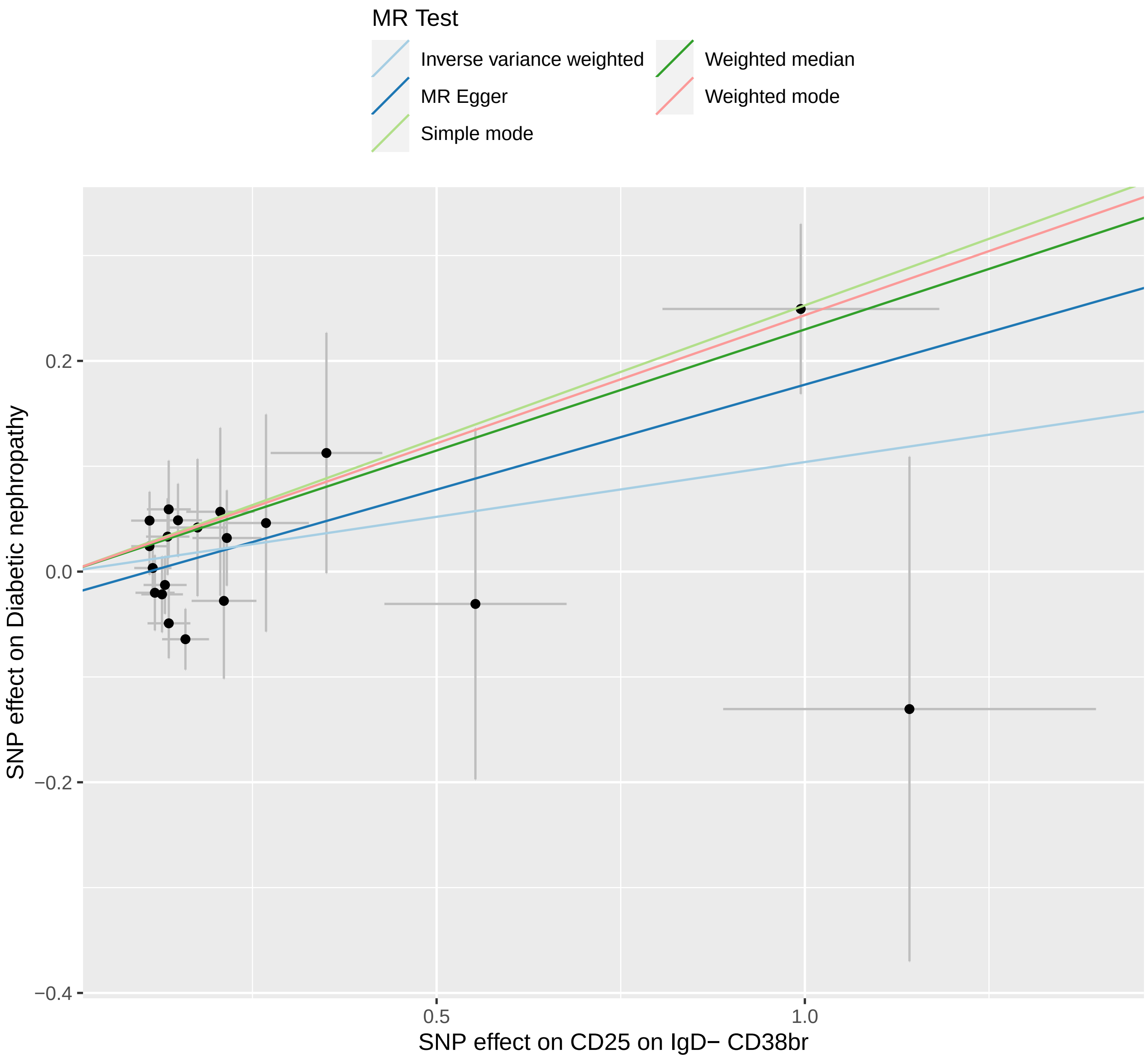

MR Test
Inverse variance weighted
MR Egger
Simple mode
Weighted median
Weighted mode
0.2
0.0
−0.2
−0.4
0.5
1.0
SNP effect on Diabetic nephropathy
SNP effect on CD25 on IgD− CD38br

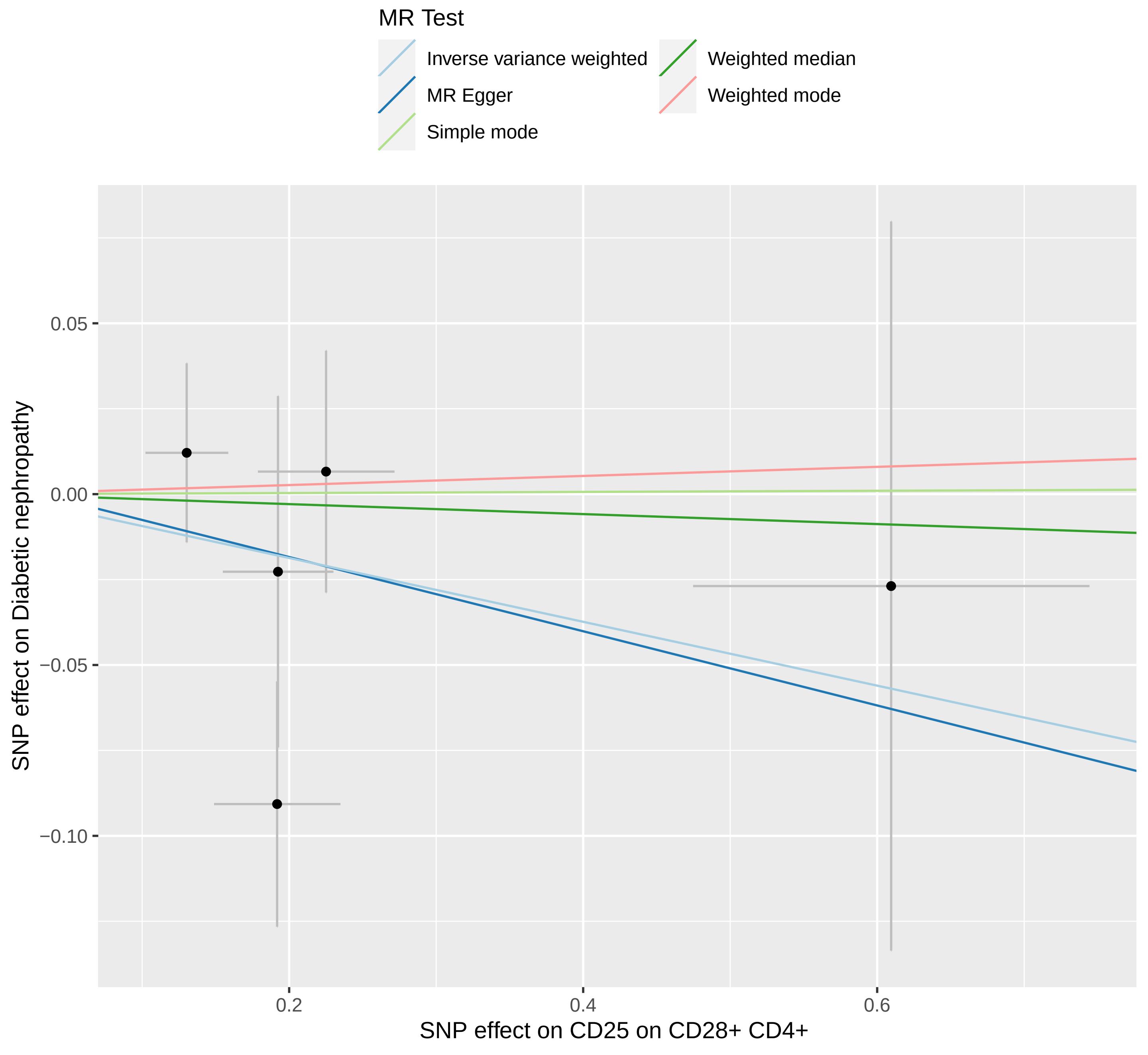

MR Test
Inverse variance weighted
MR Egger
Simple mode
Weighted median
Weighted mode
0.05
0.00
−0.05
−0.10
0.2
0.4
0.6
SNP effect on Diabetic nephropathy
SNP effect on CD25 on CD28+ CD4+

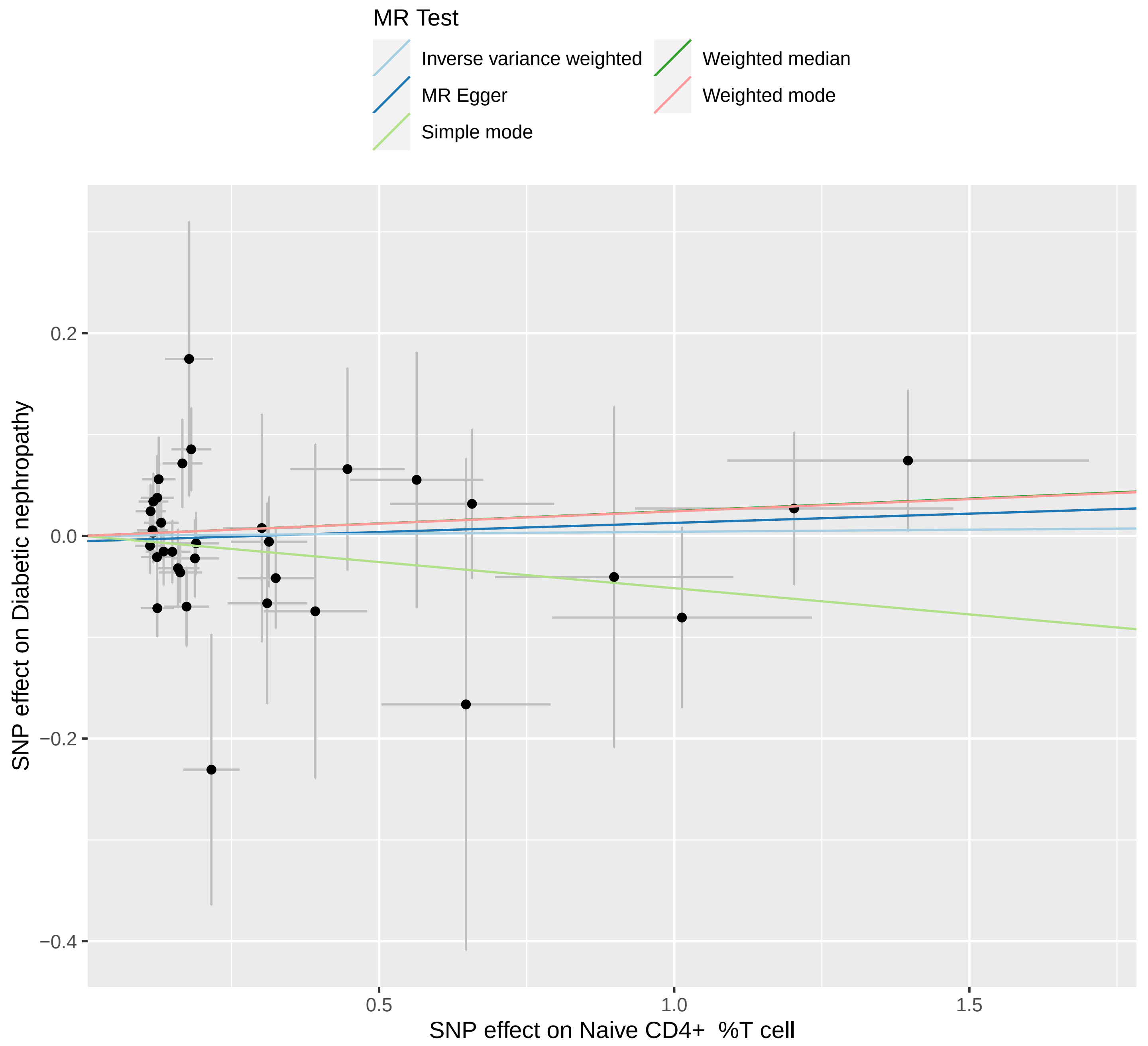
MR Test
Inverse variance weighted
MR Egger
Simple mode
Weighted median
Weighted mode
0.2
0.0
−0.2
−0.4
0.5
1.0
1.5
SNP effect on Diabetic nephropathy
SNP effect on Naive CD4+ %T cell

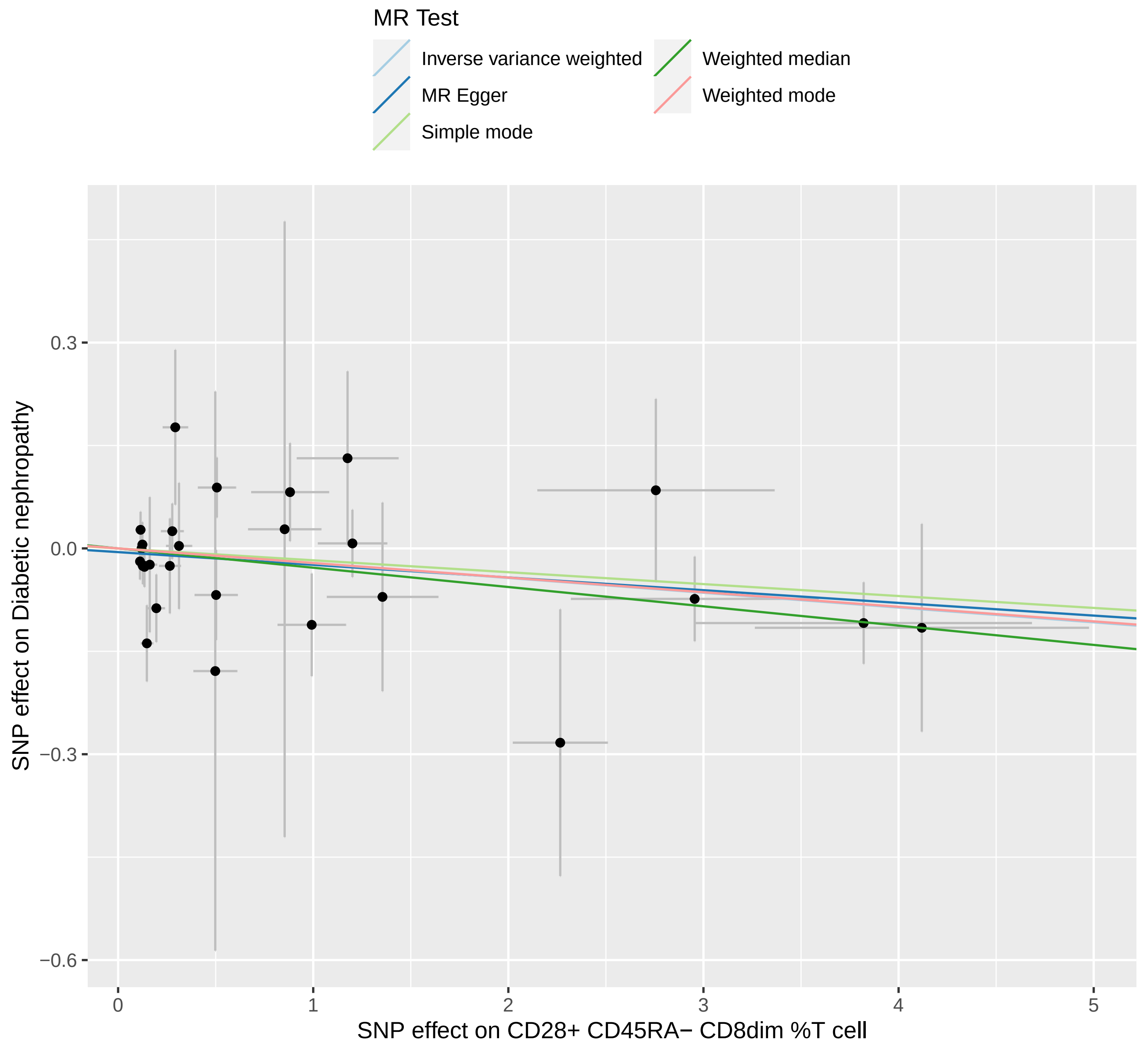

MR Test
Inverse variance weighted
MR Egger
Simple mode
Weighted median
Weighted mode
0.3
0.0
−0.3
−0.6
0
1
2
3
4
5
SNP effect on Diabetic nephropathy
SNP effect on CD28+ CD45RA− CD8dim %T cell

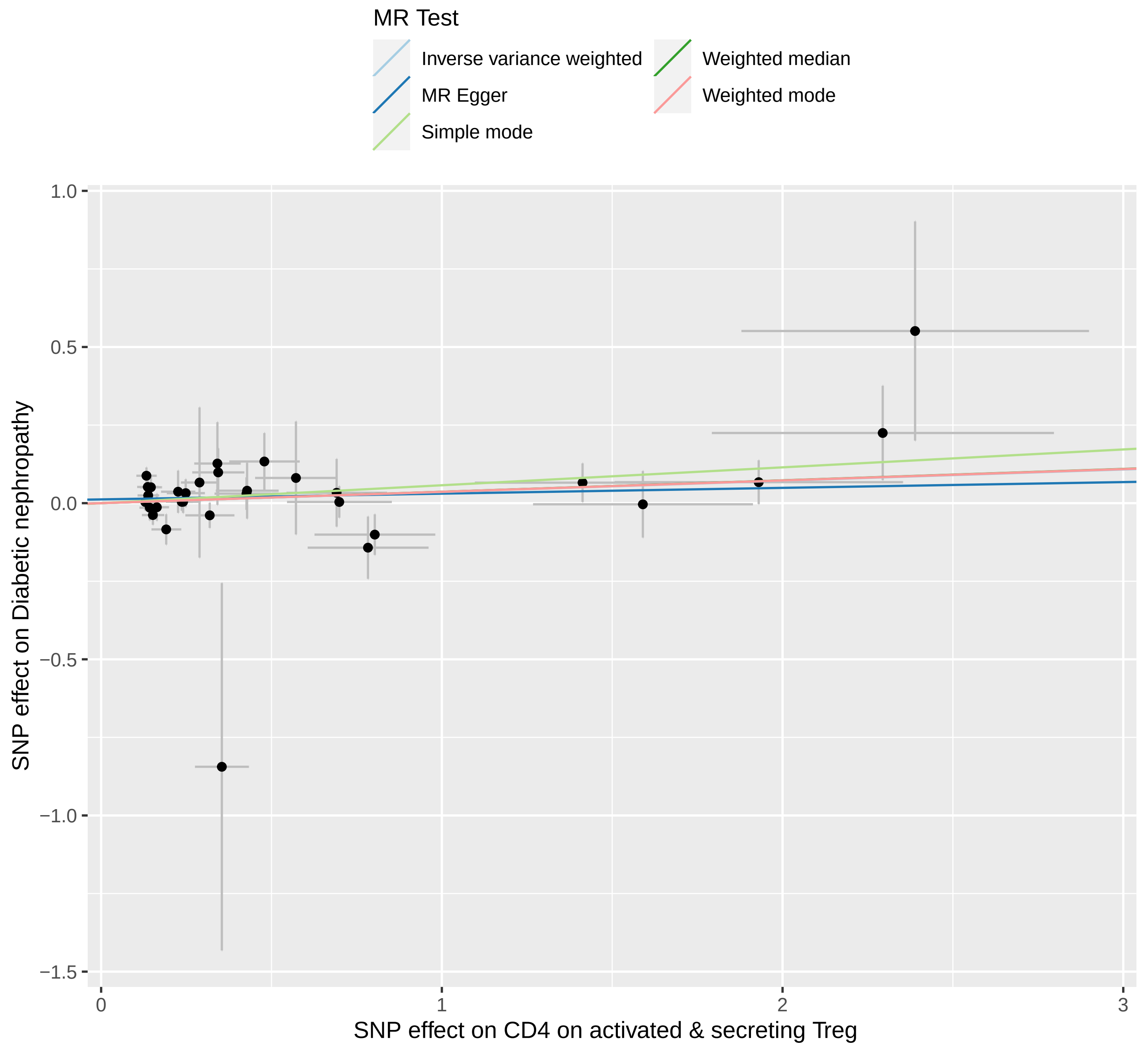
MR Test
Inverse variance weighted
MR Egger
Simple mode
Weighted median
Weighted mode
1.0
0.5
0.0
−0.5
−1.0
−1.5
0
1
2
3
SNP effect on Diabetic nephropathy
SNP effect on CD4 on activated & secreting Treg

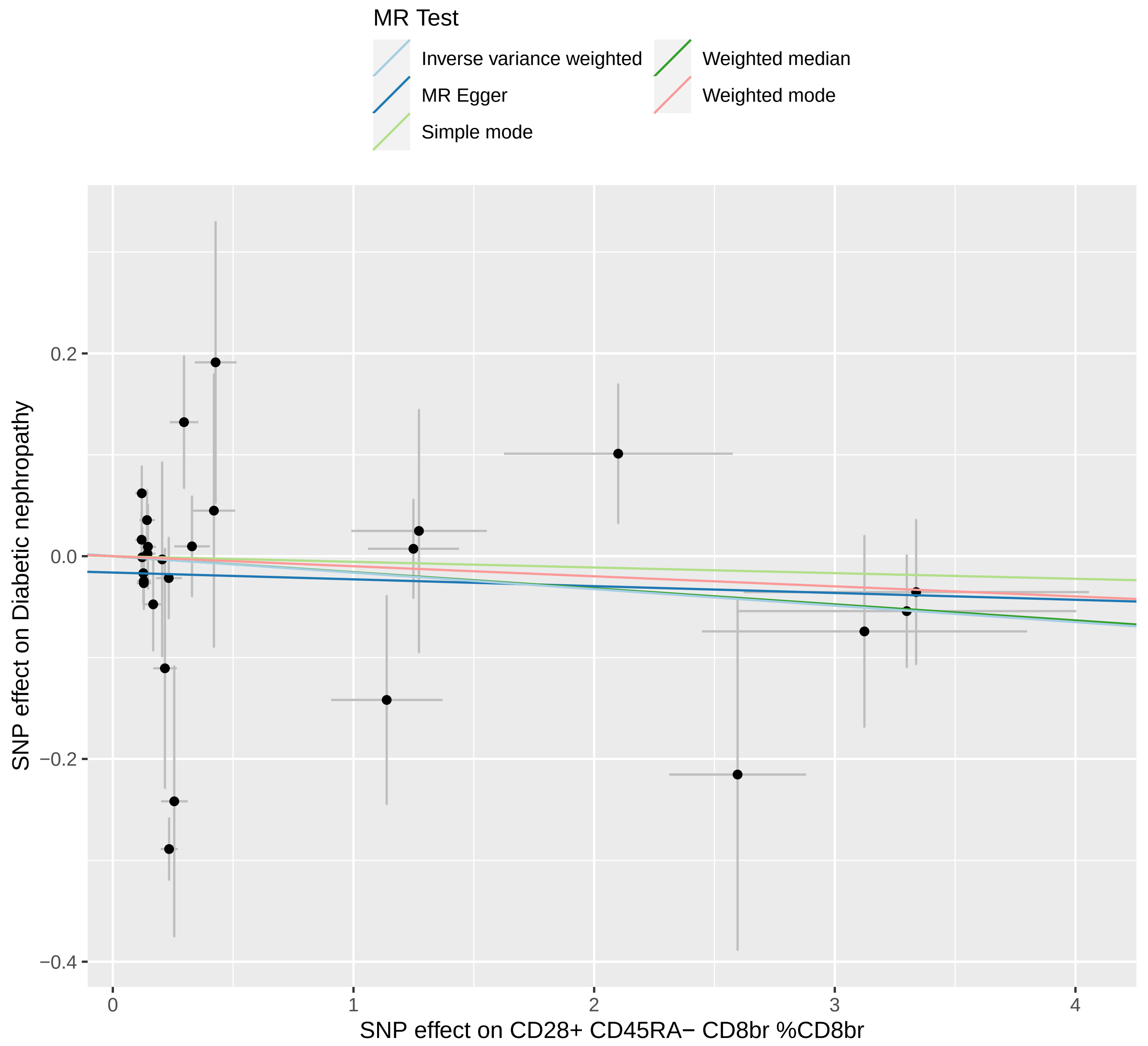

MR Test
Inverse variance weighted
MR Egger
Simple mode
Weighted median
Weighted mode
0.2
0.0
−0.2
−0.4
0
1
2
3
4
SNP effect on Diabetic nephropathy
SNP effect on CD28+ CD45RA− CD8br %CD8br

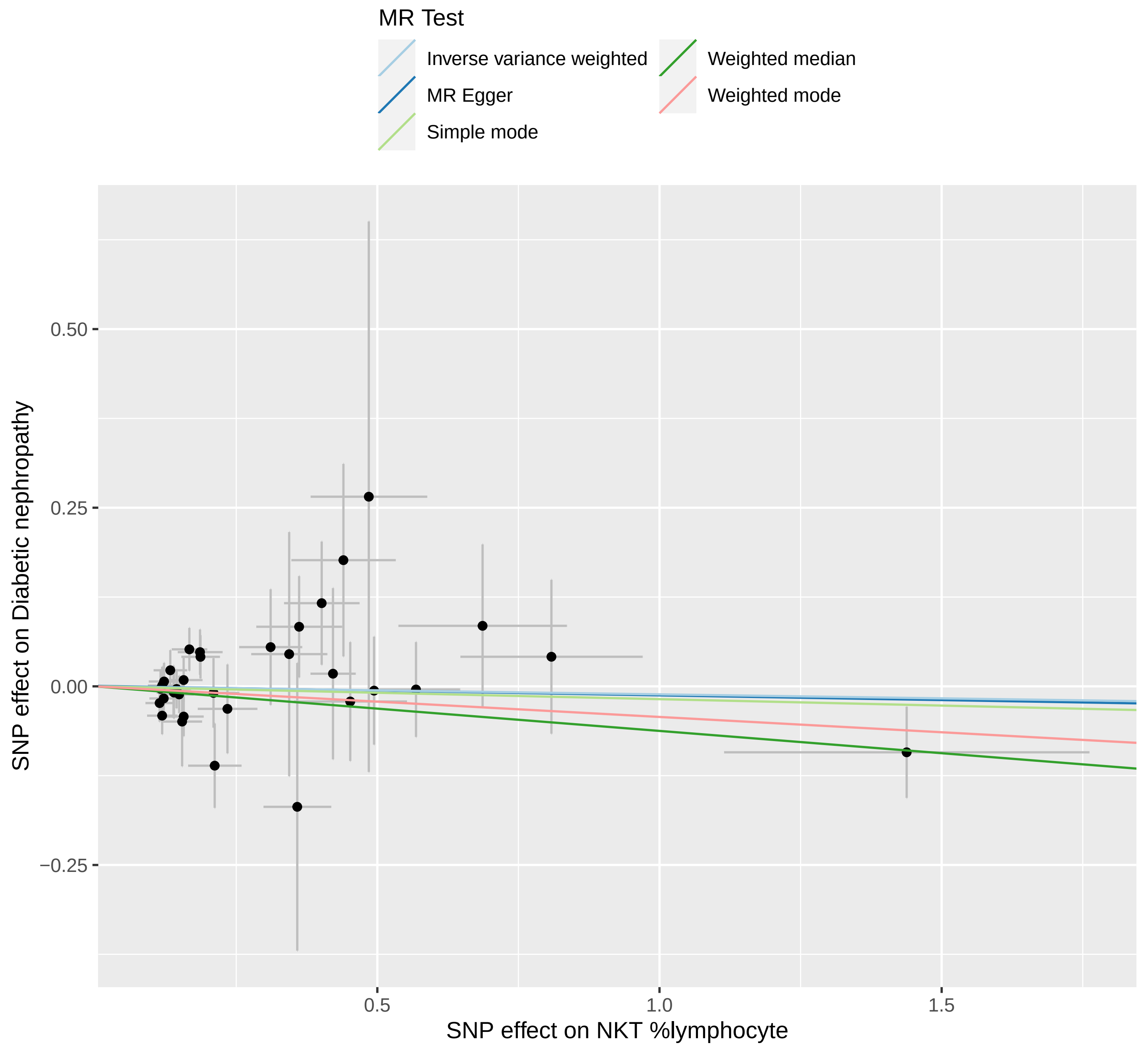
MR Test
Inverse variance weighted
MR Egger
Simple mode
Weighted median
Weighted mode
SNP effect on Diabetic nephropathy
0.50
0.25
0.00
−0.25
0.5
1.0
1.5
SNP effect on NKT %lymphocyte

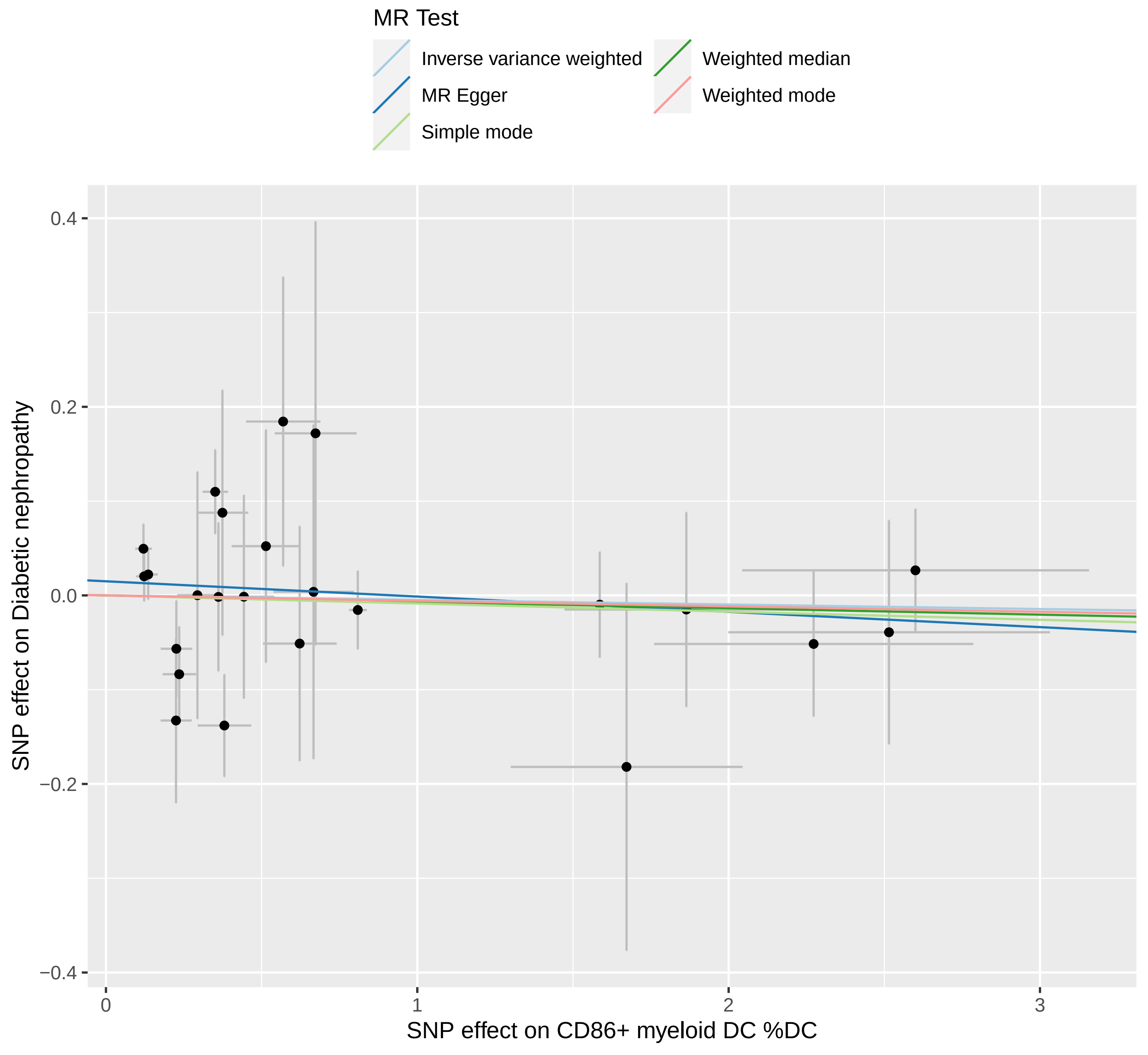

MR Test
Inverse variance weighted
MR Egger
Simple mode
Weighted median
Weighted mode
SNP effect on Diabetic nephropathy
0.4
0.2
0.0
−0.2
−0.4
0
1
2
3
SNP effect on CD86+ myeloid DC %DC

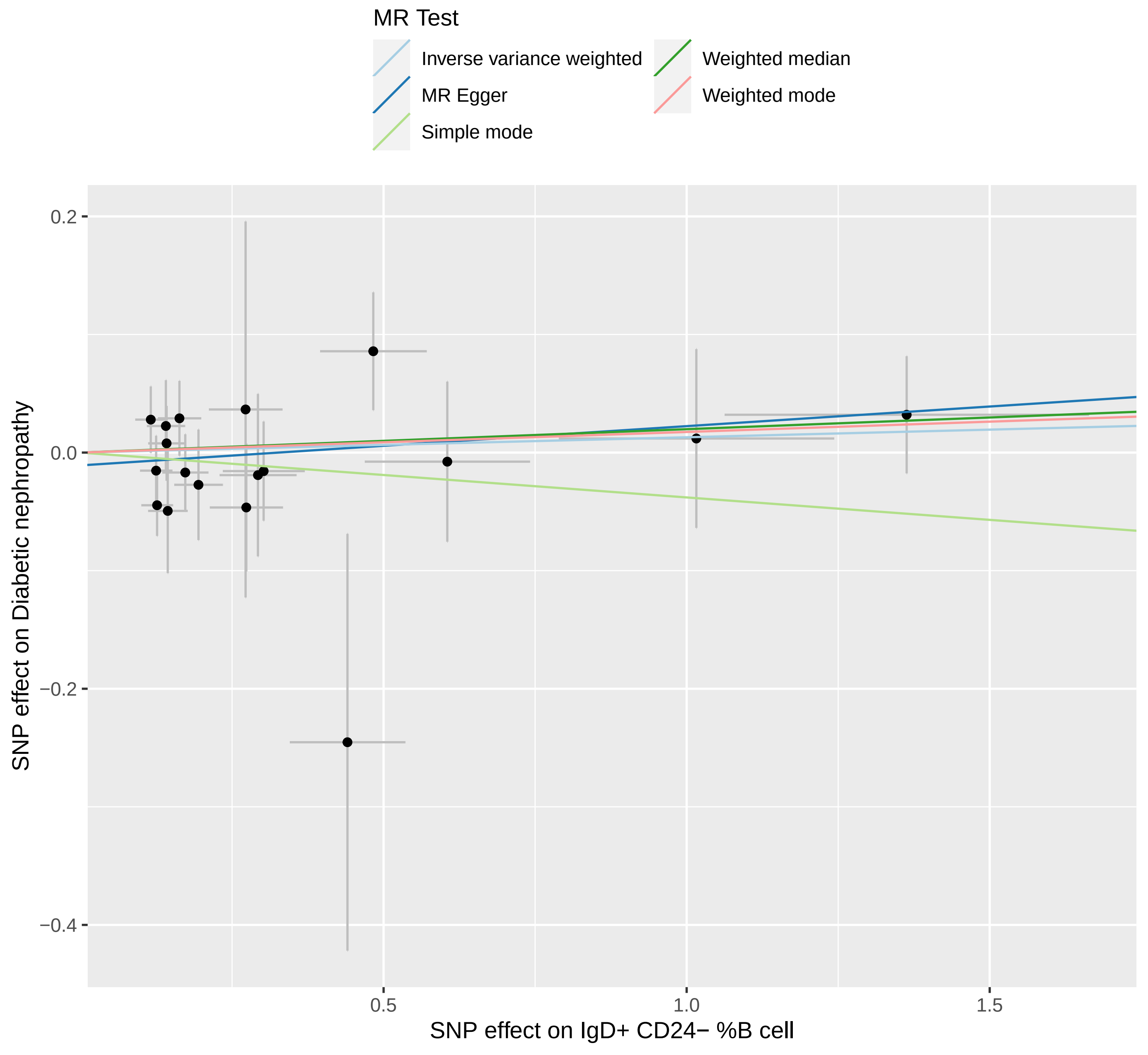
MR Test
Inverse variance weighted
MR Egger
Simple mode
Weighted median
Weighted mode
0.2
0.0
−0.2
−0.4
0.5
1.0
1.5
SNP effect on Diabetic nephropathy
SNP effect on IgD+ CD24− %B cell

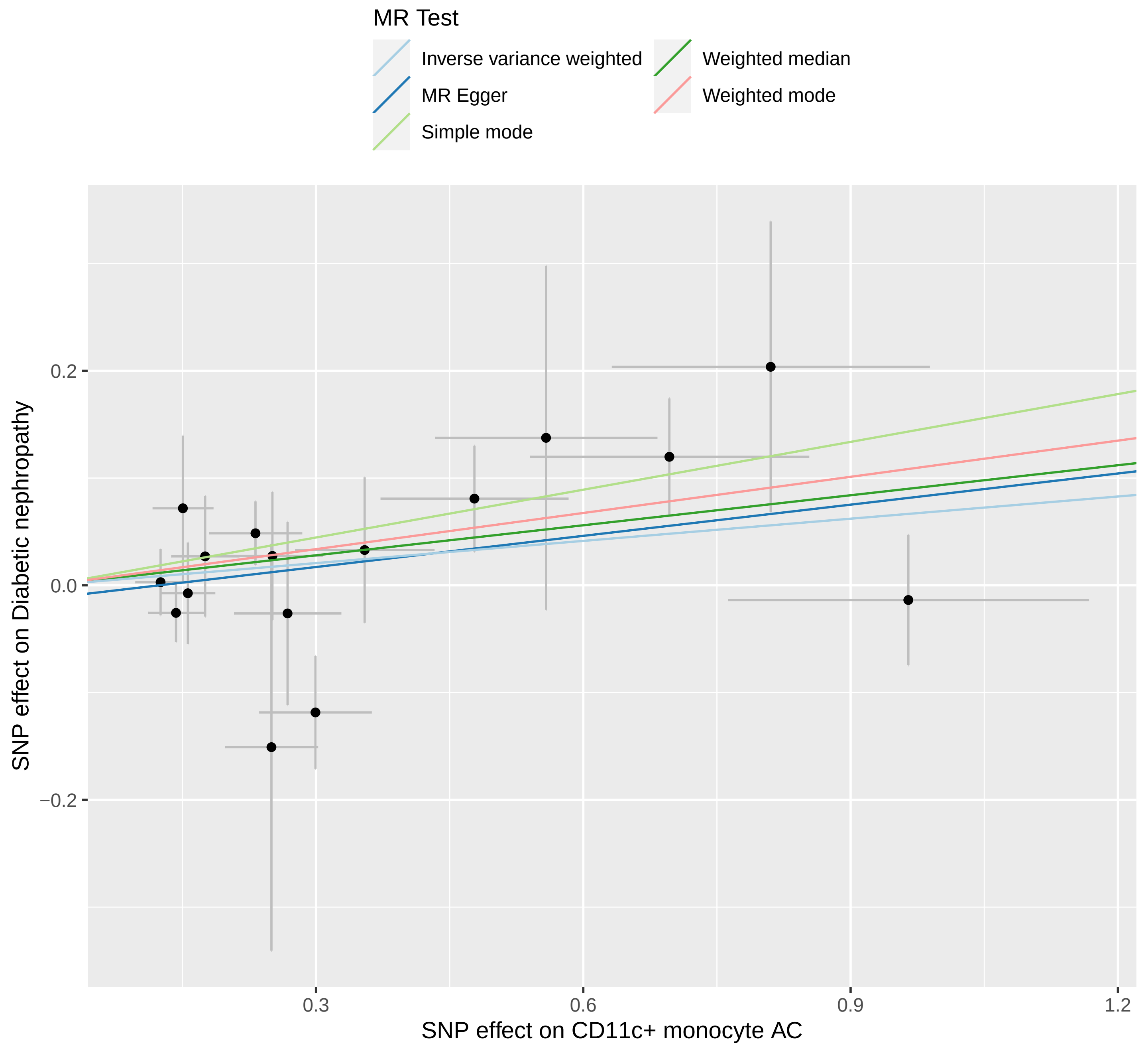
MR Test
Inverse variance weighted
MR Egger
Simple mode
Weighted median
Weighted mode
SNP effect on Diabetic nephropathy
0.2
0.0
−0.2
0.3
0.6
0.9
1.2
SNP effect on CD11c+ monocyte AC

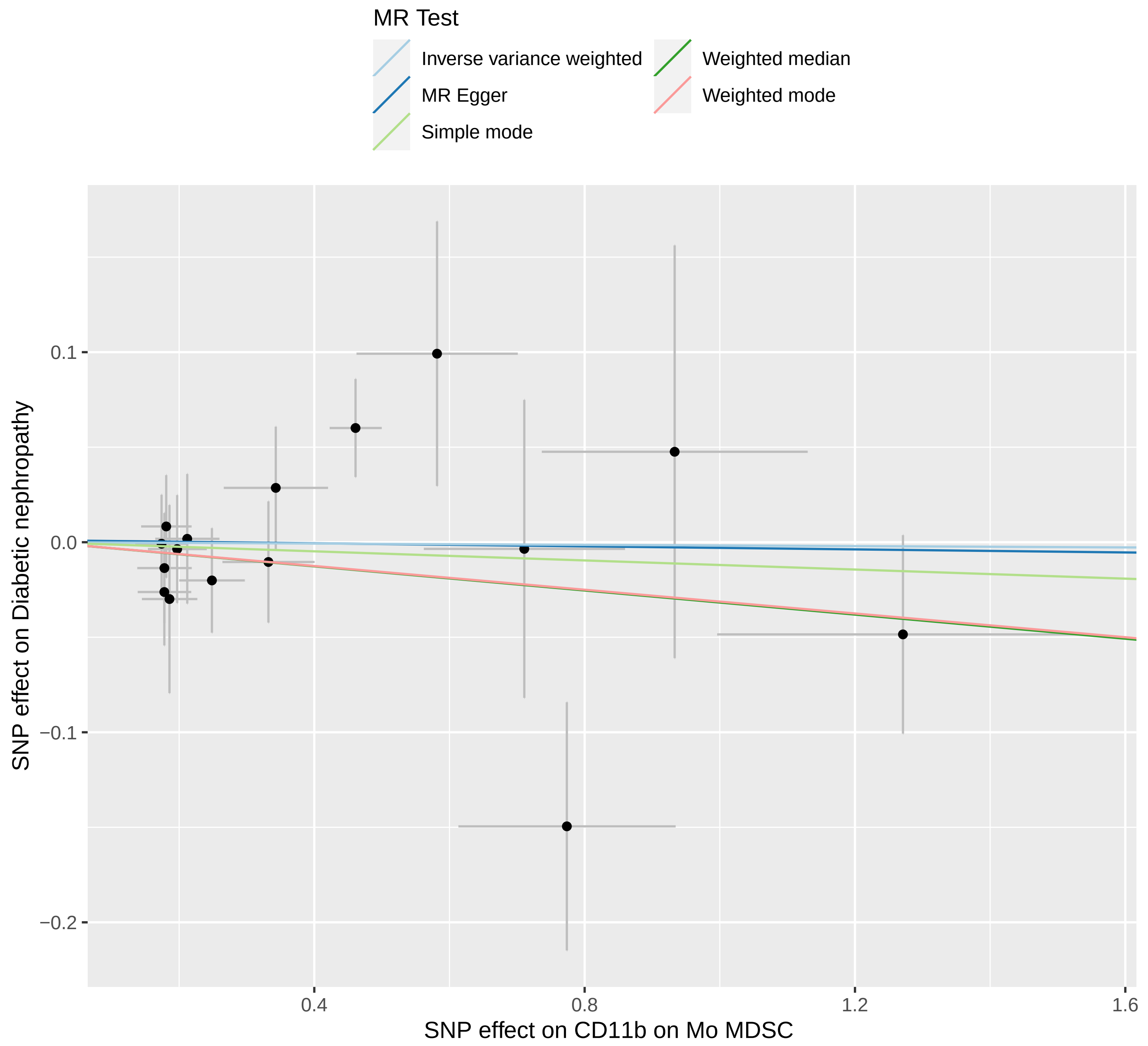

MR Test
Inverse variance weighted
MR Egger
Simple mode
Weighted median
Weighted mode
0.1
0.0
−0.1
−0.2
0.4
0.8
1.2
1.6
SNP effect on Diabetic nephropathy
SNP effect on CD11b on Mo MDSC

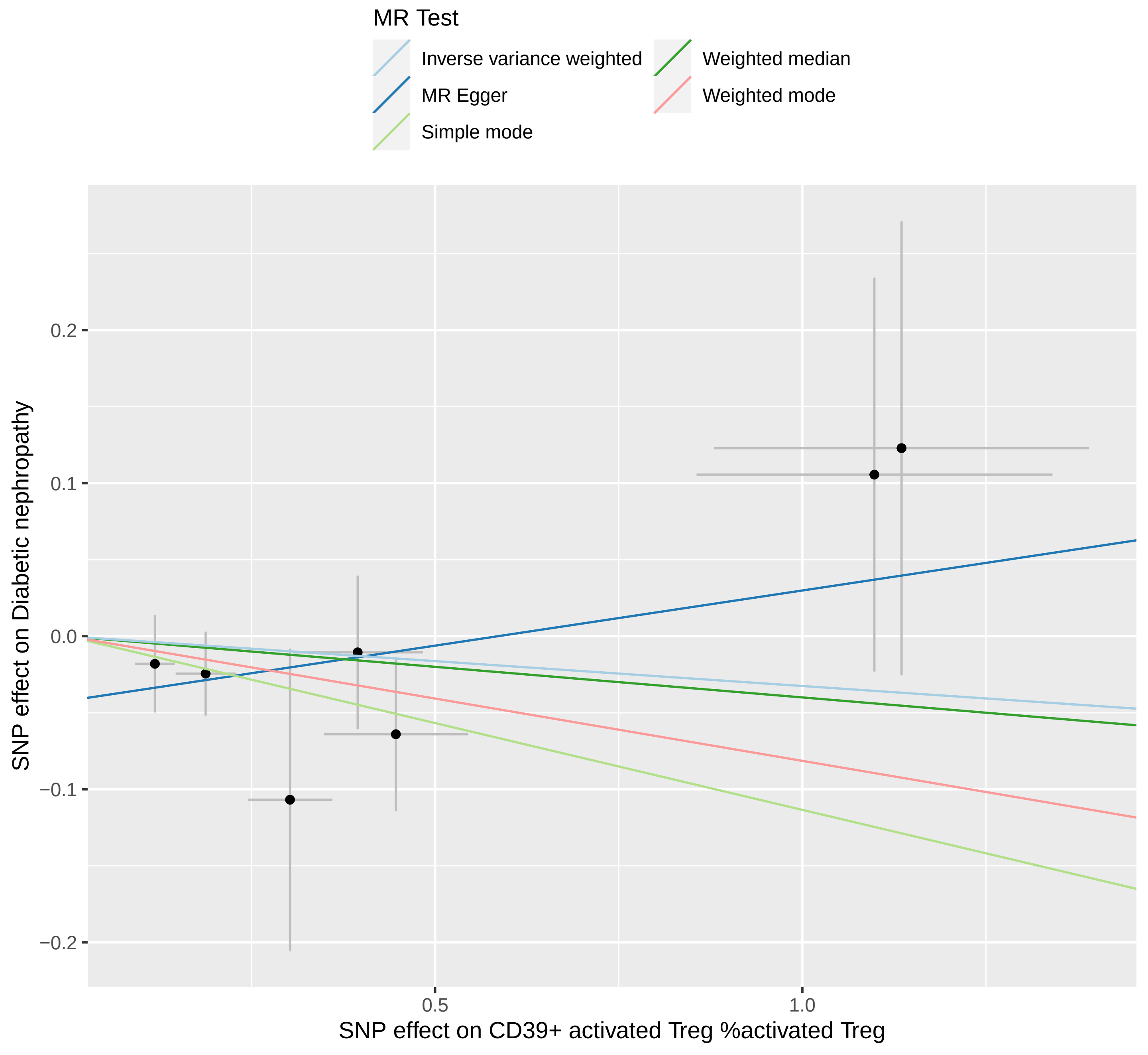

MR Test
Inverse variance weighted
MR Egger
Simple mode
Weighted median
Weighted mode
0.2
0.1
0.0
−0.1
−0.2
SNP effect on Diabetic nephropathy
0.5
1.0
SNP effect on CD39+ activated Treg %activated Treg

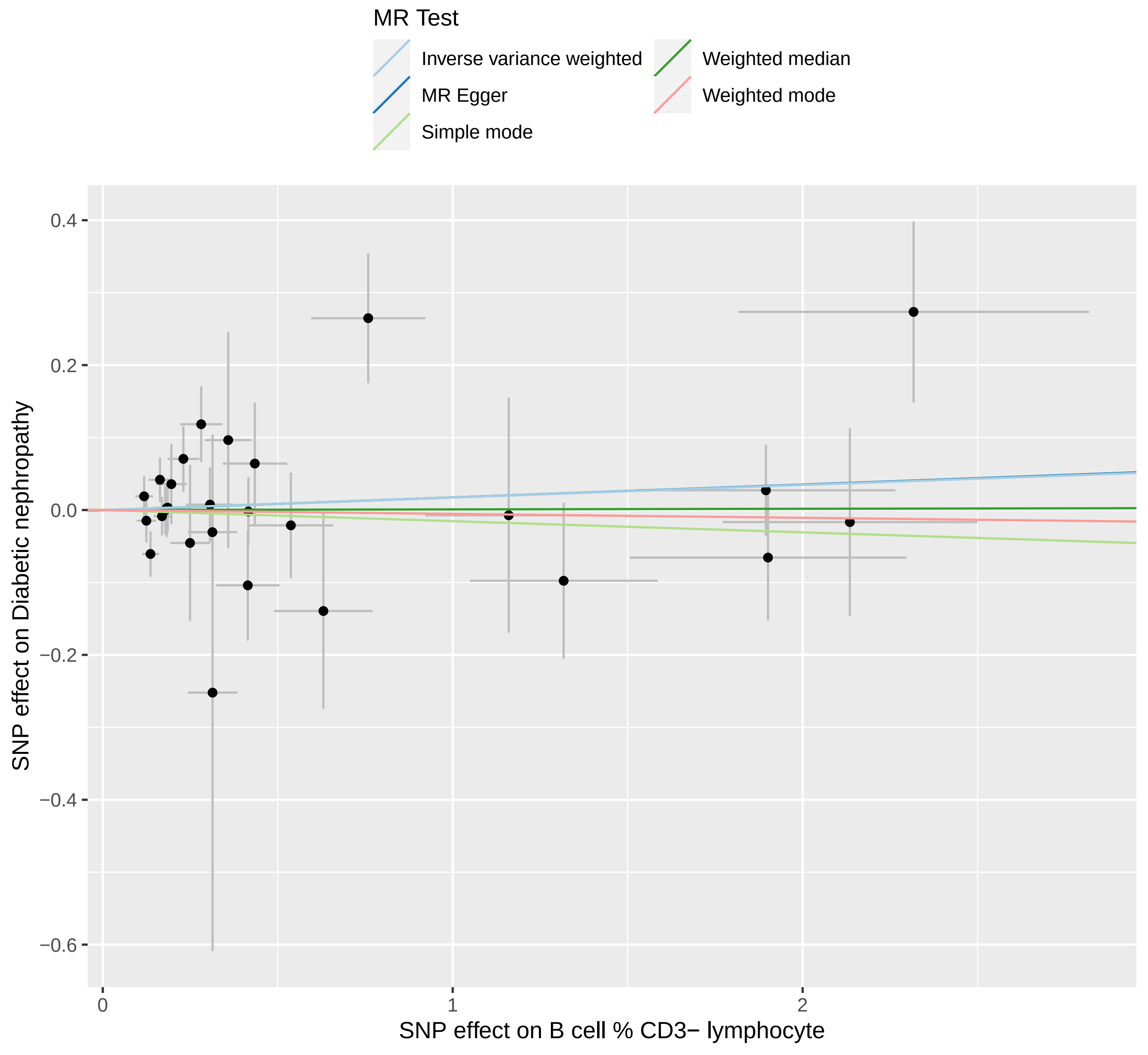

MR Test
Inverse variance weighted
MR Egger
Simple mode
Weighted median
Weighted mode
0.4
0.2
0.0
−0.2
−0.4
−0.6
0
1
2
SNP effect on Diabetic nephropathy
SNP effect on B cell % CD3− lymphocyte

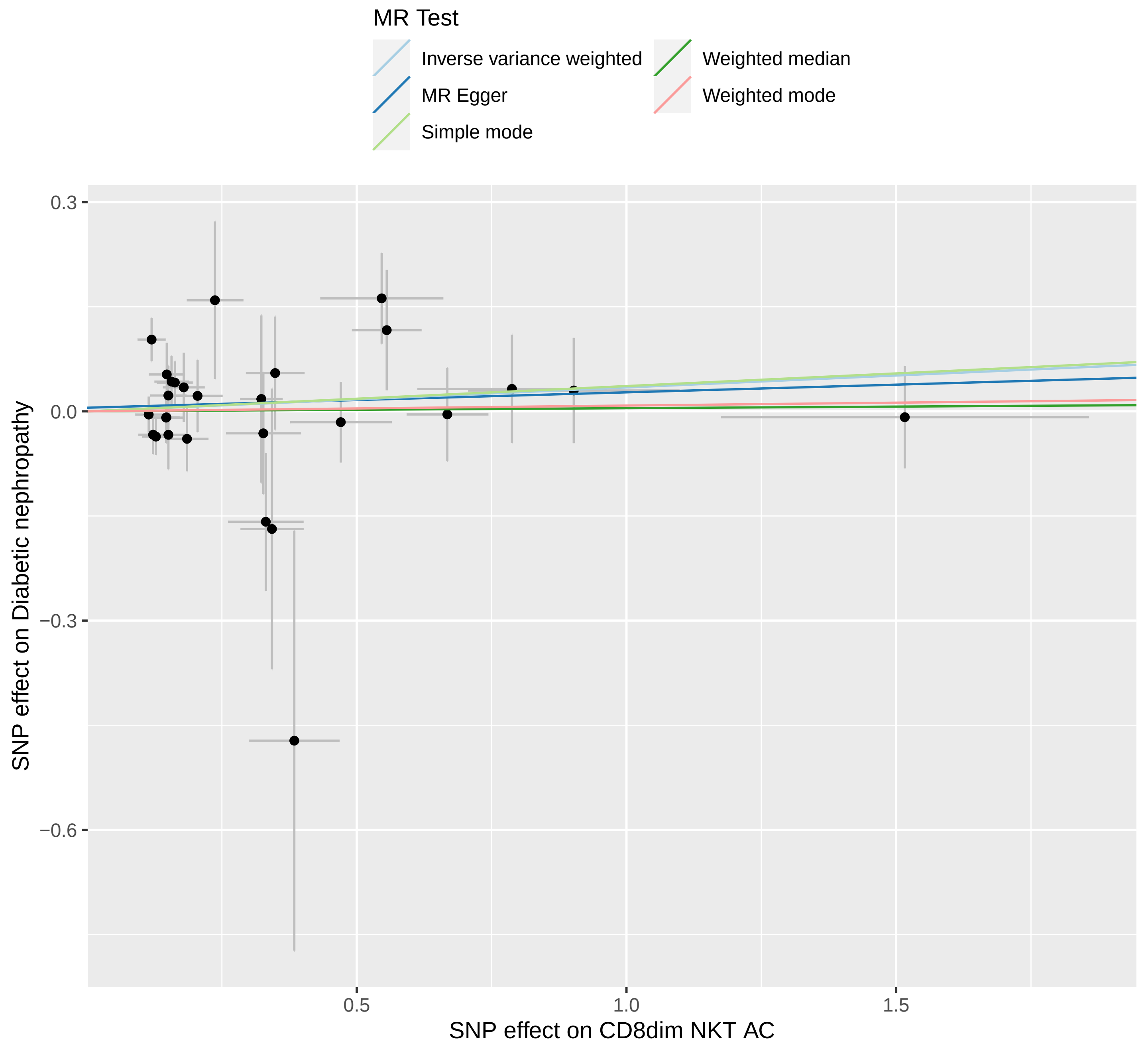
MR Test
Inverse variance weighted
MR Egger
Simple mode
Weighted median
Weighted mode
0.3
0.0
−0.3
−0.6
0.5
1.0
1.5
SNP effect on Diabetic nephropathy
SNP effect on CD8dim NKT AC

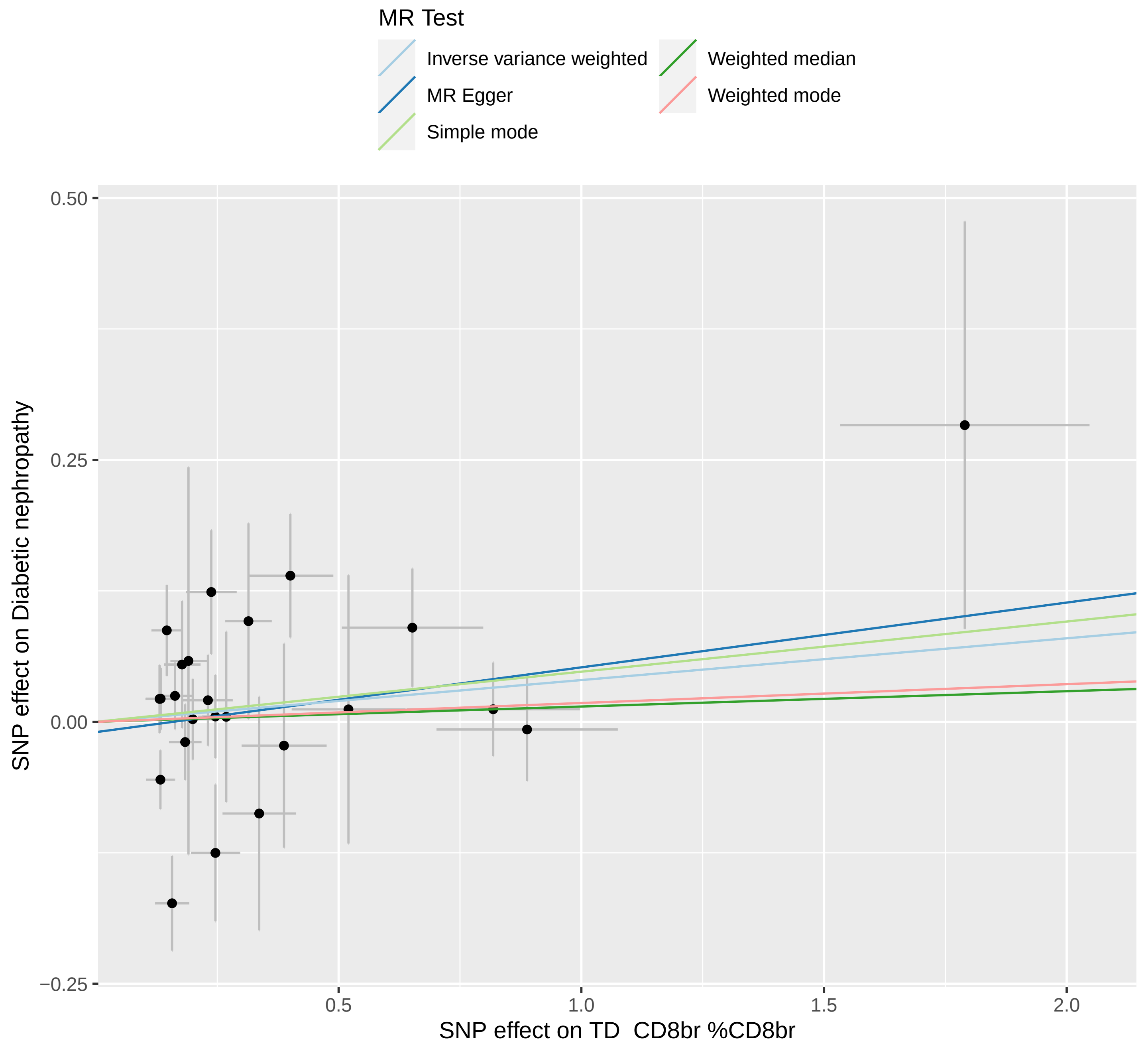

MR Test
Inverse variance weighted
MR Egger
Simple mode
Weighted median
Weighted mode
0.50
0.25
0.00
−0.25
0.5
1.0
1.5
2.0
SNP effect on Diabetic nephropathy
SNP effect on TD CD8br %CD8br

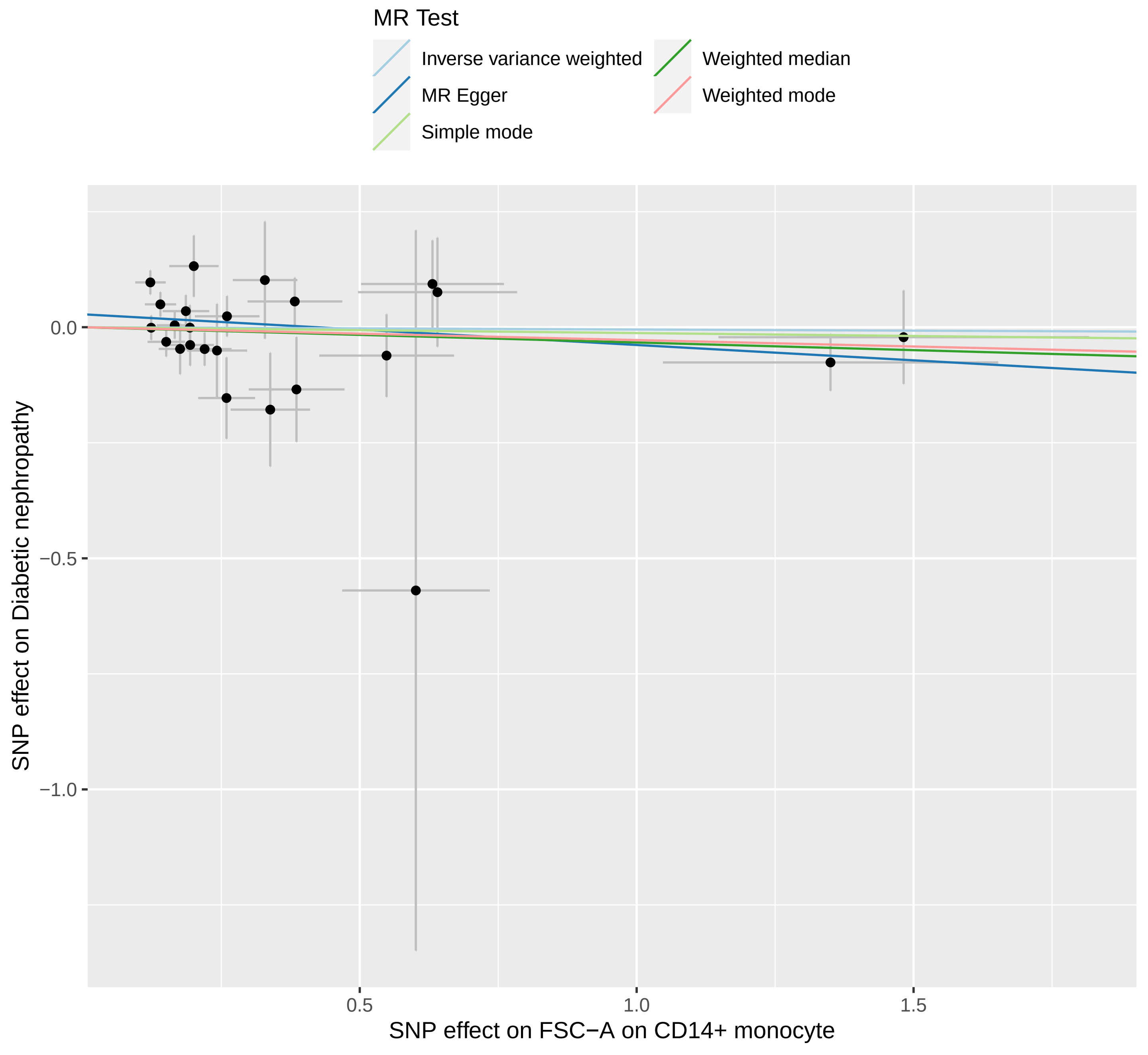

MR Test
Inverse variance weighted
MR Egger
Simple mode
Weighted median
Weighted mode
0.0
−0.5
−1.0
0.5
1.0
1.5
SNP effect on Diabetic nephropathy
SNP effect on FSC−A on CD14+ monocyte

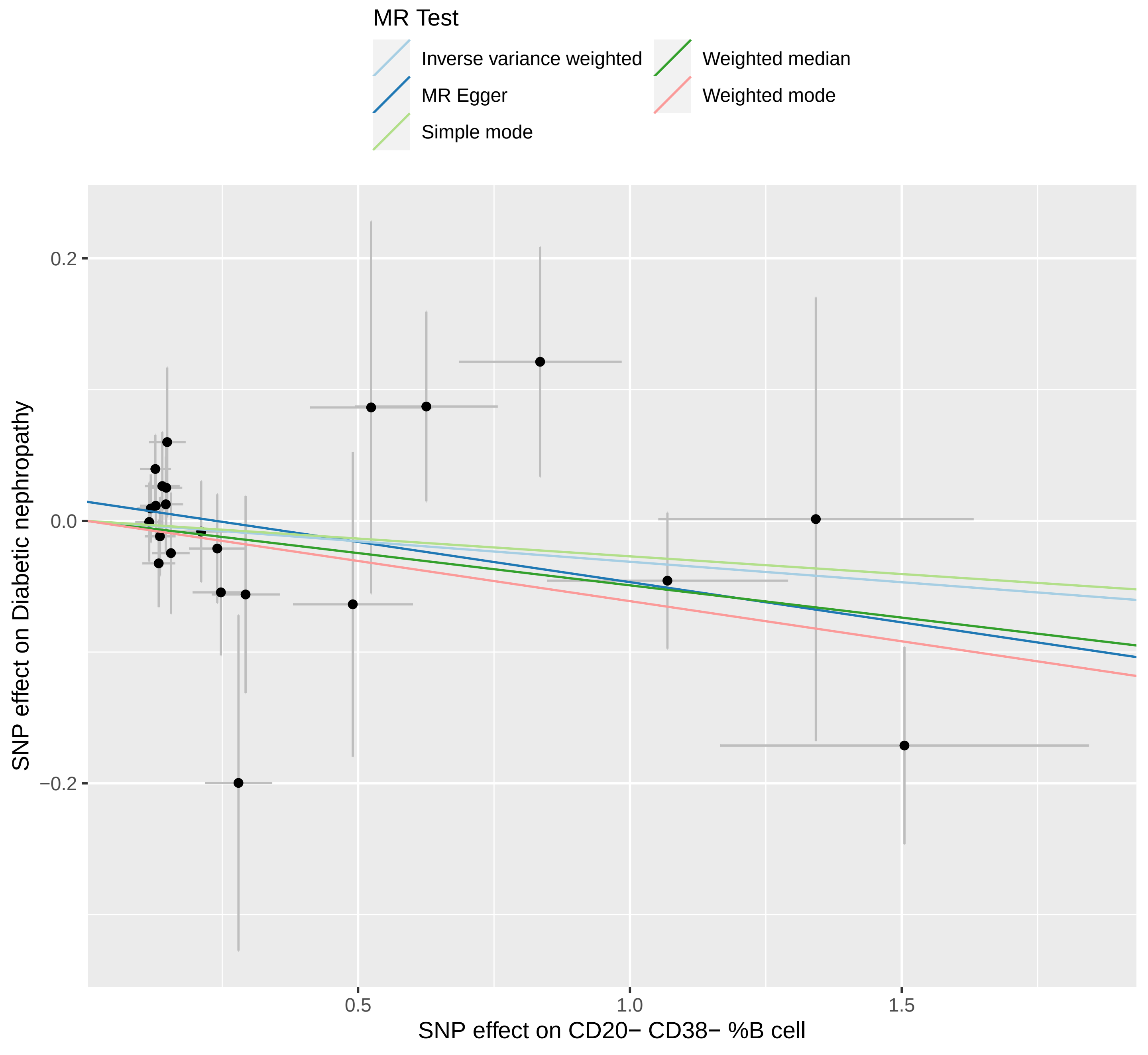
MR Test
Inverse variance weighted
MR Egger
Simple mode
Weighted median
Weighted mode
0.2
0.0
−0.2
SNP effect on Diabetic nephropathy
0.5
1.0
1.5
SNP effect on CD20− CD38− %B cell

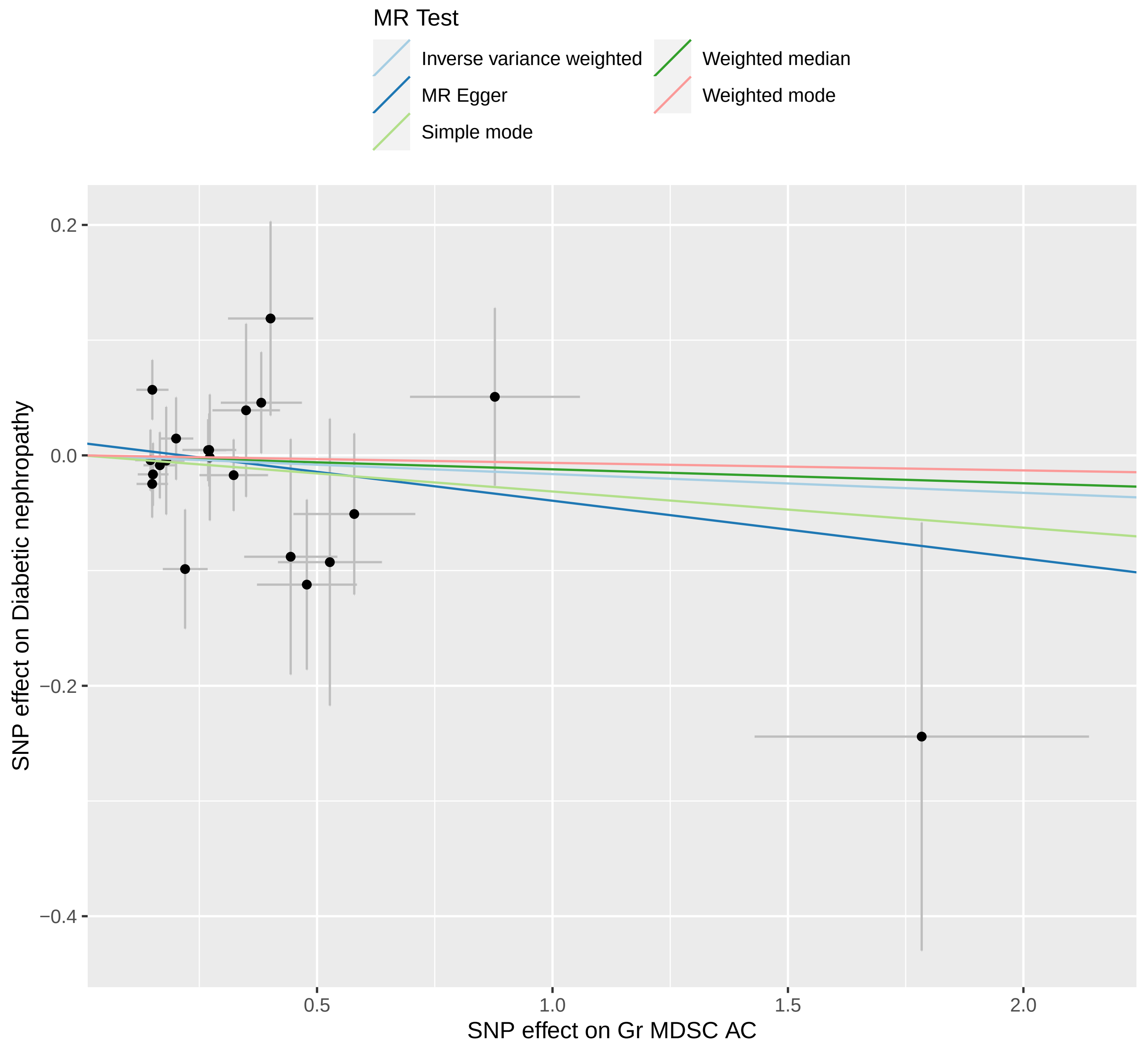

MR Test
Inverse variance weighted
MR Egger
Simple mode
Weighted median
Weighted mode
0.2
0.0
−0.2
−0.4
SNP effect on Diabetic nephropathy
0.5
1.0
1.5
2.0
SNP effect on Gr MDSC AC

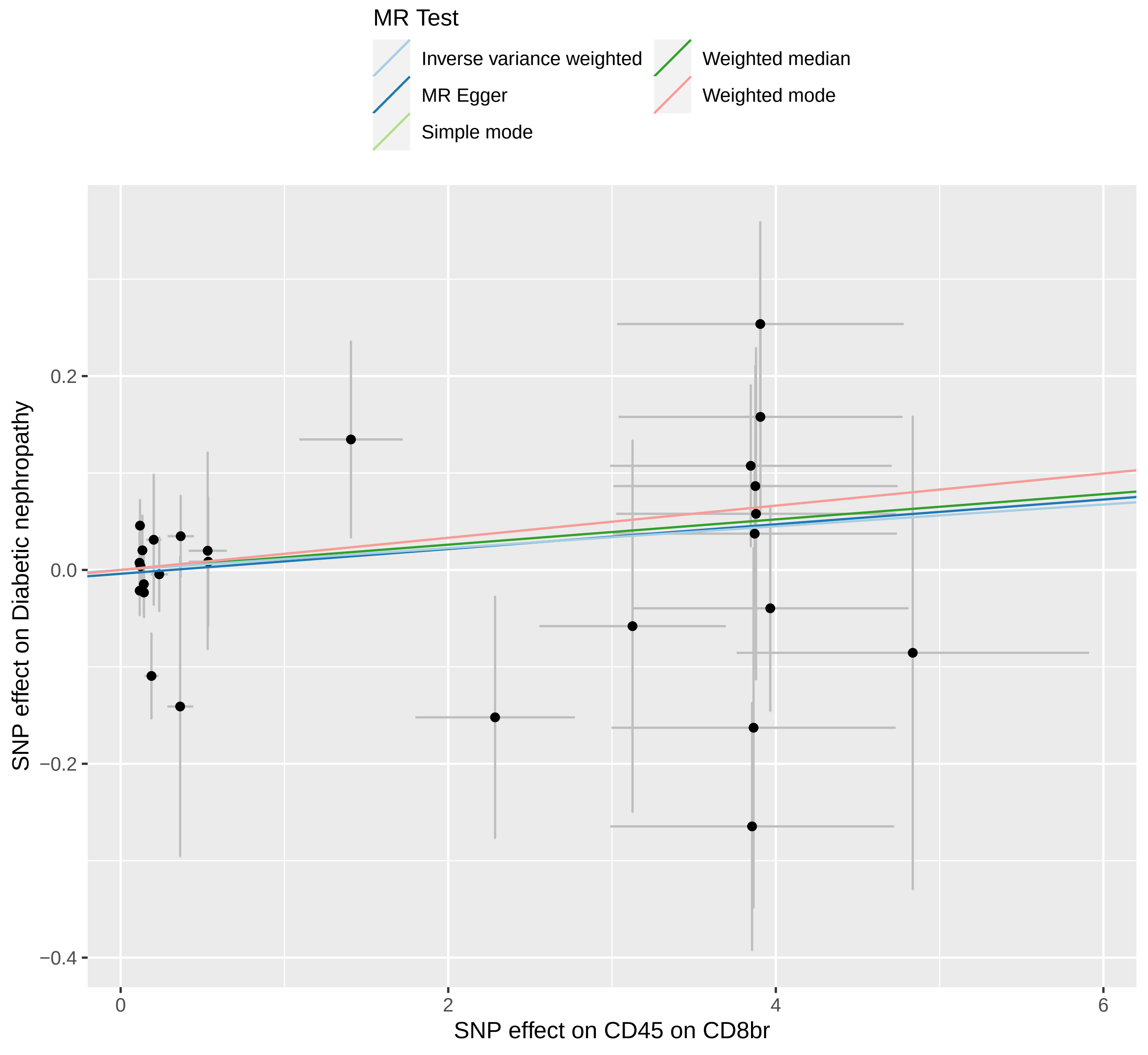

MR Test
Inverse variance weighted
MR Egger
Simple mode
Weighted median
Weighted mode
SNP effect on Diabetic nephropathy
0.2
0.0
−0.2
−0.4
0
2
4
6
SNP effect on CD45 on CD8br

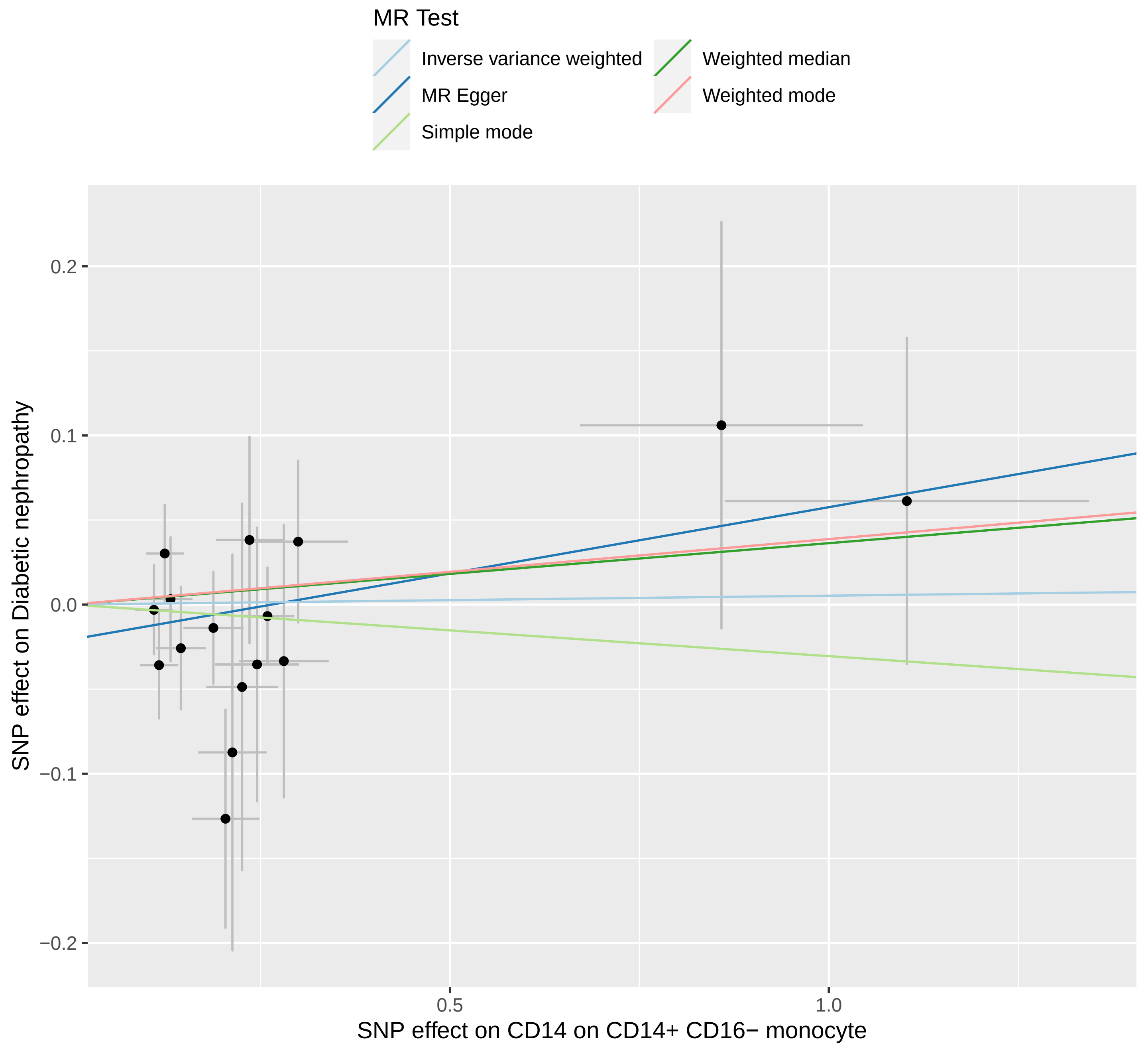
MR Test
Inverse variance weighted
MR Egger
Simple mode
Weighted median
Weighted mode
0.2
0.1
0.0
−0.1
−0.2
SNP effect on Diabetic nephropathy
0.5
1.0
SNP effect on CD14 on CD14+ CD16− monocyte

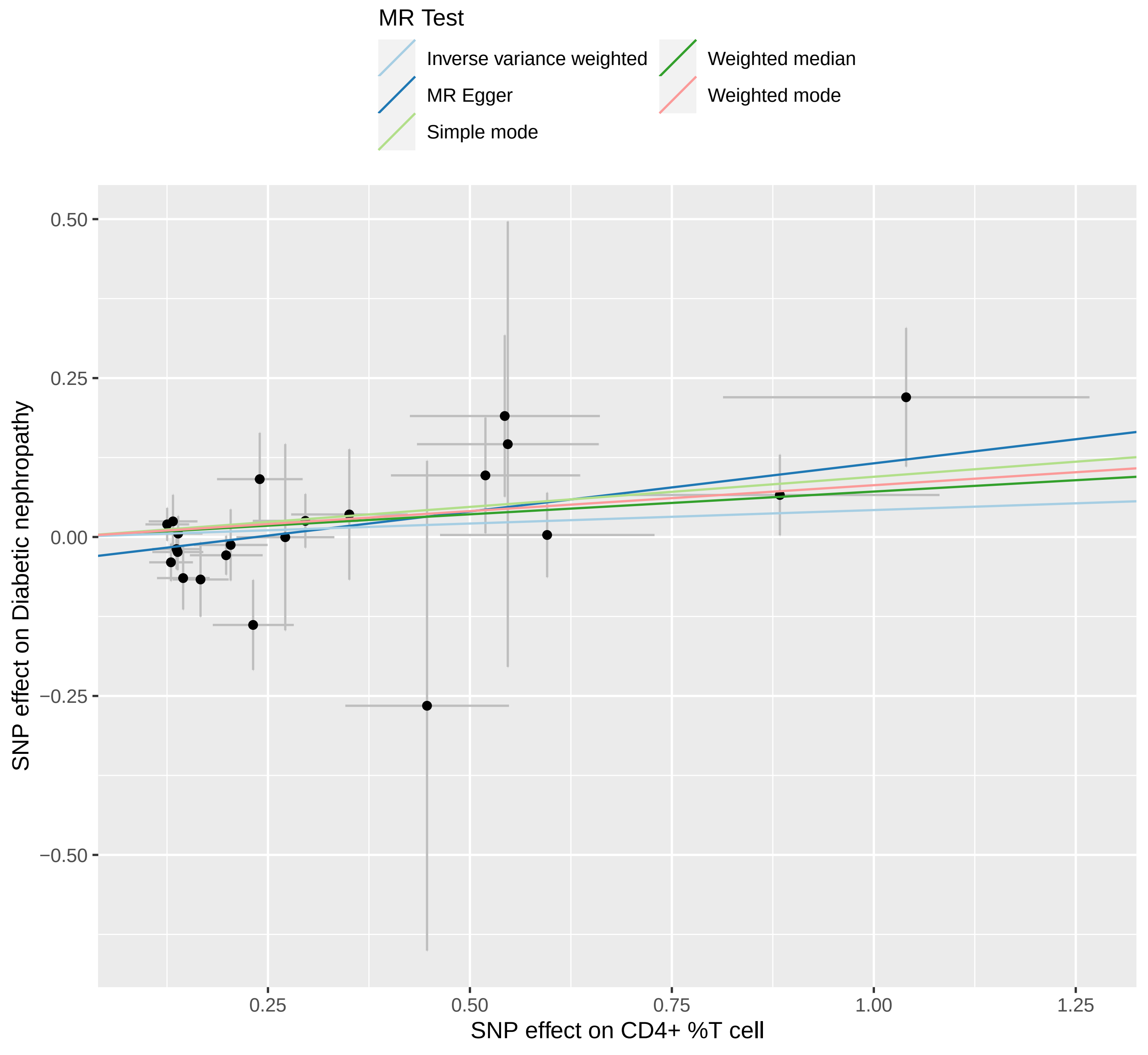

MR Test
Inverse variance weighted
MR Egger
Simple mode
Weighted median
Weighted mode
0.50
0.25
0.00
−0.25
−0.50
SNP effect on Diabetic nephropathy
0.25
0.50
0.75
1.00
1.25
SNP effect on CD4+ %T cell

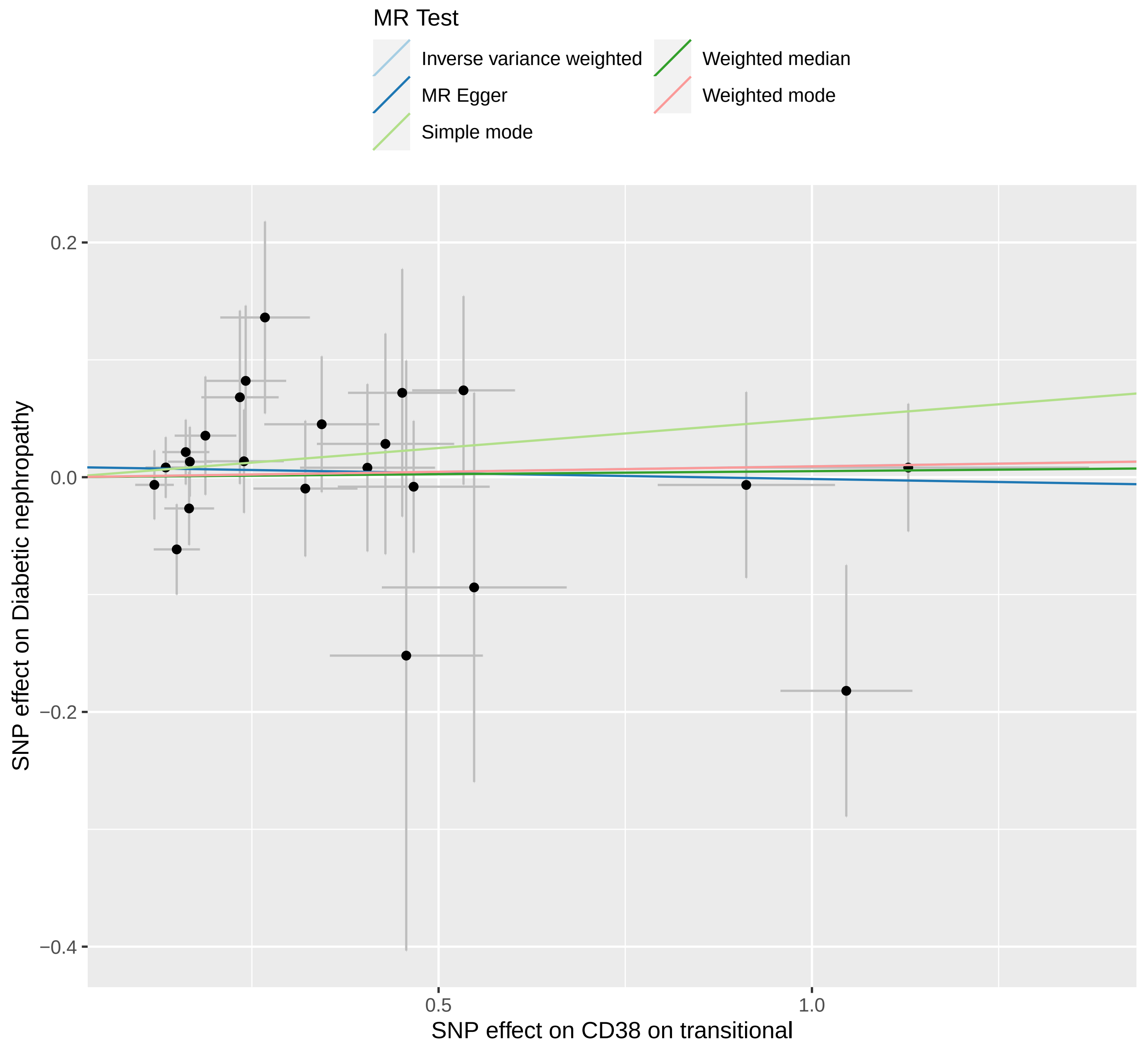
MR Test
Inverse variance weighted
MR Egger
Simple mode
Weighted median
Weighted mode
0.2
0.0
−0.2
−0.4
0.5
1.0
SNP effect on Diabetic nephropathy
SNP effect on CD38 on transitional

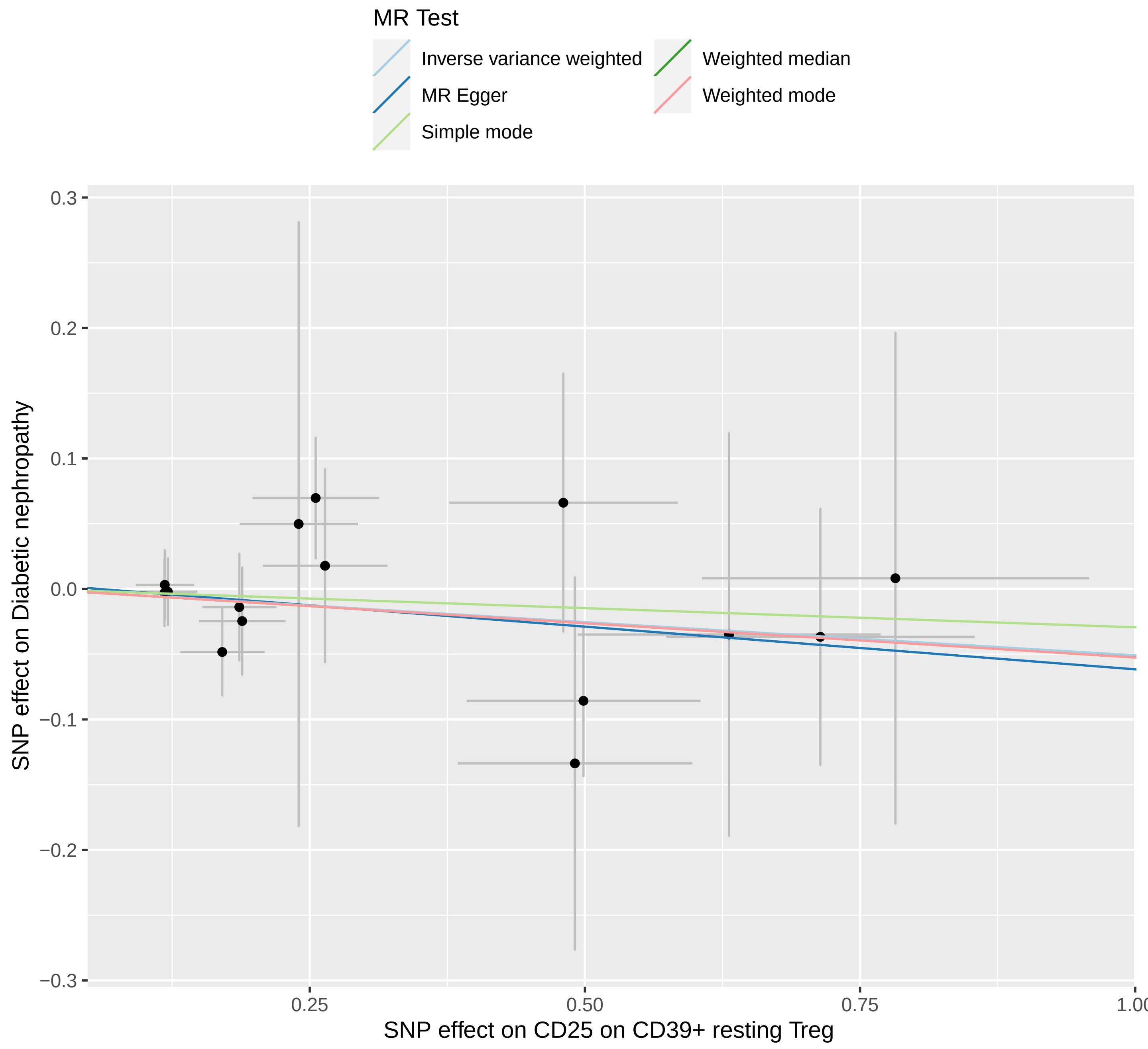

MR Test
Inverse variance weighted
MR Egger
Simple mode
Weighted median
Weighted mode
0.3
0.2
0.1
0.0
−0.1
−0.2
−0.3
0.25
0.50
0.75
1.00
SNP effect on Diabetic nephropathy
SNP effect on CD25 on CD39+ resting Treg

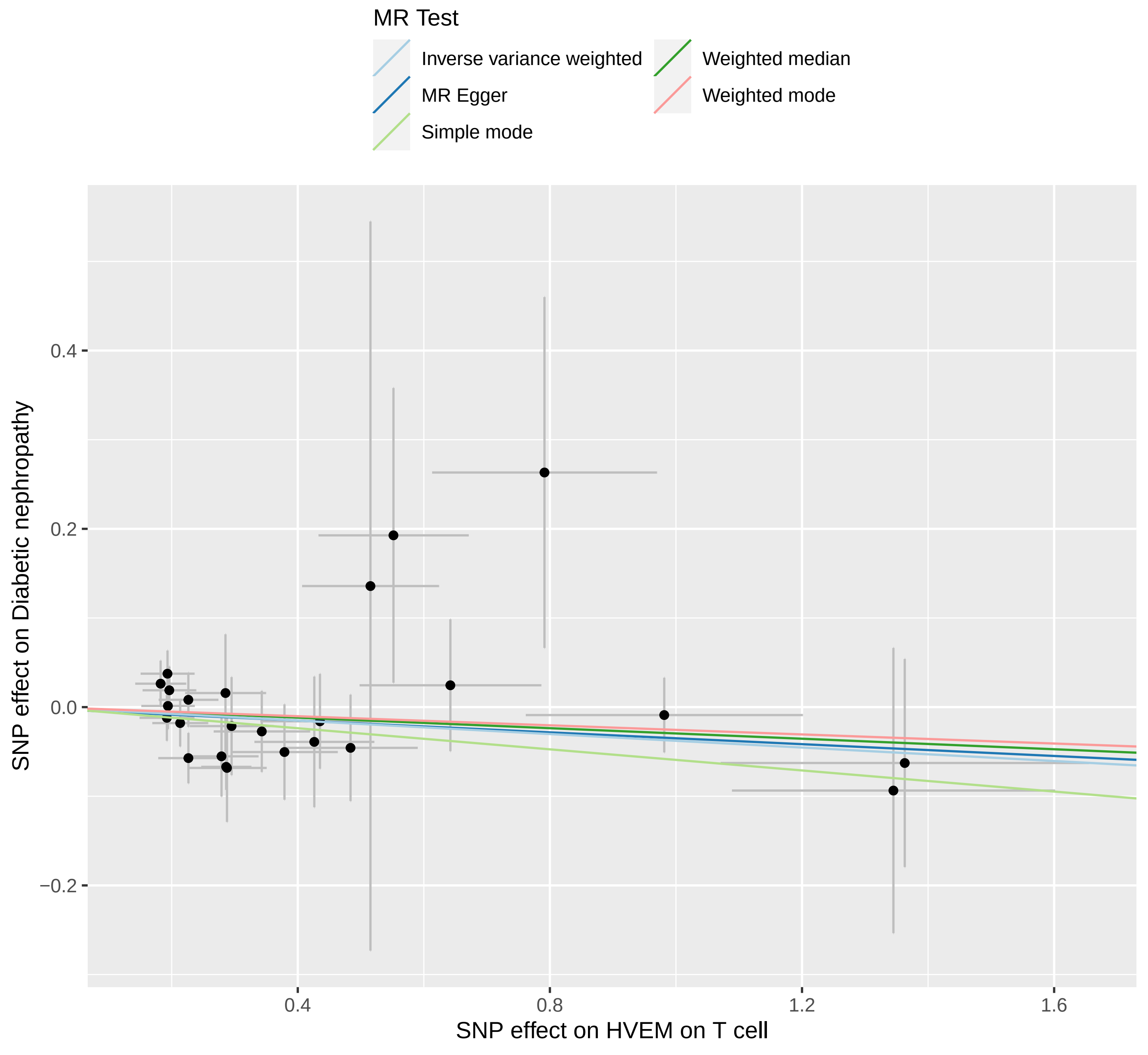
MR Test
Inverse variance weighted
MR Egger
Simple mode
Weighted median
Weighted mode
SNP effect on Diabetic nephropathy
0.4
0.2
0.0
−0.2
0.4
0.8
1.2
1.6
SNP effect on HVEM on T cell

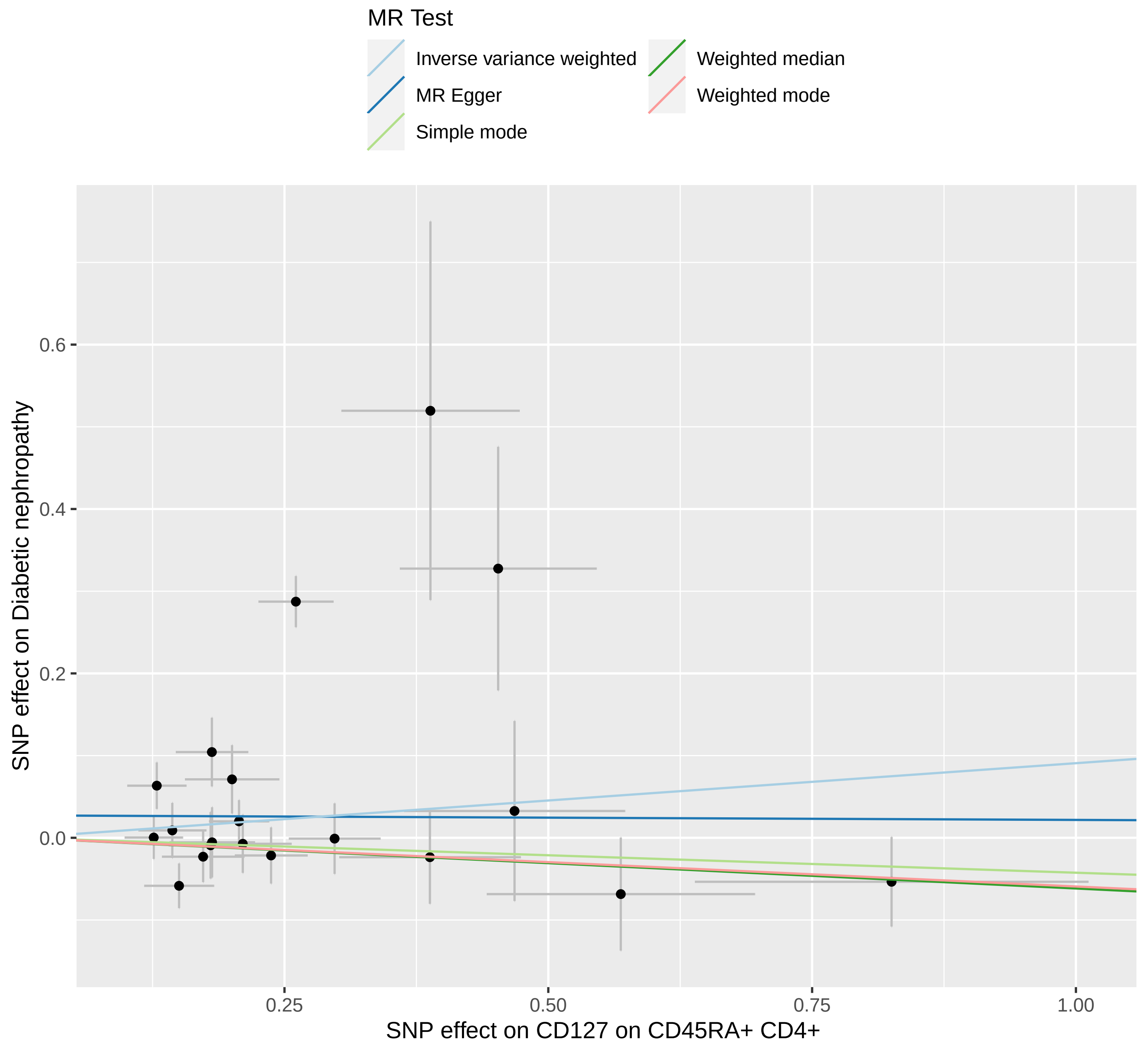
MR Test
Inverse variance weighted
MR Egger
Simple mode
Weighted median
Weighted mode
SNP effect on Diabetic nephropathy
0.6
0.4
0.2
0.0
0.25
0.50
0.75
1.00
SNP effect on CD127 on CD45RA+ CD4+

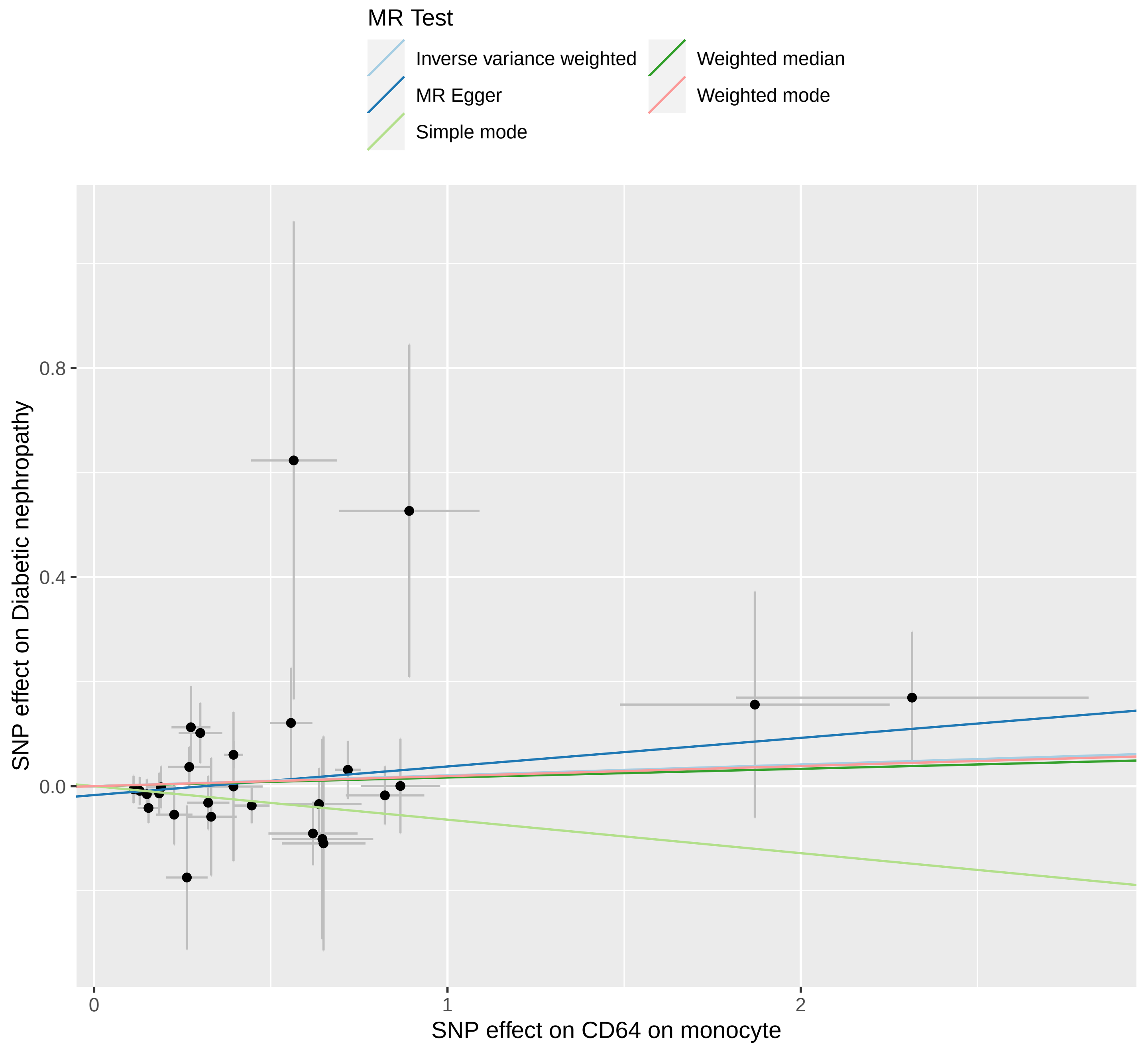
MR Test
Inverse variance weighted
MR Egger
Simple mode
Weighted median
Weighted mode
SNP effect on Diabetic nephropathy
0.8
0.4
0.0
0
1
2
SNP effect on CD64 on monocyte

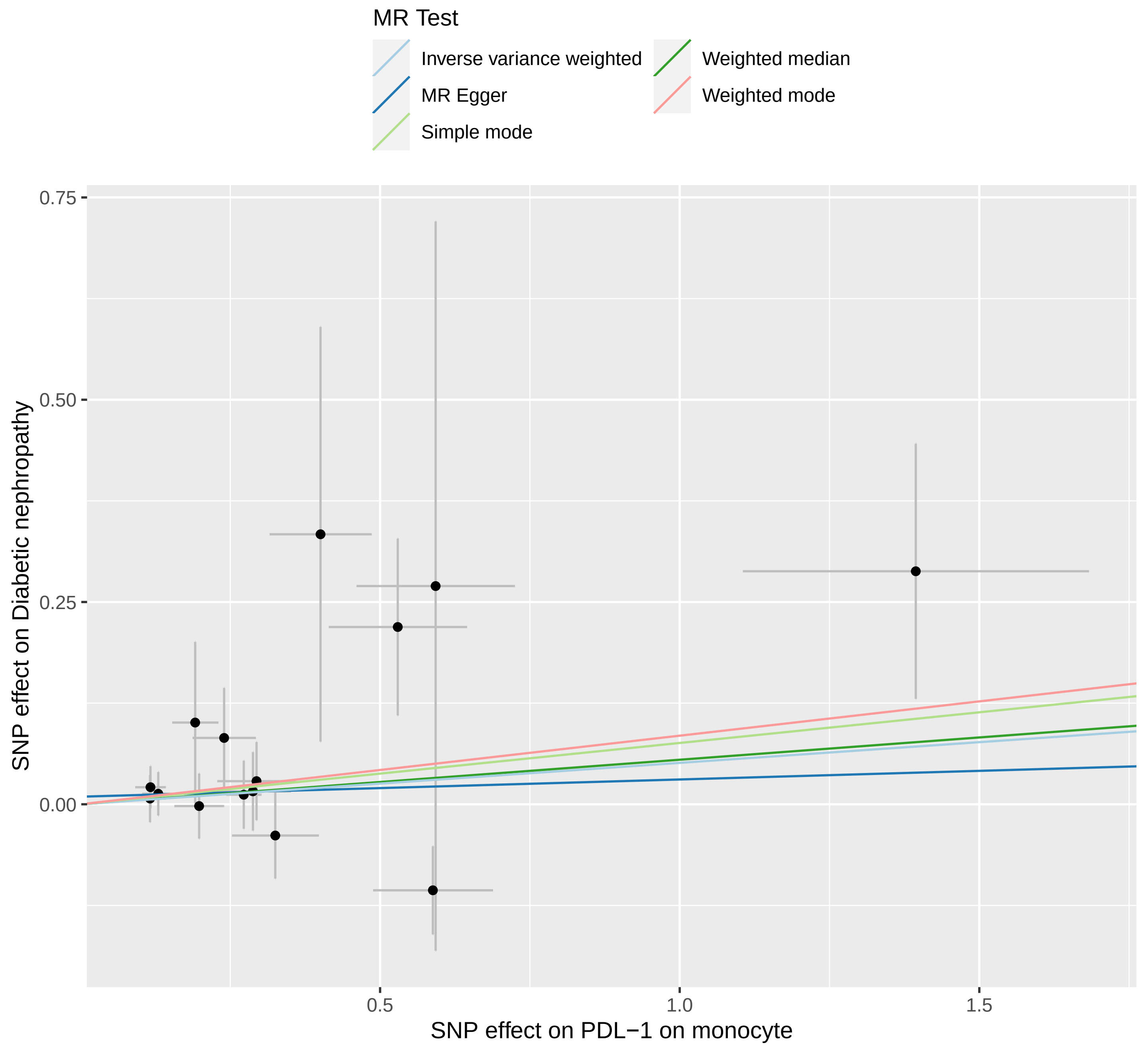
MR Test
Inverse variance weighted
MR Egger
Simple mode
Weighted median
Weighted mode
0.75
0.50
0.25
0.00
SNP effect on Diabetic nephropathy
0.5
1.0
1.5
SNP effect on PDL−1 on monocyte

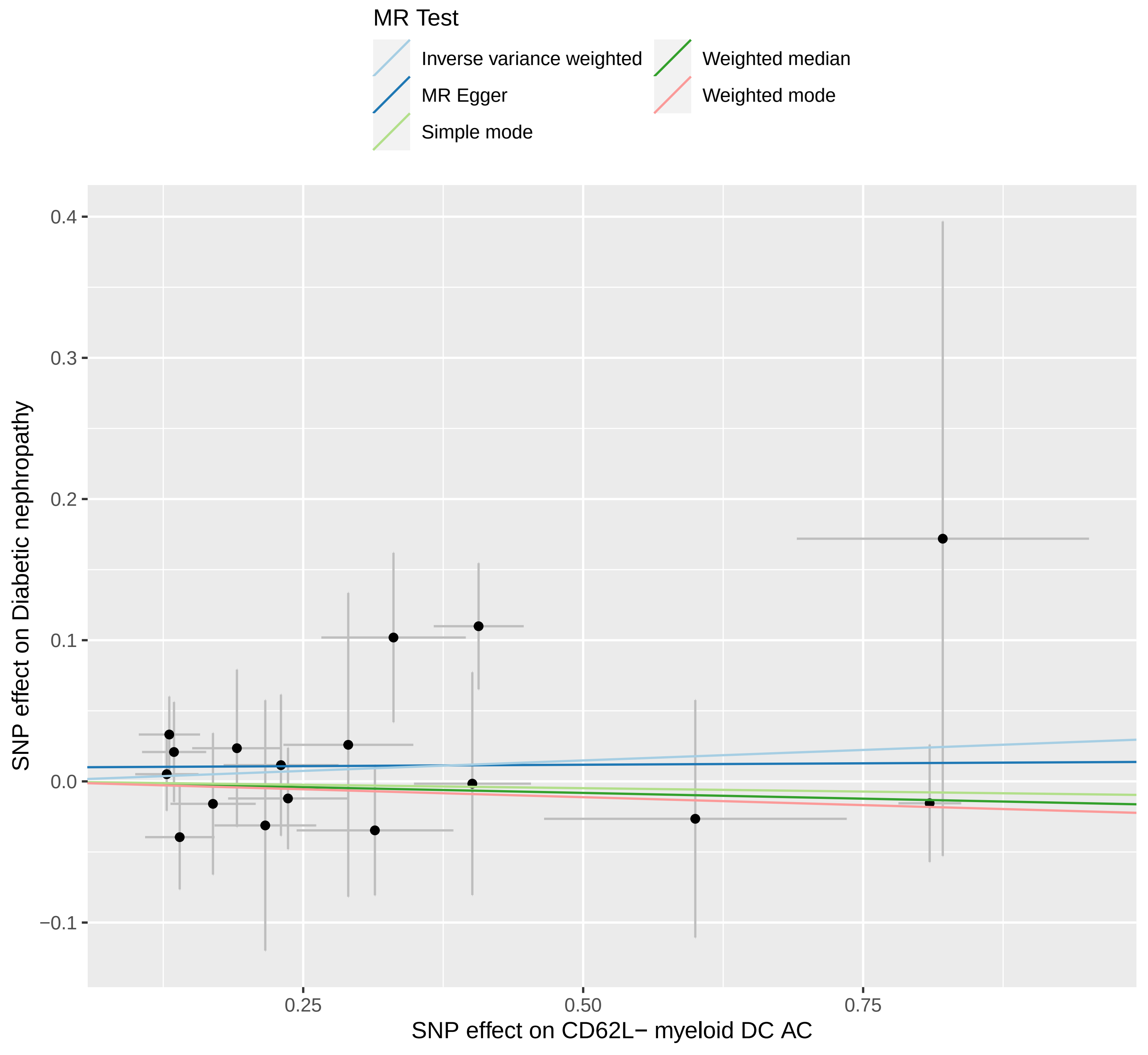
MR Test
Inverse variance weighted
MR Egger
Simple mode
Weighted median
Weighted mode
SNP effect on Diabetic nephropathy
0.4
0.3
0.2
0.1
0.0
−0.1
0.25
0.50
0.75
SNP effect on CD62L− myeloid DC AC

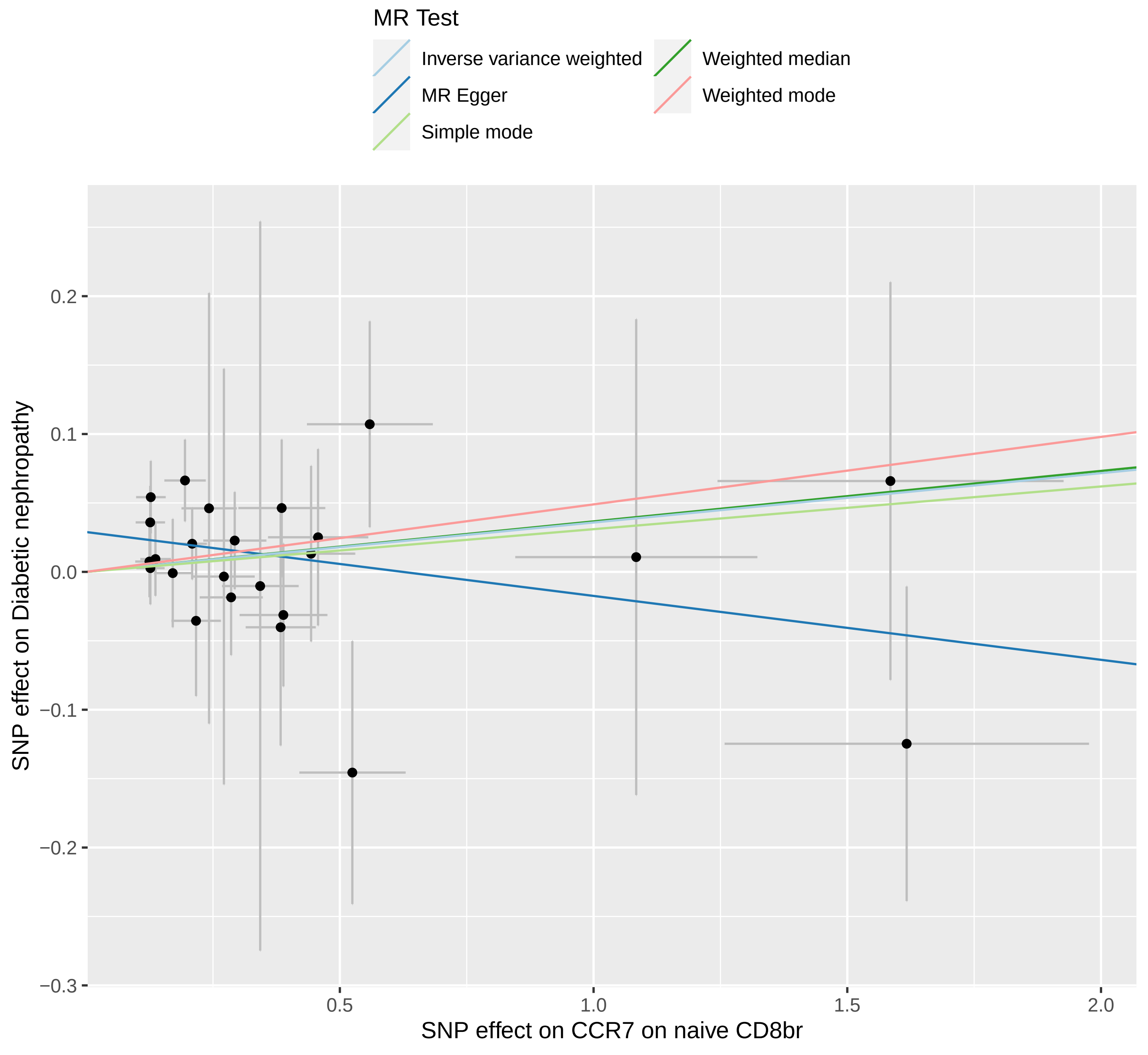

MR Test
Inverse variance weighted
MR Egger
Simple mode
Weighted median
Weighted mode
0.2
0.1
0.0
−0.1
−0.2
−0.3
SNP effect on Diabetic nephropathy
0.5
1.0
1.5
2.0
SNP effect on CCR7 on naive CD8br

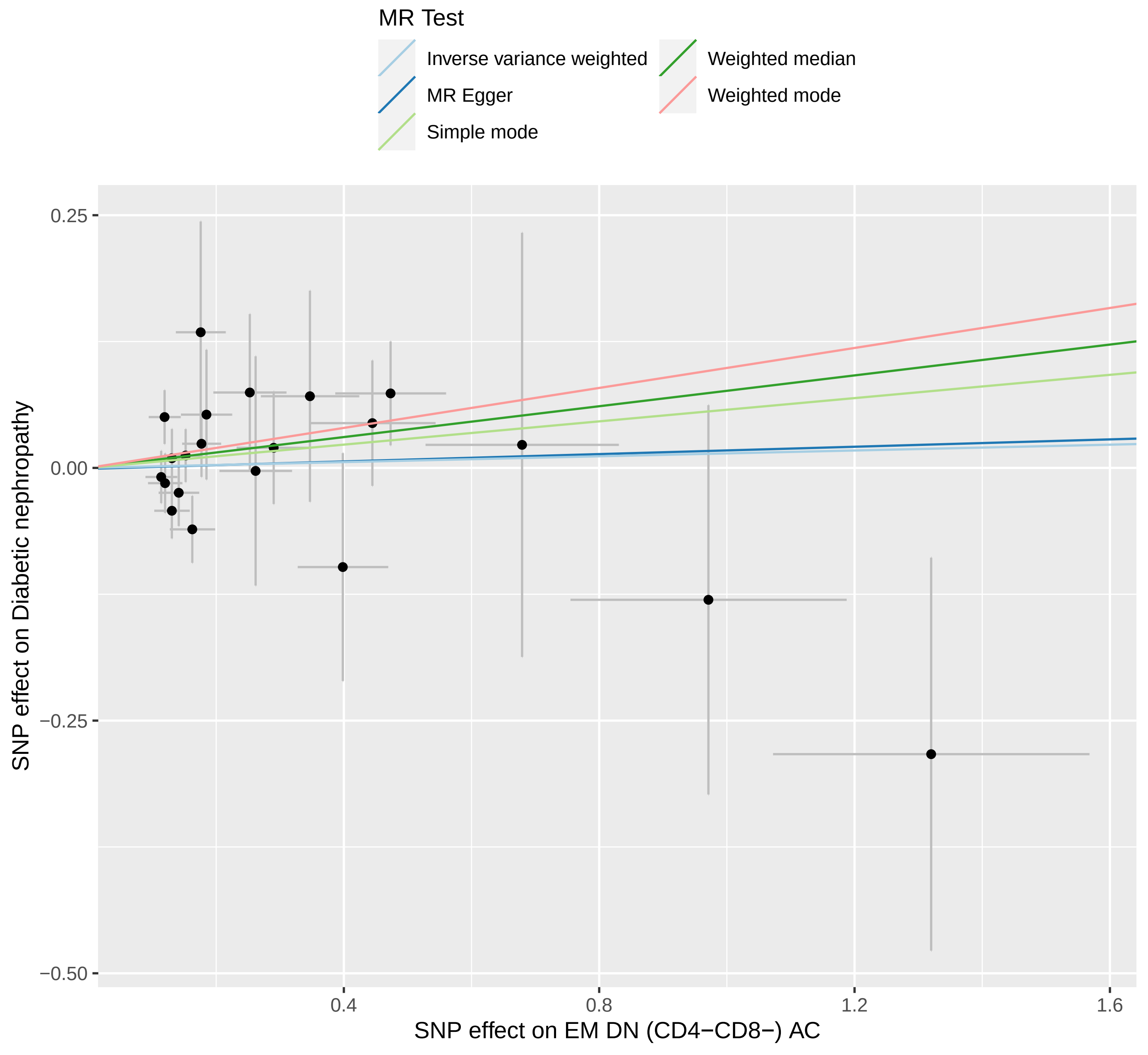
MR Test
Inverse variance weighted
MR Egger
Simple mode
Weighted median
Weighted mode
SNP effect on Diabetic nephropathy
0.25
0.00
−0.25
−0.50
0.4
0.8
1.2
1.6
SNP effect on EM DN (CD4−CD8−) AC

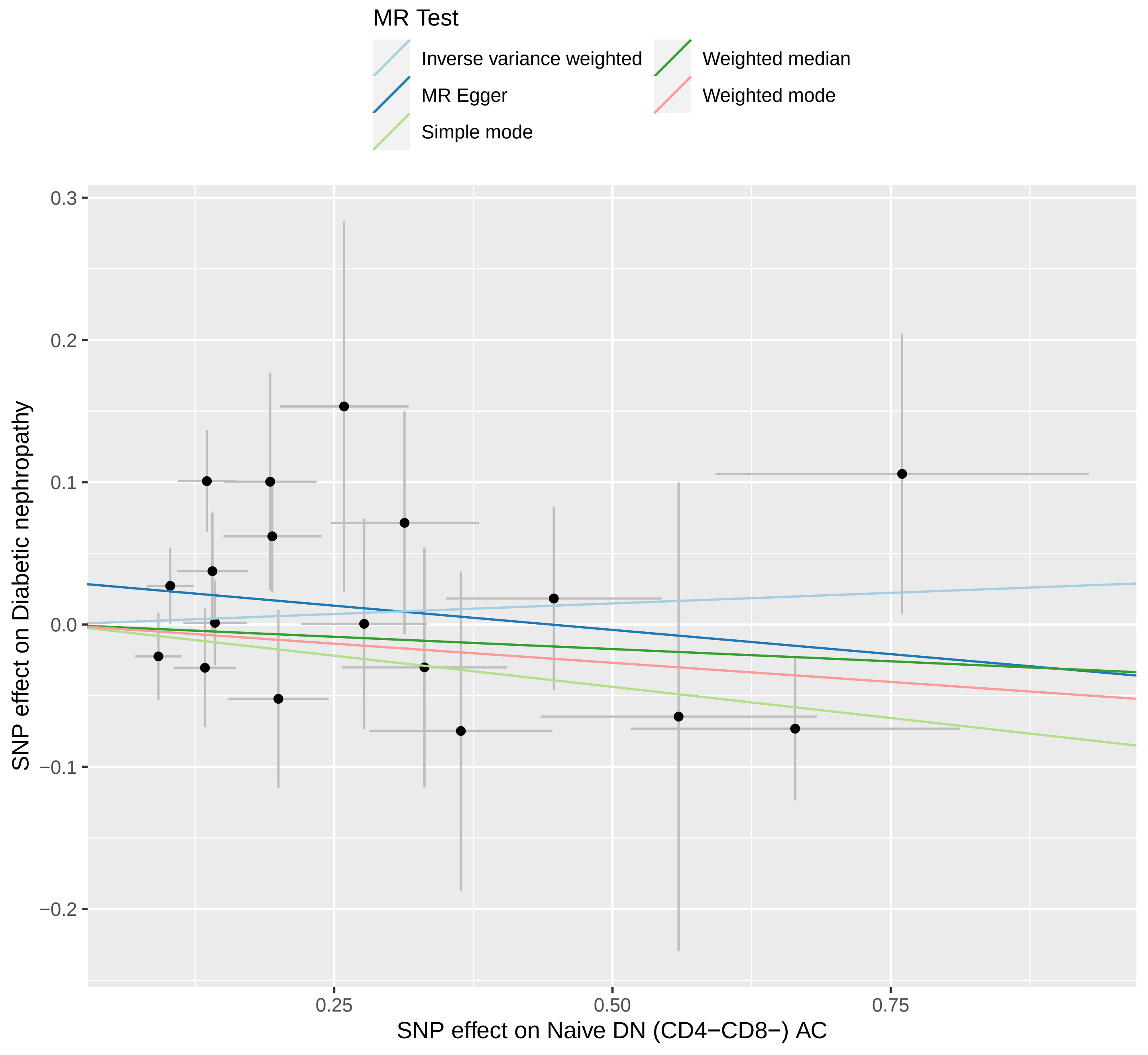

MR Test
Inverse variance weighted
MR Egger
Simple mode
Weighted median
Weighted mode
SNP effect on Diabetic nephropathy
0.3
0.2
0.1
0.0
−0.1
−0.2
0.25
0.50
0.75
SNP effect on Naive DN (CD4−CD8−) AC

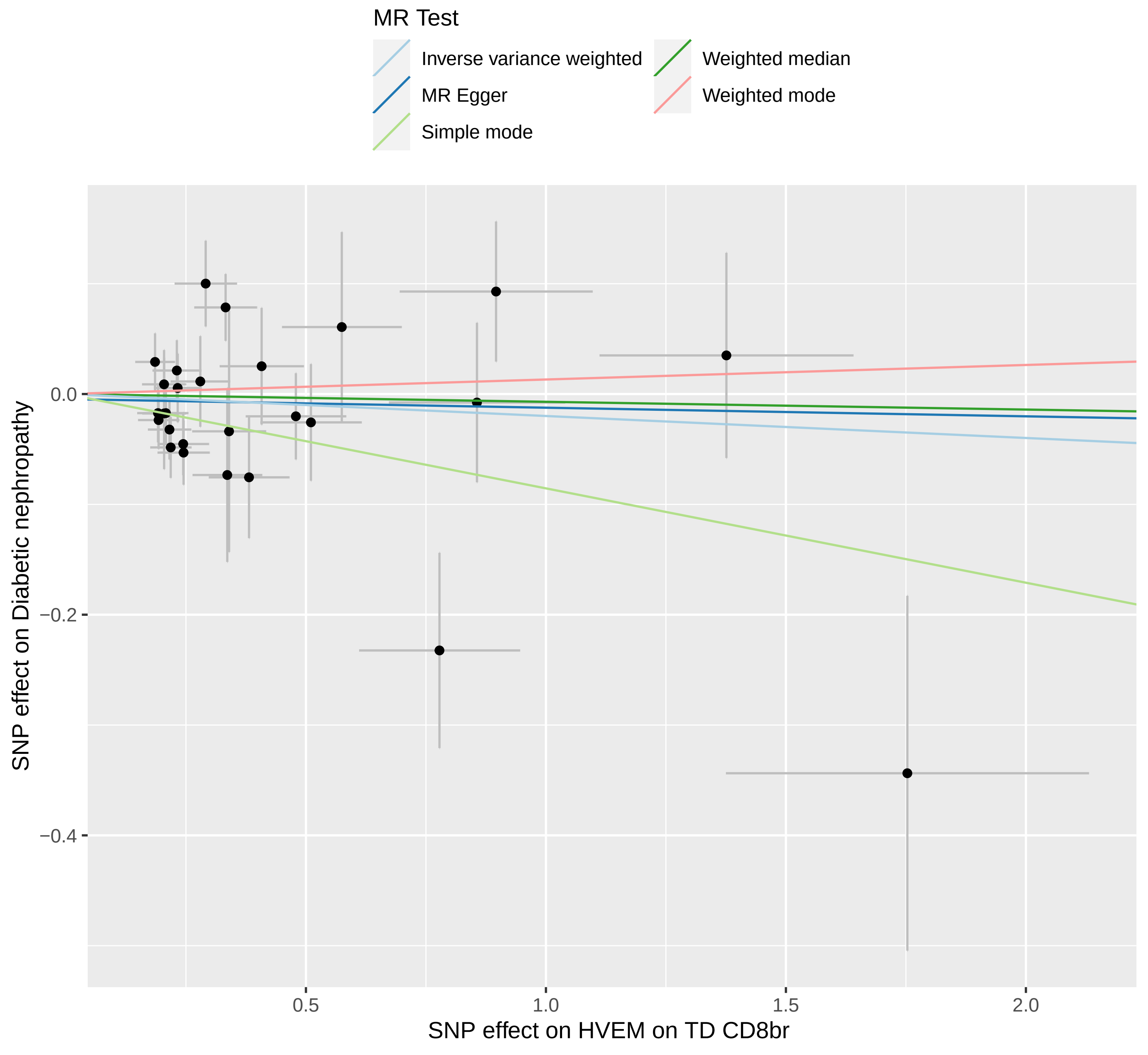

MR Test
Inverse variance weighted
MR Egger
Simple mode
Weighted median
Weighted mode
SNP effect on Diabetic nephropathy
0.0
−0.2
−0.4
0.5
1.0
1.5
2.0
SNP effect on HVEM on TD CD8br

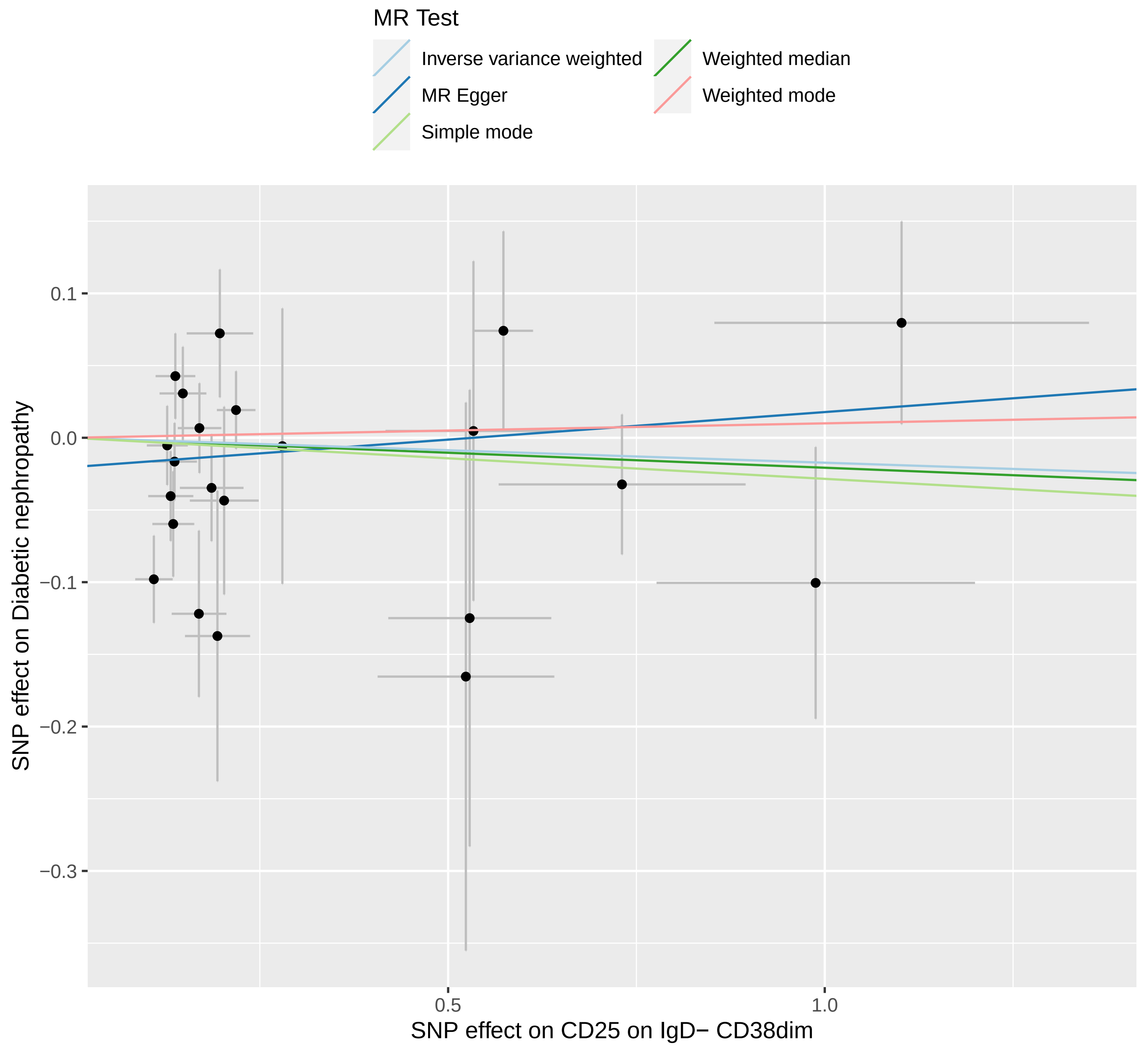
MR Test
Inverse variance weighted
MR Egger
Simple mode
Weighted median
Weighted mode
0.1
0.0
−0.1
−0.2
−0.3
0.5
1.0
SNP effect on Diabetic nephropathy
SNP effect on CD25 on IgD− CD38dim

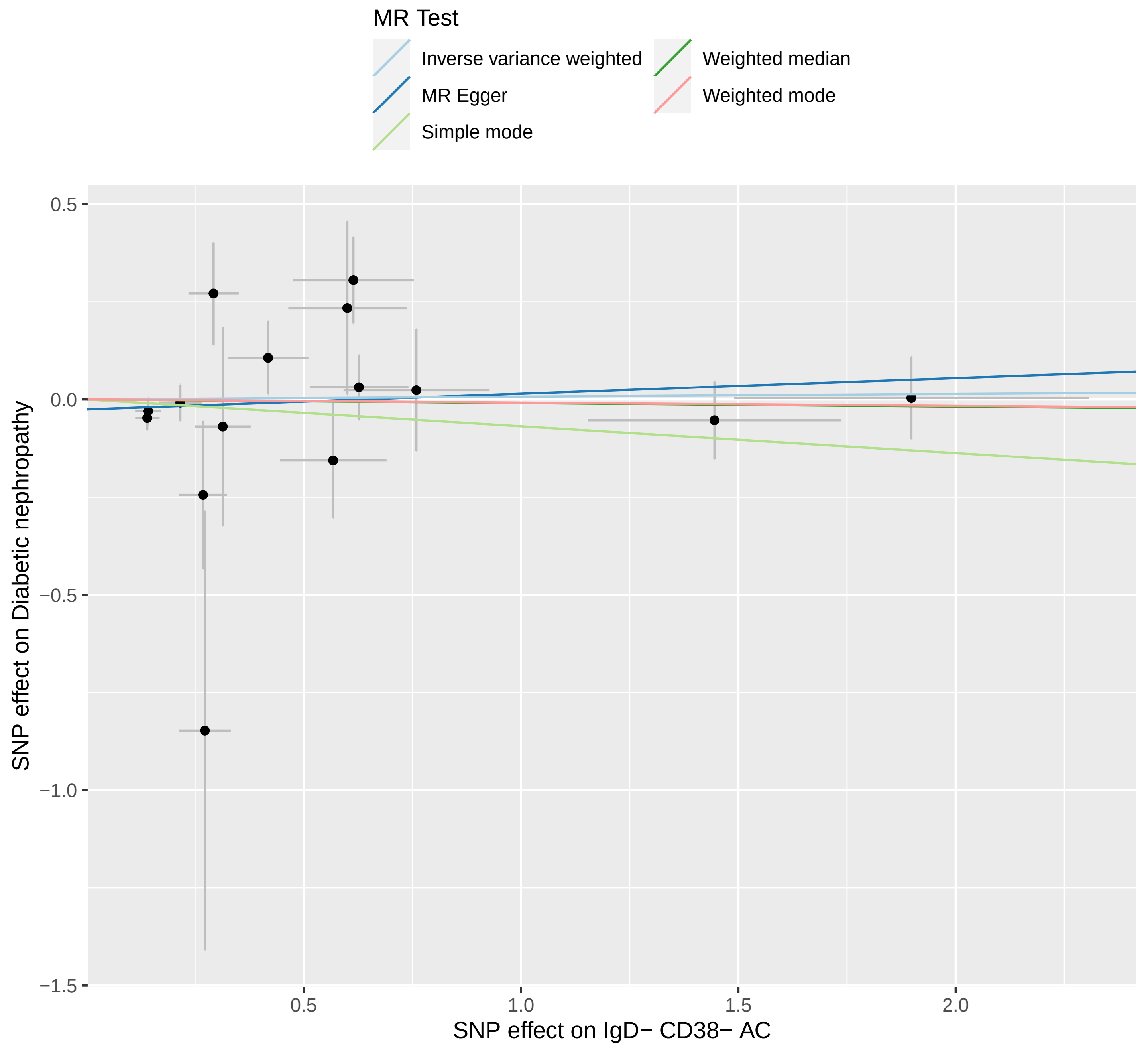
MR Test
Inverse variance weighted
MR Egger
Simple mode
Weighted median
Weighted mode
SNP effect on Diabetic nephropathy
SNP effect on IgD− CD38− AC
0.5
0.0
−0.5
−1.0
−1.5
0.5
1.0
1.5
2.0

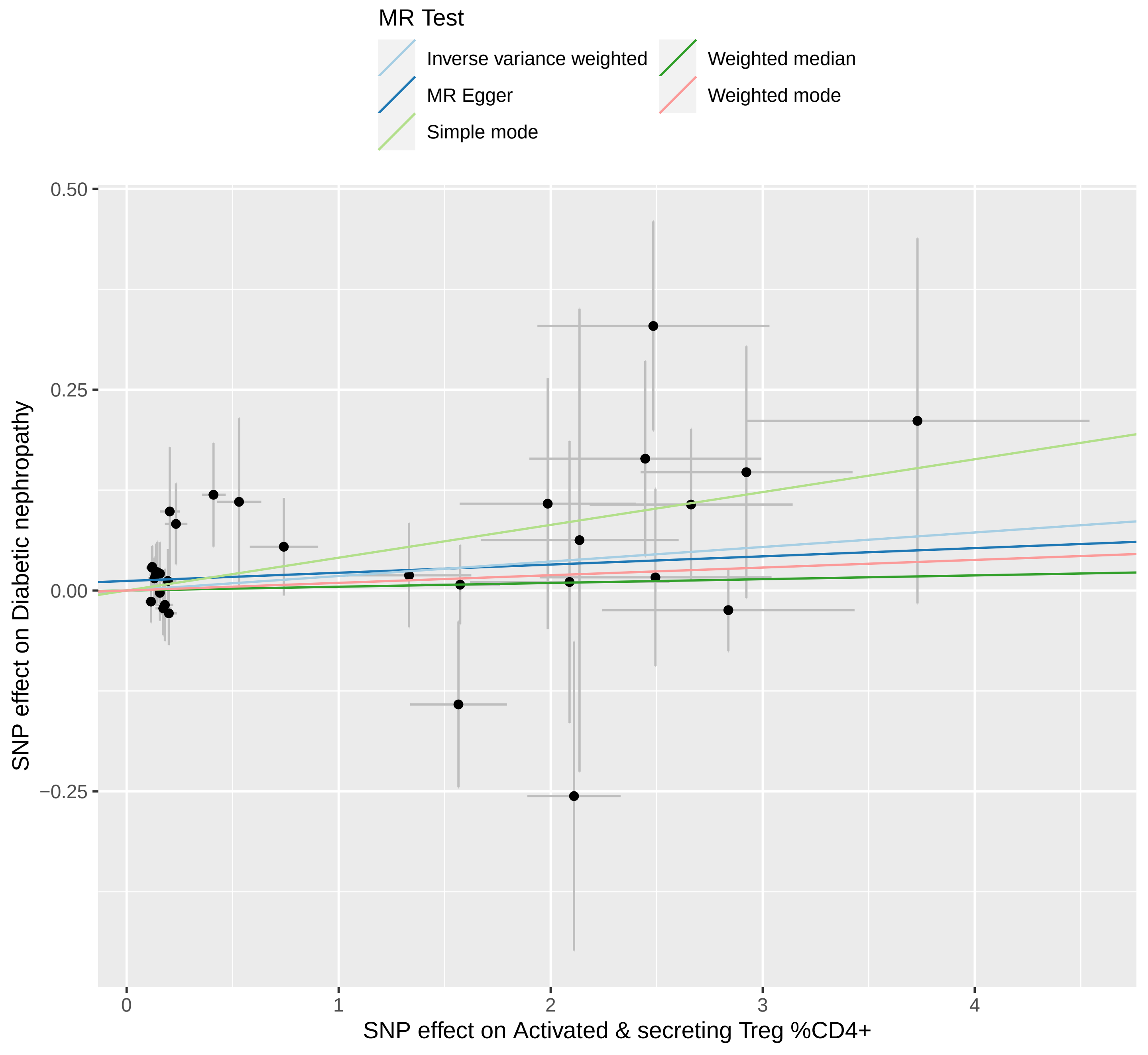
MR Test
Inverse variance weighted
MR Egger
Simple mode
Weighted median
Weighted mode
0.50
0.25
0.00
−0.25
0
1
2
3
4
SNP effect on Diabetic nephropathy
SNP effect on Activated & secreting Treg %CD4+

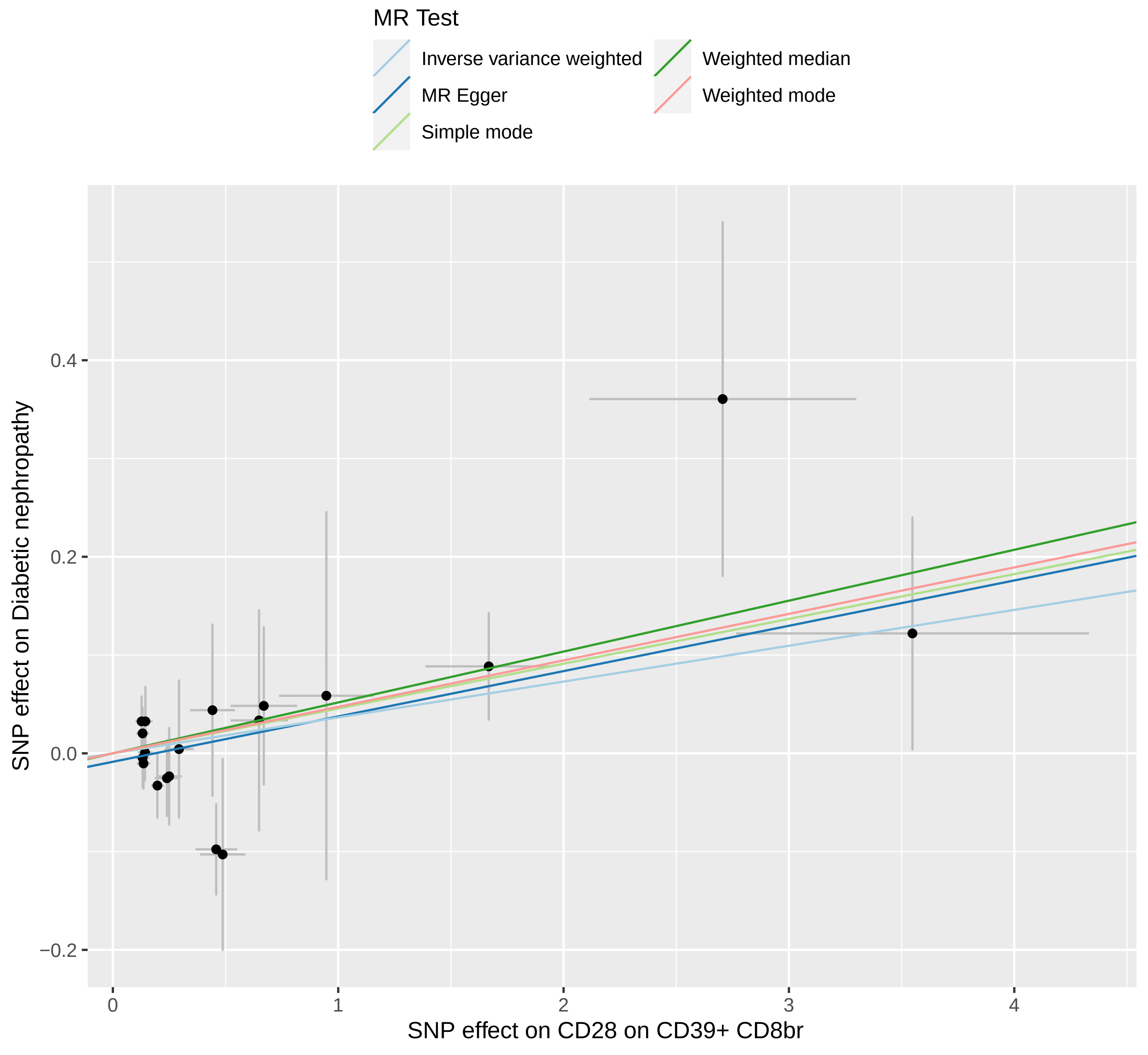

MR Test
Inverse variance weighted
MR Egger
Simple mode
Weighted median
Weighted mode
SNP effect on Diabetic nephropathy
0.4
0.2
0.0
−0.2
0
1
2
3
4
SNP effect on CD28 on CD39+ CD8br

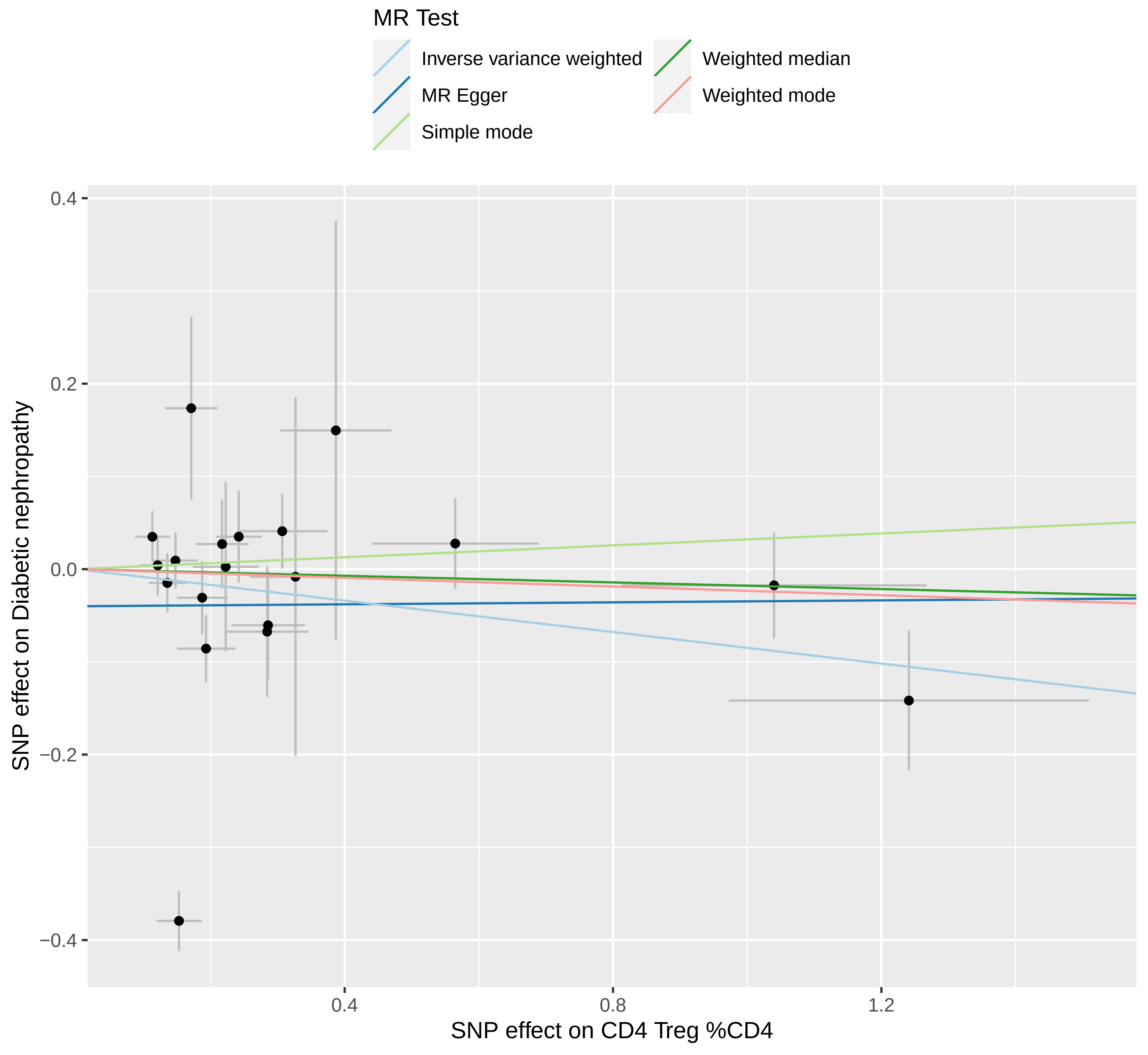
MR Test
Inverse variance weighted
MR Egger
Simple mode
Weighted median
Weighted mode
SNP effect on Diabetic nephropathy
0.4
0.2
0.0
−0.2
−0.4
0.4
0.8
1.2
SNP effect on CD4 Treg %CD4

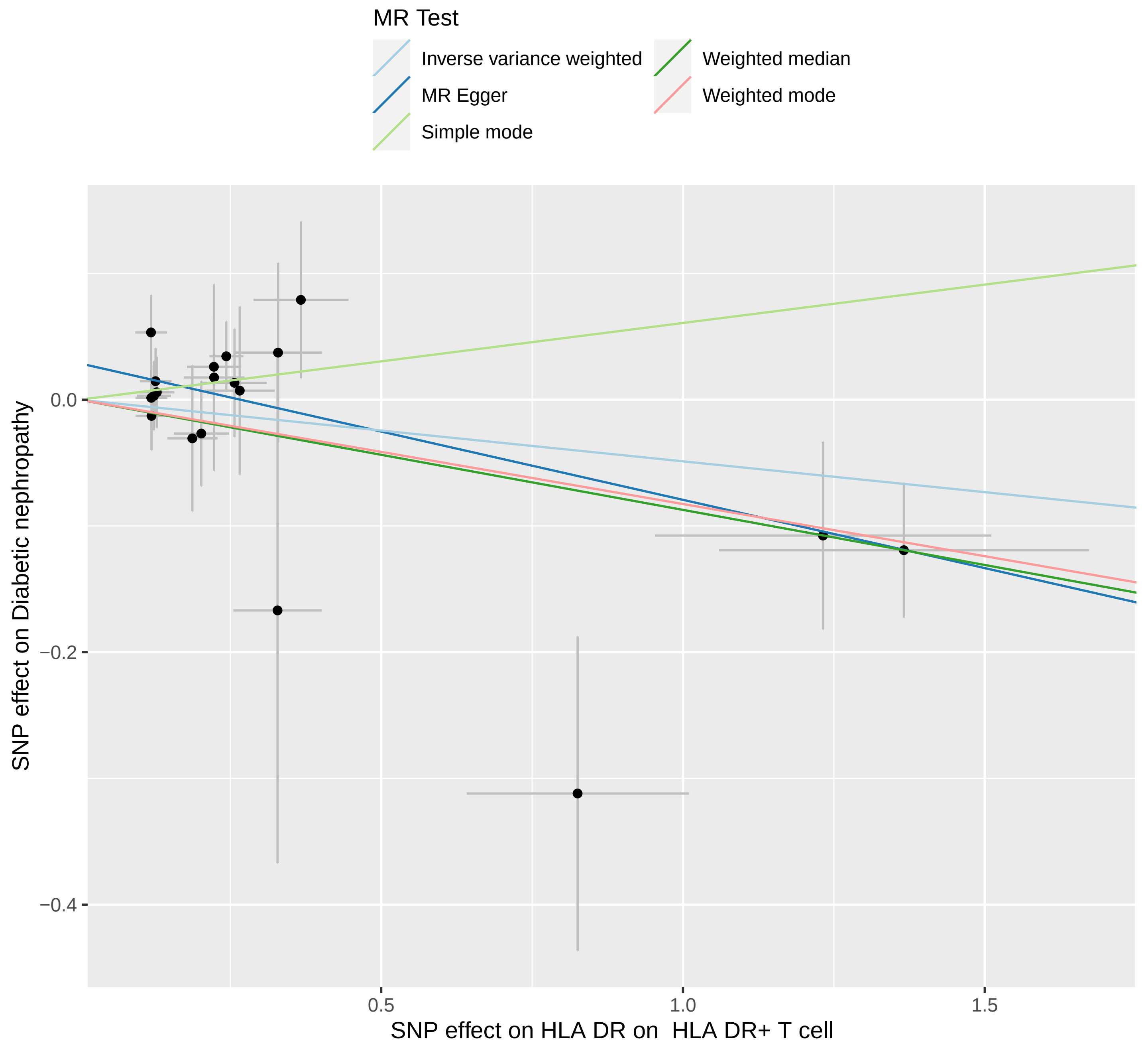
MR Test
Inverse variance weighted
MR Egger
Simple mode
Weighted median
Weighted mode
0.0
−0.2
−0.4
0.5
1.0
1.5
SNP effect on Diabetic nephropathy
SNP effect on HLA DR on HLA DR+ T cell

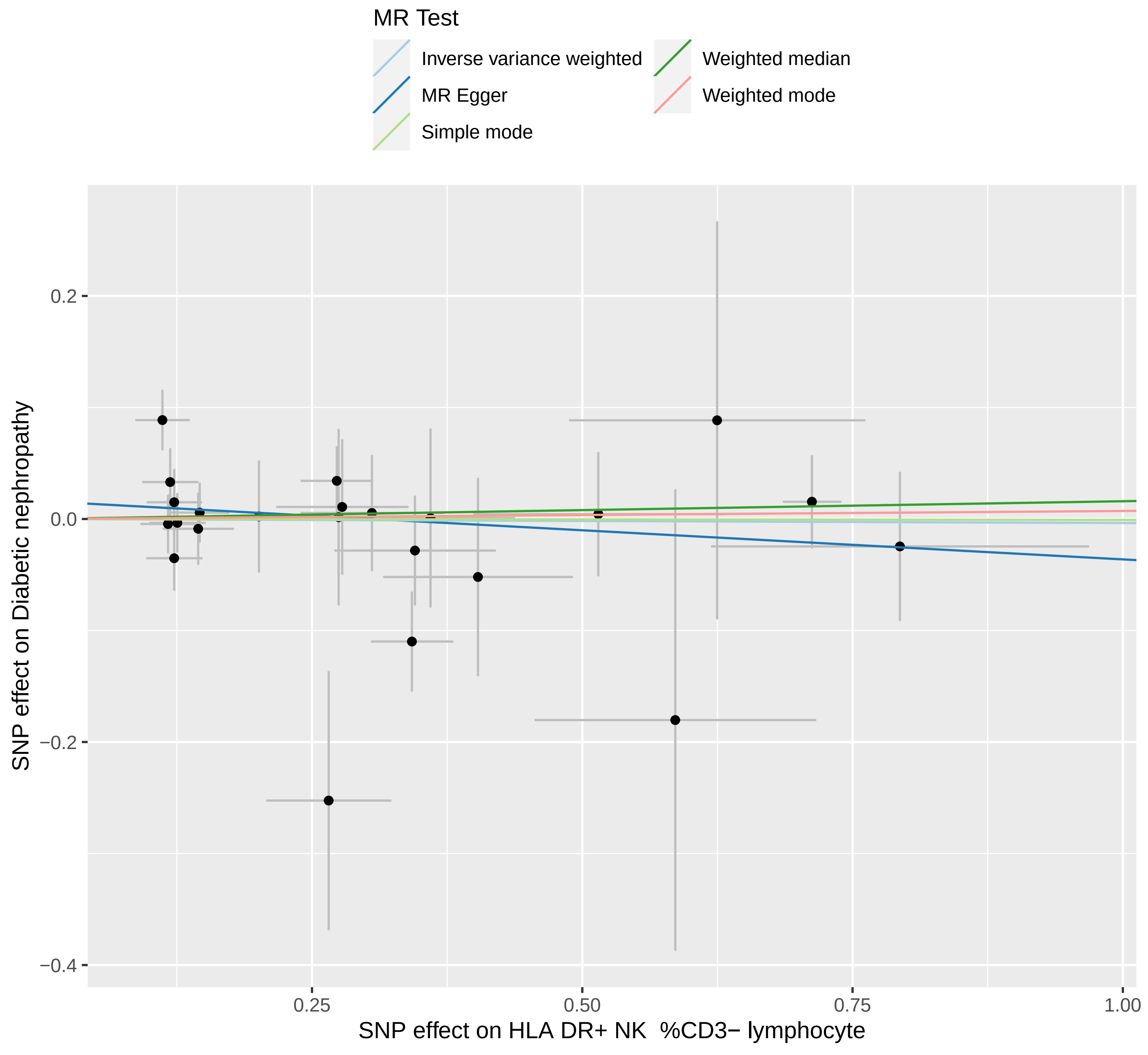
MR Test
Inverse variance weighted
MR Egger
Simple mode
Weighted median
Weighted mode
0.2
0.0
−0.2
−0.4
0.25
0.50
0.75
1.00
SNP effect on Diabetic nephropathy
SNP effect on HLA DR+ NK %CD3− lymphocyte

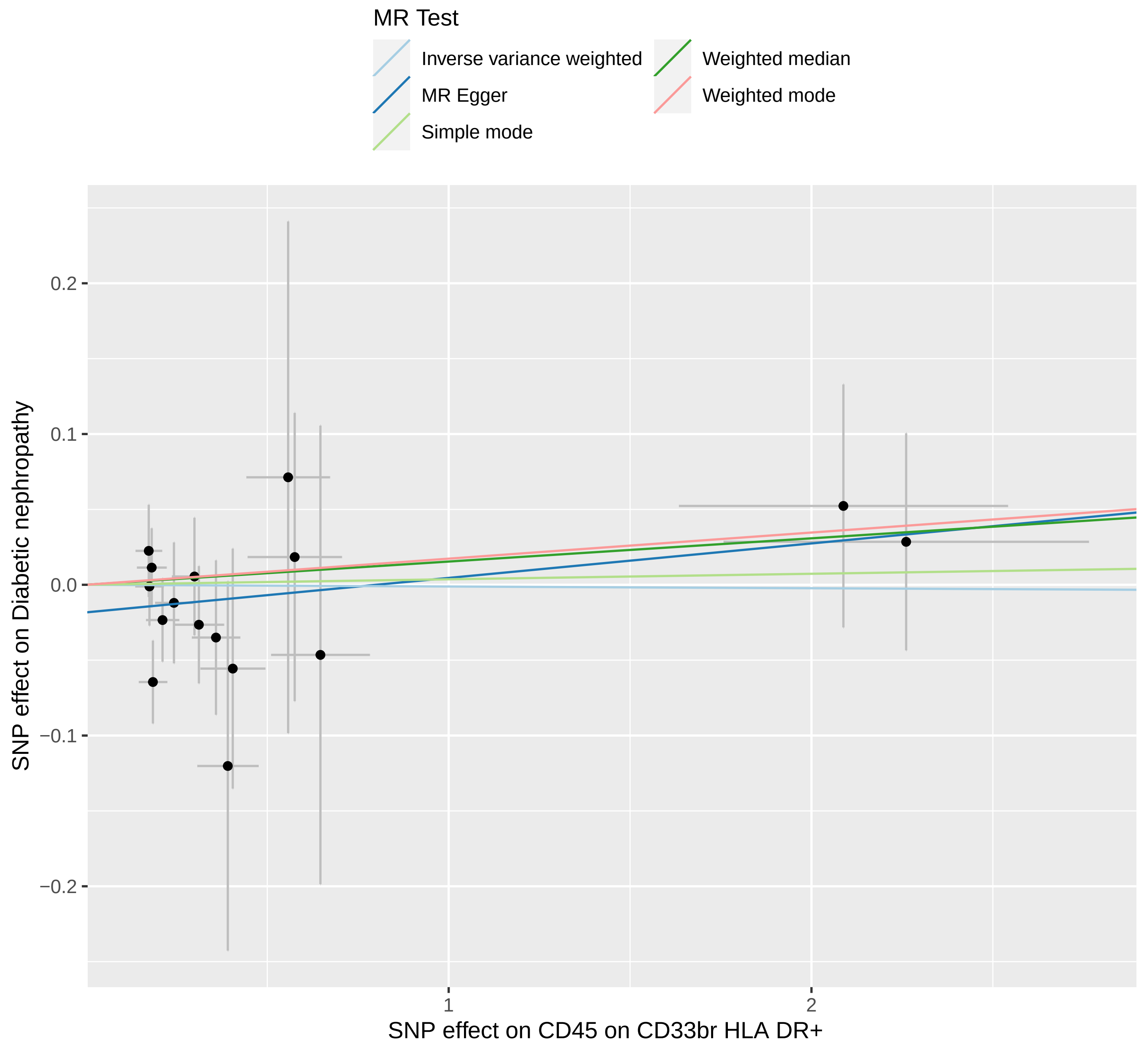

MR Test
Inverse variance weighted
MR Egger
Simple mode
Weighted median
Weighted mode
0.2
0.1
0.0
−0.1
−0.2
1
2
SNP effect on Diabetic nephropathy
SNP effect on CD45 on CD33br HLA DR+

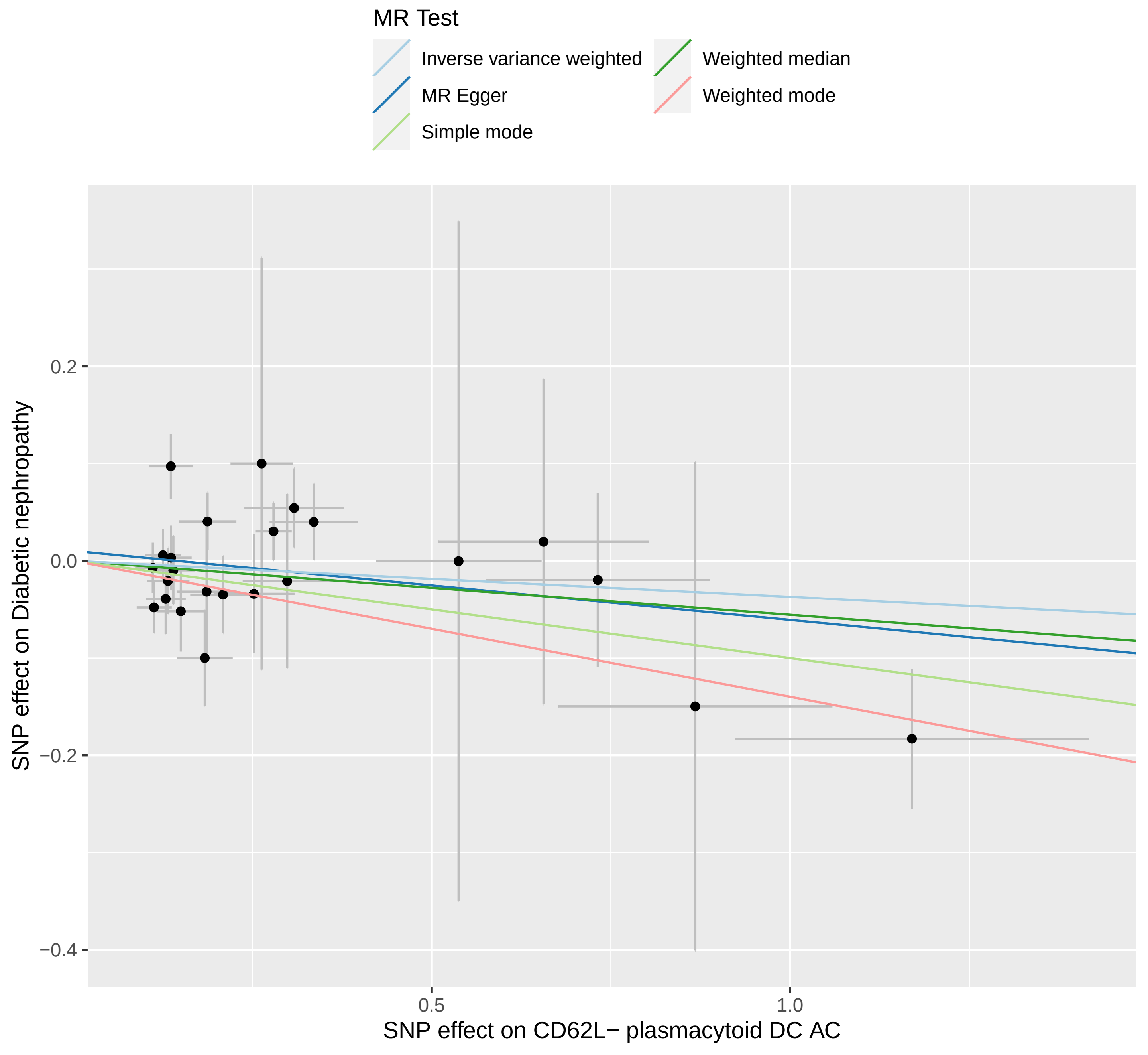
MR Test
Inverse variance weighted
MR Egger
Simple mode
Weighted median
Weighted mode
SNP effect on Diabetic nephropathy
0.2
0.0
−0.2
−0.4
0.5
1.0
SNP effect on CD62L− plasmacytoid DC AC

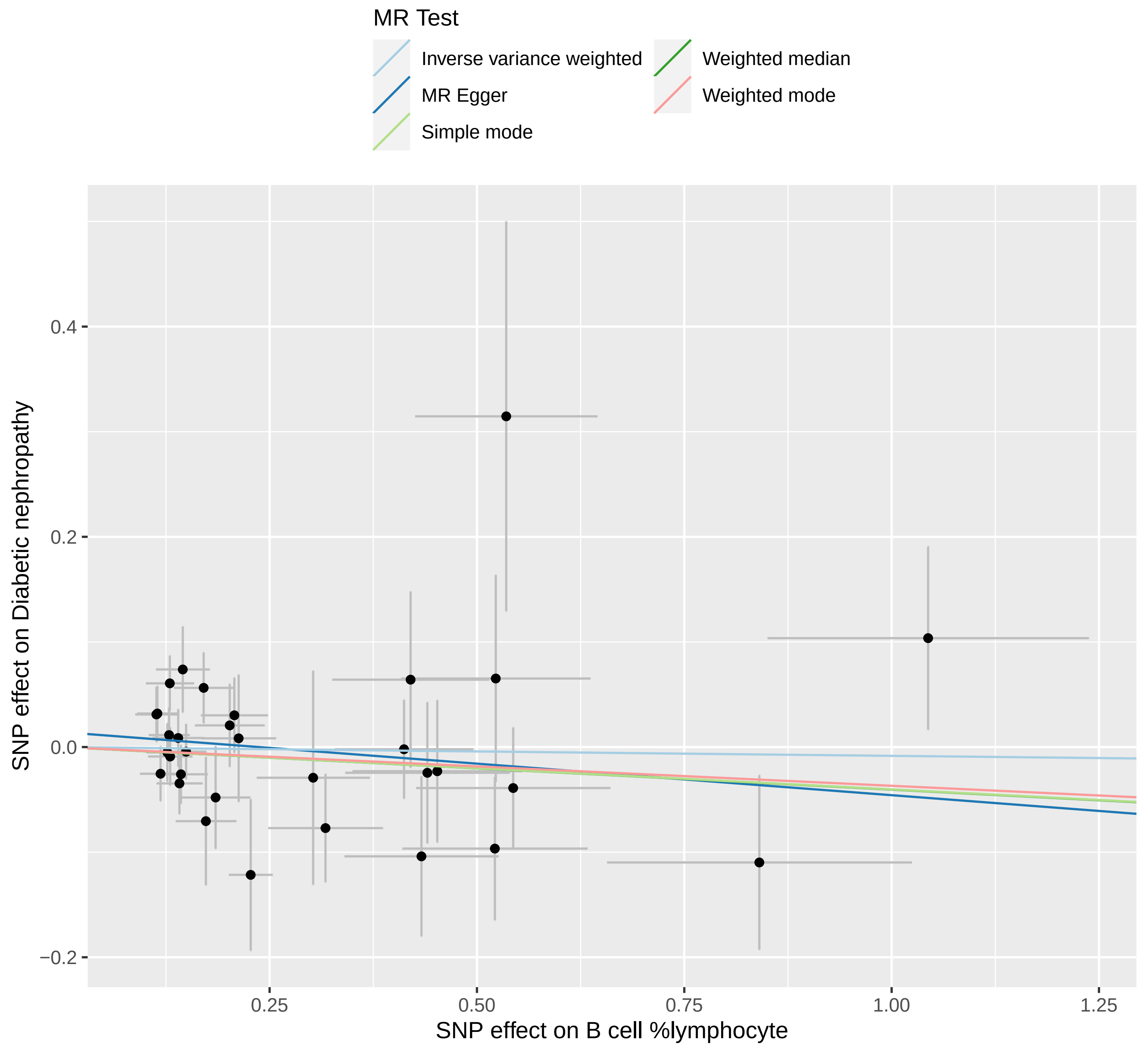
MR Test
Inverse variance weighted
MR Egger
Simple mode
Weighted median
Weighted mode
SNP effect on Diabetic nephropathy
0.4
0.2
0.0
−0.2
0.25
0.50
0.75
1.00
1.25
SNP effect on B cell %lymphocyte

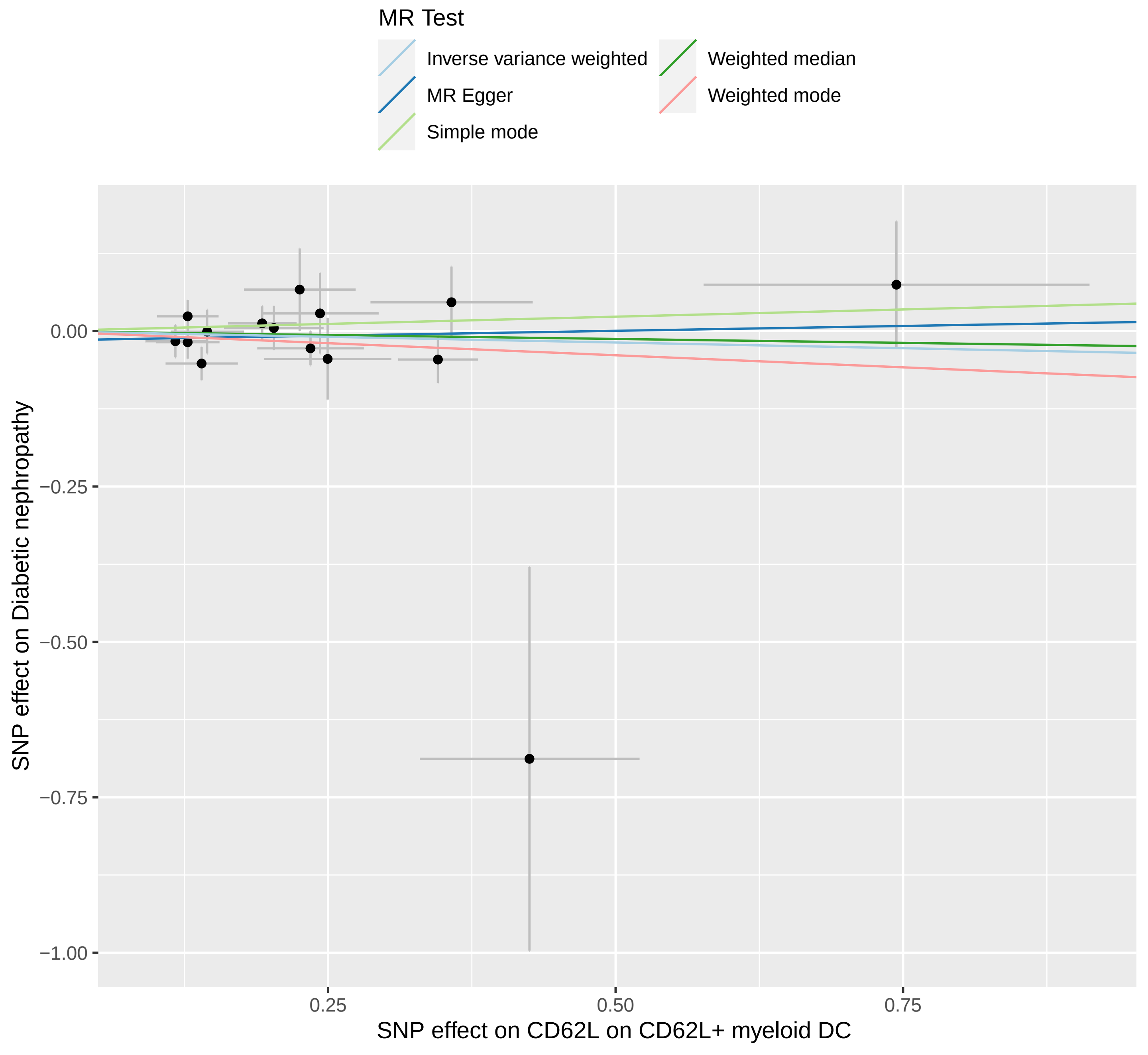

MR Test
Inverse variance weighted
MR Egger
Simple mode
Weighted median
Weighted mode
SNP effect on Diabetic nephropathy
0.00
−0.25
−0.50
−0.75
−1.00
0.25
0.50
0.75
SNP effect on CD62L on CD62L+ myeloid DC

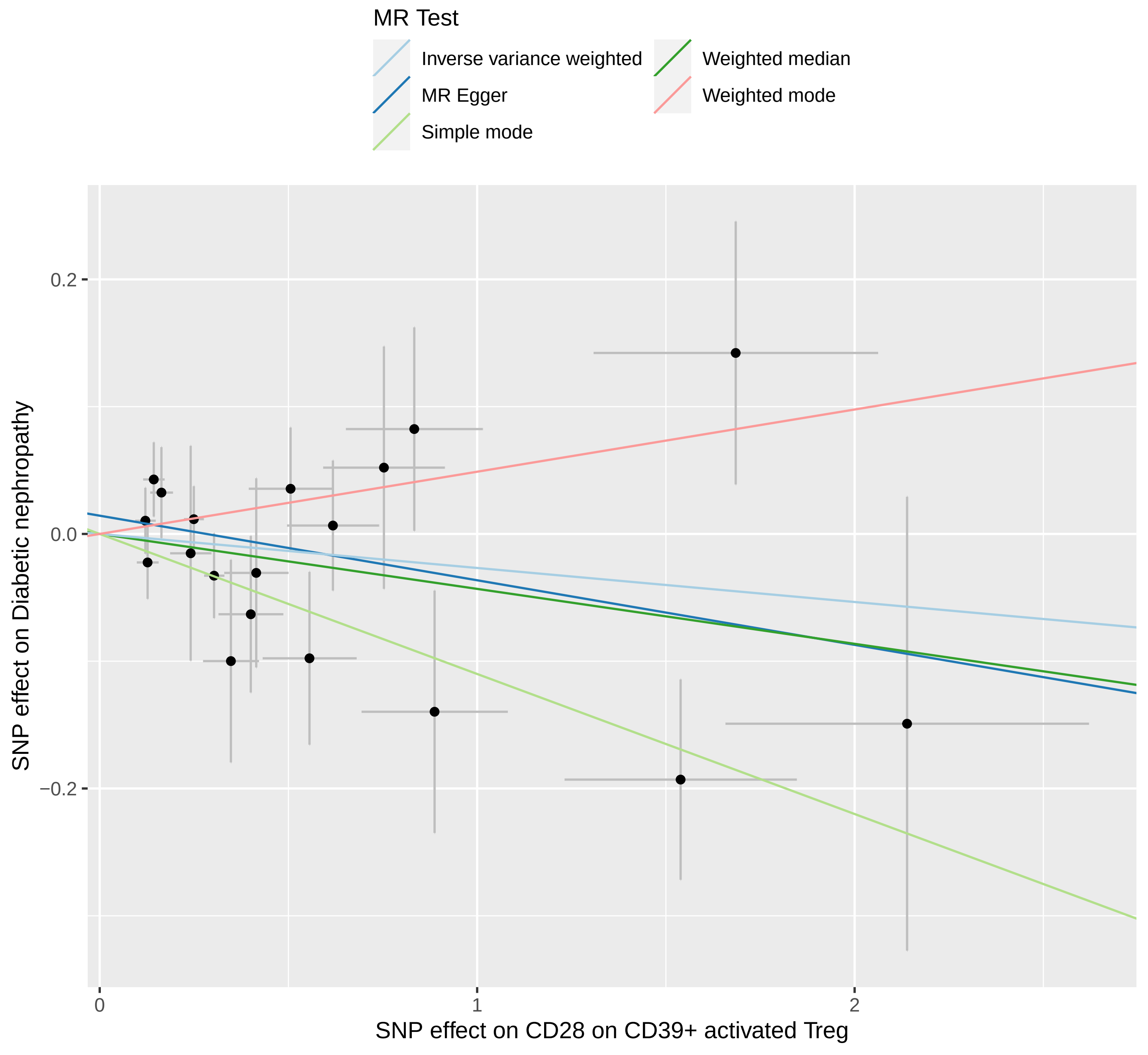

MR Test
Inverse variance weighted
MR Egger
Simple mode
Weighted median
Weighted mode
0.2
0.0
−0.2
0
1
2
SNP effect on Diabetic nephropathy
SNP effect on CD28 on CD39+ activated Treg

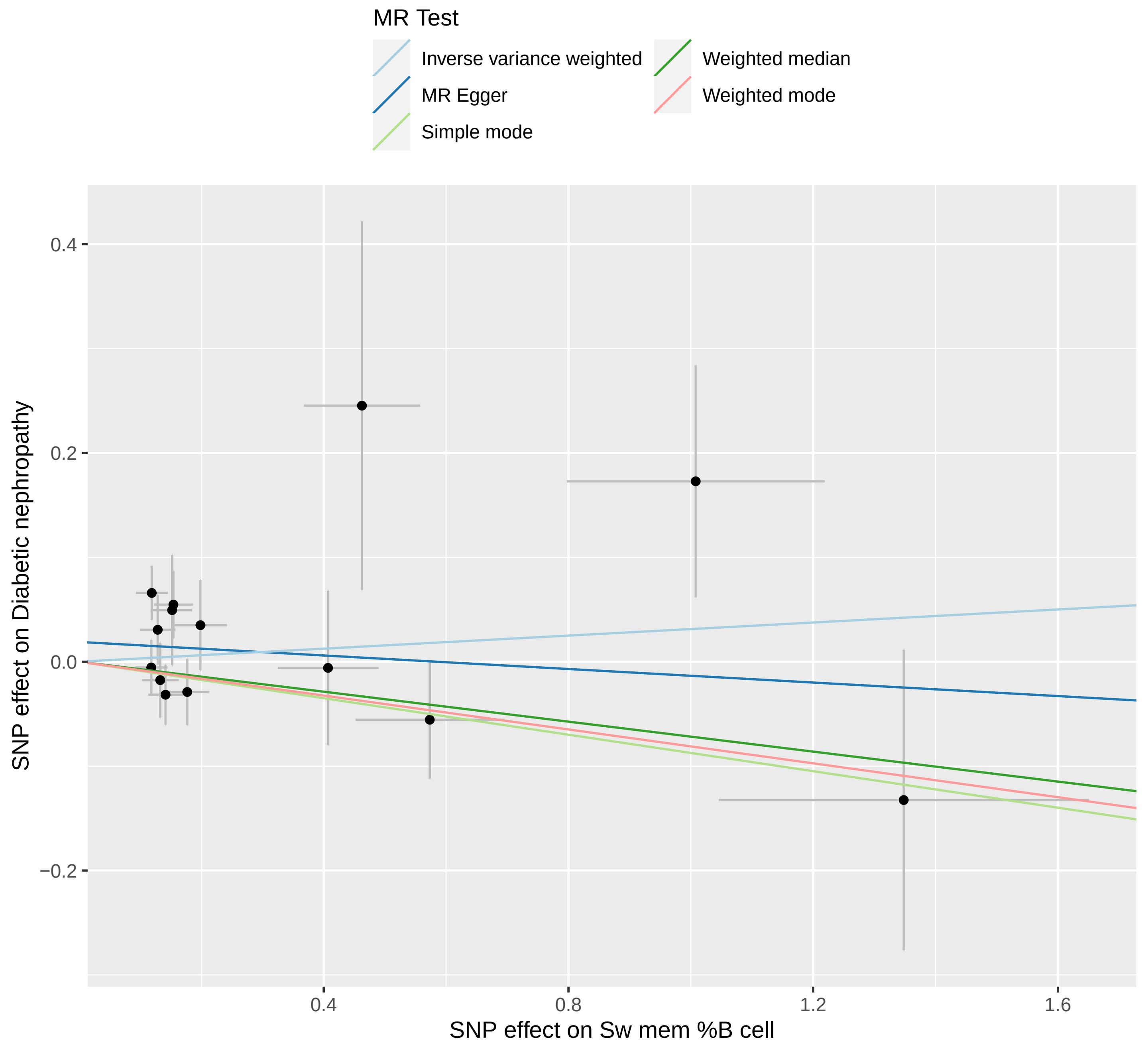

MR Test
Inverse variance weighted
MR Egger
Simple mode
Weighted median
Weighted mode
0.4
0.2
0.0
−0.2
SNP effect on Diabetic nephropathy
0.4
0.8
1.2
1.6
SNP effect on Sw mem %B cell

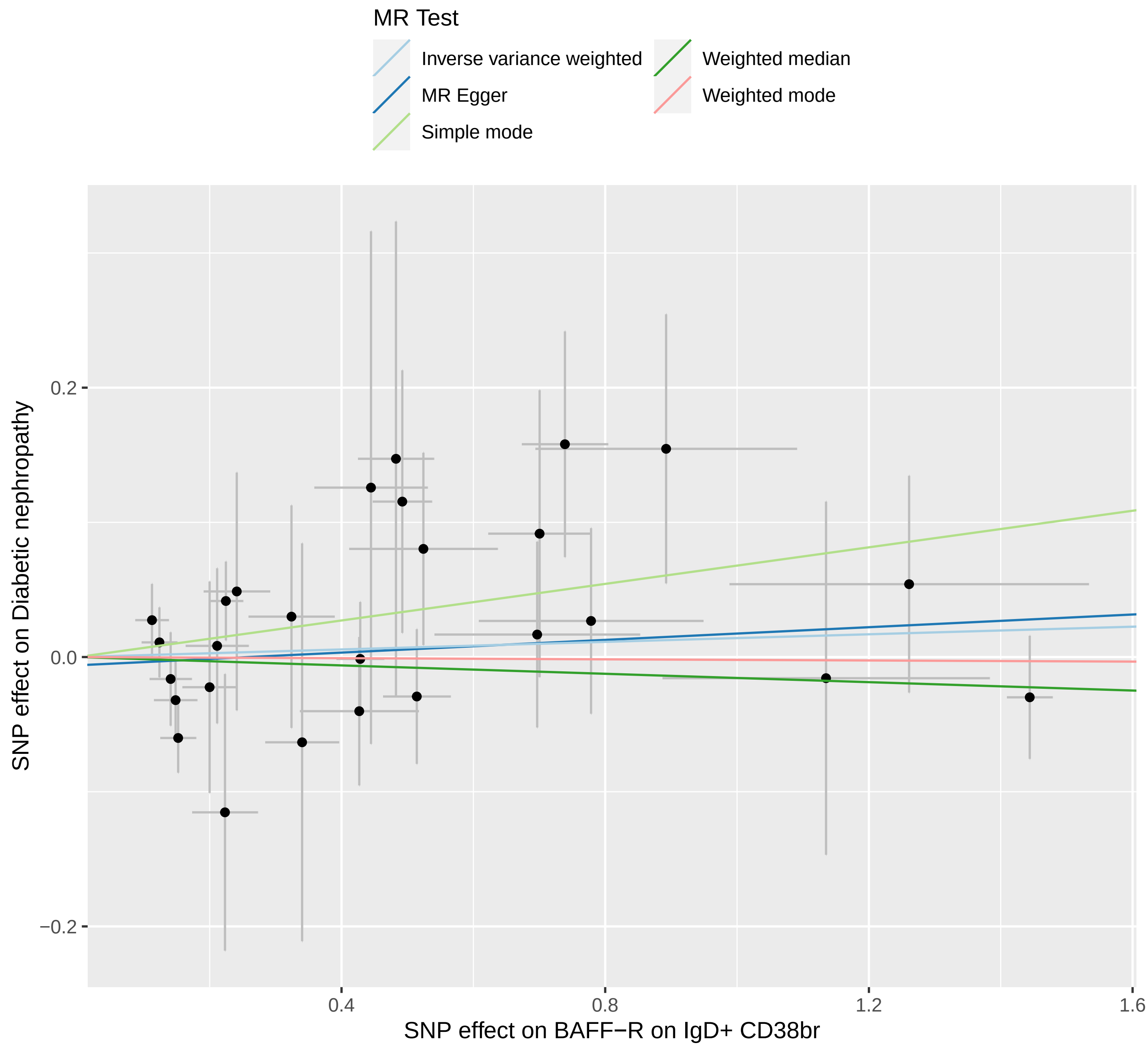

MR Test
Inverse variance weighted
MR Egger
Simple mode
Weighted median
Weighted mode
0.2
0.0
−0.2
0.4
0.8
1.2
1.6
SNP effect on Diabetic nephropathy
SNP effect on BAFF−R on IgD+ CD38br

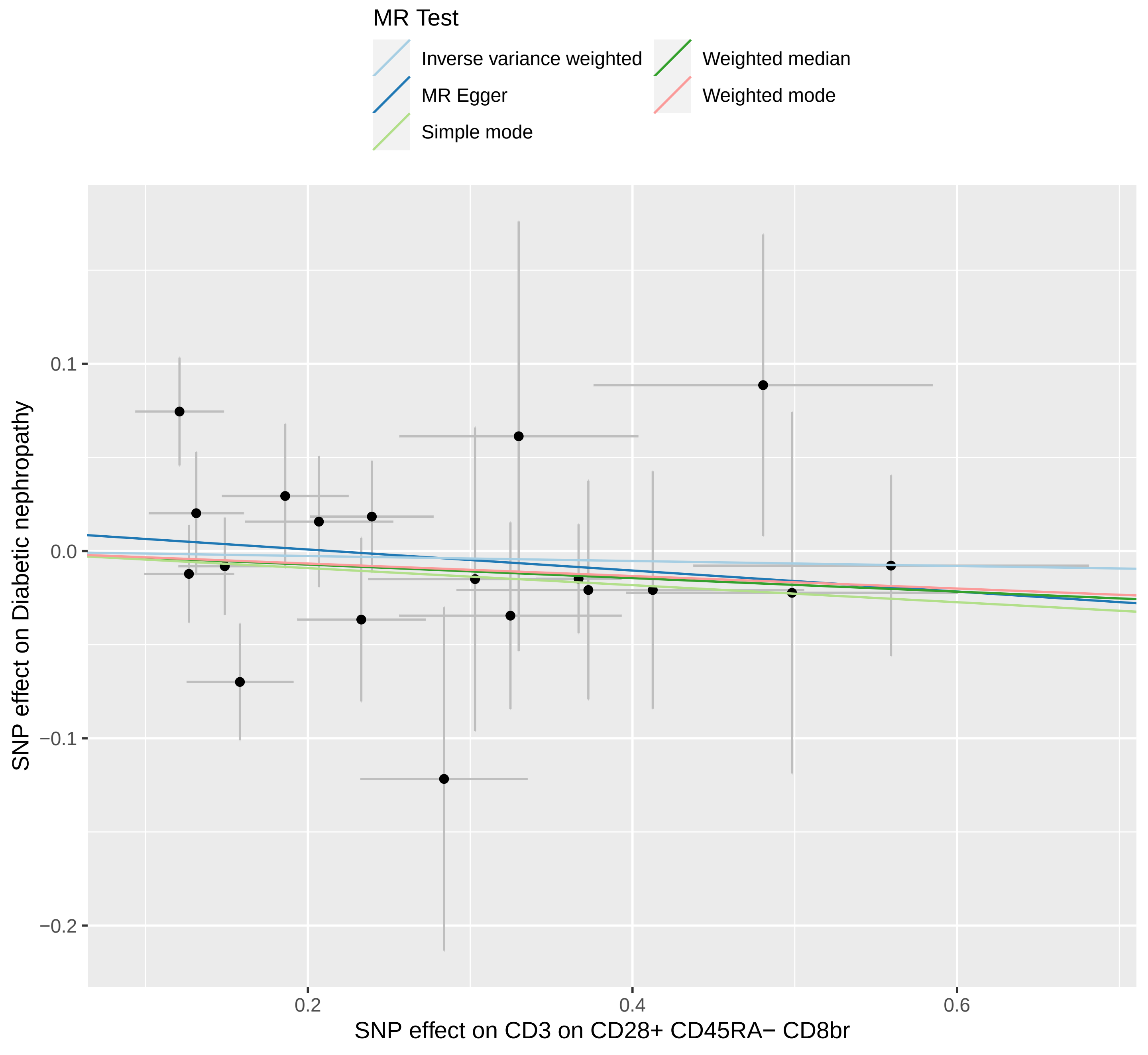

MR Test
Inverse variance weighted
MR Egger
Simple mode
Weighted median
Weighted mode
0.1
0.0
−0.1
−0.2
SNP effect on Diabetic nephropathy
0.2
0.4
0.6
SNP effect on CD3 on CD28+ CD45RA− CD8br

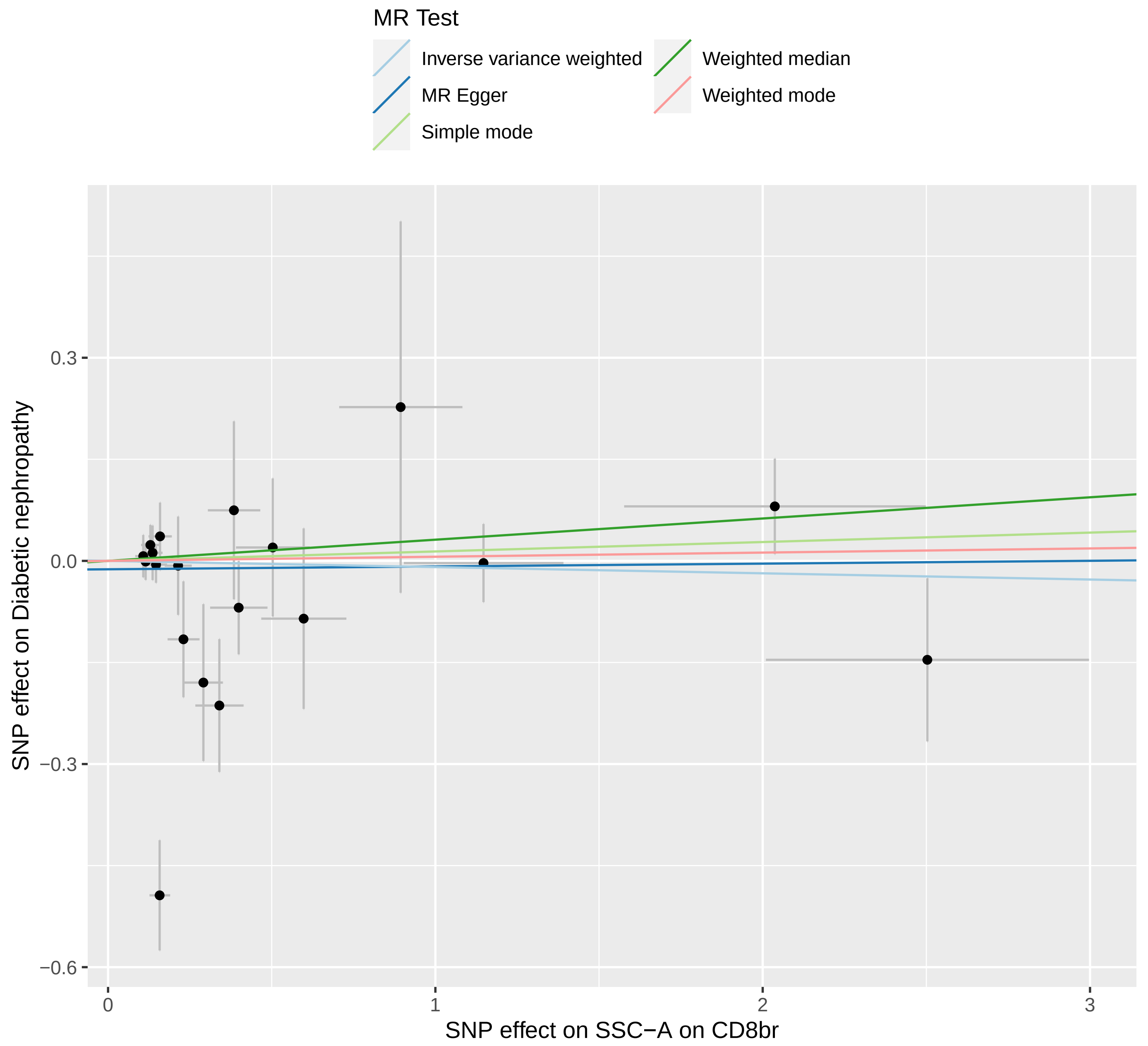
MR Test
Inverse variance weighted
MR Egger
Simple mode
Weighted median
Weighted mode
SNP effect on Diabetic nephropathy
0.3
0.0
−0.3
−0.6
0
1
2
3
SNP effect on SSC−A on CD8br

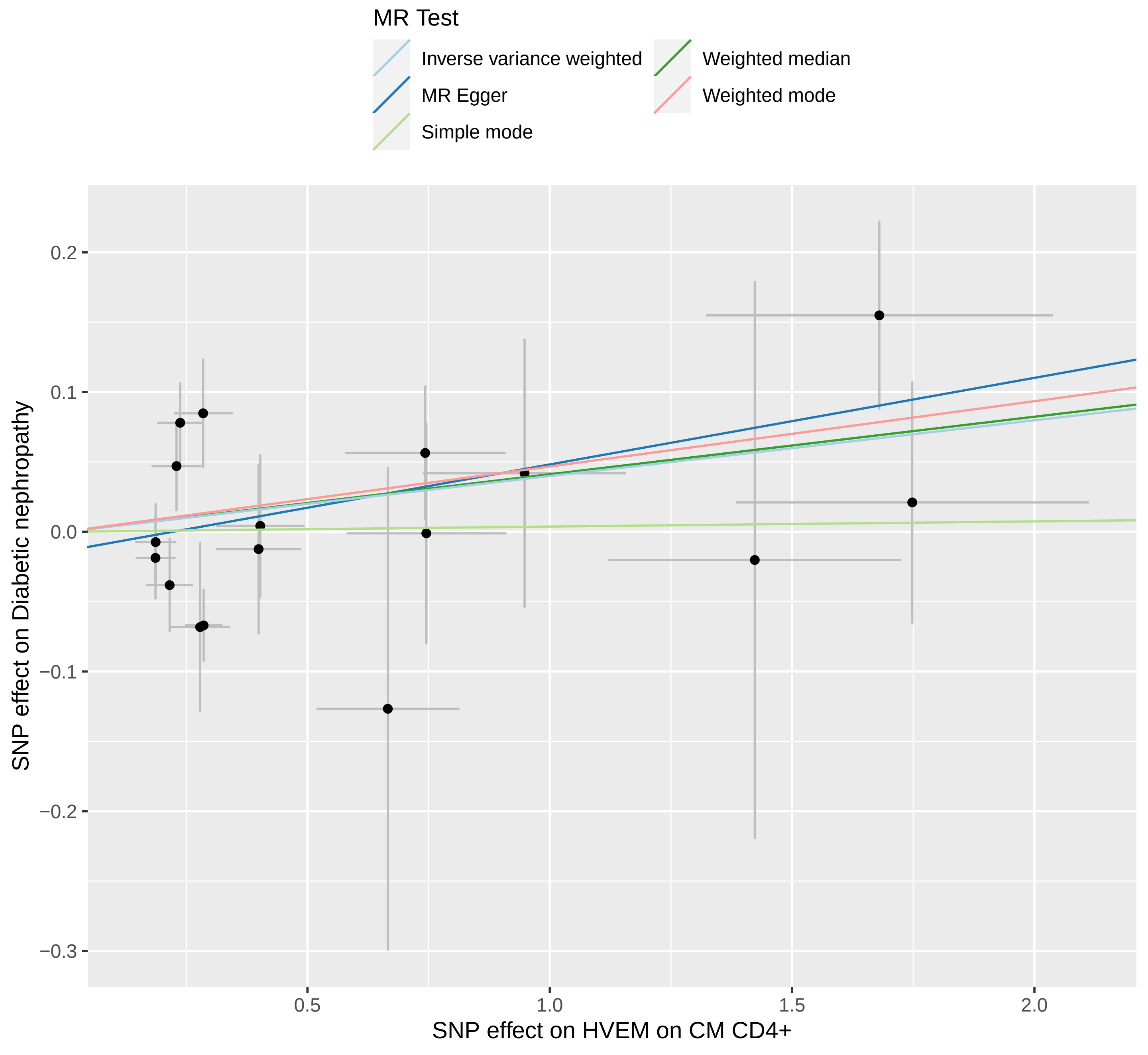

MR Test
Inverse variance weighted
MR Egger
Simple mode
Weighted median
Weighted mode
0.2
0.1
0.0
−0.1
−0.2
−0.3
0.5
1.0
1.5
2.0
SNP effect on Diabetic nephropathy
SNP effect on HVEM on CM CD4+

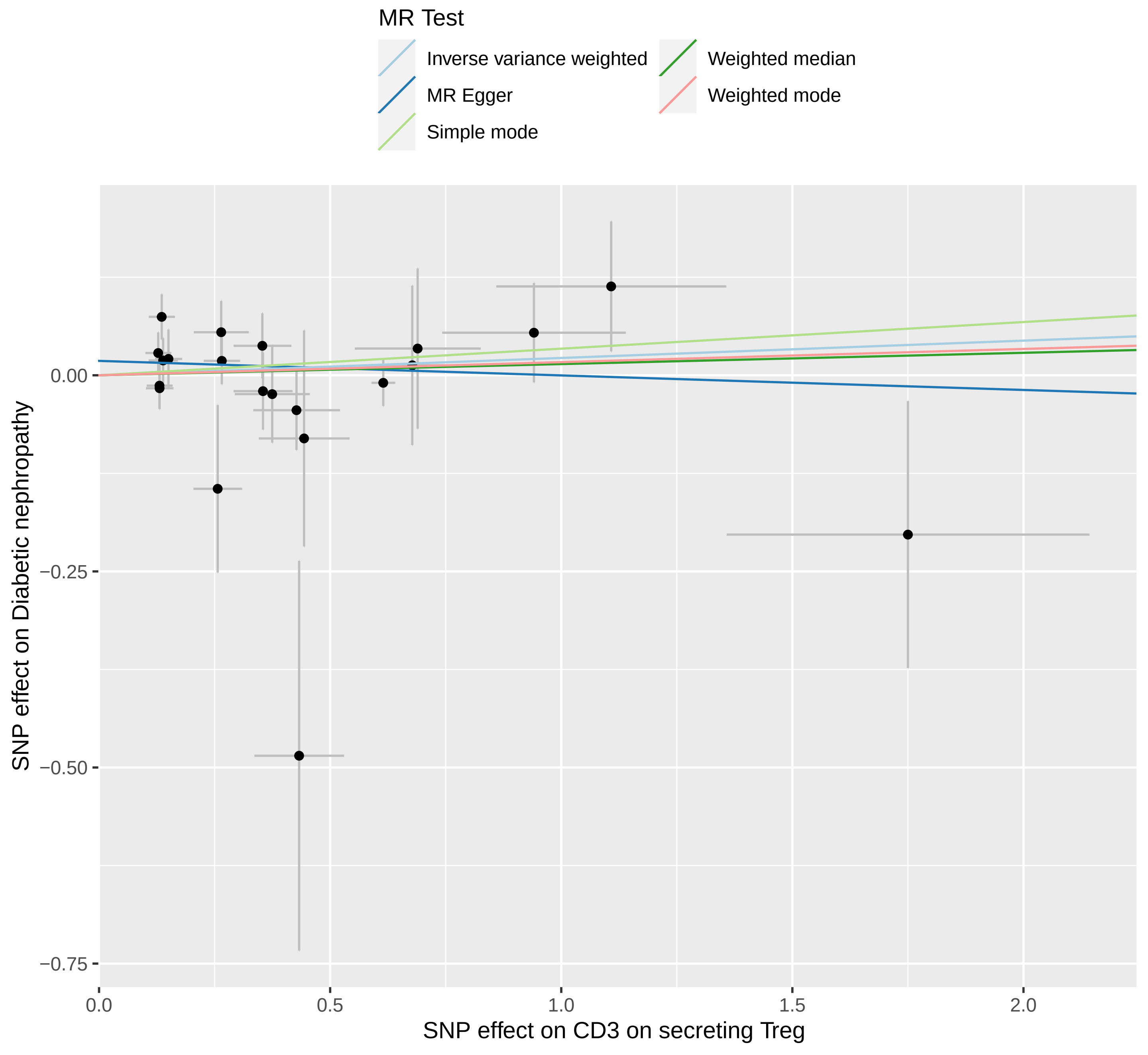
MR Test
Inverse variance weighted
MR Egger
Simple mode
Weighted median
Weighted mode
SNP effect on Diabetic nephropathy
0.00
−0.25
−0.50
−0.75
0.0
0.5
1.0
1.5
2.0
SNP effect on CD3 on secreting Treg

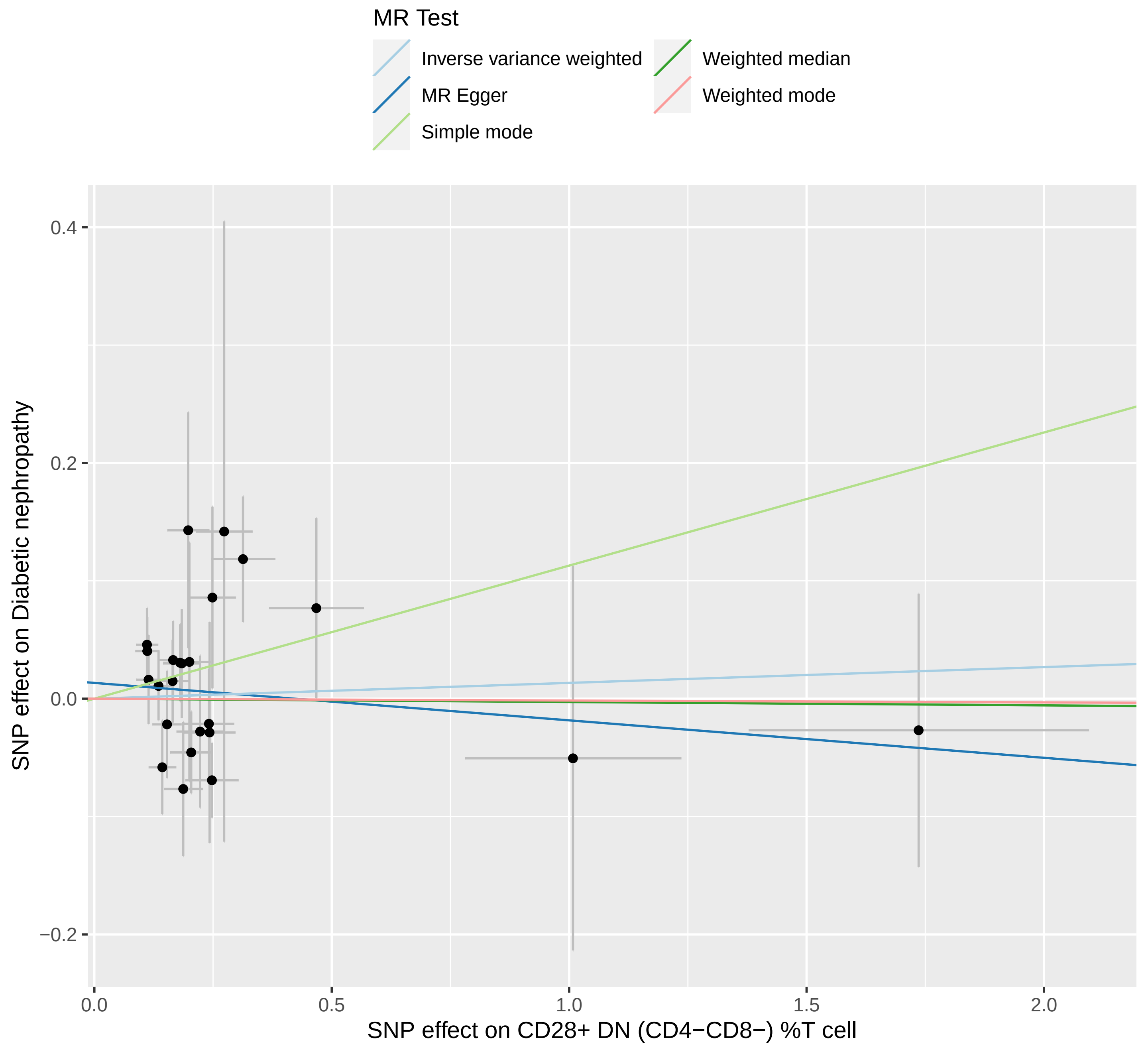
MR Test
Inverse variance weighted
MR Egger
Simple mode
Weighted median
Weighted mode
0.4
0.2
0.0
−0.2
0.0
0.5
1.0
1.5
2.0
SNP effect on Diabetic nephropathy
SNP effect on CD28+ DN (CD4−CD8−) %T cell

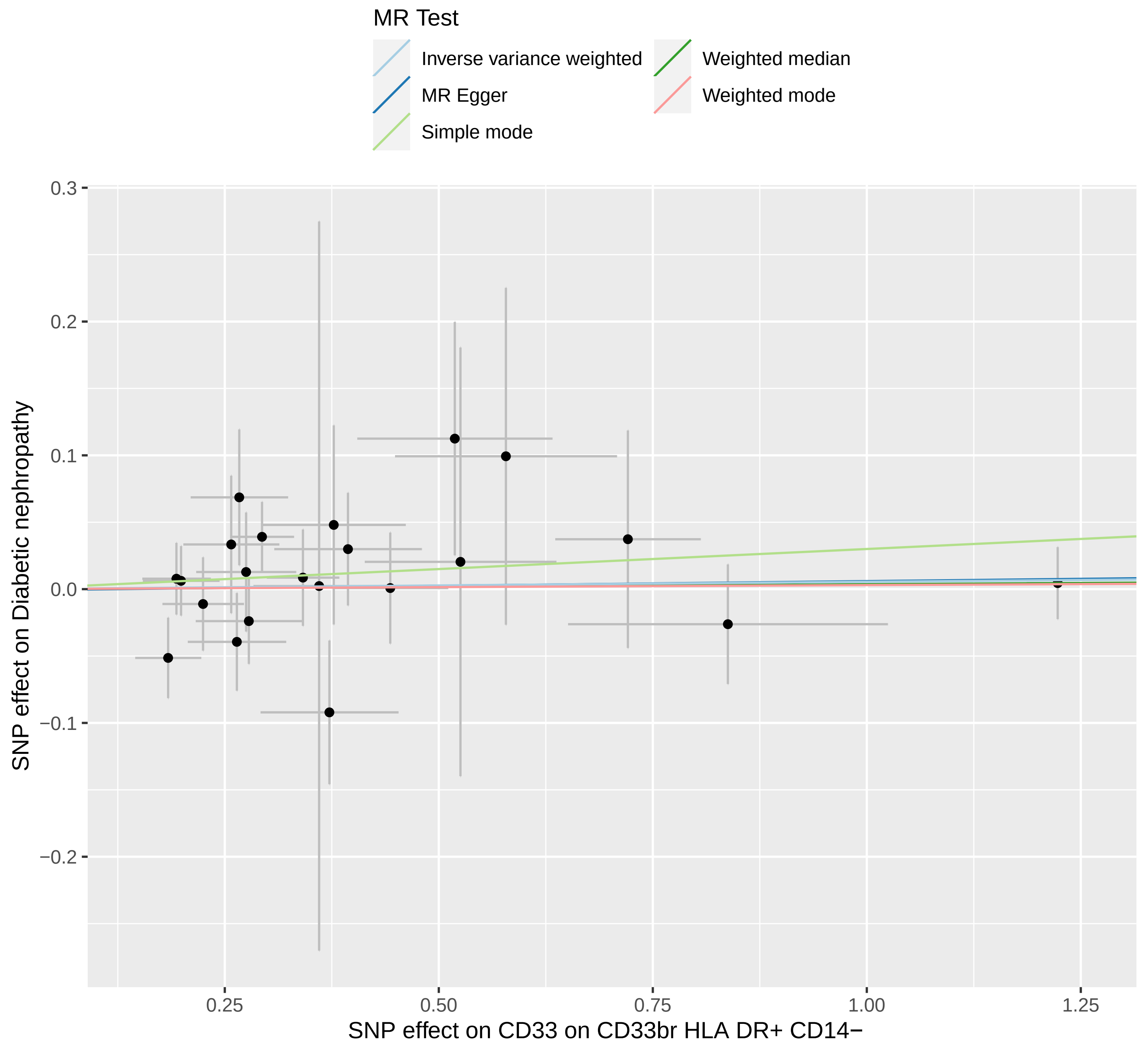
MR Test
Inverse variance weighted
MR Egger
Simple mode
Weighted median
Weighted mode
0.3
0.2
0.1
0.0
−0.1
−0.2
0.25
0.50
0.75
1.00
1.25
SNP effect on Diabetic nephropathy
SNP effect on CD33 on CD33br HLA DR+ CD14−

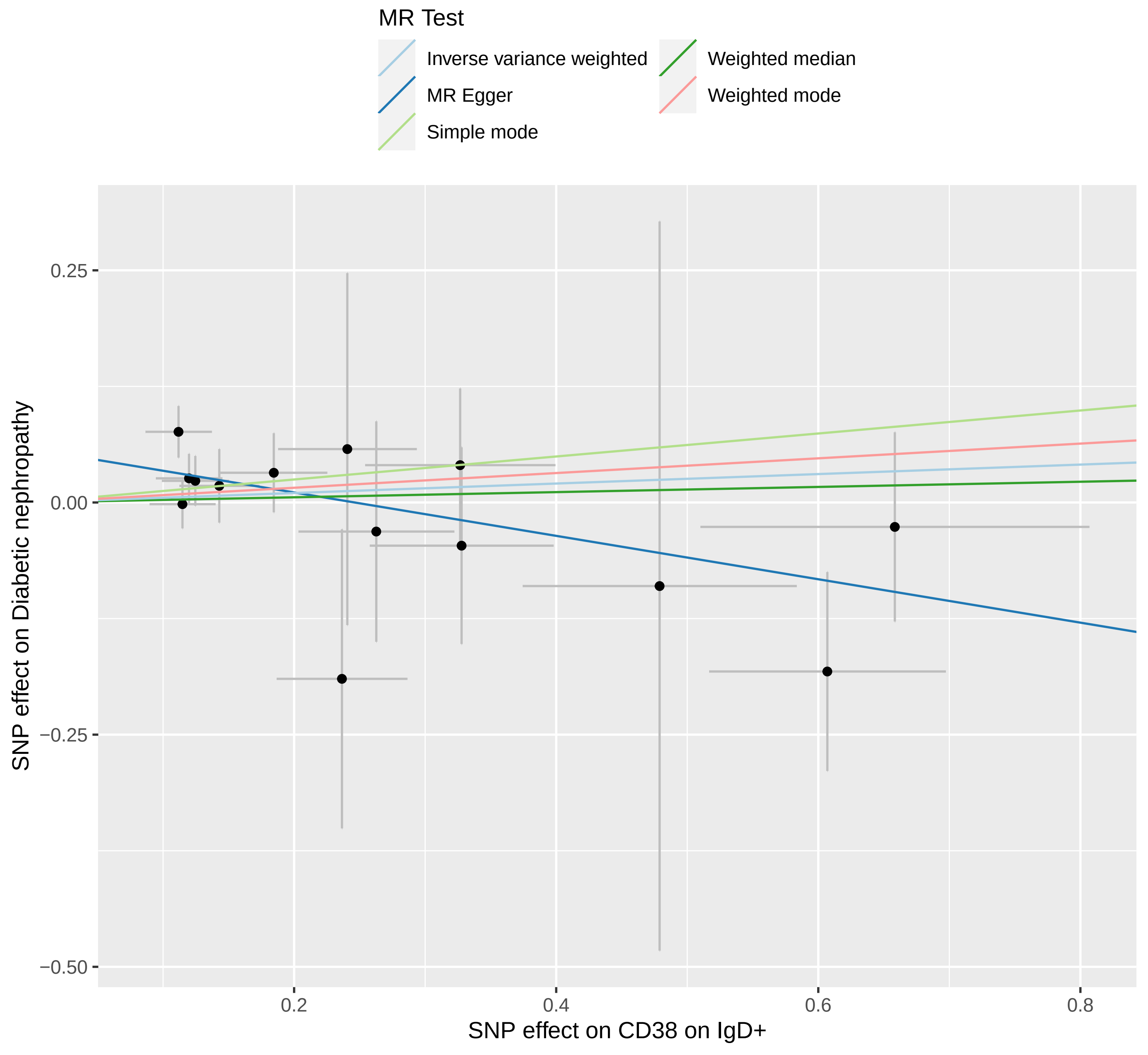
MR Test
Inverse variance weighted
MR Egger
Simple mode
Weighted median
Weighted mode
0.25
0.00
−0.25
−0.50
0.2
0.4
0.6
0.8
SNP effect on Diabetic nephropathy
SNP effect on CD38 on IgD+

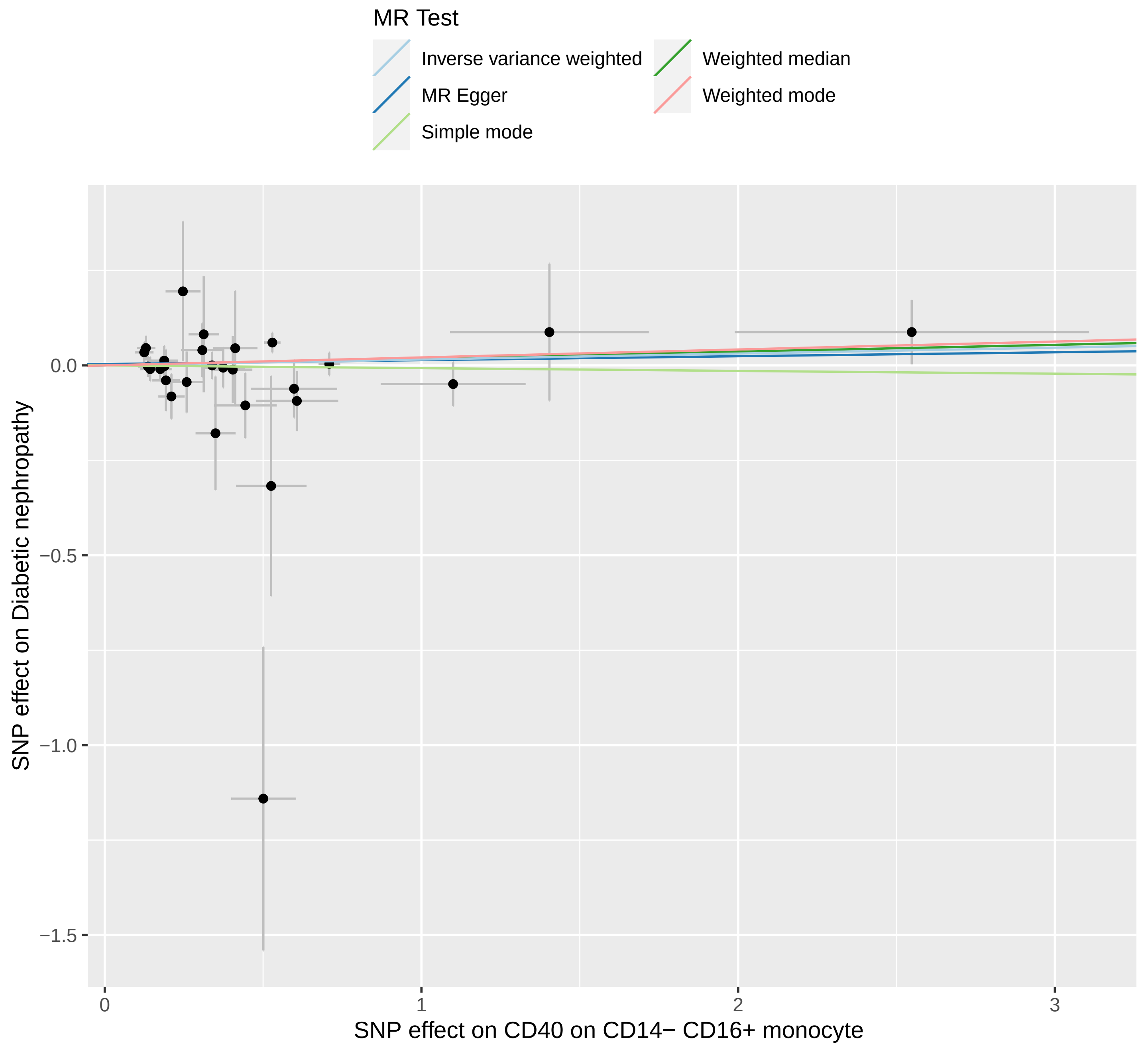
MR Test
Inverse variance weighted
MR Egger
Simple mode
Weighted median
Weighted mode
SNP effect on Diabetic nephropathy
0.0
−0.5
−1.0
−1.5
0
1
2
3
SNP effect on CD40 on CD14− CD16+ monocyte

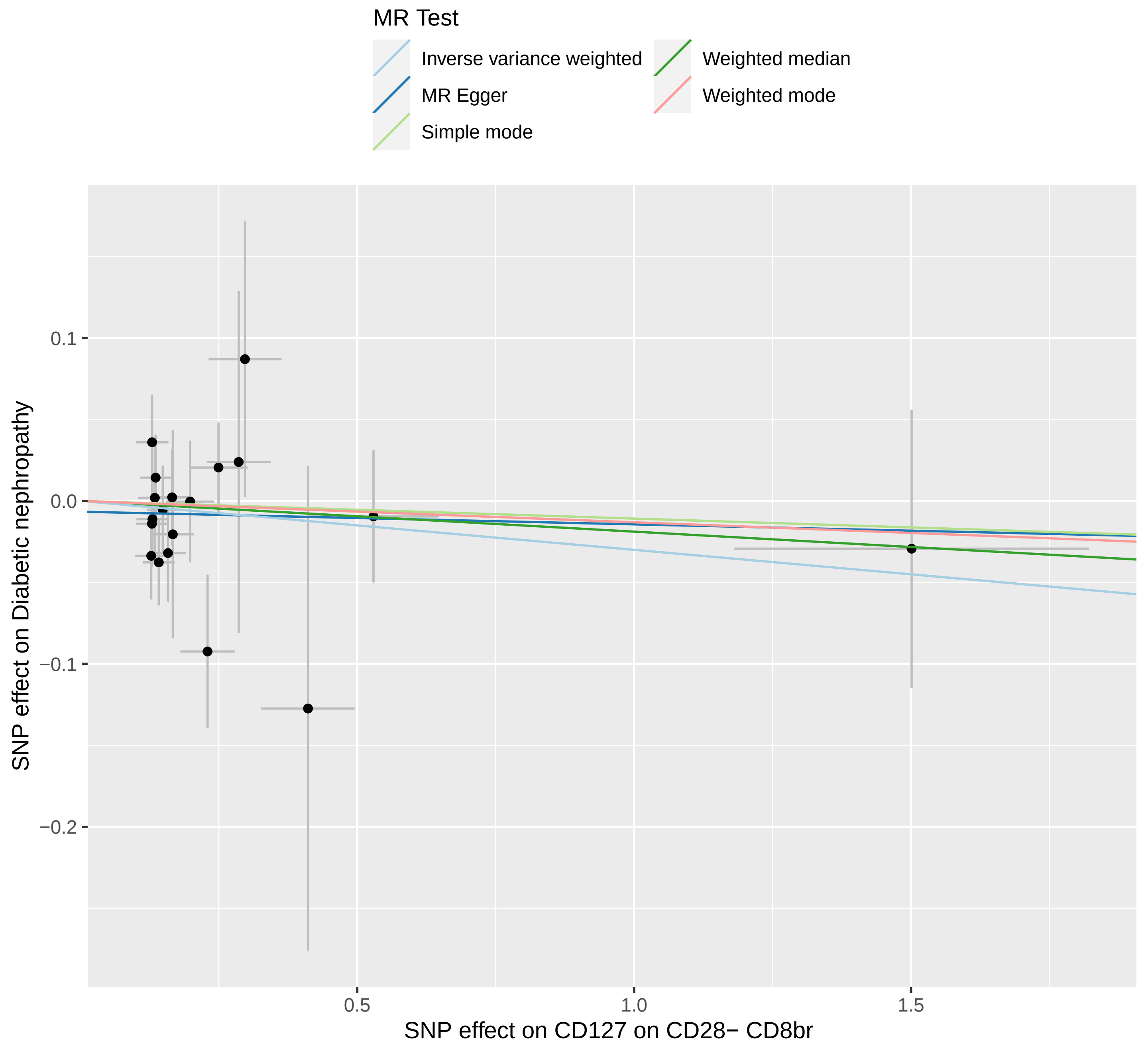
MR Test
Inverse variance weighted
MR Egger
Simple mode
Weighted median
Weighted mode
0.1
0.0
−0.1
−0.2
0.5
1.0
1.5
SNP effect on Diabetic nephropathy
SNP effect on CD127 on CD28− CD8br

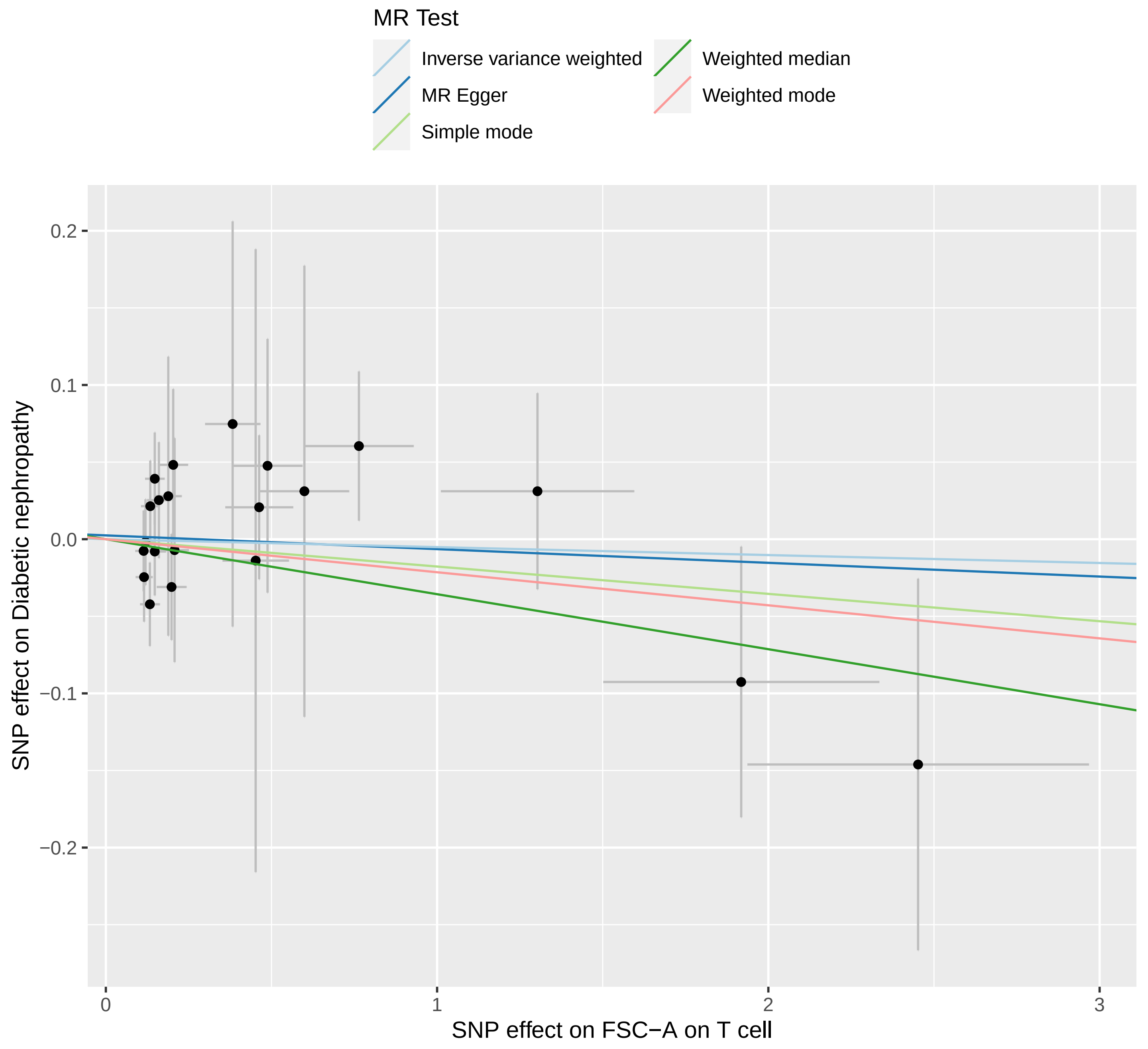
MR Test
Inverse variance weighted
MR Egger
Simple mode
Weighted median
Weighted mode
0.2
0.1
0.0
−0.1
−0.2
0
1
2
3
SNP effect on Diabetic nephropathy
SNP effect on FSC−A on T cell

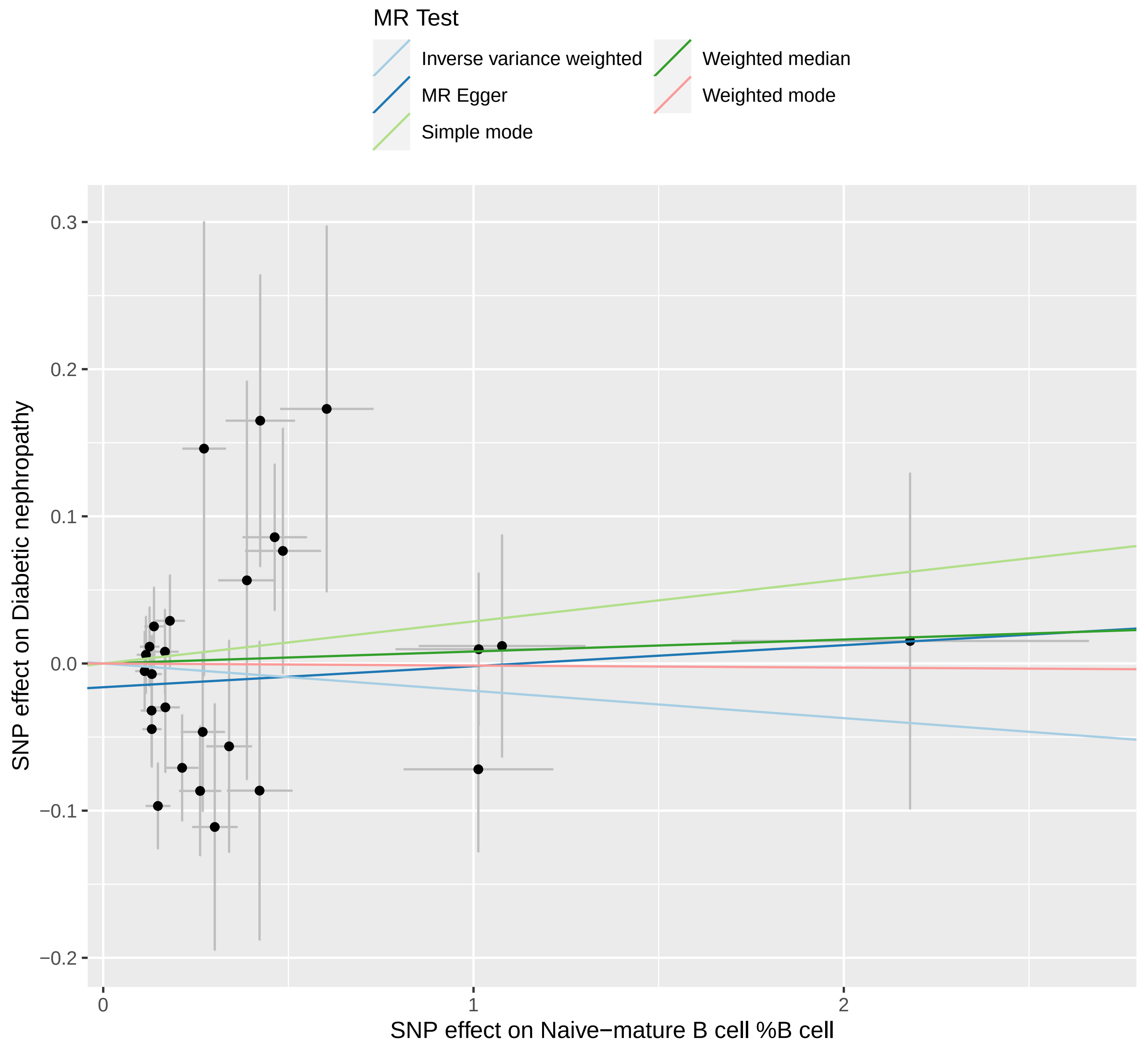
MR Test
Inverse variance weighted
Weighted median
MR Egger
Weighted mode
Simple mode
SNP effect on Diabetic nephropathy
0.3
0.2
0.1
0.0
−0.1
−0.2
0
1
2
SNP effect on Naive−mature B cell %B cell

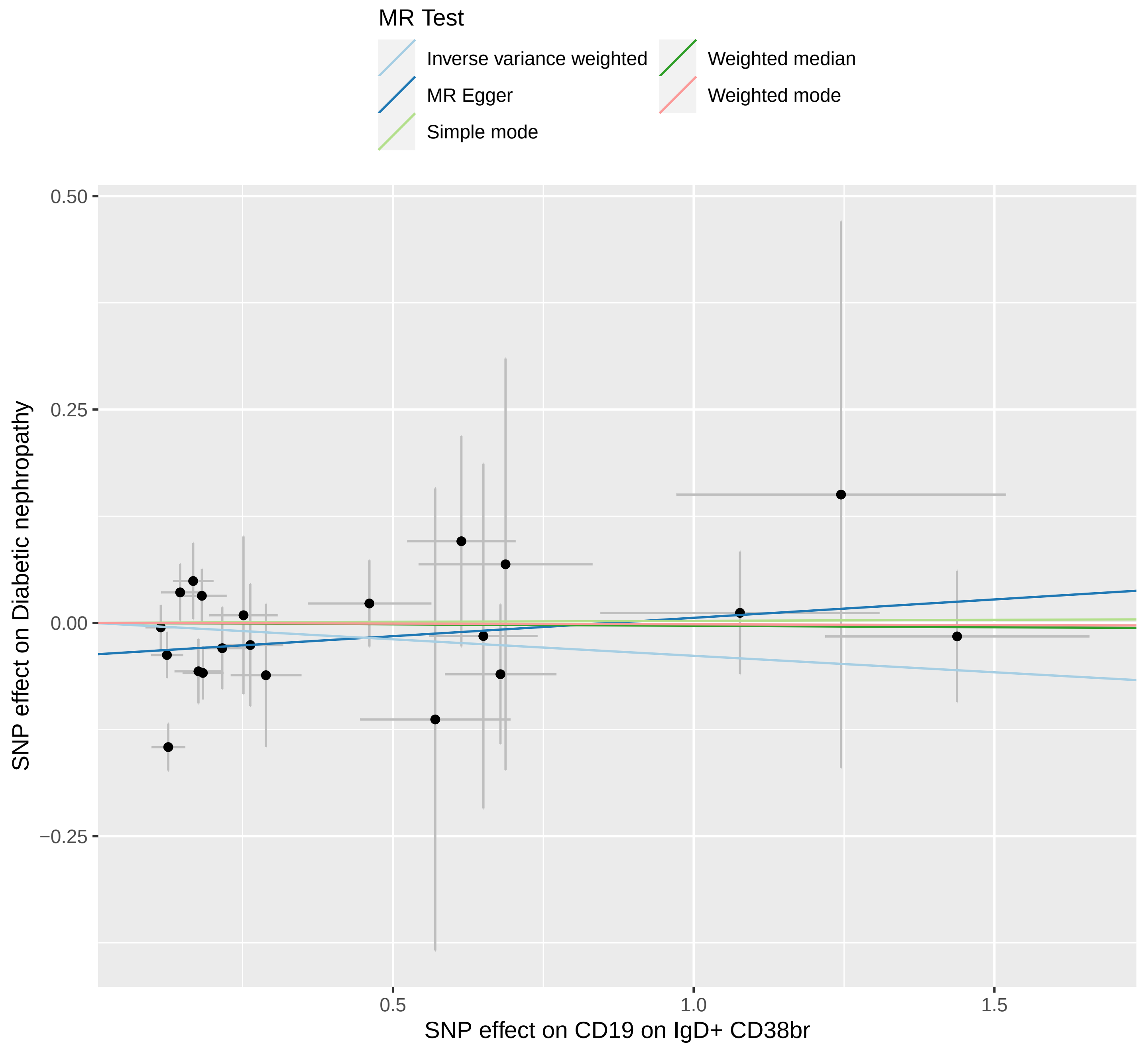

MR Test
Inverse variance weighted
MR Egger
Simple mode
Weighted median
Weighted mode
0.50
0.25
0.00
−0.25
0.5
1.0
1.5
SNP effect on Diabetic nephropathy
SNP effect on CD19 on IgD+ CD38br

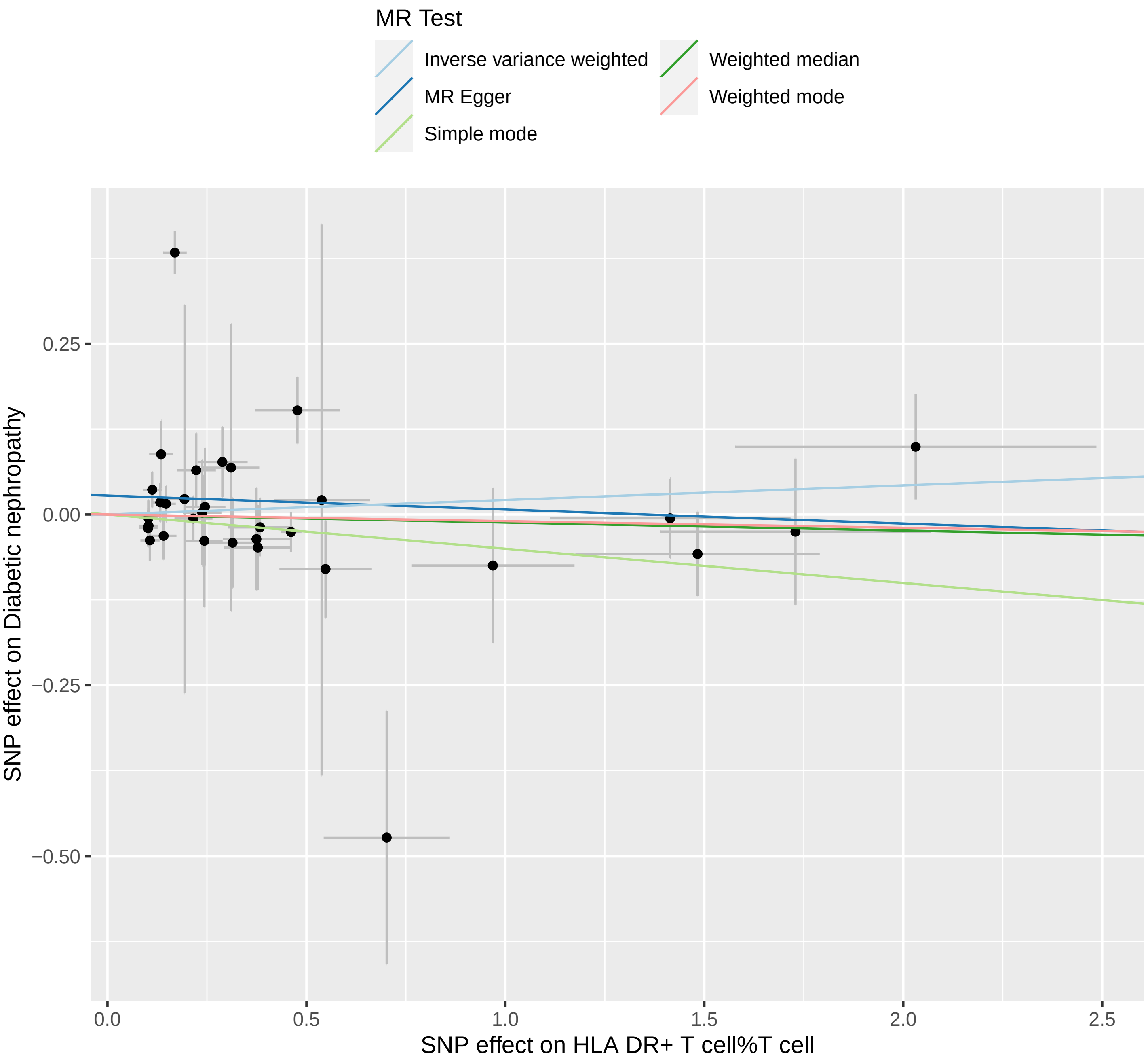
MR Test
Inverse variance weighted
MR Egger
Simple mode
Weighted median
Weighted mode
0.25
0.00
−0.25
−0.50
0.0
0.5
1.0
1.5
2.0
2.5
SNP effect on HLA DR+ T cell%T cell
SNP effect on Diabetic nephropathy

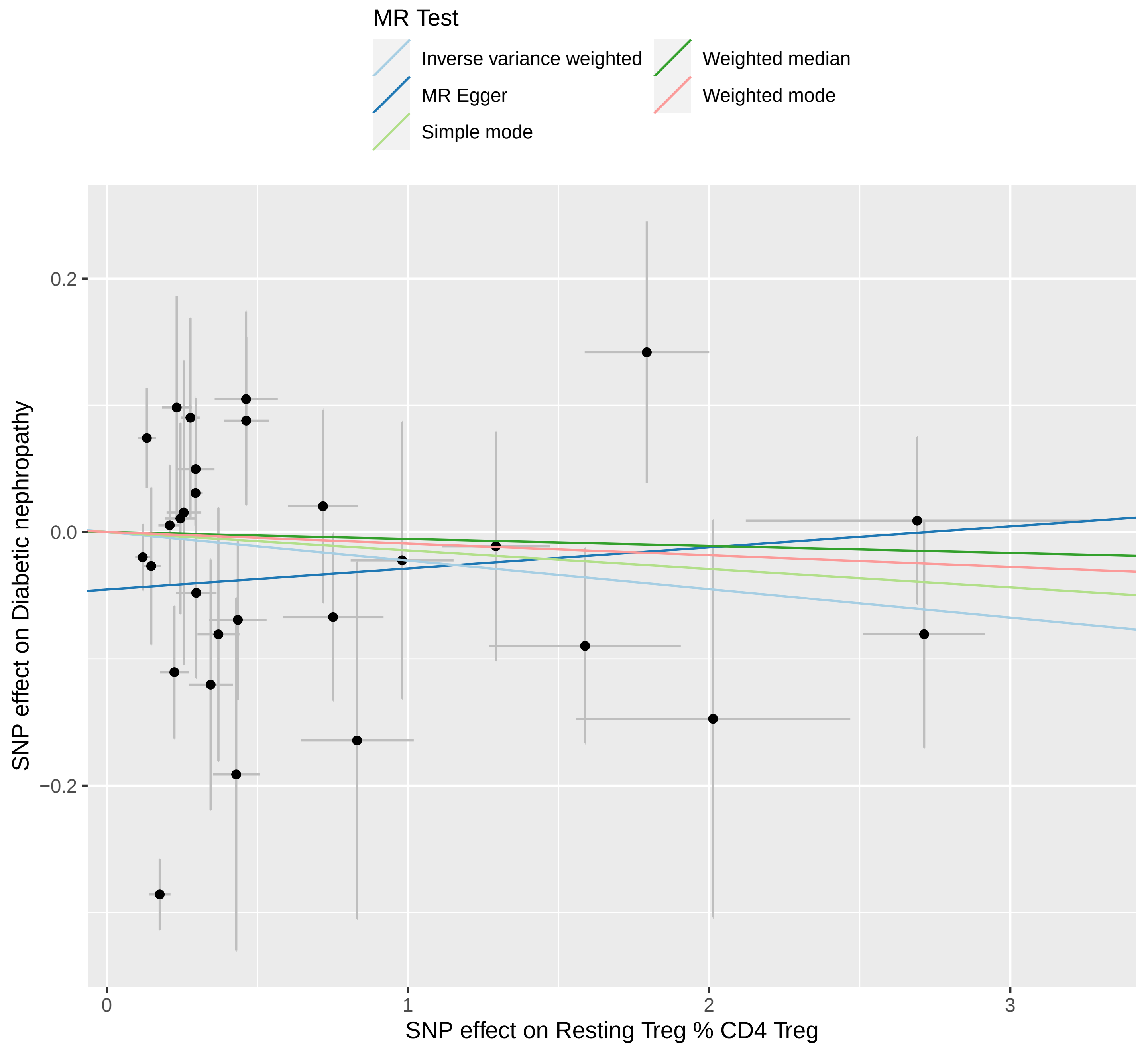

MR Test
Inverse variance weighted
MR Egger
Simple mode
Weighted median
Weighted mode
0.2
0.0
−0.2
0
1
2
3
SNP effect on Diabetic nephropathy
SNP effect on Resting Treg % CD4 Treg

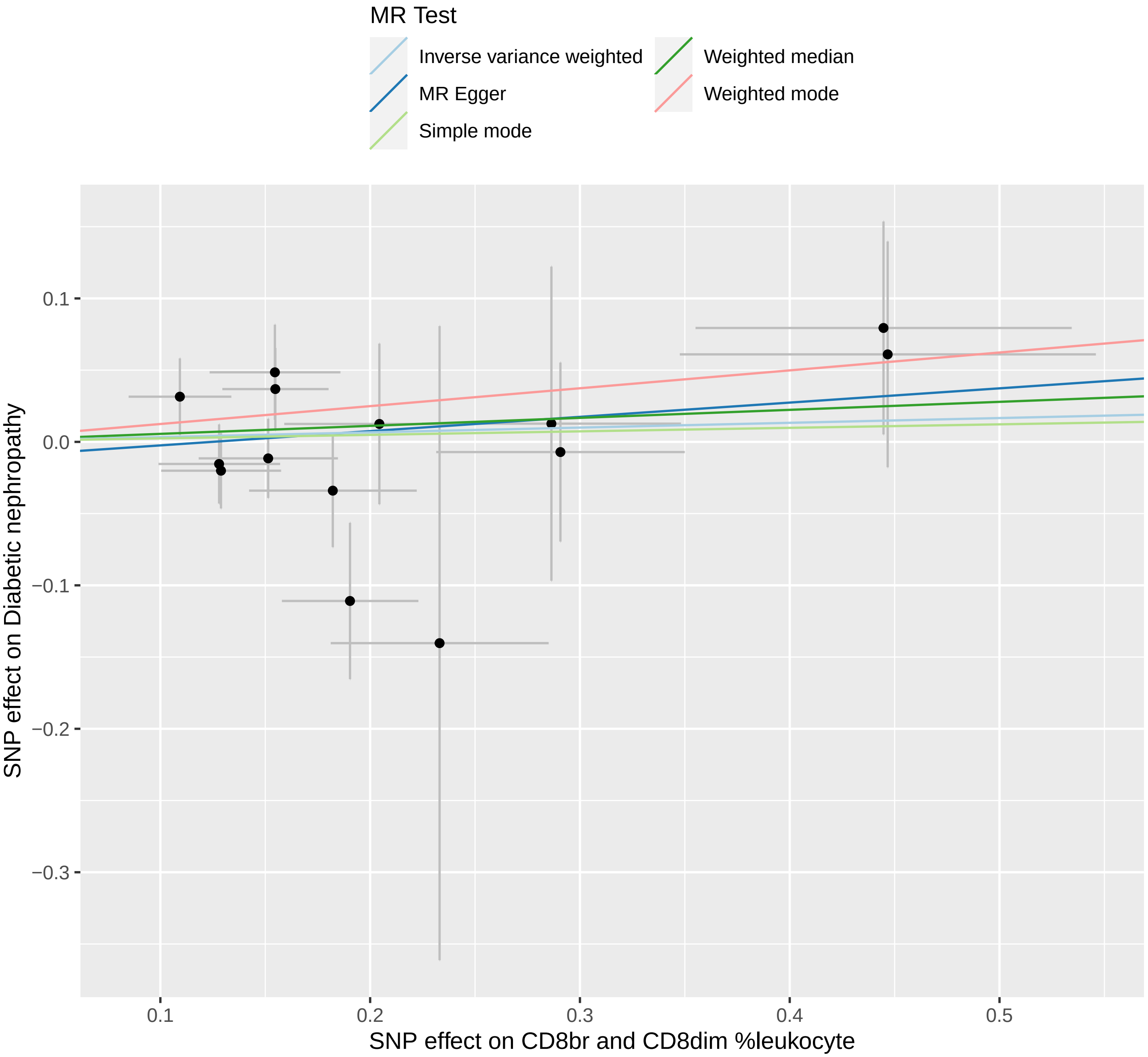

MR Test
Inverse variance weighted
MR Egger
Simple mode
Weighted median
Weighted mode
0.1
0.0
−0.1
−0.2
−0.3
SNP effect on Diabetic nephropathy
0.1
0.2
0.3
0.4
0.5
SNP effect on CD8br and CD8dim %leukocyte

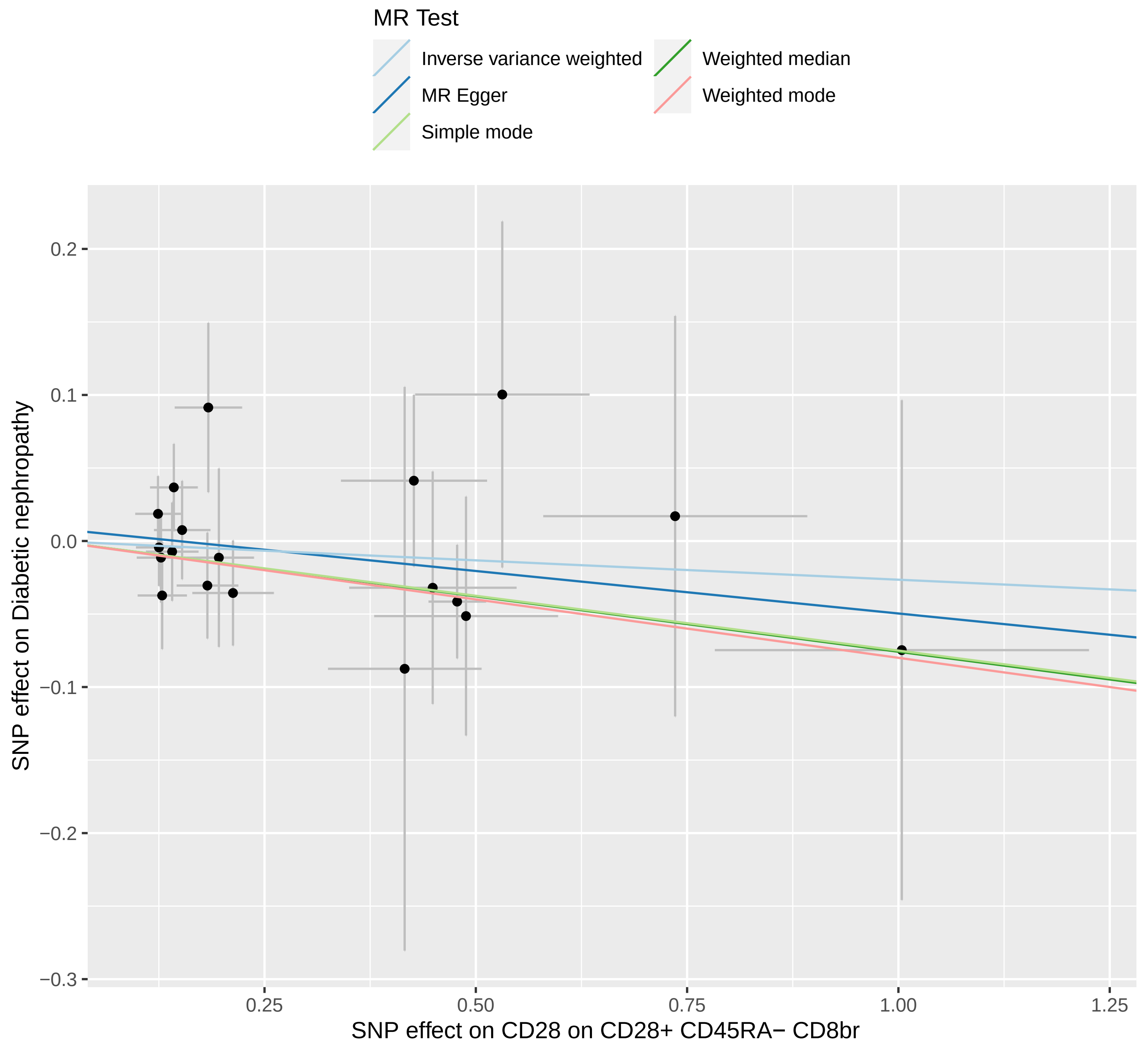

MR Test
Inverse variance weighted
MR Egger
Simple mode
Weighted median
Weighted mode
0.2
0.1
0.0
−0.1
−0.2
−0.3
0.25
0.50
0.75
1.00
1.25
SNP effect on Diabetic nephropathy
SNP effect on CD28 on CD28+ CD45RA− CD8br

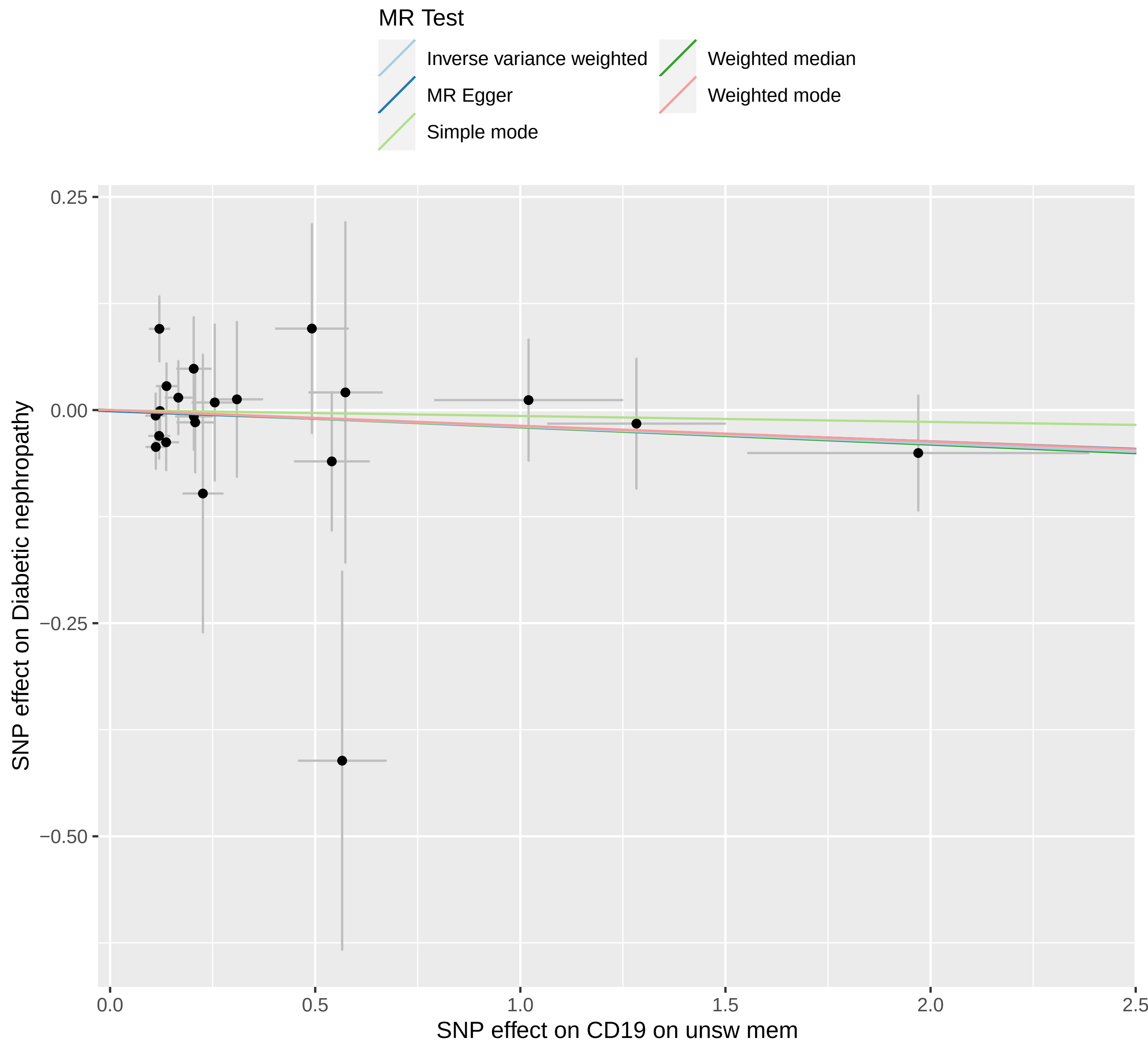

MR Test
Inverse variance weighted
MR Egger
Simple mode
Weighted median
Weighted mode
0.25
0.00
−0.25
−0.50
0.0
0.5
1.0
1.5
2.0
2.5
SNP effect on CD19 on unsw mem
SNP effect on Diabetic nephropathy

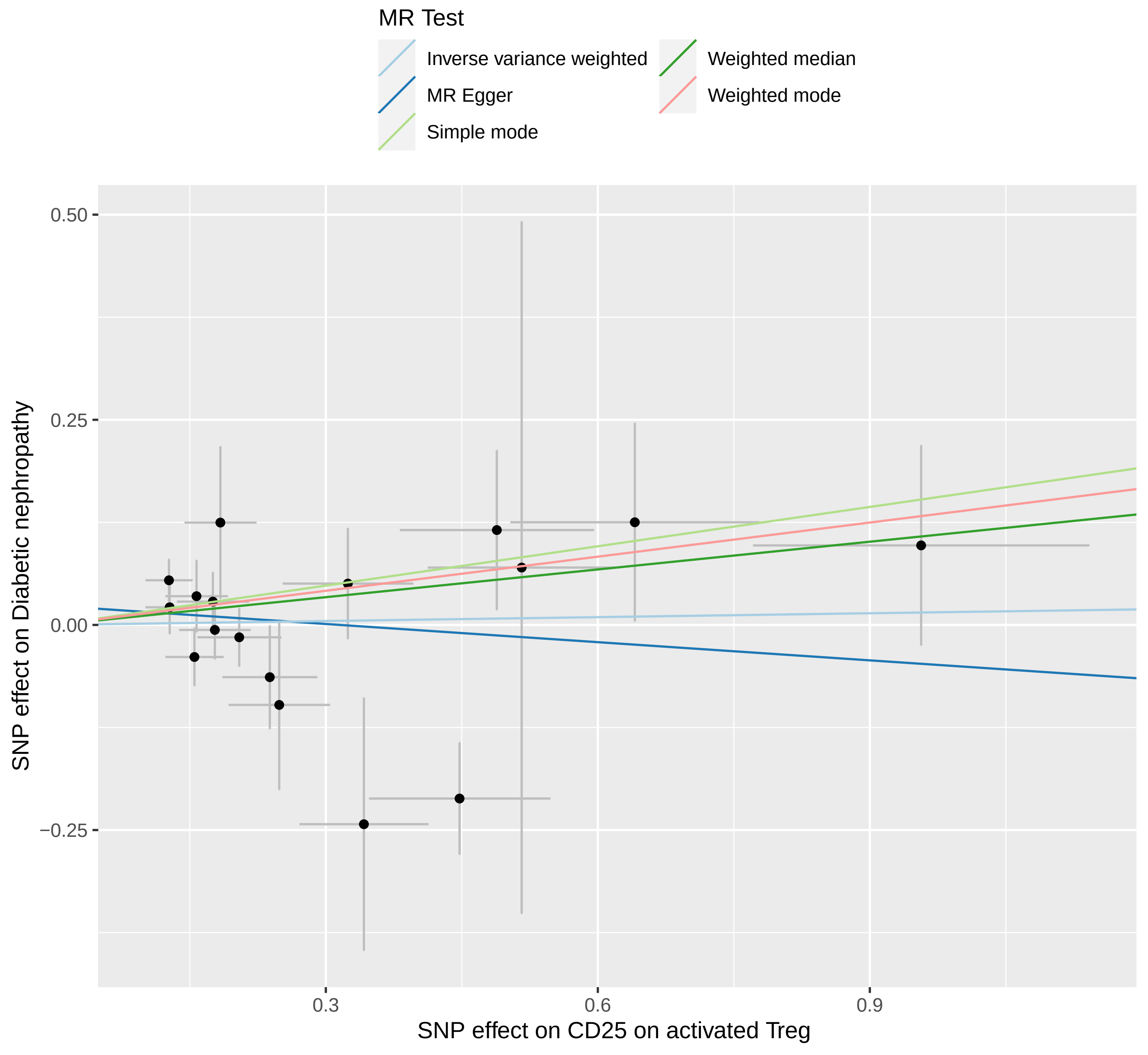
MR Test
Inverse variance weighted
MR Egger
Simple mode
Weighted median
Weighted mode
0.50
0.25
0.00
−0.25
SNP effect on Diabetic nephropathy
0.3
0.6
0.9
SNP effect on CD25 on activated Treg

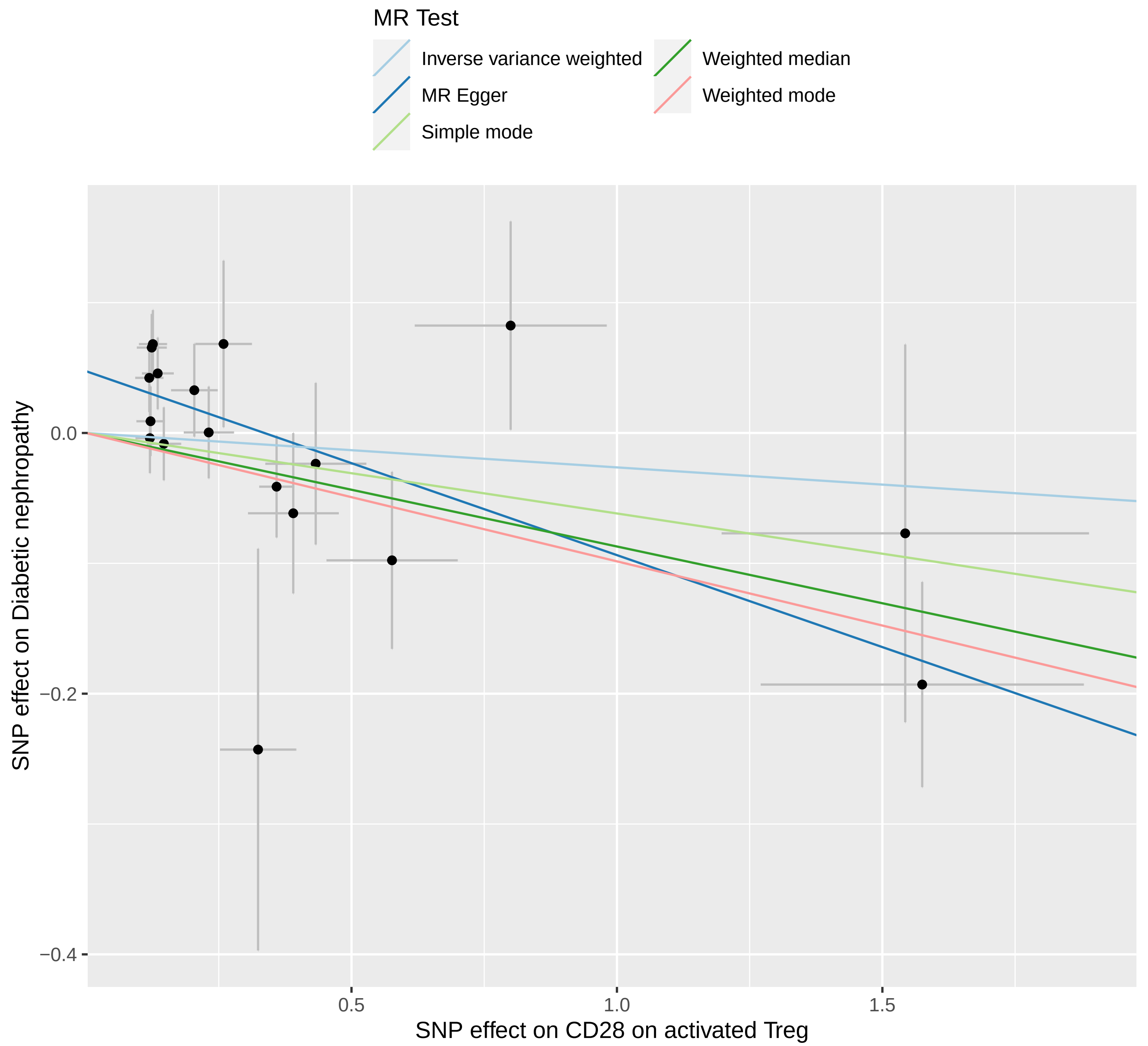

MR Test
Inverse variance weighted
MR Egger
Simple mode
Weighted median
Weighted mode
0.0
−0.2
−0.4
0.5
1.0
1.5
SNP effect on Diabetic nephropathy
SNP effect on CD28 on activated Treg

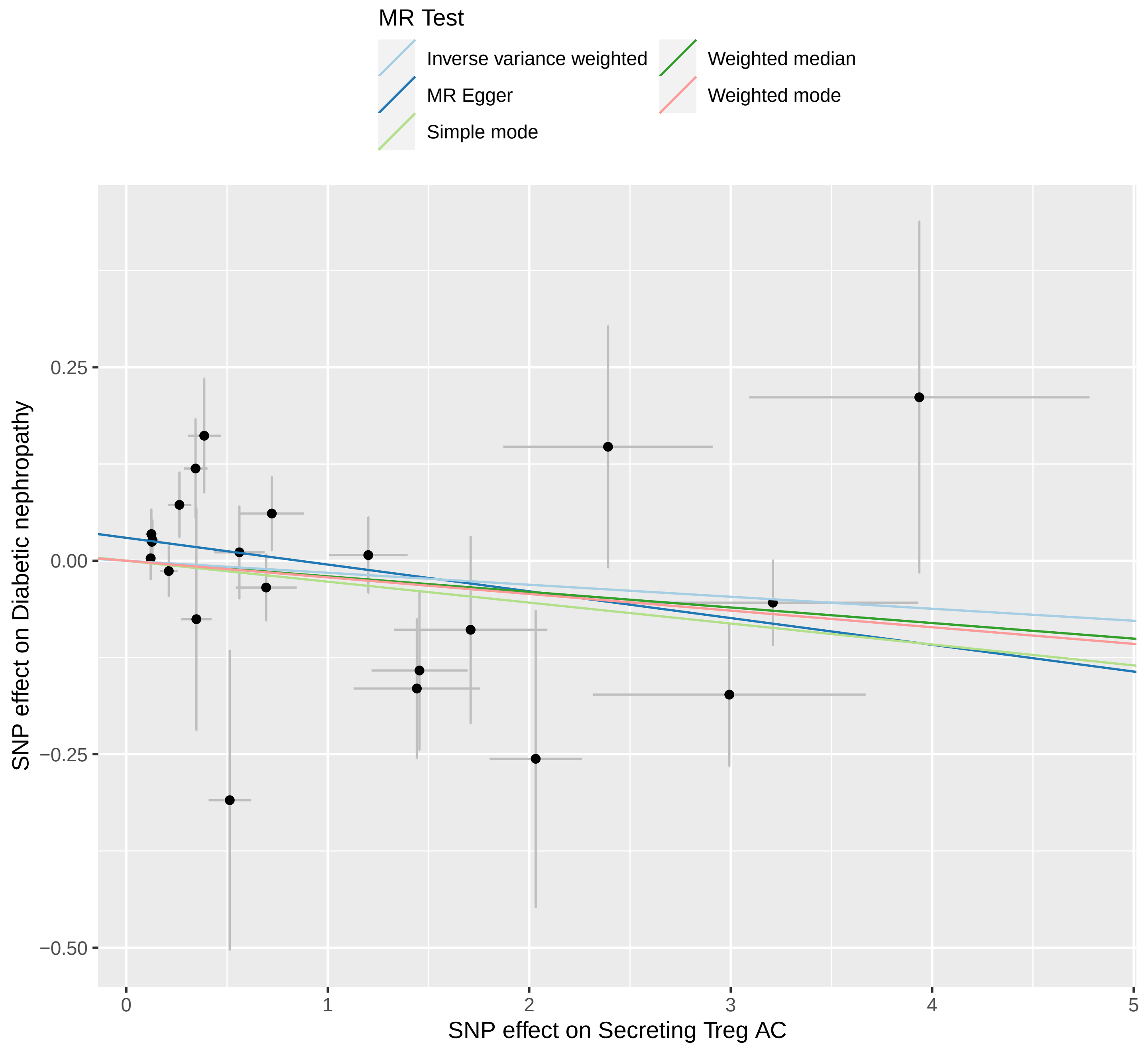

MR Test
Inverse variance weighted
MR Egger
Simple mode
Weighted median
Weighted mode
SNP effect on Diabetic nephropathy
0.25
0.00
−0.25
−0.50
0
1
2
3
4
5
SNP effect on Secreting Treg AC

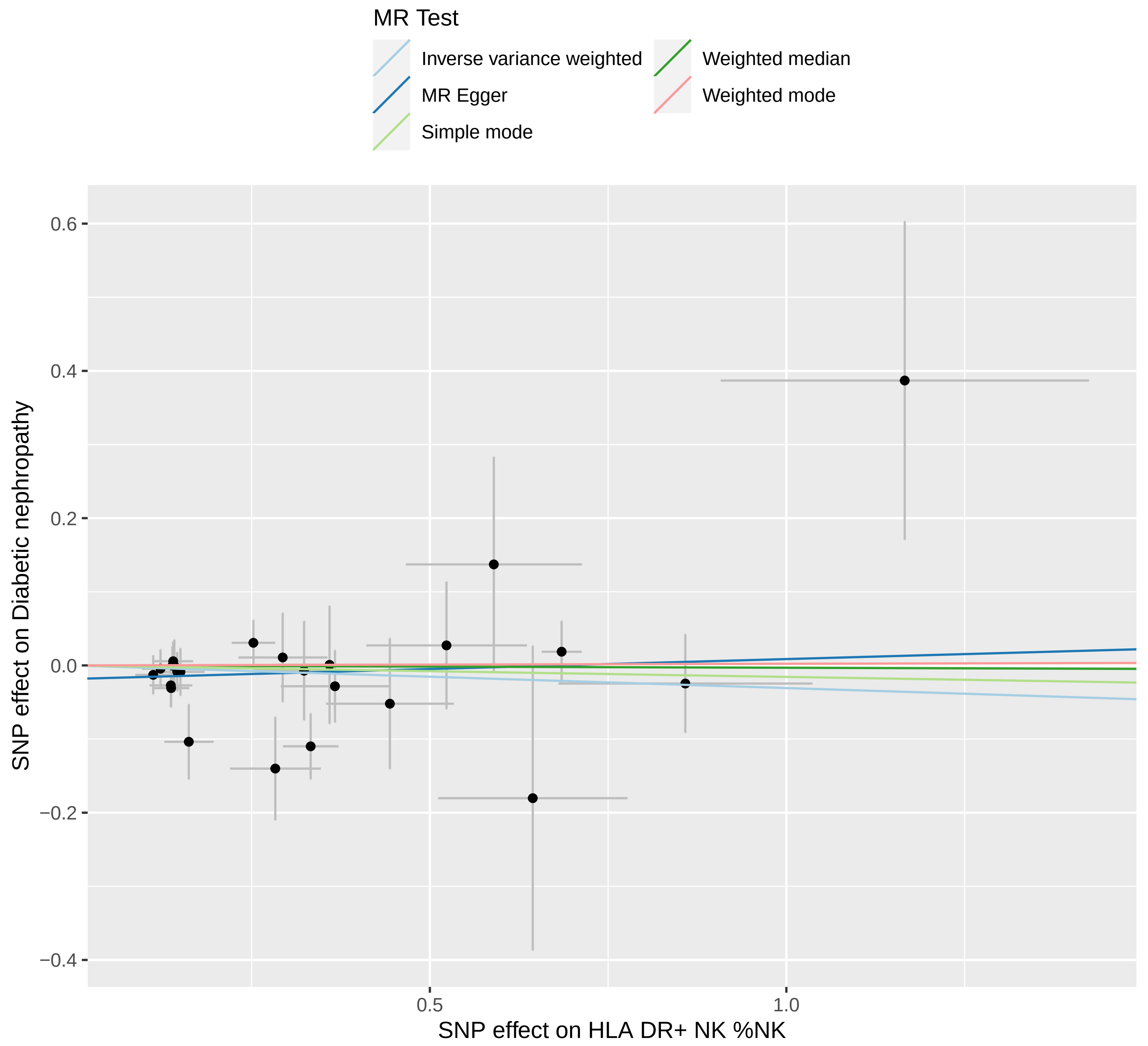

MR Test
Inverse variance weighted
MR Egger
Simple mode
Weighted median
Weighted mode
0.6
0.4
0.2
0.0
−0.2
−0.4
0.5
1.0
SNP effect on Diabetic nephropathy
SNP effect on HLA DR+ NK %NK

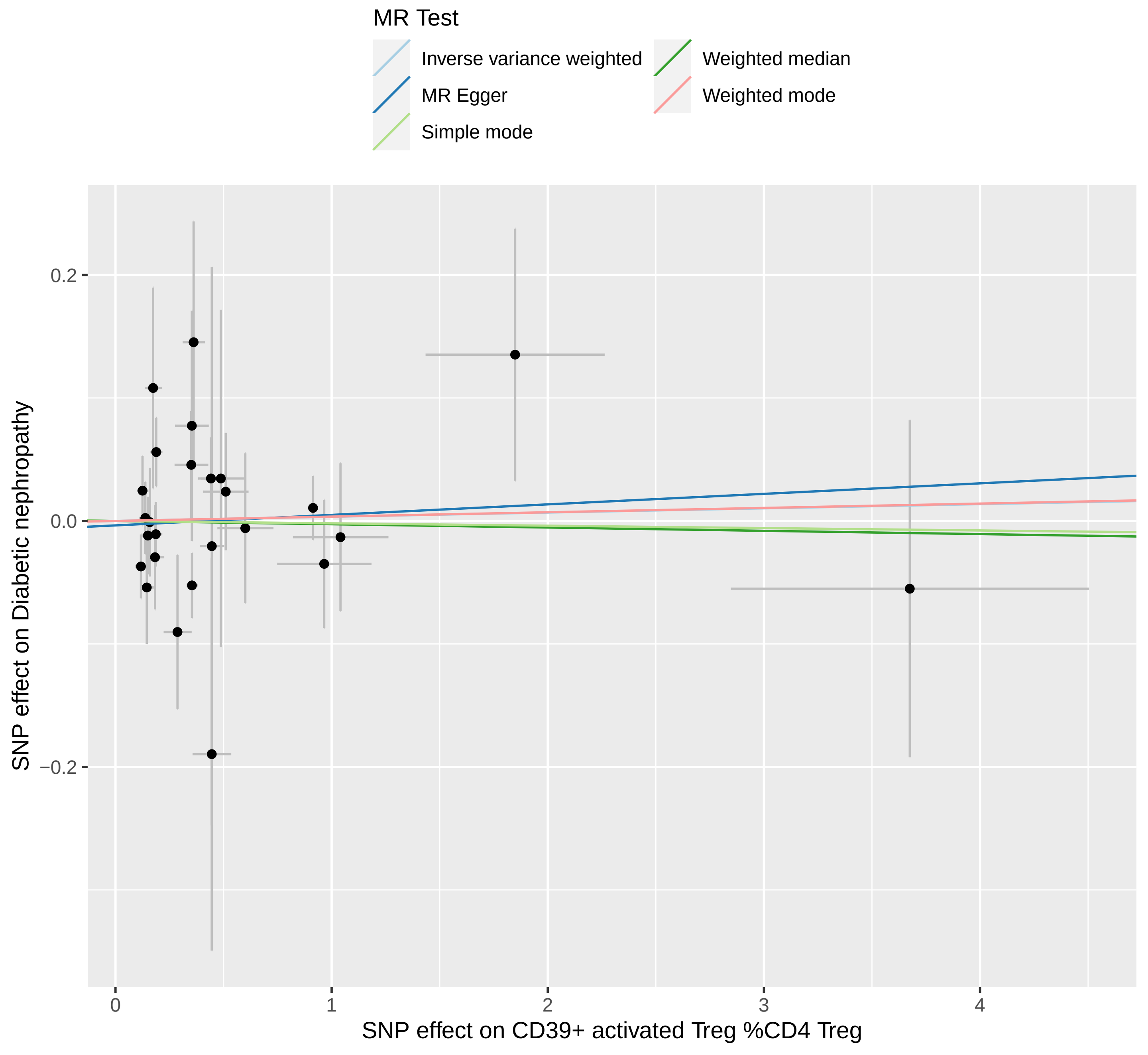
MR Test
Inverse variance weighted
MR Egger
Simple mode
Weighted median
Weighted mode
0.2
0.0
−0.2
0
1
2
3
4
SNP effect on CD39+ activated Treg %CD4 Treg
SNP effect on Diabetic nephropathy

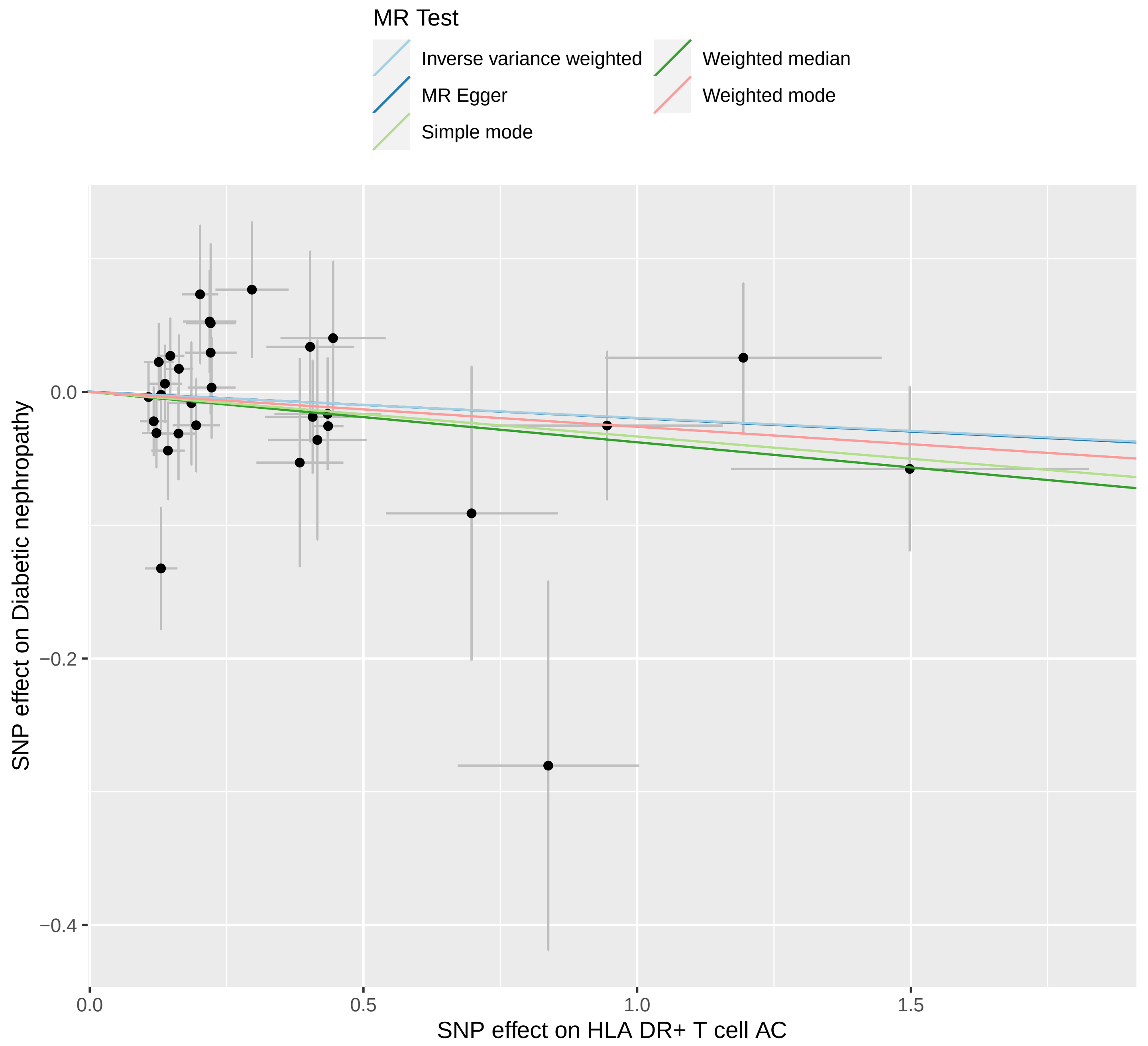
MR Test
Inverse variance weighted
MR Egger
Simple mode
Weighted median
Weighted mode
SNP effect on Diabetic nephropathy
0.0
−0.2
−0.4
0.0
0.5
1.0
1.5
SNP effect on HLA DR+ T cell AC

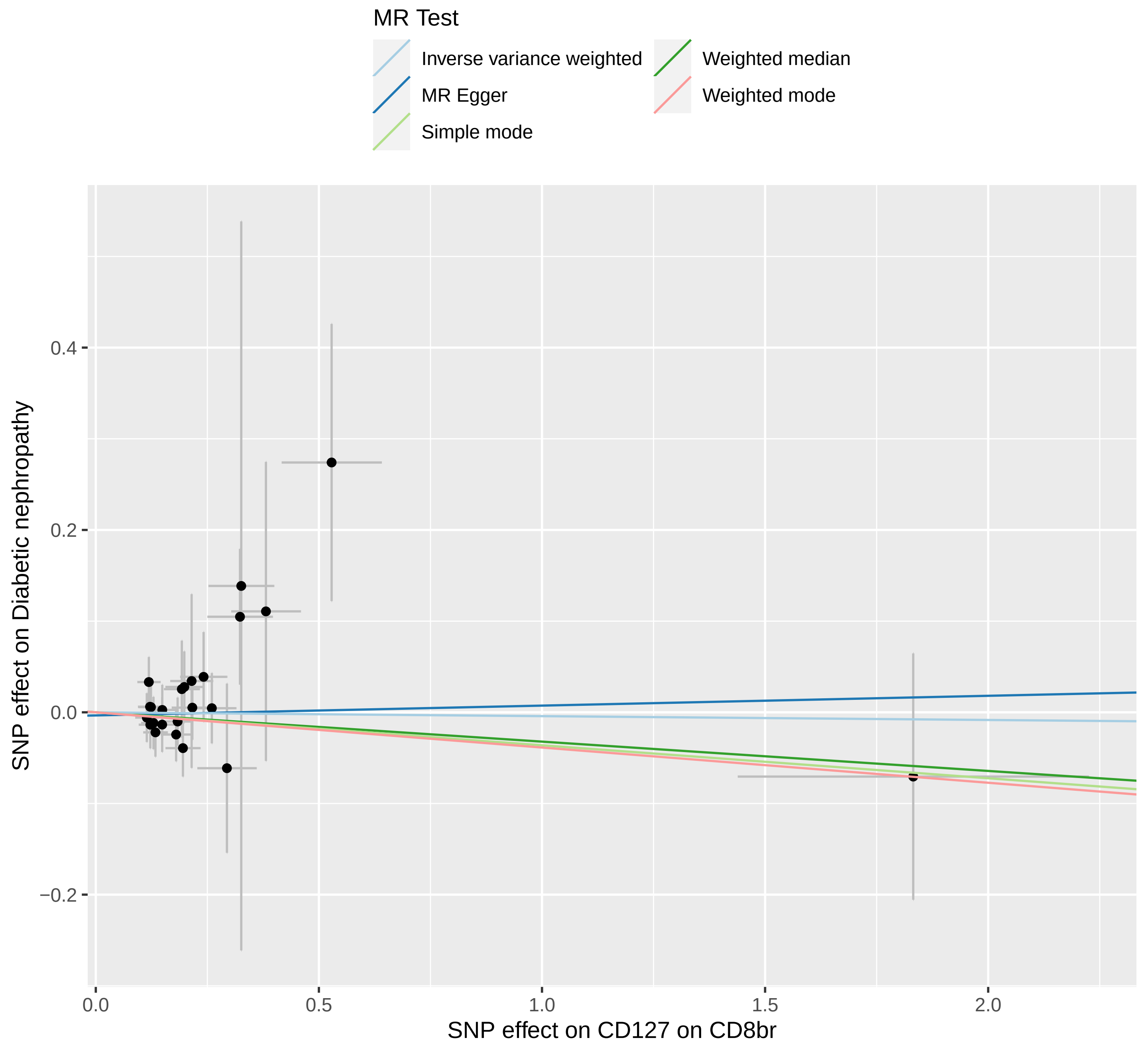

MR Test
Inverse variance weighted
MR Egger
Simple mode
Weighted median
Weighted mode
SNP effect on Diabetic nephropathy
0.4
0.2
0.0
−0.2
0.0
0.5
1.0
1.5
2.0
SNP effect on CD127 on CD8br

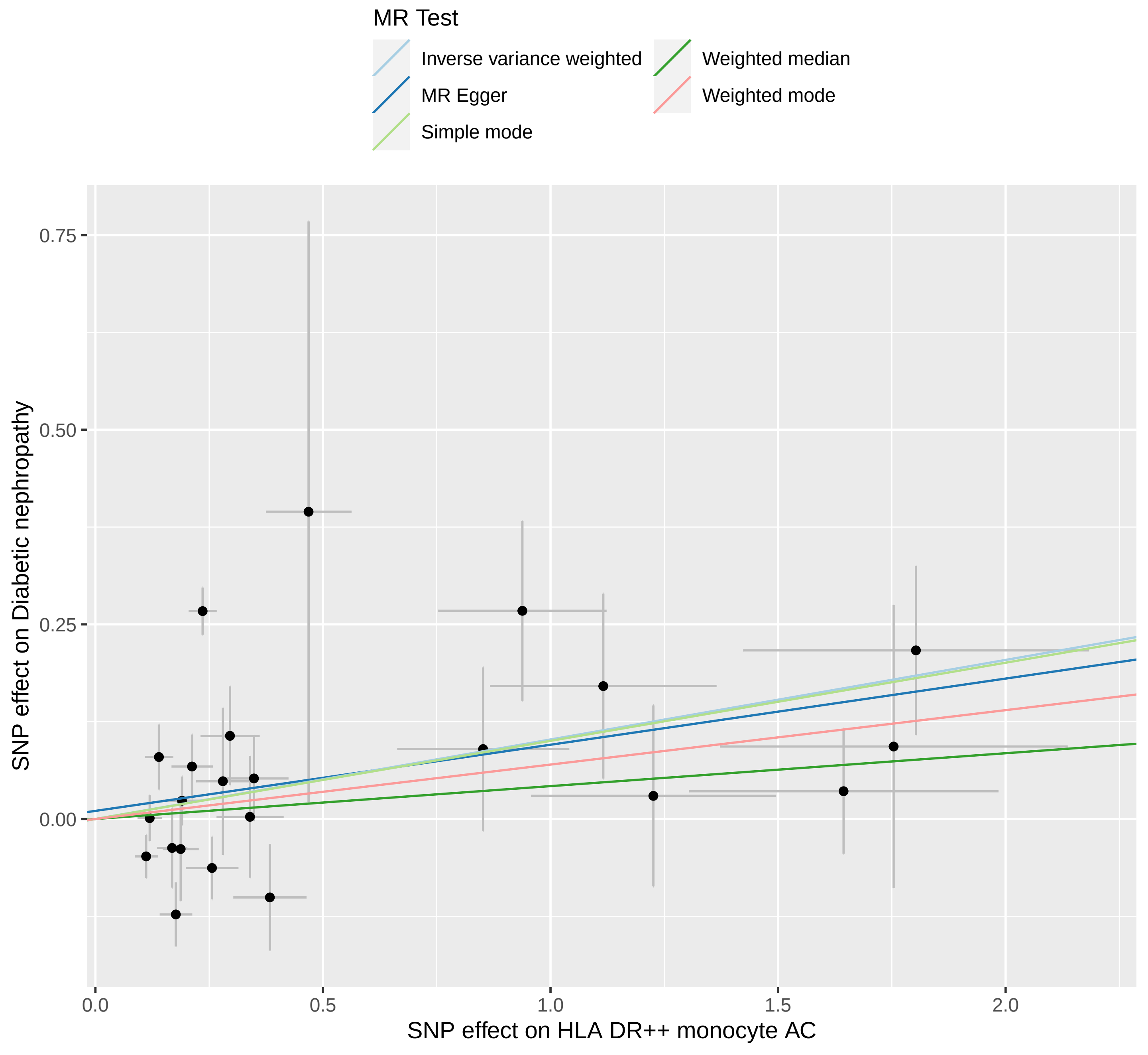
MR Test
Inverse variance weighted
MR Egger
Simple mode
Weighted median
Weighted mode
0.75
0.50
0.25
0.00
SNP effect on Diabetic nephropathy
0.0
0.5
1.0
1.5
2.0
SNP effect on HLA DR++ monocyte AC

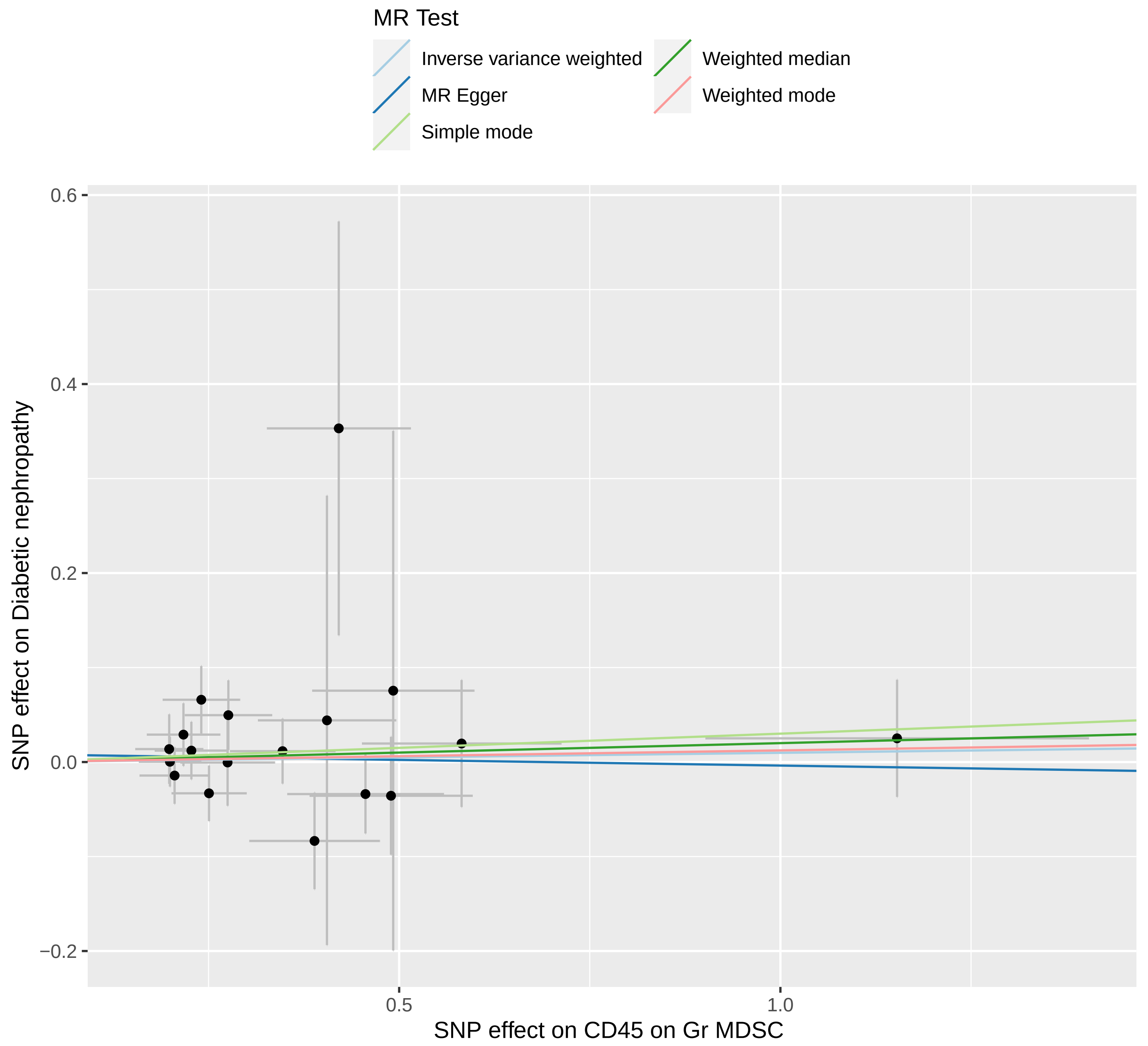
MR Test
Inverse variance weighted
MR Egger
Simple mode
Weighted median
Weighted mode
0.6
0.4
0.2
0.0
−0.2
0.5
1.0
SNP effect on Diabetic nephropathy
SNP effect on CD45 on Gr MDSC

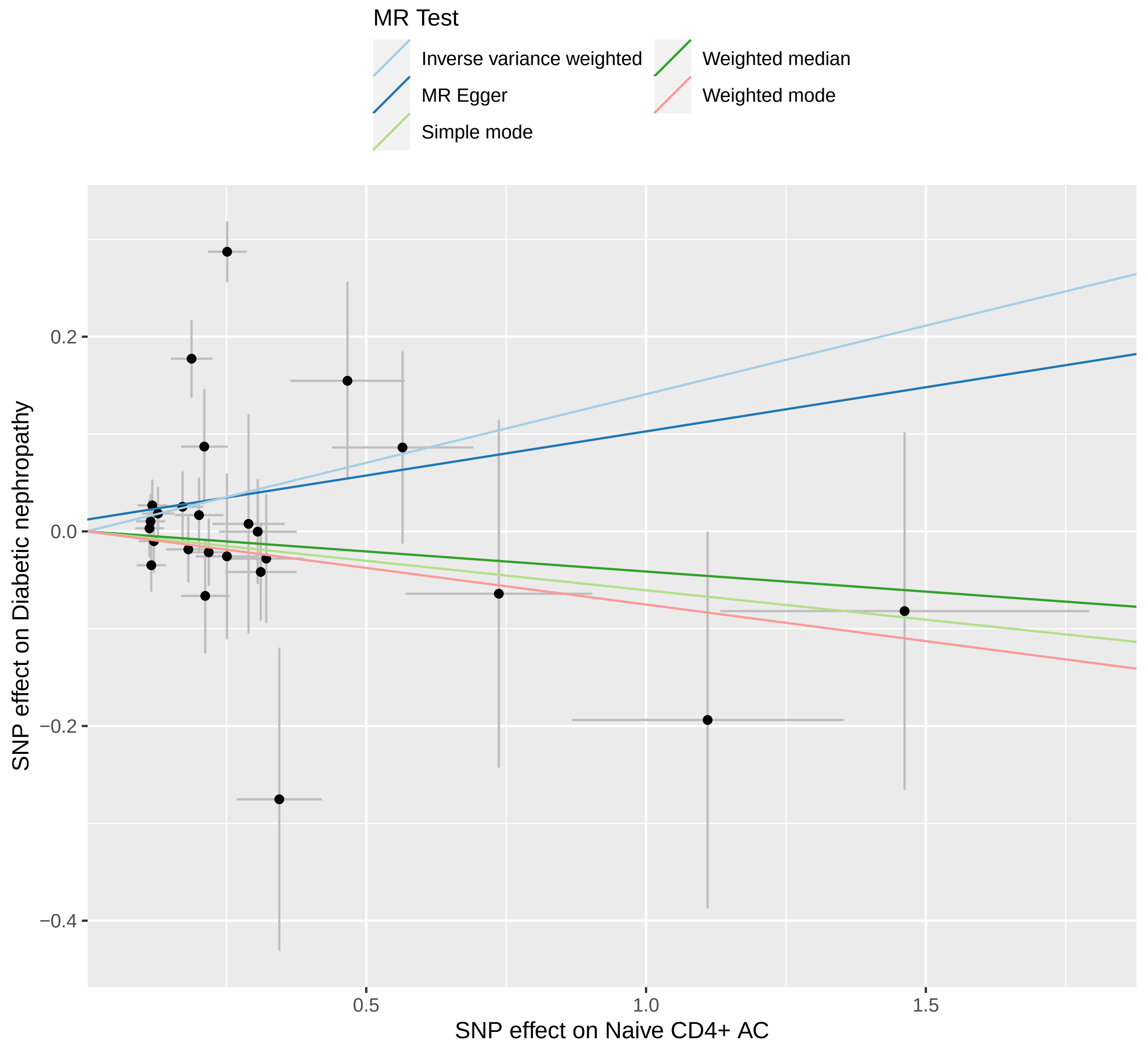
MR Test
Inverse variance weighted
MR Egger
Simple mode
Weighted median
Weighted mode
0.2
0.0
−0.2
−0.4
0.5
1.0
1.5
SNP effect on Diabetic nephropathy
SNP effect on Naive CD4+ AC

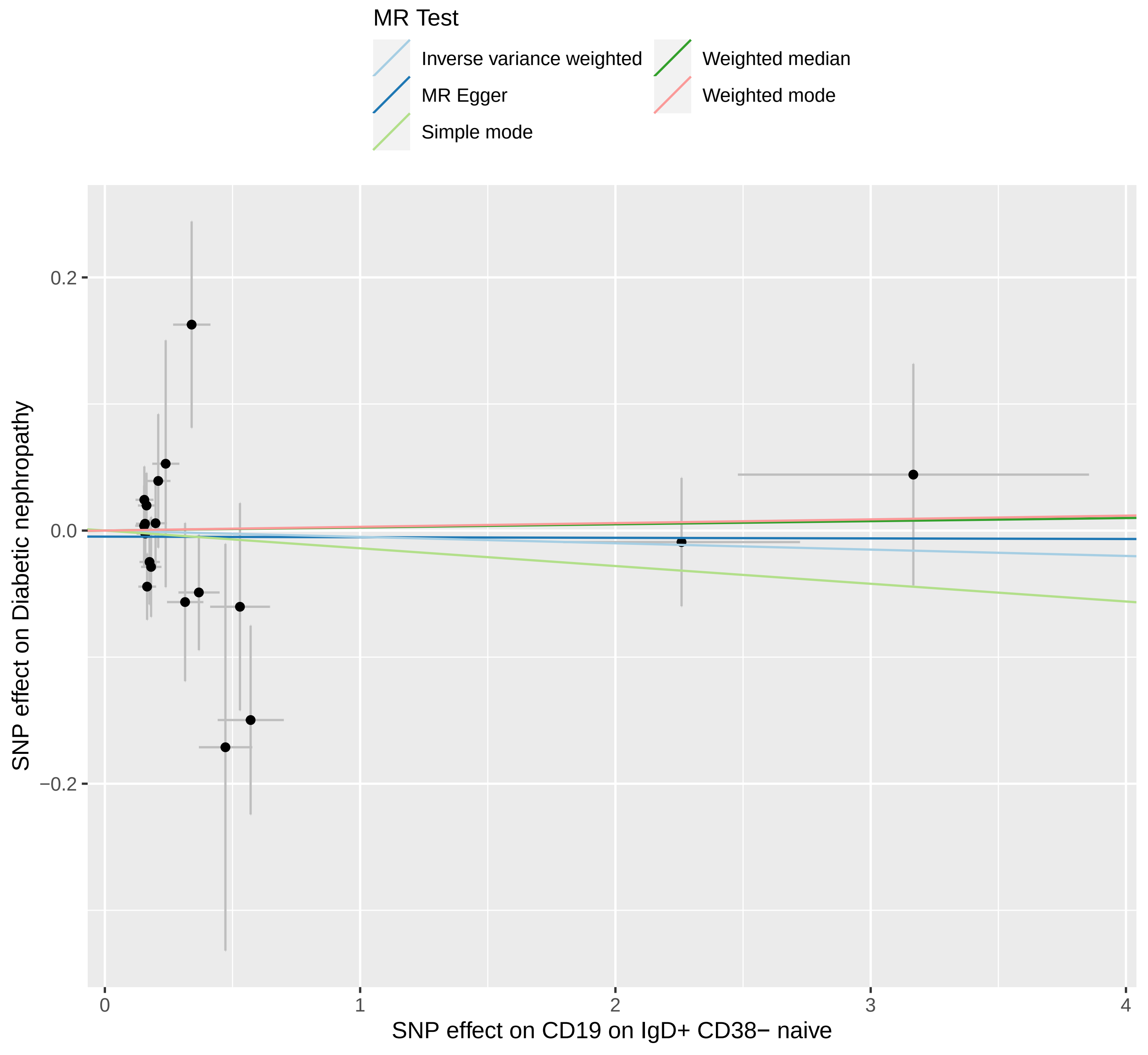
MR Test
Inverse variance weighted
MR Egger
Simple mode
Weighted median
Weighted mode
SNP effect on Diabetic nephropathy
0.2
0.0
−0.2
0
1
2
3
4
SNP effect on CD19 on IgD+ CD38− naive

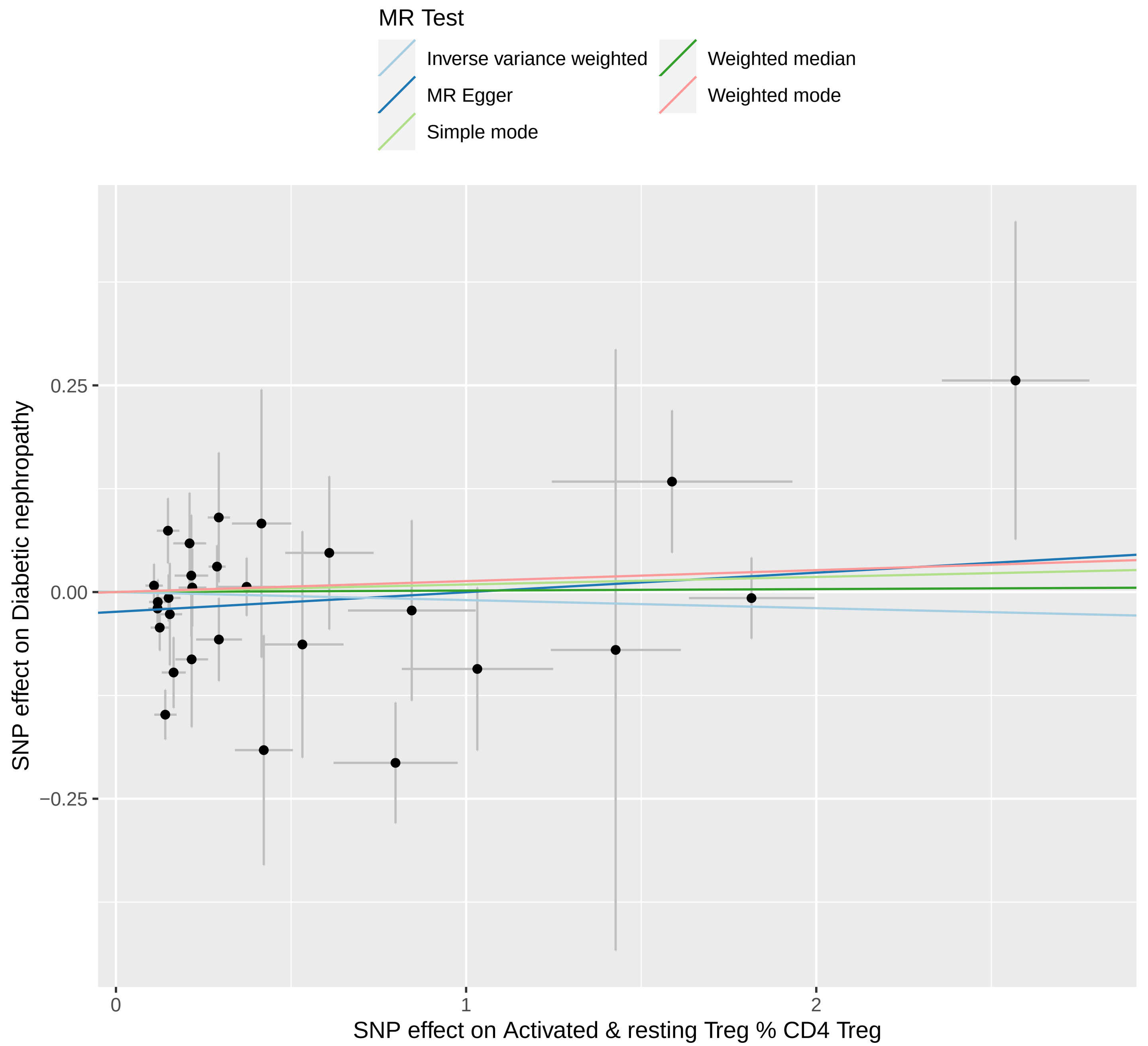
MR Test
Inverse variance weighted
MR Egger
Simple mode
Weighted median
Weighted mode
0.25
0.00
−0.25
0
1
2
SNP effect on Diabetic nephropathy
SNP effect on Activated & resting Treg % CD4 Treg

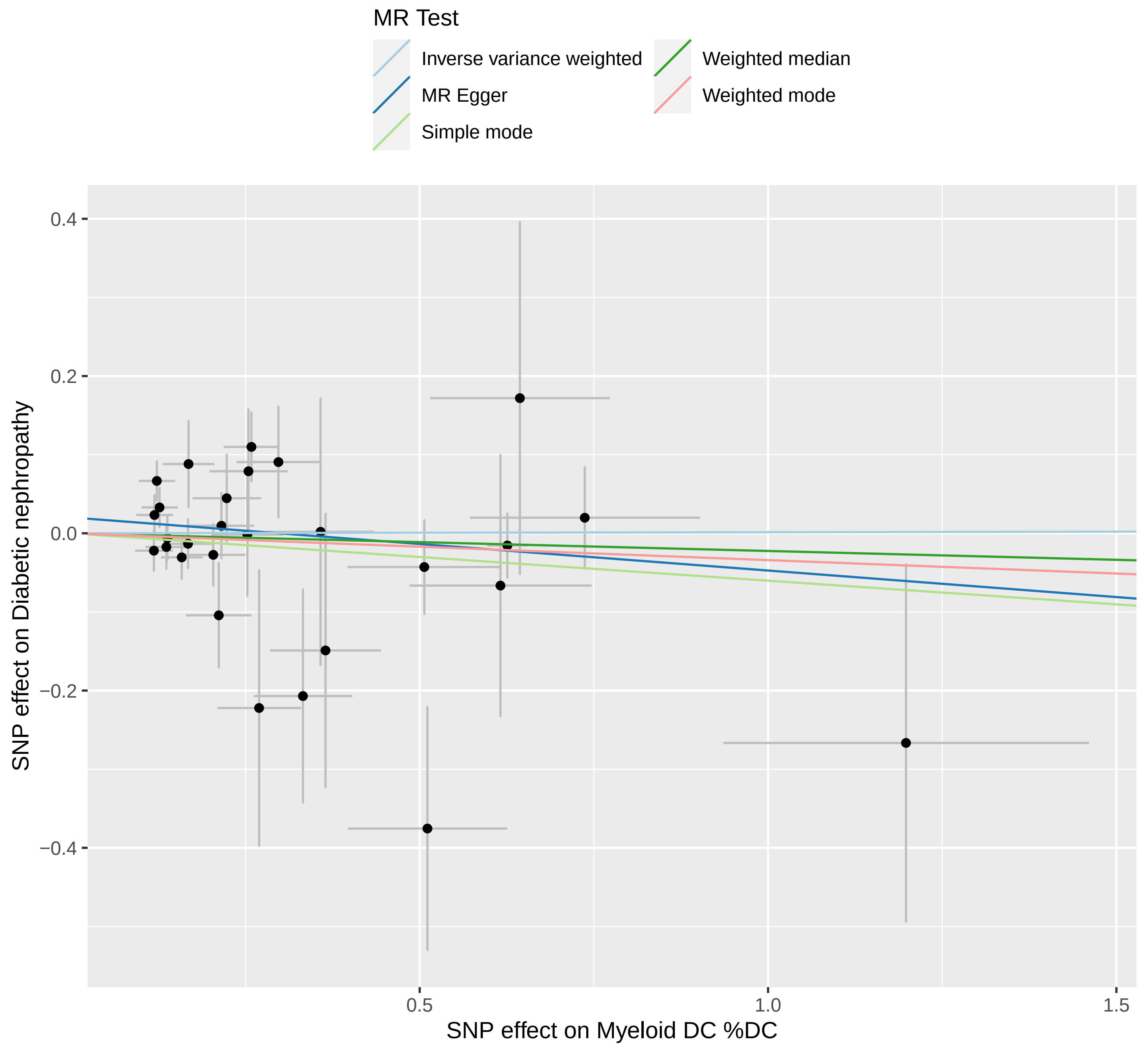

MR Test
Inverse variance weighted
MR Egger
Simple mode
Weighted median
Weighted mode
0.4
0.2
0.0
−0.2
−0.4
0.5
1.0
1.5
SNP effect on Diabetic nephropathy
SNP effect on Myeloid DC %DC

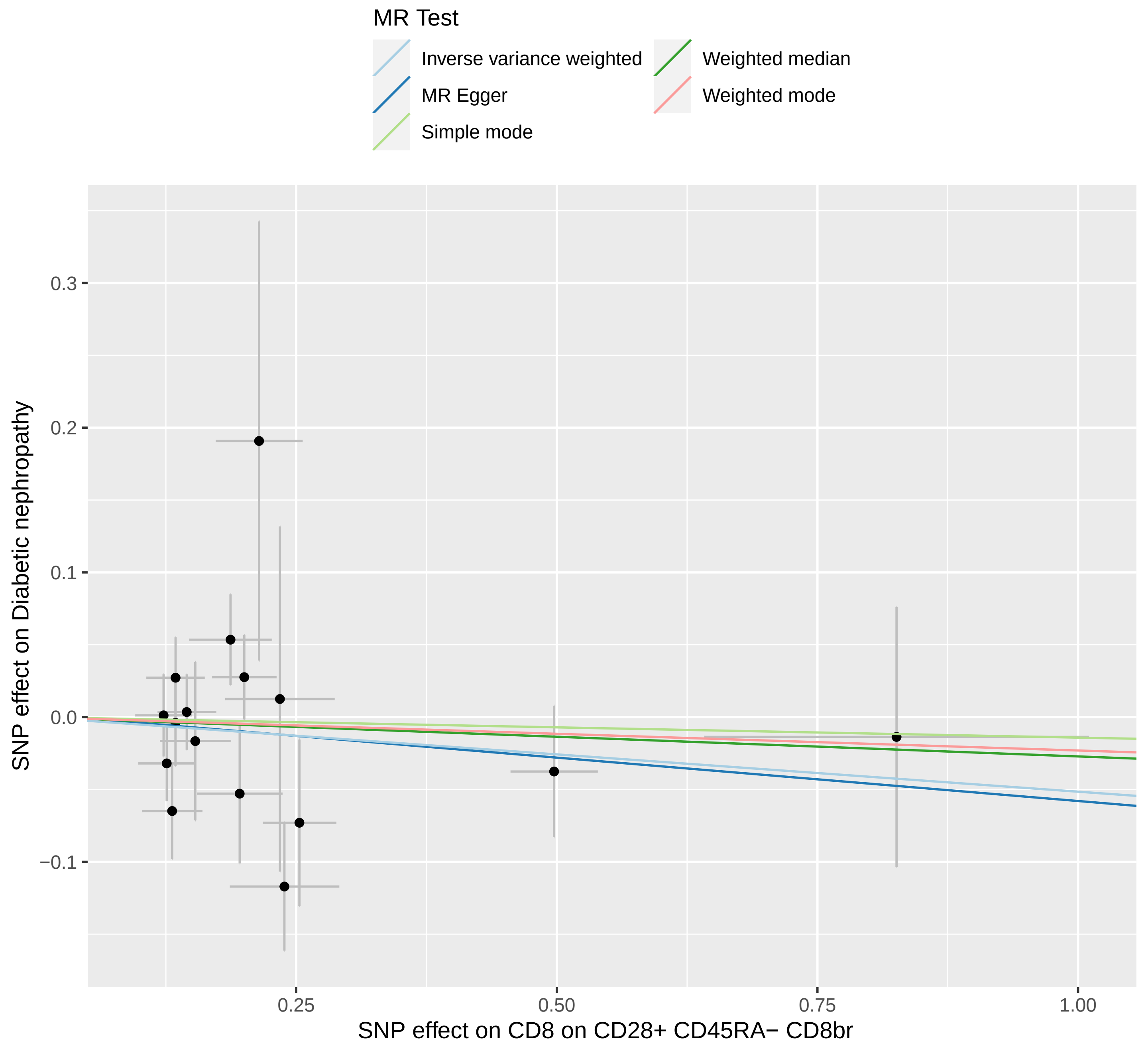
MR Test
Inverse variance weighted
MR Egger
Simple mode
Weighted median
Weighted mode
0.3
0.2
0.1
0.0
−0.1
0.25
0.50
0.75
1.00
SNP effect on Diabetic nephropathy
SNP effect on CD8 on CD28+ CD45RA− CD8br

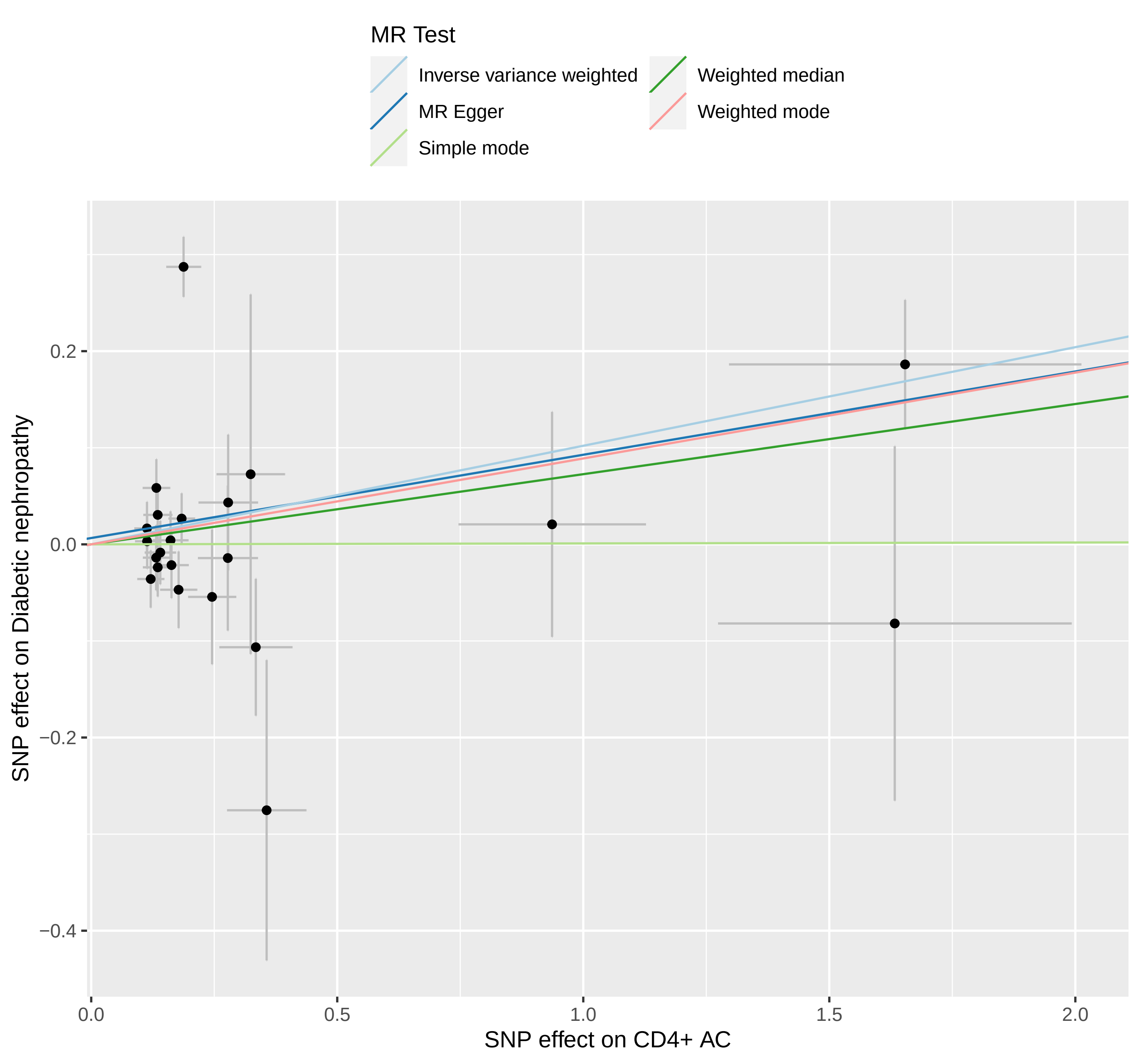
MR Test
Inverse variance weighted
MR Egger
Simple mode
Weighted median
Weighted mode
SNP effect on Diabetic nephropathy
0.2
0.0
−0.2
−0.4
0.0
0.5
1.0
1.5
2.0
SNP effect on CD4+ AC

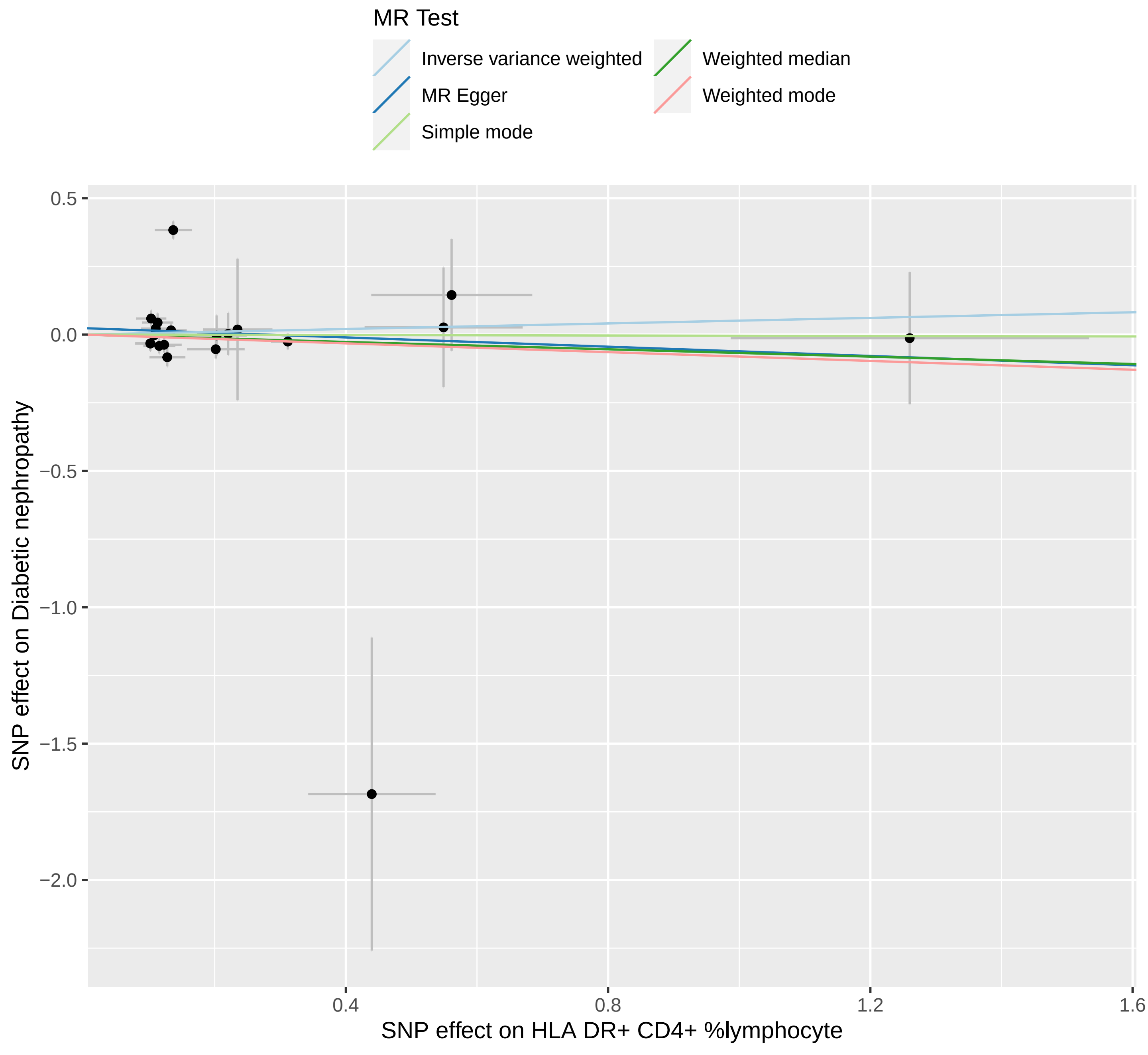

MR Test
Inverse variance weighted
MR Egger
Simple mode
Weighted median
Weighted mode
0.5
0.0
−0.5
−1.0
−1.5
−2.0
SNP effect on Diabetic nephropathy
0.4
0.8
1.2
1.6
SNP effect on HLA DR+ CD4+ %lymphocyte

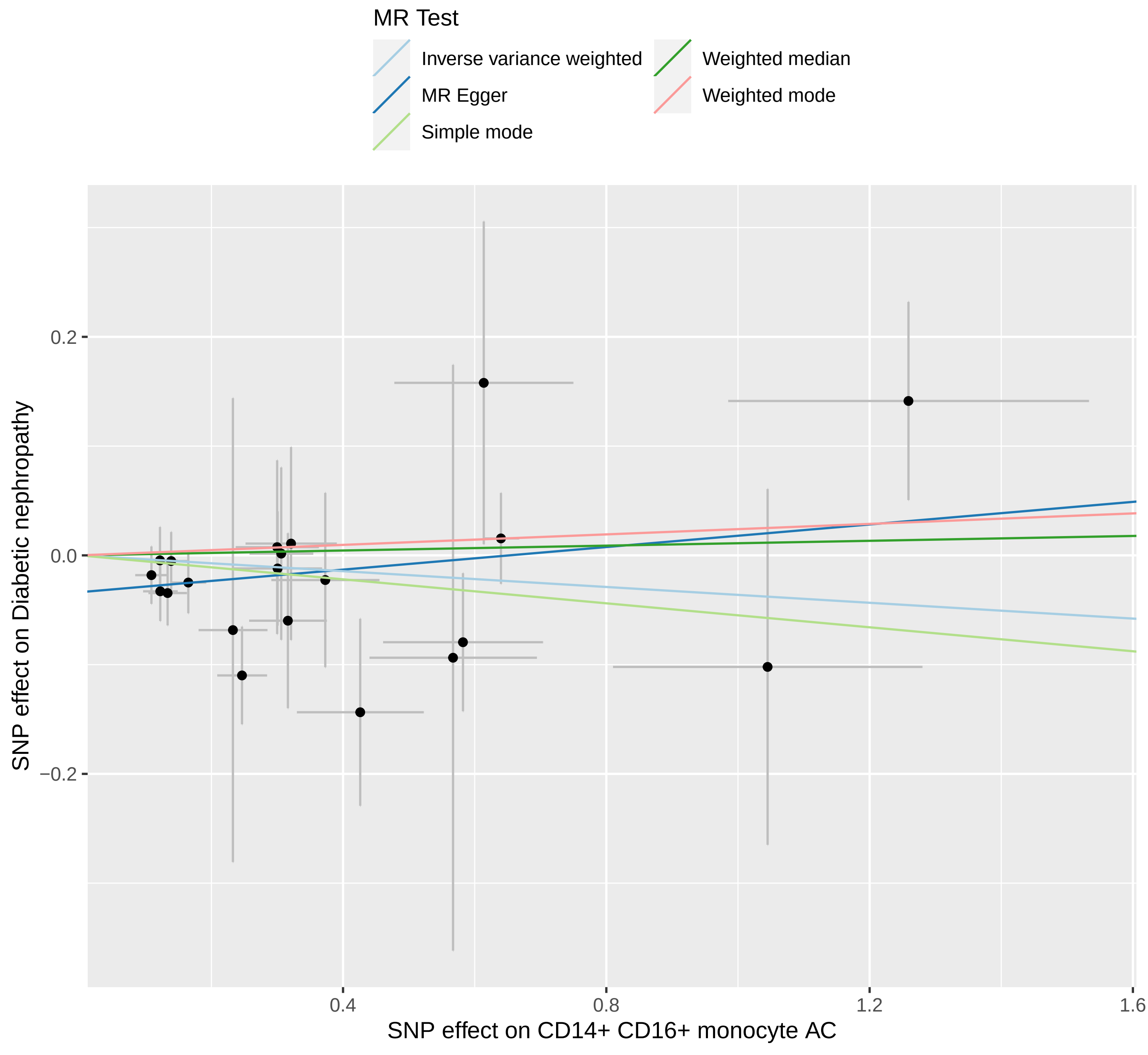

MR Test
Inverse variance weighted
MR Egger
Simple mode
Weighted median
Weighted mode
0.2
0.0
−0.2
0.4
0.8
1.2
1.6
SNP effect on CD14+ CD16+ monocyte AC
SNP effect on Diabetic nephropathy

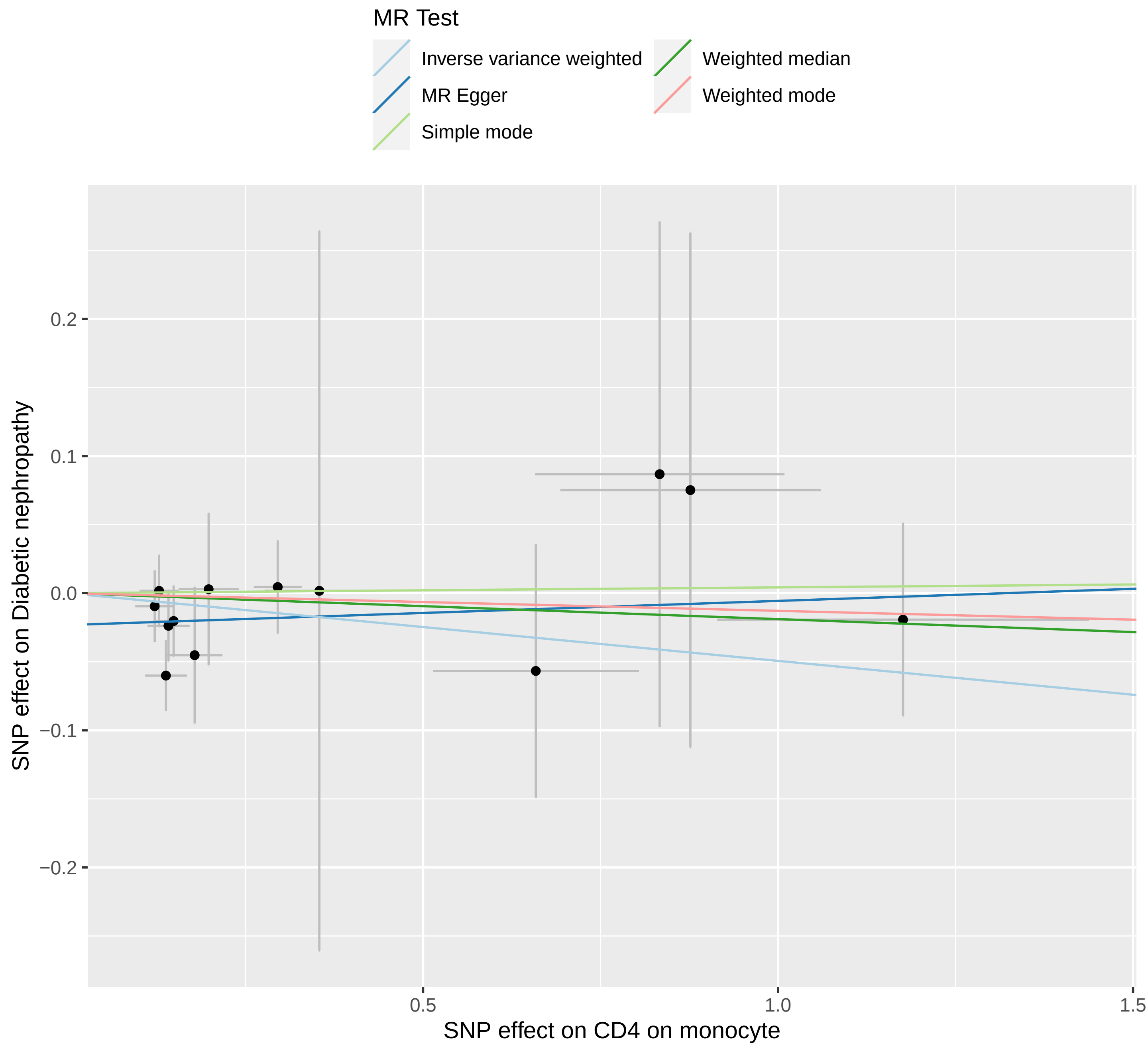

MR Test
Inverse variance weighted
MR Egger
Simple mode
Weighted median
Weighted mode
0.2
0.1
0.0
−0.1
−0.2
SNP effect on Diabetic nephropathy
0.5
1.0
1.5
SNP effect on CD4 on monocyte

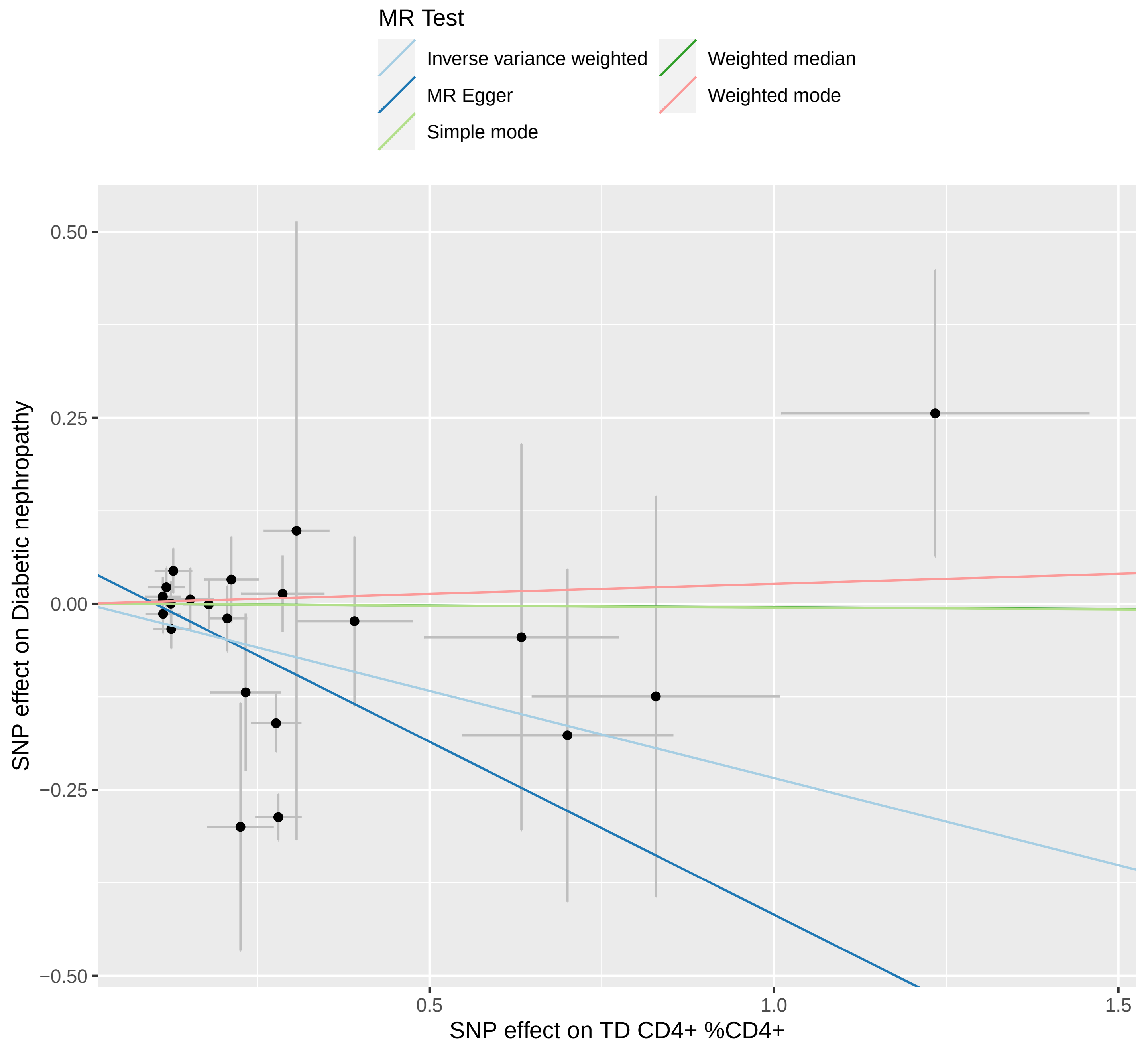
MR Test
Inverse variance weighted
MR Egger
Simple mode
Weighted median
Weighted mode
0.50
0.25
0.00
−0.25
−0.50
0.5
1.0
1.5
SNP effect on Diabetic nephropathy
SNP effect on TD CD4+ %CD4+

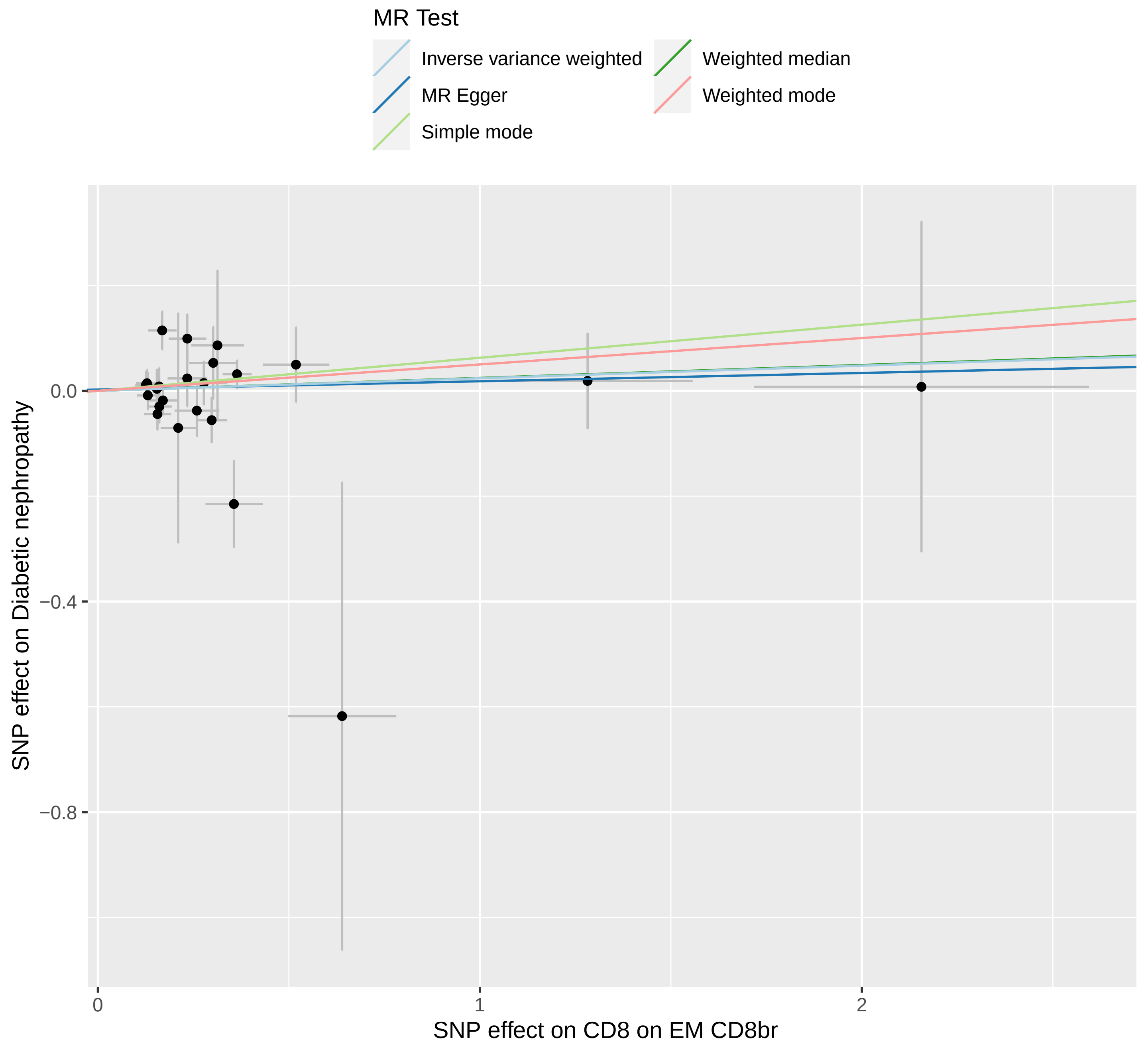
MR Test
Inverse variance weighted
MR Egger
Simple mode
Weighted median
Weighted mode
SNP effect on Diabetic nephropathy
0.0
−0.4
−0.8
0
1
2
SNP effect on CD8 on EM CD8br

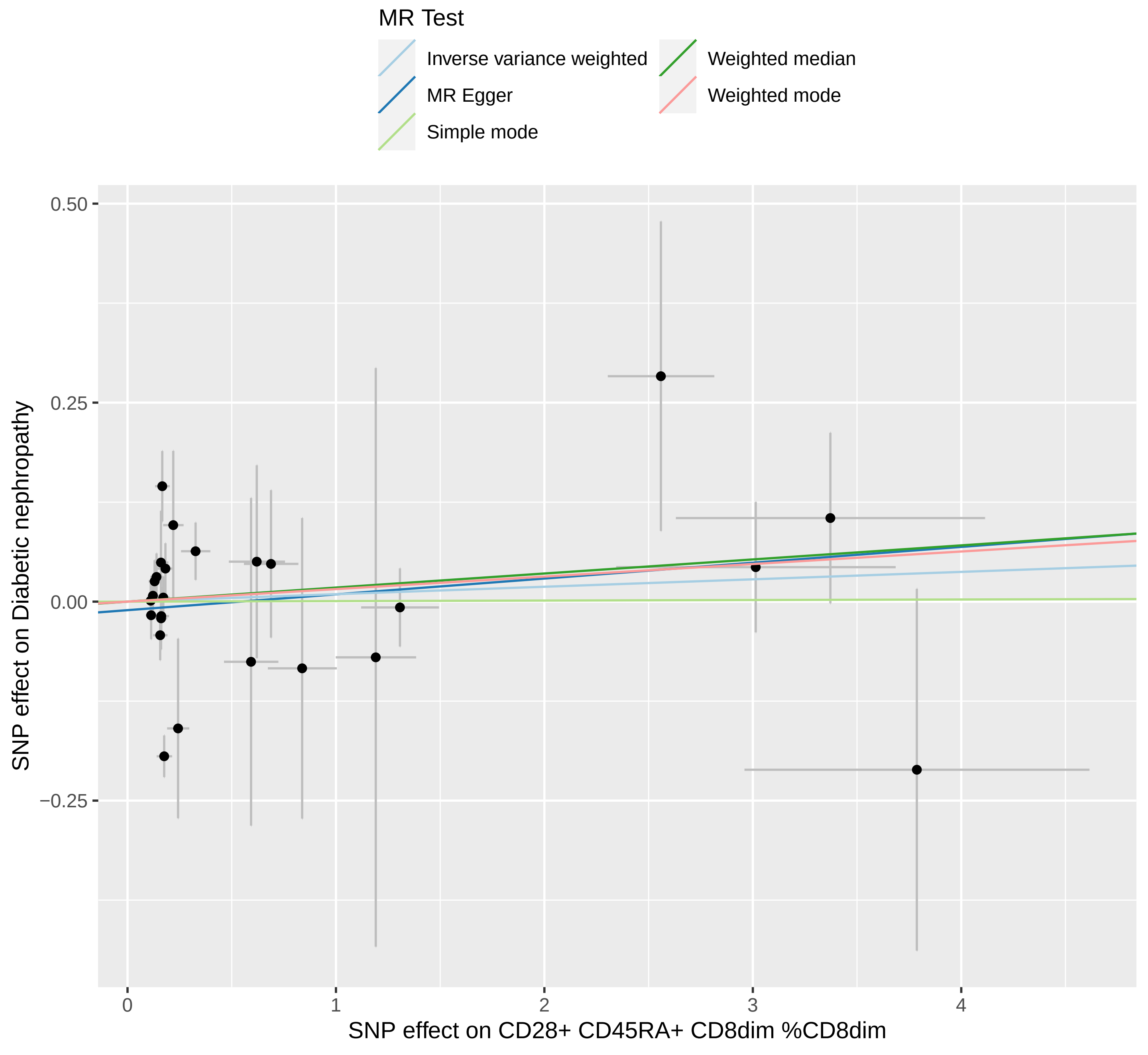
MR Test
Inverse variance weighted
MR Egger
Simple mode
Weighted median
Weighted mode
0.50
0.25
0.00
−0.25
0
1
2
3
4
SNP effect on Diabetic nephropathy
SNP effect on CD28+ CD45RA+ CD8dim %CD8dim

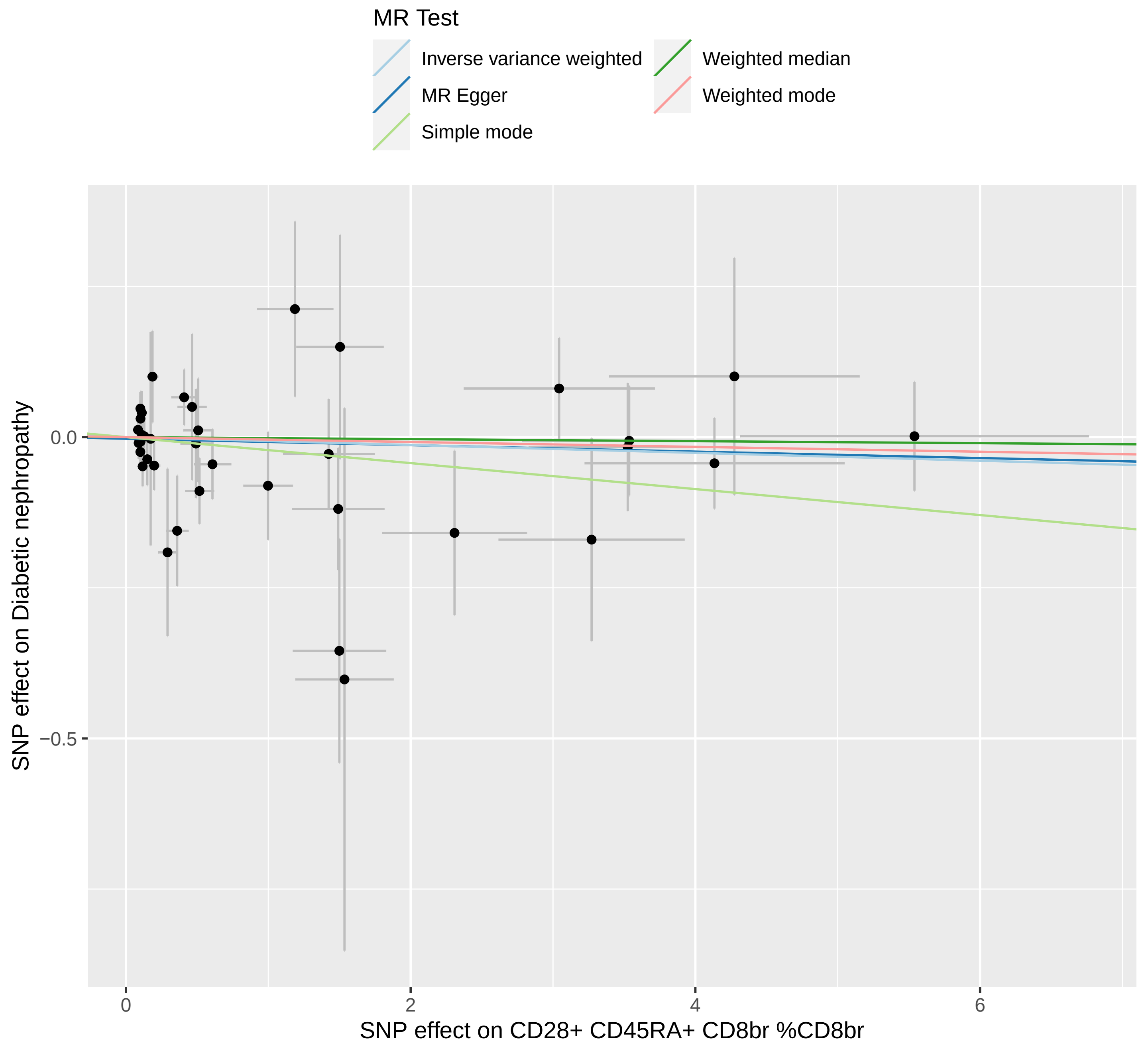
MR Test
Inverse variance weighted
Weighted median
MR Egger
Weighted mode
Simple mode
0.0
−0.5
0
2
4
6
SNP effect on Diabetic nephropathy
SNP effect on CD28+ CD45RA+ CD8br %CD8br

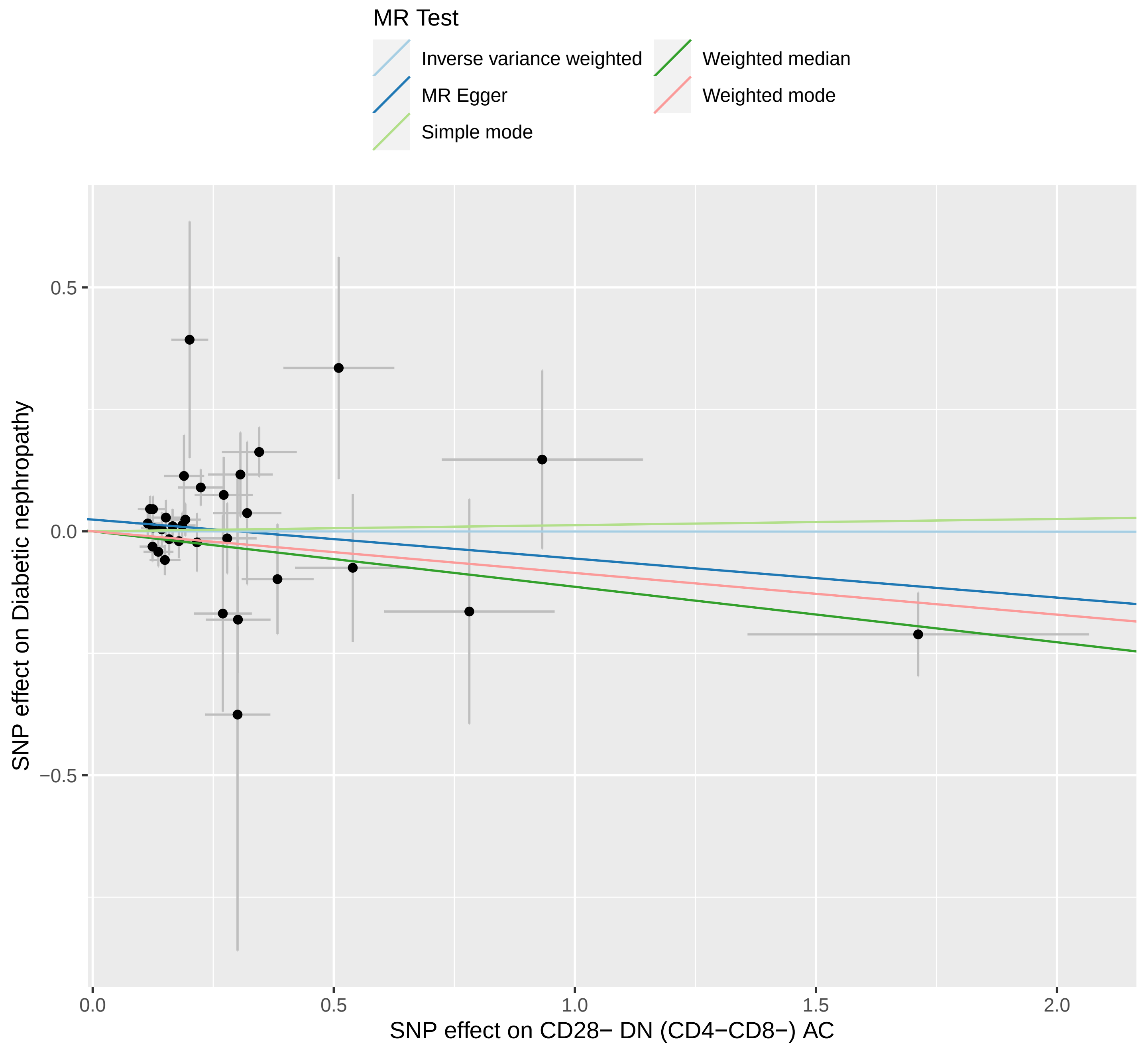

MR Test
Inverse variance weighted
MR Egger
Simple mode
Weighted median
Weighted mode
0.5
0.0
−0.5
0.0
0.5
1.0
1.5
2.0
SNP effect on Diabetic nephropathy
SNP effect on CD28− DN (CD4−CD8−) AC

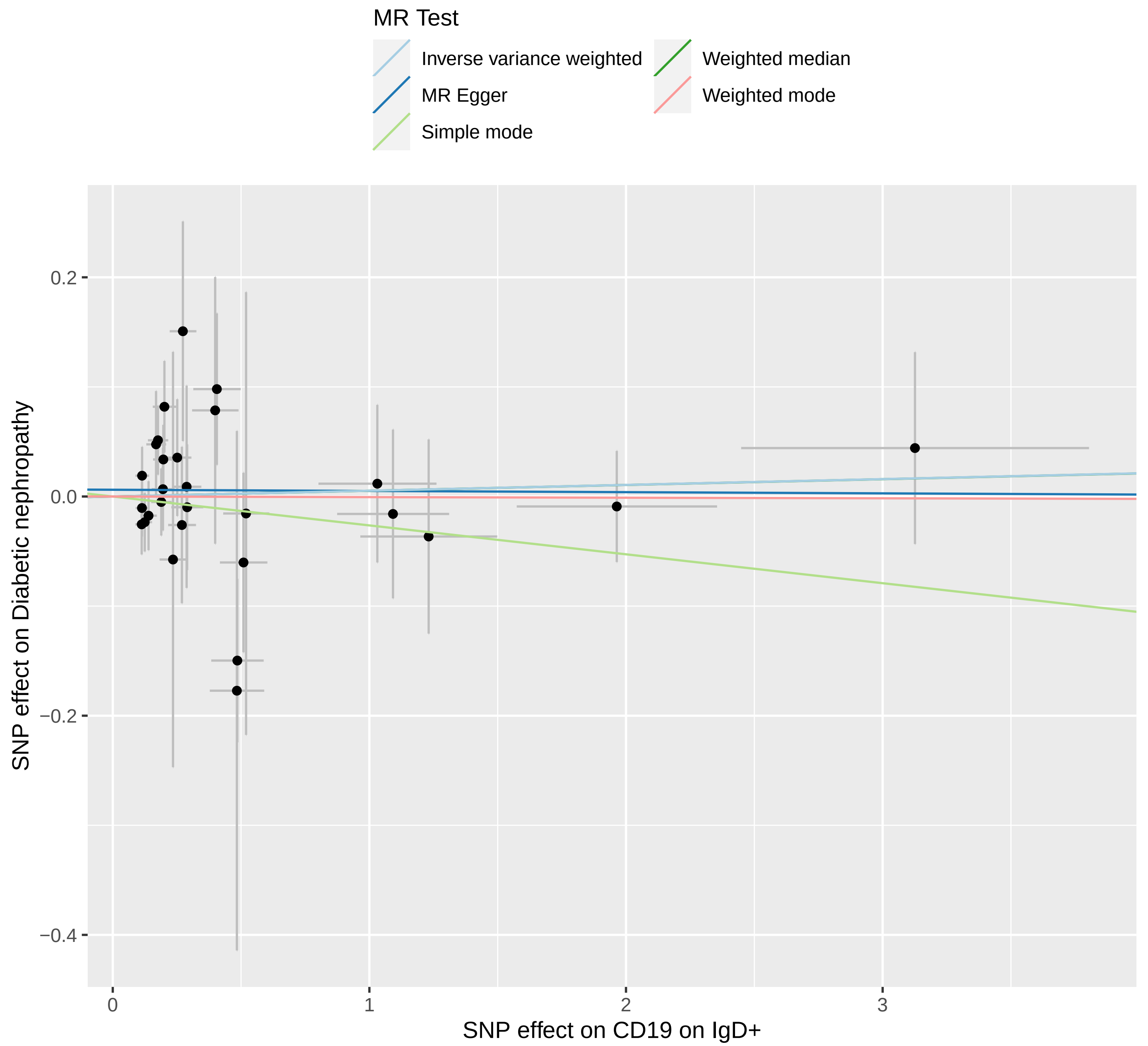

MR Test
Inverse variance weighted
MR Egger
Simple mode
Weighted median
Weighted mode
0.2
0.0
−0.2
−0.4
0
1
2
3
SNP effect on Diabetic nephropathy
SNP effect on CD19 on IgD+

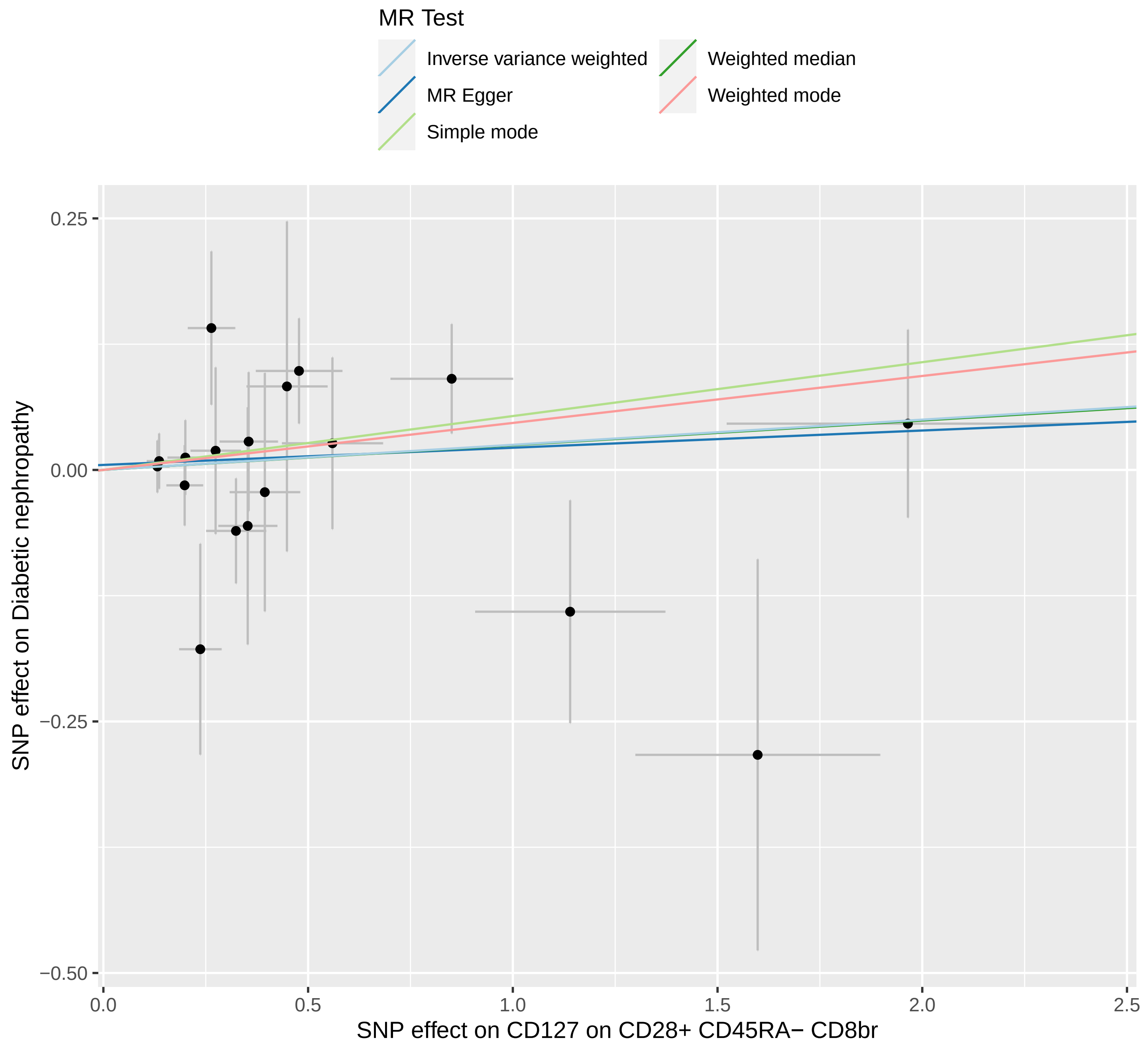

MR Test
Inverse variance weighted
MR Egger
Simple mode
Weighted median
Weighted mode
0.25
0.00
−0.25
−0.50
0.0
0.5
1.0
1.5
2.0
2.5
SNP effect on Diabetic nephropathy
SNP effect on CD127 on CD28+ CD45RA− CD8br

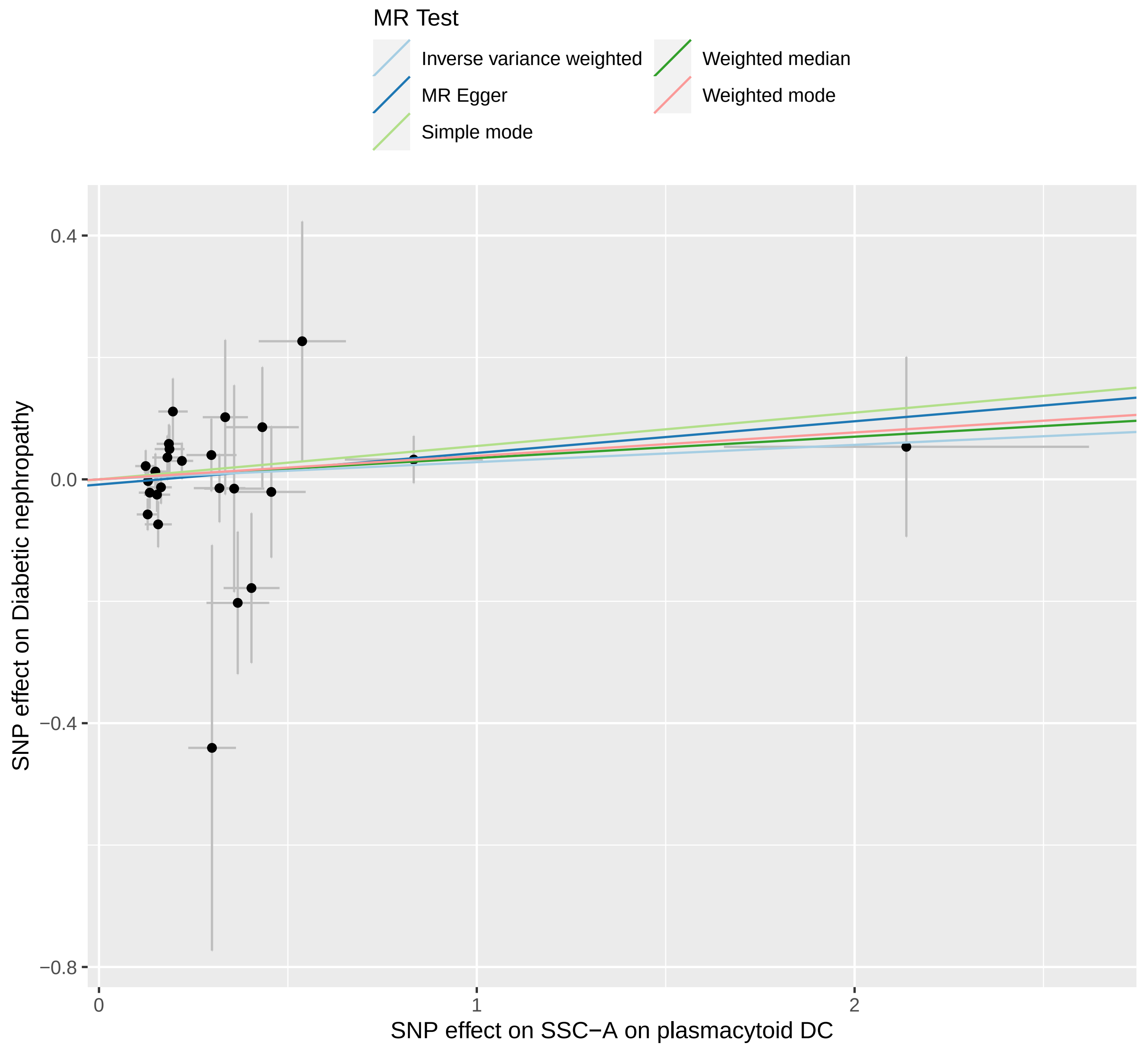
MR Test
Inverse variance weighted
MR Egger
Simple mode
Weighted median
Weighted mode
0.4
0.0
−0.4
−0.8
SNP effect on Diabetic nephropathy
0
1
2
SNP effect on SSC−A on plasmacytoid DC

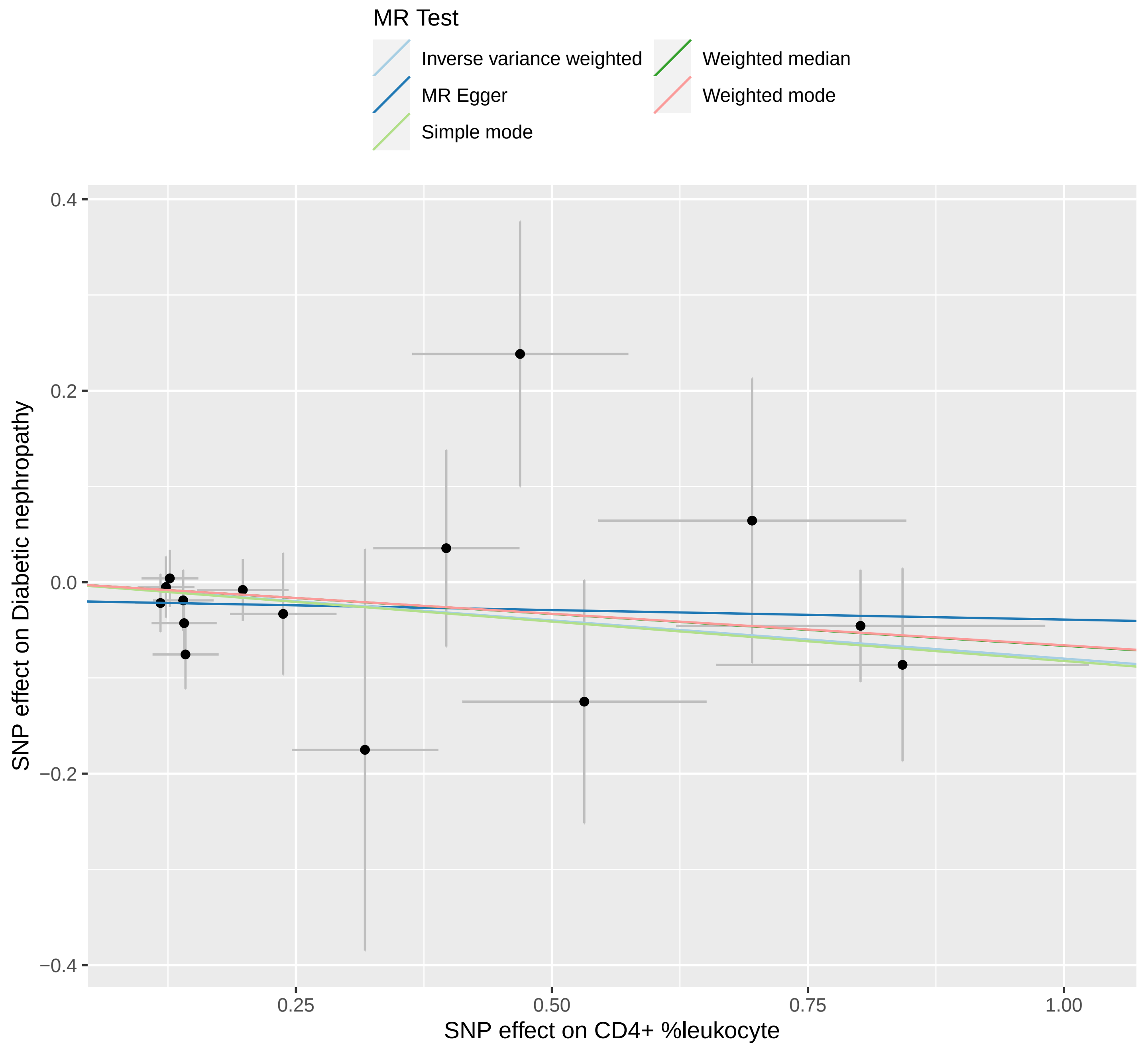

MR Test
Inverse variance weighted
MR Egger
Simple mode
Weighted median
Weighted mode
0.4
0.2
0.0
−0.2
−0.4
0.25
0.50
0.75
1.00
SNP effect on Diabetic nephropathy
SNP effect on CD4+ %leukocyte

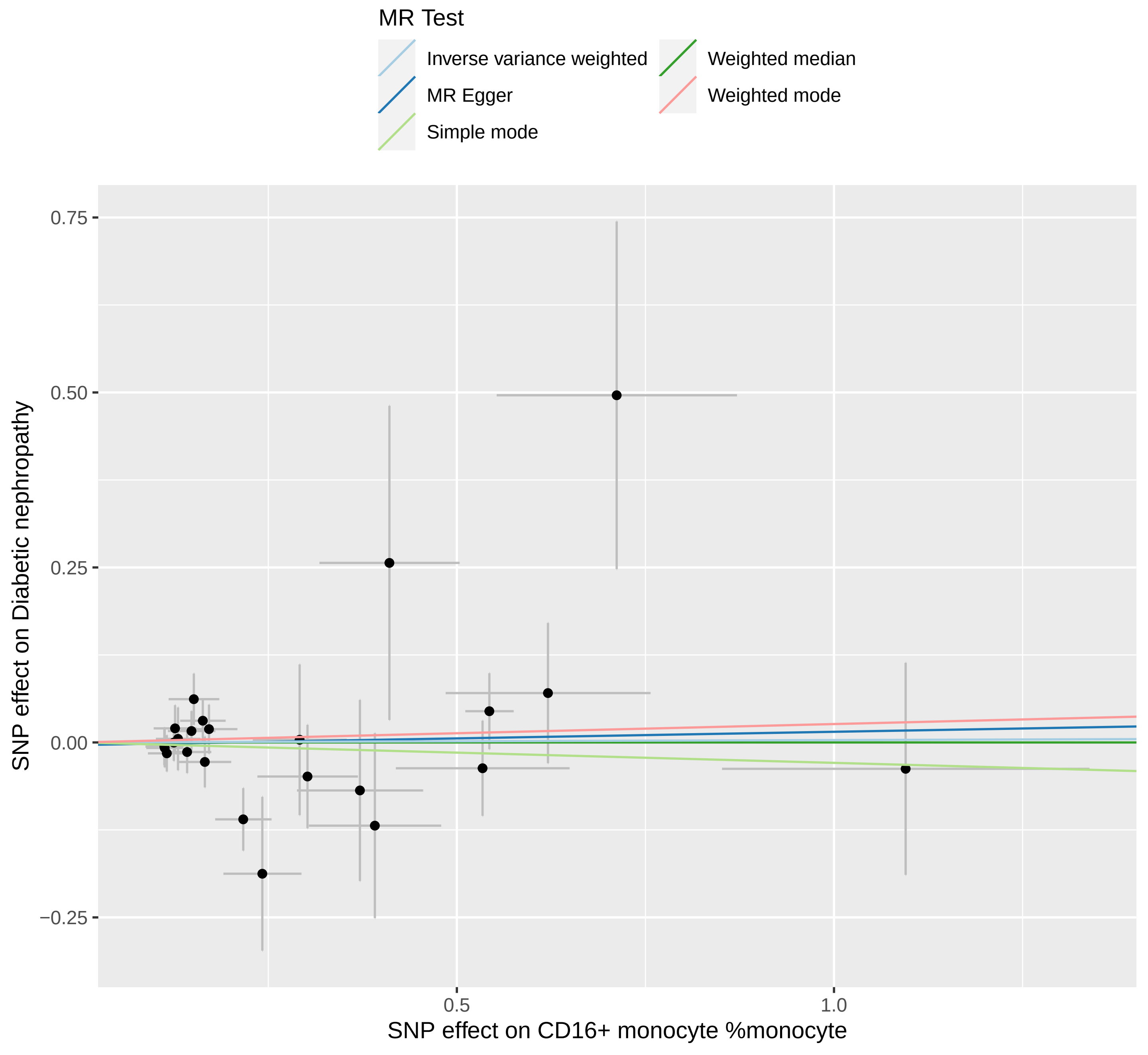

MR Test
Inverse variance weighted
MR Egger
Simple mode
Weighted median
Weighted mode
0.75
0.50
0.25
0.00
−0.25
SNP effect on Diabetic nephropathy
0.5
1.0
SNP effect on CD16+ monocyte %monocyte

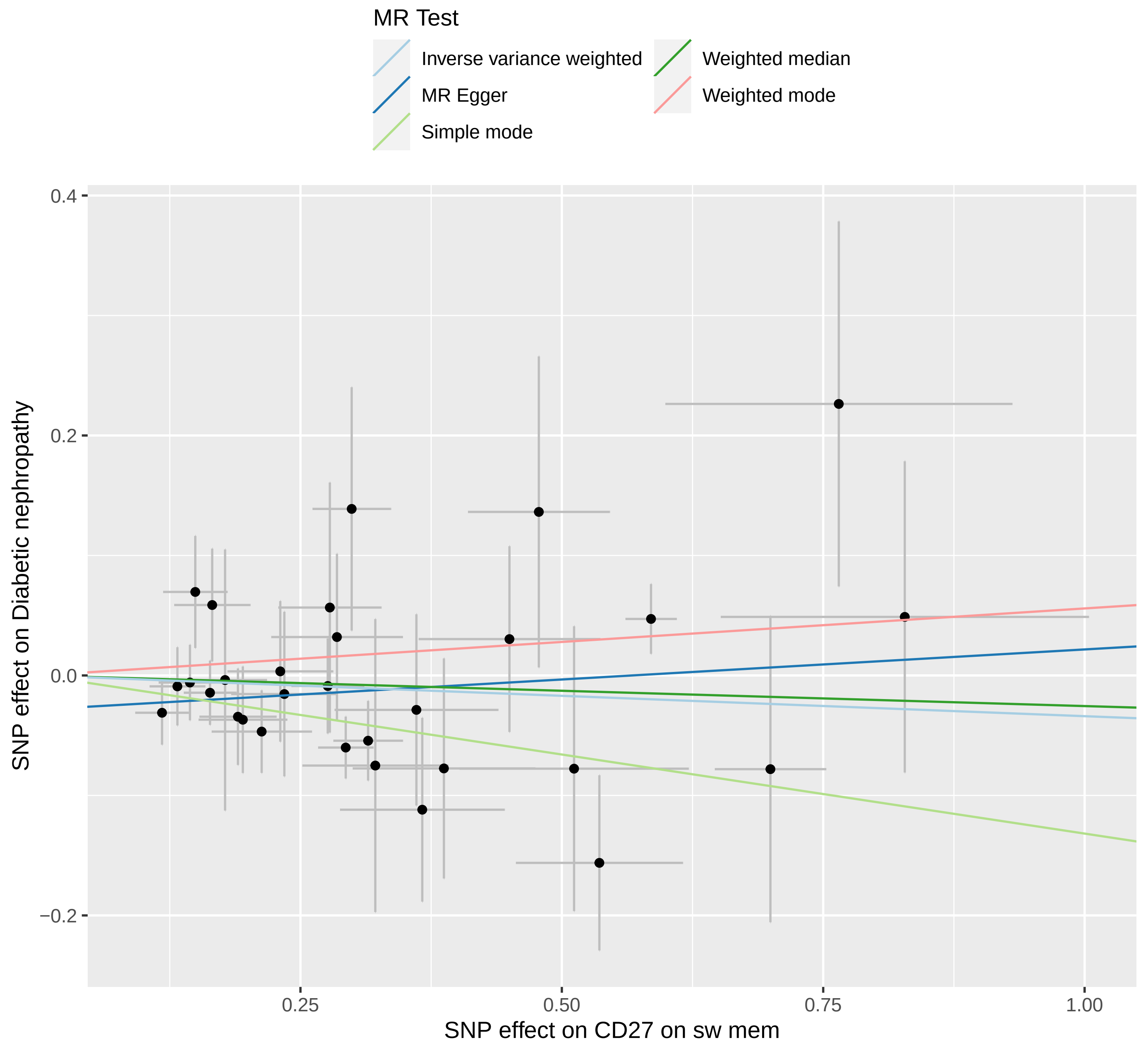
MR Test
Inverse variance weighted
MR Egger
Simple mode
Weighted median
Weighted mode
0.4
0.2
0.0
−0.2
0.25
0.50
0.75
1.00
SNP effect on Diabetic nephropathy
SNP effect on CD27 on sw mem

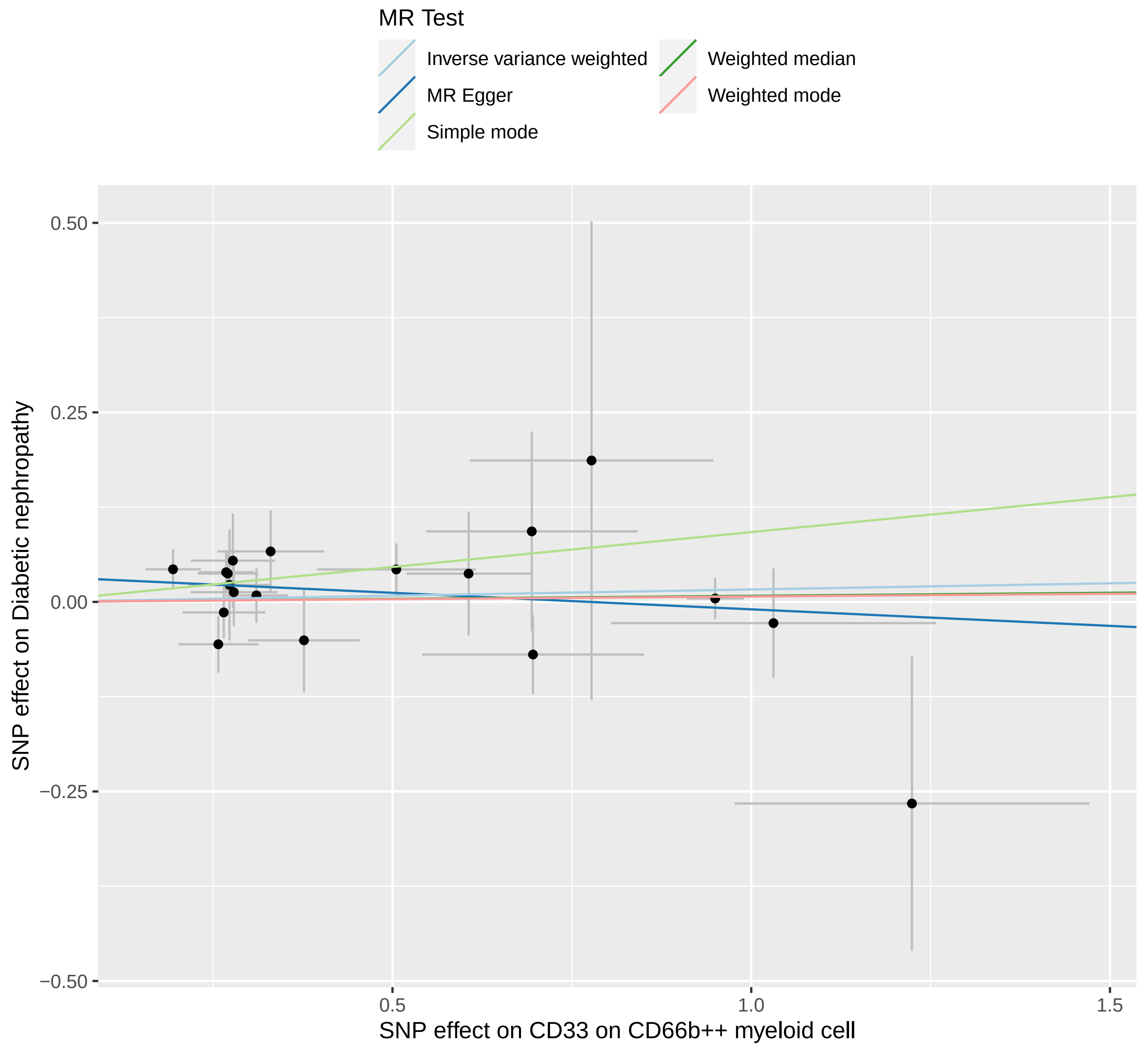
MR Test
Inverse variance weighted
MR Egger
Simple mode
Weighted median
Weighted mode
0.50
0.25
0.00
−0.25
−0.50
0.5
1.0
1.5
SNP effect on Diabetic nephropathy
SNP effect on CD33 on CD66b++ myeloid cell

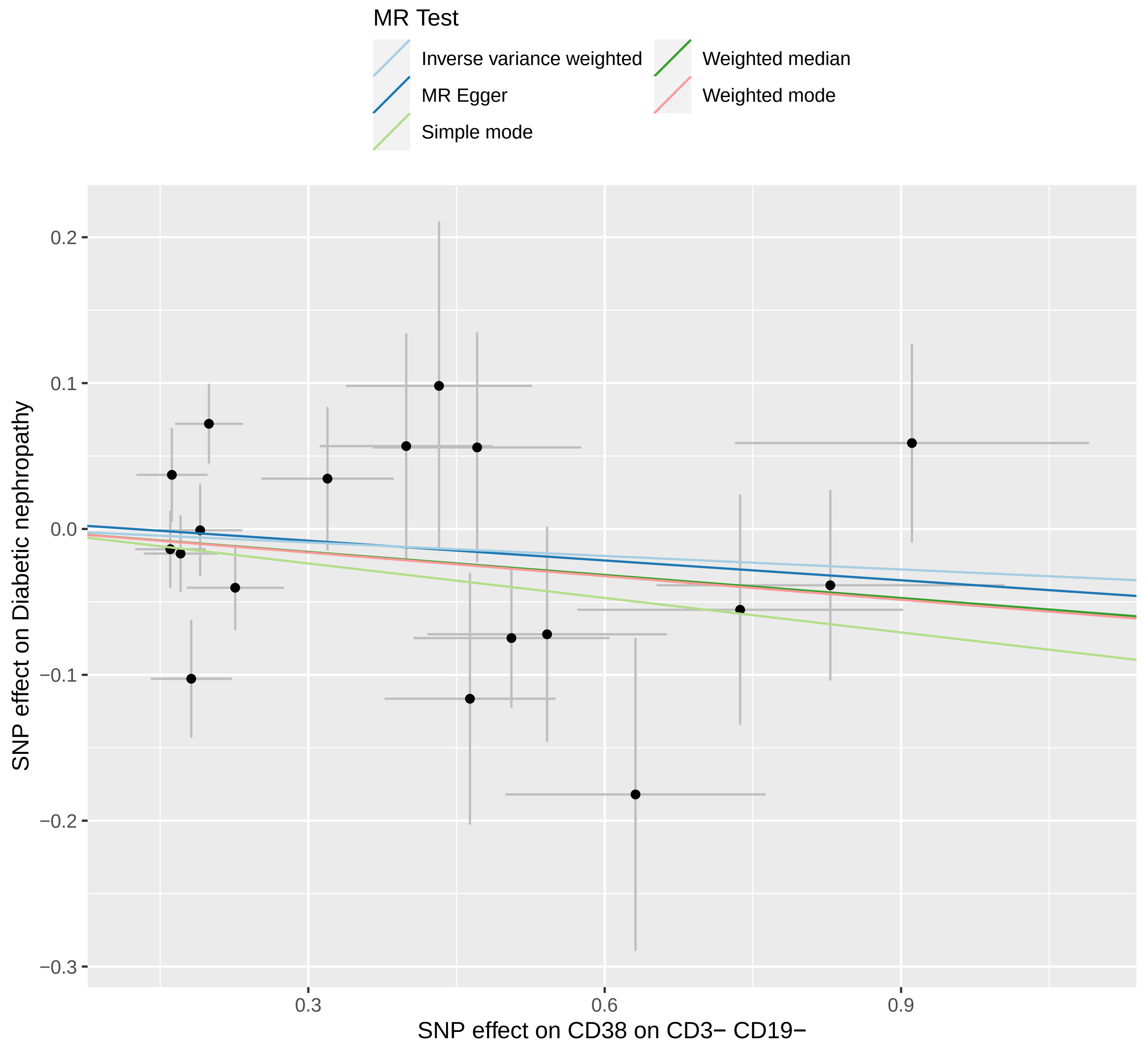
MR Test
Inverse variance weighted
MR Egger
Simple mode
Weighted median
Weighted mode
0.2
0.1
0.0
−0.1
−0.2
−0.3
SNP effect on Diabetic nephropathy
0.3
0.6
0.9
SNP effect on CD38 on CD3− CD19−

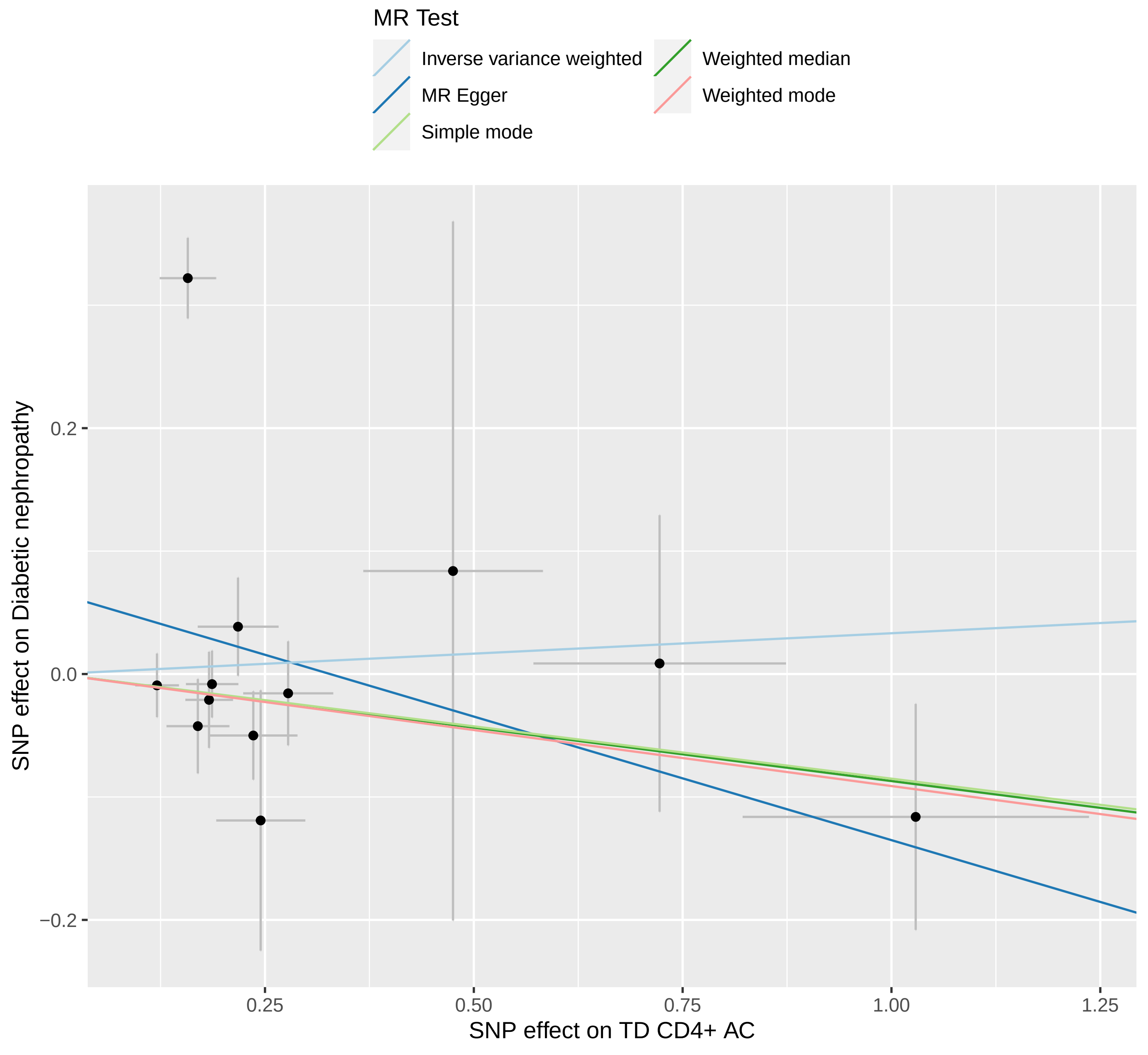
MR Test
Inverse variance weighted
MR Egger
Simple mode
Weighted median
Weighted mode
SNP effect on Diabetic nephropathy
0.2
0.0
−0.2
0.25
0.50
0.75
1.00
1.25
SNP effect on TD CD4+ AC

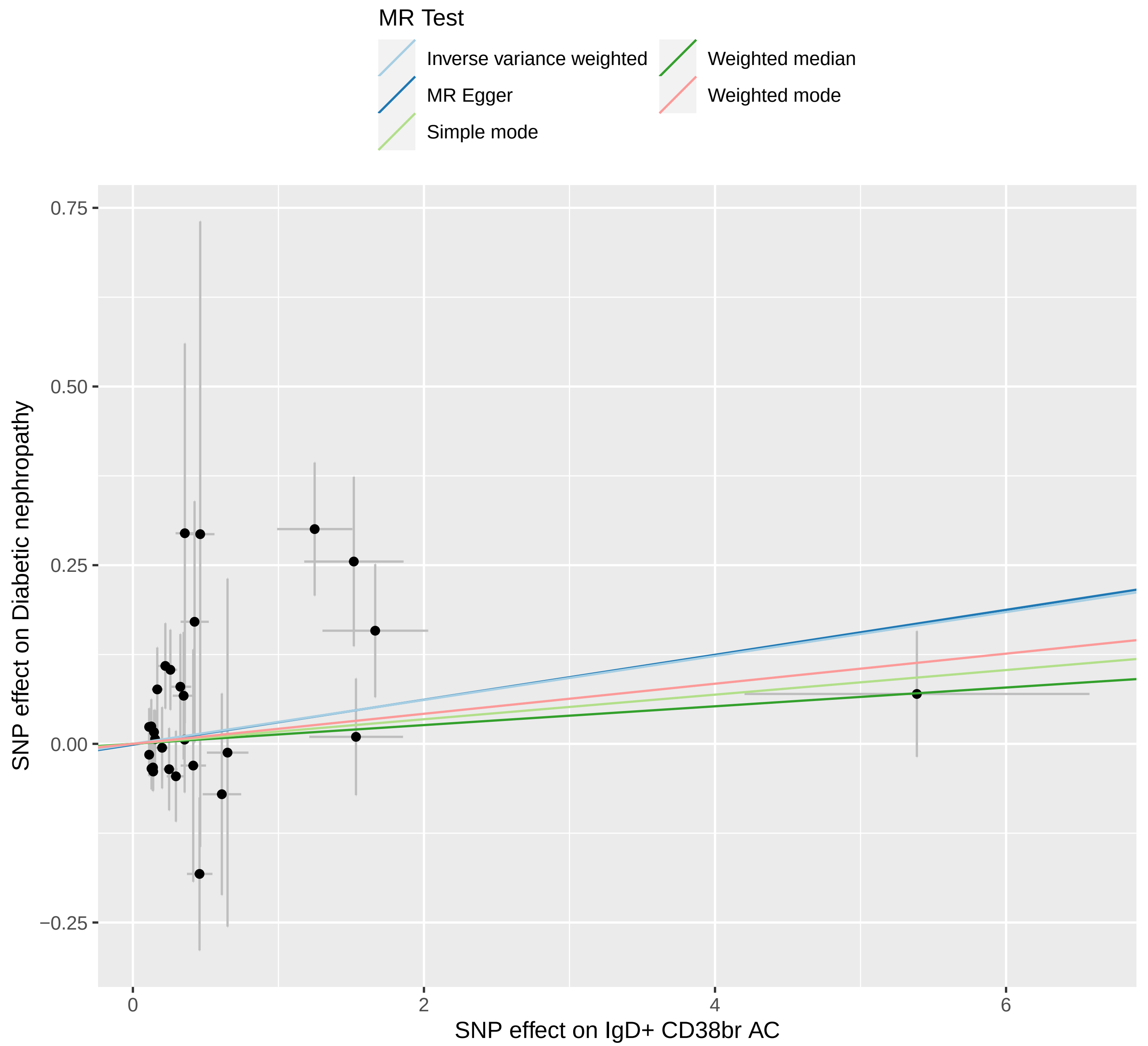
MR Test
Inverse variance weighted
MR Egger
Simple mode
Weighted median
Weighted mode
0.75
0.50
0.25
0.00
−0.25
0
2
4
6
SNP effect on Diabetic nephropathy
SNP effect on IgD+ CD38br AC

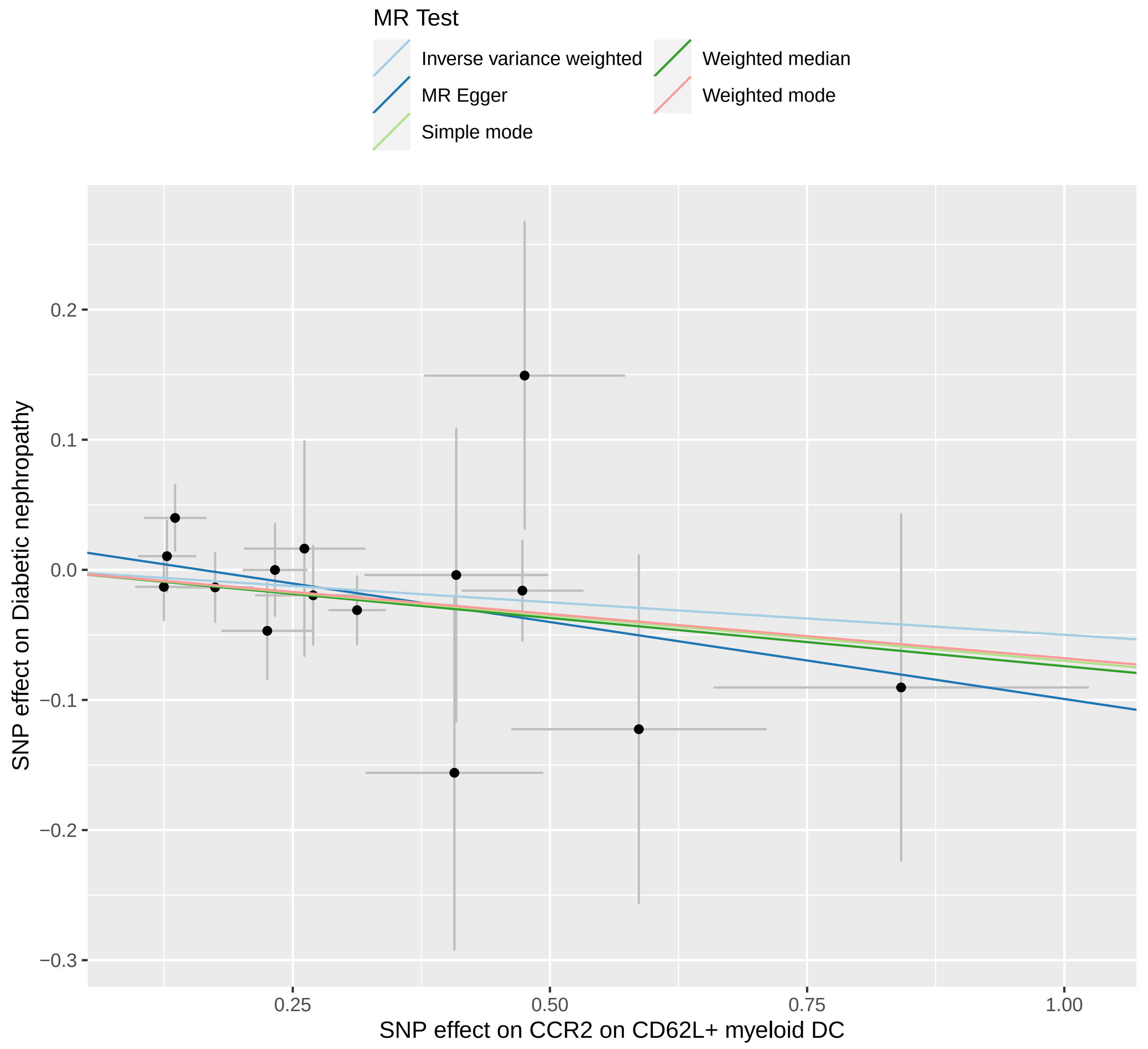

MR Test
Inverse variance weighted
MR Egger
Simple mode
Weighted median
Weighted mode
0.2
0.1
0.0
−0.1
−0.2
−0.3
SNP effect on Diabetic nephropathy
0.25
0.50
0.75
1.00
SNP effect on CCR2 on CD62L+ myeloid DC

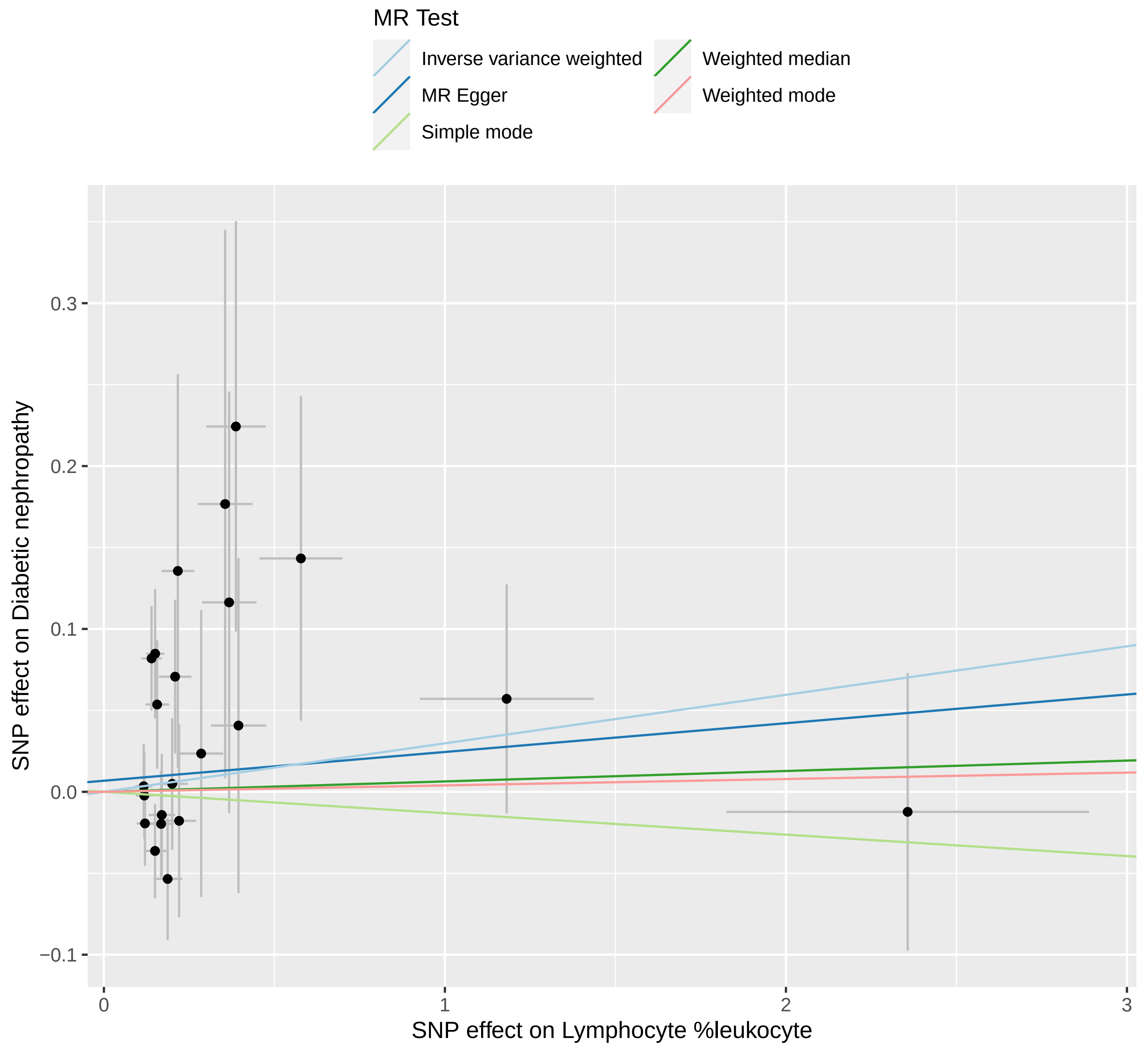
MR Test
Inverse variance weighted
MR Egger
Simple mode
Weighted median
Weighted mode
SNP effect on Diabetic nephropathy
0.3
0.2
0.1
0.0
−0.1
0
1
2
3
SNP effect on Lymphocyte %leukocyte

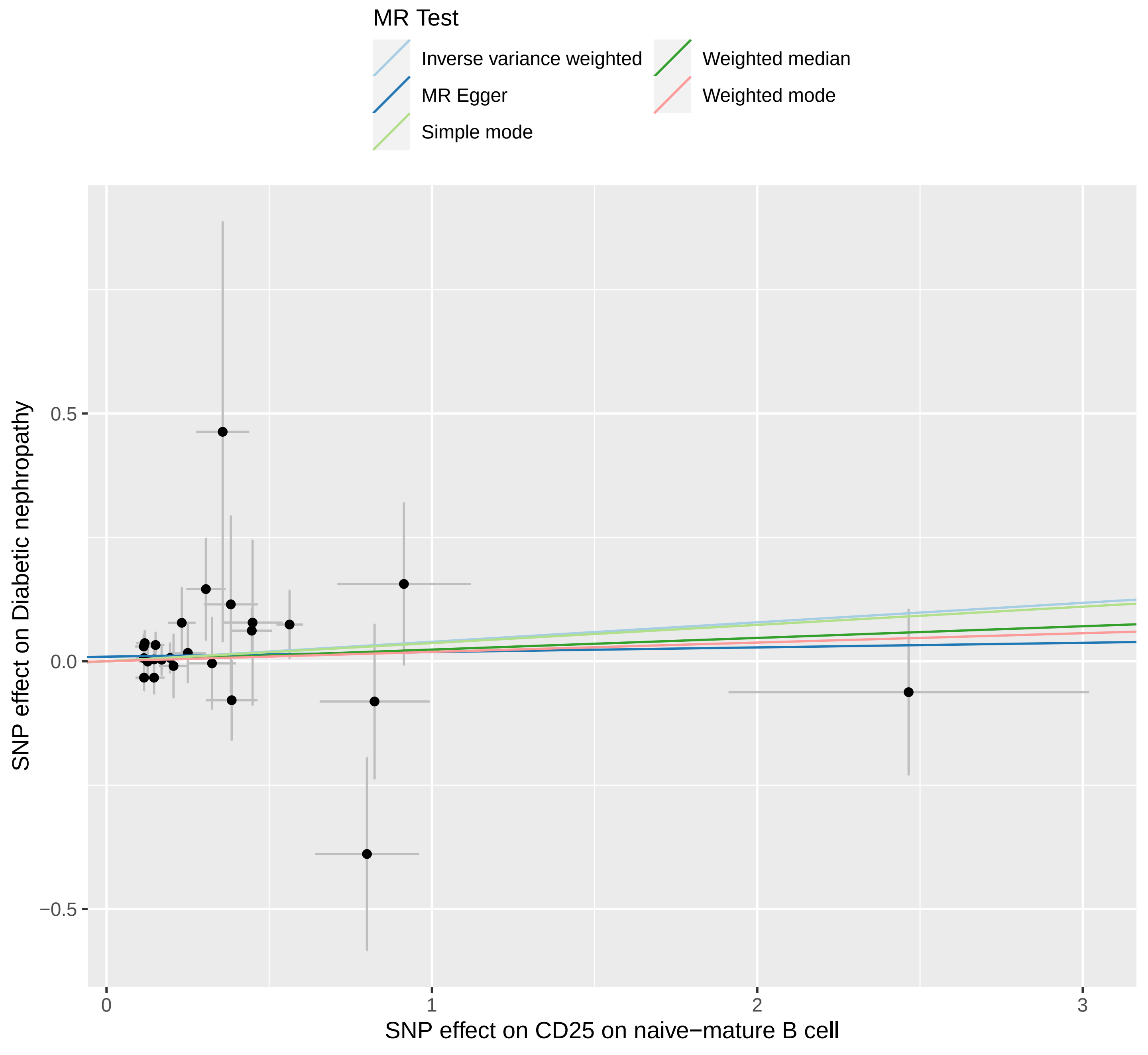

MR Test
Inverse variance weighted
MR Egger
Simple mode
Weighted median
Weighted mode
SNP effect on Diabetic nephropathy
0.5
0.0
−0.5
0
1
2
3
SNP effect on CD25 on naive−mature B cell

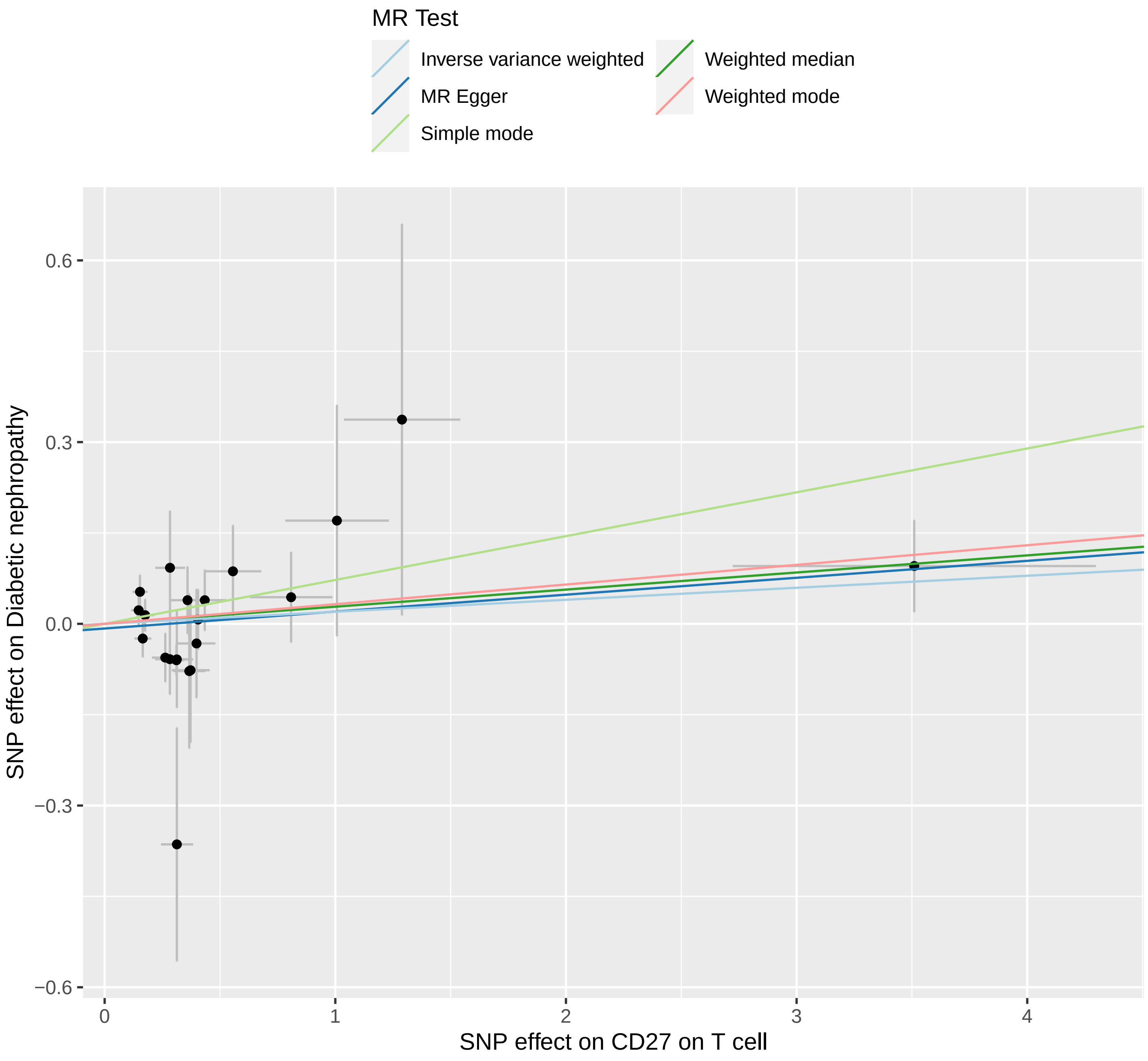

MR Test
Inverse variance weighted
MR Egger
Simple mode
Weighted median
Weighted mode
0.6
0.3
0.0
−0.3
−0.6
0
1
2
3
4
SNP effect on Diabetic nephropathy
SNP effect on CD27 on T cell

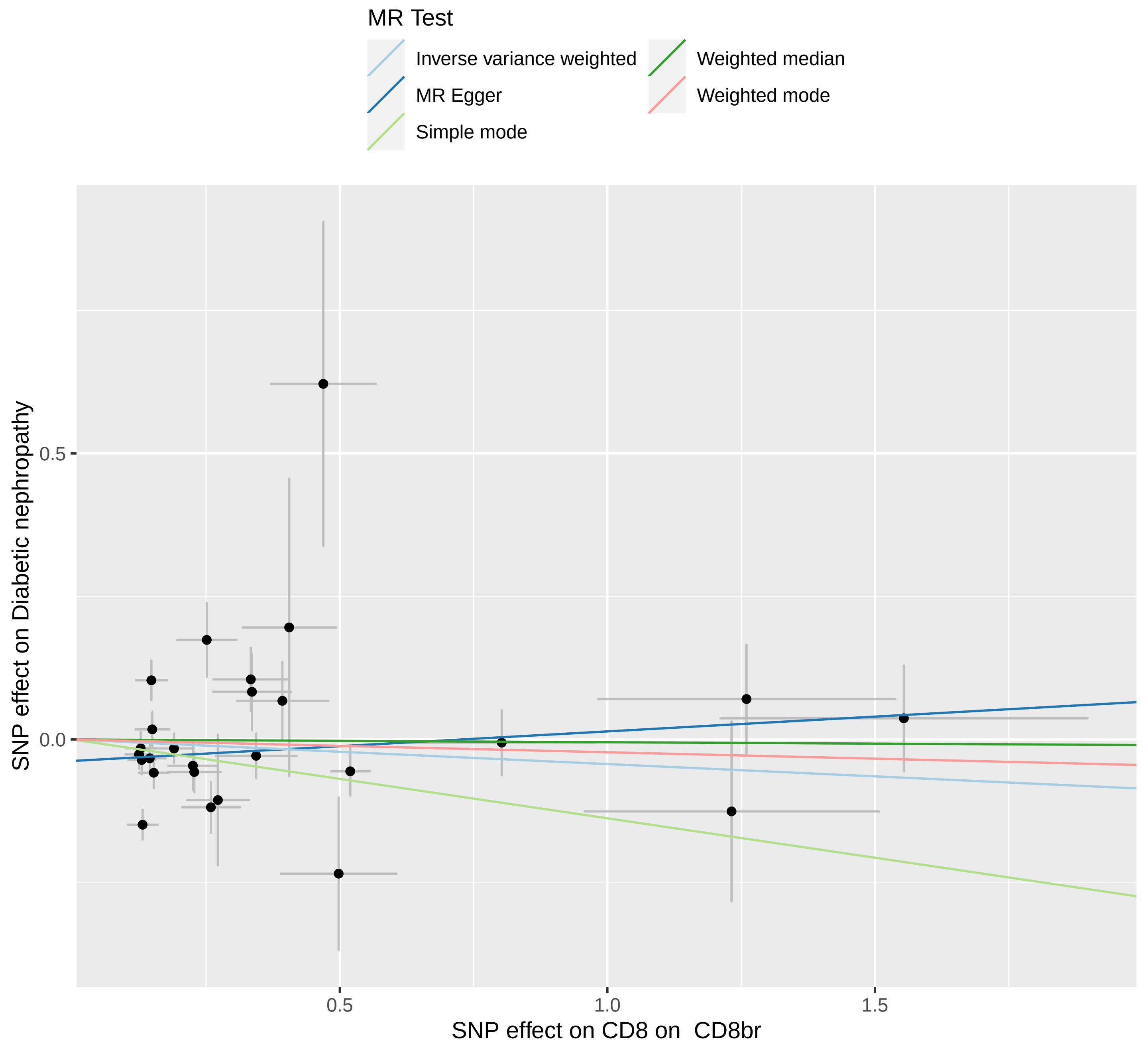
MR Test
Inverse variance weighted
MR Egger
Simple mode
Weighted median
Weighted mode
SNP effect on Diabetic nephropathy
0.5
0.0
0.5
1.0
1.5
SNP effect on CD8 on CD8br

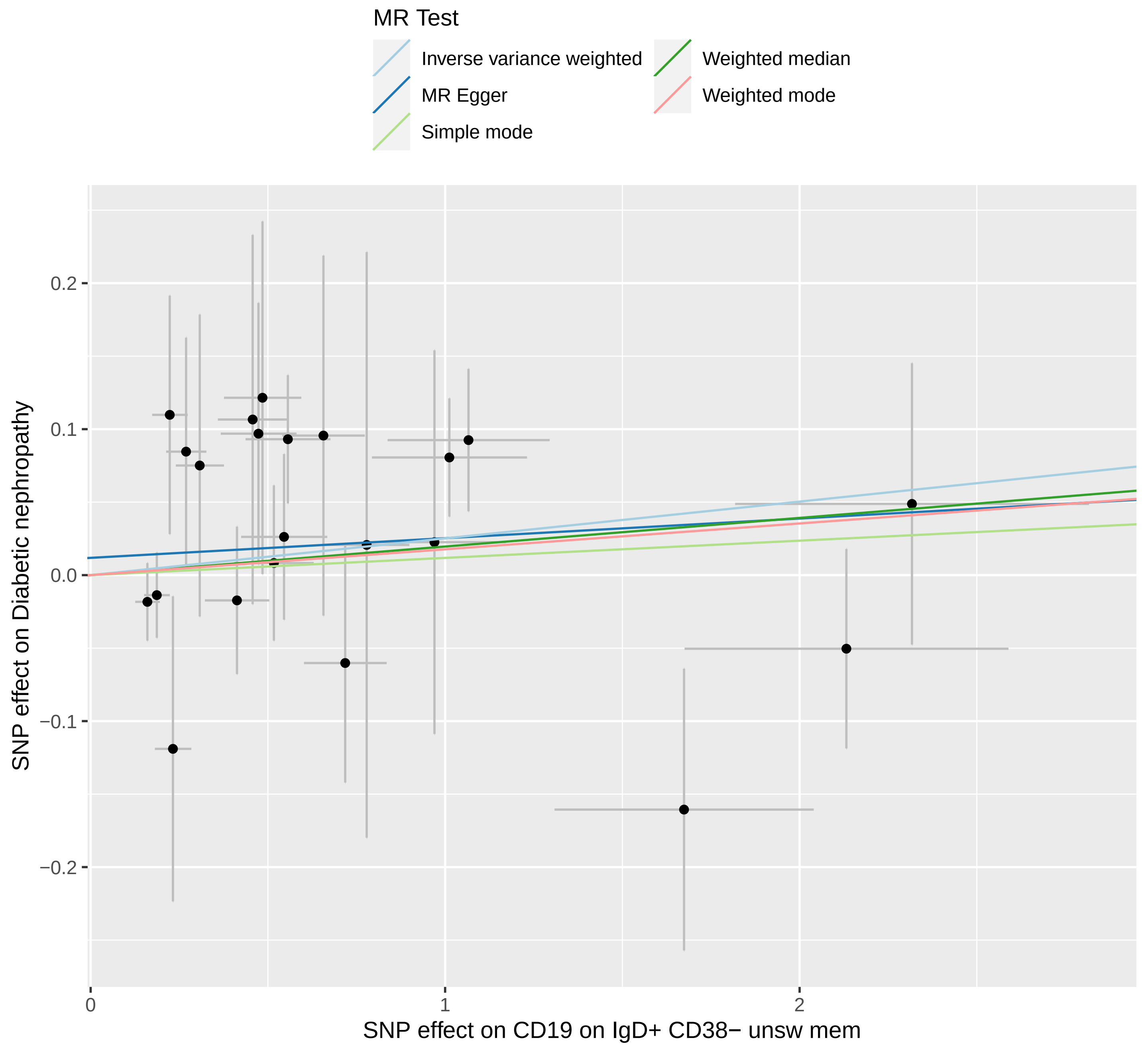

MR Test
Inverse variance weighted
MR Egger
Simple mode
Weighted median
Weighted mode
0.2
0.1
0.0
−0.1
−0.2
0
1
2
SNP effect on Diabetic nephropathy
SNP effect on CD19 on IgD+ CD38− unsw mem

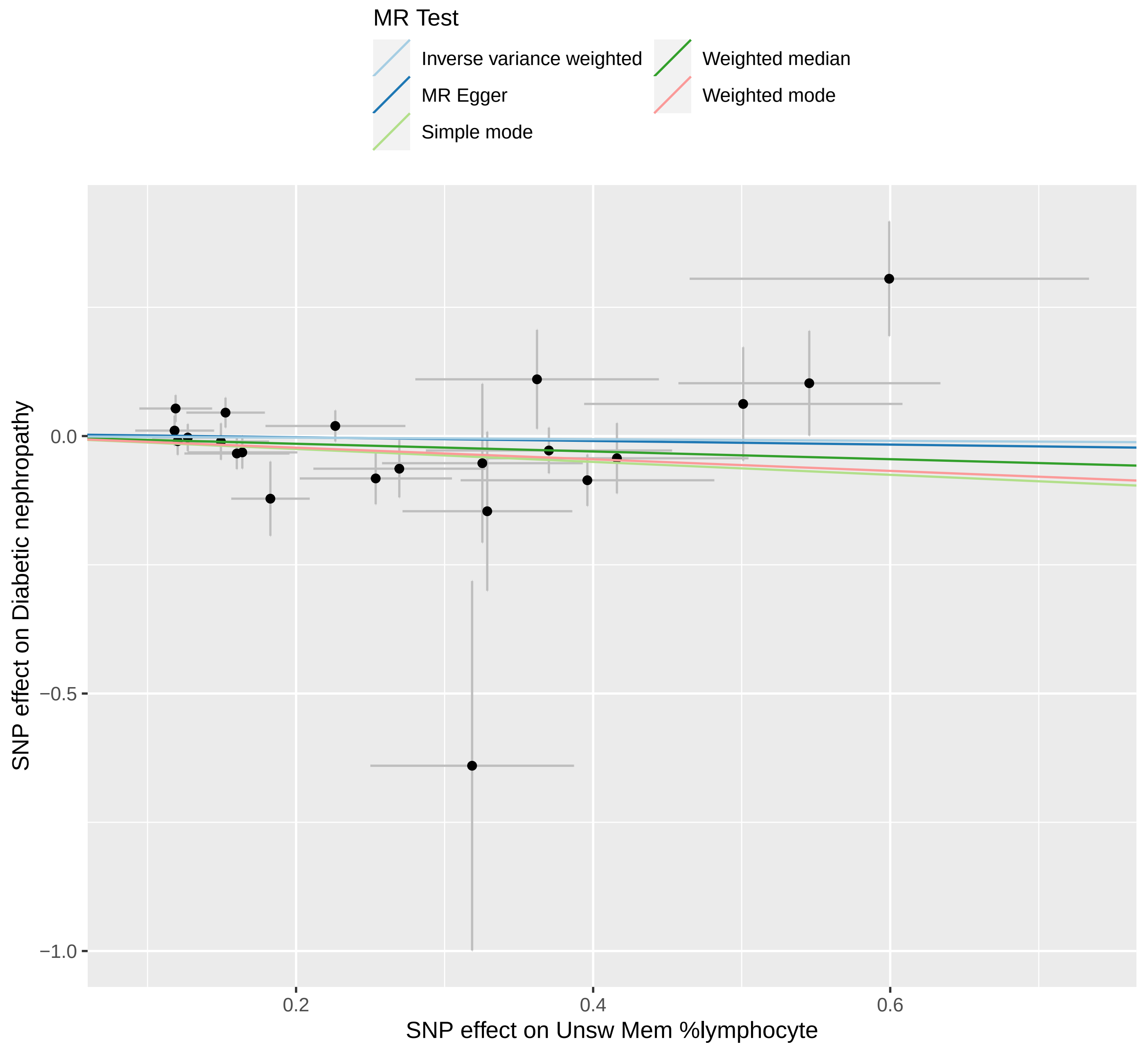
MR Test
Inverse variance weighted
MR Egger
Simple mode
Weighted median
Weighted mode
0.0
−0.5
−1.0
0.2
0.4
0.6
SNP effect on Diabetic nephropathy
SNP effect on Unsw Mem %lymphocyte

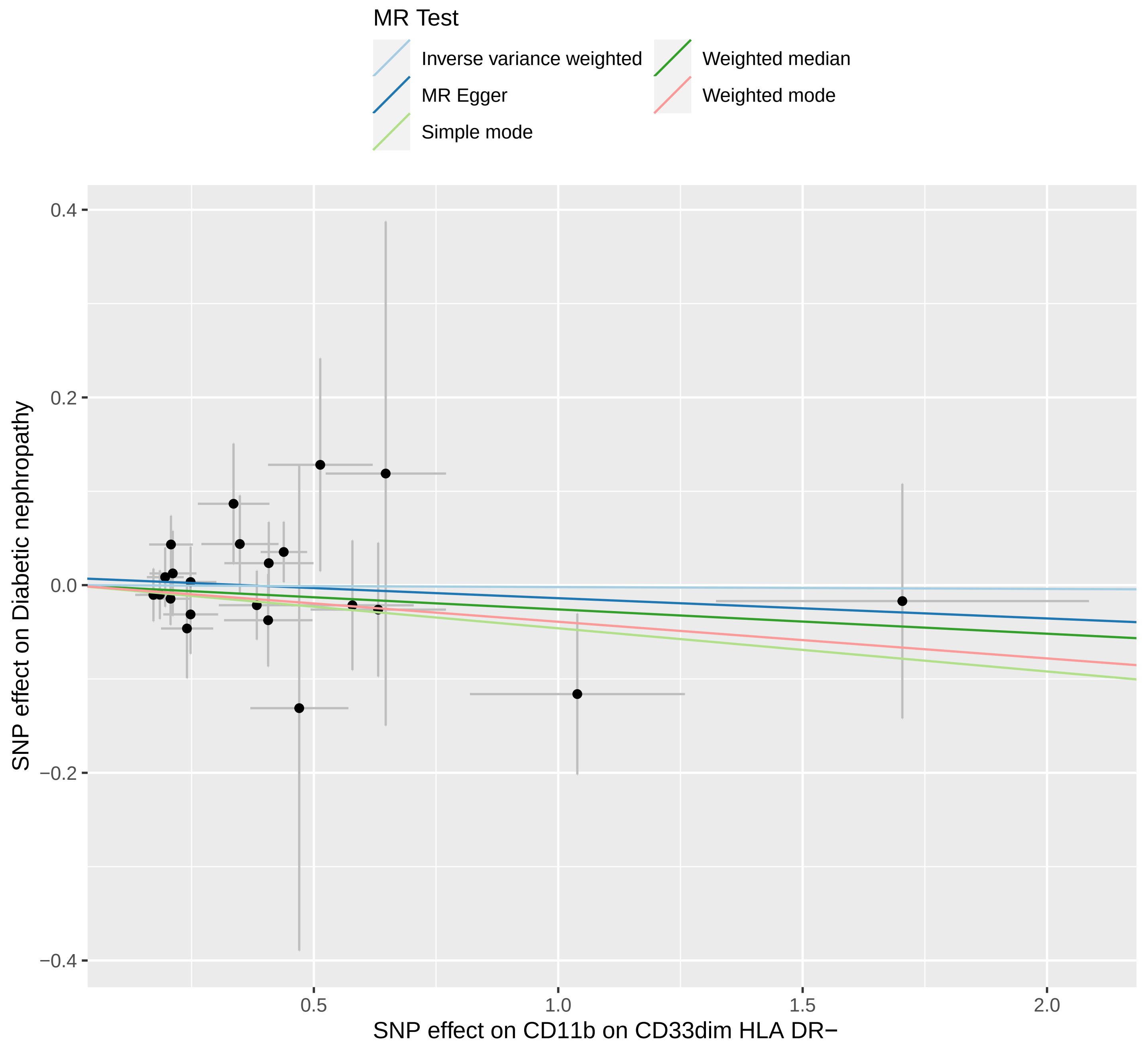
MR Test
Inverse variance weighted
MR Egger
Simple mode
Weighted median
Weighted mode
0.4
0.2
0.0
−0.2
−0.4
0.5
1.0
1.5
2.0
SNP effect on Diabetic nephropathy
SNP effect on CD11b on CD33dim HLA DR−

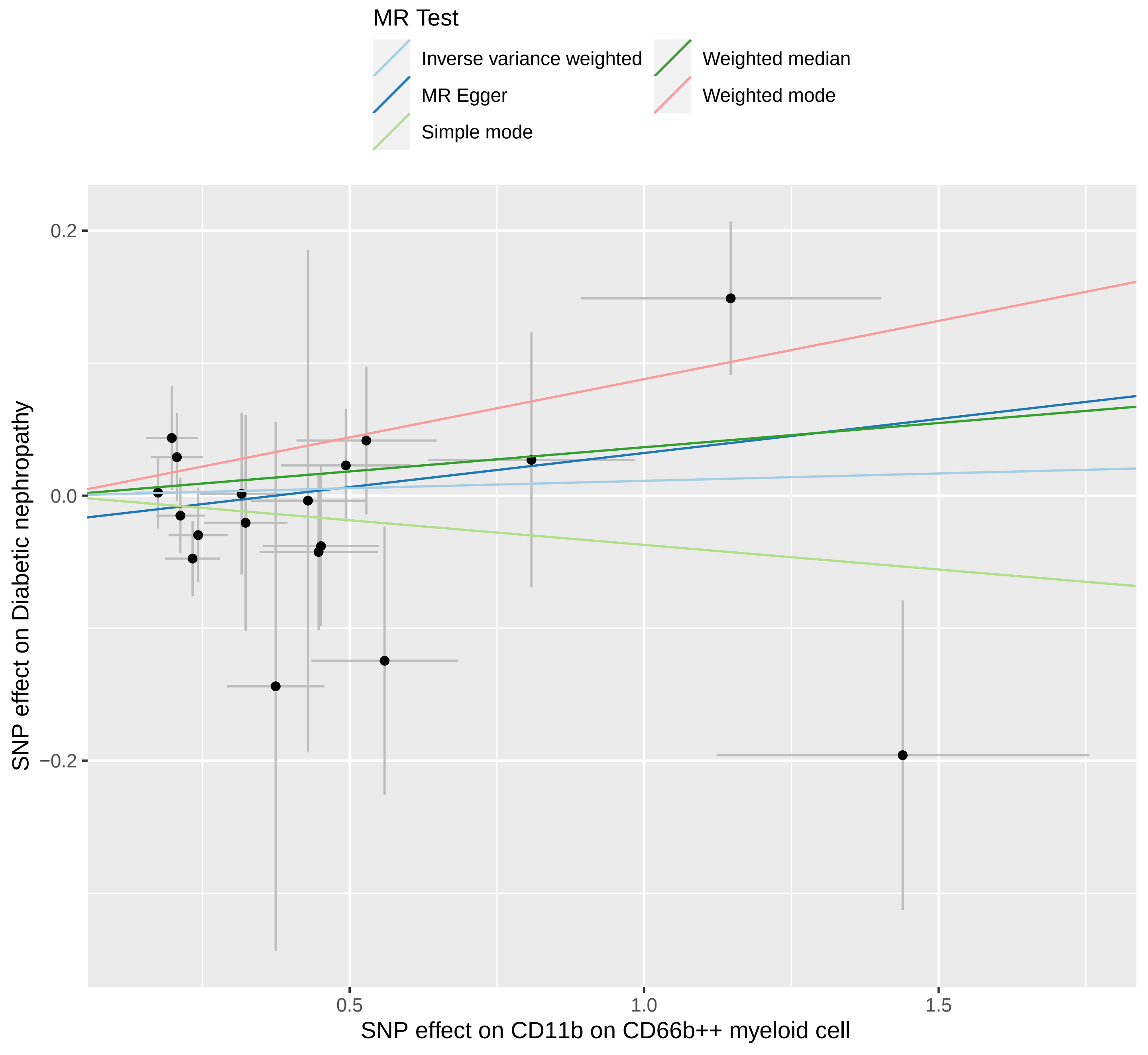

MR Test
Inverse variance weighted
MR Egger
Simple mode
Weighted median
Weighted mode
0.2
0.0
−0.2
0.5
1.0
1.5
SNP effect on Diabetic nephropathy
SNP effect on CD11b on CD66b++ myeloid cell

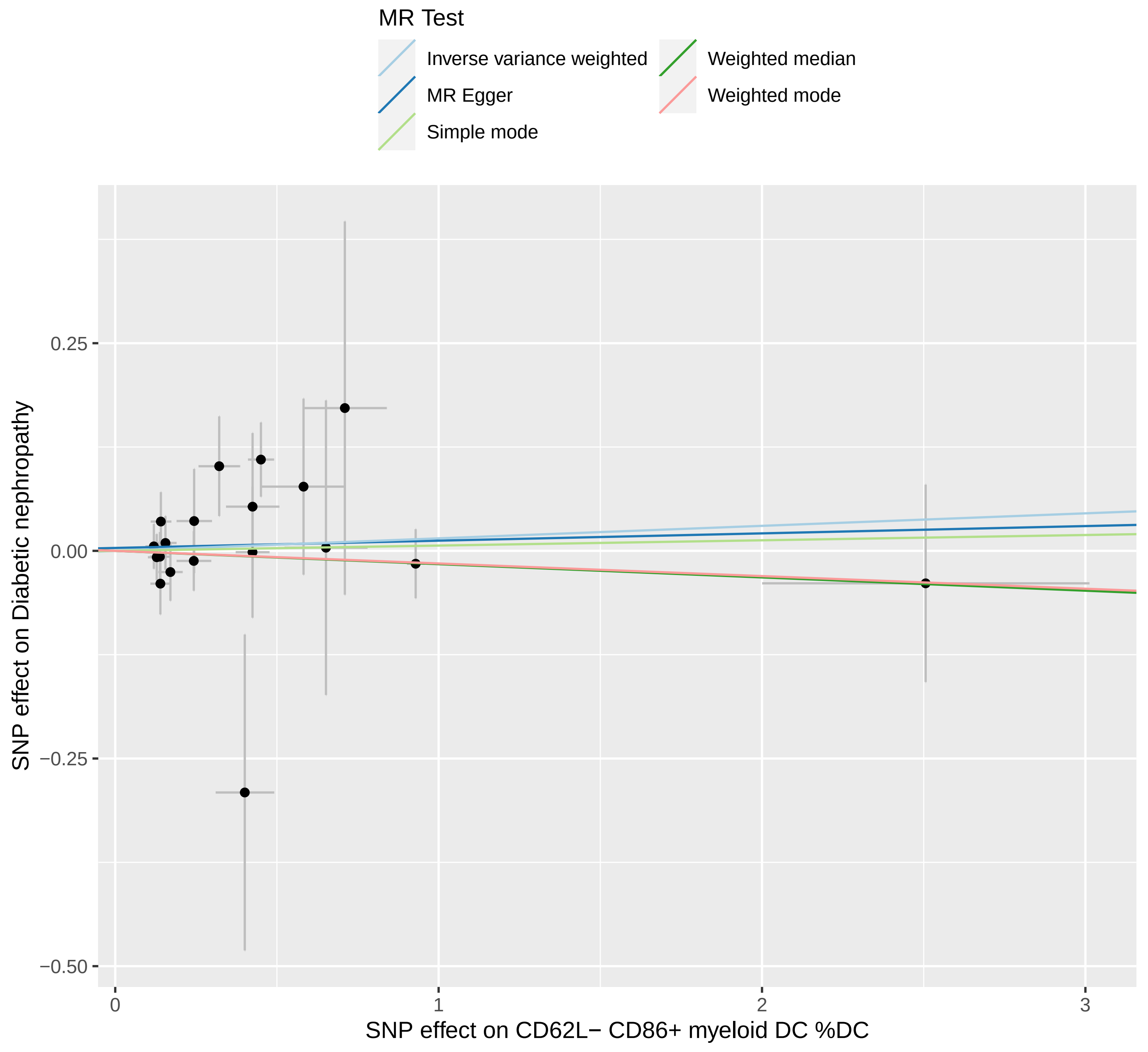

MR Test
Inverse variance weighted
MR Egger
Simple mode
Weighted median
Weighted mode
0.25
0.00
−0.25
−0.50
0
1
2
3
SNP effect on Diabetic nephropathy
SNP effect on CD62L− CD86+ myeloid DC %DC

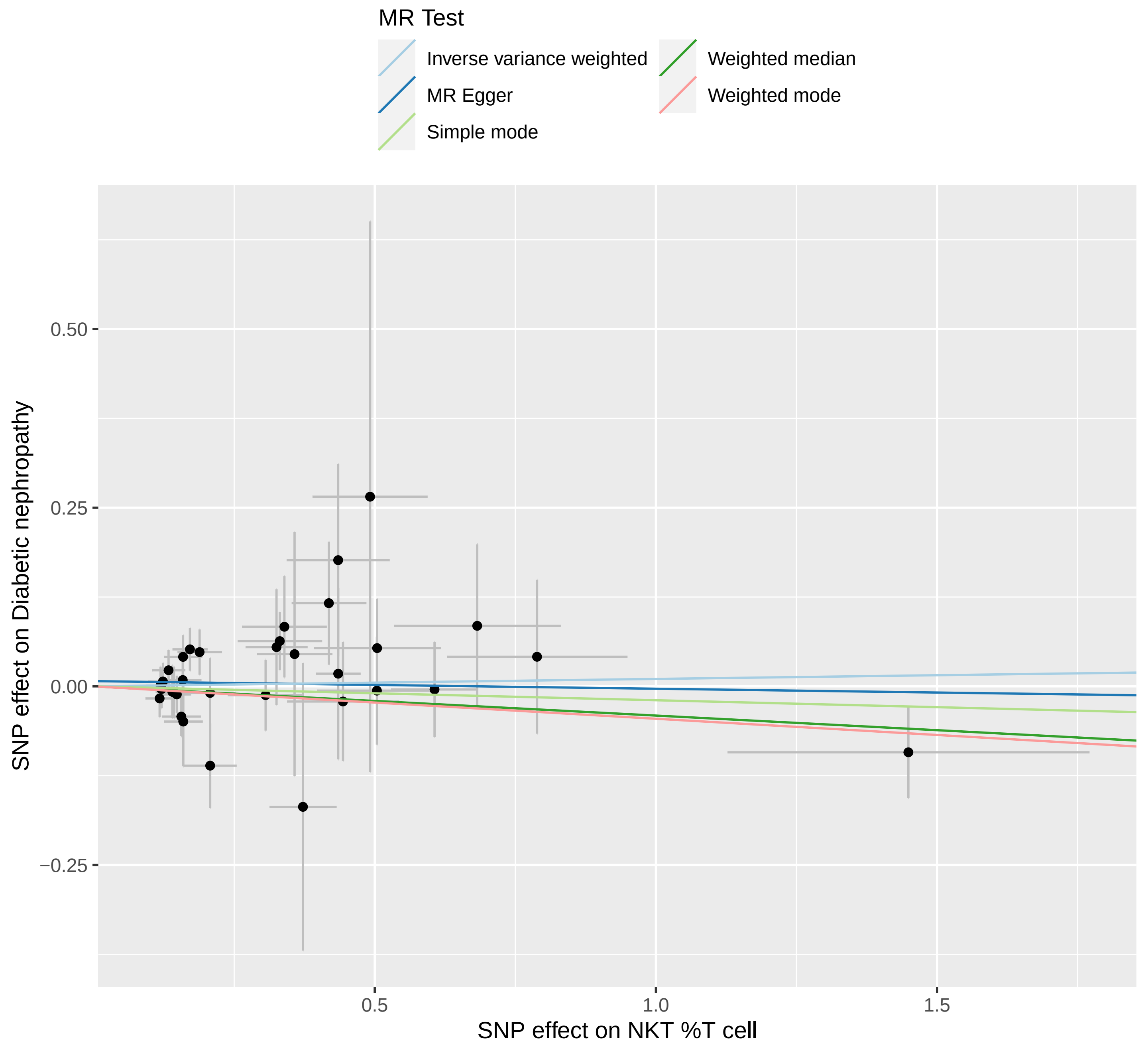
MR Test
Inverse variance weighted
MR Egger
Simple mode
Weighted median
Weighted mode
SNP effect on Diabetic nephropathy
0.50
0.25
0.00
−0.25
0.5
1.0
1.5
SNP effect on NKT %T cell

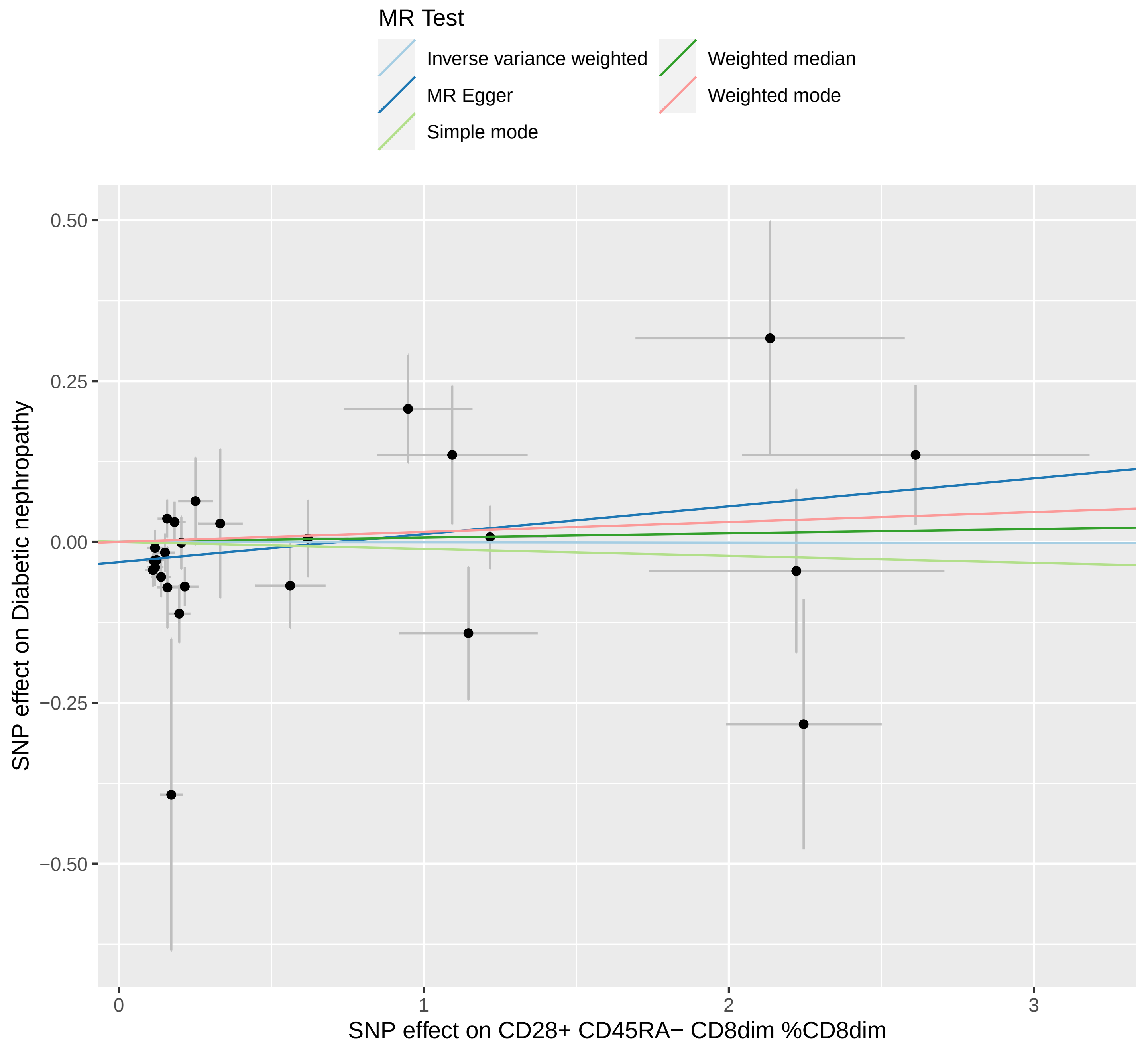
MR Test
Inverse variance weighted
MR Egger
Simple mode
Weighted median
Weighted mode
0.50
0.25
0.00
−0.25
−0.50
SNP effect on Diabetic nephropathy
0
1
2
3
SNP effect on CD28+ CD45RA− CD8dim %CD8dim

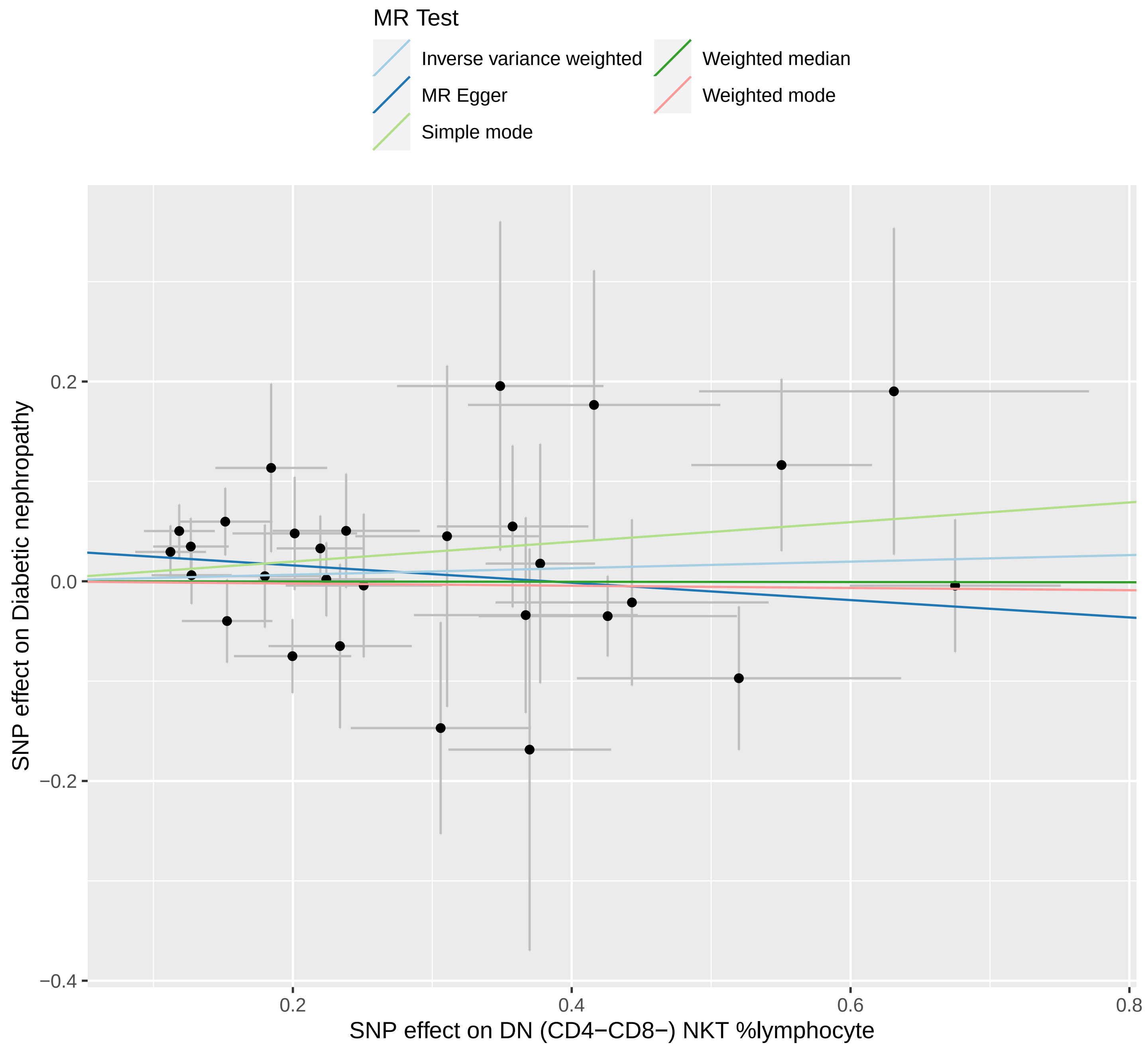

MR Test
Inverse variance weighted
MR Egger
Simple mode
Weighted median
Weighted mode
SNP effect on Diabetic nephropathy
0.2
0.0
−0.2
−0.4
0.2
0.4
0.6
0.8
SNP effect on DN (CD4−CD8−) NKT %lymphocyte

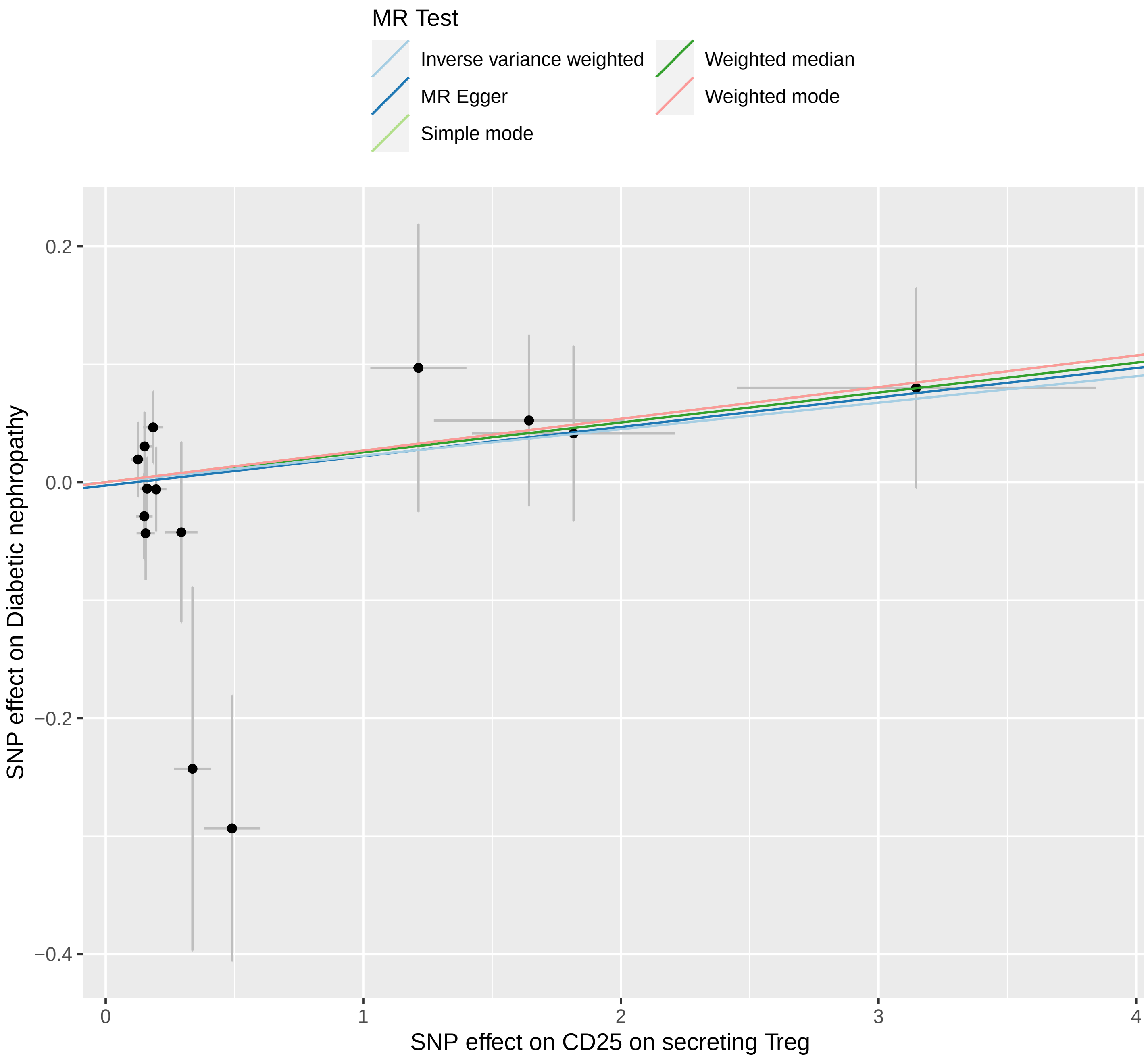

MR Test
Inverse variance weighted
MR Egger
Simple mode
Weighted median
Weighted mode
0.2
0.0
−0.2
−0.4
0
1
2
3
4
SNP effect on Diabetic nephropathy
SNP effect on CD25 on secreting Treg

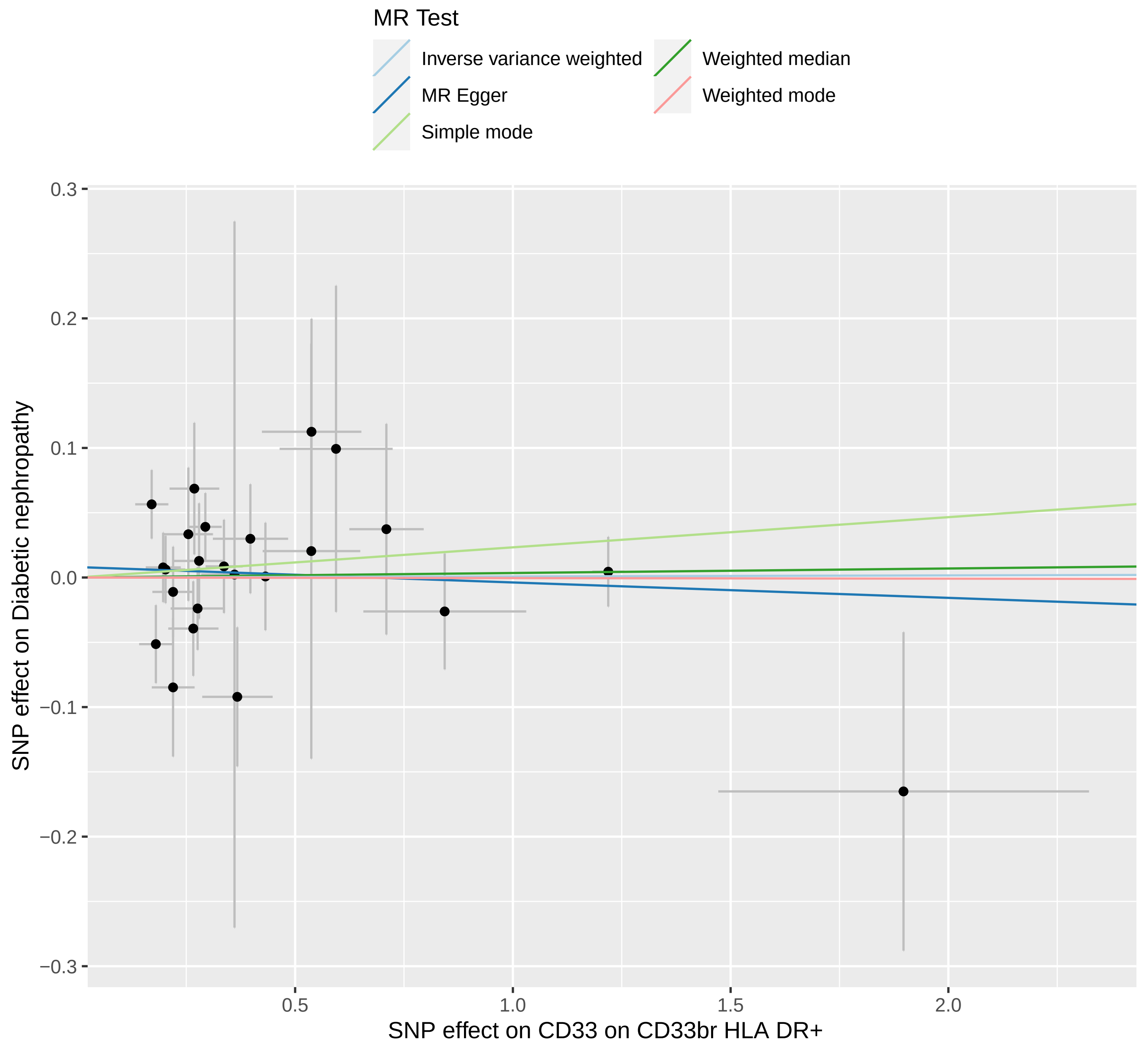
MR Test
Inverse variance weighted
MR Egger
Simple mode
Weighted median
Weighted mode
SNP effect on Diabetic nephropathy
0.3
0.2
0.1
0.0
−0.1
−0.2
−0.3
0.5
1.0
1.5
2.0
SNP effect on CD33 on CD33br HLA DR+

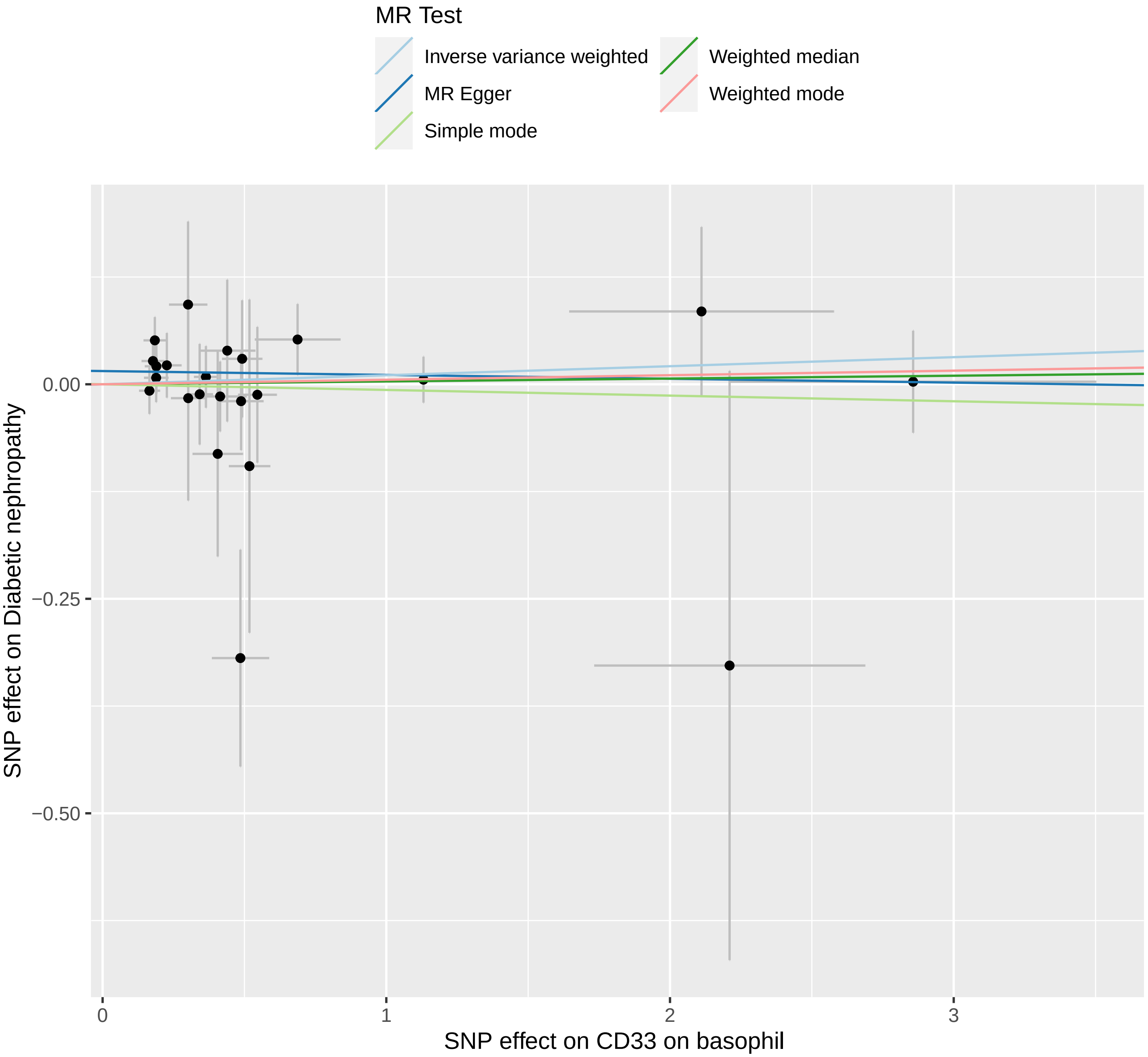

MR Test
Inverse variance weighted
MR Egger
Simple mode
Weighted median
Weighted mode
0.00
−0.25
−0.50
0
1
2
3
SNP effect on Diabetic nephropathy
SNP effect on CD33 on basophil

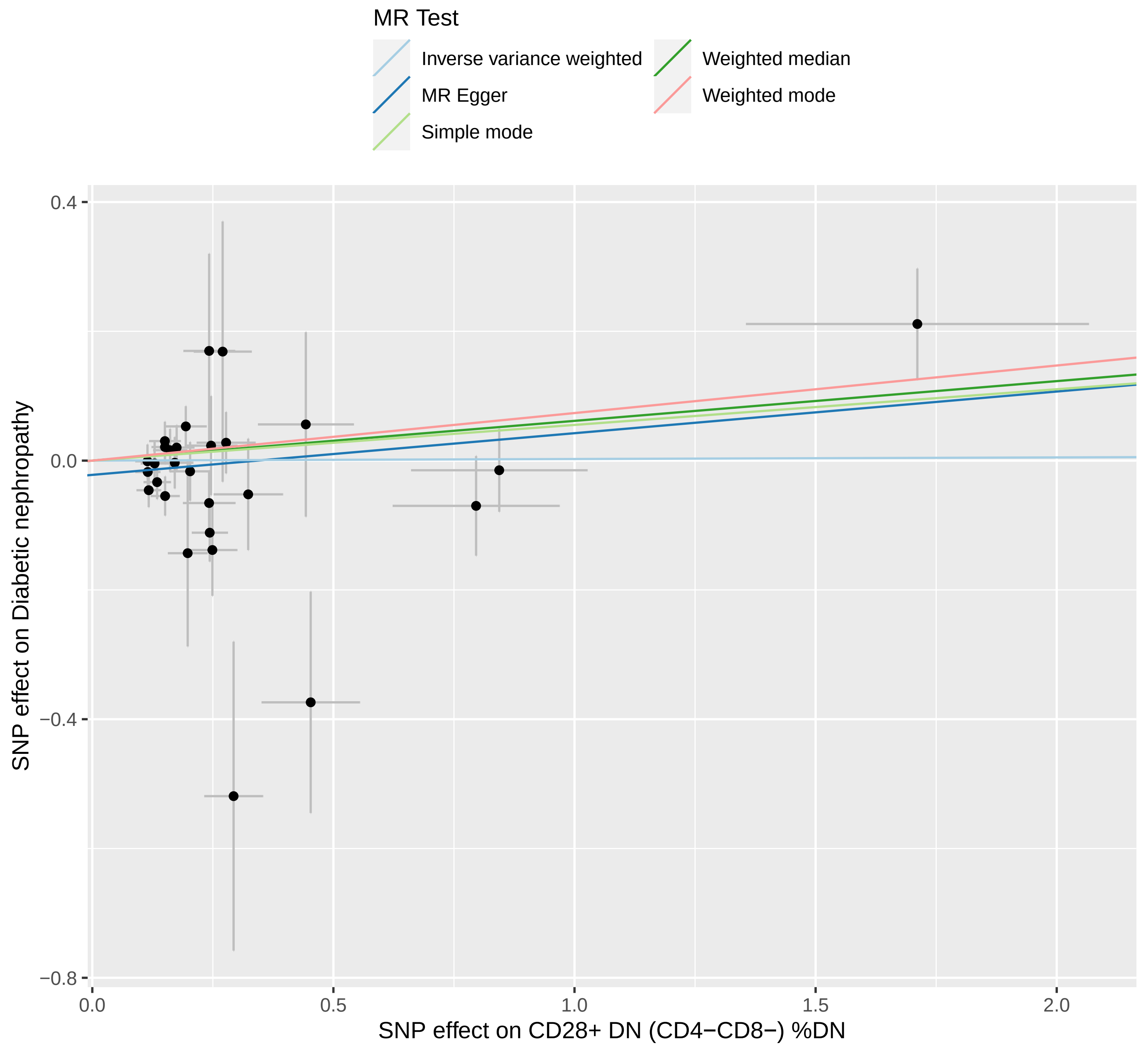
MR Test
Inverse variance weighted
MR Egger
Simple mode
Weighted median
Weighted mode
SNP effect on Diabetic nephropathy
0.4
0.0
−0.4
−0.8
0.0
0.5
1.0
1.5
2.0
SNP effect on CD28+ DN (CD4−CD8−) %DN

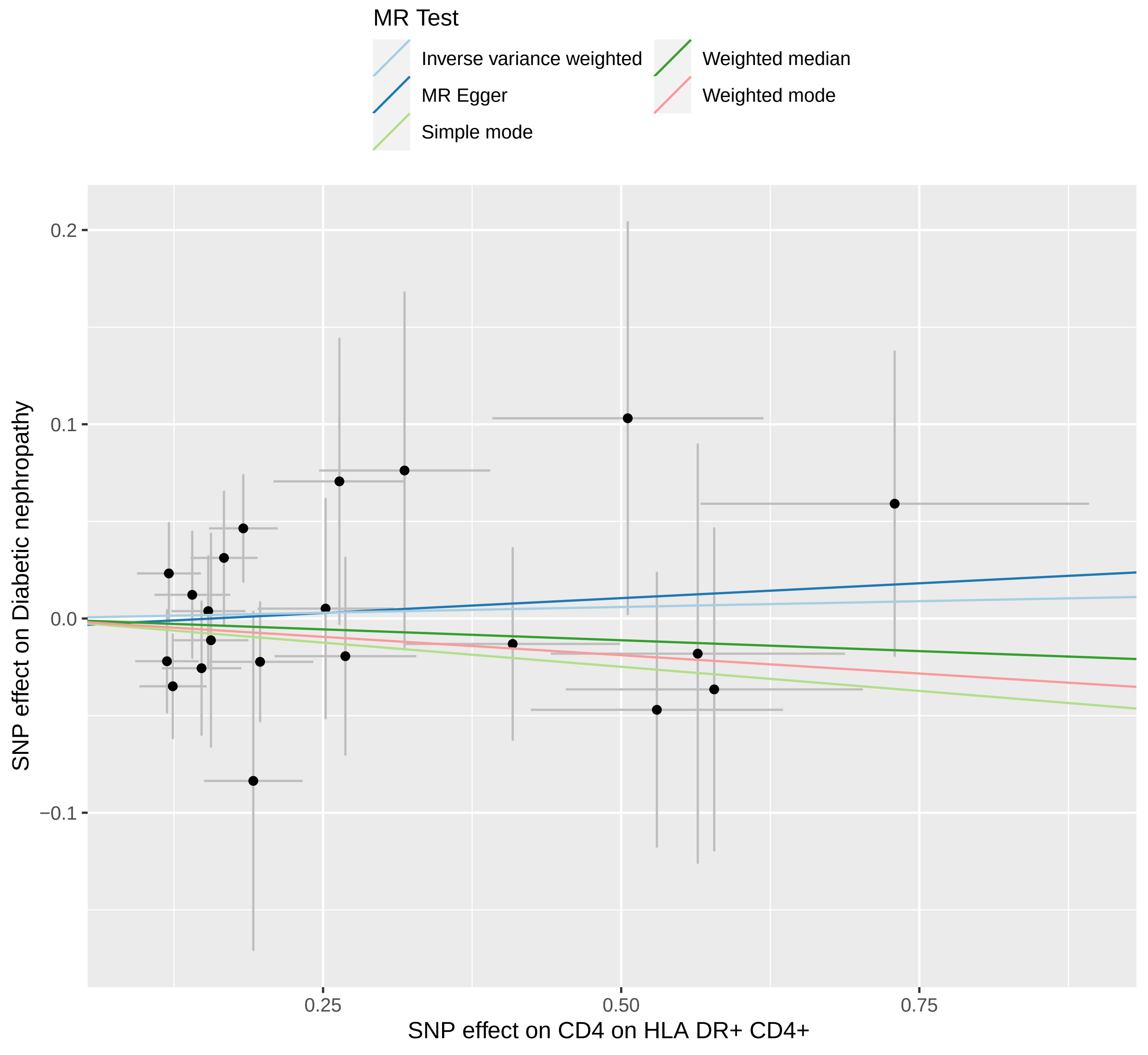
MR Test
Inverse variance weighted
MR Egger
Simple mode
Weighted median
Weighted mode
SNP effect on Diabetic nephropathy
0.2
0.1
0.0
−0.1
0.25
0.50
0.75
SNP effect on CD4 on HLA DR+ CD4+

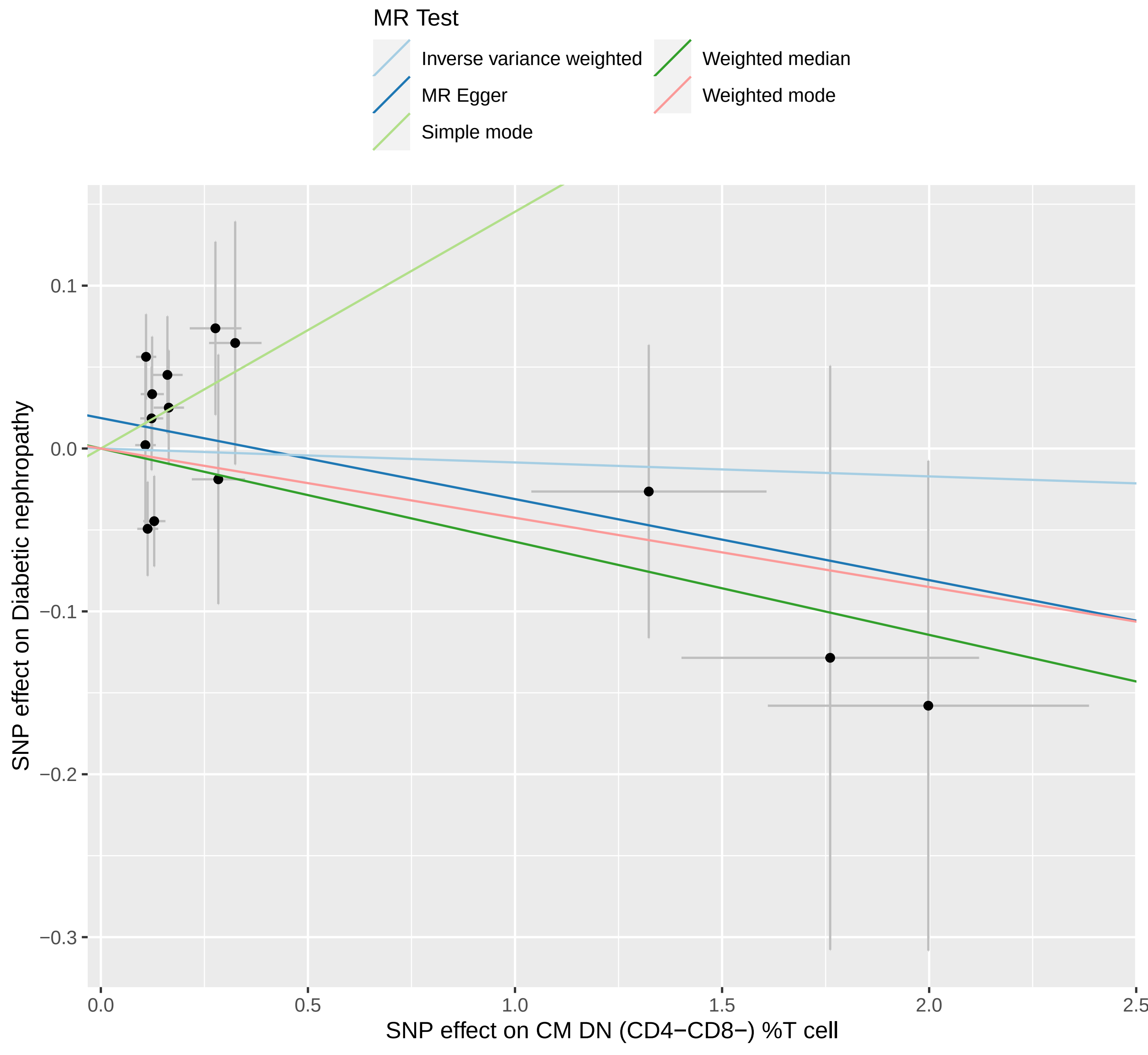

MR Test
Inverse variance weighted
MR Egger
Simple mode
Weighted median
Weighted mode
0.1
0.0
−0.1
−0.2
−0.3
SNP effect on Diabetic nephropathy
0.0
0.5
1.0
1.5
2.0
2.5
SNP effect on CM DN (CD4−CD8−) %T cell

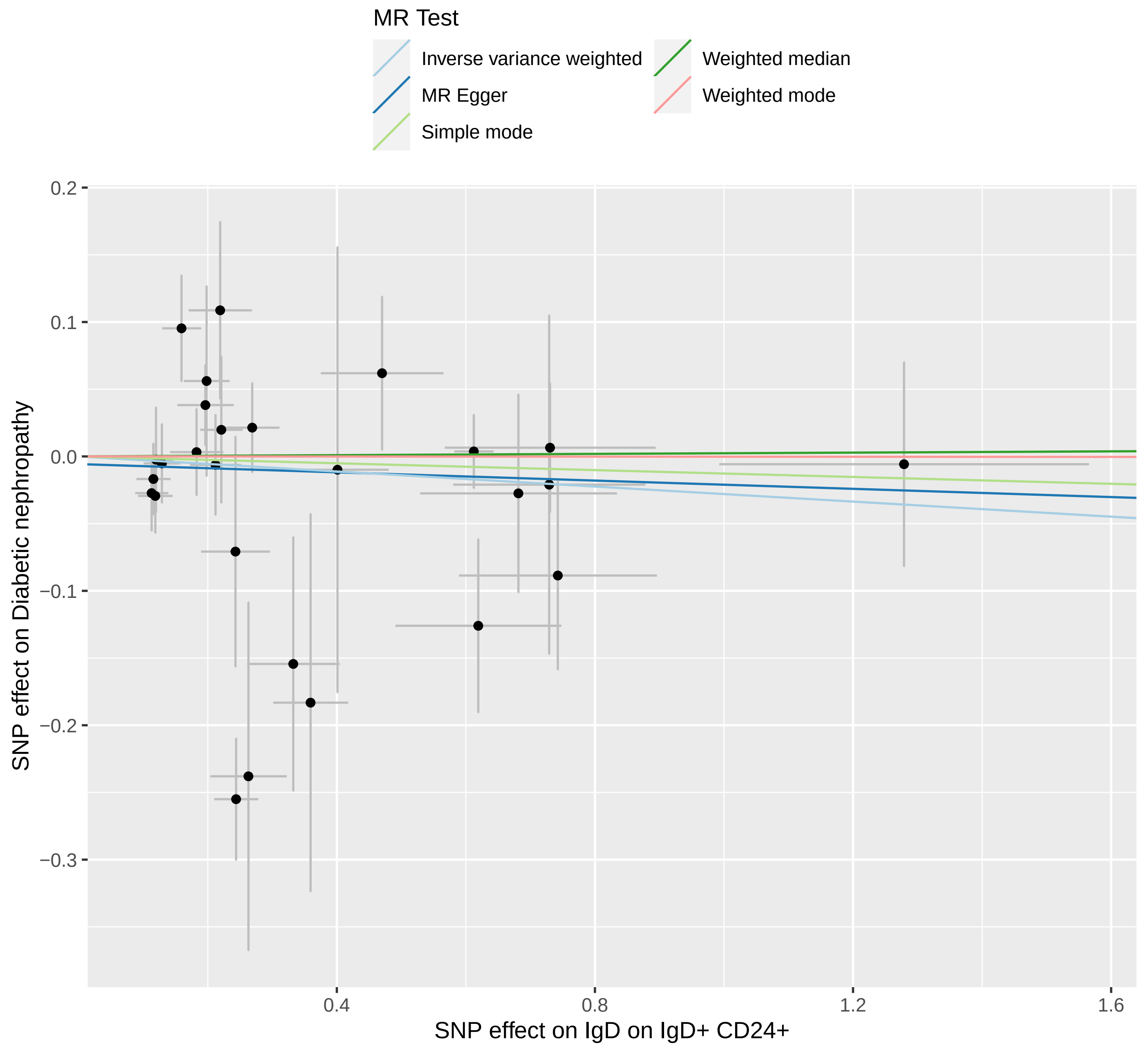
MR Test
Inverse variance weighted
MR Egger
Simple mode
Weighted median
Weighted mode
0.2
0.1
0.0
−0.1
−0.2
−0.3
SNP effect on Diabetic nephropathy
0.4
0.8
1.2
1.6
SNP effect on IgD on IgD+ CD24+

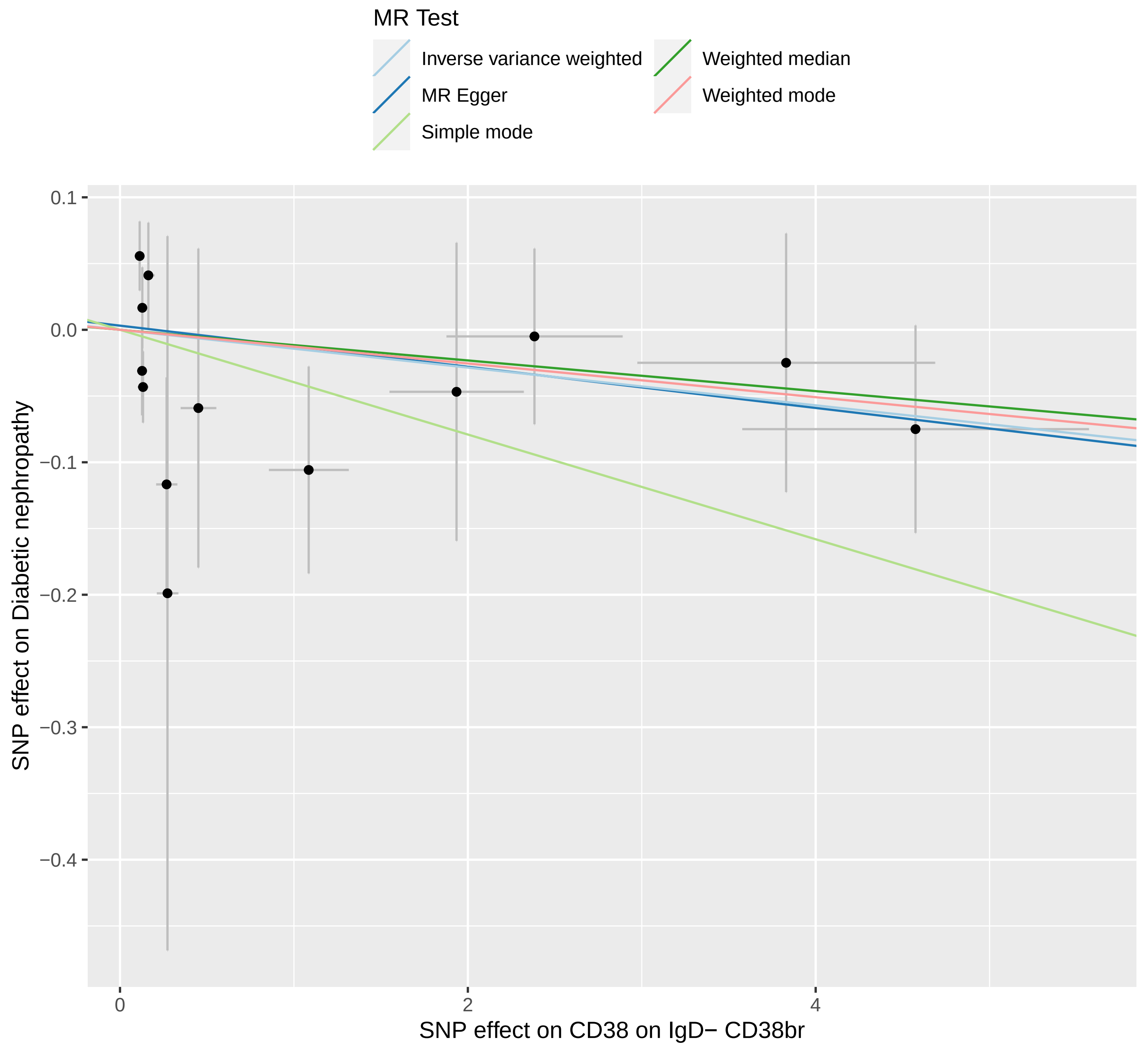

MR Test
Inverse variance weighted
MR Egger
Simple mode
Weighted median
Weighted mode
0.1
0.0
−0.1
−0.2
−0.3
−0.4
0
2
4
SNP effect on Diabetic nephropathy
SNP effect on CD38 on IgD− CD38br

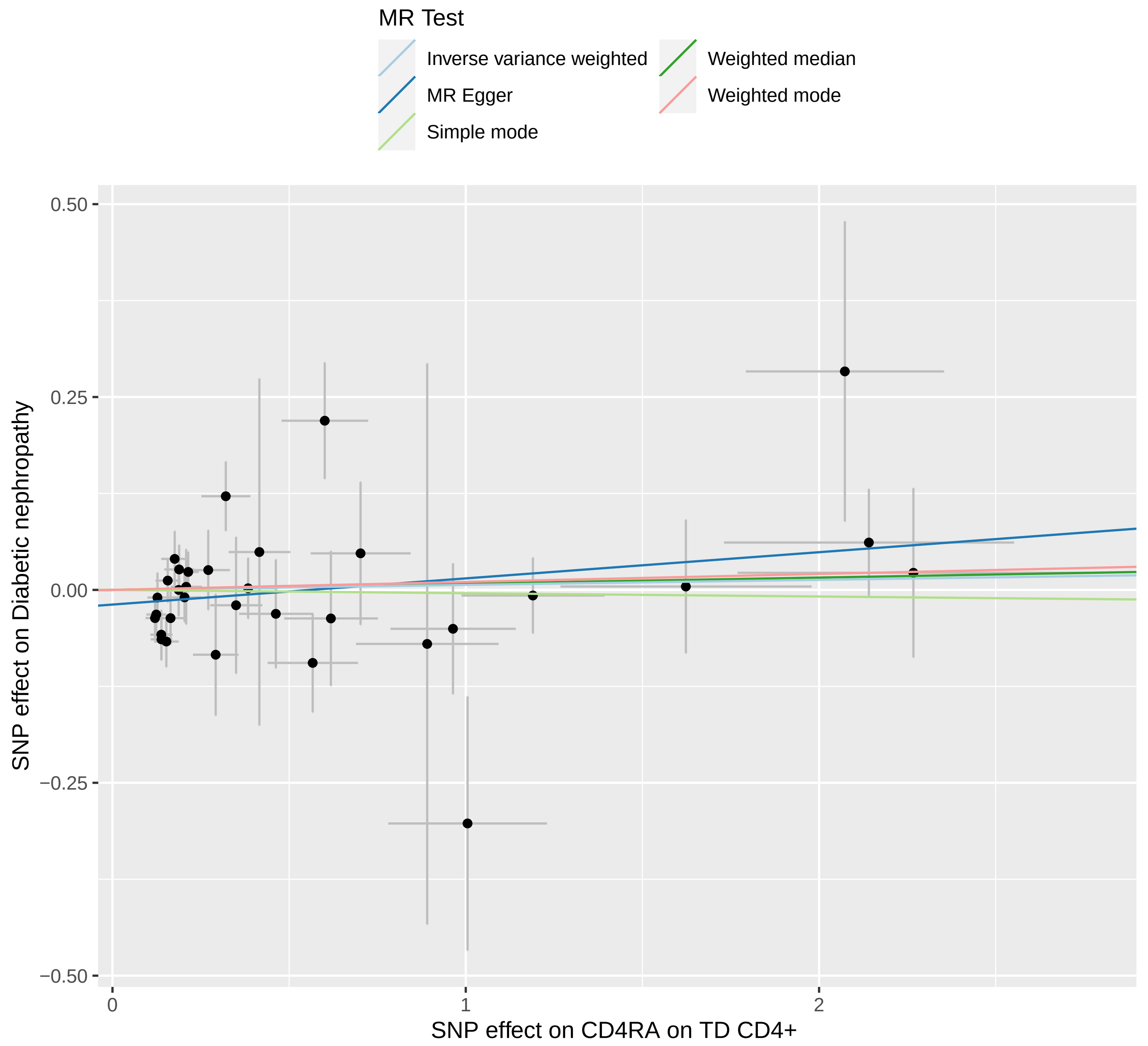
MR Test
Inverse variance weighted
MR Egger
Simple mode
Weighted median
Weighted mode
0.50
0.25
0.00
−0.25
−0.50
SNP effect on Diabetic nephropathy
0
1
2
SNP effect on CD4RA on TD CD4+

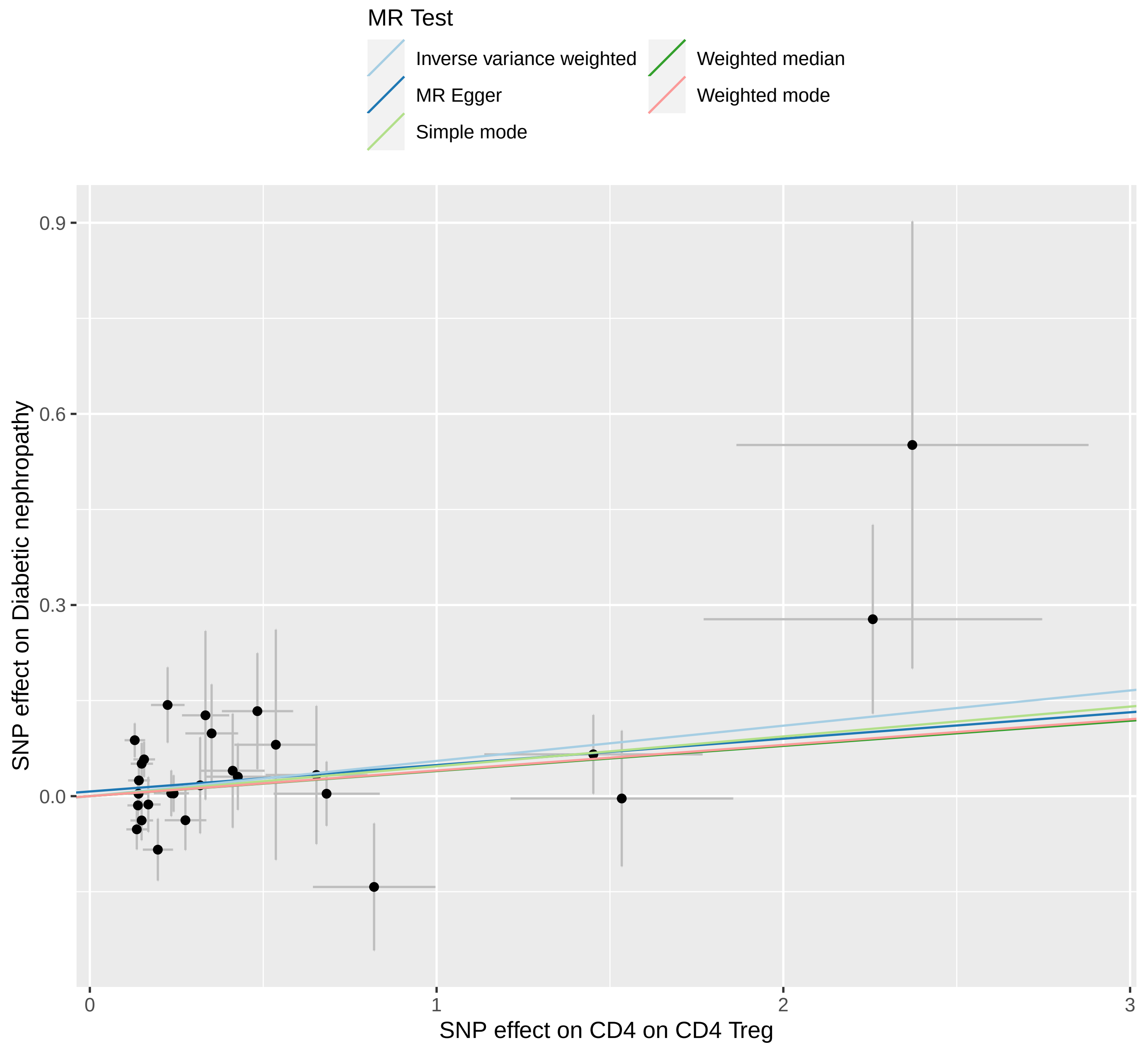

MR Test
Inverse variance weighted
MR Egger
Simple mode
Weighted median
Weighted mode
SNP effect on Diabetic nephropathy
0.9
0.6
0.3
0.0
0
1
2
3
SNP effect on CD4 on CD4 Treg

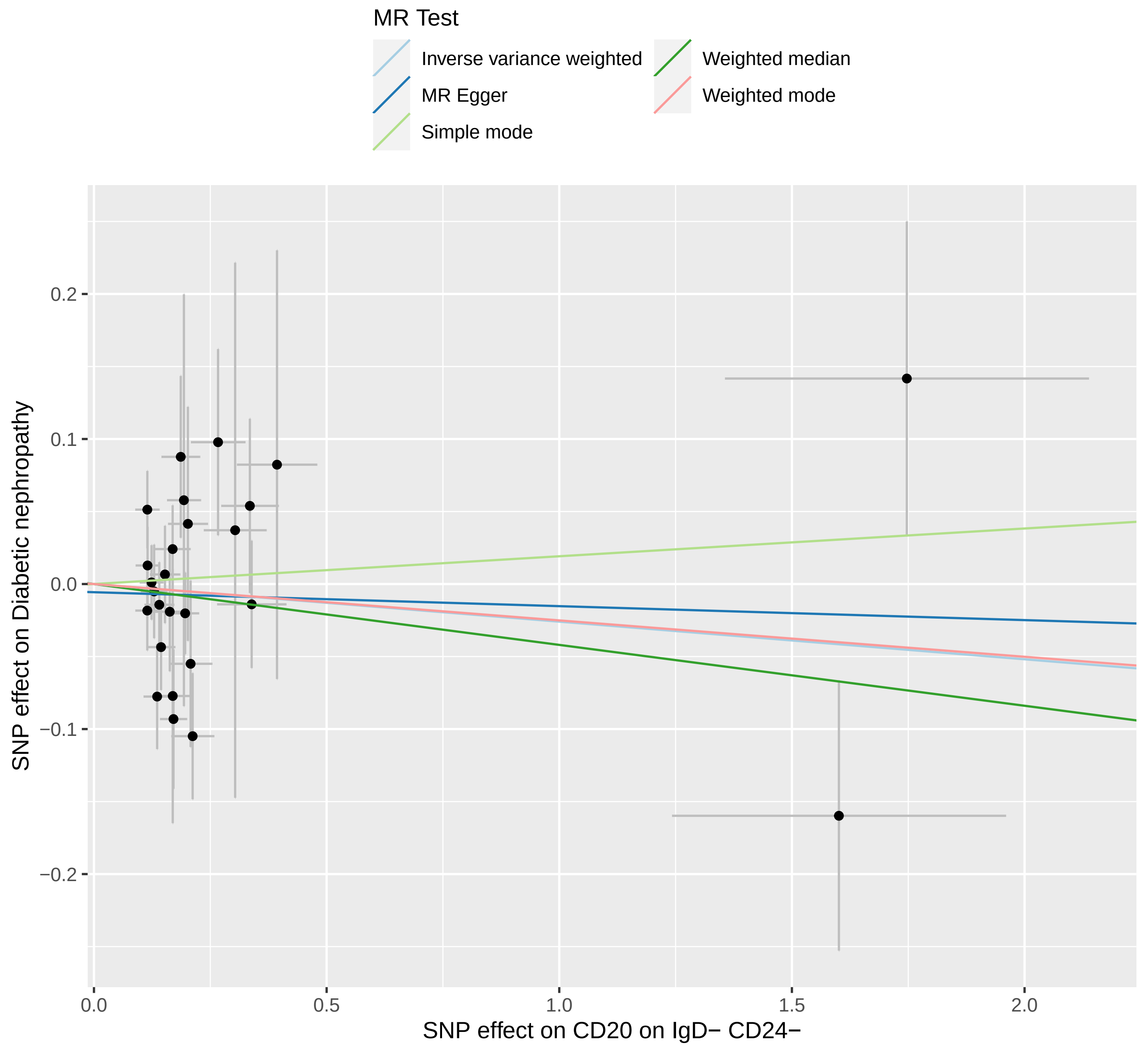
MR Test
Inverse variance weighted
MR Egger
Simple mode
Weighted median
Weighted mode
0.2
0.1
0.0
−0.1
−0.2
SNP effect on Diabetic nephropathy
0.0
0.5
1.0
1.5
2.0
SNP effect on CD20 on IgD− CD24−

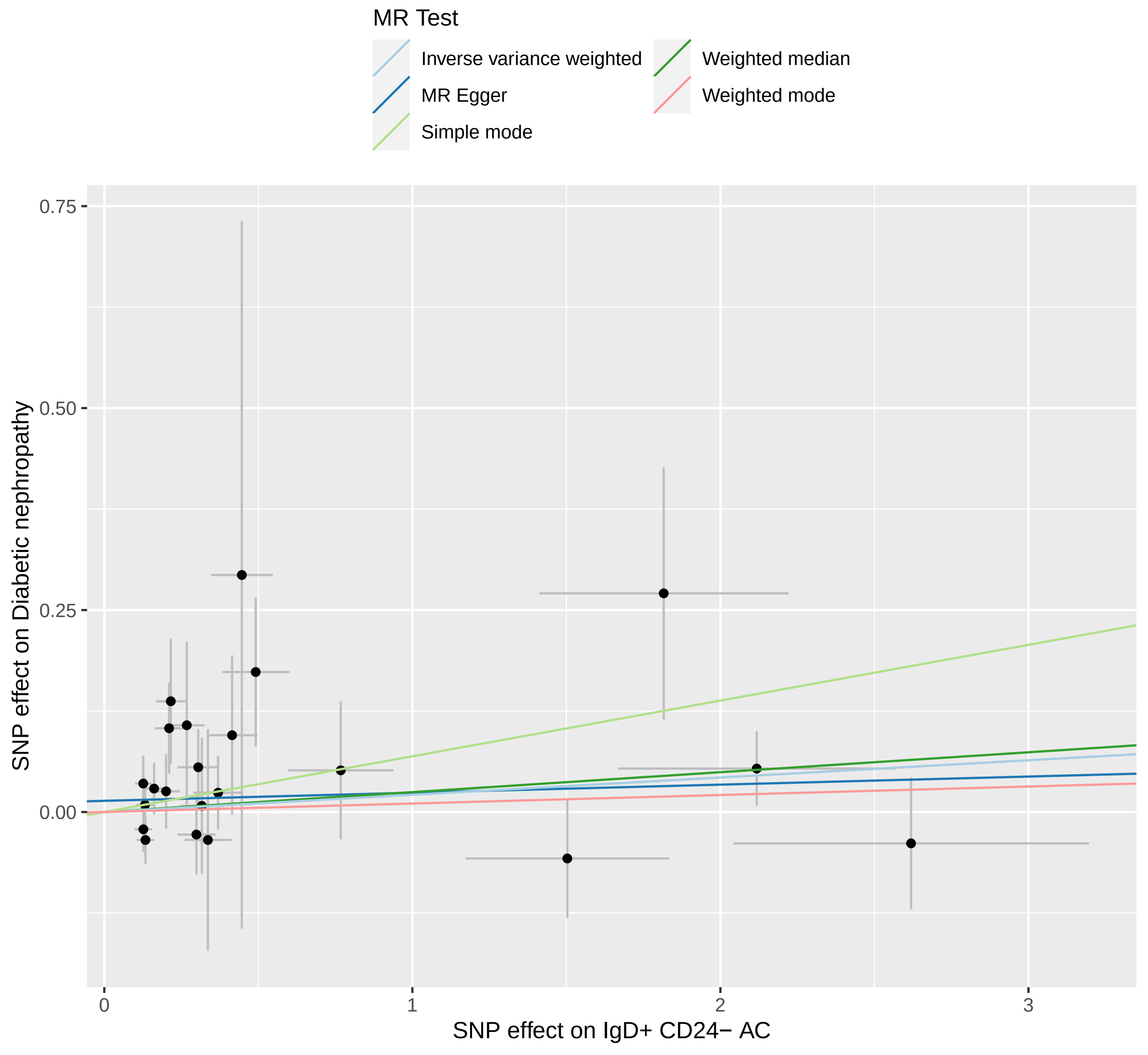
MR Test
Inverse variance weighted
MR Egger
Simple mode
Weighted median
Weighted mode
SNP effect on Diabetic nephropathy
0.75
0.50
0.25
0.00
0
1
2
3
SNP effect on IgD+ CD24− AC

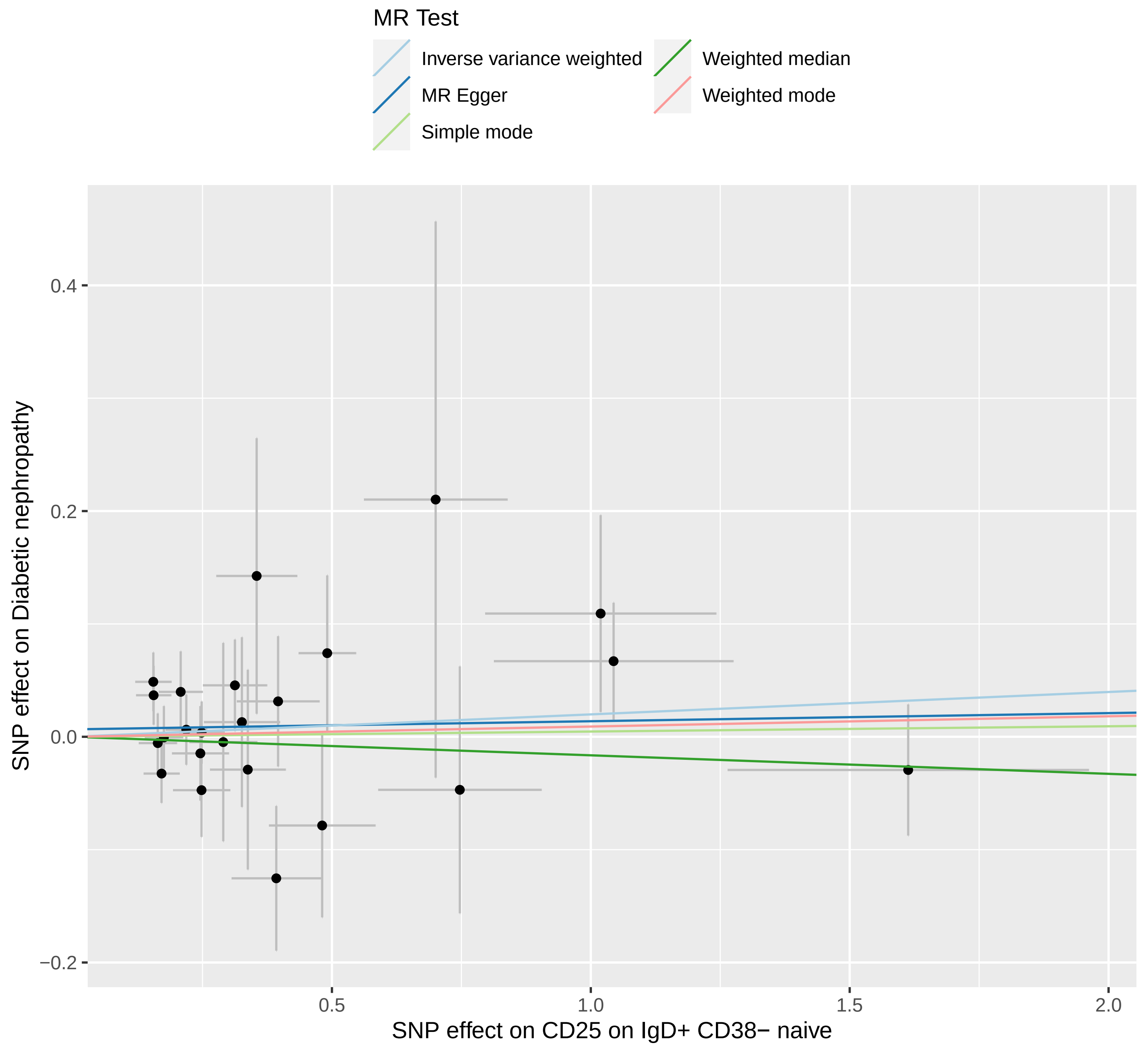
MR Test
Inverse variance weighted
MR Egger
Simple mode
Weighted median
Weighted mode
SNP effect on Diabetic nephropathy
0.4
0.2
0.0
−0.2
0.5
1.0
1.5
2.0
SNP effect on CD25 on IgD+ CD38− naive

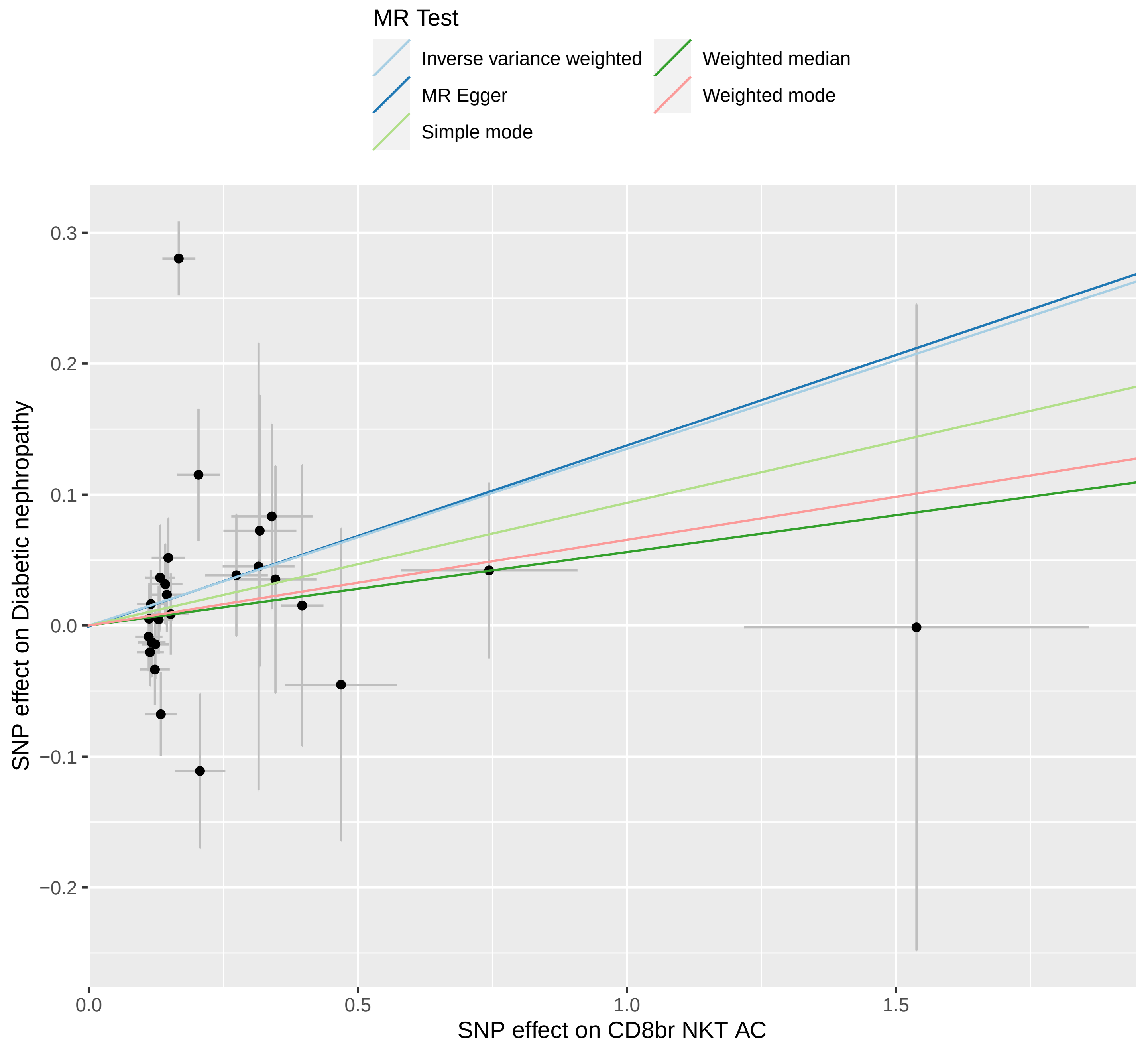
MR Test
Inverse variance weighted
MR Egger
Simple mode
Weighted median
Weighted mode
0.3
0.2
0.1
0.0
−0.1
−0.2
0.0
0.5
1.0
1.5
SNP effect on Diabetic nephropathy
SNP effect on CD8br NKT AC

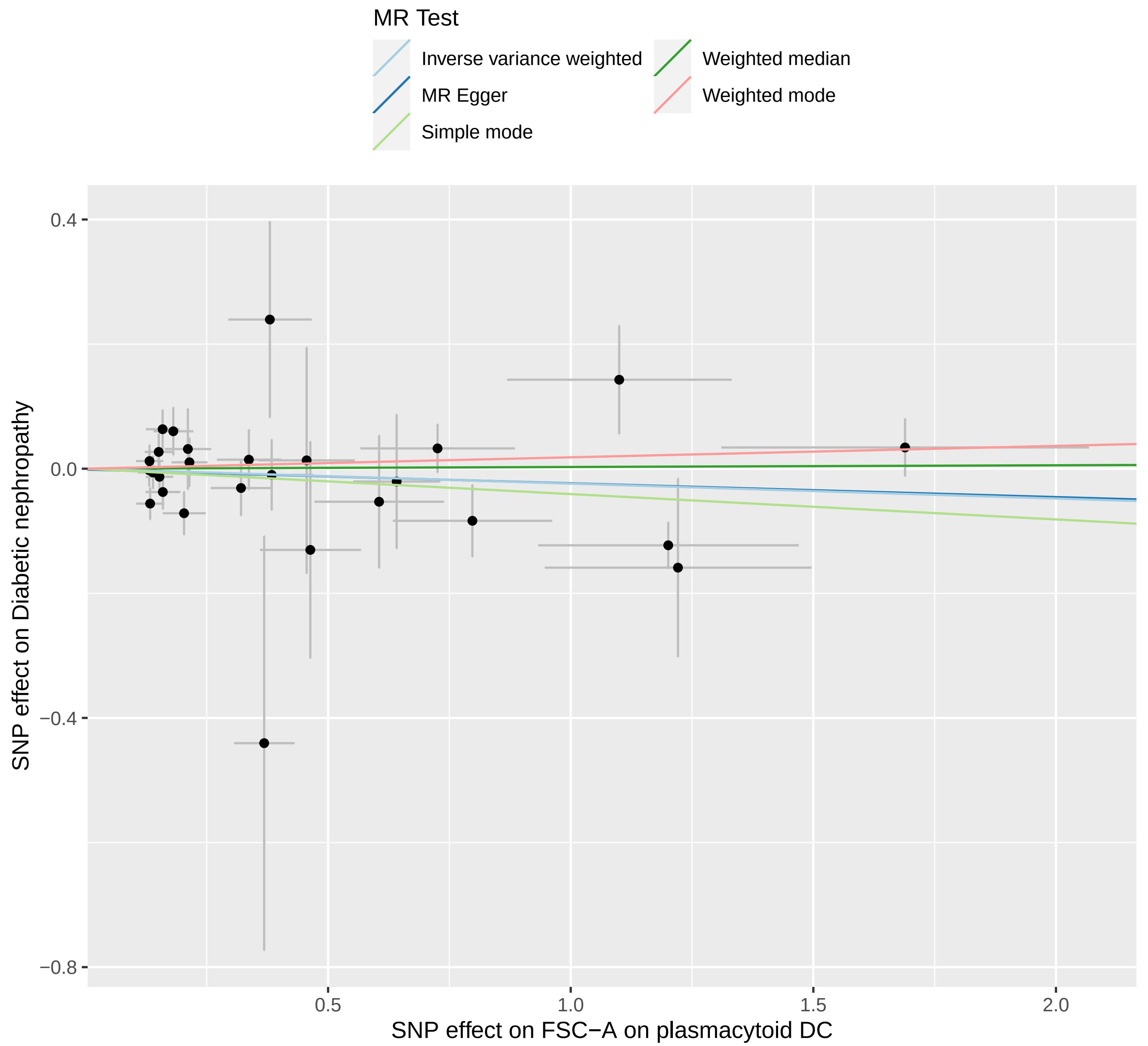

MR Test
Inverse variance weighted
MR Egger
Simple mode
Weighted median
Weighted mode
0.4
0.0
−0.4
−0.8
0.5
1.0
1.5
2.0
SNP effect on Diabetic nephropathy
SNP effect on FSC−A on plasmacytoid DC

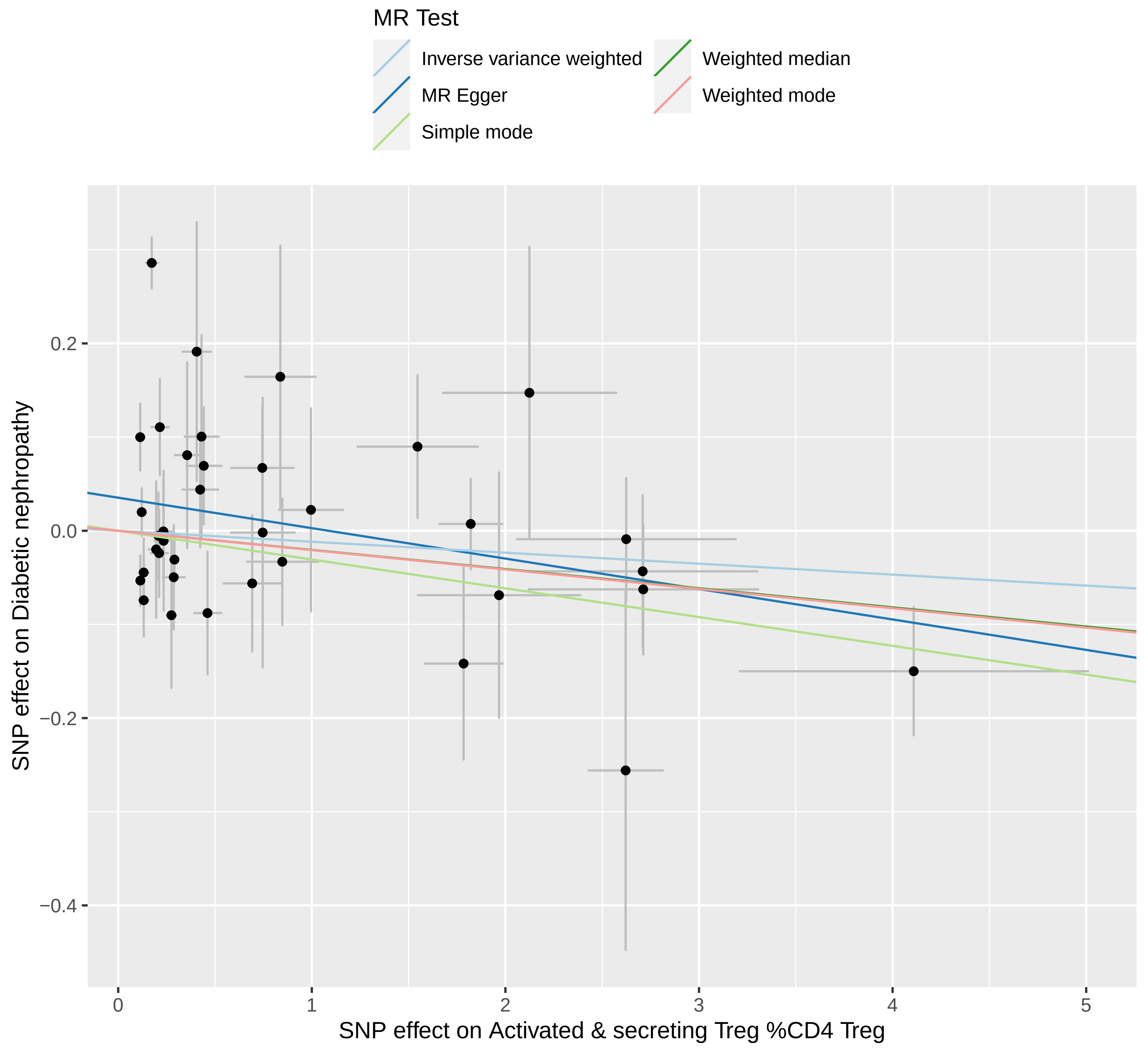
MR Test
Inverse variance weighted
MR Egger
Simple mode
Weighted median
Weighted mode
0.2
0.0
−0.2
−0.4
0
1
2
3
4
5
SNP effect on Diabetic nephropathy
SNP effect on Activated & secreting Treg %CD4 Treg

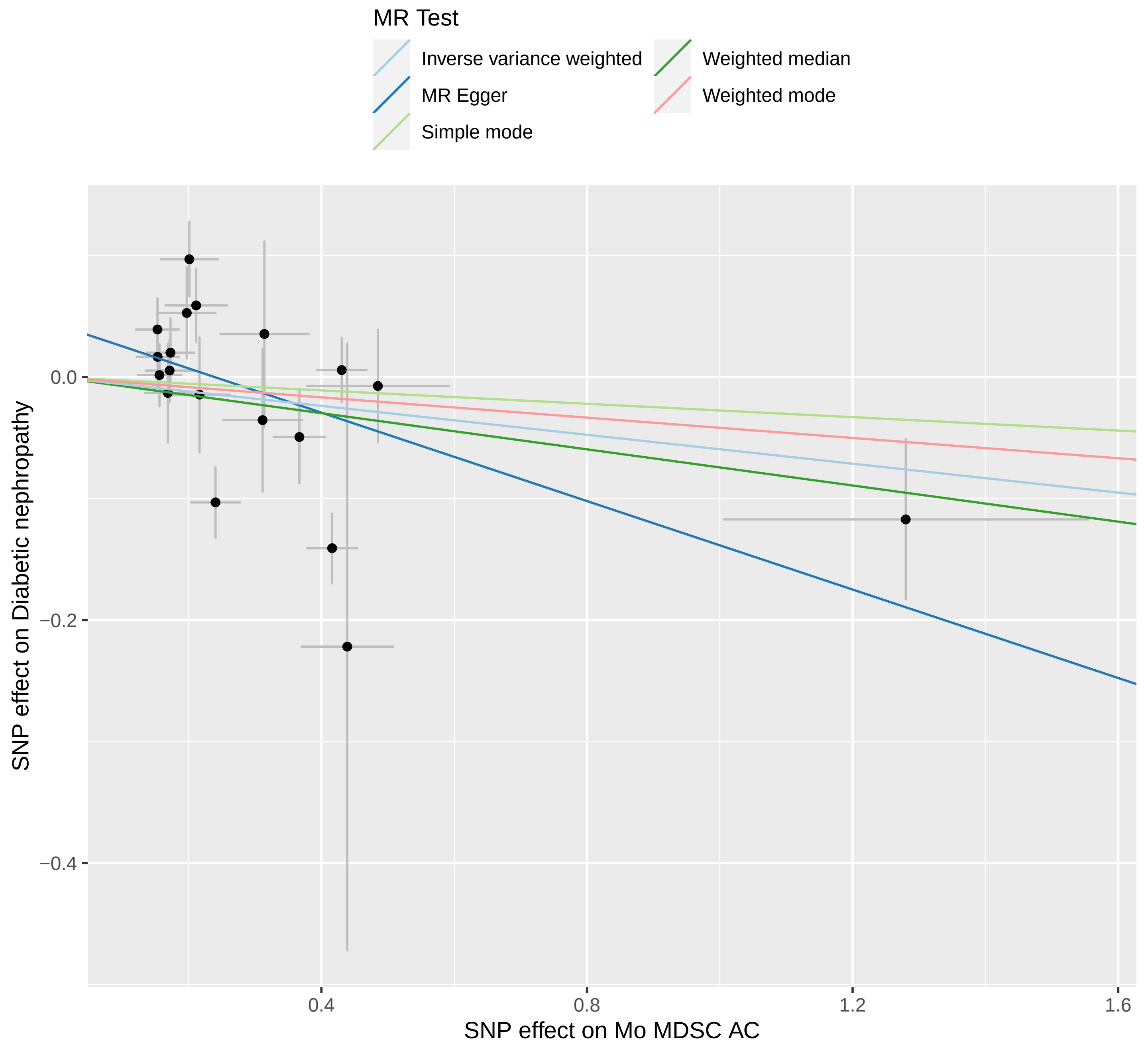
MR Test
Inverse variance weighted
MR Egger
Simple mode
Weighted median
Weighted mode
0.0
−0.2
−0.4
0.4
0.8
1.2
1.6
SNP effect on Diabetic nephropathy
SNP effect on Mo MDSC AC

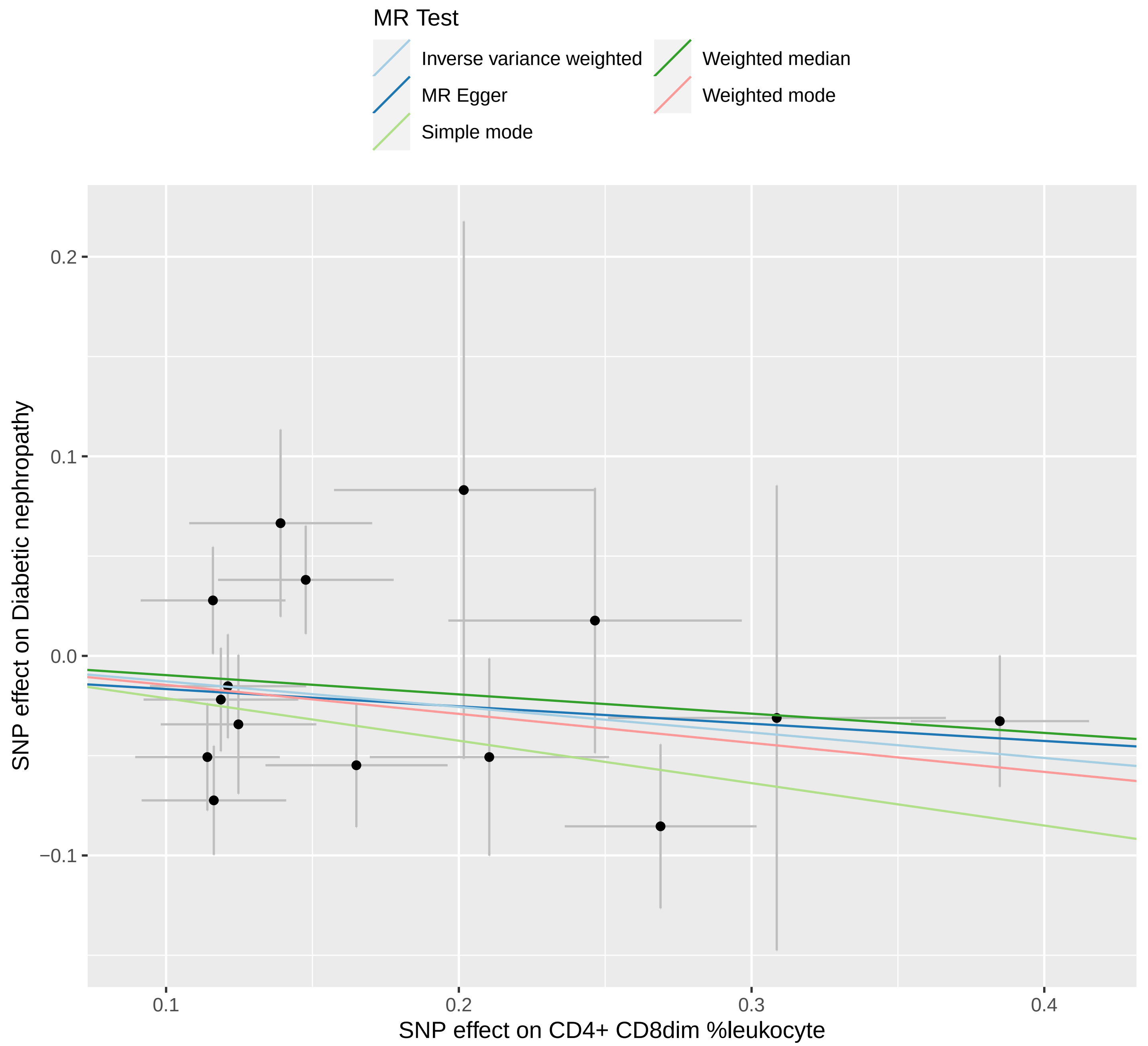

MR Test
Inverse variance weighted
Weighted median
MR Egger
Weighted mode
Simple mode
0.2
0.1
0.0
−0.1
0.1
0.2
0.3
0.4
SNP effect on Diabetic nephropathy
SNP effect on CD4+ CD8dim %leukocyte

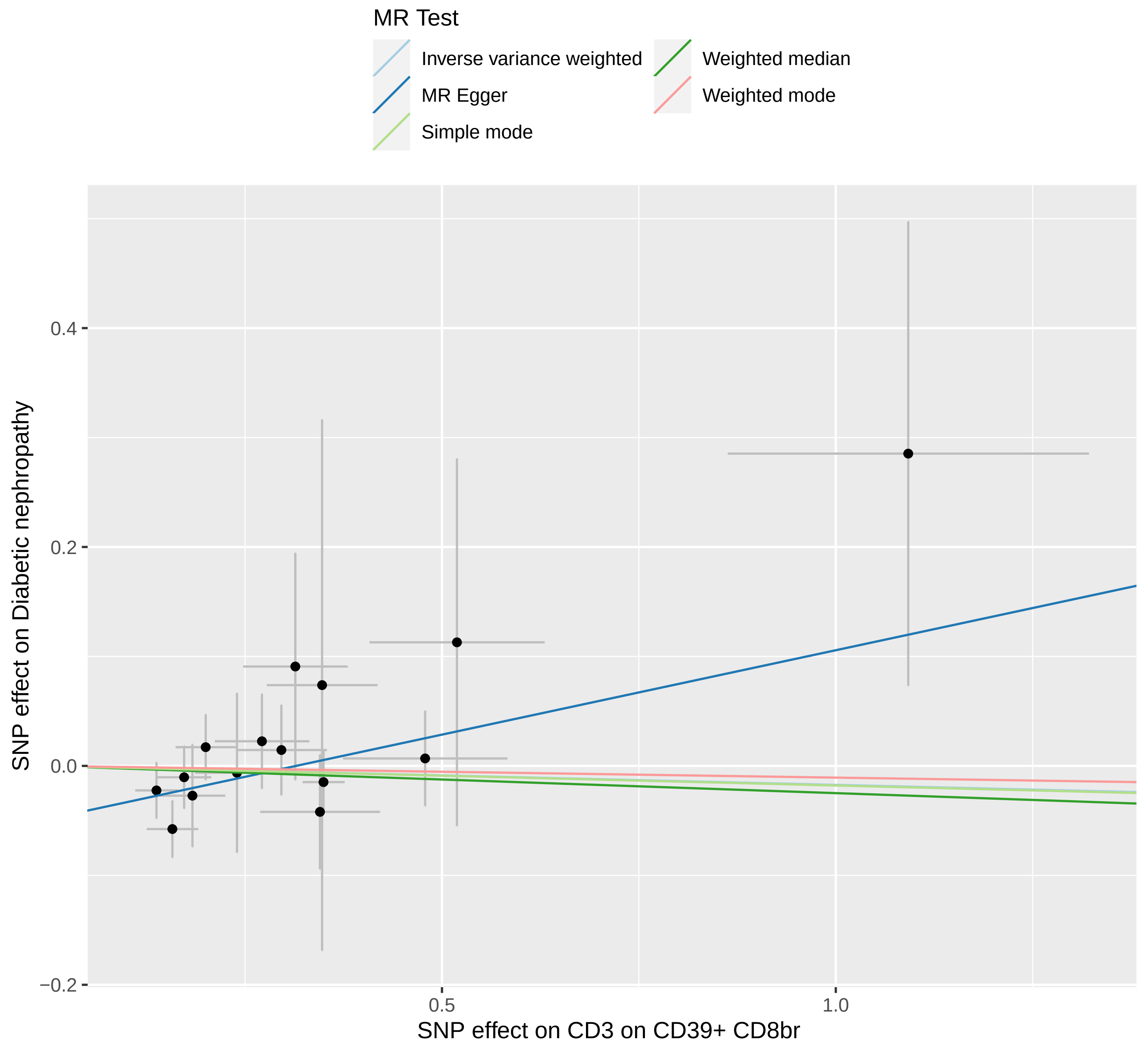
MR Test
Inverse variance weighted
MR Egger
Simple mode
Weighted median
Weighted mode
0.4
0.2
0.0
−0.2
0.5
1.0
SNP effect on Diabetic nephropathy
SNP effect on CD3 on CD39+ CD8br

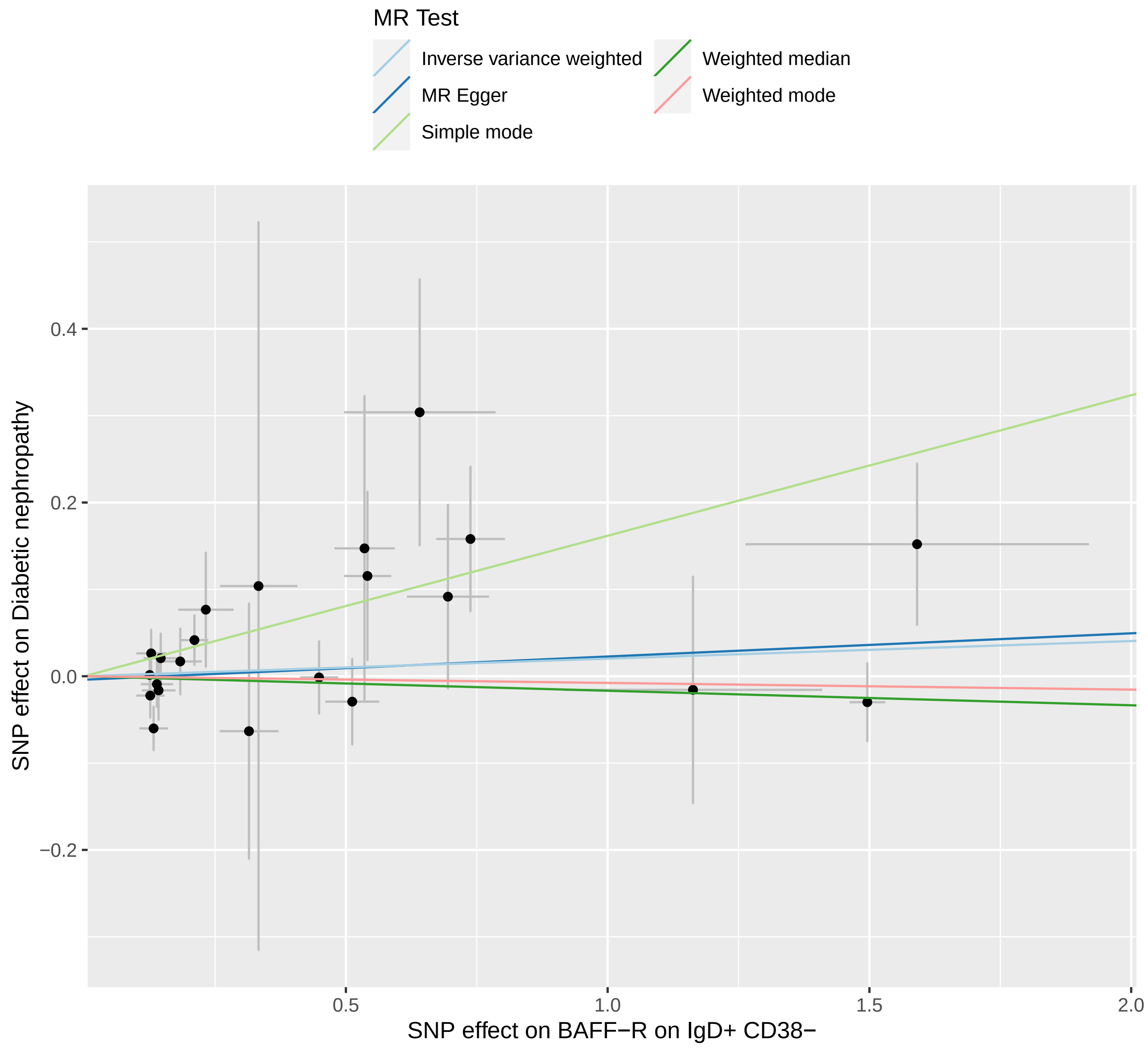
MR Test
Inverse variance weighted
MR Egger
Simple mode
Weighted median
Weighted mode
0.4
0.2
0.0
−0.2
0.5
1.0
1.5
2.0
SNP effect on Diabetic nephropathy
SNP effect on BAFF−R on IgD+ CD38−

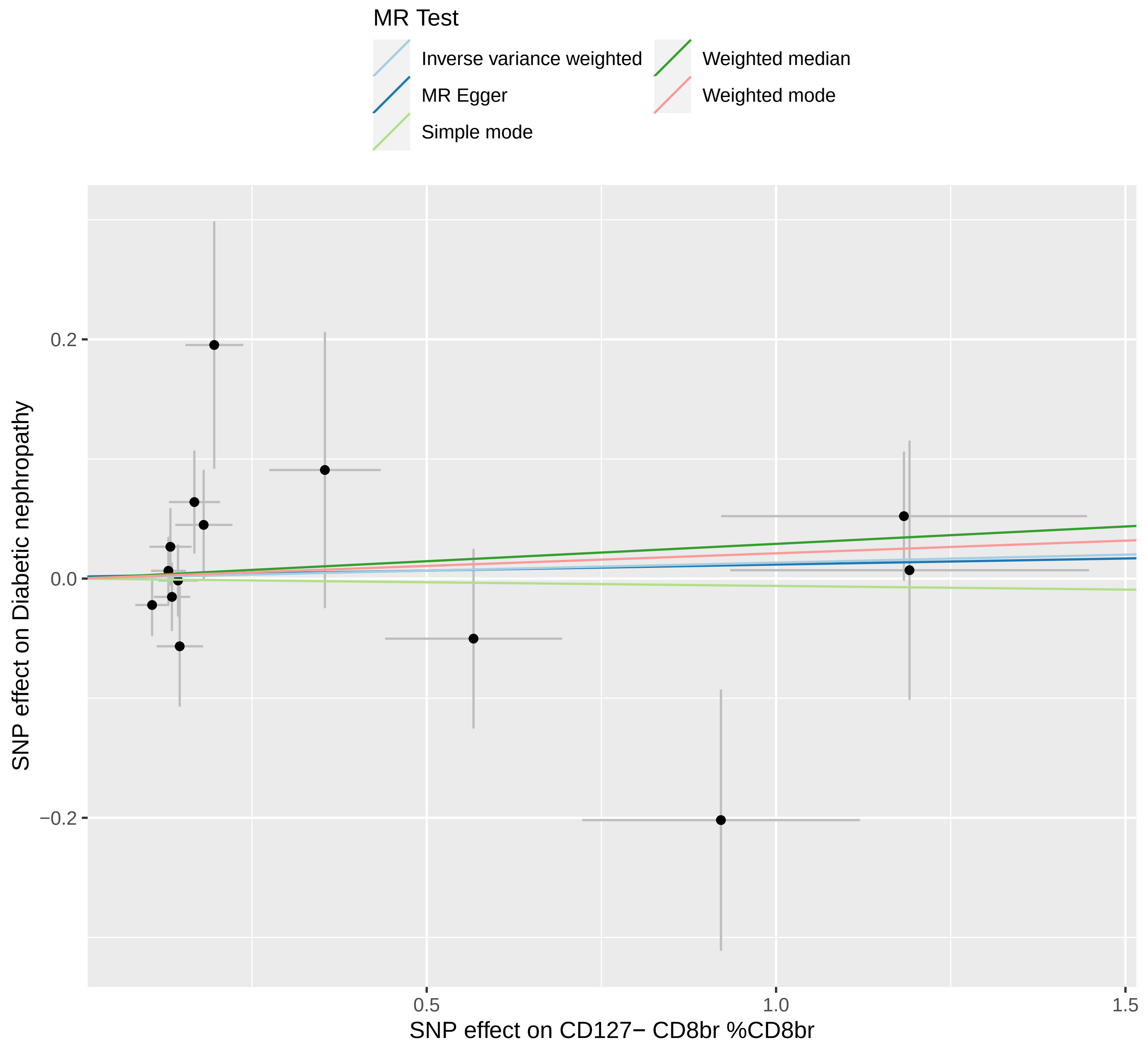

MR Test
Inverse variance weighted
MR Egger
Simple mode
Weighted median
Weighted mode
0.2
0.0
−0.2
0.5
1.0
1.5
SNP effect on Diabetic nephropathy
SNP effect on CD127− CD8br %CD8br

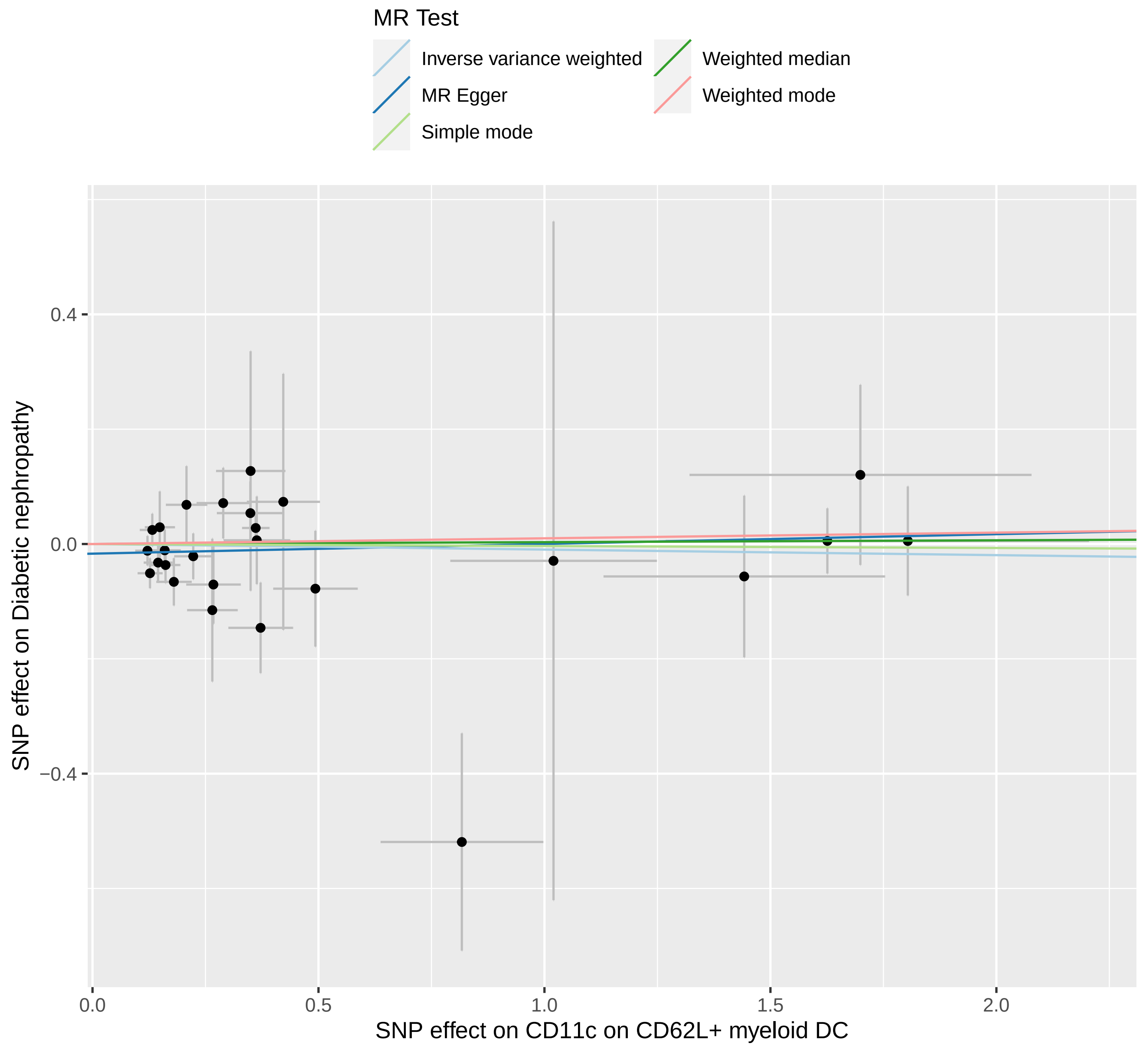
MR Test
Inverse variance weighted
MR Egger
Simple mode
Weighted median
Weighted mode
0.4
0.0
−0.4
SNP effect on Diabetic nephropathy
0.0
0.5
1.0
1.5
2.0
SNP effect on CD11c on CD62L+ myeloid DC

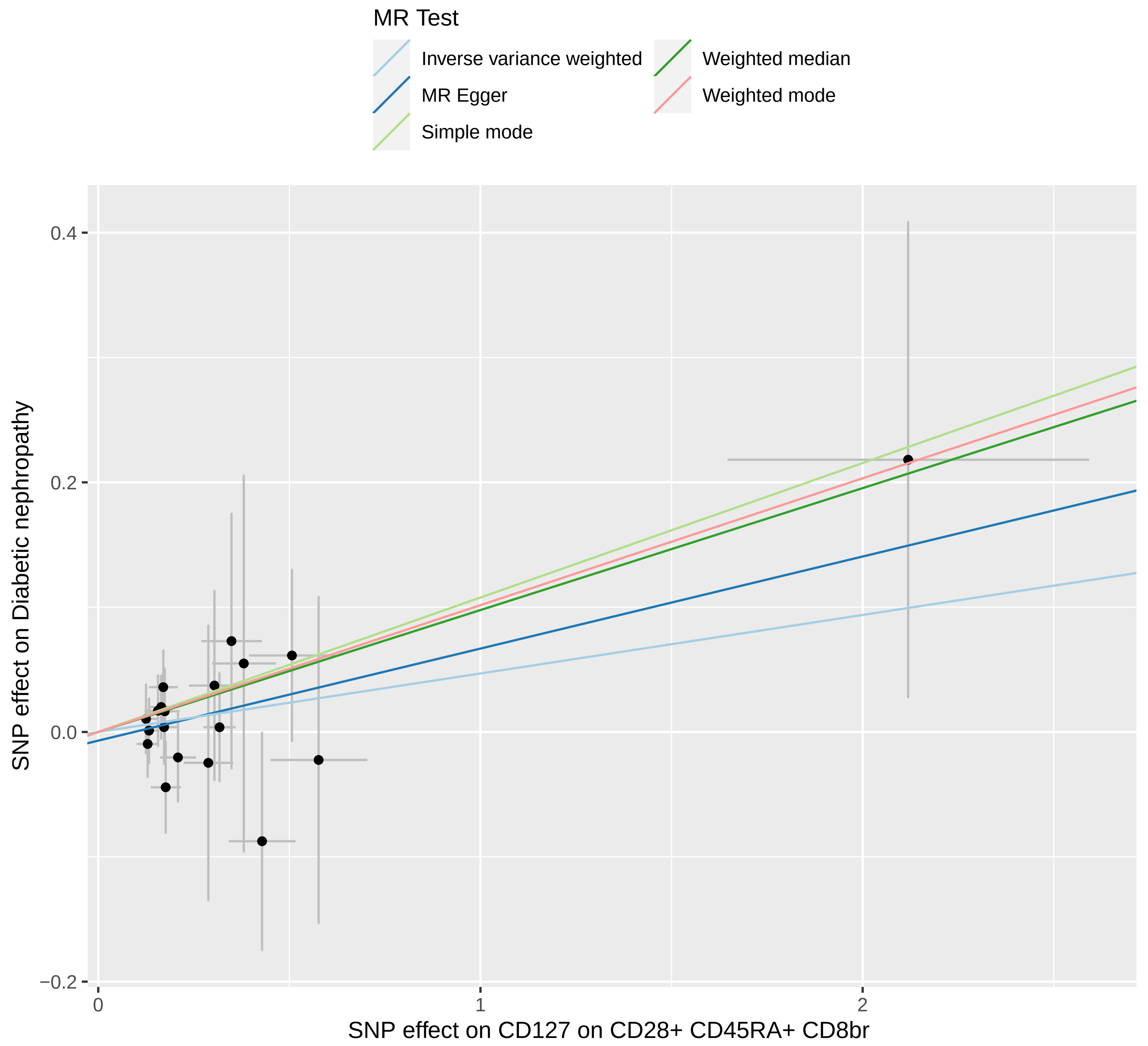

MR Test
Inverse variance weighted
MR Egger
Simple mode
Weighted median
Weighted mode
0.4
0.2
0.0
−0.2
0
1
2
SNP effect on Diabetic nephropathy
SNP effect on CD127 on CD28+ CD45RA+ CD8br

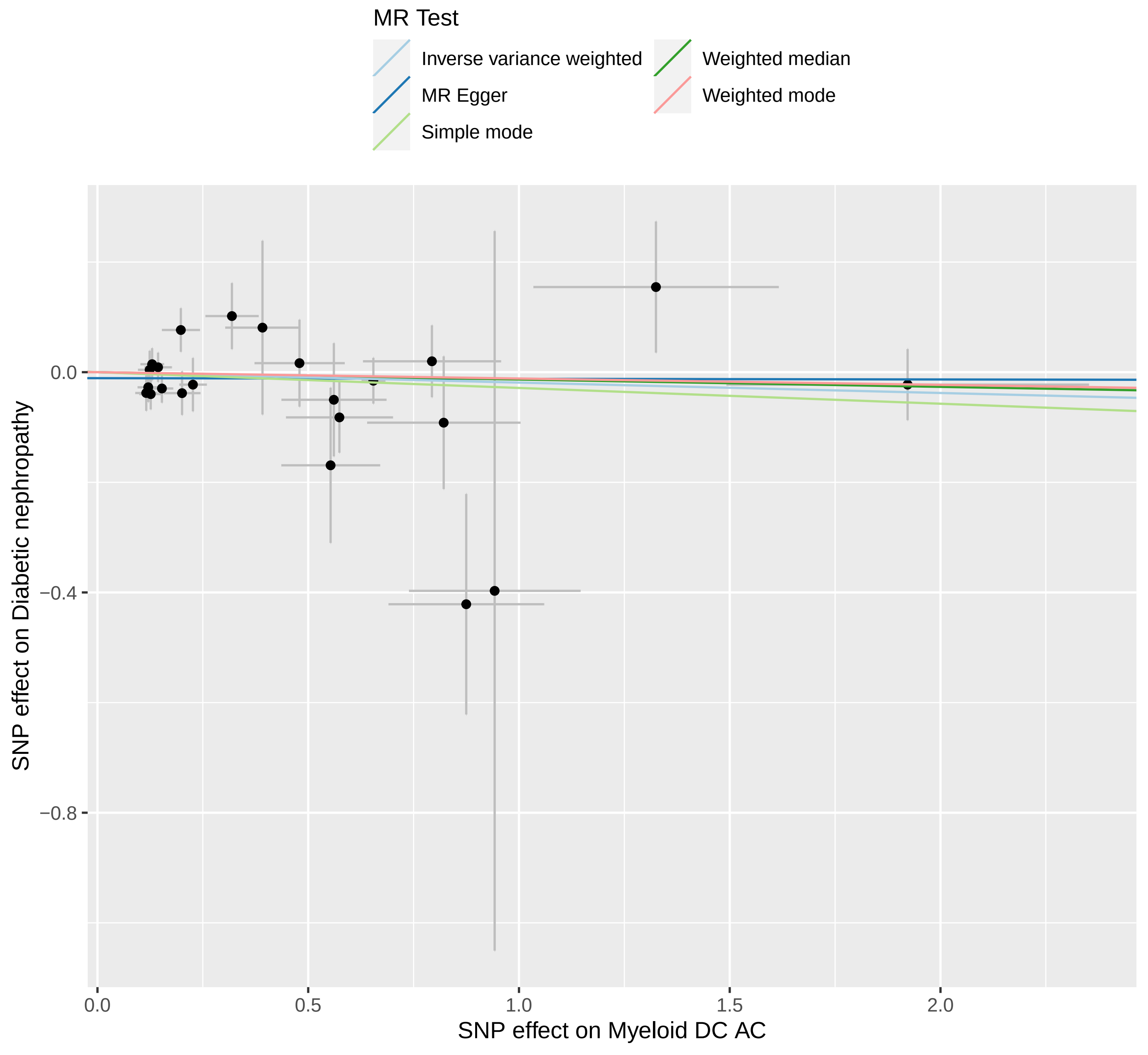
MR Test
Inverse variance weighted
MR Egger
Simple mode
Weighted median
Weighted mode
SNP effect on Diabetic nephropathy
0.0
−0.4
−0.8
0.0
0.5
1.0
1.5
2.0
SNP effect on Myeloid DC AC

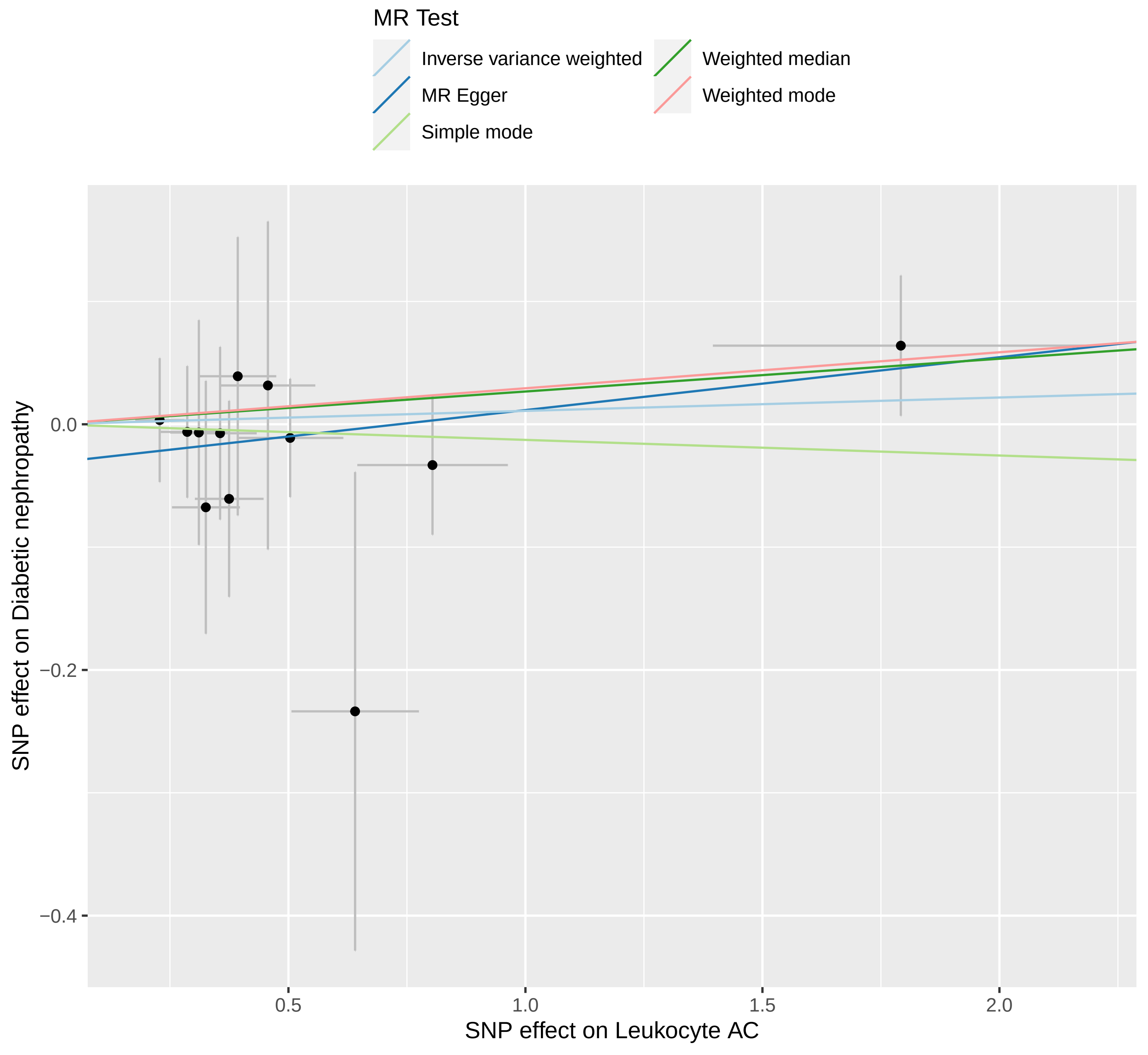
MR Test
Inverse variance weighted
MR Egger
Simple mode
Weighted median
Weighted mode
SNP effect on Diabetic nephropathy
0.0
−0.2
−0.4
0.5
1.0
1.5
2.0
SNP effect on Leukocyte AC

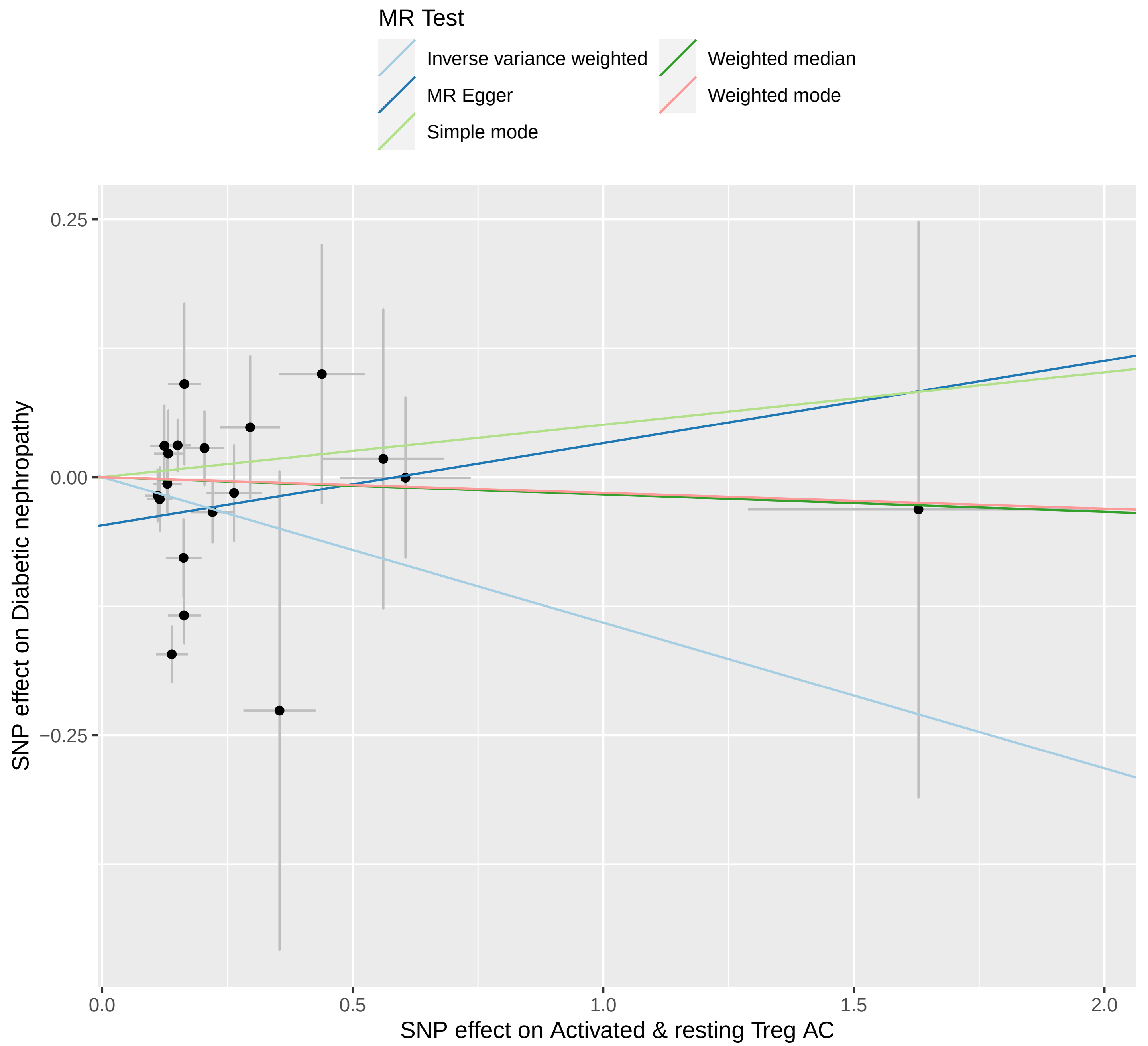
MR Test
Inverse variance weighted
MR Egger
Simple mode
Weighted median
Weighted mode
0.25
0.00
−0.25
0.0
0.5
1.0
1.5
2.0
SNP effect on Diabetic nephropathy
SNP effect on Activated & resting Treg AC

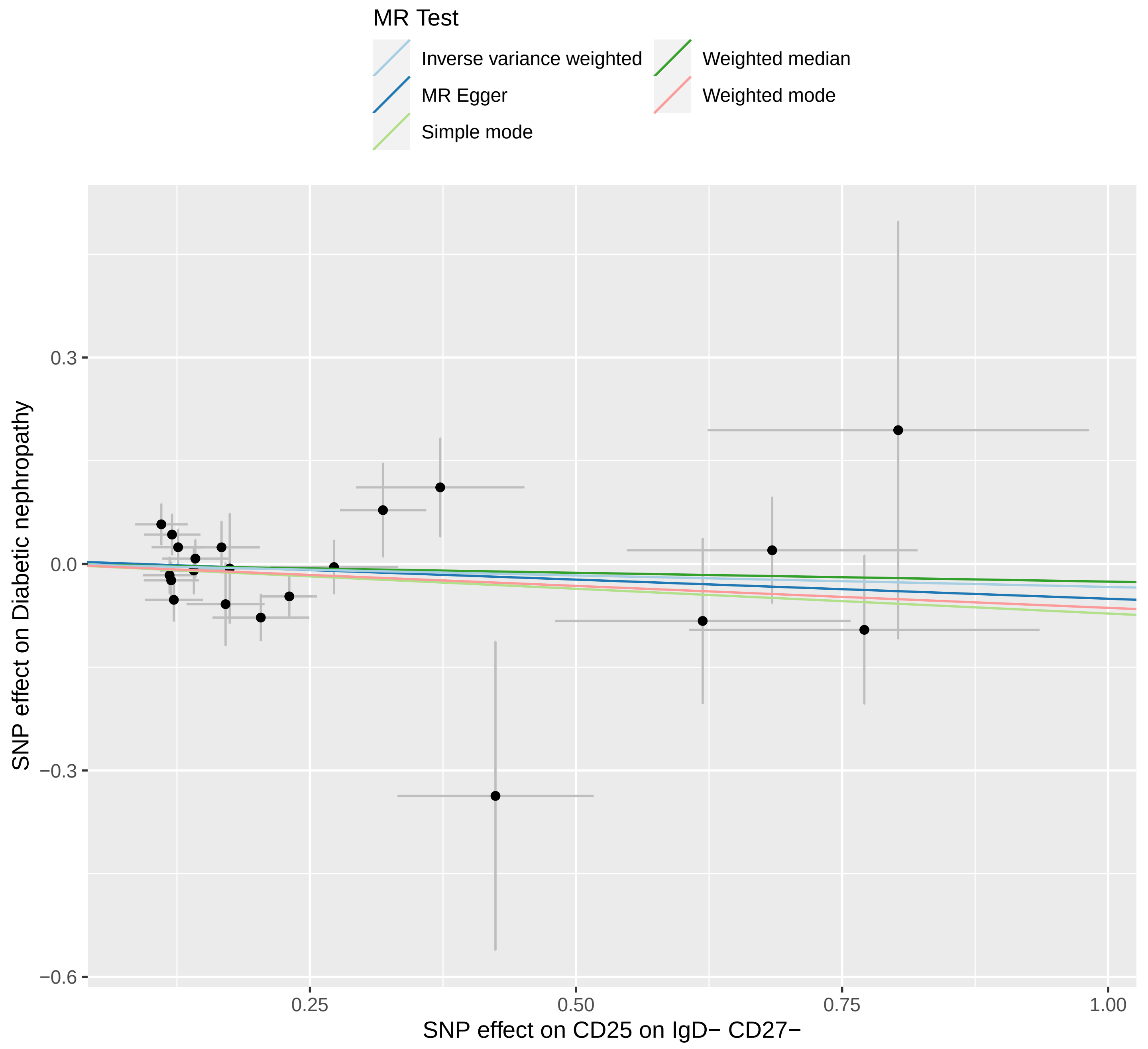
MR Test
Inverse variance weighted
MR Egger
Simple mode
Weighted median
Weighted mode
0.3
0.0
−0.3
−0.6
0.25
0.50
0.75
1.00
SNP effect on Diabetic nephropathy
SNP effect on CD25 on IgD− CD27−

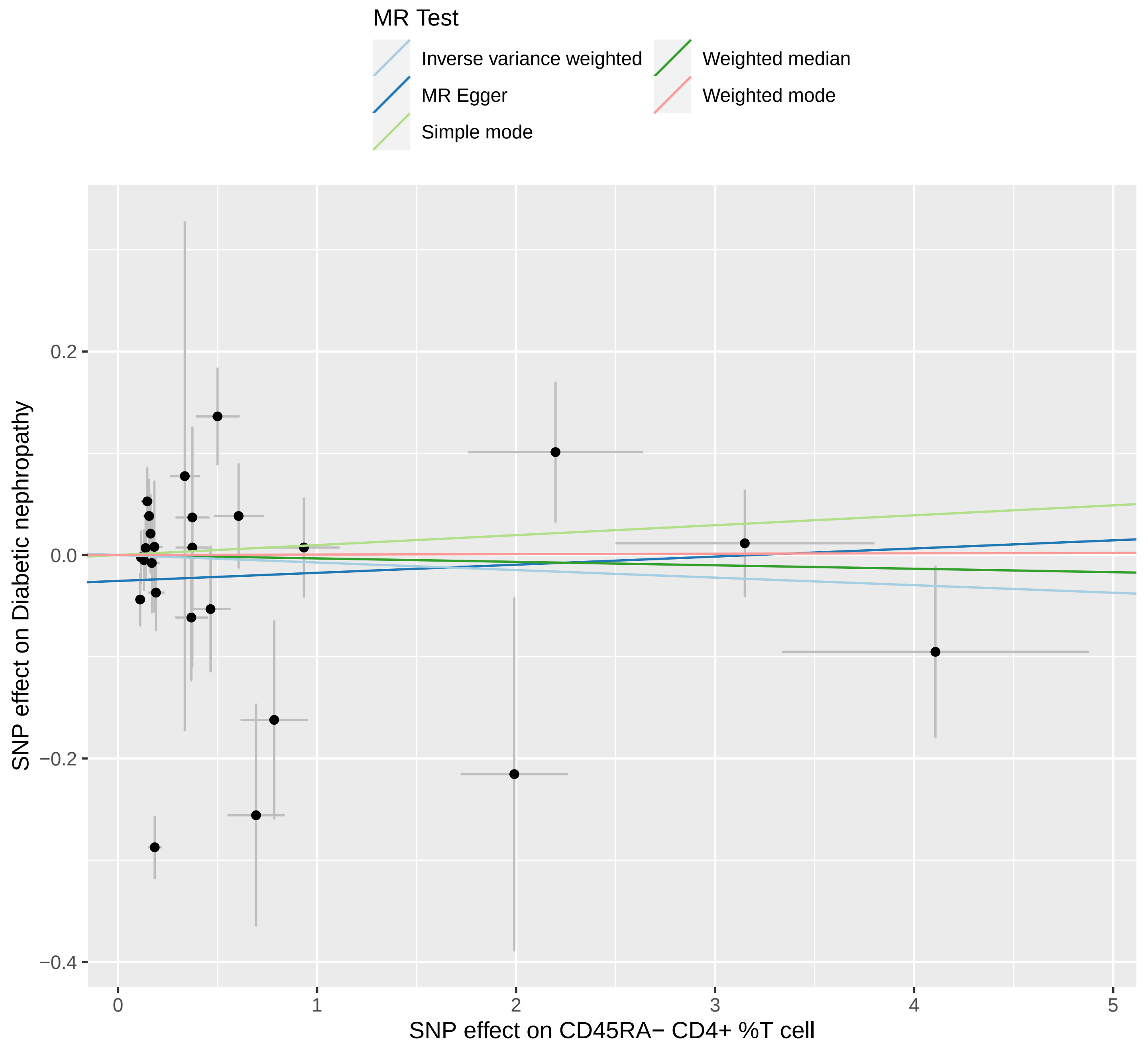

MR Test
Inverse variance weighted
MR Egger
Simple mode
Weighted median
Weighted mode
SNP effect on Diabetic nephropathy
0.2
0.0
−0.2
−0.4
0
1
2
3
4
5
SNP effect on CD45RA− CD4+ %T cell

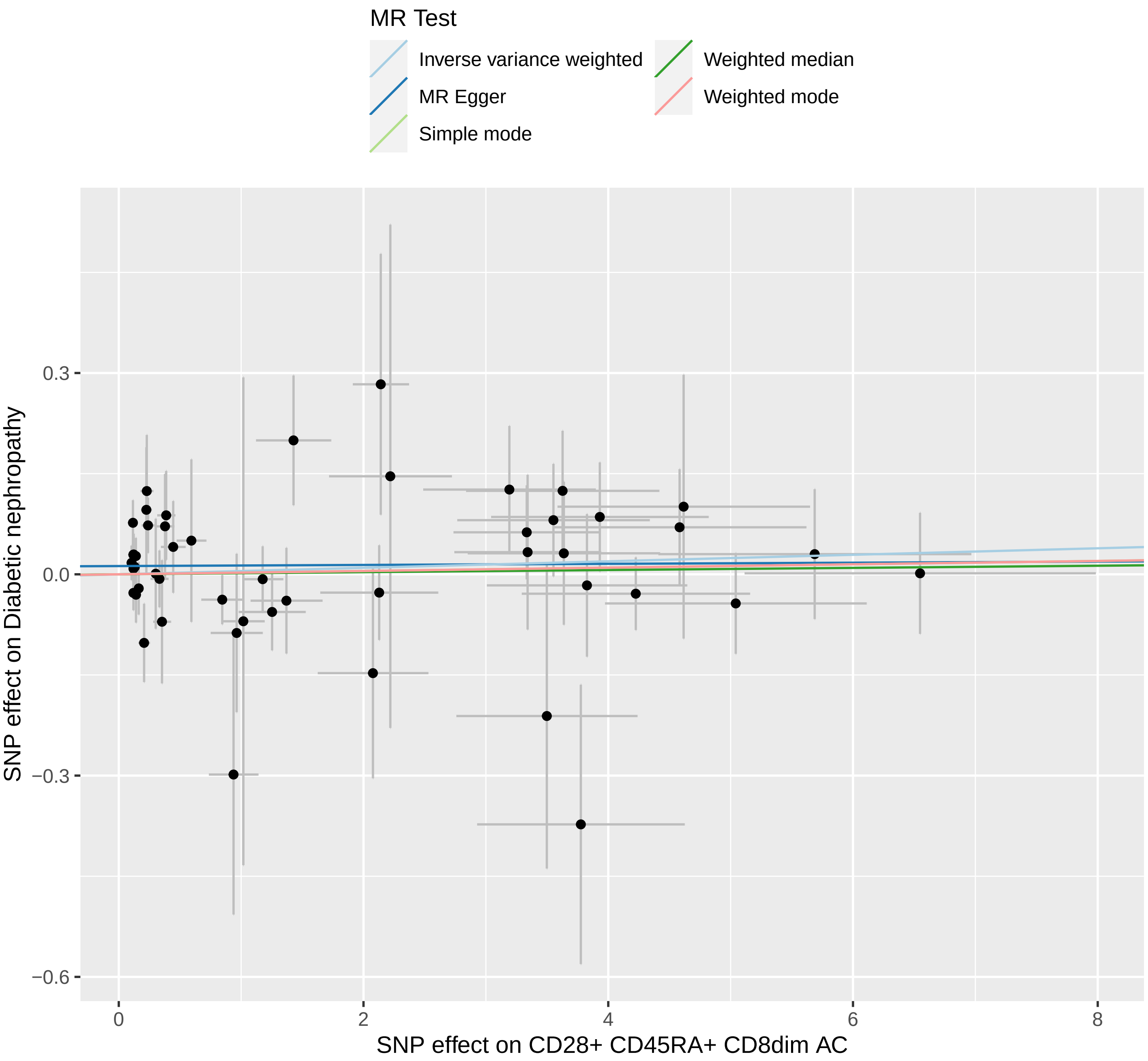

MR Test
Inverse variance weighted
MR Egger
Simple mode
Weighted median
Weighted mode
SNP effect on Diabetic nephropathy
0.3
0.0
−0.3
−0.6
0
2
4
6
8
SNP effect on CD28+ CD45RA+ CD8dim AC

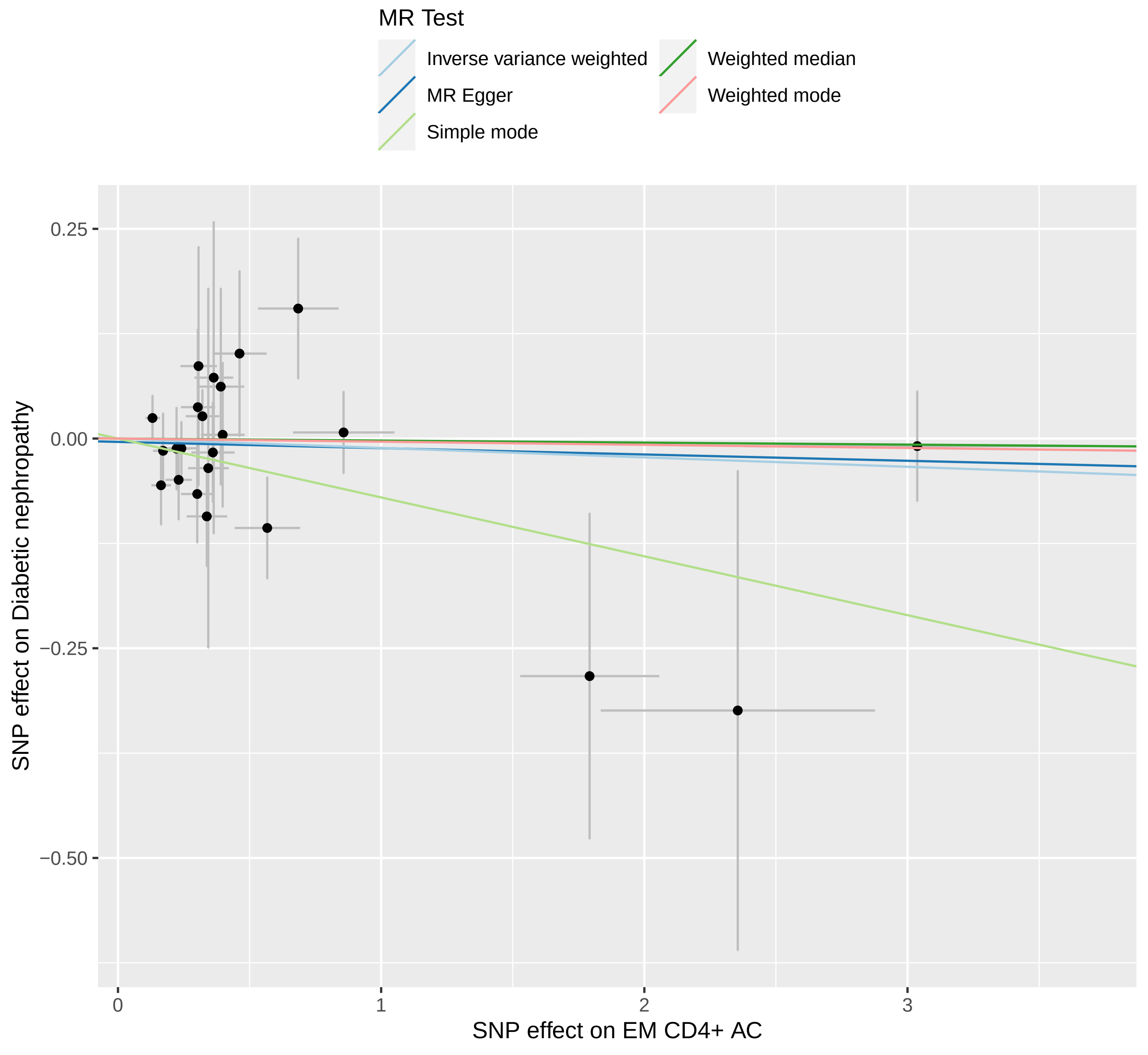
MR Test
Inverse variance weighted
MR Egger
Simple mode
Weighted median
Weighted mode
0.25
0.00
−0.25
−0.50
SNP effect on Diabetic nephropathy
0
1
2
3
SNP effect on EM CD4+ AC

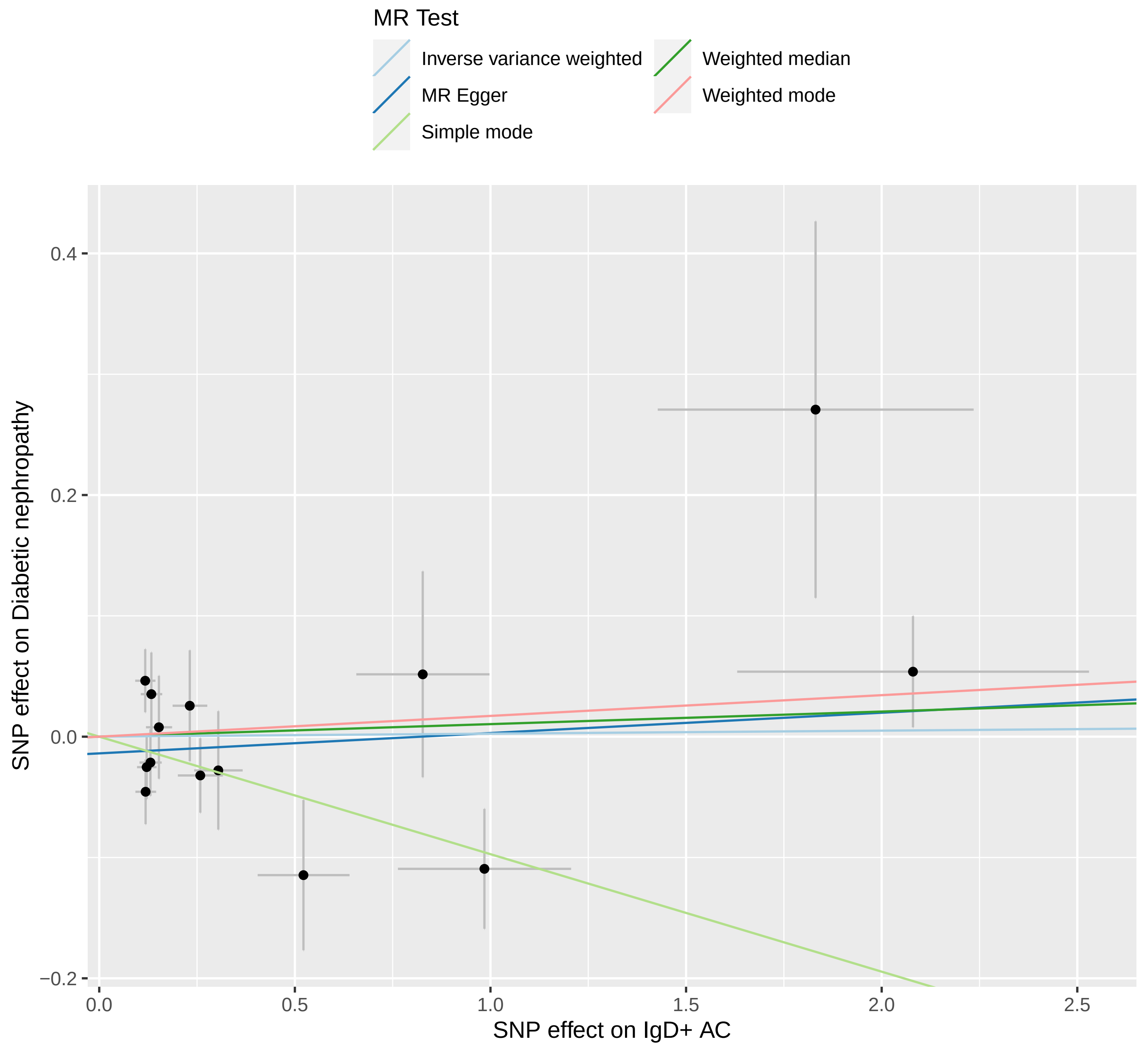
MR Test
Inverse variance weighted
MR Egger
Simple mode
Weighted median
Weighted mode
SNP effect on Diabetic nephropathy
0.4
0.2
0.0
−0.2
0.0
0.5
1.0
1.5
2.0
2.5
SNP effect on IgD+ AC

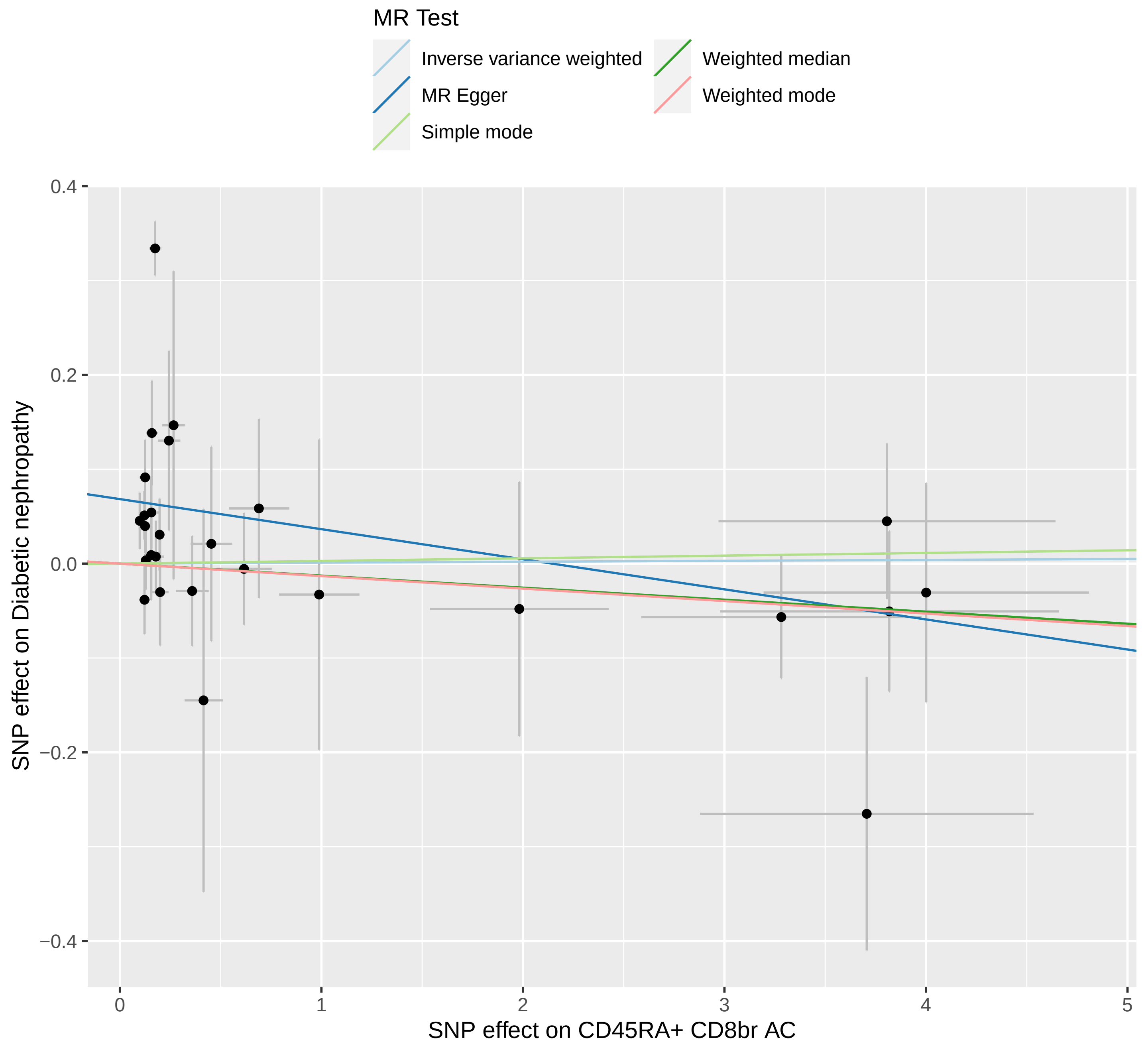

MR Test
Inverse variance weighted
MR Egger
Simple mode
Weighted median
Weighted mode
0.4
0.2
0.0
−0.2
−0.4
0
1
2
3
4
5
SNP effect on Diabetic nephropathy
SNP effect on CD45RA+ CD8br AC

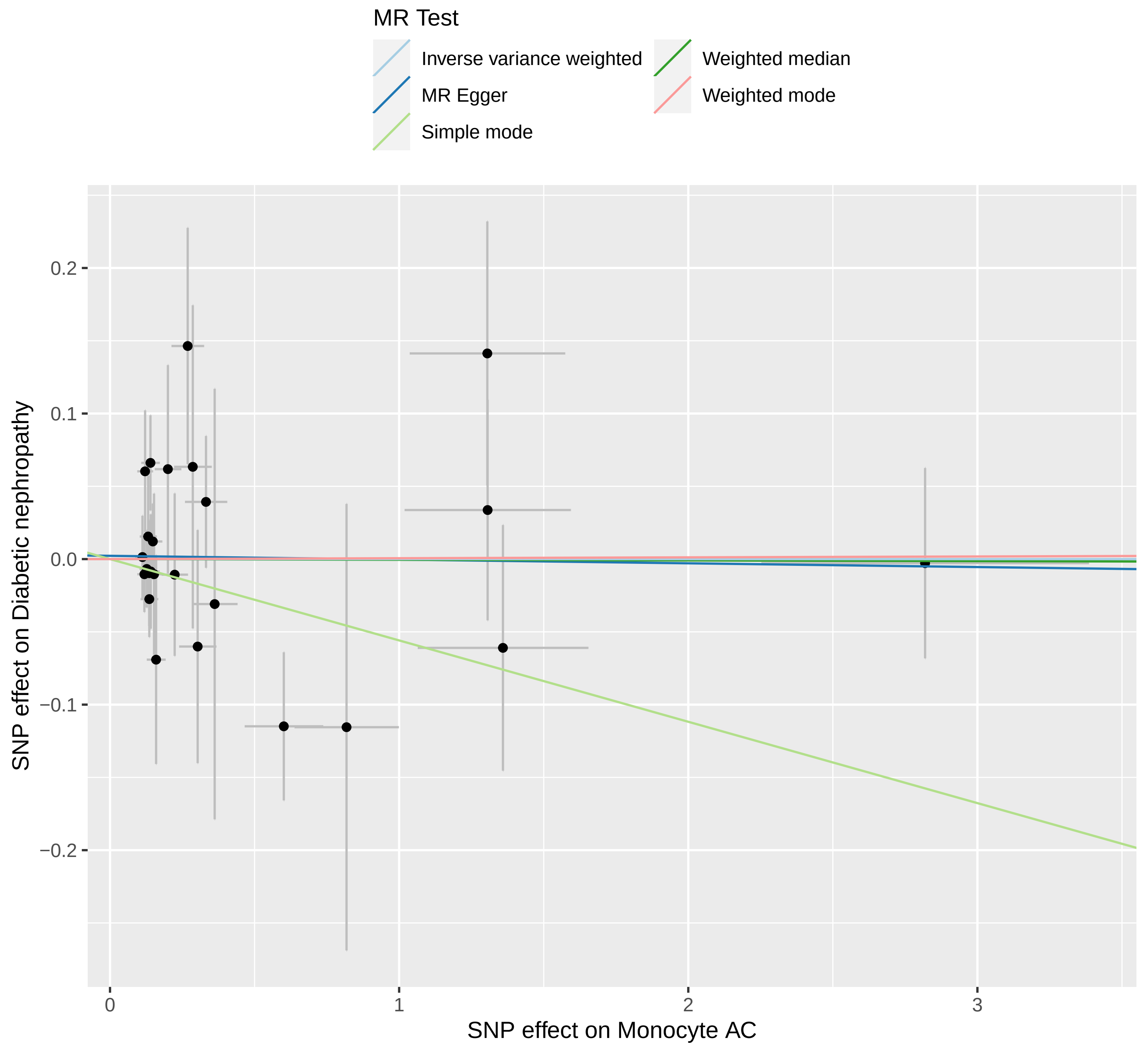
MR Test
Inverse variance weighted
MR Egger
Simple mode
Weighted median
Weighted mode
0.2
0.1
0.0
−0.1
−0.2
SNP effect on Diabetic nephropathy
0
1
2
3
SNP effect on Monocyte AC

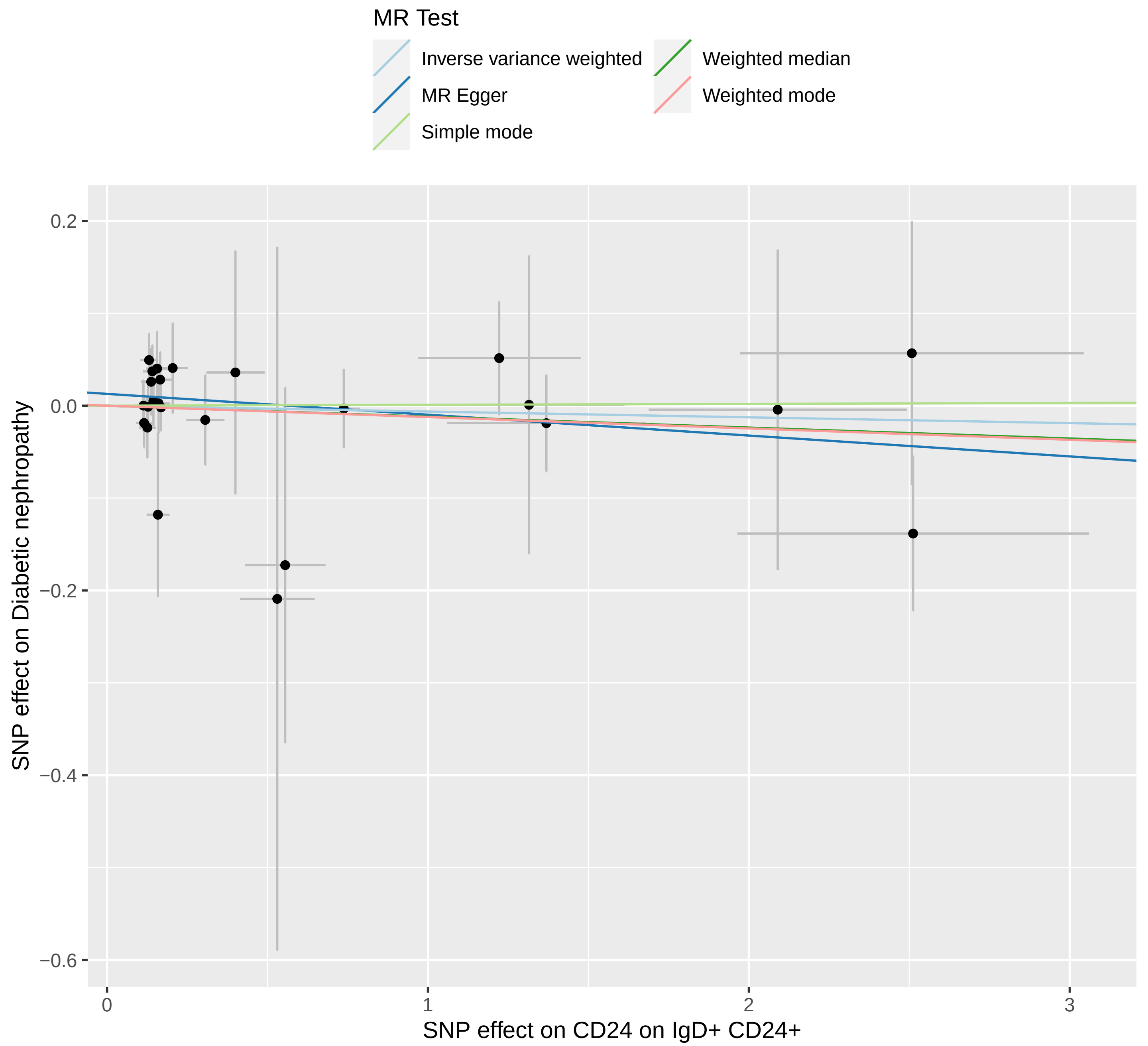

MR Test
Inverse variance weighted
Weighted median
MR Egger
Weighted mode
Simple mode
0.2
0.0
−0.2
−0.4
−0.6
SNP effect on Diabetic nephropathy
0
1
2
3
SNP effect on CD24 on IgD+ CD24+

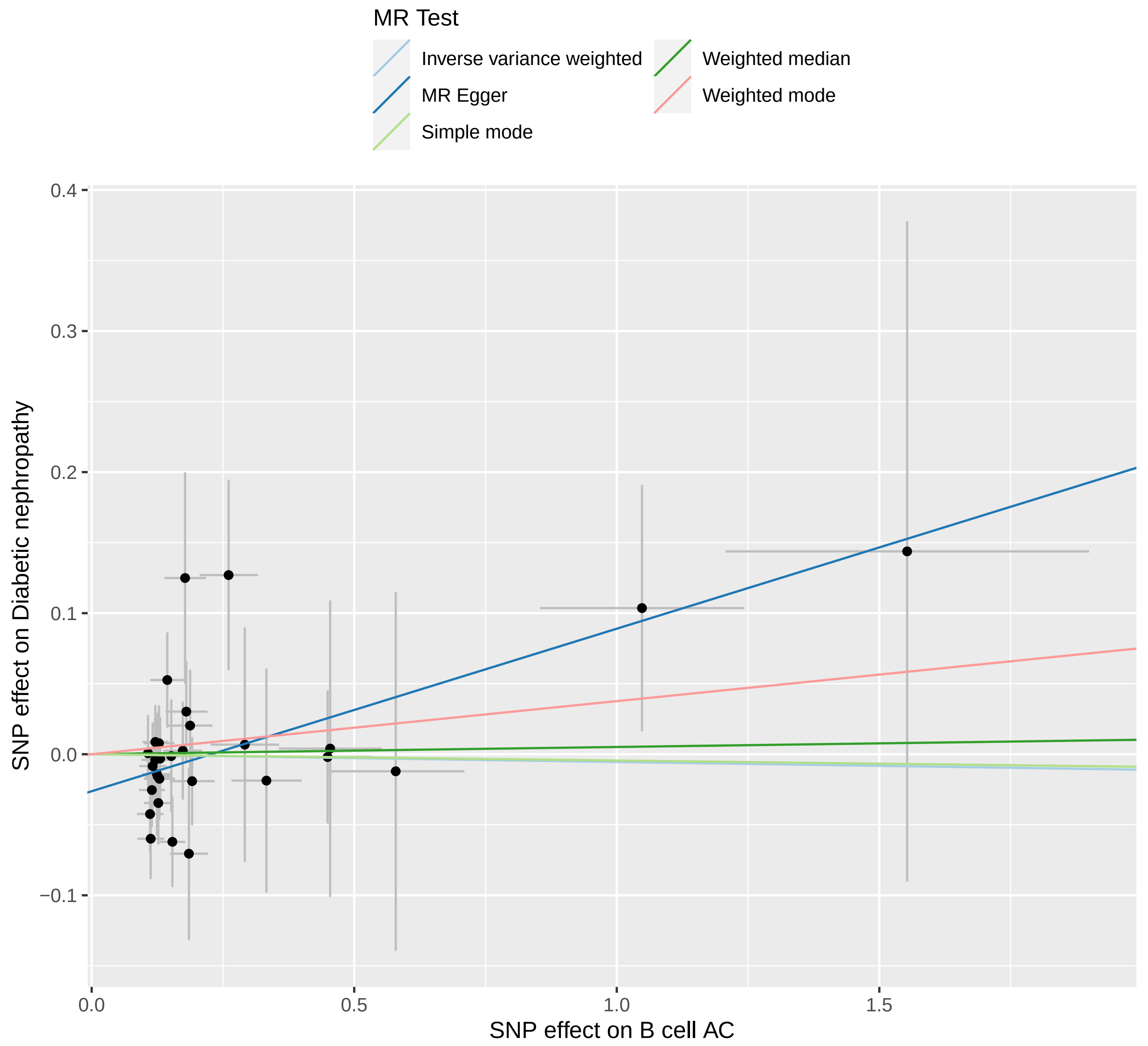

MR Test
Inverse variance weighted
MR Egger
Simple mode
Weighted median
Weighted mode
0.4
0.3
0.2
0.1
0.0
−0.1
SNP effect on Diabetic nephropathy
0.0
0.5
1.0
1.5
SNP effect on B cell AC

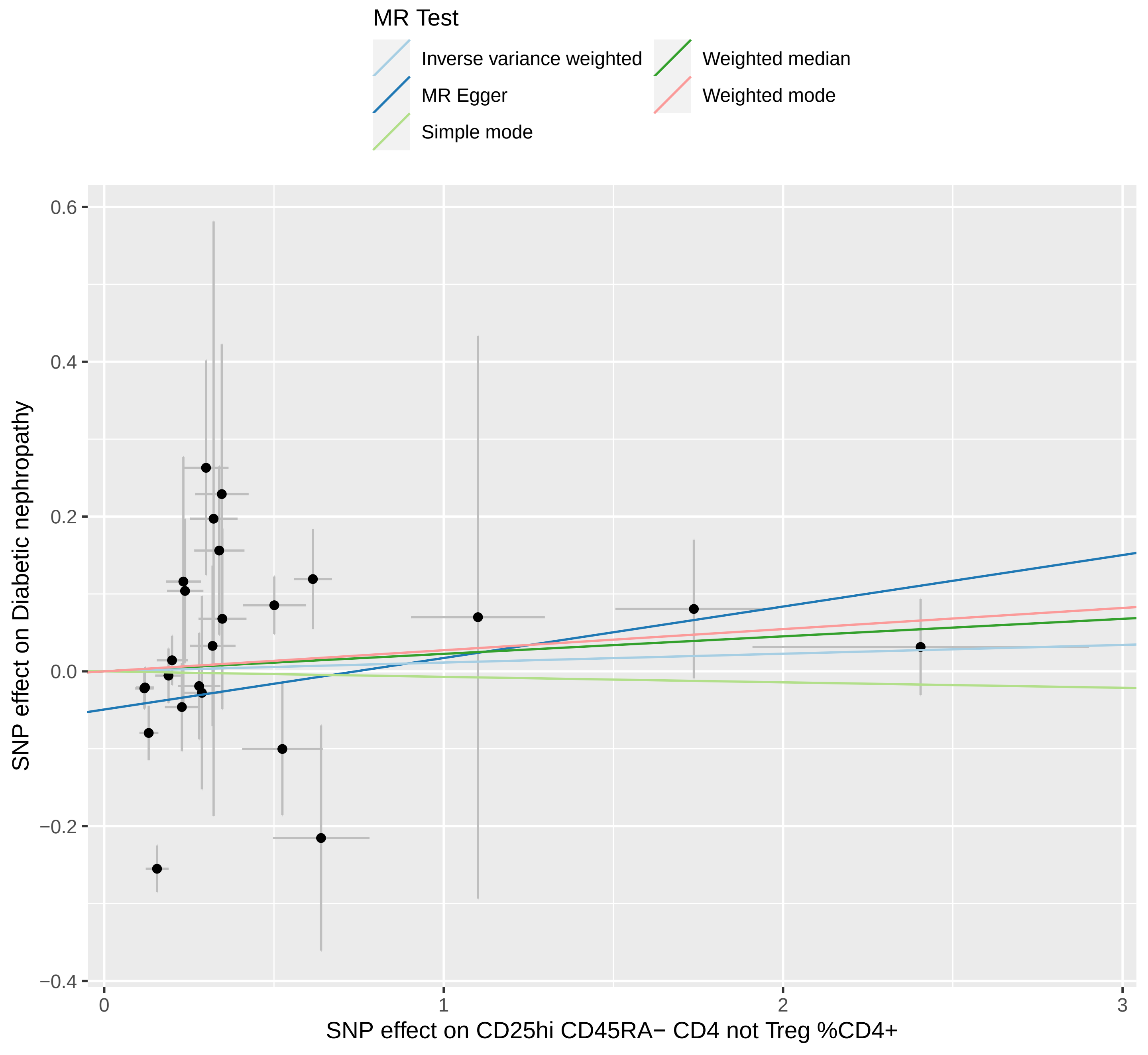
MR Test
Inverse variance weighted
MR Egger
Simple mode
Weighted median
Weighted mode
0.6
0.4
0.2
0.0
−0.2
−0.4
0
1
2
3
SNP effect on Diabetic nephropathy
SNP effect on CD25hi CD45RA− CD4 not Treg %CD4+

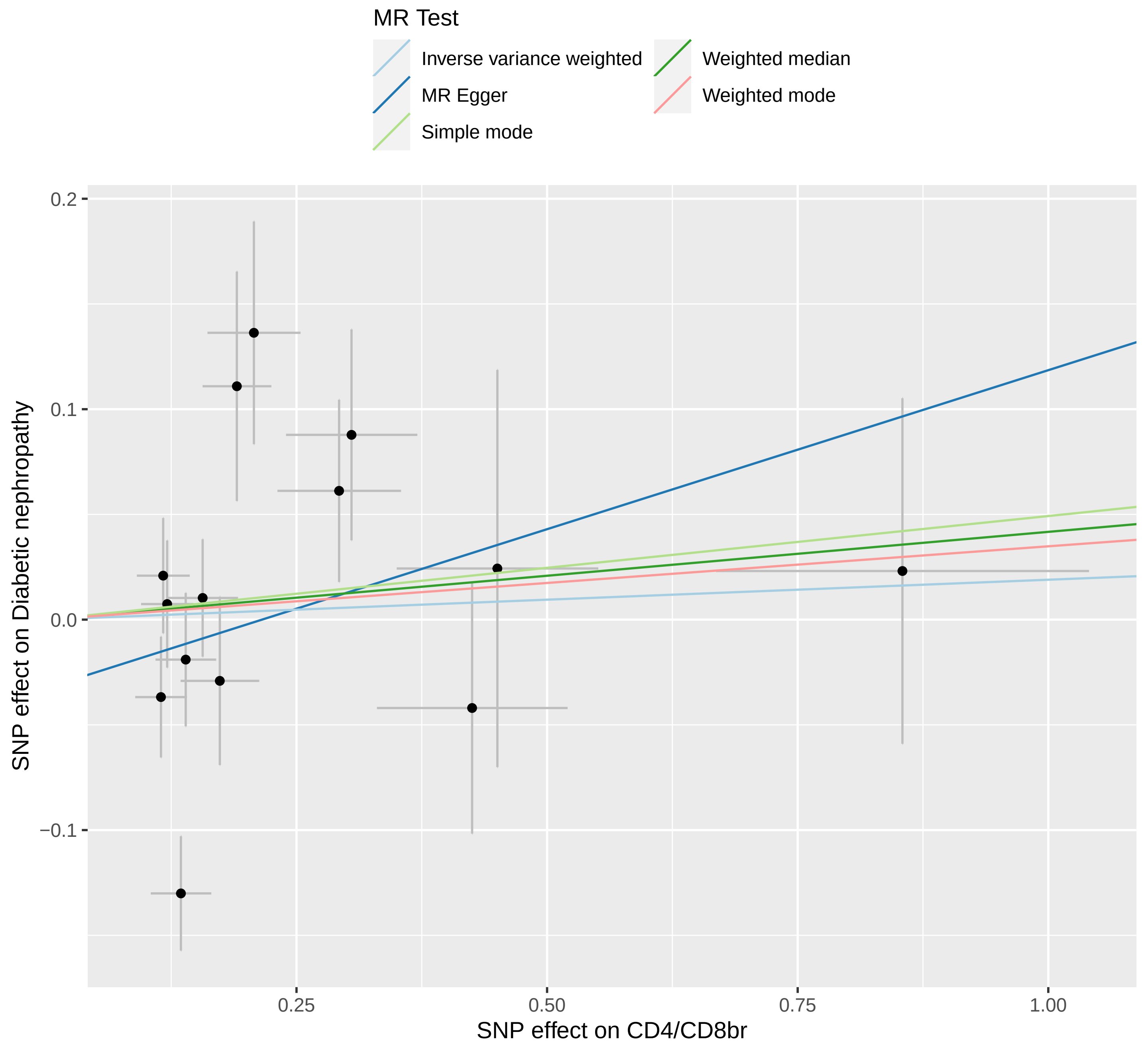

MR Test
Inverse variance weighted
MR Egger
Simple mode
Weighted median
Weighted mode
0.2
0.1
0.0
−0.1
0.25
0.50
0.75
1.00
SNP effect on CD4/CD8br
SNP effect on Diabetic nephropathy

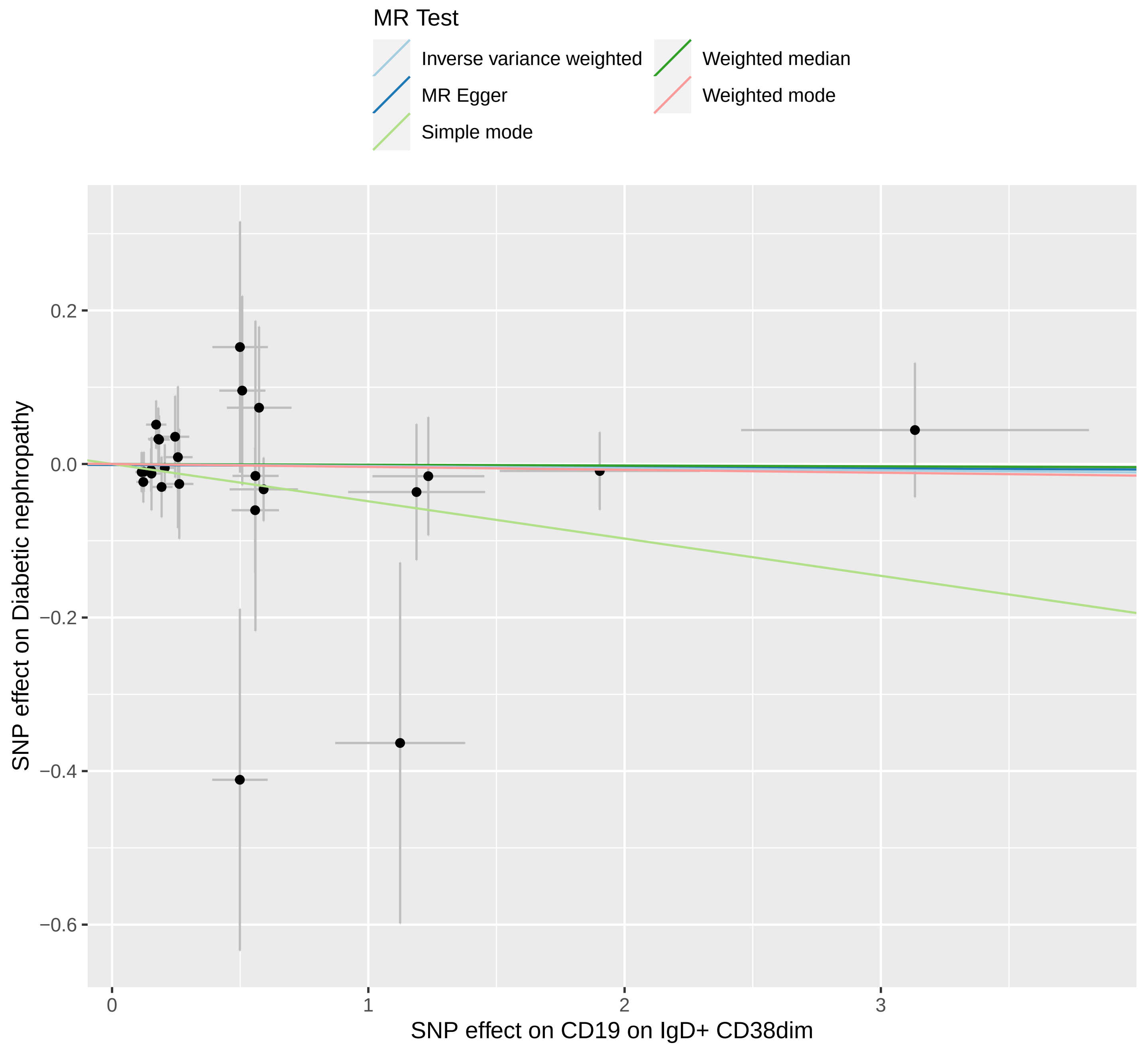

MR Test
Inverse variance weighted
MR Egger
Simple mode
Weighted median
Weighted mode
0.2
0.0
−0.2
−0.4
−0.6
SNP effect on Diabetic nephropathy
0
1
2
3
SNP effect on CD19 on IgD+ CD38dim

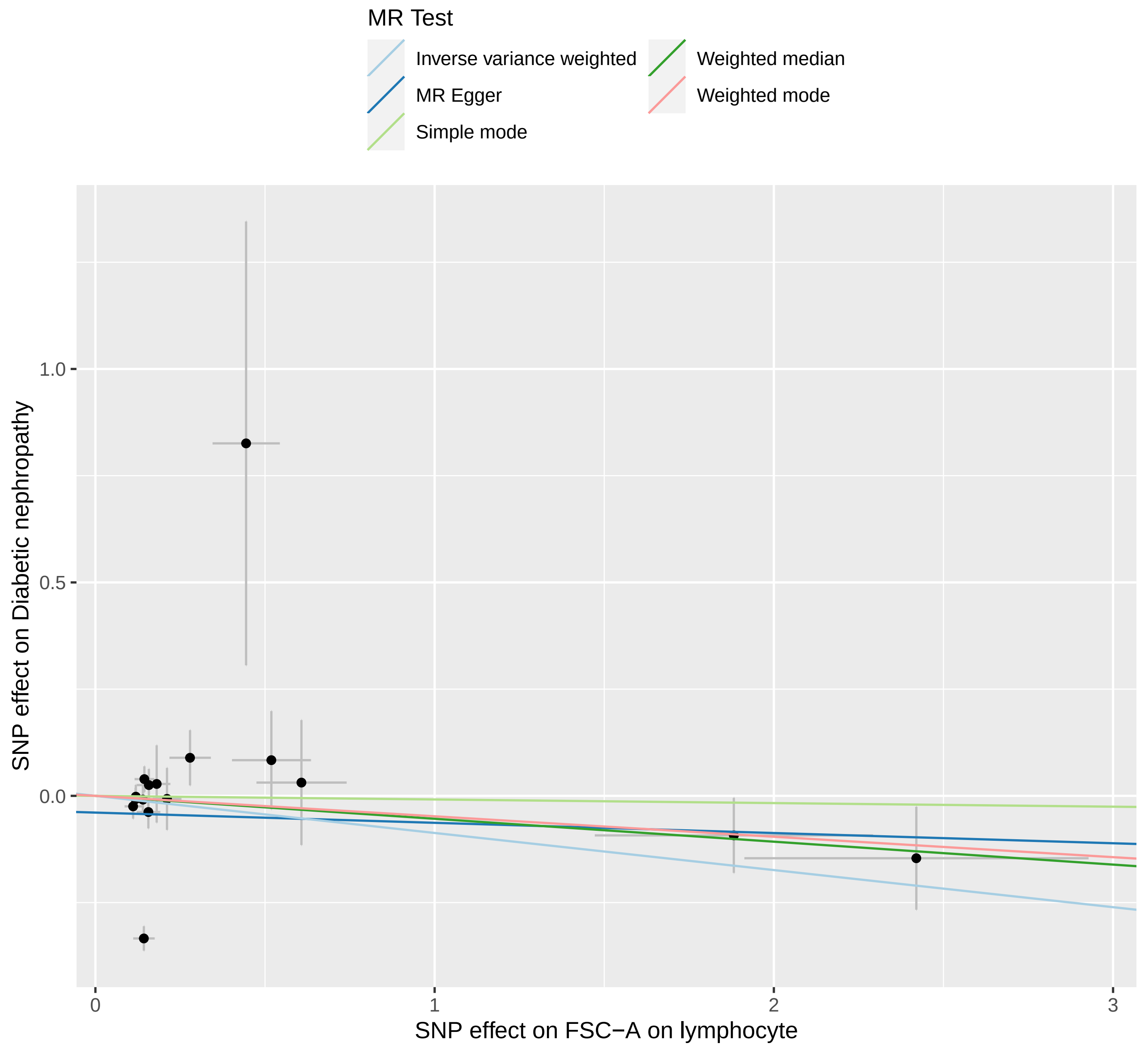
MR Test
Inverse variance weighted
MR Egger
Simple mode
Weighted median
Weighted mode
1.0
0.5
0.0
0
1
2
3
SNP effect on Diabetic nephropathy
SNP effect on FSC−A on lymphocyte

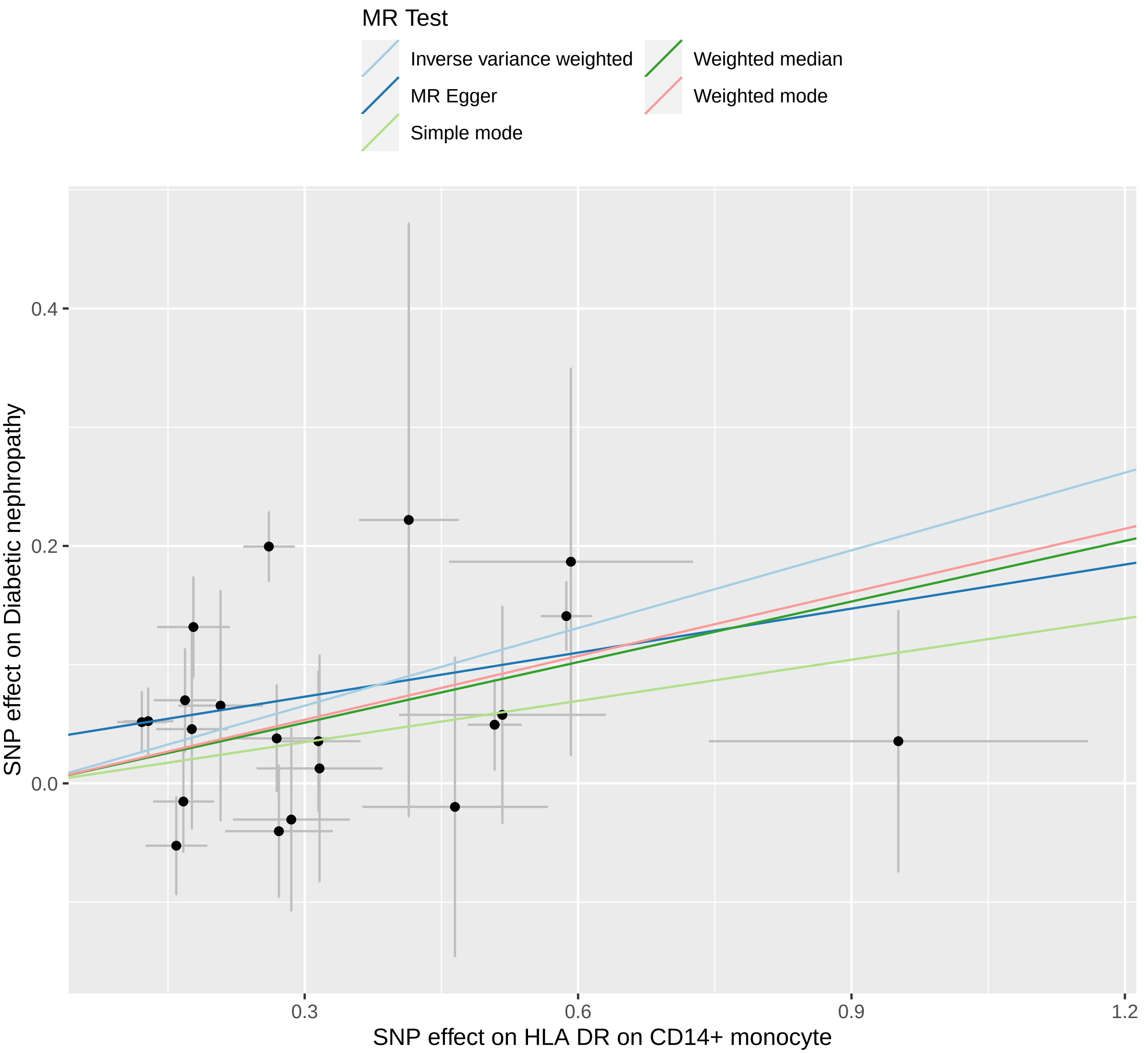
MR Test
Inverse variance weighted
MR Egger
Simple mode
Weighted median
Weighted mode
0.4
0.2
0.0
0.3
0.6
0.9
1.2
SNP effect on HLA DR on CD14+ monocyte
SNP effect on Diabetic nephropathy

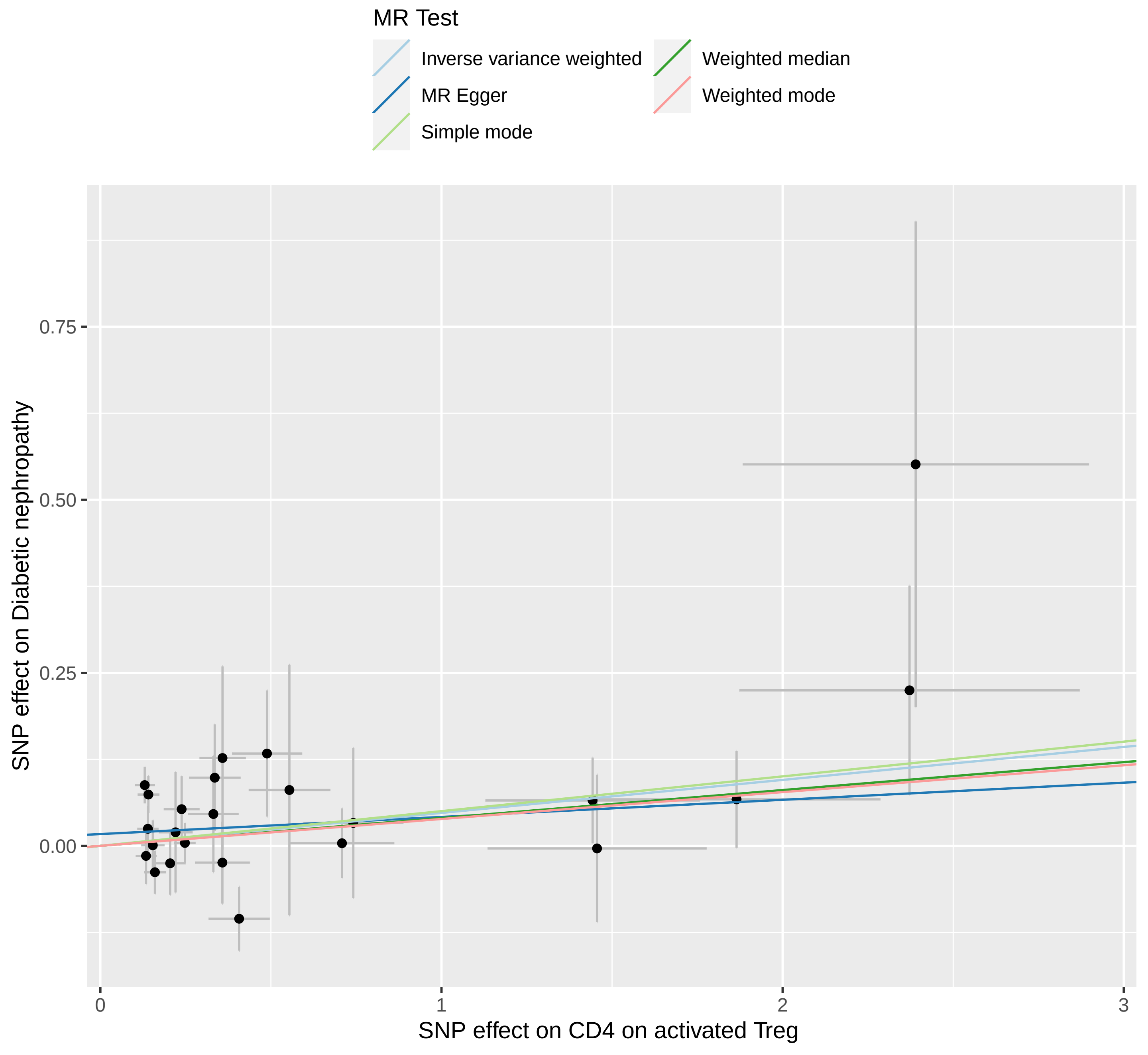
MR Test
Inverse variance weighted
MR Egger
Simple mode
Weighted median
Weighted mode
SNP effect on Diabetic nephropathy
0.75
0.50
0.25
0.00
0
1
2
3
SNP effect on CD4 on activated Treg

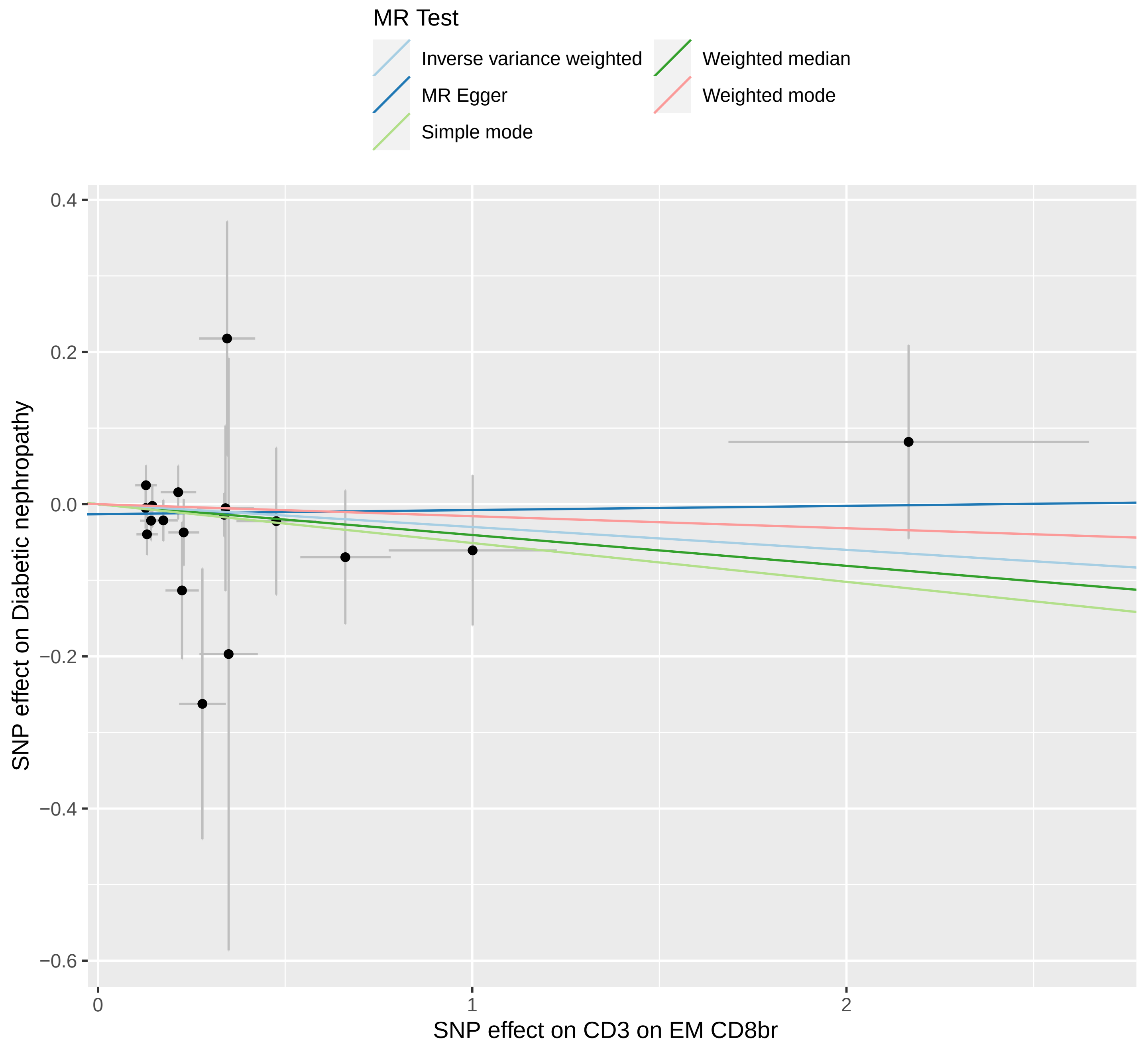

MR Test
Inverse variance weighted
MR Egger
Simple mode
Weighted median
Weighted mode
0.4
0.2
0.0
−0.2
−0.4
−0.6
SNP effect on Diabetic nephropathy
0
1
2
SNP effect on CD3 on EM CD8br

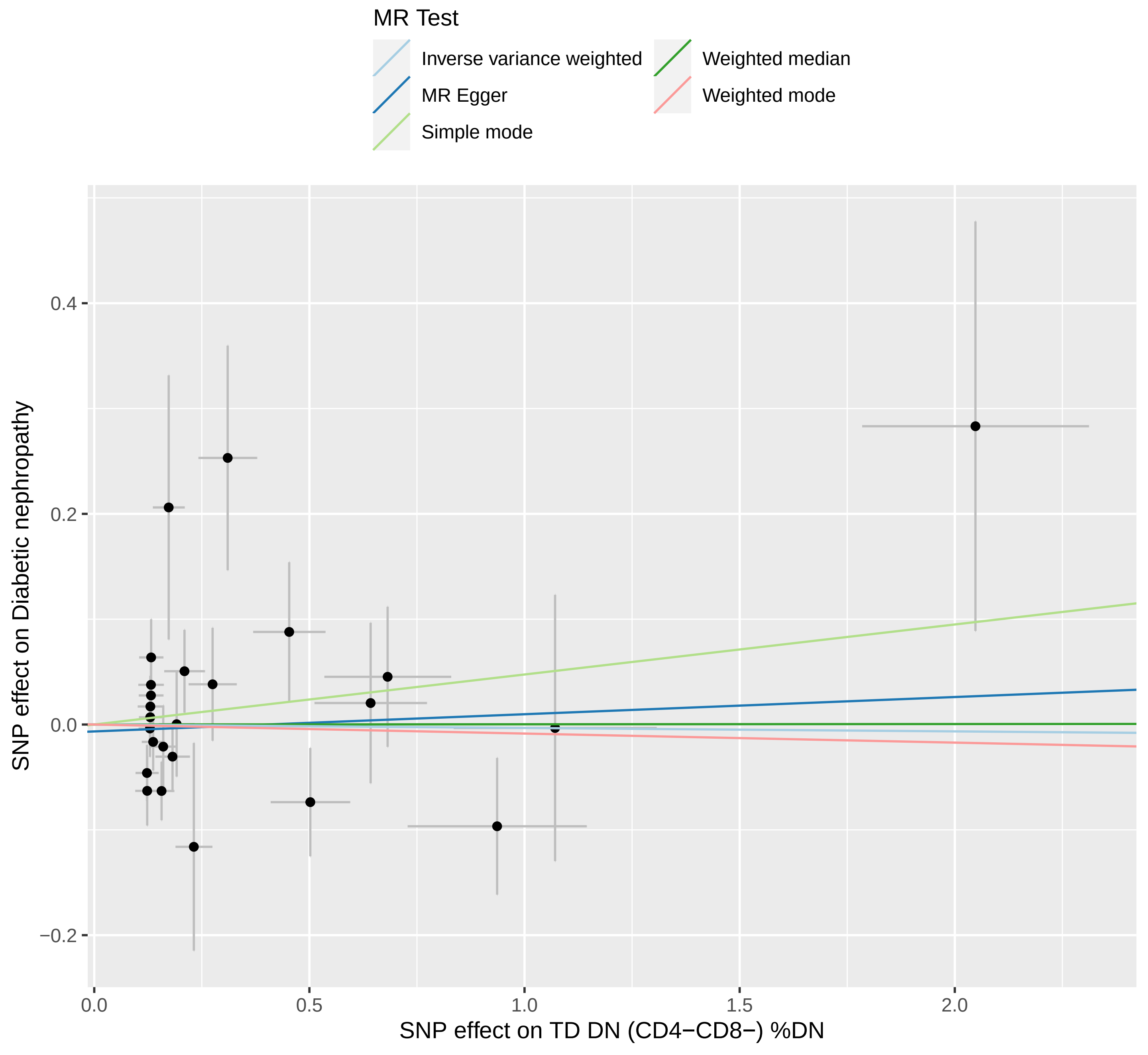
MR Test
Inverse variance weighted
MR Egger
Simple mode
Weighted median
Weighted mode
SNP effect on Diabetic nephropathy
0.4
0.2
0.0
−0.2
0.0
0.5
1.0
1.5
2.0
SNP effect on TD DN (CD4−CD8−) %DN

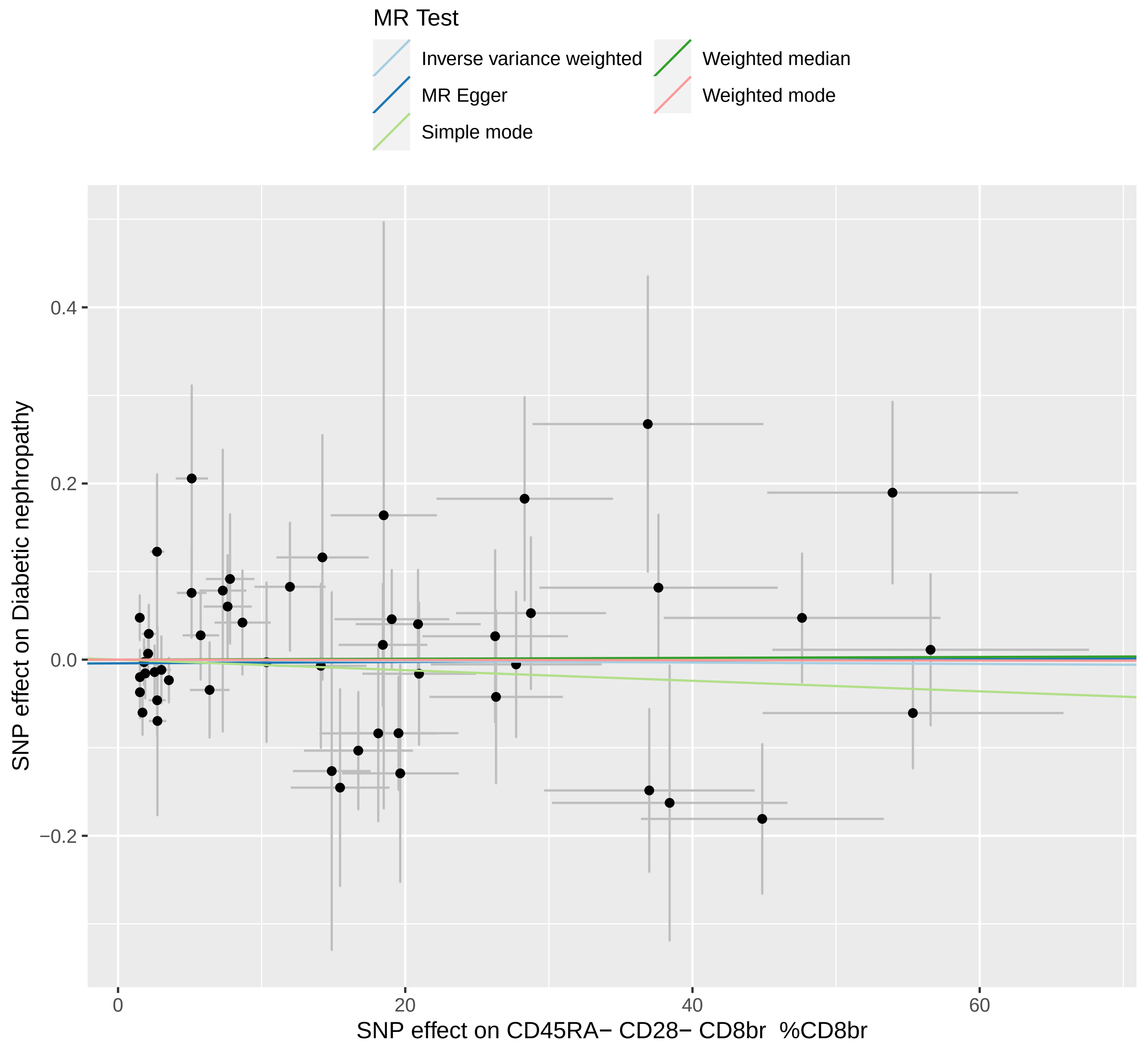

MR Test
Inverse variance weighted
MR Egger
Simple mode
Weighted median
Weighted mode
0.4
0.2
0.0
−0.2
SNP effect on Diabetic nephropathy
0
20
40
60
SNP effect on CD45RA− CD28− CD8br %CD8br

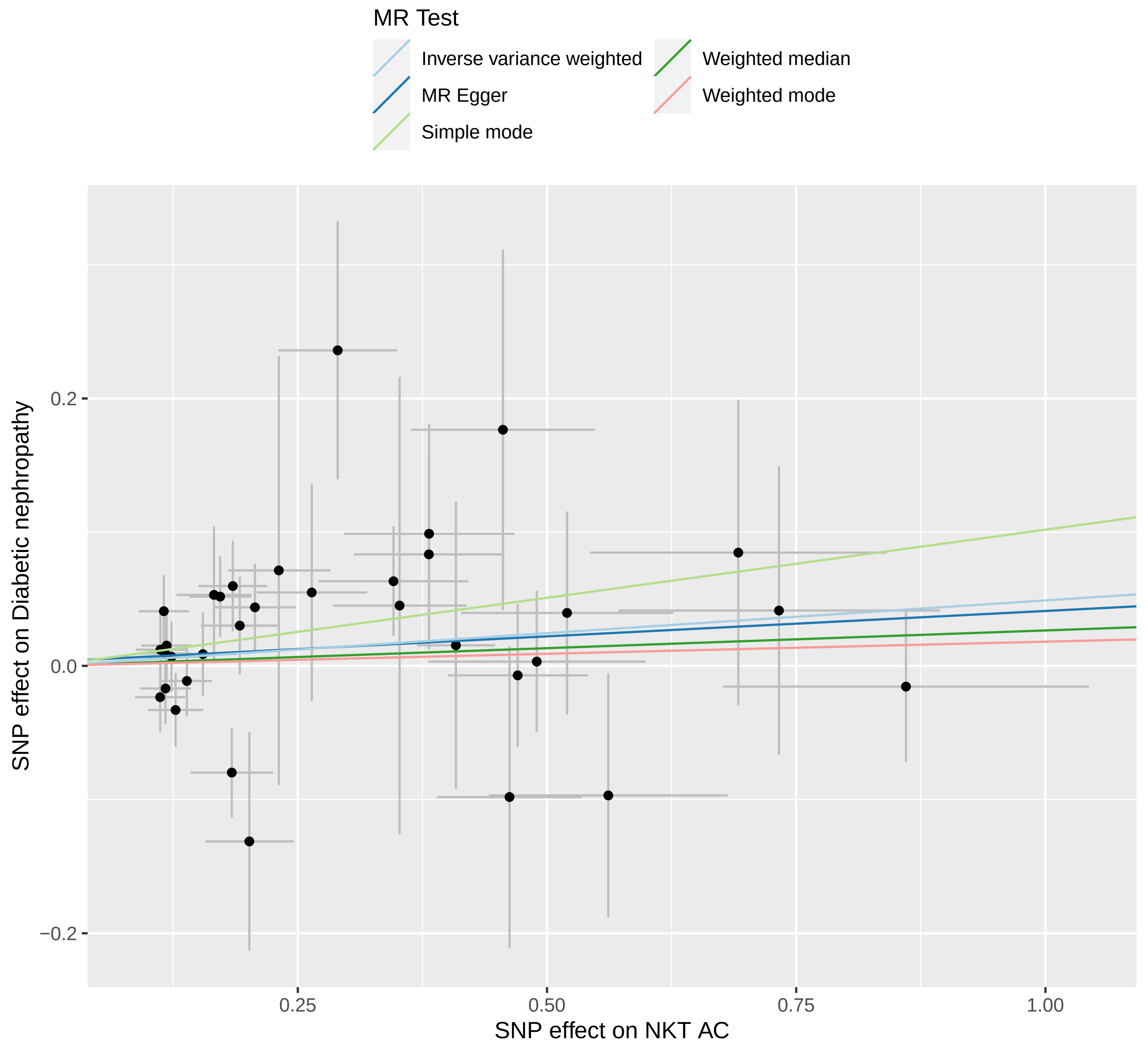

MR Test
Inverse variance weighted
MR Egger
Simple mode
Weighted median
Weighted mode
SNP effect on Diabetic nephropathy
0.2
0.0
−0.2
0.25
0.50
0.75
1.00
SNP effect on NKT AC

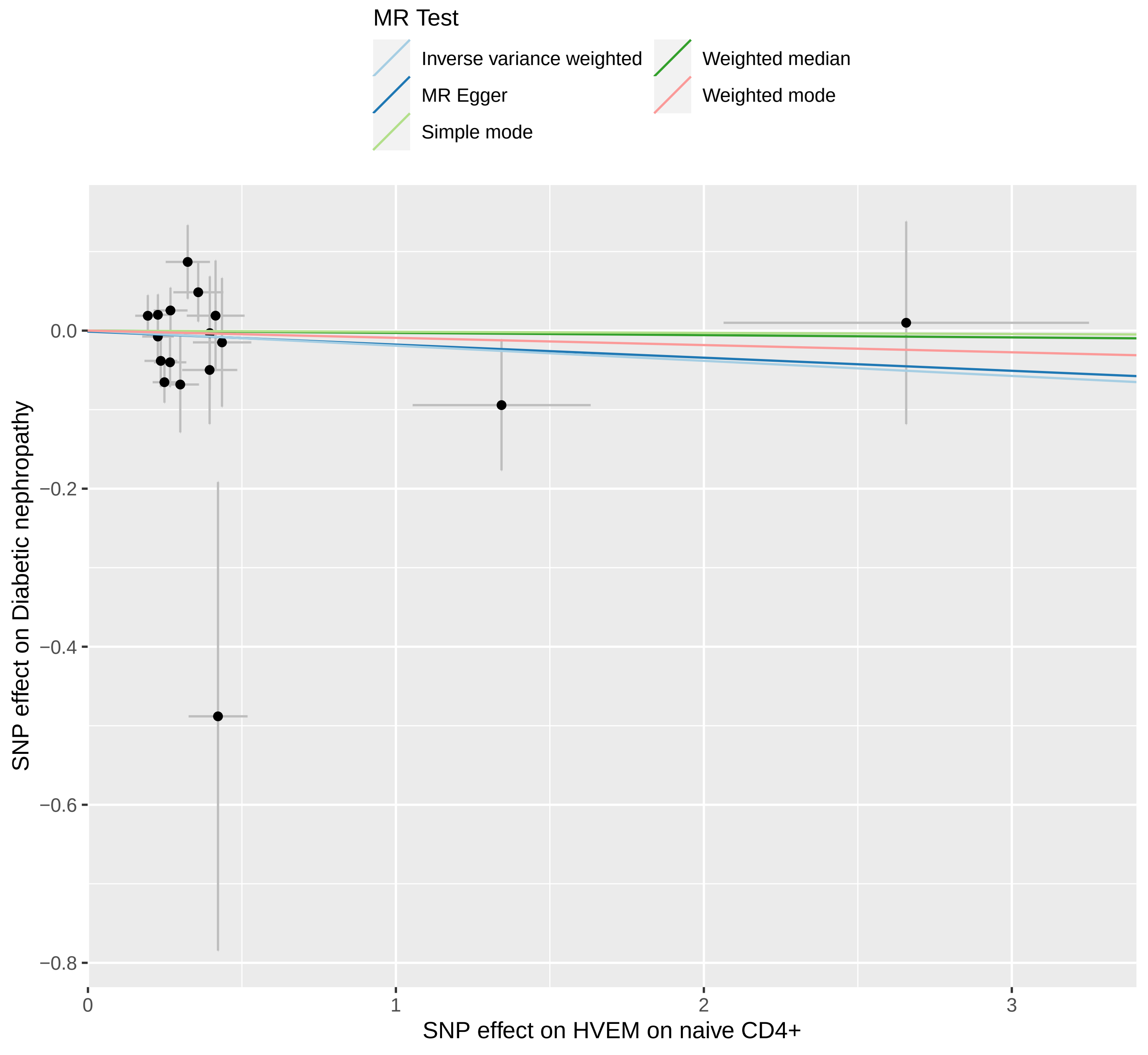

MR Test
Inverse variance weighted
MR Egger
Simple mode
Weighted median
Weighted mode
SNP effect on Diabetic nephropathy
0.0
−0.2
−0.4
−0.6
−0.8
0
1
2
3
SNP effect on HVEM on naive CD4+

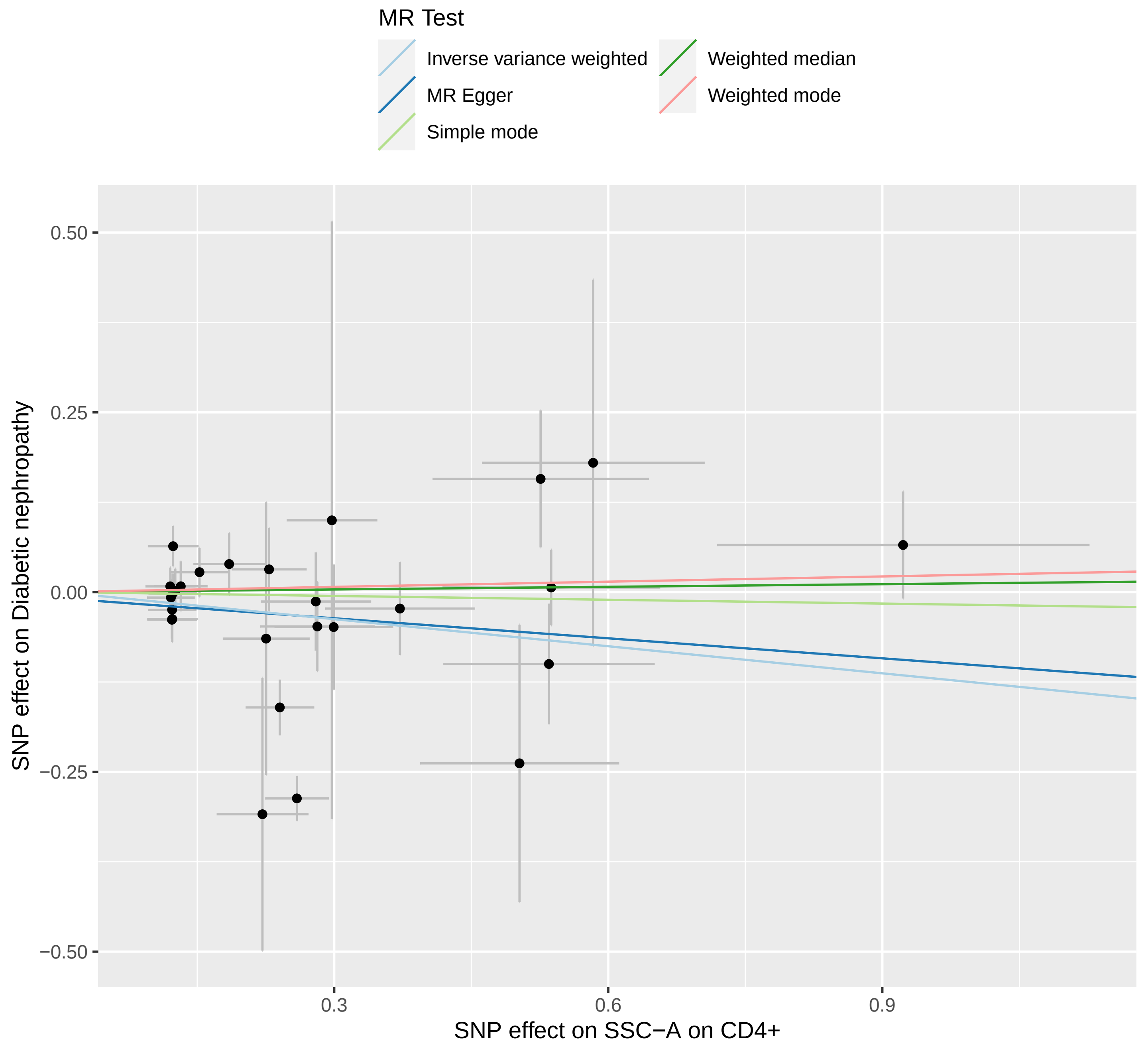
MR Test
Inverse variance weighted
MR Egger
Simple mode
Weighted median
Weighted mode
0.50
0.25
0.00
−0.25
−0.50
0.3
0.6
0.9
SNP effect on Diabetic nephropathy
SNP effect on SSC−A on CD4+

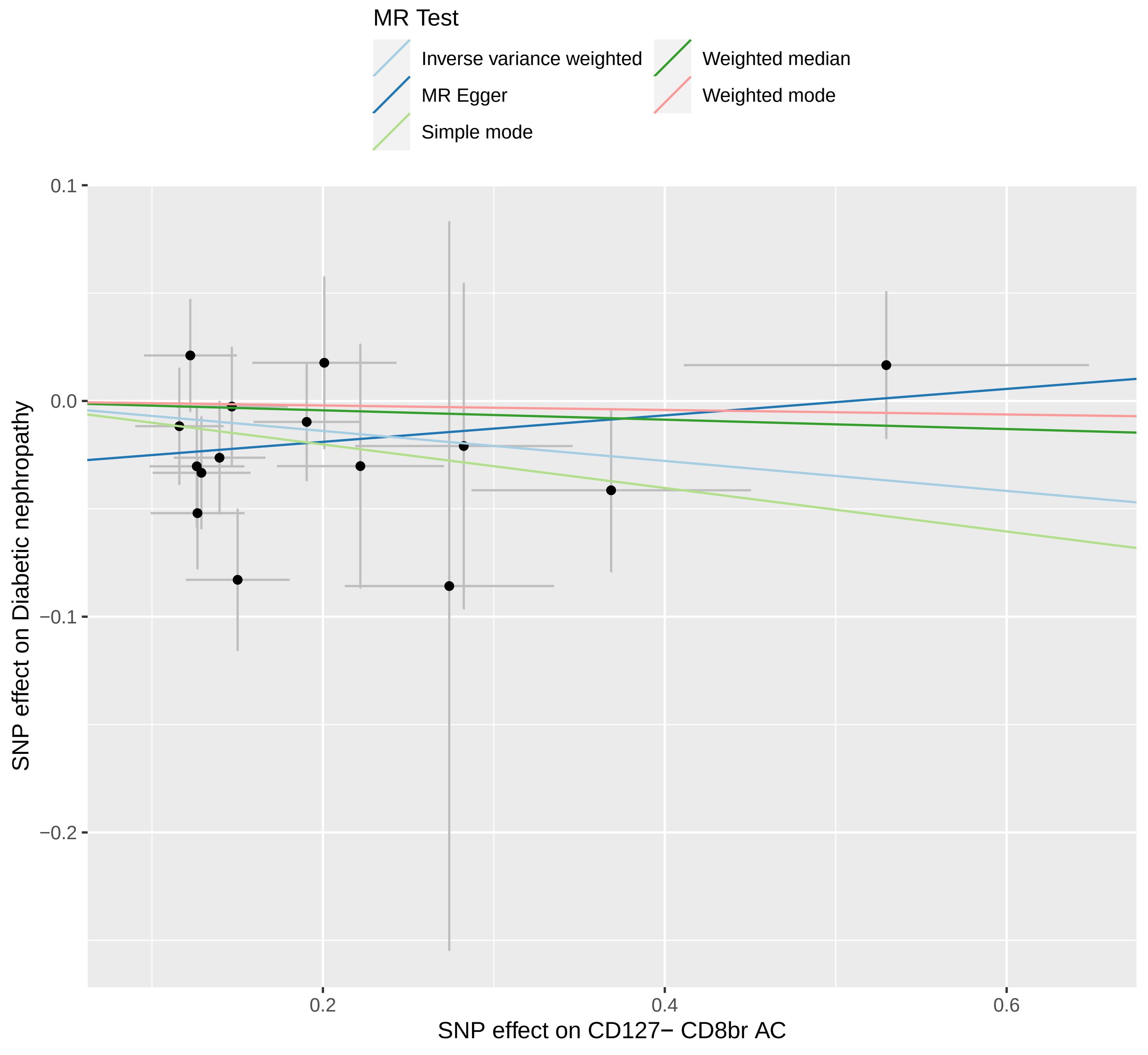

MR Test
Inverse variance weighted
MR Egger
Simple mode
Weighted median
Weighted mode
0.1
0.0
−0.1
−0.2
0.2
0.4
0.6
SNP effect on Diabetic nephropathy
SNP effect on CD127− CD8br AC

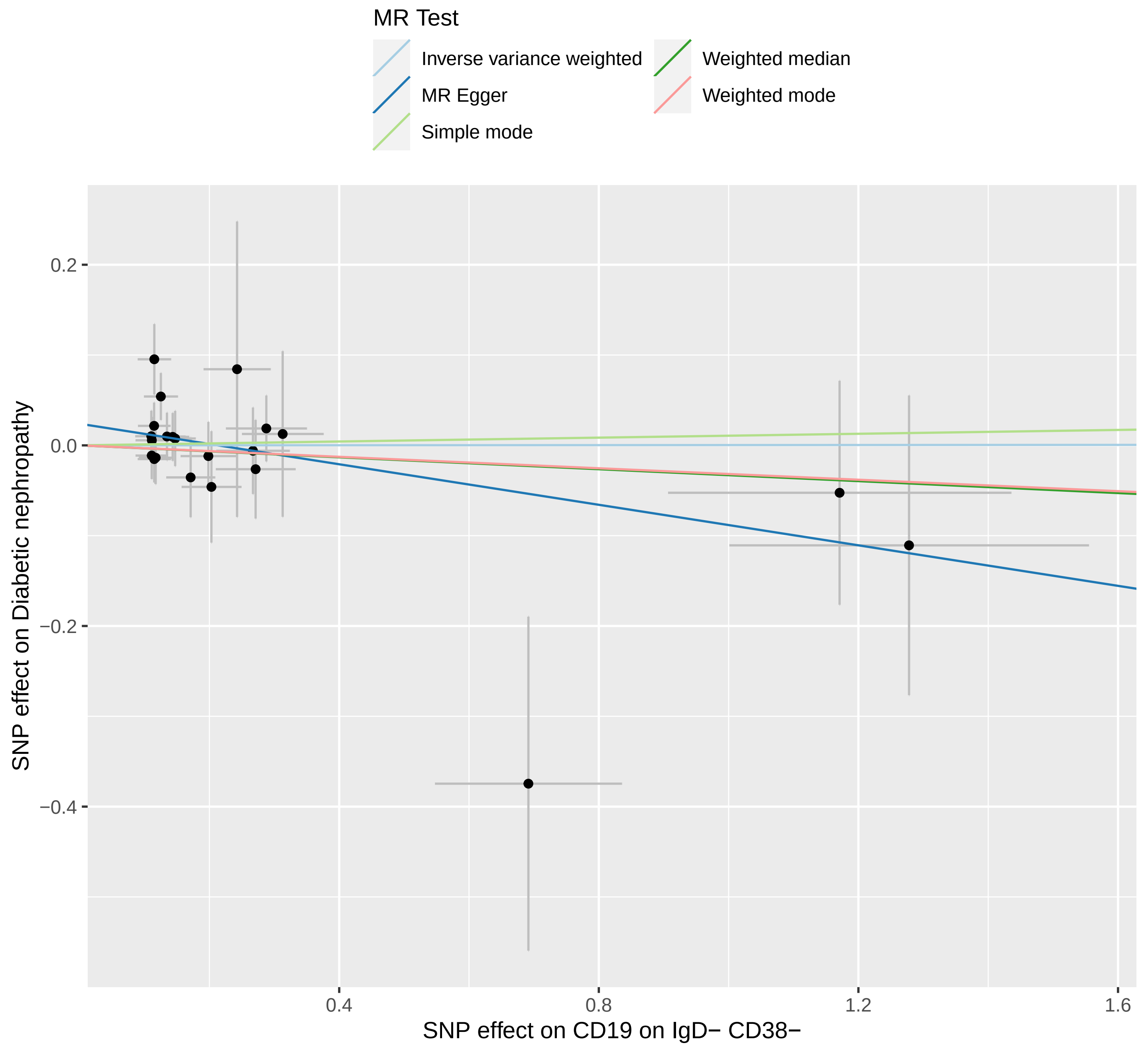
MR Test
Inverse variance weighted
MR Egger
Simple mode
Weighted median
Weighted mode
0.2
0.0
−0.2
−0.4
0.4
0.8
1.2
1.6
SNP effect on Diabetic nephropathy
SNP effect on CD19 on IgD− CD38−

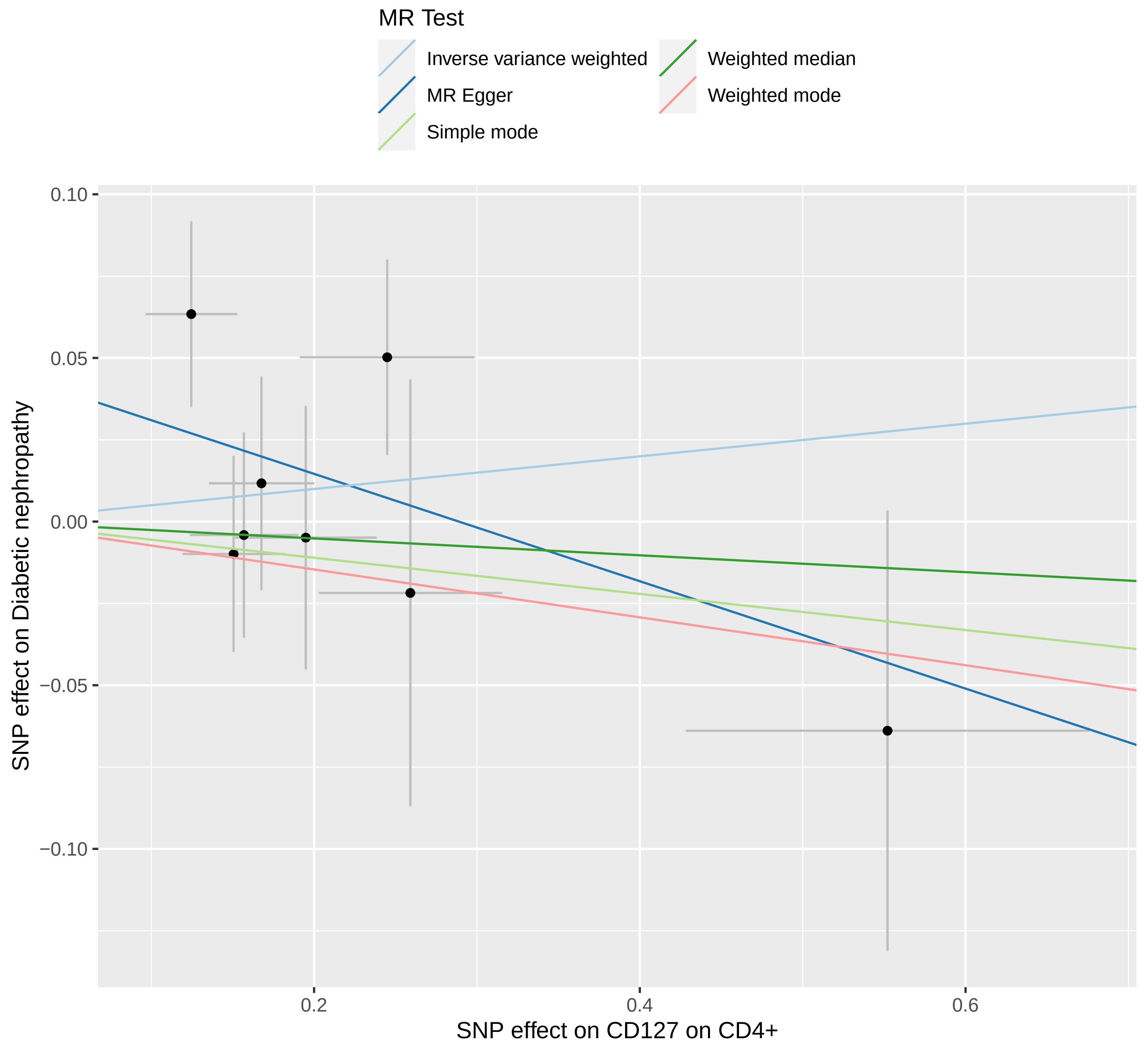

MR Test
Inverse variance weighted
MR Egger
Simple mode
Weighted median
Weighted mode
0.10
0.05
0.00
−0.05
−0.10
SNP effect on Diabetic nephropathy
0.2
0.4
0.6
SNP effect on CD127 on CD4+

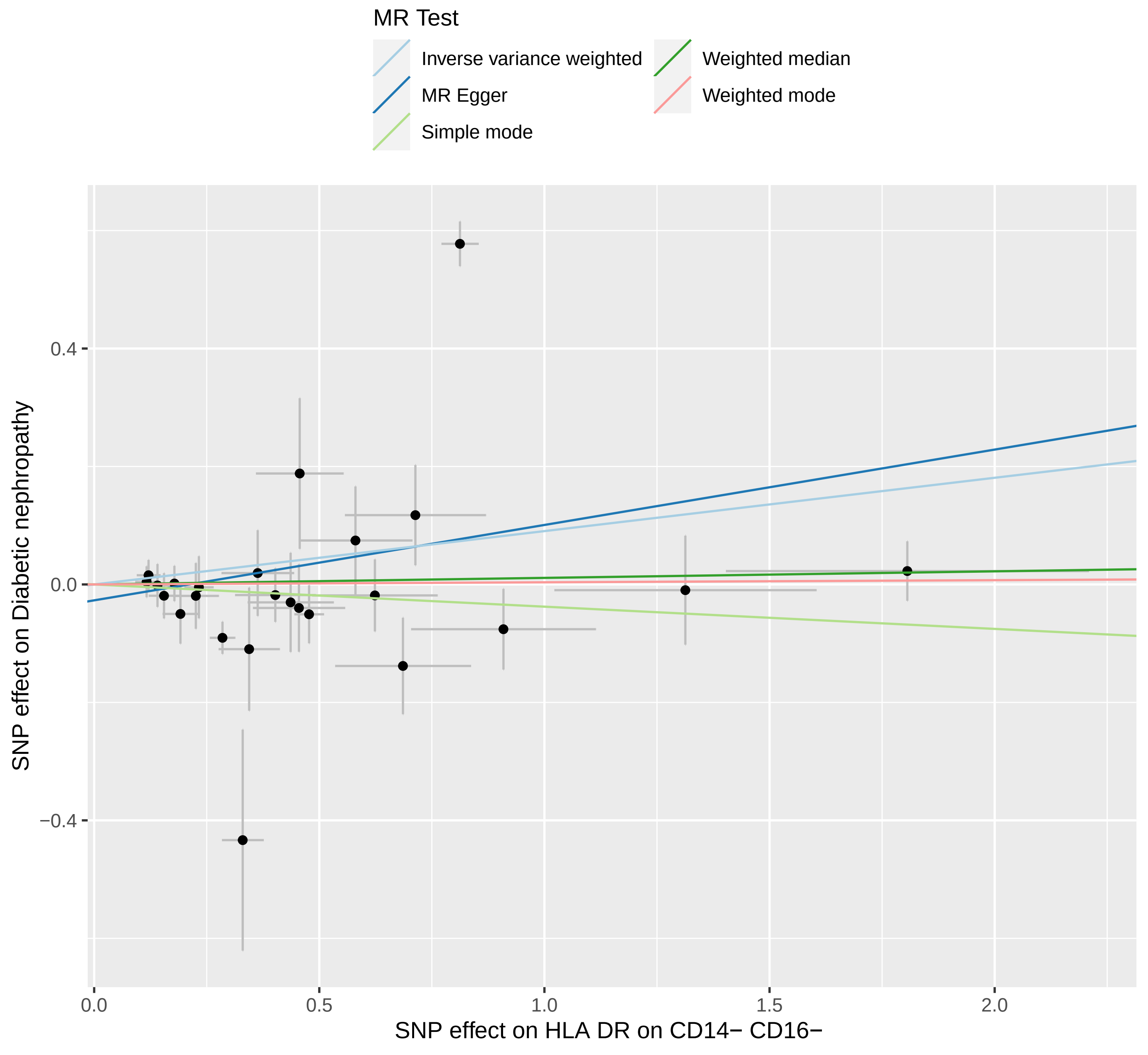
MR Test
Inverse variance weighted
MR Egger
Simple mode
Weighted median
Weighted mode
SNP effect on Diabetic nephropathy
0.4
0.0
−0.4
0.0
0.5
1.0
1.5
2.0
SNP effect on HLA DR on CD14− CD16−

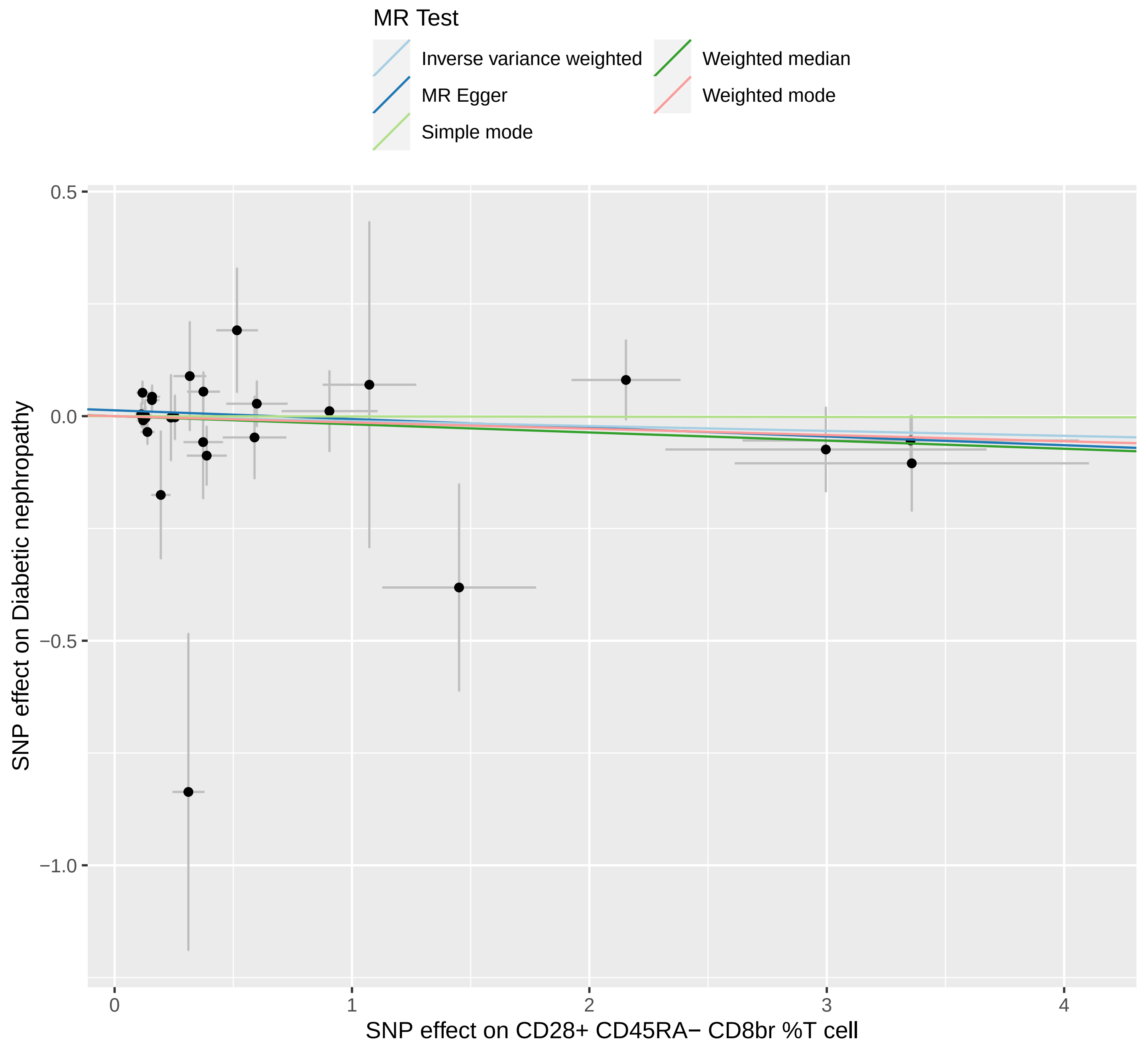
MR Test
Inverse variance weighted
MR Egger
Simple mode
Weighted median
Weighted mode
0.5
0.0
−0.5
−1.0
0
1
2
3
4
SNP effect on Diabetic nephropathy
SNP effect on CD28+ CD45RA− CD8br %T cell

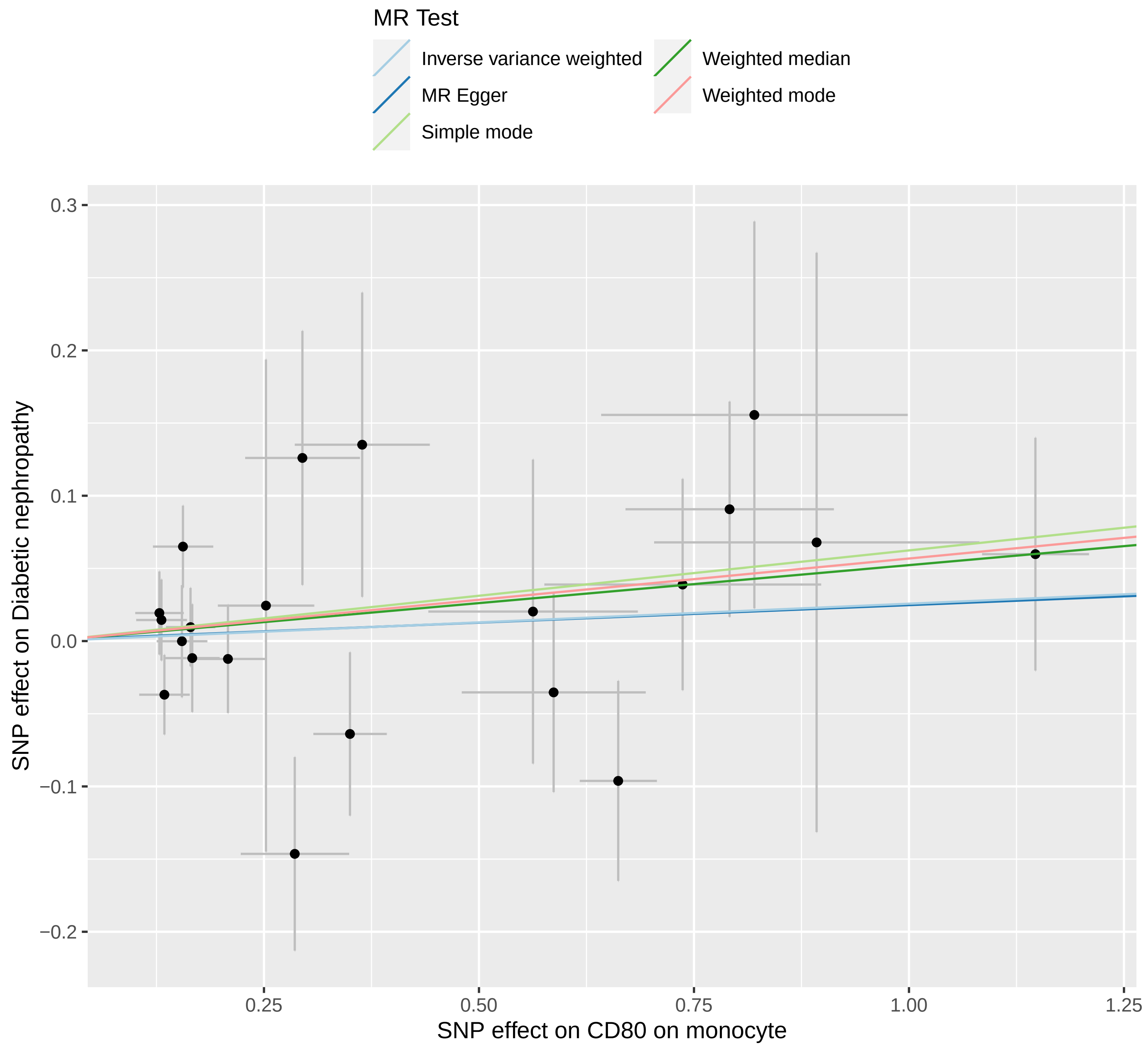
MR Test
Inverse variance weighted
MR Egger
Simple mode
Weighted median
Weighted mode
0.3
0.2
0.1
0.0
−0.1
−0.2
SNP effect on Diabetic nephropathy
0.25
0.50
0.75
1.00
1.25
SNP effect on CD80 on monocyte

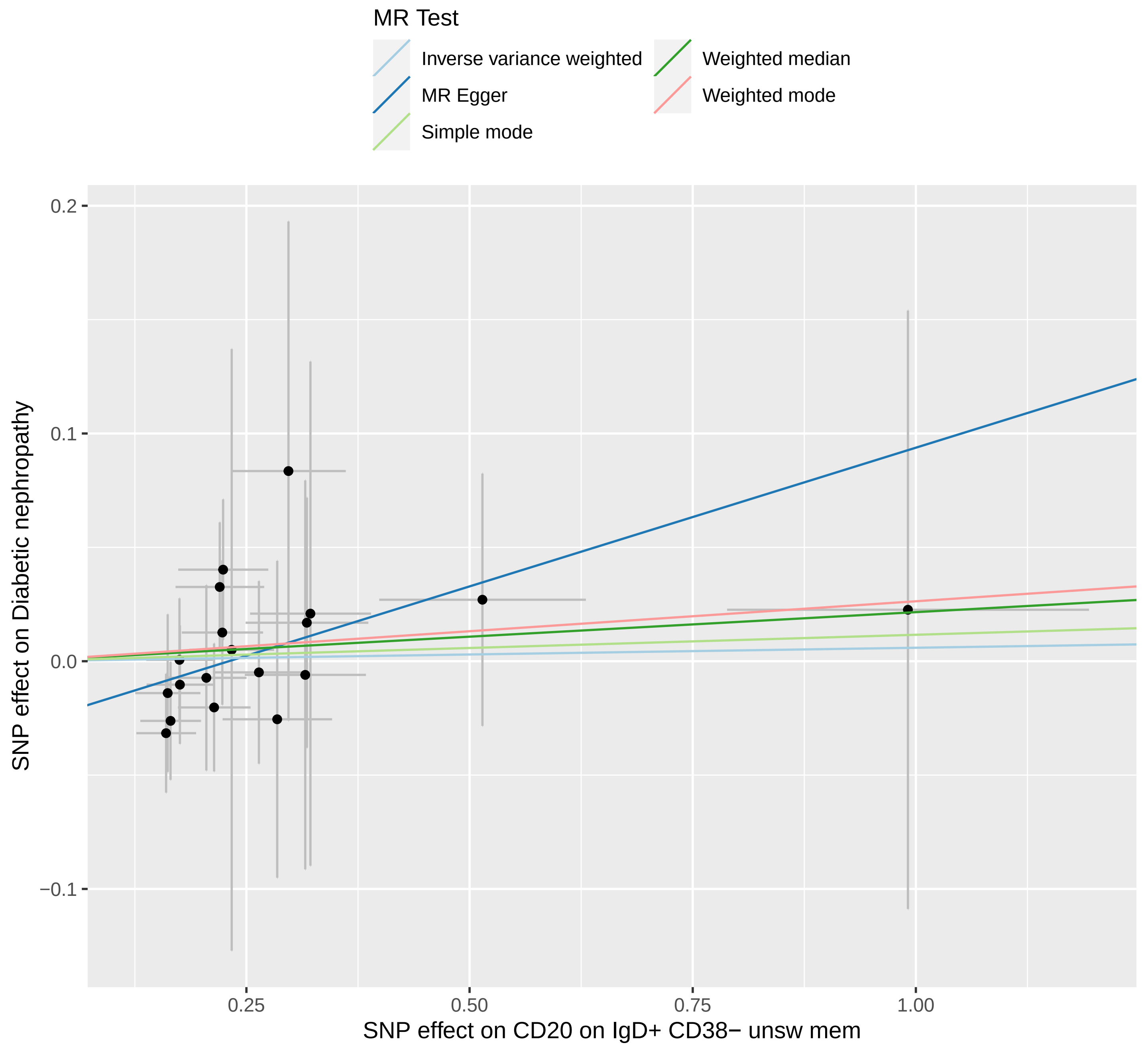
MR Test
Inverse variance weighted
MR Egger
Simple mode
Weighted median
Weighted mode
0.2
0.1
0.0
−0.1
0.25
0.50
0.75
1.00
SNP effect on Diabetic nephropathy
SNP effect on CD20 on IgD+ CD38− unsw mem

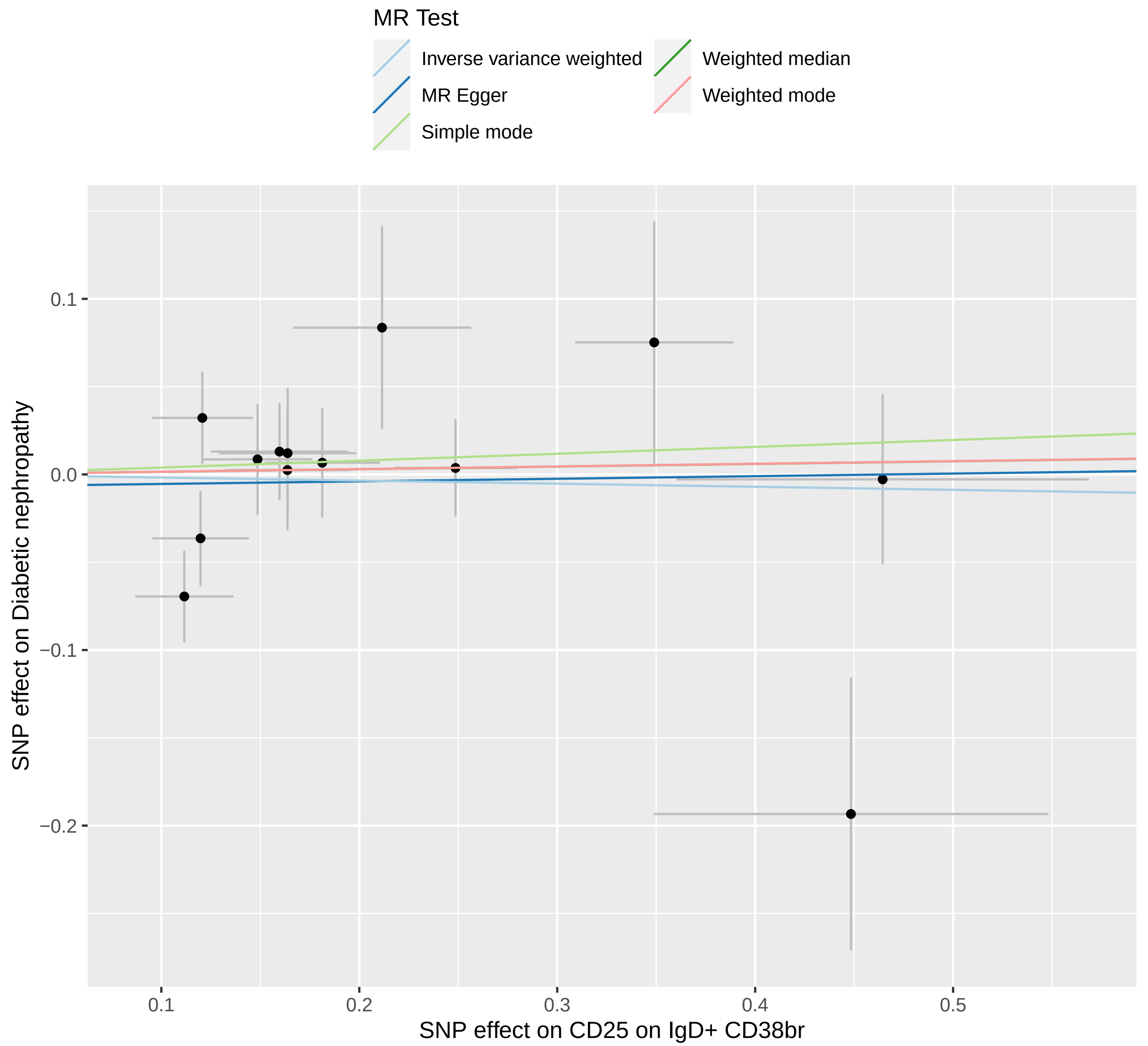

MR Test
Inverse variance weighted
MR Egger
Simple mode
Weighted median
Weighted mode
SNP effect on Diabetic nephropathy
0.1
0.0
−0.1
−0.2
0.1
0.2
0.3
0.4
0.5
SNP effect on CD25 on IgD+ CD38br

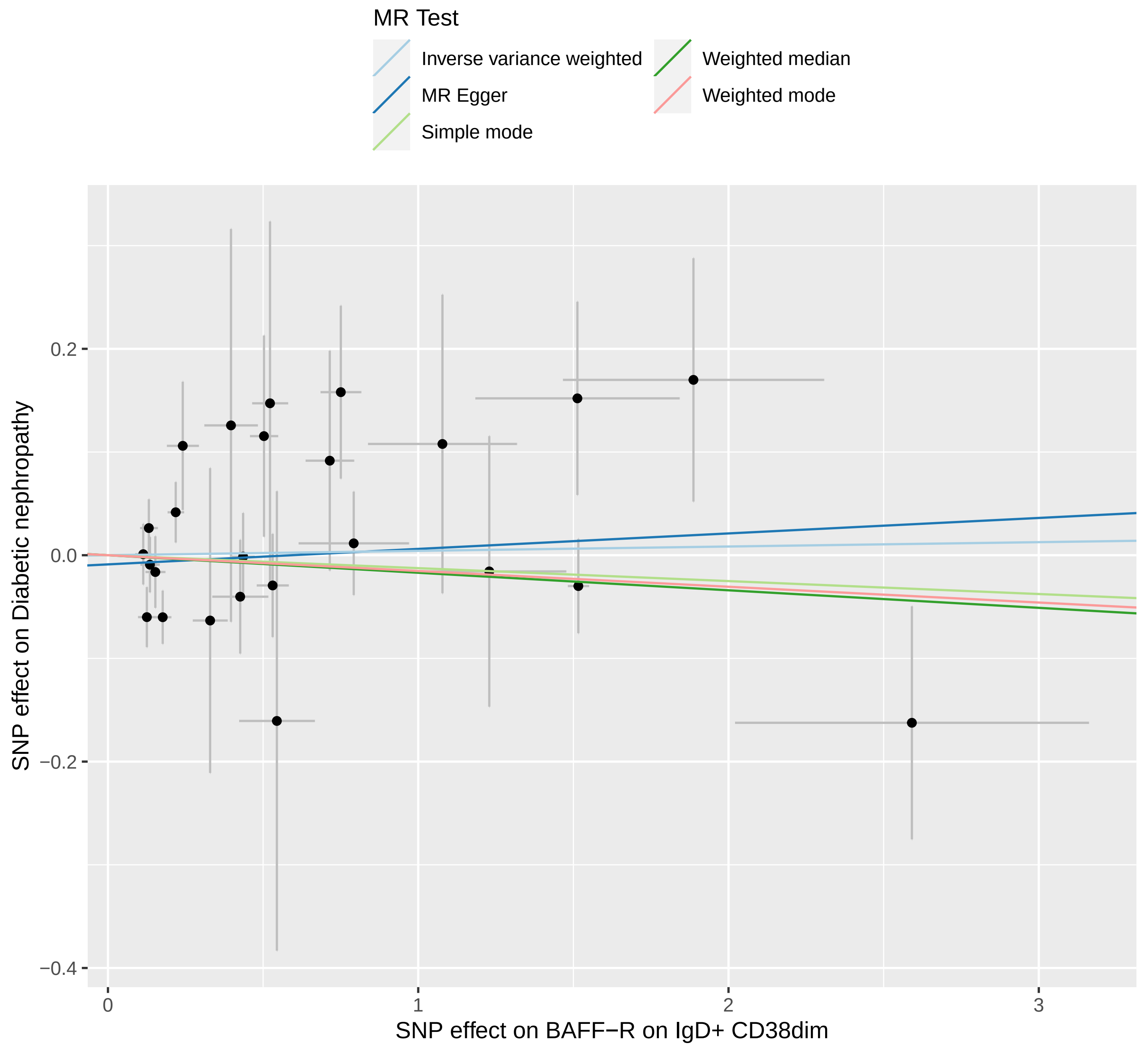

MR Test
Inverse variance weighted
MR Egger
Simple mode
Weighted median
Weighted mode
0.2
0.0
−0.2
−0.4
0
1
2
3
SNP effect on Diabetic nephropathy
SNP effect on BAFF−R on IgD+ CD38dim

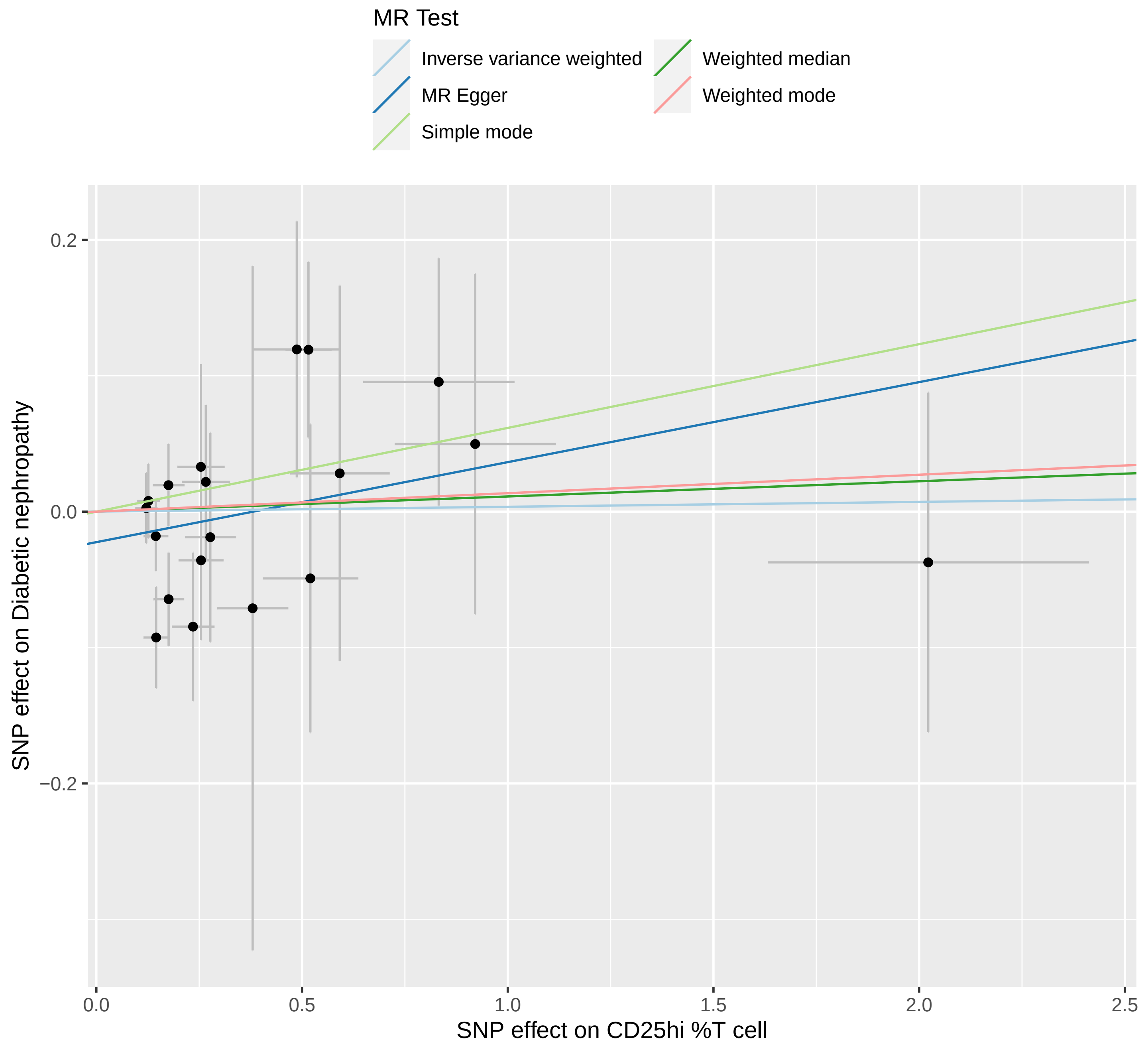
MR Test
Inverse variance weighted
Weighted median
MR Egger
Weighted mode
Simple mode
0.2
0.0
−0.2
SNP effect on Diabetic nephropathy
0.0
0.5
1.0
1.5
2.0
2.5
SNP effect on CD25hi %T cell

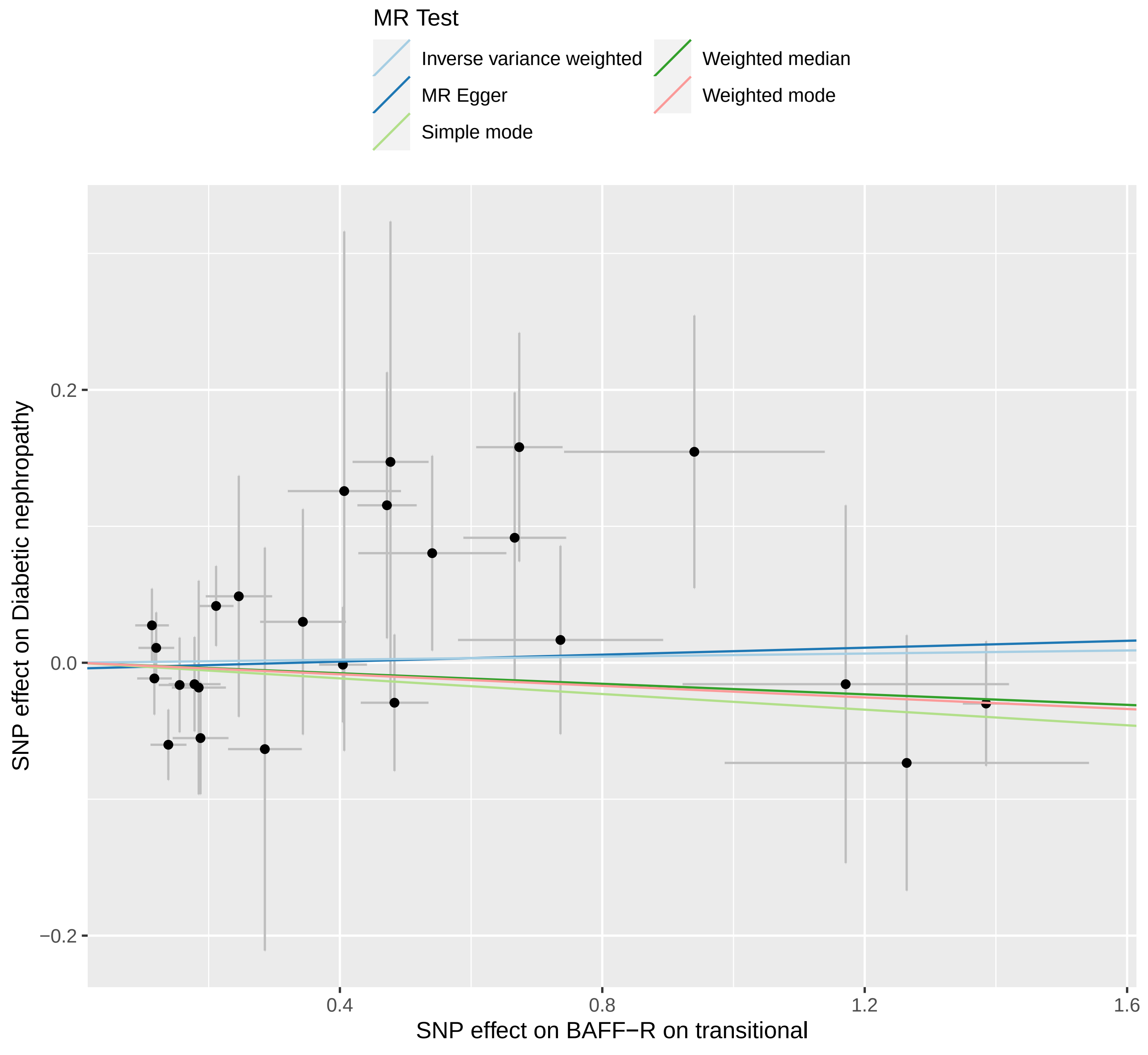
MR Test
Inverse variance weighted
MR Egger
Simple mode
Weighted median
Weighted mode
0.2
0.0
−0.2
0.4
0.8
1.2
1.6
SNP effect on Diabetic nephropathy
SNP effect on BAFF−R on transitional

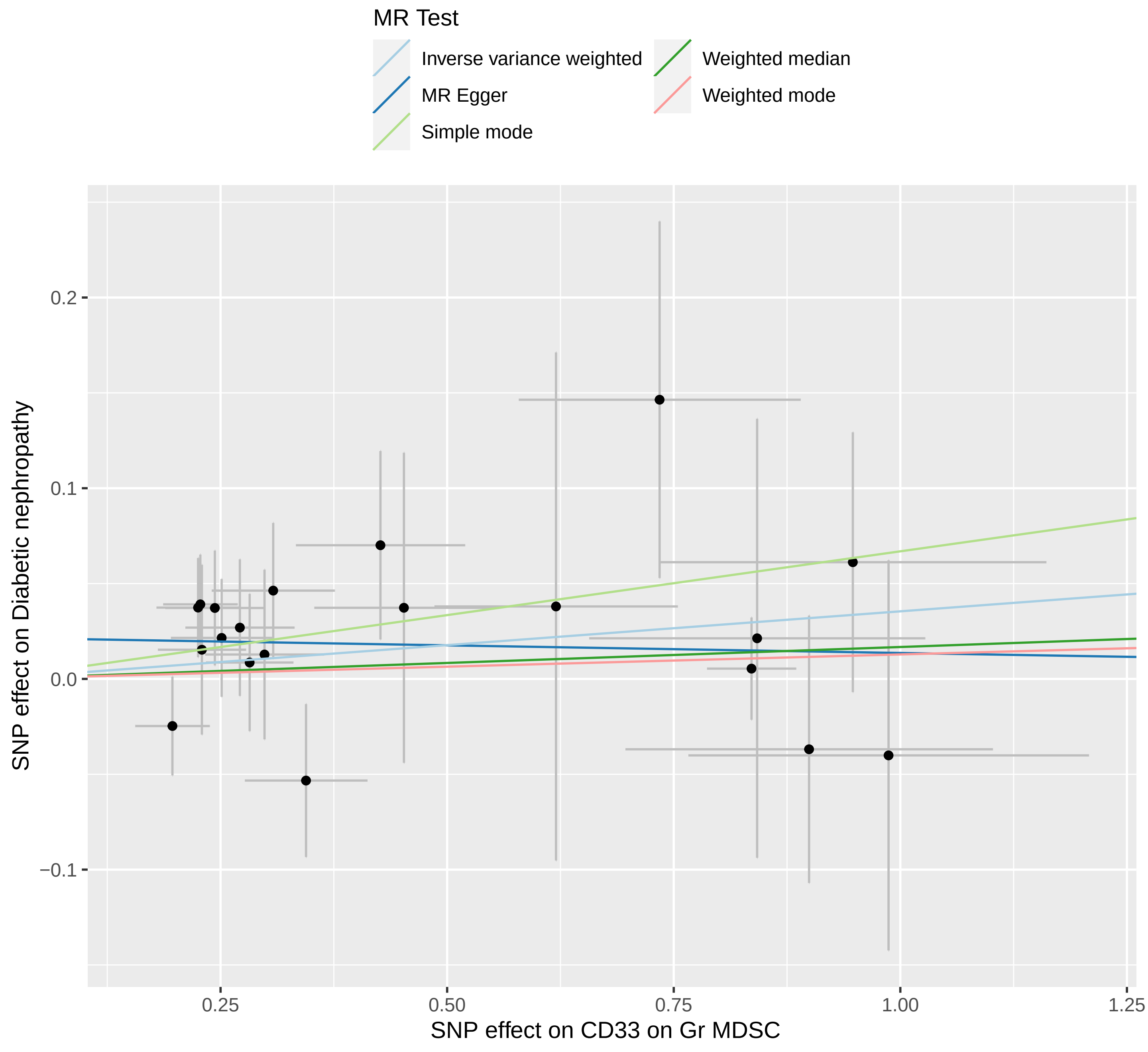
MR Test
Inverse variance weighted
MR Egger
Simple mode
Weighted median
Weighted mode
SNP effect on Diabetic nephropathy
0.2
0.1
0.0
−0.1
0.25
0.50
0.75
1.00
1.25
SNP effect on CD33 on Gr MDSC

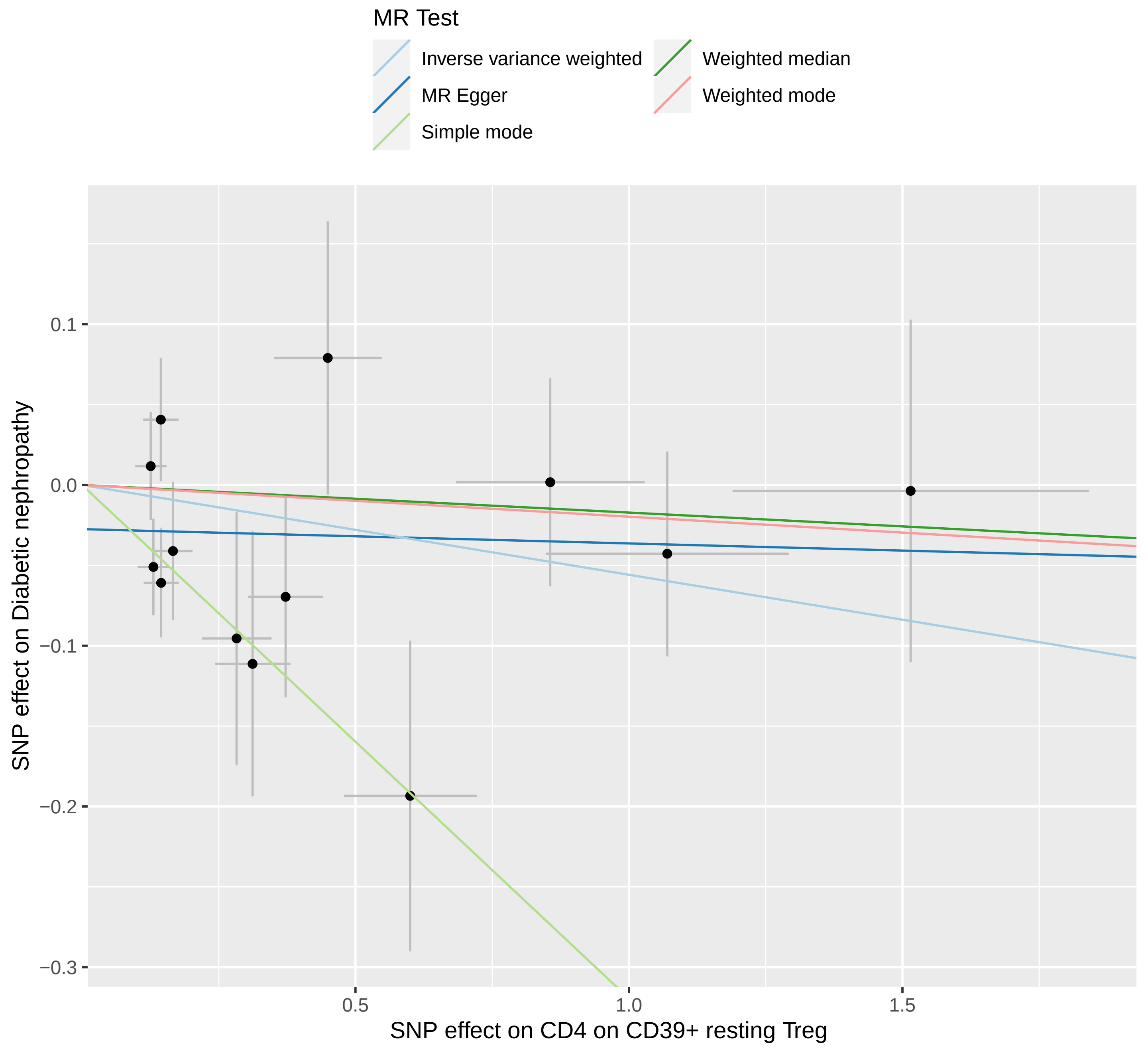

MR Test
Inverse variance weighted
MR Egger
Simple mode
Weighted median
Weighted mode
0.1
0.0
−0.1
−0.2
−0.3
SNP effect on Diabetic nephropathy
0.5
1.0
1.5
SNP effect on CD4 on CD39+ resting Treg

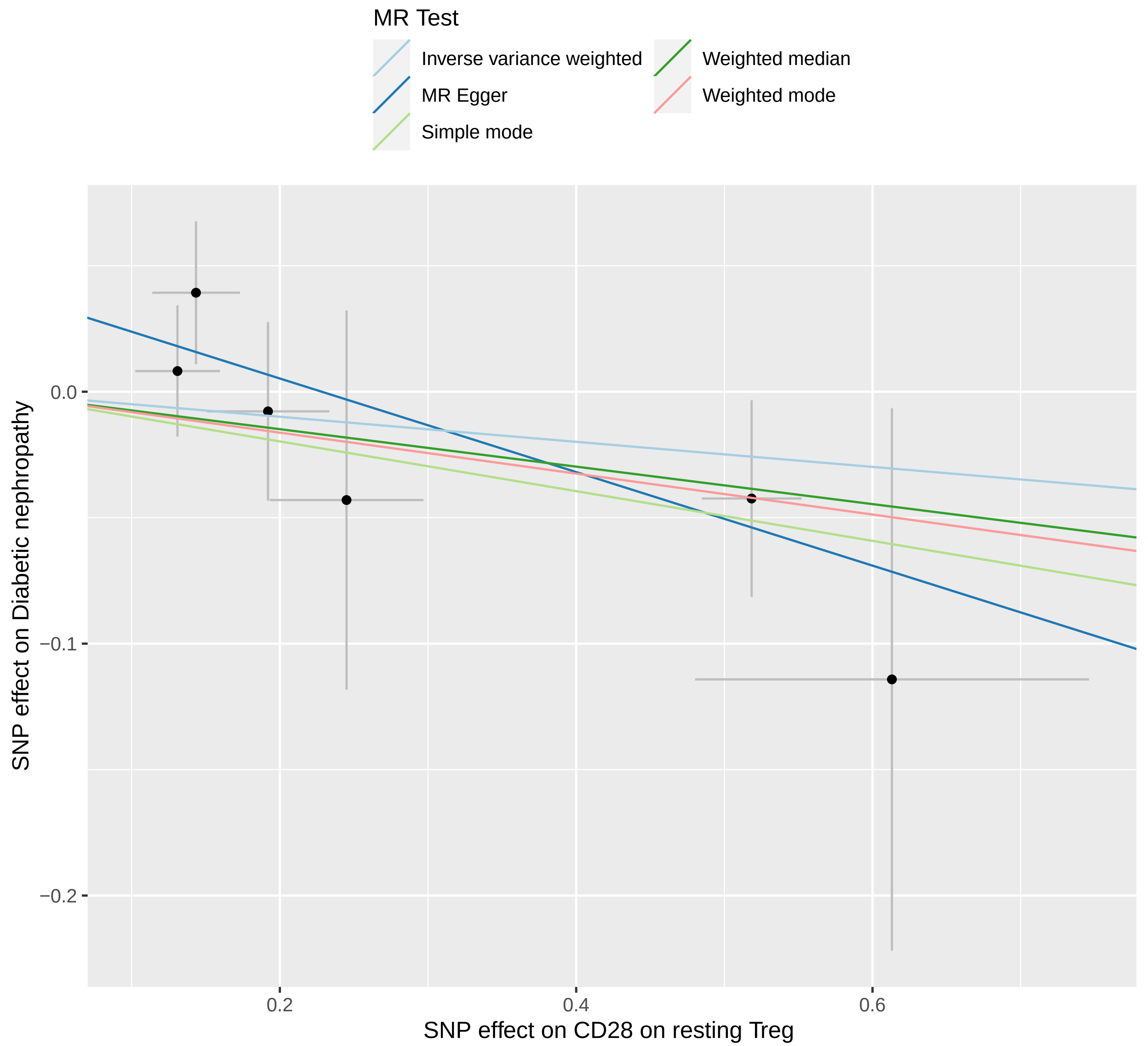

MR Test
Inverse variance weighted
MR Egger
Simple mode
Weighted median
Weighted mode
0.0
−0.1
−0.2
0.2
0.4
0.6
SNP effect on Diabetic nephropathy
SNP effect on CD28 on resting Treg

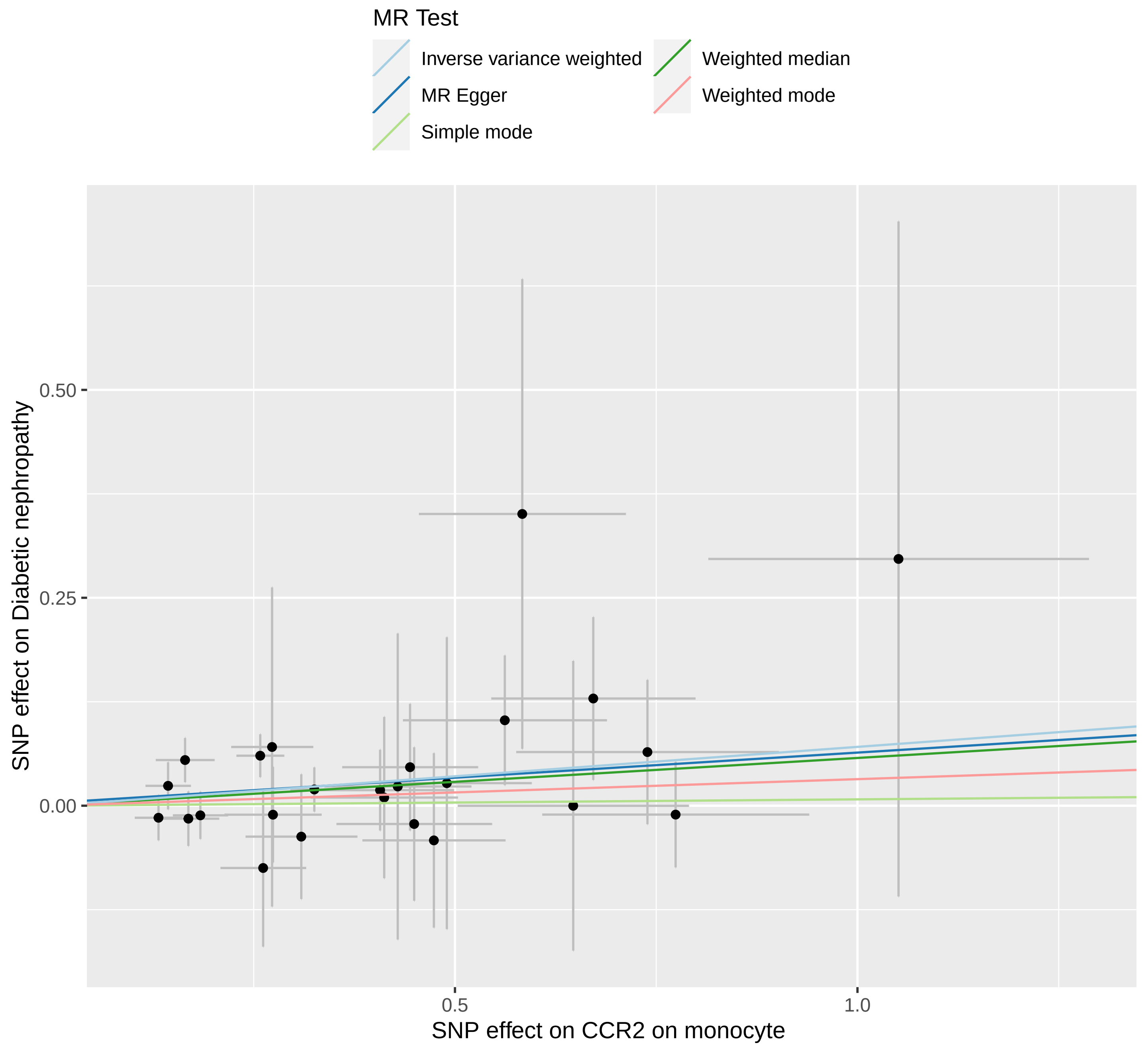
MR Test
Inverse variance weighted
MR Egger
Simple mode
Weighted median
Weighted mode
SNP effect on Diabetic nephropathy
0.50
0.25
0.00
0.5
1.0
SNP effect on CCR2 on monocyte

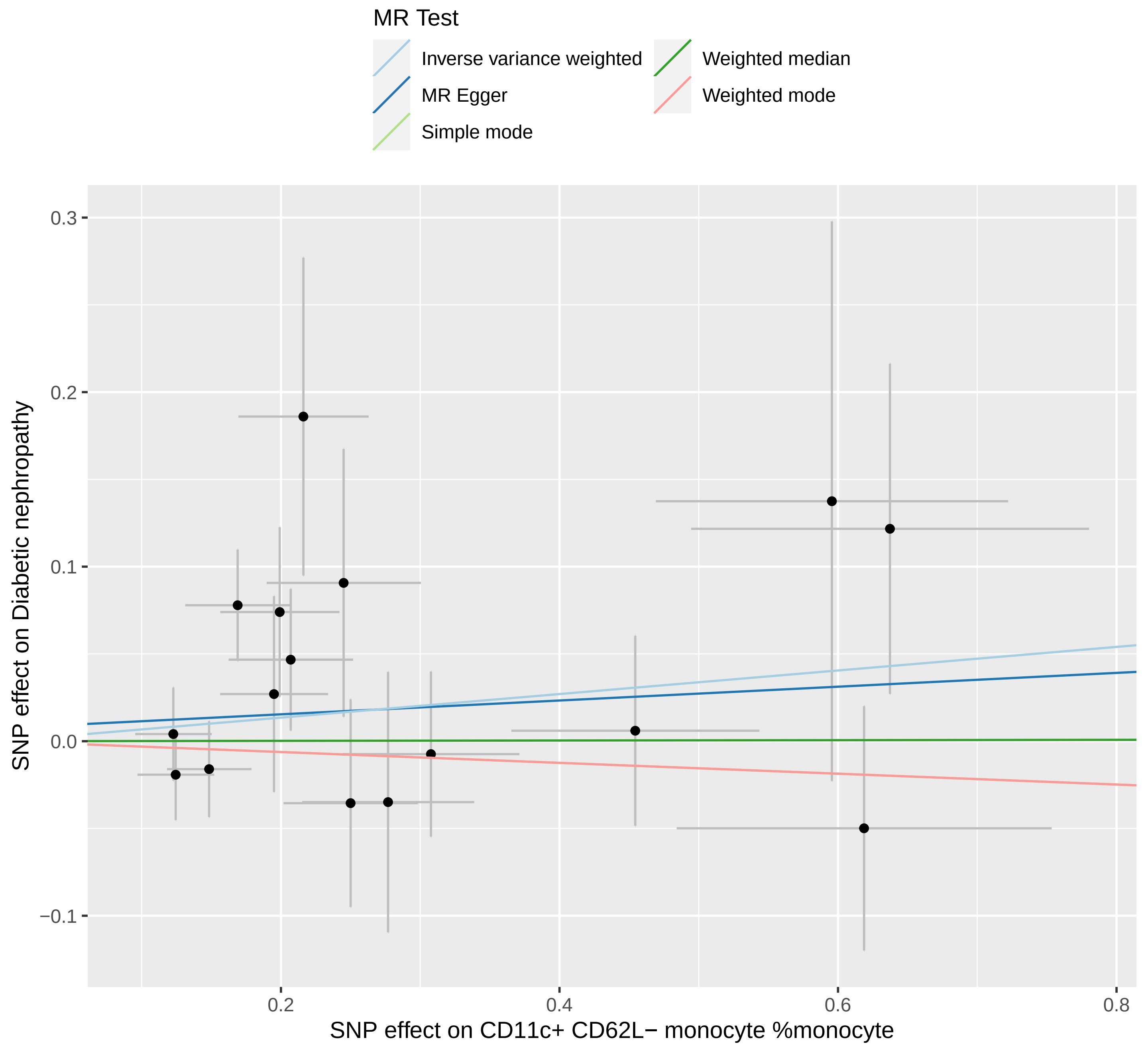
MR Test
Inverse variance weighted
MR Egger
Simple mode
Weighted median
Weighted mode
0.3
0.2
0.1
0.0
−0.1
SNP effect on Diabetic nephropathy
0.2
0.4
0.6
0.8
SNP effect on CD11c+ CD62L− monocyte %monocyte

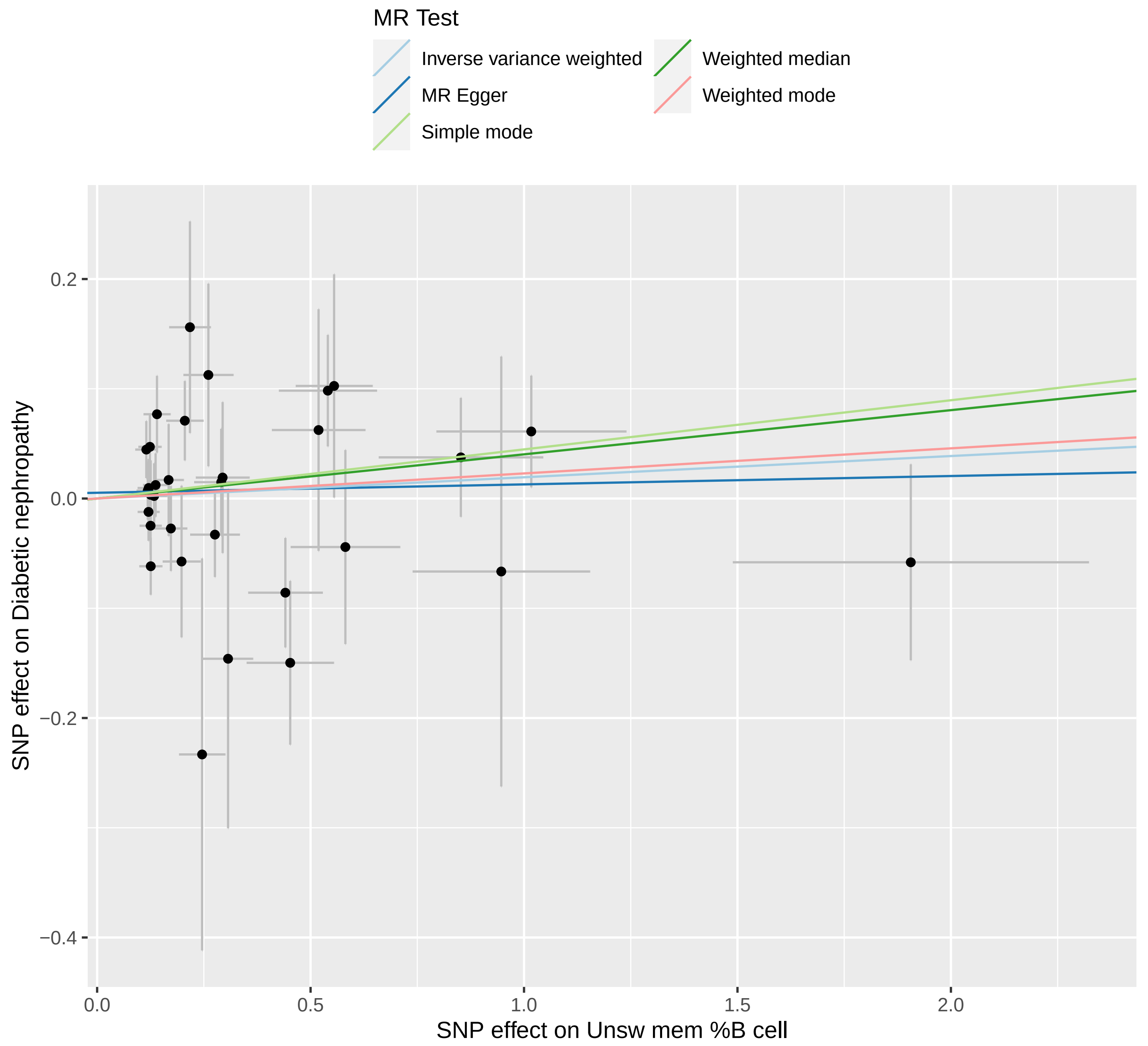
MR Test
Inverse variance weighted
MR Egger
Simple mode
Weighted median
Weighted mode
0.2
0.0
−0.2
−0.4
SNP effect on Diabetic nephropathy
0.0
0.5
1.0
1.5
2.0
SNP effect on Unsw mem %B cell

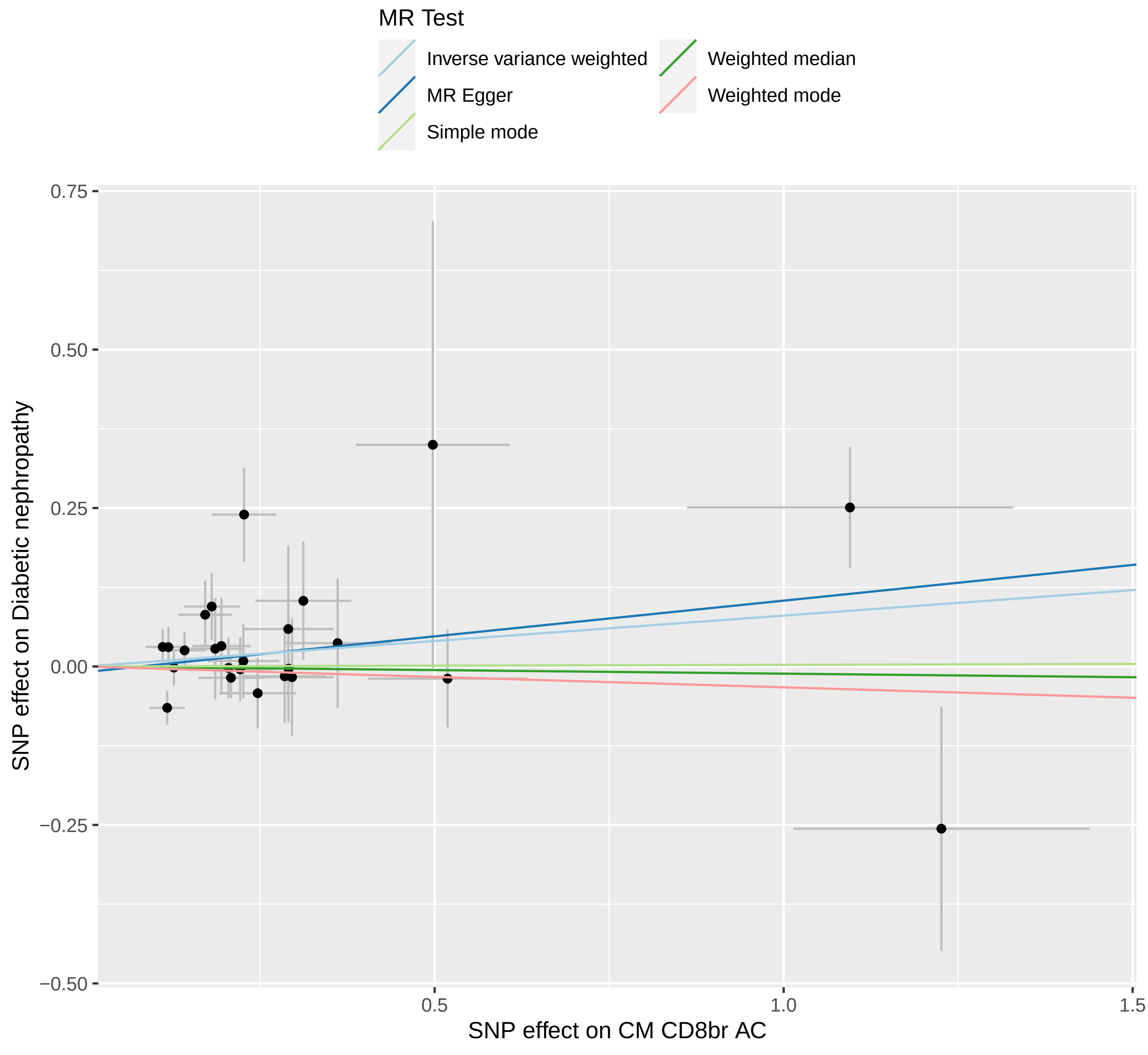

MR Test
Inverse variance weighted
MR Egger
Simple mode
Weighted median
Weighted mode
0.75
0.50
0.25
0.00
−0.25
−0.50
0.5
1.0
1.5
SNP effect on CM CD8br AC
SNP effect on Diabetic nephropathy

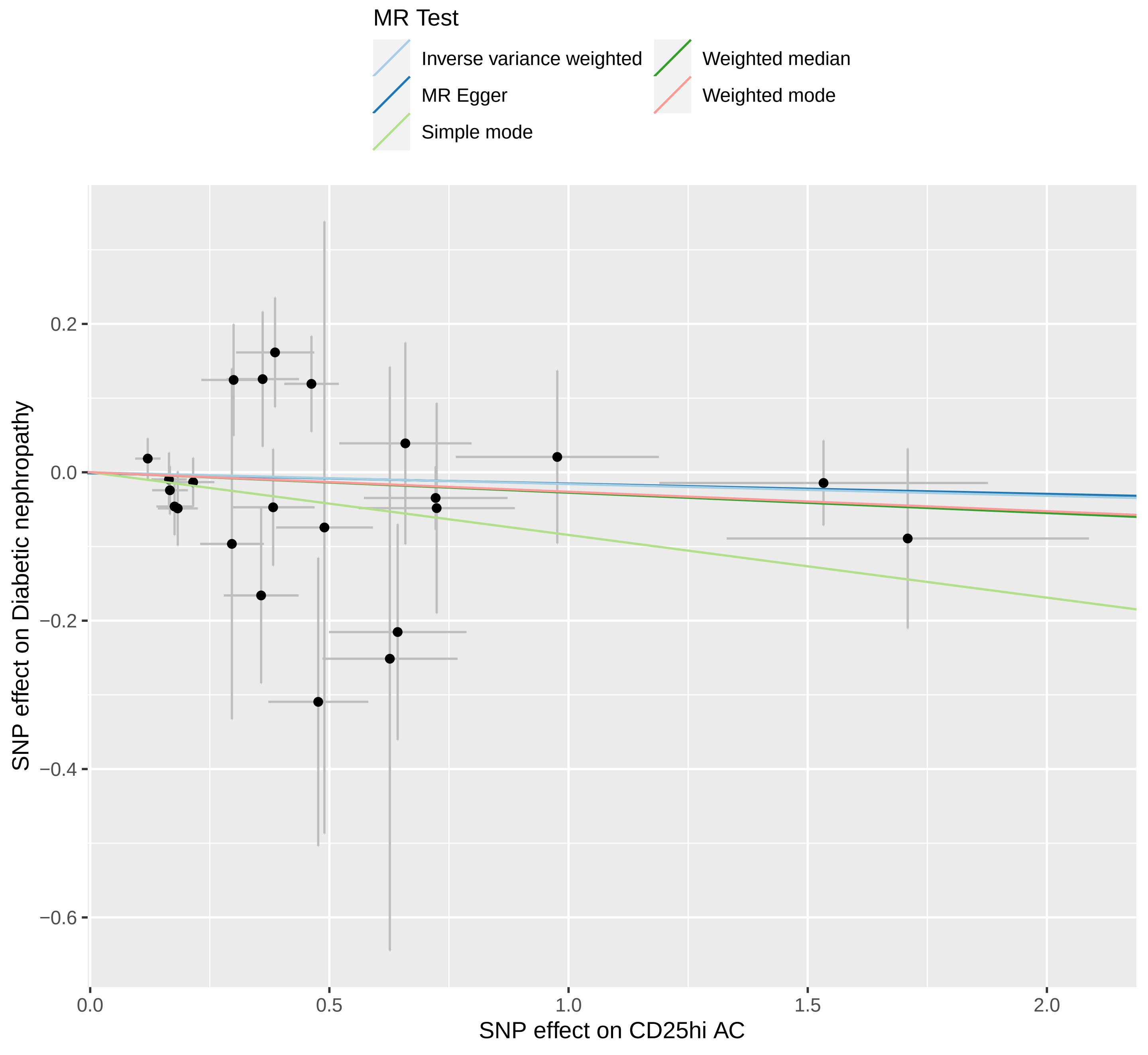

MR Test
Inverse variance weighted
MR Egger
Simple mode
Weighted median
Weighted mode
0.2
0.0
−0.2
−0.4
−0.6
SNP effect on Diabetic nephropathy
0.0
0.5
1.0
1.5
2.0
SNP effect on CD25hi AC

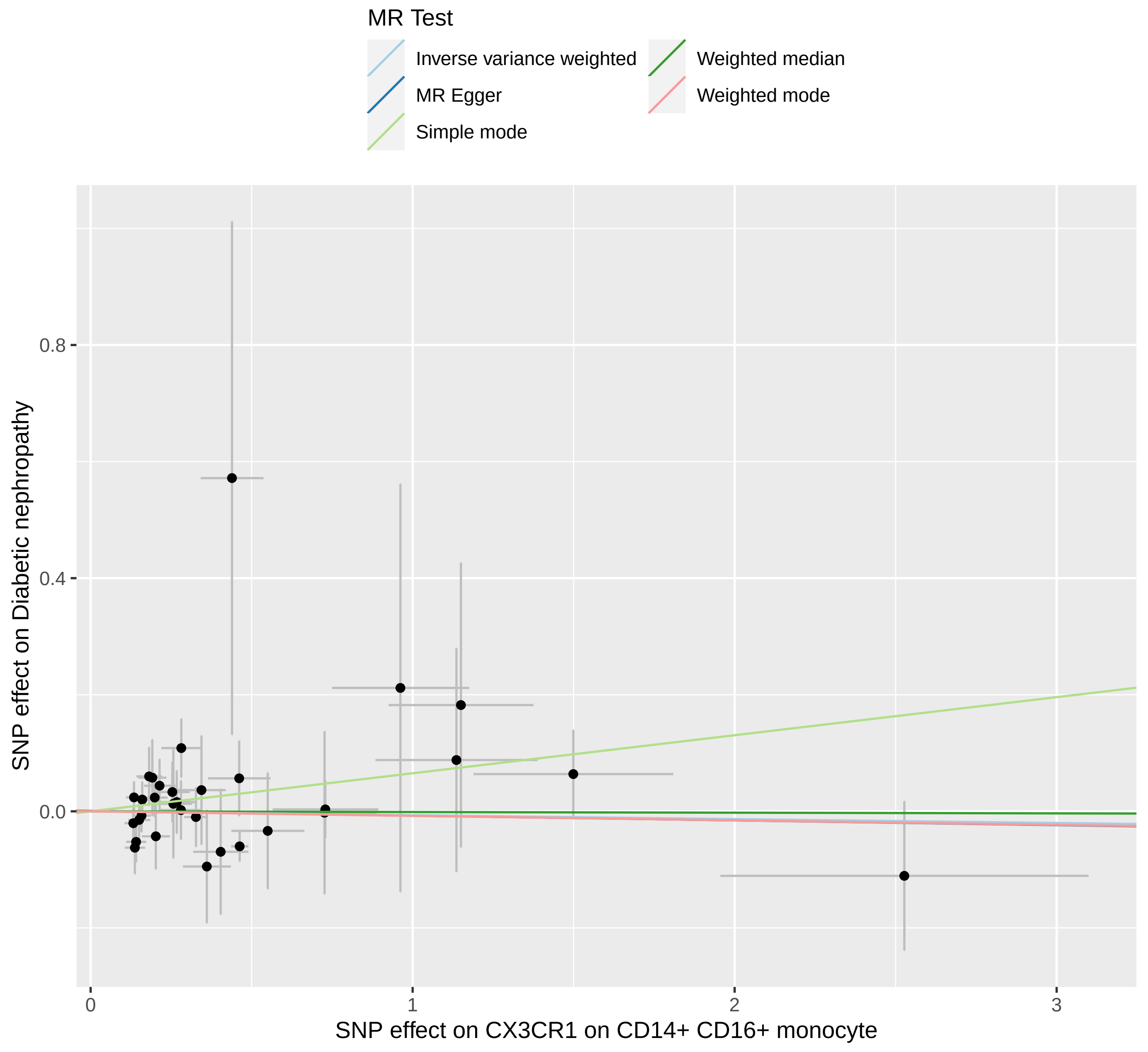
MR Test
Inverse variance weighted
MR Egger
Simple mode
Weighted median
Weighted mode
0.8
0.4
0.0
SNP effect on Diabetic nephropathy
0
1
2
3
SNP effect on CX3CR1 on CD14+ CD16+ monocyte

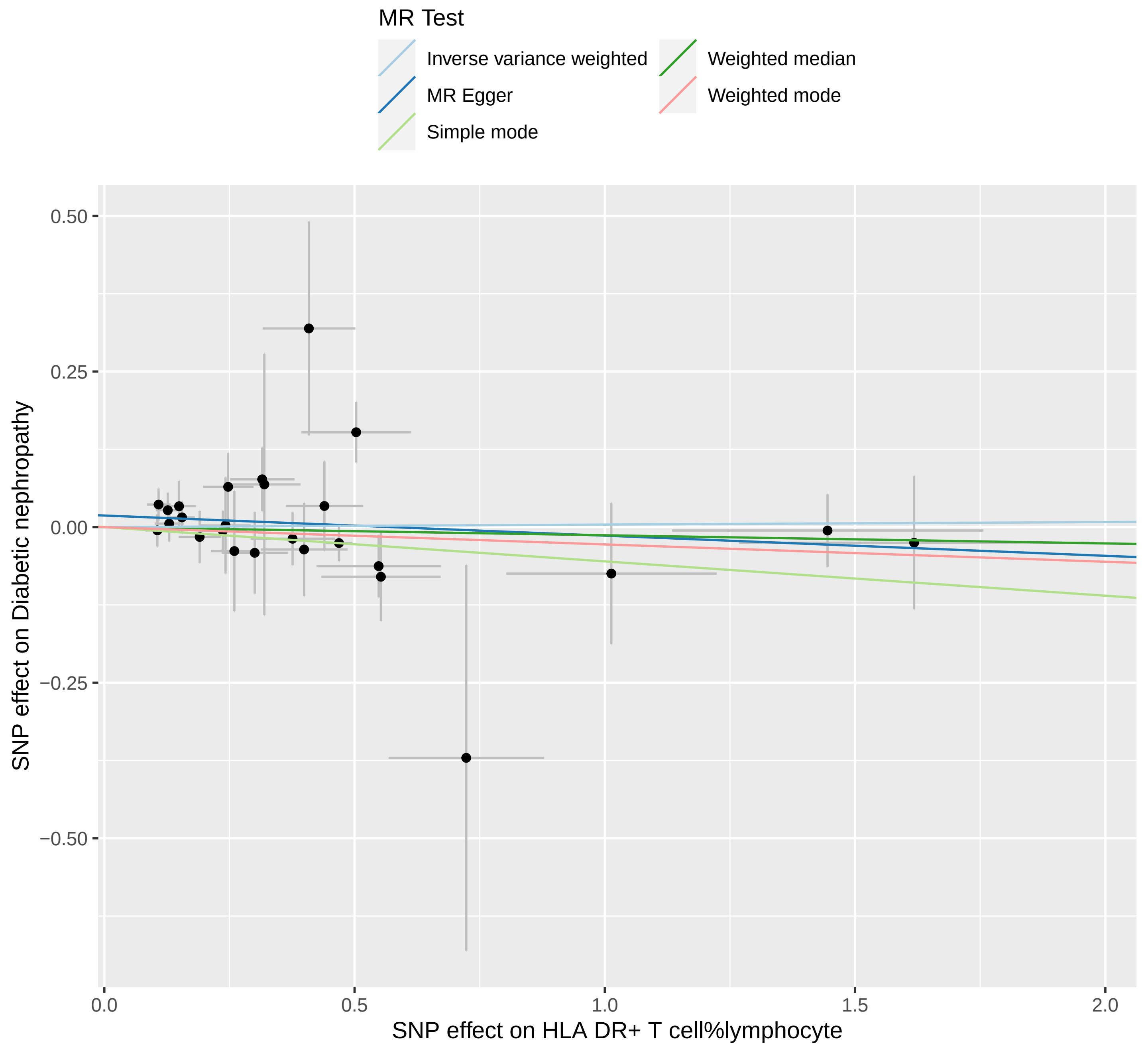

MR Test
Inverse variance weighted
MR Egger
Simple mode
Weighted median
Weighted mode
0.50
0.25
0.00
−0.25
−0.50
SNP effect on Diabetic nephropathy
0.0
0.5
1.0
1.5
2.0
SNP effect on HLA DR+ T cell%lymphocyte

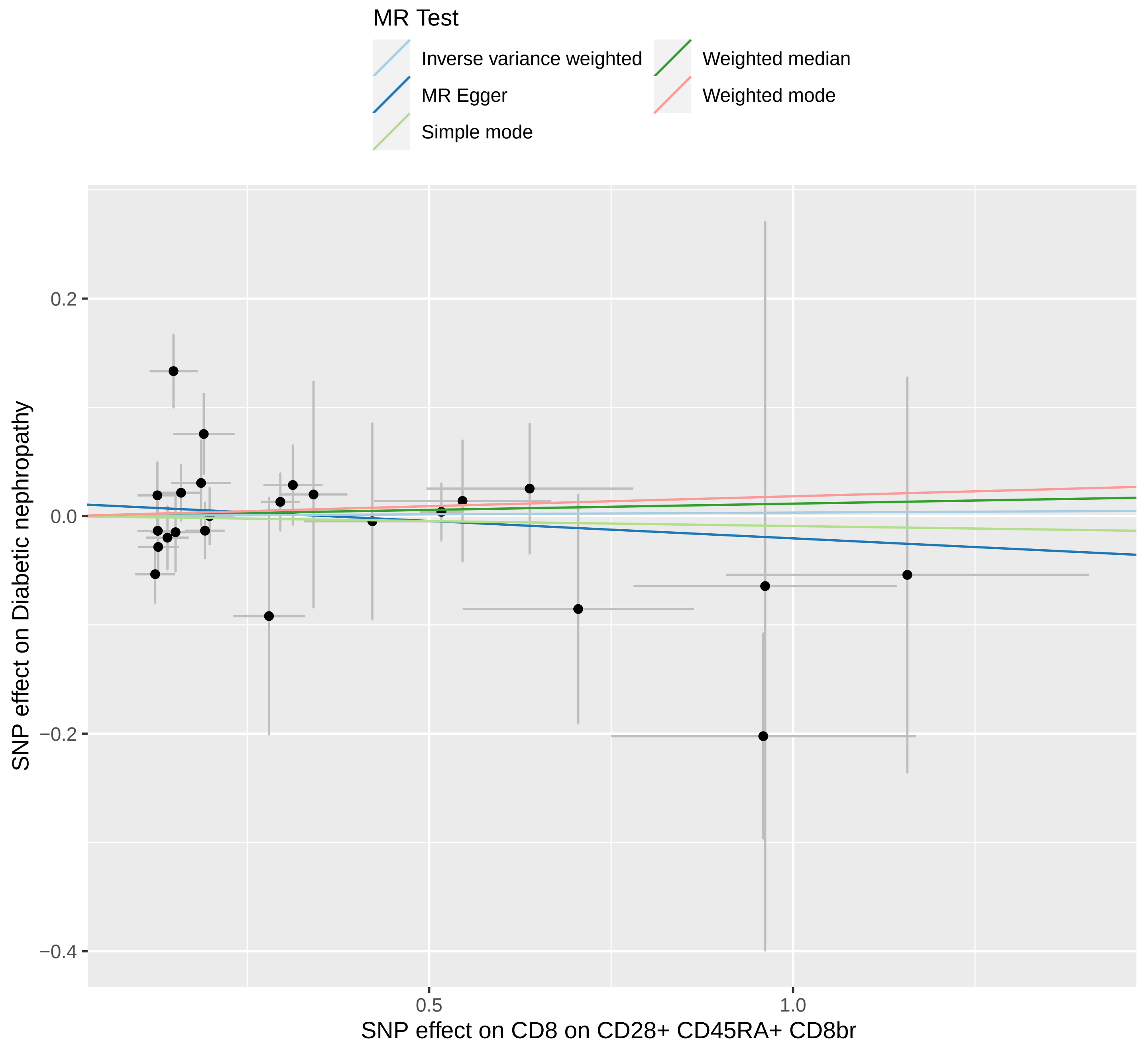

MR Test
Inverse variance weighted
MR Egger
Simple mode
Weighted median
Weighted mode
0.2
0.0
−0.2
−0.4
0.5
1.0
SNP effect on Diabetic nephropathy
SNP effect on CD8 on CD28+ CD45RA+ CD8br

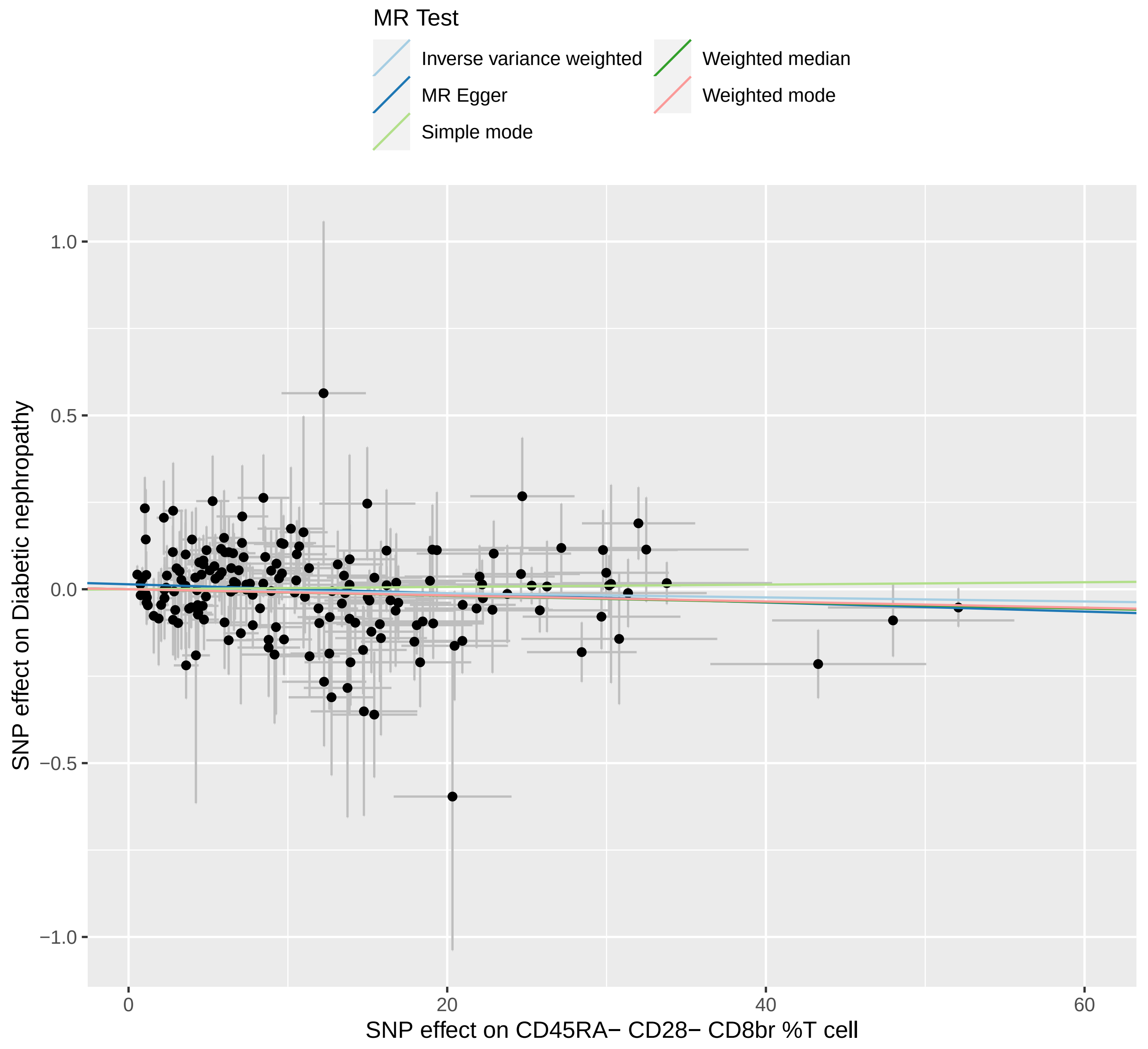
MR Test
Inverse variance weighted
Weighted median
MR Egger
Weighted mode
Simple mode
1.0
0.5
0.0
−0.5
−1.0
0
20
40
60
SNP effect on Diabetic nephropathy
SNP effect on CD45RA− CD28− CD8br %T cell

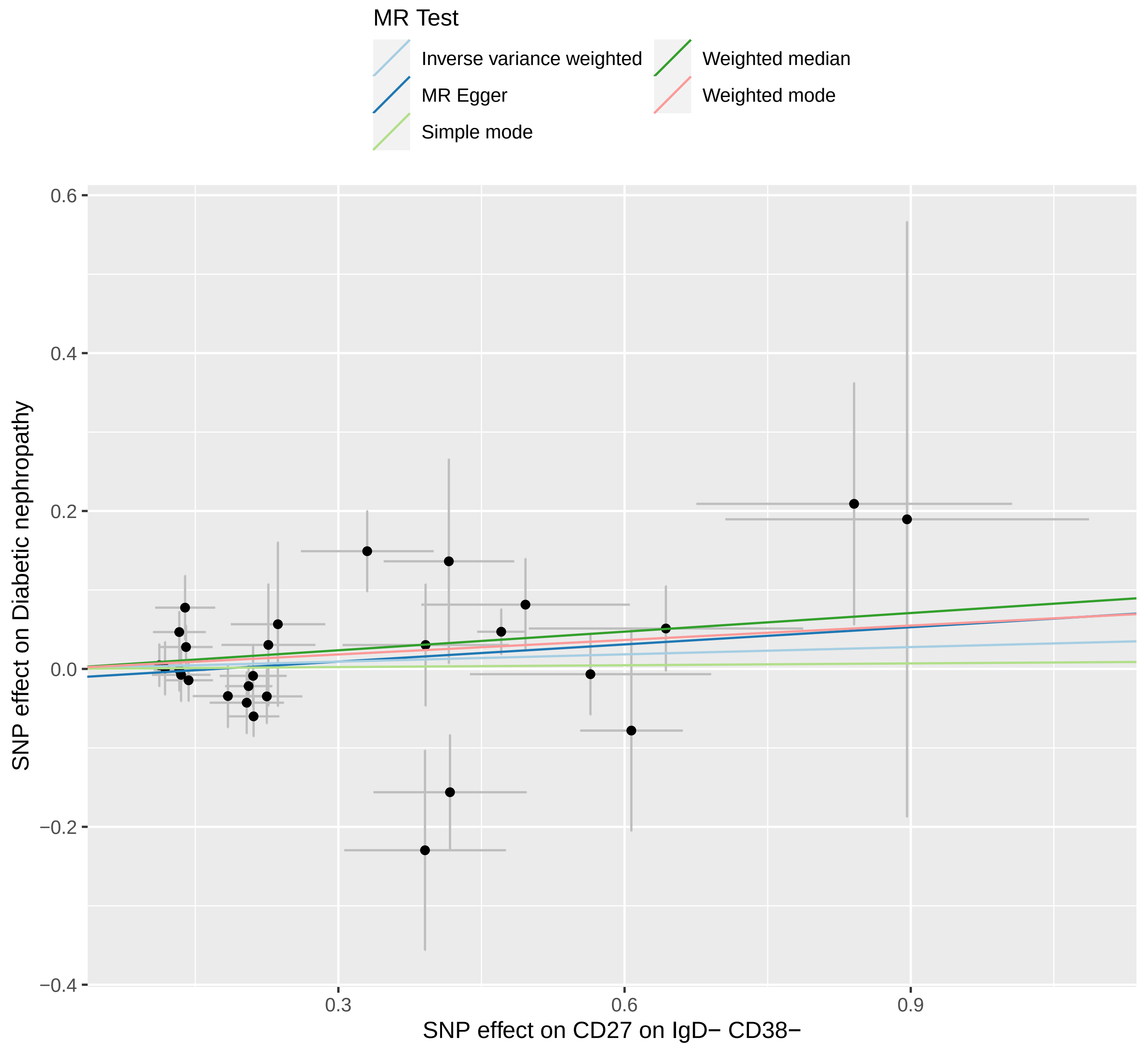
MR Test
Inverse variance weighted
MR Egger
Simple mode
Weighted median
Weighted mode
SNP effect on Diabetic nephropathy
0.6
0.4
0.2
0.0
−0.2
−0.4
0.3
0.6
0.9
SNP effect on CD27 on IgD− CD38−

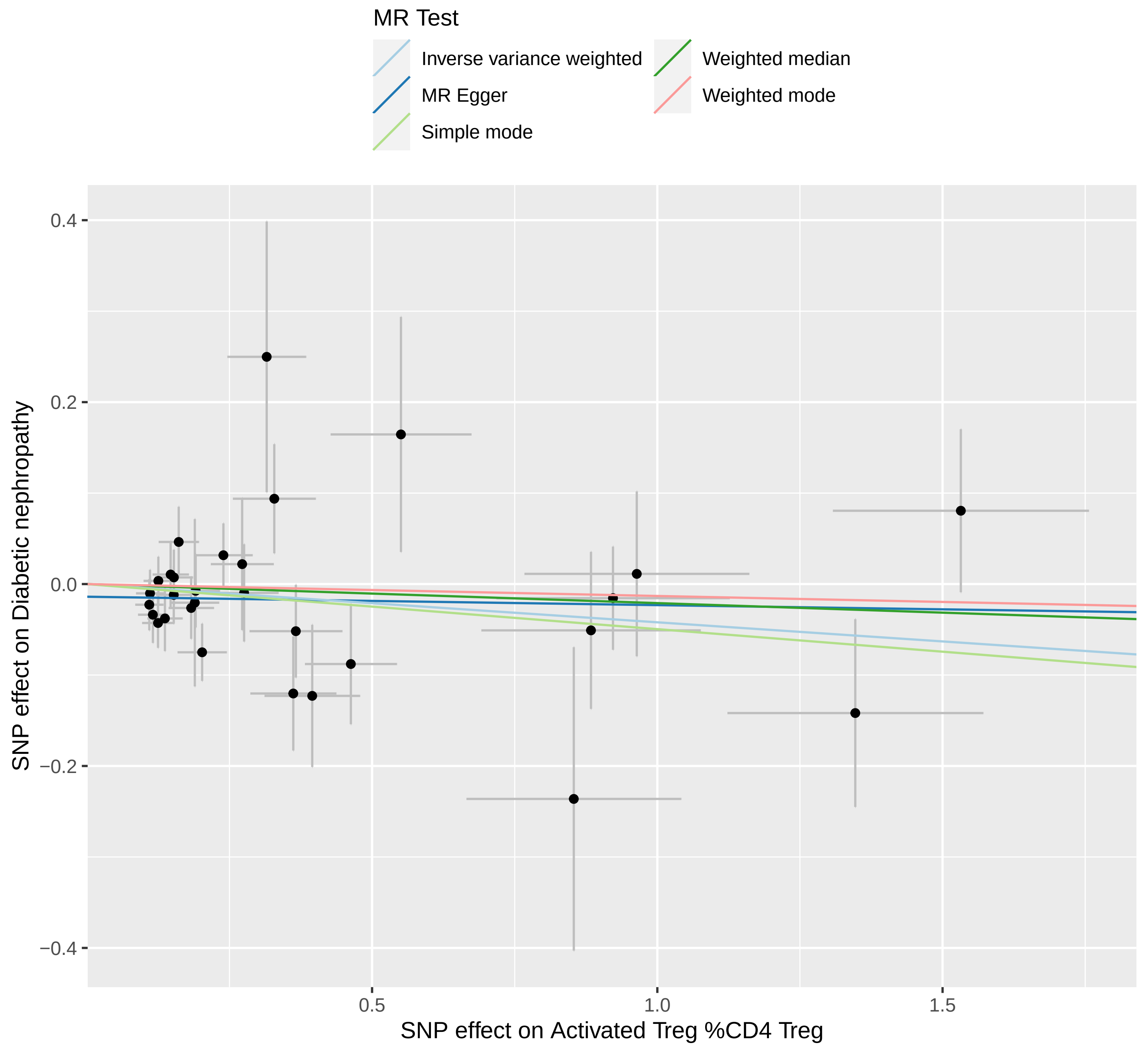

MR Test
Inverse variance weighted
MR Egger
Simple mode
Weighted median
Weighted mode
0.4
0.2
0.0
−0.2
−0.4
0.5
1.0
1.5
SNP effect on Diabetic nephropathy
SNP effect on Activated Treg %CD4 Treg

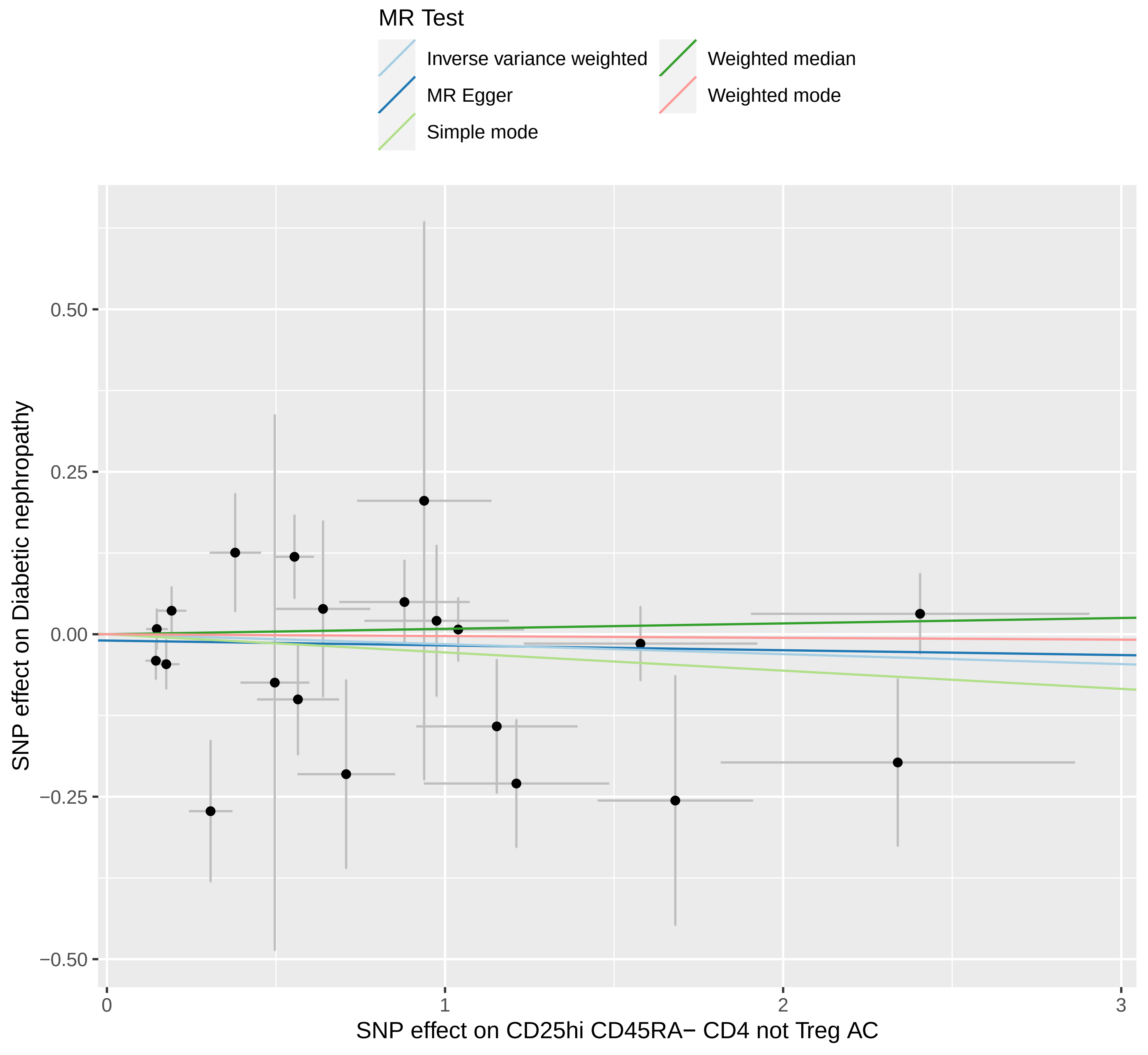
MR Test
Inverse variance weighted
MR Egger
Simple mode
Weighted median
Weighted mode
SNP effect on Diabetic nephropathy
0.50
0.25
0.00
−0.25
−0.50
0
1
2
3
SNP effect on CD25hi CD45RA− CD4 not Treg AC

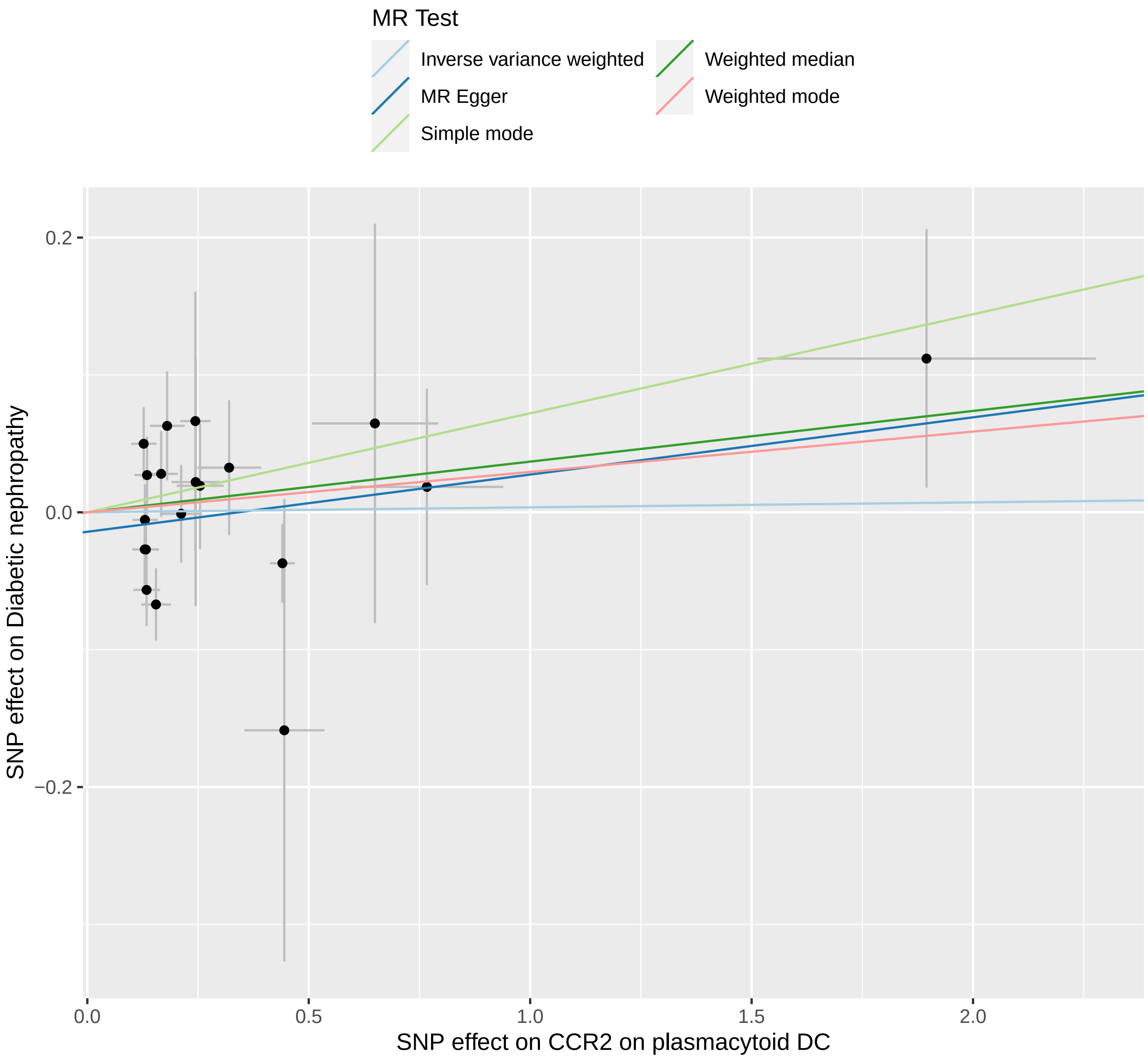
MR Test
Inverse variance weighted
MR Egger
Simple mode
Weighted median
Weighted mode
SNP effect on Diabetic nephropathy
SNP effect on CCR2 on plasmacytoid DC
0.2
0.0
−0.2
0.0
0.5
1.0
1.5
2.0

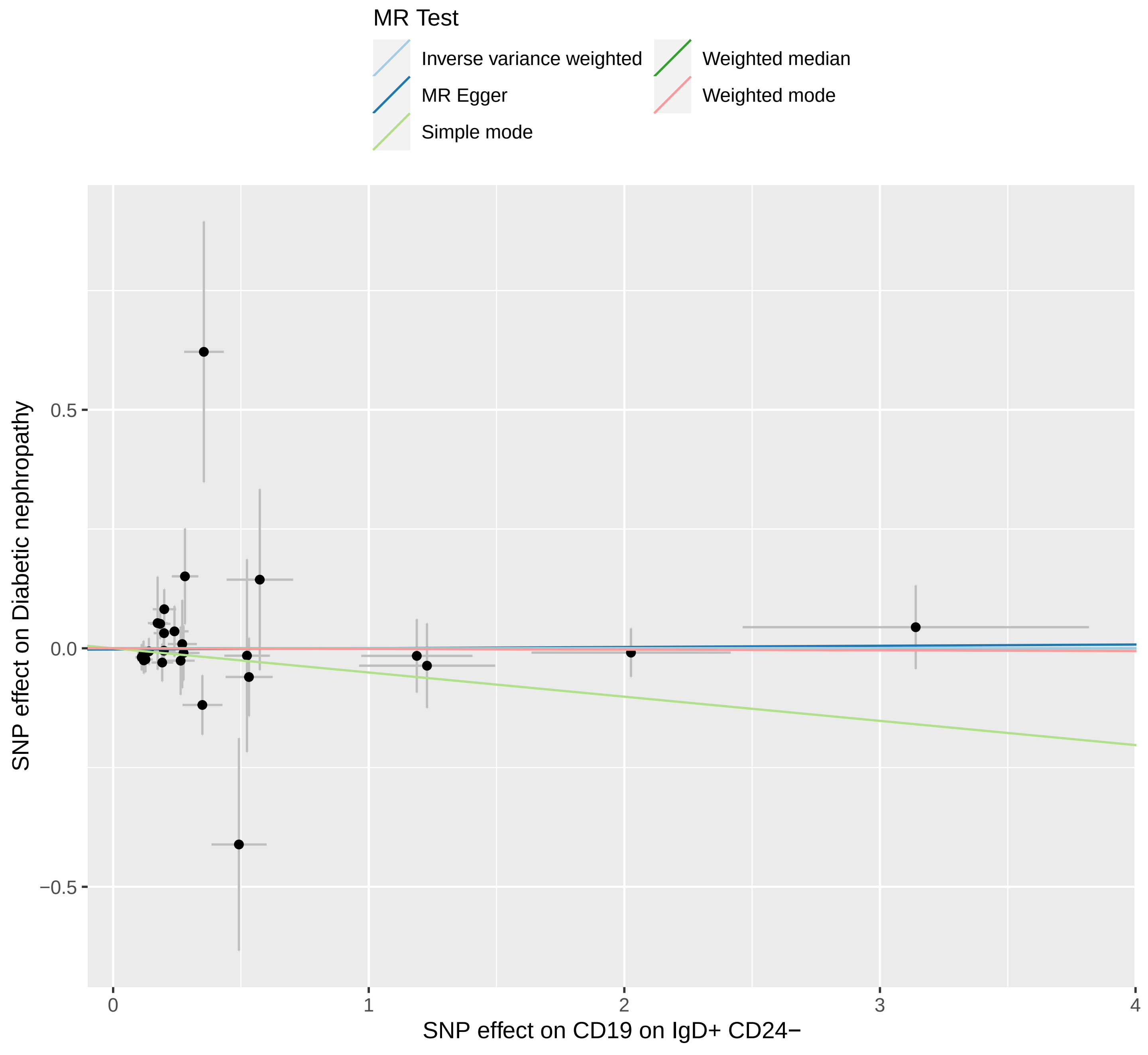

MR Test
Inverse variance weighted
MR Egger
Simple mode
Weighted median
Weighted mode
0.5
0.0
−0.5
0
1
2
3
4
SNP effect on CD19 on IgD+ CD24−
SNP effect on Diabetic nephropathy

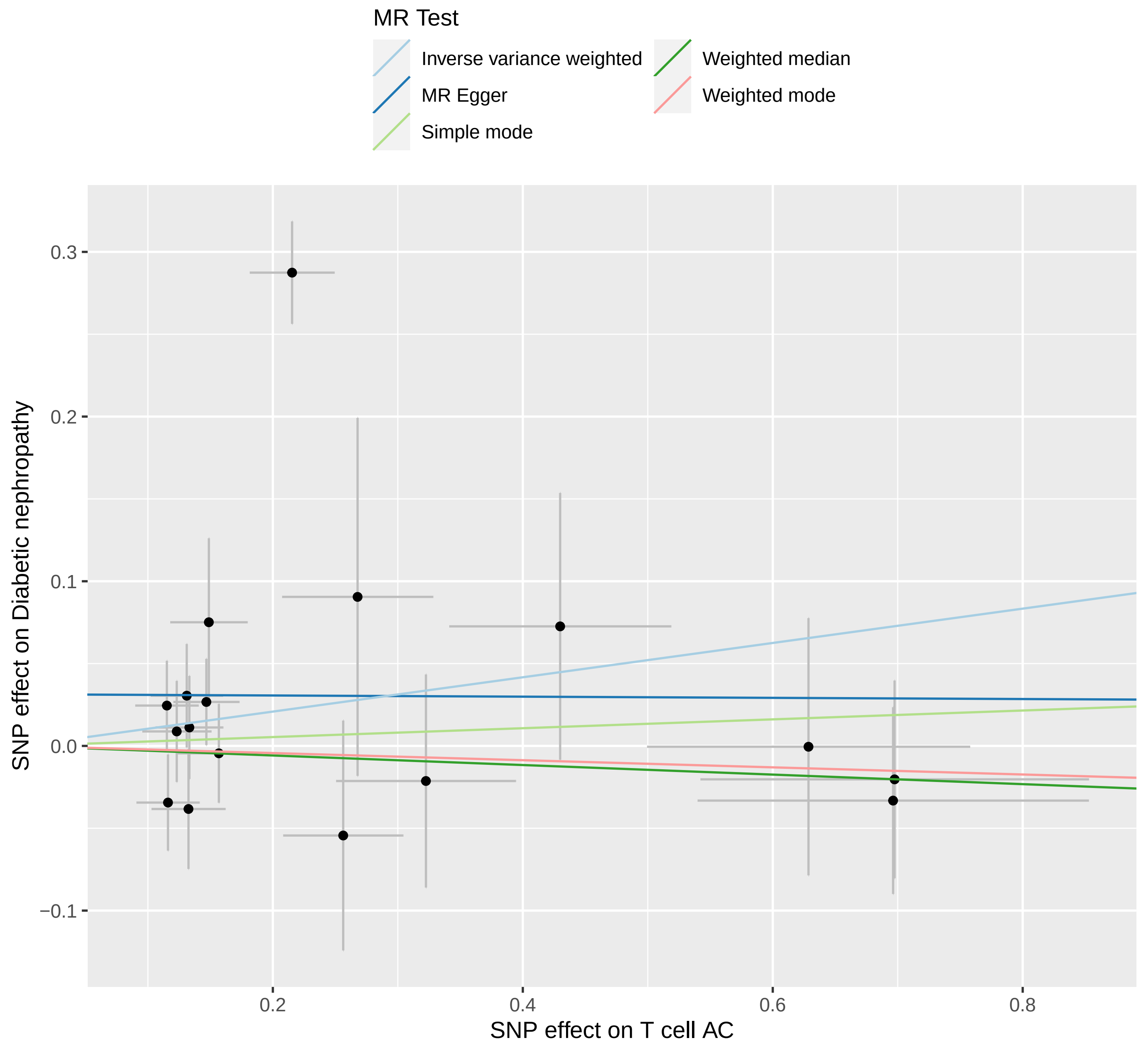
MR Test
Inverse variance weighted
MR Egger
Simple mode
Weighted median
Weighted mode
0.3
0.2
0.1
0.0
−0.1
0.2
0.4
0.6
0.8
SNP effect on Diabetic nephropathy
SNP effect on T cell AC

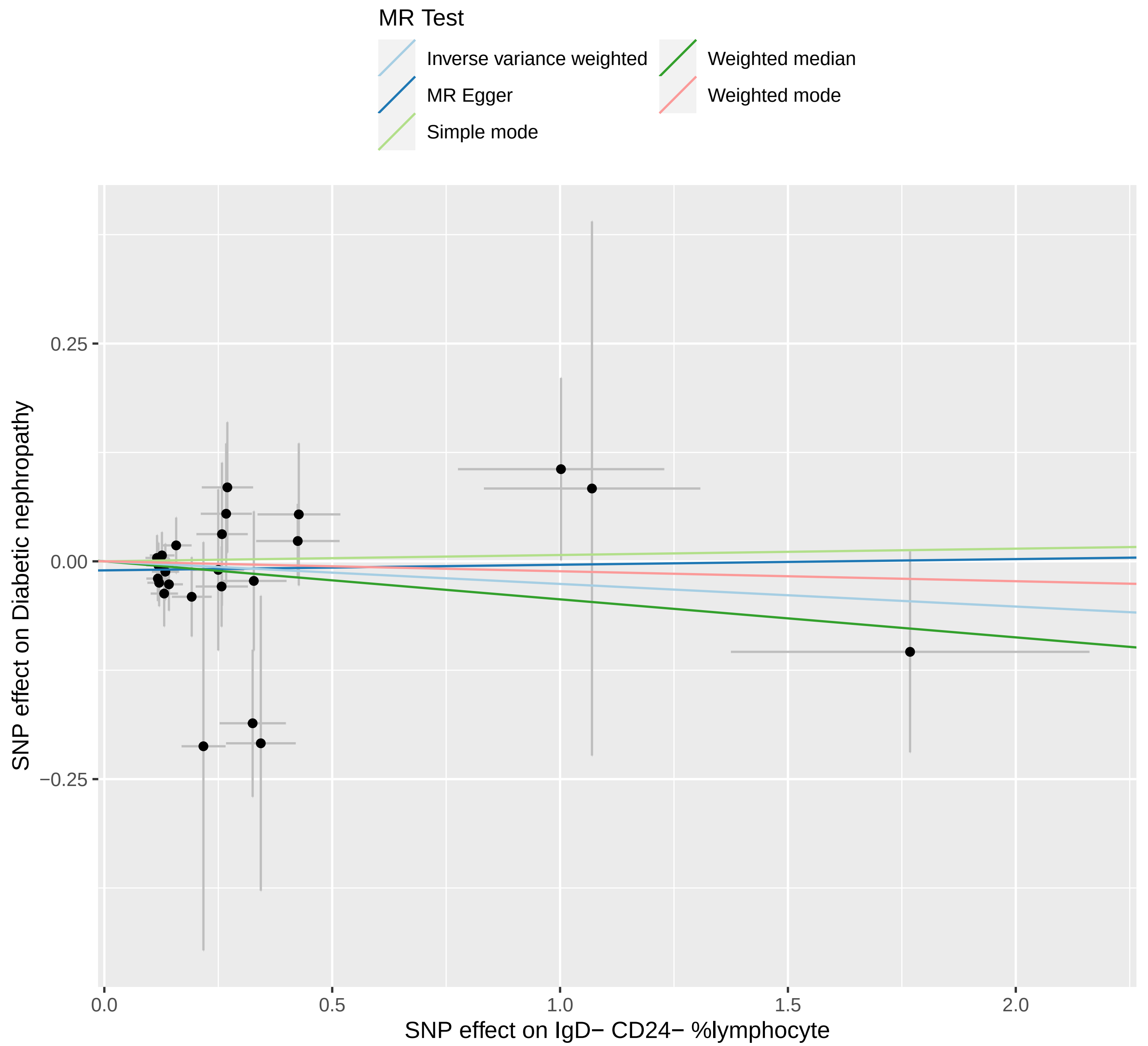
MR Test
Inverse variance weighted
MR Egger
Simple mode
Weighted median
Weighted mode
0.25
0.00
−0.25
SNP effect on Diabetic nephropathy
0.0
0.5
1.0
1.5
2.0
SNP effect on IgD− CD24− %lymphocyte

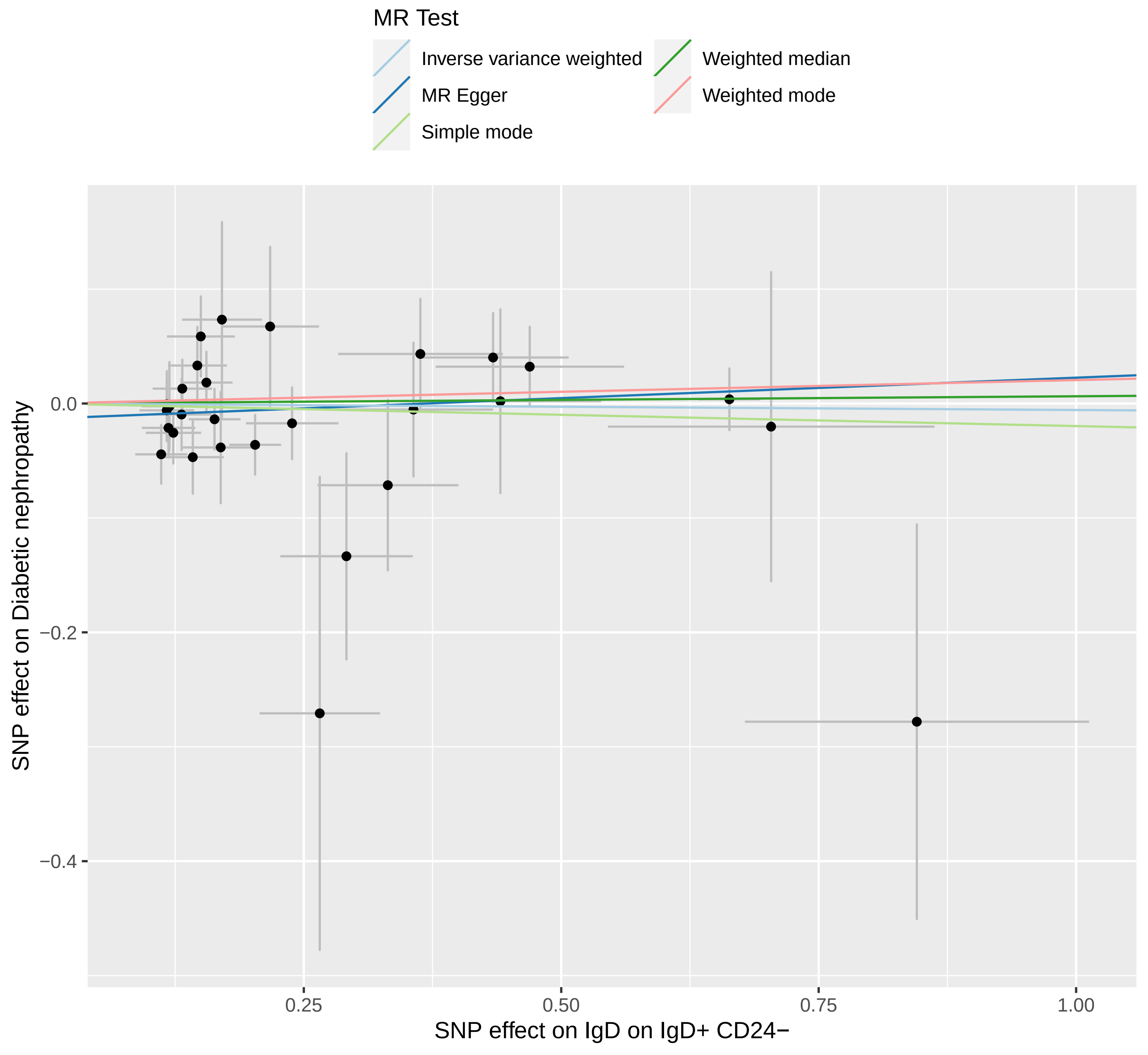

MR Test
Inverse variance weighted
MR Egger
Simple mode
Weighted median
Weighted mode
0.0
−0.2
−0.4
0.25
0.50
0.75
1.00
SNP effect on Diabetic nephropathy
SNP effect on IgD on IgD+ CD24−

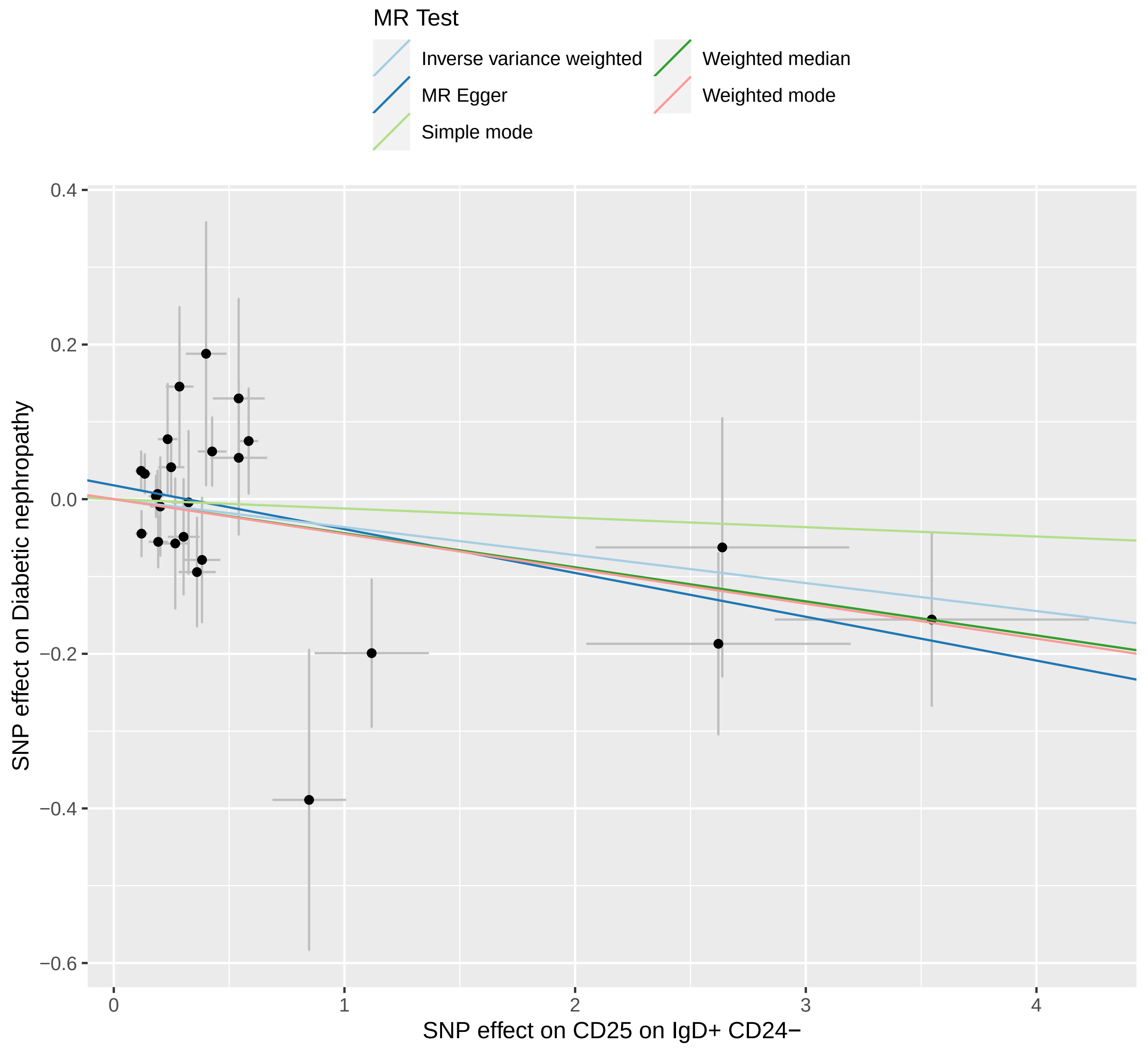
MR Test
Inverse variance weighted
MR Egger
Simple mode
Weighted median
Weighted mode
0.4
0.2
0.0
−0.2
−0.4
−0.6
SNP effect on Diabetic nephropathy
0
1
2
3
4
SNP effect on CD25 on IgD+ CD24−

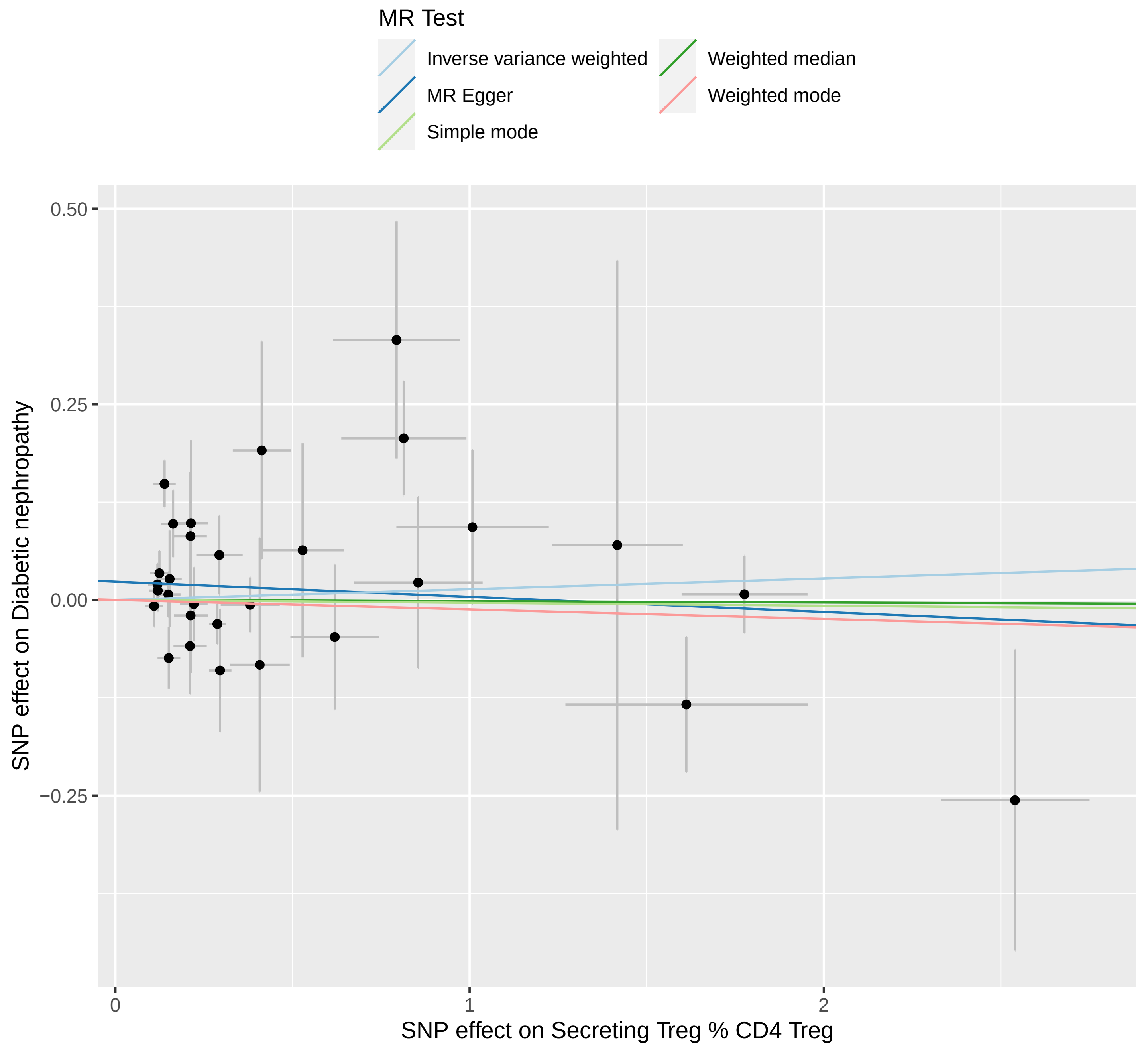
MR Test
Inverse variance weighted
MR Egger
Simple mode
Weighted median
Weighted mode
0.50
0.25
0.00
−0.25
SNP effect on Diabetic nephropathy
0
1
2
SNP effect on Secreting Treg % CD4 Treg

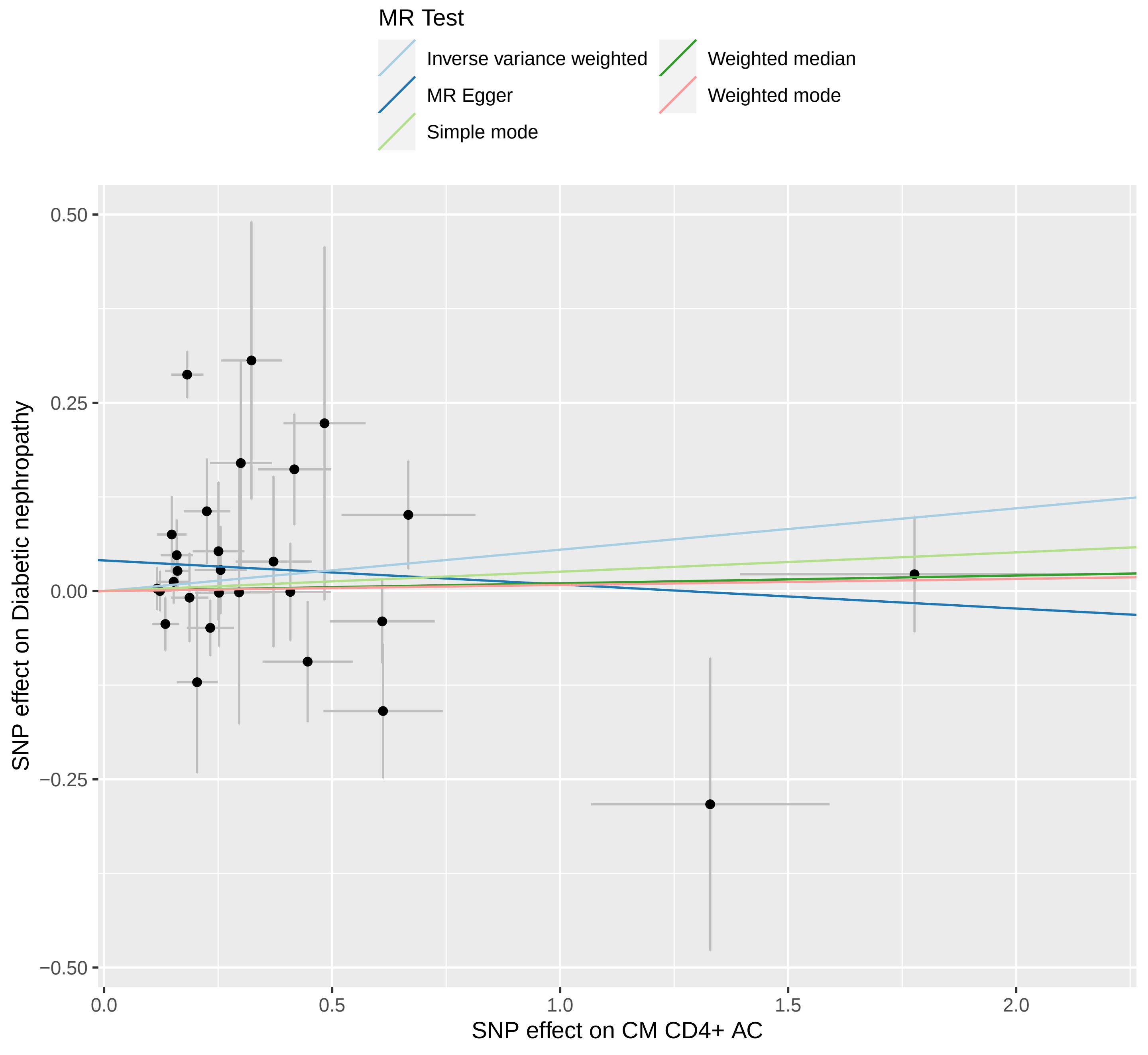

MR Test
Inverse variance weighted
MR Egger
Simple mode
Weighted median
Weighted mode
0.50
0.25
0.00
−0.25
−0.50
0.0
0.5
1.0
1.5
2.0
SNP effect on Diabetic nephropathy
SNP effect on CM CD4+ AC

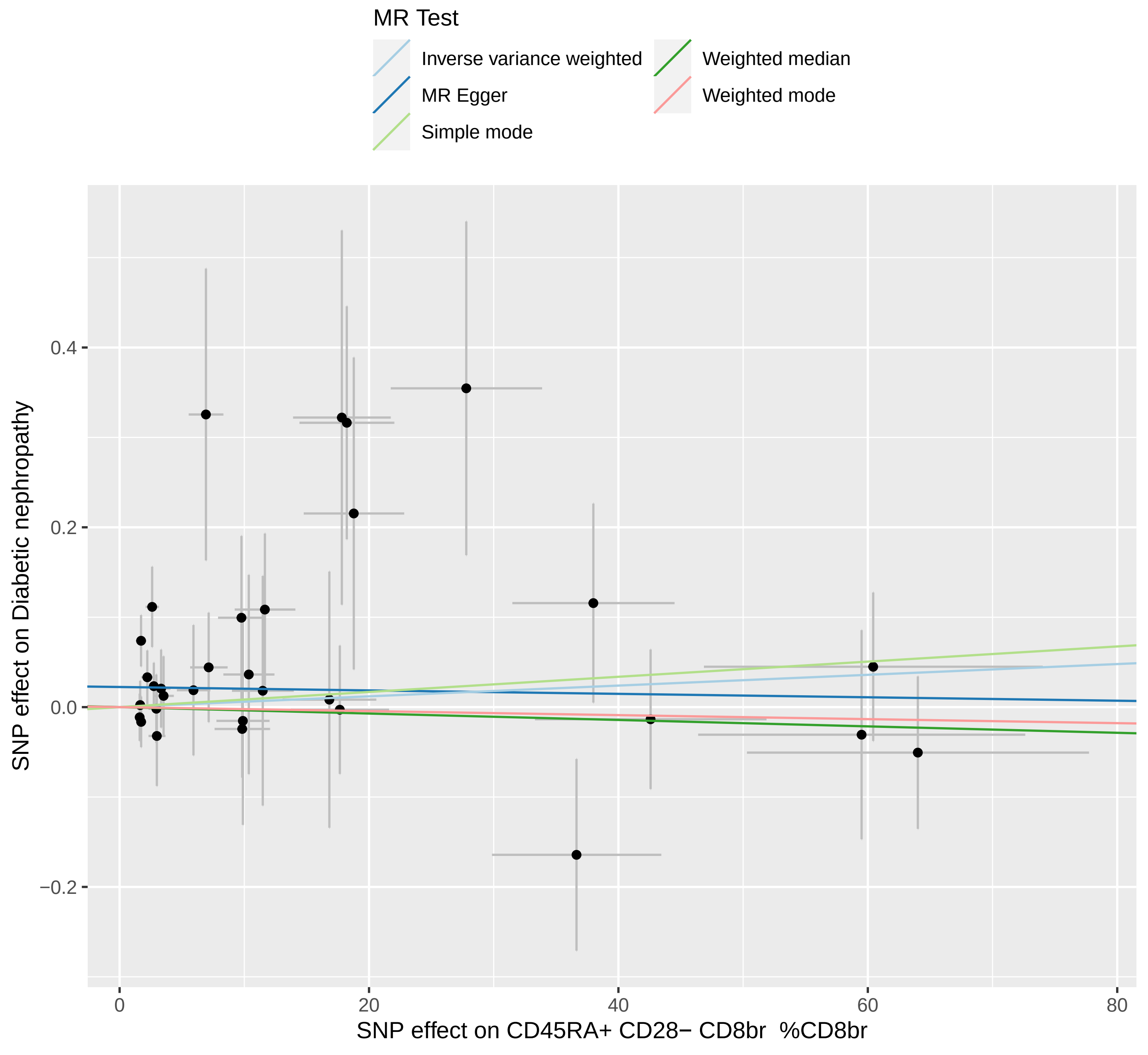
MR Test
Inverse variance weighted
MR Egger
Simple mode
Weighted median
Weighted mode
SNP effect on Diabetic nephropathy
0.4
0.2
0.0
−0.2
0
20
40
60
80
SNP effect on CD45RA+ CD28− CD8br %CD8br

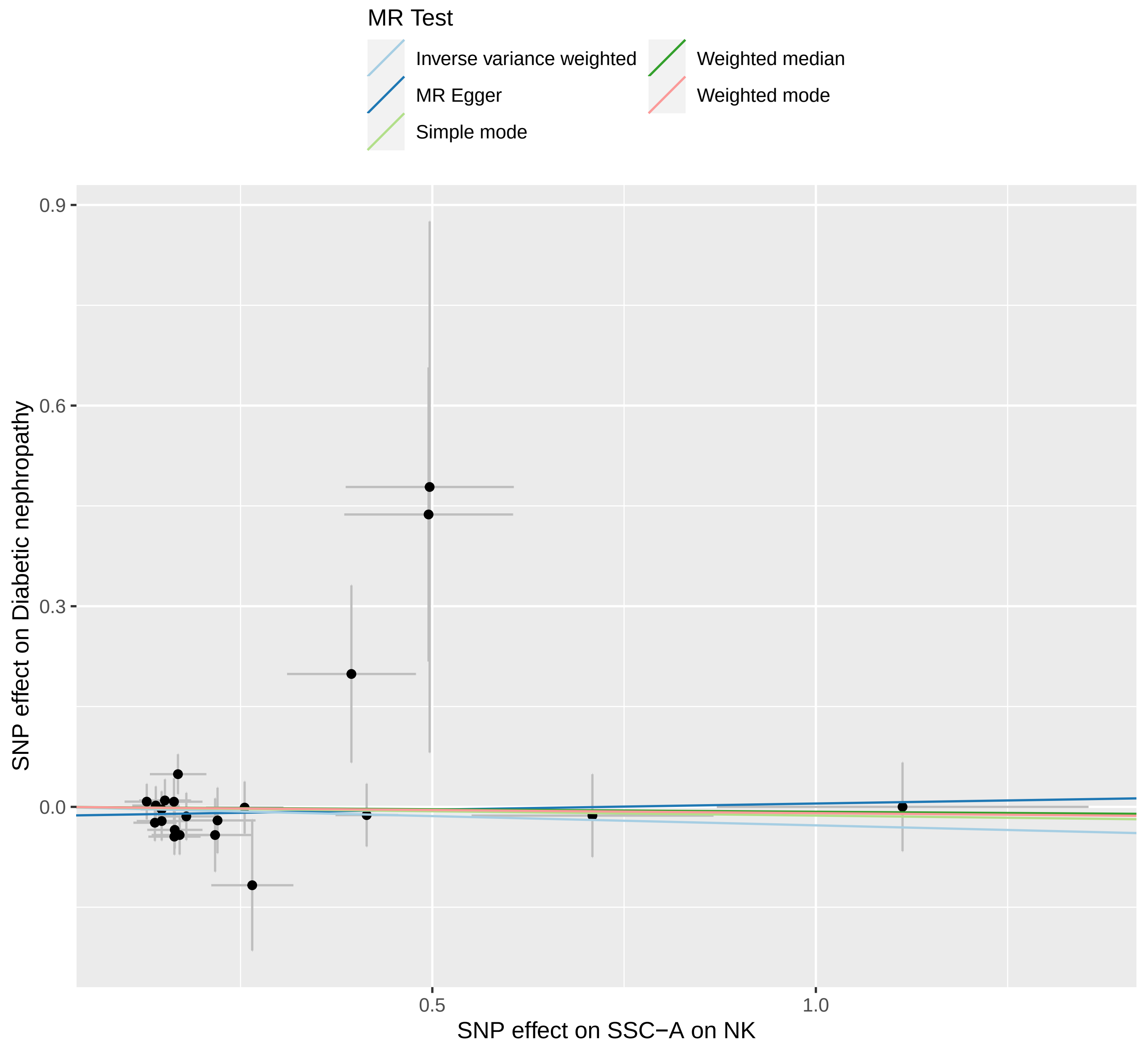

MR Test
Inverse variance weighted
MR Egger
Simple mode
Weighted median
Weighted mode
0.9
0.6
0.3
0.0
0.5
1.0
SNP effect on Diabetic nephropathy
SNP effect on SSC−A on NK

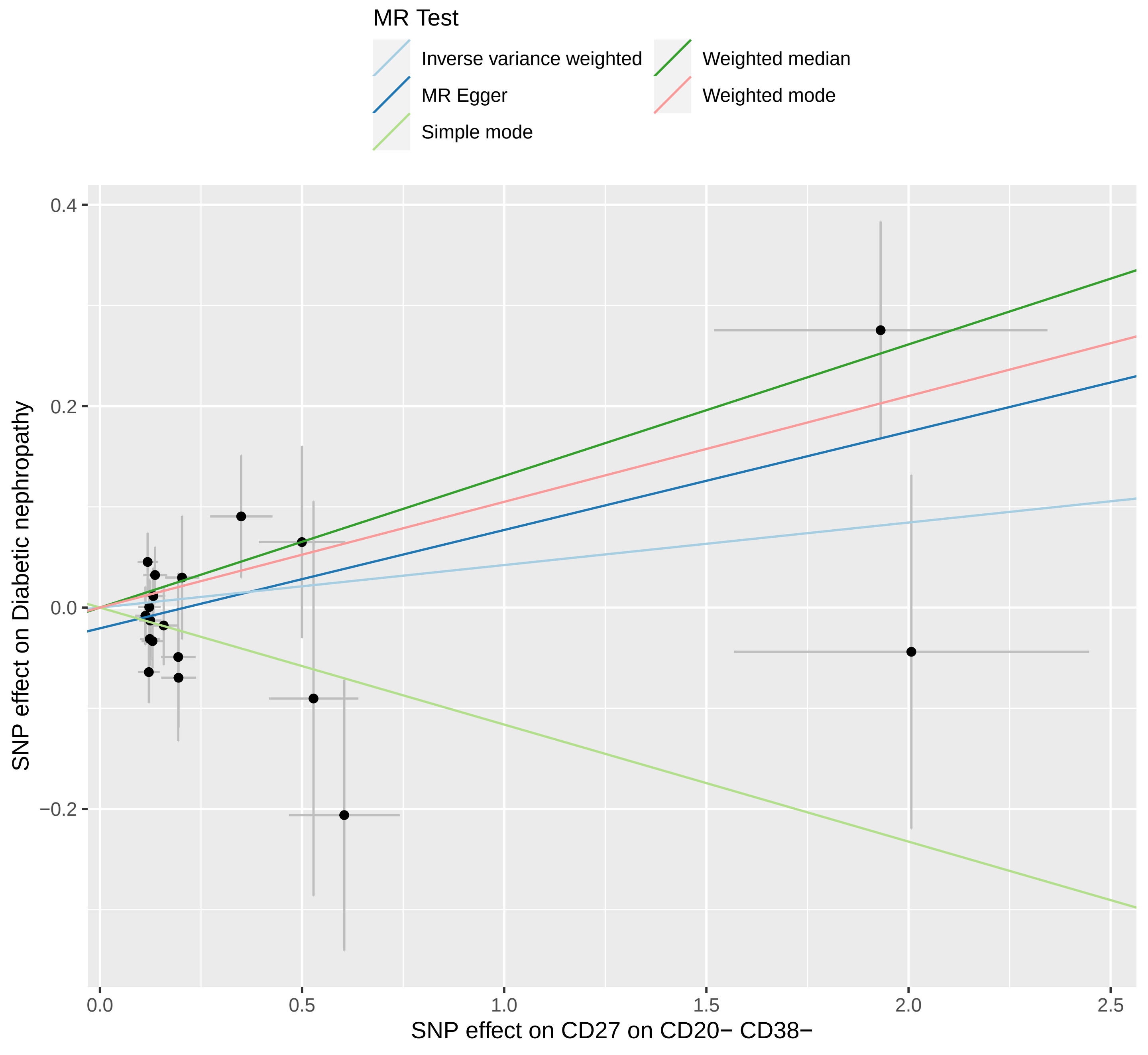
MR Test
Inverse variance weighted
MR Egger
Simple mode
Weighted median
Weighted mode
0.4
0.2
0.0
−0.2
0.0
0.5
1.0
1.5
2.0
2.5
SNP effect on Diabetic nephropathy
SNP effect on CD27 on CD20− CD38−

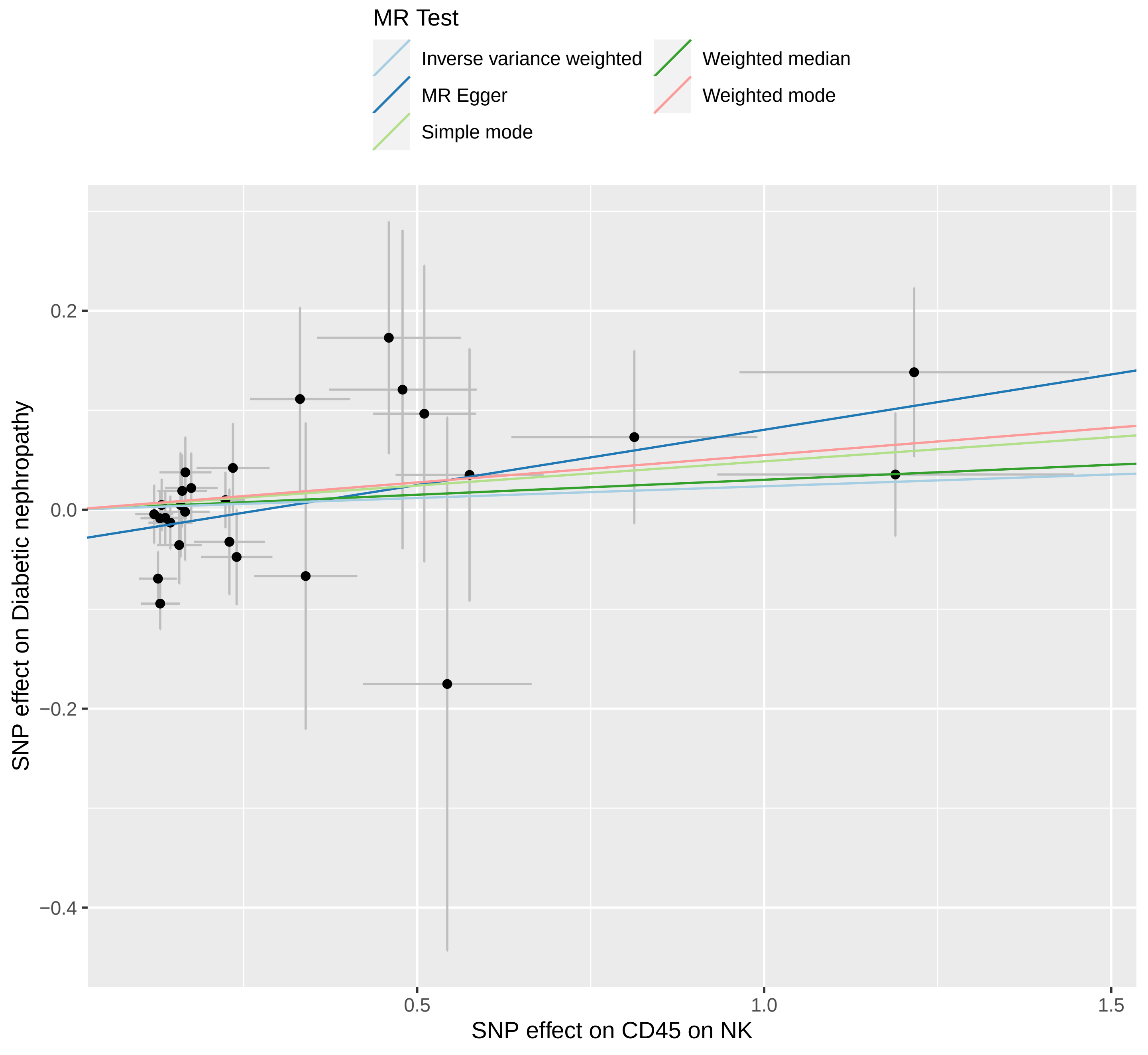
MR Test
Inverse variance weighted
MR Egger
Simple mode
Weighted median
Weighted mode
SNP effect on Diabetic nephropathy
0.2
0.0
−0.2
−0.4
0.5
1.0
1.5
SNP effect on CD45 on NK

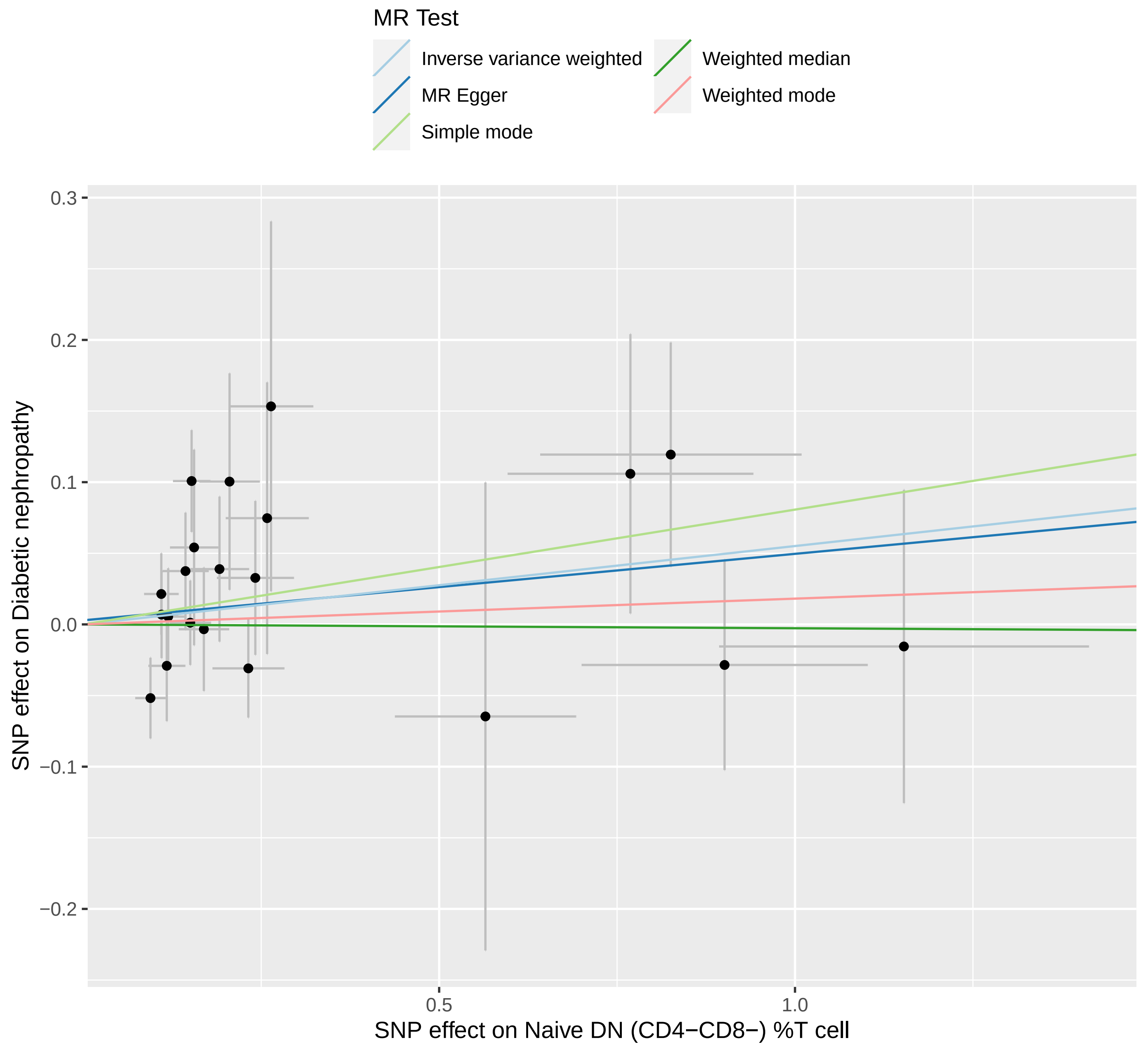
MR Test
Inverse variance weighted
MR Egger
Simple mode
Weighted median
Weighted mode
0.3
0.2
0.1
0.0
−0.1
−0.2
0.5
1.0
SNP effect on Diabetic nephropathy
SNP effect on Naive DN (CD4−CD8−) %T cell

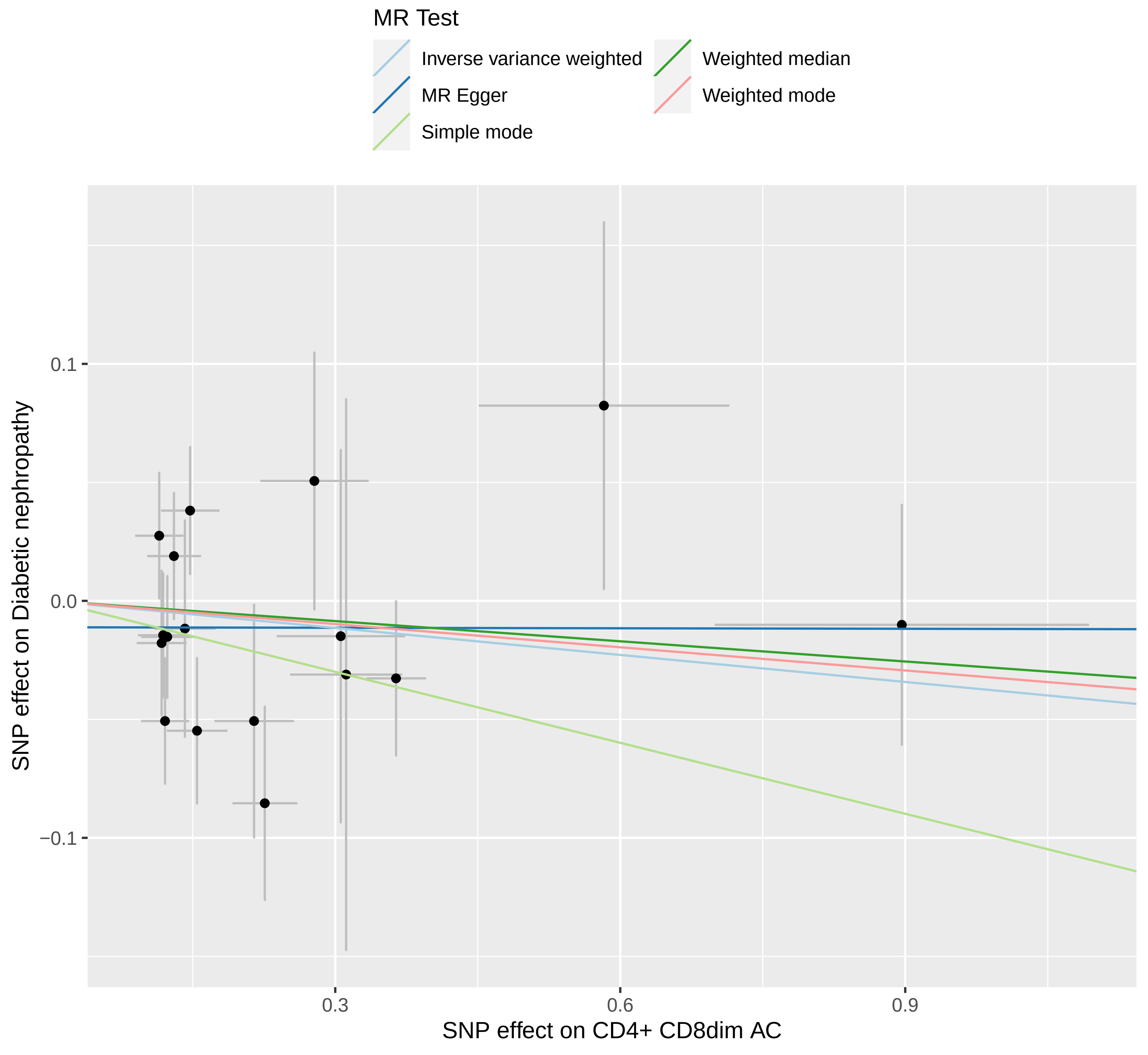

MR Test
Inverse variance weighted
MR Egger
Simple mode
Weighted median
Weighted mode
0.1
0.0
−0.1
0.3
0.6
0.9
SNP effect on Diabetic nephropathy
SNP effect on CD4+ CD8dim AC

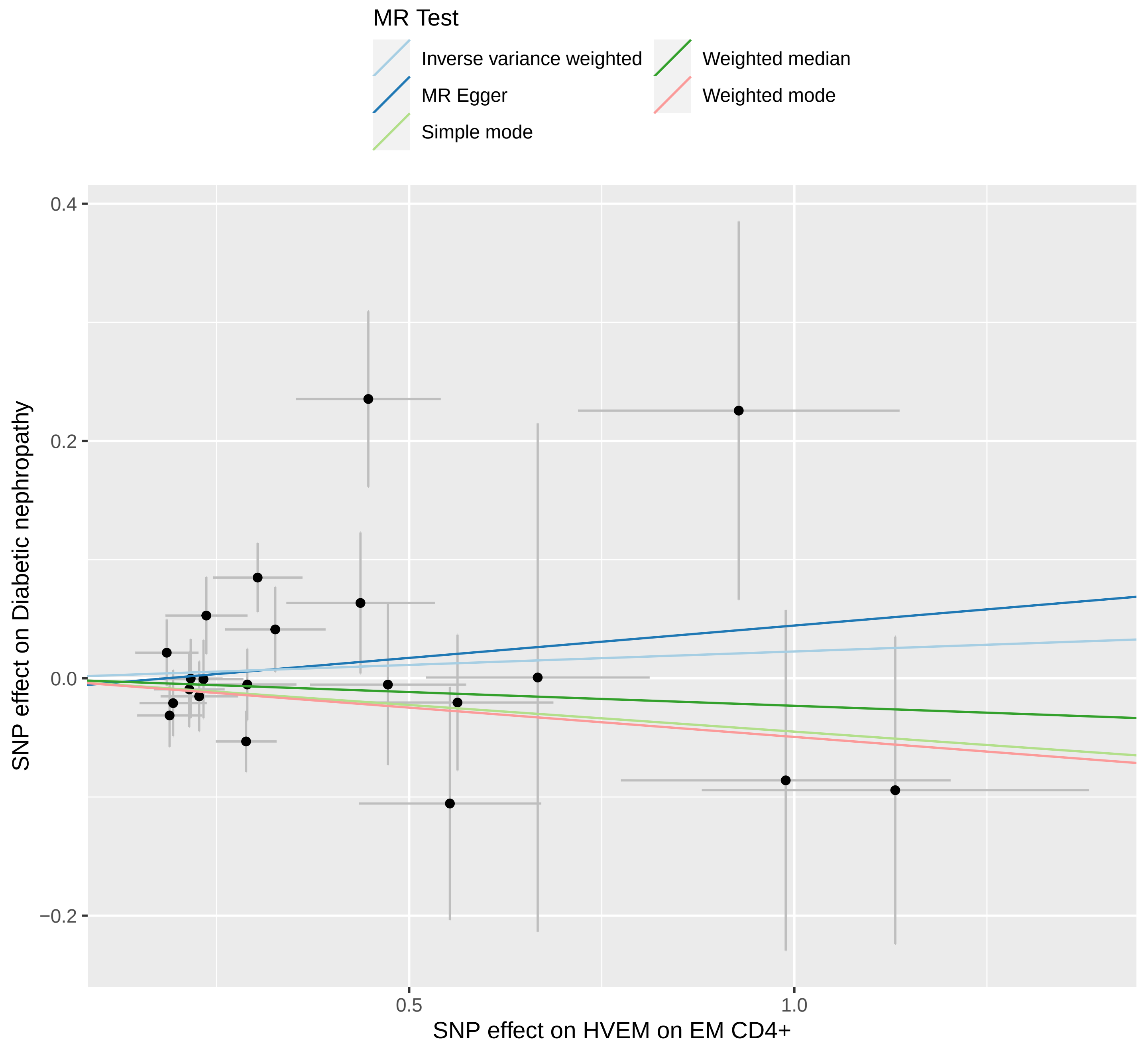

MR Test
Inverse variance weighted
MR Egger
Simple mode
Weighted median
Weighted mode
0.4
0.2
0.0
−0.2
0.5
1.0
SNP effect on Diabetic nephropathy
SNP effect on HVEM on EM CD4+

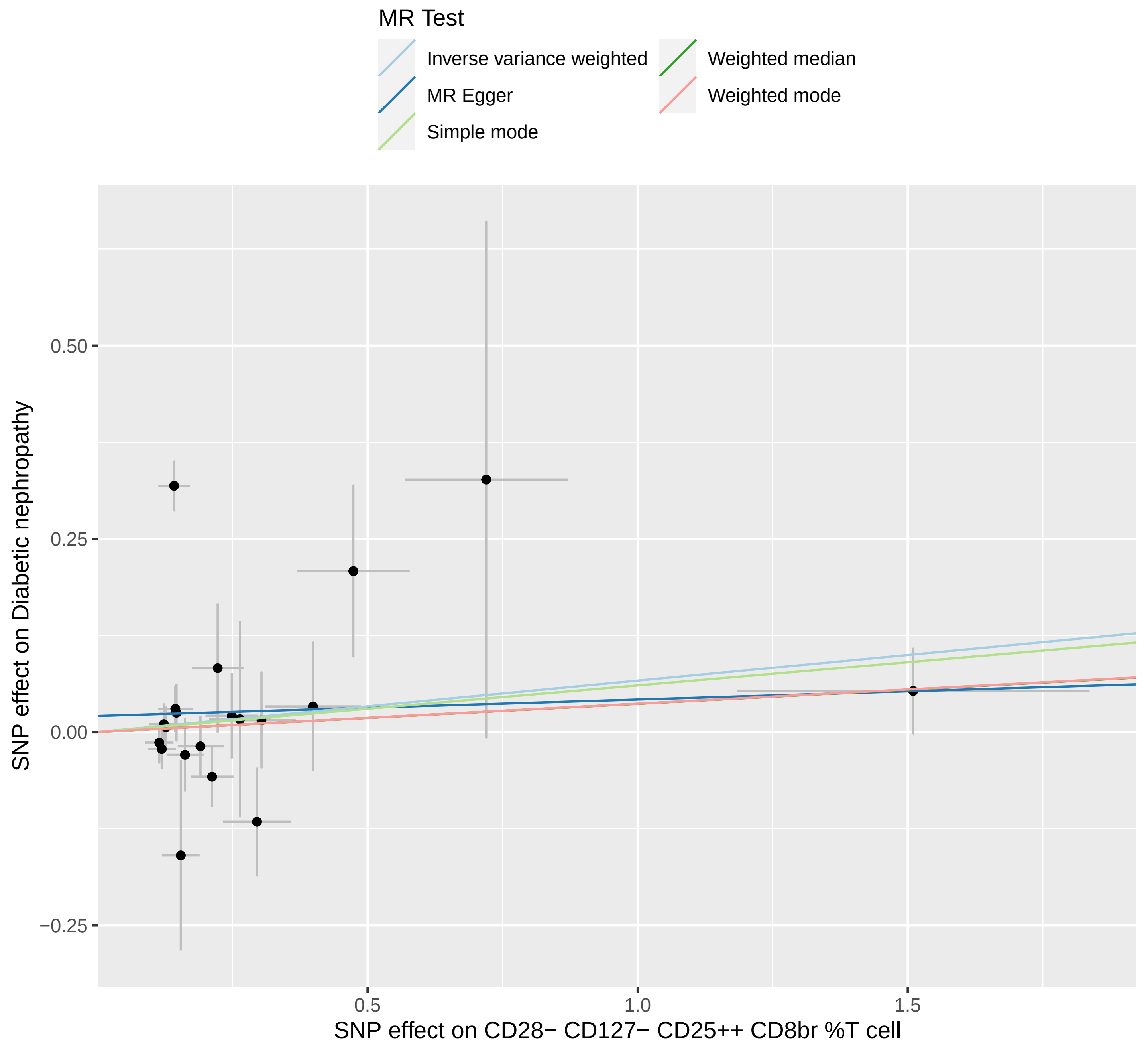

MR Test
Inverse variance weighted
MR Egger
Simple mode
Weighted median
Weighted mode
0.50
0.25
0.00
−0.25
0.5
1.0
1.5
SNP effect on Diabetic nephropathy
SNP effect on CD28− CD127− CD25++ CD8br %T cell

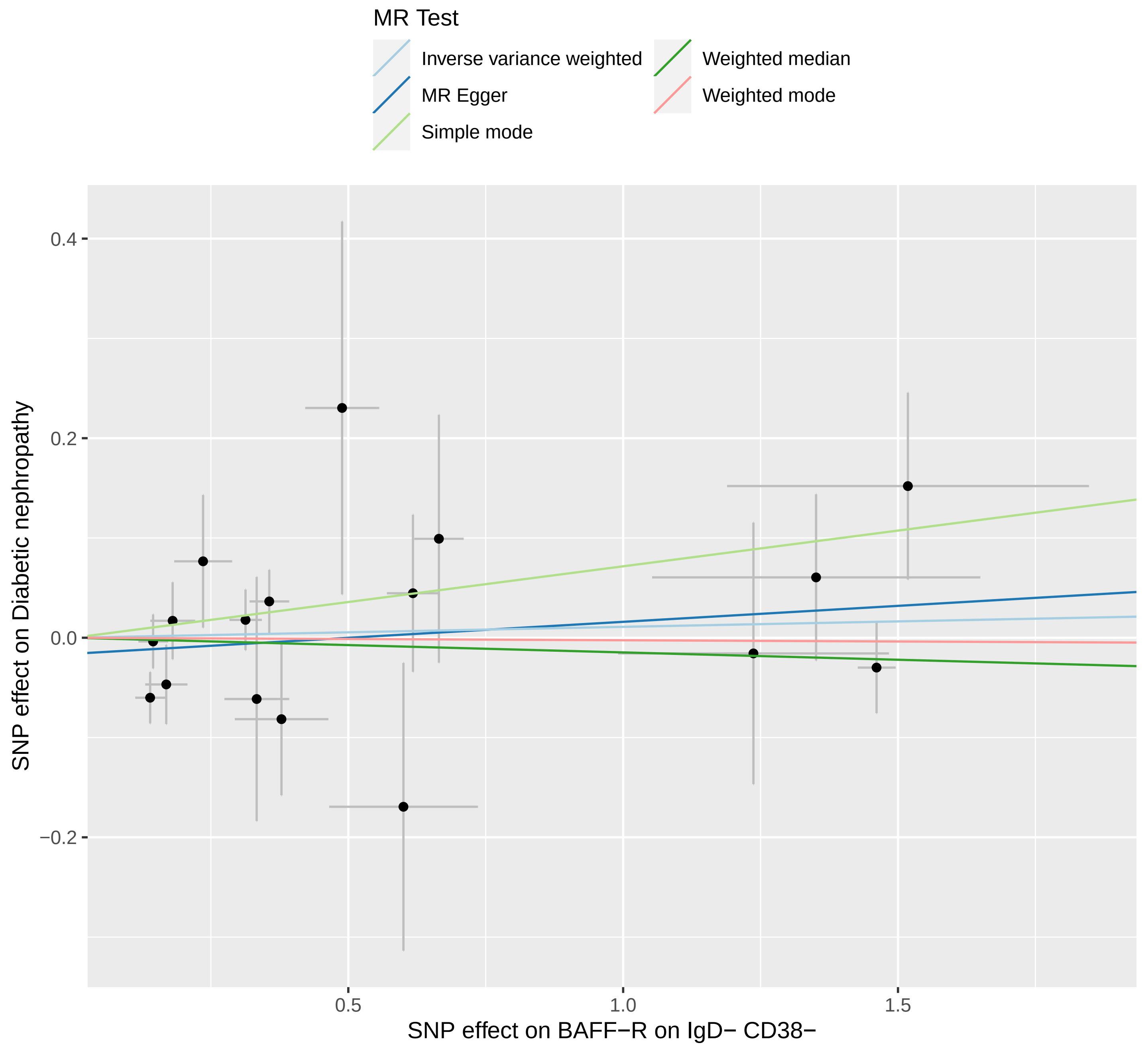
MR Test
Inverse variance weighted
MR Egger
Simple mode
Weighted median
Weighted mode
0.4
0.2
0.0
−0.2
0.5
1.0
1.5
SNP effect on Diabetic nephropathy
SNP effect on BAFF−R on IgD− CD38−

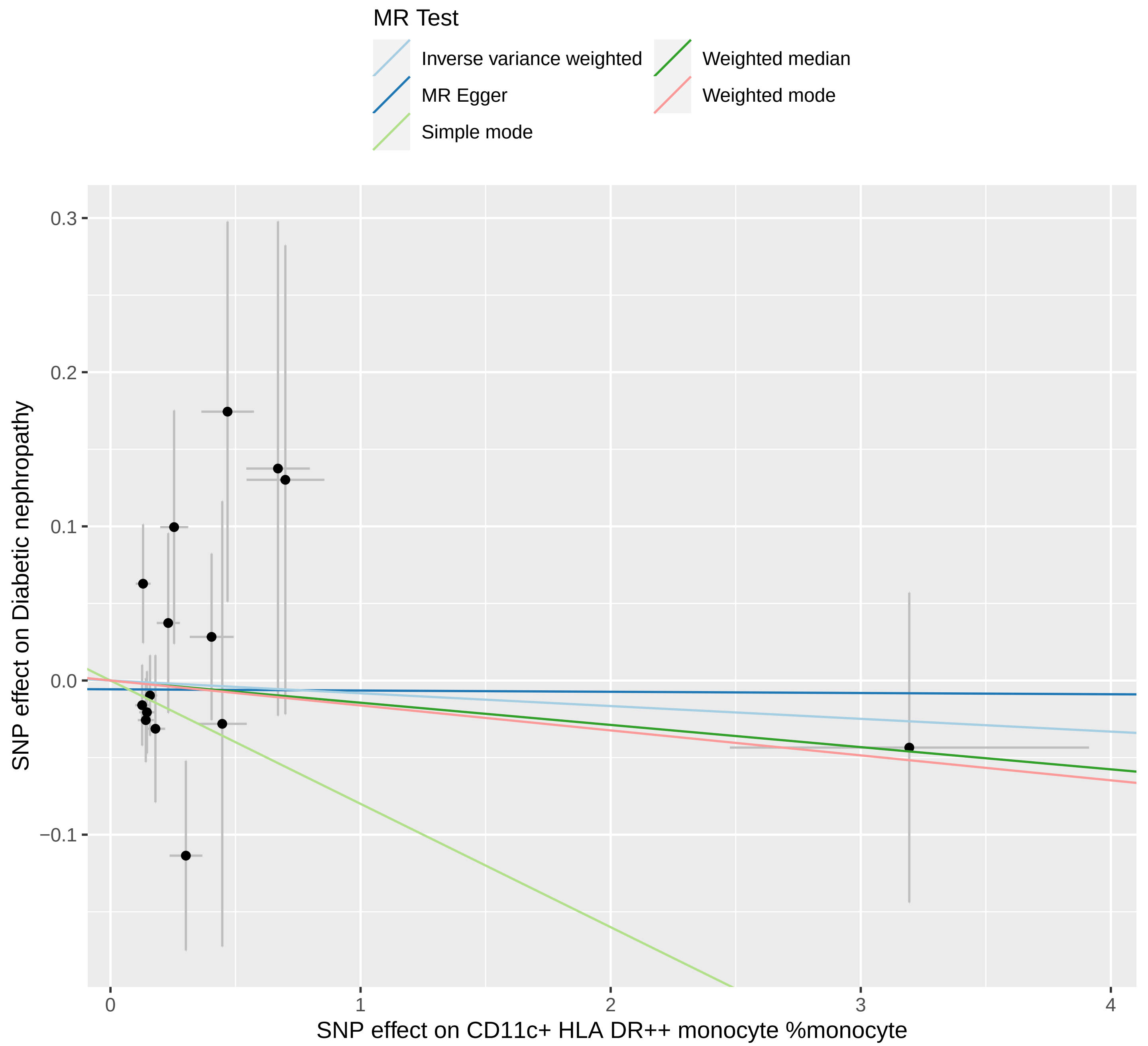
MR Test
Inverse variance weighted
MR Egger
Simple mode
Weighted median
Weighted mode
0.3
0.2
0.1
0.0
−0.1
SNP effect on Diabetic nephropathy
0
1
2
3
4
SNP effect on CD11c+ HLA DR++ monocyte %monocyte

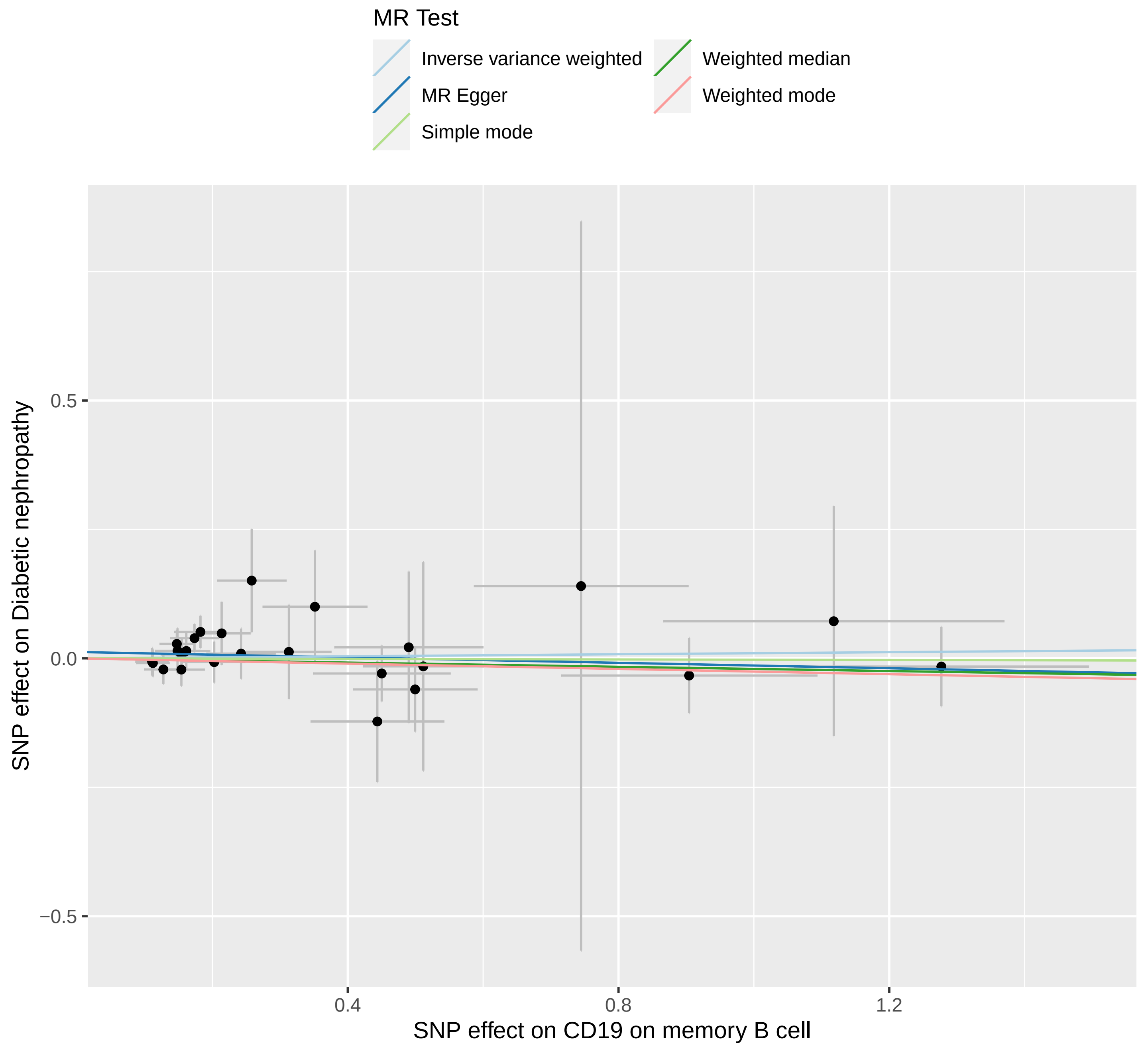
MR Test
Inverse variance weighted
MR Egger
Simple mode
Weighted median
Weighted mode
0.5
0.0
−0.5
0.4
0.8
1.2
SNP effect on Diabetic nephropathy
SNP effect on CD19 on memory B cell

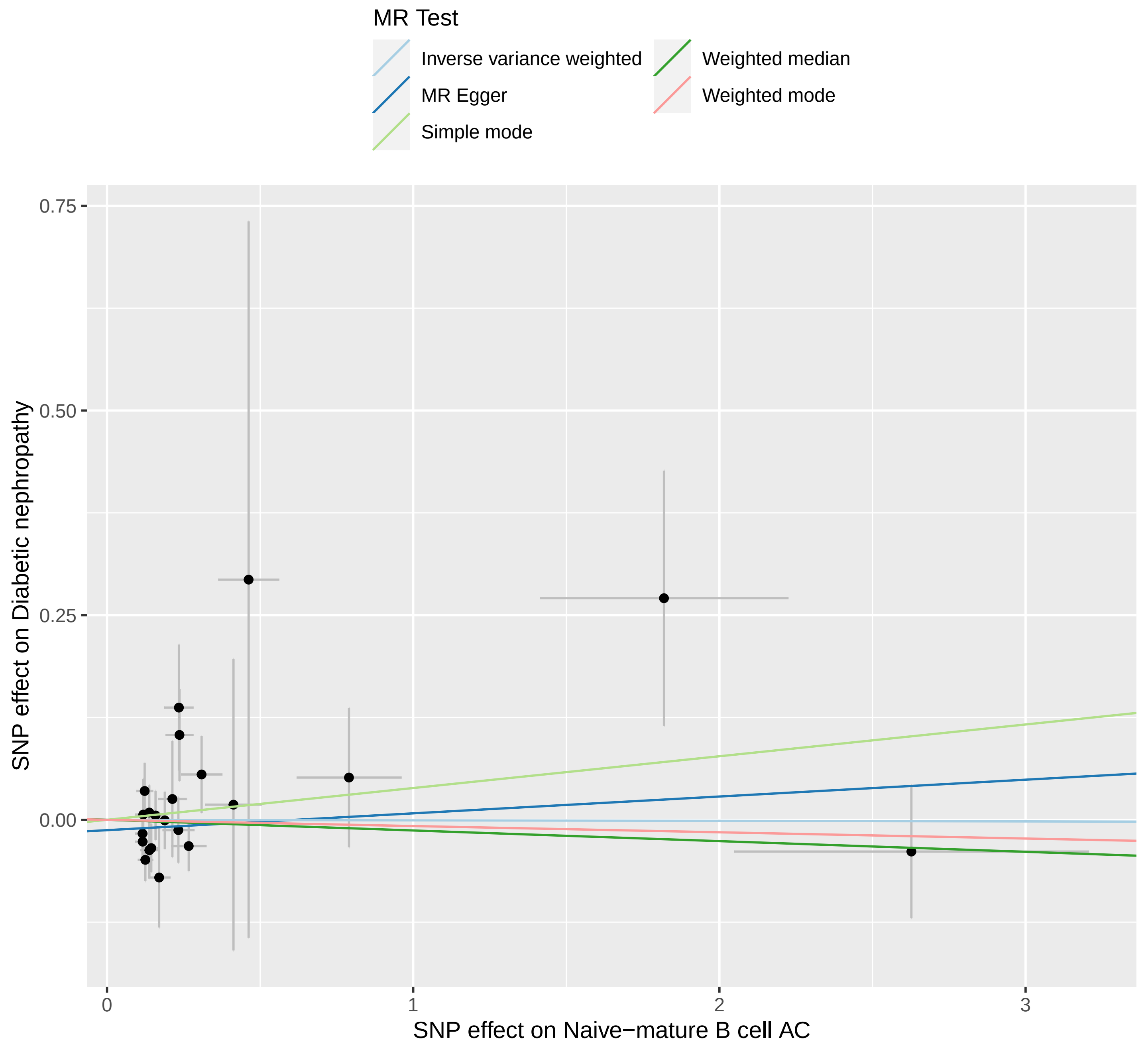

MR Test
Inverse variance weighted
MR Egger
Simple mode
Weighted median
Weighted mode
0.75
0.50
0.25
0.00
SNP effect on Diabetic nephropathy
0
1
2
3
SNP effect on Naive−mature B cell AC

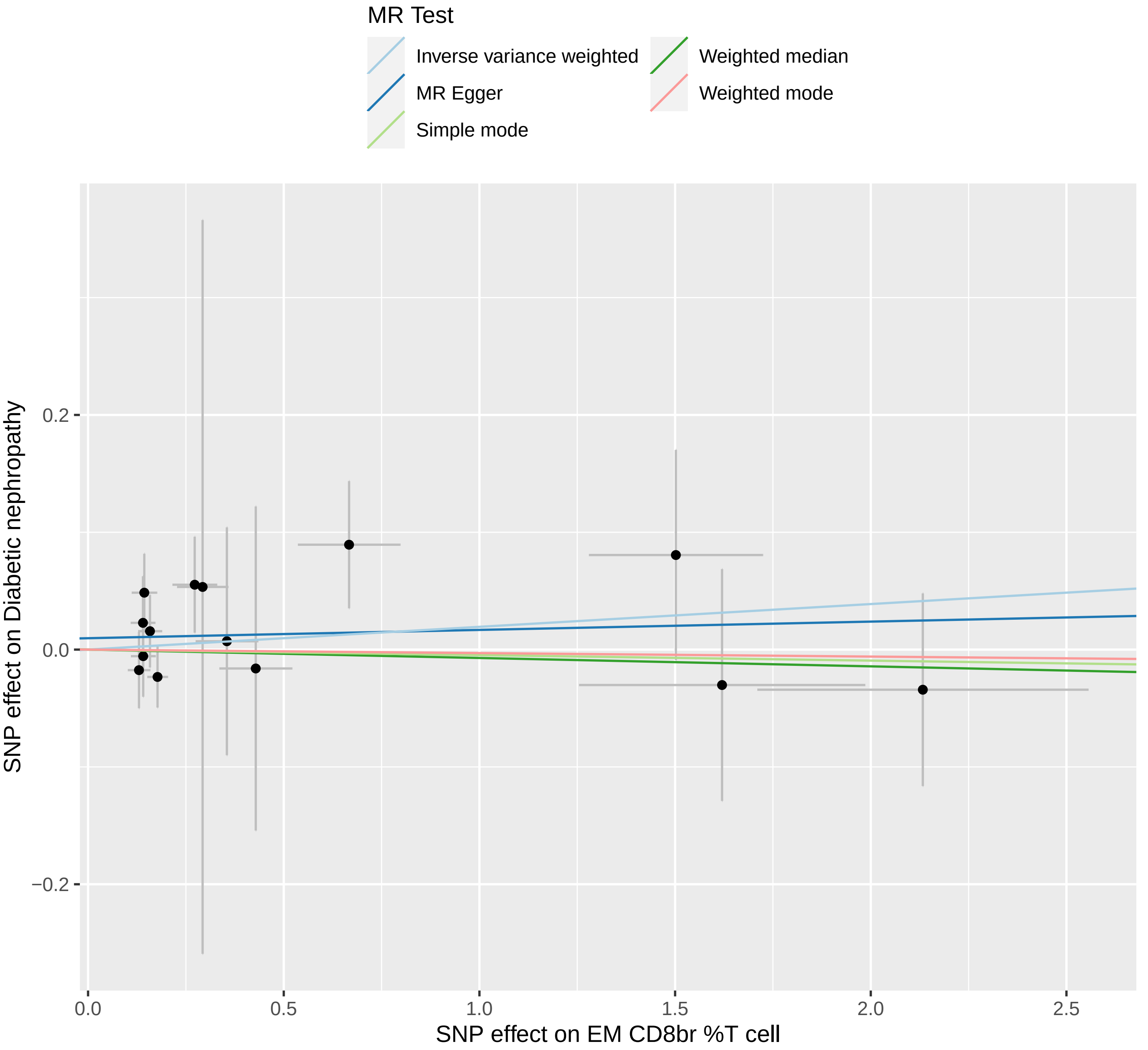

MR Test
Inverse variance weighted
MR Egger
Simple mode
Weighted median
Weighted mode
SNP effect on Diabetic nephropathy
0.2
0.0
−0.2
0.0
0.5
1.0
1.5
2.0
2.5
SNP effect on EM CD8br %T cell

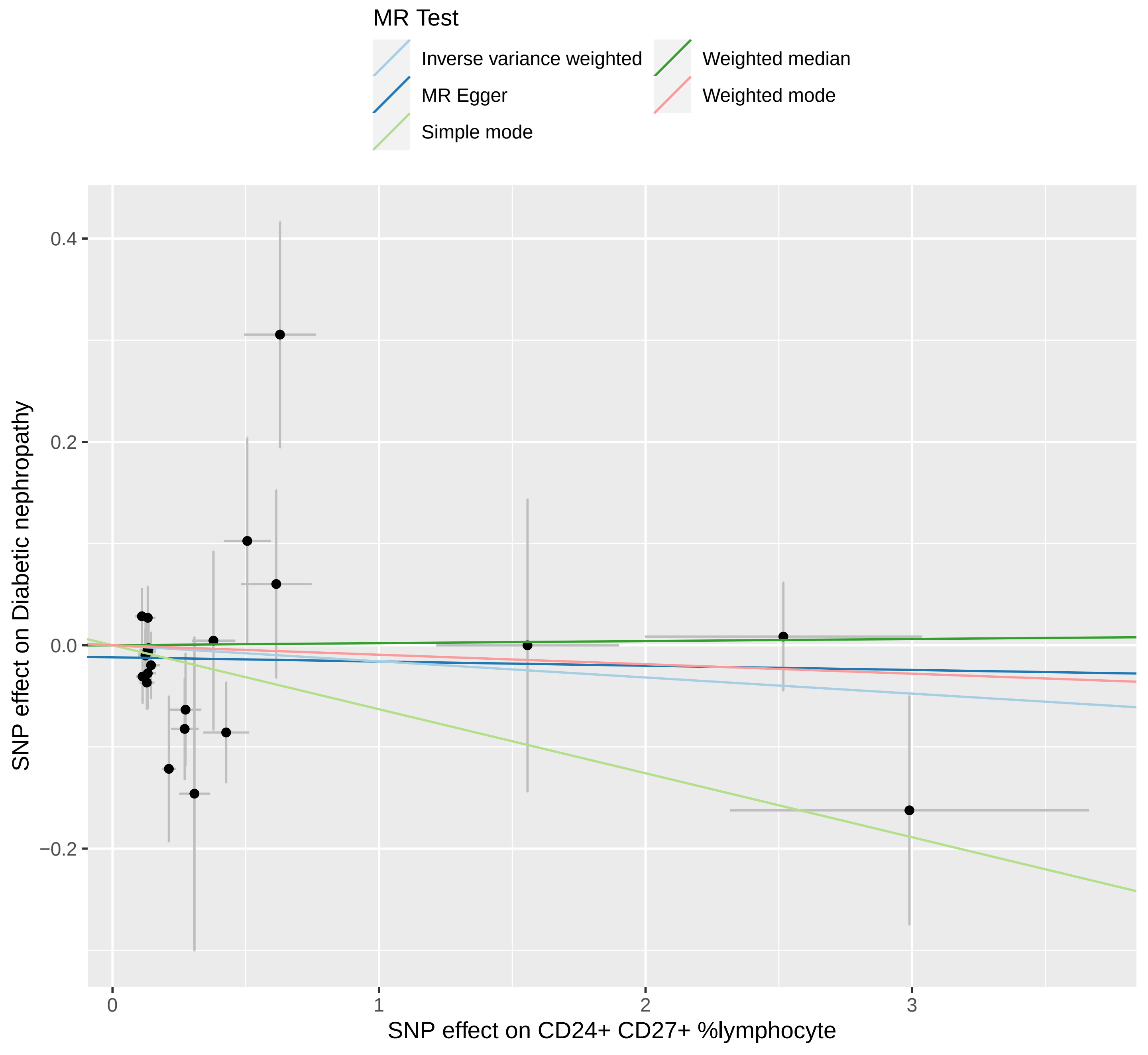

MR Test
Inverse variance weighted
MR Egger
Simple mode
Weighted median
Weighted mode
SNP effect on Diabetic nephropathy
0.4
0.2
0.0
−0.2
0
1
2
3
SNP effect on CD24+ CD27+ %lymphocyte

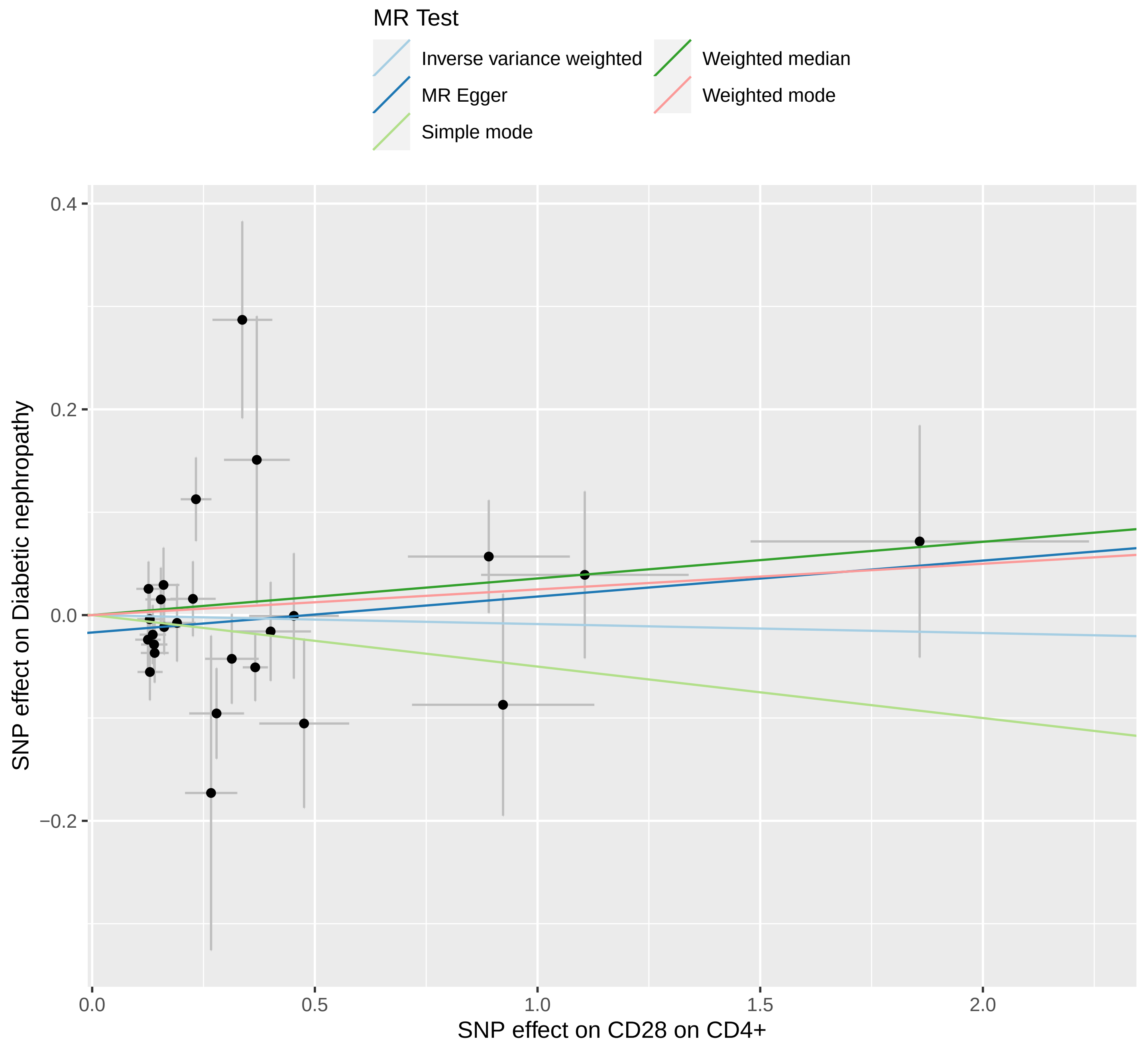
MR Test
Inverse variance weighted
MR Egger
Simple mode
Weighted median
Weighted mode
0.4
0.2
0.0
−0.2
SNP effect on Diabetic nephropathy
0.0
0.5
1.0
1.5
2.0
SNP effect on CD28 on CD4+

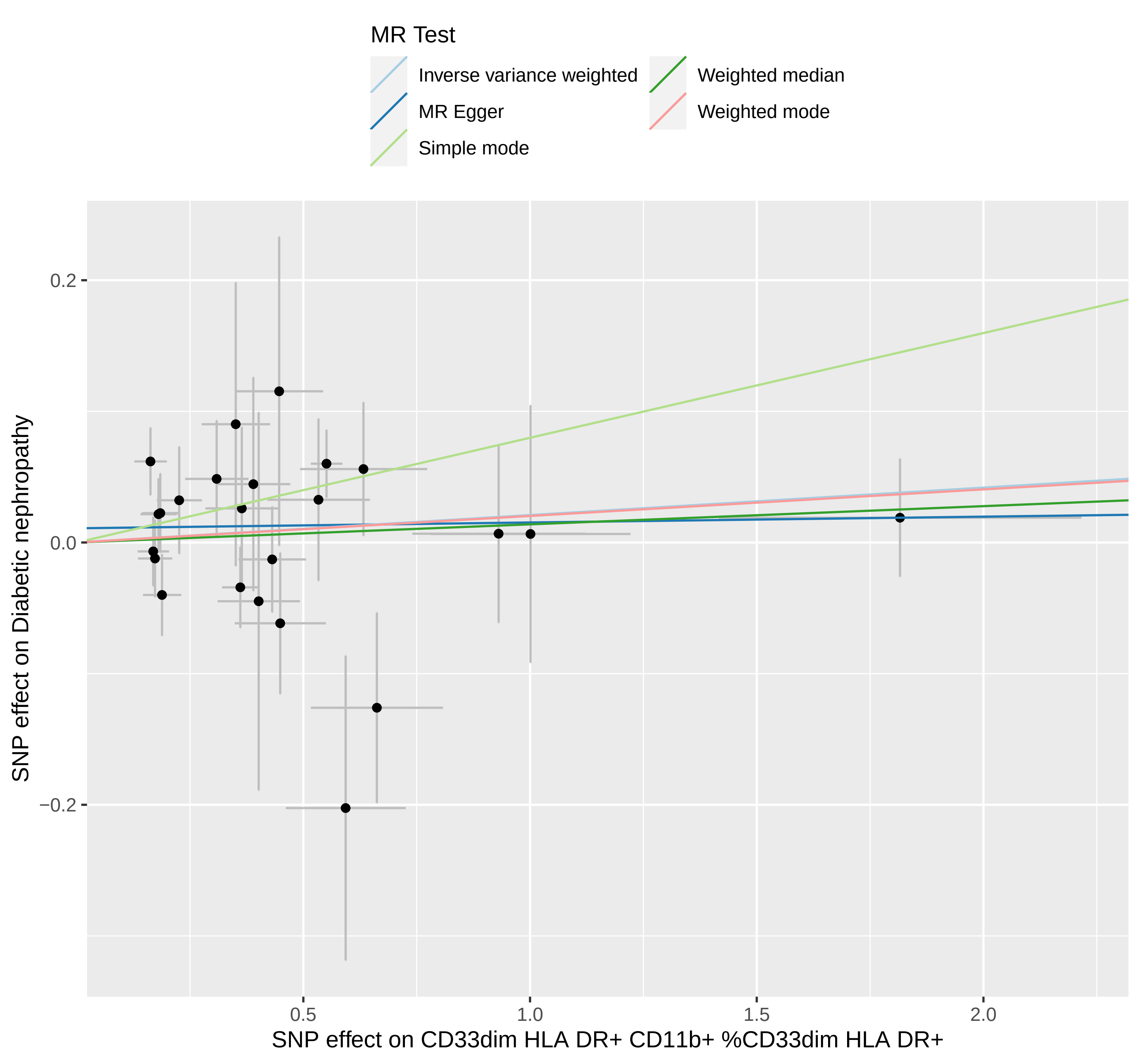

MR Test
Inverse variance weighted
MR Egger
Simple mode
Weighted median
Weighted mode
SNP effect on Diabetic nephropathy
0.2
0.0
−0.2
0.5
1.0
1.5
2.0
SNP effect on CD33dim HLA DR+ CD11b+ %CD33dim HLA DR+

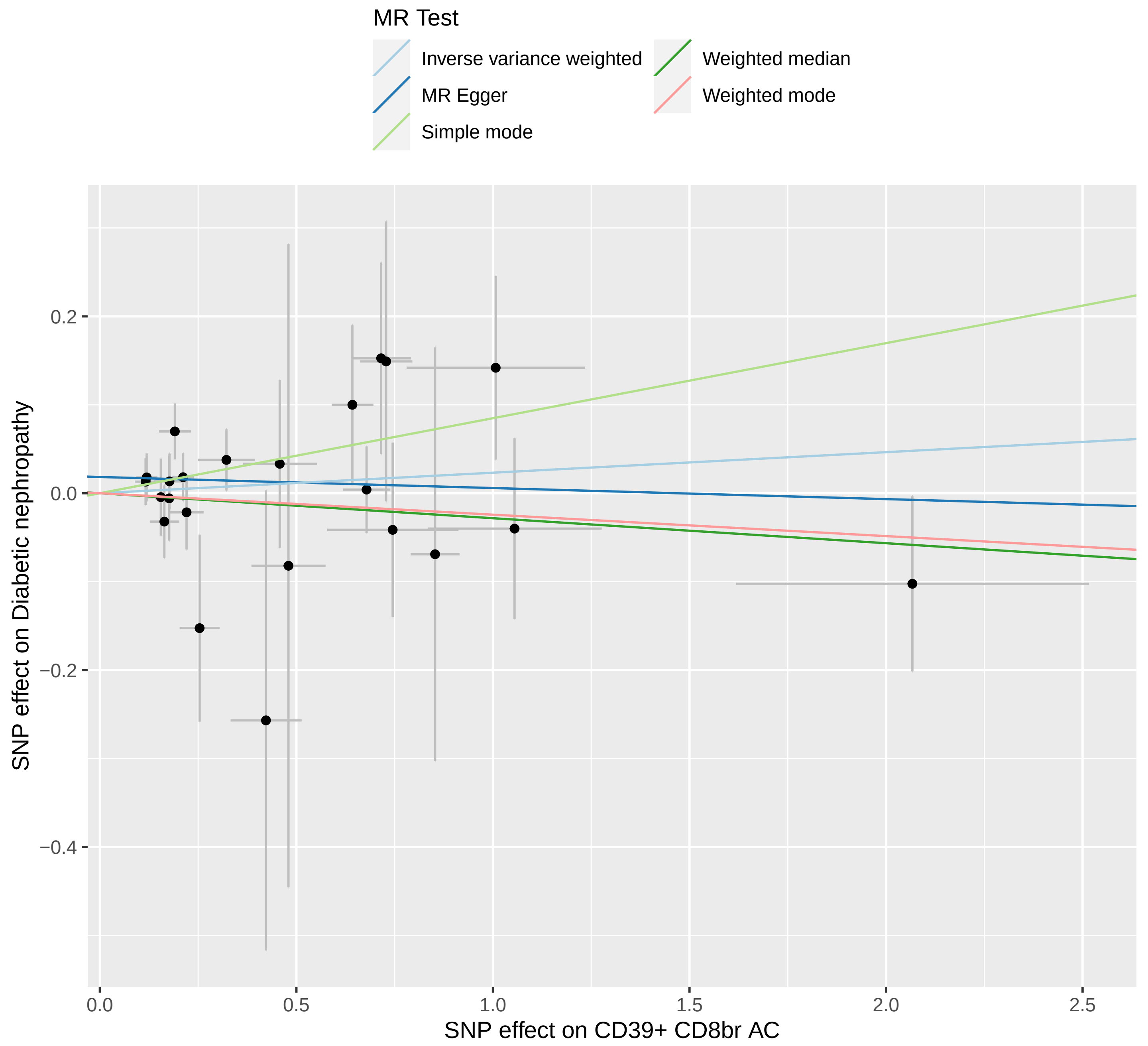
MR Test
Inverse variance weighted
MR Egger
Simple mode
Weighted median
Weighted mode
SNP effect on Diabetic nephropathy
0.2
0.0
−0.2
−0.4
0.0
0.5
1.0
1.5
2.0
2.5
SNP effect on CD39+ CD8br AC

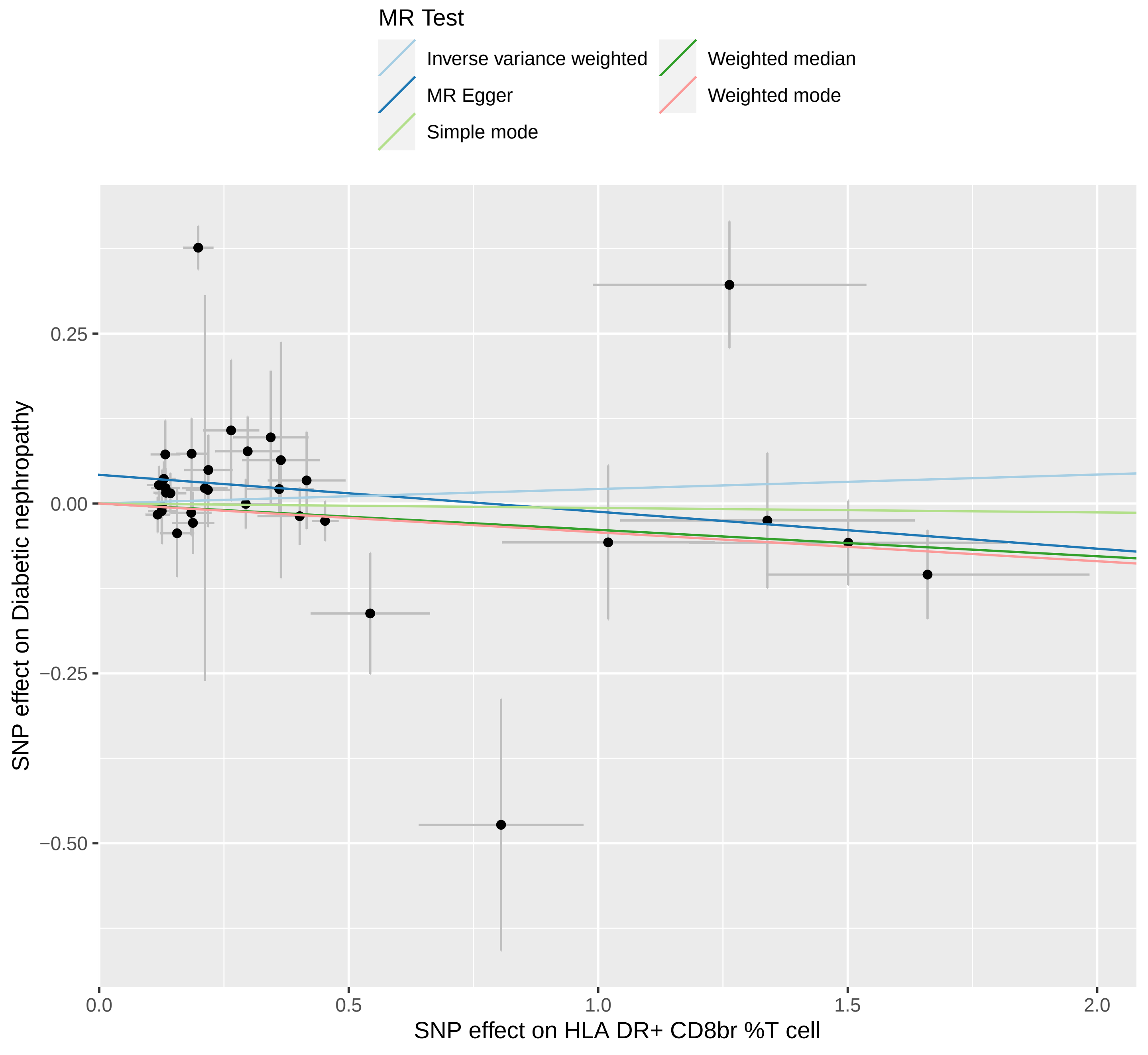
MR Test
Inverse variance weighted
MR Egger
Simple mode
Weighted median
Weighted mode
SNP effect on Diabetic nephropathy
0.25
0.00
−0.25
−0.50
0.0
0.5
1.0
1.5
2.0
SNP effect on HLA DR+ CD8br %T cell

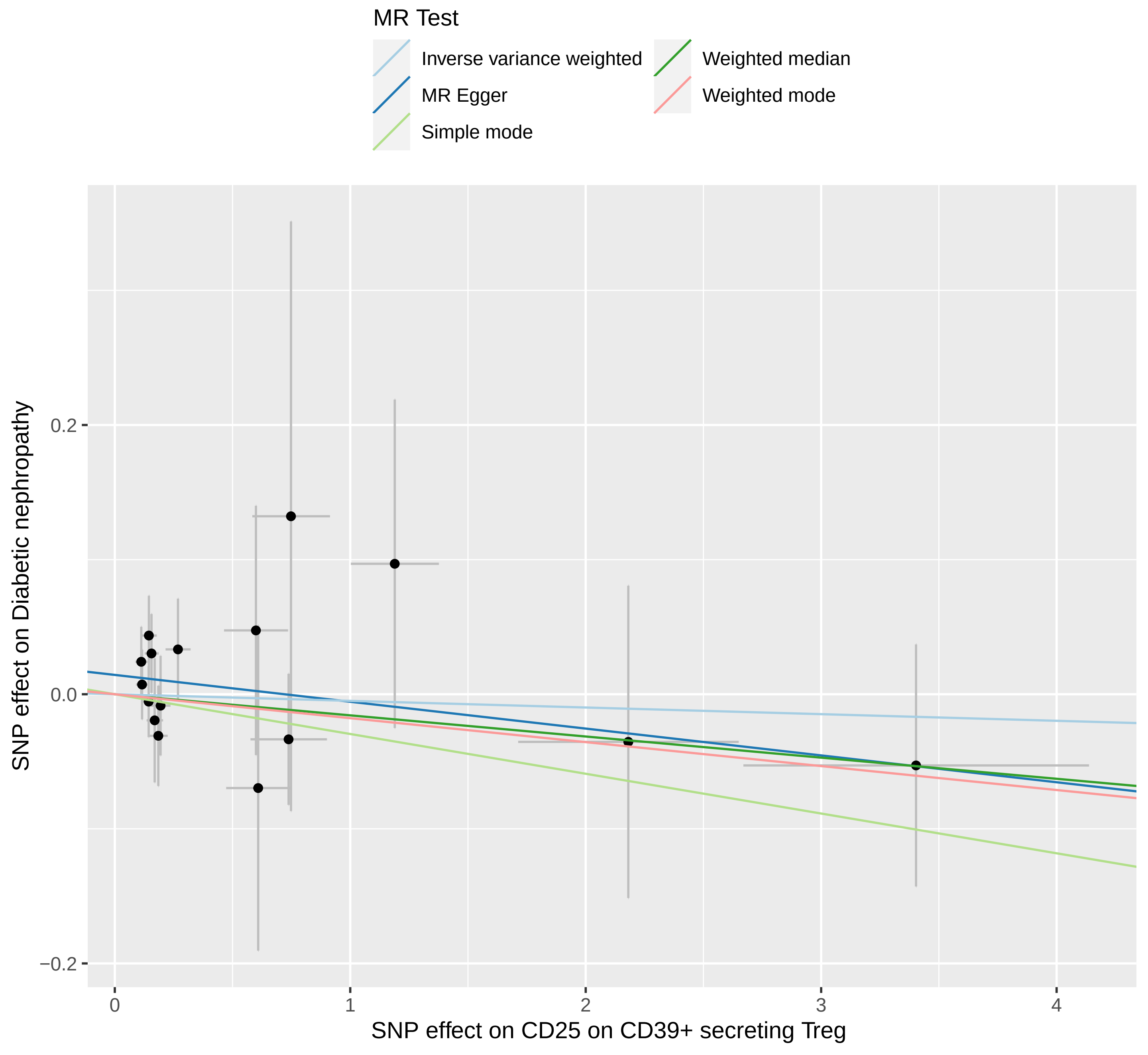
MR Test
Inverse variance weighted
MR Egger
Simple mode
Weighted median
Weighted mode
SNP effect on Diabetic nephropathy
0.2
0.0
−0.2
0
1
2
3
4
SNP effect on CD25 on CD39+ secreting Treg

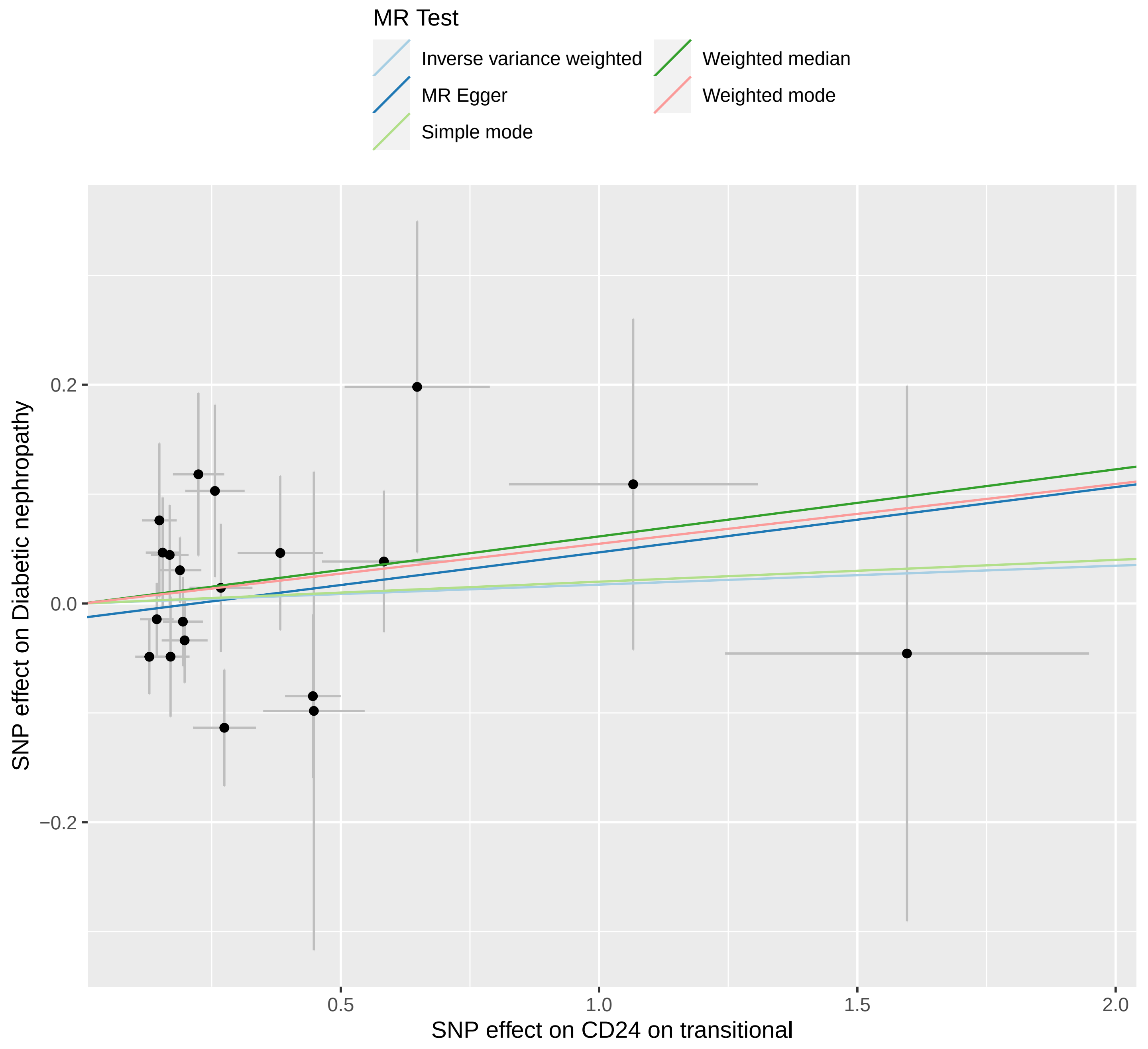

MR Test
Inverse variance weighted
MR Egger
Simple mode
Weighted median
Weighted mode
SNP effect on Diabetic nephropathy
0.2
0.0
−0.2
0.5
1.0
1.5
2.0
SNP effect on CD24 on transitional

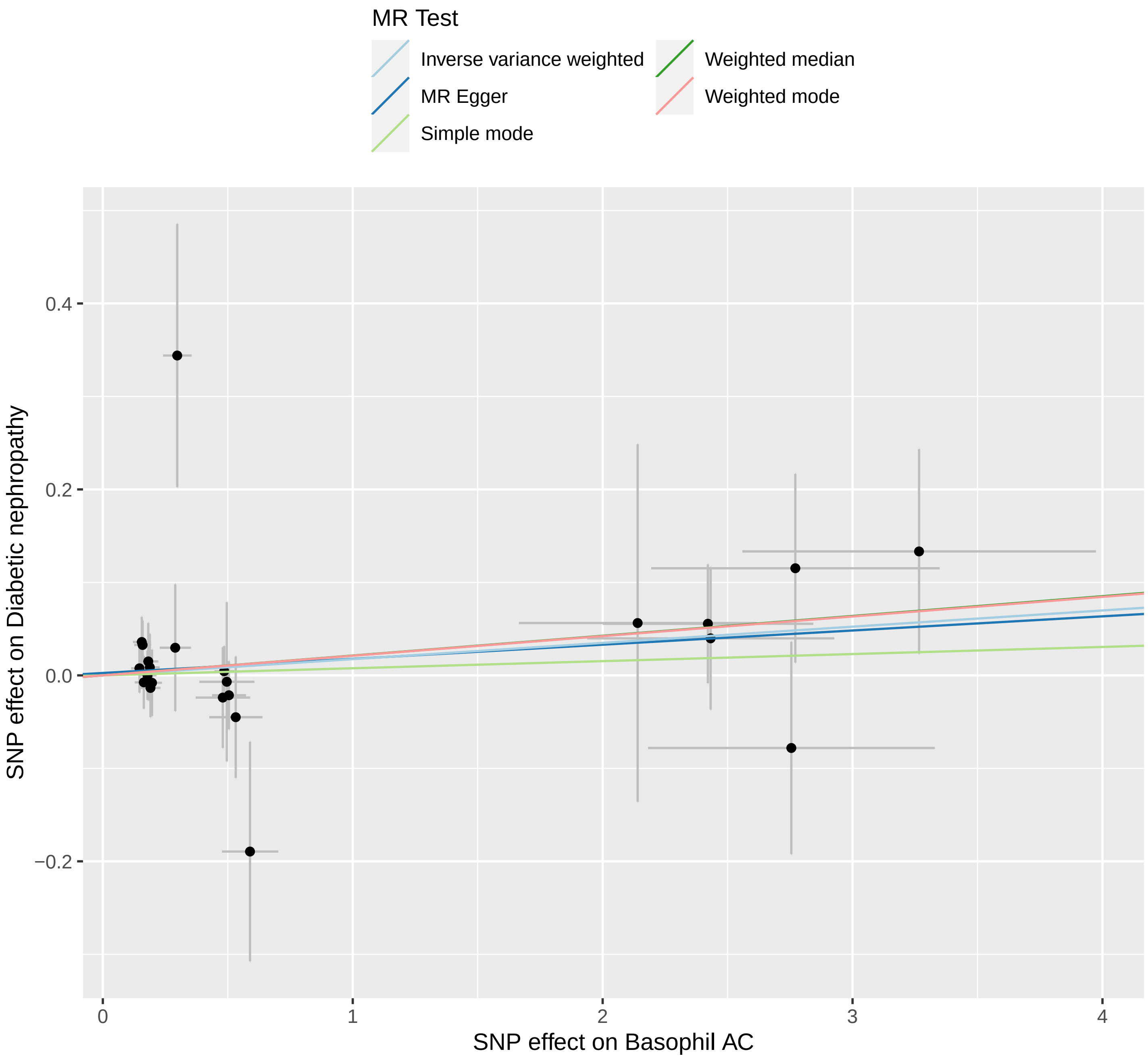

MR Test
Inverse variance weighted
MR Egger
Simple mode
Weighted median
Weighted mode
0.4
0.2
0.0
−0.2
0
1
2
3
4
SNP effect on Diabetic nephropathy
SNP effect on Basophil AC

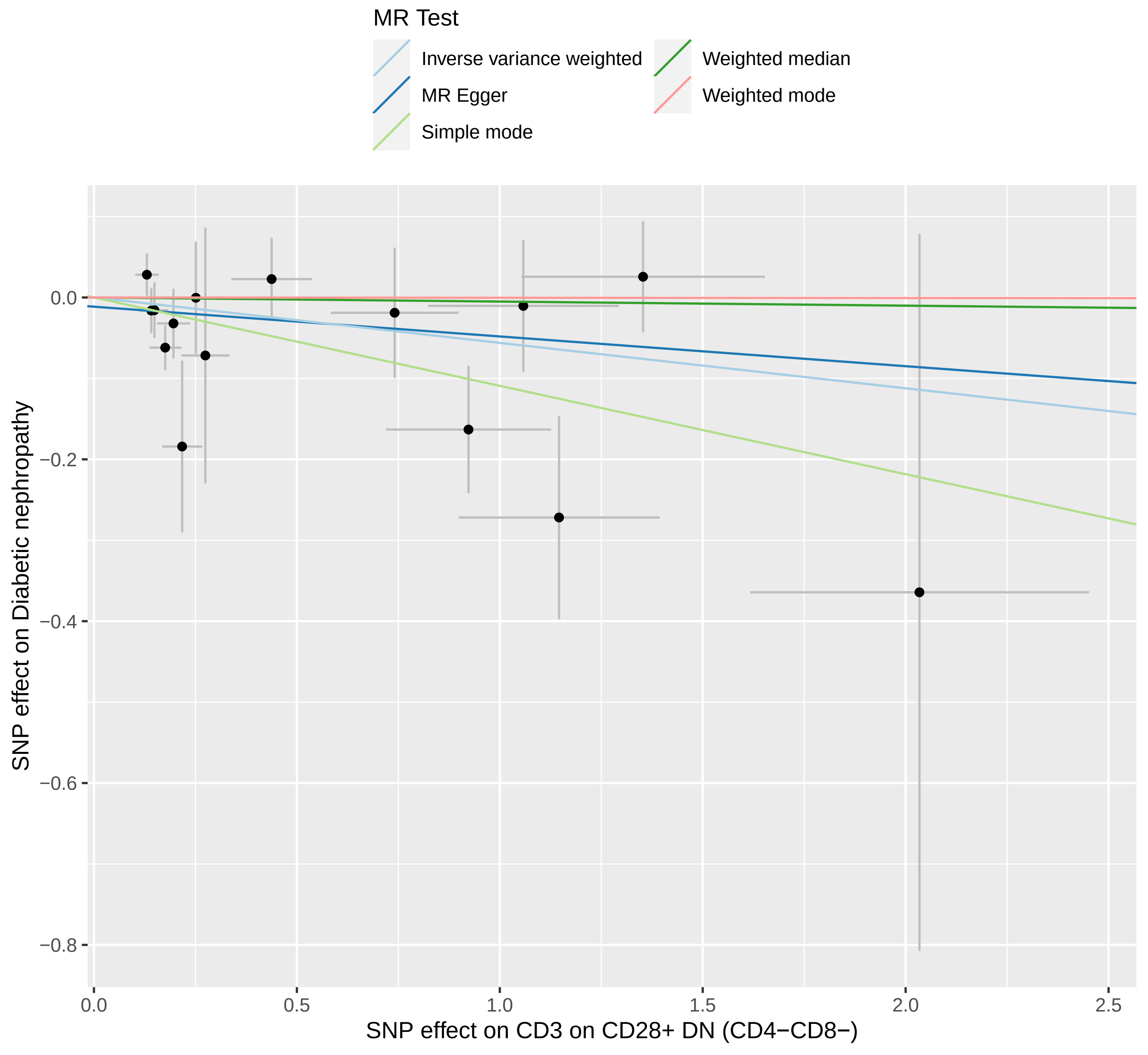
MR Test
Inverse variance weighted
MR Egger
Simple mode
Weighted median
Weighted mode
SNP effect on Diabetic nephropathy
0.0
−0.2
−0.4
−0.6
−0.8
0.0
0.5
1.0
1.5
2.0
2.5
SNP effect on CD3 on CD28+ DN (CD4−CD8−)

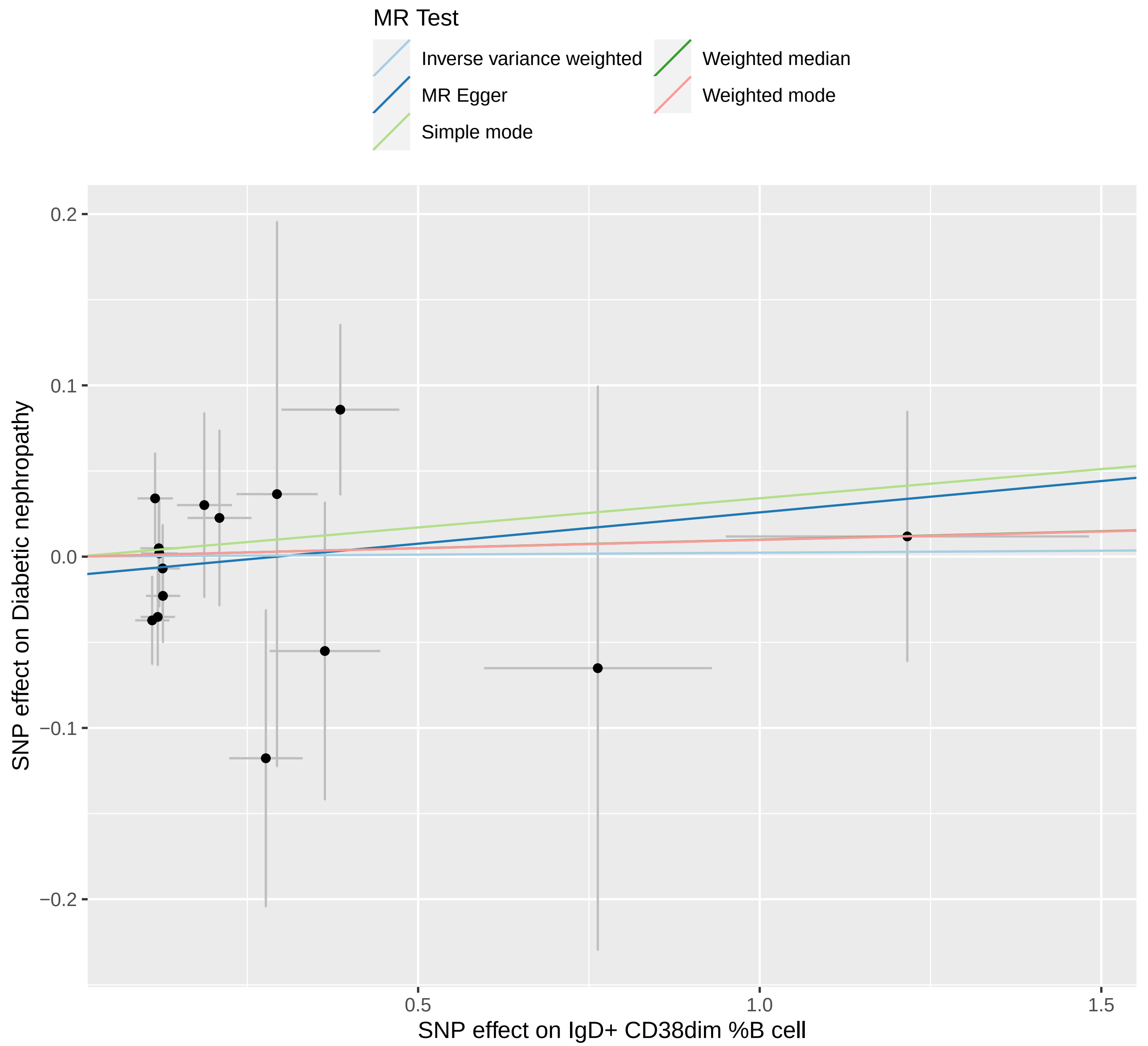

MR Test
Inverse variance weighted
MR Egger
Simple mode
Weighted median
Weighted mode
0.2
0.1
0.0
−0.1
−0.2
SNP effect on Diabetic nephropathy
0.5
1.0
1.5
SNP effect on IgD+ CD38dim %B cell

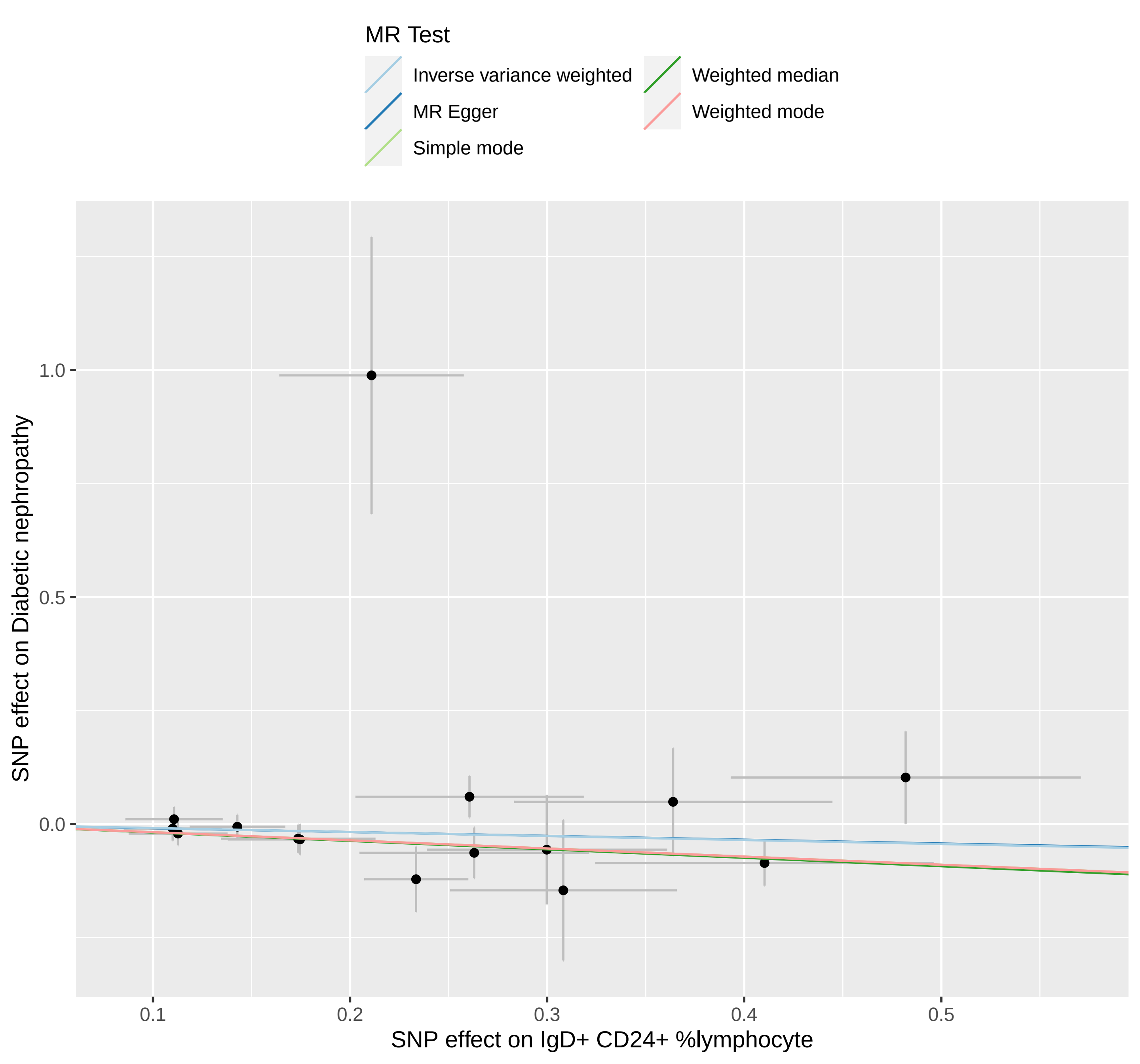
MR Test
Inverse variance weighted
MR Egger
Simple mode
Weighted median
Weighted mode
SNP effect on Diabetic nephropathy
1.0
0.5
0.0
0.1
0.2
0.3
0.4
0.5
SNP effect on IgD+ CD24+ %lymphocyte

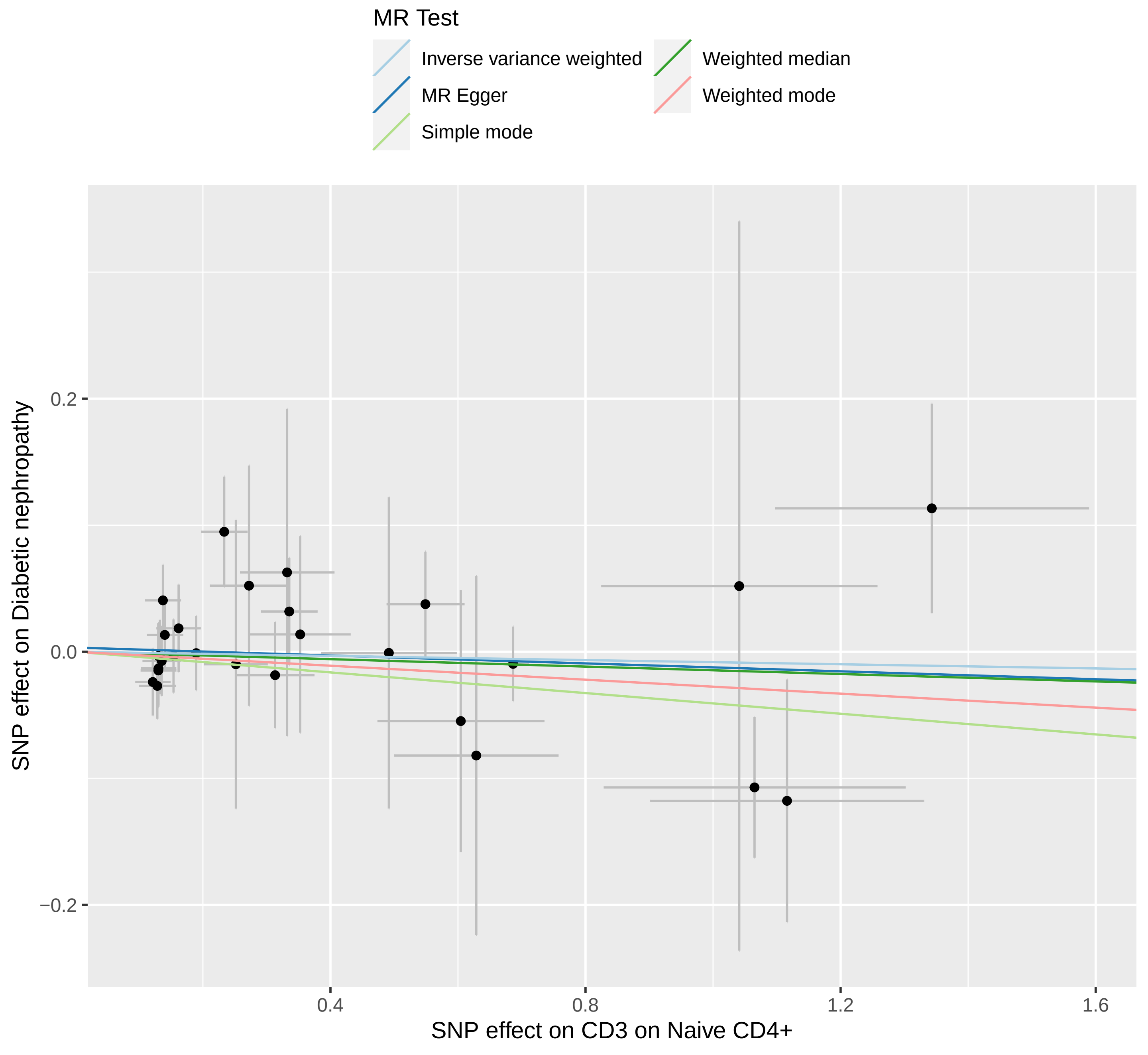
MR Test
Inverse variance weighted
MR Egger
Simple mode
Weighted median
Weighted mode
SNP effect on Diabetic nephropathy
0.2
0.0
−0.2
0.4
0.8
1.2
1.6
SNP effect on CD3 on Naive CD4+

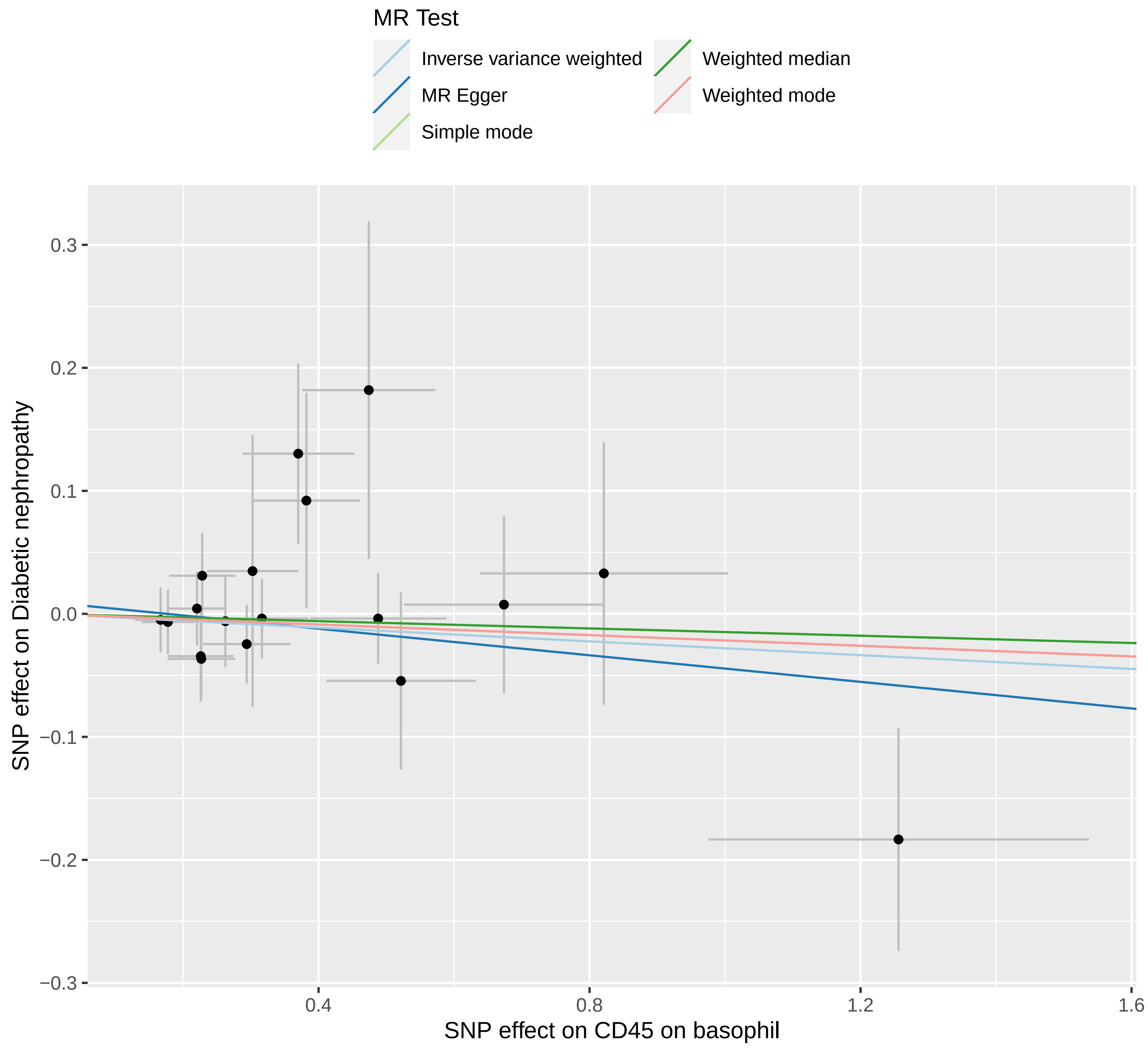
MR Test
Inverse variance weighted
MR Egger
Simple mode
Weighted median
Weighted mode
0.3
0.2
0.1
0.0
−0.1
−0.2
−0.3
SNP effect on Diabetic nephropathy
0.4
0.8
1.2
1.6
SNP effect on CD45 on basophil

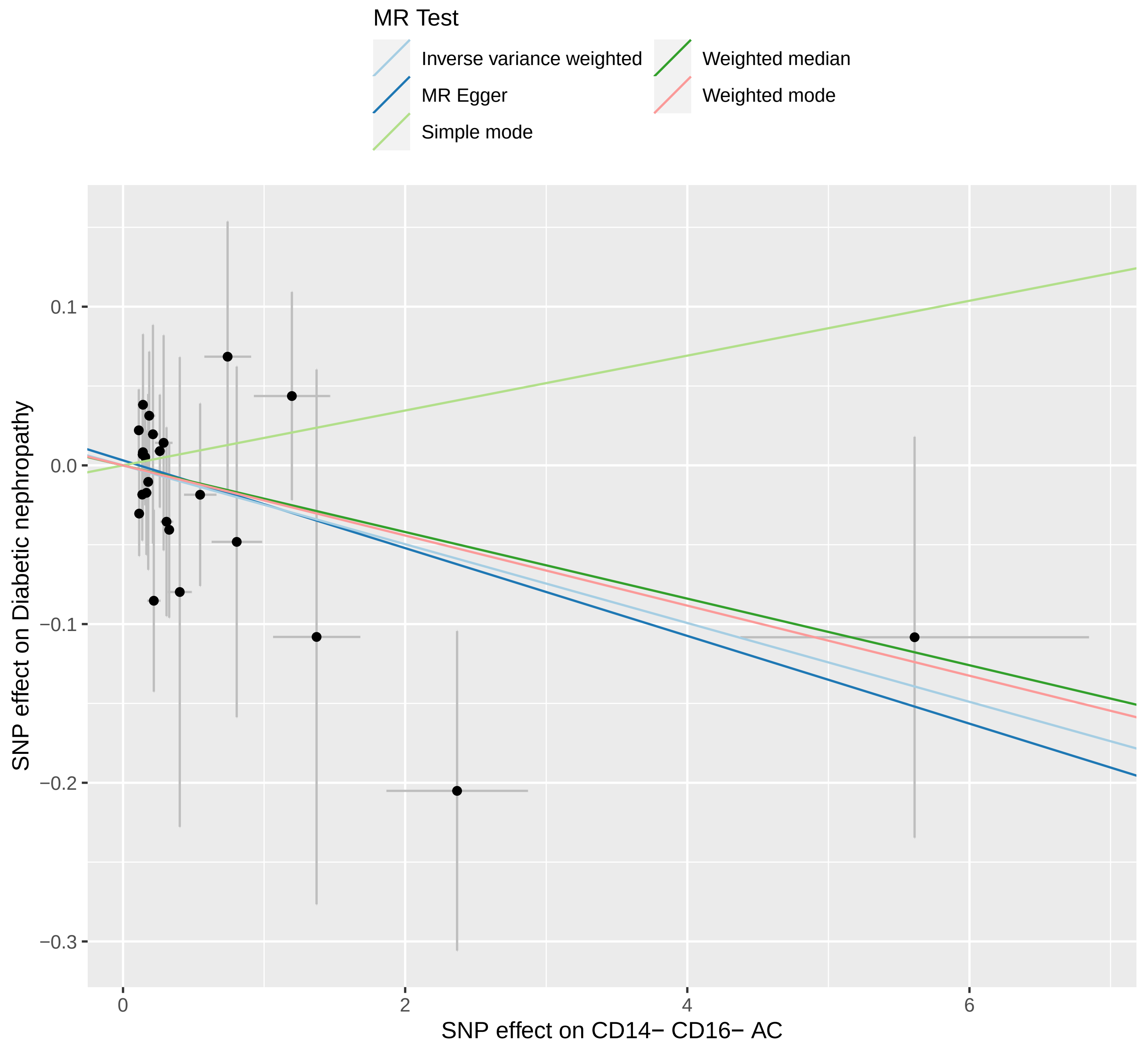

MR Test
Inverse variance weighted
MR Egger
Simple mode
Weighted median
Weighted mode
0.1
0.0
−0.1
−0.2
−0.3
SNP effect on Diabetic nephropathy
0
2
4
6
SNP effect on CD14− CD16− AC

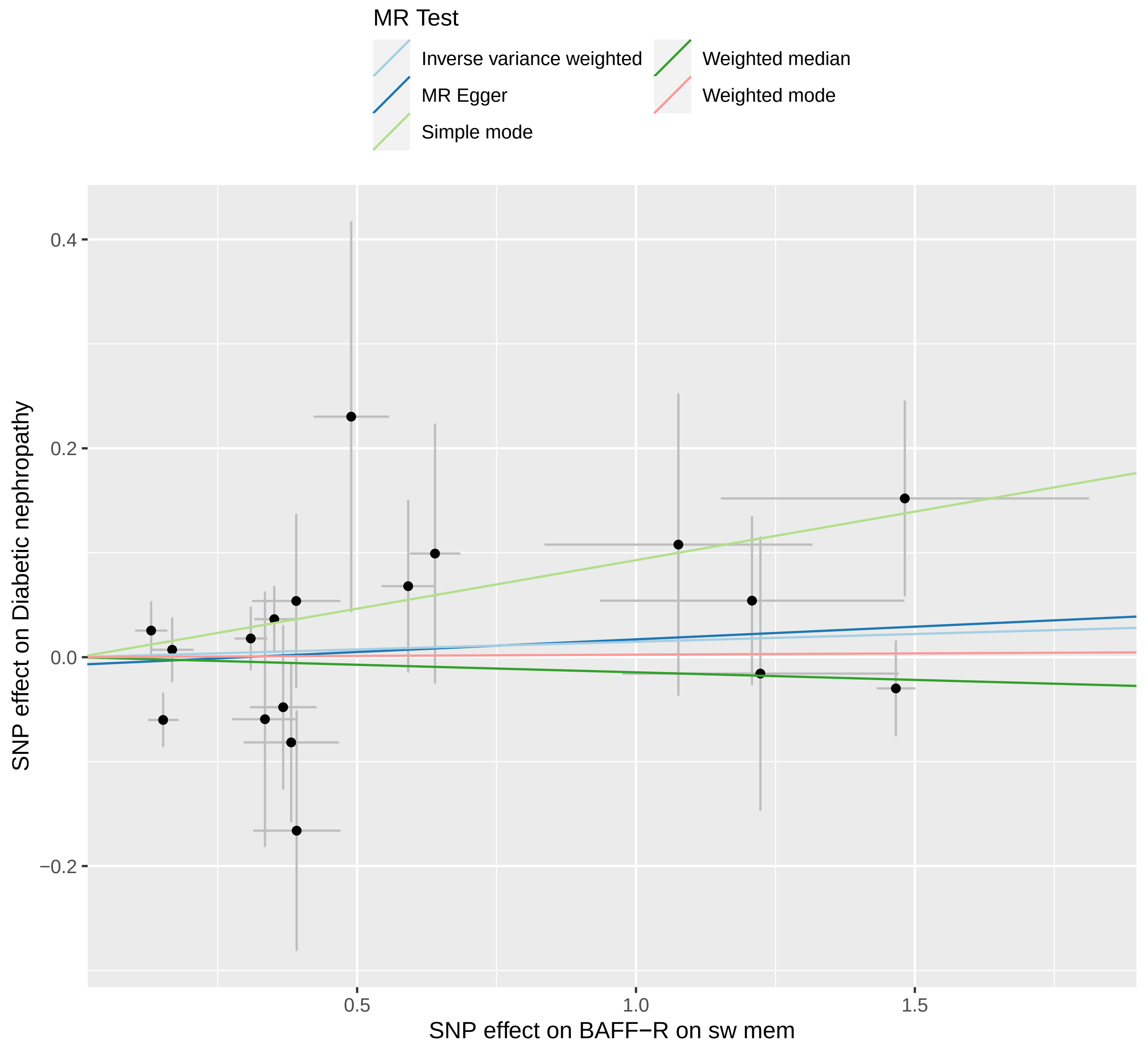
MR Test
Inverse variance weighted
MR Egger
Simple mode
Weighted median
Weighted mode
SNP effect on Diabetic nephropathy
0.4
0.2
0.0
−0.2
0.5
1.0
1.5
SNP effect on BAFF−R on sw mem

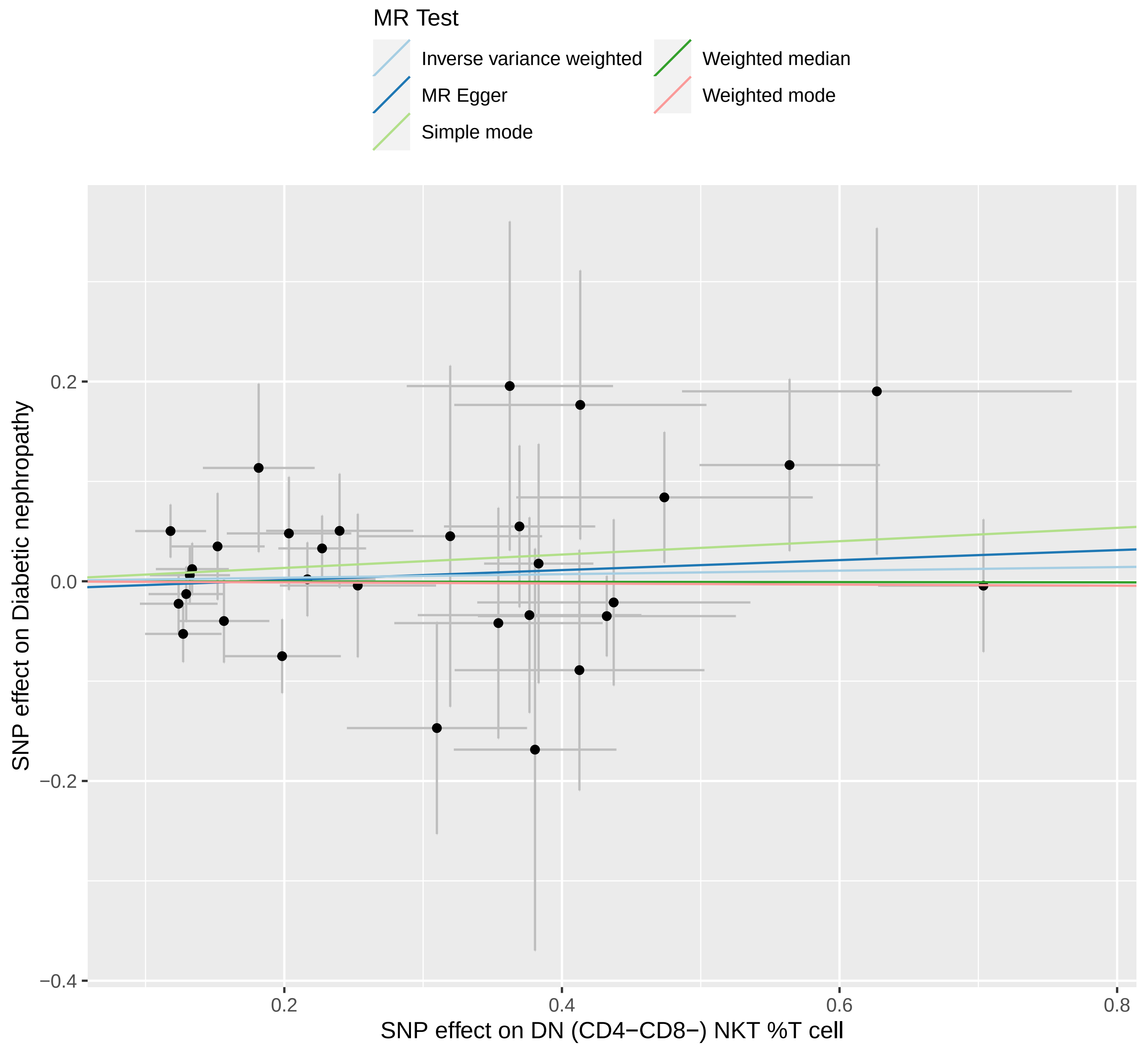
MR Test
Inverse variance weighted
MR Egger
Simple mode
Weighted median
Weighted mode
SNP effect on Diabetic nephropathy
0.2
0.0
−0.2
−0.4
0.2
0.4
0.6
0.8
SNP effect on DN (CD4−CD8−) NKT %T cell

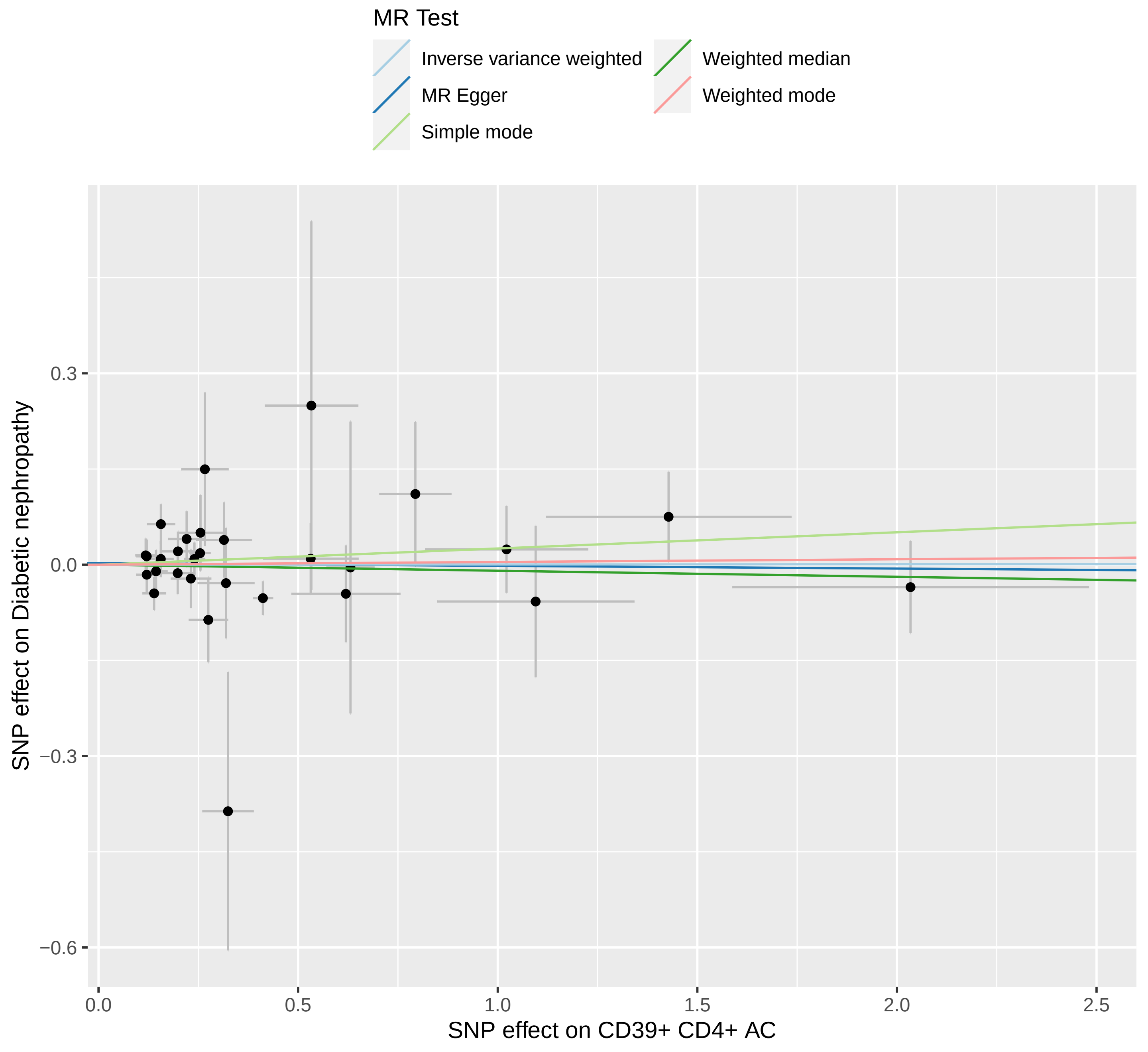

MR Test
Inverse variance weighted
MR Egger
Simple mode
Weighted median
Weighted mode
0.3
0.0
−0.3
−0.6
SNP effect on Diabetic nephropathy
0.0
0.5
1.0
1.5
2.0
2.5
SNP effect on CD39+ CD4+ AC

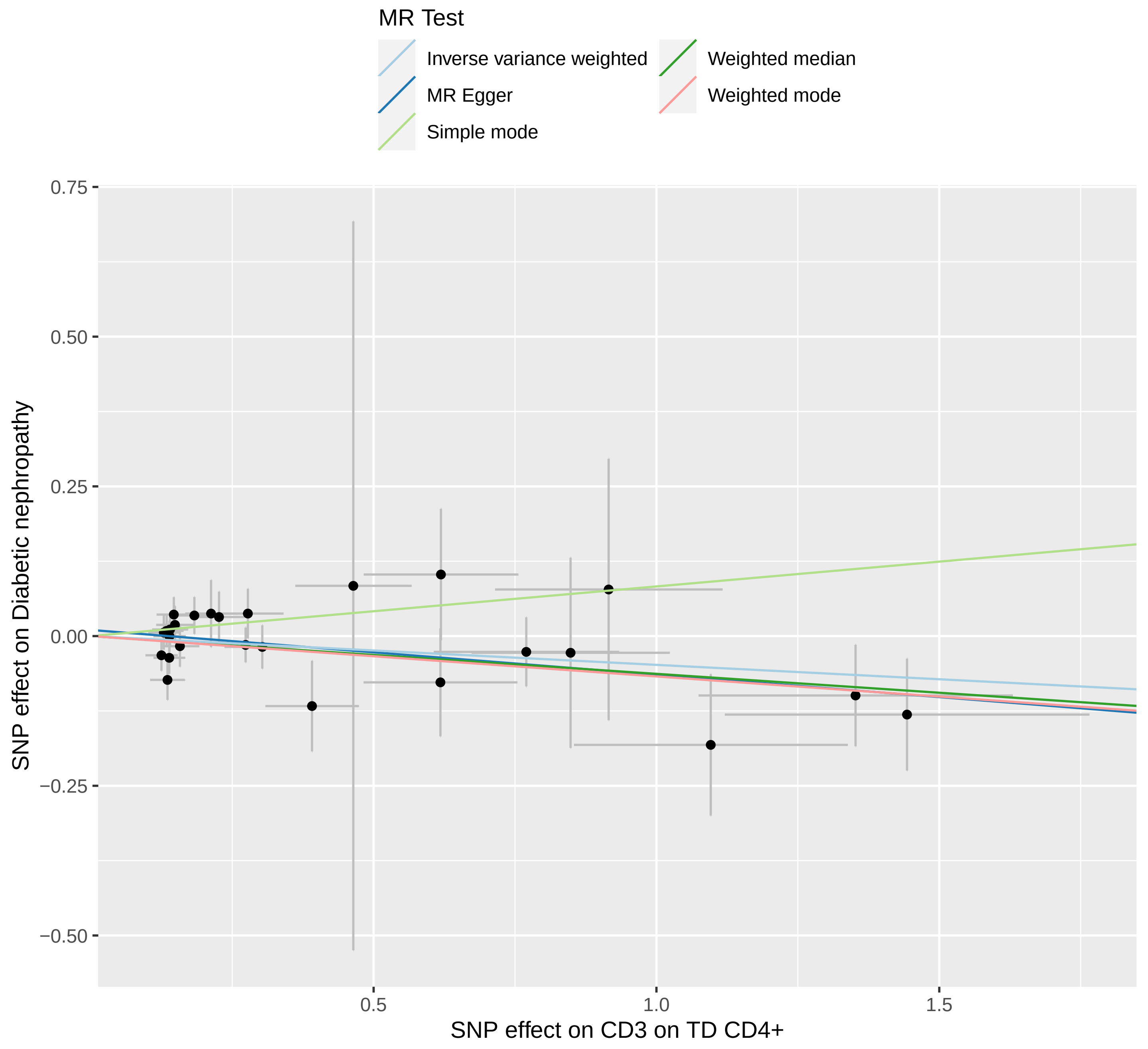

MR Test
Inverse variance weighted
MR Egger
Simple mode
Weighted median
Weighted mode
0.75
0.50
0.25
0.00
−0.25
−0.50
SNP effect on Diabetic nephropathy
0.5
1.0
1.5
SNP effect on CD3 on TD CD4+

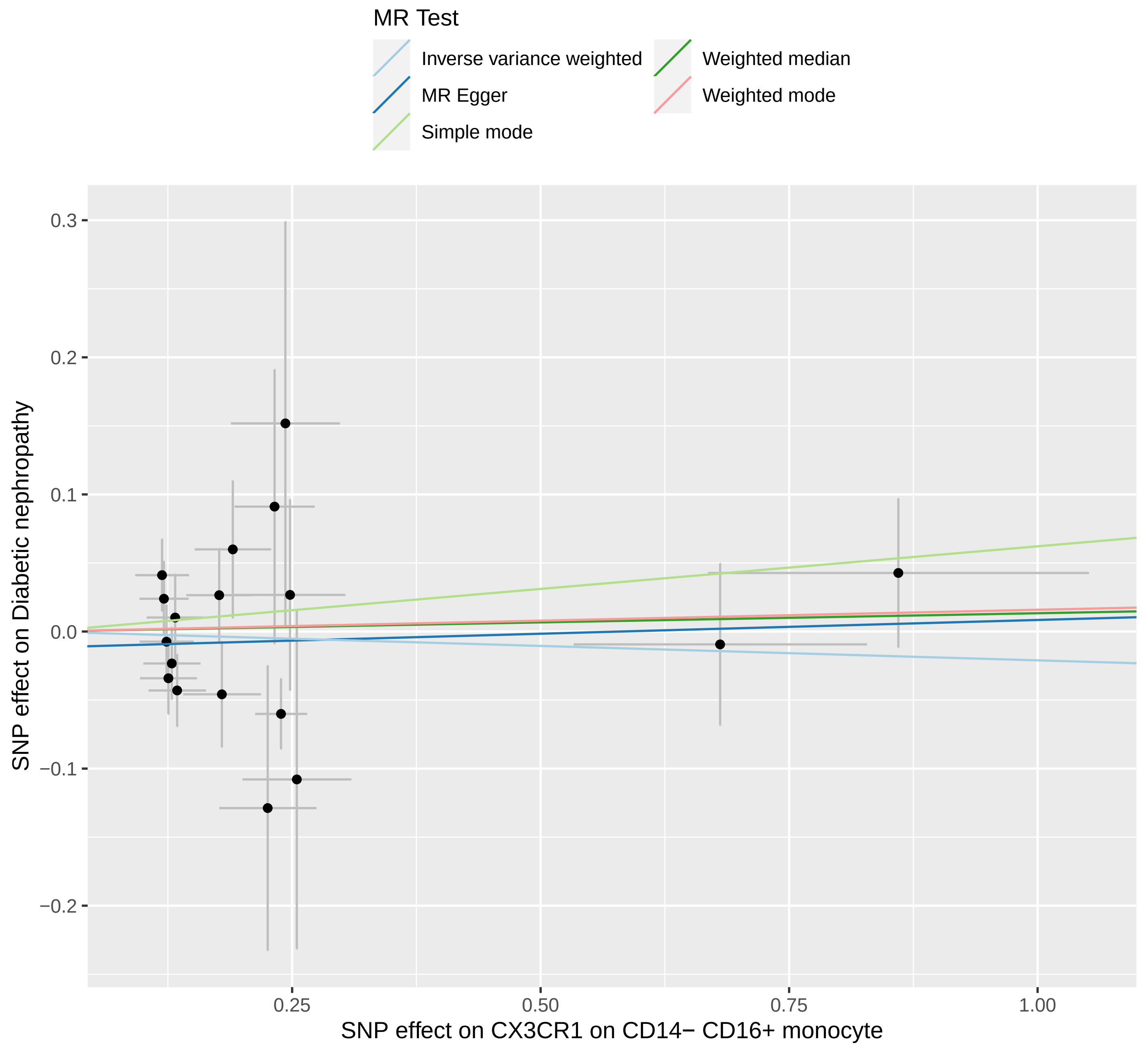
MR Test
Inverse variance weighted
MR Egger
Simple mode
Weighted median
Weighted mode
0.3
0.2
0.1
0.0
−0.1
−0.2
SNP effect on Diabetic nephropathy
0.25
0.50
0.75
1.00
SNP effect on CX3CR1 on CD14− CD16+ monocyte

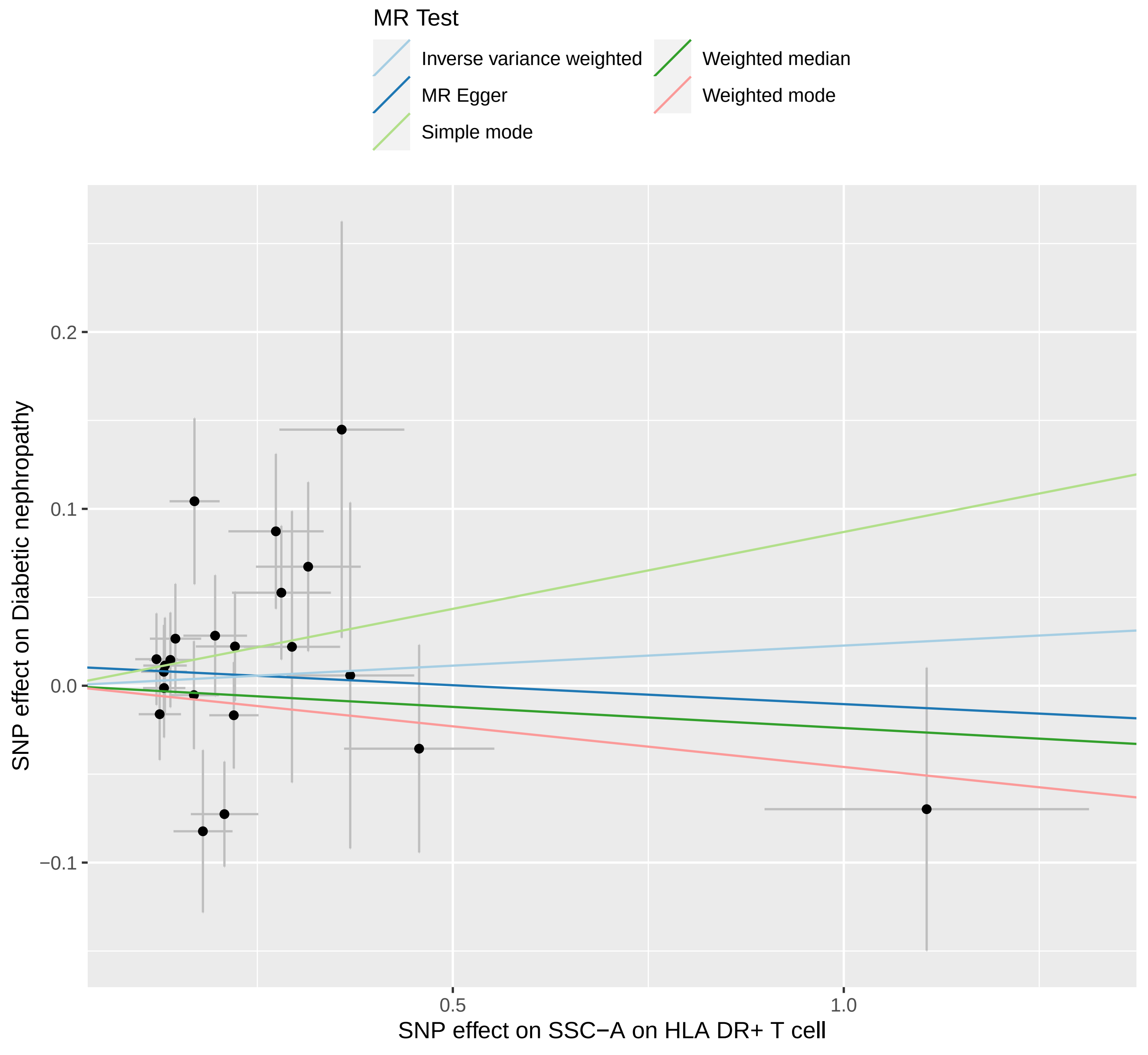
MR Test
Inverse variance weighted
MR Egger
Simple mode
Weighted median
Weighted mode
0.2
0.1
0.0
−0.1
0.5
1.0
SNP effect on Diabetic nephropathy
SNP effect on SSC−A on HLA DR+ T cell

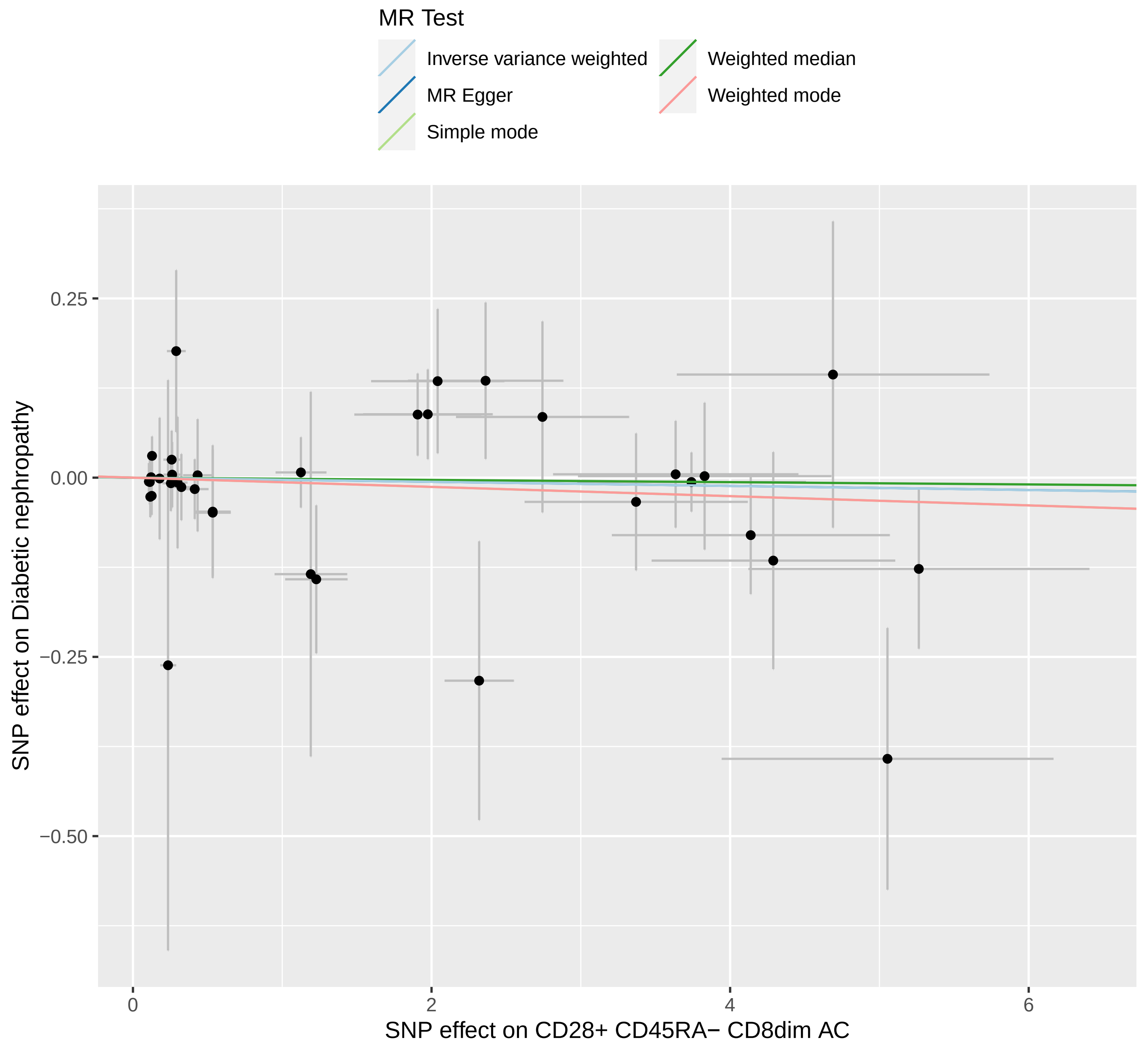
MR Test
Inverse variance weighted
MR Egger
Simple mode
Weighted median
Weighted mode
SNP effect on Diabetic nephropathy
0.25
0.00
−0.25
−0.50
0
2
4
6
SNP effect on CD28+ CD45RA− CD8dim AC

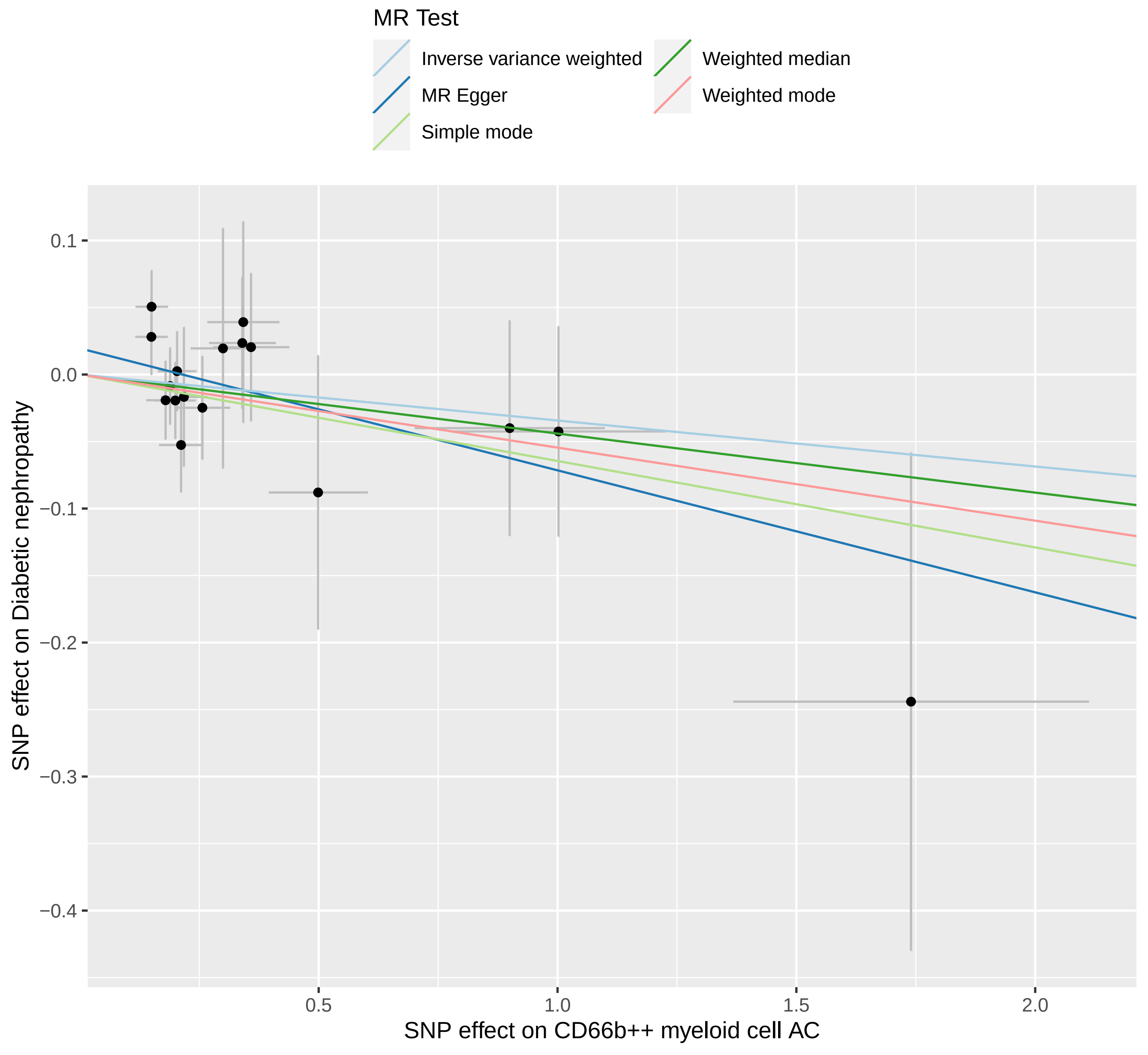

MR Test
Inverse variance weighted
MR Egger
Simple mode
Weighted median
Weighted mode
0.1
0.0
−0.1
−0.2
−0.3
−0.4
SNP effect on Diabetic nephropathy
0.5
1.0
1.5
2.0
SNP effect on CD66b++ myeloid cell AC

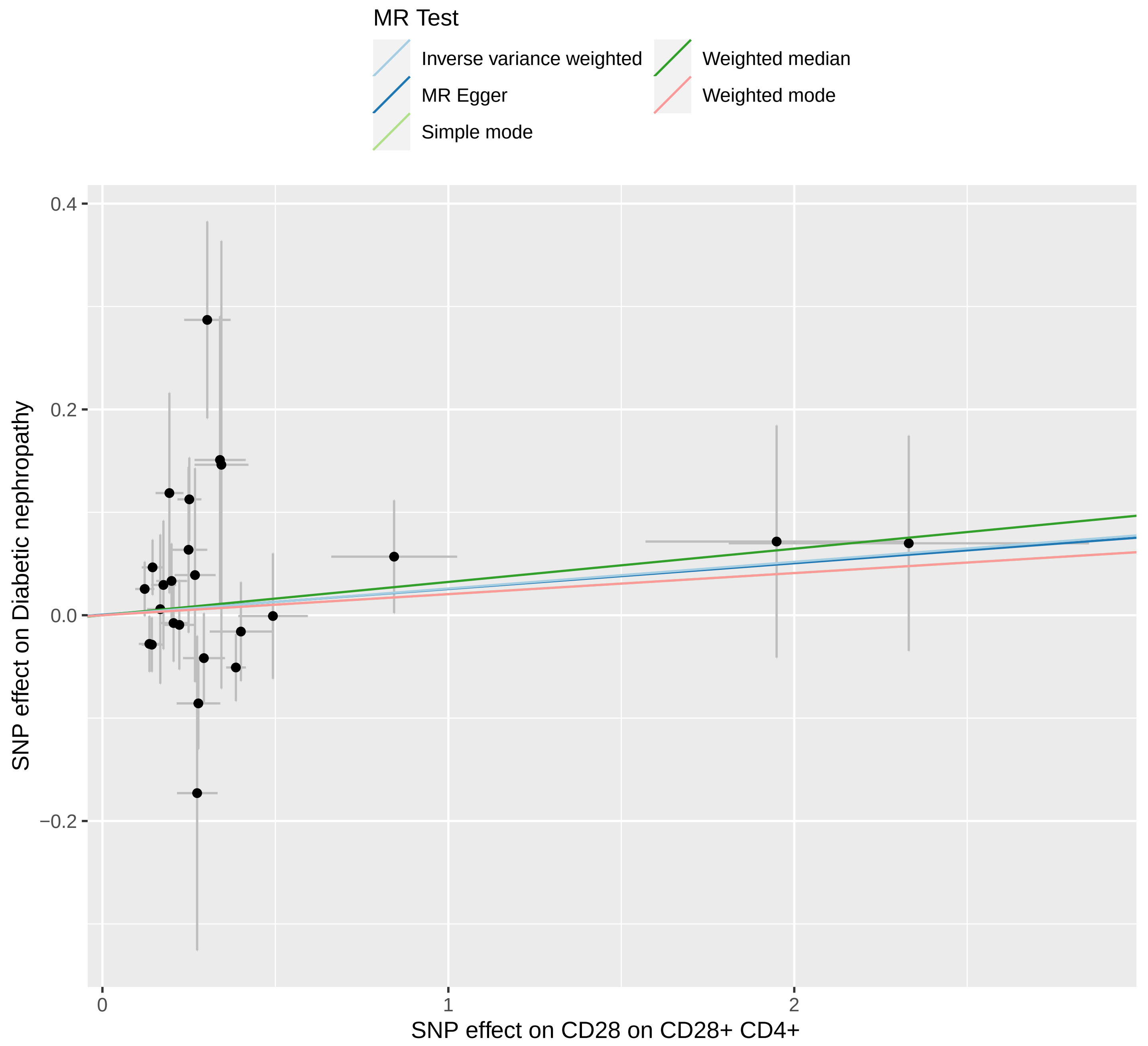
MR Test
Inverse variance weighted
MR Egger
Simple mode
Weighted median
Weighted mode
0.4
0.2
0.0
−0.2
0
1
2
SNP effect on Diabetic nephropathy
SNP effect on CD28 on CD28+ CD4+

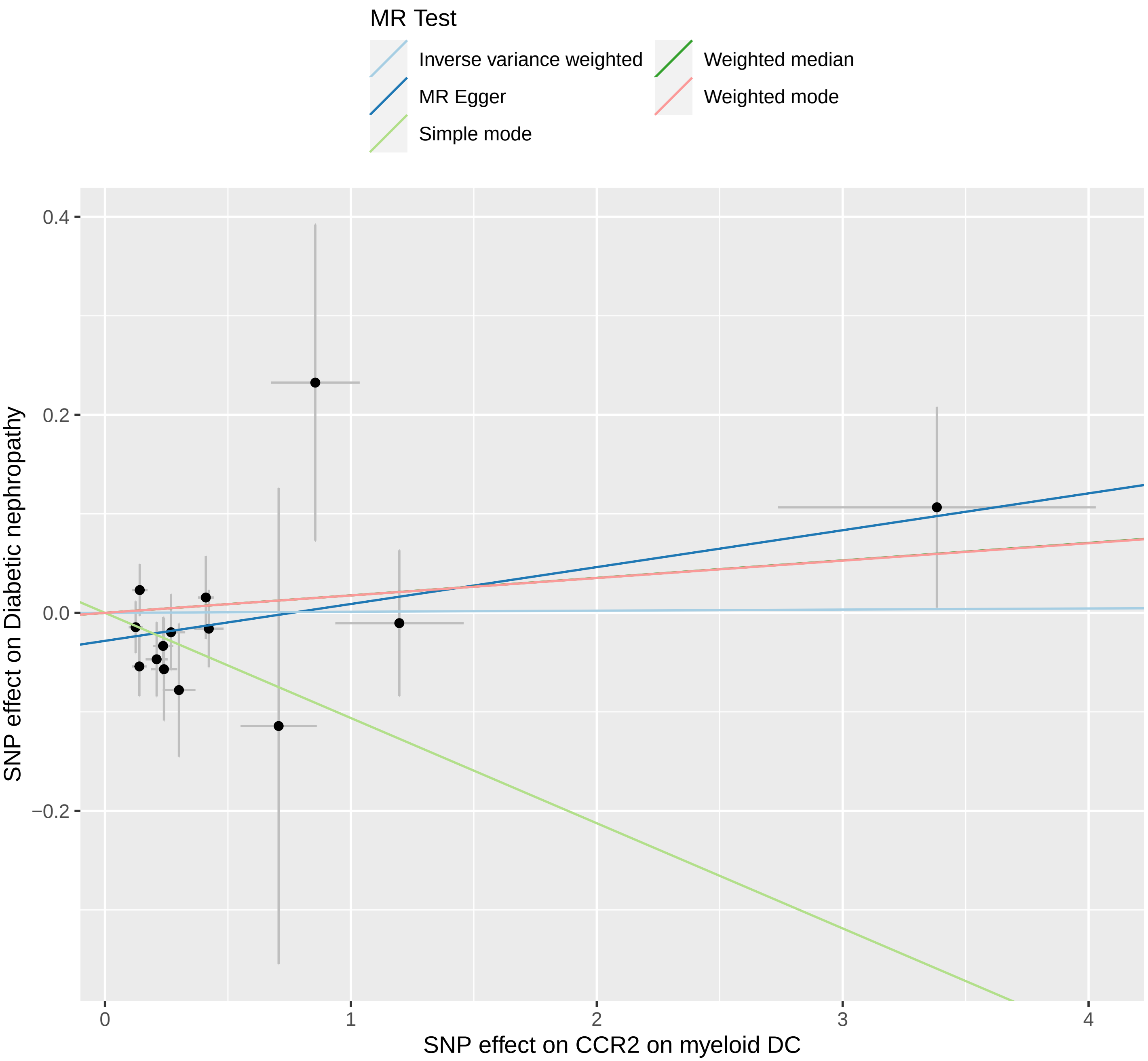

MR Test
Inverse variance weighted
MR Egger
Simple mode
Weighted median
Weighted mode
0.4
0.2
0.0
−0.2
0
1
2
3
4
SNP effect on Diabetic nephropathy
SNP effect on CCR2 on myeloid DC

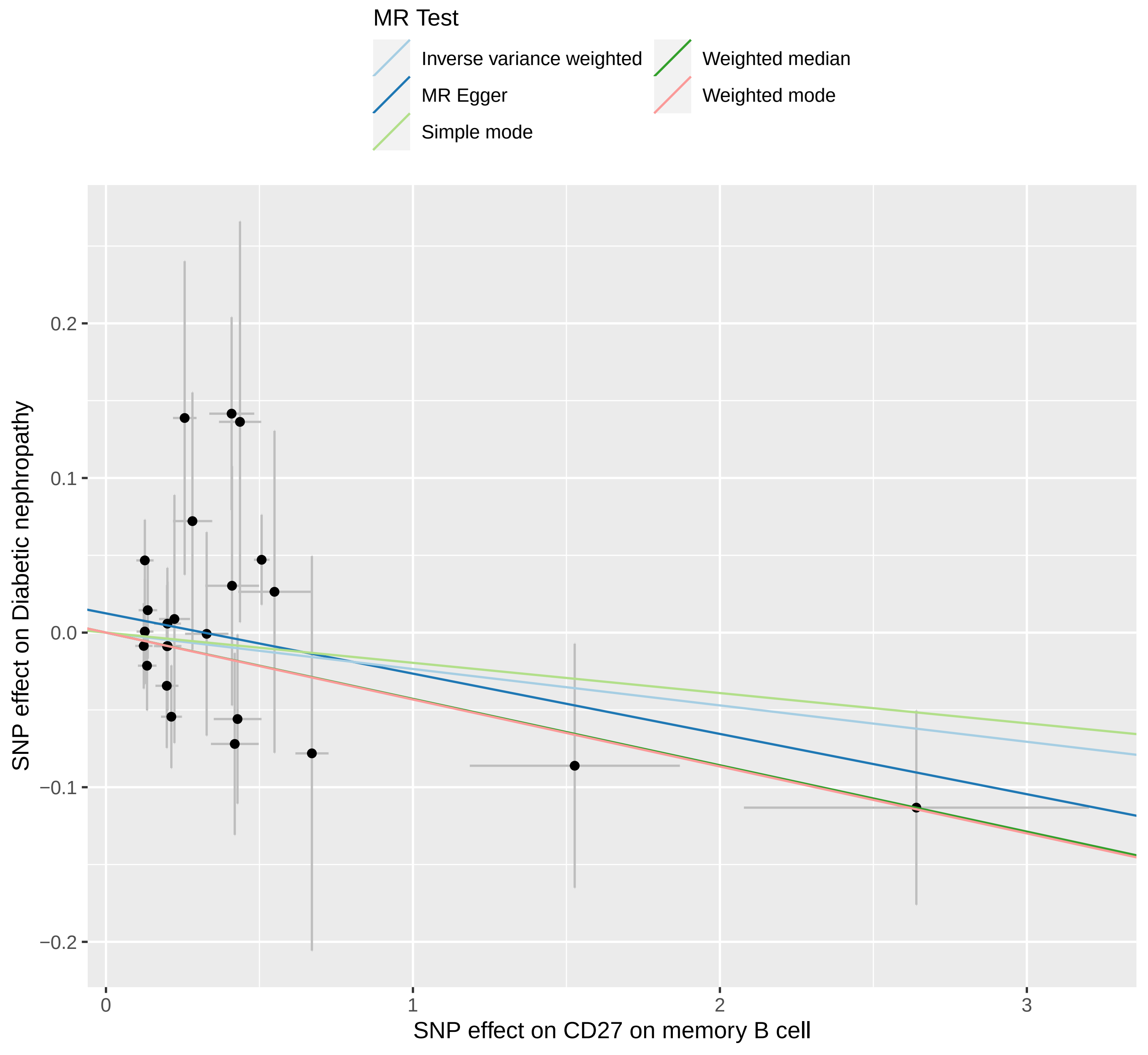

MR Test
Inverse variance weighted
MR Egger
Simple mode
Weighted median
Weighted mode
SNP effect on Diabetic nephropathy
0.2
0.1
0.0
−0.1
−0.2
0
1
2
3
SNP effect on CD27 on memory B cell

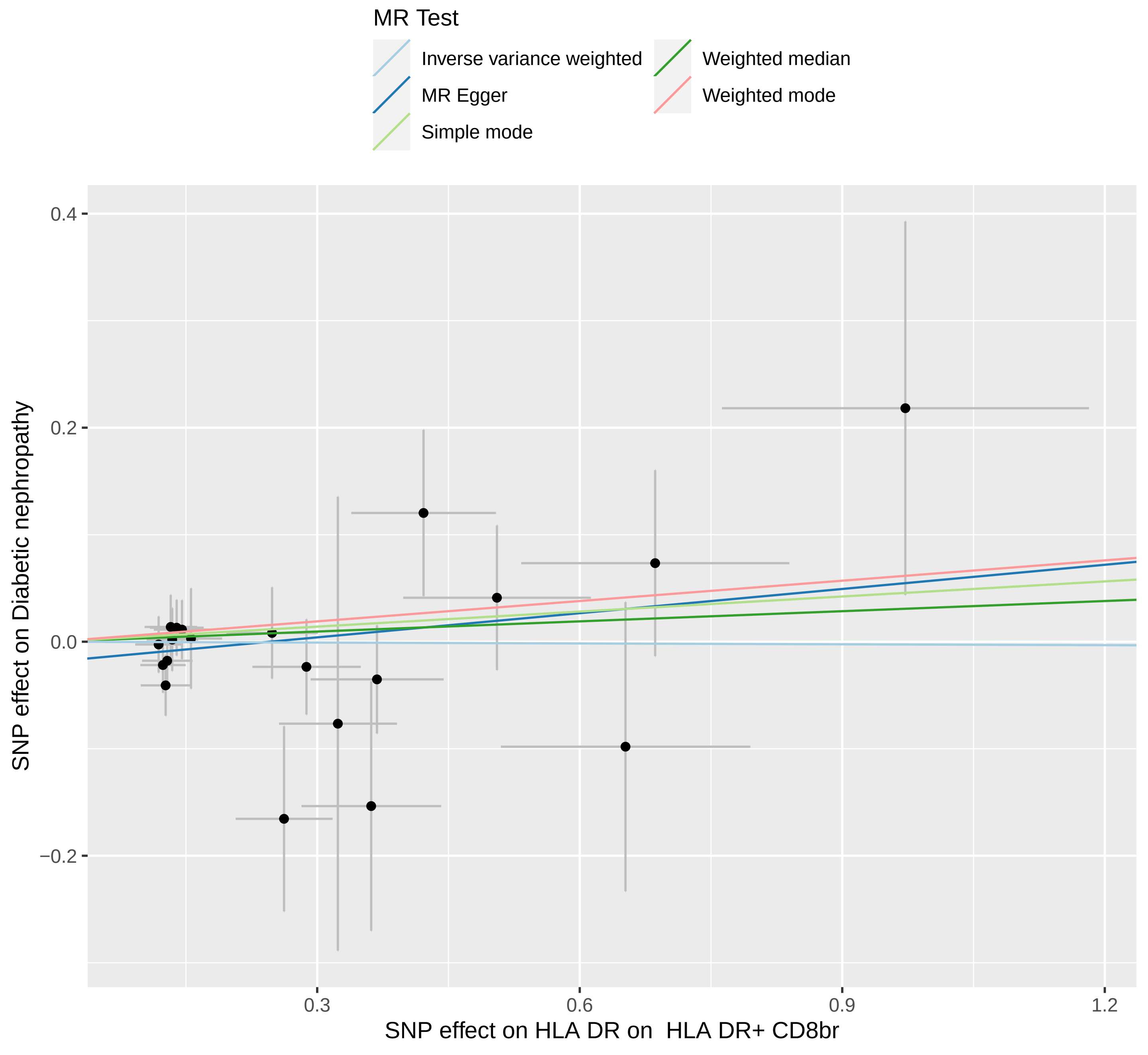
MR Test
Inverse variance weighted
MR Egger
Simple mode
Weighted median
Weighted mode
0.4
0.2
0.0
−0.2
0.3
0.6
0.9
1.2
SNP effect on Diabetic nephropathy
SNP effect on HLA DR on HLA DR+ CD8br

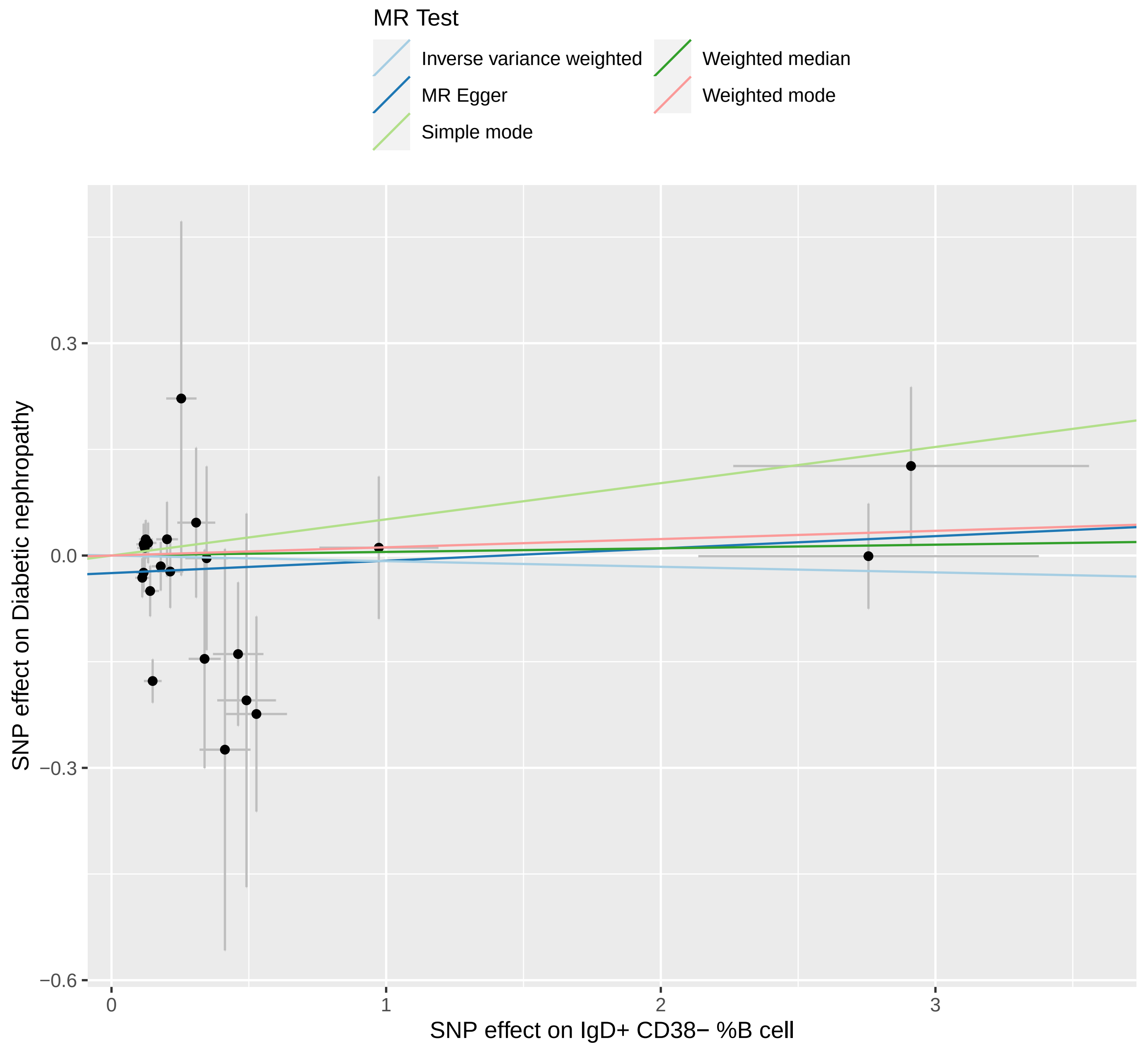
MR Test
Inverse variance weighted
Weighted median
MR Egger
Weighted mode
Simple mode
SNP effect on Diabetic nephropathy
0.3
0.0
−0.3
−0.6
0
1
2
3
SNP effect on IgD+ CD38− %B cell

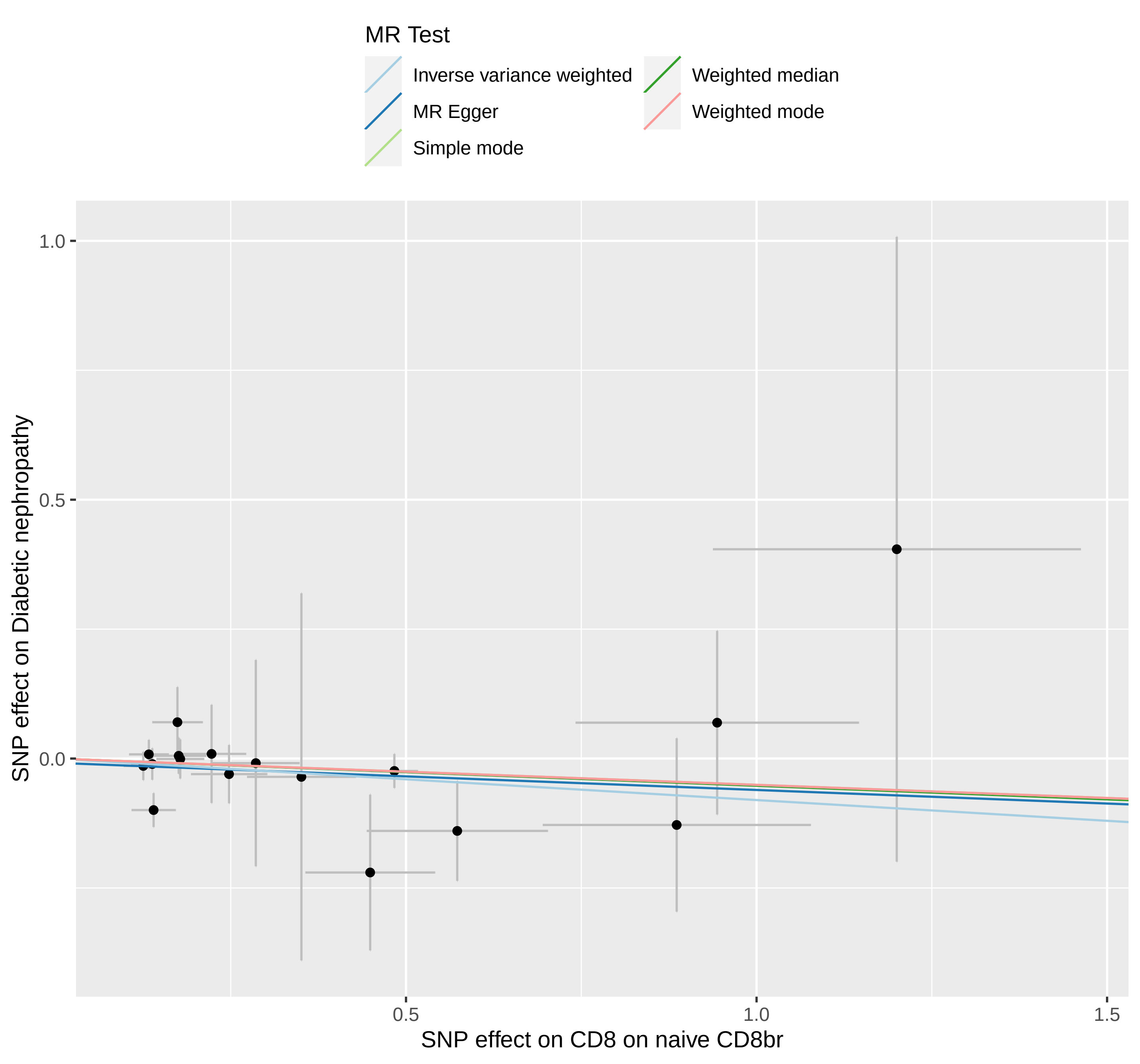
MR Test
Inverse variance weighted
MR Egger
Simple mode
Weighted median
Weighted mode
1.0
0.5
0.0
SNP effect on Diabetic nephropathy
0.5
1.0
1.5
SNP effect on CD8 on naive CD8br

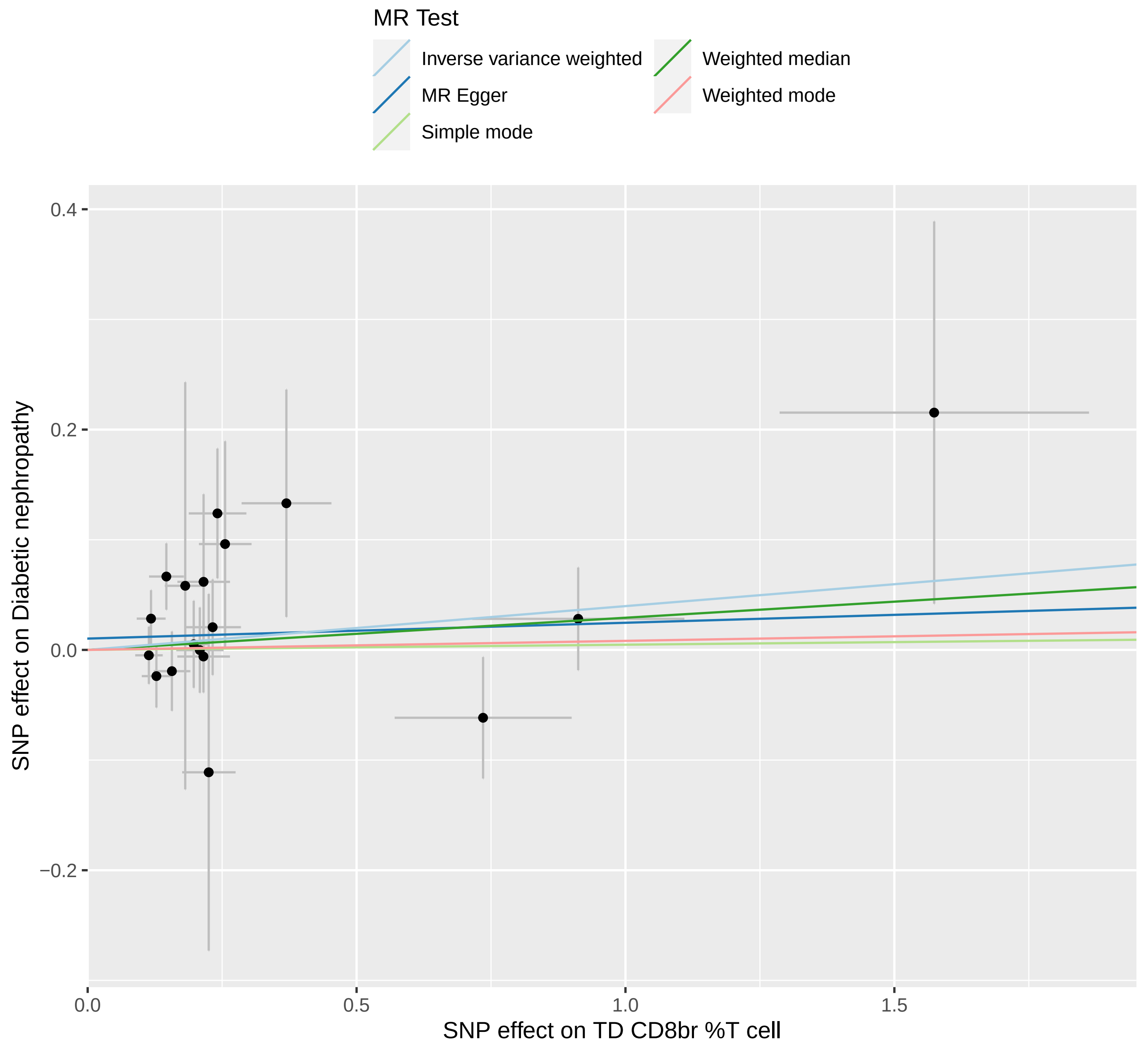
MR Test
Inverse variance weighted
MR Egger
Simple mode
Weighted median
Weighted mode
0.4
0.2
0.0
−0.2
SNP effect on Diabetic nephropathy
0.0
0.5
1.0
1.5
SNP effect on TD CD8br %T cell

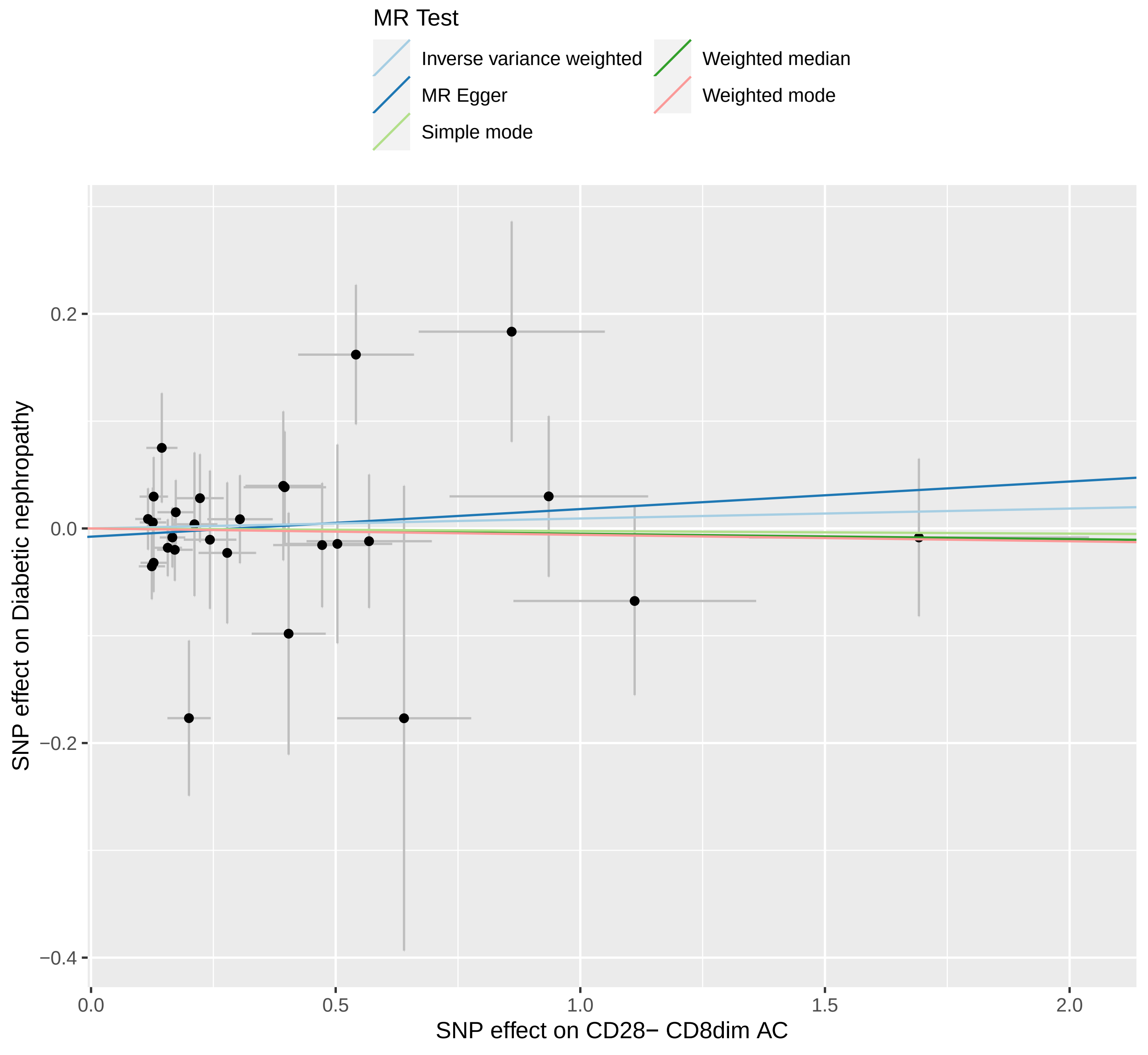
MR Test
Inverse variance weighted
MR Egger
Simple mode
Weighted median
Weighted mode
0.2
0.0
−0.2
−0.4
SNP effect on Diabetic nephropathy
0.0
0.5
1.0
1.5
2.0
SNP effect on CD28− CD8dim AC

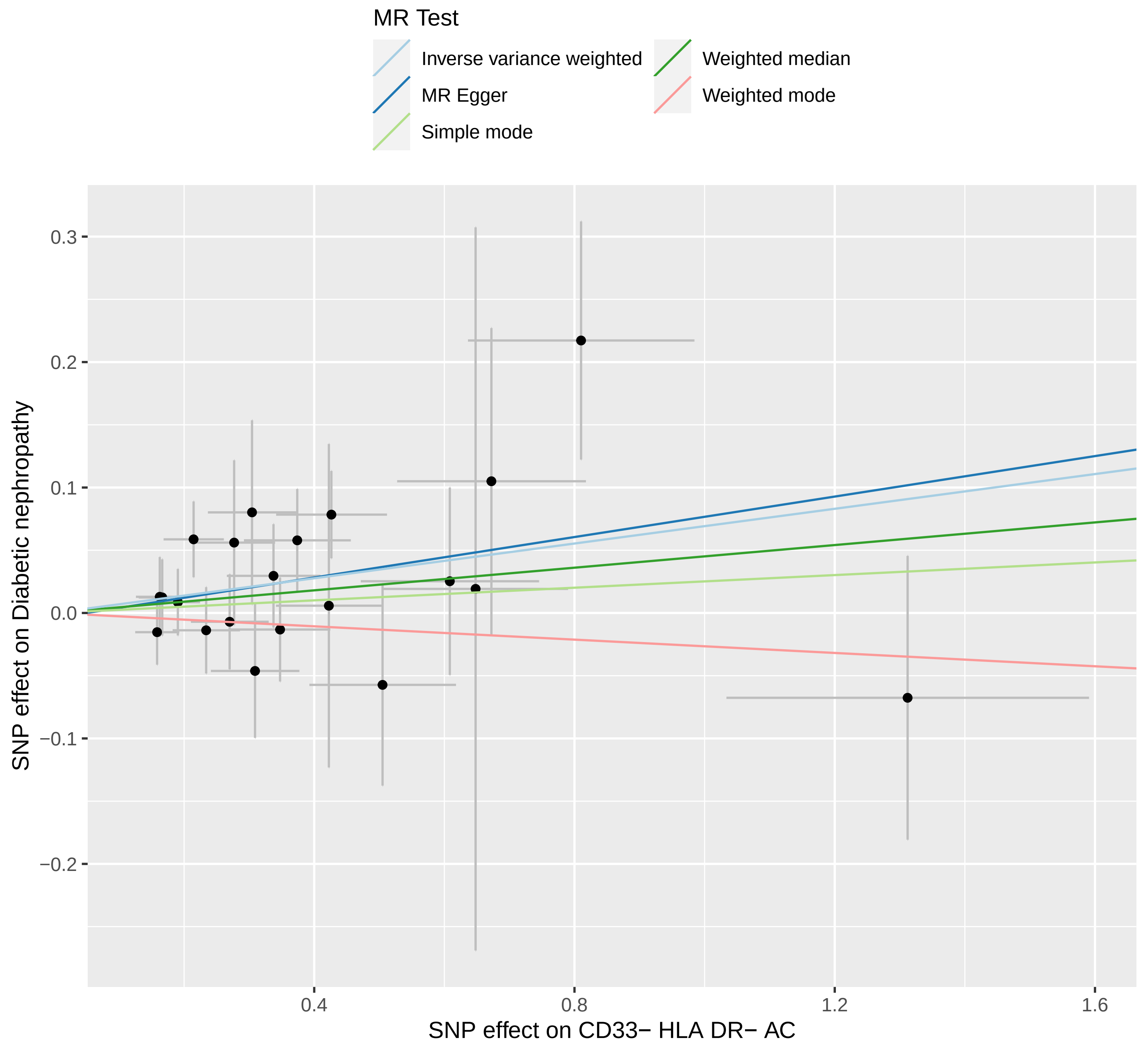

MR Test
Inverse variance weighted
MR Egger
Simple mode
Weighted median
Weighted mode
0.3
0.2
0.1
0.0
−0.1
−0.2
SNP effect on Diabetic nephropathy
0.4
0.8
1.2
1.6
SNP effect on CD33− HLA DR− AC

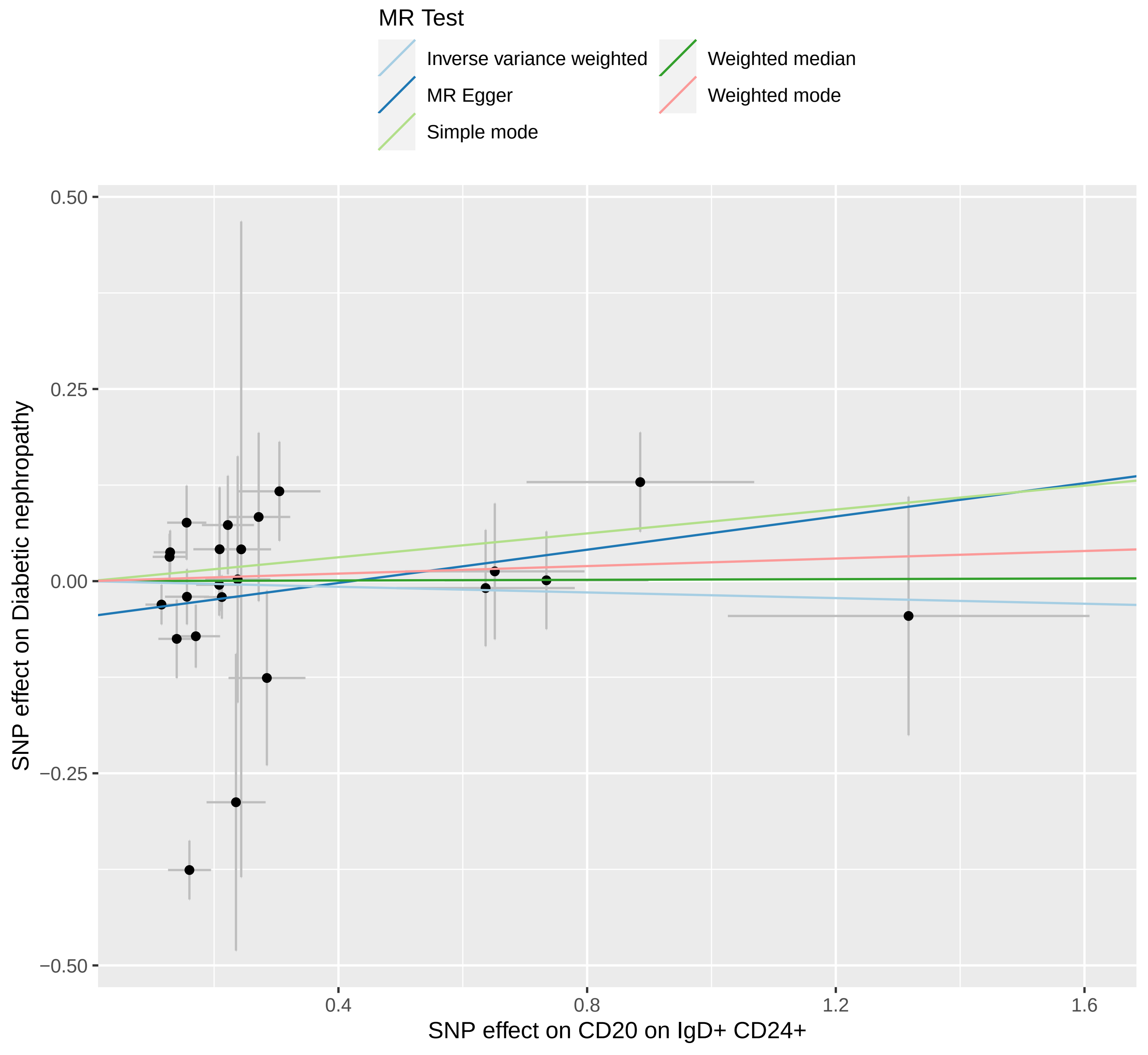

MR Test
Inverse variance weighted
MR Egger
Simple mode
Weighted median
Weighted mode
0.50
0.25
0.00
−0.25
−0.50
SNP effect on Diabetic nephropathy
0.4
0.8
1.2
1.6
SNP effect on CD20 on IgD+ CD24+

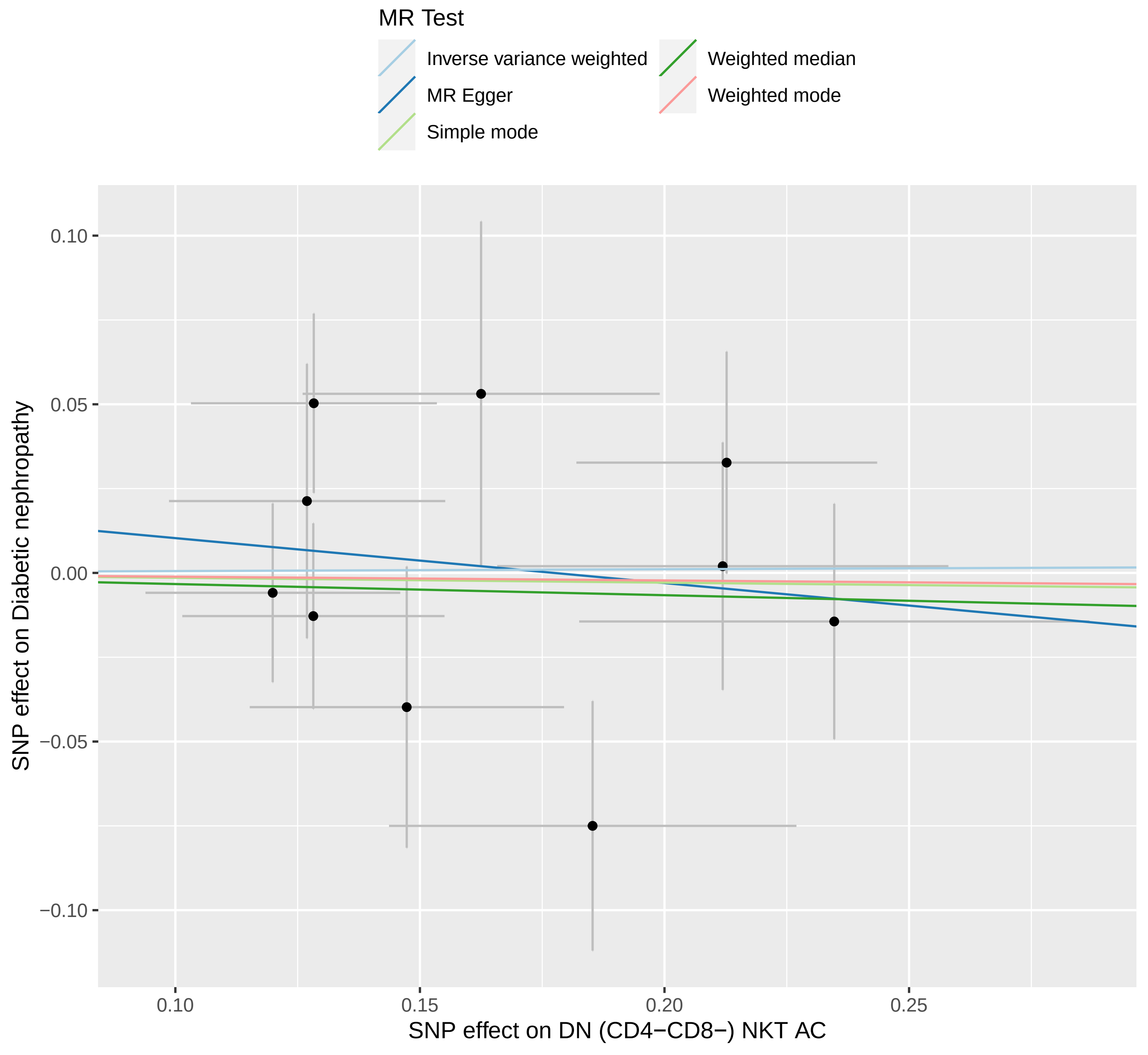
MR Test
Inverse variance weighted
MR Egger
Simple mode
Weighted median
Weighted mode
SNP effect on Diabetic nephropathy
0.10
0.05
0.00
−0.05
−0.10
0.10
0.15
0.20
0.25
SNP effect on DN (CD4−CD8−) NKT AC

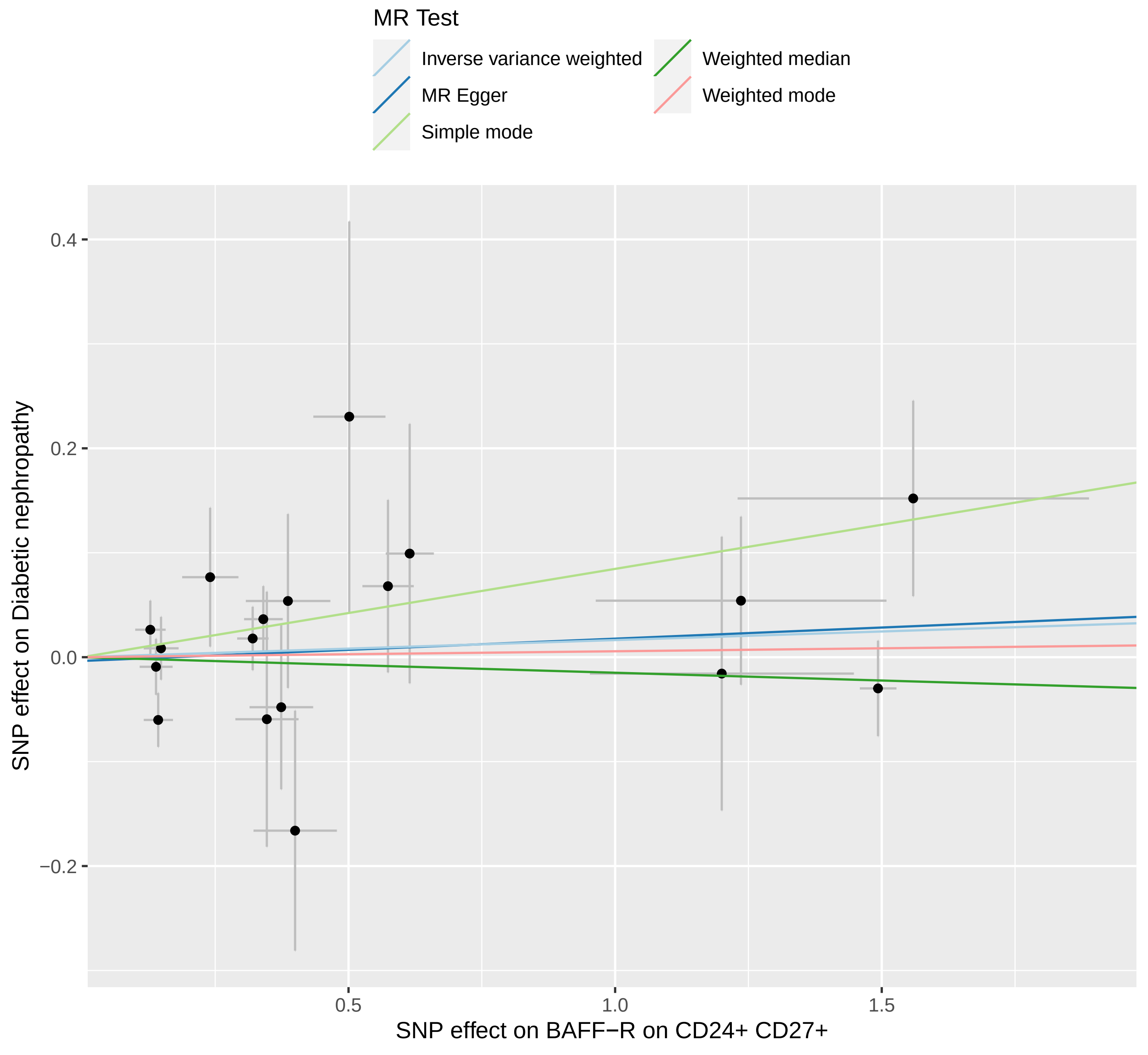

MR Test
Inverse variance weighted
MR Egger
Simple mode
Weighted median
Weighted mode
0.4
0.2
0.0
−0.2
0.5
1.0
1.5
SNP effect on Diabetic nephropathy
SNP effect on BAFF−R on CD24+ CD27+

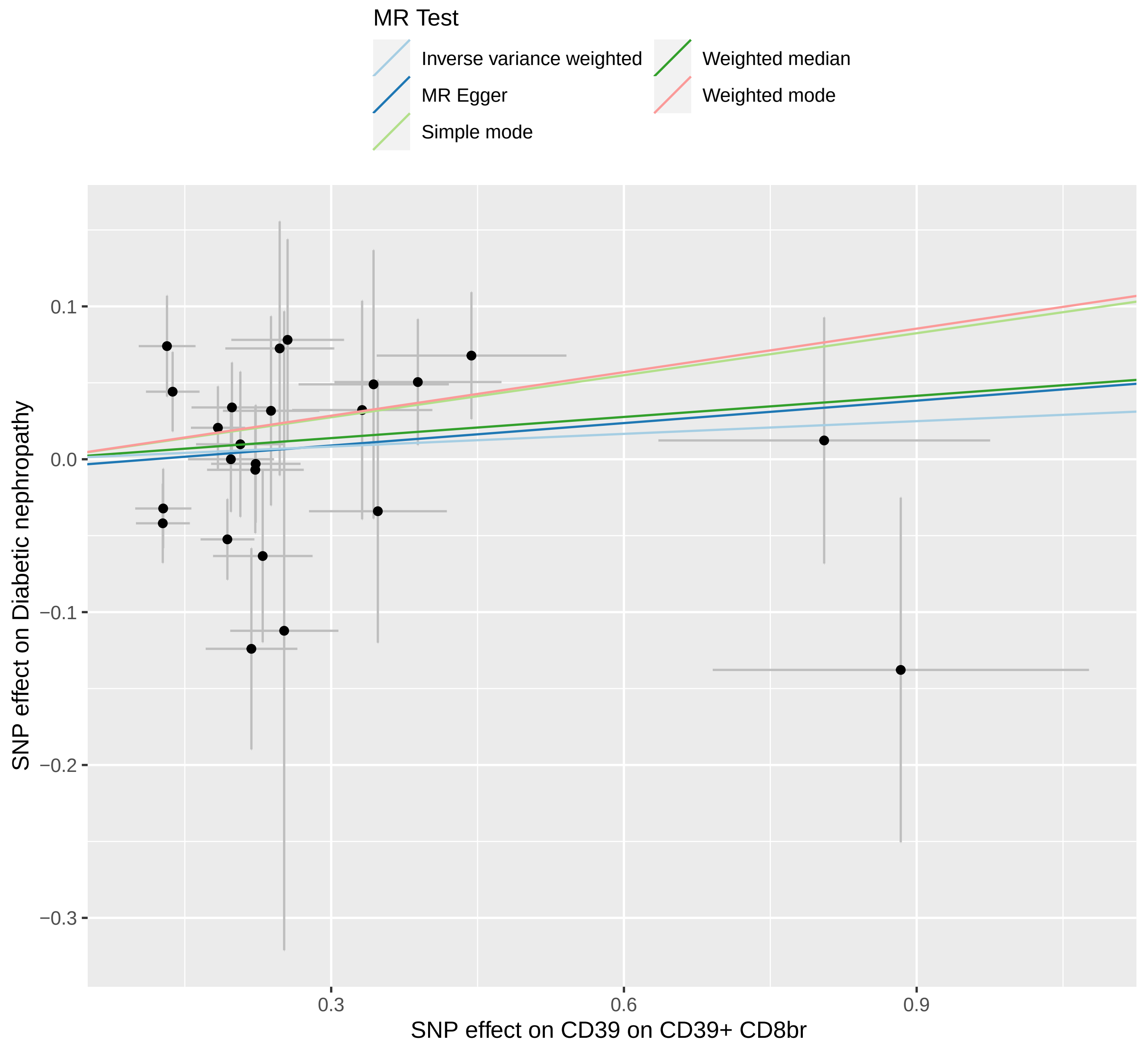

MR Test
Inverse variance weighted
MR Egger
Simple mode
Weighted median
Weighted mode
0.1
0.0
−0.1
−0.2
−0.3
0.3
0.6
0.9
SNP effect on Diabetic nephropathy
SNP effect on CD39 on CD39+ CD8br

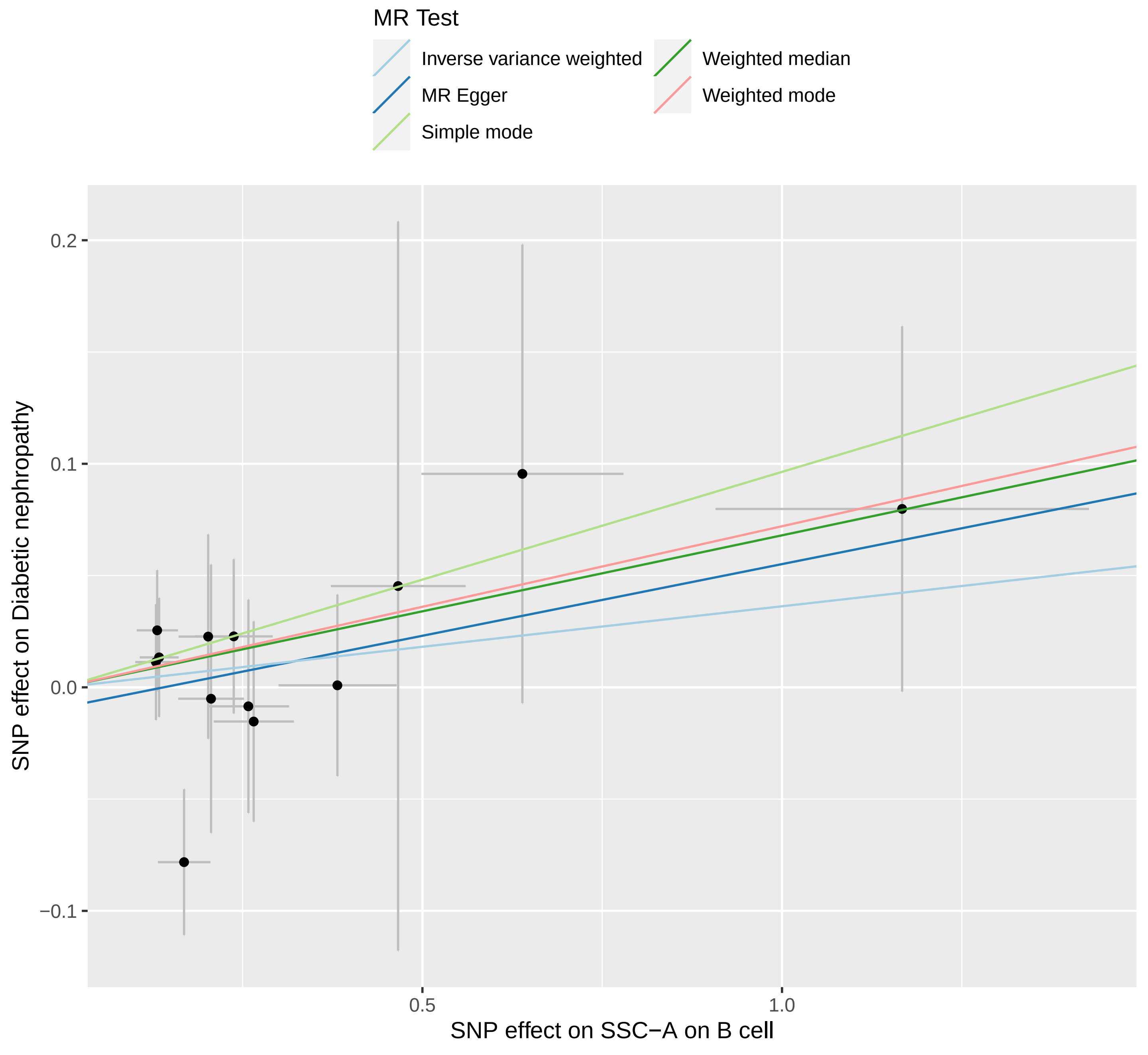

MR Test
Inverse variance weighted
MR Egger
Simple mode
Weighted median
Weighted mode
0.2
0.1
0.0
−0.1
0.5
1.0
SNP effect on Diabetic nephropathy
SNP effect on SSC−A on B cell

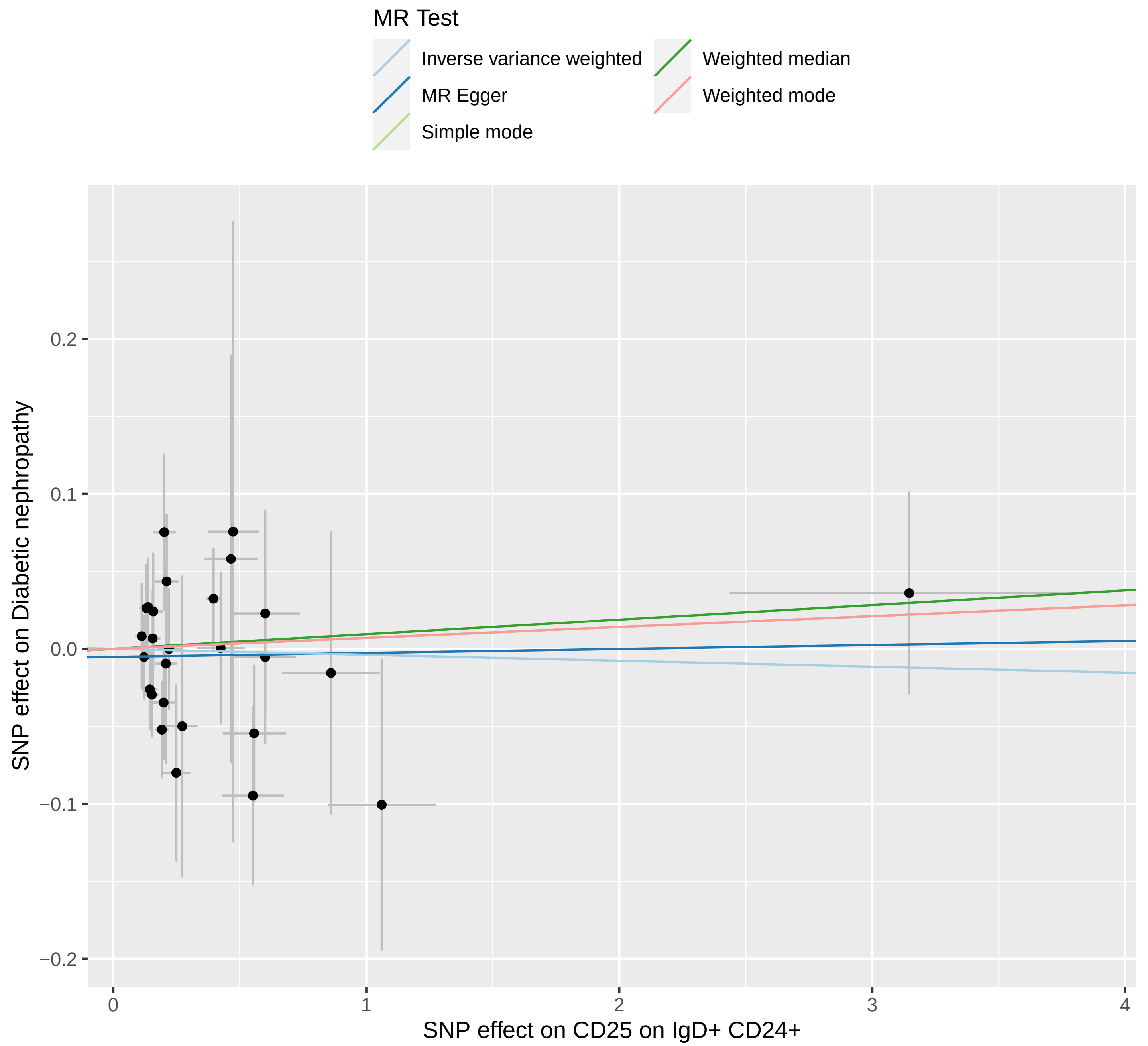

MR Test
Inverse variance weighted
MR Egger
Simple mode
Weighted median
Weighted mode
SNP effect on Diabetic nephropathy
0.2
0.1
0.0
−0.1
−0.2
0
1
2
3
4
SNP effect on CD25 on IgD+ CD24+

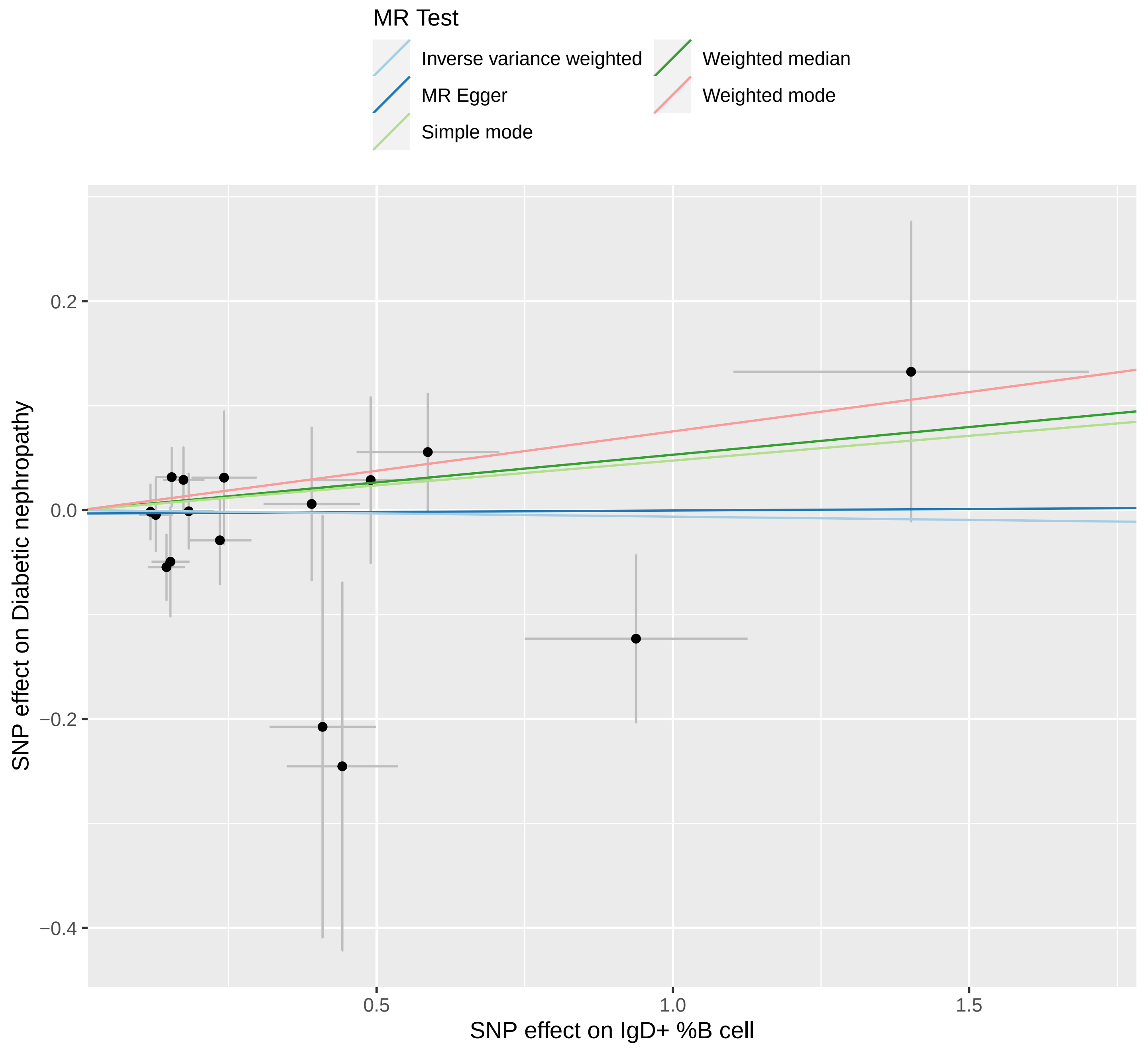
MR Test
Inverse variance weighted
MR Egger
Simple mode
Weighted median
Weighted mode
0.2
0.0
−0.2
−0.4
0.5
1.0
1.5
SNP effect on Diabetic nephropathy
SNP effect on IgD+ %B cell

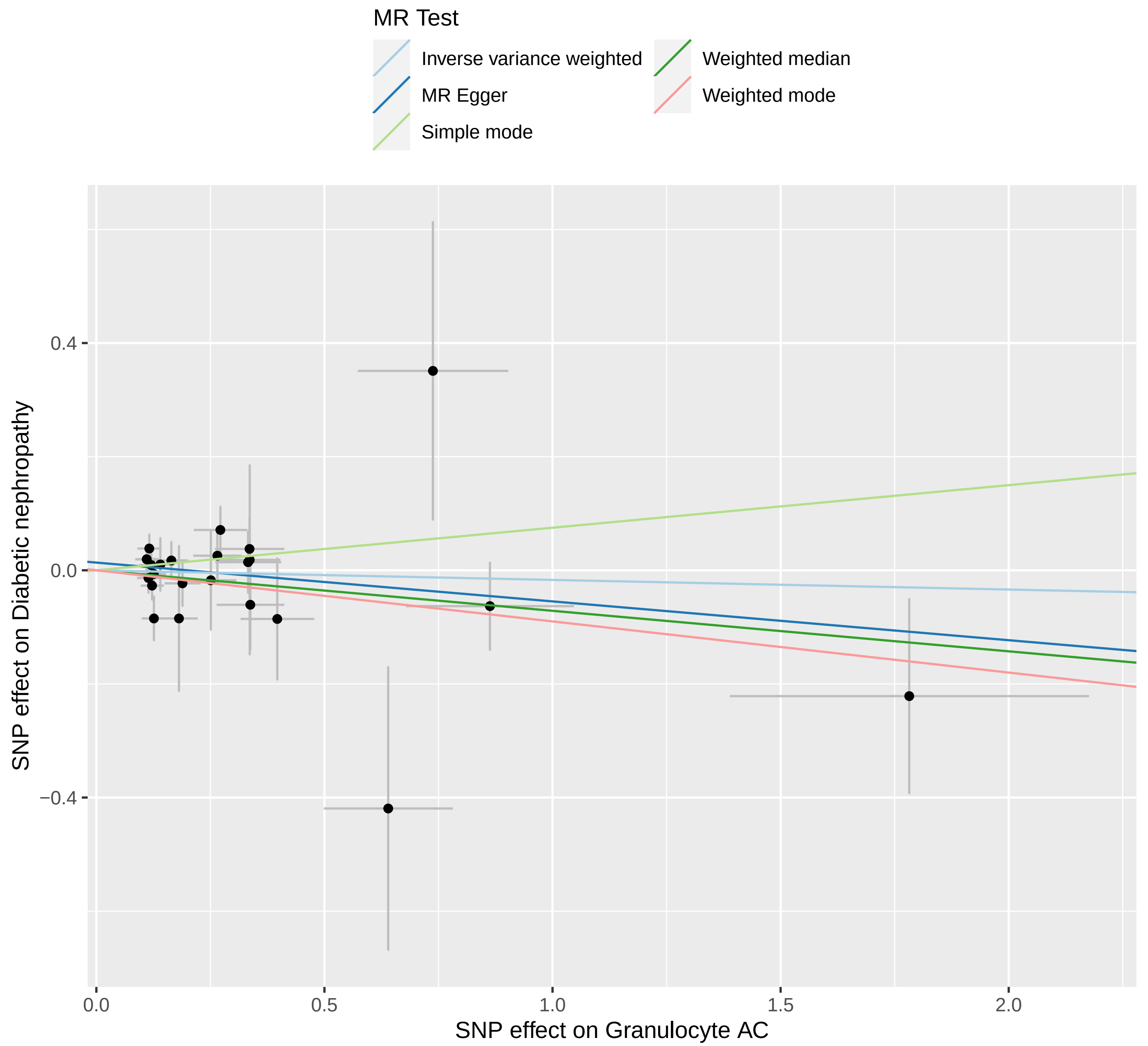
MR Test
Inverse variance weighted
MR Egger
Simple mode
Weighted median
Weighted mode
SNP effect on Diabetic nephropathy
0.4
0.0
−0.4
0.0
0.5
1.0
1.5
2.0
SNP effect on Granulocyte AC

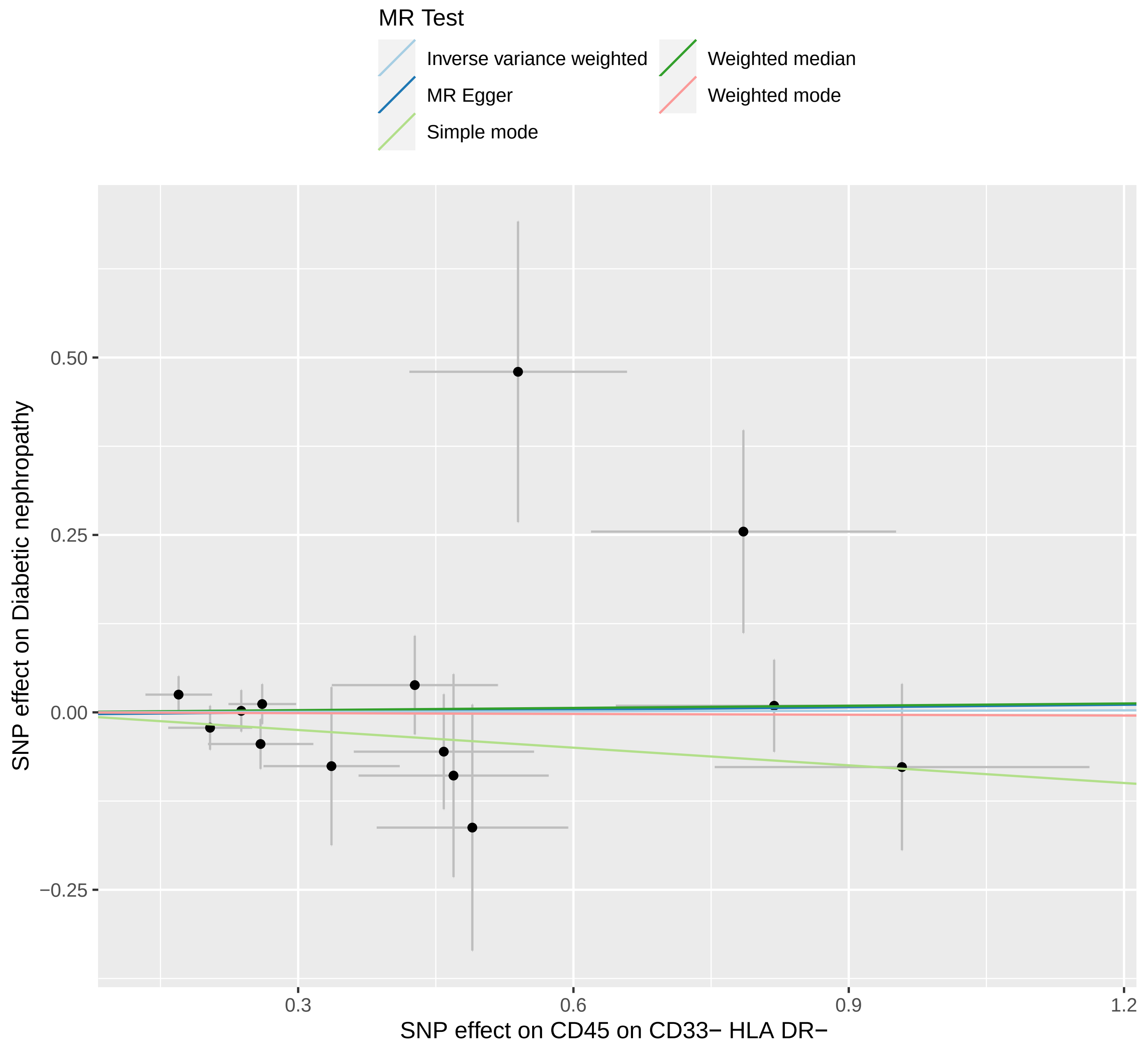

MR Test
Inverse variance weighted
MR Egger
Simple mode
Weighted median
Weighted mode
SNP effect on Diabetic nephropathy
0.50
0.25
0.00
−0.25
0.3
0.6
0.9
1.2
SNP effect on CD45 on CD33− HLA DR−

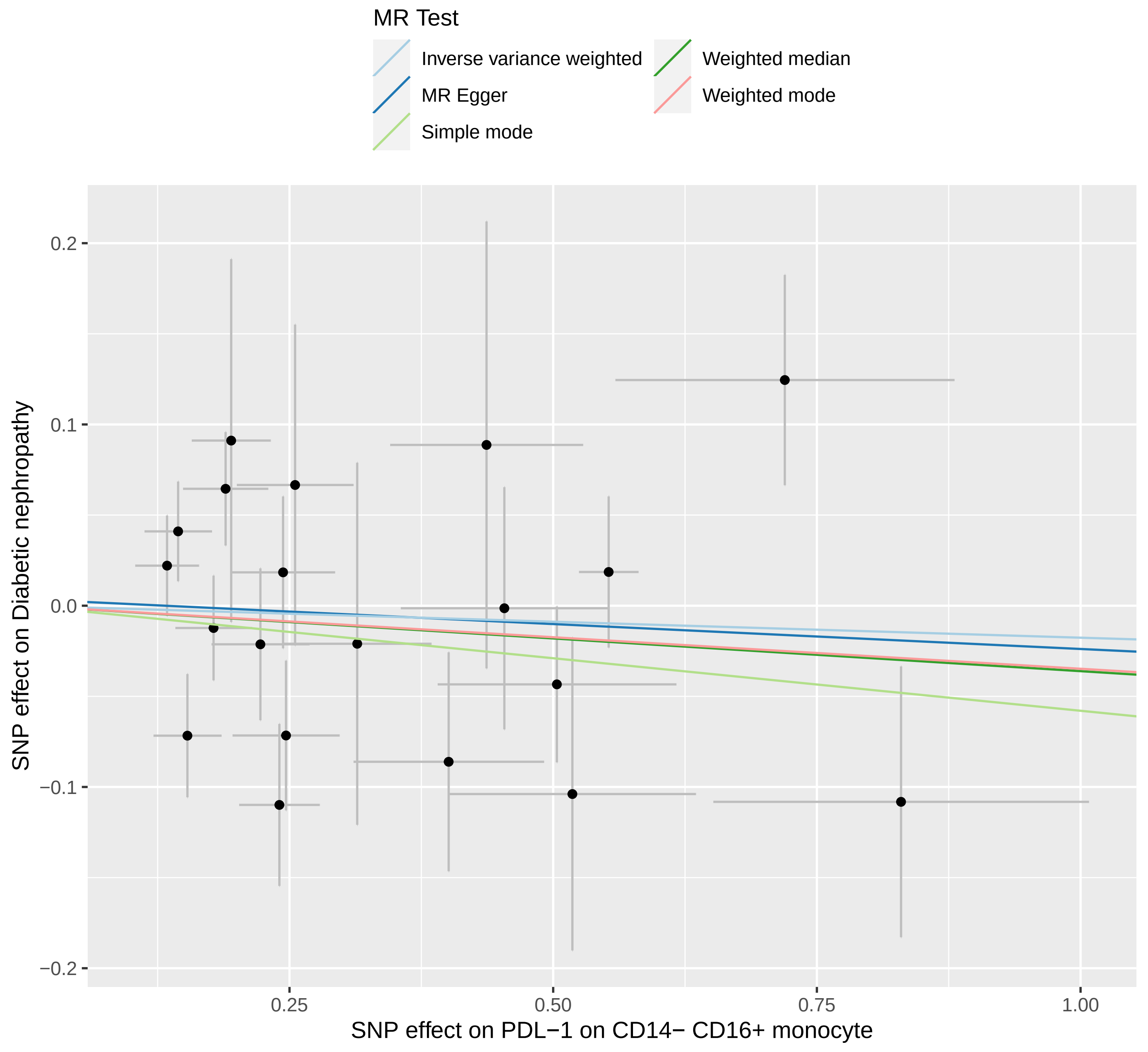
MR Test
Inverse variance weighted
MR Egger
Simple mode
Weighted median
Weighted mode
0.2
0.1
0.0
−0.1
−0.2
SNP effect on Diabetic nephropathy
0.25
0.50
0.75
1.00
SNP effect on PDL−1 on CD14− CD16+ monocyte

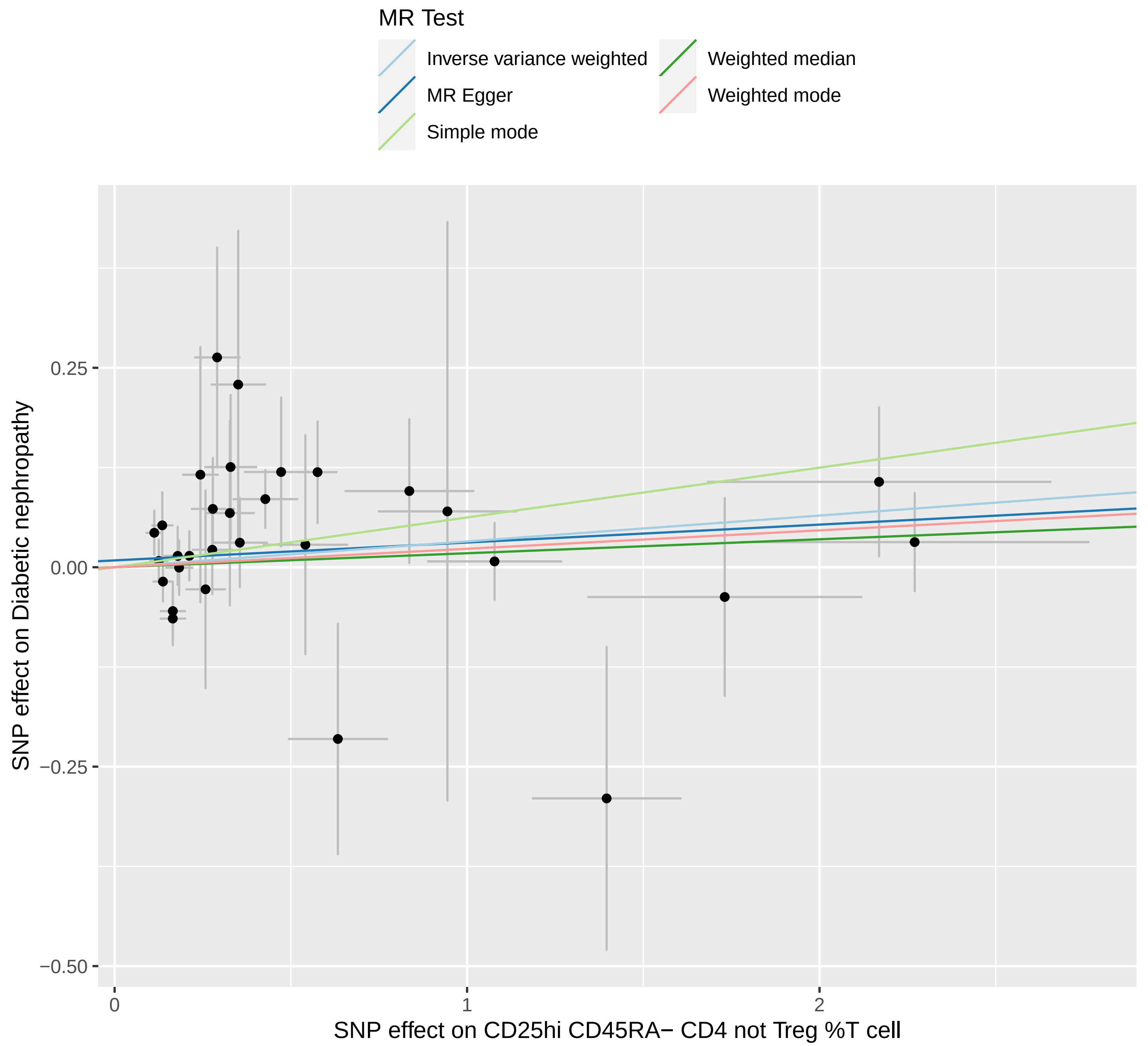

MR Test
Inverse variance weighted
MR Egger
Simple mode
Weighted median
Weighted mode
0.25
0.00
−0.25
−0.50
0
1
2
SNP effect on Diabetic nephropathy
SNP effect on CD25hi CD45RA− CD4 not Treg %T cell

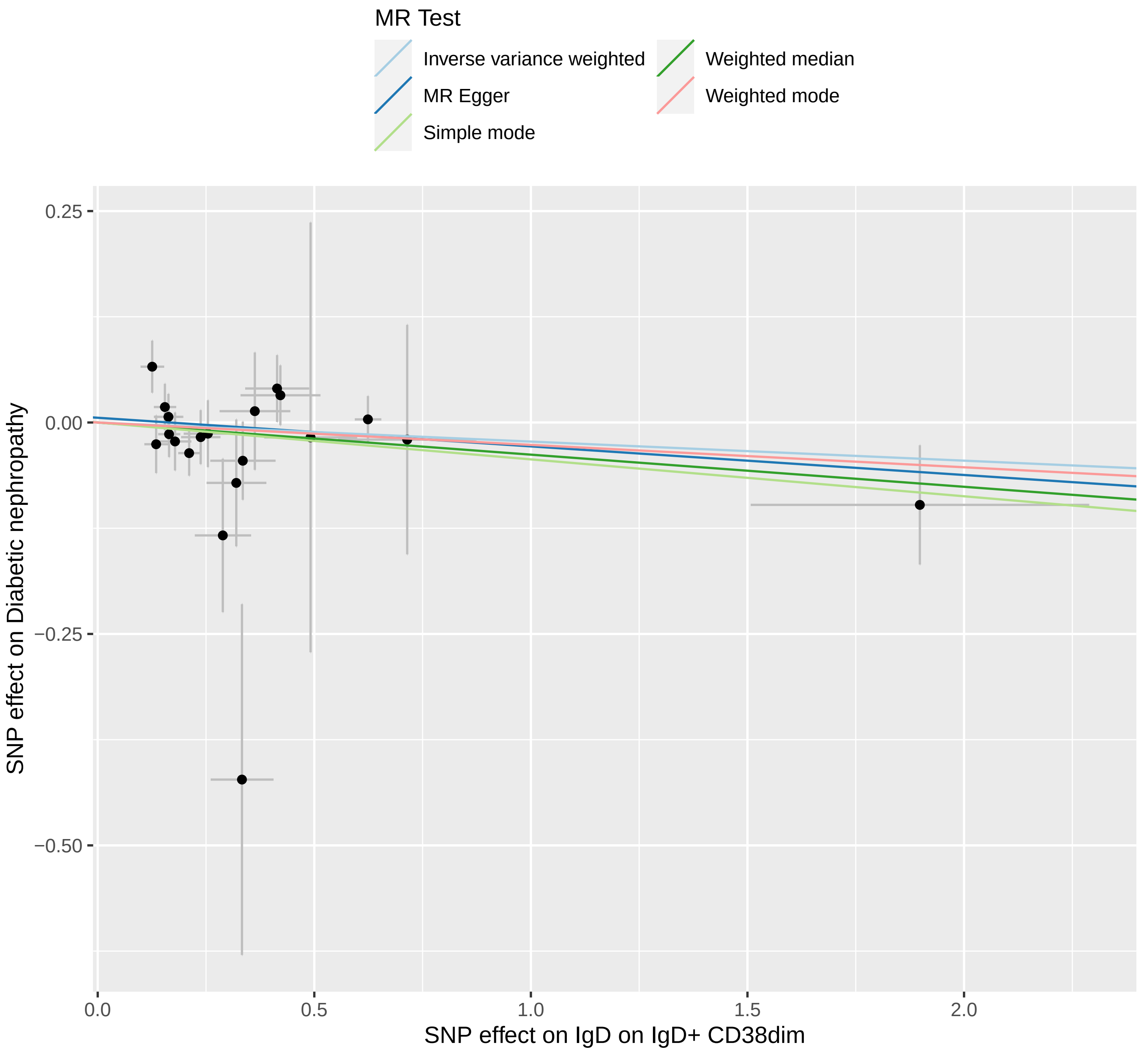

MR Test
Inverse variance weighted
MR Egger
Simple mode
Weighted median
Weighted mode
0.25
0.00
−0.25
−0.50
0.0
0.5
1.0
1.5
2.0
SNP effect on Diabetic nephropathy
SNP effect on IgD on IgD+ CD38dim

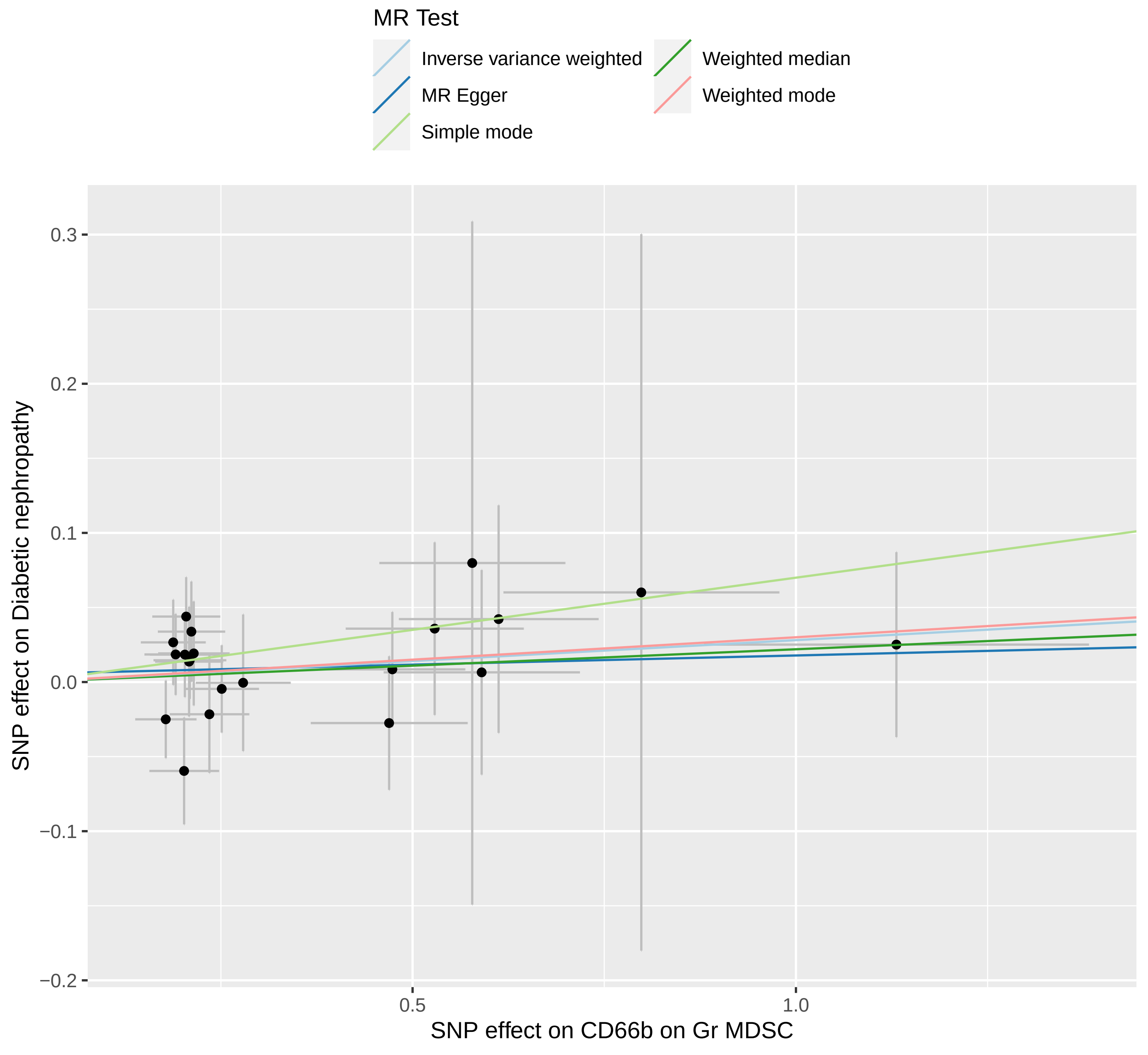

MR Test
Inverse variance weighted
MR Egger
Simple mode
Weighted median
Weighted mode
0.3
0.2
0.1
0.0
−0.1
−0.2
0.5
1.0
SNP effect on Diabetic nephropathy
SNP effect on CD66b on Gr MDSC

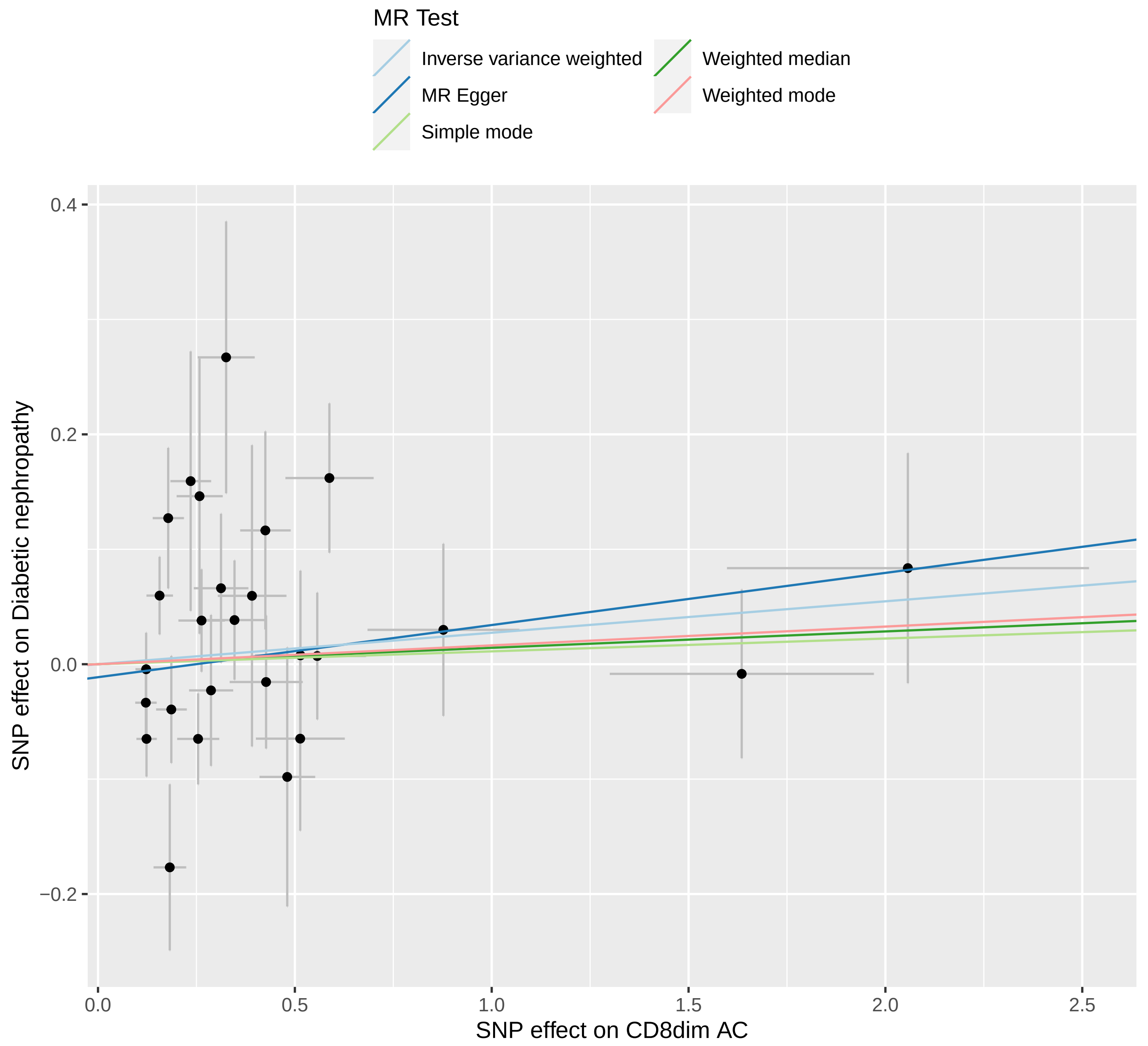

MR Test
Inverse variance weighted
MR Egger
Simple mode
Weighted median
Weighted mode
SNP effect on Diabetic nephropathy
SNP effect on CD8dim AC
0.4
0.2
0.0
−0.2
0.0
0.5
1.0
1.5
2.0
2.5

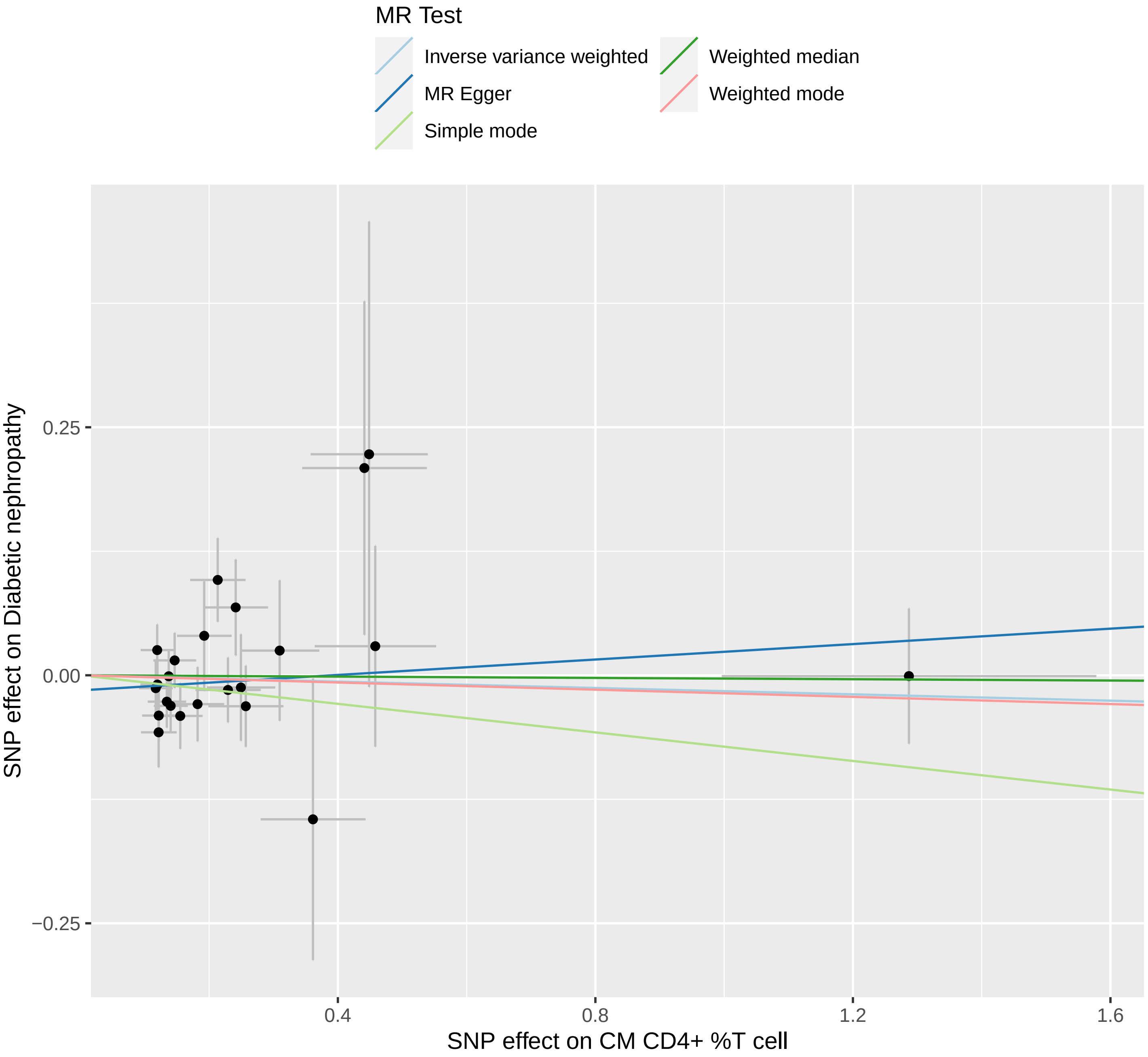
MR Test
Inverse variance weighted
MR Egger
Simple mode
Weighted median
Weighted mode
0.25
0.00
−0.25
0.4
0.8
1.2
1.6
SNP effect on Diabetic nephropathy
SNP effect on CM CD4+ %T cell

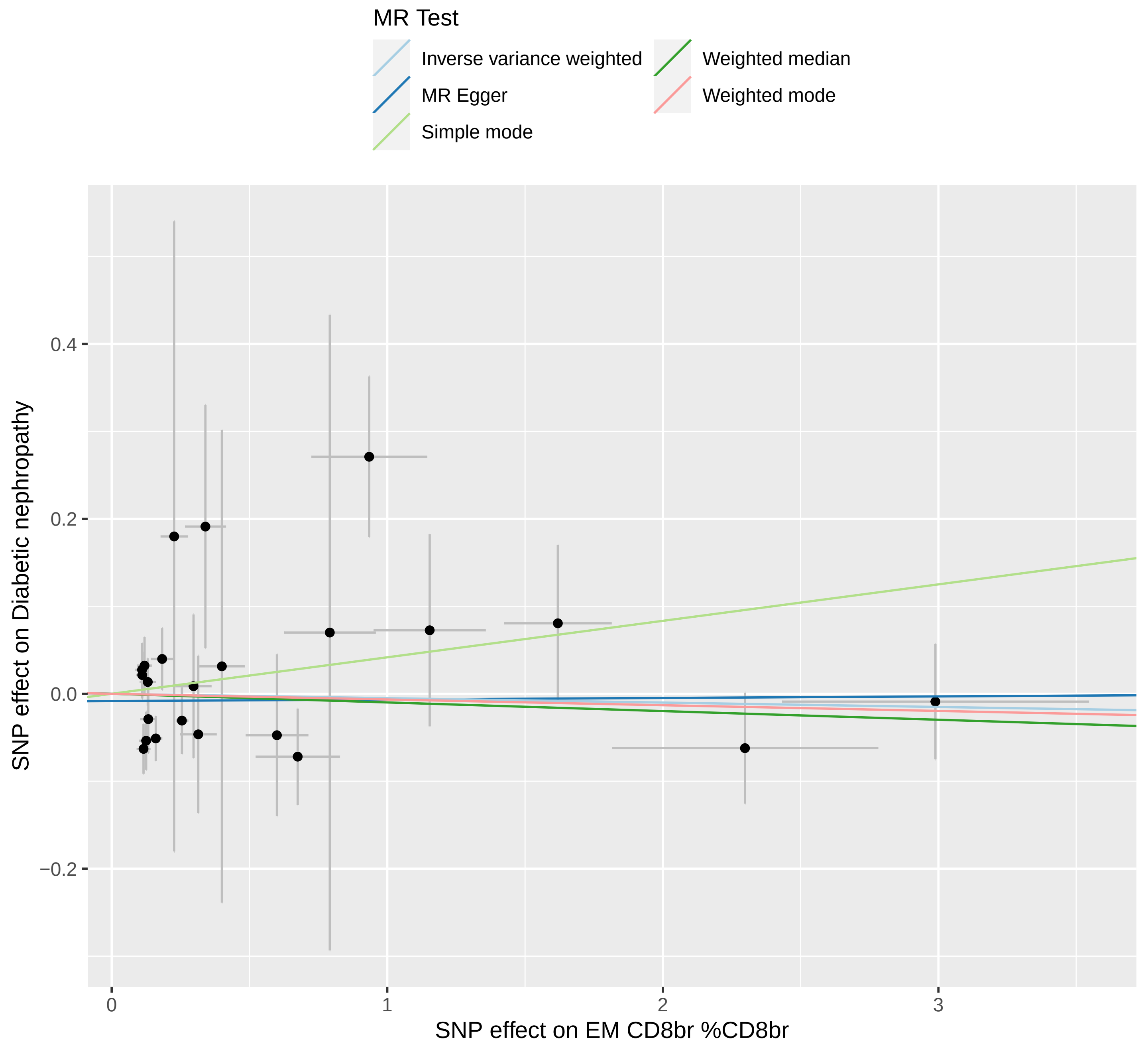

MR Test
Inverse variance weighted
MR Egger
Simple mode
Weighted median
Weighted mode
SNP effect on Diabetic nephropathy
0.4
0.2
0.0
−0.2
0
1
2
3
SNP effect on EM CD8br %CD8br

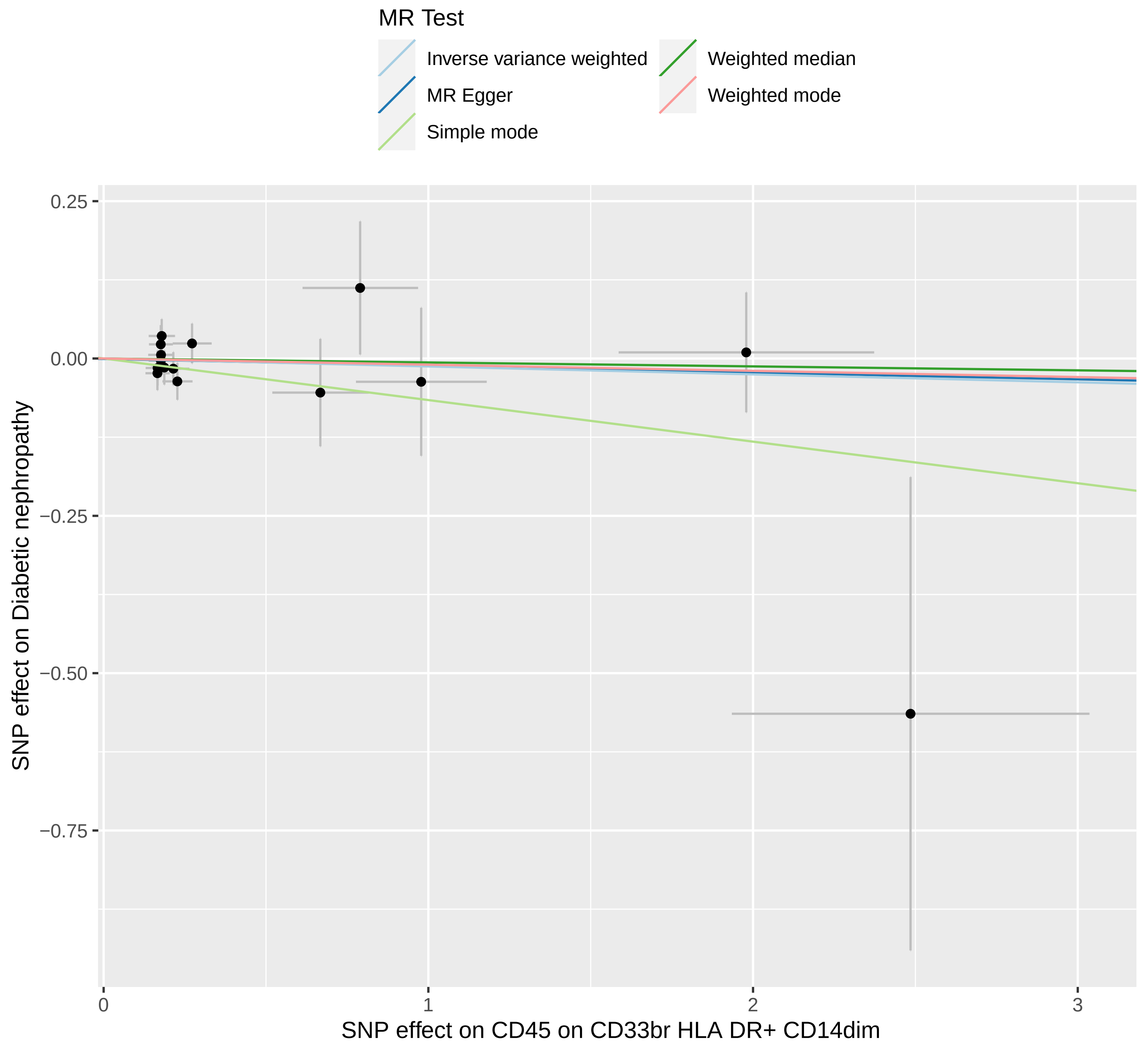
MR Test
Inverse variance weighted
MR Egger
Simple mode
Weighted median
Weighted mode
SNP effect on Diabetic nephropathy
0.25
0.00
−0.25
−0.50
−0.75
0
1
2
3
SNP effect on CD45 on CD33br HLA DR+ CD14dim

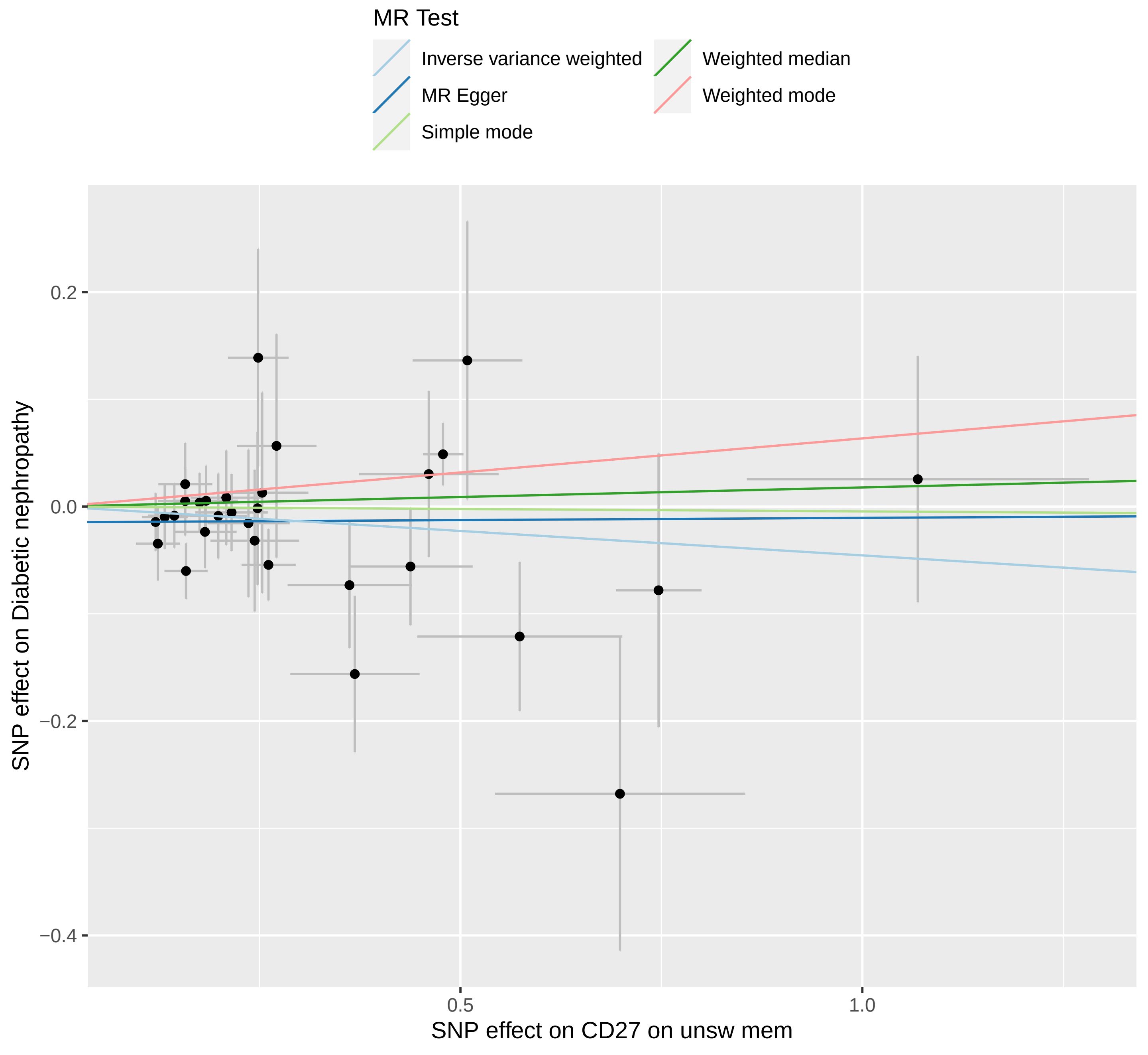
MR Test
Inverse variance weighted
MR Egger
Simple mode
Weighted median
Weighted mode
0.2
0.0
−0.2
−0.4
0.5
1.0
SNP effect on Diabetic nephropathy
SNP effect on CD27 on unsw mem

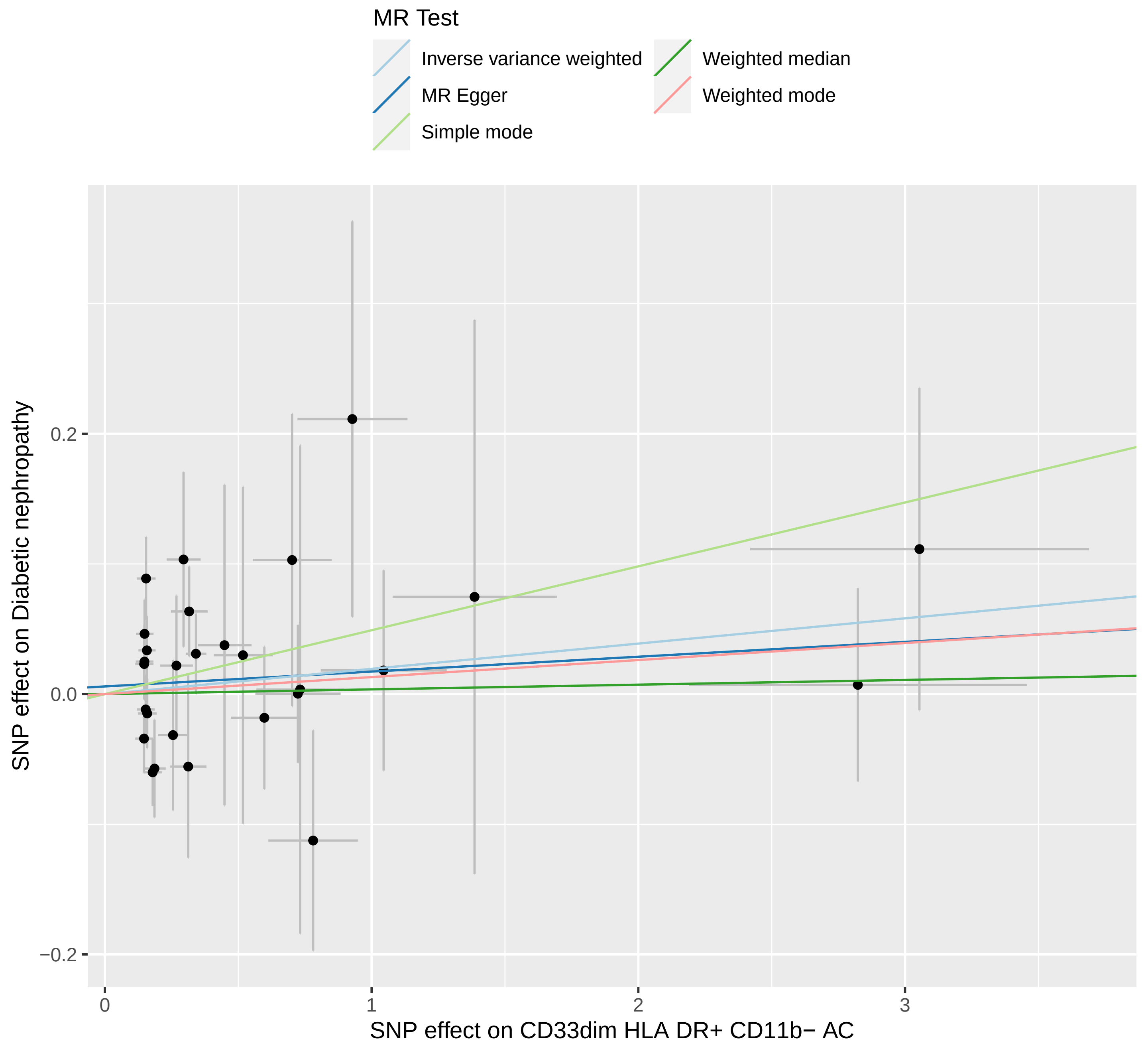

MR Test
Inverse variance weighted
MR Egger
Simple mode
Weighted median
Weighted mode
0.2
0.0
−0.2
0
1
2
3
SNP effect on Diabetic nephropathy
SNP effect on CD33dim HLA DR+ CD11b− AC

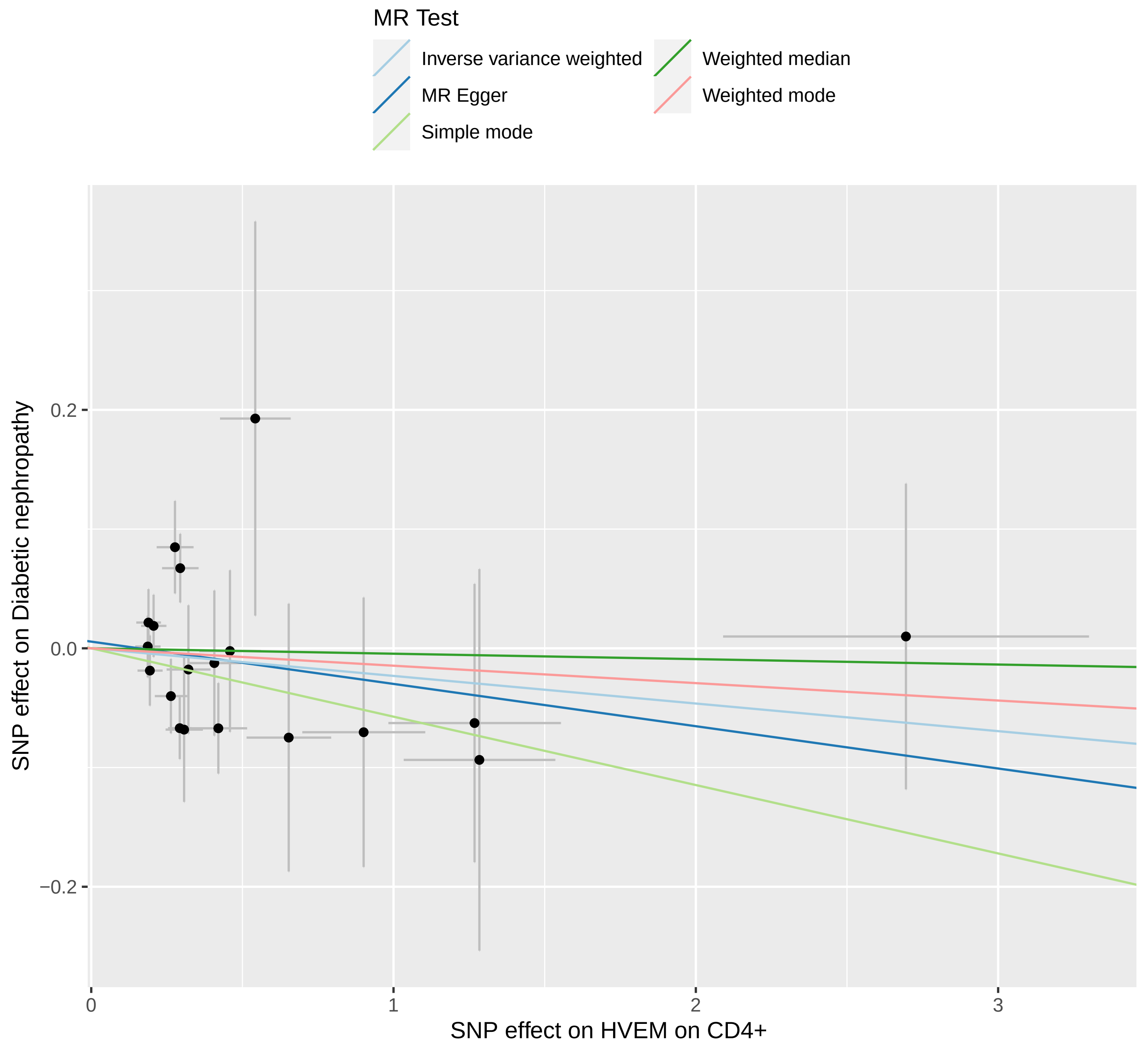

MR Test
Inverse variance weighted
MR Egger
Simple mode
Weighted median
Weighted mode
0.2
0.0
−0.2
0
1
2
3
SNP effect on Diabetic nephropathy
SNP effect on HVEM on CD4+

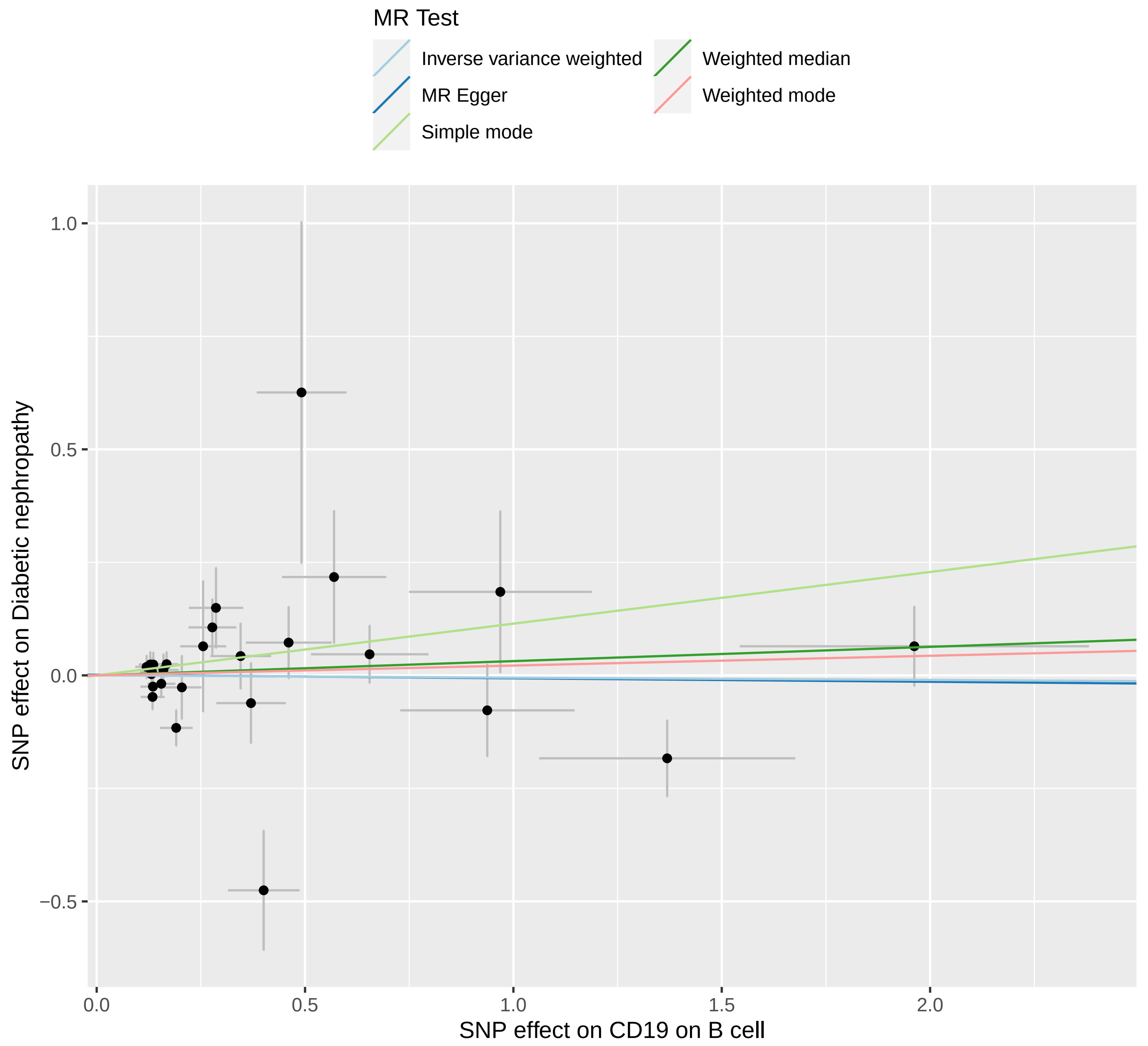

MR Test
Inverse variance weighted
MR Egger
Simple mode
Weighted median
Weighted mode
1.0
0.5
0.0
−0.5
0.0
0.5
1.0
1.5
2.0
SNP effect on Diabetic nephropathy
SNP effect on CD19 on B cell

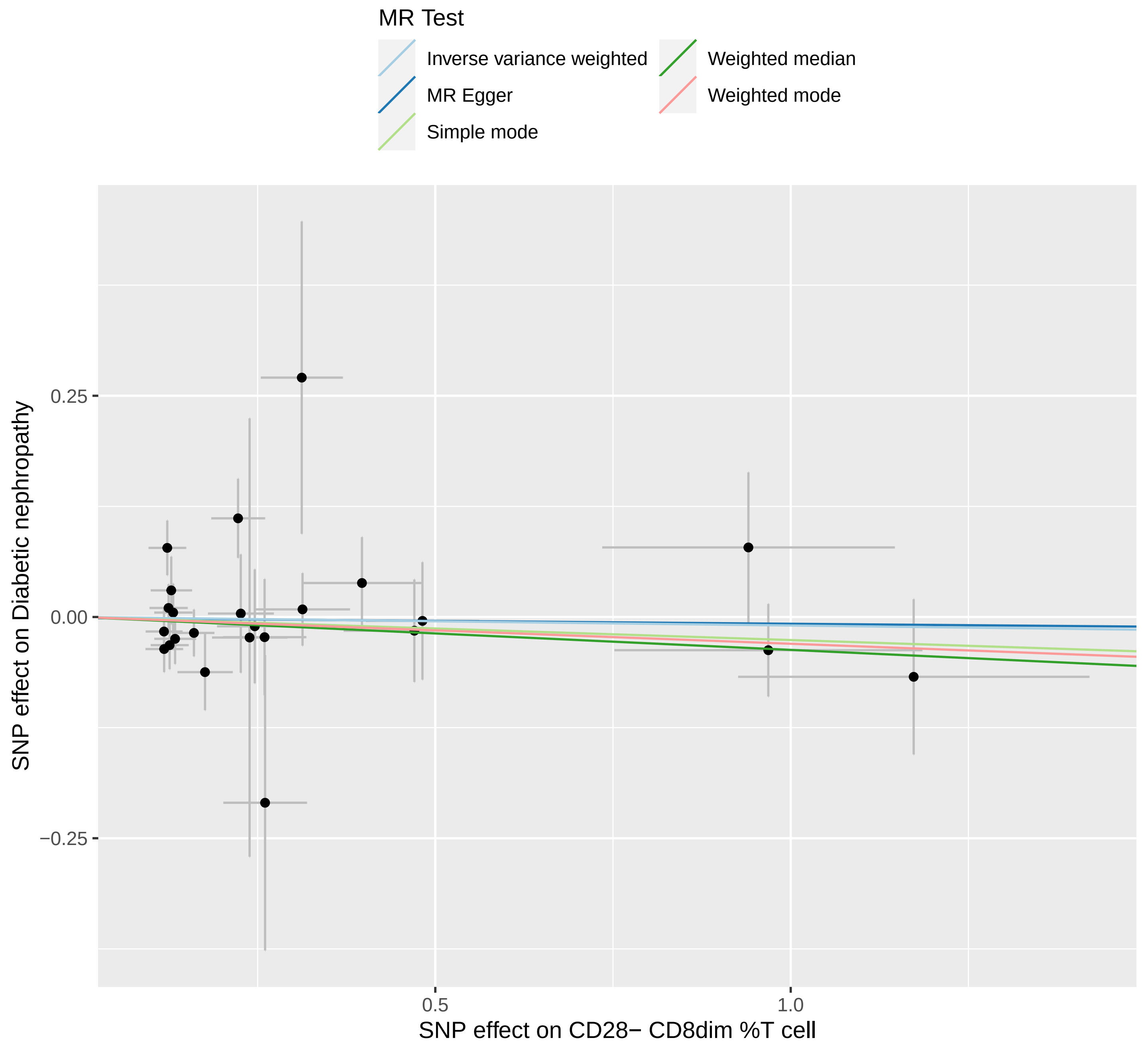

MR Test
Inverse variance weighted
MR Egger
Simple mode
Weighted median
Weighted mode
0.25
0.00
−0.25
0.5
1.0
SNP effect on Diabetic nephropathy
SNP effect on CD28− CD8dim %T cell

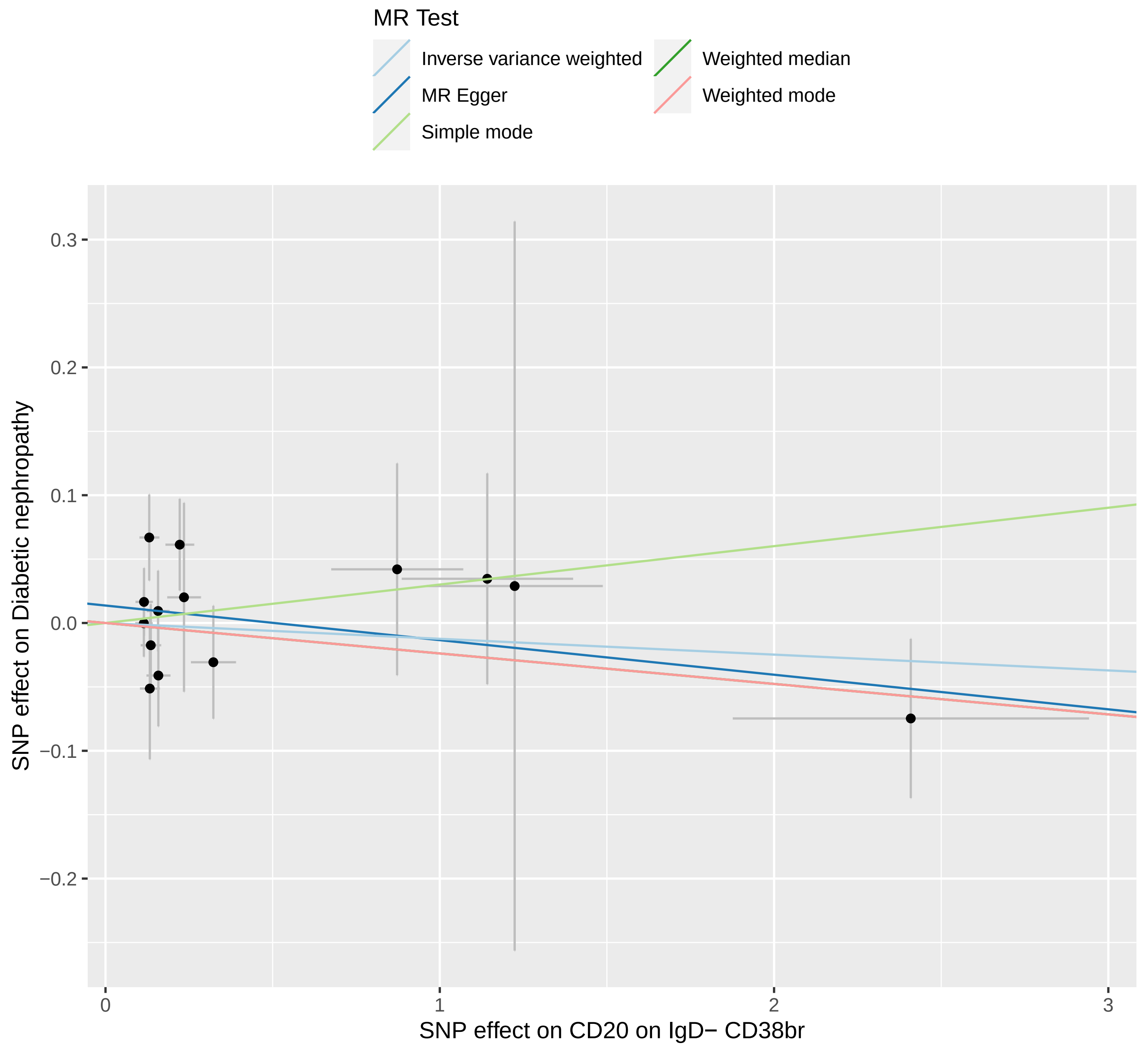
MR Test
Inverse variance weighted
MR Egger
Simple mode
Weighted median
Weighted mode
0.3
0.2
0.1
0.0
−0.1
−0.2
SNP effect on Diabetic nephropathy
0
1
2
3
SNP effect on CD20 on IgD− CD38br

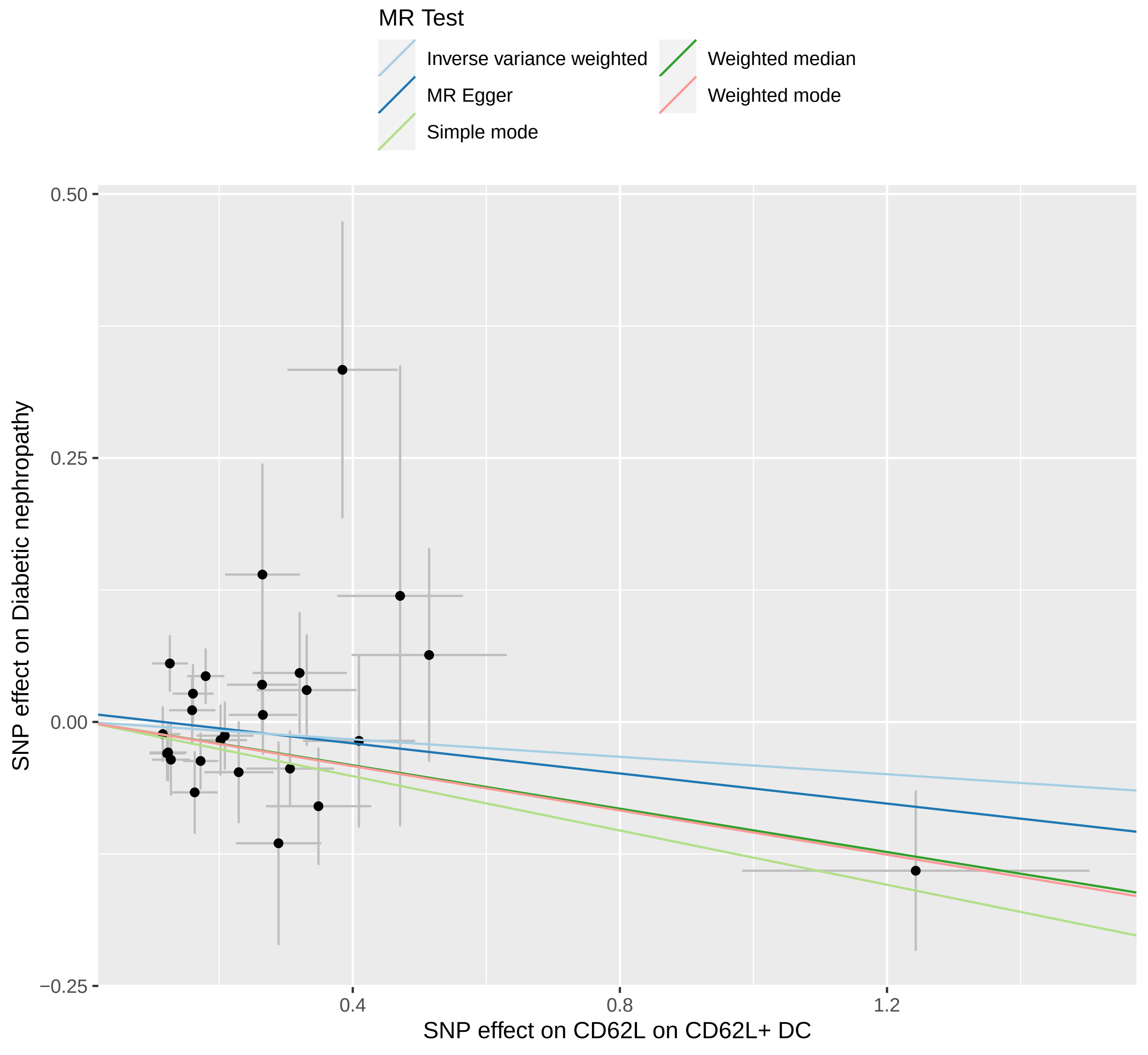
MR Test
Inverse variance weighted
MR Egger
Simple mode
Weighted median
Weighted mode
0.50
0.25
0.00
−0.25
0.4
0.8
1.2
SNP effect on Diabetic nephropathy
SNP effect on CD62L on CD62L+ DC

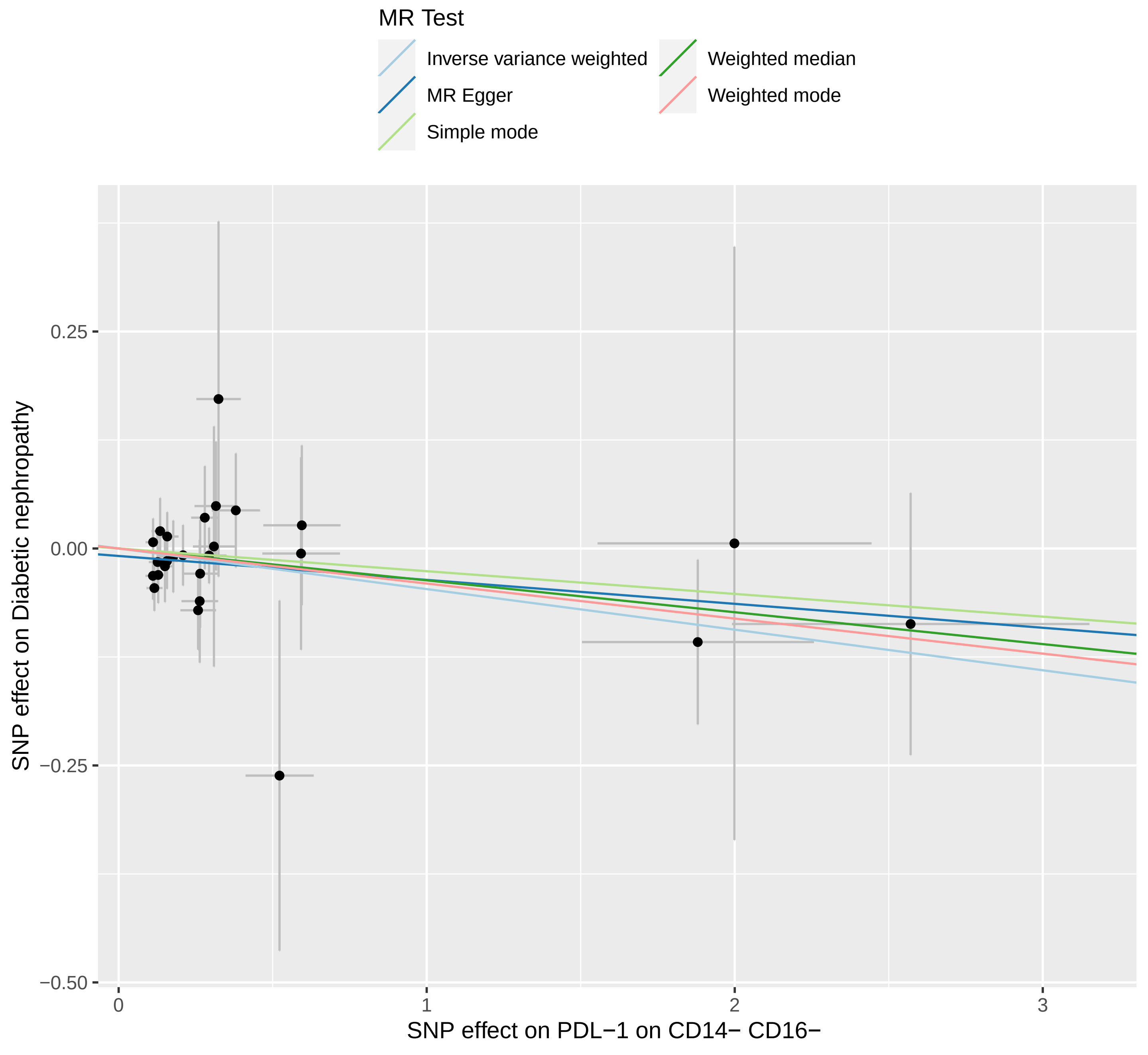
MR Test
Inverse variance weighted
MR Egger
Simple mode
Weighted median
Weighted mode
SNP effect on Diabetic nephropathy
0.25
0.00
−0.25
−0.50
0
1
2
3
SNP effect on PDL−1 on CD14− CD16−

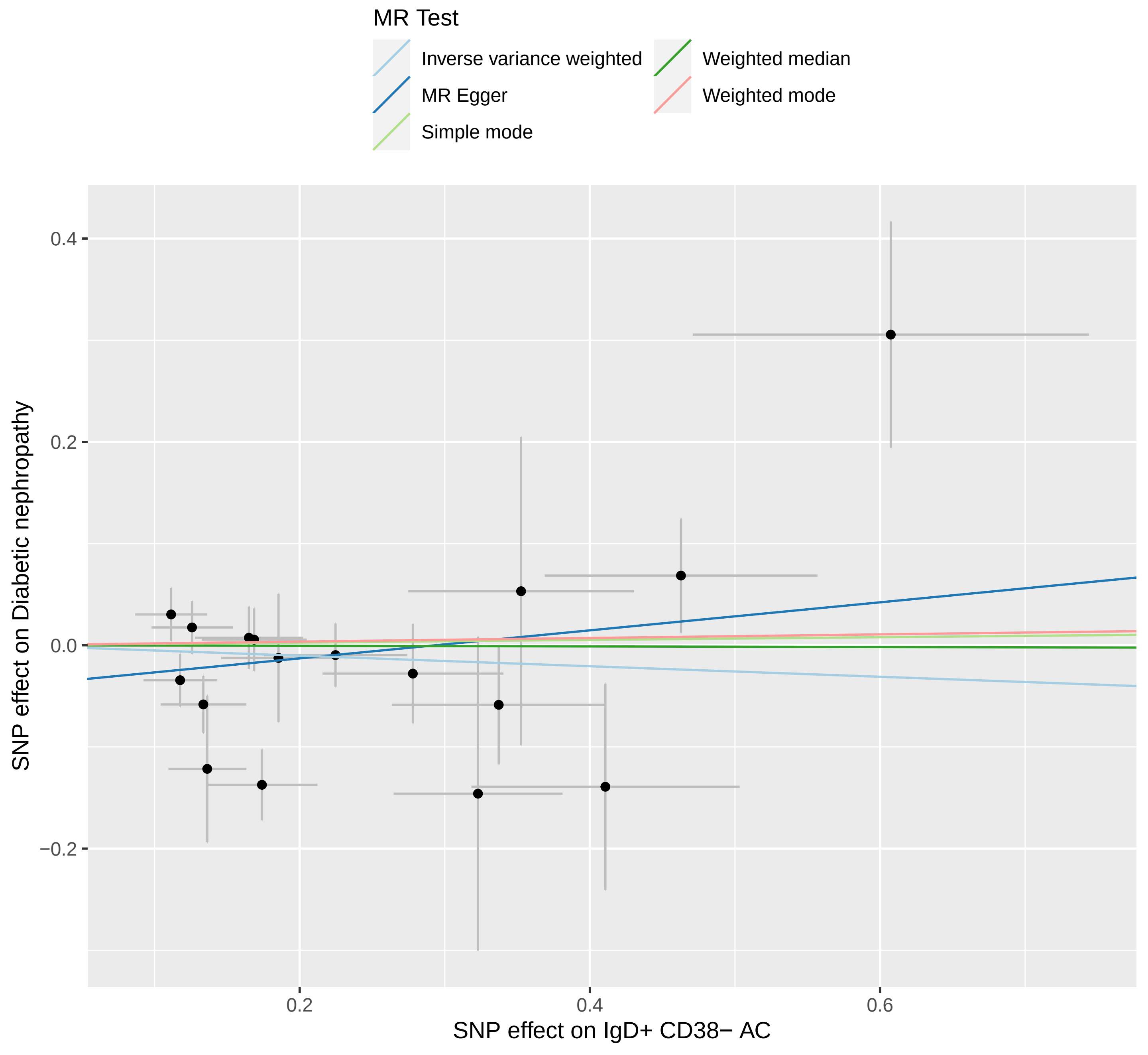

MR Test
Inverse variance weighted
MR Egger
Simple mode
Weighted median
Weighted mode
0.4
0.2
0.0
−0.2
0.2
0.4
0.6
SNP effect on Diabetic nephropathy
SNP effect on IgD+ CD38− AC

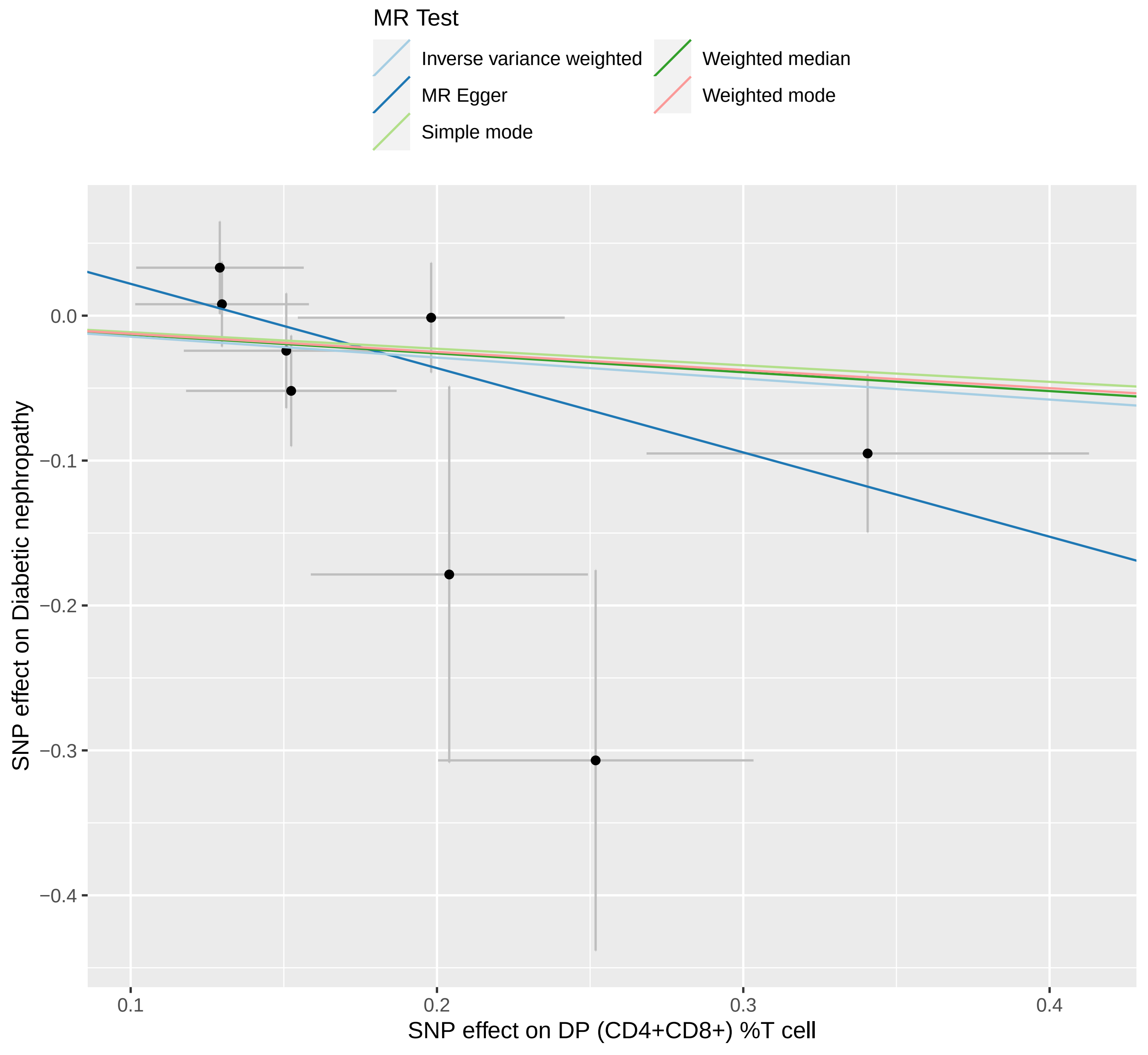
MR Test
Inverse variance weighted
MR Egger
Simple mode
Weighted median
Weighted mode
0.0
−0.1
−0.2
−0.3
−0.4
0.1
0.2
0.3
0.4
SNP effect on Diabetic nephropathy
SNP effect on DP (CD4+CD8+) %T cell

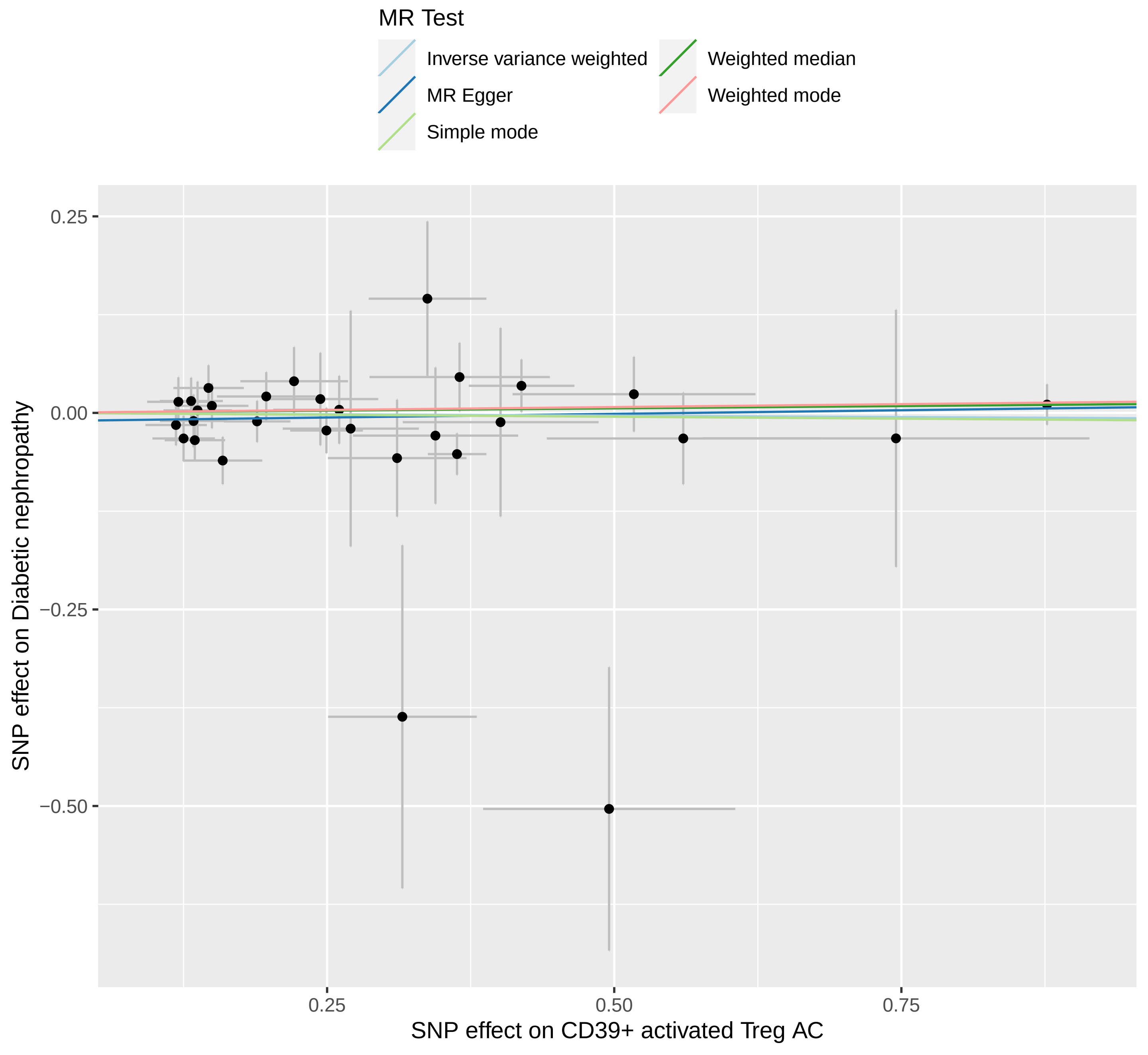
MR Test
Inverse variance weighted
MR Egger
Simple mode
Weighted median
Weighted mode
0.25
0.00
−0.25
−0.50
SNP effect on Diabetic nephropathy
0.25
0.50
0.75
SNP effect on CD39+ activated Treg AC

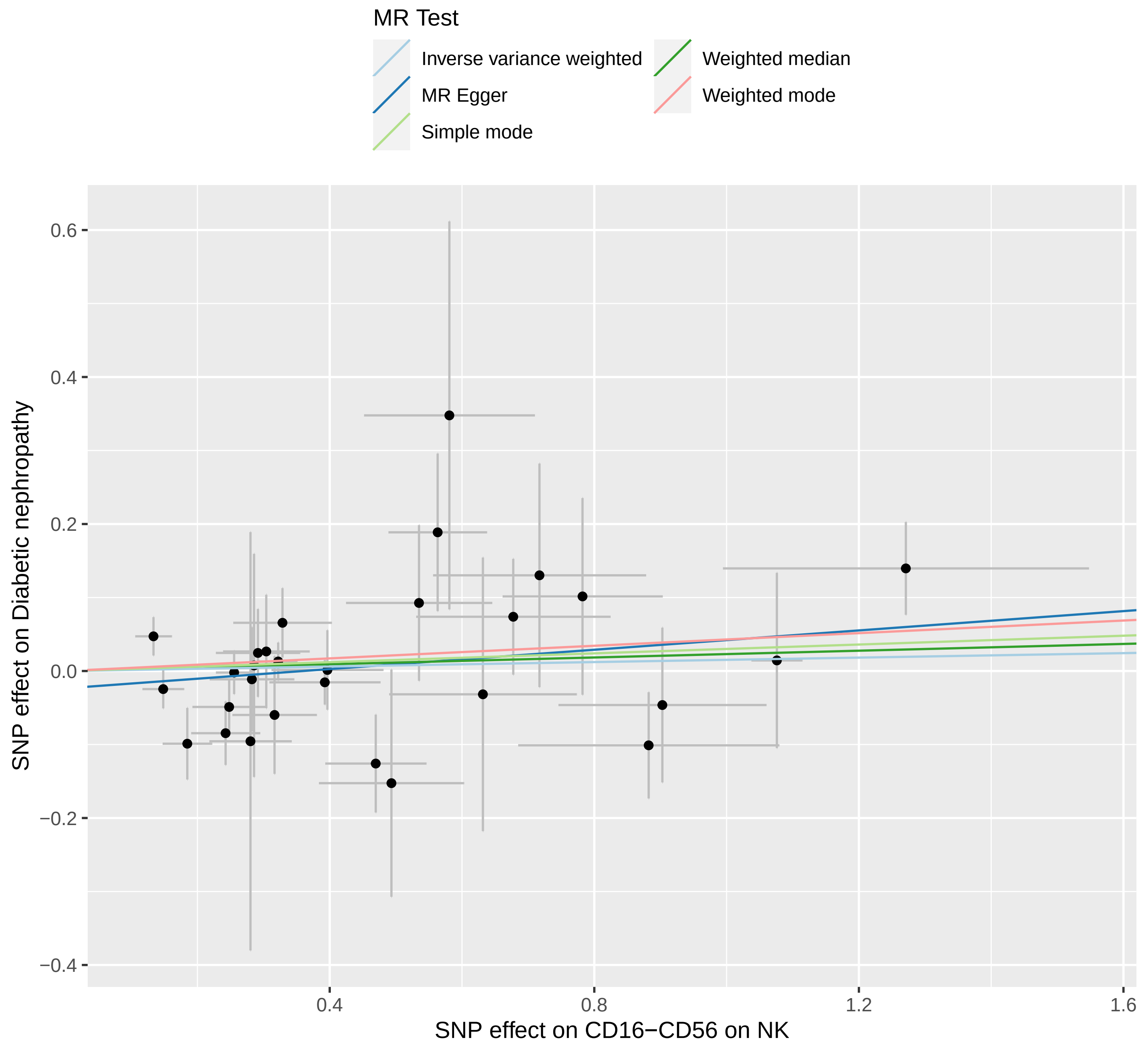

MR Test
Inverse variance weighted
MR Egger
Simple mode
Weighted median
Weighted mode
0.6
0.4
0.2
0.0
−0.2
−0.4
0.4
0.8
1.2
1.6
SNP effect on Diabetic nephropathy
SNP effect on CD16−CD56 on NK

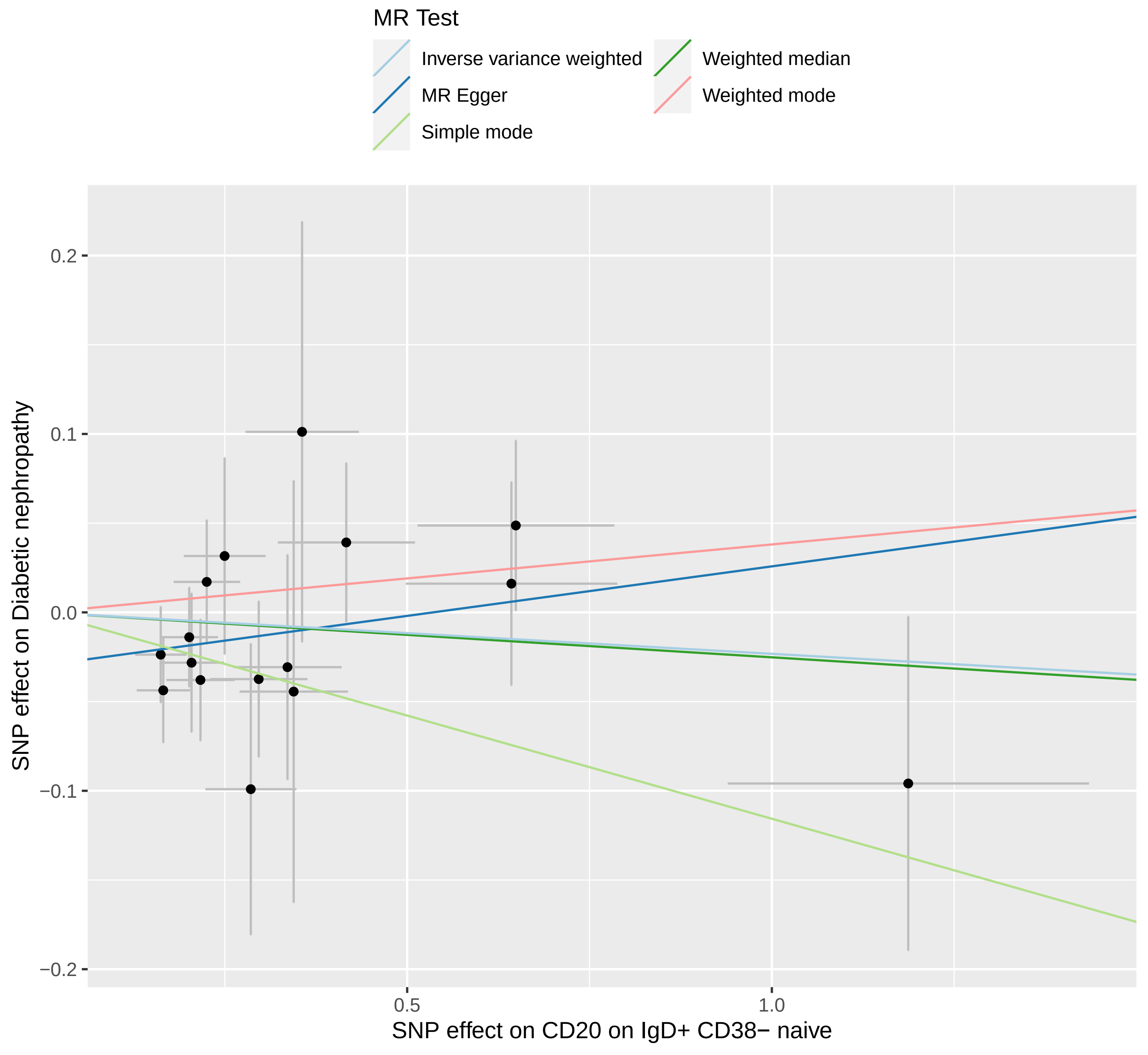

MR Test
Inverse variance weighted
MR Egger
Simple mode
Weighted median
Weighted mode
0.2
0.1
0.0
−0.1
−0.2
SNP effect on Diabetic nephropathy
0.5
1.0
SNP effect on CD20 on IgD+ CD38− naive

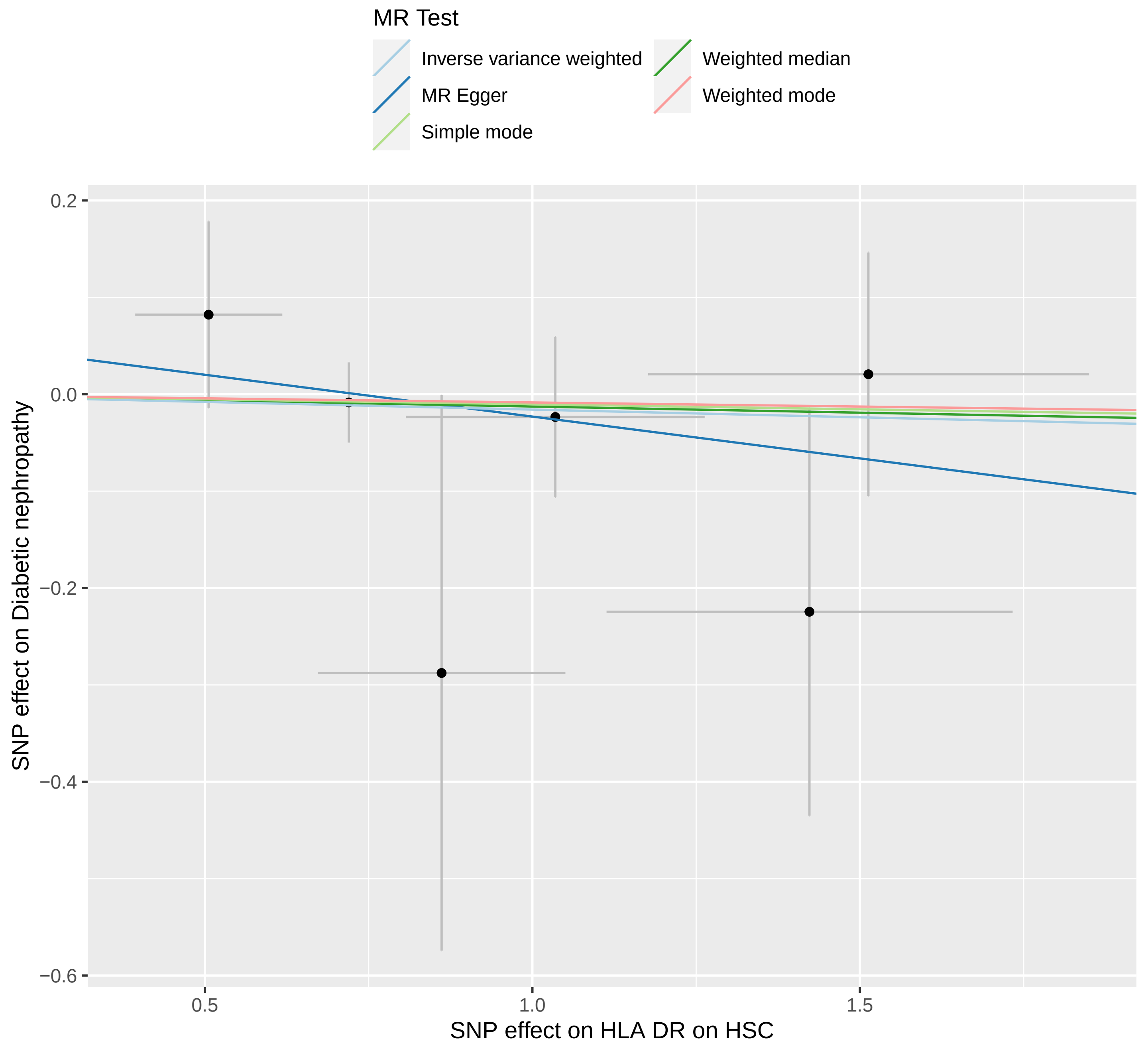
MR Test
Inverse variance weighted
MR Egger
Simple mode
Weighted median
Weighted mode
0.2
0.0
−0.2
−0.4
−0.6
0.5
1.0
1.5
SNP effect on Diabetic nephropathy
SNP effect on HLA DR on HSC

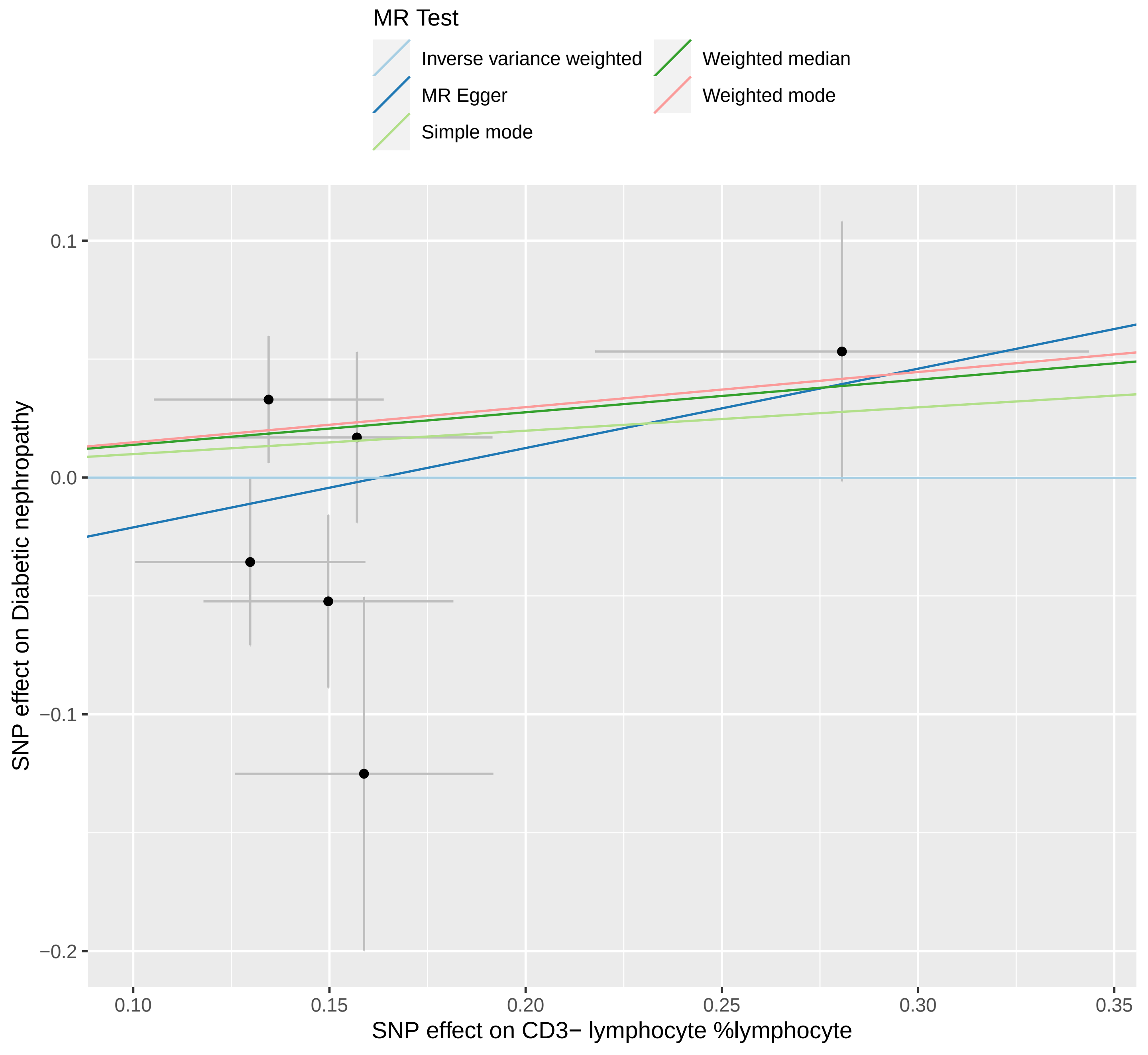
MR Test
Inverse variance weighted
MR Egger
Simple mode
Weighted median
Weighted mode
0.1
0.0
−0.1
−0.2
0.10
0.15
0.20
0.25
0.30
0.35
SNP effect on Diabetic nephropathy
SNP effect on CD3− lymphocyte %lymphocyte

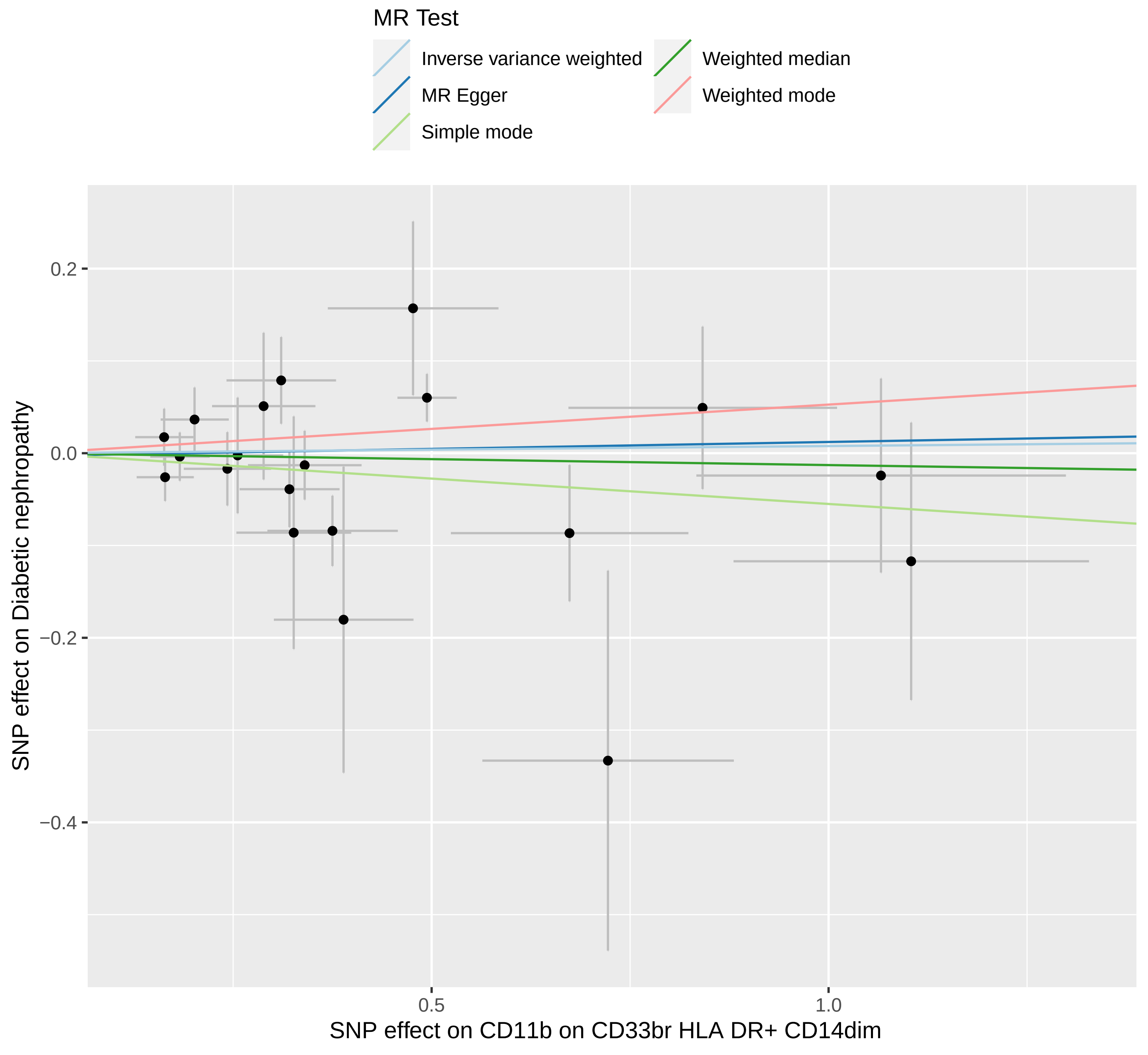

MR Test
Inverse variance weighted
MR Egger
Simple mode
Weighted median
Weighted mode
SNP effect on Diabetic nephropathy
0.2
0.0
−0.2
−0.4
0.5
1.0
SNP effect on CD11b on CD33br HLA DR+ CD14dim

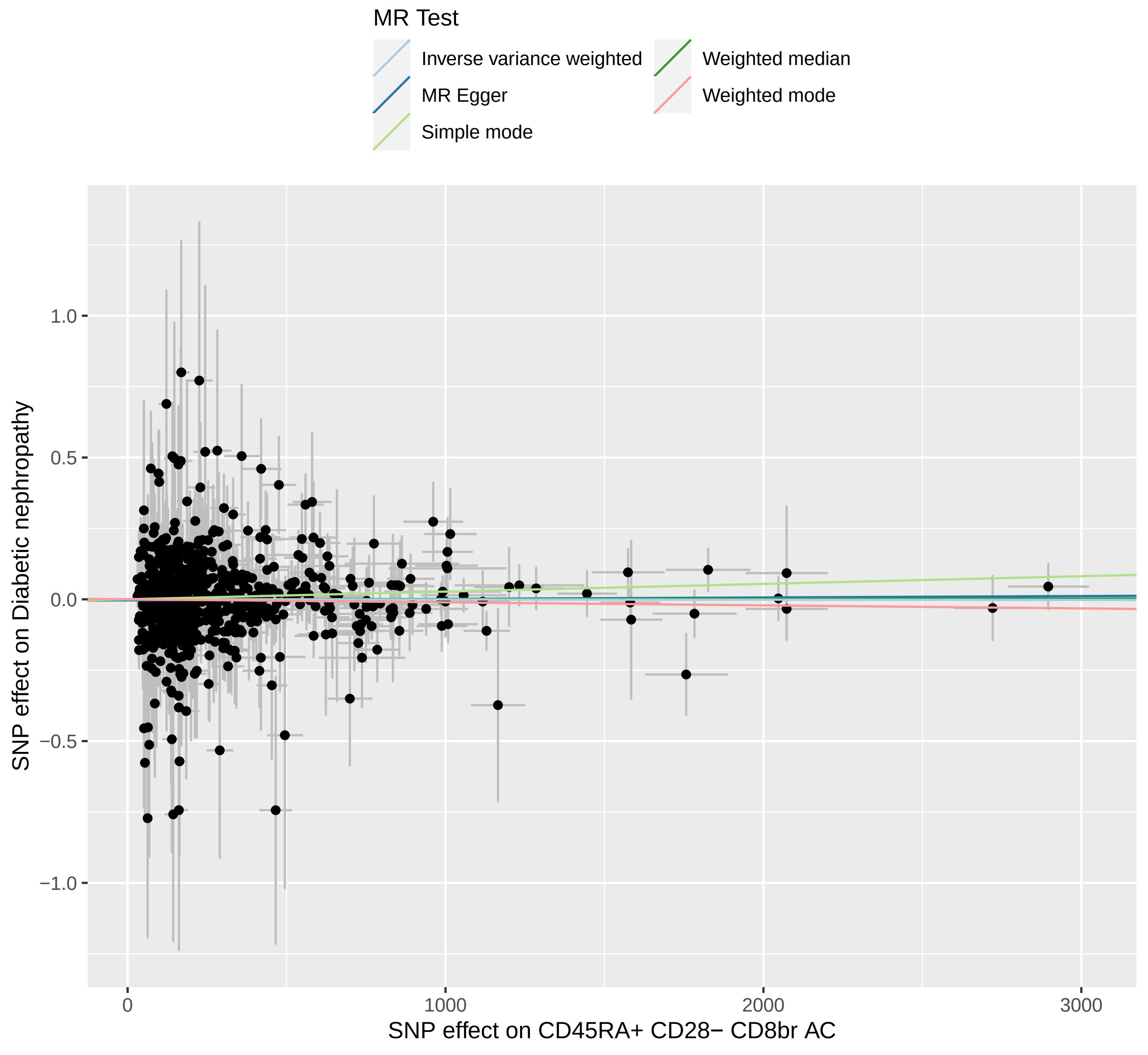

MR Test
Inverse variance weighted
MR Egger
Simple mode
Weighted median
Weighted mode
1.0
0.5
0.0
−0.5
−1.0
SNP effect on Diabetic nephropathy
0
1000
2000
3000
SNP effect on CD45RA+ CD28− CD8br AC

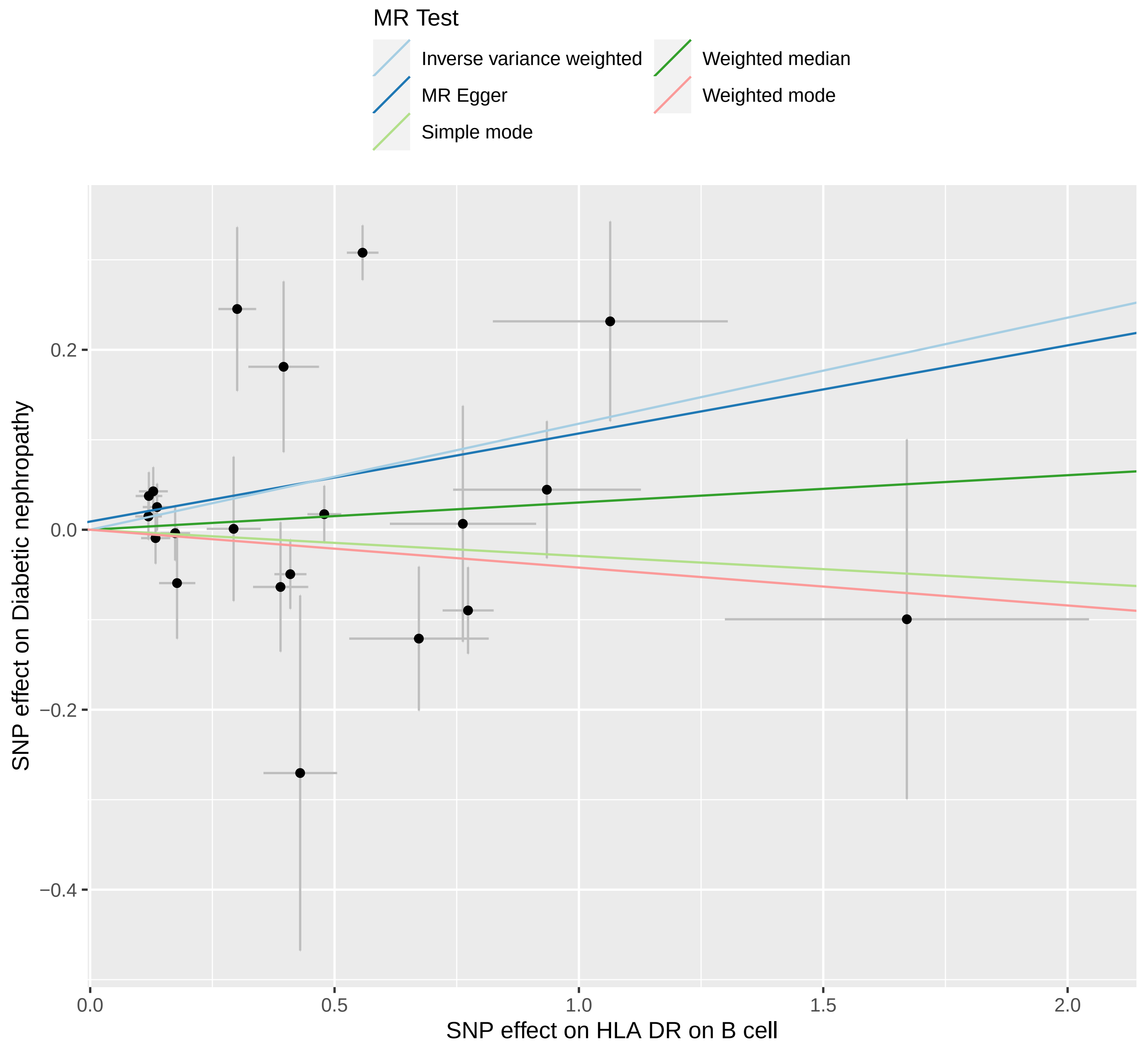
MR Test
Inverse variance weighted
MR Egger
Simple mode
Weighted median
Weighted mode
SNP effect on Diabetic nephropathy
0.2
0.0
−0.2
−0.4
0.0
0.5
1.0
1.5
2.0
SNP effect on HLA DR on B cell

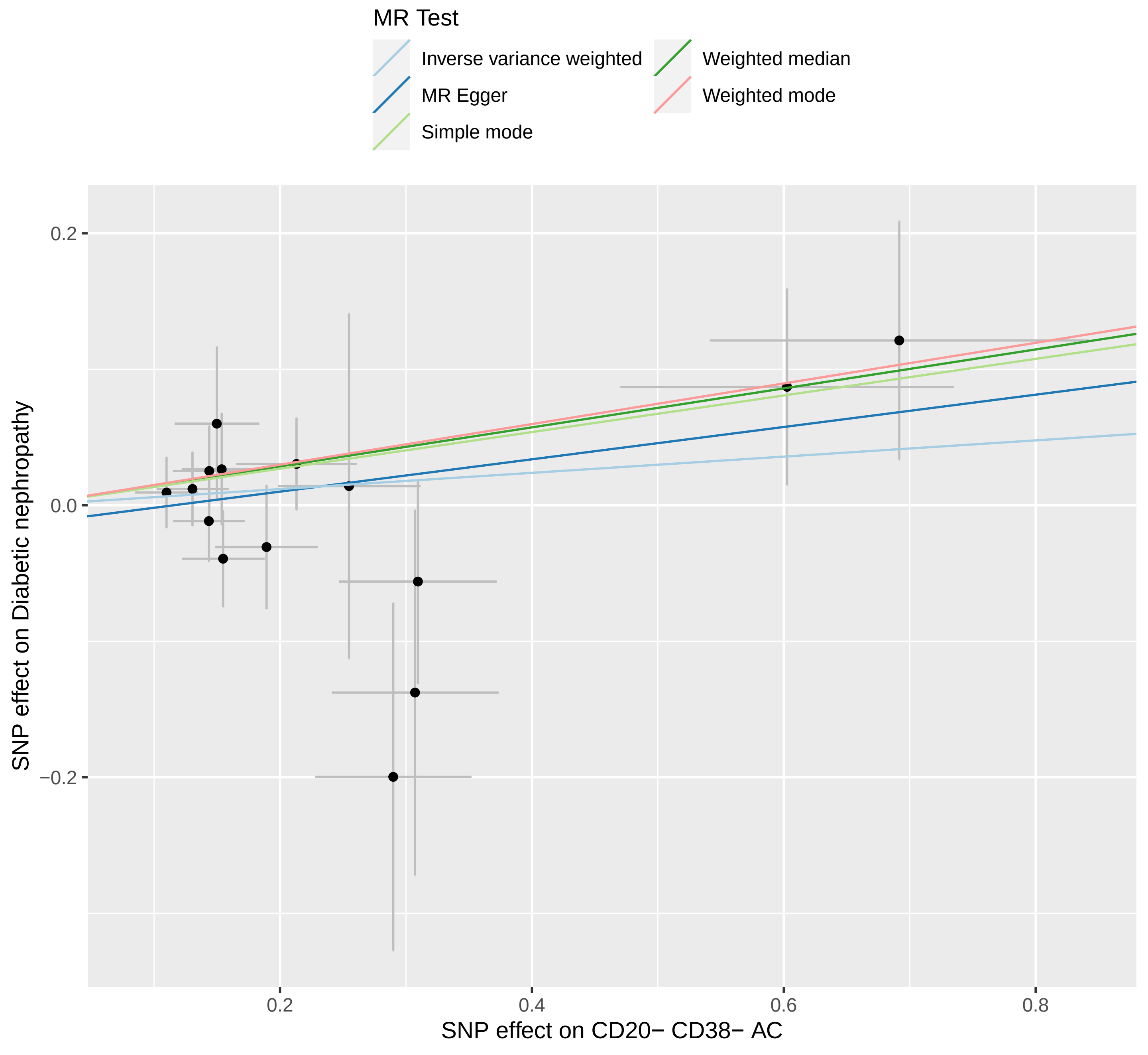

MR Test
Inverse variance weighted
MR Egger
Simple mode
Weighted median
Weighted mode
0.2
0.0
−0.2
SNP effect on Diabetic nephropathy
0.2
0.4
0.6
0.8
SNP effect on CD20− CD38− AC

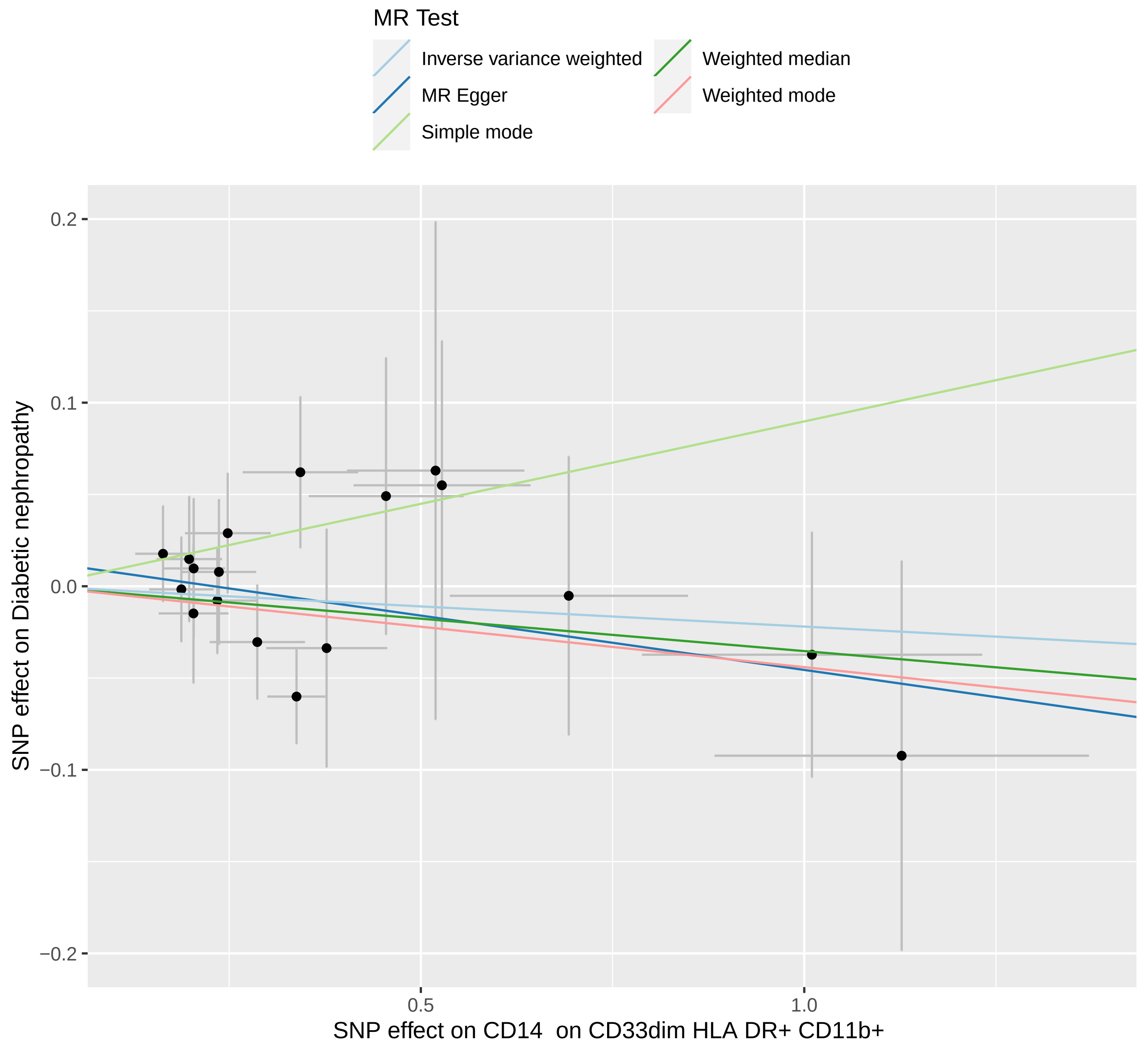

MR Test
Inverse variance weighted
MR Egger
Simple mode
Weighted median
Weighted mode
0.2
0.1
0.0
−0.1
−0.2
0.5
1.0
SNP effect on Diabetic nephropathy
SNP effect on CD14 on CD33dim HLA DR+ CD11b+

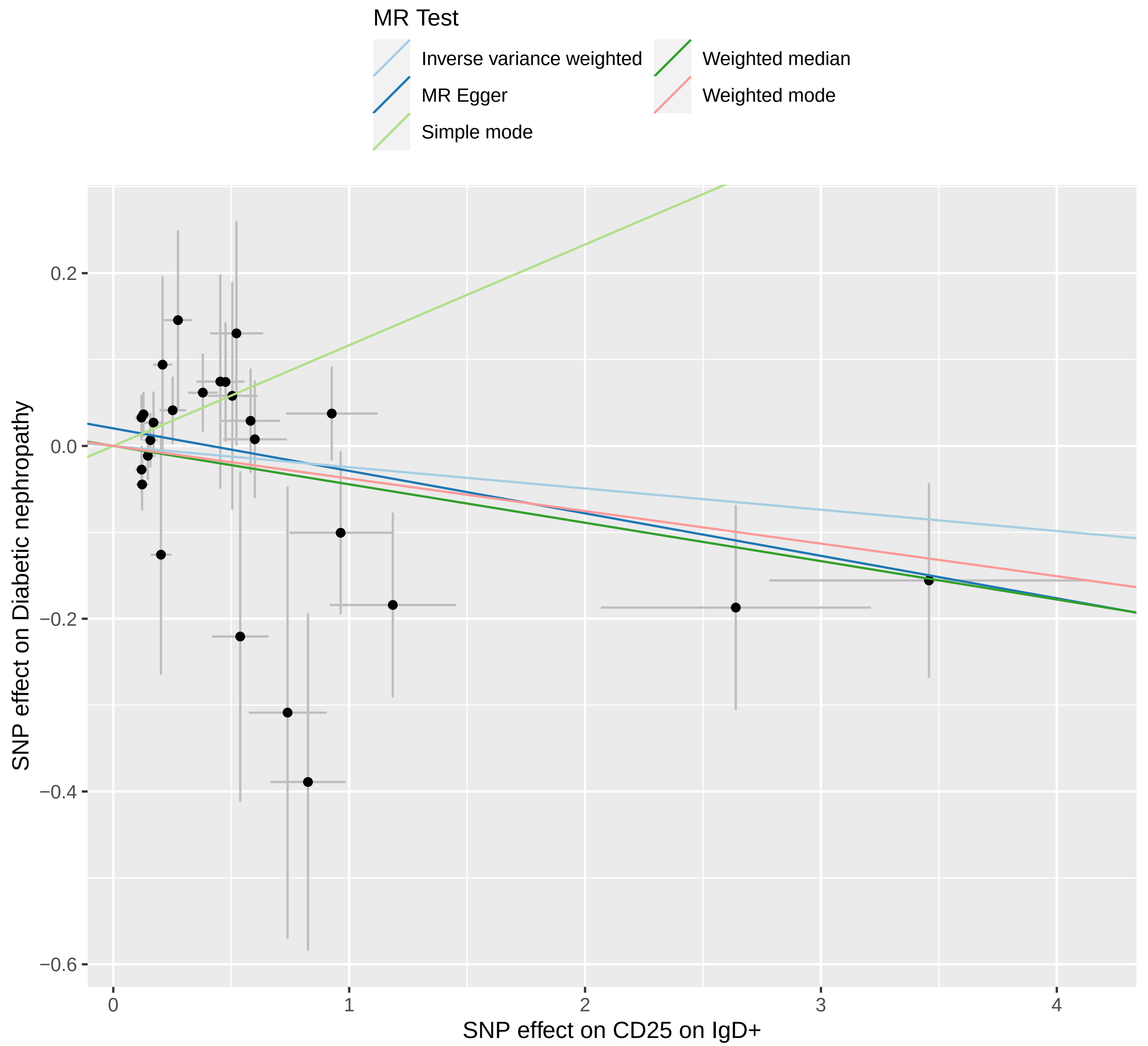
MR Test
Inverse variance weighted
MR Egger
Simple mode
Weighted median
Weighted mode
SNP effect on Diabetic nephropathy
SNP effect on CD25 on IgD+
0.2
0.0
−0.2
−0.4
−0.6
0
1
2
3
4

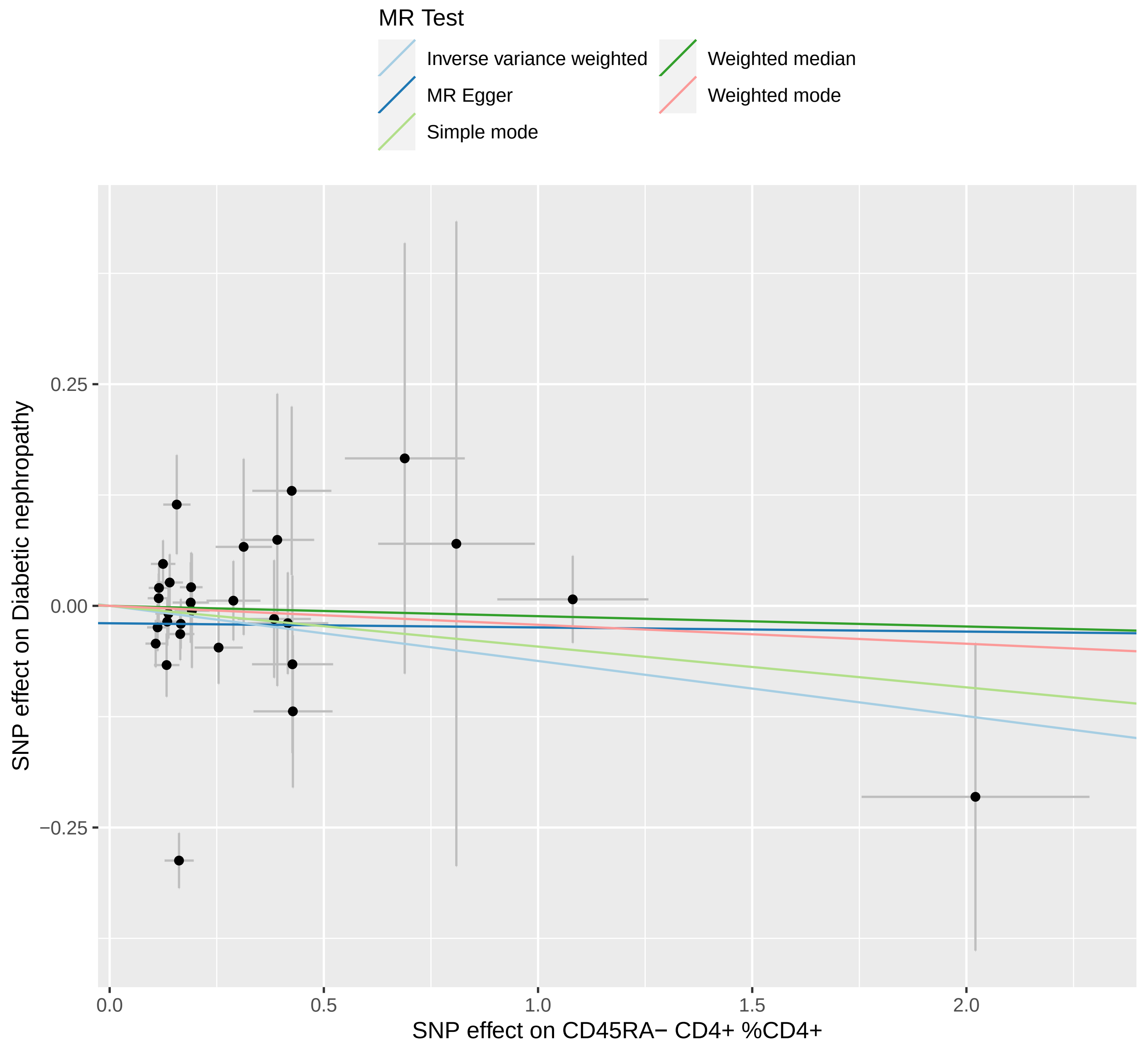

MR Test
Inverse variance weighted
MR Egger
Simple mode
Weighted median
Weighted mode
0.25
0.00
−0.25
0.0
0.5
1.0
1.5
2.0
SNP effect on Diabetic nephropathy
SNP effect on CD45RA− CD4+ %CD4+

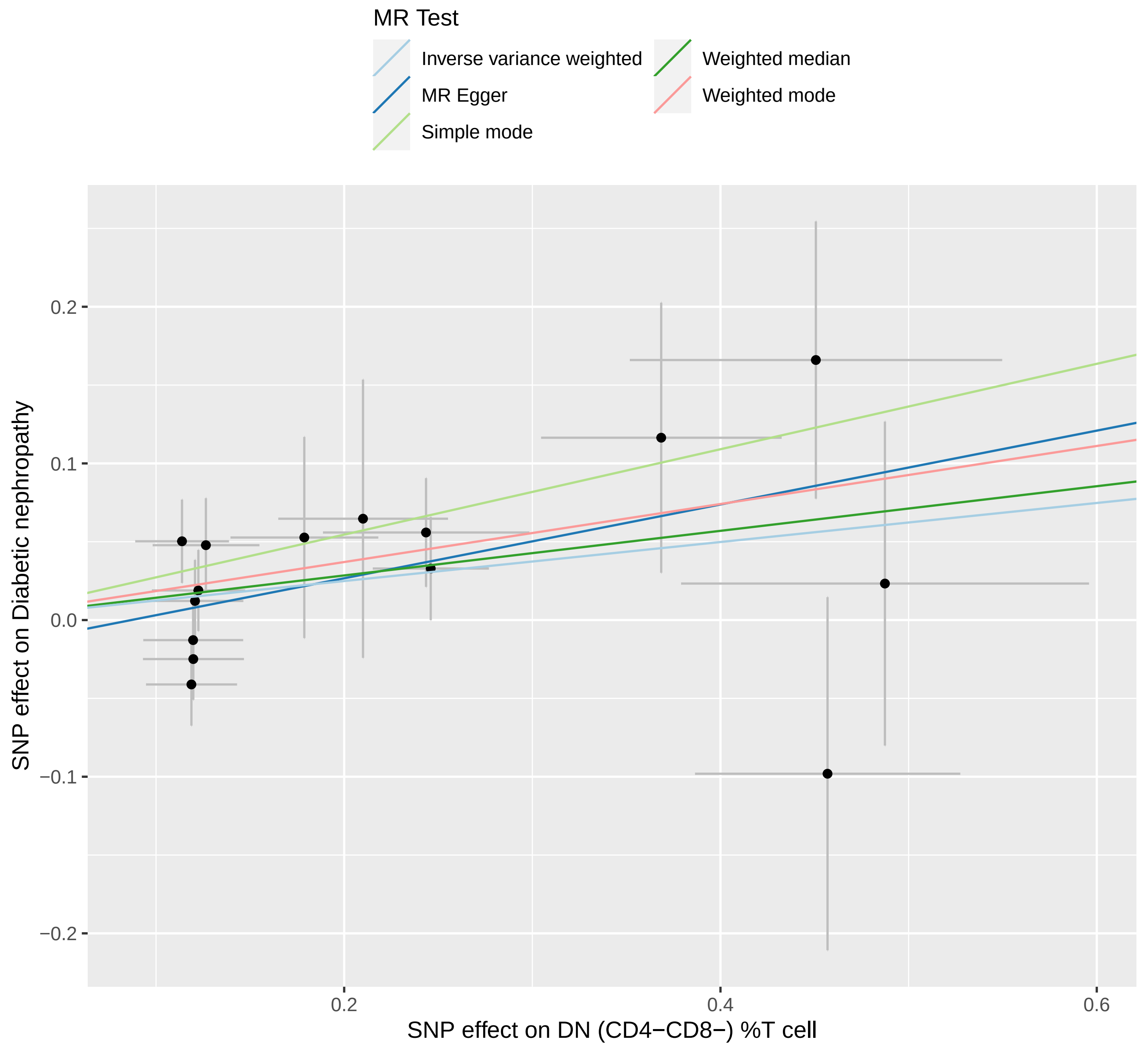

MR Test
Inverse variance weighted
MR Egger
Simple mode
Weighted median
Weighted mode
0.2
0.1
0.0
−0.1
−0.2
SNP effect on Diabetic nephropathy
0.2
0.4
0.6
SNP effect on DN (CD4−CD8−) %T cell

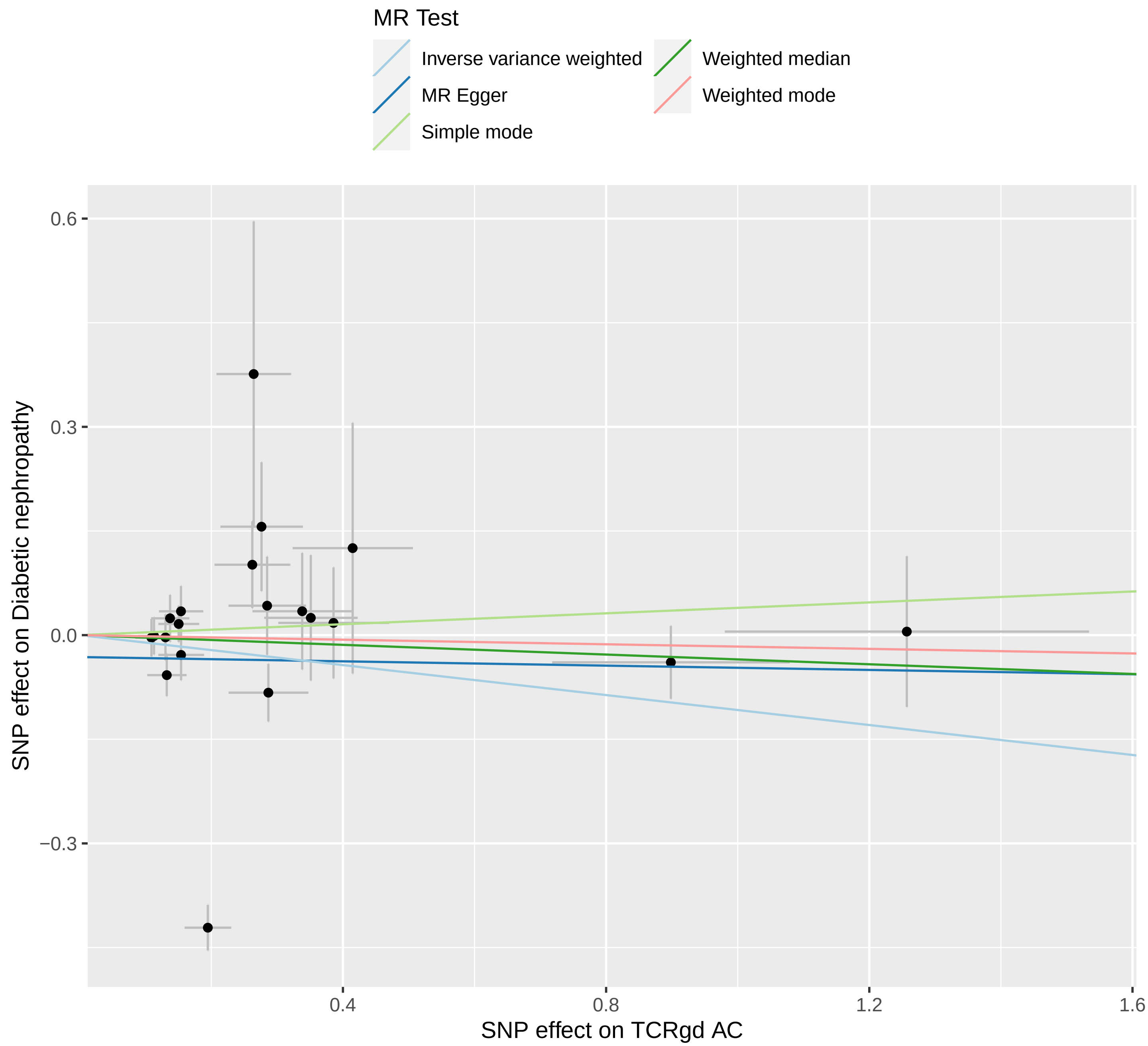

MR Test
Inverse variance weighted
MR Egger
Simple mode
Weighted median
Weighted mode
0.6
0.3
0.0
−0.3
SNP effect on Diabetic nephropathy
0.4
0.8
1.2
1.6
SNP effect on TCRgd AC

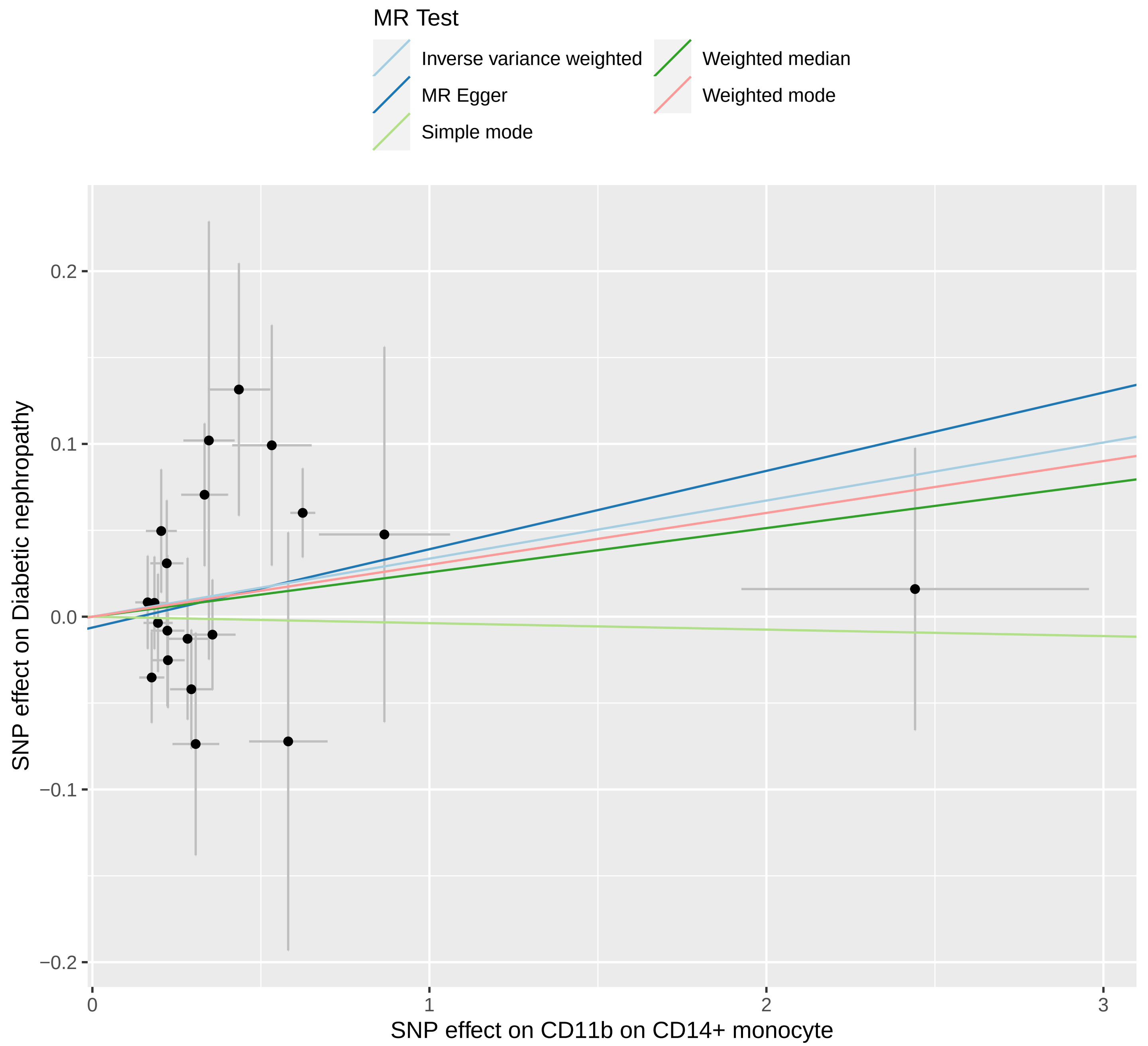
MR Test
Inverse variance weighted
MR Egger
Simple mode
Weighted median
Weighted mode
0.2
0.1
0.0
−0.1
−0.2
SNP effect on Diabetic nephropathy
0
1
2
3
SNP effect on CD11b on CD14+ monocyte

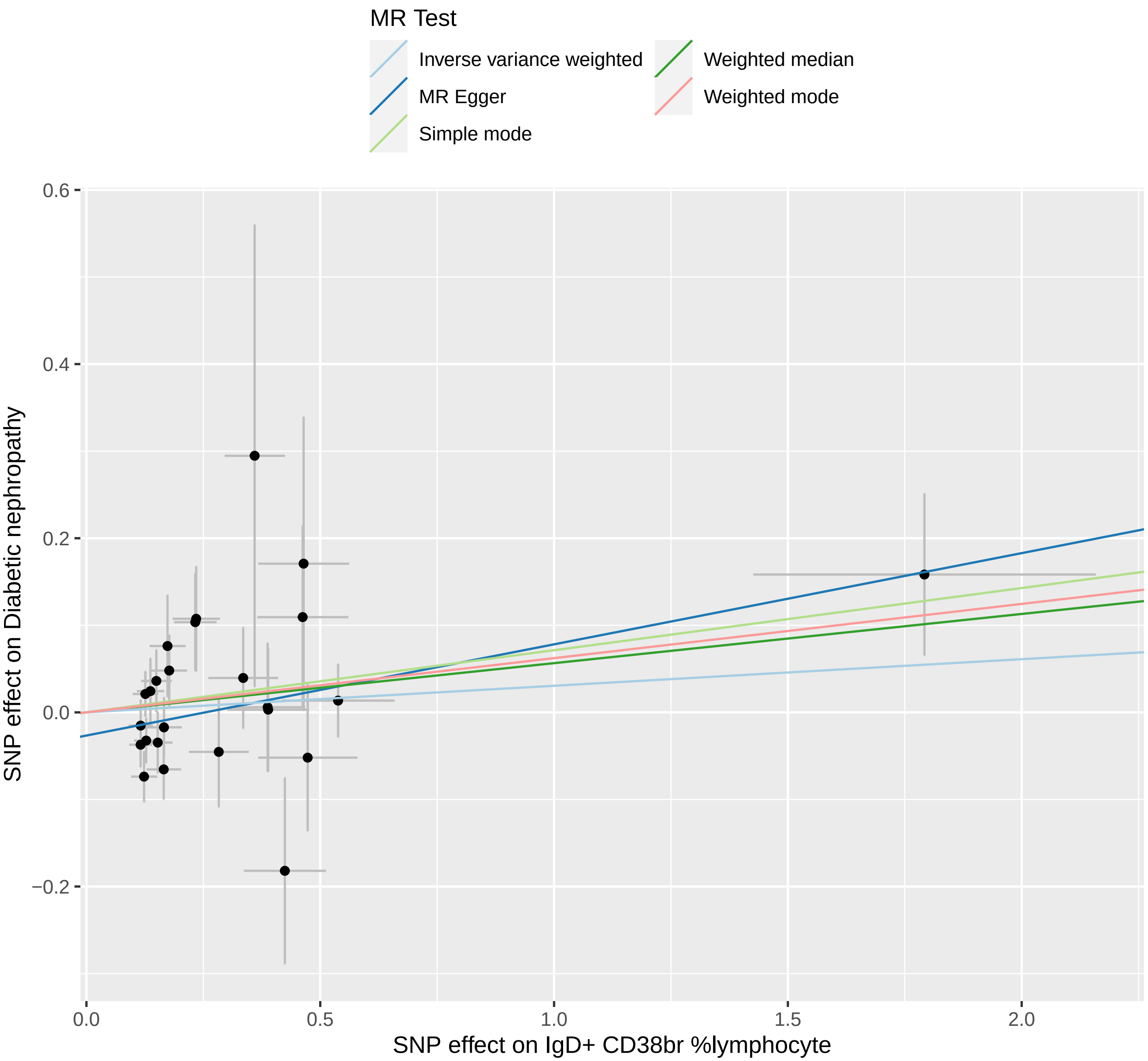
MR Test
Inverse variance weighted
MR Egger
Simple mode
Weighted median
Weighted mode
0.6
0.4
0.2
0.0
−0.2
0.0
0.5
1.0
1.5
2.0
SNP effect on Diabetic nephropathy
SNP effect on IgD+ CD38br %lymphocyte

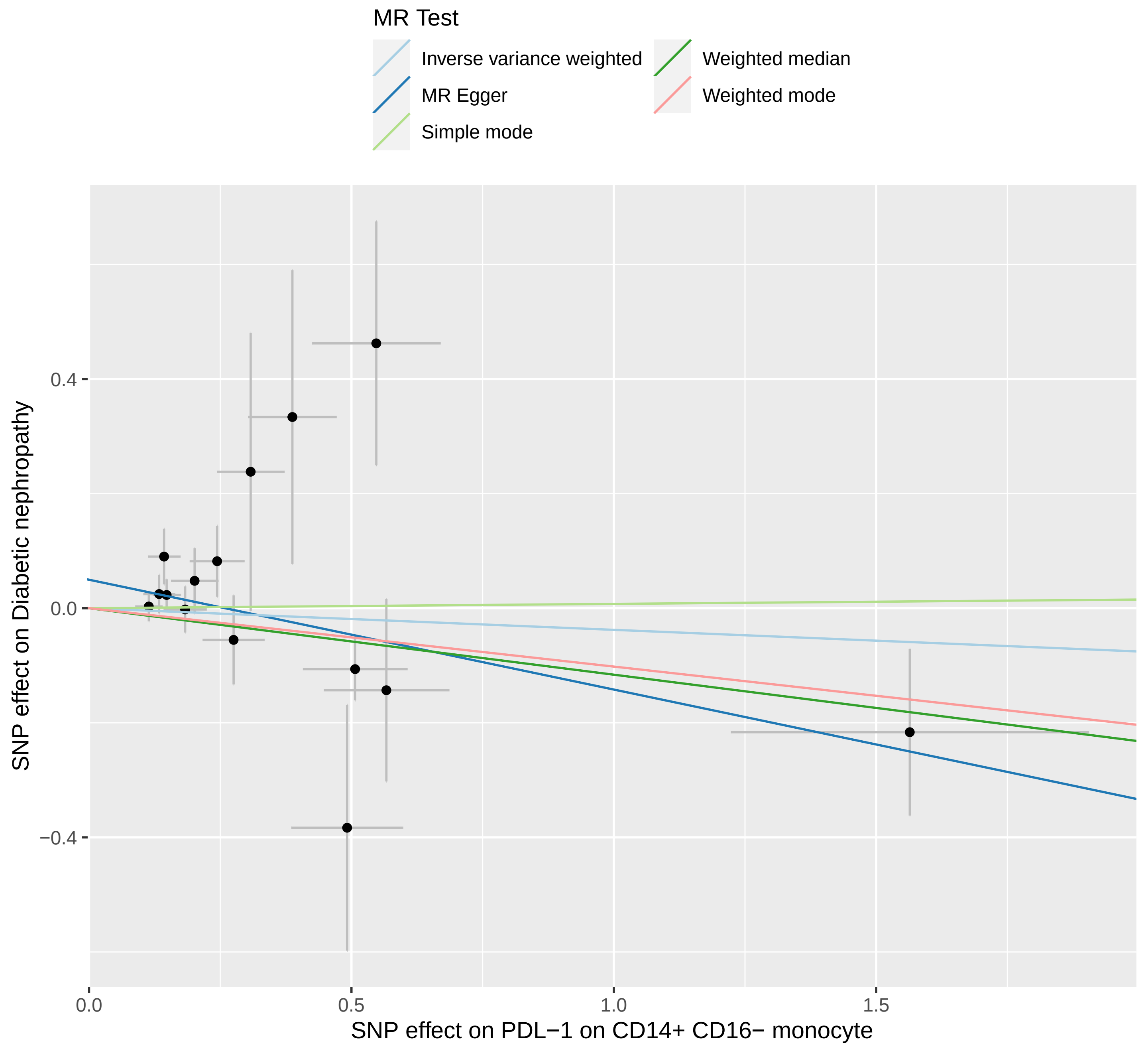
MR Test
Inverse variance weighted
MR Egger
Simple mode
Weighted median
Weighted mode
0.4
0.0
−0.4
0.0
0.5
1.0
1.5
SNP effect on Diabetic nephropathy
SNP effect on PDL−1 on CD14+ CD16− monocyte

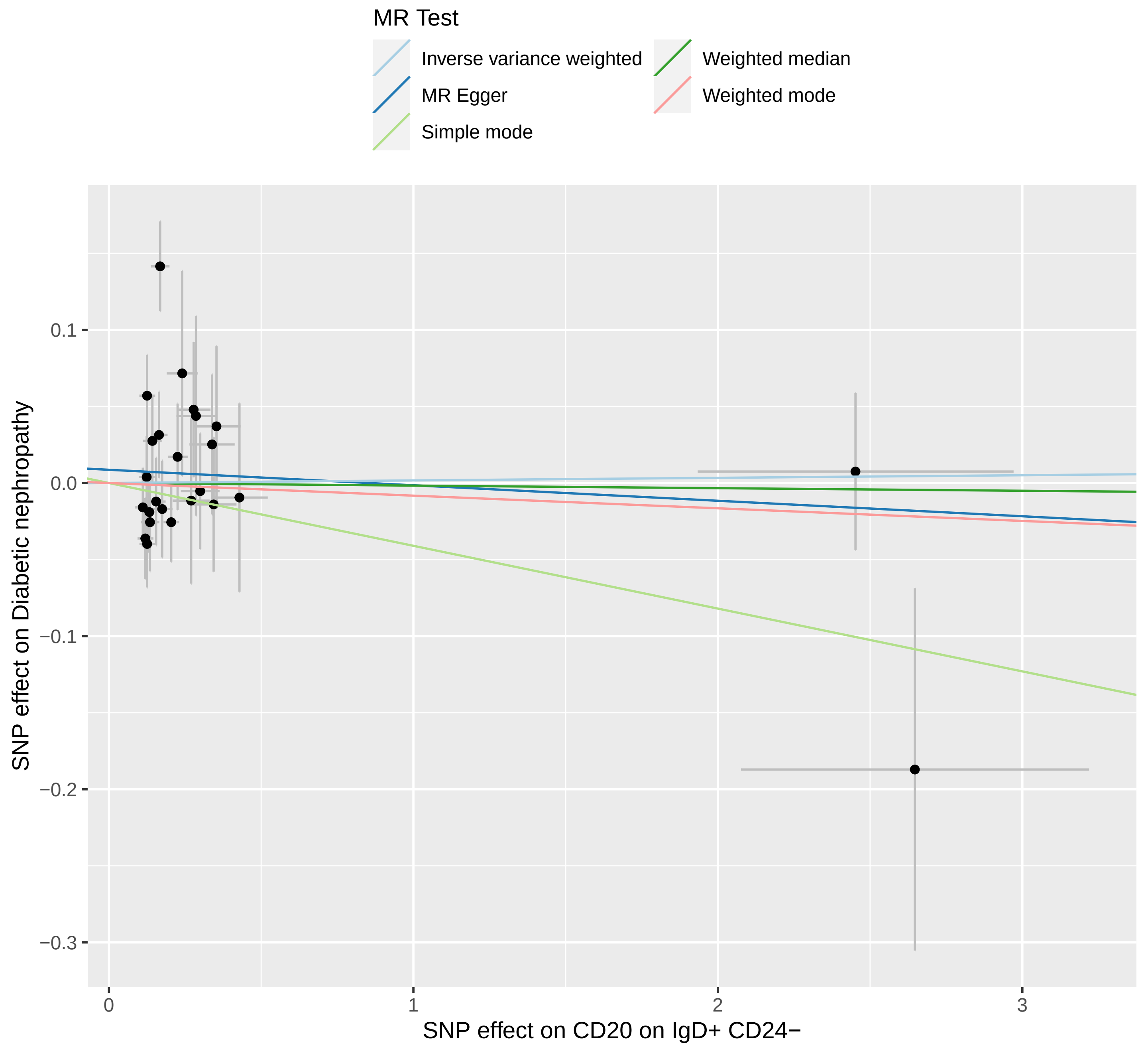

MR Test
Inverse variance weighted
MR Egger
Simple mode
Weighted median
Weighted mode
0.1
0.0
−0.1
−0.2
−0.3
0
1
2
3
SNP effect on Diabetic nephropathy
SNP effect on CD20 on IgD+ CD24−

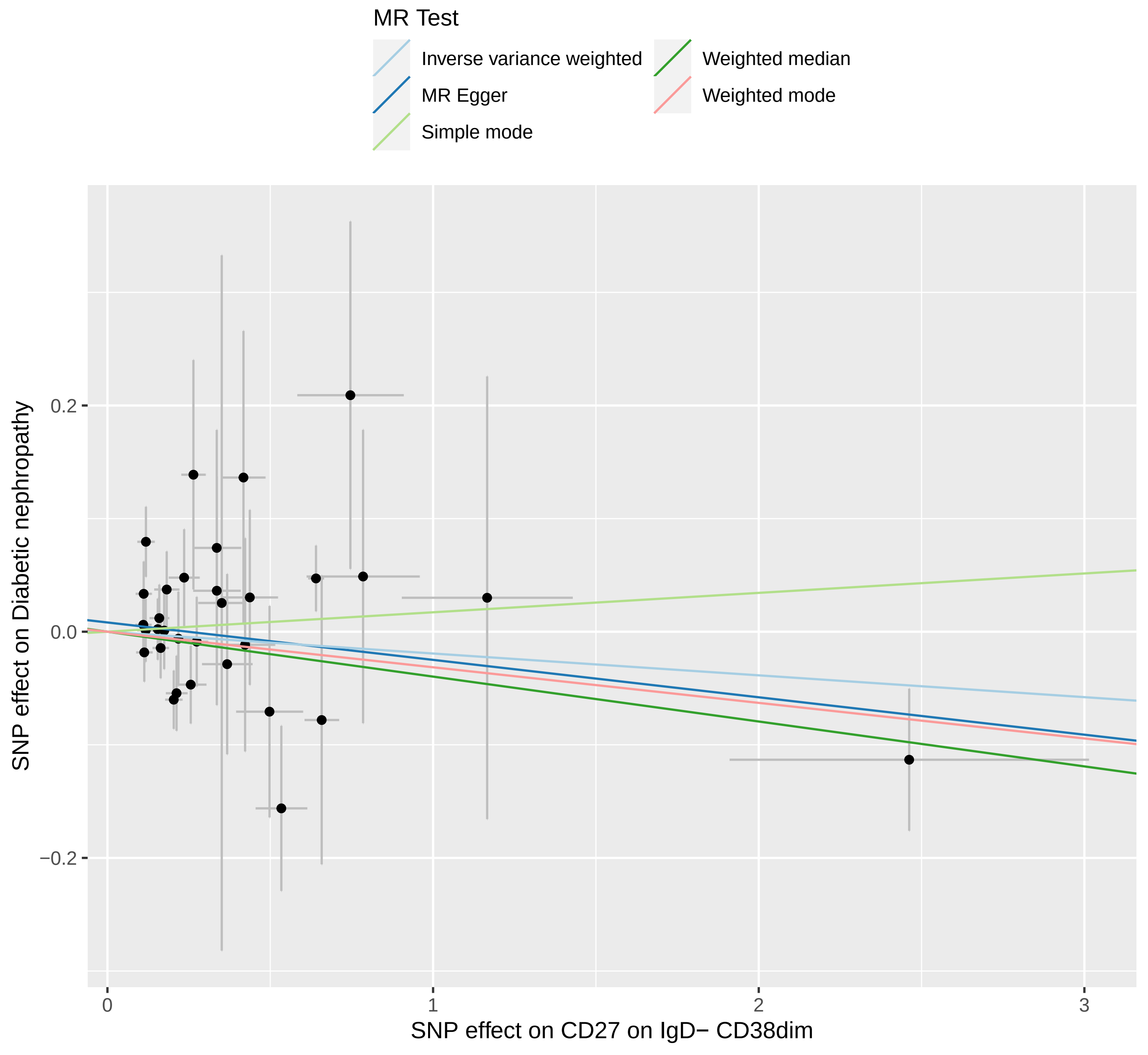

MR Test
Inverse variance weighted
MR Egger
Simple mode
Weighted median
Weighted mode
SNP effect on Diabetic nephropathy
0.2
0.0
−0.2
0
1
2
3
SNP effect on CD27 on IgD− CD38dim

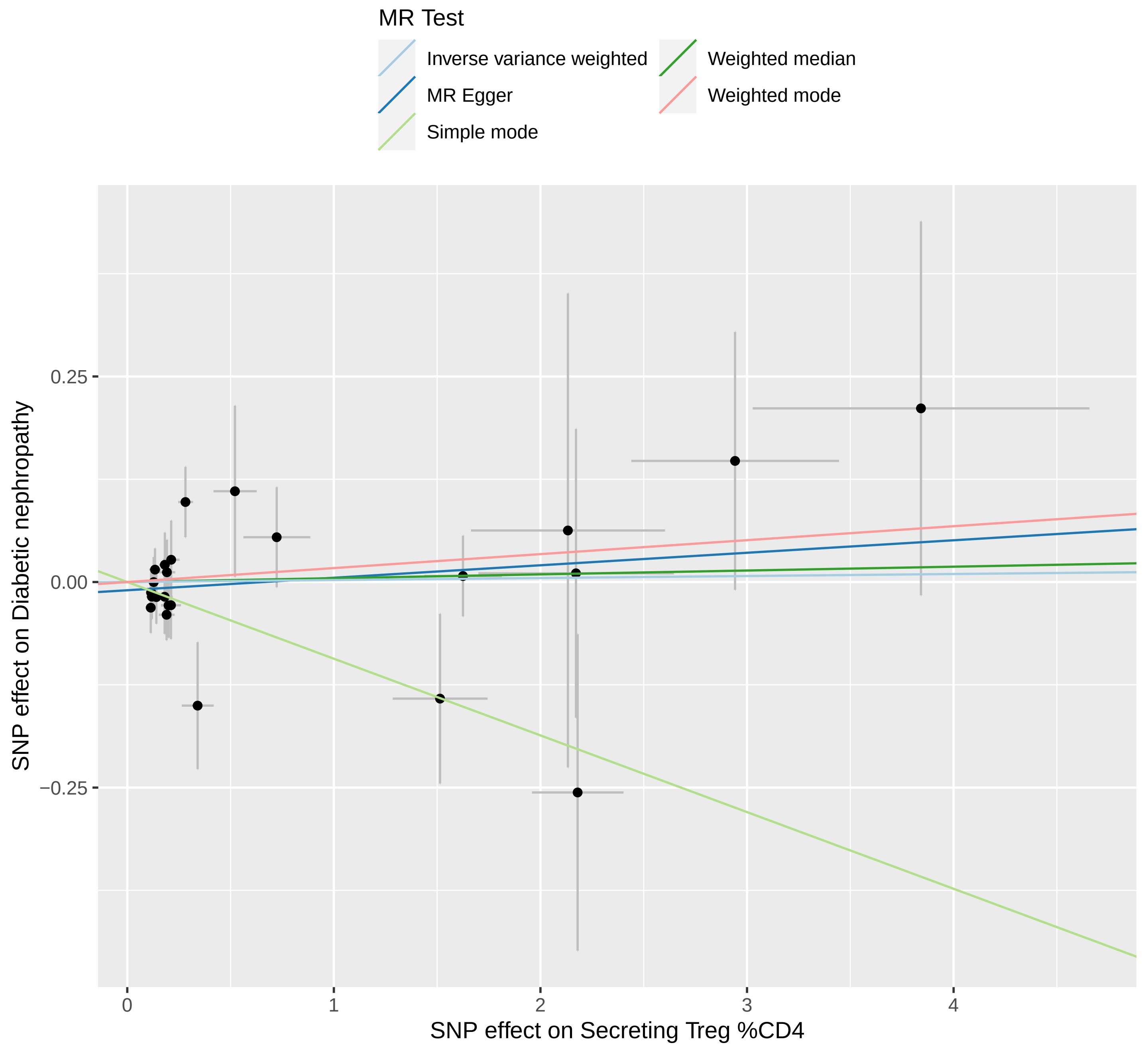
MR Test
Inverse variance weighted
MR Egger
Simple mode
Weighted median
Weighted mode
0.25
0.00
−0.25
SNP effect on Diabetic nephropathy
0
1
2
3
4
SNP effect on Secreting Treg %CD4

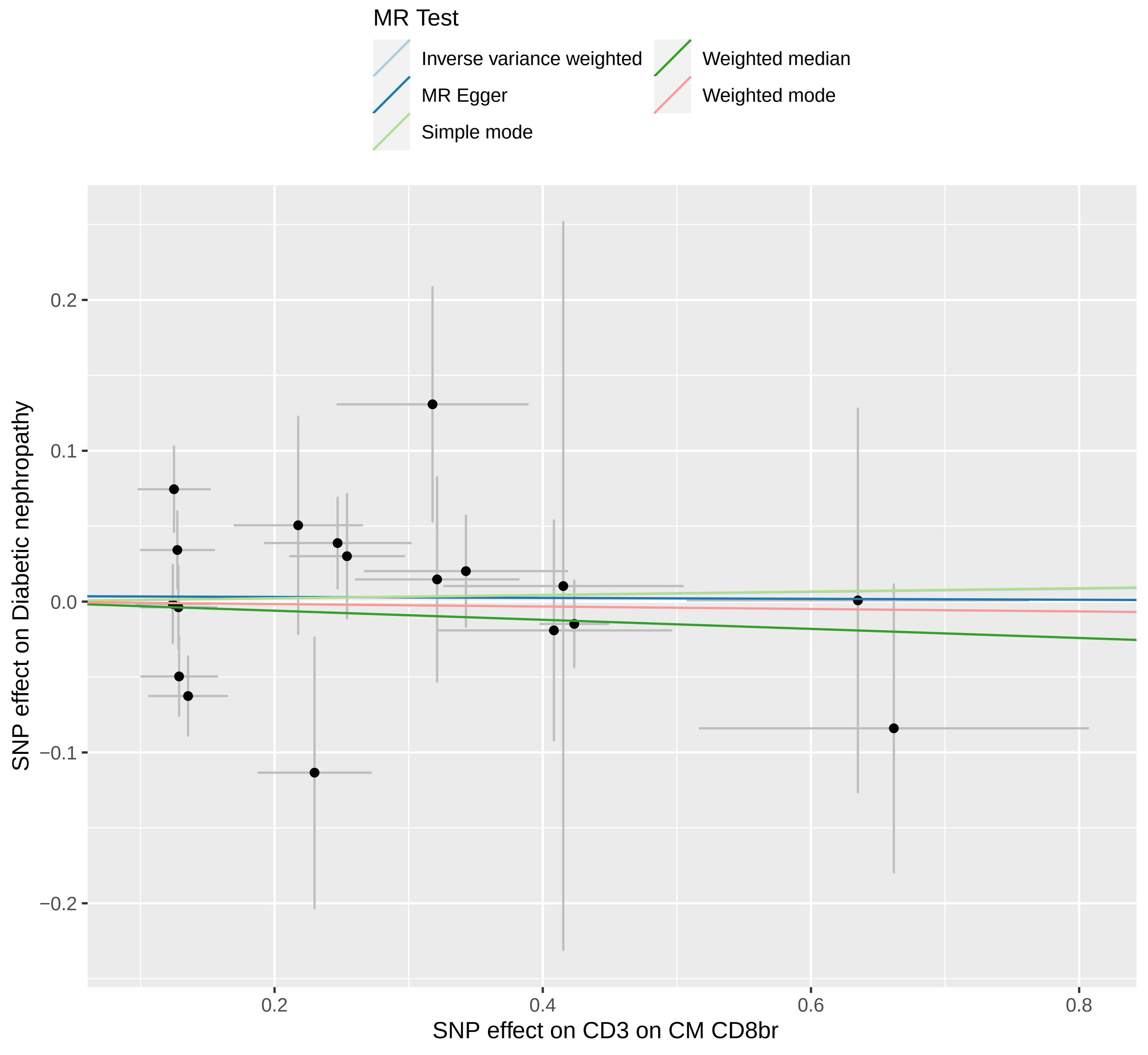
MR Test
Inverse variance weighted
MR Egger
Simple mode
Weighted median
Weighted mode
SNP effect on Diabetic nephropathy
0.2
0.1
0.0
−0.1
−0.2
0.2
0.4
0.6
0.8
SNP effect on CD3 on CM CD8br

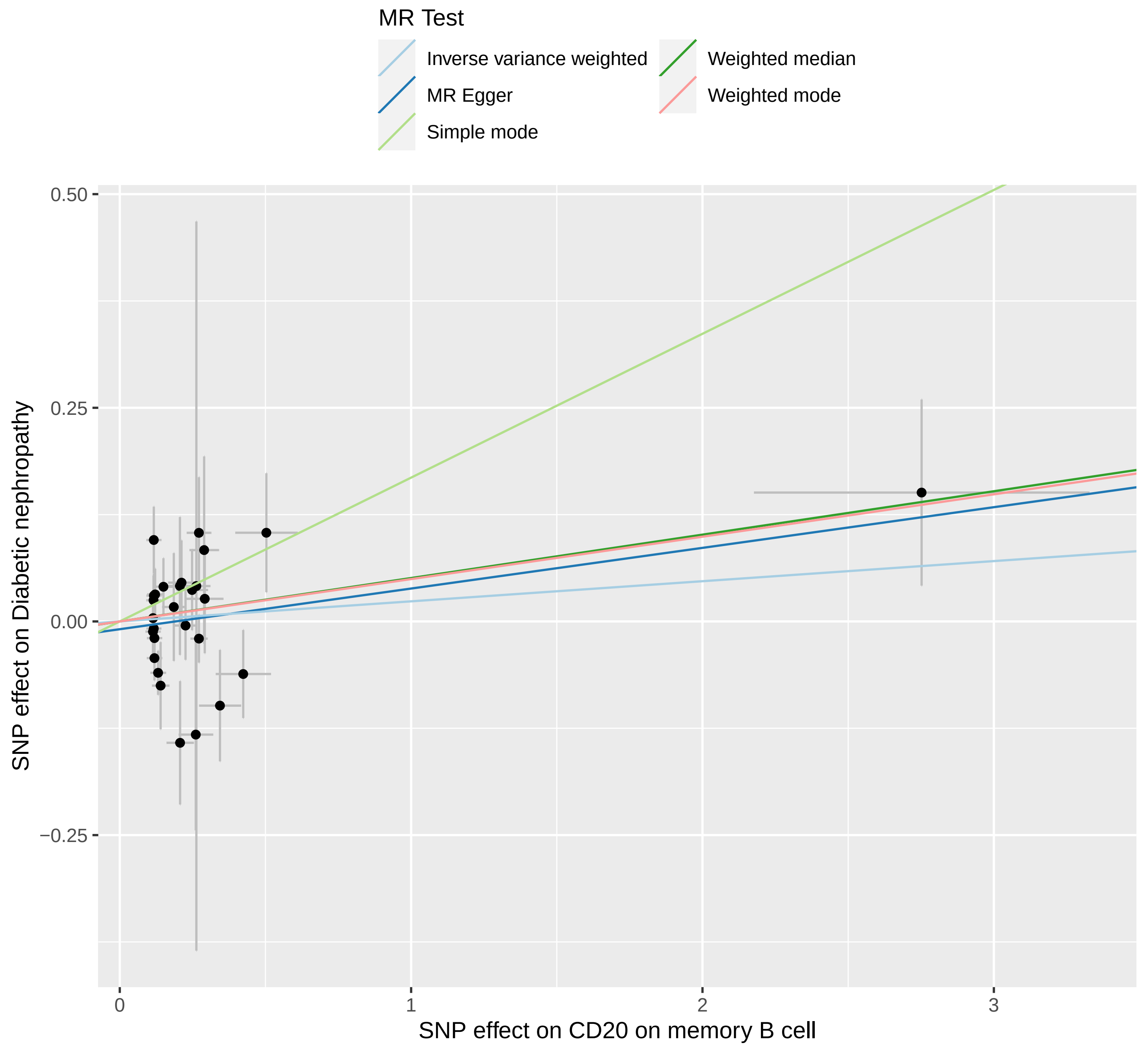
MR Test
Inverse variance weighted
MR Egger
Simple mode
Weighted median
Weighted mode
0.50
0.25
0.00
−0.25
SNP effect on Diabetic nephropathy
0
1
2
3
SNP effect on CD20 on memory B cell

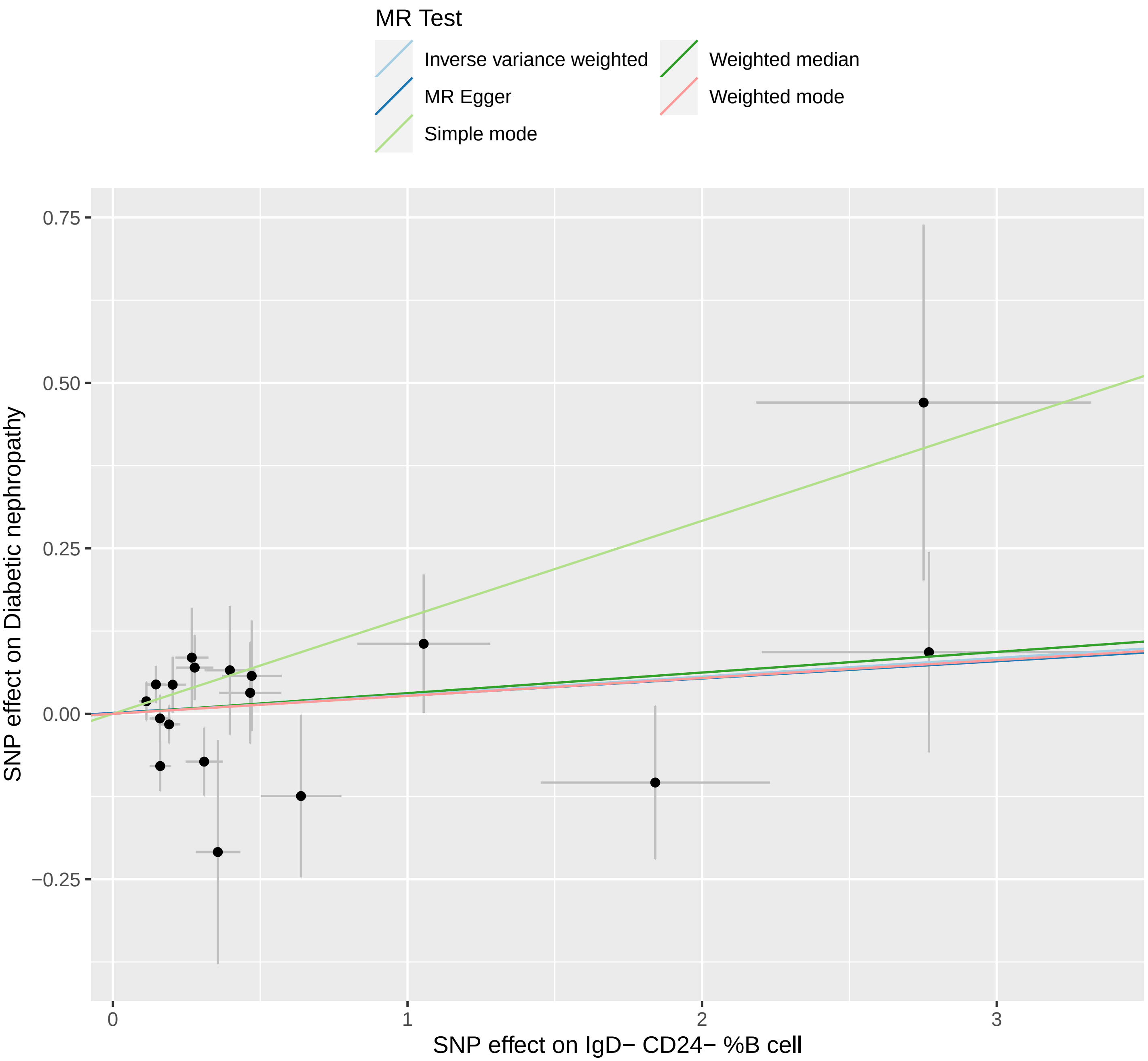
MR Test
Inverse variance weighted
MR Egger
Simple mode
Weighted median
Weighted mode
0.75
0.50
0.25
0.00
−0.25
SNP effect on Diabetic nephropathy
0
1
2
3
SNP effect on IgD− CD24− %B cell

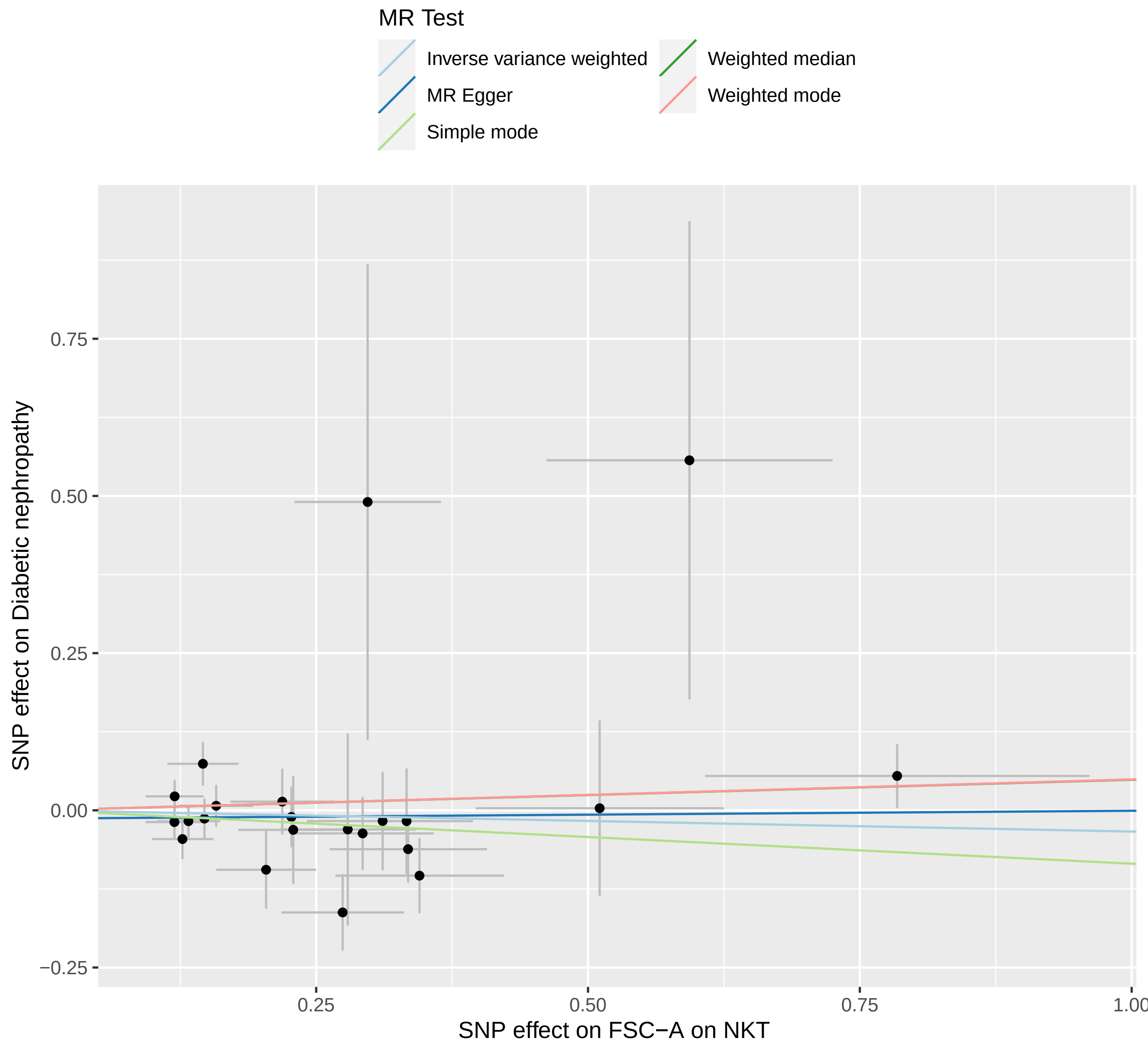
MR Test
Inverse variance weighted
MR Egger
Simple mode
Weighted median
Weighted mode
0.75
0.50
0.25
0.00
−0.25
SNP effect on Diabetic nephropathy
0.25
0.50
0.75
1.00
SNP effect on FSC−A on NKT

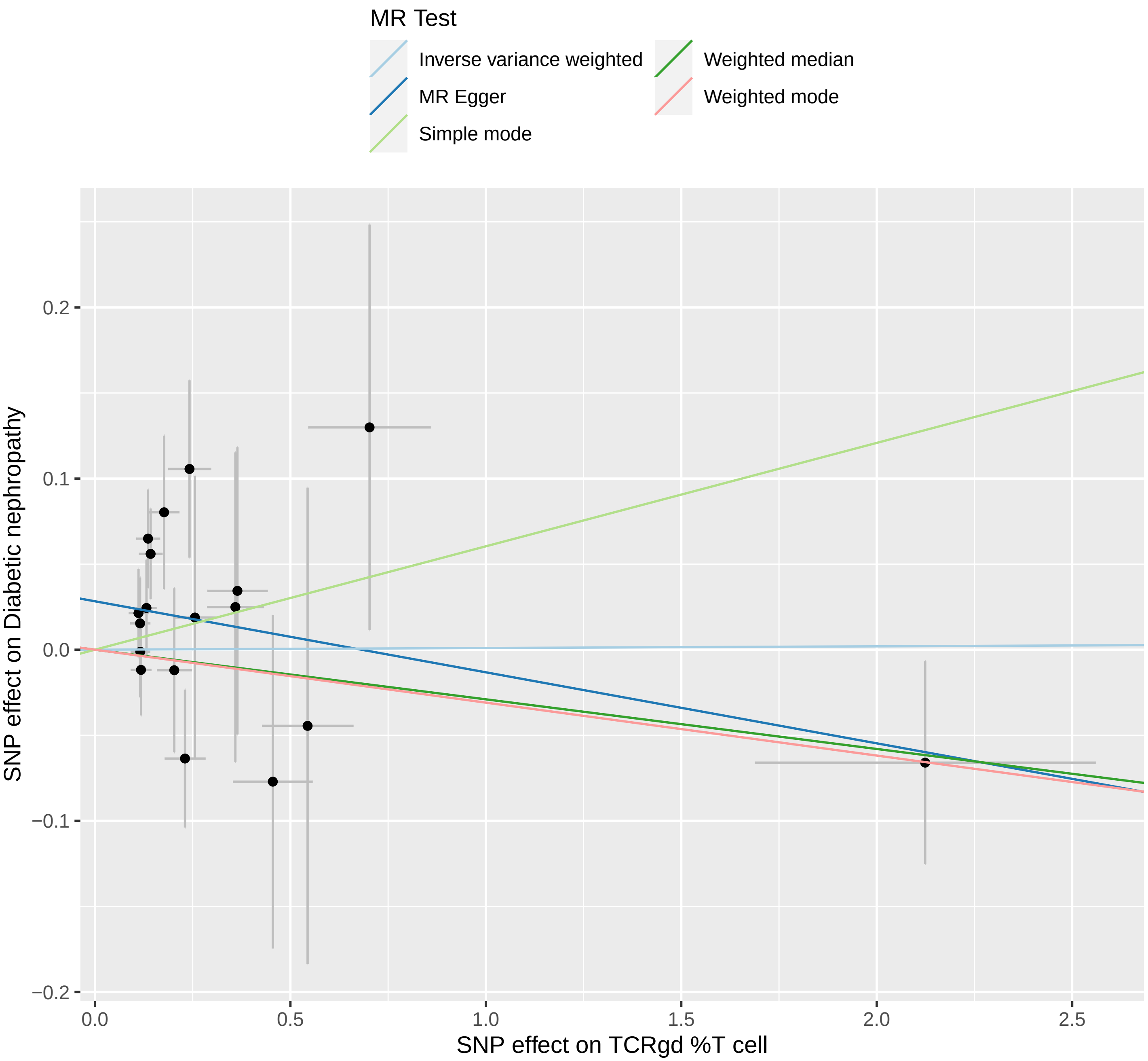
MR Test
Inverse variance weighted
MR Egger
Simple mode
Weighted median
Weighted mode
SNP effect on Diabetic nephropathy
0.2
0.1
0.0
−0.1
−0.2
0.0
0.5
1.0
1.5
2.0
2.5
SNP effect on TCRgd %T cell

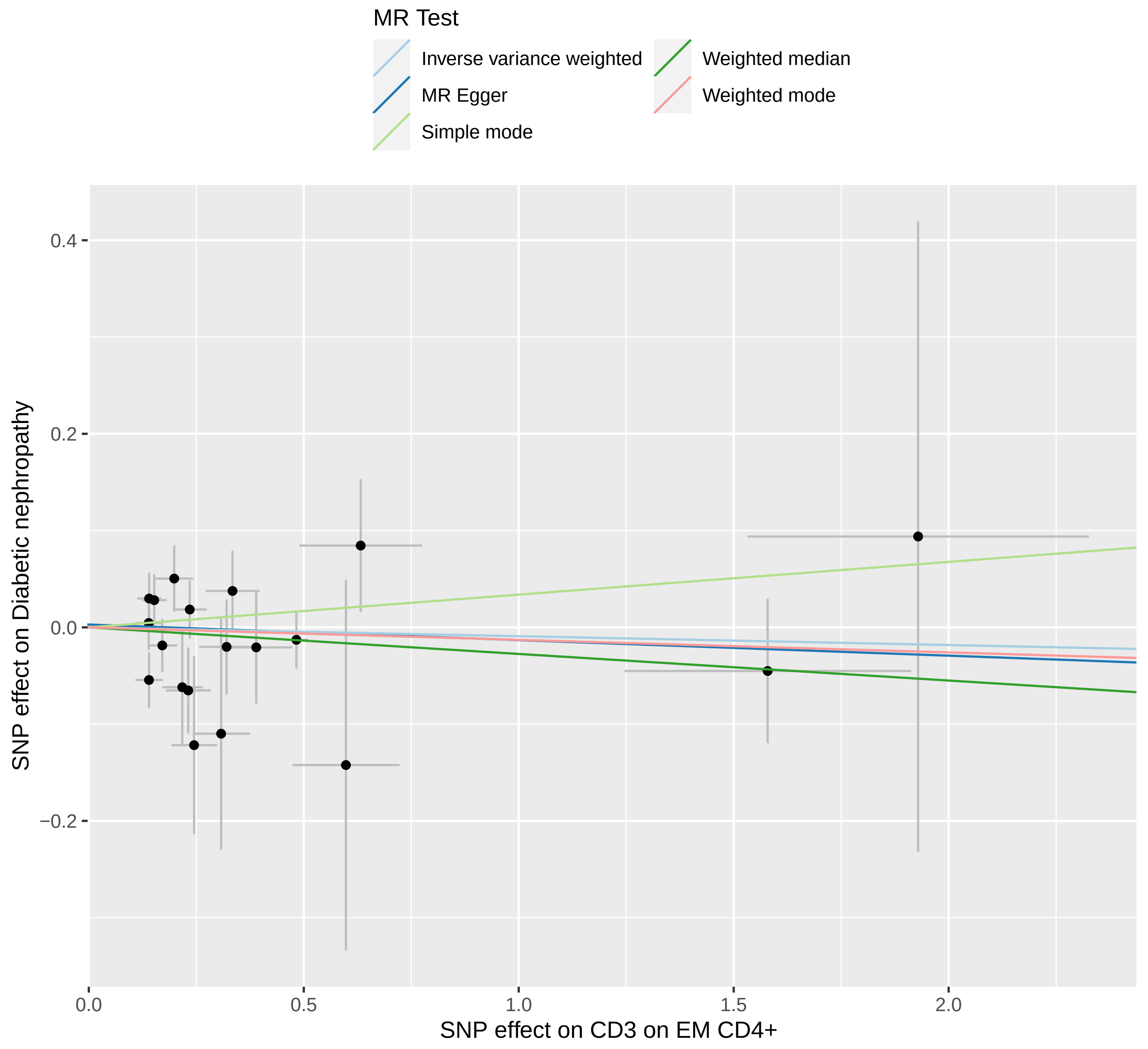
MR Test
Inverse variance weighted
MR Egger
Simple mode
Weighted median
Weighted mode
0.4
0.2
0.0
−0.2
0.0
0.5
1.0
1.5
2.0
SNP effect on CD3 on EM CD4+
SNP effect on Diabetic nephropathy

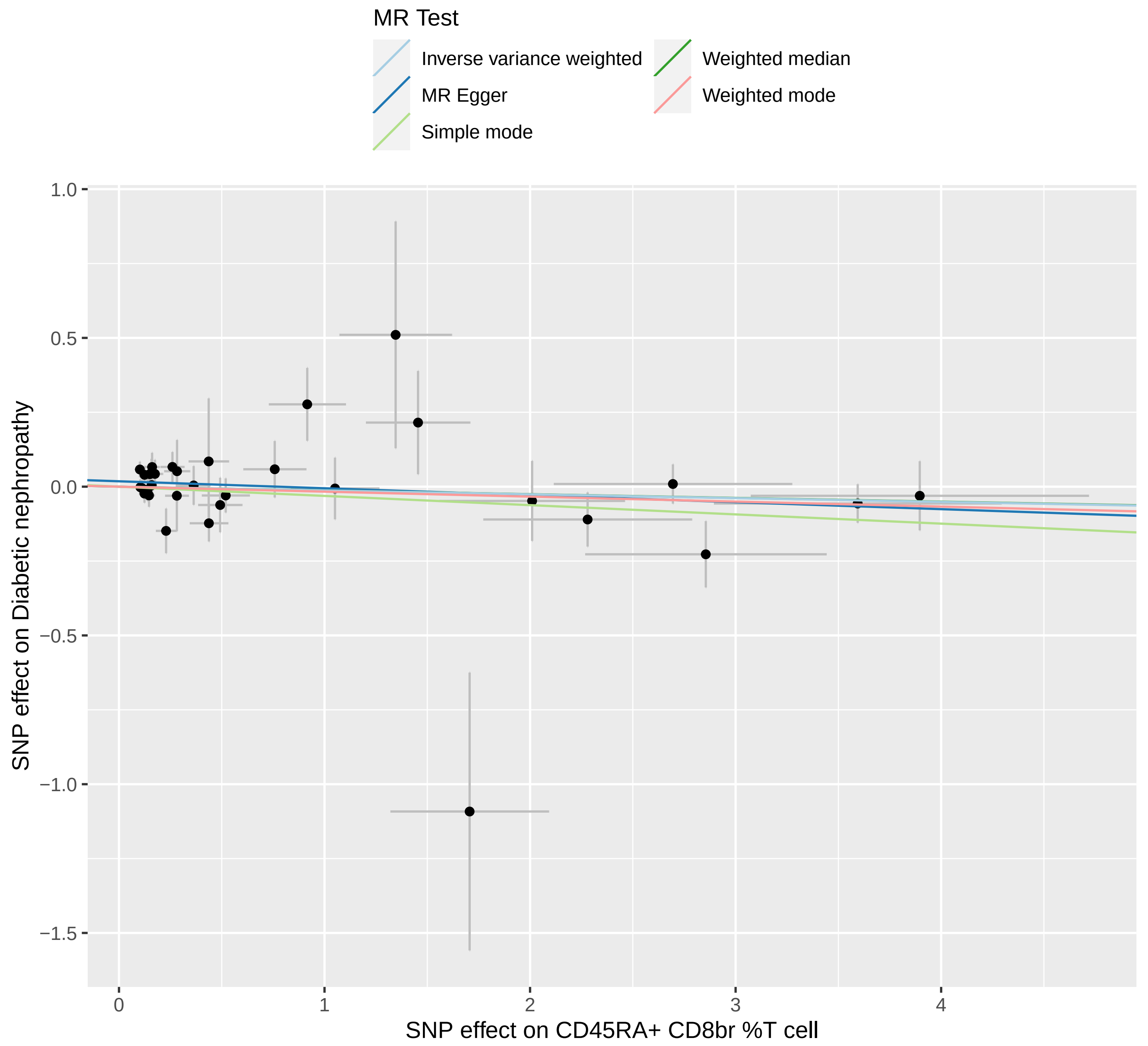
MR Test
Inverse variance weighted
MR Egger
Simple mode
Weighted median
Weighted mode
1.0
0.5
0.0
−0.5
−1.0
−1.5
SNP effect on Diabetic nephropathy
0
1
2
3
4
SNP effect on CD45RA+ CD8br %T cell

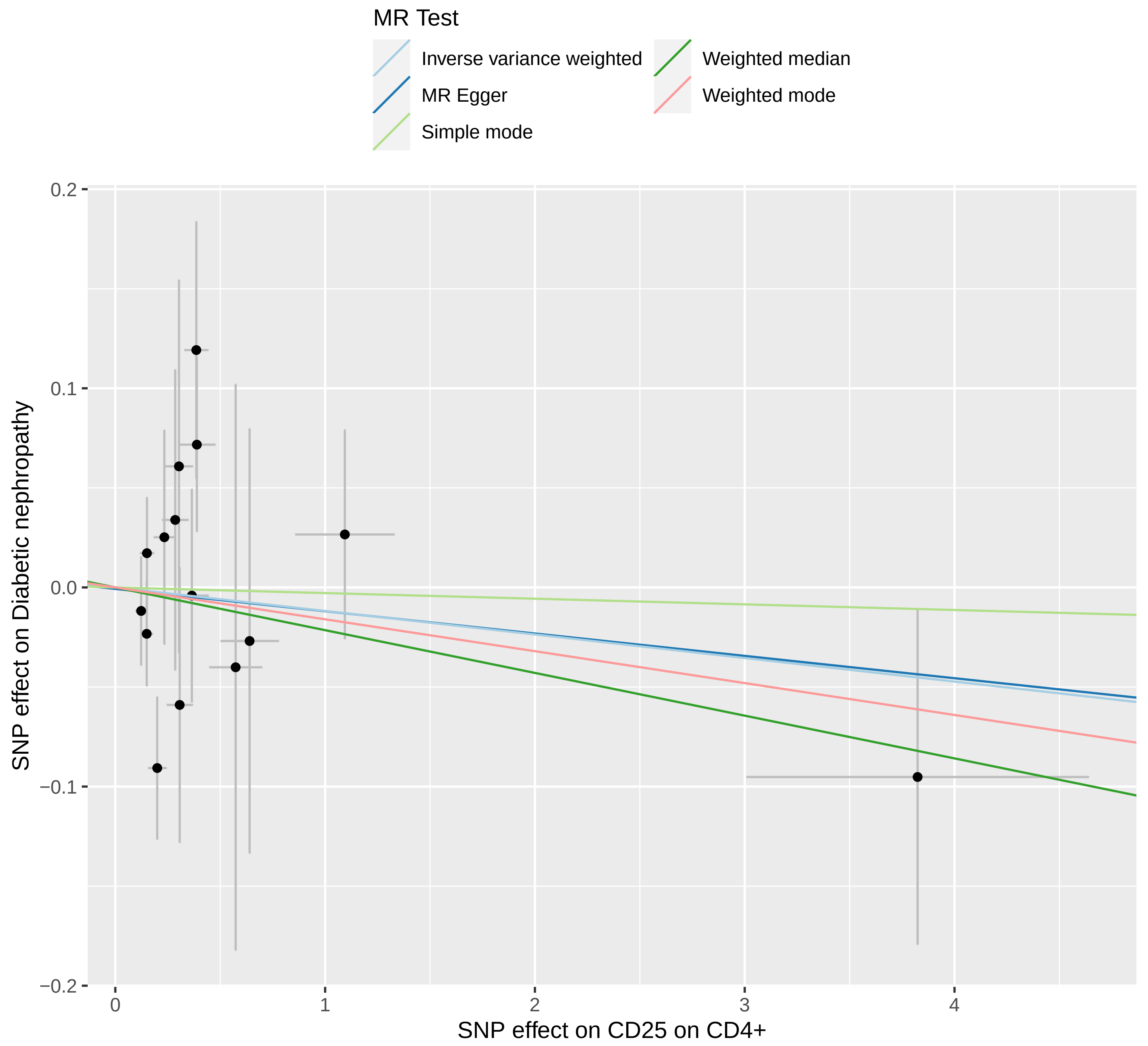
MR Test
Inverse variance weighted
MR Egger
Simple mode
Weighted median
Weighted mode
0.2
0.1
0.0
−0.1
−0.2
SNP effect on Diabetic nephropathy
0
1
2
3
4
SNP effect on CD25 on CD4+

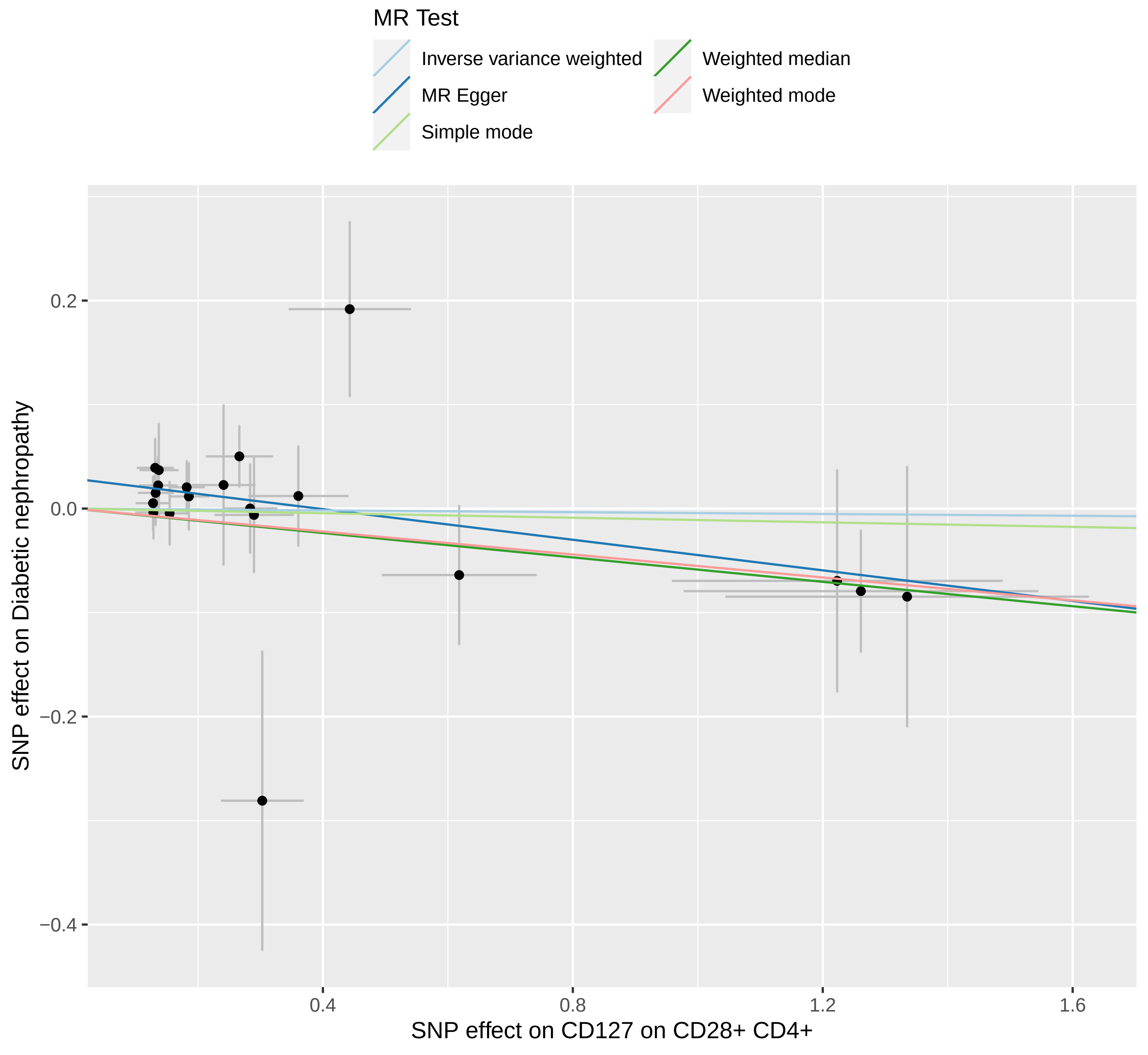
MR Test
Inverse variance weighted
MR Egger
Simple mode
Weighted median
Weighted mode
0.2
0.0
−0.2
−0.4
0.4
0.8
1.2
1.6
SNP effect on Diabetic nephropathy
SNP effect on CD127 on CD28+ CD4+

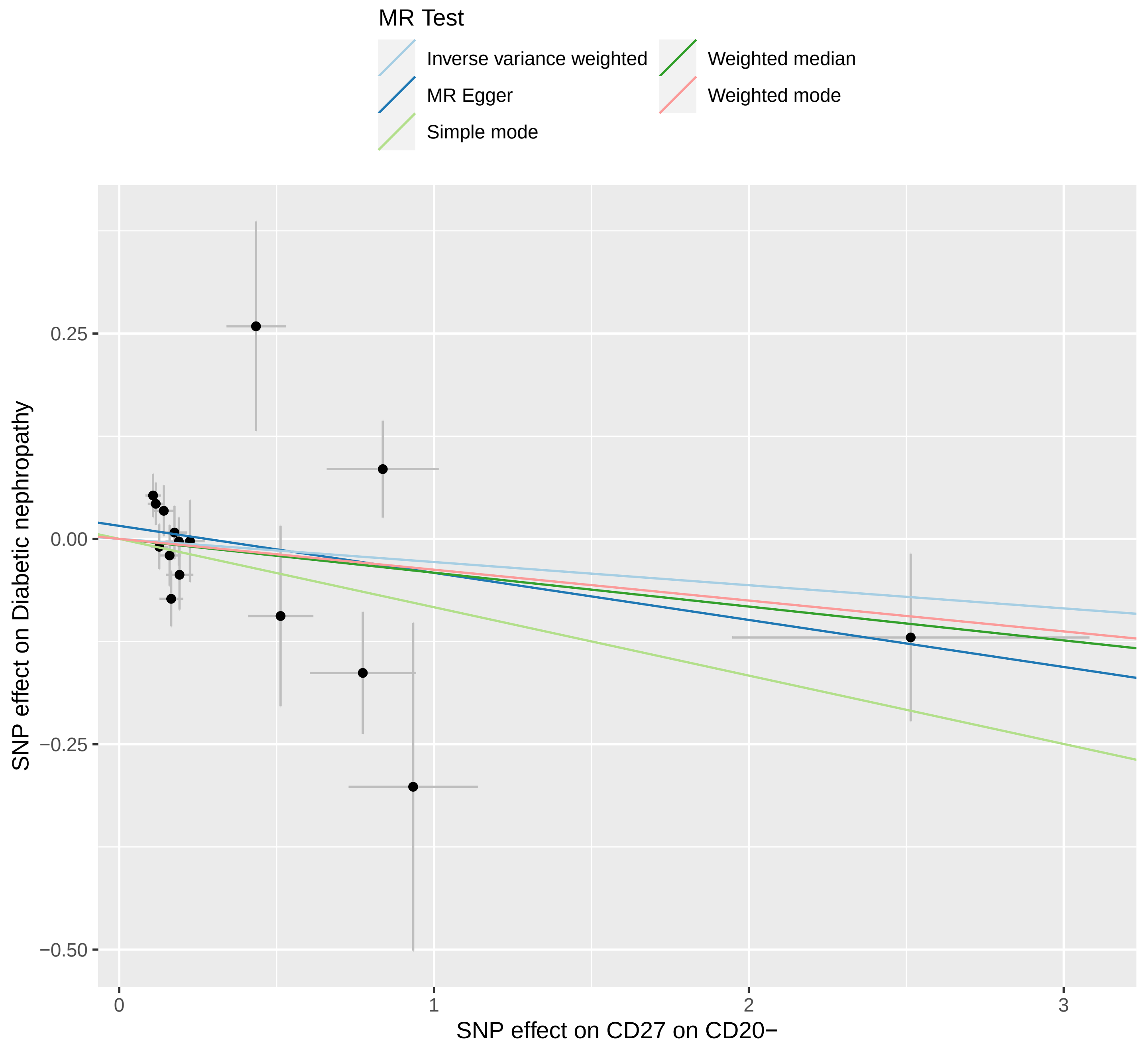

MR Test
Inverse variance weighted
MR Egger
Simple mode
Weighted median
Weighted mode
SNP effect on Diabetic nephropathy
0.25
0.00
−0.25
−0.50
0
1
2
3
SNP effect on CD27 on CD20−

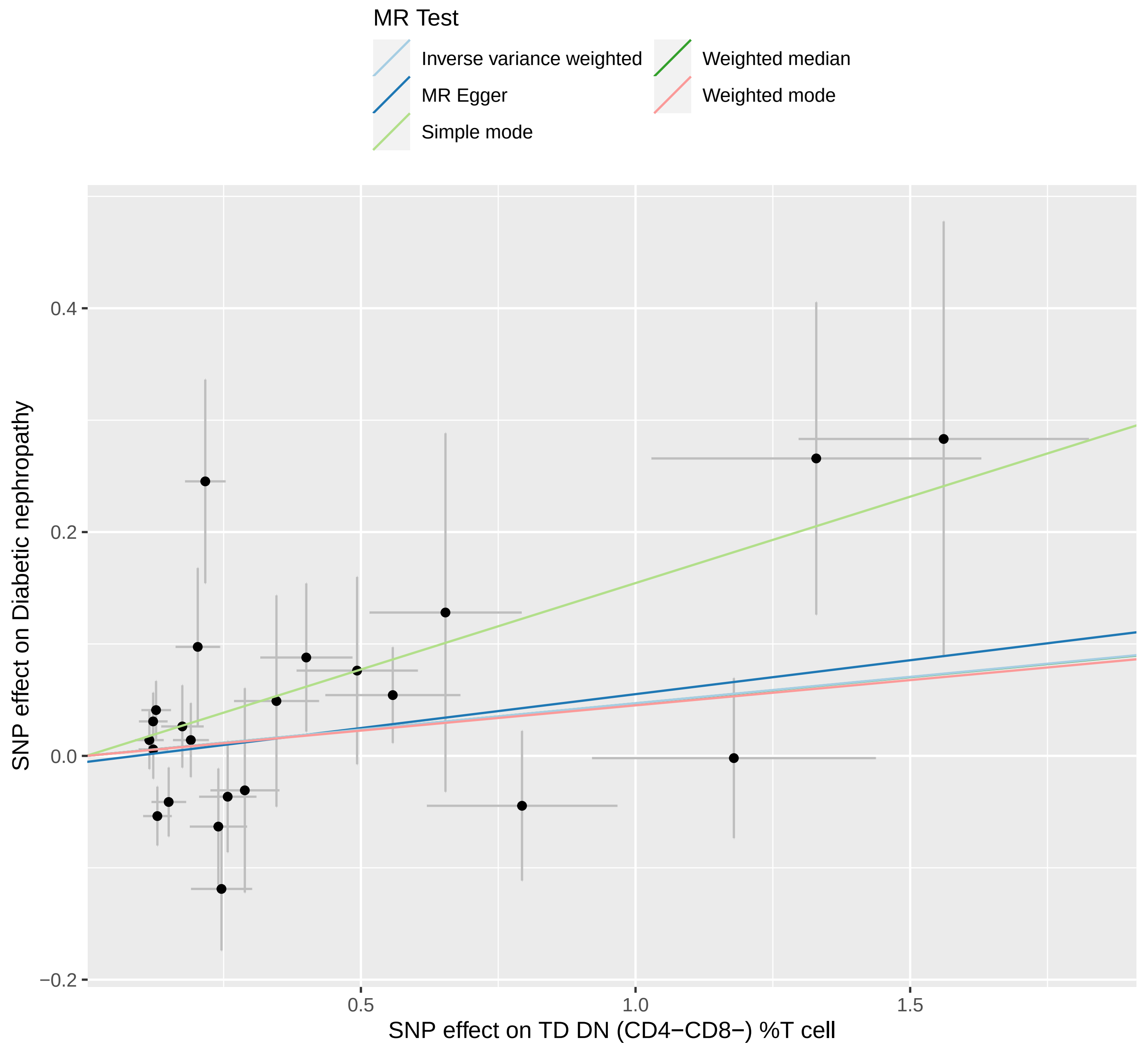
MR Test
Inverse variance weighted
MR Egger
Simple mode
Weighted median
Weighted mode
0.4
0.2
0.0
−0.2
0.5
1.0
1.5
SNP effect on Diabetic nephropathy
SNP effect on TD DN (CD4−CD8−) %T cell

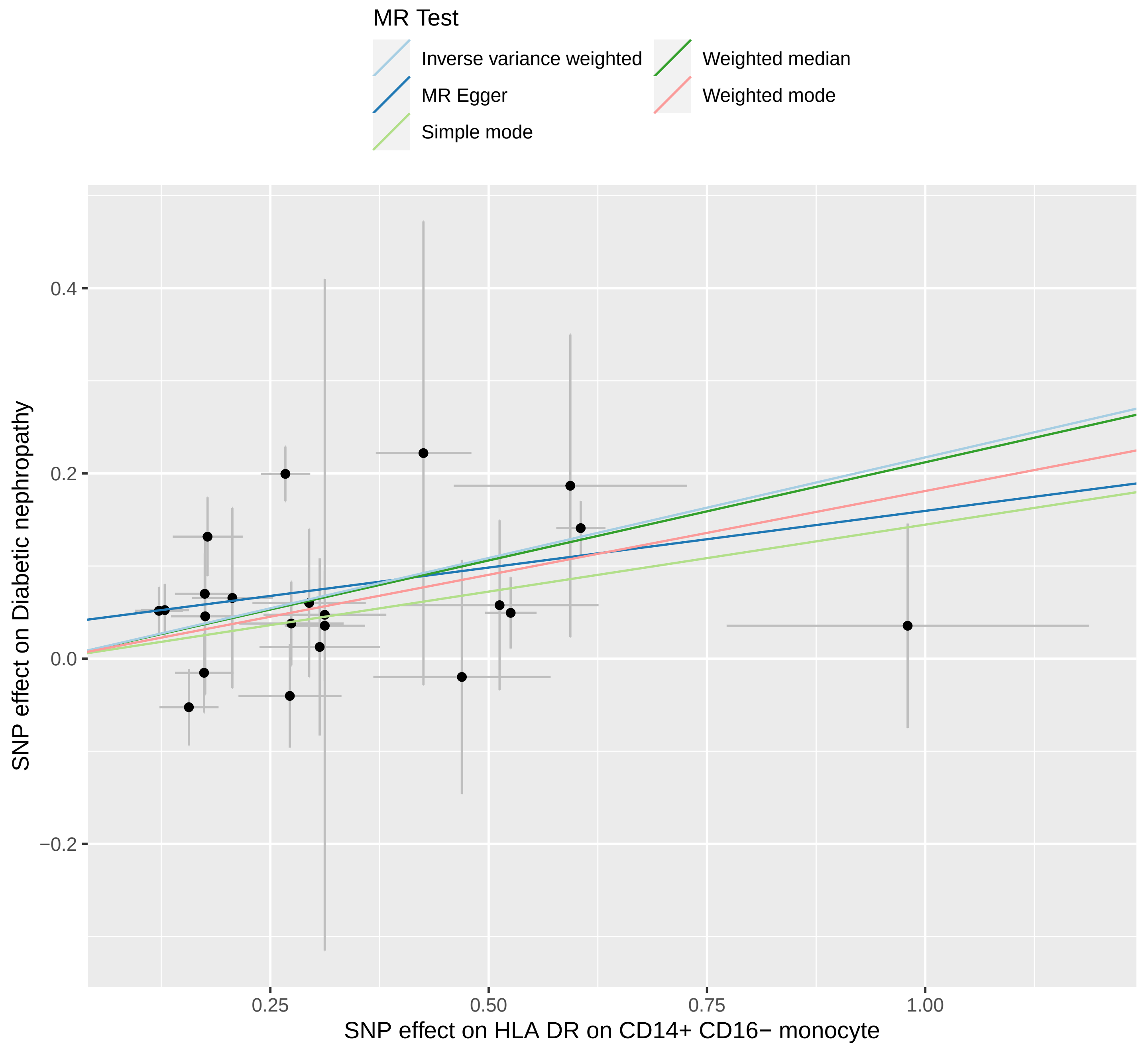

MR Test
Inverse variance weighted
MR Egger
Simple mode
Weighted median
Weighted mode
SNP effect on Diabetic nephropathy
0.4
0.2
0.0
−0.2
0.25
0.50
0.75
1.00
SNP effect on HLA DR on CD14+ CD16− monocyte

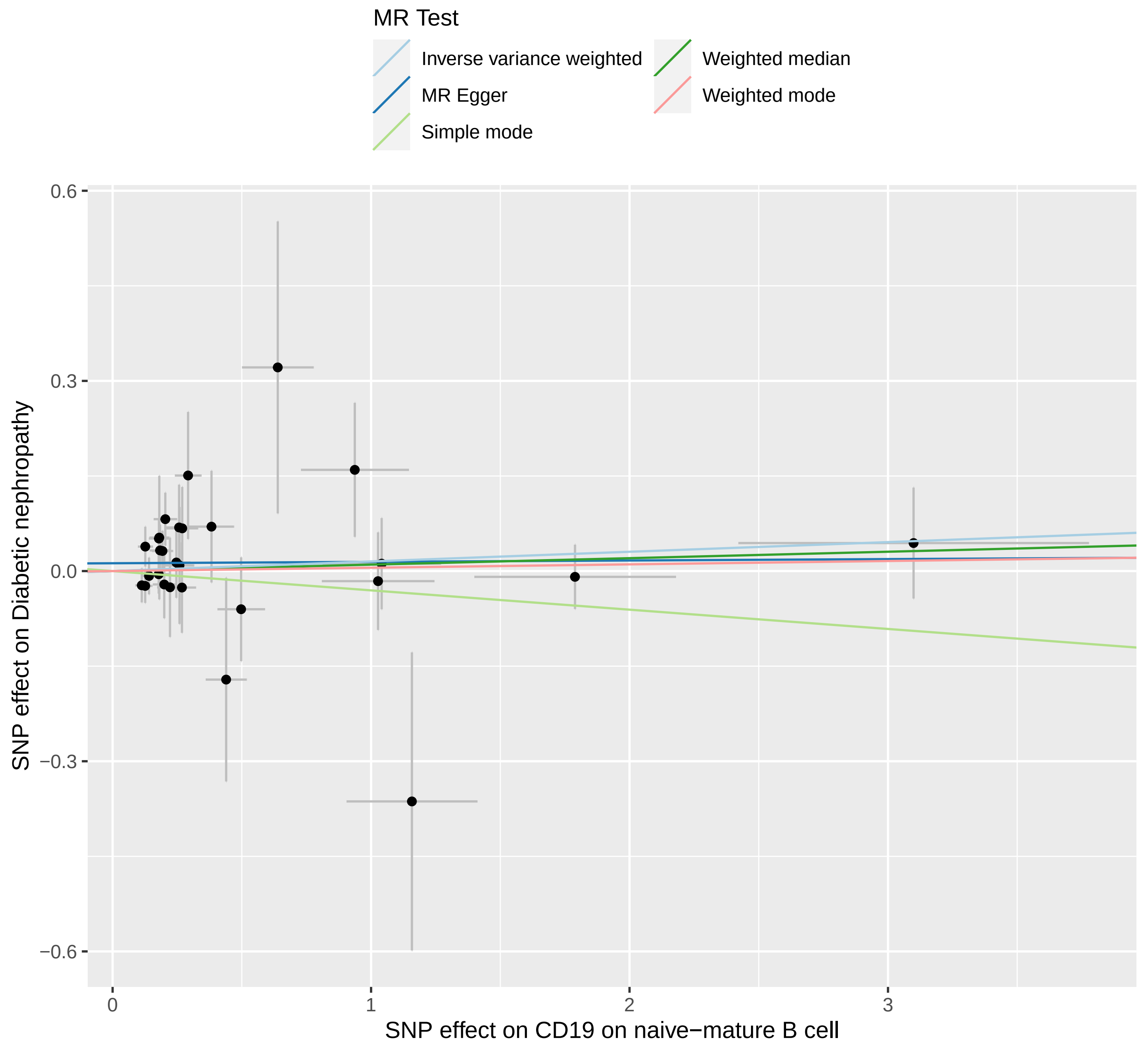
MR Test
Inverse variance weighted
MR Egger
Simple mode
Weighted median
Weighted mode
0.6
0.3
0.0
−0.3
−0.6
SNP effect on Diabetic nephropathy
0
1
2
3
SNP effect on CD19 on naive−mature B cell

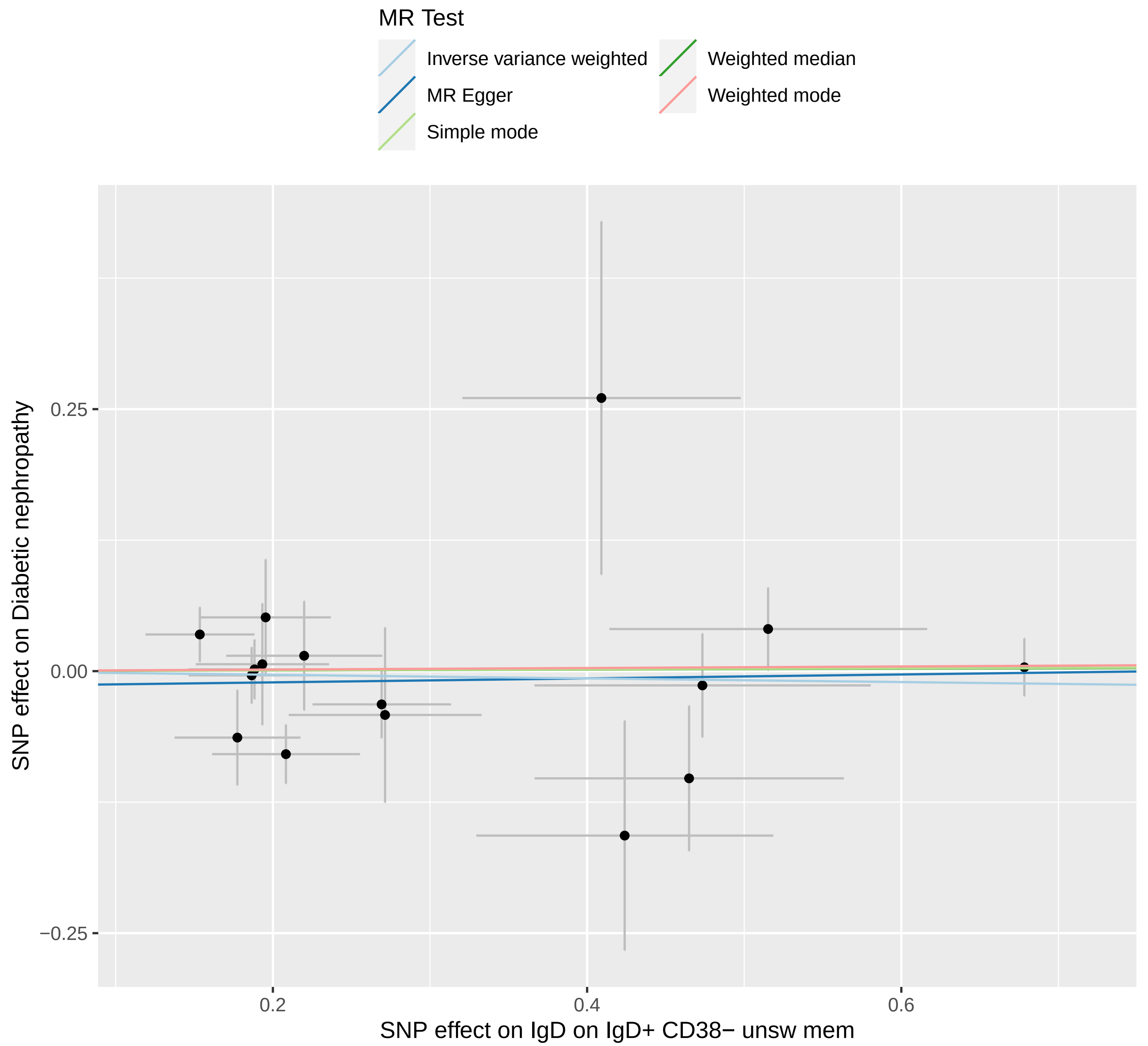
MR Test
Inverse variance weighted
MR Egger
Simple mode
Weighted median
Weighted mode
SNP effect on Diabetic nephropathy
0.25
0.00
−0.25
0.2
0.4
0.6
SNP effect on IgD on IgD+ CD38− unsw mem

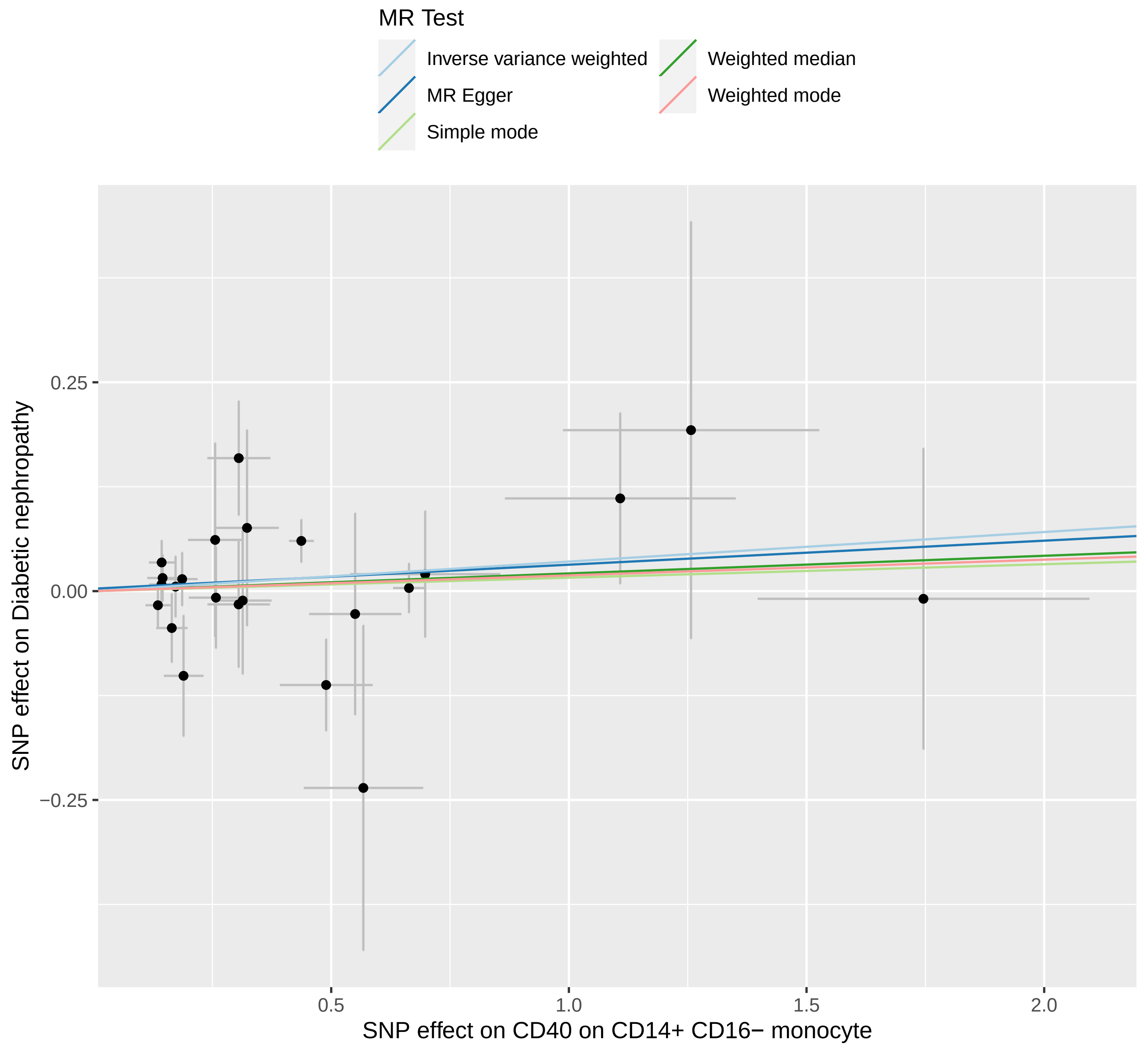
MR Test
Inverse variance weighted
MR Egger
Simple mode
Weighted median
Weighted mode
0.25
0.00
−0.25
0.5
1.0
1.5
2.0
SNP effect on Diabetic nephropathy
SNP effect on CD40 on CD14+ CD16− monocyte

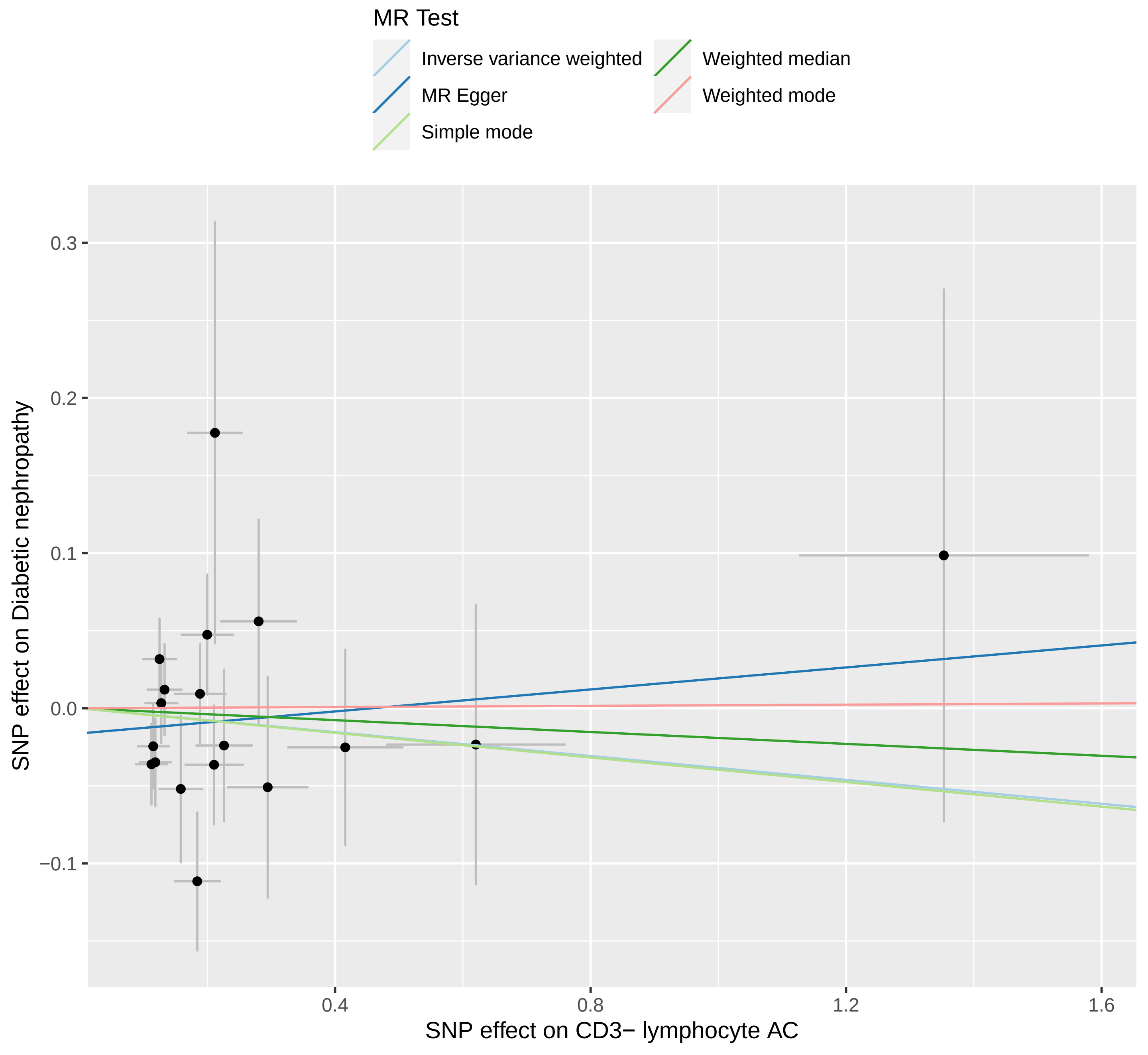
MR Test
Inverse variance weighted
MR Egger
Simple mode
Weighted median
Weighted mode
0.3
0.2
0.1
0.0
−0.1
SNP effect on Diabetic nephropathy
0.4
0.8
1.2
1.6
SNP effect on CD3− lymphocyte AC

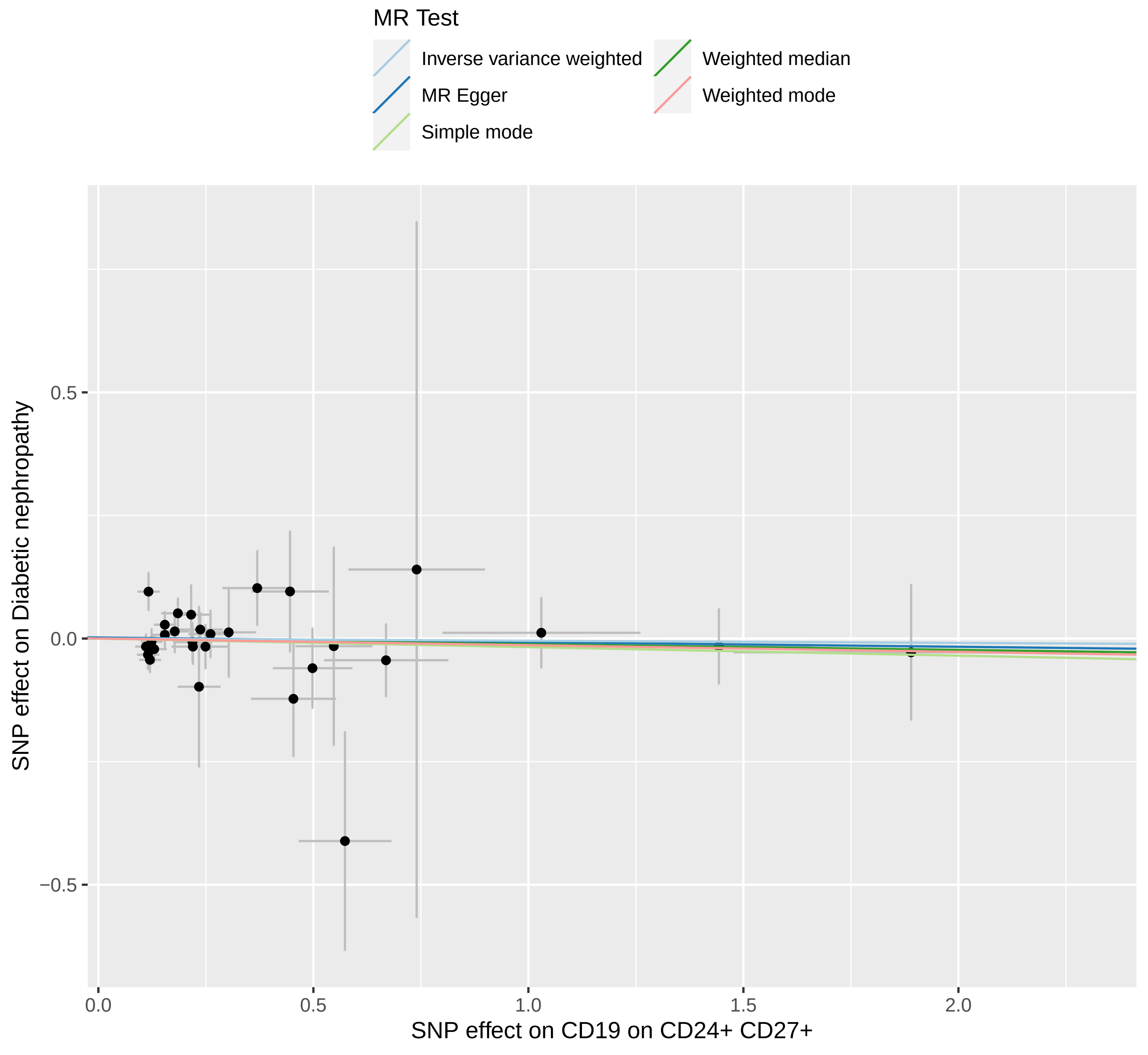
MR Test
Inverse variance weighted
Weighted median
MR Egger
Weighted mode
Simple mode
SNP effect on Diabetic nephropathy
0.5
0.0
−0.5
0.0
0.5
1.0
1.5
2.0
SNP effect on CD19 on CD24+ CD27+

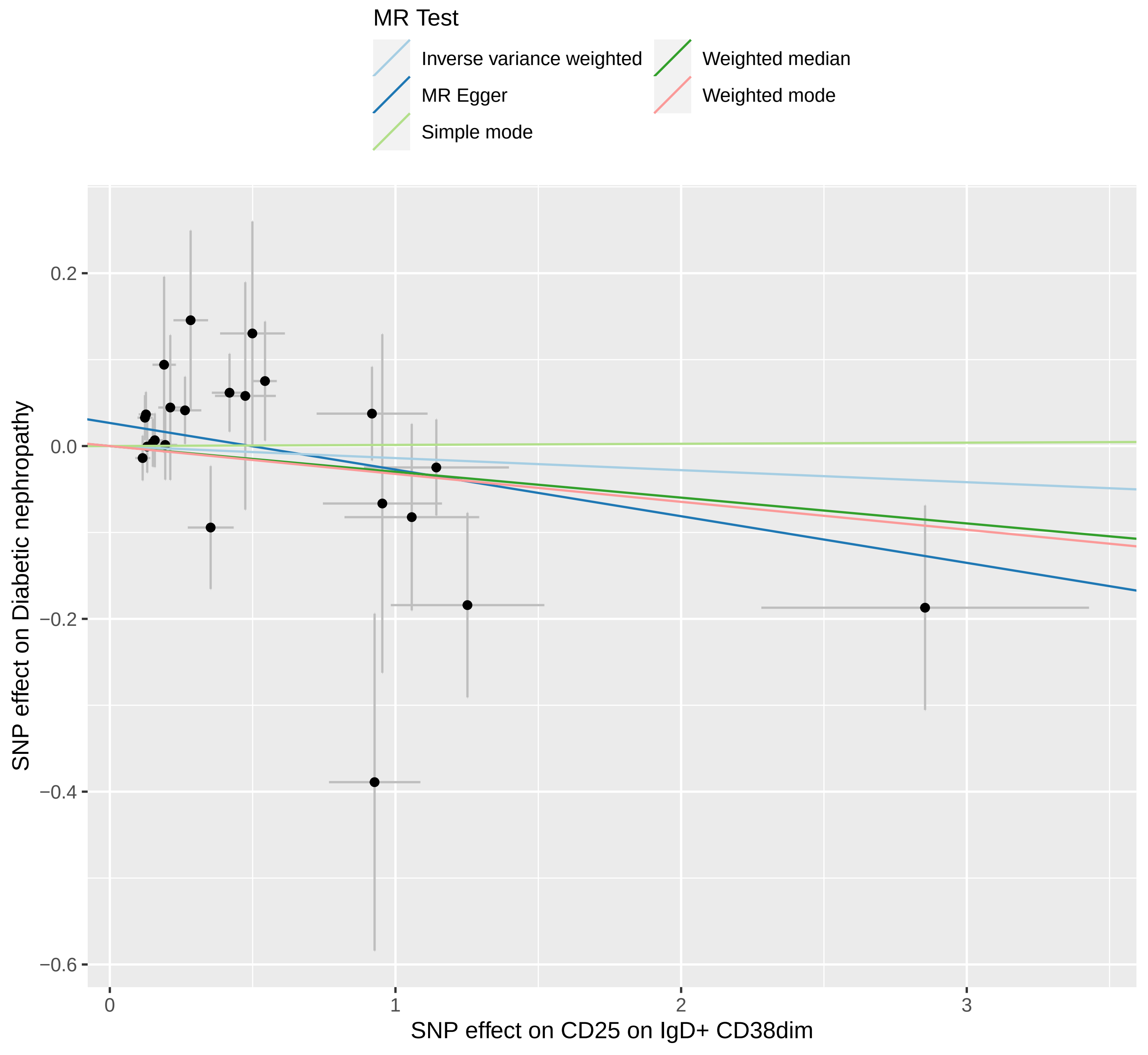

MR Test
Inverse variance weighted
MR Egger
Simple mode
Weighted median
Weighted mode
SNP effect on Diabetic nephropathy
0.2
0.0
−0.2
−0.4
−0.6
0
1
2
3
SNP effect on CD25 on IgD+ CD38dim

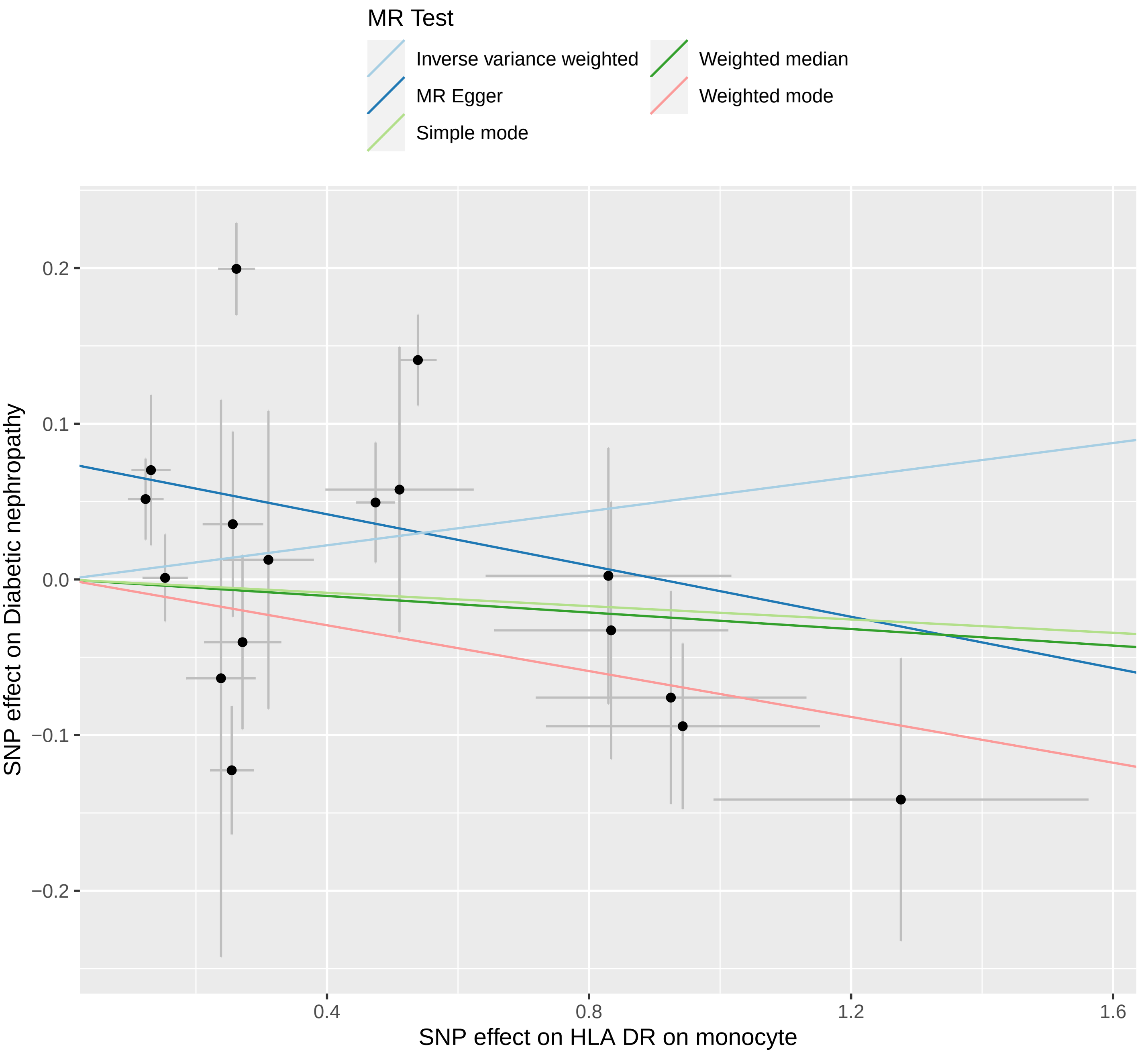

MR Test
Inverse variance weighted
MR Egger
Simple mode
Weighted median
Weighted mode
0.2
0.1
0.0
−0.1
−0.2
0.4
0.8
1.2
1.6
SNP effect on Diabetic nephropathy
SNP effect on HLA DR on monocyte

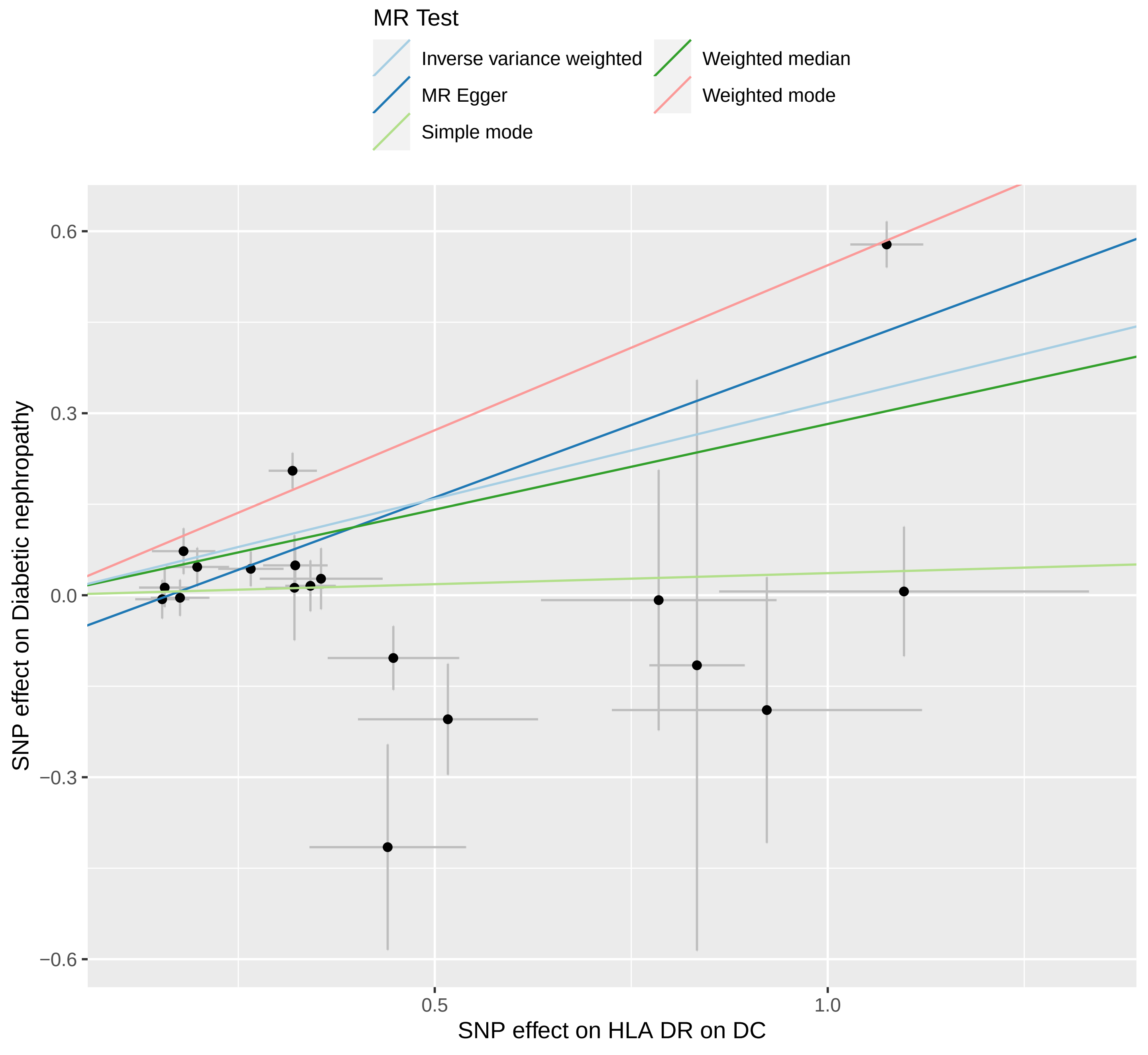
MR Test
Inverse variance weighted
MR Egger
Simple mode
Weighted median
Weighted mode
0.6
0.3
0.0
−0.3
−0.6
SNP effect on Diabetic nephropathy
0.5
1.0
SNP effect on HLA DR on DC

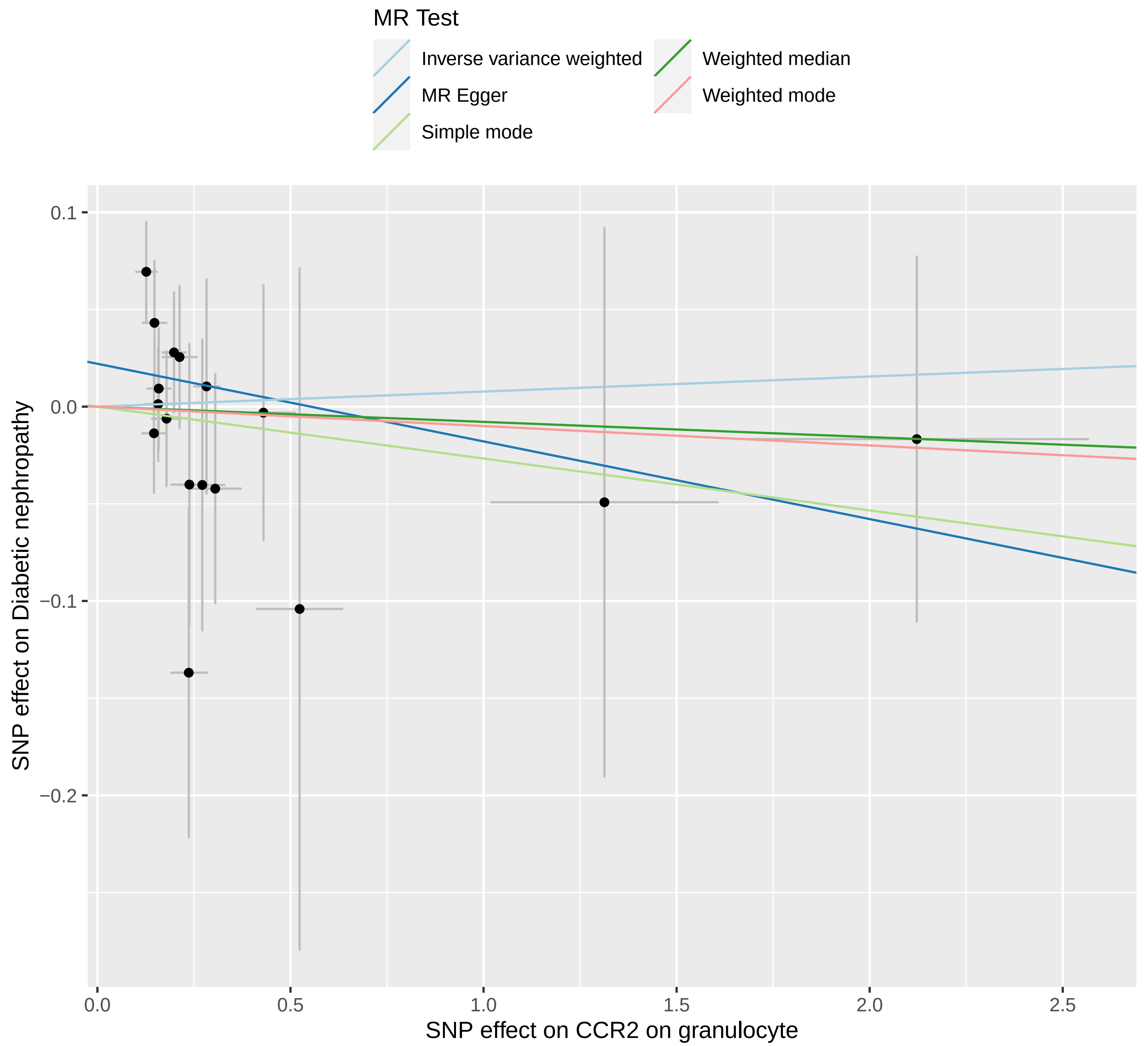

MR Test
Inverse variance weighted
MR Egger
Simple mode
Weighted median
Weighted mode
0.1
0.0
−0.1
−0.2
SNP effect on Diabetic nephropathy
0.0
0.5
1.0
1.5
2.0
2.5
SNP effect on CCR2 on granulocyte

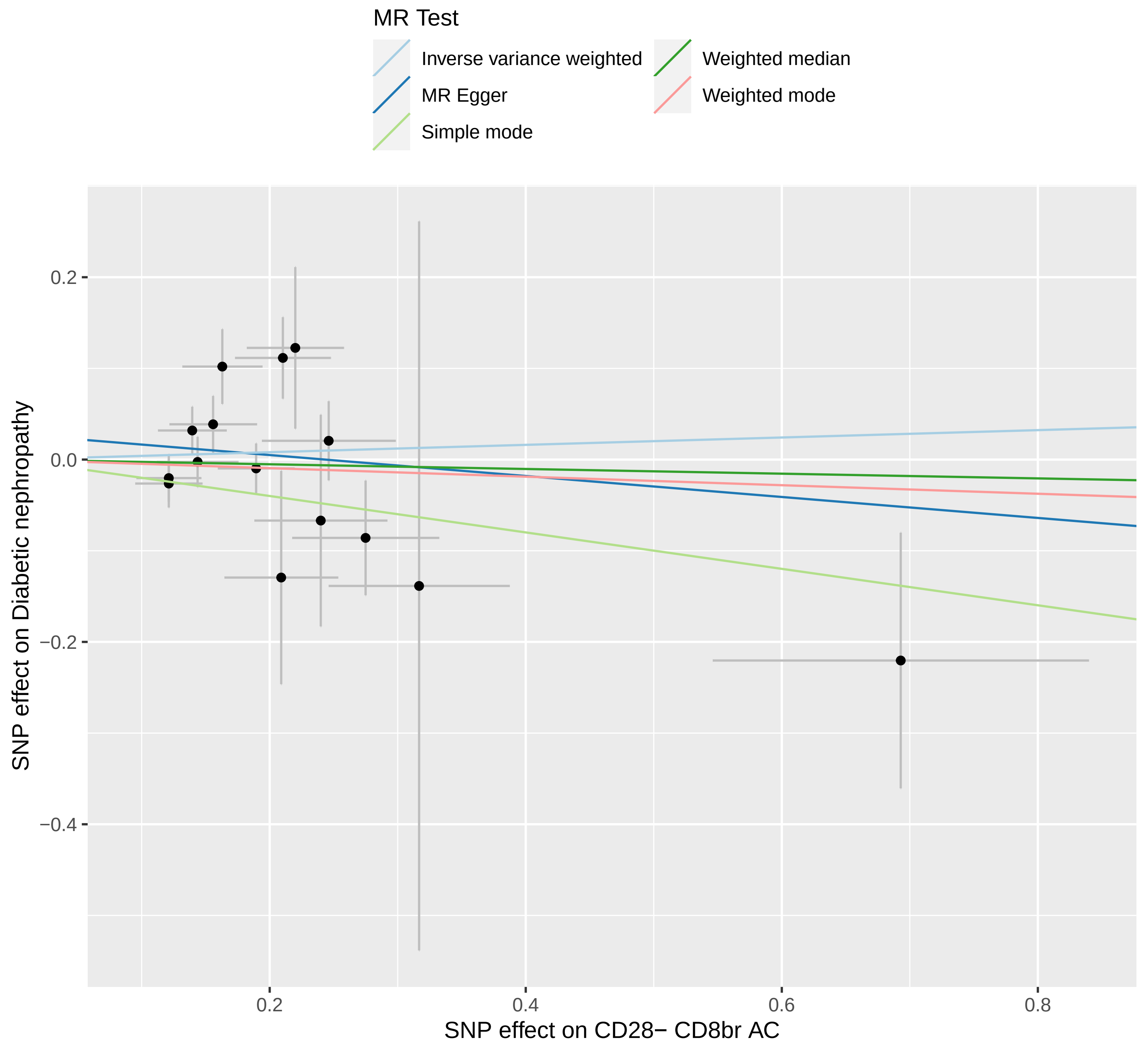

MR Test
Inverse variance weighted
MR Egger
Simple mode
Weighted median
Weighted mode
SNP effect on Diabetic nephropathy
0.2
0.0
−0.2
−0.4
0.2
0.4
0.6
0.8
SNP effect on CD28− CD8br AC

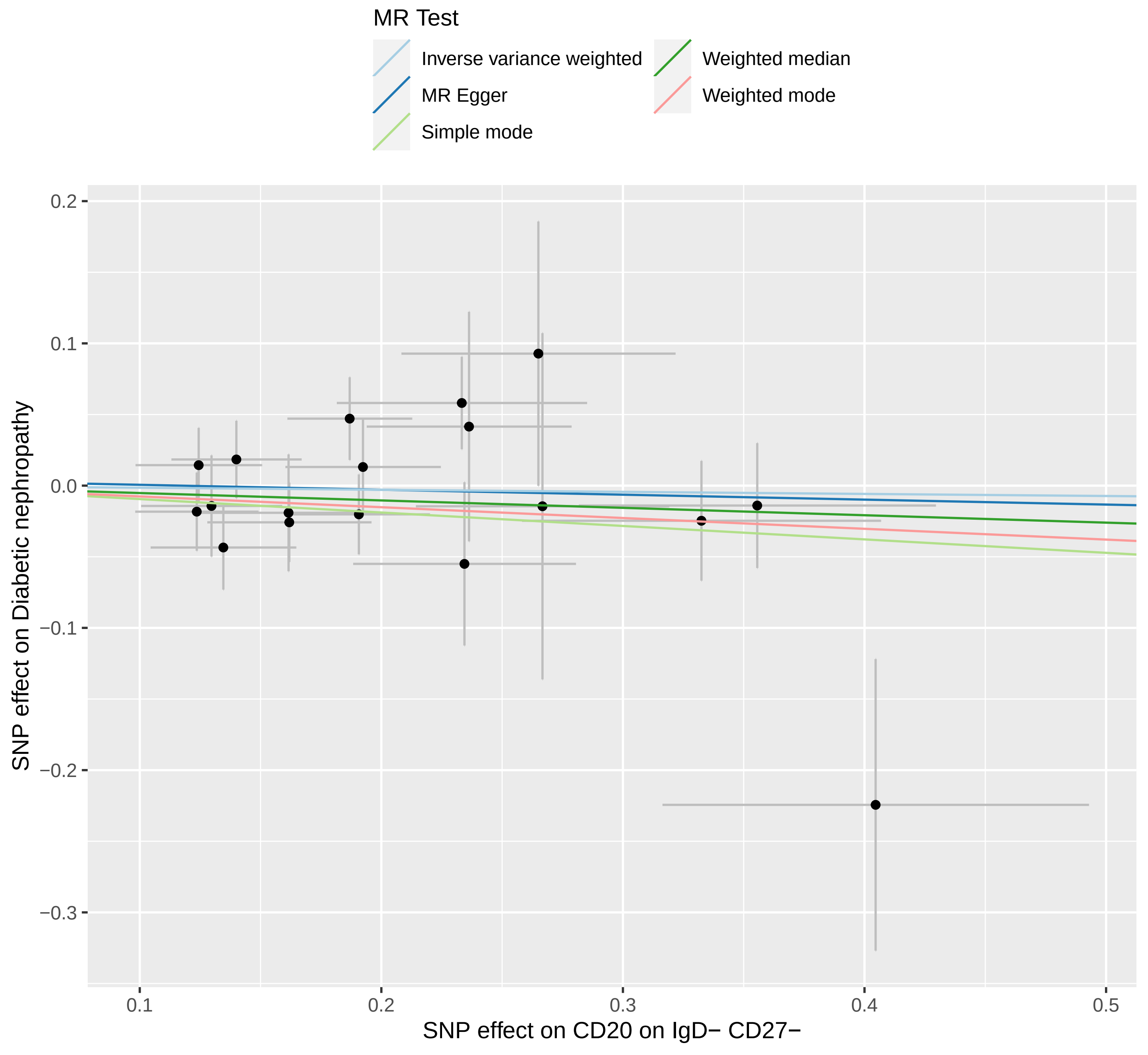

MR Test
Inverse variance weighted
MR Egger
Simple mode
Weighted median
Weighted mode
0.2
0.1
0.0
−0.1
−0.2
−0.3
0.1
0.2
0.3
0.4
0.5
SNP effect on Diabetic nephropathy
SNP effect on CD20 on IgD− CD27−

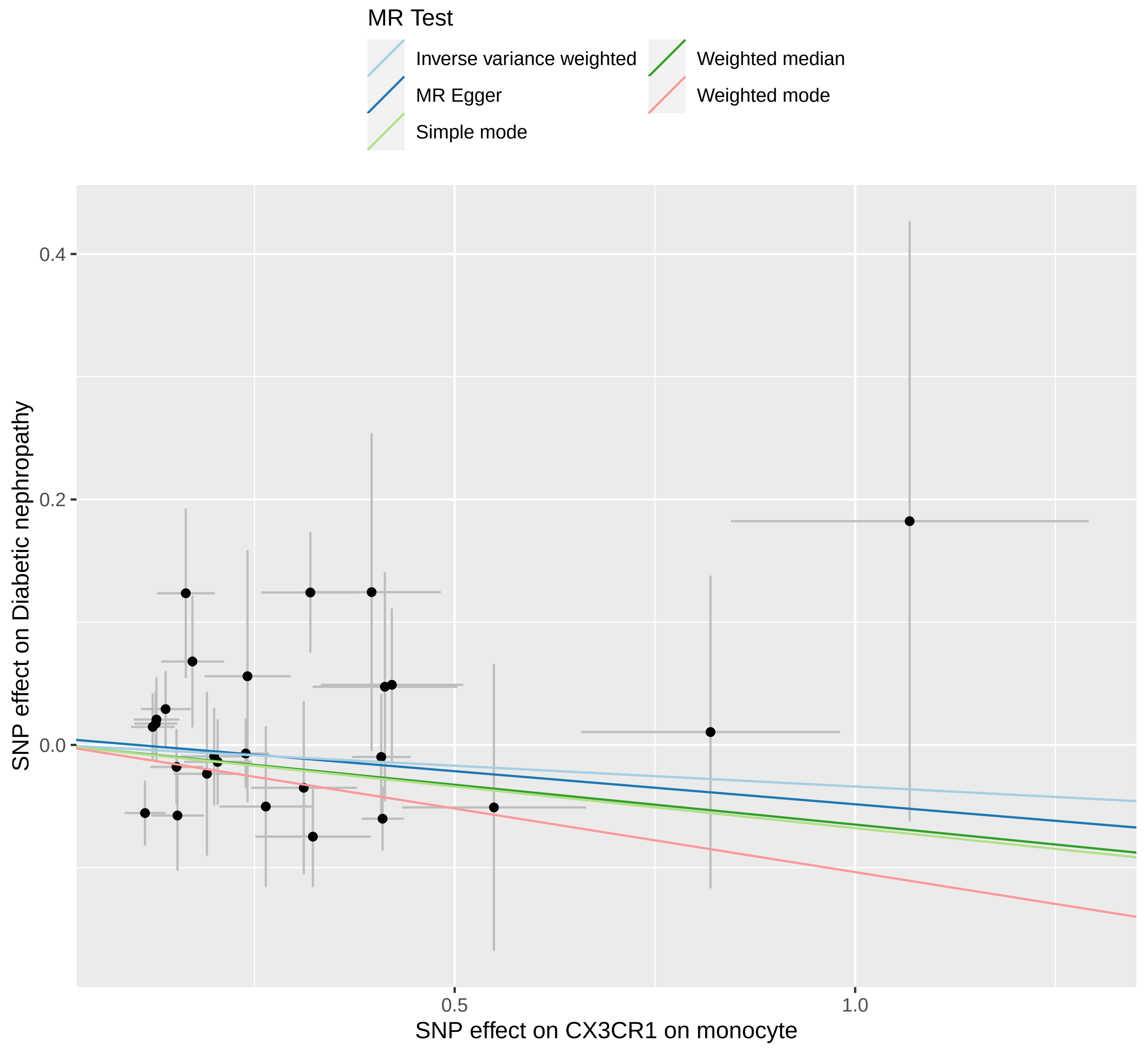

MR Test
Inverse variance weighted
MR Egger
Simple mode
Weighted median
Weighted mode
0.4
0.2
0.0
0.5
1.0
SNP effect on Diabetic nephropathy
SNP effect on CX3CR1 on monocyte

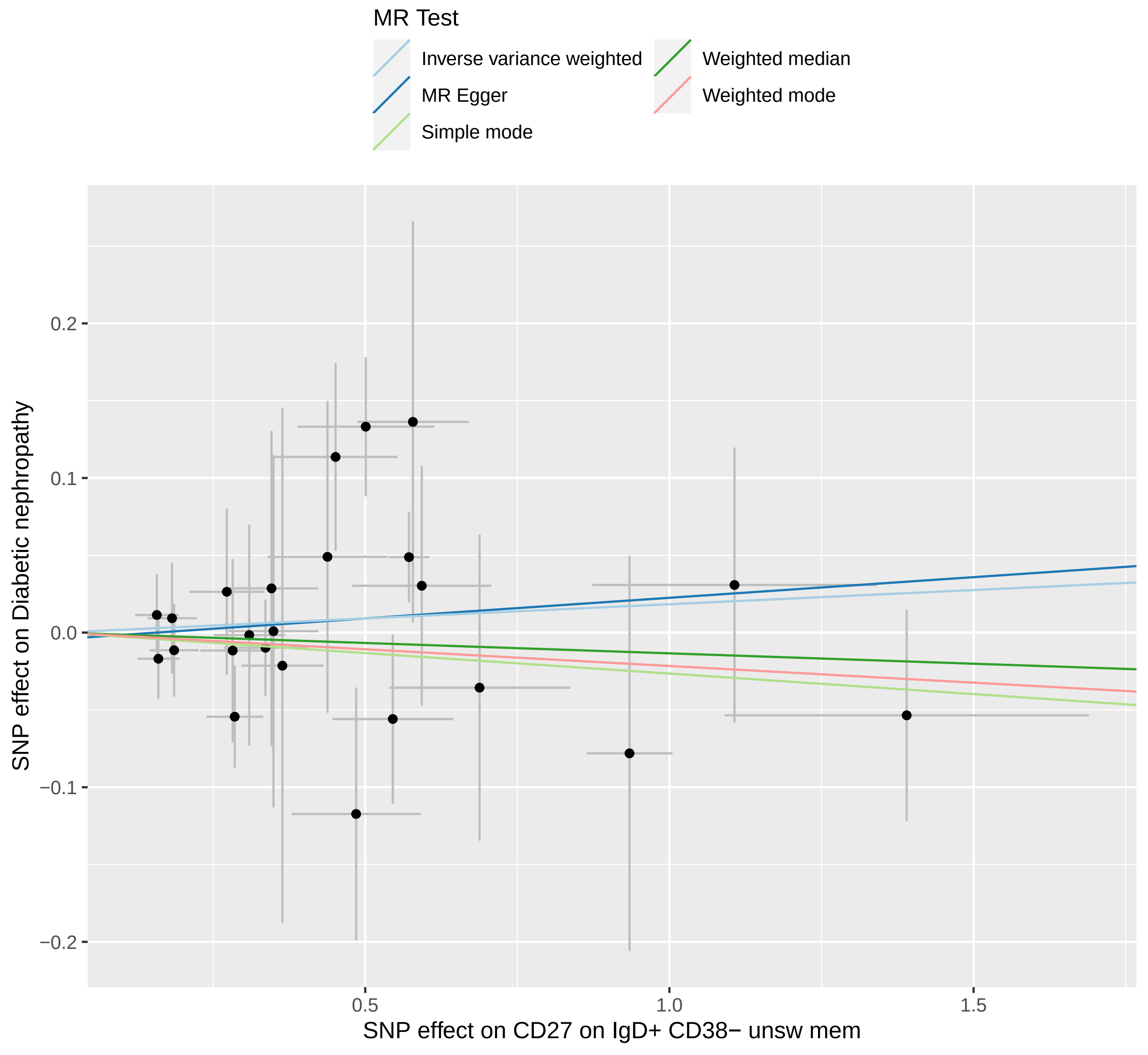
MR Test
Inverse variance weighted
MR Egger
Simple mode
Weighted median
Weighted mode
SNP effect on Diabetic nephropathy
SNP effect on CD27 on IgD+ CD38− unsw mem
0.2
0.1
0.0
−0.1
−0.2
0.5
1.0
1.5

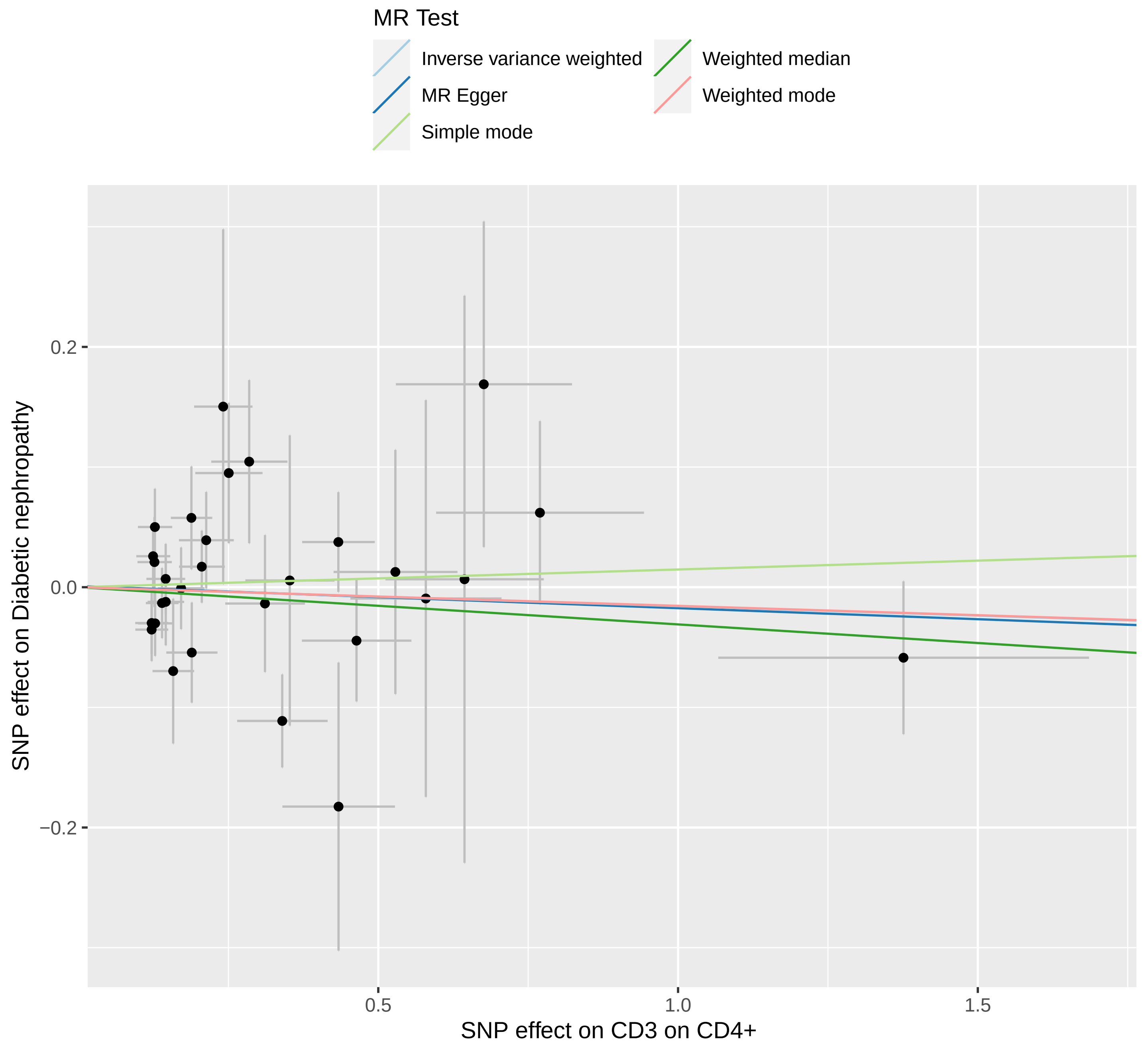
MR Test
Inverse variance weighted
MR Egger
Simple mode
Weighted median
Weighted mode
0.2
0.0
−0.2
0.5
1.0
1.5
SNP effect on Diabetic nephropathy
SNP effect on CD3 on CD4+

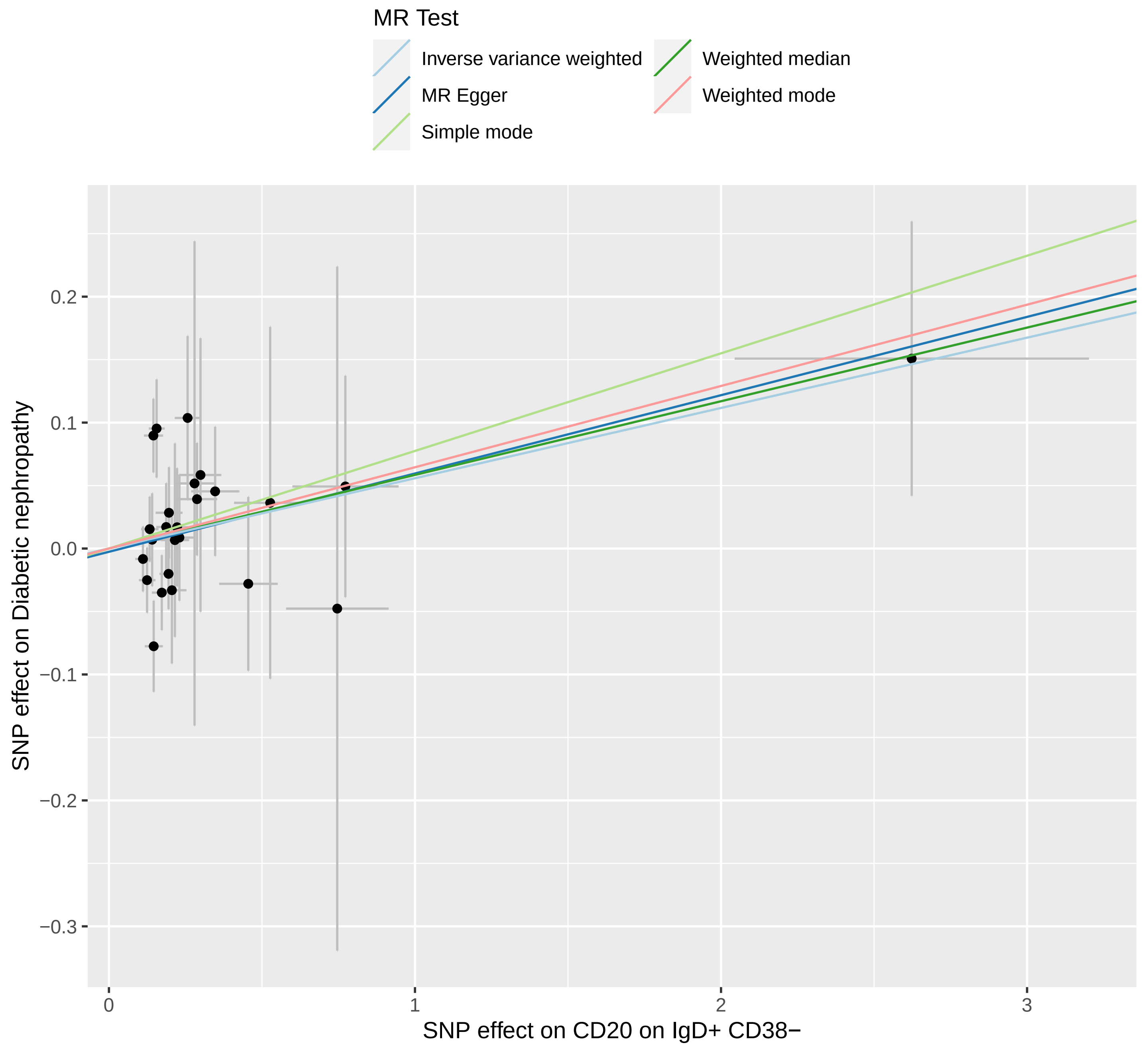

MR Test
Inverse variance weighted
MR Egger
Simple mode
Weighted median
Weighted mode
SNP effect on Diabetic nephropathy
0.2
0.1
0.0
−0.1
−0.2
−0.3
0
1
2
3
SNP effect on CD20 on IgD+ CD38−

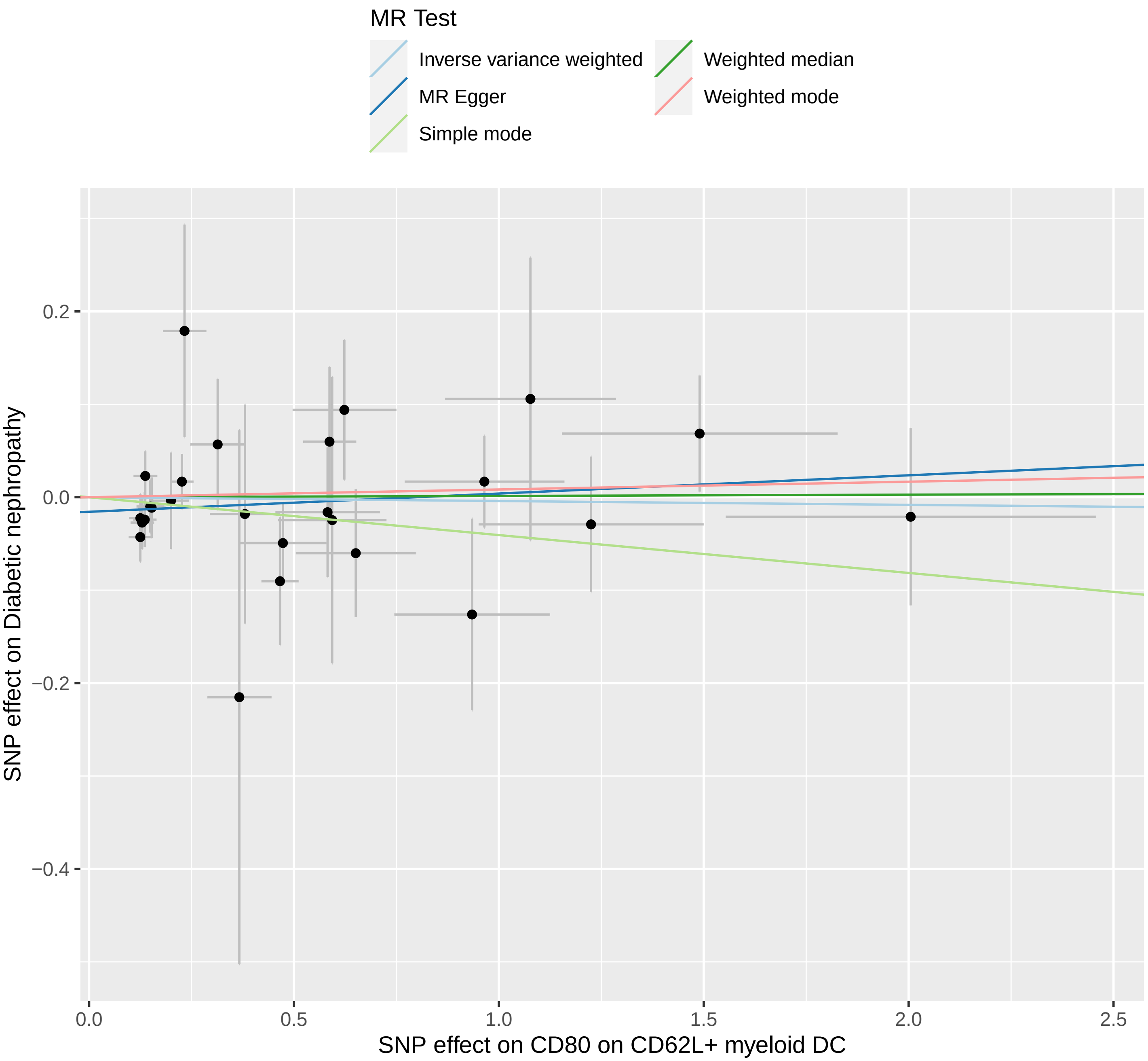
MR Test
Inverse variance weighted
MR Egger
Simple mode
Weighted median
Weighted mode
0.2
0.0
−0.2
−0.4
0.0
0.5
1.0
1.5
2.0
2.5
SNP effect on Diabetic nephropathy
SNP effect on CD80 on CD62L+ myeloid DC

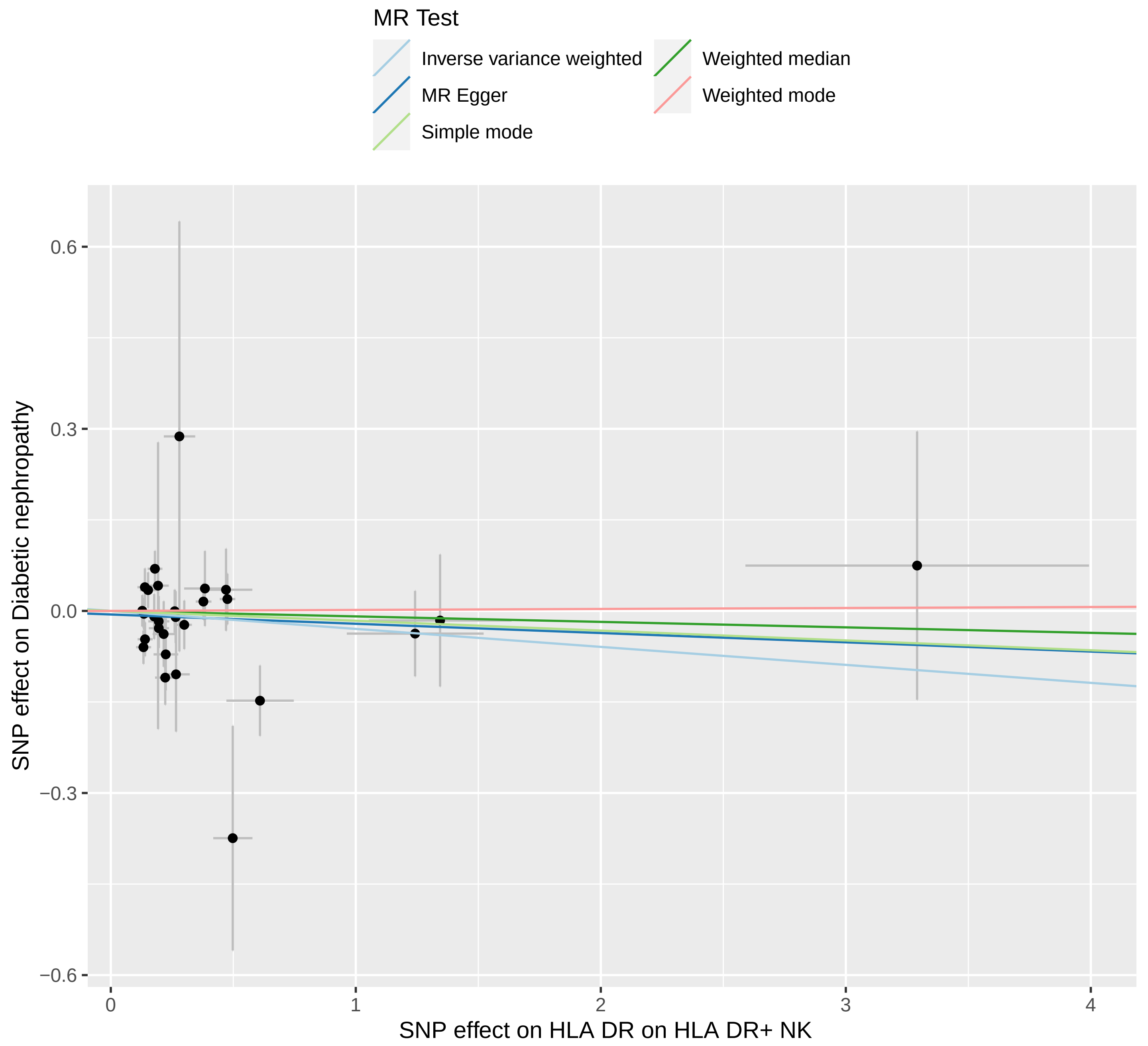

MR Test
Inverse variance weighted
MR Egger
Simple mode
Weighted median
Weighted mode
SNP effect on Diabetic nephropathy
0.6
0.3
0.0
−0.3
−0.6
0
1
2
3
4
SNP effect on HLA DR on HLA DR+ NK

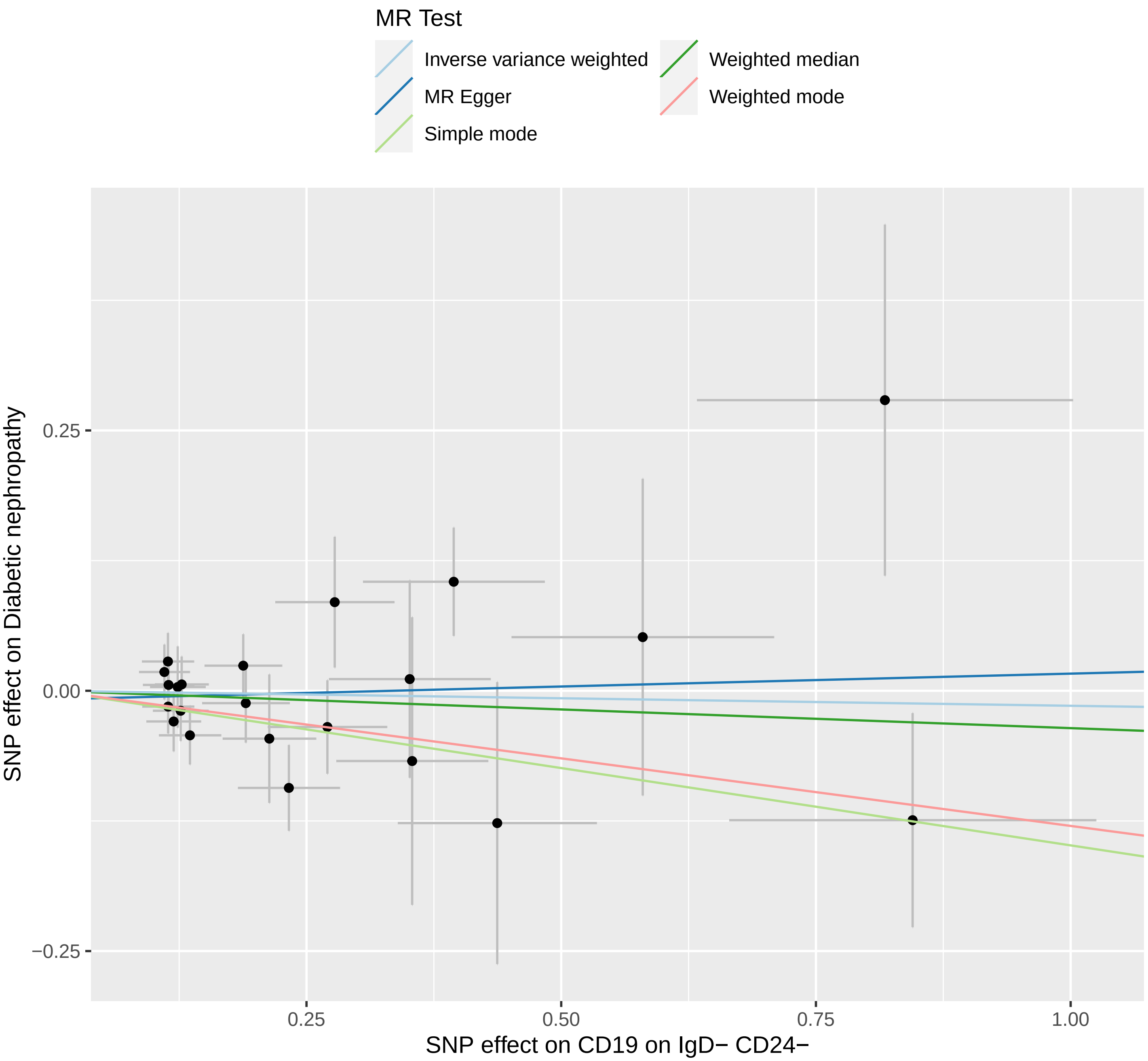
MR Test
Inverse variance weighted
MR Egger
Simple mode
Weighted median
Weighted mode
SNP effect on Diabetic nephropathy
0.25
0.00
−0.25
0.25
0.50
0.75
1.00
SNP effect on CD19 on IgD− CD24−

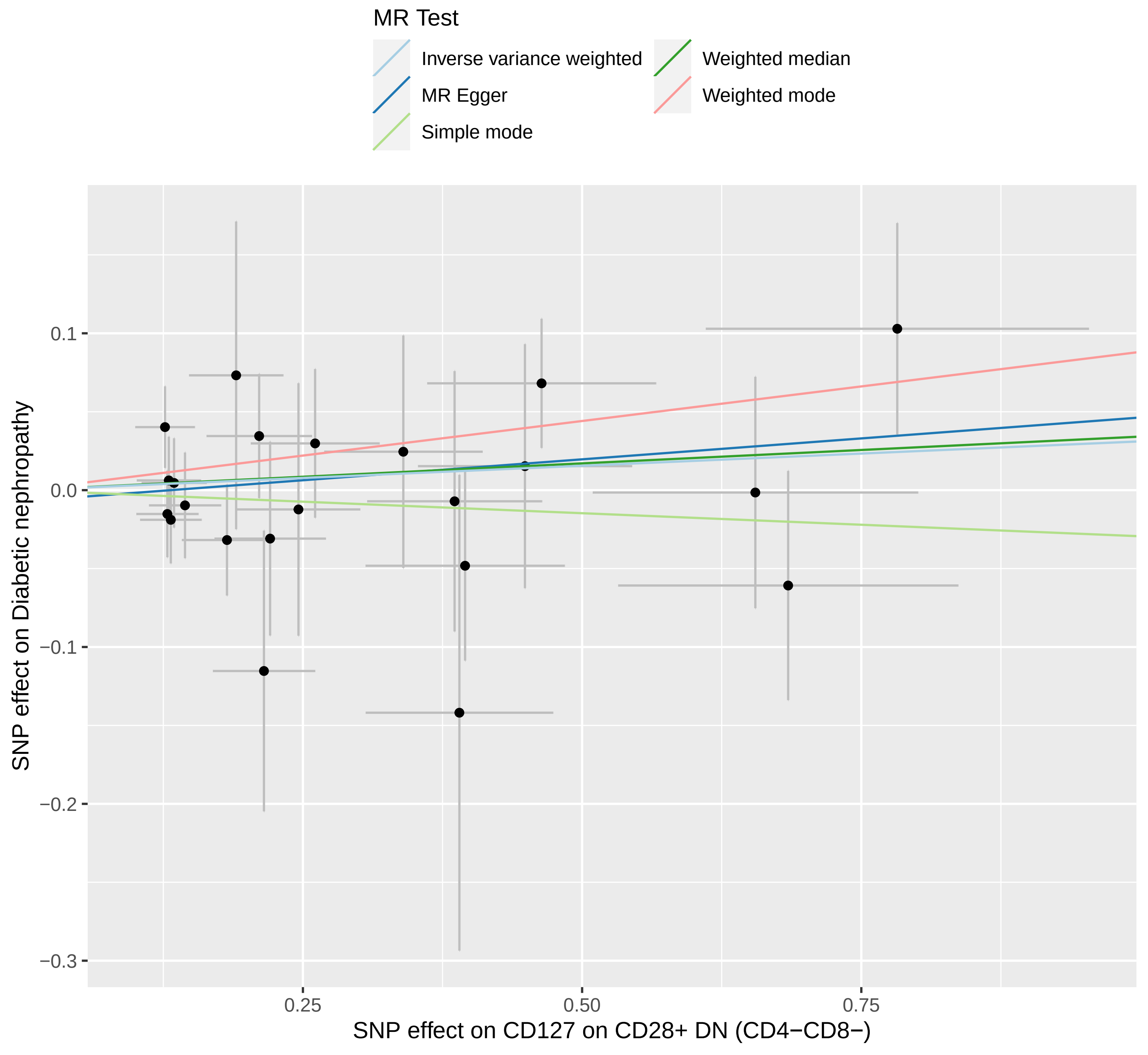
MR Test
Inverse variance weighted
MR Egger
Simple mode
Weighted median
Weighted mode
SNP effect on Diabetic nephropathy
0.1
0.0
−0.1
−0.2
−0.3
0.25
0.50
0.75
SNP effect on CD127 on CD28+ DN (CD4−CD8−)

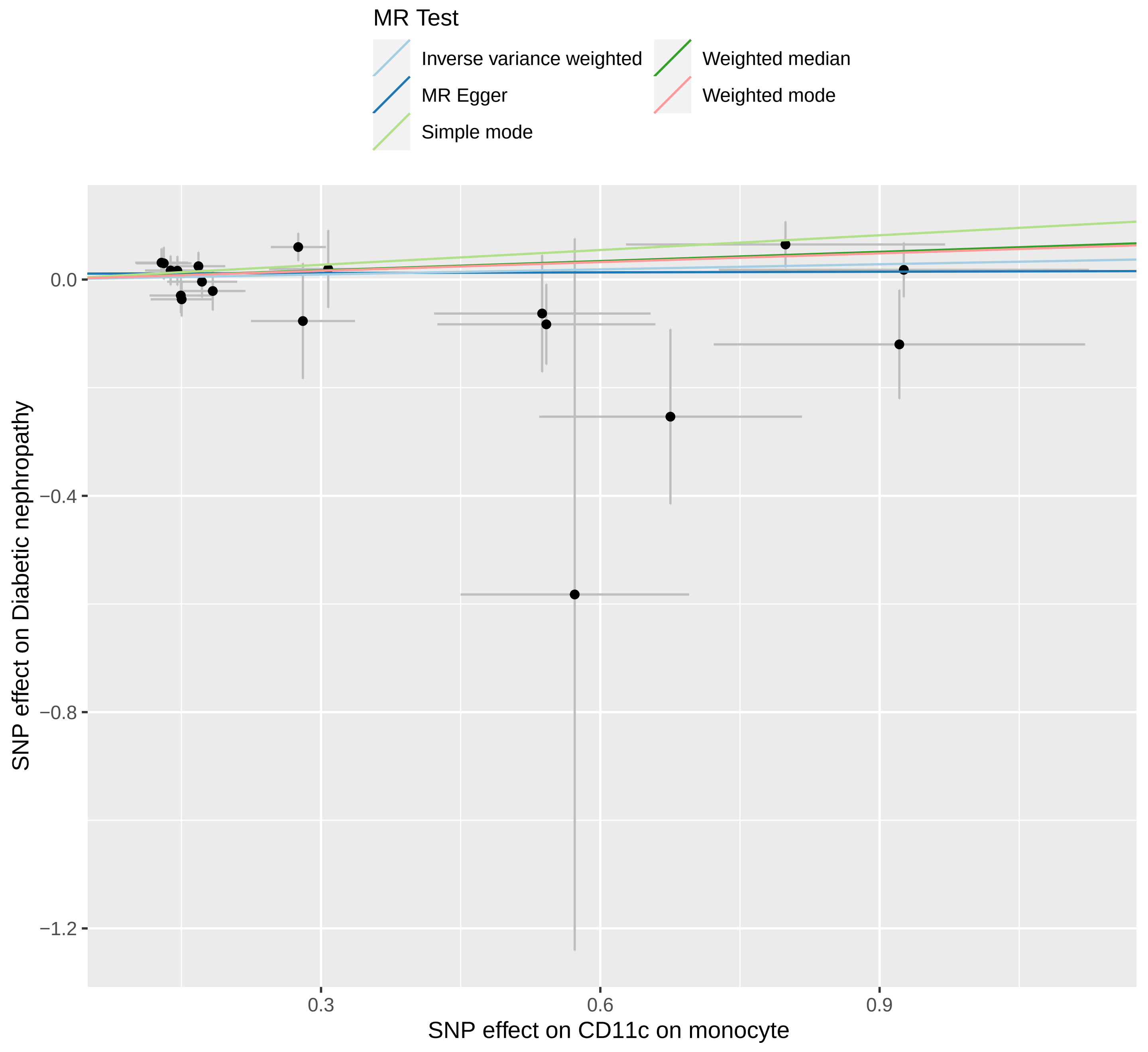
MR Test
Inverse variance weighted
MR Egger
Simple mode
Weighted median
Weighted mode
0.0
−0.4
−0.8
−1.2
0.3
0.6
0.9
SNP effect on CD11c on monocyte
SNP effect on Diabetic nephropathy

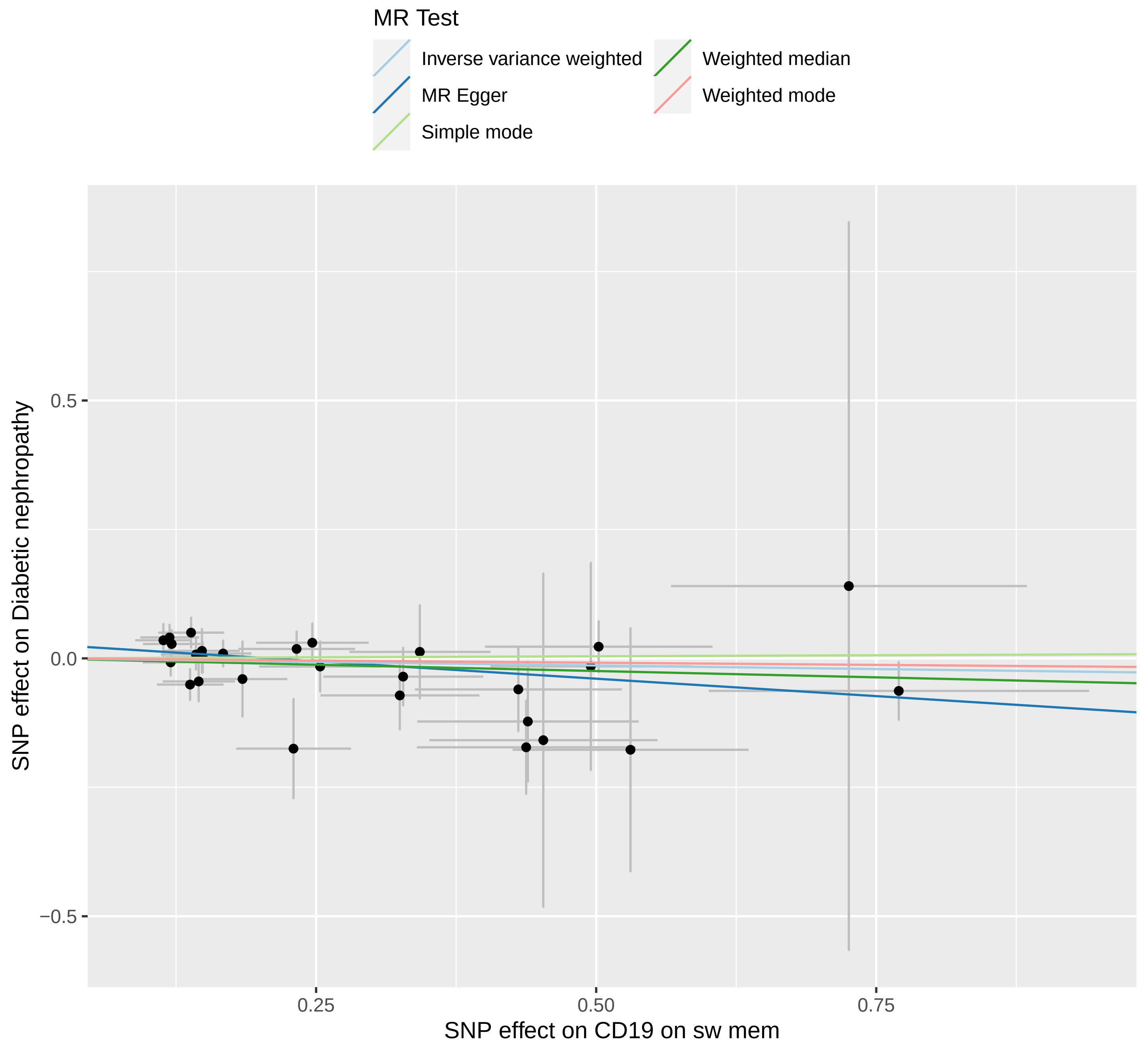
MR Test
Inverse variance weighted
MR Egger
Simple mode
Weighted median
Weighted mode
0.5
0.0
−0.5
0.25
0.50
0.75
SNP effect on Diabetic nephropathy
SNP effect on CD19 on sw mem

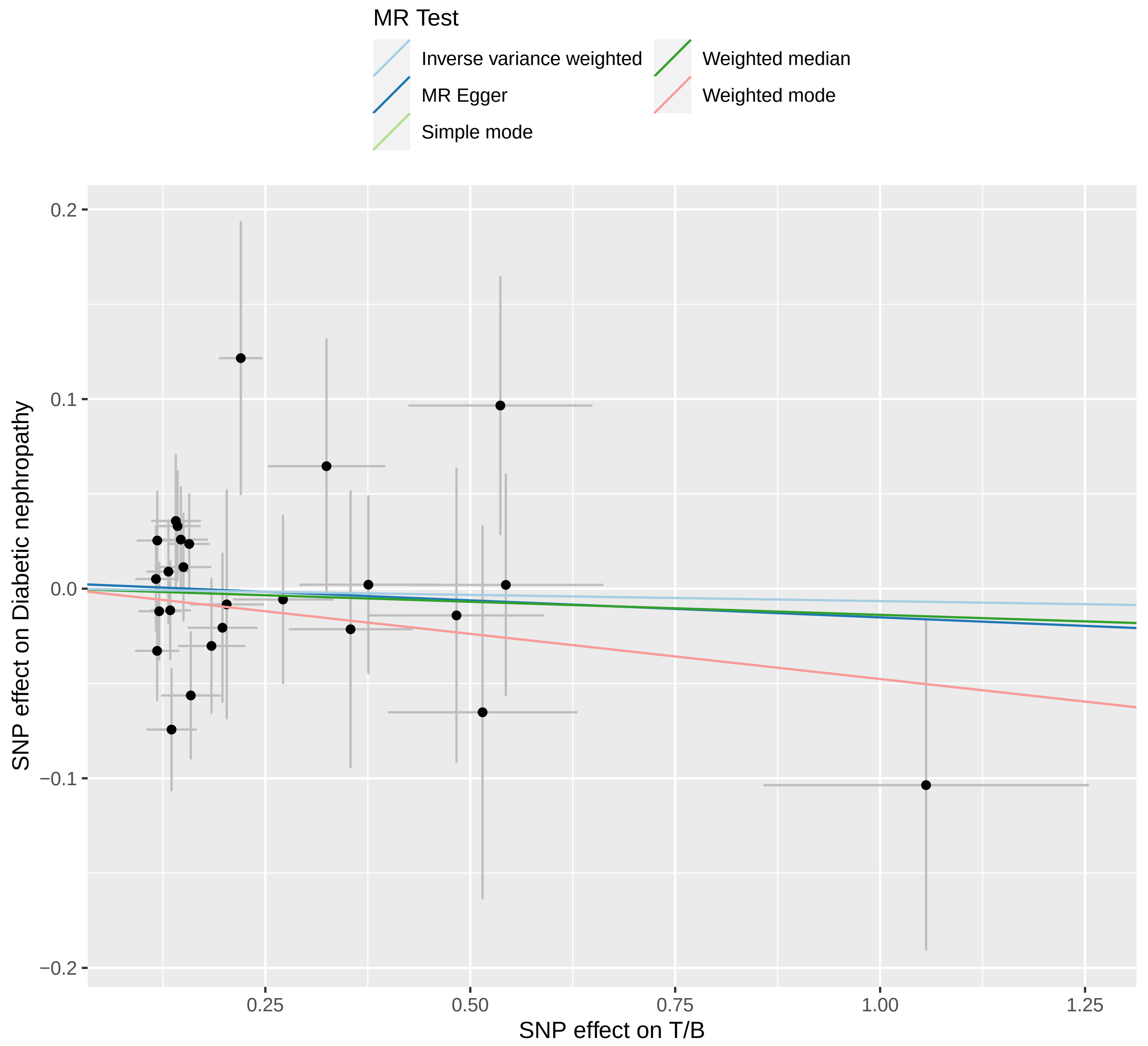
MR Test
Inverse variance weighted
MR Egger
Simple mode
Weighted median
Weighted mode
0.2
0.1
0.0
−0.1
−0.2
SNP effect on Diabetic nephropathy
0.25
0.50
0.75
1.00
1.25
SNP effect on T/B

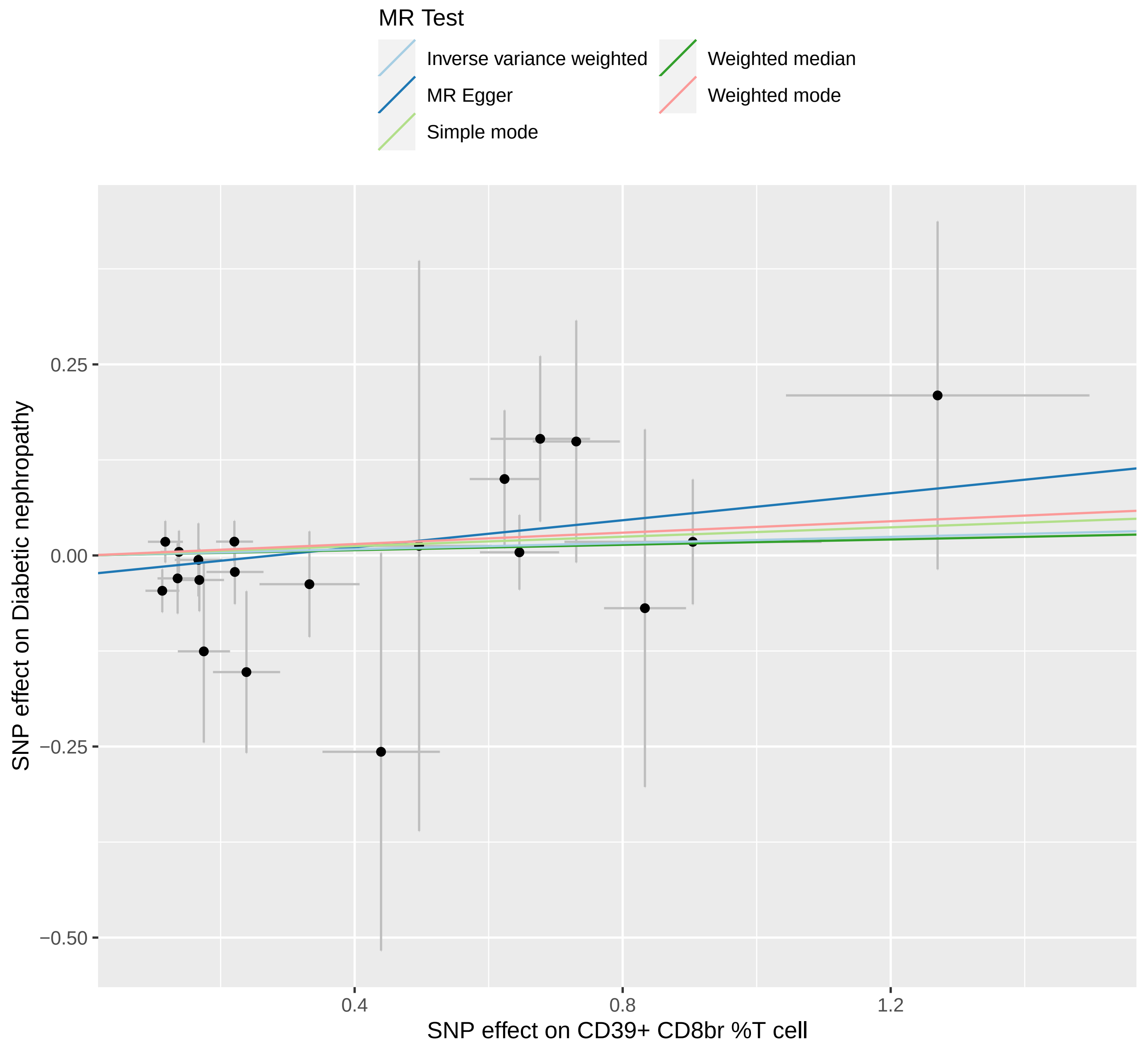
MR Test
Inverse variance weighted
MR Egger
Simple mode
Weighted median
Weighted mode
0.25
0.00
−0.25
−0.50
0.4
0.8
1.2
SNP effect on Diabetic nephropathy
SNP effect on CD39+ CD8br %T cell

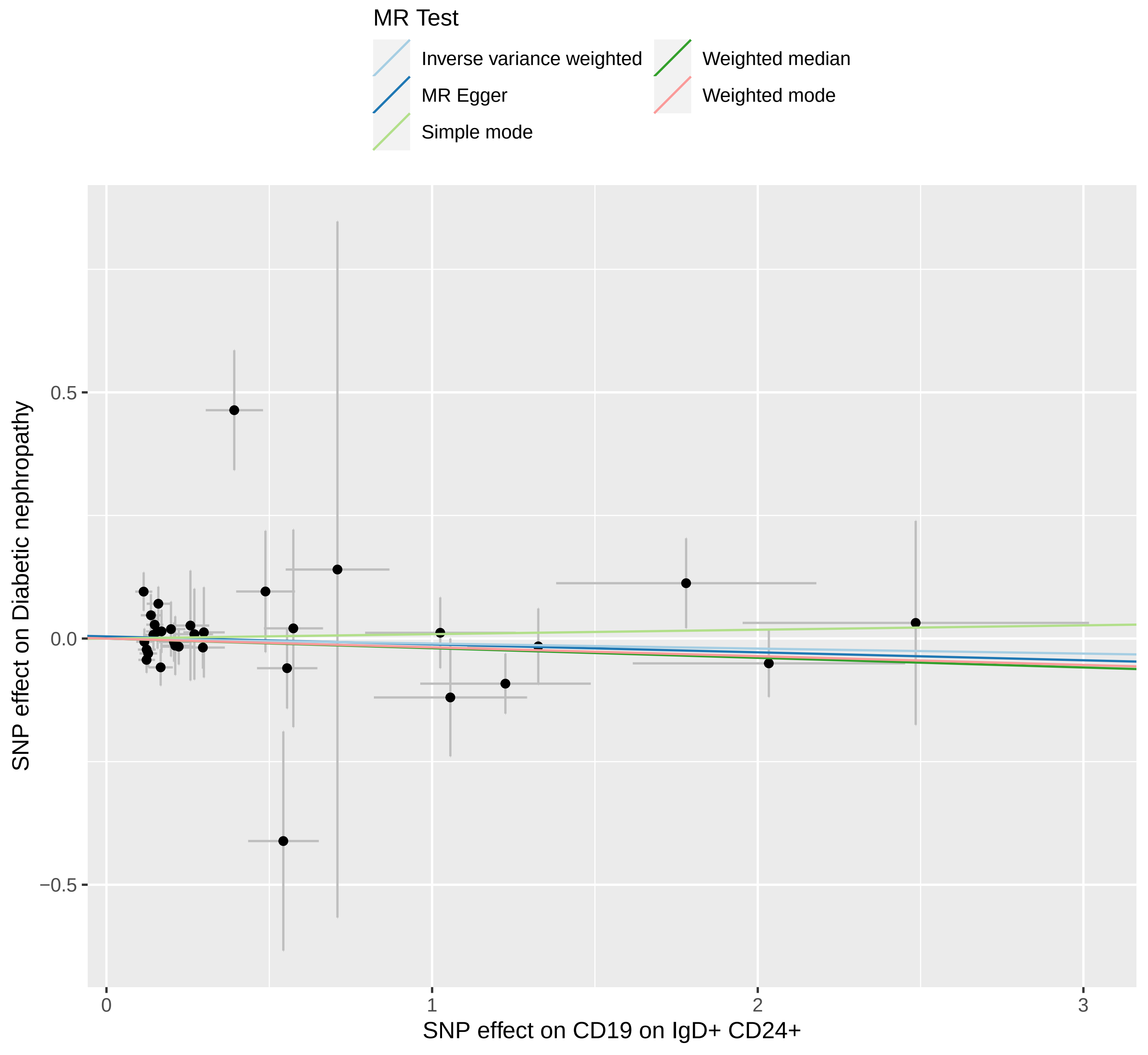
MR Test
Inverse variance weighted
MR Egger
Simple mode
Weighted median
Weighted mode
0.5
0.0
−0.5
0
1
2
3
SNP effect on Diabetic nephropathy
SNP effect on CD19 on IgD+ CD24+

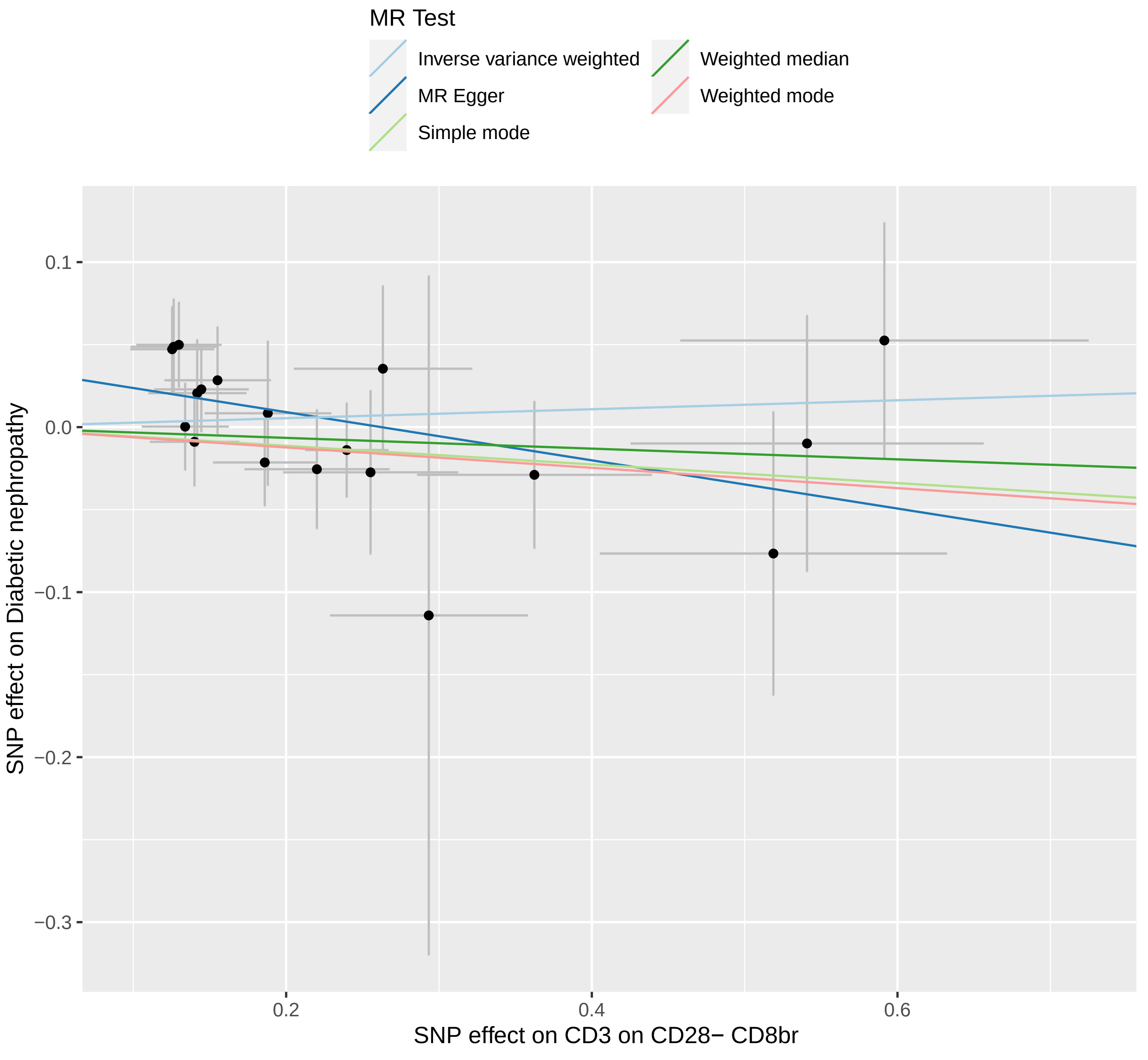
MR Test
Inverse variance weighted
MR Egger
Simple mode
Weighted median
Weighted mode
0.1
0.0
−0.1
−0.2
−0.3
0.2
0.4
0.6
SNP effect on Diabetic nephropathy
SNP effect on CD3 on CD28− CD8br

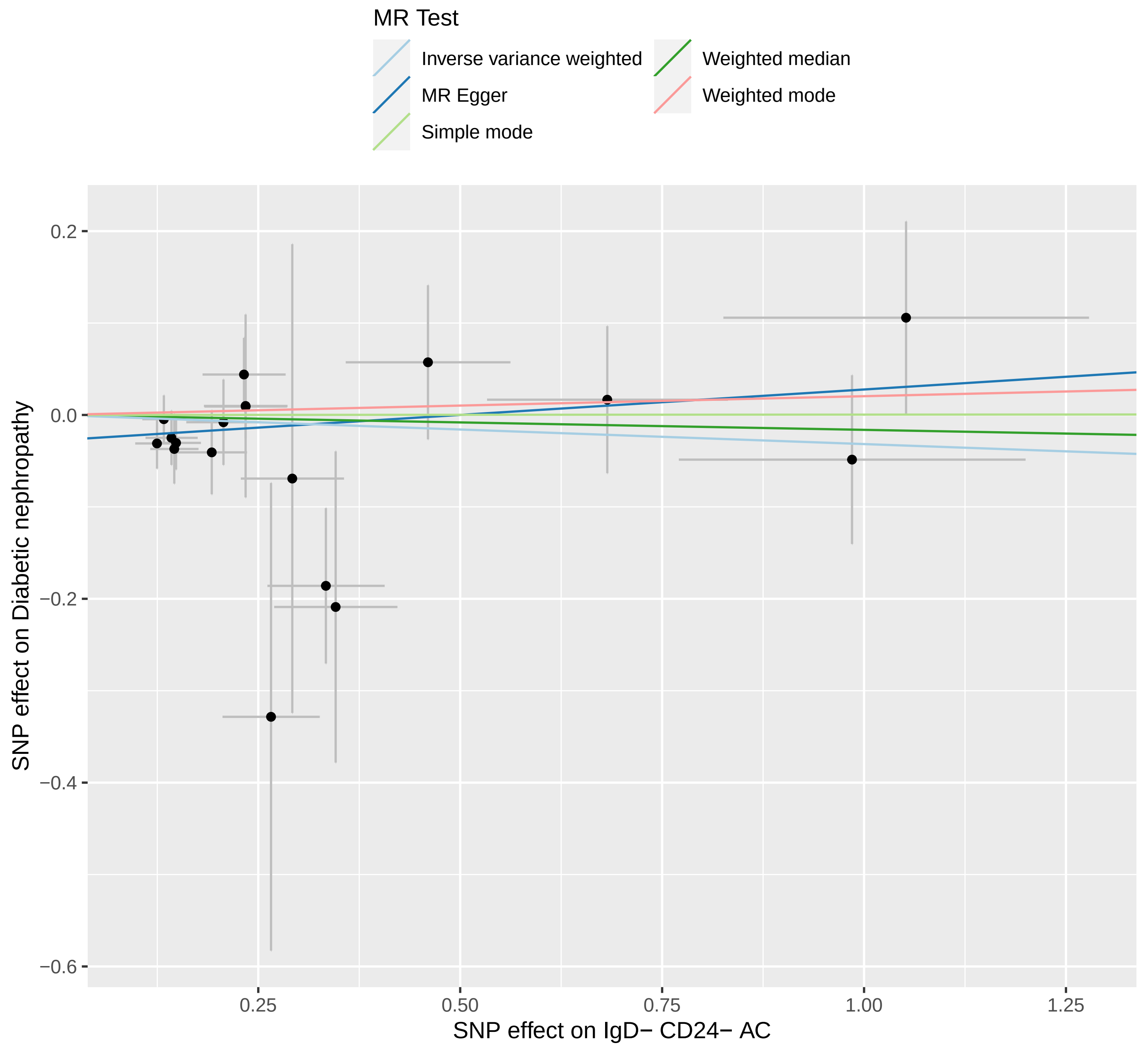
MR Test
Inverse variance weighted
MR Egger
Simple mode
Weighted median
Weighted mode
0.2
0.0
−0.2
−0.4
−0.6
SNP effect on Diabetic nephropathy
0.25
0.50
0.75
1.00
1.25
SNP effect on IgD− CD24− AC

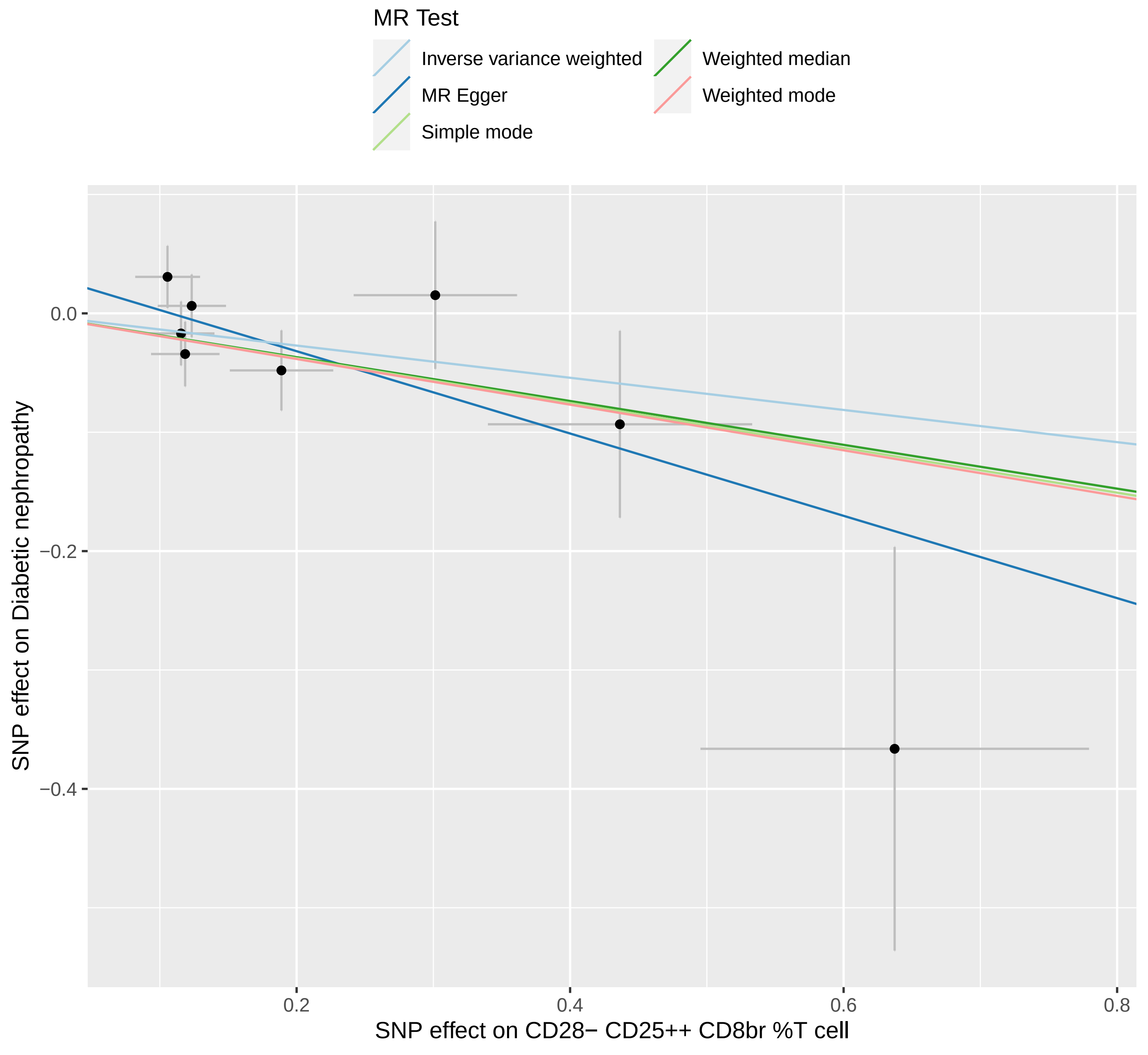
MR Test
Inverse variance weighted
MR Egger
Simple mode
Weighted median
Weighted mode
0.0
−0.2
−0.4
0.2
0.4
0.6
0.8
SNP effect on Diabetic nephropathy
SNP effect on CD28− CD25++ CD8br %T cell

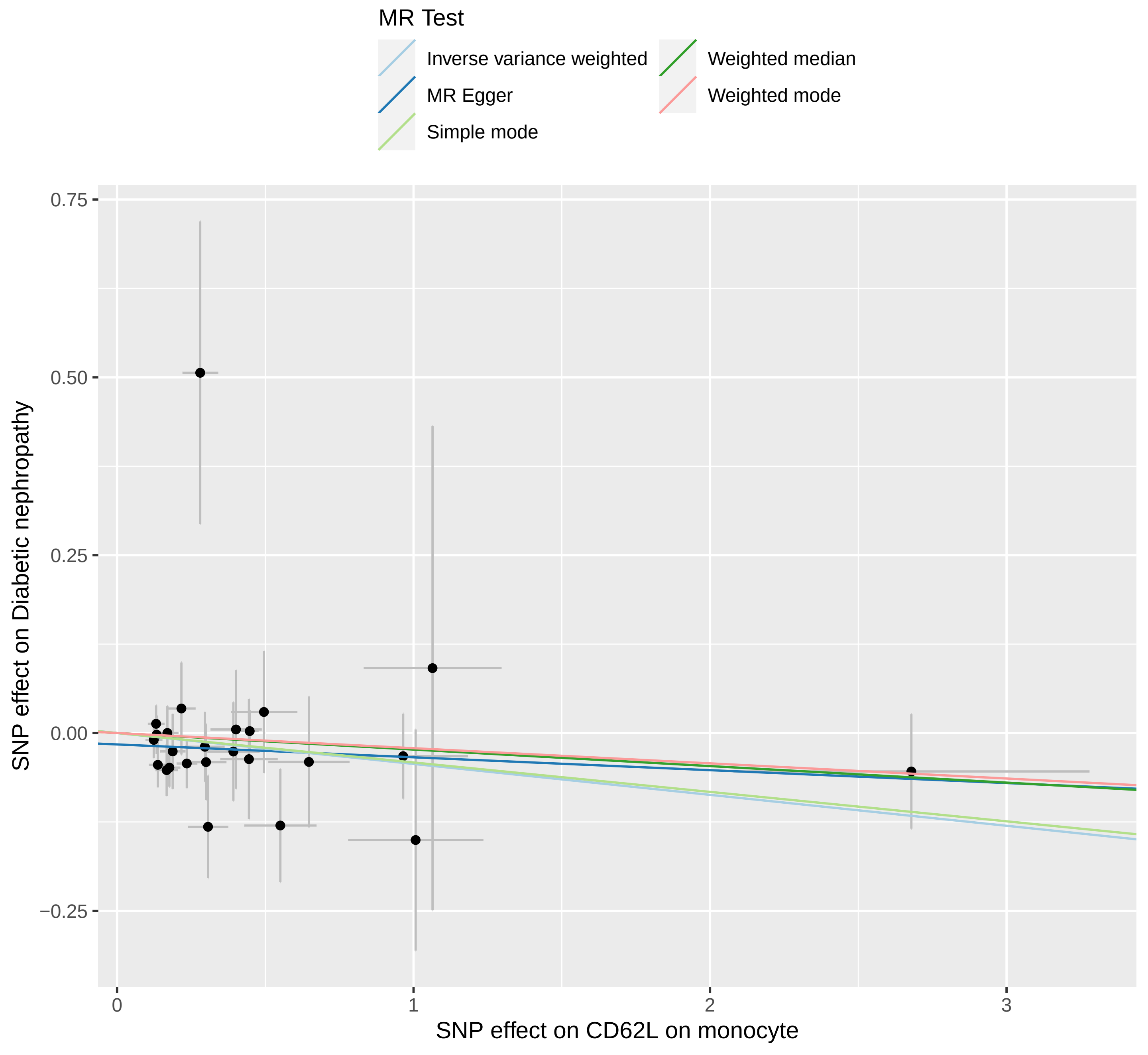
MR Test
Inverse variance weighted
MR Egger
Simple mode
Weighted median
Weighted mode
0.75
0.50
0.25
0.00
−0.25
SNP effect on Diabetic nephropathy
0
1
2
3
SNP effect on CD62L on monocyte

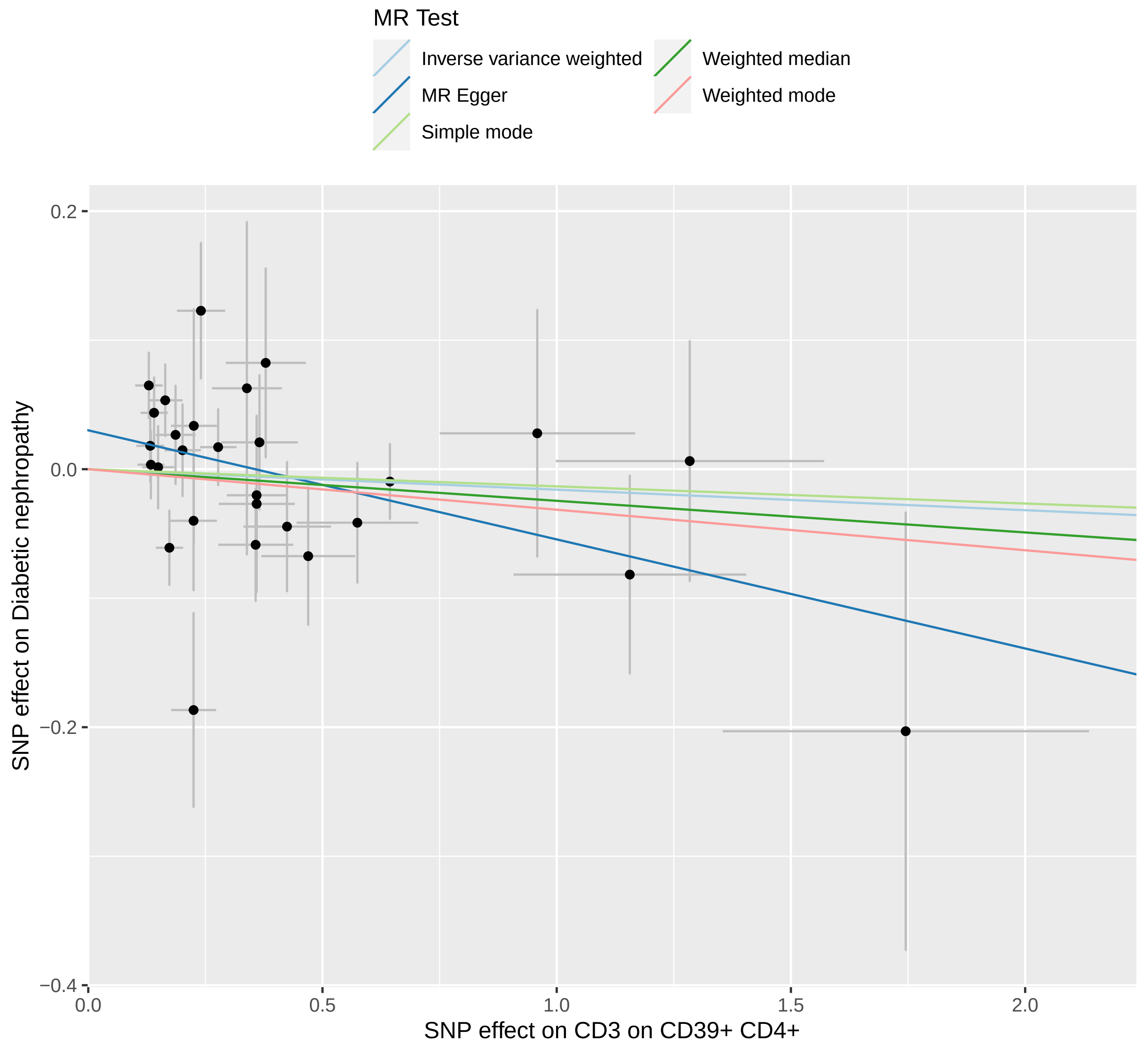

MR Test
Inverse variance weighted
MR Egger
Simple mode
Weighted median
Weighted mode
0.2
0.0
−0.2
−0.4
0.0
0.5
1.0
1.5
2.0
SNP effect on CD3 on CD39+ CD4+
SNP effect on Diabetic nephropathy

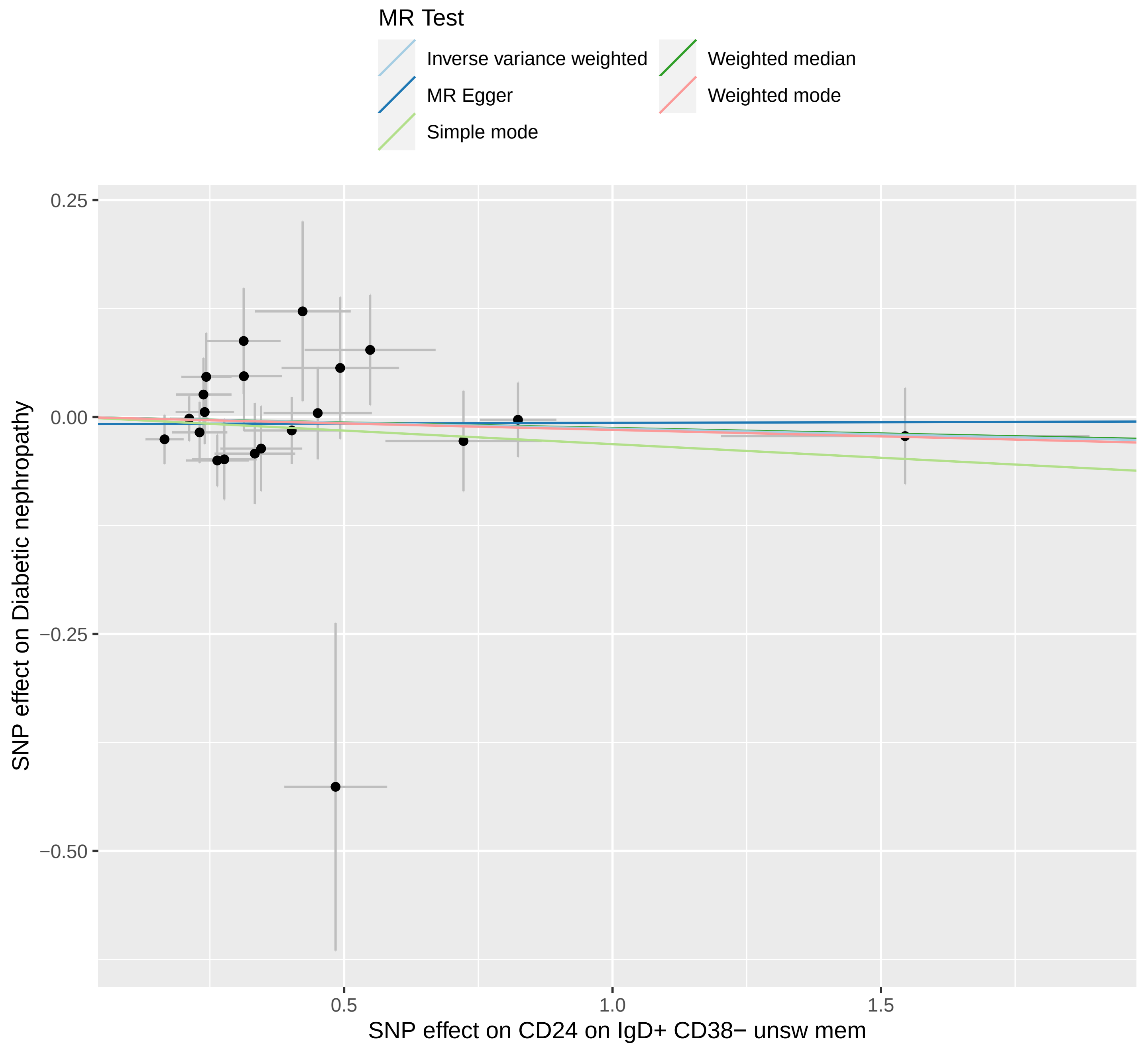

MR Test
Inverse variance weighted
MR Egger
Simple mode
Weighted median
Weighted mode
0.25
0.00
−0.25
−0.50
0.5
1.0
1.5
SNP effect on Diabetic nephropathy
SNP effect on CD24 on IgD+ CD38− unsw mem

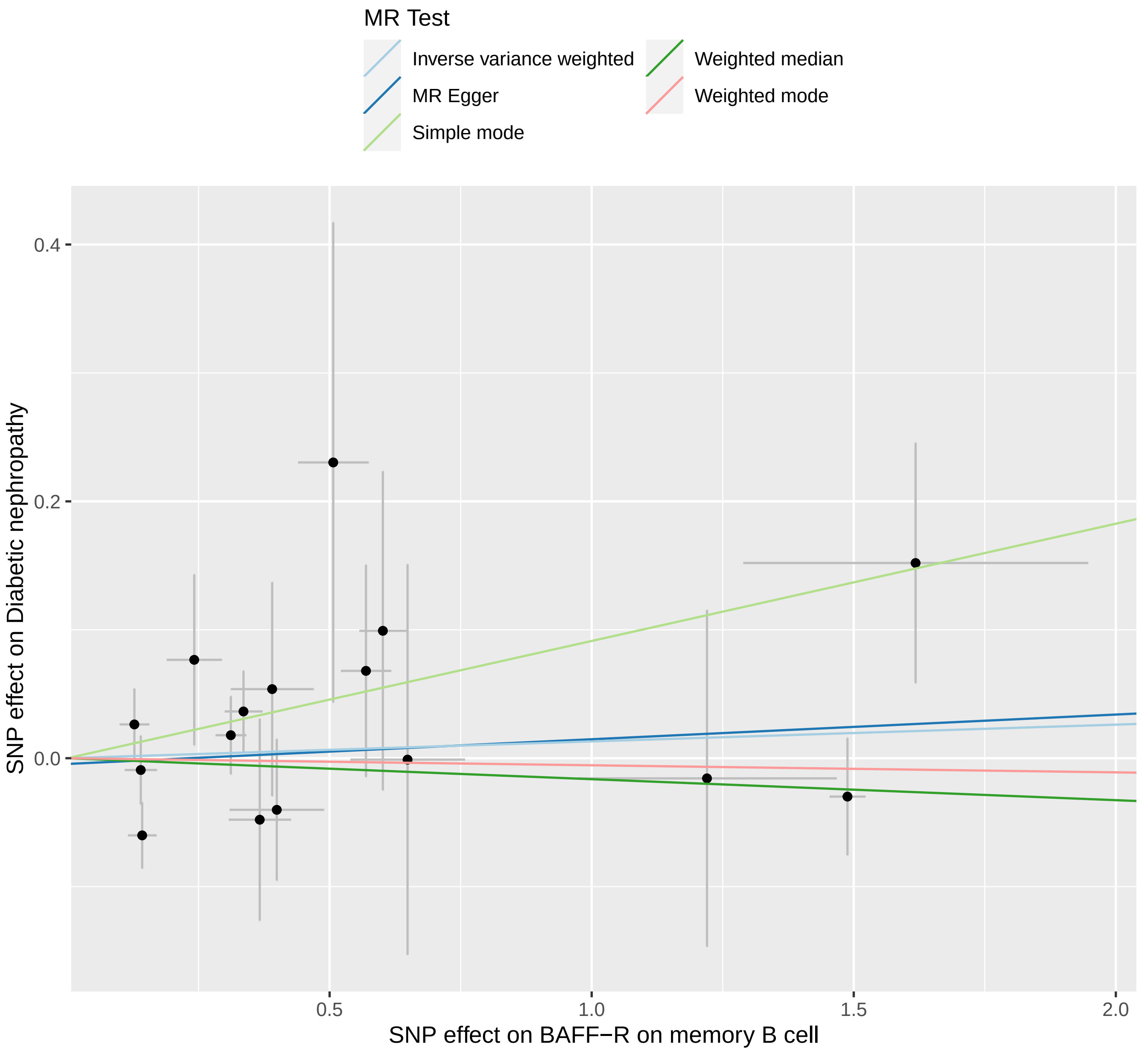
MR Test
Inverse variance weighted
MR Egger
Simple mode
Weighted median
Weighted mode
0.4
0.2
0.0
0.5
1.0
1.5
2.0
SNP effect on Diabetic nephropathy
SNP effect on BAFF−R on memory B cell

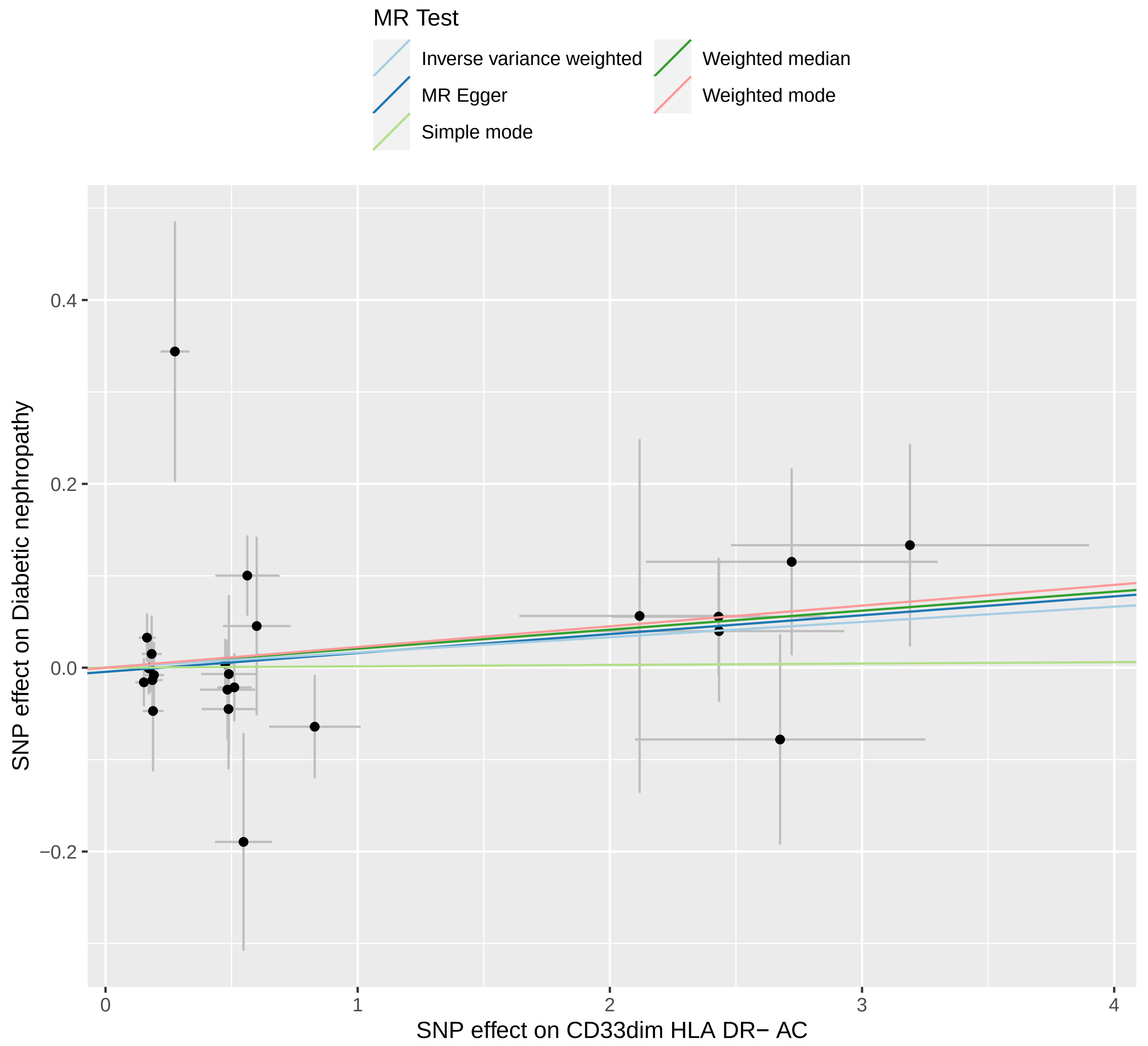

MR Test
Inverse variance weighted
MR Egger
Simple mode
Weighted median
Weighted mode
SNP effect on Diabetic nephropathy
0.4
0.2
0.0
−0.2
0
1
2
3
4
SNP effect on CD33dim HLA DR− AC

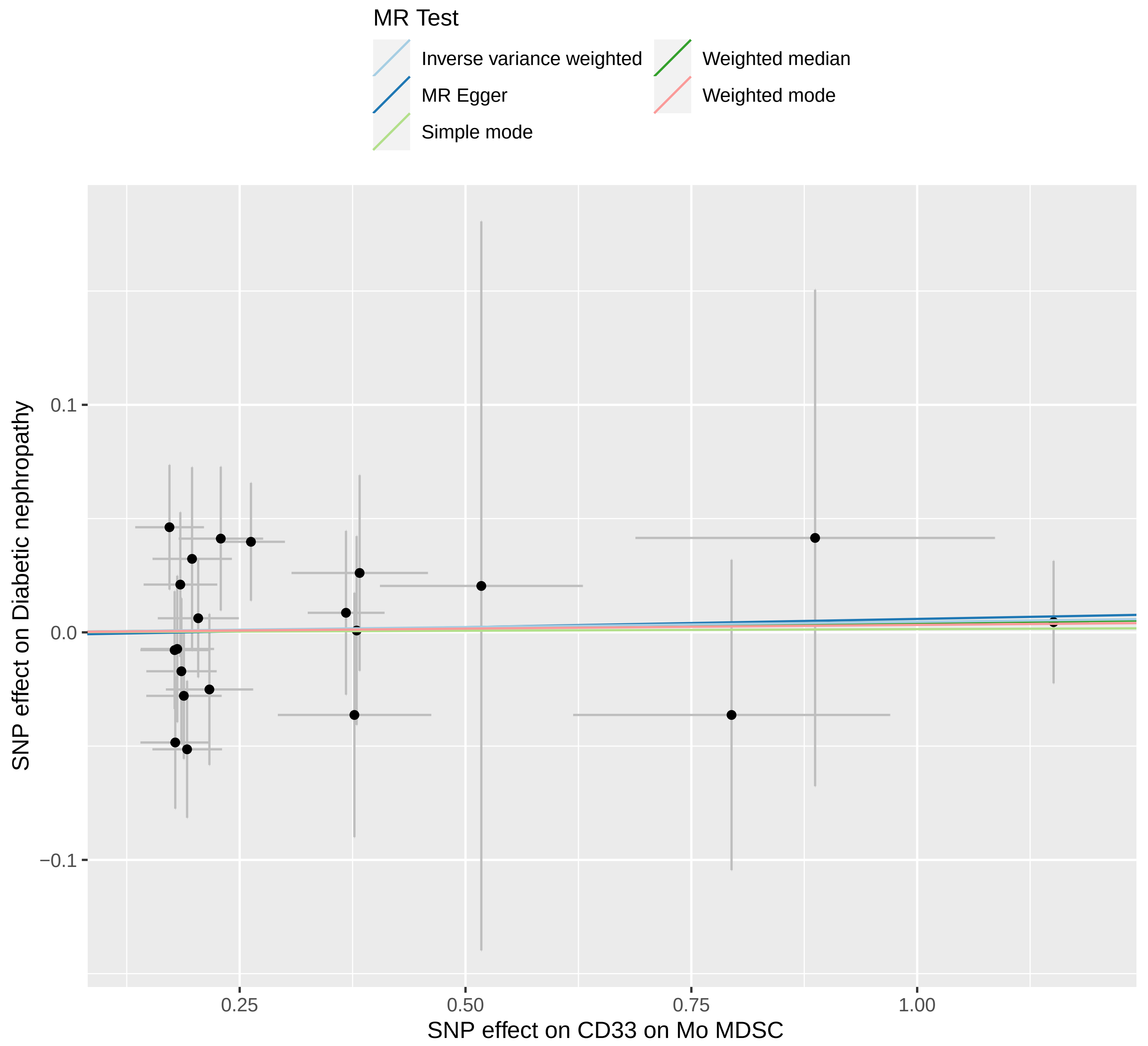
MR Test
Inverse variance weighted
MR Egger
Simple mode
Weighted median
Weighted mode
0.1
0.0
−0.1
0.25
0.50
0.75
1.00
SNP effect on Diabetic nephropathy
SNP effect on CD33 on Mo MDSC

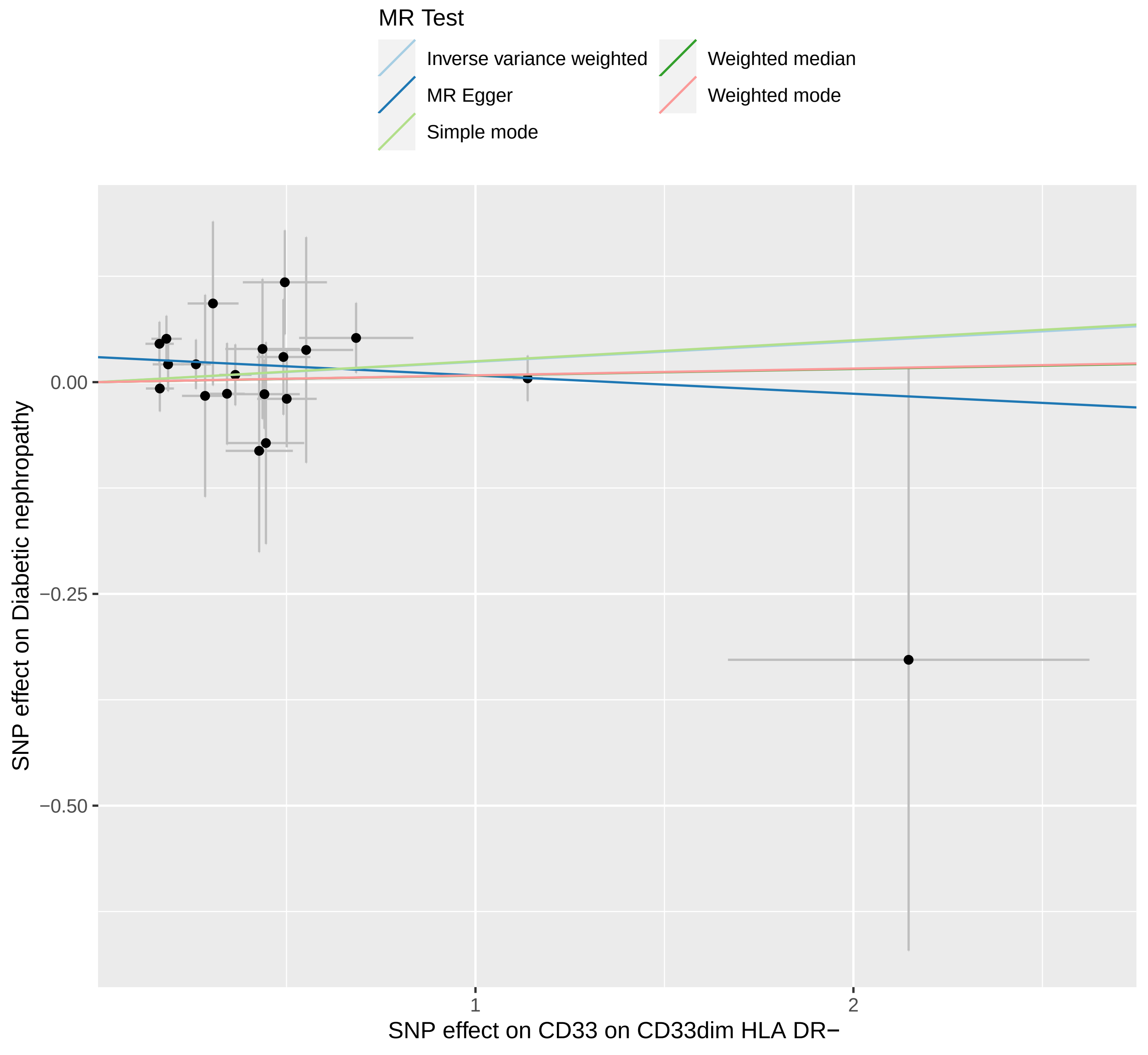
MR Test
Inverse variance weighted
MR Egger
Simple mode
Weighted median
Weighted mode
0.00
−0.25
−0.50
1
2
SNP effect on CD33 on CD33dim HLA DR−
SNP effect on Diabetic nephropathy

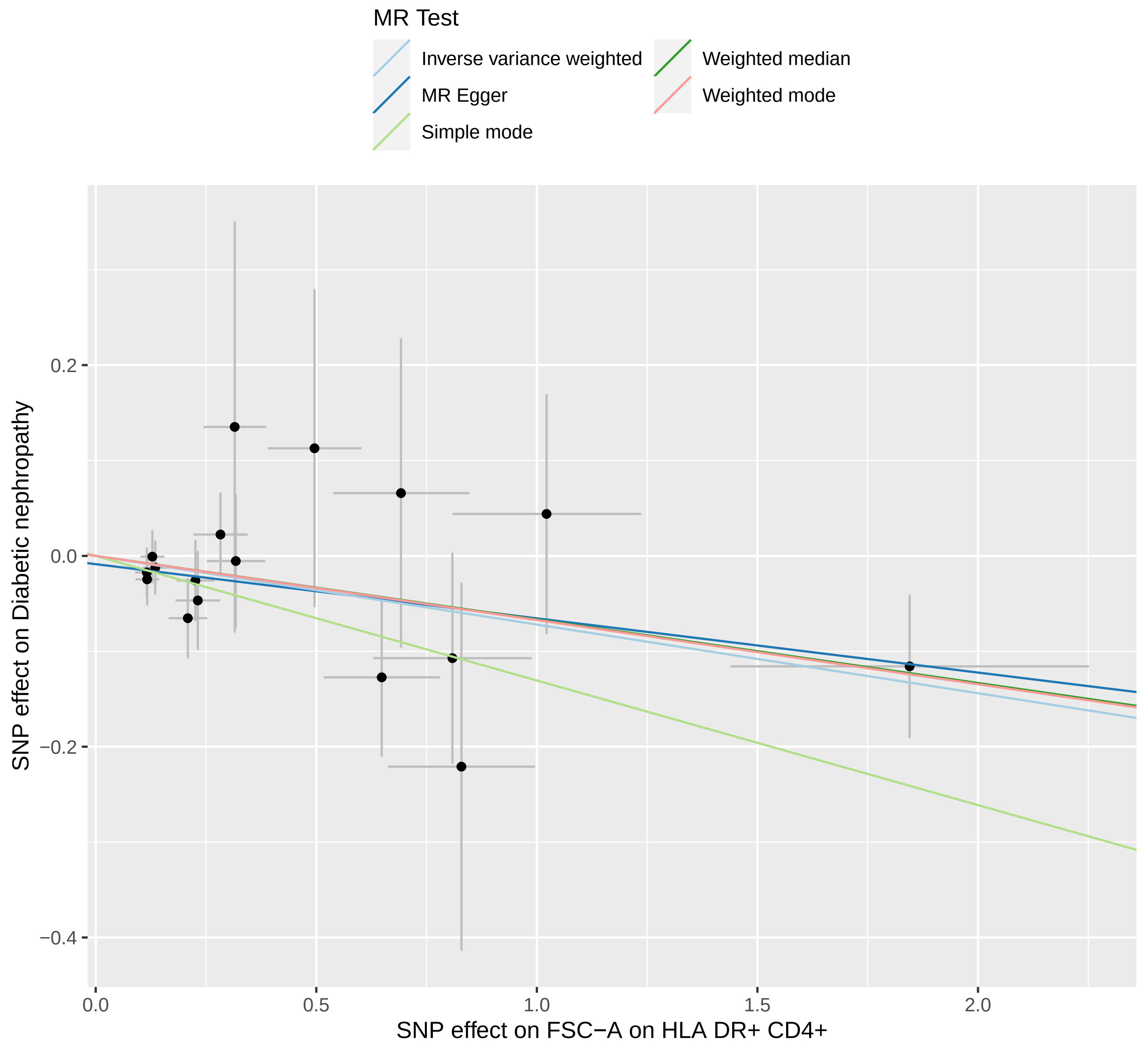
MR Test
Inverse variance weighted
MR Egger
Simple mode
Weighted median
Weighted mode
0.2
0.0
−0.2
−0.4
SNP effect on Diabetic nephropathy
0.0
0.5
1.0
1.5
2.0
SNP effect on FSC−A on HLA DR+ CD4+

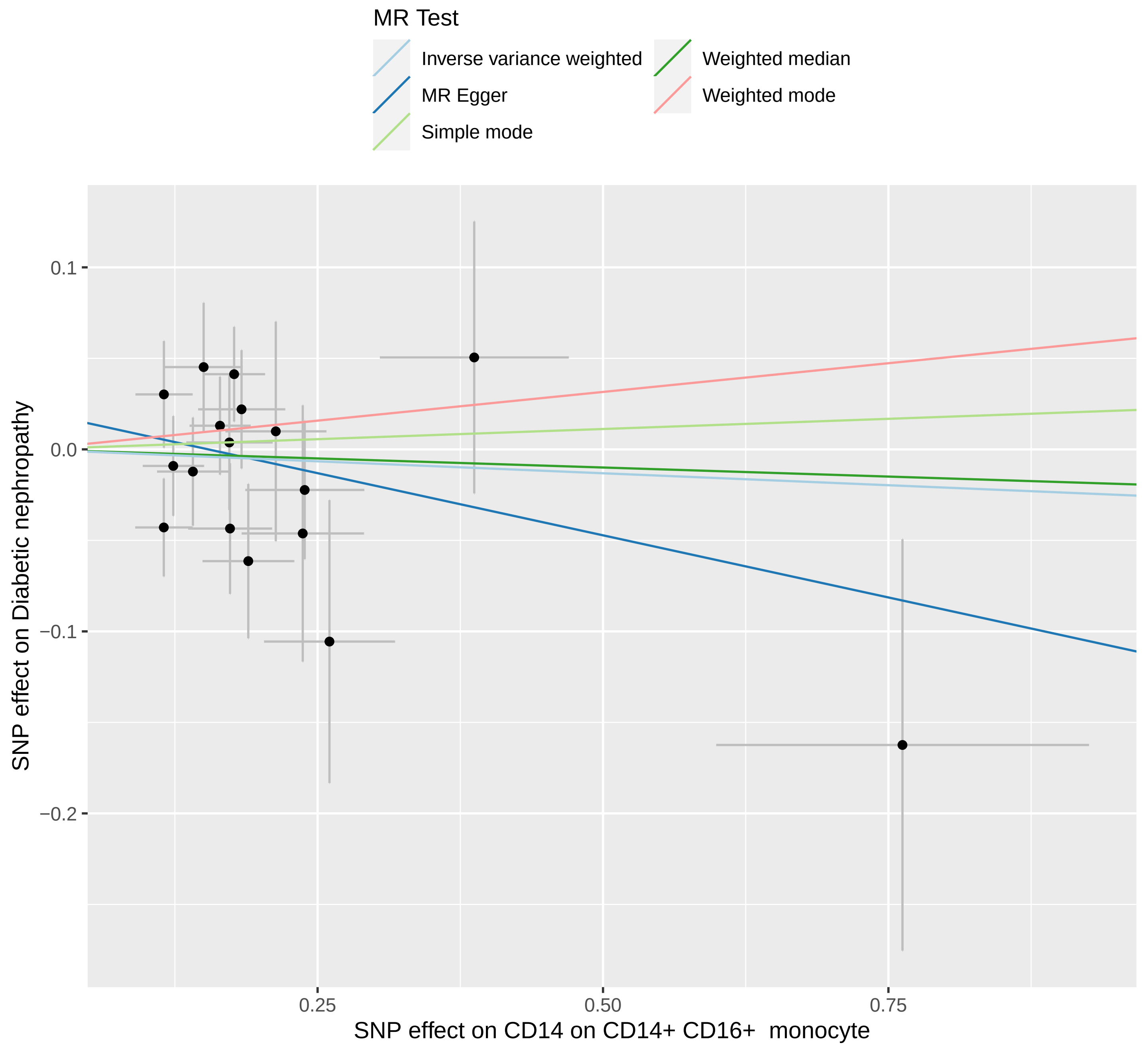
MR Test
Inverse variance weighted
MR Egger
Simple mode
Weighted median
Weighted mode
0.1
0.0
−0.1
−0.2
0.25
0.50
0.75
SNP effect on Diabetic nephropathy
SNP effect on CD14 on CD14+ CD16+ monocyte

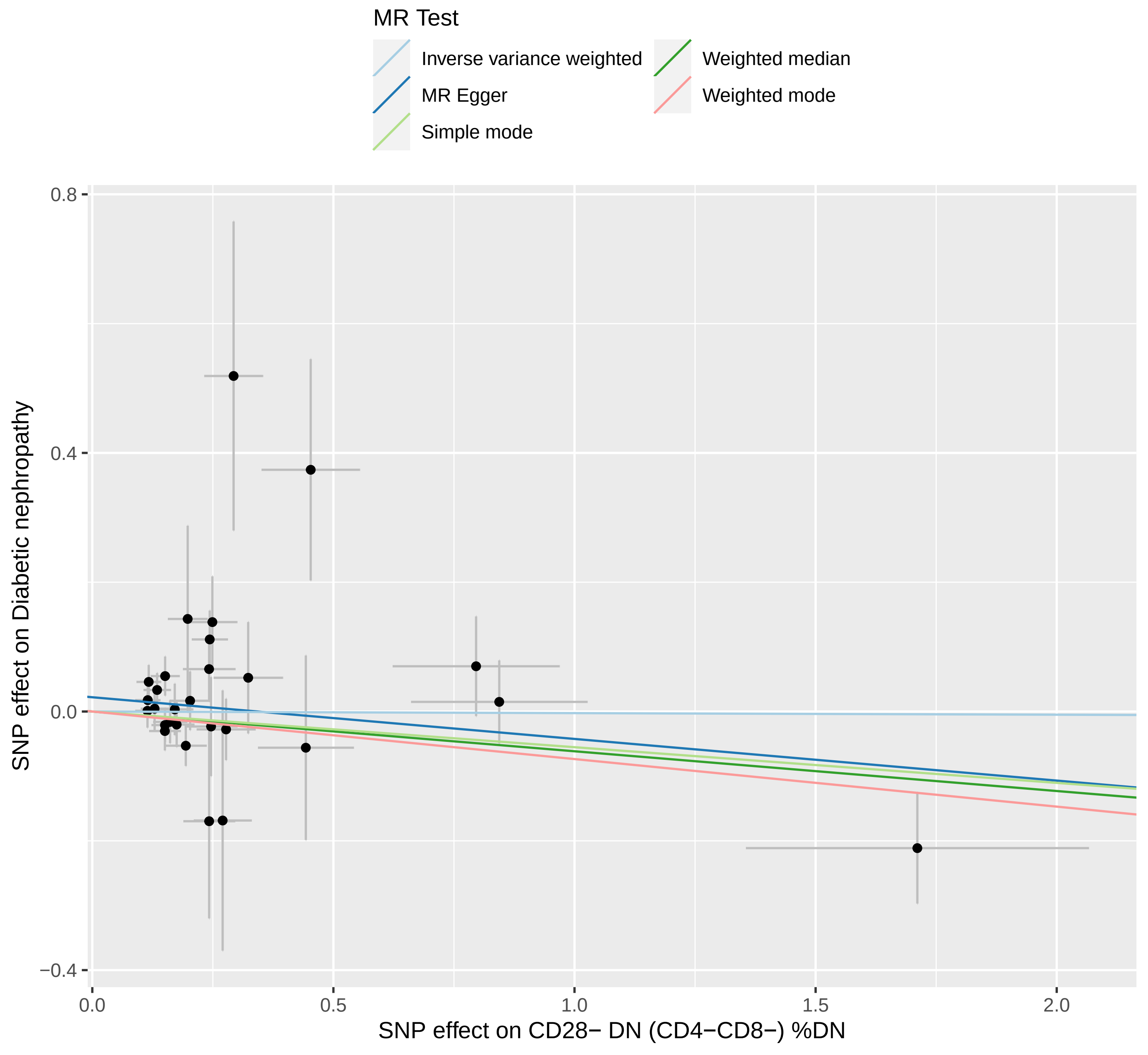

MR Test
Inverse variance weighted
Weighted median
MR Egger
Weighted mode
Simple mode
0.8
0.4
0.0
−0.4
0.0
0.5
1.0
1.5
2.0
SNP effect on Diabetic nephropathy
SNP effect on CD28− DN (CD4−CD8−) %DN

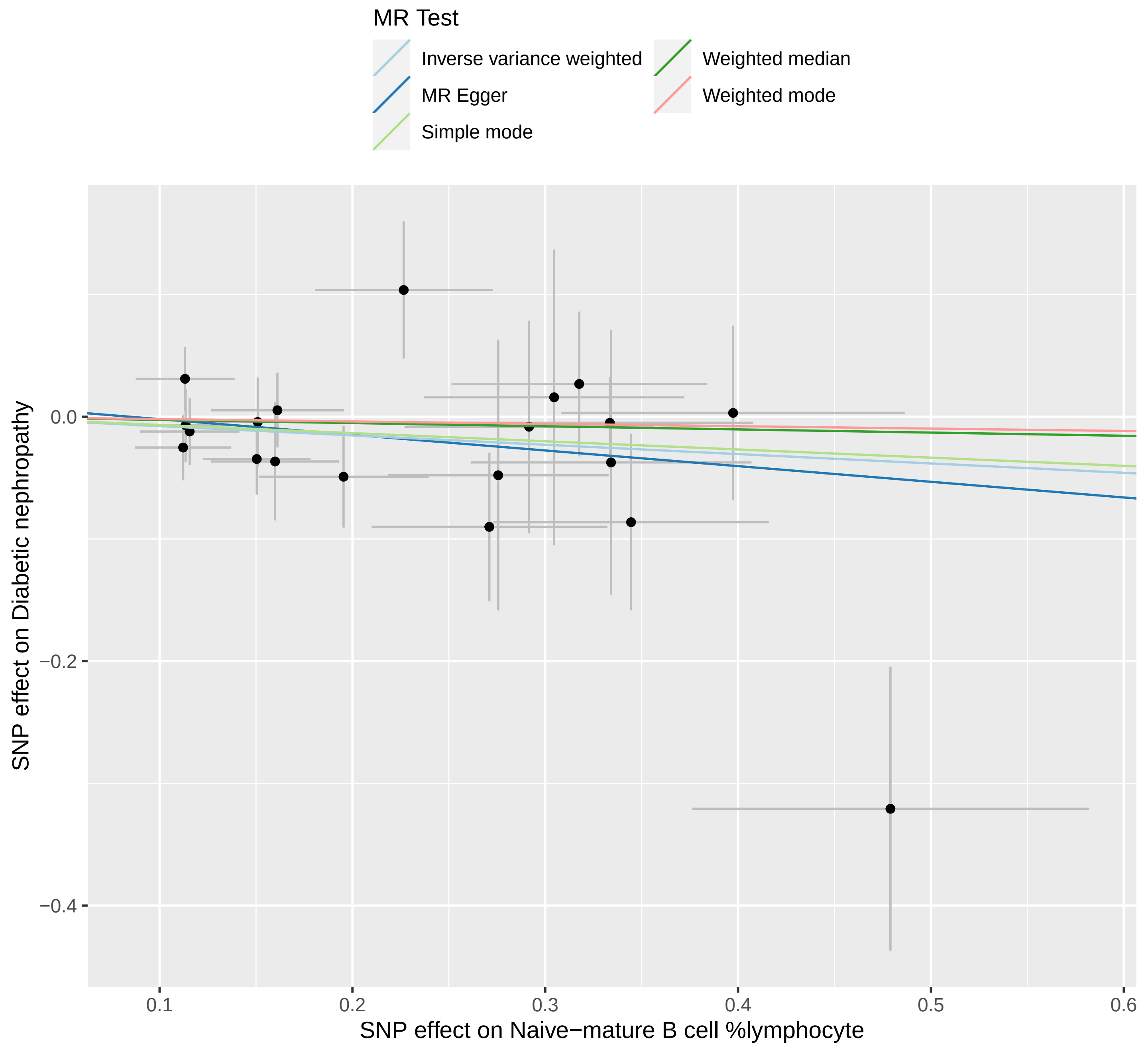

MR Test
Inverse variance weighted
MR Egger
Simple mode
Weighted median
Weighted mode
SNP effect on Diabetic nephropathy
0.0
−0.2
−0.4
0.1
0.2
0.3
0.4
0.5
0.6
SNP effect on Naive−mature B cell %lymphocyte

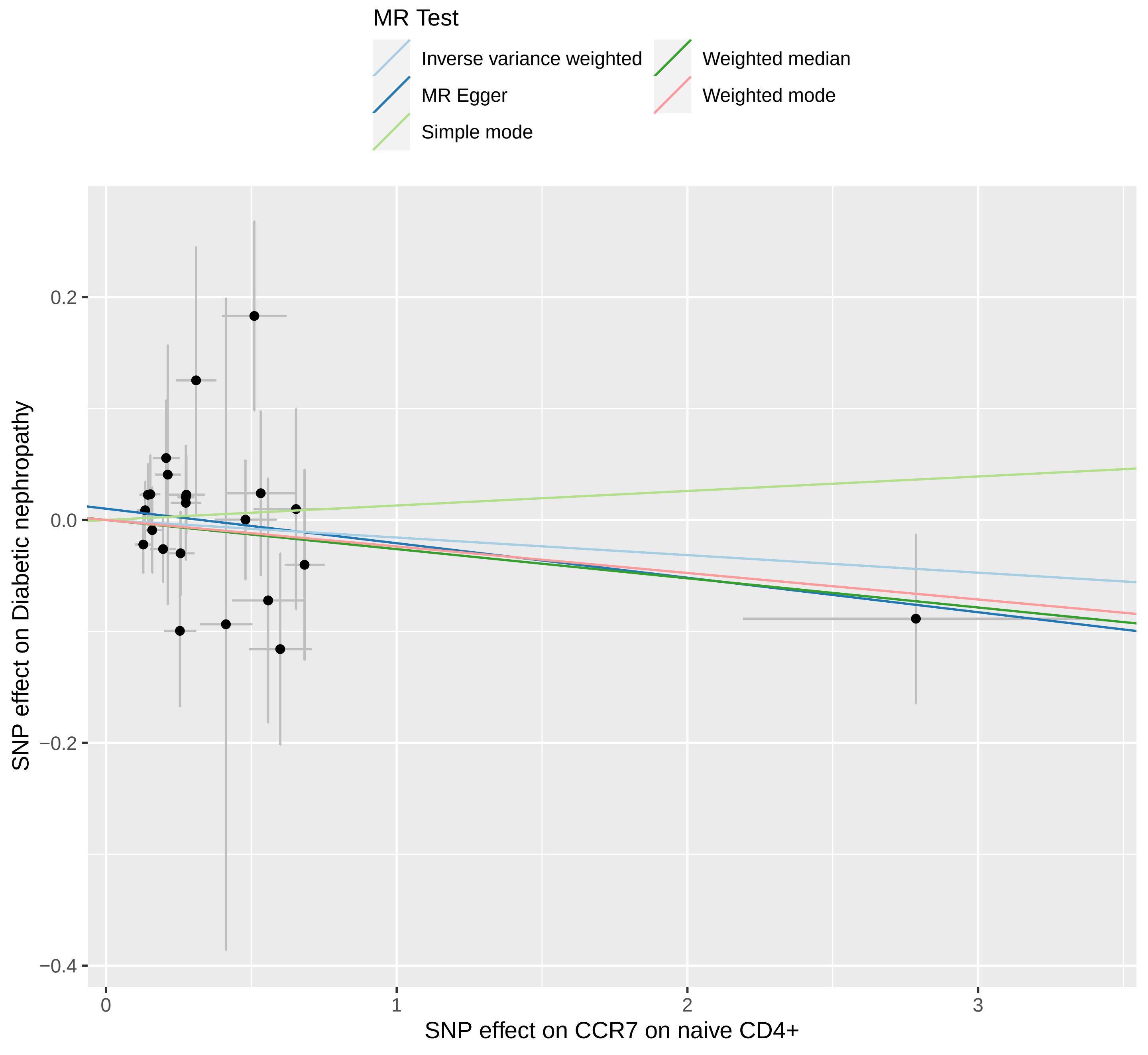

MR Test
Inverse variance weighted
MR Egger
Simple mode
Weighted median
Weighted mode
0.2
0.0
−0.2
−0.4
SNP effect on Diabetic nephropathy
0
1
2
3
SNP effect on CCR7 on naive CD4+

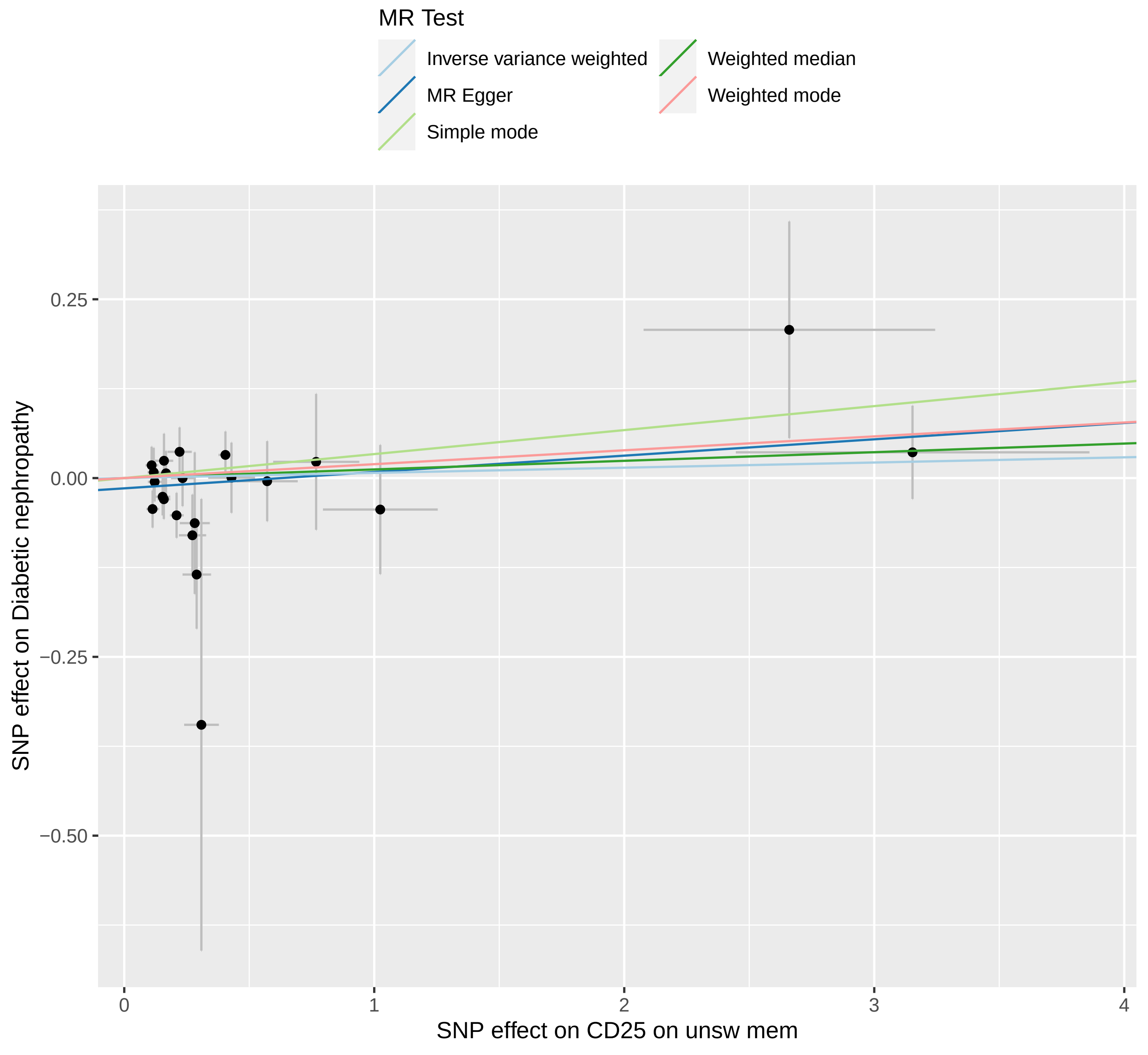
MR Test
Inverse variance weighted
MR Egger
Simple mode
Weighted median
Weighted mode
0.25
0.00
−0.25
−0.50
SNP effect on Diabetic nephropathy
0
1
2
3
4
SNP effect on CD25 on unsw mem

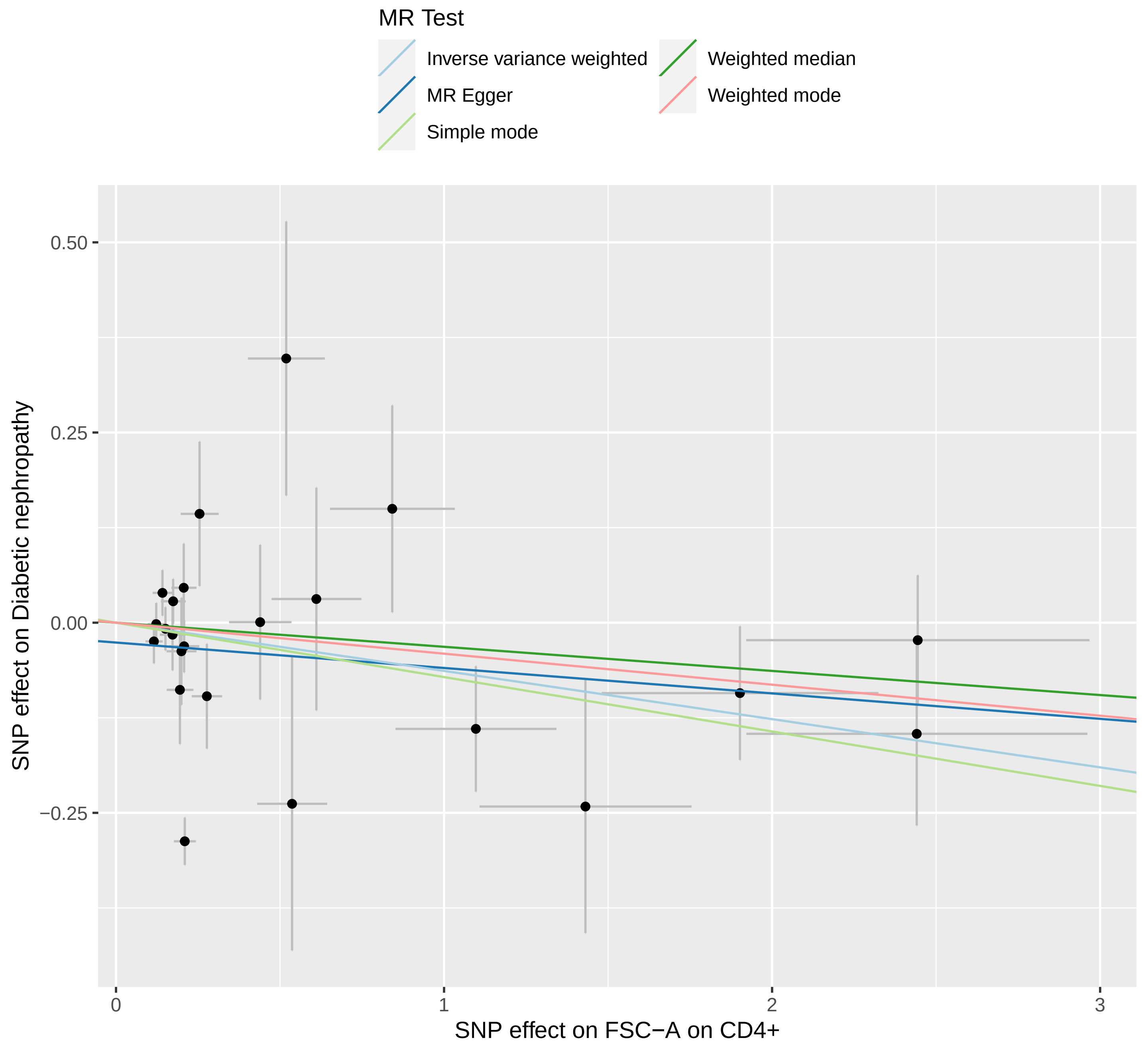

MR Test
Inverse variance weighted
MR Egger
Simple mode
Weighted median
Weighted mode
SNP effect on Diabetic nephropathy
0.50
0.25
0.00
−0.25
0
1
2
3
SNP effect on FSC−A on CD4+

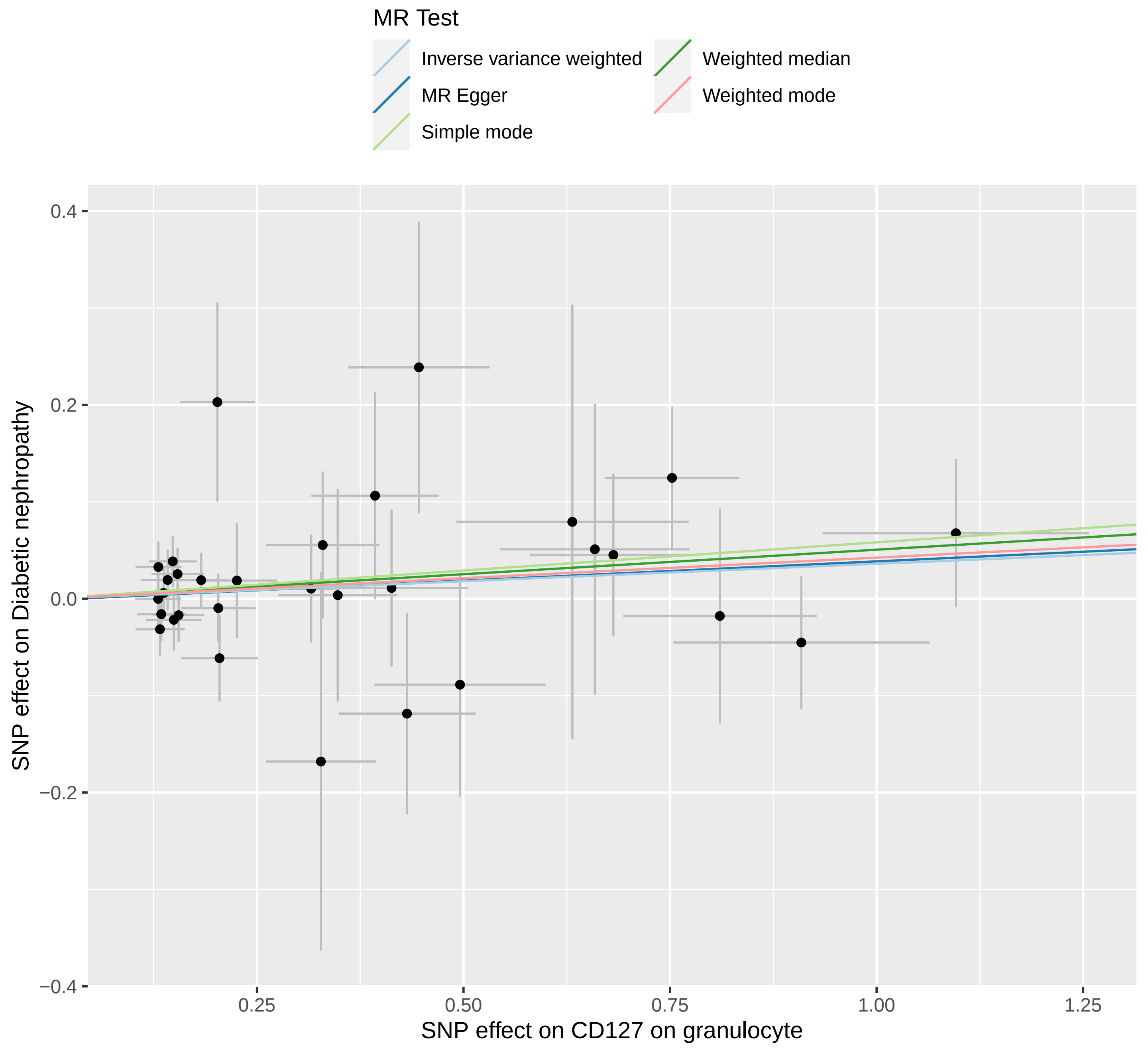
MR Test
Inverse variance weighted
MR Egger
Simple mode
Weighted median
Weighted mode
0.4
0.2
0.0
−0.2
−0.4
SNP effect on Diabetic nephropathy
0.25
0.50
0.75
1.00
1.25
SNP effect on CD127 on granulocyte

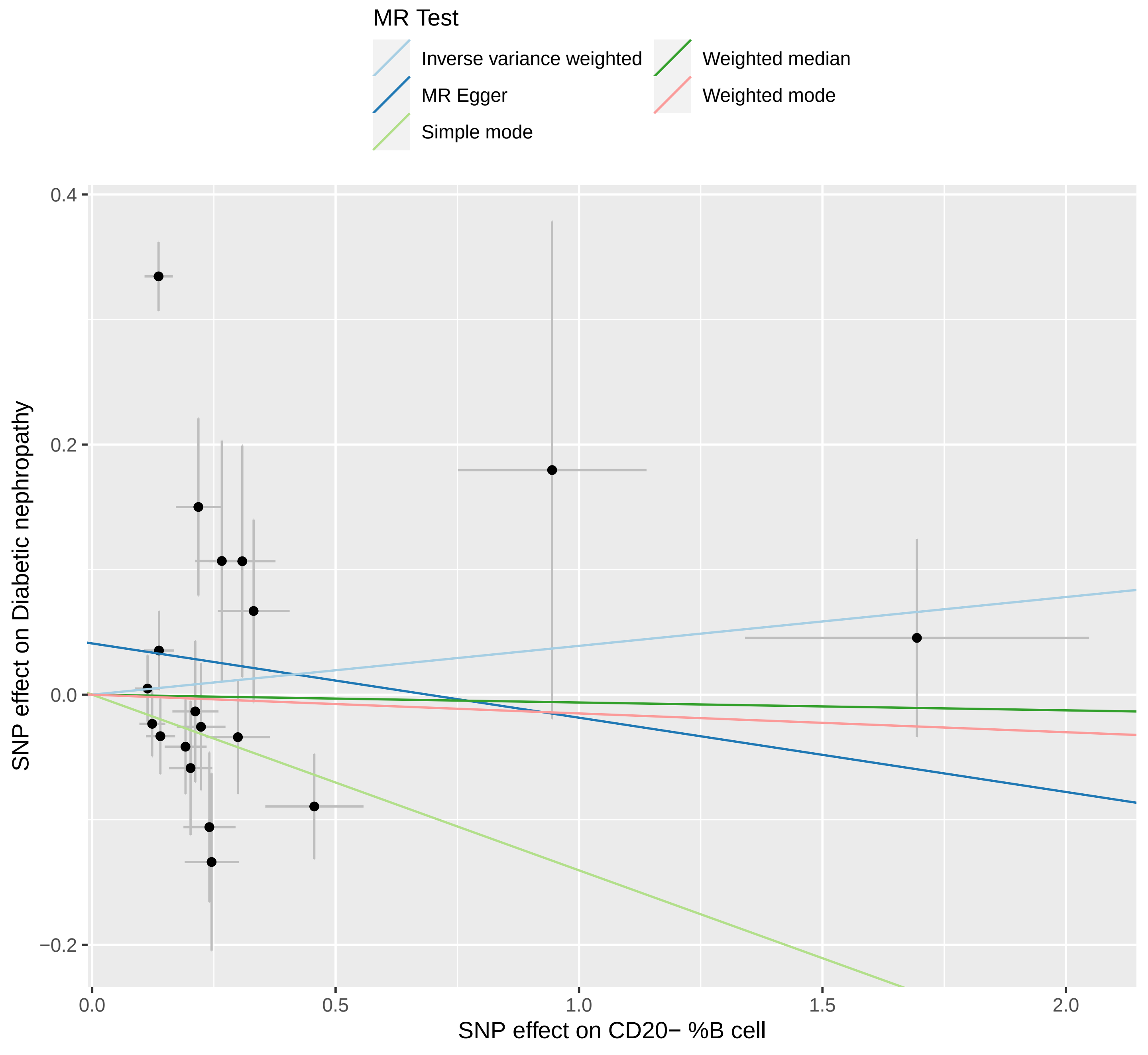

MR Test
Inverse variance weighted
MR Egger
Simple mode
Weighted median
Weighted mode
0.4
0.2
0.0
−0.2
SNP effect on Diabetic nephropathy
0.0
0.5
1.0
1.5
2.0
SNP effect on CD20− %B cell

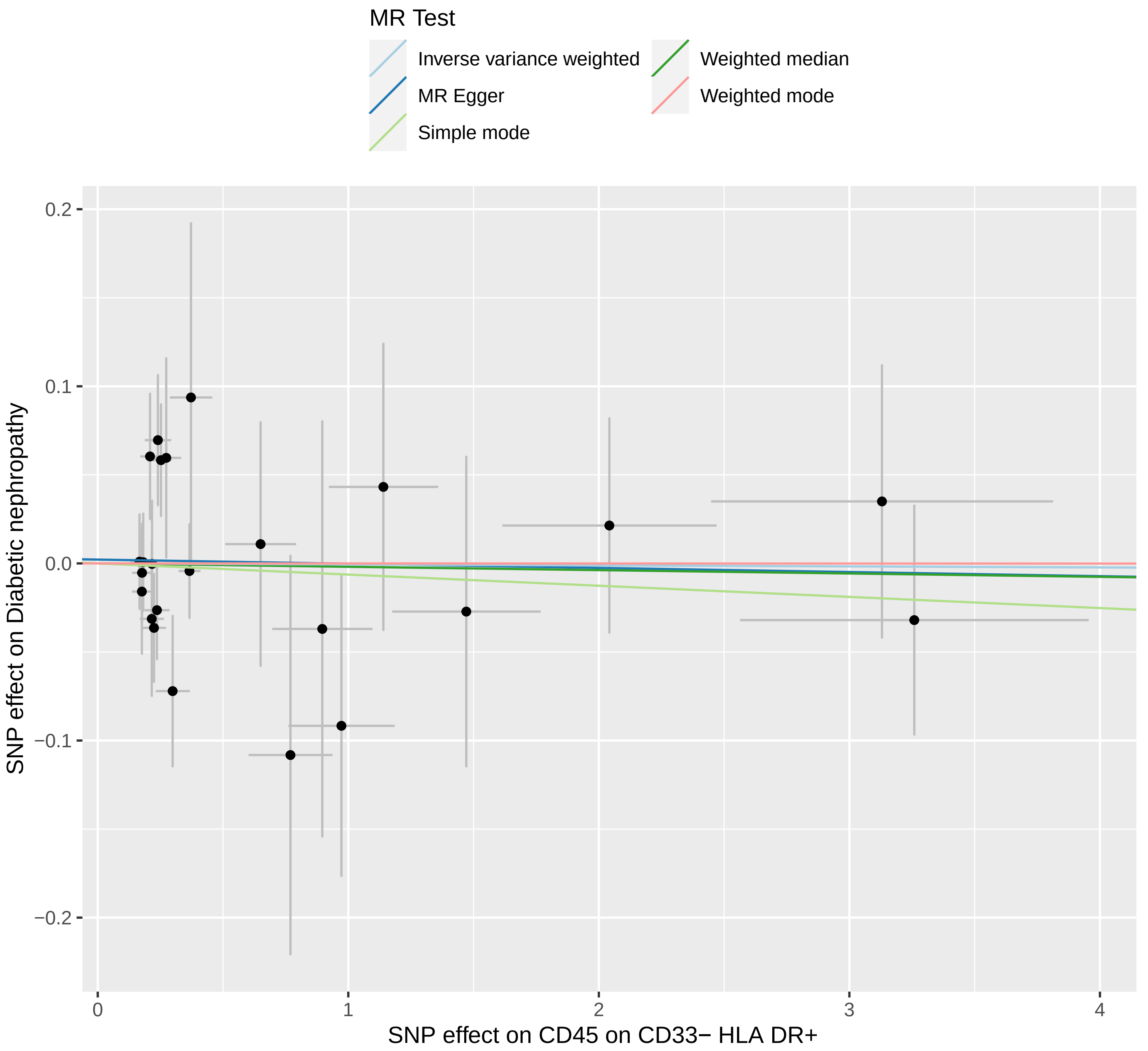

MR Test
Inverse variance weighted
MR Egger
Simple mode
Weighted median
Weighted mode
0.2
0.1
0.0
−0.1
−0.2
0
1
2
3
4
SNP effect on Diabetic nephropathy
SNP effect on CD45 on CD33− HLA DR+

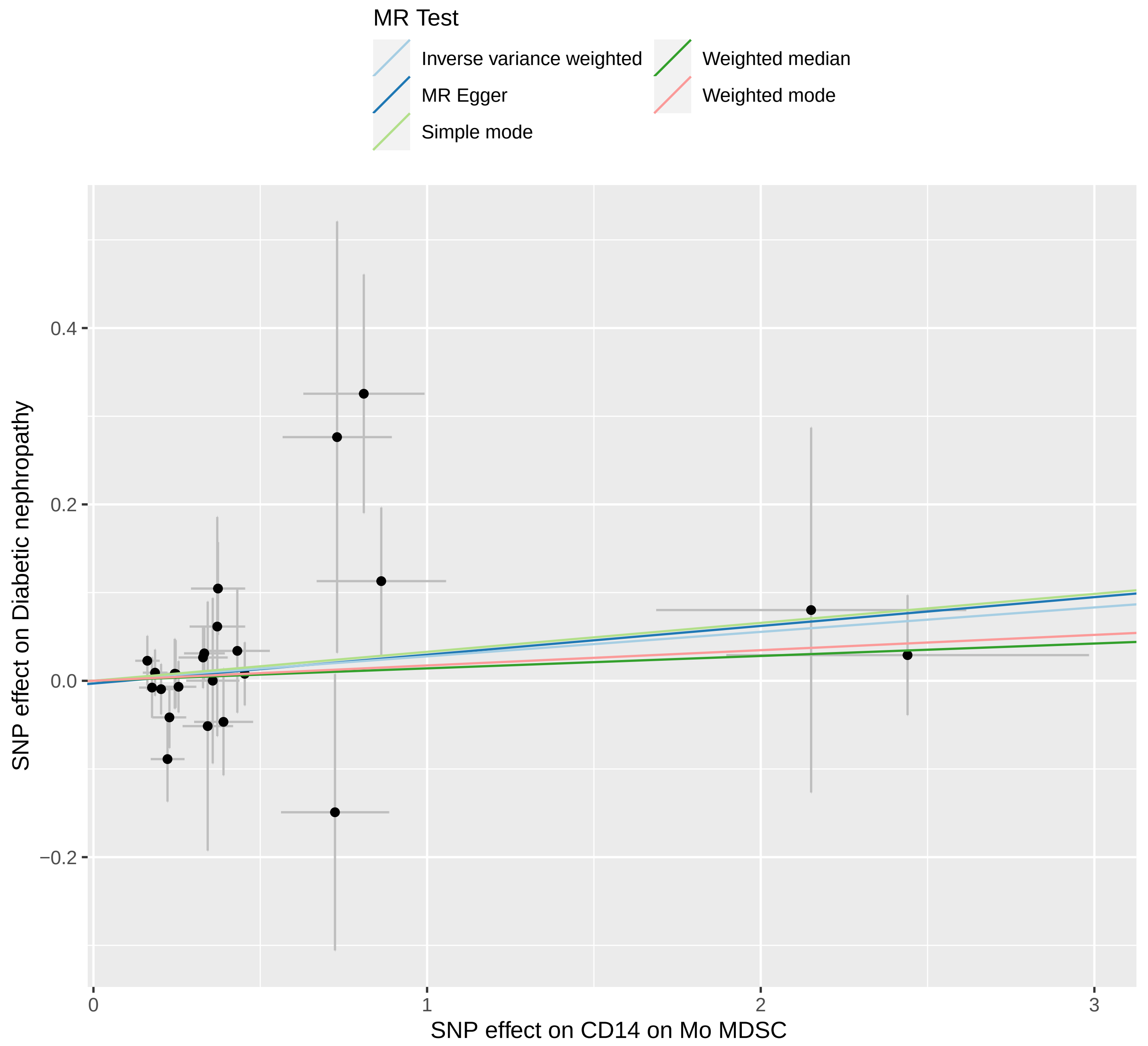
MR Test
Inverse variance weighted
MR Egger
Simple mode
Weighted median
Weighted mode
SNP effect on Diabetic nephropathy
0.4
0.2
0.0
−0.2
0
1
2
3
SNP effect on CD14 on Mo MDSC

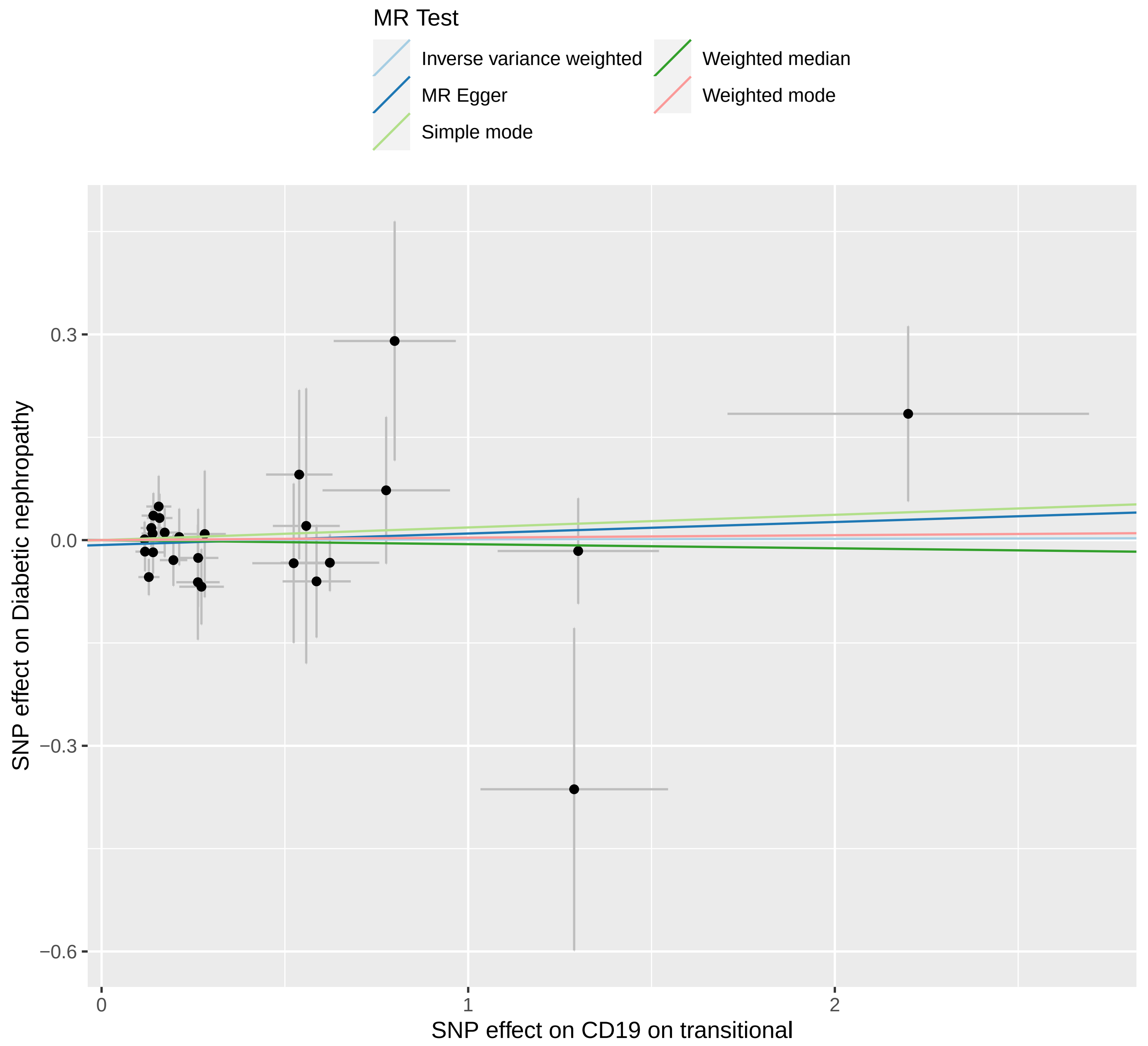
MR Test
Inverse variance weighted
MR Egger
Simple mode
Weighted median
Weighted mode
0.3
0.0
−0.3
−0.6
0
1
2
SNP effect on Diabetic nephropathy
SNP effect on CD19 on transitional

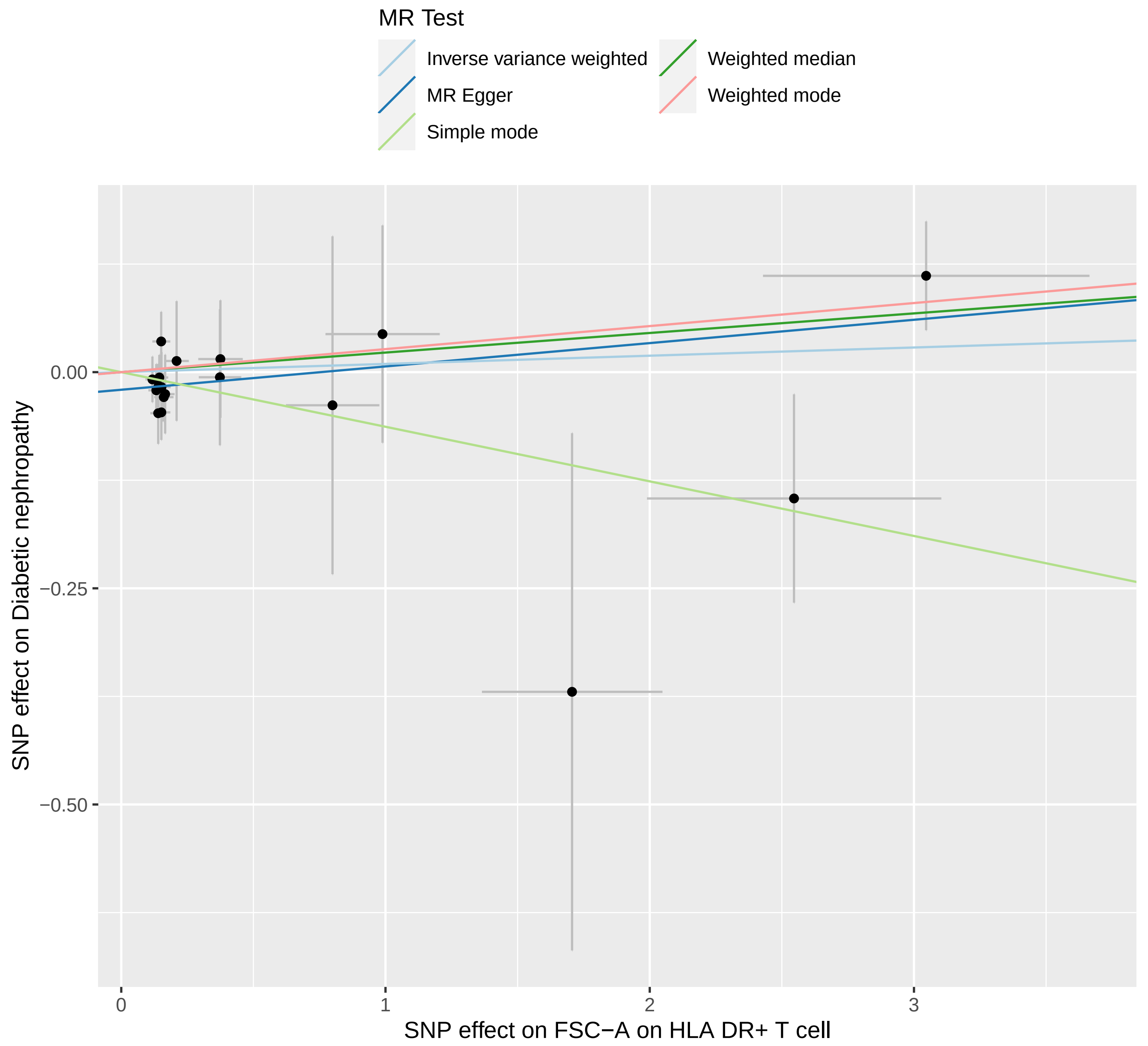
MR Test
Inverse variance weighted
MR Egger
Simple mode
Weighted median
Weighted mode
0.00
−0.25
−0.50
0
1
2
3
SNP effect on Diabetic nephropathy
SNP effect on FSC−A on HLA DR+ T cell

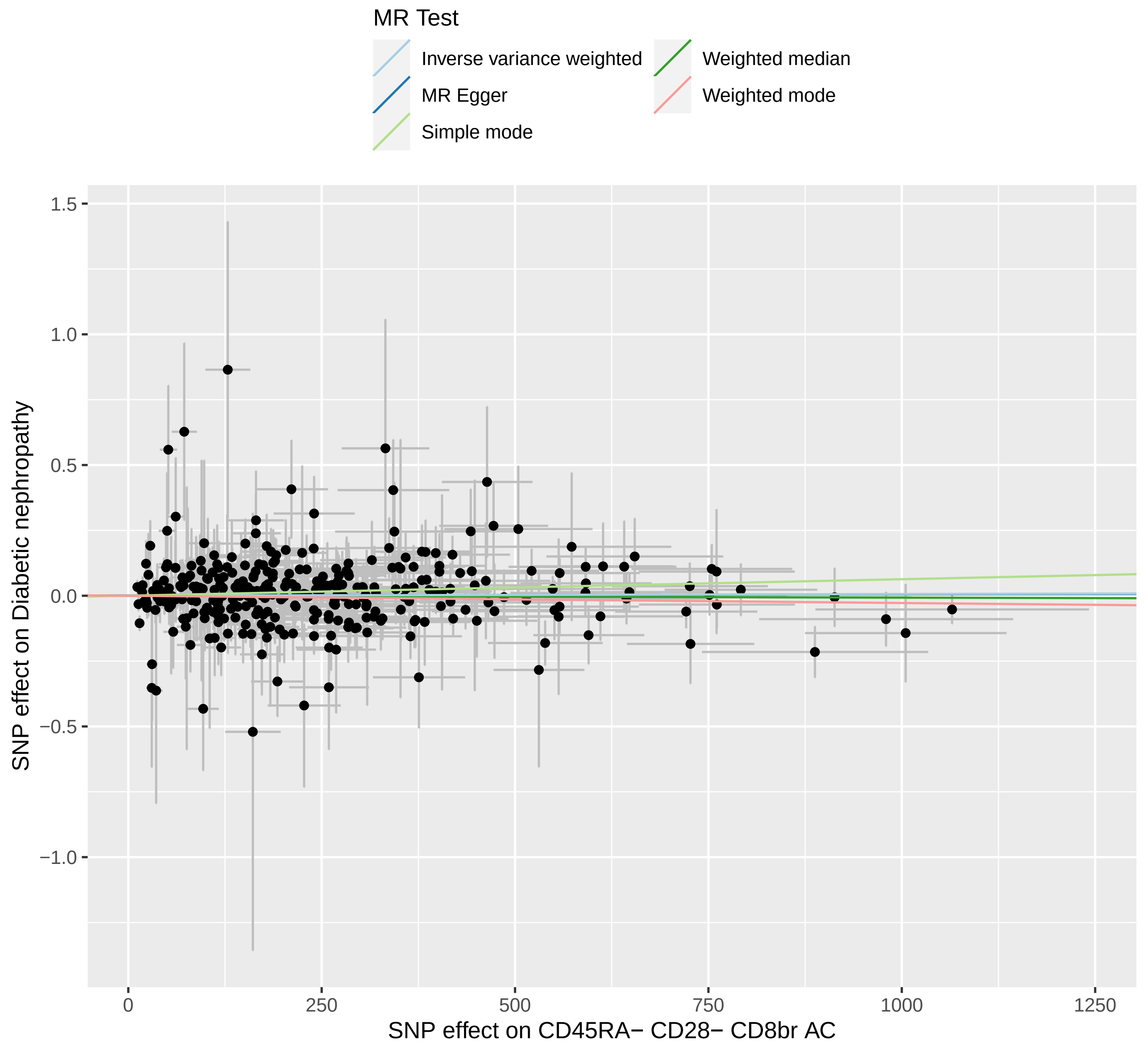
MR Test
Inverse variance weighted
MR Egger
Simple mode
Weighted median
Weighted mode
1.5
1.0
0.5
0.0
−0.5
−1.0
0
250
500
750
1000
1250
SNP effect on Diabetic nephropathy
SNP effect on CD45RA− CD28− CD8br AC

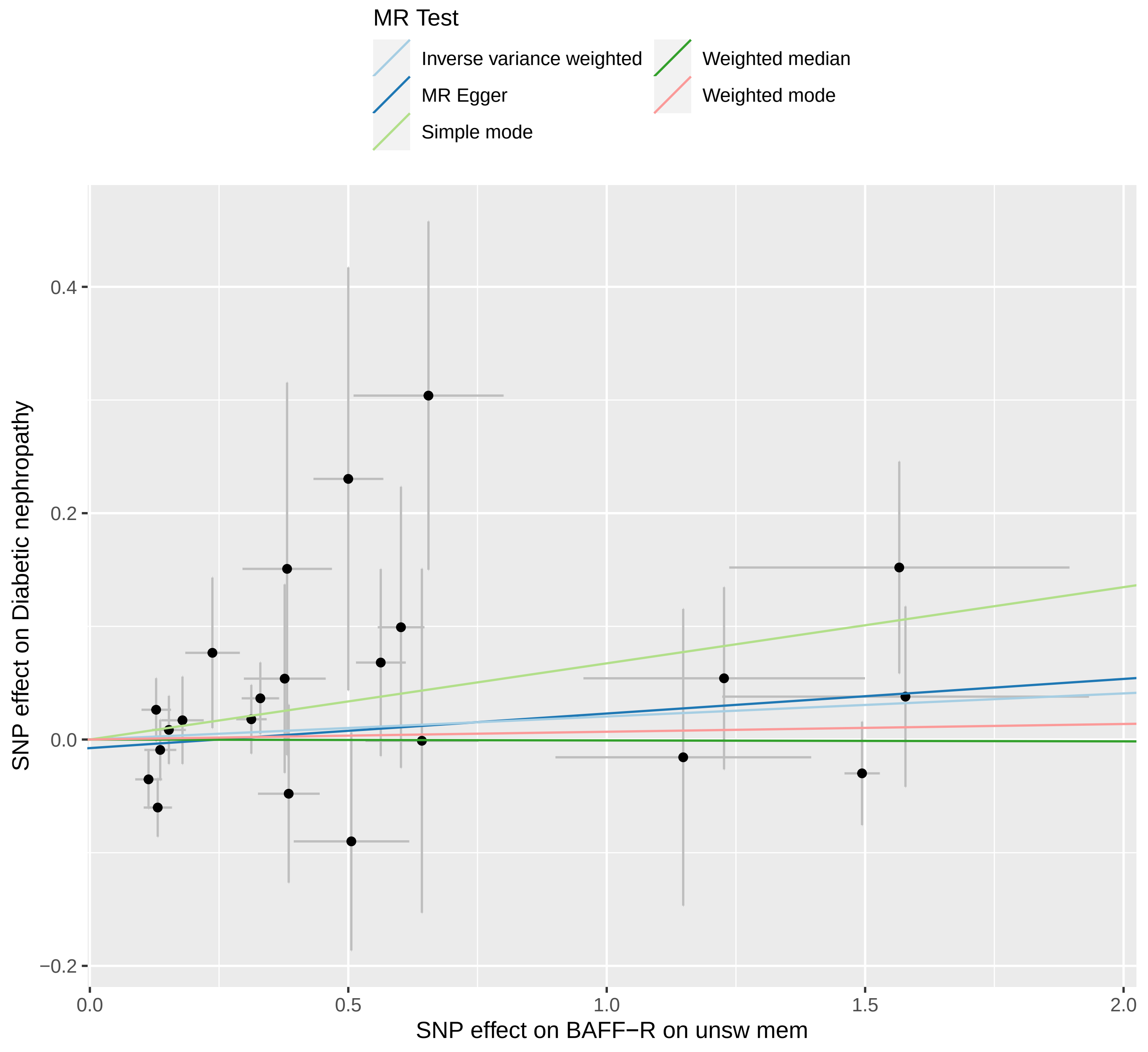

MR Test
Inverse variance weighted
MR Egger
Simple mode
Weighted median
Weighted mode
0.4
0.2
0.0
−0.2
0.0
0.5
1.0
1.5
2.0
SNP effect on Diabetic nephropathy
SNP effect on BAFF−R on unsw mem

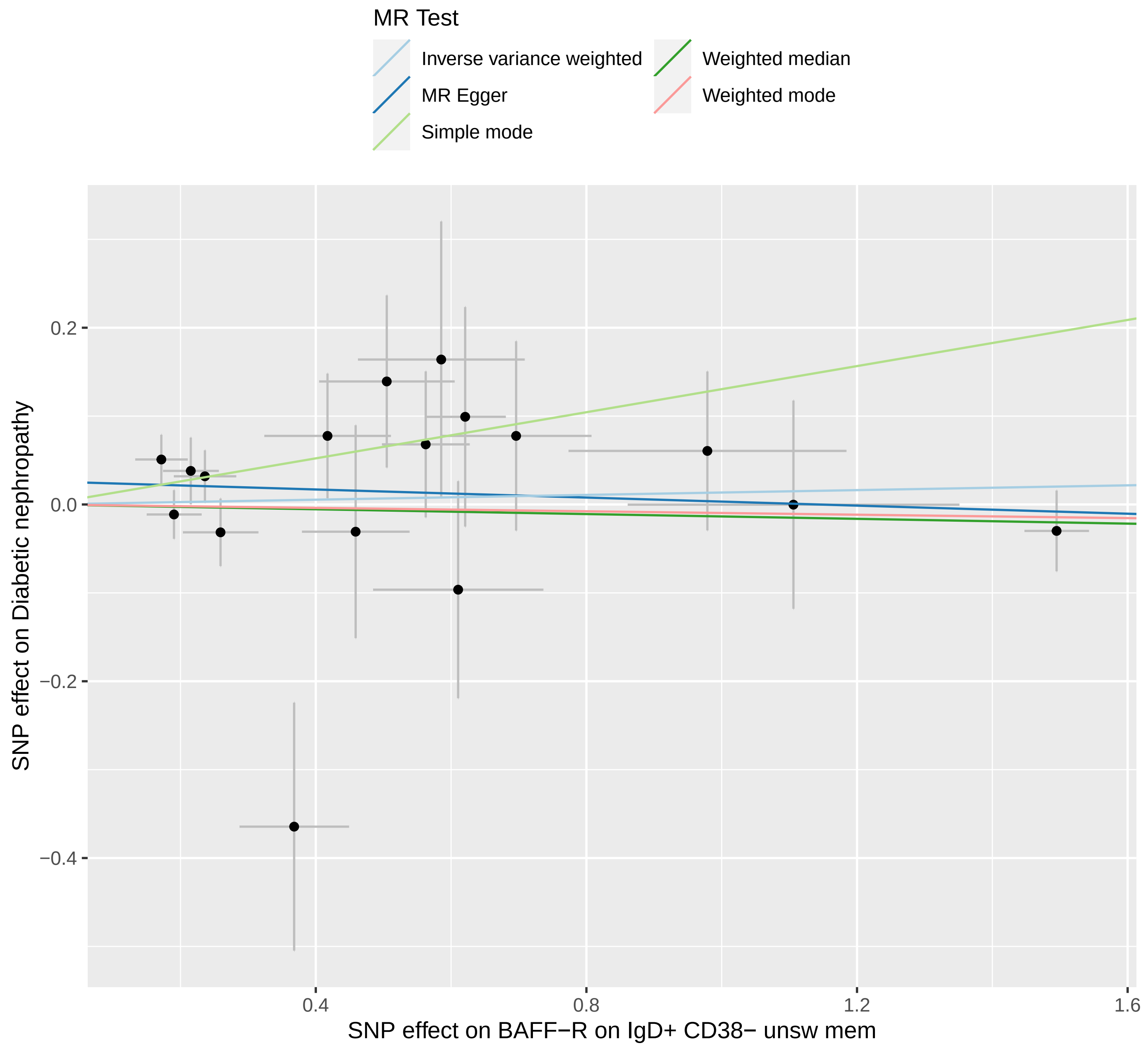
MR Test
Inverse variance weighted
MR Egger
Simple mode
Weighted median
Weighted mode
0.2
0.0
−0.2
−0.4
0.4
0.8
1.2
1.6
SNP effect on Diabetic nephropathy
SNP effect on BAFF−R on IgD+ CD38− unsw mem

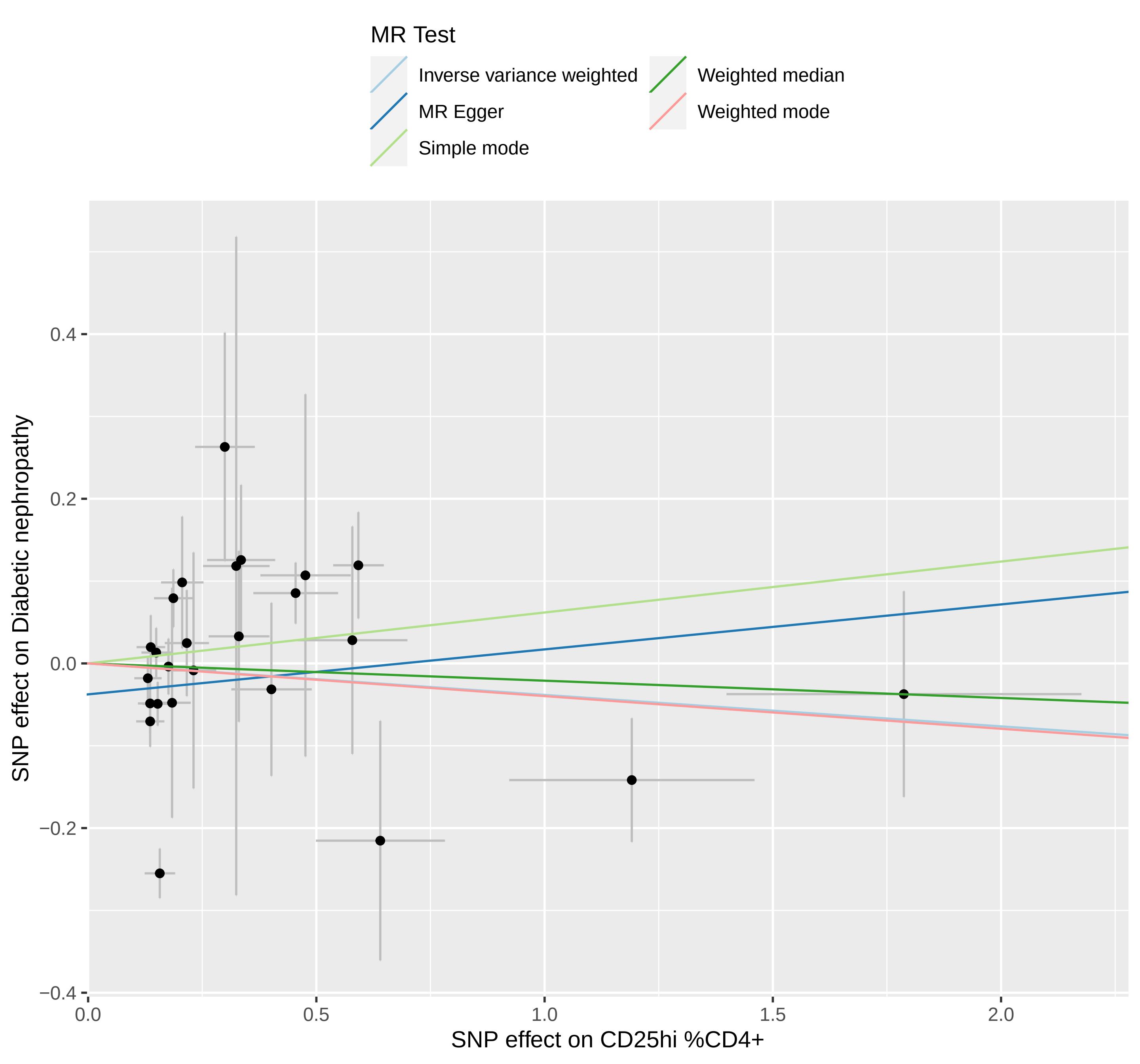

MR Test
Inverse variance weighted
MR Egger
Simple mode
Weighted median
Weighted mode
SNP effect on Diabetic nephropathy
0.4
0.2
0.0
−0.2
−0.4
0.0
0.5
1.0
1.5
2.0
SNP effect on CD25hi %CD4+

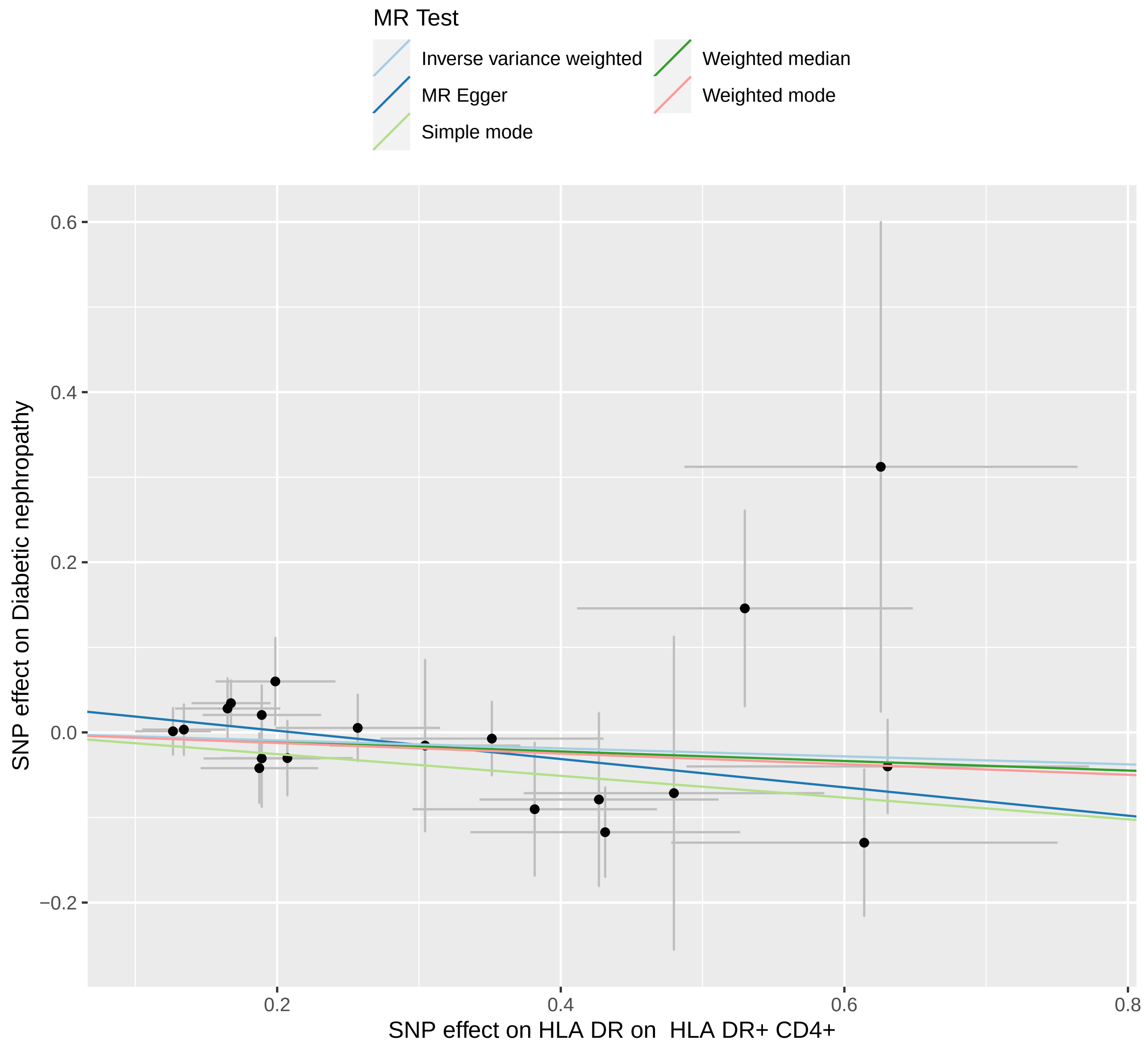
MR Test
Inverse variance weighted
MR Egger
Simple mode
Weighted median
Weighted mode
0.6
0.4
0.2
0.0
−0.2
0.2
0.4
0.6
0.8
SNP effect on Diabetic nephropathy
SNP effect on HLA DR on HLA DR+ CD4+

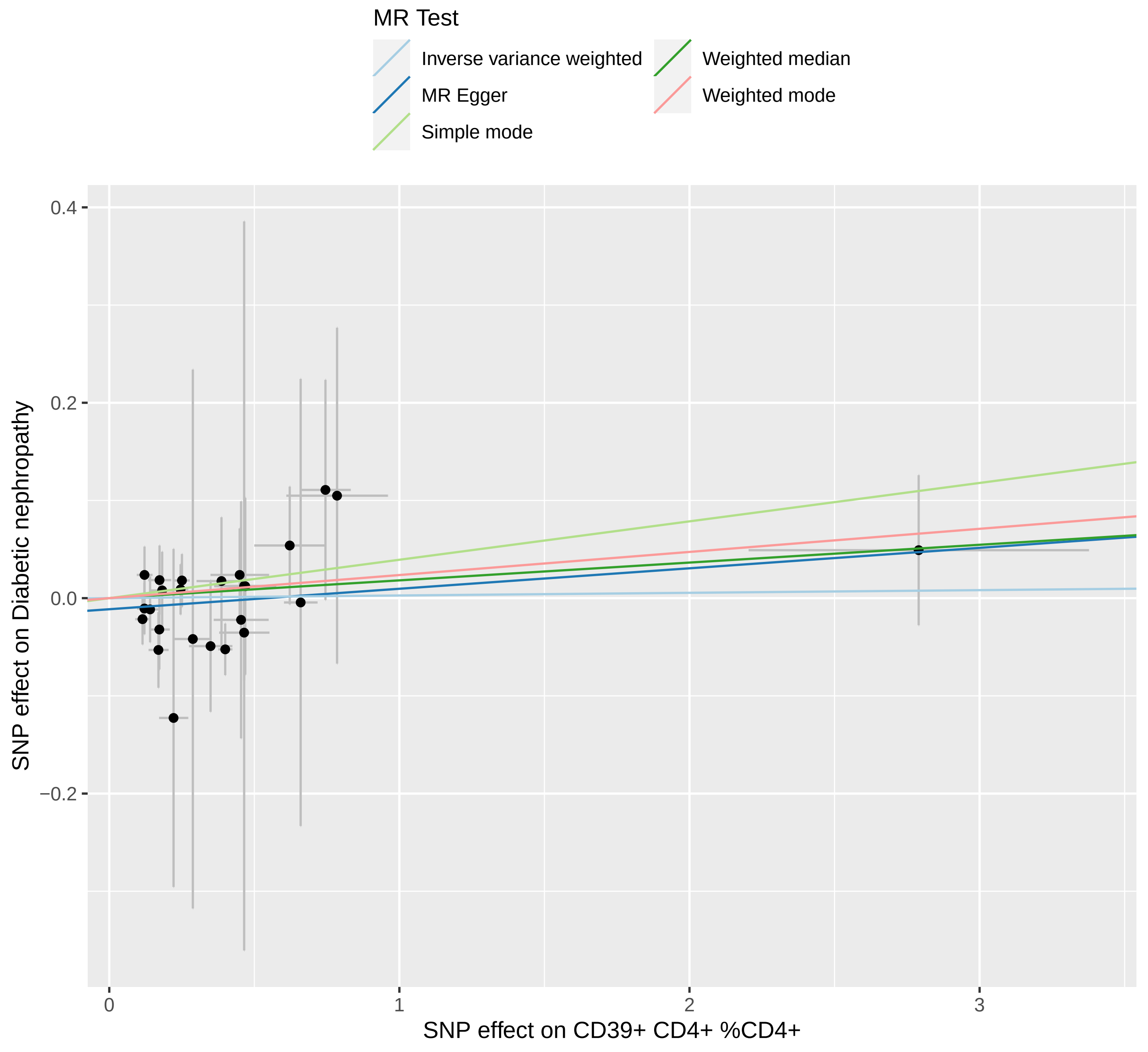

MR Test
Inverse variance weighted
MR Egger
Simple mode
Weighted median
Weighted mode
SNP effect on Diabetic nephropathy
0.4
0.2
0.0
−0.2
0
1
2
3
SNP effect on CD39+ CD4+ %CD4+

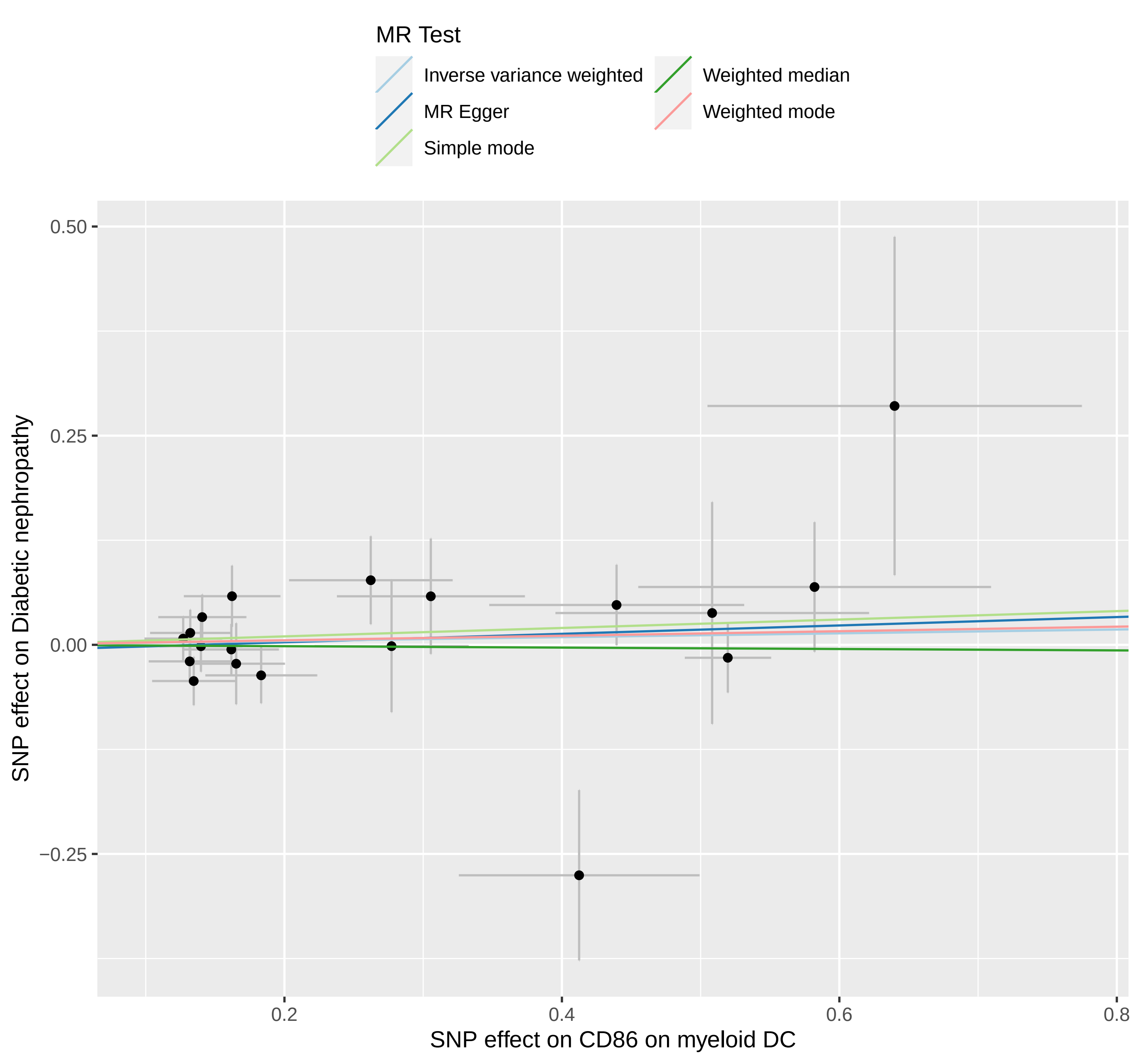

MR Test
Inverse variance weighted
MR Egger
Simple mode
Weighted median
Weighted mode
0.50
0.25
0.00
−0.25
0.2
0.4
0.6
0.8
SNP effect on Diabetic nephropathy
SNP effect on CD86 on myeloid DC

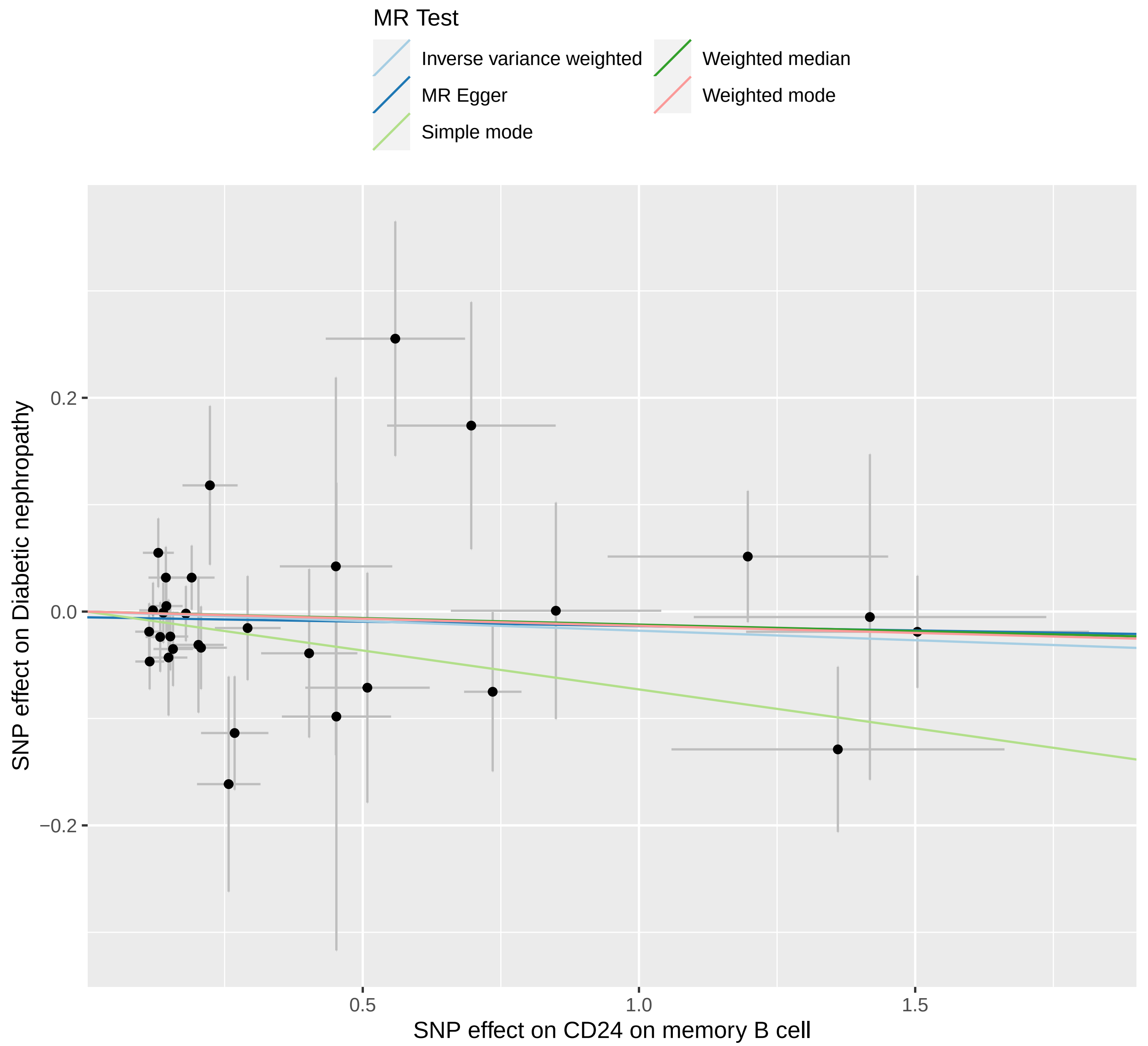
MR Test
Inverse variance weighted
MR Egger
Simple mode
Weighted median
Weighted mode
0.2
0.0
−0.2
0.5
1.0
1.5
SNP effect on Diabetic nephropathy
SNP effect on CD24 on memory B cell

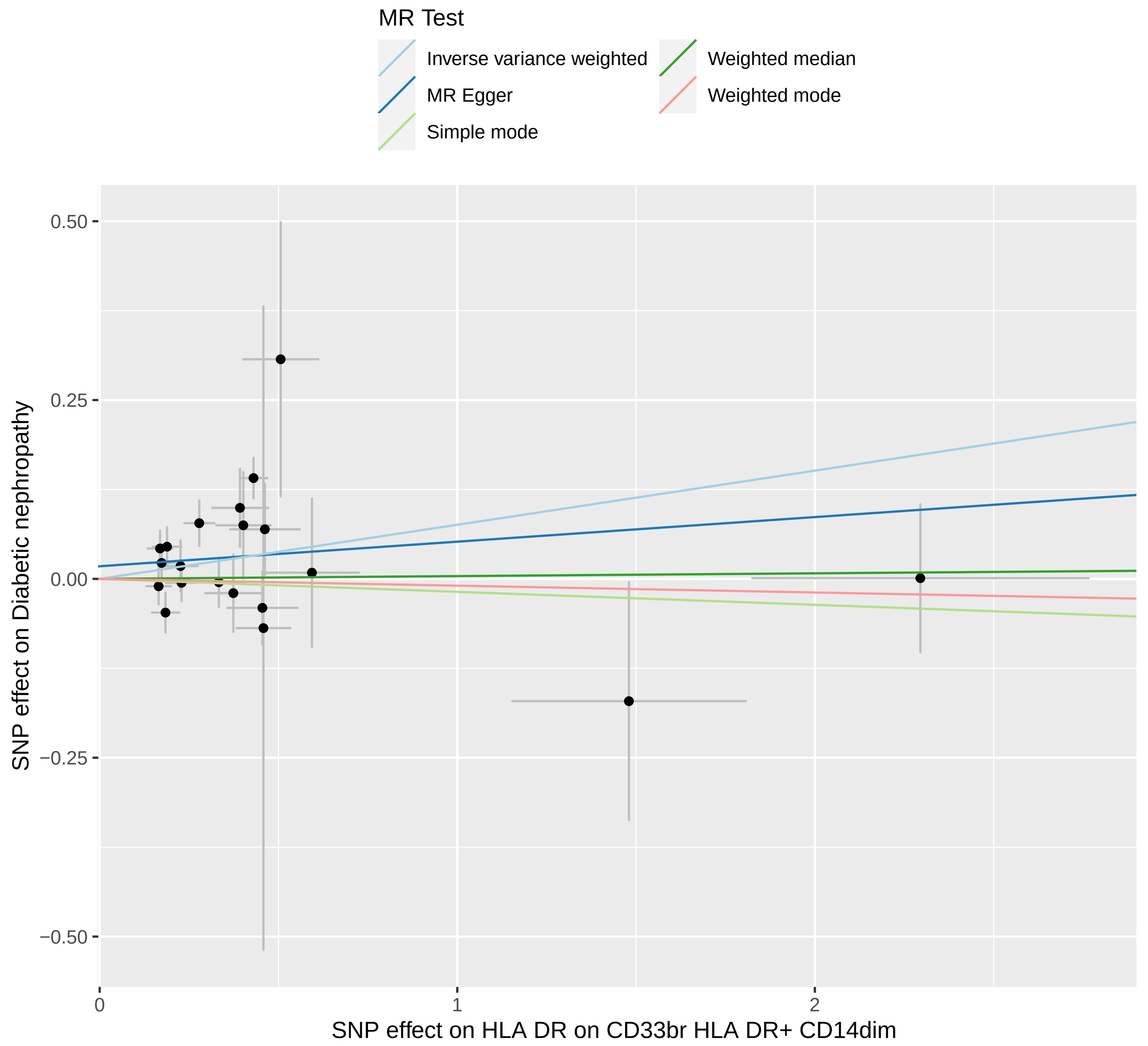
MR Test
Inverse variance weighted
MR Egger
Simple mode
Weighted median
Weighted mode
0.50
0.25
0.00
−0.25
−0.50
0
1
2
SNP effect on Diabetic nephropathy
SNP effect on HLA DR on CD33br HLA DR+ CD14dim

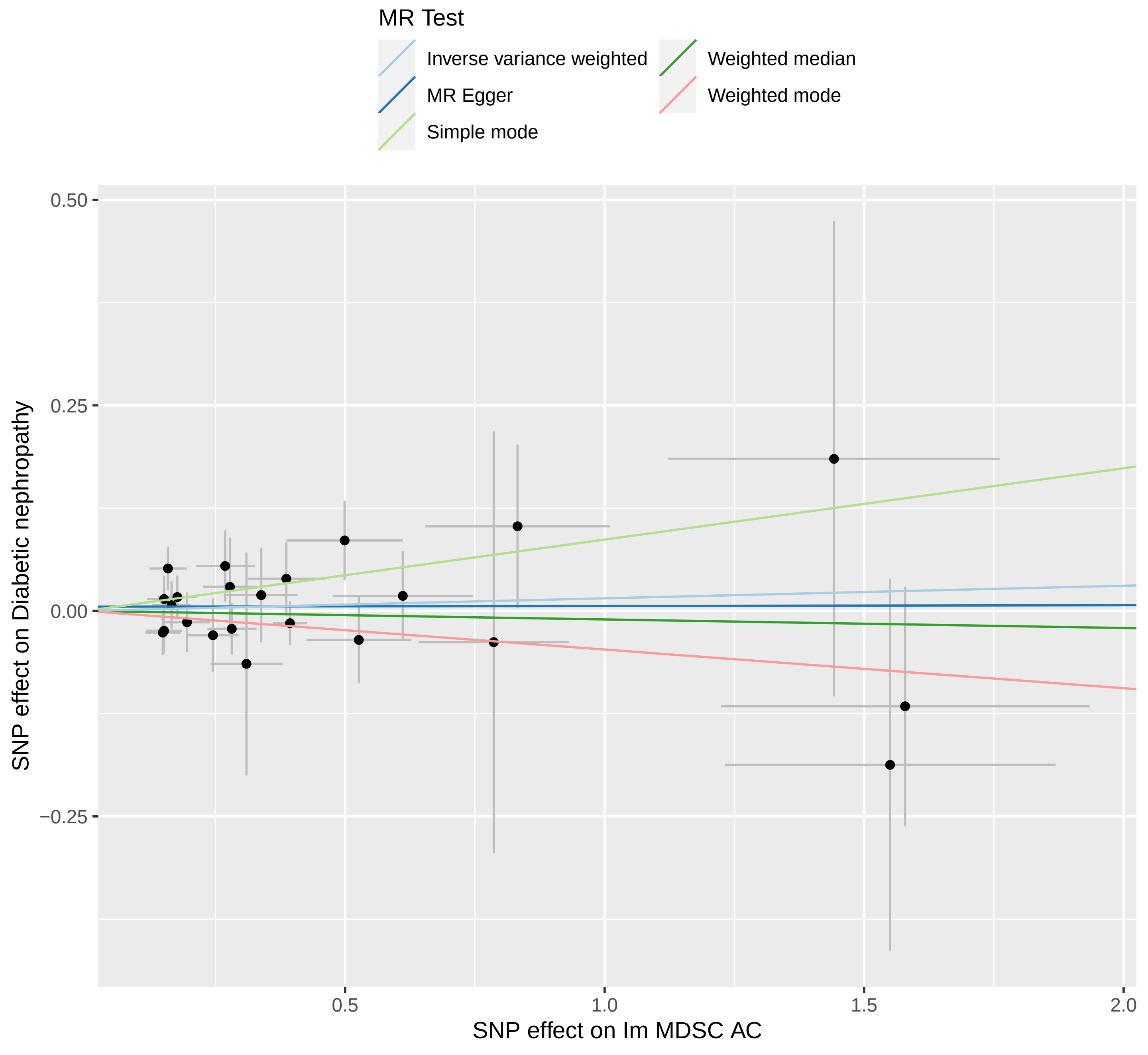

MR Test
Inverse variance weighted
MR Egger
Simple mode
Weighted median
Weighted mode
0.50
0.25
0.00
−0.25
0.5
1.0
1.5
2.0
SNP effect on Diabetic nephropathy
SNP effect on Im MDSC AC

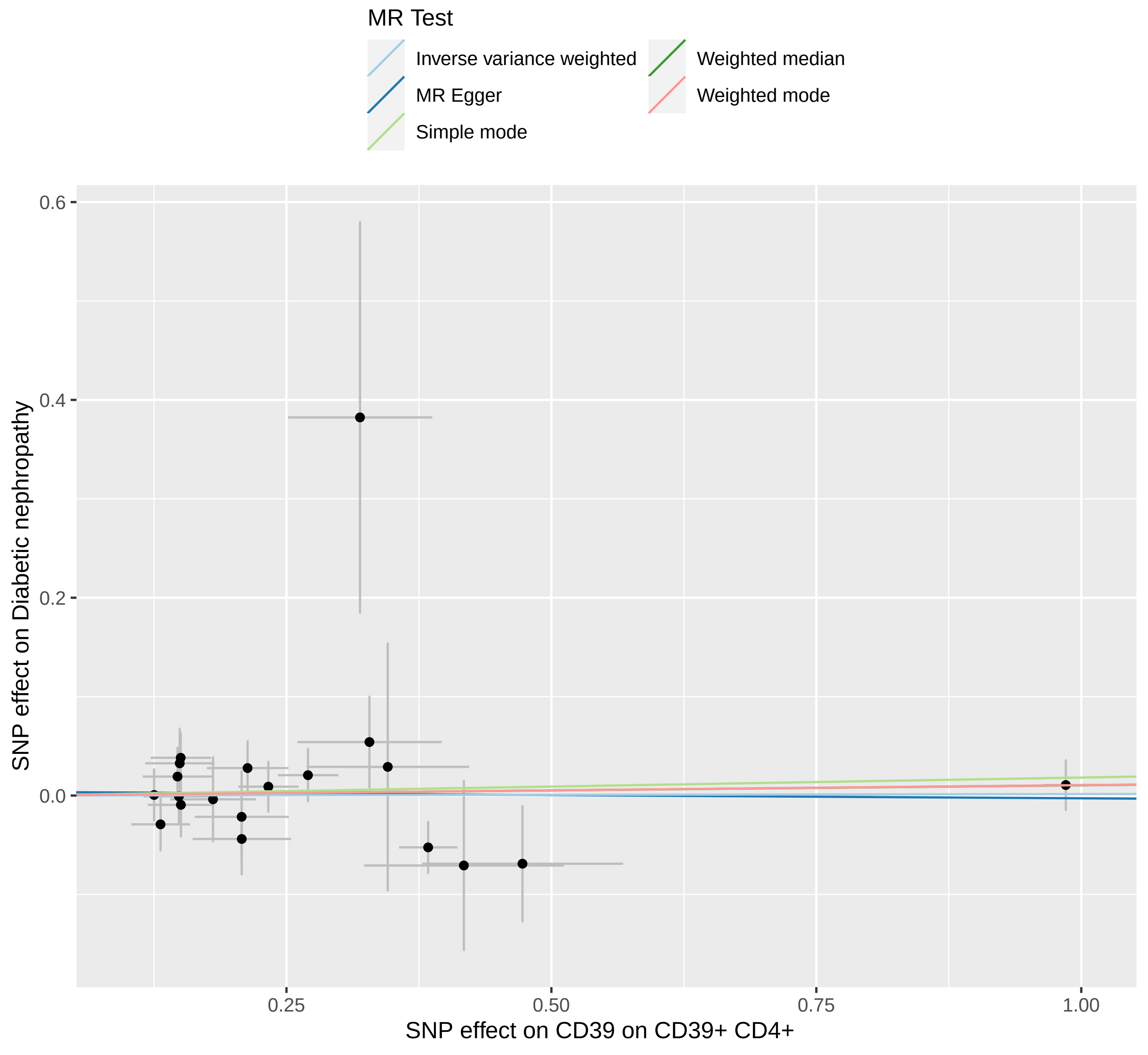

MR Test
Inverse variance weighted
MR Egger
Simple mode
Weighted median
Weighted mode
0.6
0.4
0.2
0.0
0.25
0.50
0.75
1.00
SNP effect on Diabetic nephropathy
SNP effect on CD39 on CD39+ CD4+

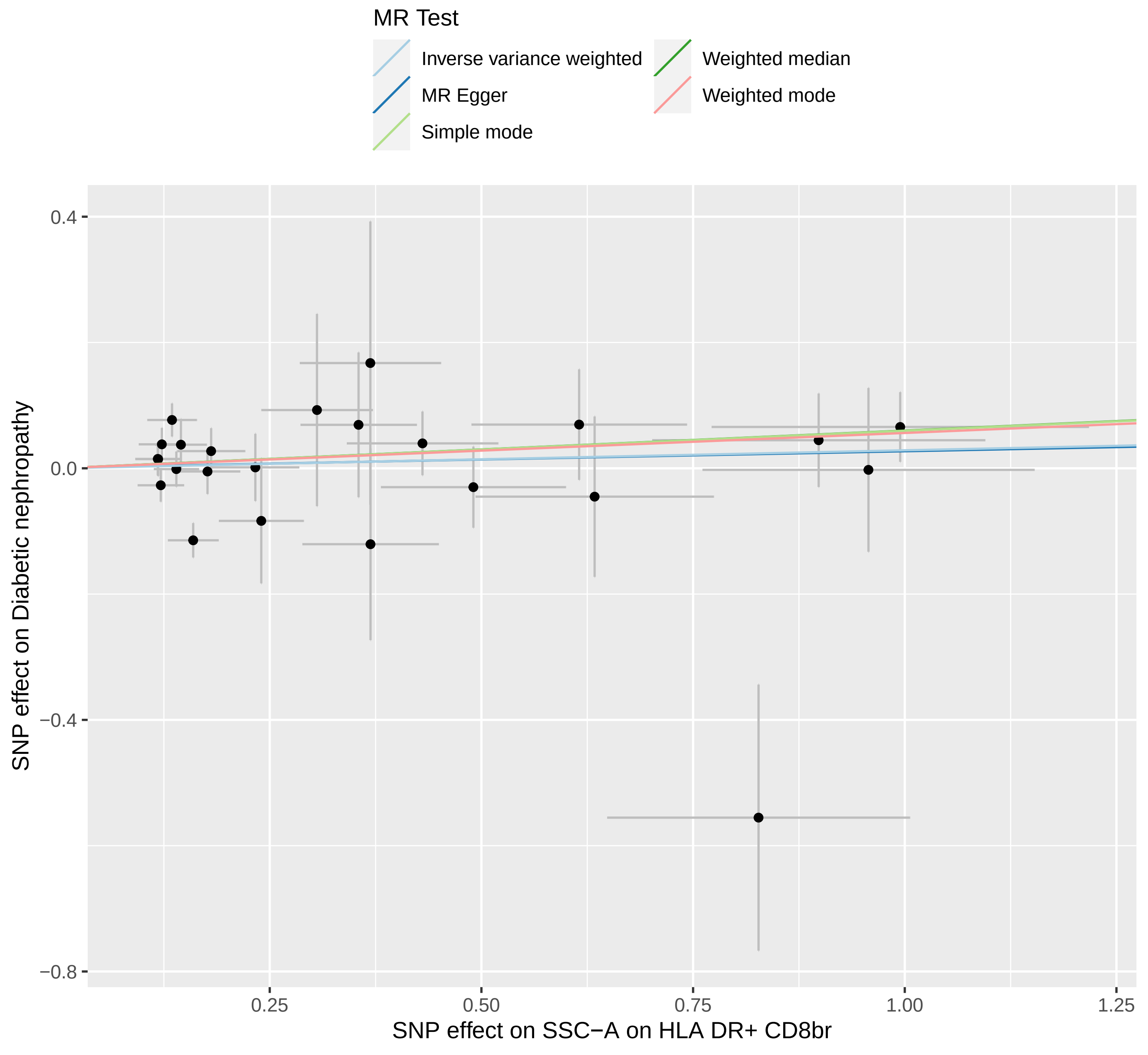
MR Test
Inverse variance weighted
MR Egger
Simple mode
Weighted median
Weighted mode
0.4
0.0
−0.4
−0.8
0.25
0.50
0.75
1.00
1.25
SNP effect on Diabetic nephropathy
SNP effect on SSC−A on HLA DR+ CD8br

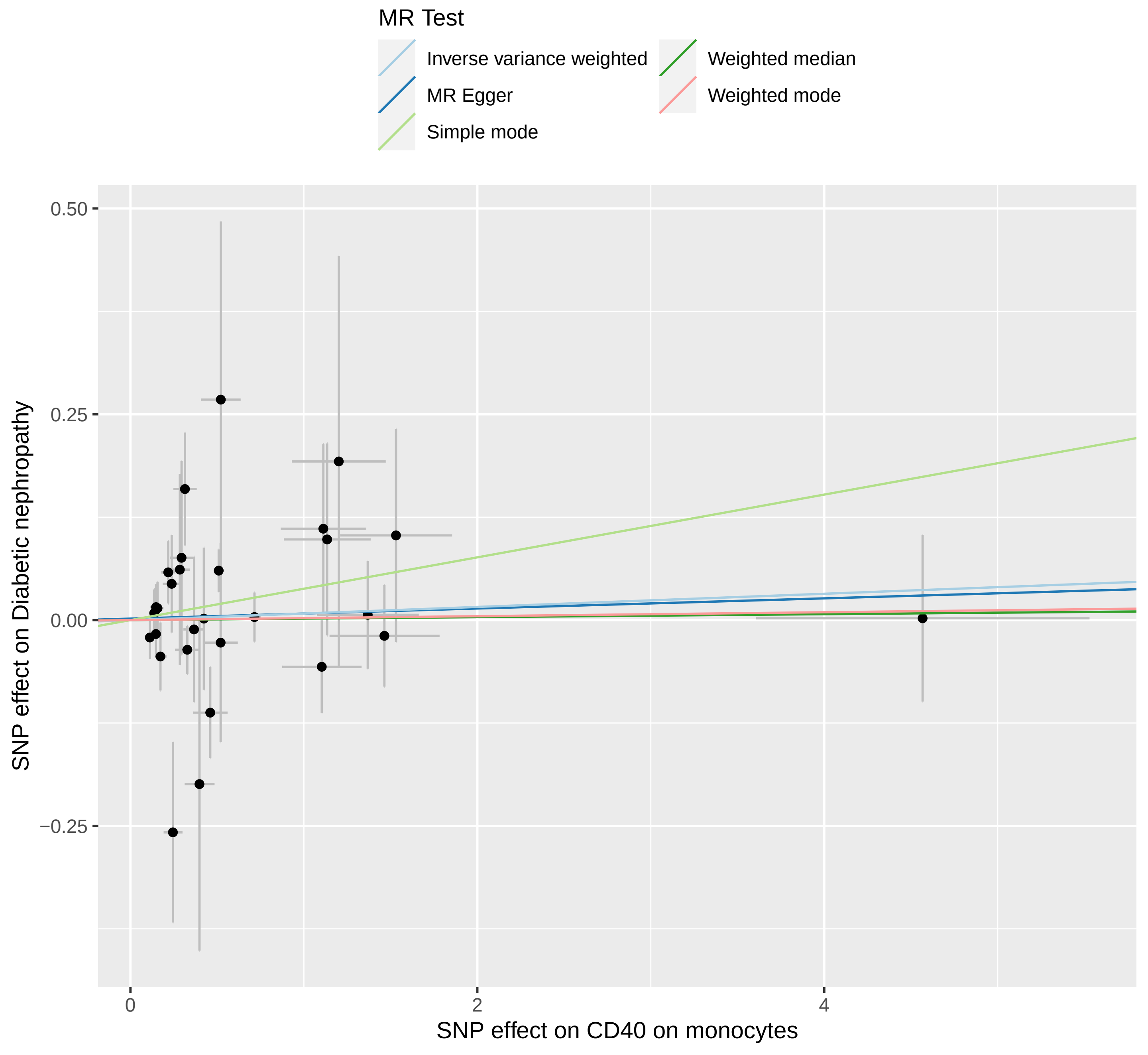
MR Test
Inverse variance weighted
MR Egger
Simple mode
Weighted median
Weighted mode
0.50
0.25
0.00
−0.25
0
2
4
SNP effect on Diabetic nephropathy
SNP effect on CD40 on monocytes

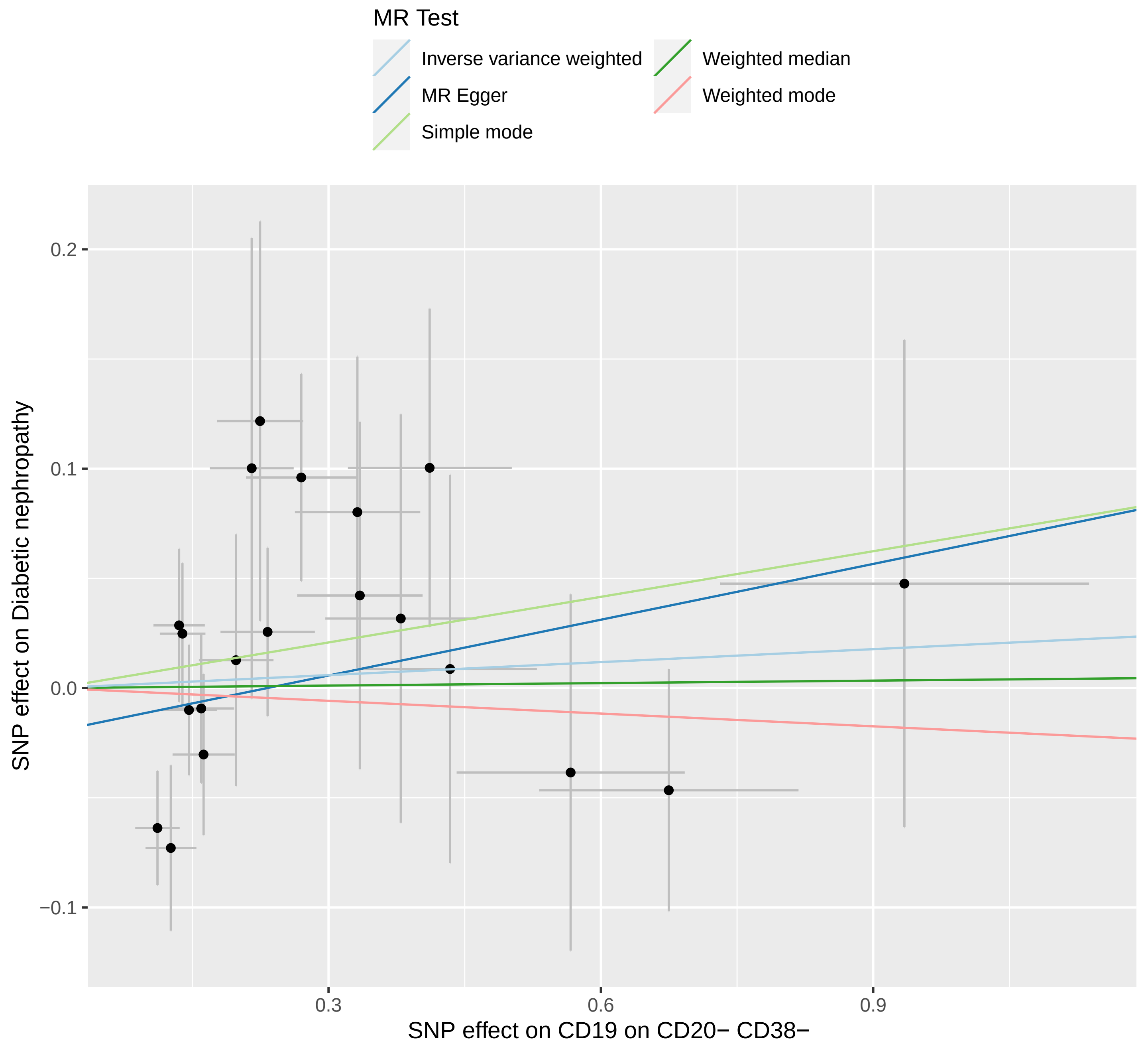
MR Test
Inverse variance weighted
MR Egger
Simple mode
Weighted median
Weighted mode
0.2
0.1
0.0
−0.1
0.3
0.6
0.9
SNP effect on Diabetic nephropathy
SNP effect on CD19 on CD20− CD38−

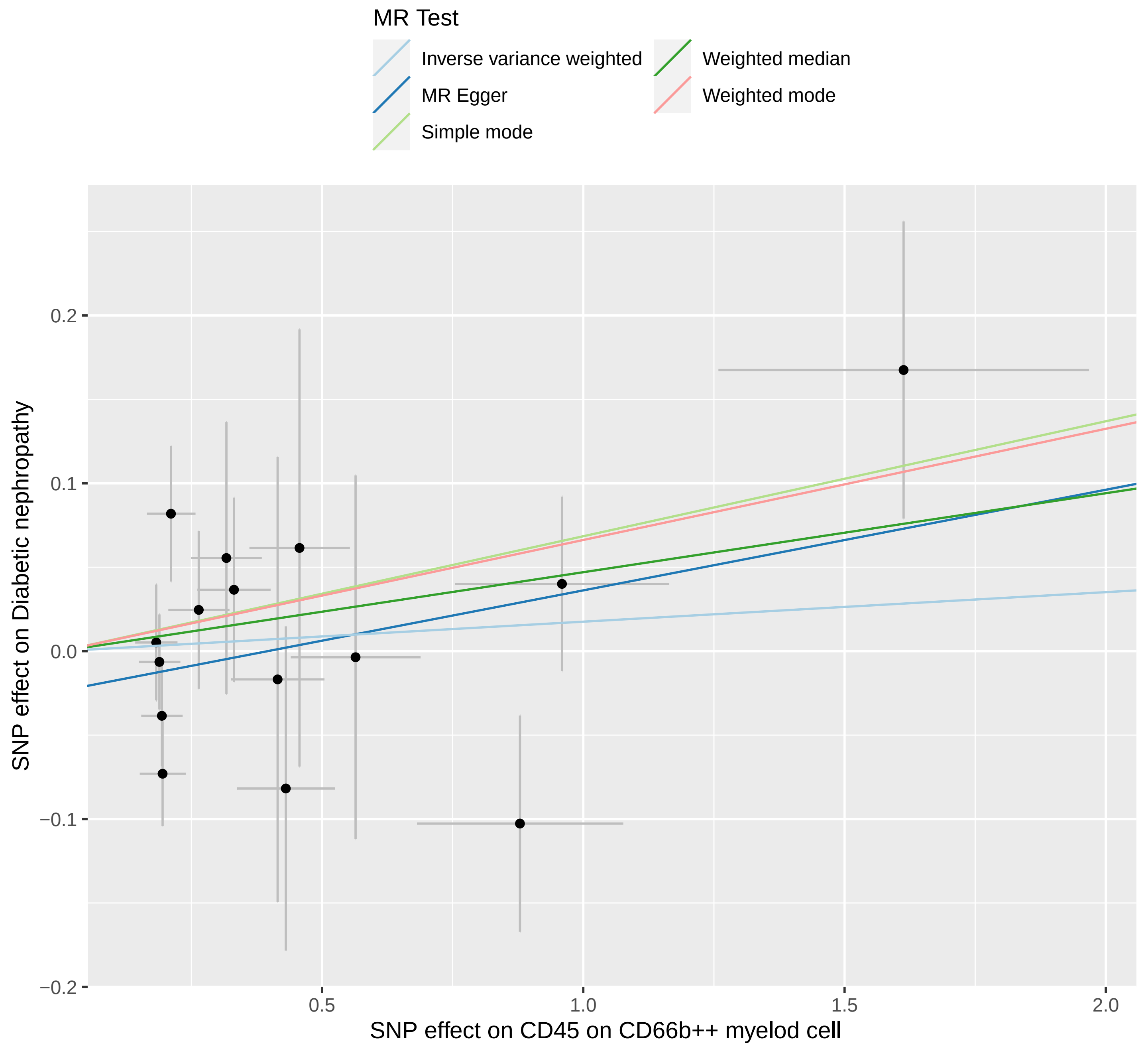

MR Test
Inverse variance weighted
MR Egger
Simple mode
Weighted median
Weighted mode
0.2
0.1
0.0
−0.1
−0.2
0.5
1.0
1.5
2.0
SNP effect on Diabetic nephropathy
SNP effect on CD45 on CD66b++ myelod cell

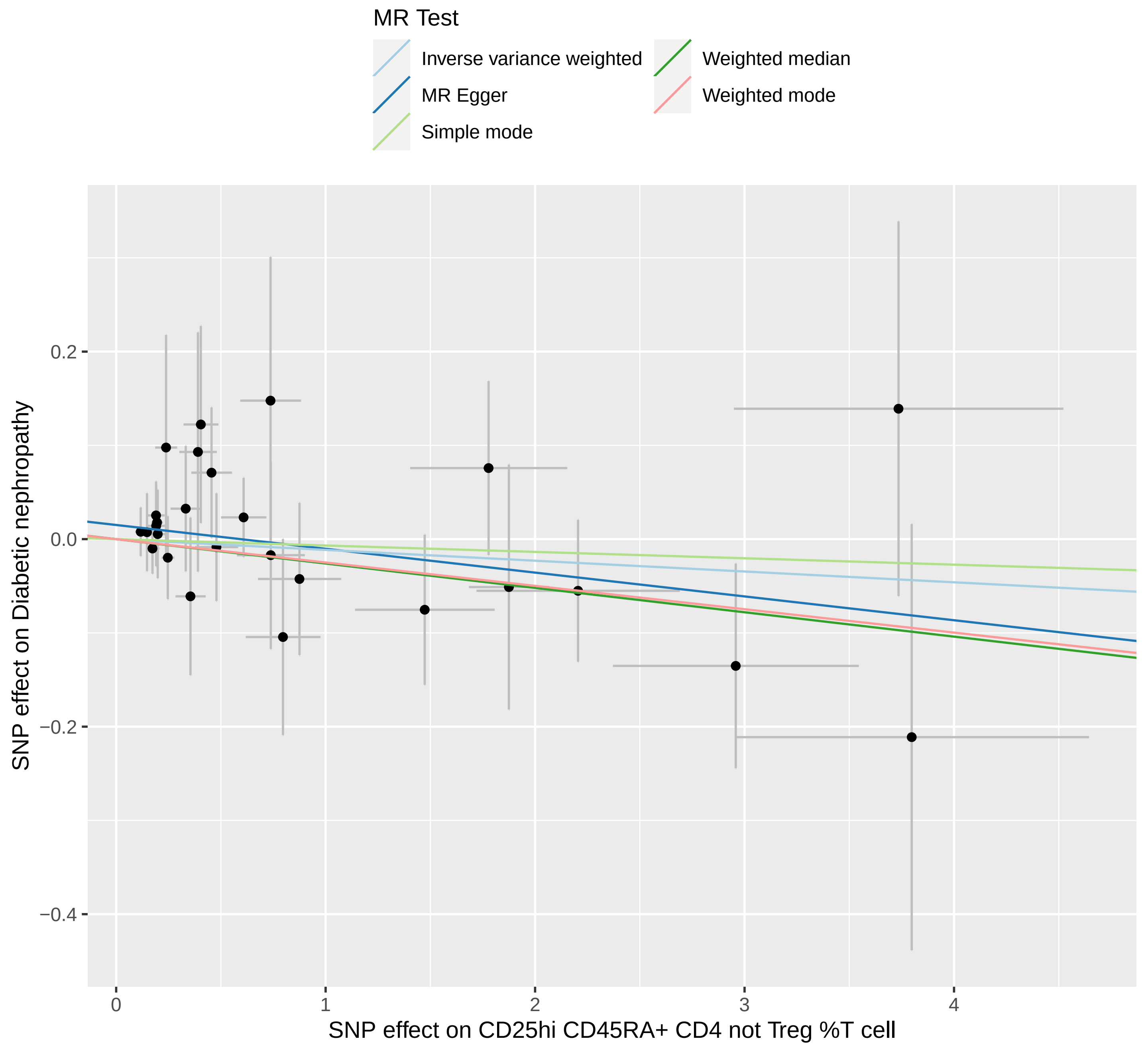

MR Test
Inverse variance weighted
MR Egger
Simple mode
Weighted median
Weighted mode
0.2
0.0
−0.2
−0.4
0
1
2
3
4
SNP effect on Diabetic nephropathy
SNP effect on CD25hi CD45RA+ CD4 not Treg %T cell

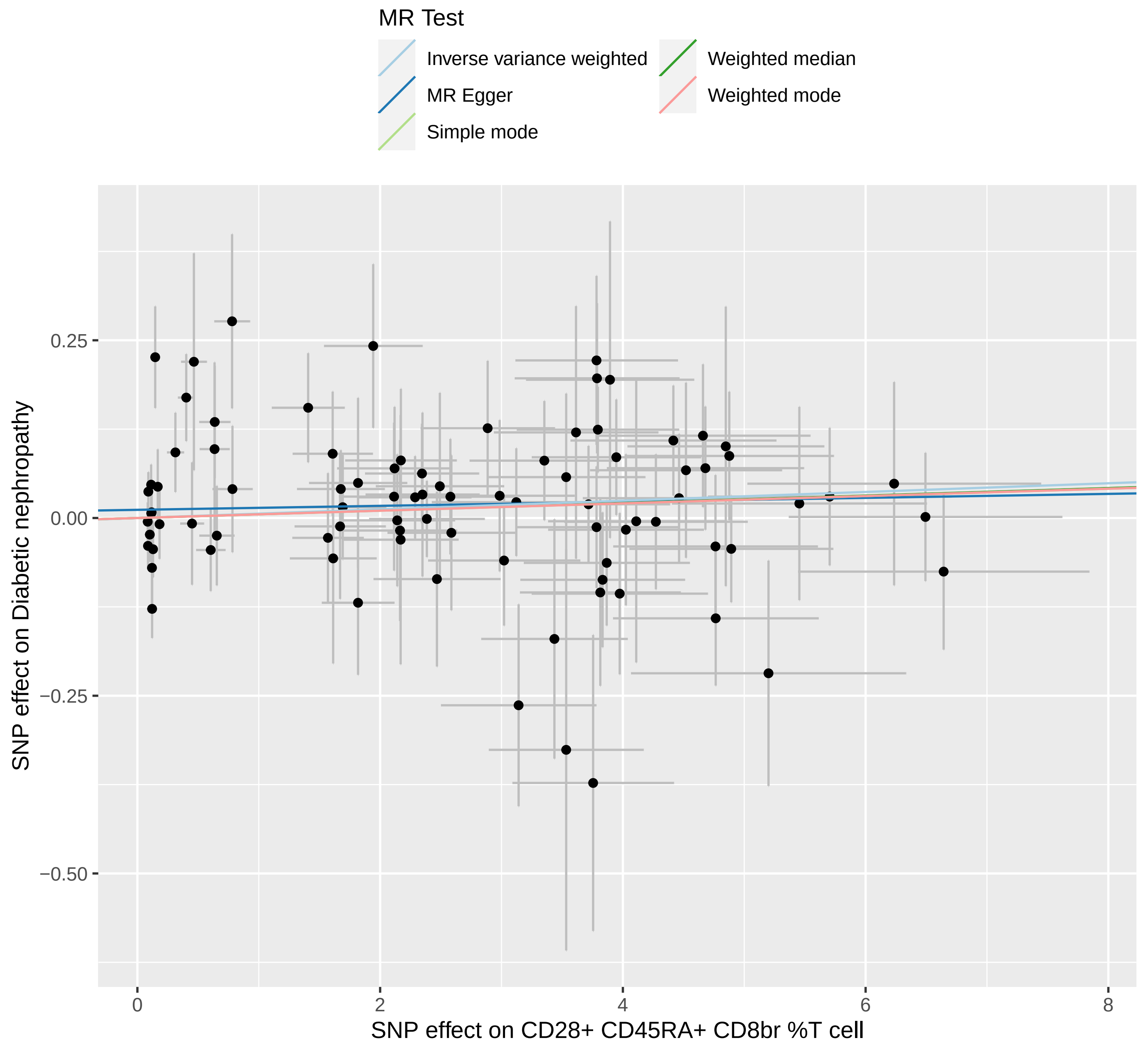

MR Test
Inverse variance weighted
MR Egger
Simple mode
Weighted median
Weighted mode
0.25
0.00
−0.25
−0.50
0
2
4
6
8
SNP effect on Diabetic nephropathy
SNP effect on CD28+ CD45RA+ CD8br %T cell

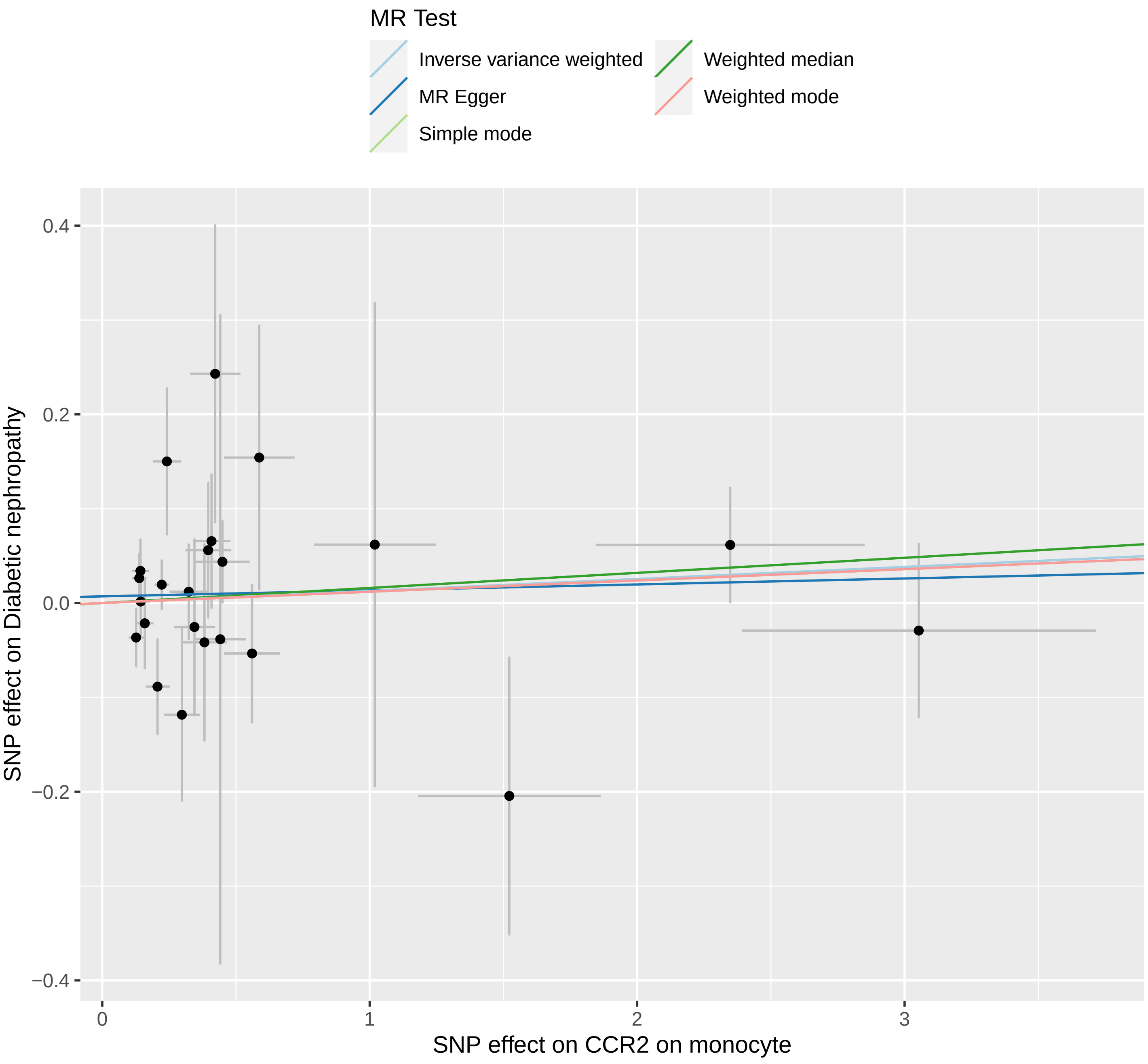
MR Test
Inverse variance weighted
MR Egger
Simple mode
Weighted median
Weighted mode
0.4
0.2
0.0
−0.2
−0.4
0
1
2
3
SNP effect on Diabetic nephropathy
SNP effect on CCR2 on monocyte

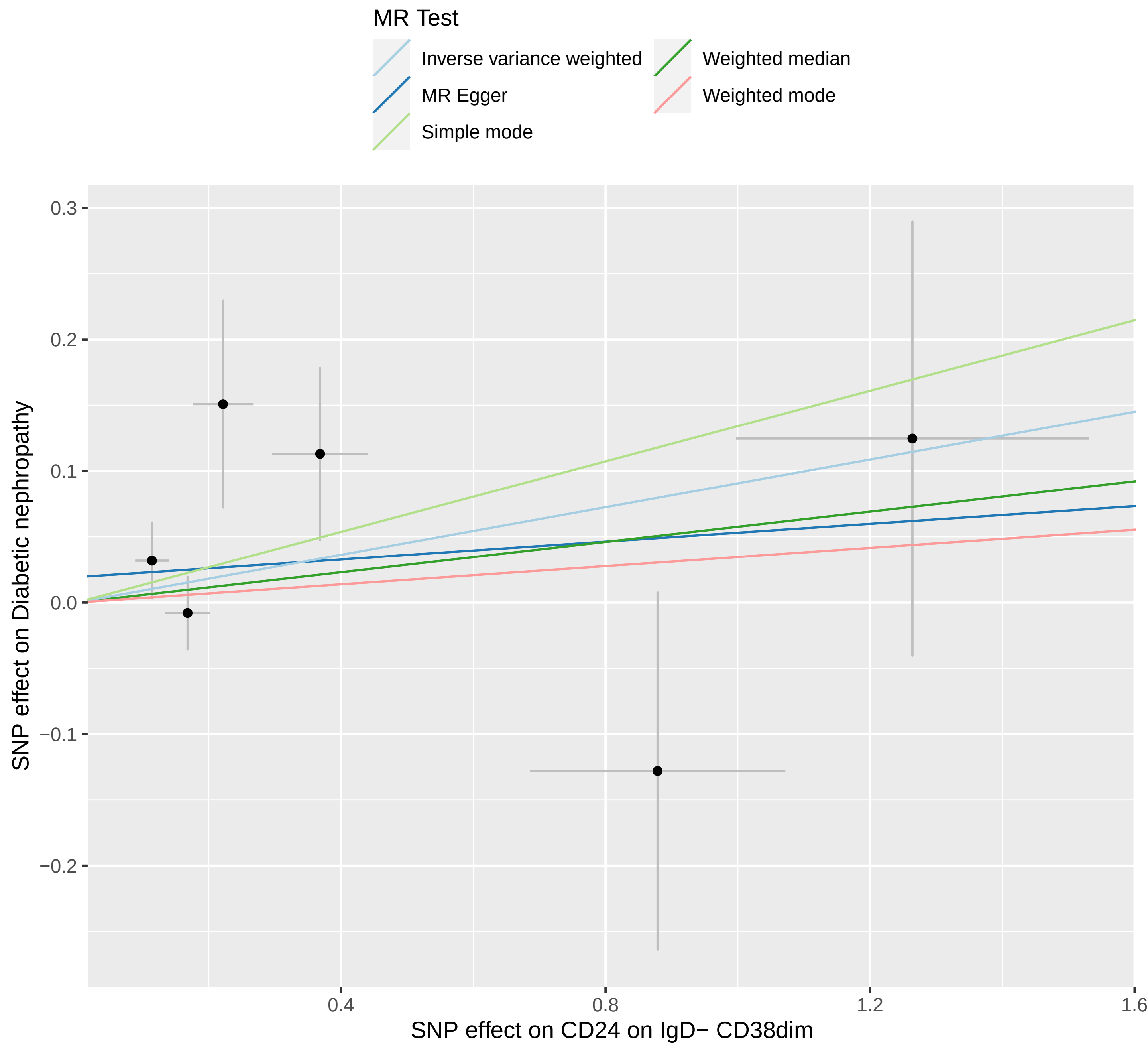
MR Test
Inverse variance weighted
MR Egger
Simple mode
Weighted median
Weighted mode
0.3
0.2
0.1
0.0
−0.1
−0.2
0.4
0.8
1.2
1.6
SNP effect on Diabetic nephropathy
SNP effect on CD24 on IgD− CD38dim

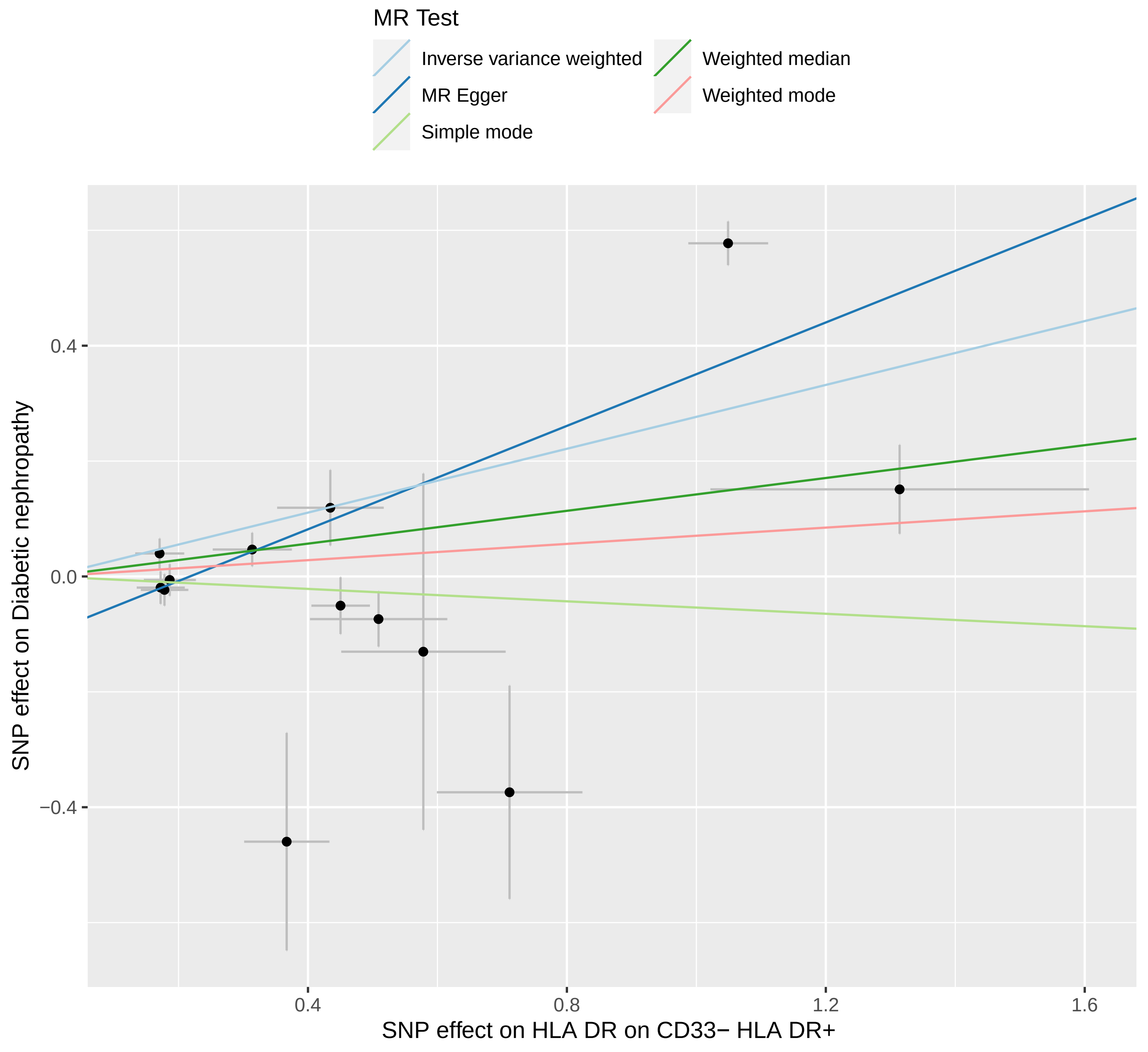
MR Test
Inverse variance weighted
MR Egger
Simple mode
Weighted median
Weighted mode
0.4
0.0
−0.4
SNP effect on Diabetic nephropathy
0.4
0.8
1.2
1.6
SNP effect on HLA DR on CD33− HLA DR+

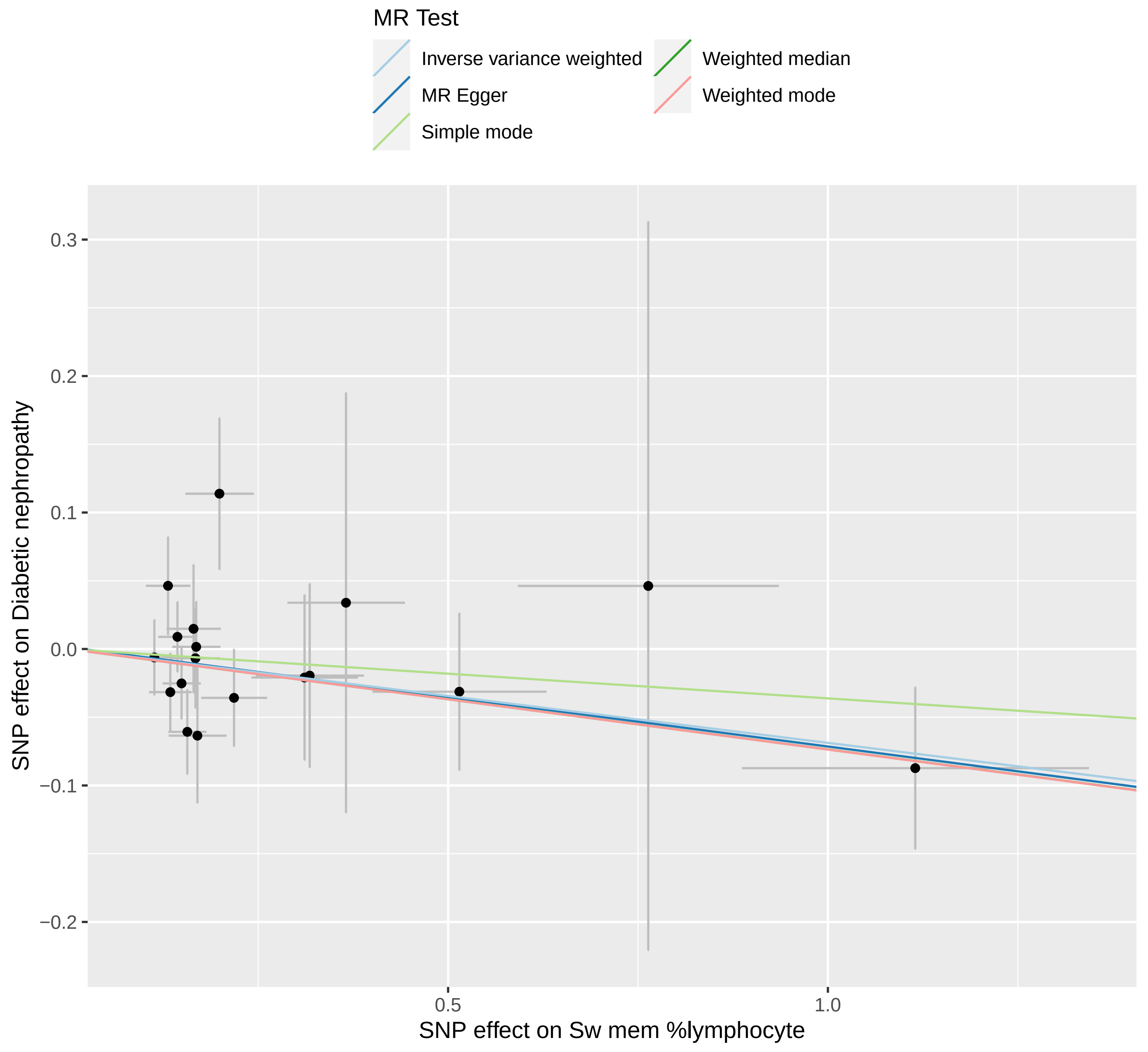
MR Test
Inverse variance weighted
MR Egger
Simple mode
Weighted median
Weighted mode
0.3
0.2
0.1
0.0
−0.1
−0.2
0.5
1.0
SNP effect on Diabetic nephropathy
SNP effect on Sw mem %lymphocyte

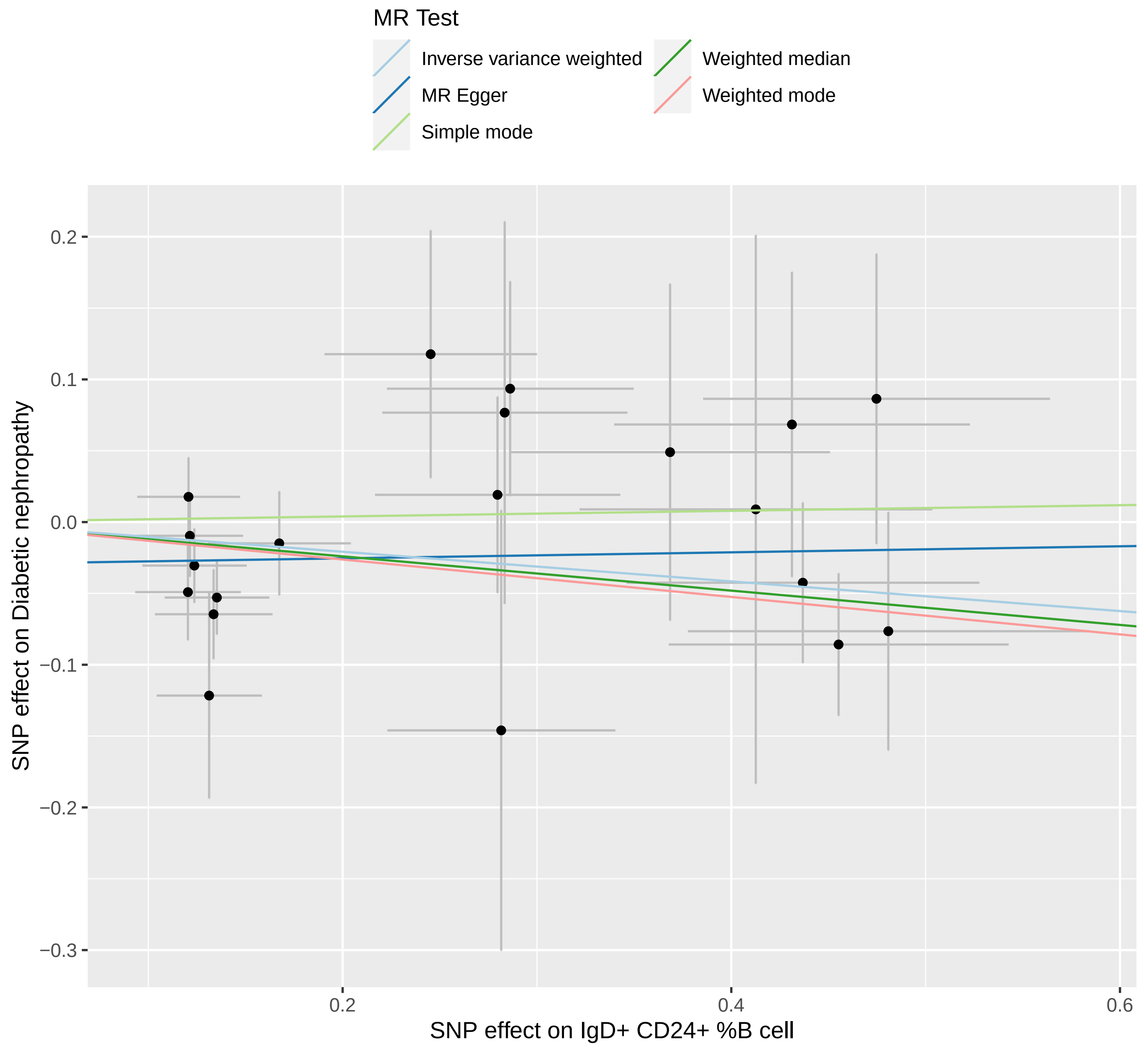
MR Test
Inverse variance weighted
MR Egger
Simple mode
Weighted median
Weighted mode
0.2
0.1
0.0
−0.1
−0.2
−0.3
SNP effect on Diabetic nephropathy
0.2
0.4
0.6
SNP effect on IgD+ CD24+ %B cell

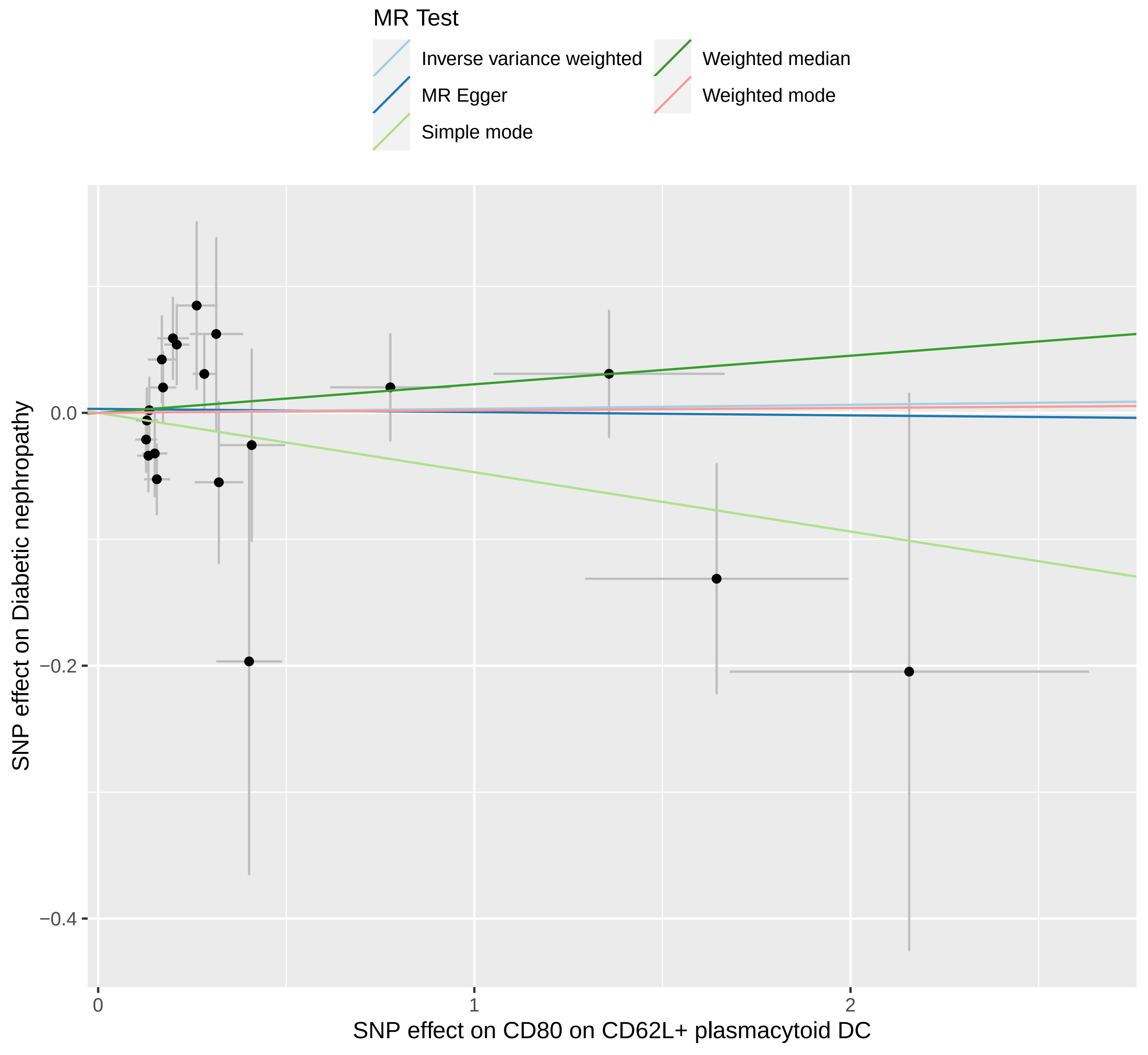
MR Test
Inverse variance weighted
MR Egger
Simple mode
Weighted median
Weighted mode
0.0
−0.2
−0.4
0
1
2
SNP effect on Diabetic nephropathy
SNP effect on CD80 on CD62L+ plasmacytoid DC

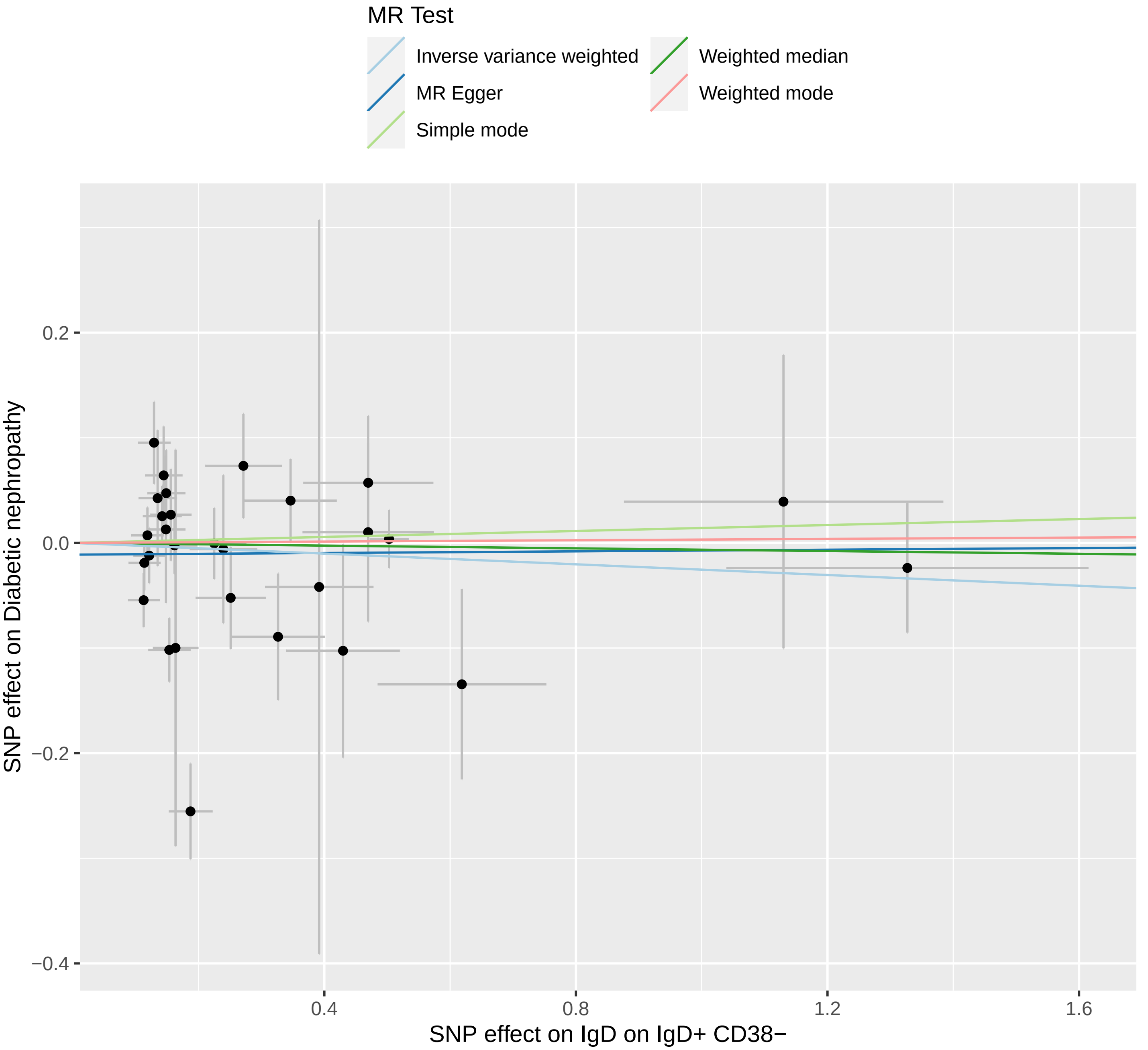
MR Test
Inverse variance weighted
MR Egger
Simple mode
Weighted median
Weighted mode
0.2
0.0
−0.2
−0.4
0.4
0.8
1.2
1.6
SNP effect on Diabetic nephropathy
SNP effect on IgD on IgD+ CD38−

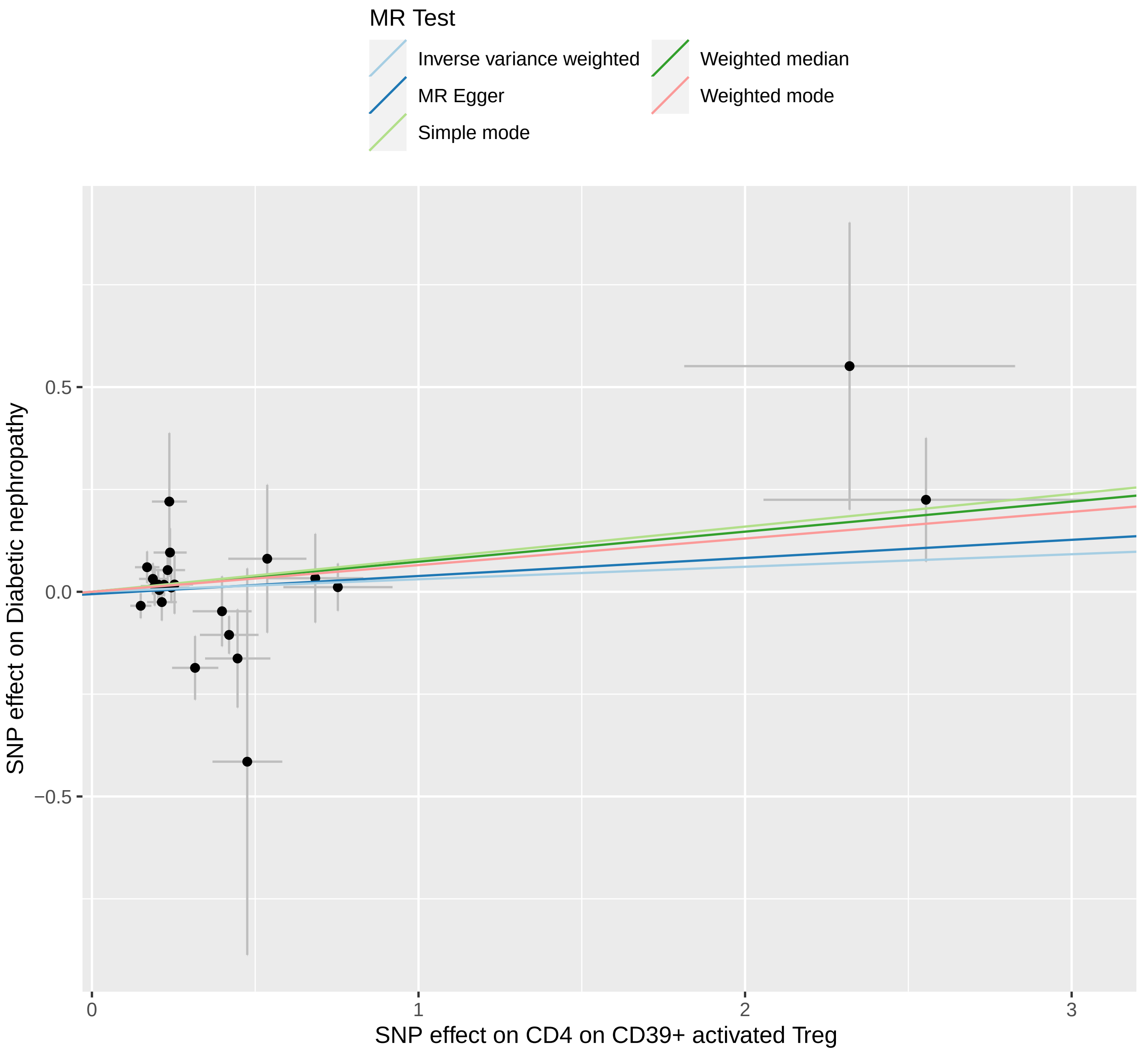
MR Test
Inverse variance weighted
MR Egger
Simple mode
Weighted median
Weighted mode
0.5
0.0
−0.5
SNP effect on Diabetic nephropathy
0
1
2
3
SNP effect on CD4 on CD39+ activated Treg

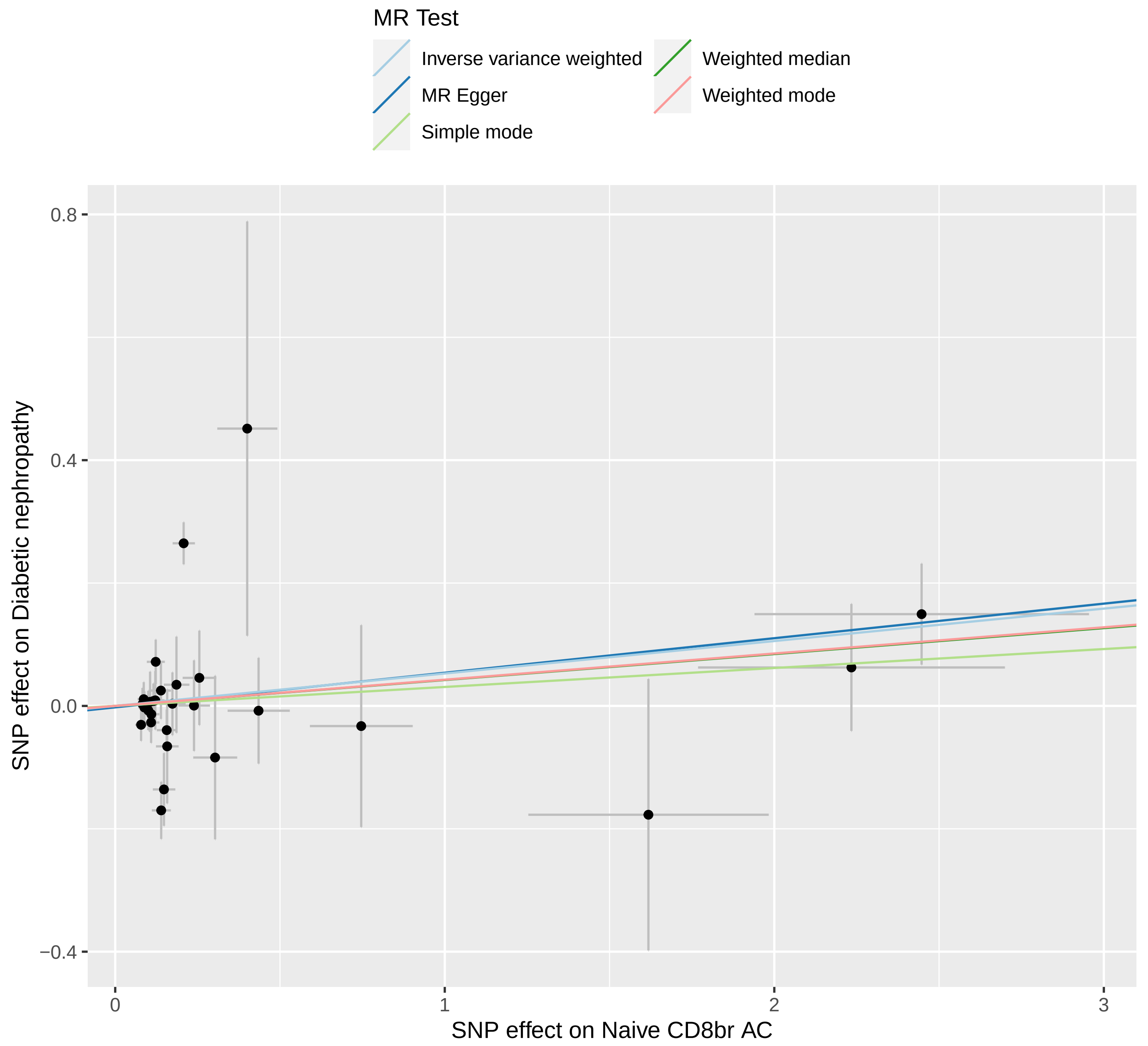

MR Test
Inverse variance weighted
MR Egger
Simple mode
Weighted median
Weighted mode
0.8
0.4
0.0
−0.4
0
1
2
3
SNP effect on Diabetic nephropathy
SNP effect on Naive CD8br AC

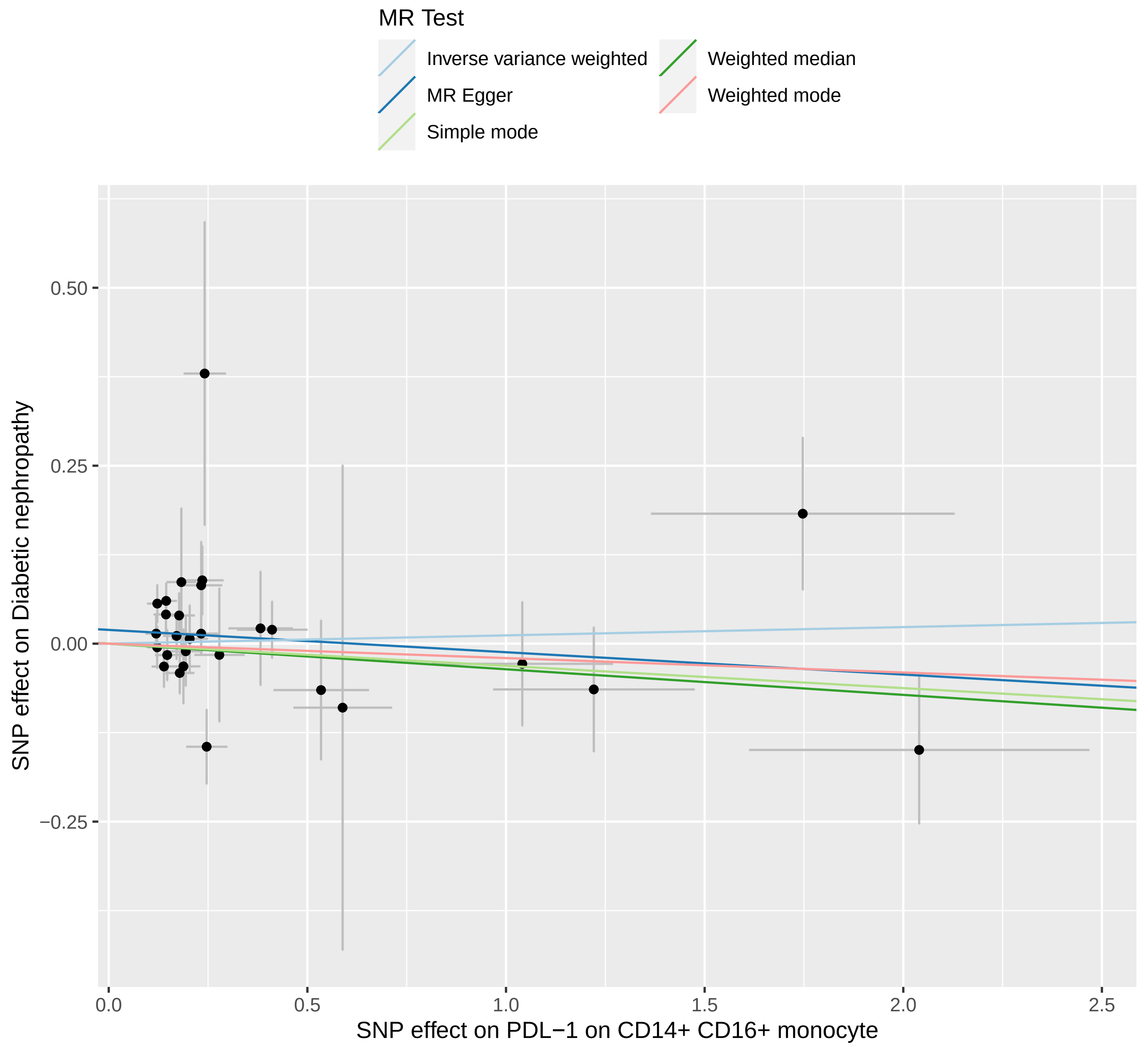
MR Test
Inverse variance weighted
MR Egger
Simple mode
Weighted median
Weighted mode
0.50
0.25
0.00
−0.25
0.0
0.5
1.0
1.5
2.0
2.5
SNP effect on Diabetic nephropathy
SNP effect on PDL−1 on CD14+ CD16+ monocyte

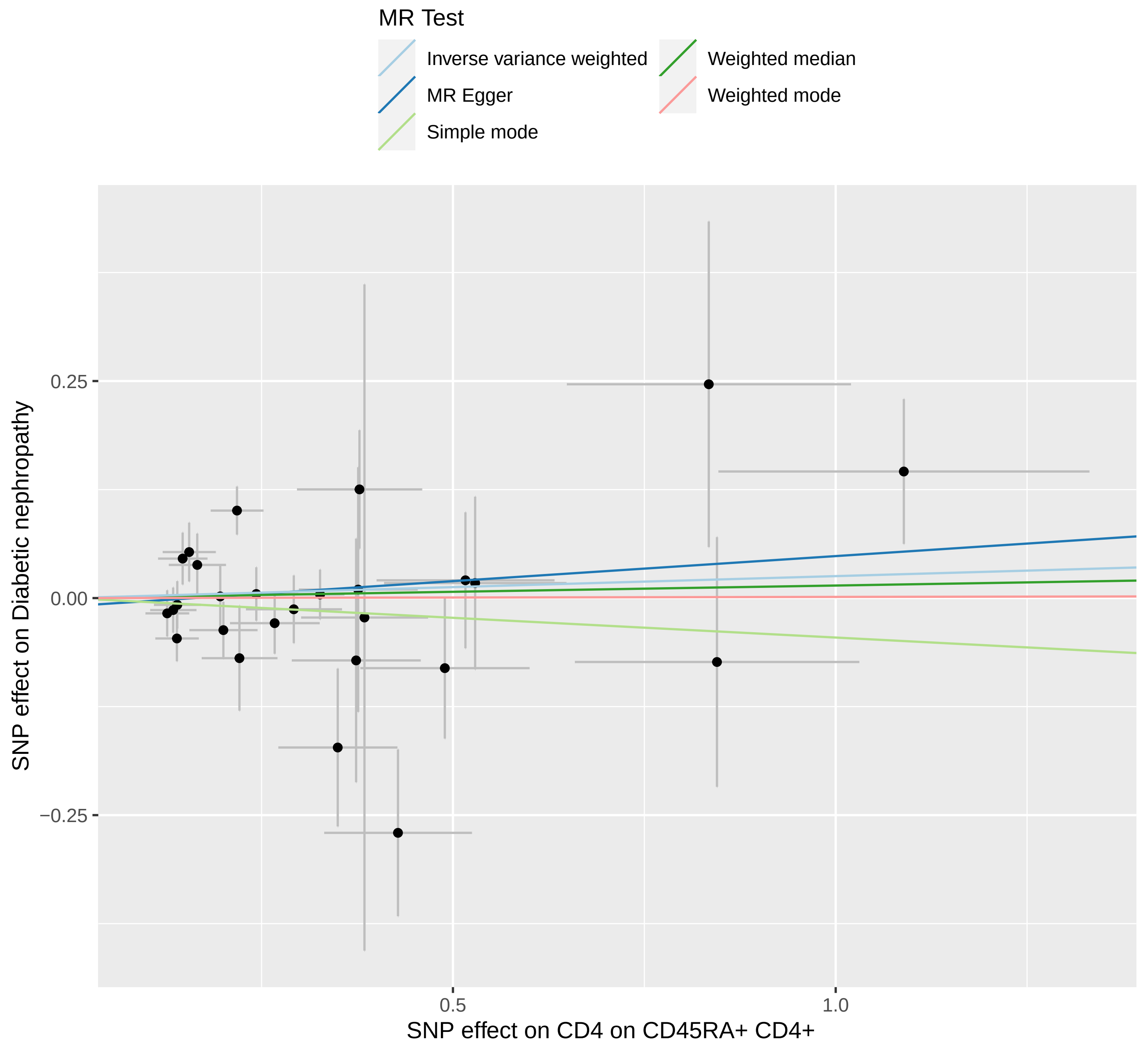

MR Test
Inverse variance weighted
MR Egger
Simple mode
Weighted median
Weighted mode
0.25
0.00
−0.25
0.5
1.0
SNP effect on Diabetic nephropathy
SNP effect on CD4 on CD45RA+ CD4+

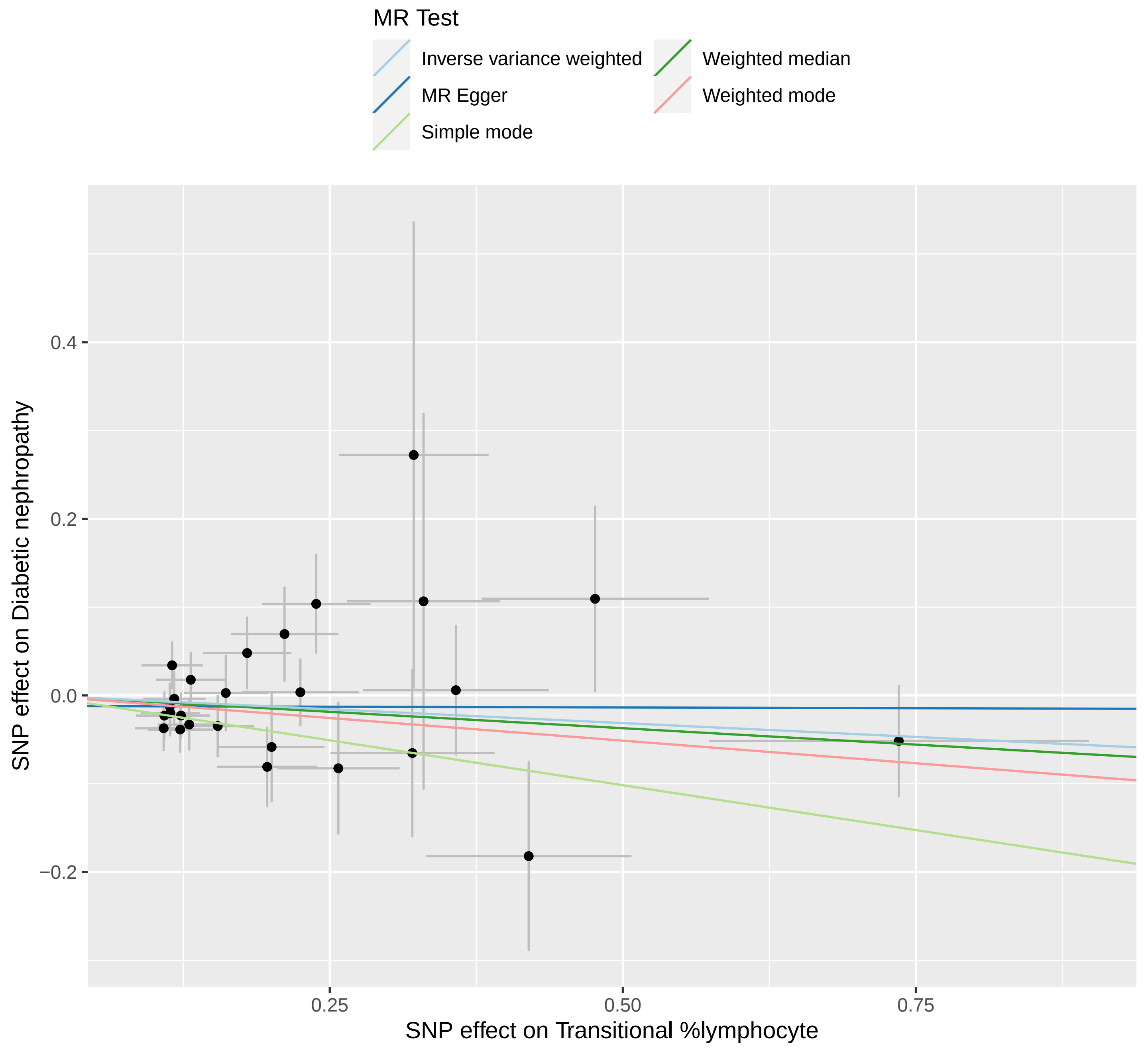
MR Test
Inverse variance weighted
MR Egger
Simple mode
Weighted median
Weighted mode
0.4
0.2
0.0
−0.2
0.25
0.50
0.75
SNP effect on Diabetic nephropathy
SNP effect on Transitional %lymphocyte

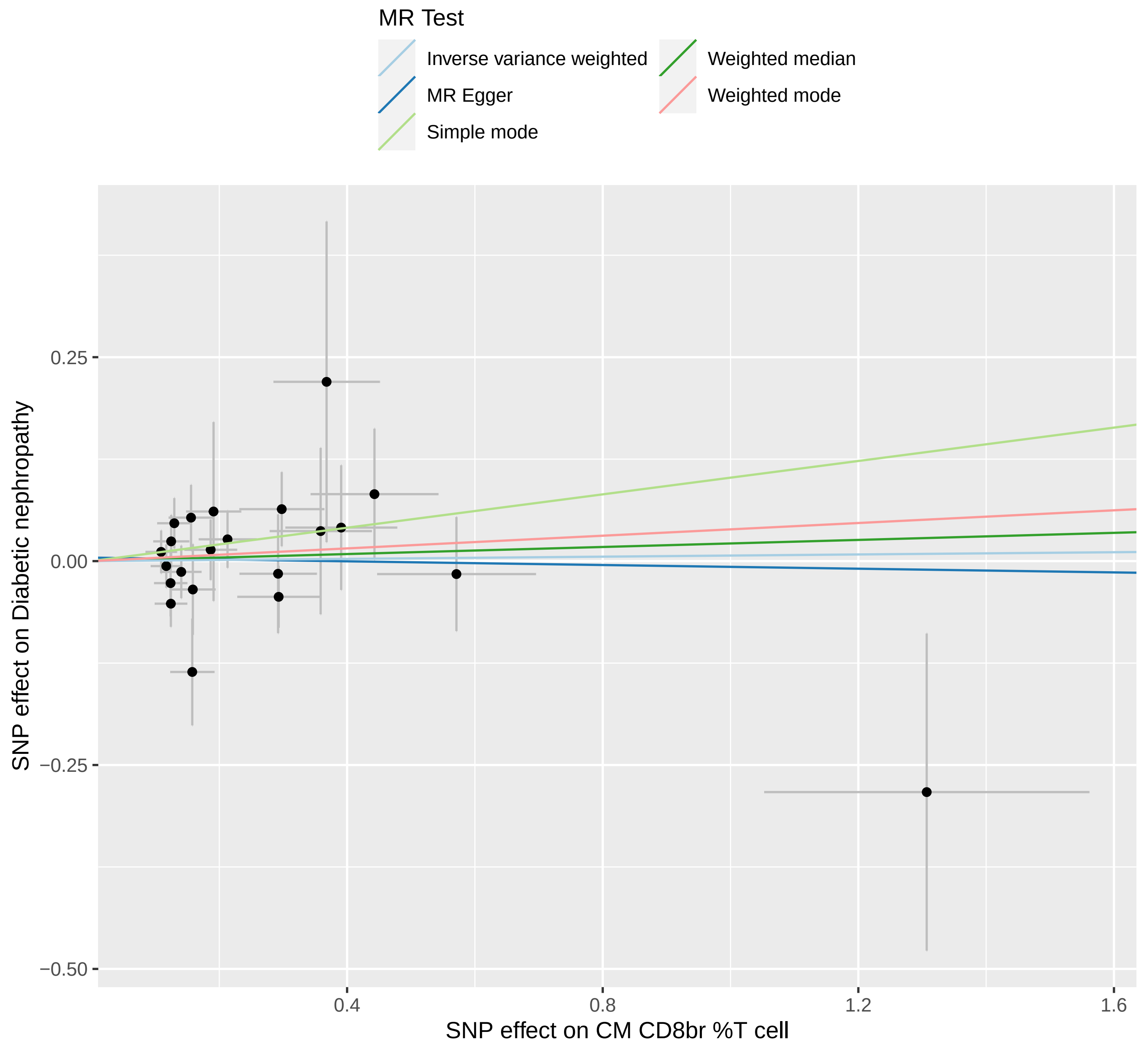
MR Test
Inverse variance weighted
MR Egger
Simple mode
Weighted median
Weighted mode
0.25
0.00
−0.25
−0.50
0.4
0.8
1.2
1.6
SNP effect on Diabetic nephropathy
SNP effect on CM CD8br %T cell

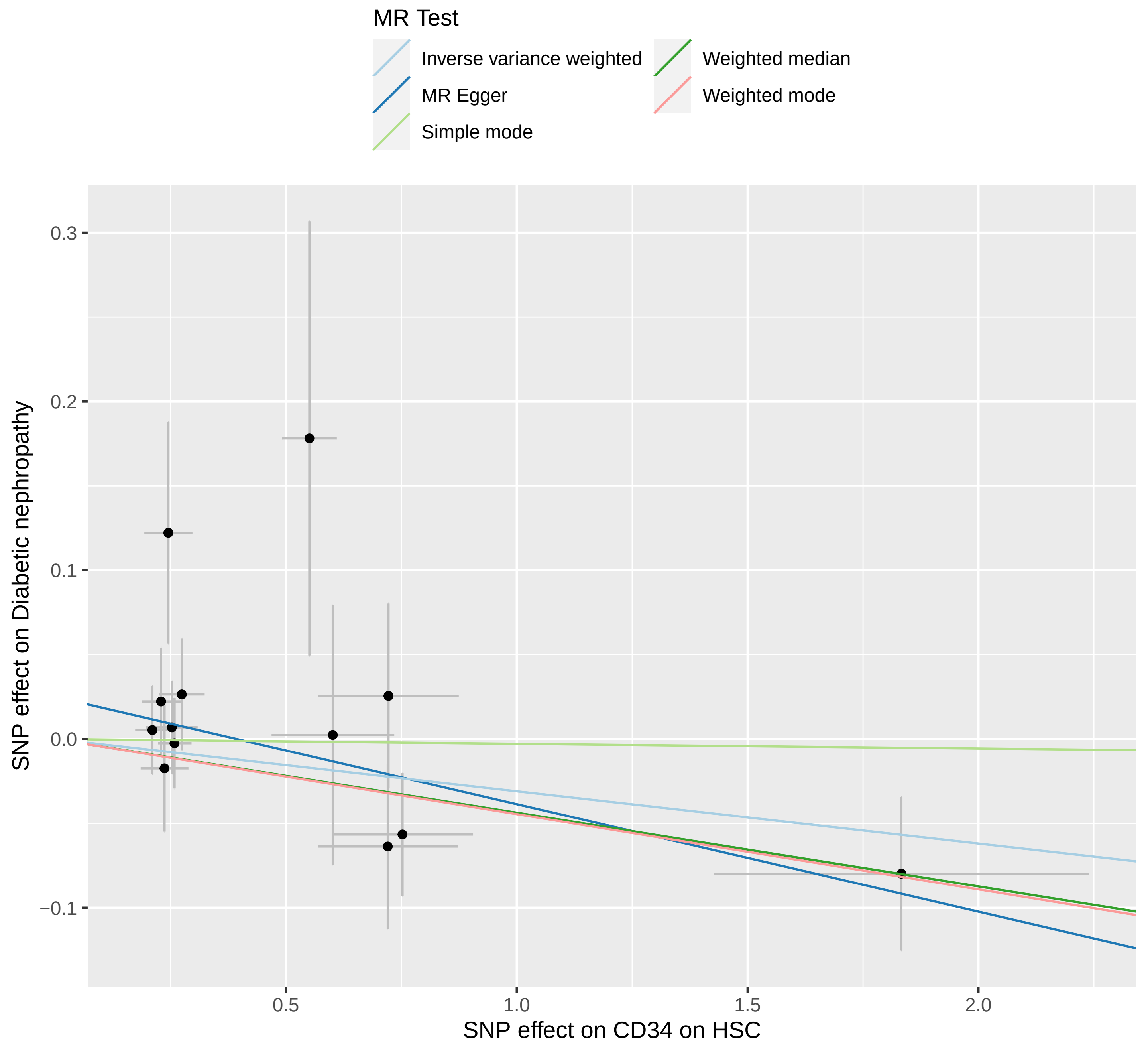
MR Test
Inverse variance weighted
MR Egger
Simple mode
Weighted median
Weighted mode
SNP effect on Diabetic nephropathy
SNP effect on CD34 on HSC
0.3
0.2
0.1
0.0
−0.1
0.5
1.0
1.5
2.0

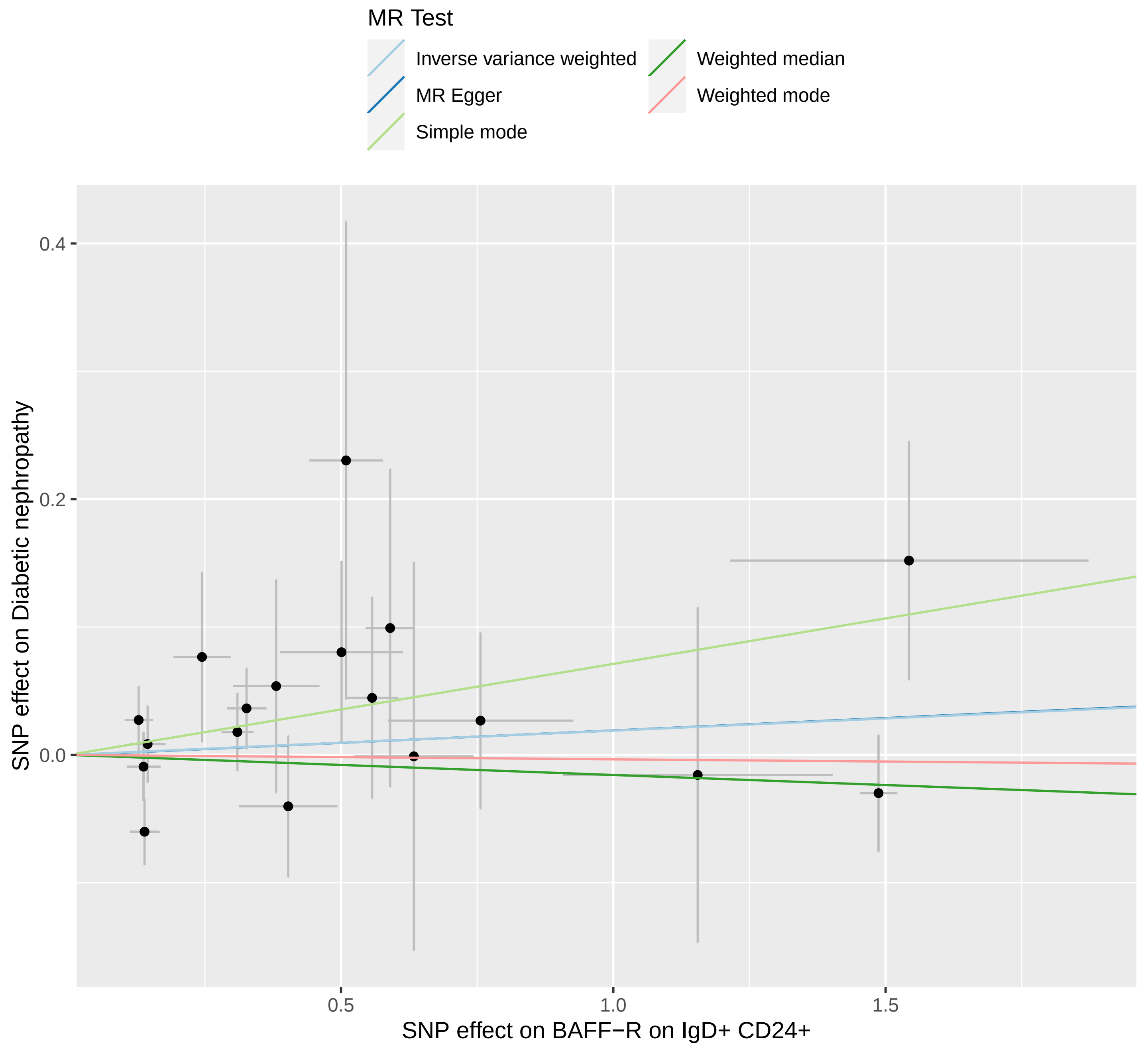
MR Test
Inverse variance weighted
MR Egger
Simple mode
Weighted median
Weighted mode
0.4
0.2
0.0
0.5
1.0
1.5
SNP effect on Diabetic nephropathy
SNP effect on BAFF−R on IgD+ CD24+

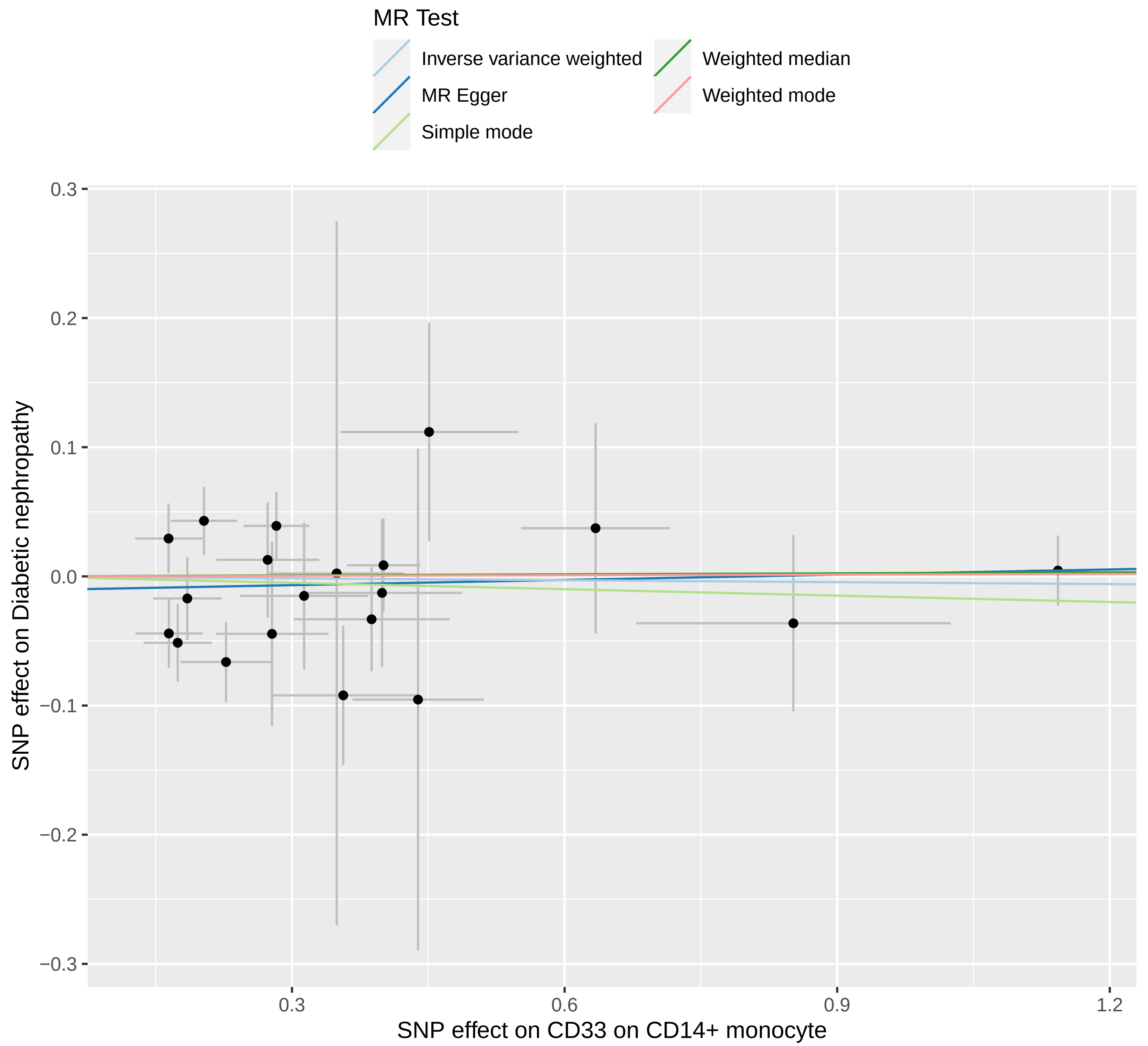

MR Test
Inverse variance weighted
MR Egger
Simple mode
Weighted median
Weighted mode
0.3
0.2
0.1
0.0
−0.1
−0.2
−0.3
0.3
0.6
0.9
1.2
SNP effect on Diabetic nephropathy
SNP effect on CD33 on CD14+ monocyte

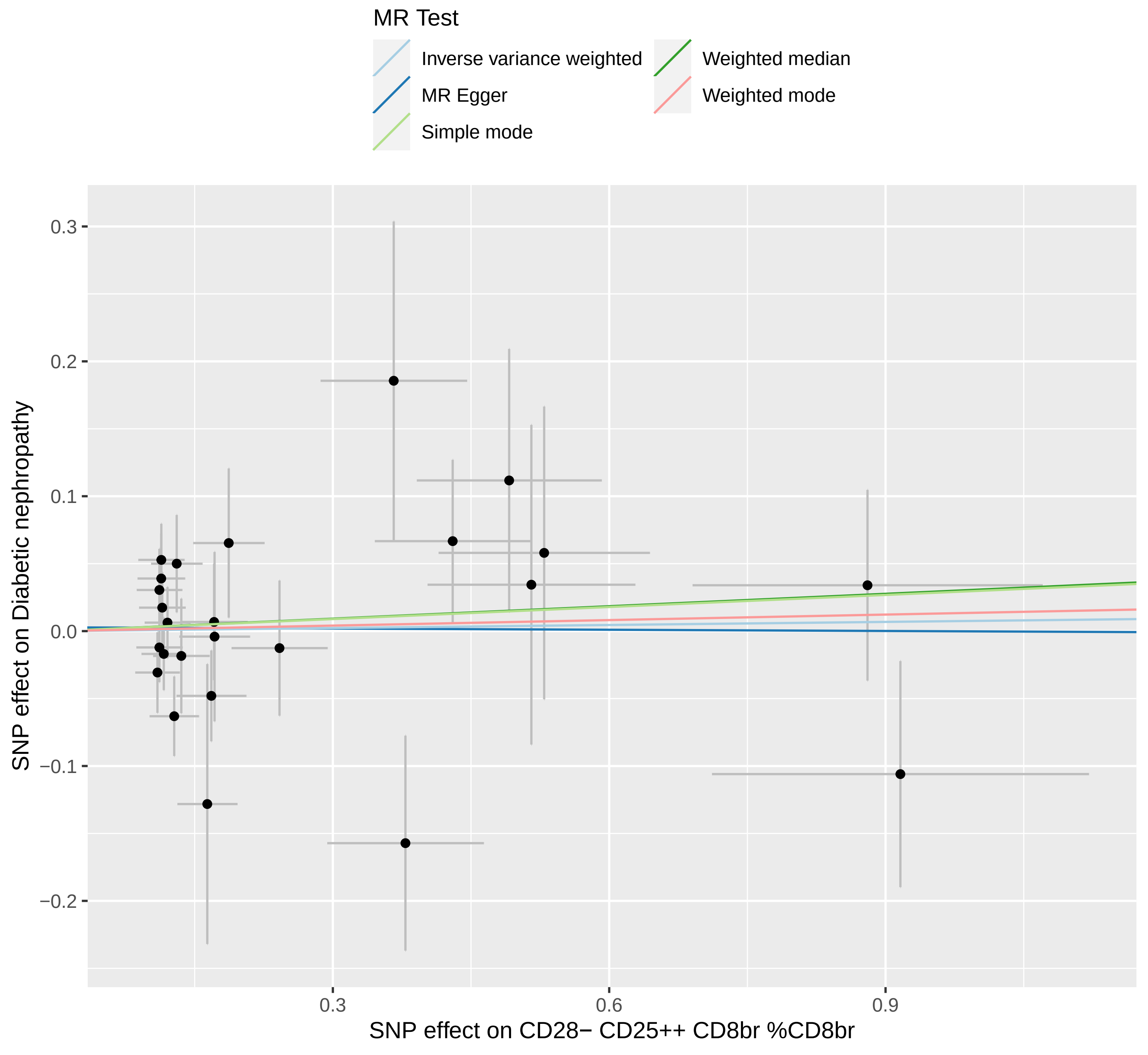
MR Test
Inverse variance weighted
MR Egger
Simple mode
Weighted median
Weighted mode
0.3
0.2
0.1
0.0
−0.1
−0.2
0.3
0.6
0.9
SNP effect on Diabetic nephropathy
SNP effect on CD28− CD25++ CD8br %CD8br

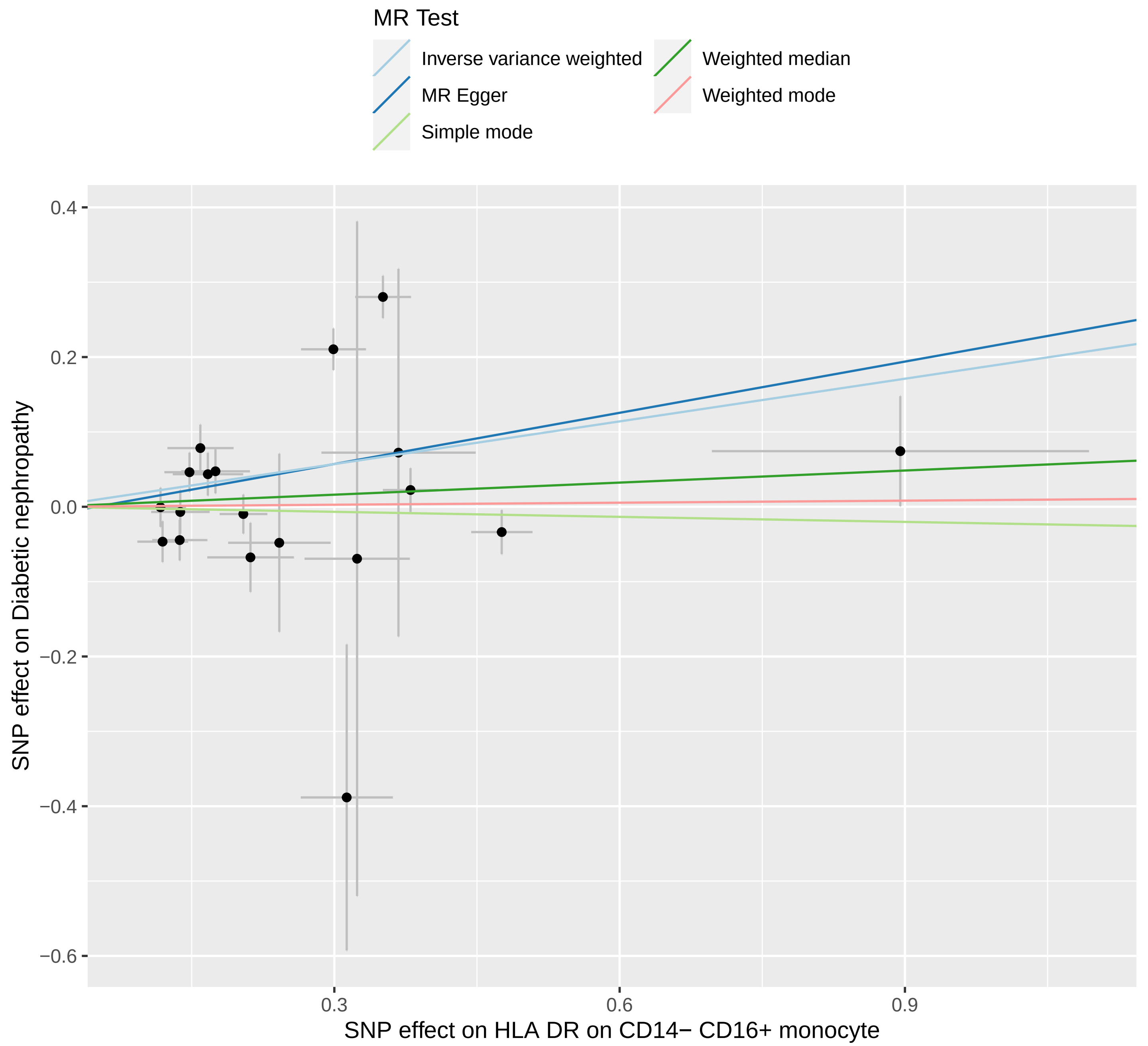
MR Test
Inverse variance weighted
MR Egger
Simple mode
Weighted median
Weighted mode
0.4
0.2
0.0
−0.2
−0.4
−0.6
0.3
0.6
0.9
SNP effect on Diabetic nephropathy
SNP effect on HLA DR on CD14− CD16+ monocyte

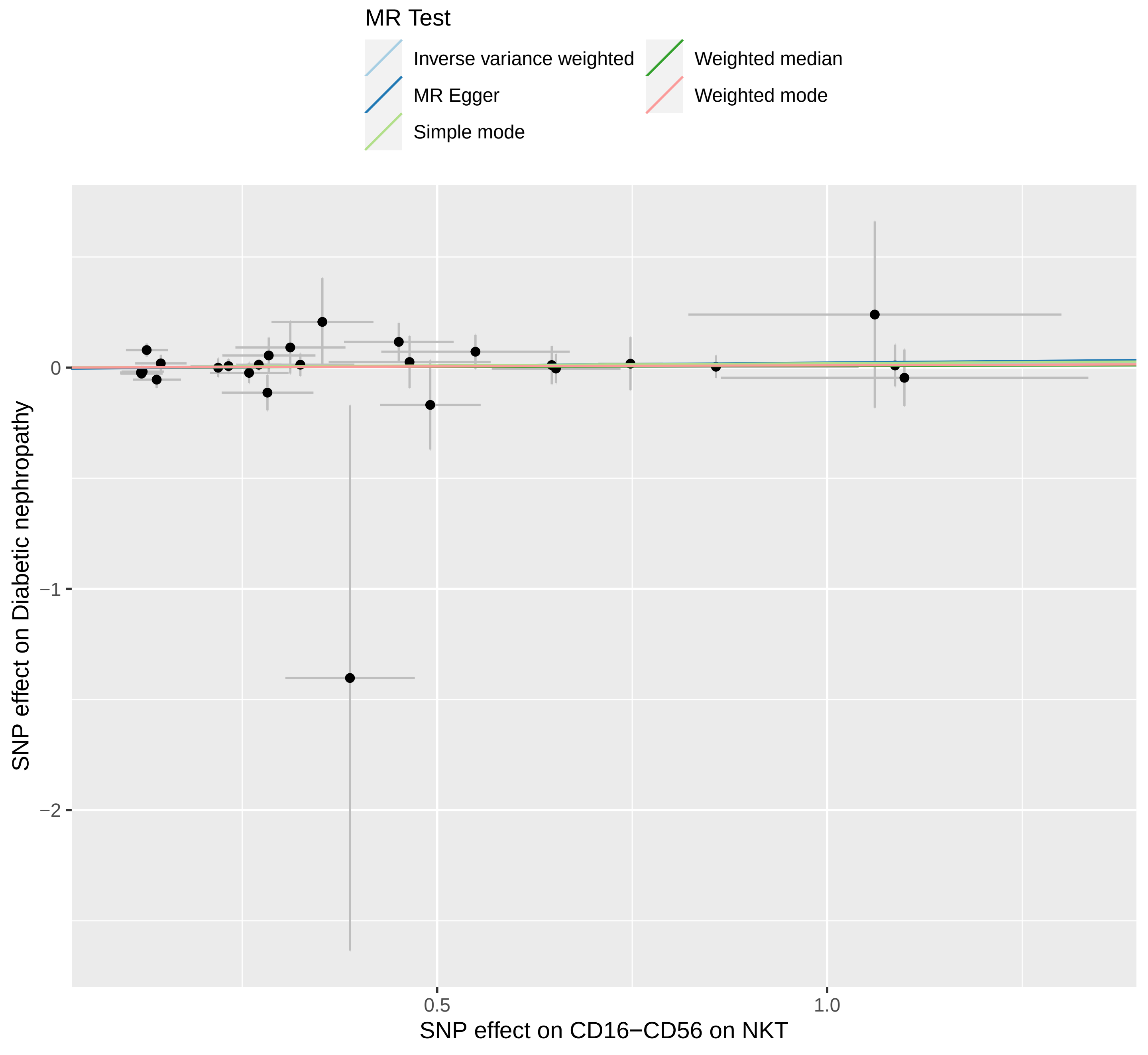
MR Test
Inverse variance weighted
MR Egger
Simple mode
Weighted median
Weighted mode
0
−1
−2
0.5
1.0
SNP effect on Diabetic nephropathy
SNP effect on CD16−CD56 on NKT

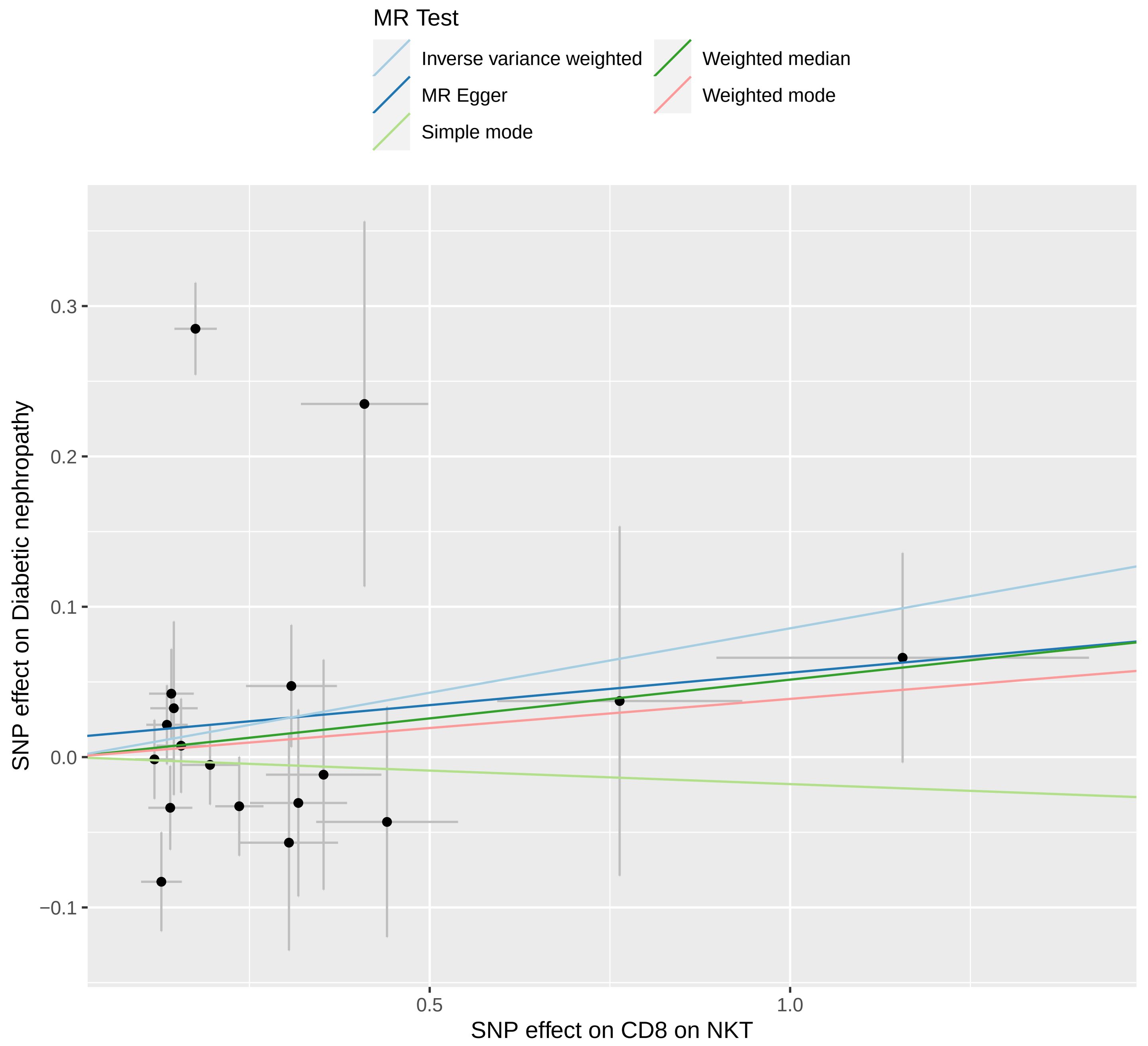
MR Test
Inverse variance weighted
MR Egger
Simple mode
Weighted median
Weighted mode
SNP effect on Diabetic nephropathy
0.3
0.2
0.1
0.0
−0.1
0.5
1.0
SNP effect on CD8 on NKT

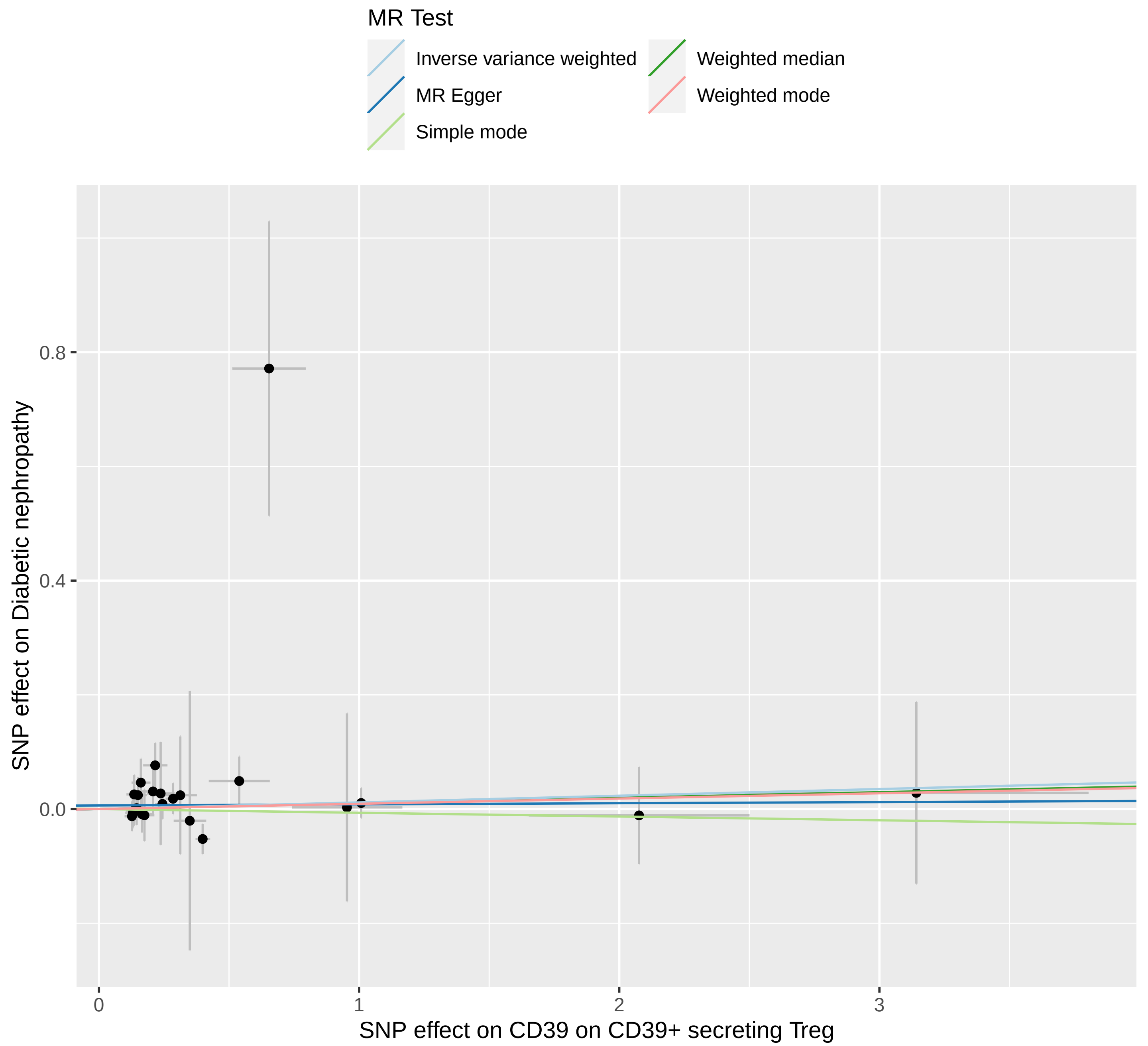
MR Test
Inverse variance weighted
MR Egger
Simple mode
Weighted median
Weighted mode
0.8
0.4
0.0
0
1
2
3
SNP effect on Diabetic nephropathy
SNP effect on CD39 on CD39+ secreting Treg

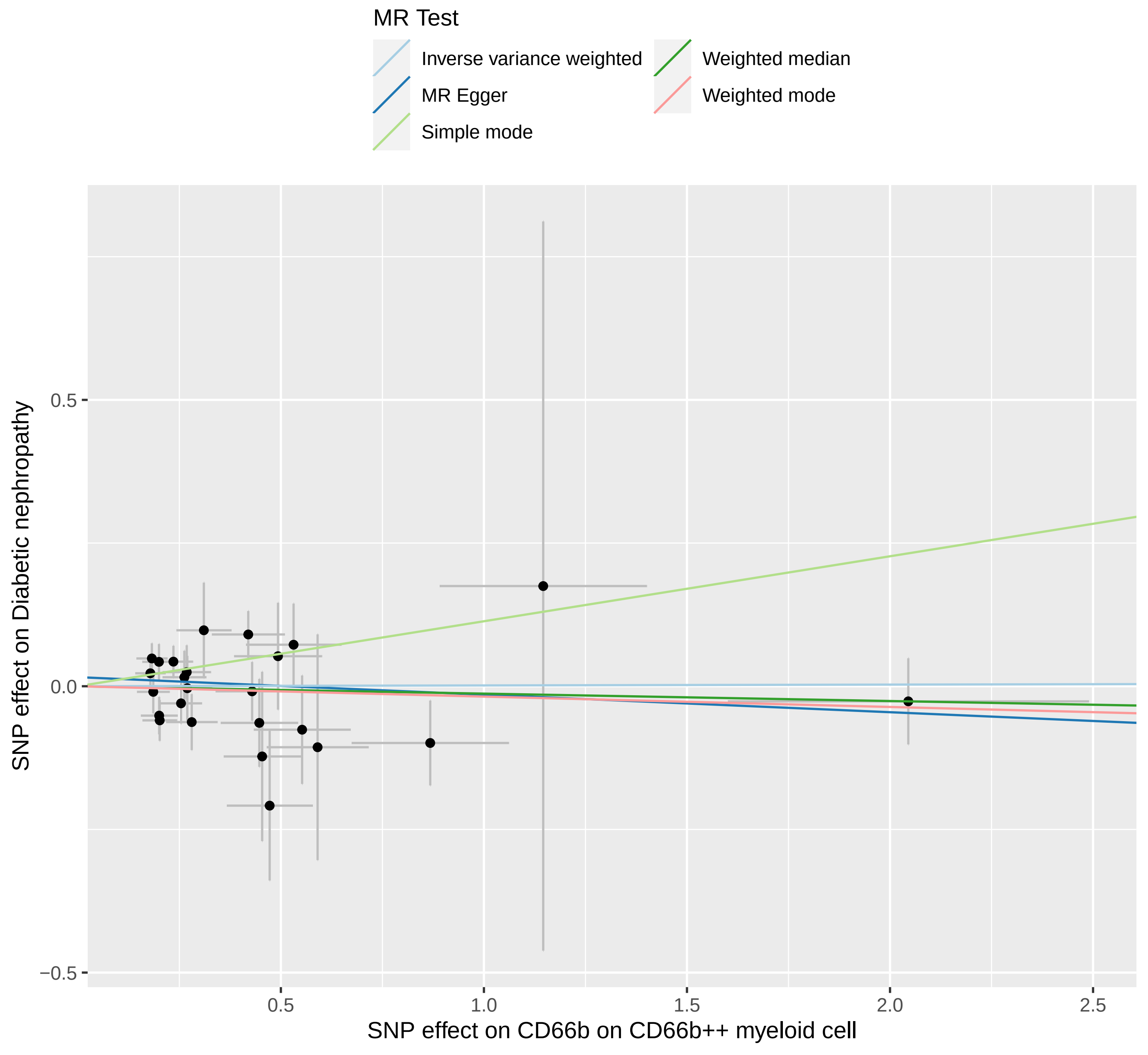

MR Test
Inverse variance weighted
MR Egger
Simple mode
Weighted median
Weighted mode
SNP effect on Diabetic nephropathy
0.5
0.0
−0.5
0.5
1.0
1.5
2.0
2.5
SNP effect on CD66b on CD66b++ myeloid cell

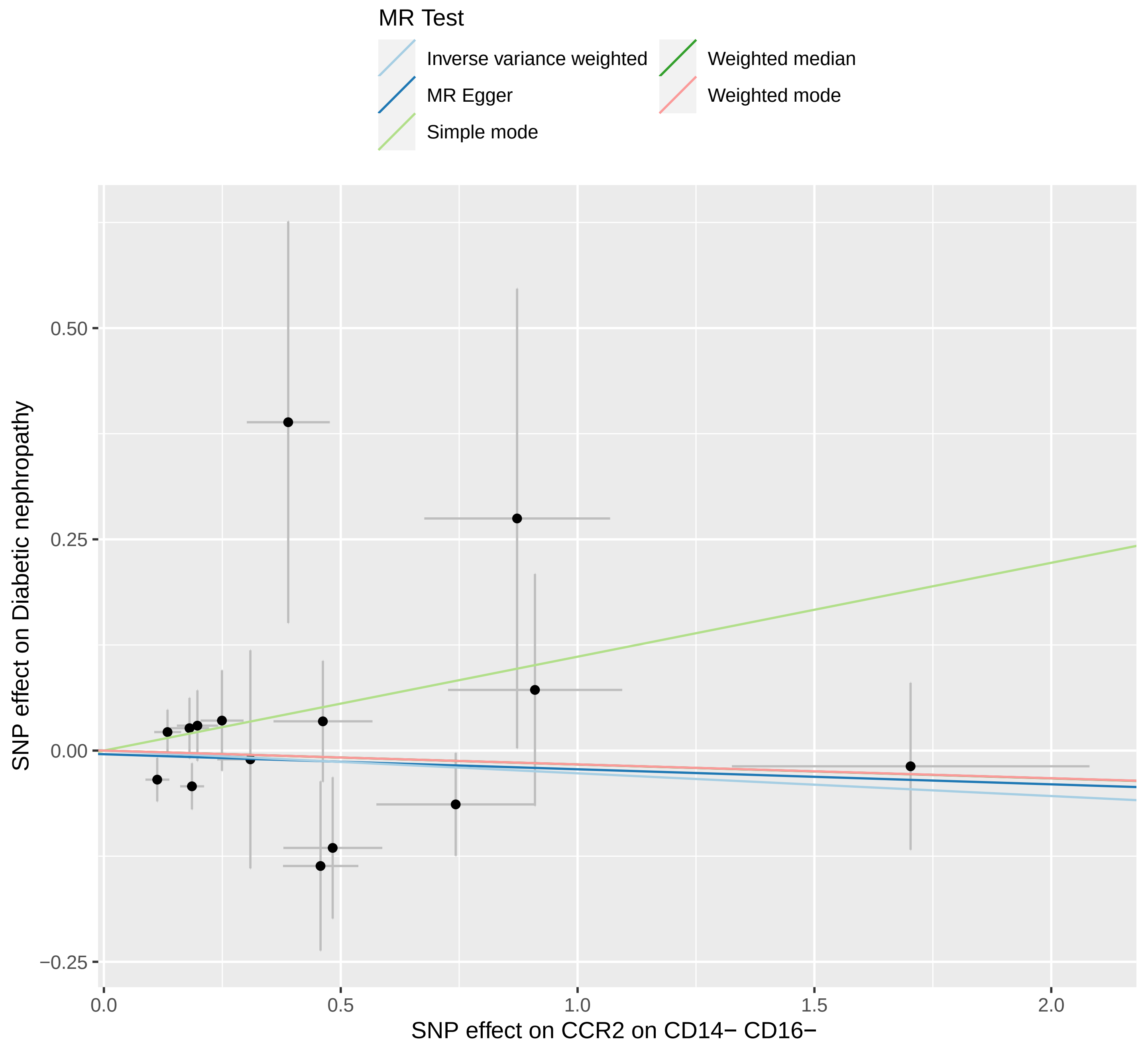
MR Test
Inverse variance weighted
MR Egger
Simple mode
Weighted median
Weighted mode
SNP effect on Diabetic nephropathy
0.50
0.25
0.00
−0.25
0.0
0.5
1.0
1.5
2.0
SNP effect on CCR2 on CD14− CD16−

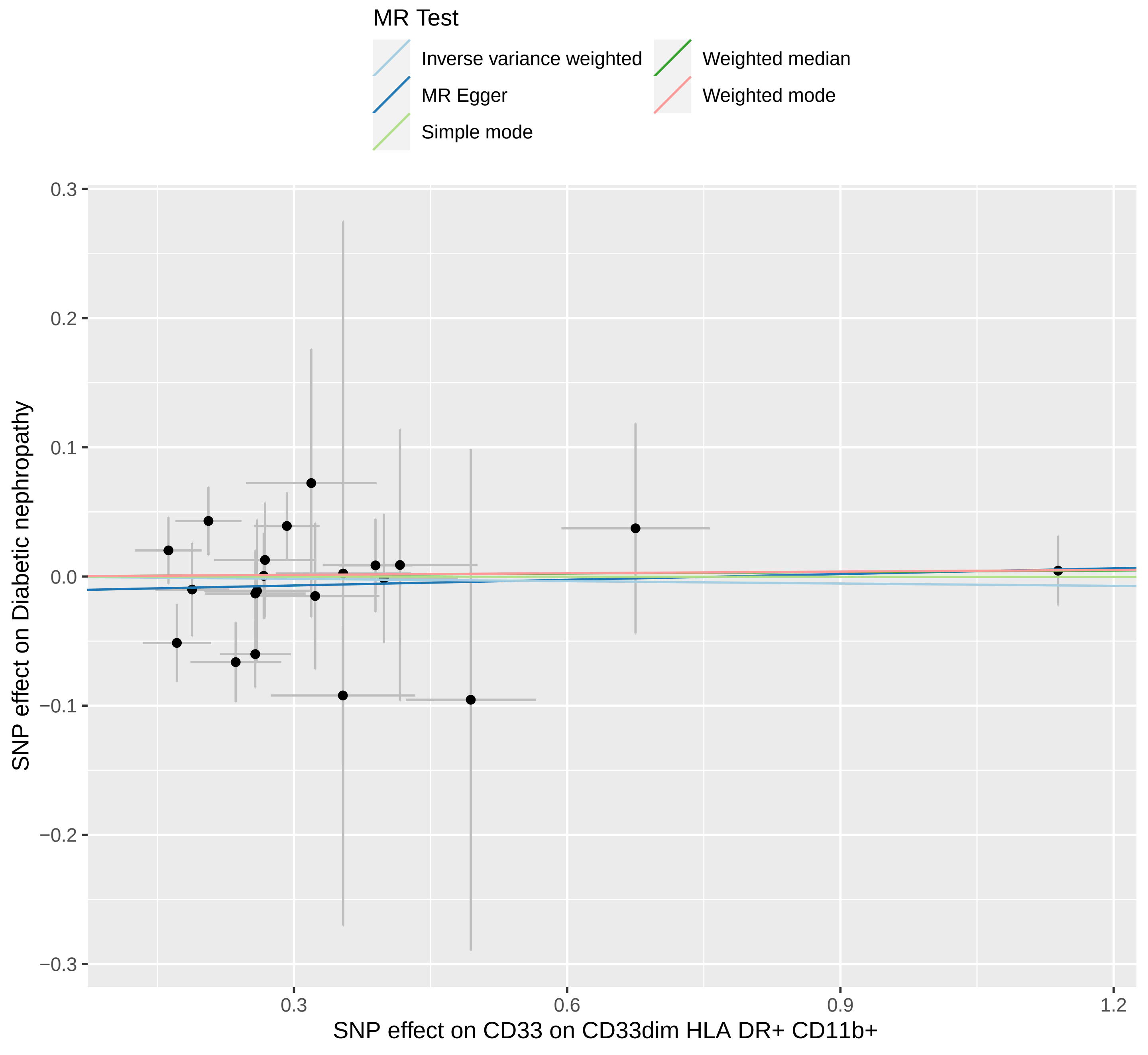

MR Test
Inverse variance weighted
MR Egger
Simple mode
Weighted median
Weighted mode
0.3
0.2
0.1
0.0
−0.1
−0.2
−0.3
0.3
0.6
0.9
1.2
SNP effect on Diabetic nephropathy
SNP effect on CD33 on CD33dim HLA DR+ CD11b+

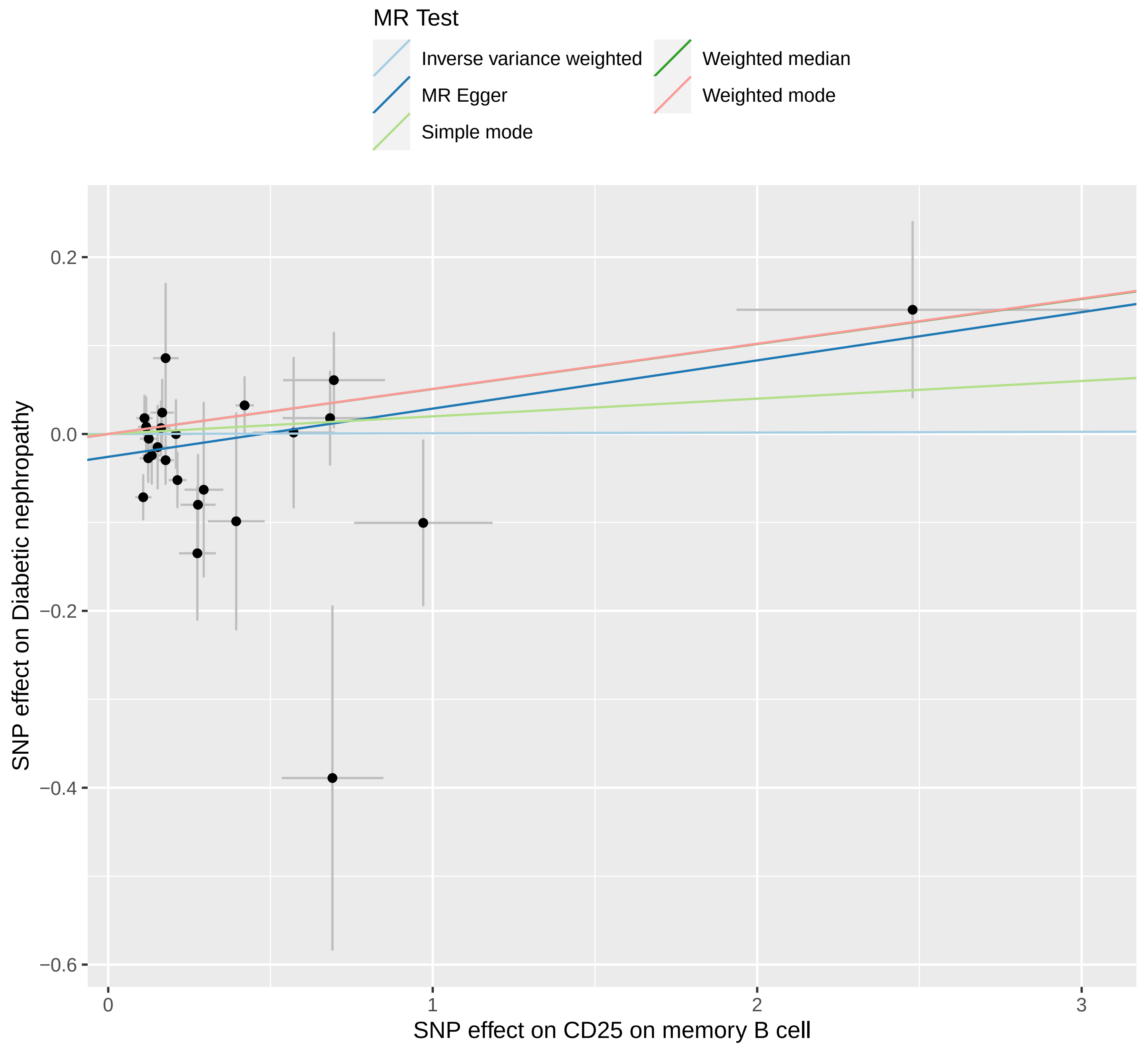

MR Test
Inverse variance weighted
MR Egger
Simple mode
Weighted median
Weighted mode
0.2
0.0
−0.2
−0.4
−0.6
0
1
2
3
SNP effect on Diabetic nephropathy
SNP effect on CD25 on memory B cell

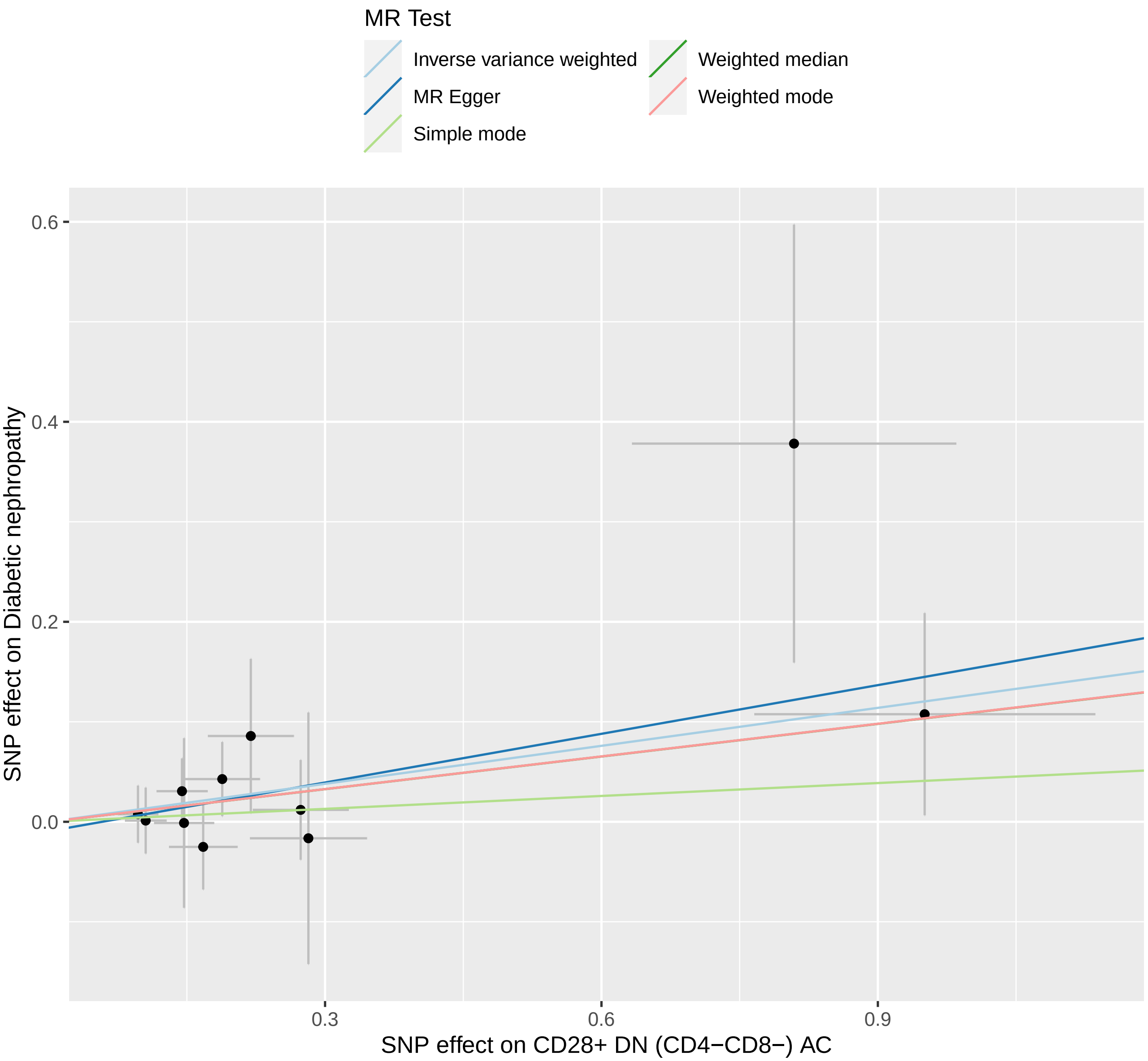
MR Test
Inverse variance weighted
MR Egger
Simple mode
Weighted median
Weighted mode
0.6
0.4
0.2
0.0
0.3
0.6
0.9
SNP effect on Diabetic nephropathy
SNP effect on CD28+ DN (CD4−CD8−) AC

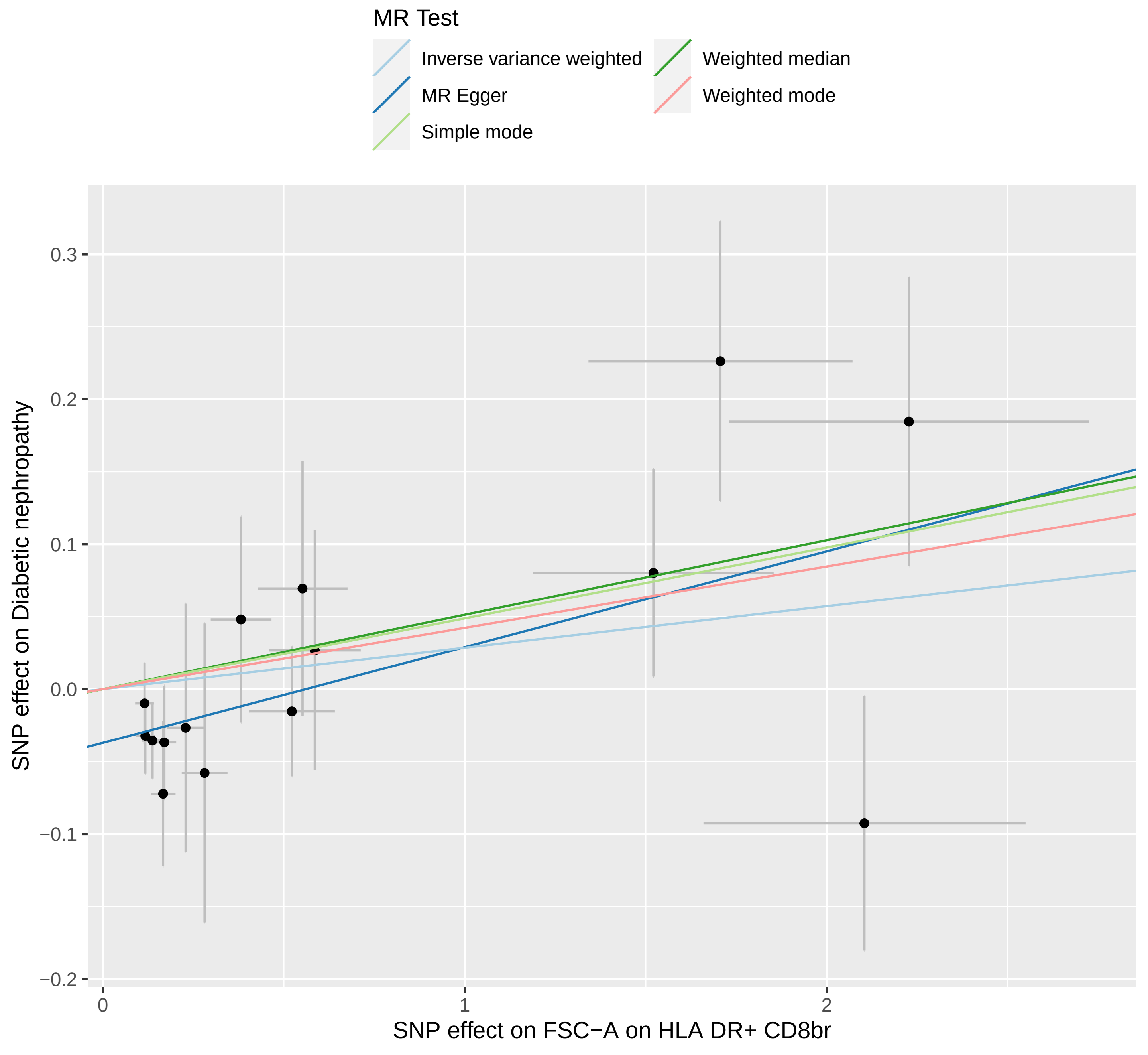
MR Test
Inverse variance weighted
MR Egger
Simple mode
Weighted median
Weighted mode
SNP effect on Diabetic nephropathy
SNP effect on FSC−A on HLA DR+ CD8br
0.3
0.2
0.1
0.0
−0.1
−0.2
0
1
2

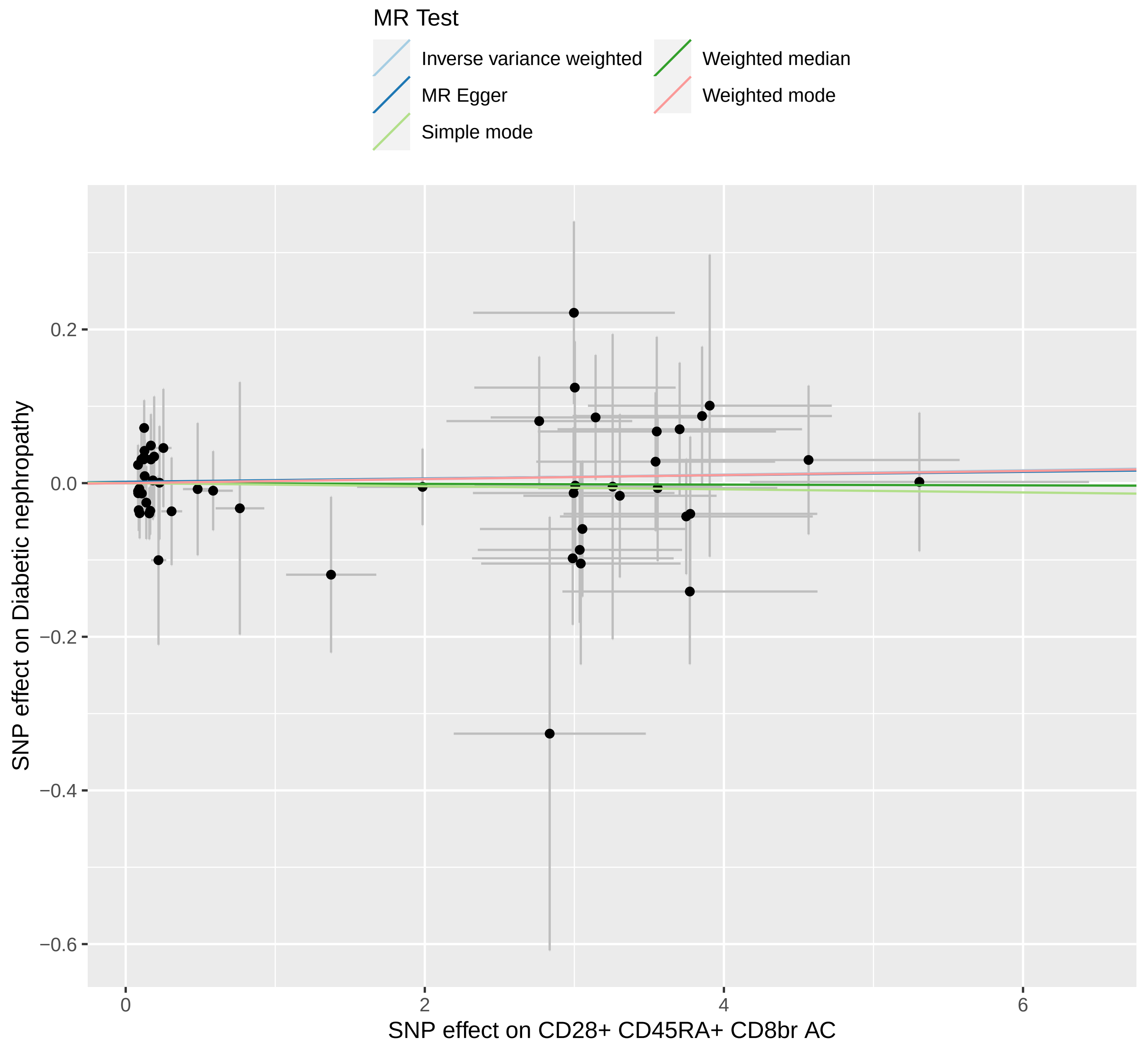

MR Test
Inverse variance weighted
MR Egger
Simple mode
Weighted median
Weighted mode
SNP effect on Diabetic nephropathy
0.2
0.0
−0.2
−0.4
−0.6
0
2
4
6
SNP effect on CD28+ CD45RA+ CD8br AC

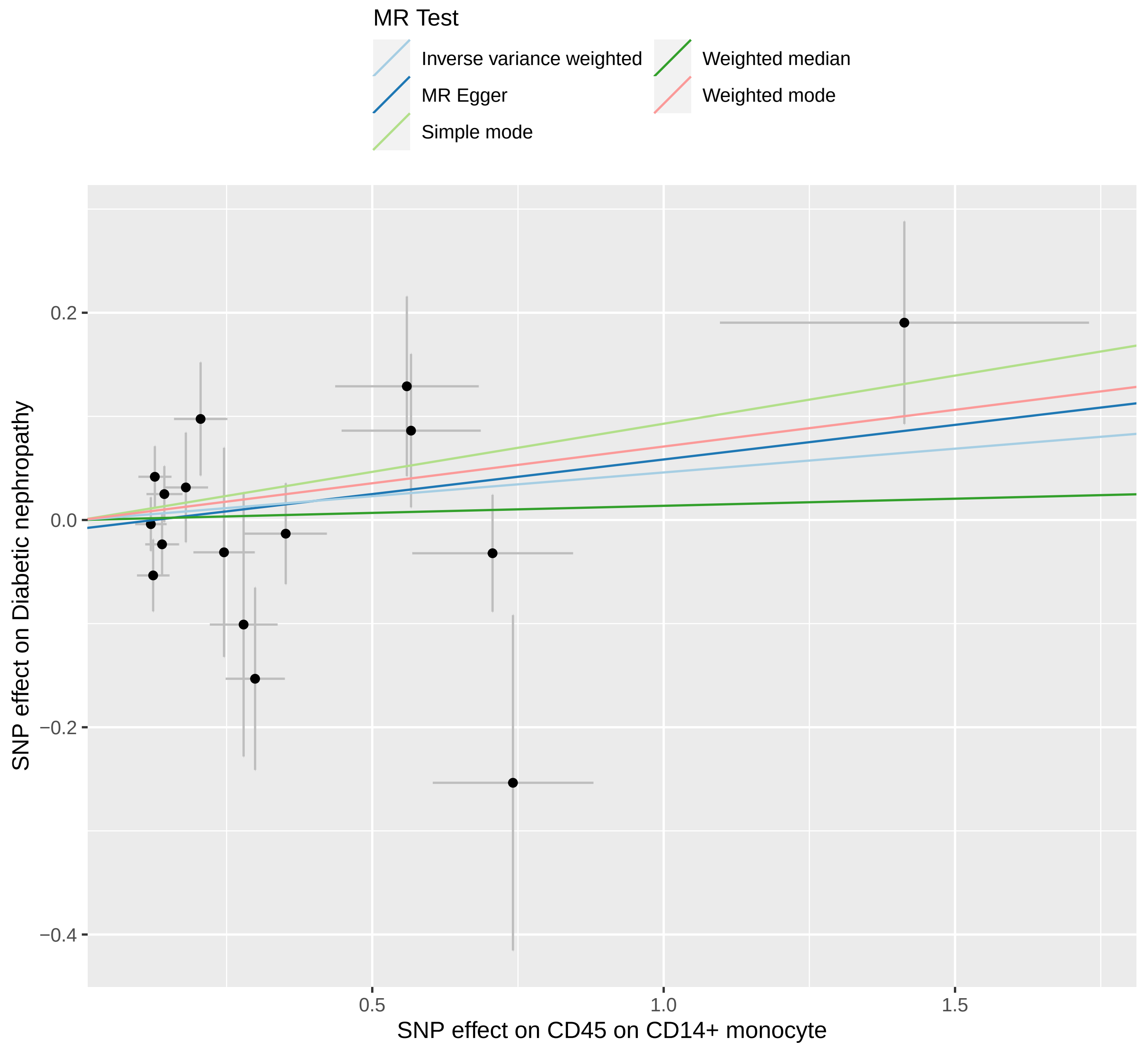

MR Test
Inverse variance weighted
MR Egger
Simple mode
Weighted median
Weighted mode
0.2
0.0
−0.2
−0.4
0.5
1.0
1.5
SNP effect on Diabetic nephropathy
SNP effect on CD45 on CD14+ monocyte

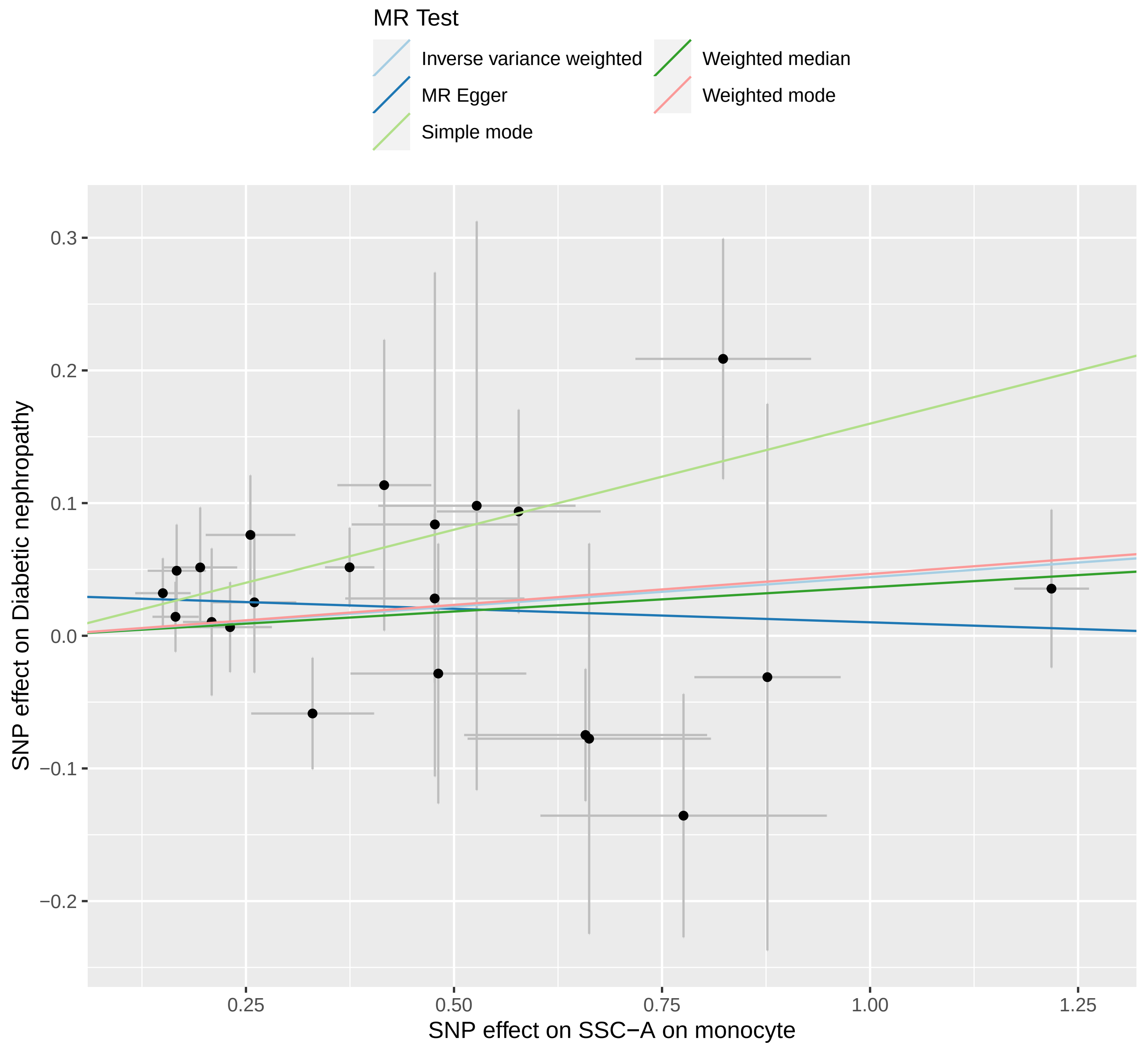

MR Test
Inverse variance weighted
MR Egger
Simple mode
Weighted median
Weighted mode
0.3
0.2
0.1
0.0
−0.1
−0.2
0.25
0.50
0.75
1.00
1.25
SNP effect on Diabetic nephropathy
SNP effect on SSC−A on monocyte

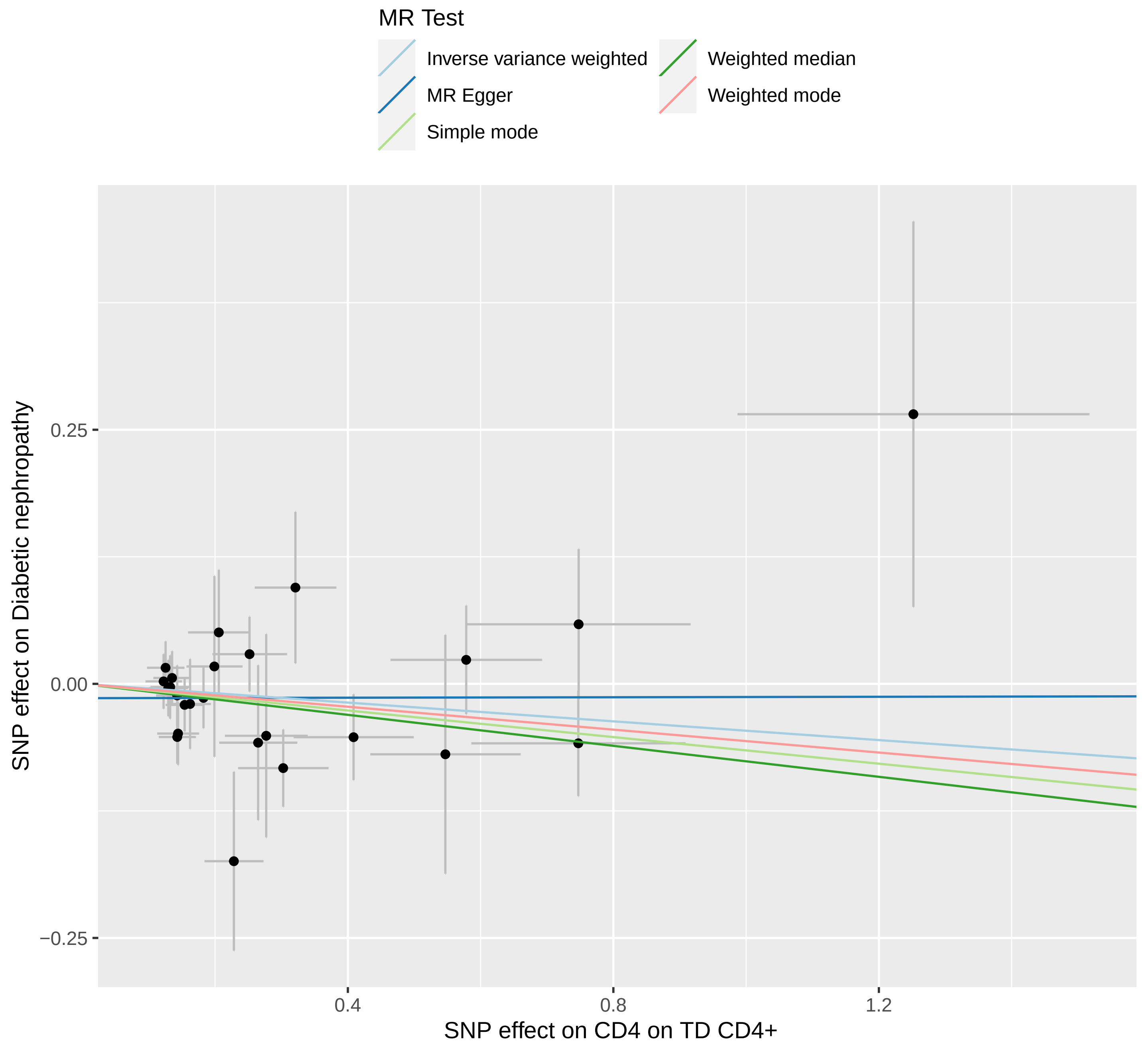

MR Test
Inverse variance weighted
MR Egger
Simple mode
Weighted median
Weighted mode
SNP effect on Diabetic nephropathy
0.25
0.00
−0.25
0.4
0.8
1.2
SNP effect on CD4 on TD CD4+

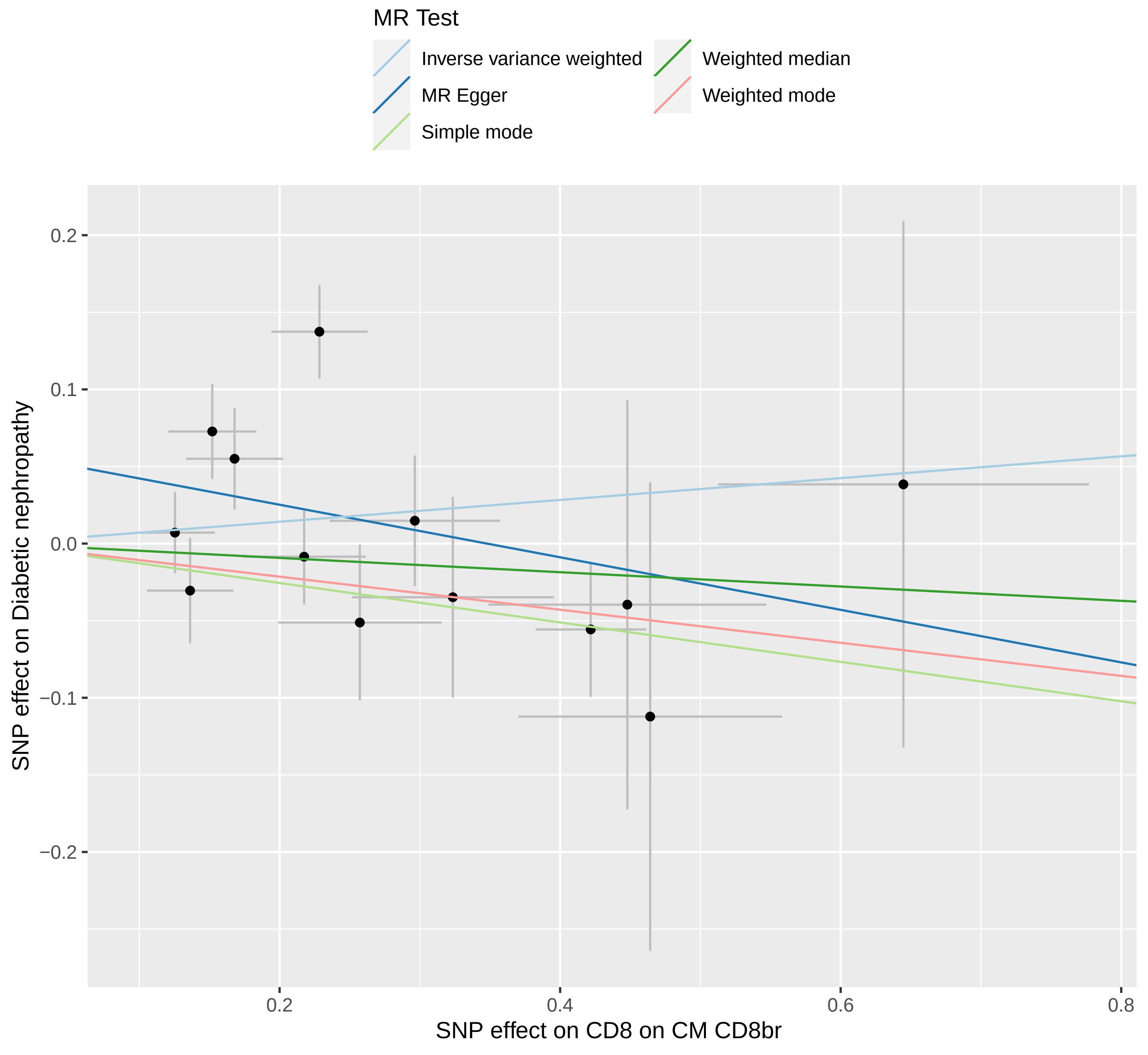

MR Test
Inverse variance weighted
MR Egger
Simple mode
Weighted median
Weighted mode
0.2
0.1
0.0
−0.1
−0.2
SNP effect on Diabetic nephropathy
0.2
0.4
0.6
0.8
SNP effect on CD8 on CM CD8br

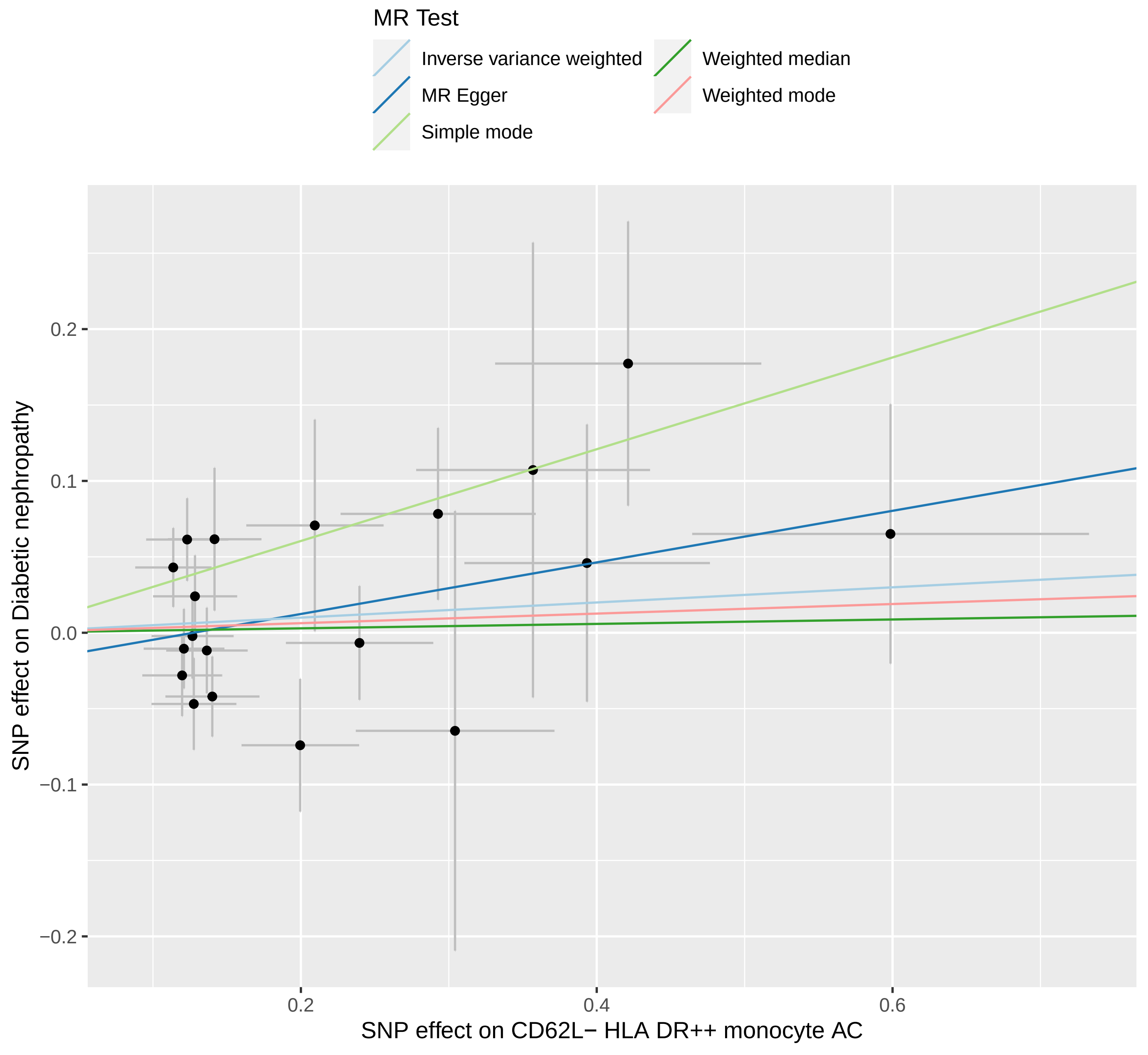

MR Test
Inverse variance weighted
MR Egger
Simple mode
Weighted median
Weighted mode
0.2
0.1
0.0
−0.1
−0.2
0.2
0.4
0.6
SNP effect on Diabetic nephropathy
SNP effect on CD62L− HLA DR++ monocyte AC

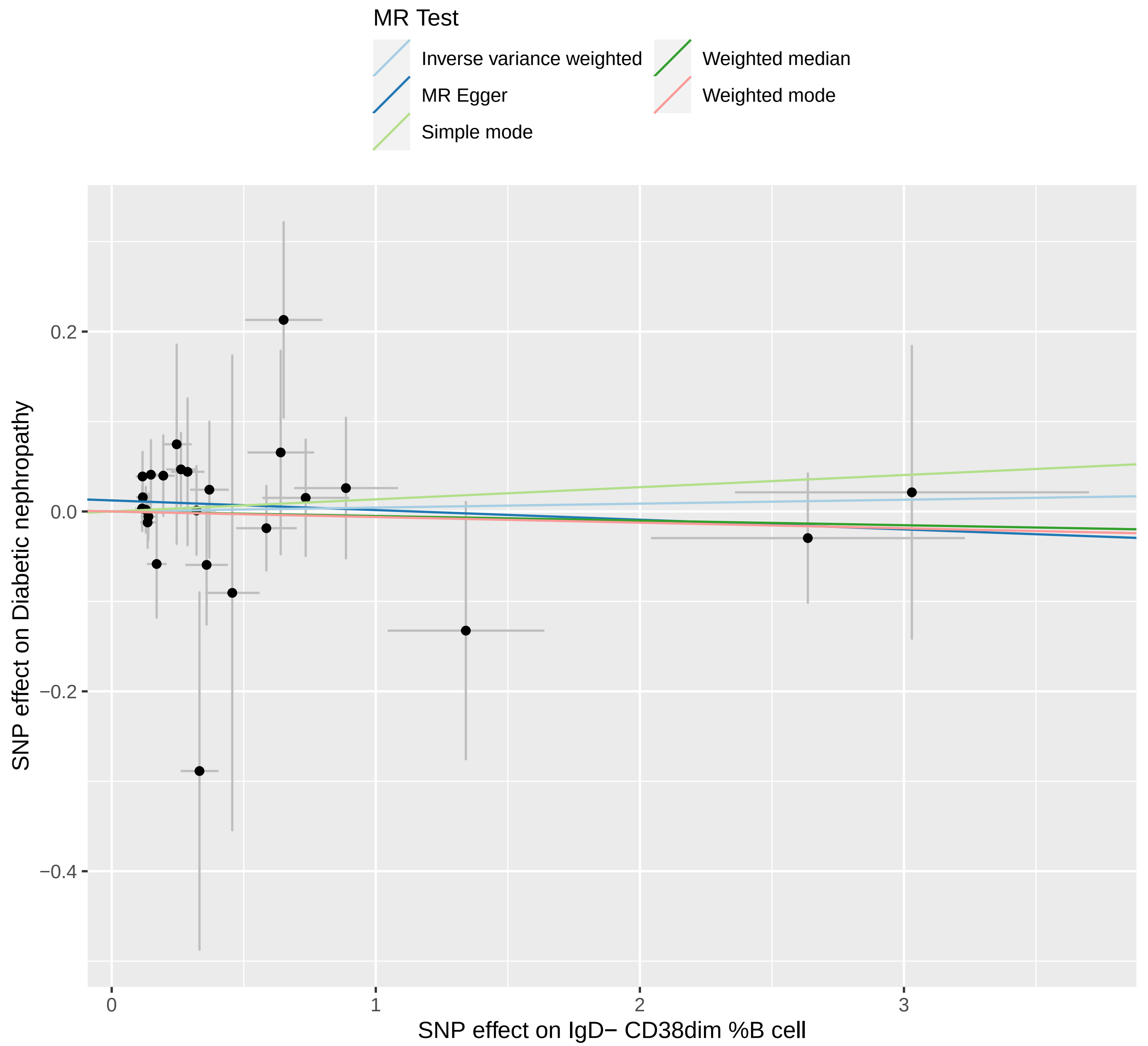

MR Test
Inverse variance weighted
MR Egger
Simple mode
Weighted median
Weighted mode
0.2
0.0
−0.2
−0.4
SNP effect on Diabetic nephropathy
0
1
2
3
SNP effect on IgD− CD38dim %B cell

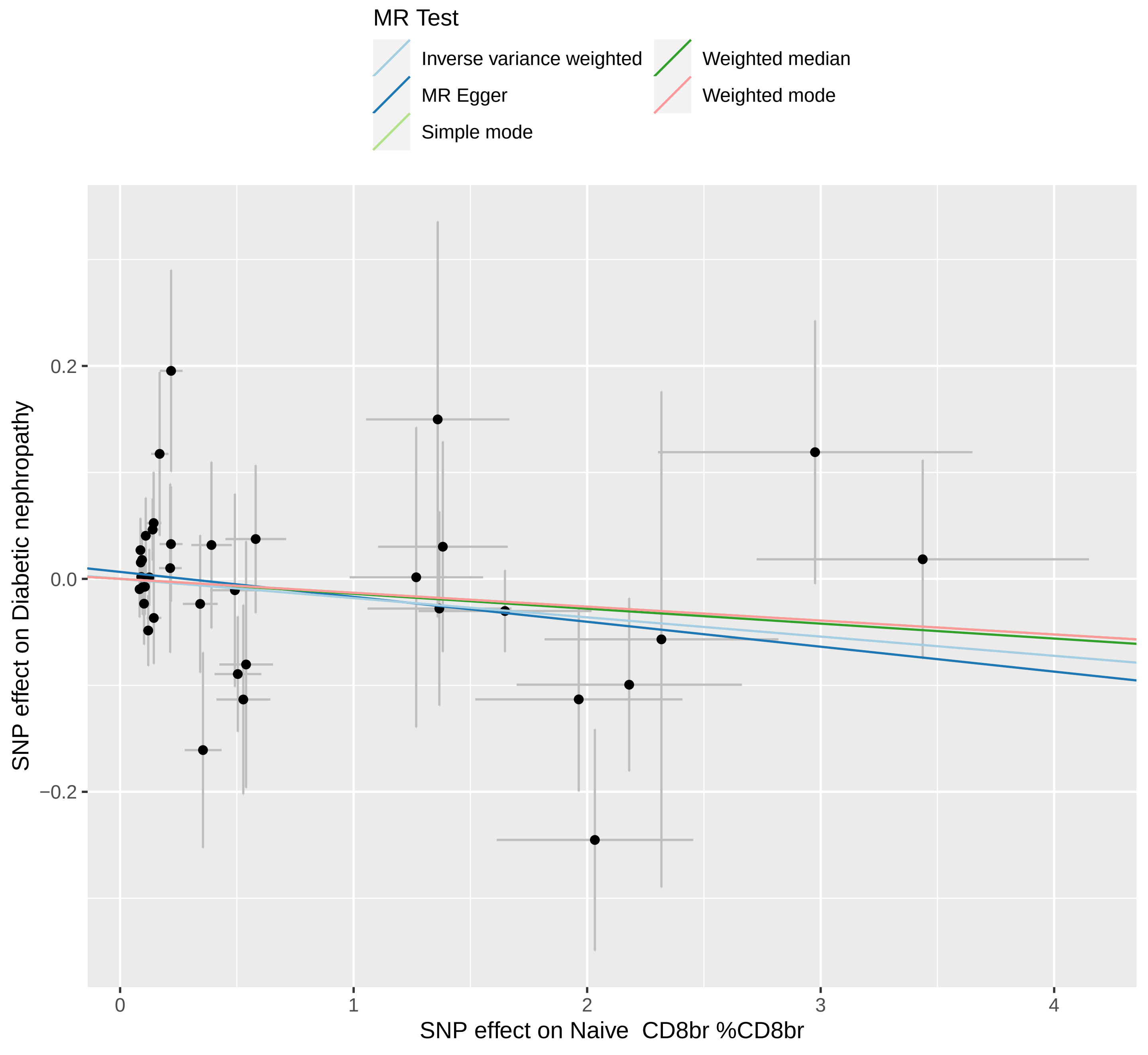
MR Test
Inverse variance weighted
MR Egger
Simple mode
Weighted median
Weighted mode
0.2
0.0
−0.2
0
1
2
3
4
SNP effect on Diabetic nephropathy
SNP effect on Naive CD8br %CD8br

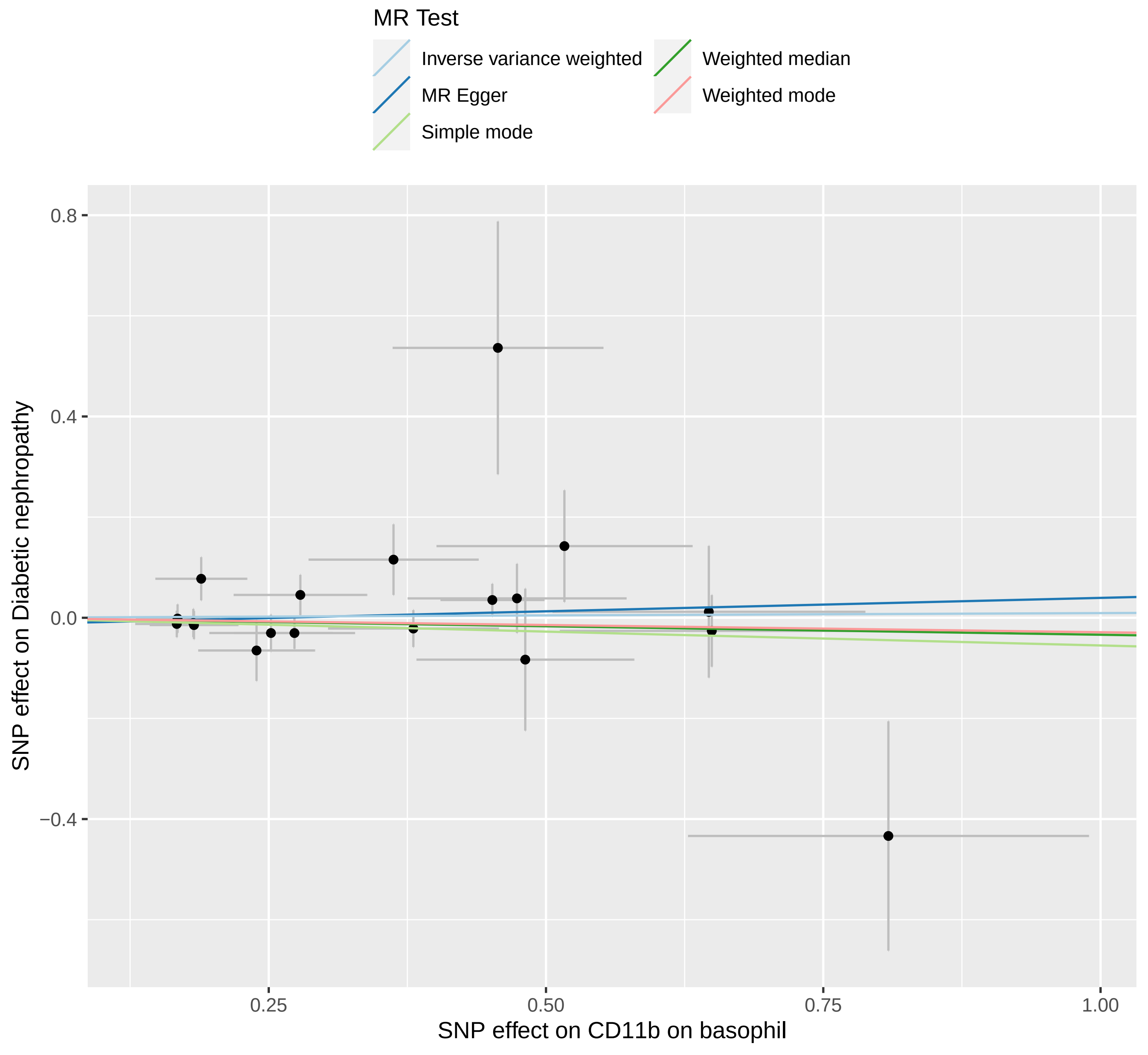
MR Test
Inverse variance weighted
MR Egger
Simple mode
Weighted median
Weighted mode
0.8
0.4
0.0
−0.4
0.25
0.50
0.75
1.00
SNP effect on CD11b on basophil
SNP effect on Diabetic nephropathy

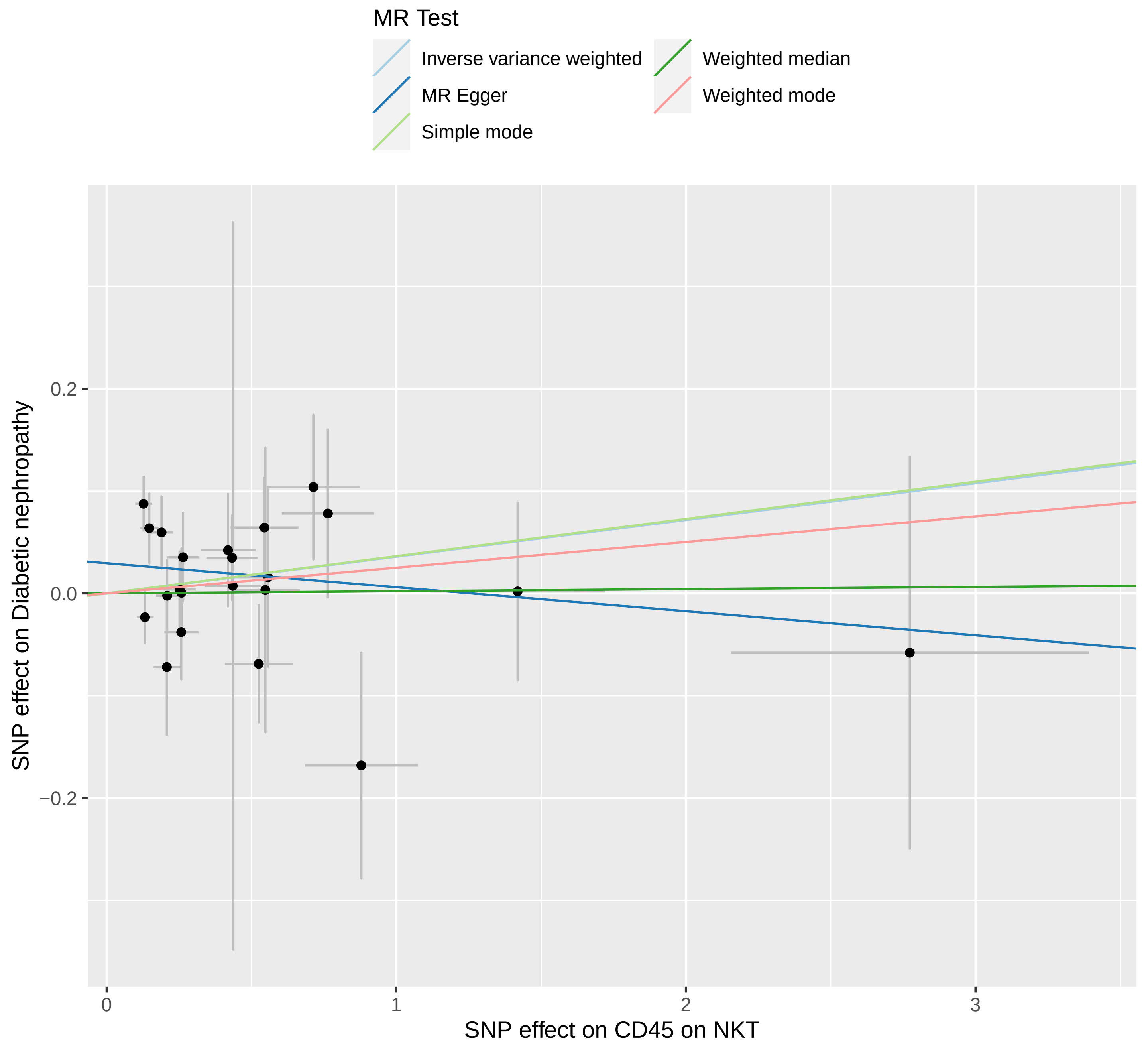

MR Test
Inverse variance weighted
MR Egger
Simple mode
Weighted median
Weighted mode
0.2
0.0
−0.2
0
1
2
3
SNP effect on Diabetic nephropathy
SNP effect on CD45 on NKT

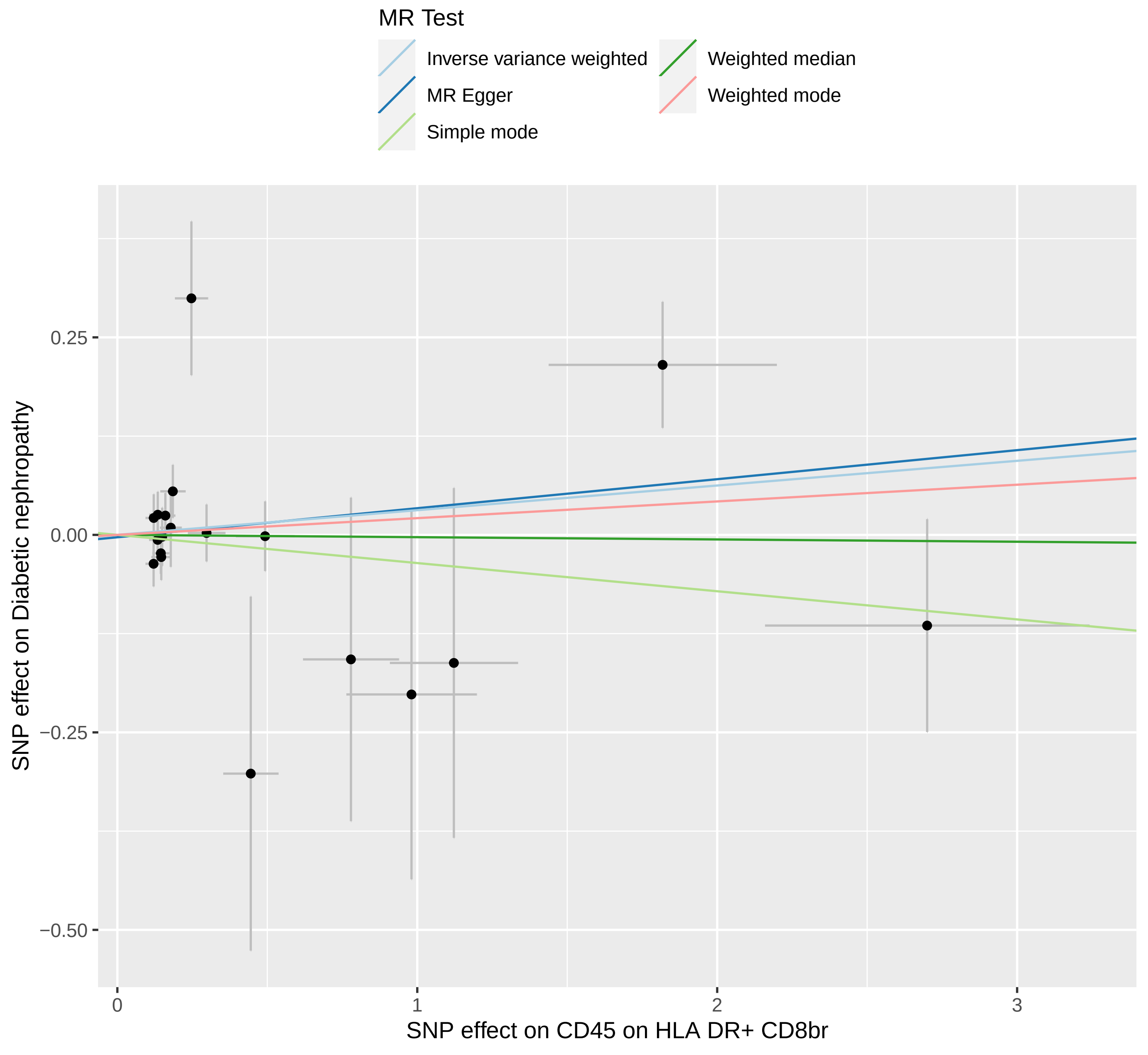

MR Test
Inverse variance weighted
MR Egger
Simple mode
Weighted median
Weighted mode
SNP effect on Diabetic nephropathy
0.25
0.00
−0.25
−0.50
0
1
2
3
SNP effect on CD45 on HLA DR+ CD8br

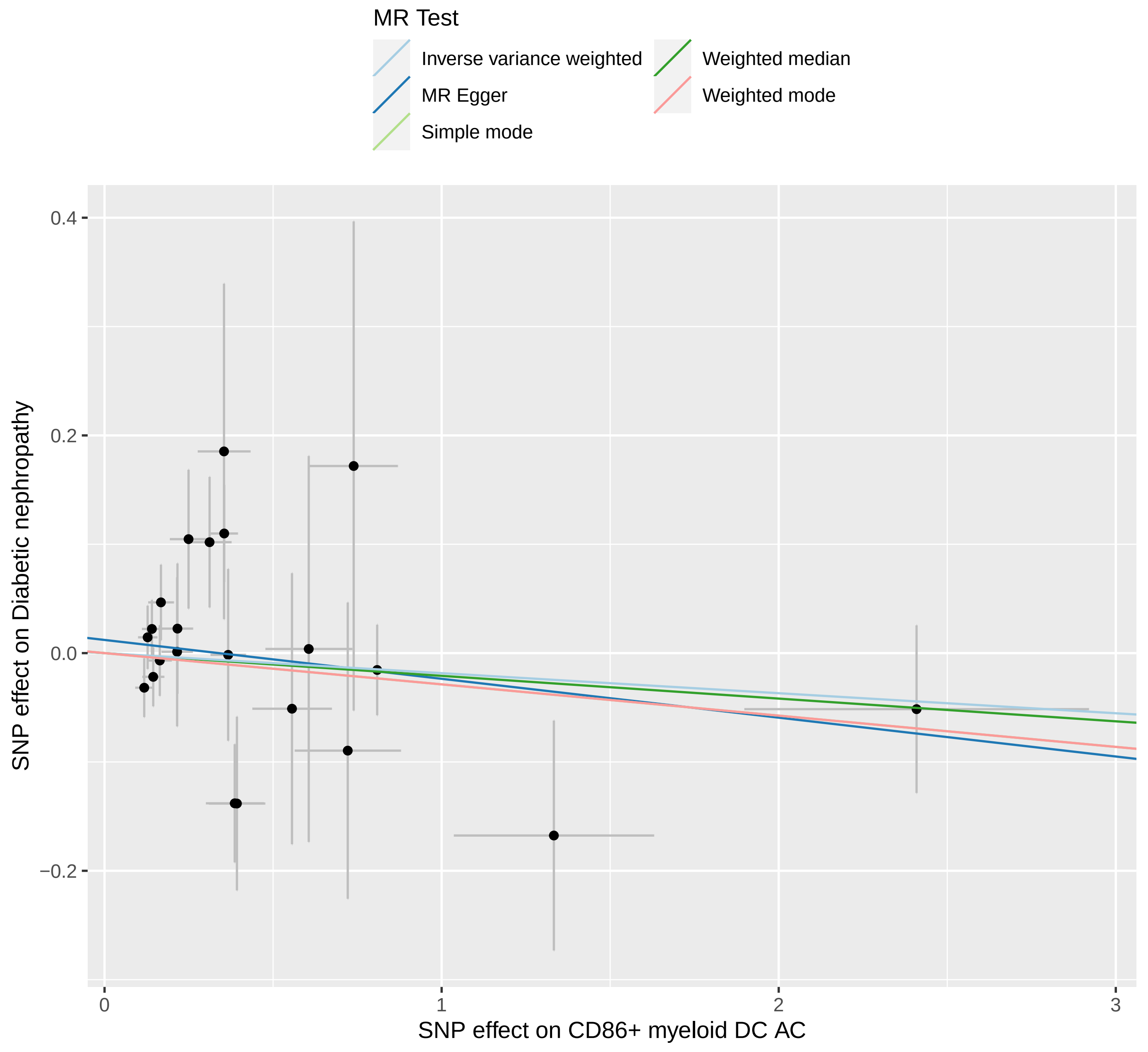

MR Test
Inverse variance weighted
MR Egger
Simple mode
Weighted median
Weighted mode
0.4
0.2
0.0
−0.2
0
1
2
3
SNP effect on Diabetic nephropathy
SNP effect on CD86+ myeloid DC AC

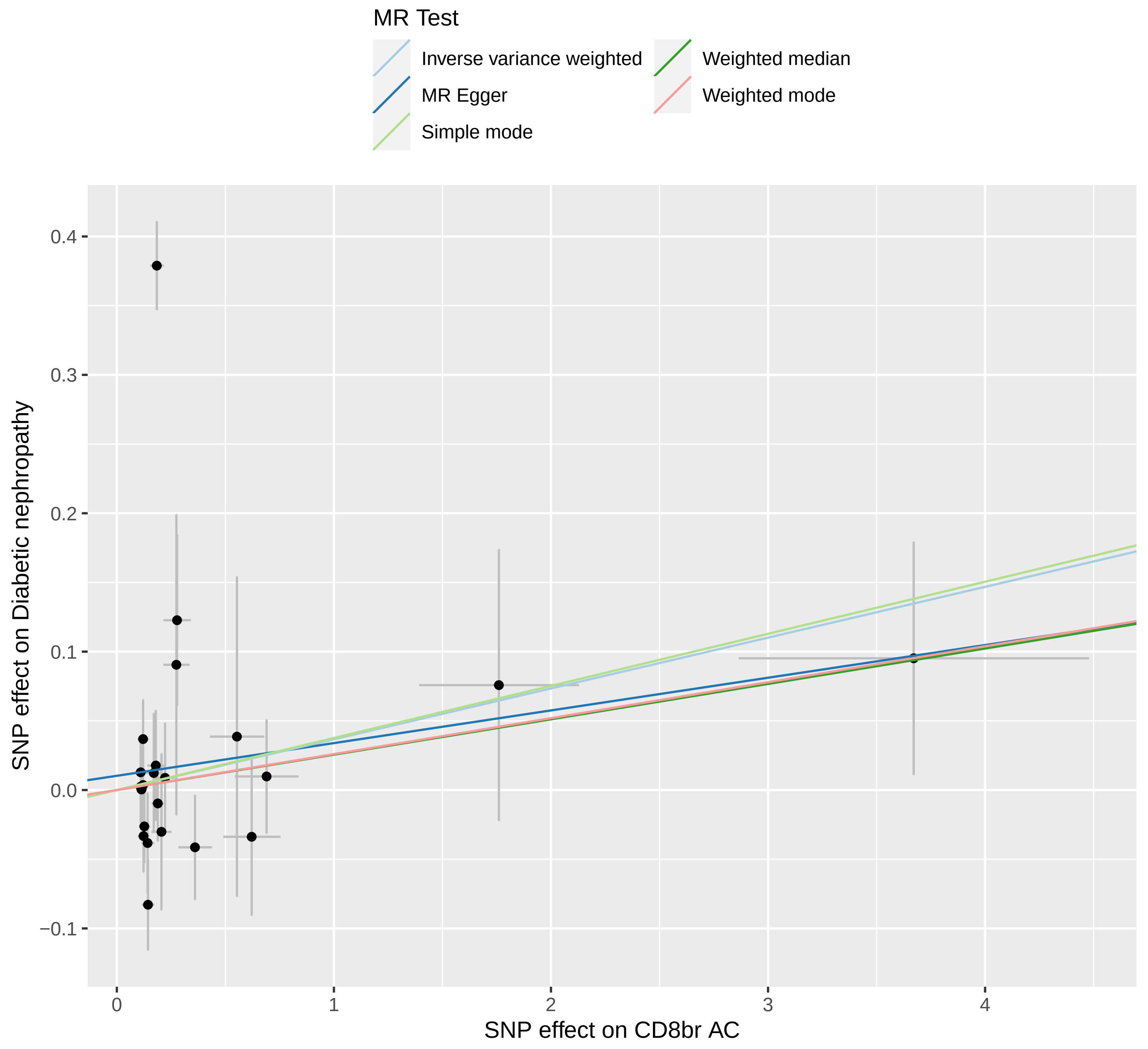

MR Test
Inverse variance weighted
MR Egger
Simple mode
Weighted median
Weighted mode
SNP effect on Diabetic nephropathy
0.4
0.3
0.2
0.1
0.0
−0.1
0
1
2
3
4
SNP effect on CD8br AC

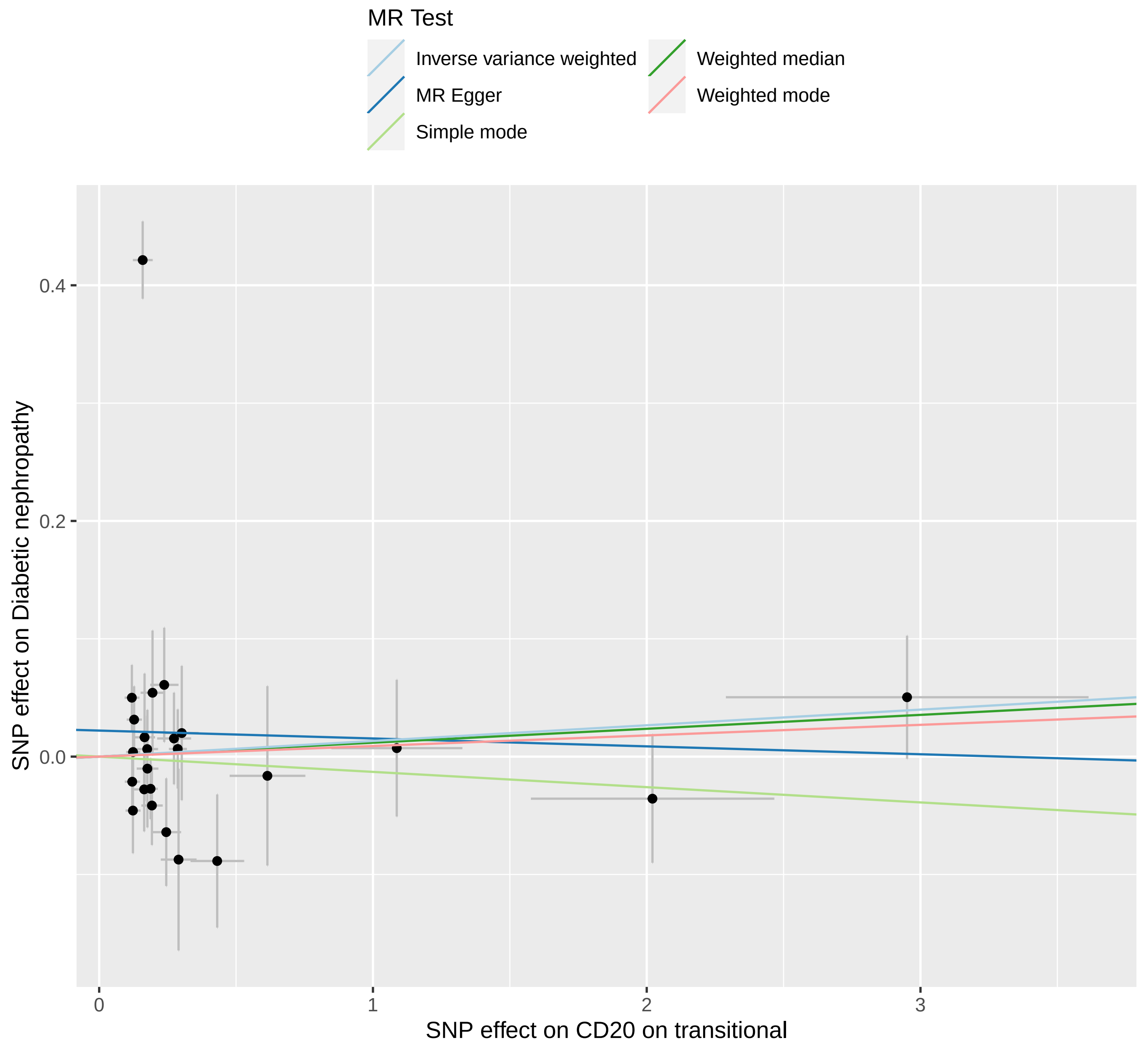
MR Test
Inverse variance weighted
MR Egger
Simple mode
Weighted median
Weighted mode
SNP effect on Diabetic nephropathy
0.4
0.2
0.0
0
1
2
3
SNP effect on CD20 on transitional

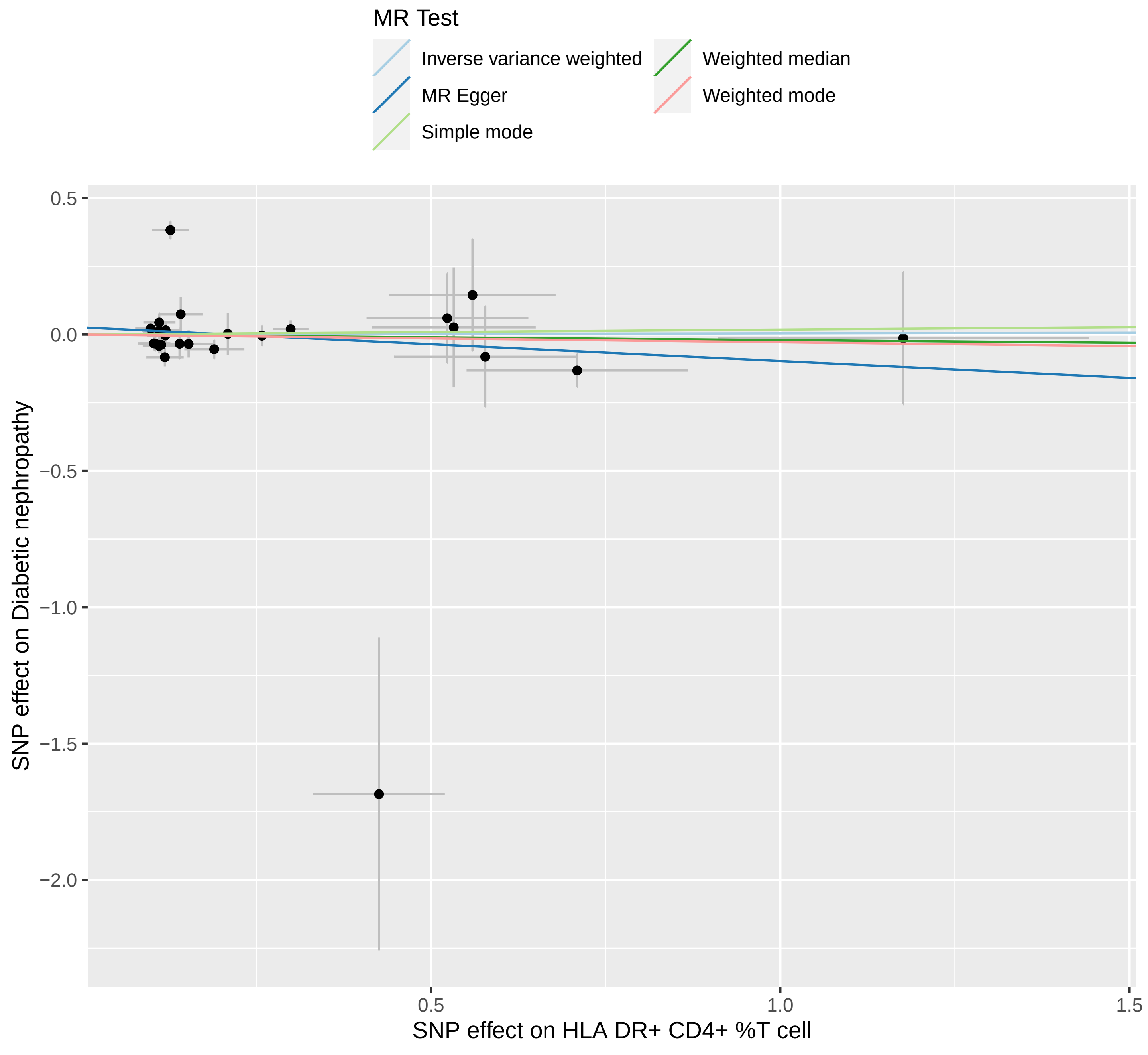
MR Test
Inverse variance weighted
MR Egger
Simple mode
Weighted median
Weighted mode
0.5
0.0
−0.5
−1.0
−1.5
−2.0
SNP effect on Diabetic nephropathy
0.5
1.0
1.5
SNP effect on HLA DR+ CD4+ %T cell

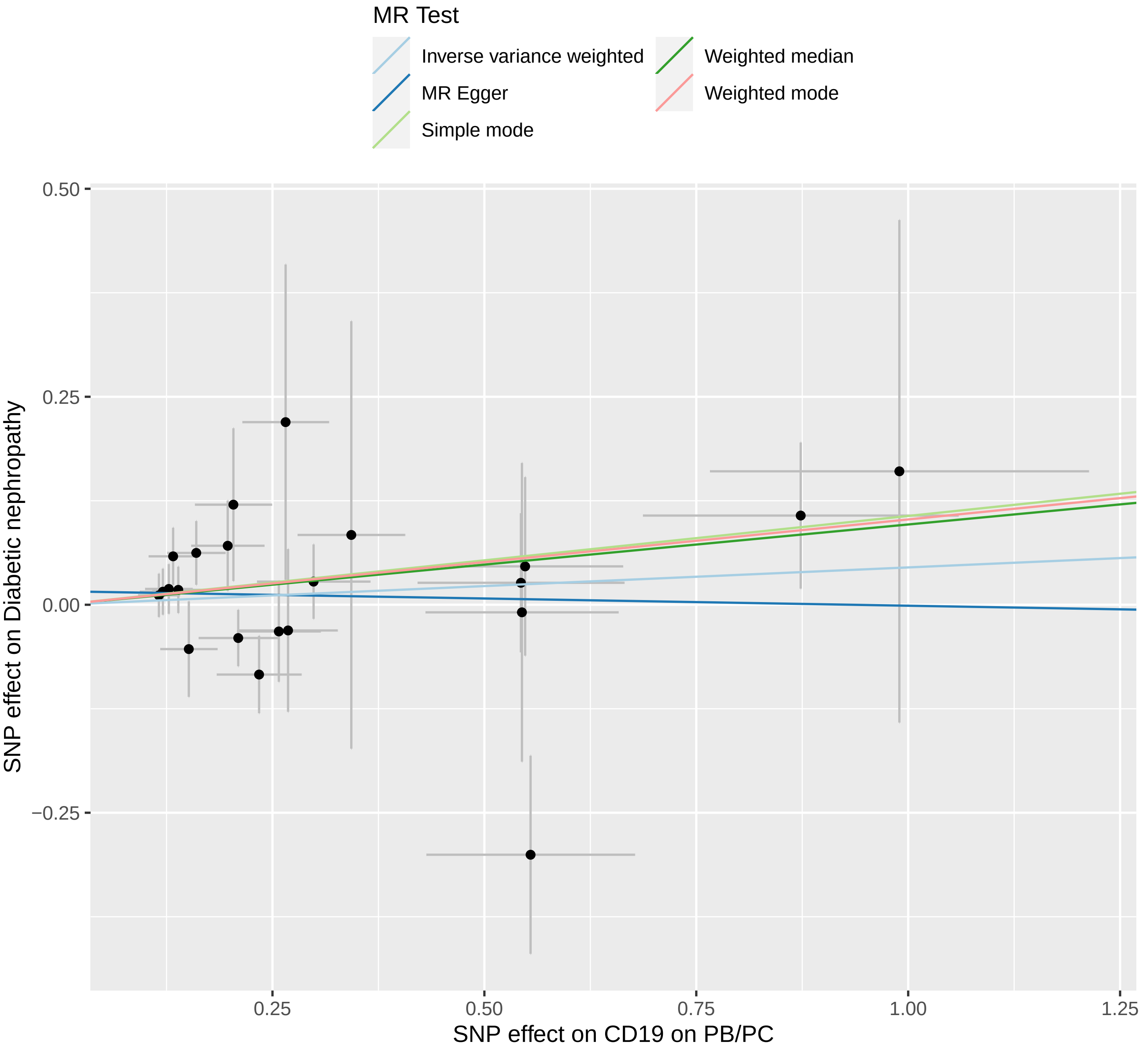

MR Test
Inverse variance weighted
MR Egger
Simple mode
Weighted median
Weighted mode
0.50
0.25
0.00
−0.25
SNP effect on Diabetic nephropathy
0.25
0.50
0.75
1.00
1.25
SNP effect on CD19 on PB/PC

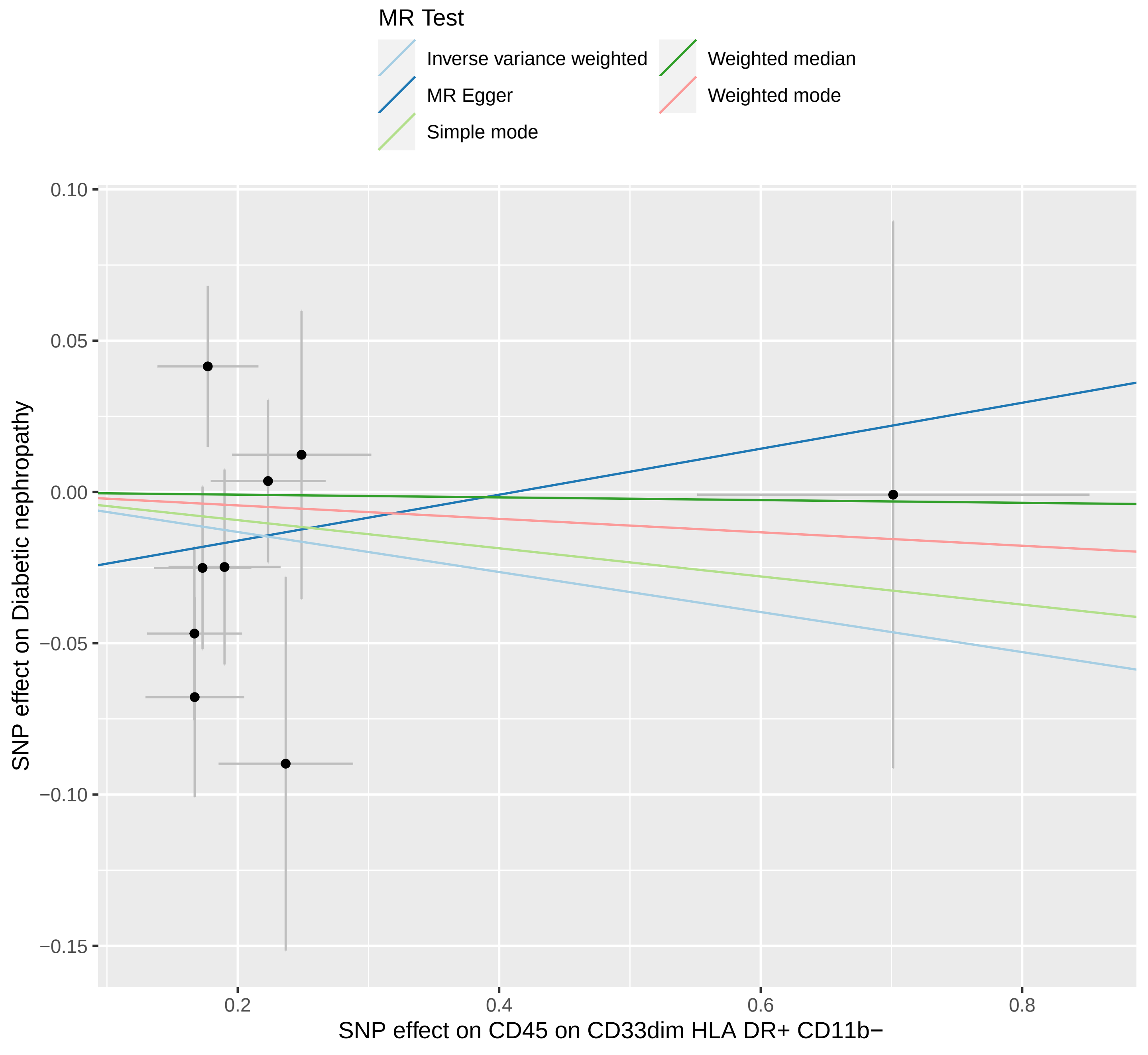
MR Test
Inverse variance weighted
MR Egger
Simple mode
Weighted median
Weighted mode
0.10
0.05
0.00
−0.05
−0.10
−0.15
0.2
0.4
0.6
0.8
SNP effect on Diabetic nephropathy
SNP effect on CD45 on CD33dim HLA DR+ CD11b−

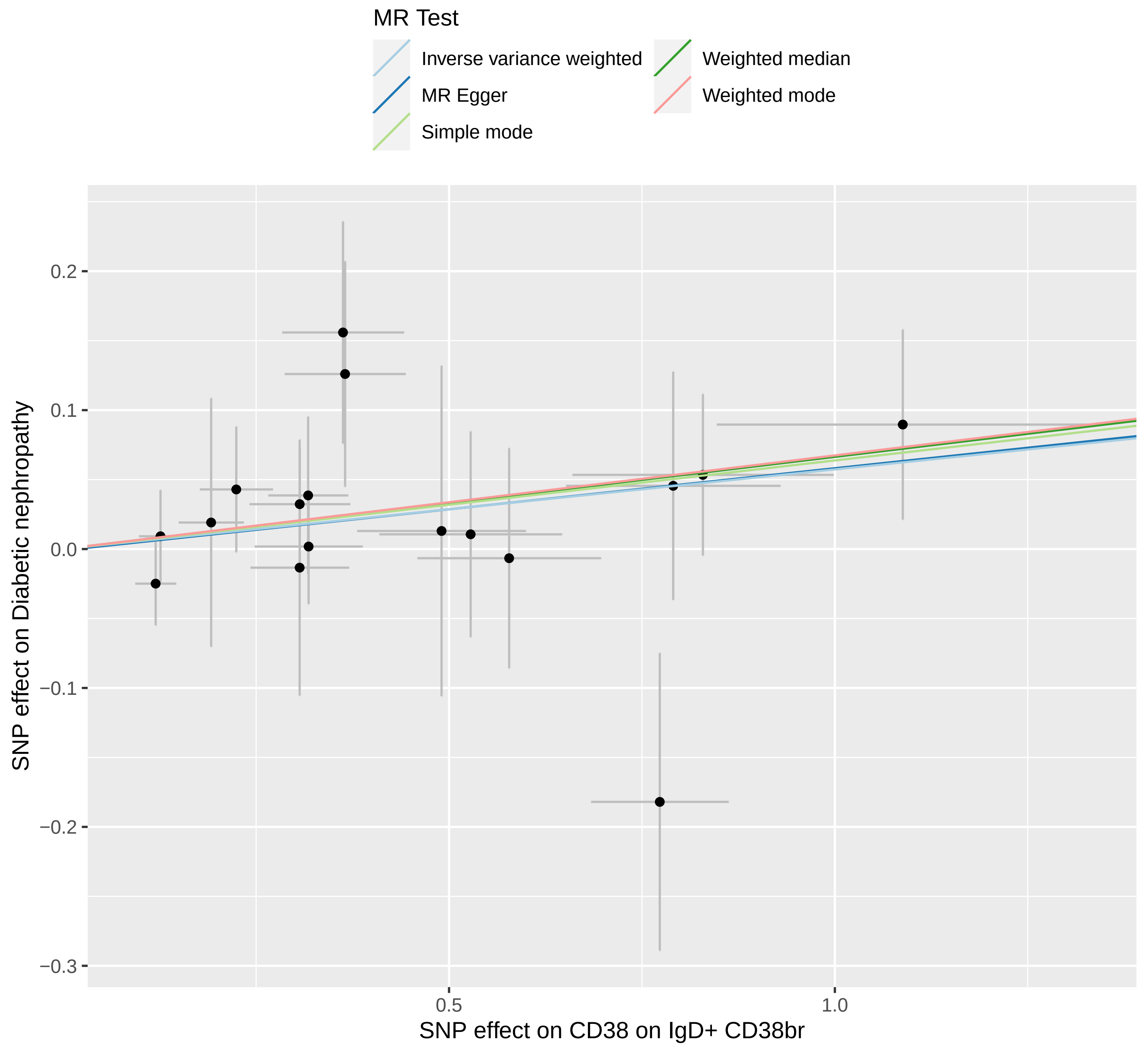

MR Test
Inverse variance weighted
MR Egger
Simple mode
Weighted median
Weighted mode
0.2
0.1
0.0
−0.1
−0.2
−0.3
SNP effect on Diabetic nephropathy
0.5
1.0
SNP effect on CD38 on IgD+ CD38br

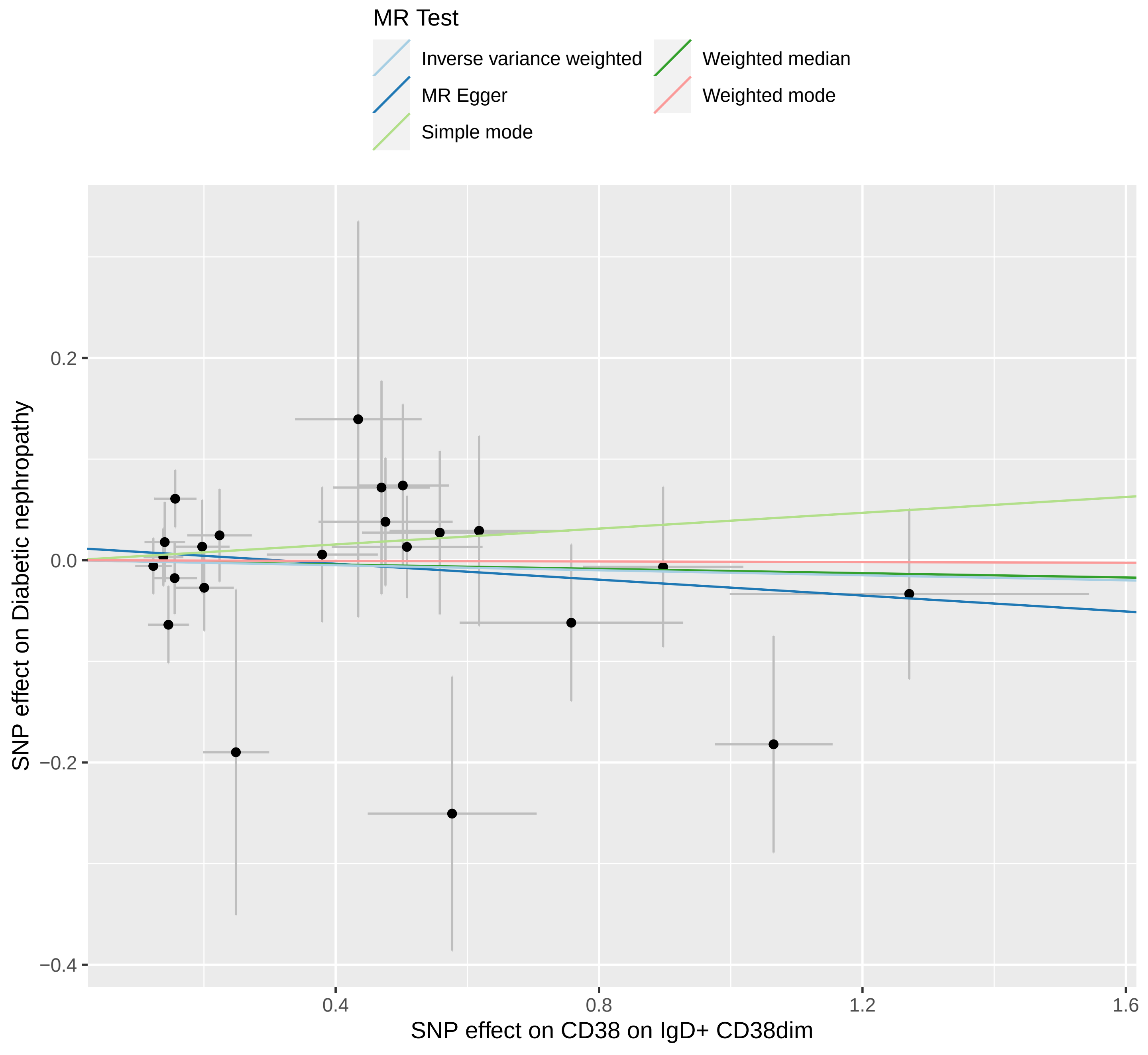
MR Test
Inverse variance weighted
MR Egger
Simple mode
Weighted median
Weighted mode
0.2
0.0
−0.2
−0.4
0.4
0.8
1.2
1.6
SNP effect on Diabetic nephropathy
SNP effect on CD38 on IgD+ CD38dim

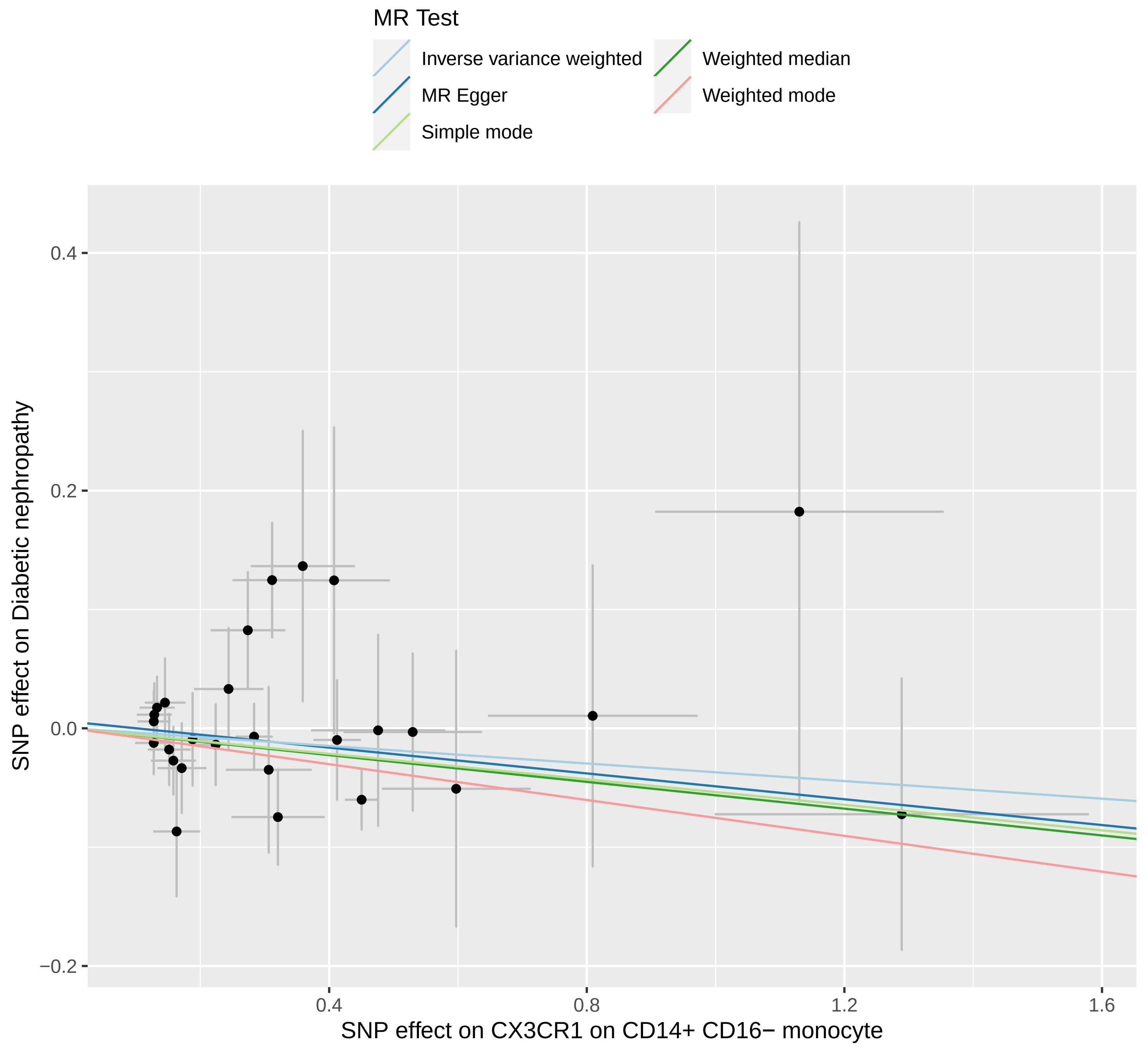

MR Test
Inverse variance weighted
Weighted median
MR Egger
Weighted mode
Simple mode
0.4
0.2
0.0
−0.2
0.4
0.8
1.2
1.6
SNP effect on Diabetic nephropathy
SNP effect on CX3CR1 on CD14+ CD16− monocyte

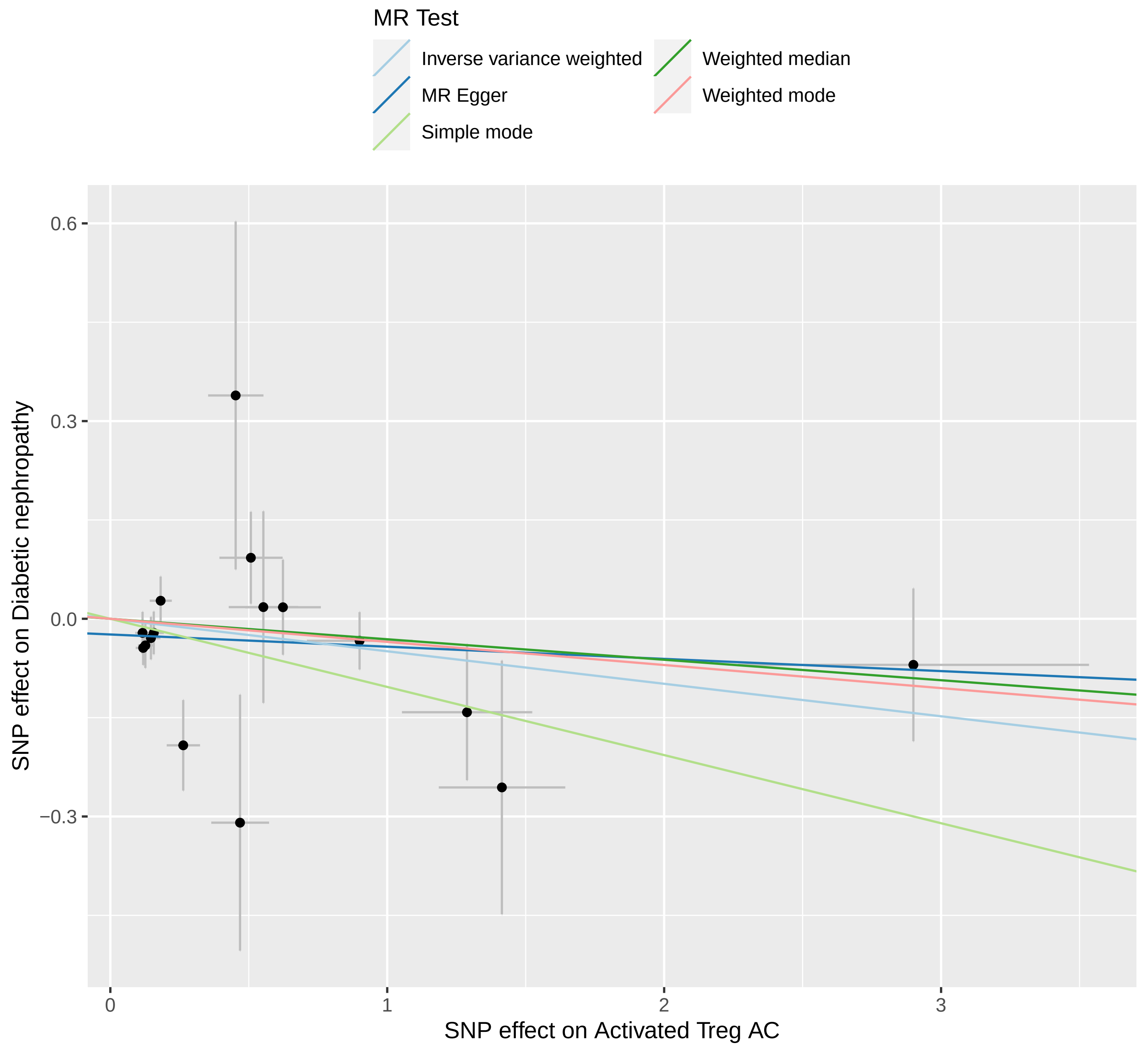

MR Test
Inverse variance weighted
MR Egger
Simple mode
Weighted median
Weighted mode
0.6
0.3
0.0
−0.3
SNP effect on Diabetic nephropathy
0
1
2
3
SNP effect on Activated Treg AC

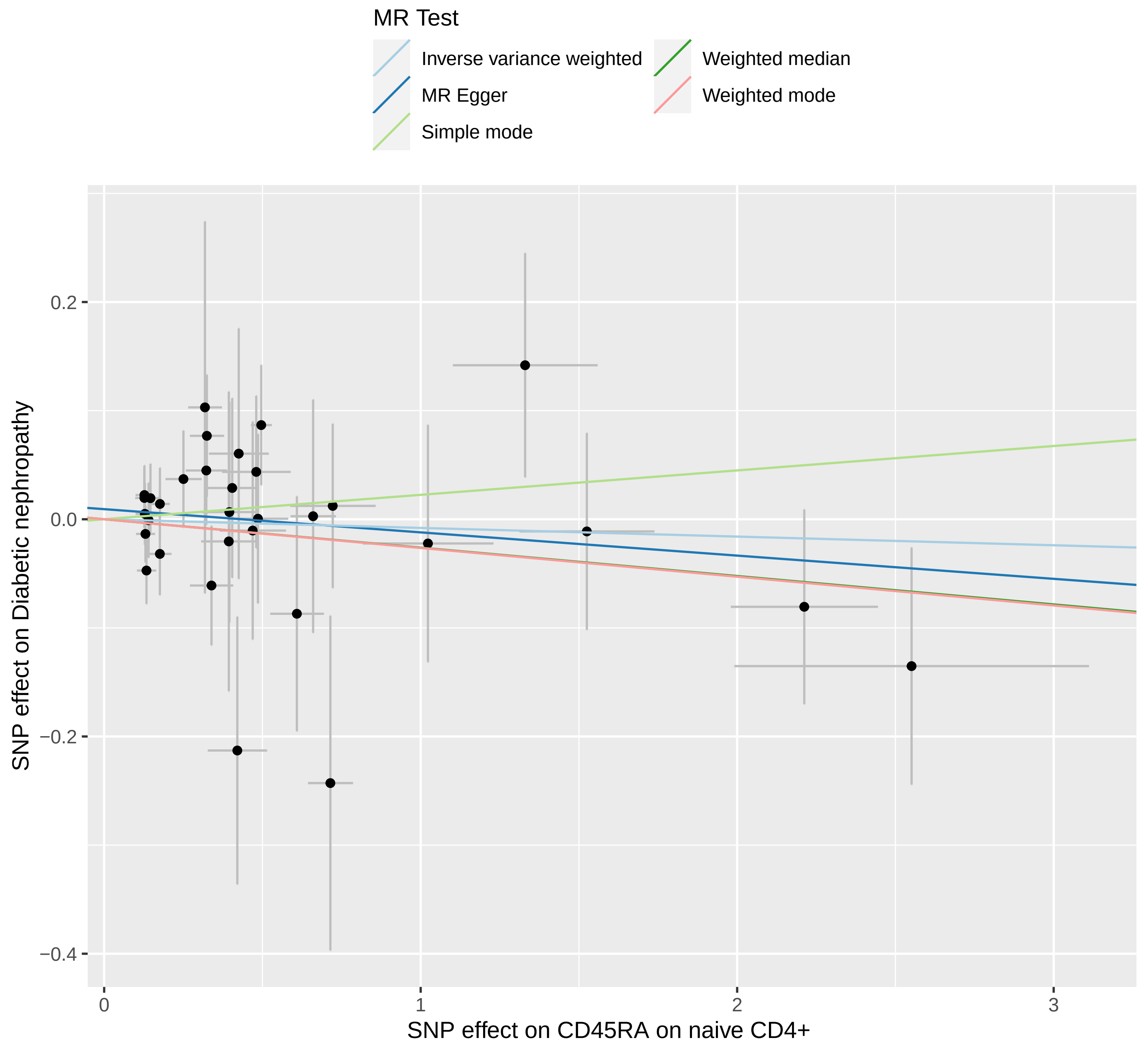

MR Test
Inverse variance weighted
MR Egger
Simple mode
Weighted median
Weighted mode
0.2
0.0
−0.2
−0.4
0
1
2
3
SNP effect on Diabetic nephropathy
SNP effect on CD45RA on naive CD4+

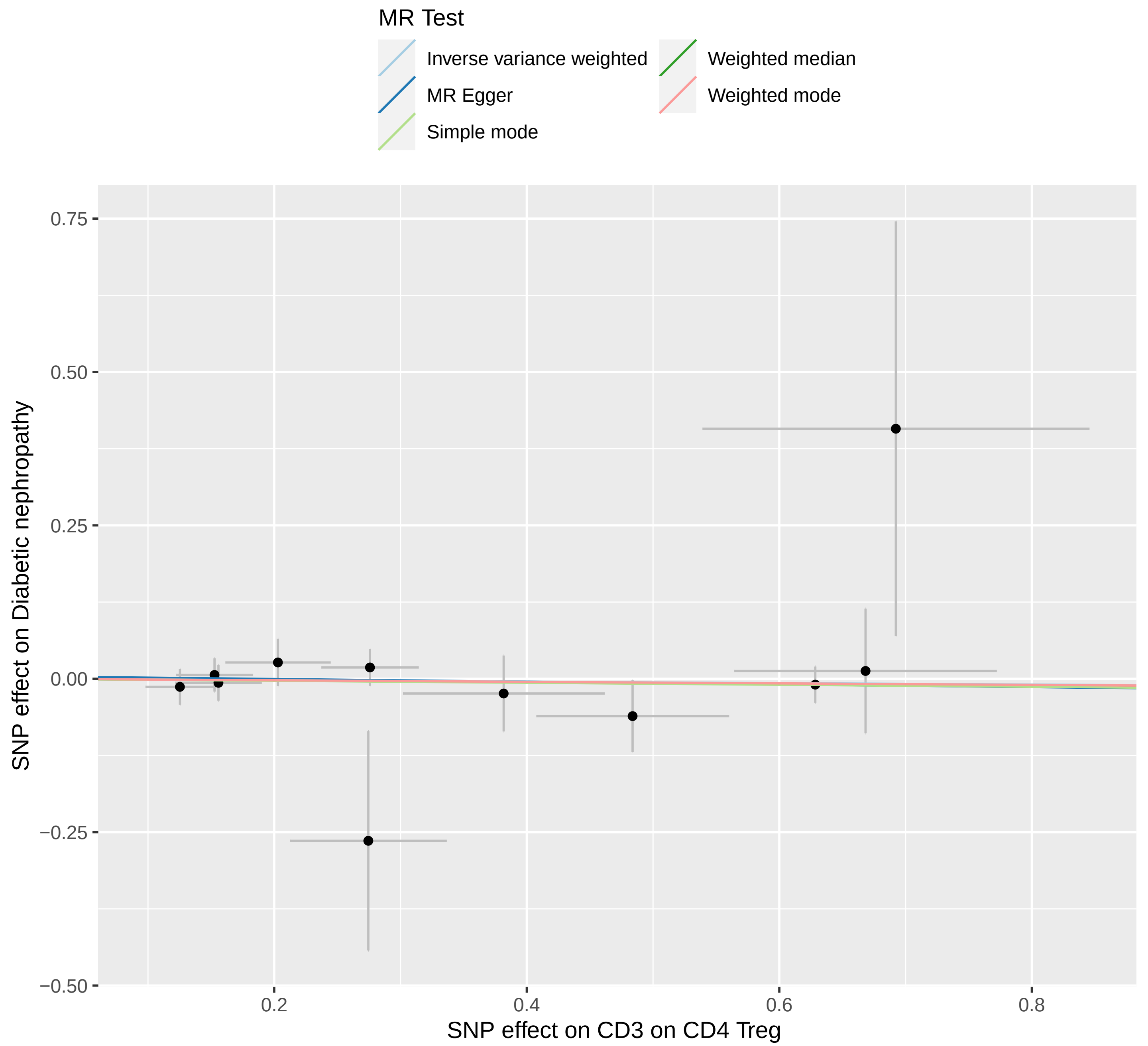

MR Test
Inverse variance weighted
MR Egger
Simple mode
Weighted median
Weighted mode
0.75
0.50
0.25
0.00
−0.25
−0.50
0.2
0.4
0.6
0.8
SNP effect on Diabetic nephropathy
SNP effect on CD3 on CD4 Treg

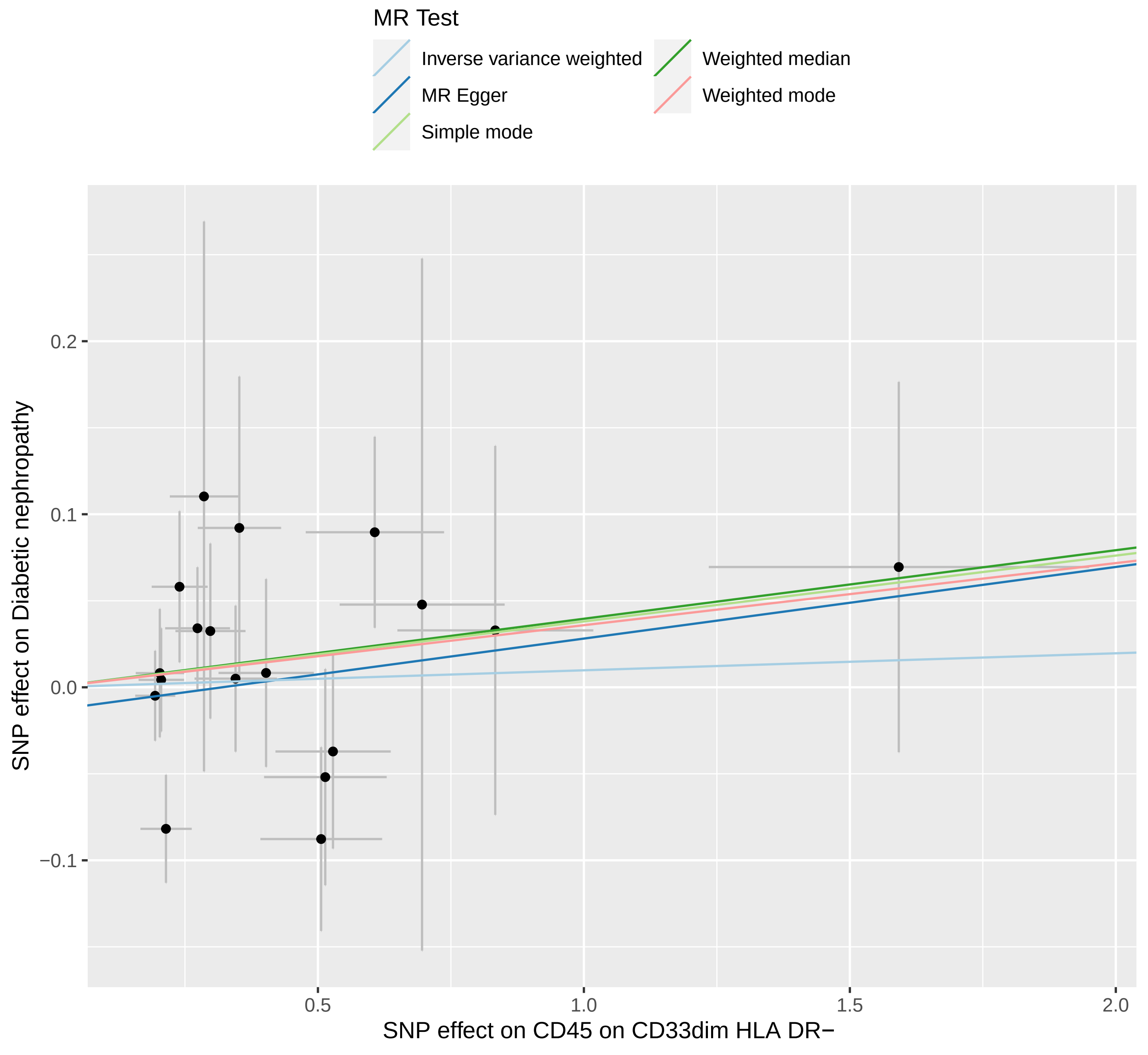
MR Test
Inverse variance weighted
MR Egger
Simple mode
Weighted median
Weighted mode
0.2
0.1
0.0
−0.1
0.5
1.0
1.5
2.0
SNP effect on Diabetic nephropathy
SNP effect on CD45 on CD33dim HLA DR−

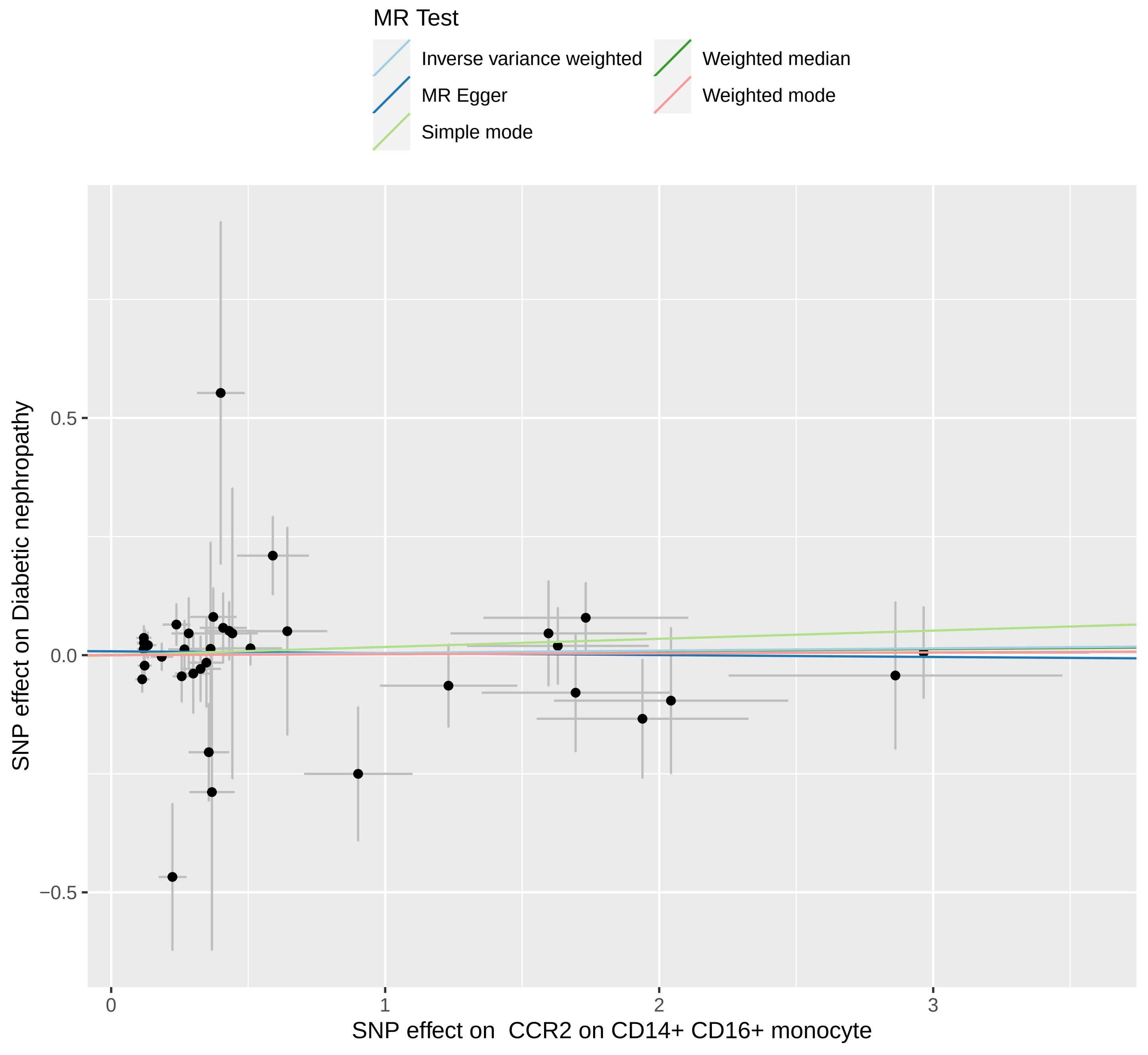
MR Test
Inverse variance weighted
Weighted median
MR Egger
Weighted mode
Simple mode
SNP effect on Diabetic nephropathy
0.5
0.0
−0.5
0
1
2
3
SNP effect on CCR2 on CD14+ CD16+ monocyte

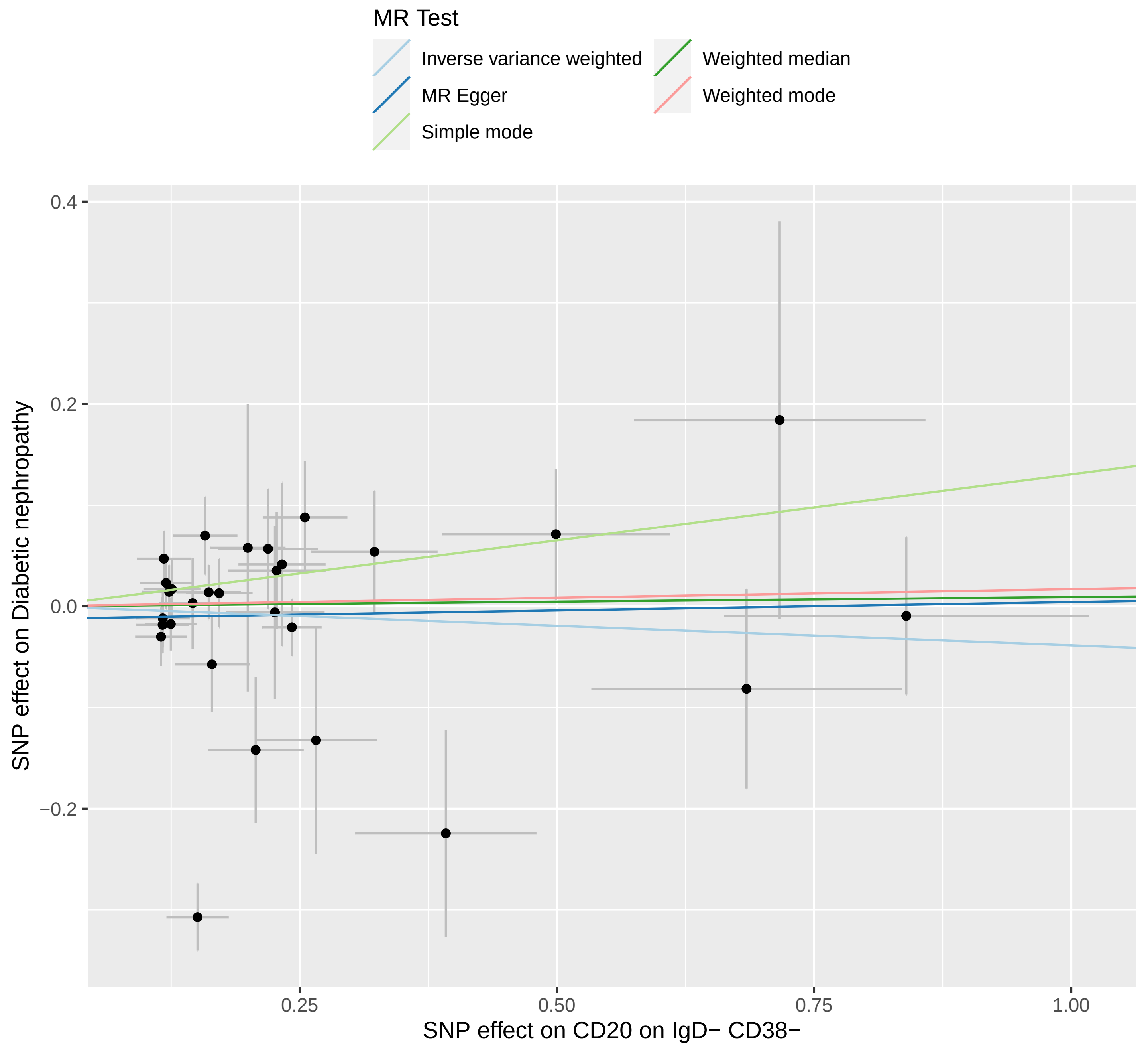
MR Test
Inverse variance weighted
MR Egger
Simple mode
Weighted median
Weighted mode
0.4
0.2
0.0
−0.2
SNP effect on Diabetic nephropathy
0.25
0.50
0.75
1.00
SNP effect on CD20 on IgD− CD38−

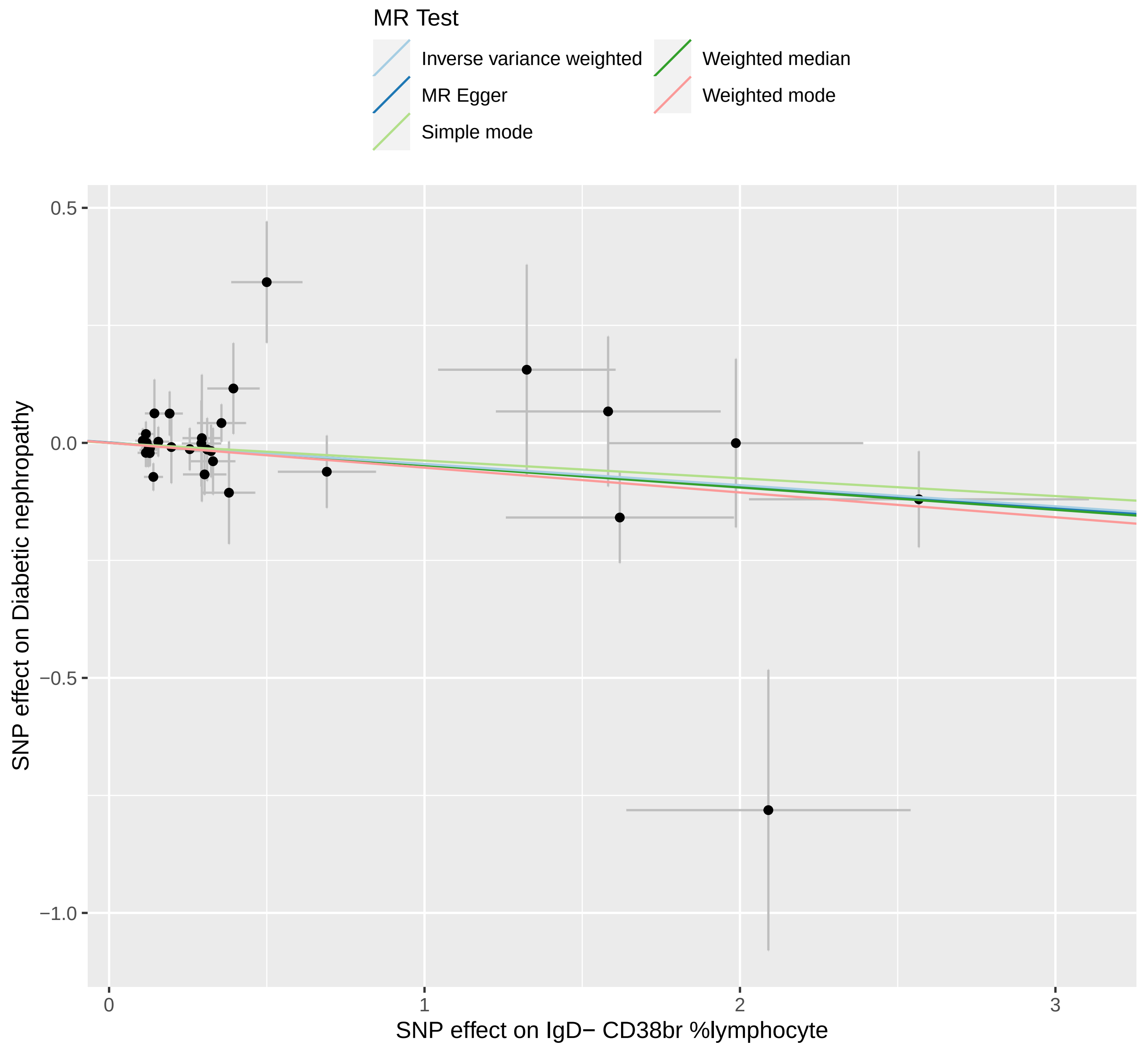

MR Test
Inverse variance weighted
MR Egger
Simple mode
Weighted median
Weighted mode
0.5
0.0
−0.5
−1.0
0
1
2
3
SNP effect on Diabetic nephropathy
SNP effect on IgD− CD38br %lymphocyte

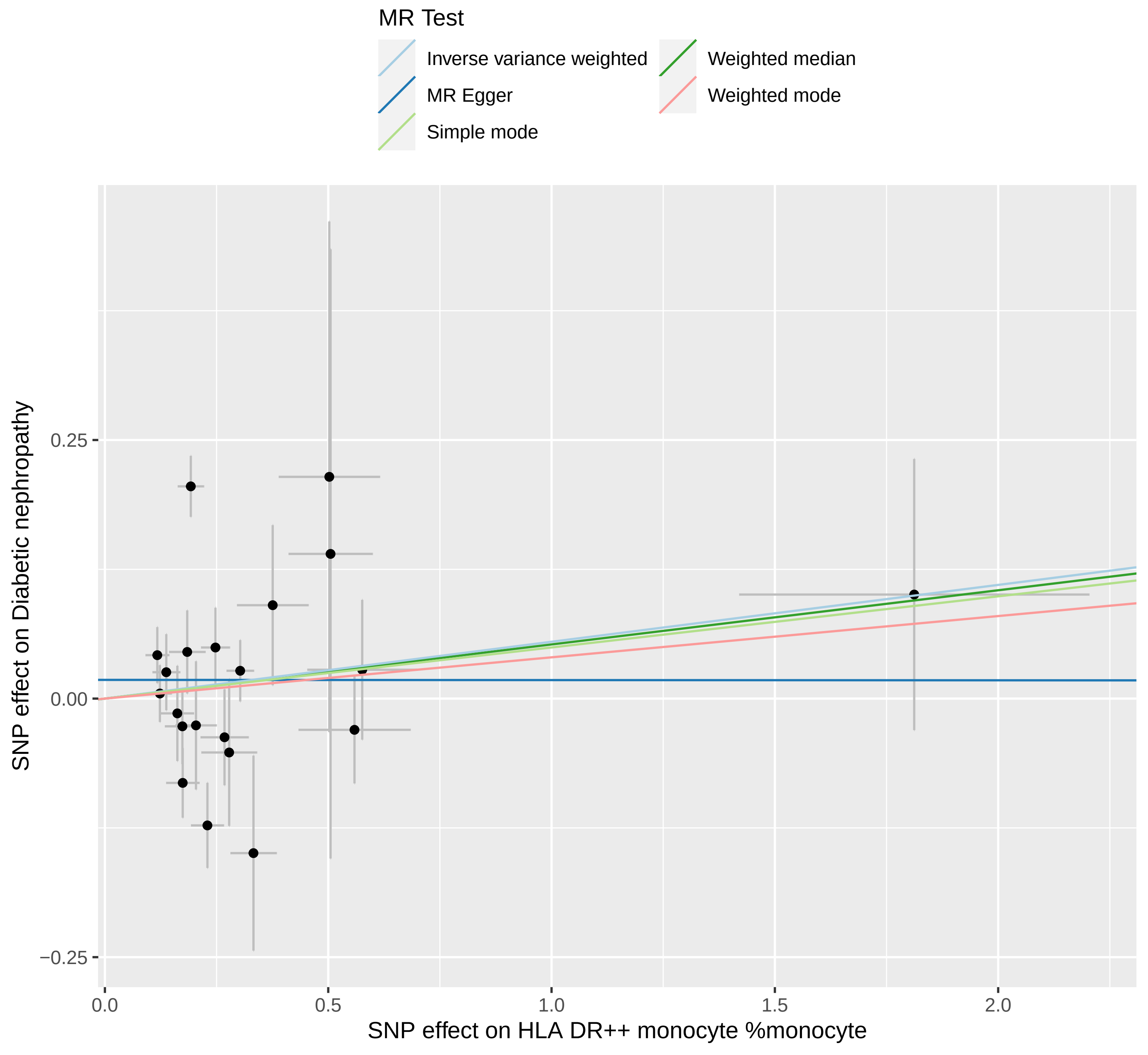
MR Test
Inverse variance weighted
MR Egger
Simple mode
Weighted median
Weighted mode
0.25
0.00
−0.25
0.0
0.5
1.0
1.5
2.0
SNP effect on Diabetic nephropathy
SNP effect on HLA DR++ monocyte %monocyte

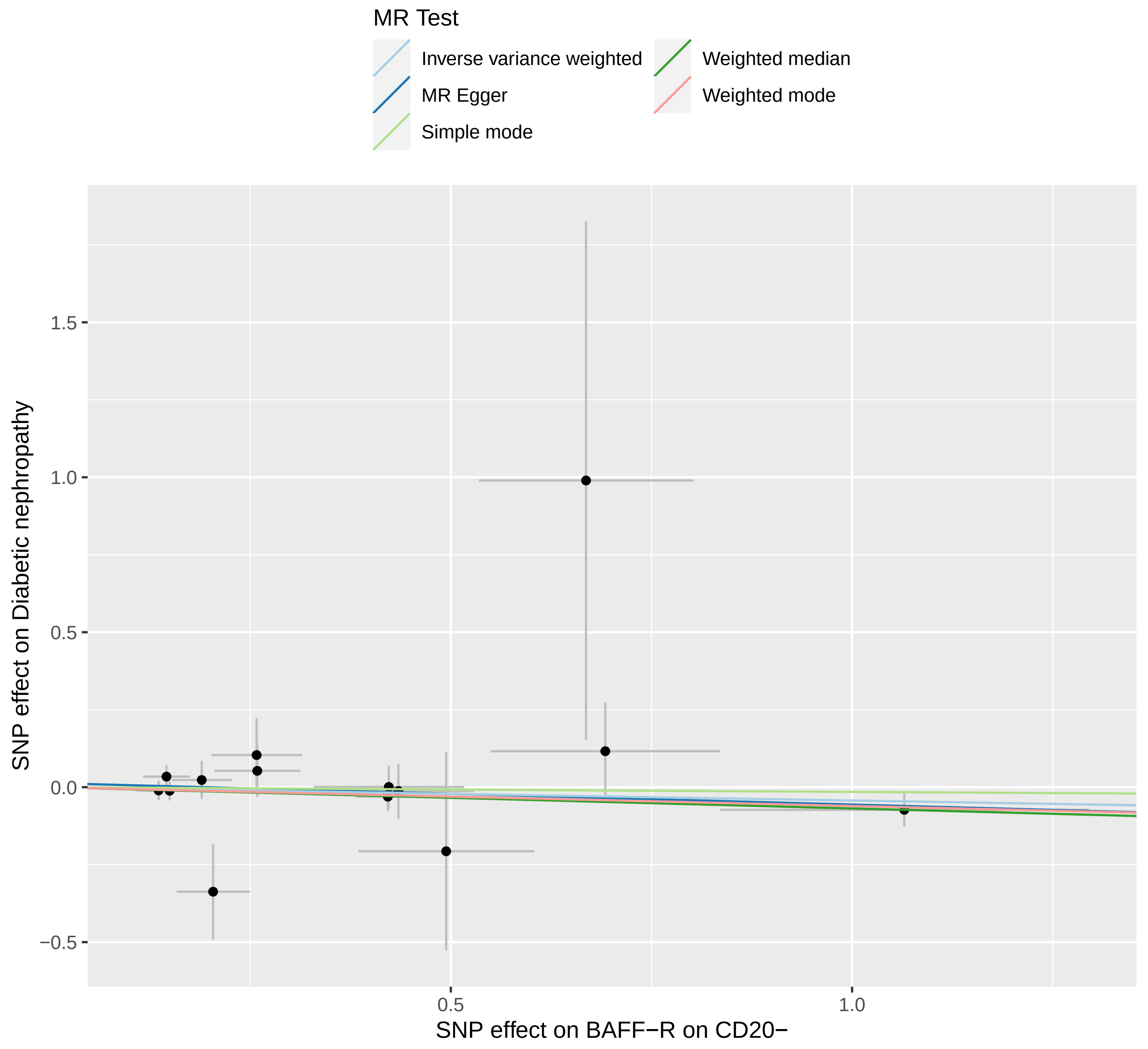
MR Test
Inverse variance weighted
MR Egger
Simple mode
Weighted median
Weighted mode
1.5
1.0
0.5
0.0
−0.5
0.5
1.0
SNP effect on Diabetic nephropathy
SNP effect on BAFF−R on CD20−

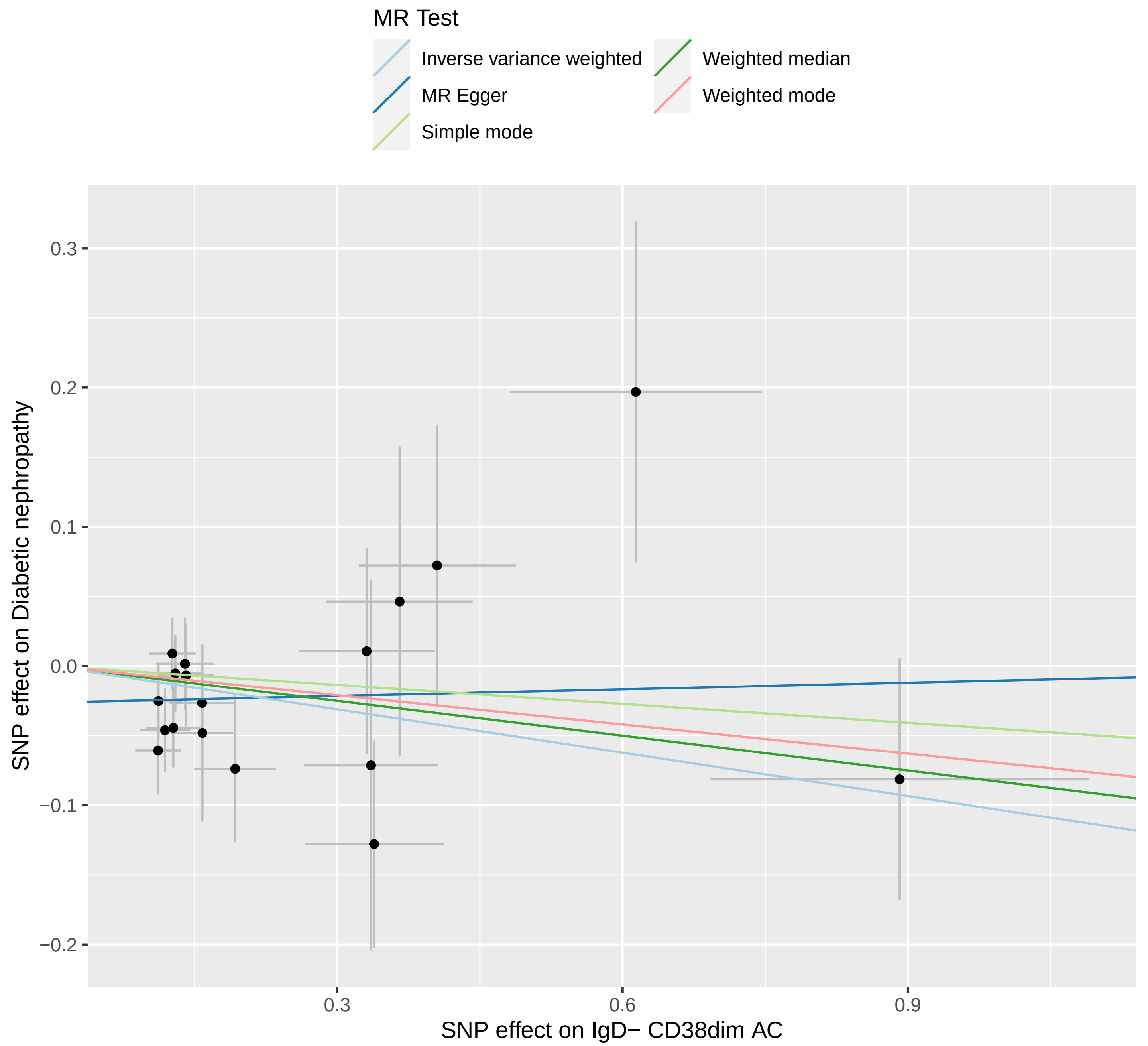

MR Test
Inverse variance weighted
MR Egger
Simple mode
Weighted median
Weighted mode
SNP effect on Diabetic nephropathy
0.3
0.2
0.1
0.0
−0.1
−0.2
0.3
0.6
0.9
SNP effect on IgD− CD38dim AC

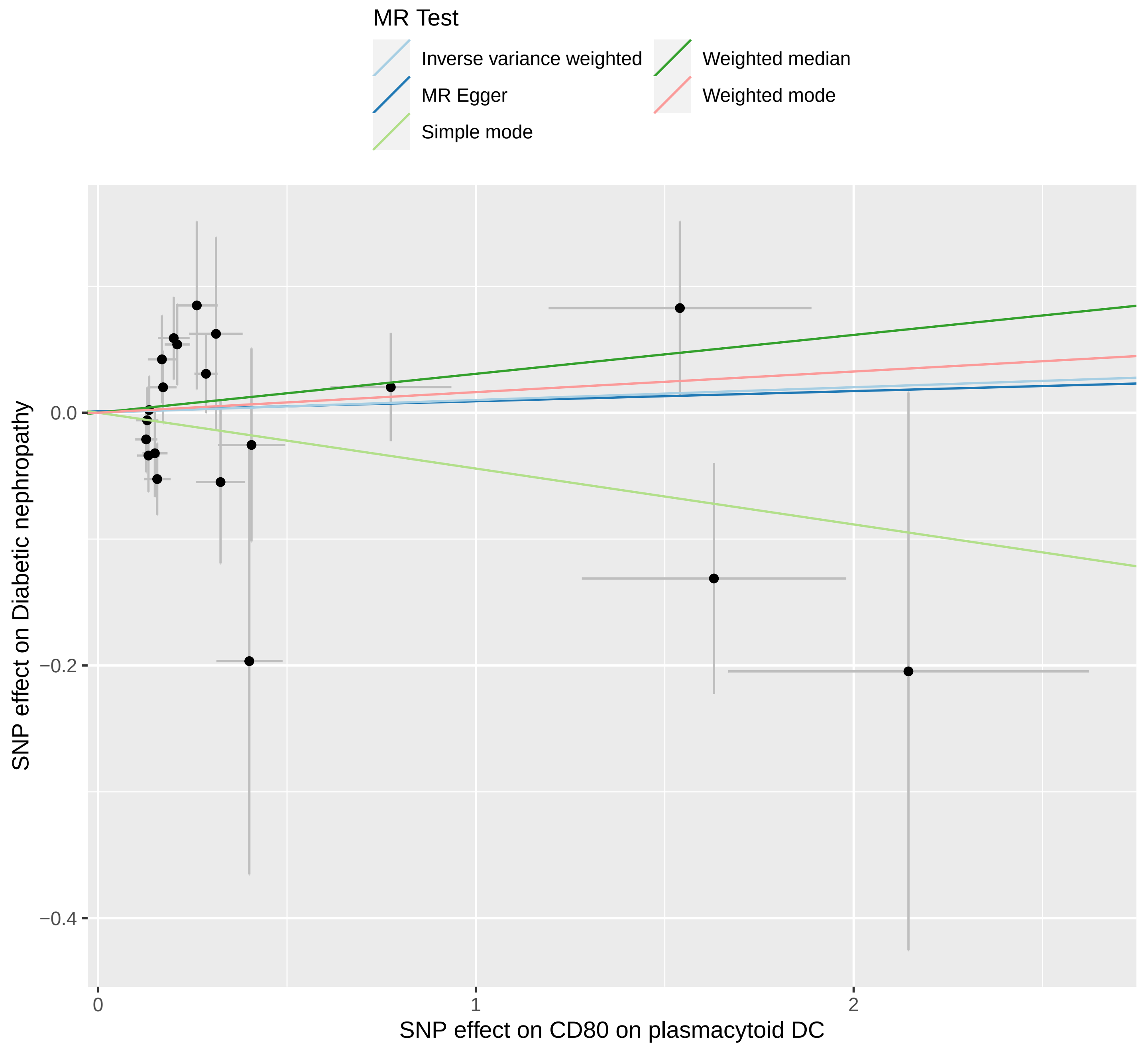
MR Test
Inverse variance weighted
MR Egger
Simple mode
Weighted median
Weighted mode
SNP effect on Diabetic nephropathy
0.0
−0.2
−0.4
0
1
2
SNP effect on CD80 on plasmacytoid DC

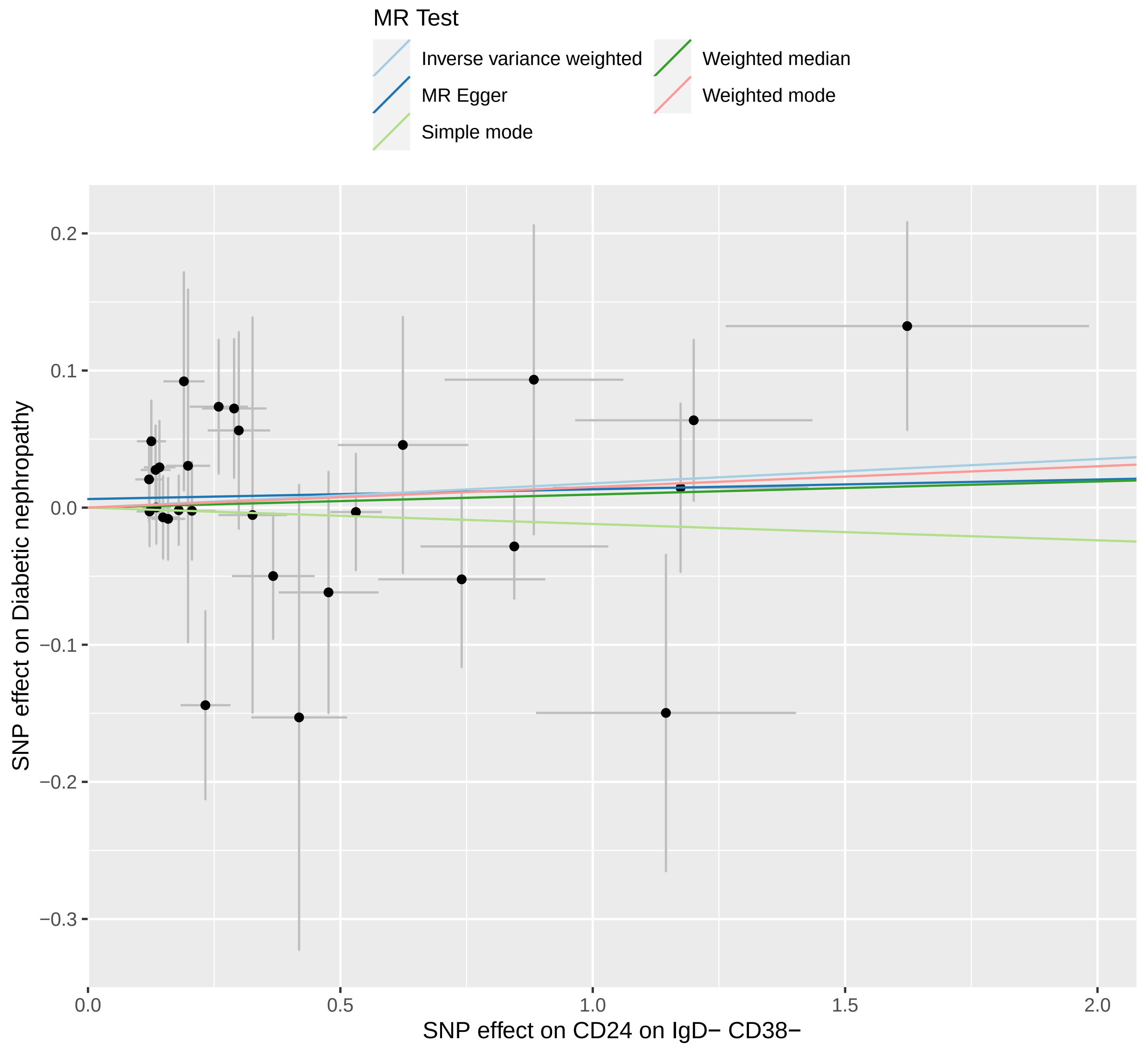

MR Test
Inverse variance weighted
MR Egger
Simple mode
Weighted median
Weighted mode
SNP effect on Diabetic nephropathy
0.2
0.1
0.0
−0.1
−0.2
−0.3
0.0
0.5
1.0
1.5
2.0
SNP effect on CD24 on IgD− CD38−

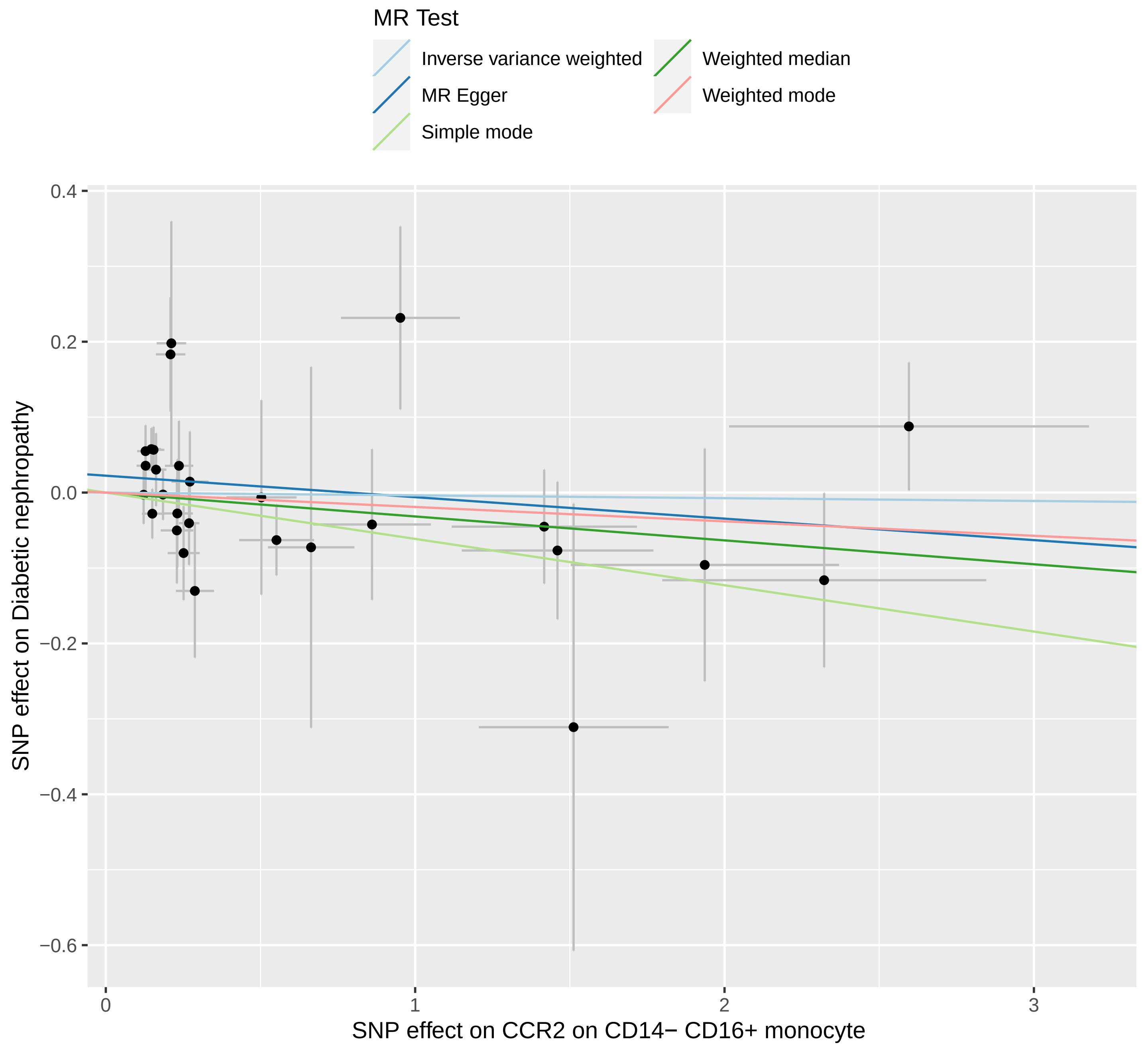
MR Test
Inverse variance weighted
MR Egger
Simple mode
Weighted median
Weighted mode
0.4
0.2
0.0
−0.2
−0.4
−0.6
0
1
2
3
SNP effect on Diabetic nephropathy
SNP effect on CCR2 on CD14− CD16+ monocyte

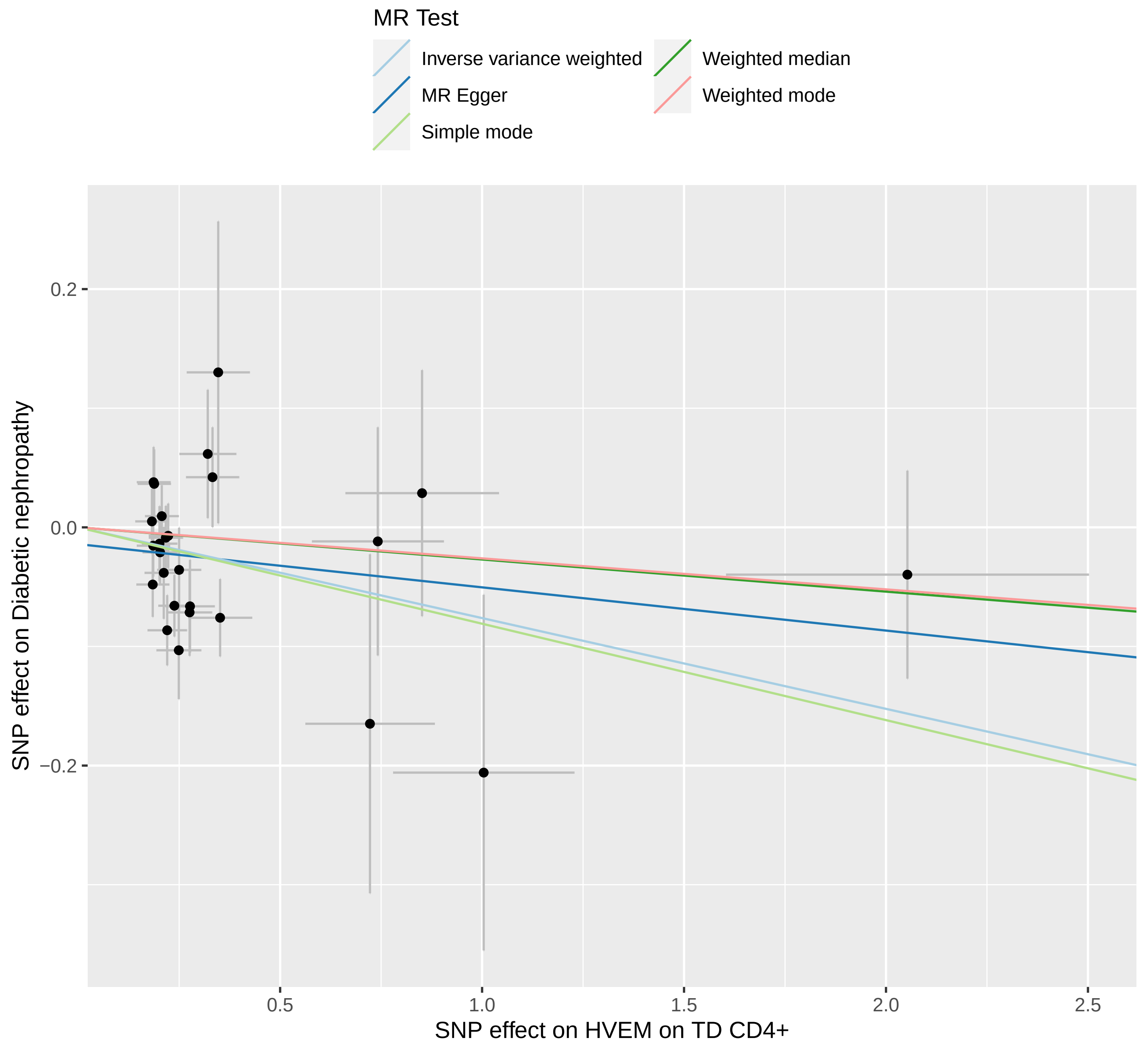
MR Test
Inverse variance weighted
MR Egger
Simple mode
Weighted median
Weighted mode
0.2
0.0
−0.2
0.5
1.0
1.5
2.0
2.5
SNP effect on Diabetic nephropathy
SNP effect on HVEM on TD CD4+

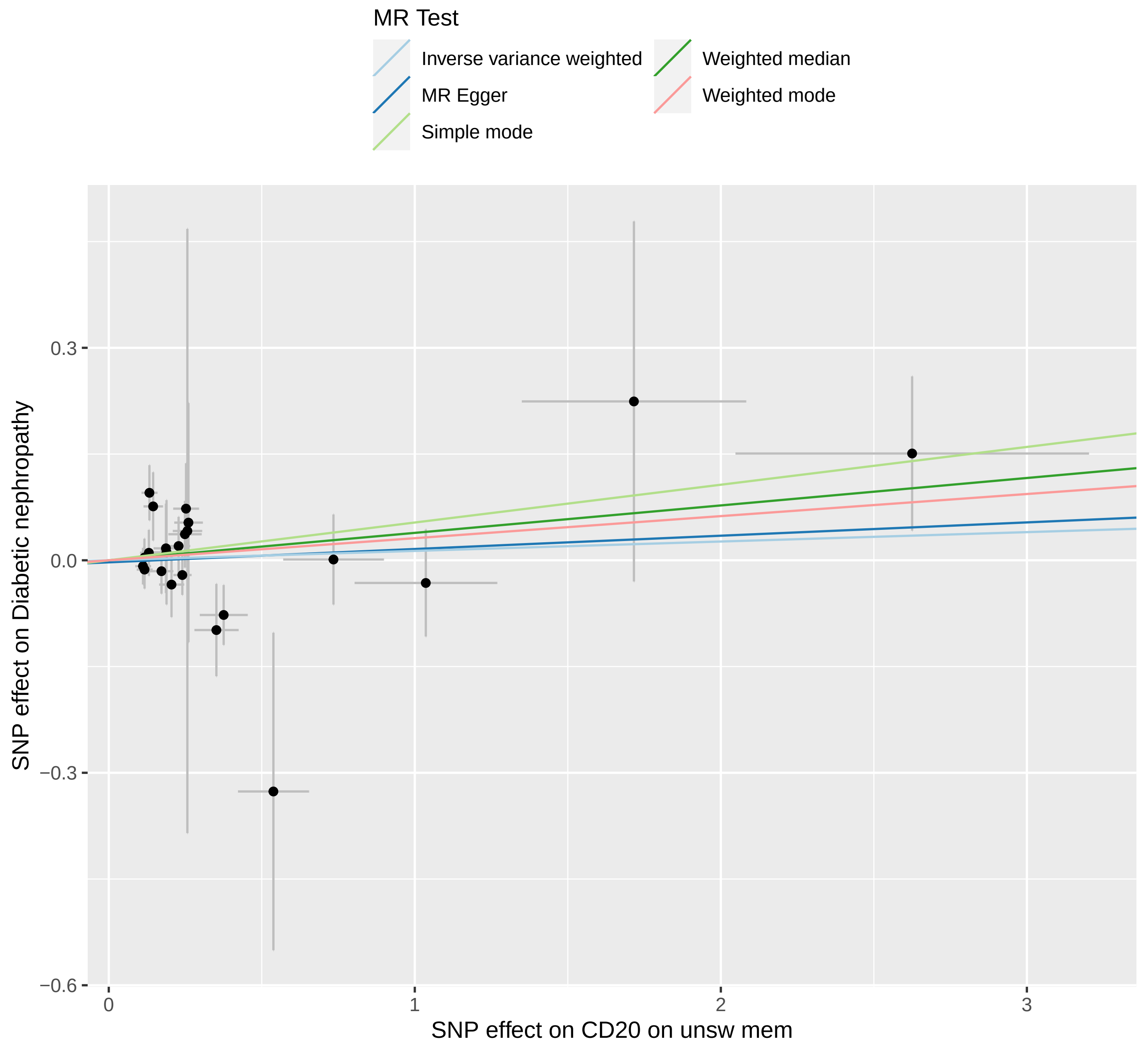
MR Test
Inverse variance weighted
MR Egger
Simple mode
Weighted median
Weighted mode
0.3
0.0
−0.3
−0.6
0
1
2
3
SNP effect on Diabetic nephropathy
SNP effect on CD20 on unsw mem

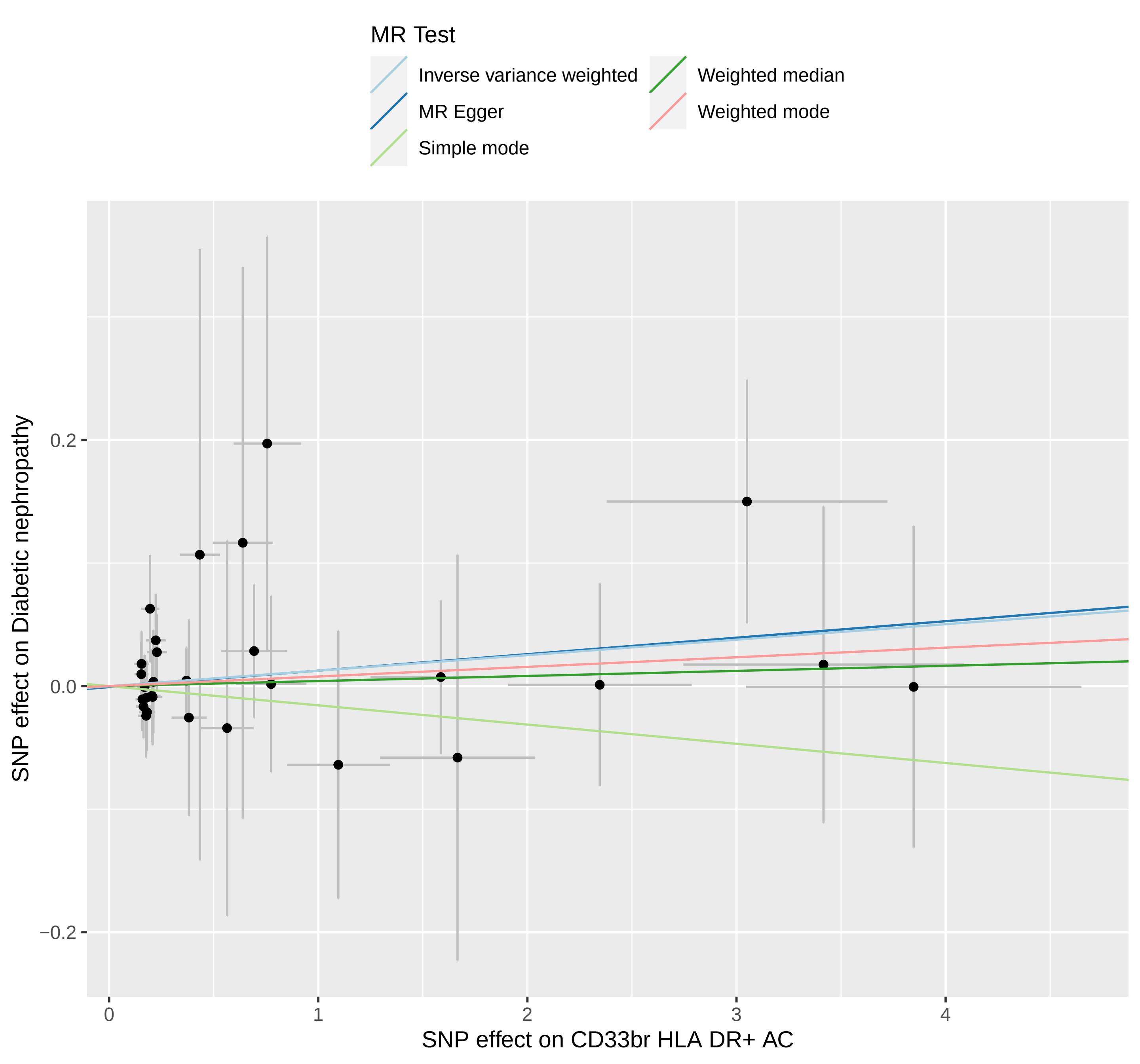
MR Test
Inverse variance weighted
MR Egger
Simple mode
Weighted median
Weighted mode
SNP effect on Diabetic nephropathy
0.2
0.0
−0.2
0
1
2
3
4
SNP effect on CD33br HLA DR+ AC

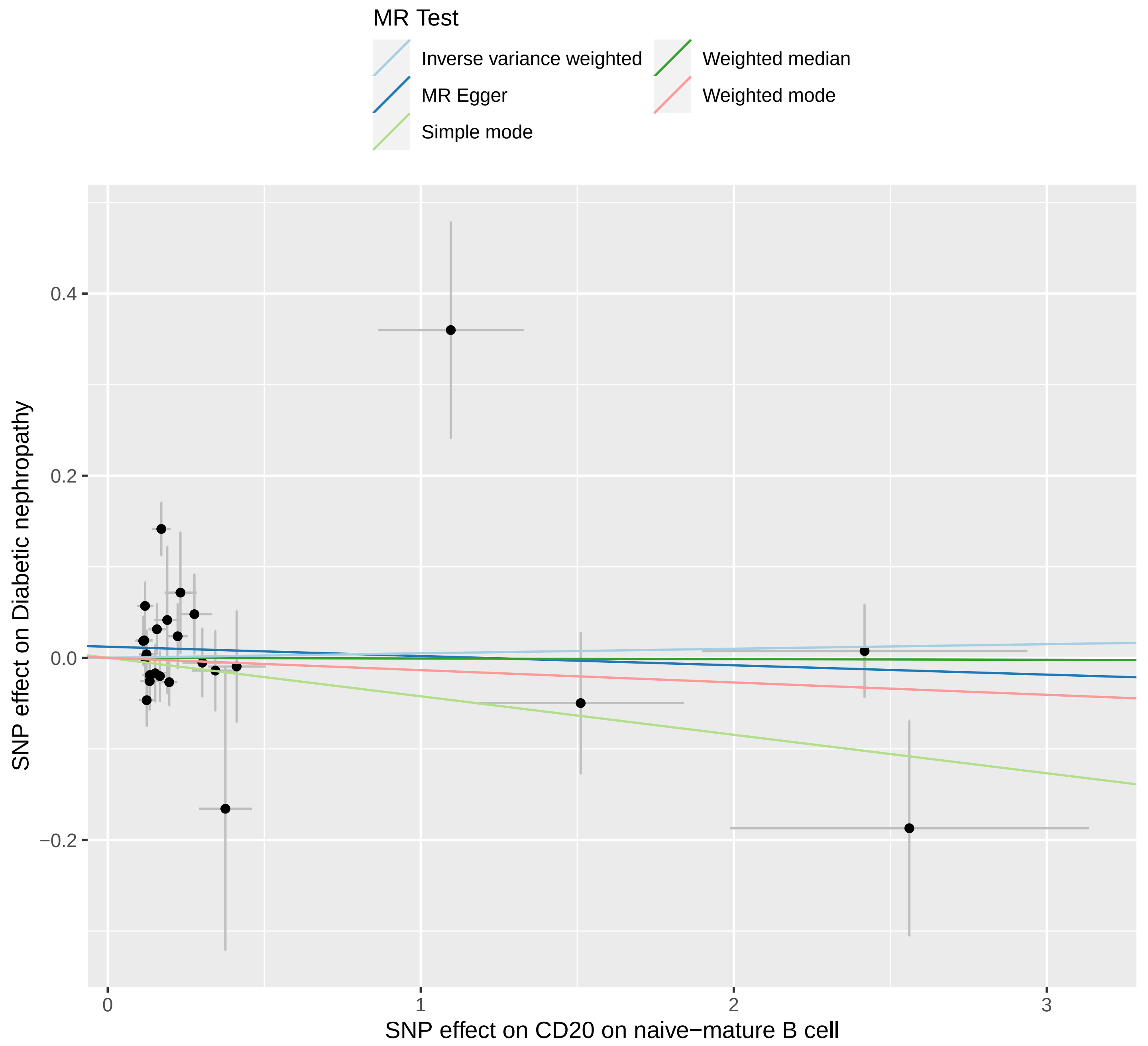

MR Test
Inverse variance weighted
MR Egger
Simple mode
Weighted median
Weighted mode
SNP effect on Diabetic nephropathy
0.4
0.2
0.0
−0.2
0
1
2
3
SNP effect on CD20 on naive−mature B cell

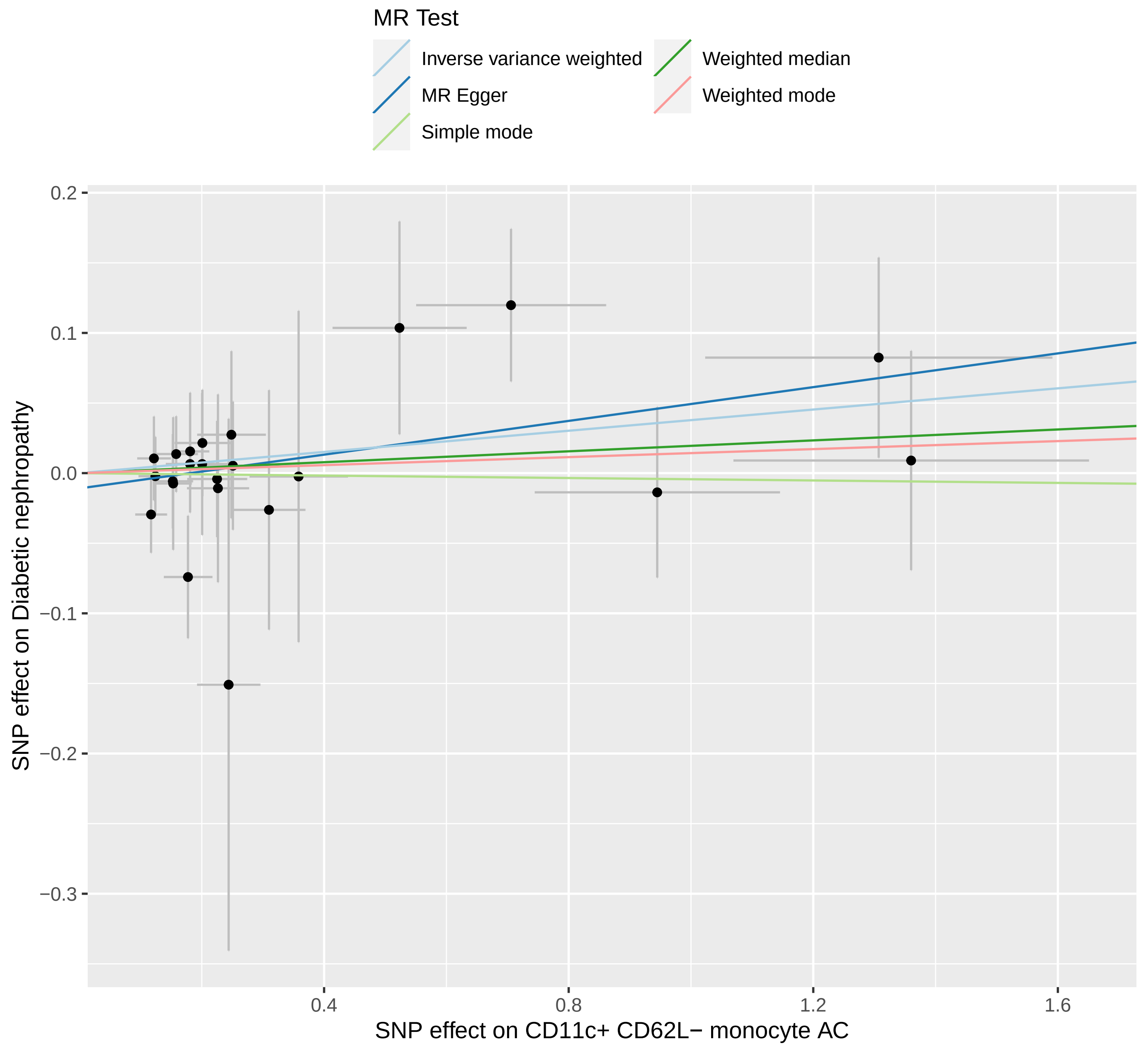
MR Test
Inverse variance weighted
MR Egger
Simple mode
Weighted median
Weighted mode
0.2
0.1
0.0
−0.1
−0.2
−0.3
SNP effect on Diabetic nephropathy
0.4
0.8
1.2
1.6
SNP effect on CD11c+ CD62L− monocyte AC

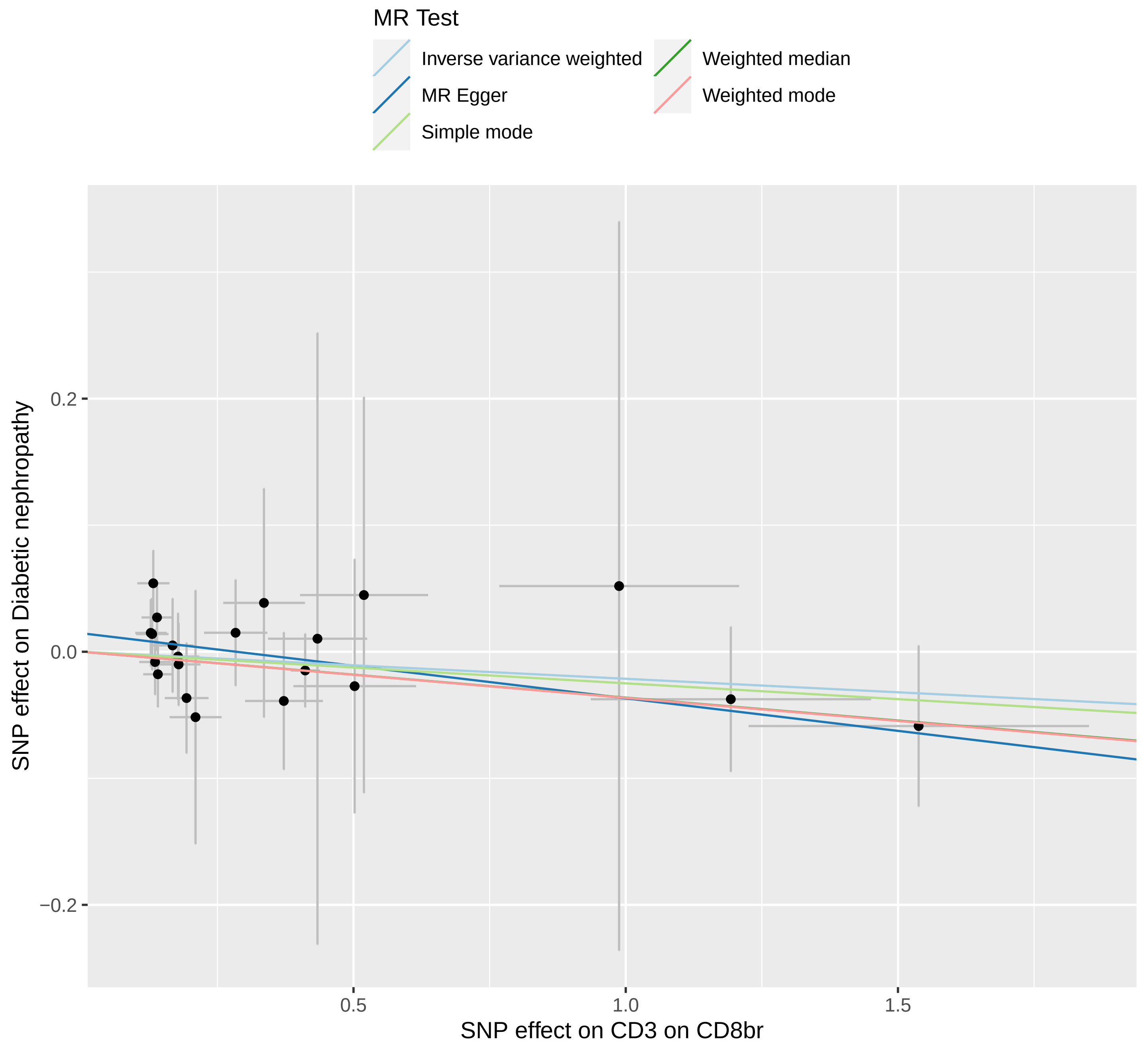
MR Test
Inverse variance weighted
MR Egger
Simple mode
Weighted median
Weighted mode
SNP effect on Diabetic nephropathy
0.2
0.0
−0.2
0.5
1.0
1.5
SNP effect on CD3 on CD8br

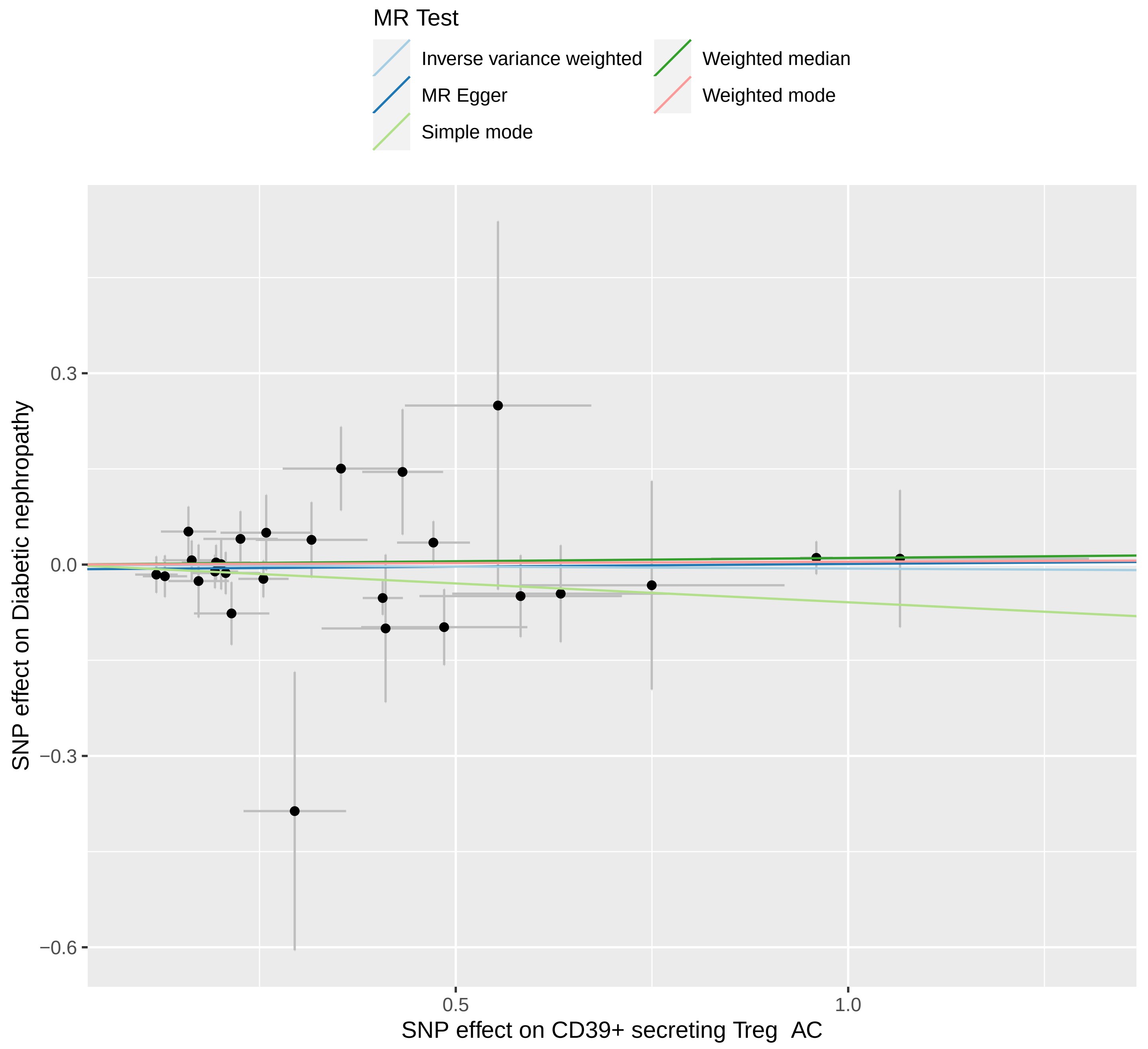

MR Test
Inverse variance weighted
MR Egger
Simple mode
Weighted median
Weighted mode
0.3
0.0
−0.3
−0.6
0.5
1.0
SNP effect on Diabetic nephropathy
SNP effect on CD39+ secreting Treg AC

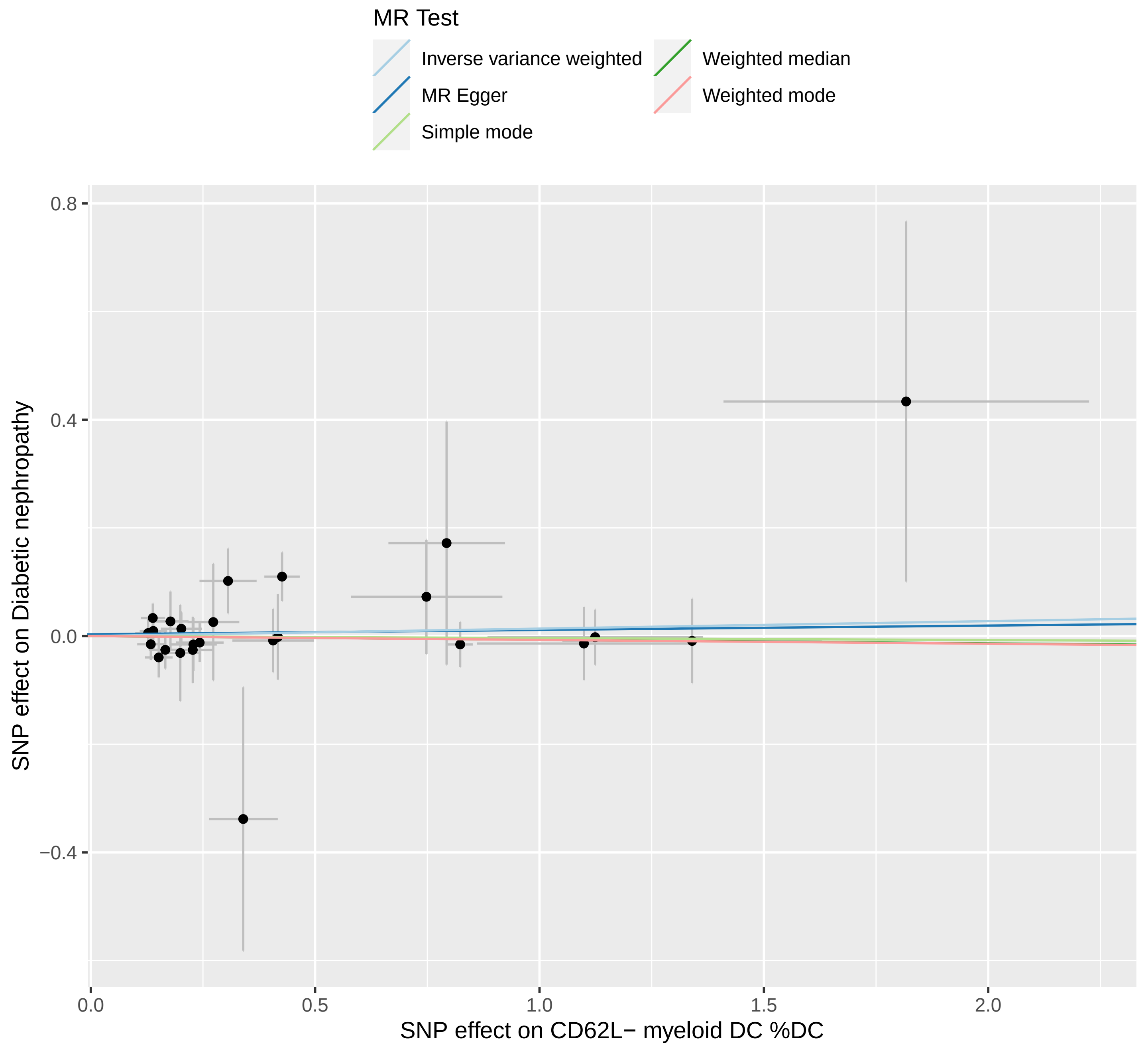

MR Test
Inverse variance weighted
MR Egger
Simple mode
Weighted median
Weighted mode
0.8
0.4
0.0
−0.4
SNP effect on Diabetic nephropathy
0.0
0.5
1.0
1.5
2.0
SNP effect on CD62L− myeloid DC %DC

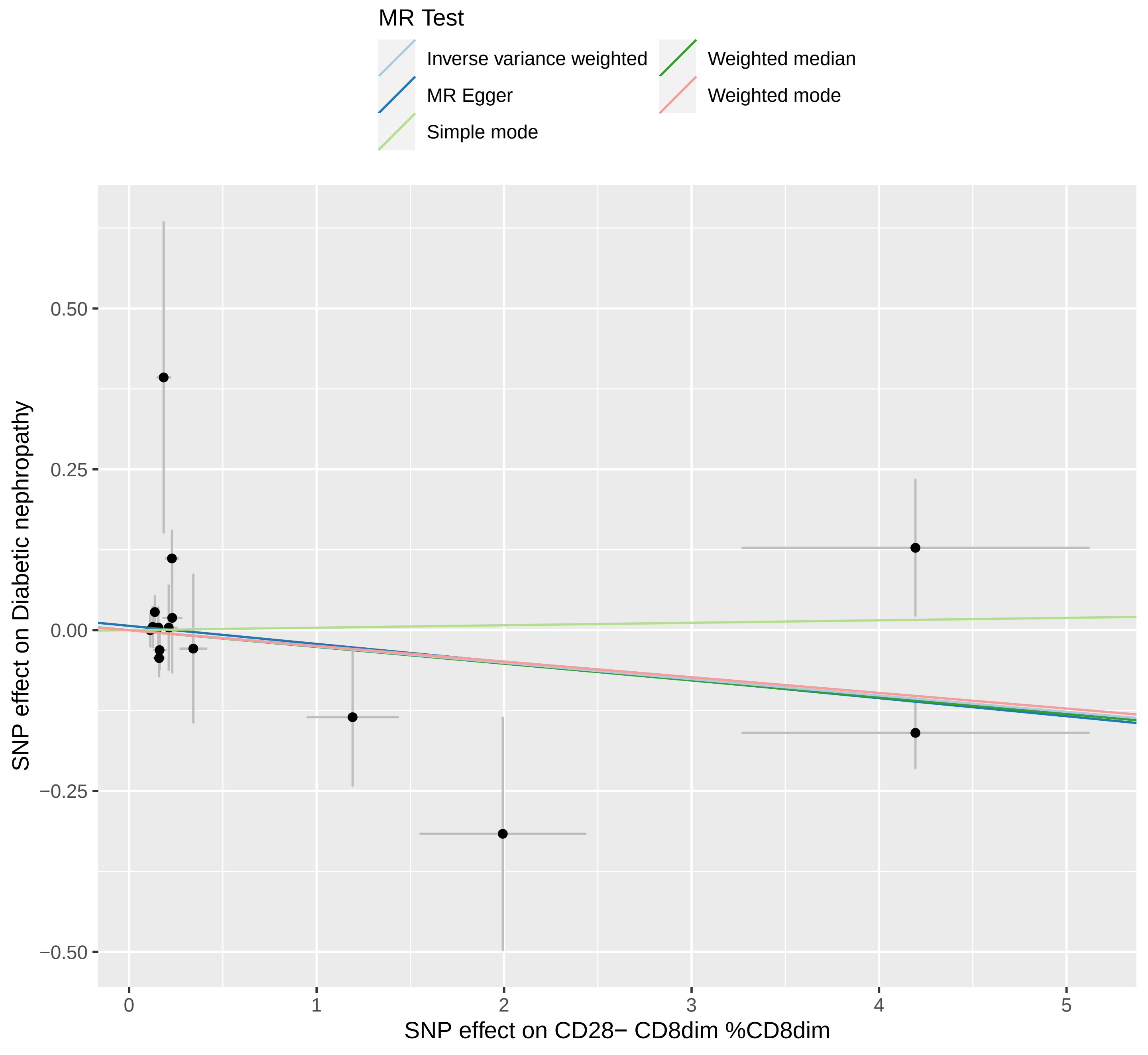
MR Test
Inverse variance weighted
MR Egger
Simple mode
Weighted median
Weighted mode
SNP effect on Diabetic nephropathy
0.50
0.25
0.00
−0.25
−0.50
0
1
2
3
4
5
SNP effect on CD28− CD8dim %CD8dim

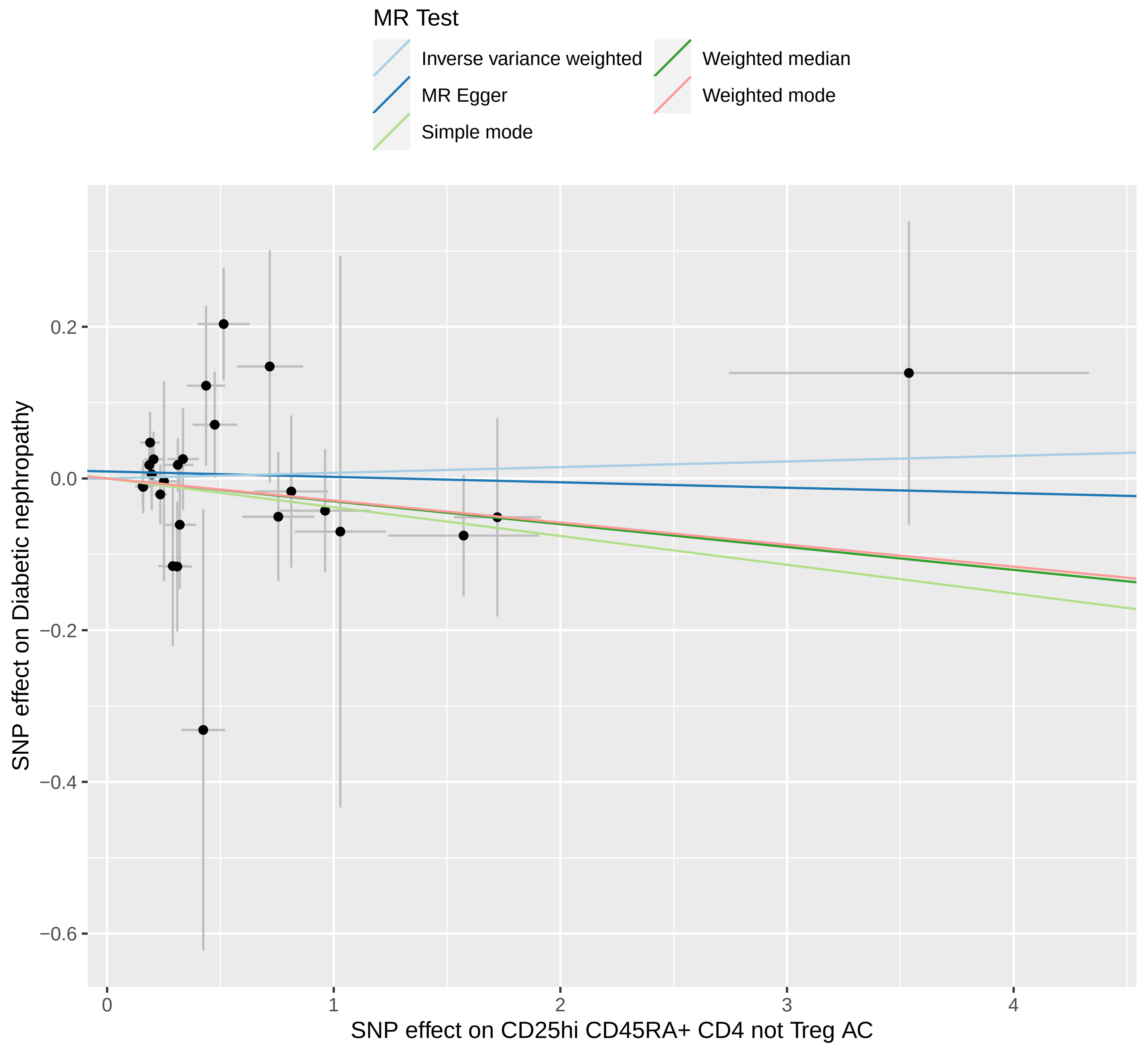
MR Test
Inverse variance weighted
MR Egger
Simple mode
Weighted median
Weighted mode
0.2
0.0
−0.2
−0.4
−0.6
SNP effect on Diabetic nephropathy
0
1
2
3
4
SNP effect on CD25hi CD45RA+ CD4 not Treg AC

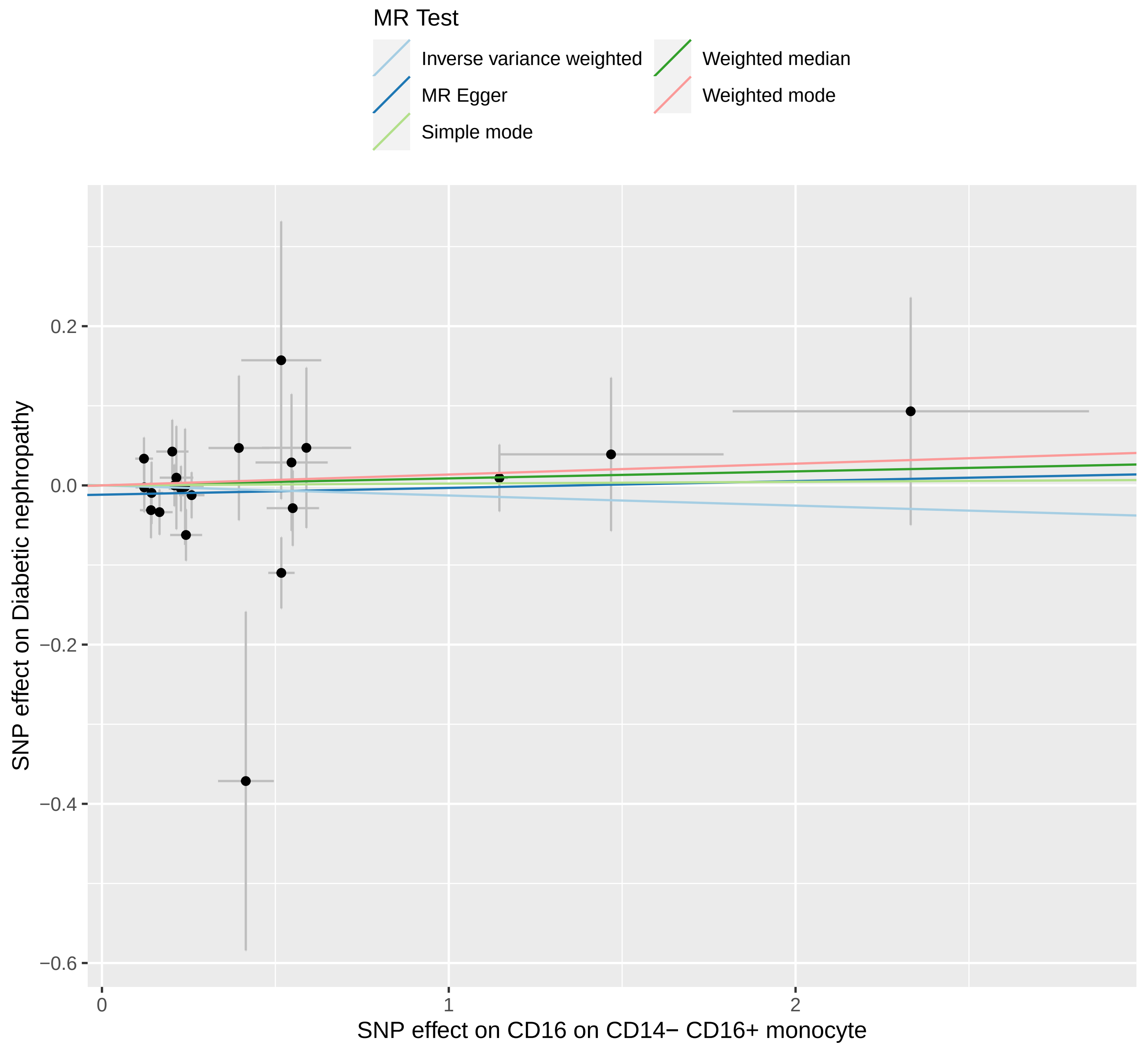

MR Test
Inverse variance weighted
MR Egger
Simple mode
Weighted median
Weighted mode
SNP effect on Diabetic nephropathy
0.2
0.0
−0.2
−0.4
−0.6
0
1
2
SNP effect on CD16 on CD14− CD16+ monocyte

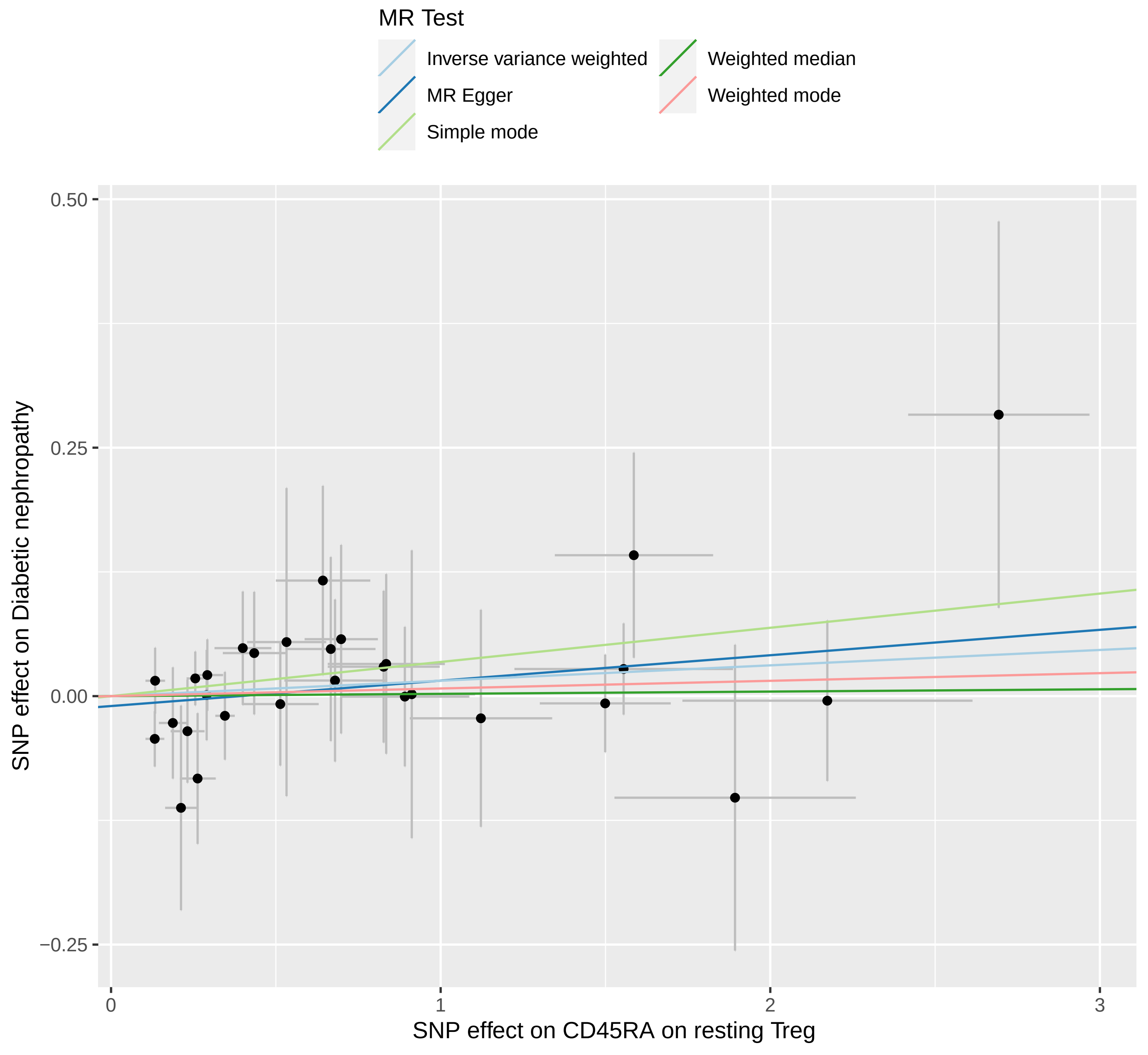
MR Test
Inverse variance weighted
MR Egger
Simple mode
Weighted median
Weighted mode
0.50
0.25
0.00
−0.25
0
1
2
3
SNP effect on Diabetic nephropathy
SNP effect on CD45RA on resting Treg

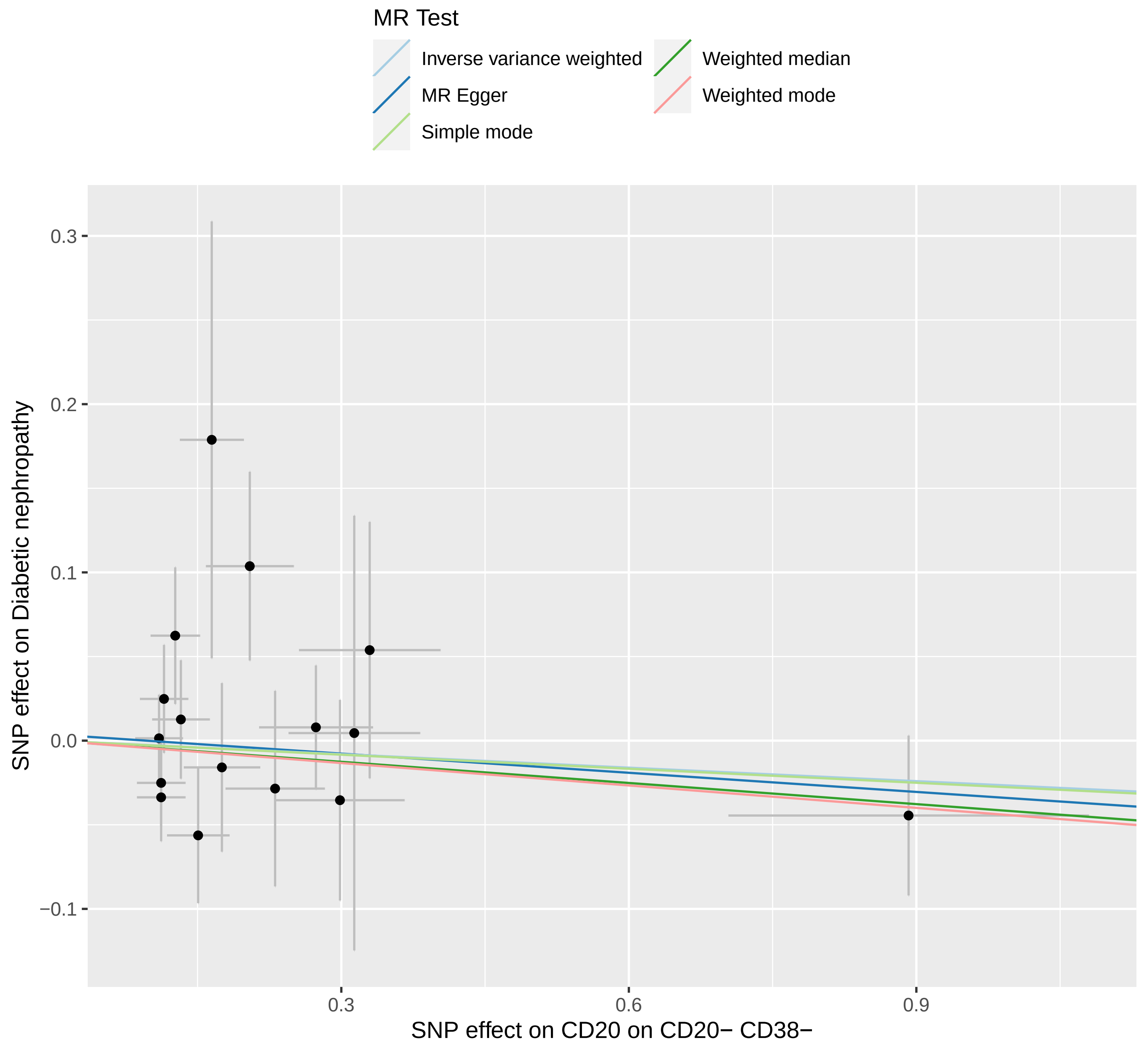
MR Test
Inverse variance weighted
MR Egger
Simple mode
Weighted median
Weighted mode
0.3
0.2
0.1
0.0
−0.1
SNP effect on Diabetic nephropathy
0.3
0.6
0.9
SNP effect on CD20 on CD20− CD38−

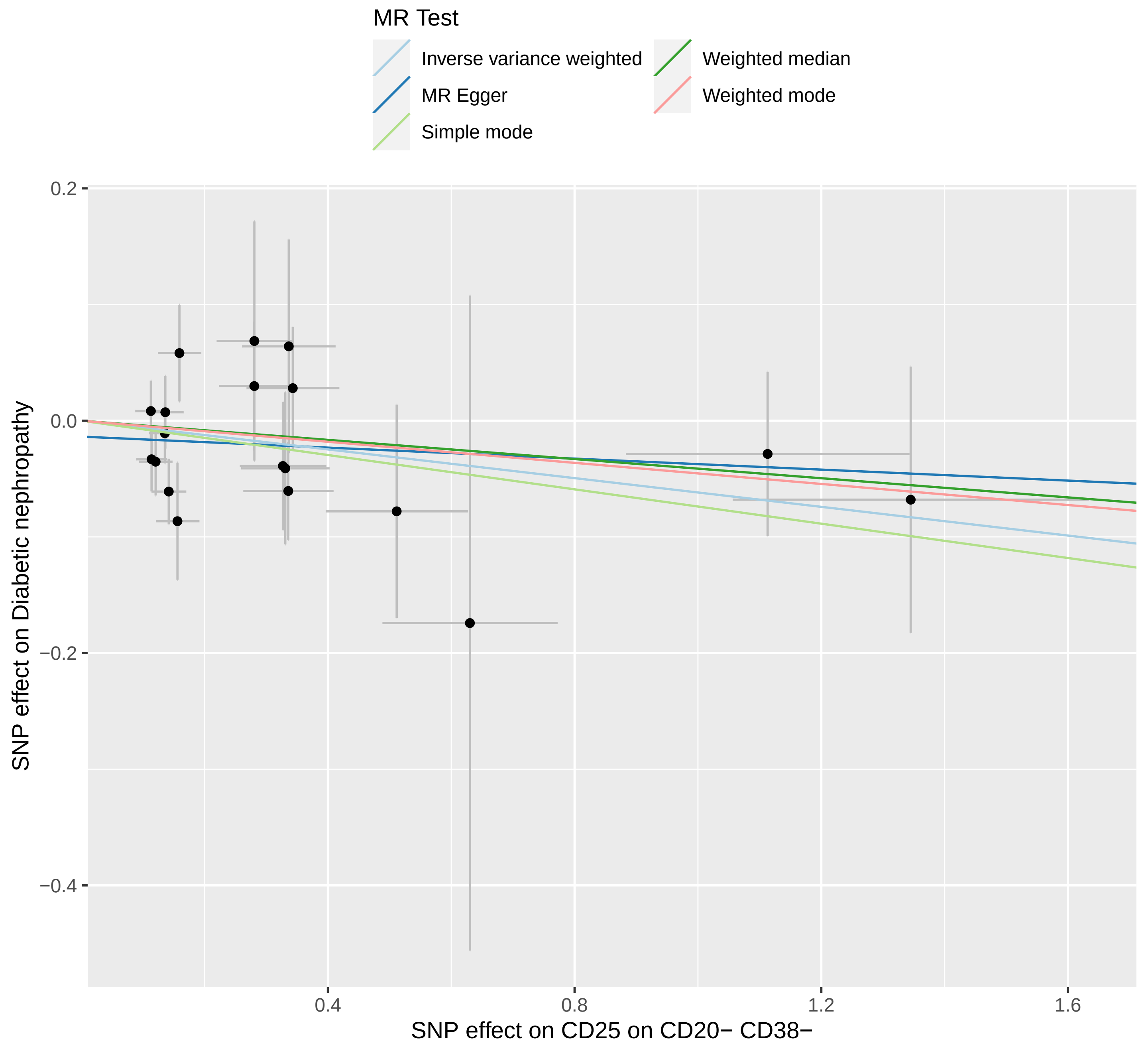

MR Test
Inverse variance weighted
MR Egger
Simple mode
Weighted median
Weighted mode
0.2
0.0
−0.2
−0.4
0.4
0.8
1.2
1.6
SNP effect on Diabetic nephropathy
SNP effect on CD25 on CD20− CD38−

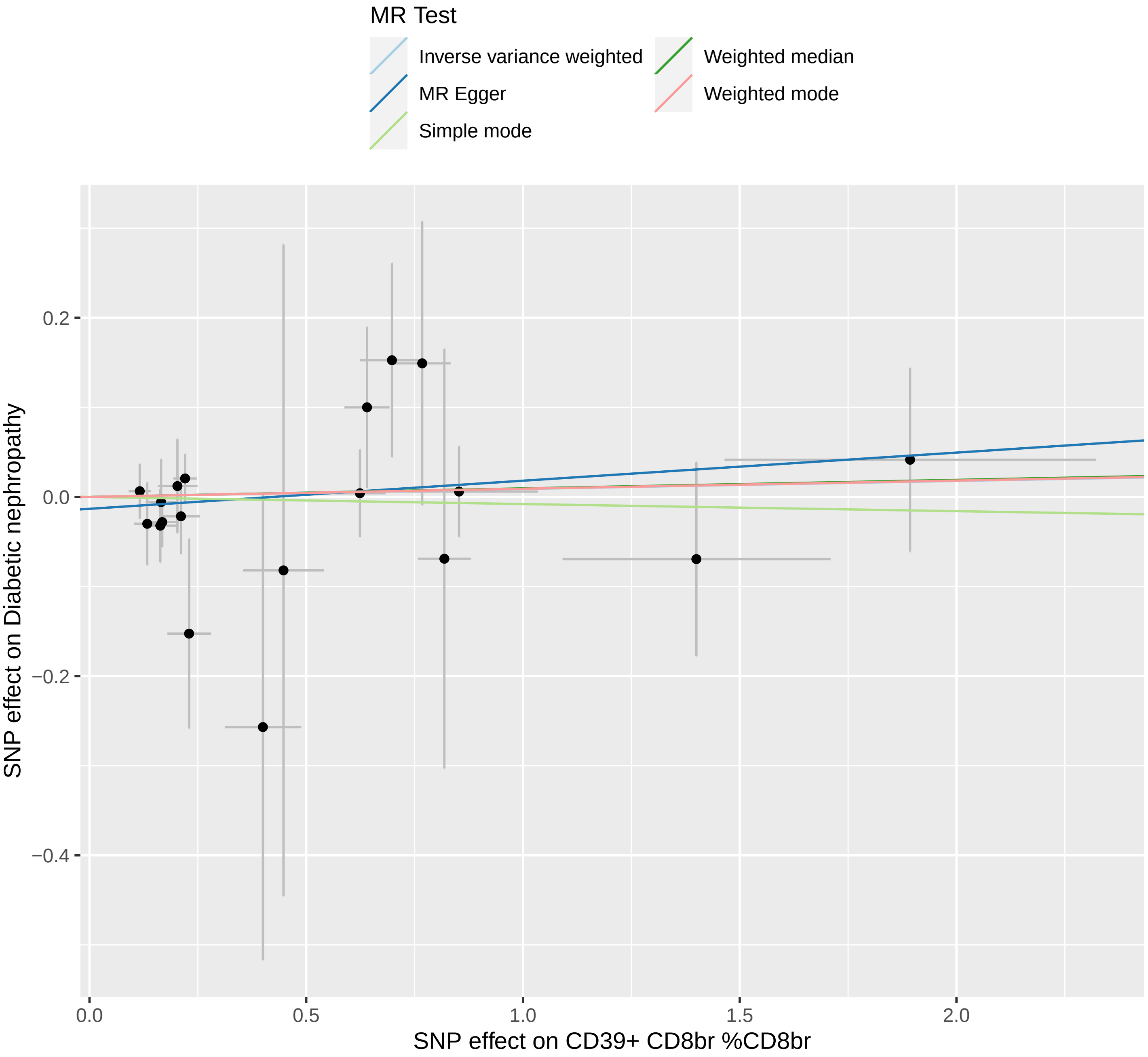

MR Test
Inverse variance weighted
MR Egger
Simple mode
Weighted median
Weighted mode
SNP effect on Diabetic nephropathy
0.2
0.0
−0.2
−0.4
0.0
0.5
1.0
1.5
2.0
SNP effect on CD39+ CD8br %CD8br

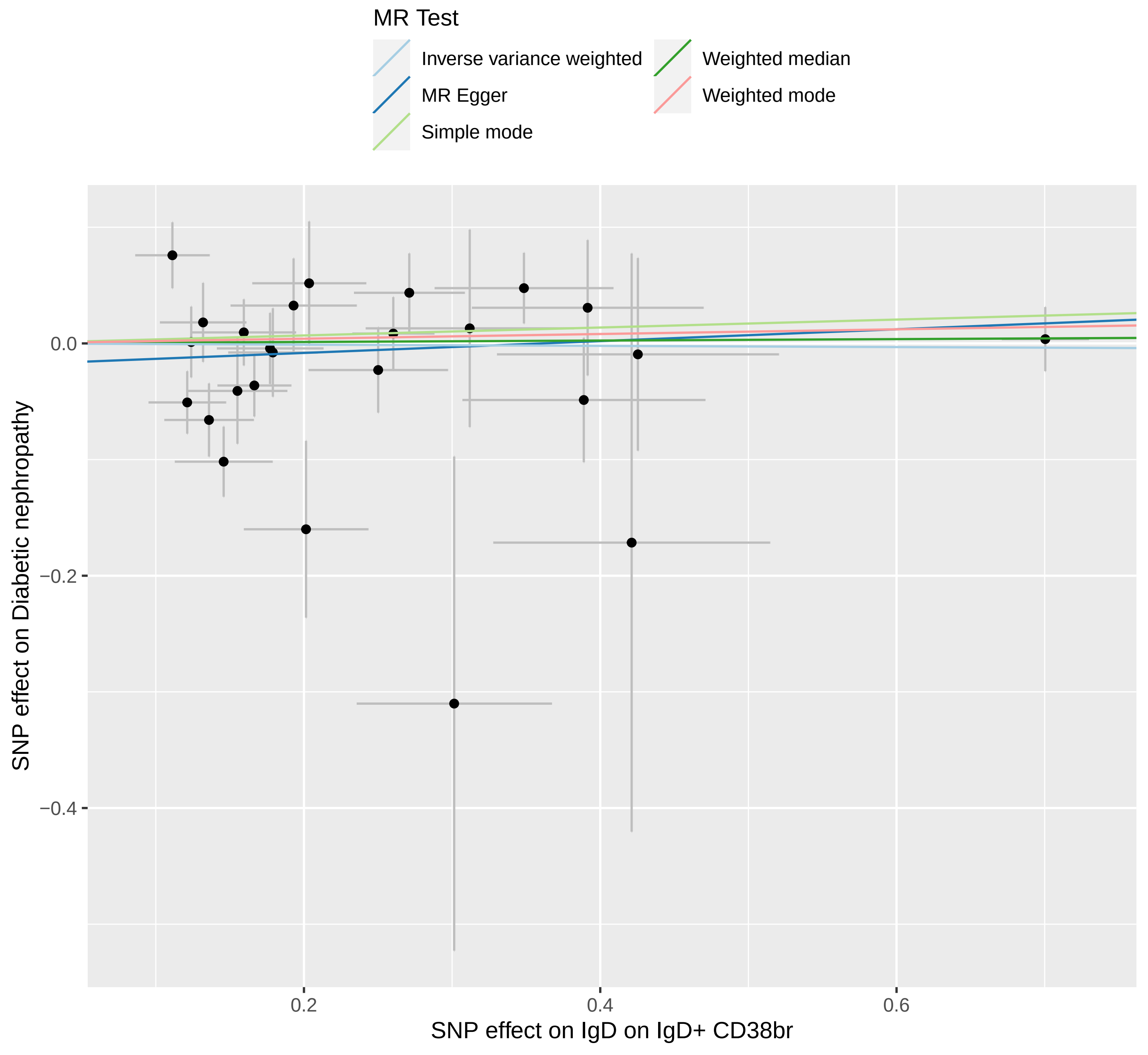

MR Test
Inverse variance weighted
MR Egger
Simple mode
Weighted median
Weighted mode
SNP effect on Diabetic nephropathy
0.0
−0.2
−0.4
0.2
0.4
0.6
SNP effect on IgD on IgD+ CD38br

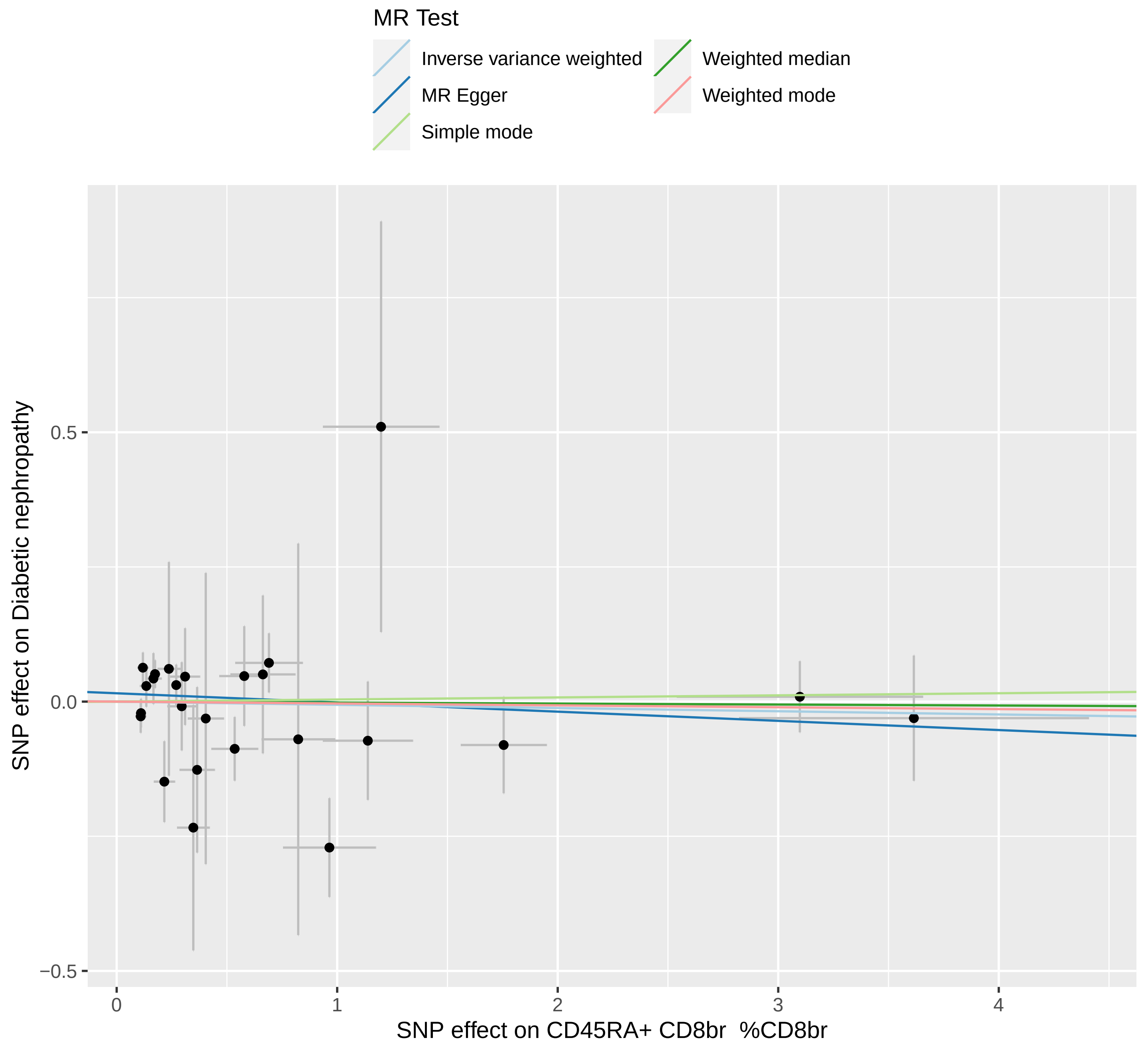
MR Test
Inverse variance weighted
MR Egger
Simple mode
Weighted median
Weighted mode
SNP effect on Diabetic nephropathy
0.5
0.0
−0.5
0
1
2
3
4
SNP effect on CD45RA+ CD8br %CD8br

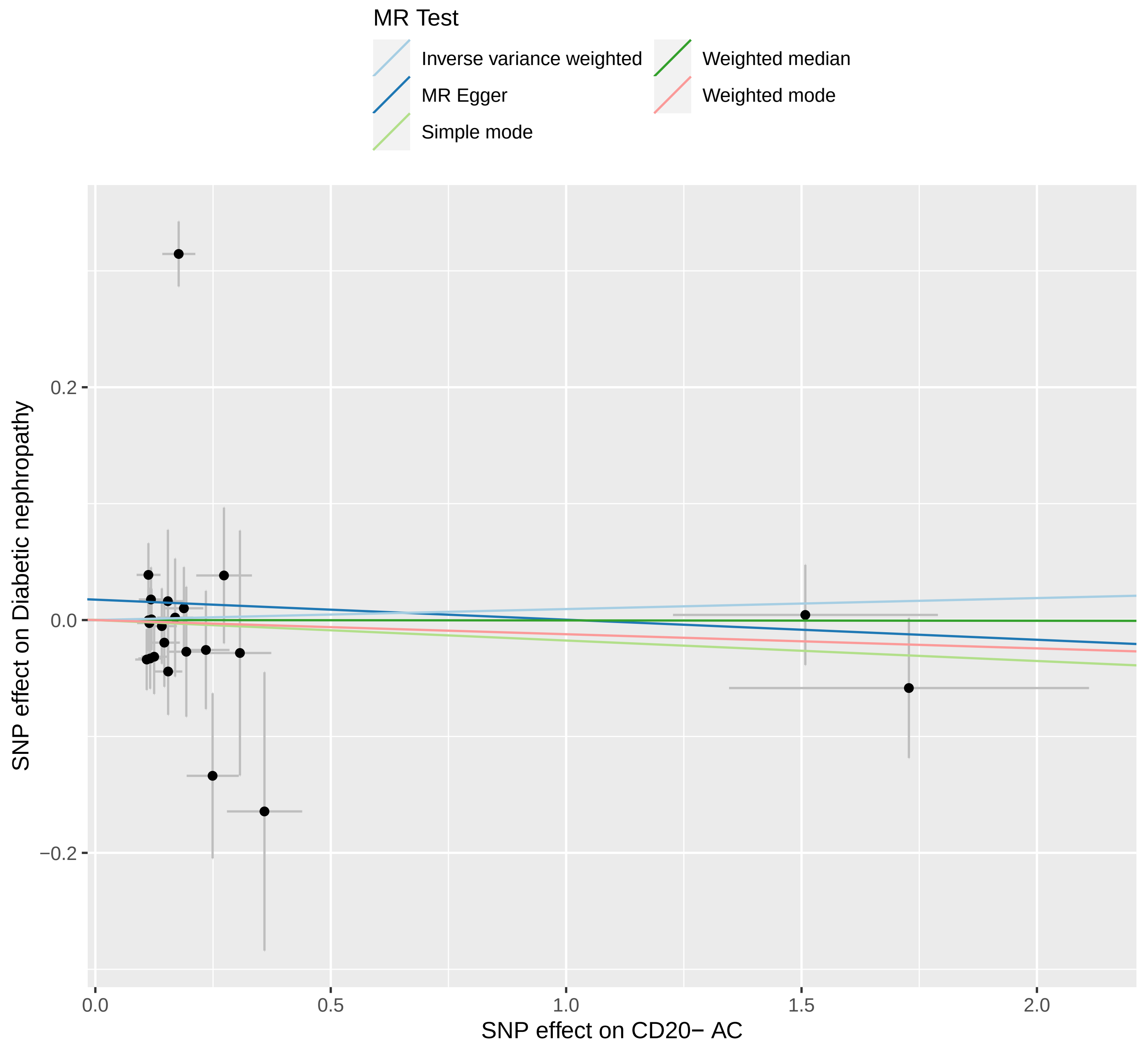
MR Test
Inverse variance weighted
MR Egger
Simple mode
Weighted median
Weighted mode
0.2
0.0
−0.2
SNP effect on Diabetic nephropathy
0.0
0.5
1.0
1.5
2.0
SNP effect on CD20− AC

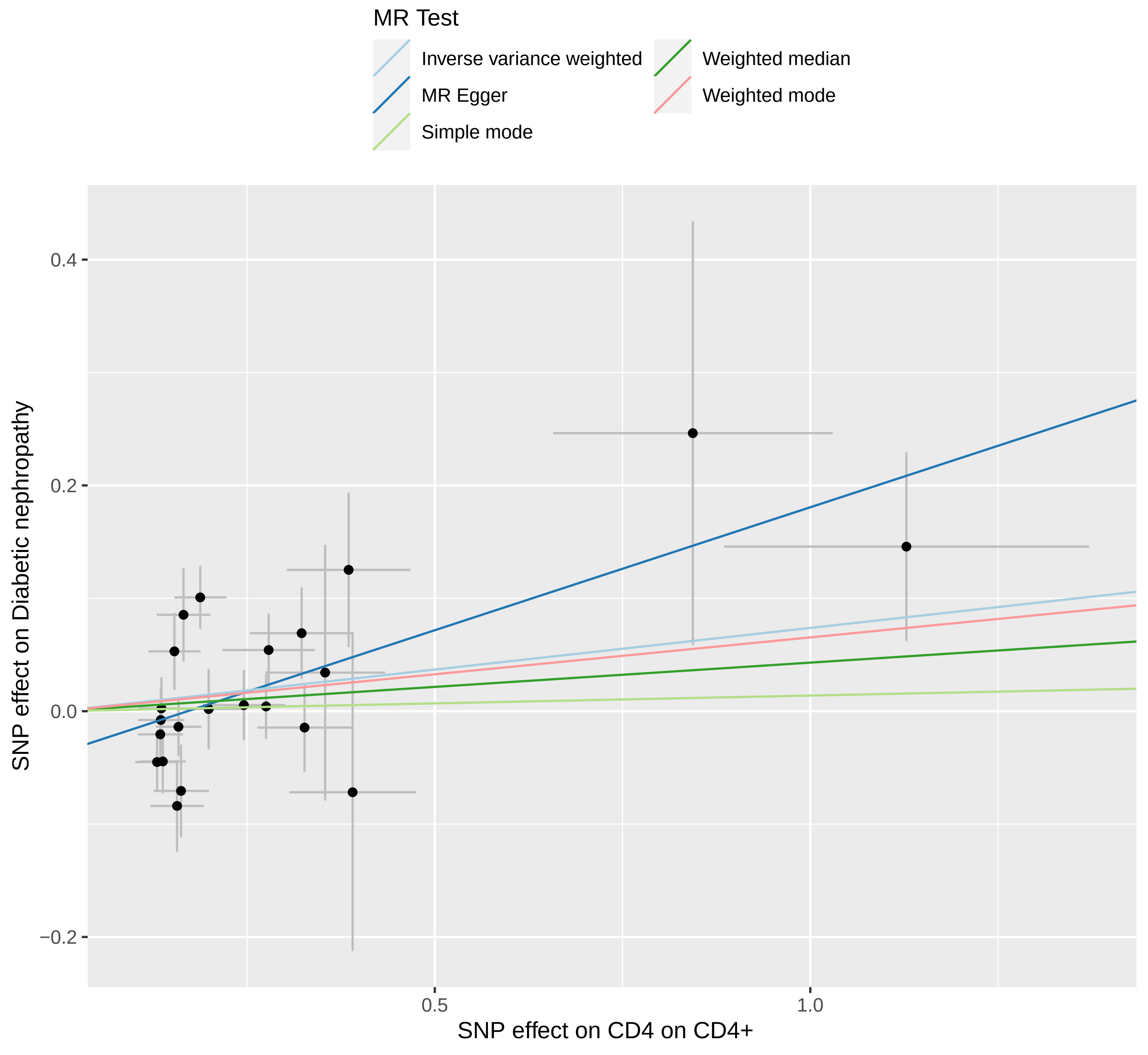

MR Test
Inverse variance weighted
MR Egger
Simple mode
Weighted median
Weighted mode
0.4
0.2
0.0
−0.2
0.5
1.0
SNP effect on Diabetic nephropathy
SNP effect on CD4 on CD4+

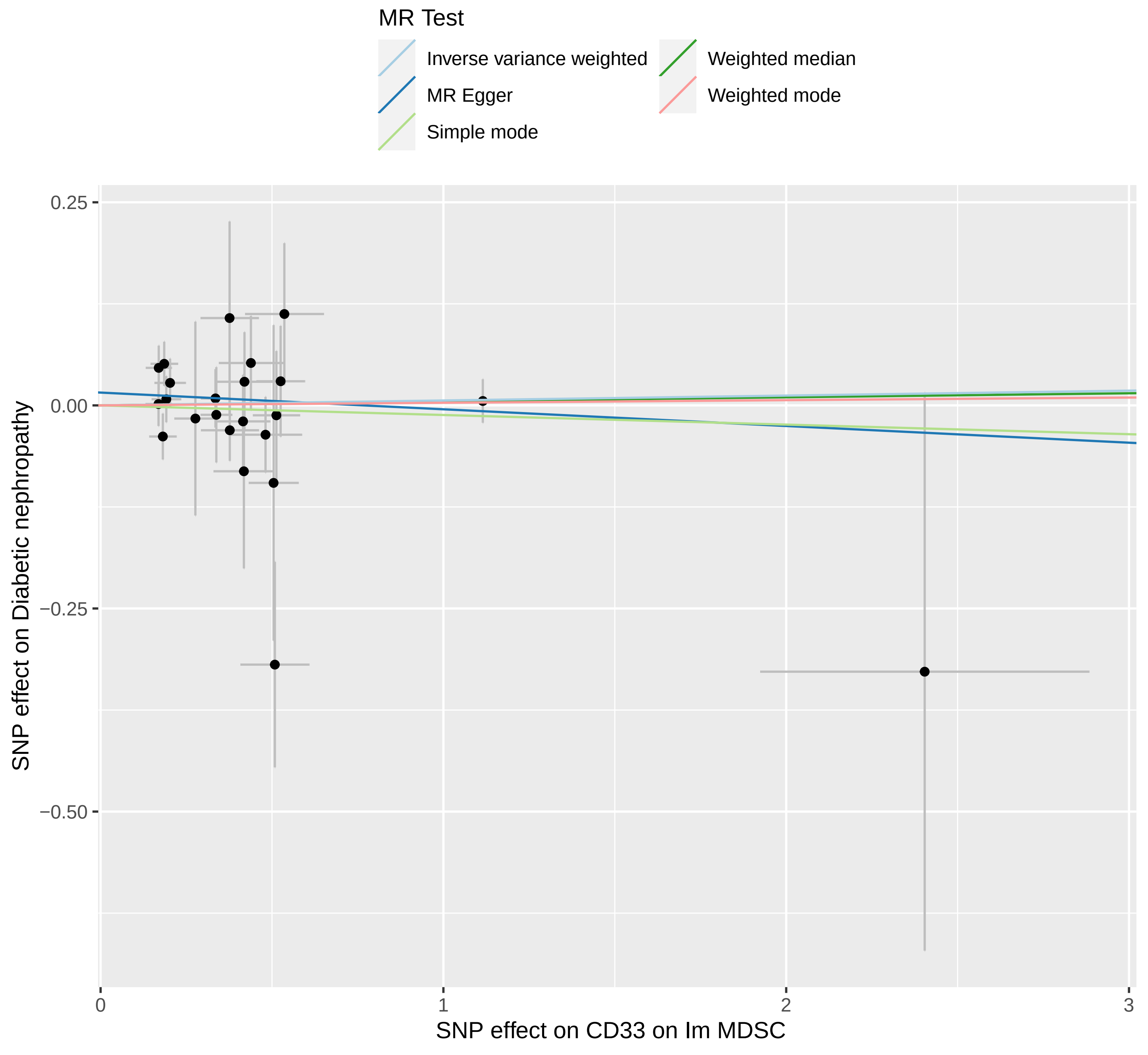
MR Test
Inverse variance weighted
MR Egger
Simple mode
Weighted median
Weighted mode
0.25
0.00
−0.25
−0.50
0
1
2
3
SNP effect on Diabetic nephropathy
SNP effect on CD33 on Im MDSC

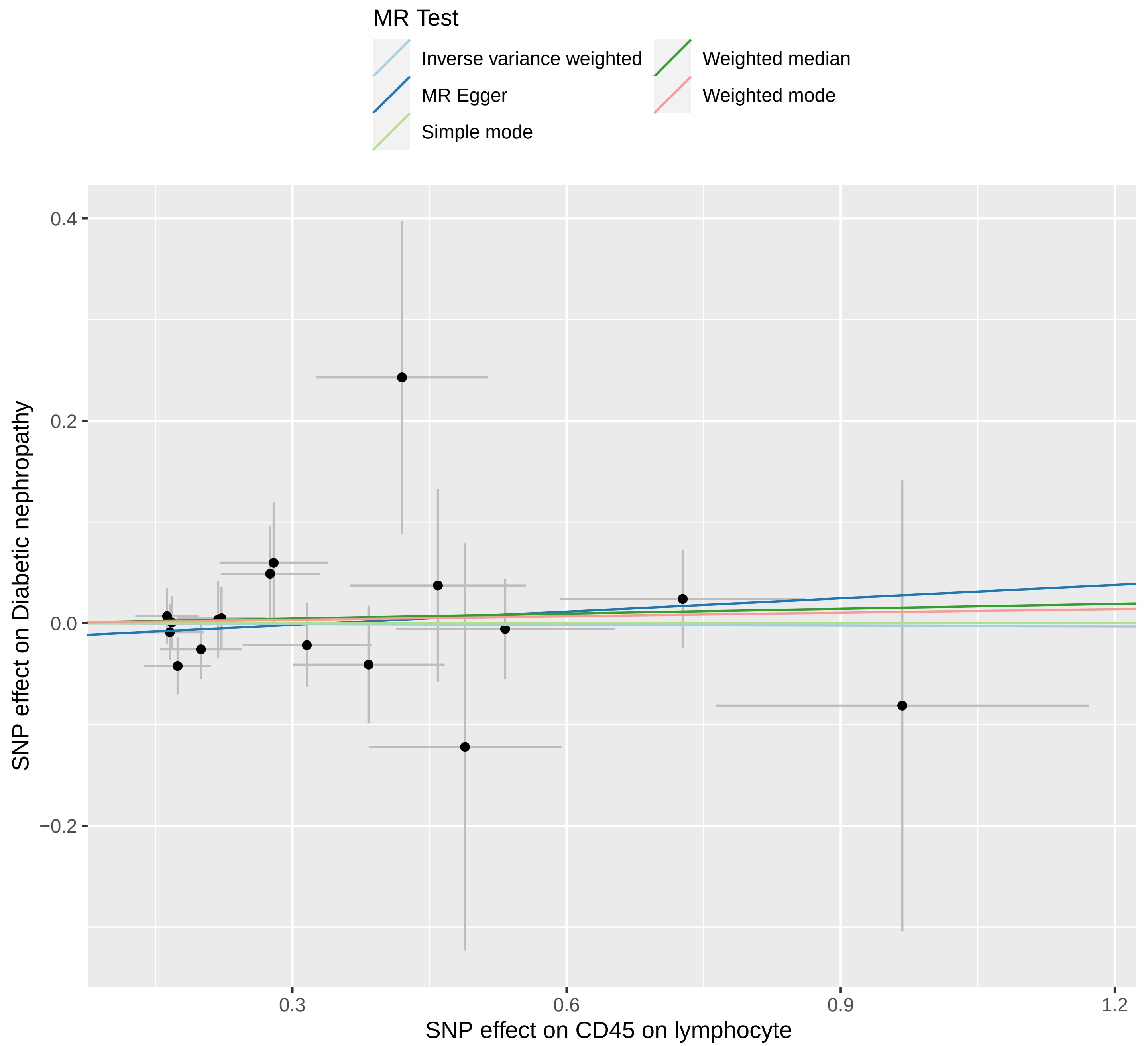

MR Test
Inverse variance weighted
MR Egger
Simple mode
Weighted median
Weighted mode
0.4
0.2
0.0
−0.2
0.3
0.6
0.9
1.2
SNP effect on Diabetic nephropathy
SNP effect on CD45 on lymphocyte

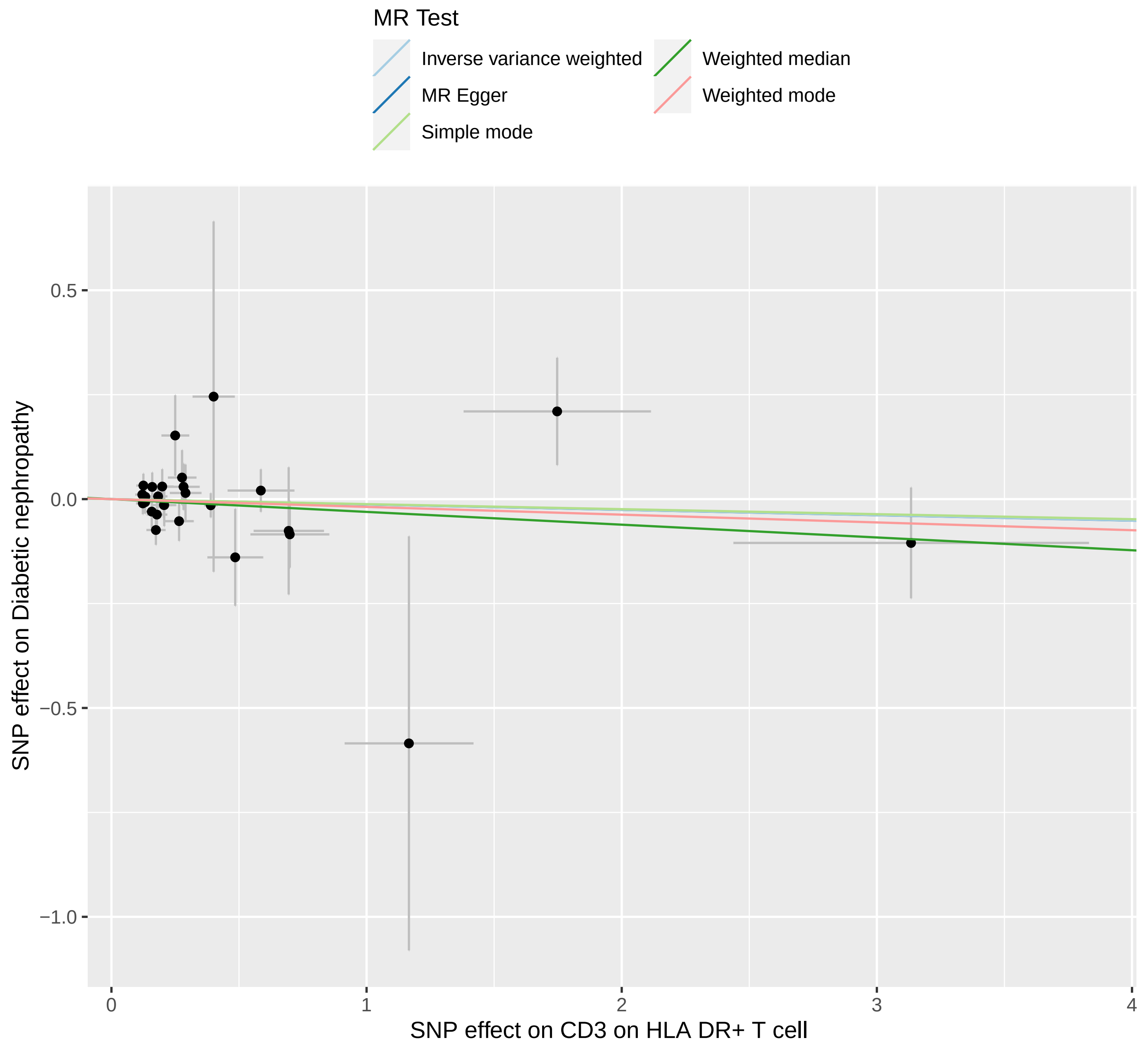
MR Test
Inverse variance weighted
MR Egger
Simple mode
Weighted median
Weighted mode
0.5
0.0
−0.5
−1.0
SNP effect on Diabetic nephropathy
0
1
2
3
4
SNP effect on CD3 on HLA DR+ T cell

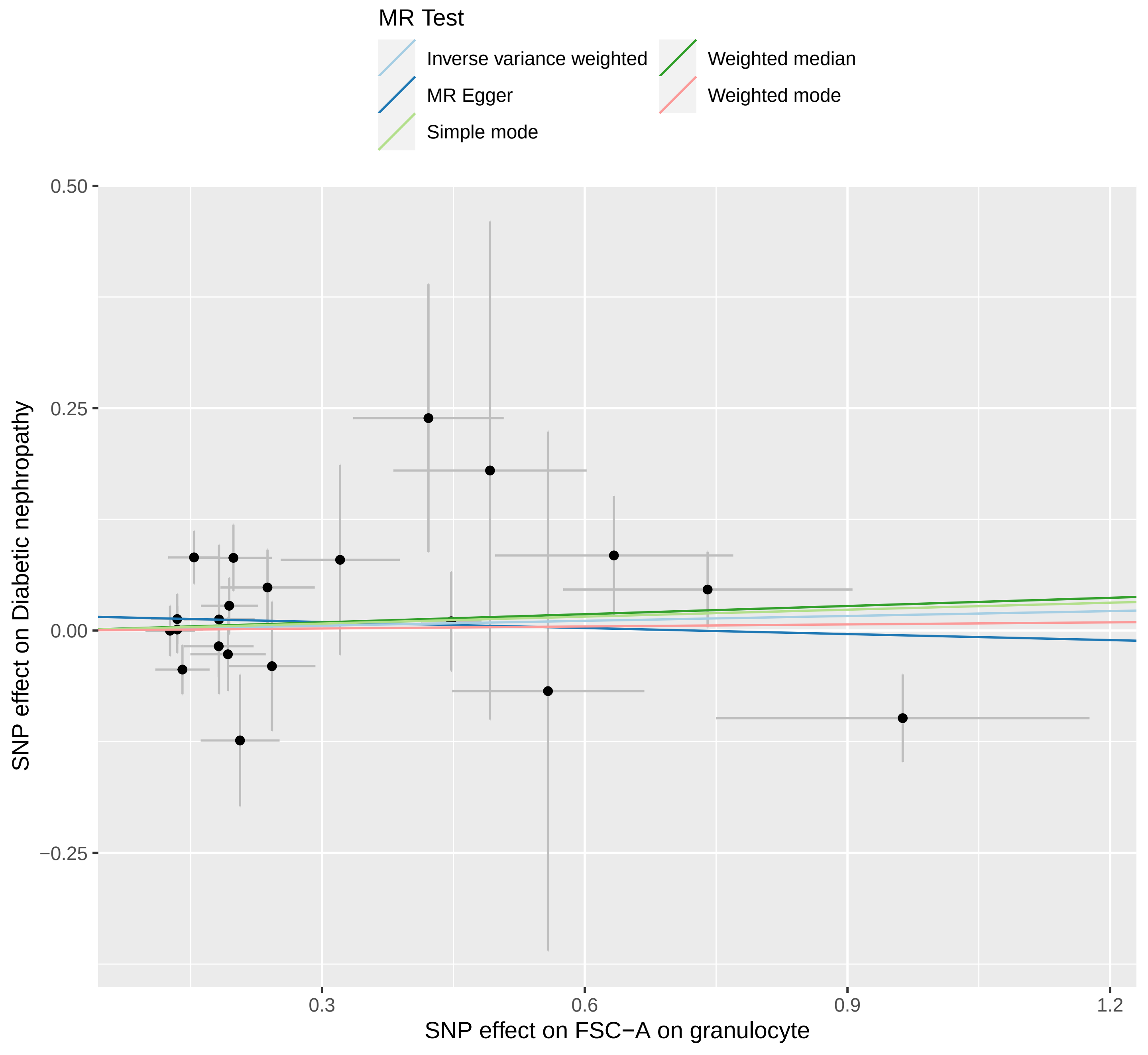

MR Test
Inverse variance weighted
MR Egger
Simple mode
Weighted median
Weighted mode
0.50
0.25
0.00
−0.25
0.3
0.6
0.9
1.2
SNP effect on Diabetic nephropathy
SNP effect on FSC−A on granulocyte

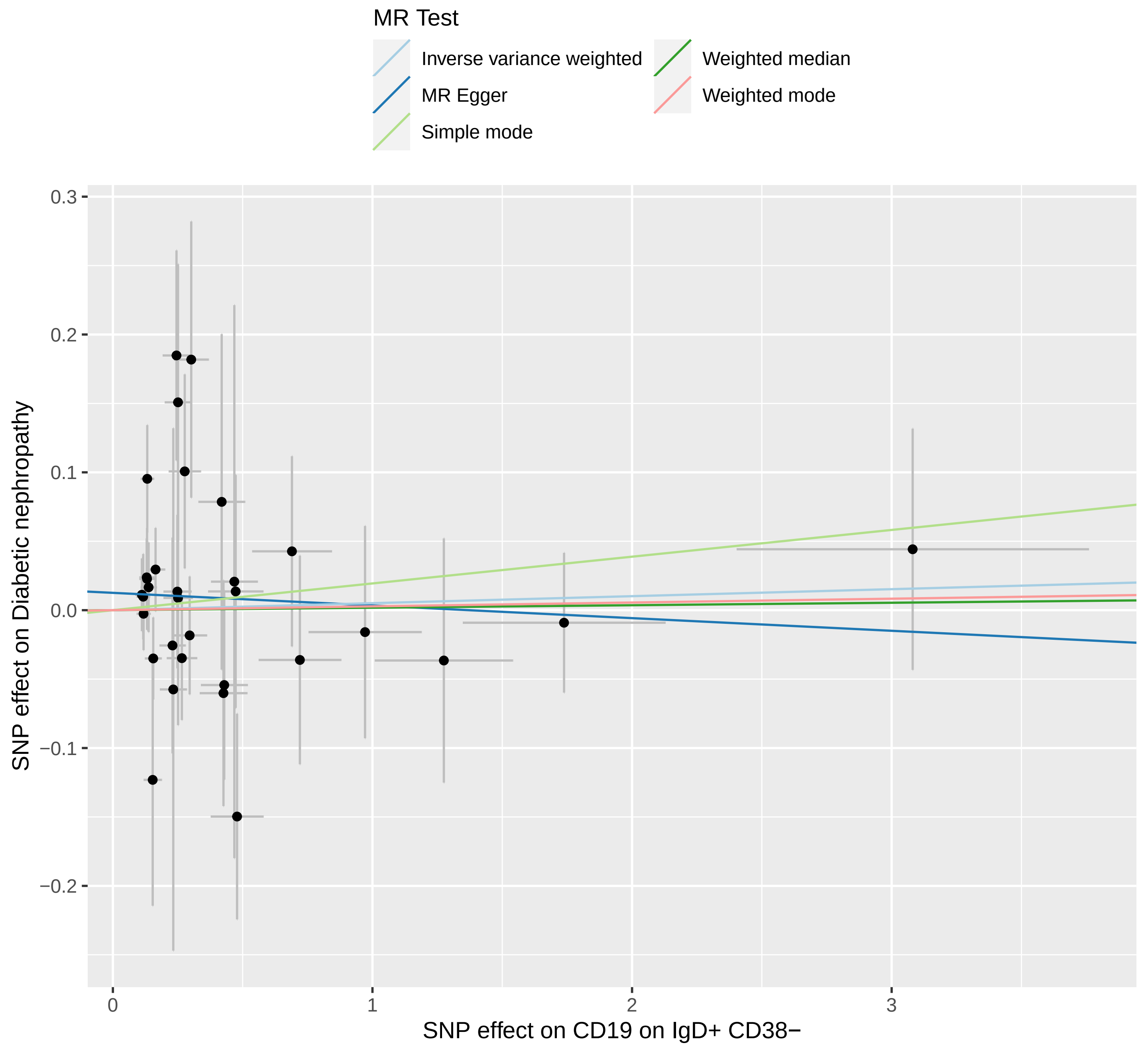
MR Test
Inverse variance weighted
MR Egger
Simple mode
Weighted median
Weighted mode
0.3
0.2
0.1
0.0
−0.1
−0.2
SNP effect on Diabetic nephropathy
0
1
2
3
SNP effect on CD19 on IgD+ CD38−

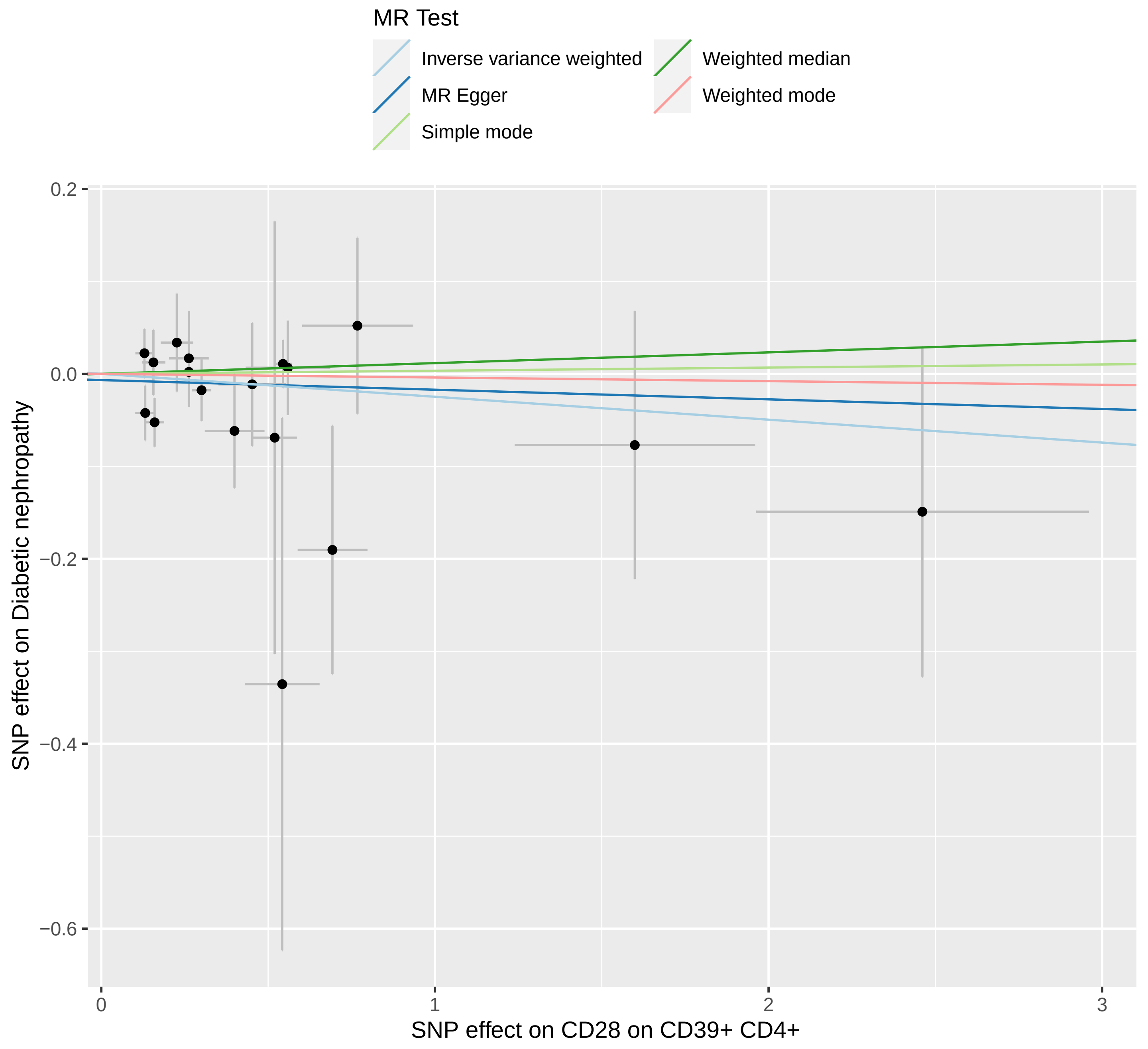

MR Test
Inverse variance weighted
MR Egger
Simple mode
Weighted median
Weighted mode
0.2
0.0
−0.2
−0.4
−0.6
SNP effect on Diabetic nephropathy
0
1
2
3
SNP effect on CD28 on CD39+ CD4+

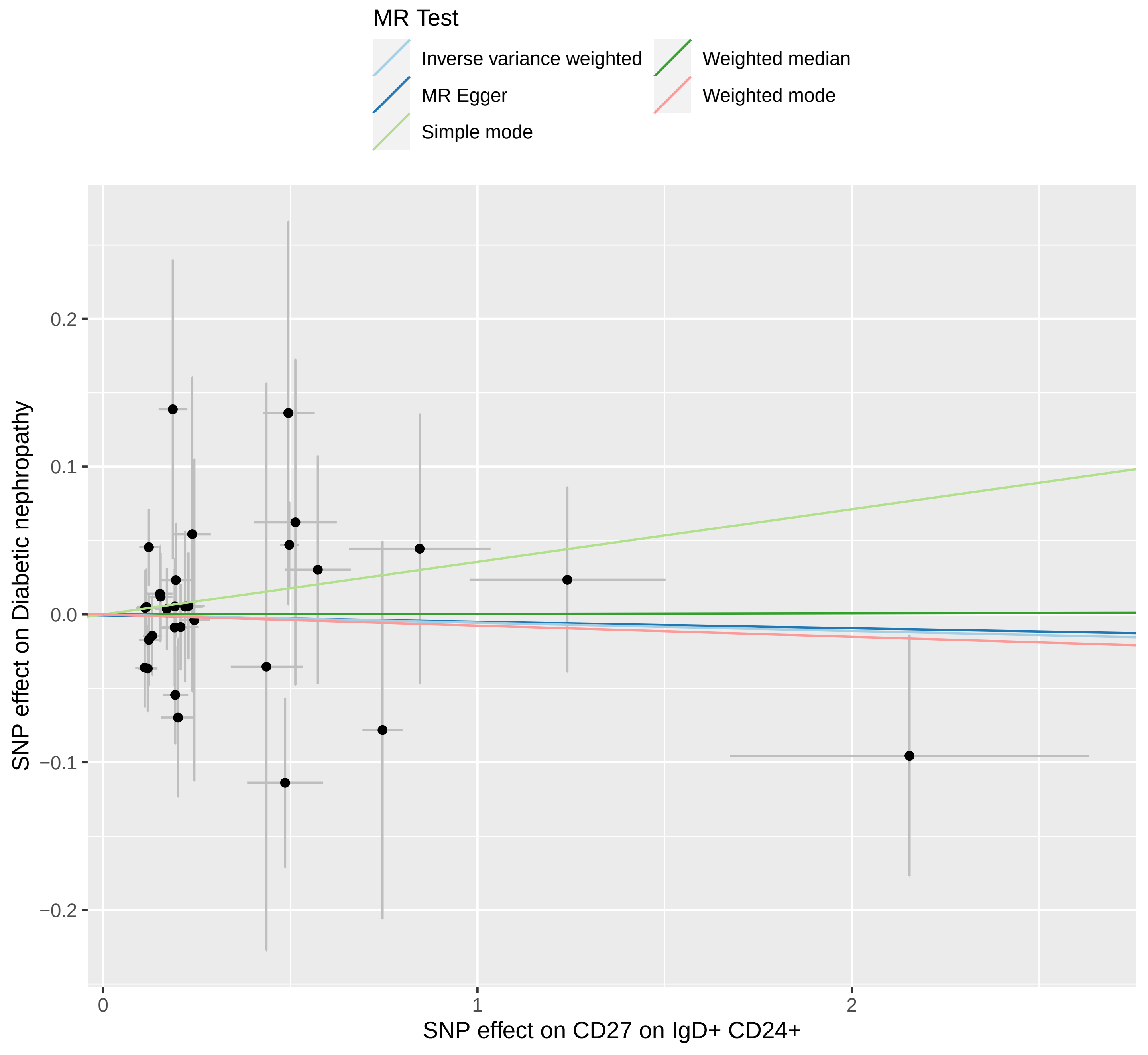

MR Test
Inverse variance weighted
MR Egger
Simple mode
Weighted median
Weighted mode
SNP effect on Diabetic nephropathy
0.2
0.1
0.0
−0.1
−0.2
0
1
2
SNP effect on CD27 on IgD+ CD24+

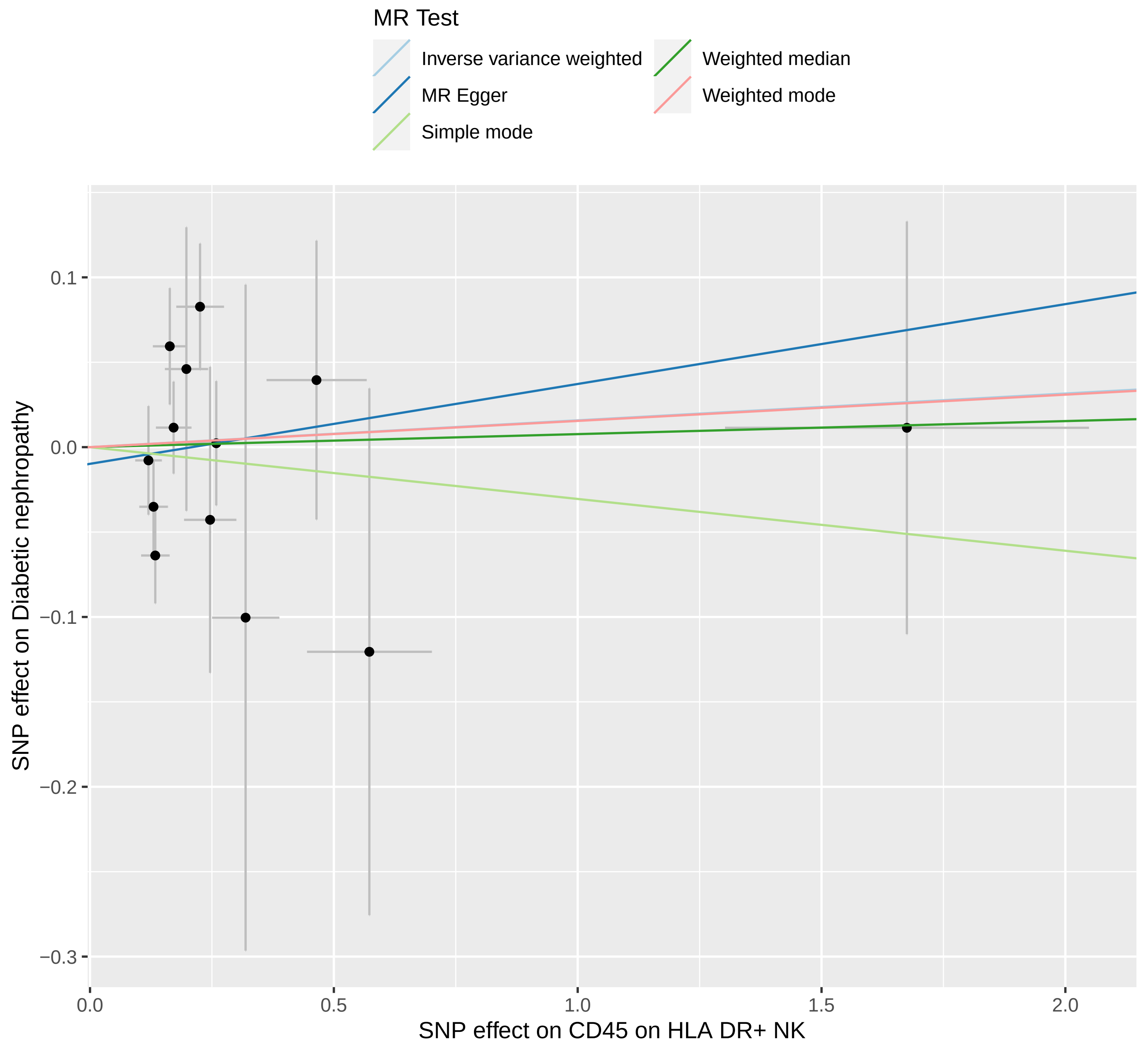
MR Test
Inverse variance weighted
MR Egger
Simple mode
Weighted median
Weighted mode
0.1
0.0
−0.1
−0.2
−0.3
0.0
0.5
1.0
1.5
2.0
SNP effect on Diabetic nephropathy
SNP effect on CD45 on HLA DR+ NK

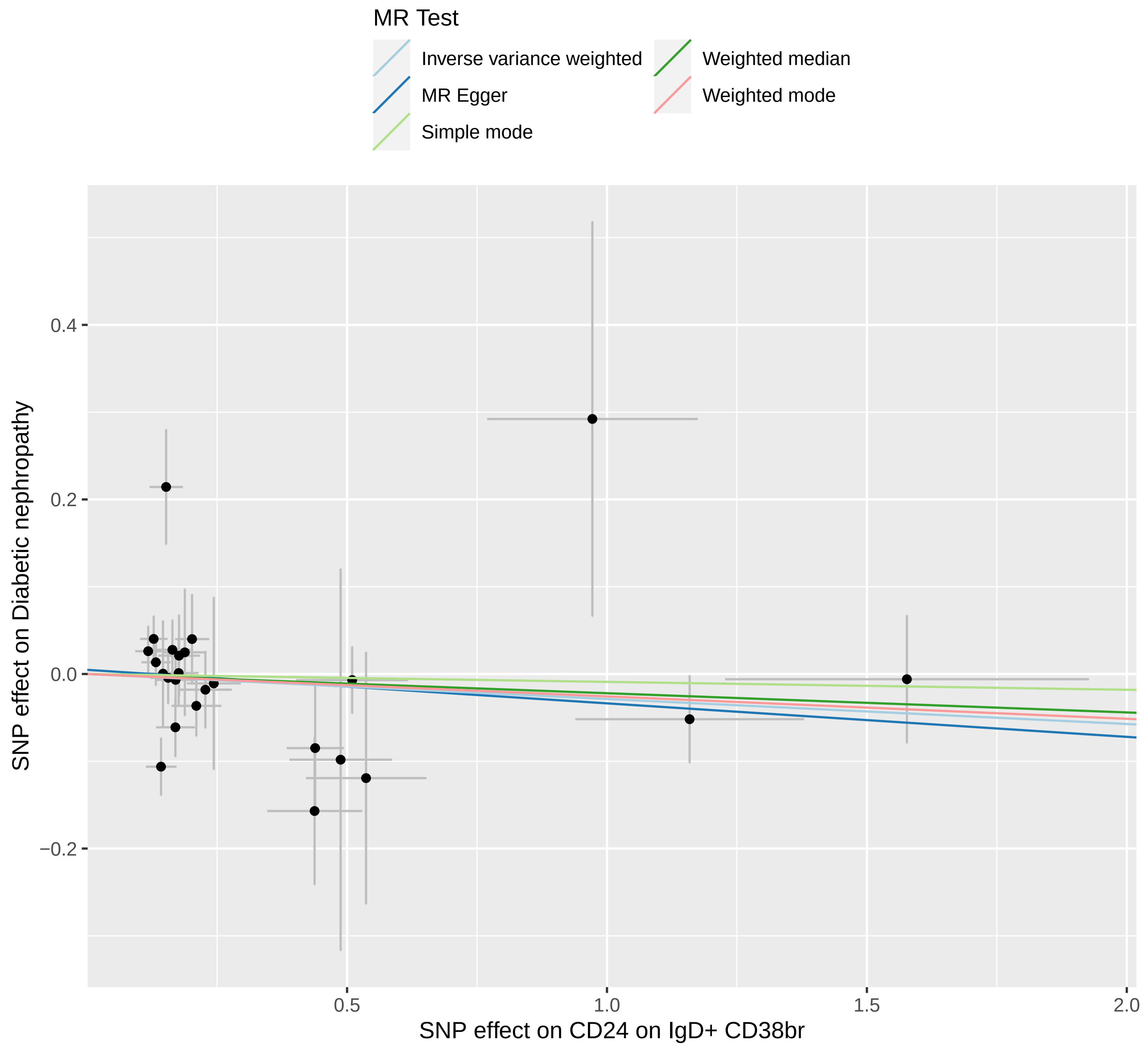
MR Test
Inverse variance weighted
MR Egger
Simple mode
Weighted median
Weighted mode
SNP effect on Diabetic nephropathy
0.4
0.2
0.0
−0.2
0.5
1.0
1.5
2.0
SNP effect on CD24 on IgD+ CD38br

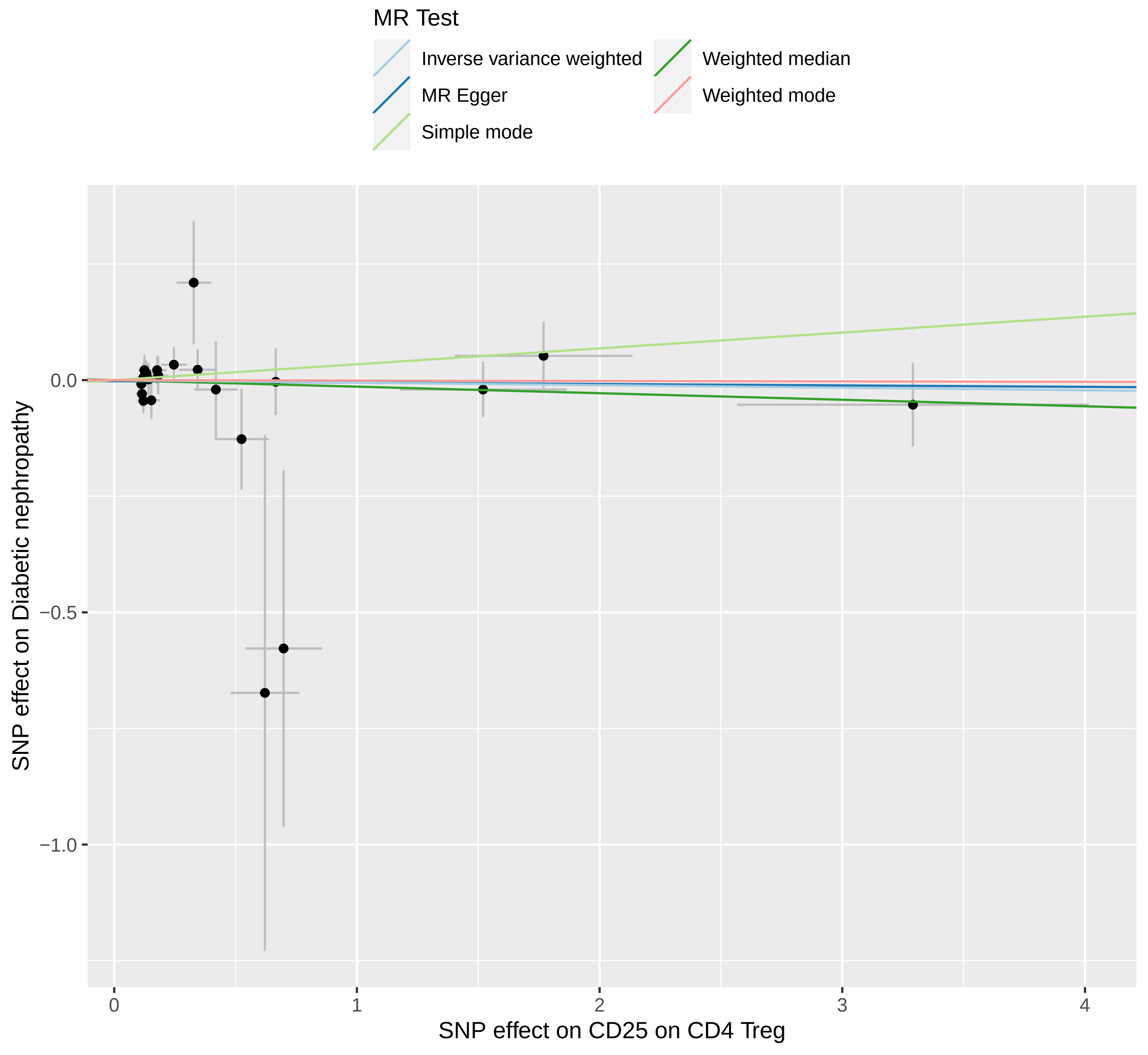
MR Test
Inverse variance weighted
MR Egger
Simple mode
Weighted median
Weighted mode
0.0
−0.5
−1.0
0
1
2
3
4
SNP effect on Diabetic nephropathy
SNP effect on CD25 on CD4 Treg

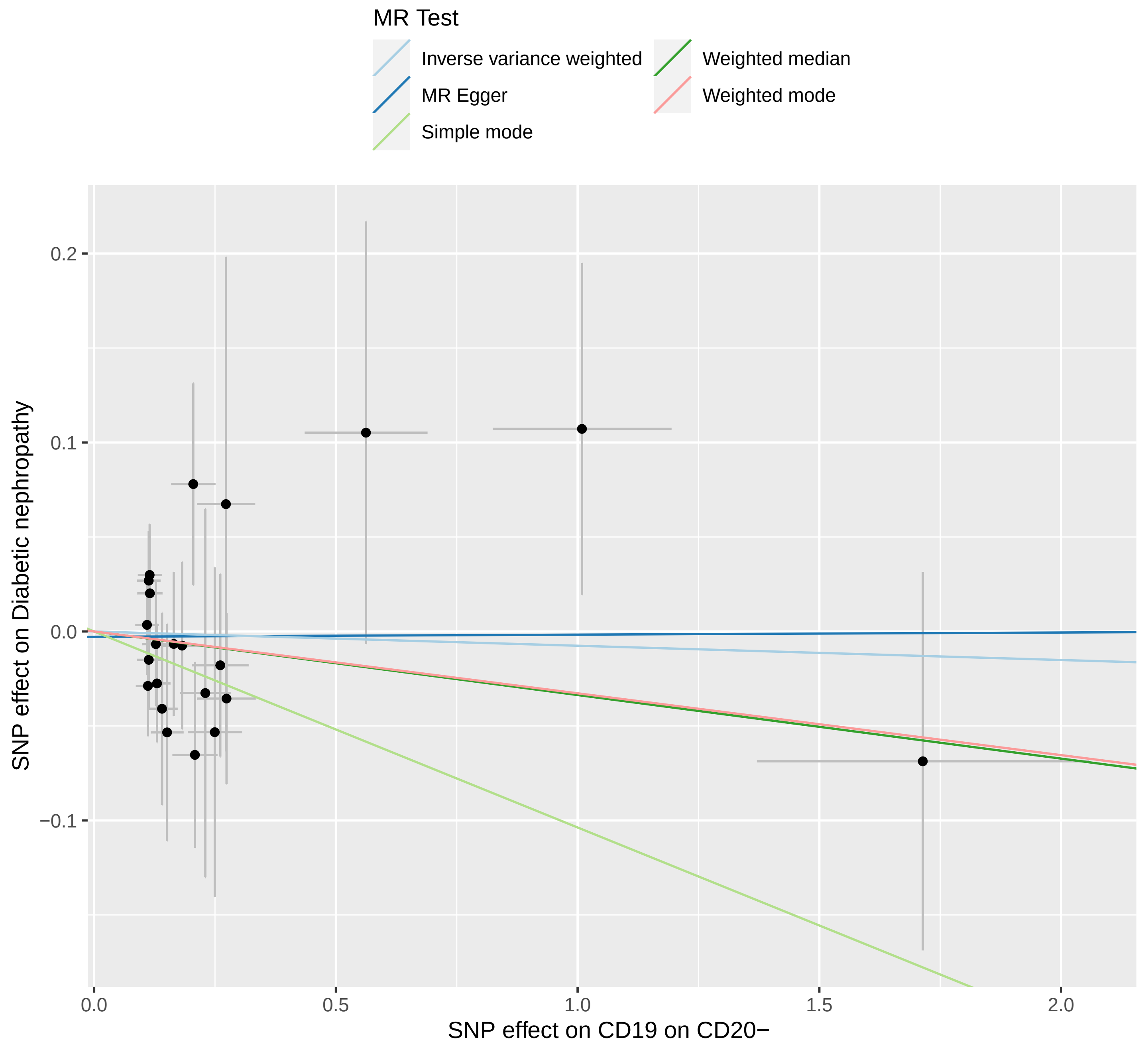
MR Test
Inverse variance weighted
MR Egger
Simple mode
Weighted median
Weighted mode
0.2
0.1
0.0
−0.1
0.0
0.5
1.0
1.5
2.0
SNP effect on Diabetic nephropathy
SNP effect on CD19 on CD20−

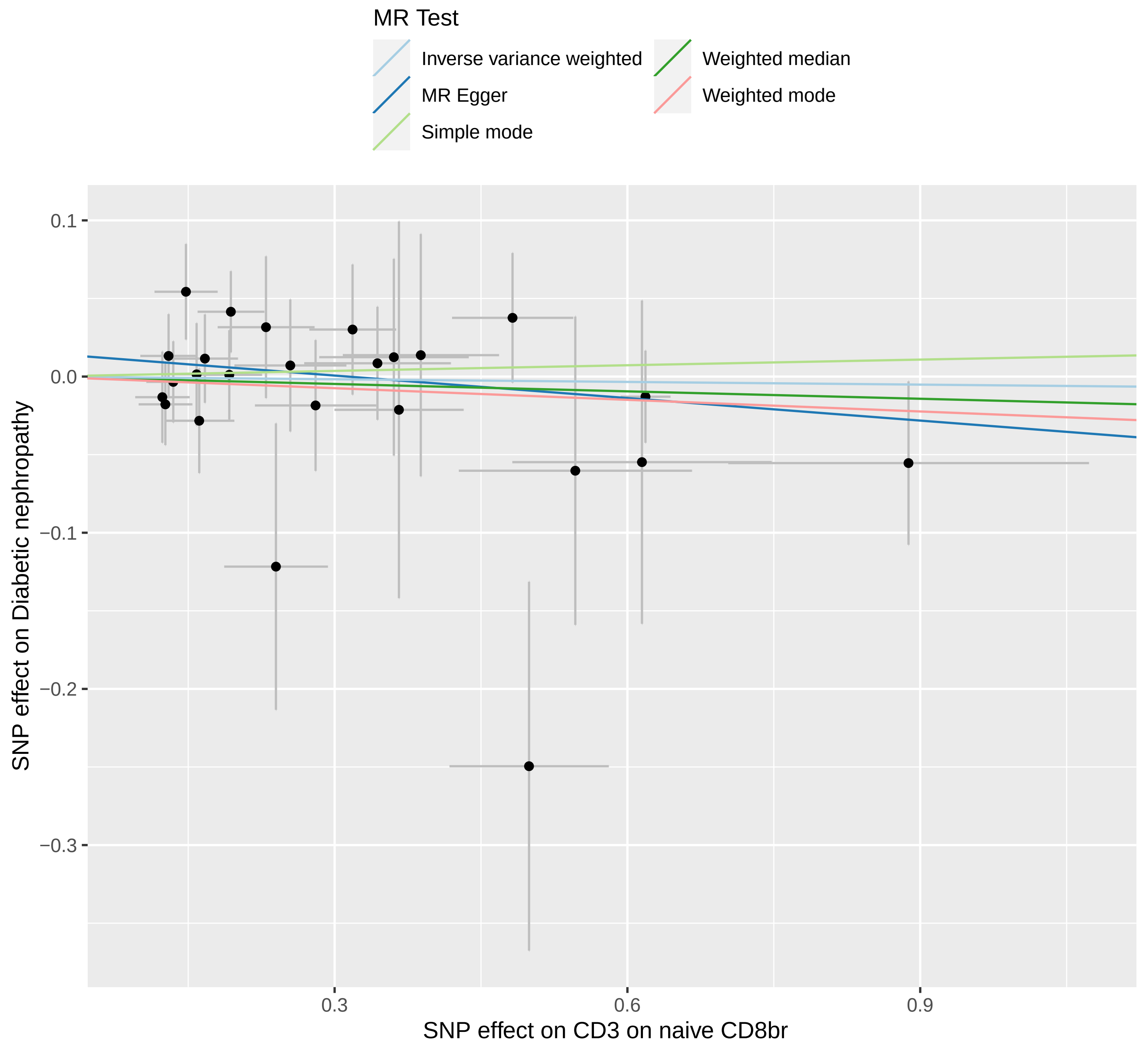
MR Test
Inverse variance weighted
MR Egger
Simple mode
Weighted median
Weighted mode
0.1
0.0
−0.1
−0.2
−0.3
0.3
0.6
0.9
SNP effect on Diabetic nephropathy
SNP effect on CD3 on naive CD8br

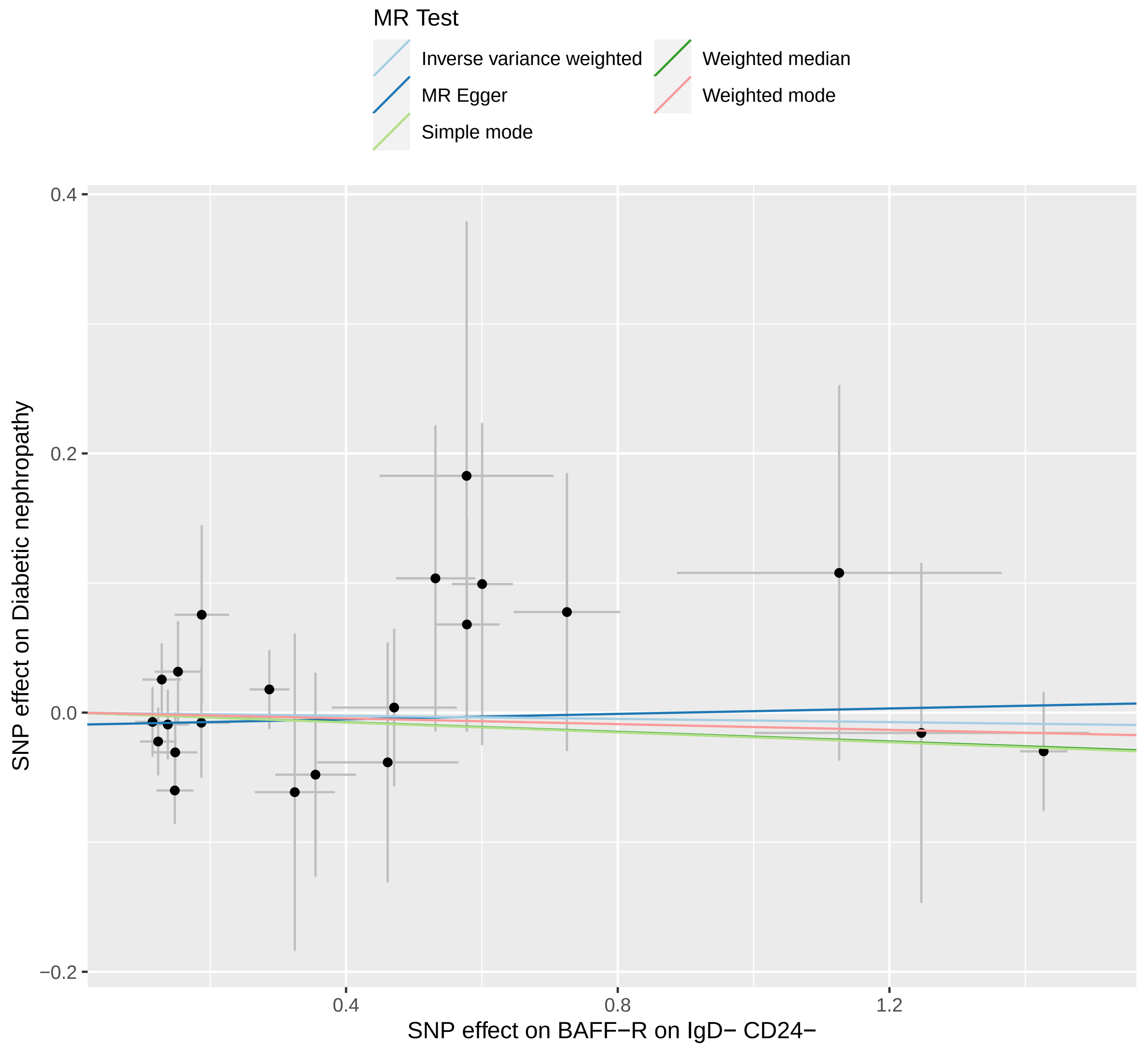
MR Test
Inverse variance weighted
MR Egger
Simple mode
Weighted median
Weighted mode
0.4
0.2
0.0
−0.2
0.4
0.8
1.2
SNP effect on Diabetic nephropathy
SNP effect on BAFF−R on IgD− CD24−

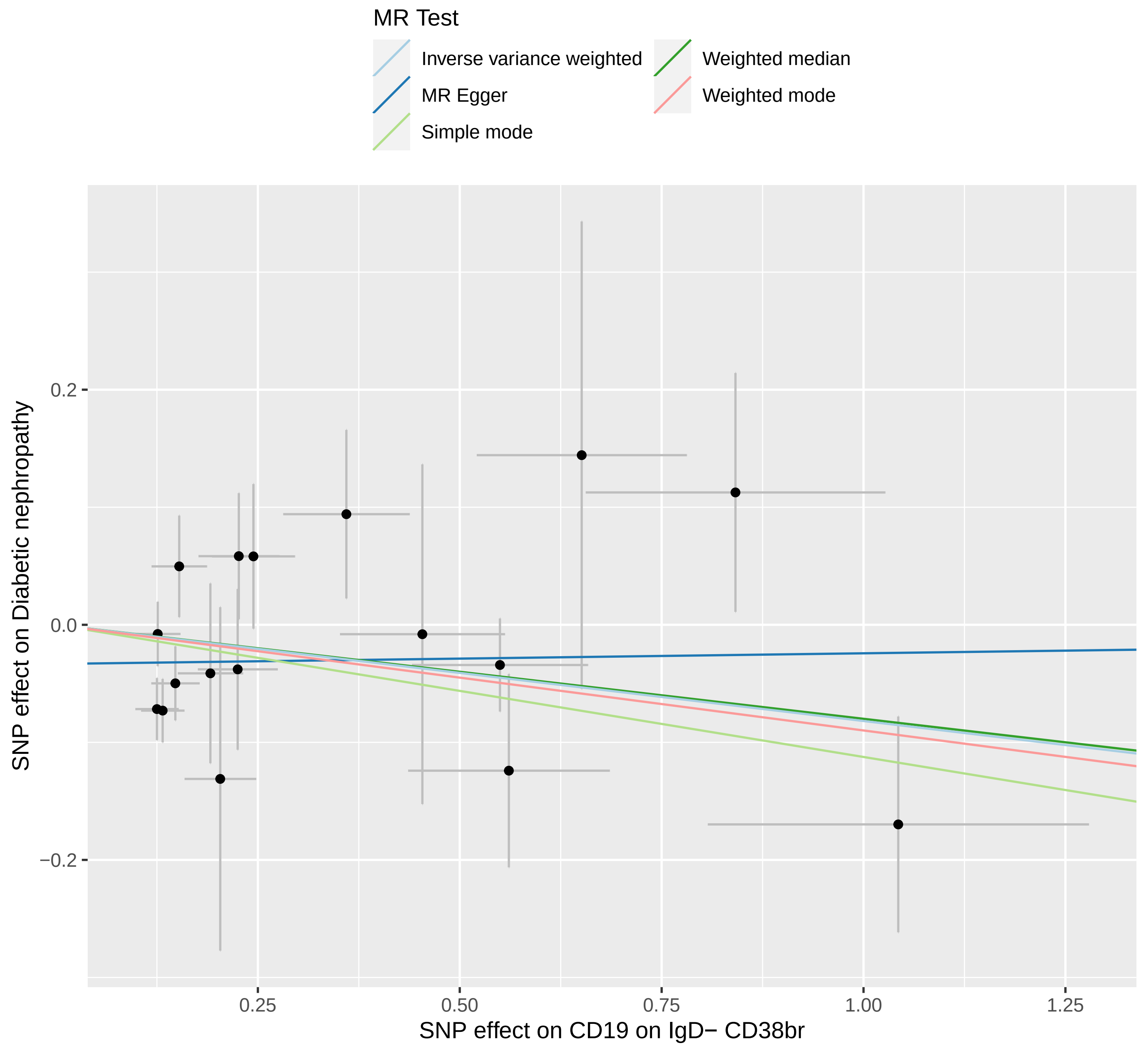
MR Test
Inverse variance weighted
MR Egger
Simple mode
Weighted median
Weighted mode
0.2
0.0
−0.2
0.25
0.50
0.75
1.00
1.25
SNP effect on Diabetic nephropathy
SNP effect on CD19 on IgD− CD38br

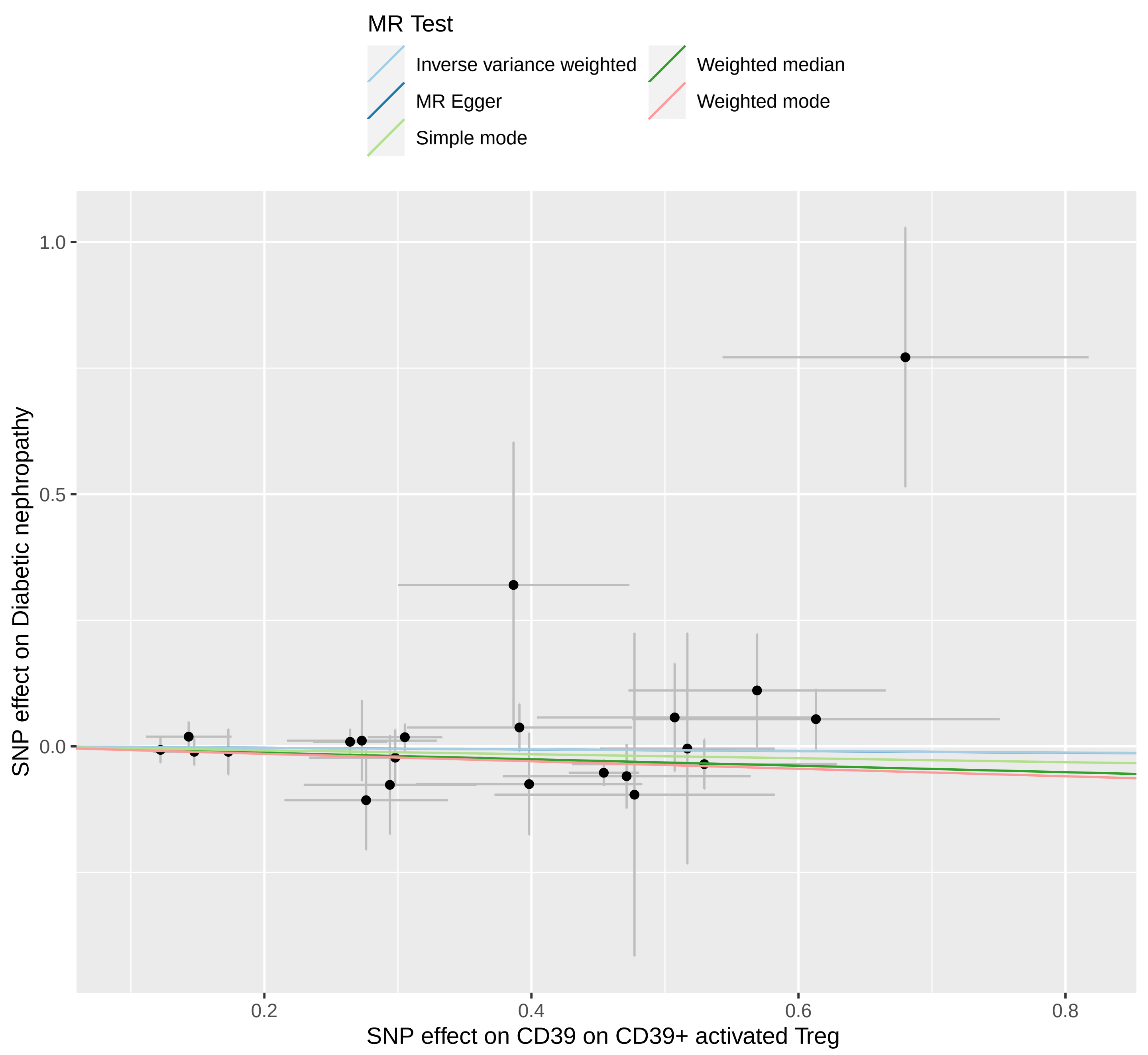

MR Test
Inverse variance weighted
MR Egger
Simple mode
Weighted median
Weighted mode
1.0
0.5
0.0
0.2
0.4
0.6
0.8
SNP effect on Diabetic nephropathy
SNP effect on CD39 on CD39+ activated Treg

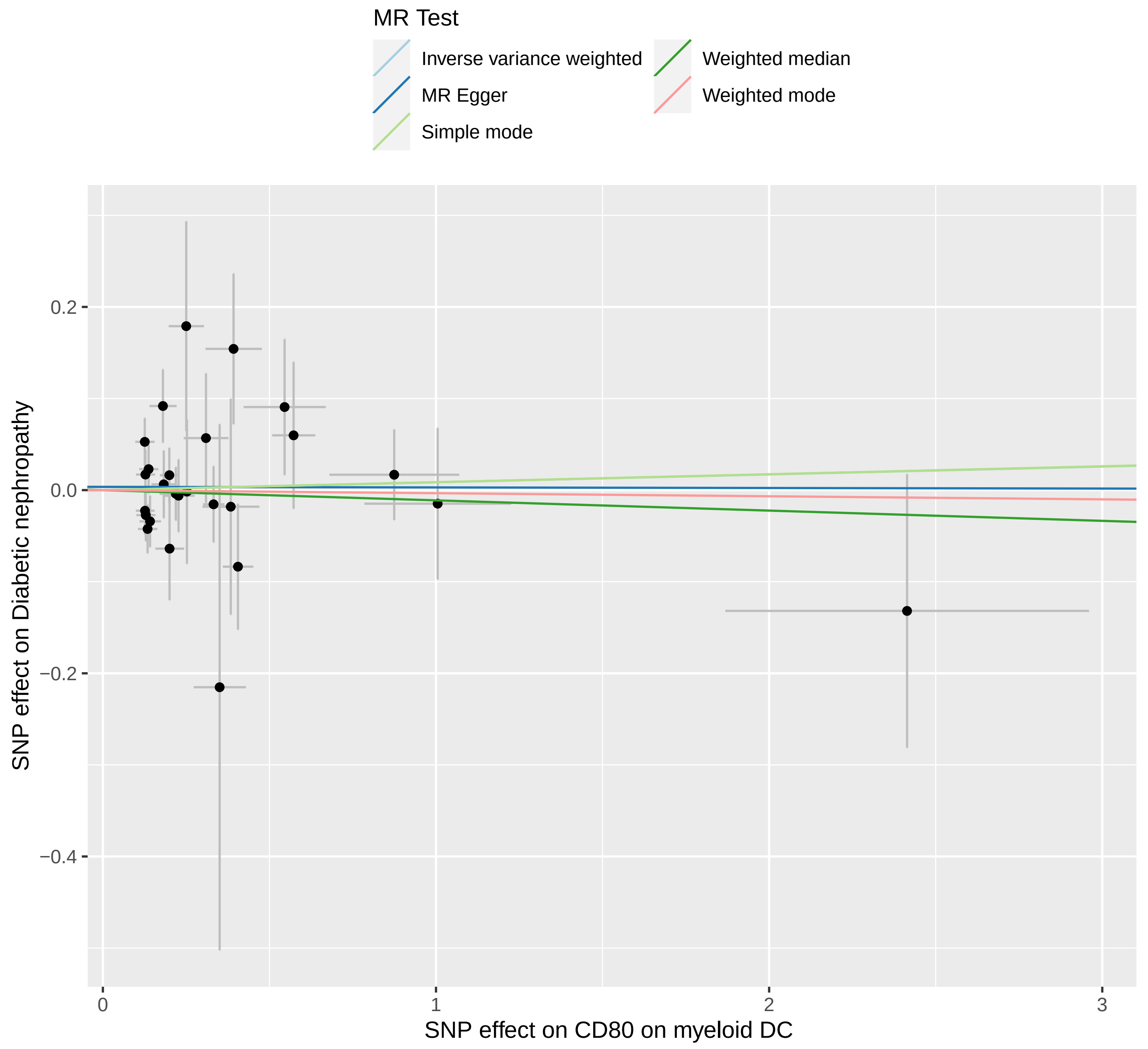

MR Test
Inverse variance weighted
MR Egger
Simple mode
Weighted median
Weighted mode
0.2
0.0
−0.2
−0.4
0
1
2
3
SNP effect on Diabetic nephropathy
SNP effect on CD80 on myeloid DC

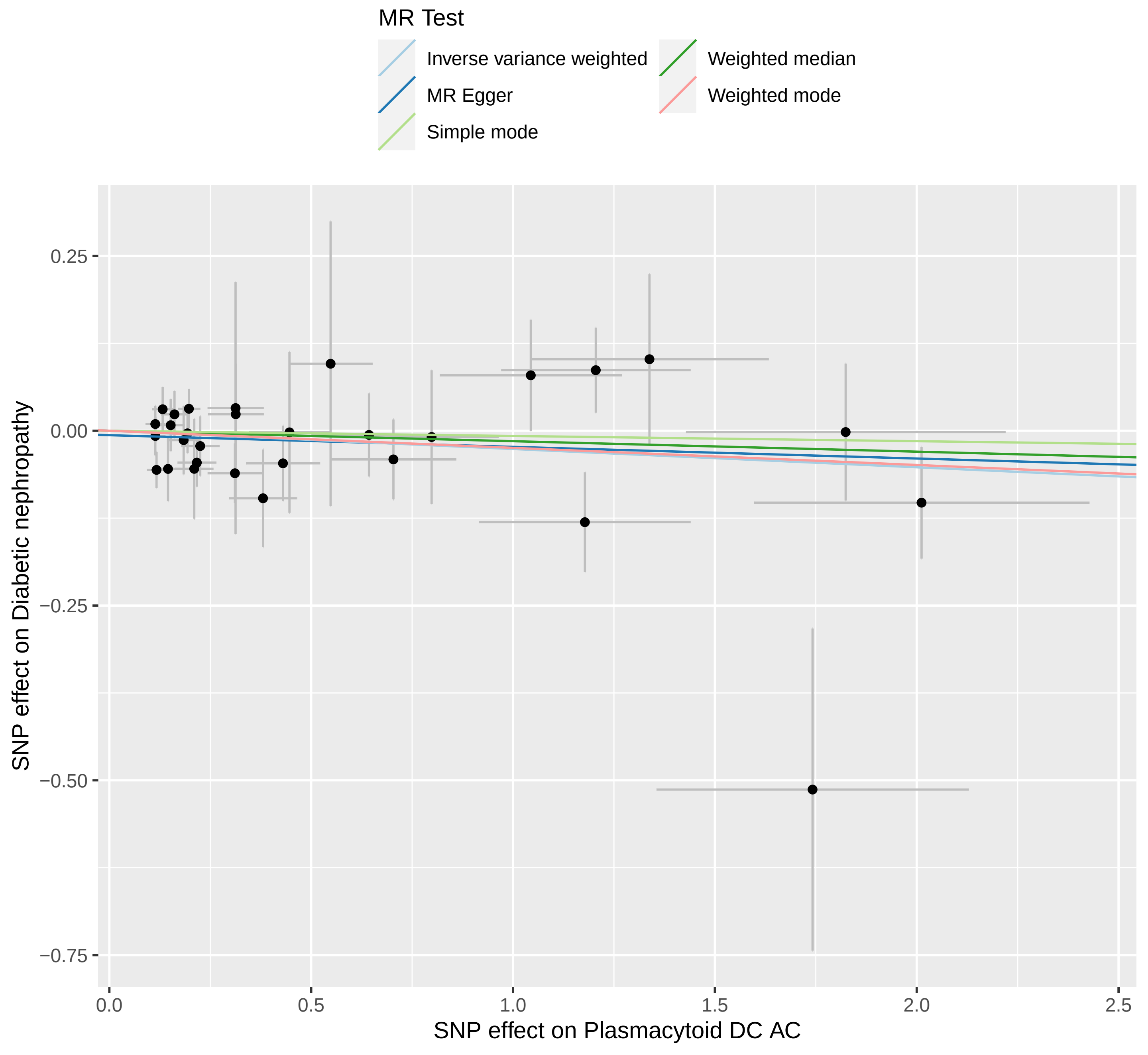

MR Test
Inverse variance weighted
MR Egger
Simple mode
Weighted median
Weighted mode
0.25
0.00
−0.25
−0.50
−0.75
0.0
0.5
1.0
1.5
2.0
2.5
SNP effect on Diabetic nephropathy
SNP effect on Plasmacytoid DC AC

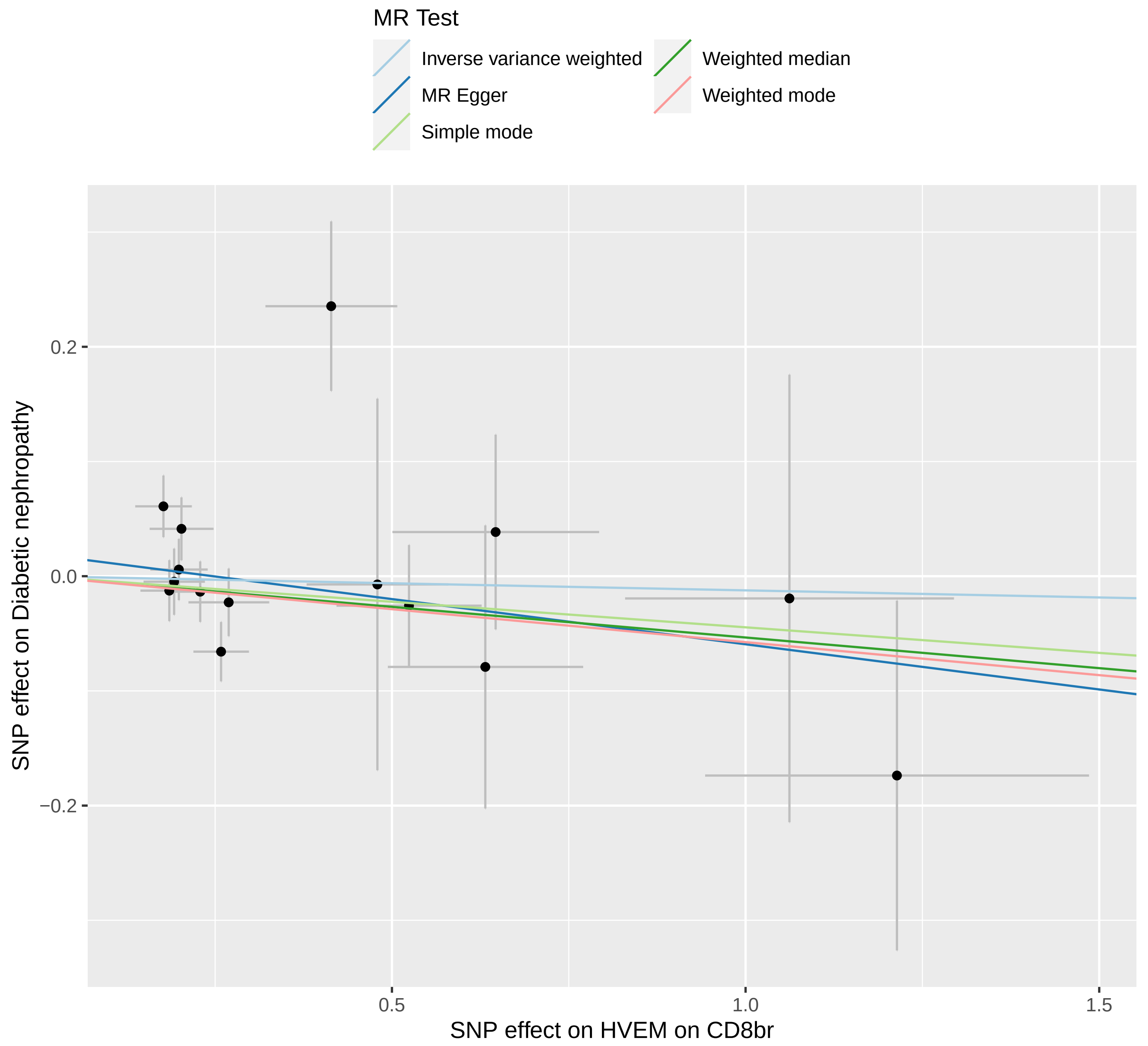
MR Test
Inverse variance weighted
MR Egger
Simple mode
Weighted median
Weighted mode
0.2
0.0
−0.2
0.5
1.0
1.5
SNP effect on Diabetic nephropathy
SNP effect on HVEM on CD8br

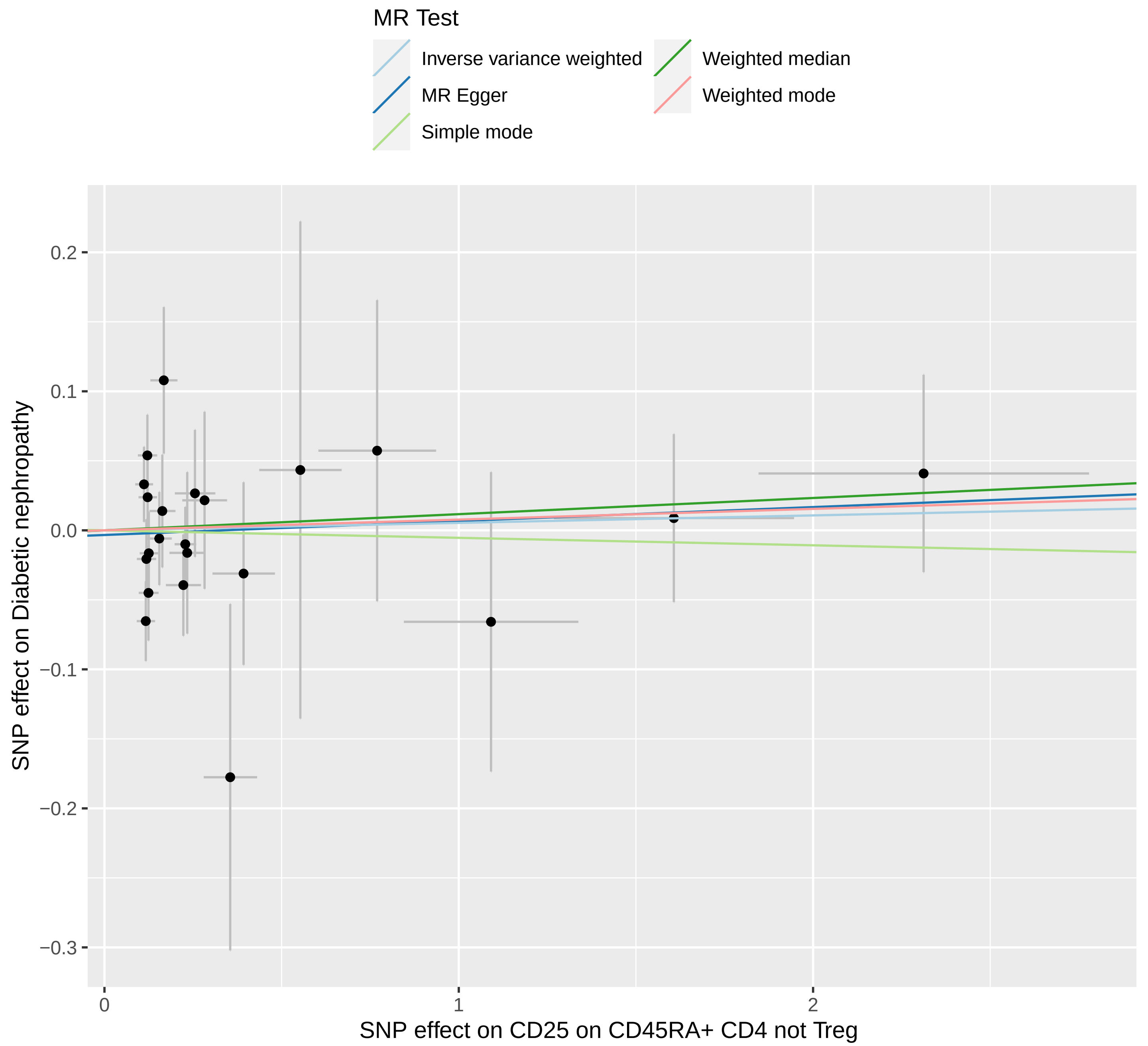
MR Test
Inverse variance weighted
MR Egger
Simple mode
Weighted median
Weighted mode
0.2
0.1
0.0
−0.1
−0.2
−0.3
SNP effect on Diabetic nephropathy
0
1
2
SNP effect on CD25 on CD45RA+ CD4 not Treg

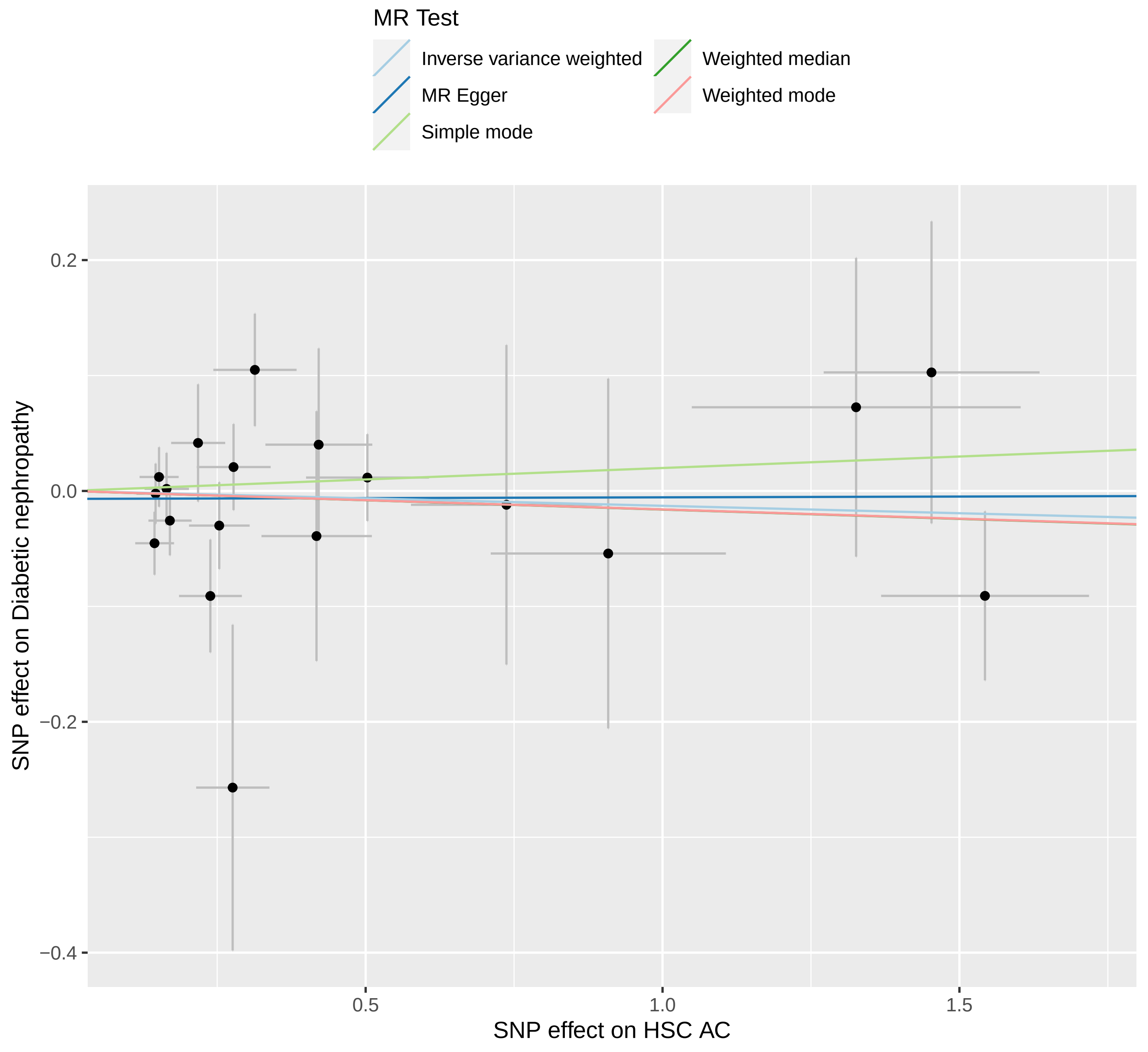

MR Test
Inverse variance weighted
MR Egger
Simple mode
Weighted median
Weighted mode
0.2
0.0
−0.2
−0.4
0.5
1.0
1.5
SNP effect on Diabetic nephropathy
SNP effect on HSC AC

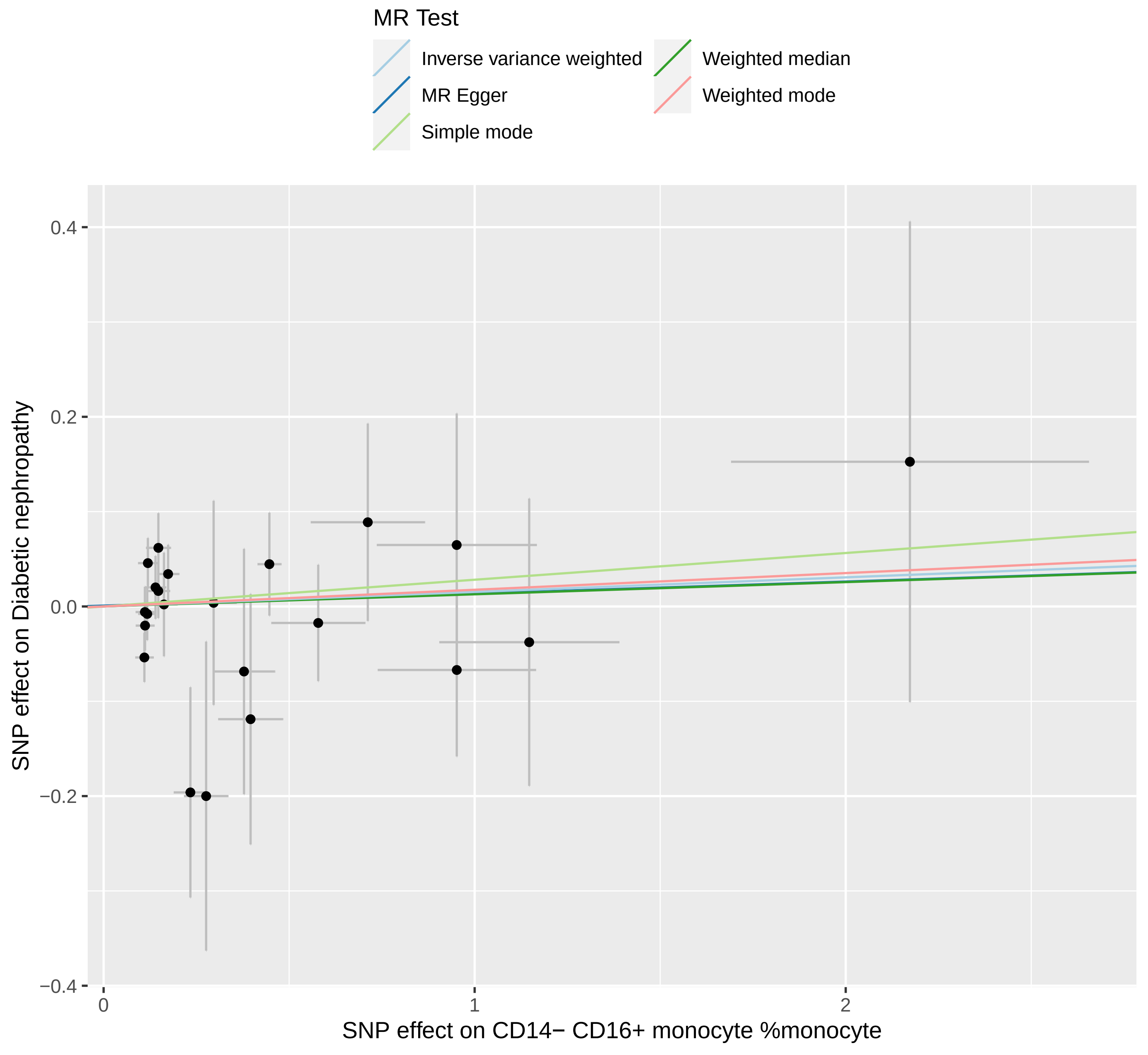

MR Test
Inverse variance weighted
MR Egger
Simple mode
Weighted median
Weighted mode
SNP effect on Diabetic nephropathy
0.4
0.2
0.0
−0.2
−0.4
0
1
2
SNP effect on CD14− CD16+ monocyte %monocyte

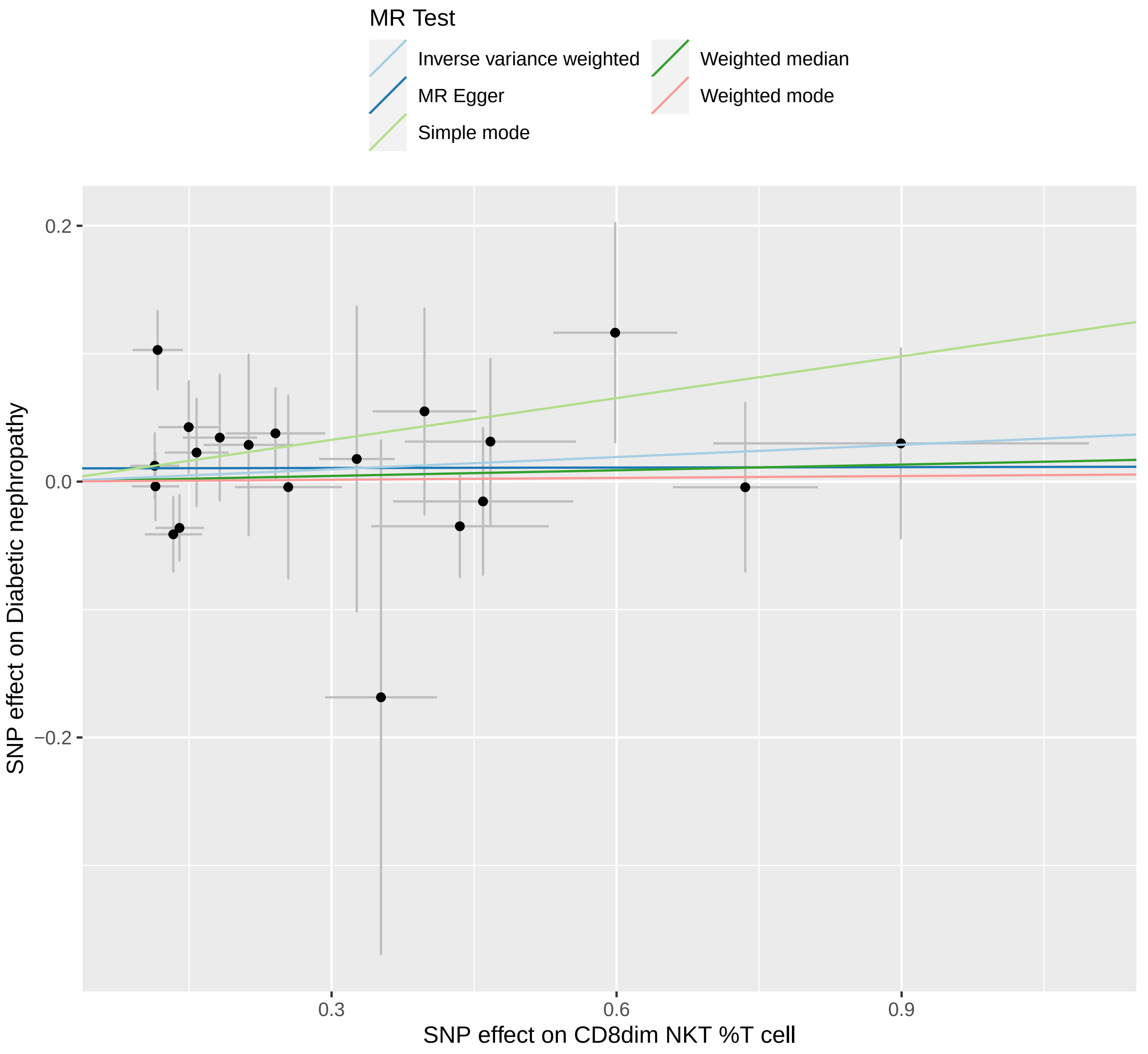
MR Test
Inverse variance weighted
MR Egger
Simple mode
Weighted median
Weighted mode
0.2
0.0
−0.2
0.3
0.6
0.9
SNP effect on Diabetic nephropathy
SNP effect on CD8dim NKT %T cell

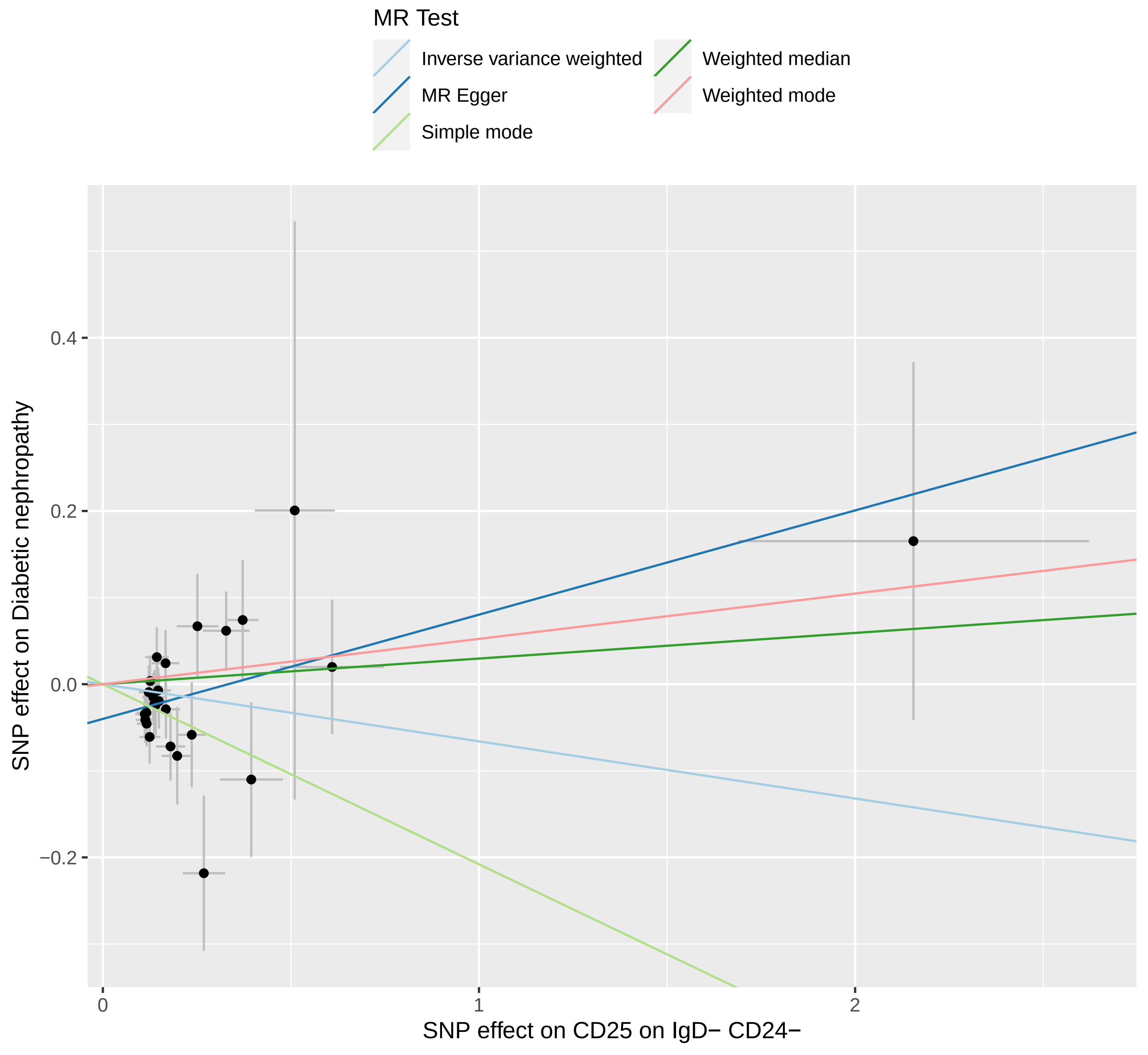
MR Test
Inverse variance weighted
MR Egger
Simple mode
Weighted median
Weighted mode
0.4
0.2
0.0
−0.2
0
1
2
SNP effect on Diabetic nephropathy
SNP effect on CD25 on IgD− CD24−

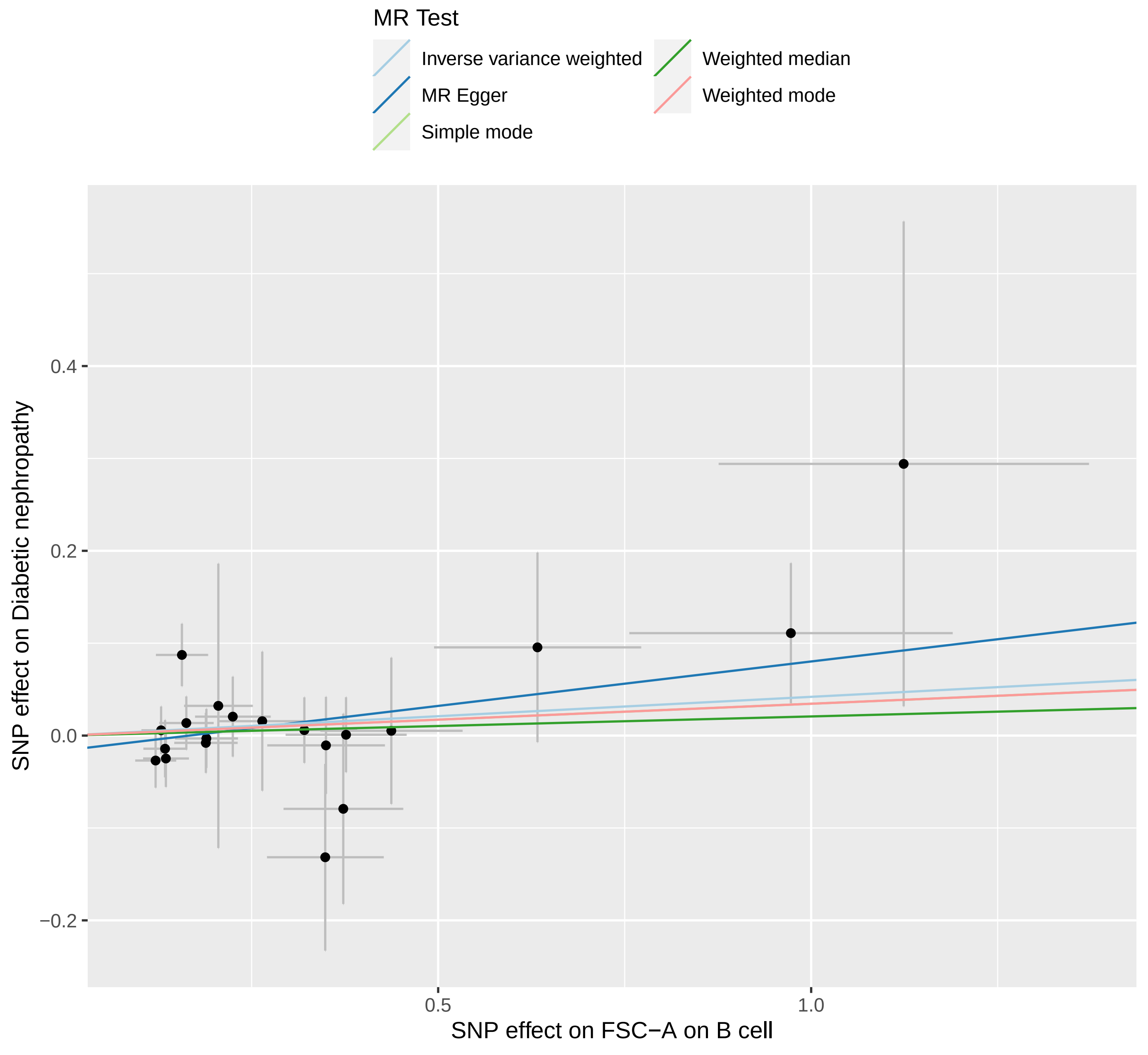

MR Test
Inverse variance weighted
MR Egger
Simple mode
Weighted median
Weighted mode
0.4
0.2
0.0
−0.2
0.5
1.0
SNP effect on Diabetic nephropathy
SNP effect on FSC−A on B cell

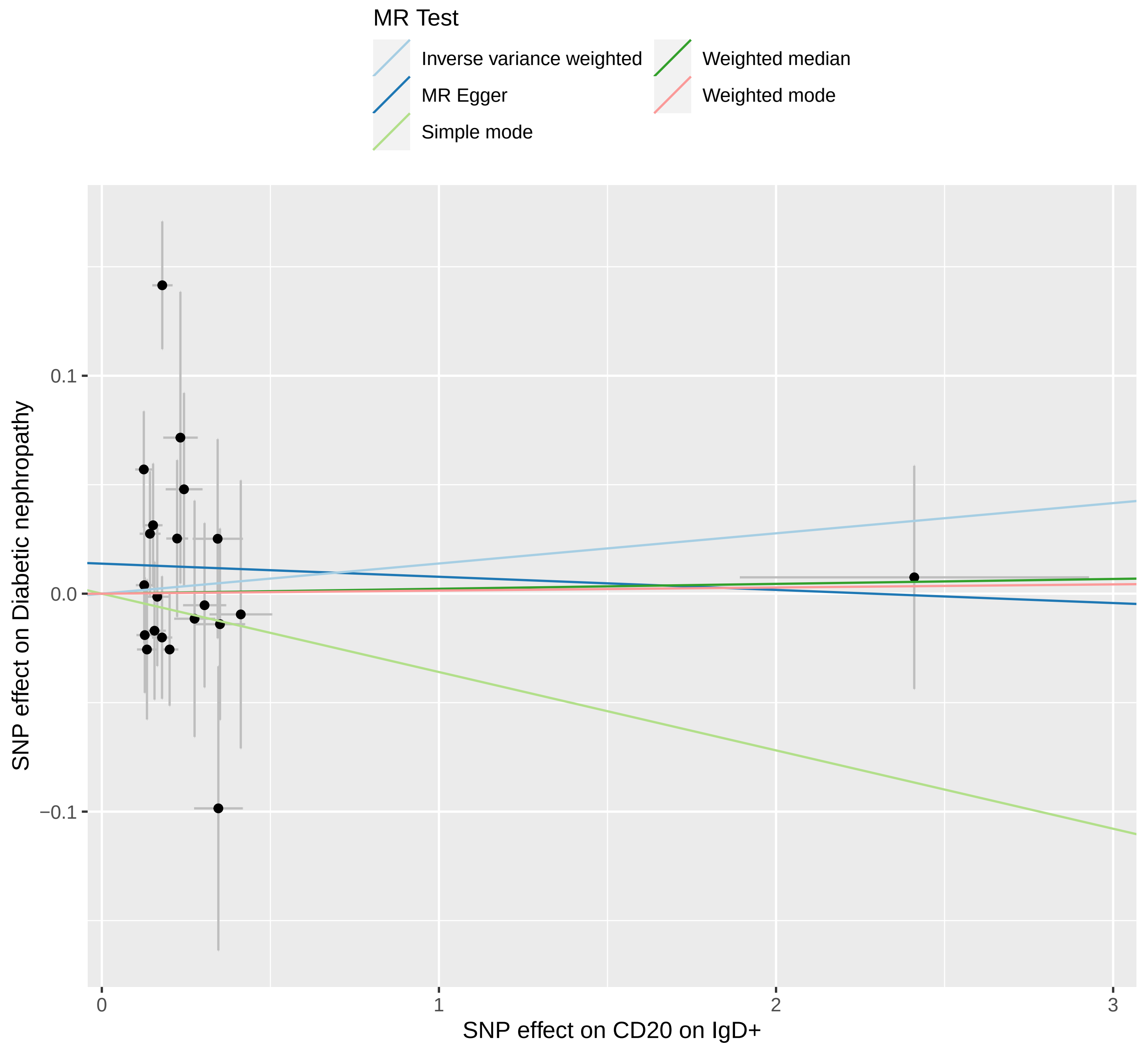

MR Test
Inverse variance weighted
MR Egger
Simple mode
Weighted median
Weighted mode
0.1
0.0
−0.1
SNP effect on Diabetic nephropathy
0
1
2
3
SNP effect on CD20 on IgD+

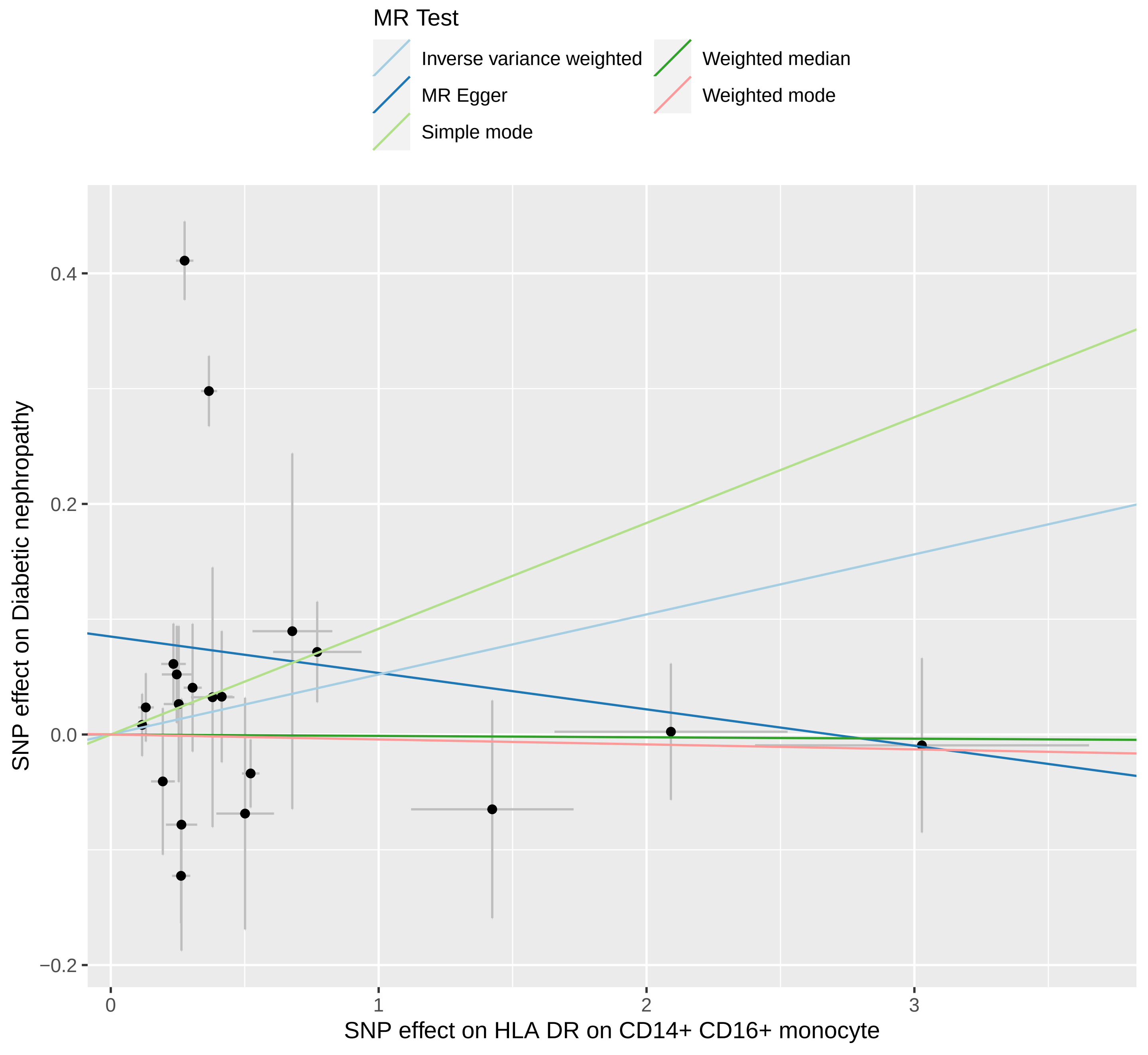

MR Test
Inverse variance weighted
MR Egger
Simple mode
Weighted median
Weighted mode
0.4
0.2
0.0
−0.2
0
1
2
3
SNP effect on Diabetic nephropathy
SNP effect on HLA DR on CD14+ CD16+ monocyte

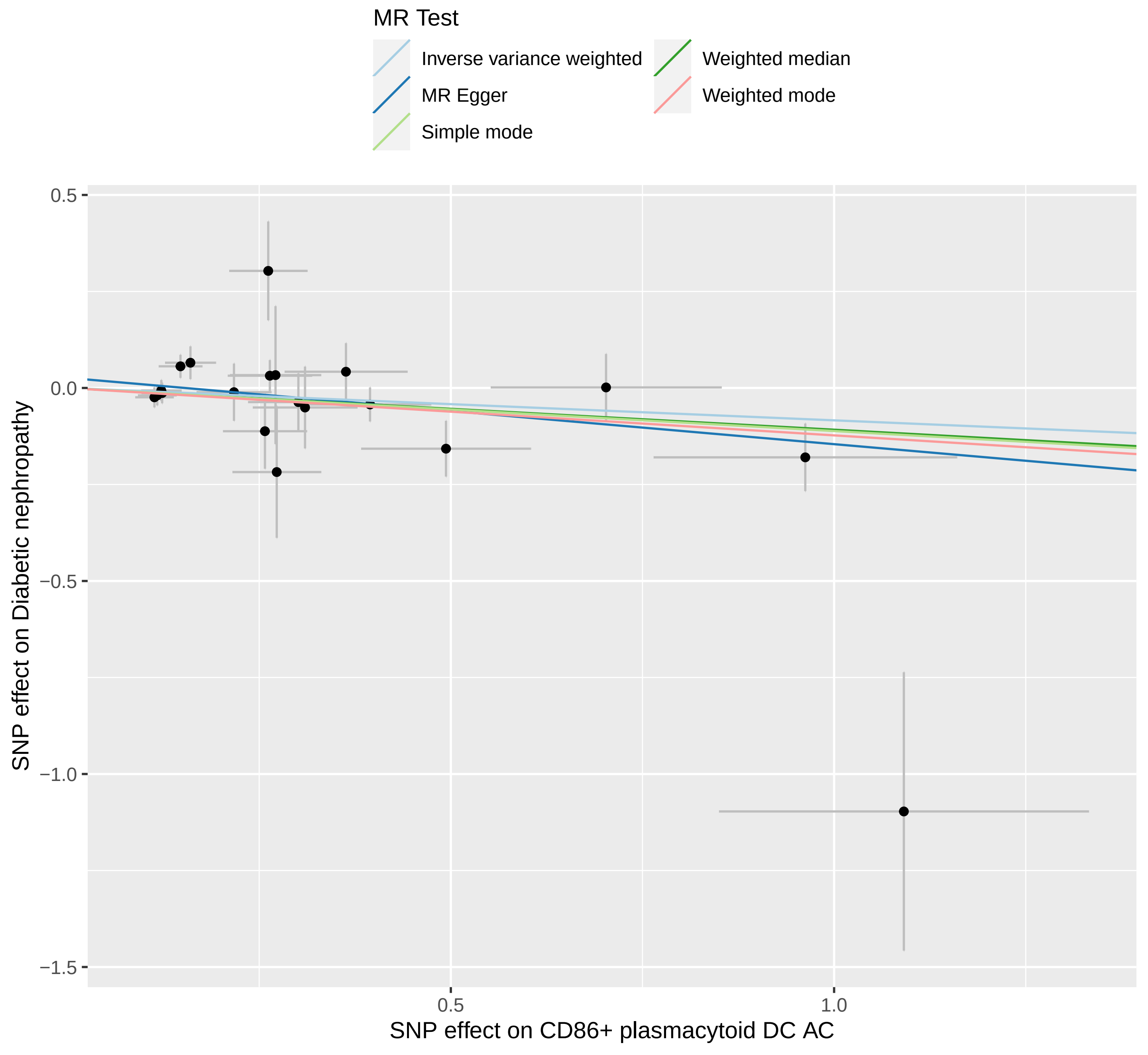

MR Test
Inverse variance weighted
MR Egger
Simple mode
Weighted median
Weighted mode
0.5
0.0
−0.5
−1.0
−1.5
0.5
1.0
SNP effect on Diabetic nephropathy
SNP effect on CD86+ plasmacytoid DC AC

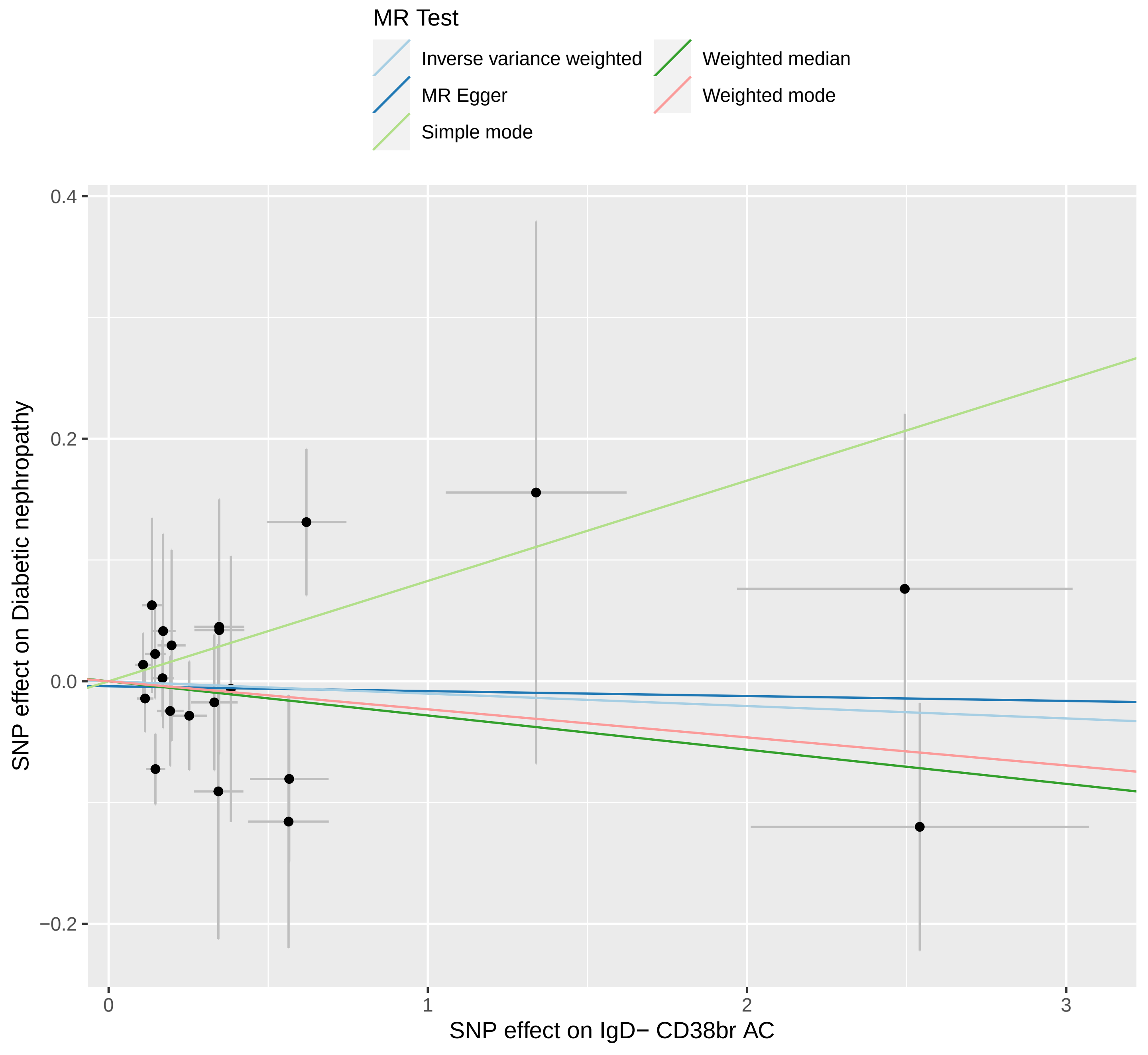
MR Test
Inverse variance weighted
MR Egger
Simple mode
Weighted median
Weighted mode
0.4
0.2
0.0
−0.2
SNP effect on Diabetic nephropathy
0
1
2
3
SNP effect on IgD− CD38br AC

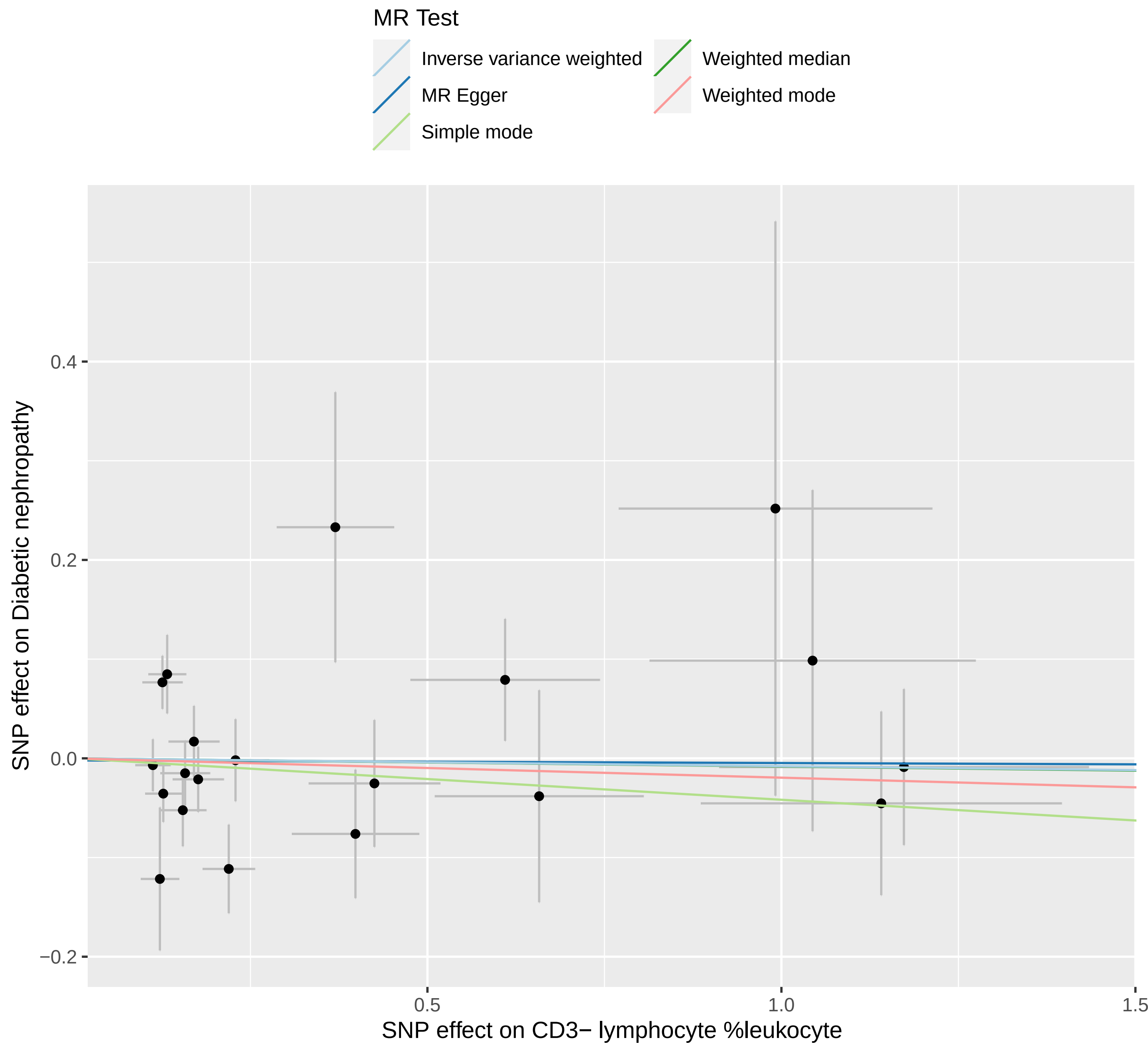

MR Test
Inverse variance weighted
MR Egger
Simple mode
Weighted median
Weighted mode
SNP effect on Diabetic nephropathy
0.4
0.2
0.0
−0.2
0.5
1.0
1.5
SNP effect on CD3− lymphocyte %leukocyte

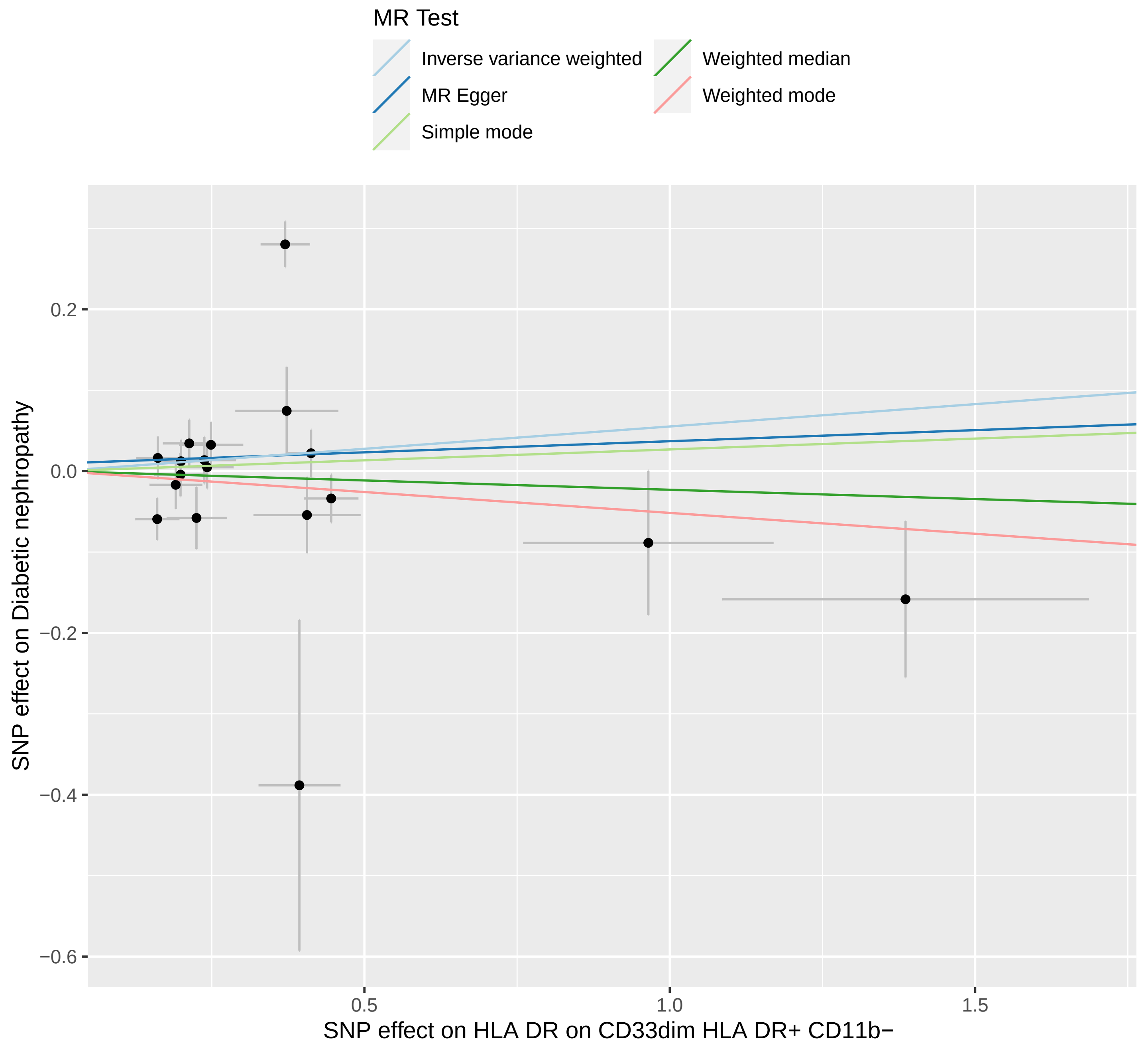
MR Test
Inverse variance weighted
MR Egger
Simple mode
Weighted median
Weighted mode
0.2
0.0
−0.2
−0.4
−0.6
SNP effect on Diabetic nephropathy
0.5
1.0
1.5
SNP effect on HLA DR on CD33dim HLA DR+ CD11b−

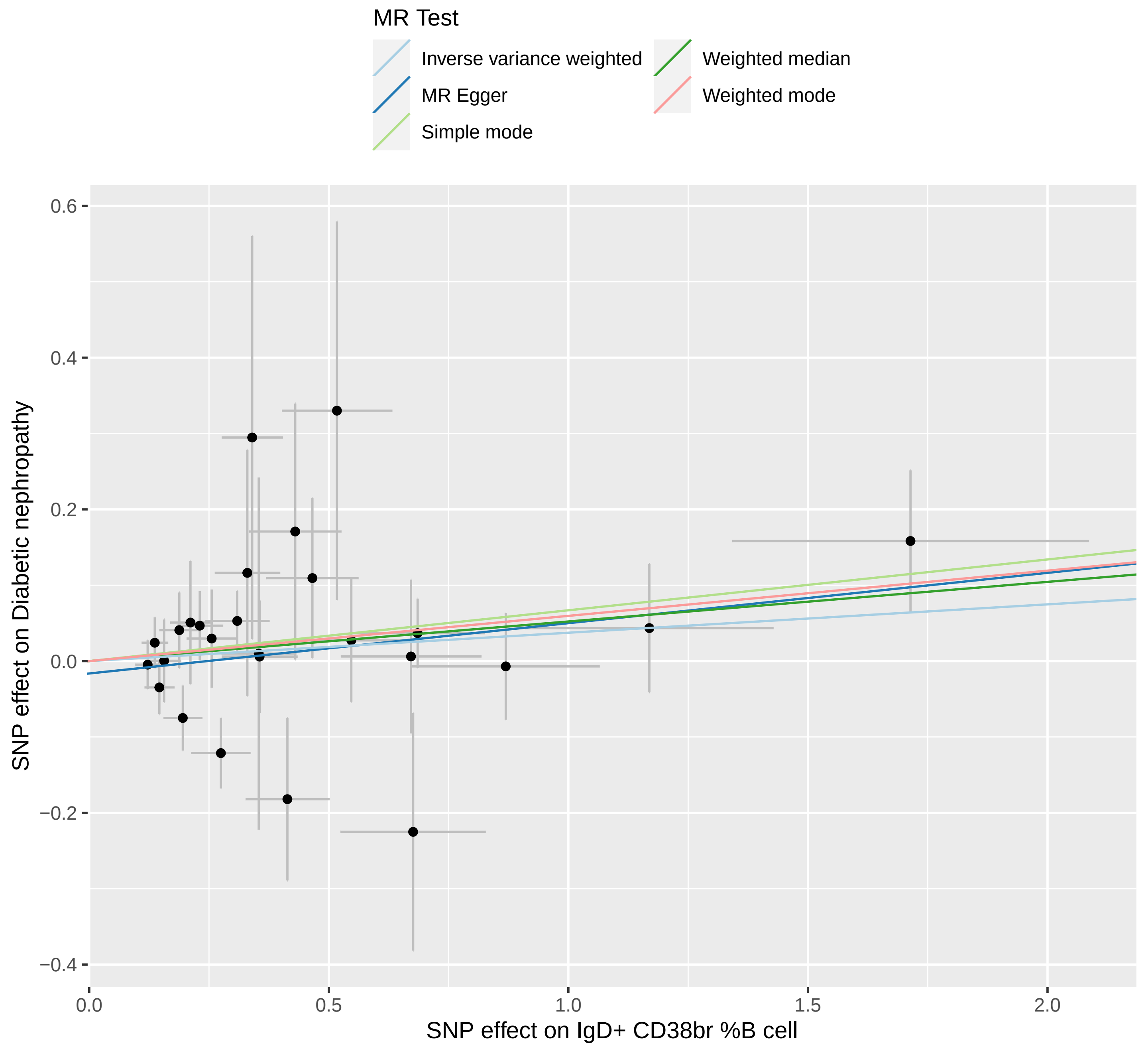
MR Test
Inverse variance weighted
MR Egger
Simple mode
Weighted median
Weighted mode
SNP effect on Diabetic nephropathy
0.6
0.4
0.2
0.0
−0.2
−0.4
0.0
0.5
1.0
1.5
2.0
SNP effect on IgD+ CD38br %B cell

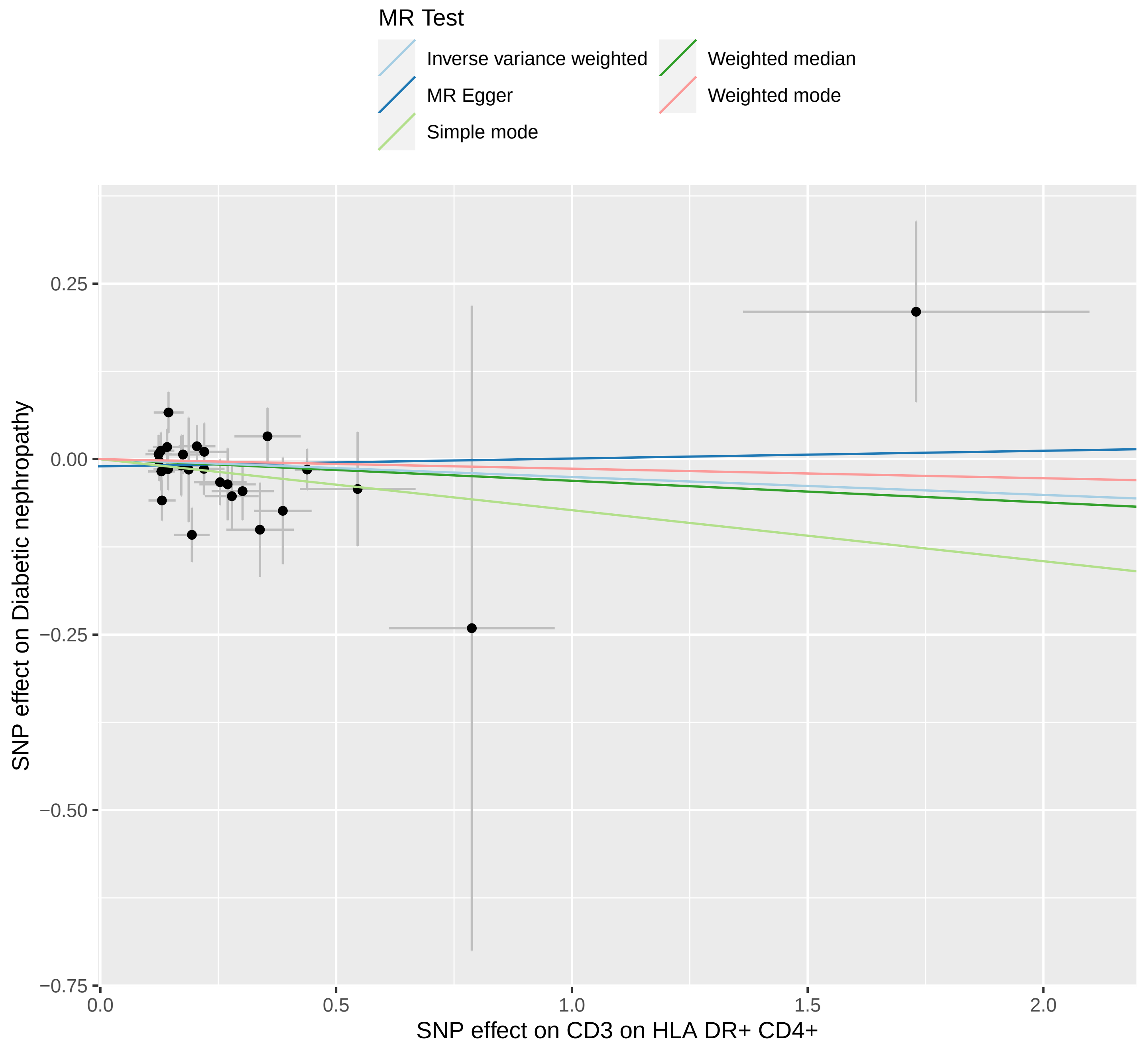
MR Test
Inverse variance weighted
MR Egger
Simple mode
Weighted median
Weighted mode
0.25
0.00
−0.25
−0.50
−0.75
0.0
0.5
1.0
1.5
2.0
SNP effect on Diabetic nephropathy
SNP effect on CD3 on HLA DR+ CD4+

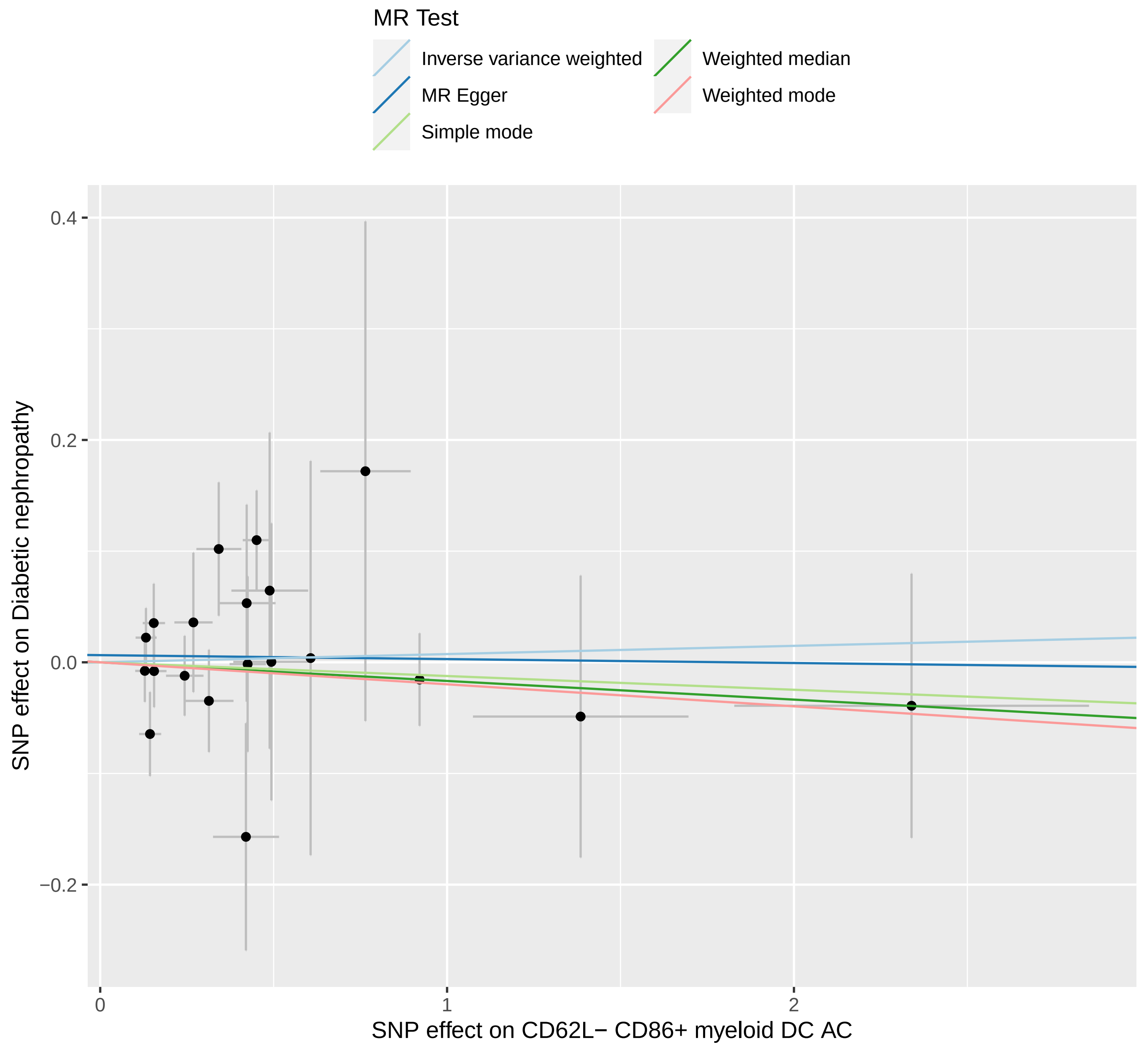
MR Test
Inverse variance weighted
MR Egger
Simple mode
Weighted median
Weighted mode
0.4
0.2
0.0
−0.2
0
1
2
SNP effect on Diabetic nephropathy
SNP effect on CD62L− CD86+ myeloid DC AC

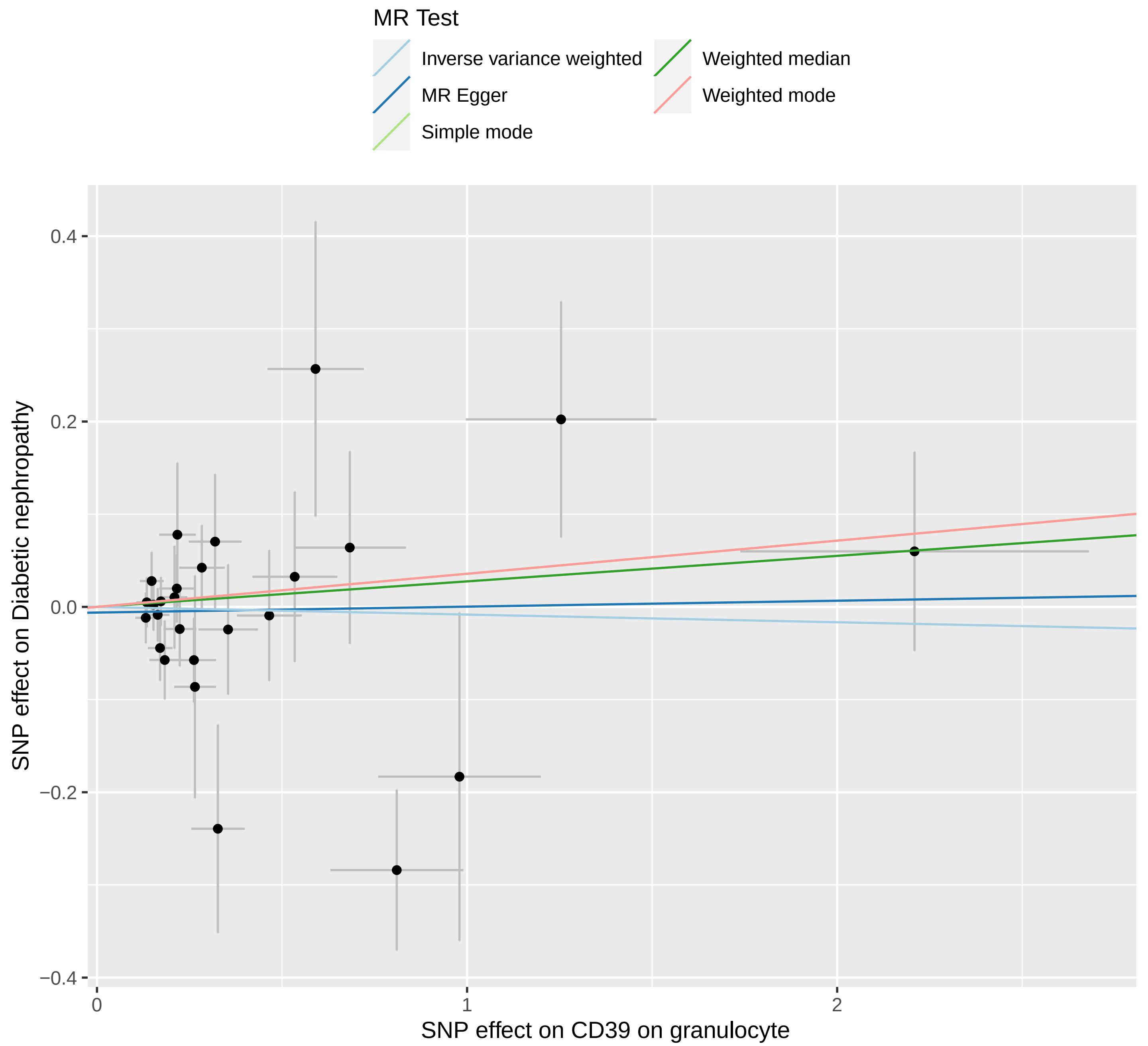

MR Test
Inverse variance weighted
MR Egger
Simple mode
Weighted median
Weighted mode
0.4
0.2
0.0
−0.2
−0.4
SNP effect on Diabetic nephropathy
0
1
2
SNP effect on CD39 on granulocyte

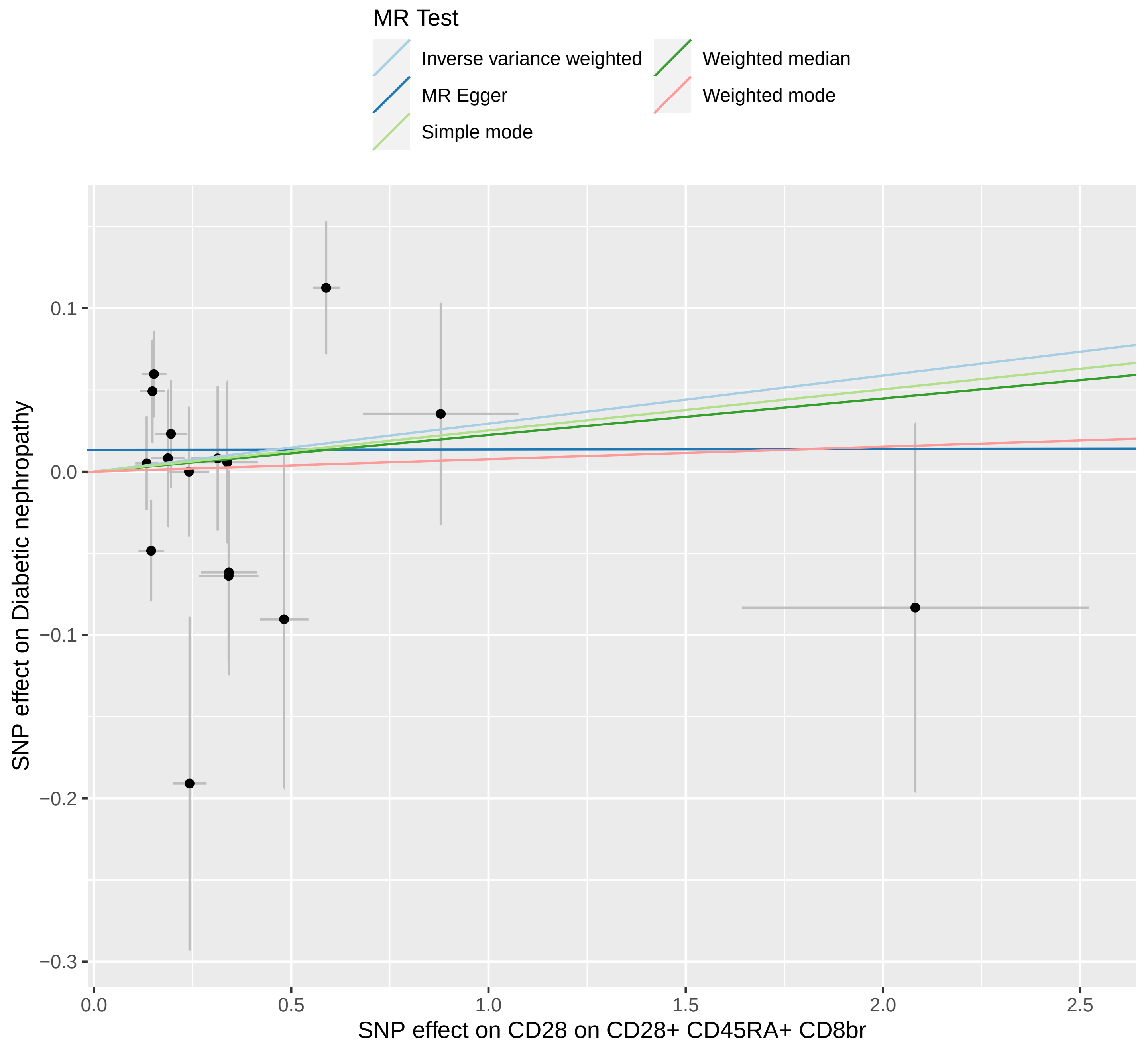

MR Test
Inverse variance weighted
MR Egger
Simple mode
Weighted median
Weighted mode
0.1
0.0
−0.1
−0.2
−0.3
SNP effect on Diabetic nephropathy
0.0
0.5
1.0
1.5
2.0
2.5
SNP effect on CD28 on CD28+ CD45RA+ CD8br

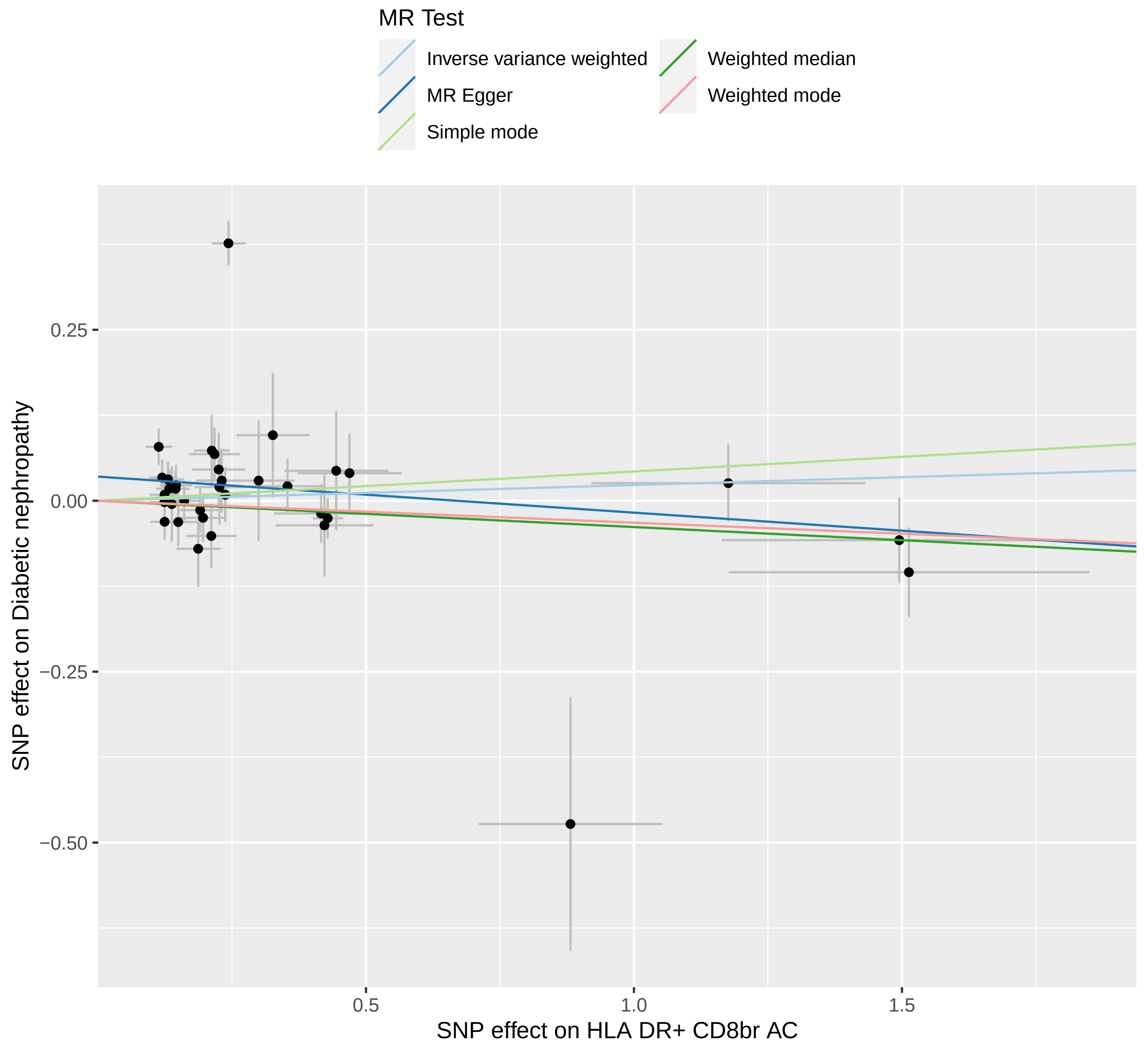

MR Test
Inverse variance weighted
MR Egger
Simple mode
Weighted median
Weighted mode
0.25
0.00
−0.25
−0.50
0.5
1.0
1.5
SNP effect on Diabetic nephropathy
SNP effect on HLA DR+ CD8br AC

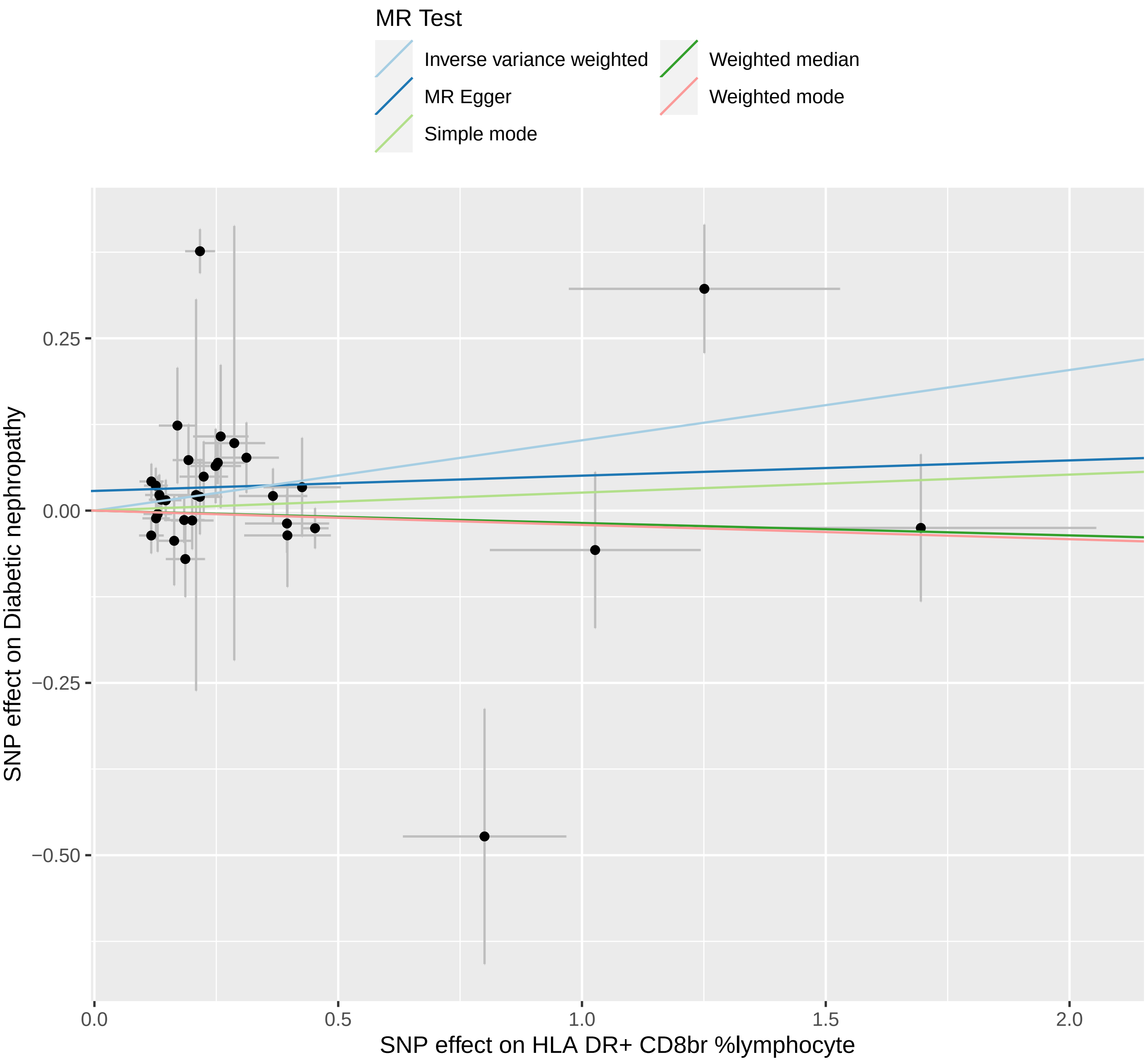

MR Test
Inverse variance weighted
MR Egger
Simple mode
Weighted median
Weighted mode
0.25
0.00
−0.25
−0.50
0.0
0.5
1.0
1.5
2.0
SNP effect on Diabetic nephropathy
SNP effect on HLA DR+ CD8br %lymphocyte

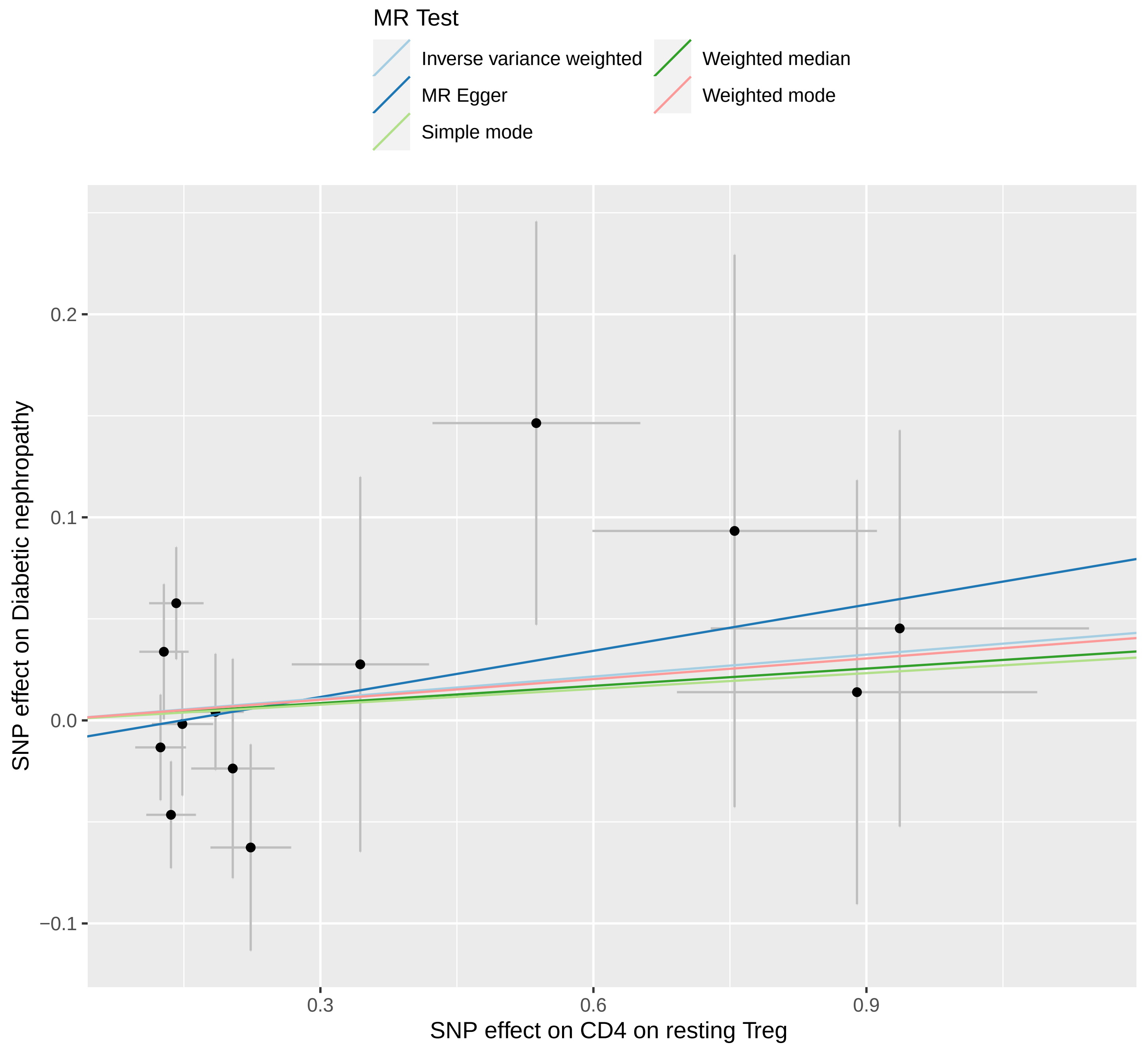
MR Test
Inverse variance weighted
MR Egger
Simple mode
Weighted median
Weighted mode
SNP effect on Diabetic nephropathy
0.2
0.1
0.0
−0.1
0.3
0.6
0.9
SNP effect on CD4 on resting Treg

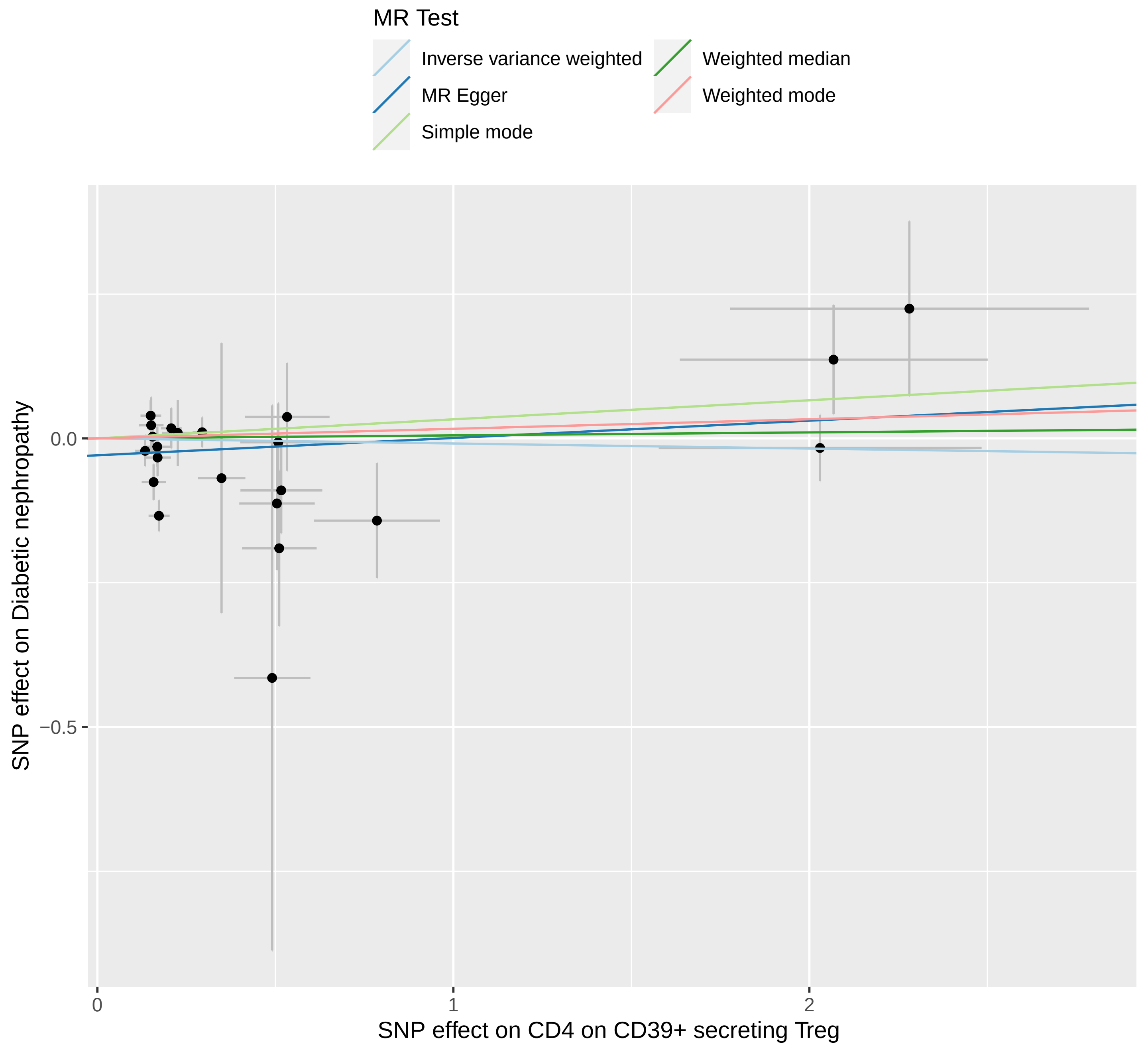
MR Test
Inverse variance weighted
MR Egger
Simple mode
Weighted median
Weighted mode
0.0
−0.5
0
1
2
SNP effect on CD4 on CD39+ secreting Treg
SNP effect on Diabetic nephropathy

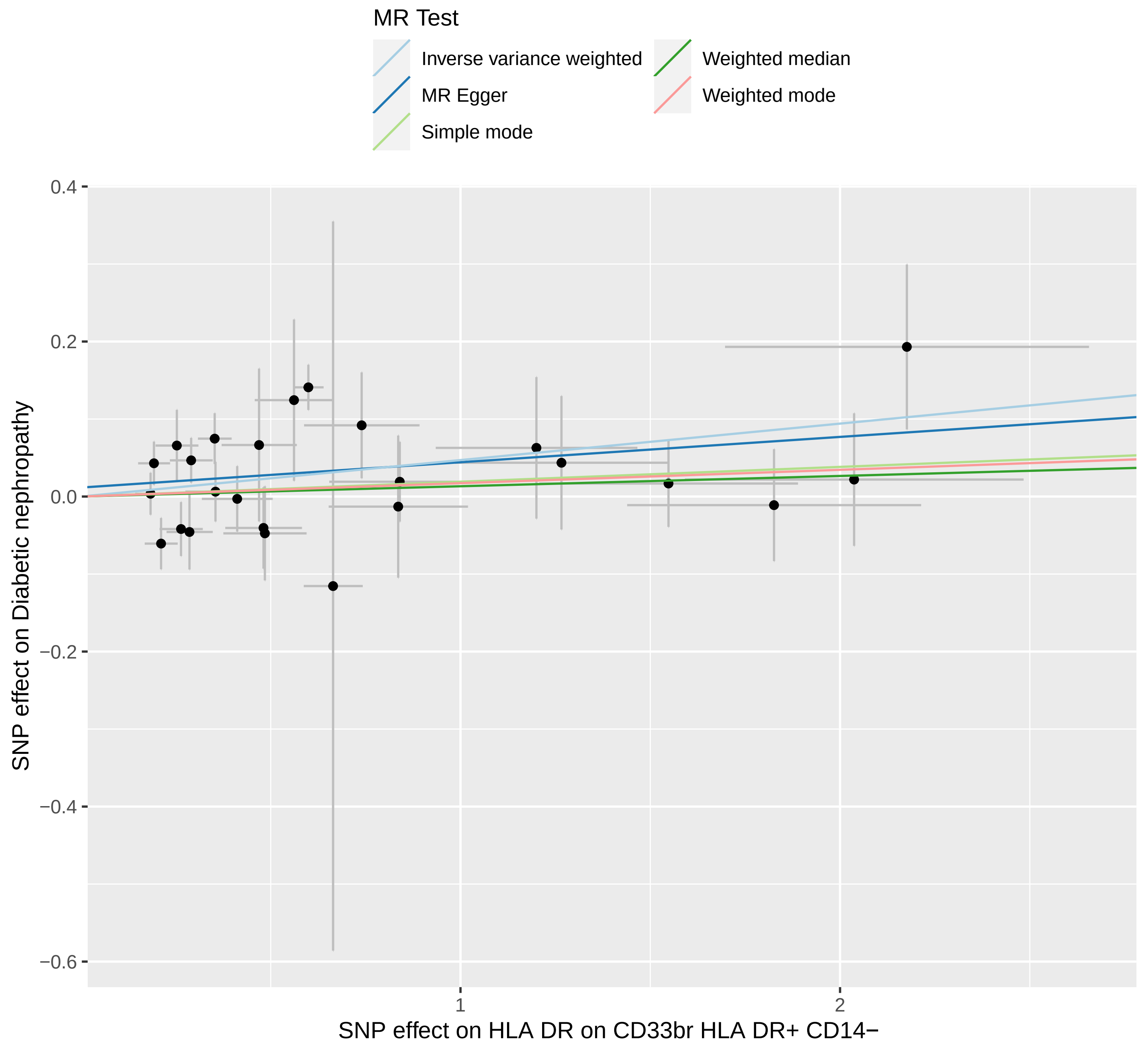

MR Test
Inverse variance weighted
MR Egger
Simple mode
Weighted median
Weighted mode
0.4
0.2
0.0
−0.2
−0.4
−0.6
1
2
SNP effect on Diabetic nephropathy
SNP effect on HLA DR on CD33br HLA DR+ CD14−

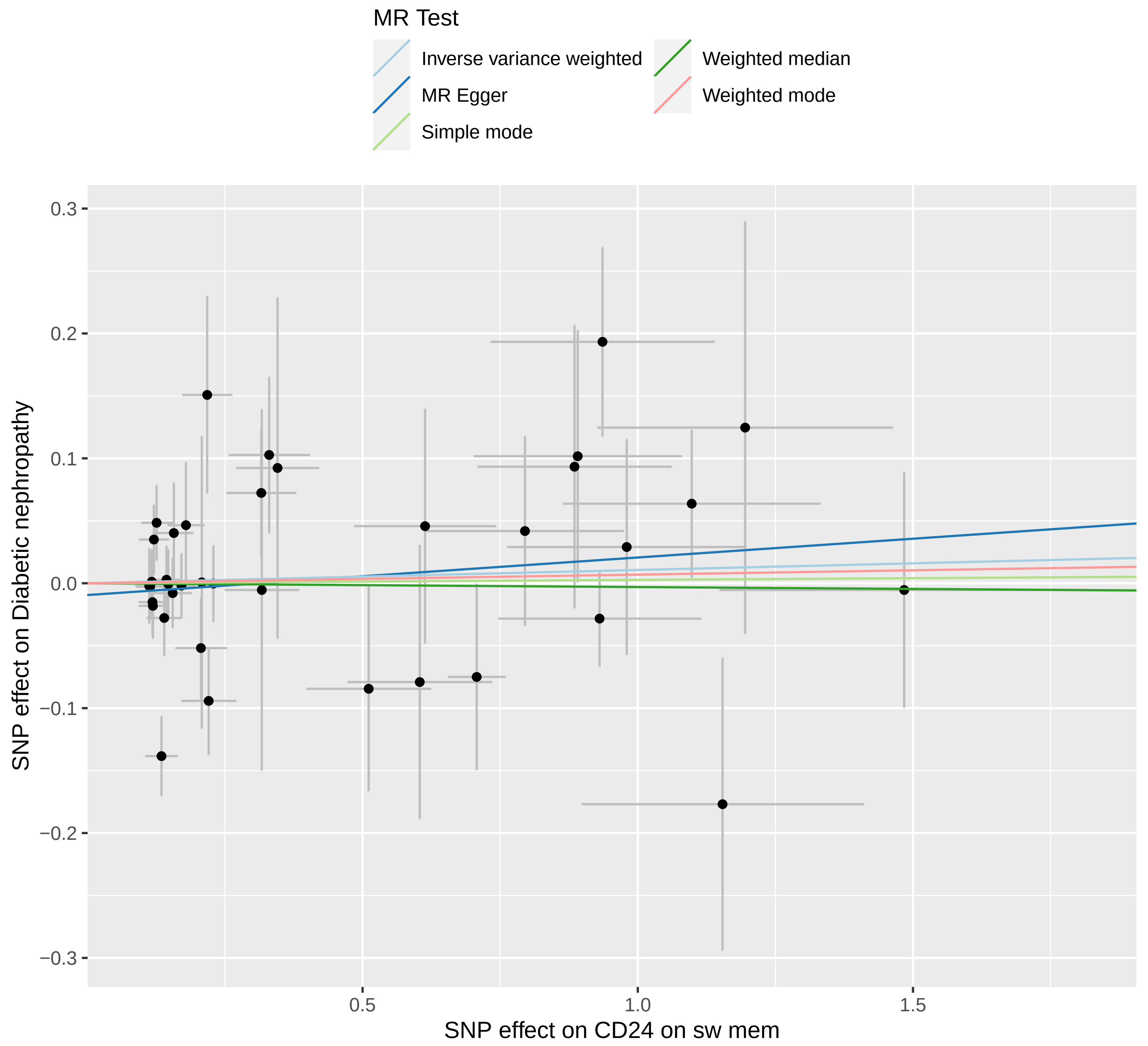
MR Test
Inverse variance weighted
MR Egger
Simple mode
Weighted median
Weighted mode
0.3
0.2
0.1
0.0
−0.1
−0.2
−0.3
SNP effect on Diabetic nephropathy
0.5
1.0
1.5
SNP effect on CD24 on sw mem

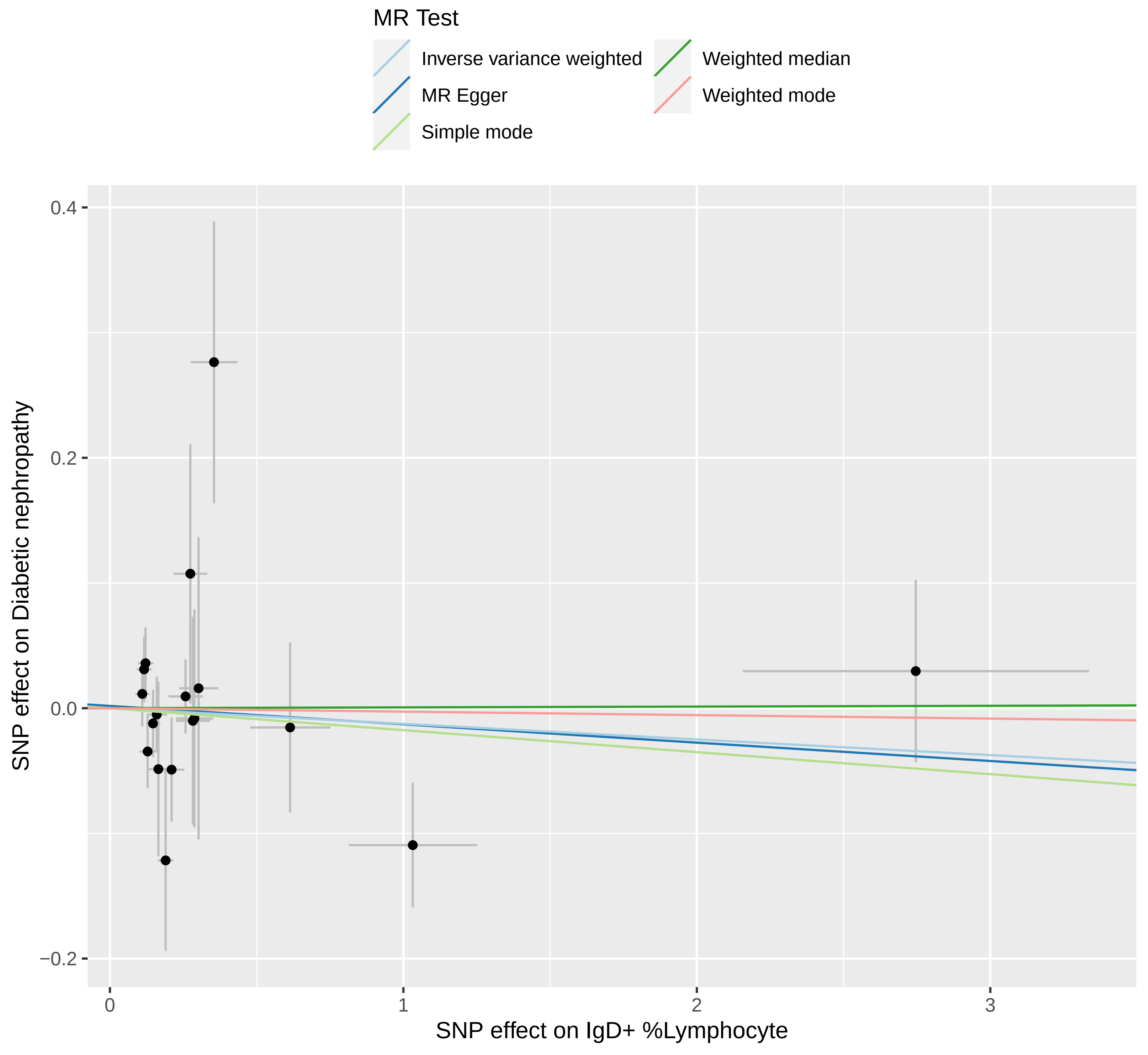

MR Test
Inverse variance weighted
MR Egger
Simple mode
Weighted median
Weighted mode
0.4
0.2
0.0
−0.2
SNP effect on Diabetic nephropathy
0
1
2
3
SNP effect on IgD+ %Lymphocyte

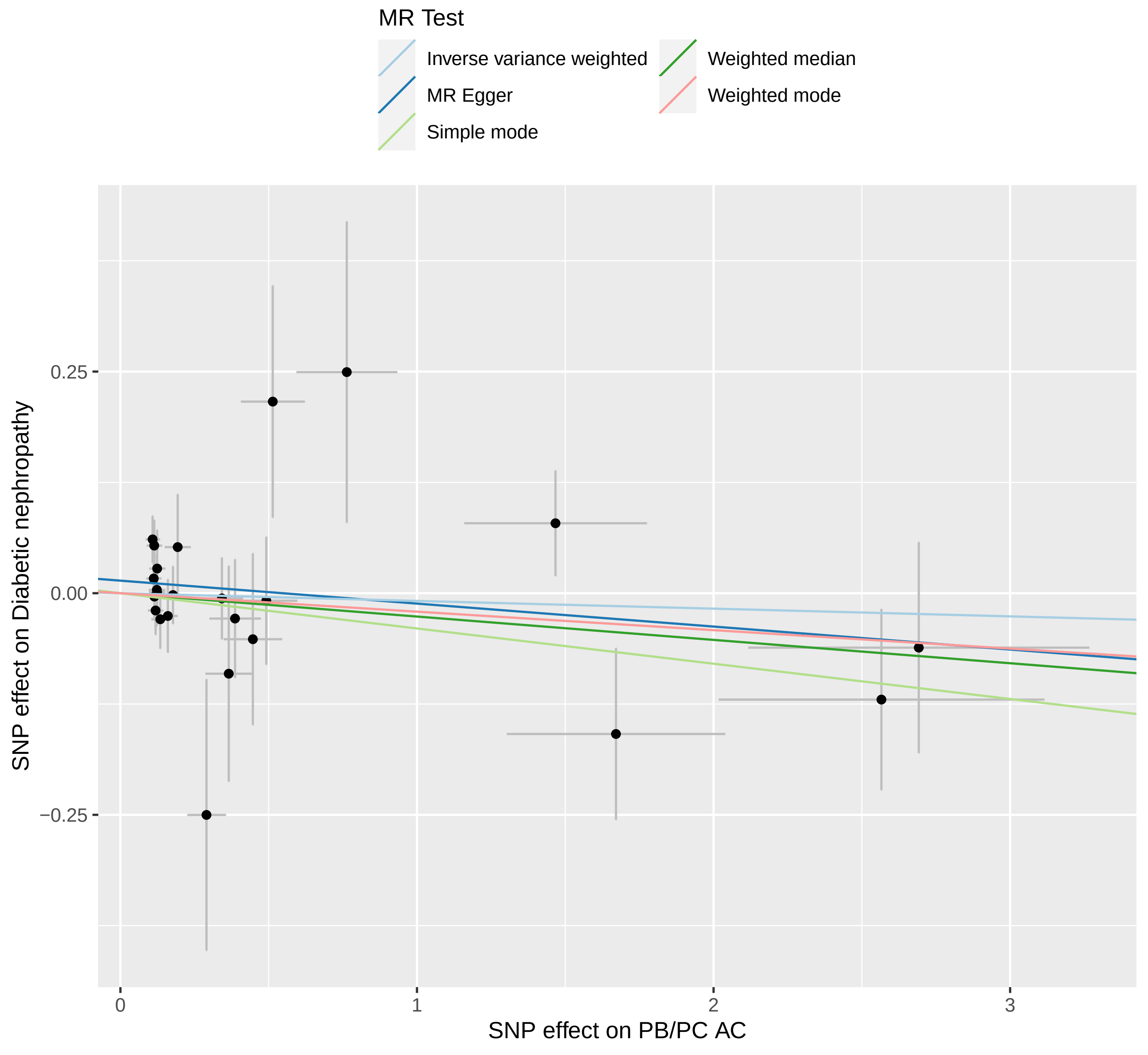

MR Test
Inverse variance weighted
MR Egger
Simple mode
Weighted median
Weighted mode
0.25
0.00
−0.25
0
1
2
3
SNP effect on Diabetic nephropathy
SNP effect on PB/PC AC

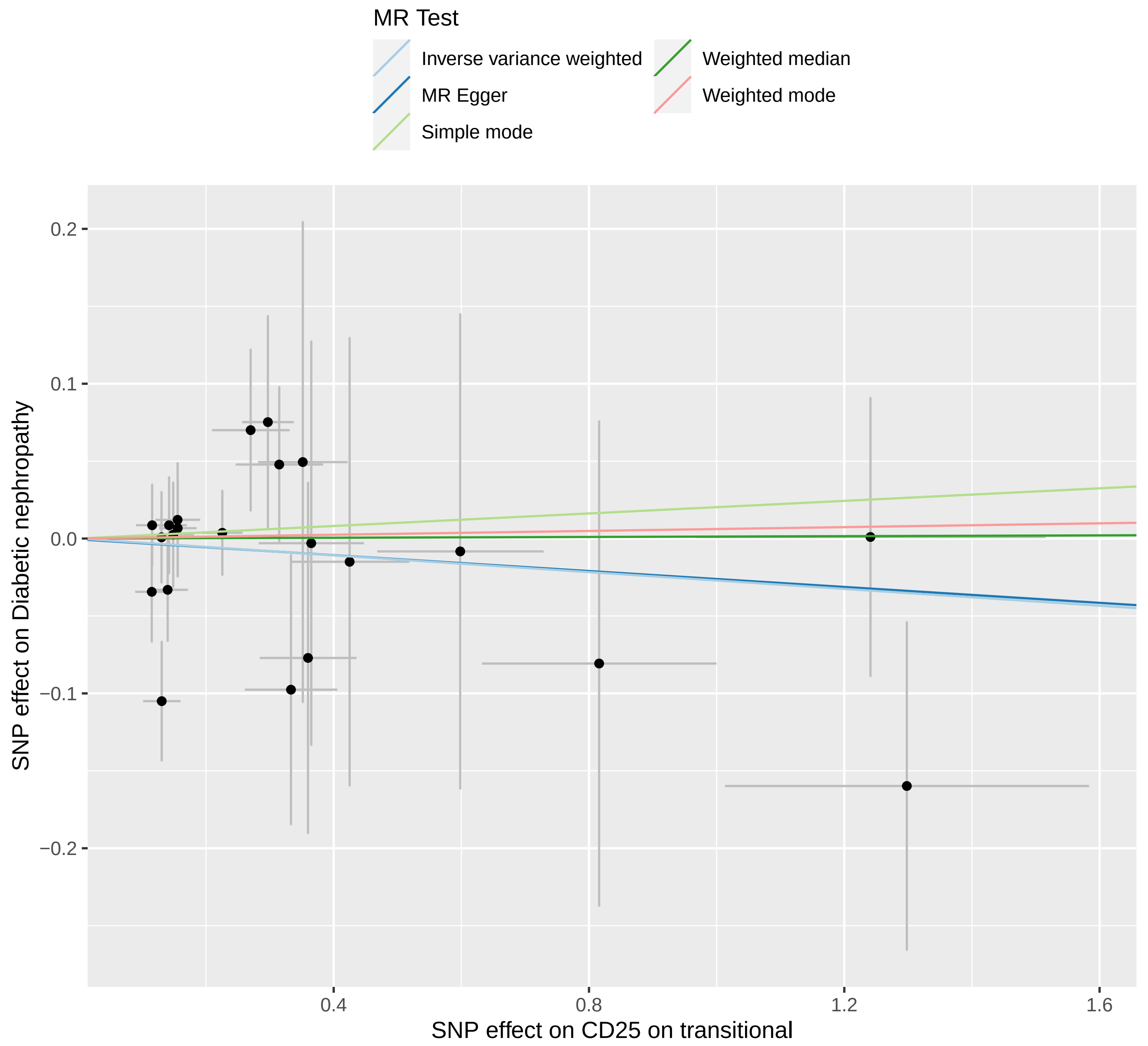

MR Test
Inverse variance weighted
MR Egger
Simple mode
Weighted median
Weighted mode
SNP effect on Diabetic nephropathy
0.2
0.1
0.0
−0.1
−0.2
0.4
0.8
1.2
1.6
SNP effect on CD25 on transitional

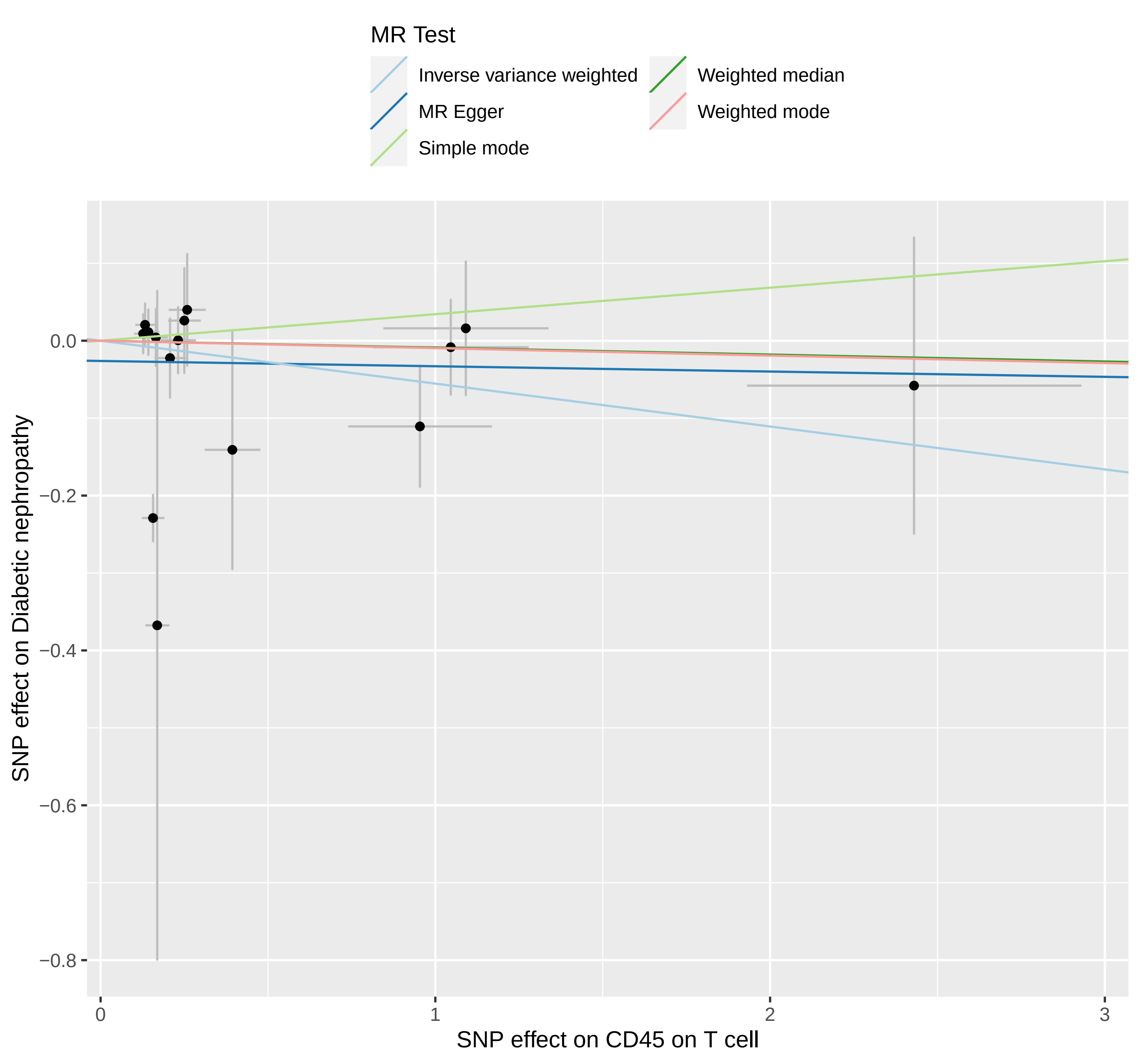
MR Test
Inverse variance weighted
MR Egger
Simple mode
Weighted median
Weighted mode
0.0
−0.2
−0.4
−0.6
−0.8
0
1
2
3
SNP effect on Diabetic nephropathy
SNP effect on CD45 on T cell

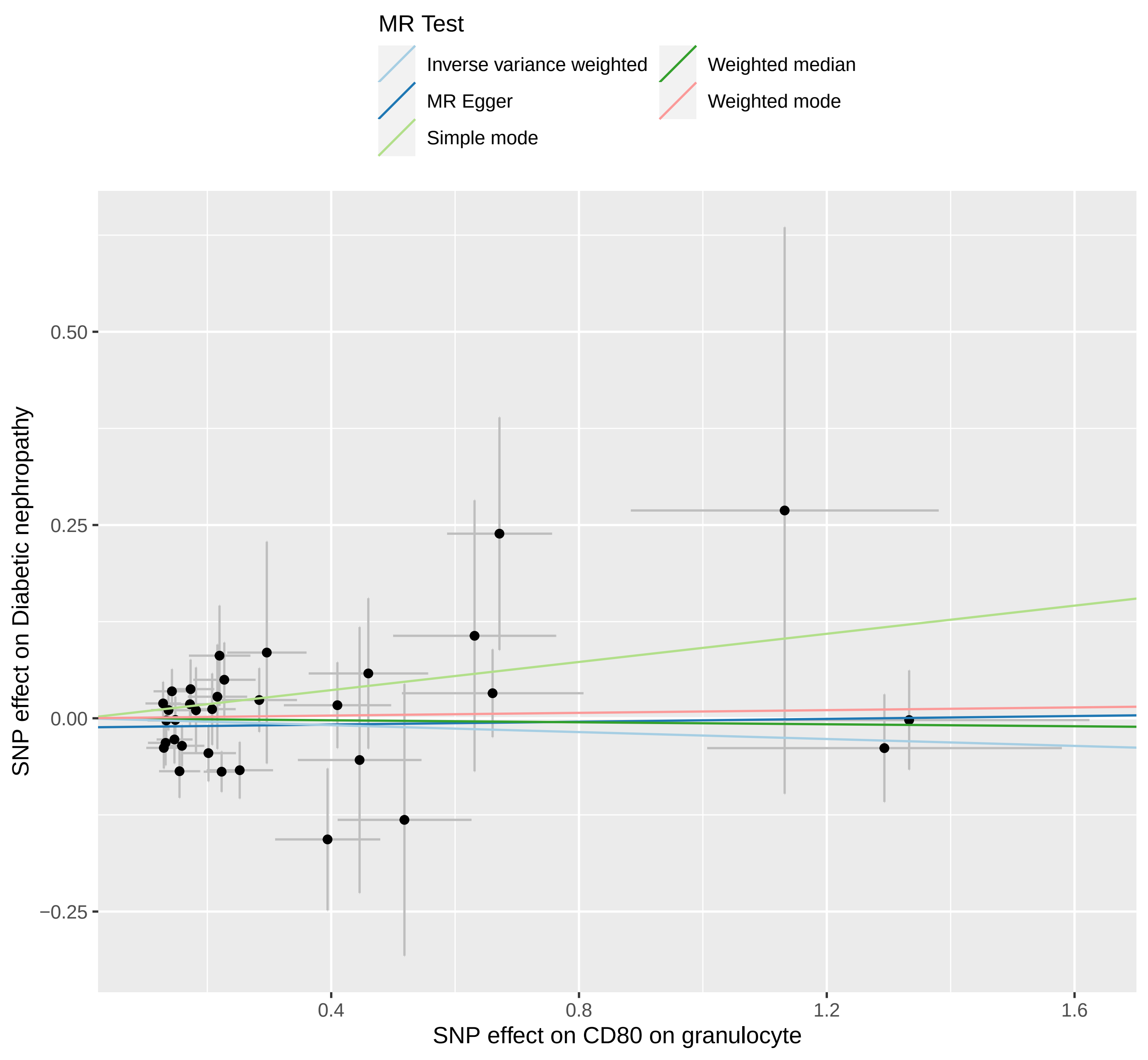

MR Test
Inverse variance weighted
MR Egger
Simple mode
Weighted median
Weighted mode
0.50
0.25
0.00
−0.25
0.4
0.8
1.2
1.6
SNP effect on Diabetic nephropathy
SNP effect on CD80 on granulocyte

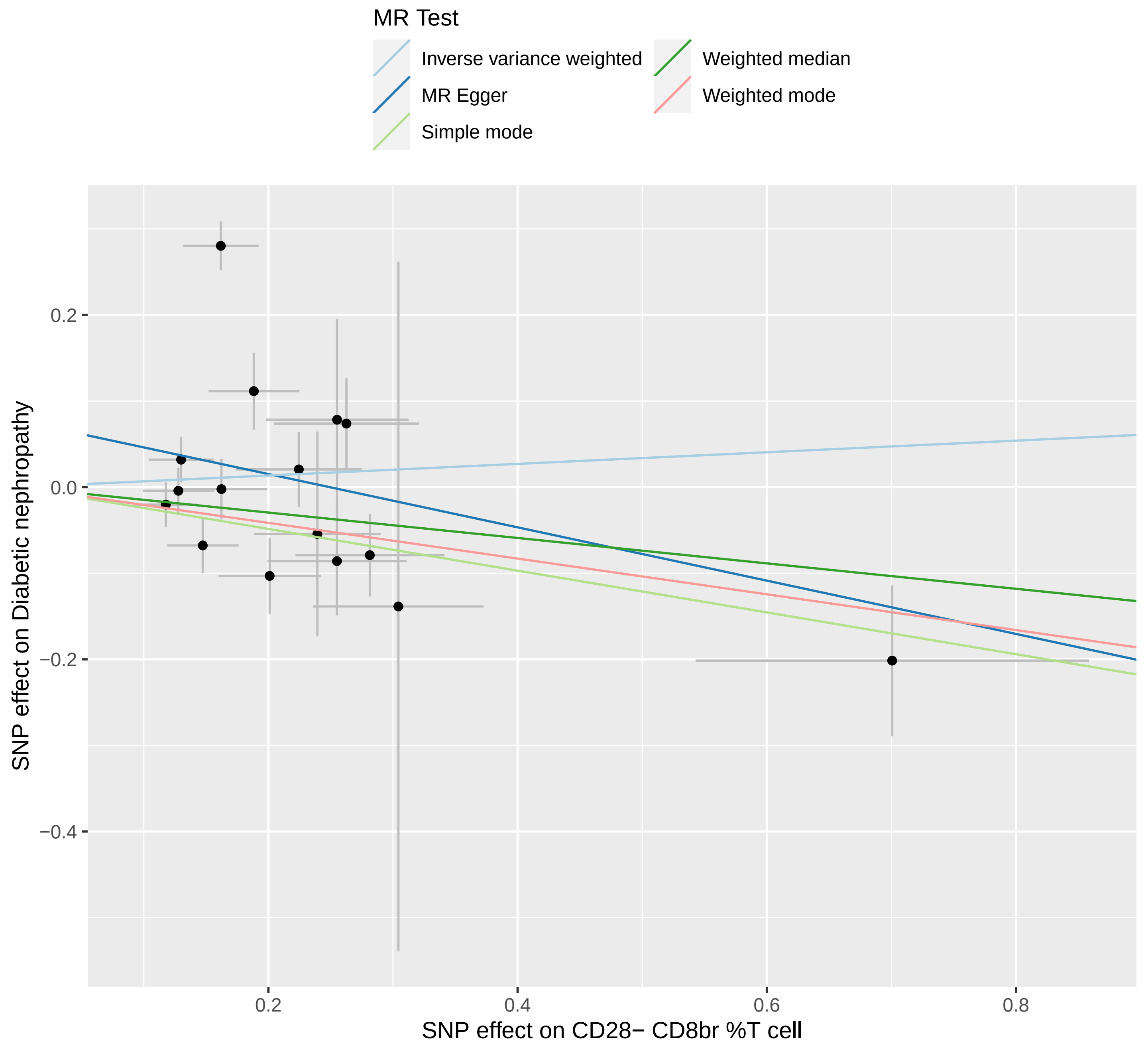

MR Test
Inverse variance weighted
MR Egger
Simple mode
Weighted median
Weighted mode
0.2
0.0
−0.2
−0.4
0.2
0.4
0.6
0.8
SNP effect on Diabetic nephropathy
SNP effect on CD28− CD8br %T cell

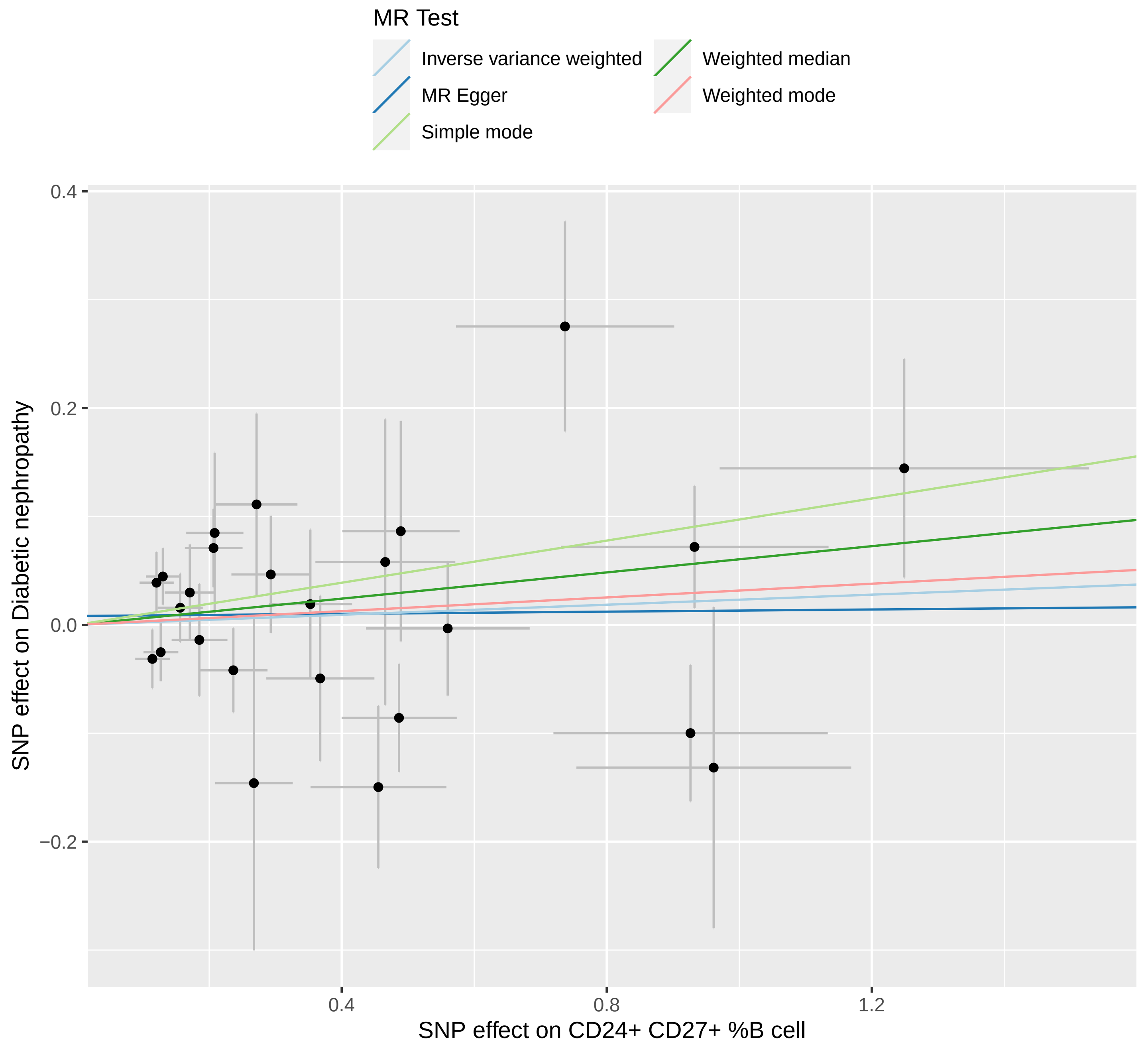
MR Test
Inverse variance weighted
MR Egger
Simple mode
Weighted median
Weighted mode
0.4
0.2
0.0
−0.2
0.4
0.8
1.2
SNP effect on Diabetic nephropathy
SNP effect on CD24+ CD27+ %B cell

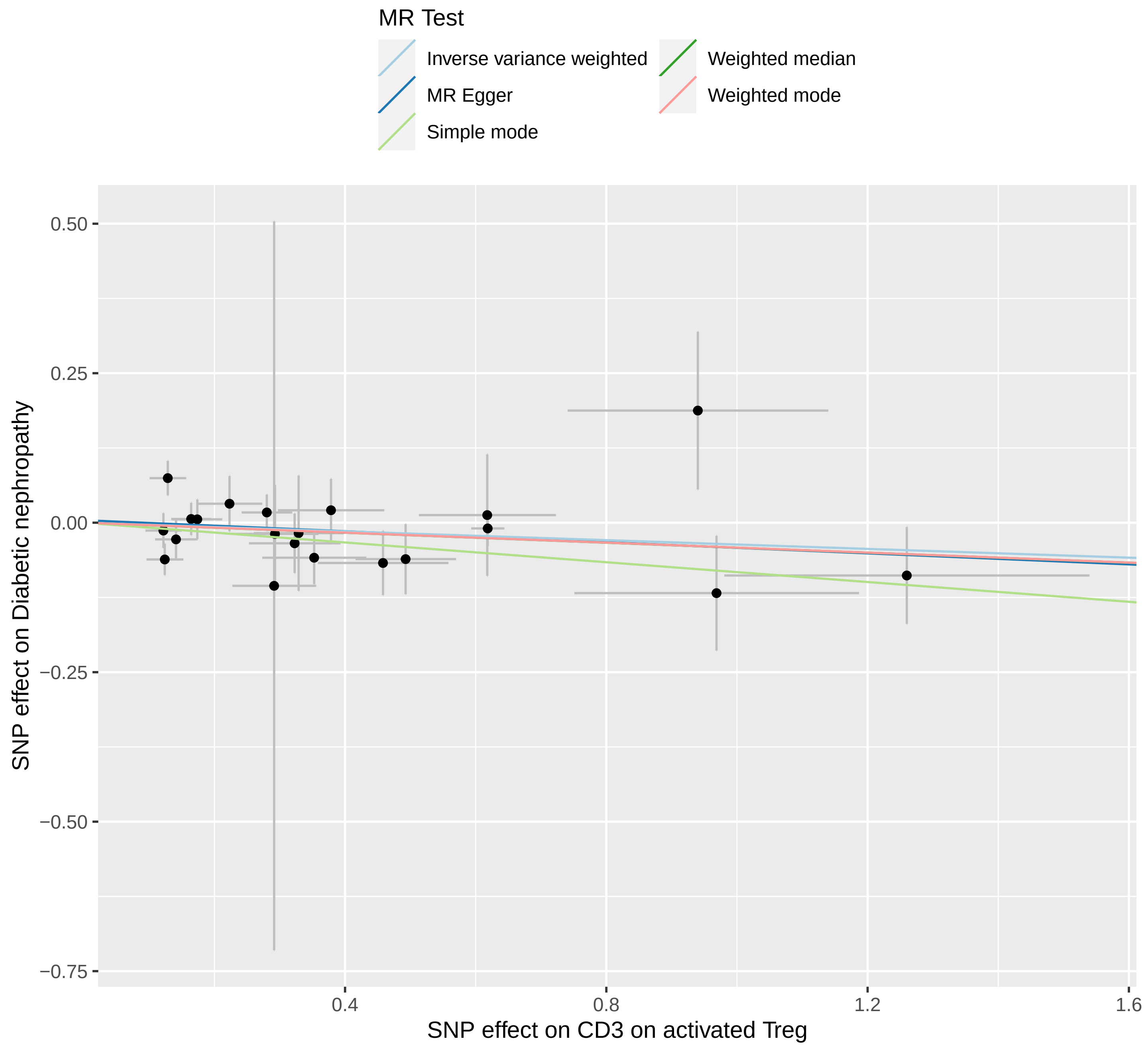

MR Test
Inverse variance weighted
MR Egger
Simple mode
Weighted median
Weighted mode
0.50
0.25
0.00
−0.25
−0.50
−0.75
0.4
0.8
1.2
1.6
SNP effect on Diabetic nephropathy
SNP effect on CD3 on activated Treg

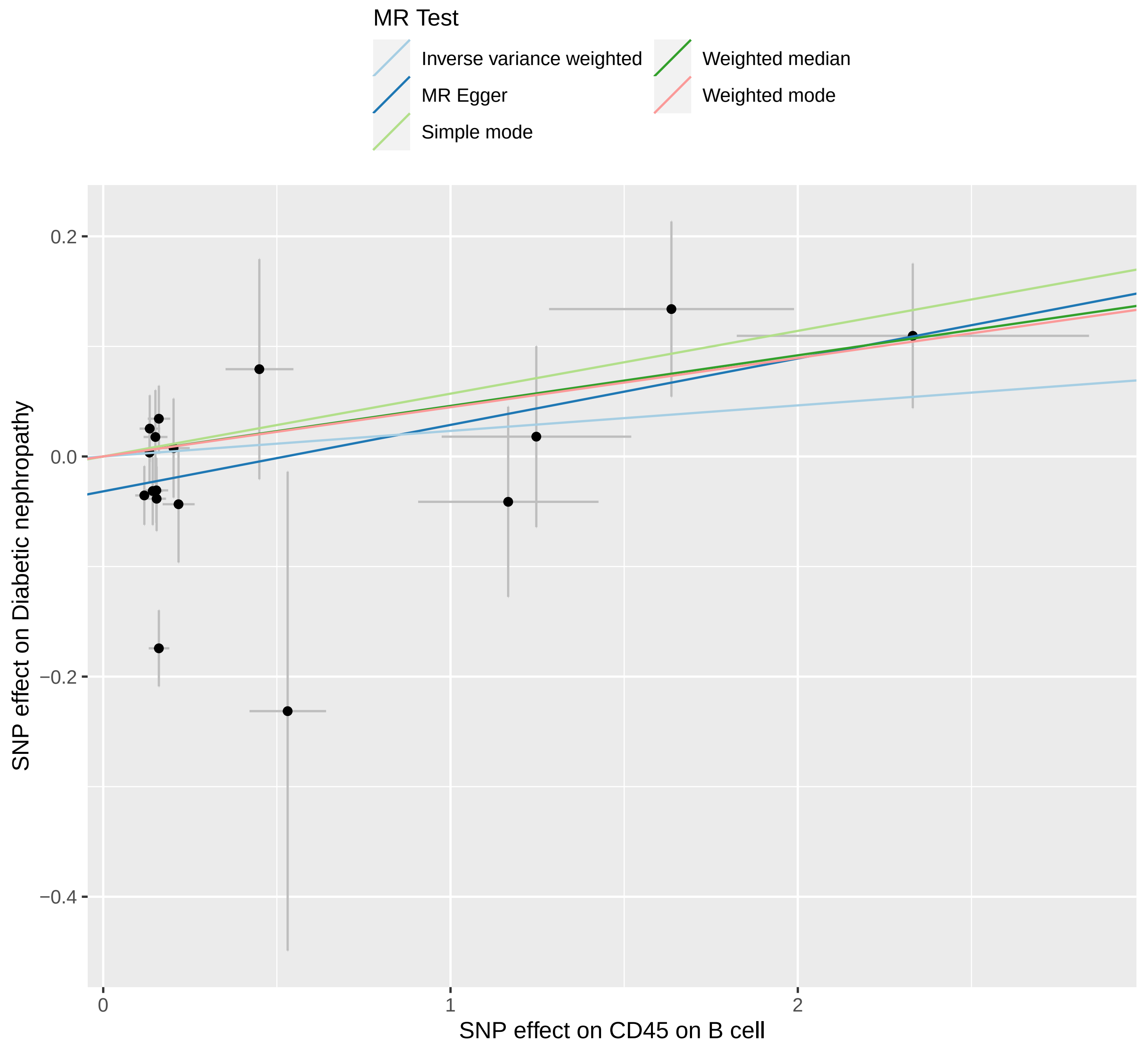
MR Test
Inverse variance weighted
MR Egger
Simple mode
Weighted median
Weighted mode
0.2
0.0
−0.2
−0.4
0
1
2
SNP effect on Diabetic nephropathy
SNP effect on CD45 on B cell

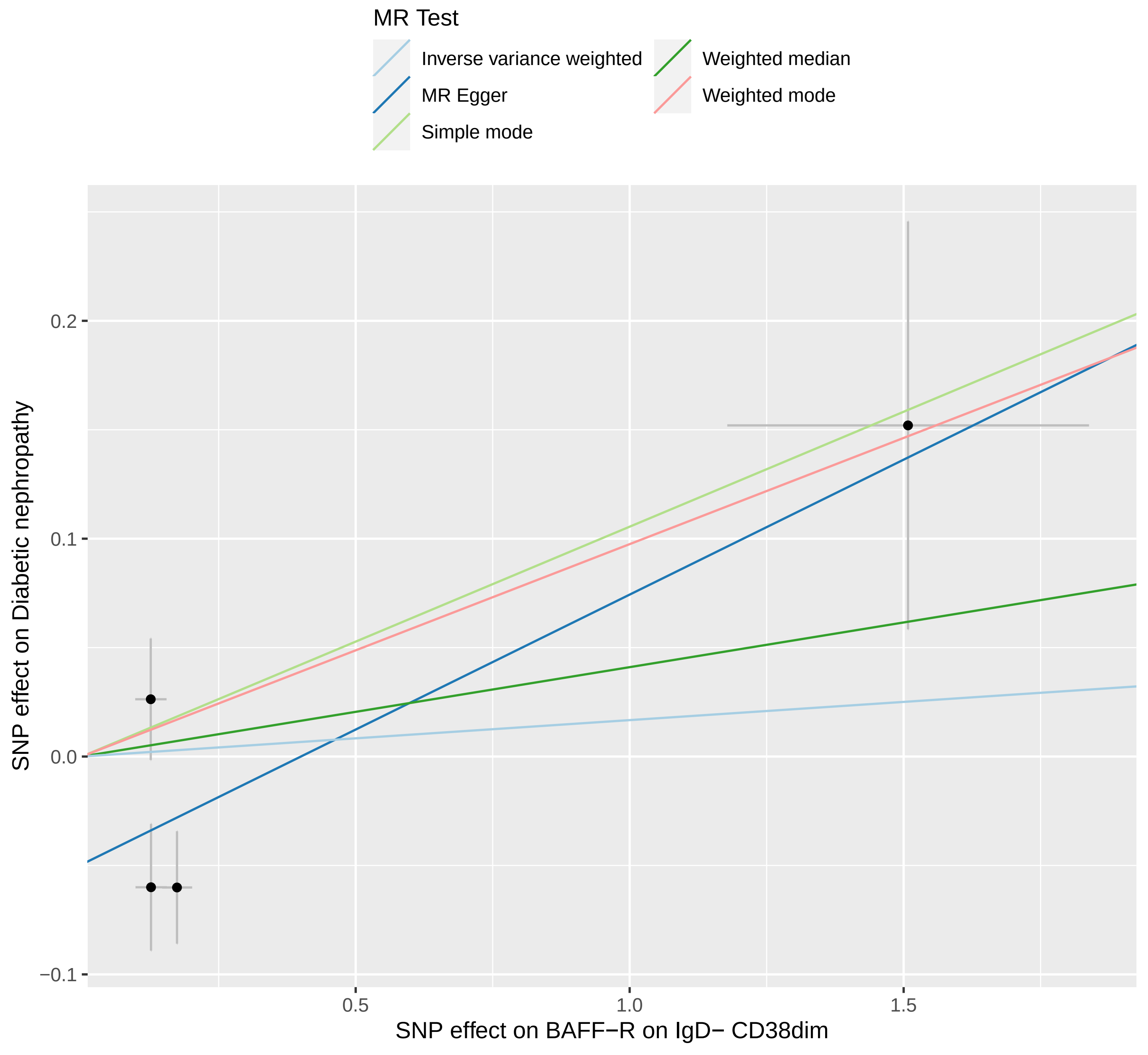
MR Test
Inverse variance weighted
MR Egger
Simple mode
Weighted median
Weighted mode
0.2
0.1
0.0
−0.1
0.5
1.0
1.5
SNP effect on Diabetic nephropathy
SNP effect on BAFF−R on IgD− CD38dim

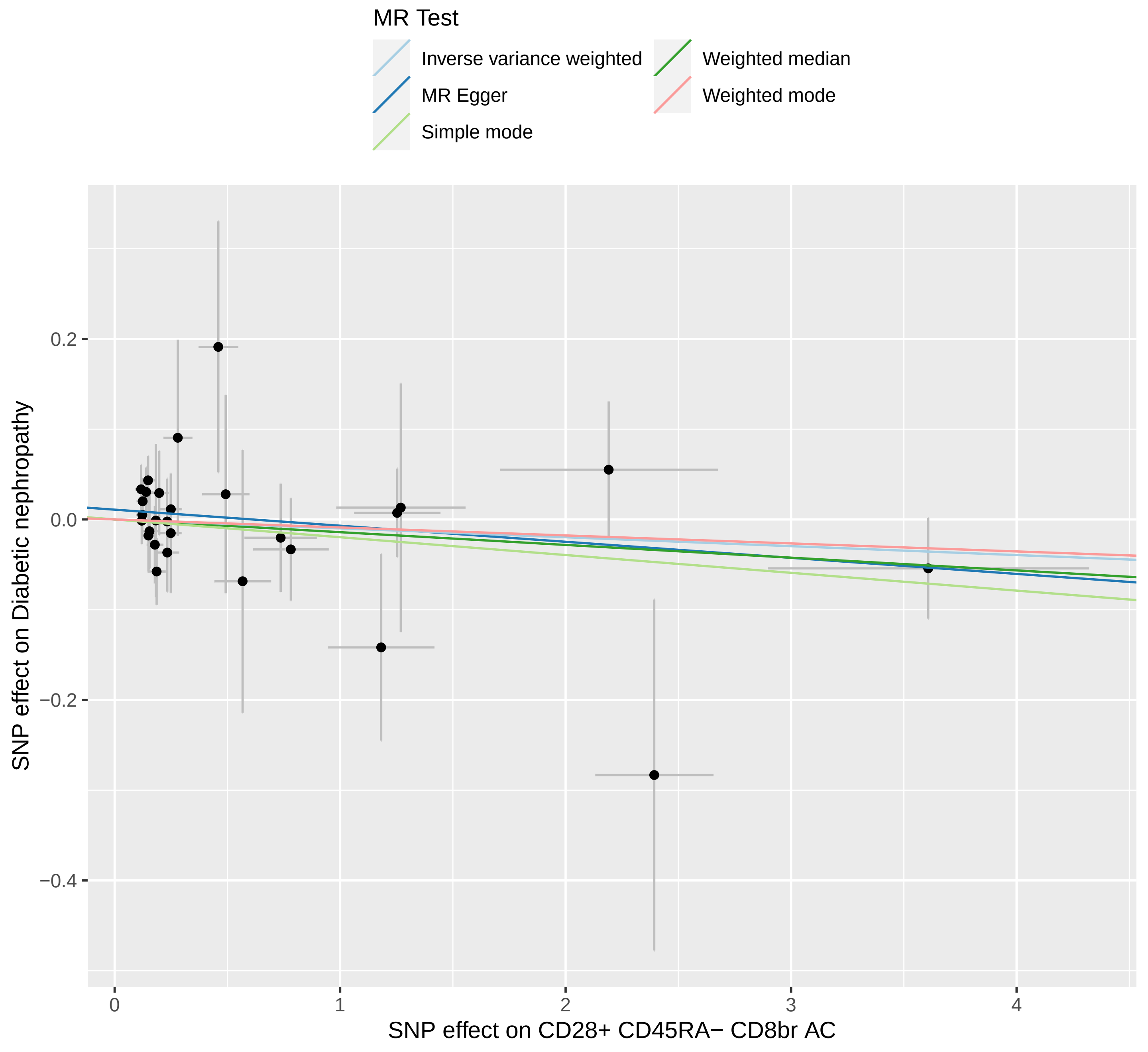
MR Test
Inverse variance weighted
MR Egger
Simple mode
Weighted median
Weighted mode
0.2
0.0
−0.2
−0.4
SNP effect on Diabetic nephropathy
0
1
2
3
4
SNP effect on CD28+ CD45RA− CD8br AC

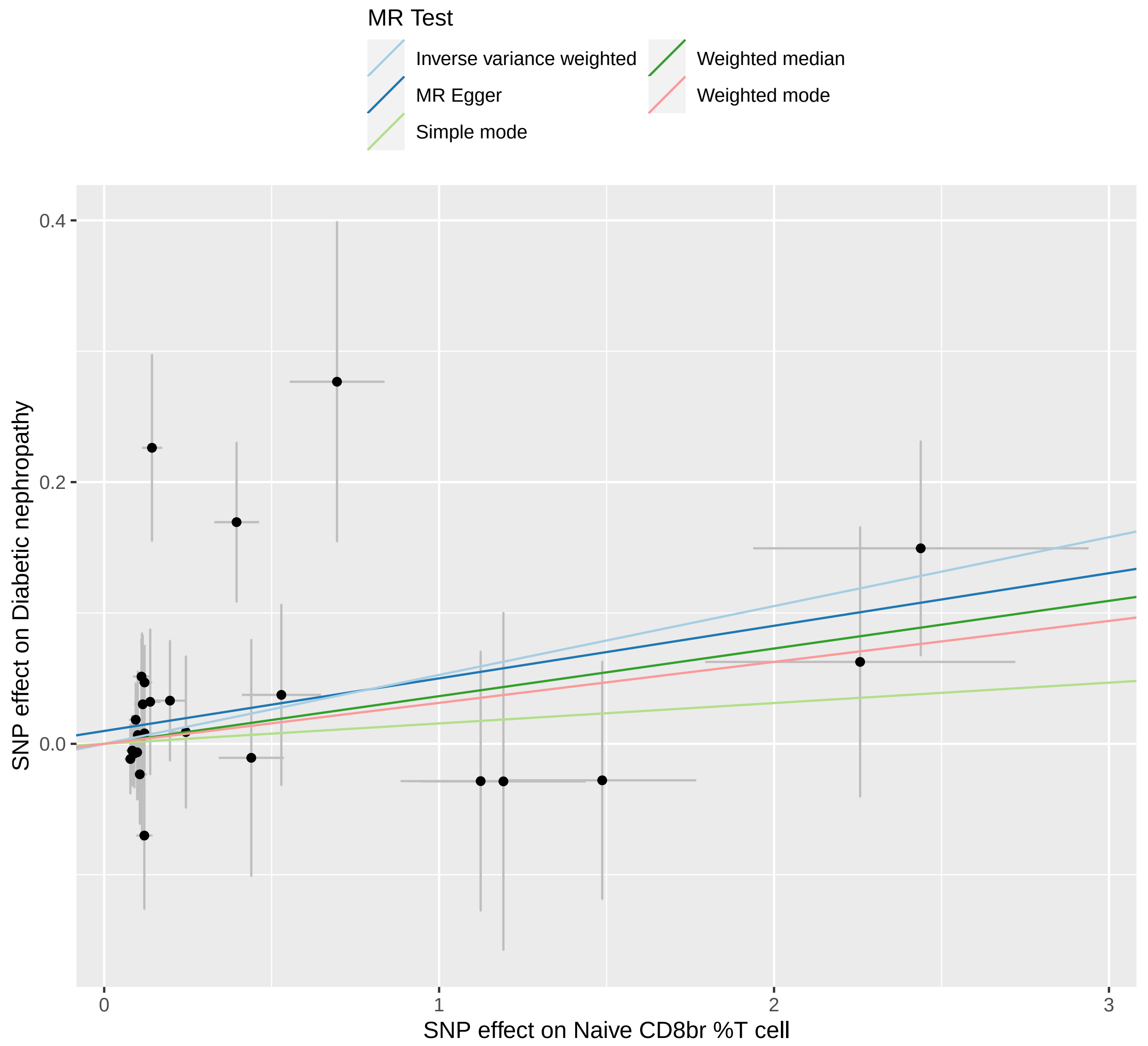
MR Test
Inverse variance weighted
MR Egger
Simple mode
Weighted median
Weighted mode
0.4
0.2
0.0
SNP effect on Diabetic nephropathy
0
1
2
3
SNP effect on Naive CD8br %T cell

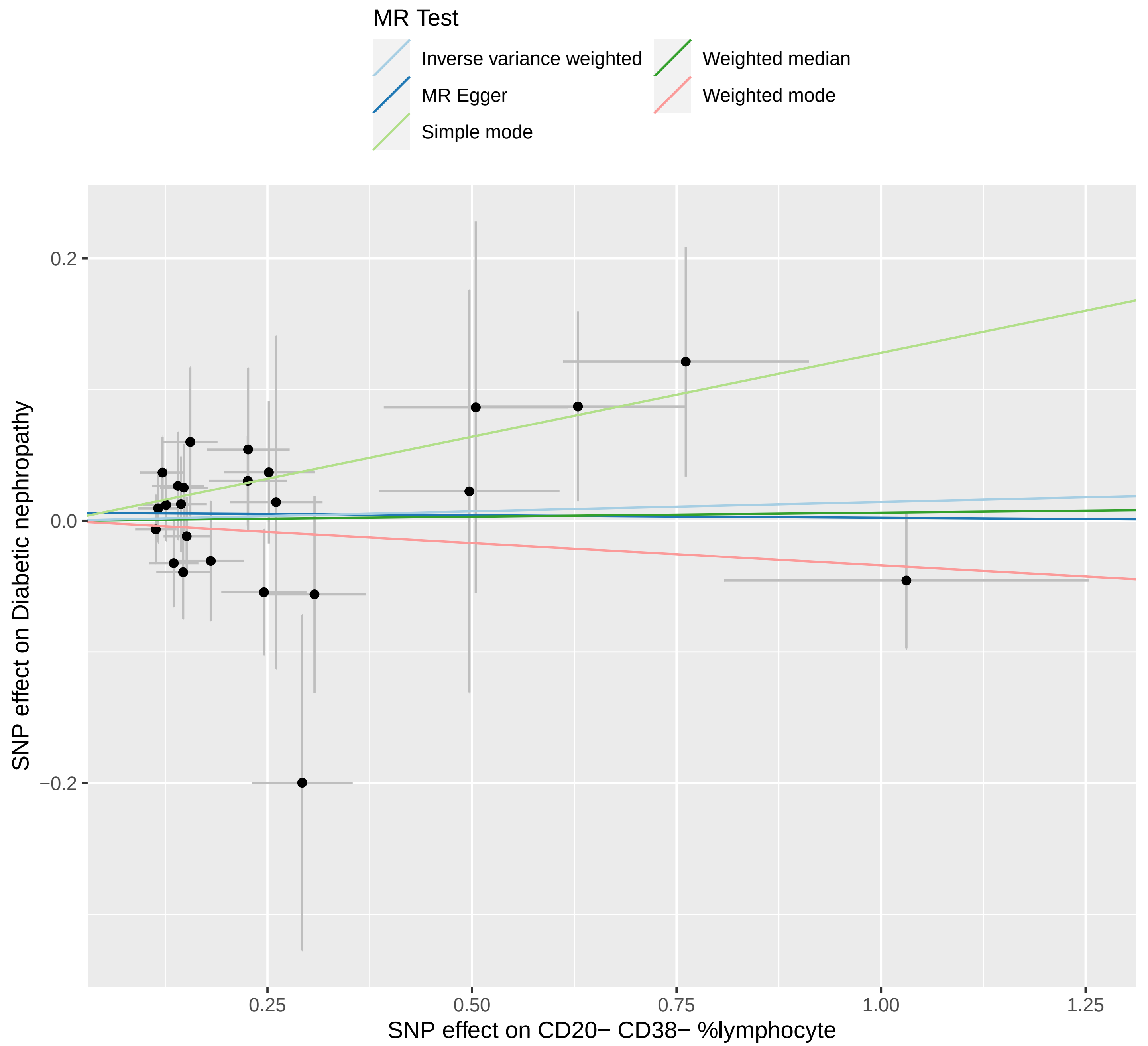
MR Test
Inverse variance weighted
MR Egger
Simple mode
Weighted median
Weighted mode
SNP effect on Diabetic nephropathy
0.2
0.0
−0.2
0.25
0.50
0.75
1.00
1.25
SNP effect on CD20− CD38− %lymphocyte

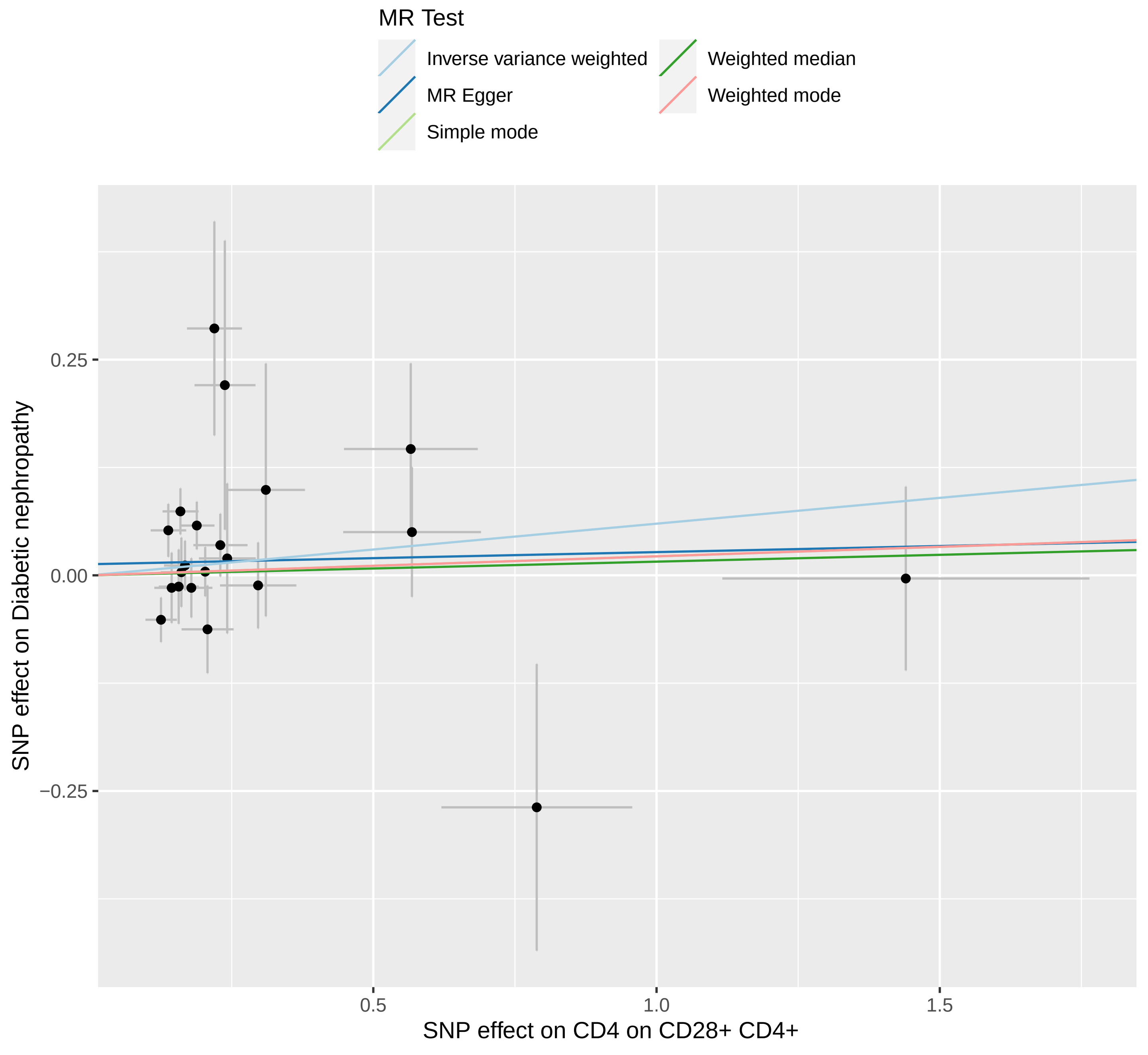
MR Test
Inverse variance weighted
MR Egger
Simple mode
Weighted median
Weighted mode
0.25
0.00
−0.25
0.5
1.0
1.5
SNP effect on Diabetic nephropathy
SNP effect on CD4 on CD28+ CD4+

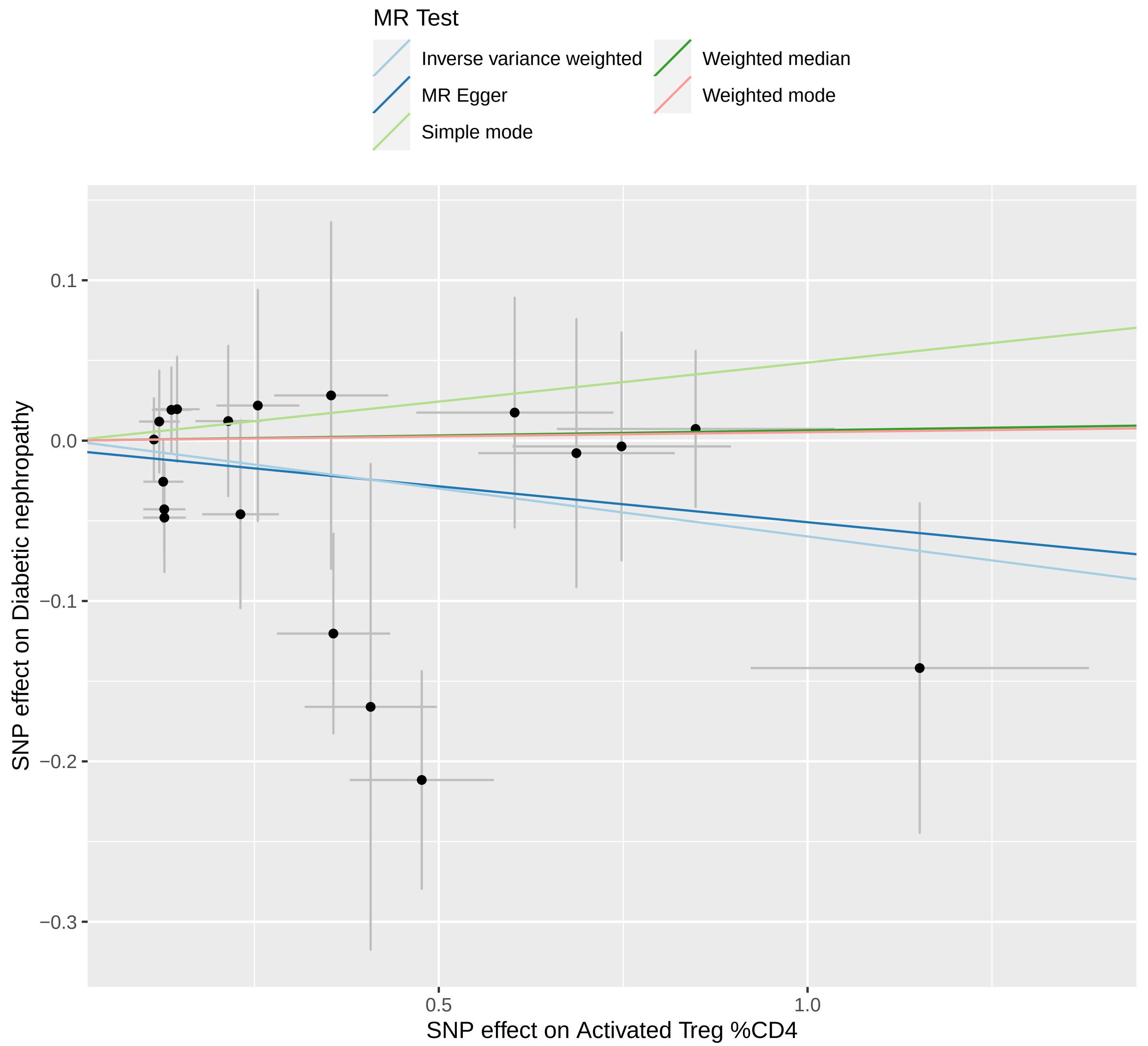
MR Test
Inverse variance weighted
MR Egger
Simple mode
Weighted median
Weighted mode
0.1
0.0
−0.1
−0.2
−0.3
0.5
1.0
SNP effect on Diabetic nephropathy
SNP effect on Activated Treg %CD4

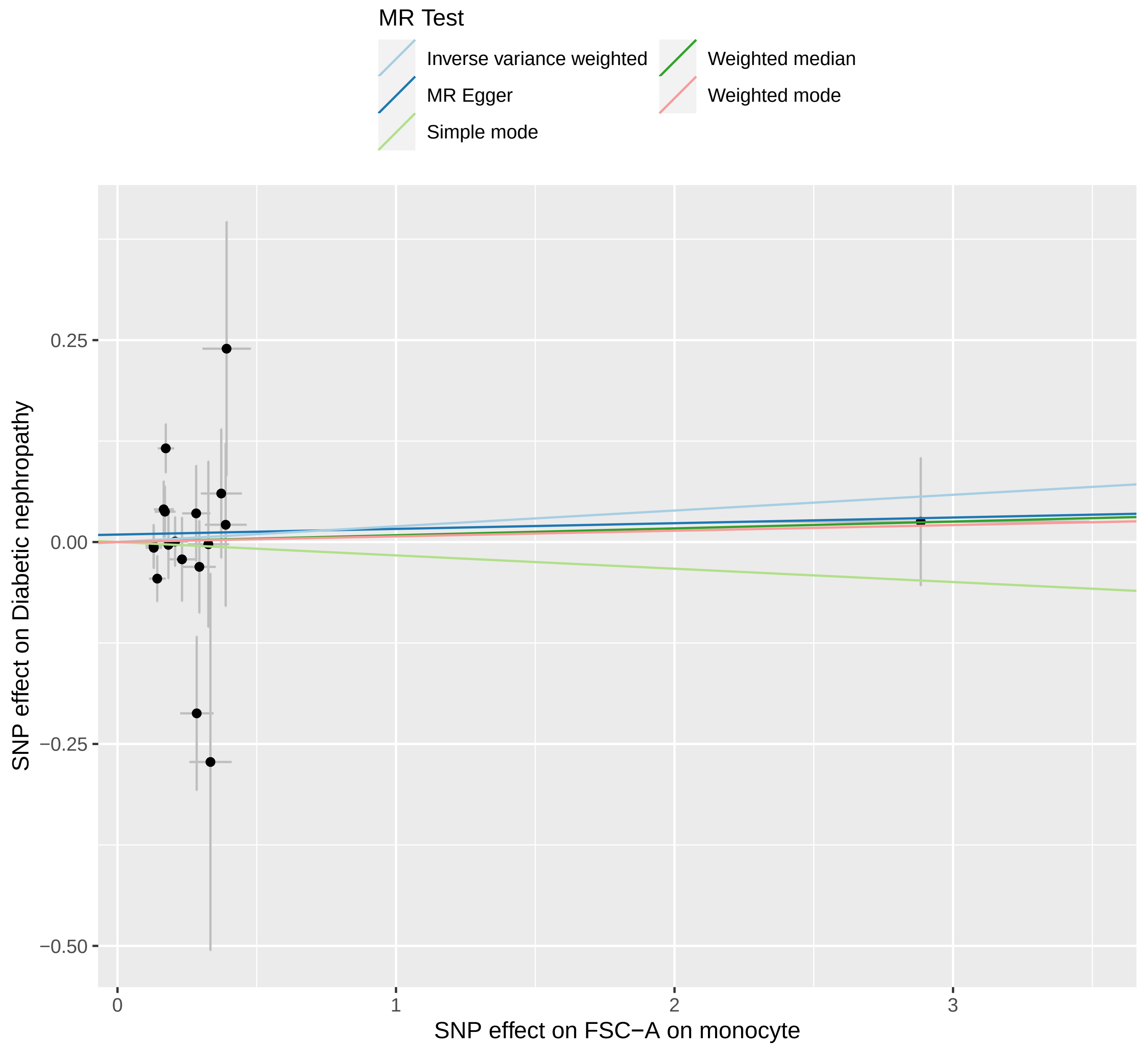

MR Test
Inverse variance weighted
MR Egger
Simple mode
Weighted median
Weighted mode
SNP effect on Diabetic nephropathy
0.25
0.00
−0.25
−0.50
0
1
2
3
SNP effect on FSC−A on monocyte

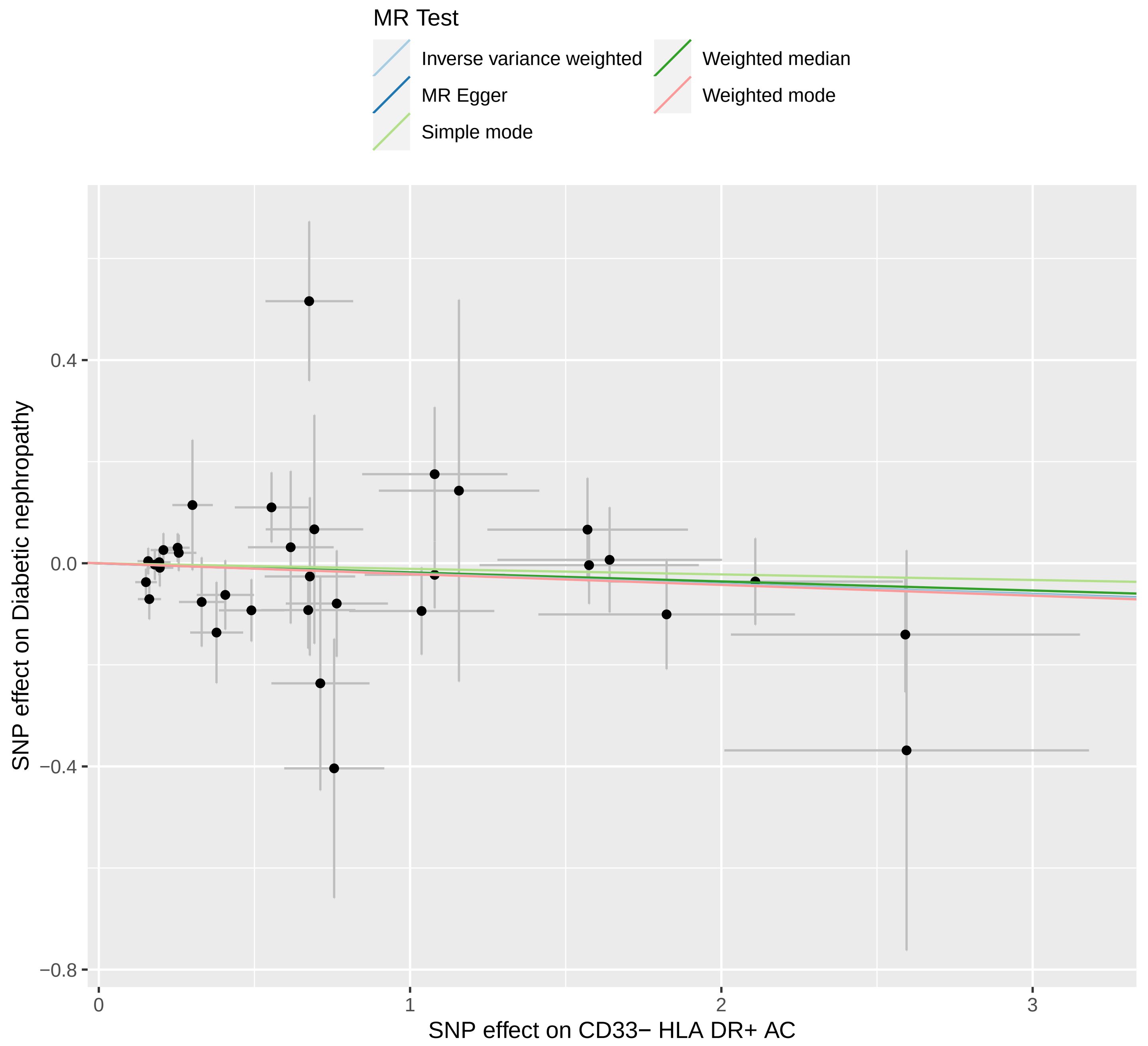

MR Test
Inverse variance weighted
MR Egger
Simple mode
Weighted median
Weighted mode
0.4
0.0
−0.4
−0.8
0
1
2
3
SNP effect on Diabetic nephropathy
SNP effect on CD33− HLA DR+ AC

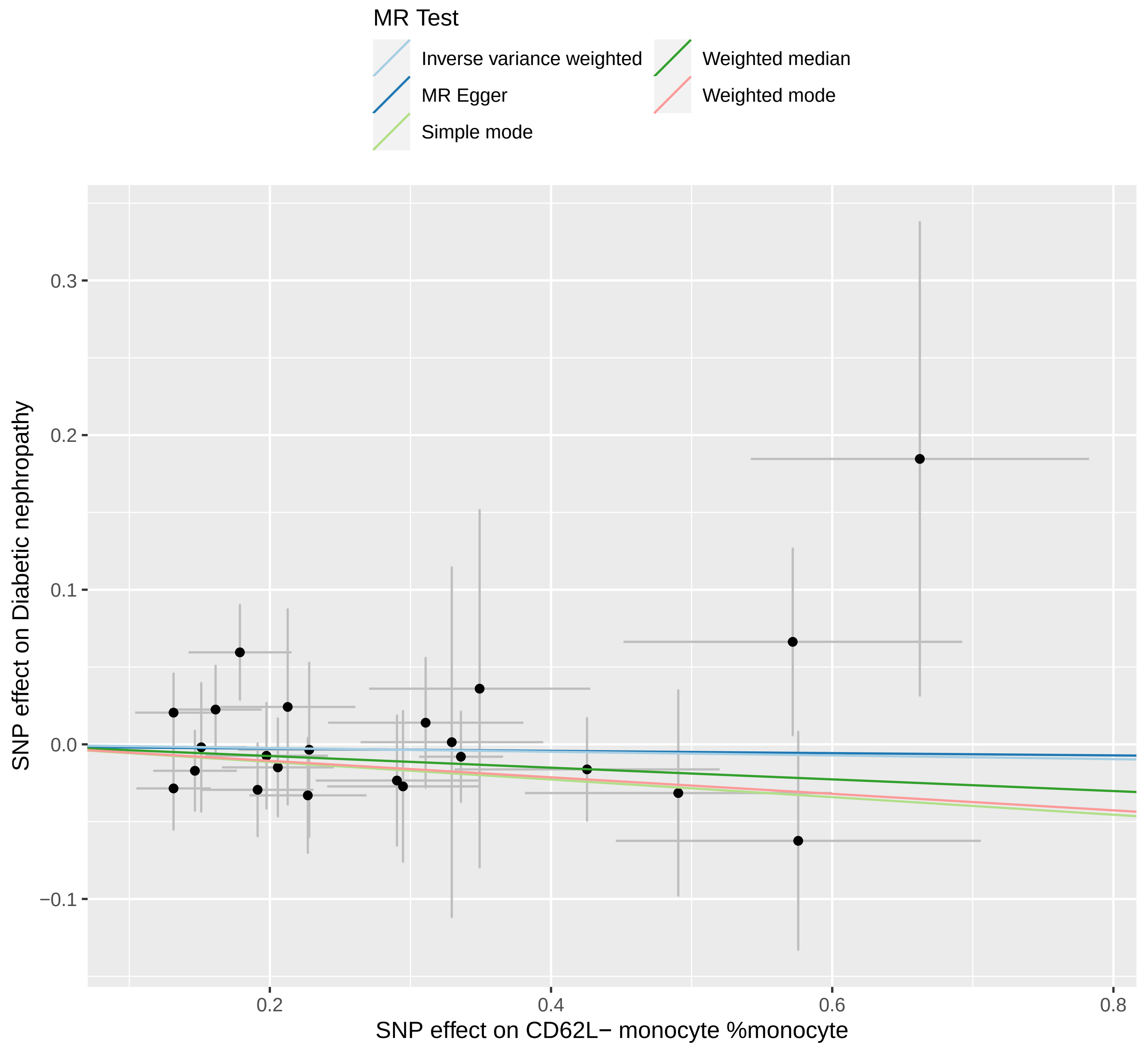

MR Test
Inverse variance weighted
MR Egger
Simple mode
Weighted median
Weighted mode
0.3
0.2
0.1
0.0
−0.1
0.2
0.4
0.6
0.8
SNP effect on Diabetic nephropathy
SNP effect on CD62L− monocyte %monocyte

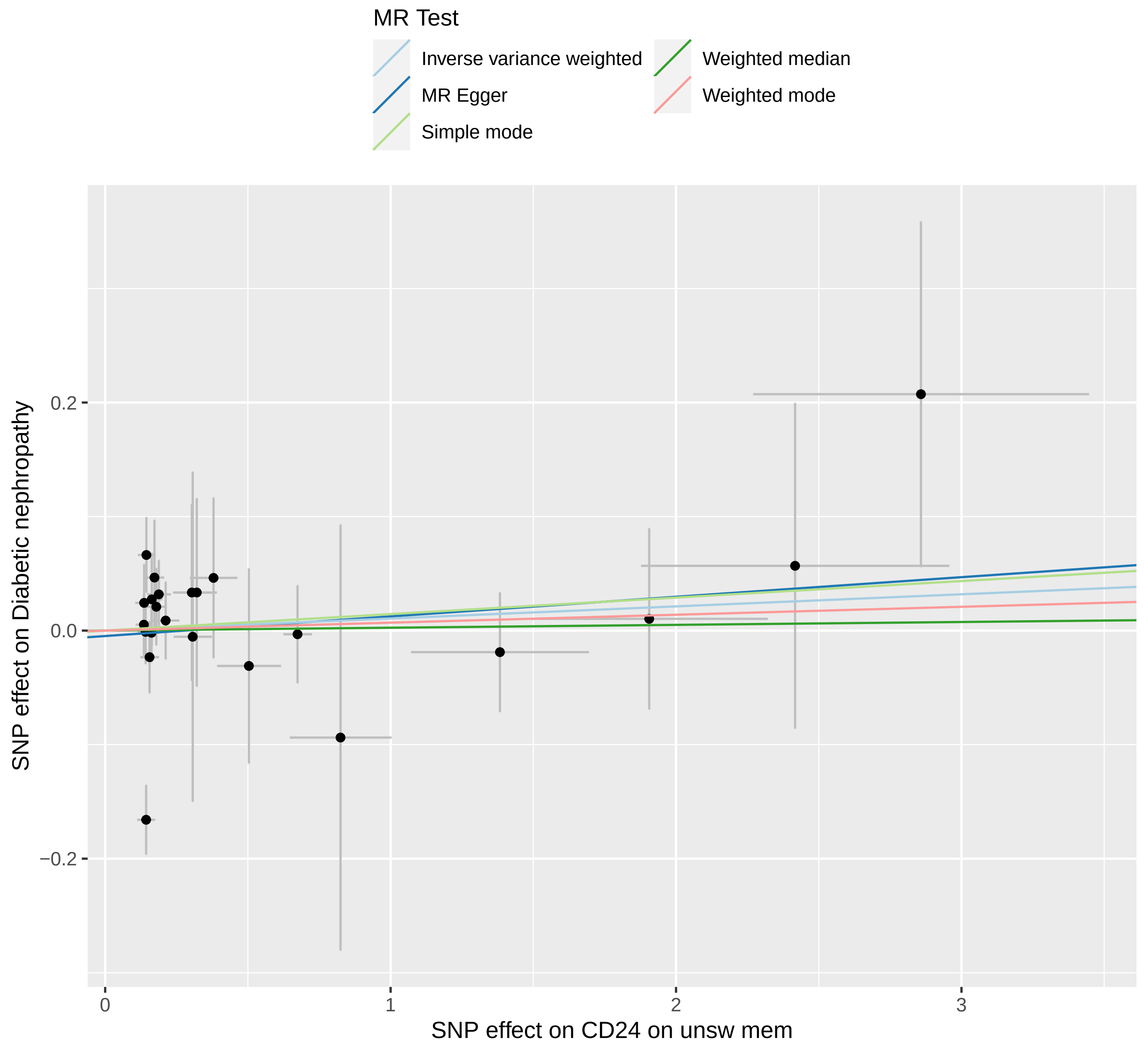
MR Test
Inverse variance weighted
MR Egger
Simple mode
Weighted median
Weighted mode
SNP effect on Diabetic nephropathy
0.2
0.0
−0.2
0
1
2
3
SNP effect on CD24 on unsw mem

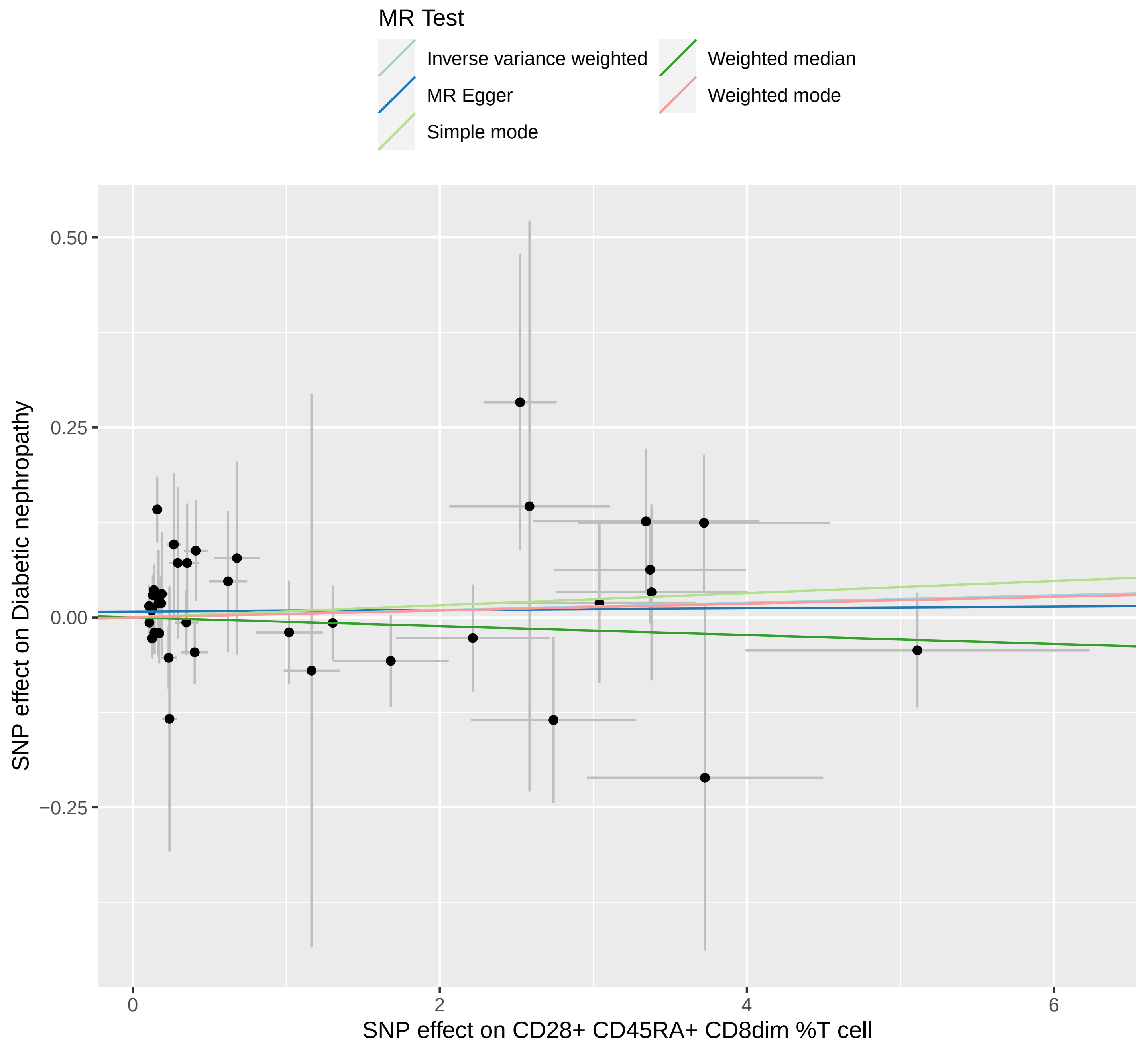

MR Test
Inverse variance weighted
MR Egger
Simple mode
Weighted median
Weighted mode
0.50
0.25
0.00
−0.25
0
2
4
6
SNP effect on Diabetic nephropathy
SNP effect on CD28+ CD45RA+ CD8dim %T cell

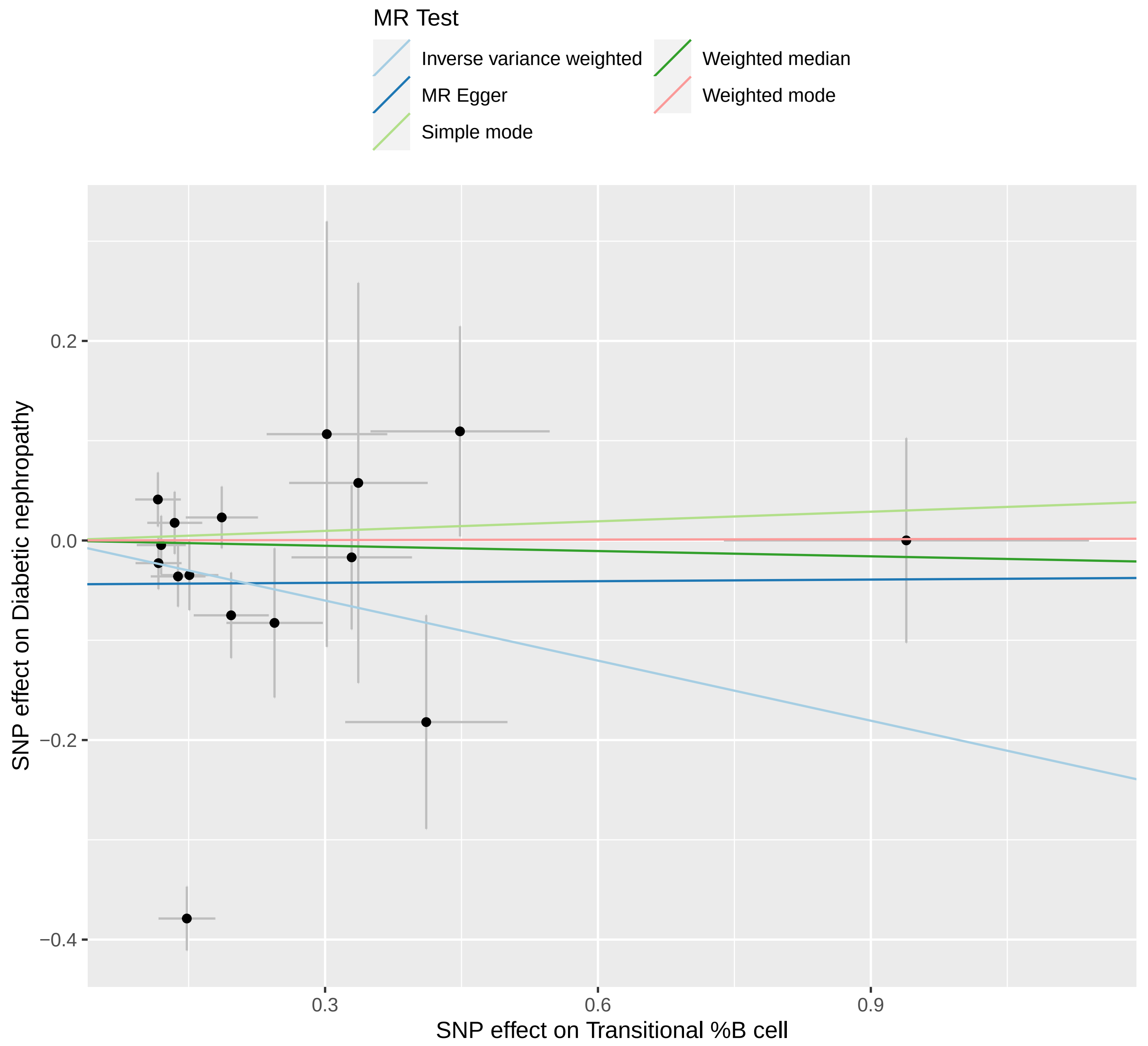

MR Test
Inverse variance weighted
MR Egger
Simple mode
Weighted median
Weighted mode
0.2
0.0
−0.2
−0.4
0.3
0.6
0.9
SNP effect on Diabetic nephropathy
SNP effect on Transitional %B cell

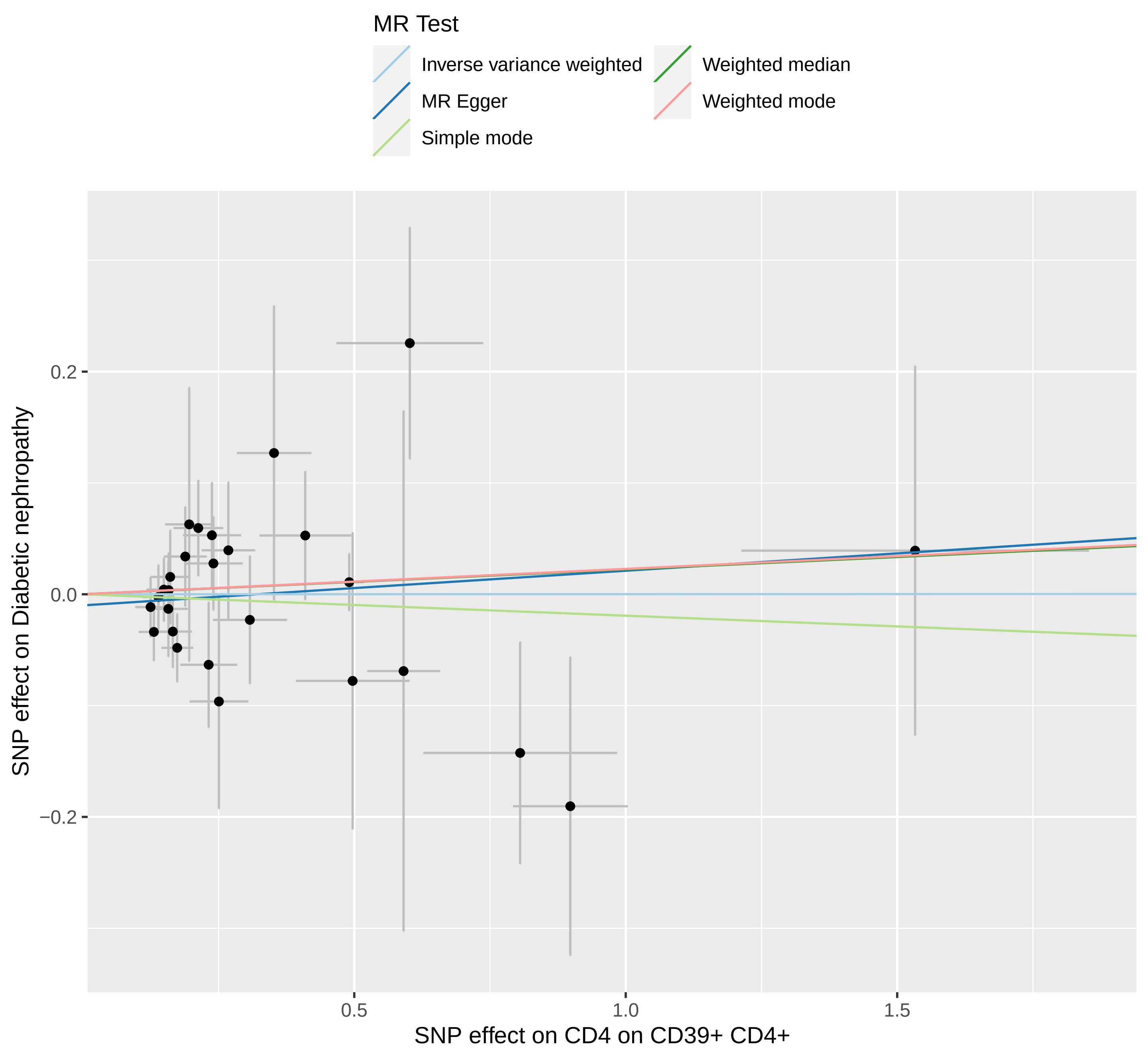
MR Test
Inverse variance weighted
MR Egger
Simple mode
Weighted median
Weighted mode
0.2
0.0
−0.2
0.5
1.0
1.5
SNP effect on Diabetic nephropathy
SNP effect on CD4 on CD39+ CD4+

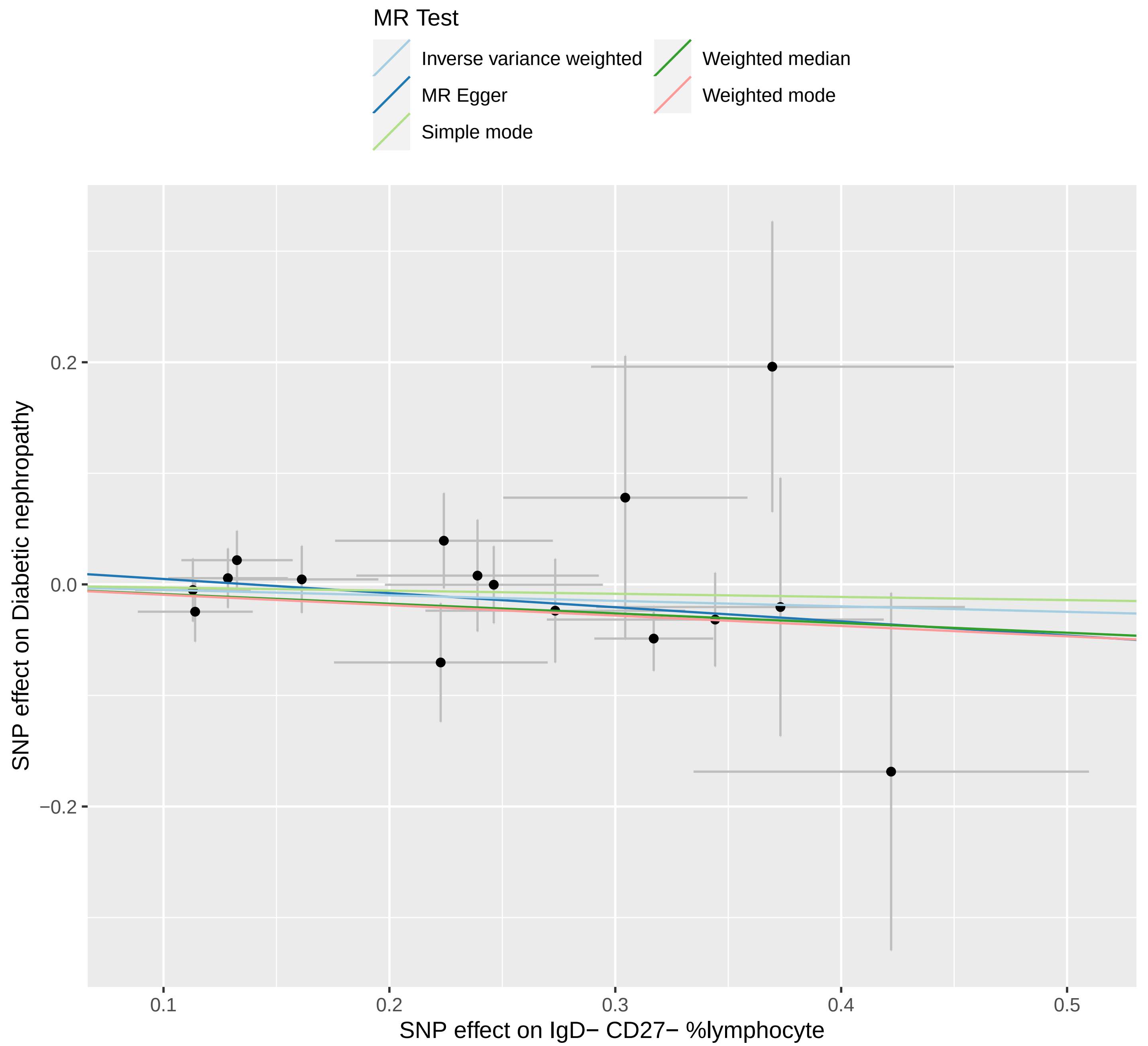

MR Test
Inverse variance weighted
MR Egger
Simple mode
Weighted median
Weighted mode
0.2
0.0
−0.2
SNP effect on Diabetic nephropathy
0.1
0.2
0.3
0.4
0.5
SNP effect on IgD− CD27− %lymphocyte

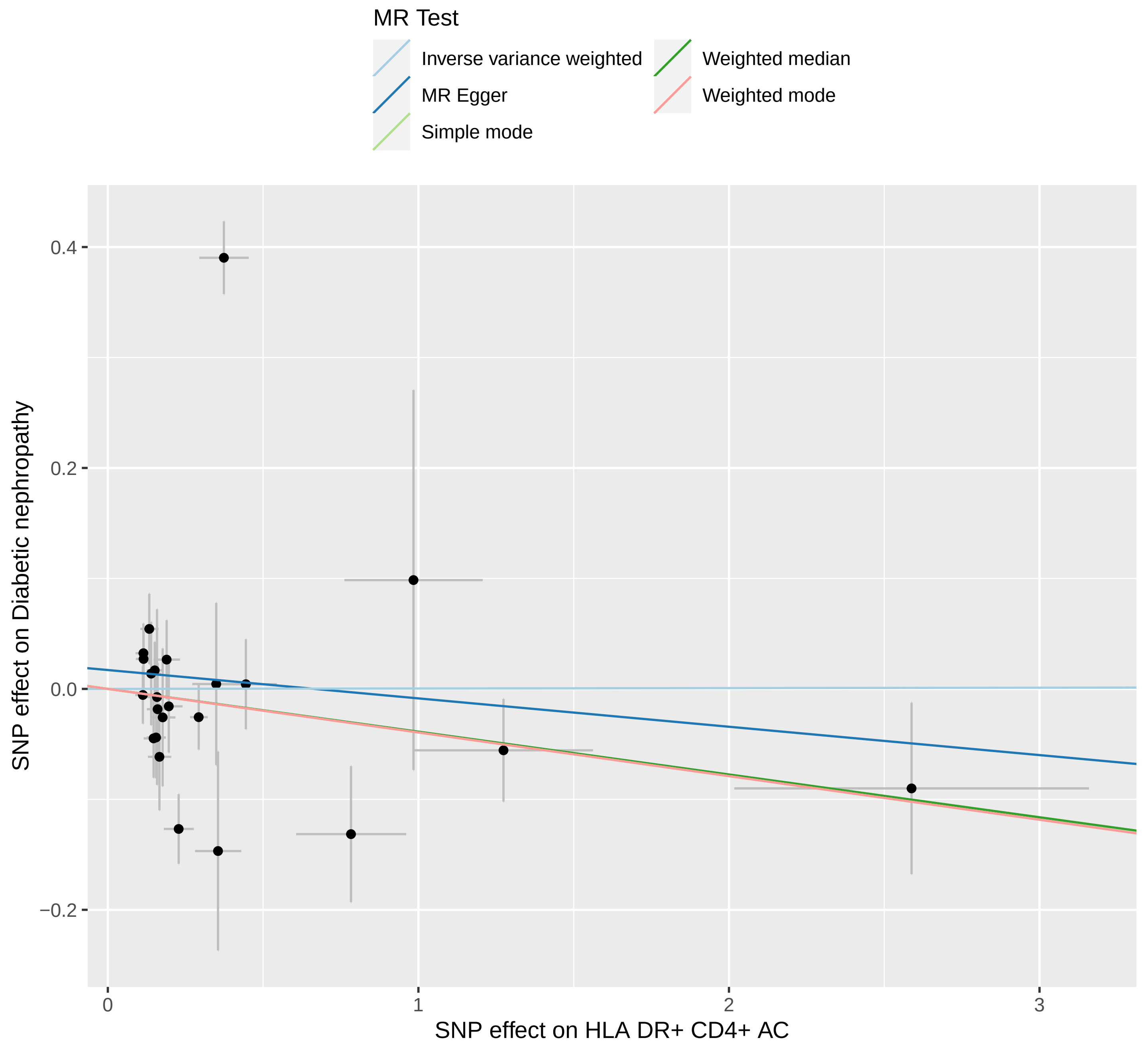

MR Test
Inverse variance weighted
MR Egger
Simple mode
Weighted median
Weighted mode
0.4
0.2
0.0
−0.2
0
1
2
3
SNP effect on Diabetic nephropathy
SNP effect on HLA DR+ CD4+ AC

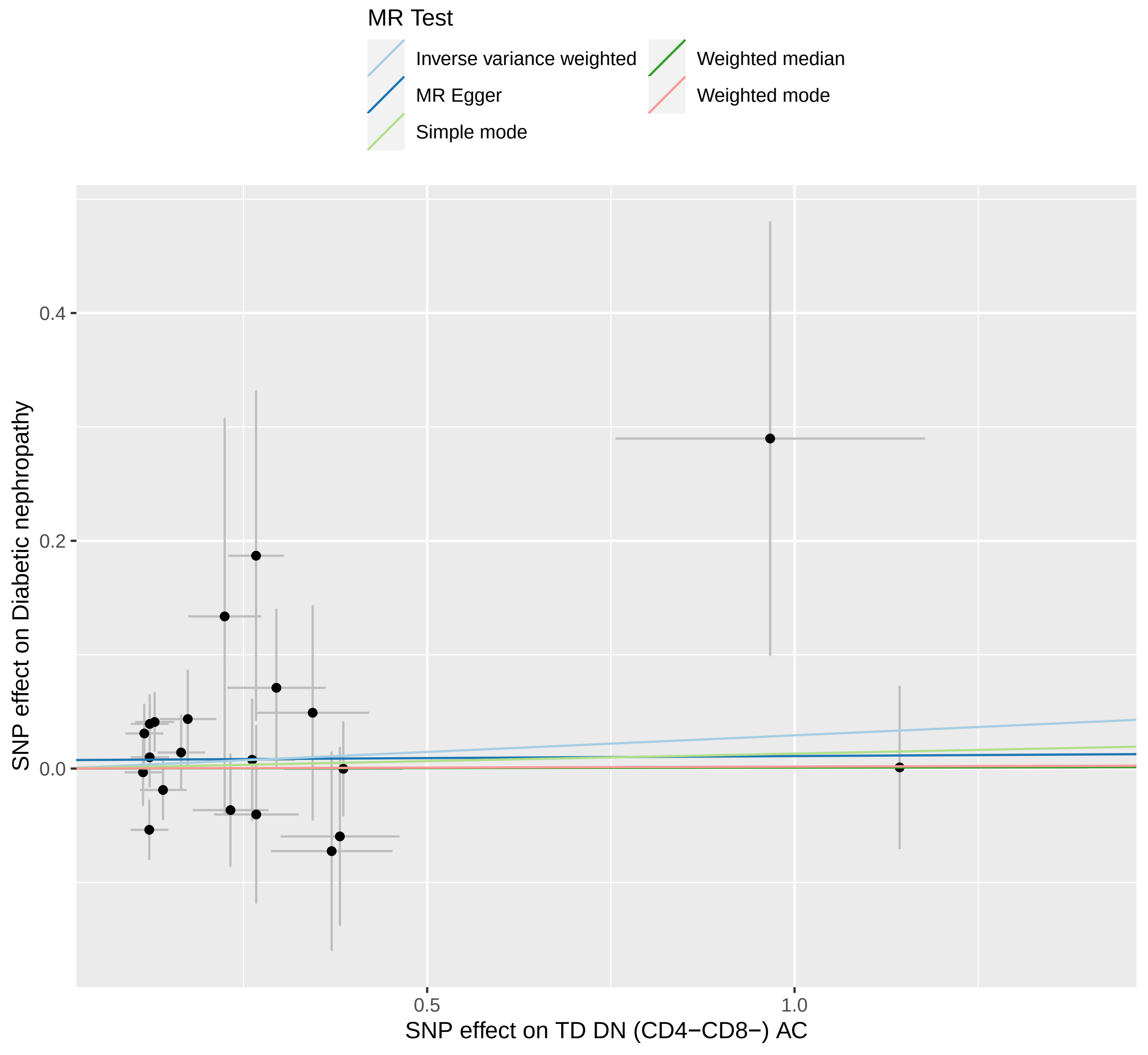
MR Test
Inverse variance weighted
MR Egger
Simple mode
Weighted median
Weighted mode
0.4
0.2
0.0
0.5
1.0
SNP effect on Diabetic nephropathy
SNP effect on TD DN (CD4−CD8−) AC

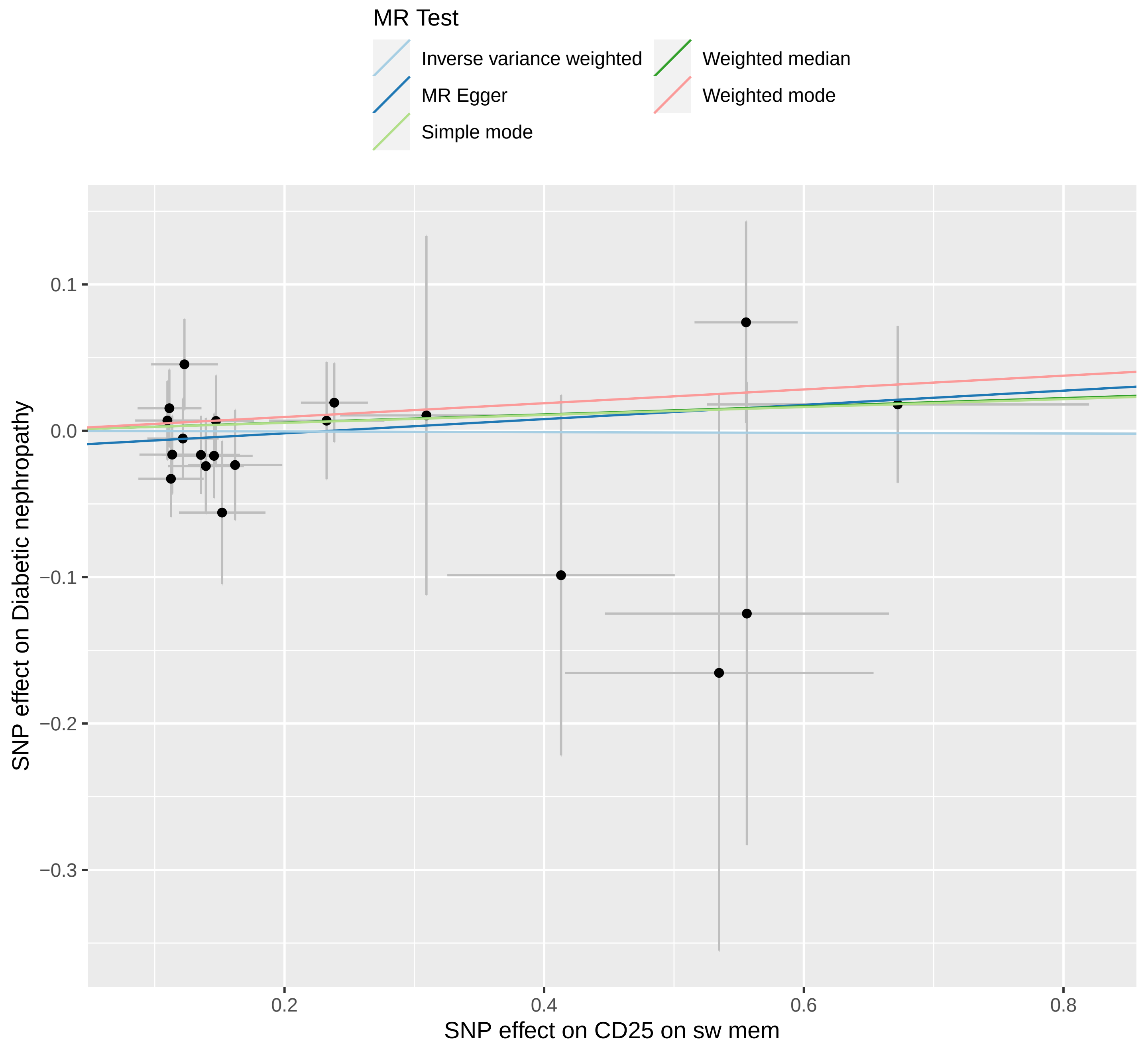

MR Test
Inverse variance weighted
MR Egger
Simple mode
Weighted median
Weighted mode
SNP effect on Diabetic nephropathy
0.1
0.0
−0.1
−0.2
−0.3
0.2
0.4
0.6
0.8
SNP effect on CD25 on sw mem

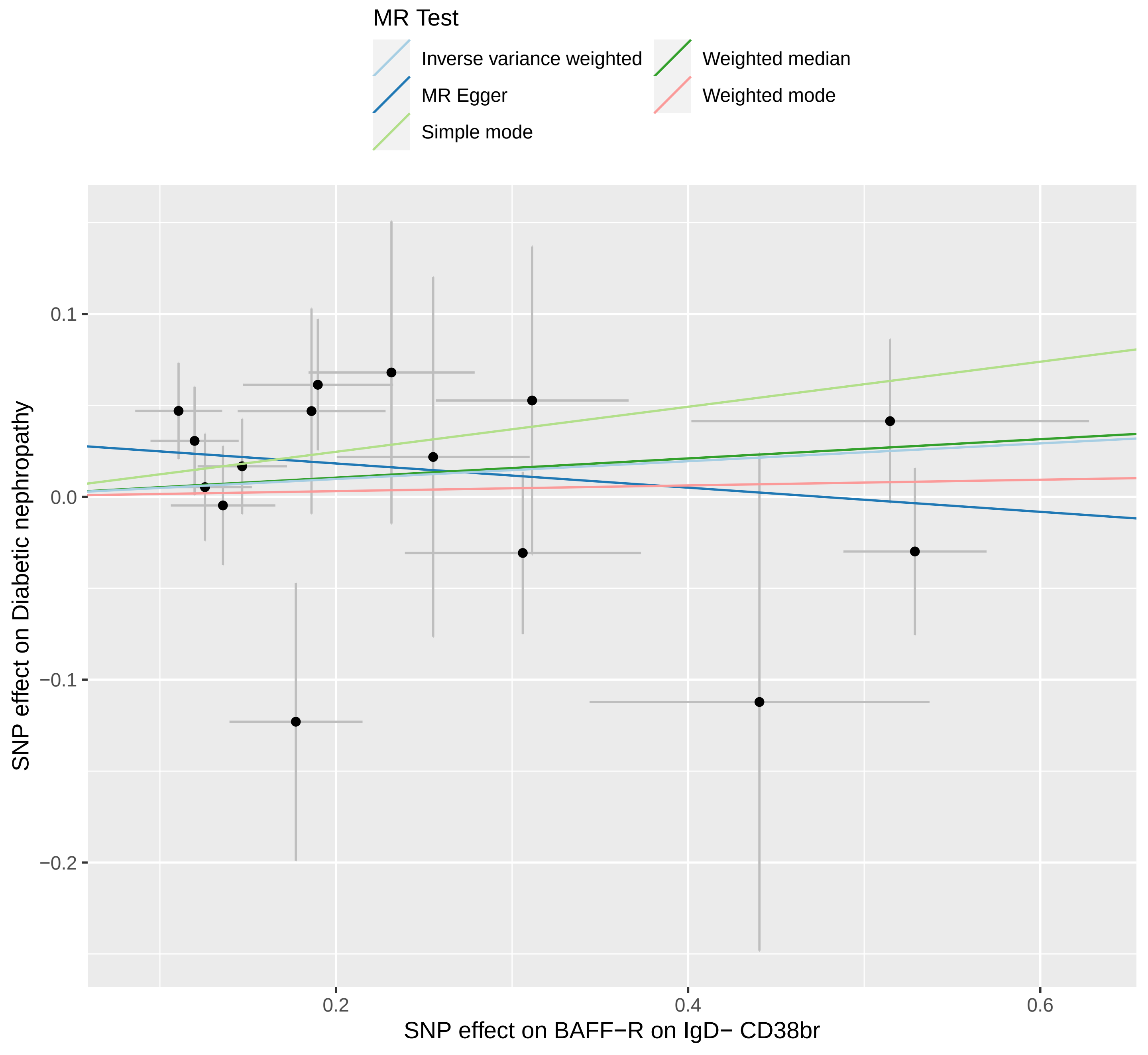
MR Test
Inverse variance weighted
MR Egger
Simple mode
Weighted median
Weighted mode
SNP effect on Diabetic nephropathy
0.1
0.0
−0.1
−0.2
0.2
0.4
0.6
SNP effect on BAFF−R on IgD− CD38br

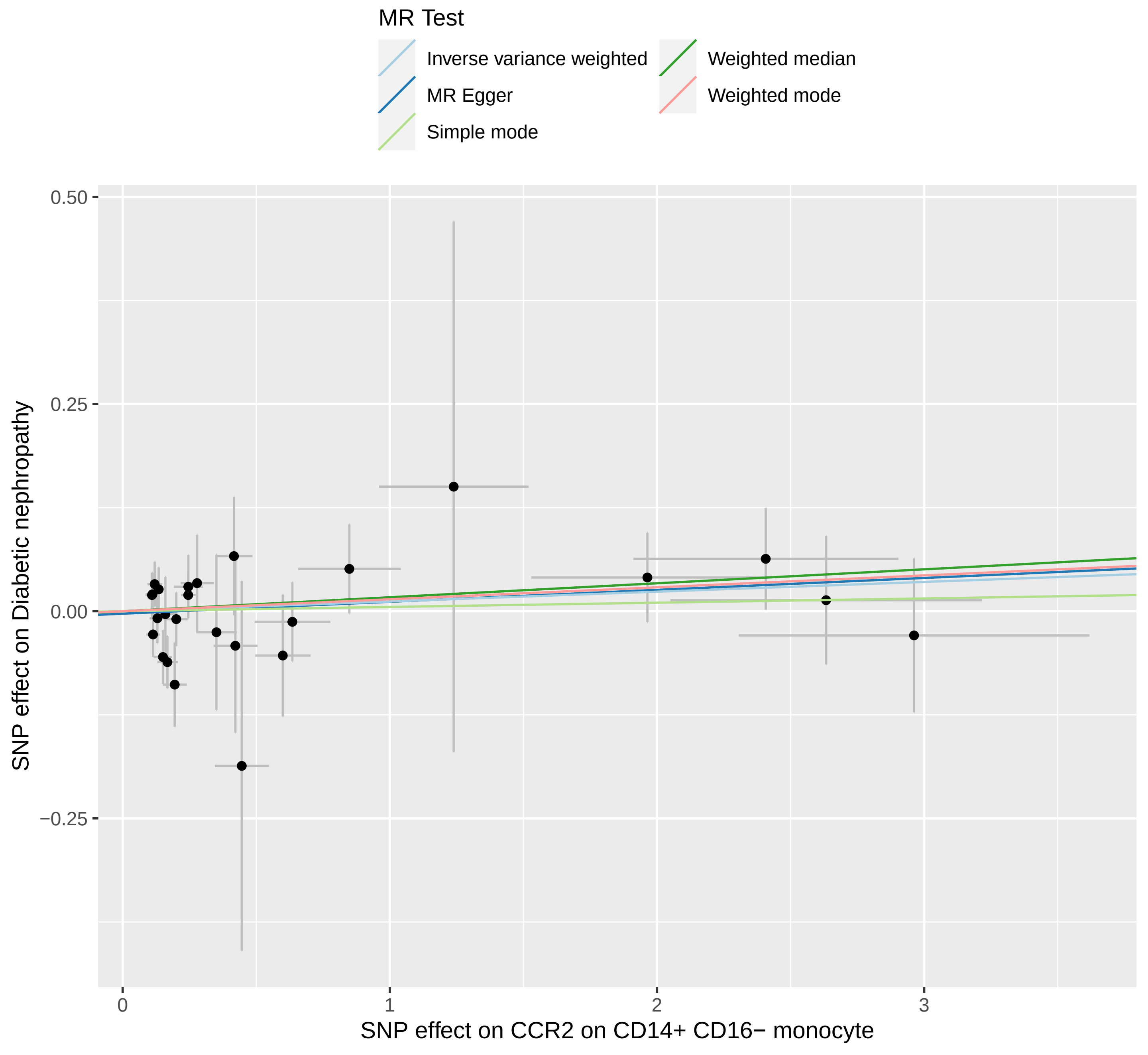
MR Test
Inverse variance weighted
MR Egger
Simple mode
Weighted median
Weighted mode
0.50
0.25
0.00
−0.25
0
1
2
3
SNP effect on Diabetic nephropathy
SNP effect on CCR2 on CD14+ CD16− monocyte

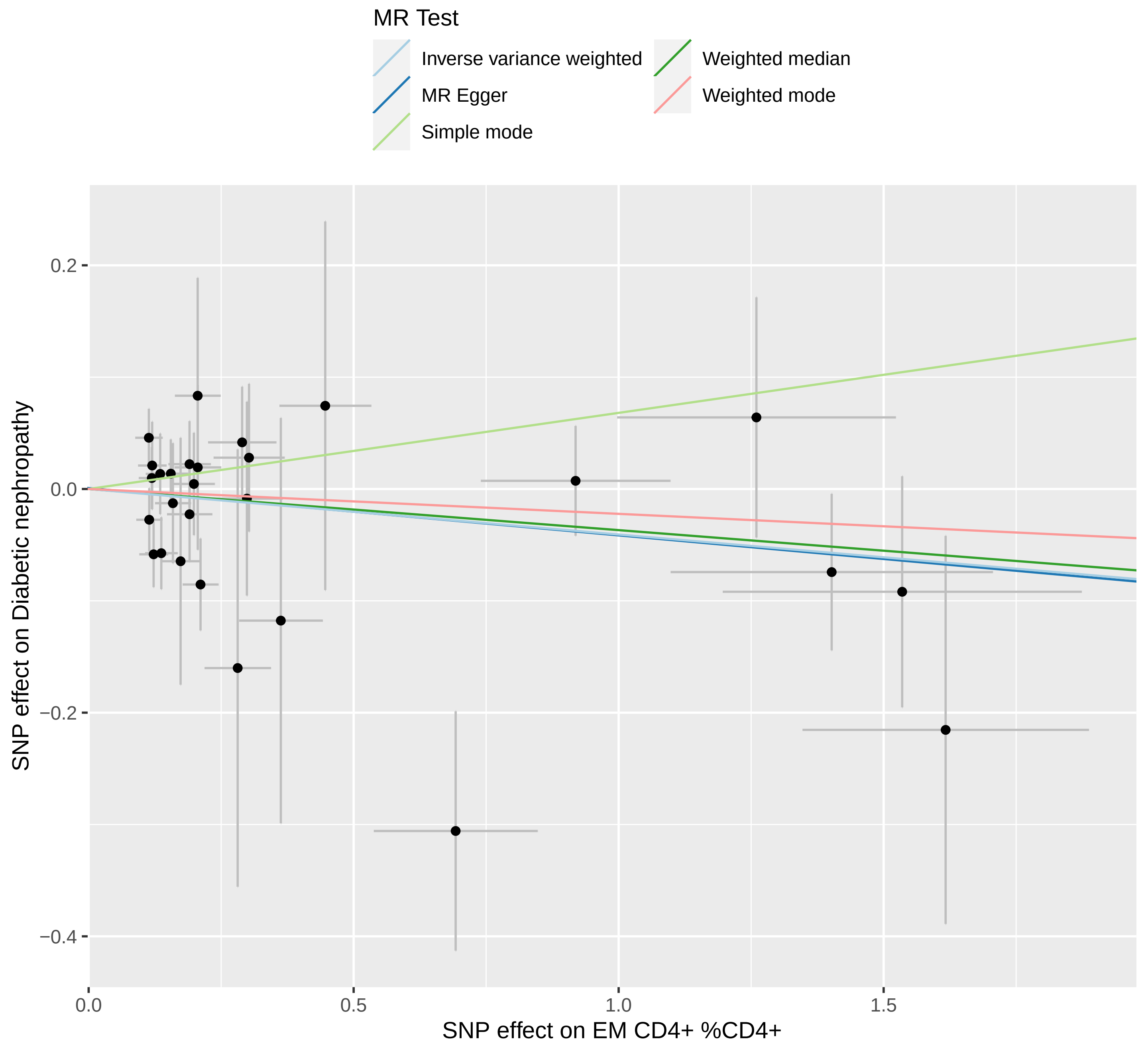
MR Test
Inverse variance weighted
MR Egger
Simple mode
Weighted median
Weighted mode
0.2
0.0
−0.2
−0.4
0.0
0.5
1.0
1.5
SNP effect on Diabetic nephropathy
SNP effect on EM CD4+ %CD4+

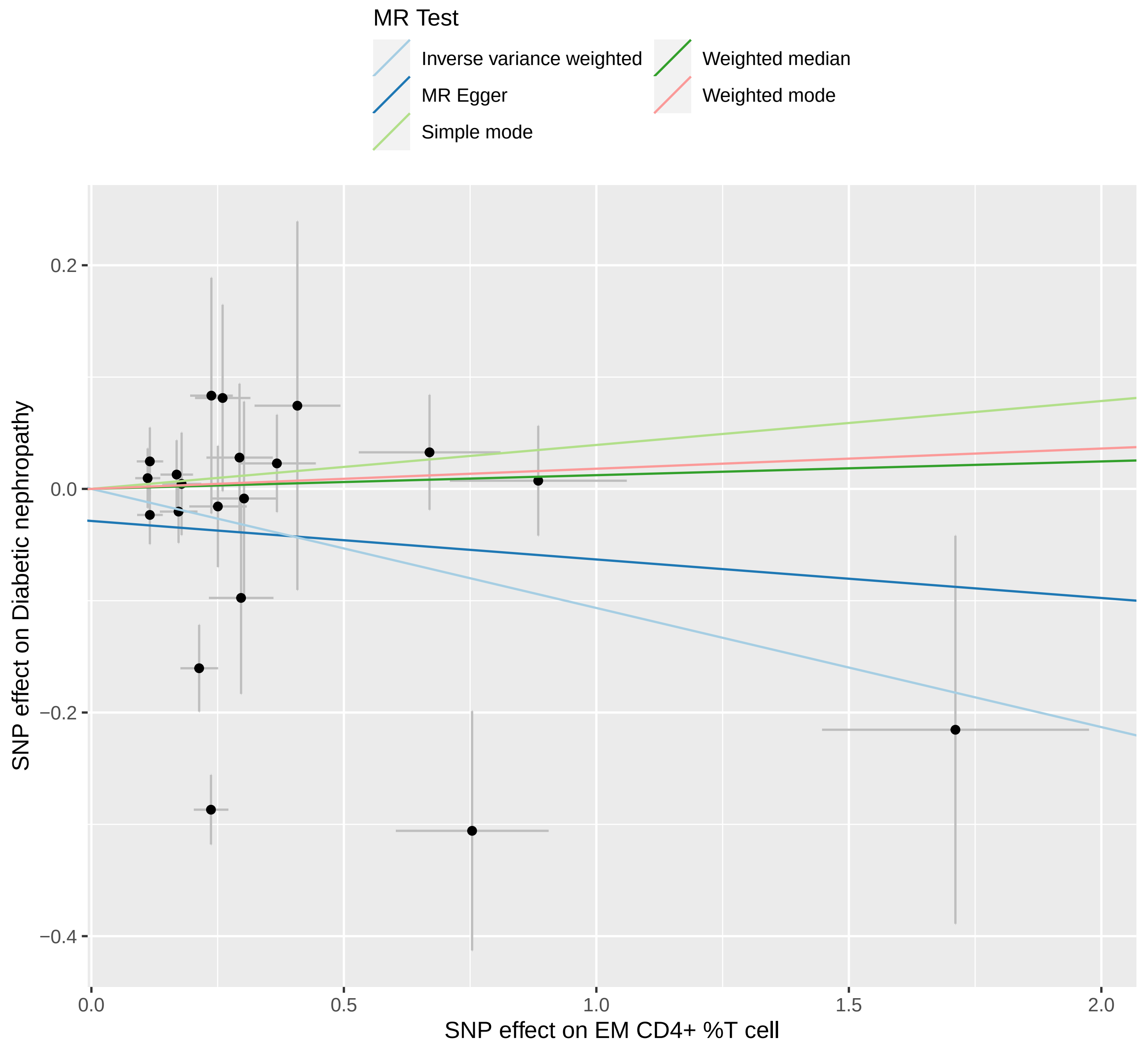
MR Test
Inverse variance weighted
MR Egger
Simple mode
Weighted median
Weighted mode
0.2
0.0
−0.2
−0.4
0.0
0.5
1.0
1.5
2.0
SNP effect on Diabetic nephropathy
SNP effect on EM CD4+ %T cell

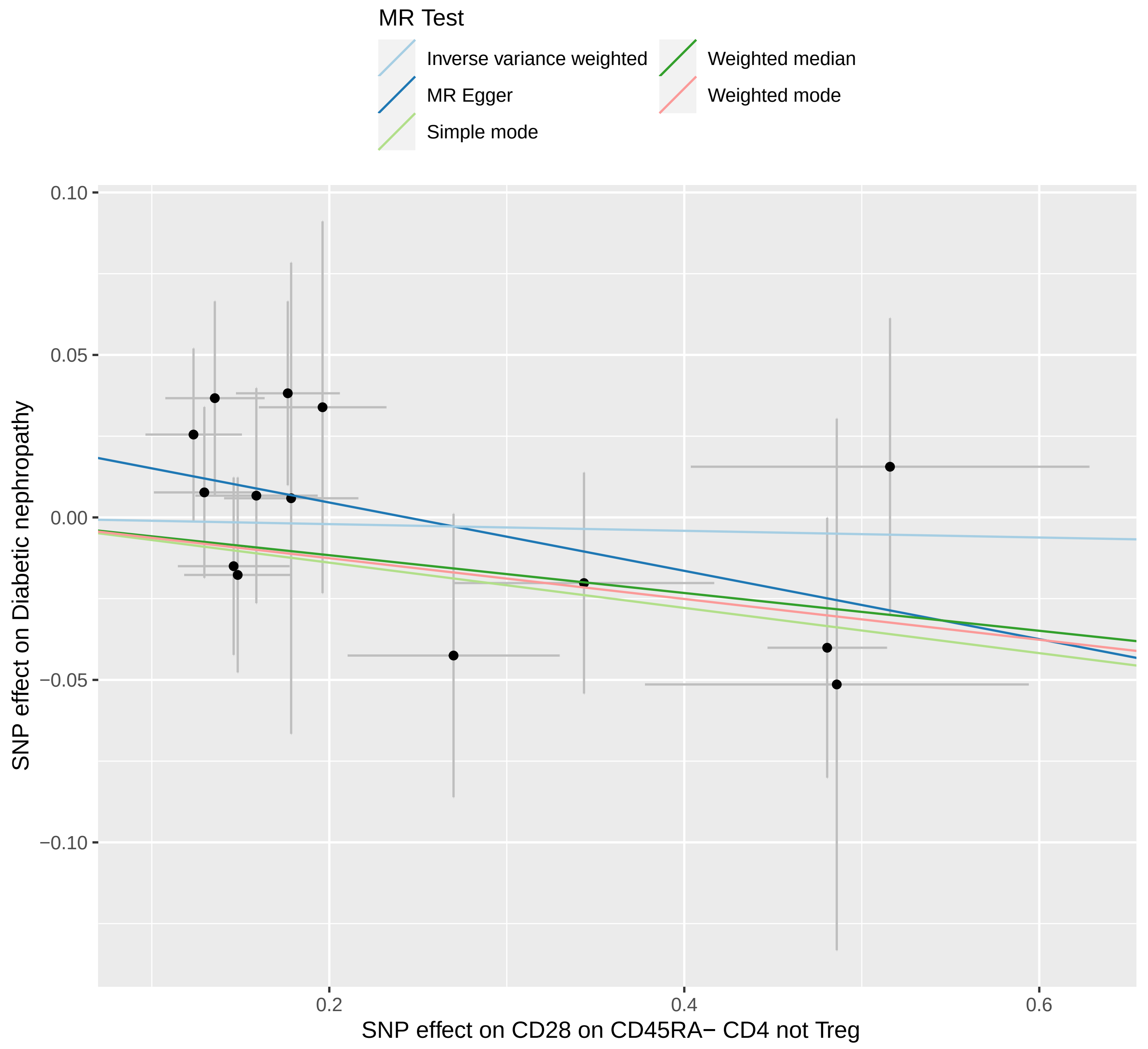

MR Test
Inverse variance weighted
MR Egger
Simple mode
Weighted median
Weighted mode
0.10
0.05
0.00
−0.05
−0.10
0.2
0.4
0.6
SNP effect on Diabetic nephropathy
SNP effect on CD28 on CD45RA− CD4 not Treg

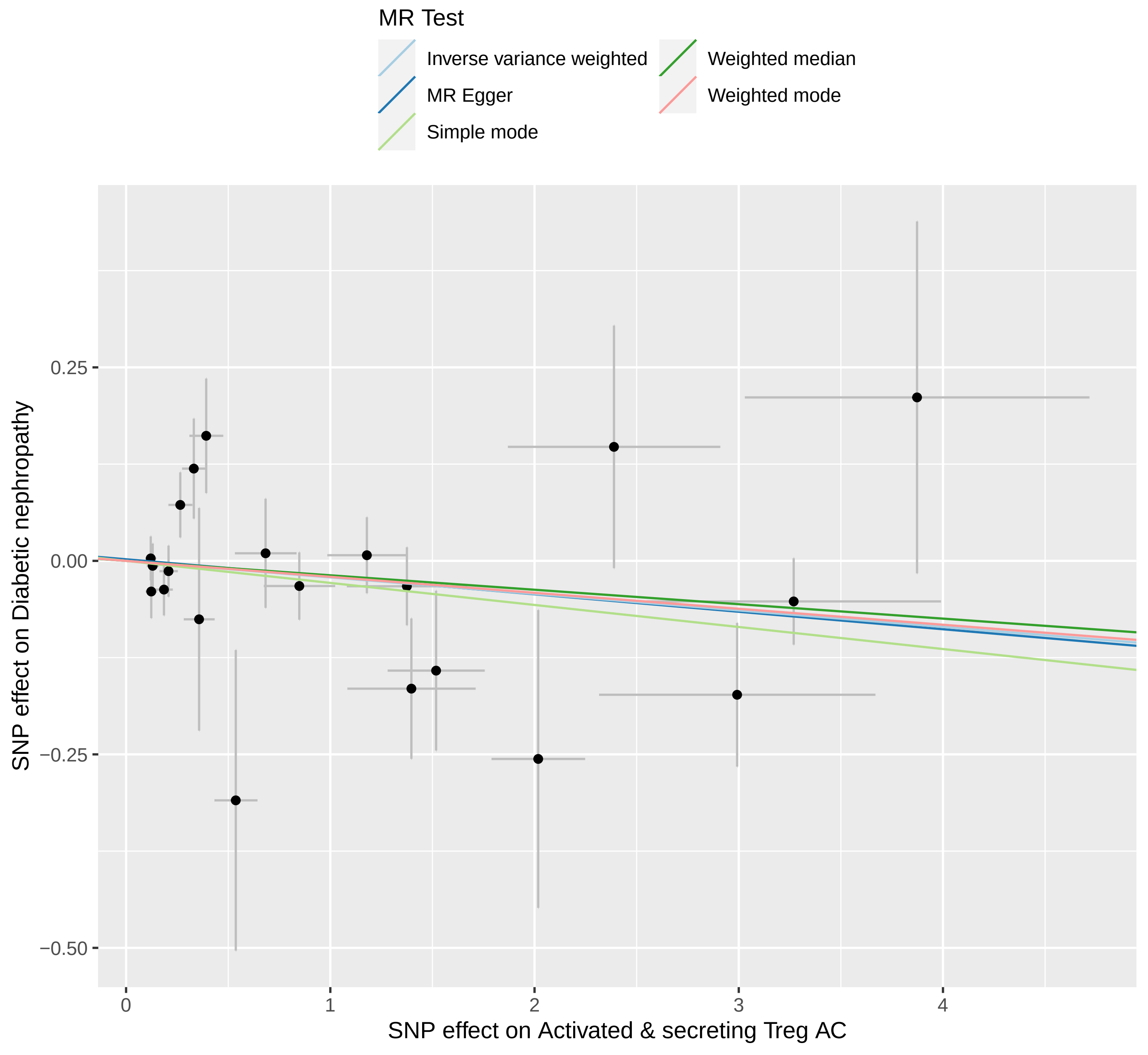
MR Test
Inverse variance weighted
MR Egger
Simple mode
Weighted median
Weighted mode
SNP effect on Diabetic nephropathy
0.25
0.00
−0.25
−0.50
0
1
2
3
4
SNP effect on Activated & secreting Treg AC

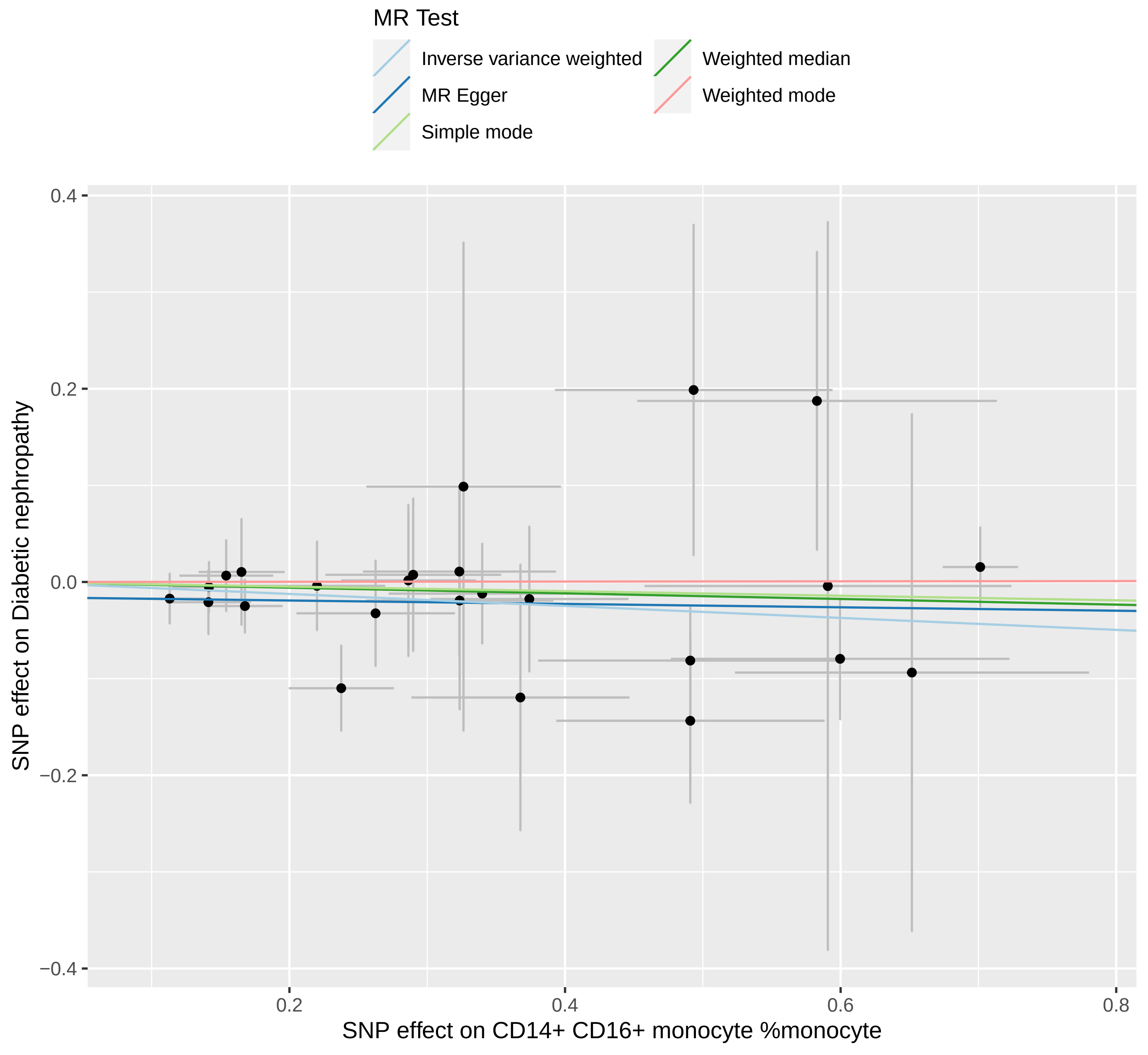

MR Test
Inverse variance weighted
MR Egger
Simple mode
Weighted median
Weighted mode
0.4
0.2
0.0
−0.2
−0.4
0.2
0.4
0.6
0.8
SNP effect on Diabetic nephropathy
SNP effect on CD14+ CD16+ monocyte %monocyte

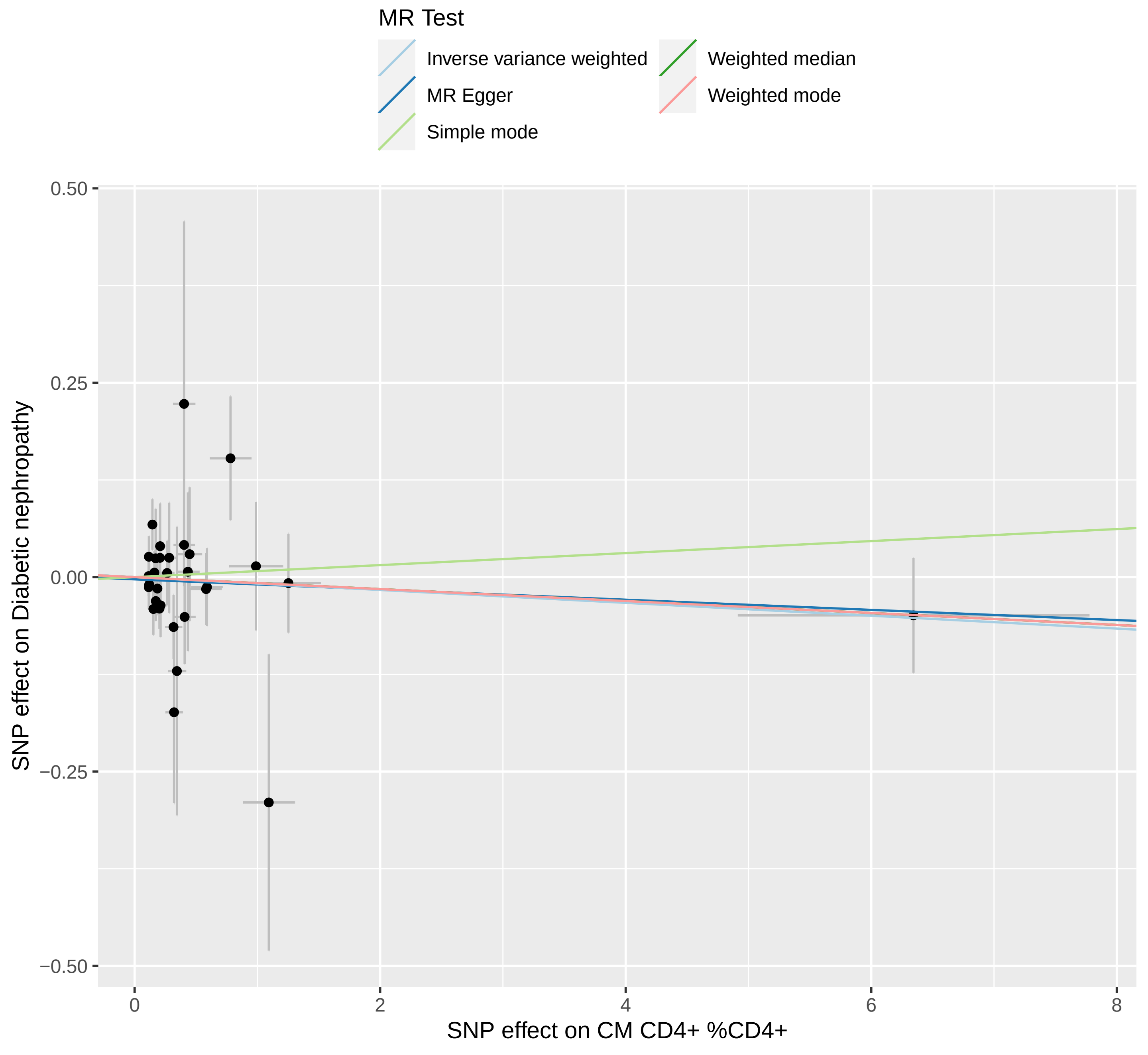

MR Test
Inverse variance weighted
MR Egger
Simple mode
Weighted median
Weighted mode
0.50
0.25
0.00
−0.25
−0.50
SNP effect on Diabetic nephropathy
0
2
4
6
8
SNP effect on CM CD4+ %CD4+

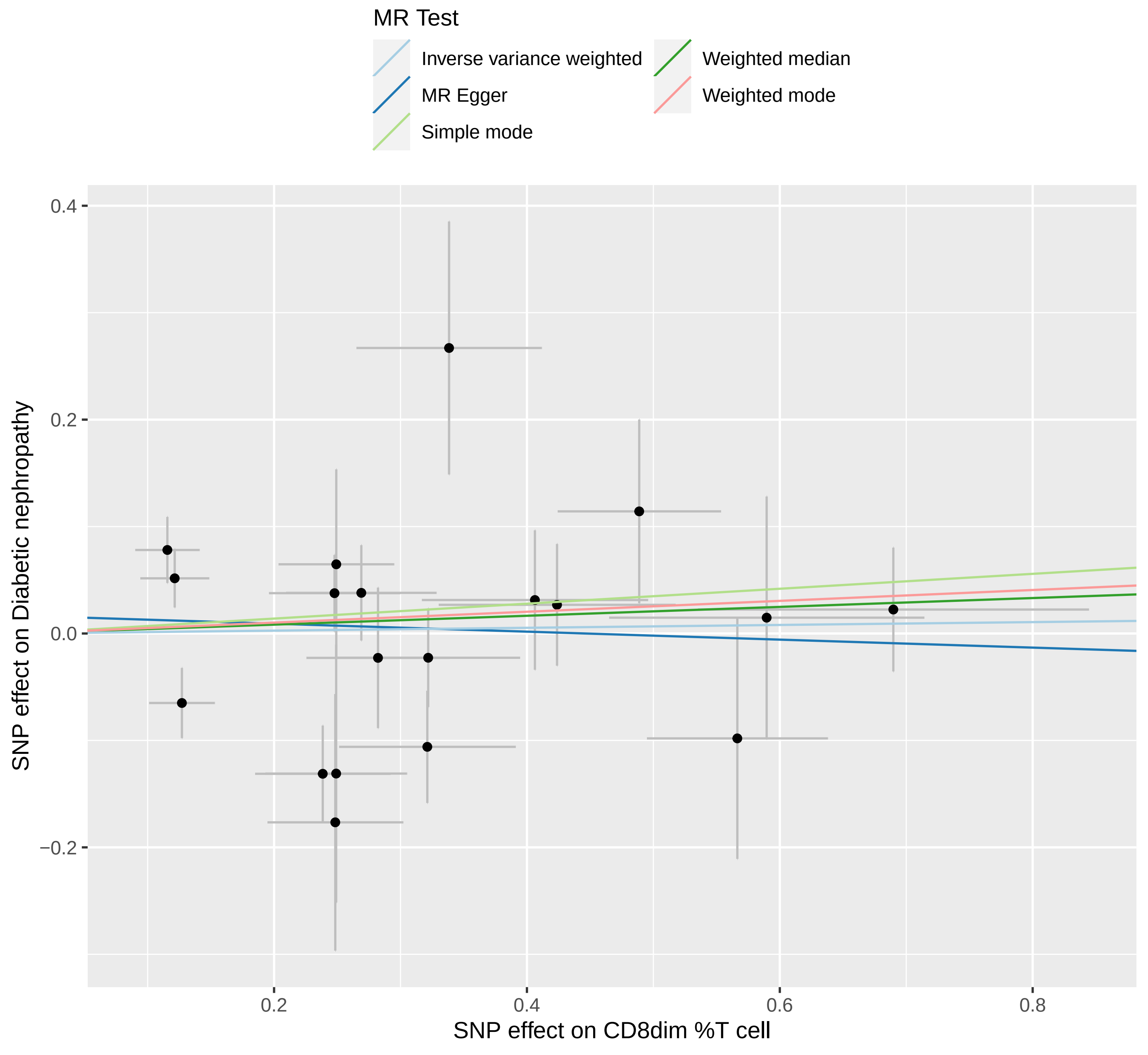

MR Test
Inverse variance weighted
MR Egger
Simple mode
Weighted median
Weighted mode
0.4
0.2
0.0
−0.2
0.2
0.4
0.6
0.8
SNP effect on Diabetic nephropathy
SNP effect on CD8dim %T cell

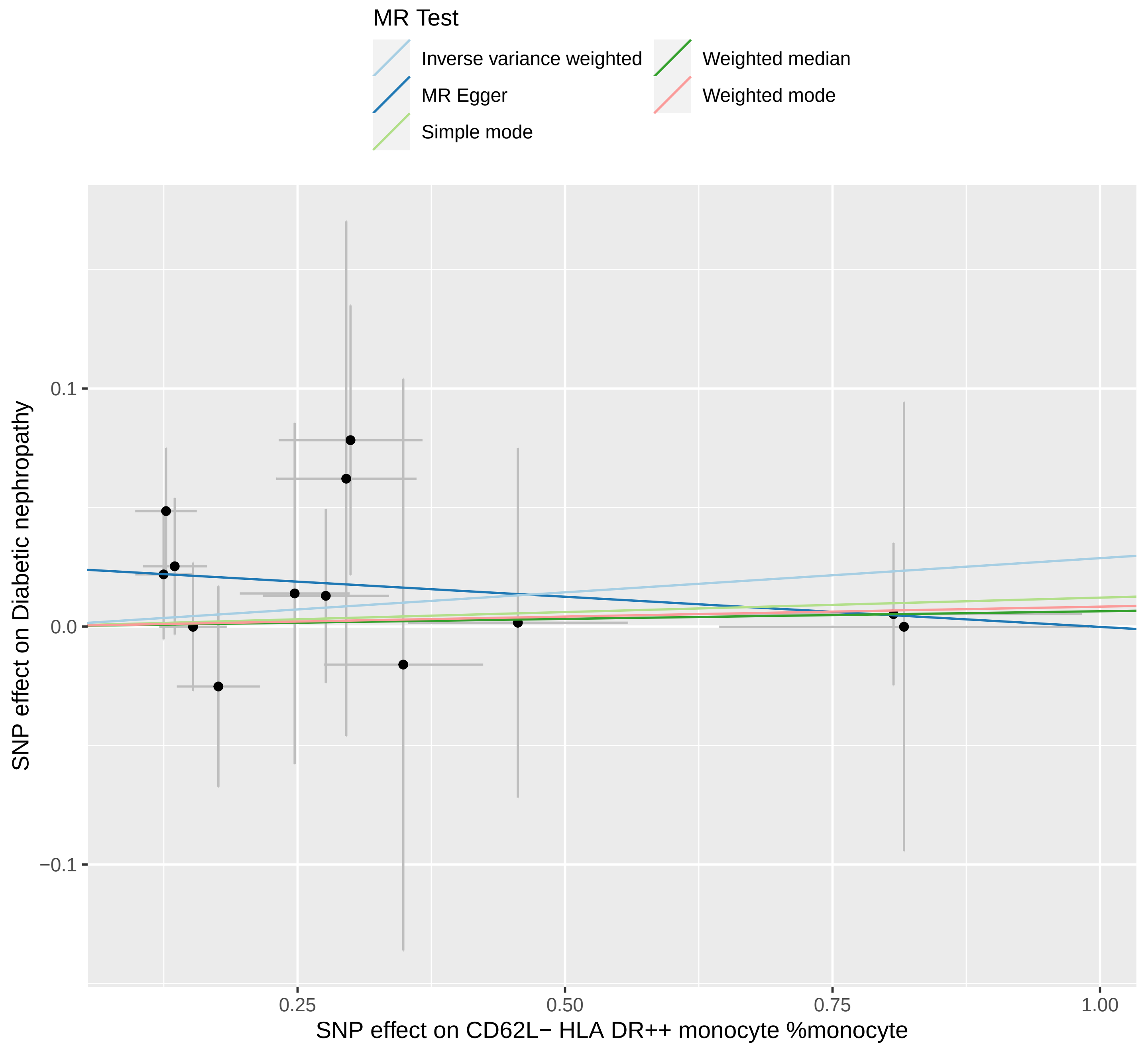
MR Test
Inverse variance weighted
MR Egger
Simple mode
Weighted median
Weighted mode
0.1
0.0
−0.1
0.25
0.50
0.75
1.00
SNP effect on Diabetic nephropathy
SNP effect on CD62L− HLA DR++ monocyte %monocyte

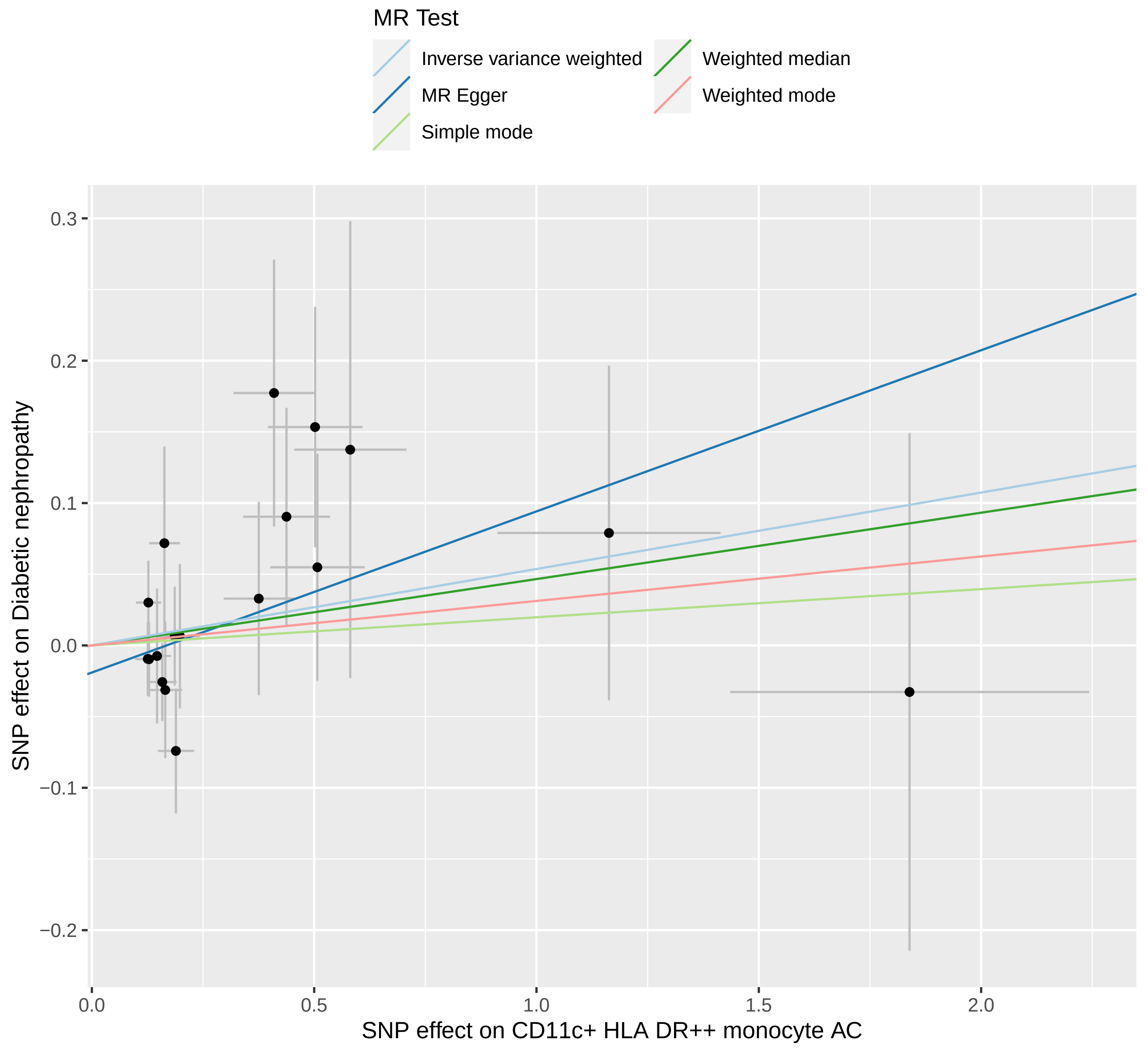
MR Test
Inverse variance weighted
MR Egger
Simple mode
Weighted median
Weighted mode
0.3
0.2
0.1
0.0
−0.1
−0.2
SNP effect on Diabetic nephropathy
0.0
0.5
1.0
1.5
2.0
SNP effect on CD11c+ HLA DR++ monocyte AC

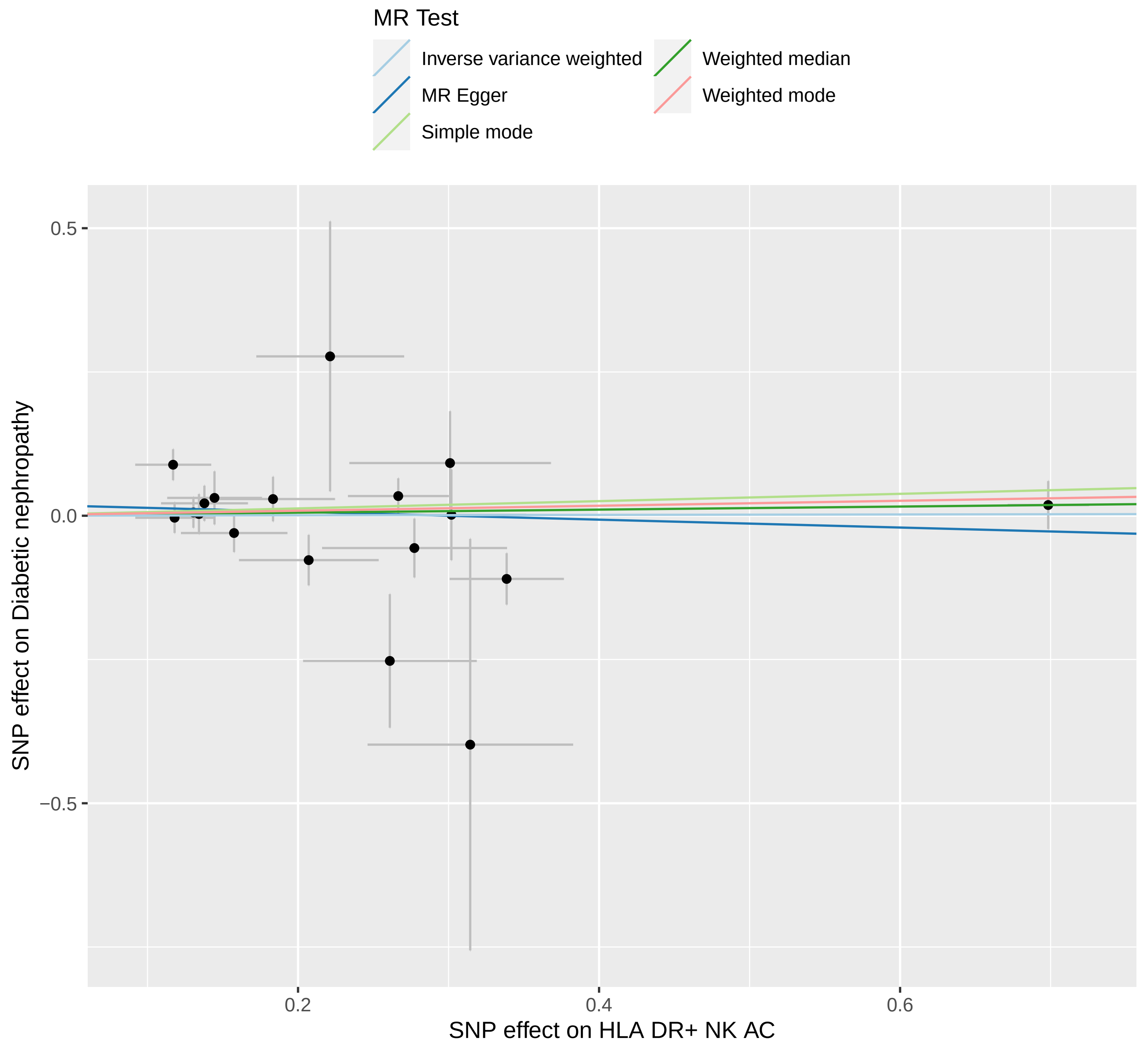

MR Test
Inverse variance weighted
MR Egger
Simple mode
Weighted median
Weighted mode
0.5
0.0
−0.5
SNP effect on Diabetic nephropathy
0.2
0.4
0.6
SNP effect on HLA DR+ NK AC

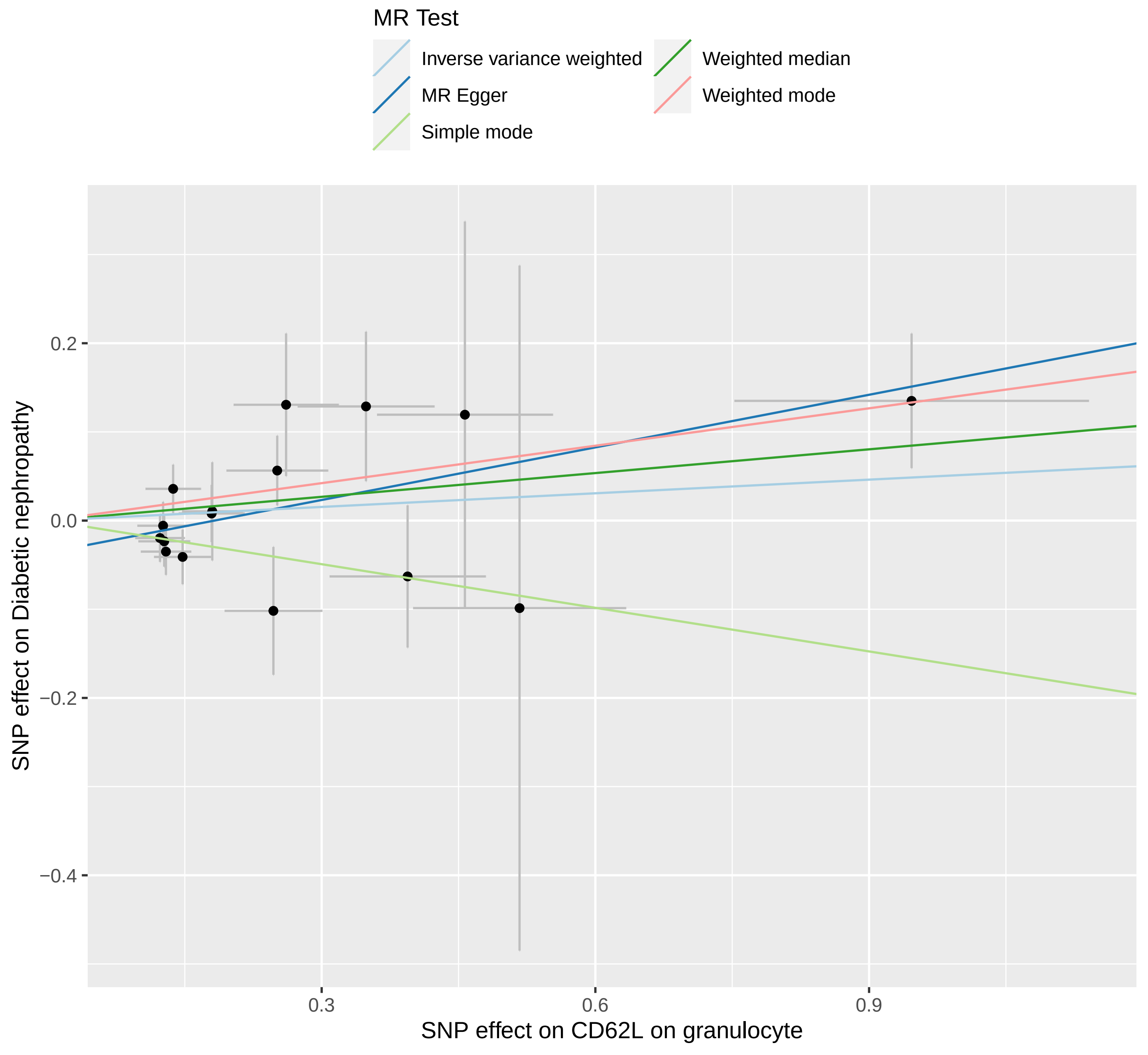

MR Test
Inverse variance weighted
MR Egger
Simple mode
Weighted median
Weighted mode
0.2
0.0
−0.2
−0.4
0.3
0.6
0.9
SNP effect on Diabetic nephropathy
SNP effect on CD62L on granulocyte

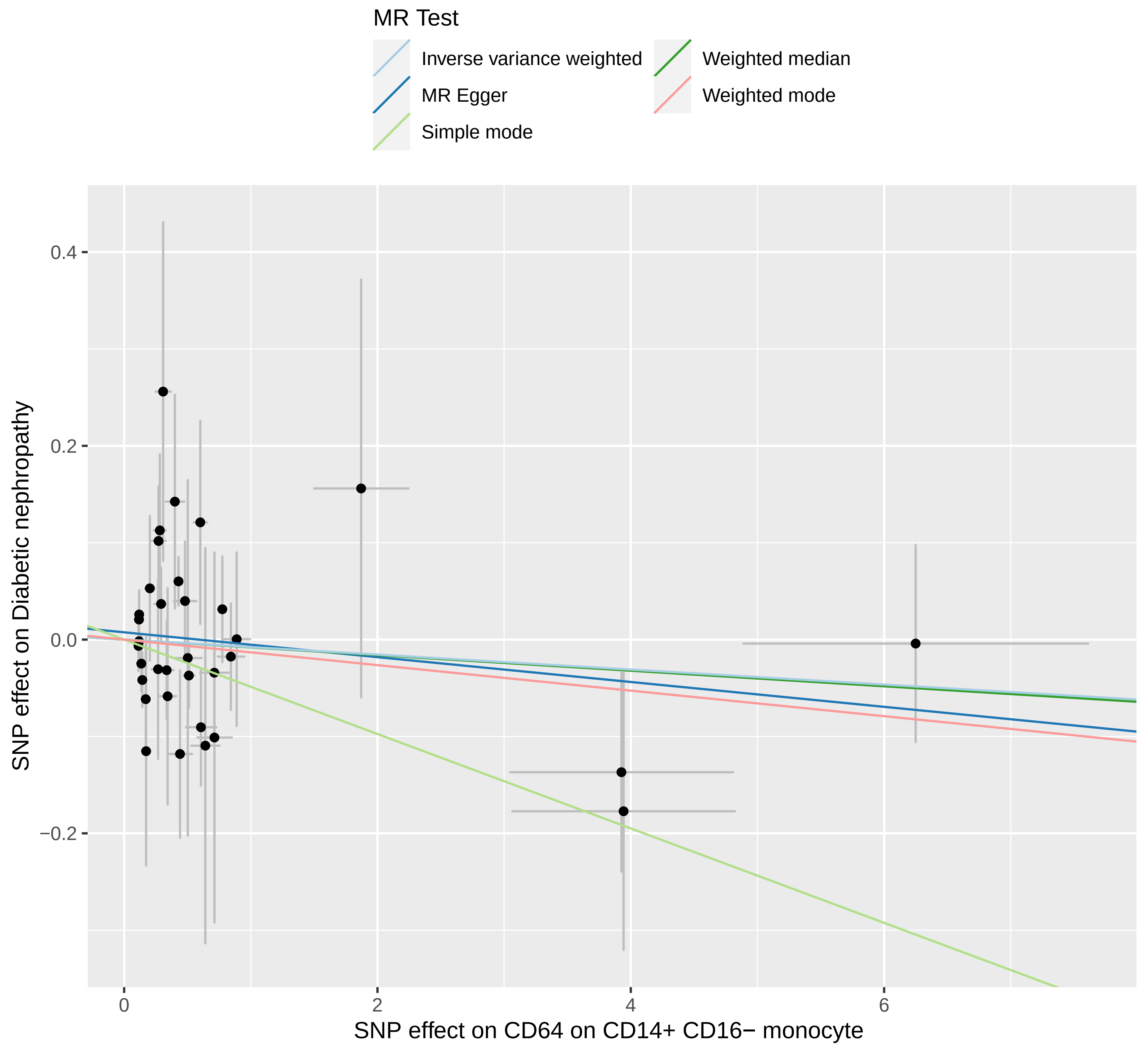
MR Test
Inverse variance weighted
MR Egger
Simple mode
Weighted median
Weighted mode
0.4
0.2
0.0
−0.2
0
2
4
6
SNP effect on Diabetic nephropathy
SNP effect on CD64 on CD14+ CD16− monocyte

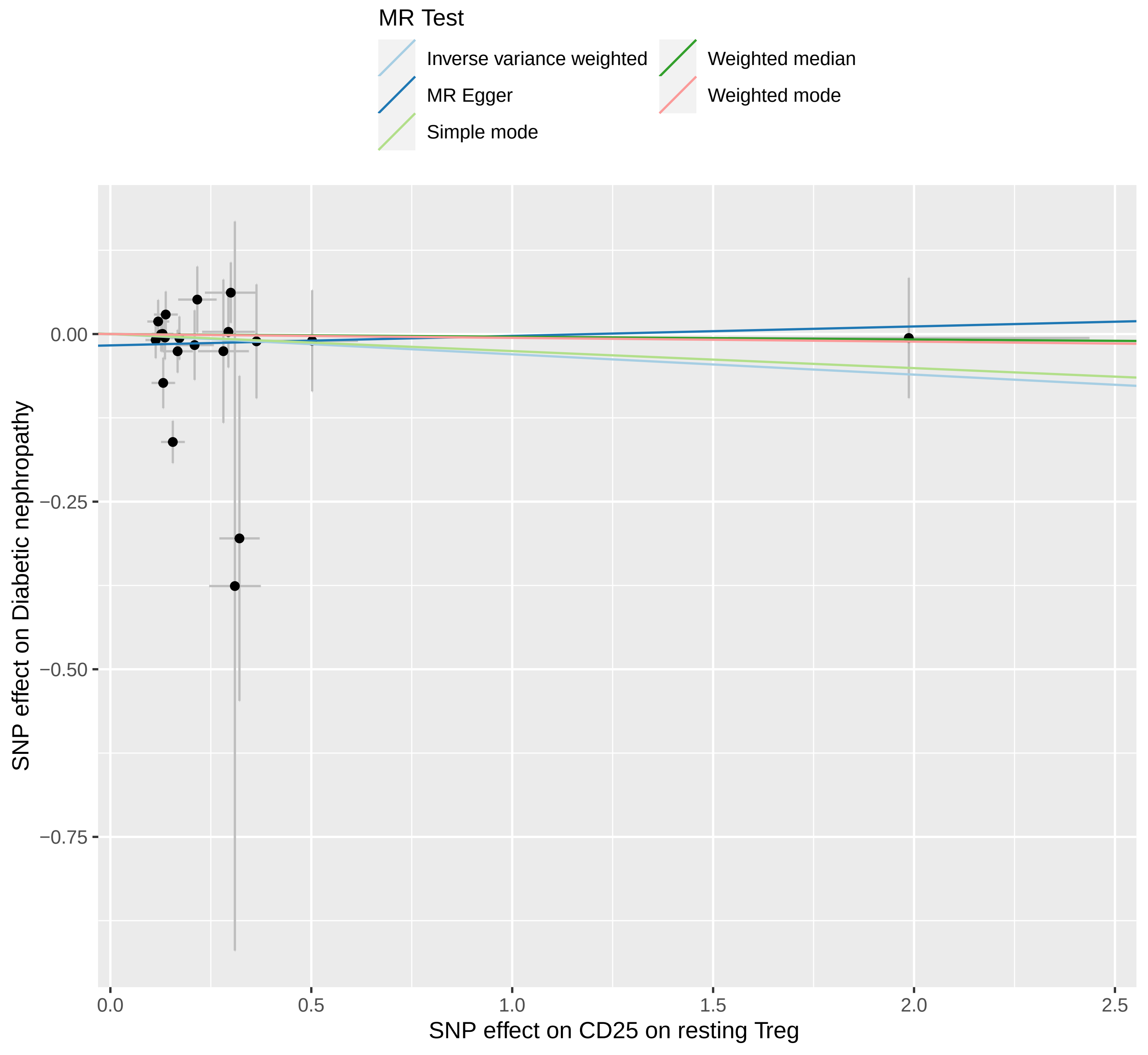

MR Test
Inverse variance weighted
MR Egger
Simple mode
Weighted median
Weighted mode
0.00
−0.25
−0.50
−0.75
SNP effect on Diabetic nephropathy
0.0
0.5
1.0
1.5
2.0
2.5
SNP effect on CD25 on resting Treg

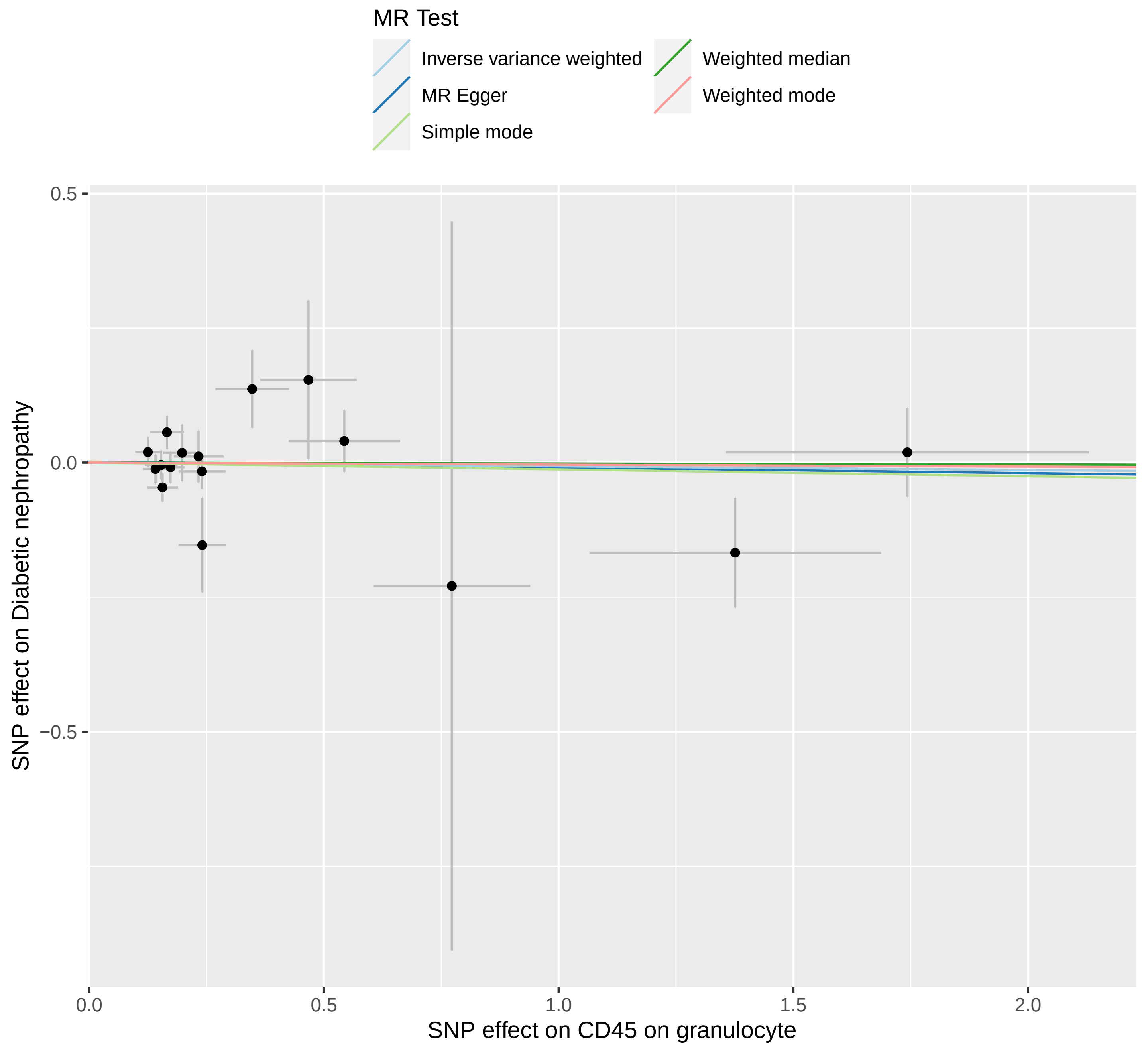
MR Test
Inverse variance weighted
MR Egger
Simple mode
Weighted median
Weighted mode
0.5
0.0
−0.5
SNP effect on Diabetic nephropathy
0.0
0.5
1.0
1.5
2.0
SNP effect on CD45 on granulocyte

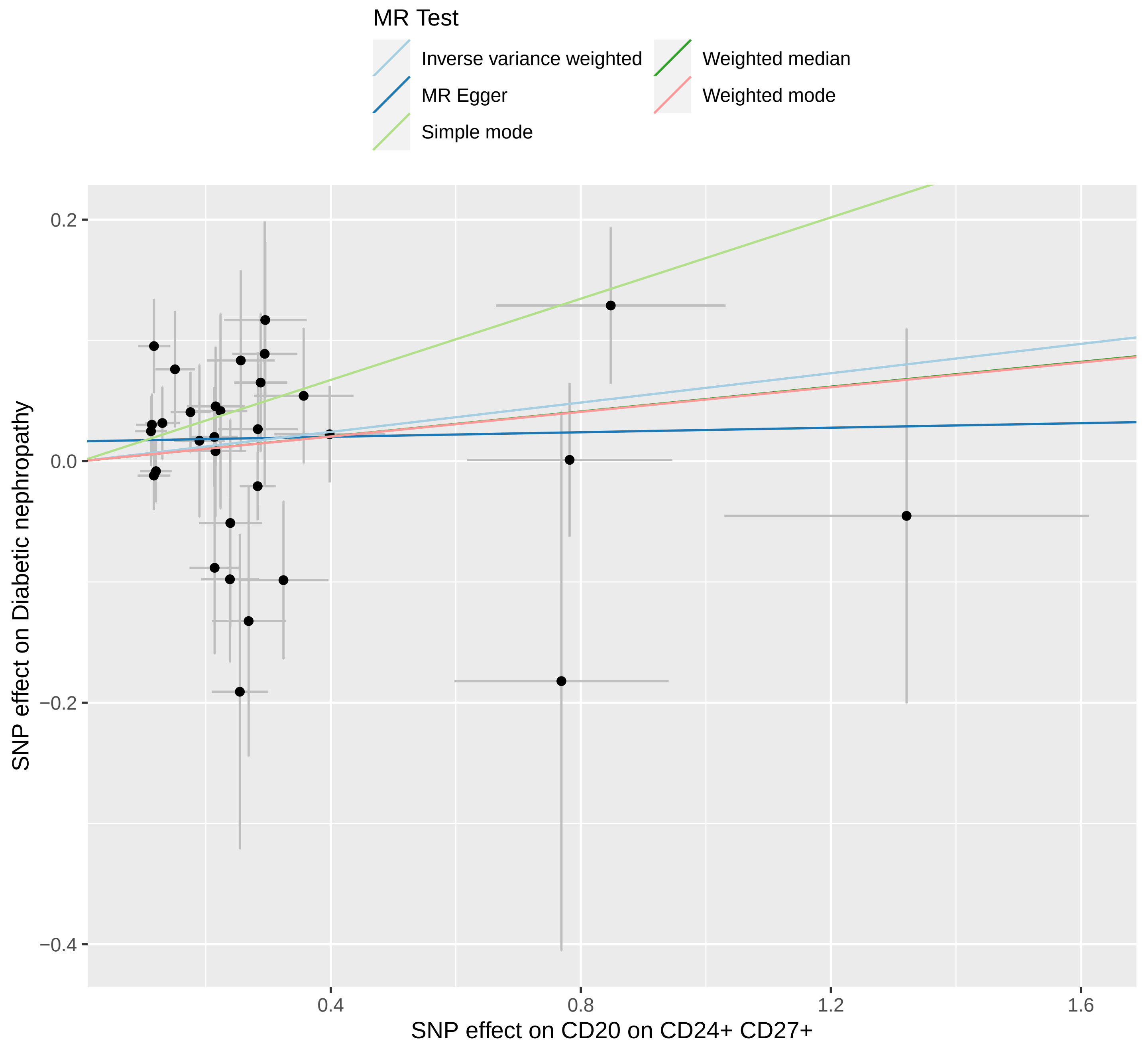

MR Test
Inverse variance weighted
MR Egger
Simple mode
Weighted median
Weighted mode
0.2
0.0
−0.2
−0.4
0.4
0.8
1.2
1.6
SNP effect on Diabetic nephropathy
SNP effect on CD20 on CD24+ CD27+

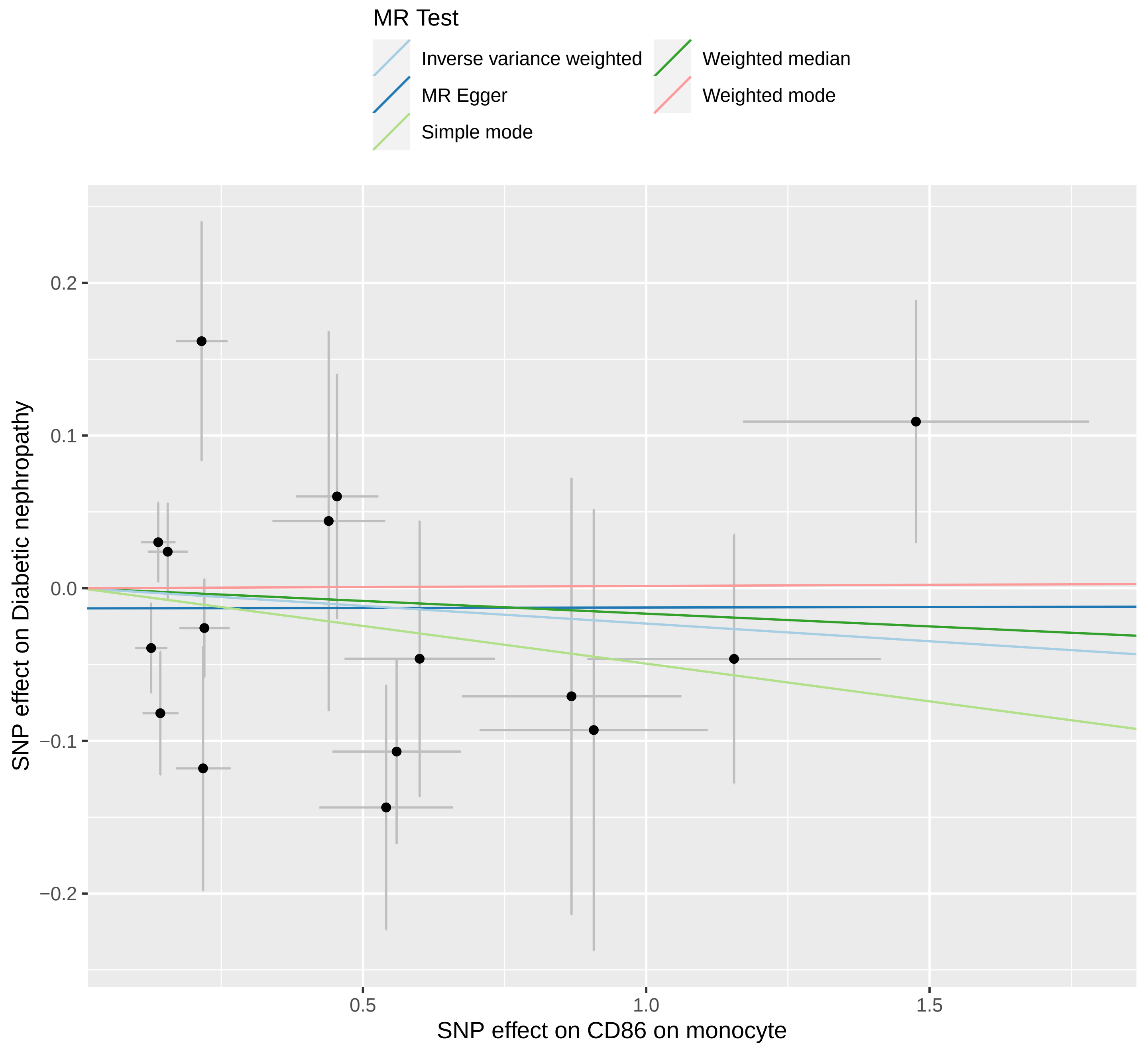
MR Test
Inverse variance weighted
MR Egger
Simple mode
Weighted median
Weighted mode
SNP effect on Diabetic nephropathy
0.2
0.1
0.0
−0.1
−0.2
0.5
1.0
1.5
SNP effect on CD86 on monocyte

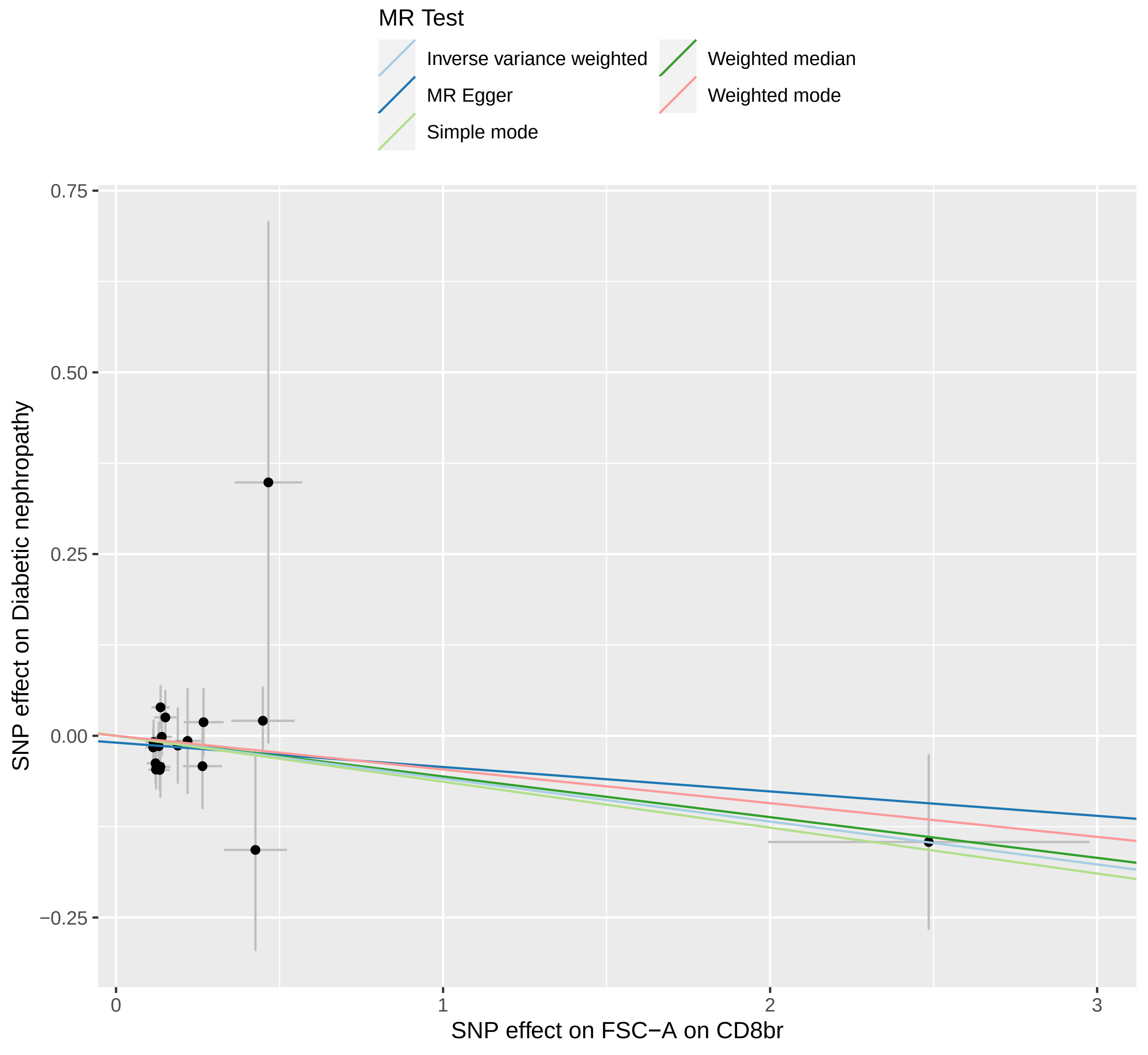

MR Test
Inverse variance weighted
MR Egger
Simple mode
Weighted median
Weighted mode
0.75
0.50
0.25
0.00
−0.25
SNP effect on Diabetic nephropathy
0
1
2
3
SNP effect on FSC−A on CD8br

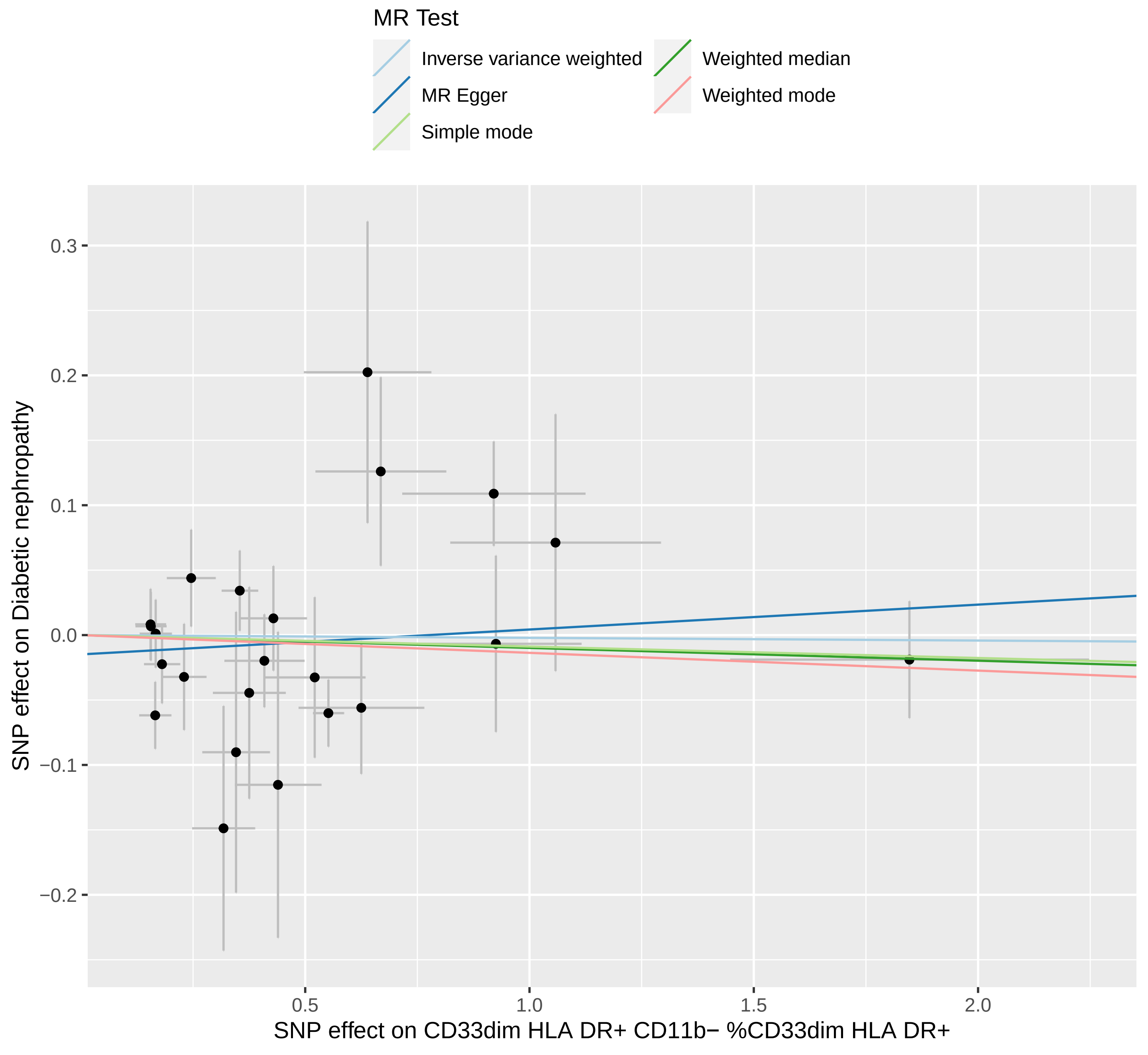
MR Test
Inverse variance weighted
MR Egger
Simple mode
Weighted median
Weighted mode
0.3
0.2
0.1
0.0
−0.1
−0.2
SNP effect on Diabetic nephropathy
0.5
1.0
1.5
2.0
SNP effect on CD33dim HLA DR+ CD11b− %CD33dim HLA DR+

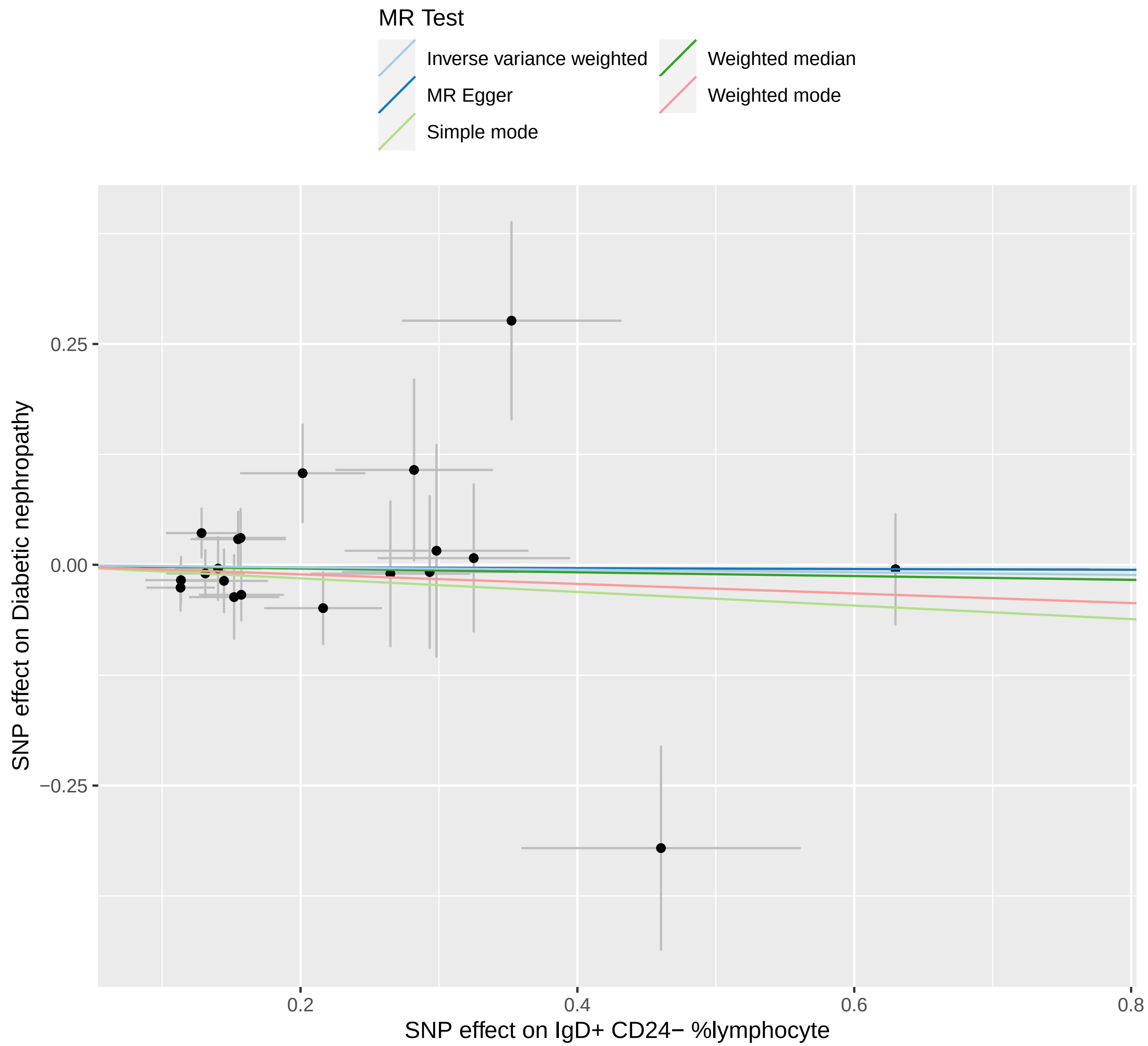

MR Test
Inverse variance weighted
MR Egger
Simple mode
Weighted median
Weighted mode
0.25
0.00
−0.25
SNP effect on Diabetic nephropathy
0.2
0.4
0.6
0.8
SNP effect on IgD+ CD24− %lymphocyte

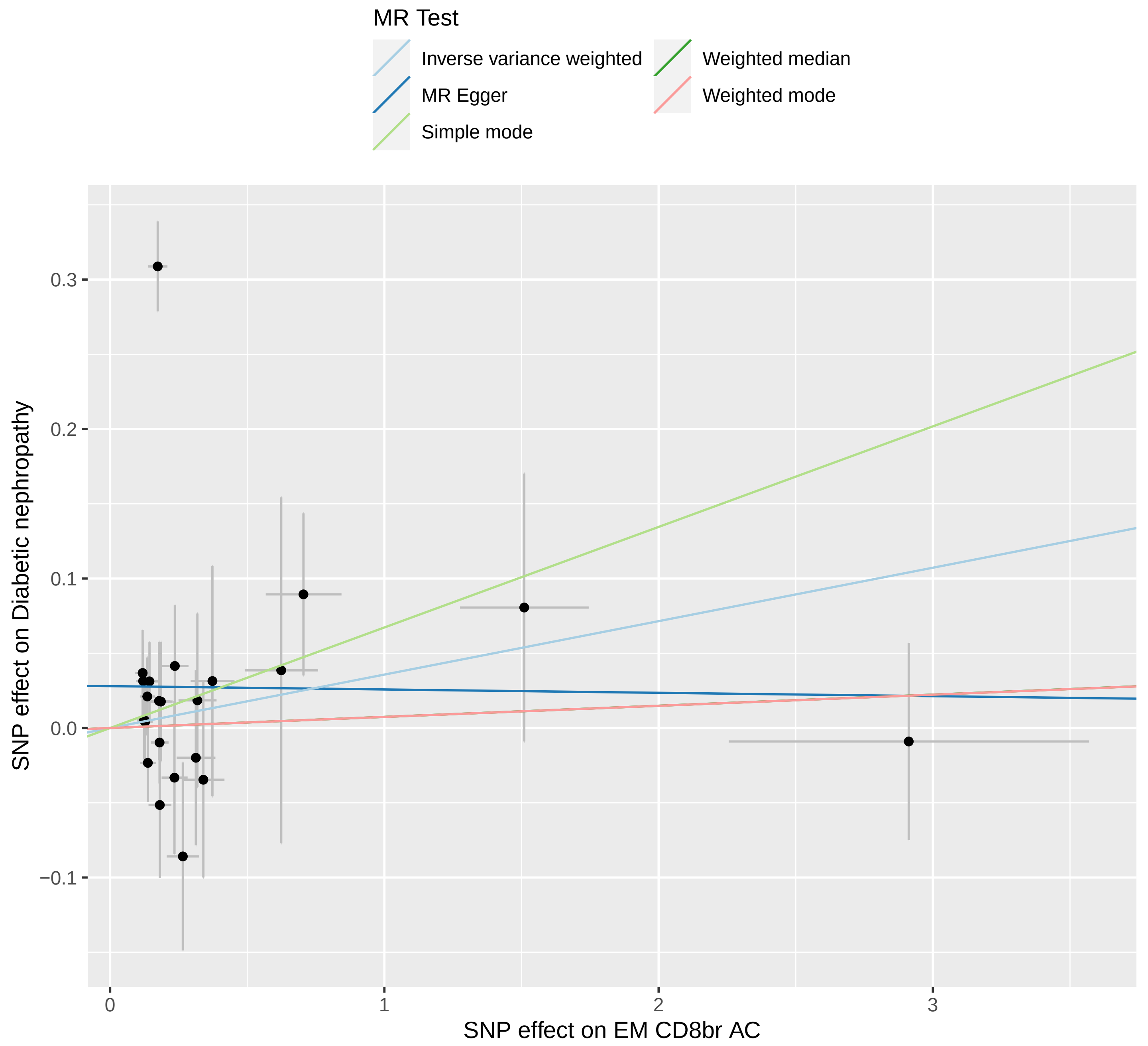
MR Test
Inverse variance weighted
MR Egger
Simple mode
Weighted median
Weighted mode
SNP effect on Diabetic nephropathy
0.3
0.2
0.1
0.0
−0.1
0
1
2
3
SNP effect on EM CD8br AC

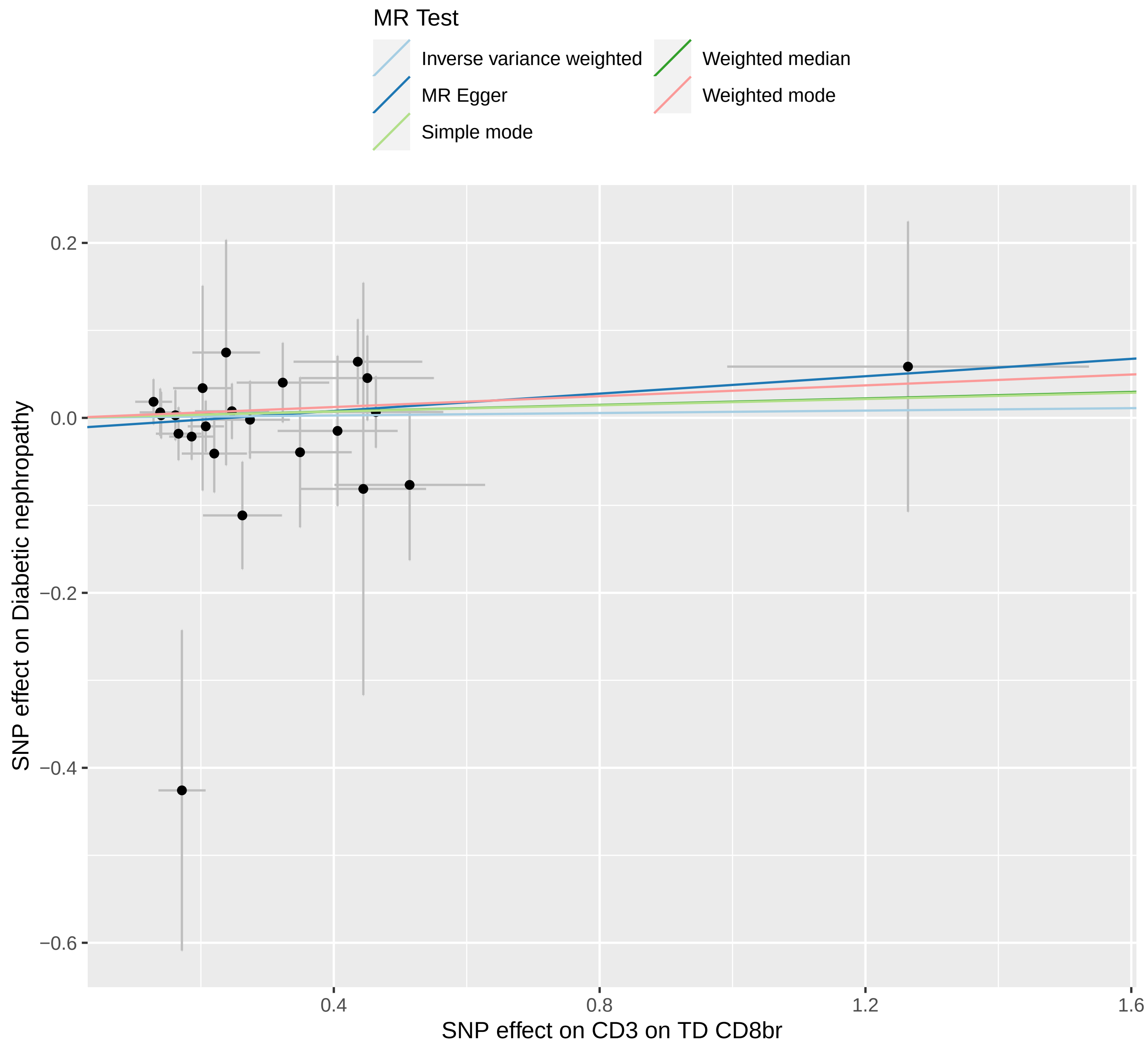

MR Test
Inverse variance weighted
MR Egger
Simple mode
Weighted median
Weighted mode
0.2
0.0
−0.2
−0.4
−0.6
0.4
0.8
1.2
1.6
SNP effect on Diabetic nephropathy
SNP effect on CD3 on TD CD8br

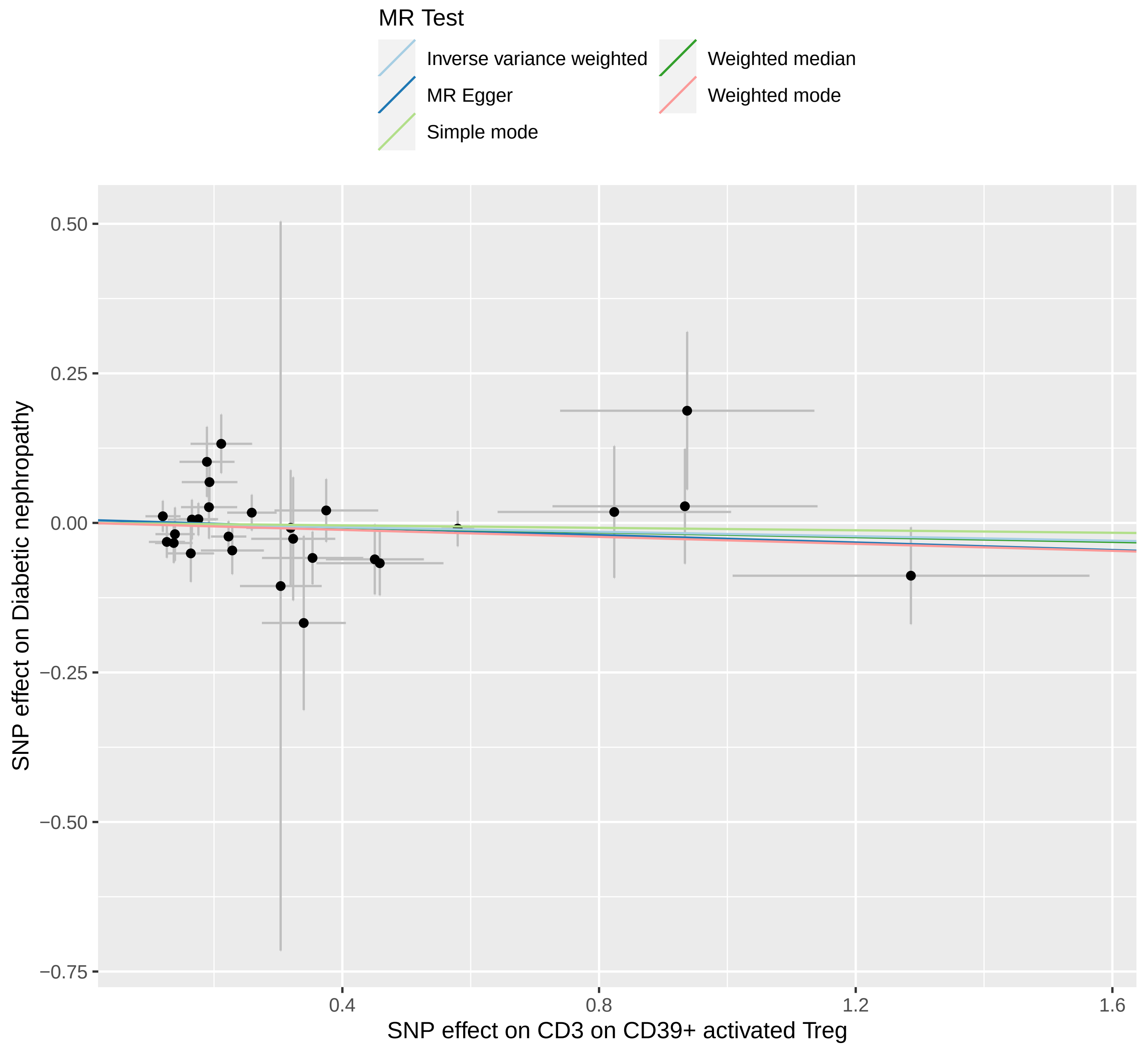
MR Test
Inverse variance weighted
MR Egger
Simple mode
Weighted median
Weighted mode
0.50
0.25
0.00
−0.25
−0.50
−0.75
0.4
0.8
1.2
1.6
SNP effect on Diabetic nephropathy
SNP effect on CD3 on CD39+ activated Treg

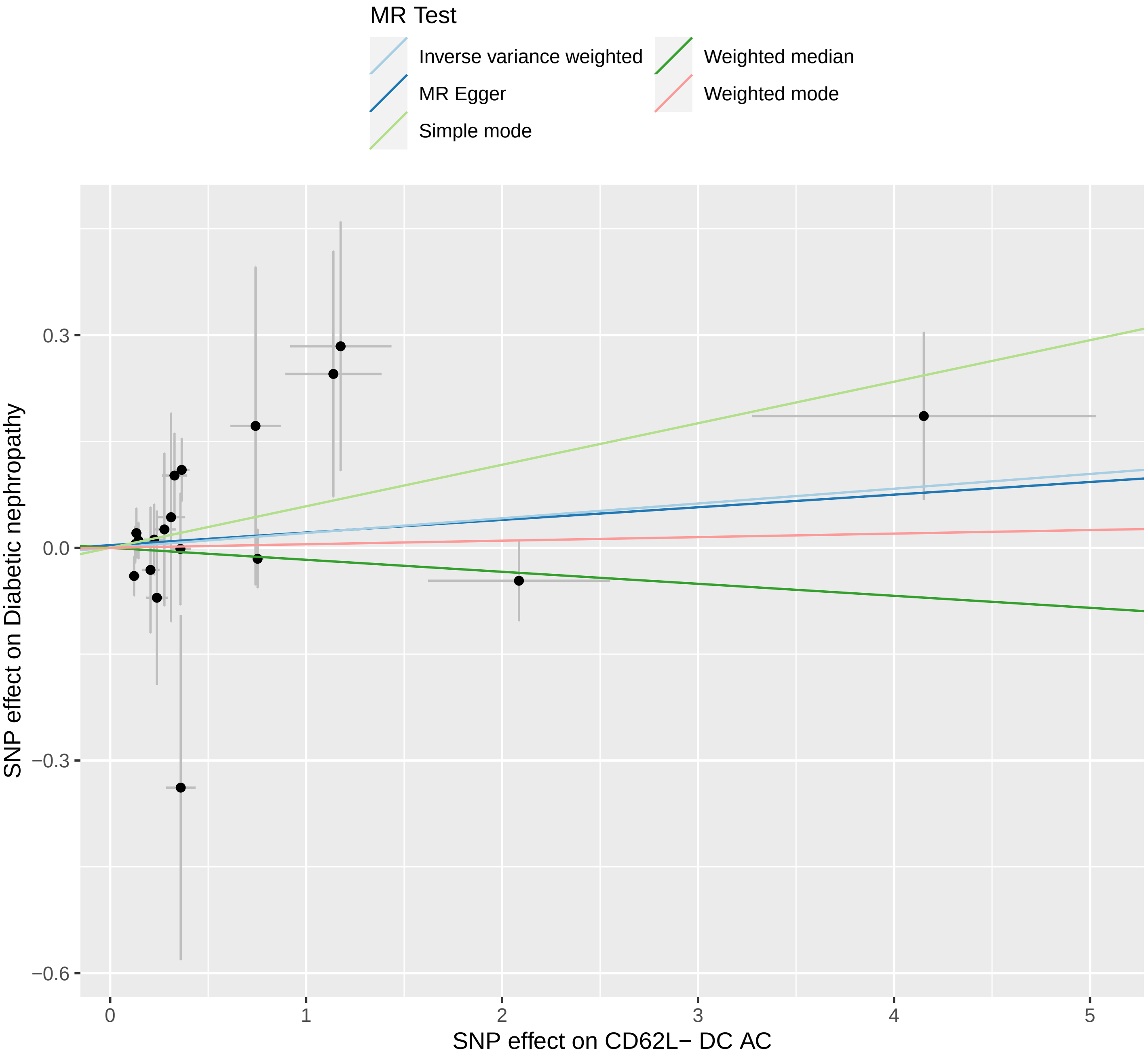
MR Test
Inverse variance weighted
MR Egger
Simple mode
Weighted median
Weighted mode
0.3
0.0
−0.3
−0.6
0
1
2
3
4
5
SNP effect on CD62L− DC AC
SNP effect on Diabetic nephropathy

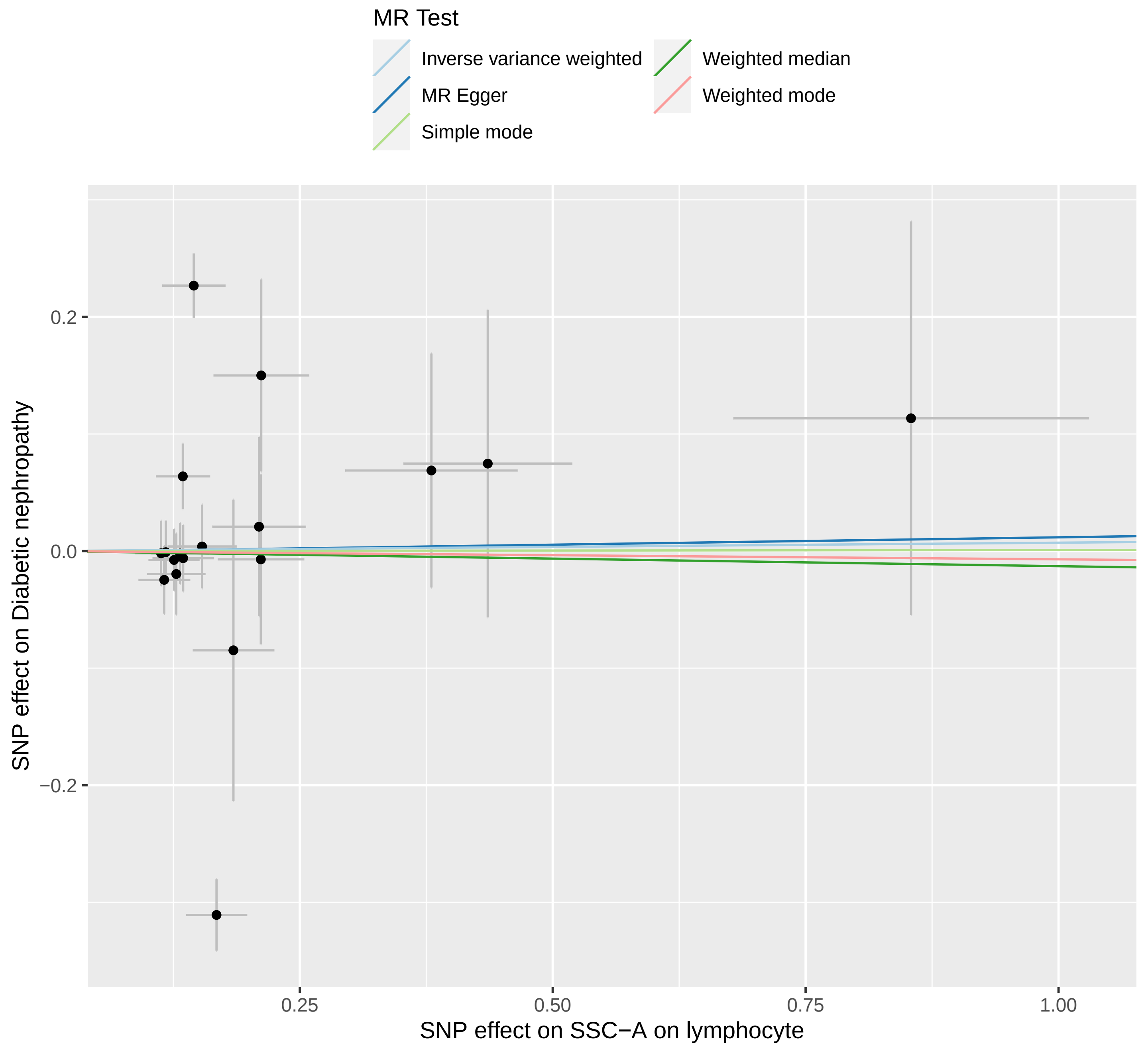
MR Test
Inverse variance weighted
MR Egger
Simple mode
Weighted median
Weighted mode
0.2
0.0
−0.2
SNP effect on Diabetic nephropathy
0.25
0.50
0.75
1.00
SNP effect on SSC−A on lymphocyte

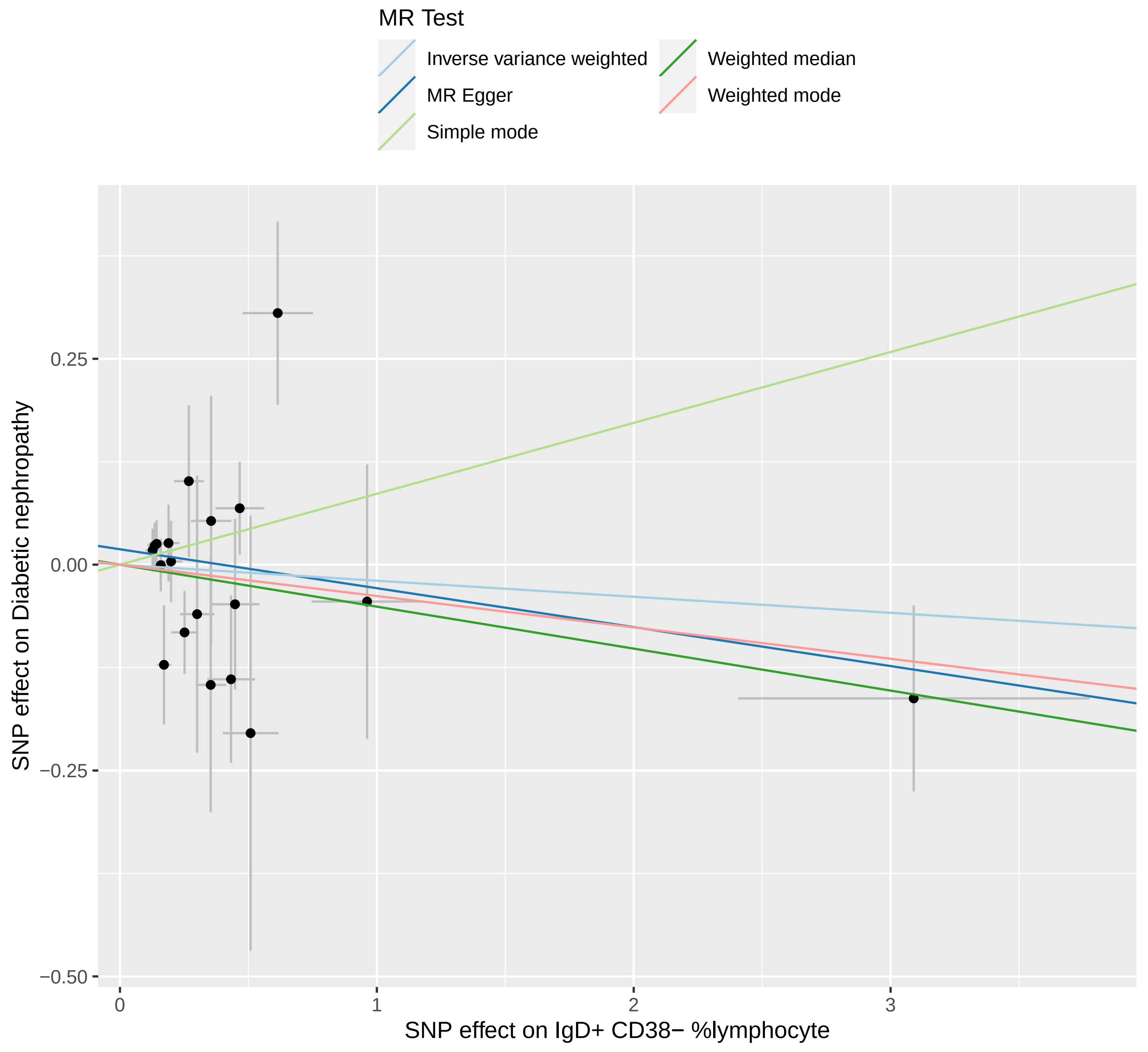

MR Test
Inverse variance weighted
MR Egger
Simple mode
Weighted median
Weighted mode
0.25
0.00
−0.25
−0.50
0
1
2
3
SNP effect on Diabetic nephropathy
SNP effect on IgD+ CD38− %lymphocyte

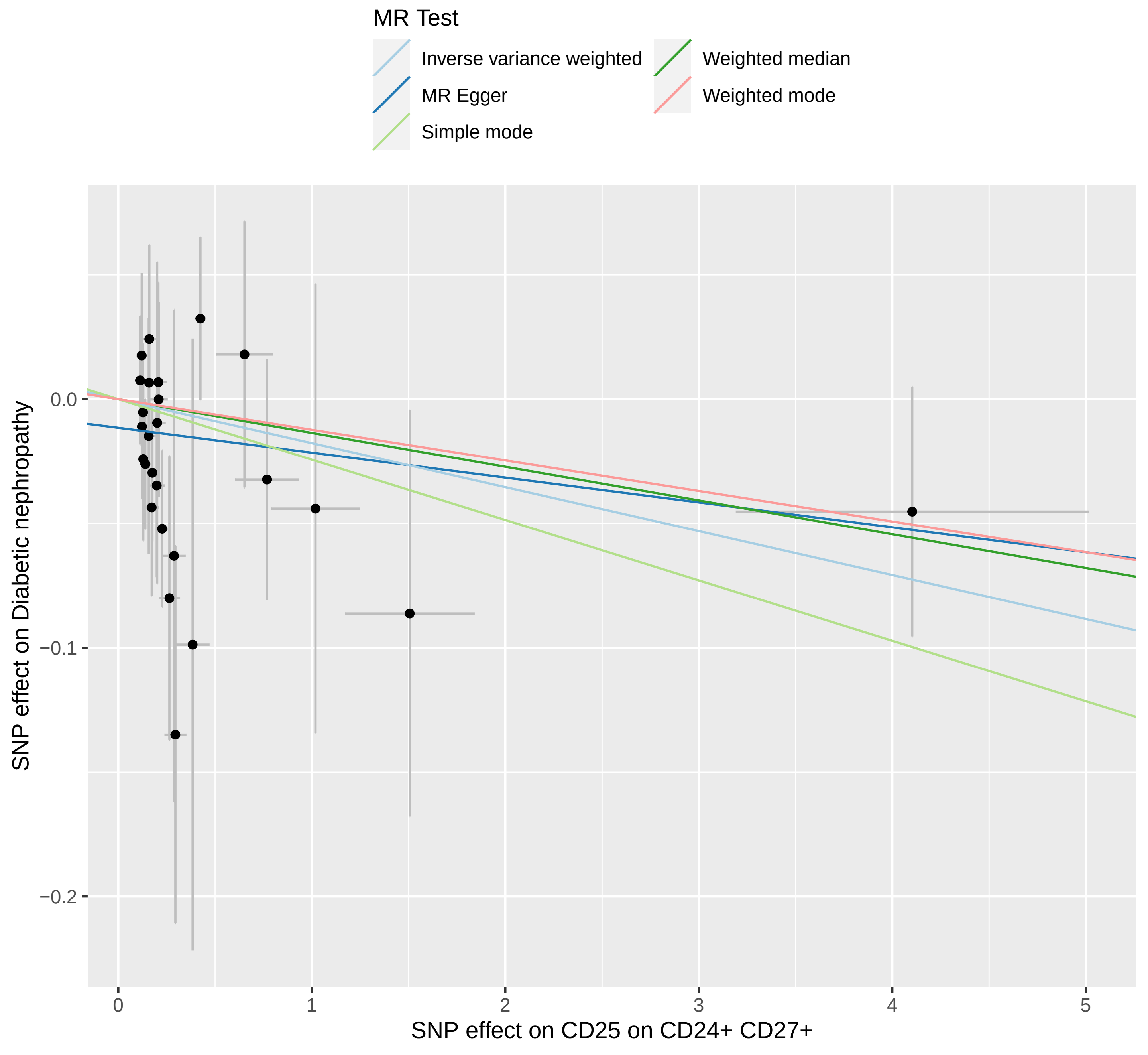

MR Test
Inverse variance weighted
MR Egger
Simple mode
Weighted median
Weighted mode
SNP effect on Diabetic nephropathy
0.0
−0.1
−0.2
0
1
2
3
4
5
SNP effect on CD25 on CD24+ CD27+

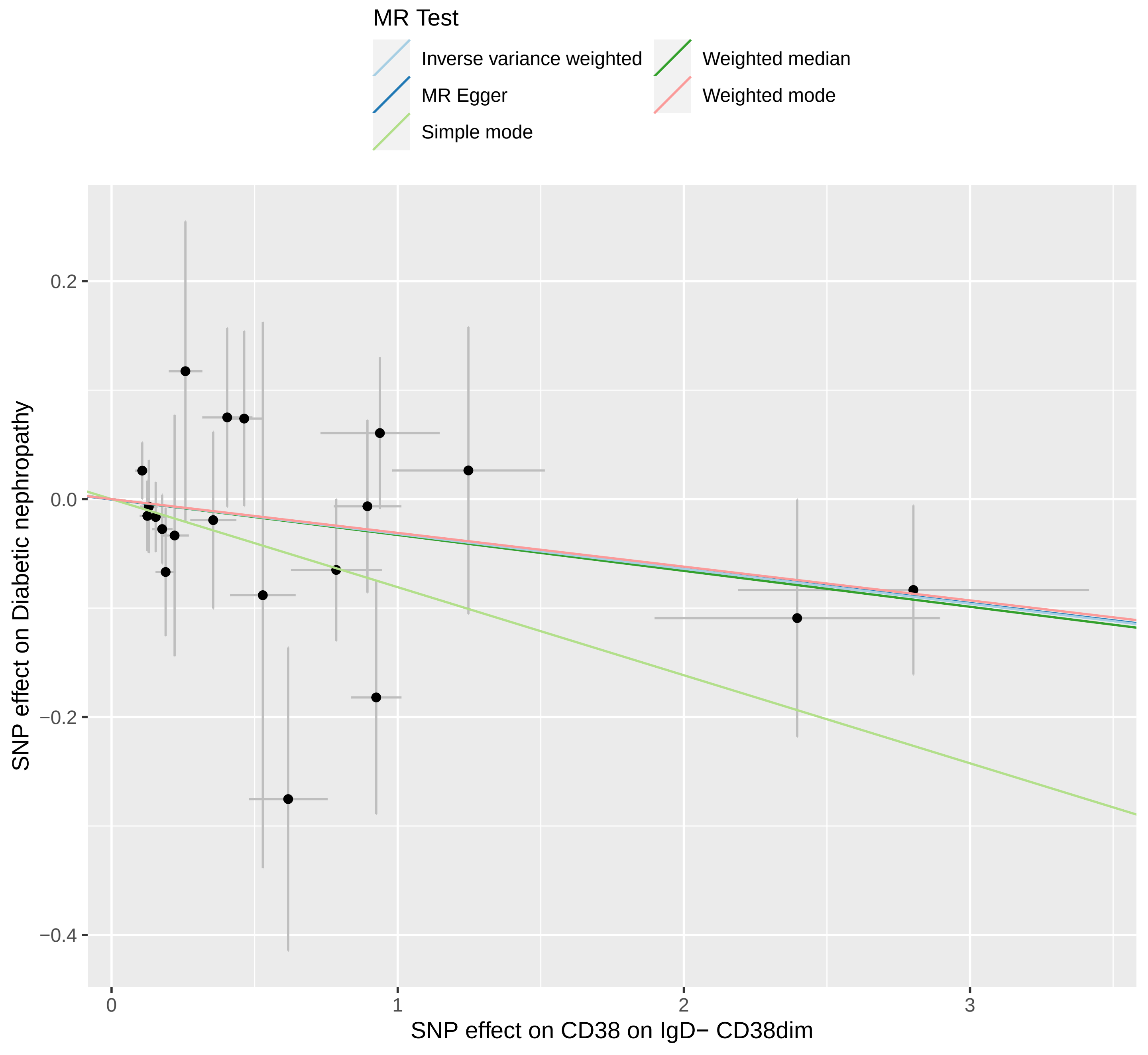

MR Test
Inverse variance weighted
MR Egger
Simple mode
Weighted median
Weighted mode
0.2
0.0
−0.2
−0.4
SNP effect on Diabetic nephropathy
0
1
2
3
SNP effect on CD38 on IgD− CD38dim

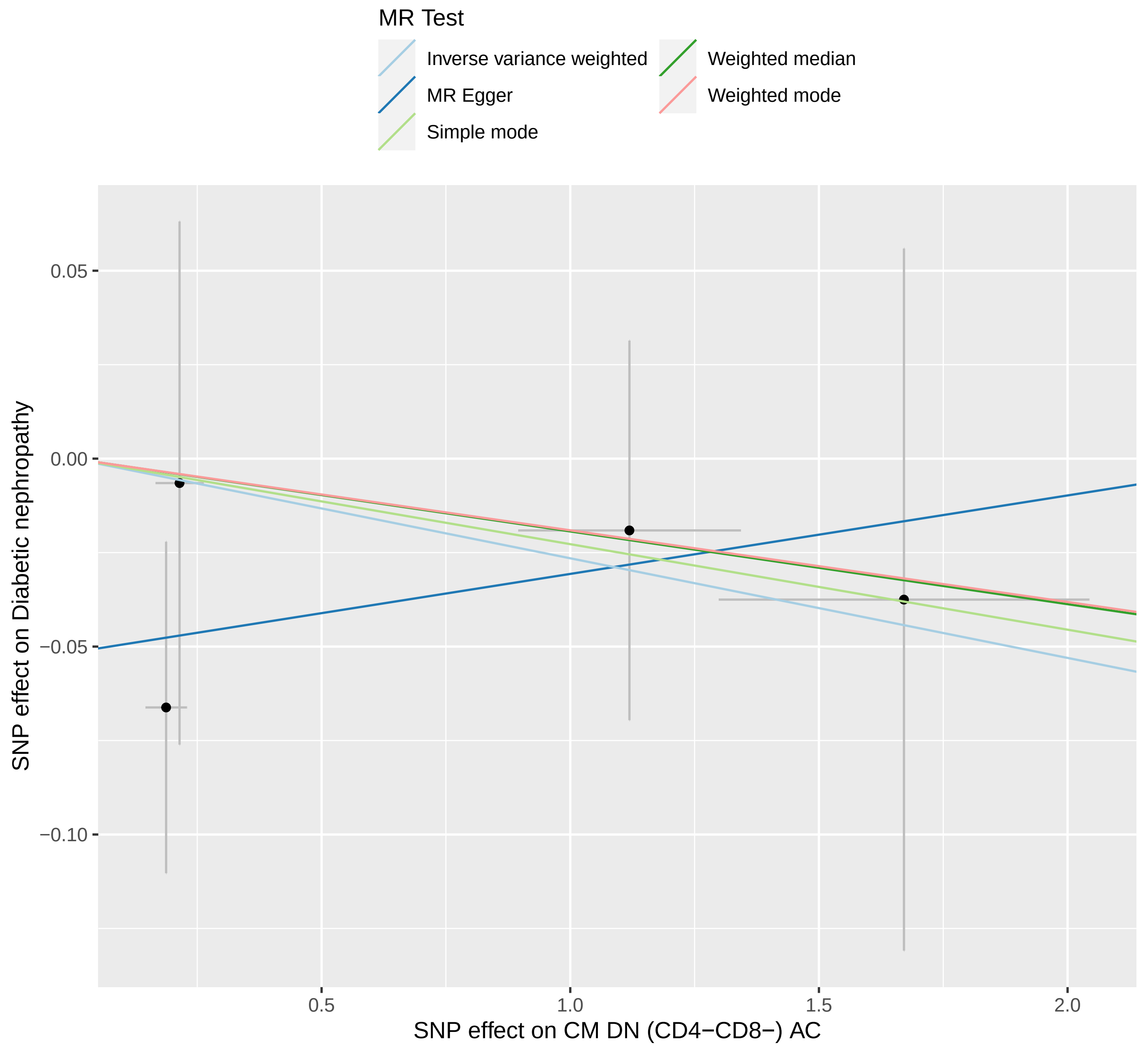
MR Test
Inverse variance weighted
MR Egger
Simple mode
Weighted median
Weighted mode
SNP effect on Diabetic nephropathy
0.05
0.00
−0.05
−0.10
0.5
1.0
1.5
2.0
SNP effect on CM DN (CD4−CD8−) AC

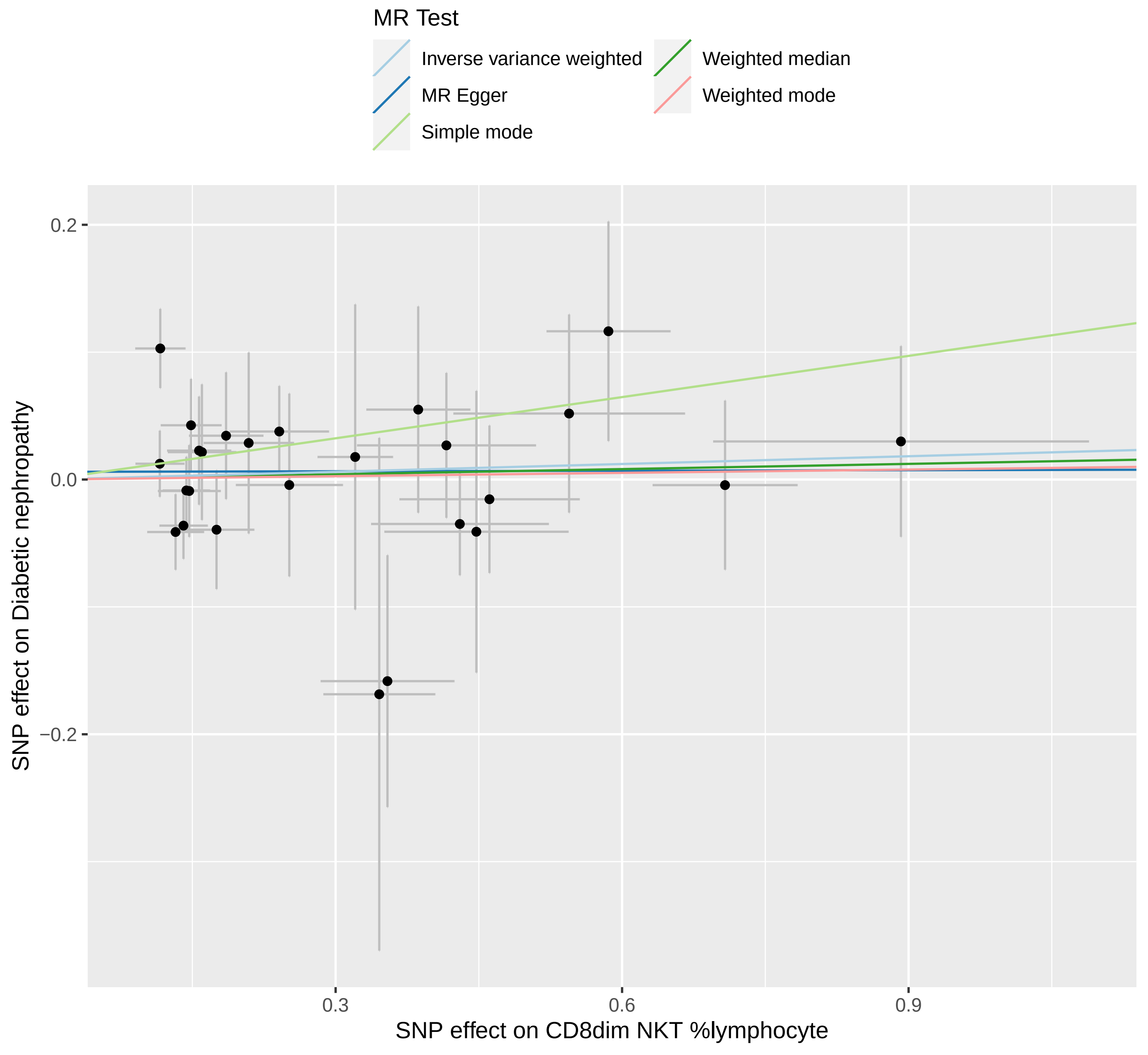

MR Test
Inverse variance weighted
MR Egger
Simple mode
Weighted median
Weighted mode
0.2
0.0
−0.2
0.3
0.6
0.9
SNP effect on Diabetic nephropathy
SNP effect on CD8dim NKT %lymphocyte

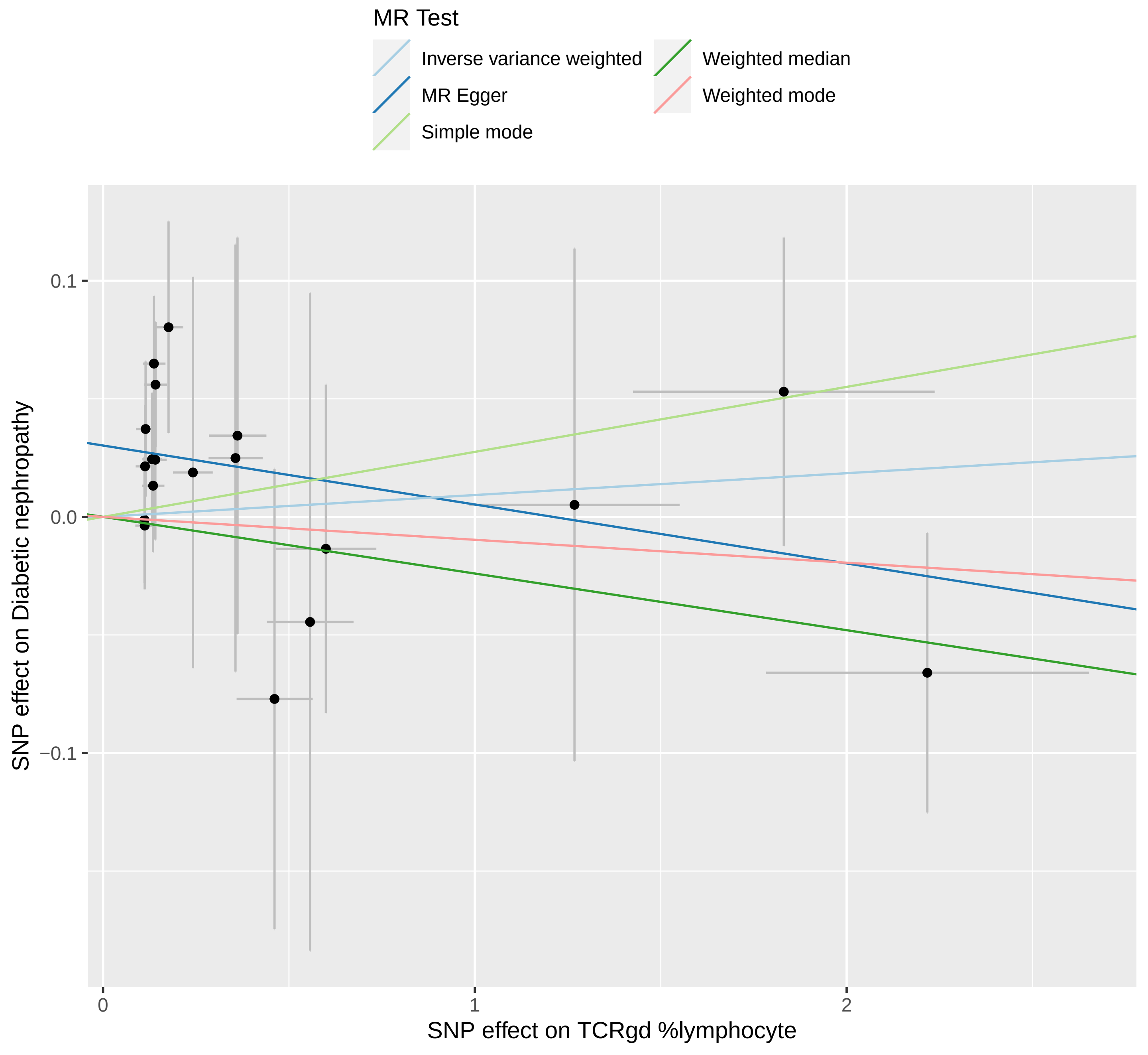

MR Test
Inverse variance weighted
MR Egger
Simple mode
Weighted median
Weighted mode
0.1
0.0
−0.1
SNP effect on Diabetic nephropathy
0
1
2
SNP effect on TCRgd %lymphocyte

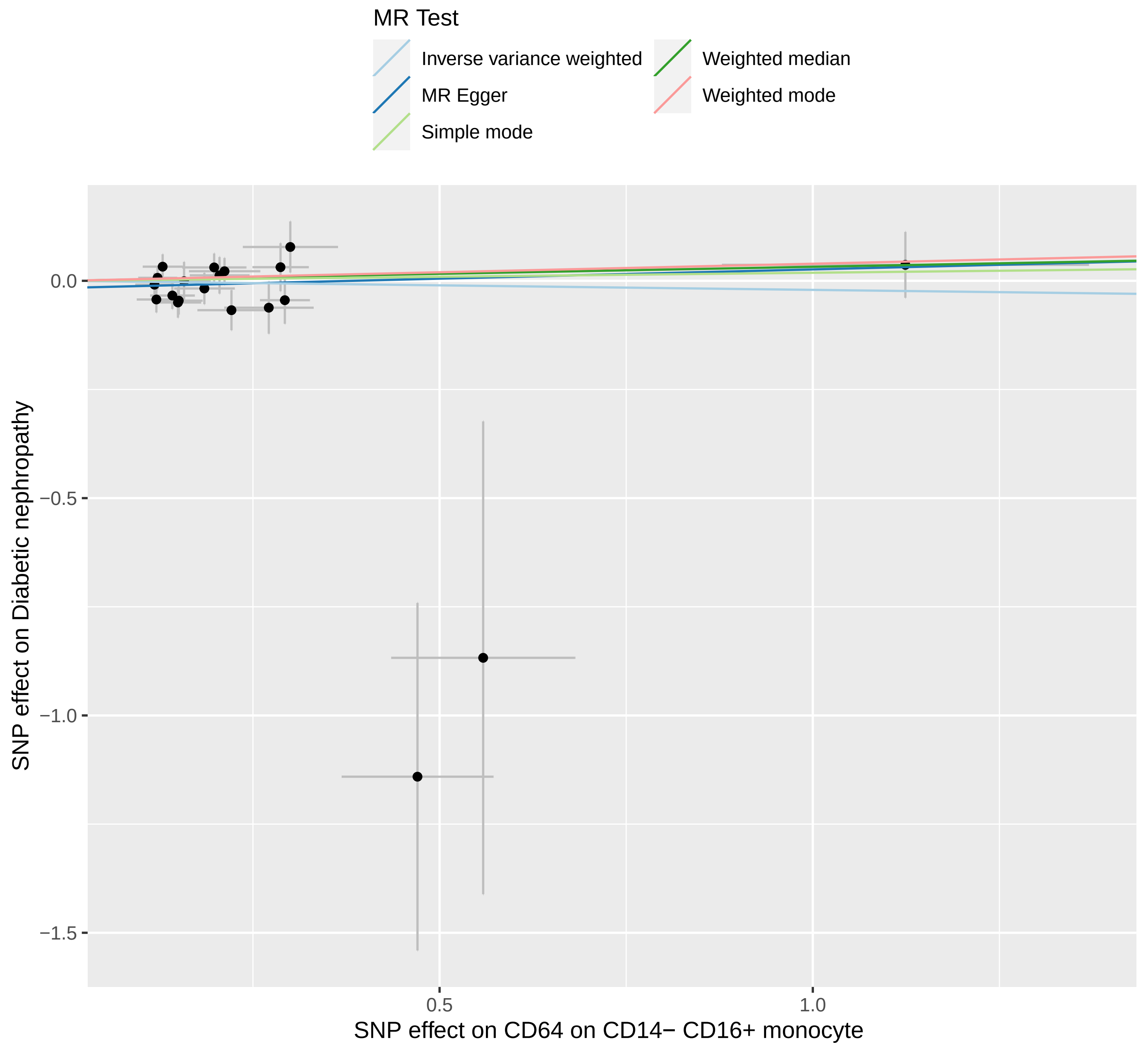
MR Test
Inverse variance weighted
MR Egger
Simple mode
Weighted median
Weighted mode
0.0
−0.5
−1.0
−1.5
0.5
1.0
SNP effect on Diabetic nephropathy
SNP effect on CD64 on CD14− CD16+ monocyte

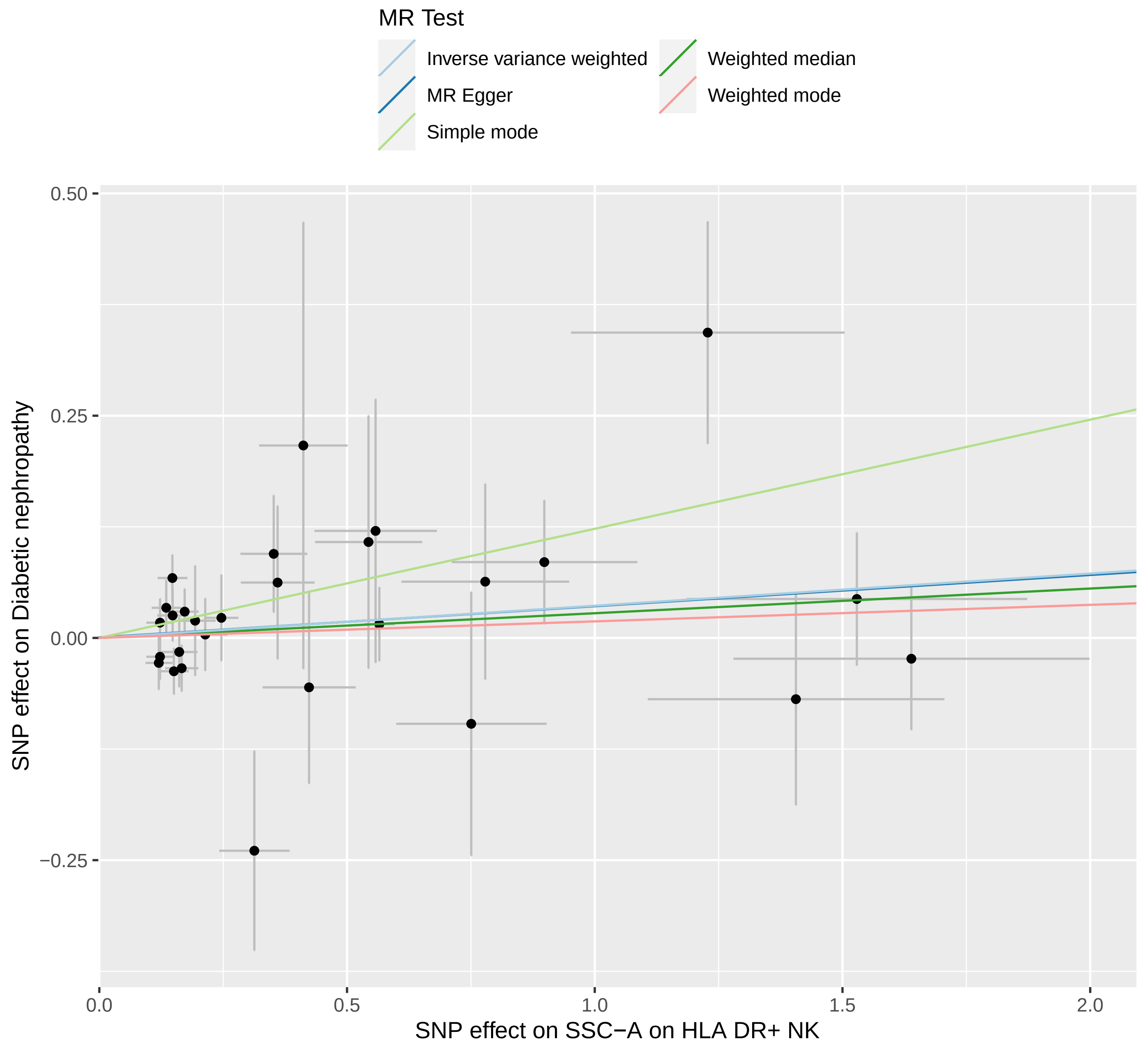
MR Test
Inverse variance weighted
MR Egger
Simple mode
Weighted median
Weighted mode
0.50
0.25
0.00
−0.25
SNP effect on Diabetic nephropathy
0.0
0.5
1.0
1.5
2.0
SNP effect on SSC−A on HLA DR+ NK

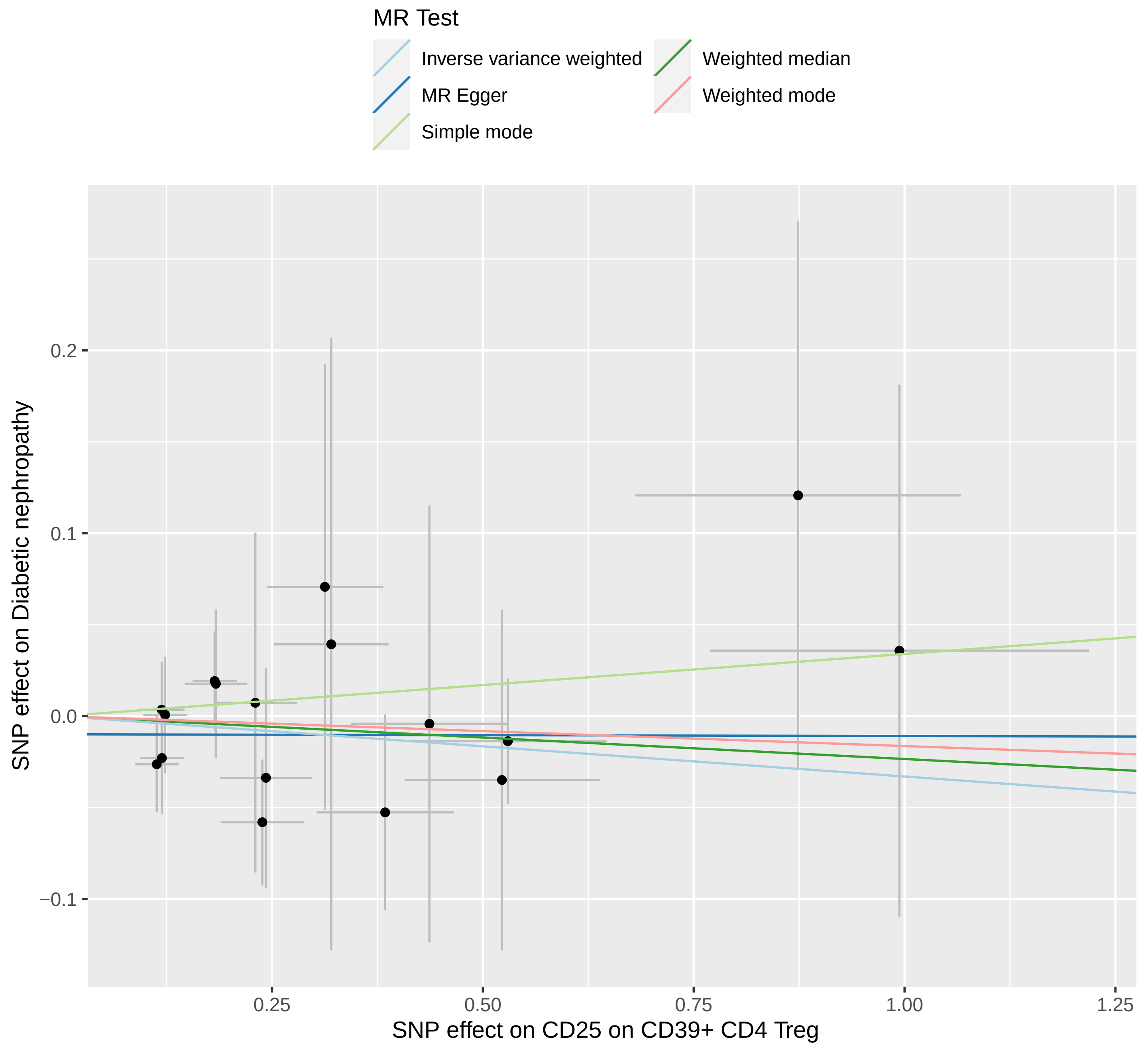

MR Test
Inverse variance weighted
MR Egger
Simple mode
Weighted median
Weighted mode
0.2
0.1
0.0
−0.1
SNP effect on Diabetic nephropathy
0.25
0.50
0.75
1.00
1.25
SNP effect on CD25 on CD39+ CD4 Treg

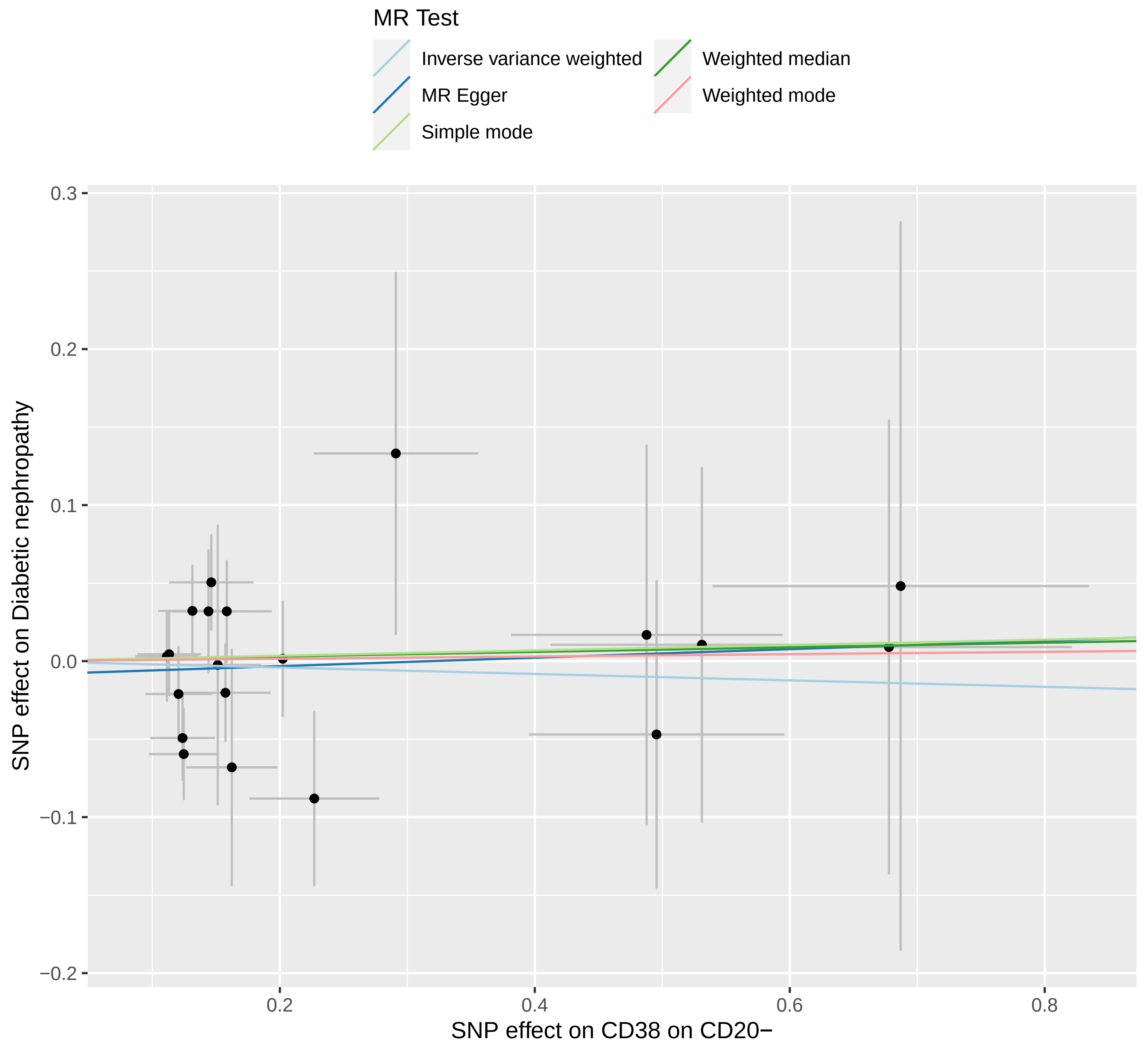
MR Test
Inverse variance weighted
MR Egger
Simple mode
Weighted median
Weighted mode
0.3
0.2
0.1
0.0
−0.1
−0.2
0.2
0.4
0.6
0.8
SNP effect on Diabetic nephropathy
SNP effect on CD38 on CD20−

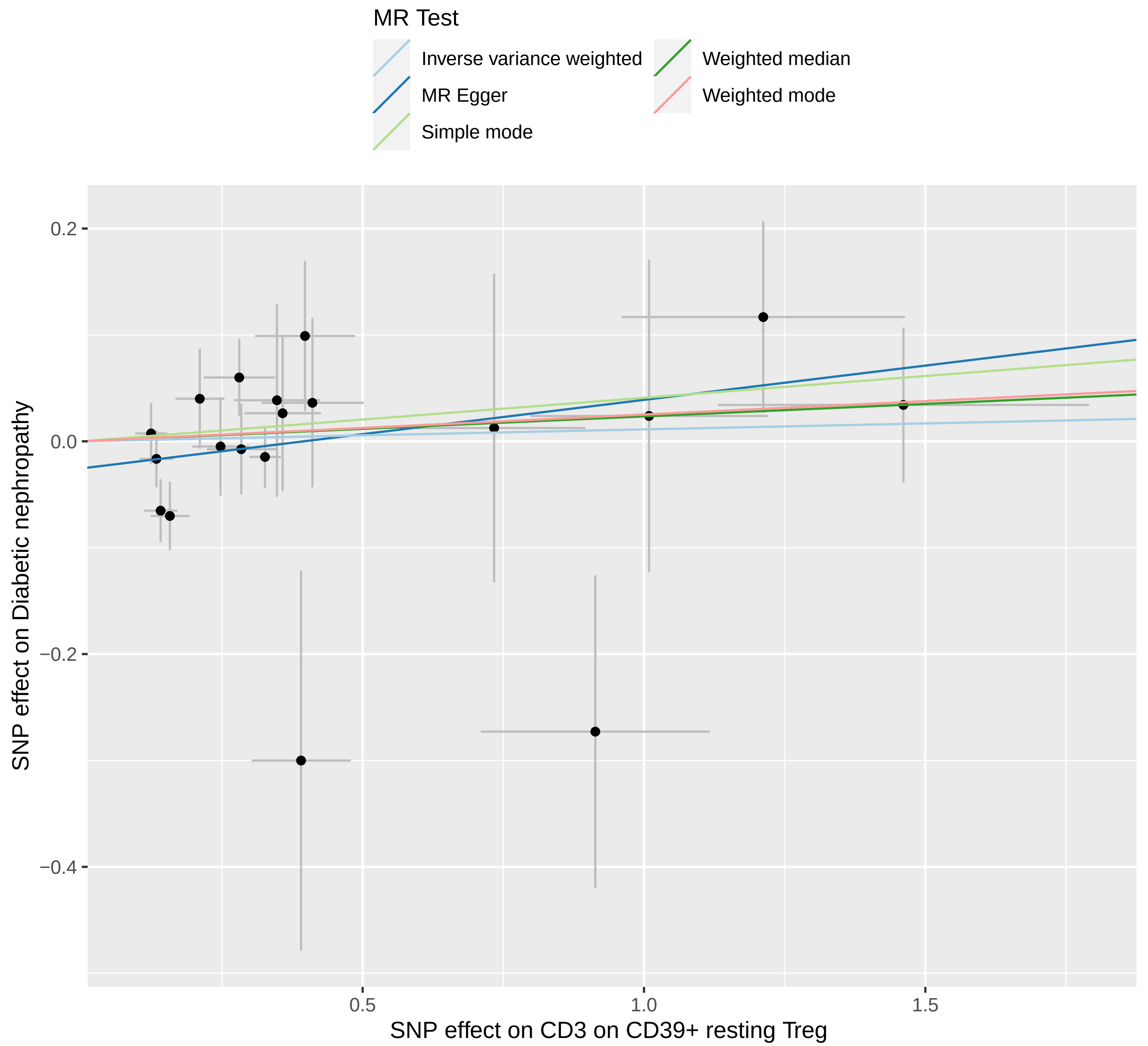
MR Test
Inverse variance weighted
MR Egger
Simple mode
Weighted median
Weighted mode
0.2
0.0
−0.2
−0.4
0.5
1.0
1.5
SNP effect on Diabetic nephropathy
SNP effect on CD3 on CD39+ resting Treg

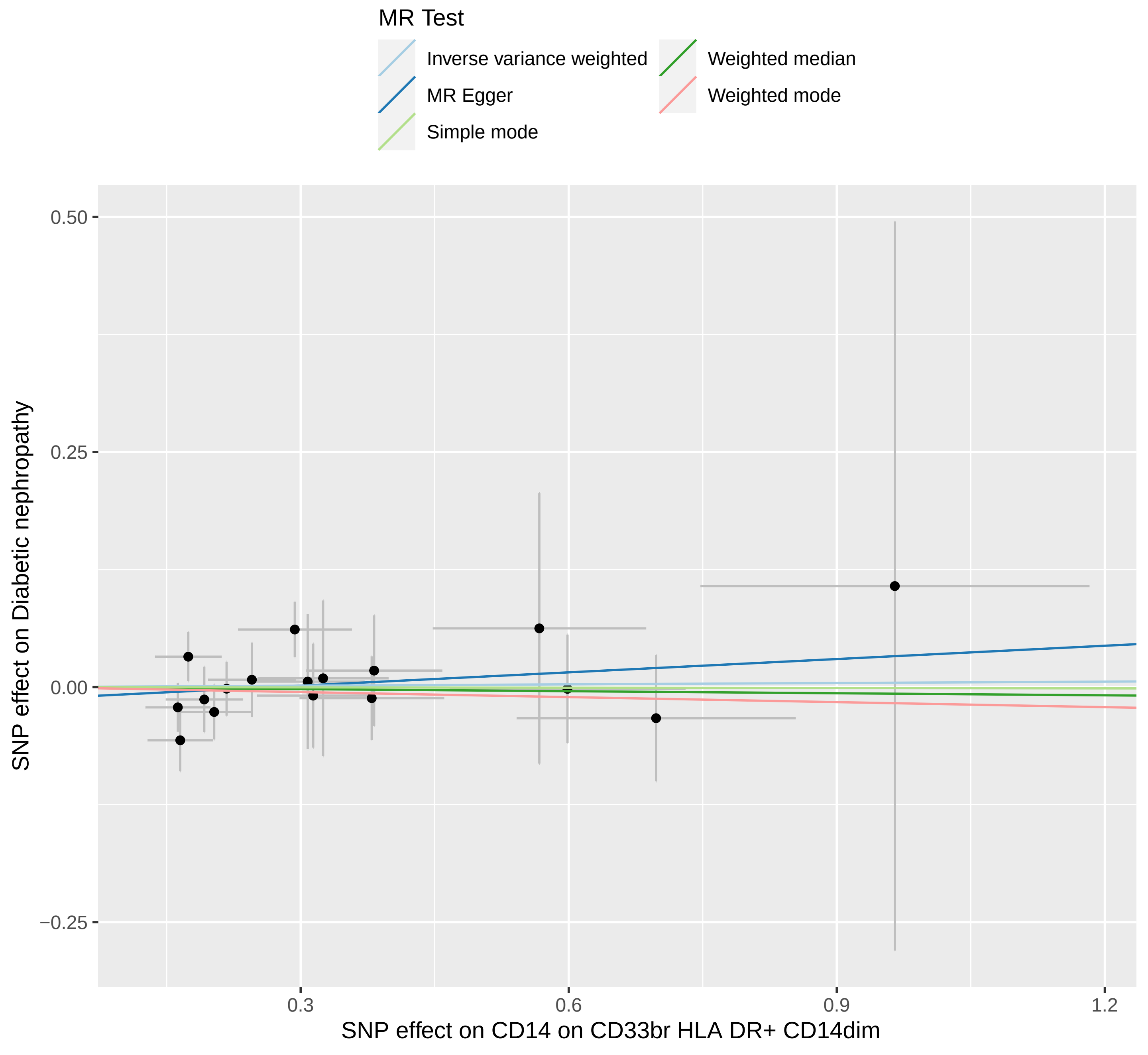

MR Test
Inverse variance weighted
MR Egger
Simple mode
Weighted median
Weighted mode
0.50
0.25
0.00
−0.25
0.3
0.6
0.9
1.2
SNP effect on Diabetic nephropathy
SNP effect on CD14 on CD33br HLA DR+ CD14dim

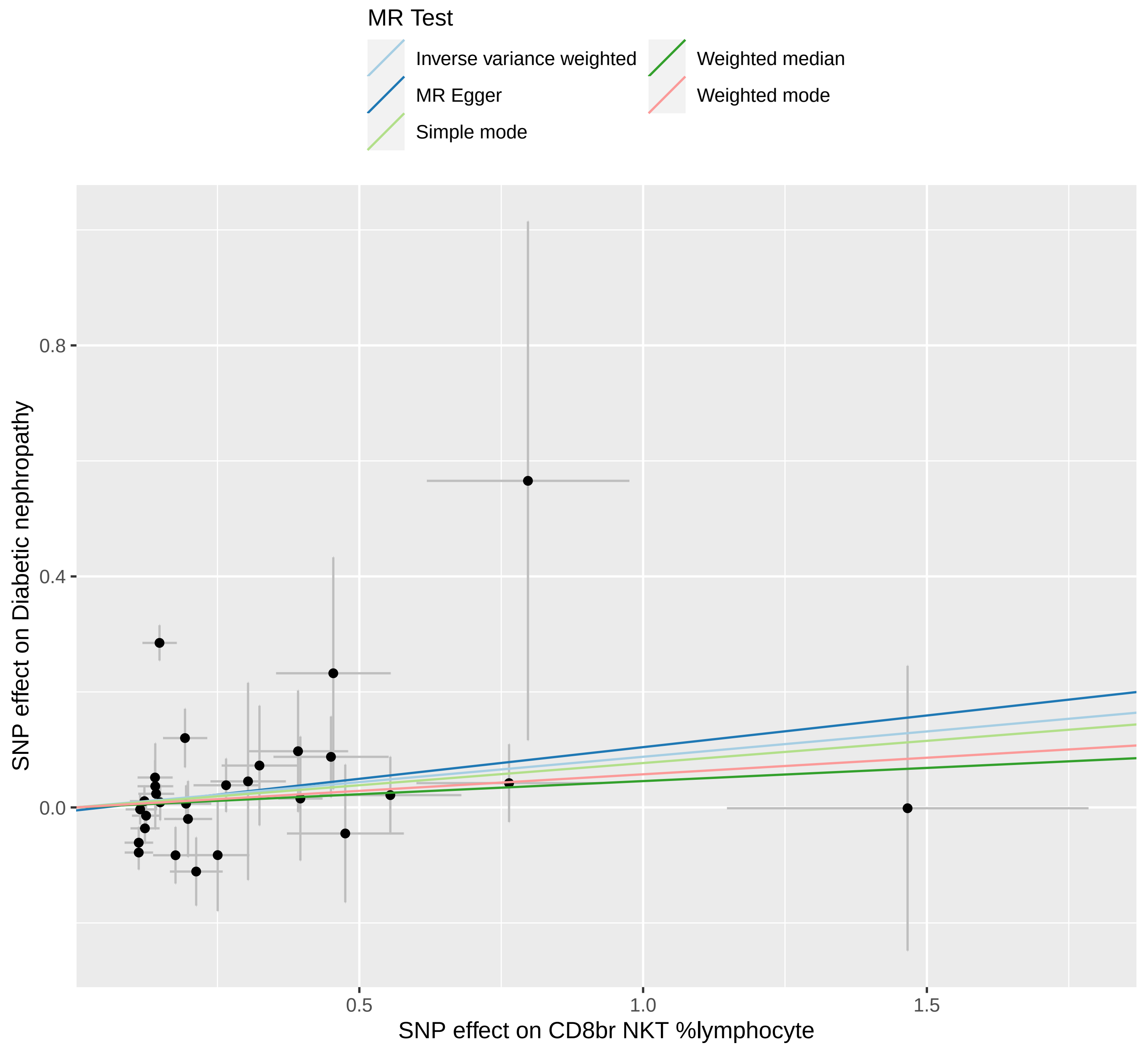
MR Test
Inverse variance weighted
MR Egger
Simple mode
Weighted median
Weighted mode
0.8
0.4
0.0
0.5
1.0
1.5
SNP effect on Diabetic nephropathy
SNP effect on CD8br NKT %lymphocyte

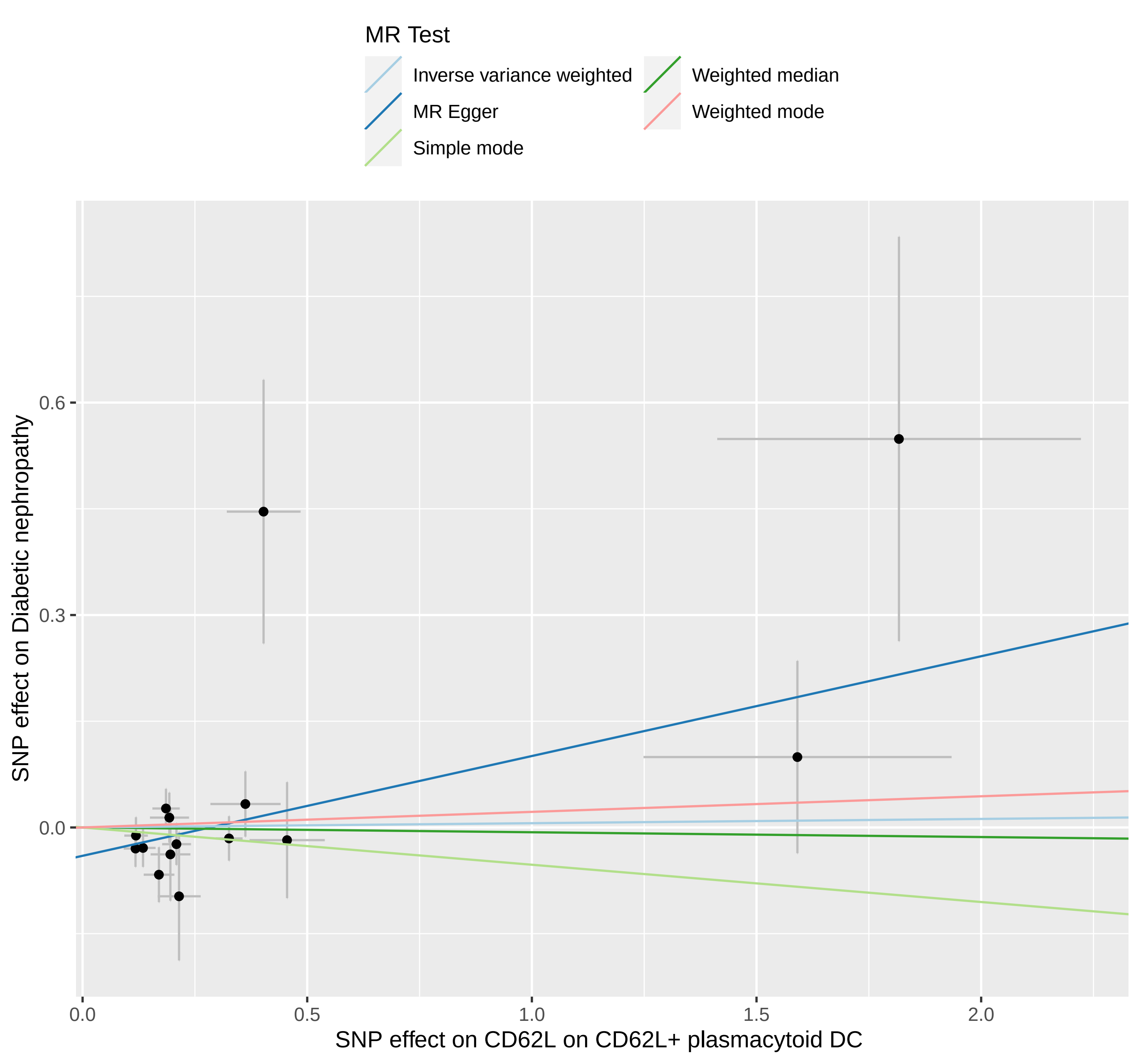

MR Test
Inverse variance weighted
MR Egger
Simple mode
Weighted median
Weighted mode
0.6
0.3
0.0
0.0
0.5
1.0
1.5
2.0
SNP effect on Diabetic nephropathy
SNP effect on CD62L on CD62L+ plasmacytoid DC

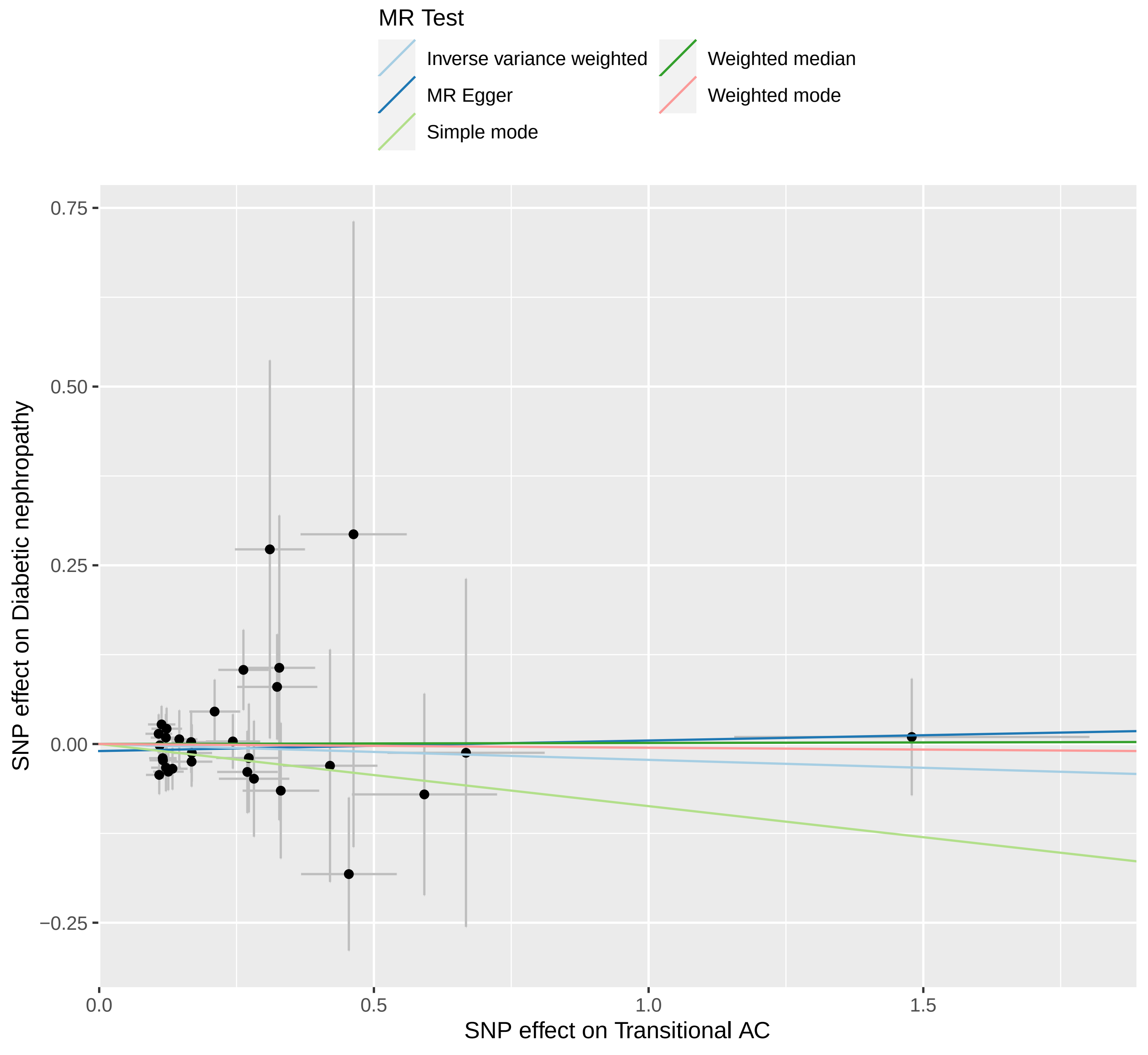

MR Test
Inverse variance weighted
MR Egger
Simple mode
Weighted median
Weighted mode
0.75
0.50
0.25
0.00
−0.25
0.0
0.5
1.0
1.5
SNP effect on Diabetic nephropathy
SNP effect on Transitional AC

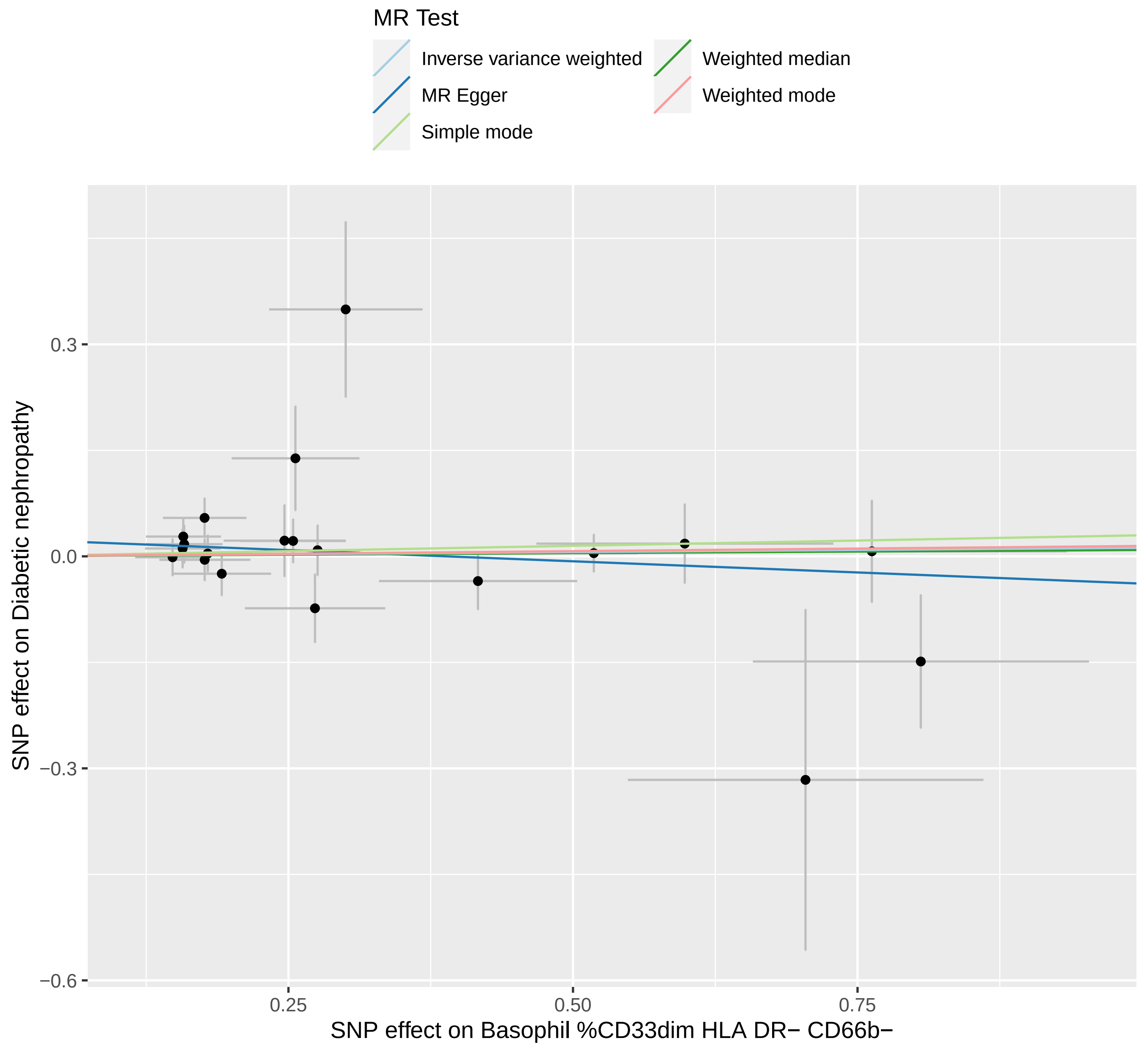

MR Test
Inverse variance weighted
MR Egger
Simple mode
Weighted median
Weighted mode
0.3
0.0
−0.3
−0.6
0.25
0.50
0.75
SNP effect on Diabetic nephropathy
SNP effect on Basophil %CD33dim HLA DR− CD66b−

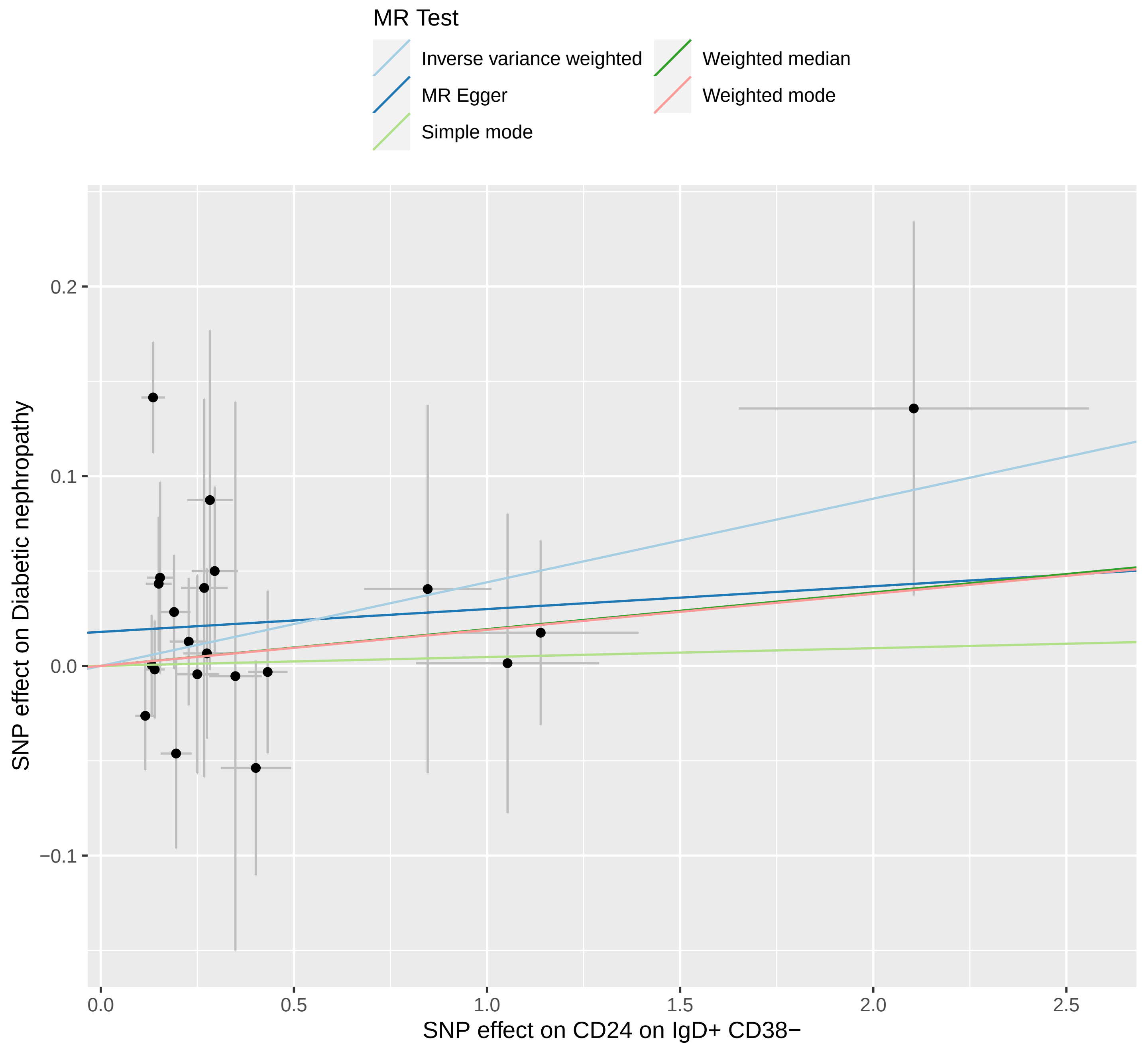

MR Test
Inverse variance weighted
MR Egger
Simple mode
Weighted median
Weighted mode
0.2
0.1
0.0
−0.1
0.0
0.5
1.0
1.5
2.0
2.5
SNP effect on Diabetic nephropathy
SNP effect on CD24 on IgD+ CD38−

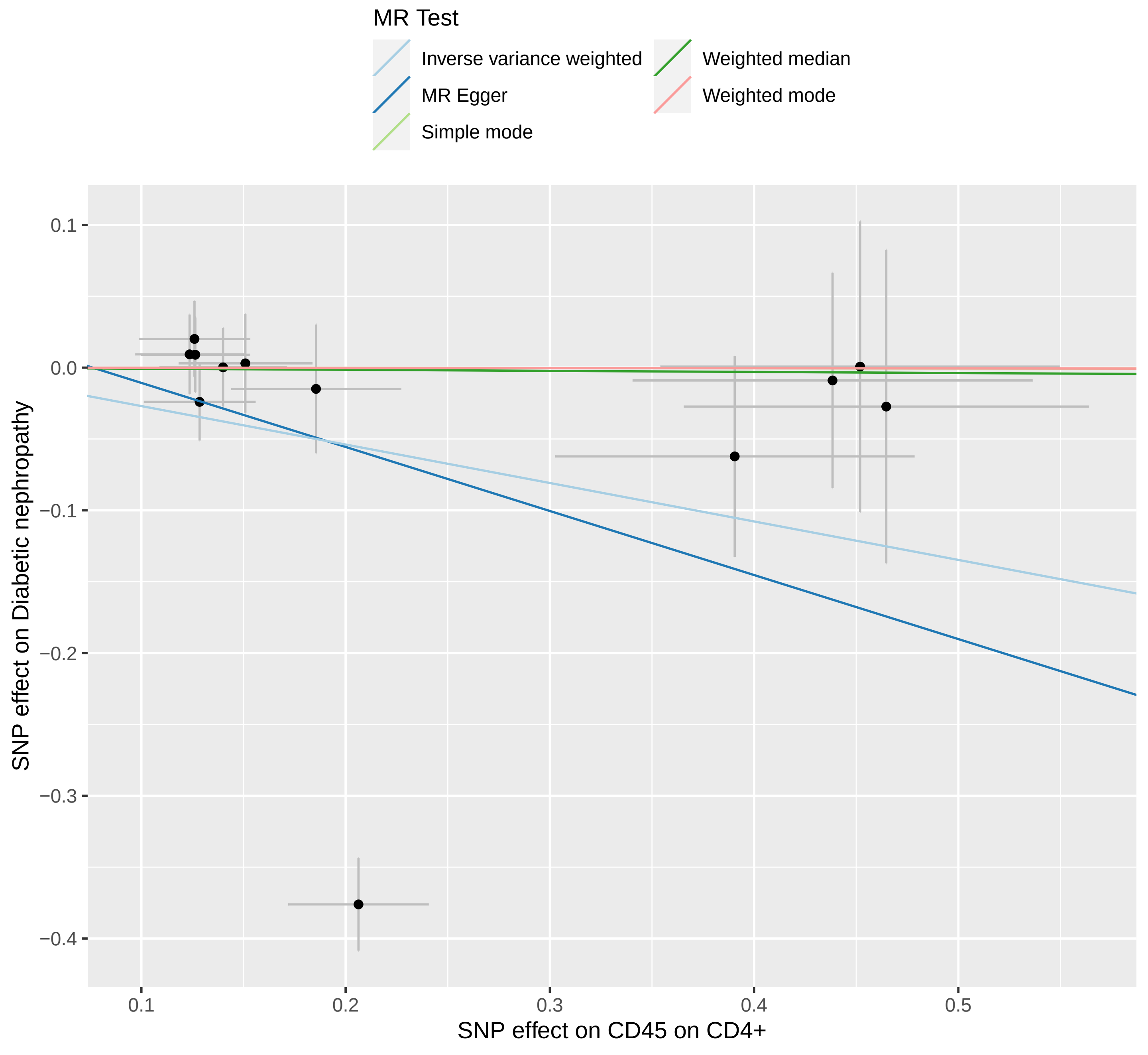

MR Test
Inverse variance weighted
MR Egger
Simple mode
Weighted median
Weighted mode
0.1
0.0
−0.1
−0.2
−0.3
−0.4
SNP effect on Diabetic nephropathy
0.1
0.2
0.3
0.4
0.5
SNP effect on CD45 on CD4+

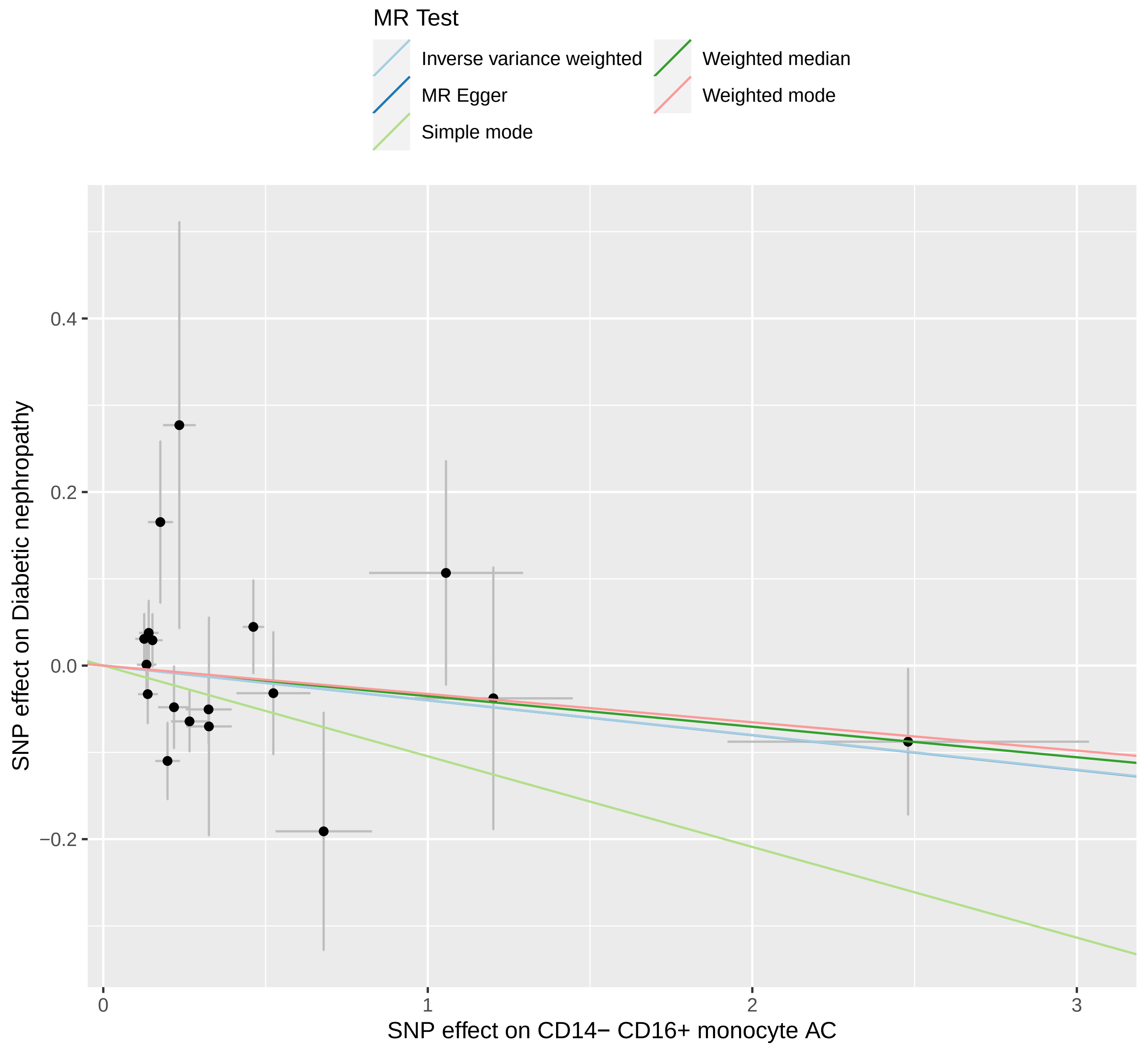

MR Test
Inverse variance weighted
MR Egger
Simple mode
Weighted median
Weighted mode
0.4
0.2
0.0
−0.2
SNP effect on Diabetic nephropathy
0
1
2
3
SNP effect on CD14− CD16+ monocyte AC

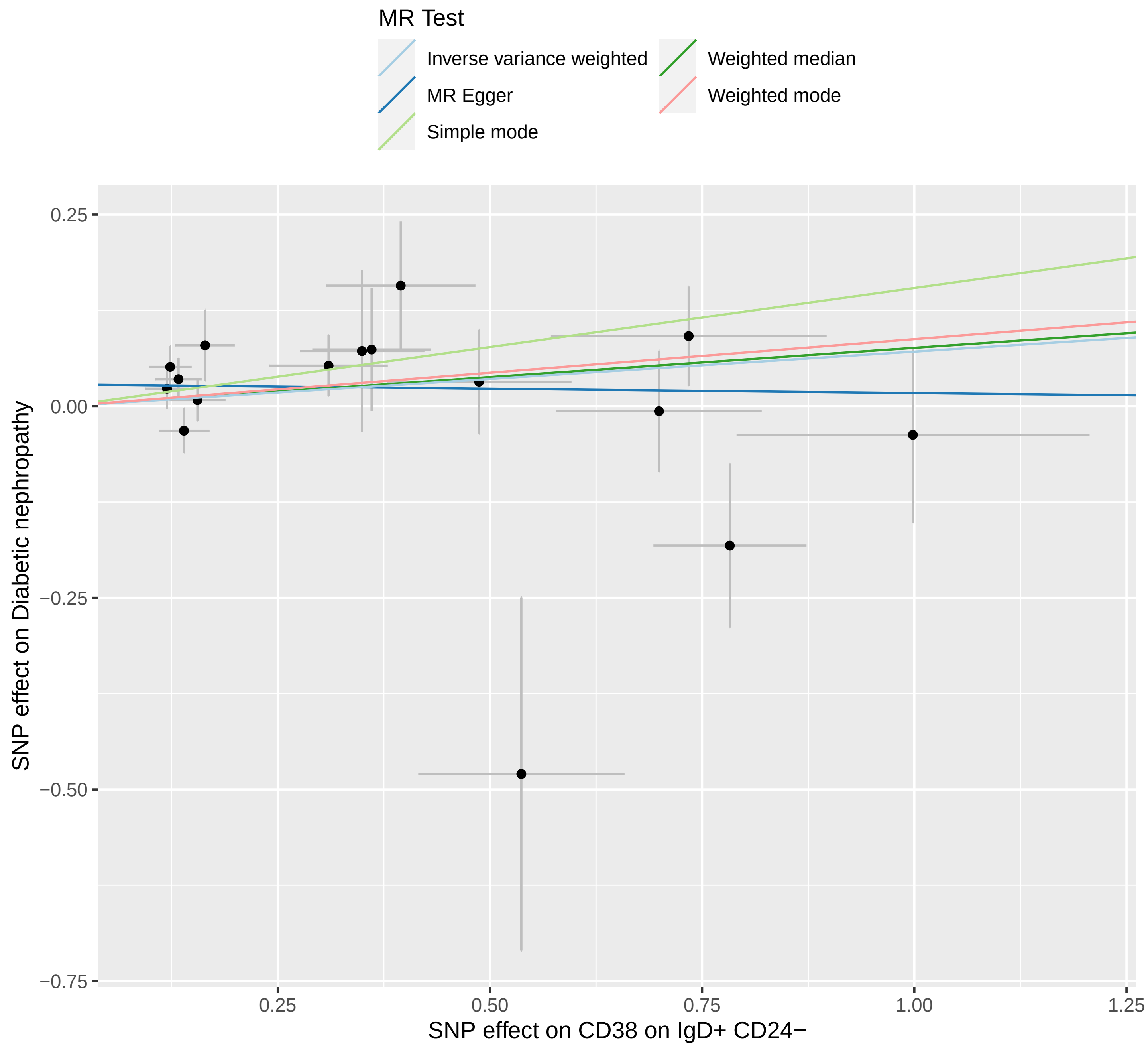
MR Test
Inverse variance weighted
MR Egger
Simple mode
Weighted median
Weighted mode
0.25
0.00
−0.25
−0.50
−0.75
SNP effect on Diabetic nephropathy
0.25
0.50
0.75
1.00
1.25
SNP effect on CD38 on IgD+ CD24−

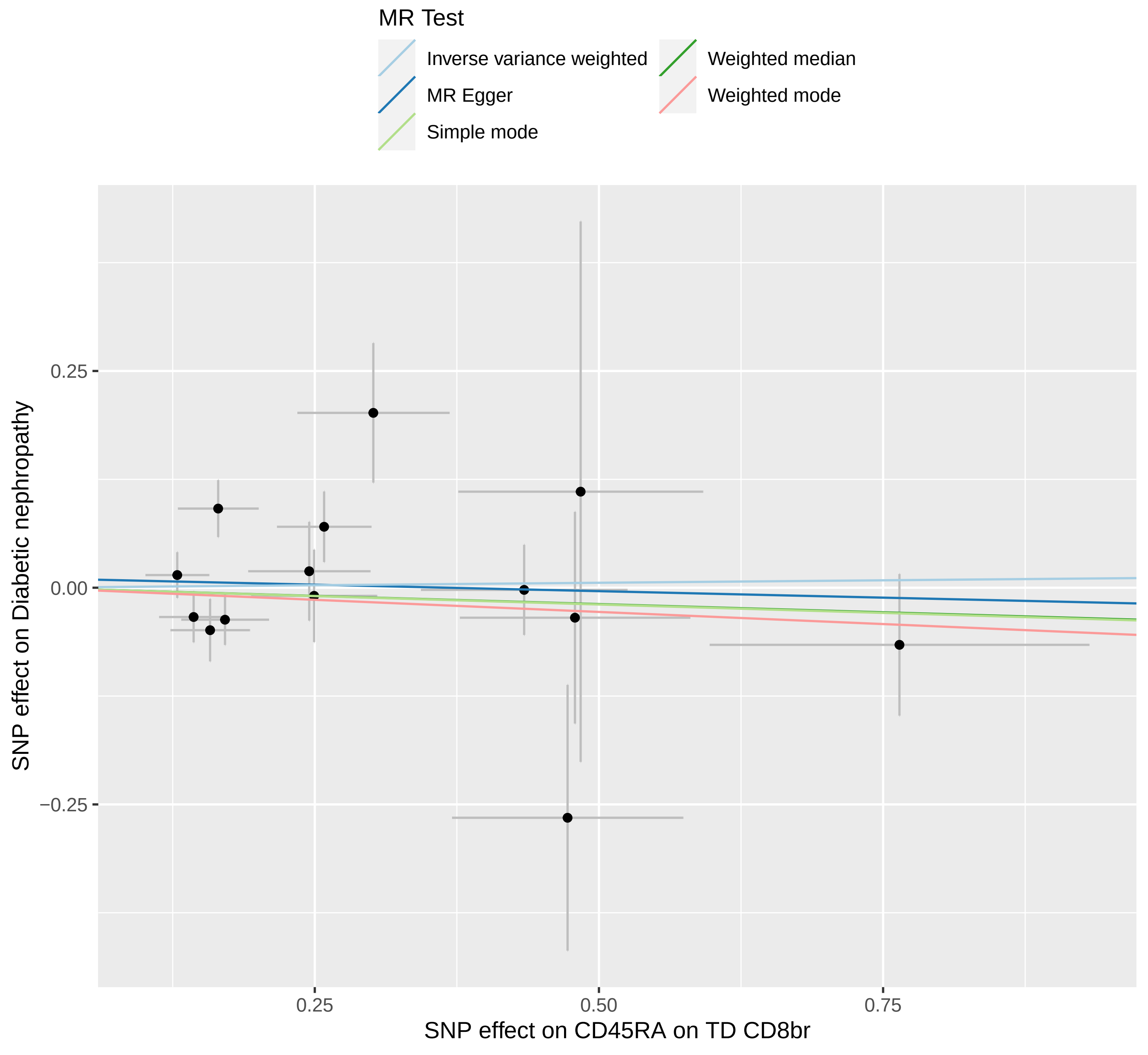

MR Test
Inverse variance weighted
MR Egger
Simple mode
Weighted median
Weighted mode
0.25
0.00
−0.25
SNP effect on Diabetic nephropathy
0.25
0.50
0.75
SNP effect on CD45RA on TD CD8br

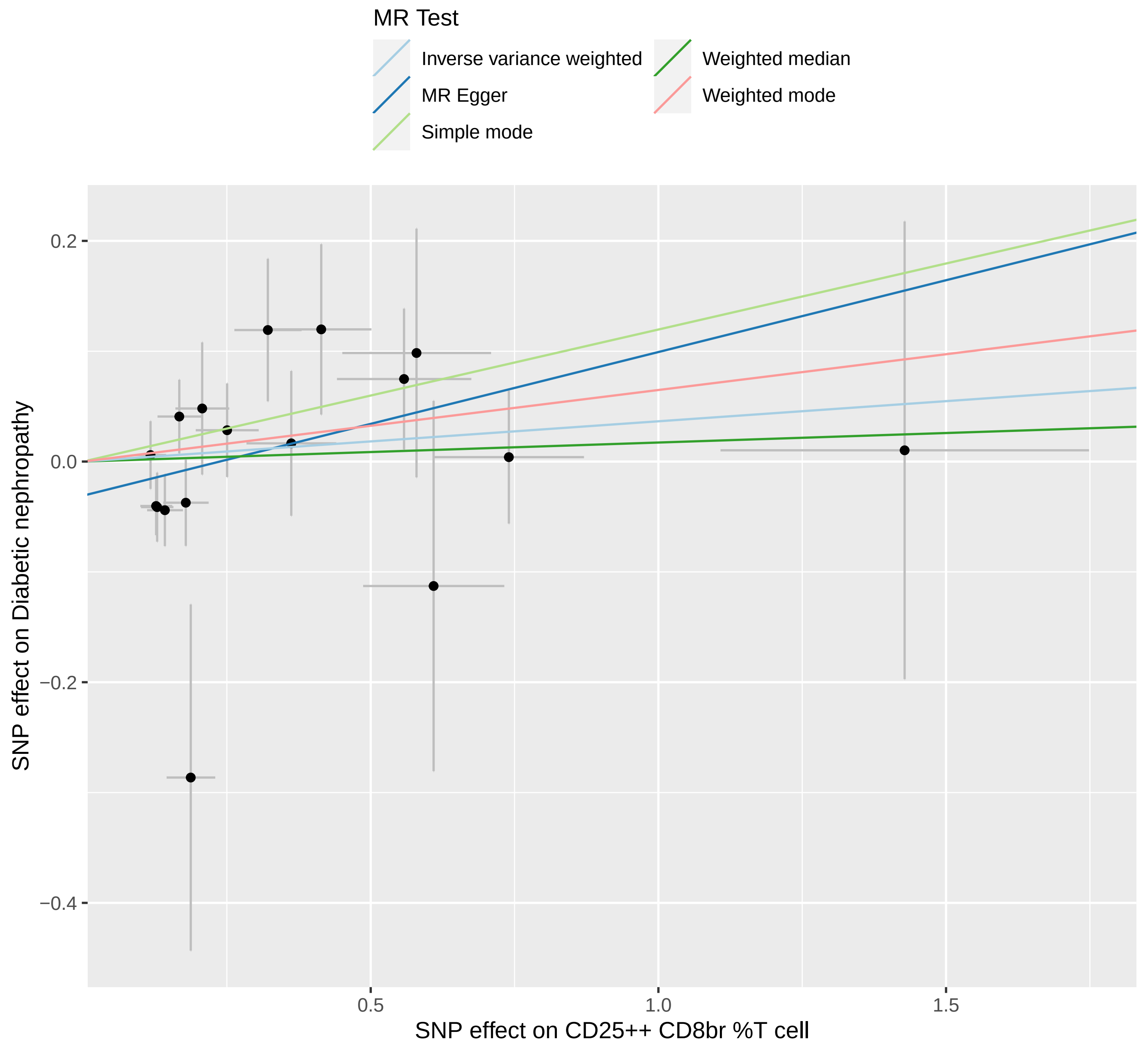

MR Test
Inverse variance weighted
MR Egger
Simple mode
Weighted median
Weighted mode
0.2
0.0
−0.2
−0.4
SNP effect on Diabetic nephropathy
0.5
1.0
1.5
SNP effect on CD25++ CD8br %T cell

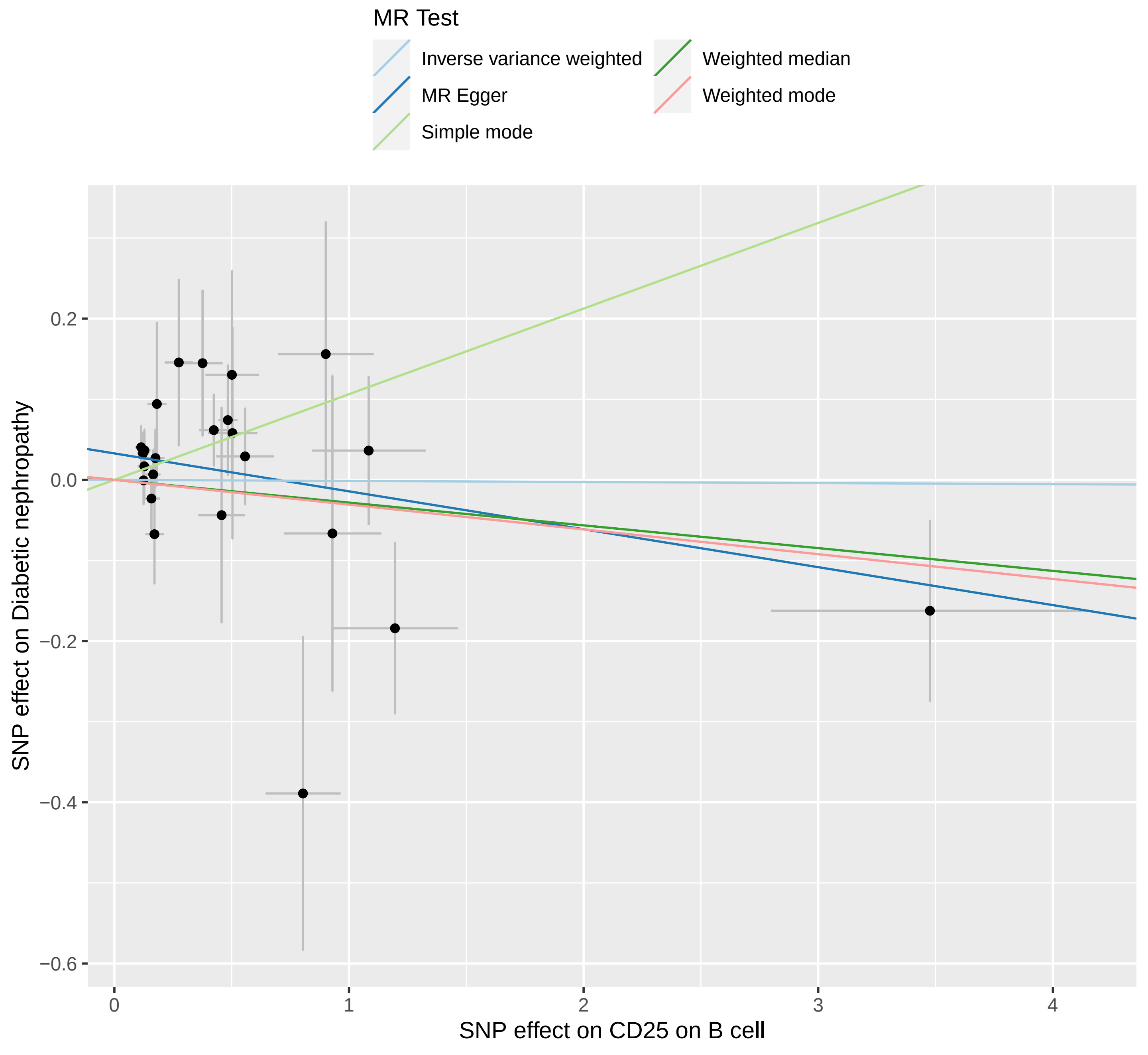

MR Test
Inverse variance weighted
MR Egger
Simple mode
Weighted median
Weighted mode
0.2
0.0
−0.2
−0.4
−0.6
0
1
2
3
4
SNP effect on Diabetic nephropathy
SNP effect on CD25 on B cell

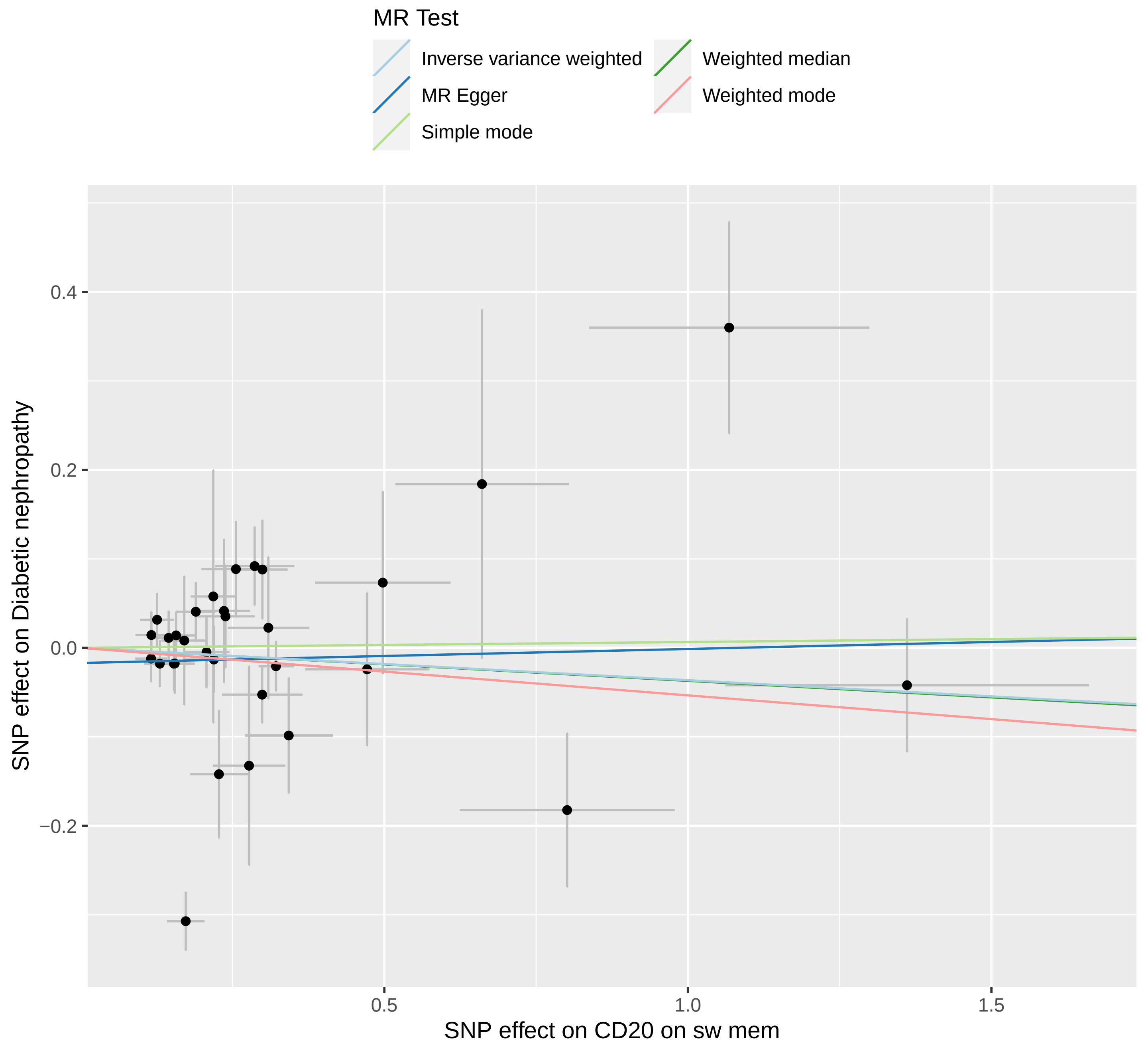

MR Test
Inverse variance weighted
MR Egger
Simple mode
Weighted median
Weighted mode
0.4
0.2
0.0
−0.2
0.5
1.0
1.5
SNP effect on Diabetic nephropathy
SNP effect on CD20 on sw mem

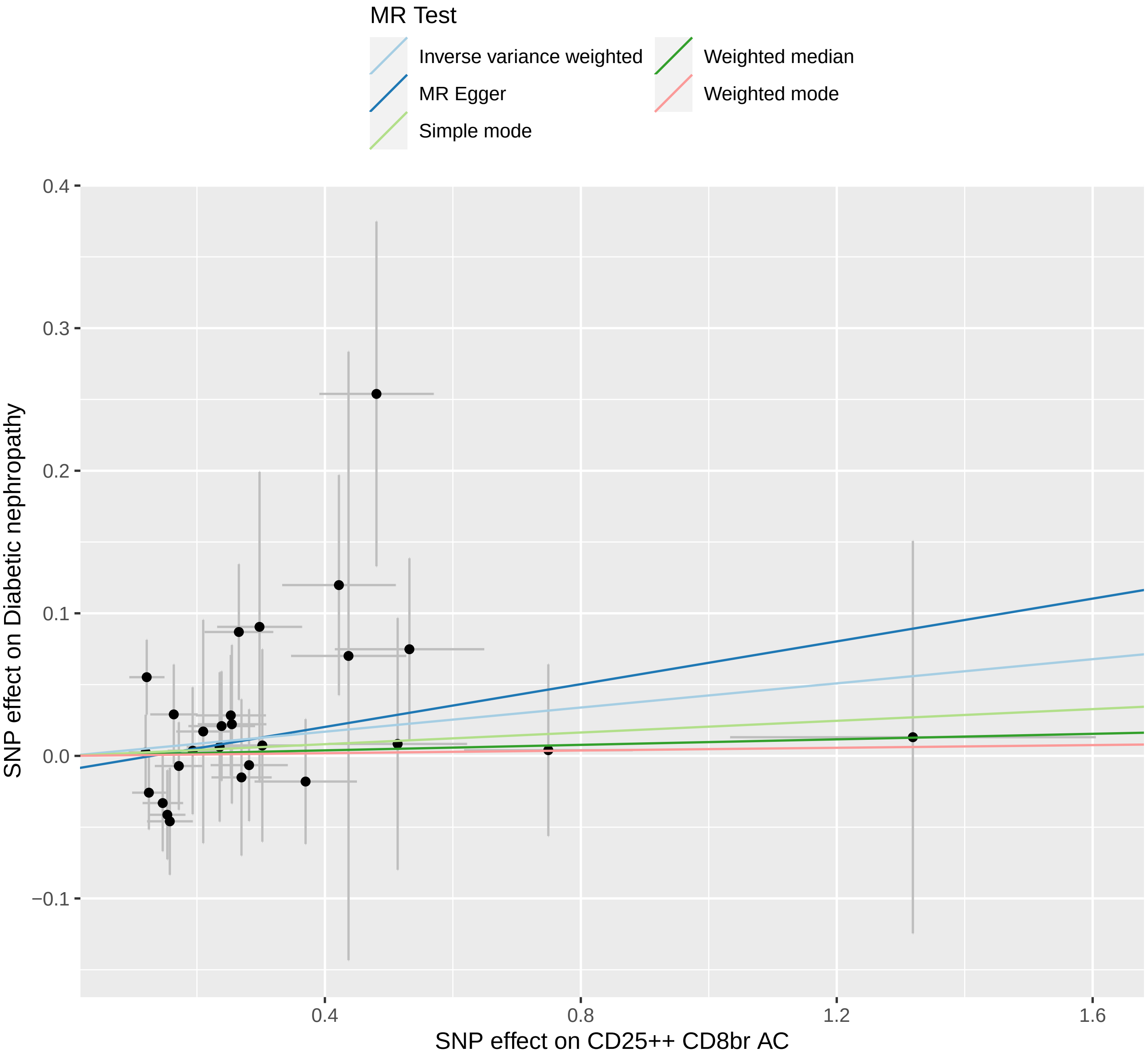

MR Test
Inverse variance weighted
MR Egger
Simple mode
Weighted median
Weighted mode
0.4
0.3
0.2
0.1
0.0
−0.1
SNP effect on Diabetic nephropathy
0.4
0.8
1.2
1.6
SNP effect on CD25++ CD8br AC

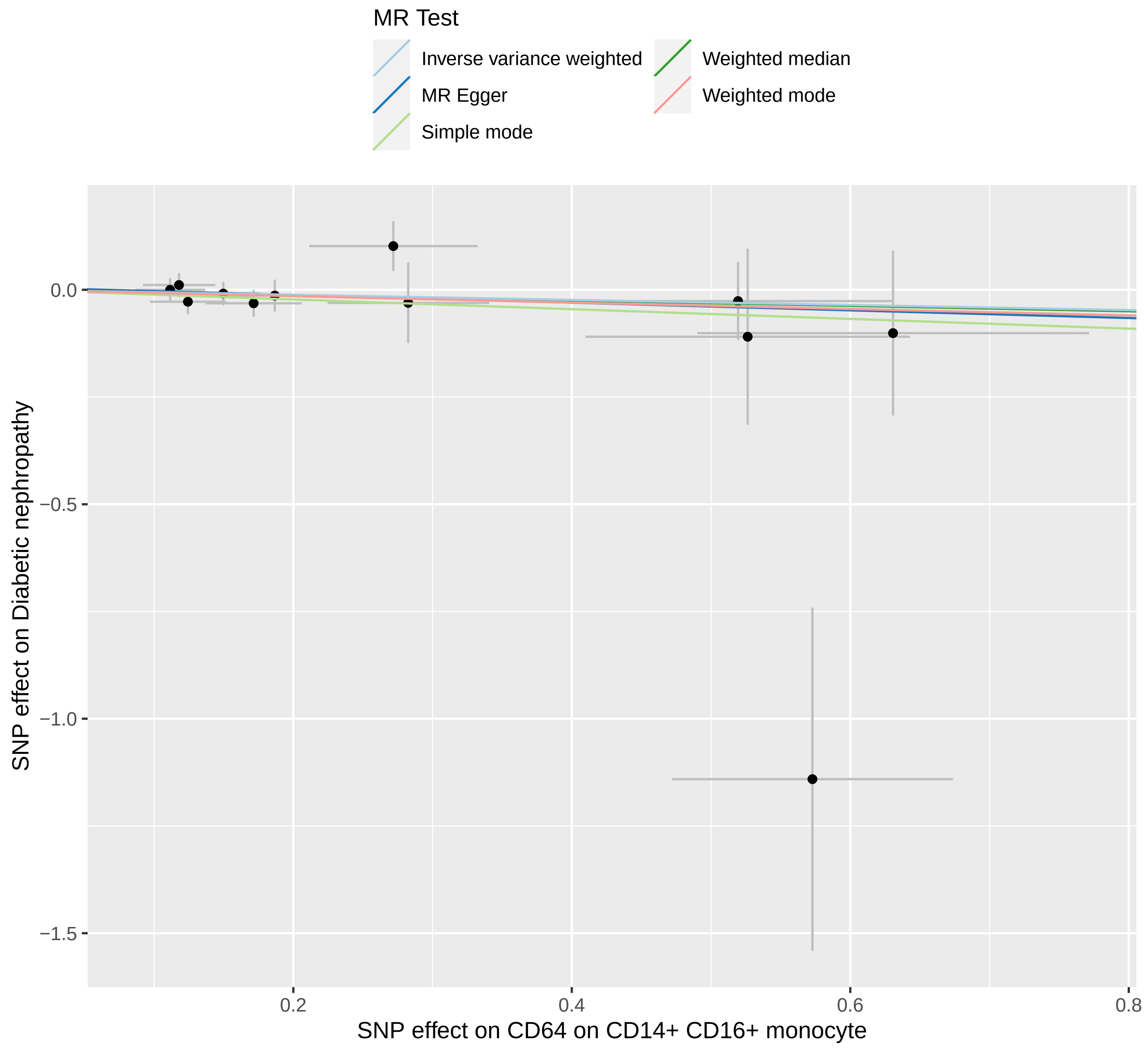

MR Test
Inverse variance weighted
MR Egger
Simple mode
Weighted median
Weighted mode
0.0
−0.5
−1.0
−1.5
0.2
0.4
0.6
0.8
SNP effect on Diabetic nephropathy
SNP effect on CD64 on CD14+ CD16+ monocyte

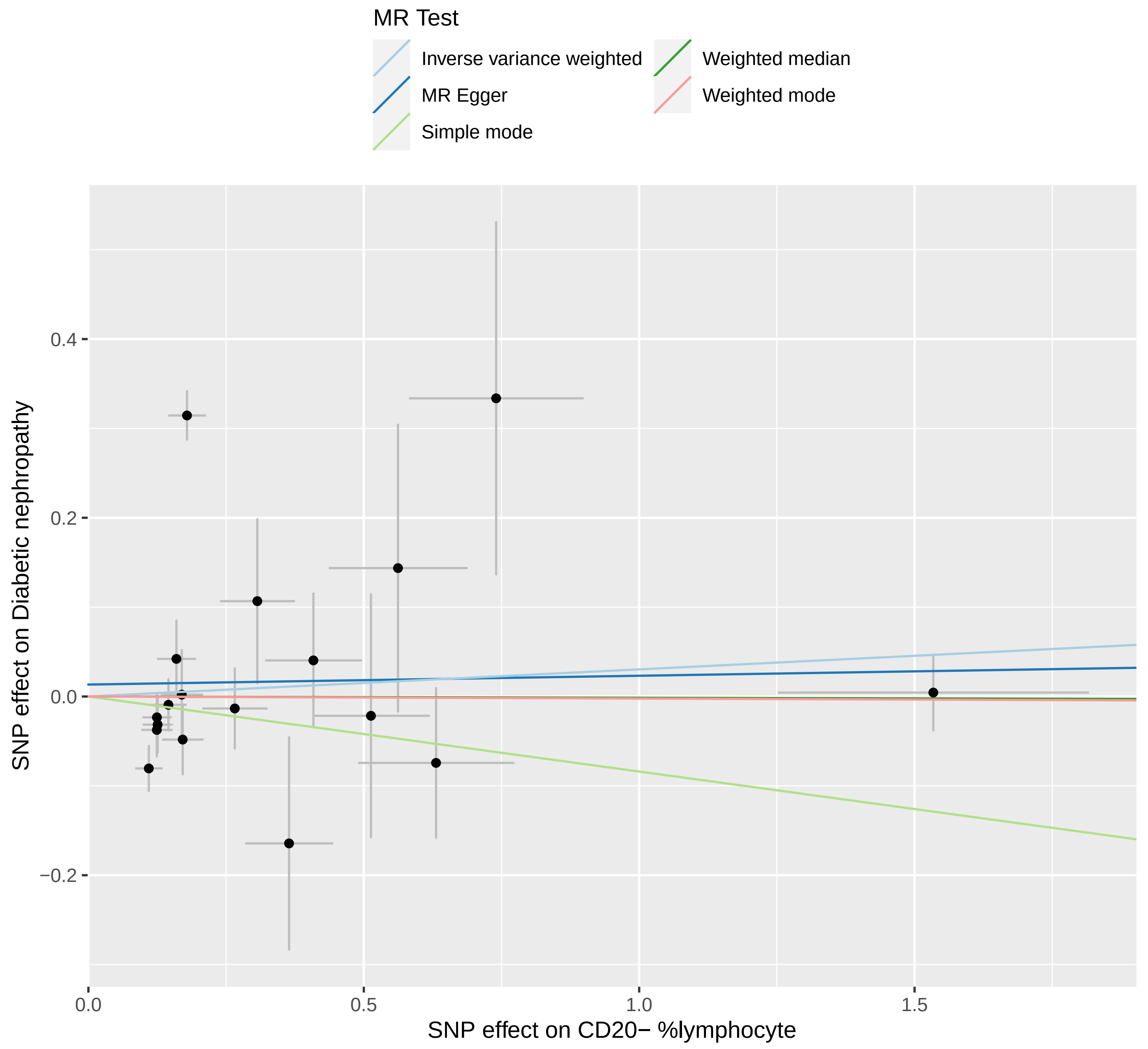
MR Test
Inverse variance weighted
MR Egger
Simple mode
Weighted median
Weighted mode
SNP effect on Diabetic nephropathy
0.4
0.2
0.0
−0.2
0.0
0.5
1.0
1.5
SNP effect on CD20− %lymphocyte

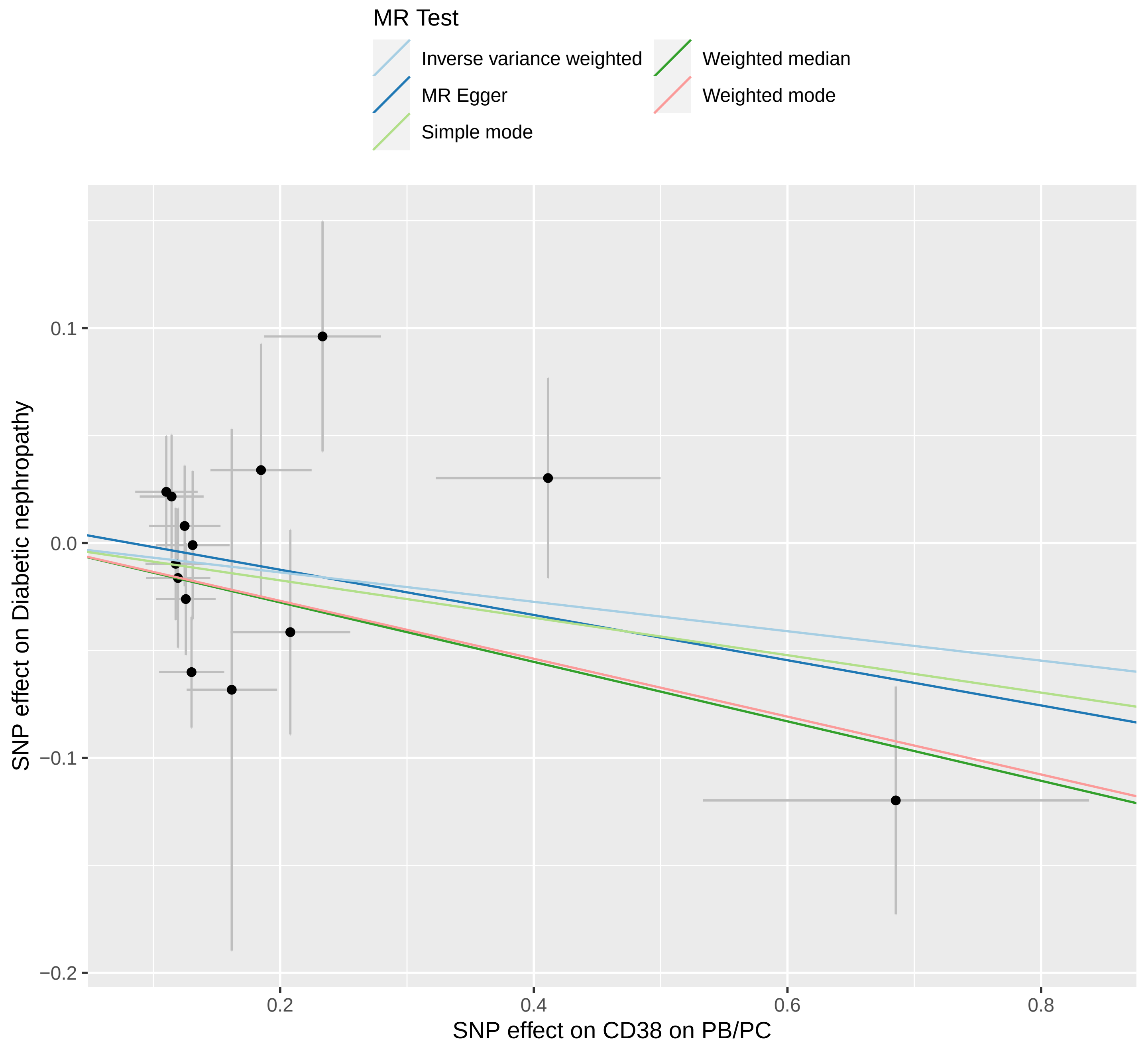

MR Test
Inverse variance weighted
MR Egger
Simple mode
Weighted median
Weighted mode
SNP effect on Diabetic nephropathy
0.1
0.0
−0.1
−0.2
0.2
0.4
0.6
0.8
SNP effect on CD38 on PB/PC

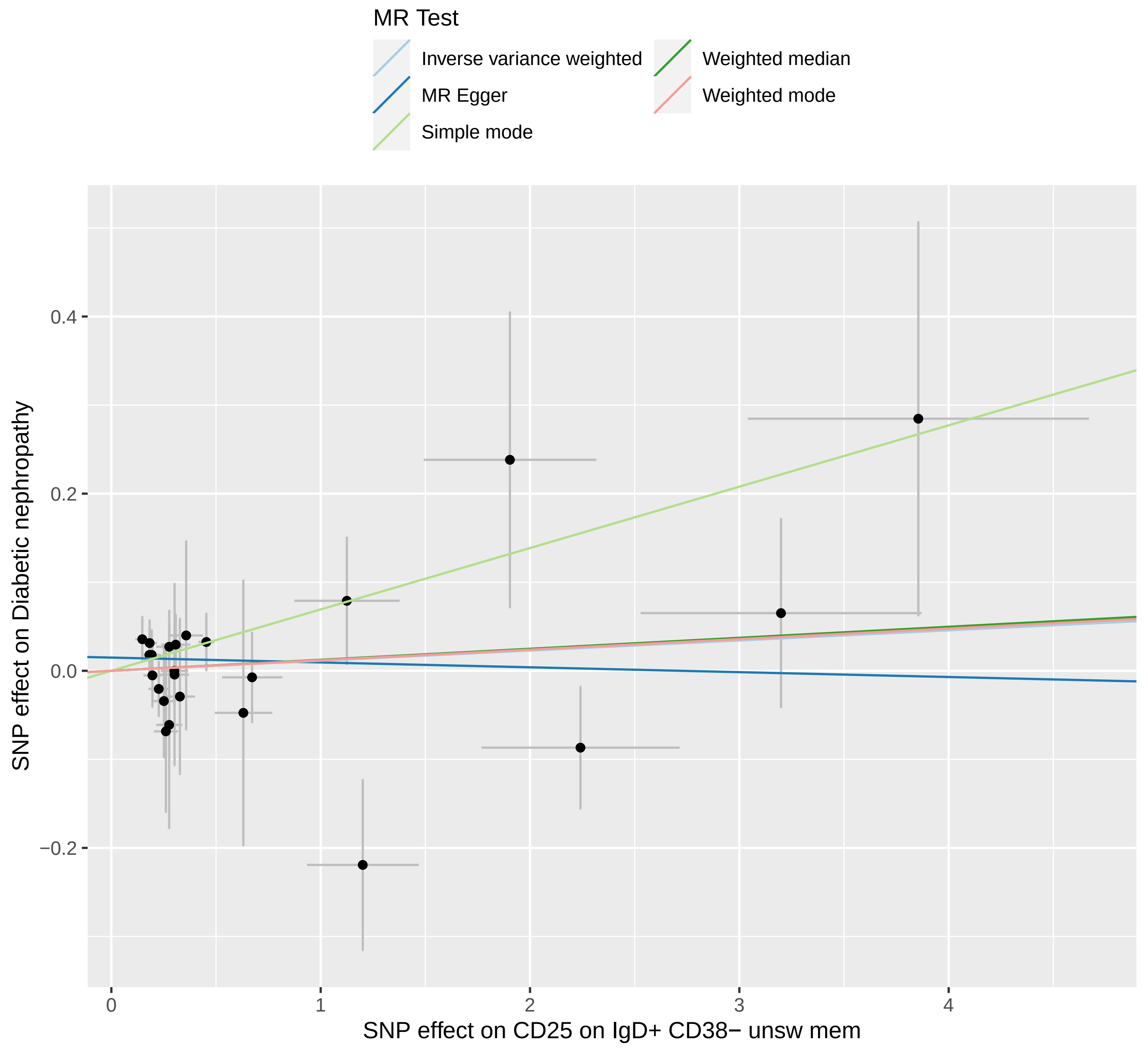

MR Test
Inverse variance weighted
MR Egger
Simple mode
Weighted median
Weighted mode
SNP effect on Diabetic nephropathy
0.4
0.2
0.0
−0.2
0
1
2
3
4
SNP effect on CD25 on IgD+ CD38− unsw mem

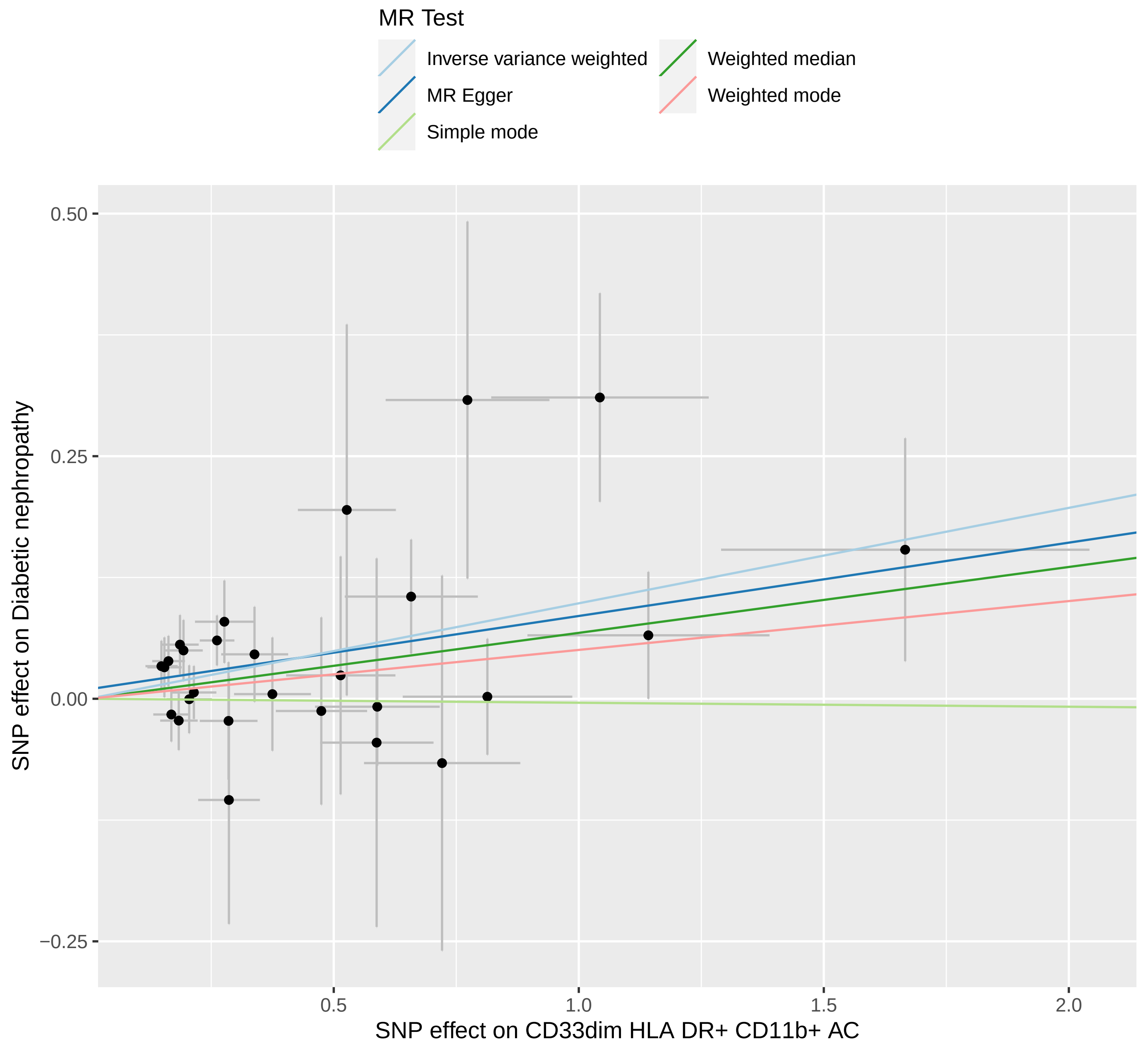

MR Test
Inverse variance weighted
MR Egger
Simple mode
Weighted median
Weighted mode
0.50
0.25
0.00
−0.25
0.5
1.0
1.5
2.0
SNP effect on Diabetic nephropathy
SNP effect on CD33dim HLA DR+ CD11b+ AC

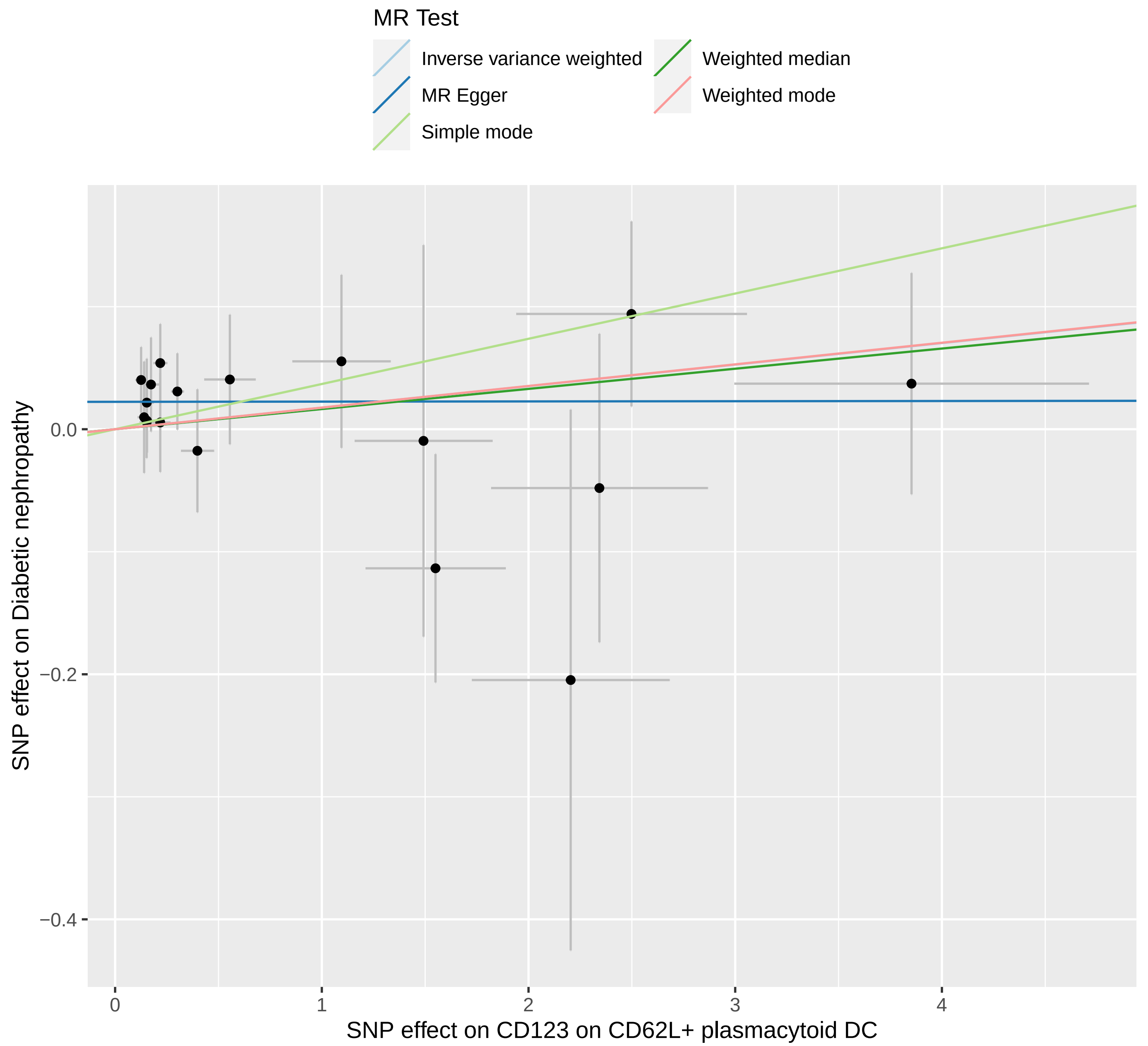
MR Test
Inverse variance weighted
MR Egger
Simple mode
Weighted median
Weighted mode
SNP effect on Diabetic nephropathy
0.0
−0.2
−0.4
0
1
2
3
4
SNP effect on CD123 on CD62L+ plasmacytoid DC

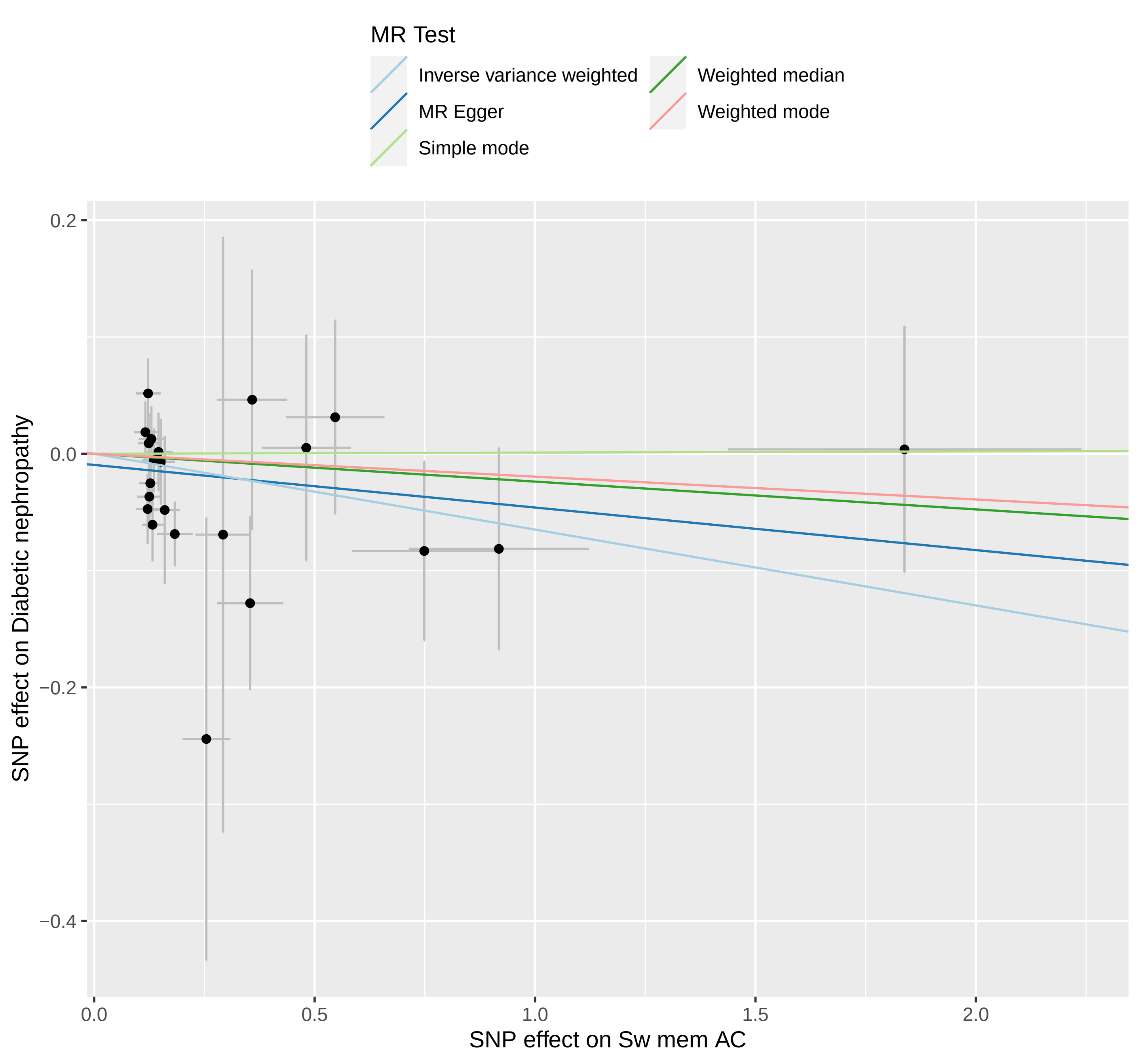
MR Test
Inverse variance weighted
MR Egger
Simple mode
Weighted median
Weighted mode
0.2
0.0
−0.2
−0.4
0.0
0.5
1.0
1.5
2.0
SNP effect on Diabetic nephropathy
SNP effect on Sw mem AC

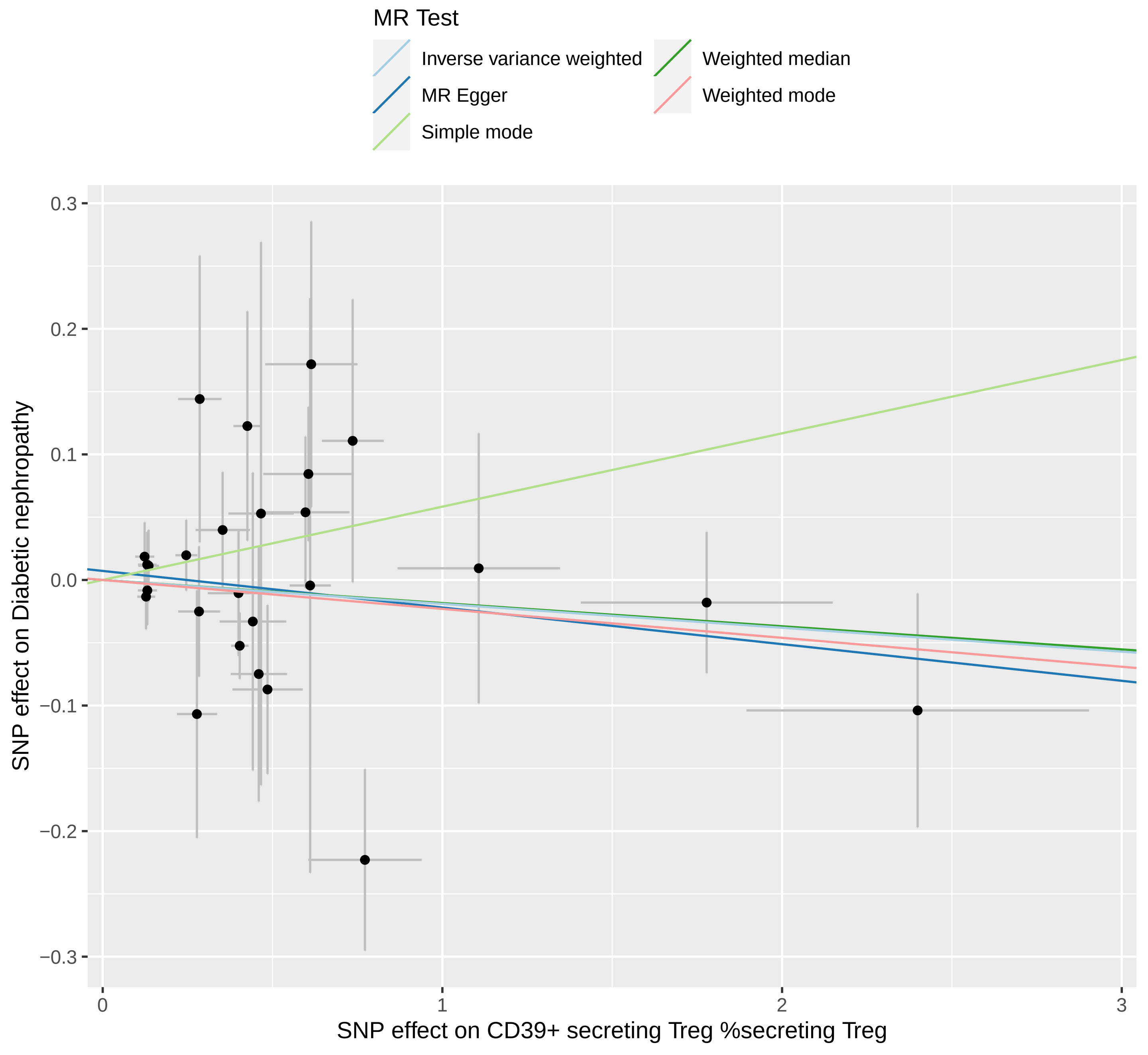
MR Test
Inverse variance weighted
MR Egger
Simple mode
Weighted median
Weighted mode
SNP effect on Diabetic nephropathy
0.3
0.2
0.1
0.0
−0.1
−0.2
−0.3
0
1
2
3
SNP effect on CD39+ secreting Treg %secreting Treg

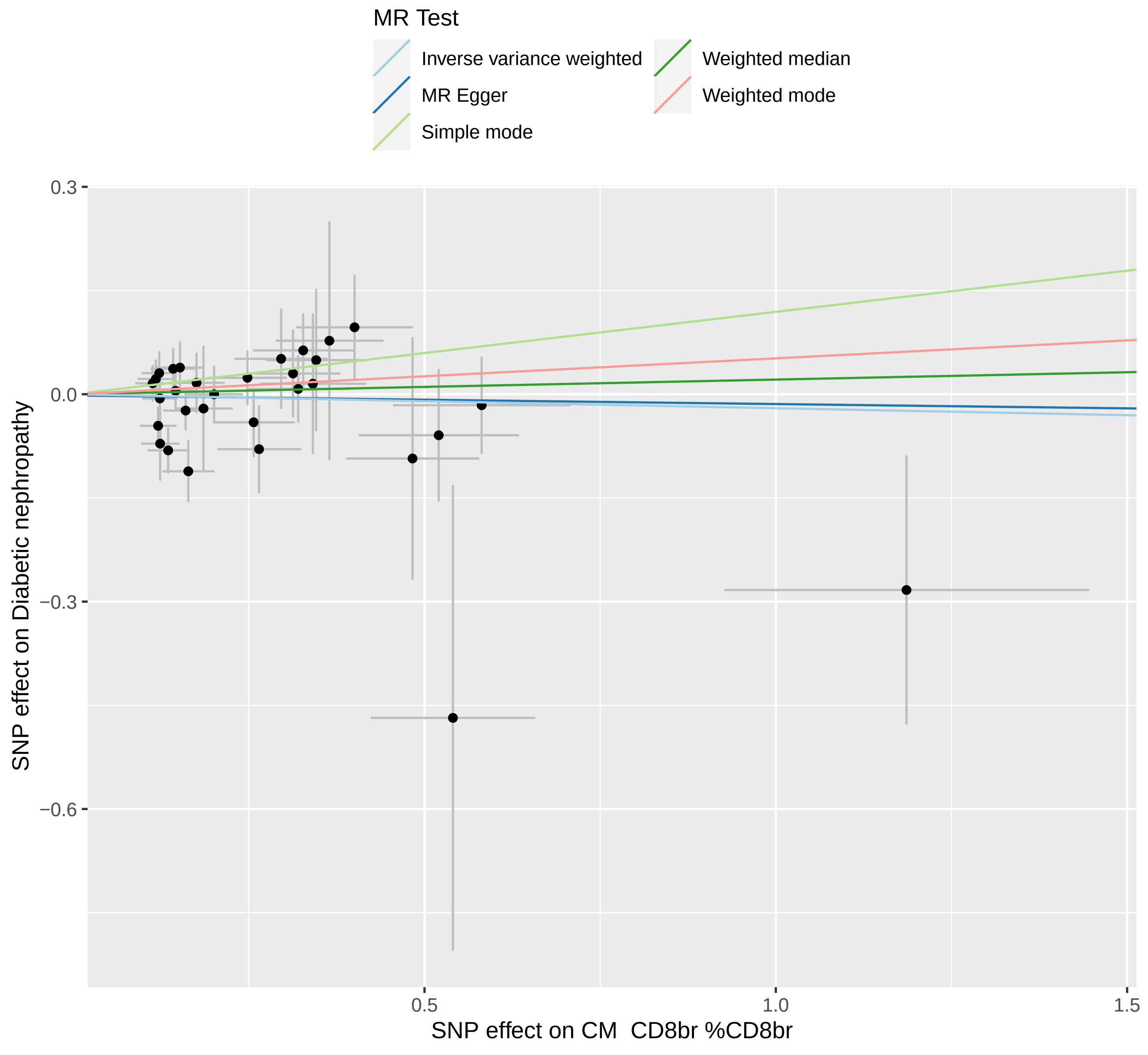

MR Test
Inverse variance weighted
MR Egger
Simple mode
Weighted median
Weighted mode
0.3
0.0
−0.3
−0.6
SNP effect on Diabetic nephropathy
0.5
1.0
1.5
SNP effect on CM CD8br %CD8br

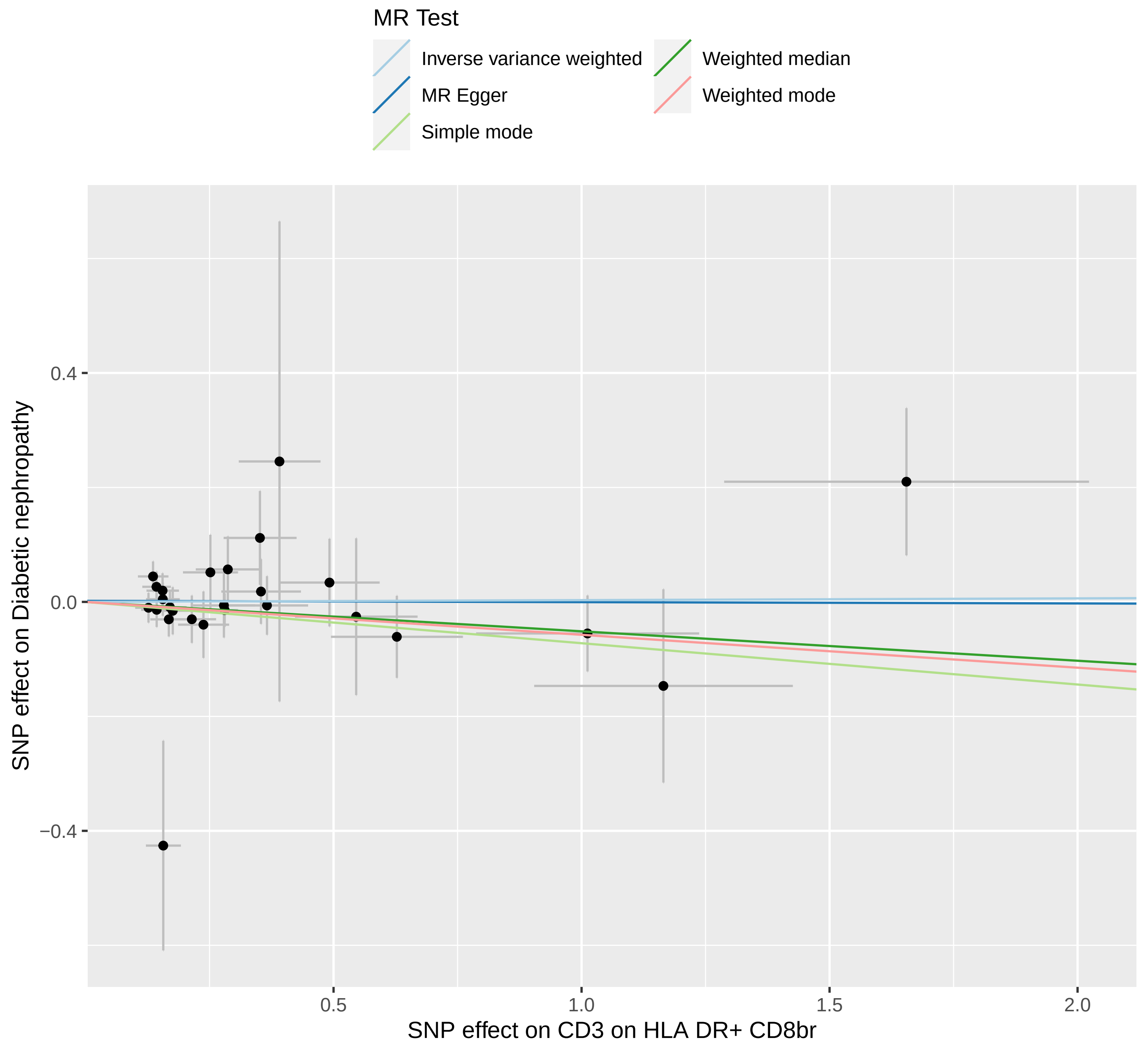
MR Test
Inverse variance weighted
MR Egger
Simple mode
Weighted median
Weighted mode
0.4
0.0
−0.4
SNP effect on Diabetic nephropathy
0.5
1.0
1.5
2.0
SNP effect on CD3 on HLA DR+ CD8br

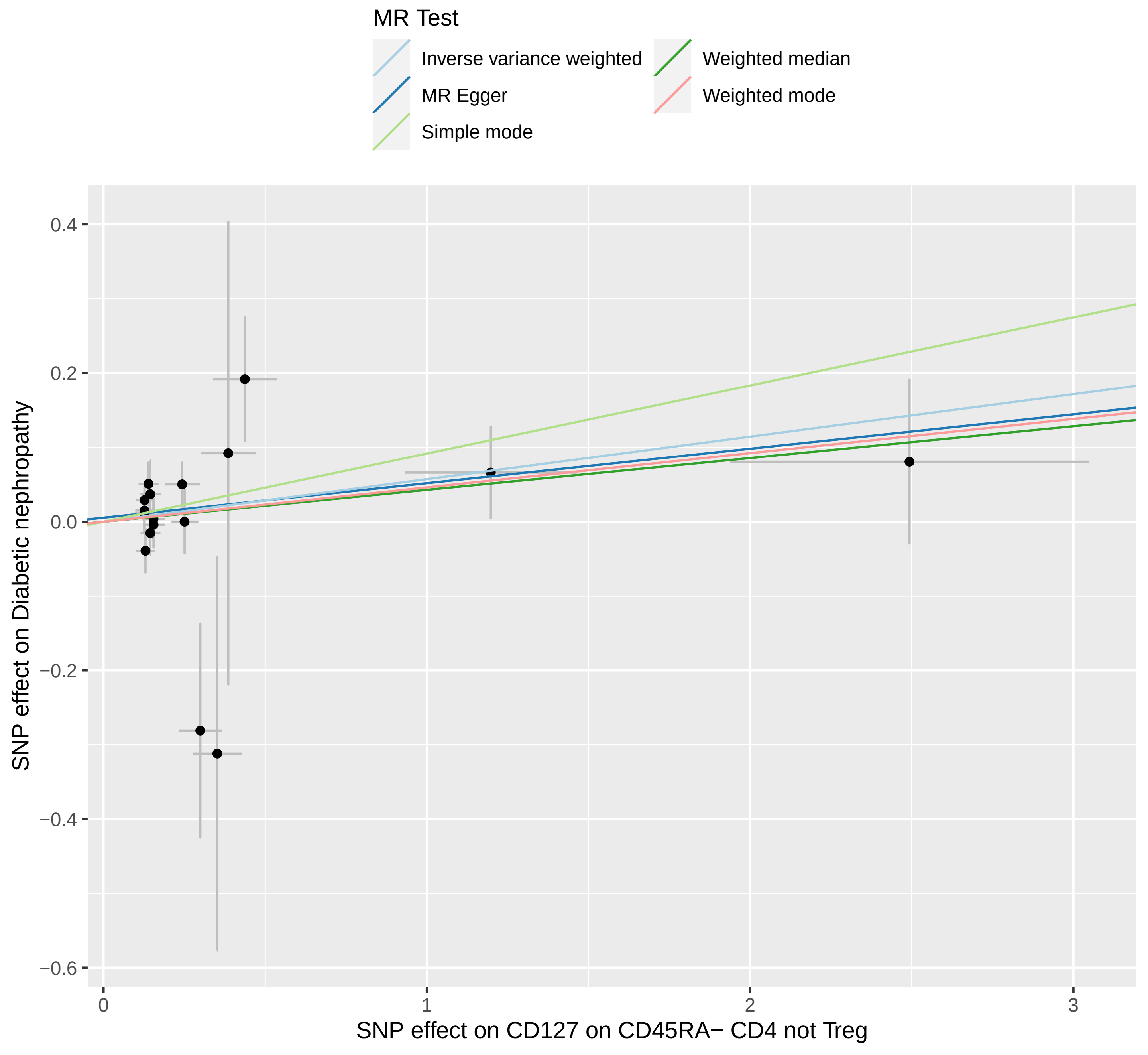

MR Test
Inverse variance weighted
MR Egger
Simple mode
Weighted median
Weighted mode
SNP effect on Diabetic nephropathy
0.4
0.2
0.0
−0.2
−0.4
−0.6
0
1
2
3
SNP effect on CD127 on CD45RA− CD4 not Treg

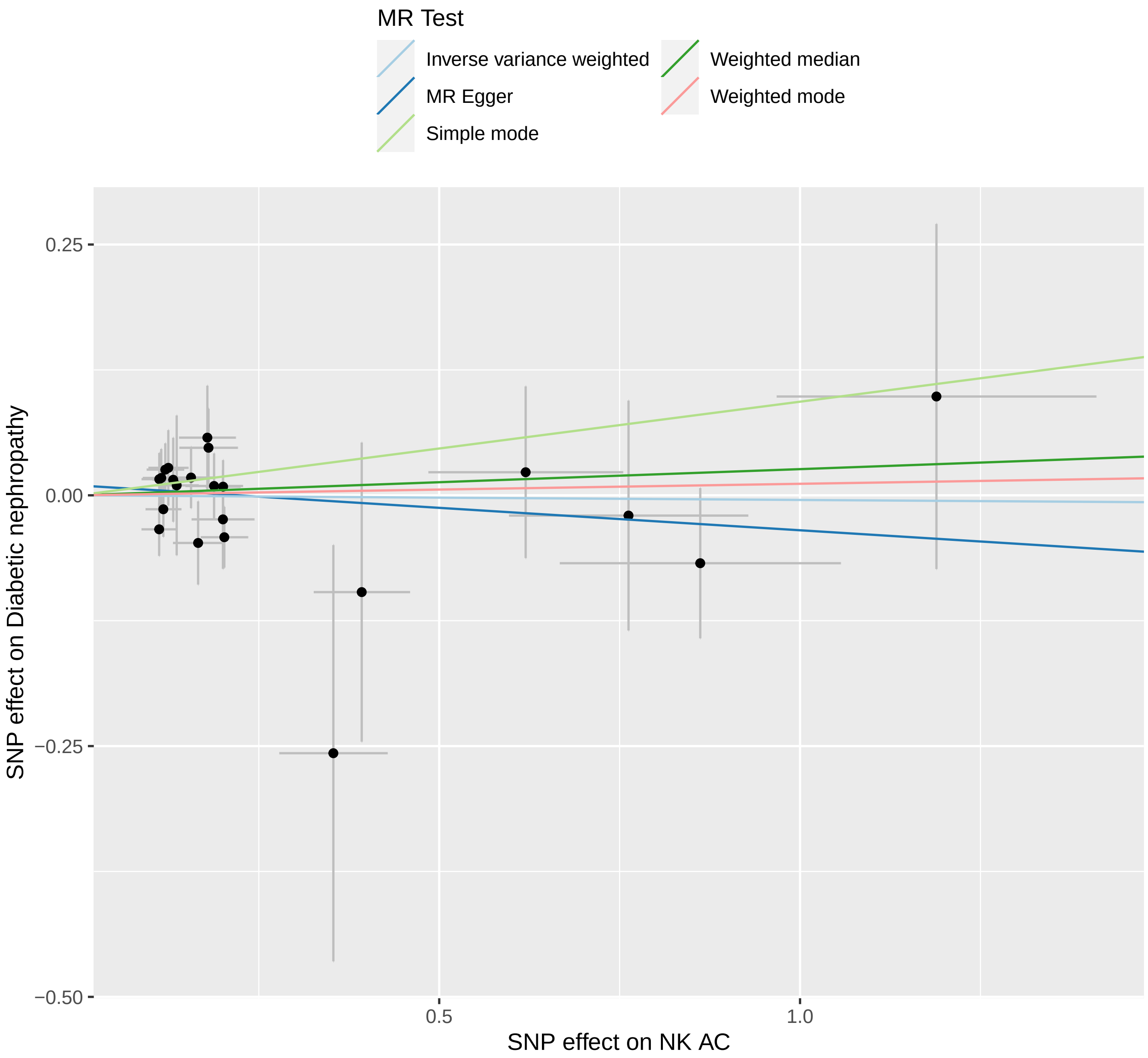

MR Test
Inverse variance weighted
MR Egger
Simple mode
Weighted median
Weighted mode
0.25
0.00
−0.25
−0.50
0.5
1.0
SNP effect on Diabetic nephropathy
SNP effect on NK AC

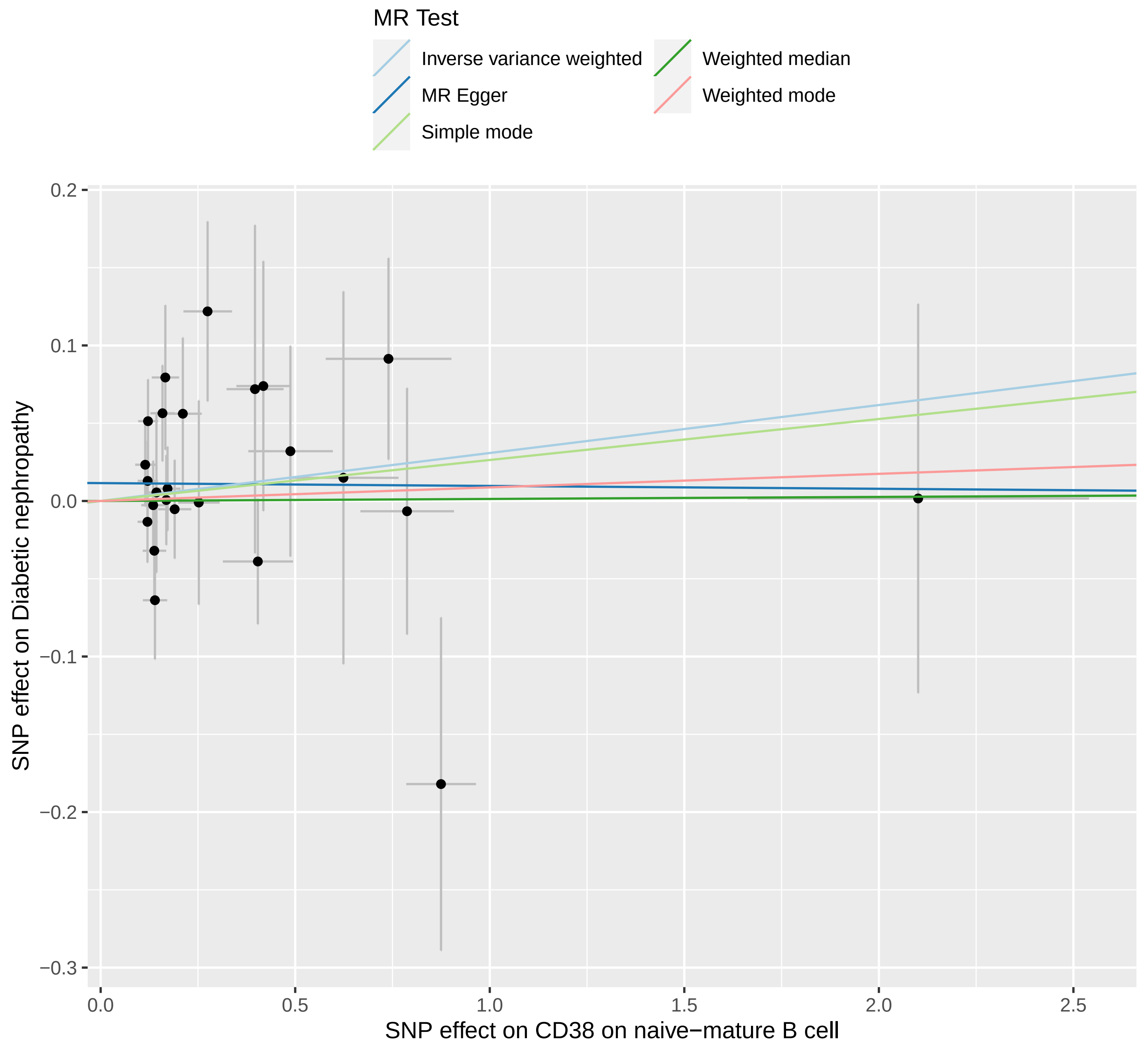
MR Test
Inverse variance weighted
MR Egger
Simple mode
Weighted median
Weighted mode
0.2
0.1
0.0
−0.1
−0.2
−0.3
SNP effect on Diabetic nephropathy
0.0
0.5
1.0
1.5
2.0
2.5
SNP effect on CD38 on naive−mature B cell

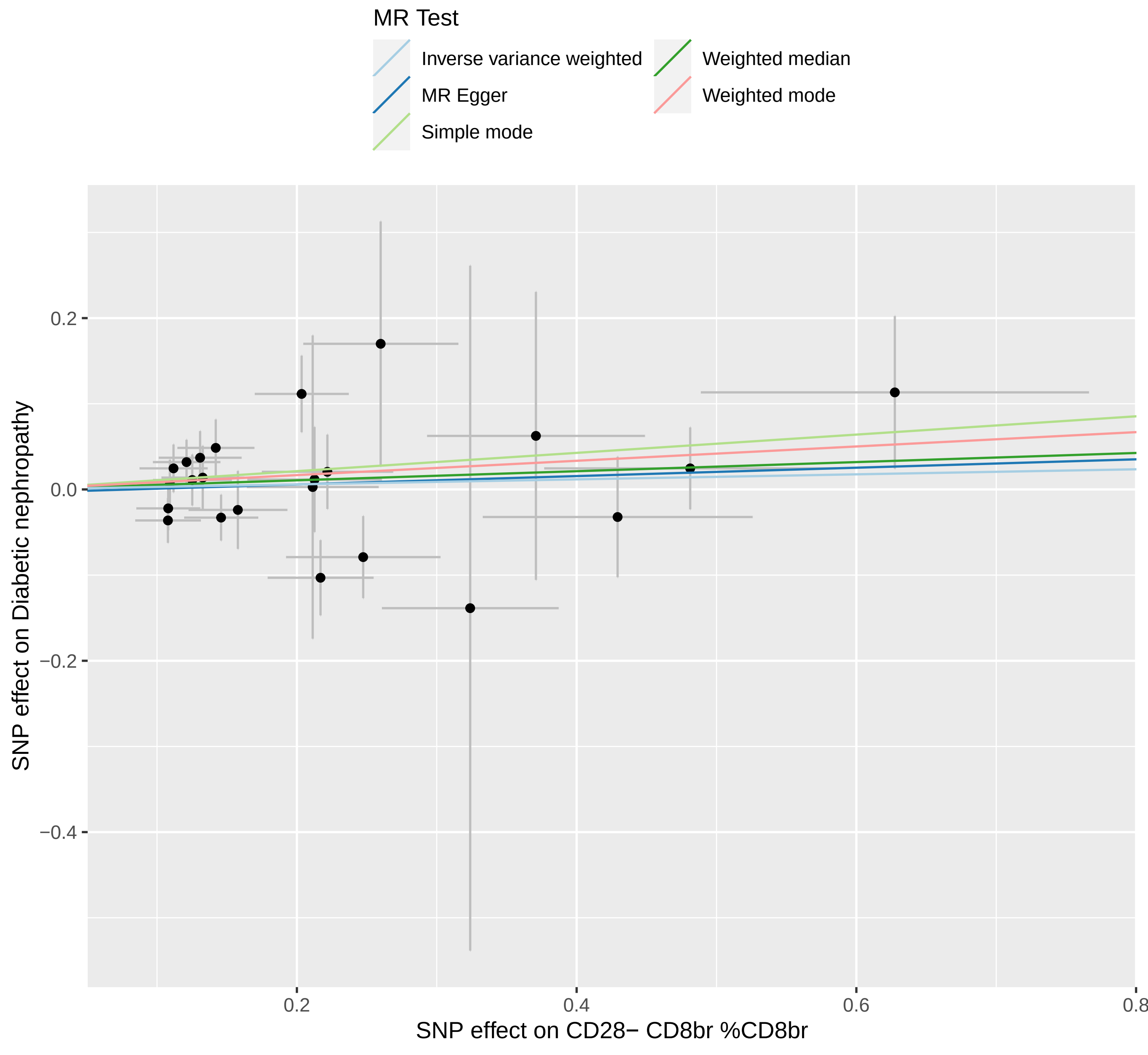
MR Test
Inverse variance weighted
MR Egger
Simple mode
Weighted median
Weighted mode
0.2
0.0
−0.2
−0.4
SNP effect on Diabetic nephropathy
0.2
0.4
0.6
0.8
SNP effect on CD28− CD8br %CD8br

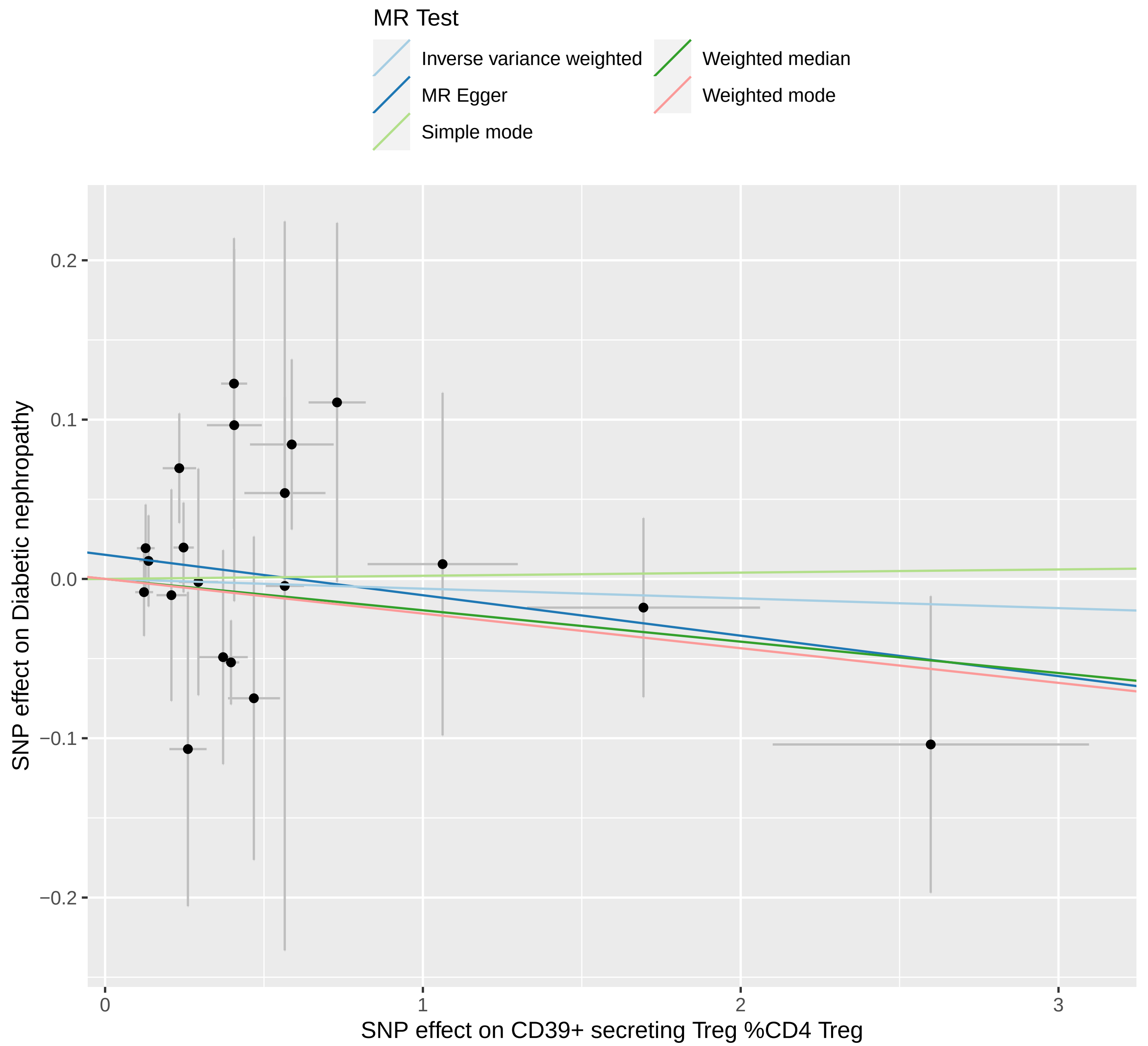
MR Test
Inverse variance weighted
MR Egger
Simple mode
Weighted median
Weighted mode
SNP effect on Diabetic nephropathy
0.2
0.1
0.0
−0.1
−0.2
0
1
2
3
SNP effect on CD39+ secreting Treg %CD4 Treg

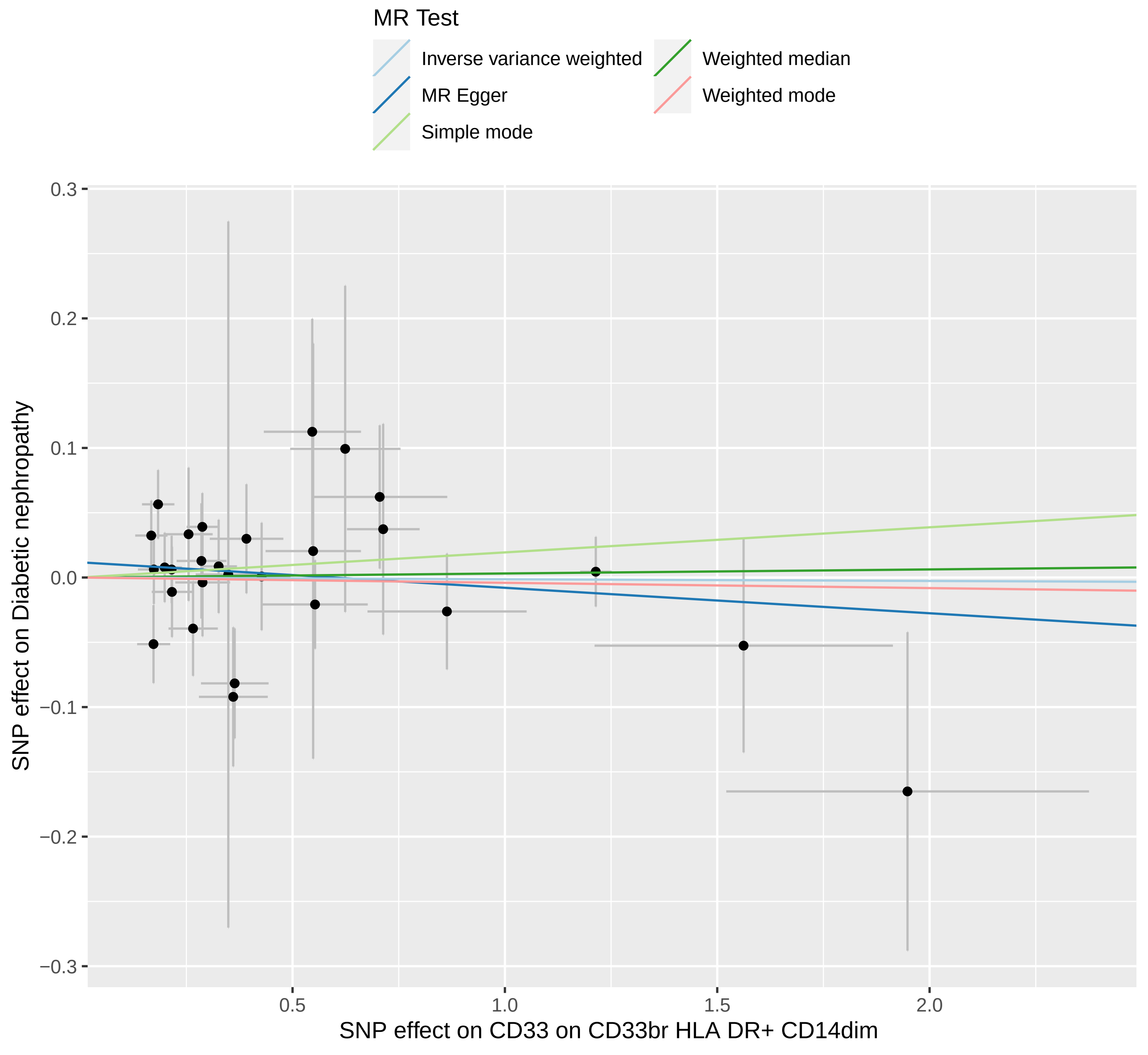

MR Test
Inverse variance weighted
MR Egger
Simple mode
Weighted median
Weighted mode
0.3
0.2
0.1
0.0
−0.1
−0.2
−0.3
SNP effect on Diabetic nephropathy
0.5
1.0
1.5
2.0
SNP effect on CD33 on CD33br HLA DR+ CD14dim

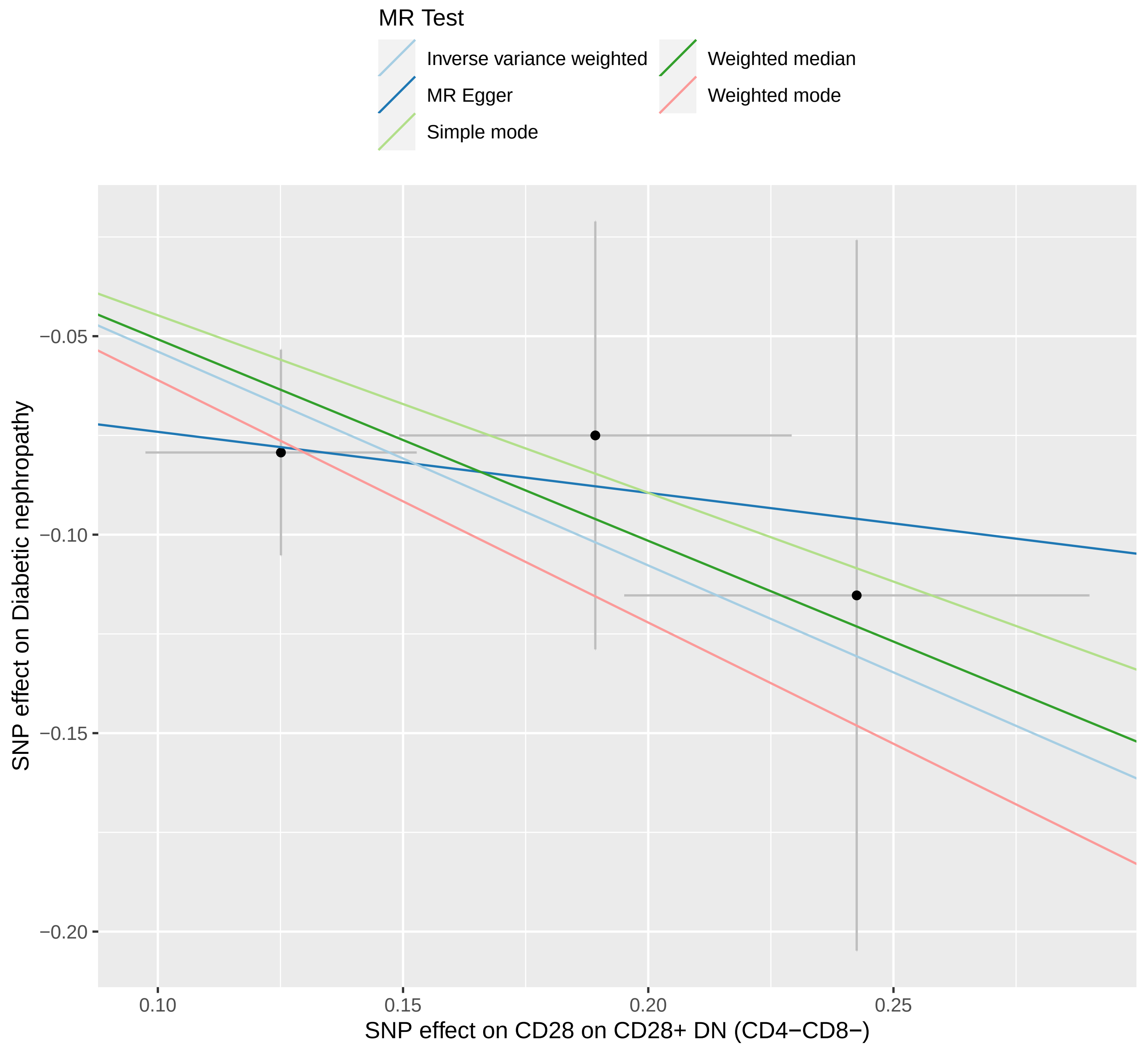

MR Test
Inverse variance weighted
MR Egger
Simple mode
Weighted median
Weighted mode
−0.05
−0.10
−0.15
−0.20
0.10
0.15
0.20
0.25
SNP effect on Diabetic nephropathy
SNP effect on CD28 on CD28+ DN (CD4−CD8−)

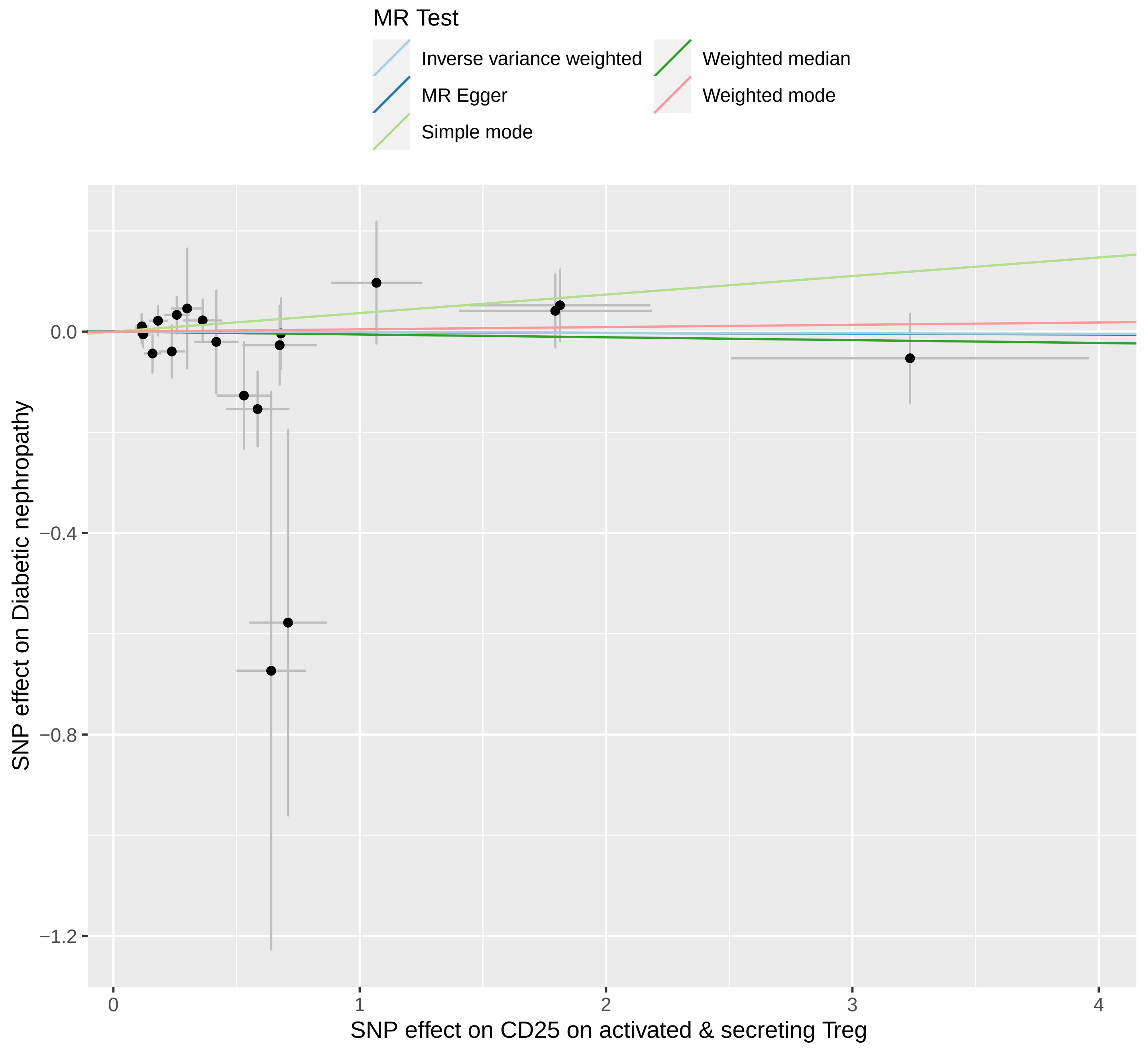
MR Test
Inverse variance weighted
MR Egger
Simple mode
Weighted median
Weighted mode
SNP effect on Diabetic nephropathy
0.0
−0.4
−0.8
−1.2
0
1
2
3
4
SNP effect on CD25 on activated & secreting Treg

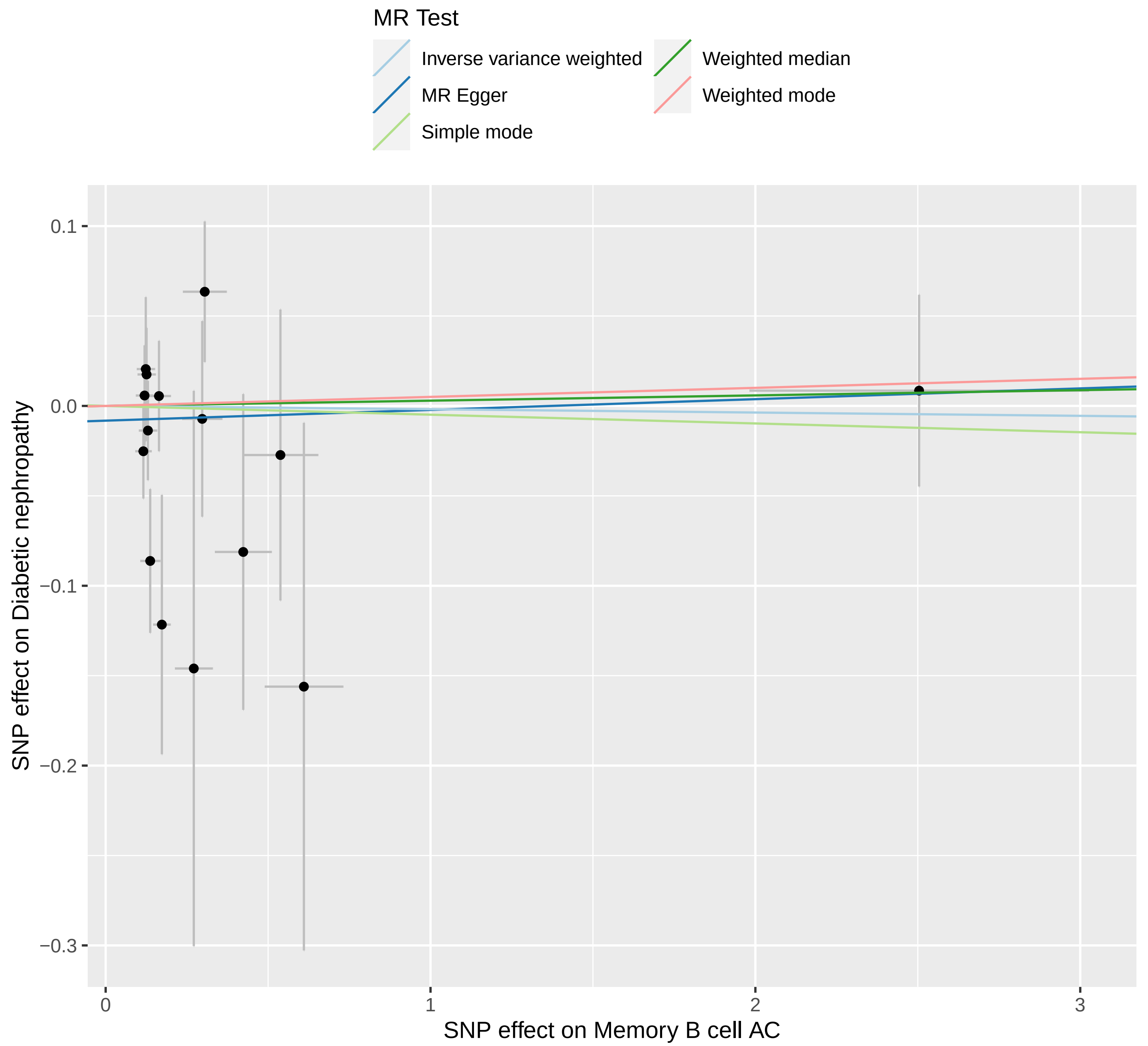
MR Test
Inverse variance weighted
MR Egger
Simple mode
Weighted median
Weighted mode
SNP effect on Diabetic nephropathy
0.1
0.0
−0.1
−0.2
−0.3
0
1
2
3
SNP effect on Memory B cell AC

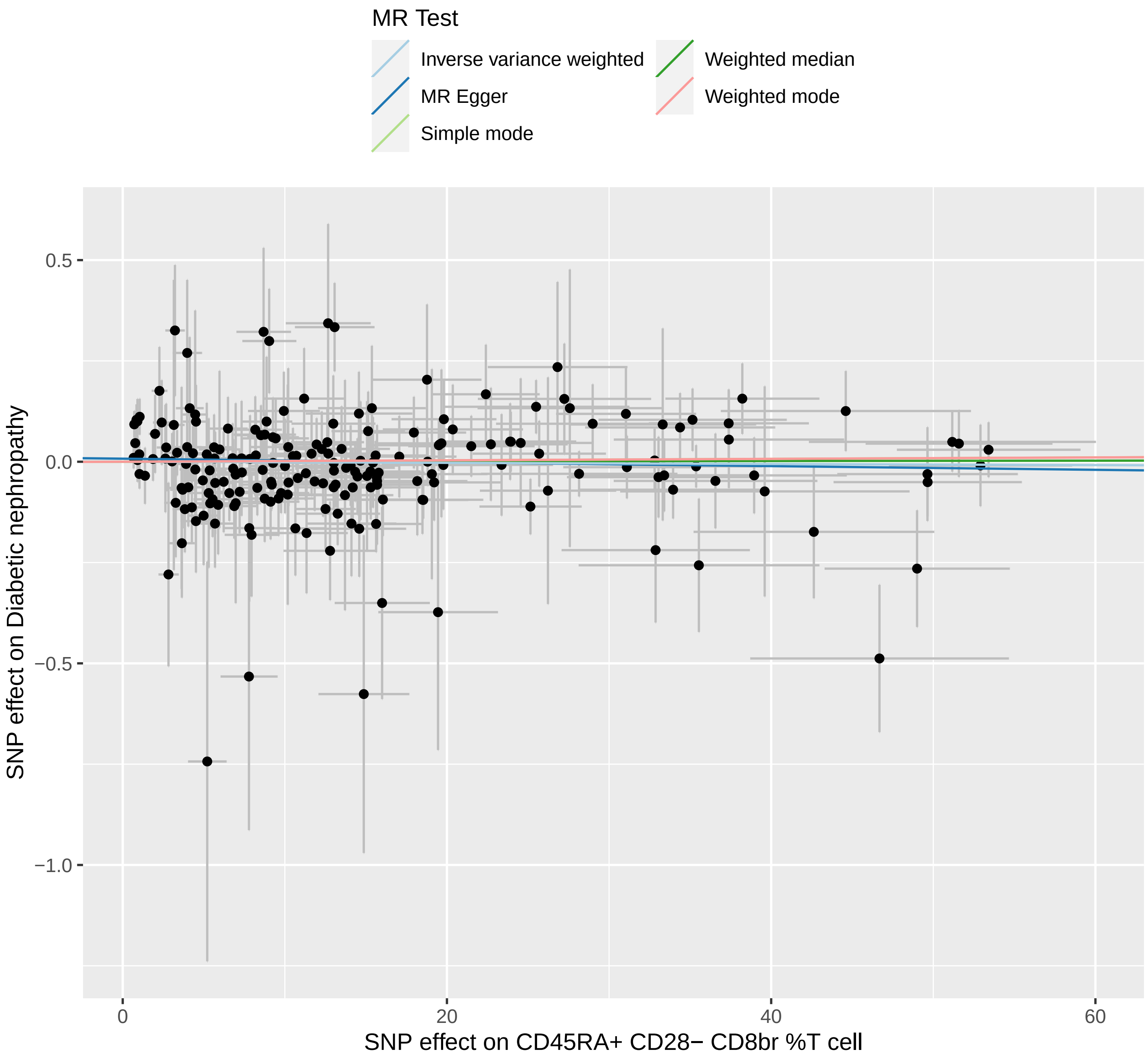
MR Test
Inverse variance weighted
Weighted median
MR Egger
Weighted mode
Simple mode
0.5
0.0
−0.5
−1.0
SNP effect on Diabetic nephropathy
0
20
40
60
SNP effect on CD45RA+ CD28− CD8br %T cell

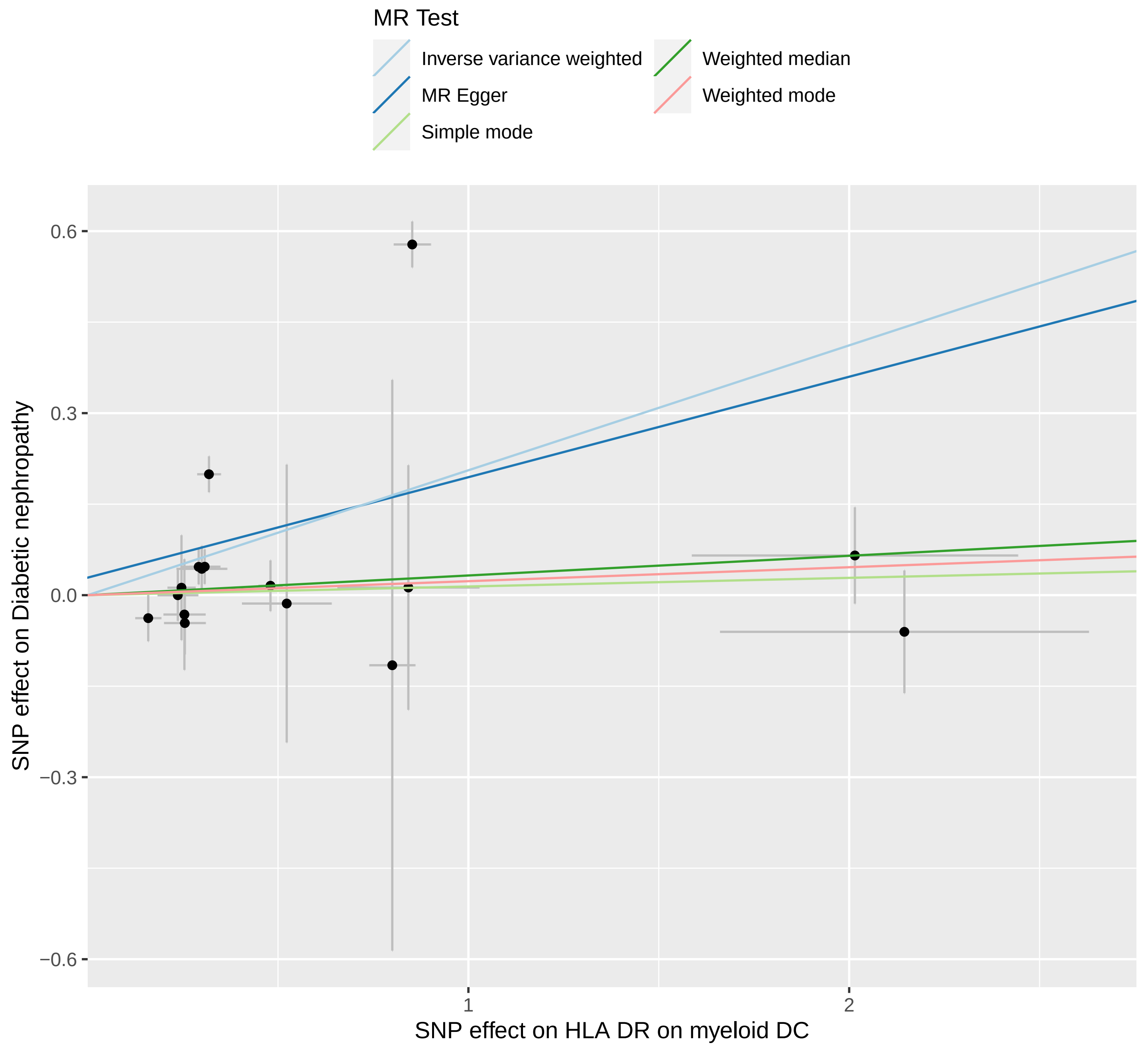
MR Test
Inverse variance weighted
MR Egger
Simple mode
Weighted median
Weighted mode
0.6
0.3
0.0
−0.3
−0.6
1
2
SNP effect on Diabetic nephropathy
SNP effect on HLA DR on myeloid DC

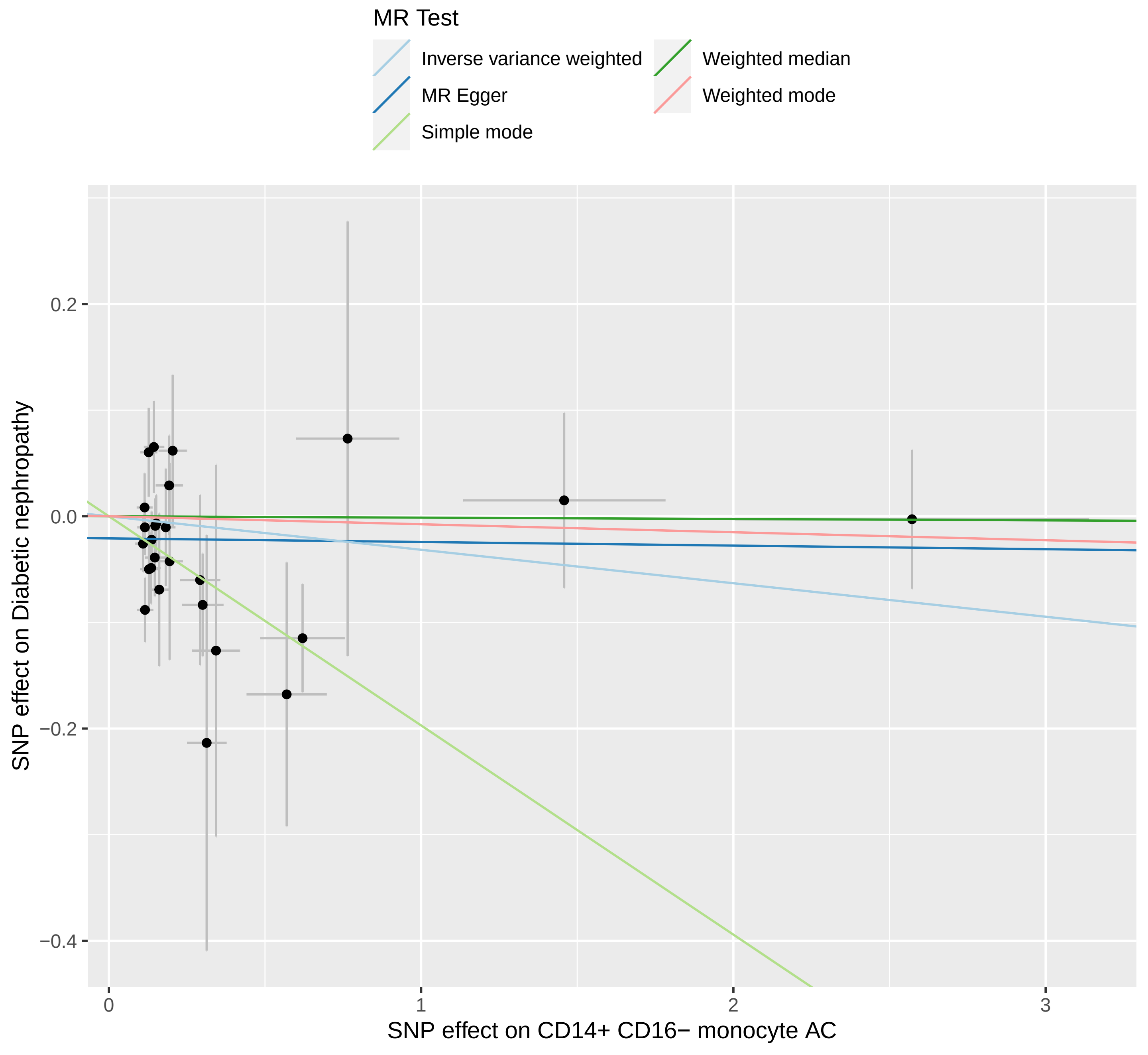

MR Test
Inverse variance weighted
MR Egger
Simple mode
Weighted median
Weighted mode
0.2
0.0
−0.2
−0.4
0
1
2
3
SNP effect on Diabetic nephropathy
SNP effect on CD14+ CD16− monocyte AC

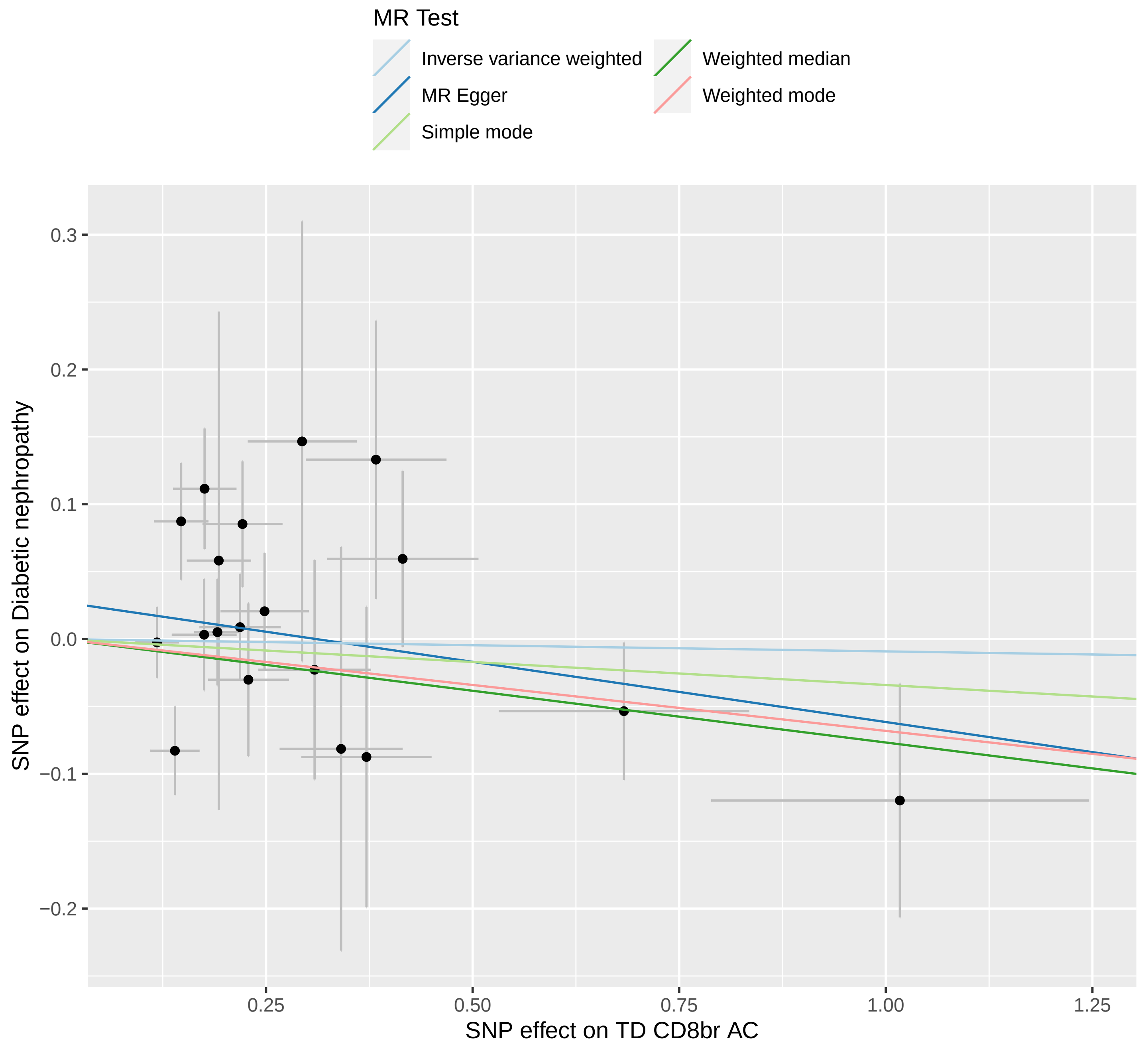

MR Test
Inverse variance weighted
MR Egger
Simple mode
Weighted median
Weighted mode
SNP effect on Diabetic nephropathy
0.3
0.2
0.1
0.0
−0.1
−0.2
0.25
0.50
0.75
1.00
1.25
SNP effect on TD CD8br AC

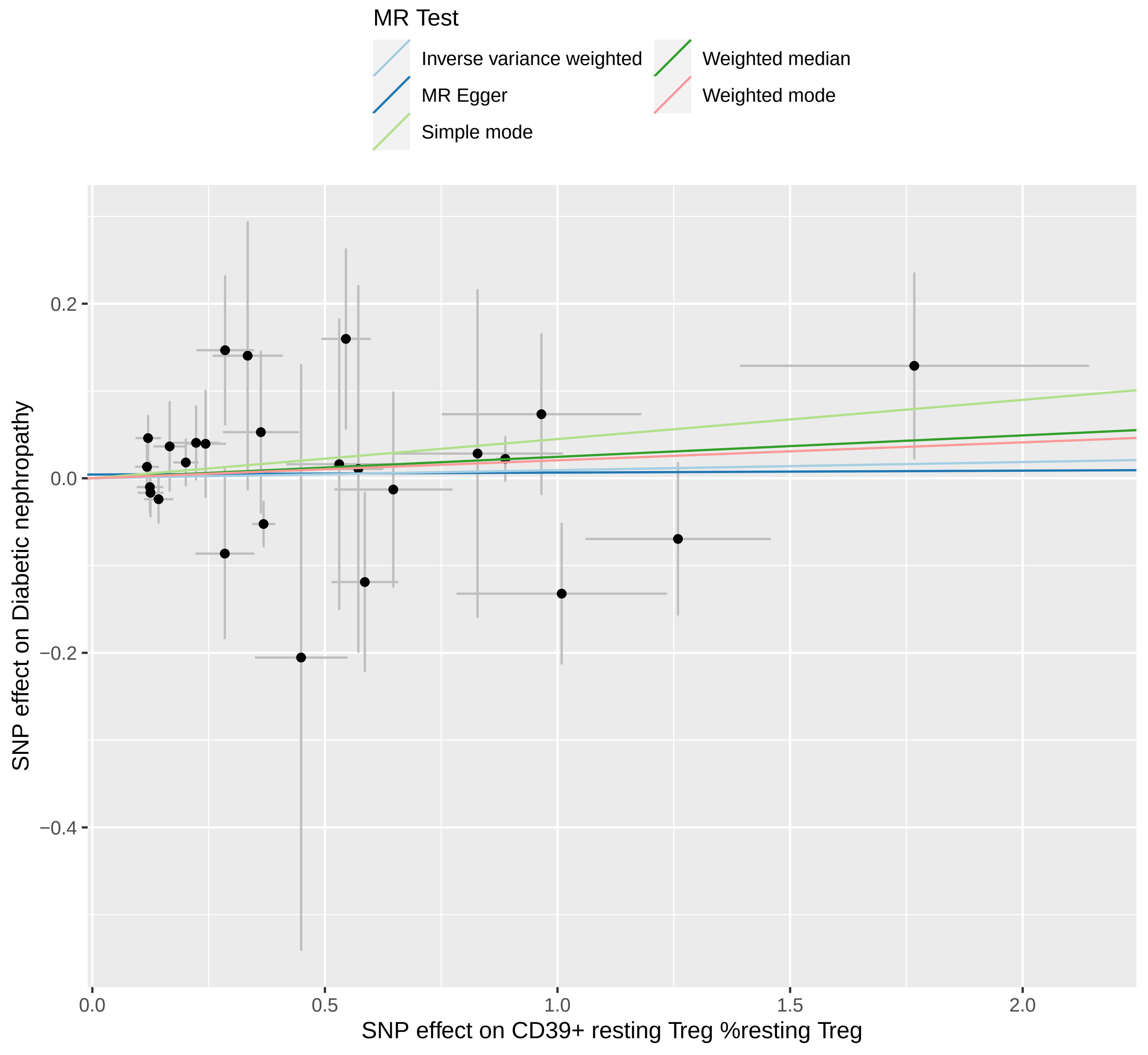
MR Test
Inverse variance weighted
MR Egger
Simple mode
Weighted median
Weighted mode
SNP effect on Diabetic nephropathy
SNP effect on CD39+ resting Treg %resting Treg
0.2
0.0
−0.2
−0.4
0.0
0.5
1.0
1.5
2.0

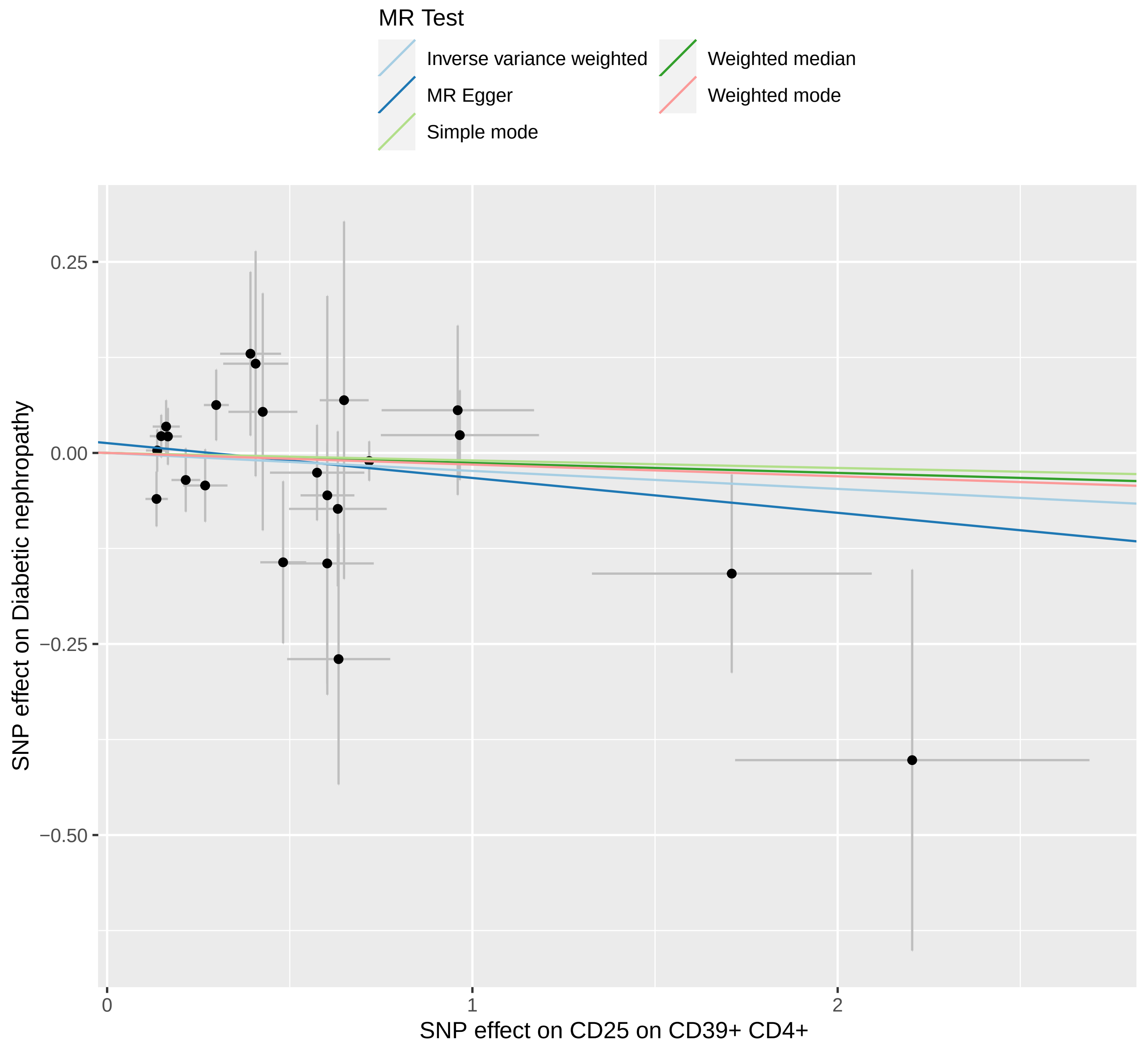
MR Test
Inverse variance weighted
MR Egger
Simple mode
Weighted median
Weighted mode
0.25
0.00
−0.25
−0.50
0
1
2
SNP effect on Diabetic nephropathy
SNP effect on CD25 on CD39+ CD4+

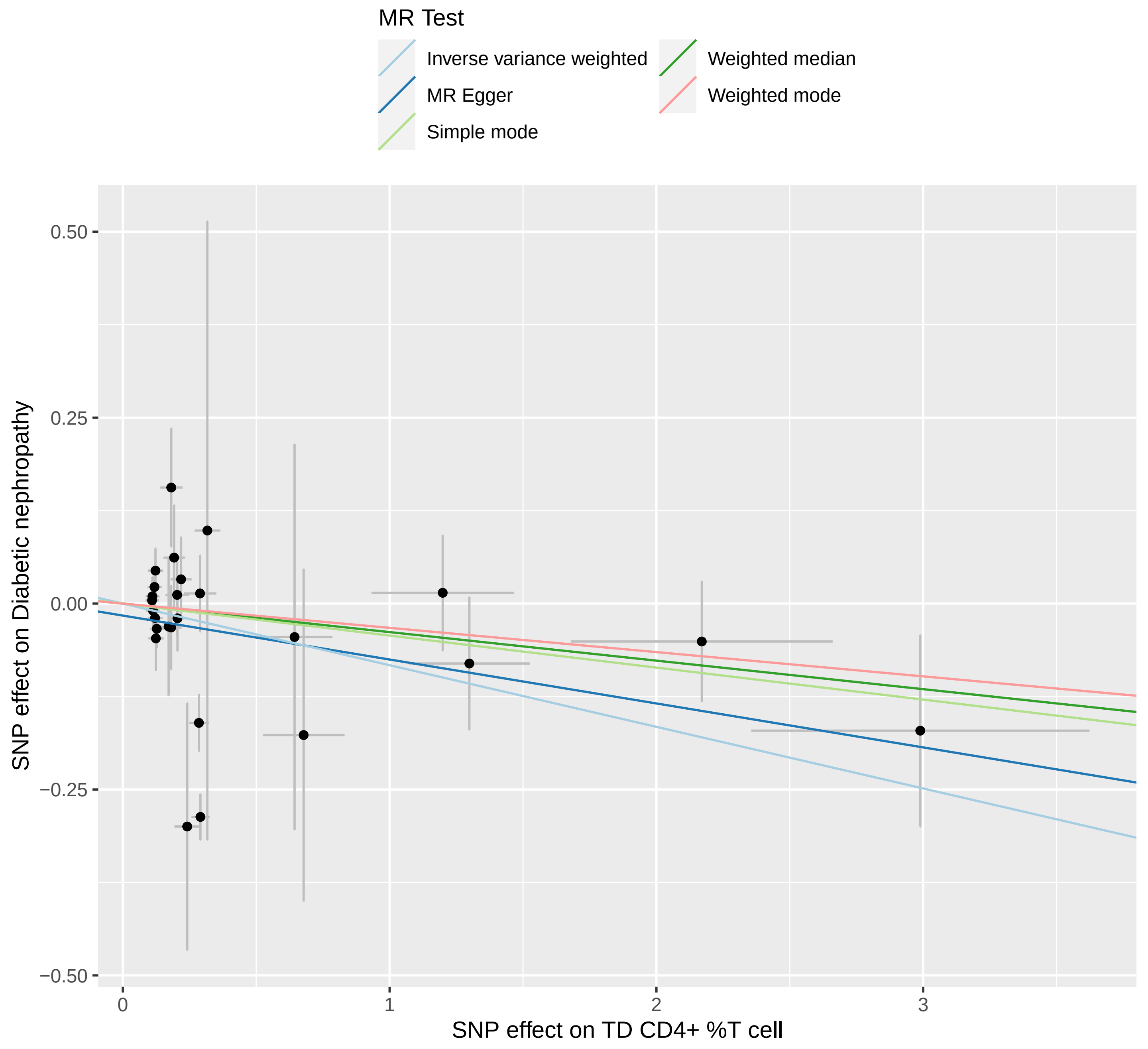

MR Test
Inverse variance weighted
MR Egger
Simple mode
Weighted median
Weighted mode
0.50
0.25
0.00
−0.25
−0.50
0
1
2
3
SNP effect on Diabetic nephropathy
SNP effect on TD CD4+ %T cell

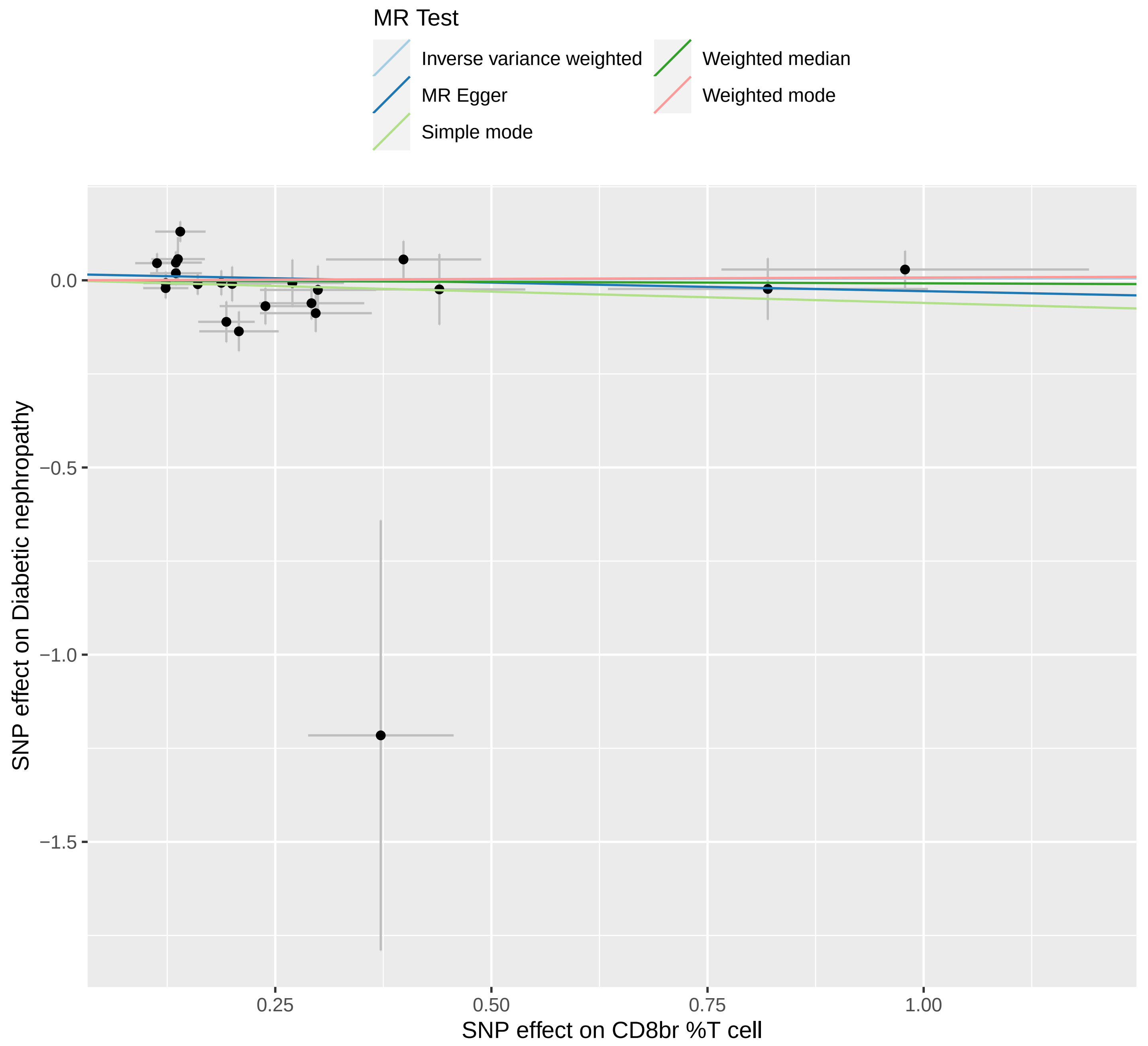
MR Test
Inverse variance weighted
MR Egger
Simple mode
Weighted median
Weighted mode
SNP effect on Diabetic nephropathy
0.0
−0.5
−1.0
−1.5
0.25
0.50
0.75
1.00
SNP effect on CD8br %T cell

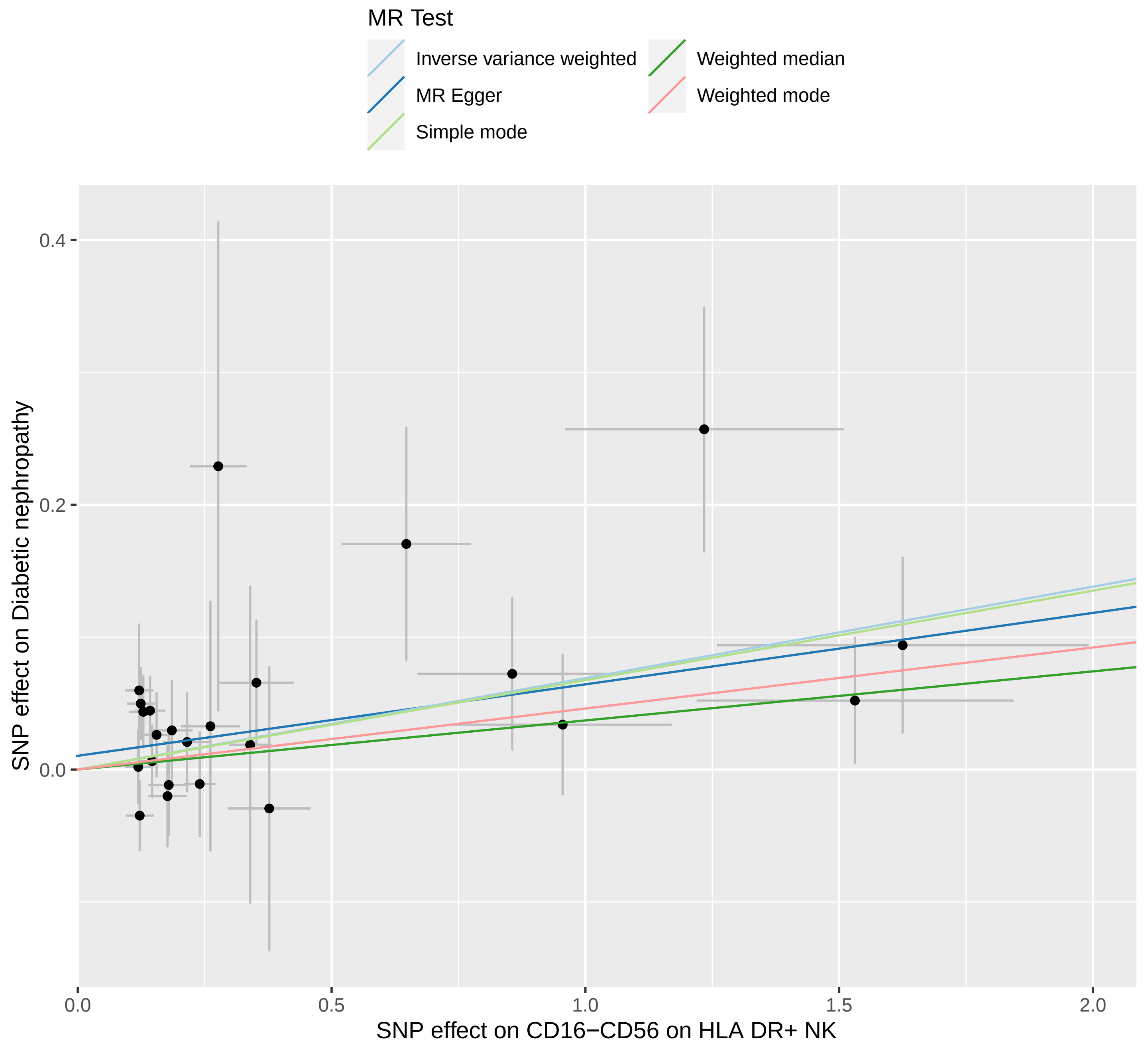
MR Test
Inverse variance weighted
MR Egger
Simple mode
Weighted median
Weighted mode
0.4
0.2
0.0
0.0
0.5
1.0
1.5
2.0
SNP effect on Diabetic nephropathy
SNP effect on CD16−CD56 on HLA DR+ NK

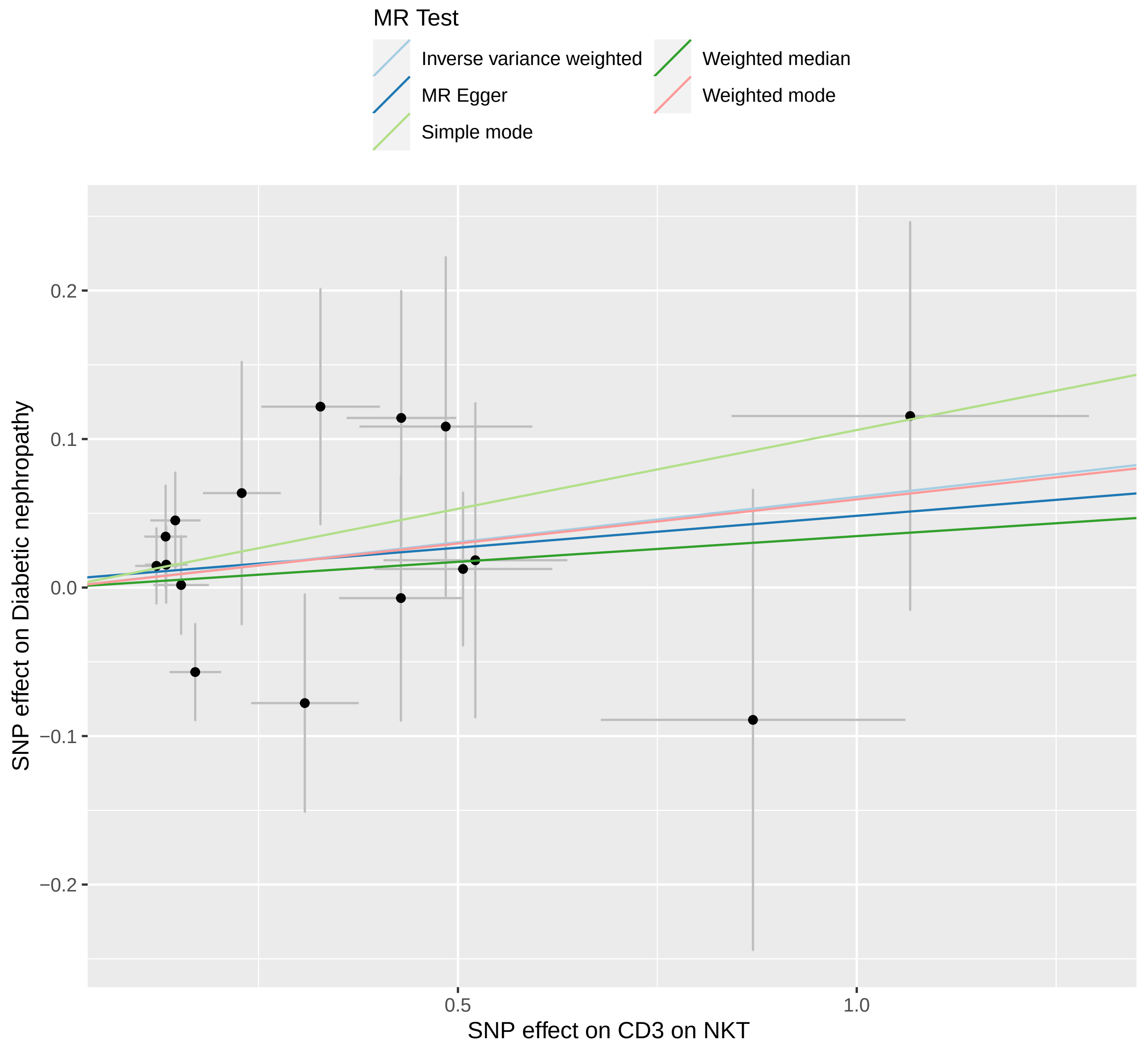

MR Test
Inverse variance weighted
MR Egger
Simple mode
Weighted median
Weighted mode
0.2
0.1
0.0
−0.1
−0.2
SNP effect on Diabetic nephropathy
0.5
1.0
SNP effect on CD3 on NKT

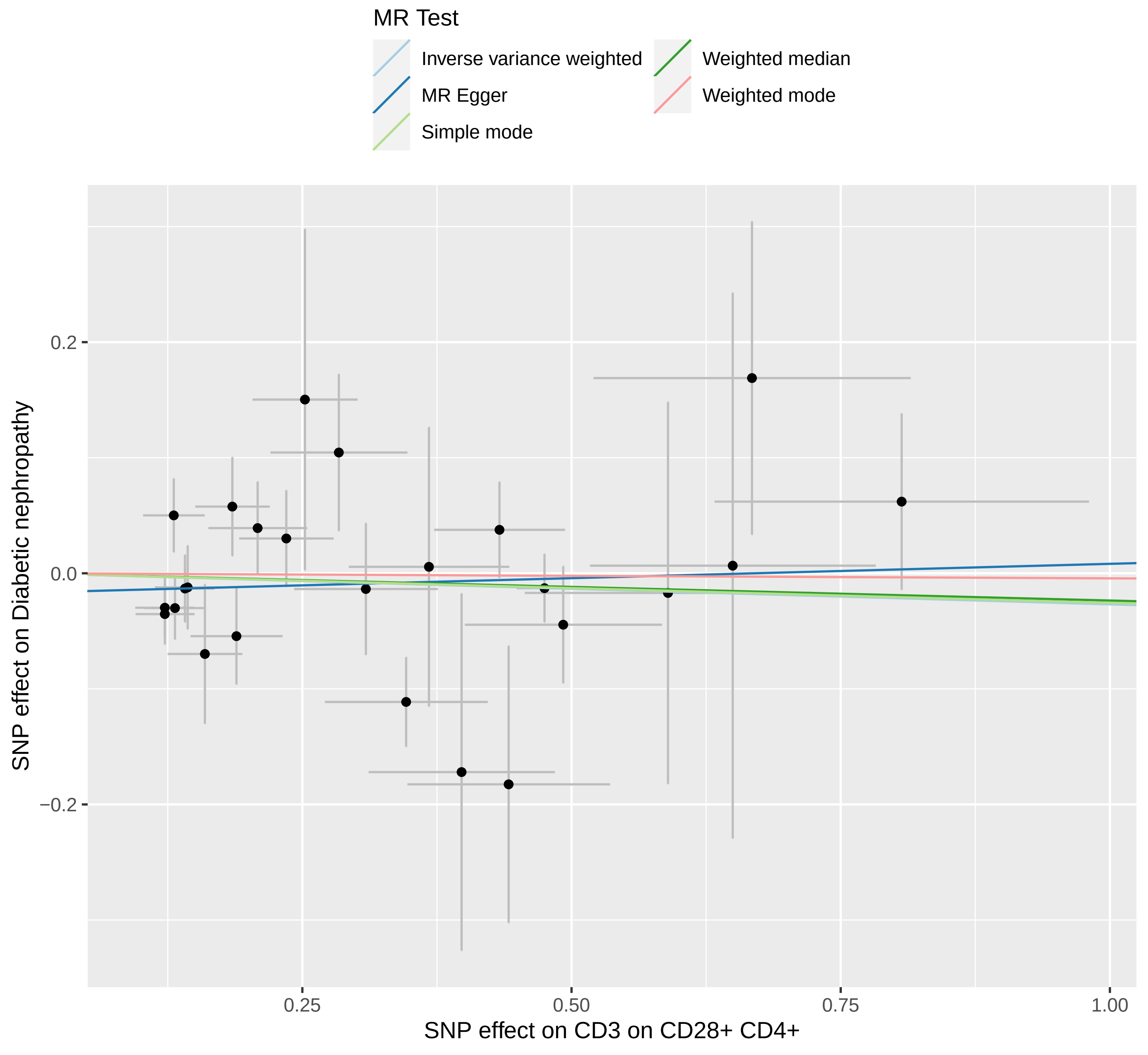
MR Test
Inverse variance weighted
MR Egger
Simple mode
Weighted median
Weighted mode
0.2
0.0
−0.2
0.25
0.50
0.75
1.00
SNP effect on Diabetic nephropathy
SNP effect on CD3 on CD28+ CD4+

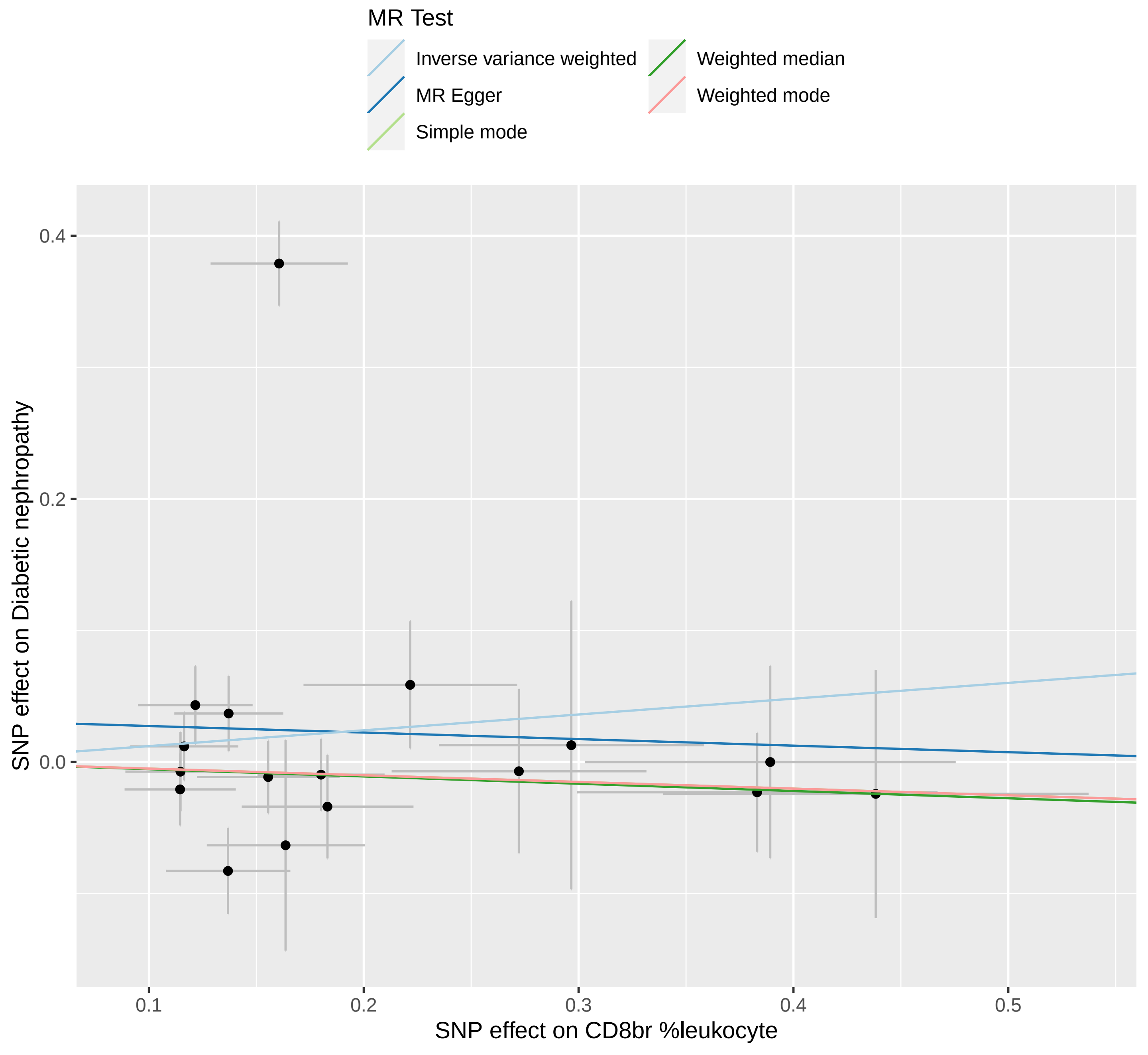

MR Test
Inverse variance weighted
MR Egger
Simple mode
Weighted median
Weighted mode
0.4
0.2
0.0
0.1
0.2
0.3
0.4
0.5
SNP effect on Diabetic nephropathy
SNP effect on CD8br %leukocyte

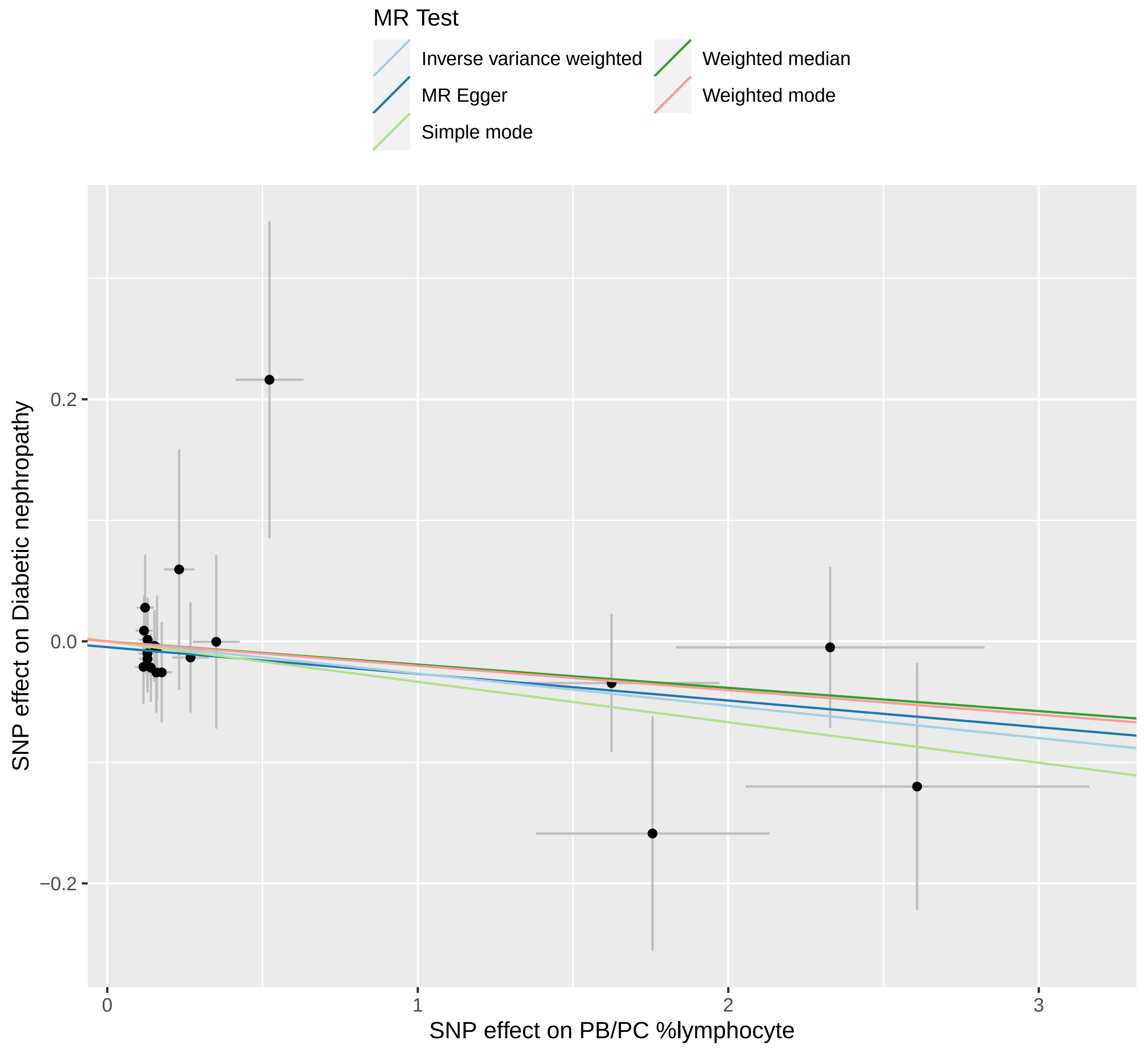
MR Test
Inverse variance weighted
MR Egger
Simple mode
Weighted median
Weighted mode
0.2
0.0
−0.2
0
1
2
3
SNP effect on Diabetic nephropathy
SNP effect on PB/PC %lymphocyte

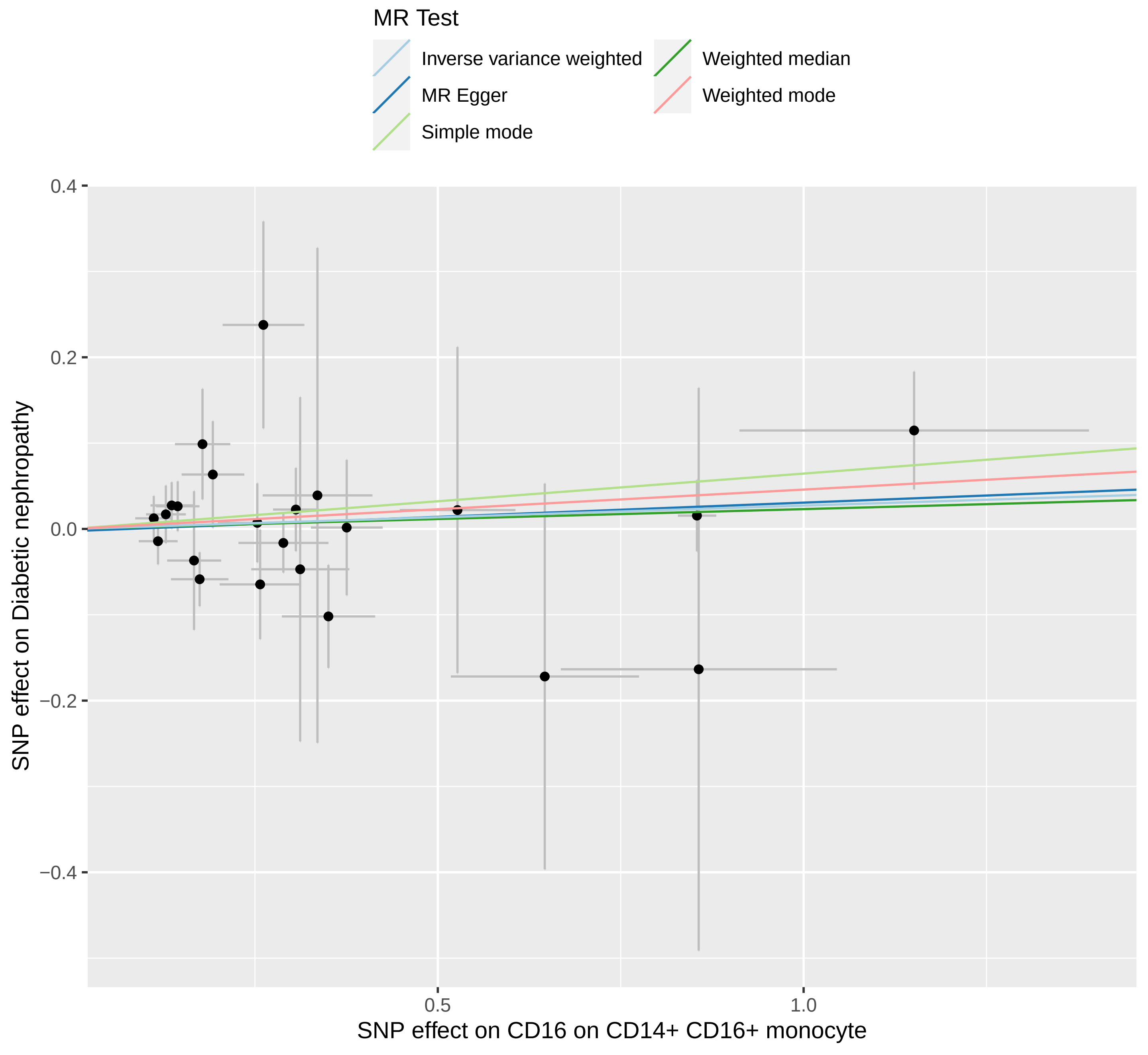

MR Test
Inverse variance weighted
MR Egger
Simple mode
Weighted median
Weighted mode
0.4
0.2
0.0
−0.2
−0.4
0.5
1.0
SNP effect on CD16 on CD14+ CD16+ monocyte
SNP effect on Diabetic nephropathy

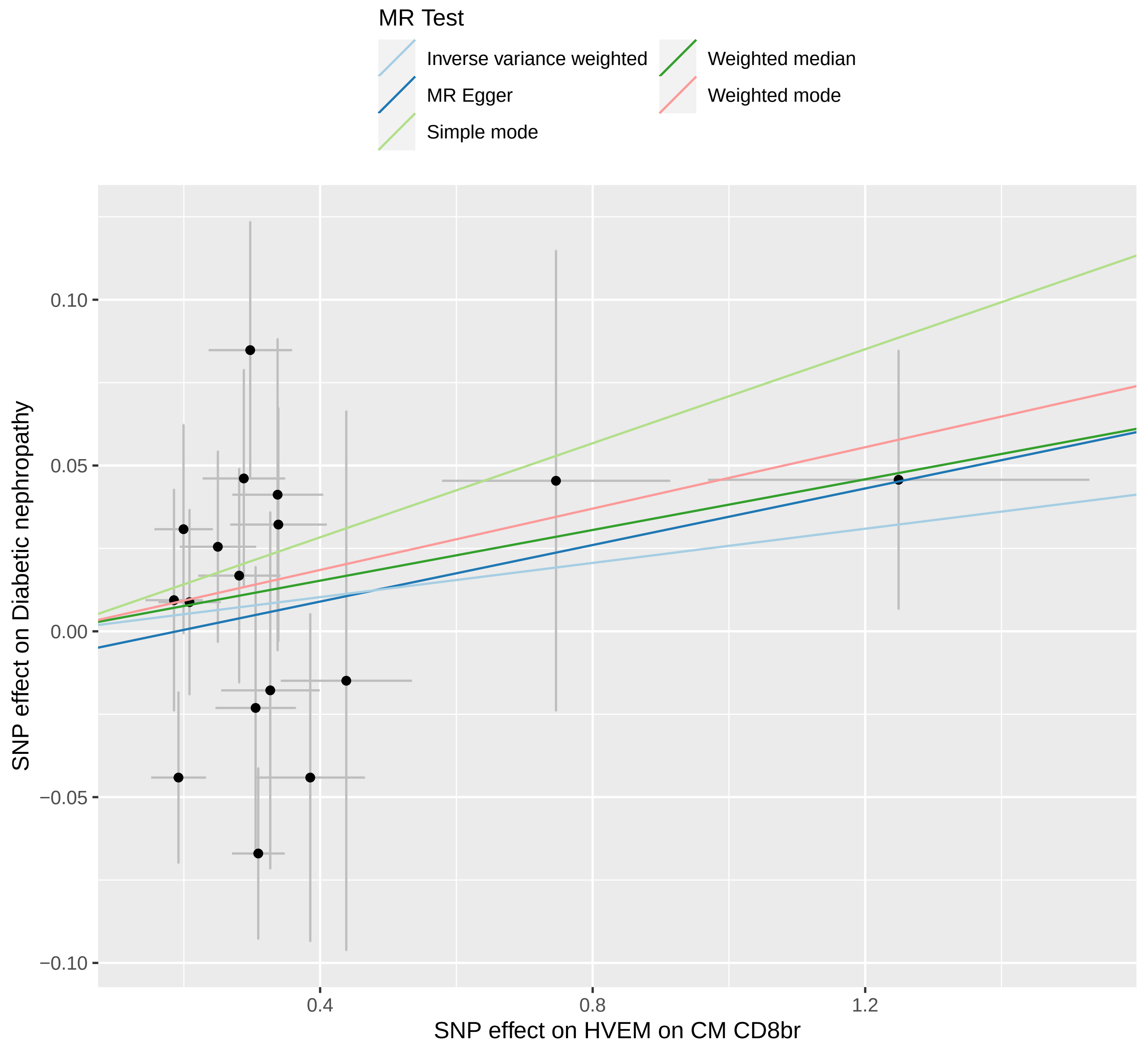
MR Test
Inverse variance weighted
MR Egger
Simple mode
Weighted median
Weighted mode
SNP effect on Diabetic nephropathy
0.10
0.05
0.00
−0.05
−0.10
0.4
0.8
1.2
SNP effect on HVEM on CM CD8br

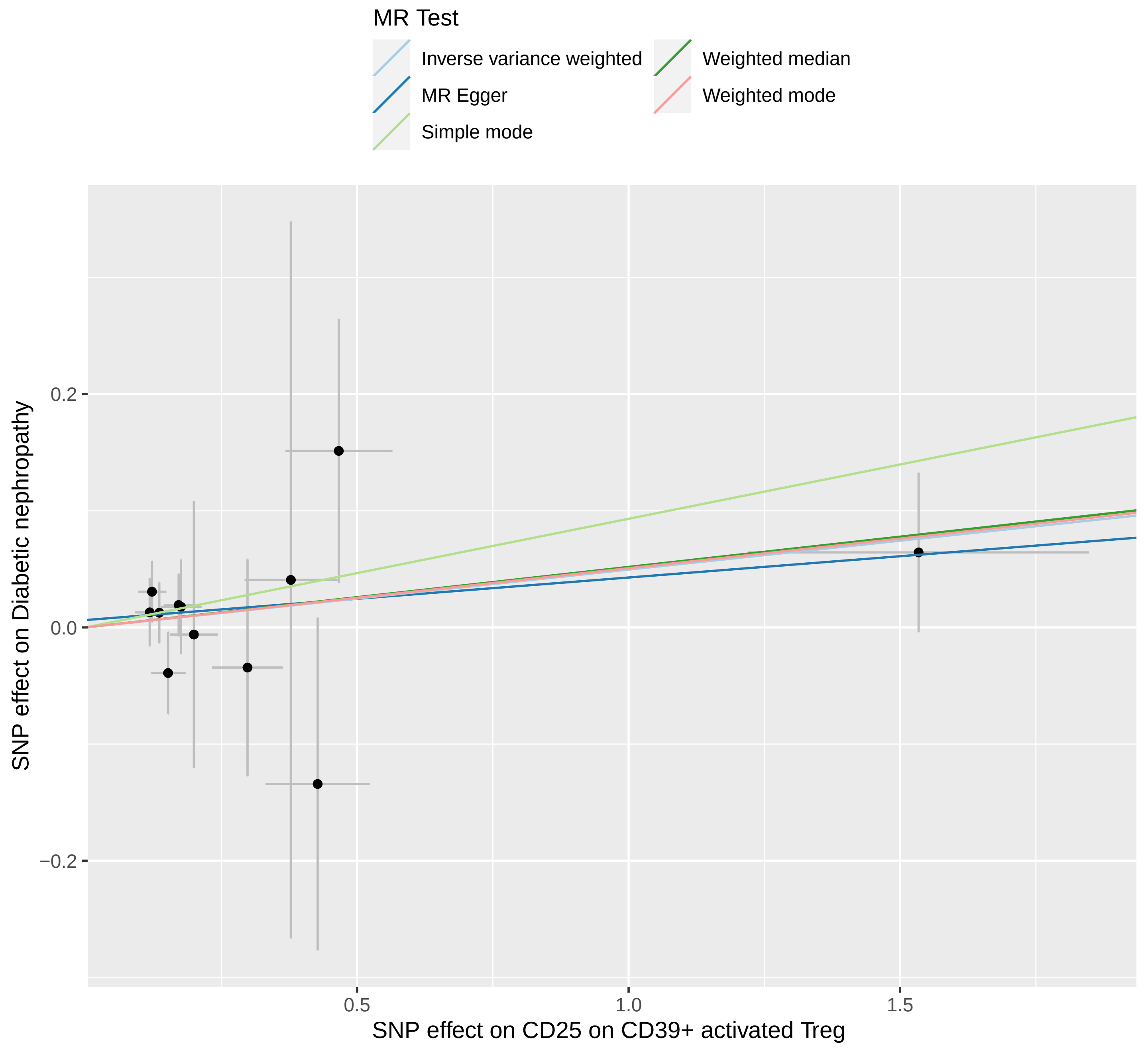
MR Test
Inverse variance weighted
MR Egger
Simple mode
Weighted median
Weighted mode
0.2
0.0
−0.2
0.5
1.0
1.5
SNP effect on Diabetic nephropathy
SNP effect on CD25 on CD39+ activated Treg

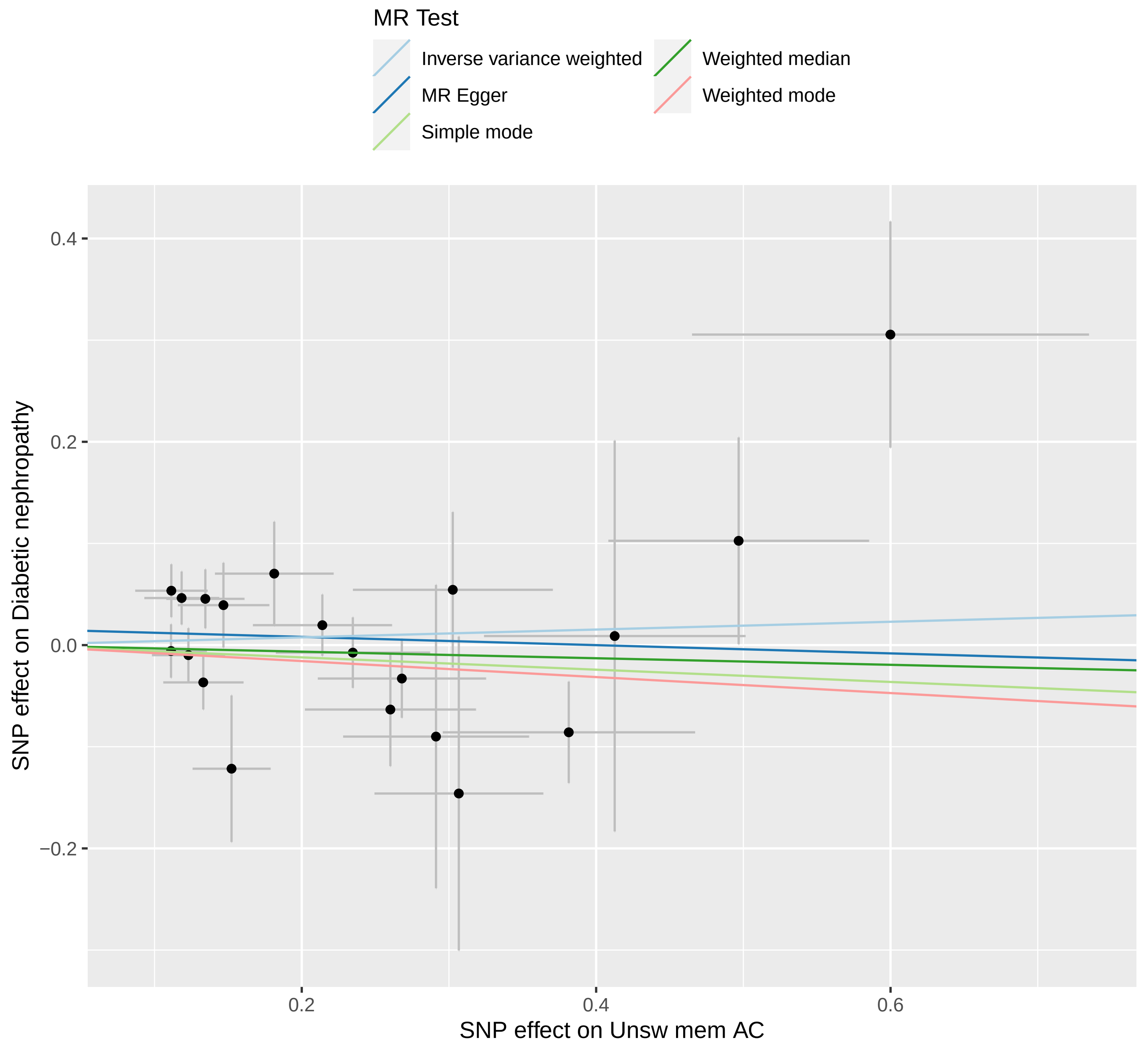
MR Test
Inverse variance weighted
MR Egger
Simple mode
Weighted median
Weighted mode
0.4
0.2
0.0
−0.2
0.2
0.4
0.6
SNP effect on Diabetic nephropathy
SNP effect on Unsw mem AC

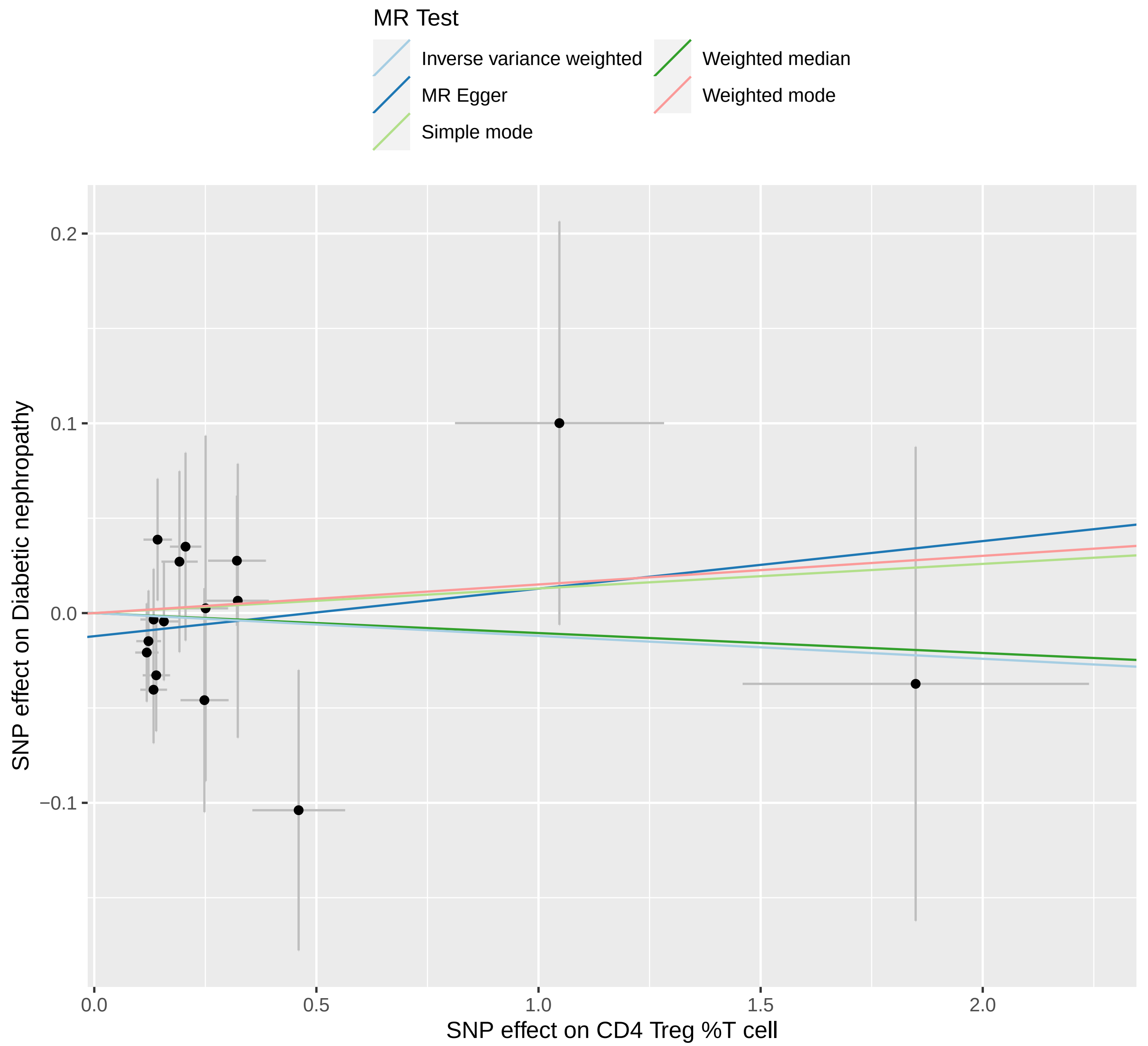

MR Test
Inverse variance weighted
MR Egger
Simple mode
Weighted median
Weighted mode
0.2
0.1
0.0
−0.1
0.0
0.5
1.0
1.5
2.0
SNP effect on Diabetic nephropathy
SNP effect on CD4 Treg %T cell

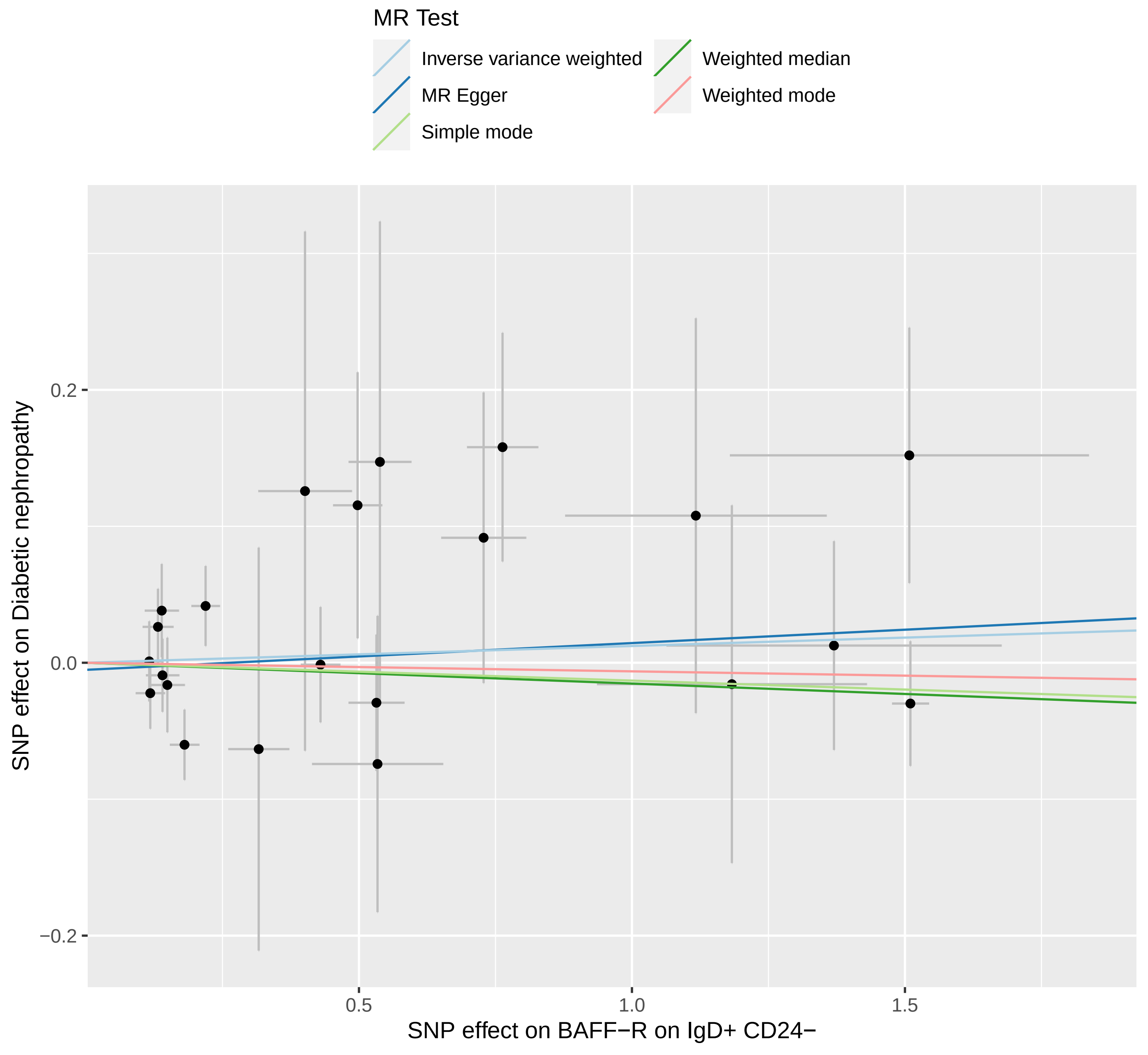

MR Test
Inverse variance weighted
MR Egger
Simple mode
Weighted median
Weighted mode
SNP effect on Diabetic nephropathy
0.2
0.0
−0.2
0.5
1.0
1.5
SNP effect on BAFF−R on IgD+ CD24−

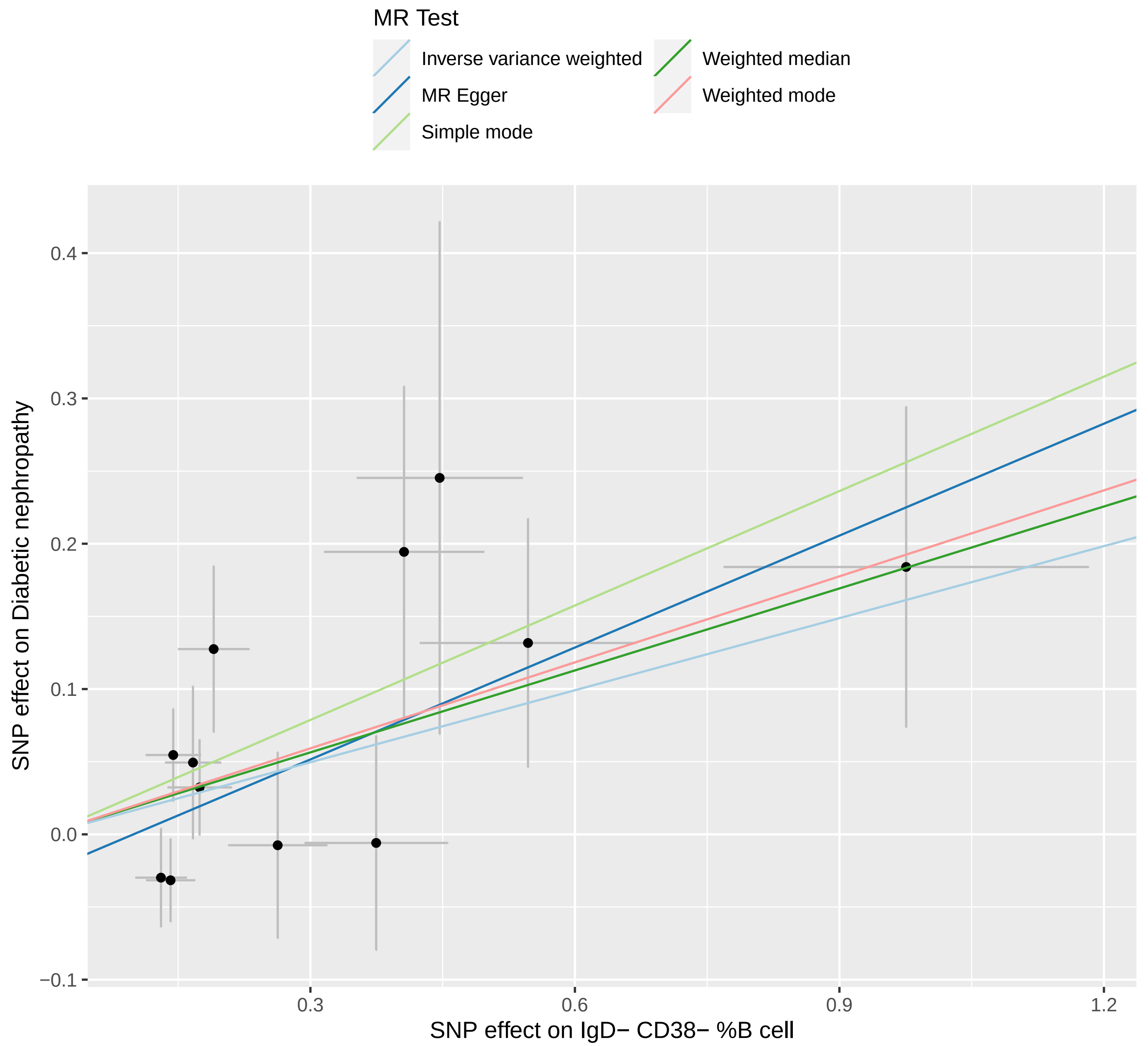
MR Test
Inverse variance weighted
MR Egger
Simple mode
Weighted median
Weighted mode
SNP effect on Diabetic nephropathy
SNP effect on IgD− CD38− %B cell
0.4
0.3
0.2
0.1
0.0
−0.1
0.3
0.6
0.9
1.2

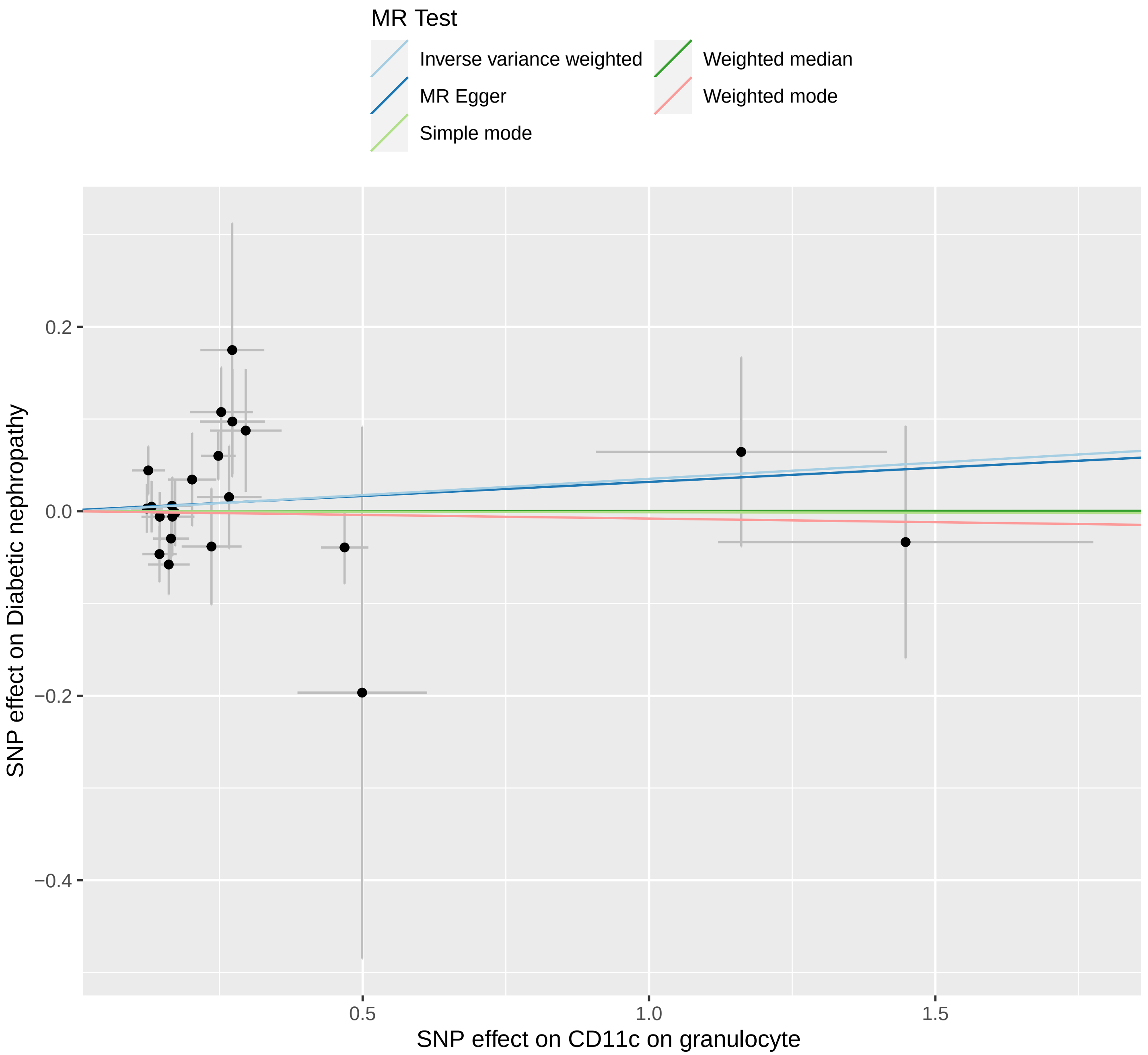
MR Test
Inverse variance weighted
MR Egger
Simple mode
Weighted median
Weighted mode
0.2
0.0
−0.2
−0.4
0.5
1.0
1.5
SNP effect on Diabetic nephropathy
SNP effect on CD11c on granulocyte

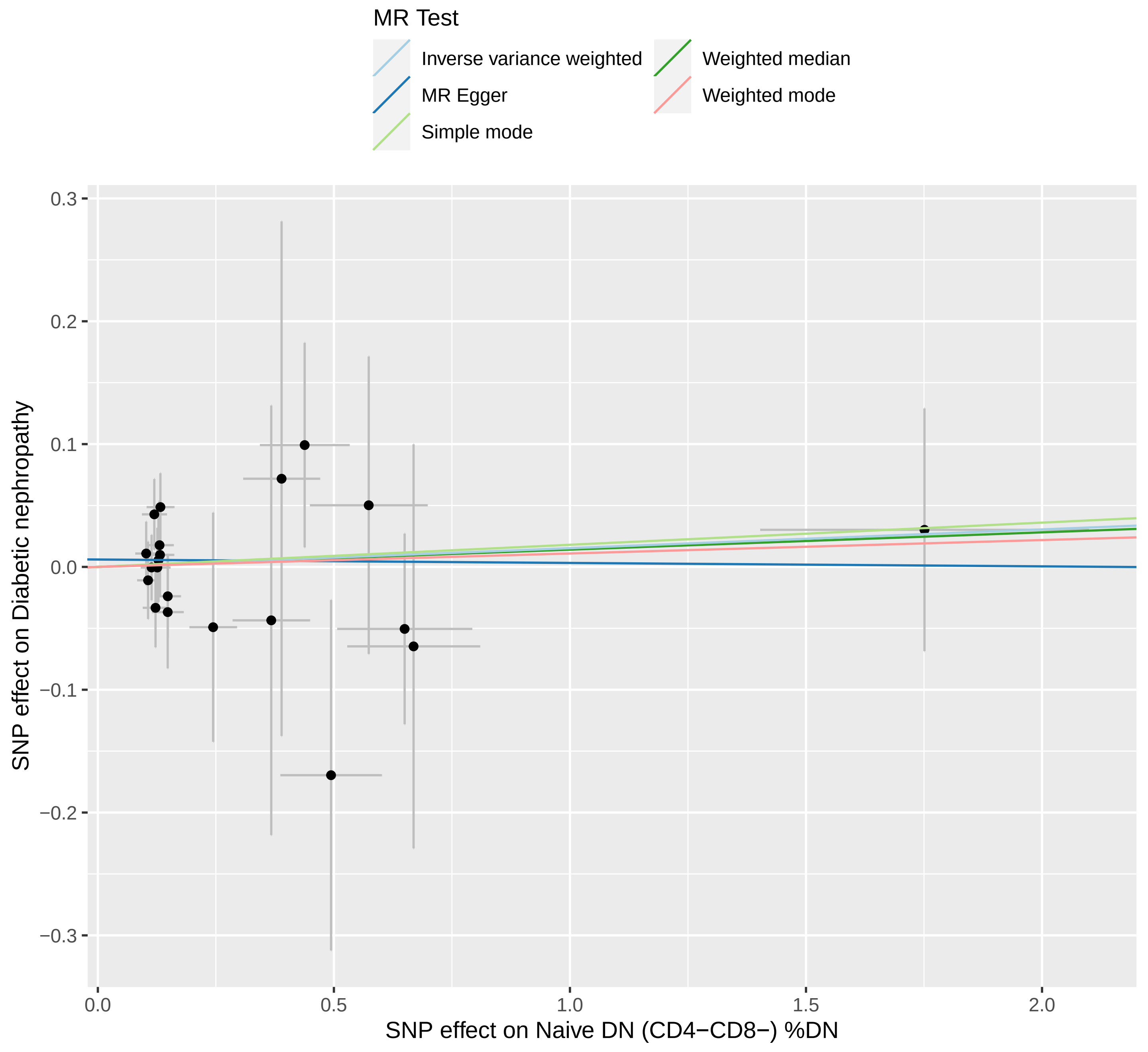

MR Test
Inverse variance weighted
MR Egger
Simple mode
Weighted median
Weighted mode
0.3
0.2
0.1
0.0
−0.1
−0.2
−0.3
SNP effect on Diabetic nephropathy
0.0
0.5
1.0
1.5
2.0
SNP effect on Naive DN (CD4−CD8−) %DN

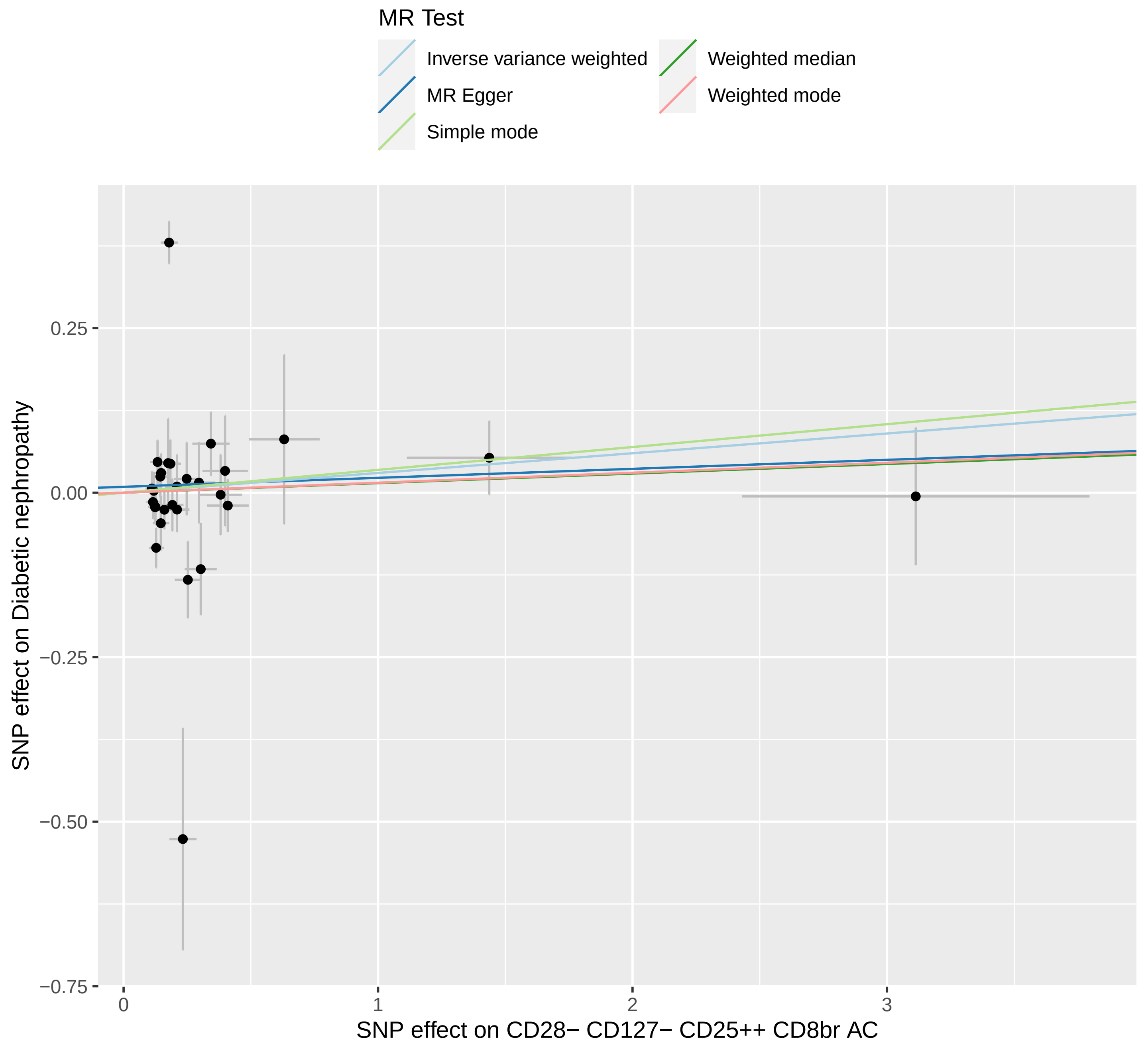

MR Test
Inverse variance weighted
MR Egger
Simple mode
Weighted median
Weighted mode
0.25
0.00
−0.25
−0.50
−0.75
0
1
2
3
SNP effect on Diabetic nephropathy
SNP effect on CD28− CD127− CD25++ CD8br AC

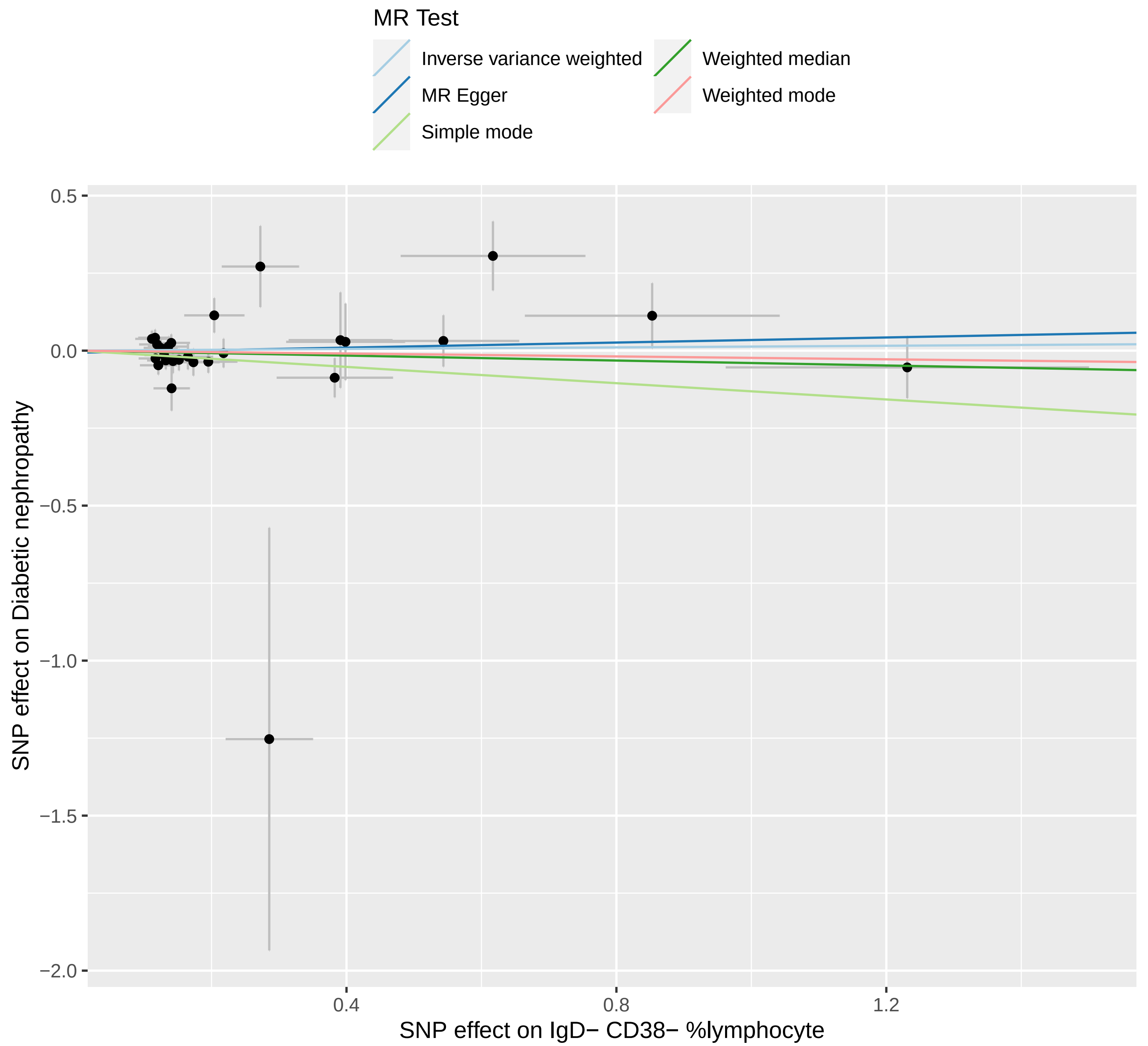
MR Test
Inverse variance weighted
MR Egger
Simple mode
Weighted median
Weighted mode
0.5
0.0
−0.5
−1.0
−1.5
−2.0
0.4
0.8
1.2
SNP effect on Diabetic nephropathy
SNP effect on IgD− CD38− %lymphocyte

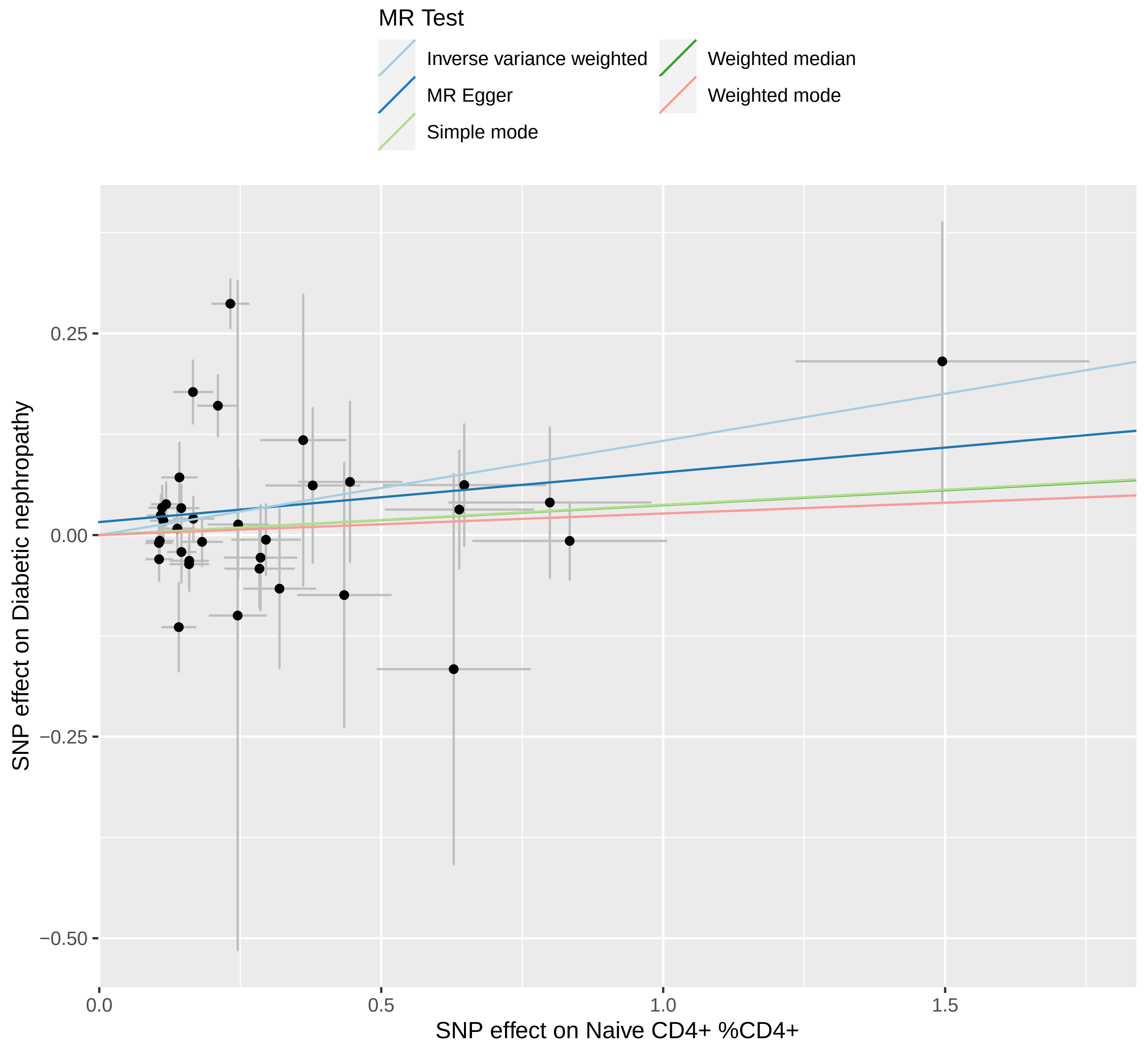
MR Test
Inverse variance weighted
MR Egger
Simple mode
Weighted median
Weighted mode
0.25
0.00
−0.25
−0.50
SNP effect on Diabetic nephropathy
0.0
0.5
1.0
1.5
SNP effect on Naive CD4+ %CD4+

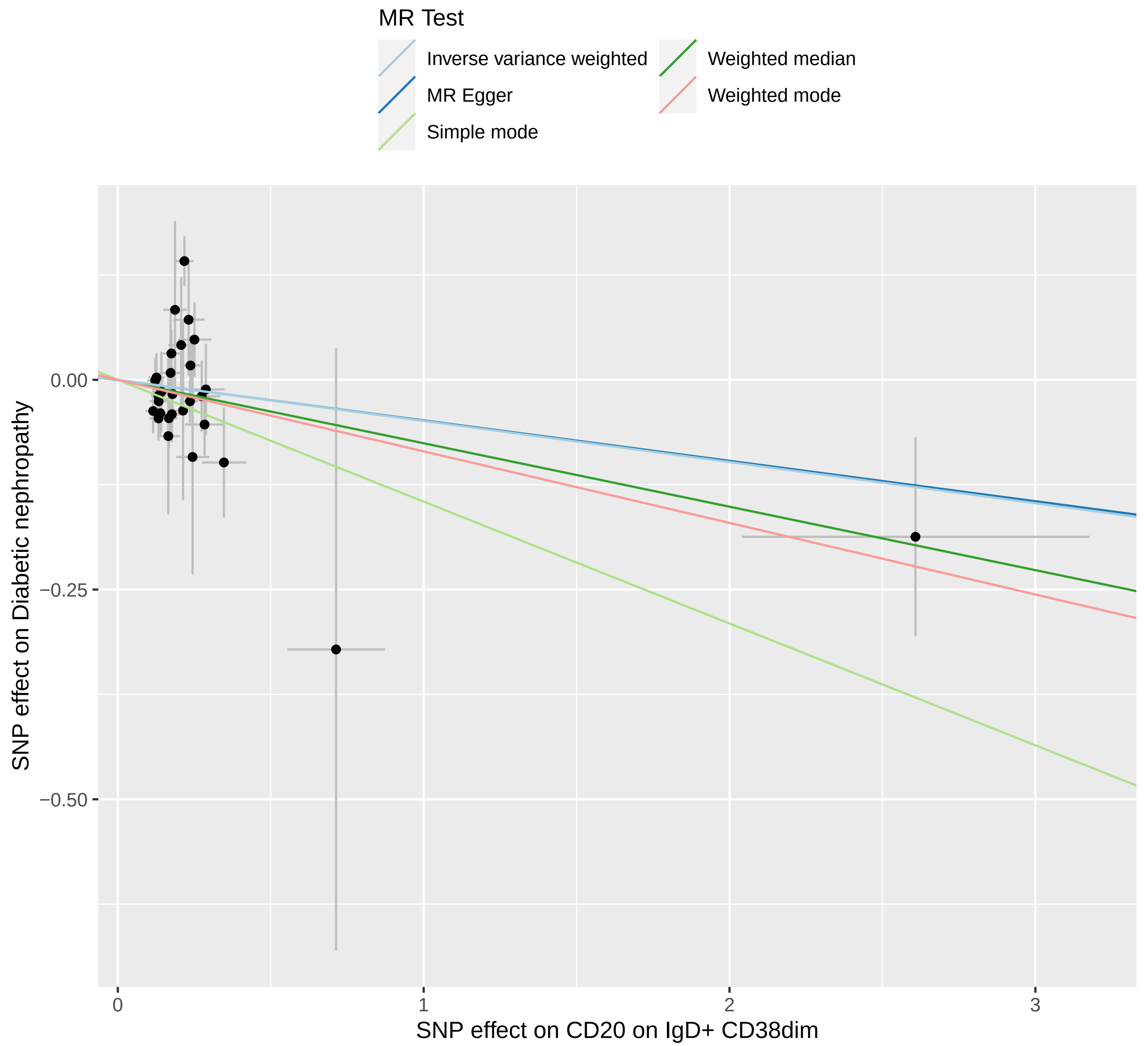

MR Test
Inverse variance weighted
MR Egger
Simple mode
Weighted median
Weighted mode
0.00
−0.25
−0.50
0
1
2
3
SNP effect on Diabetic nephropathy
SNP effect on CD20 on IgD+ CD38dim

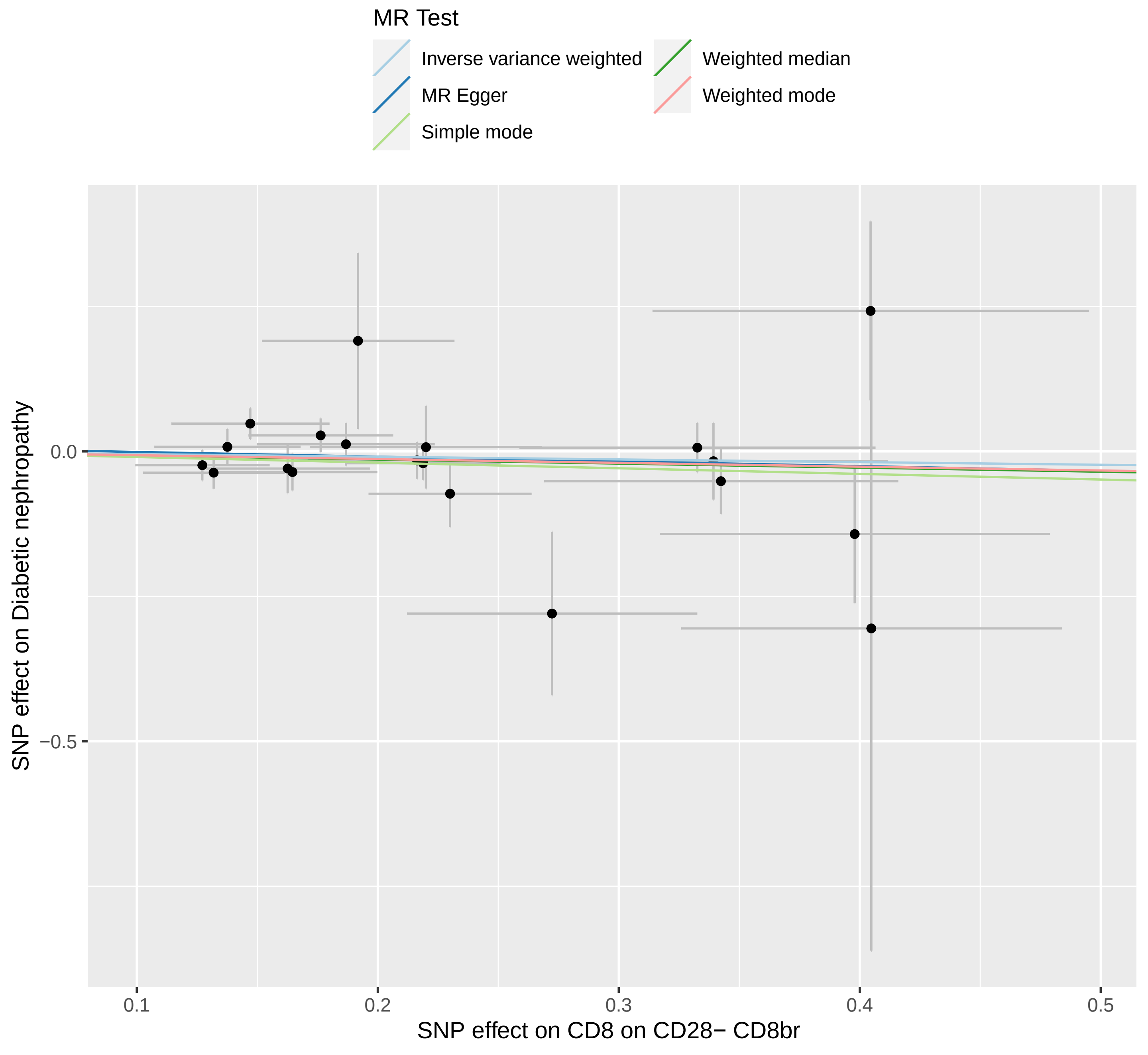

MR Test
Inverse variance weighted
MR Egger
Simple mode
Weighted median
Weighted mode
SNP effect on Diabetic nephropathy
0.0
−0.5
0.1
0.2
0.3
0.4
0.5
SNP effect on CD8 on CD28− CD8br

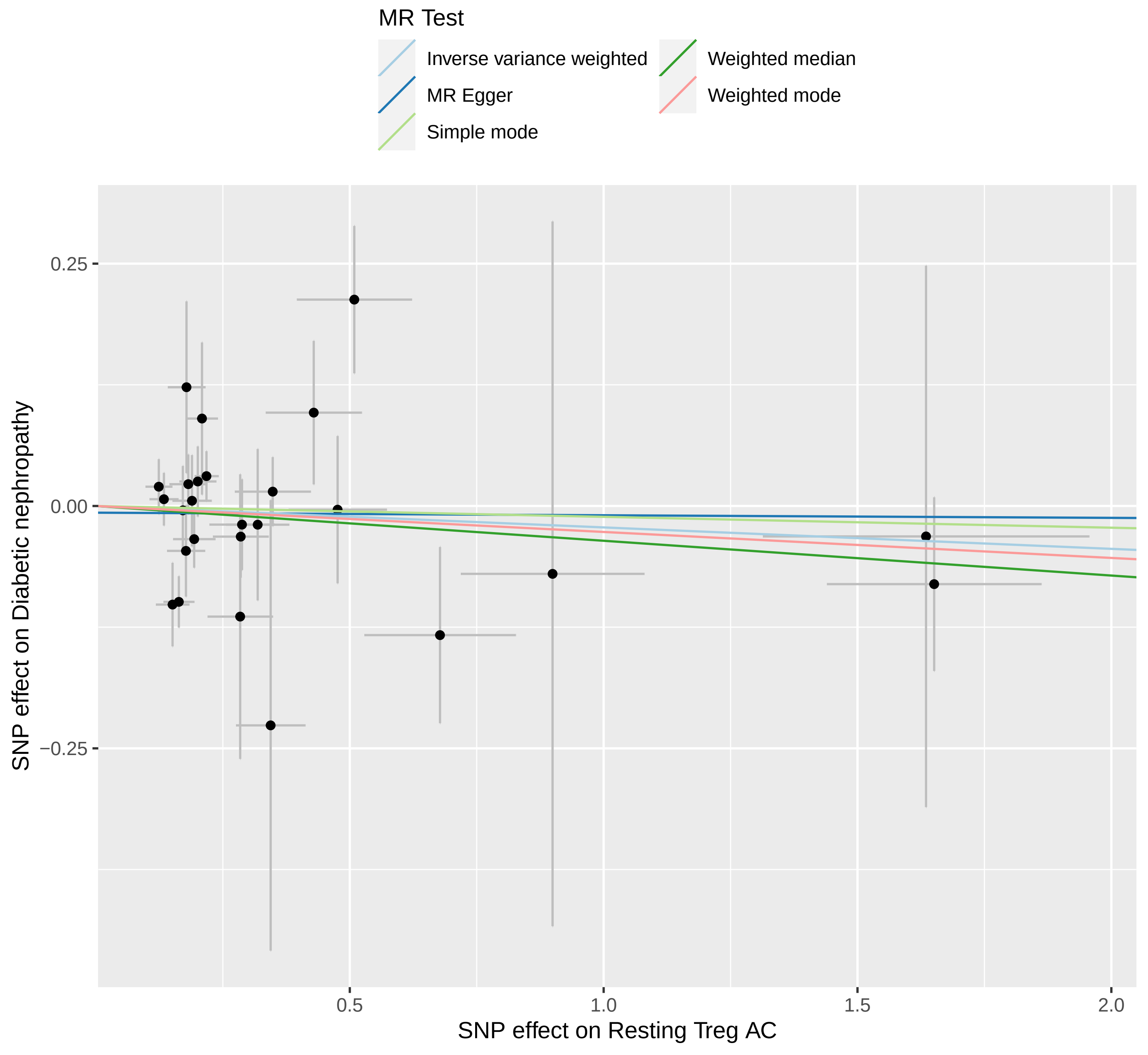

MR Test
Inverse variance weighted
MR Egger
Simple mode
Weighted median
Weighted mode
0.25
0.00
−0.25
0.5
1.0
1.5
2.0
SNP effect on Diabetic nephropathy
SNP effect on Resting Treg AC

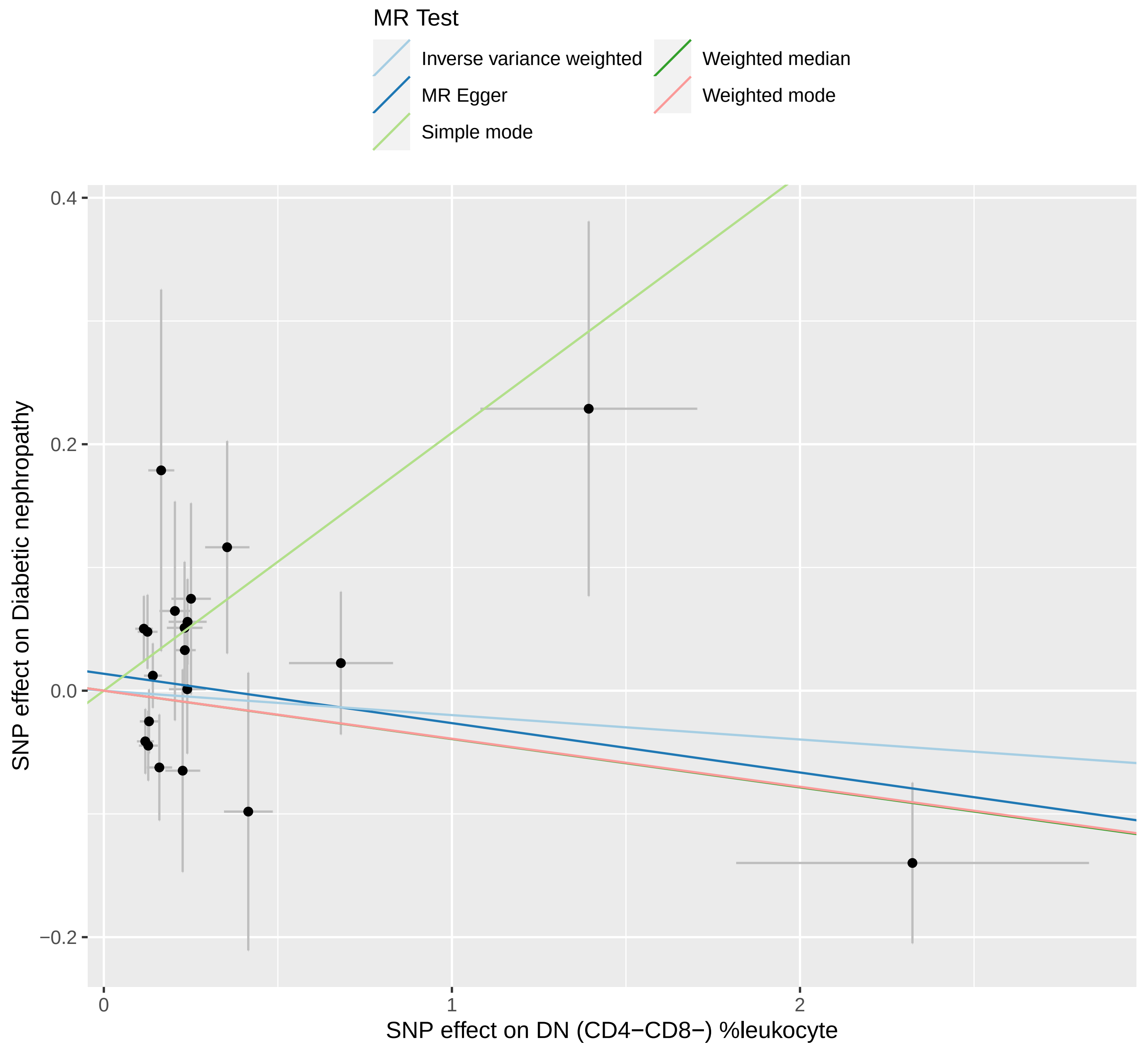
MR Test
Inverse variance weighted
MR Egger
Simple mode
Weighted median
Weighted mode
SNP effect on Diabetic nephropathy
SNP effect on DN (CD4−CD8−) %leukocyte
0.4
0.2
0.0
−0.2
0
1
2

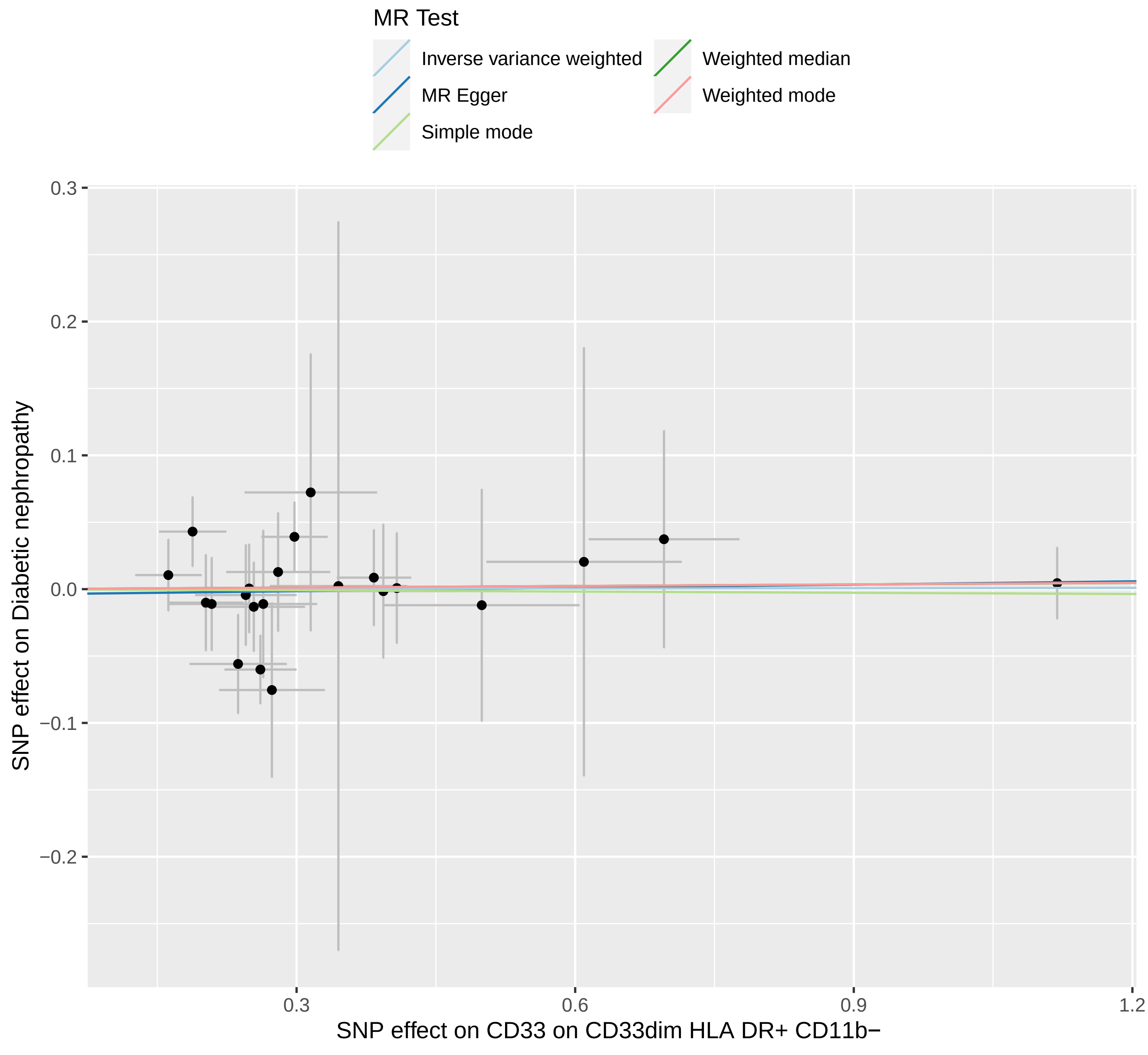

MR Test
Inverse variance weighted
MR Egger
Simple mode
Weighted median
Weighted mode
0.3
0.2
0.1
0.0
−0.1
−0.2
0.3
0.6
0.9
1.2
SNP effect on Diabetic nephropathy
SNP effect on CD33 on CD33dim HLA DR+ CD11b−

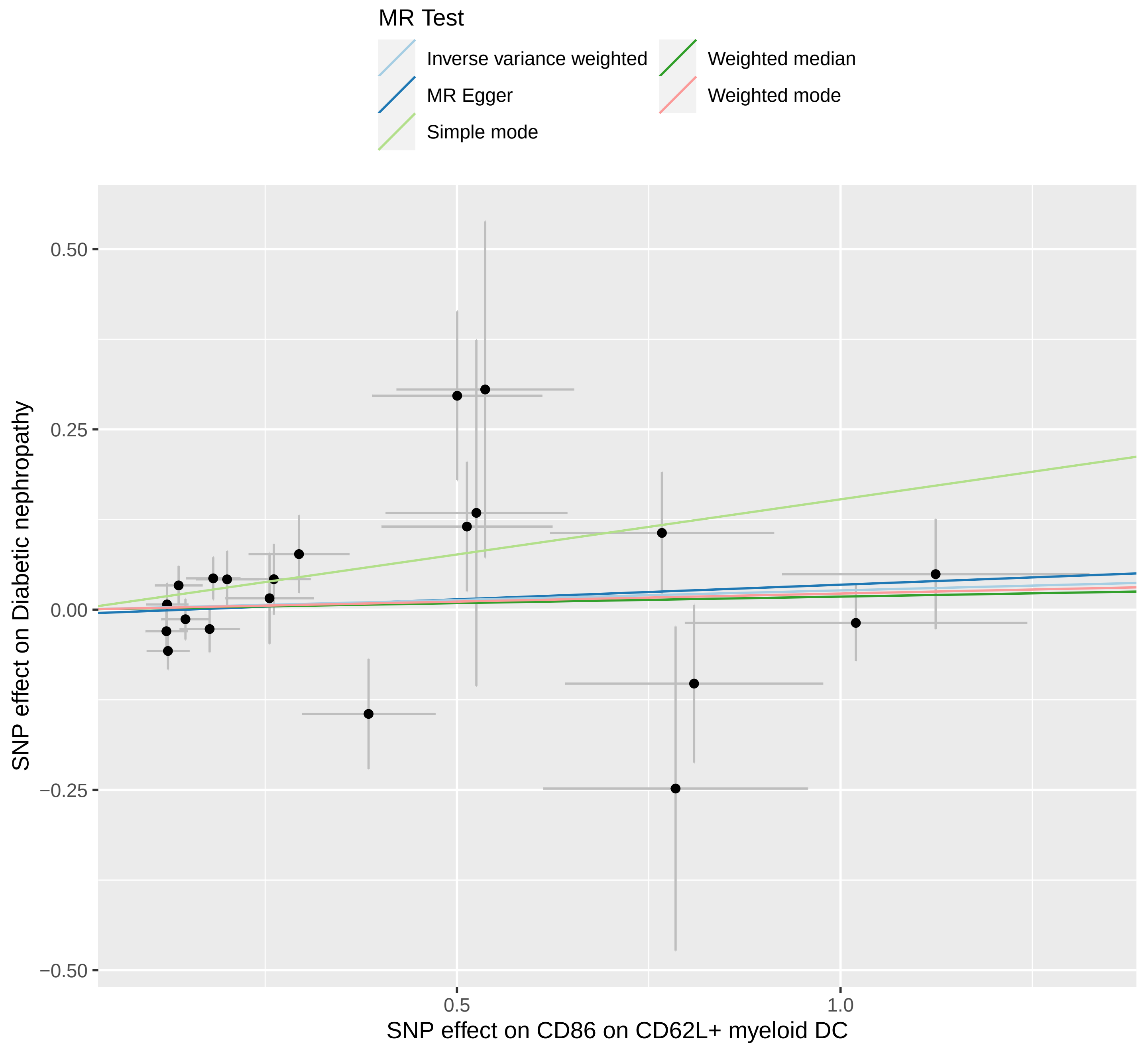
MR Test
Inverse variance weighted
MR Egger
Simple mode
Weighted median
Weighted mode
0.50
0.25
0.00
−0.25
−0.50
0.5
1.0
SNP effect on Diabetic nephropathy
SNP effect on CD86 on CD62L+ myeloid DC

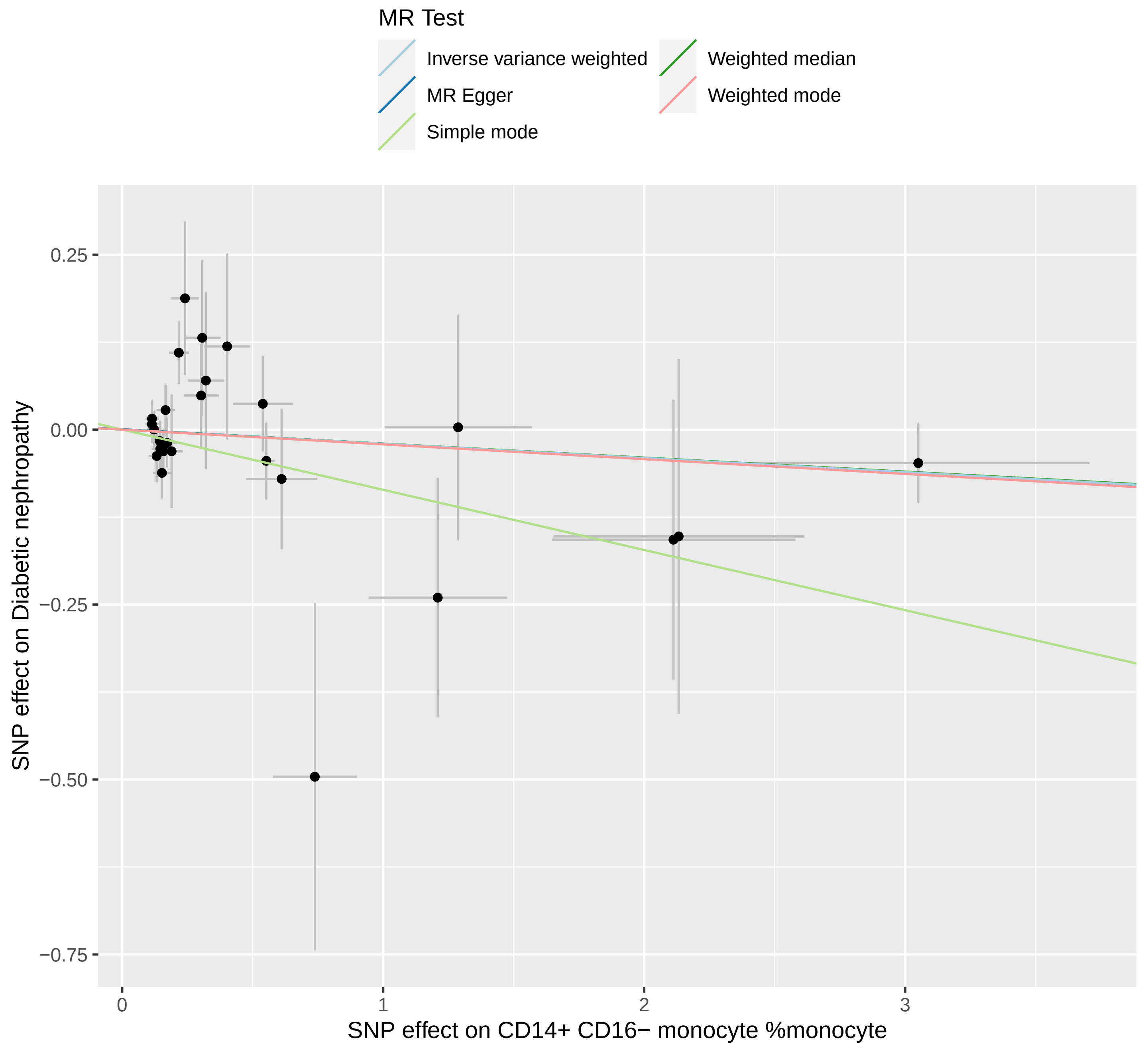

MR Test
Inverse variance weighted
MR Egger
Simple mode
Weighted median
Weighted mode
0.25
0.00
−0.25
−0.50
−0.75
0
1
2
3
SNP effect on Diabetic nephropathy
SNP effect on CD14+ CD16− monocyte %monocyte

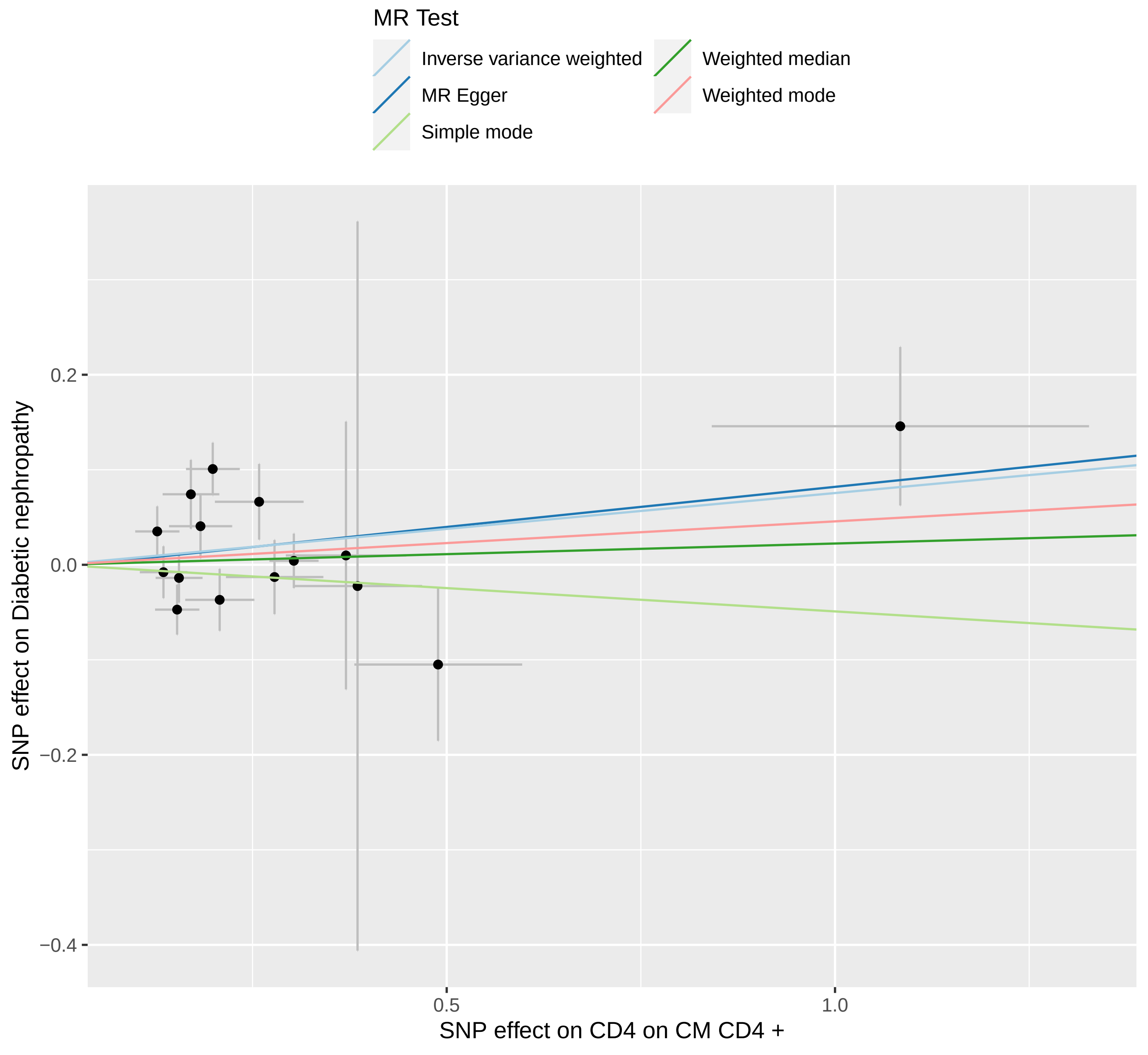
MR Test
Inverse variance weighted
MR Egger
Simple mode
Weighted median
Weighted mode
0.2
0.0
−0.2
−0.4
0.5
1.0
SNP effect on Diabetic nephropathy
SNP effect on CD4 on CM CD4 +

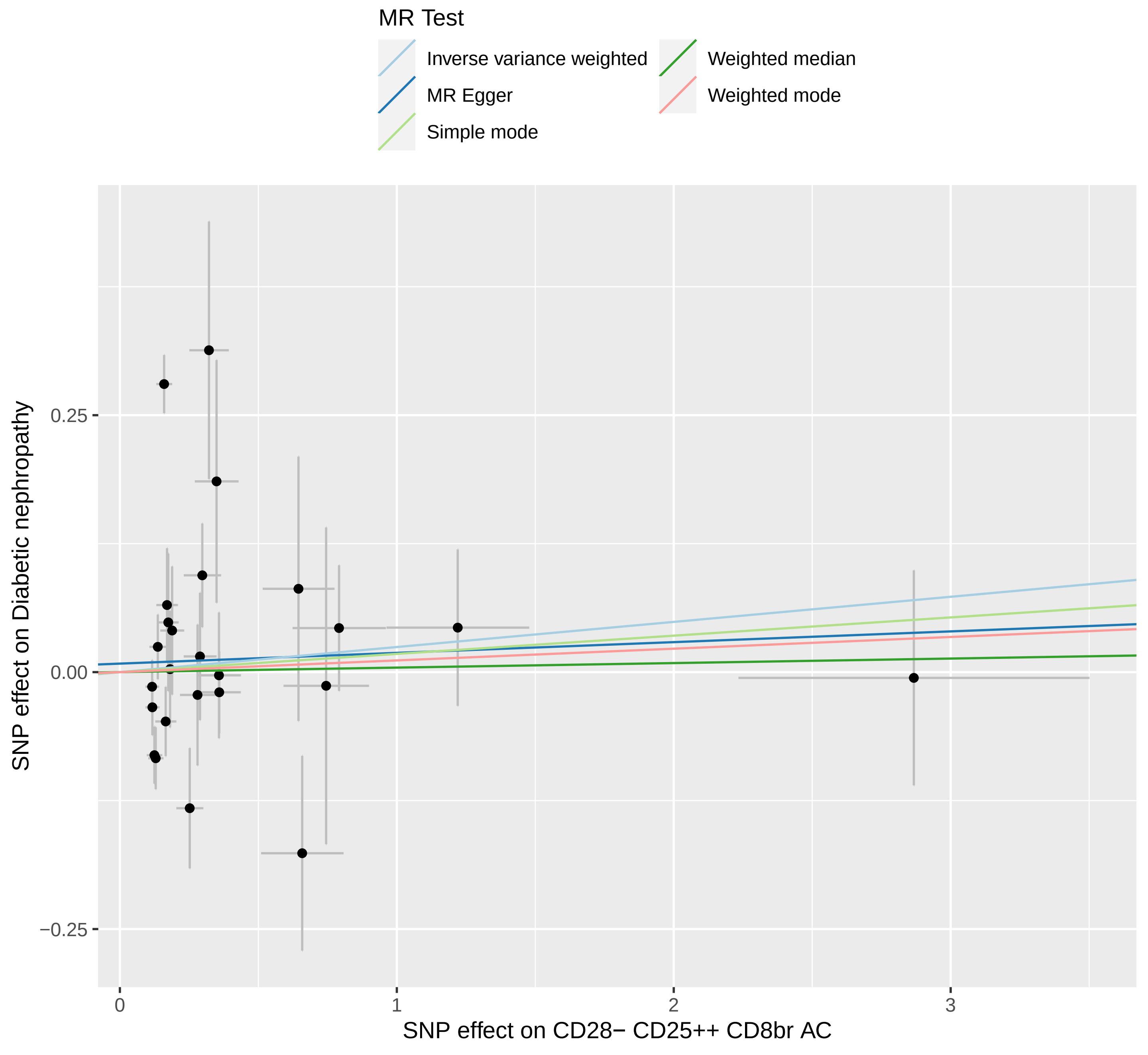

MR Test
Inverse variance weighted
MR Egger
Simple mode
Weighted median
Weighted mode
SNP effect on Diabetic nephropathy
0.25
0.00
−0.25
0
1
2
3
SNP effect on CD28− CD25++ CD8br AC

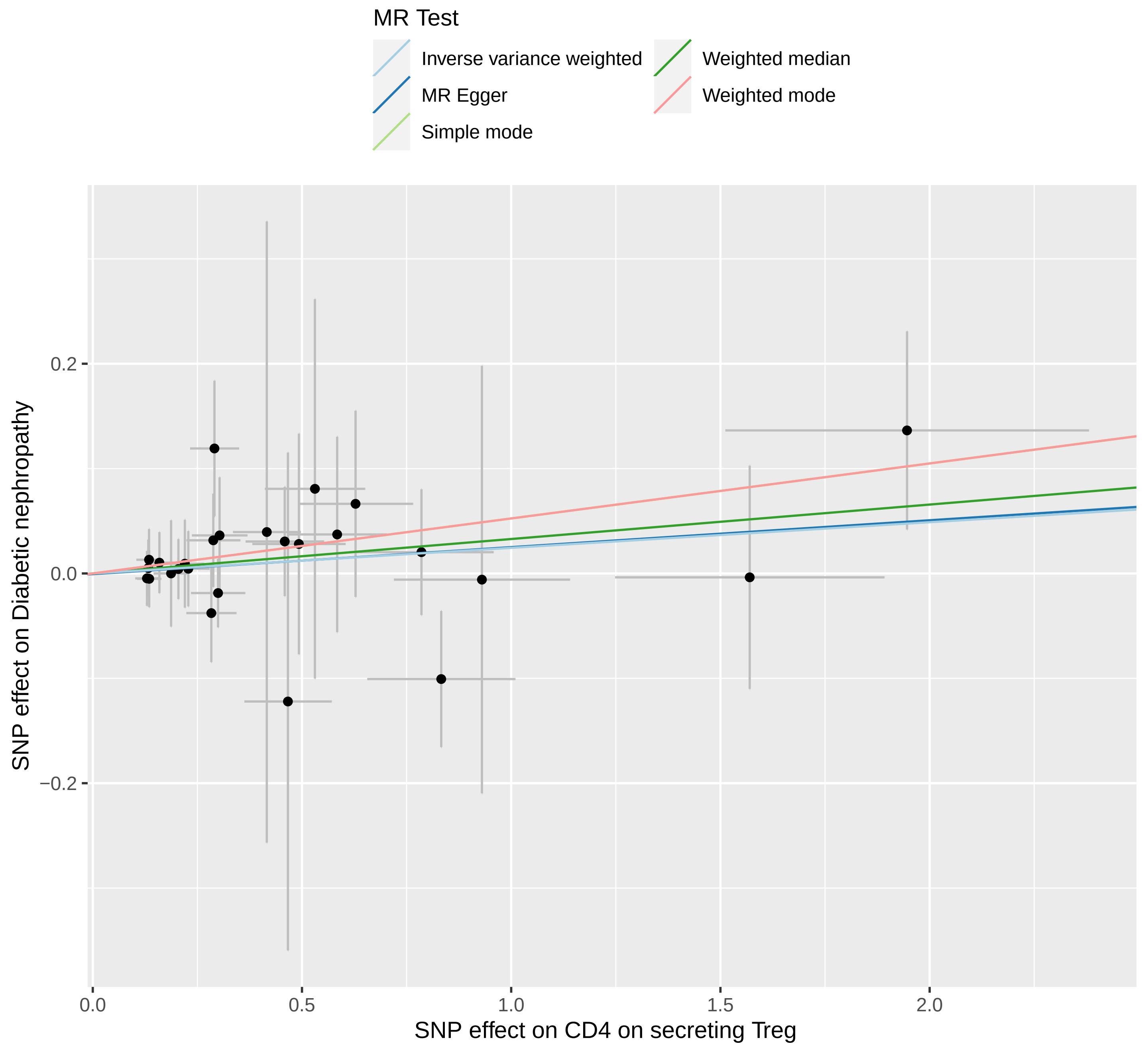
MR Test
Inverse variance weighted
MR Egger
Simple mode
Weighted median
Weighted mode
0.2
0.0
−0.2
SNP effect on Diabetic nephropathy
0.0
0.5
1.0
1.5
2.0
SNP effect on CD4 on secreting Treg

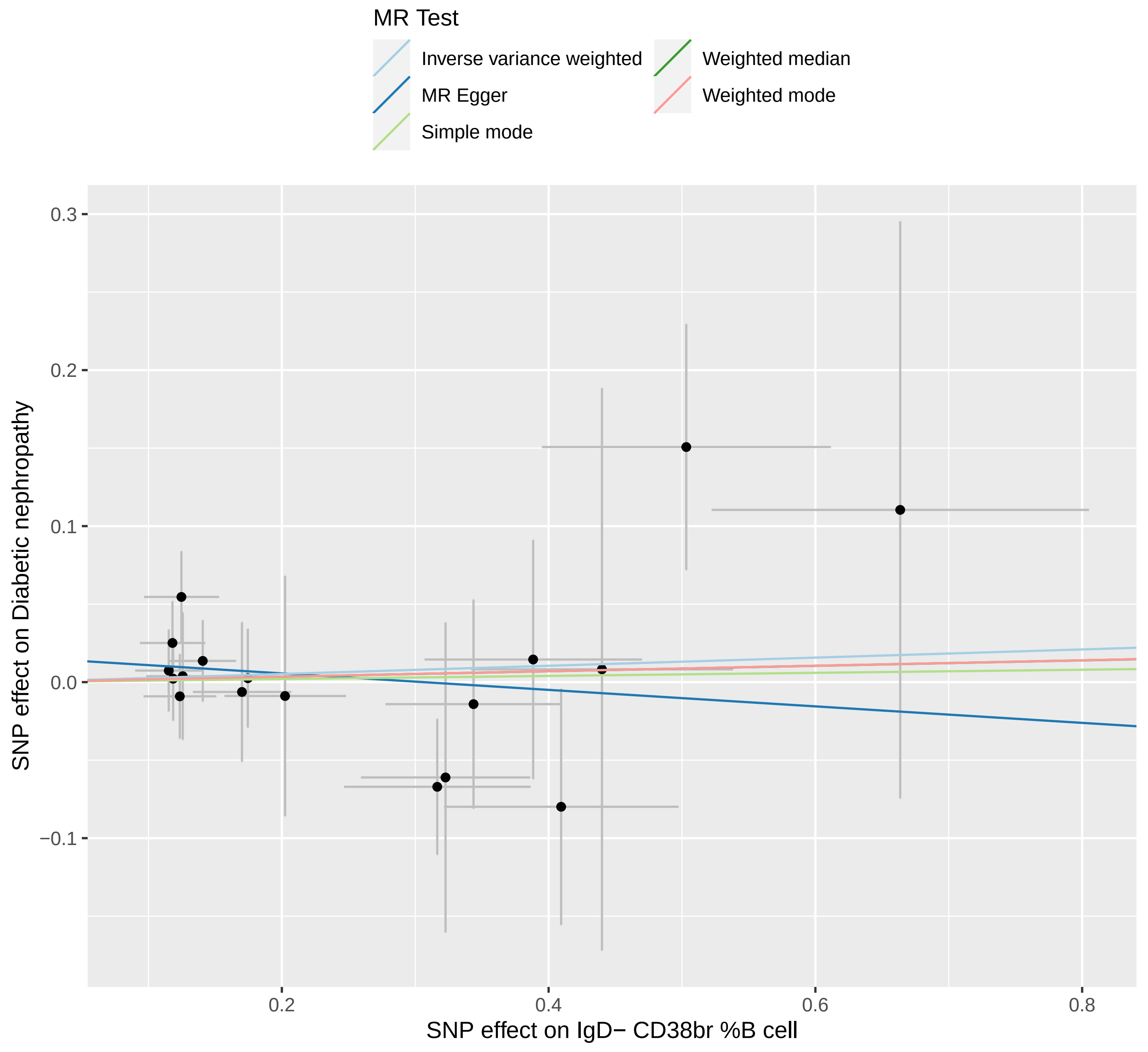

MR Test
Inverse variance weighted
MR Egger
Simple mode
Weighted median
Weighted mode
0.3
0.2
0.1
0.0
−0.1
0.2
0.4
0.6
0.8
SNP effect on Diabetic nephropathy
SNP effect on IgD− CD38br %B cell

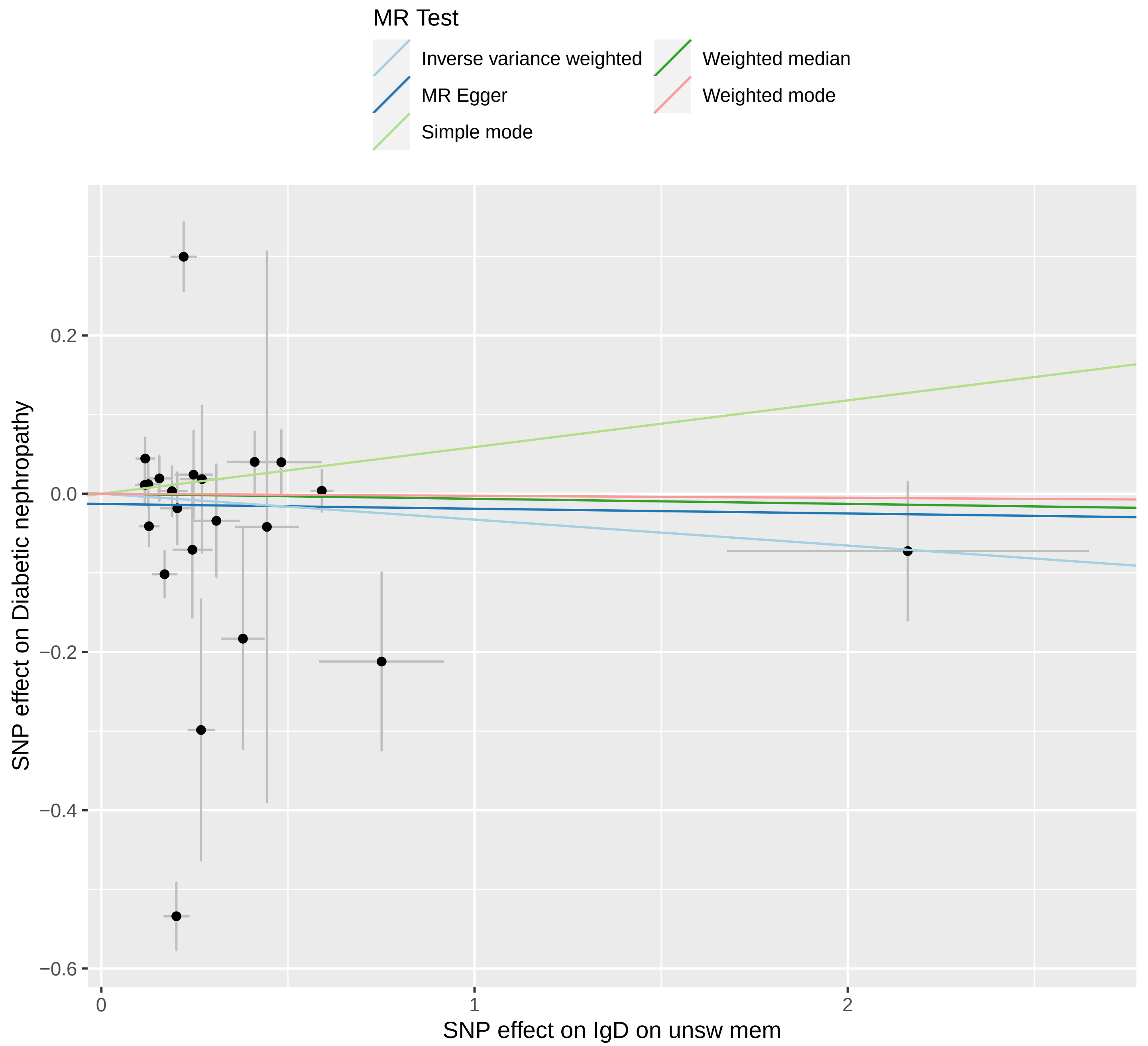
MR Test
Inverse variance weighted
MR Egger
Simple mode
Weighted median
Weighted mode
0.2
0.0
−0.2
−0.4
−0.6
0
1
2
SNP effect on Diabetic nephropathy
SNP effect on IgD on unsw mem

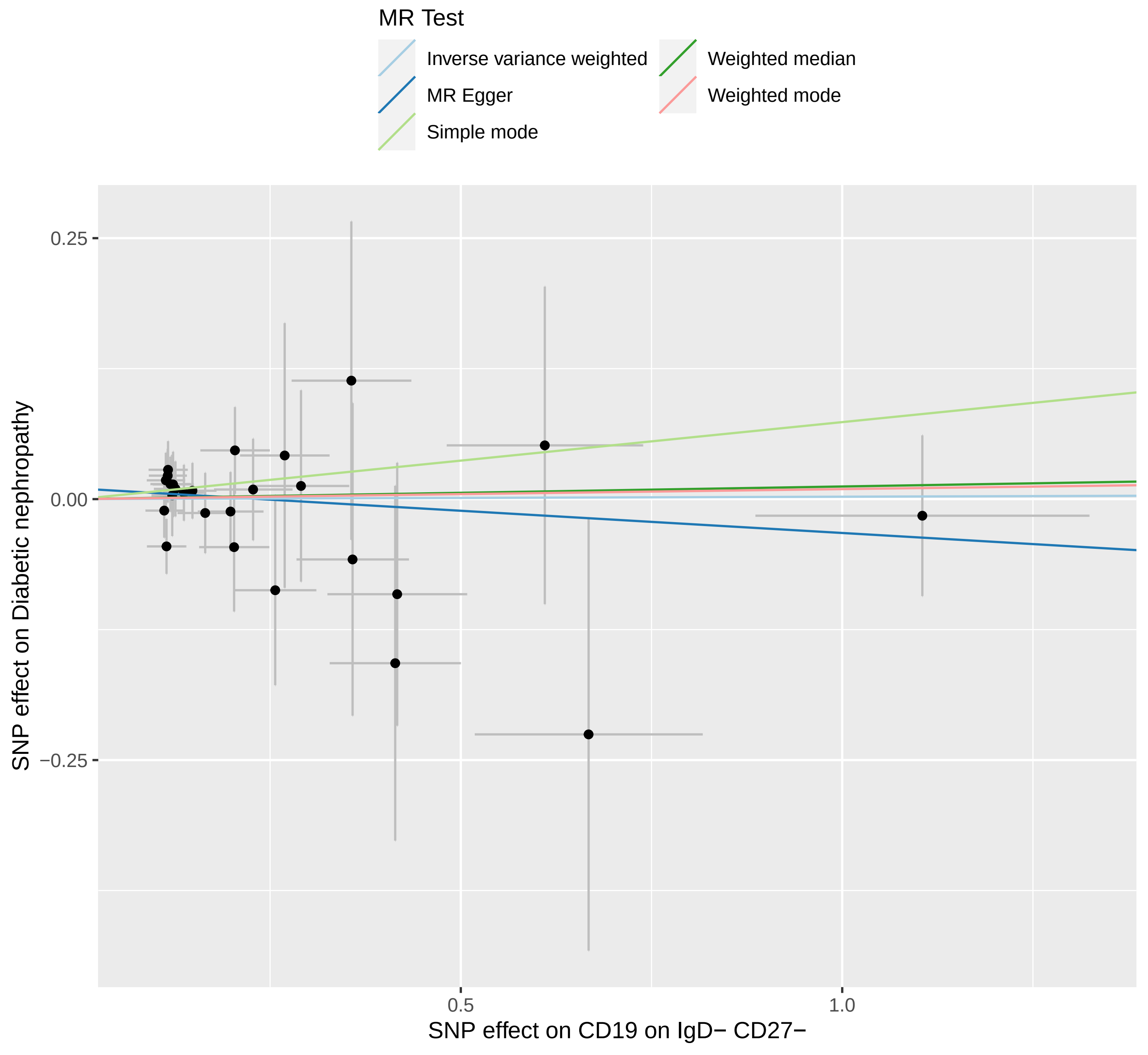
MR Test
Inverse variance weighted
MR Egger
Simple mode
Weighted median
Weighted mode
0.25
0.00
−0.25
SNP effect on Diabetic nephropathy
0.5
1.0
SNP effect on CD19 on IgD− CD27−

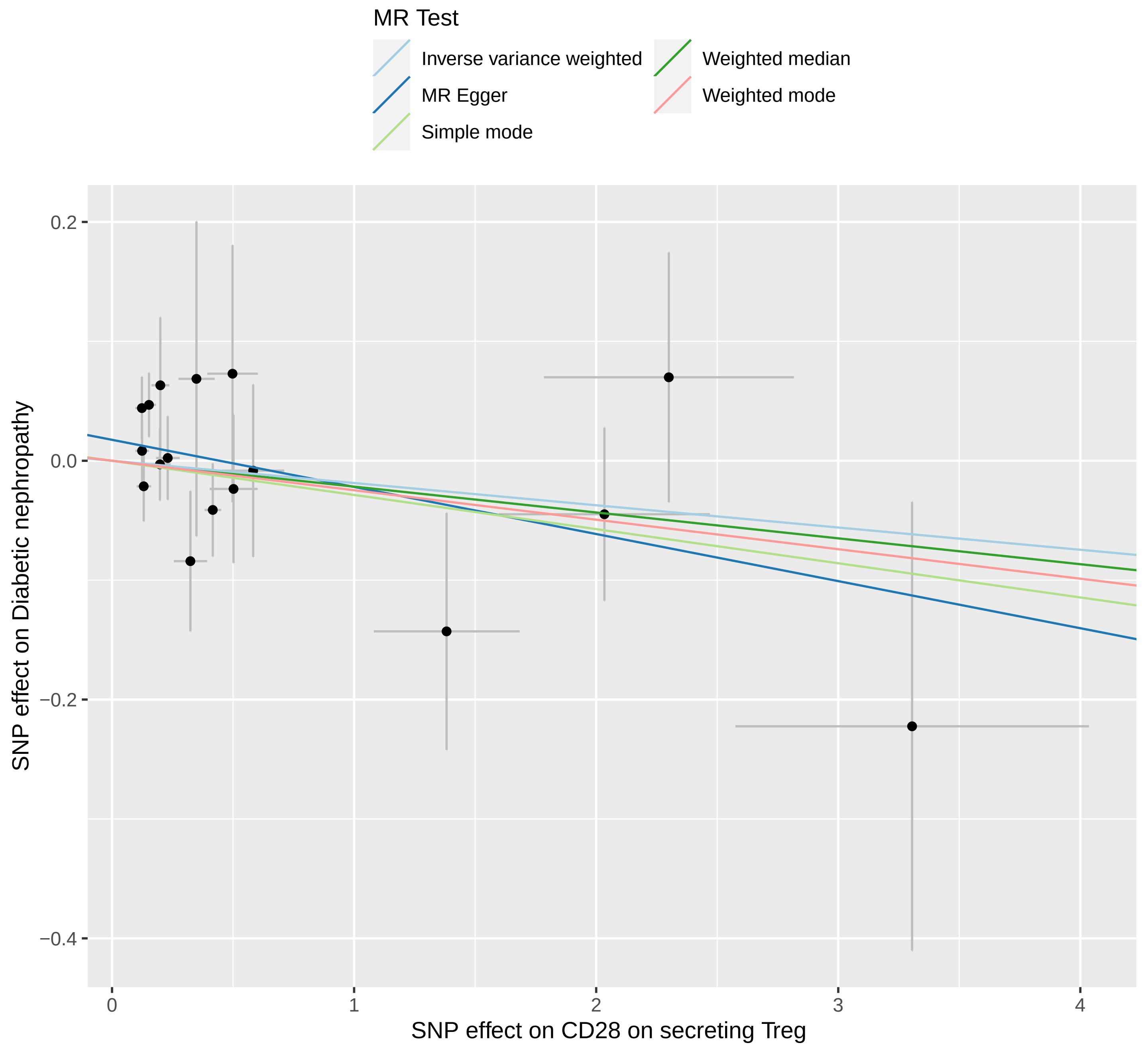
MR Test
Inverse variance weighted
MR Egger
Simple mode
Weighted median
Weighted mode
0.2
0.0
−0.2
−0.4
0
1
2
3
4
SNP effect on Diabetic nephropathy
SNP effect on CD28 on secreting Treg

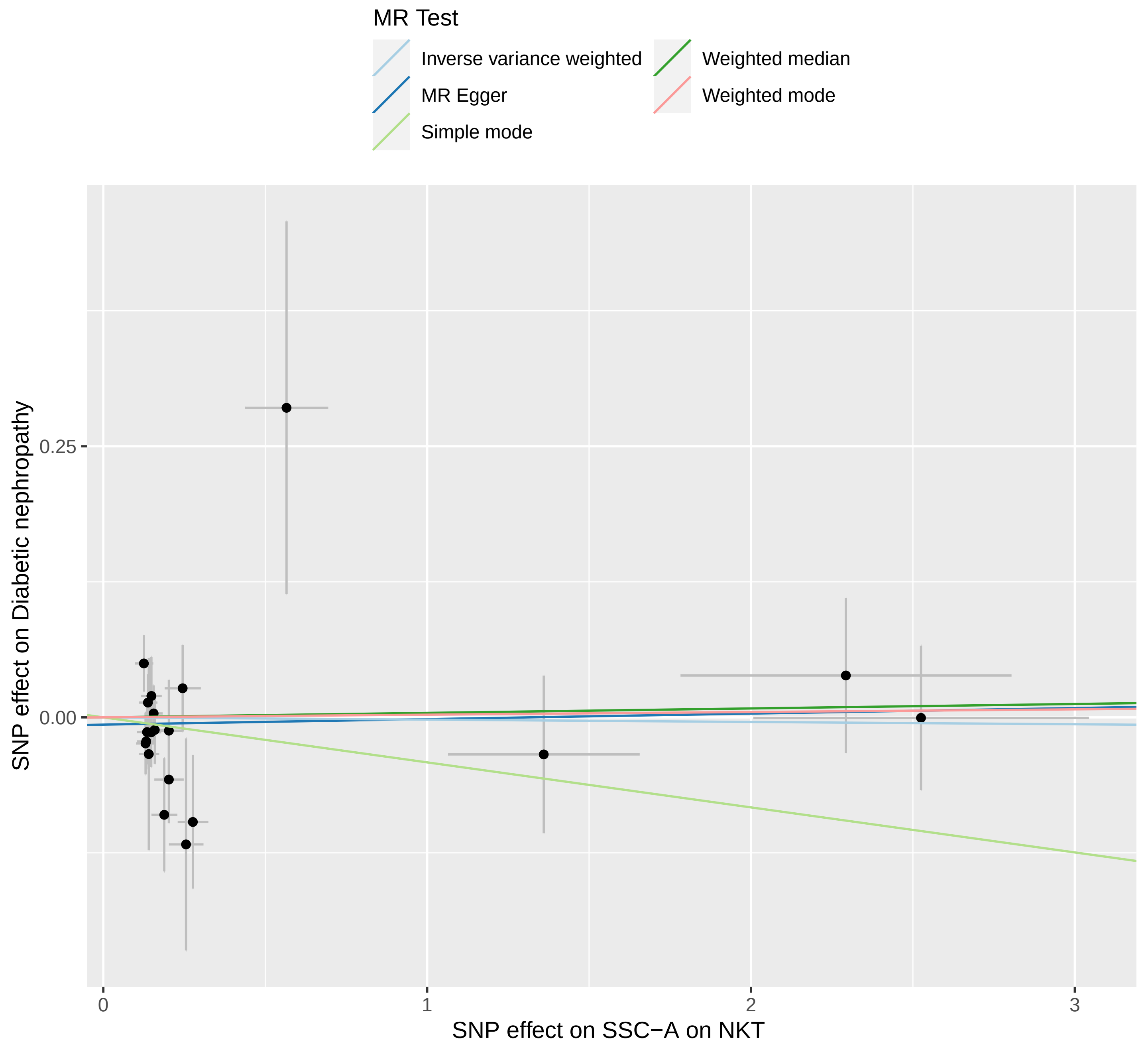

MR Test
Inverse variance weighted
MR Egger
Simple mode
Weighted median
Weighted mode
0.25
0.00
0
1
2
3
SNP effect on SSC−A on NKT
SNP effect on Diabetic nephropathy

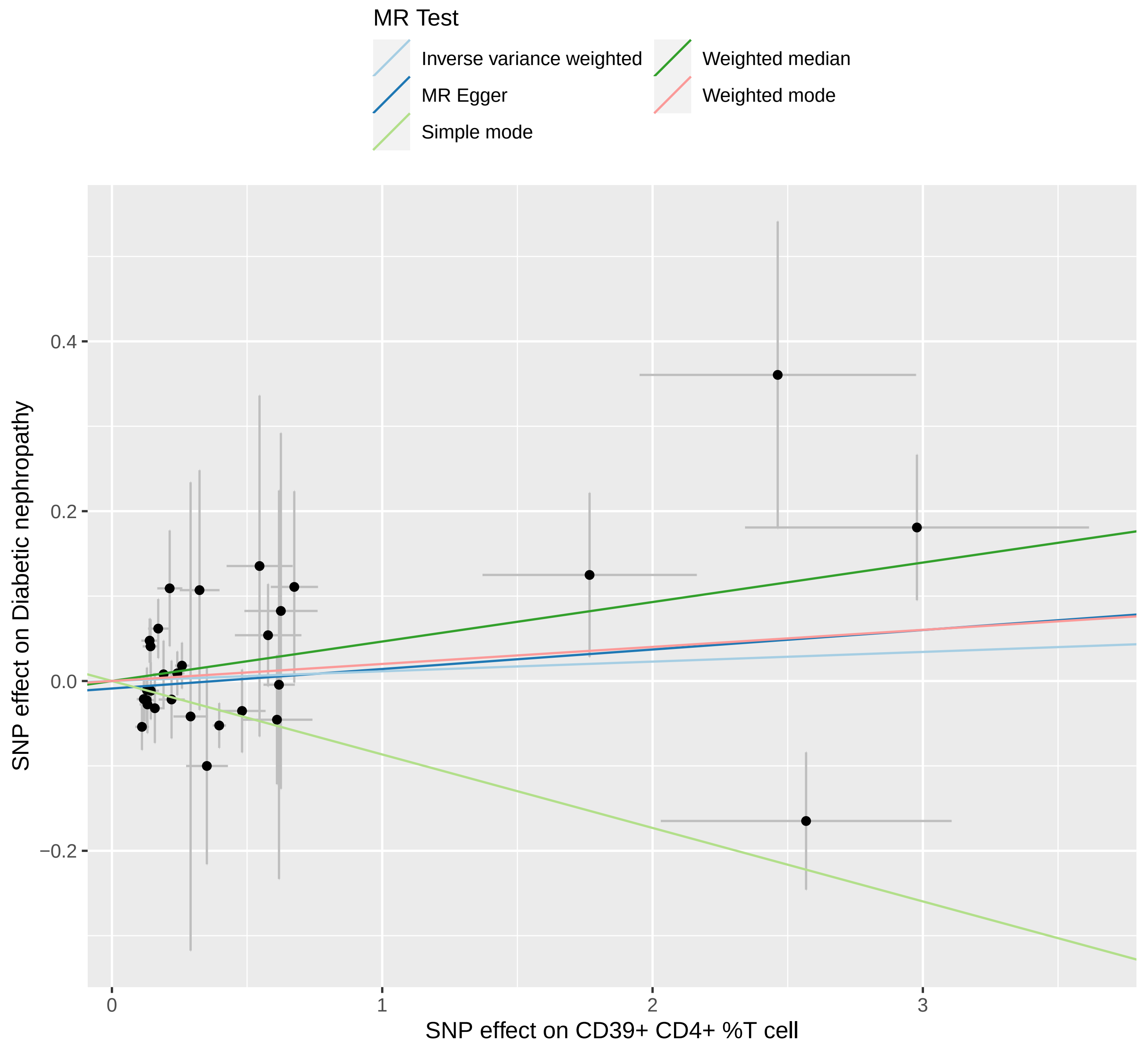
MR Test
Inverse variance weighted
MR Egger
Simple mode
Weighted median
Weighted mode
SNP effect on Diabetic nephropathy
0.4
0.2
0.0
−0.2
0
1
2
3
SNP effect on CD39+ CD4+ %T cell

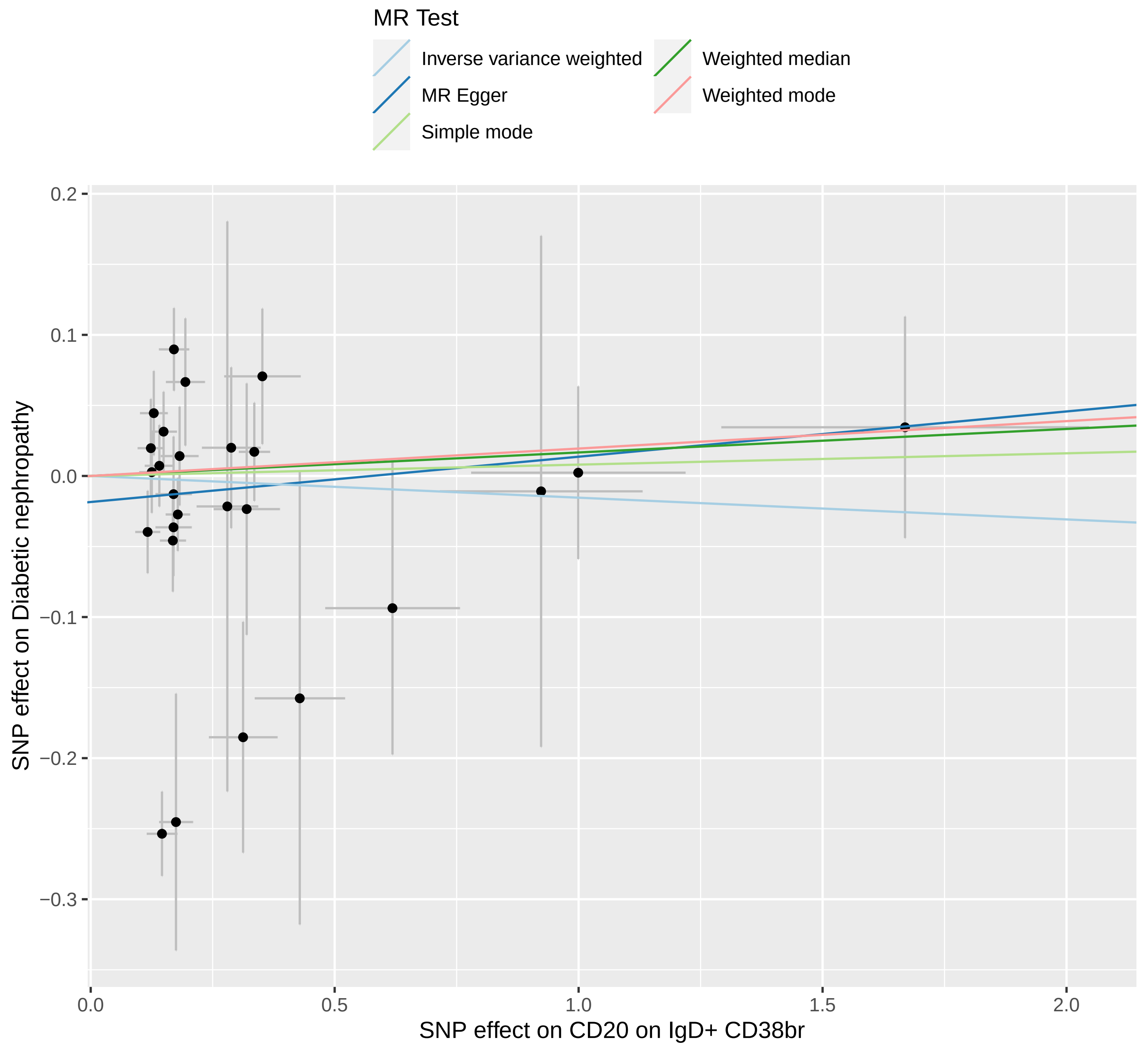
MR Test
Inverse variance weighted
MR Egger
Simple mode
Weighted median
Weighted mode
SNP effect on Diabetic nephropathy
0.2
0.1
0.0
−0.1
−0.2
−0.3
0.0
0.5
1.0
1.5
2.0
SNP effect on CD20 on IgD+ CD38br

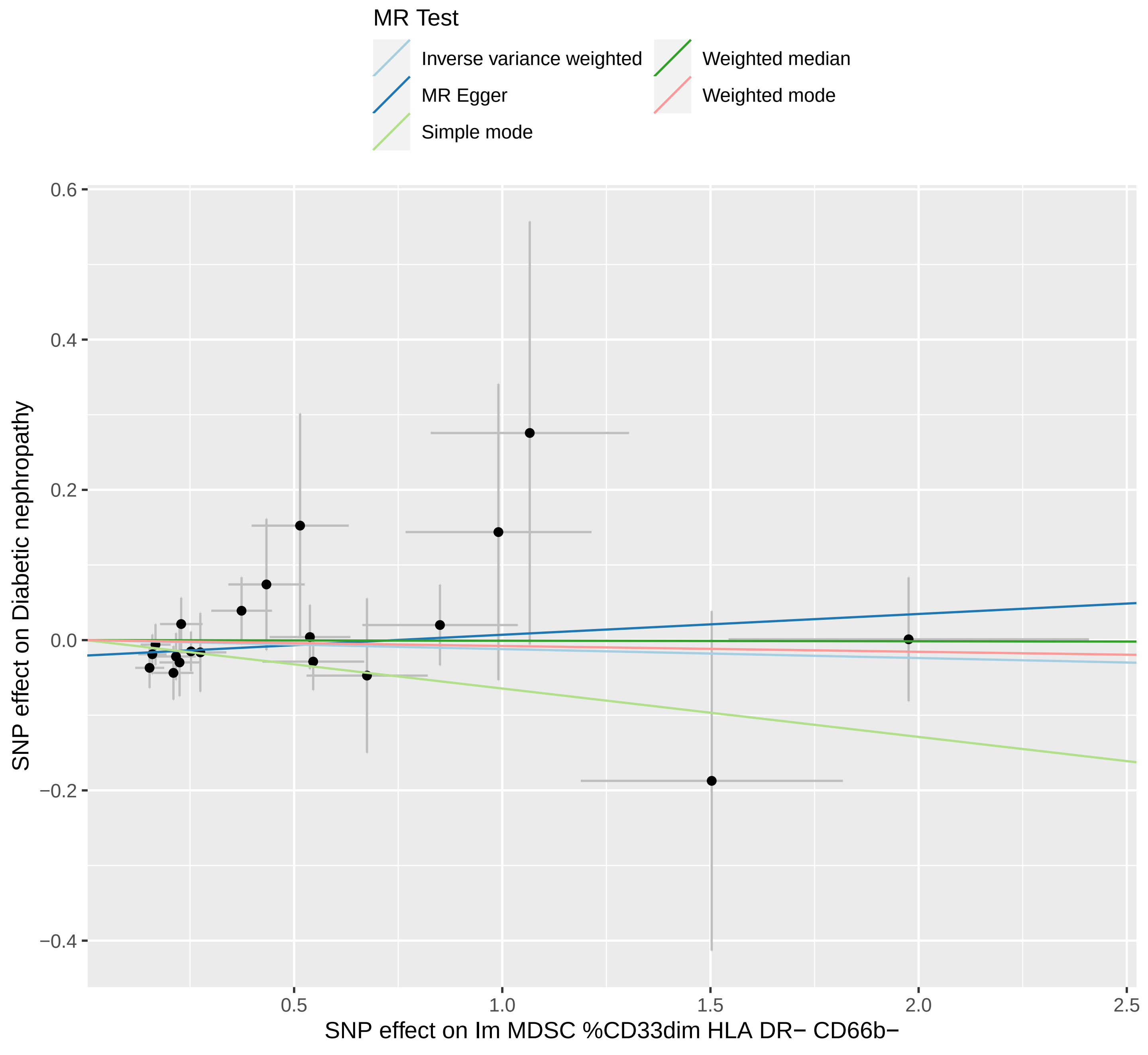

MR Test
Inverse variance weighted
MR Egger
Simple mode
Weighted median
Weighted mode
0.6
0.4
0.2
0.0
−0.2
−0.4
SNP effect on Diabetic nephropathy
0.5
1.0
1.5
2.0
2.5
SNP effect on Im MDSC %CD33dim HLA DR− CD66b−

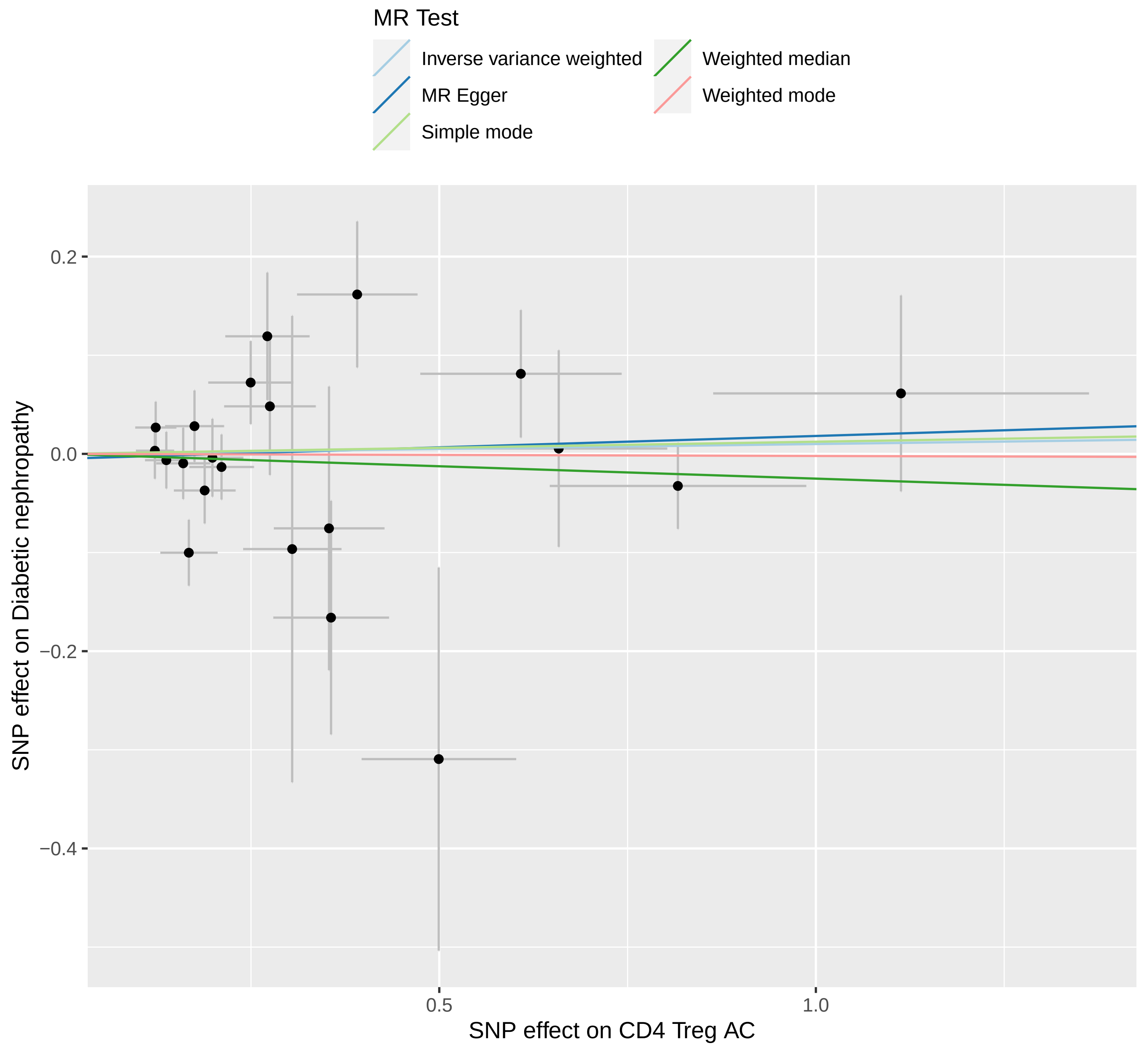

MR Test
Inverse variance weighted
MR Egger
Simple mode
Weighted median
Weighted mode
0.2
0.0
−0.2
−0.4
SNP effect on Diabetic nephropathy
0.5
1.0
SNP effect on CD4 Treg AC

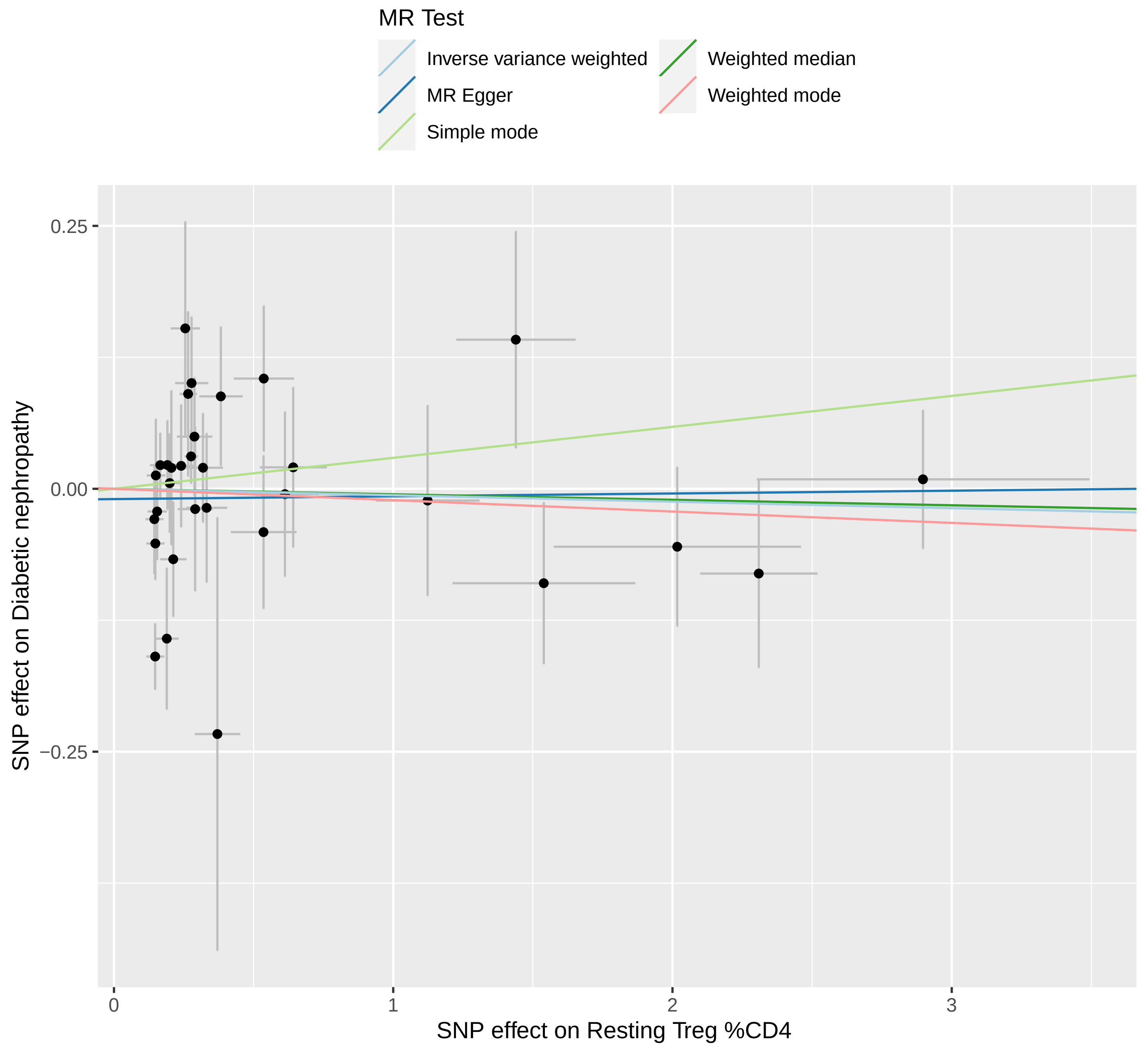

MR Test
Inverse variance weighted
MR Egger
Simple mode
Weighted median
Weighted mode
0.25
0.00
−0.25
0
1
2
3
SNP effect on Diabetic nephropathy
SNP effect on Resting Treg %CD4

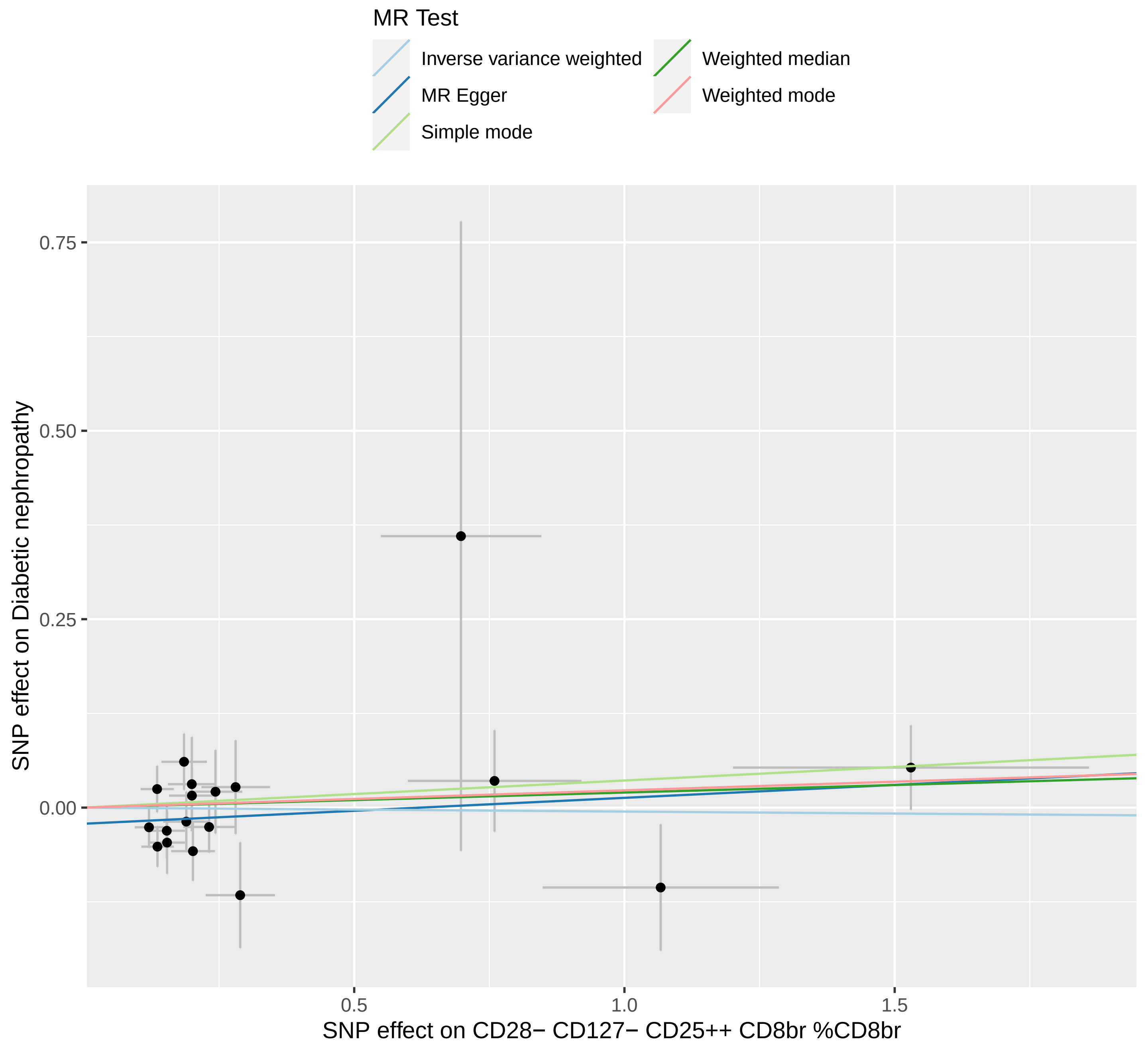
MR Test
Inverse variance weighted
MR Egger
Simple mode
Weighted median
Weighted mode
SNP effect on Diabetic nephropathy
0.75
0.50
0.25
0.00
0.5
1.0
1.5
SNP effect on CD28− CD127− CD25++ CD8br %CD8br

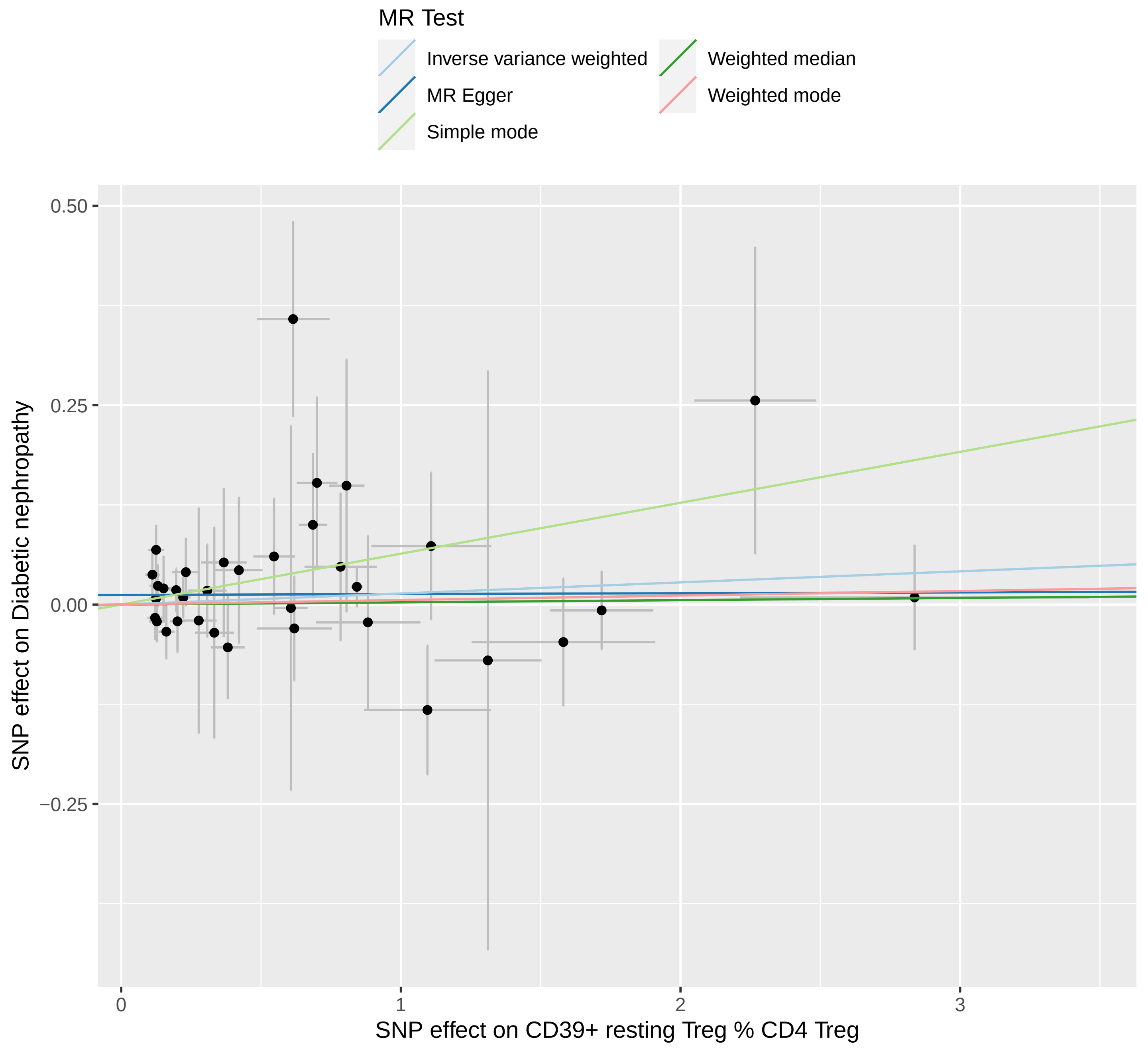

MR Test
Inverse variance weighted
MR Egger
Simple mode
Weighted median
Weighted mode
0.50
0.25
0.00
−0.25
SNP effect on Diabetic nephropathy
0
1
2
3
SNP effect on CD39+ resting Treg % CD4 Treg

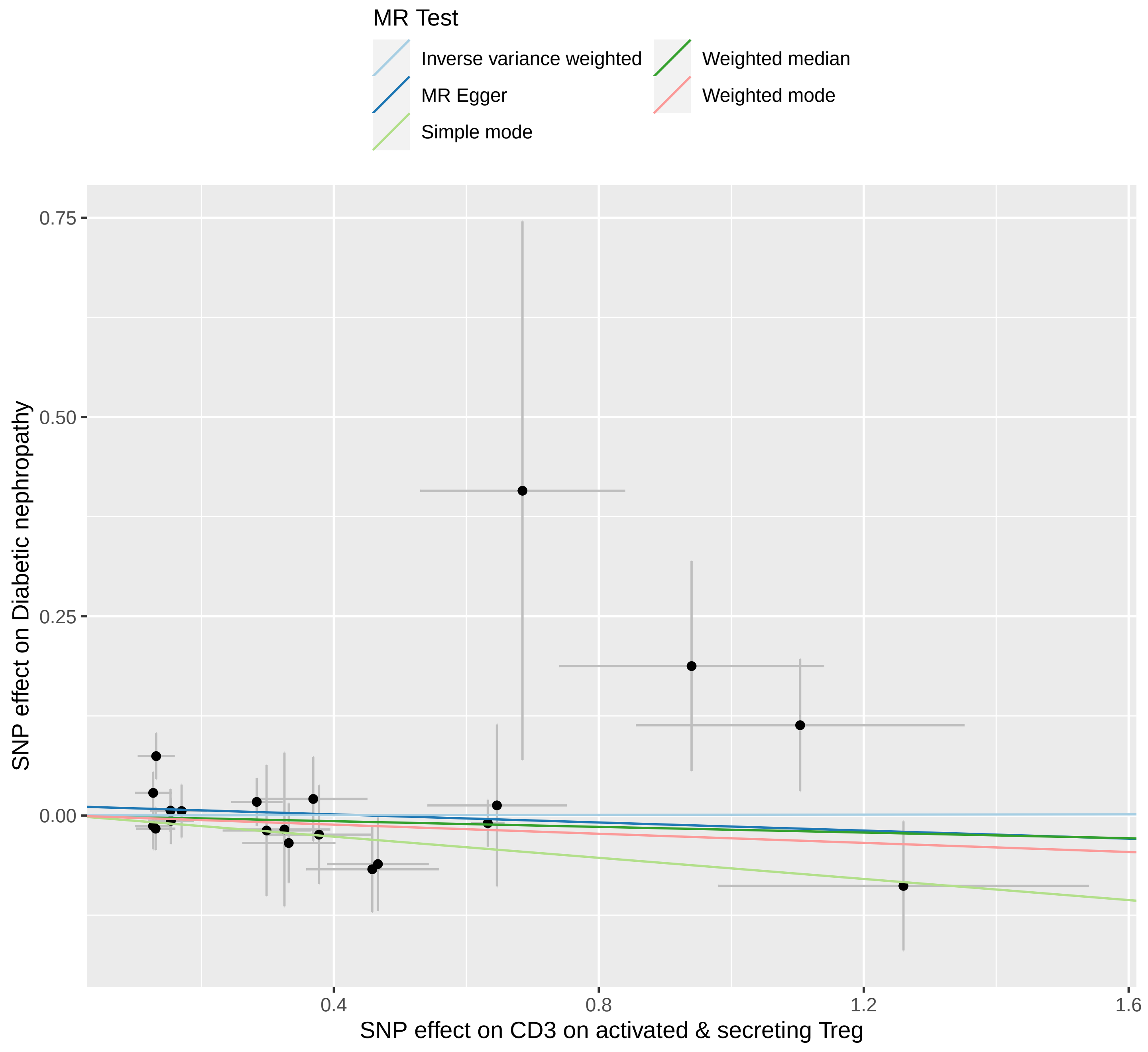
MR Test
Inverse variance weighted
MR Egger
Simple mode
Weighted median
Weighted mode
SNP effect on Diabetic nephropathy
0.75
0.50
0.25
0.00
0.4
0.8
1.2
1.6
SNP effect on CD3 on activated & secreting Treg

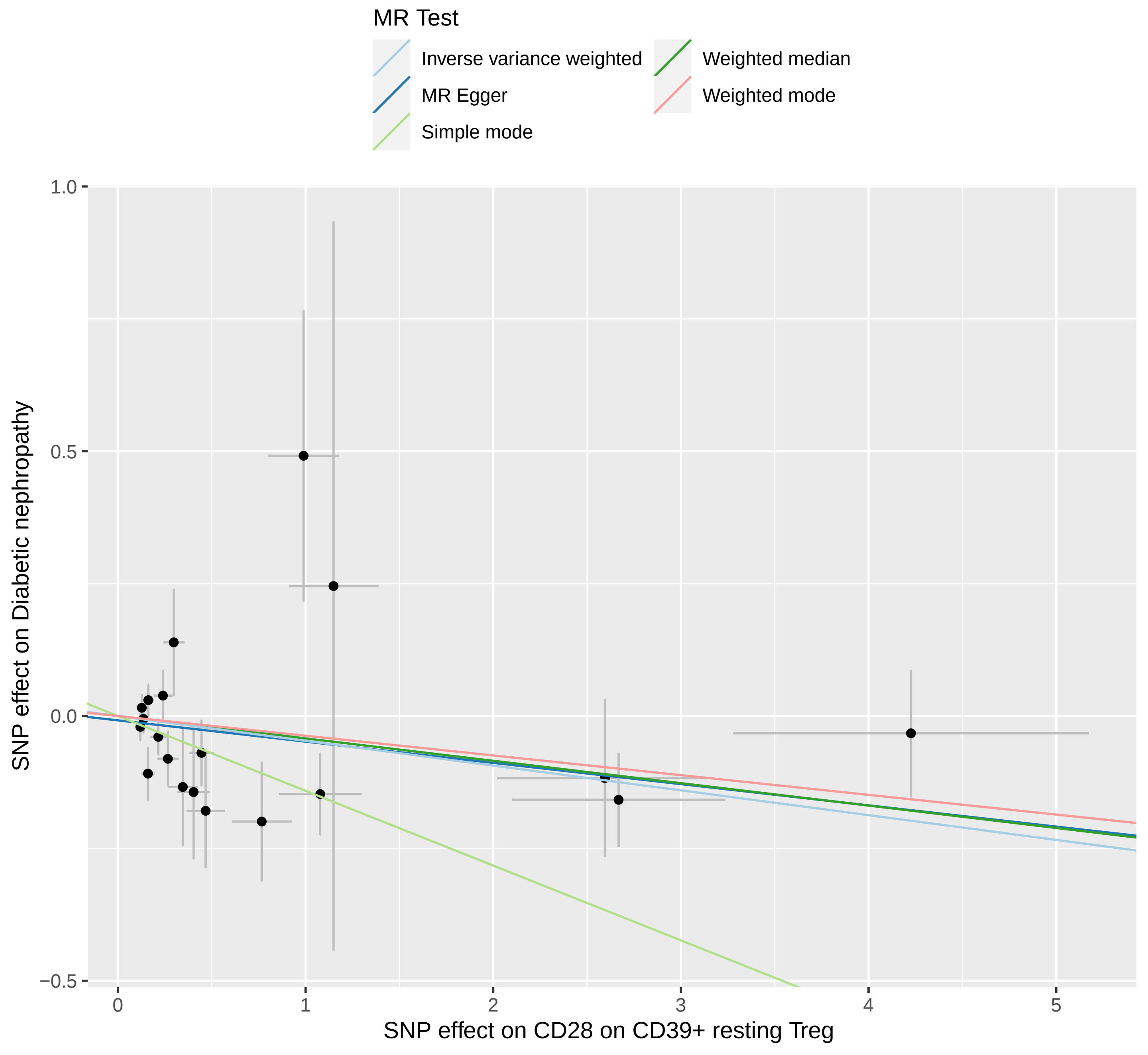

MR Test
Inverse variance weighted
MR Egger
Simple mode
Weighted median
Weighted mode
1.0
0.5
0.0
−0.5
SNP effect on Diabetic nephropathy
0
1
2
3
4
5
SNP effect on CD28 on CD39+ resting Treg

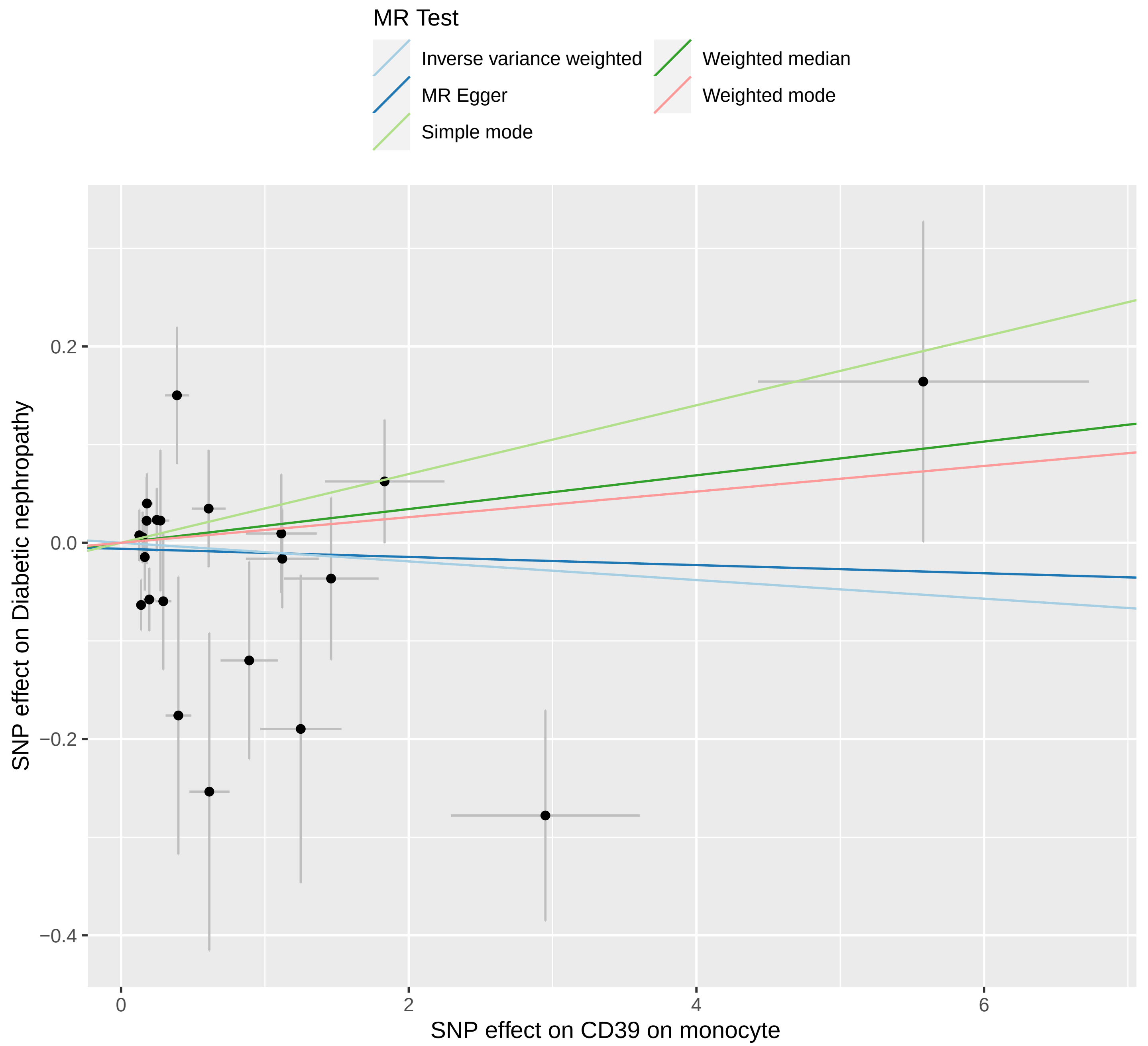

MR Test
Inverse variance weighted
MR Egger
Simple mode
Weighted median
Weighted mode
SNP effect on Diabetic nephropathy
0.2
0.0
−0.2
−0.4
0
2
4
6
SNP effect on CD39 on monocyte

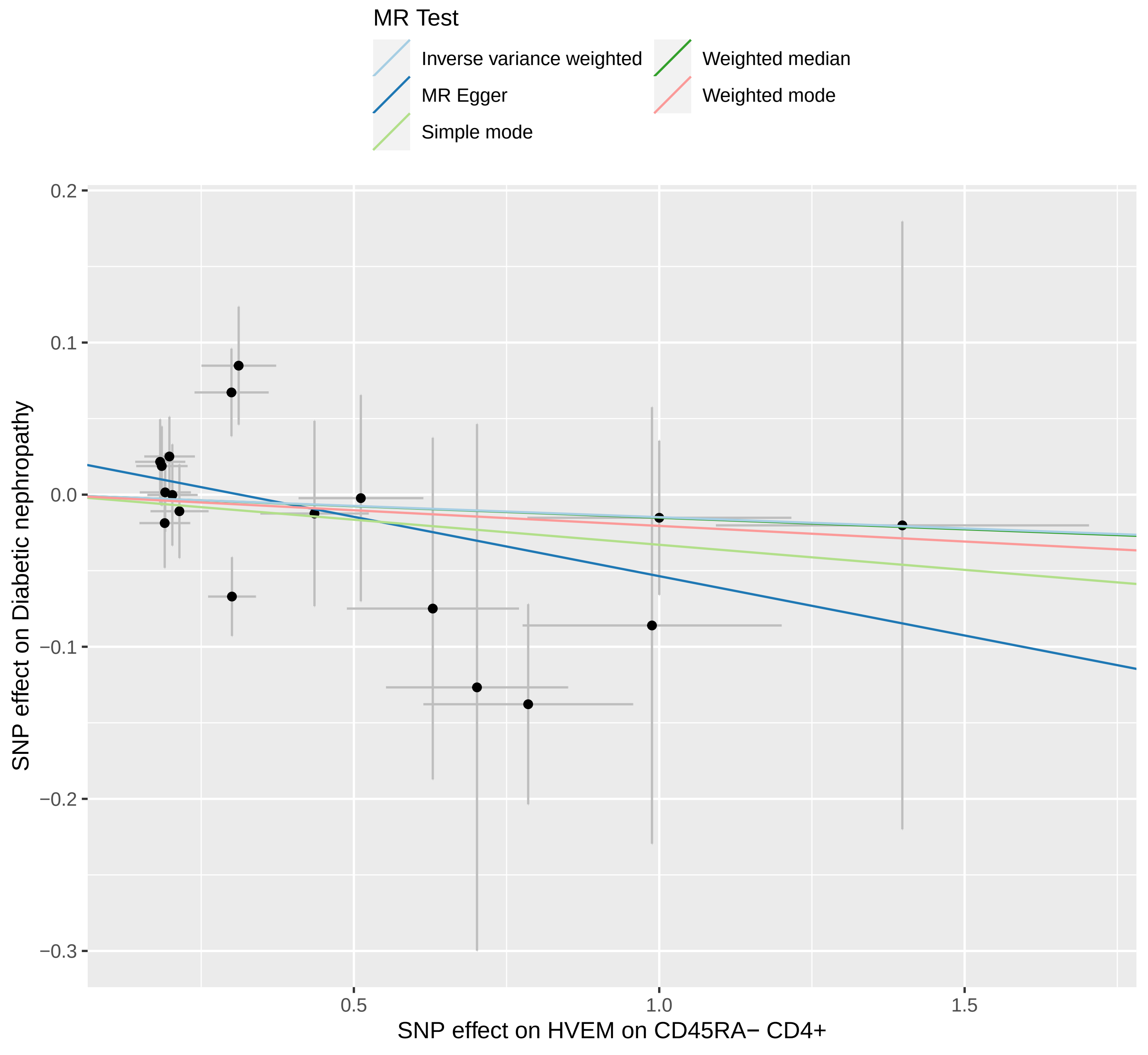
MR Test
Inverse variance weighted
MR Egger
Simple mode
Weighted median
Weighted mode
0.2
0.1
0.0
−0.1
−0.2
−0.3
0.5
1.0
1.5
SNP effect on Diabetic nephropathy
SNP effect on HVEM on CD45RA− CD4+

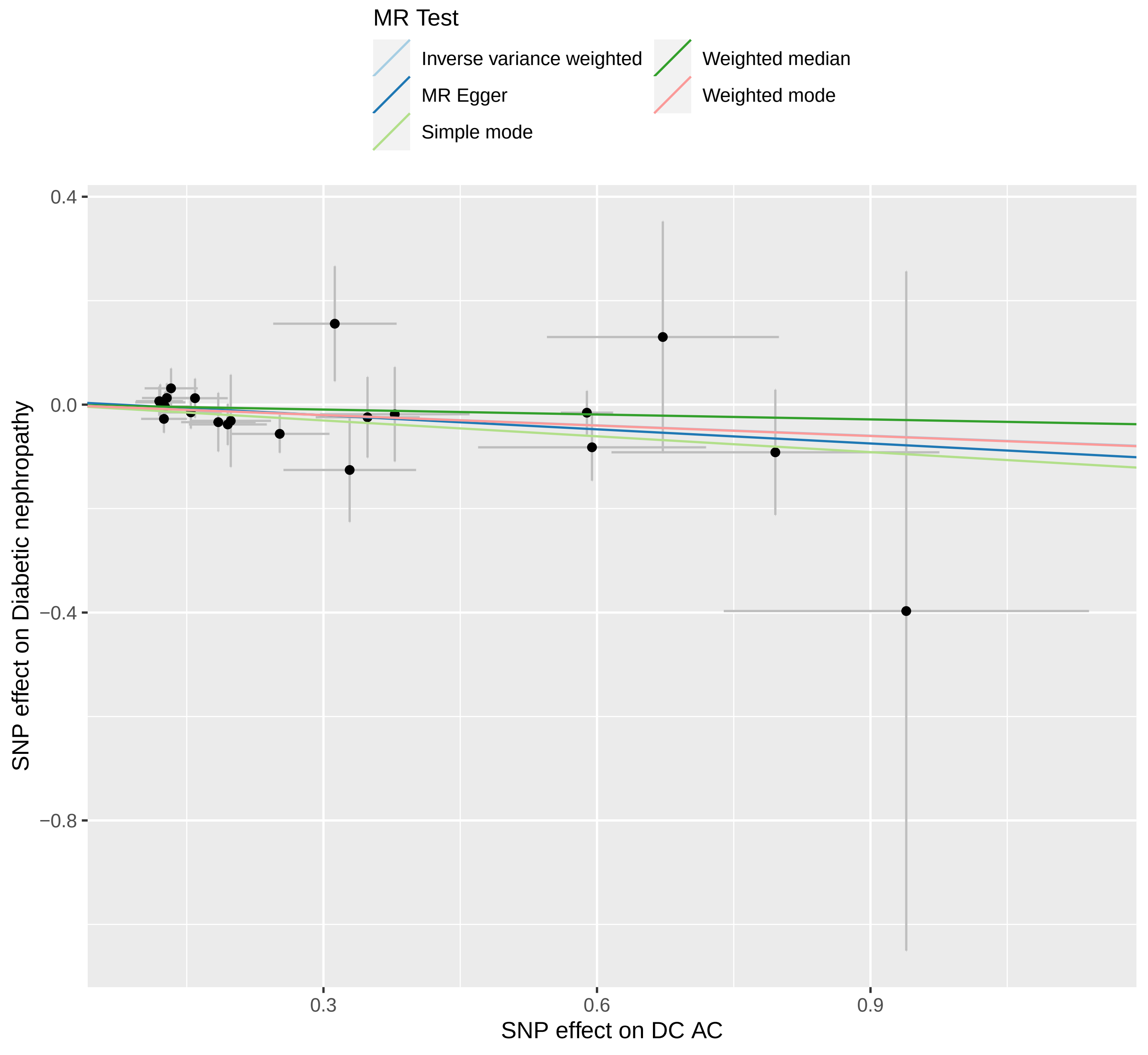

MR Test
Inverse variance weighted
MR Egger
Simple mode
Weighted median
Weighted mode
0.4
0.0
−0.4
−0.8
SNP effect on Diabetic nephropathy
0.3
0.6
0.9
SNP effect on DC AC

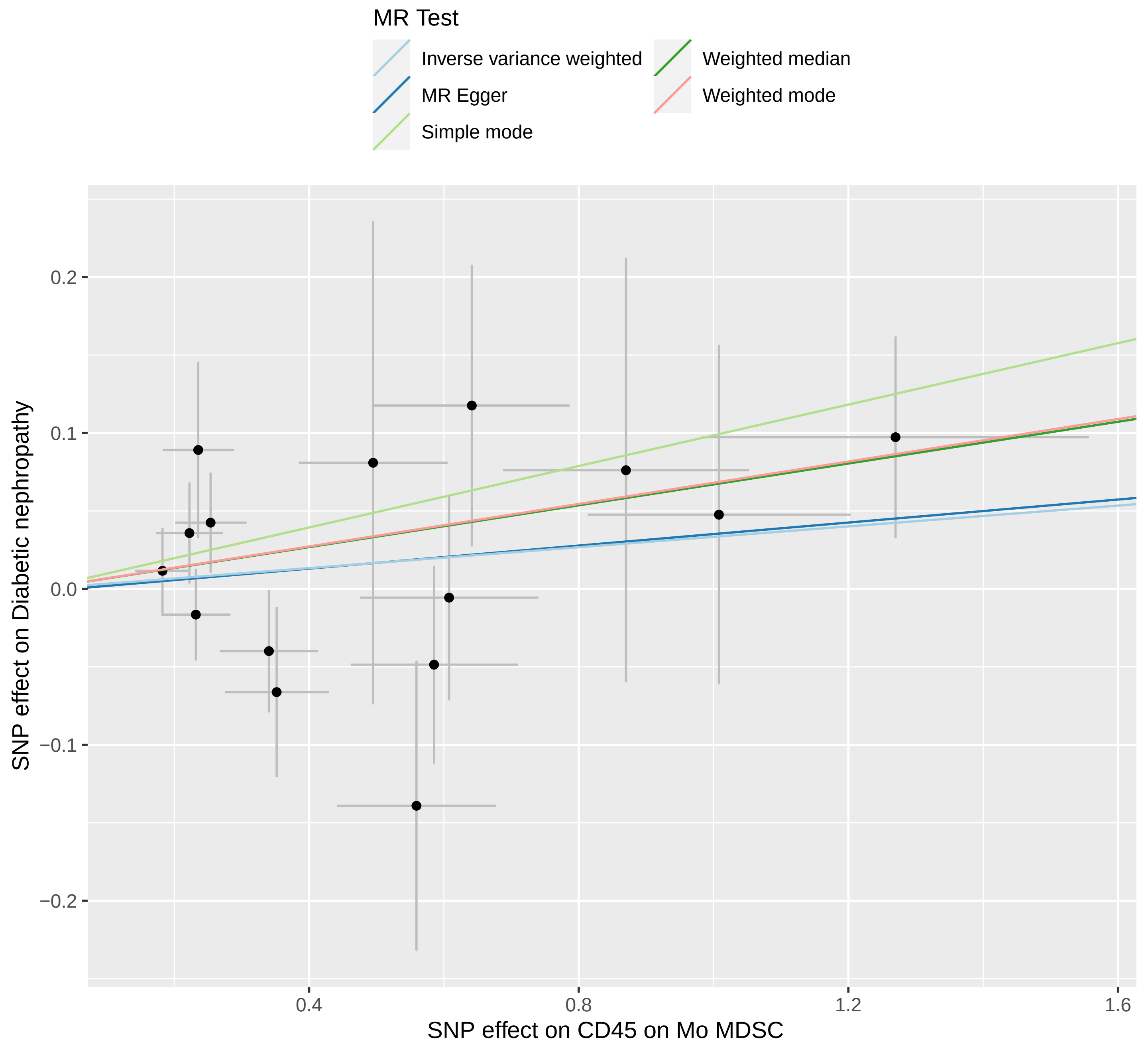
MR Test
Inverse variance weighted
MR Egger
Simple mode
Weighted median
Weighted mode
SNP effect on Diabetic nephropathy
0.2
0.1
0.0
−0.1
−0.2
0.4
0.8
1.2
1.6
SNP effect on CD45 on Mo MDSC

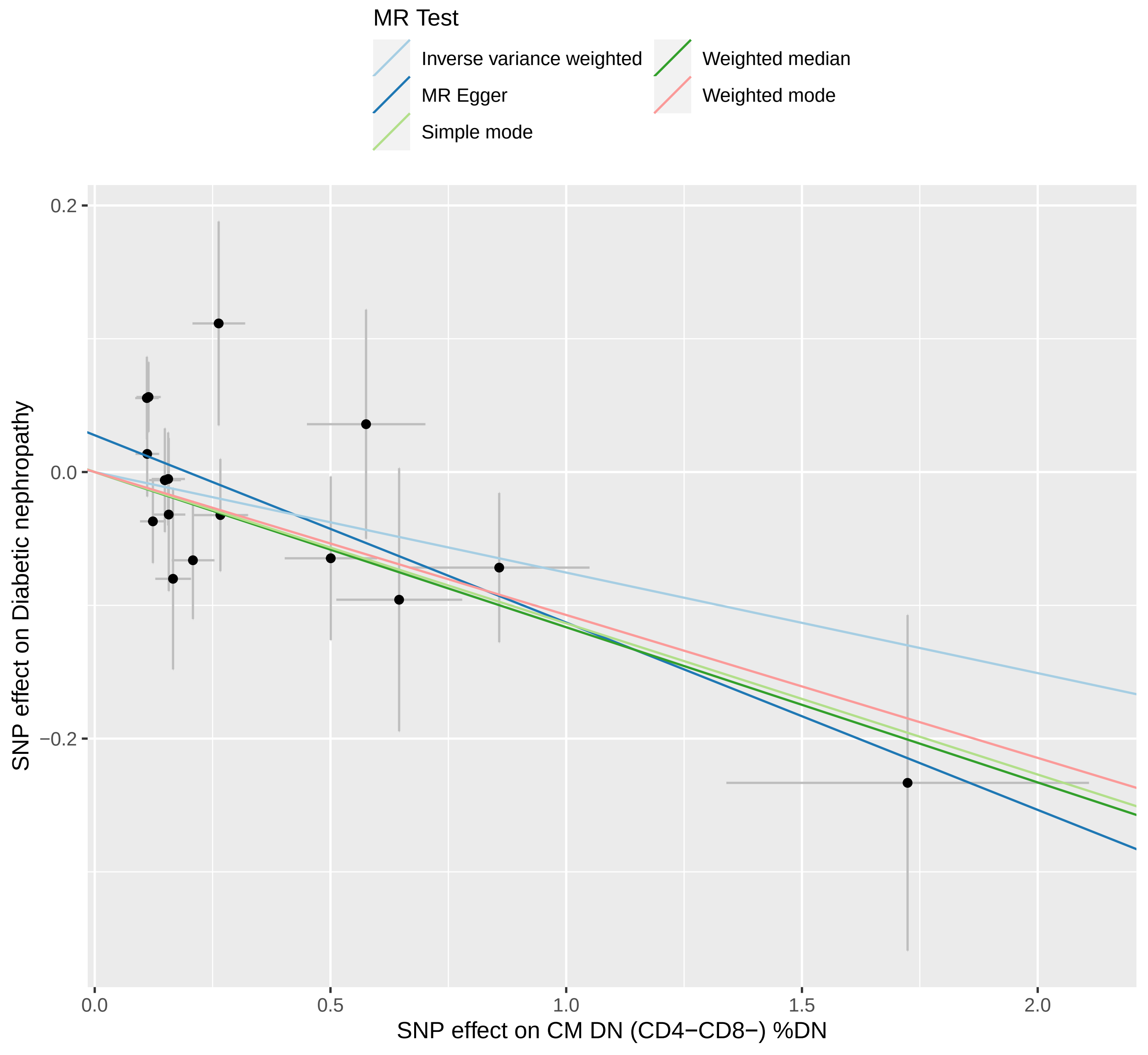
MR Test
Inverse variance weighted
MR Egger
Simple mode
Weighted median
Weighted mode
0.2
0.0
−0.2
0.0
0.5
1.0
1.5
2.0
SNP effect on Diabetic nephropathy
SNP effect on CM DN (CD4−CD8−) %DN

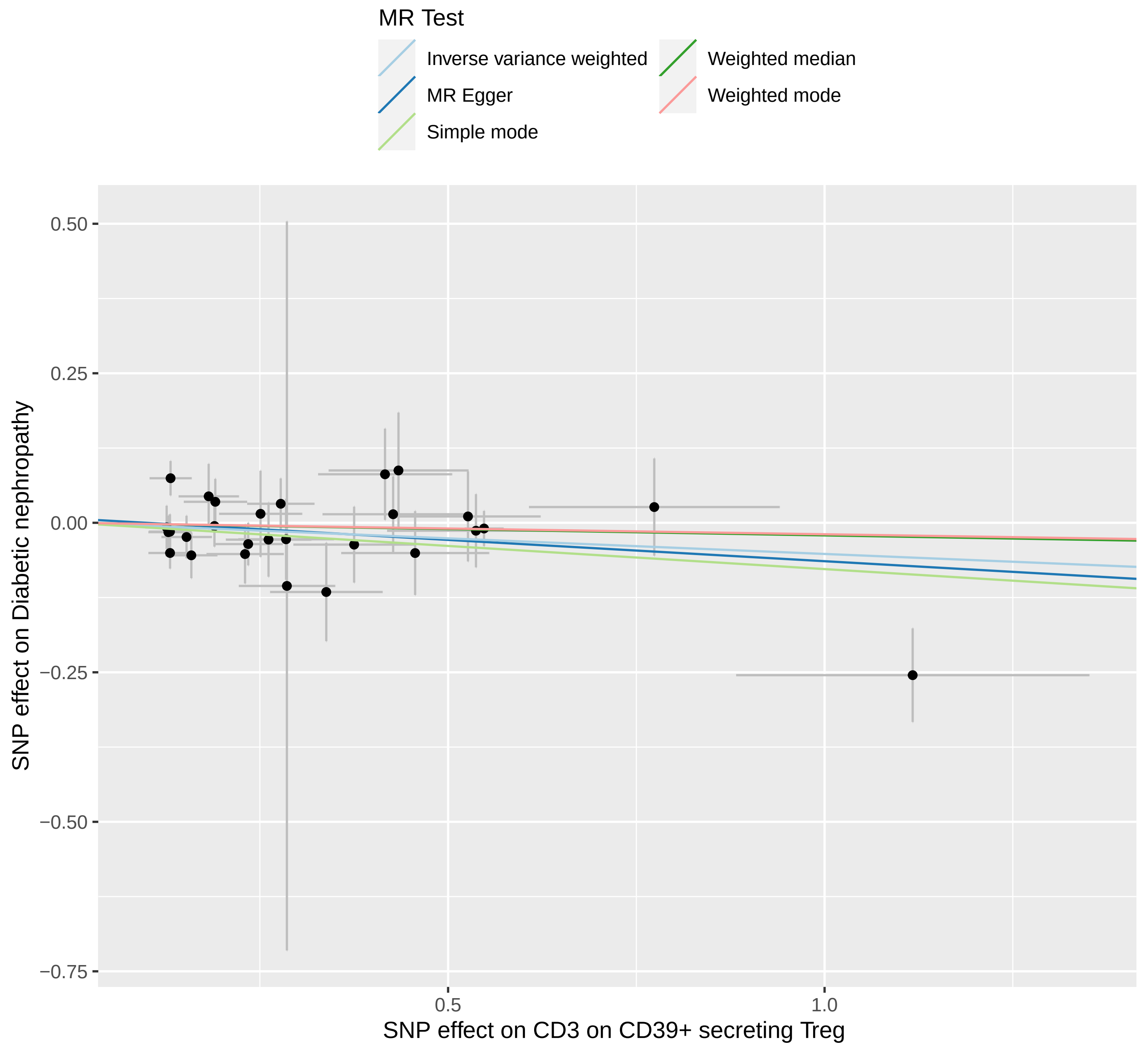
MR Test
Inverse variance weighted
MR Egger
Simple mode
Weighted median
Weighted mode
0.50
0.25
0.00
−0.25
−0.50
−0.75
SNP effect on Diabetic nephropathy
0.5
1.0
SNP effect on CD3 on CD39+ secreting Treg

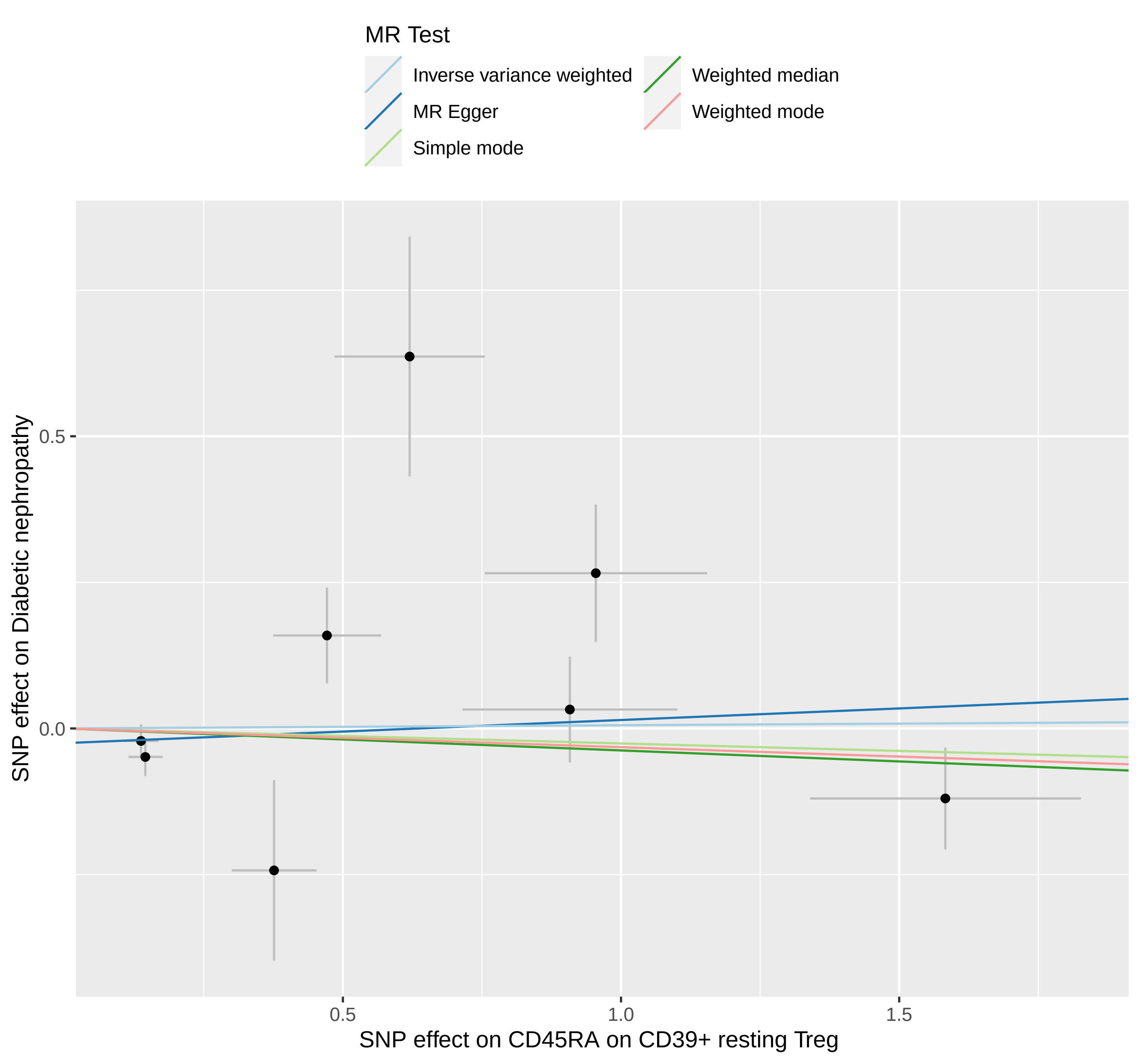

MR Test
Inverse variance weighted
MR Egger
Simple mode
Weighted median
Weighted mode
SNP effect on Diabetic nephropathy
0.5
0.0
0.5
1.0
1.5
SNP effect on CD45RA on CD39+ resting Treg

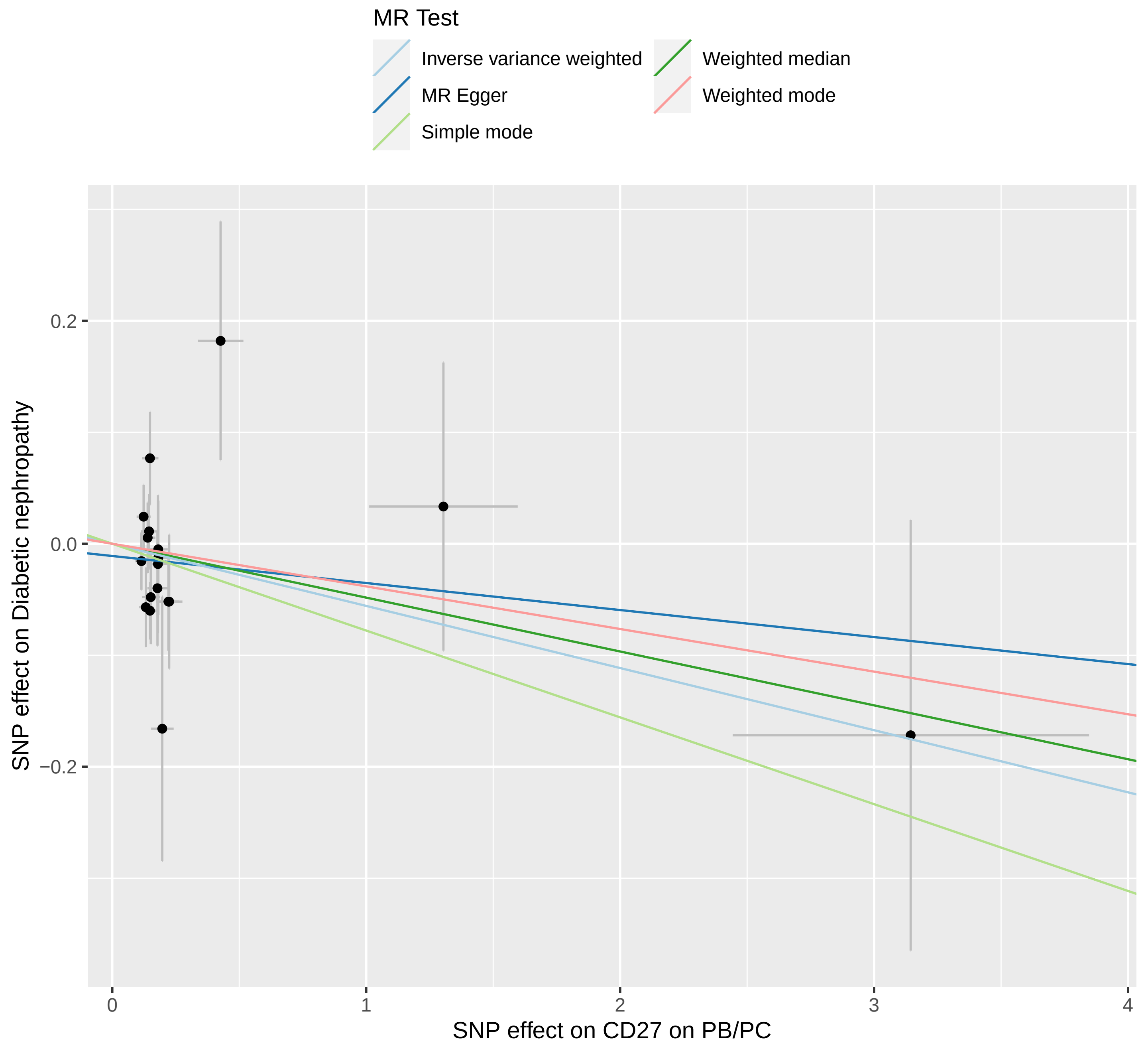

MR Test
Inverse variance weighted
MR Egger
Simple mode
Weighted median
Weighted mode
0.2
0.0
−0.2
SNP effect on Diabetic nephropathy
0
1
2
3
4
SNP effect on CD27 on PB/PC

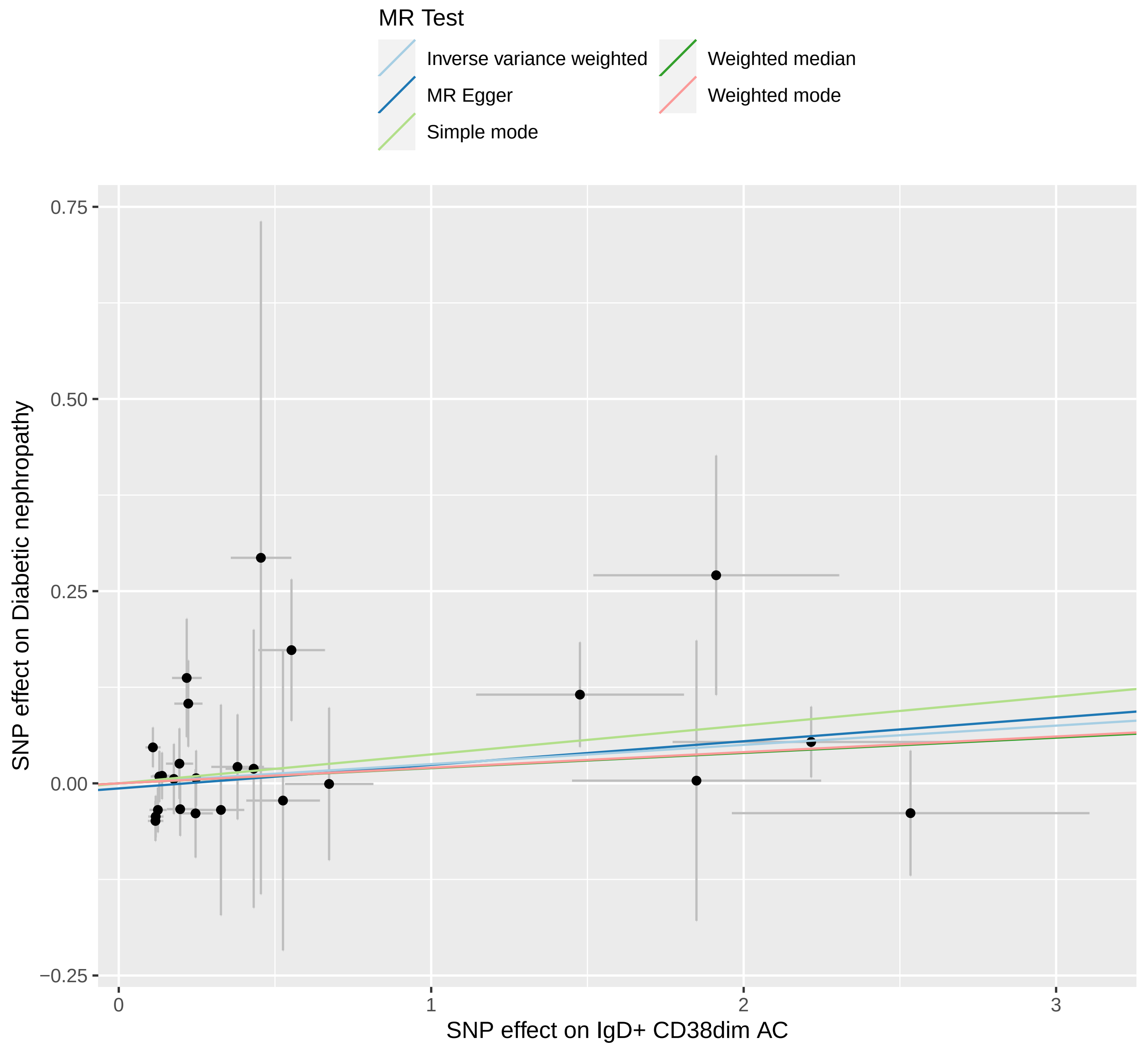
MR Test
Inverse variance weighted
MR Egger
Simple mode
Weighted median
Weighted mode
0.75
0.50
0.25
0.00
−0.25
0
1
2
3
SNP effect on Diabetic nephropathy
SNP effect on IgD+ CD38dim AC

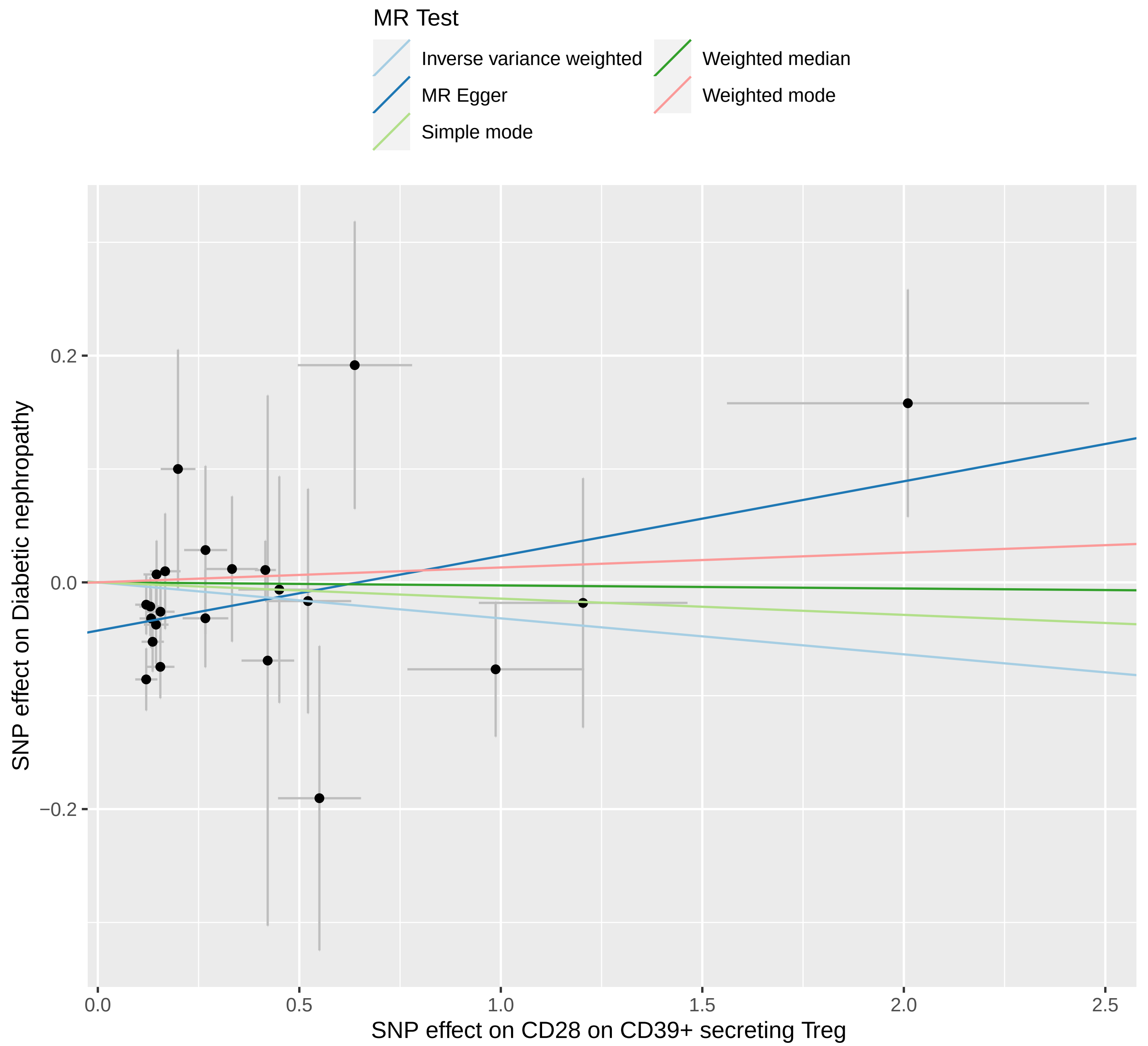
MR Test
Inverse variance weighted
MR Egger
Simple mode
Weighted median
Weighted mode
0.2
0.0
−0.2
0.0
0.5
1.0
1.5
2.0
2.5
SNP effect on Diabetic nephropathy
SNP effect on CD28 on CD39+ secreting Treg

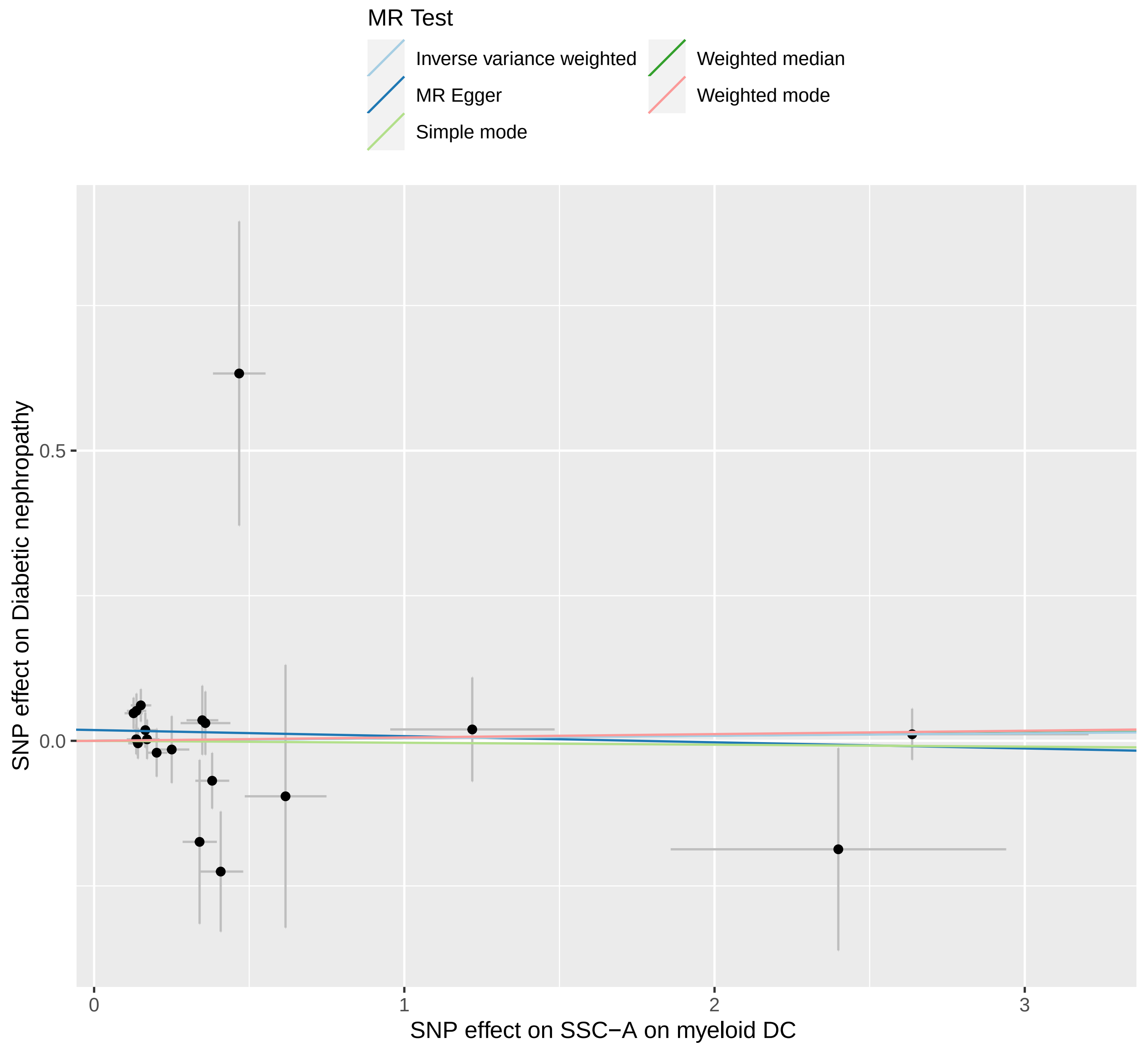
MR Test
Inverse variance weighted
MR Egger
Simple mode
Weighted median
Weighted mode
0.5
0.0
0
1
2
3
SNP effect on Diabetic nephropathy
SNP effect on SSC−A on myeloid DC

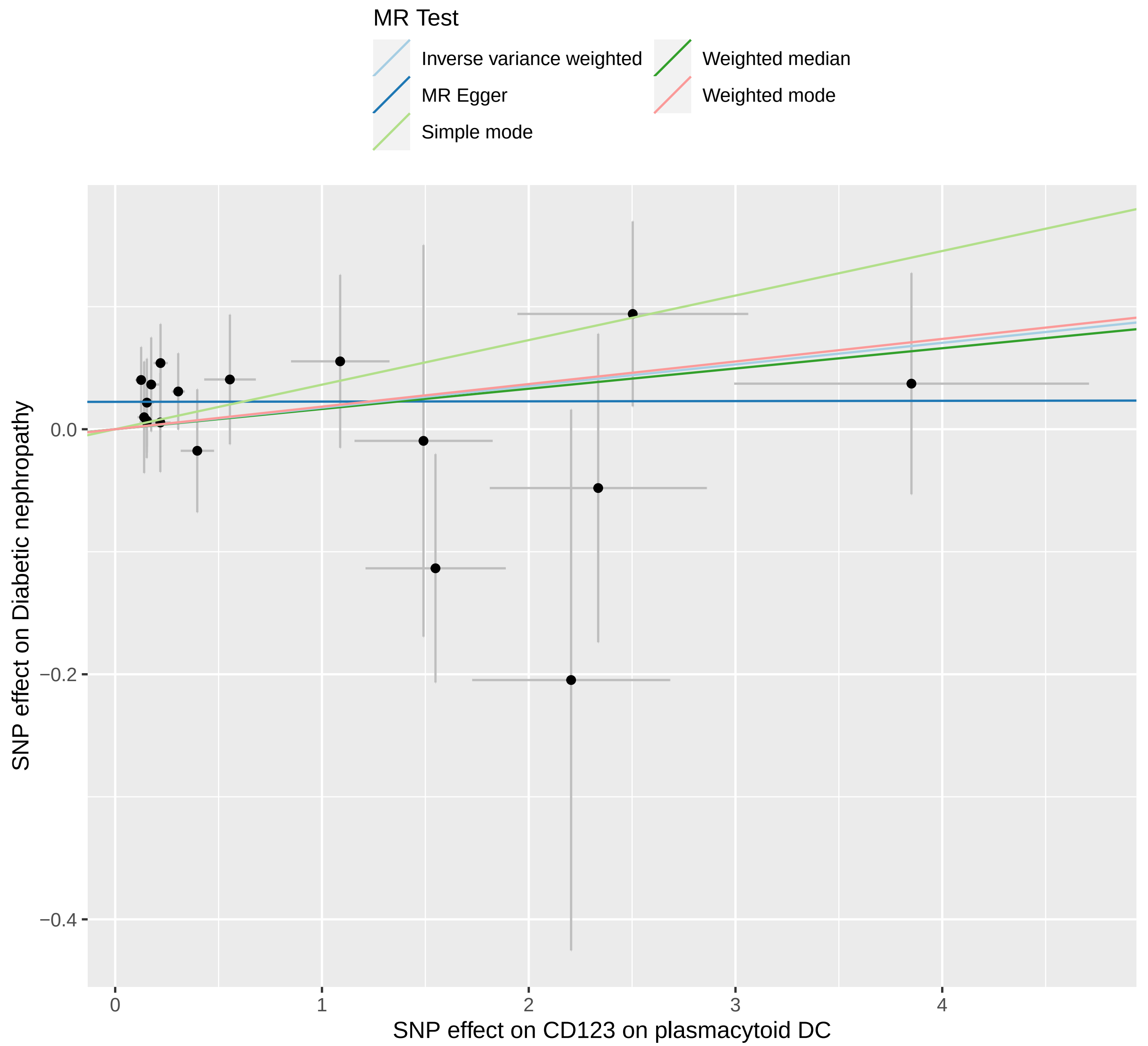

MR Test
Inverse variance weighted
MR Egger
Simple mode
Weighted median
Weighted mode
SNP effect on Diabetic nephropathy
0.0
−0.2
−0.4
0
1
2
3
4
SNP effect on CD123 on plasmacytoid DC

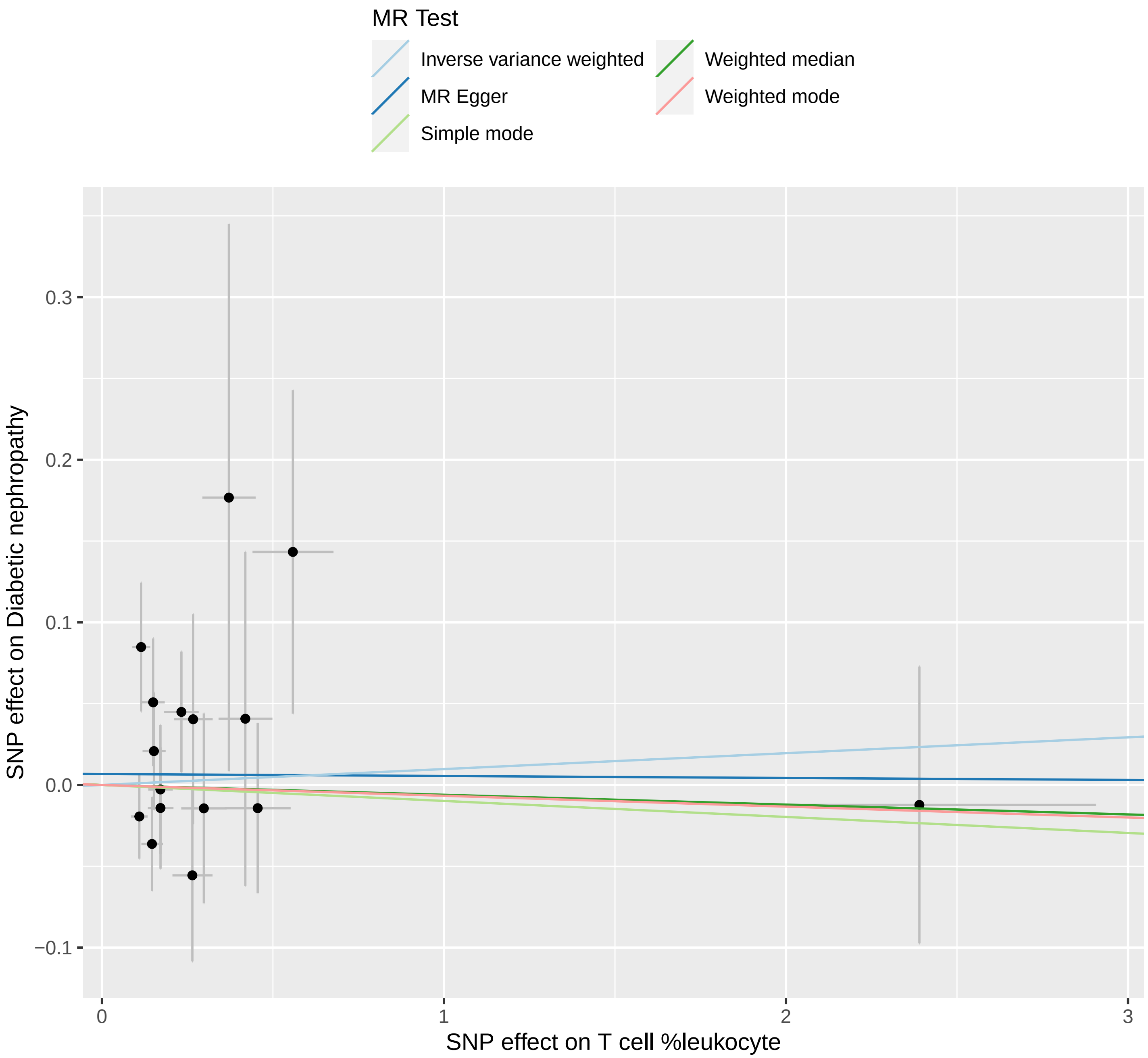

MR Test
Inverse variance weighted
MR Egger
Simple mode
Weighted median
Weighted mode
SNP effect on Diabetic nephropathy
0.3
0.2
0.1
0.0
−0.1
0
1
2
3
SNP effect on T cell %leukocyte

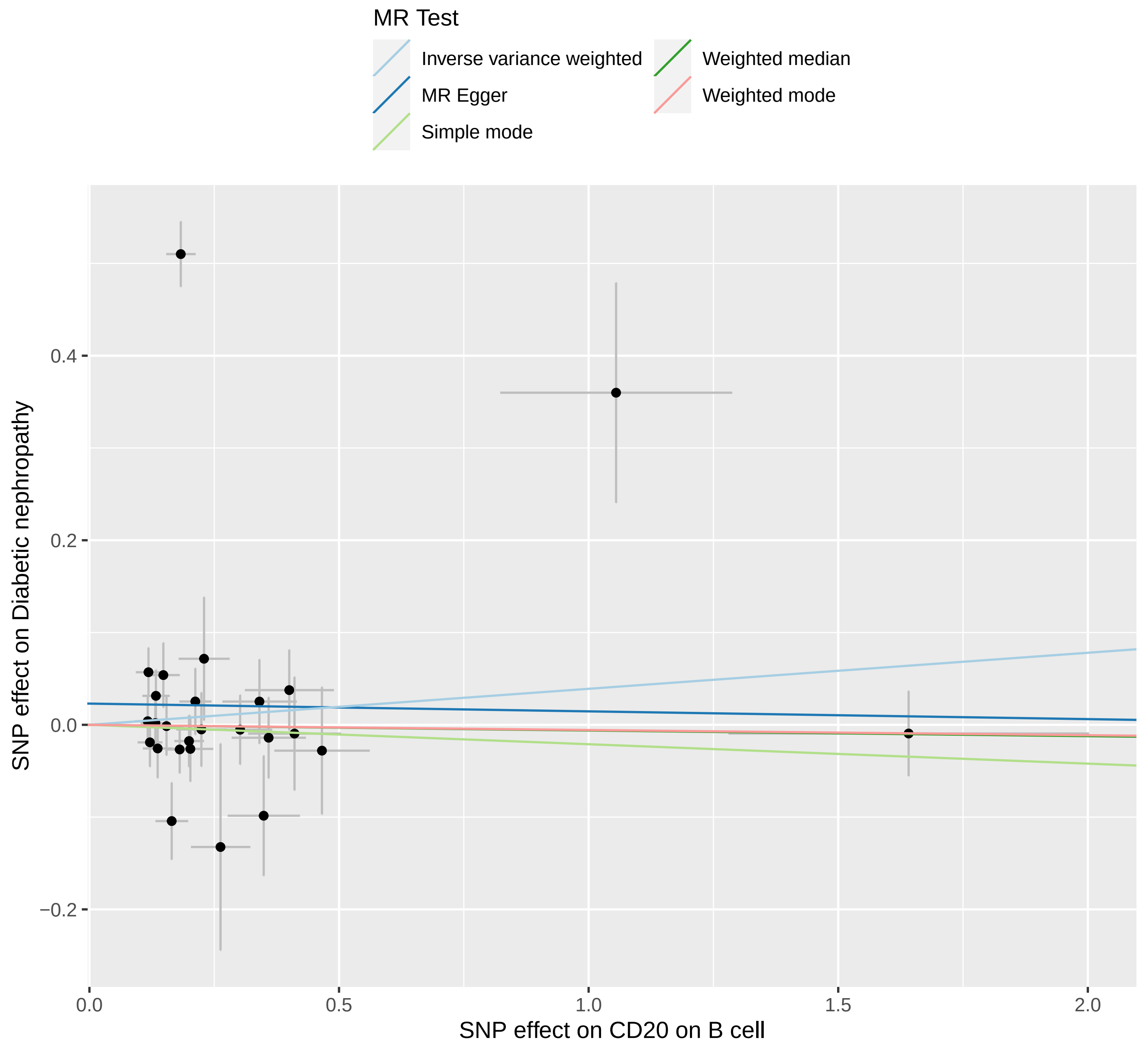
MR Test
Inverse variance weighted
MR Egger
Simple mode
Weighted median
Weighted mode
SNP effect on Diabetic nephropathy
0.4
0.2
0.0
−0.2
0.0
0.5
1.0
1.5
2.0
SNP effect on CD20 on B cell

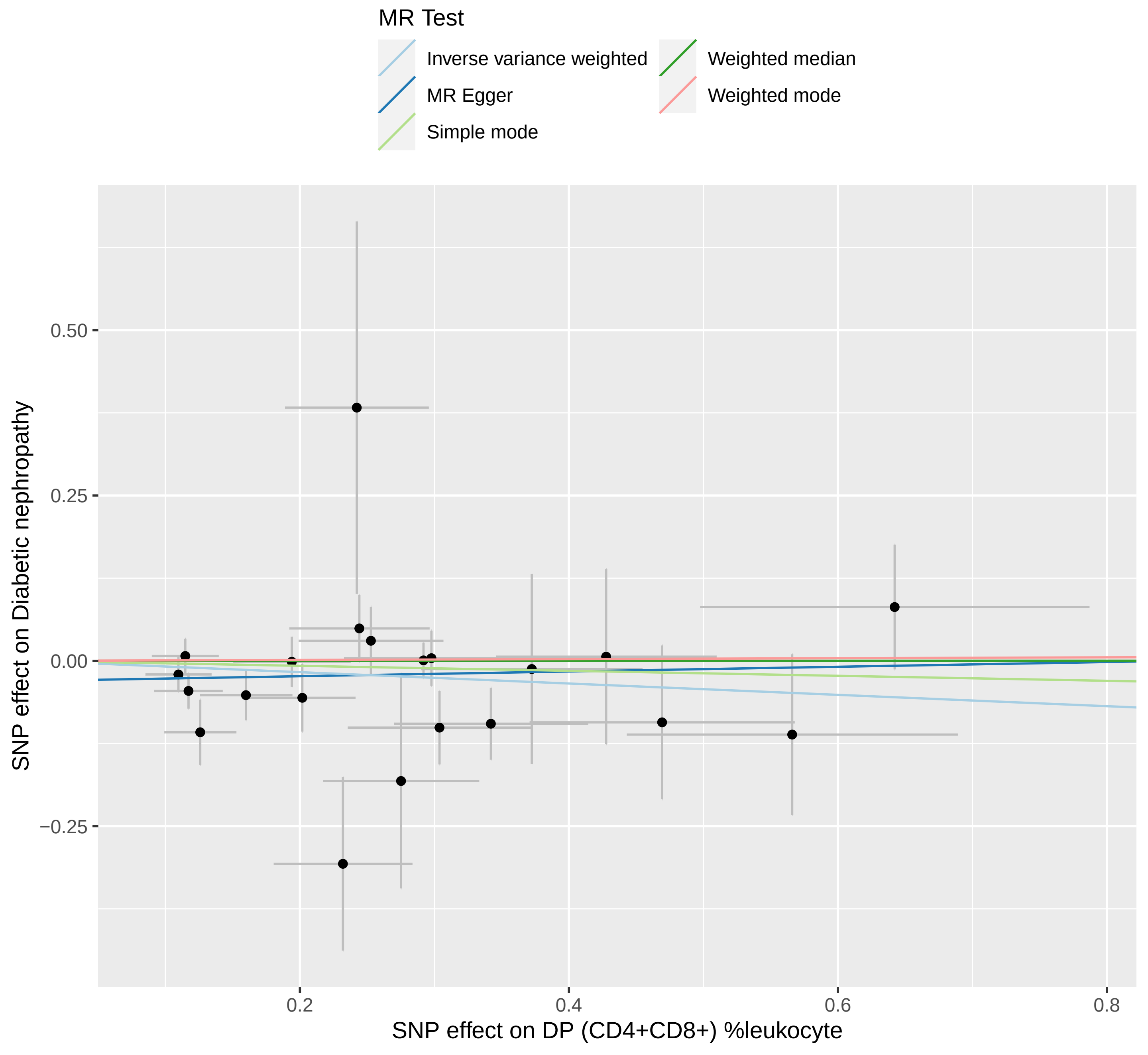
MR Test
Inverse variance weighted
MR Egger
Simple mode
Weighted median
Weighted mode
0.50
0.25
0.00
−0.25
0.2
0.4
0.6
0.8
SNP effect on Diabetic nephropathy
SNP effect on DP (CD4+CD8+) %leukocyte

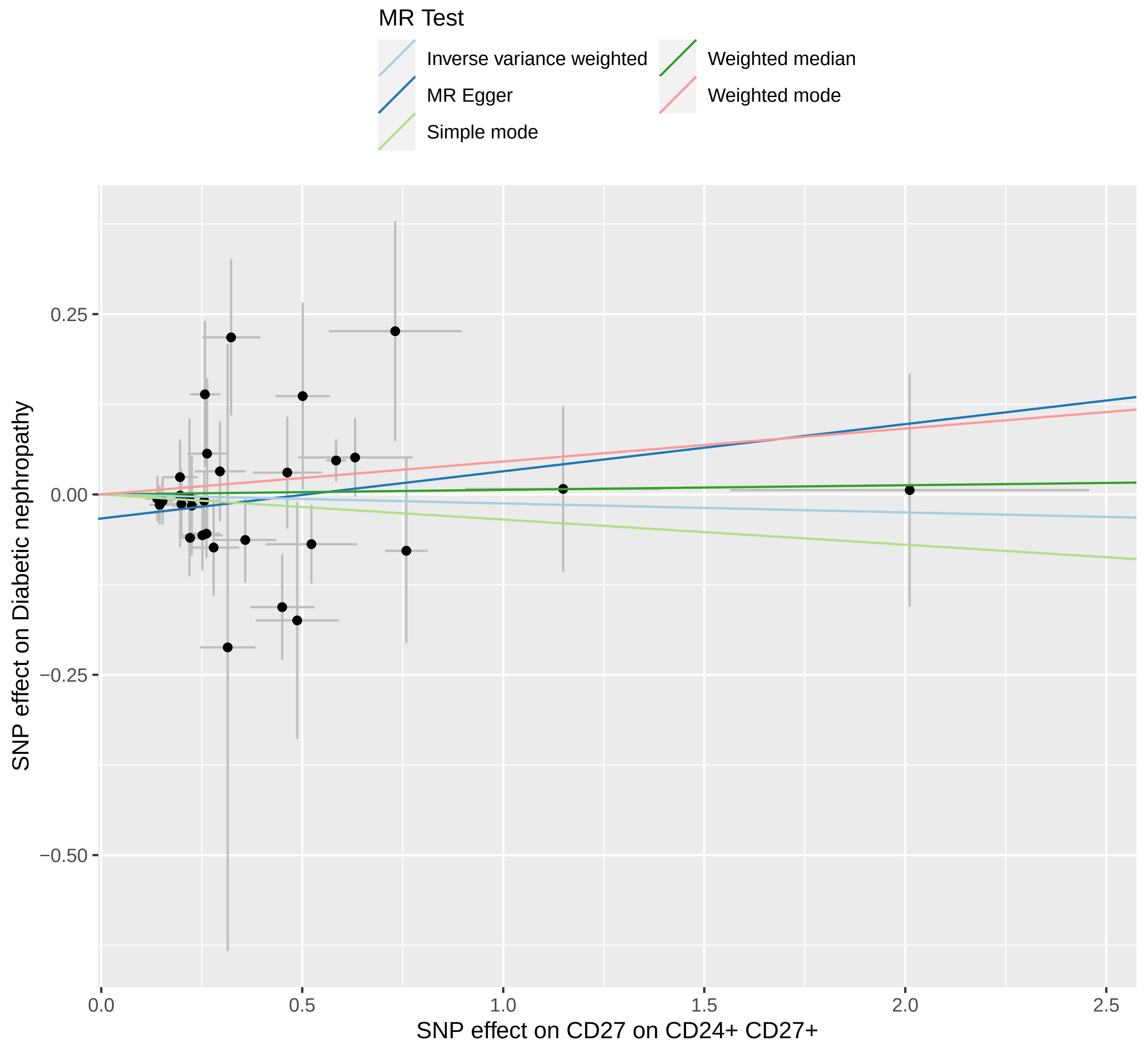
MR Test
Inverse variance weighted
MR Egger
Simple mode
Weighted median
Weighted mode
SNP effect on Diabetic nephropathy
0.25
0.00
−0.25
−0.50
0.0
0.5
1.0
1.5
2.0
2.5
SNP effect on CD27 on CD24+ CD27+

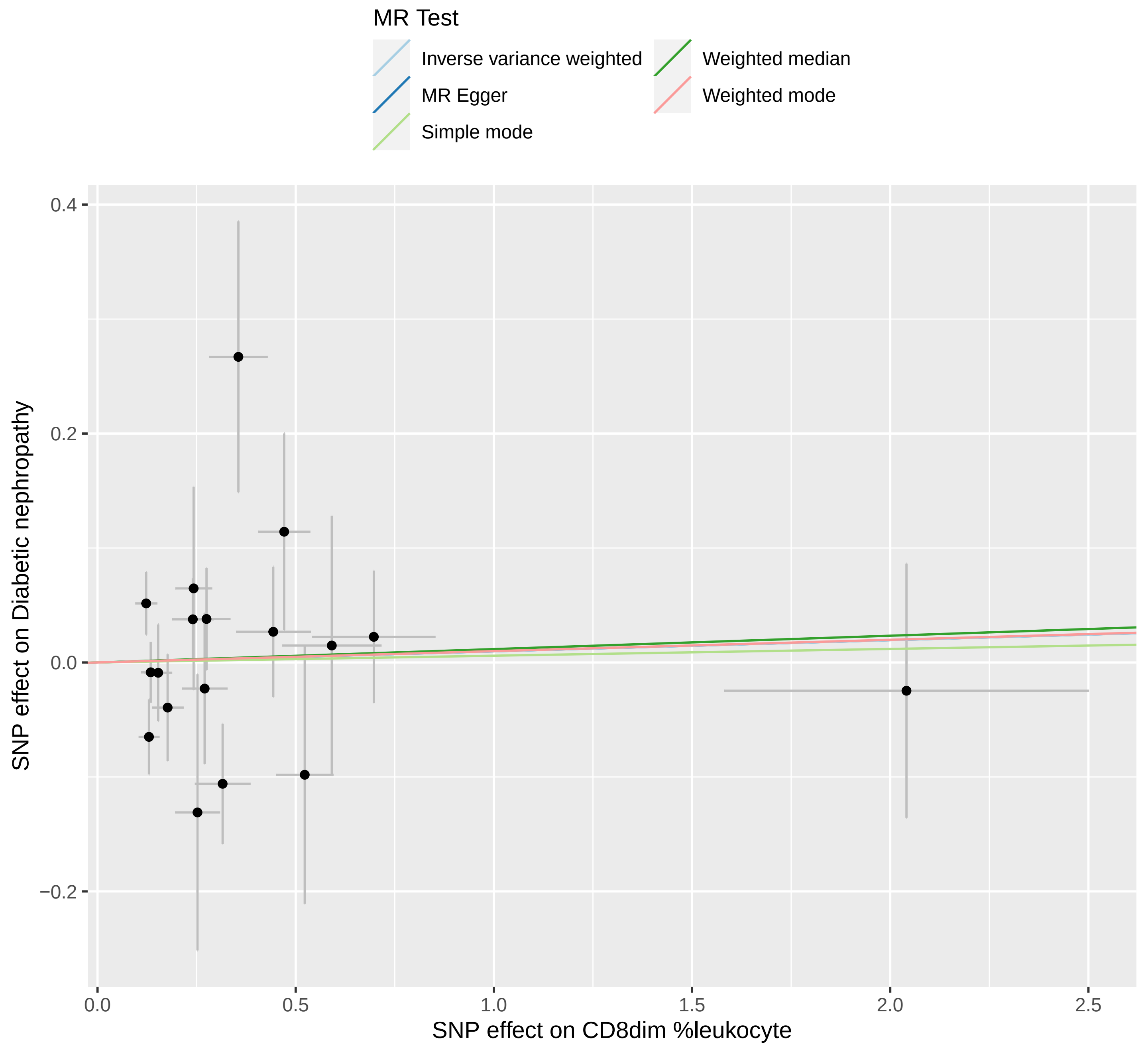

MR Test
Inverse variance weighted
MR Egger
Simple mode
Weighted median
Weighted mode
0.4
0.2
0.0
−0.2
0.0
0.5
1.0
1.5
2.0
2.5
SNP effect on Diabetic nephropathy
SNP effect on CD8dim %leukocyte

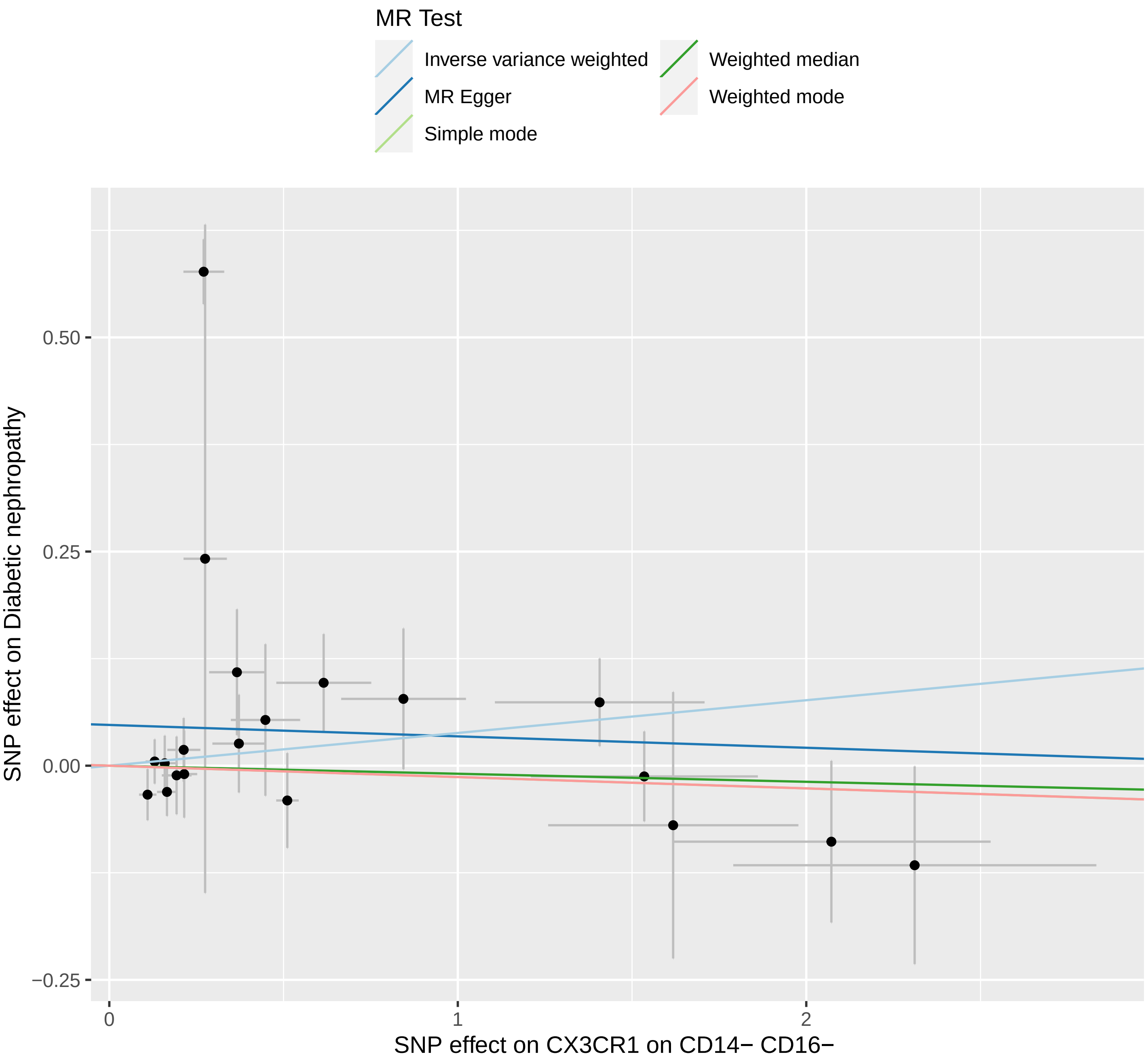
MR Test
Inverse variance weighted
MR Egger
Simple mode
Weighted median
Weighted mode
SNP effect on Diabetic nephropathy
0.50
0.25
0.00
−0.25
0
1
2
SNP effect on CX3CR1 on CD14− CD16−

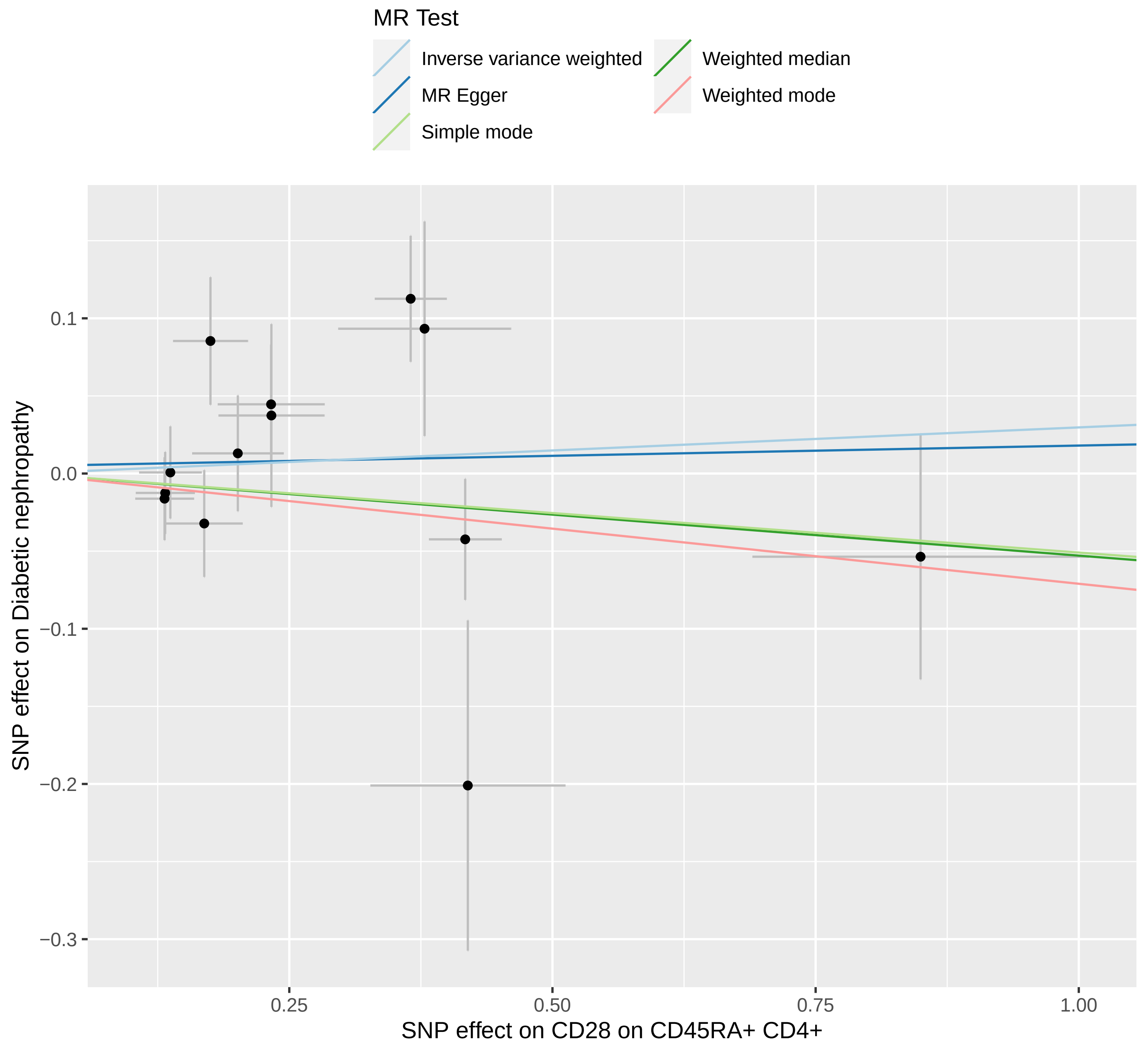
MR Test
Inverse variance weighted
MR Egger
Simple mode
Weighted median
Weighted mode
0.1
0.0
−0.1
−0.2
−0.3
0.25
0.50
0.75
1.00
SNP effect on Diabetic nephropathy
SNP effect on CD28 on CD45RA+ CD4+

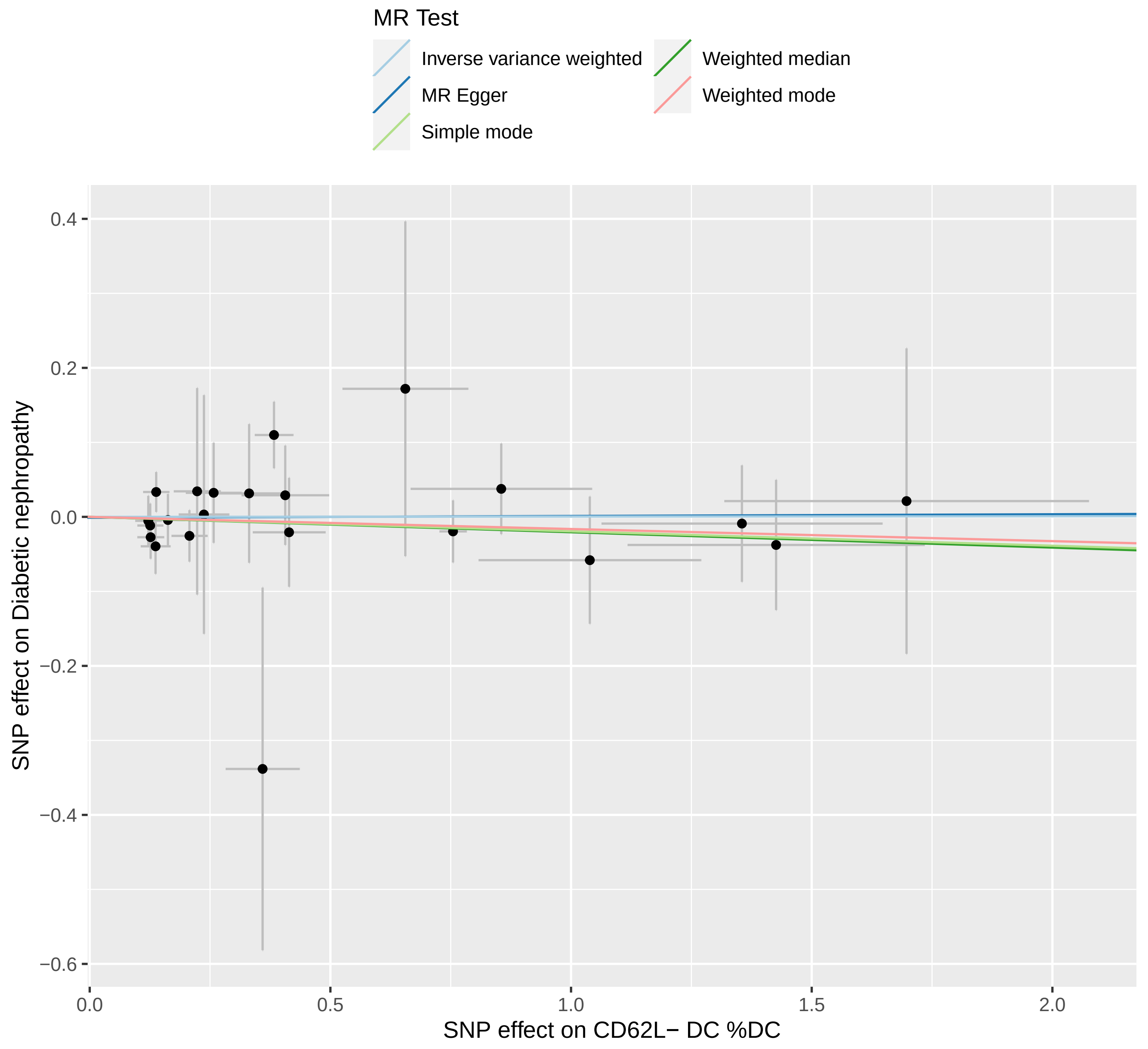
MR Test
Inverse variance weighted
MR Egger
Simple mode
Weighted median
Weighted mode
0.4
0.2
0.0
−0.2
−0.4
−0.6
SNP effect on Diabetic nephropathy
0.0
0.5
1.0
1.5
2.0
SNP effect on CD62L− DC %DC

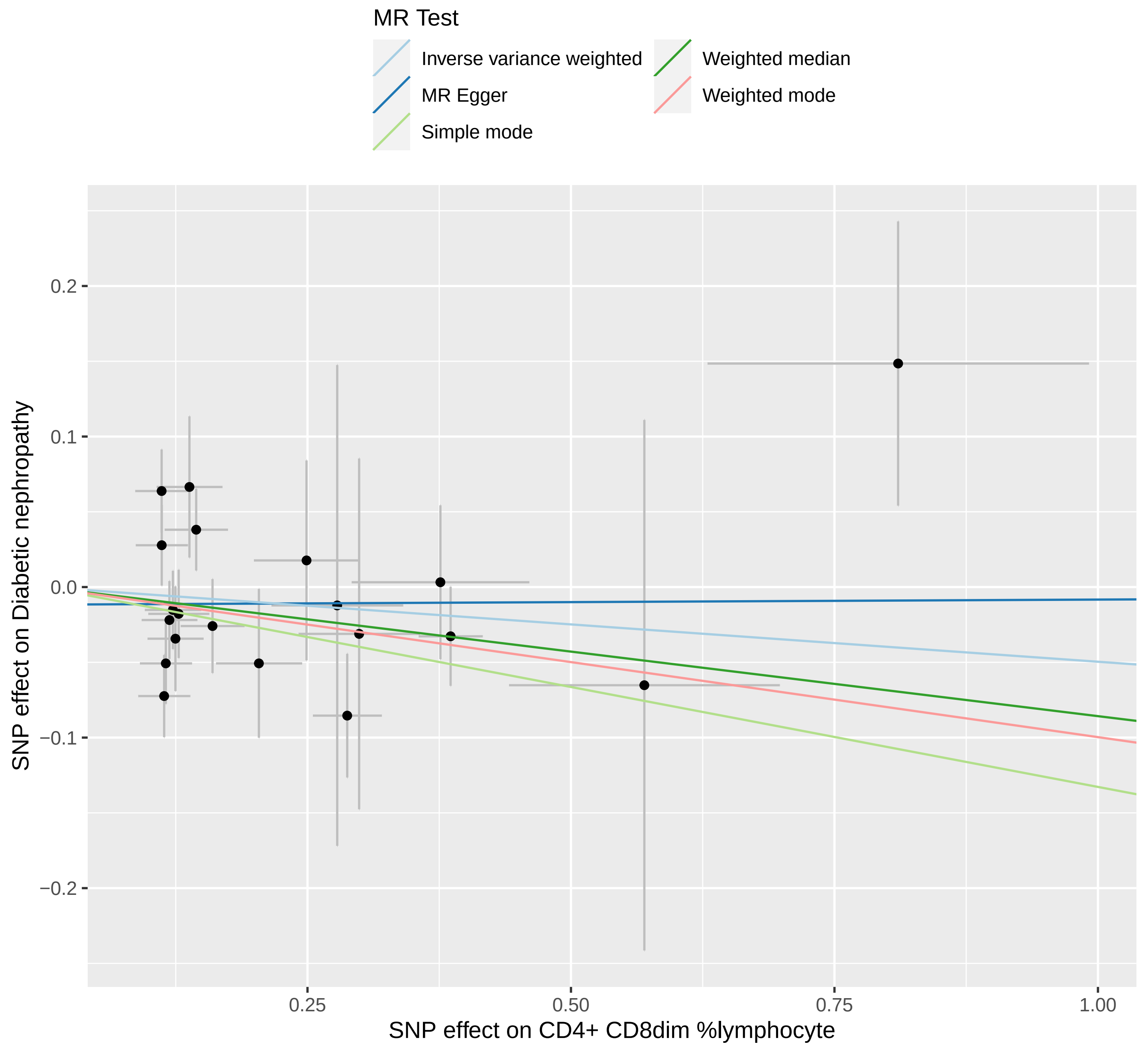
MR Test
Inverse variance weighted
Weighted median
MR Egger
Weighted mode
Simple mode
0.2
0.1
0.0
−0.1
−0.2
SNP effect on Diabetic nephropathy
0.25
0.50
0.75
1.00
SNP effect on CD4+ CD8dim %lymphocyte

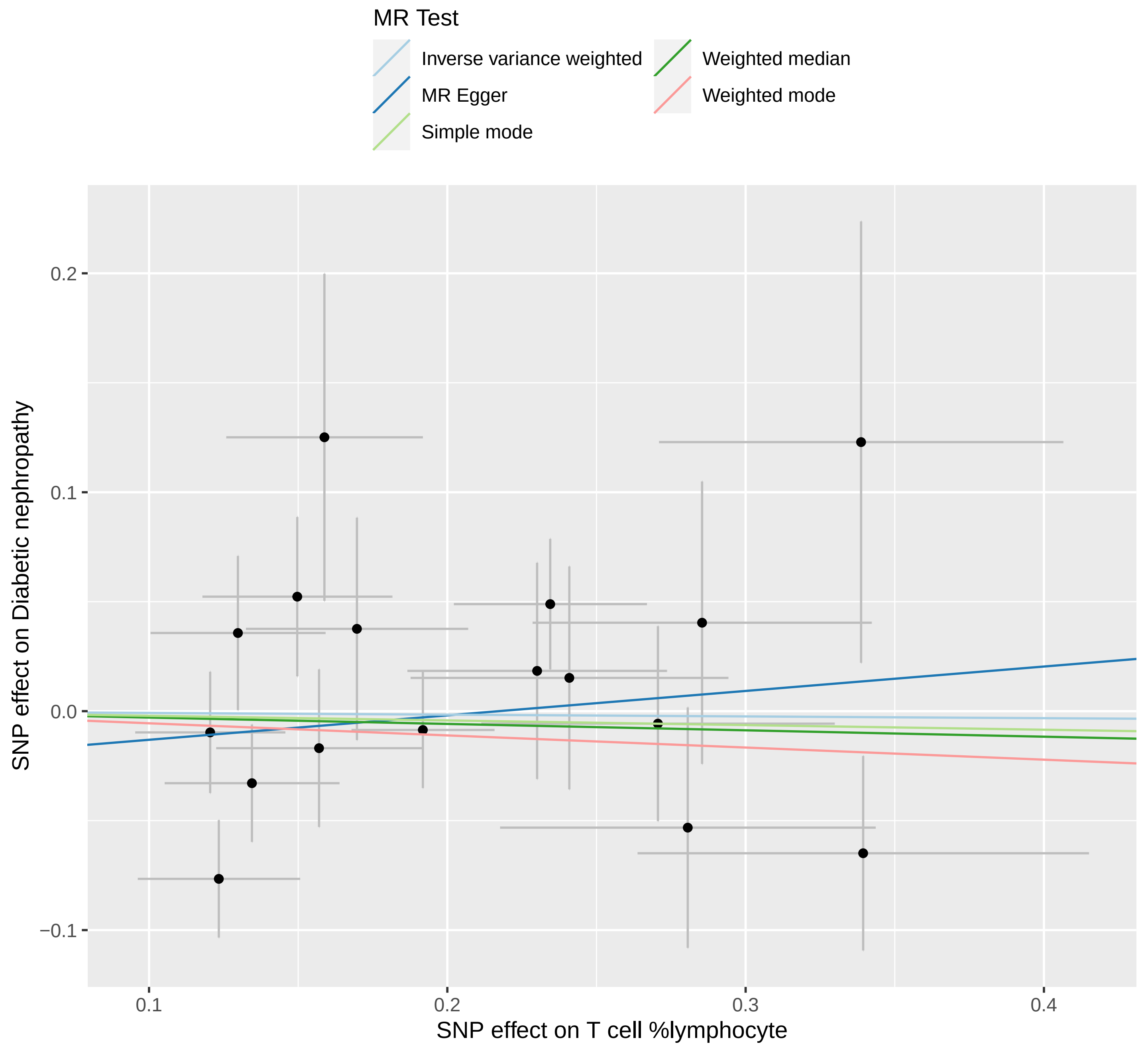
MR Test
Inverse variance weighted
MR Egger
Simple mode
Weighted median
Weighted mode
SNP effect on Diabetic nephropathy
0.2
0.1
0.0
−0.1
0.1
0.2
0.3
0.4
SNP effect on T cell %lymphocyte

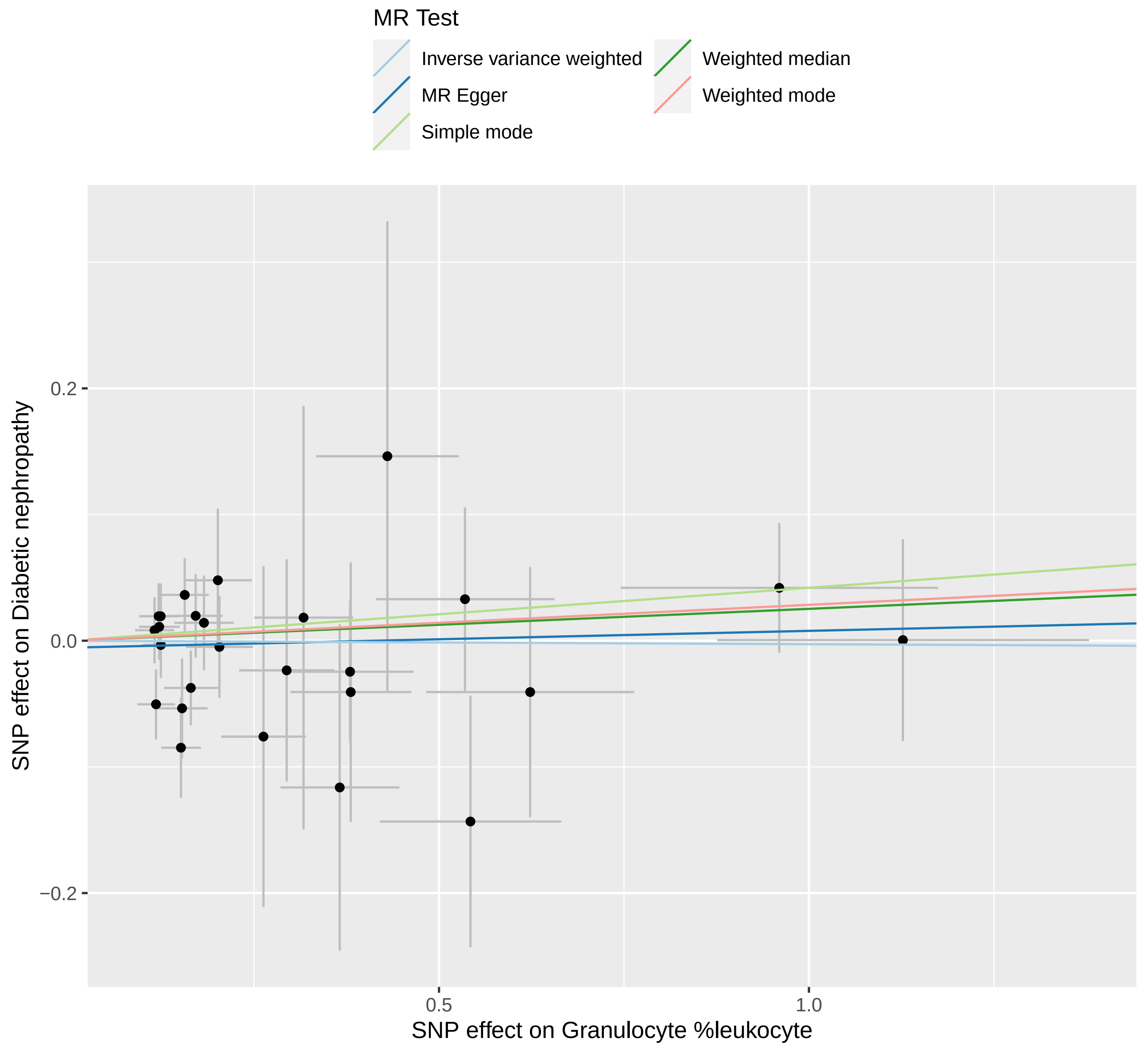
MR Test
Inverse variance weighted
MR Egger
Simple mode
Weighted median
Weighted mode
0.2
0.0
−0.2
0.5
1.0
SNP effect on Diabetic nephropathy
SNP effect on Granulocyte %leukocyte

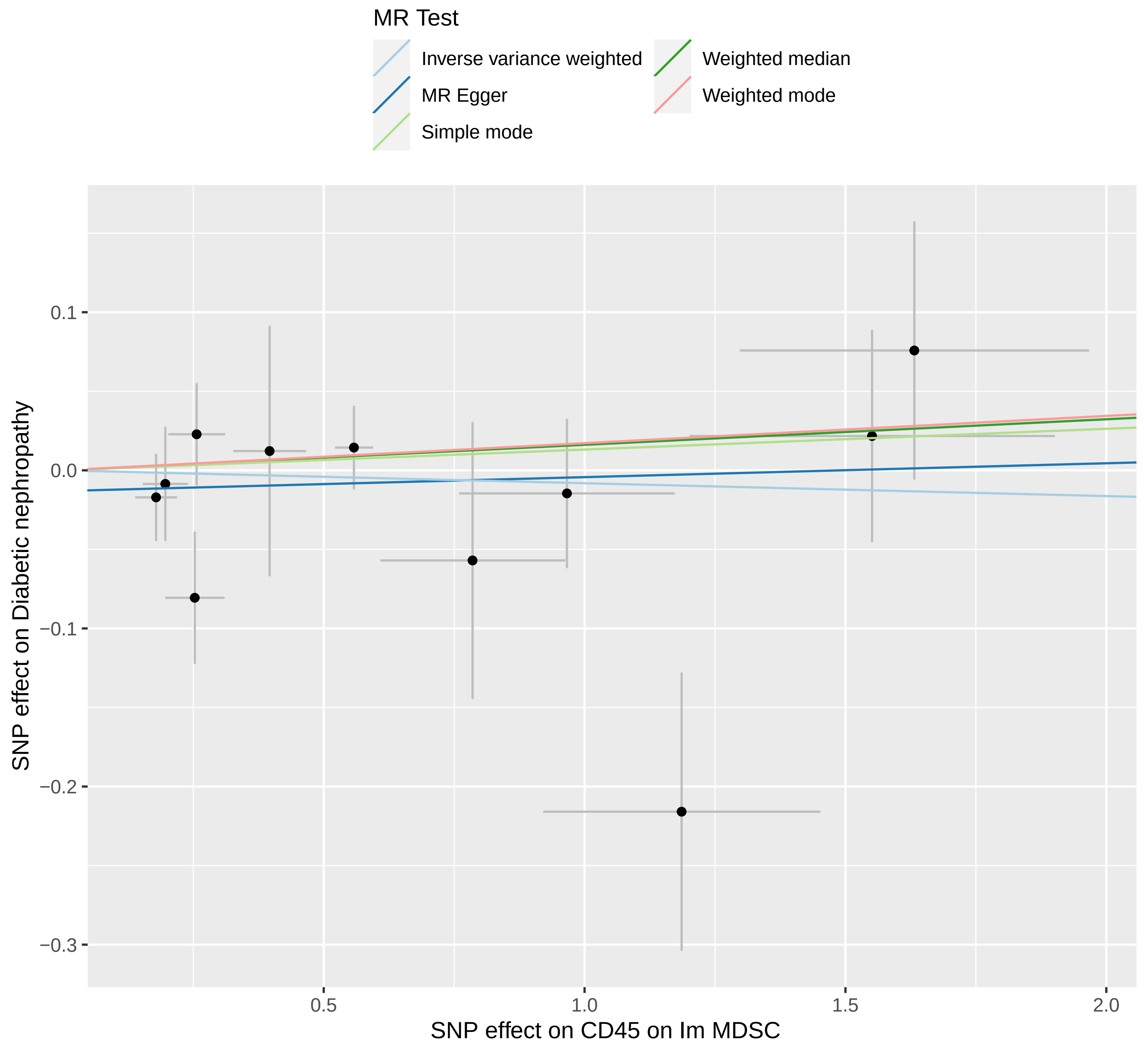
MR Test
Inverse variance weighted
MR Egger
Simple mode
Weighted median
Weighted mode
0.1
0.0
−0.1
−0.2
−0.3
0.5
1.0
1.5
2.0
SNP effect on Diabetic nephropathy
SNP effect on CD45 on Im MDSC

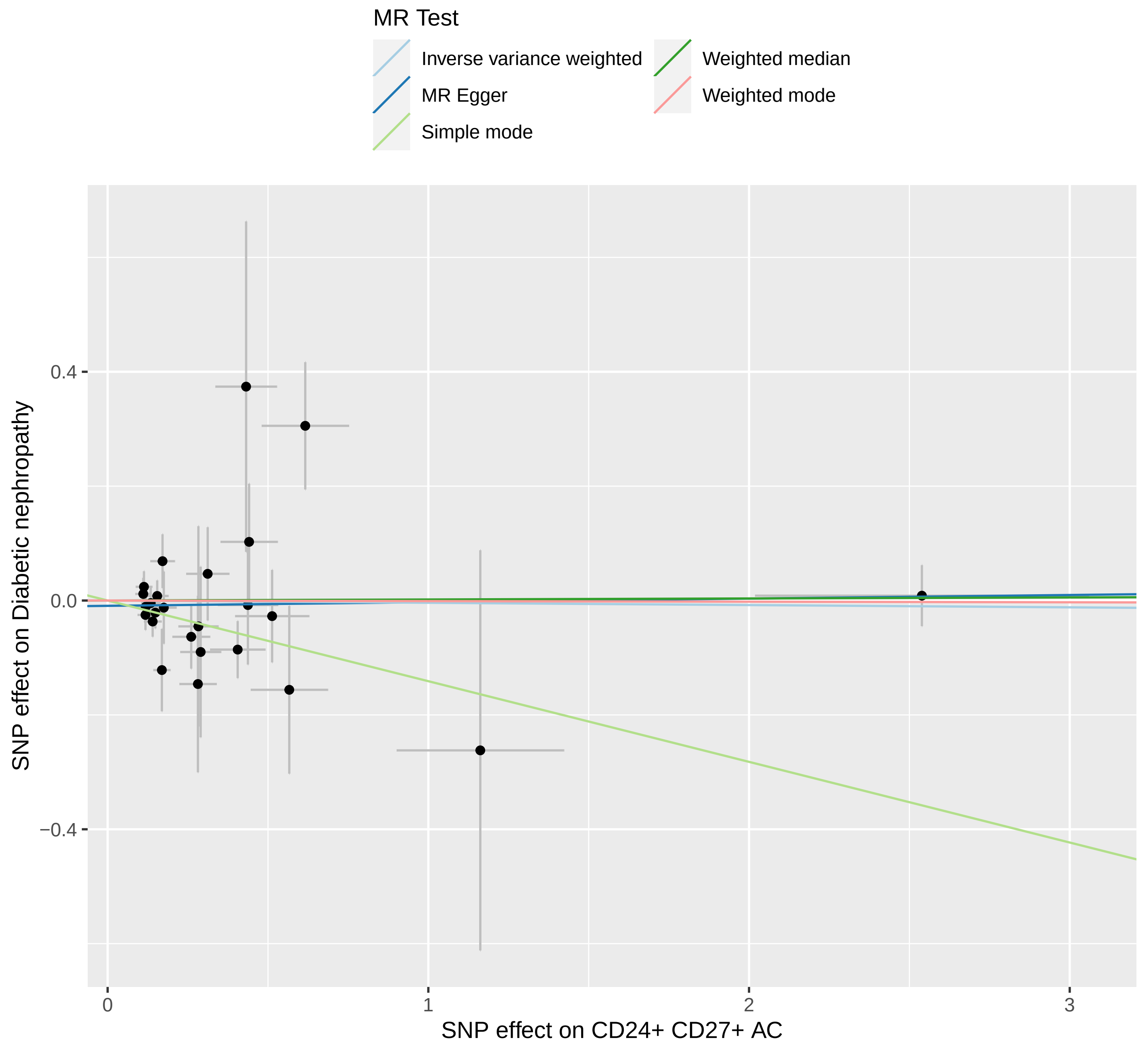

MR Test
Inverse variance weighted
MR Egger
Simple mode
Weighted median
Weighted mode
0.4
0.0
−0.4
0
1
2
3
SNP effect on Diabetic nephropathy
SNP effect on CD24+ CD27+ AC

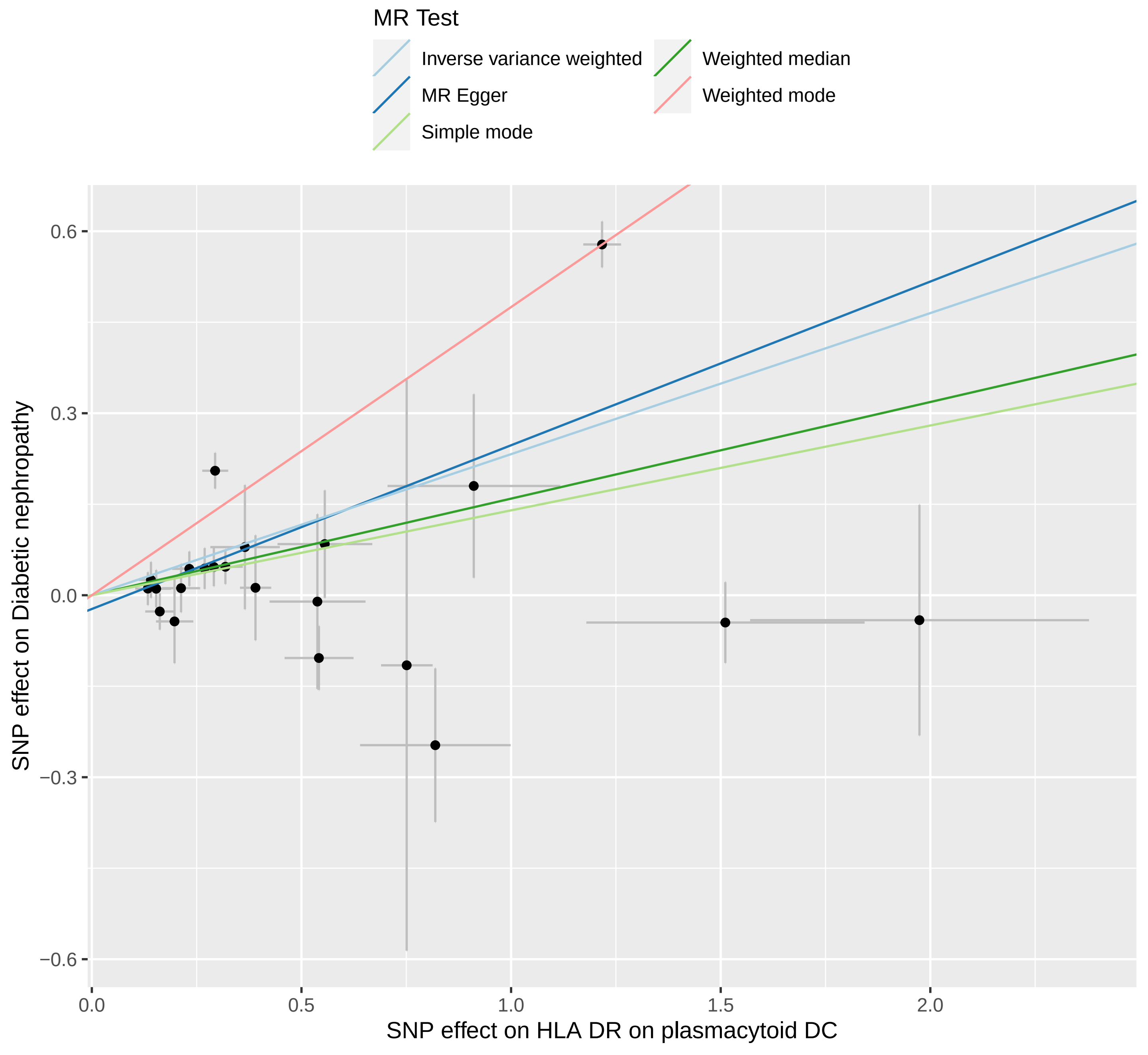
MR Test
Inverse variance weighted
MR Egger
Simple mode
Weighted median
Weighted mode
0.6
0.3
0.0
−0.3
−0.6
0.0
0.5
1.0
1.5
2.0
SNP effect on Diabetic nephropathy
SNP effect on HLA DR on plasmacytoid DC

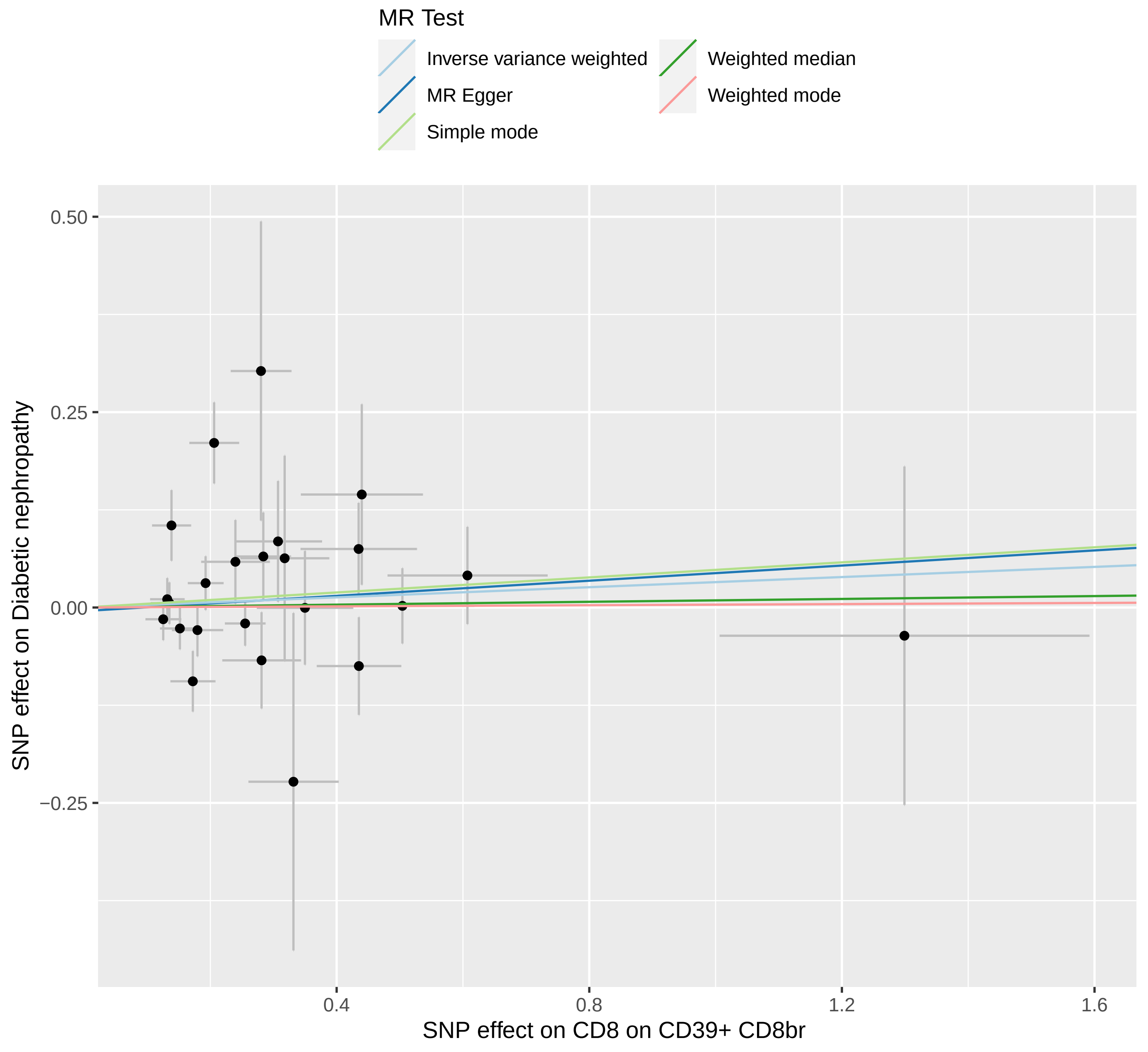

MR Test
Inverse variance weighted
MR Egger
Simple mode
Weighted median
Weighted mode
0.50
0.25
0.00
−0.25
SNP effect on Diabetic nephropathy
0.4
0.8
1.2
1.6
SNP effect on CD8 on CD39+ CD8br

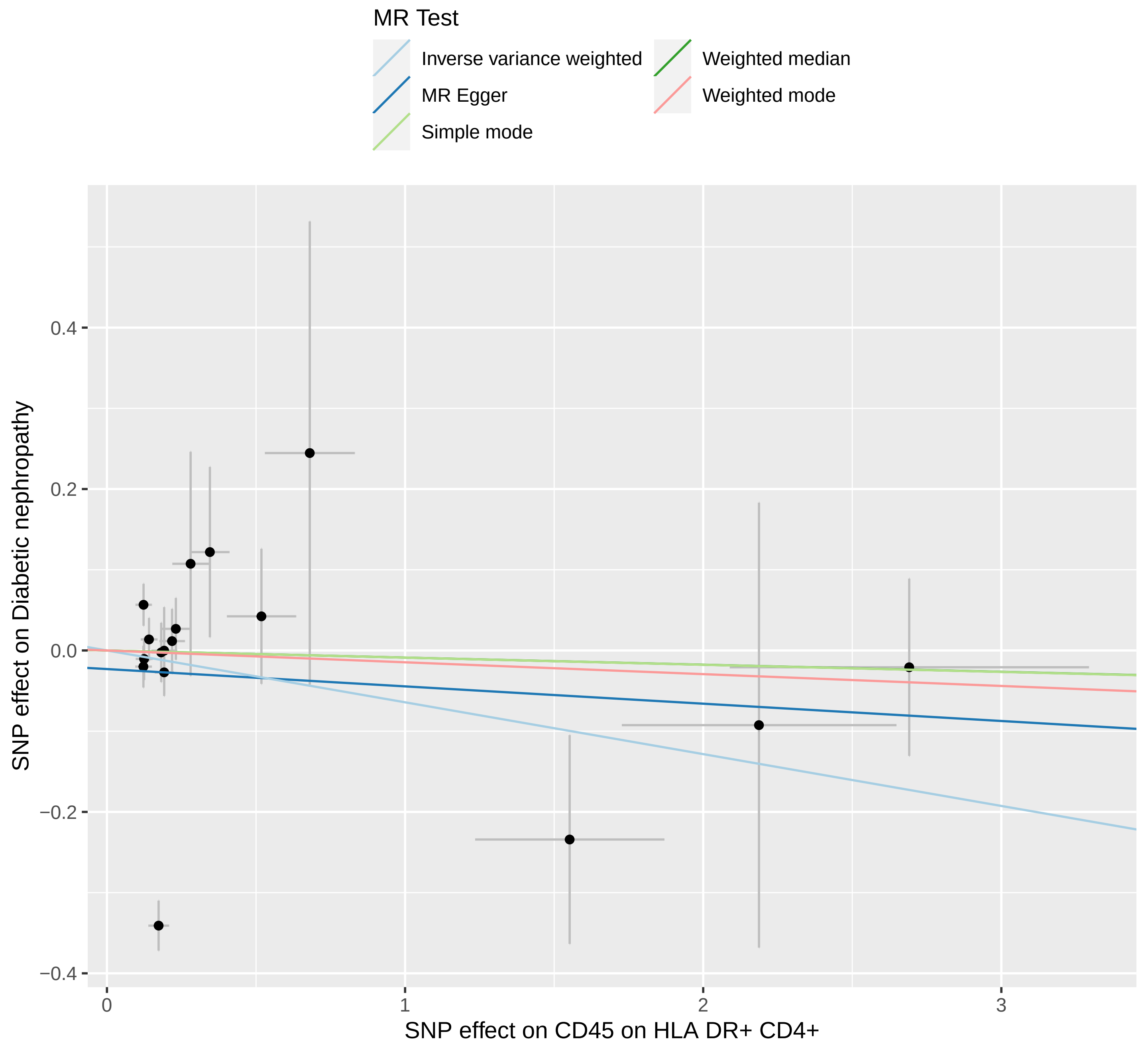

MR Test
Inverse variance weighted
MR Egger
Simple mode
Weighted median
Weighted mode
0.4
0.2
0.0
−0.2
−0.4
SNP effect on Diabetic nephropathy
0
1
2
3
SNP effect on CD45 on HLA DR+ CD4+

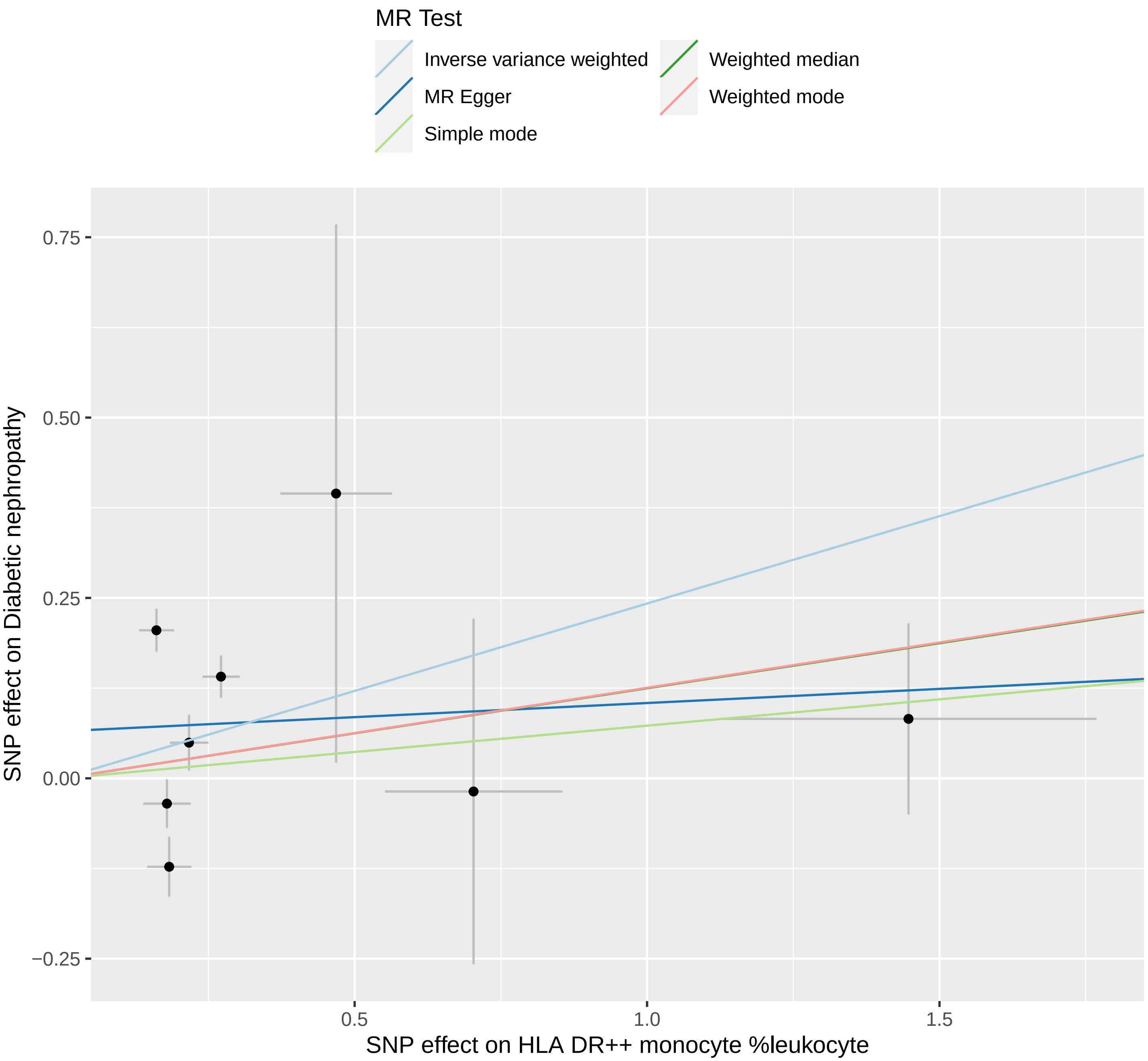
MR Test
Inverse variance weighted
MR Egger
Simple mode
Weighted median
Weighted mode
0.75
0.50
0.25
0.00
−0.25
0.5
1.0
1.5
SNP effect on Diabetic nephropathy
SNP effect on HLA DR++ monocyte %leukocyte

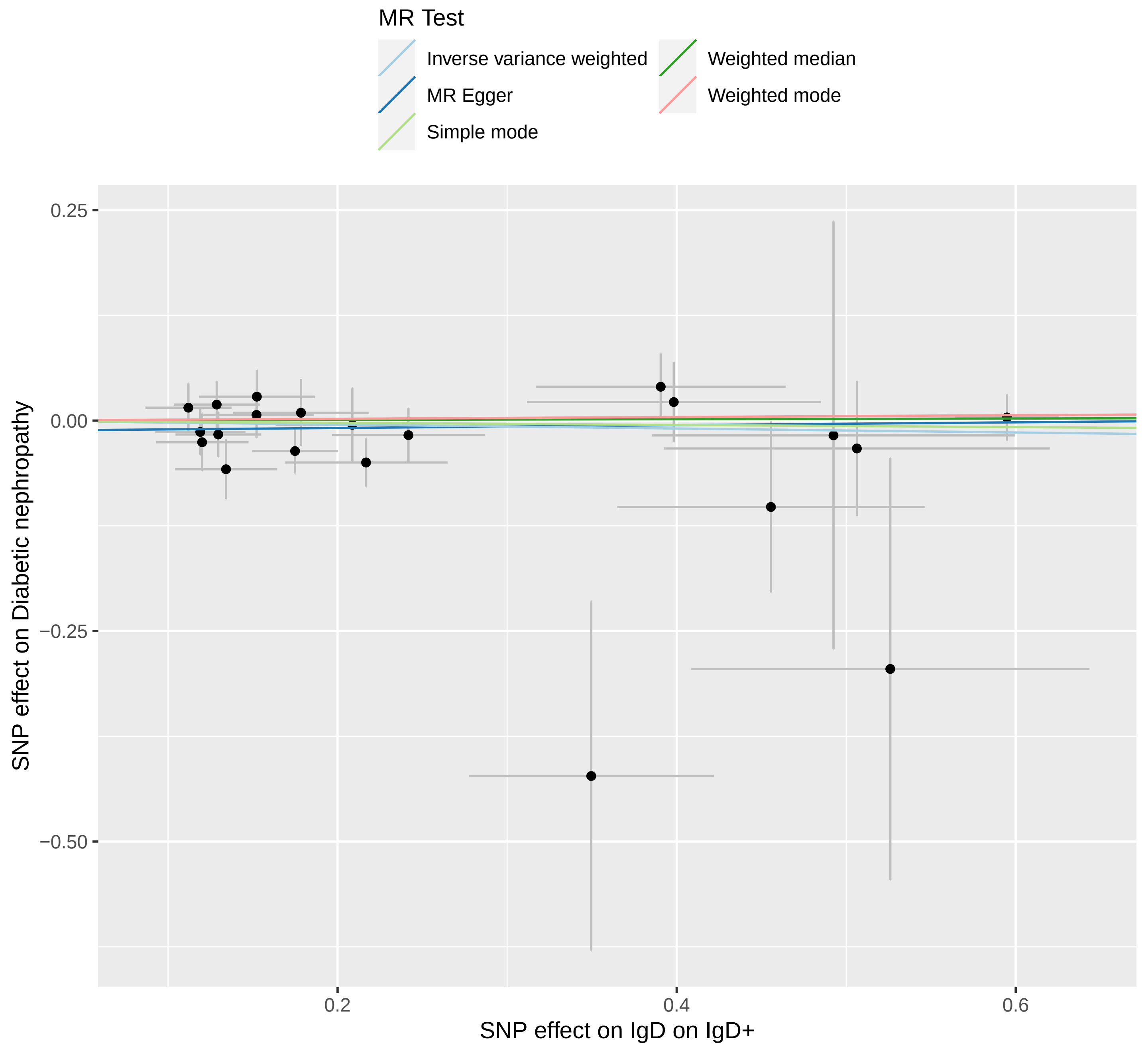

MR Test
Inverse variance weighted
MR Egger
Simple mode
Weighted median
Weighted mode
0.25
0.00
−0.25
−0.50
0.2
0.4
0.6
SNP effect on Diabetic nephropathy
SNP effect on IgD on IgD+

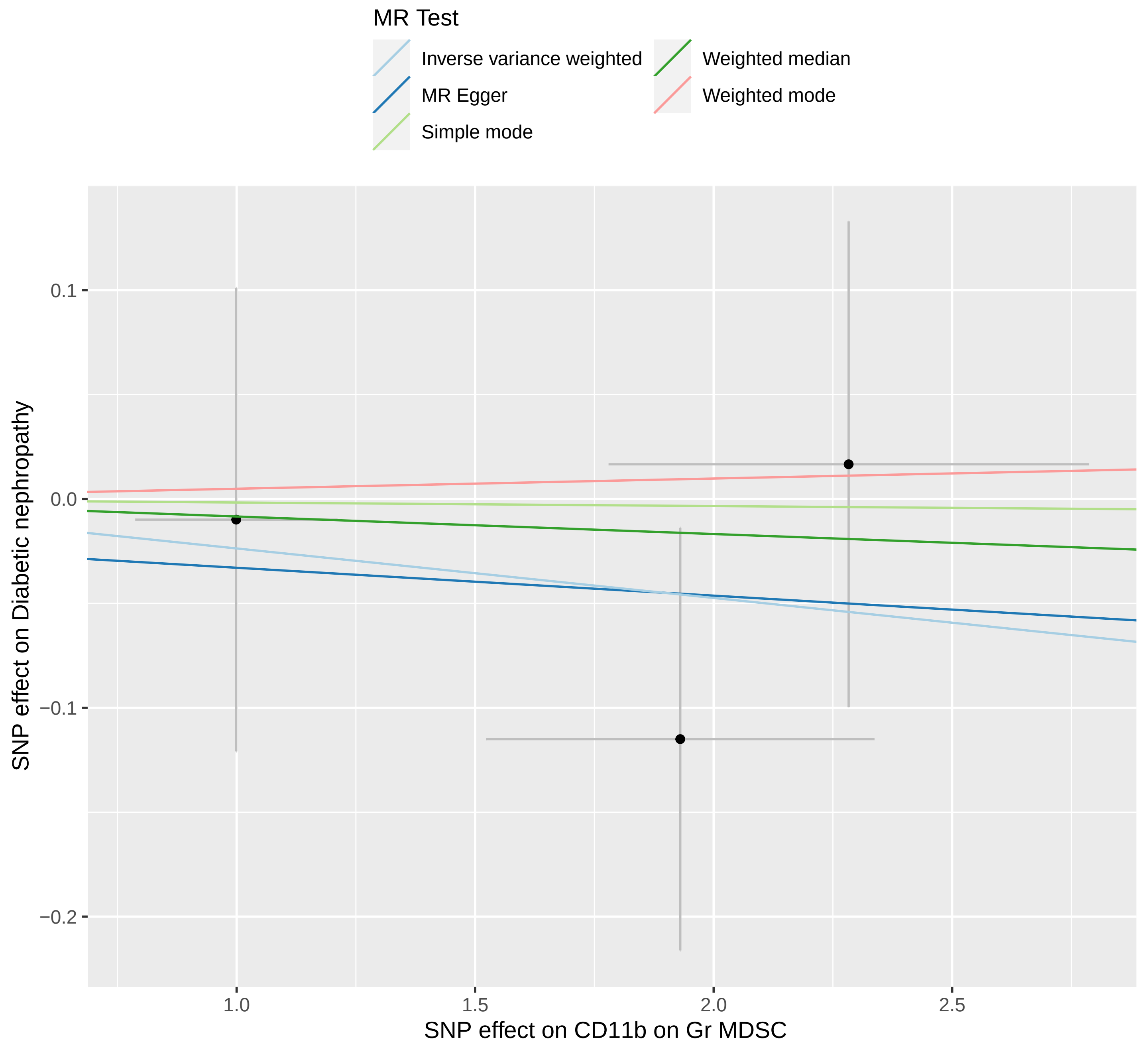

MR Test
Inverse variance weighted
MR Egger
Simple mode
Weighted median
Weighted mode
SNP effect on Diabetic nephropathy
0.1
0.0
−0.1
−0.2
1.0
1.5
2.0
2.5
SNP effect on CD11b on Gr MDSC

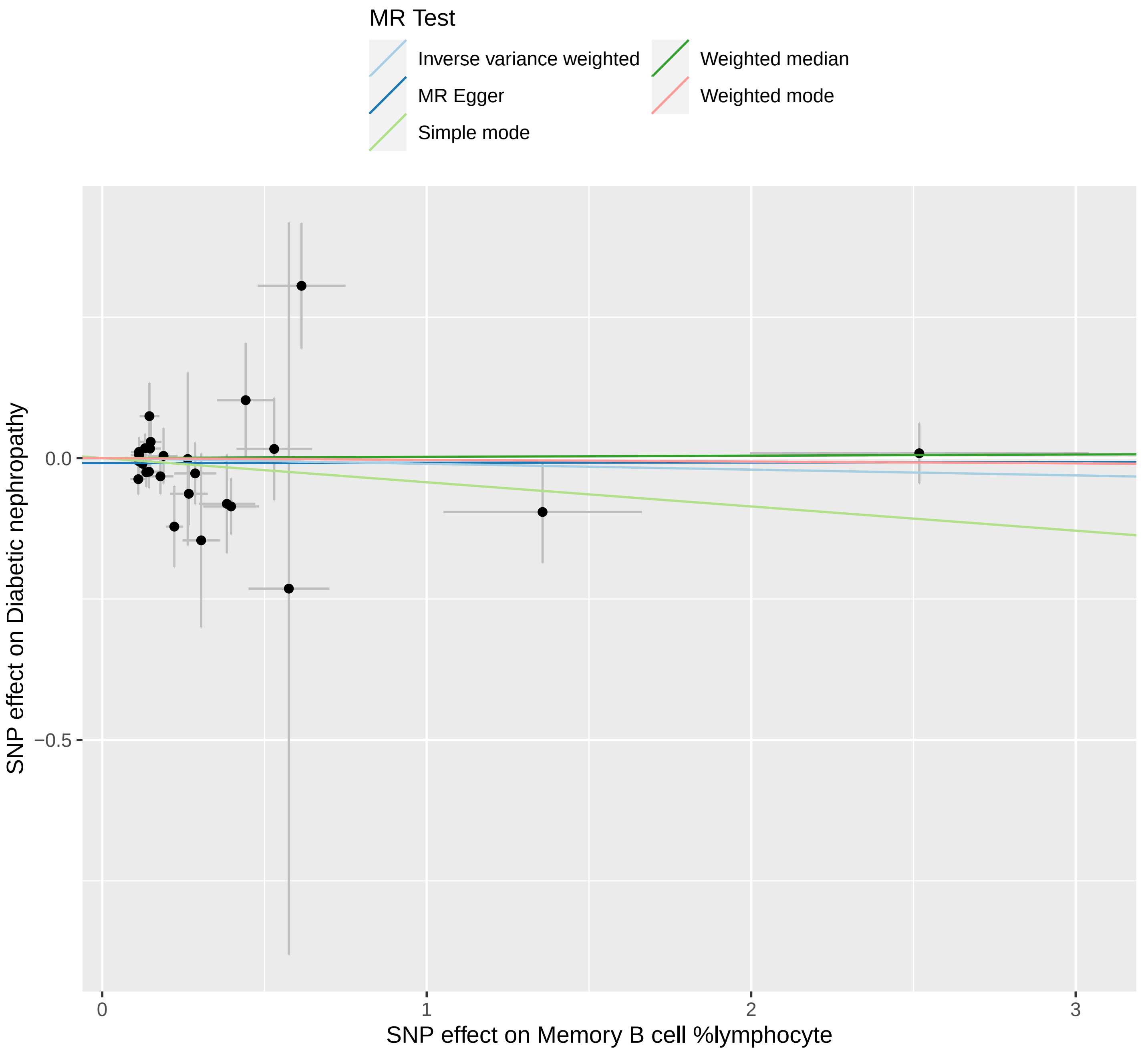
MR Test
Inverse variance weighted
MR Egger
Simple mode
Weighted median
Weighted mode
SNP effect on Diabetic nephropathy
0.0
−0.5
0
1
2
3
SNP effect on Memory B cell %lymphocyte

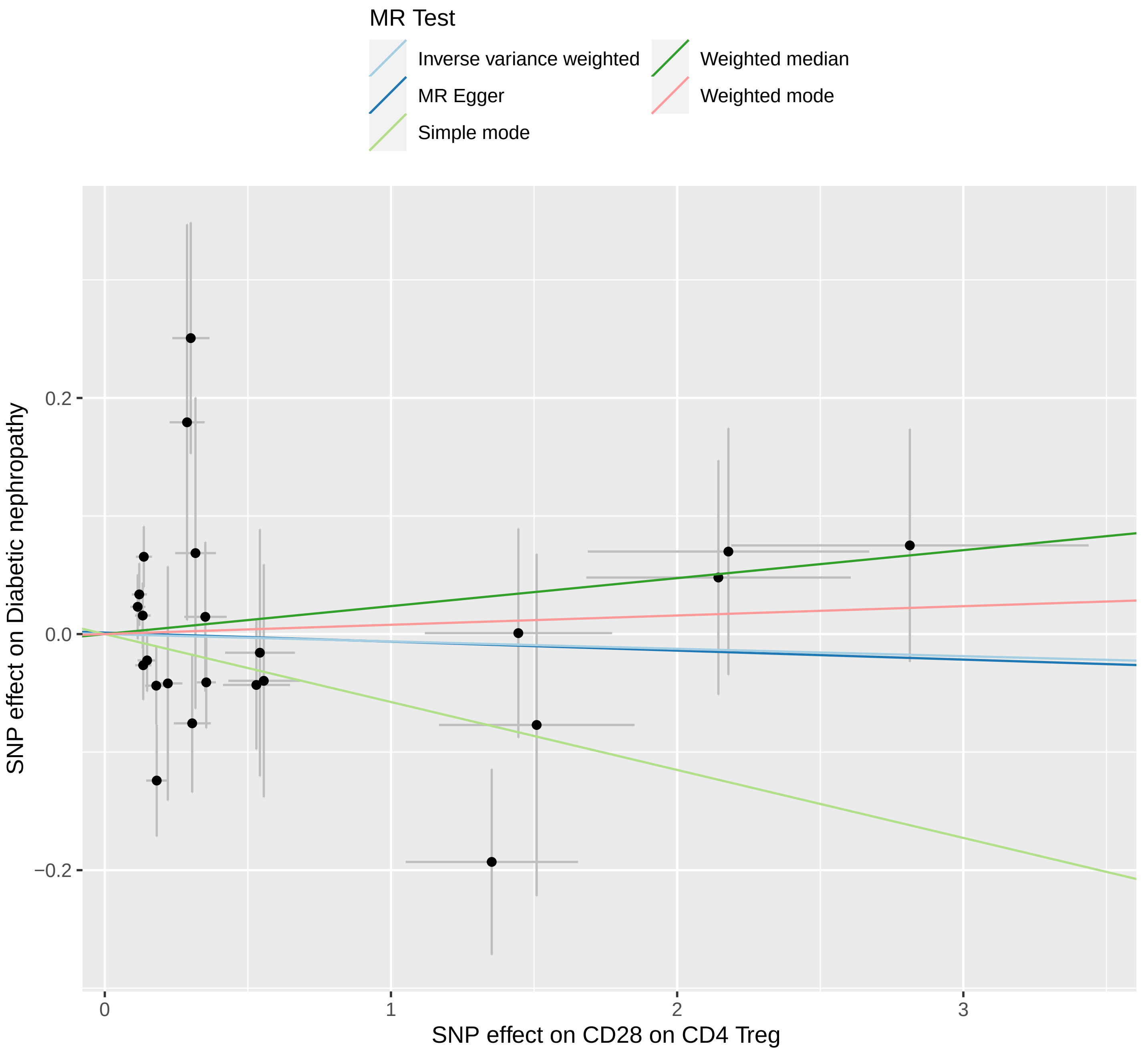
MR Test
Inverse variance weighted
MR Egger
Simple mode
Weighted median
Weighted mode
0.2
0.0
−0.2
0
1
2
3
SNP effect on Diabetic nephropathy
SNP effect on CD28 on CD4 Treg

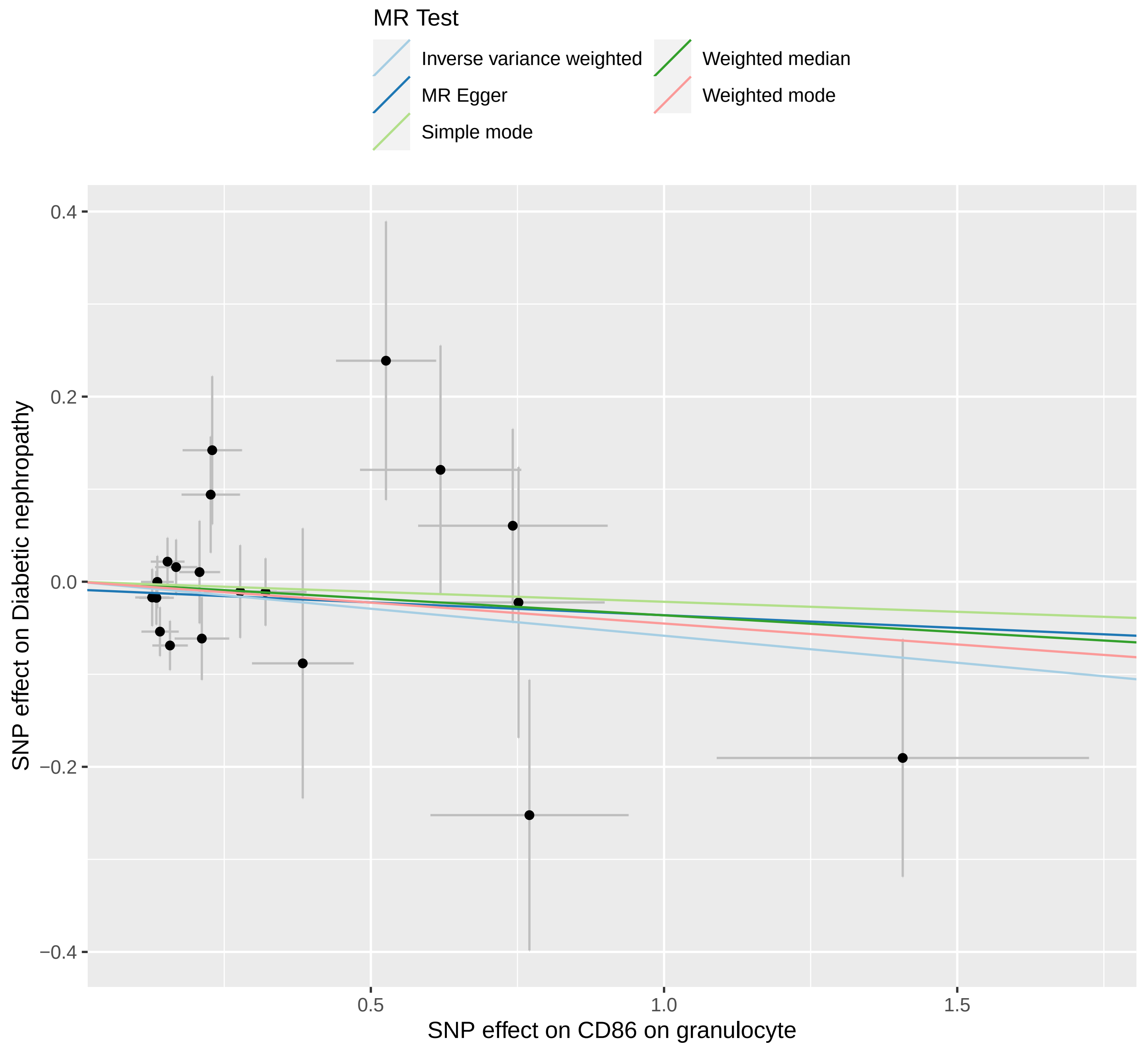

MR Test
Inverse variance weighted
MR Egger
Simple mode
Weighted median
Weighted mode
0.4
0.2
0.0
−0.2
−0.4
SNP effect on Diabetic nephropathy
0.5
1.0
1.5
SNP effect on CD86 on granulocyte

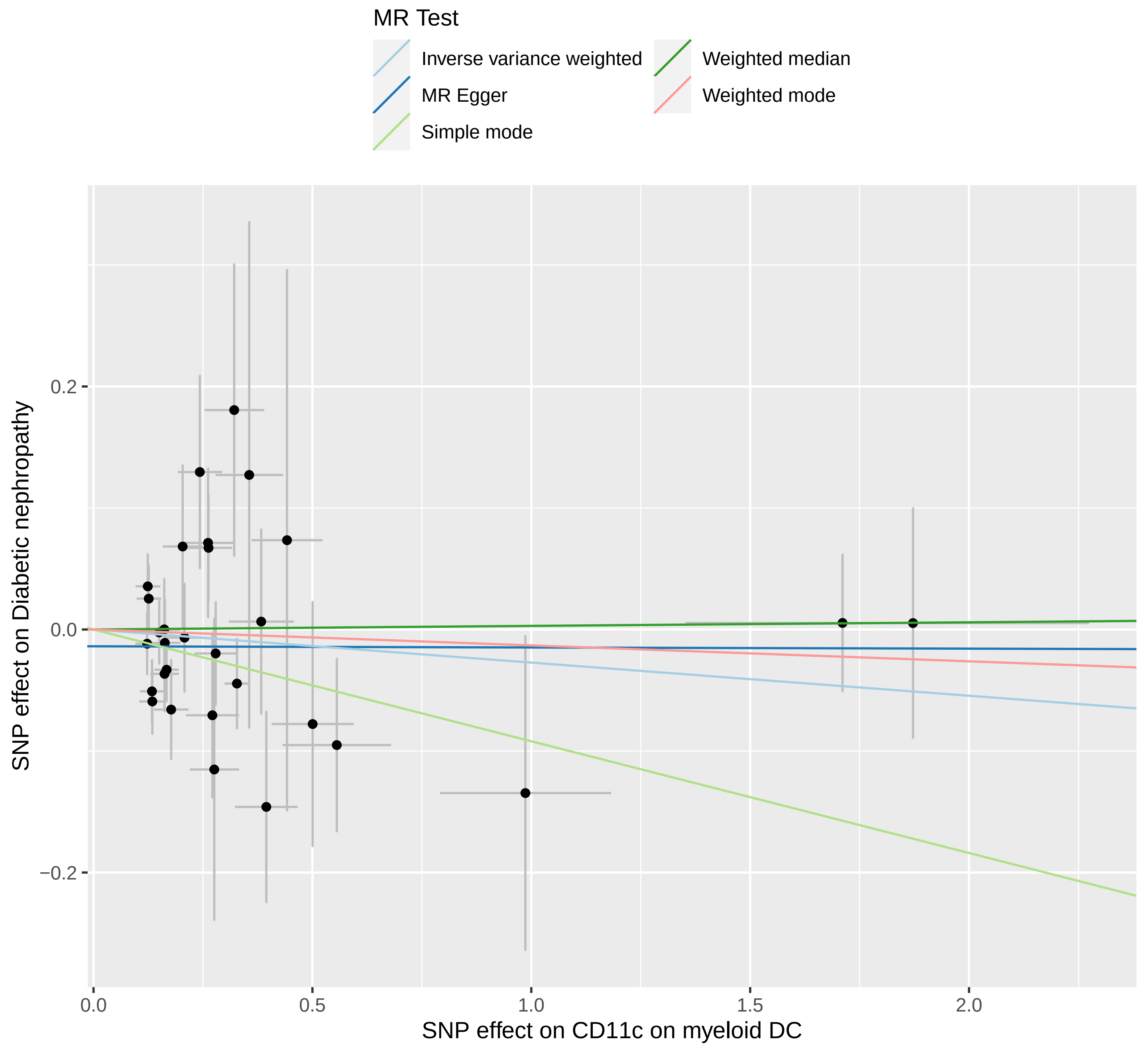
MR Test
Inverse variance weighted
MR Egger
Simple mode
Weighted median
Weighted mode
SNP effect on Diabetic nephropathy
0.2
0.0
−0.2
0.0
0.5
1.0
1.5
2.0
SNP effect on CD11c on myeloid DC

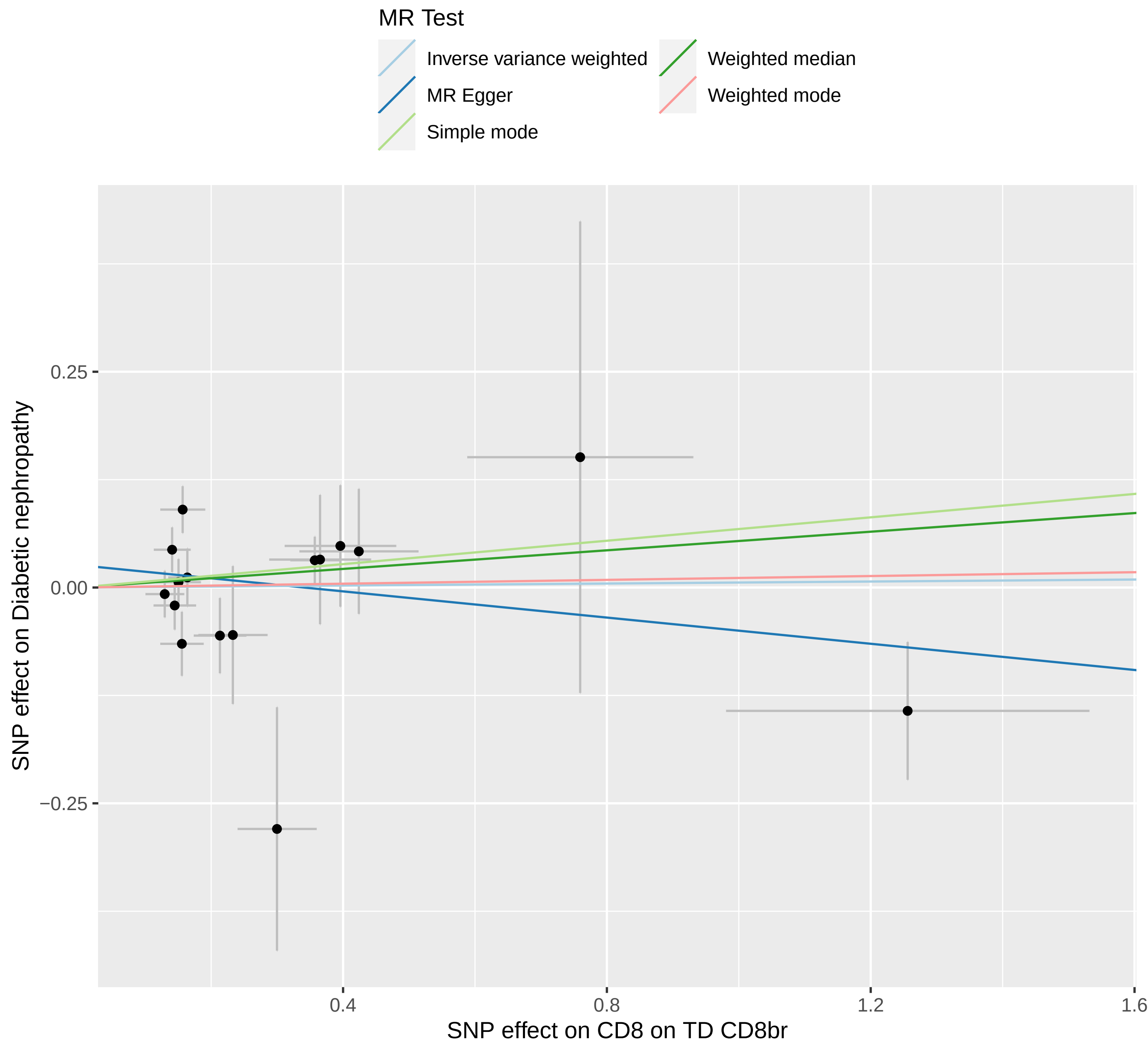

MR Test
Inverse variance weighted
MR Egger
Simple mode
Weighted median
Weighted mode
0.25
0.00
−0.25
0.4
0.8
1.2
1.6
SNP effect on Diabetic nephropathy
SNP effect on CD8 on TD CD8br

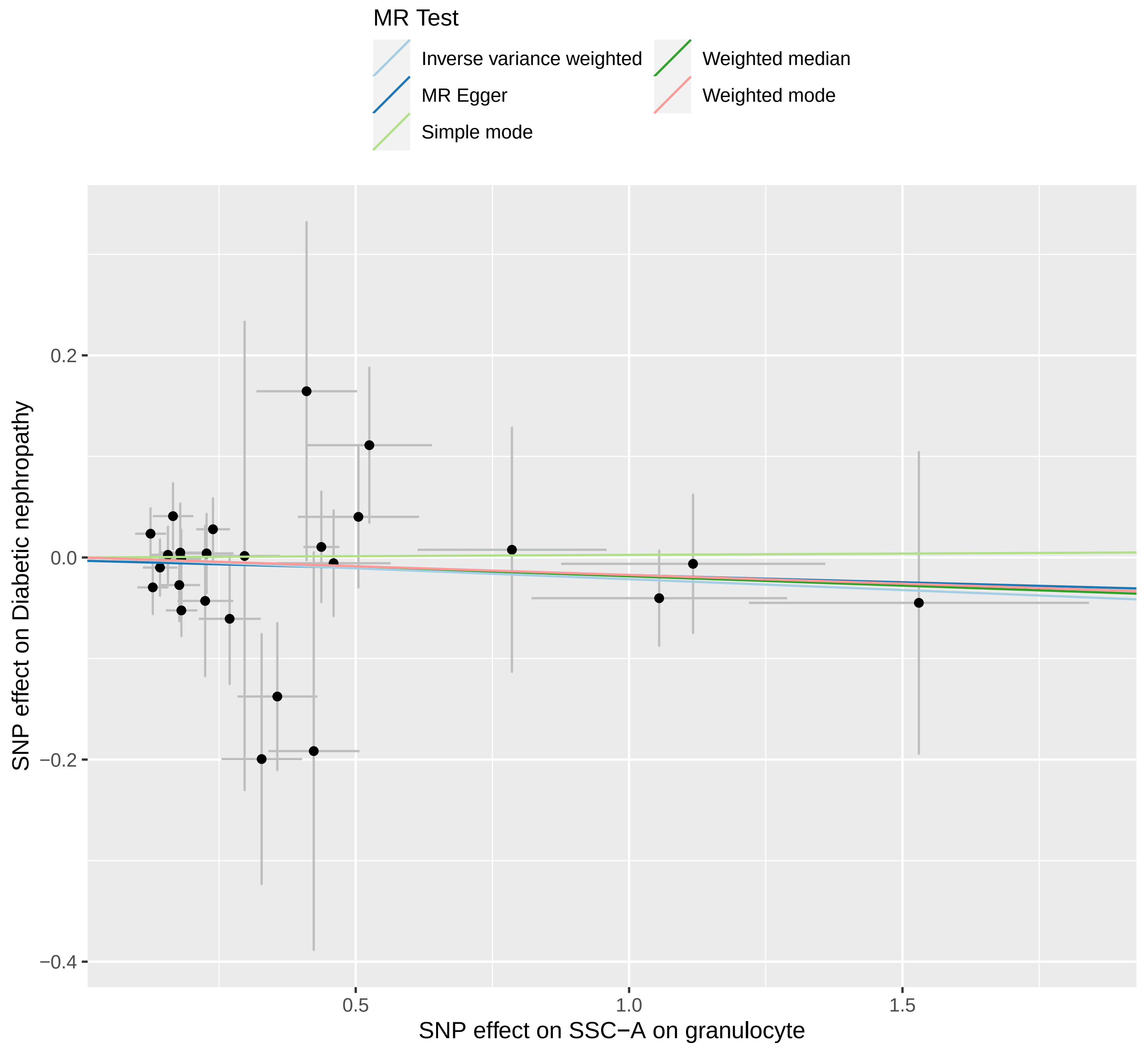
MR Test
Inverse variance weighted
MR Egger
Simple mode
Weighted median
Weighted mode
SNP effect on Diabetic nephropathy
0.2
0.0
−0.2
−0.4
0.5
1.0
1.5
SNP effect on SSC−A on granulocyte

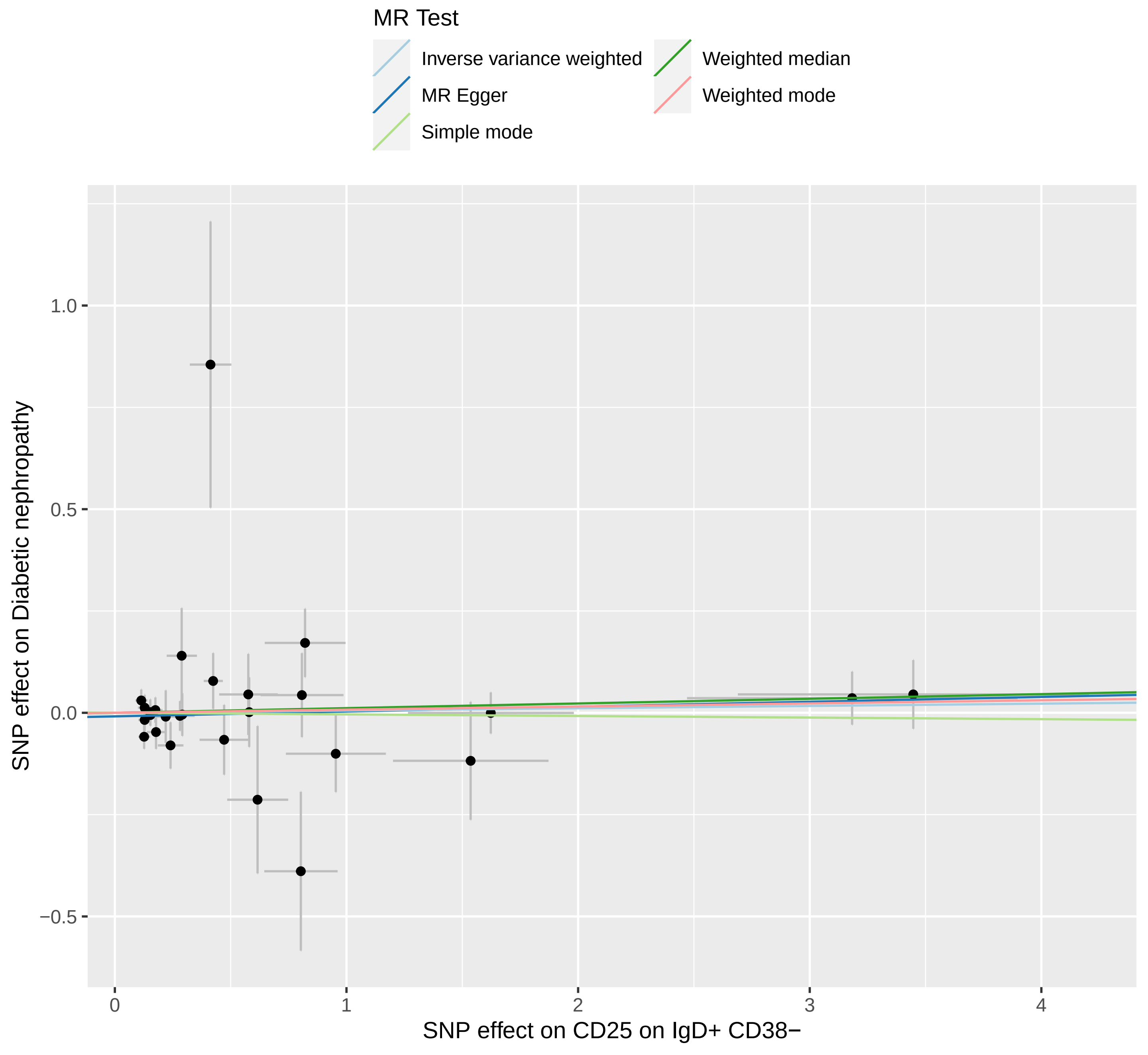
MR Test
Inverse variance weighted
MR Egger
Simple mode
Weighted median
Weighted mode
SNP effect on Diabetic nephropathy
1.0
0.5
0.0
−0.5
0
1
2
3
4
SNP effect on CD25 on IgD+ CD38−

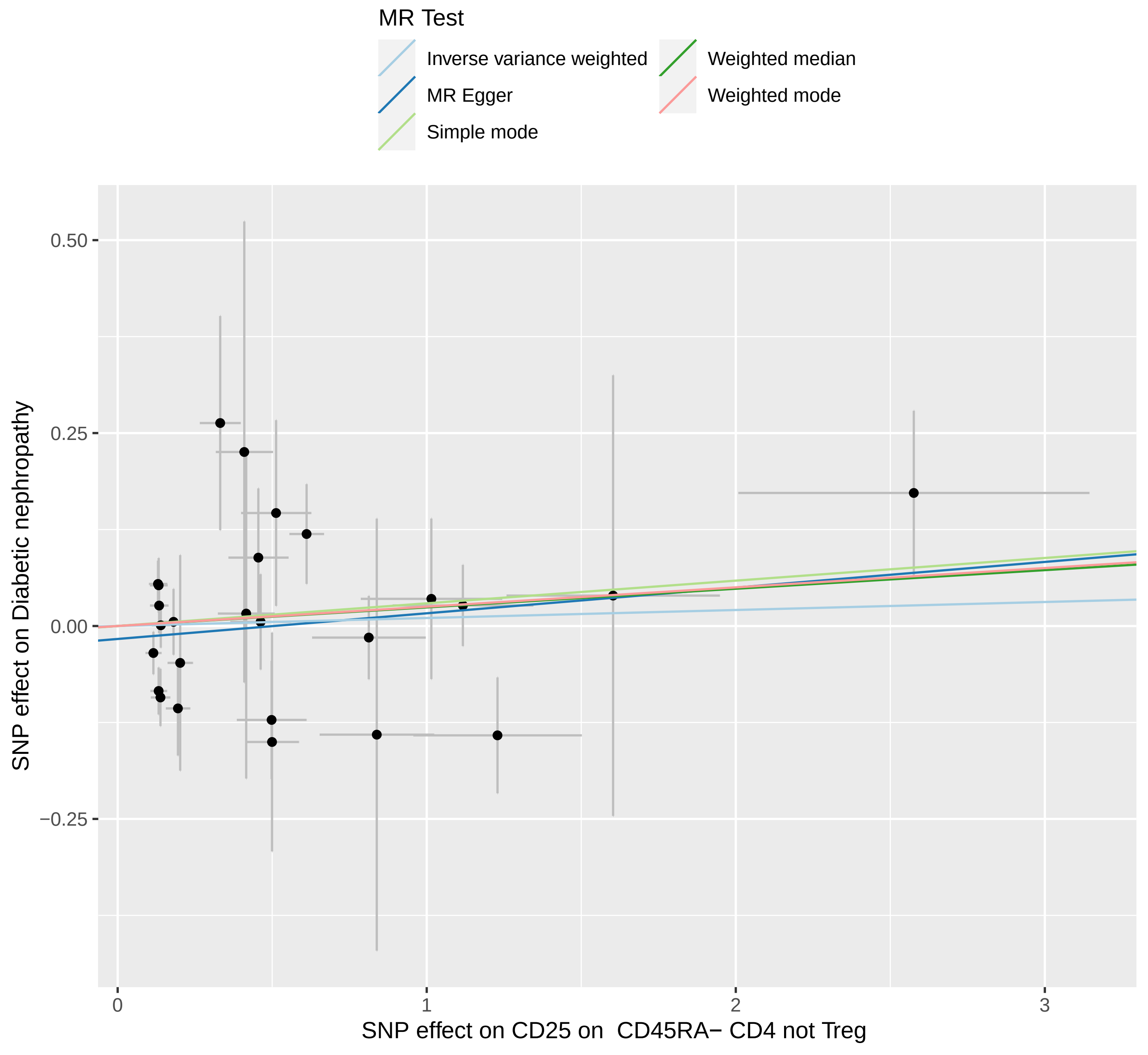
MR Test
Inverse variance weighted
MR Egger
Simple mode
Weighted median
Weighted mode
0.50
0.25
0.00
−0.25
0
1
2
3
SNP effect on Diabetic nephropathy
SNP effect on CD25 on CD45RA− CD4 not Treg
