# Supplementary material for: Monocyte human leukocyte antigen-DR-mediated diabetic nephropathy progression is a promising therapeutic target
Source: Front Endocrinol (Lausanne). 2025 Dec 9;16:1733139. doi: 10.3389/fendo.2025.1733139 (PMC12722863; doi:10.3389/fendo.2025.1733139)
Supplement: Supplementary Figure 4 — Funnel plots of all MR analyses of 731 immunophenotypes on DN. MR, mendelian randomization; DN, diabetic nephropathy. [file DataSheet4.pdf]

Supplementary Figure 4: Funnel plots of all MR analyses of 731 immunophenotypes on diabetic nephropathy

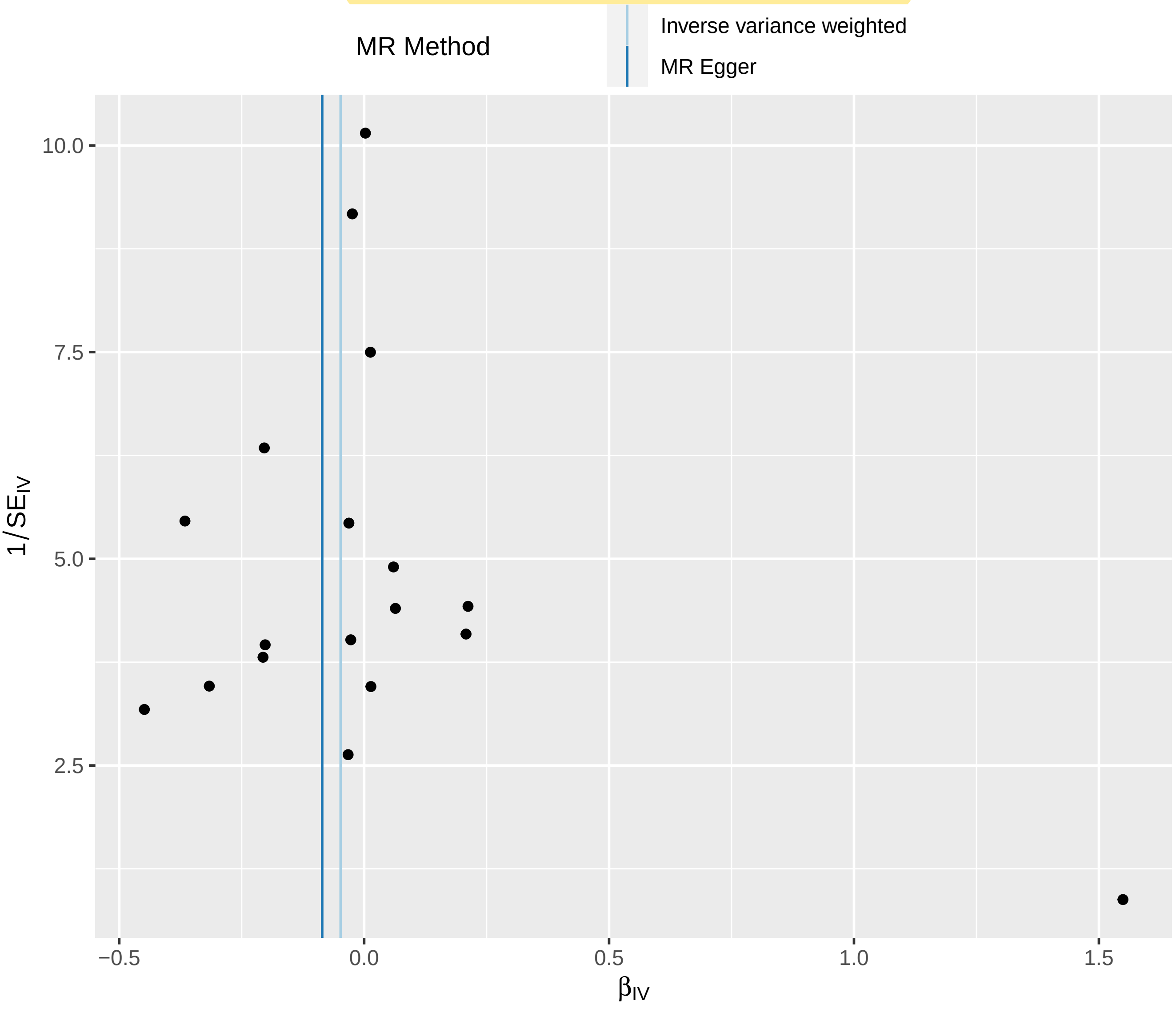

Funnel plot analyse of "CD45 on Gr MDSC " on 'Diabetic nephropathy'

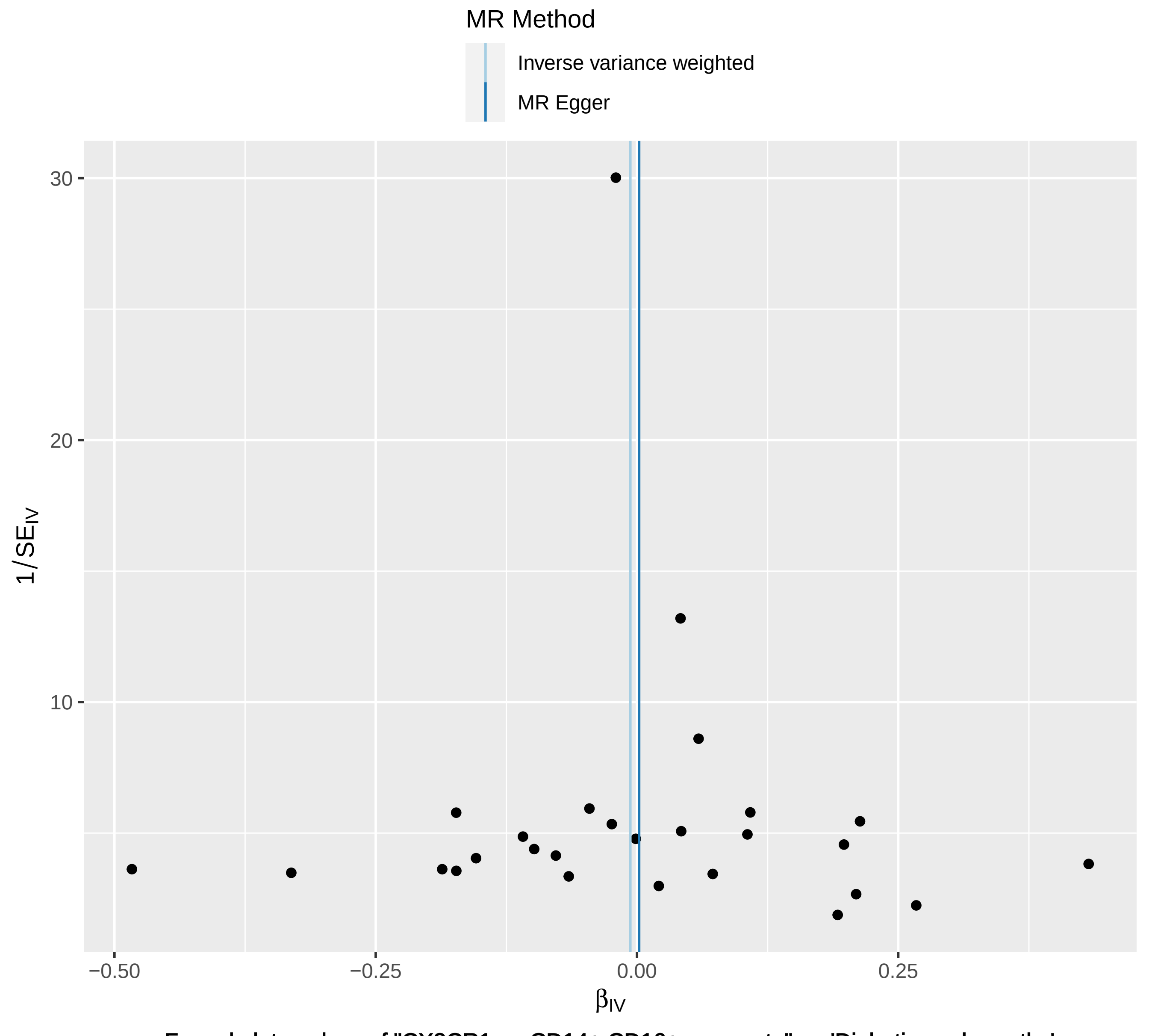

Funnel plot analyse of "CX3CR1 on CD14+ CD16+ monocyte" on 'Diabetic nephropathy'

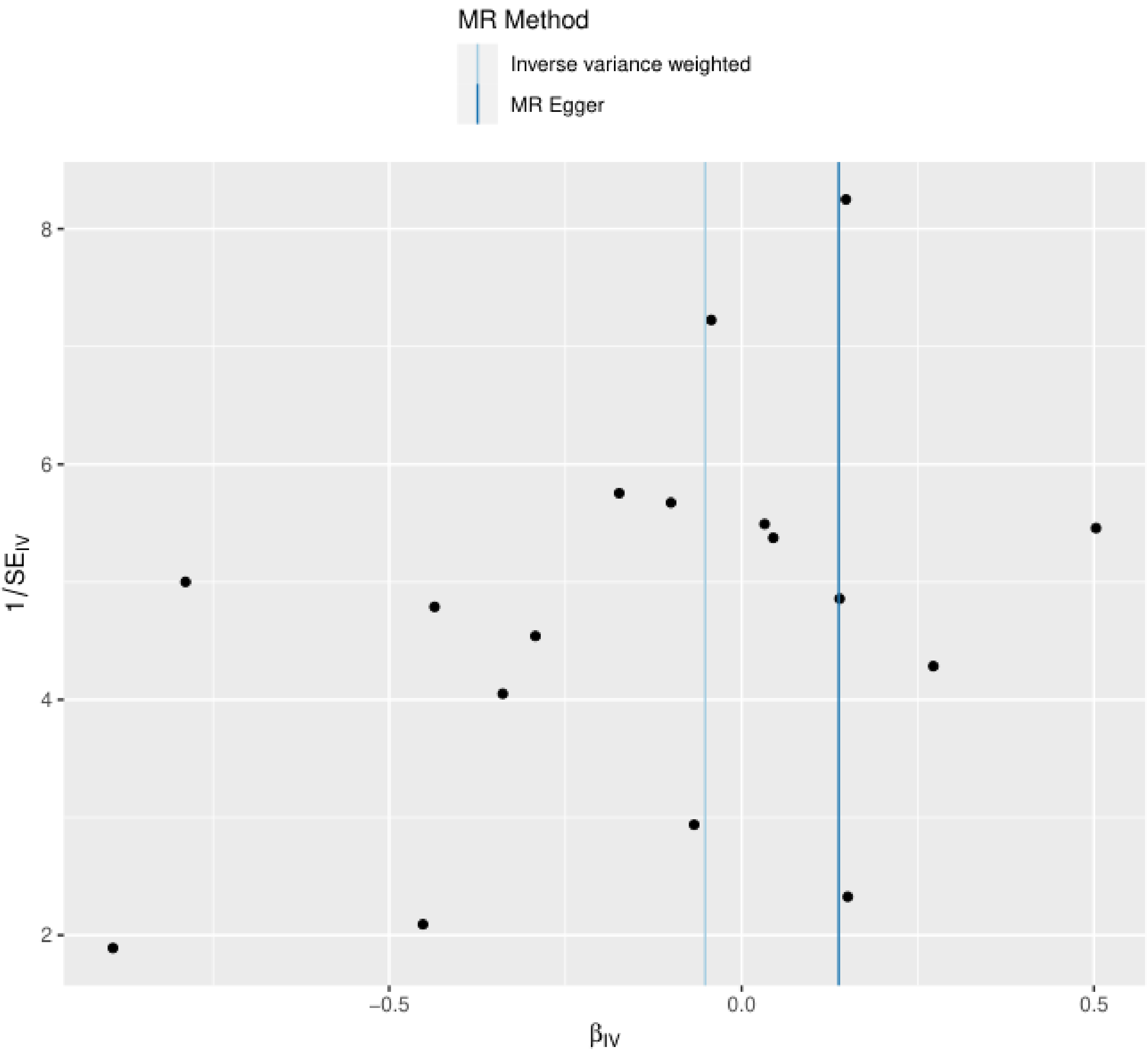

Funnel plot analyse of "IgD+ CD38- AC" on 'Diabetic nephropathy'

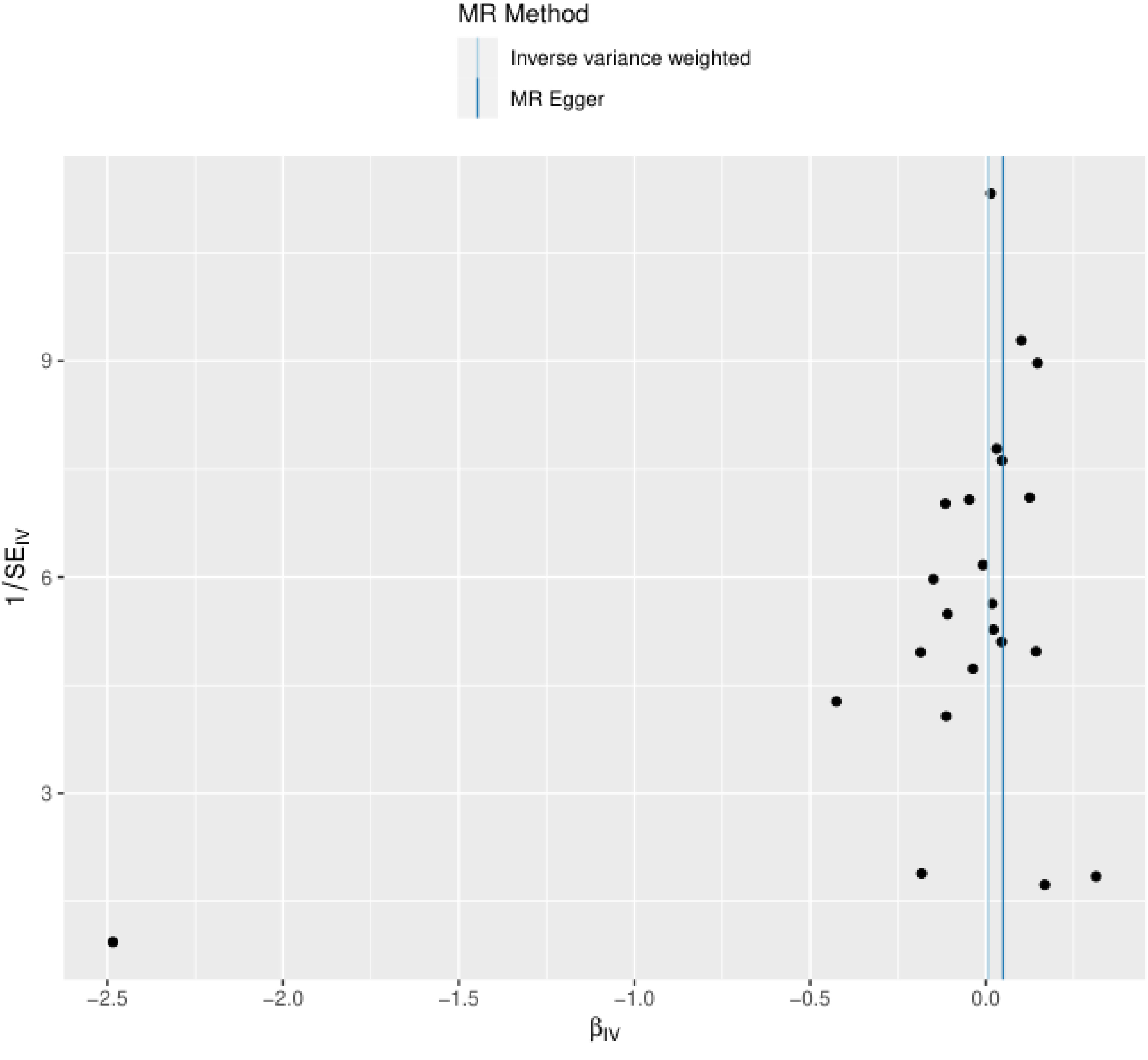

Funnel plot analyse of "CD3 on TD CD8br" on 'Diabetic nephropathy'

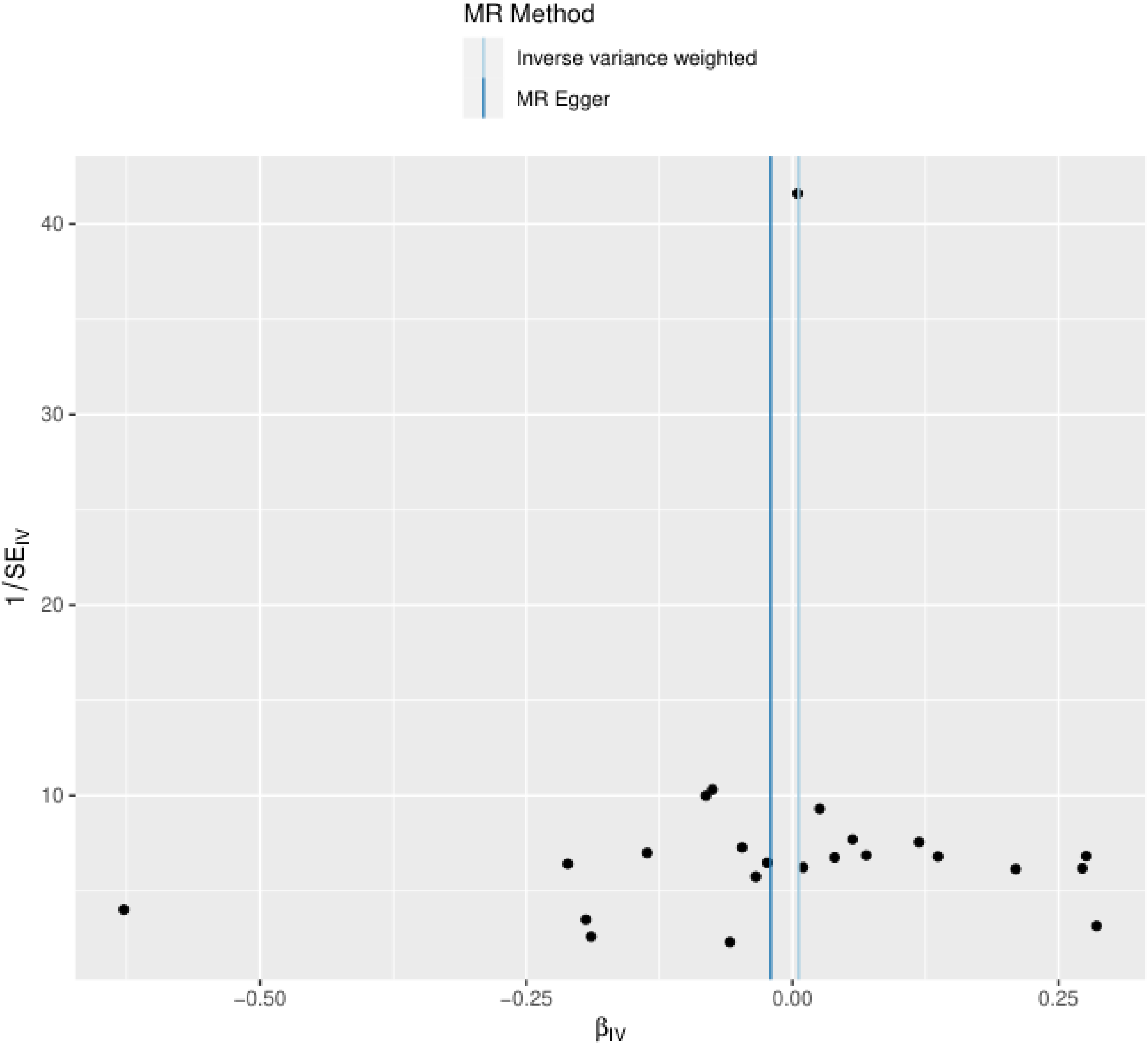

Funnel plot analyse of "CD33 on Im MDSC " on 'Diabetic nephropathy'

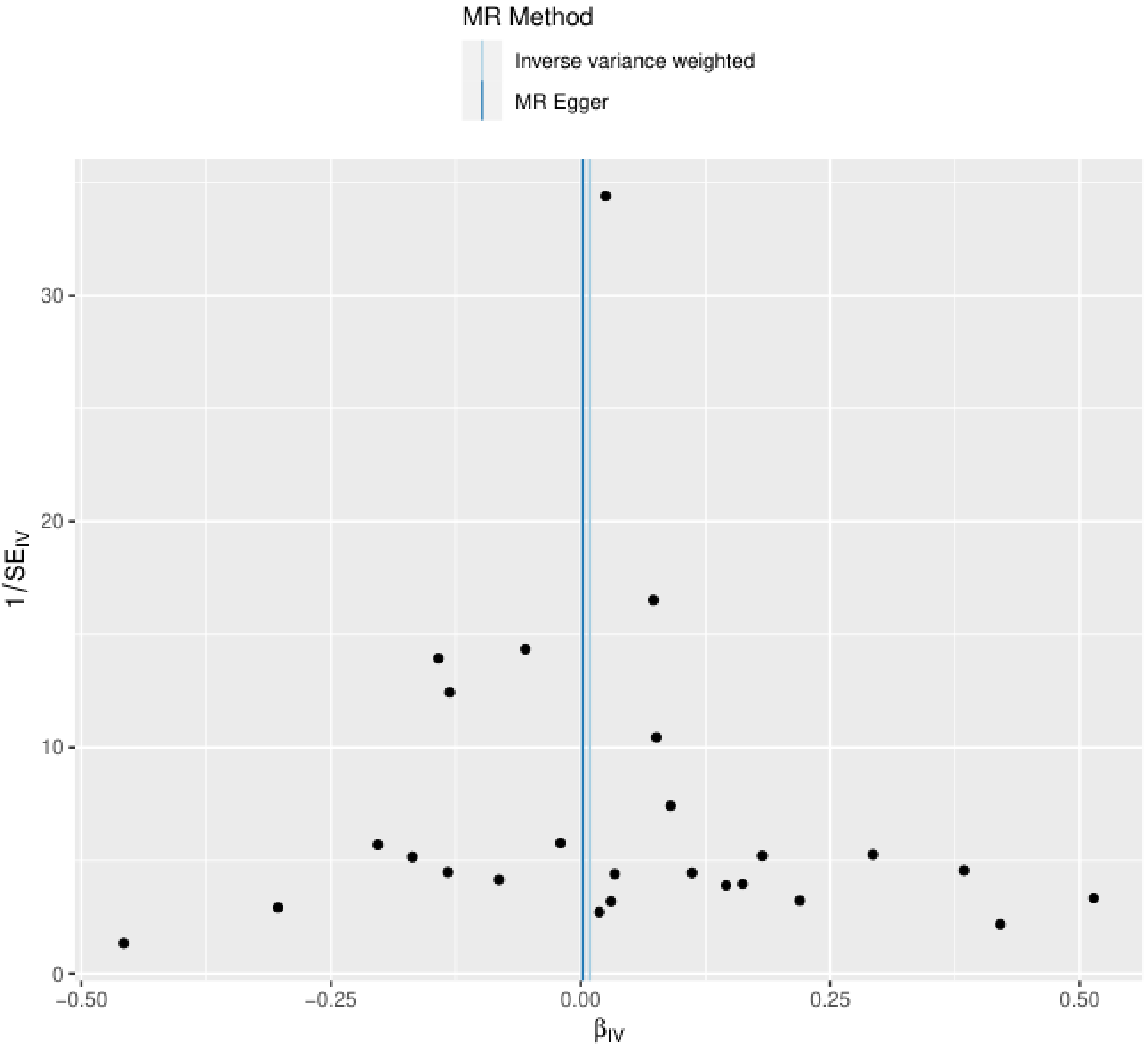

Funnel plot analyse of "CD39+ resting Treg %resting Treg" on 'Diabetic nephropathy'

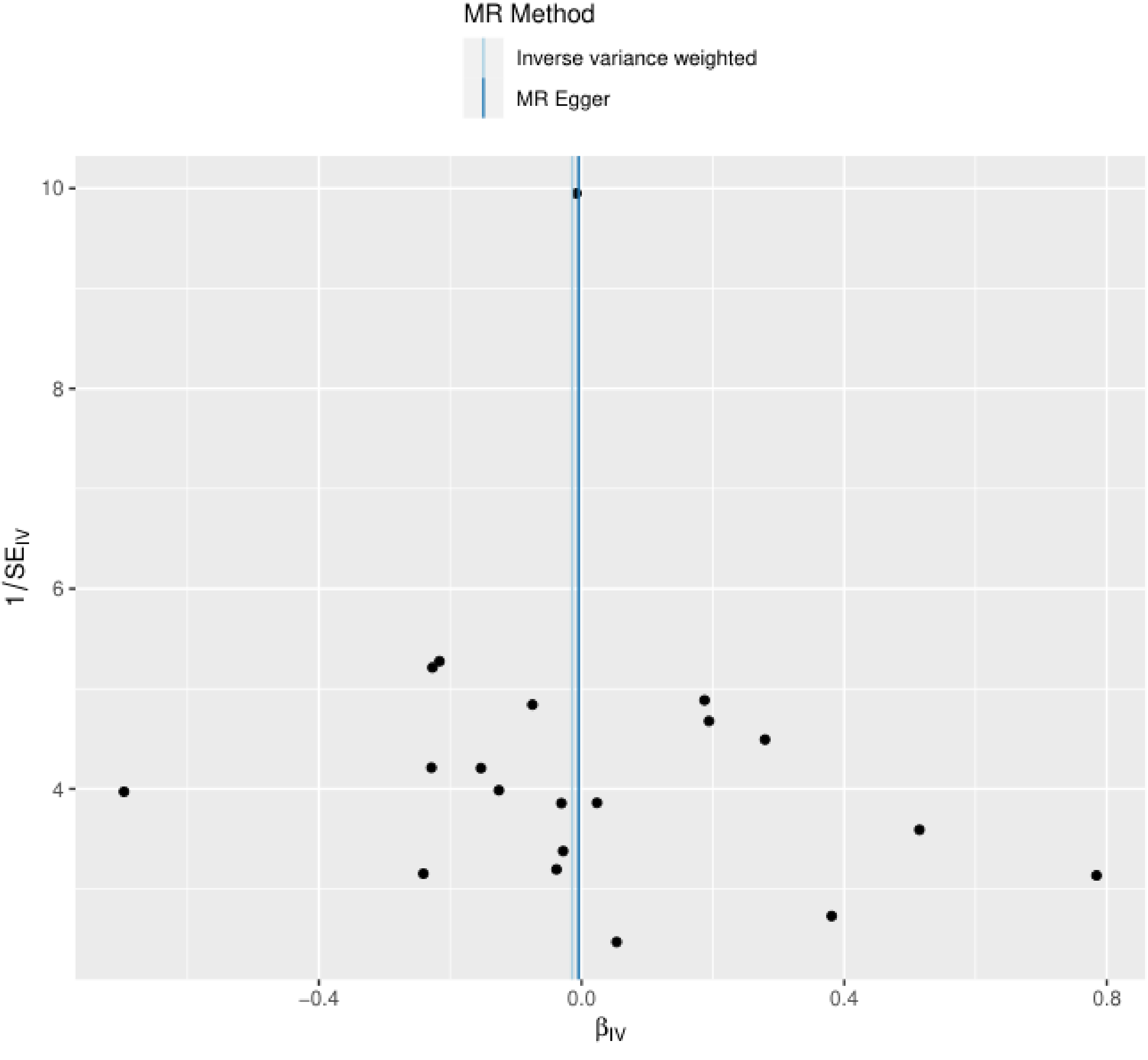

Funnel plot analyse of "IgD+ CD24- %lymphocyte" on 'Diabetic nephropathy'

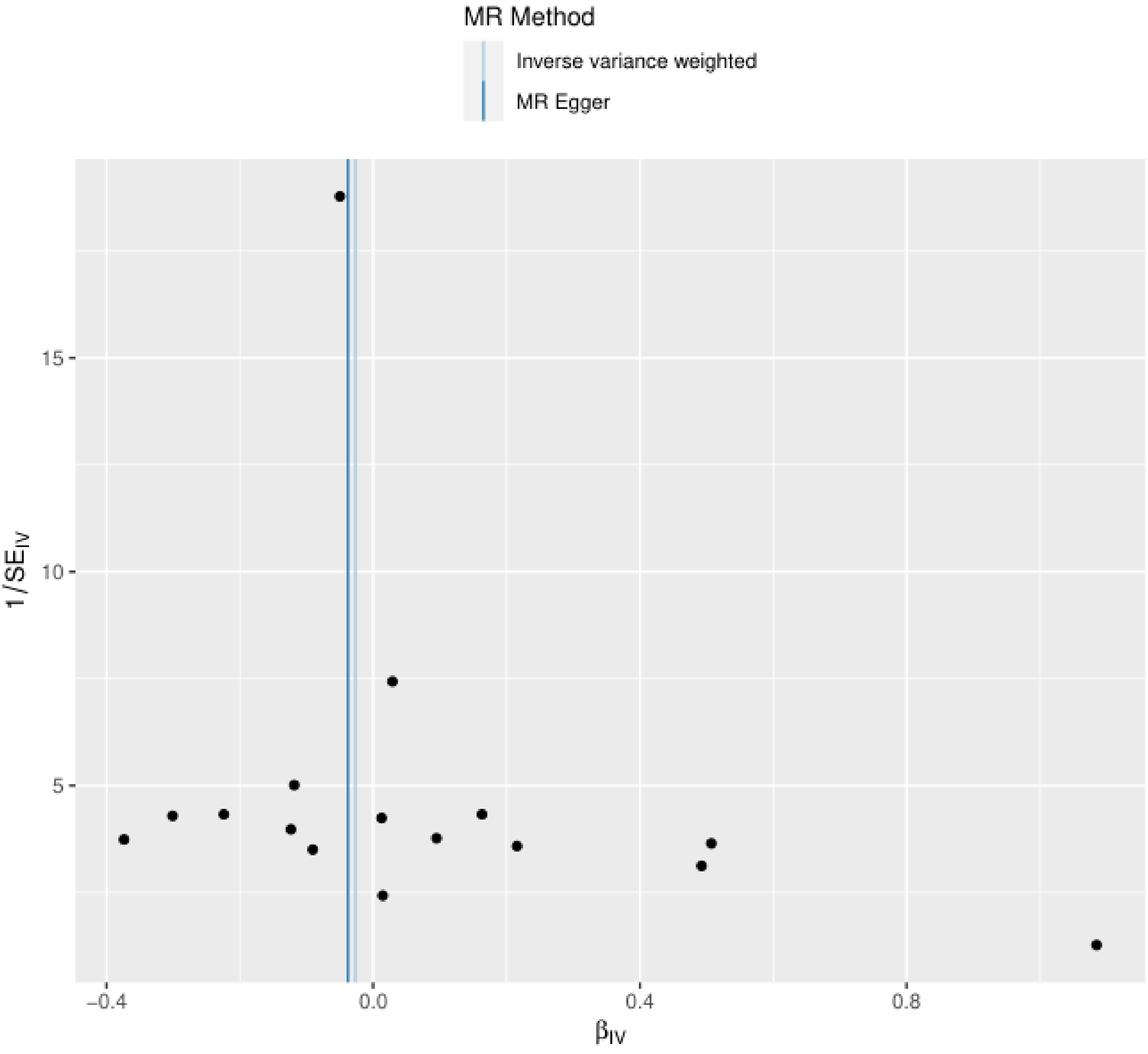

Funnel plot analyse of "CD20 on CD20- CD38-" on 'Diabetic nephropathy'

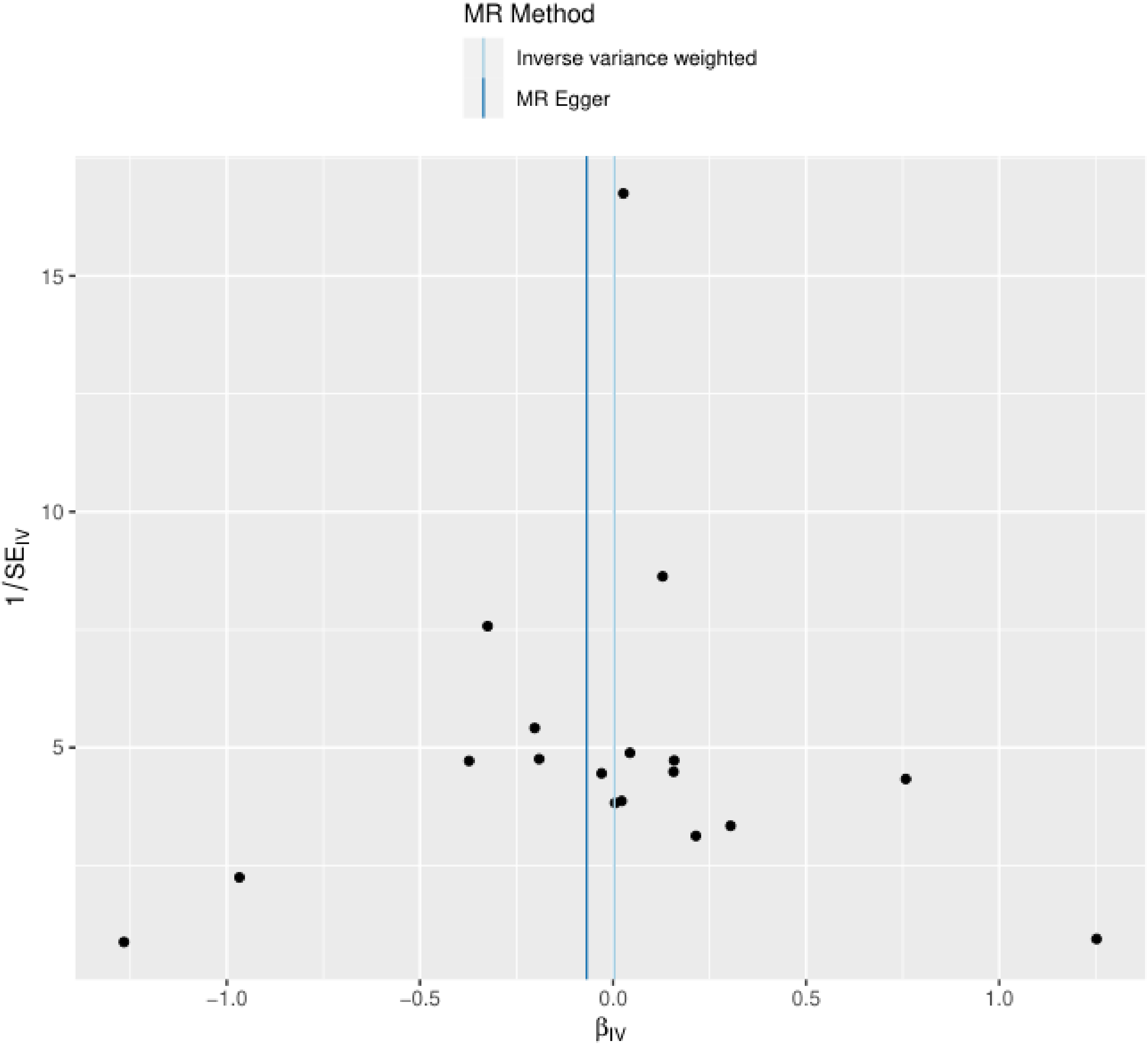

Funnel plot analyse of "HLA DR+ NK AC" on 'Diabetic nephropathy'

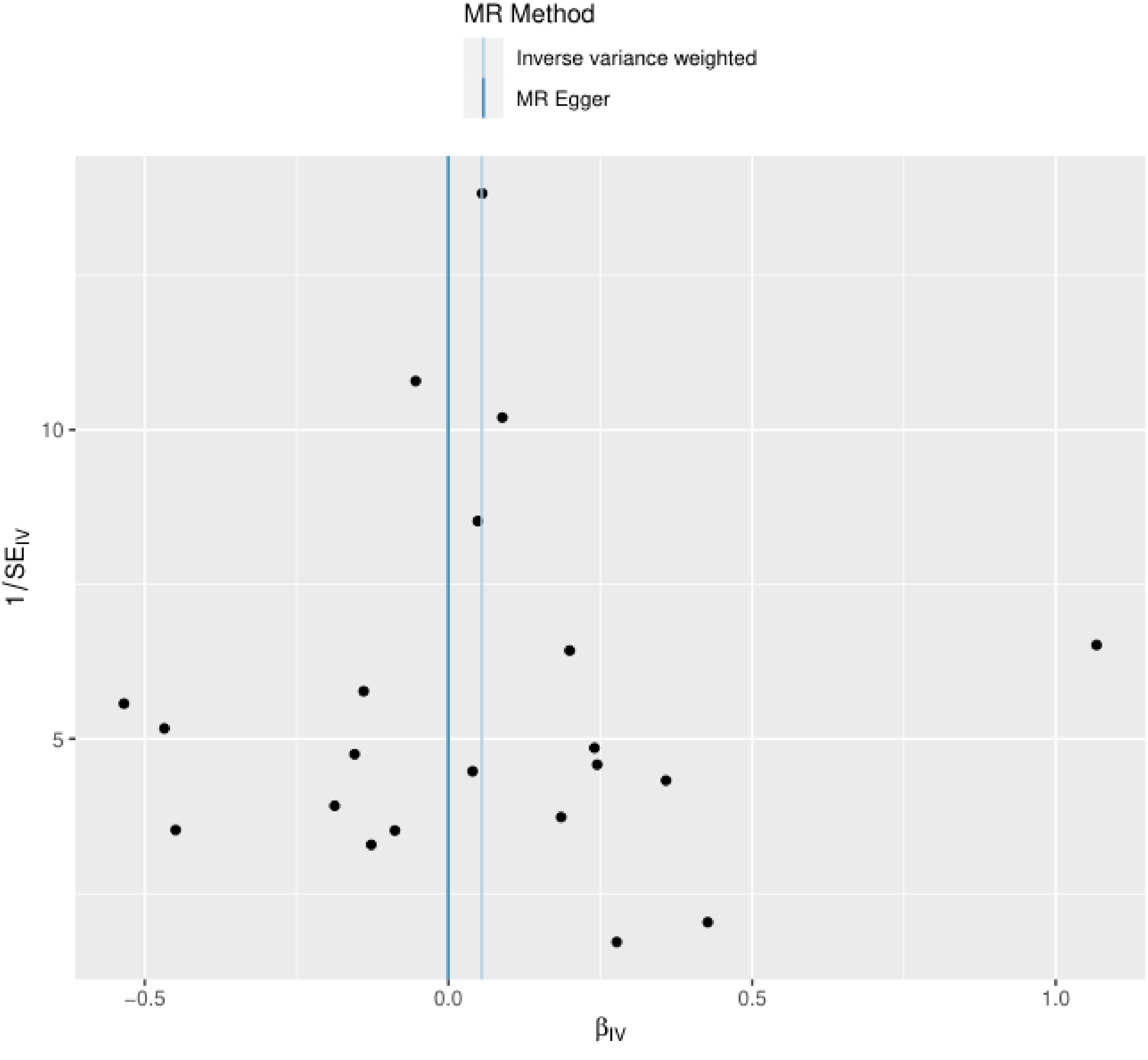

Funnel plot analyse of "HLA DR++ monocyte %monocyte" on 'Diabetic nephropathy'

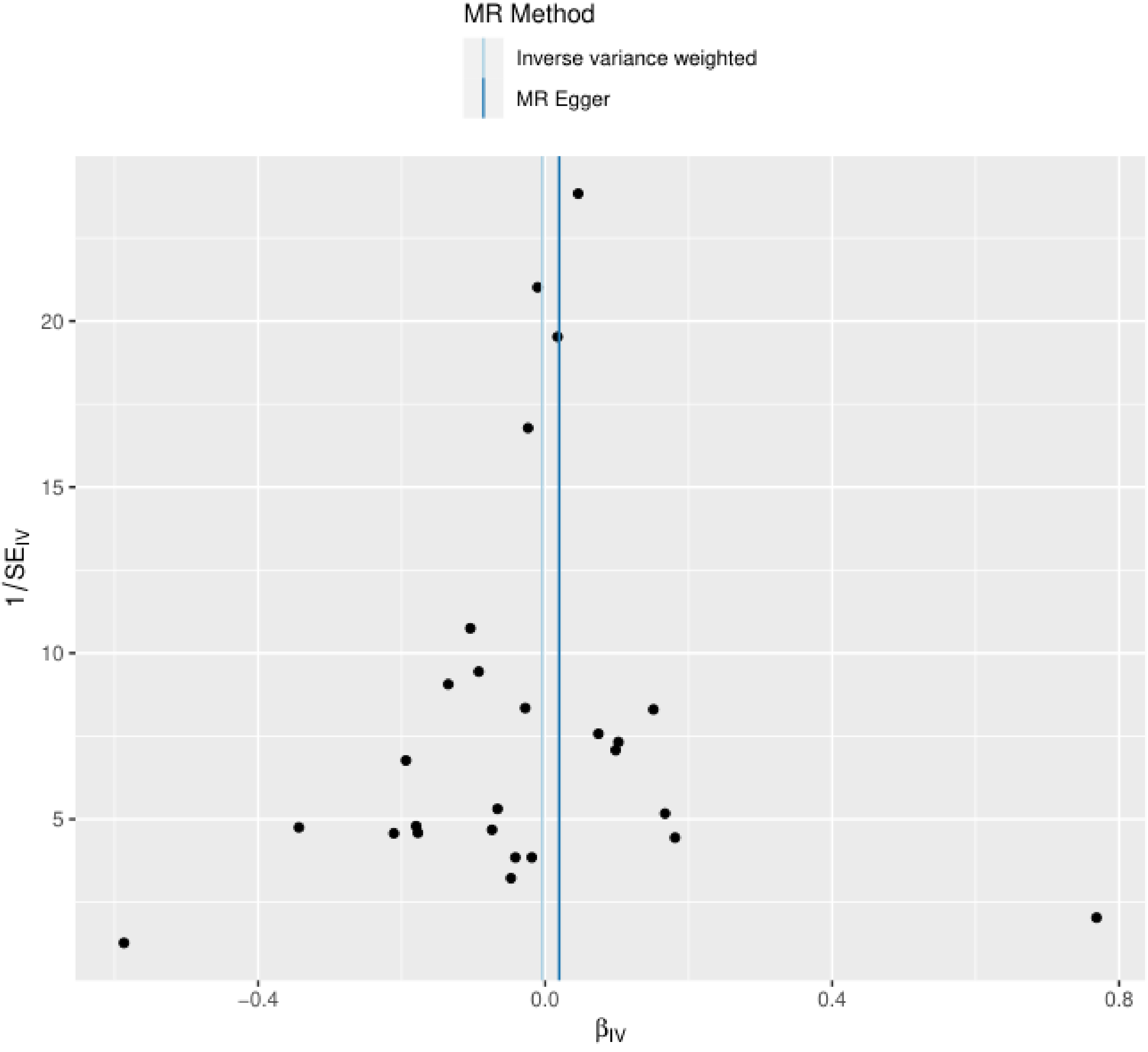

Funnel plot analyse of "CD80 on CD62L+ myeloid DC" on 'Diabetic nephropathy'

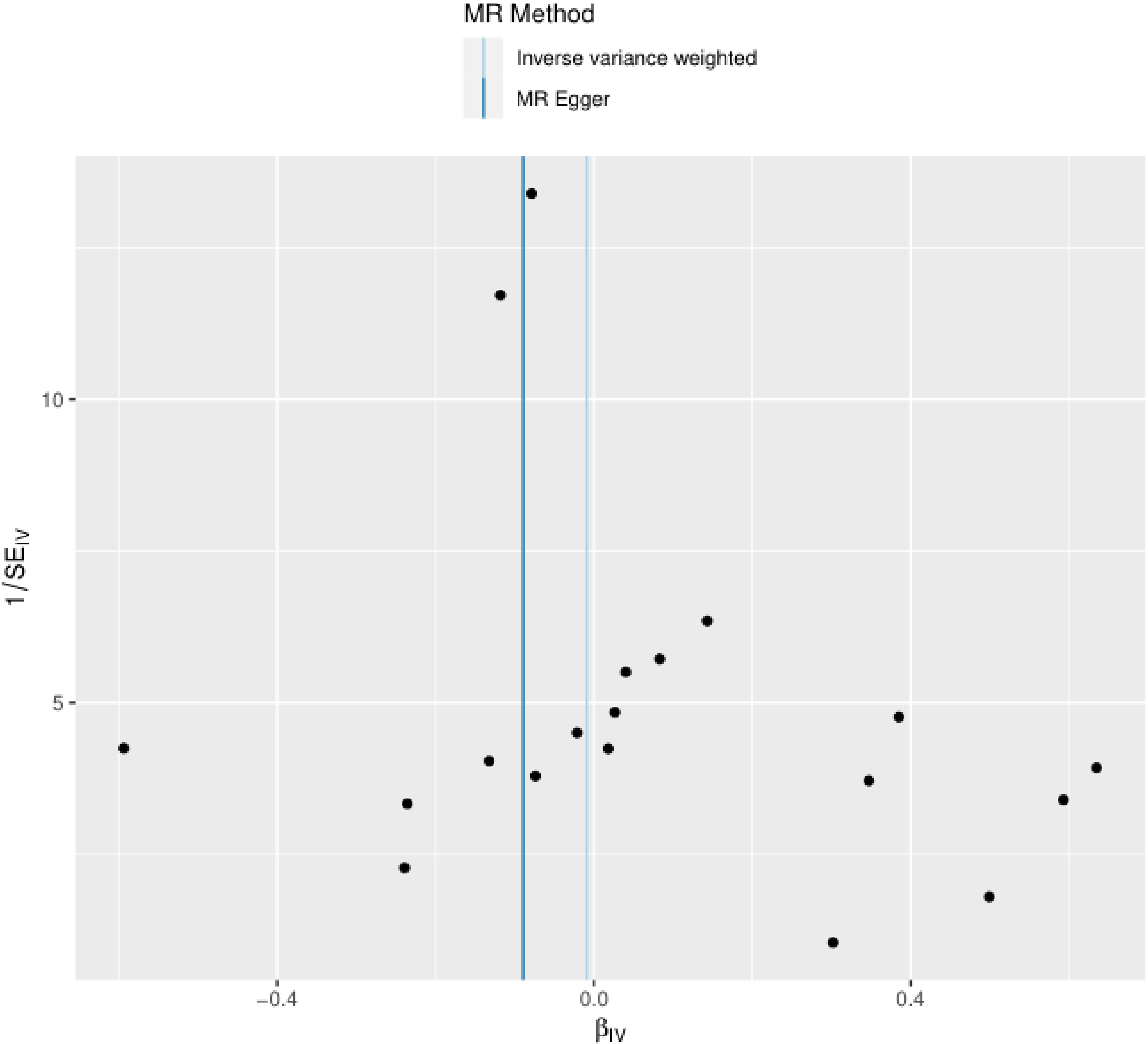

Funnel plot analyse of "TD CD8br AC" on 'Diabetic nephropathy'

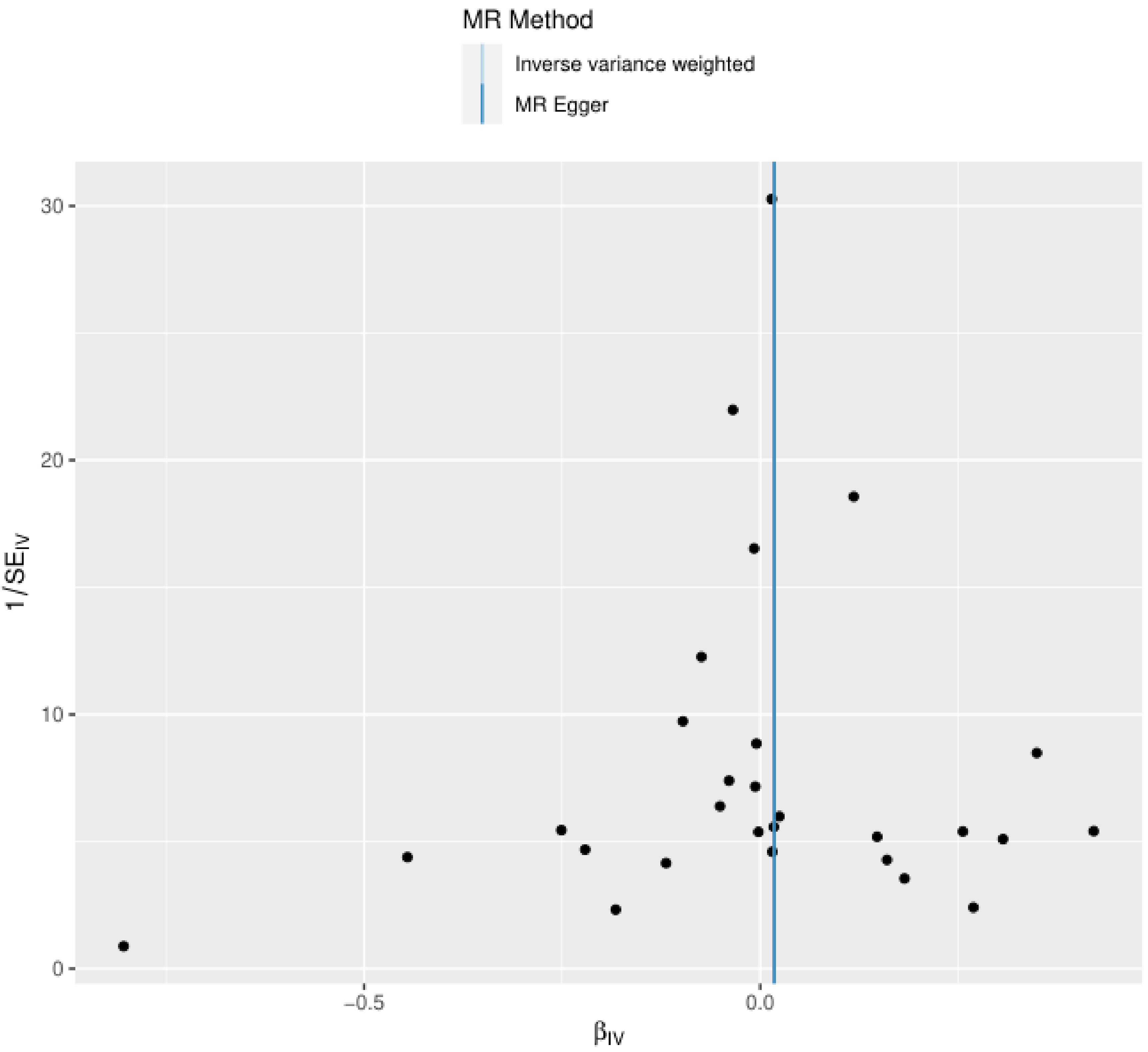

Funnel plot analyse of "B cell % CD3- lymphocyte" on 'Diabetic nephropathy'

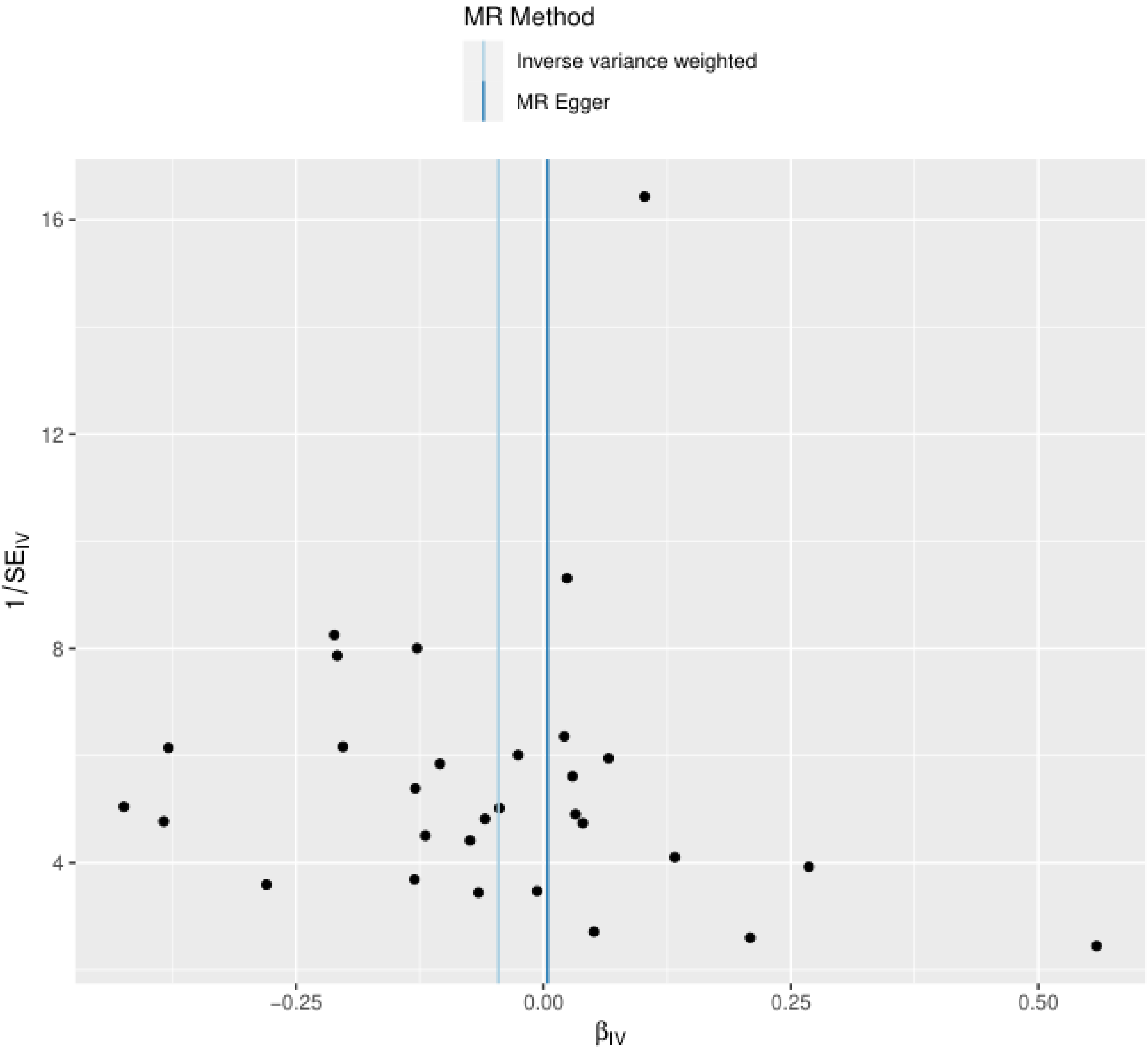

Funnel plot analyse of "CD27 on unsw mem" on 'Diabetic nephropathy'

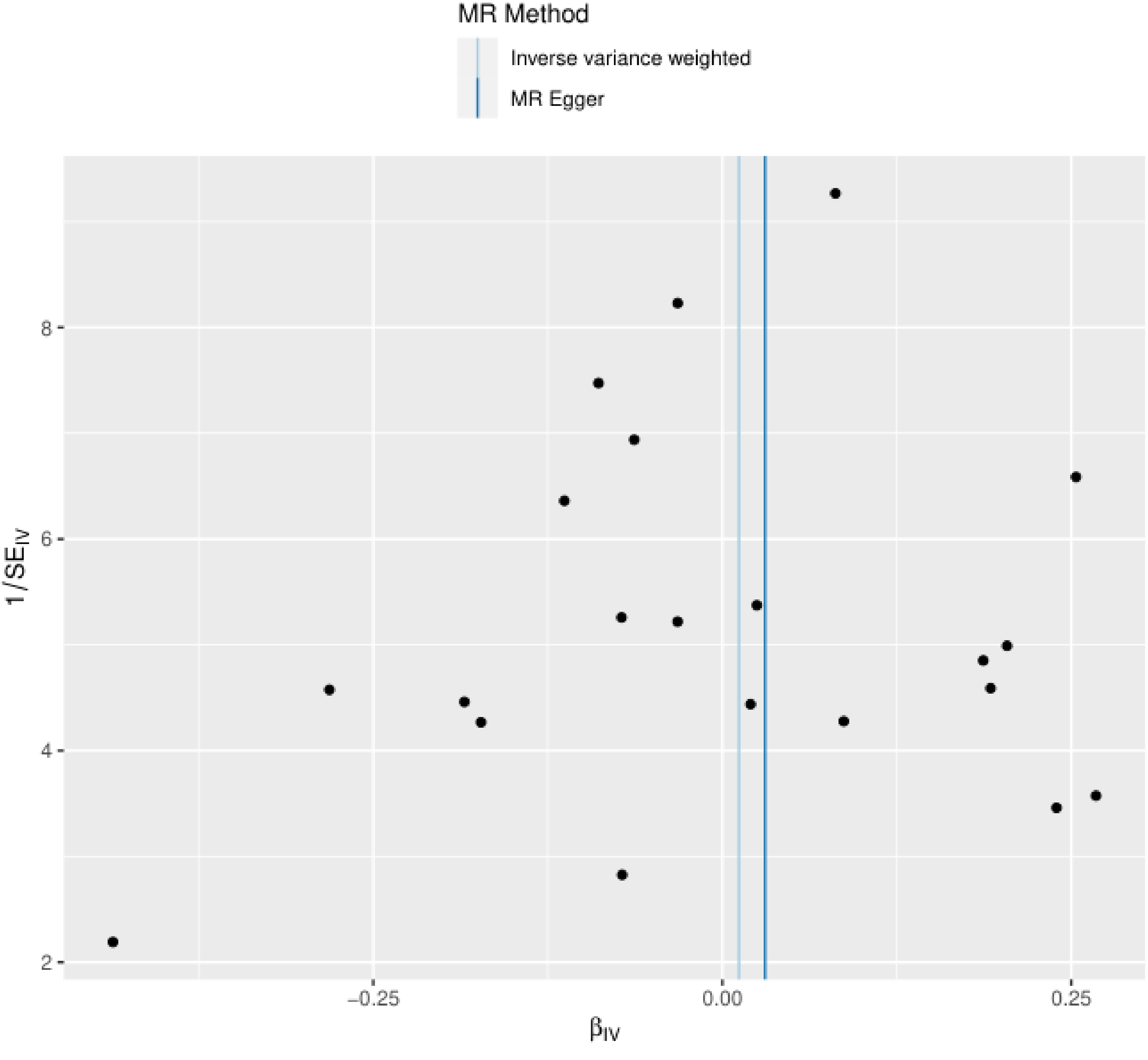

Funnel plot analyse of "CD4 on HLA DR+ CD4+ " on 'Diabetic nephropathy'

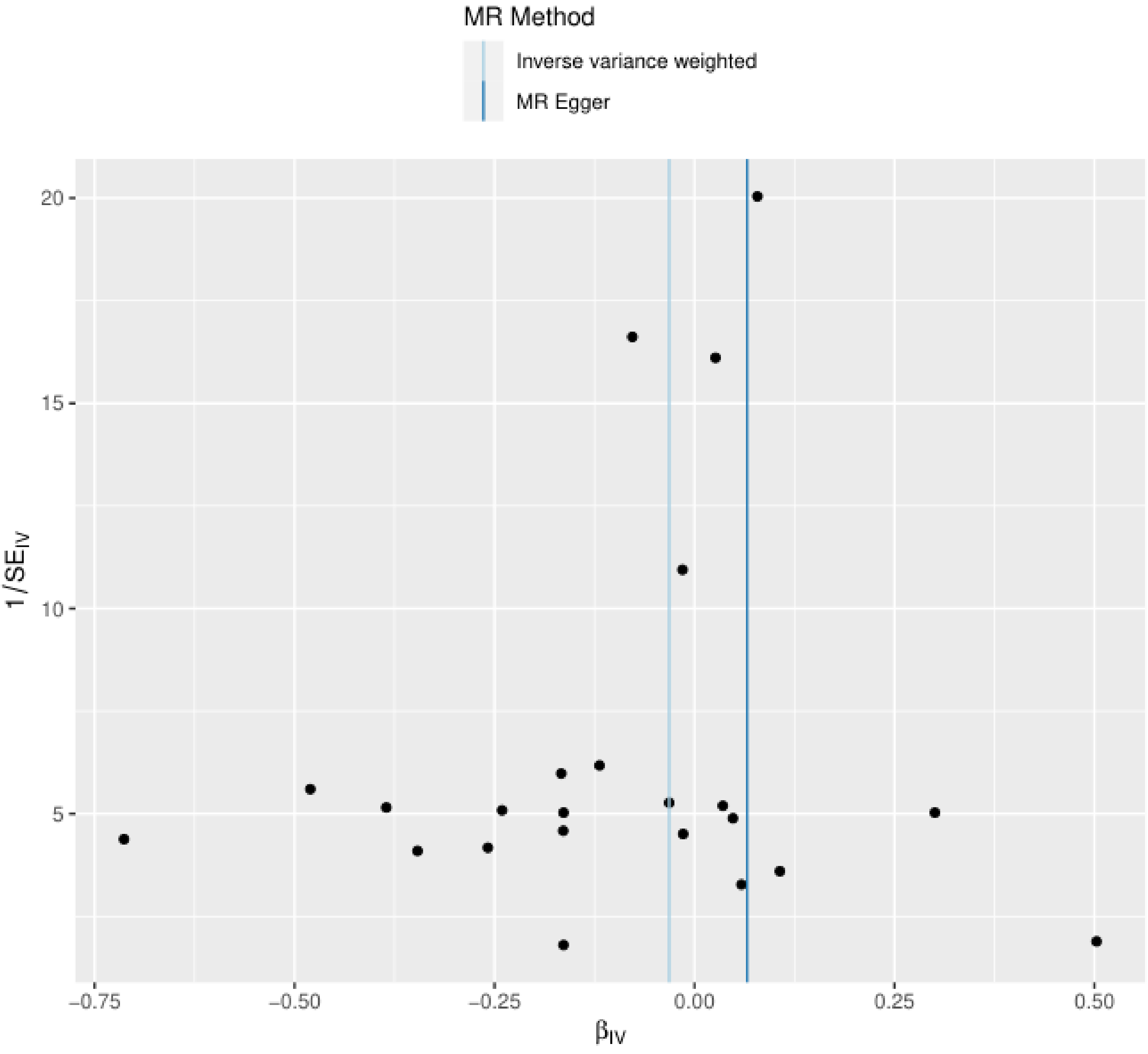

Funnel plot analyse of "CD28 on CD39+ secreting Treg " on 'Diabetic nephropathy'

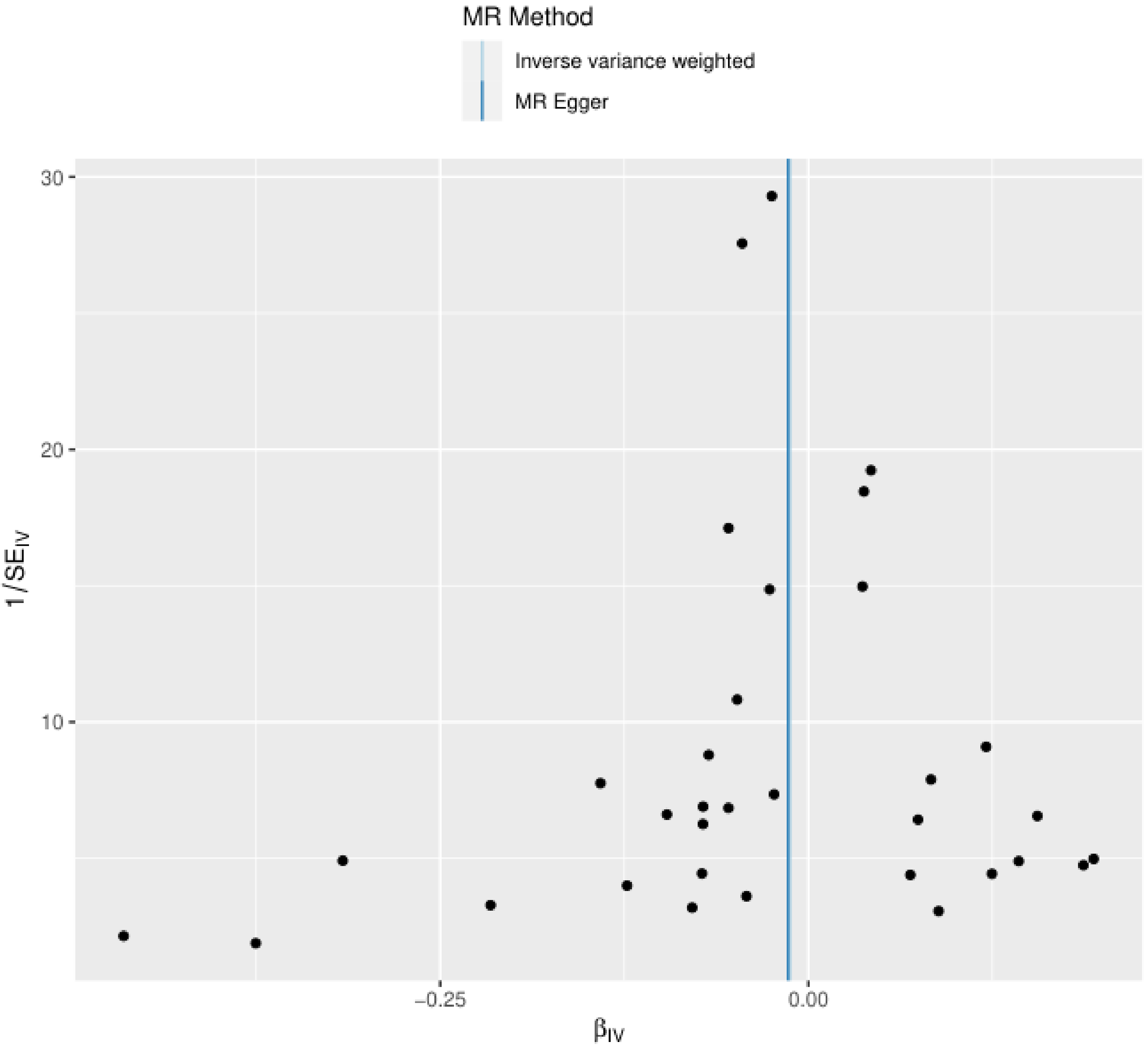

Funnel plot analyse of "CD25hi CD45RA+ CD4 not Treg %CD4+" on 'Diabetic nephropathy'

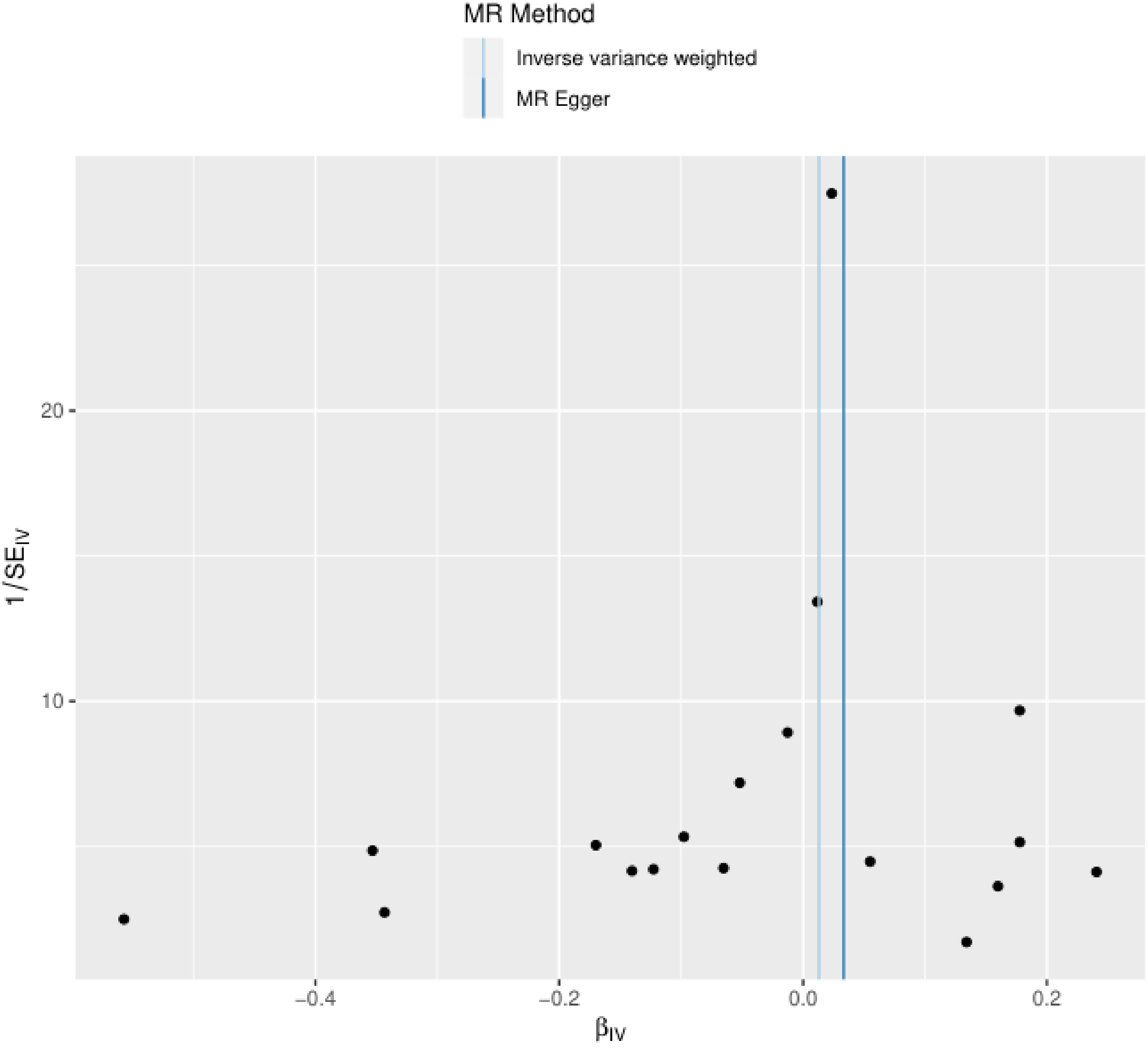

Funnel plot analyse of "IgD+ CD24- %B cell" on 'Diabetic nephropathy'

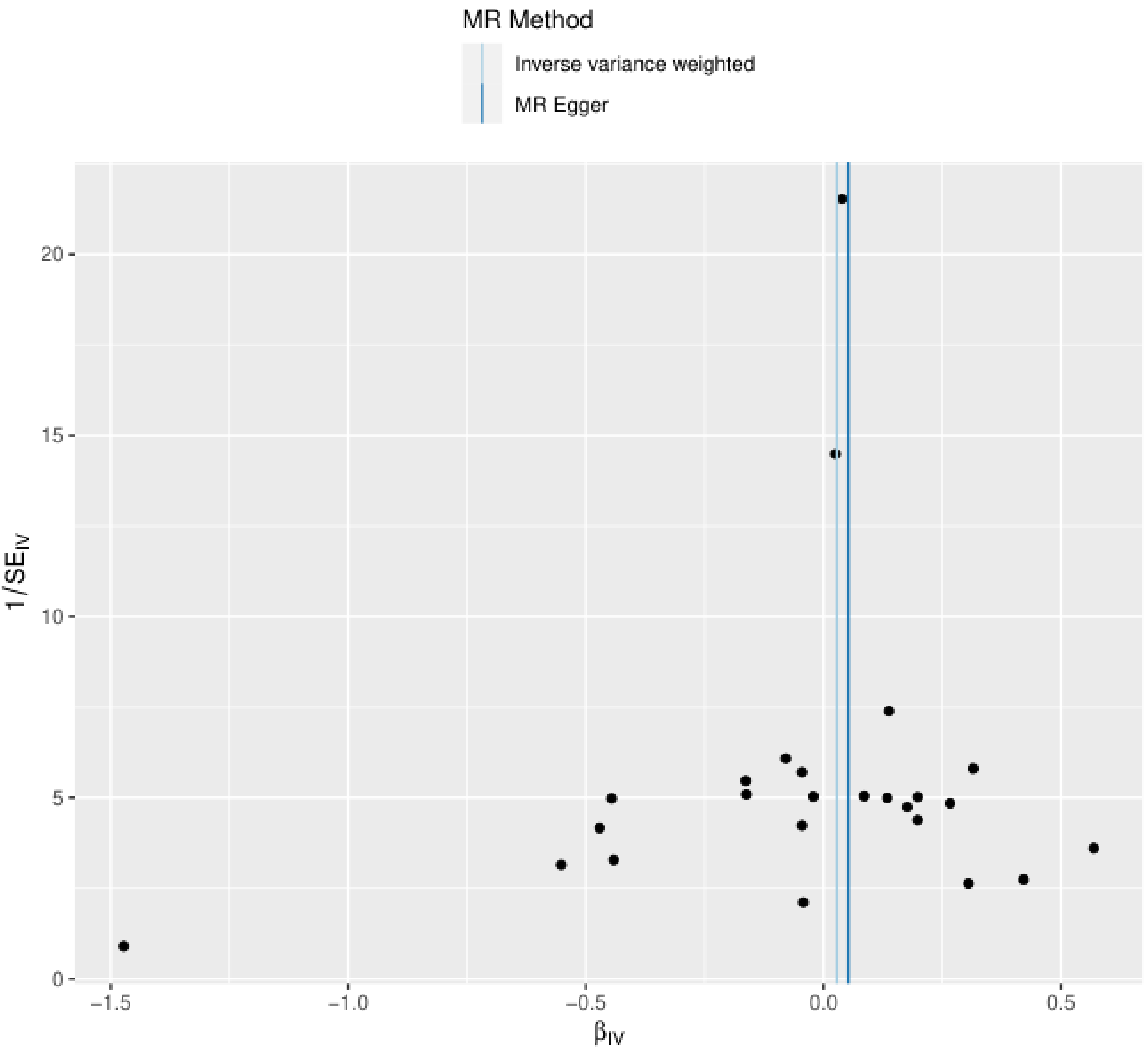

Funnel plot analyse of "SSC-A on plasmacytoid DC" on 'Diabetic nephropathy'

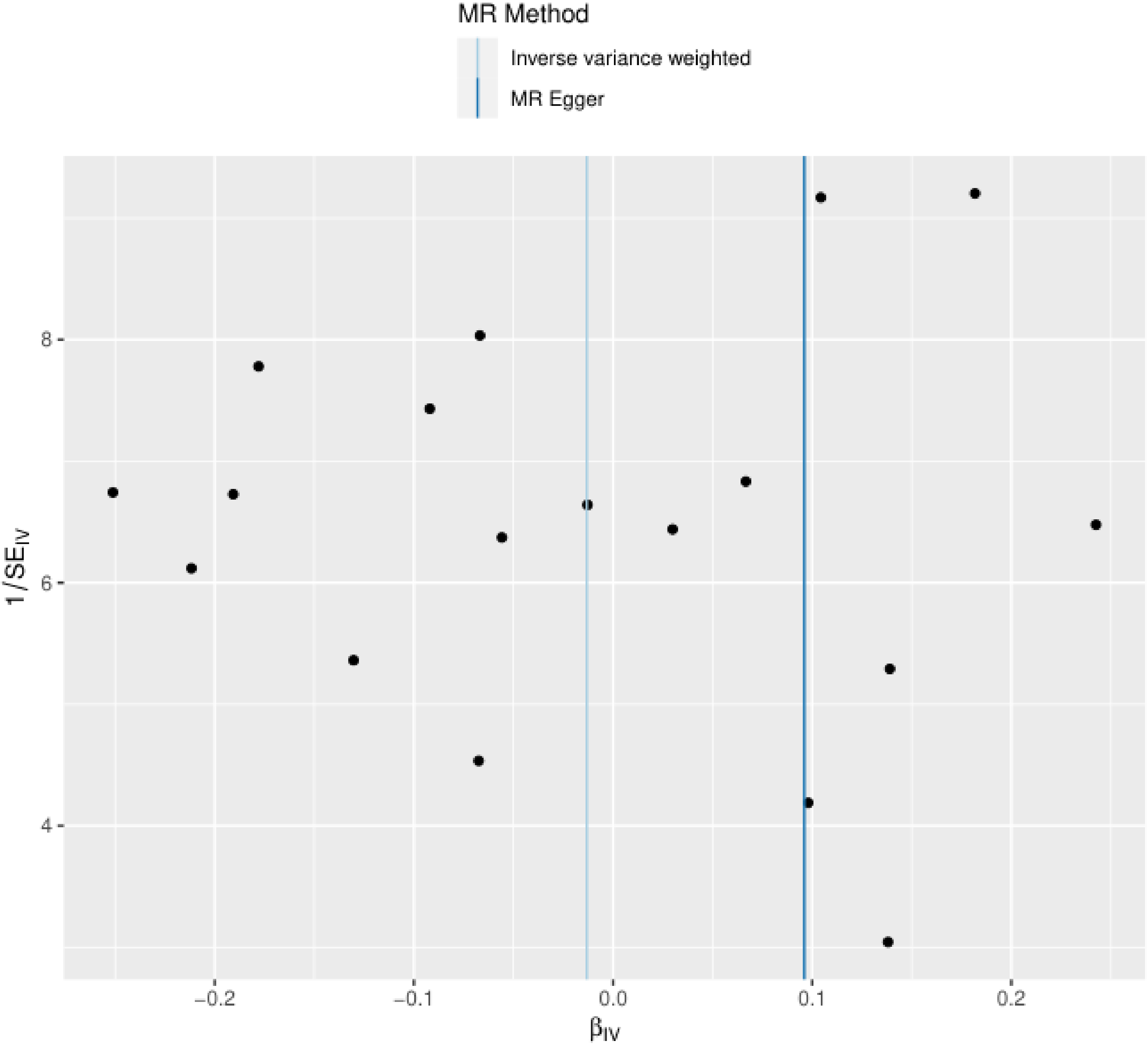

Funnel plot analyse of "CD45 on CD33br HLA DR+ CD14- " on 'Diabetic nephropathy'

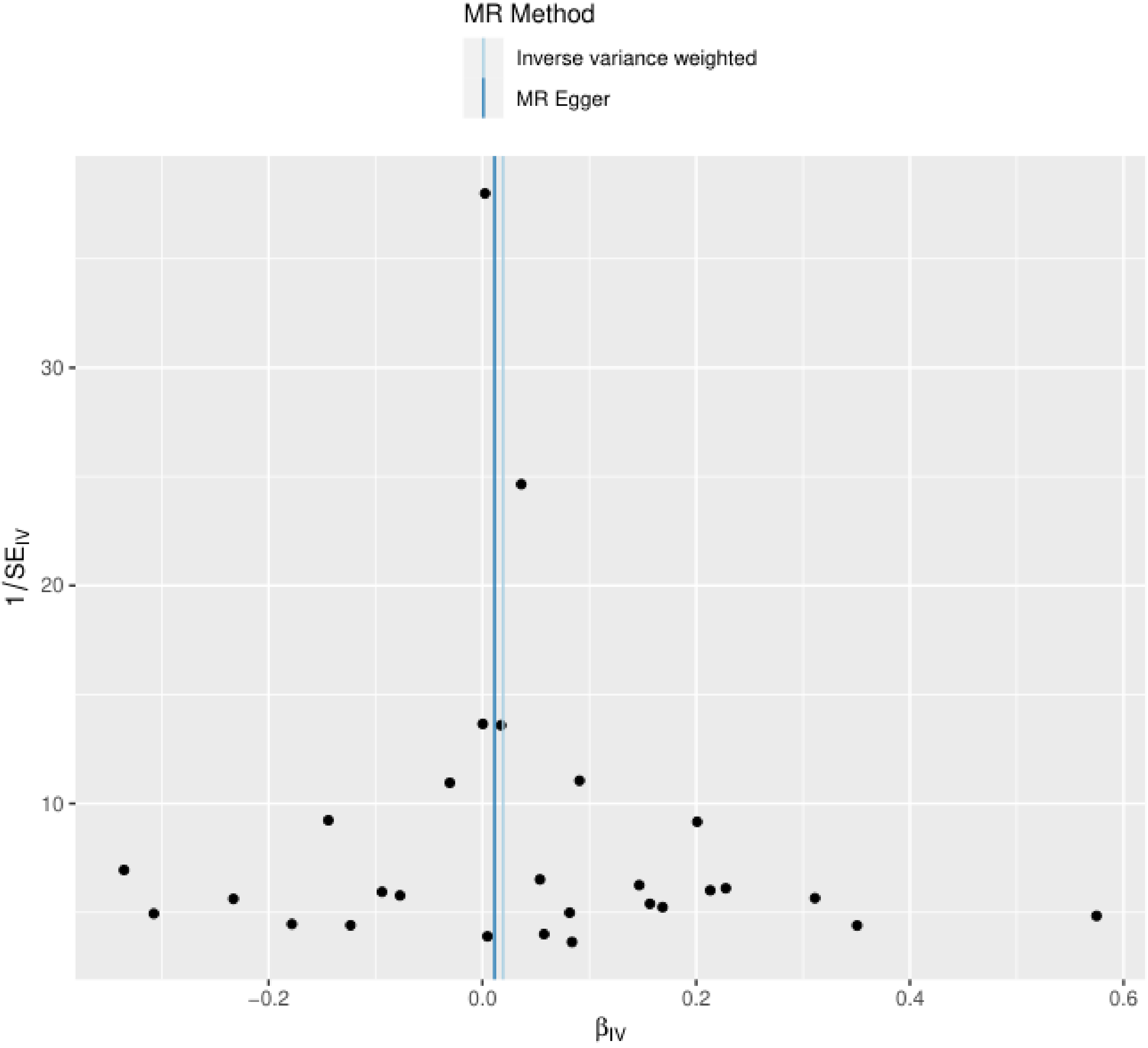

Funnel plot analyse of "CD33dim HLA DR+ CD11b- AC" on 'Diabetic nephropathy'

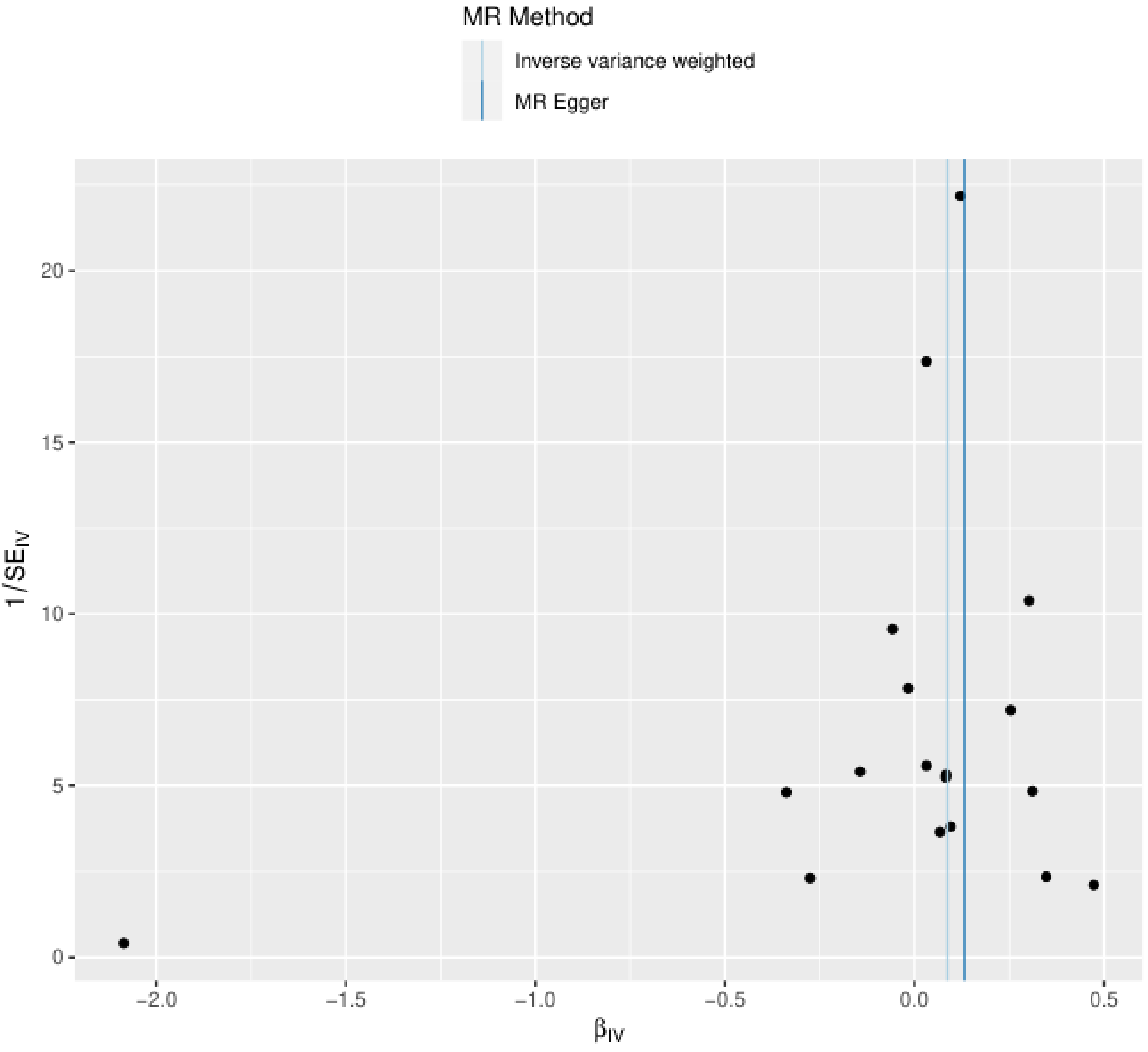

Funnel plot analyse of "CD45 on HLA DR+ T cell" on 'Diabetic nephropathy'

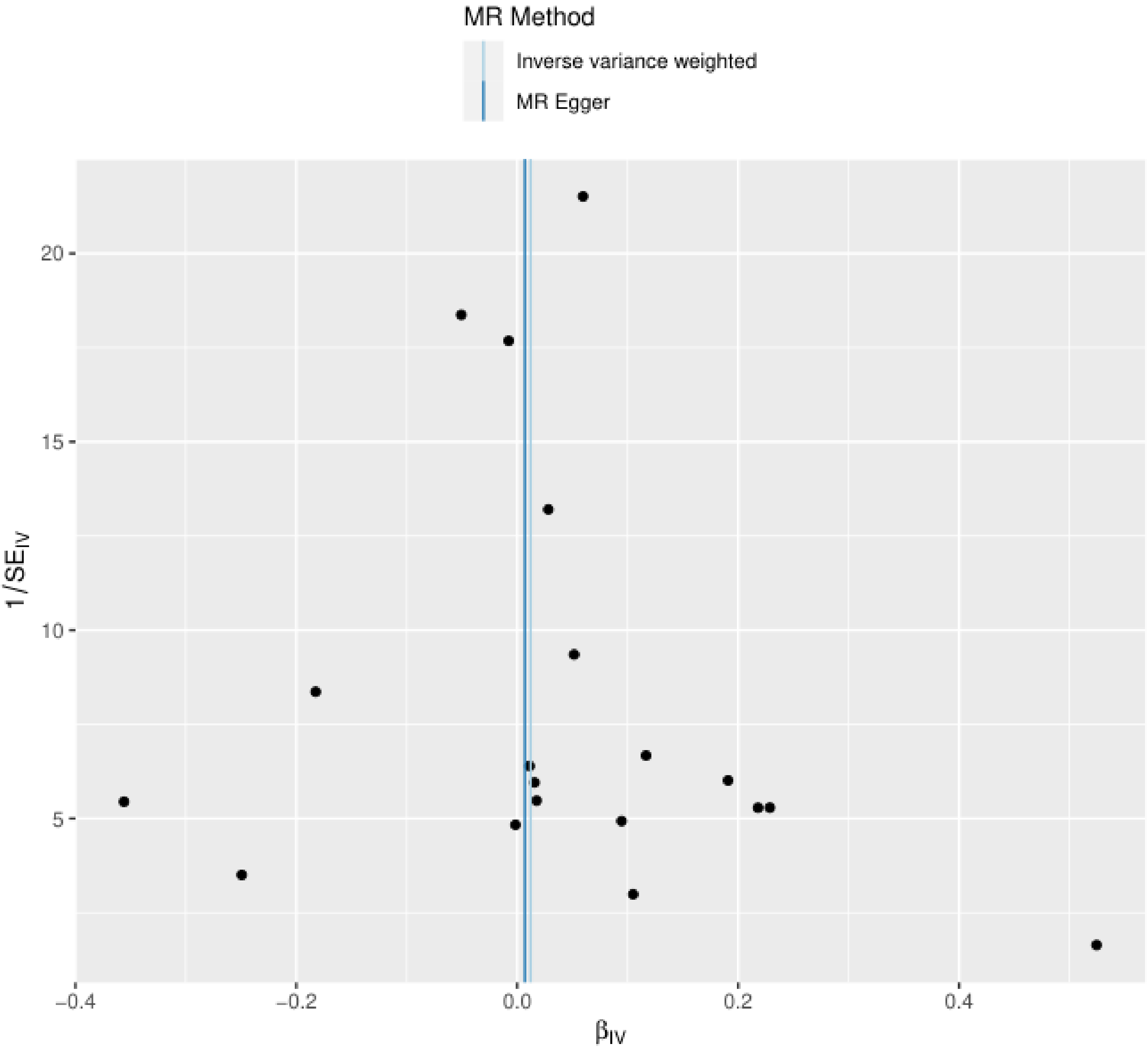

Funnel plot analyse of "FSC-A on HLA DR+ NK" on 'Diabetic nephropathy'

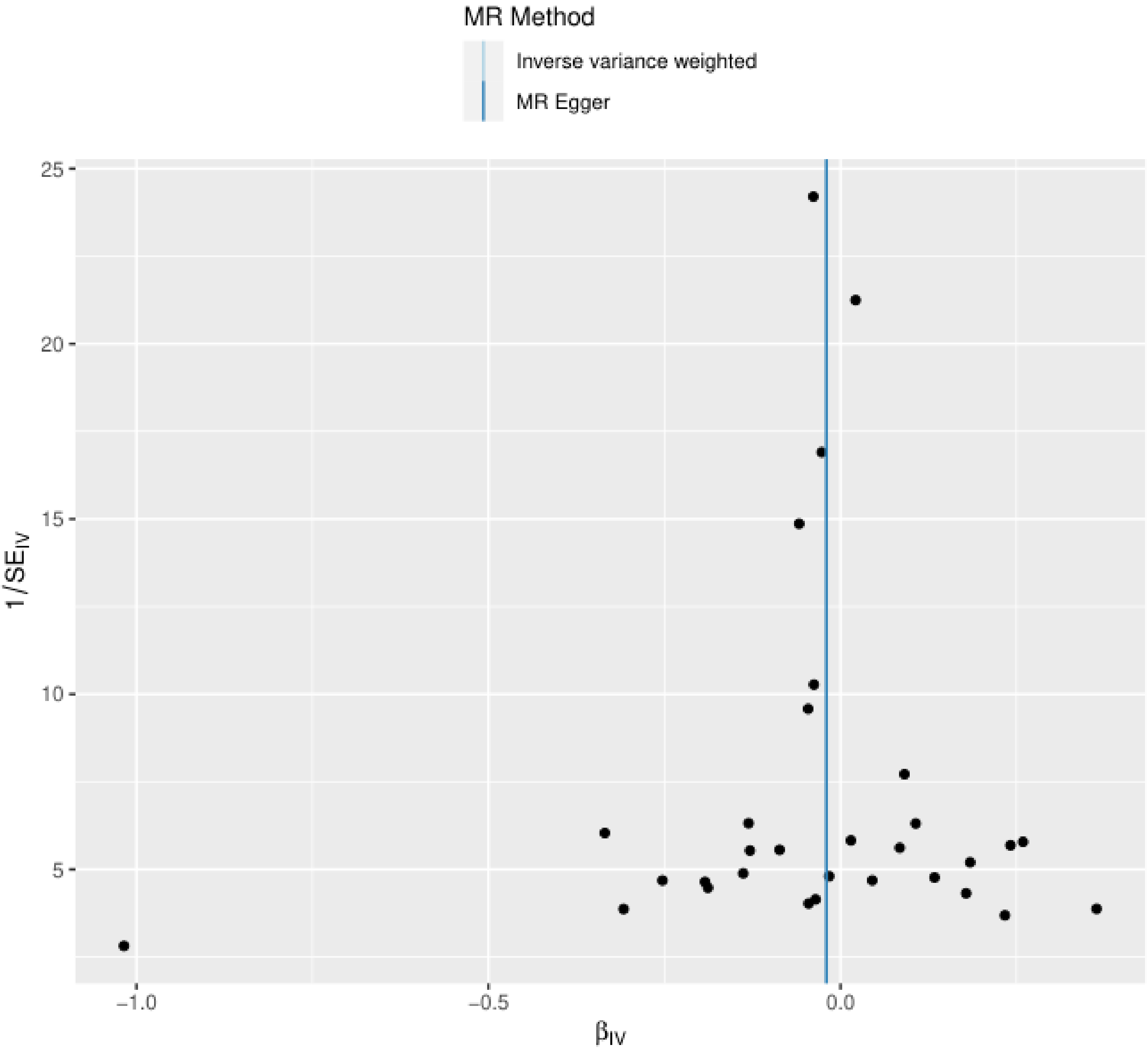

Funnel plot analyse of "HLA DR+ T cell AC" on 'Diabetic nephropathy'

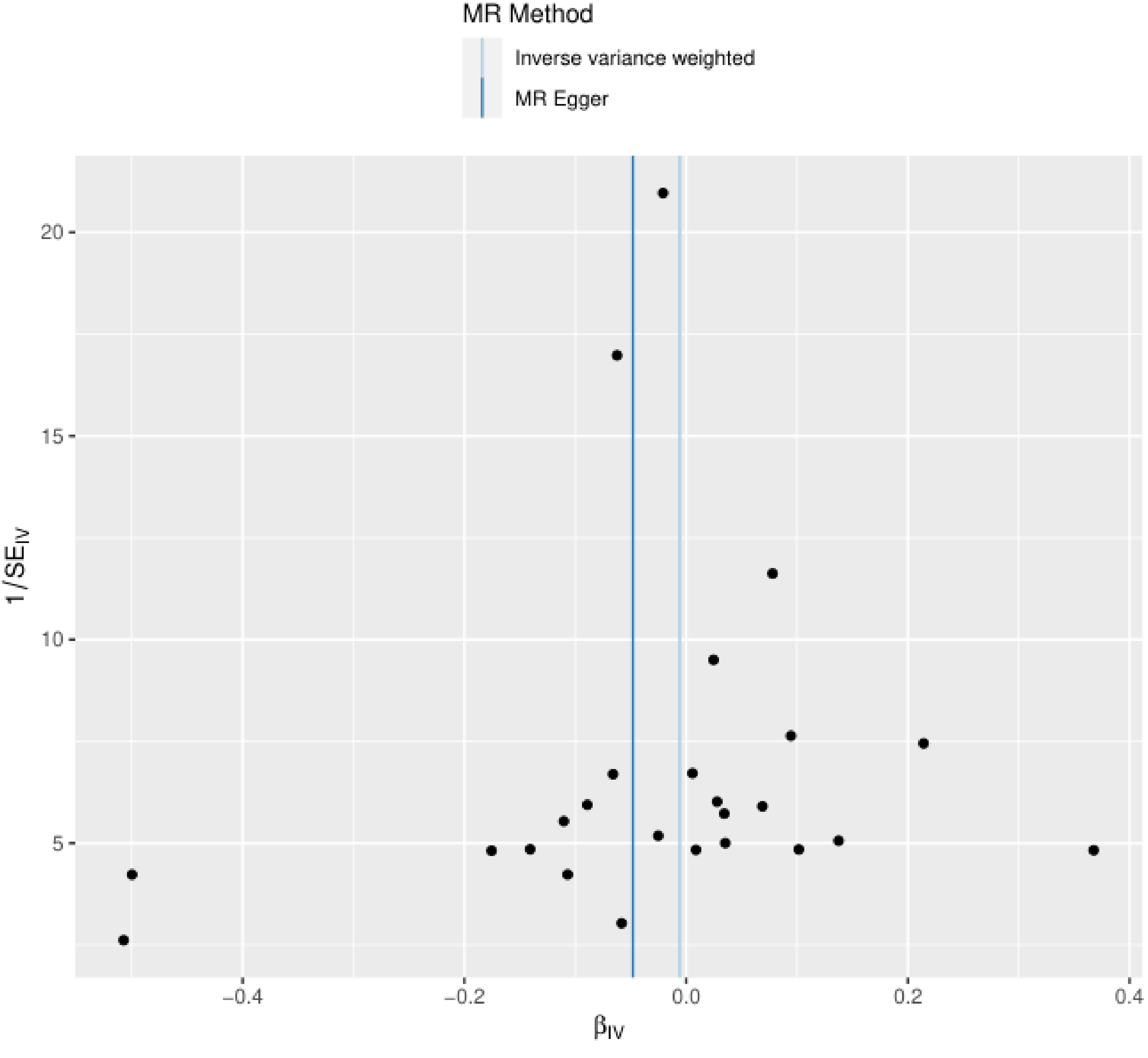

Funnel plot analyse of "CD3 on naive CD8br " on 'Diabetic nephropathy'

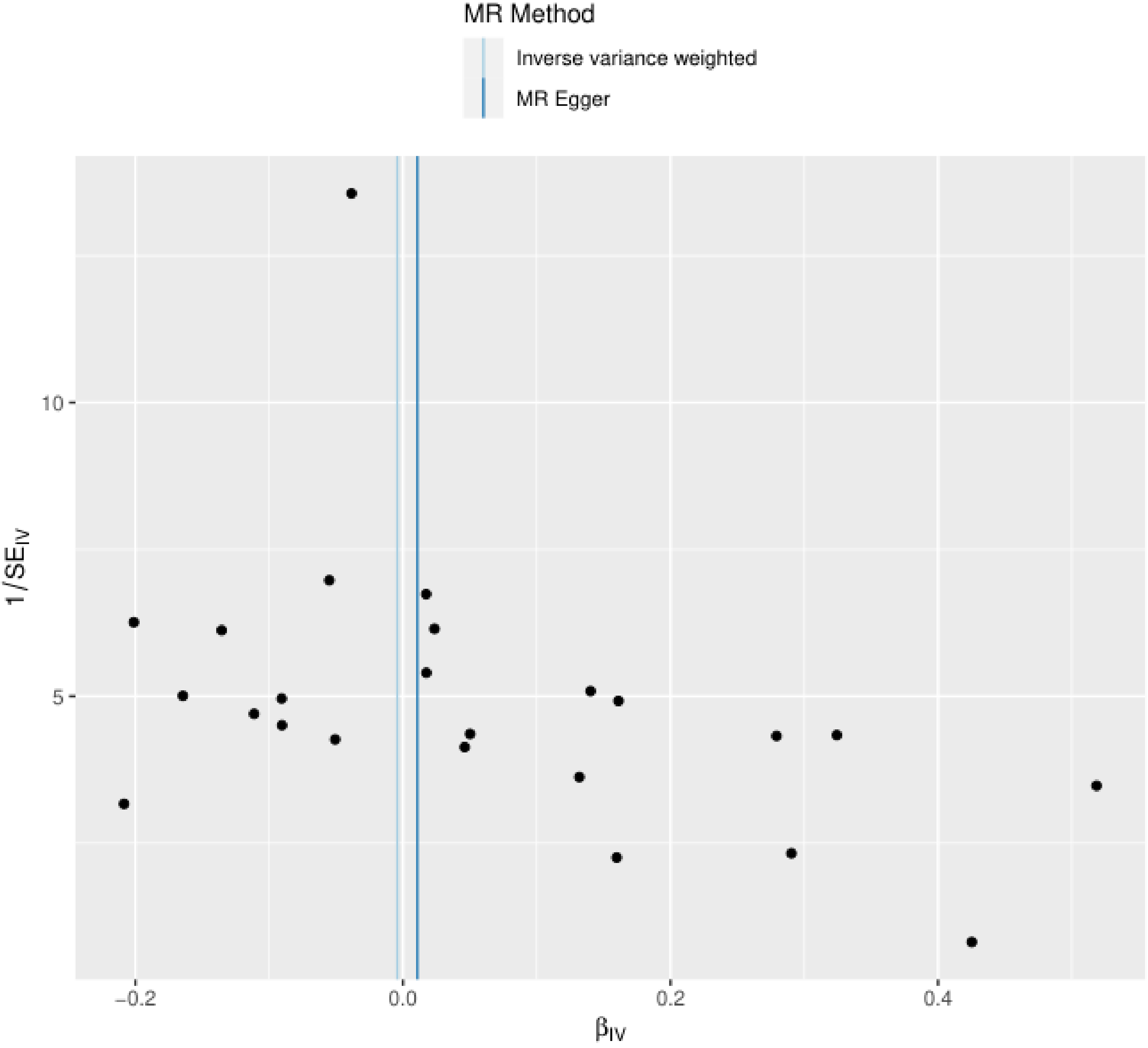

Funnel plot analyse of "CD127 on CD8br" on 'Diabetic nephropathy'

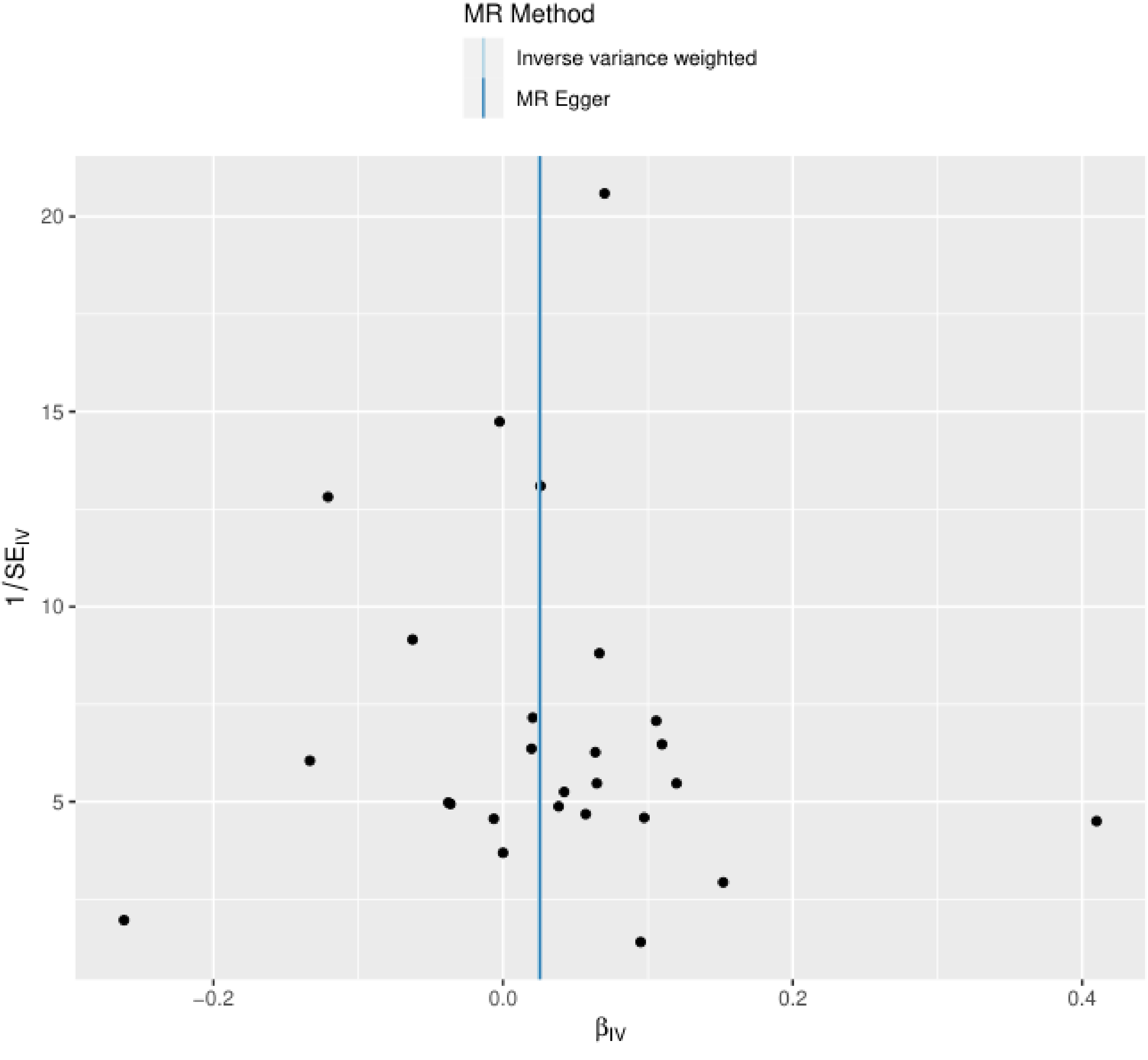

Funnel plot analyse of "CD4 on secreting Treg " on 'Diabetic nephropathy'

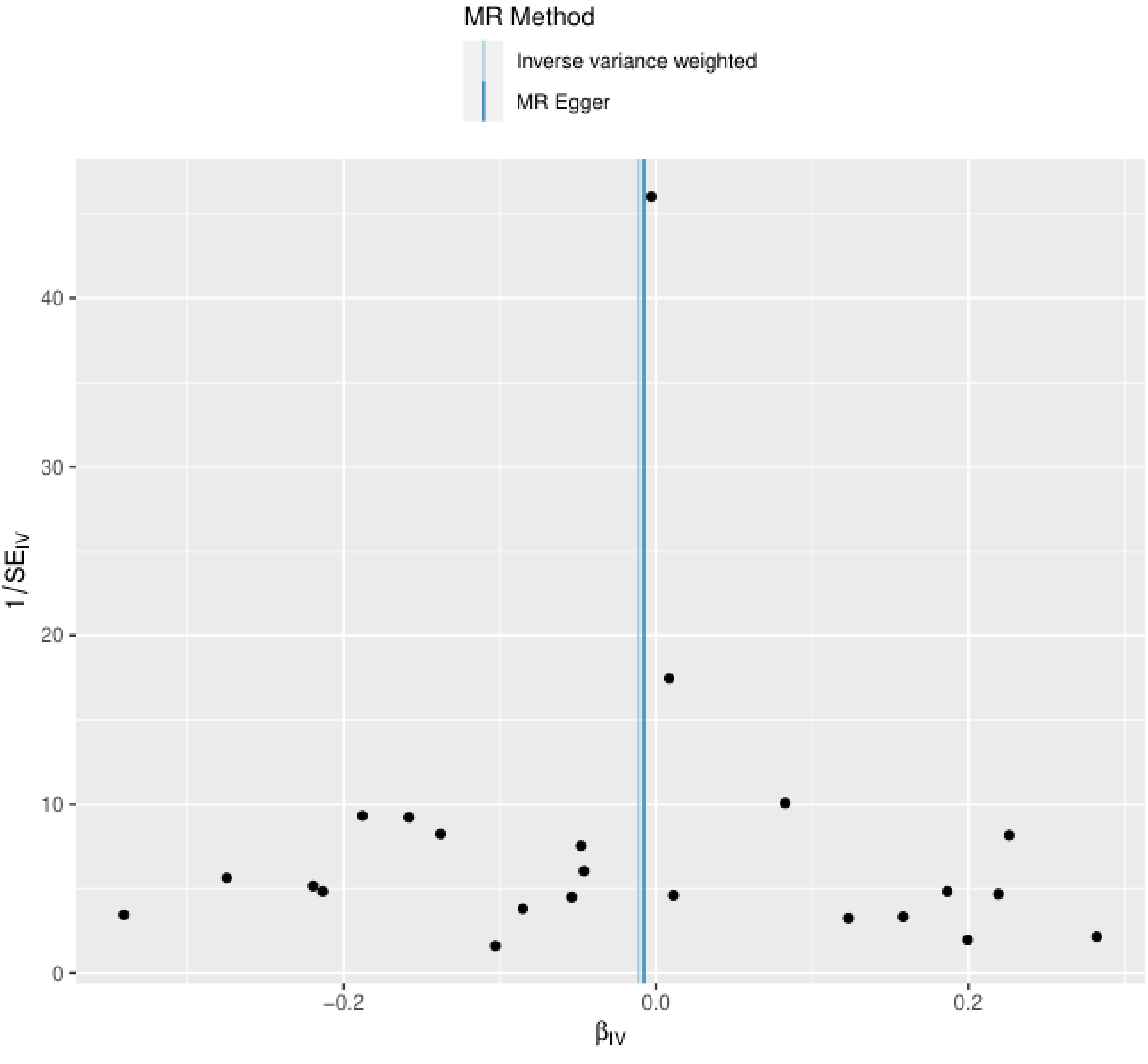

Funnel plot analyse of "EM CD4+ AC" on 'Diabetic nephropathy'

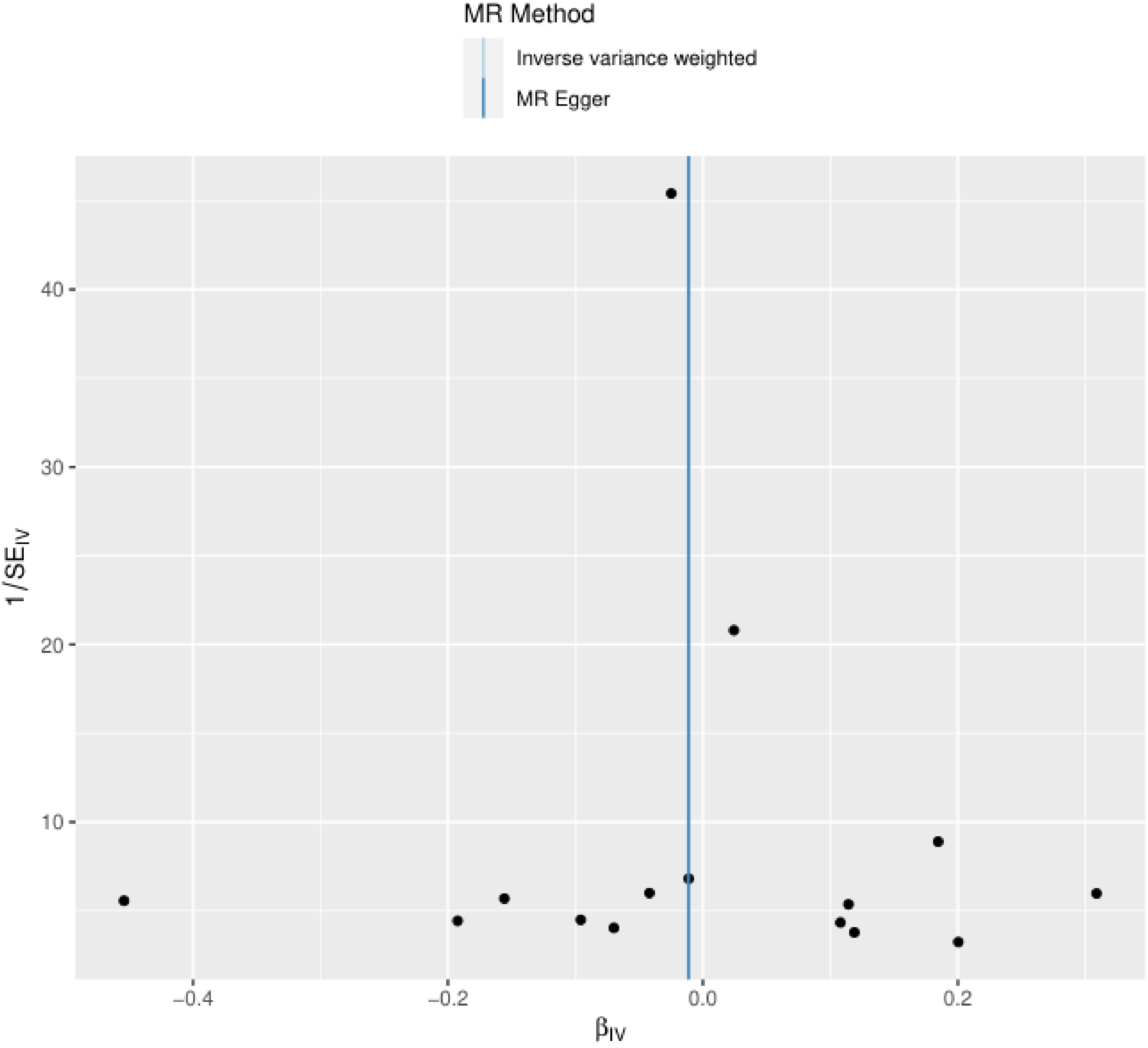

Funnel plot analyse of "CD25 on CD4+" on 'Diabetic nephropathy'

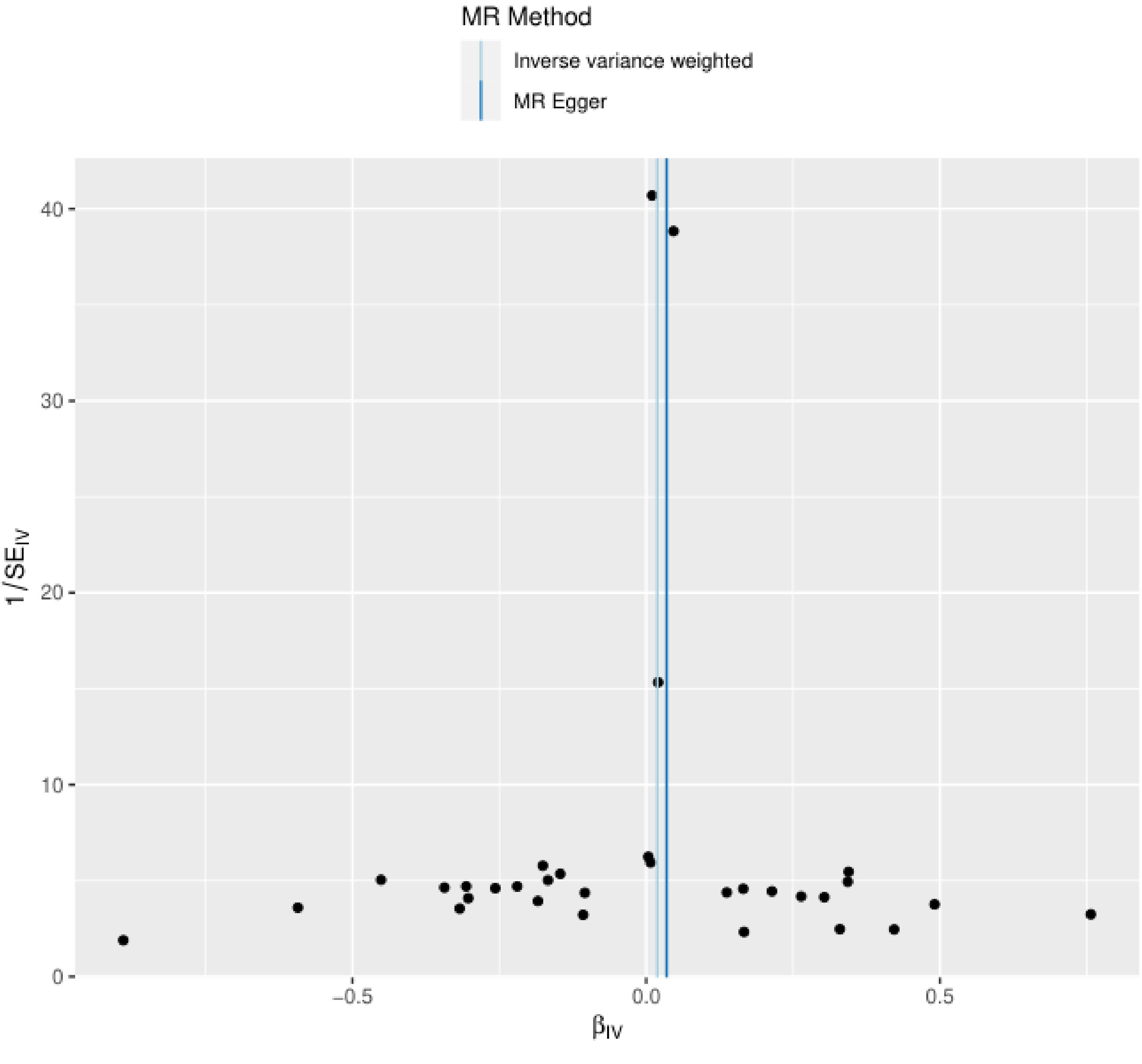

Funnel plot analyse of "IgD+ CD38dim %lymphocyte" on 'Diabetic nephropathy'

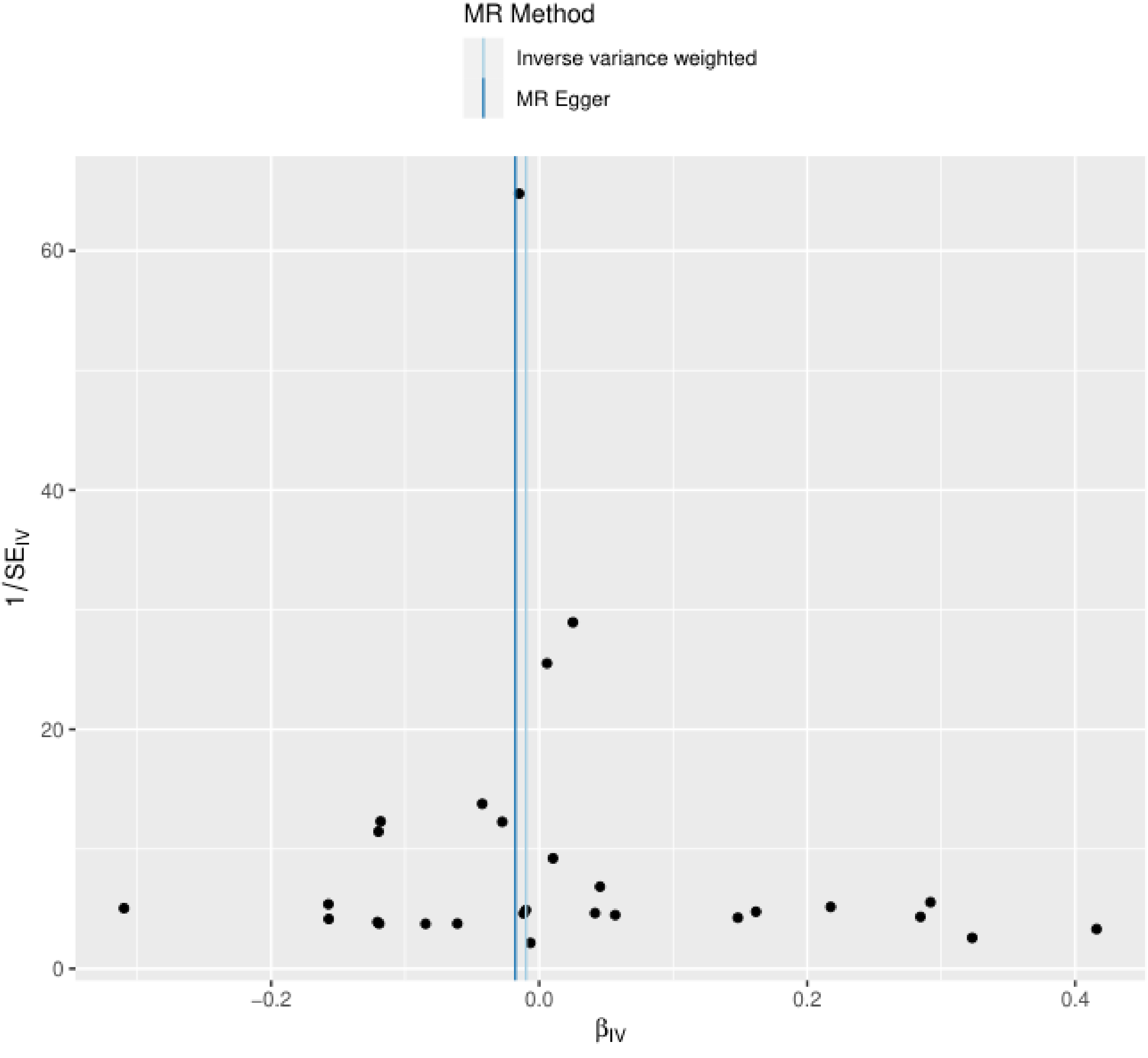

Funnel plot analyse of "CD28+ CD45RA- CD8br AC" on 'Diabetic nephropathy'

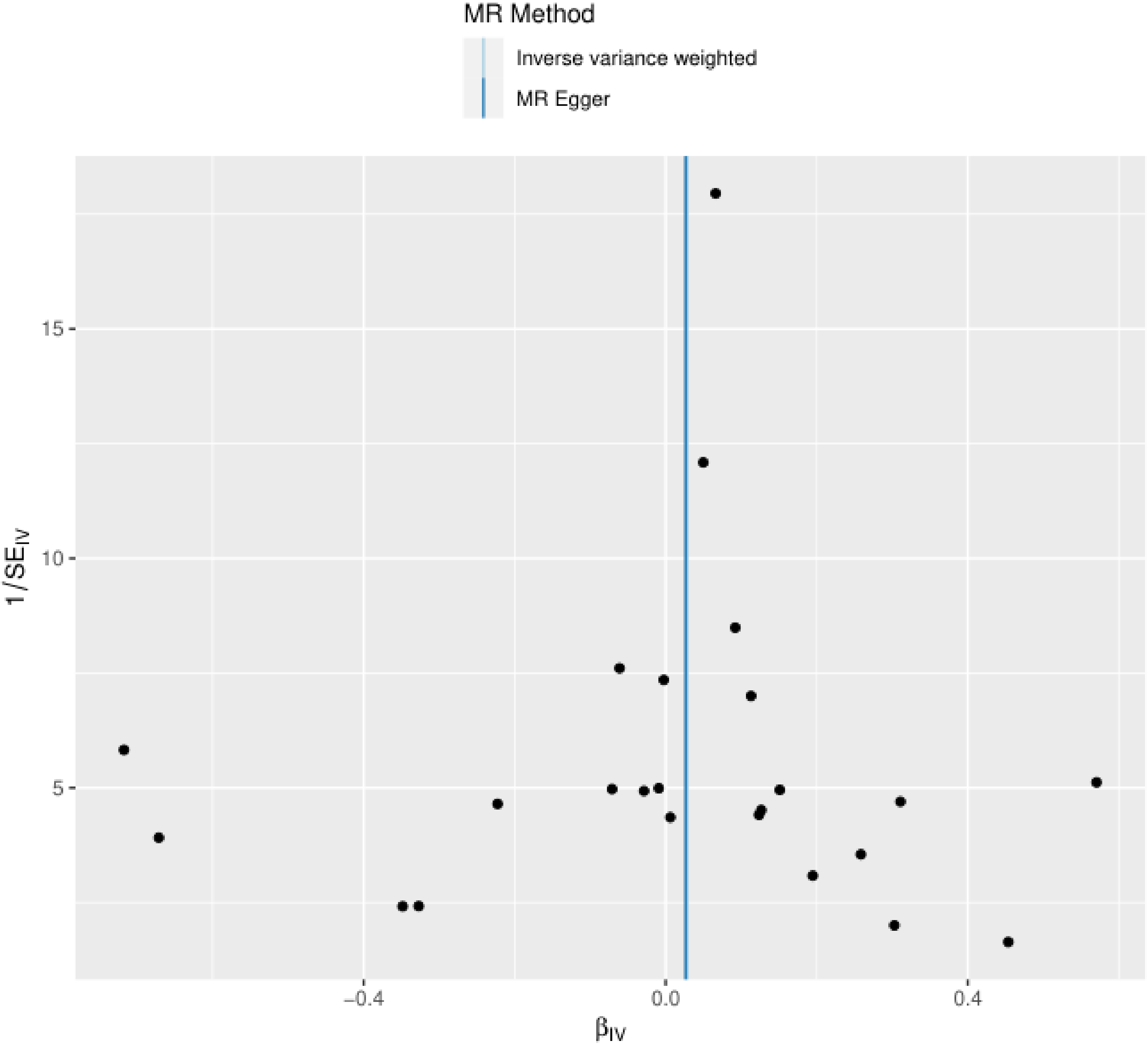

Funnel plot analyse of "SSC-A on HLA DR+ CD8br" on 'Diabetic nephropathy'

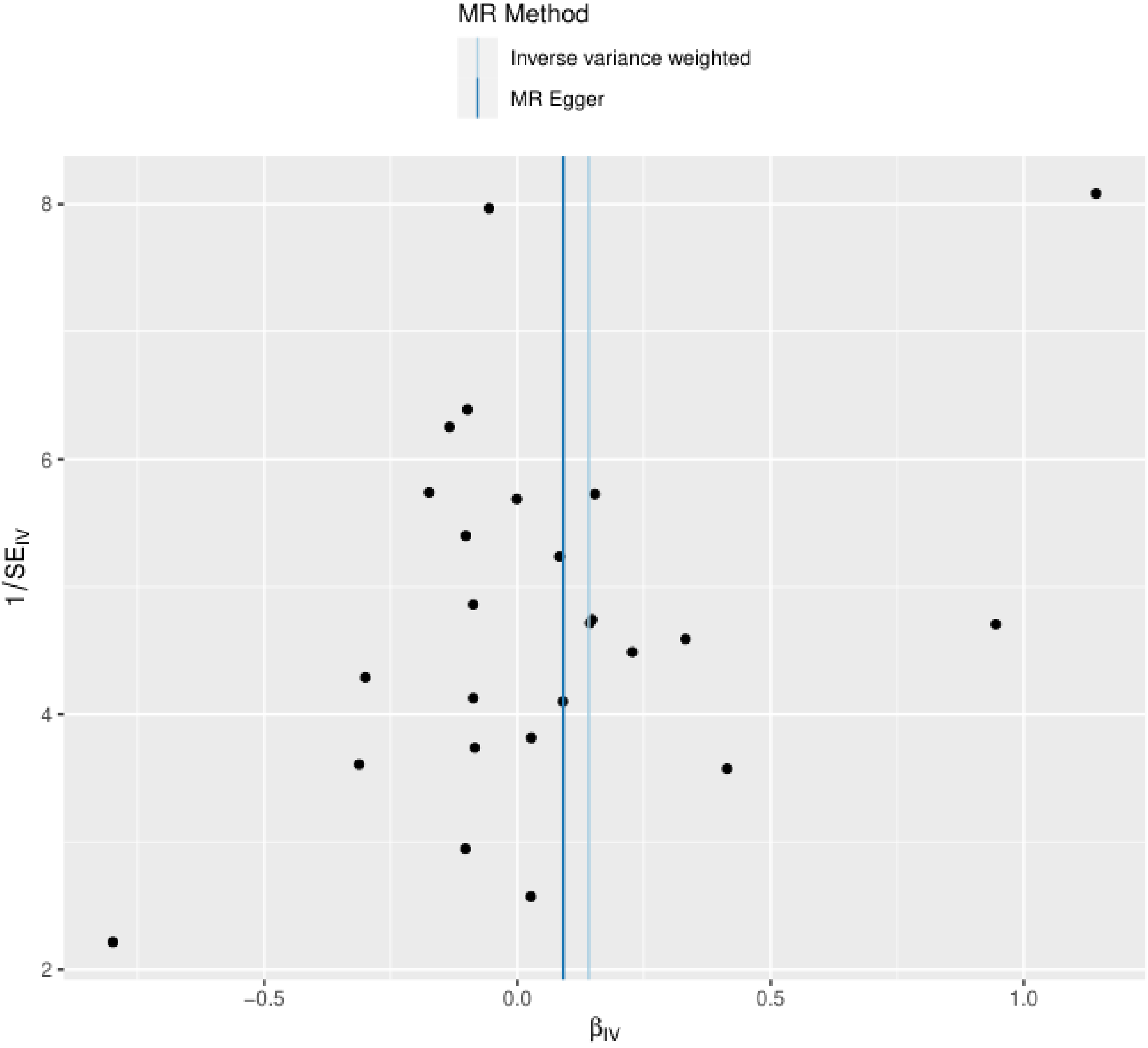

Funnel plot analyse of "Naive CD4+ AC" on 'Diabetic nephropathy'

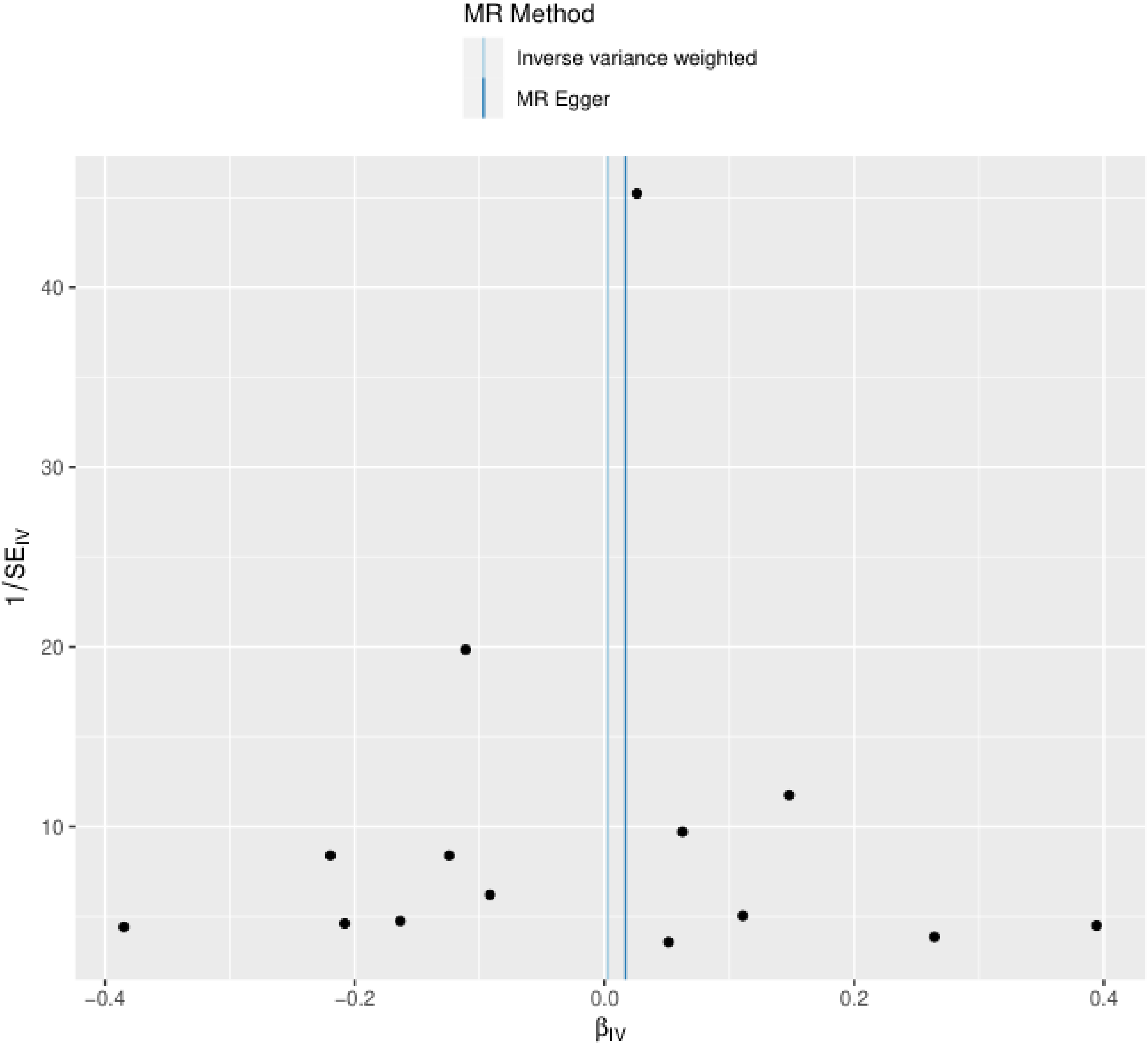

Funnel plot analyse of "IgD+ AC" on 'Diabetic nephropathy'

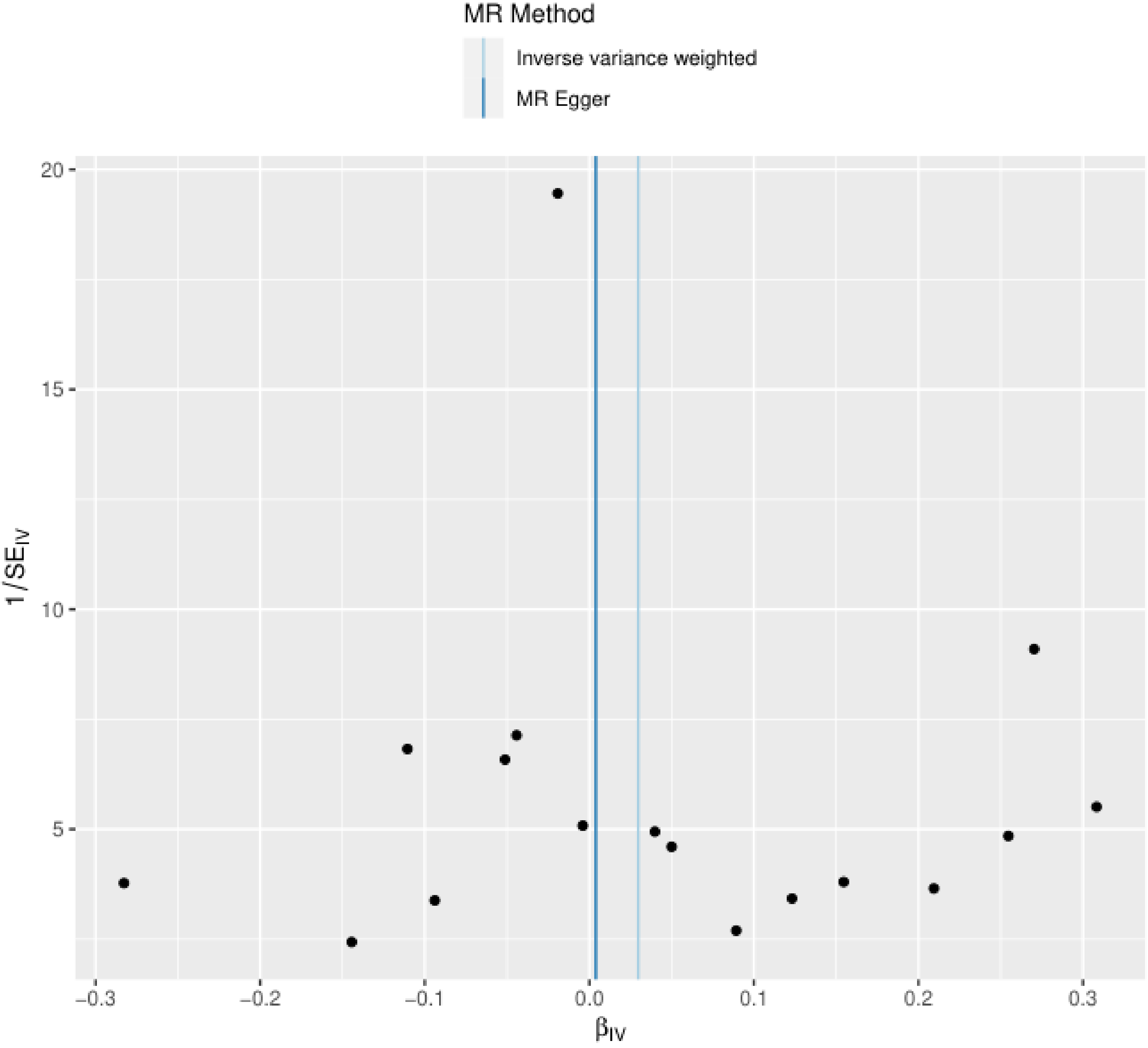

Funnel plot analyse of "CD62L- myeloid DC AC" on 'Diabetic nephropathy'

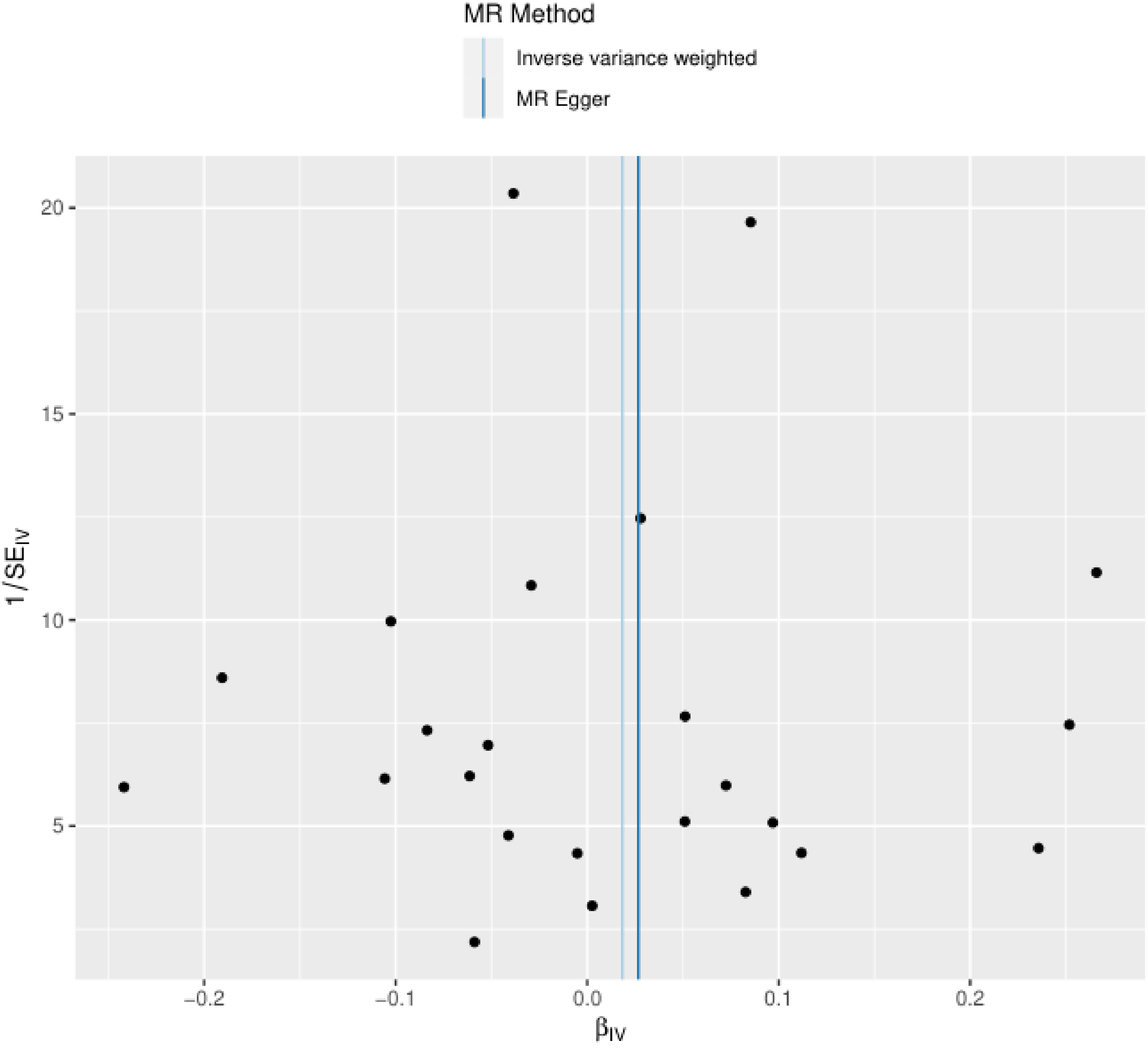

Funnel plot analyse of "CD27 on IgD+ CD38- unsw mem" on 'Diabetic nephropathy'

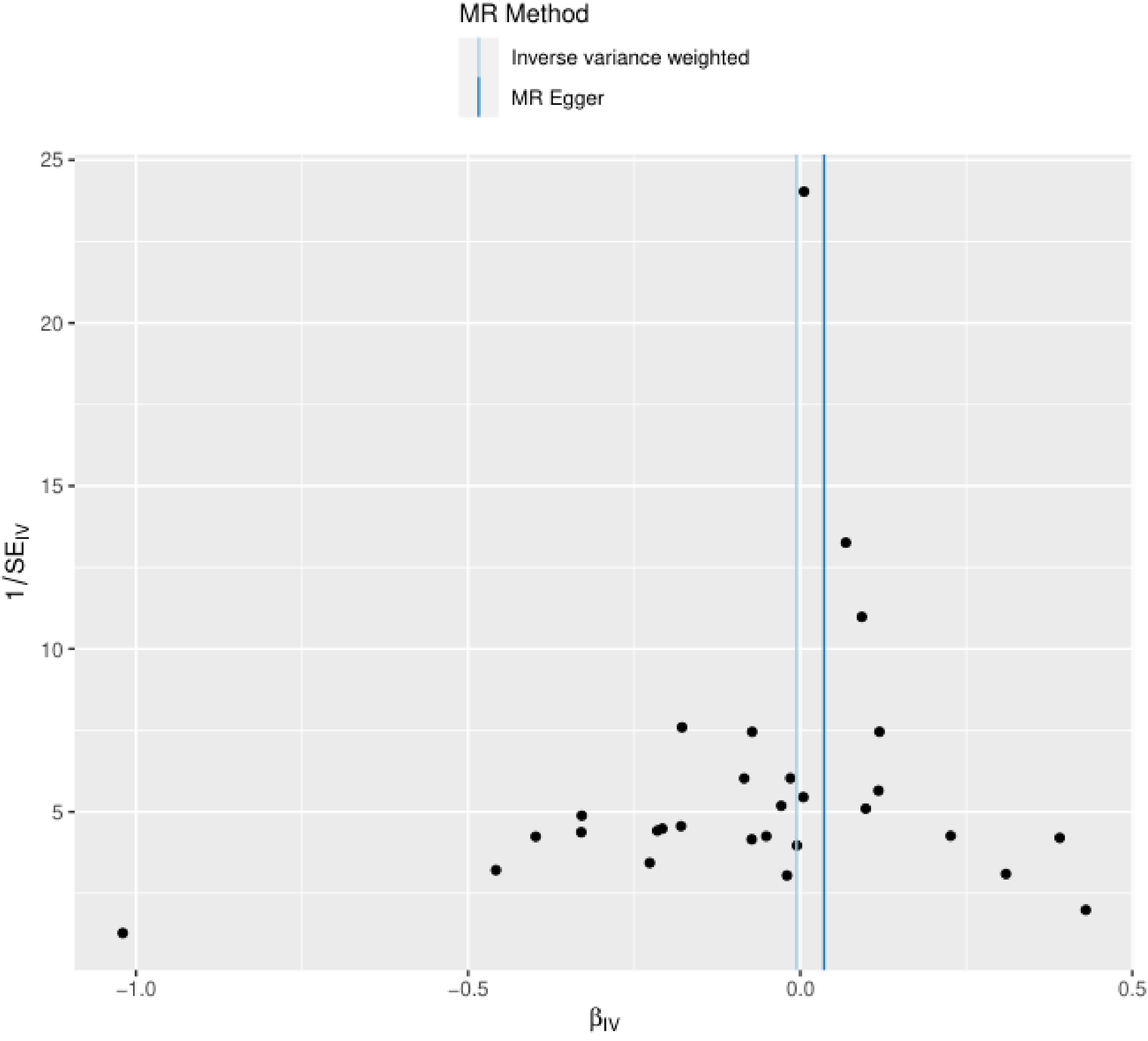

Funnel plot analyse of "IgD on IgD+ CD24-" on 'Diabetic nephropathy'

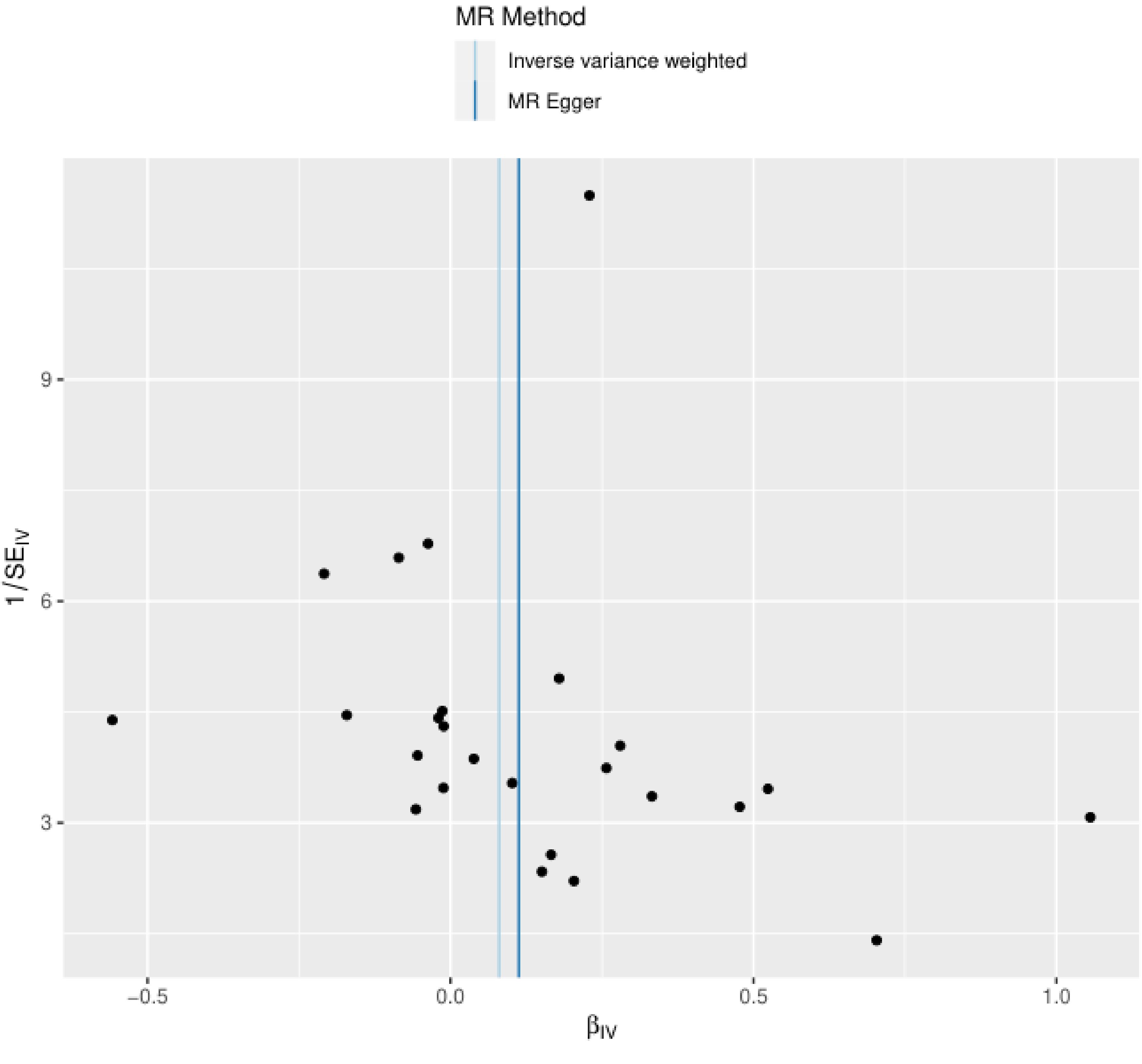

Funnel plot analyse of "CM CD8br AC" on 'Diabetic nephropathy'

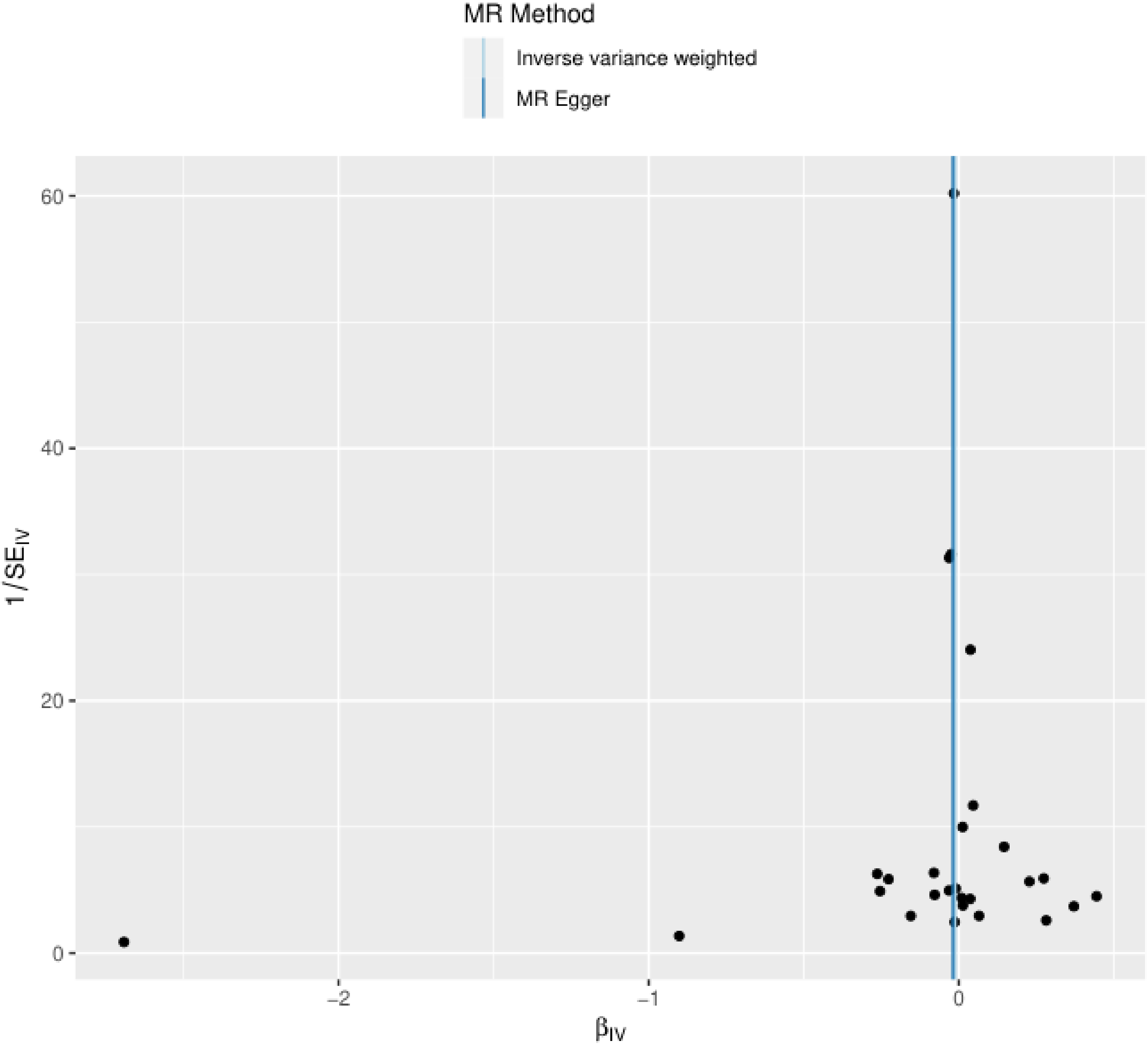

Funnel plot analyse of "CD28+ CD45RA- CD8br %T cell" on 'Diabetic nephropathy'

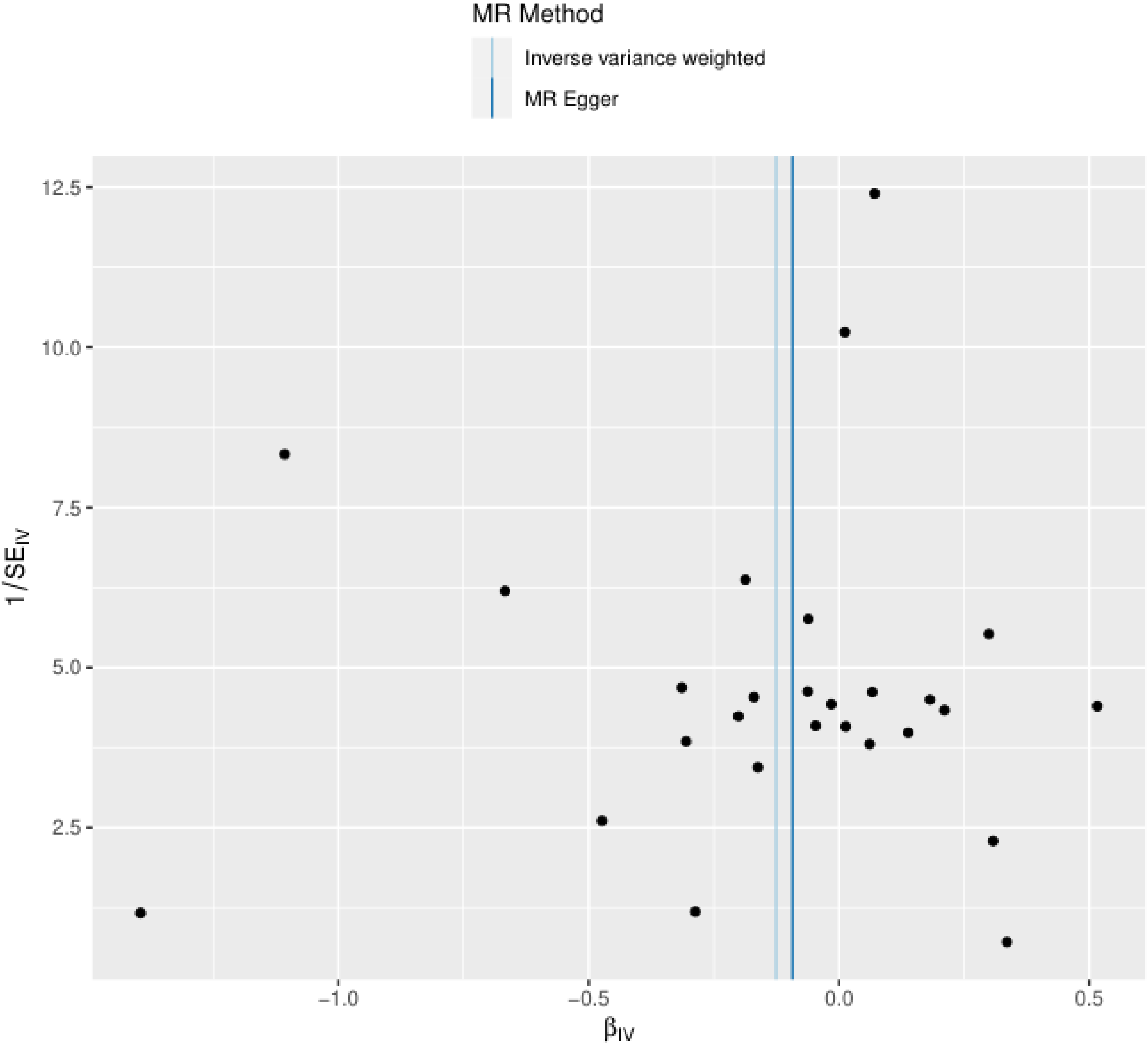

Funnel plot analyse of "SSC-A on CD4+" on 'Diabetic nephropathy'

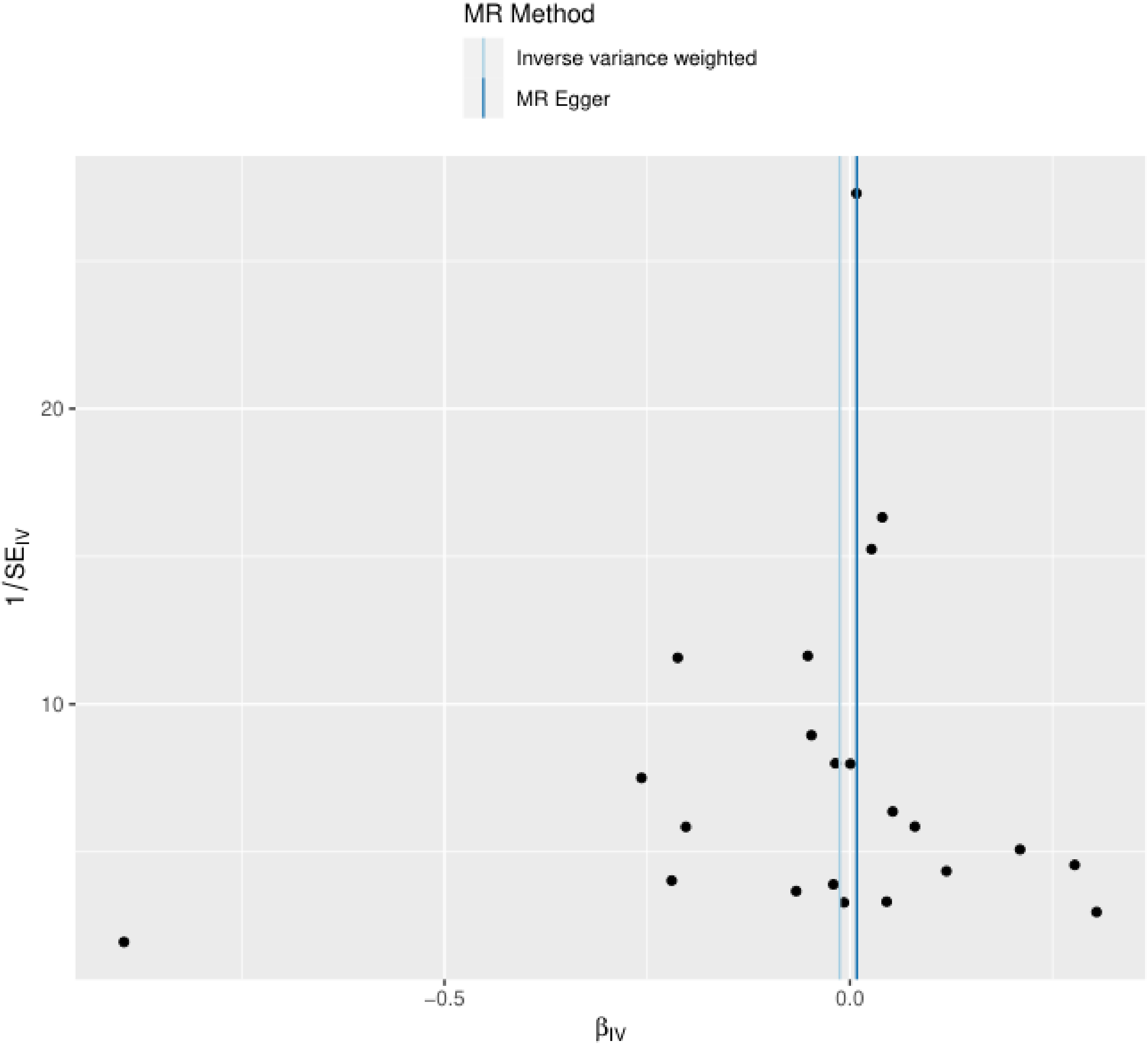

Funnel plot analyse of "CD16 on CD14- CD16+ monocyte" on 'Diabetic nephropathy'

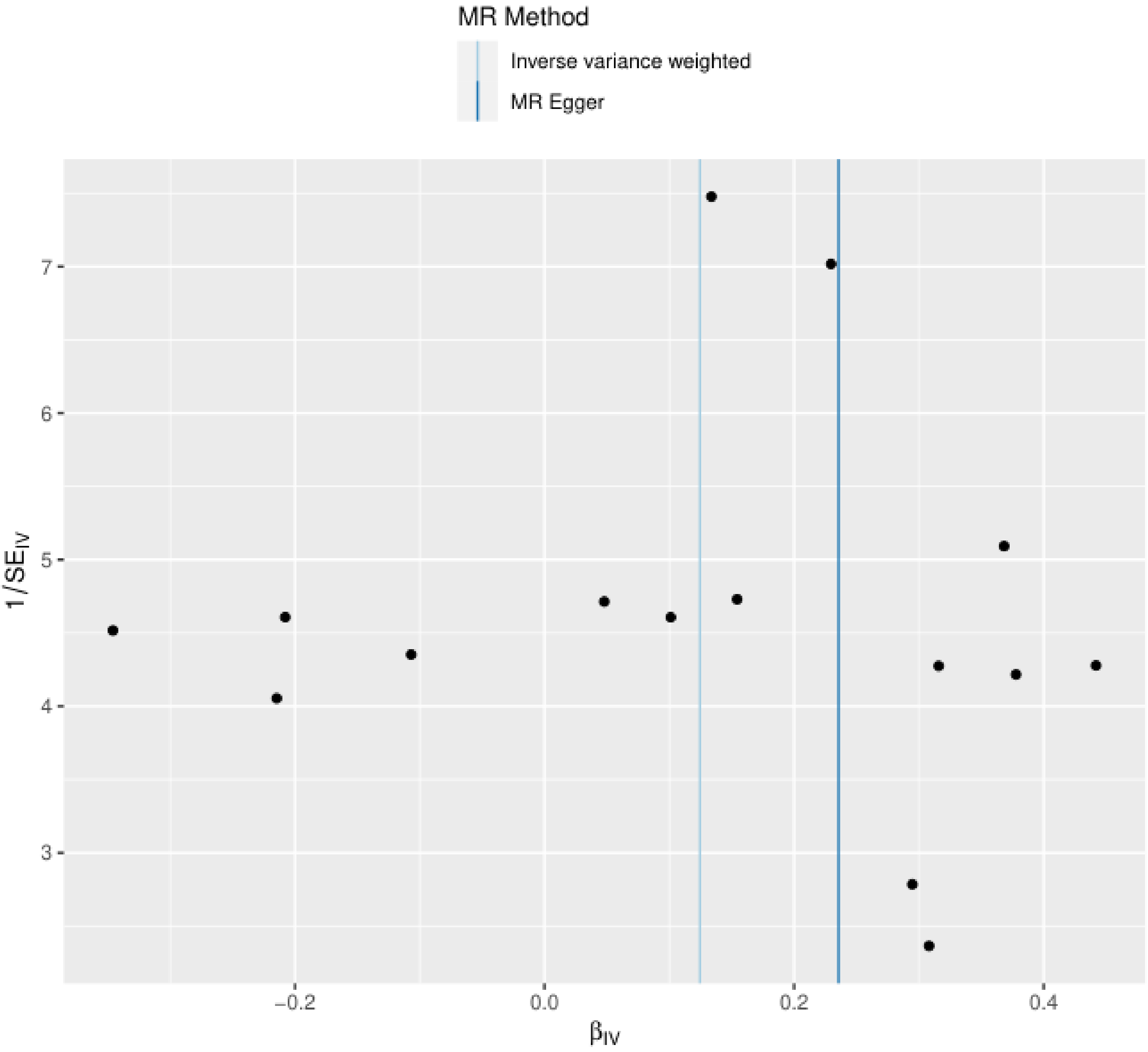

Funnel plot analyse of "DN (CD4-CD8-) %T cell" on 'Diabetic nephropathy'

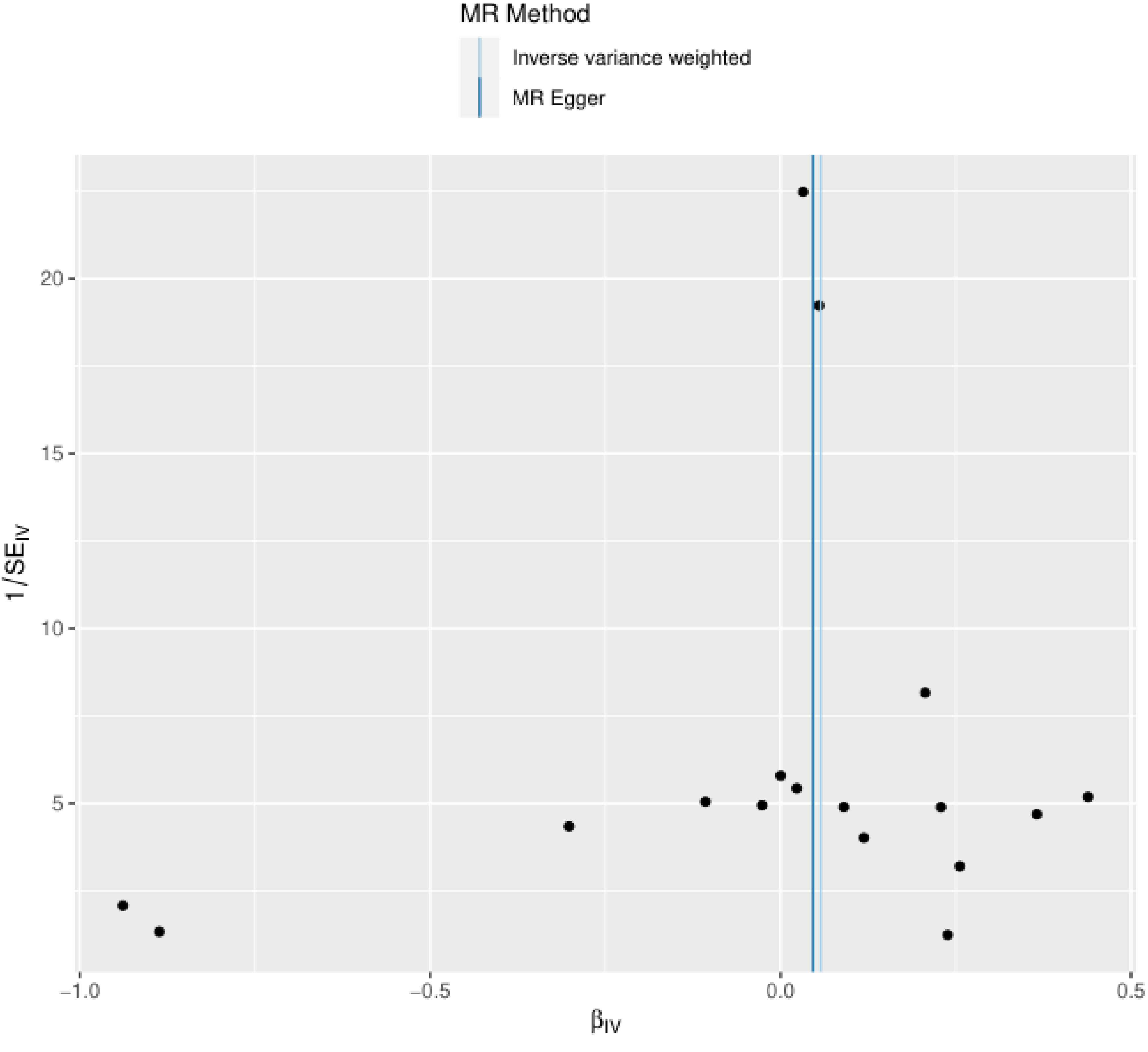

Funnel plot analyse of "CD127 on CD45RA- CD4 not Treg" on 'Diabetic nephropathy'

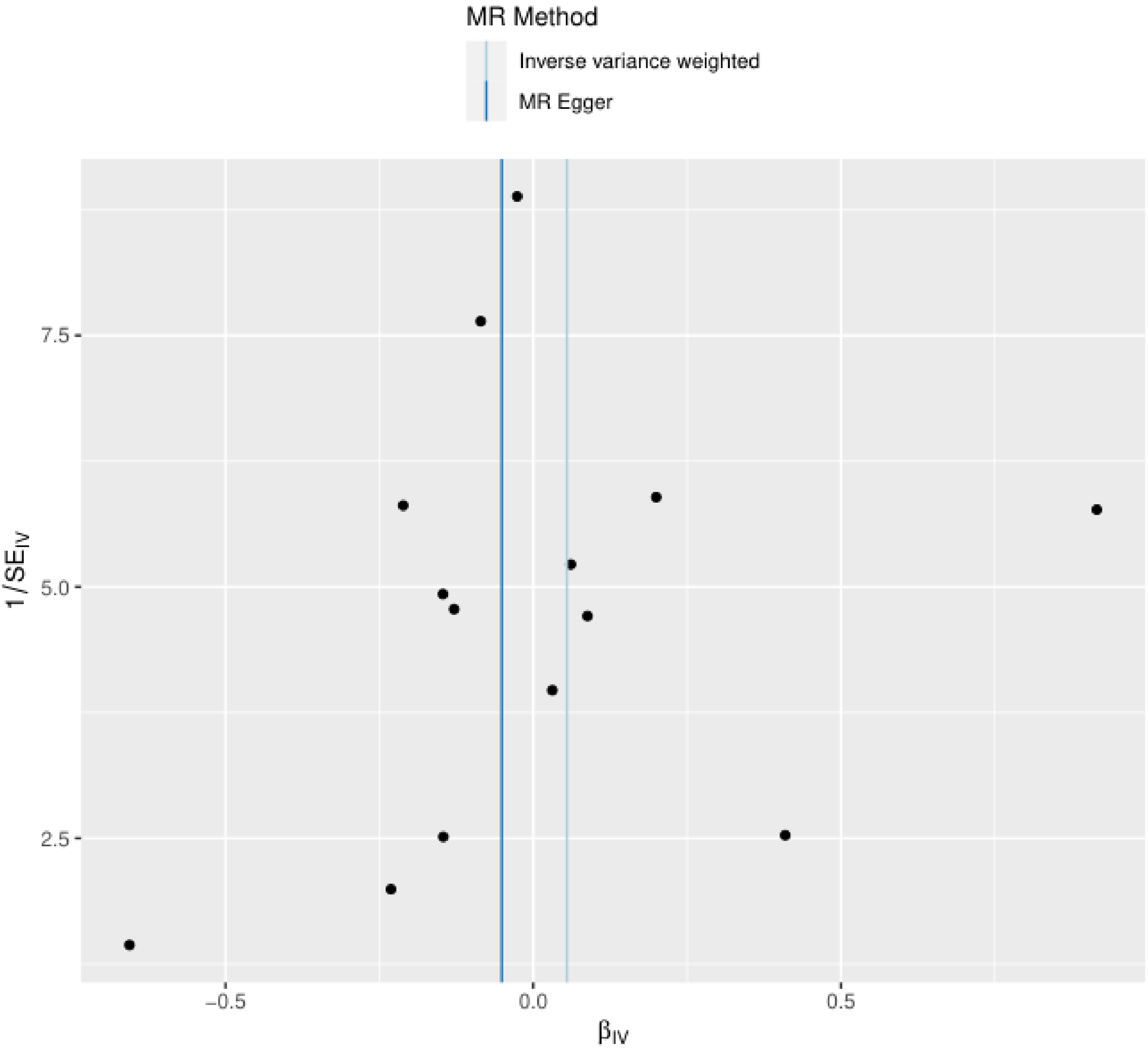

Funnel plot analyse of "CD127- CD8br %T cell" on 'Diabetic nephropathy'

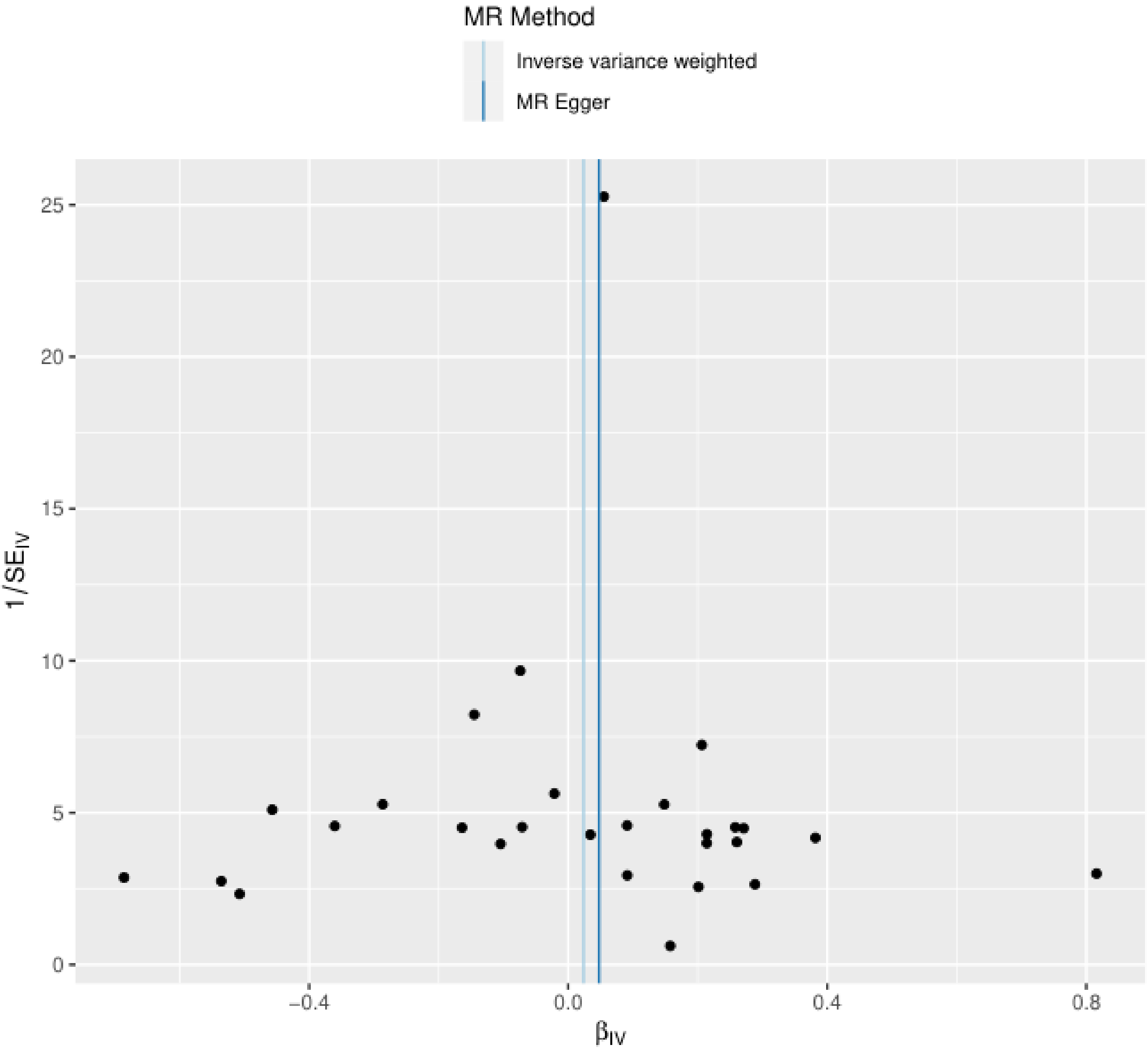

Funnel plot analyse of "CD20 on memory B cell" on 'Diabetic nephropathy'

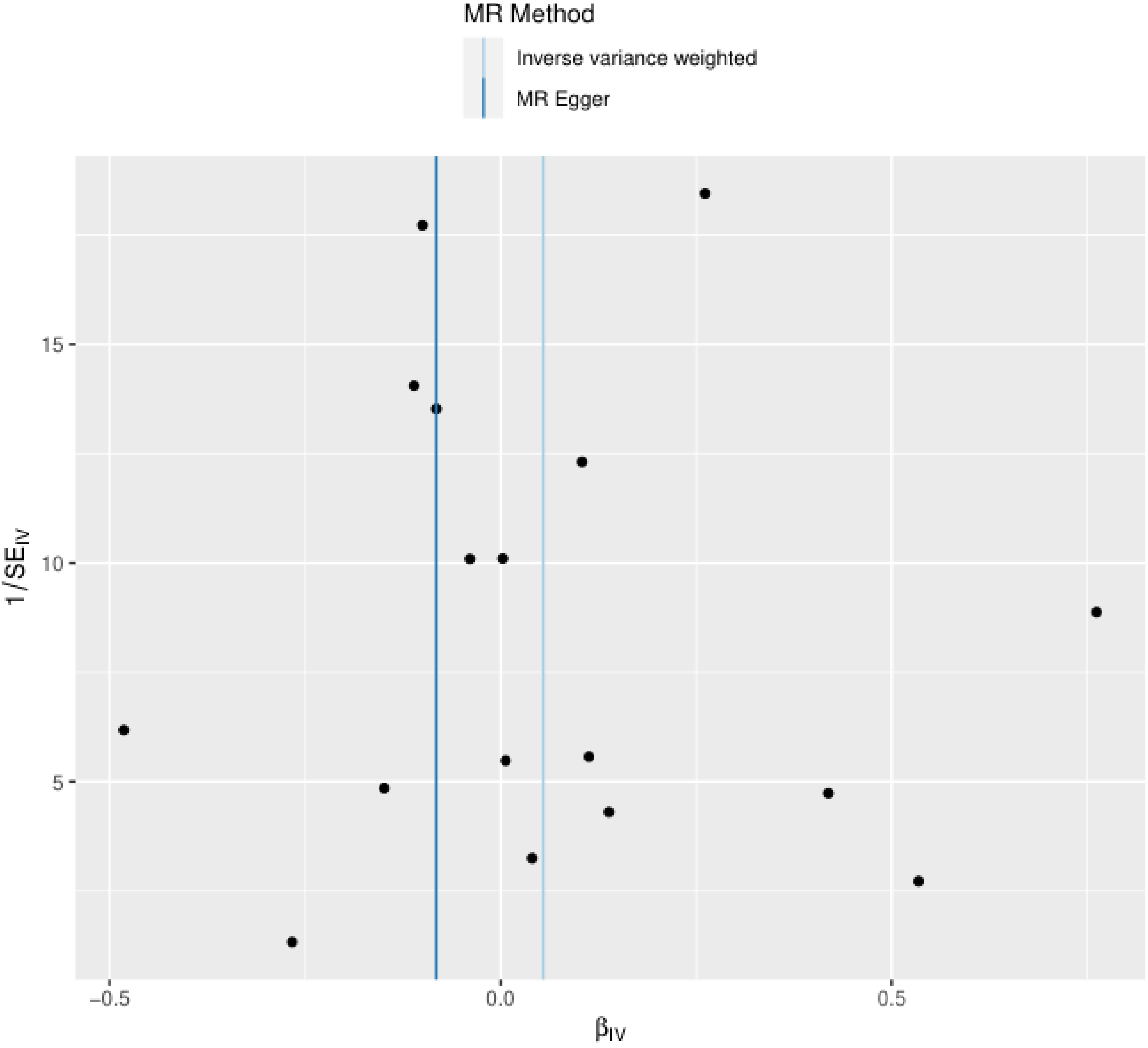

Funnel plot analyse of "HLA DR on monocyte" on 'Diabetic nephropathy'

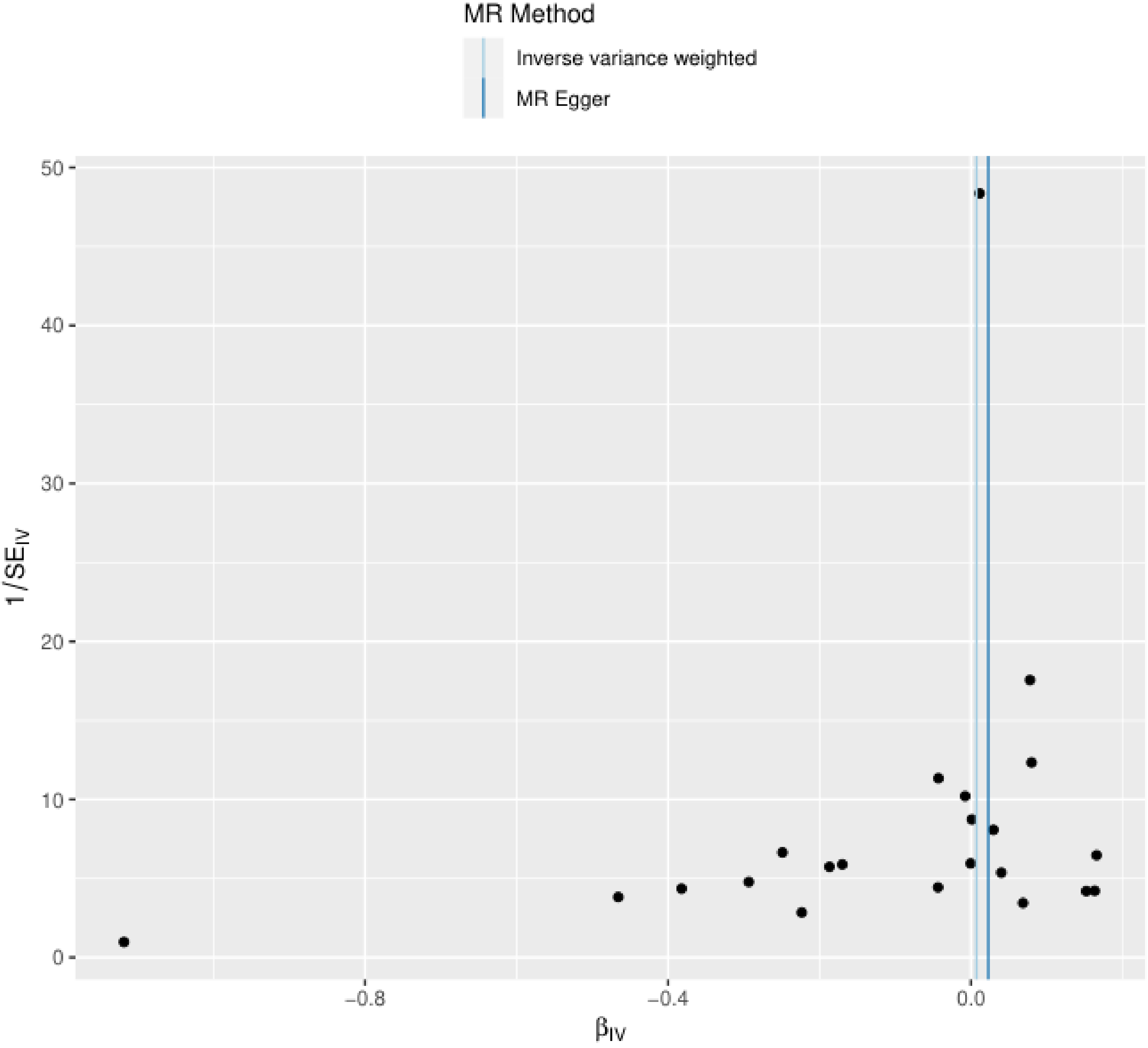

Funnel plot analyse of "CD25 on unsw mem" on 'Diabetic nephropathy'

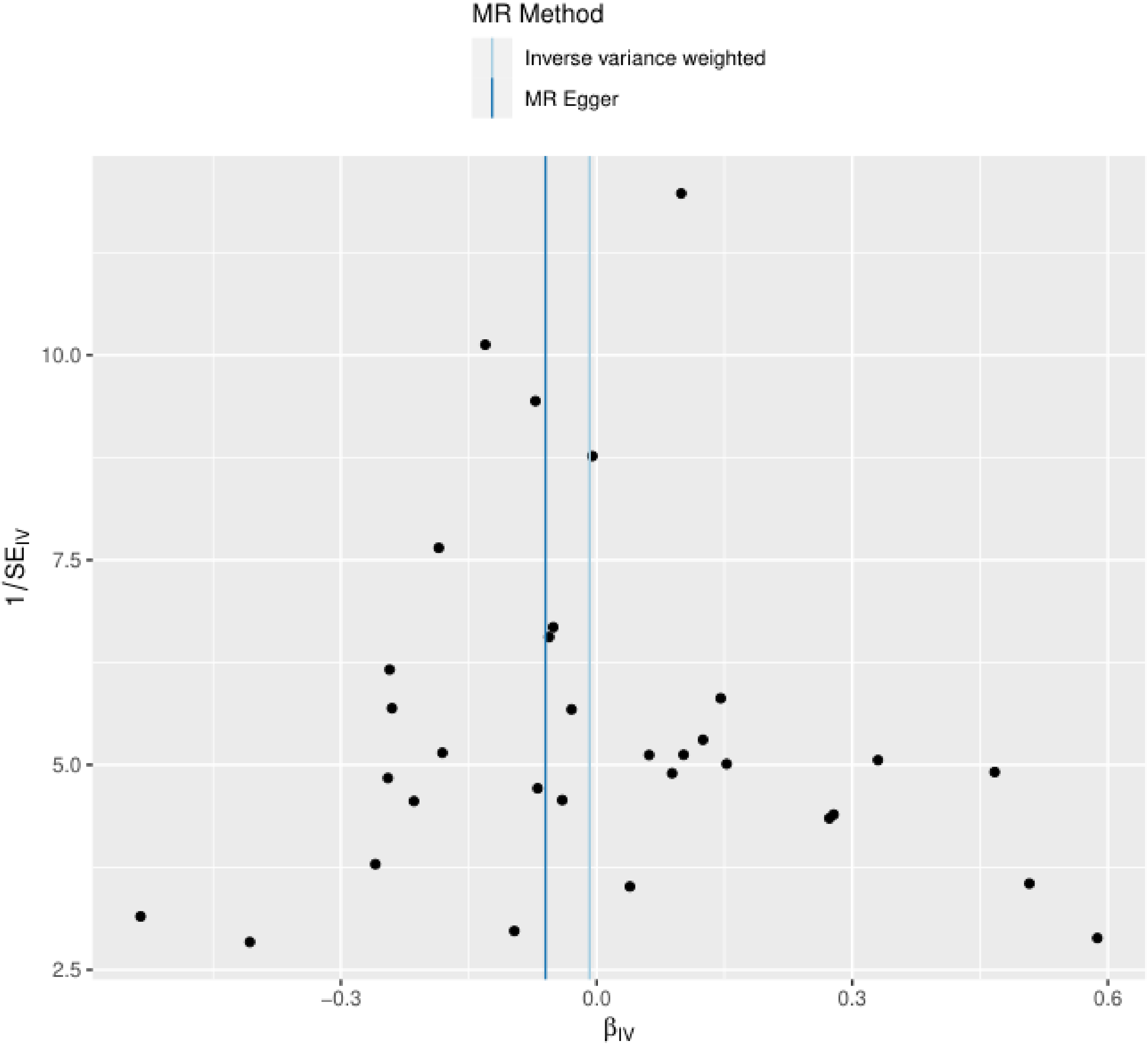

Funnel plot analyse of "B cell %lymphocyte" on 'Diabetic nephropathy'

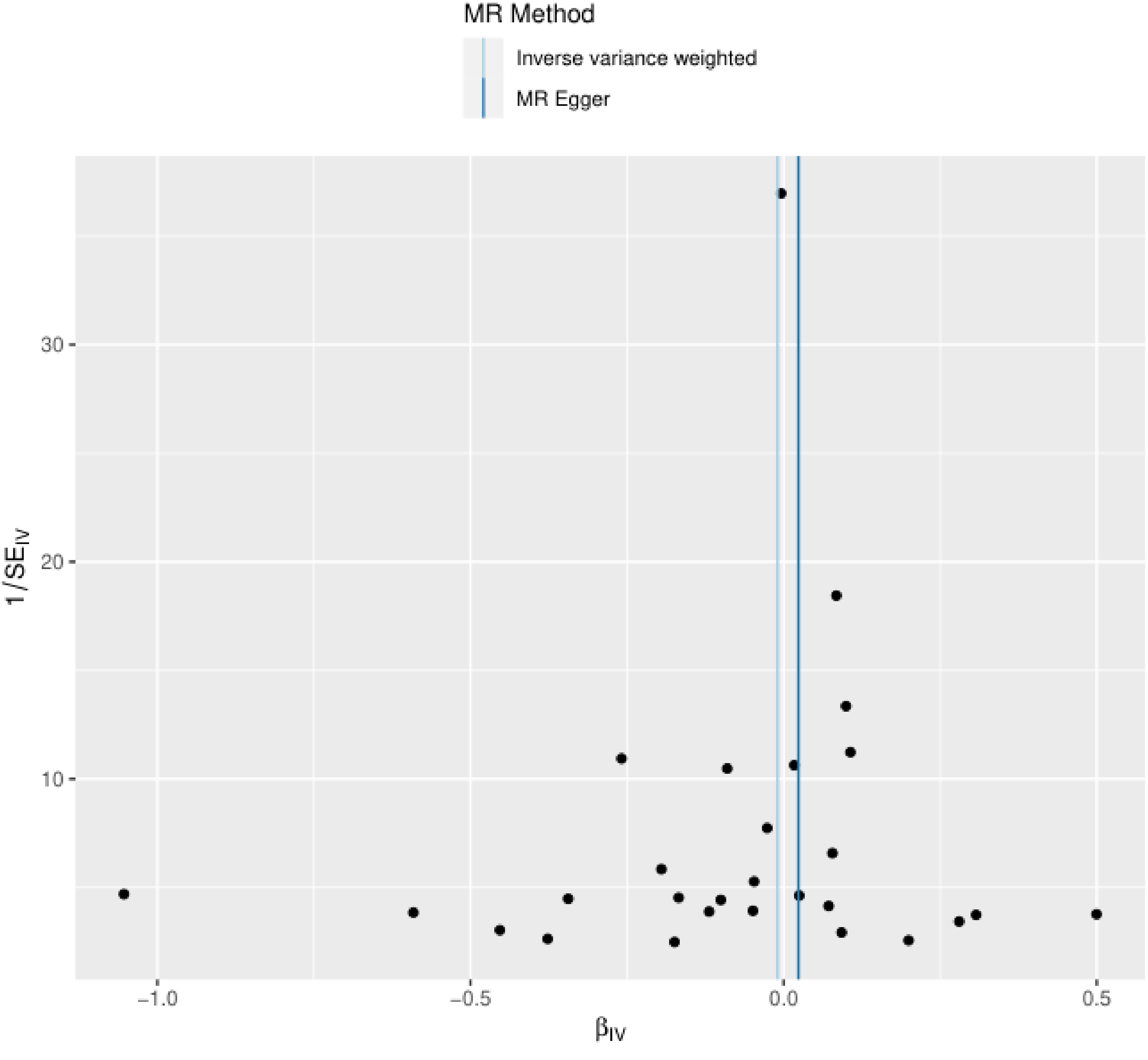

Funnel plot analyse of "Activated & resting Treg % CD4 Treg" on 'Diabetic nephropathy'

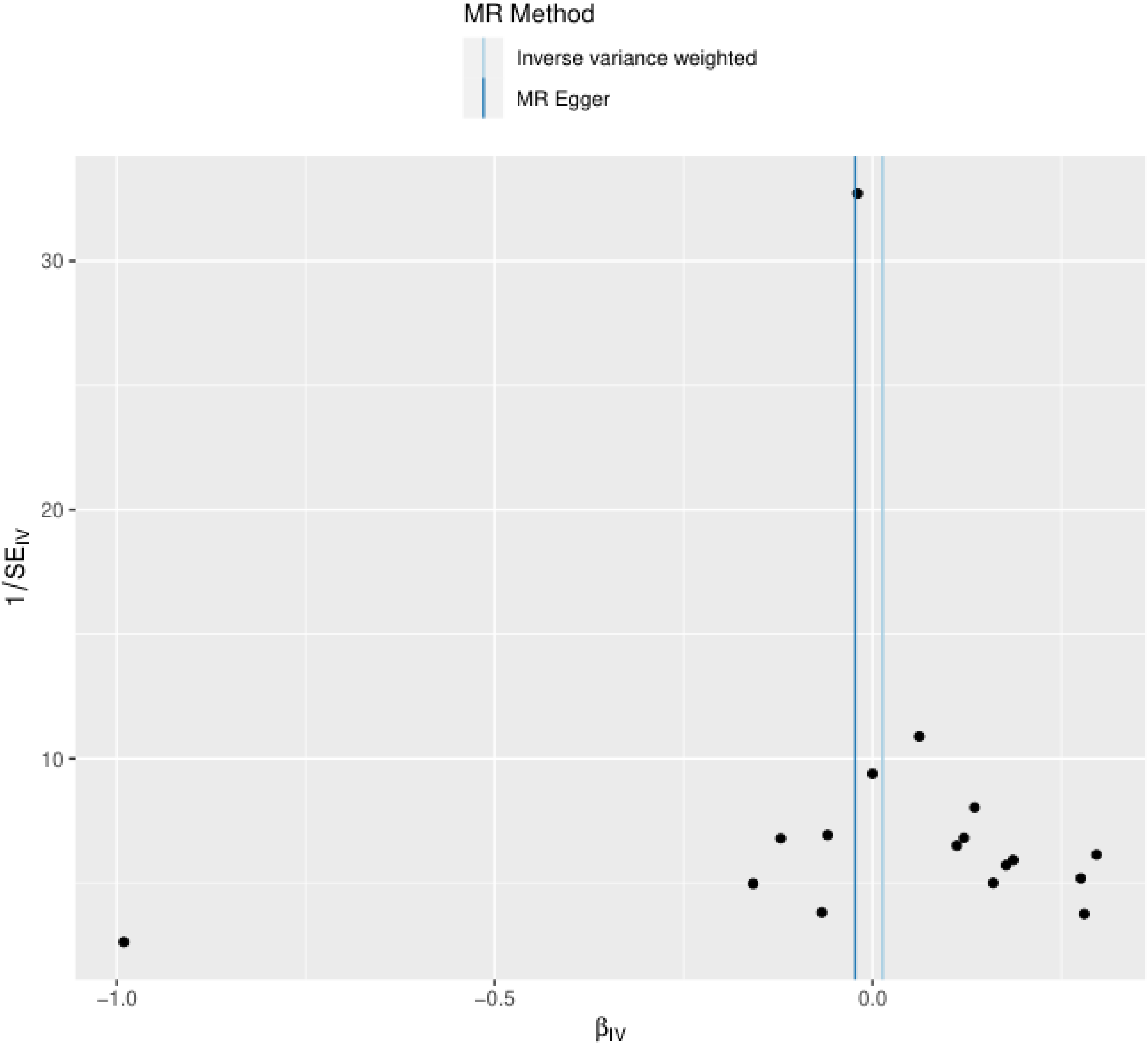

Funnel plot analyse of "BAFF-R on IgD+ CD38- unsw mem" on 'Diabetic nephropathy'

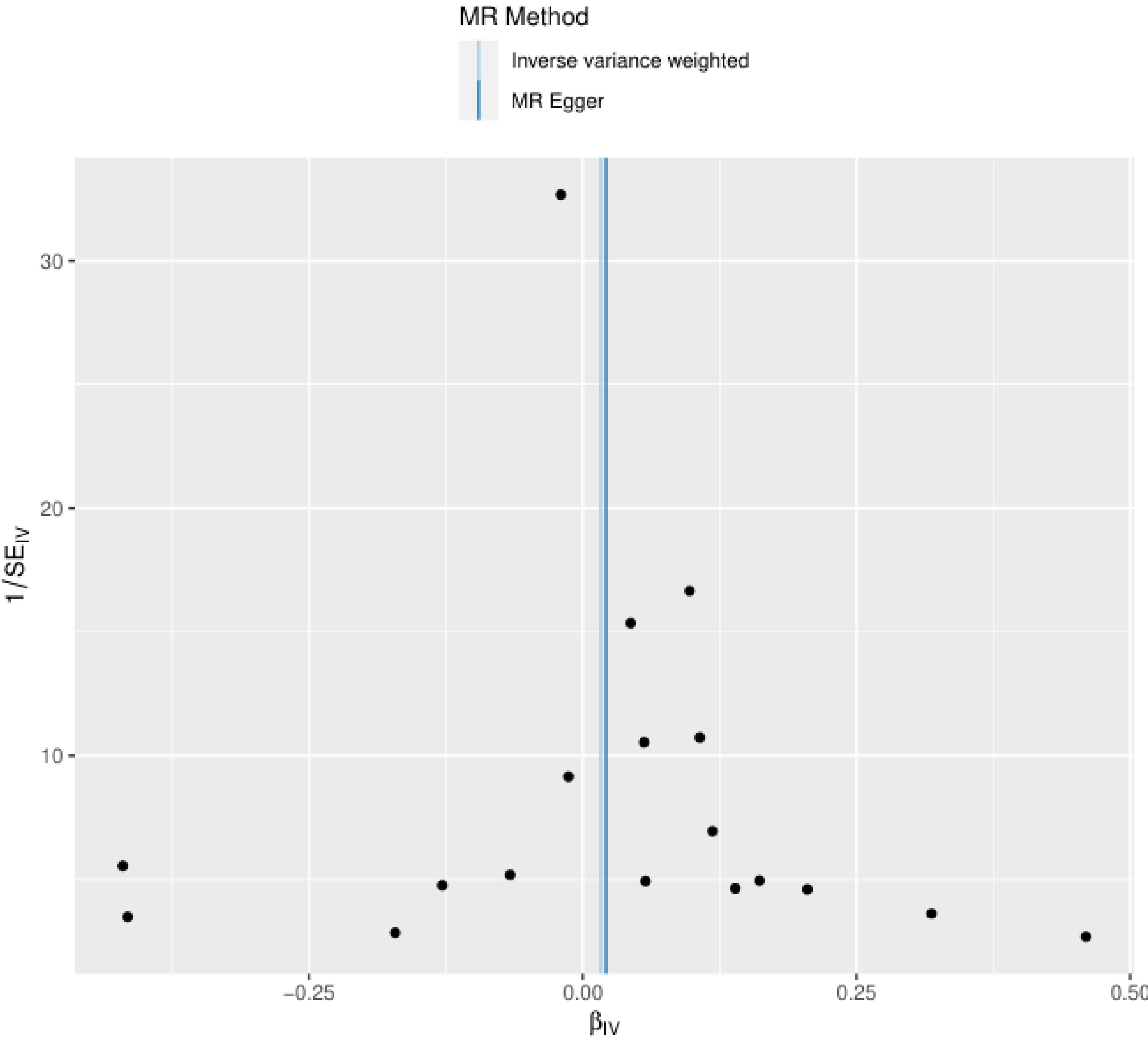

Funnel plot analyse of "BAFF-R on CD24+ CD27+" on 'Diabetic nephropathy'

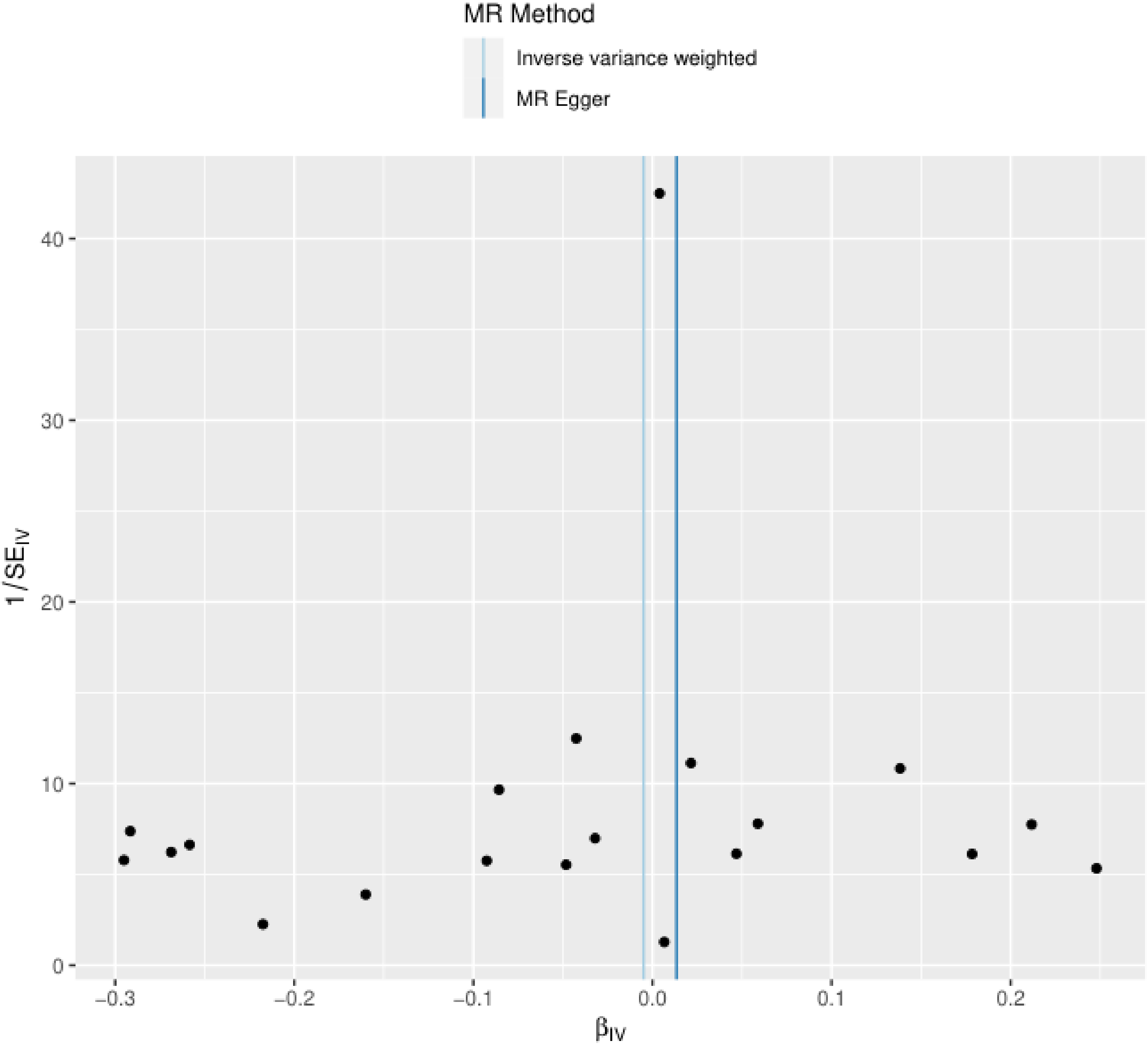

Funnel plot analyse of "CD33 on CD14+ monocyte" on 'Diabetic nephropathy'

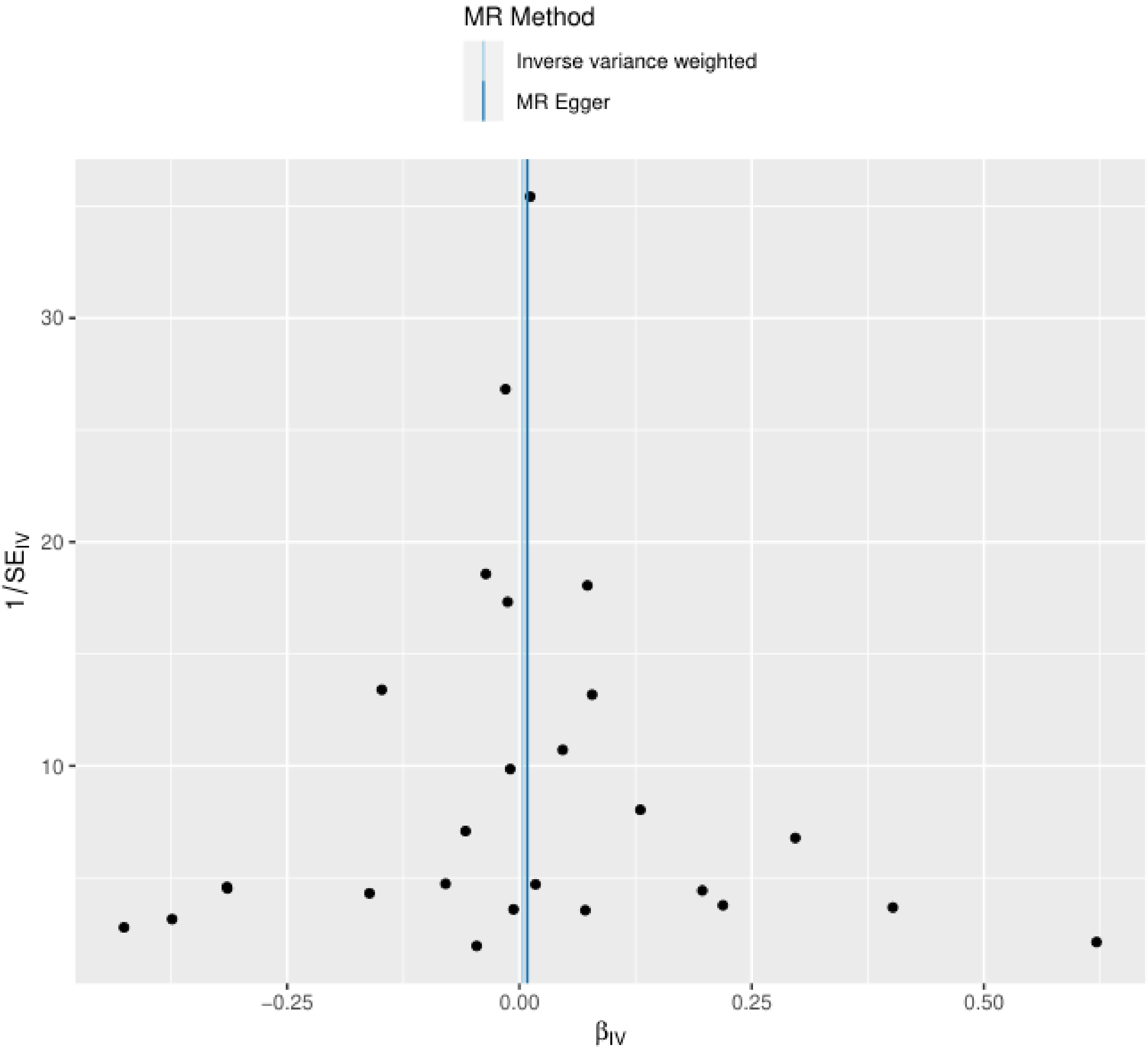

Funnel plot analyse of "CD39+ activated Treg %CD4 Treg" on 'Diabetic nephropathy'

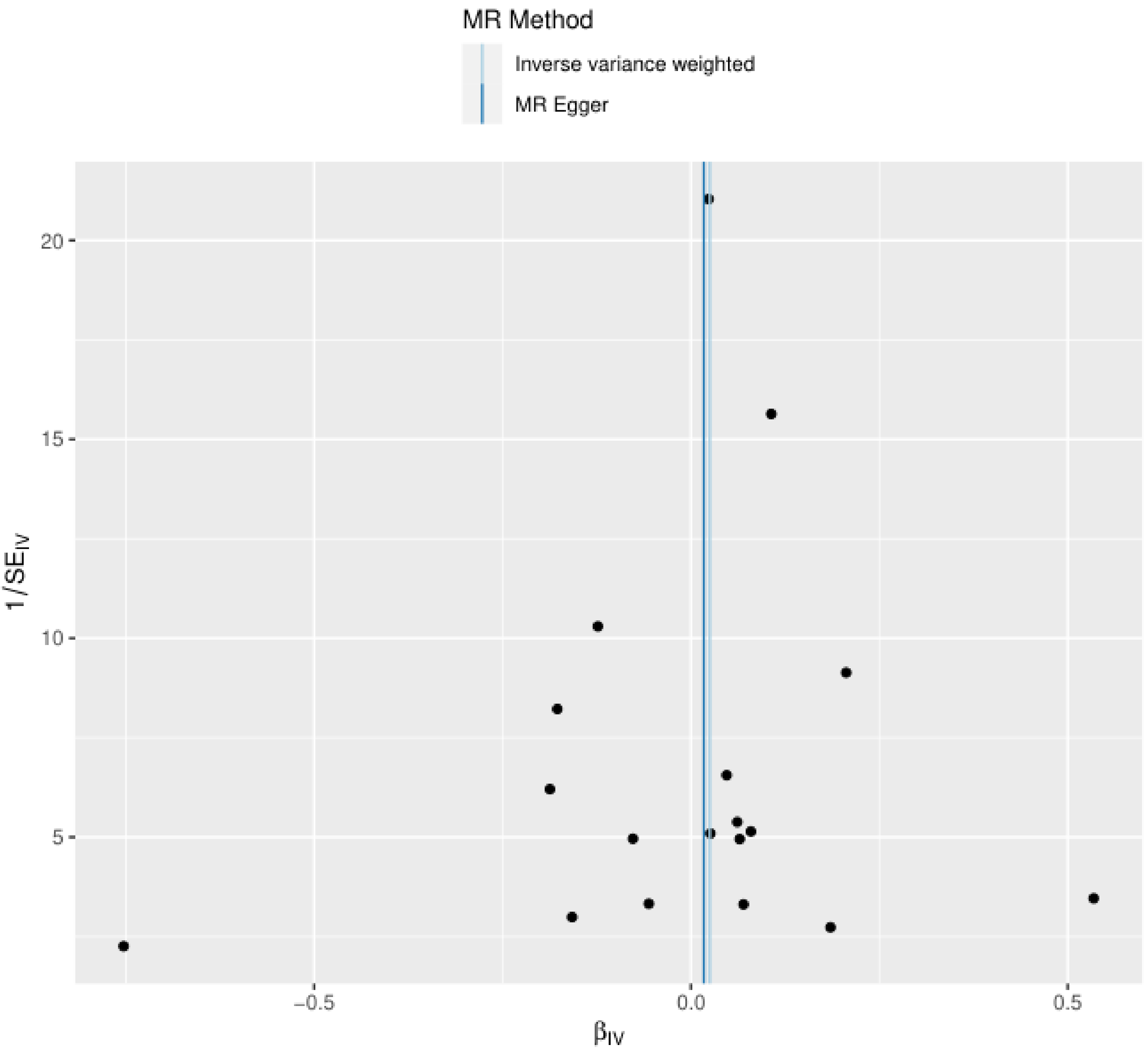

Funnel plot analyse of "CD127 on CD28+ CD45RA- CD8br" on 'Diabetic nephropathy'

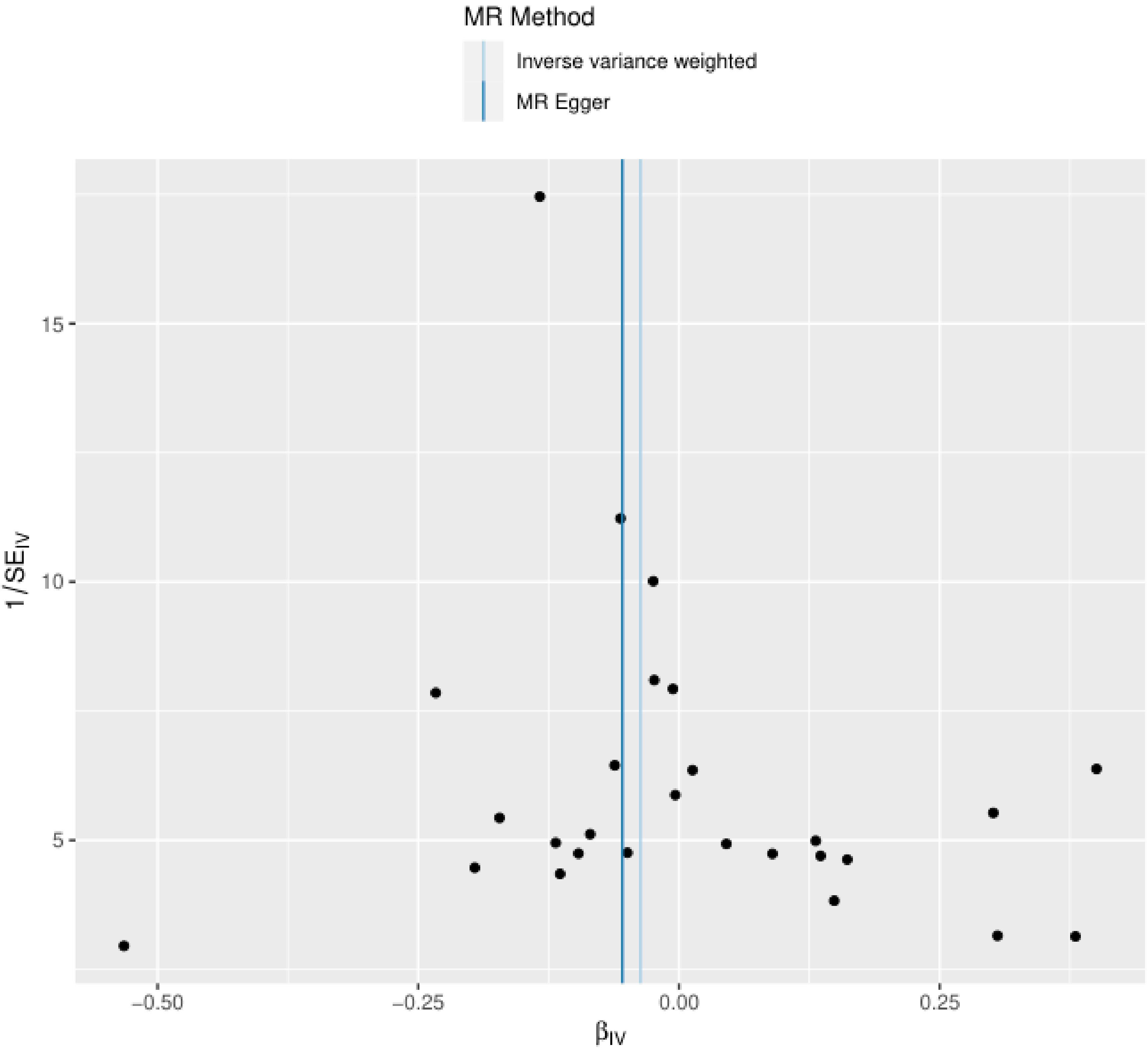

Funnel plot analyse of "CX3CR1 on CD14+ CD16- monocyte" on 'Diabetic nephropathy'

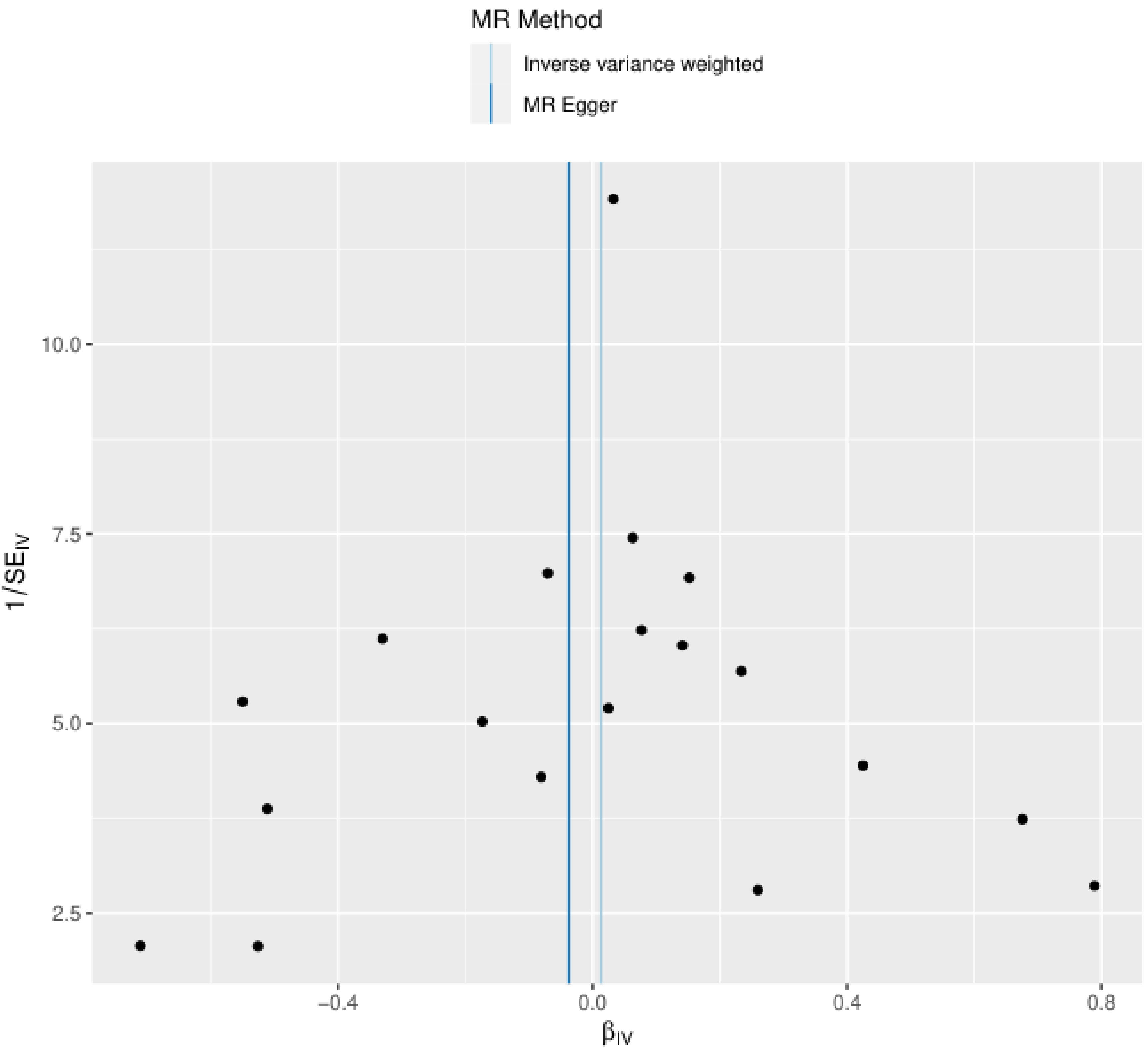

Funnel plot analyse of "CD8dim %T cell" on 'Diabetic nephropathy'

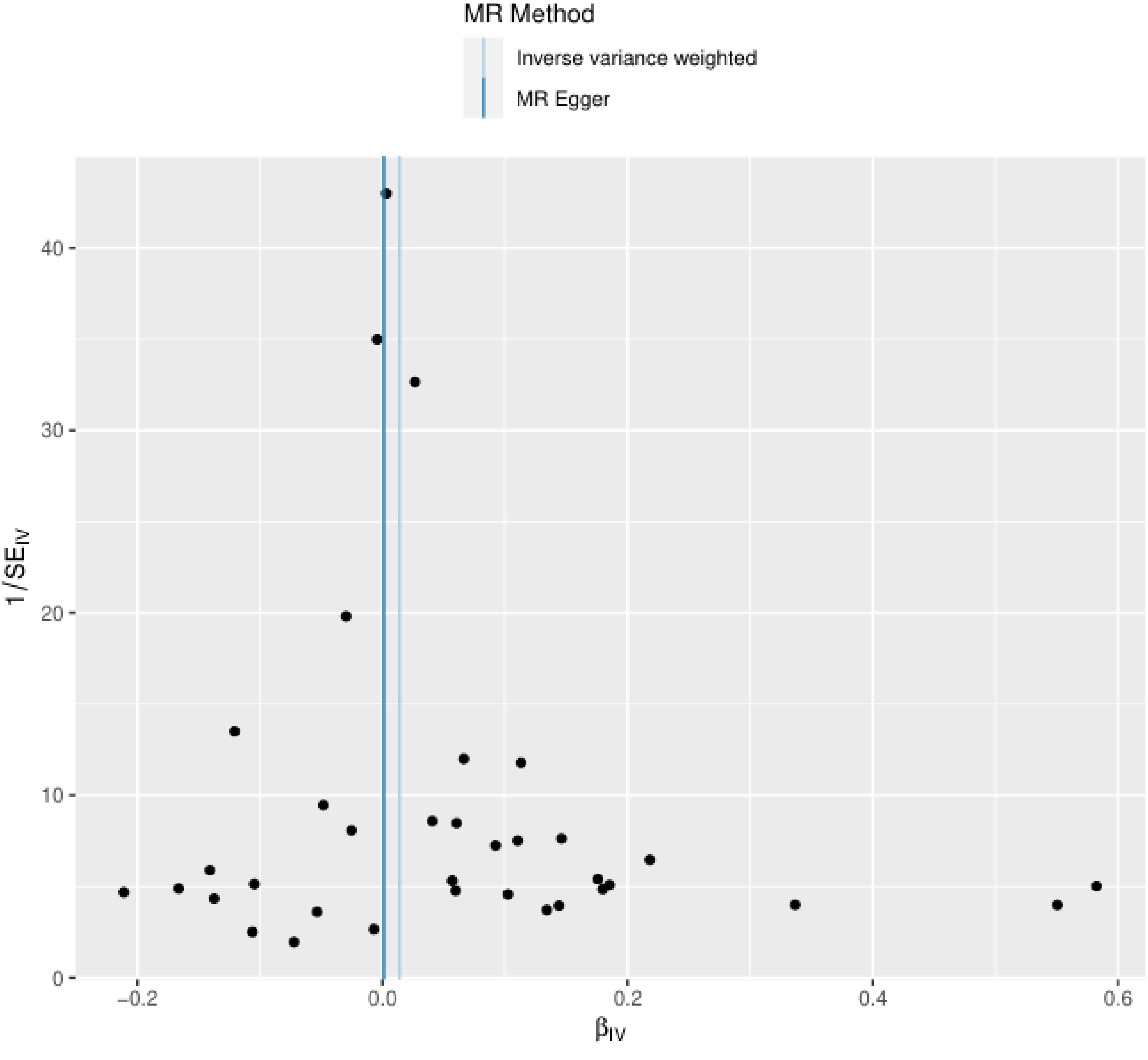

Funnel plot analyse of "CD39+ resting Treg % CD4 Treg" on 'Diabetic nephropathy'

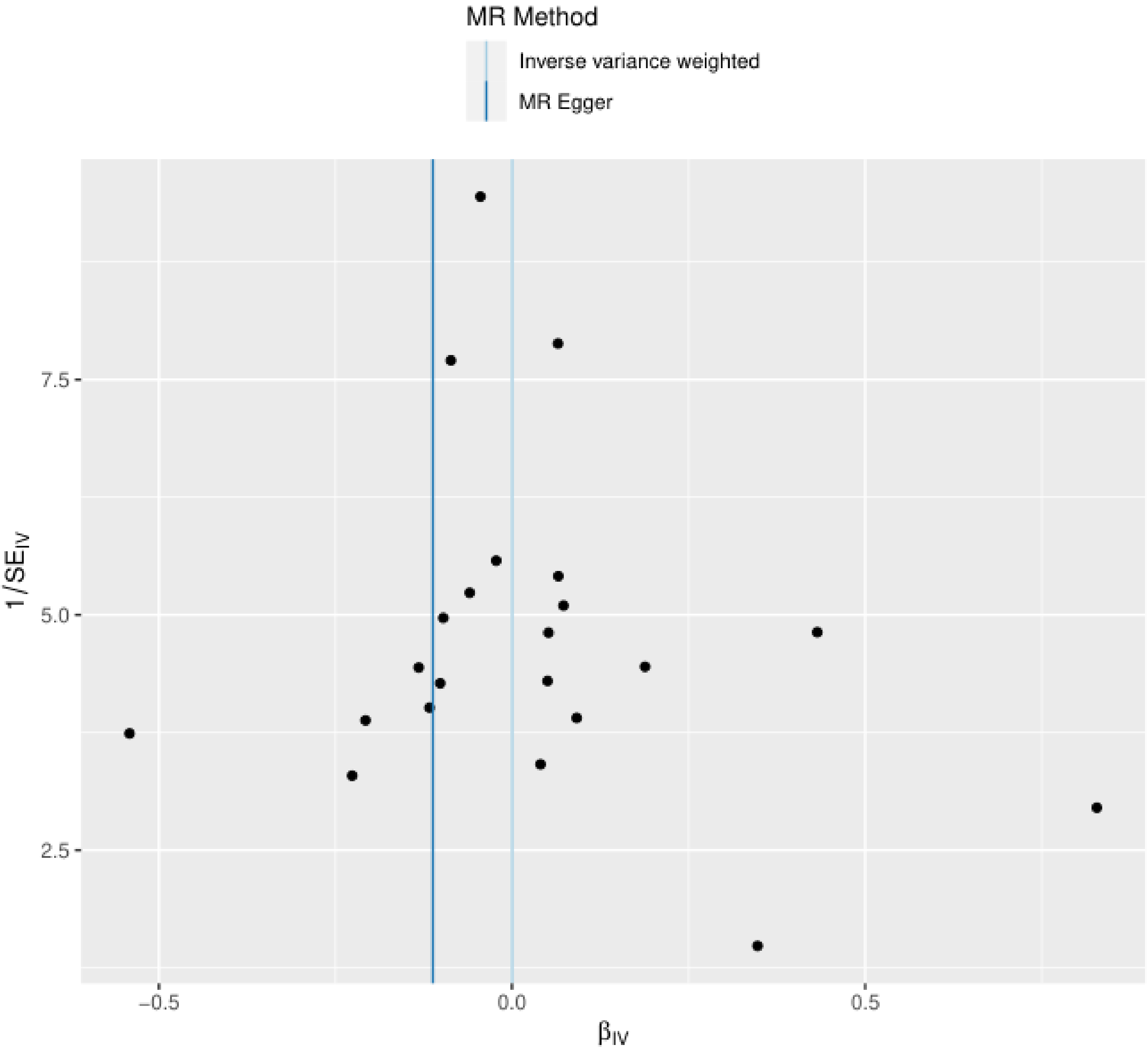

Funnel plot analyse of "CD19 on IgD- CD38-" on 'Diabetic nephropathy'

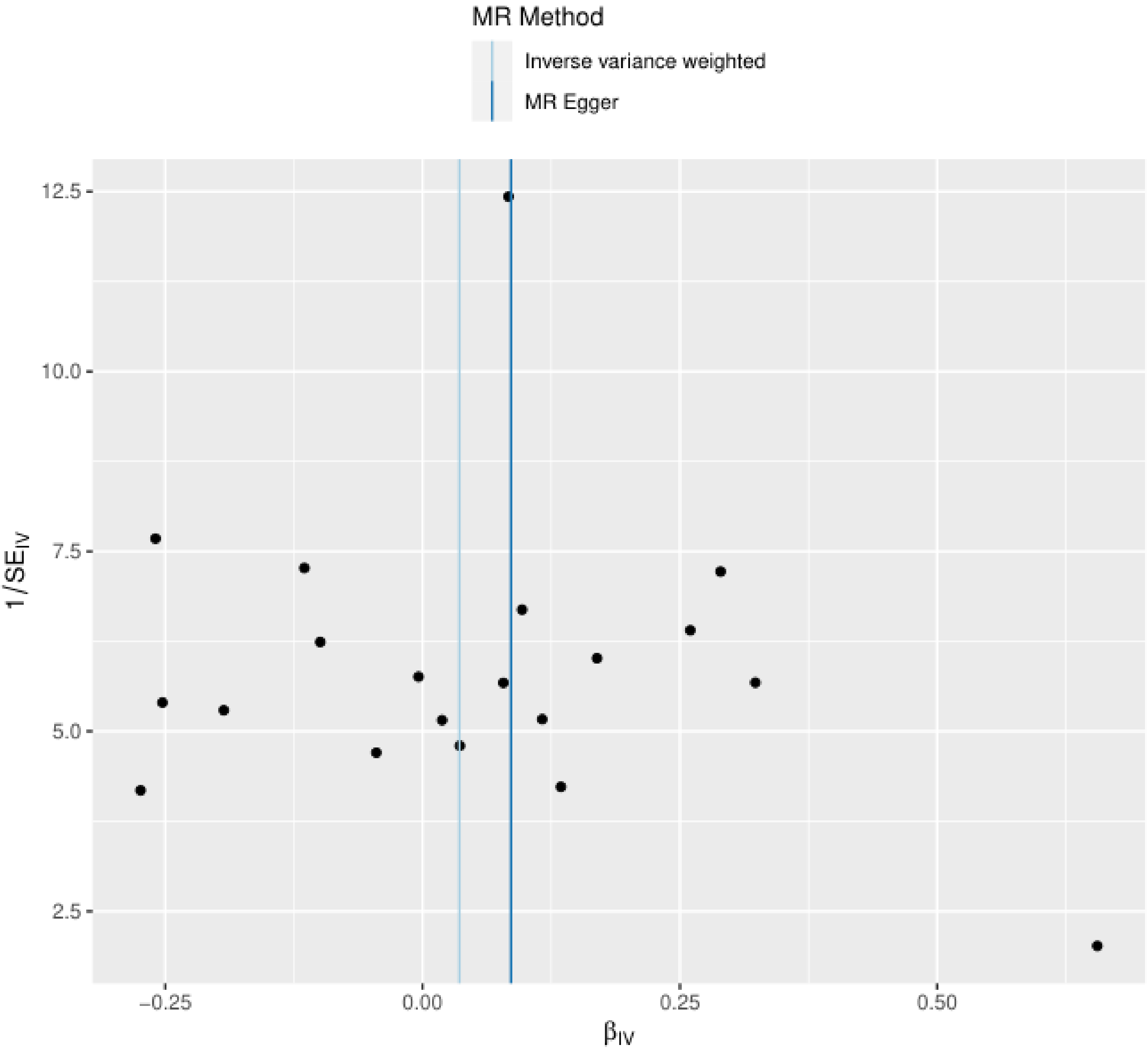

Funnel plot analyse of "CD4 on naive CD4+ " on 'Diabetic nephropathy'

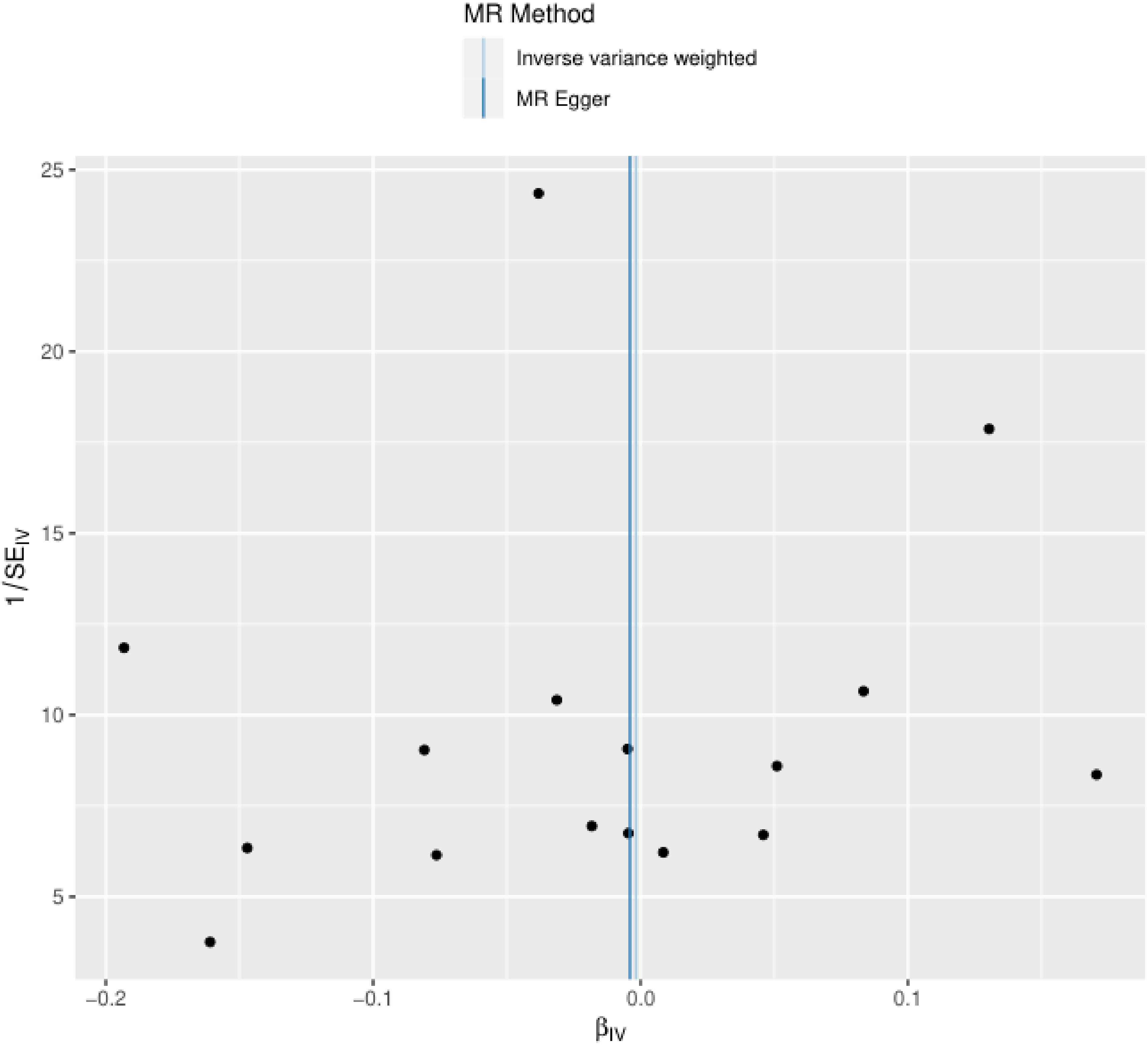

Funnel plot analyse of "CD11b on Mo MDSC " on 'Diabetic nephropathy'

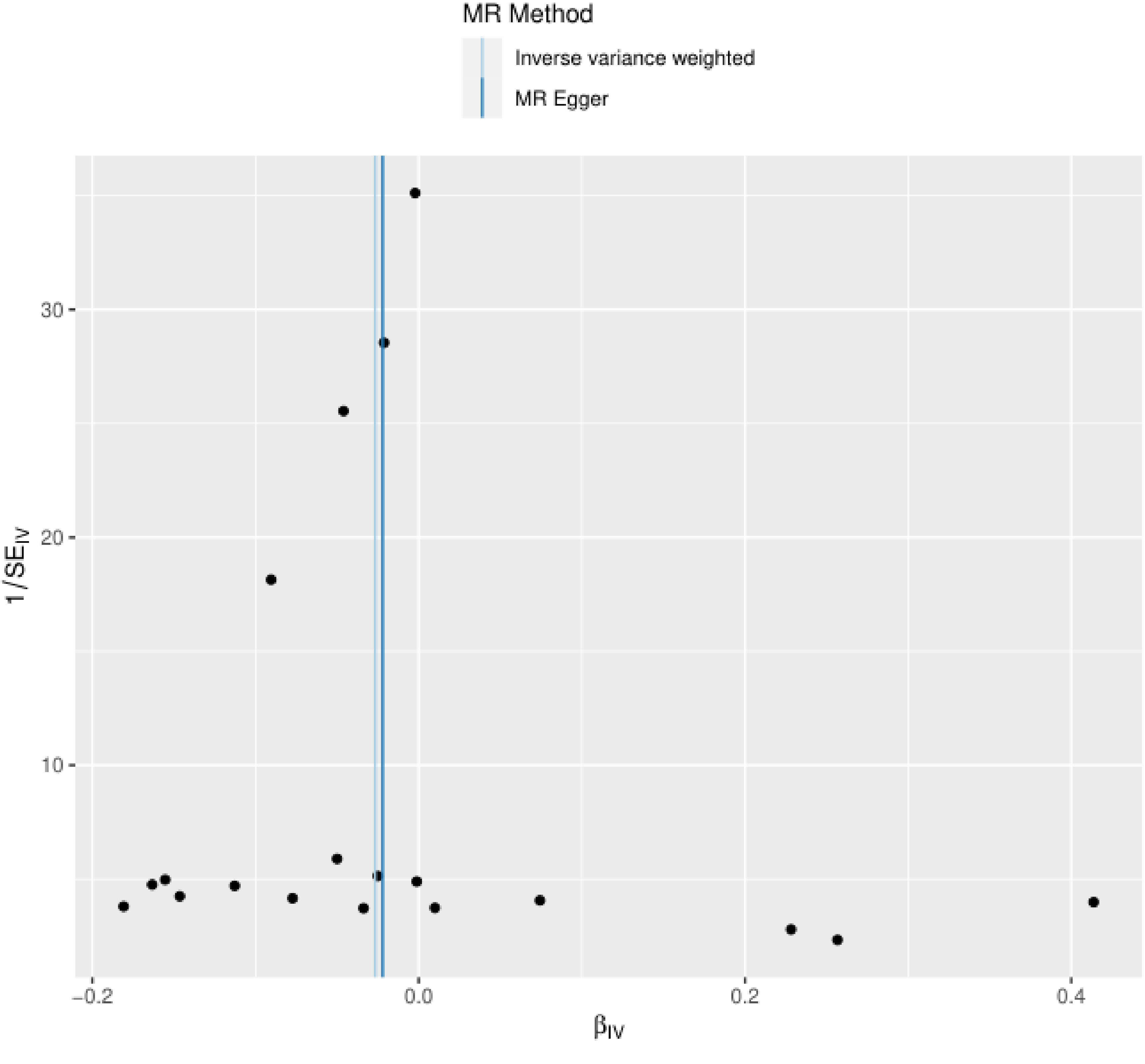

Funnel plot analyse of "PB/PC %lymphocyte" on 'Diabetic nephropathy'

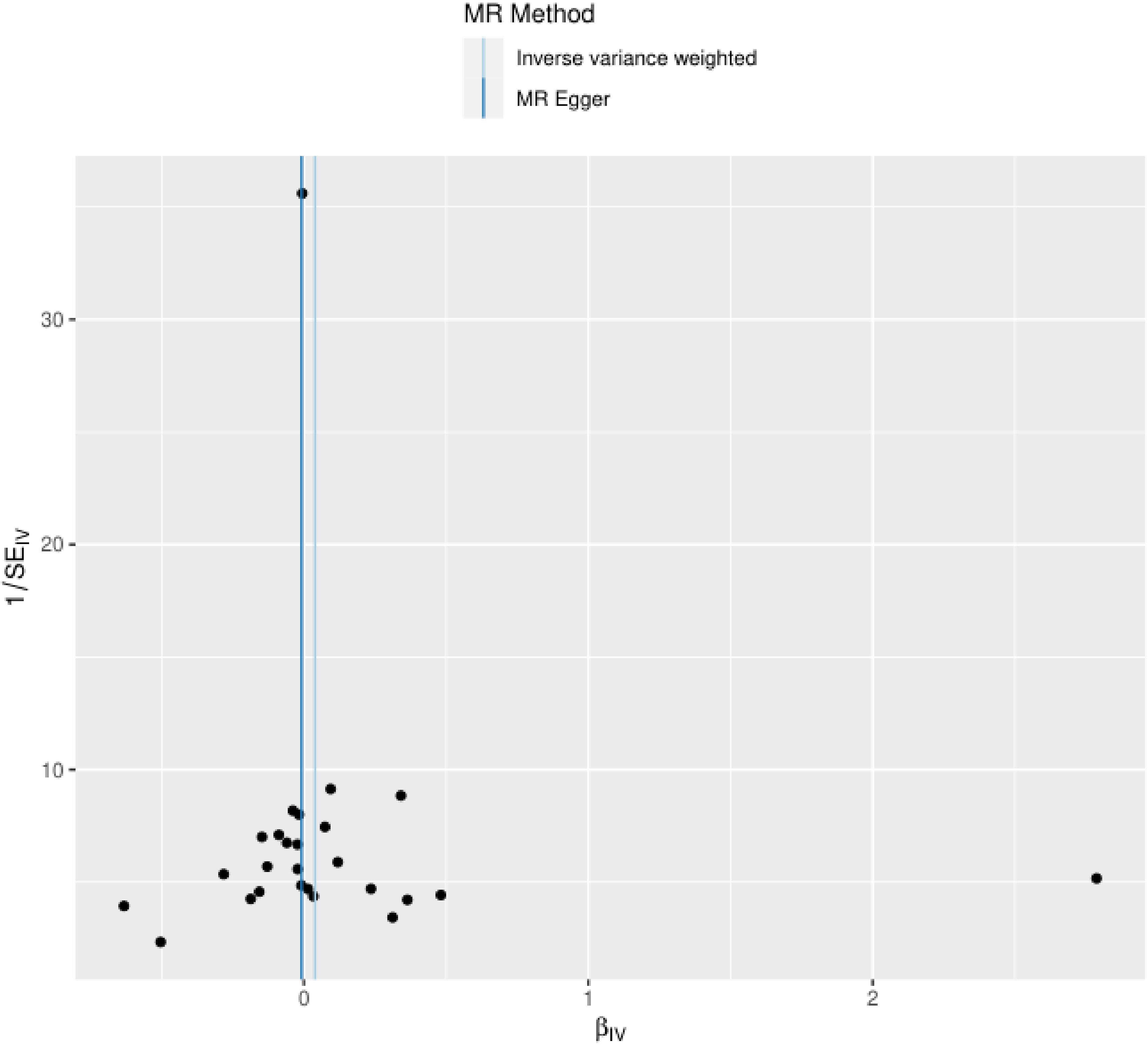

Funnel plot analyse of "CD20 on B cell" on 'Diabetic nephropathy'

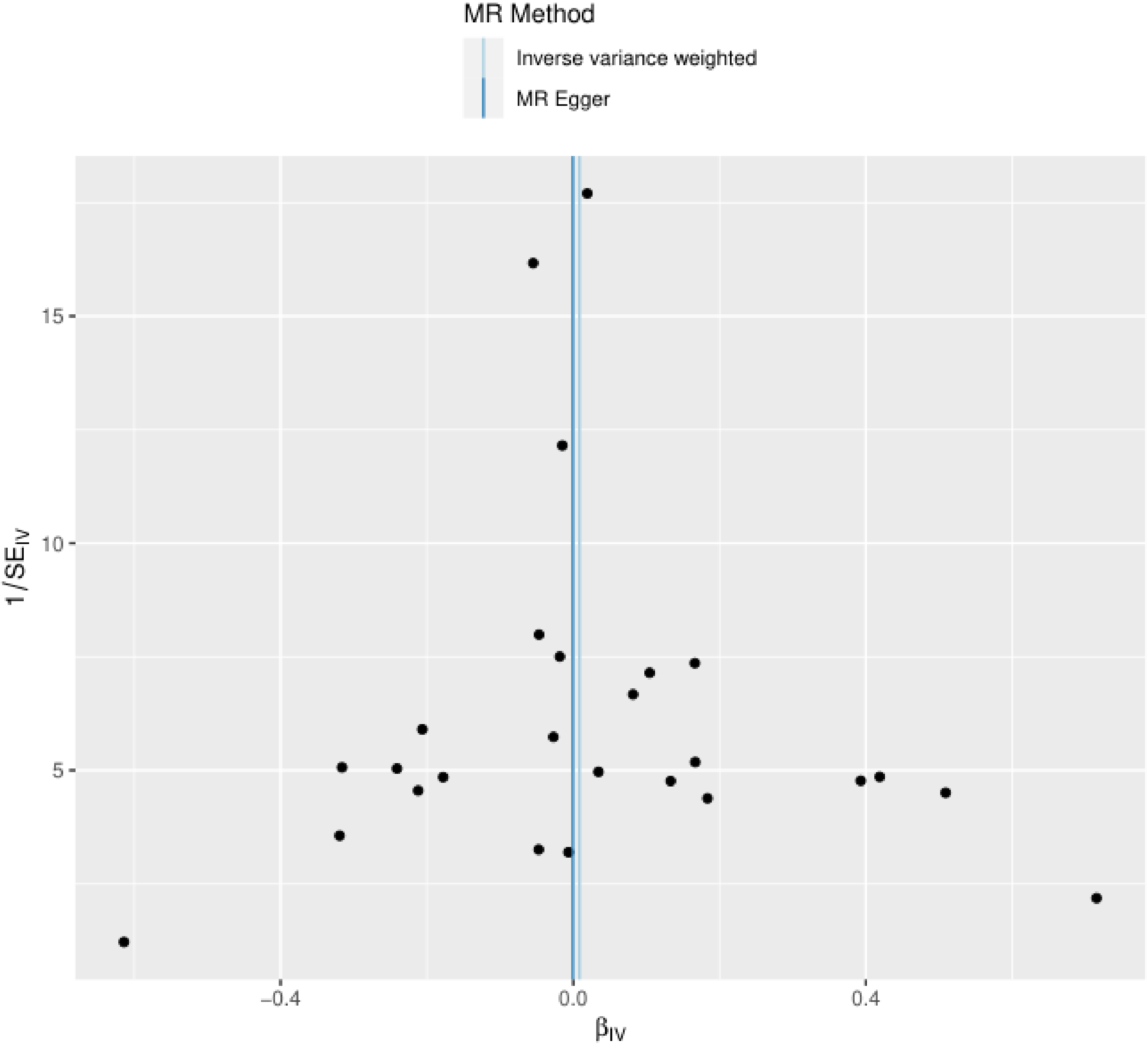

Funnel plot analyse of "CD80 on myeloid DC " on 'Diabetic nephropathy'

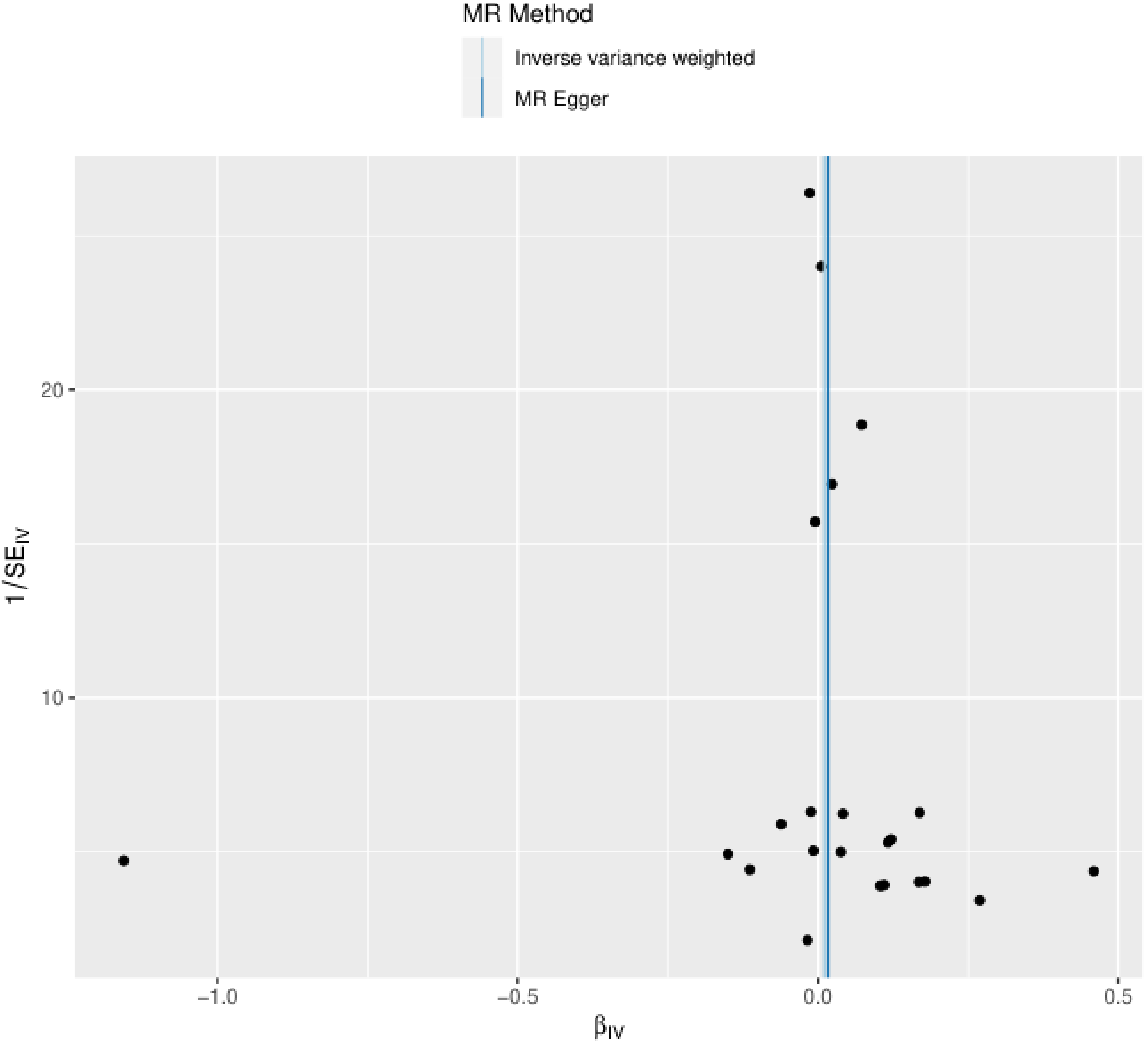

Funnel plot analyse of "CD24 on unsw mem" on 'Diabetic nephropathy'

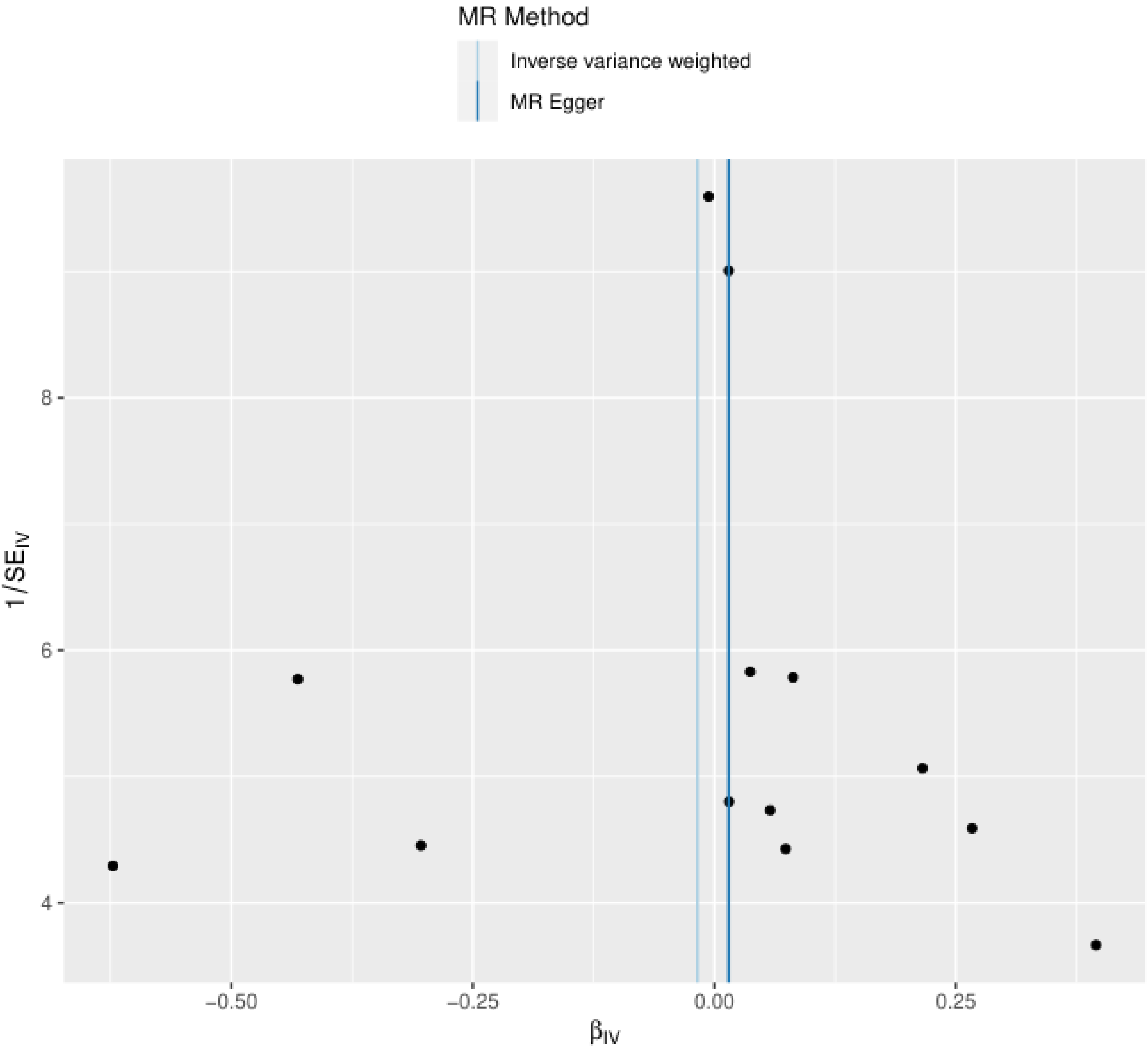

Funnel plot analyse of "CD25 on IgD+ CD38br" on 'Diabetic nephropathy'

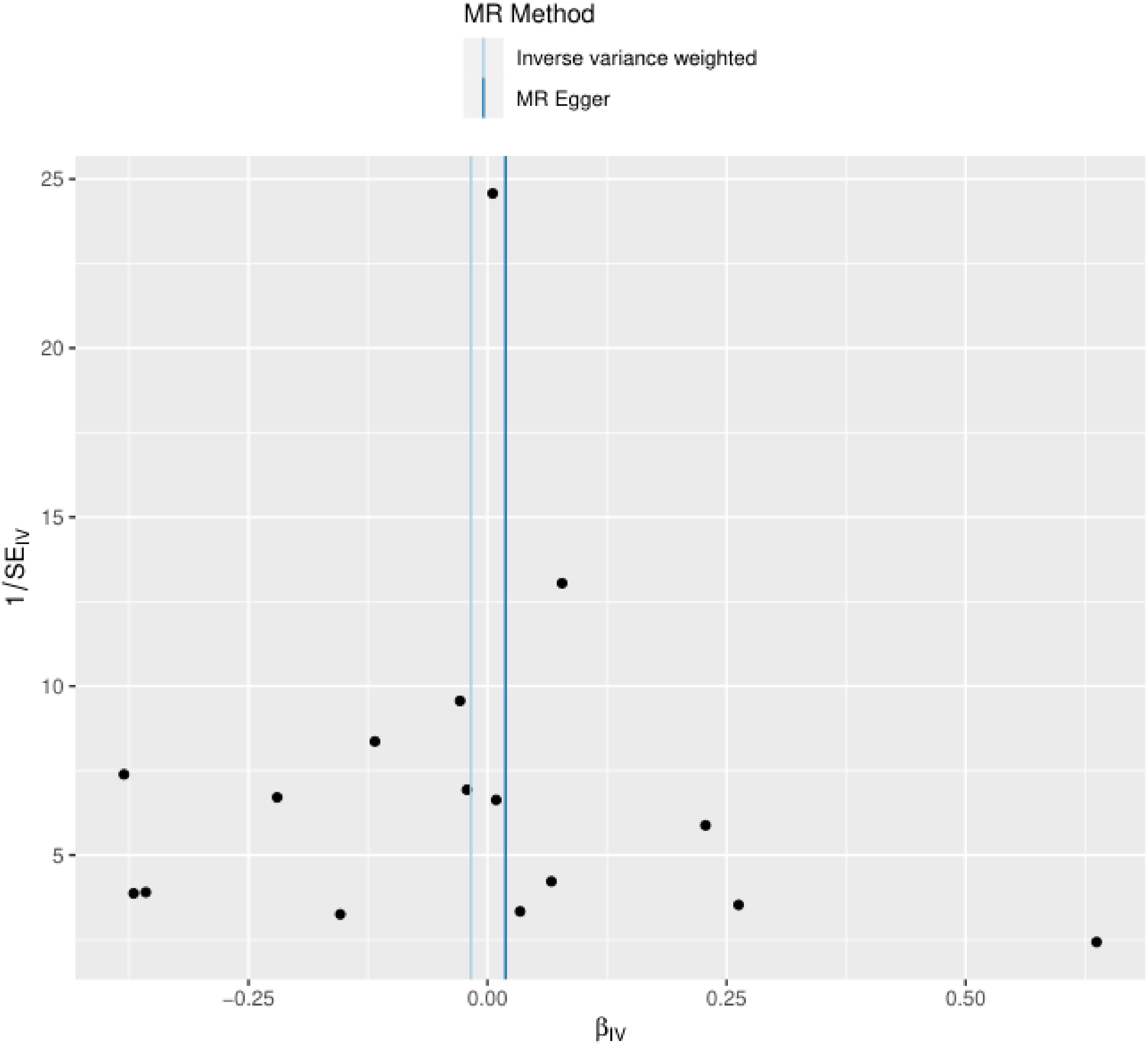

Funnel plot analyse of "IgD on IgD+ CD38- unsw mem" on 'Diabetic nephropathy'

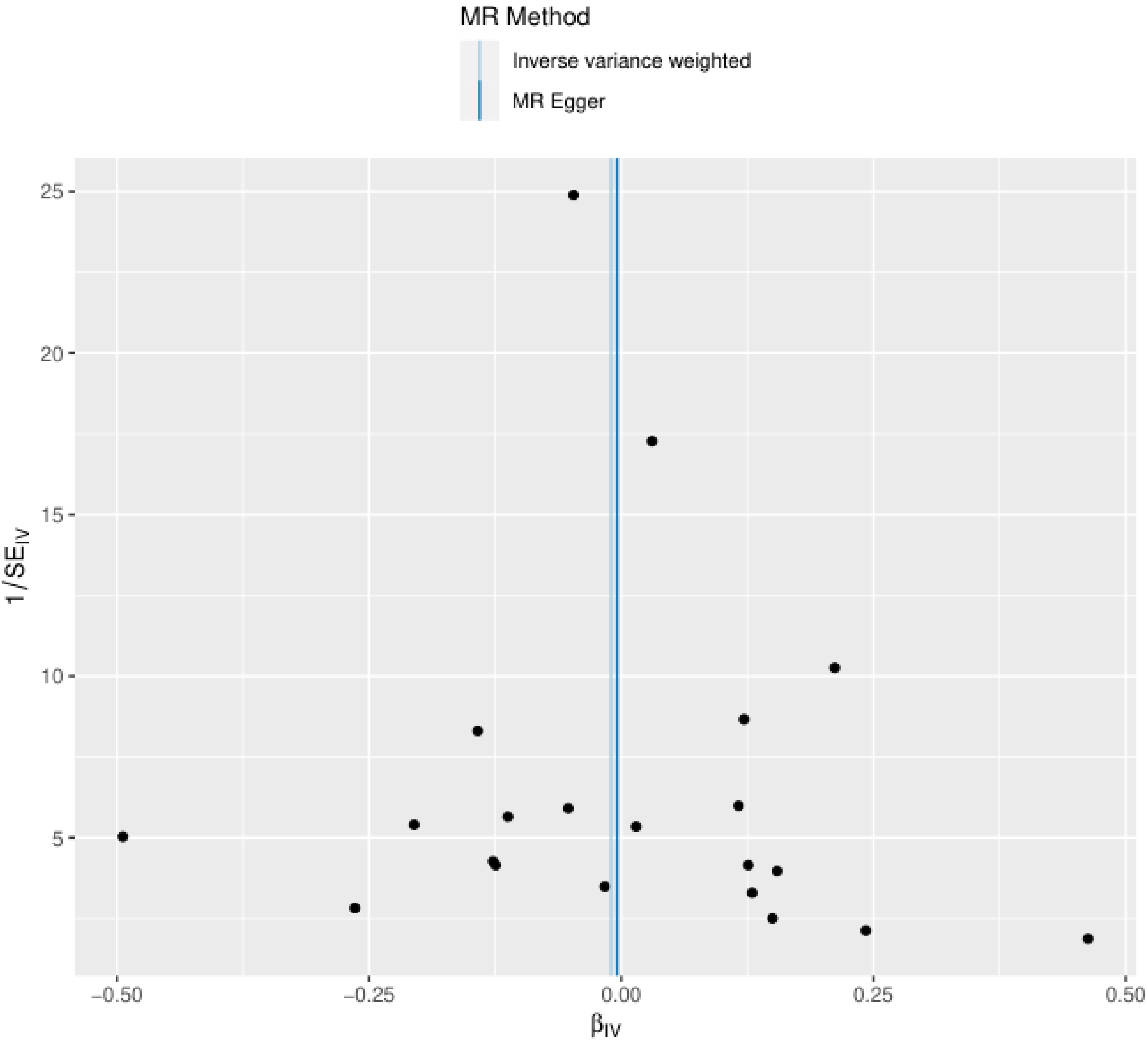

Funnel plot analyse of "IgD- CD38br AC" on 'Diabetic nephropathy'

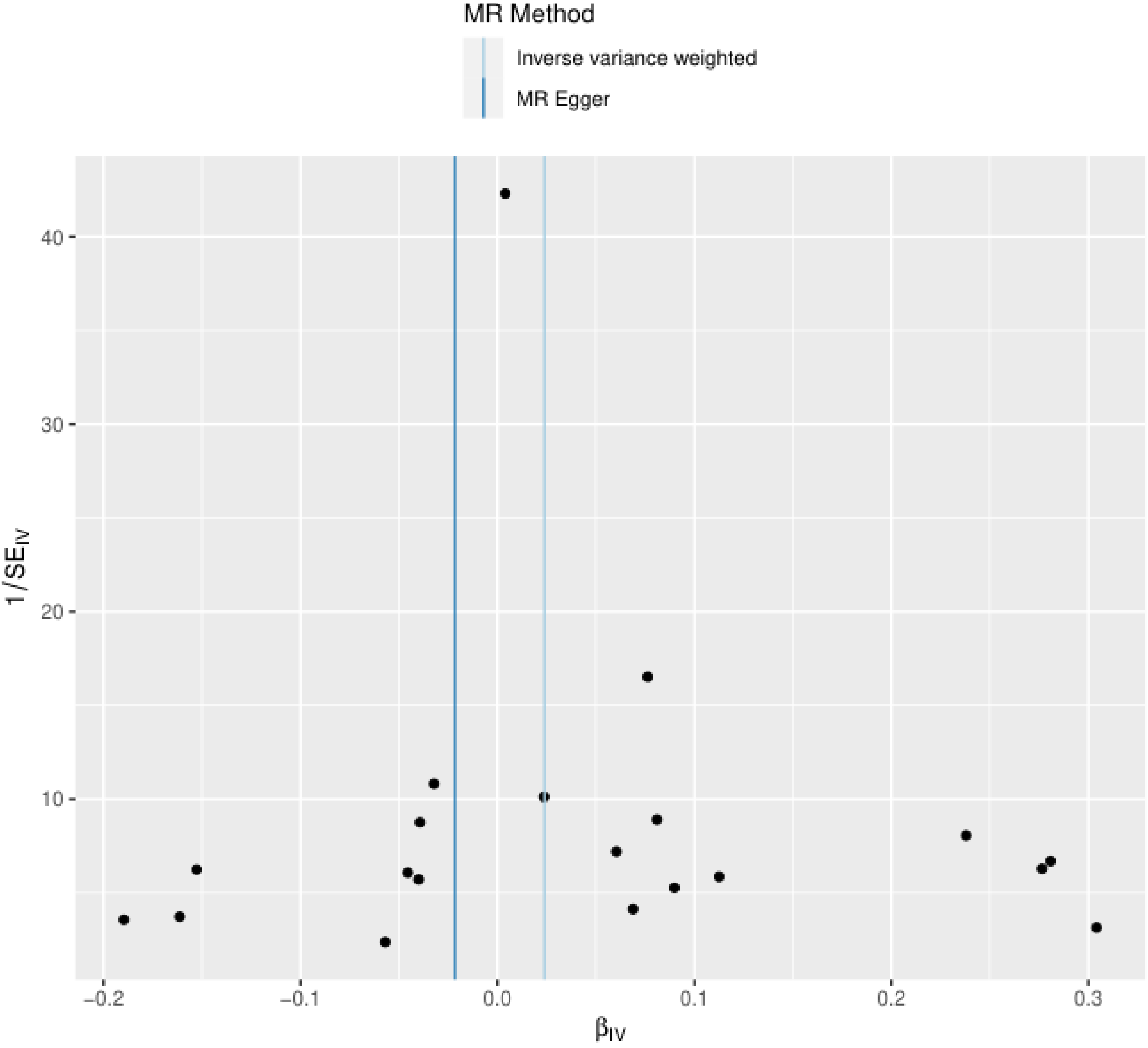

Funnel plot analyse of "CD33 on CD33dim HLA DR- " on 'Diabetic nephropathy'

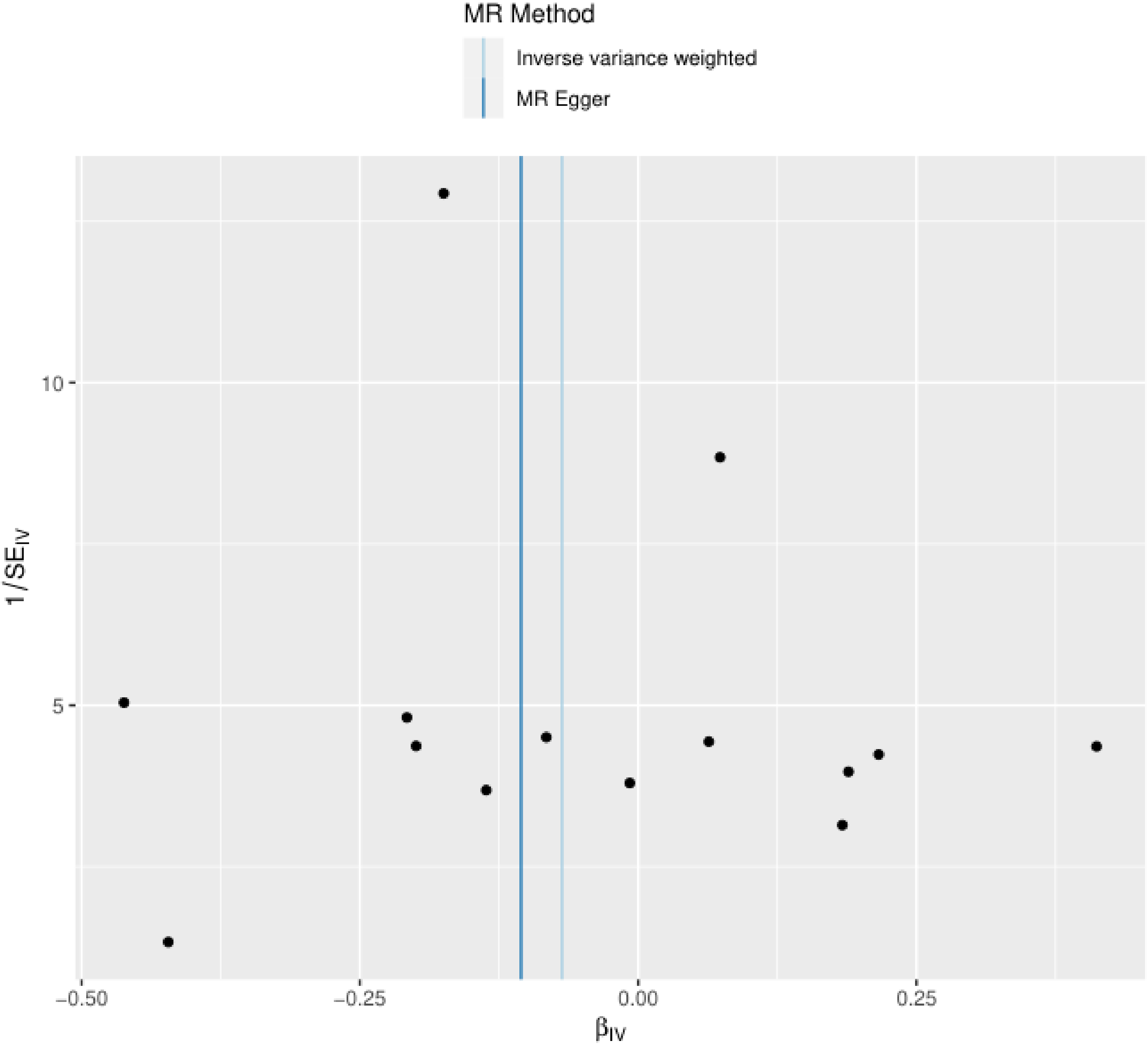

Funnel plot analyse of "CD38 on PB/PC" on 'Diabetic nephropathy'

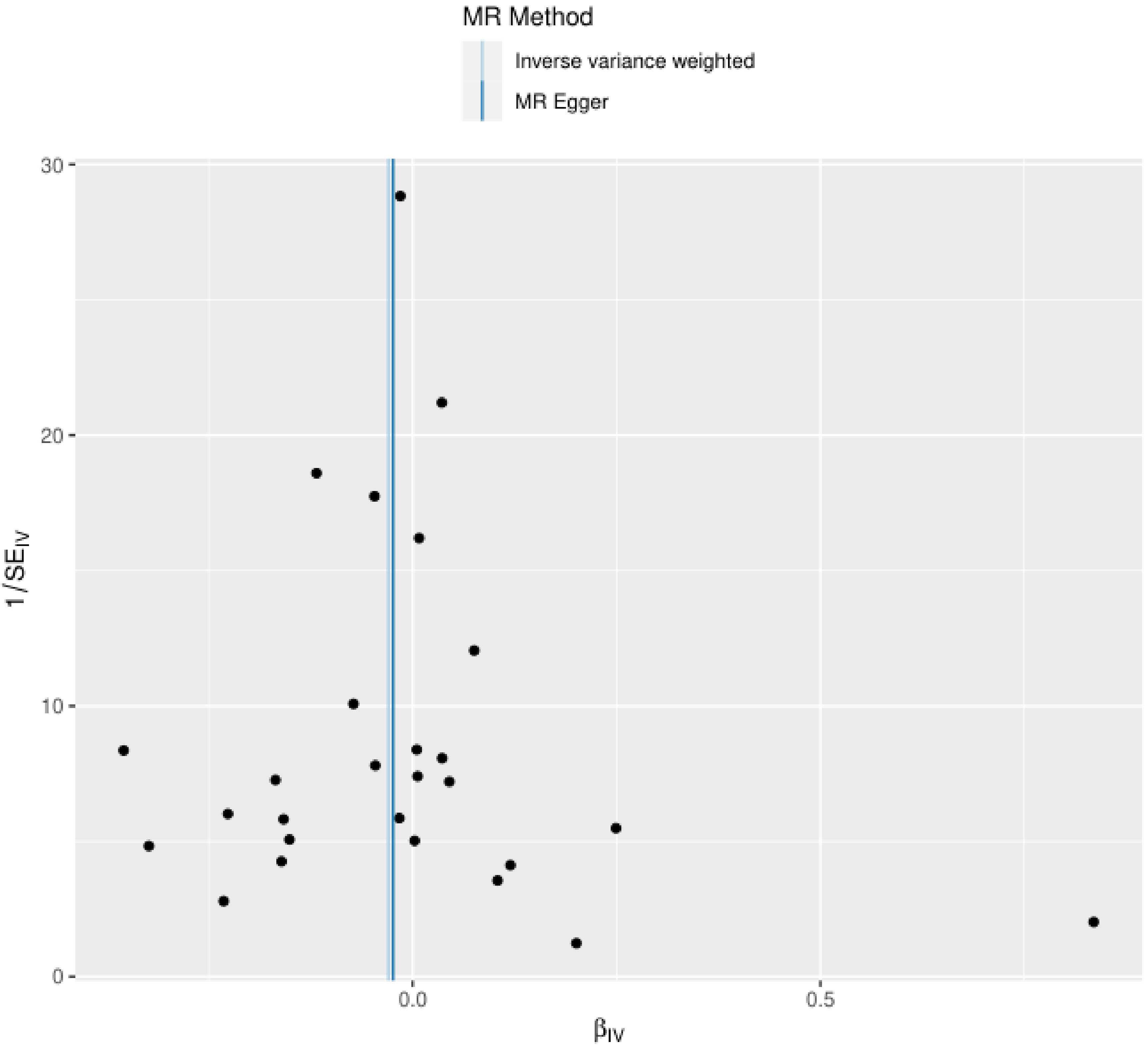

Funnel plot analyse of "NK %CD3- lymphocyte" on 'Diabetic nephropathy'

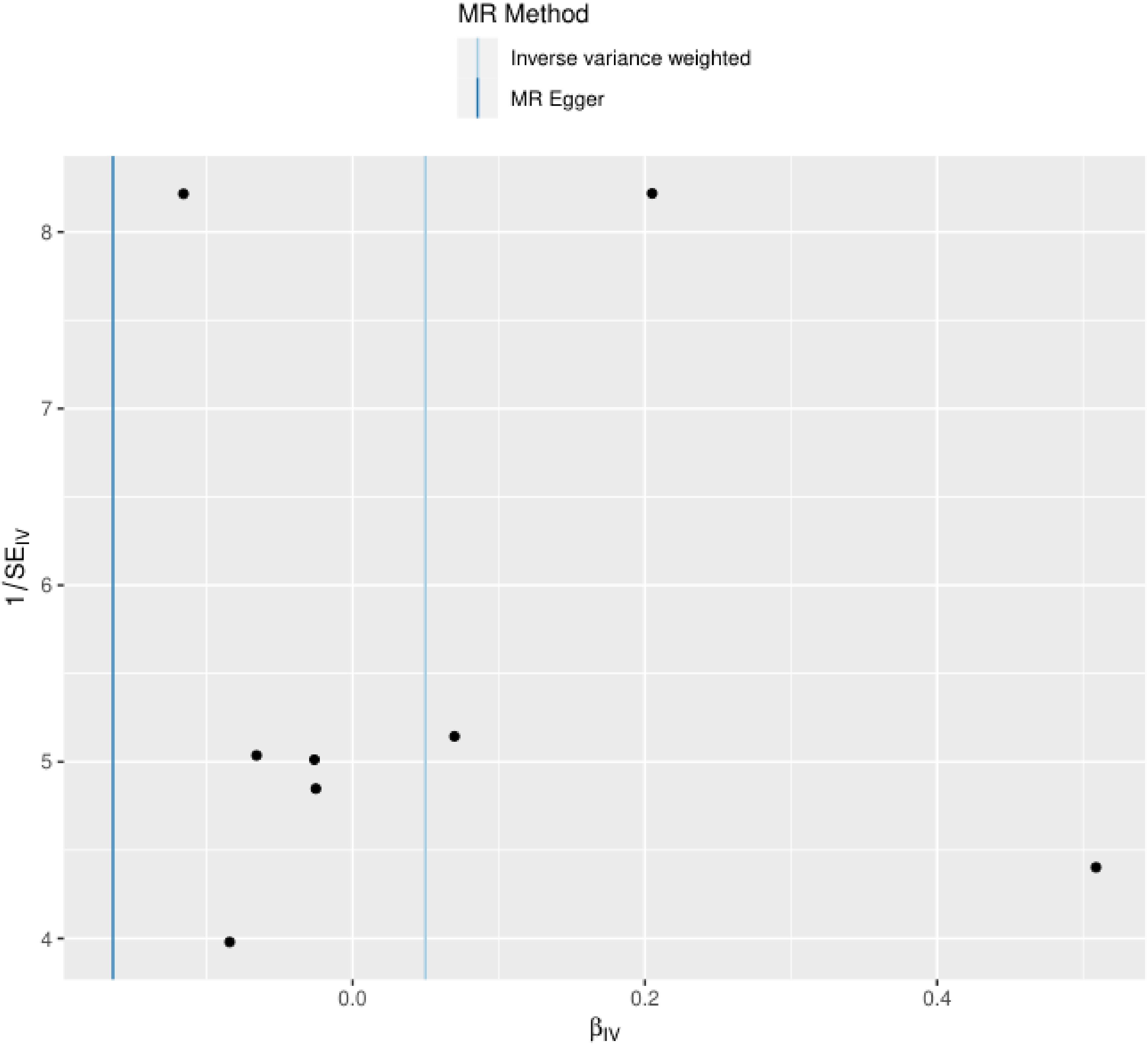

Funnel plot analyse of "CD127 on CD4+" on 'Diabetic nephropathy'

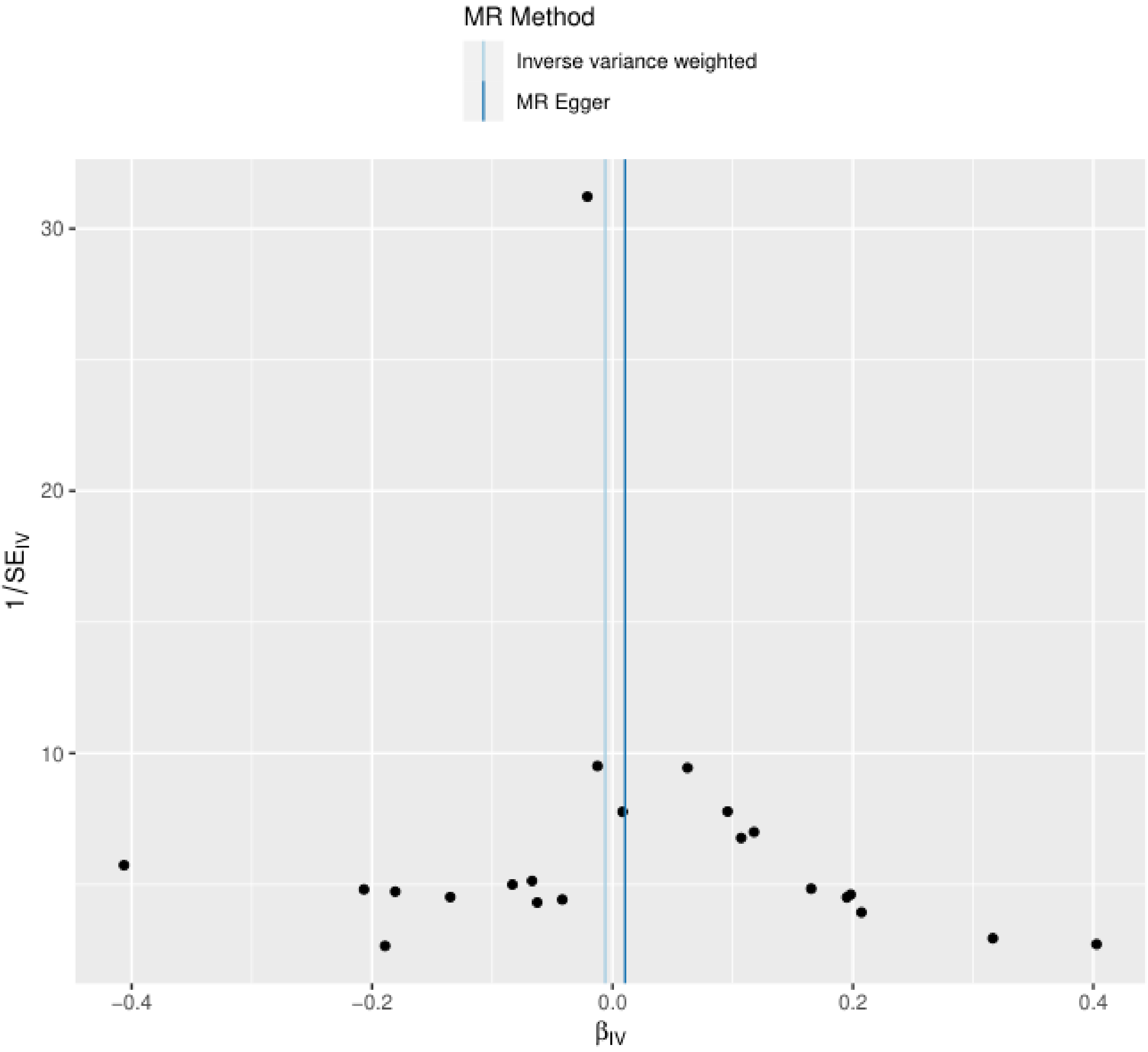

Funnel plot analyse of "BAFF-R on IgD- CD24-" on 'Diabetic nephropathy'

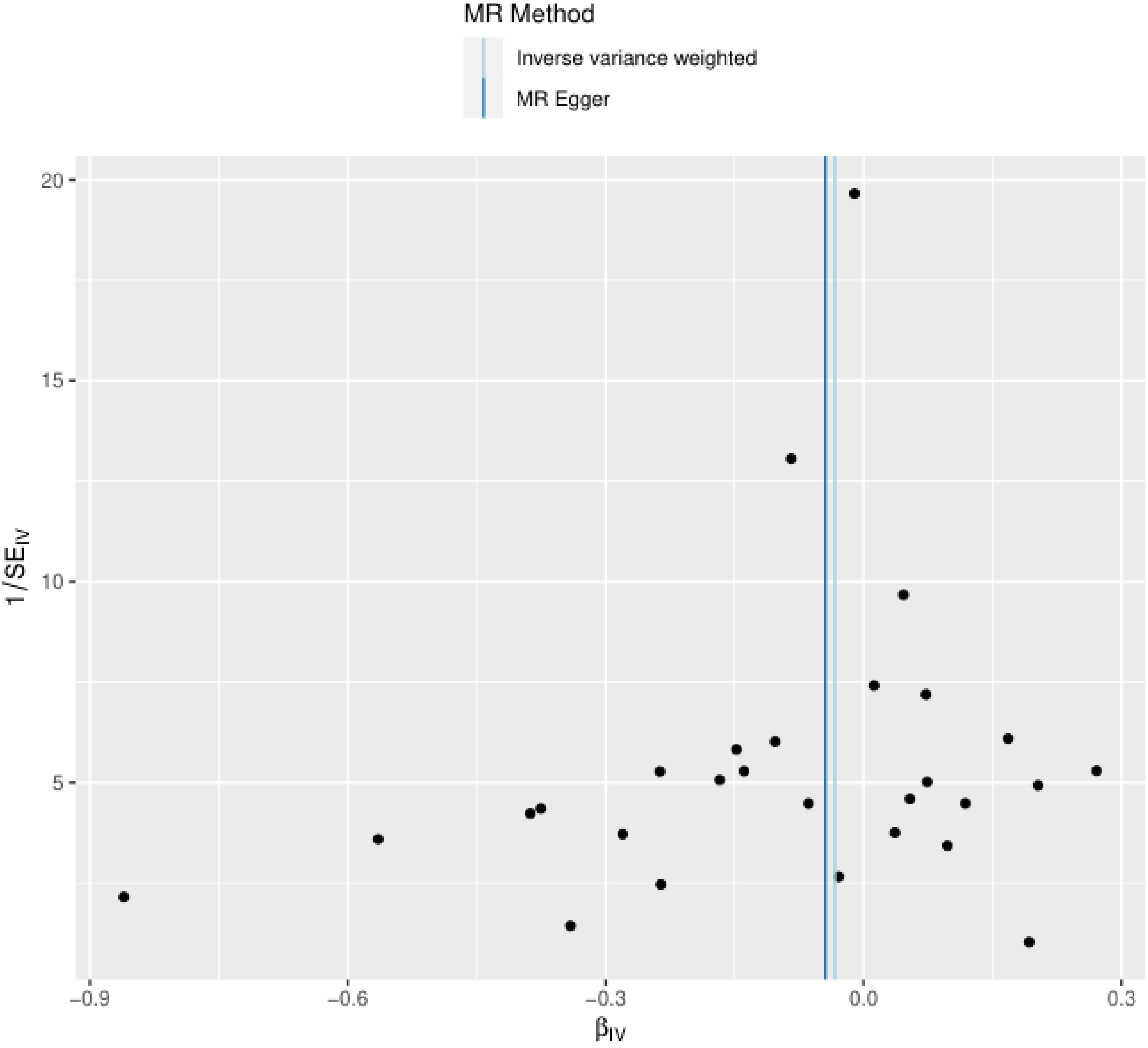

Funnel plot analyse of "CD19 on IgD- CD38dim" on 'Diabetic nephropathy'

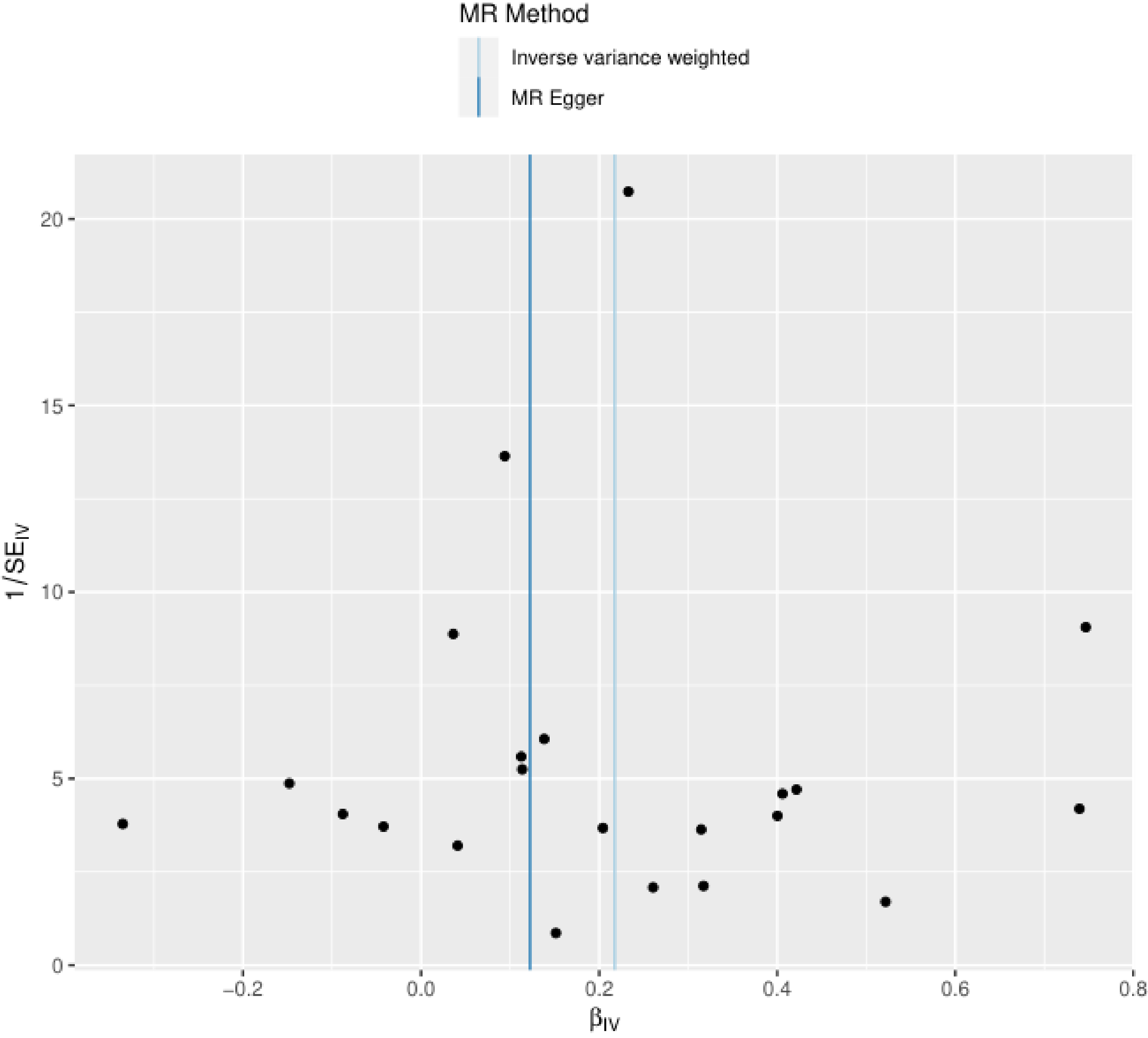

Funnel plot analyse of "HLA DR on CD14+ CD16- monocyte" on 'Diabetic nephropathy'

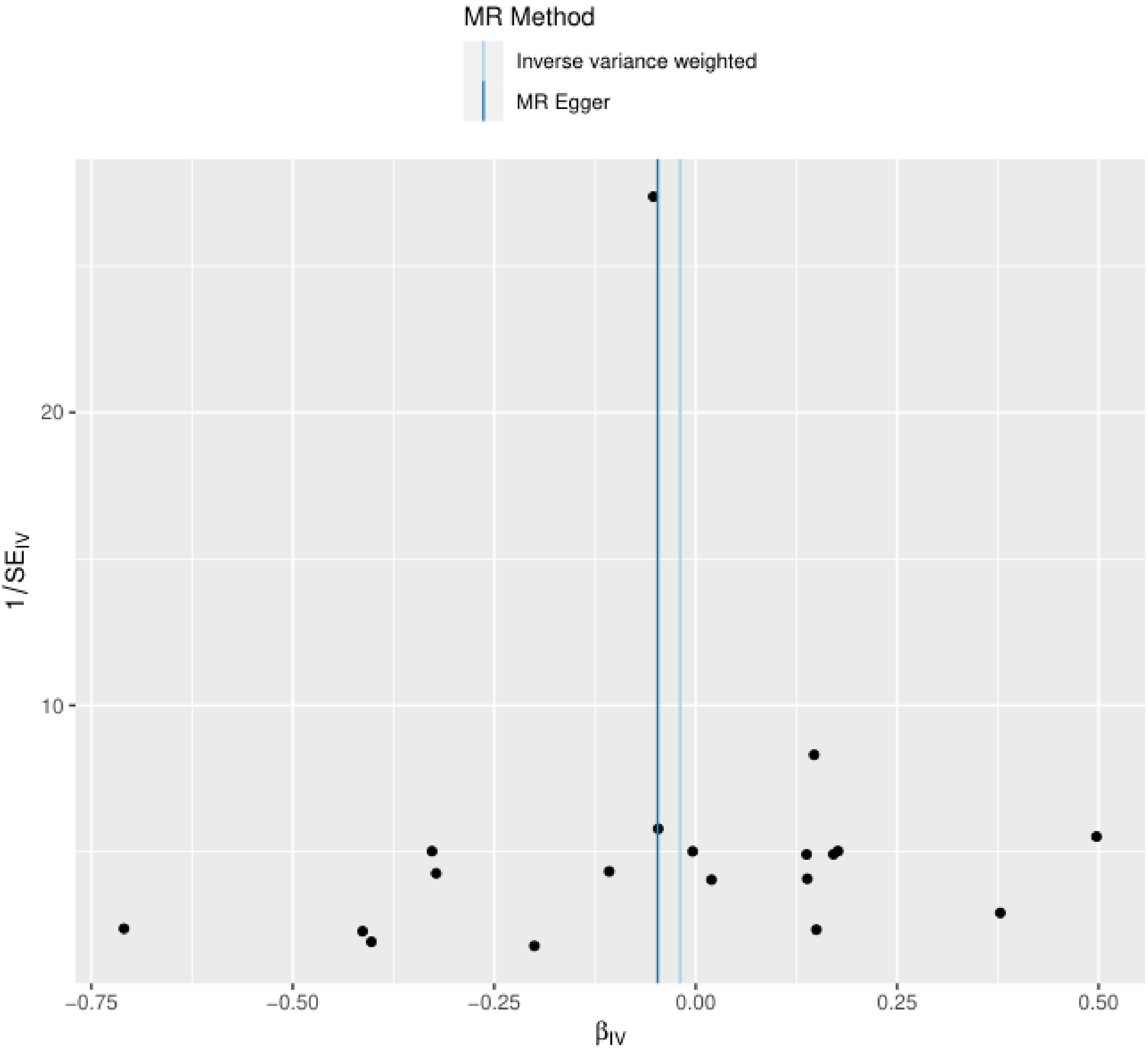

Funnel plot analyse of "IgD+ CD38- %lymphocyte" on 'Diabetic nephropathy'

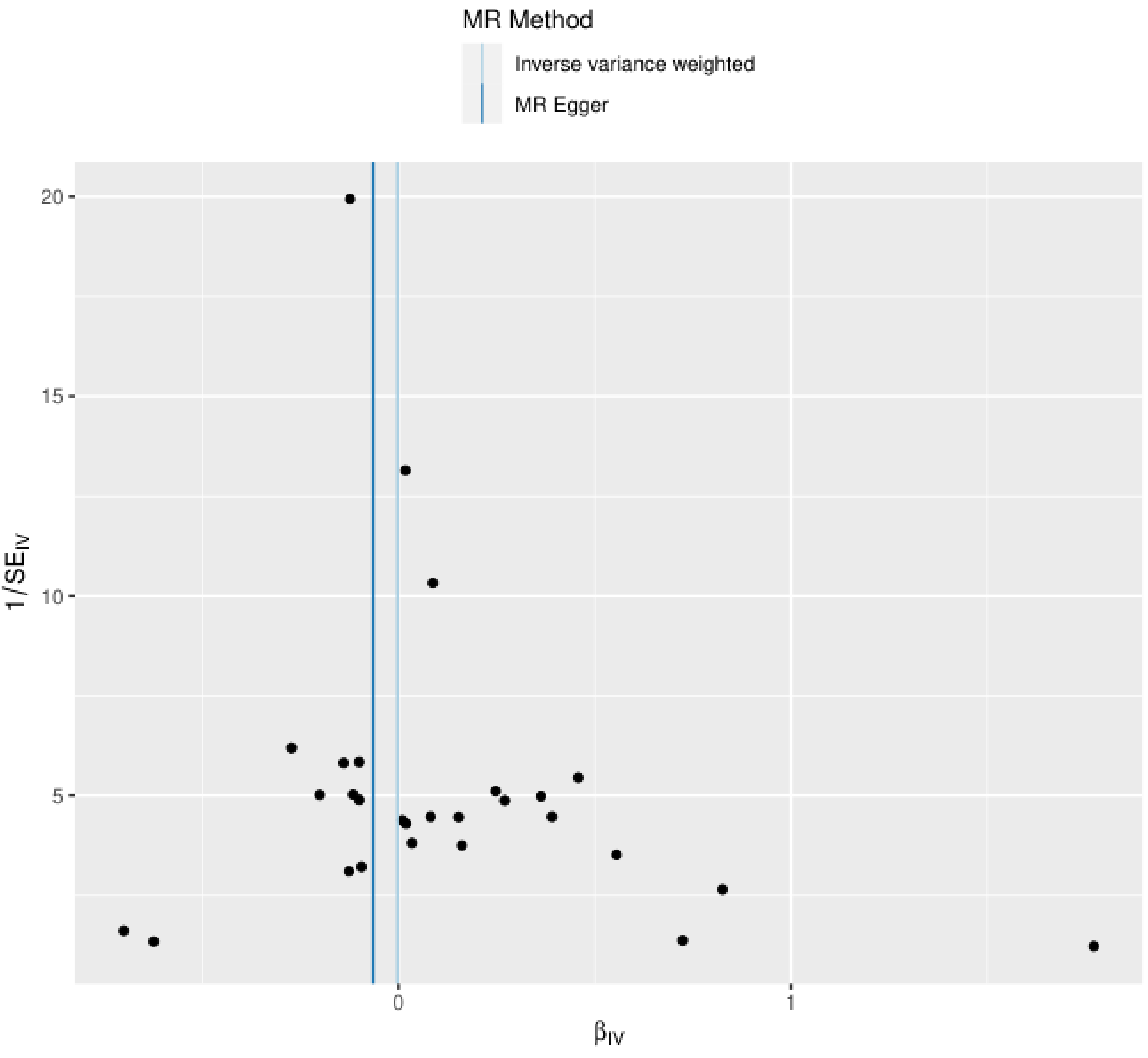

Funnel plot analyse of "CD28- DN (CD4-CD8-) %DN" on 'Diabetic nephropathy'

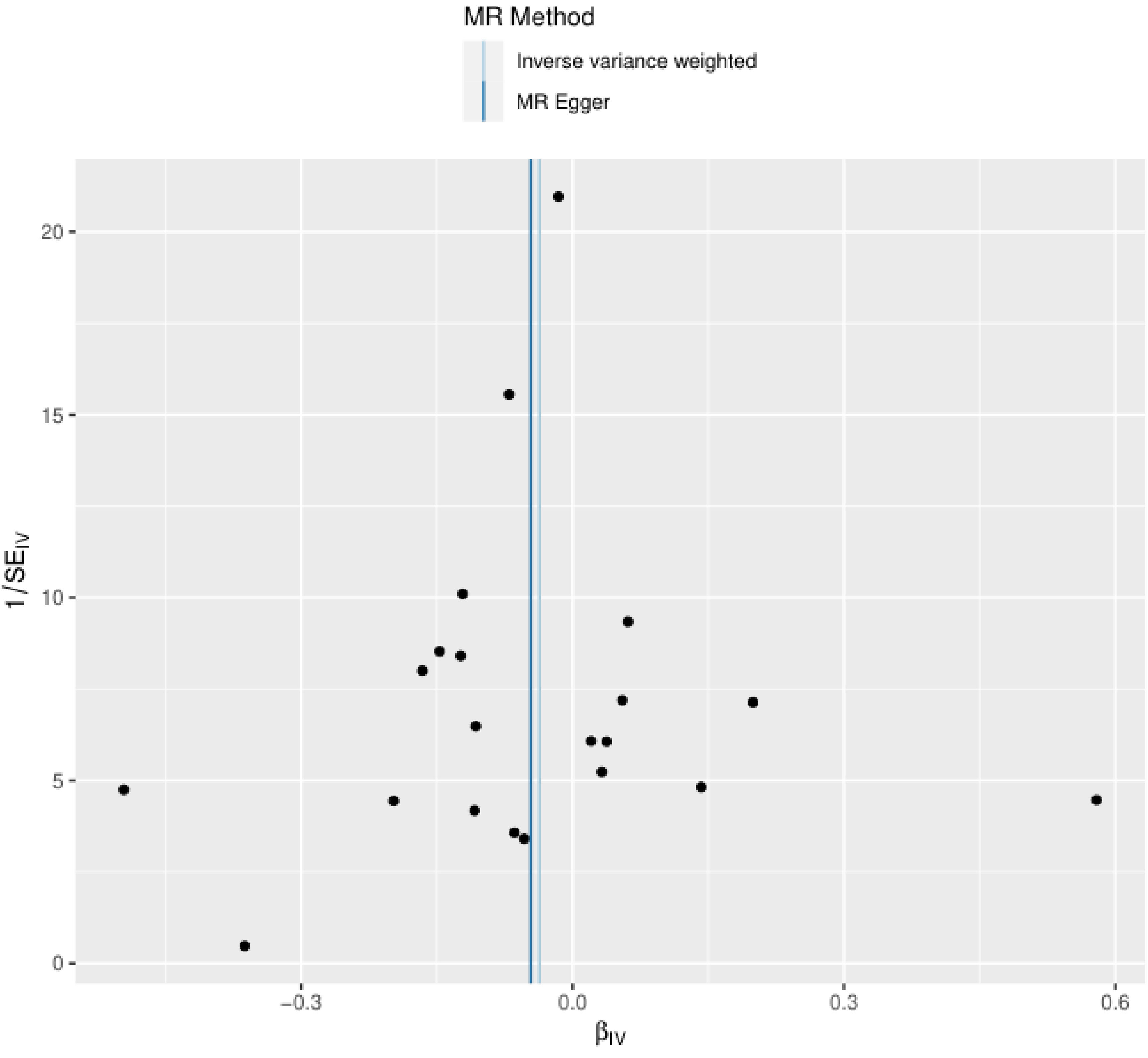

Funnel plot analyse of "CD3 on activated Treg" on 'Diabetic nephropathy'

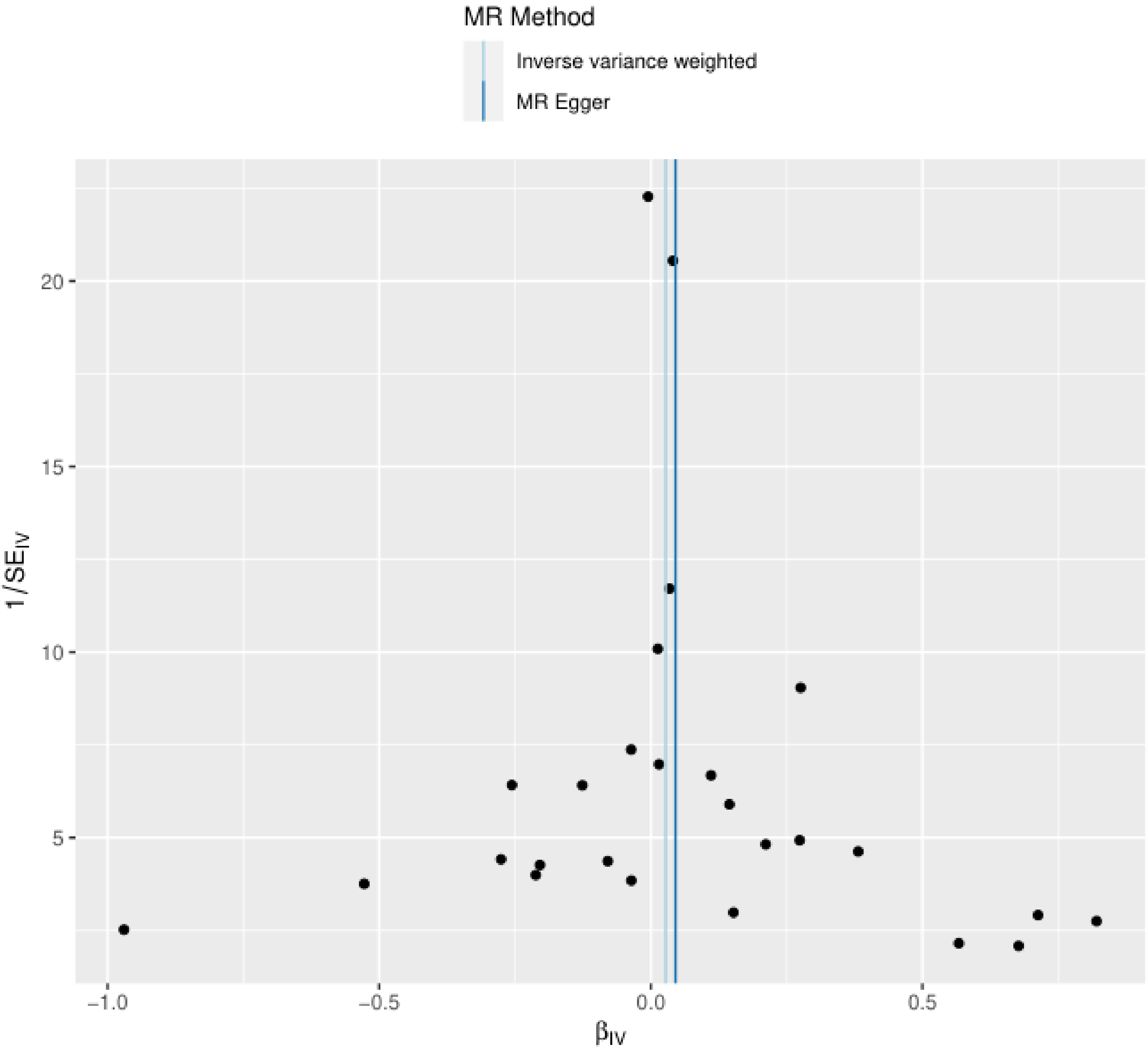

Funnel plot analyse of "CD8dim AC" on 'Diabetic nephropathy'

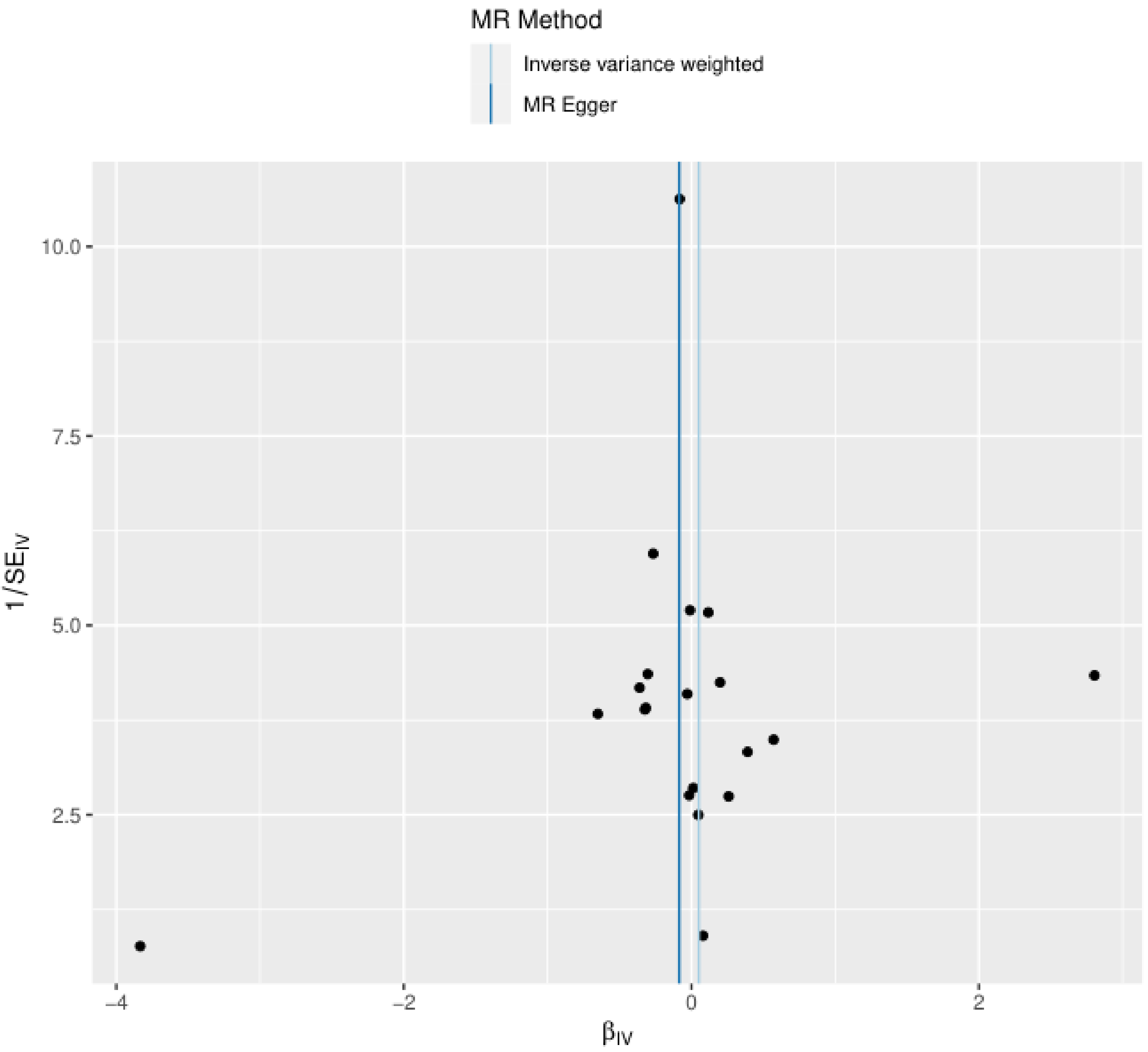

Funnel plot analyse of "HLA DR+ CD4+ %lymphocyte" on 'Diabetic nephropathy'

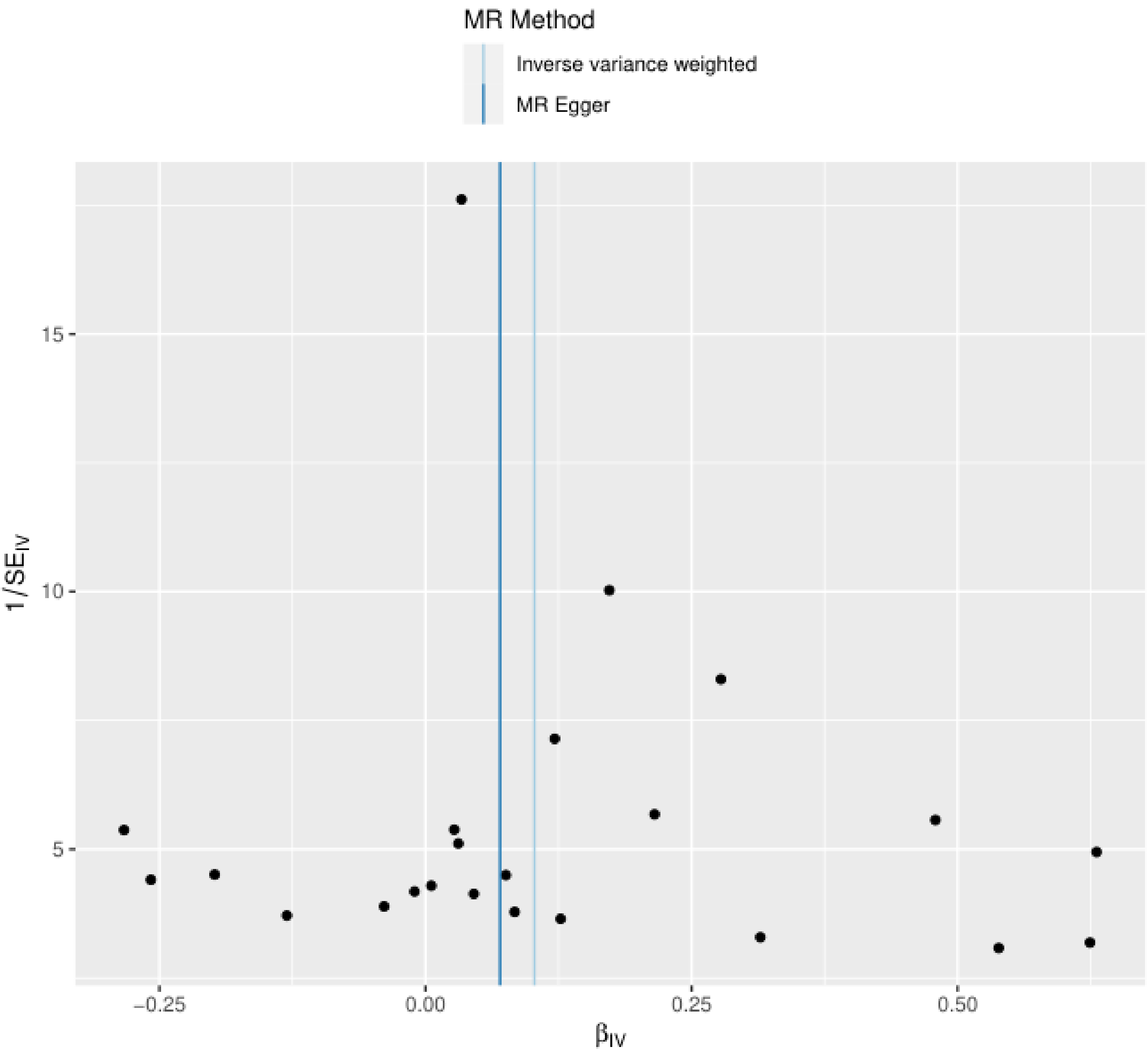

Funnel plot analyse of "SSC-A on CD14+ monocyte" on 'Diabetic nephropathy'

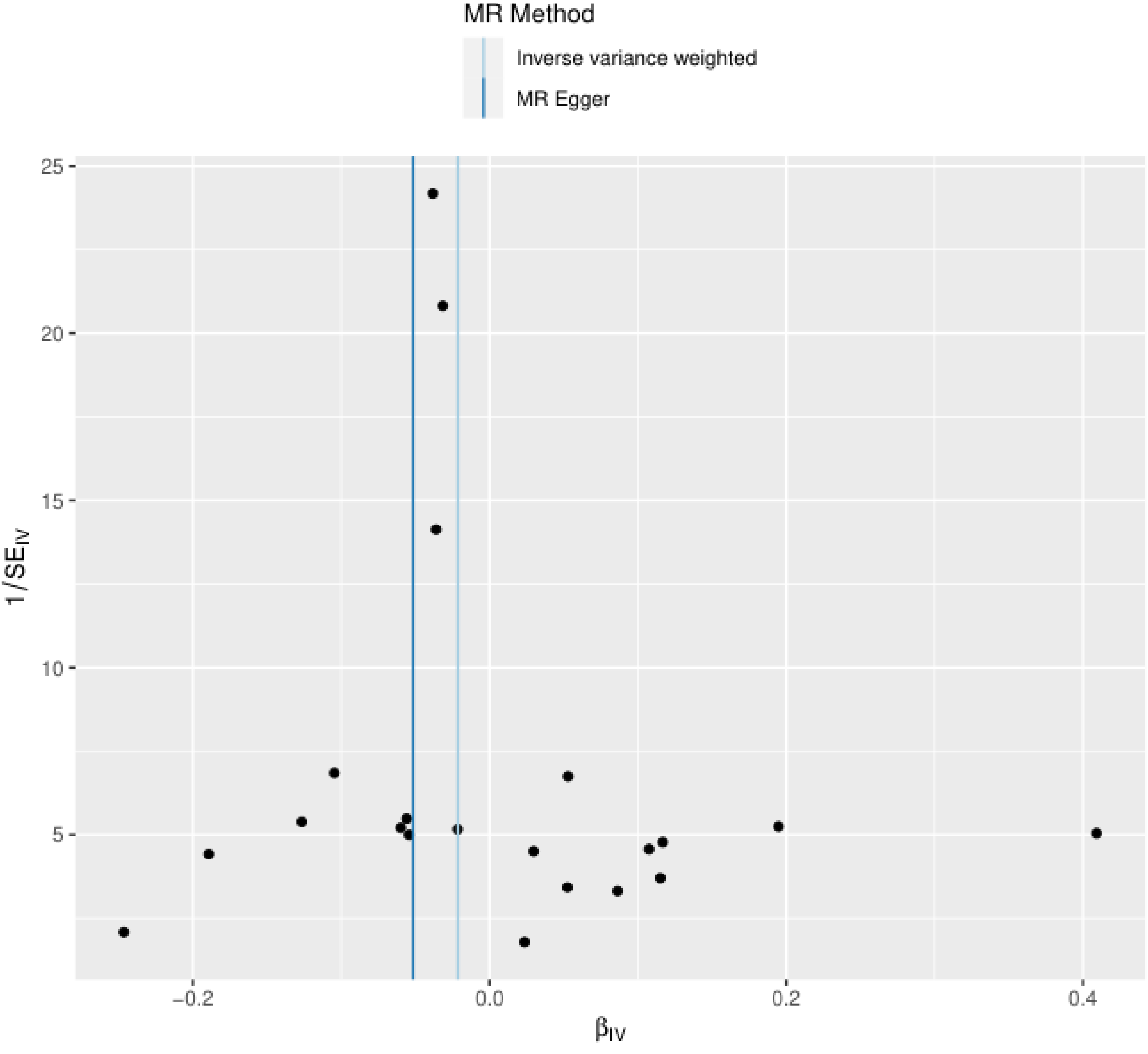

Funnel plot analyse of "CD3 on CD8br" on 'Diabetic nephropathy'

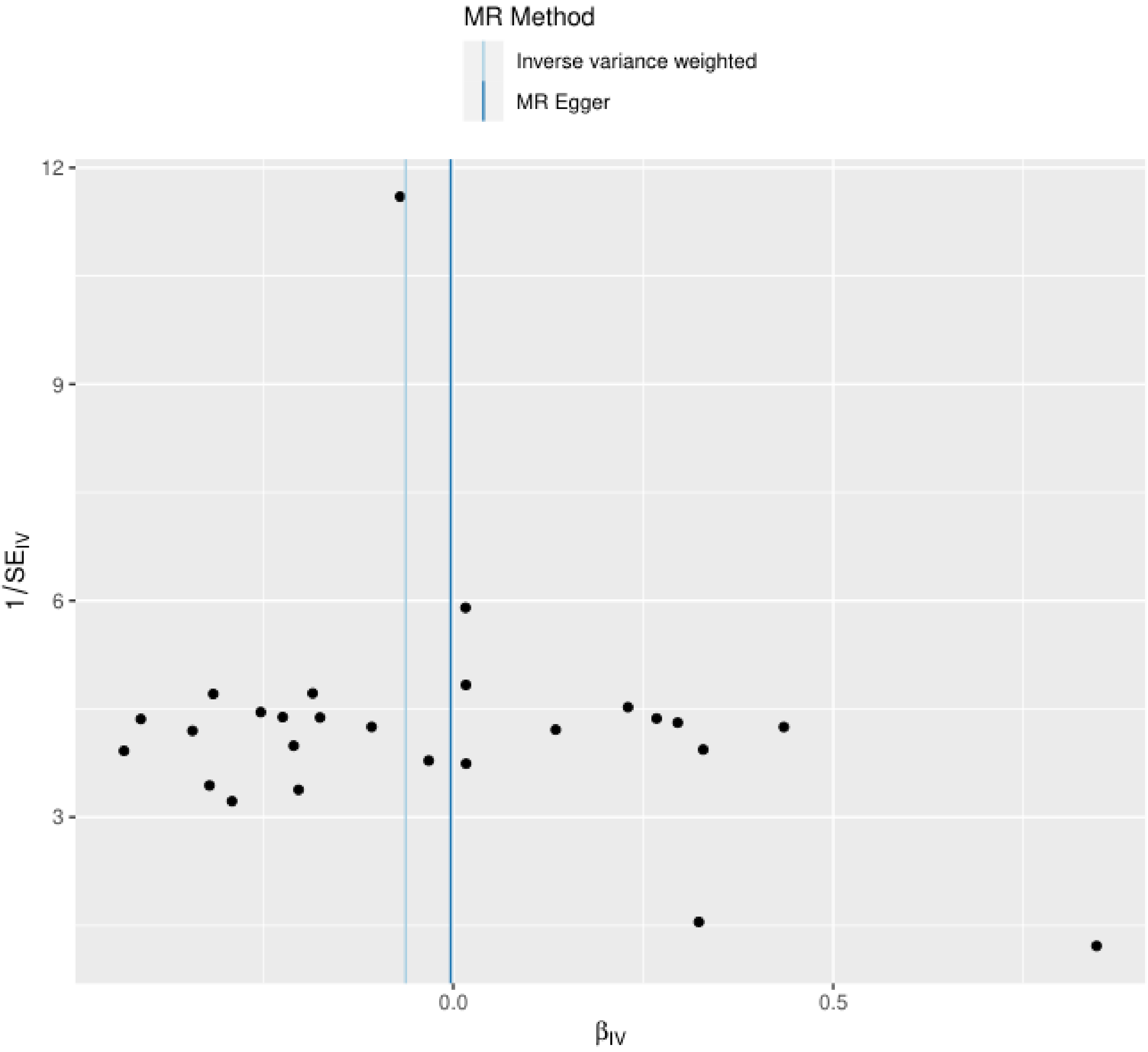

Funnel plot analyse of "Transitional %lymphocyte" on 'Diabetic nephropathy'

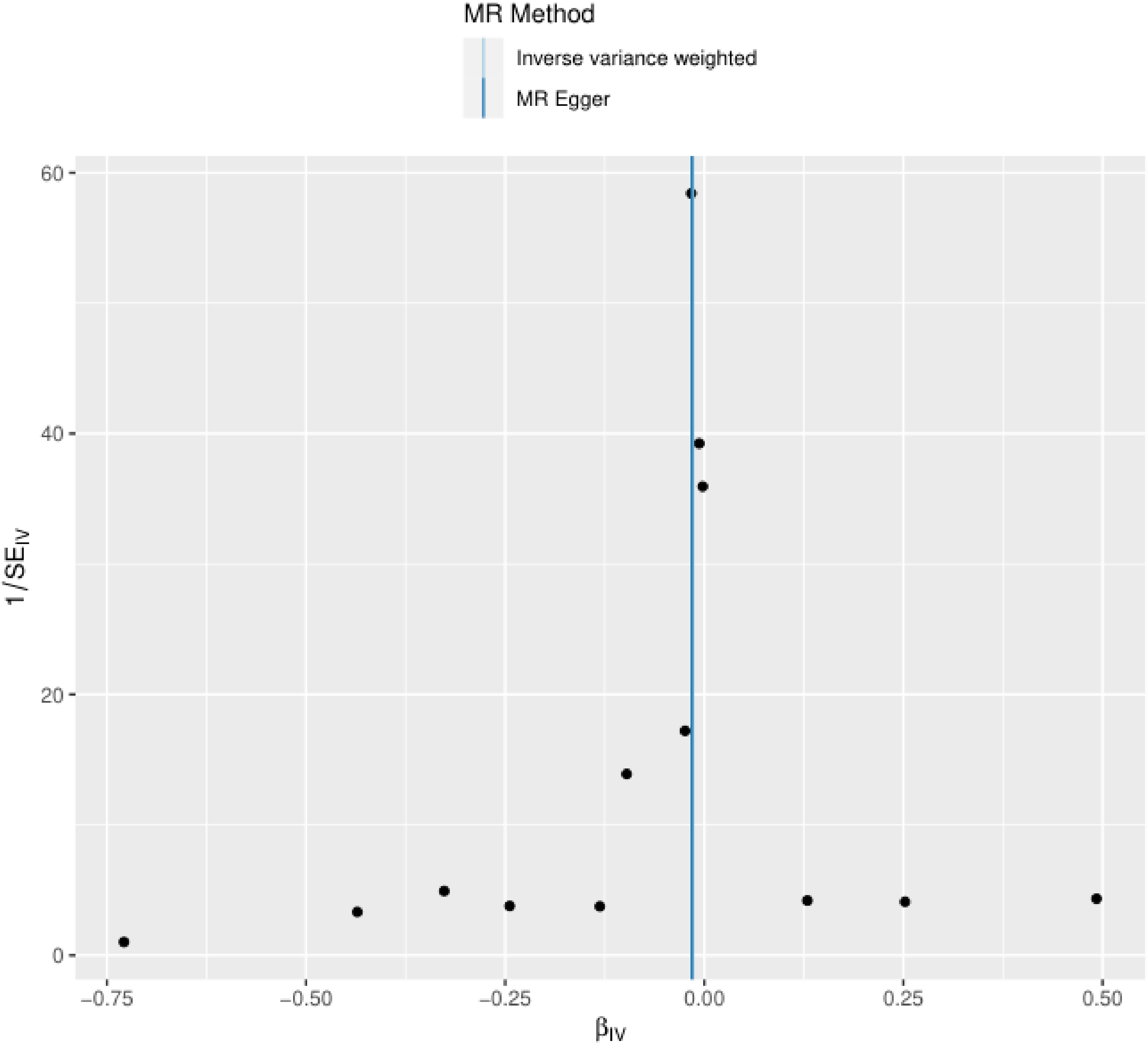

Funnel plot analyse of "CD38 on IgD- CD38br" on 'Diabetic nephropathy'

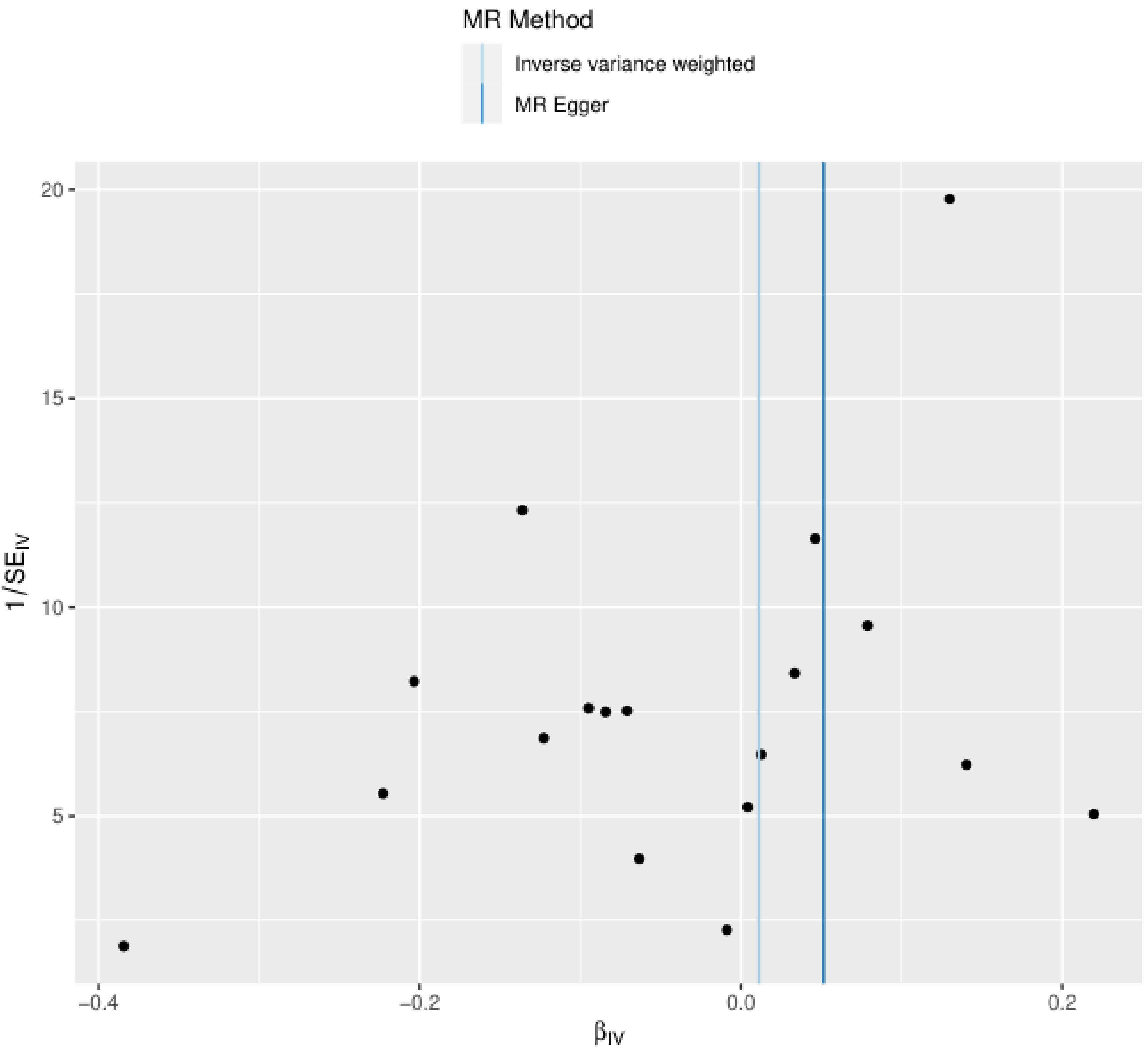

Funnel plot analyse of "CD11b on CD66b++ myeloid cell " on 'Diabetic nephropathy'

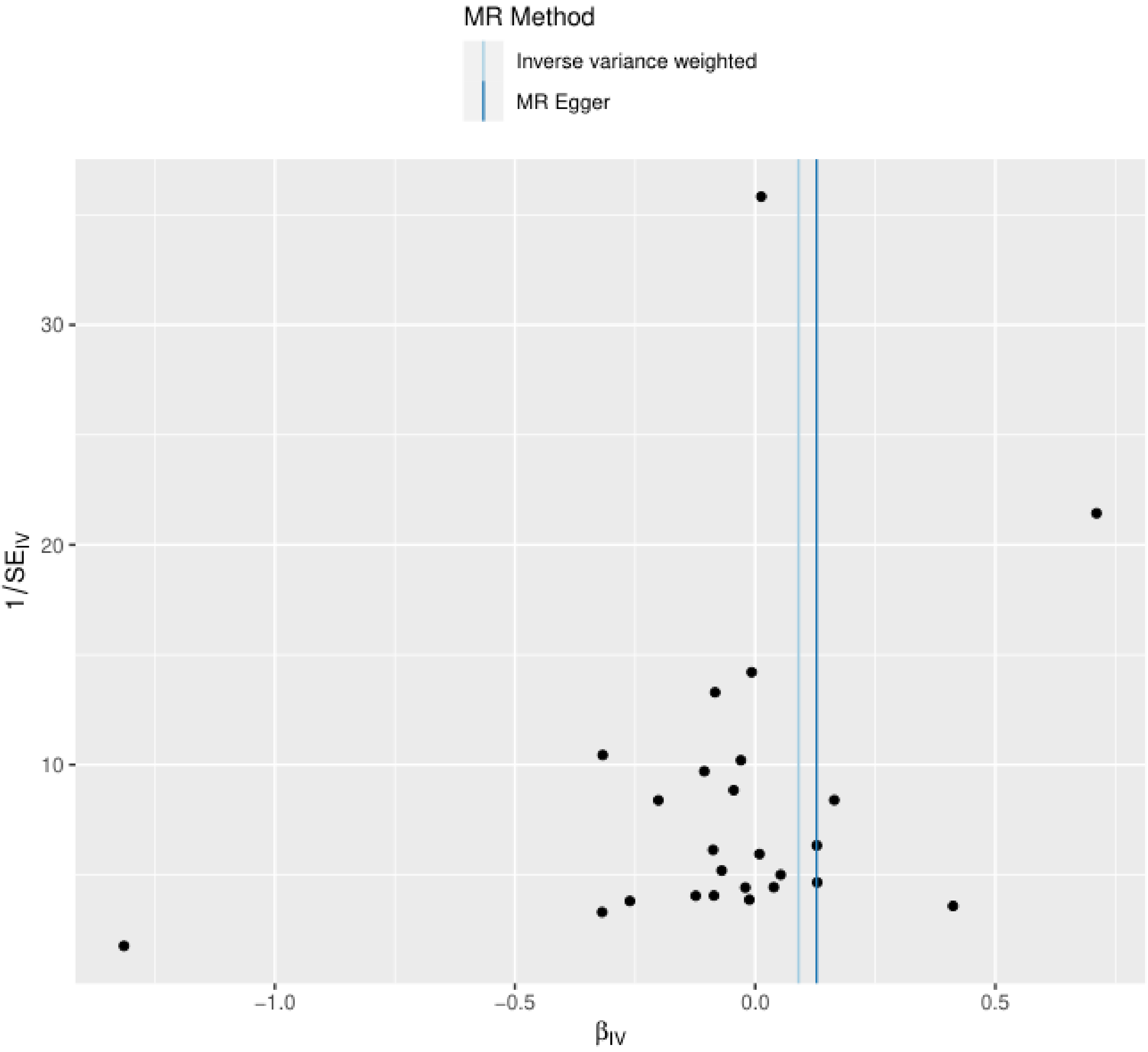

Funnel plot analyse of "HLA DR on CD14- CD16- " on 'Diabetic nephropathy'

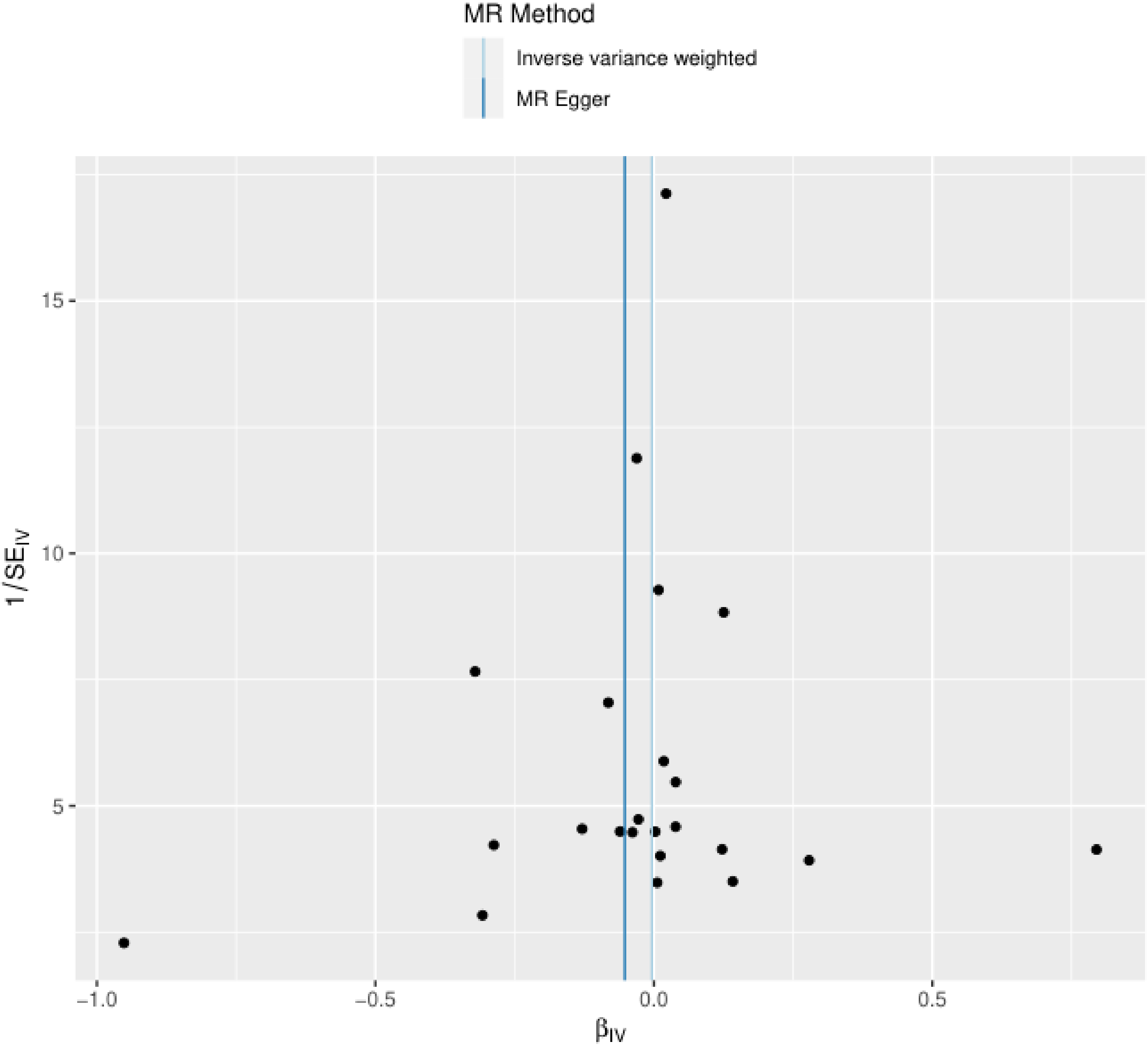

Funnel plot analyse of "HLA DR+ NK %CD3- lymphocyte" on 'Diabetic nephropathy'

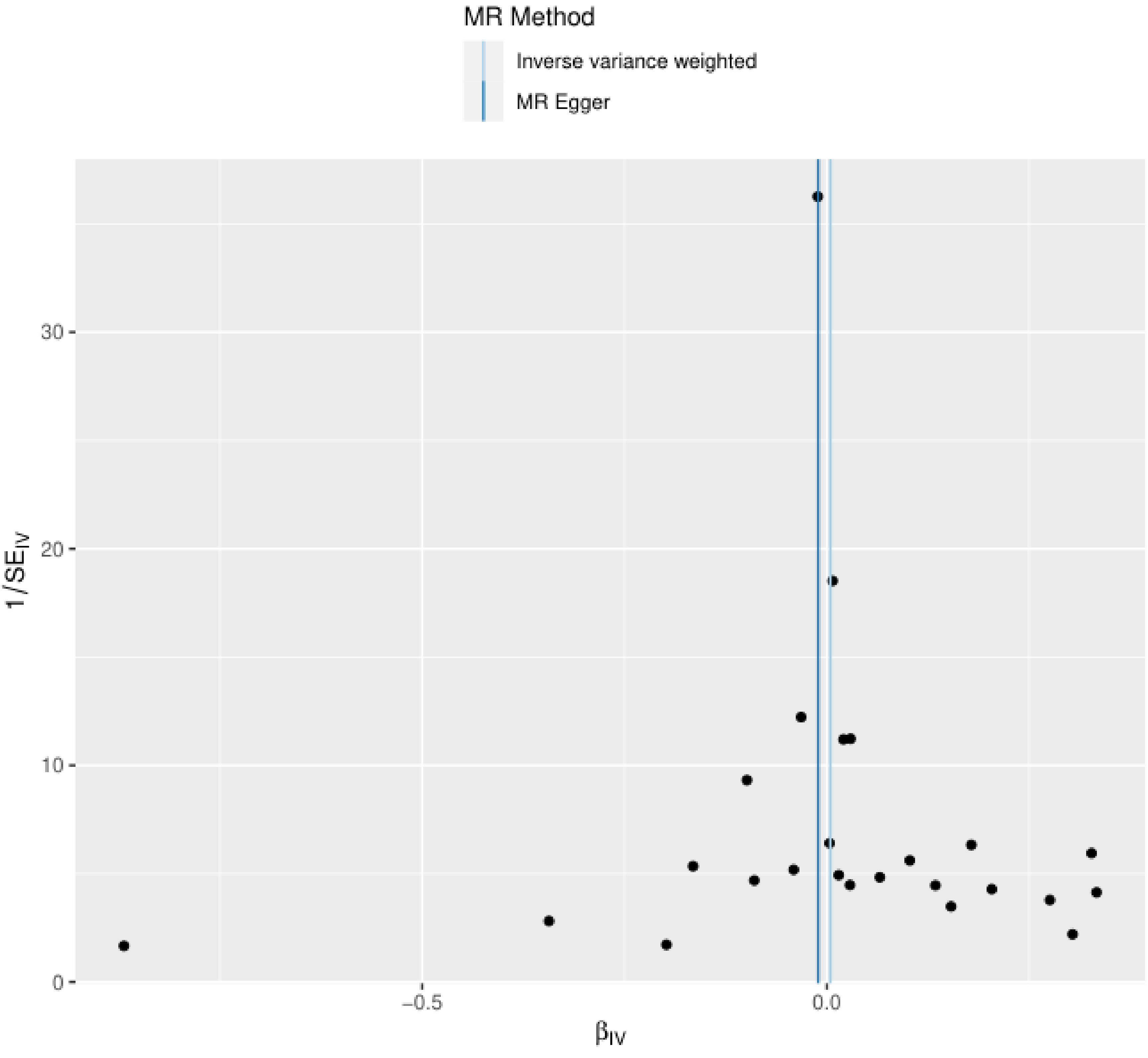

Funnel plot analyse of "IgD- CD38dim %B cell" on 'Diabetic nephropathy'

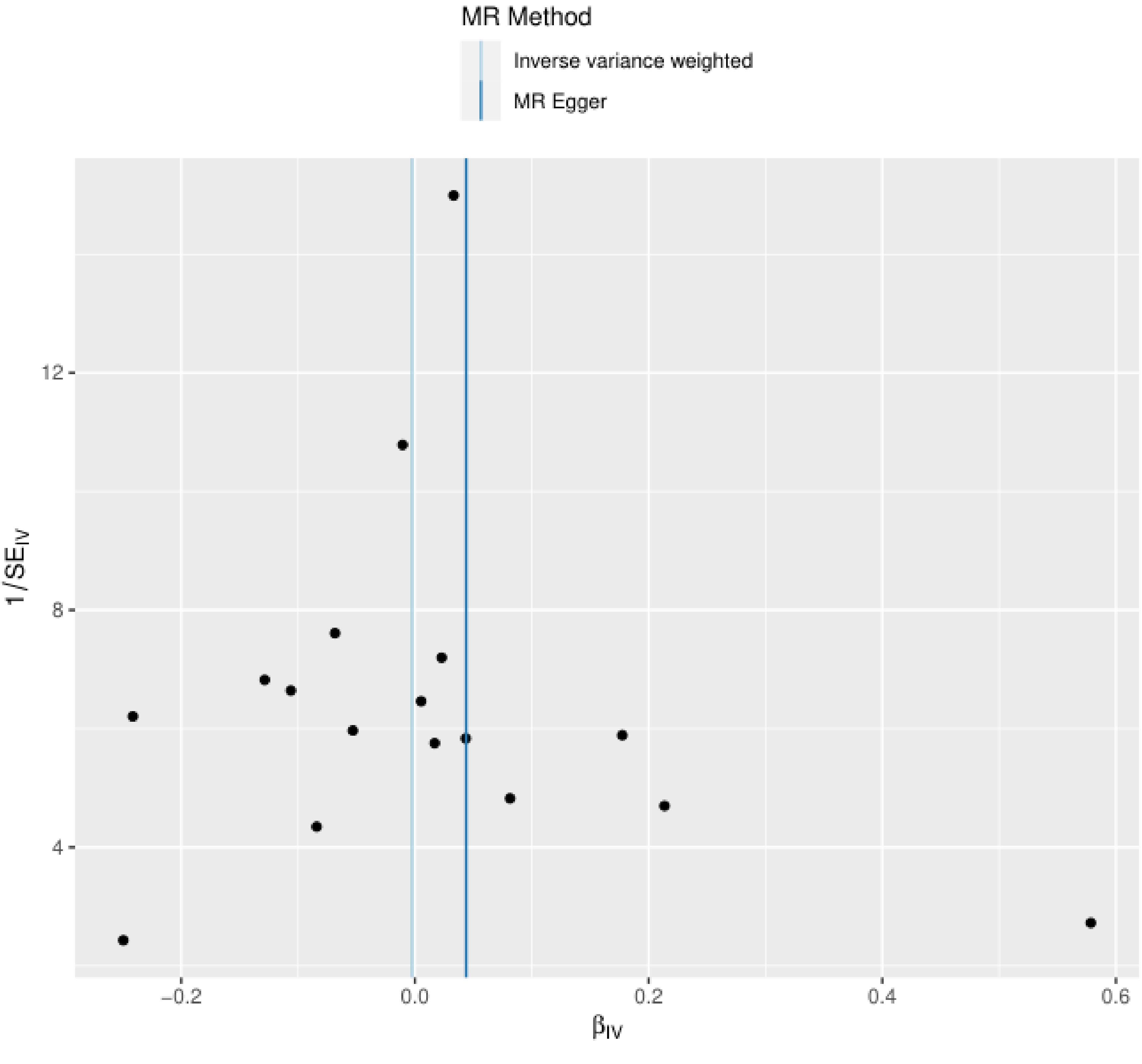

Funnel plot analyse of "CD45 on lymphocyte " on 'Diabetic nephropathy'

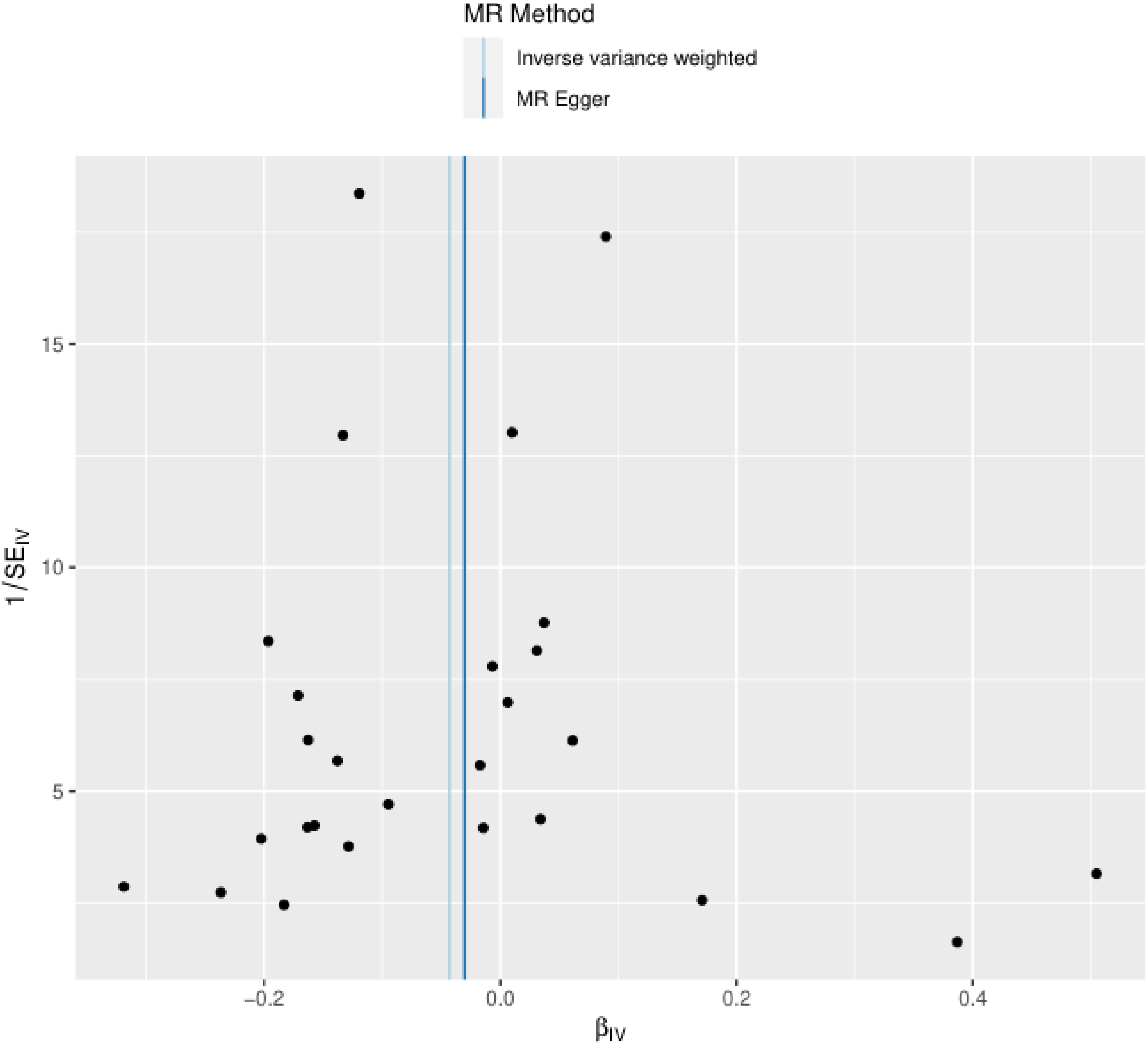

Funnel plot analyse of "NK %lymphocyte" on 'Diabetic nephropathy'

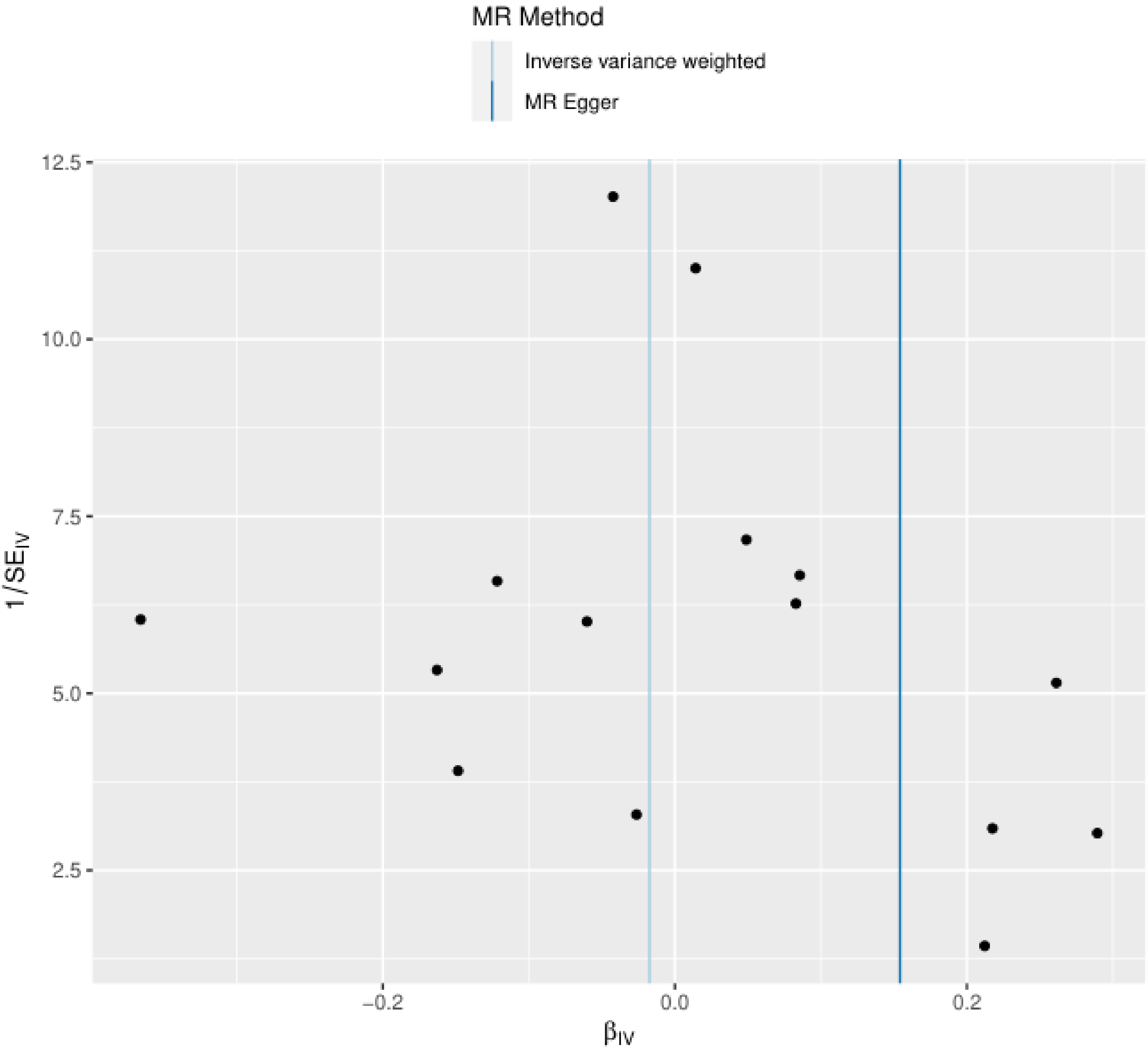

Funnel plot analyse of "CD3 on CD39+ CD8br " on 'Diabetic nephropathy'

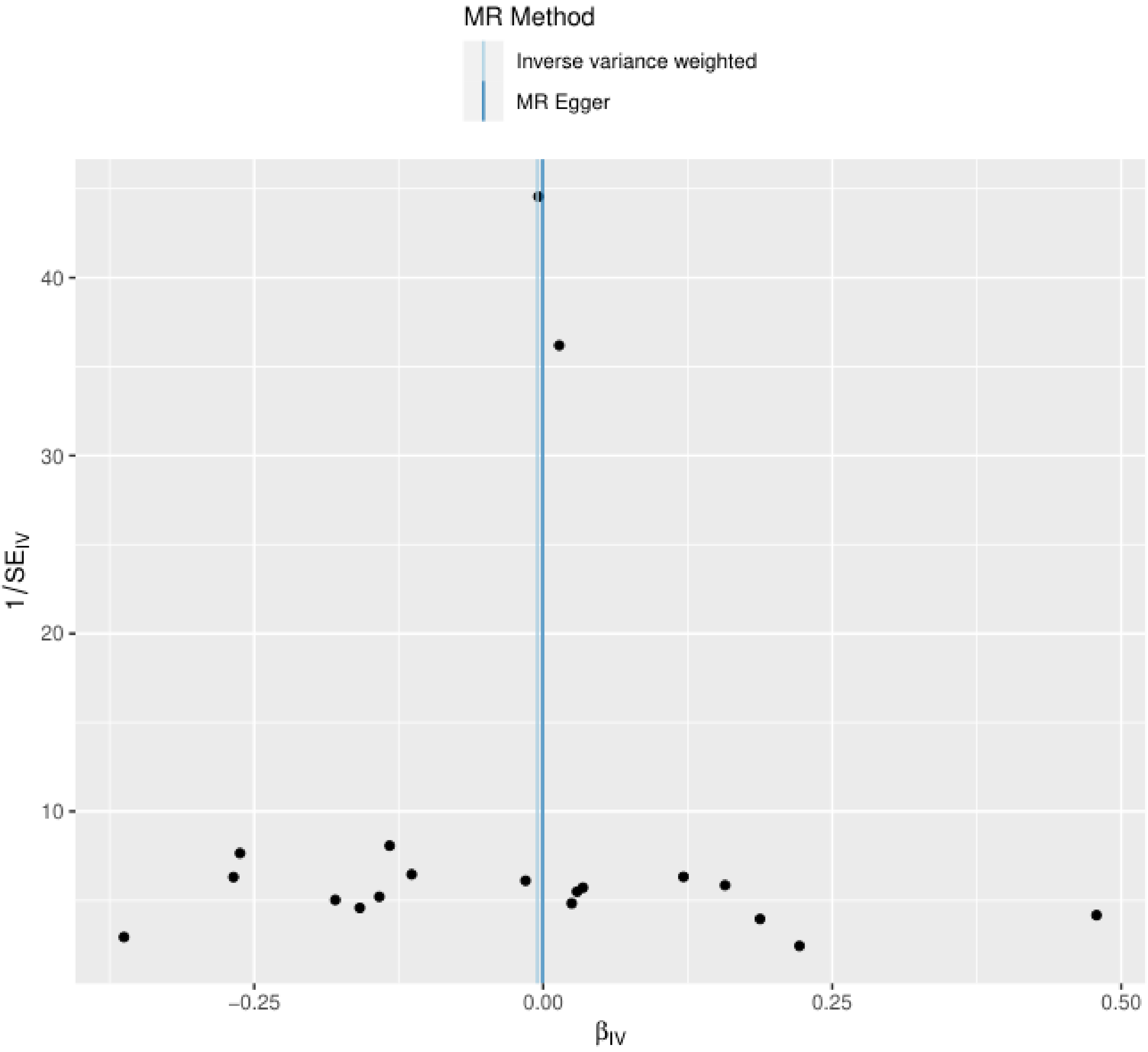

Funnel plot analyse of "CD19 on IgD+ CD38- naive" on 'Diabetic nephropathy'

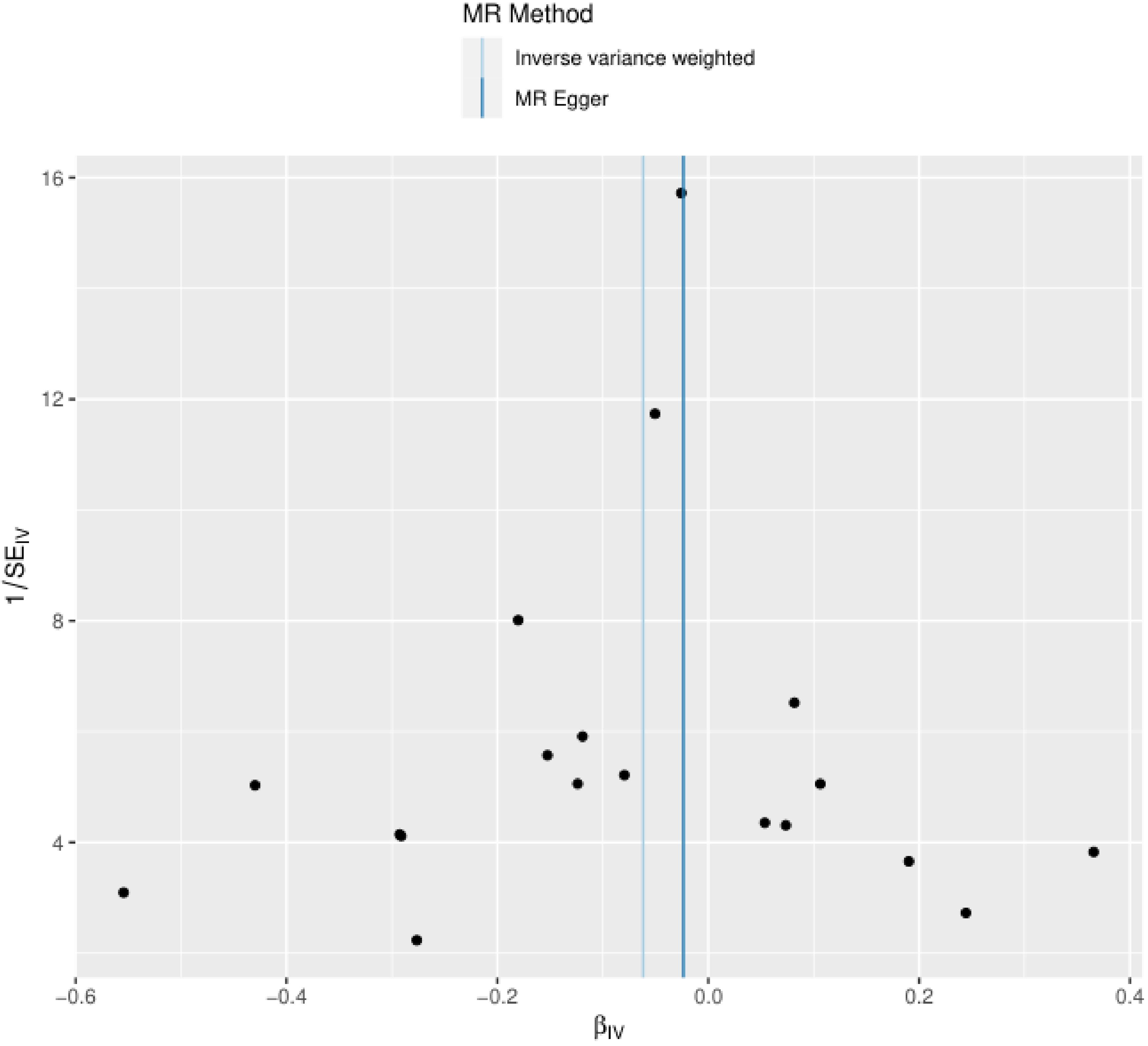

Funnel plot analyse of "CD25 on CD20- CD38-" on 'Diabetic nephropathy'

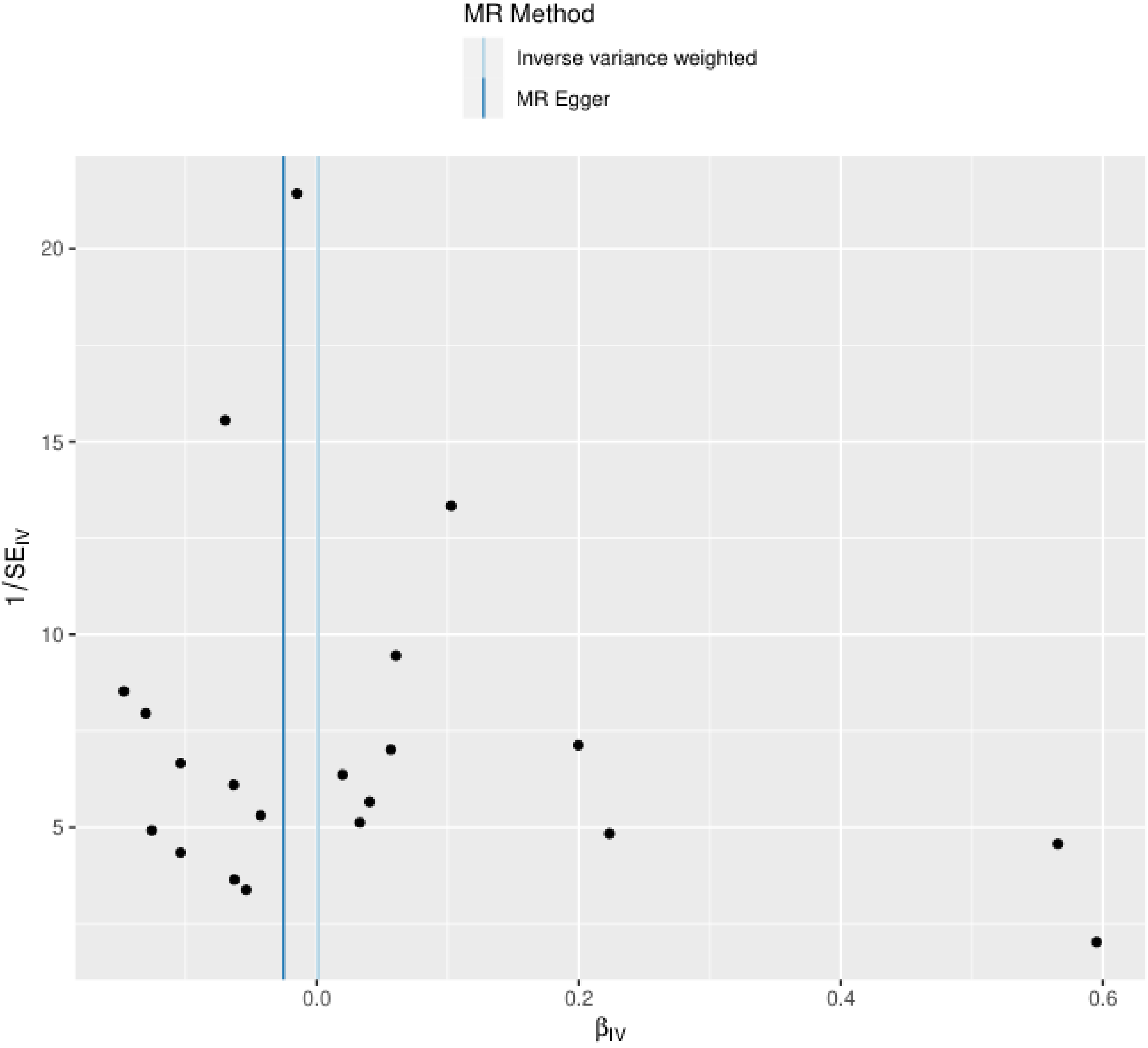

Funnel plot analyse of "CD3 on activated & secreting Treg" on 'Diabetic nephropathy'

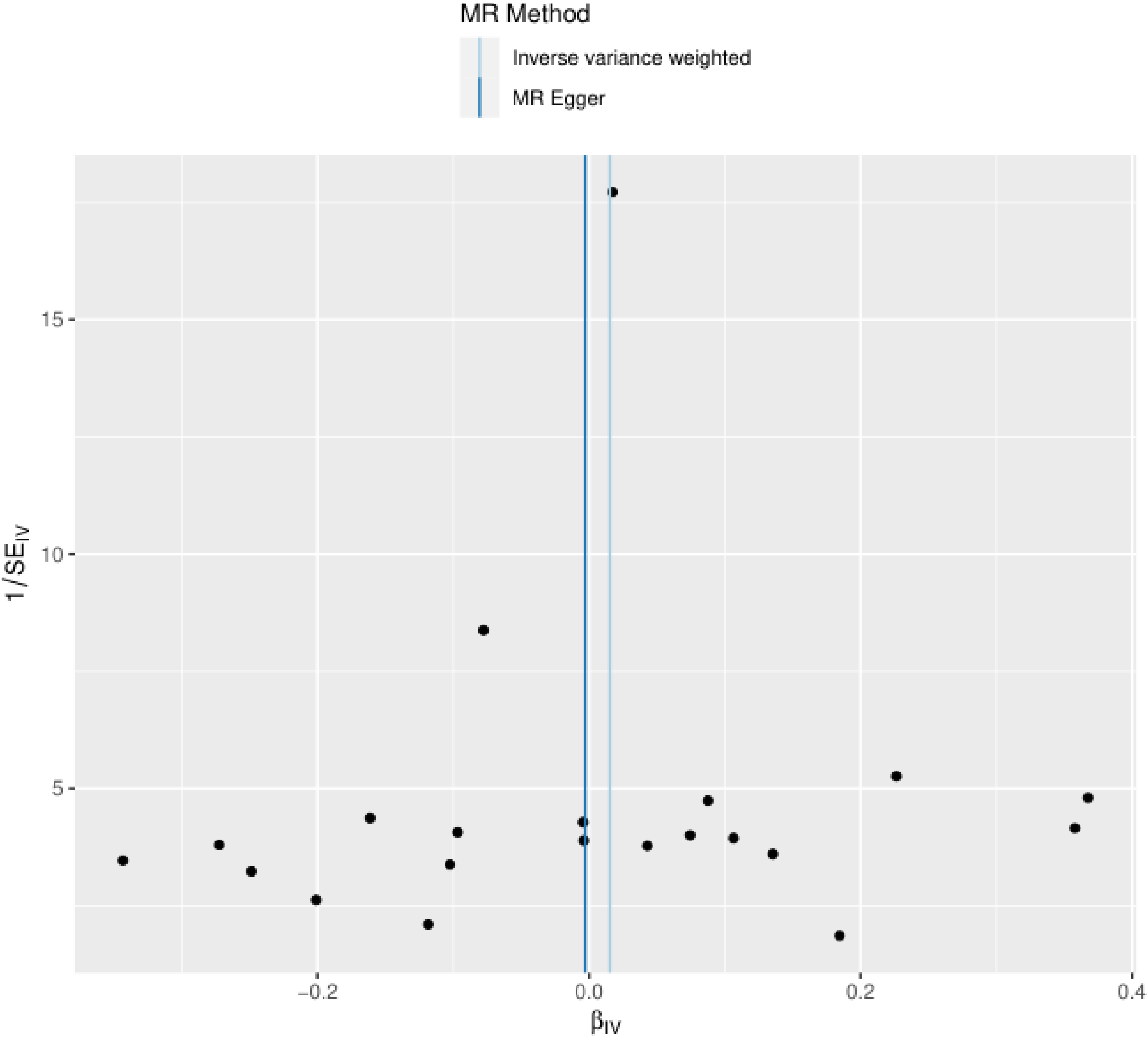

Funnel plot analyse of "Naive DN (CD4-CD8-) %DN" on 'Diabetic nephropathy'

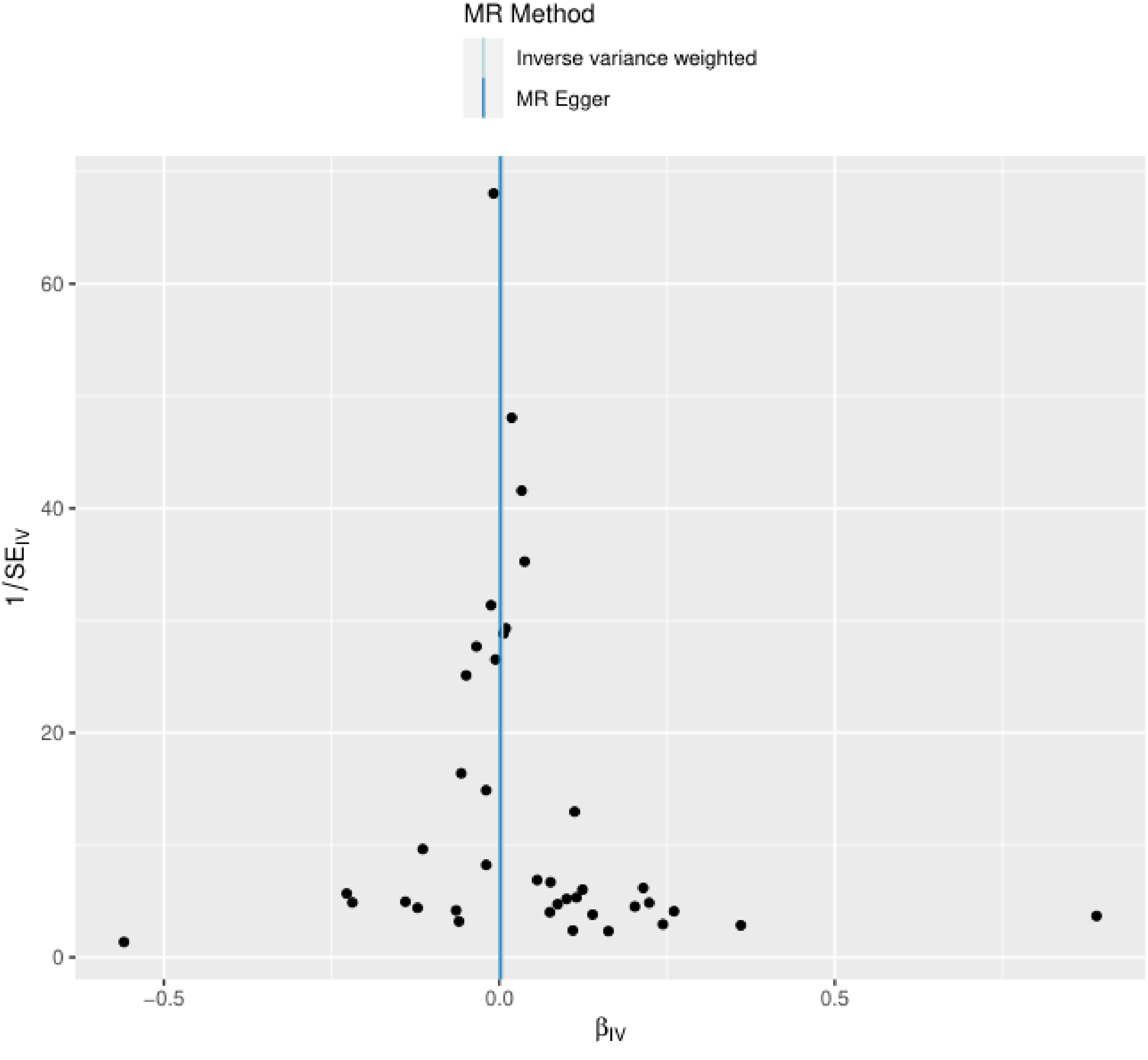

Funnel plot analyse of "CD28+ CD45RA+ CD8dim %T cell" on 'Diabetic nephropathy'

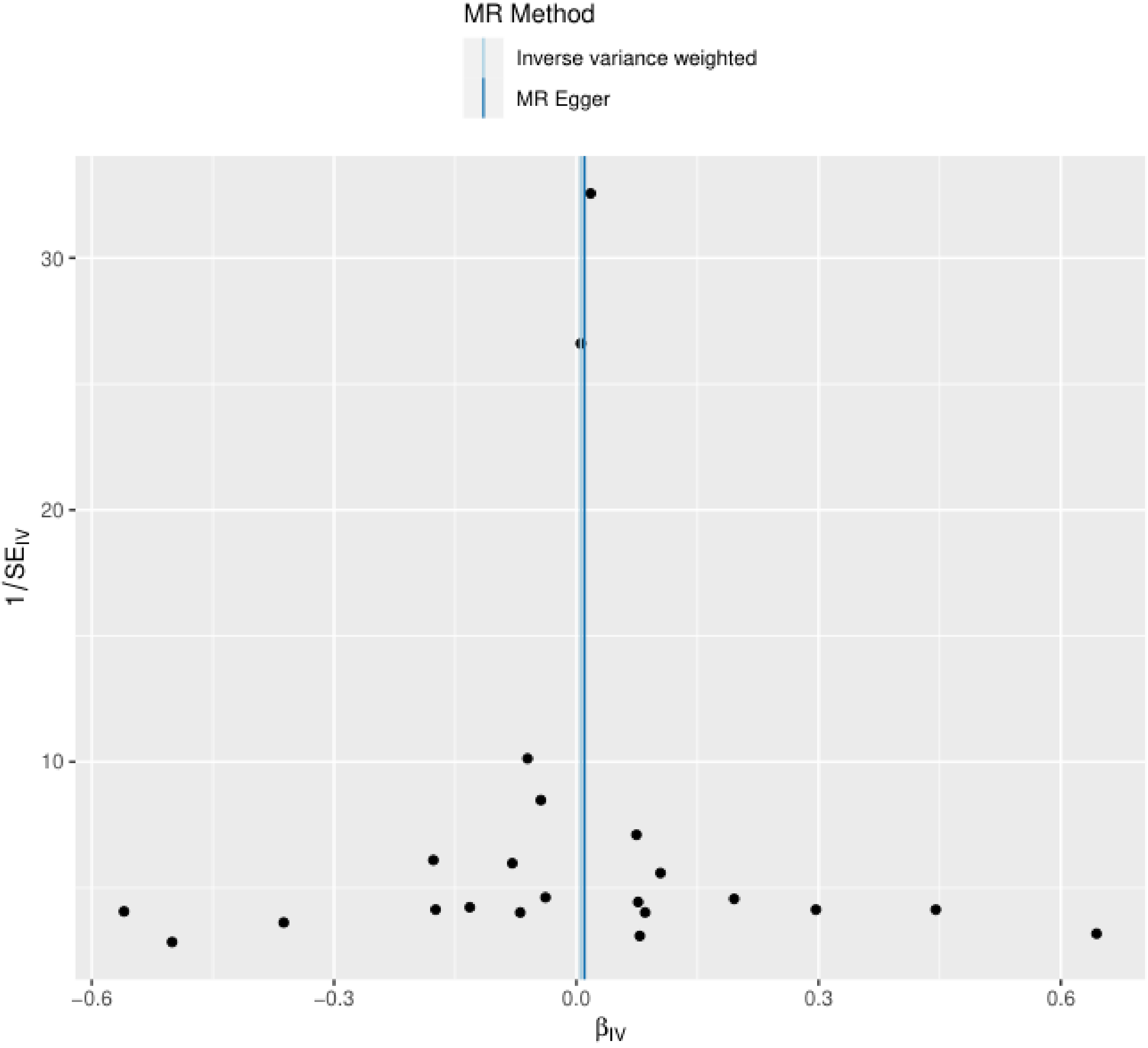

Funnel plot analyse of "CD25 on CD45RA+ CD4 not Treg " on 'Diabetic nephropathy'

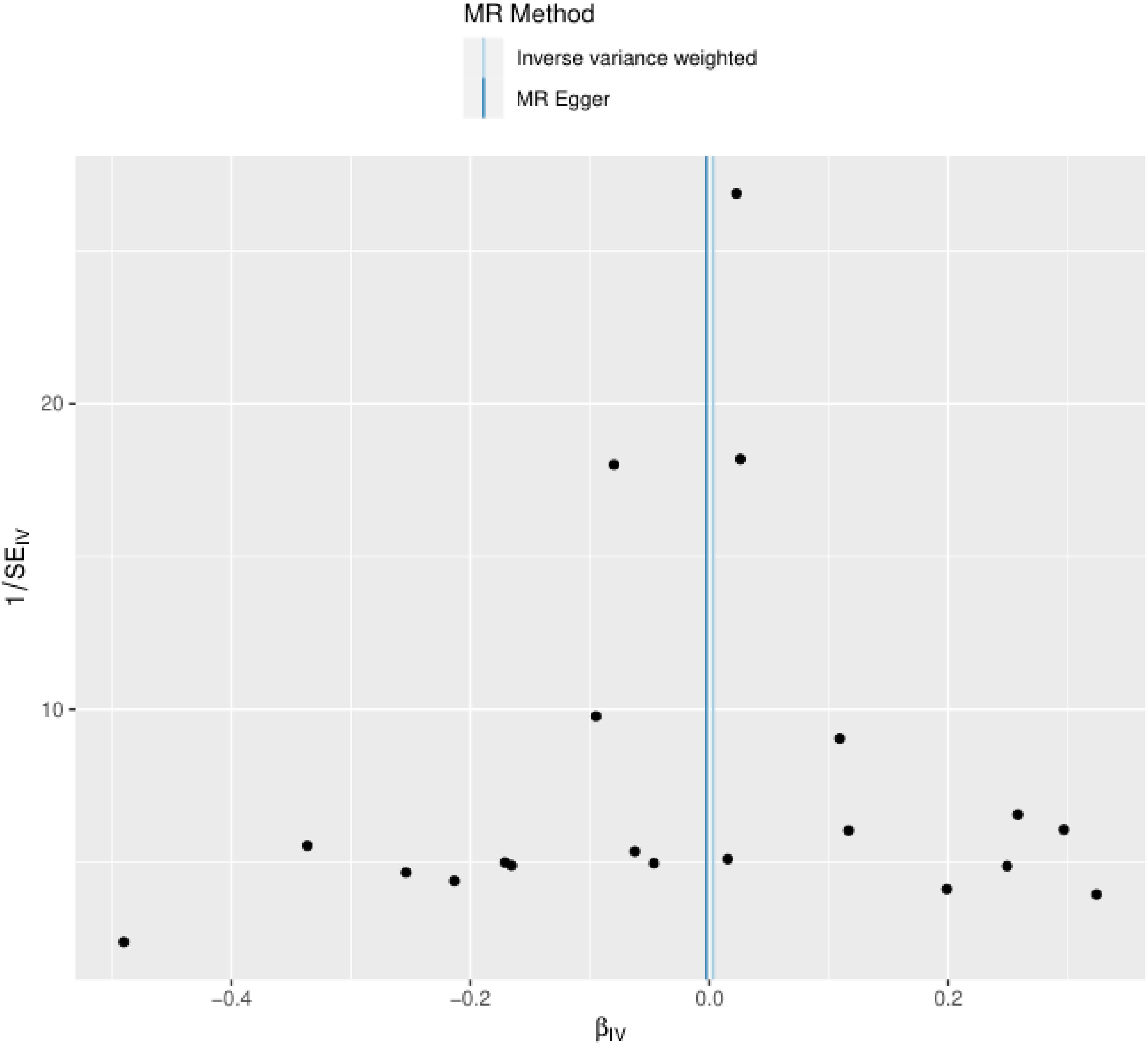

Funnel plot analyse of "CD80 on CD62L+ plasmacytoid DC" on 'Diabetic nephropathy'

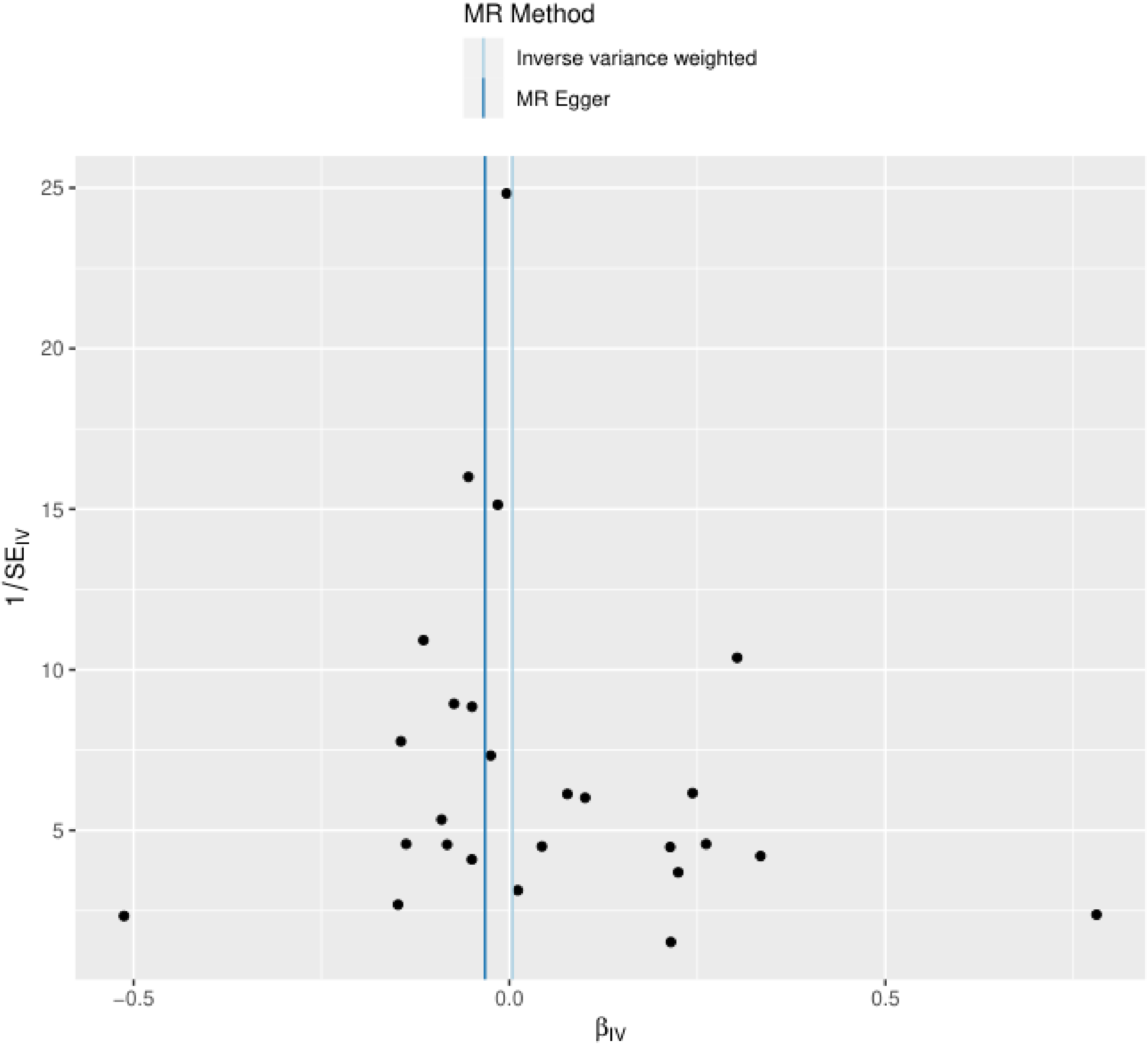

Funnel plot analyse of "HLA DR+ T cell%lymphocyte" on 'Diabetic nephropathy'

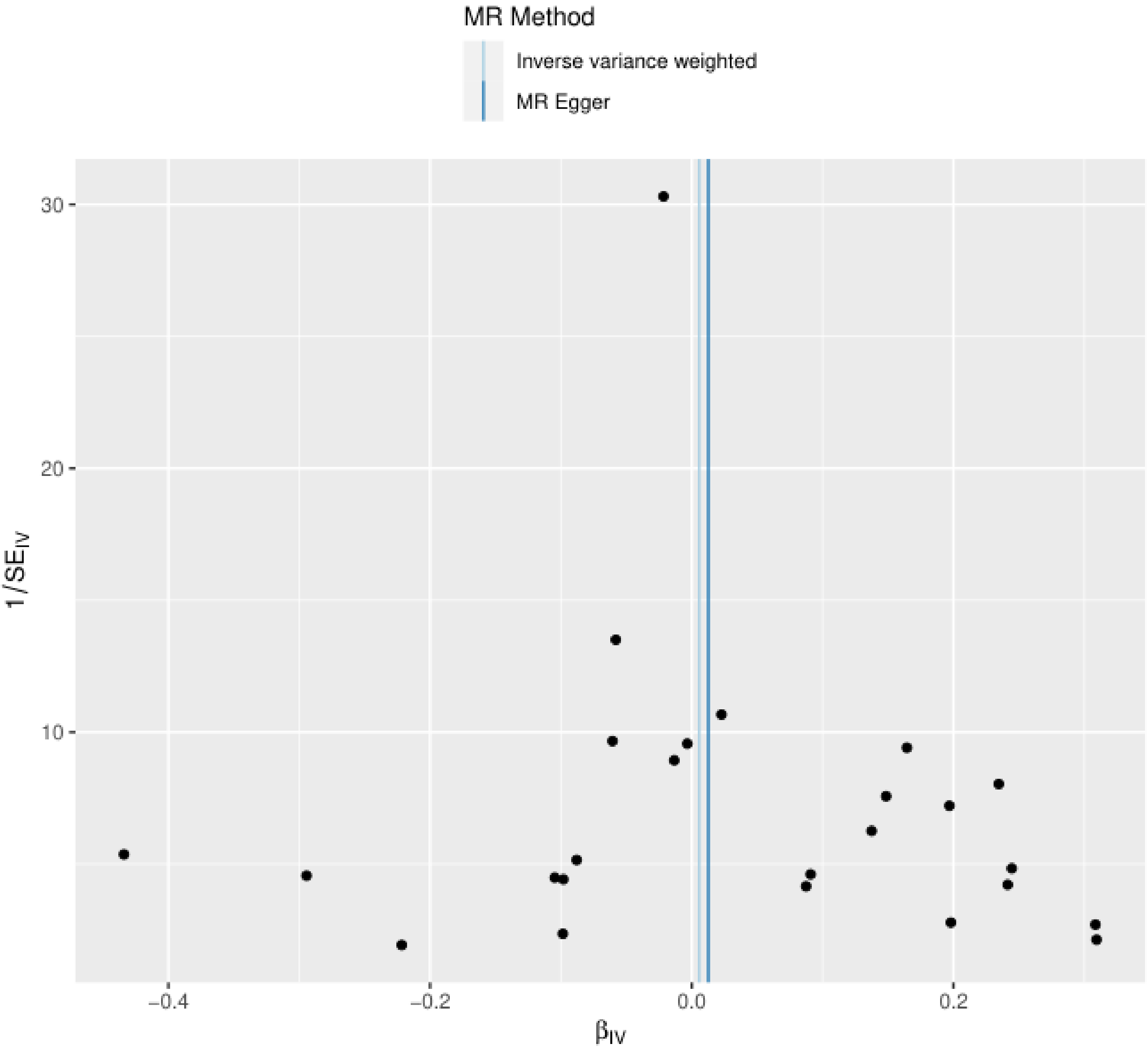

Funnel plot analyse of "BAFF-R on transitional" on 'Diabetic nephropathy'

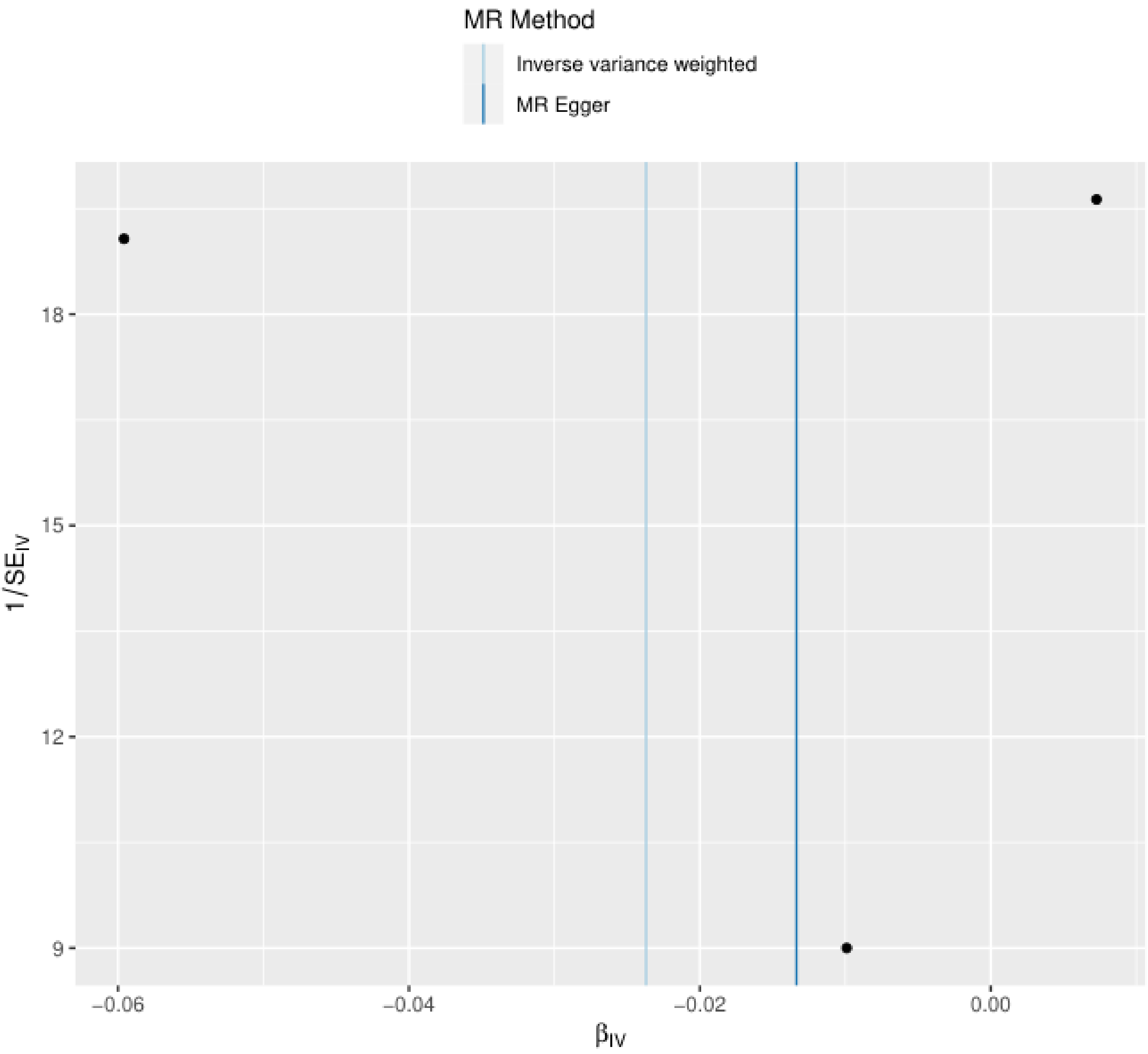

Funnel plot analyse of "CD11b on Gr MDSC " on 'Diabetic nephropathy'

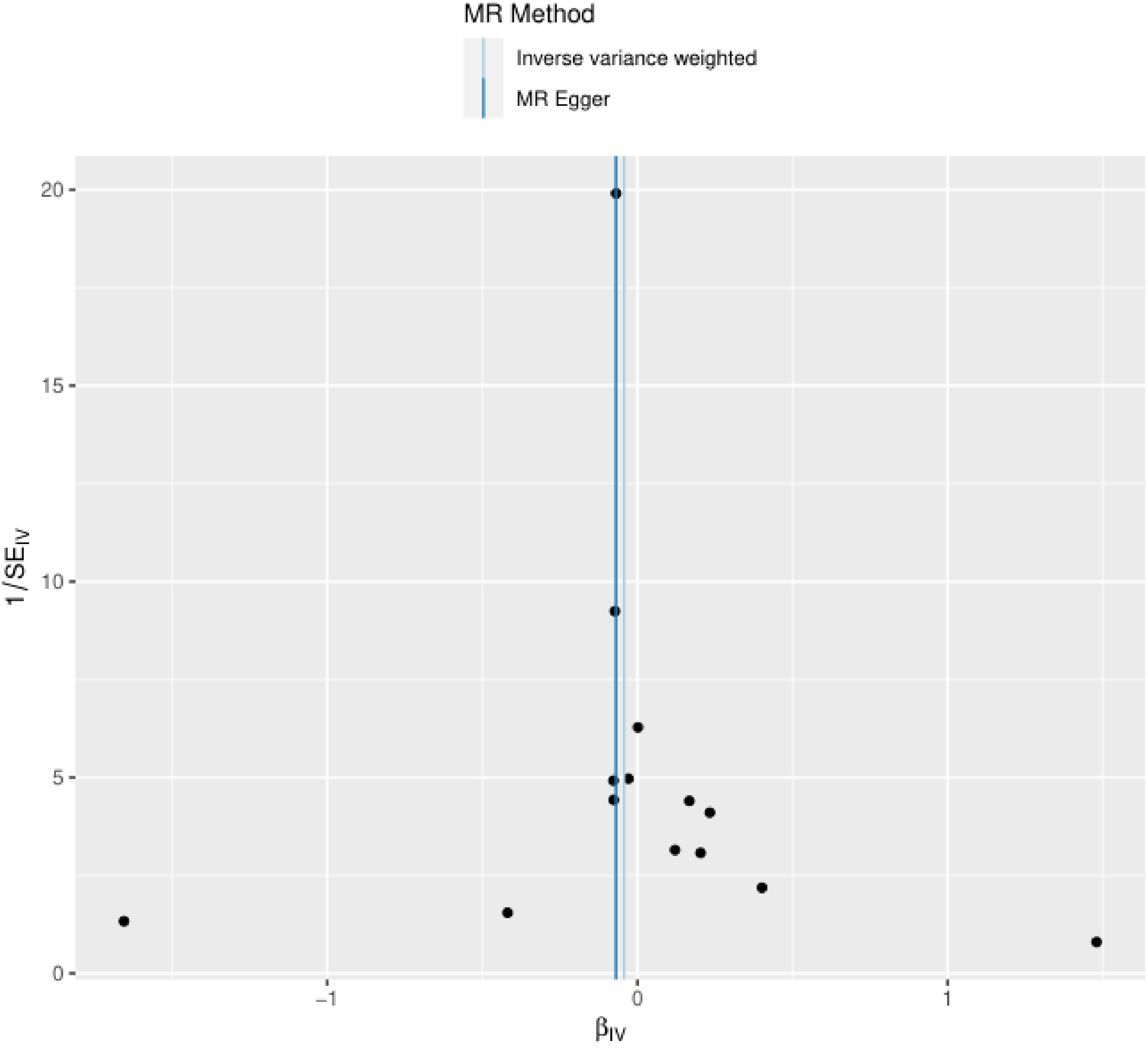

Funnel plot analyse of "BAFF-R on CD20-" on 'Diabetic nephropathy'

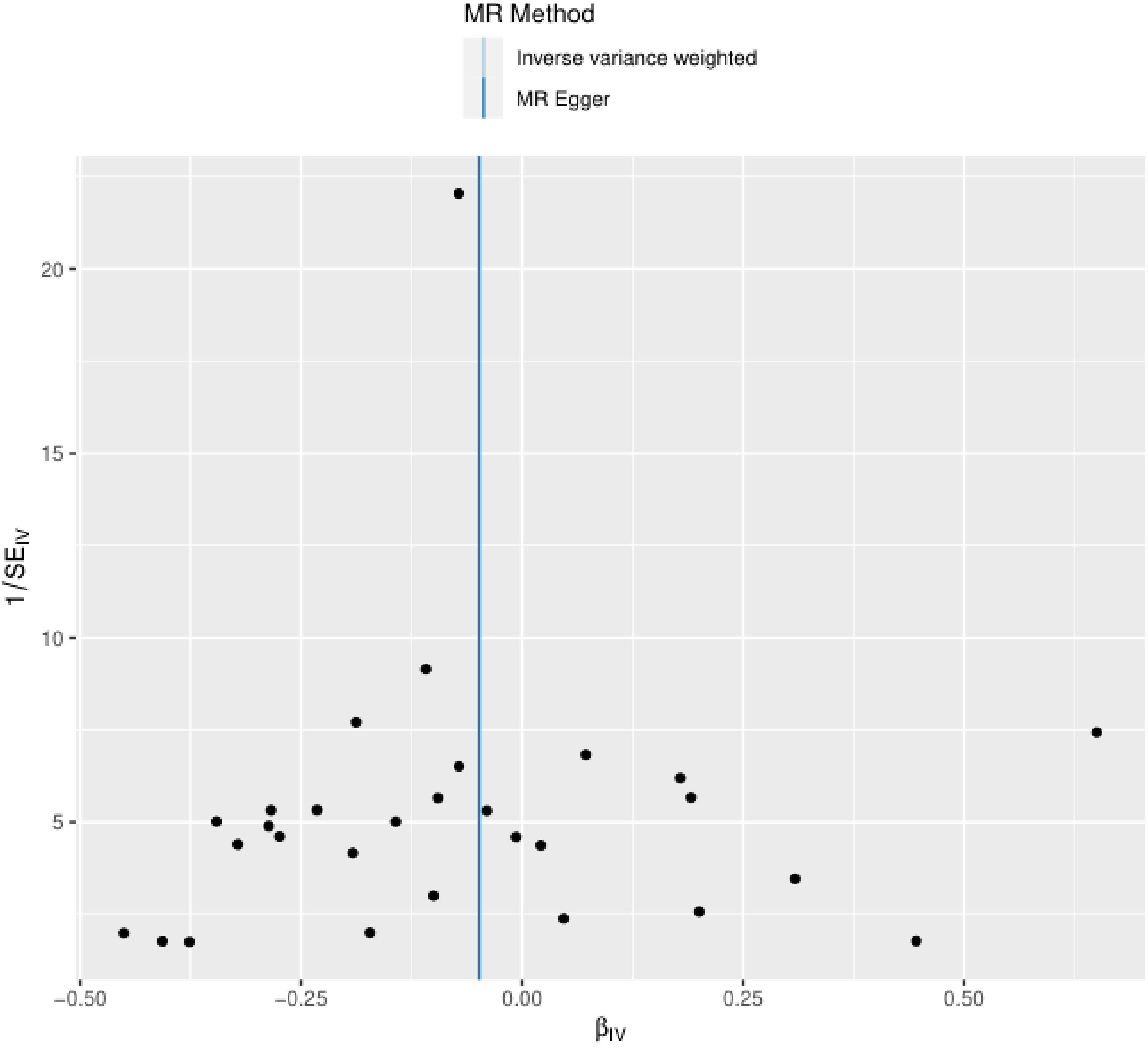

Funnel plot analyse of "CD20 on IgD+ CD38dim" on 'Diabetic nephropathy'

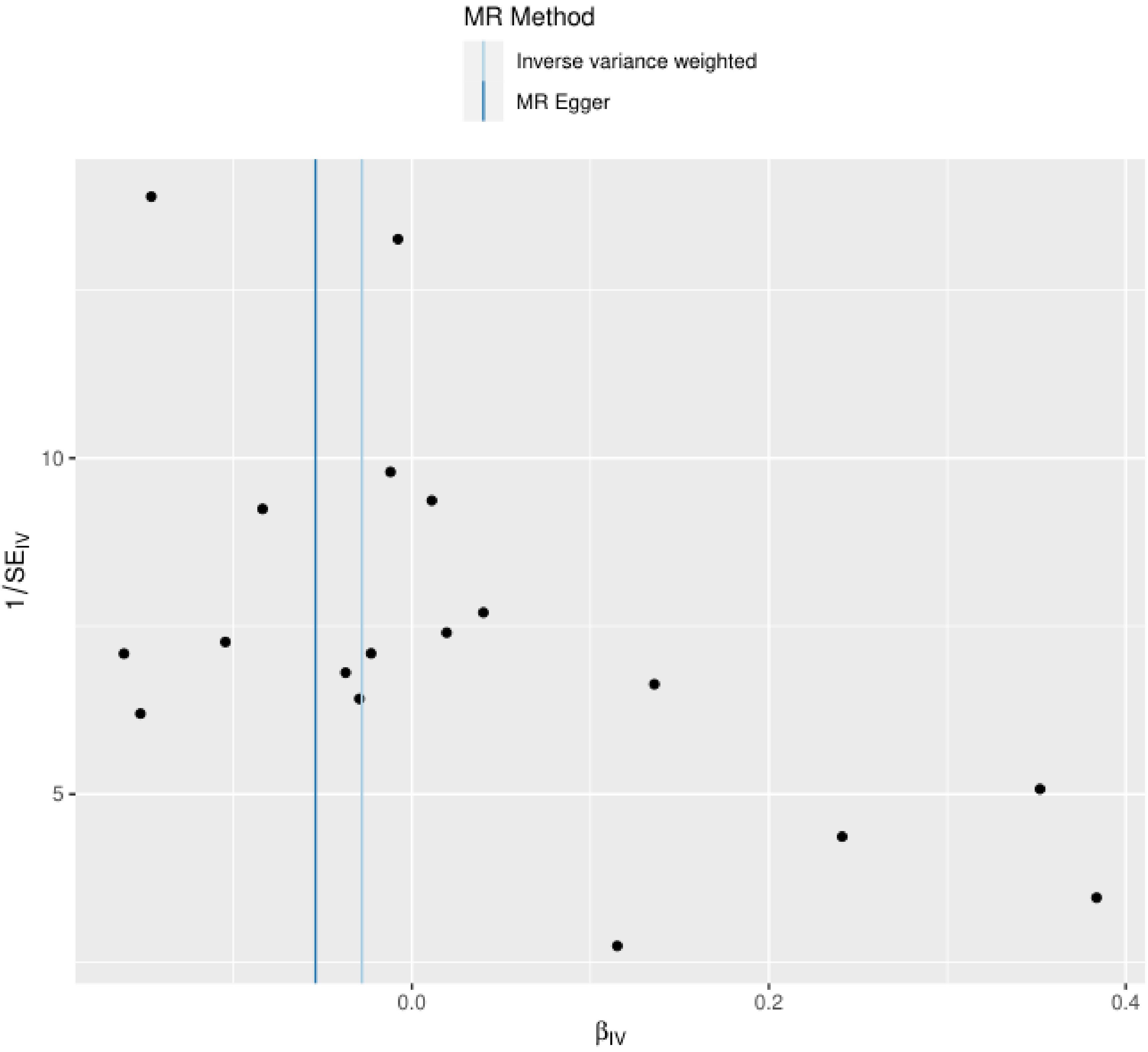

Funnel plot analyse of "CD45 on basophil" on 'Diabetic nephropathy'

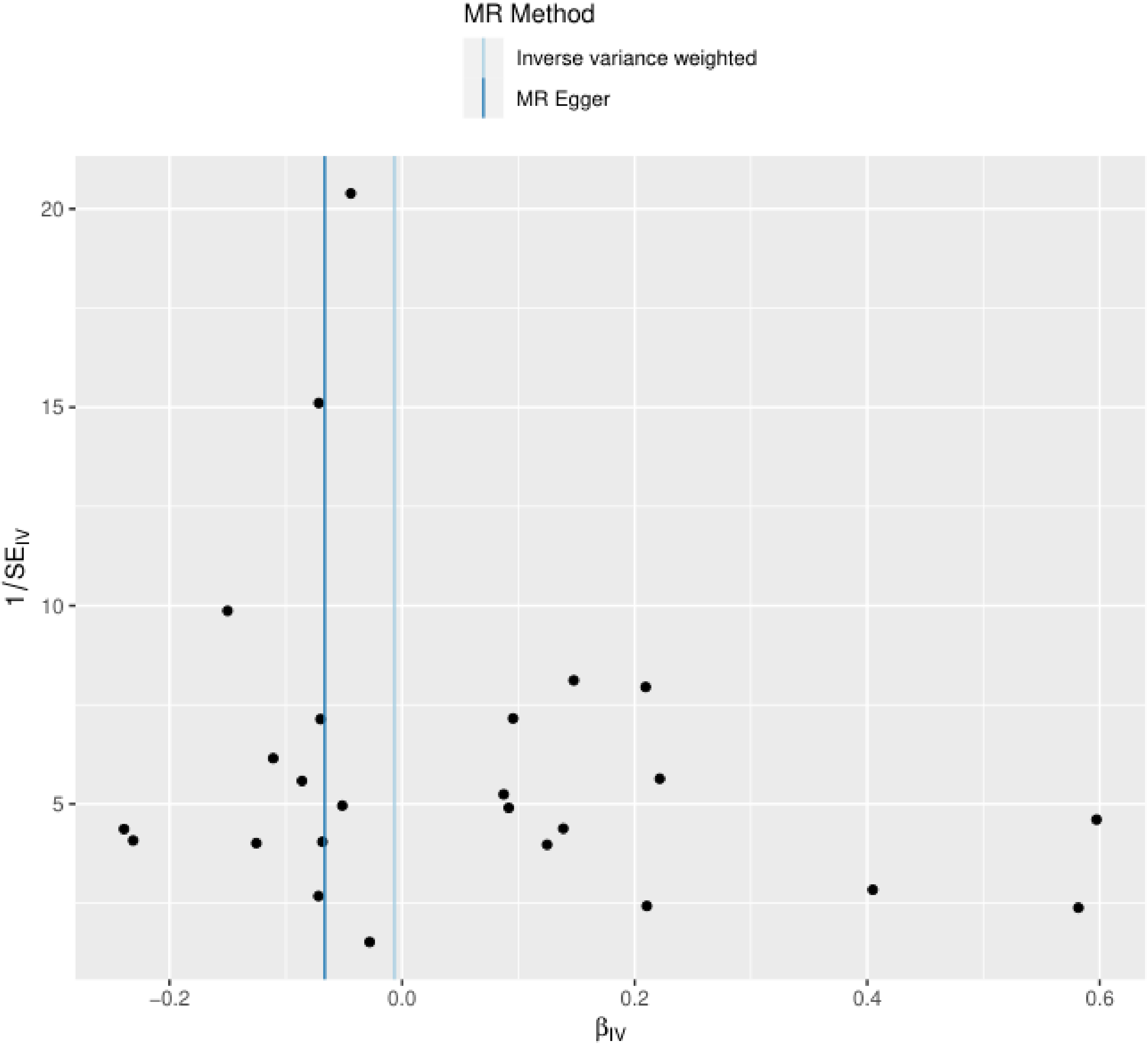

Funnel plot analyse of "Activated & resting Treg %CD4+" on 'Diabetic nephropathy'

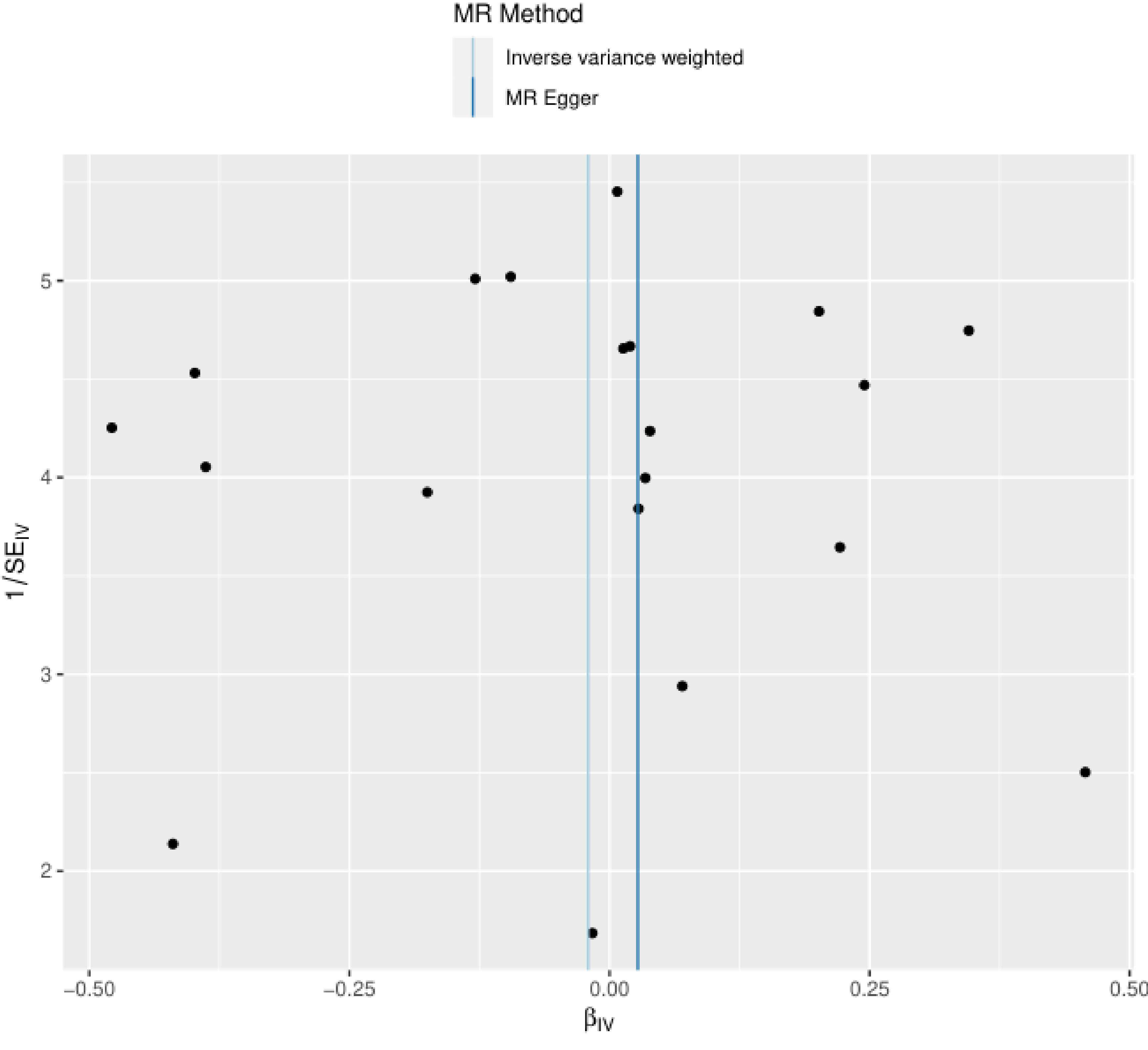

Funnel plot analyse of "CD38 on CD20-" on 'Diabetic nephropathy'

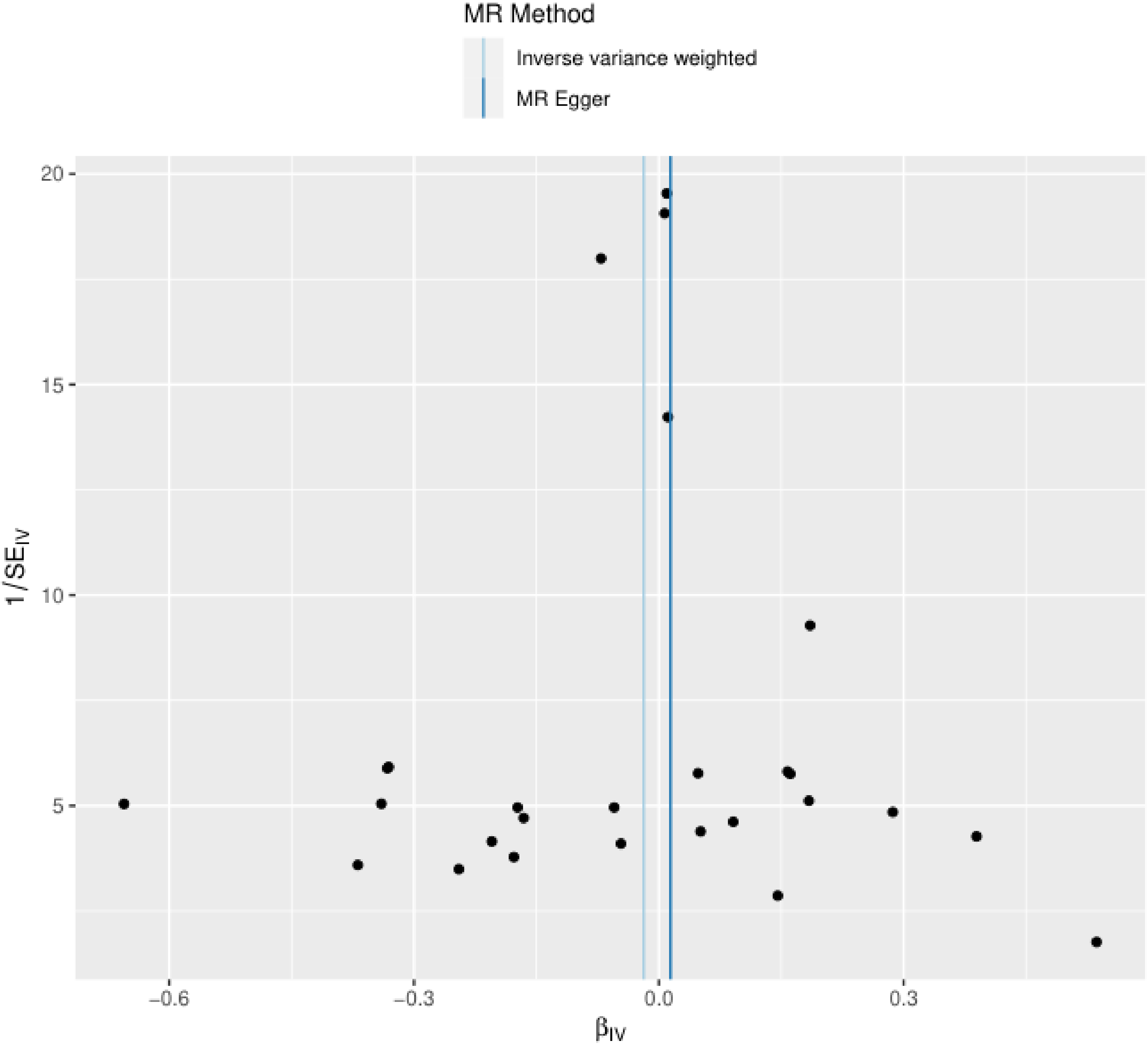

Funnel plot analyse of ''Naive-mature B cell %B cell'' on 'Diabetic nephropathy'

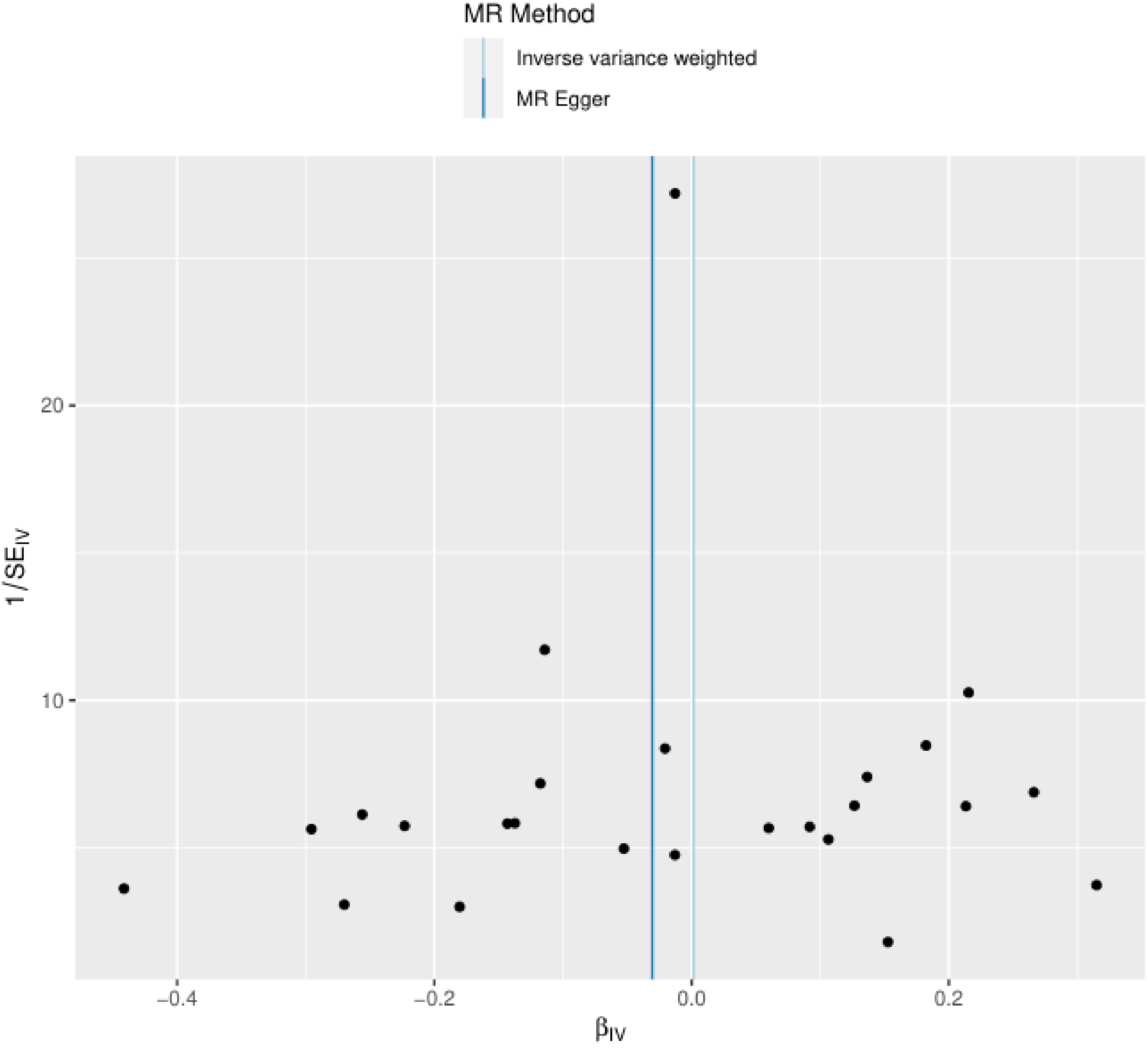

Funnel plot analyse of "CD66b on CD66b++ myeloid cell" on 'Diabetic nephropathy'

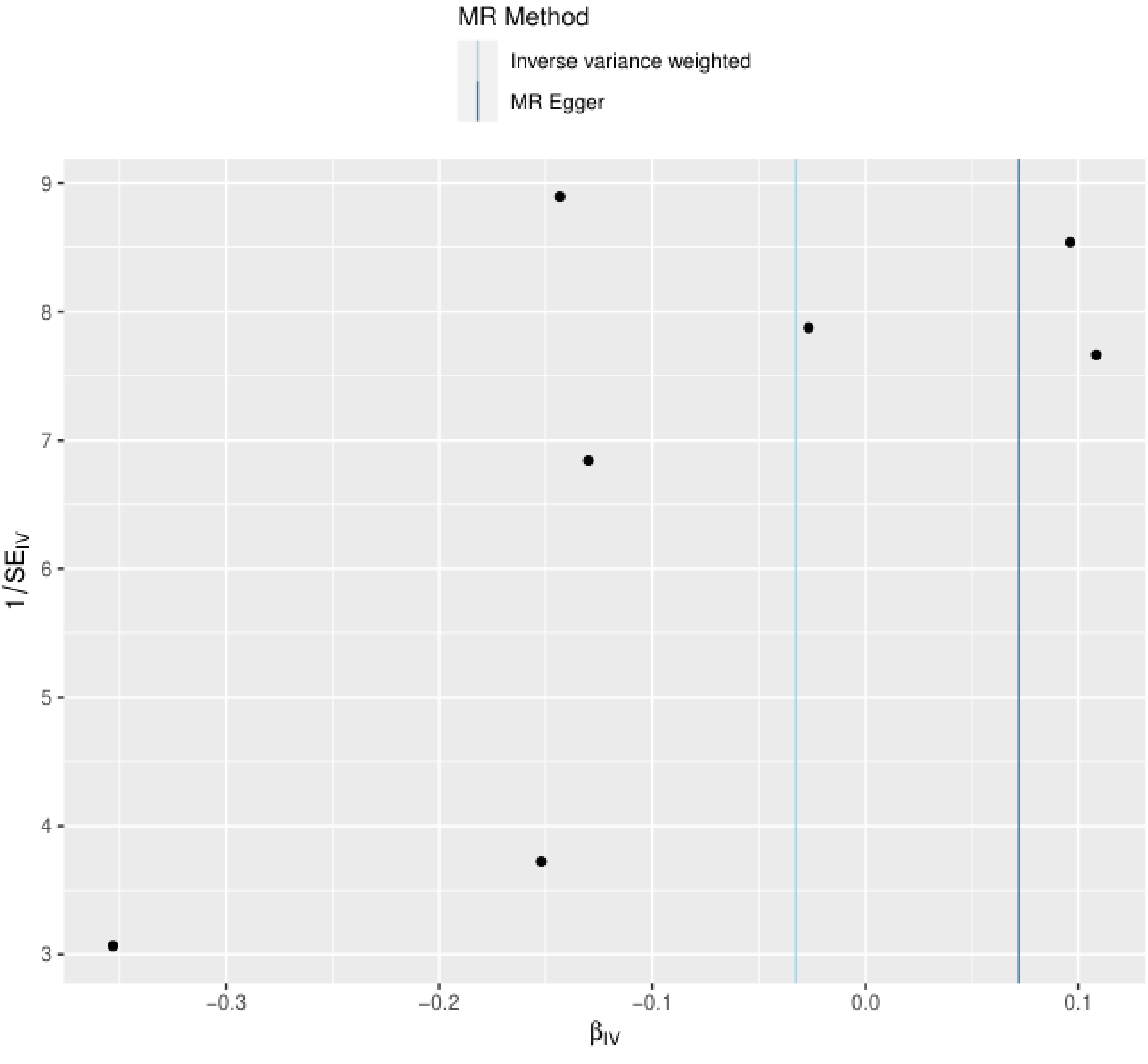

Funnel plot analyse of "CD39+ activated Treg %activated Treg" on 'Diabetic nephropathy'

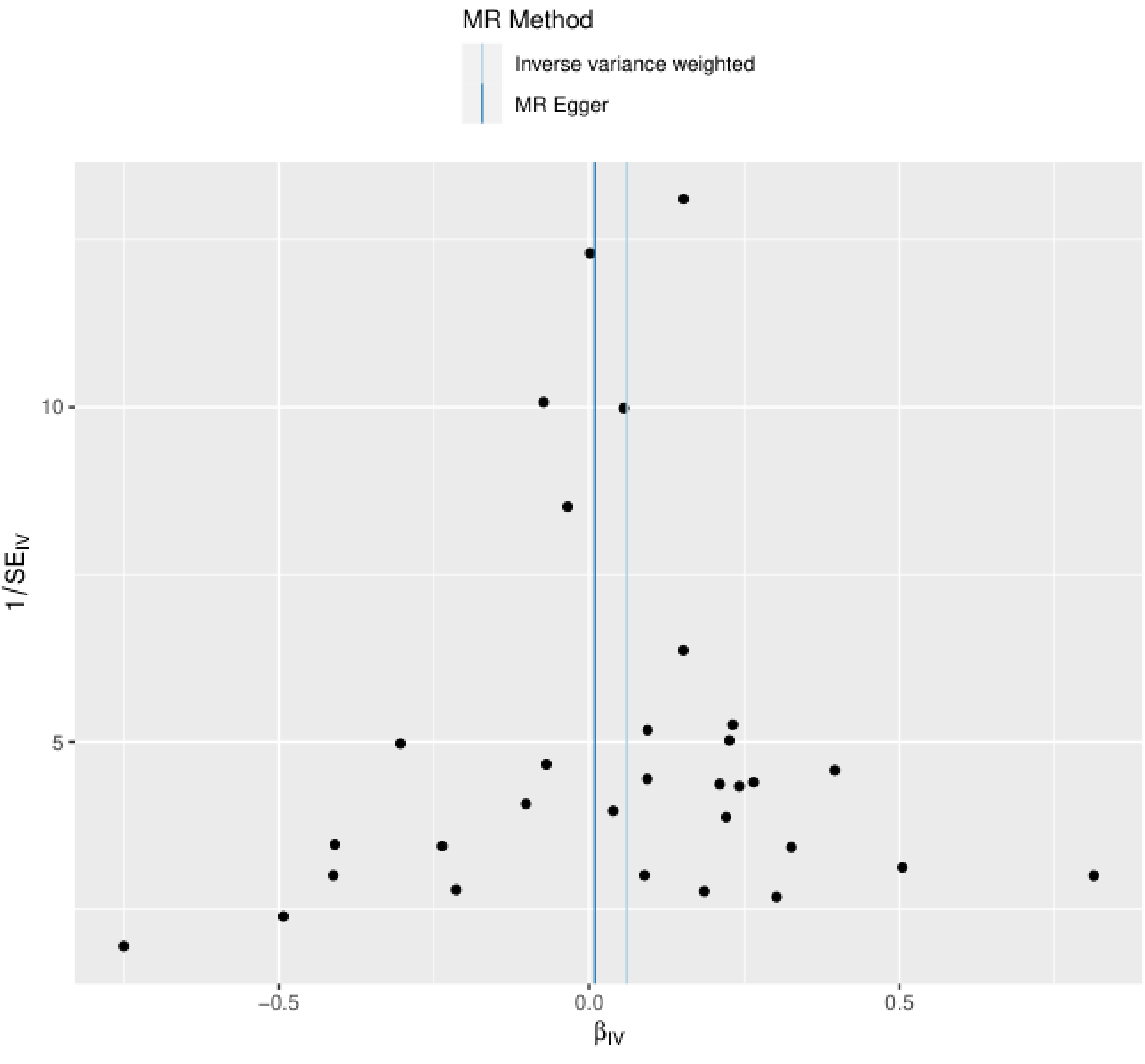

Funnel plot analyse of "CD20 on CD24+ CD27+" on 'Diabetic nephropathy'

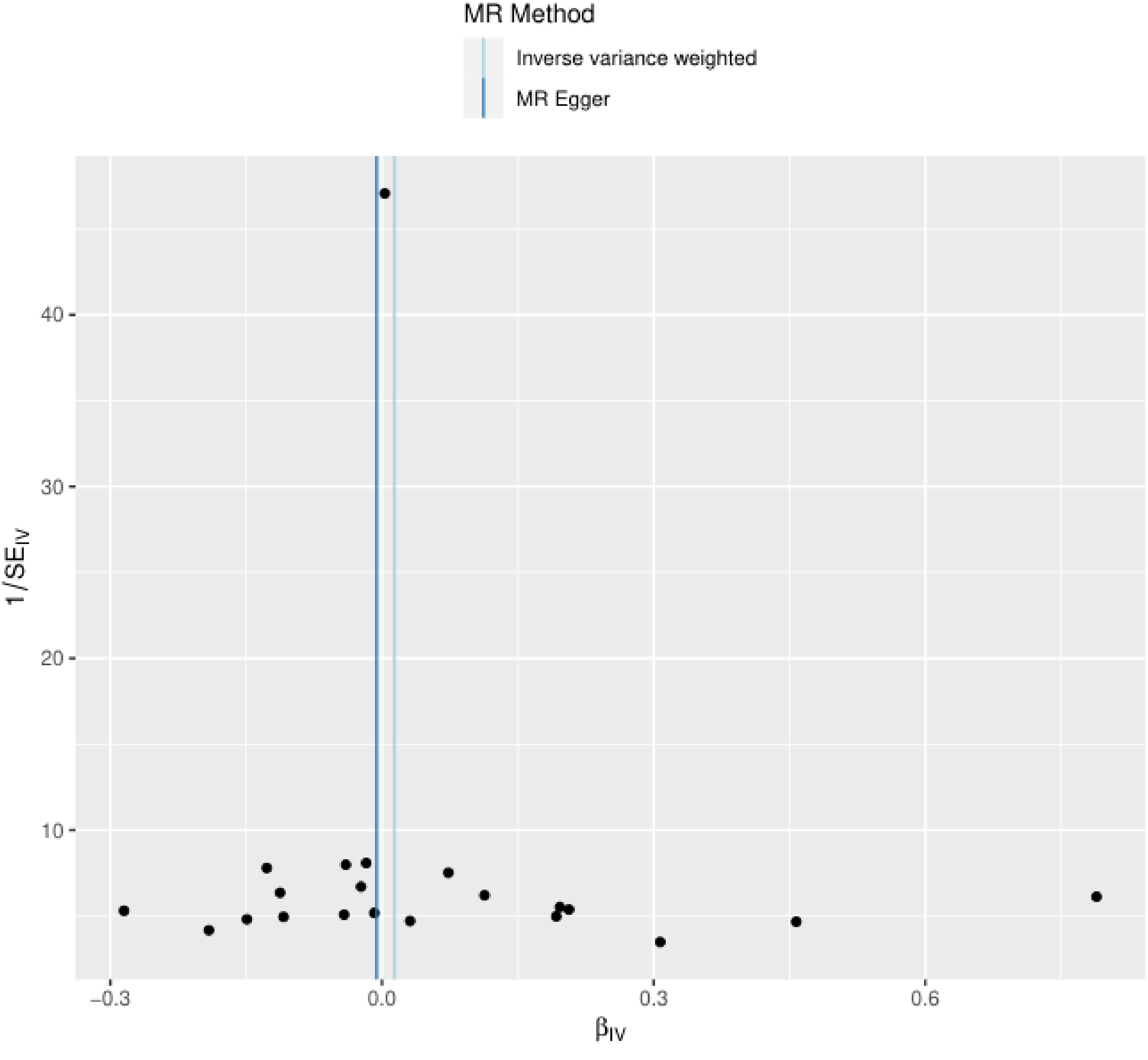

Funnel plot analyse of "CD20 on IgD+" on 'Diabetic nephropathy'

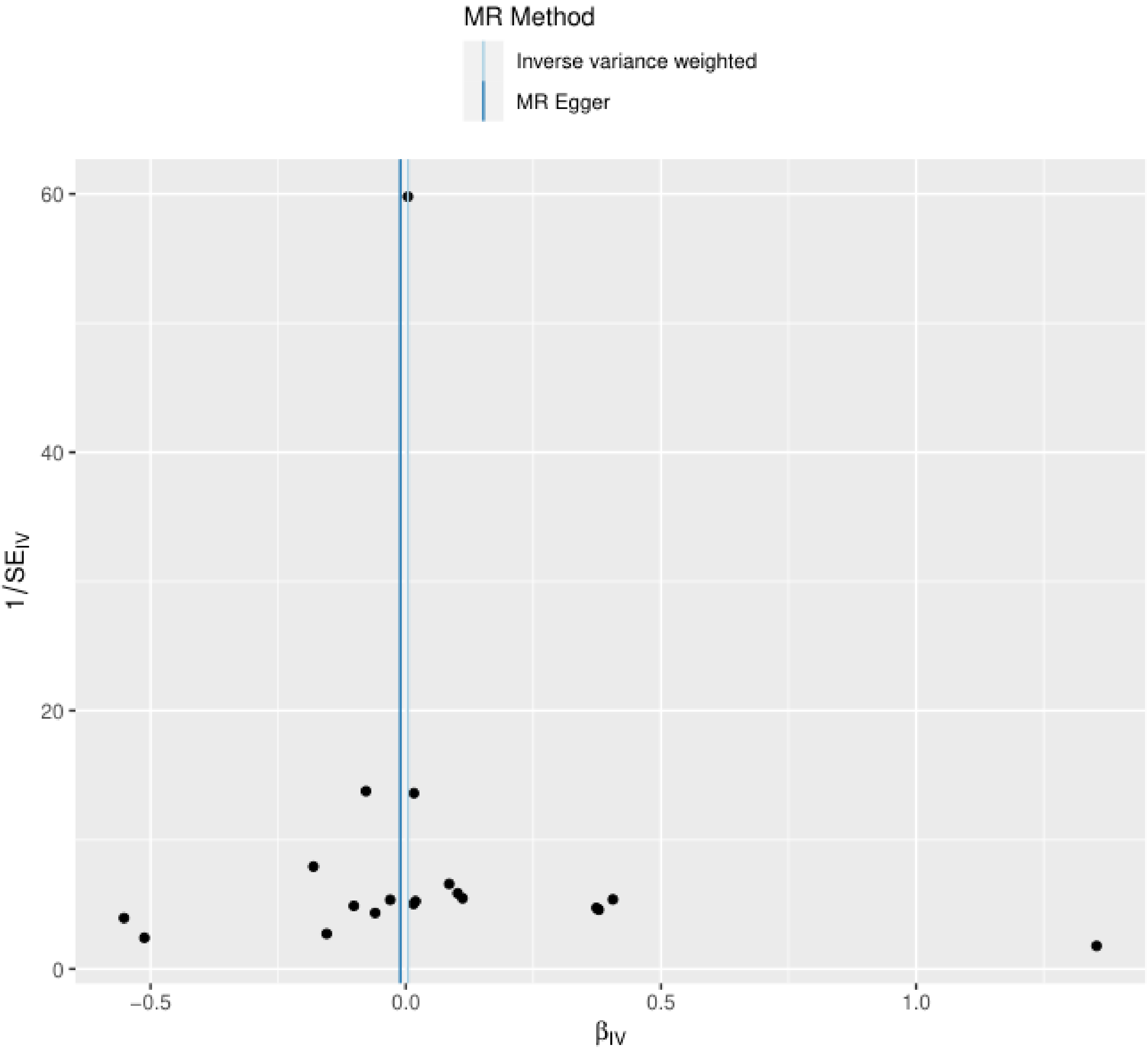

Funnel plot analyse of "SSC-A on myeloid DC " on 'Diabetic nephropathy'

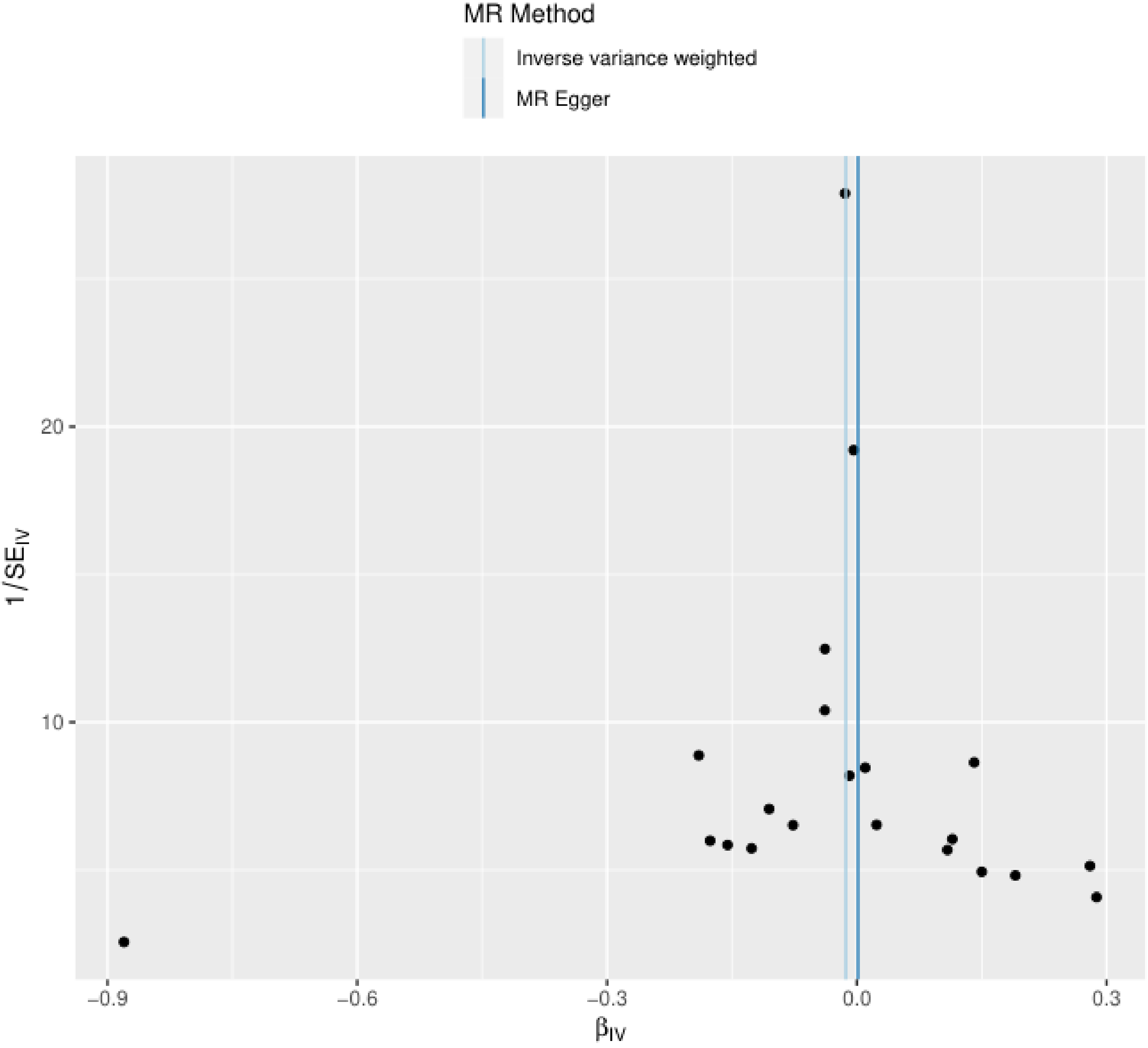

Funnel plot analyse of "CD24 on IgD+ CD38- unsw mem" on 'Diabetic nephropathy'

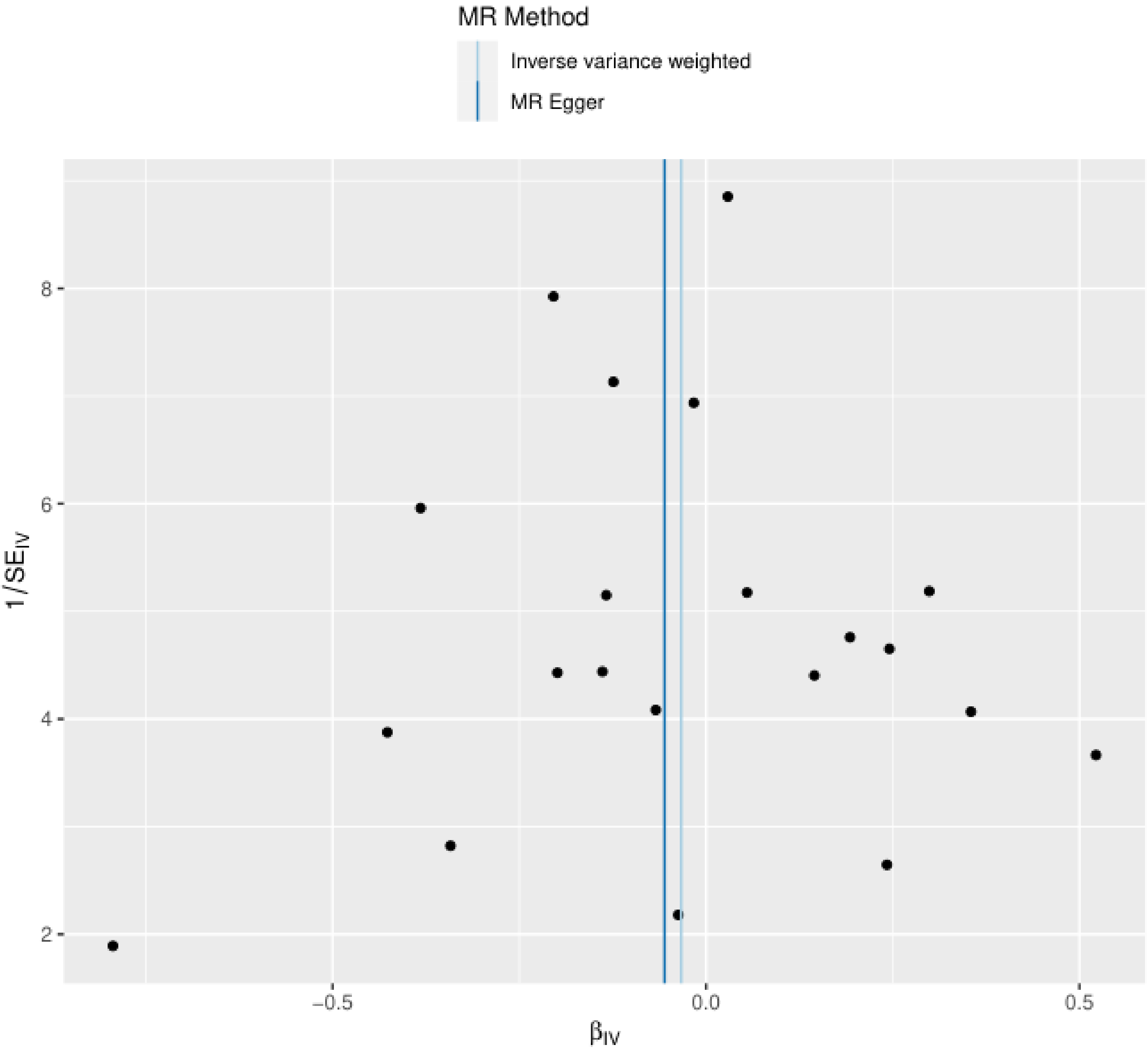

Funnel plot analyse of "CD25 on IgD- CD27-" on 'Diabetic nephropathy'

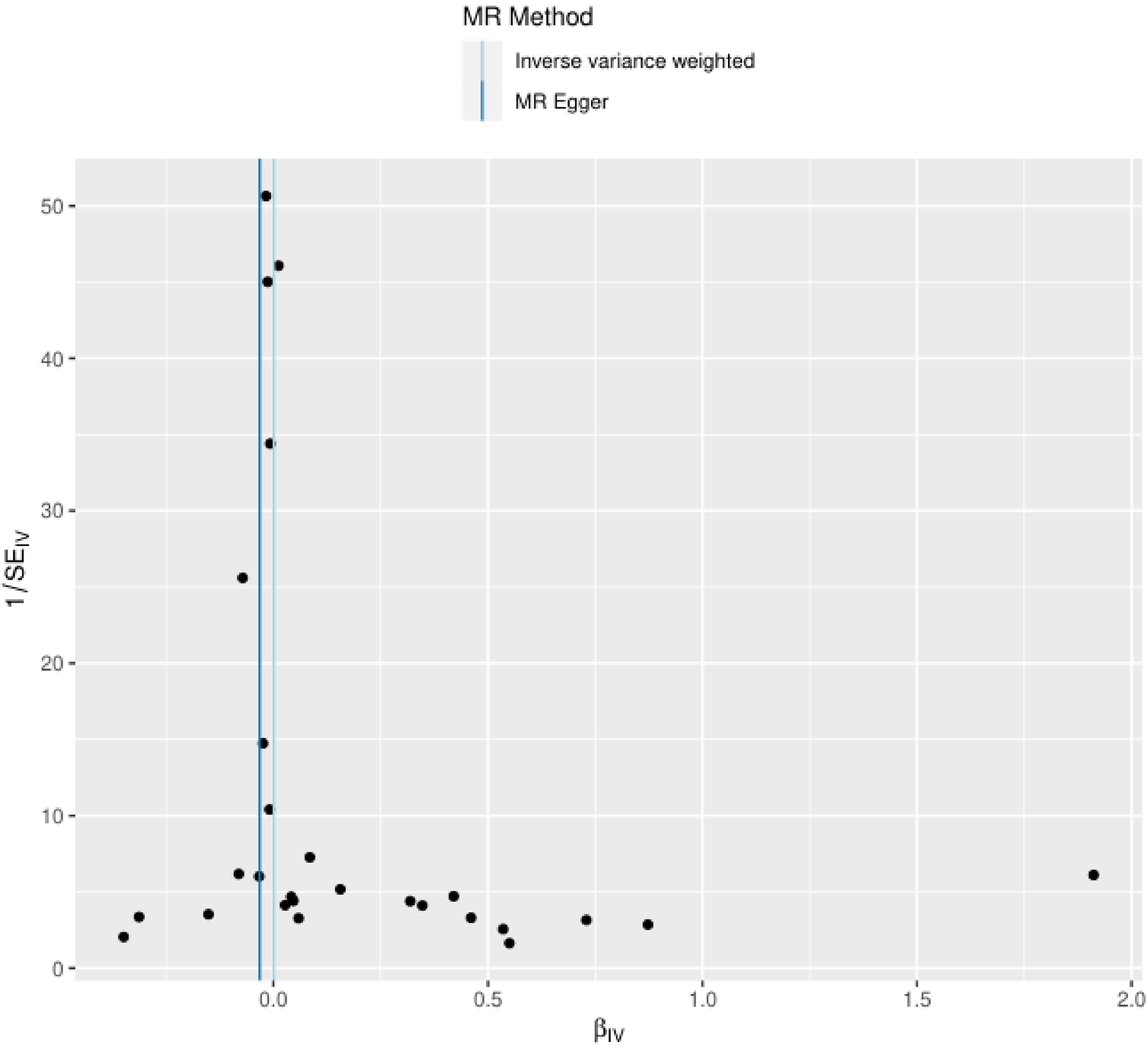

Funnel plot analyse of "CD45RA+ CD8br AC" on 'Diabetic nephropathy'

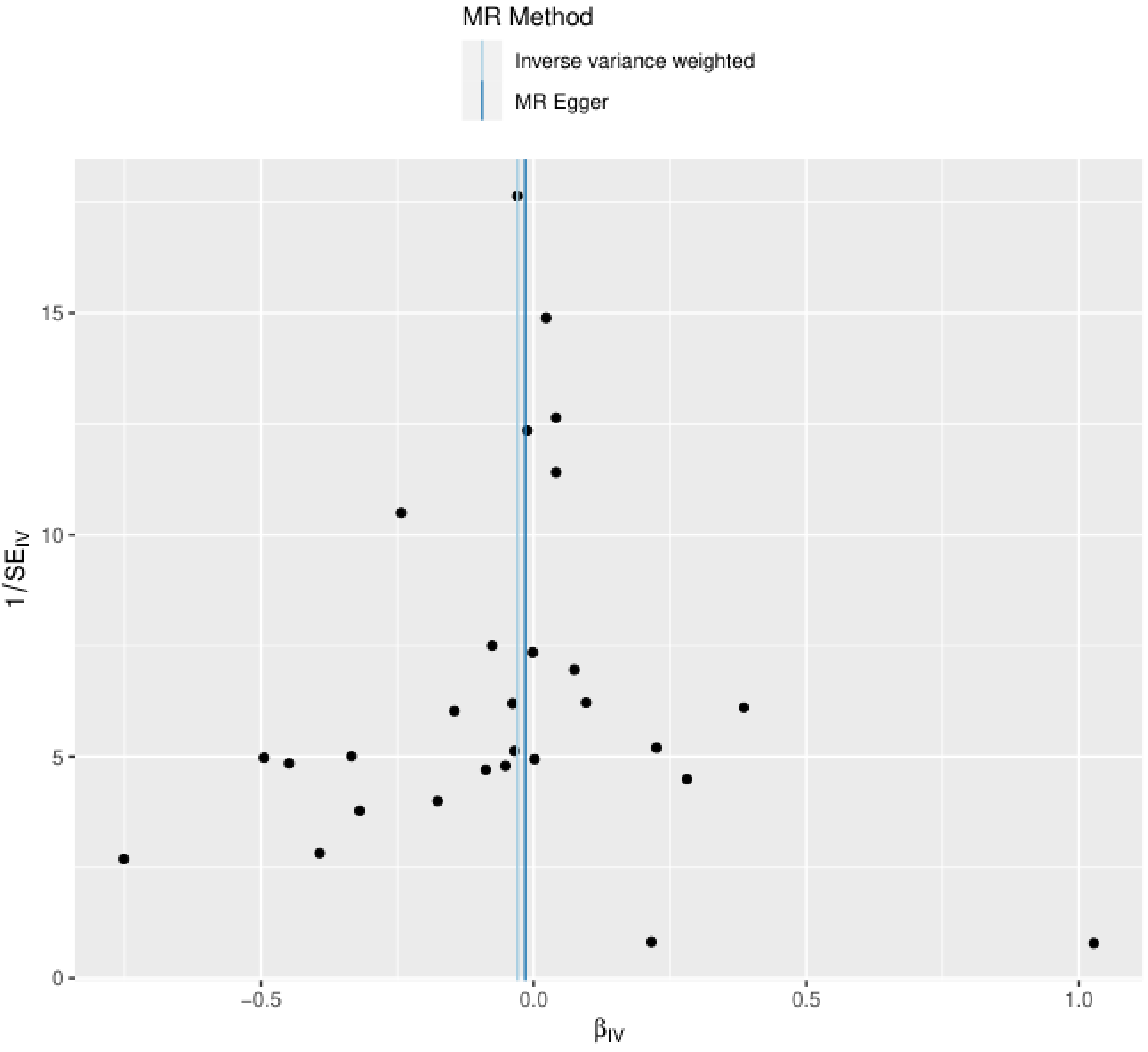

Funnel plot analyse of "HLA DR on HLA DR+ NK " on 'Diabetic nephropathy'

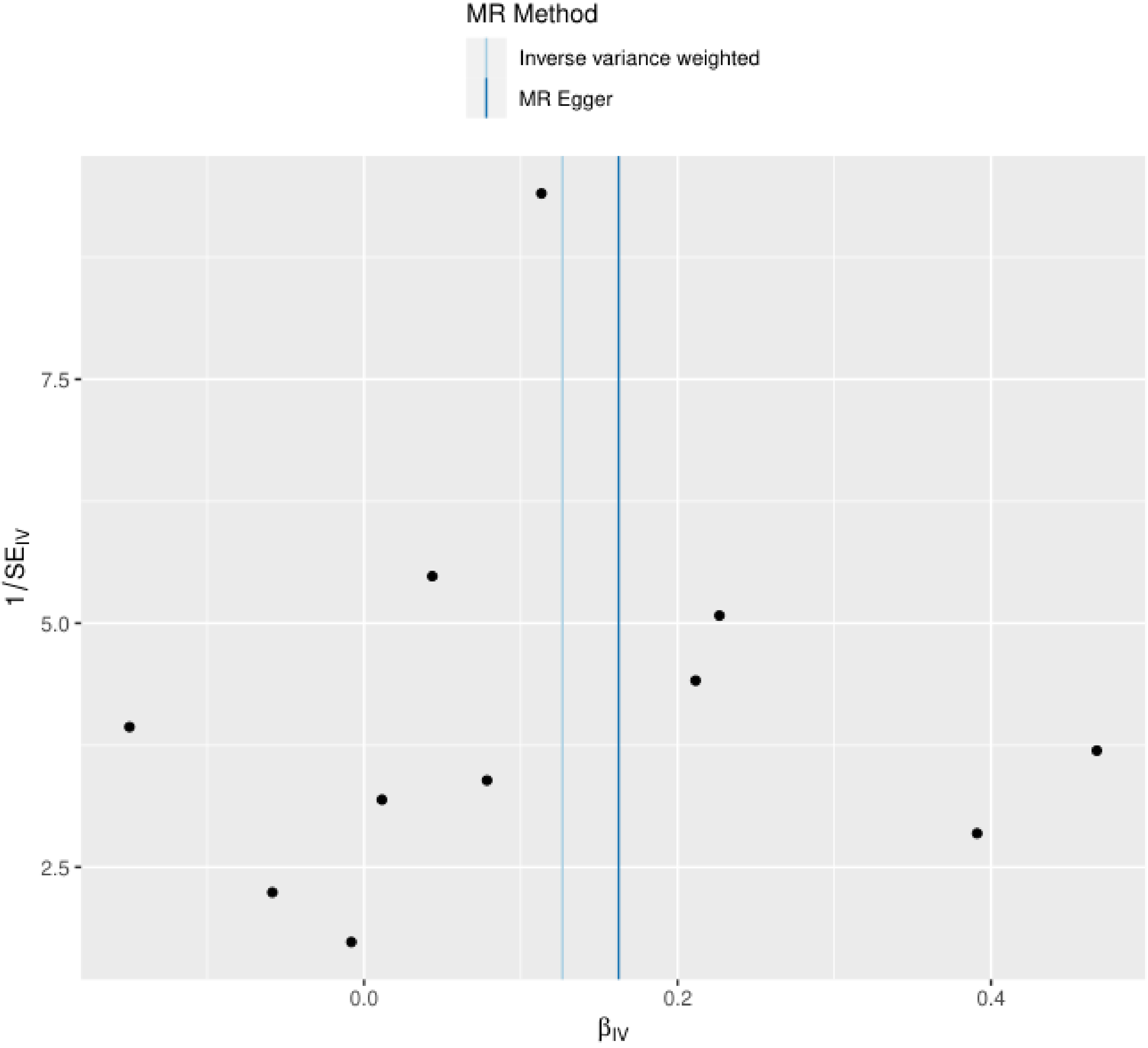

Funnel plot analyse of "CD28+ DN (CD4-CD8-) AC" on 'Diabetic nephropathy'

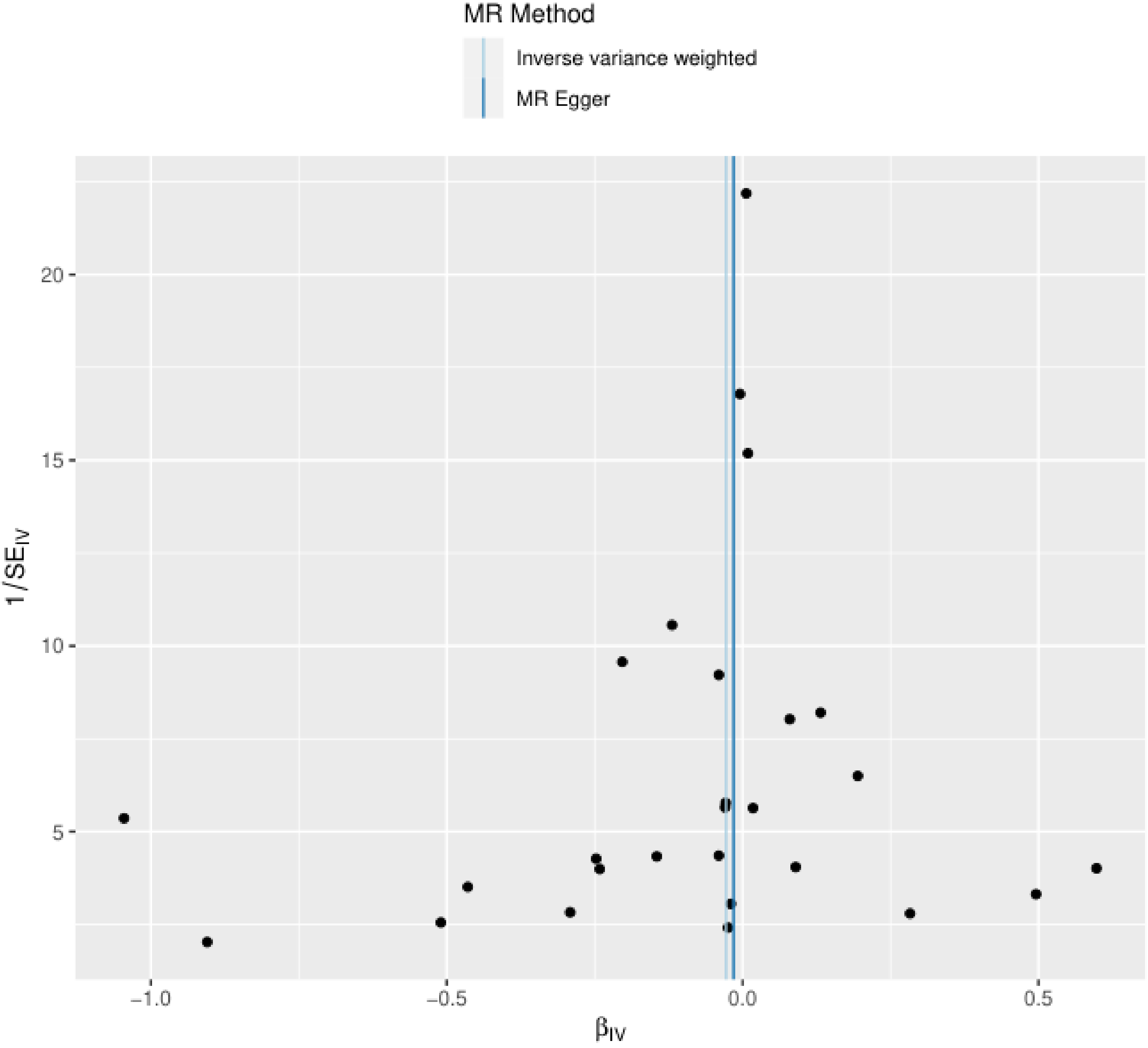

Funnel plot analyse of "IgD on IgD+ CD24+" on 'Diabetic nephropathy'

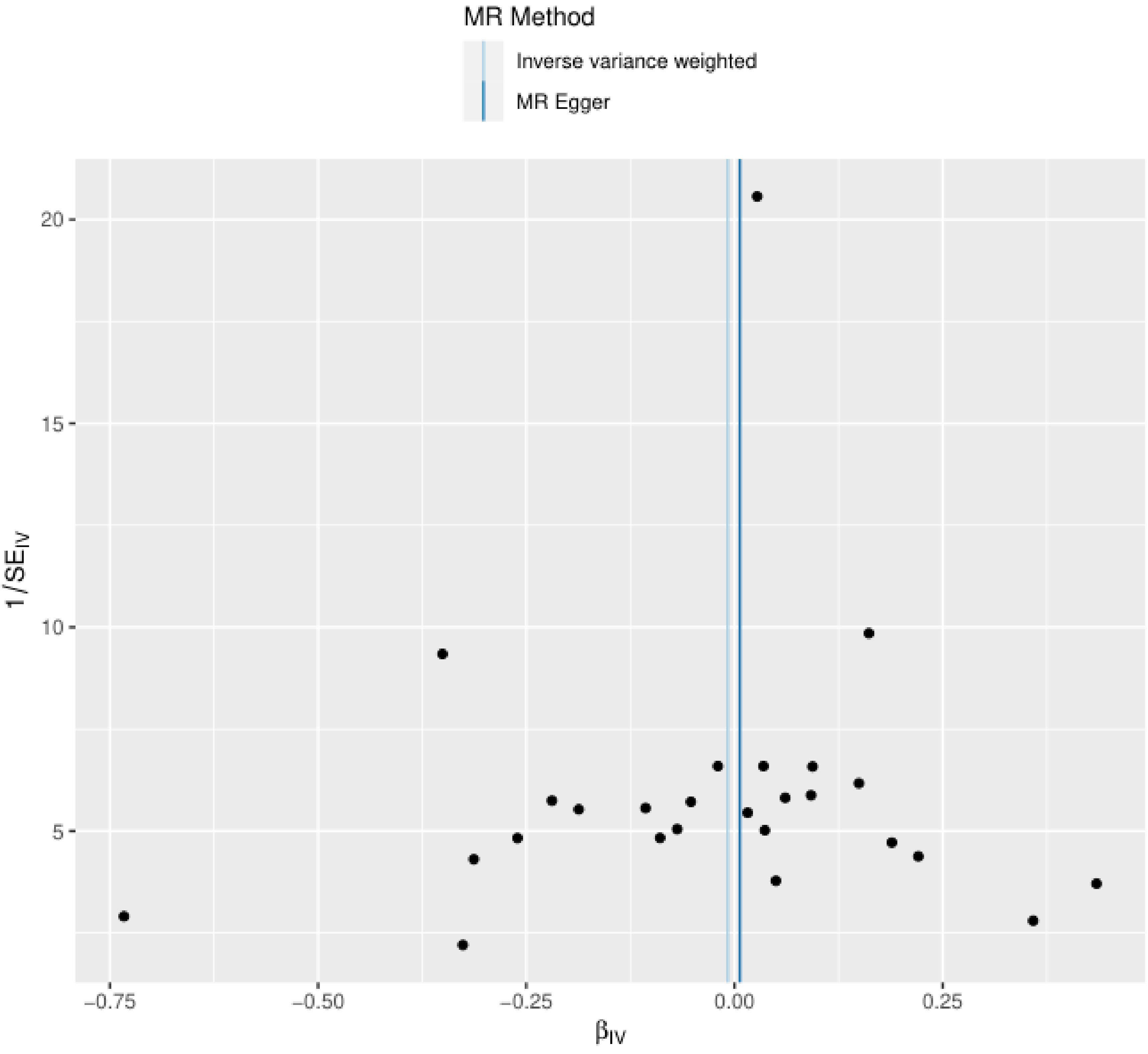

Funnel plot analyse of "CD39 on granulocyte " on 'Diabetic nephropathy'

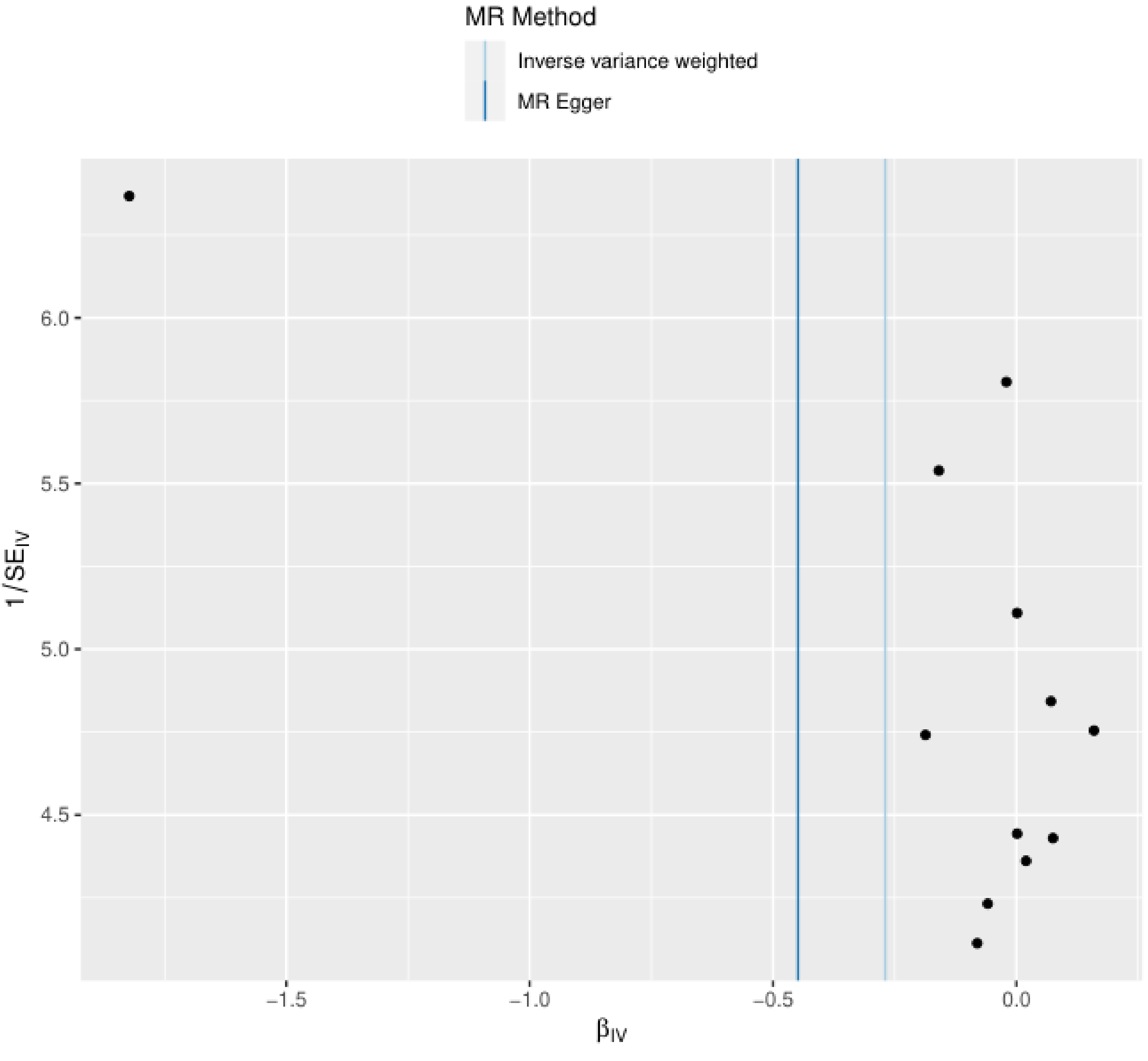

Funnel plot analyse of "CD45 on CD4+" on 'Diabetic nephropathy'

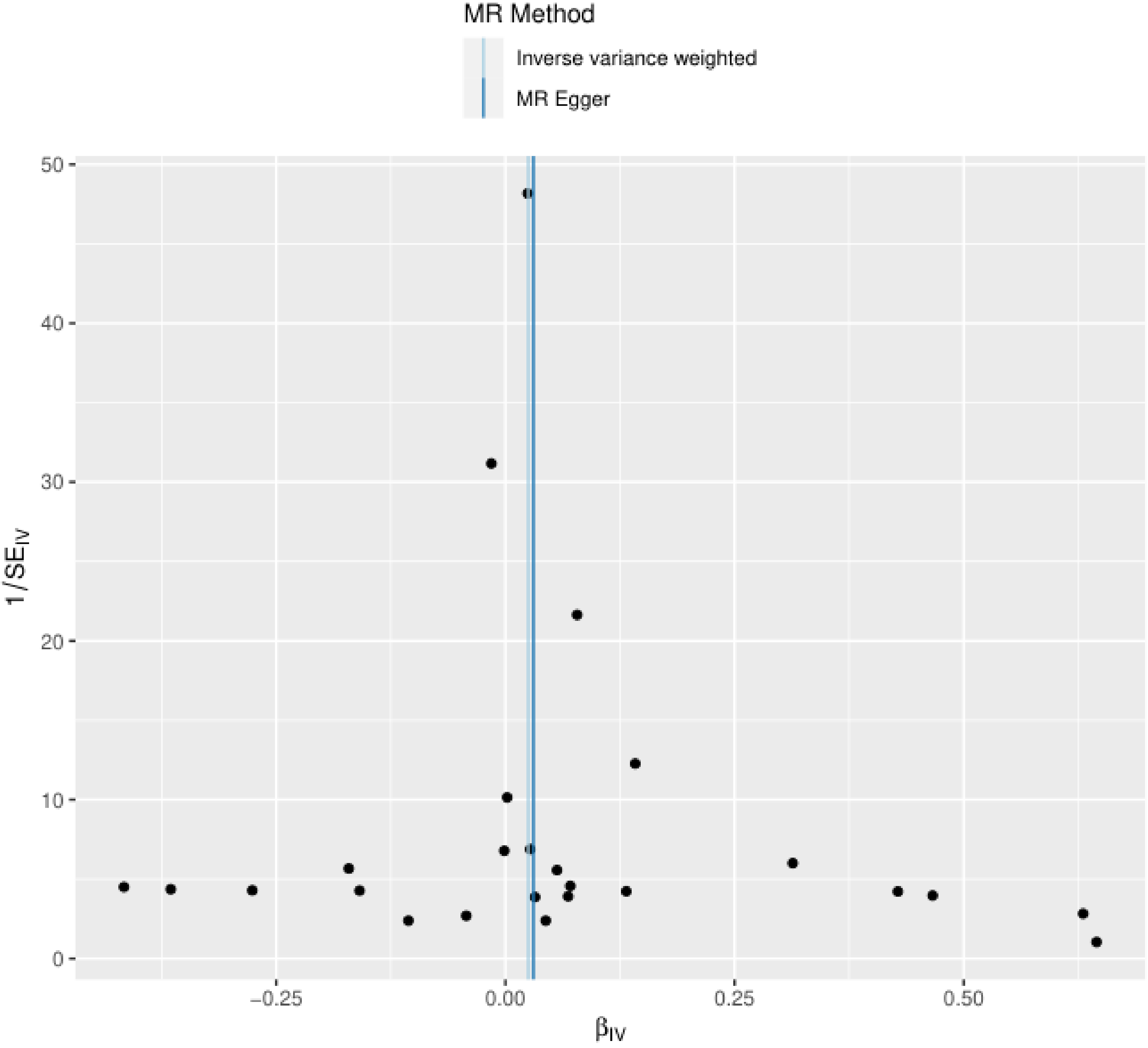

Funnel plot analyse of "IgD+ CD38dim AC" on 'Diabetic nephropathy'

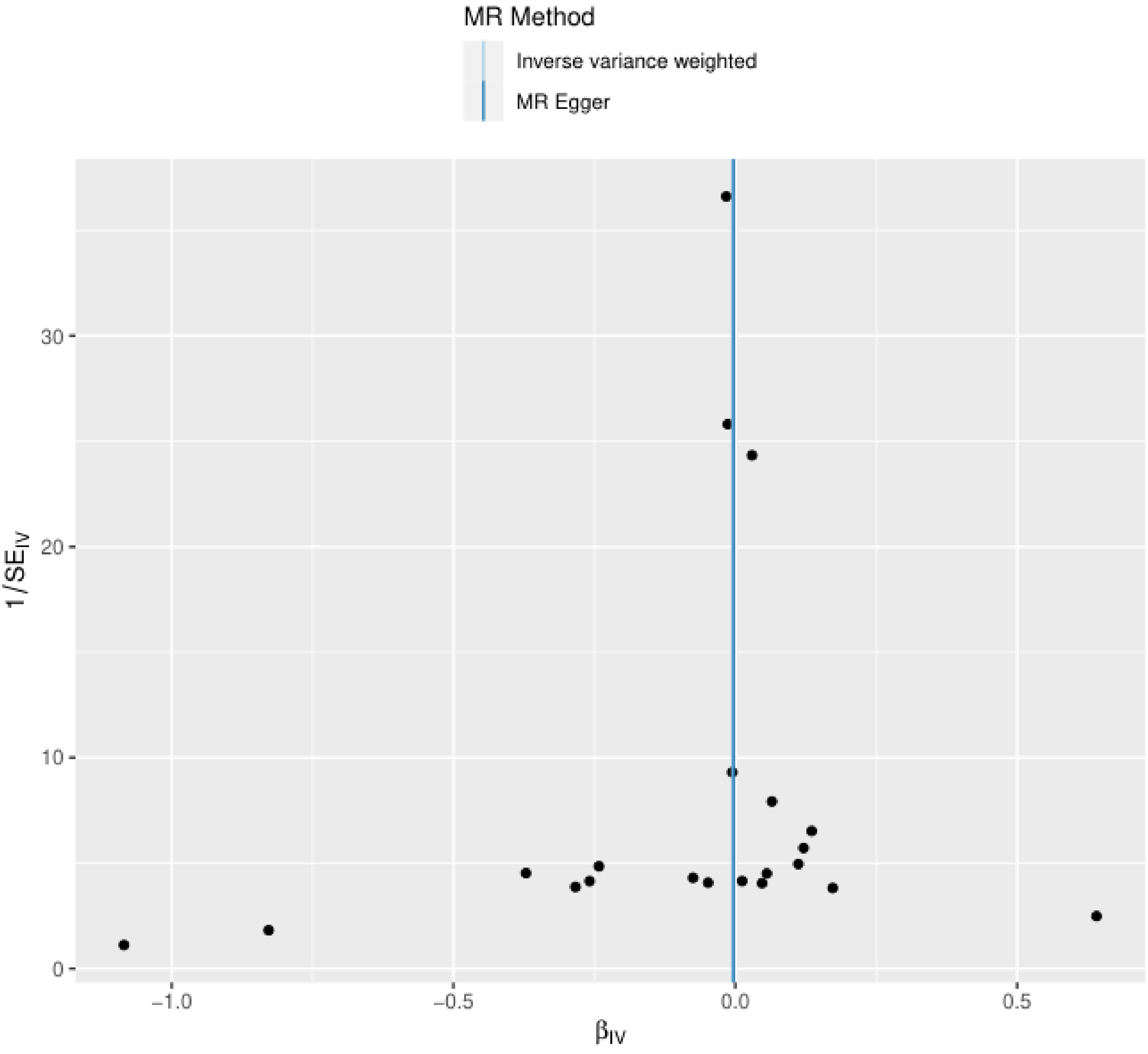

Funnel plot analyse of "CD25 on CD4 Treg" on 'Diabetic nephropathy'

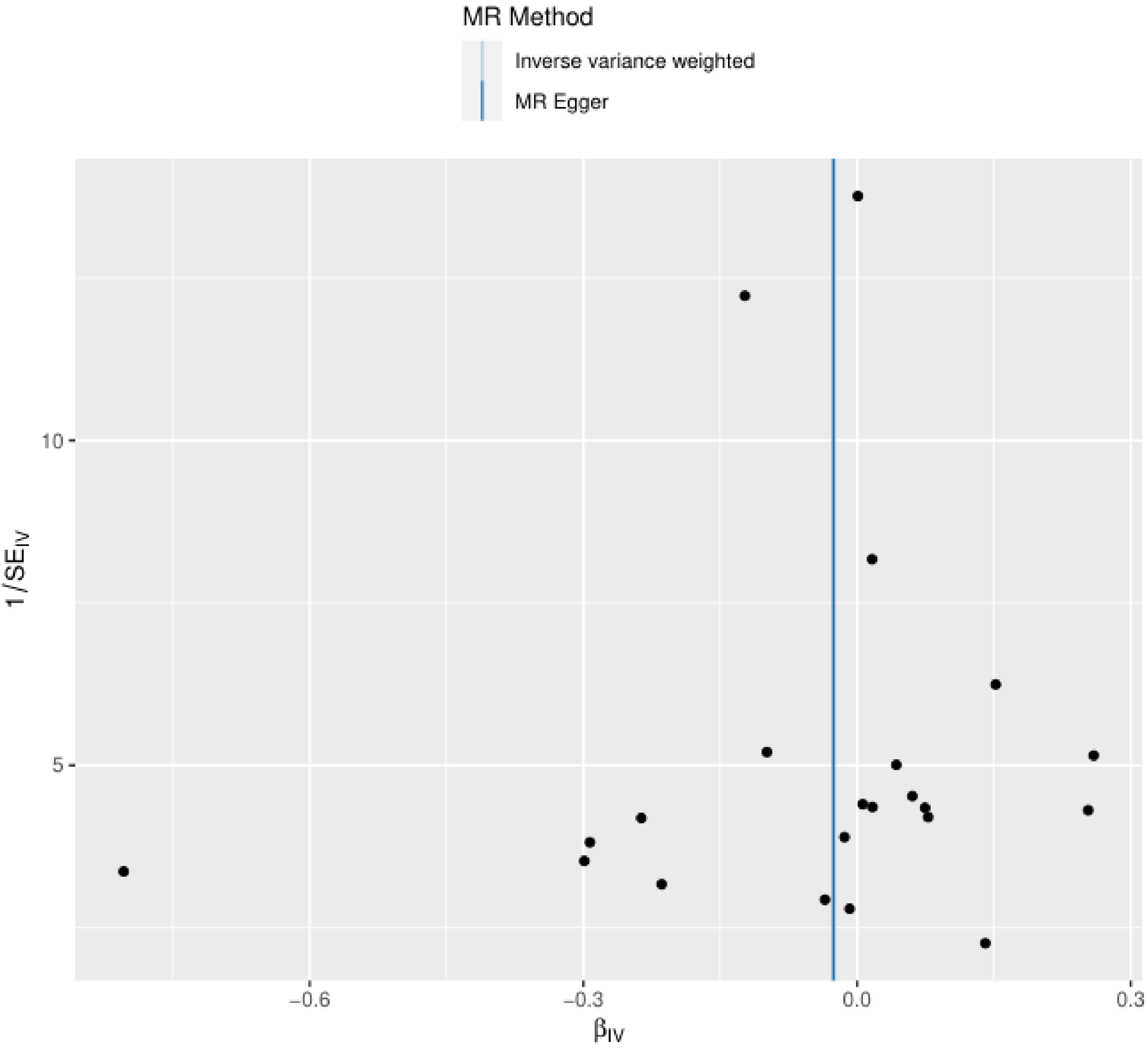

Funnel plot analyse of "CD25 on transitional" on 'Diabetic nephropathy'

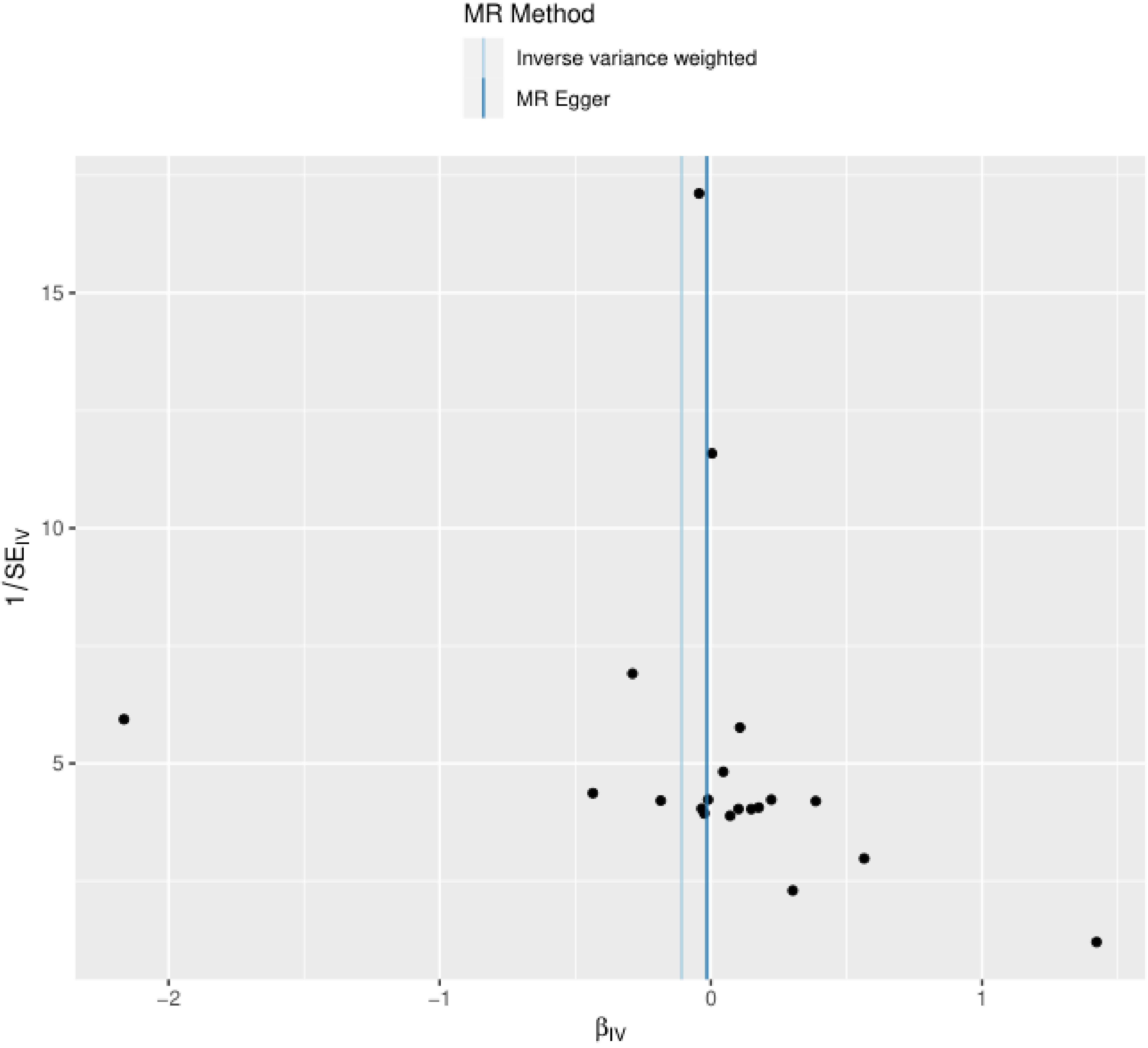

Funnel plot analyse of "TCRgd AC" on 'Diabetic nephropathy'

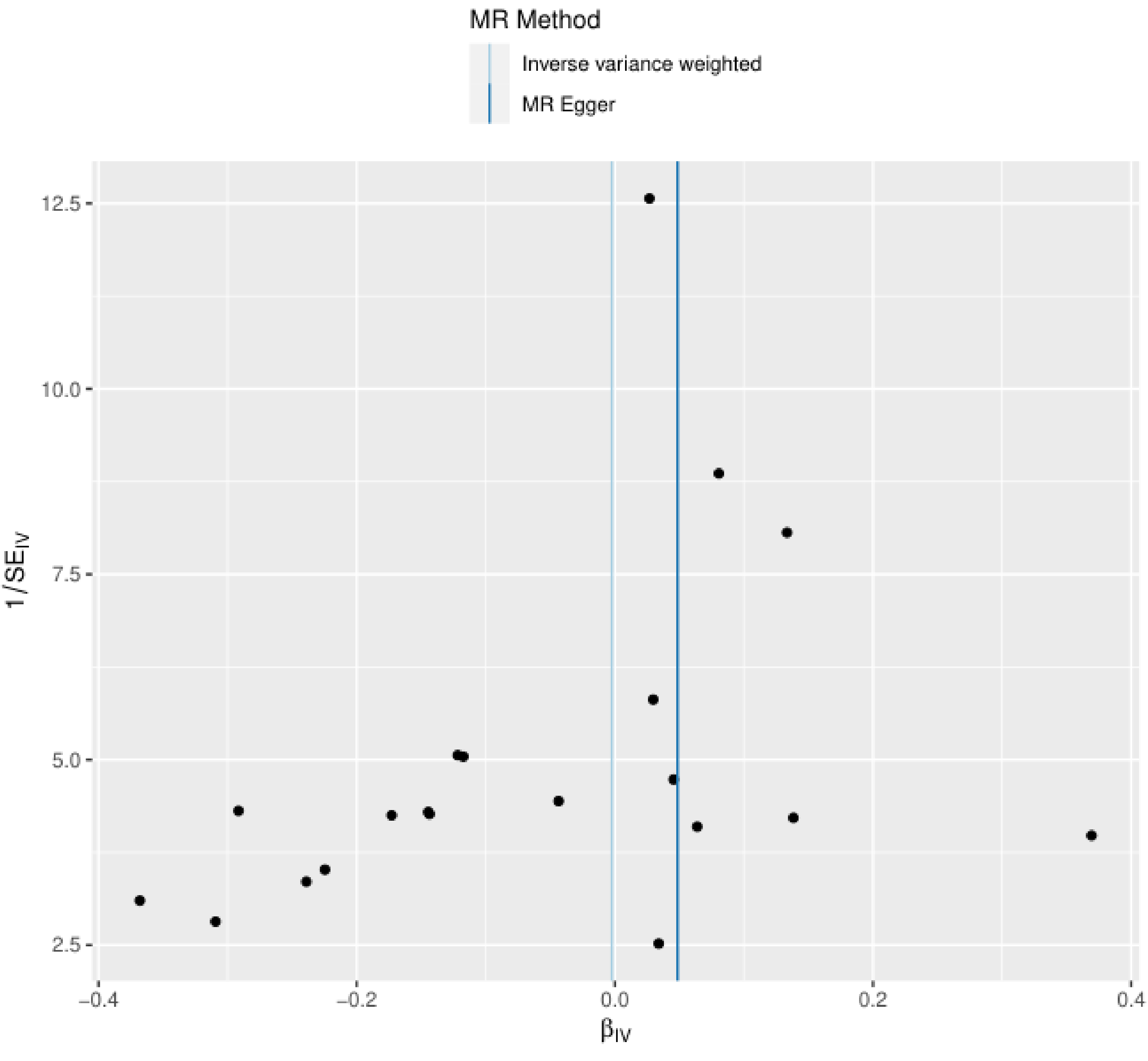

Funnel plot analyse of "CD25 on sw mem" on 'Diabetic nephropathy'

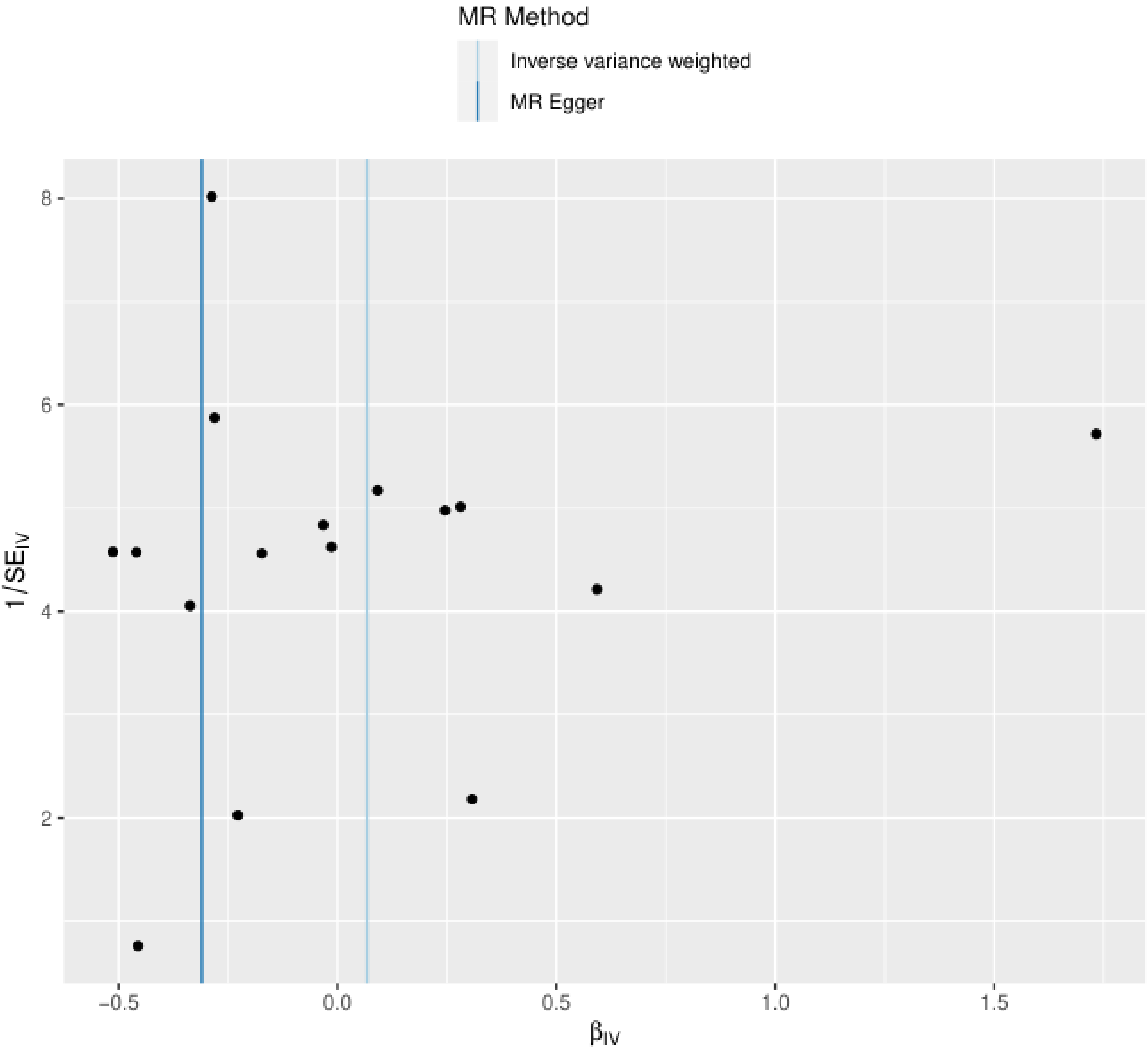

Funnel plot analyse of "CD28- CD8br %T cell" on 'Diabetic nephropathy'

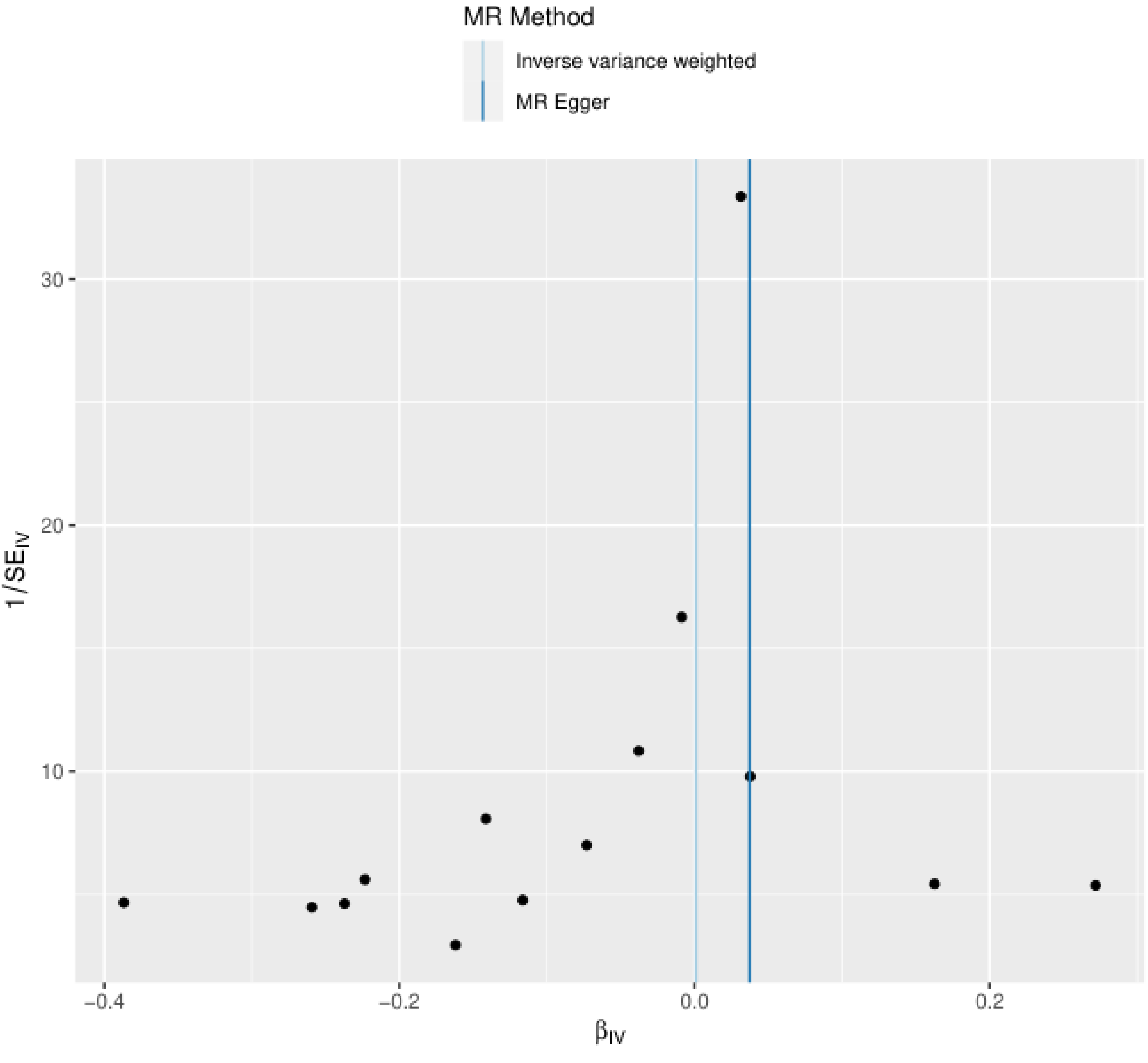

Funnel plot analyse of "CCR2 on myeloid DC" on 'Diabetic nephropathy'

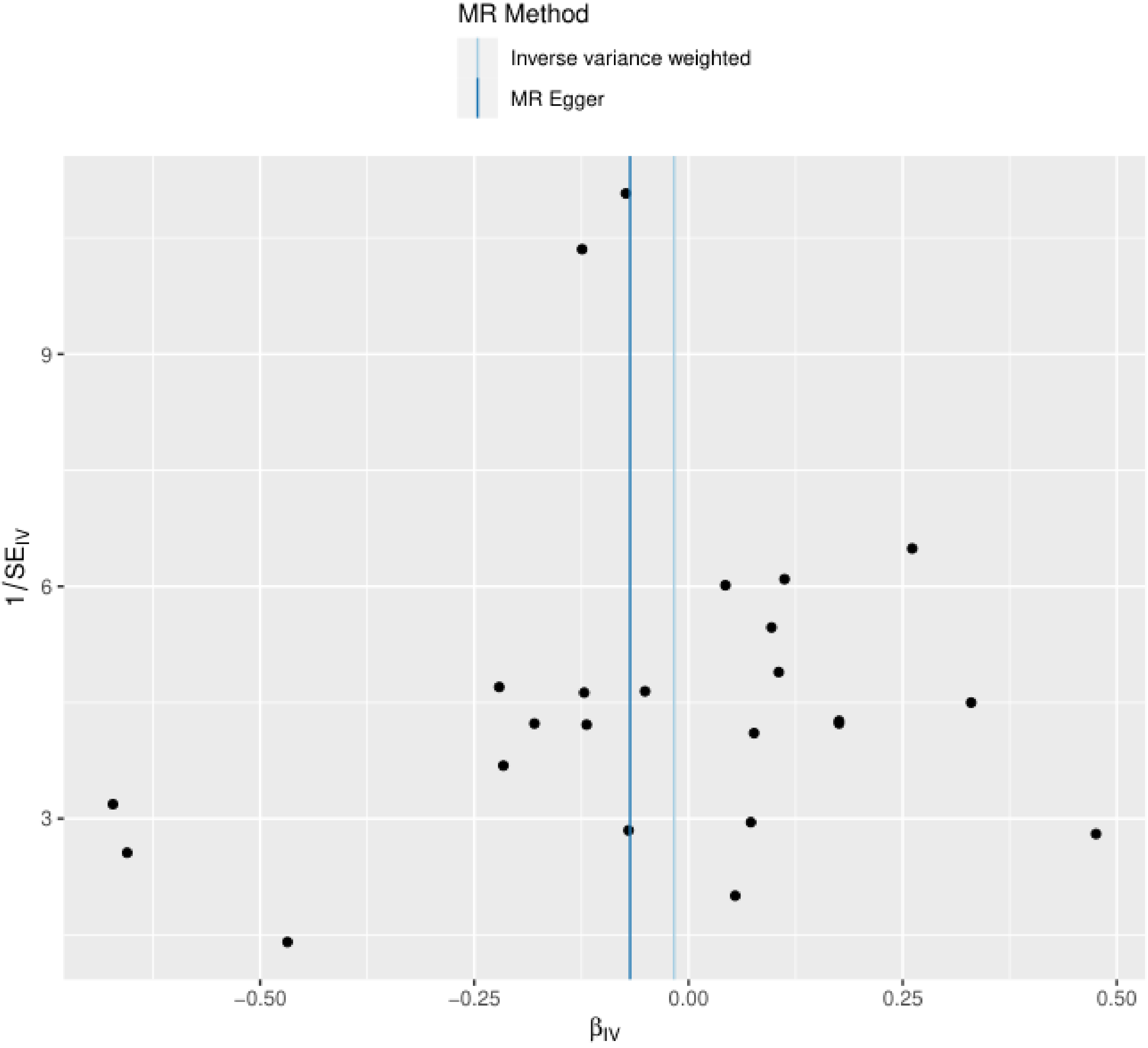

Funnel plot analyse of "Granulocyte AC" on 'Diabetic nephropathy'

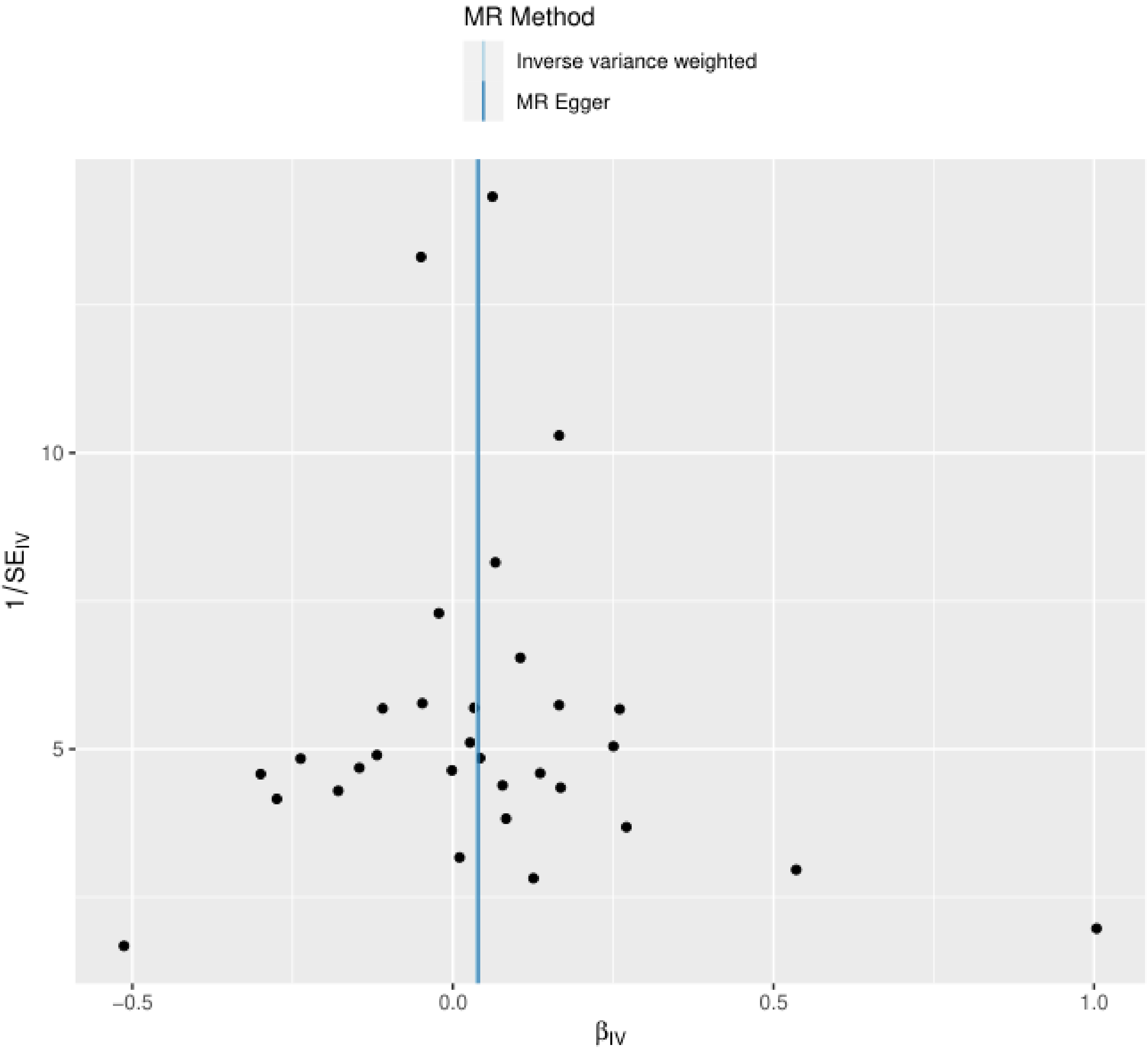

Funnel plot analyse of "CD127 on granulocyte" on 'Diabetic nephropathy'

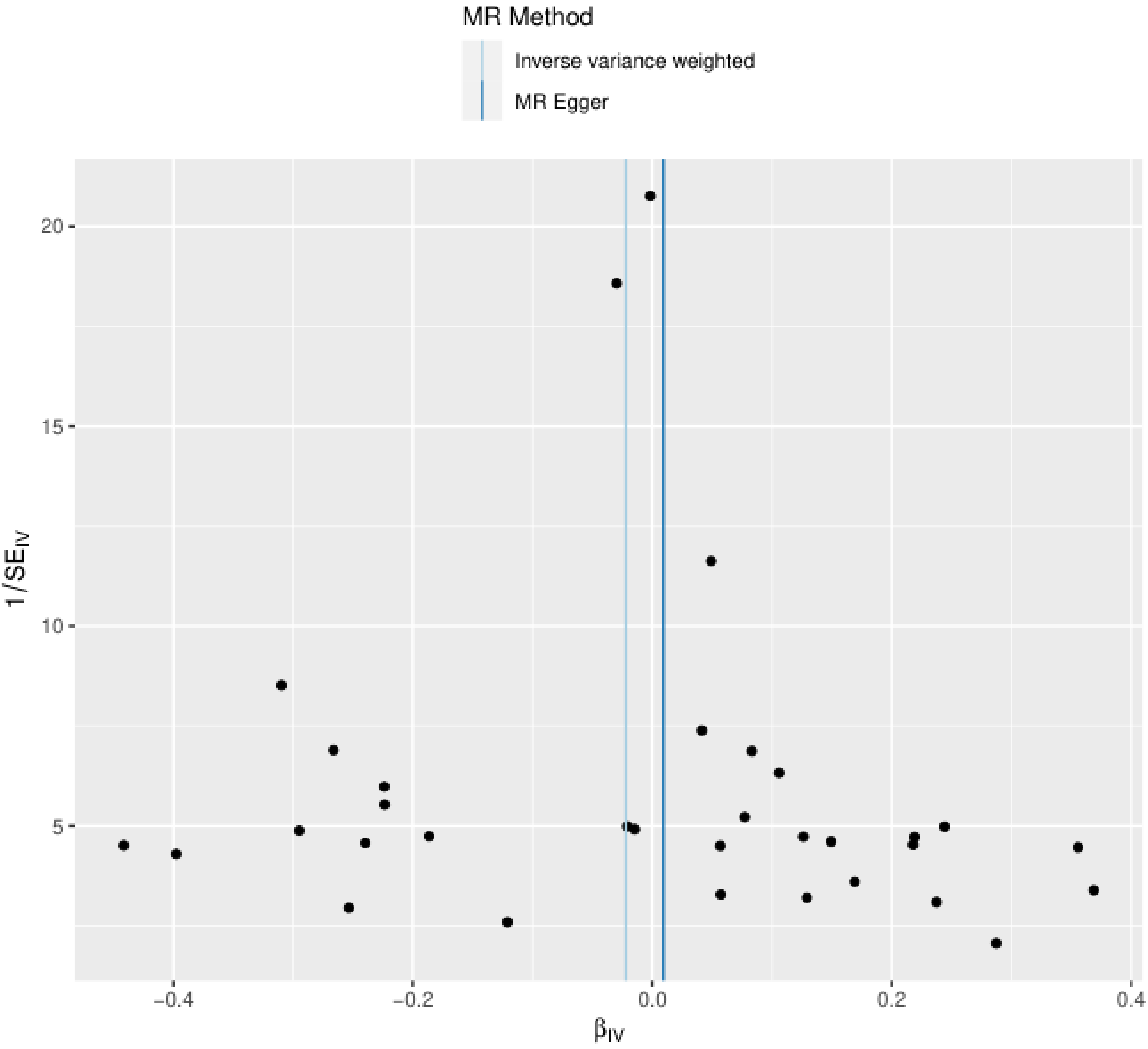

Funnel plot analyse of "CD80 on granulocyte" on 'Diabetic nephropathy'

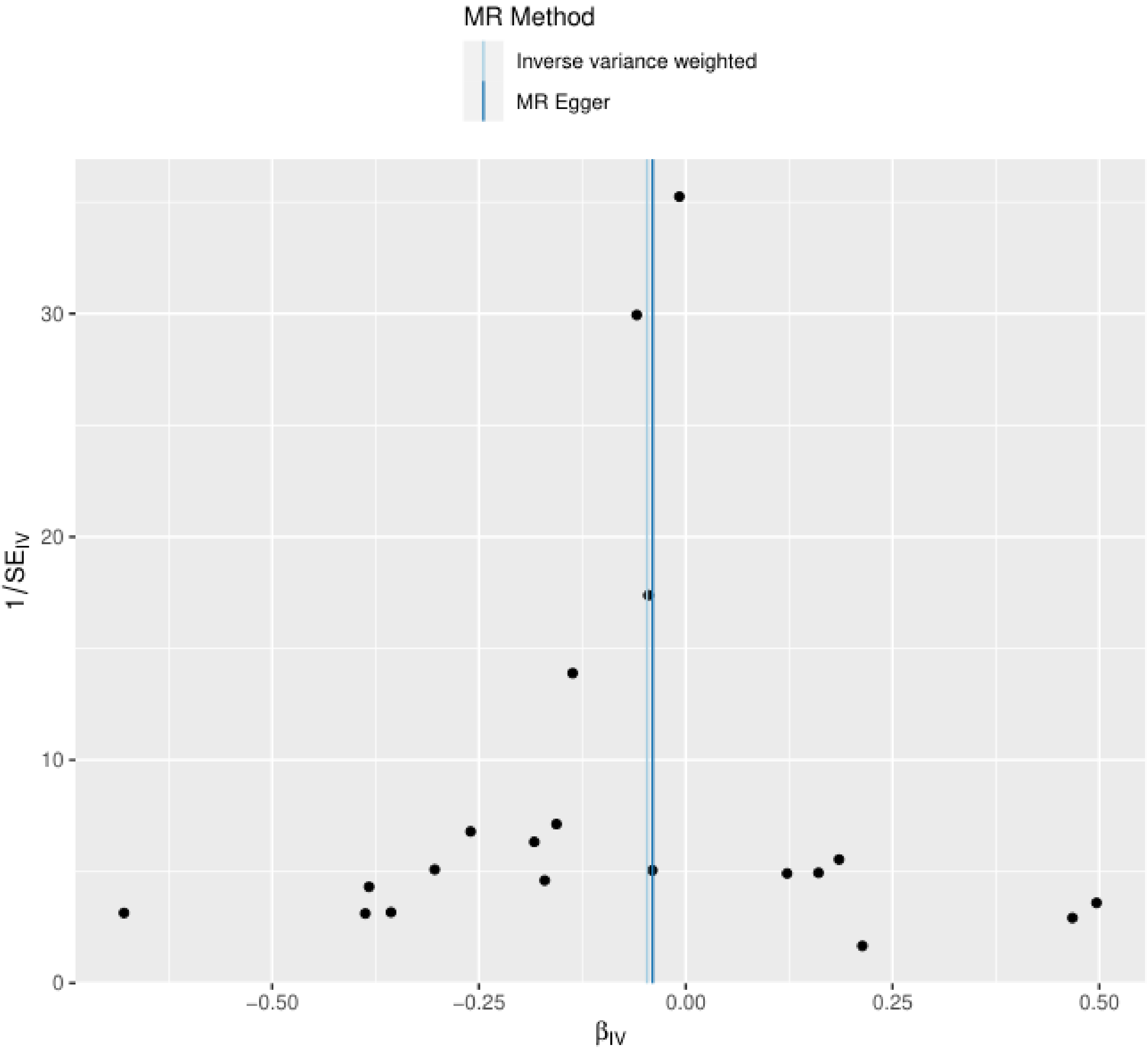

Funnel plot analyse of "CD28 on CD39+ resting Treg " on 'Diabetic nephropathy'

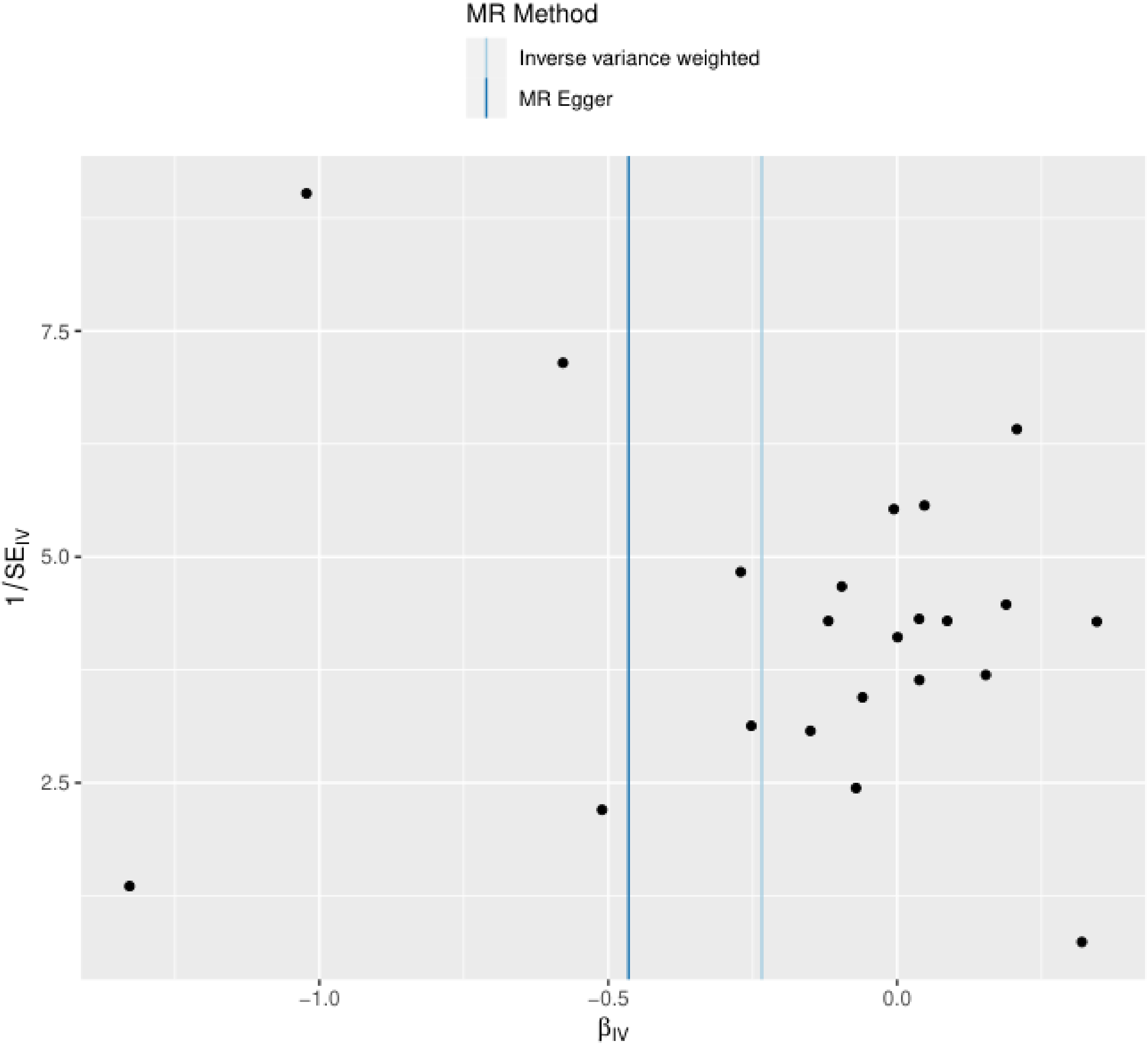

Funnel plot analyse of "TD CD4+ %CD4+" on 'Diabetic nephropathy'

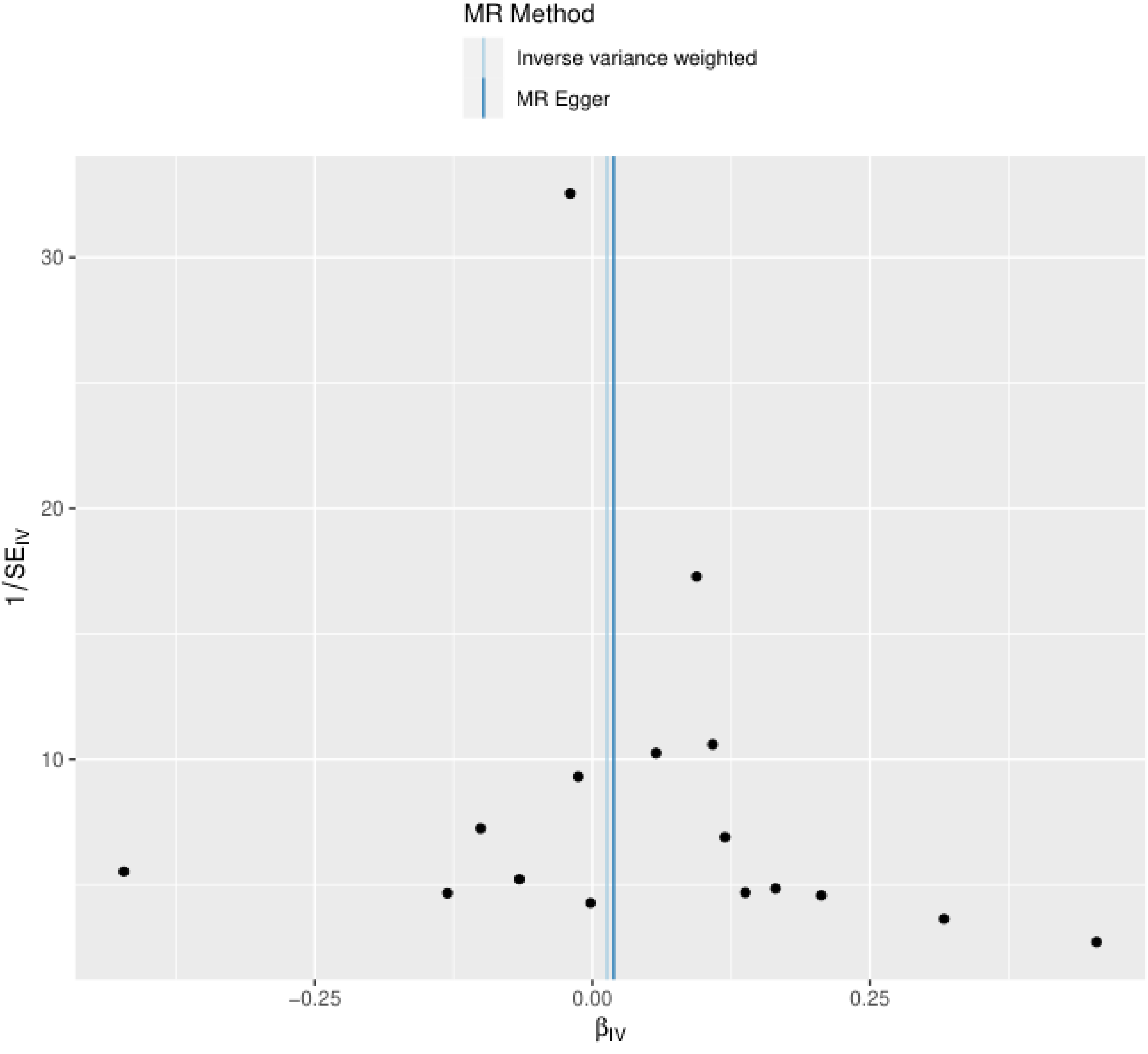

Funnel plot analyse of "BAFF-R on memory B cell" on 'Diabetic nephropathy'

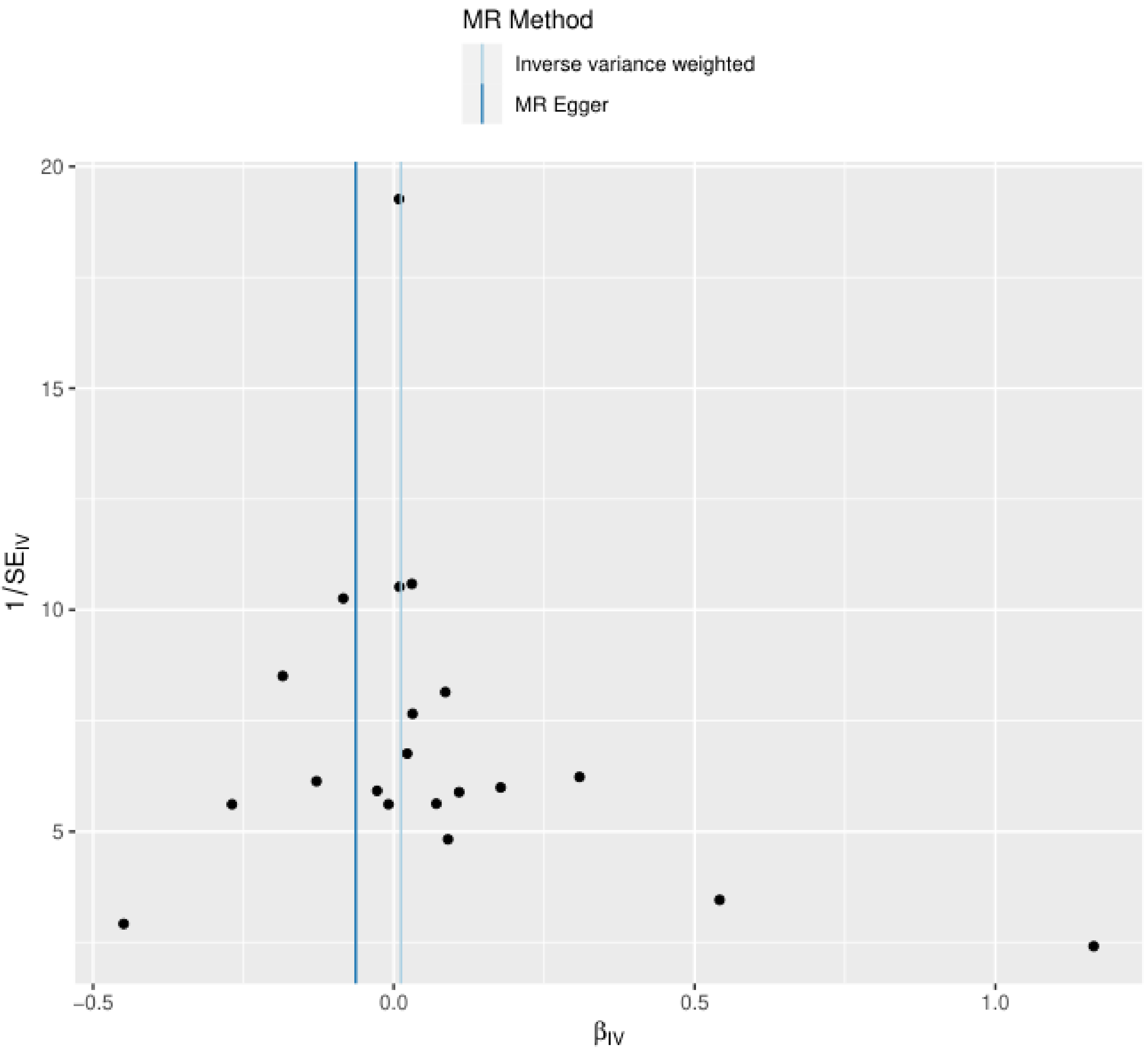

Funnel plot analyse of "Basophil %CD33dim HLA DR- CD66b-" on 'Diabetic nephropathy'

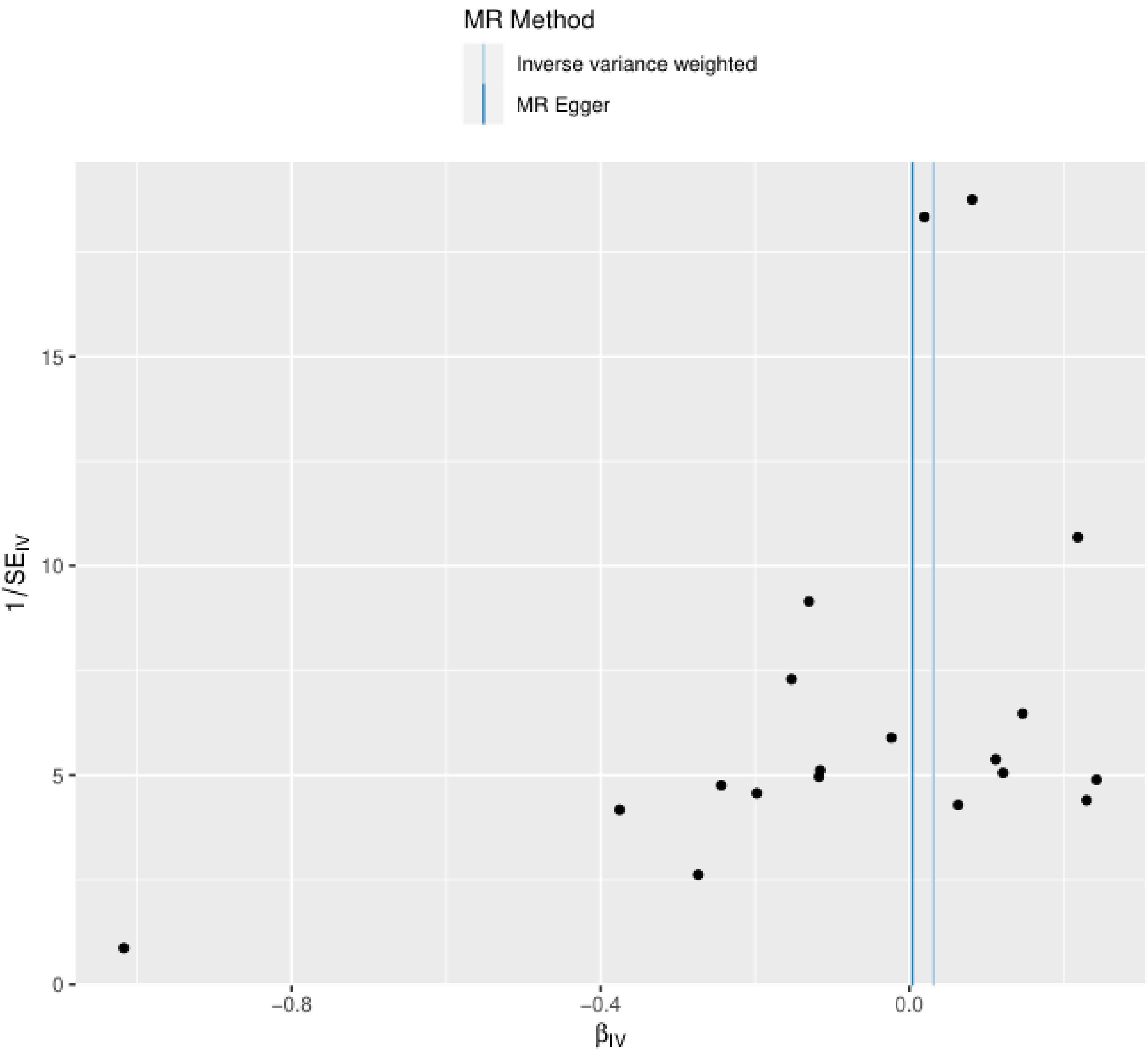

Funnel plot analyse of "CD11c on monocyte " on 'Diabetic nephropathy'

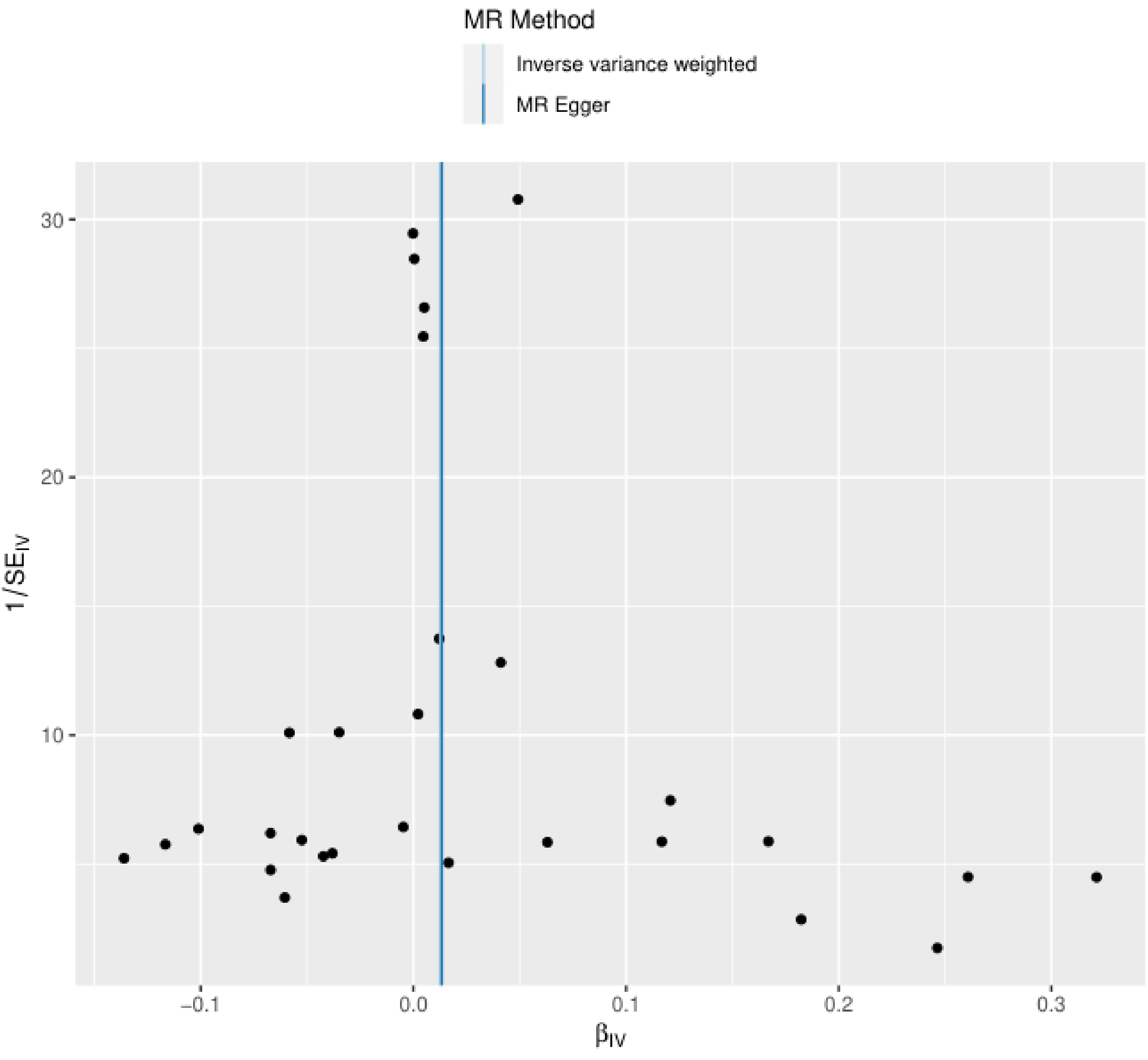

Funnel plot analyse of "CD33br HLA DR+ AC" on 'Diabetic nephropathy'

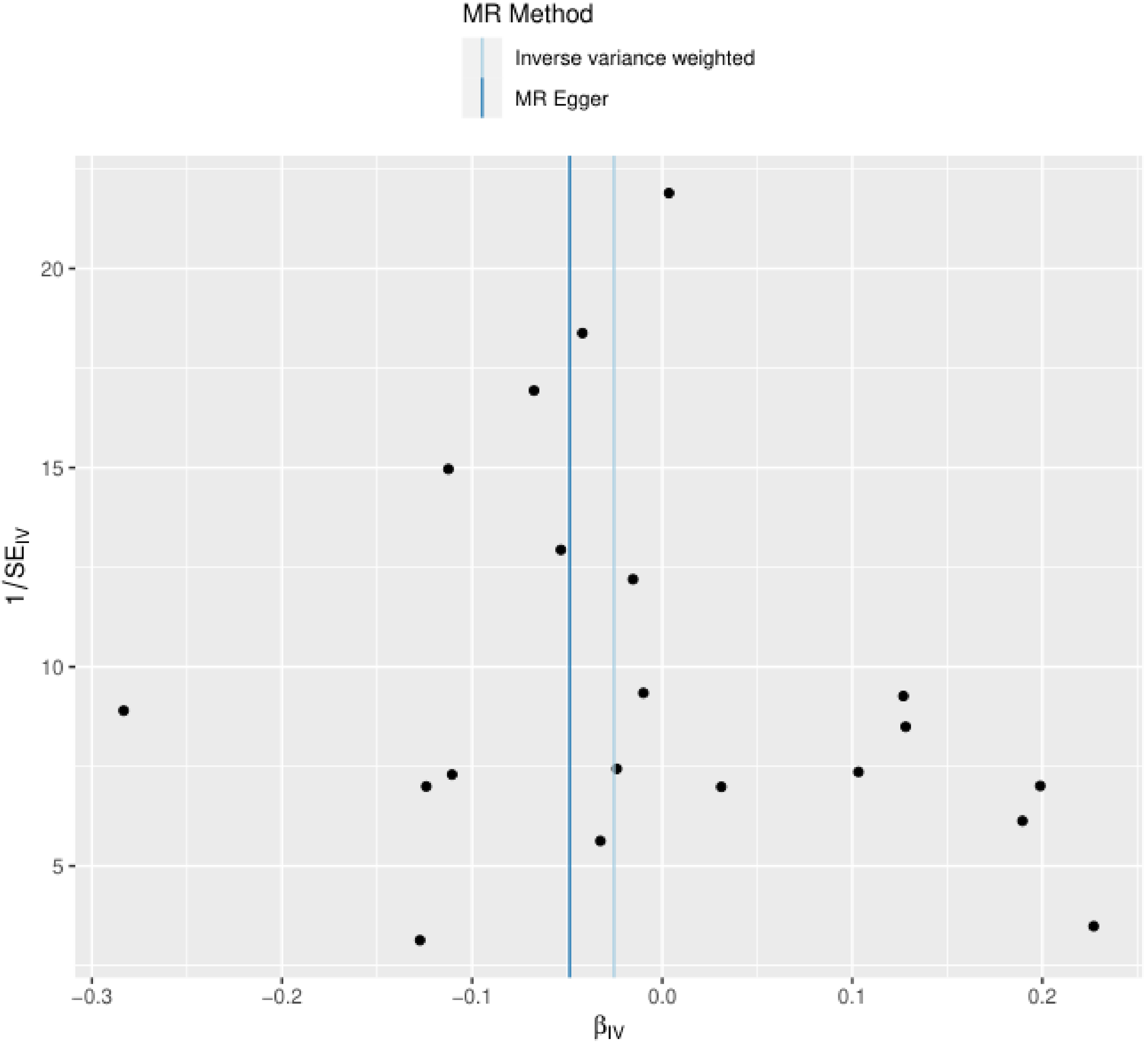

Funnel plot analyse of "HVEM on naive CD8br " on 'Diabetic nephropathy'

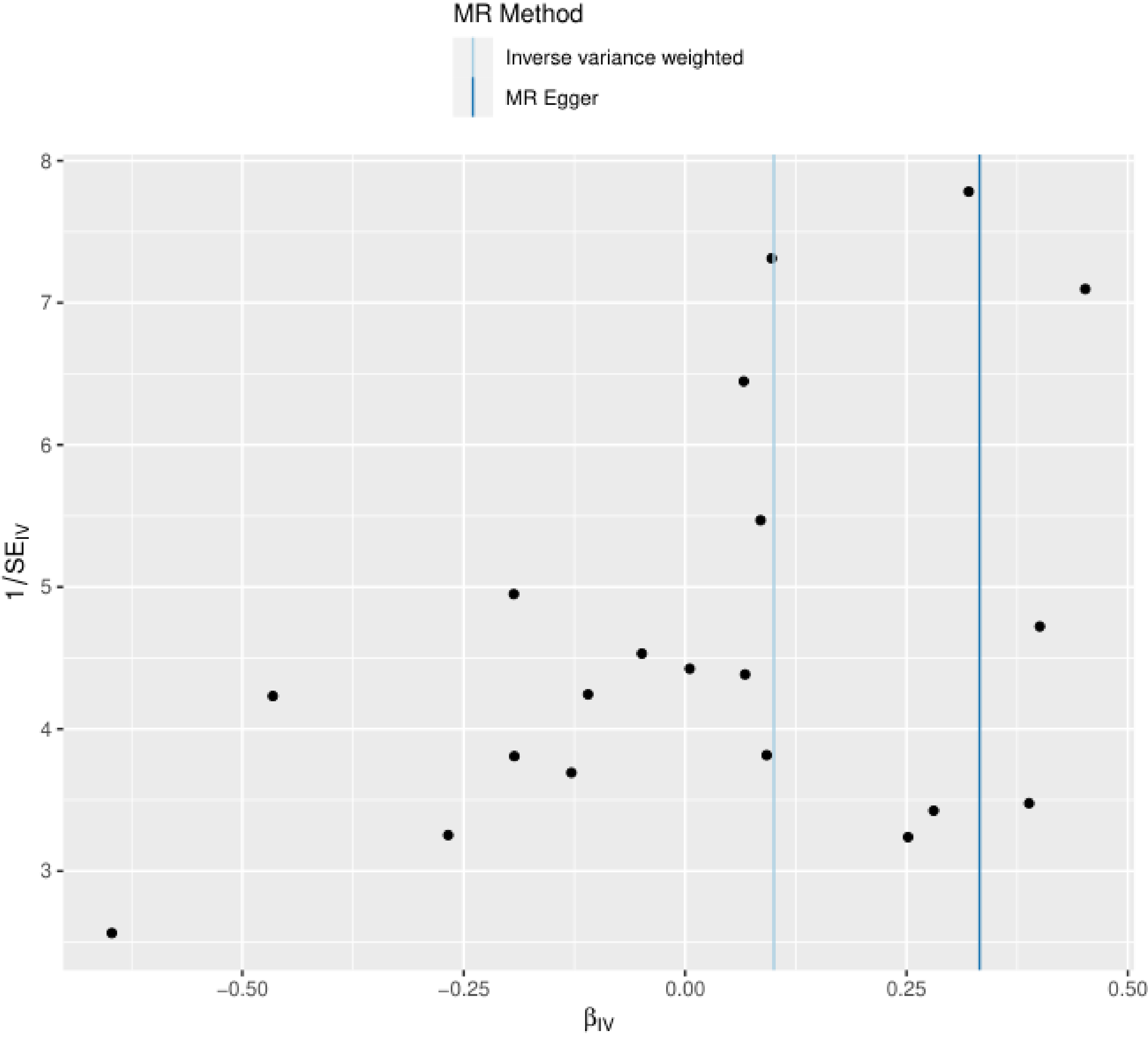

Funnel plot analyse of "DN (CD4-CD8-) AC" on 'Diabetic nephropathy'

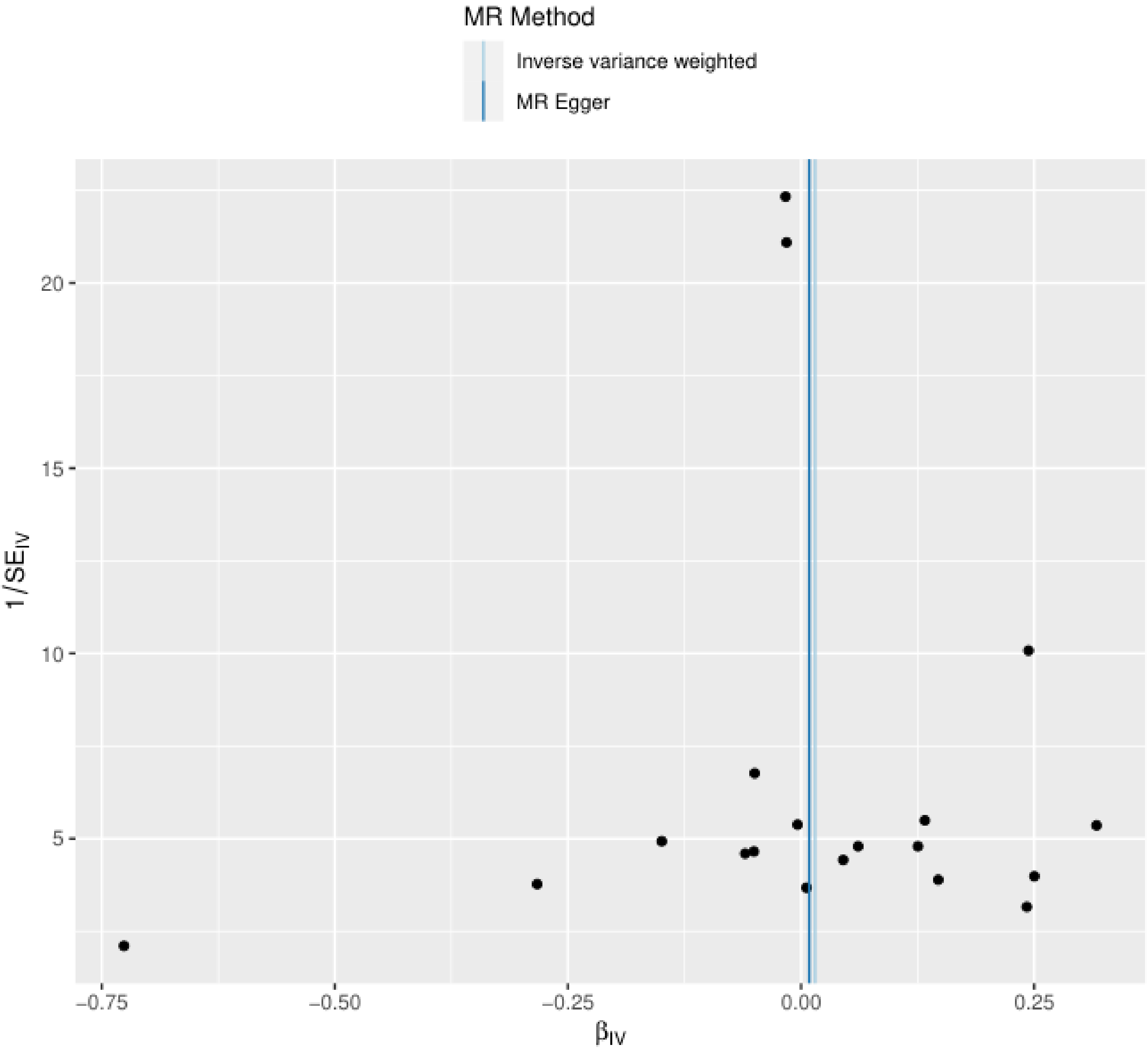

Funnel plot analyse of "CD62L- CD86+ myeloid DC %DC" on 'Diabetic nephropathy'

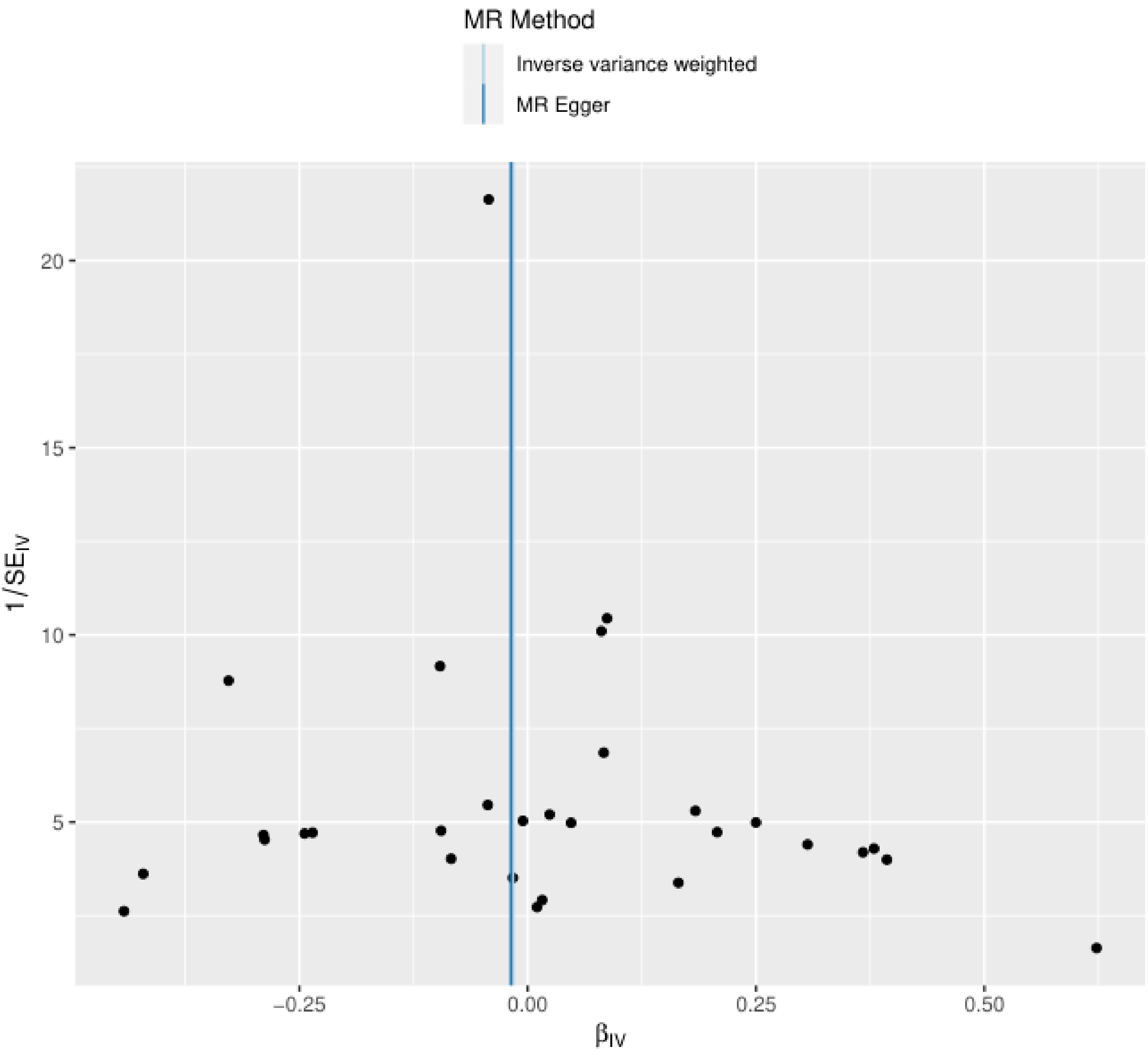

Funnel plot analyse of "CD3 on CD4+ " on 'Diabetic nephropathy'

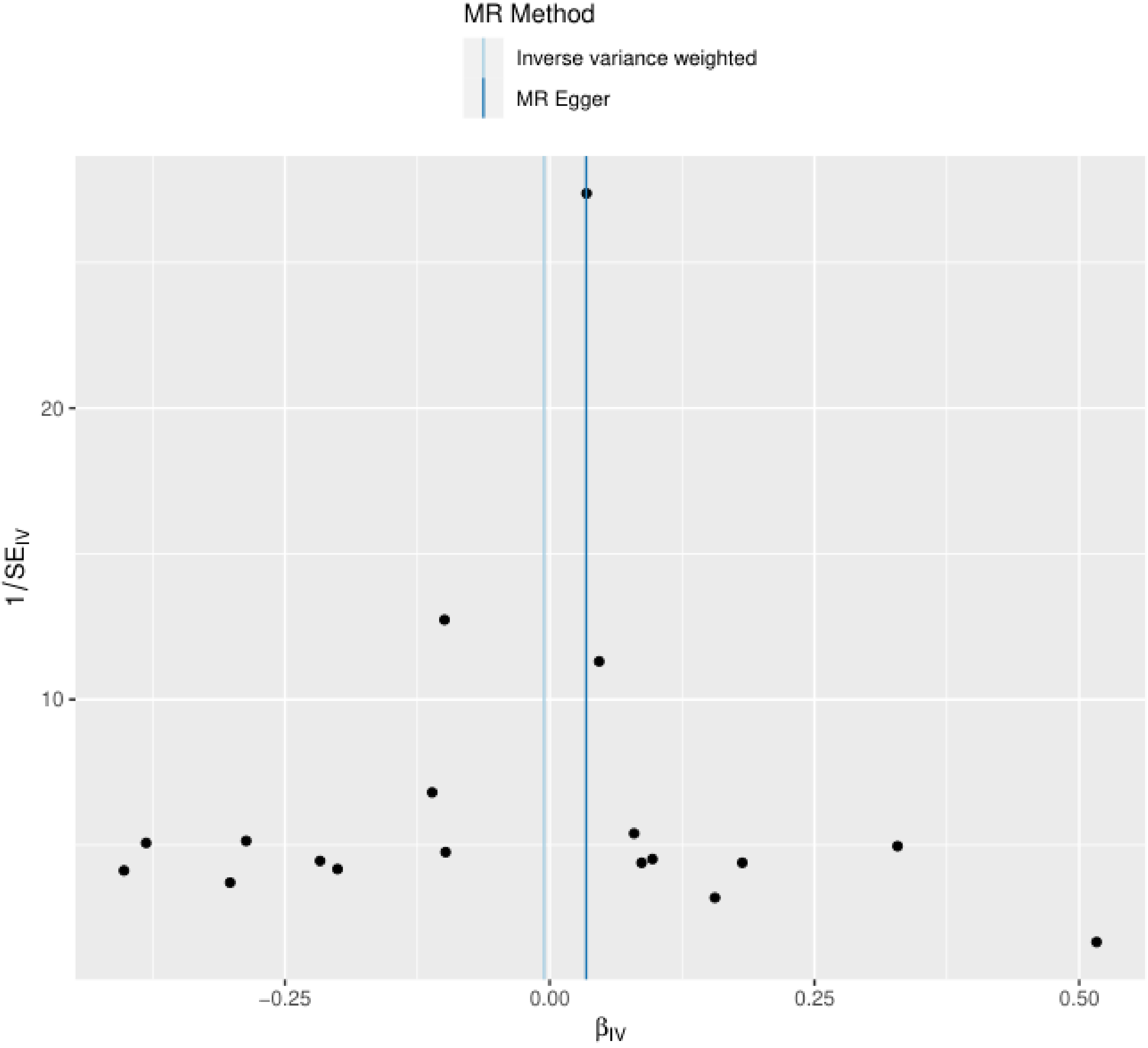

Funnel plot analyse of "CD28- CD127- CD25++ CD8br %CD8br" on 'Diabetic nephropathy'

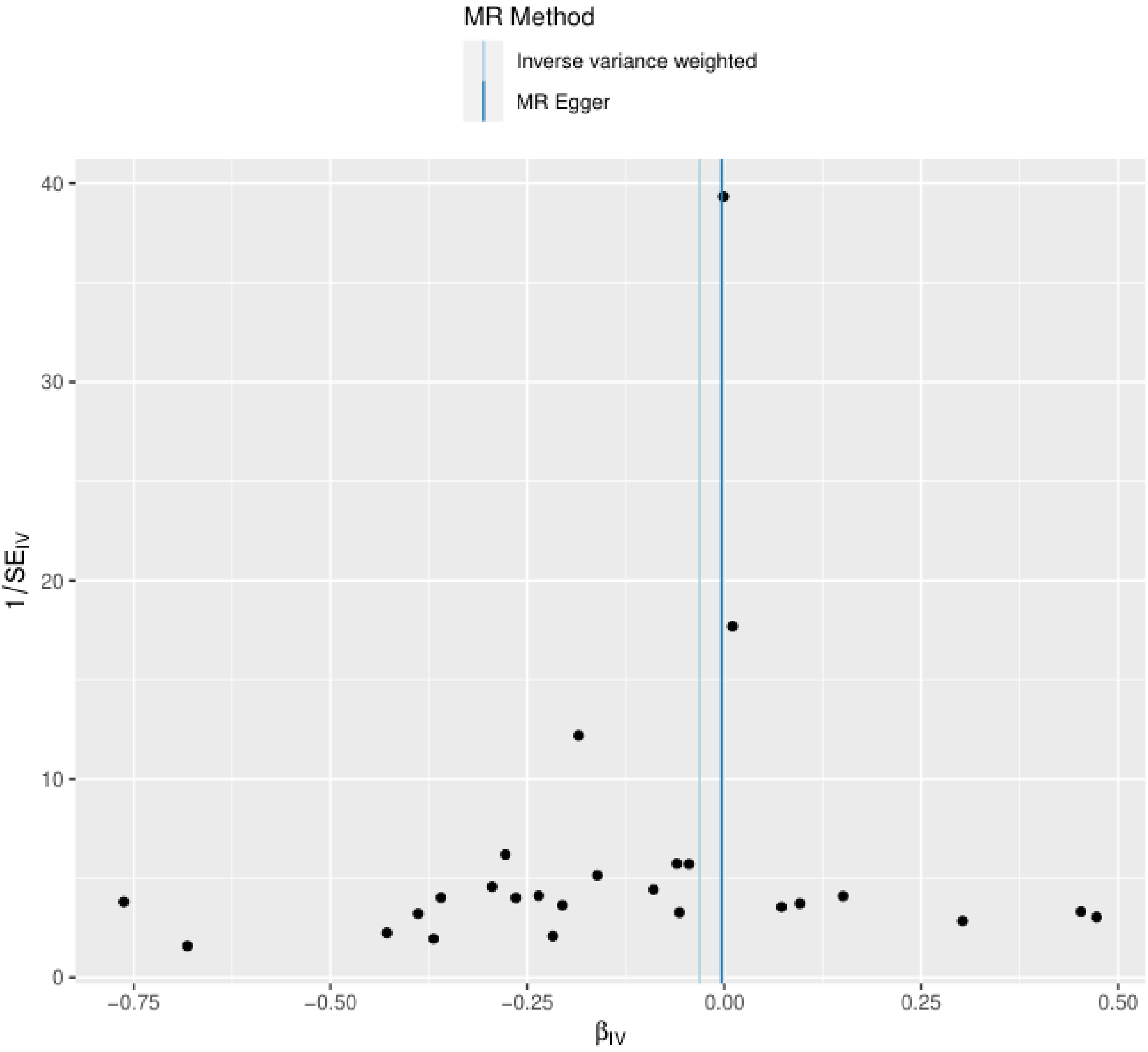

Funnel plot analyse of "CD14+ CD16- monocyte AC" on 'Diabetic nephropathy'

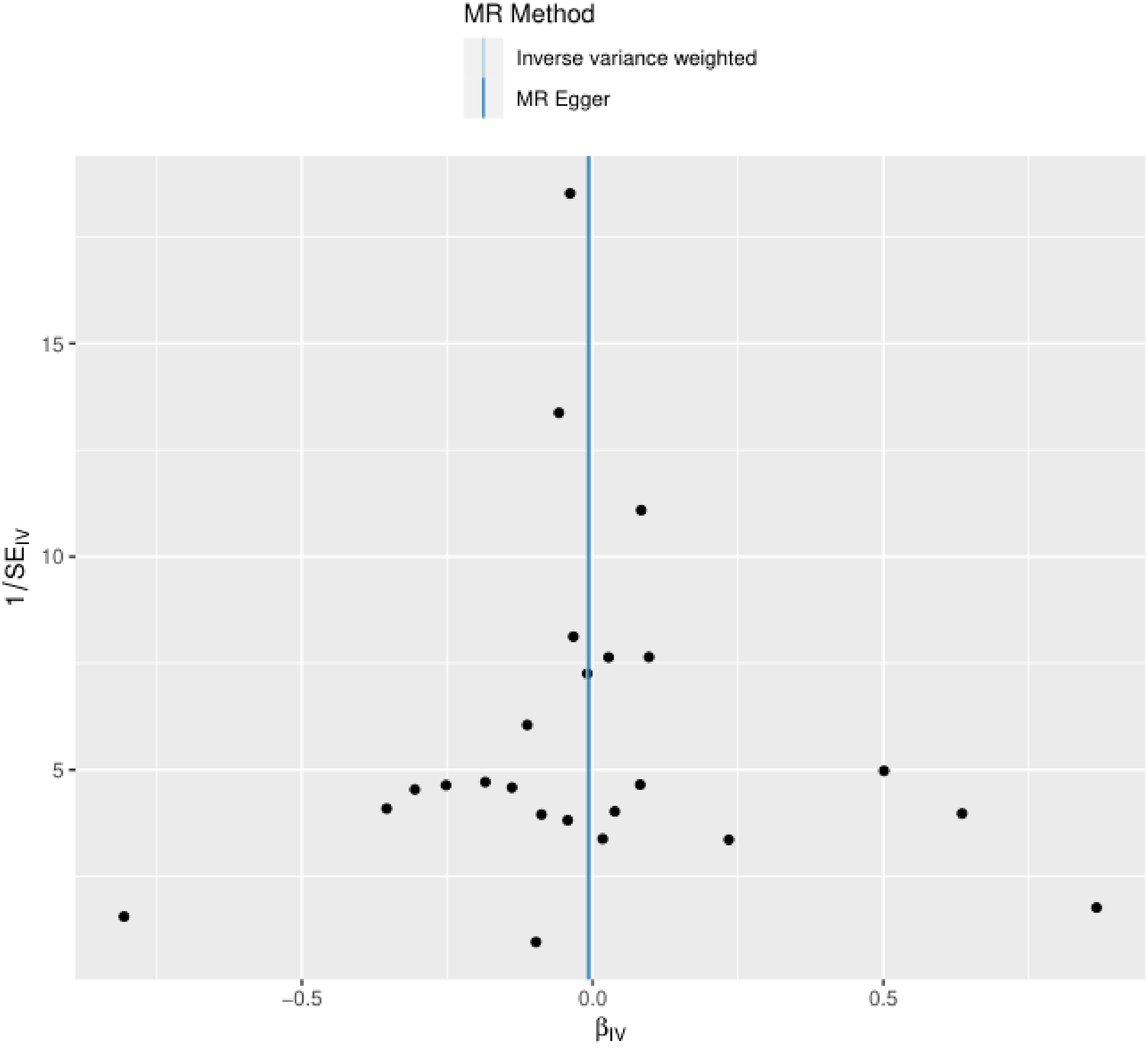

Funnel plot analyse of "CD28- CD8dim %T cell" on 'Diabetic nephropathy'

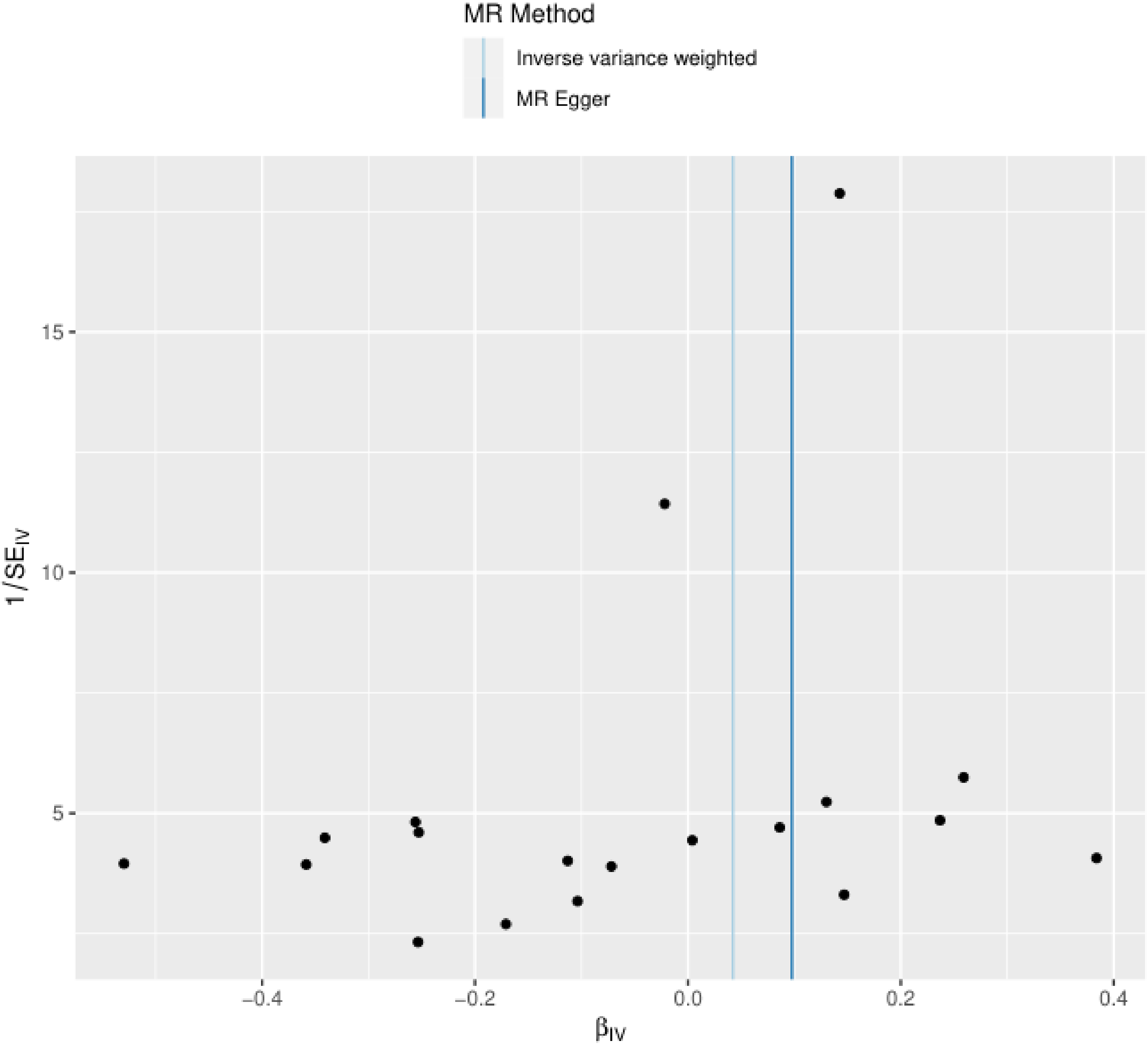

Funnel plot analyse of "CD27 on CD20- CD38-" on 'Diabetic nephropathy'

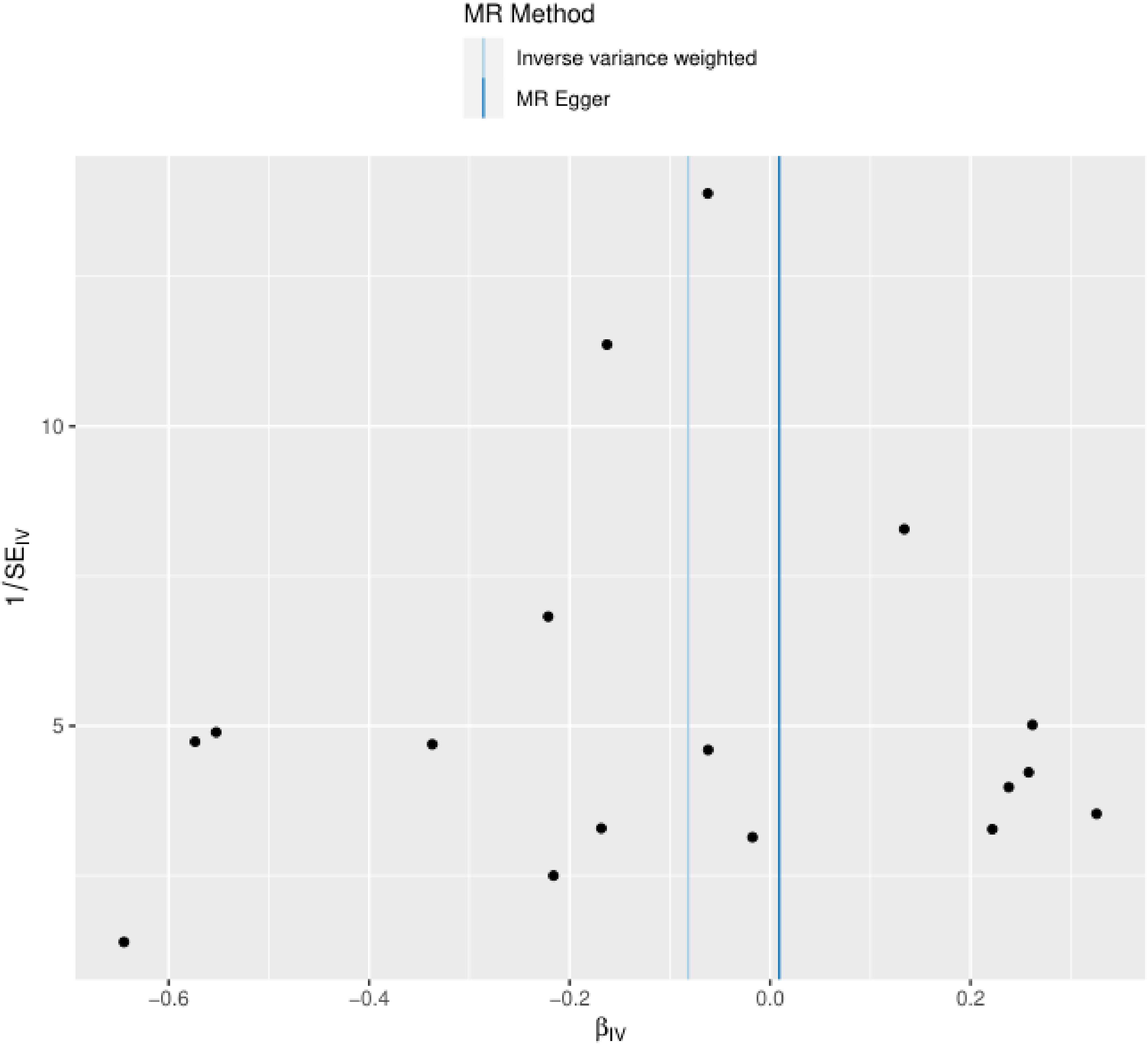

Funnel plot analyse of "CD19 on IgD- CD38br" on 'Diabetic nephropathy'

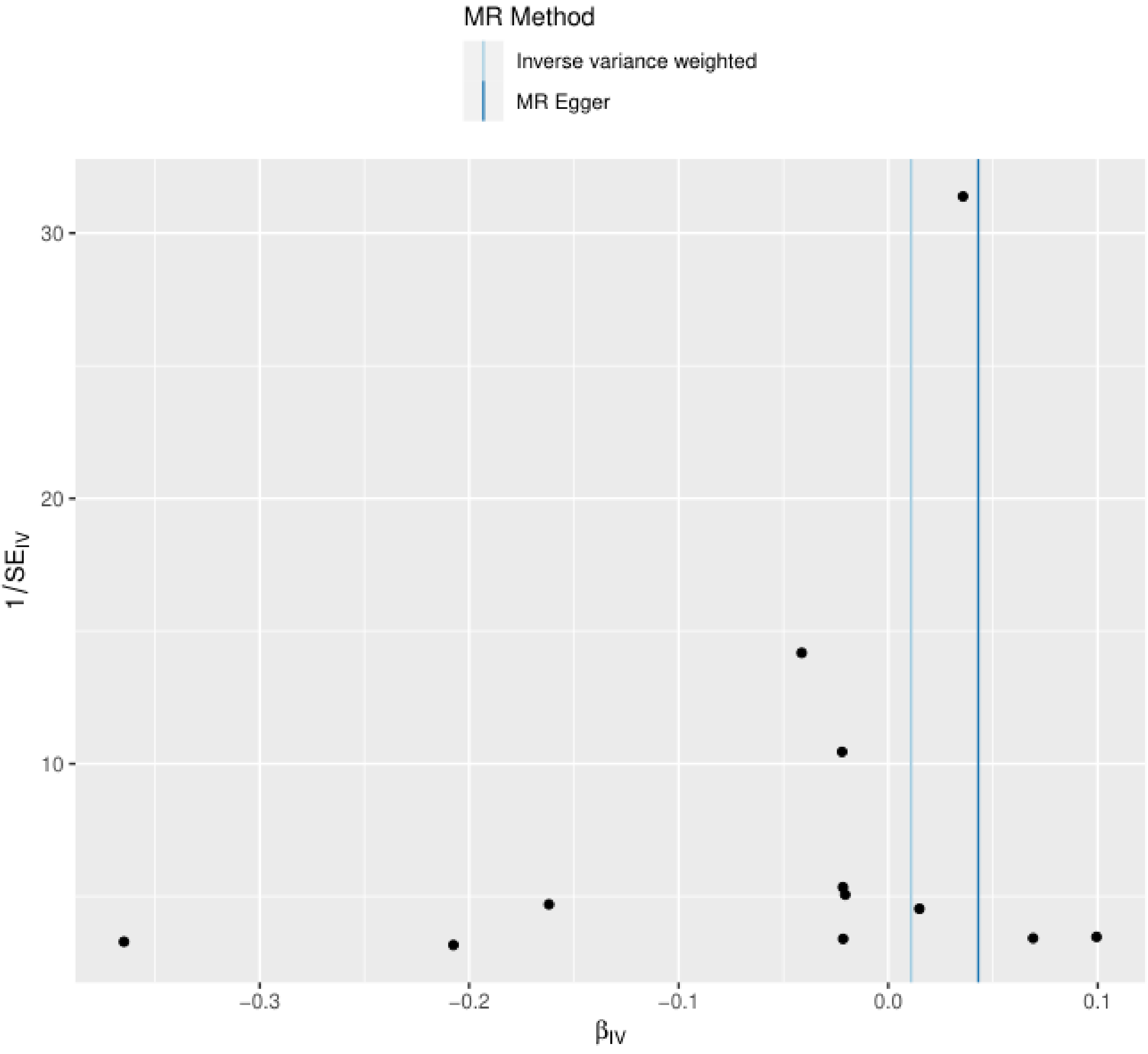

Funnel plot analyse of "Leukocyte AC" on 'Diabetic nephropathy'

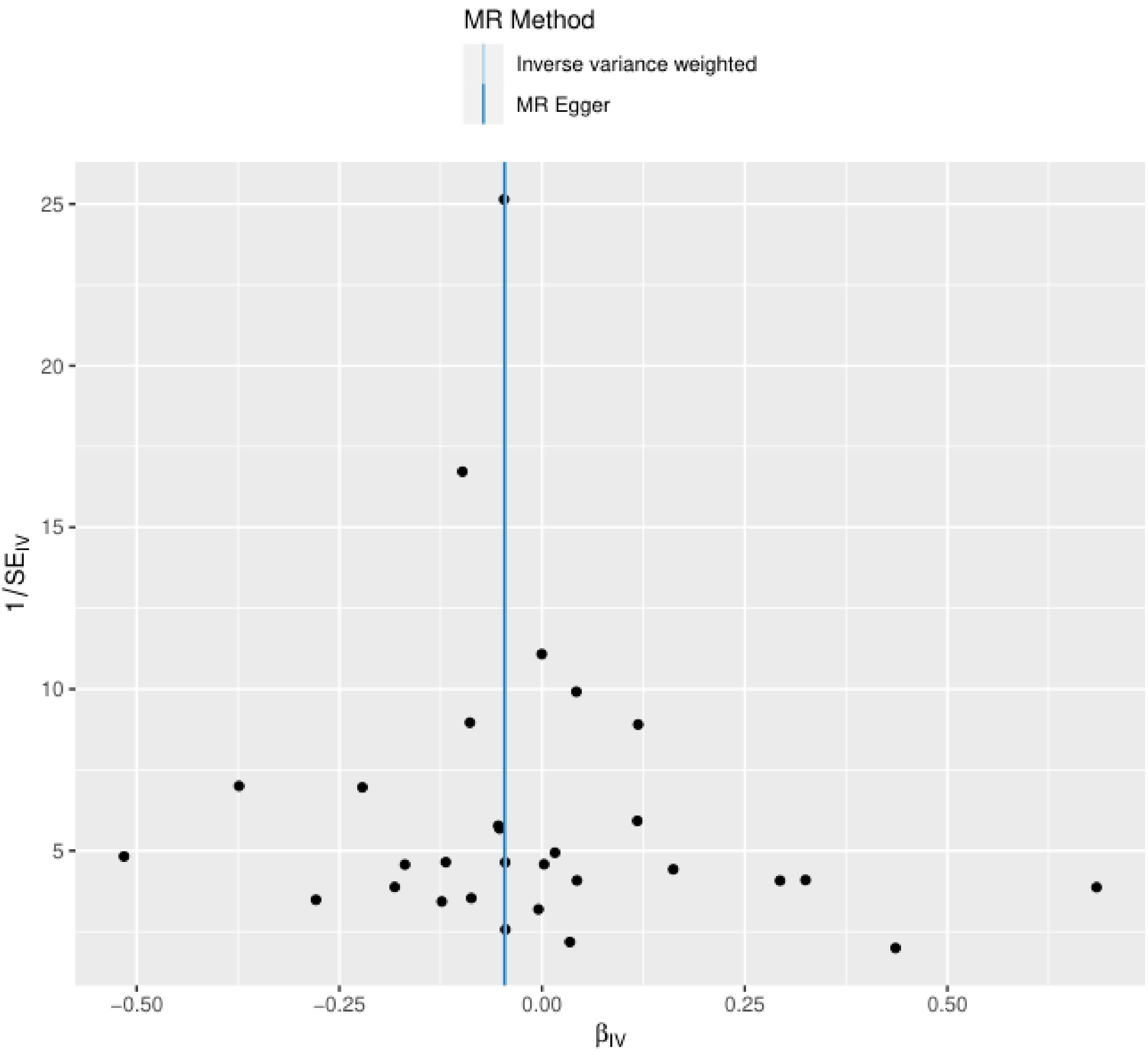

Funnel plot analyse of ''IgD- CD38br %lymphocyte'' on 'Diabetic nephropathy'

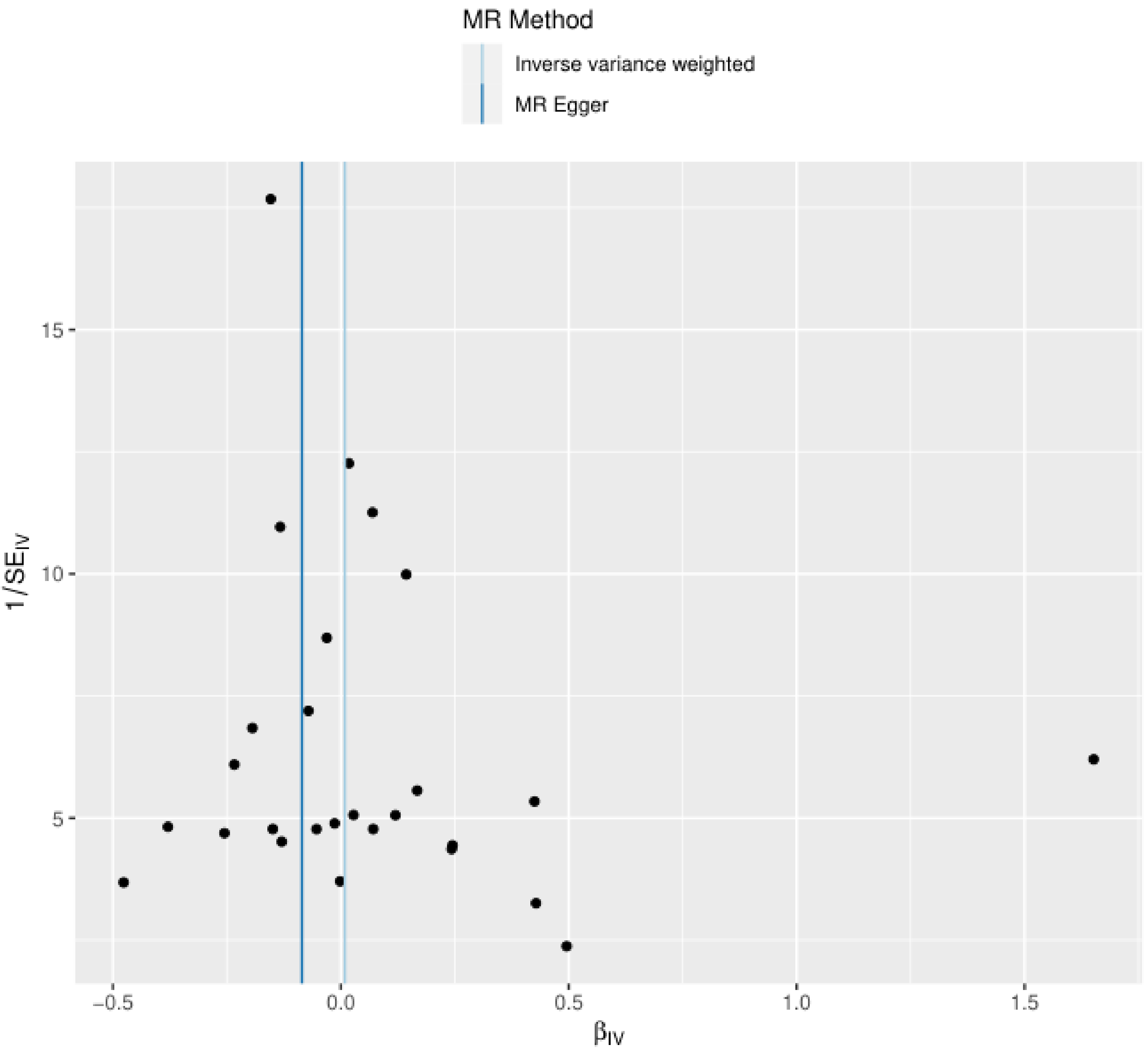

Funnel plot analyse of "EM DN (CD4-CD8-) %DN" on 'Diabetic nephropathy'

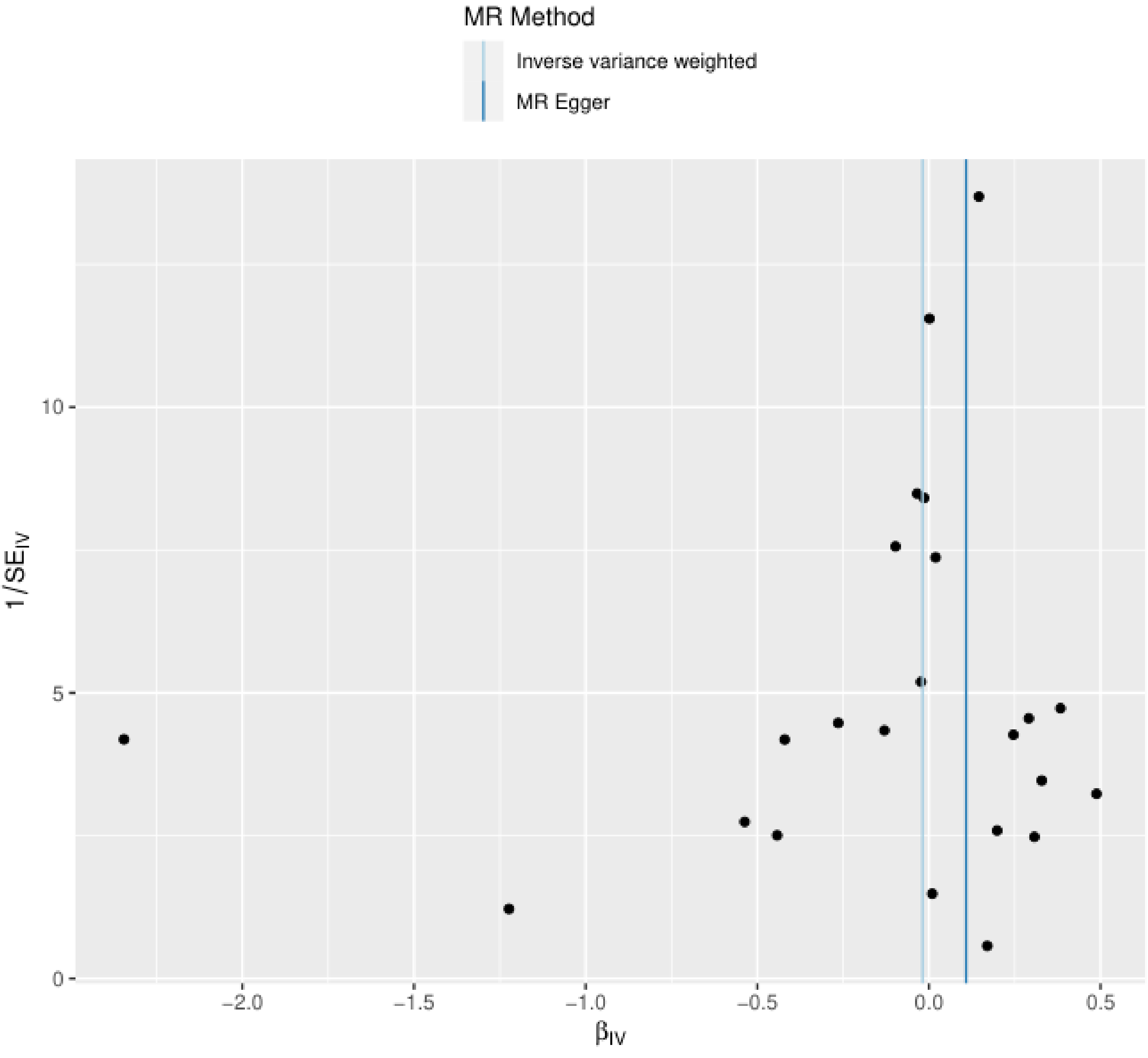

Funnel plot analyse of "CD20 on IgD+ CD24+" on 'Diabetic nephropathy'

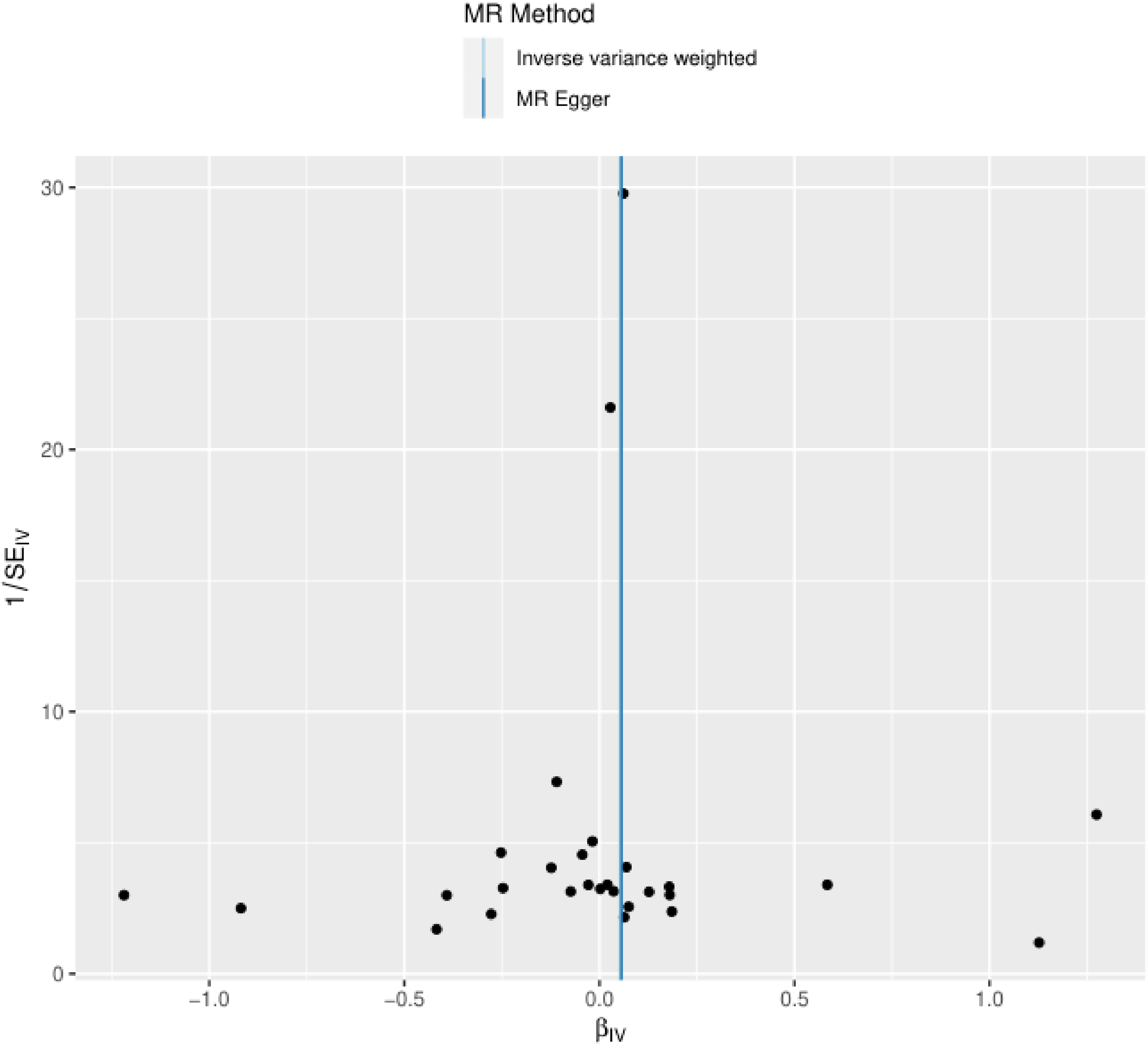

Funnel plot analyse of "Naive CD8br AC" on 'Diabetic nephropathy'

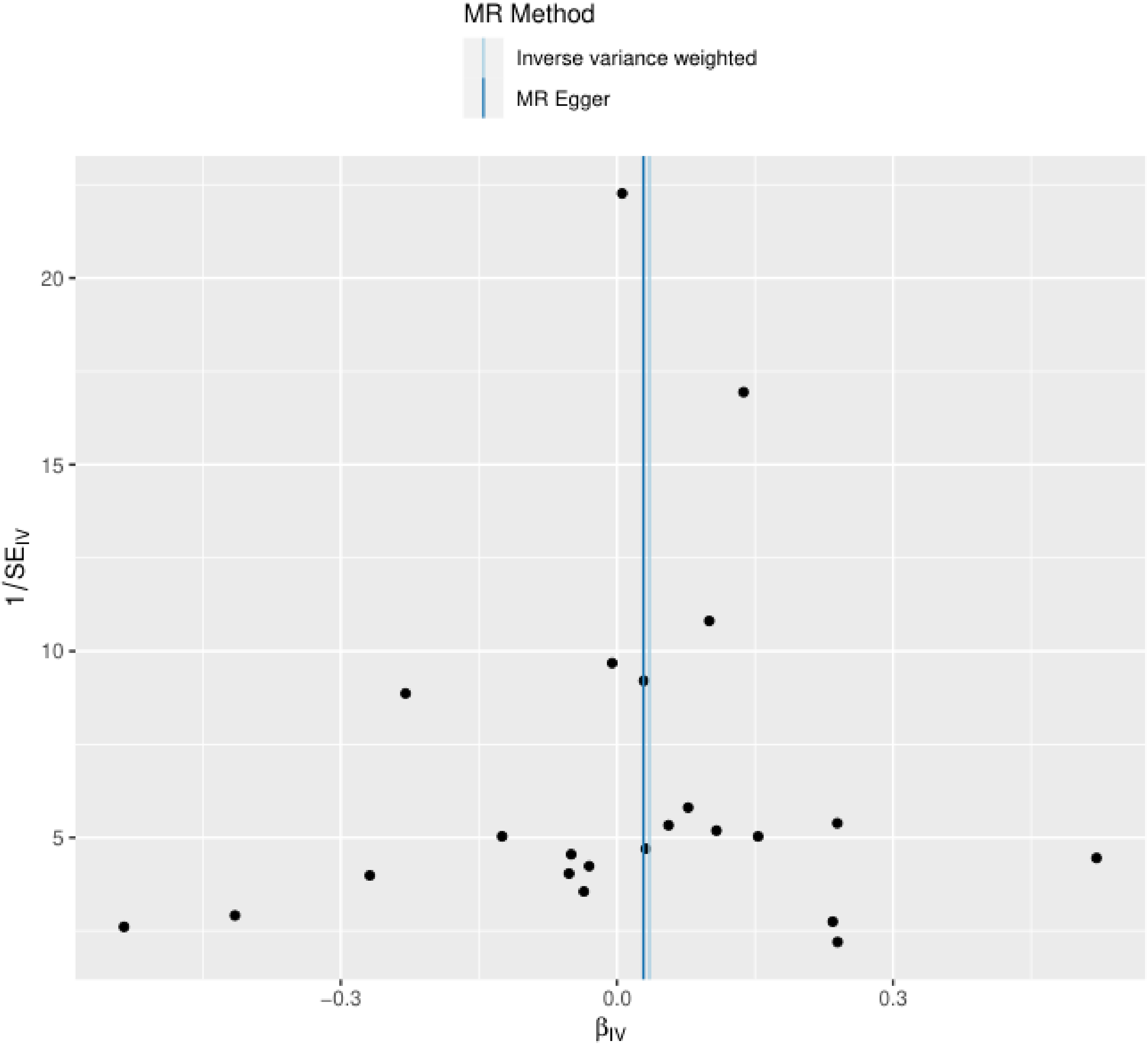

Funnel plot analyse of "CD40 on CD14+ CD16- monocyte" on 'Diabetic nephropathy'

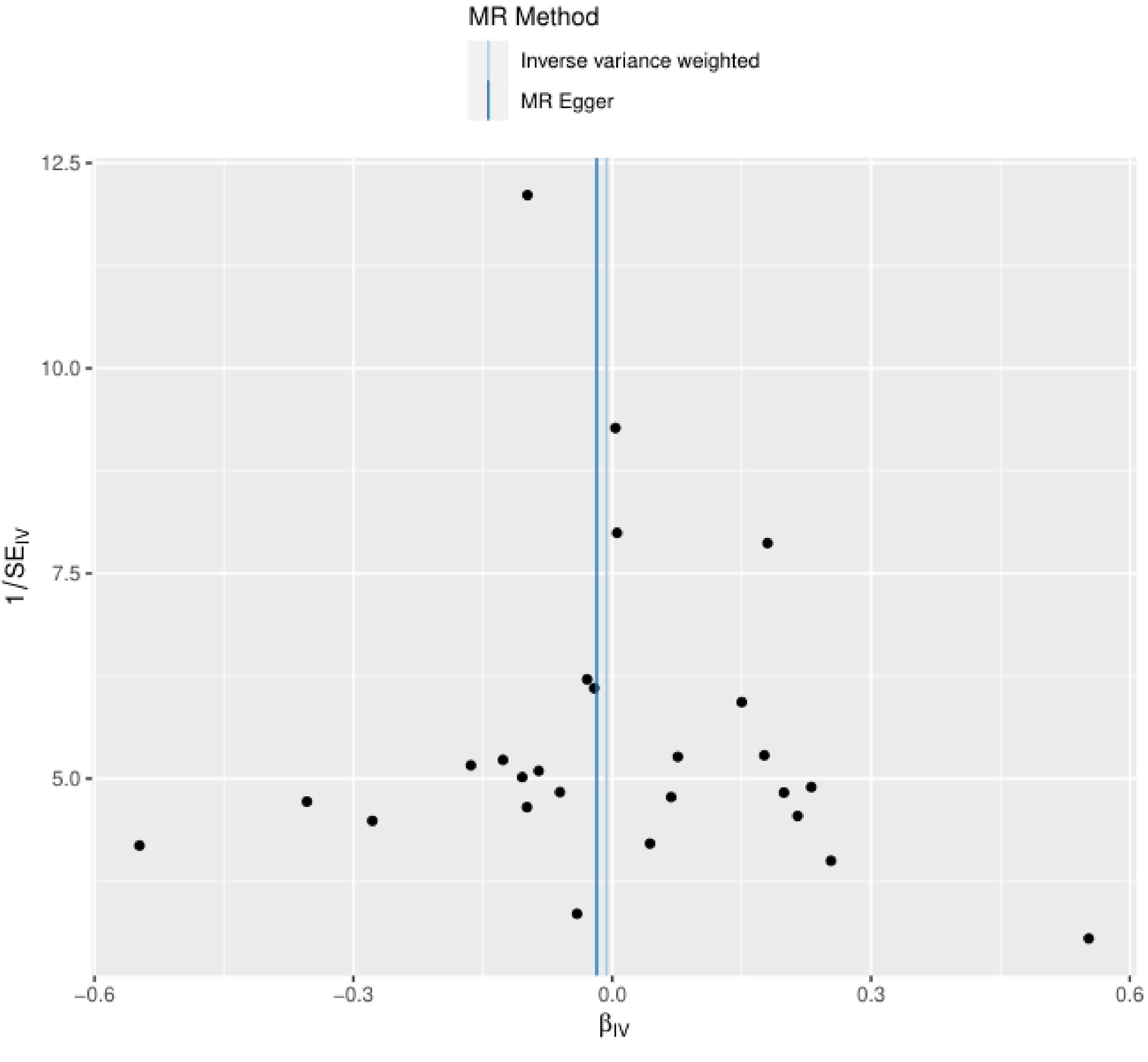

Funnel plot analyse of "T/B" on 'Diabetic nephropathy'

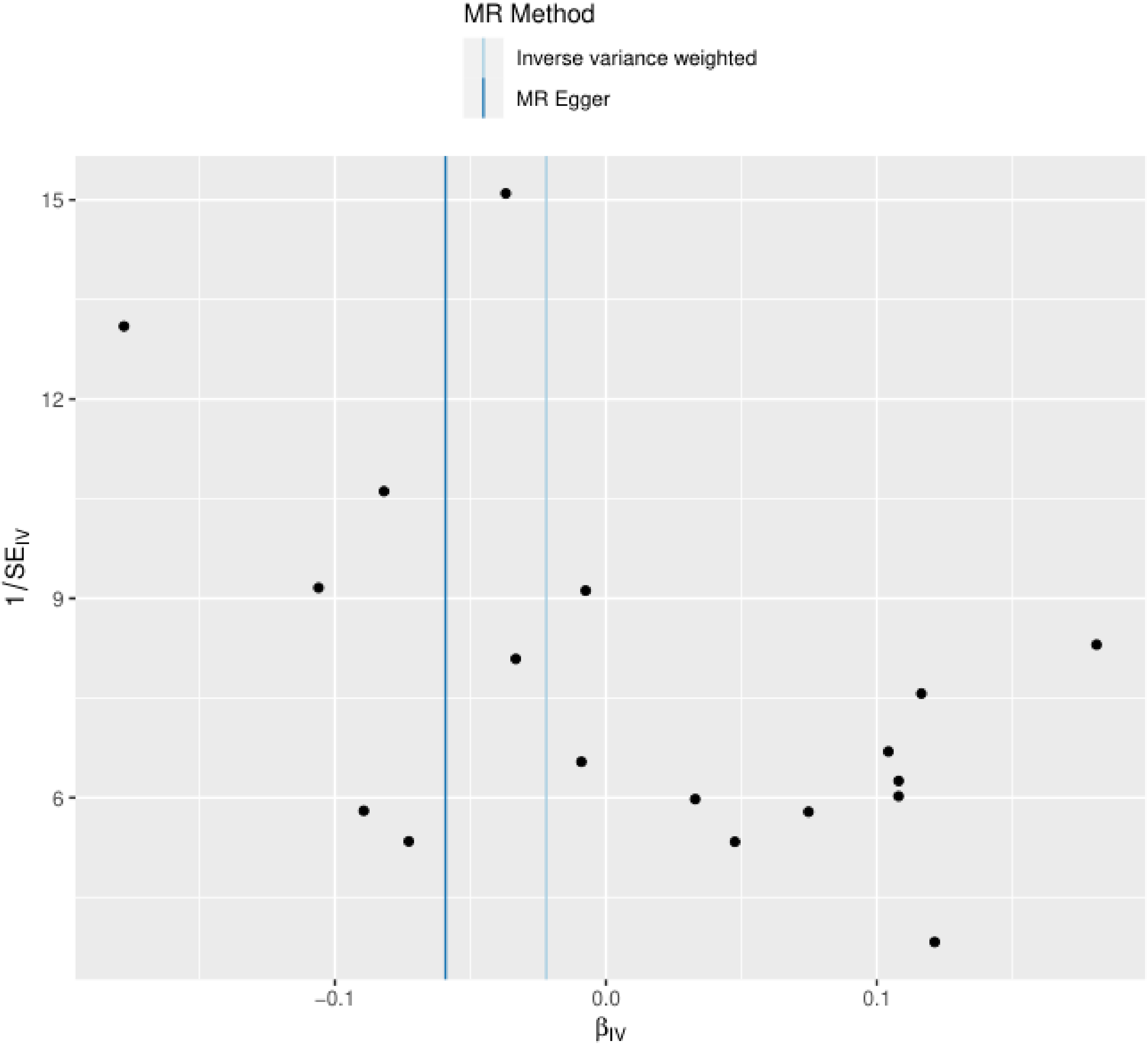

Funnel plot analyse of "CD14 on CD33dim HLA DR+ CD11b+ " on 'Diabetic nephropathy'

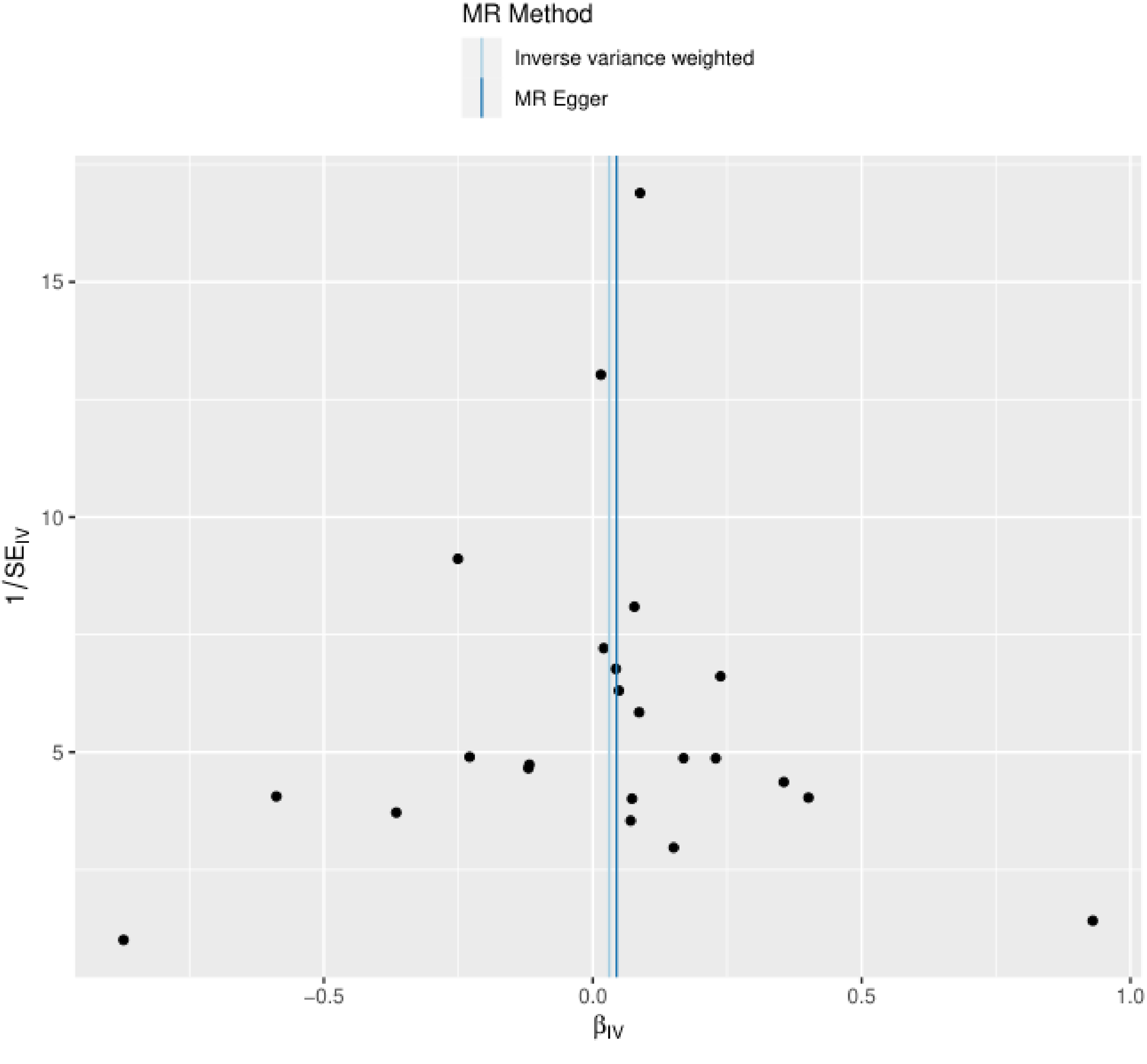

Funnel plot analyse of ''CD4 on CD39+ activated Treg '' on 'Diabetic nephropathy'

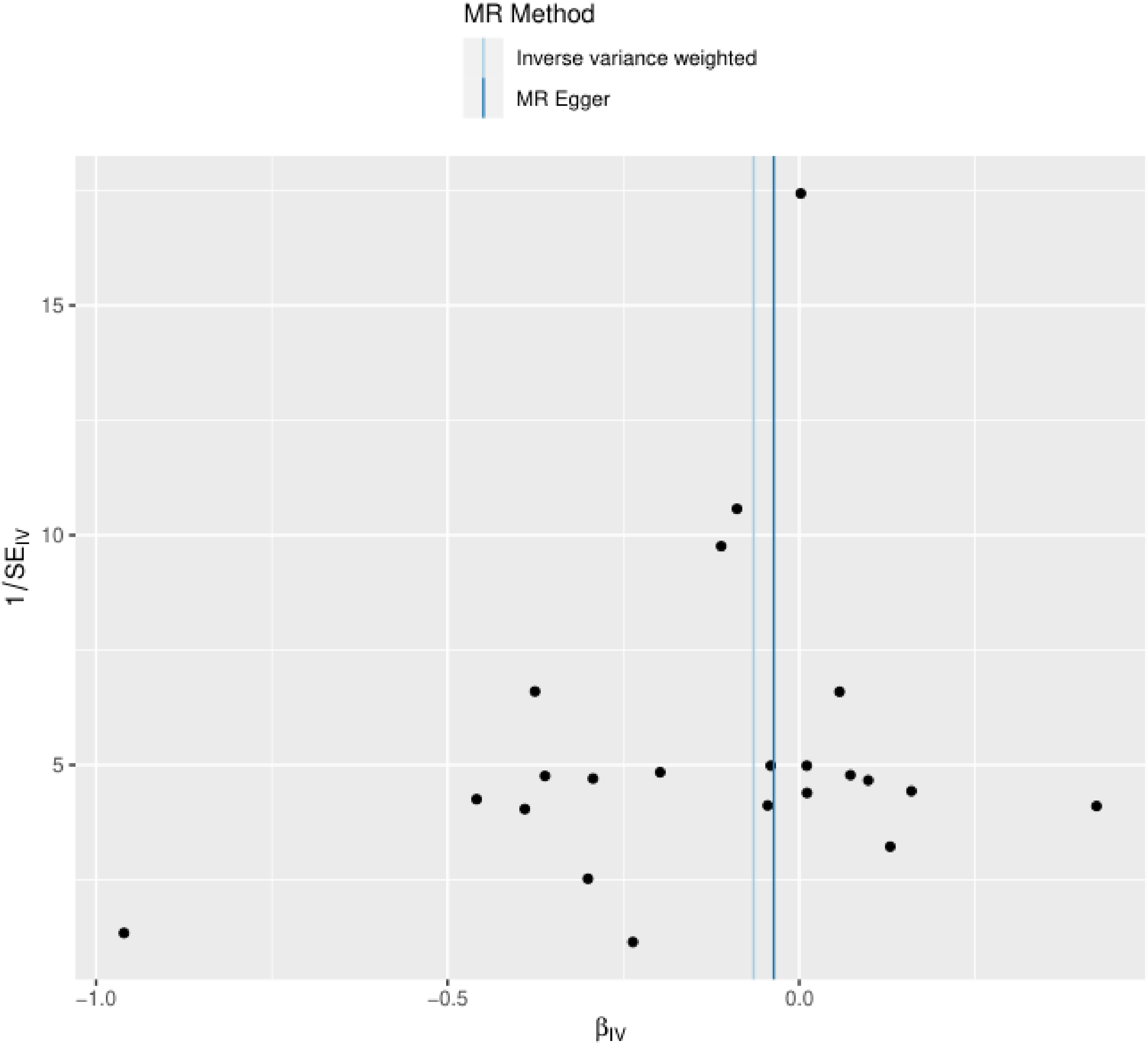

Funnel plot analyse of "Sw mem AC" on 'Diabetic nephropathy'

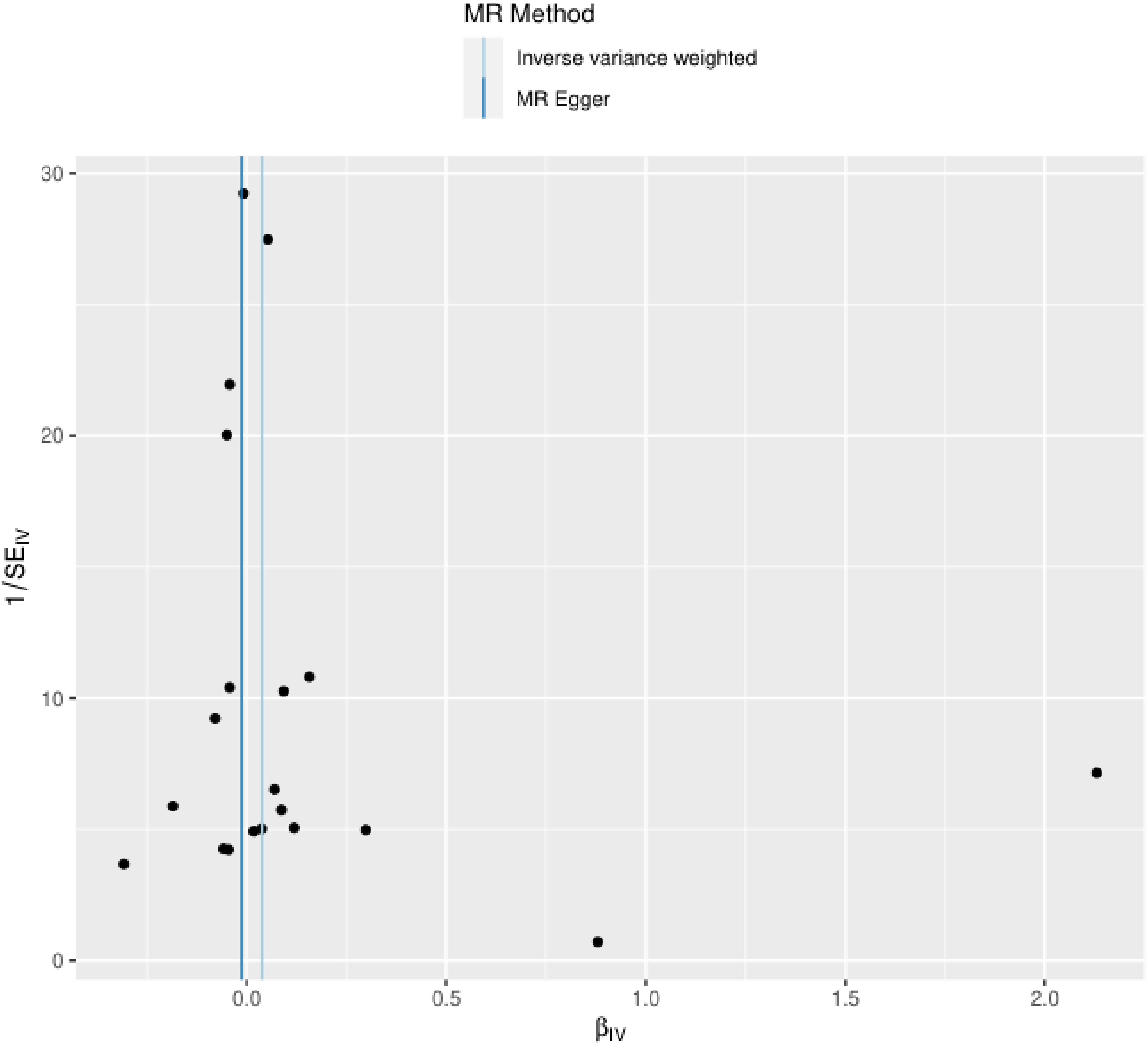

Funnel plot analyse of "CX3CR1 on CD14- CD16- " on 'Diabetic nephropathy'

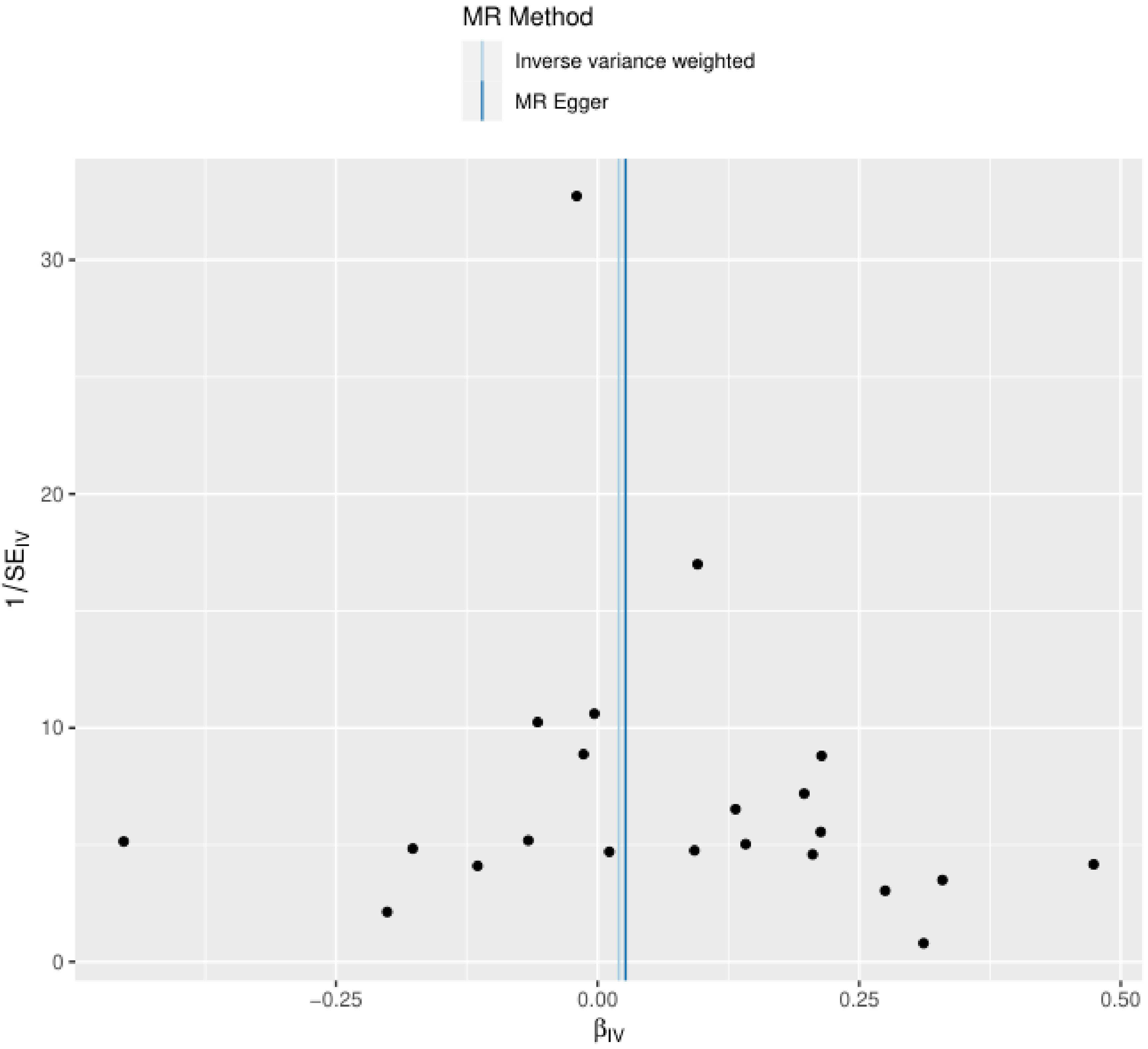

Funnel plot analyse of "BAFF-R on IgD+ CD38-" on 'Diabetic nephropathy'

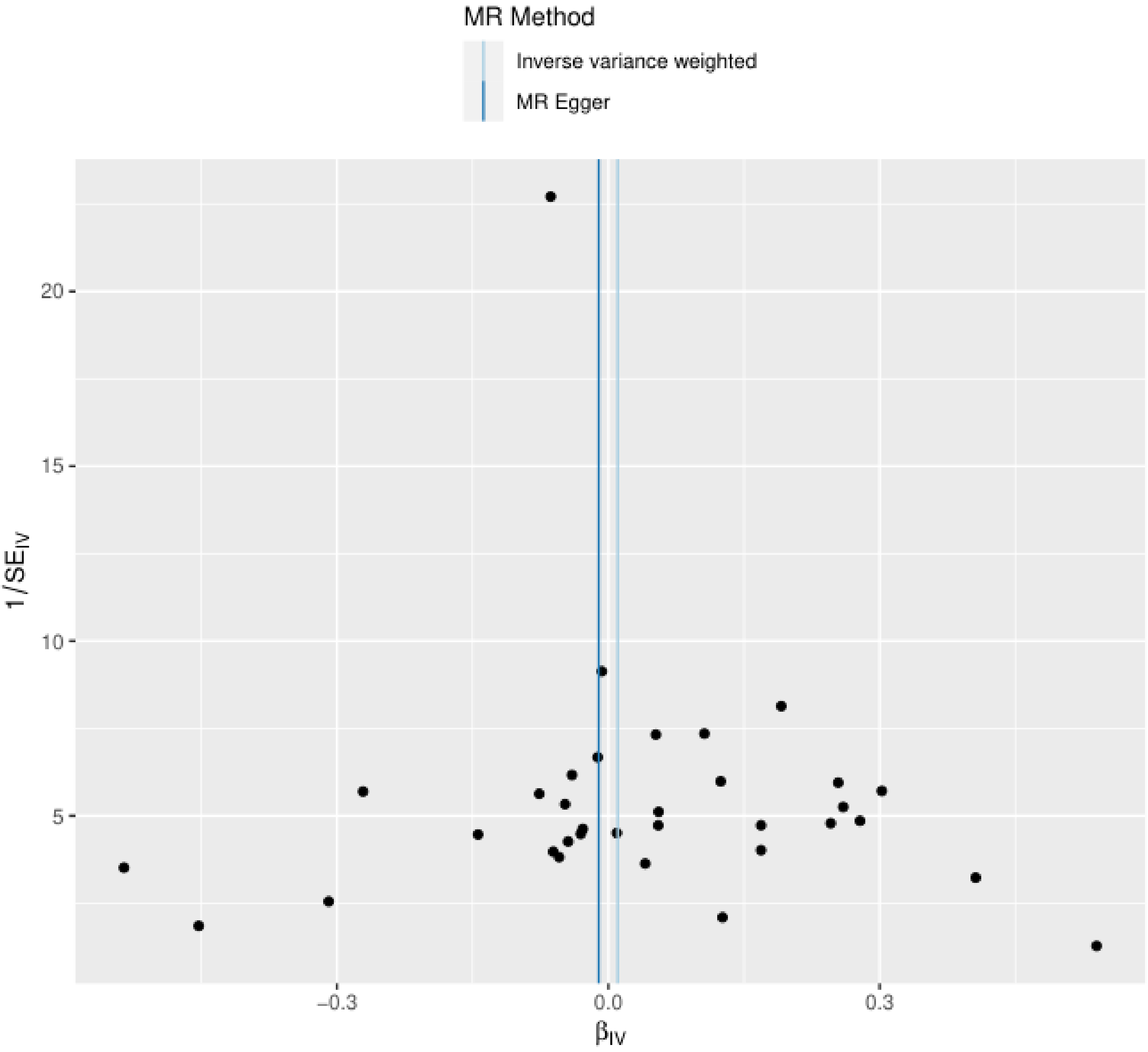

Funnel plot analyse of "NKT %T cell" on 'Diabetic nephropathy'

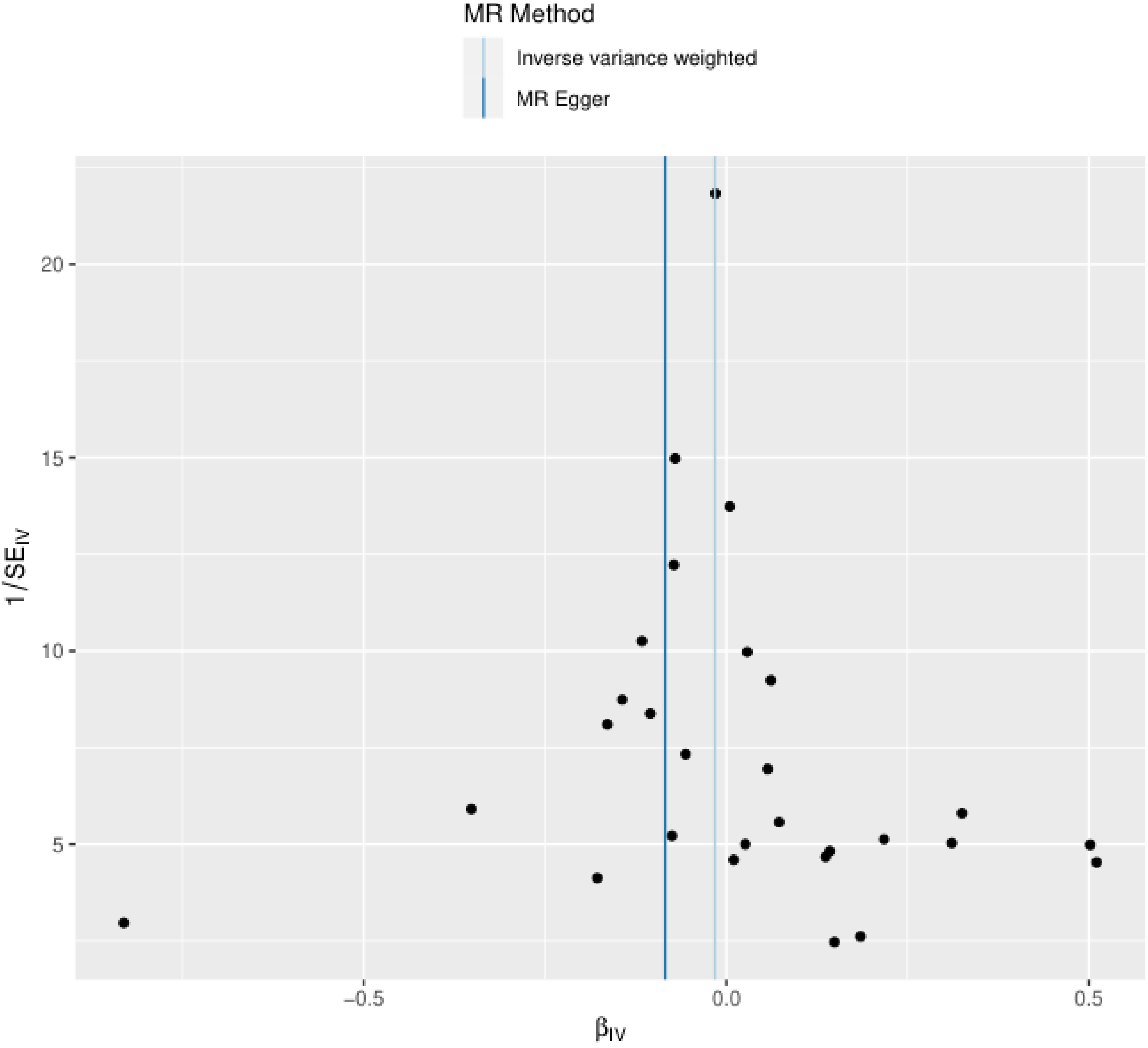

Funnel plot analyse of "CD3 on CD39+ CD4+" on 'Diabetic nephropathy'

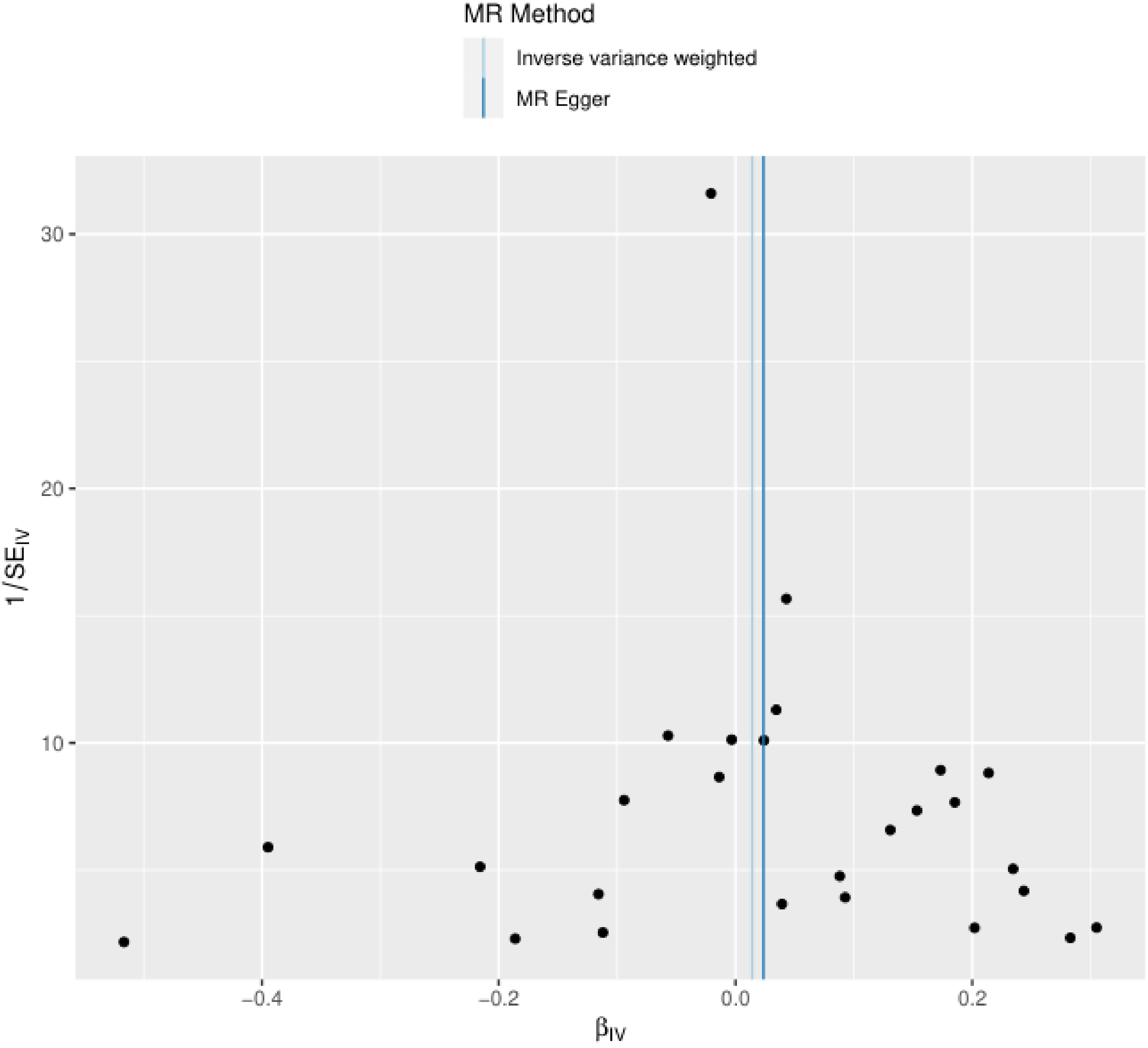

Funnel plot analyse of "BAFF-R on IgD+ CD38br" on 'Diabetic nephropathy'

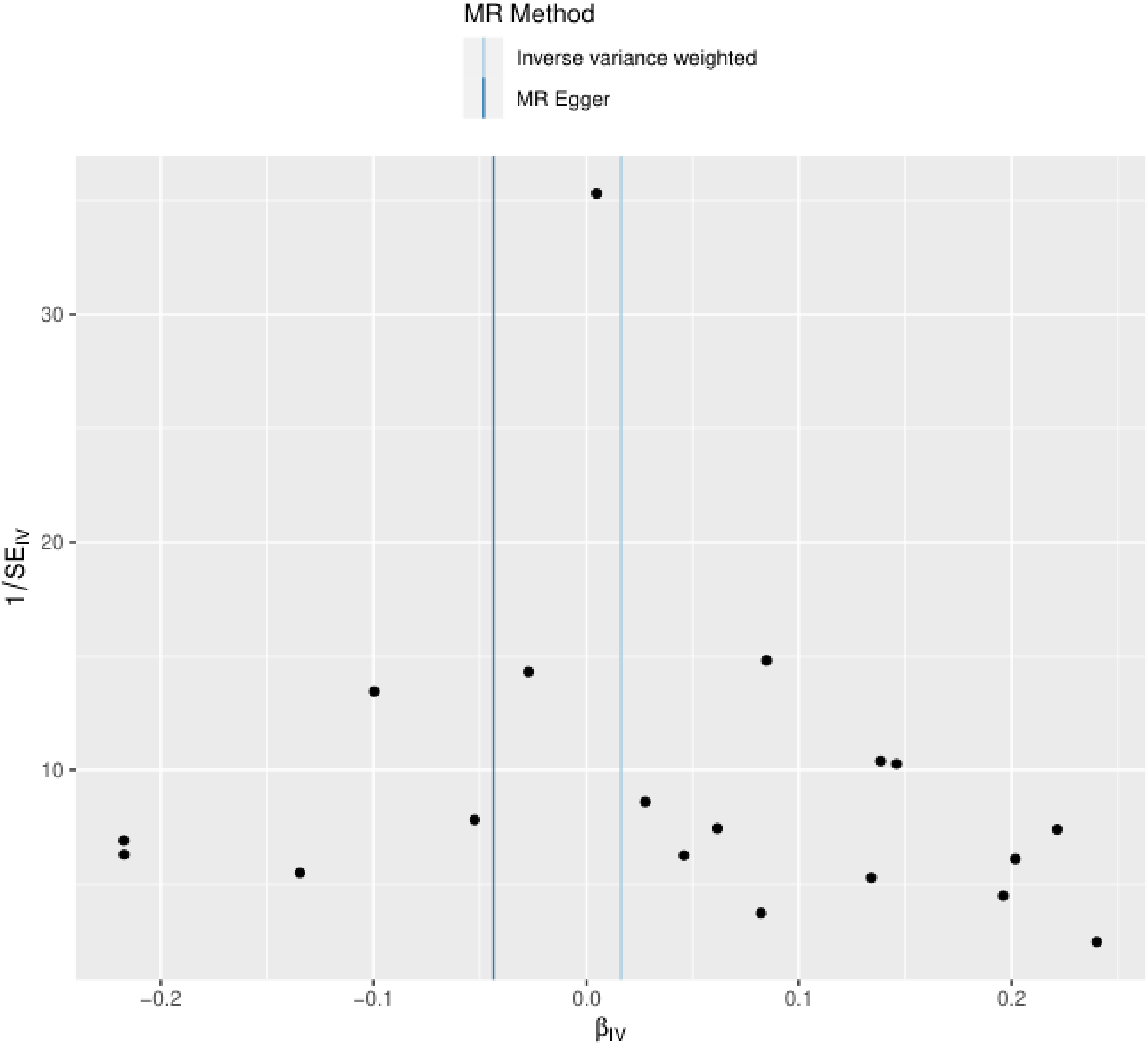

Funnel plot analyse of "CD33 on CD66b++ myeloid cell" on 'Diabetic nephropathy'

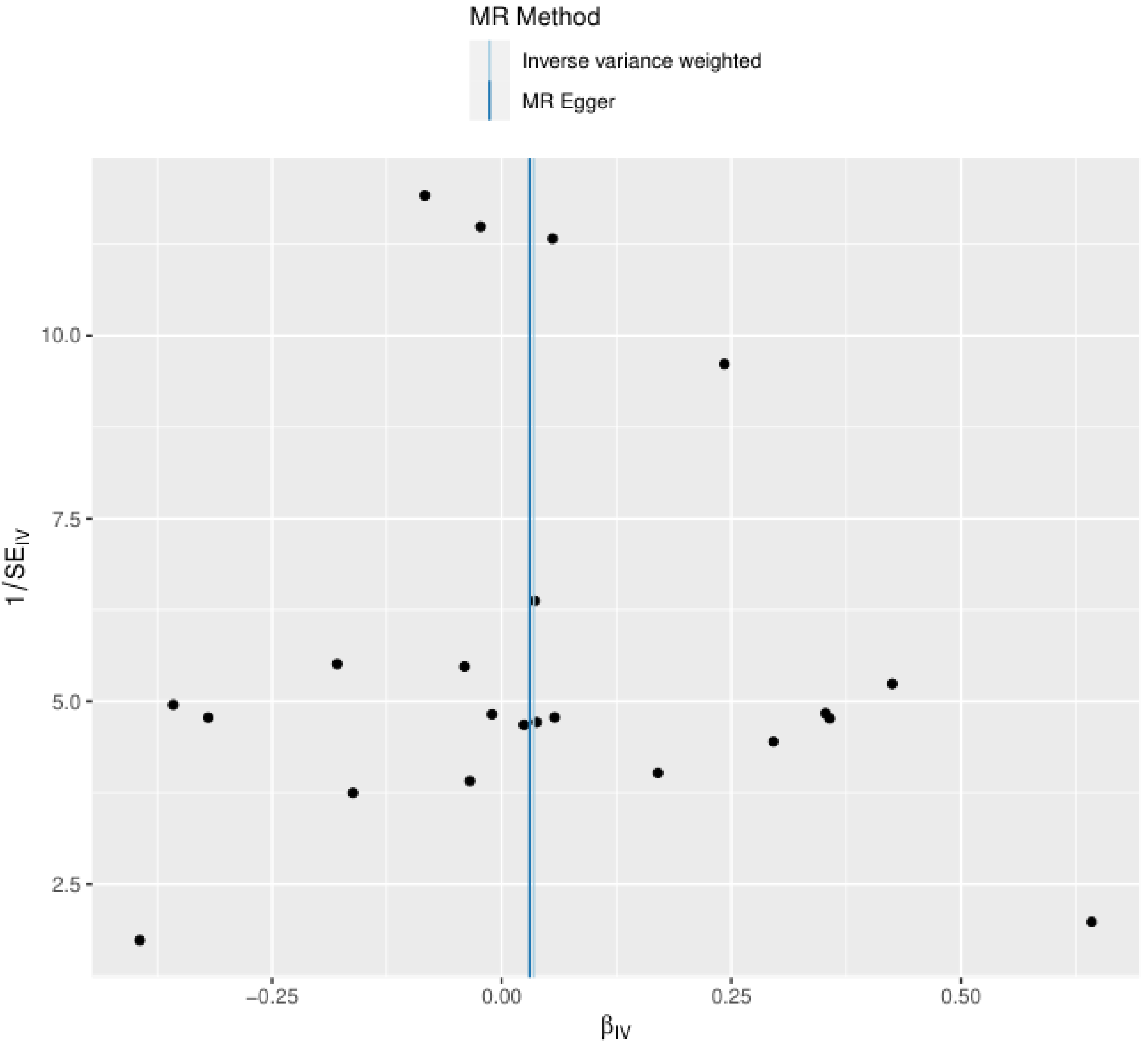

Funnel plot analyse of "CD11c on granulocyte " on 'Diabetic nephropathy'

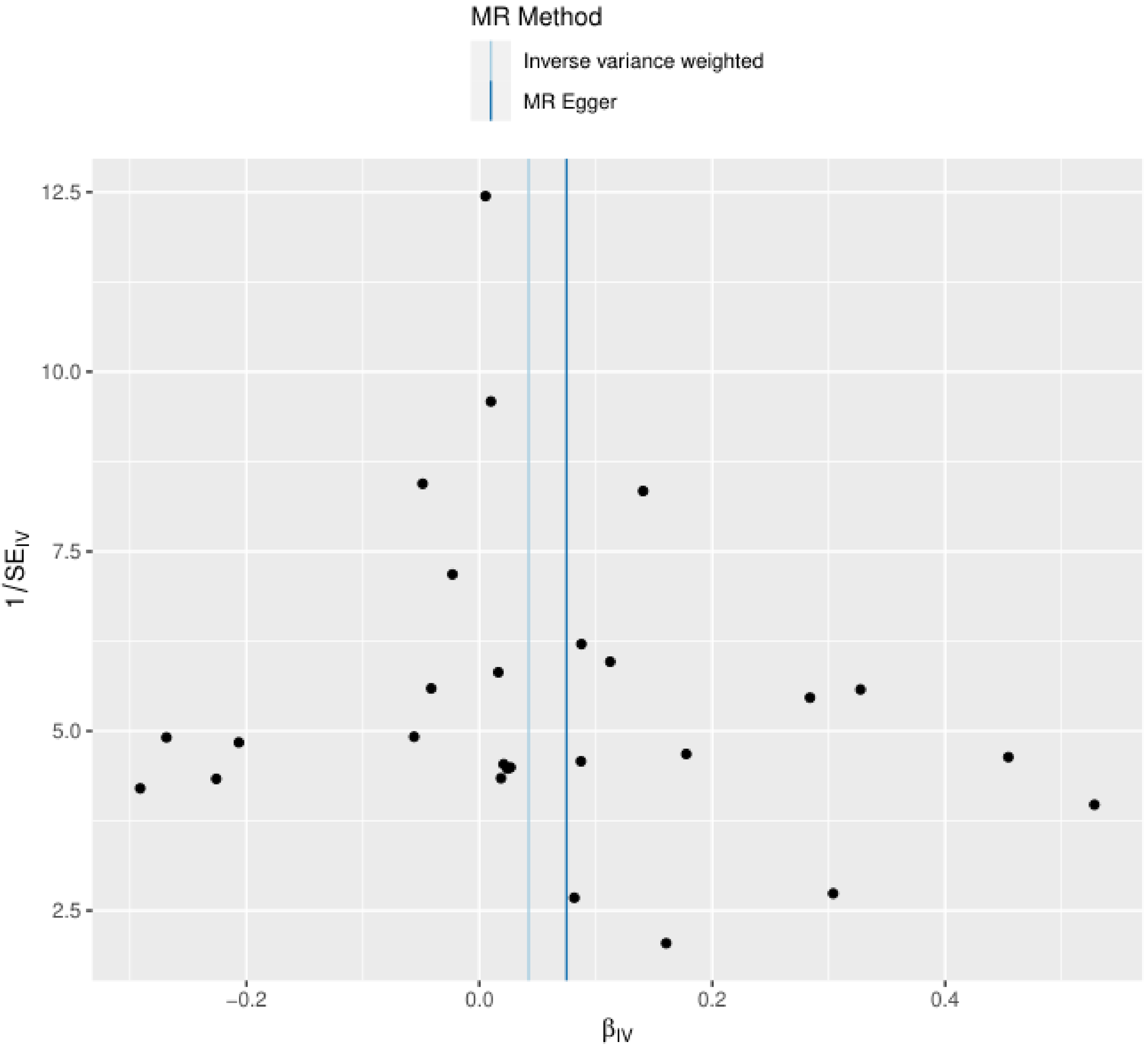

Funnel plot analyse of "CD25++ CD8br AC" on 'Diabetic nephropathy'

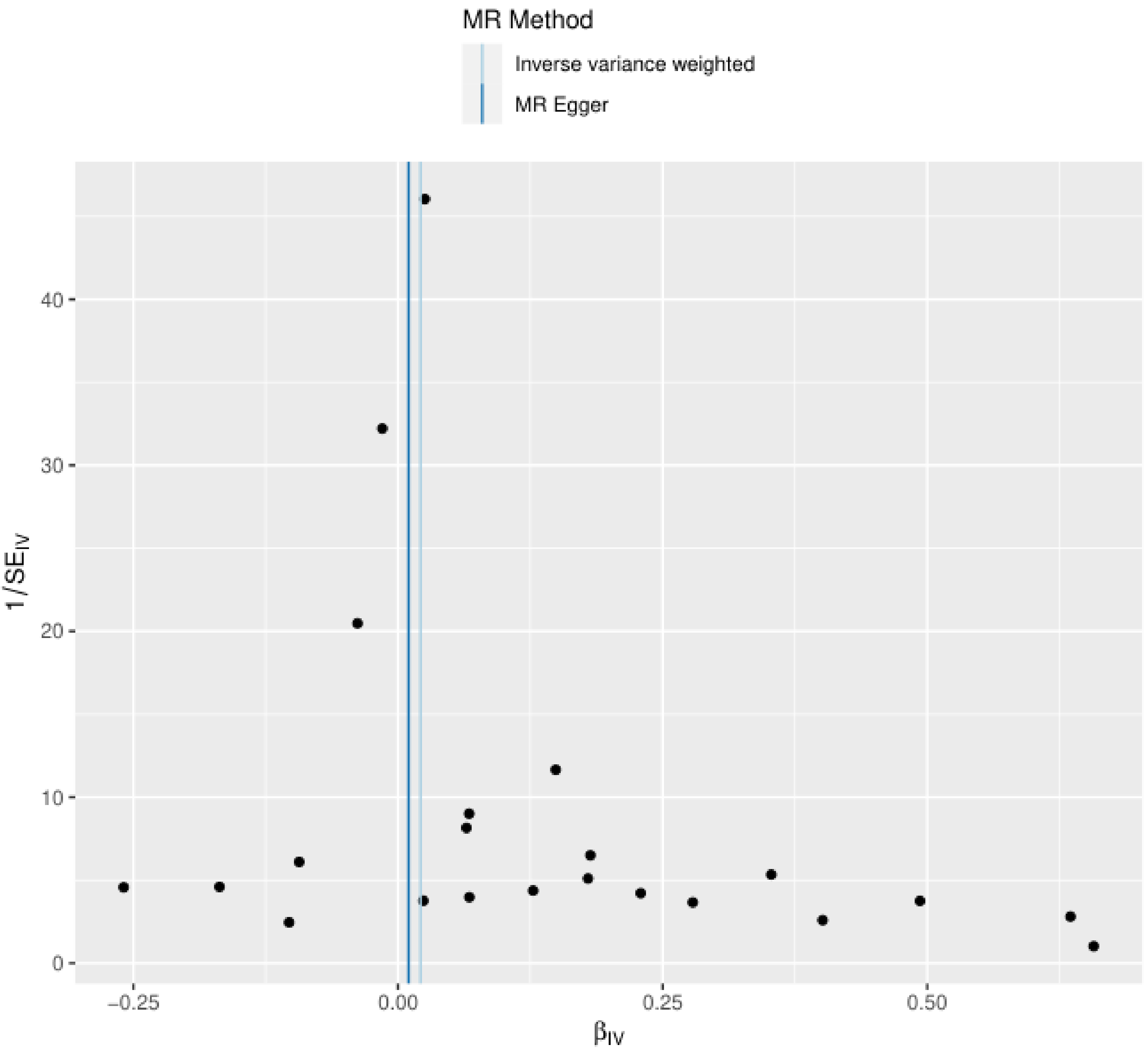

Funnel plot analyse of "IgD+ CD24- AC" on 'Diabetic nephropathy'

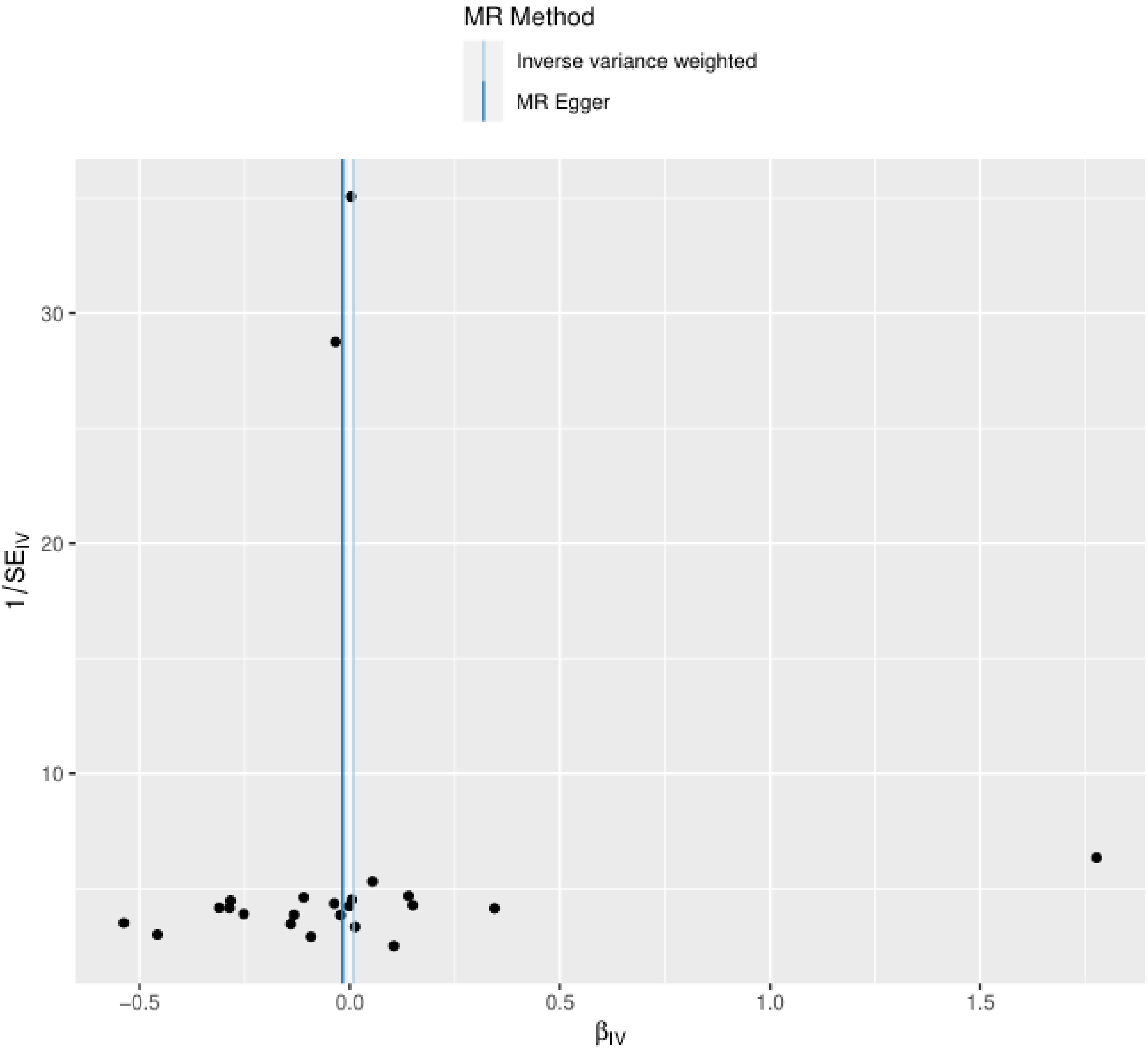

Funnel plot analyse of "CD20- AC" on 'Diabetic nephropathy'

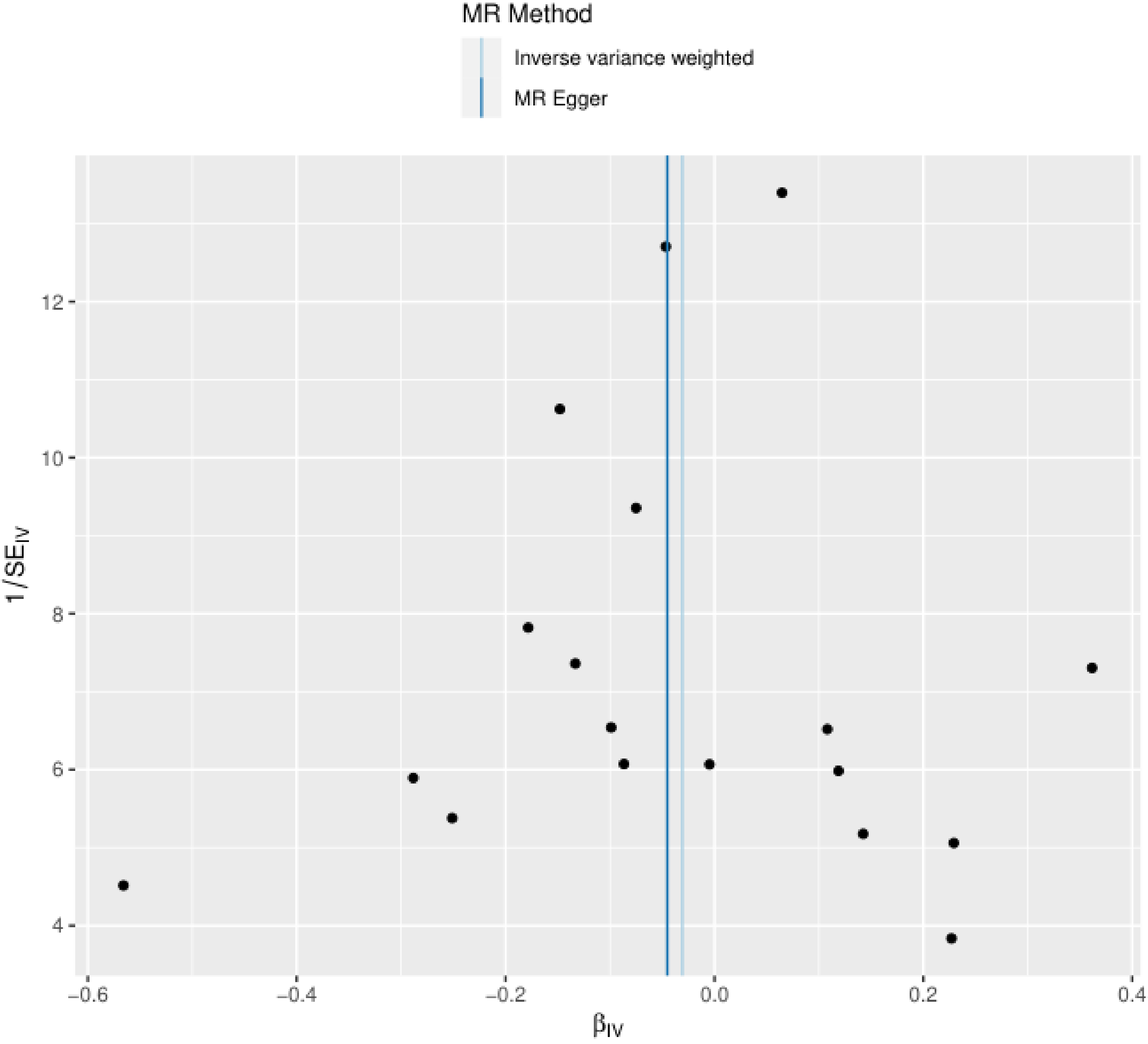

Funnel plot analyse of "CD38 on CD3- CD19-" on 'Diabetic nephropathy'

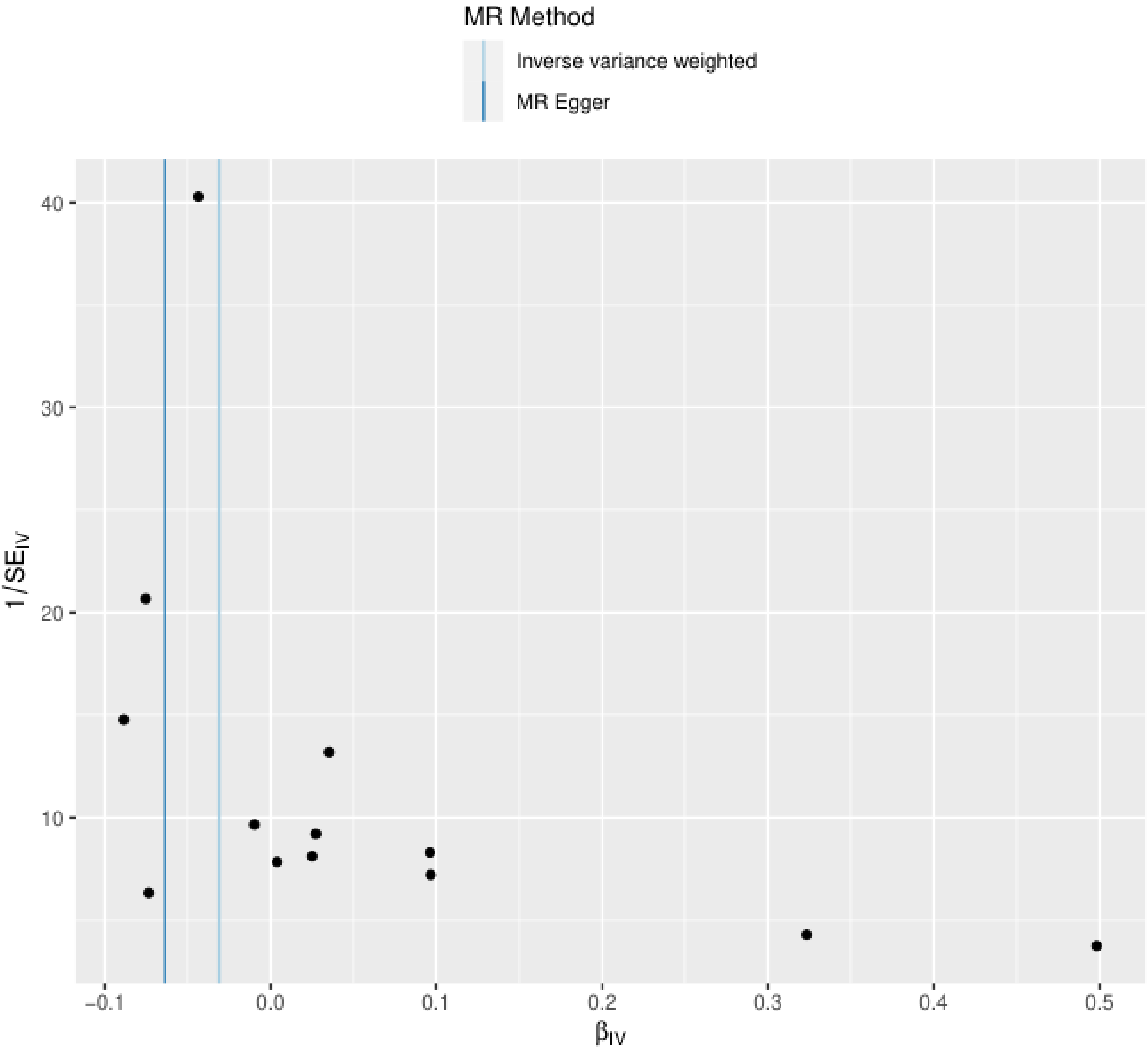

Funnel plot analyse of "CD34 on HSC" on 'Diabetic nephropathy'

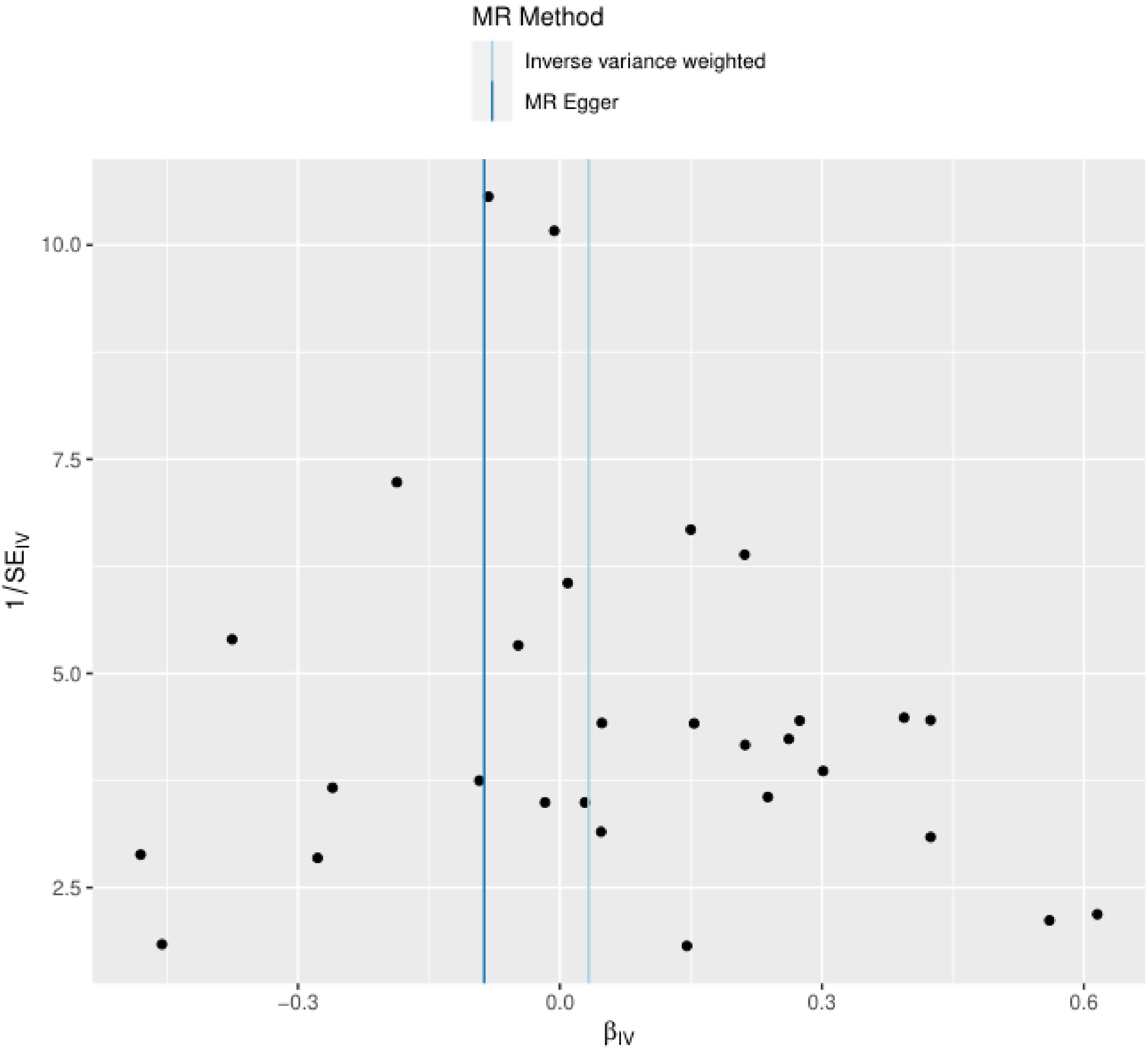

Funnel plot analyse of "DN (CD4-CD8-) NKT %lymphocyte" on 'Diabetic nephropathy'

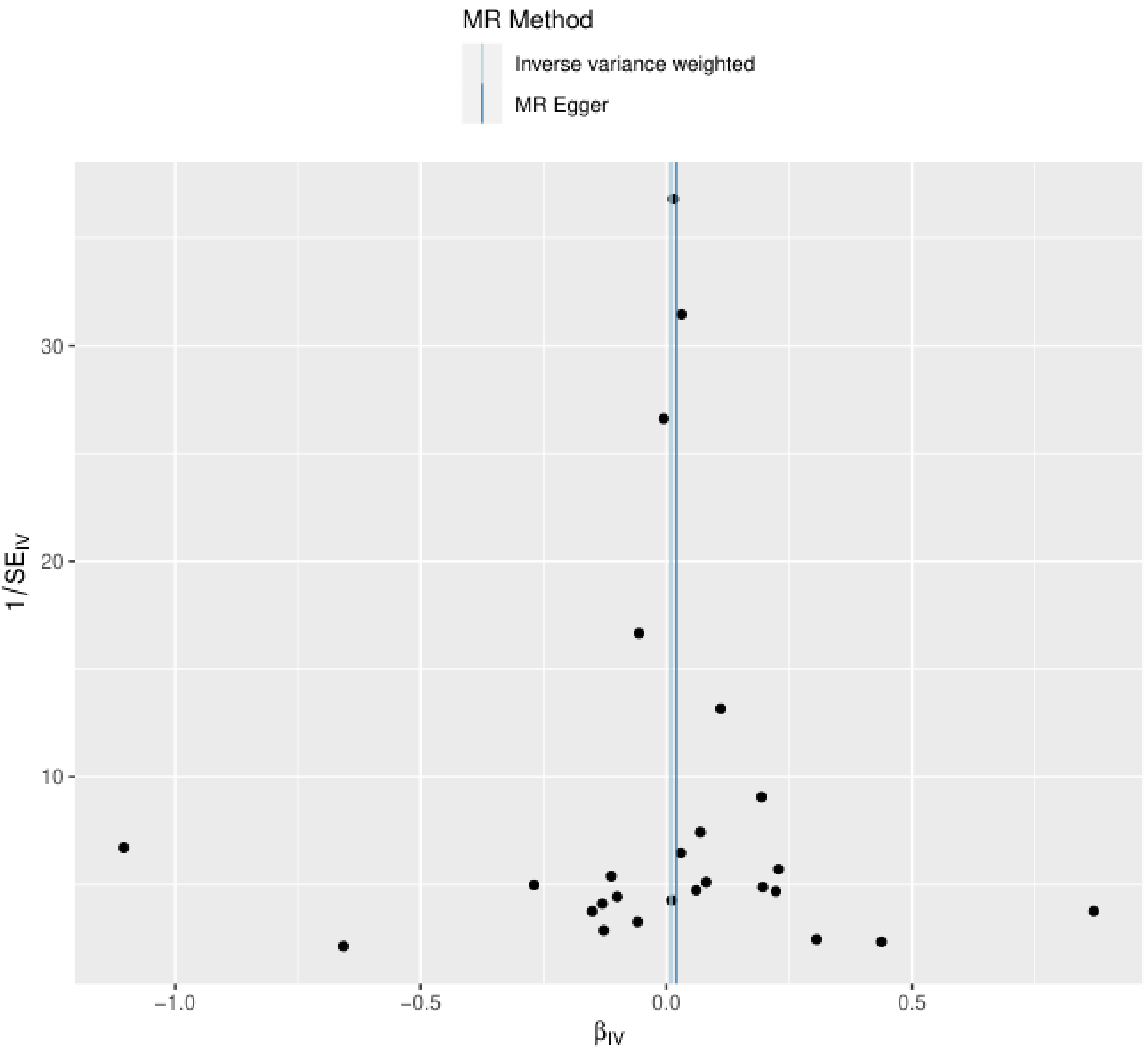

Funnel plot analyse of "CD28+ CD45RA+ CD8dim %CD8dim" on 'Diabetic nephropathy'

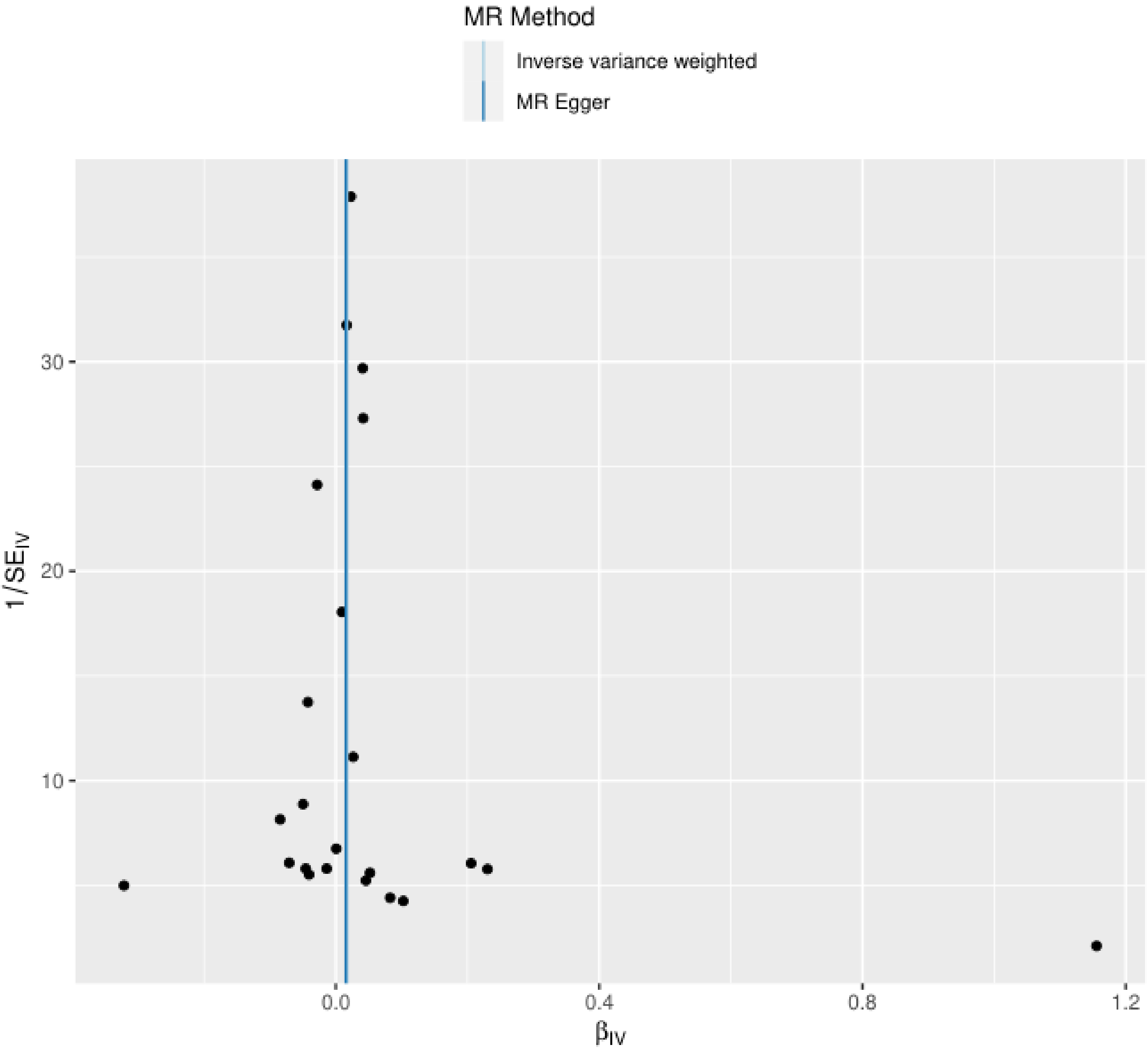

Funnel plot analyse of "Basophil AC" on 'Diabetic nephropathy'

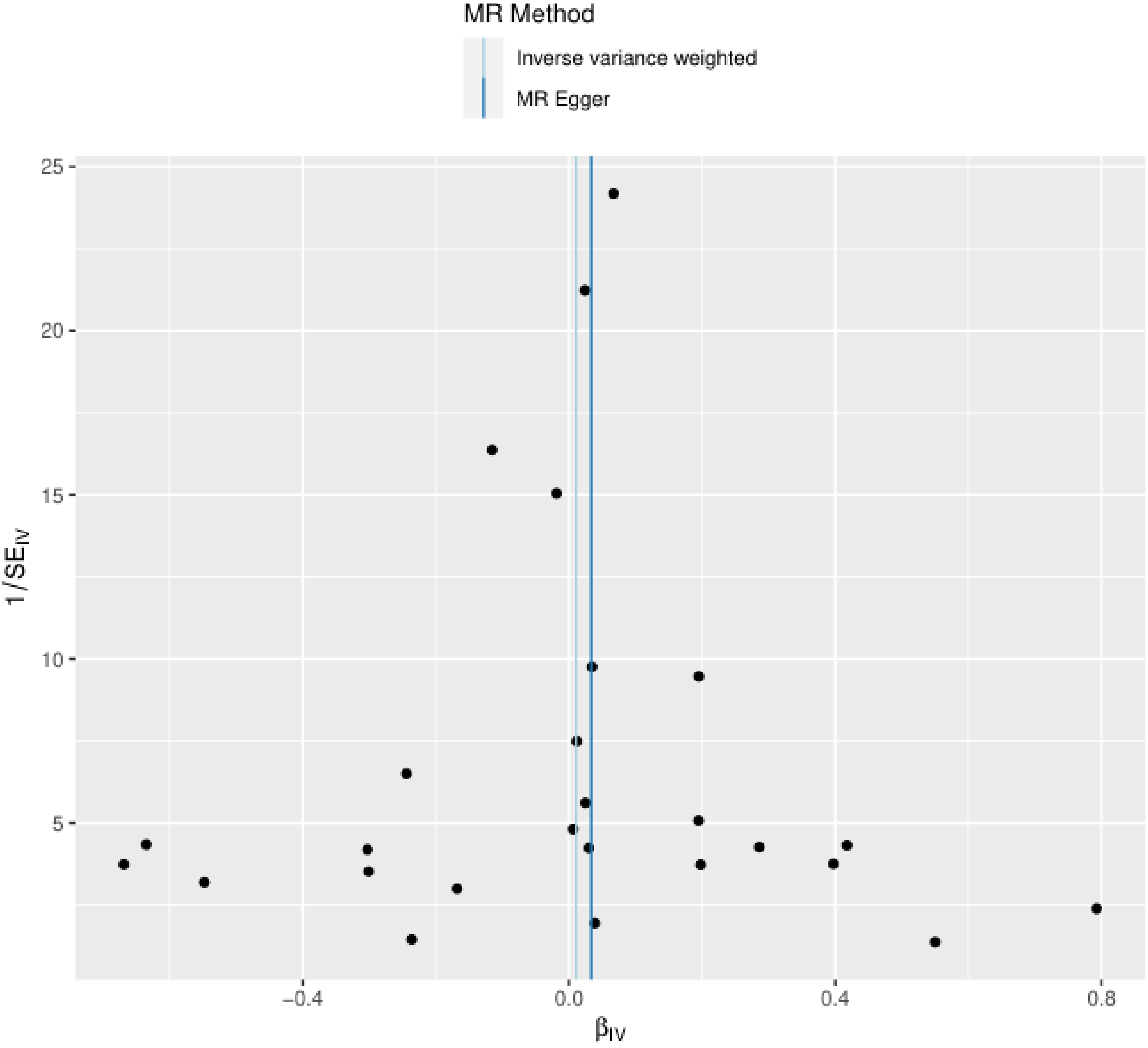

Funnel plot analyse of "CD25 on CD45RA- CD4 not Treg " on 'Diabetic nephropathy'

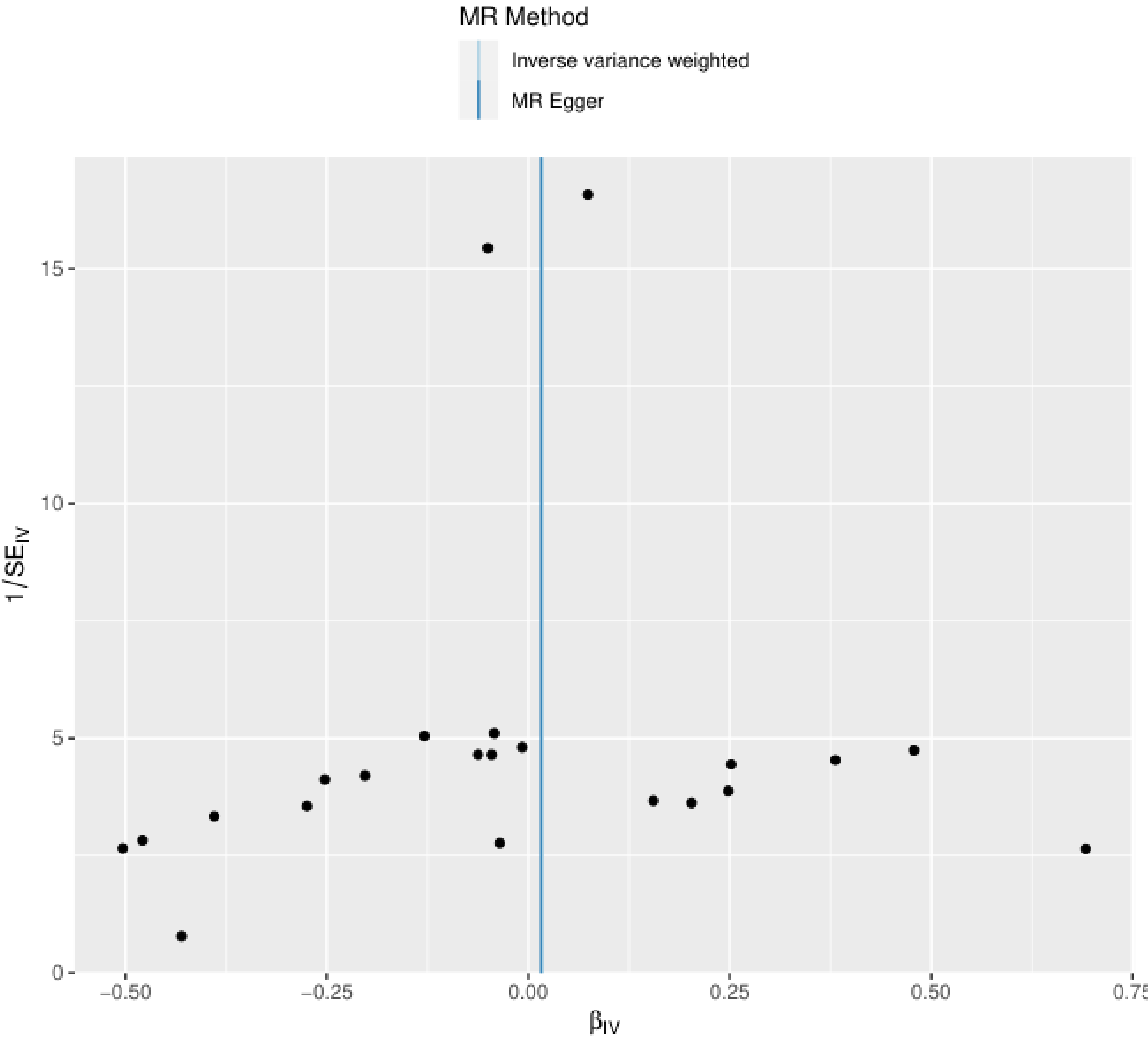

Funnel plot analyse of "SSC-A on T cell" on 'Diabetic nephropathy'

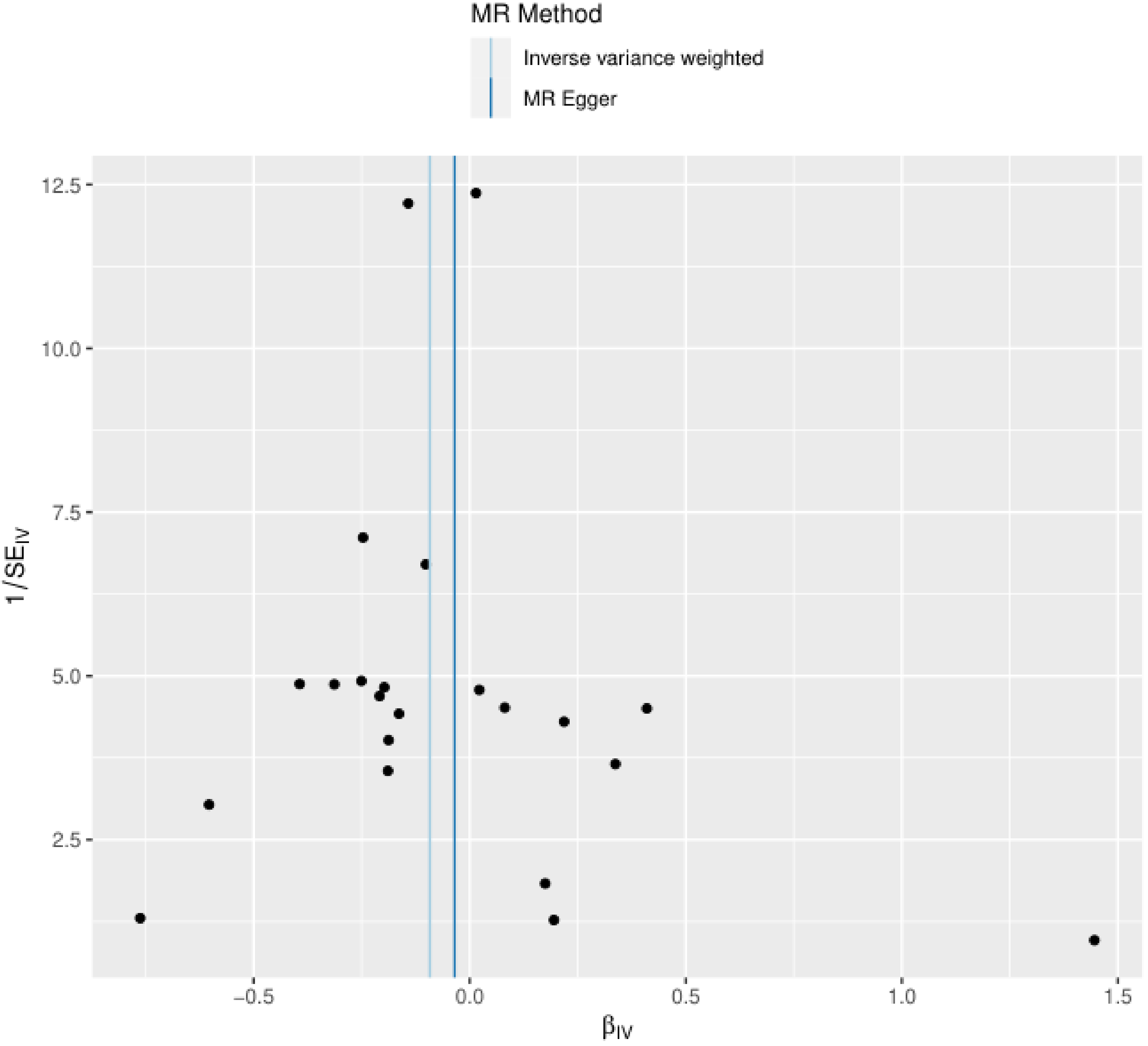

Funnel plot analyse of "CD25++ CD8br %CD8br" on 'Diabetic nephropathy'

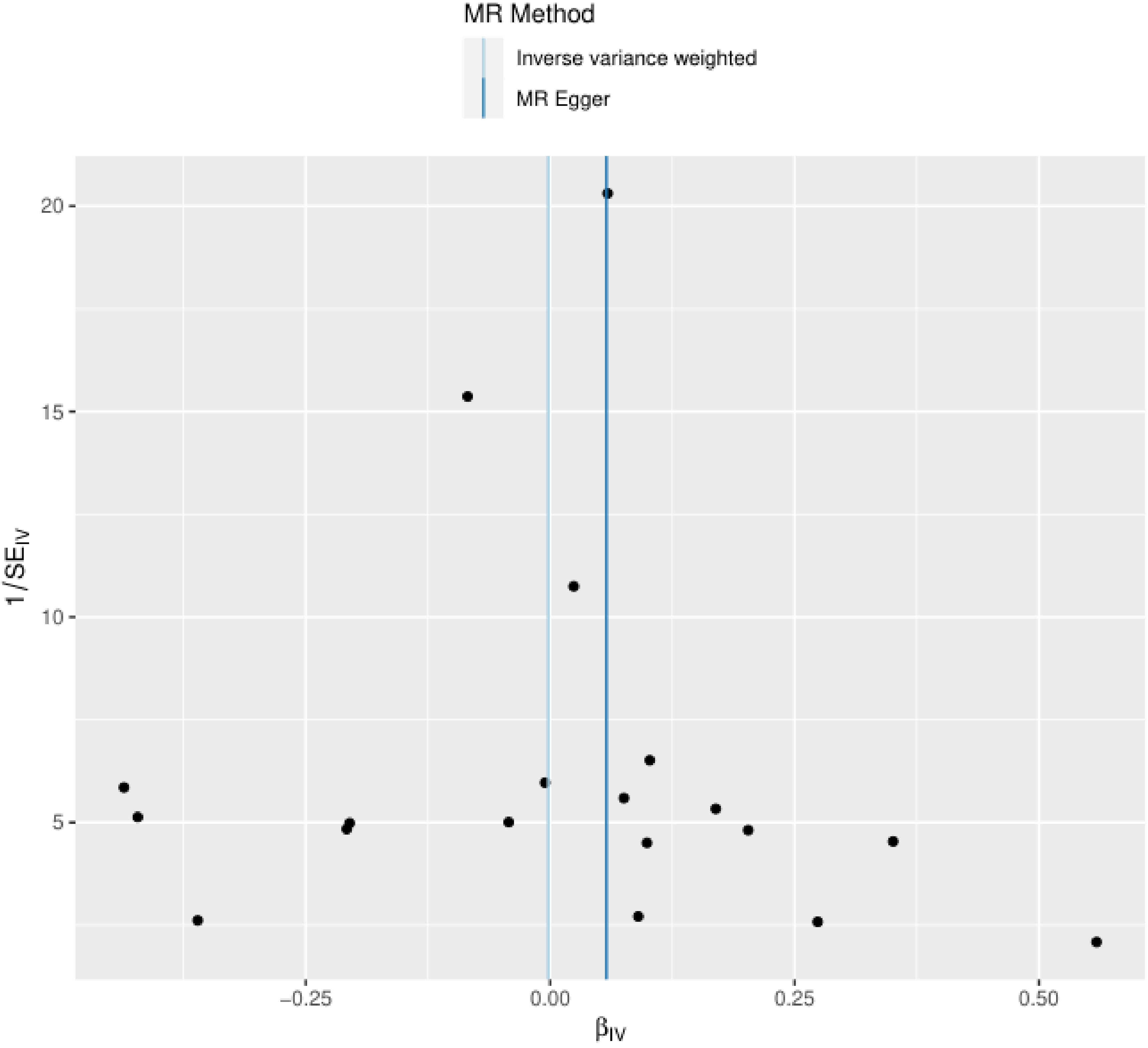

Funnel plot analyse of "CCR2 on CD62L+ plasmacytoid DC" on 'Diabetic nephropathy'

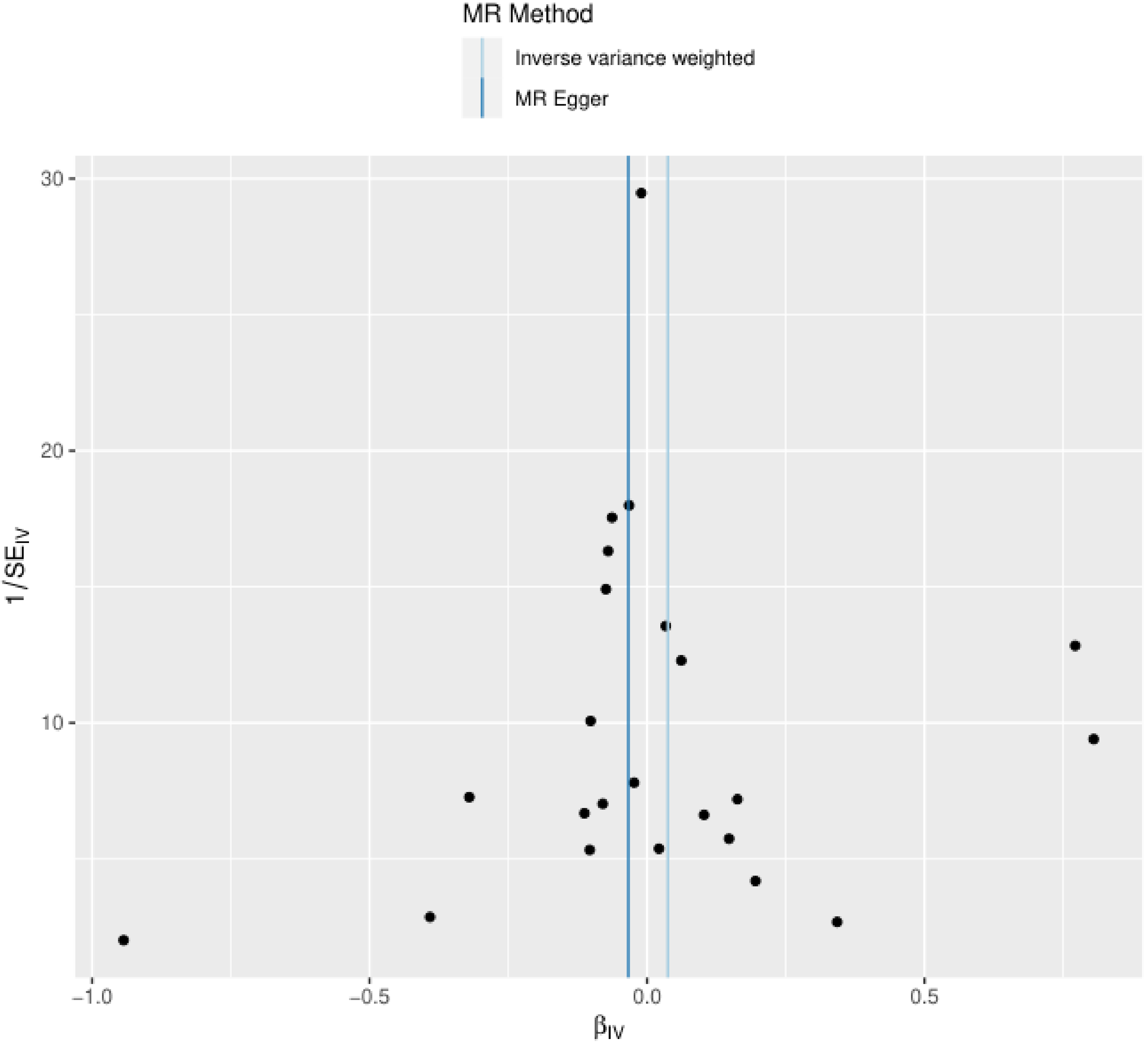

Funnel plot analyse of "HLA DR on CD33dim HLA DR+ CD11b+ " on 'Diabetic nephropathy'

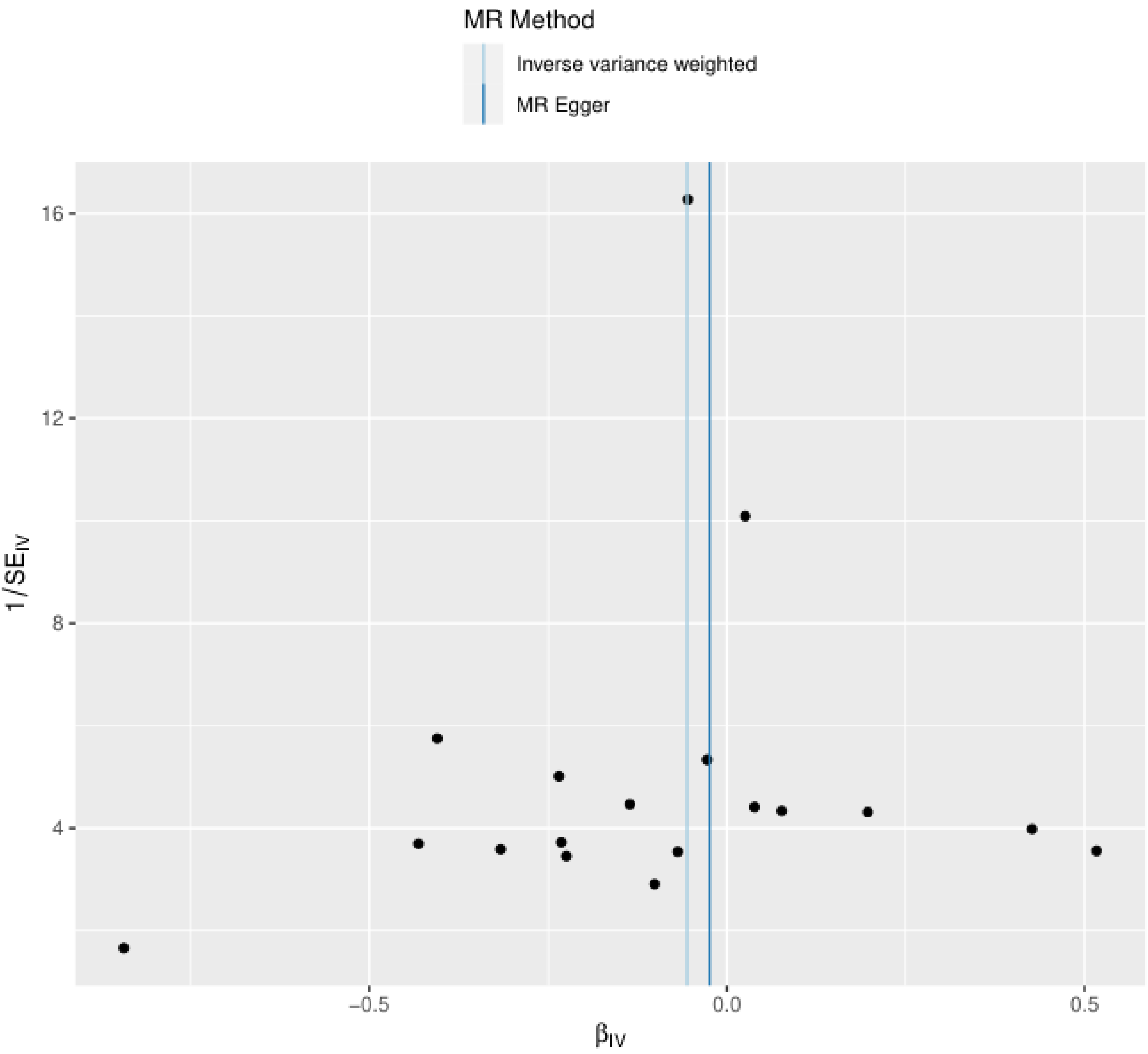

Funnel plot analyse of "CD27 on PB/PC" on 'Diabetic nephropathy'

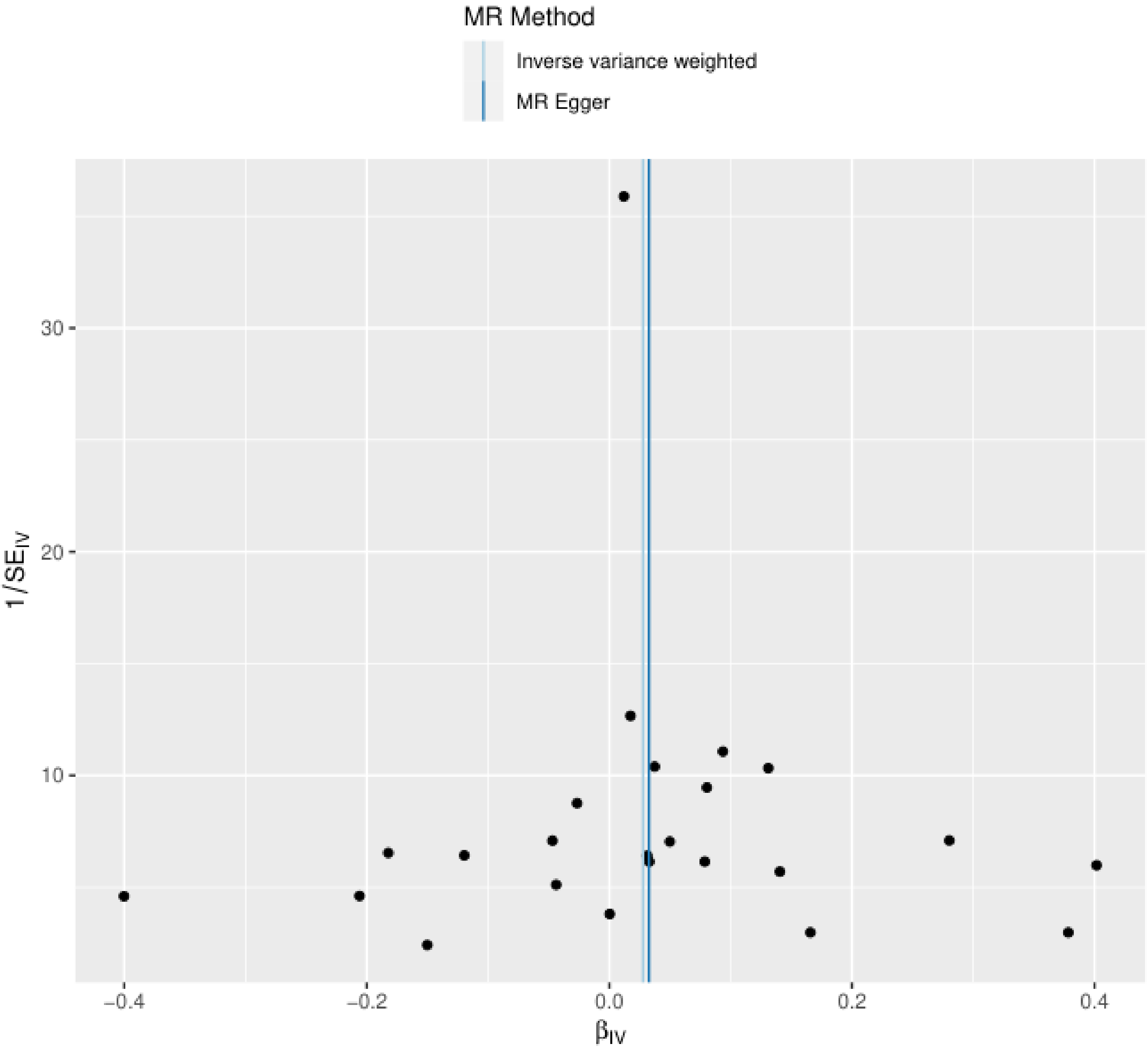

Funnel plot analyse of "CD14 on Mo MDSC " on 'Diabetic nephropathy'

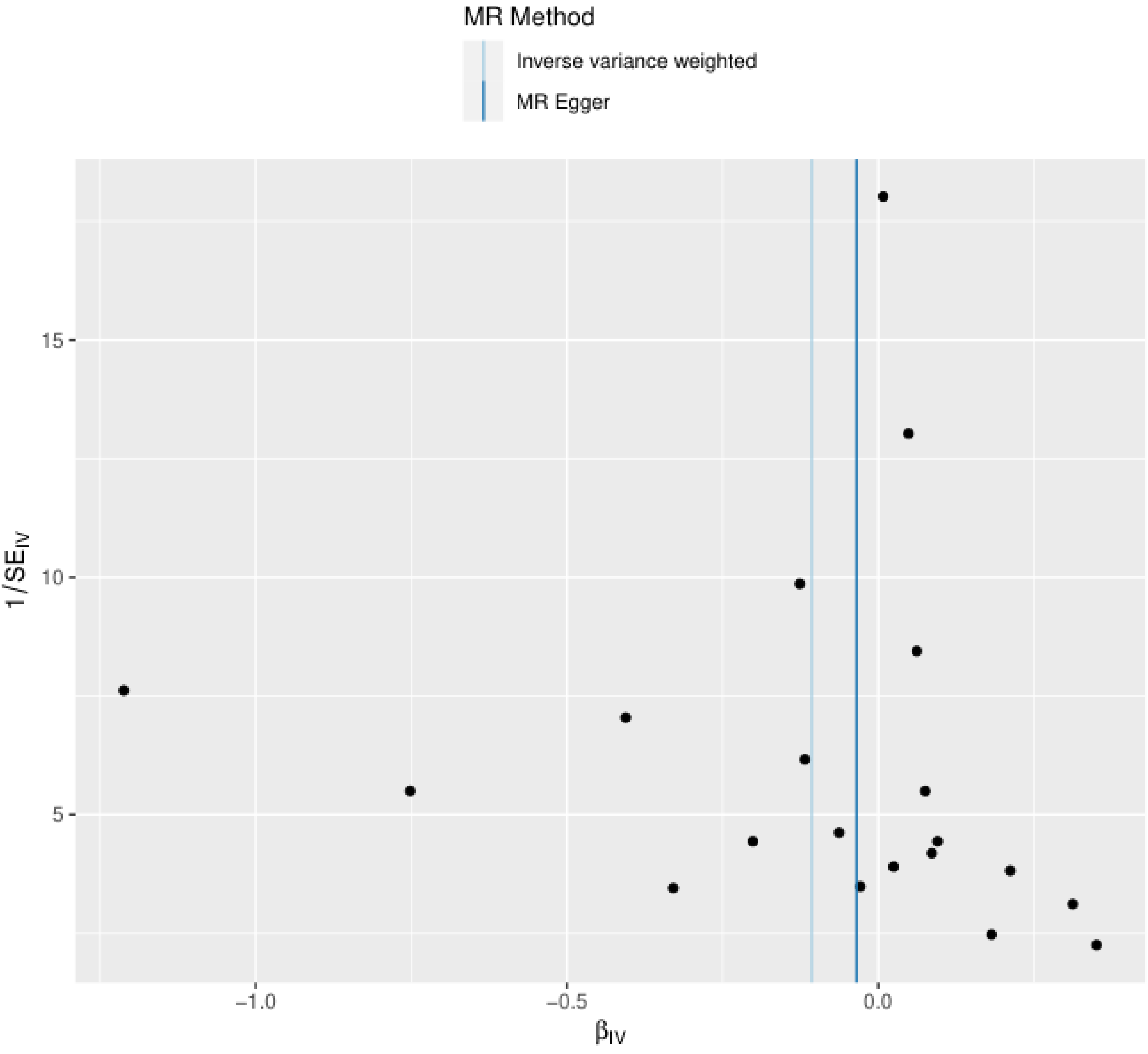

Funnel plot analyse of "EM CD4+ %T cell" on 'Diabetic nephropathy'

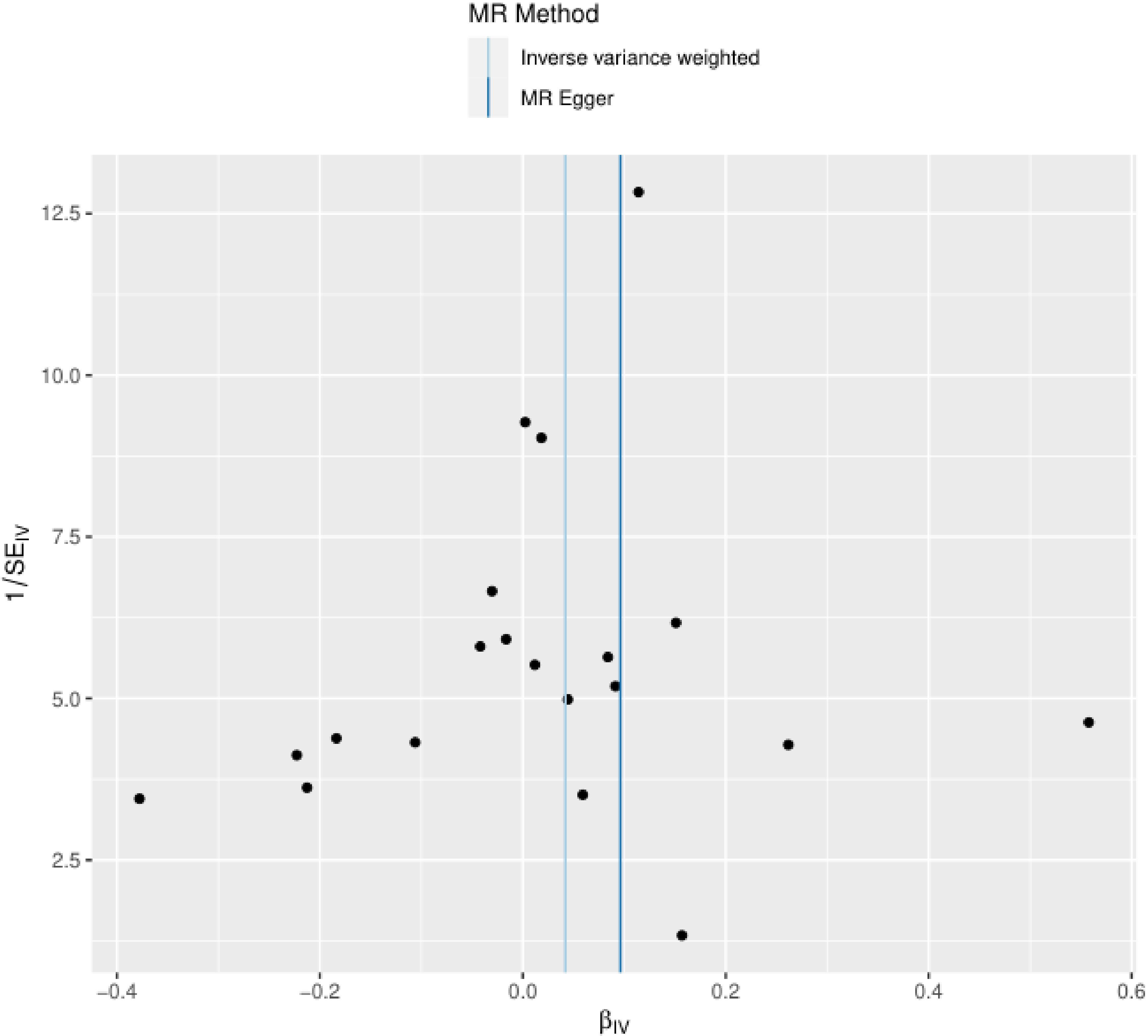

Funnel plot analyse of "FSC-A on B cell" on 'Diabetic nephropathy'

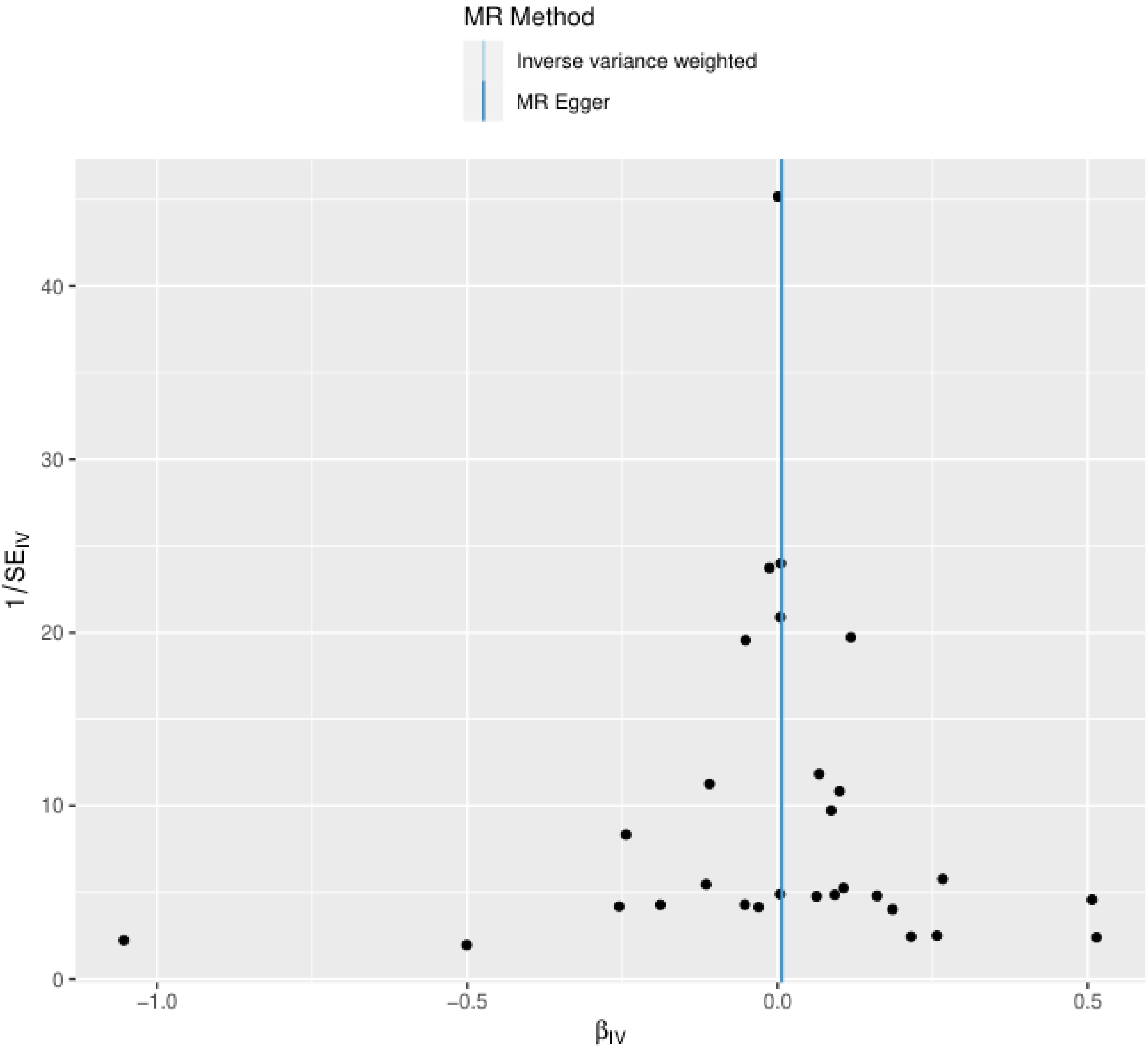

Funnel plot analyse of "CD40 on monocytes" on 'Diabetic nephropathy'

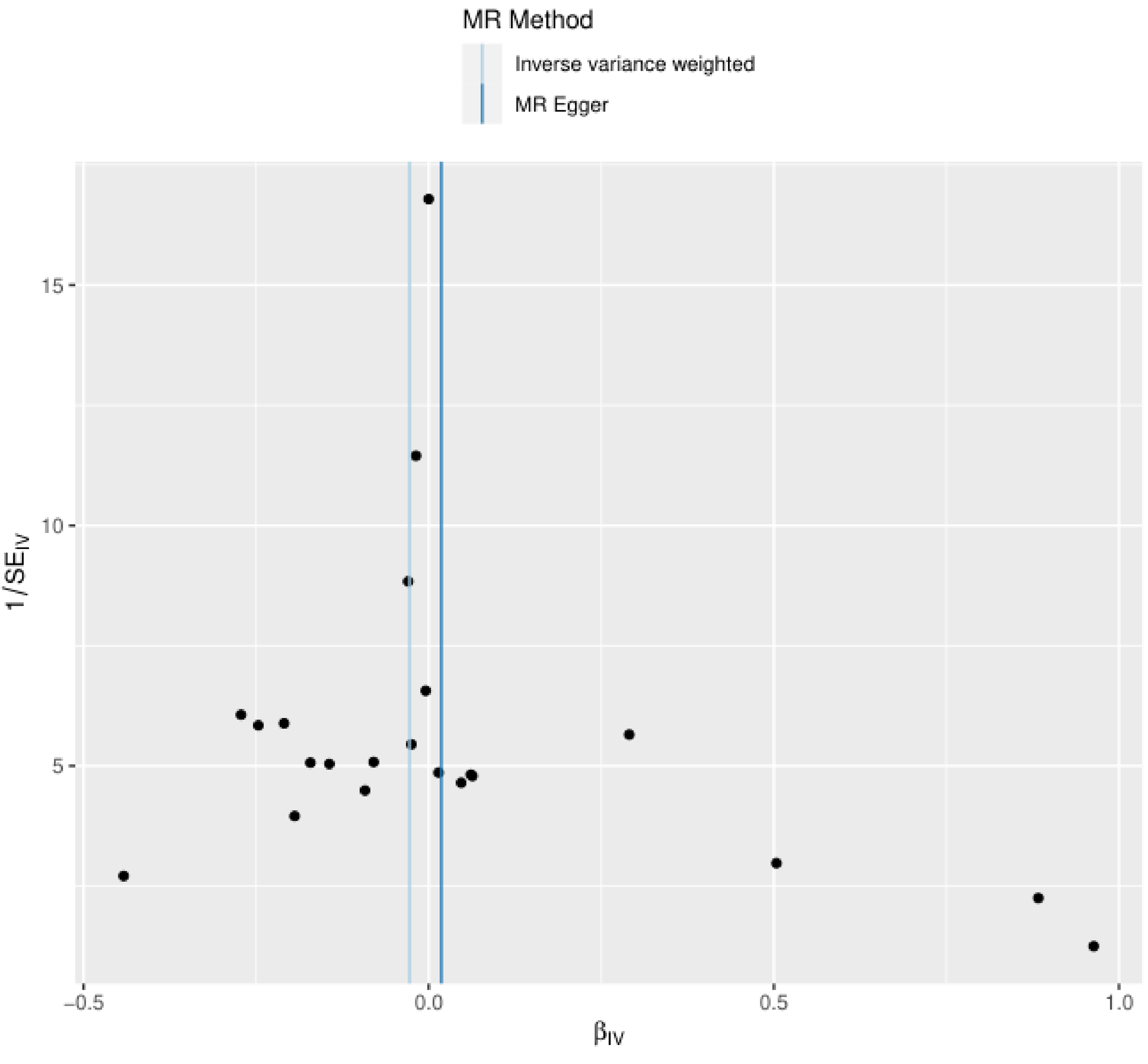

Funnel plot analyse of "SSC-A on NK" on 'Diabetic nephropathy'

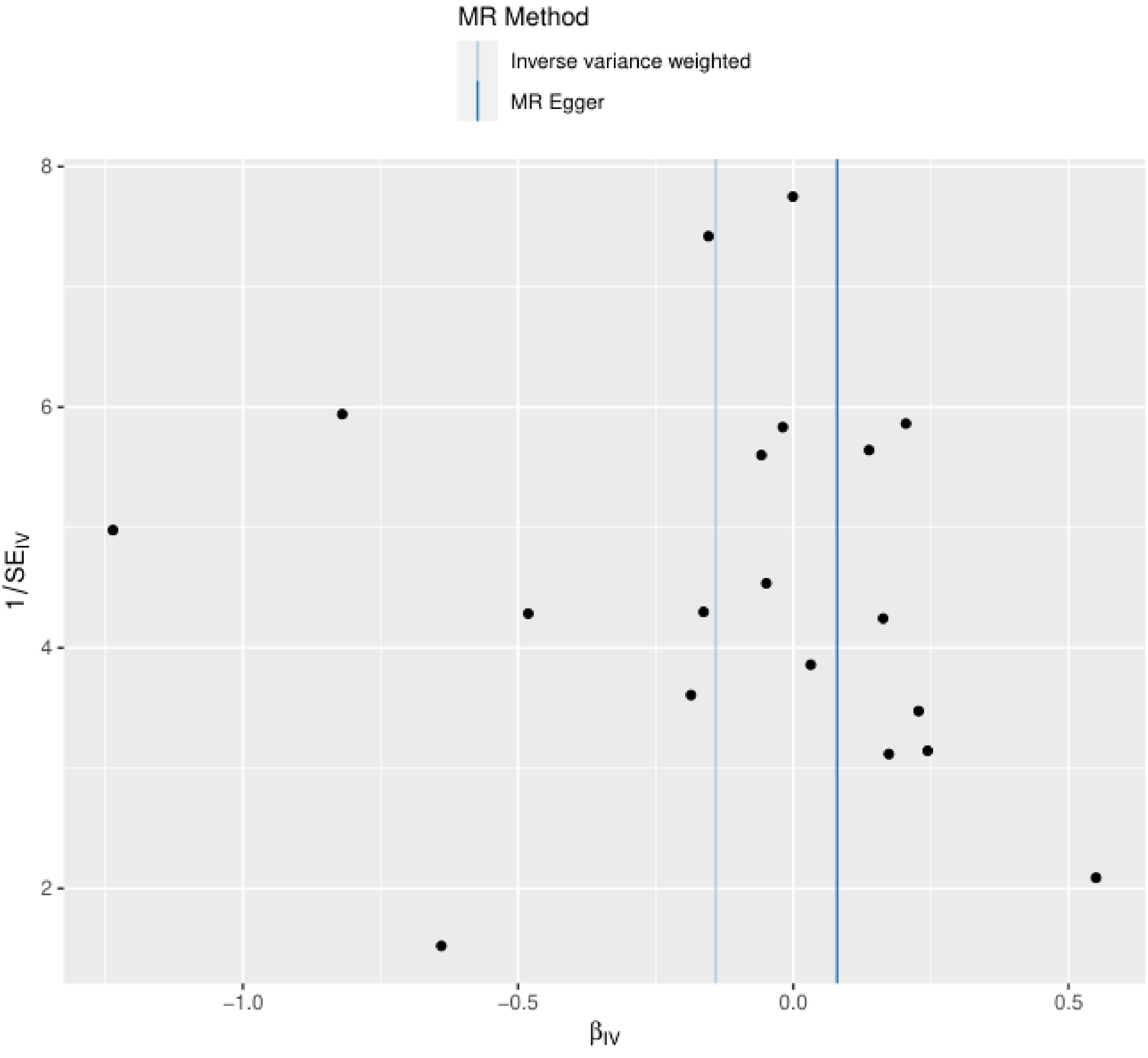

Funnel plot analyse of "Activated & resting Treg AC" on 'Diabetic nephropathy'

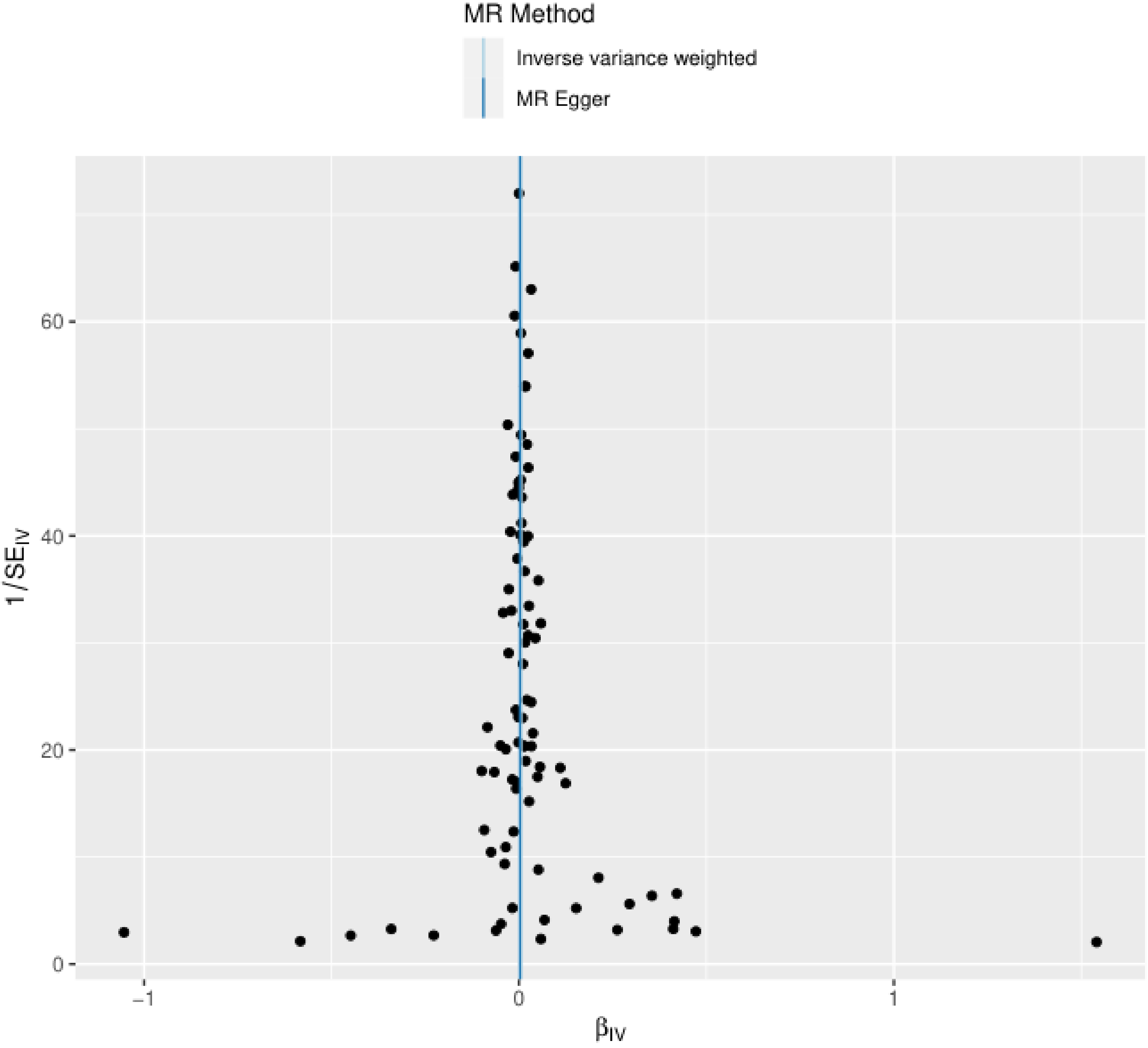

Funnel plot analyse of "CD28+ CD45RA+ CD8br %T cell" on 'Diabetic nephropathy'

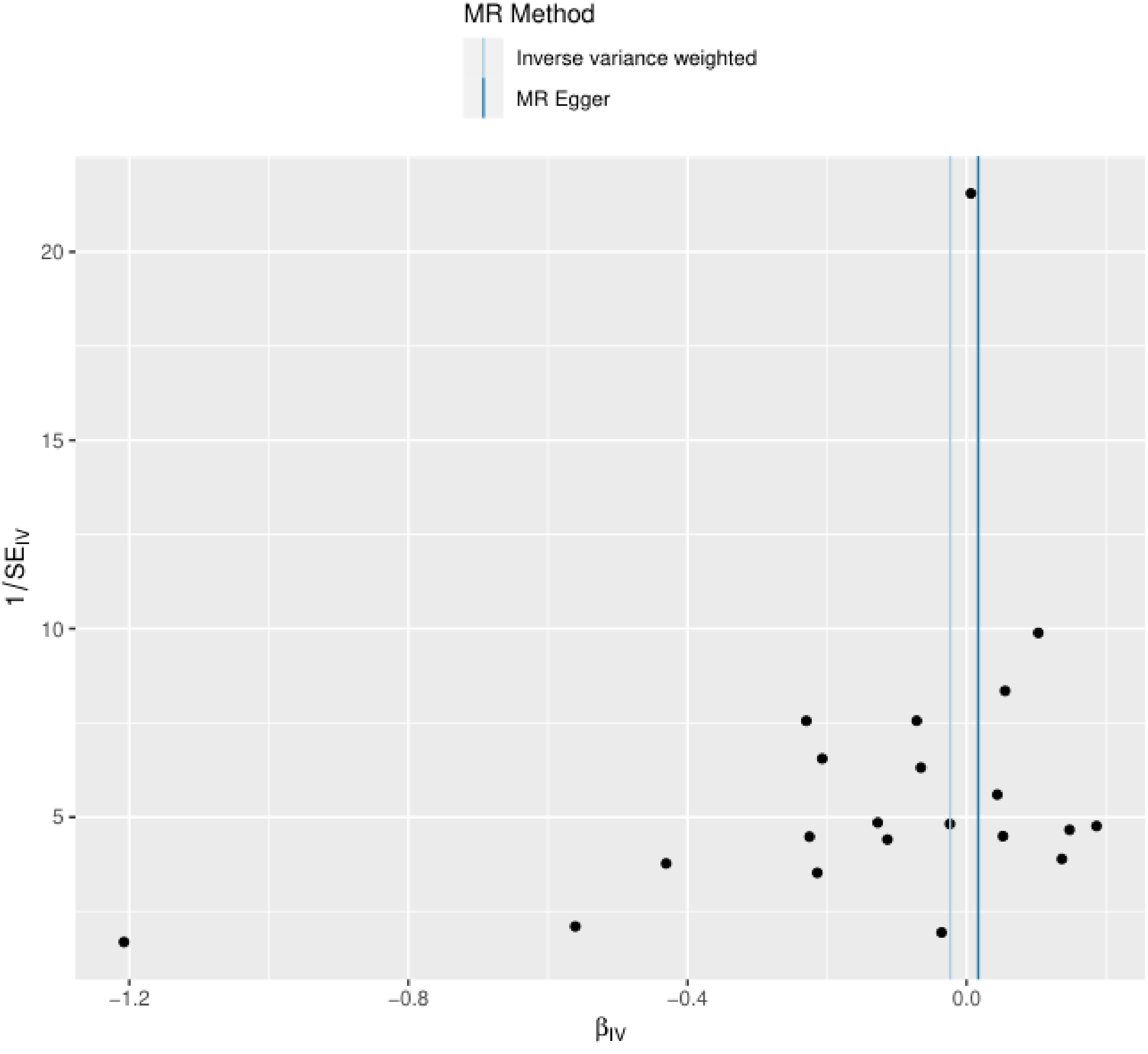

Funnel plot analyse of "IgD on IgD+" on 'Diabetic nephropathy'

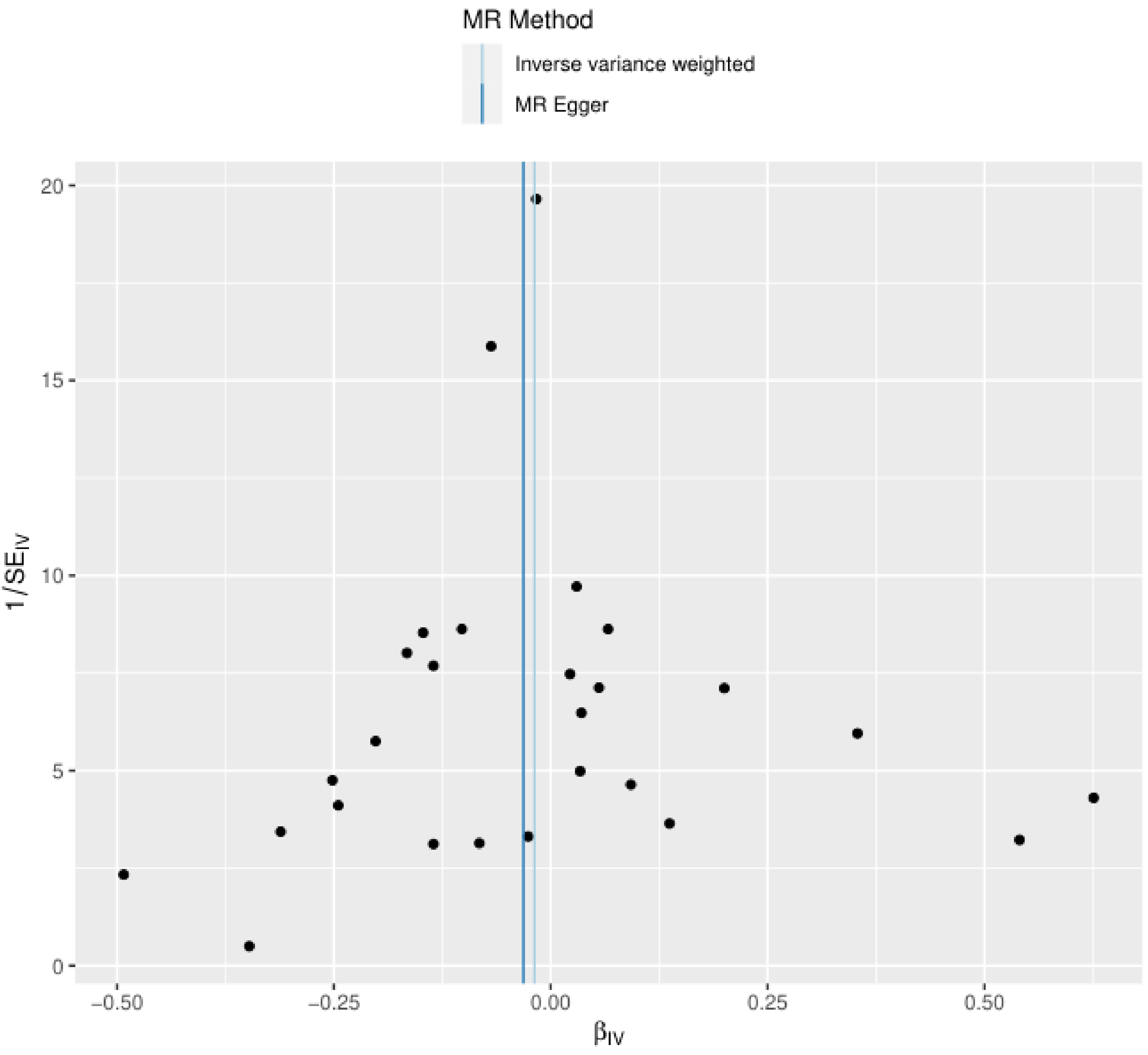

Funnel plot analyse of "CD3 on CD39+ activated Treg" on 'Diabetic nephropathy'

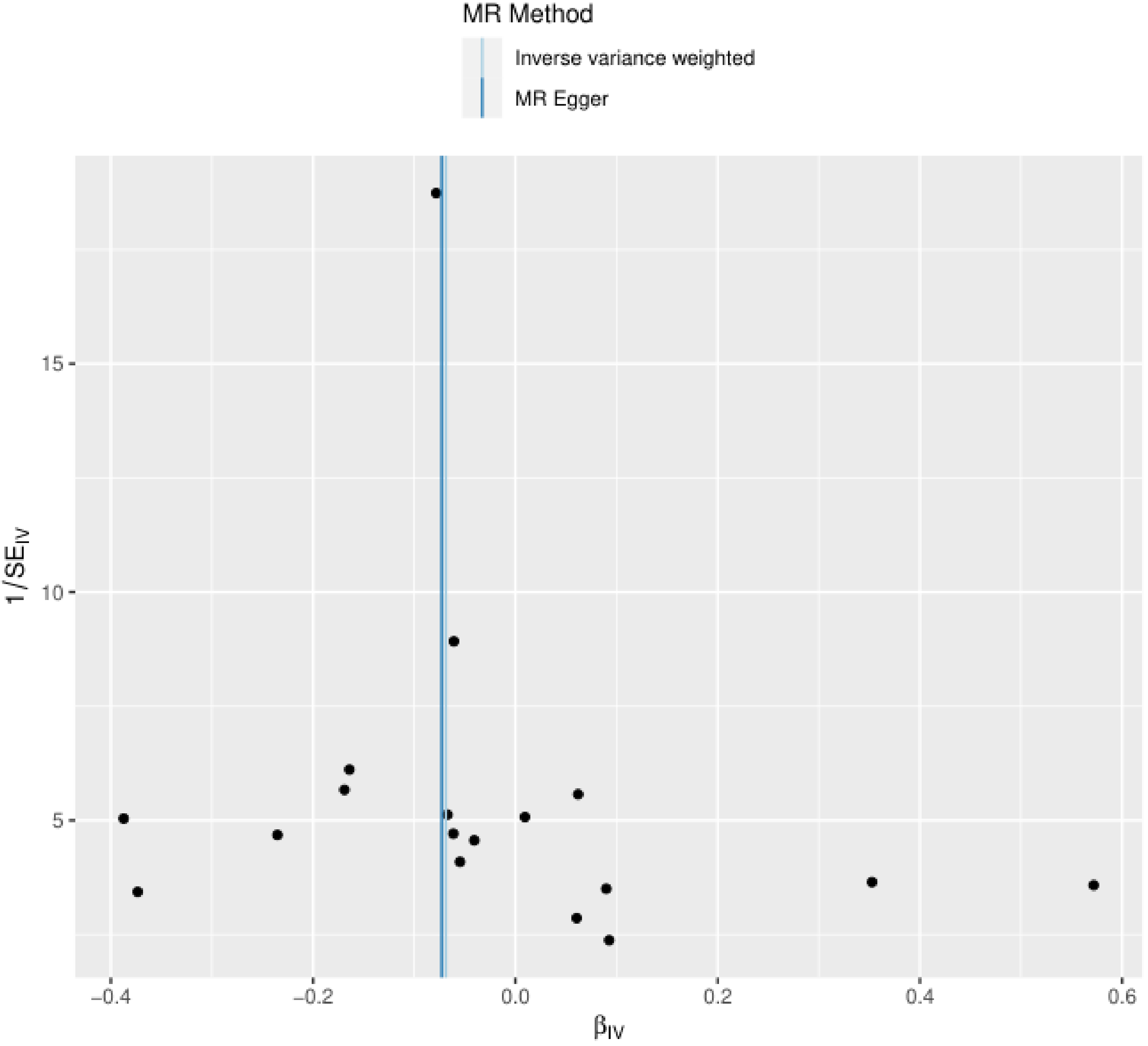

Funnel plot analyse of "Sw mem %lymphocyte" on 'Diabetic nephropathy'

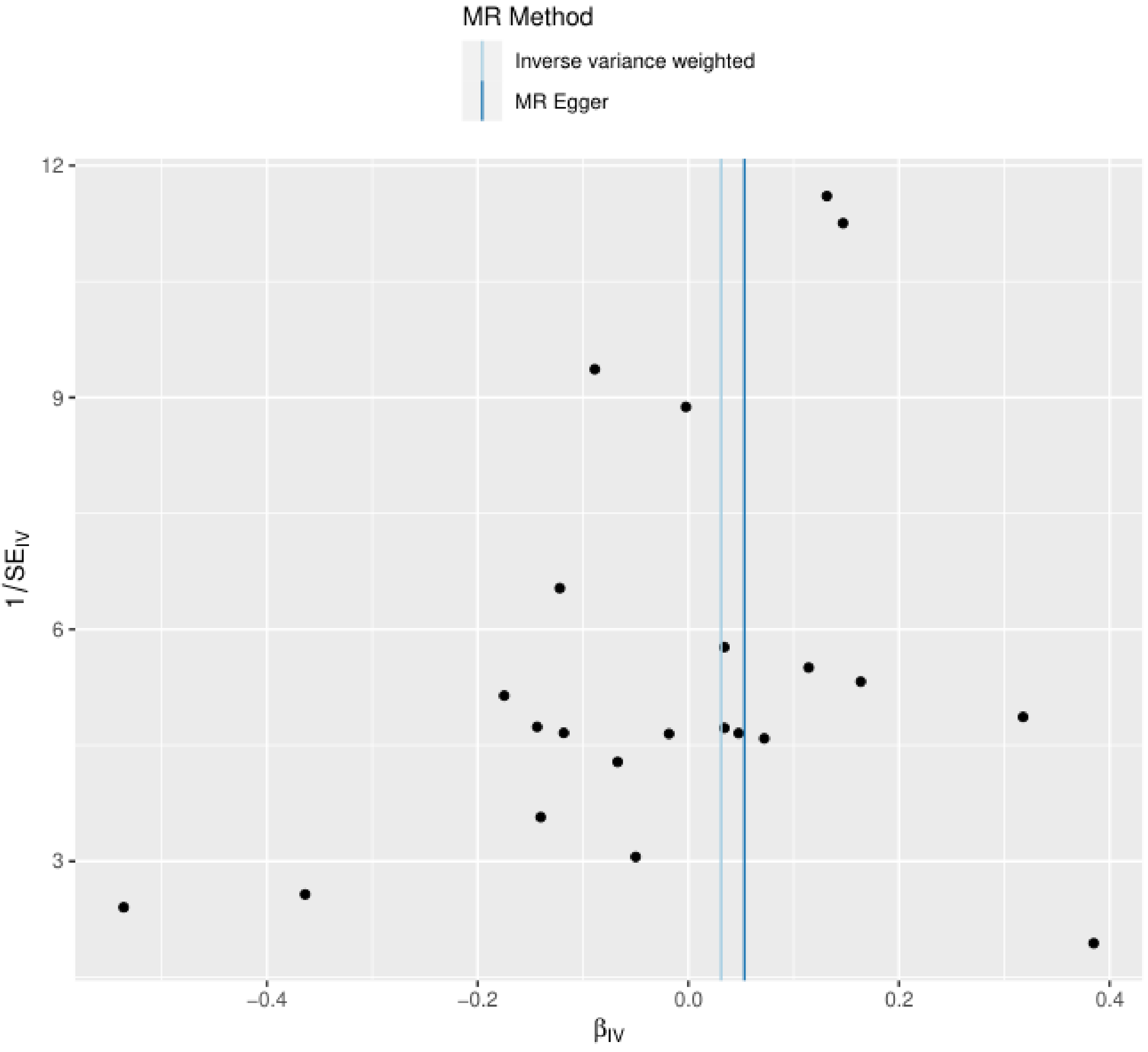

Funnel plot analyse of "CD127 on CD28+ DN (CD4-CD8-)" on 'Diabetic nephropathy'

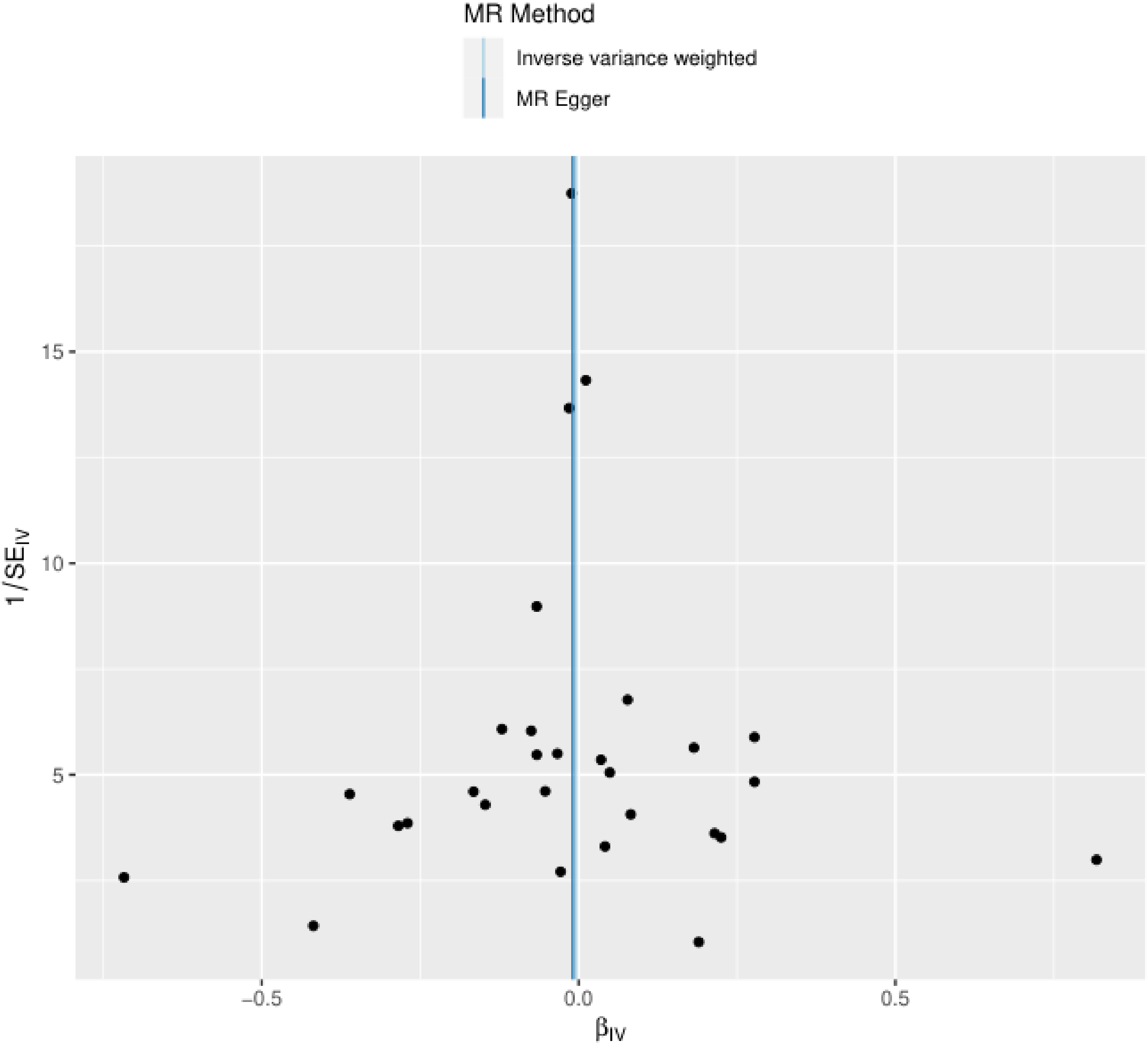

Funnel plot analyse of "CD19 on CD24+ CD27+" on 'Diabetic nephropathy'

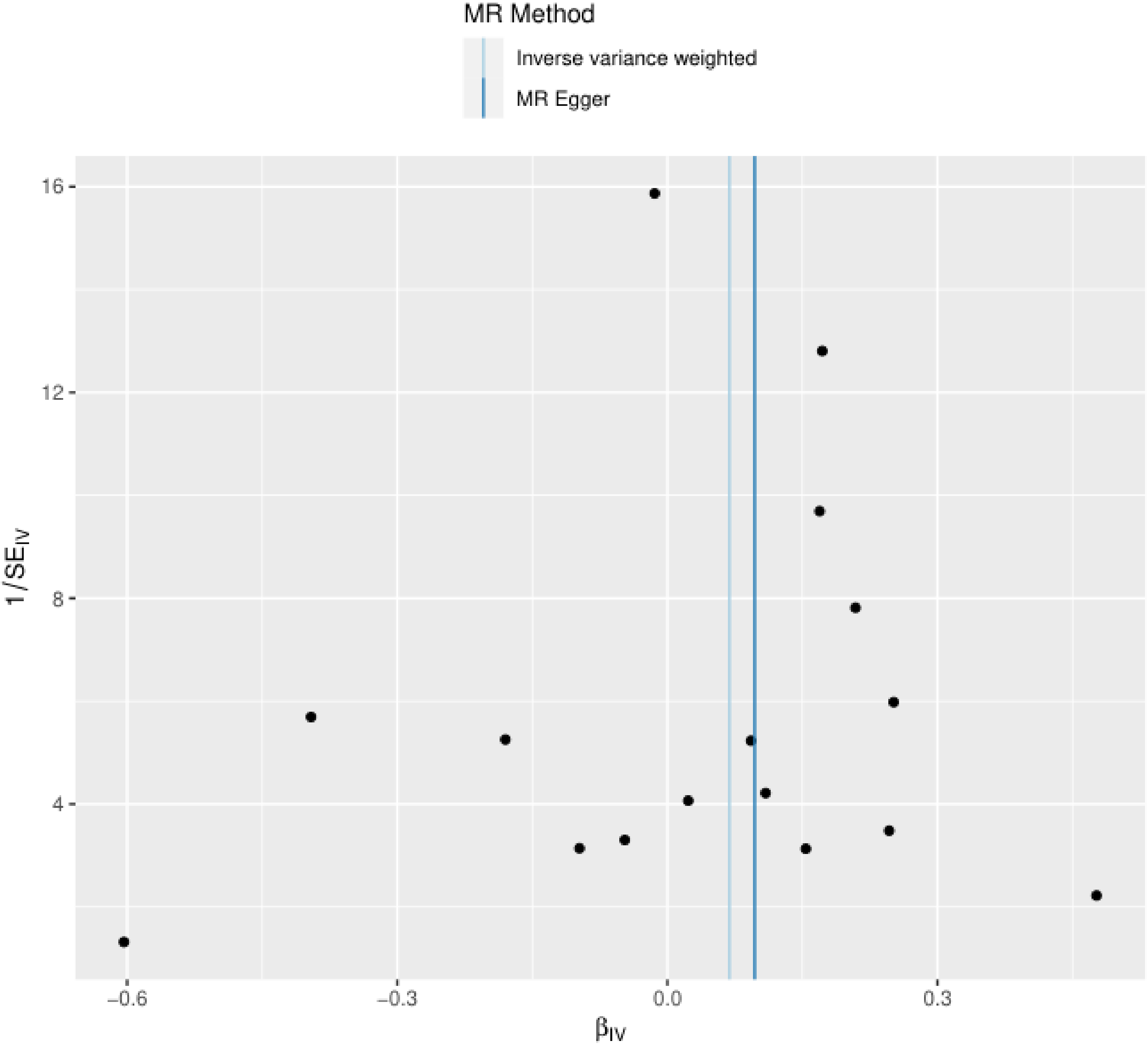

Funnel plot analyse of "CD11c+ monocyte AC" on 'Diabetic nephropathy'

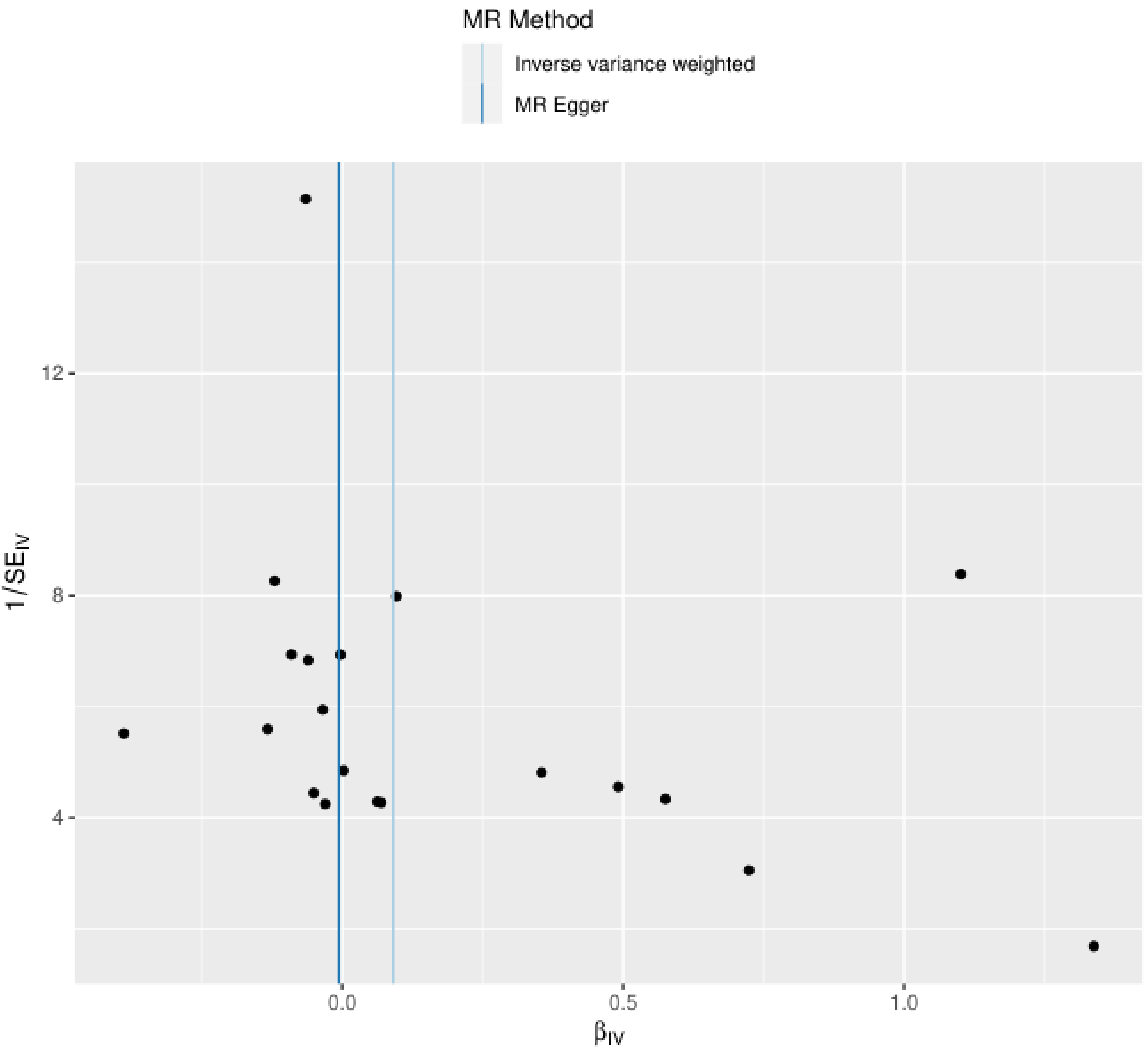

Funnel plot analyse of "CD127 on CD45RA+ CD4+ " on 'Diabetic nephropathy'

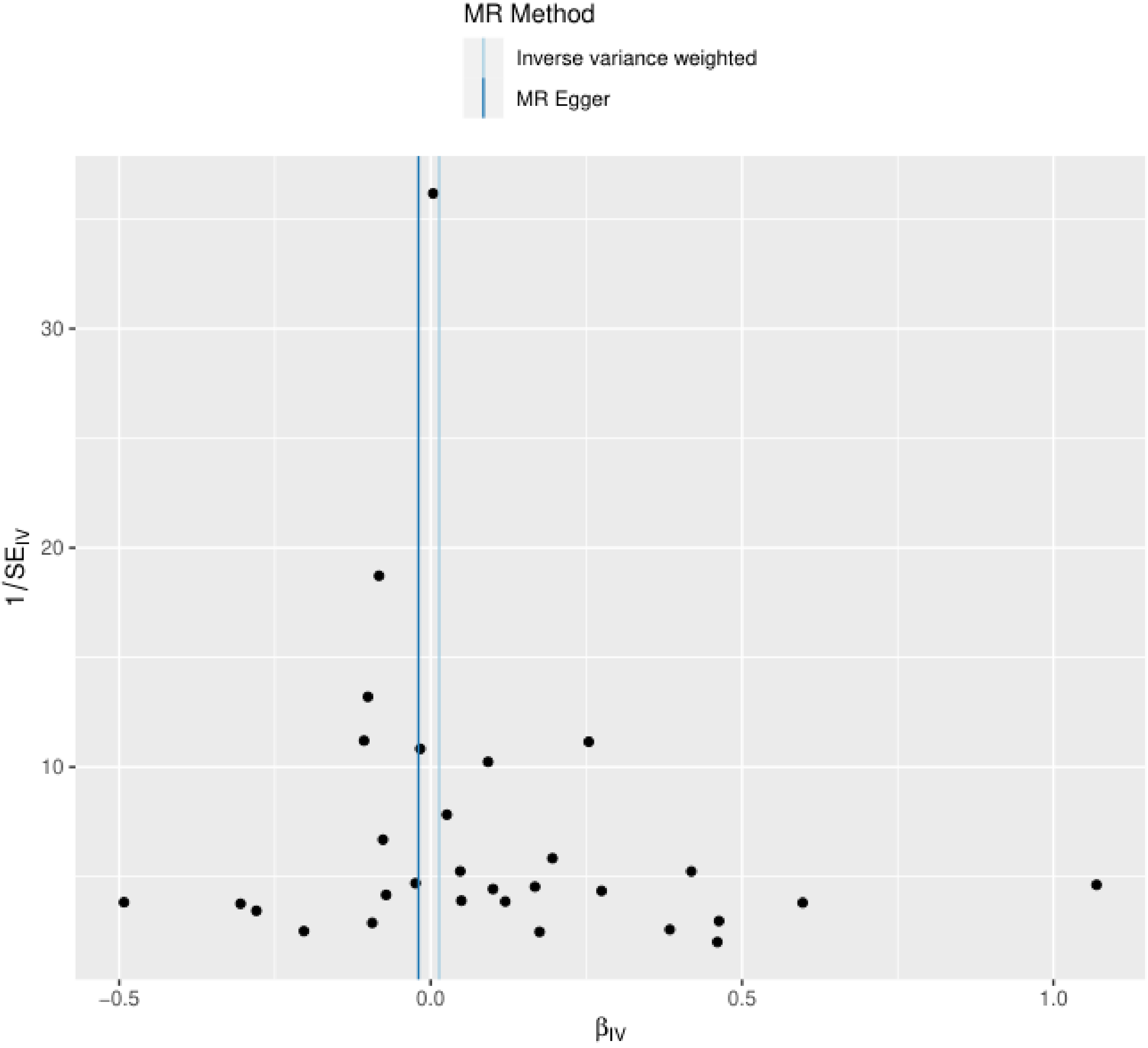

Funnel plot analyse of "Secreting Treg % CD4 Treg" on 'Diabetic nephropathy'

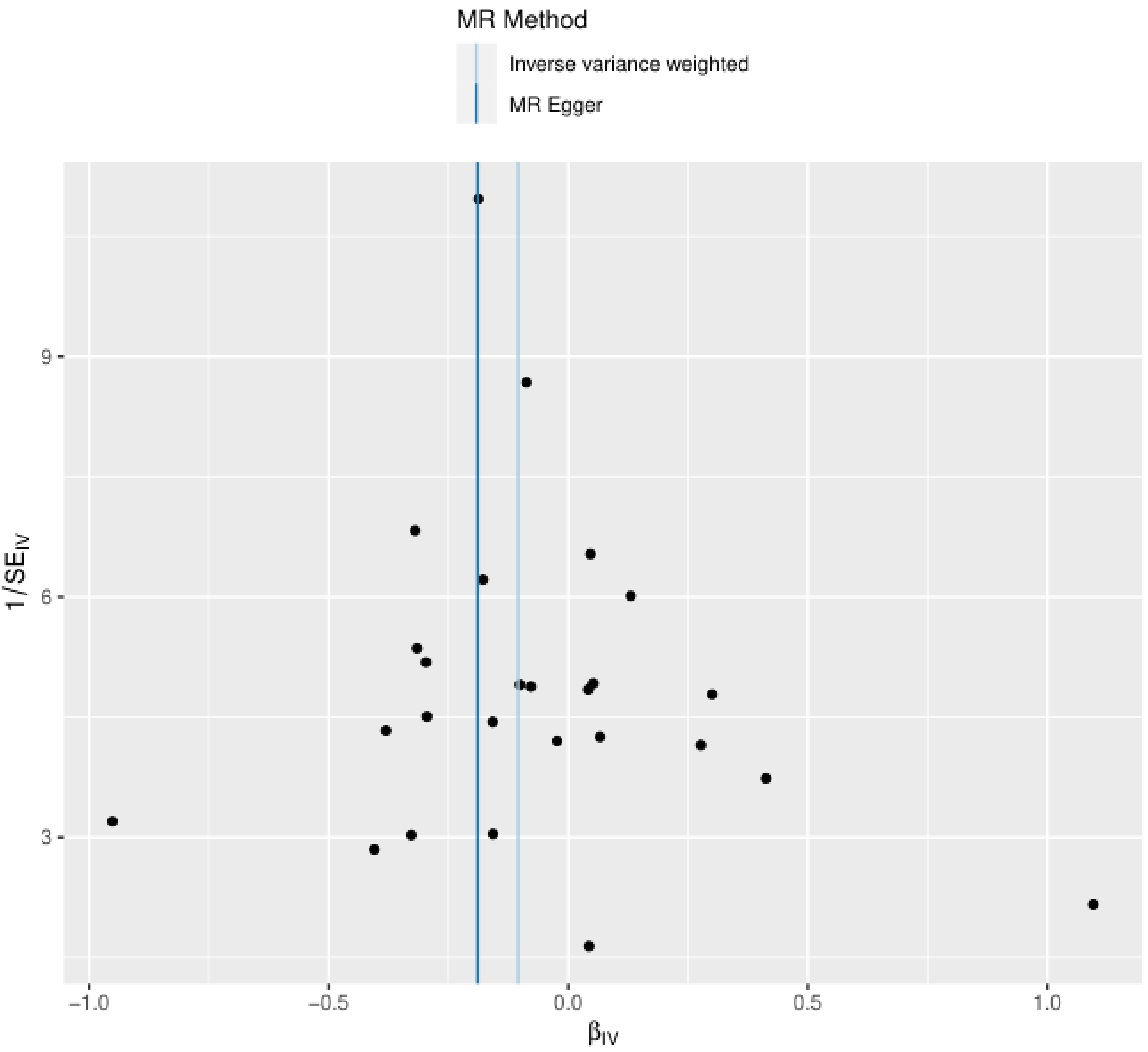

Funnel plot analyse of "CD86+ plasmacytoid DC %DC" on 'Diabetic nephropathy'

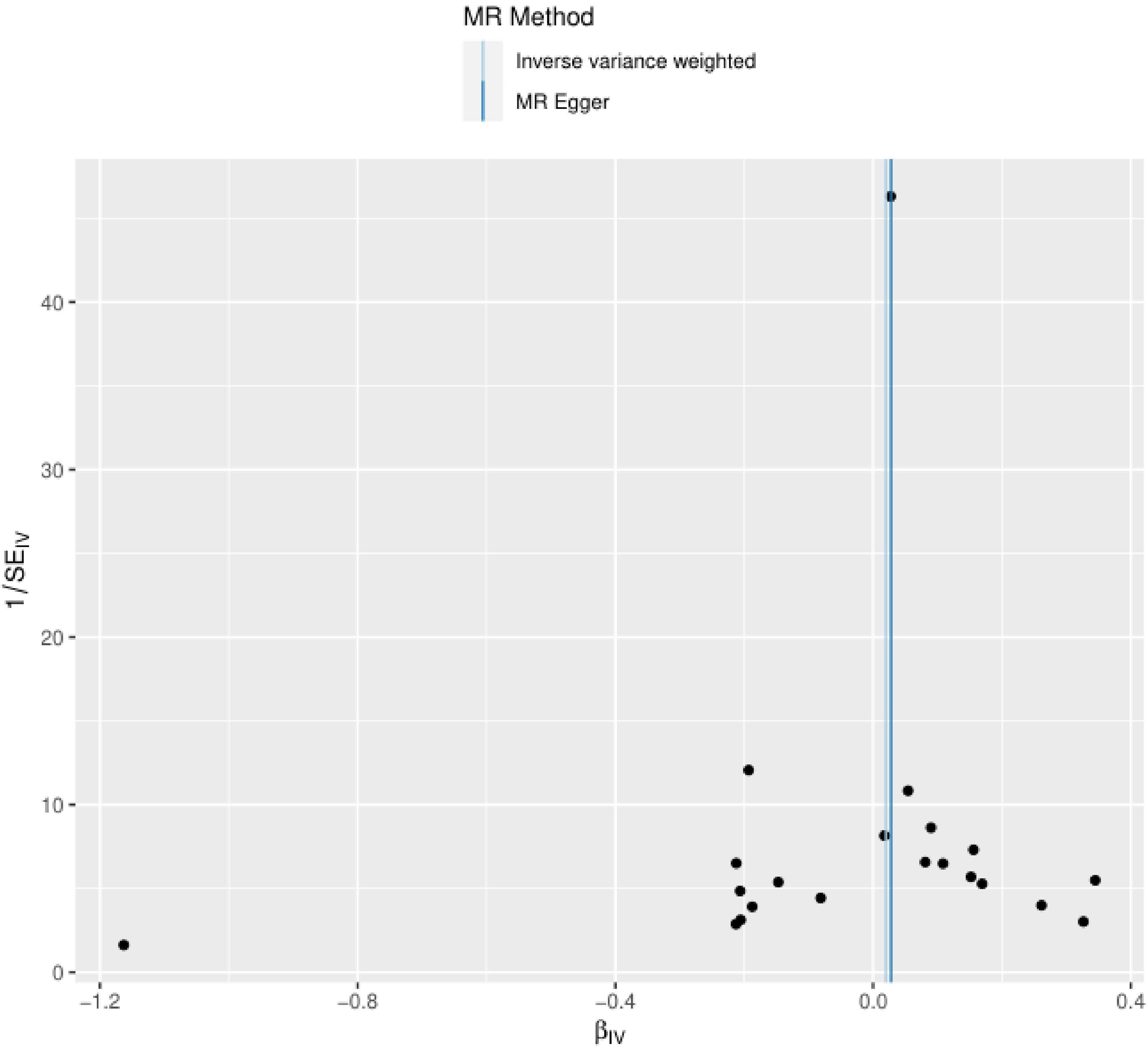

Funnel plot analyse of "CD27 on T cell" on 'Diabetic nephropathy'

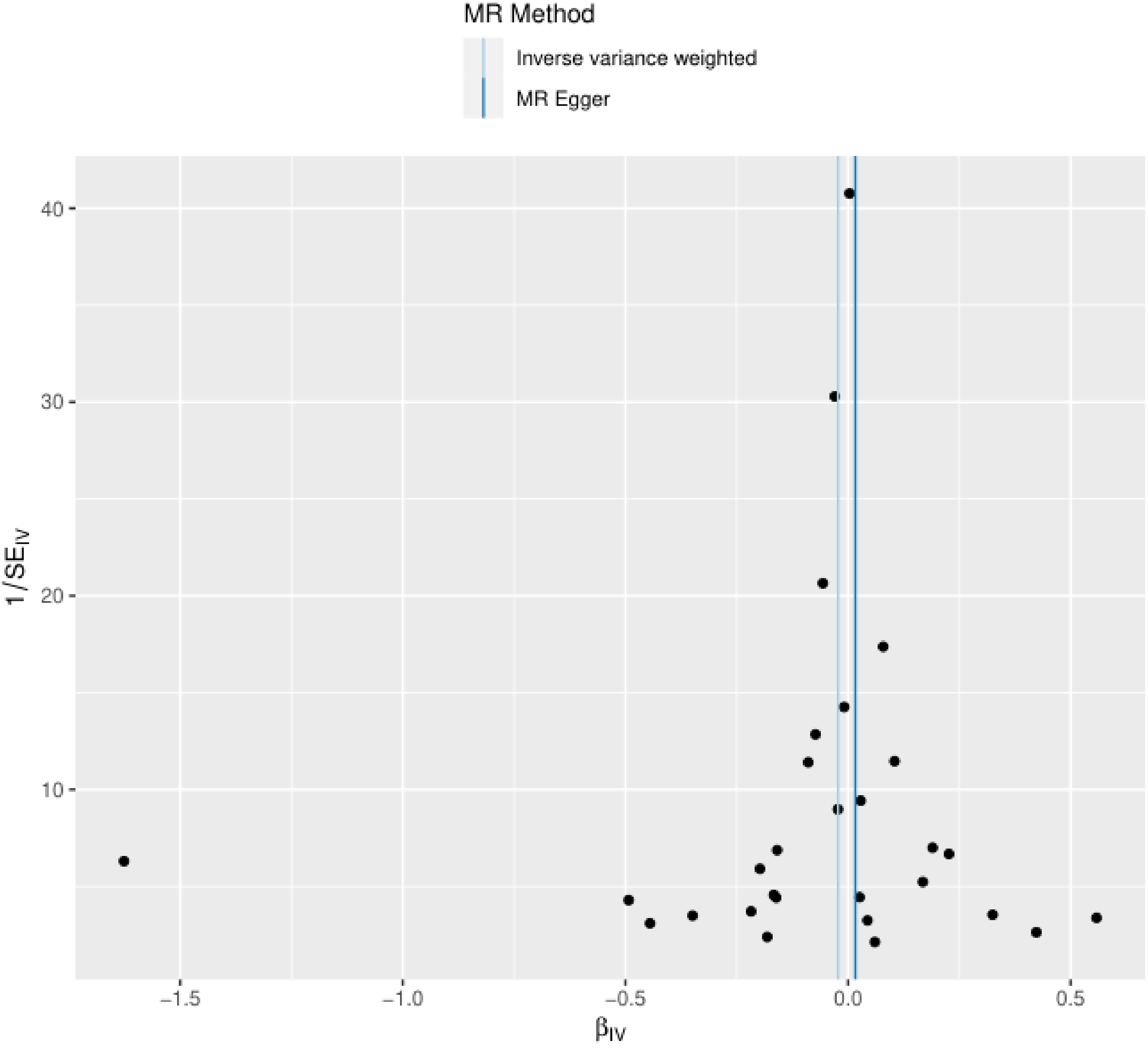

Funnel plot analyse of "Resting Treg % CD4 Treg" on 'Diabetic nephropathy'

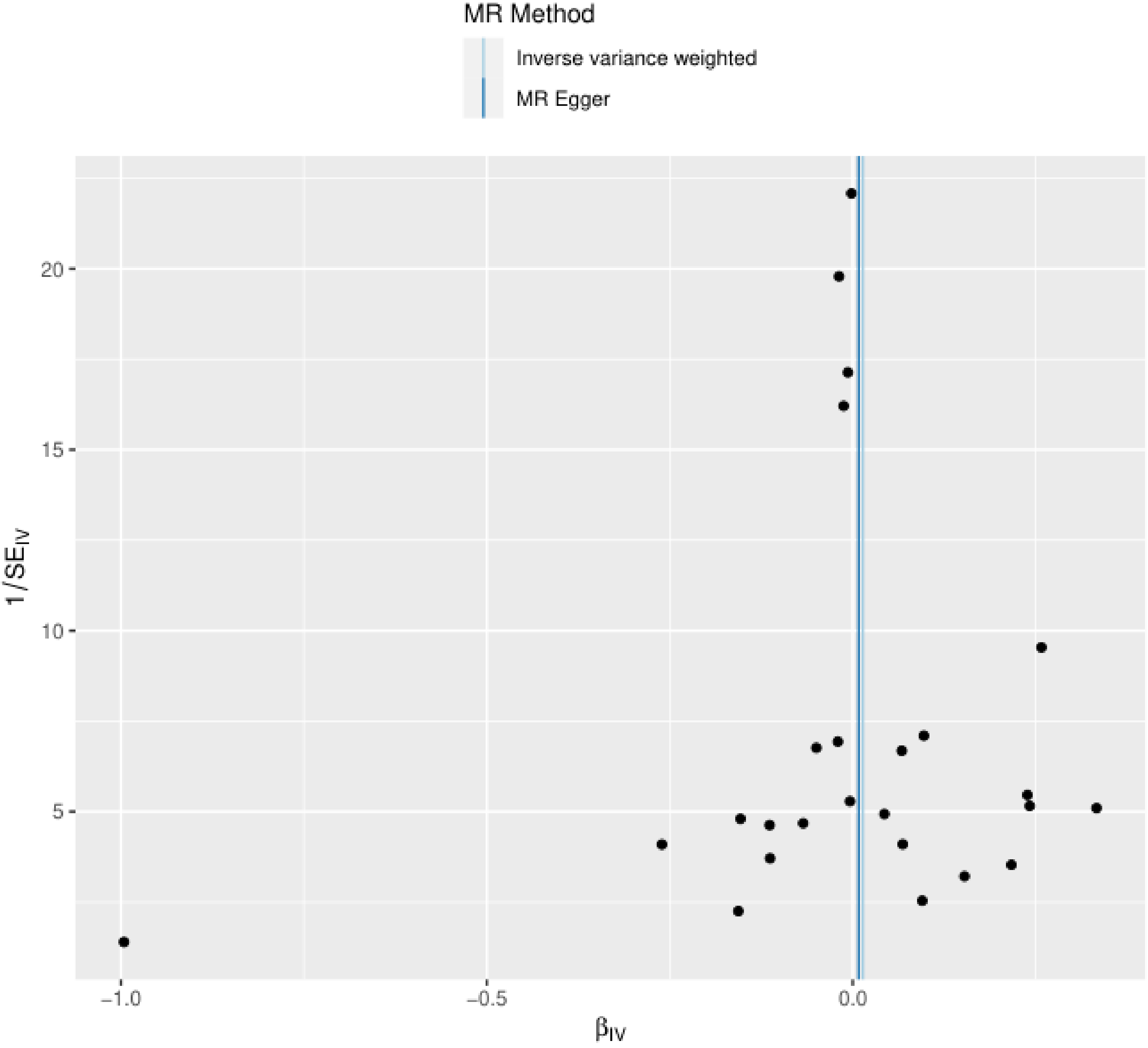

Funnel plot analyse of "CD62L- myeloid DC %DC" on 'Diabetic nephropathy'

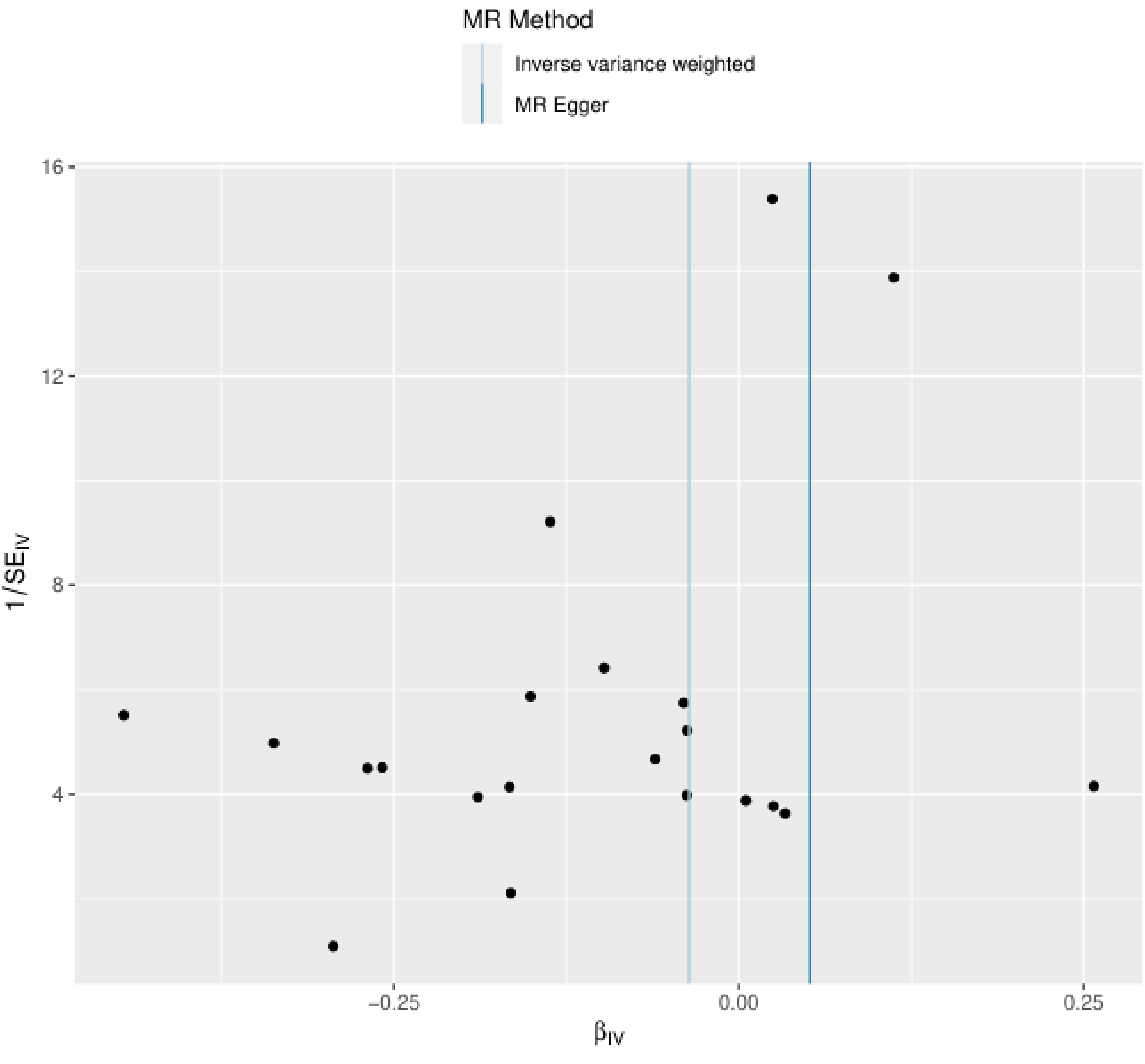

Funnel plot analyse of "CD14+ CD16+ monocyte AC" on 'Diabetic nephropathy'

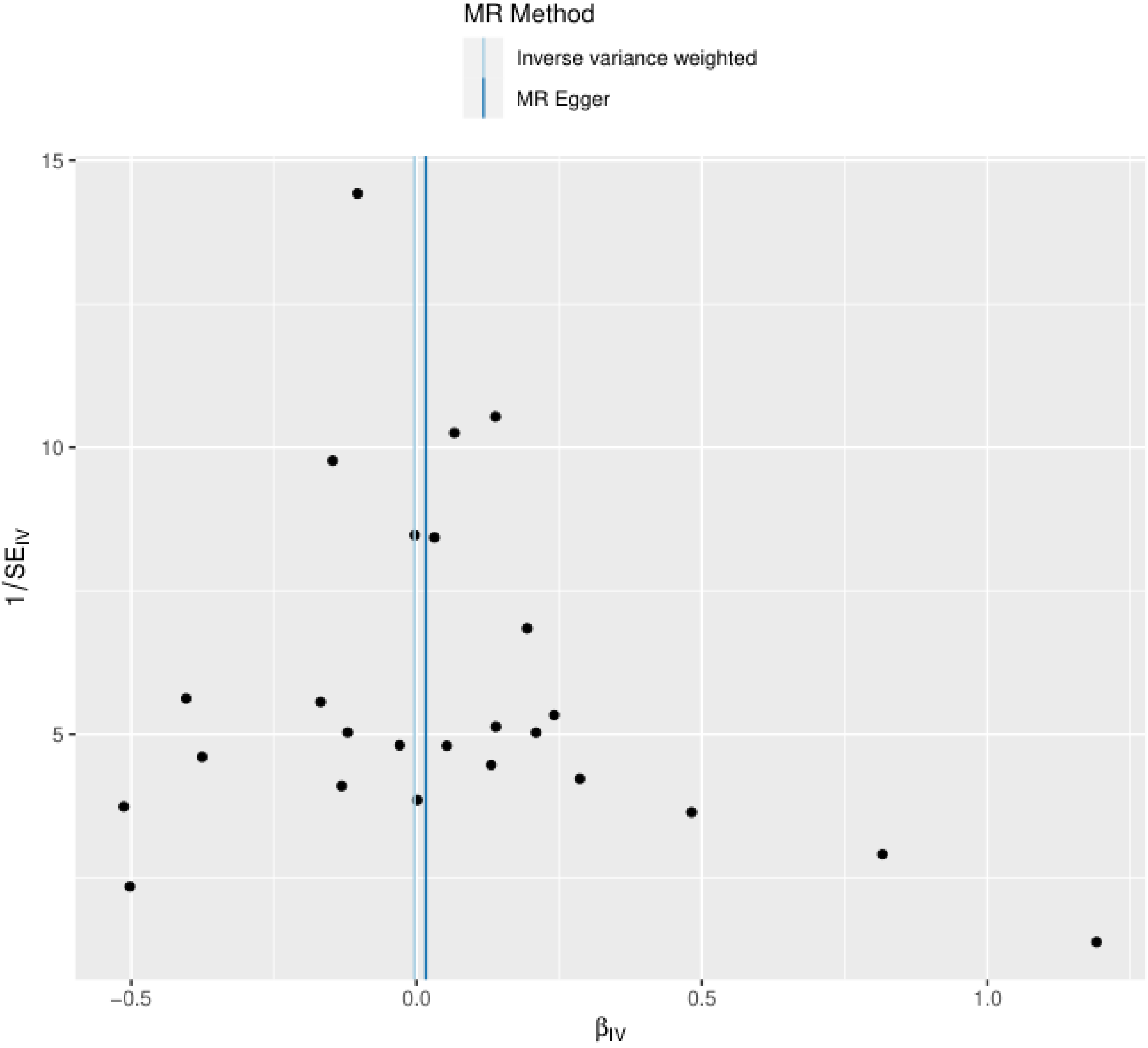

Funnel plot analyse of "TD DN (CD4-CD8-) %DN" on 'Diabetic nephropathy'

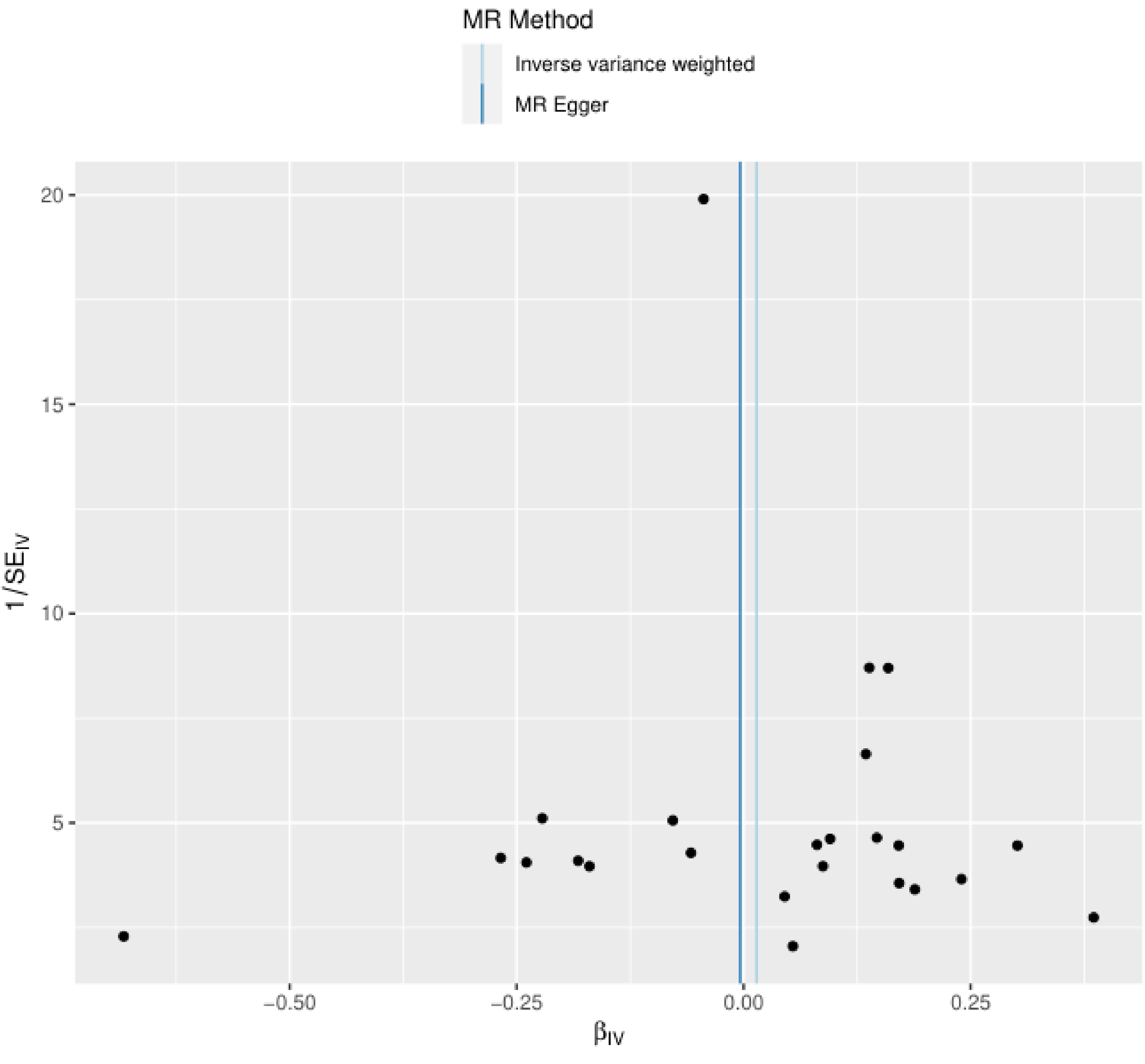

Funnel plot analyse of "CD20- CD38- %lymphocyte" on 'Diabetic nephropathy'

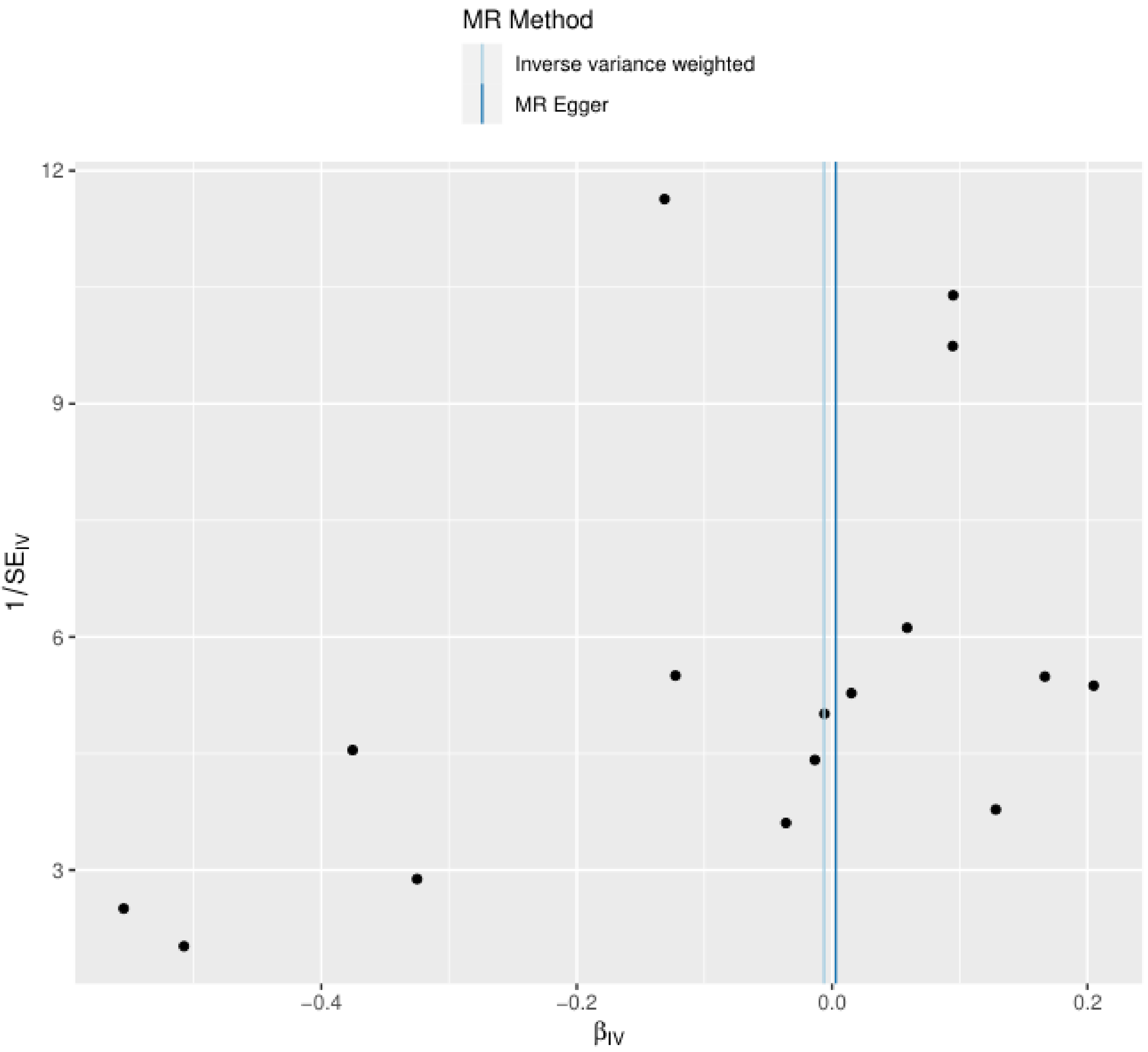

Funnel plot analyse of "IgD+ %B cell" on 'Diabetic nephropathy'

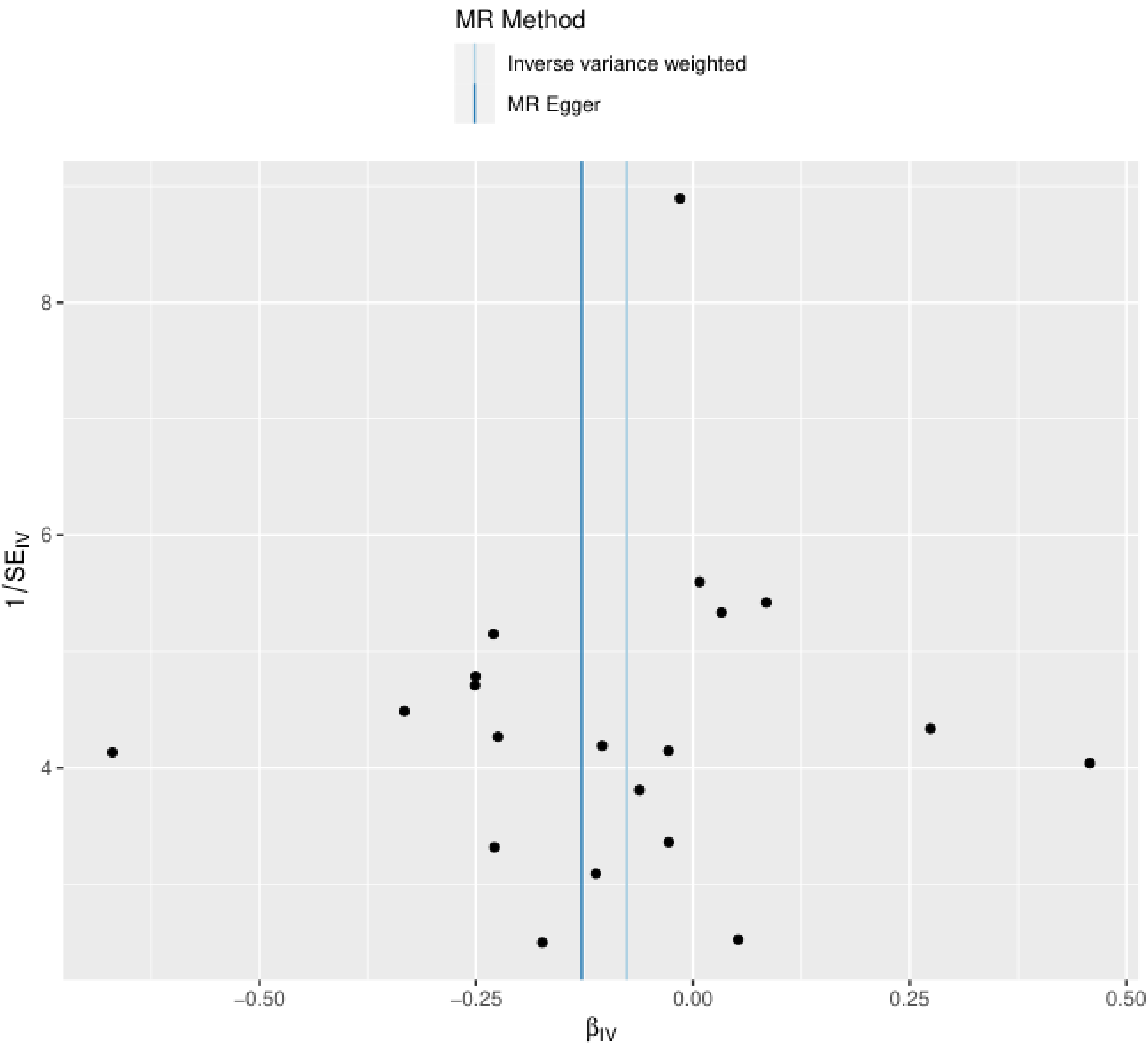

Funnel plot analyse of "Naive-mature B cell %lymphocyte" on 'Diabetic nephropathy'

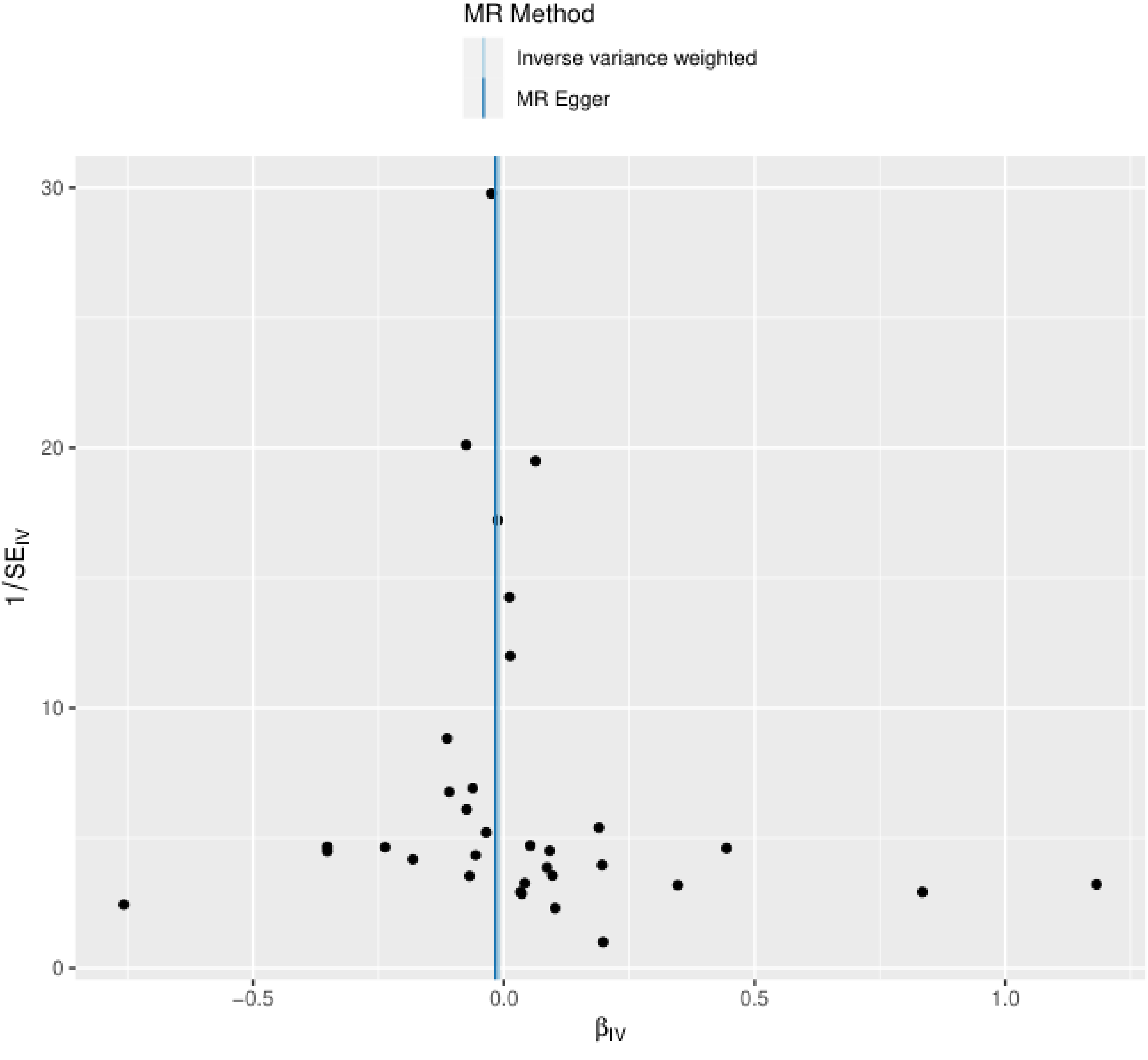

Funnel plot analyse of "CD19 on IgD+ CD24+" on 'Diabetic nephropathy'

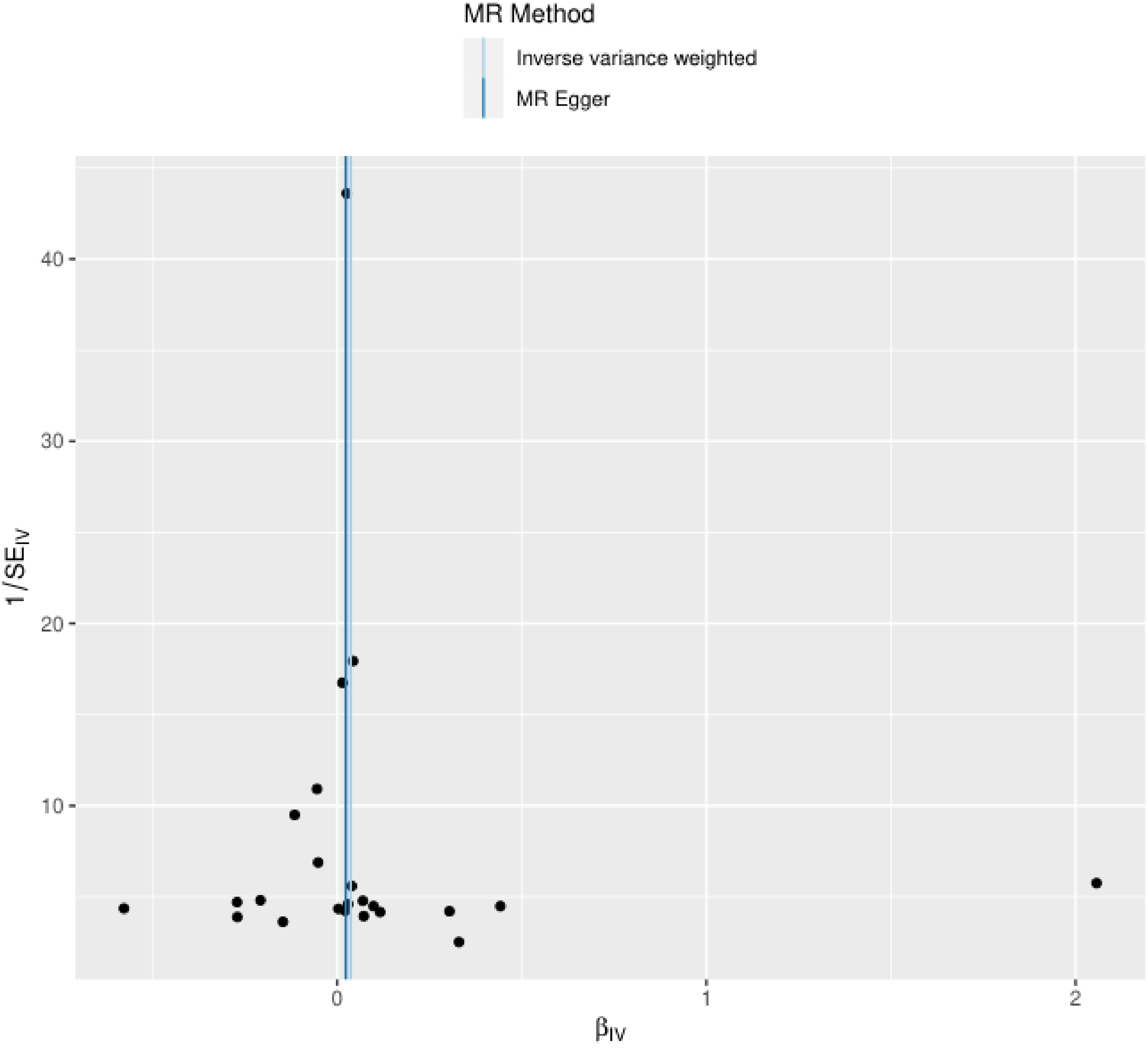

Funnel plot analyse of "CD8br AC" on 'Diabetic nephropathy'

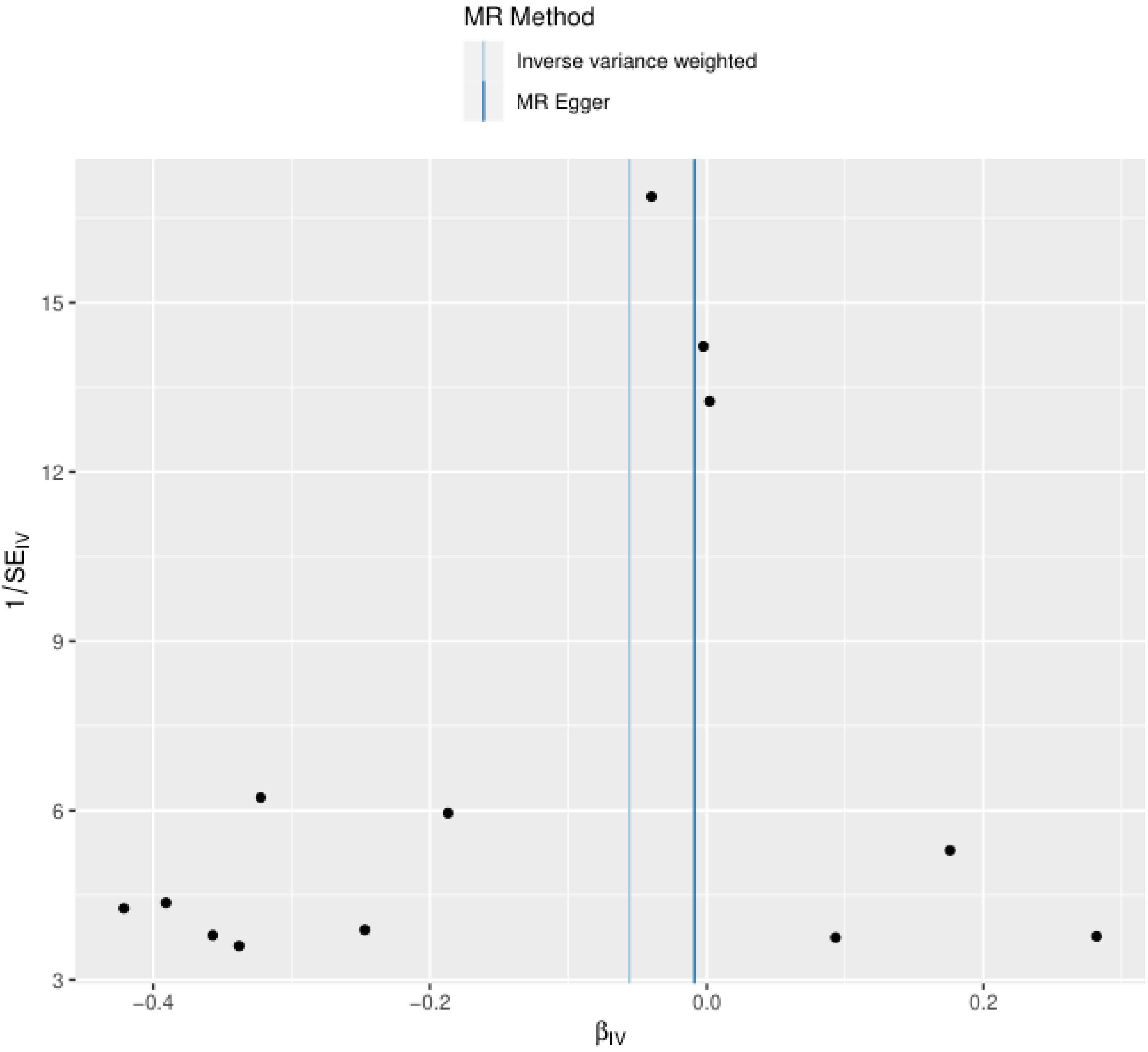

Funnel plot analyse of "CD4 on CD39+ resting Treg " on 'Diabetic nephropathy'

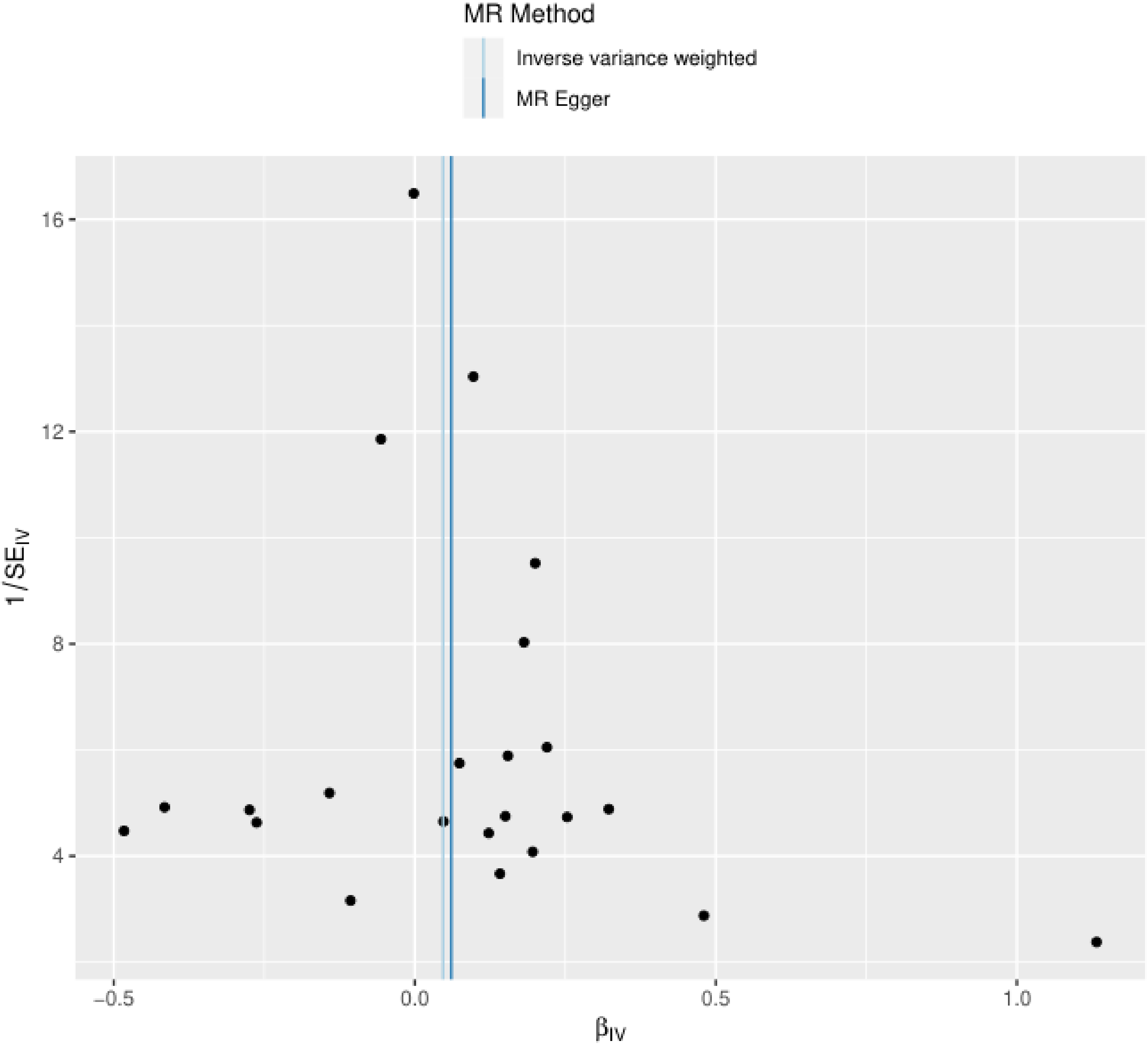

Funnel plot analyse of "TD DN (CD4-CD8-) %T cell" on 'Diabetic nephropathy'

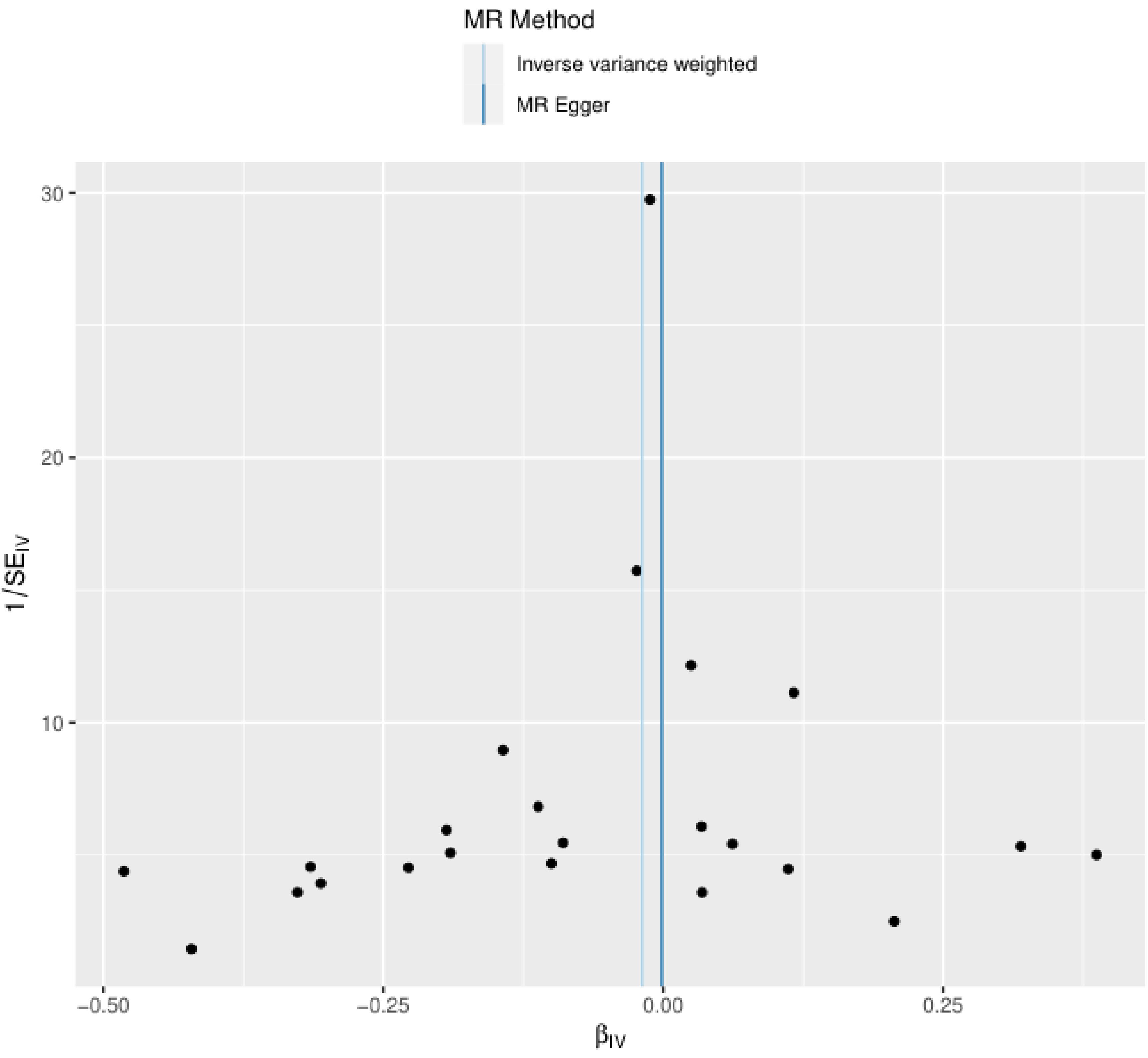

Funnel plot analyse of "Myeloid DC AC" on 'Diabetic nephropathy'

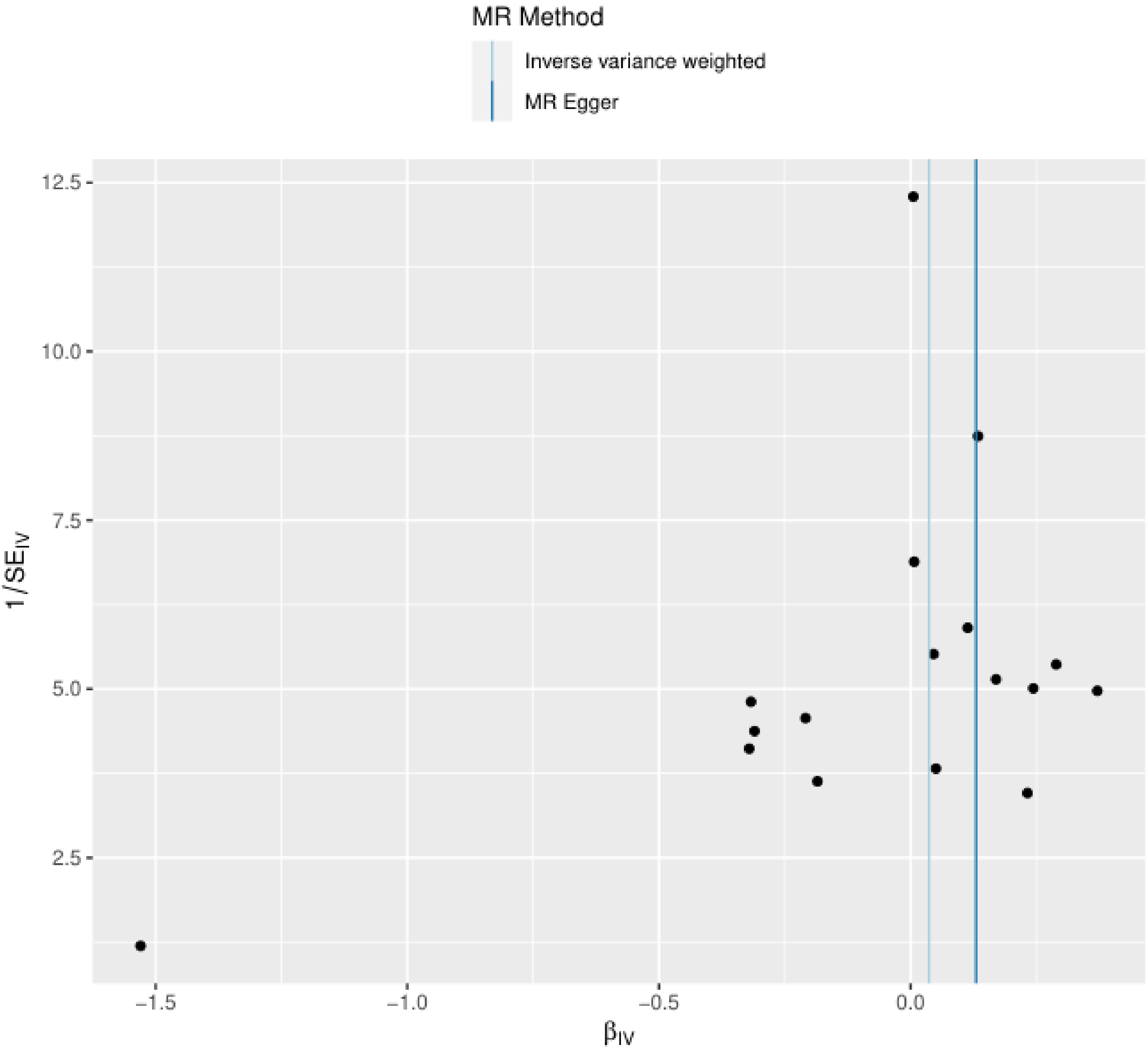

Funnel plot analyse of "CD25++ CD8br %T cell" on 'Diabetic nephropathy'

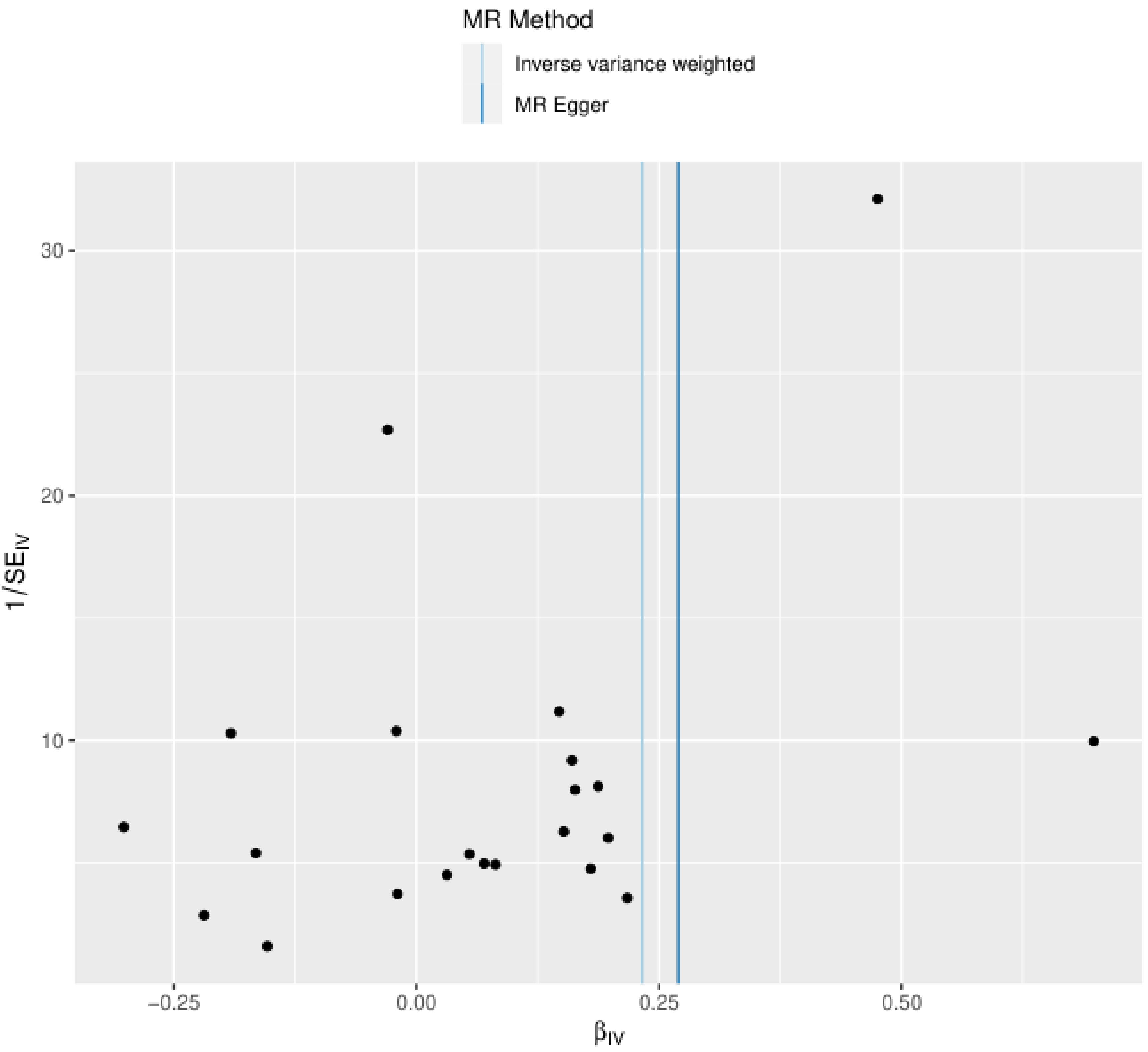

Funnel plot analyse of "HLA DR on plasmacytoid DC" on 'Diabetic nephropathy'

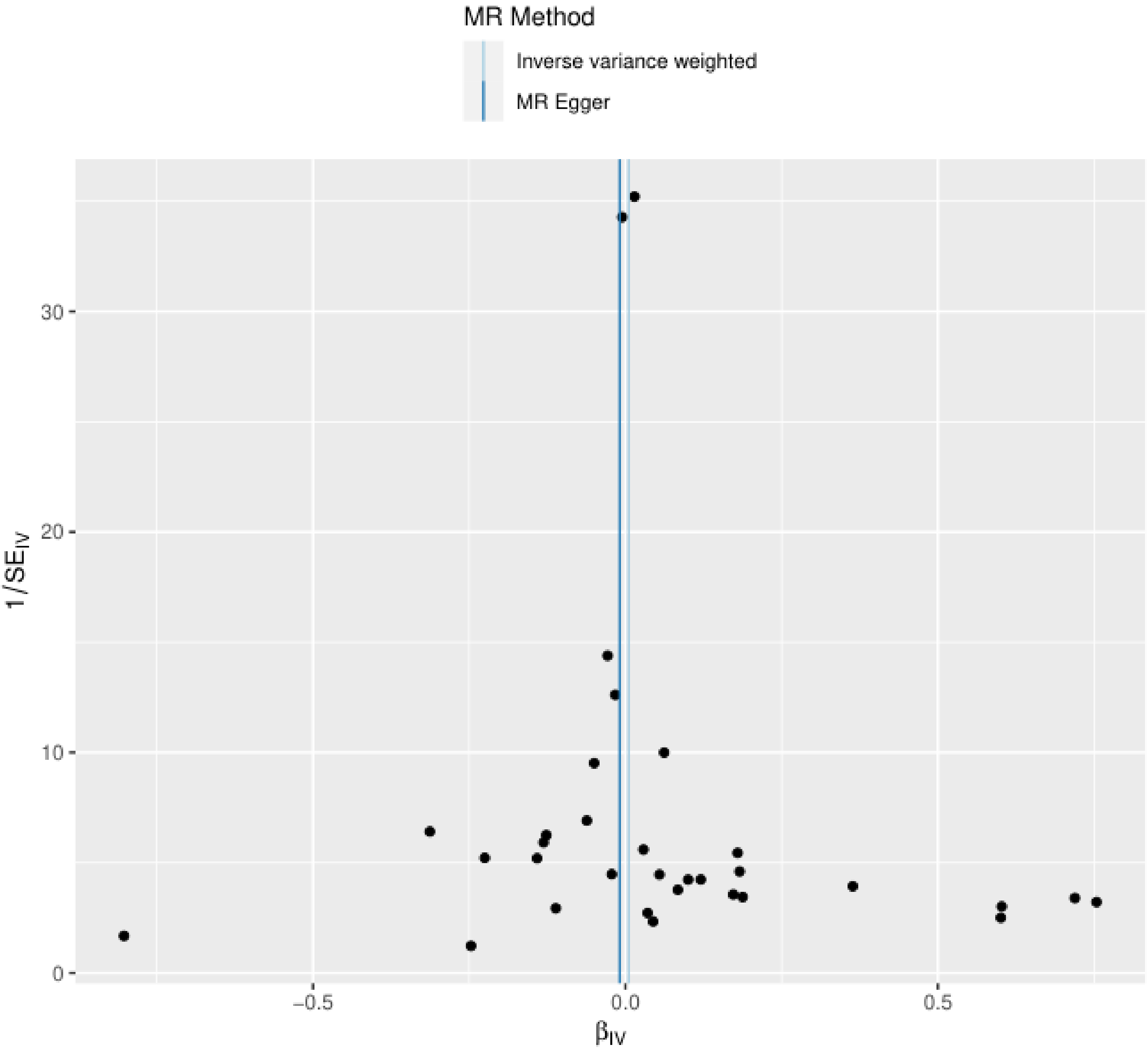

Funnel plot analyse of "CD19 on IgD+ CD38-" on 'Diabetic nephropathy'

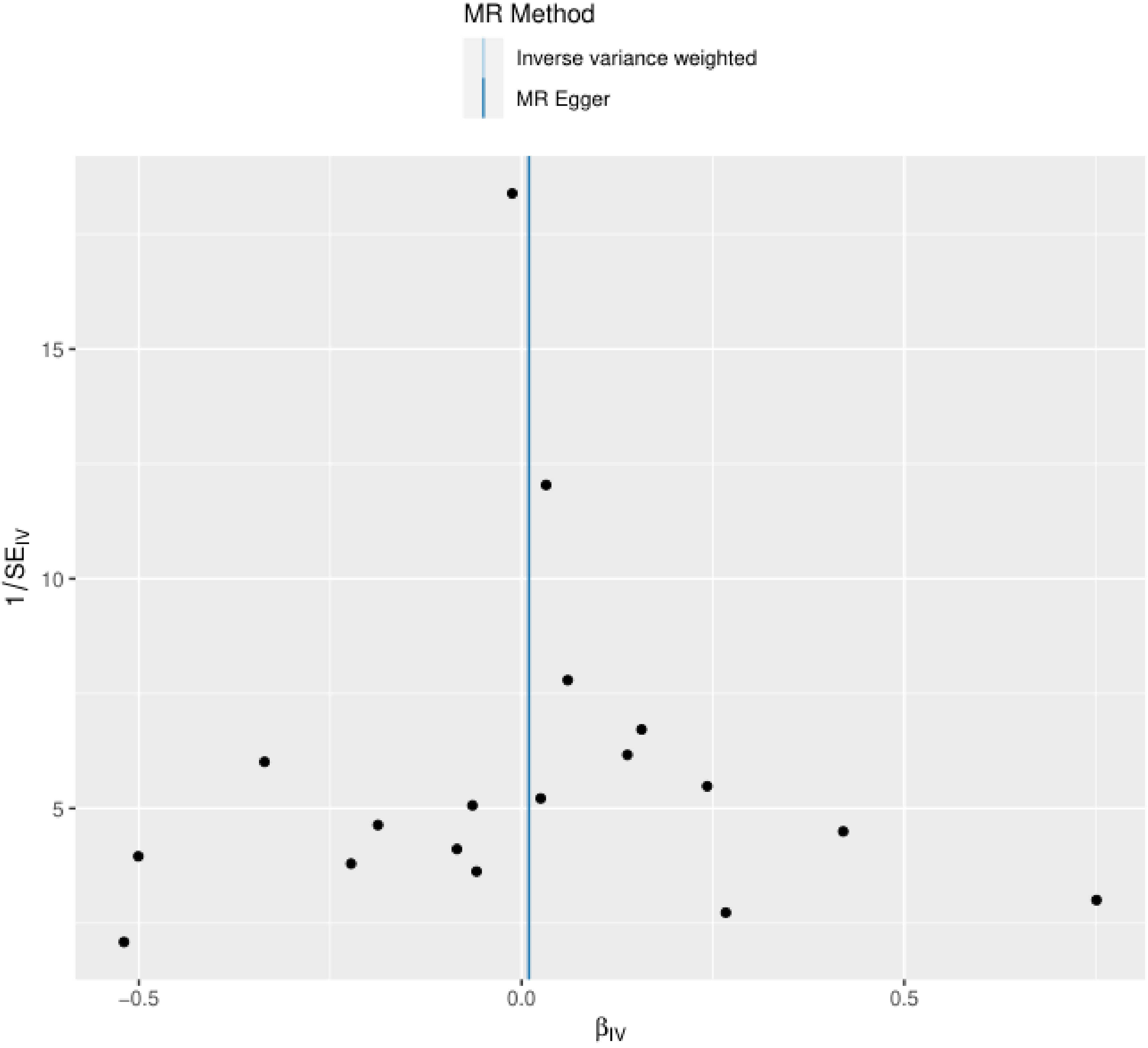

Funnel plot analyse of "CD8dim %leukocyte" on 'Diabetic nephropathy'

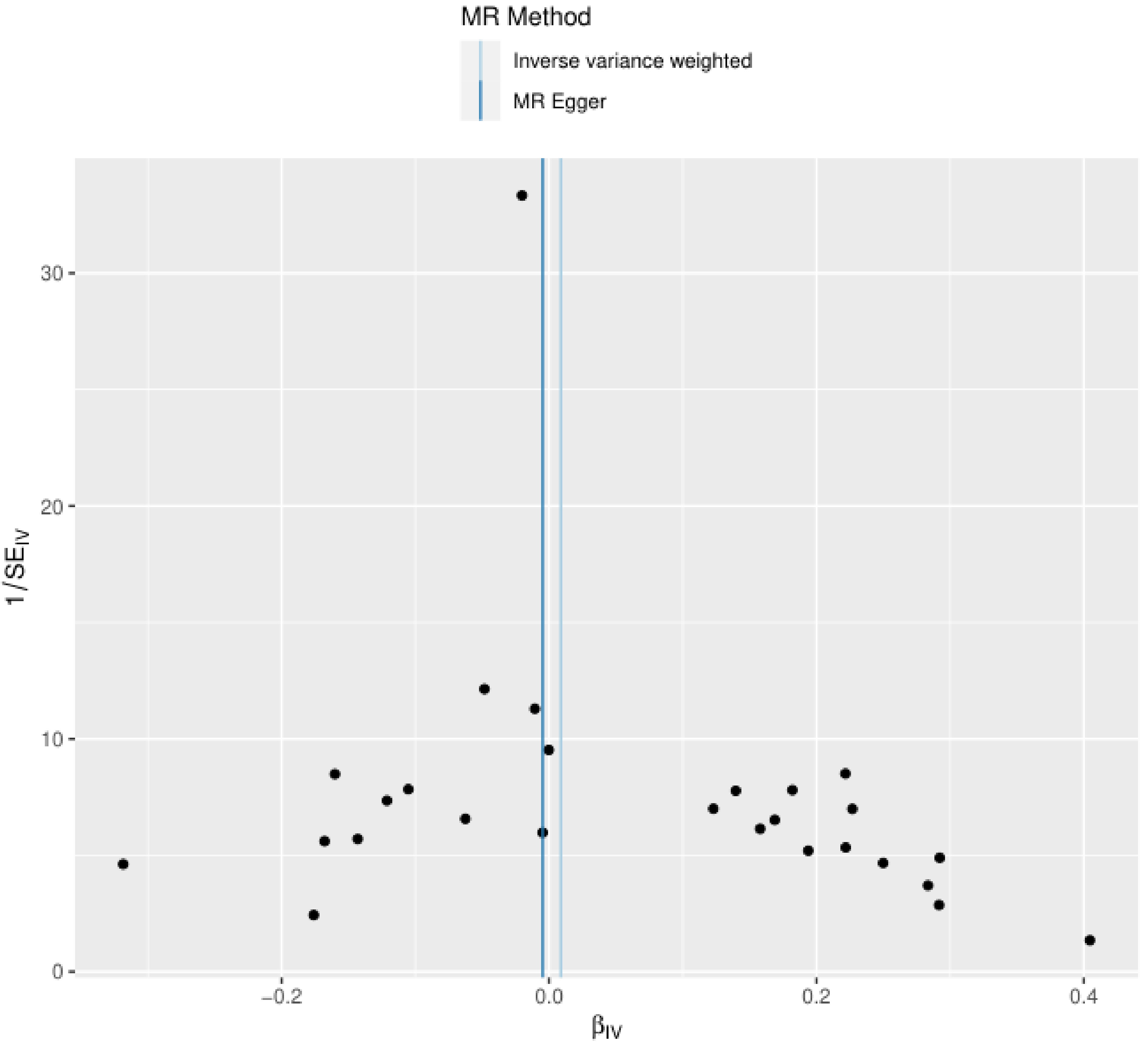

Funnel plot analyse of "BAFF-R on IgD+ CD38- naive" on 'Diabetic nephropathy'

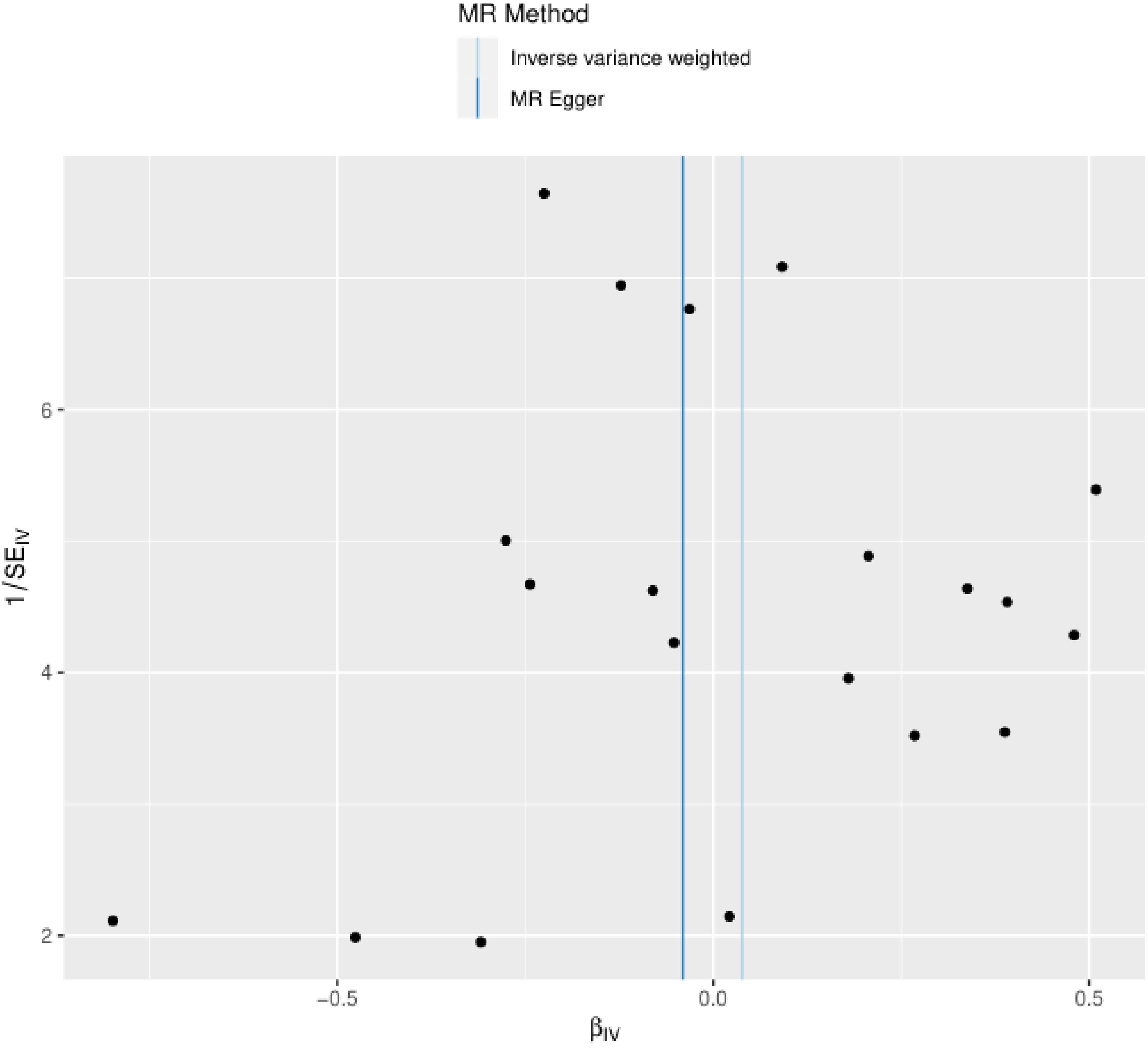

Funnel plot analyse of "Unsw mem AC" on 'Diabetic nephropathy'

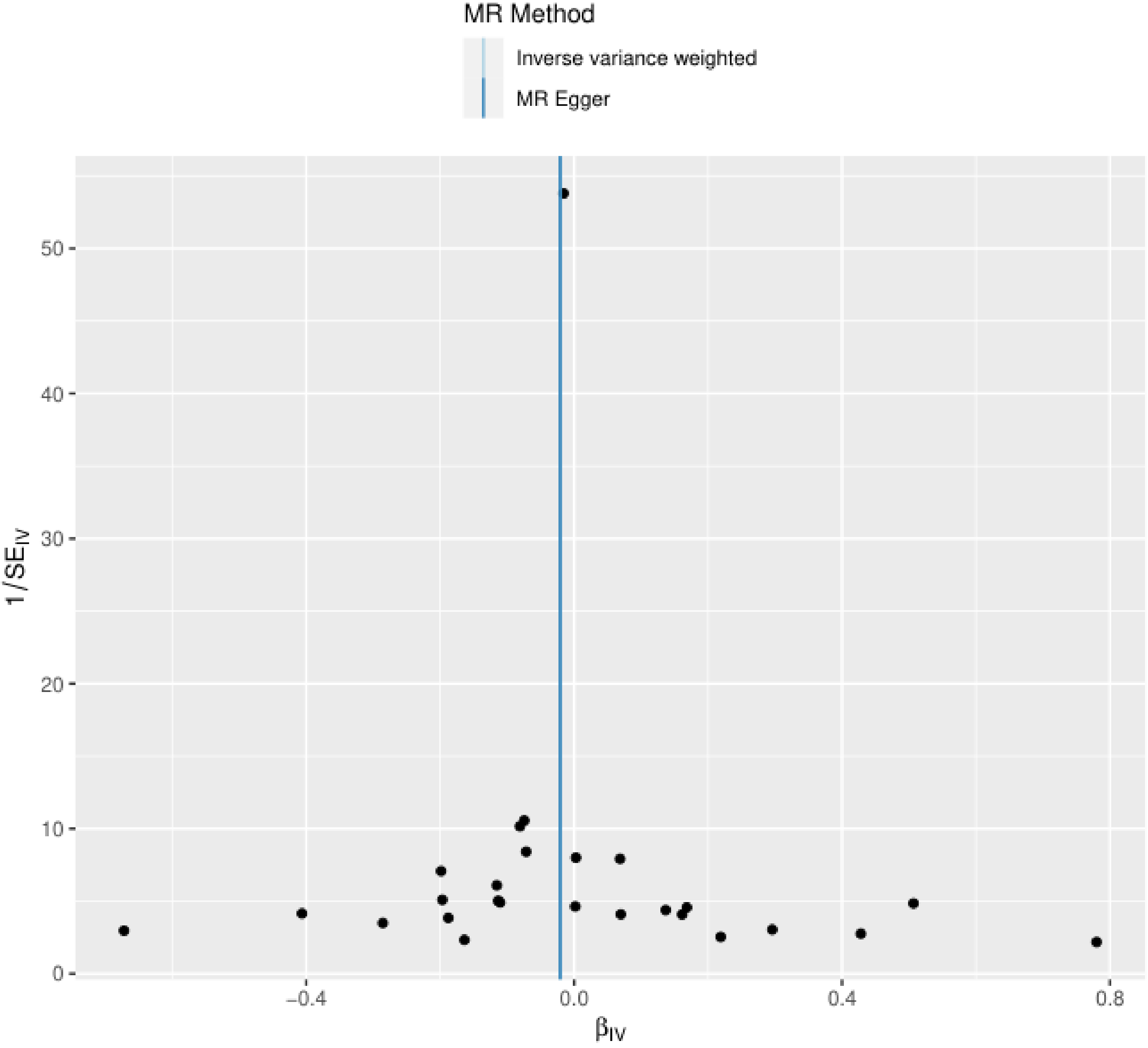

Funnel plot analyse of "CD14+ CD16- monocyte %monocyte" on 'Diabetic nephropathy'

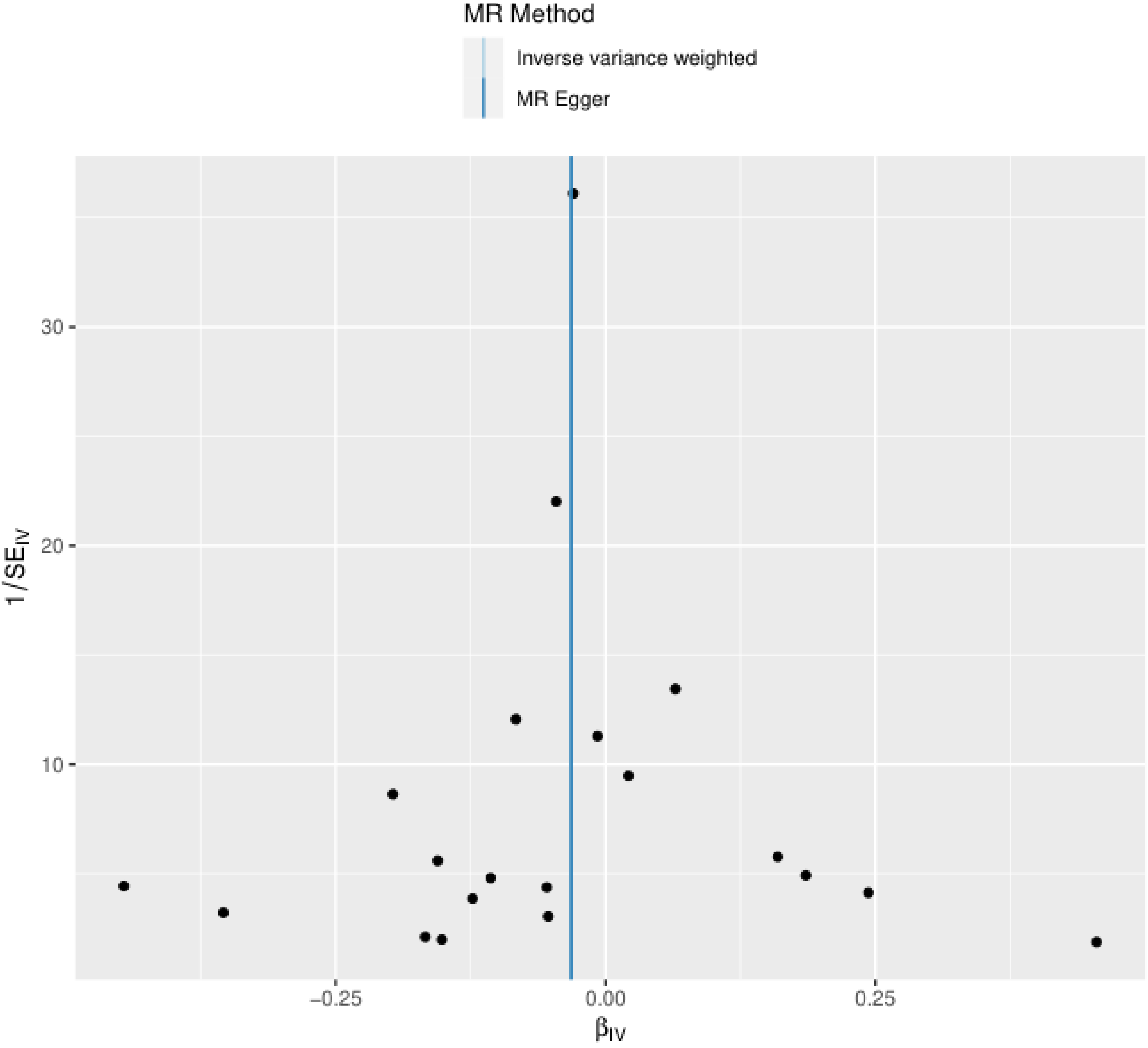

Funnel plot analyse of "CD38 on IgD- CD38dim" on 'Diabetic nephropathy'

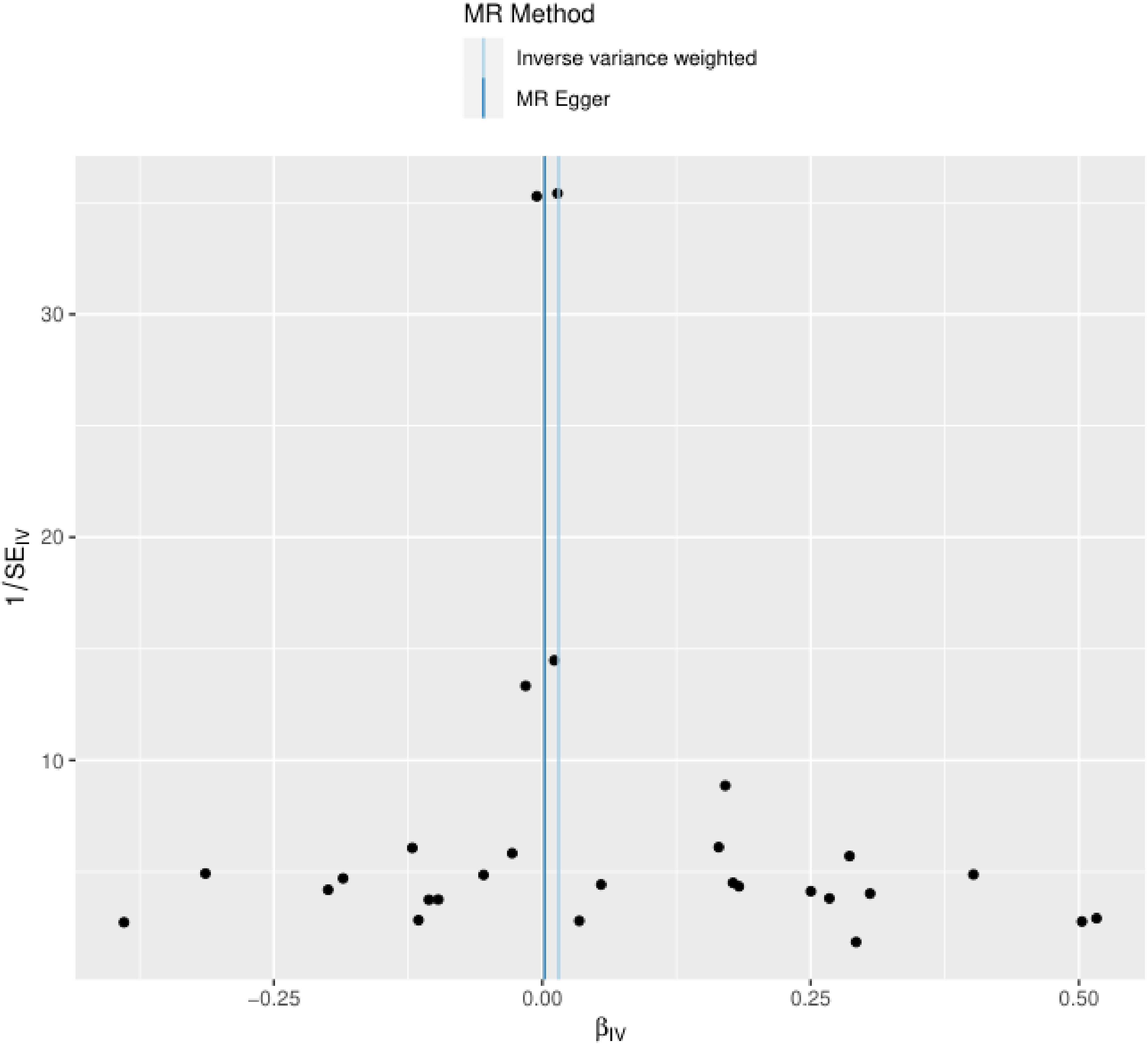

Funnel plot analyse of "CD19 on naive-mature B cell" on 'Diabetic nephropathy'

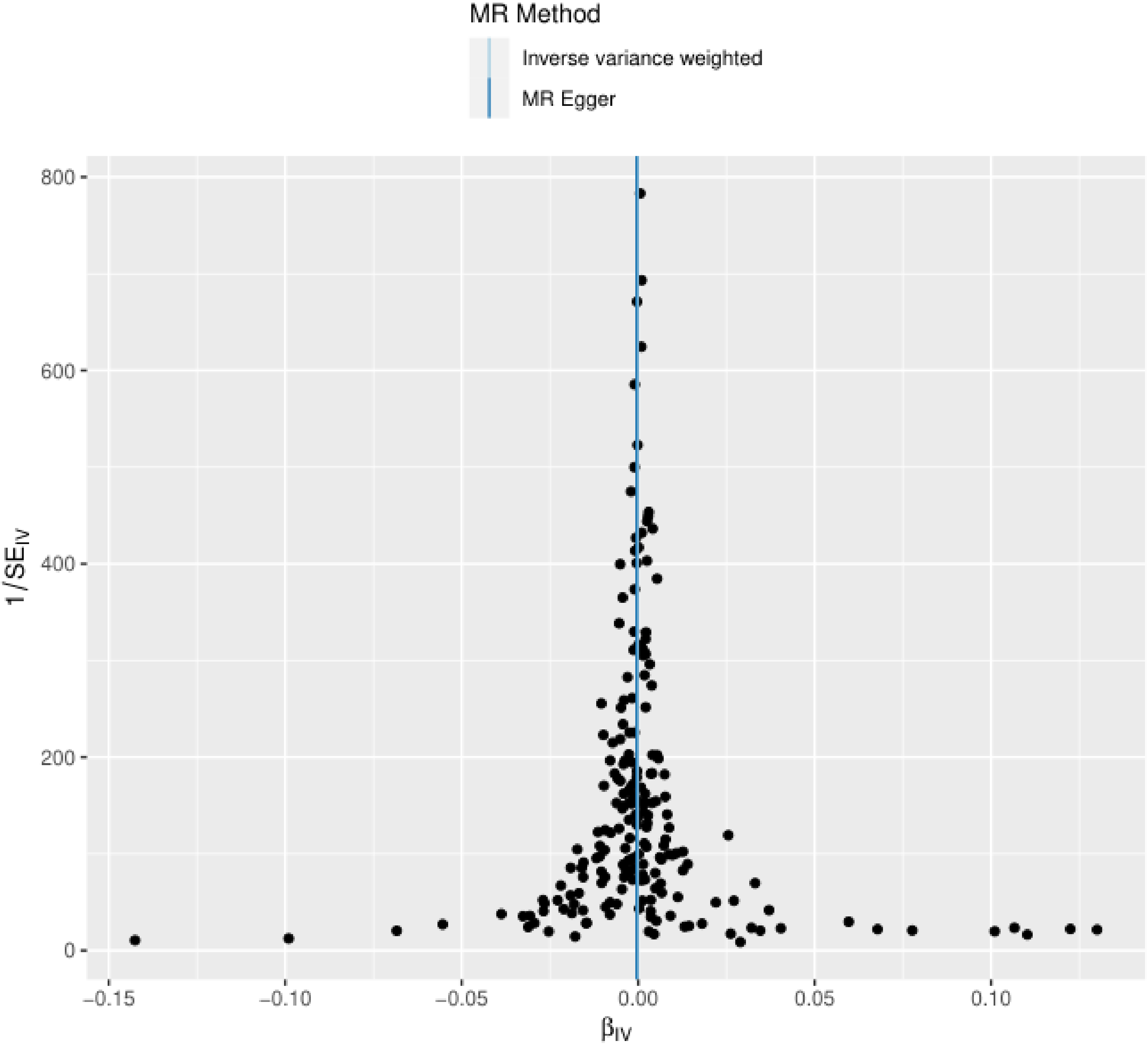

Funnel plot analyse of "CD45RA+ CD28- CD8br %T cell" on 'Diabetic nephropathy'

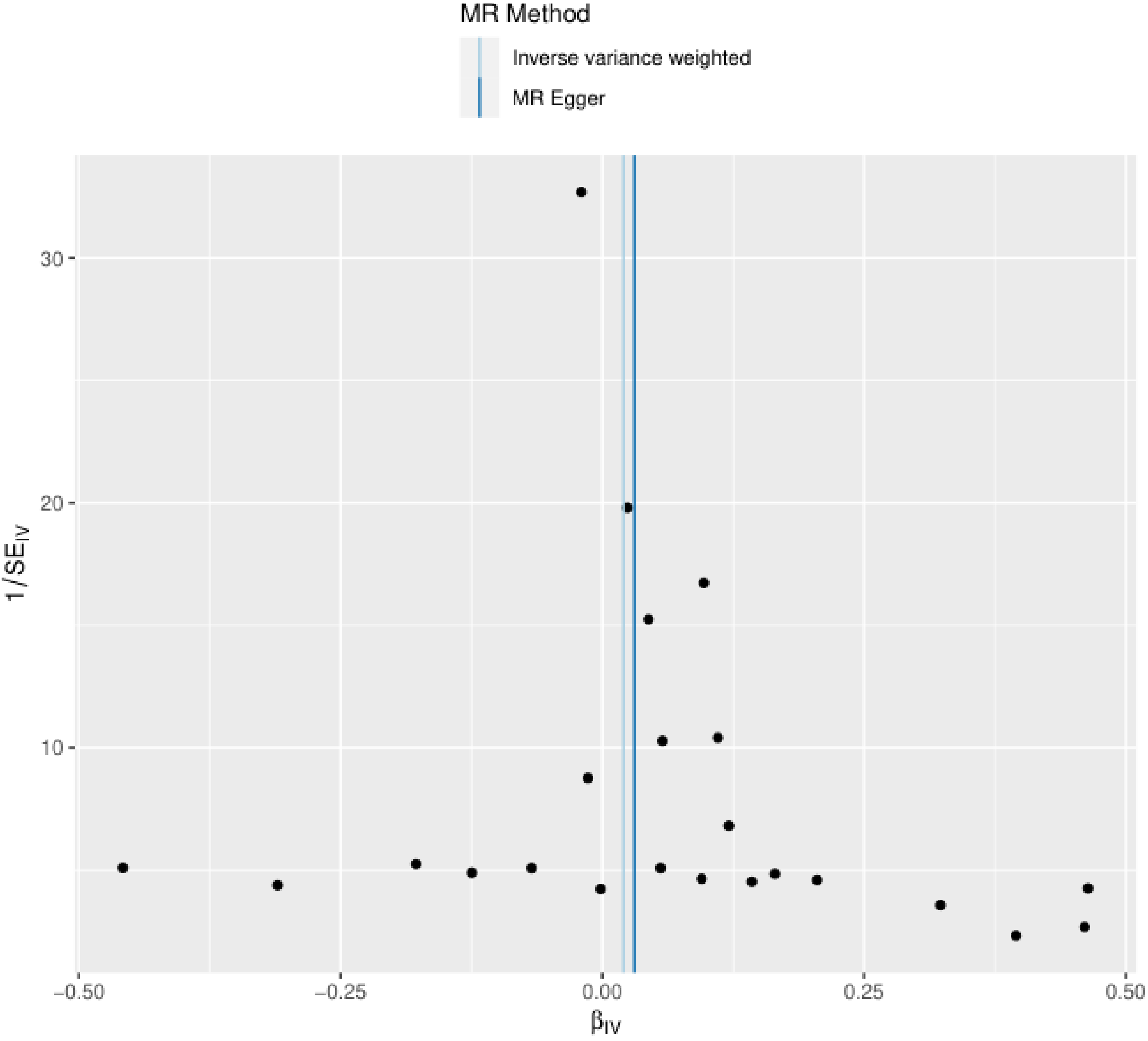

Funnel plot analyse of "BAFF-R on unsw mem" on 'Diabetic nephropathy'

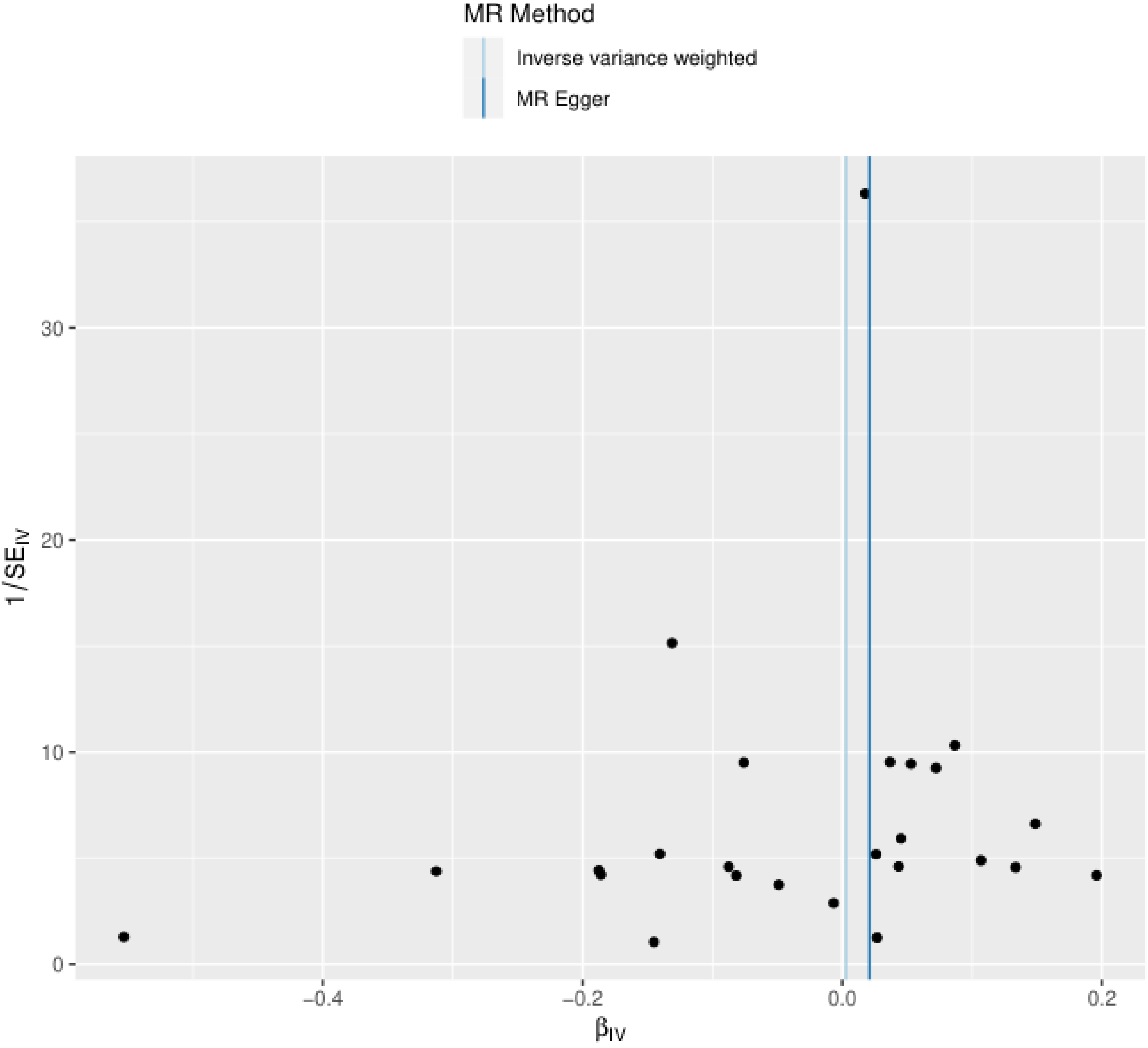

Funnel plot analyse of "CD39+ CD4+ %CD4+" on 'Diabetic nephropathy'

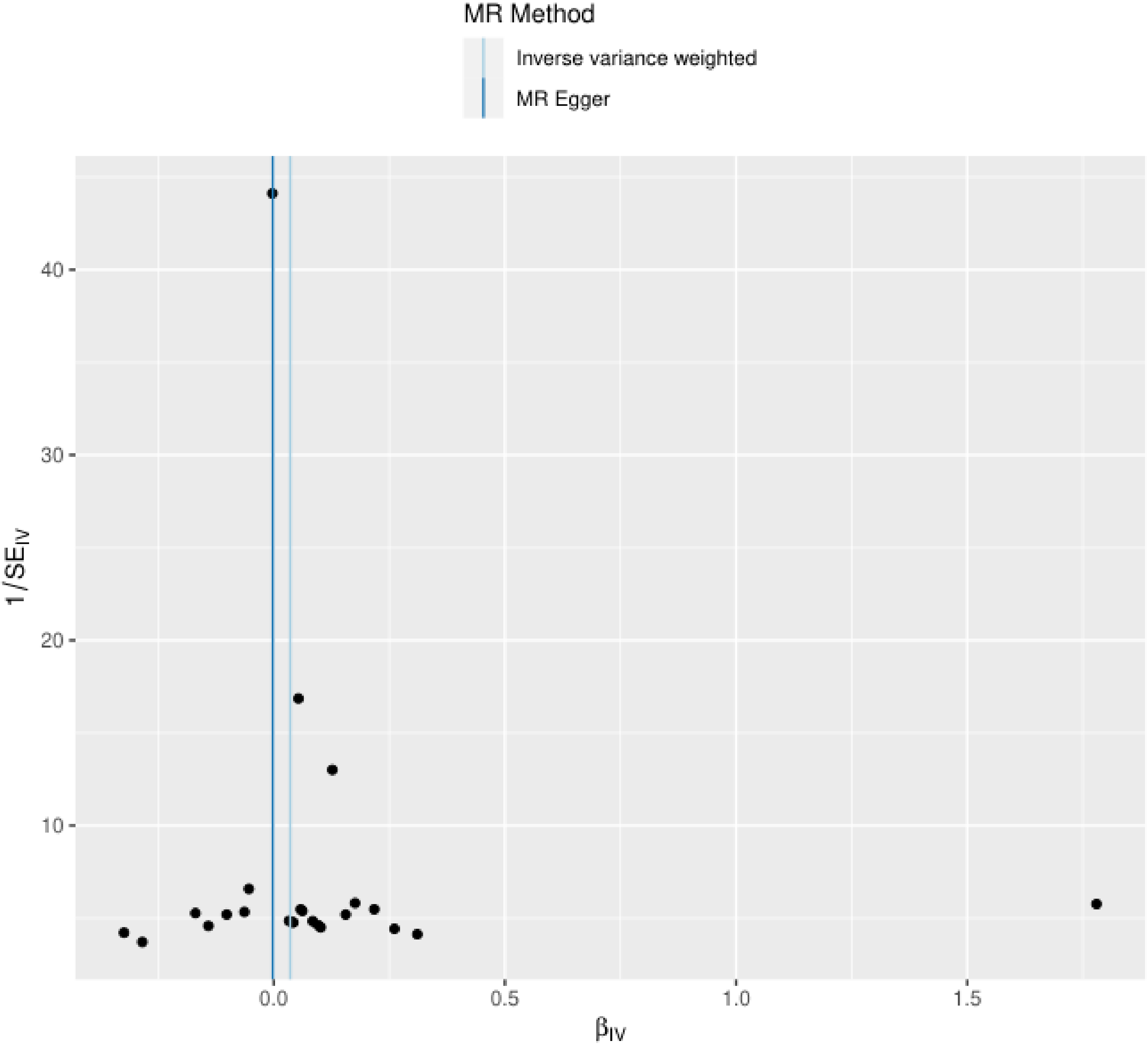

Funnel plot analyse of "EM CD8br AC" on 'Diabetic nephropathy'

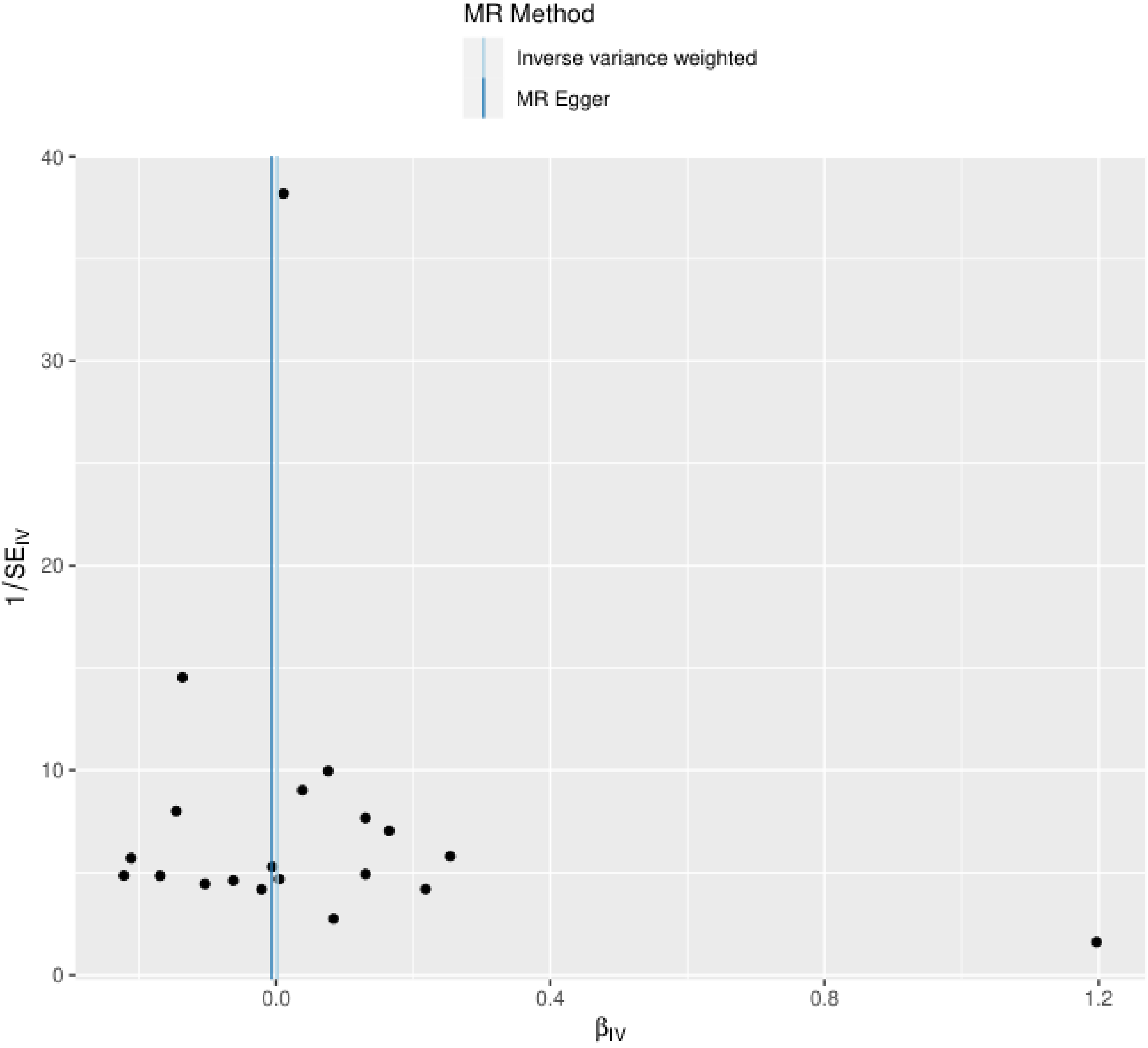

Funnel plot analyse of "CD39 on CD39+ CD4+ " on 'Diabetic nephropathy'

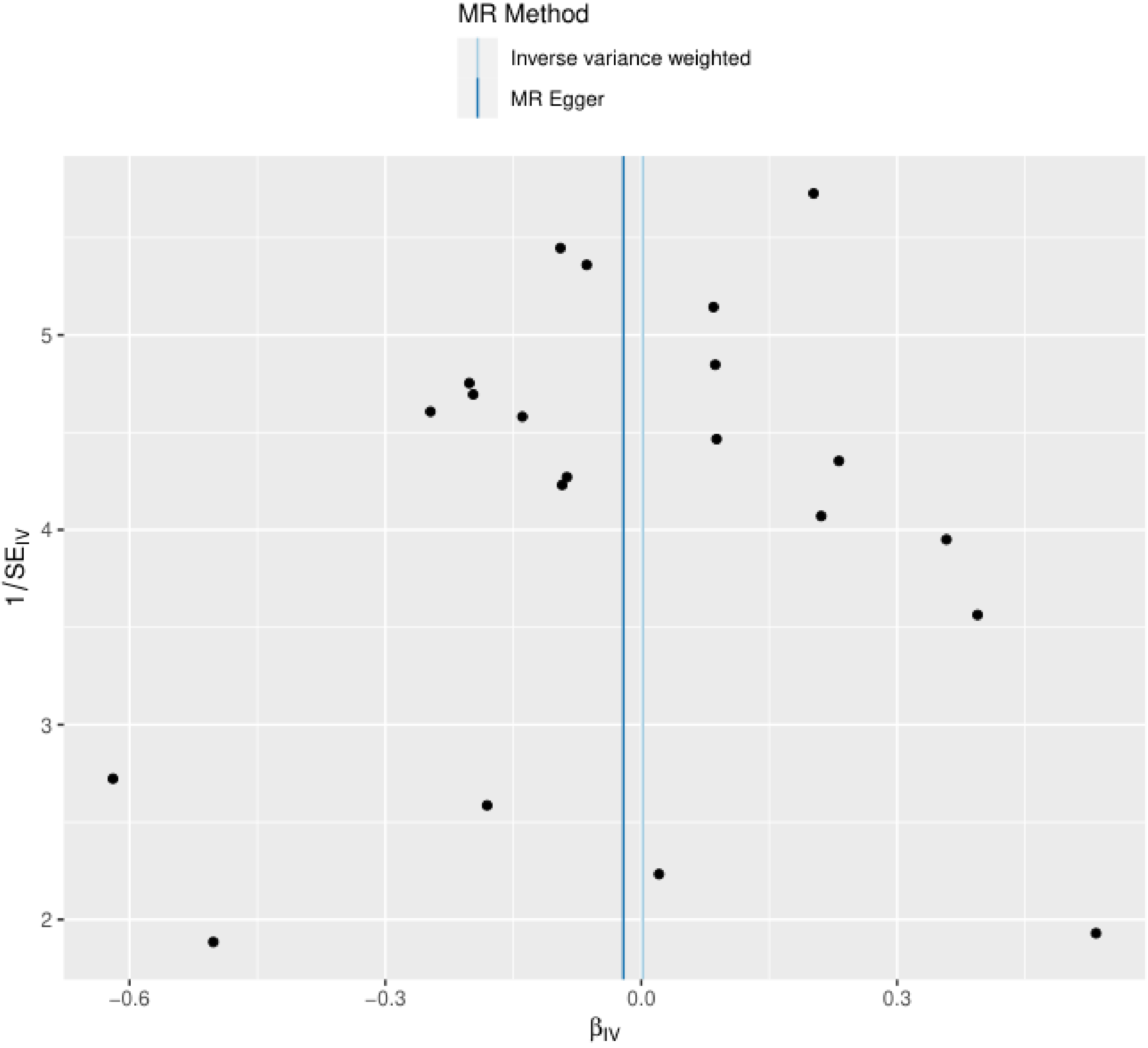

Funnel plot analyse of "IgD+ CD24+ AC" on 'Diabetic nephropathy'

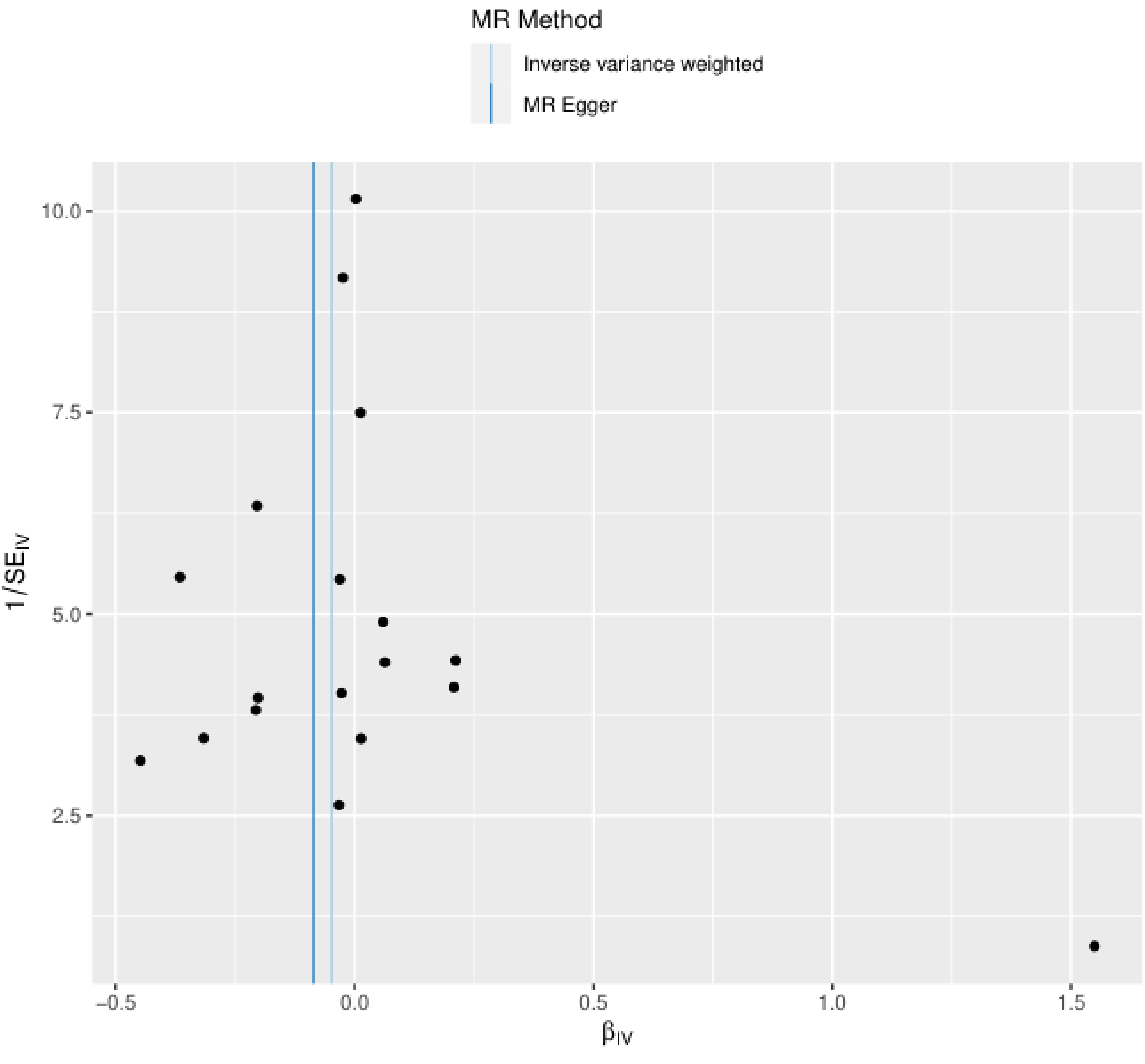

Funnel plot analyse of "DP (CD4+CD8+) AC" on 'Diabetic nephropathy'

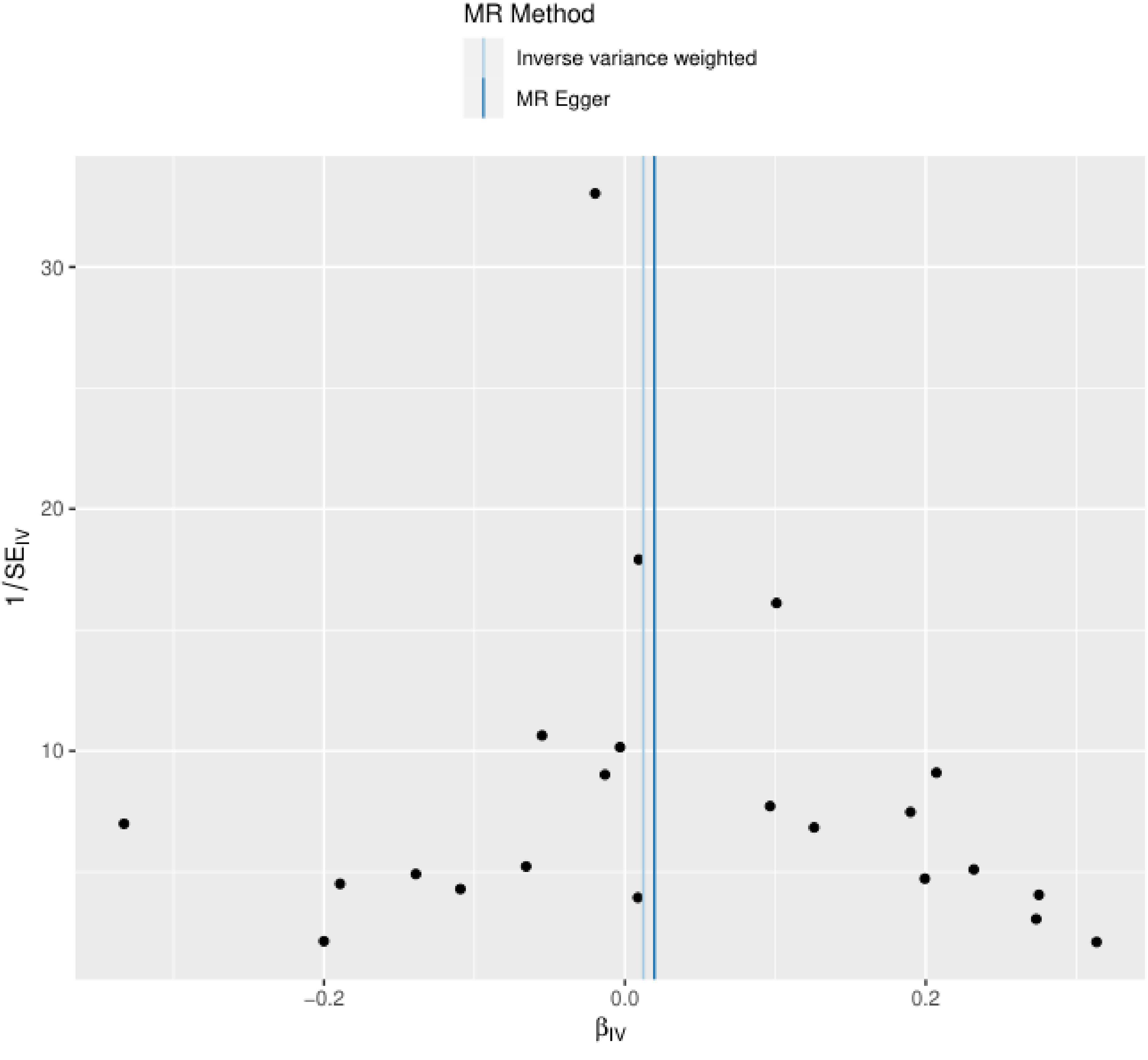

Funnel plot analyse of "BAFF-R on IgD+ CD24-" on 'Diabetic nephropathy'

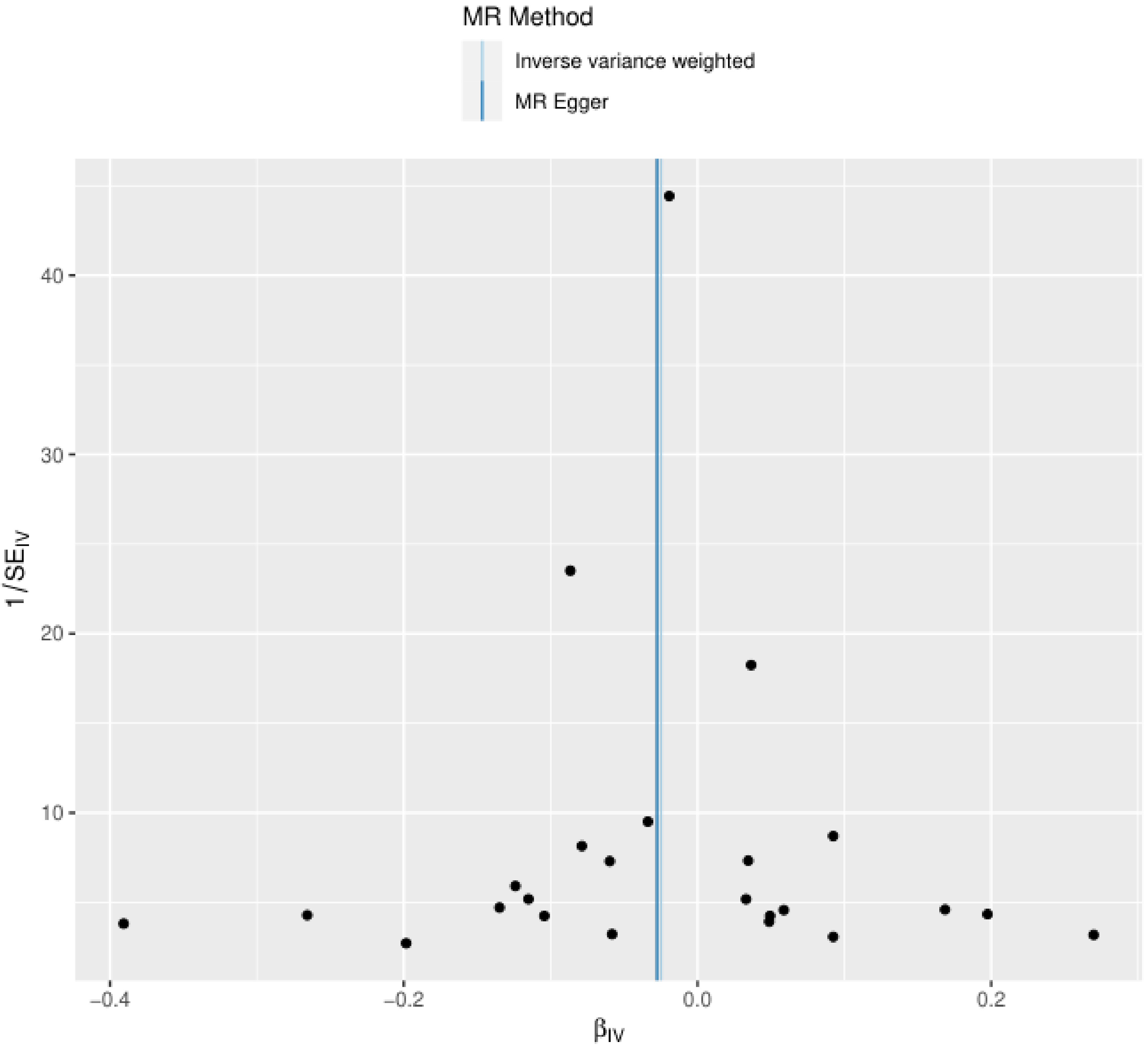

Funnel plot analyse of "CD14- CD16- AC" on 'Diabetic nephropathy'

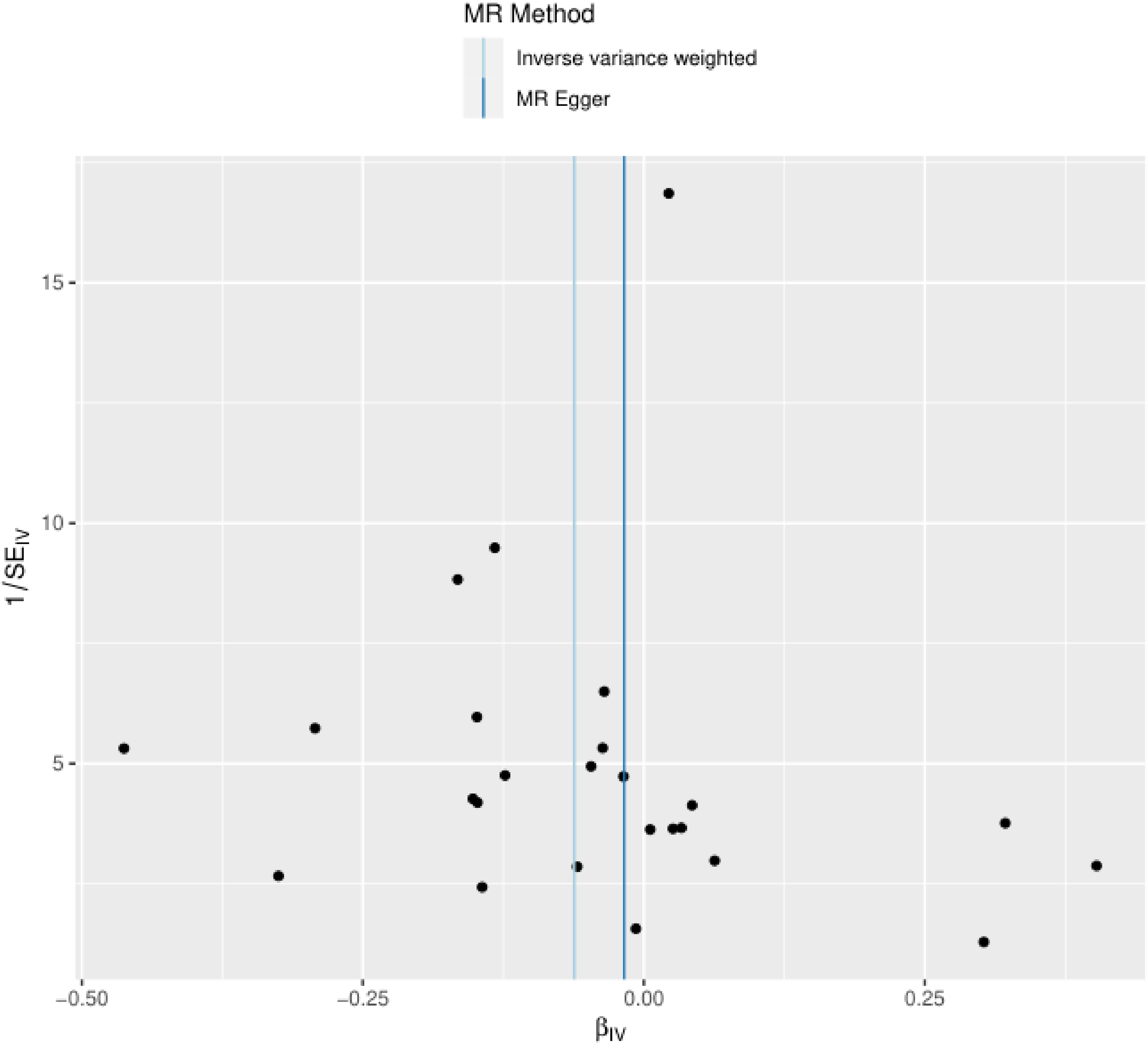

Funnel plot analyse of "CD14+ CD16+ monocyte %monocyte" on 'Diabetic nephropathy'

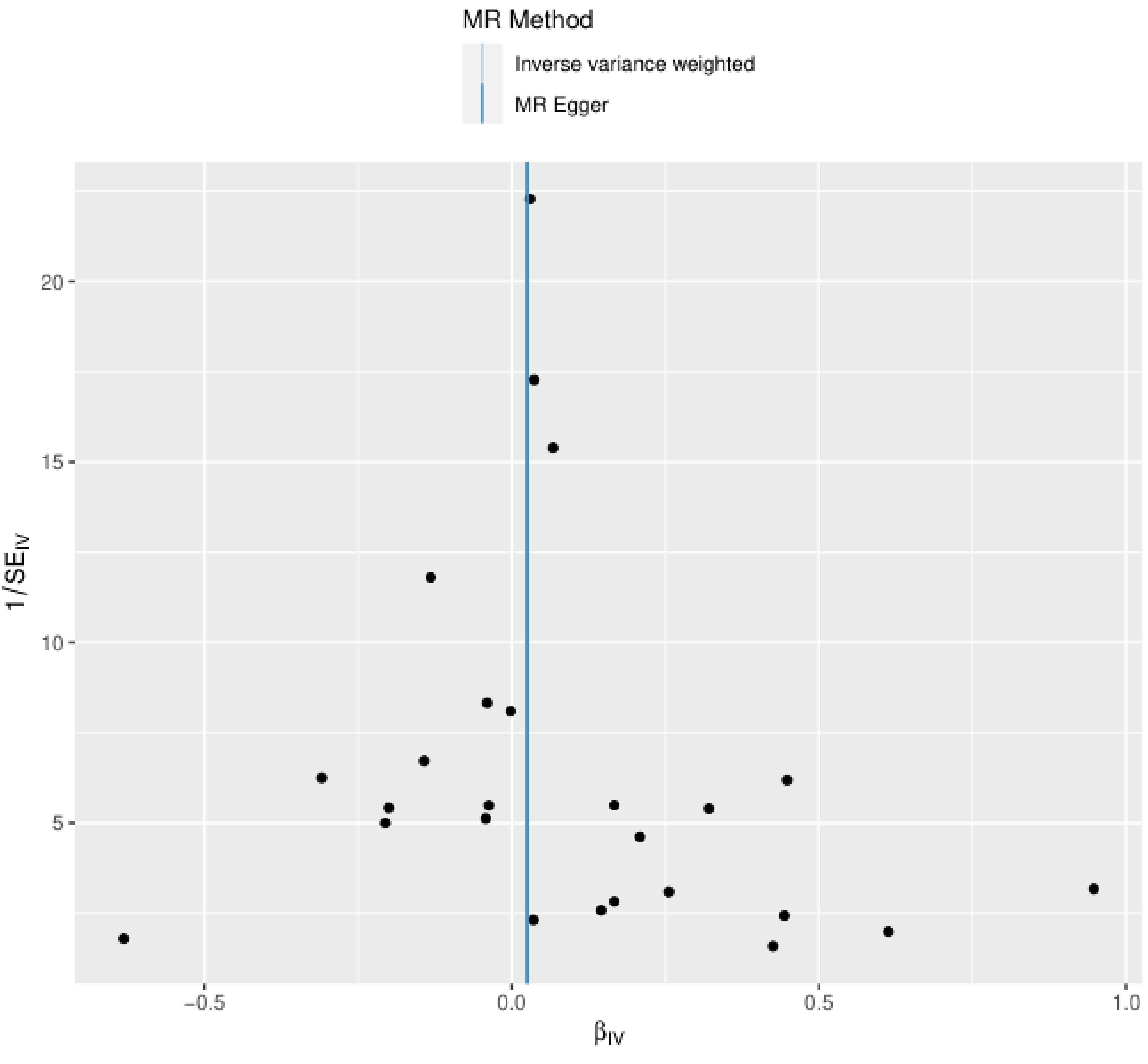

Funnel plot analyse of "CD28 on CD28+ CD4+ " on 'Diabetic nephropathy'

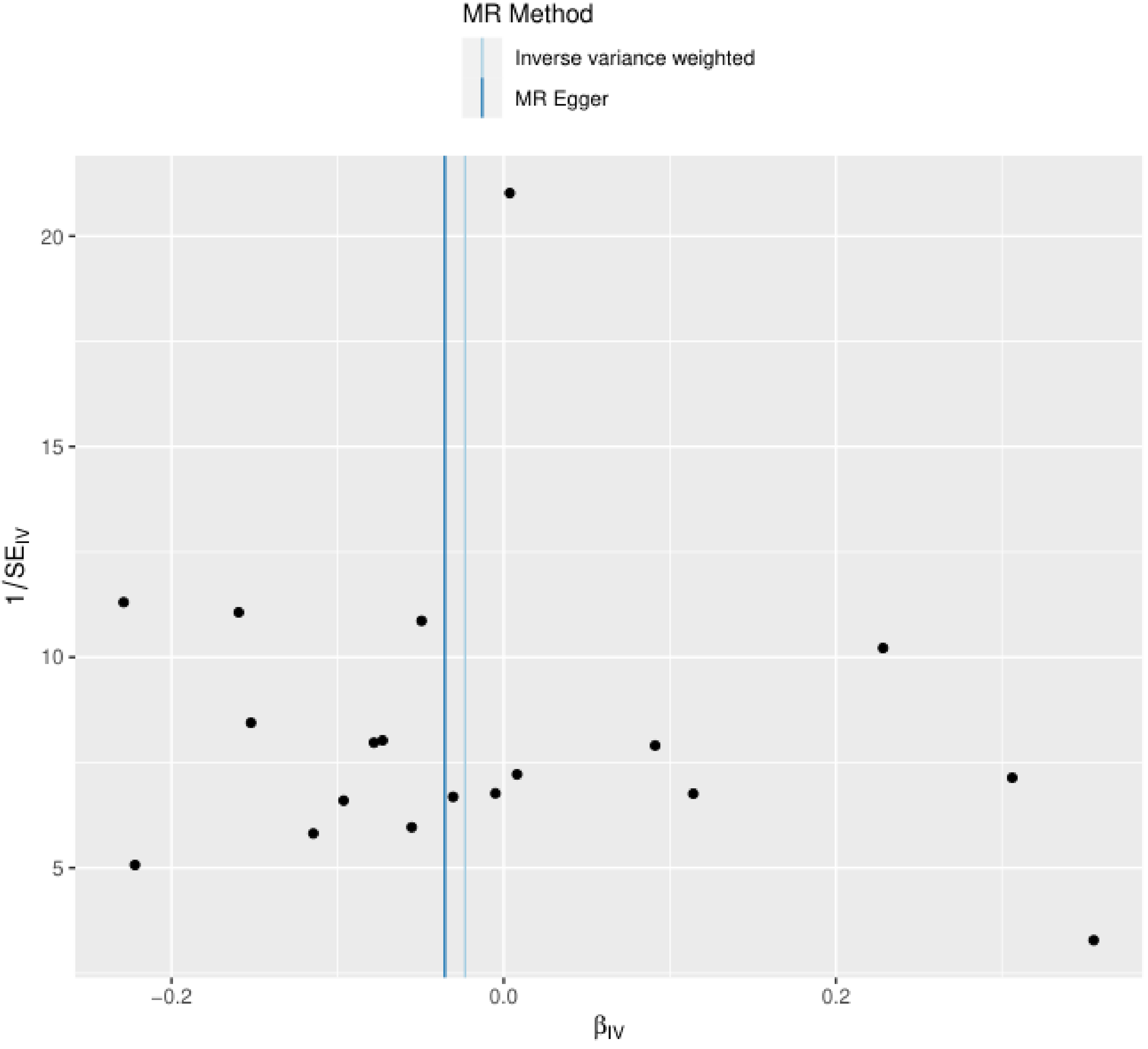

Funnel plot analyse of "HVEM on CD4+ " on 'Diabetic nephropathy'

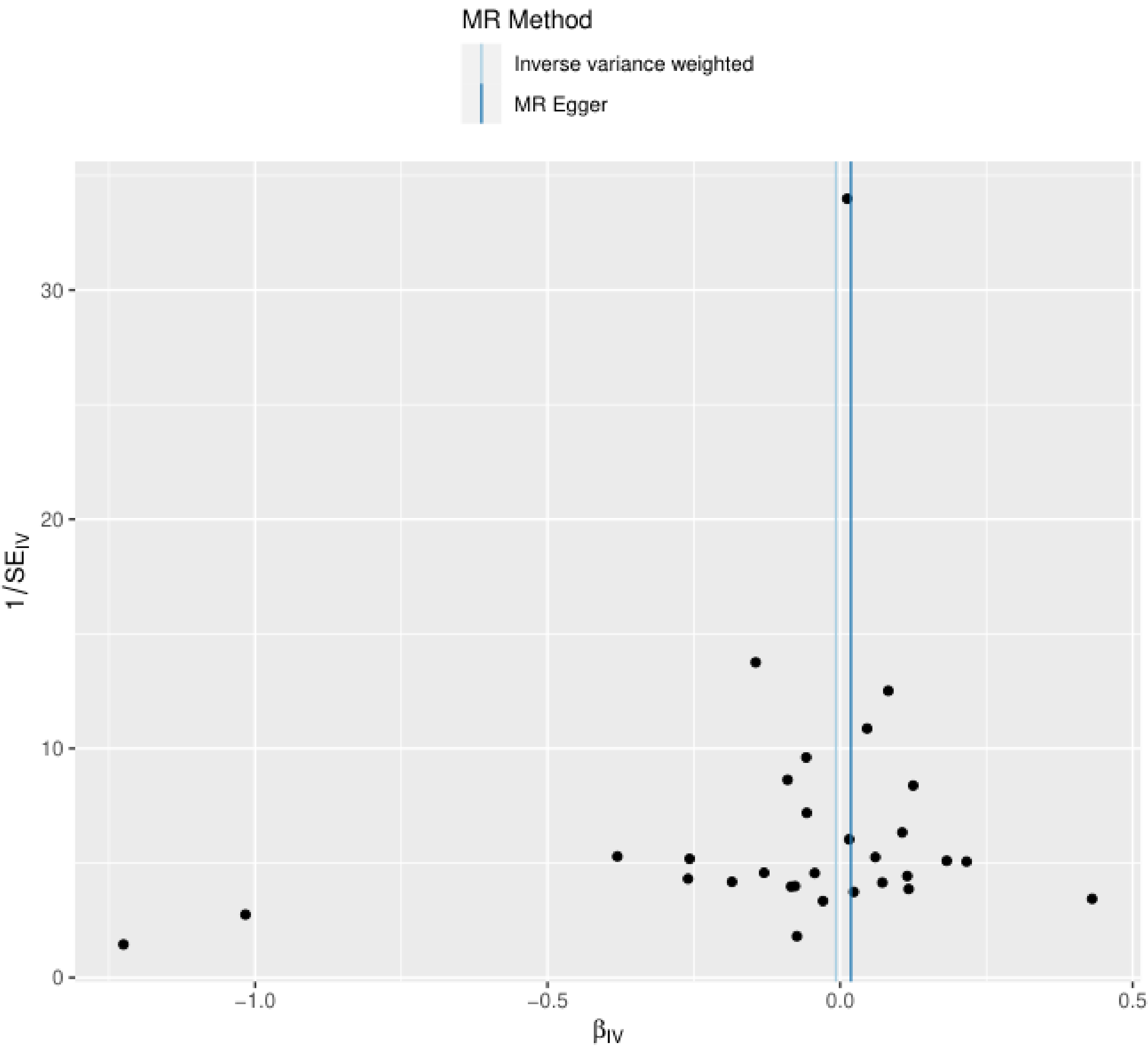

Funnel plot analyse of "CD39+ activated Treg AC" on 'Diabetic nephropathy'

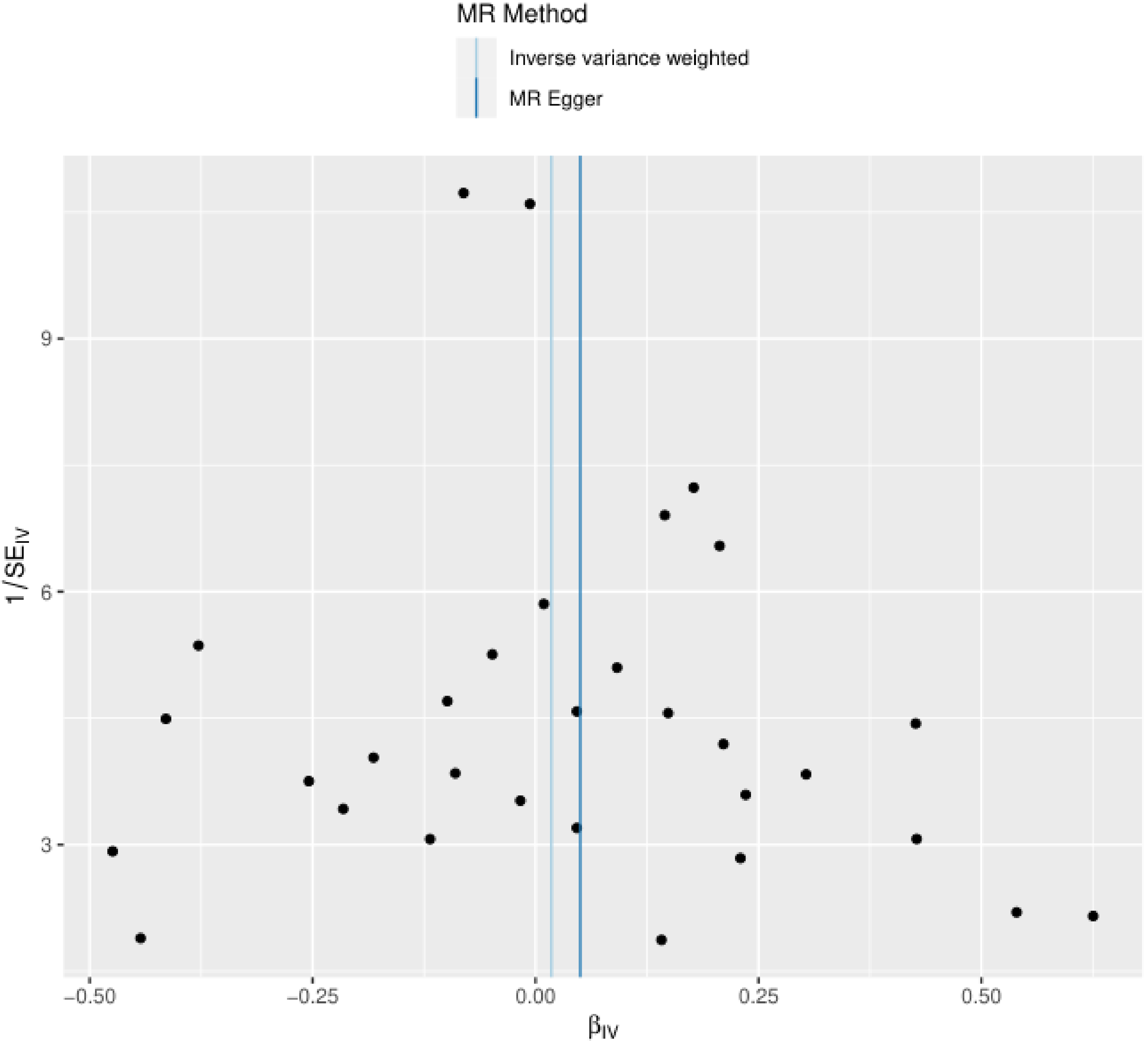

Funnel plot analyse of "DN (CD4-CD8-) NKT %T cell" on 'Diabetic nephropathy'

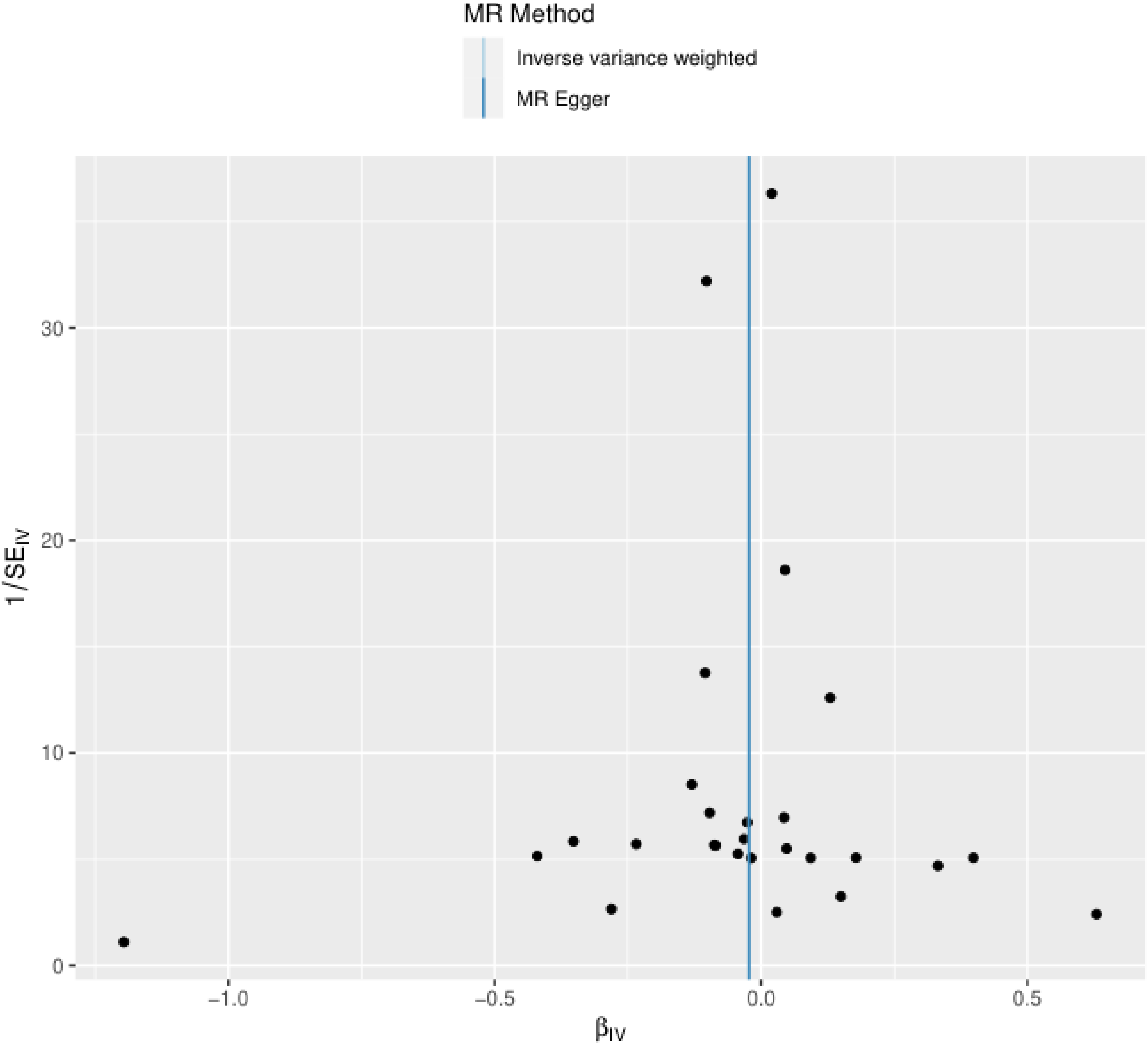

Funnel plot analyse of "FSC-A on plasmacytoid DC" on 'Diabetic nephropathy'

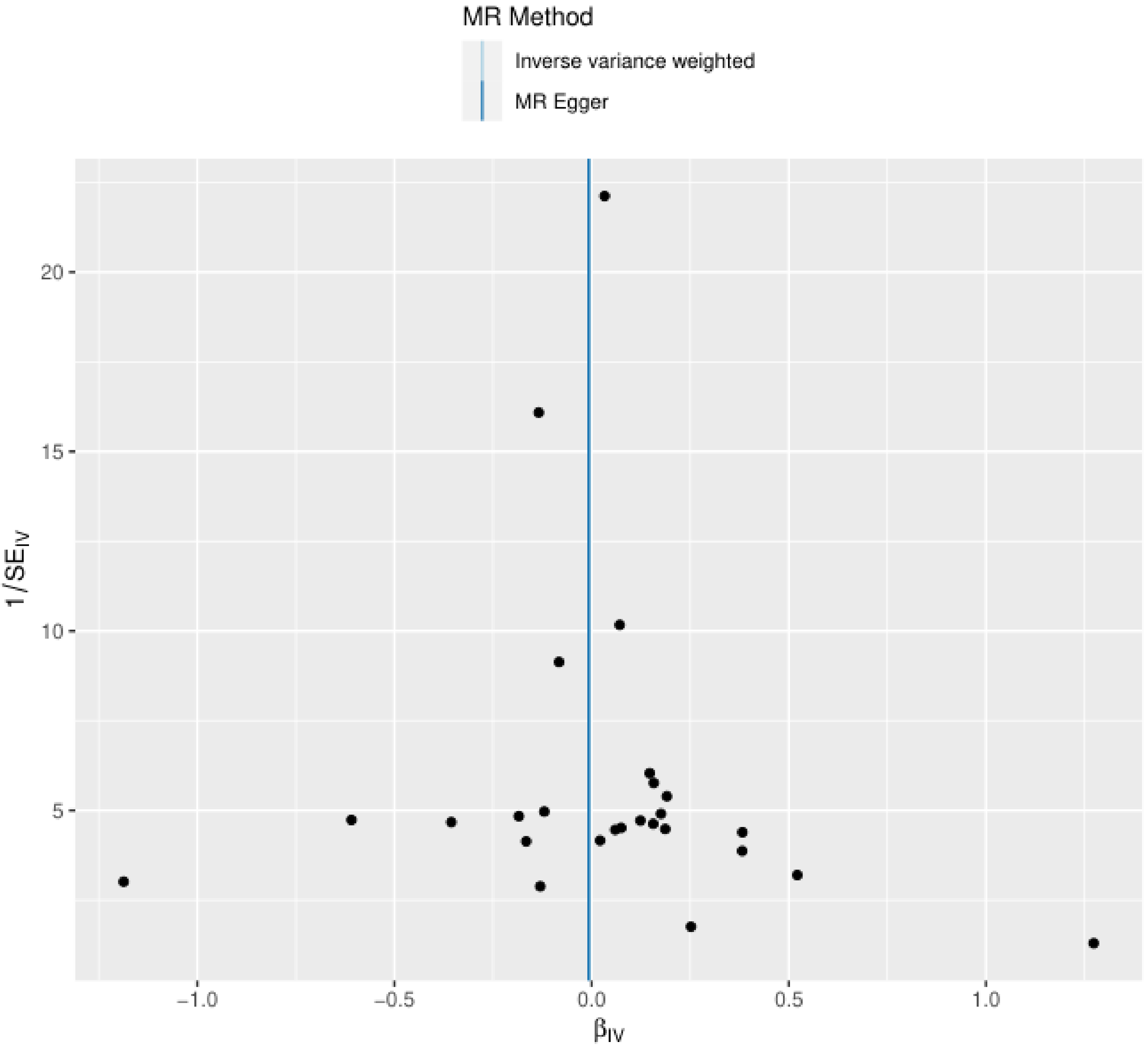

Funnel plot analyse of "CD19 on B cell" on 'Diabetic nephropathy'

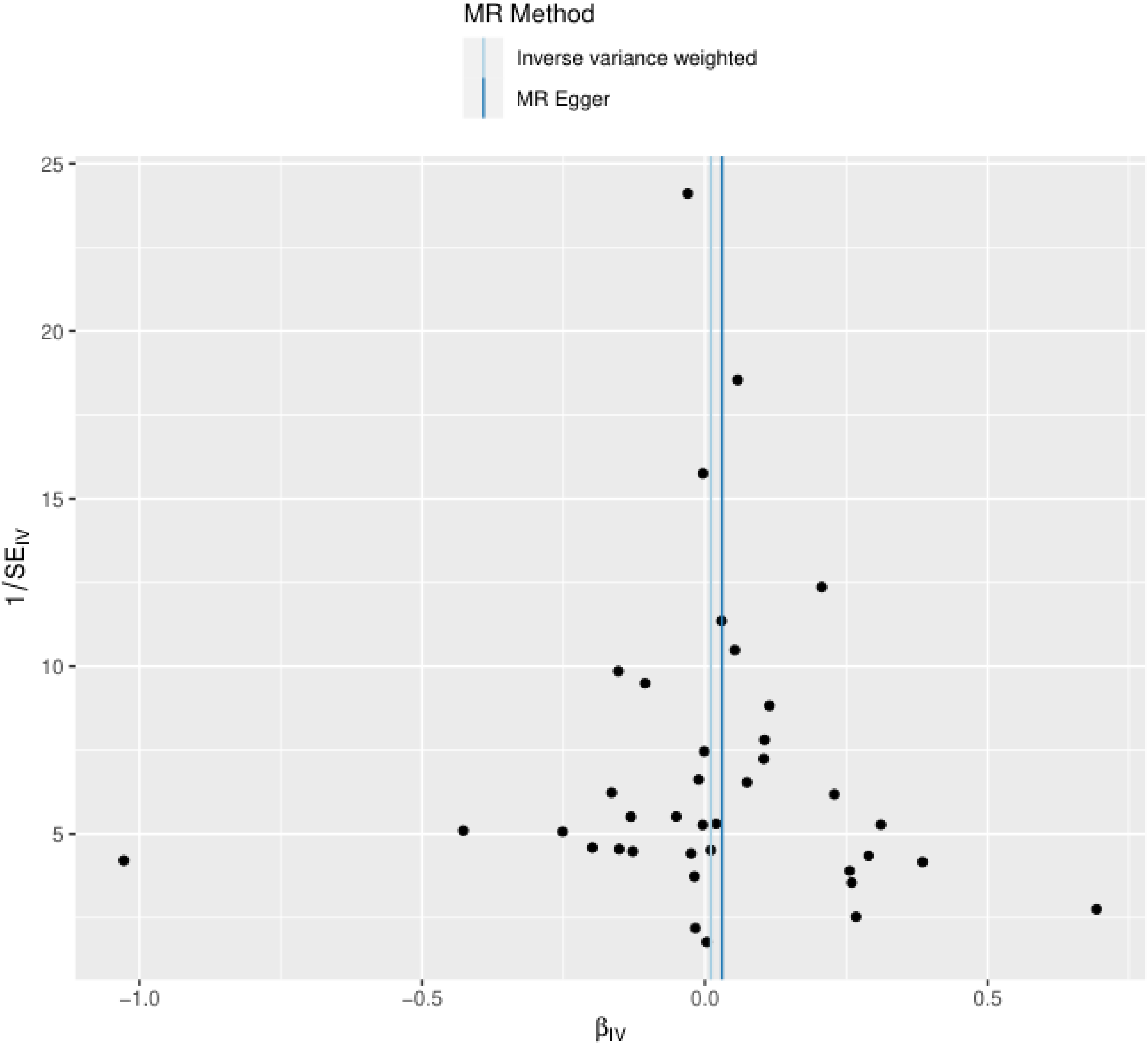

Funnel plot analyse of "CD24 on sw mem" on 'Diabetic nephropathy'

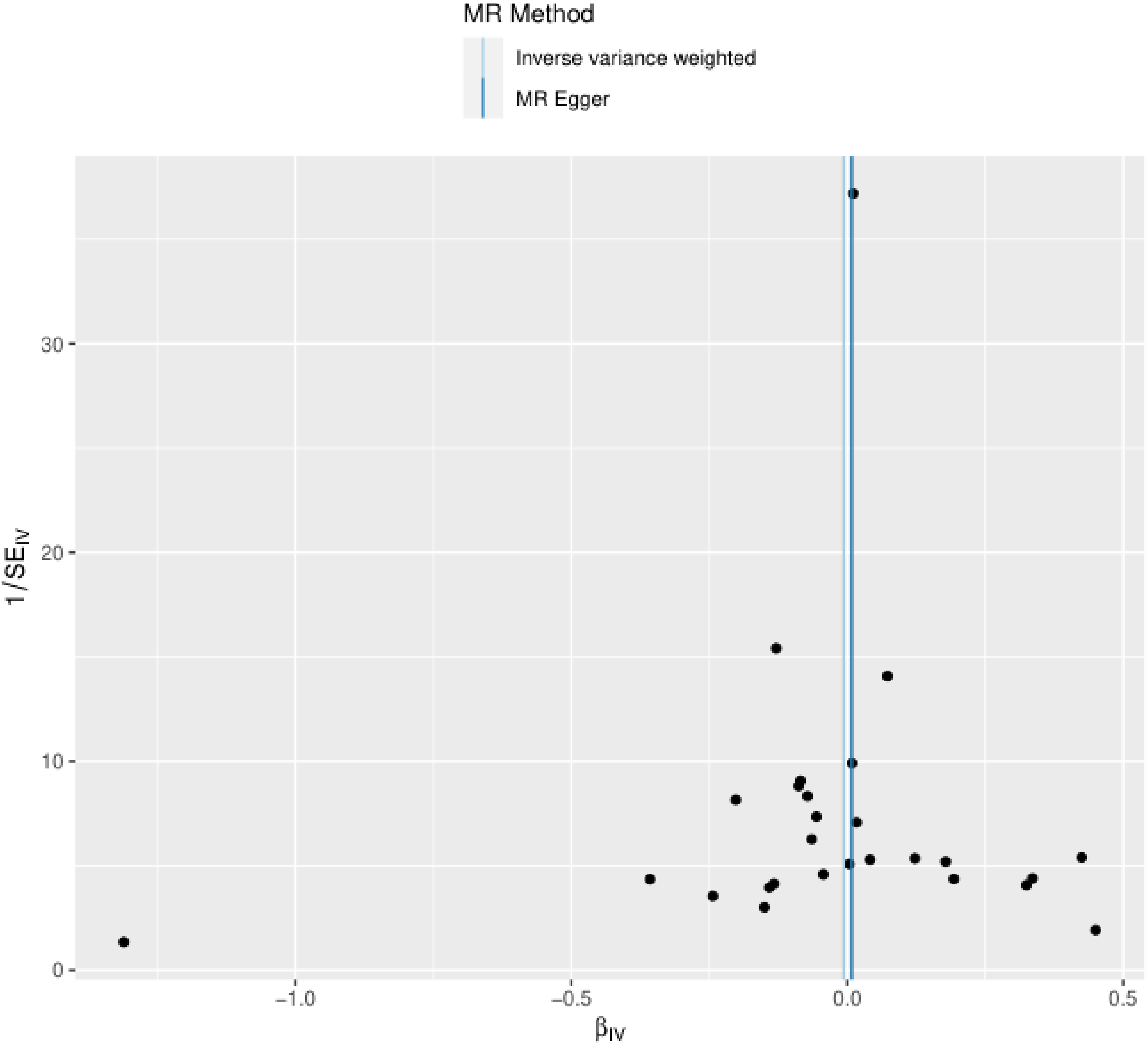

Funnel plot analyse of "CD39+ secreting Treg AC" on 'Diabetic nephropathy'

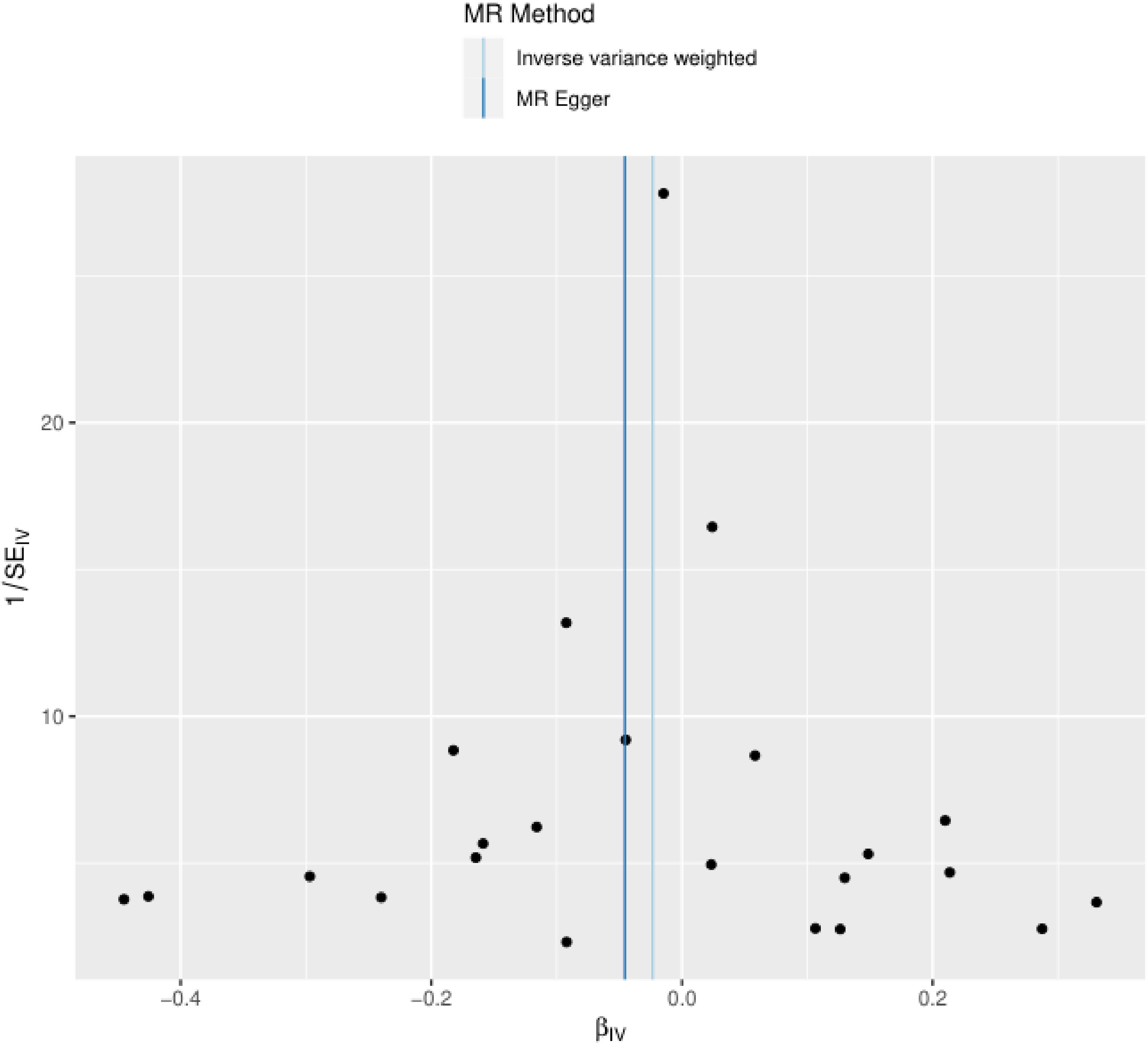

Funnel plot analyse of "CD25 on CD39+ CD4+ " on 'Diabetic nephropathy'

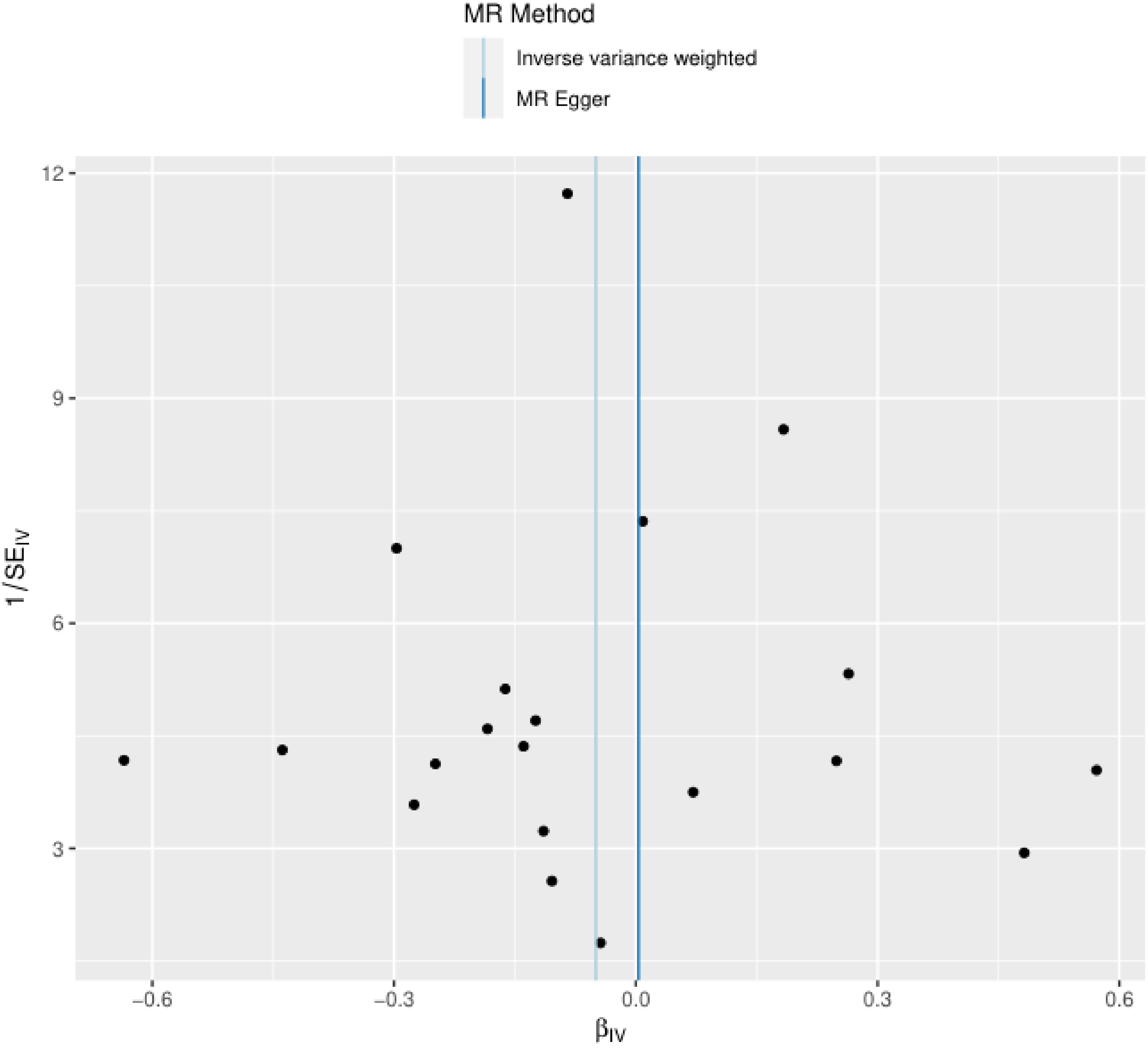

Funnel plot analyse of "CD4+ CD8dim %lymphocyte" on 'Diabetic nephropathy'

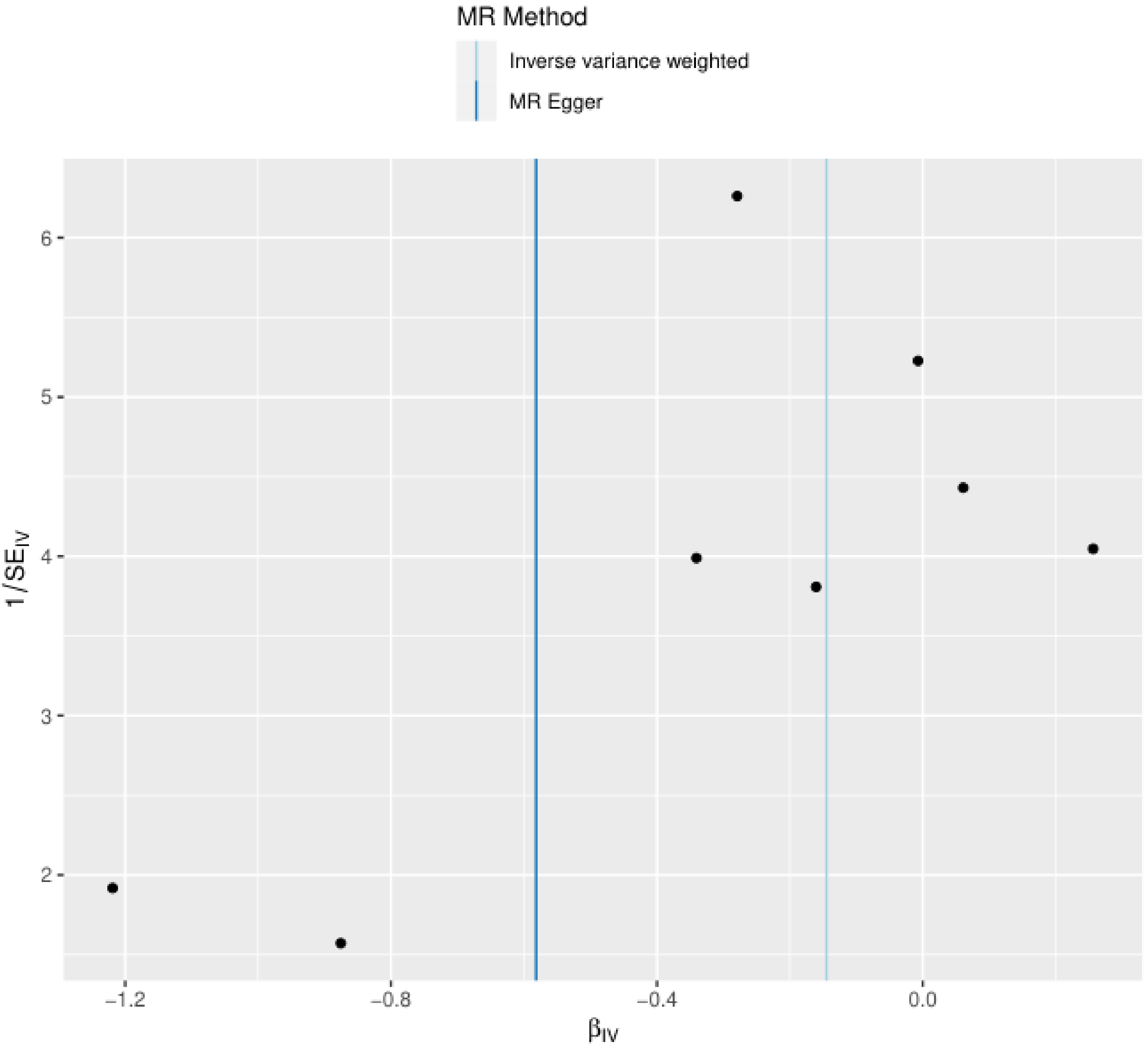

Funnel plot analyse of "DP (CD4+CD8+) %T cell" on 'Diabetic nephropathy'

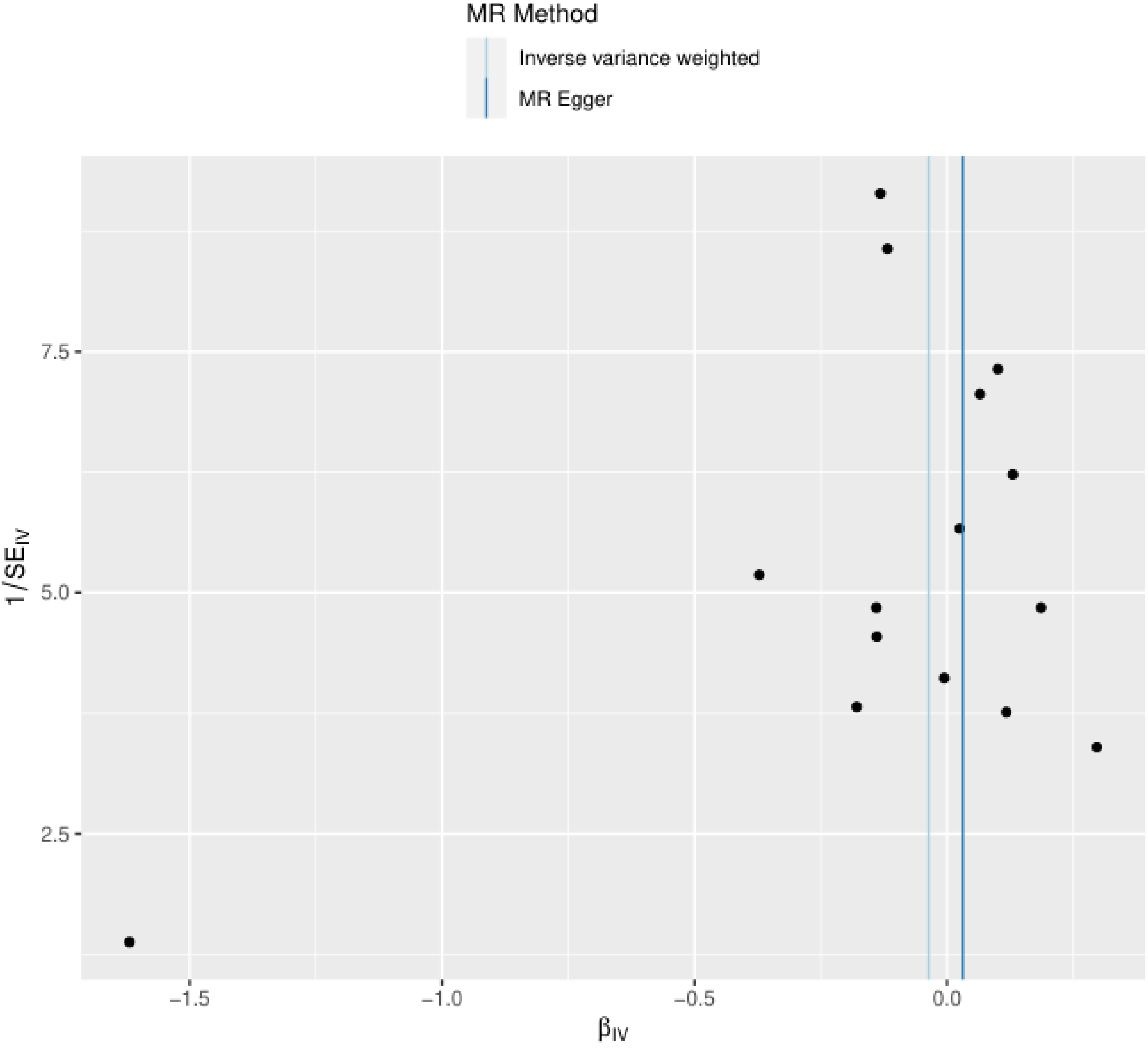

Funnel plot analyse of "CD62L on CD62L+ myeloid DC " on 'Diabetic nephropathy'

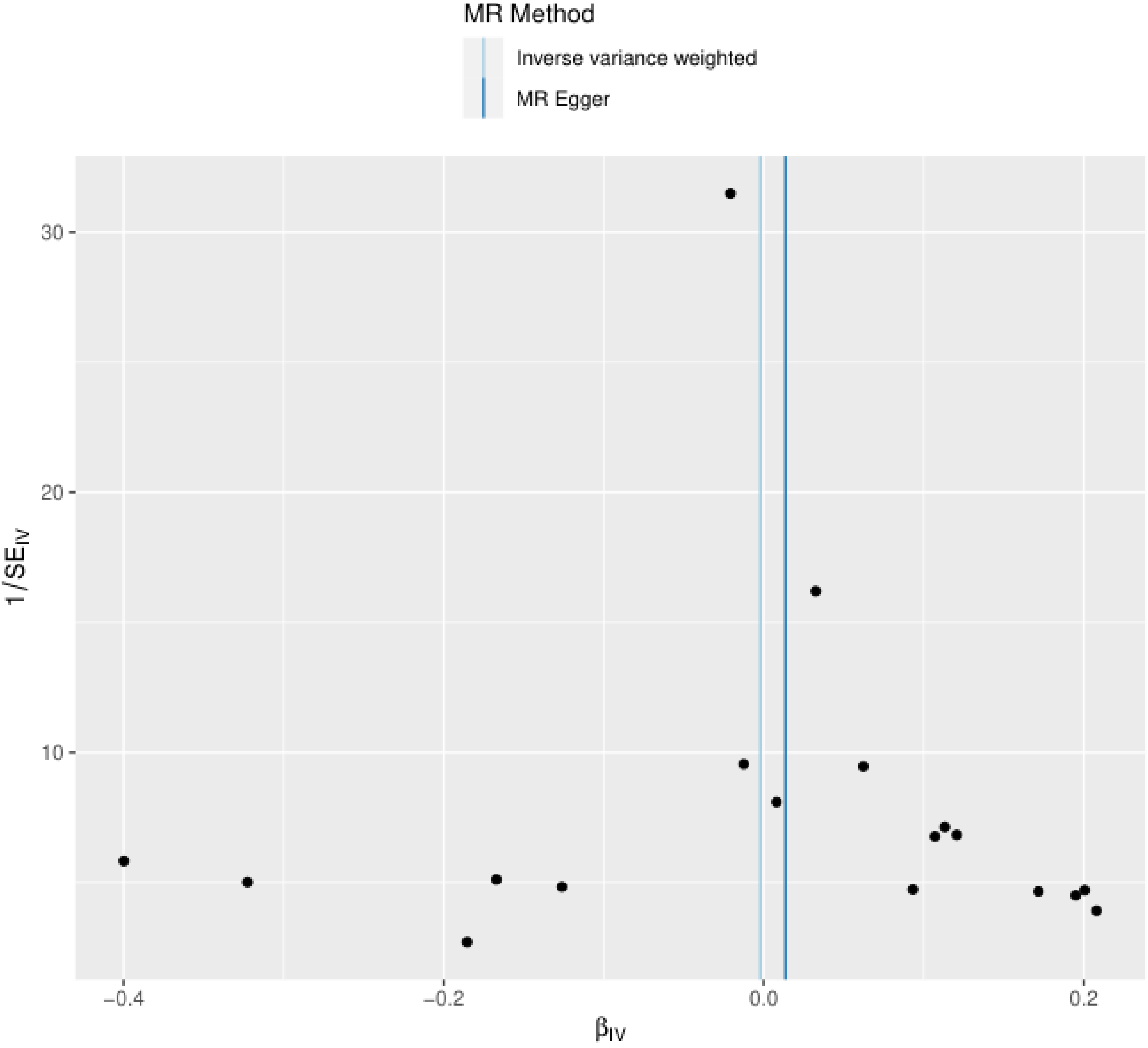

Funnel plot analyse of "BAFF-R on IgD- CD27-" on 'Diabetic nephropathy'

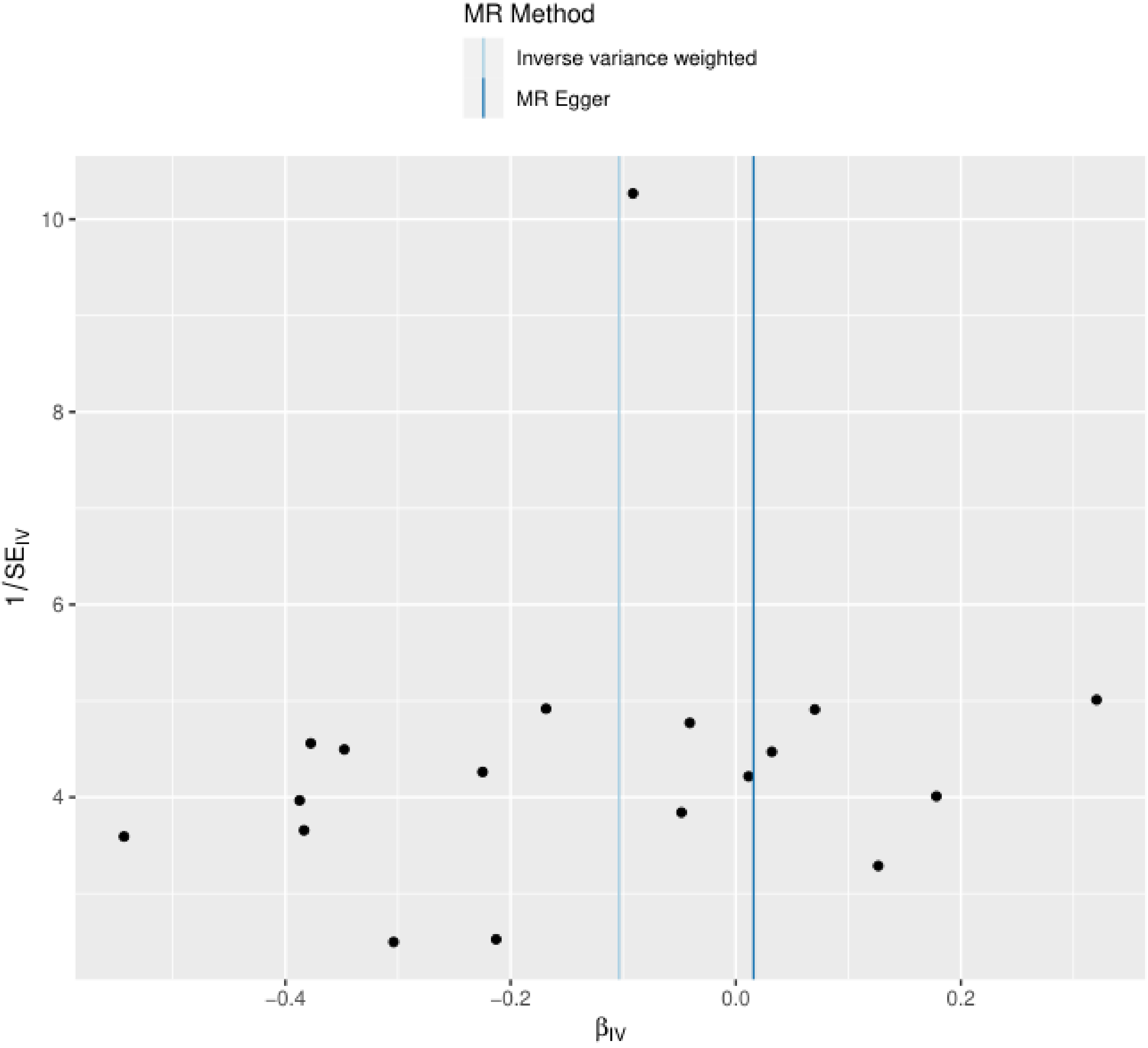

Funnel plot analyse of "IgD- CD38dim AC" on 'Diabetic nephropathy'

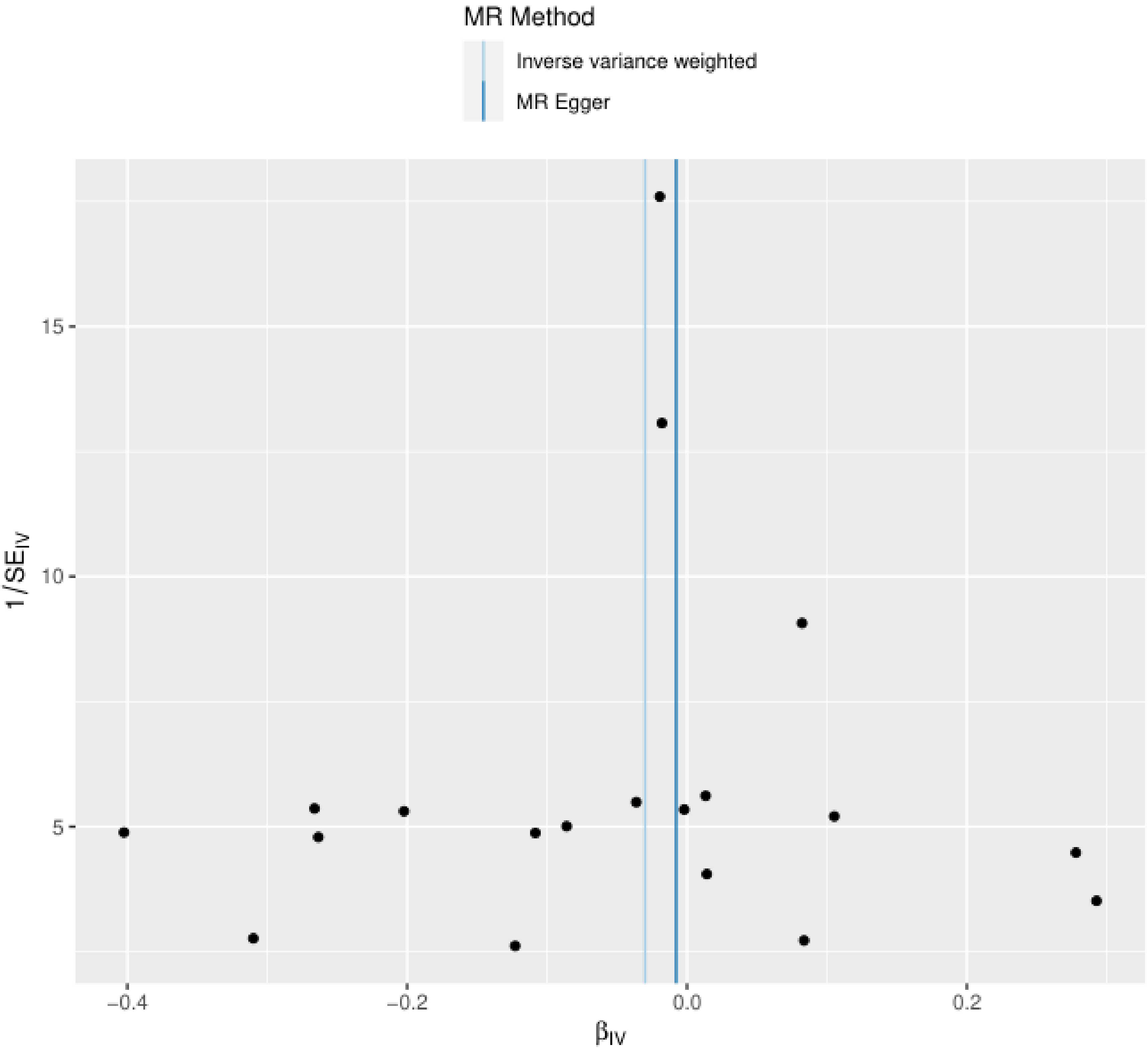

Funnel plot analyse of "CD127 on CD28- CD8br " on 'Diabetic nephropathy'

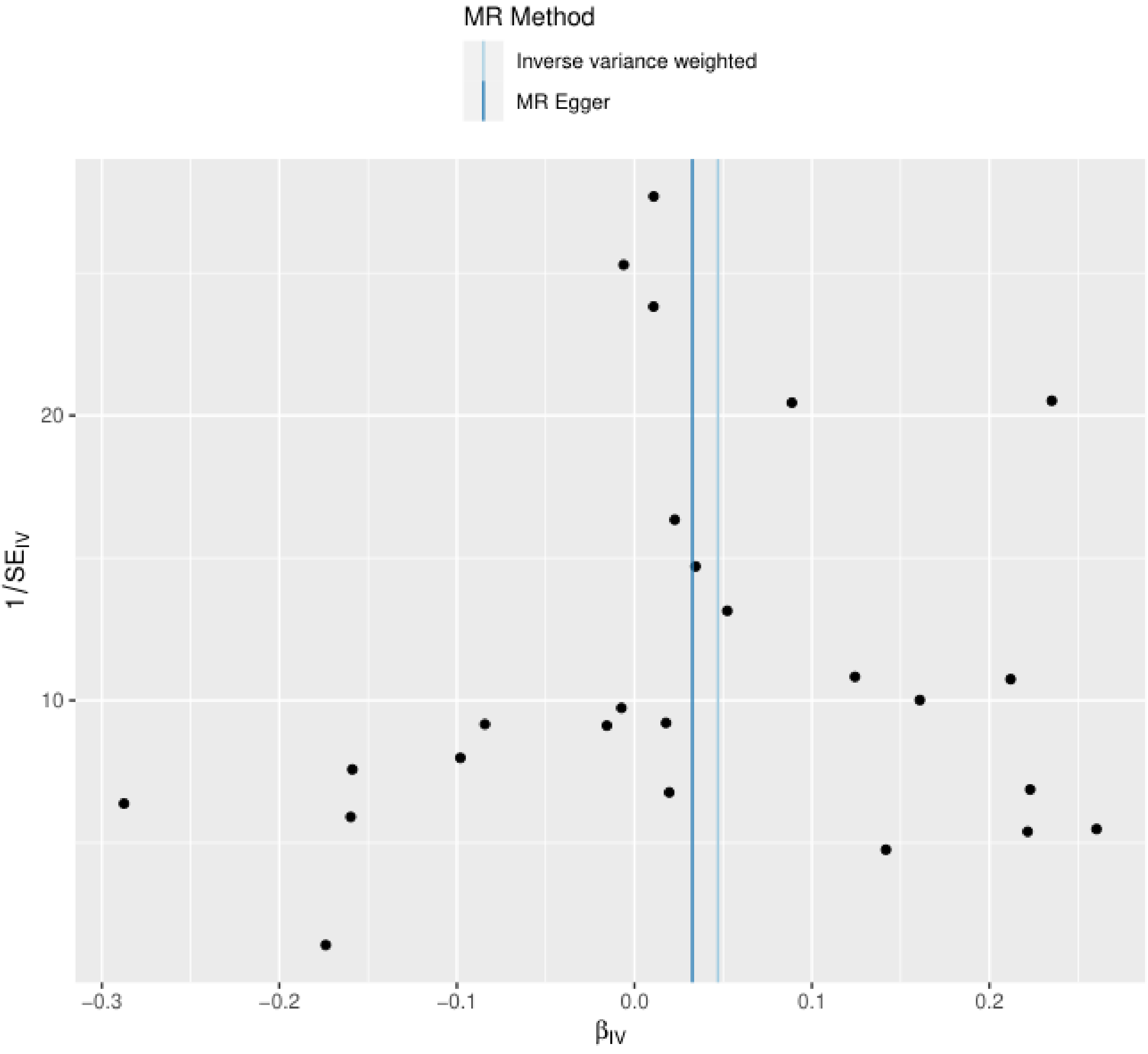

Funnel plot analyse of "HLA DR on CD33br HLA DR+ CD14- " on 'Diabetic nephropathy'

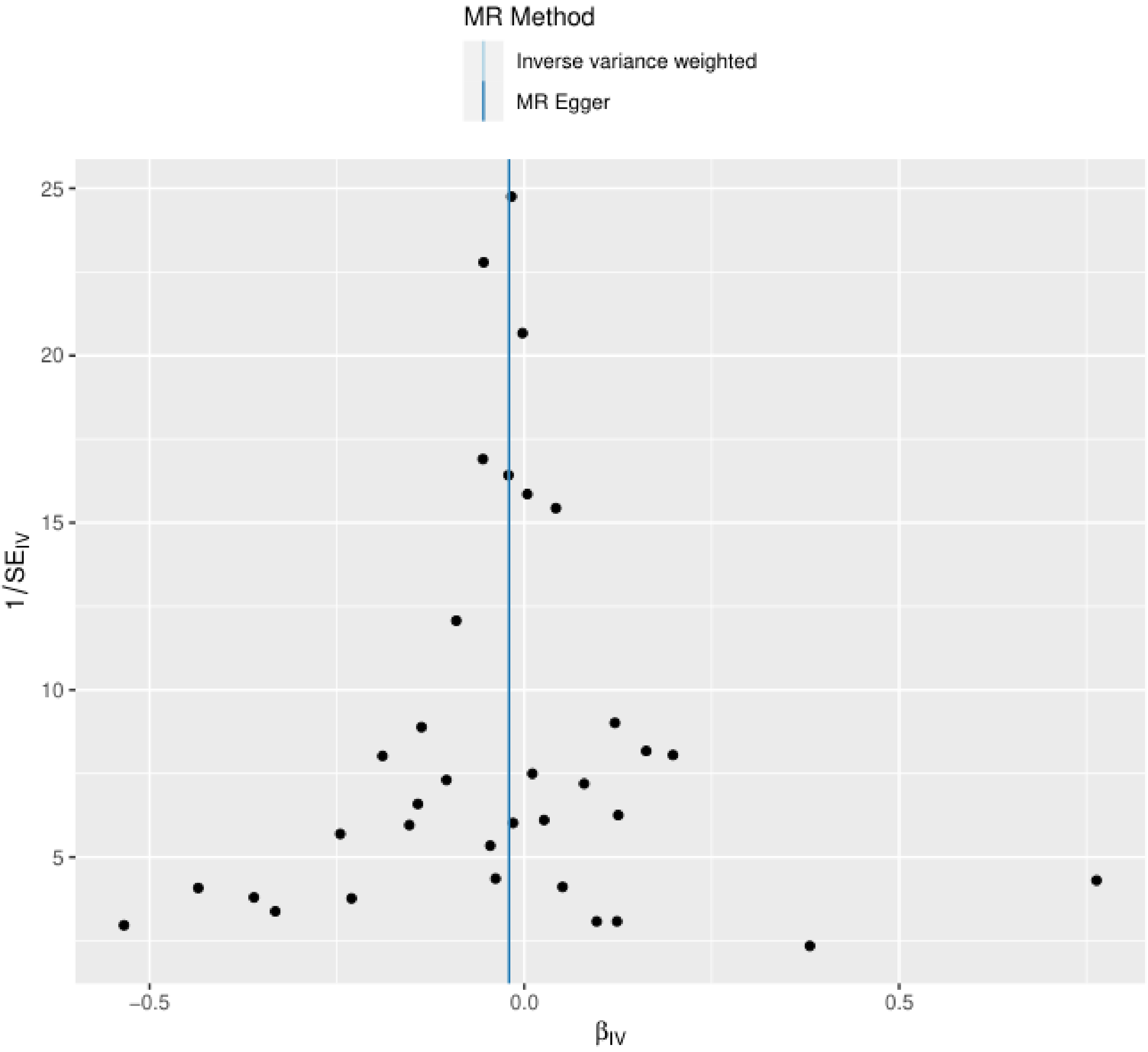

Funnel plot analyse of "CD33- HLA DR+ AC" on 'Diabetic nephropathy'

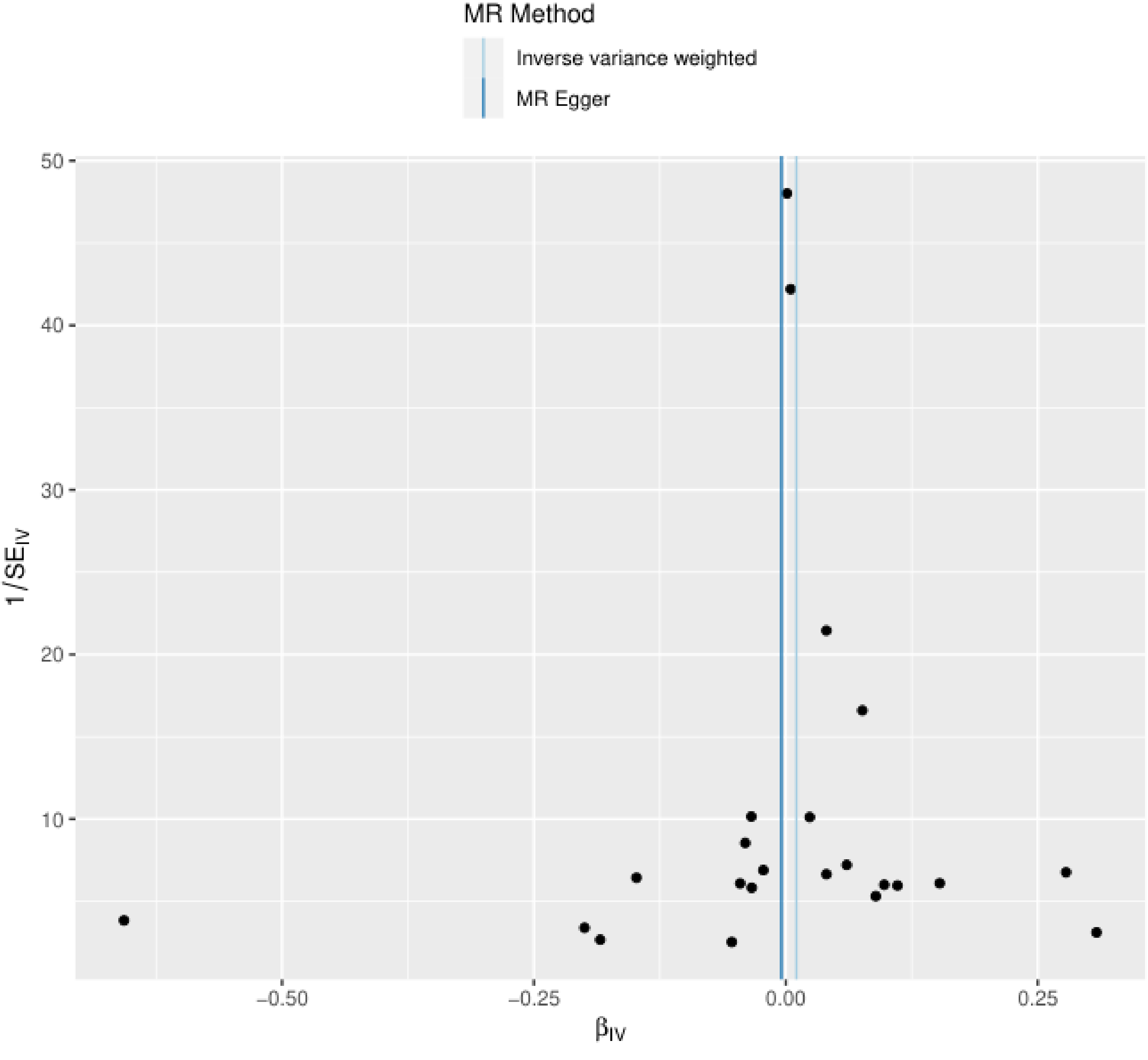

Funnel plot analyse of "CD33 on basophil " on 'Diabetic nephropathy'

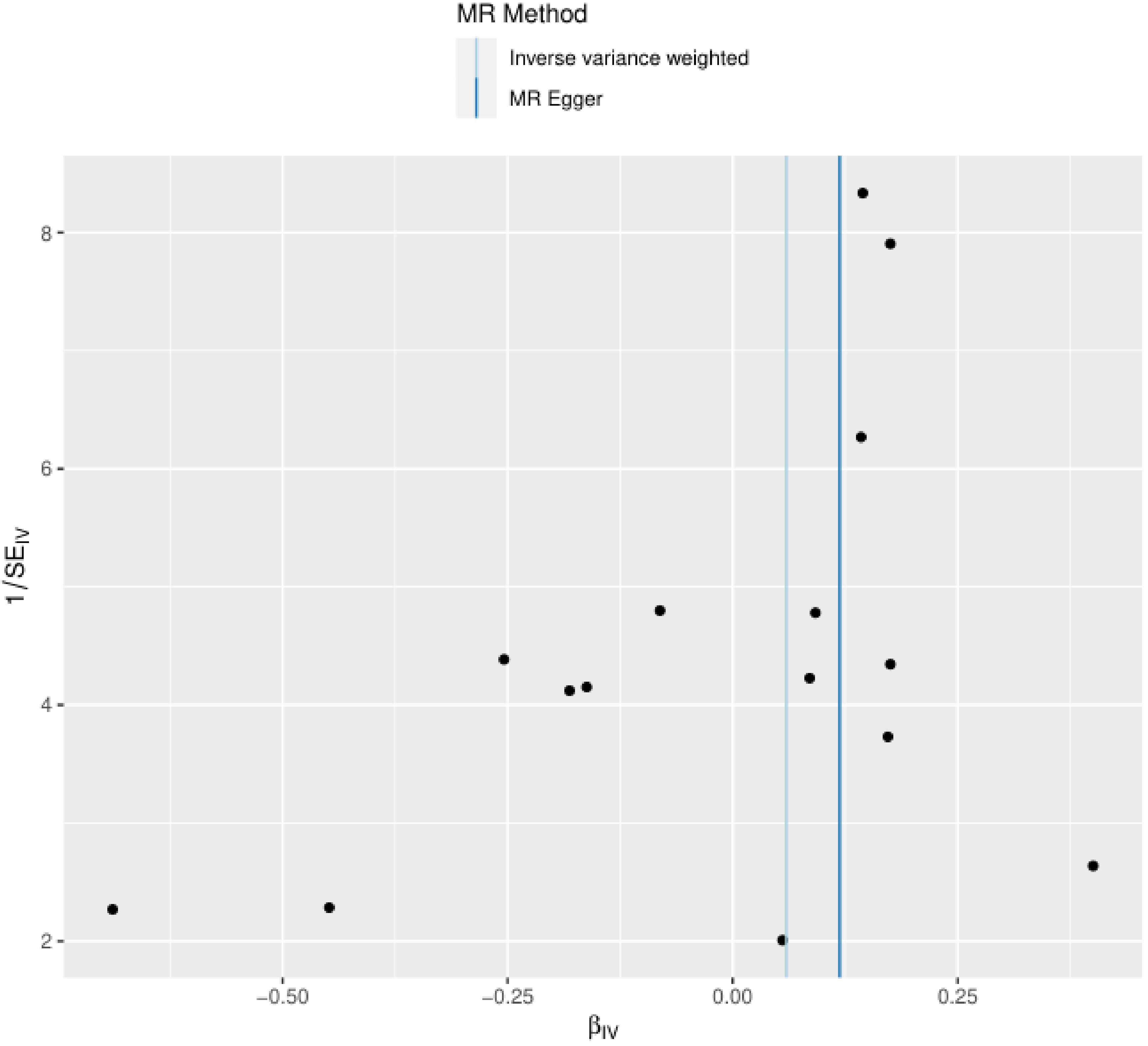

Funnel plot analyse of "CD20- CD38- AC" on 'Diabetic nephropathy'

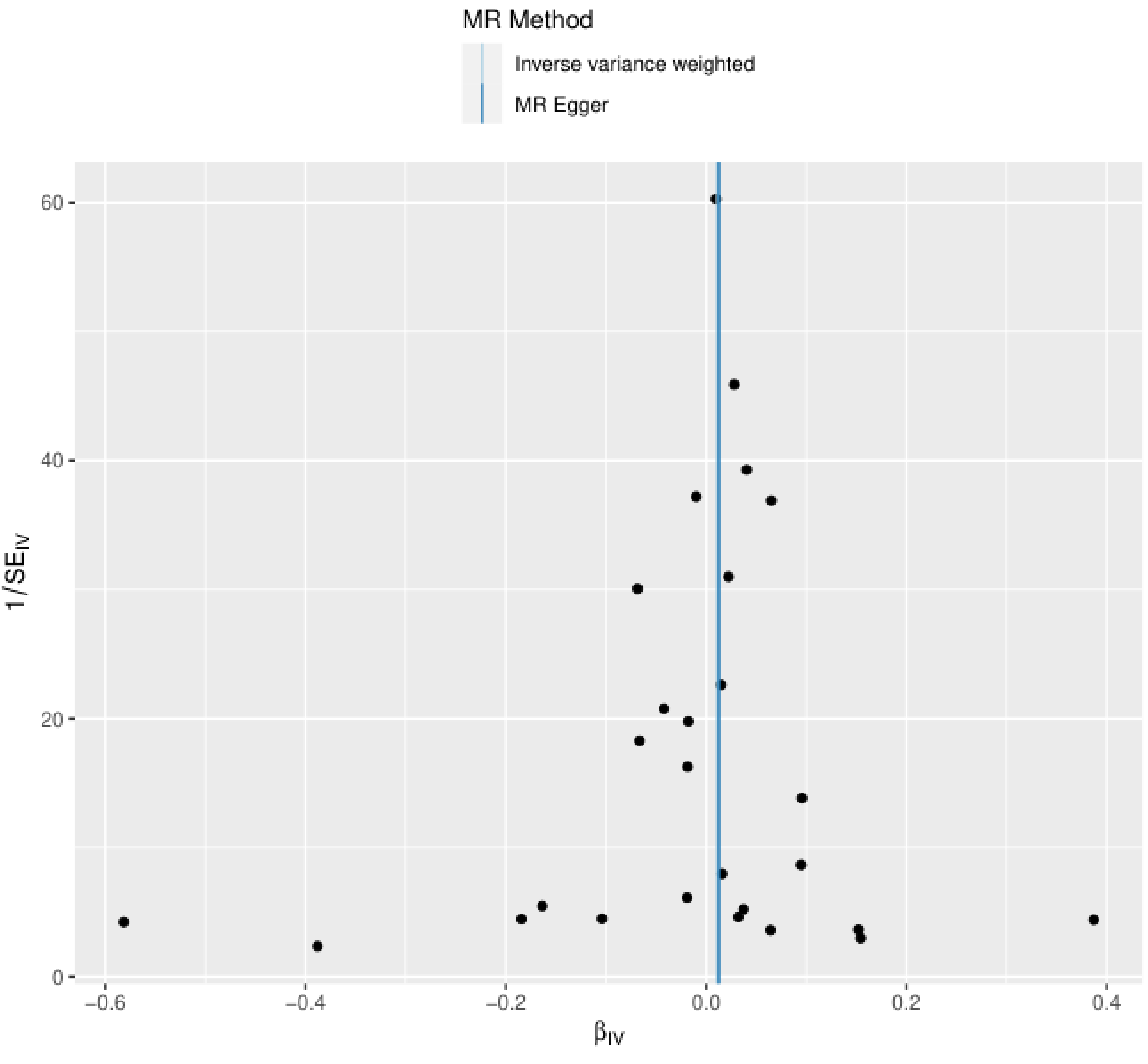

Funnel plot analyse of "CD45 on CD8br" on 'Diabetic nephropathy'

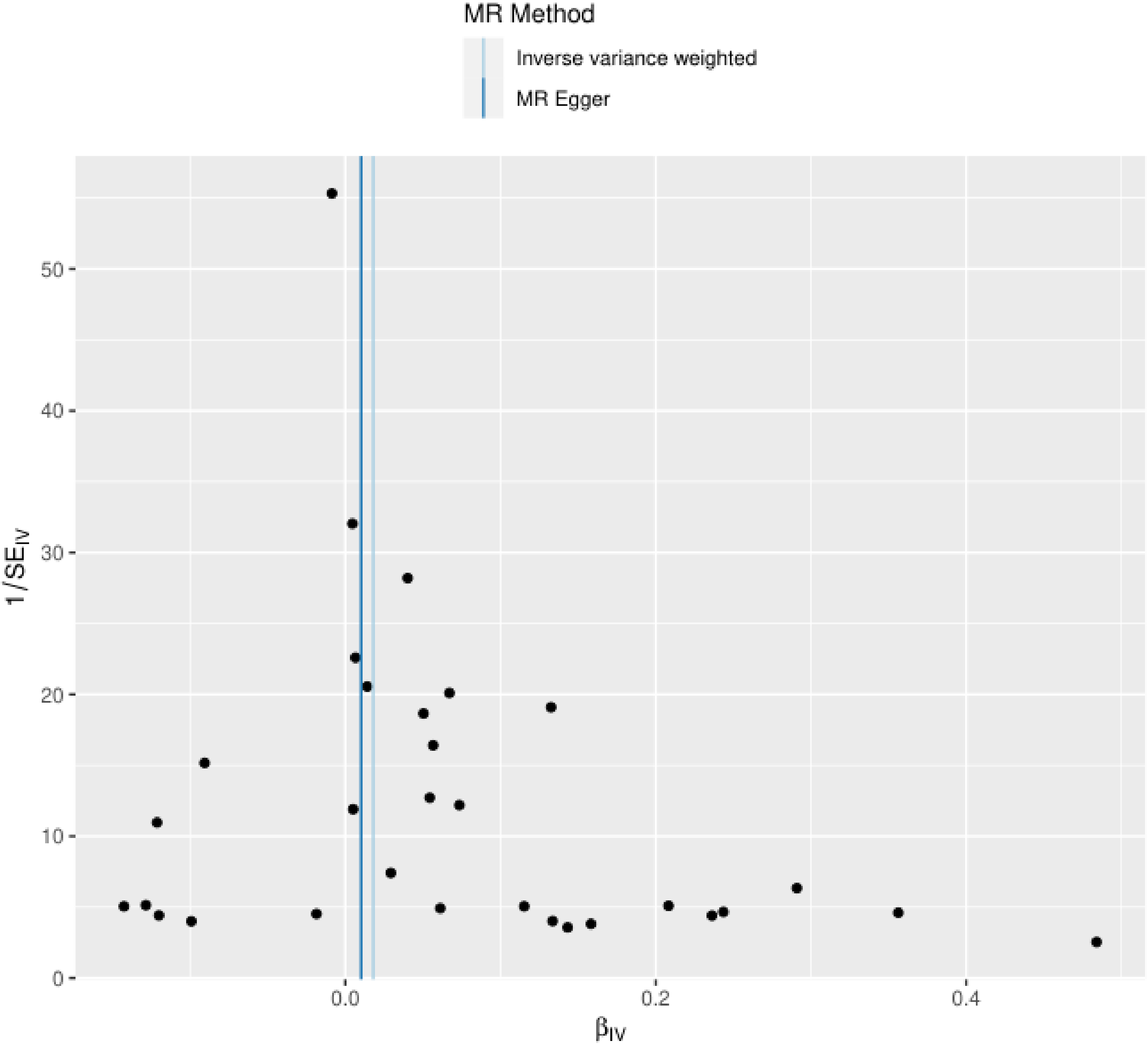

Funnel plot analyse of "Activated & secreting Treg %CD4+" on 'Diabetic nephropathy'

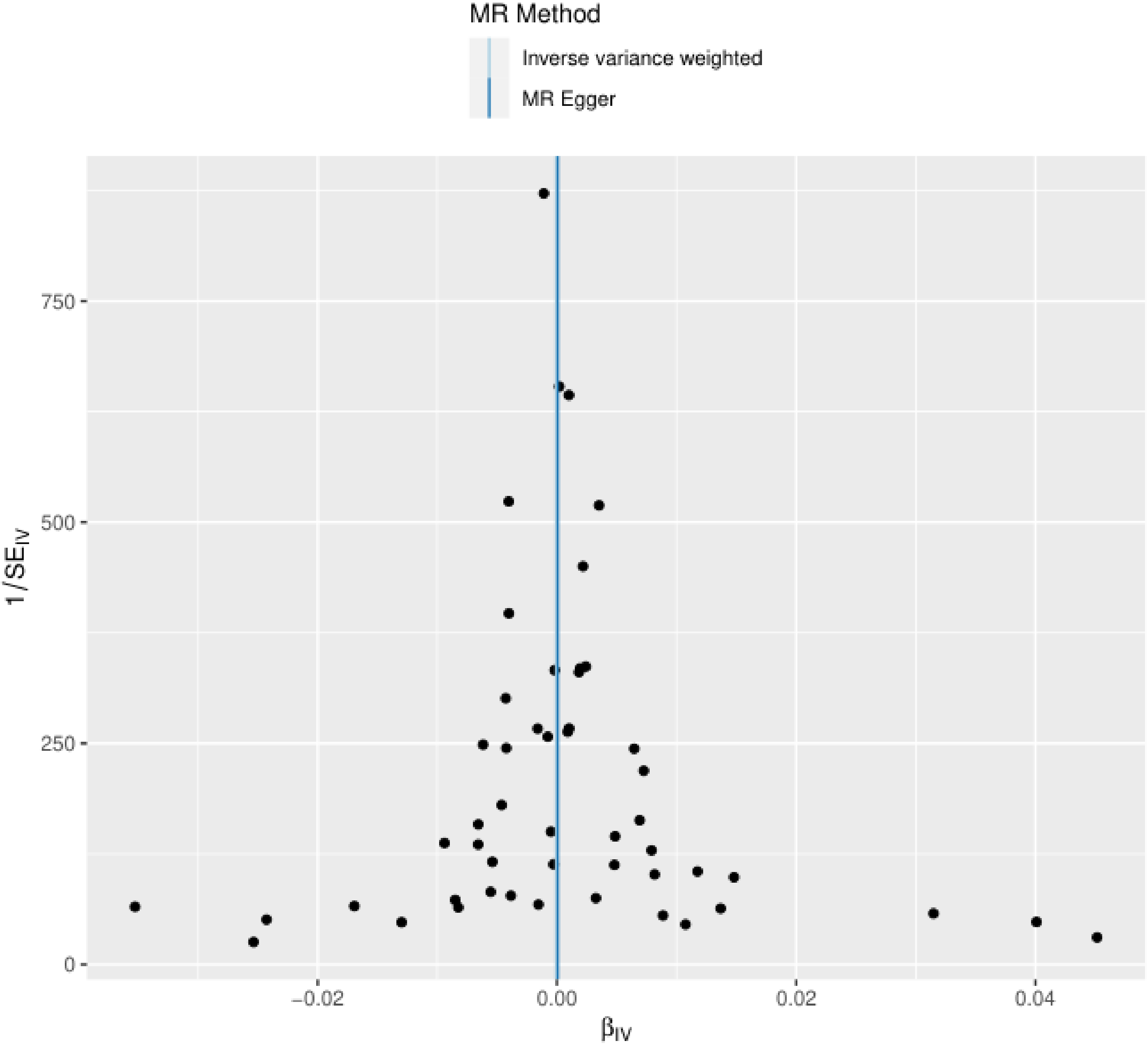

Funnel plot analyse of "CD45RA- CD28- CD8br %CD8br" on 'Diabetic nephropathy'

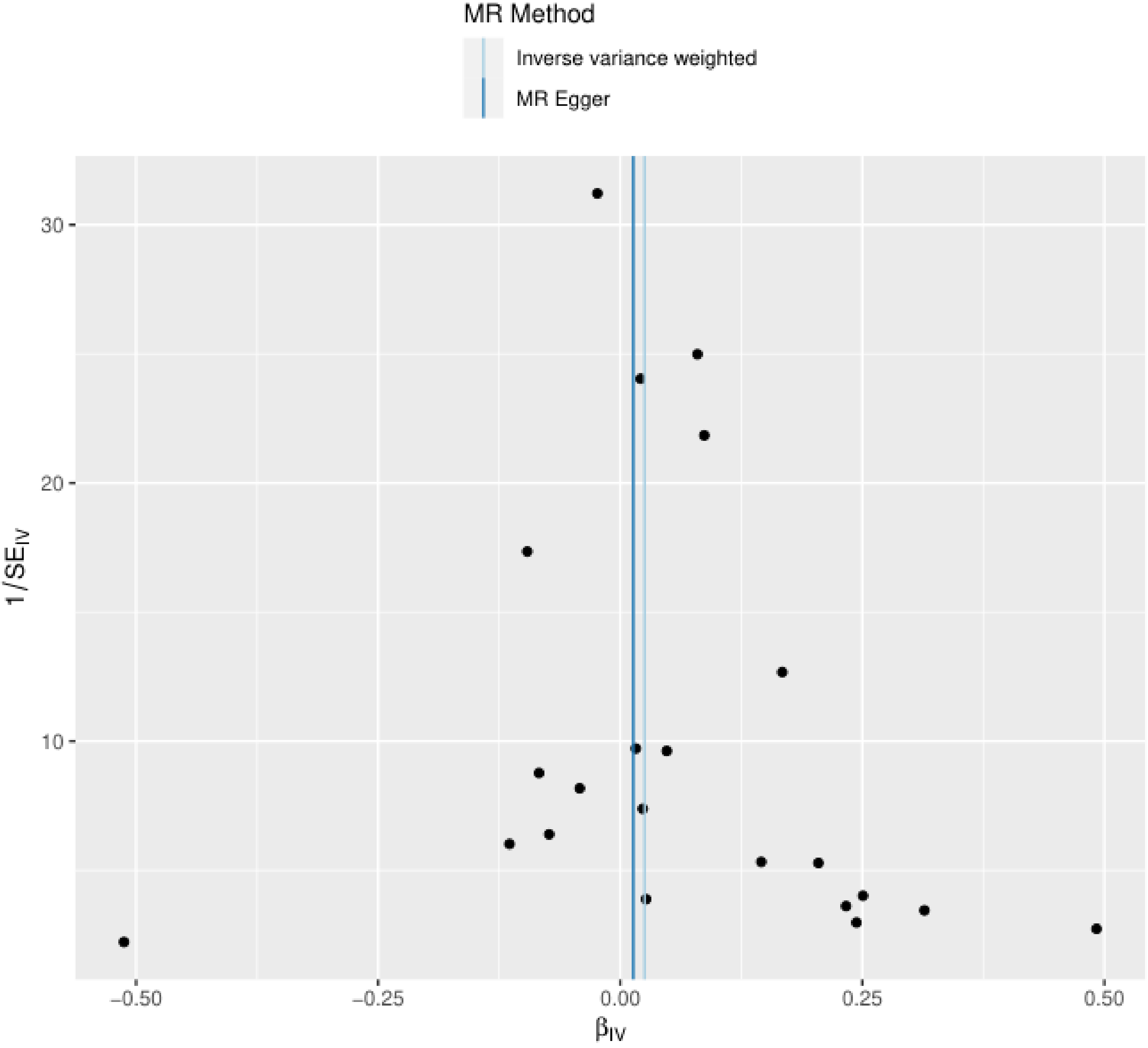

Funnel plot analyse of "CD19 on IgD+ CD38- unsw mem" on 'Diabetic nephropathy'

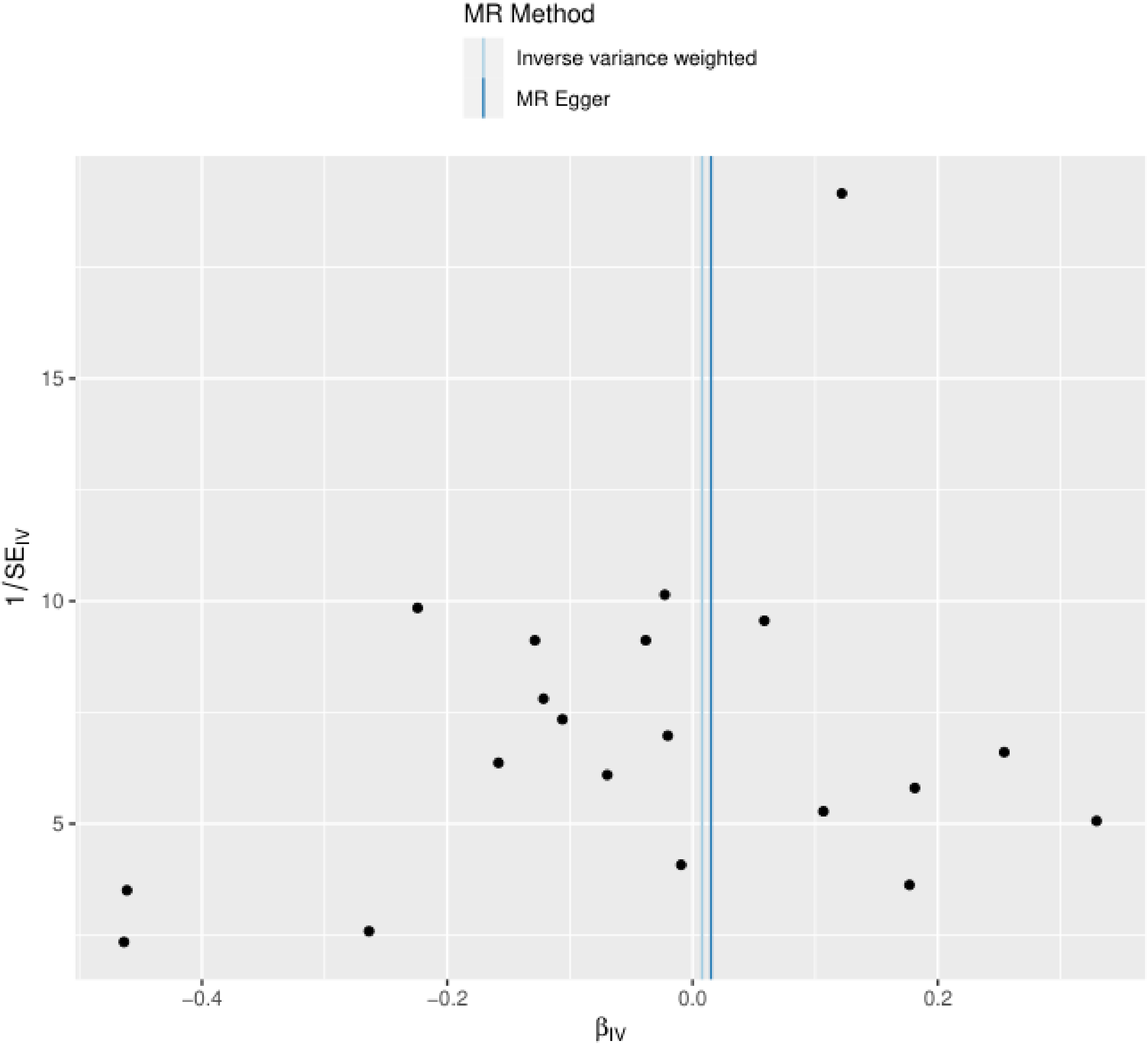

Funnel plot analyse of "CD11b on CD33br HLA DR+ CD14dim " on 'Diabetic nephropathy'

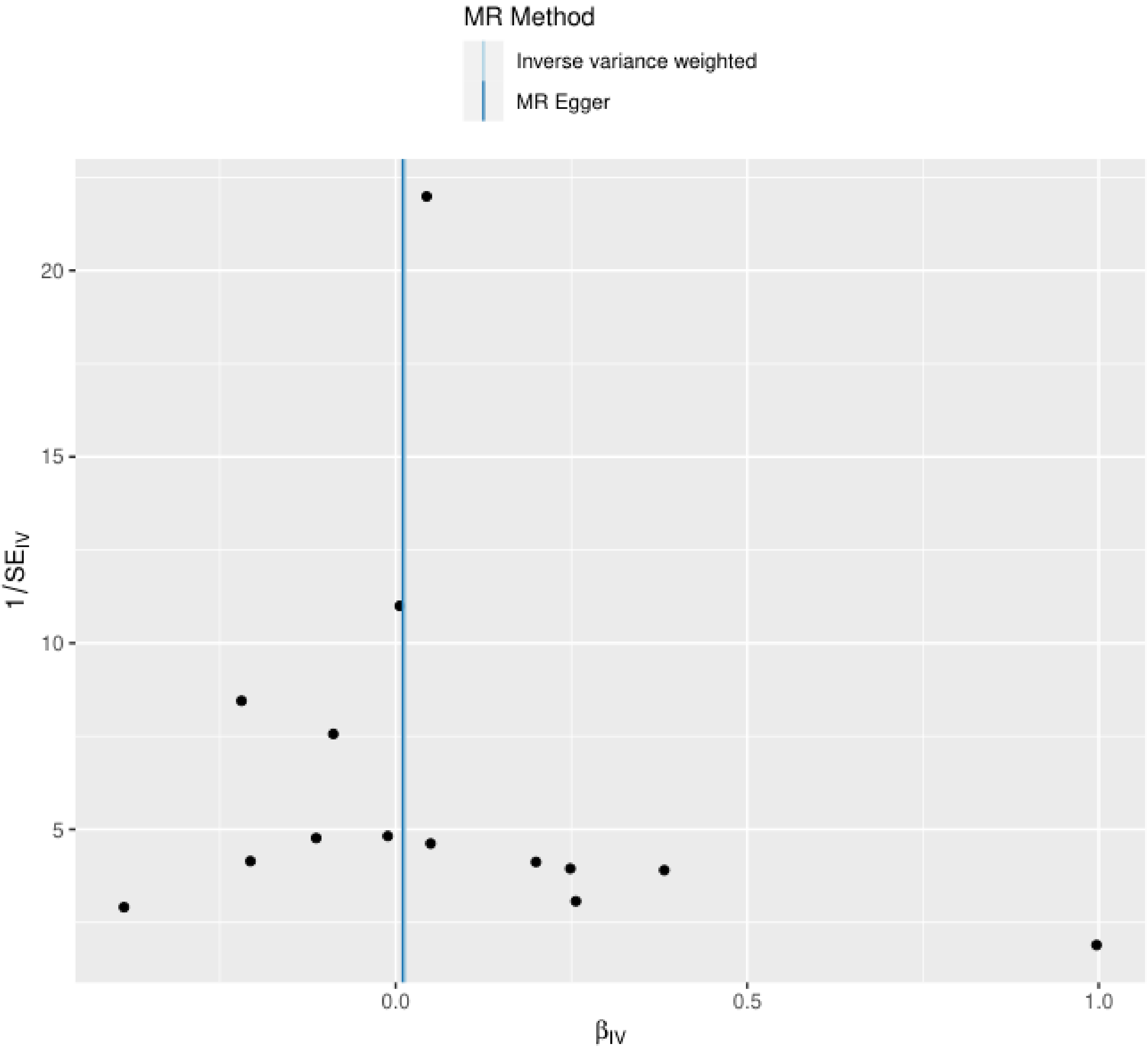

Funnel plot analyse of "CD127- CD8br %CD8br" on 'Diabetic nephropathy'

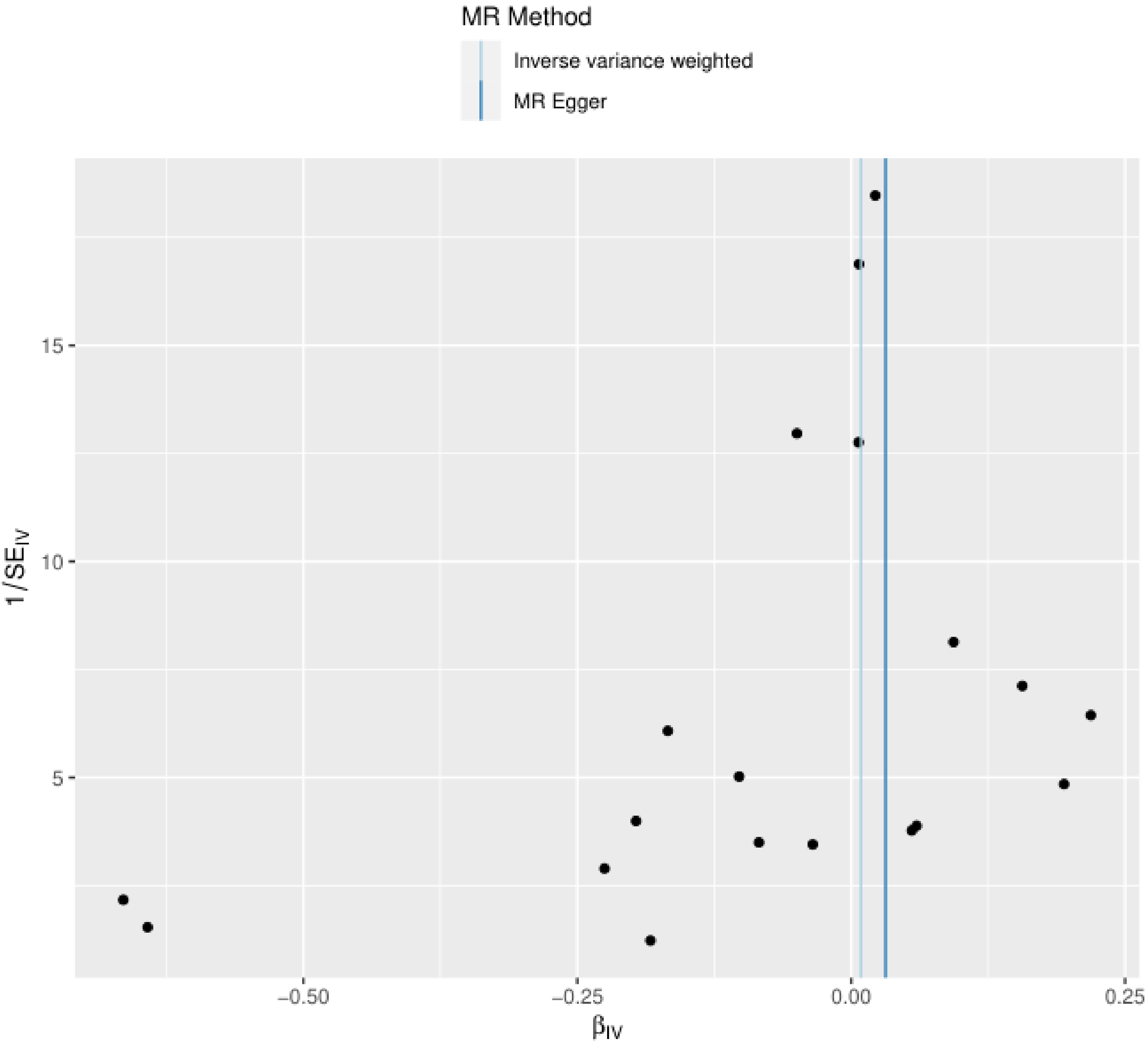

Funnel plot analyse of "CD39+ CD8br %CD8br" on 'Diabetic nephropathy'

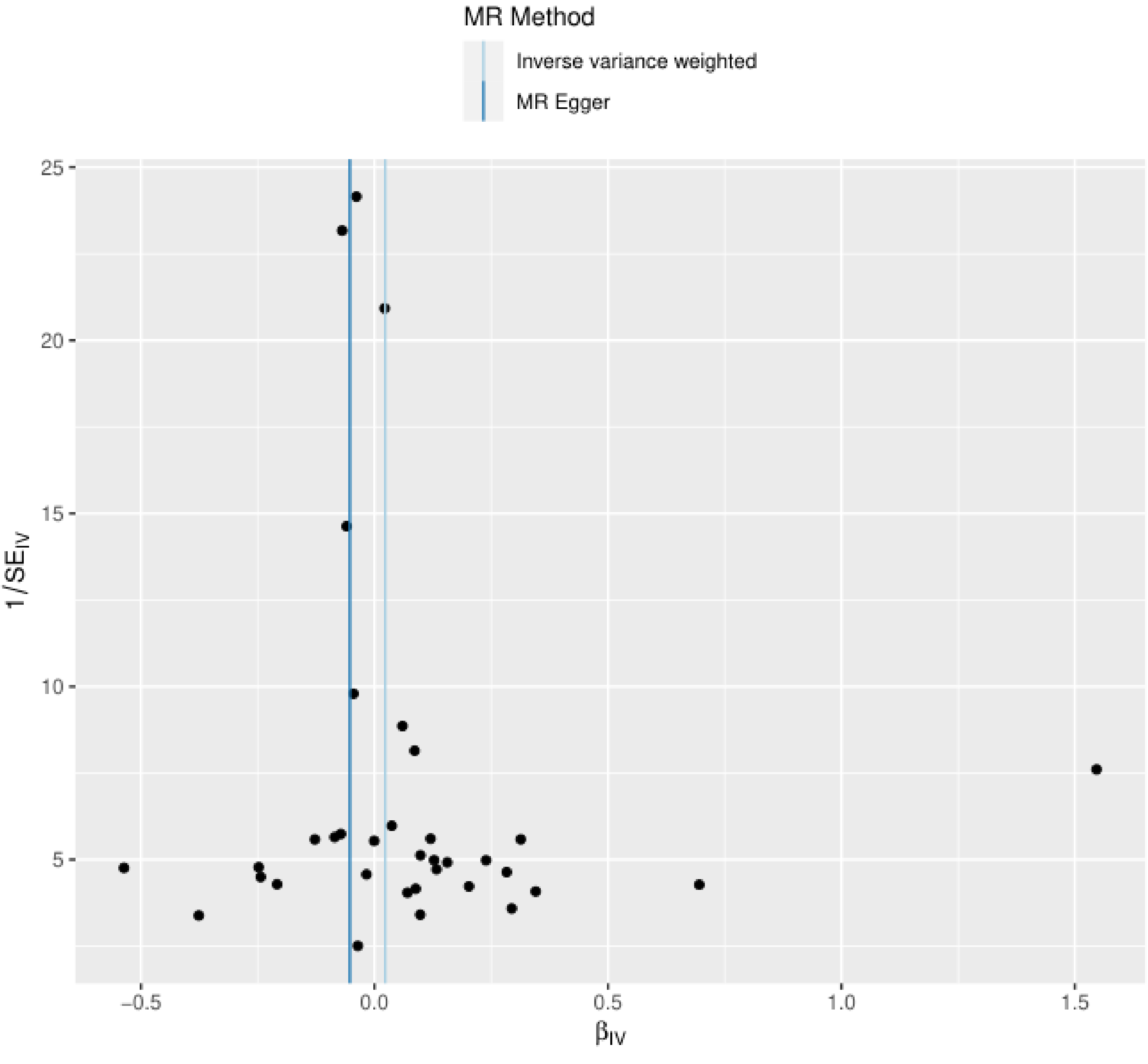

Funnel plot analyse of "HLA DR+ CD8br AC" on 'Diabetic nephropathy'

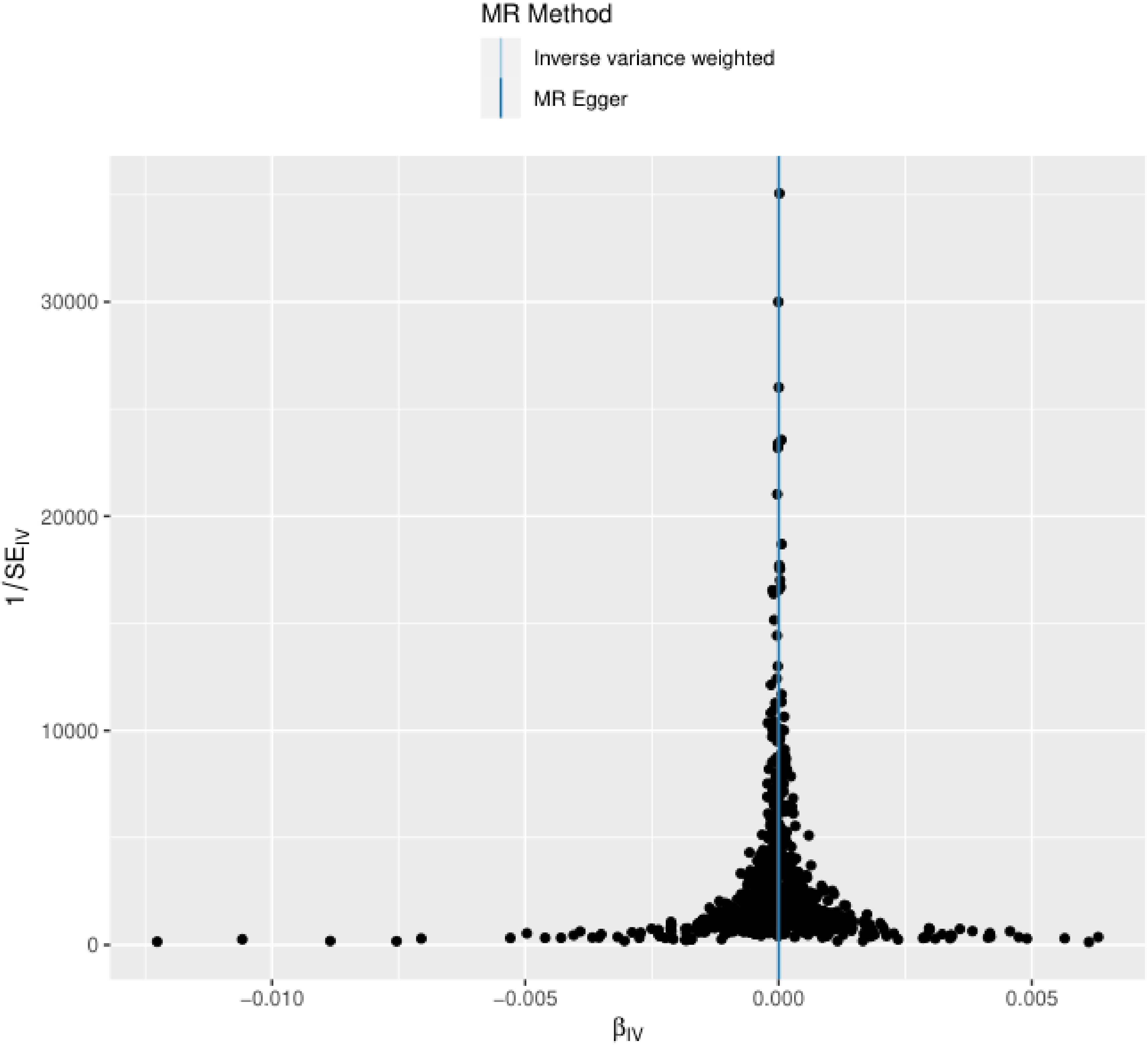

Funnel plot analyse of ''CD45RA+ CD28- CD8br AC'' on 'Diabetic nephropathy'

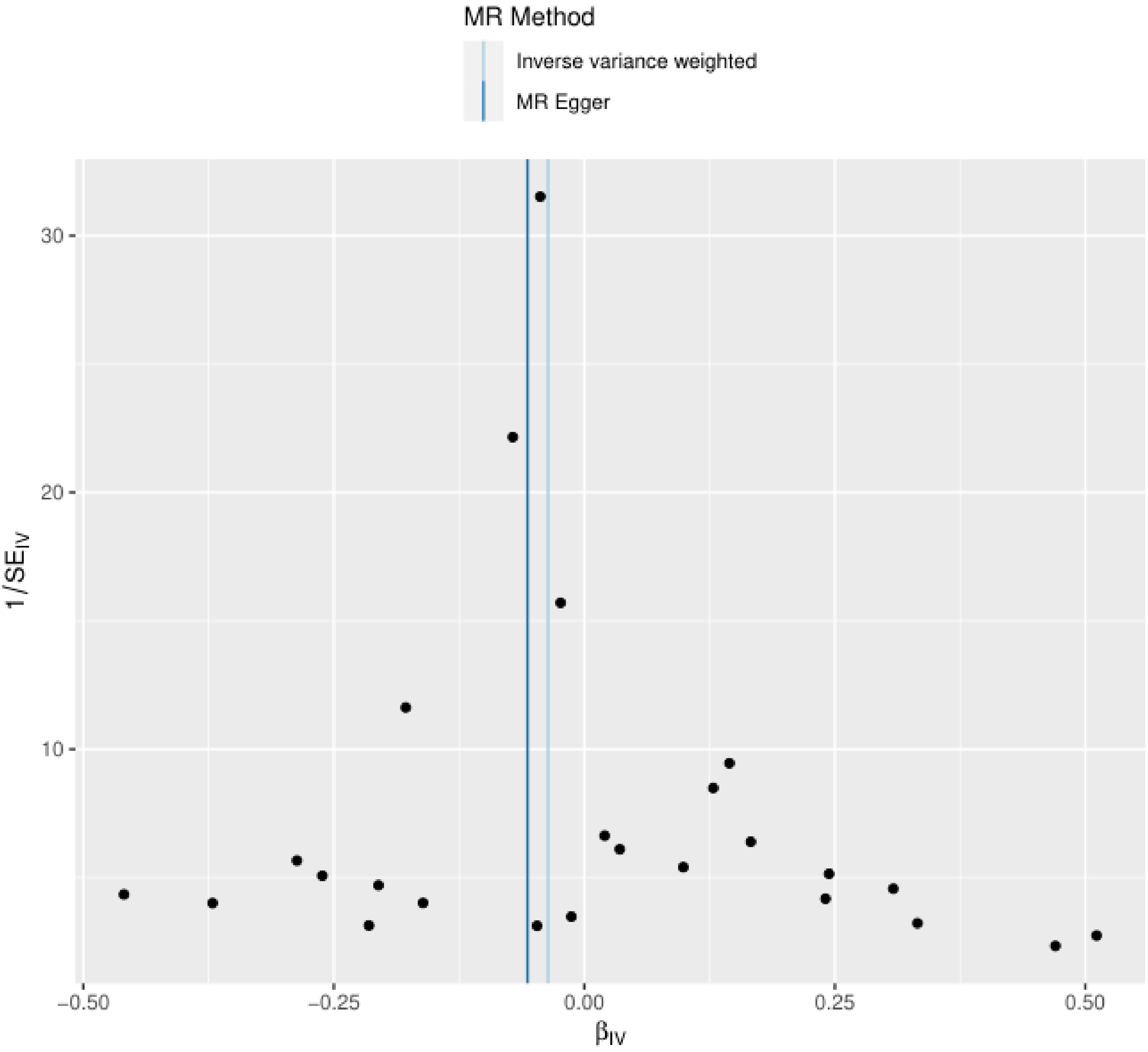

Funnel plot analyse of "CD25 on IgD+ CD24-" on 'Diabetic nephropathy'

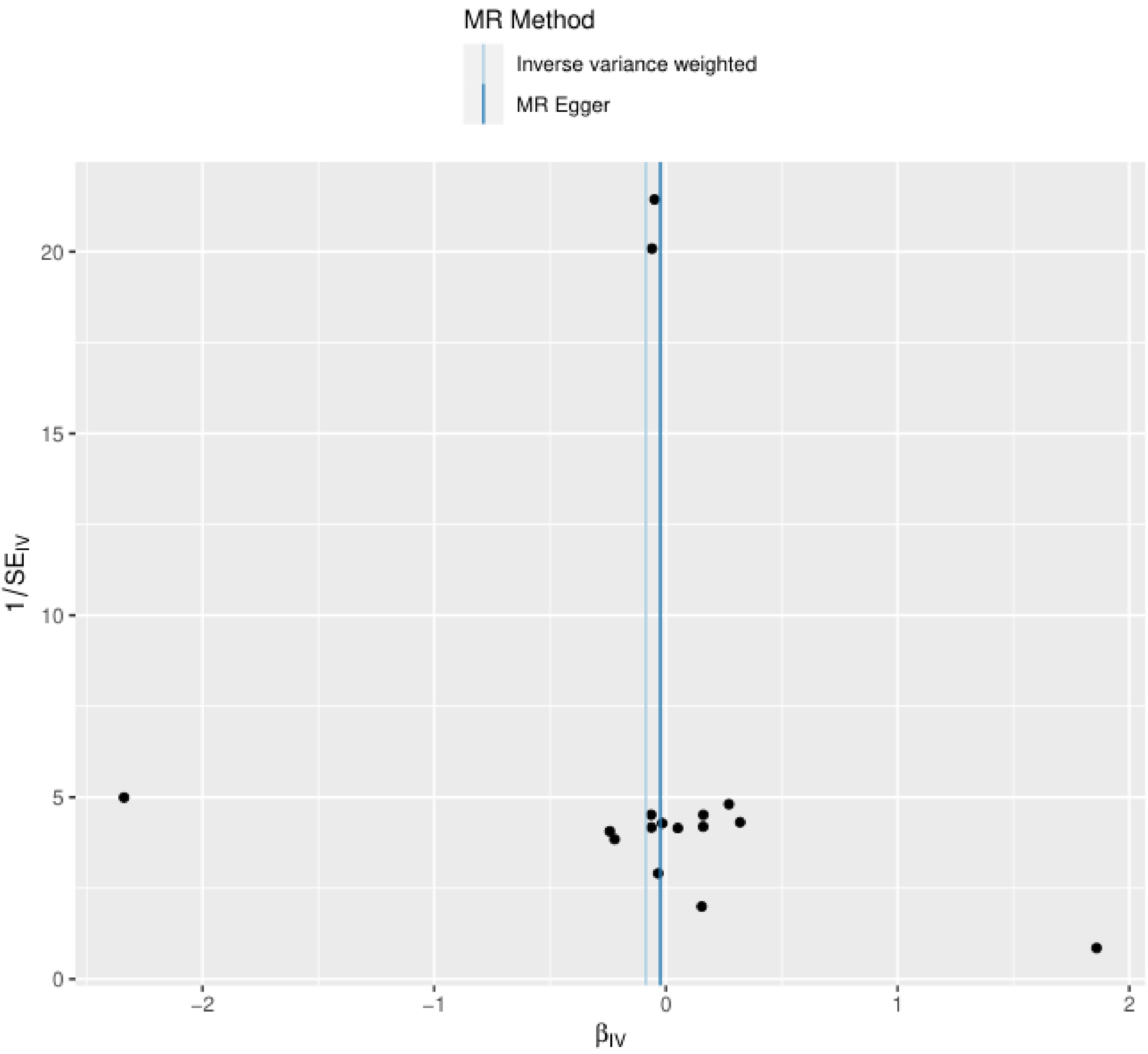

Funnel plot analyse of "FSC-A on lymphocyte" on 'Diabetic nephropathy'

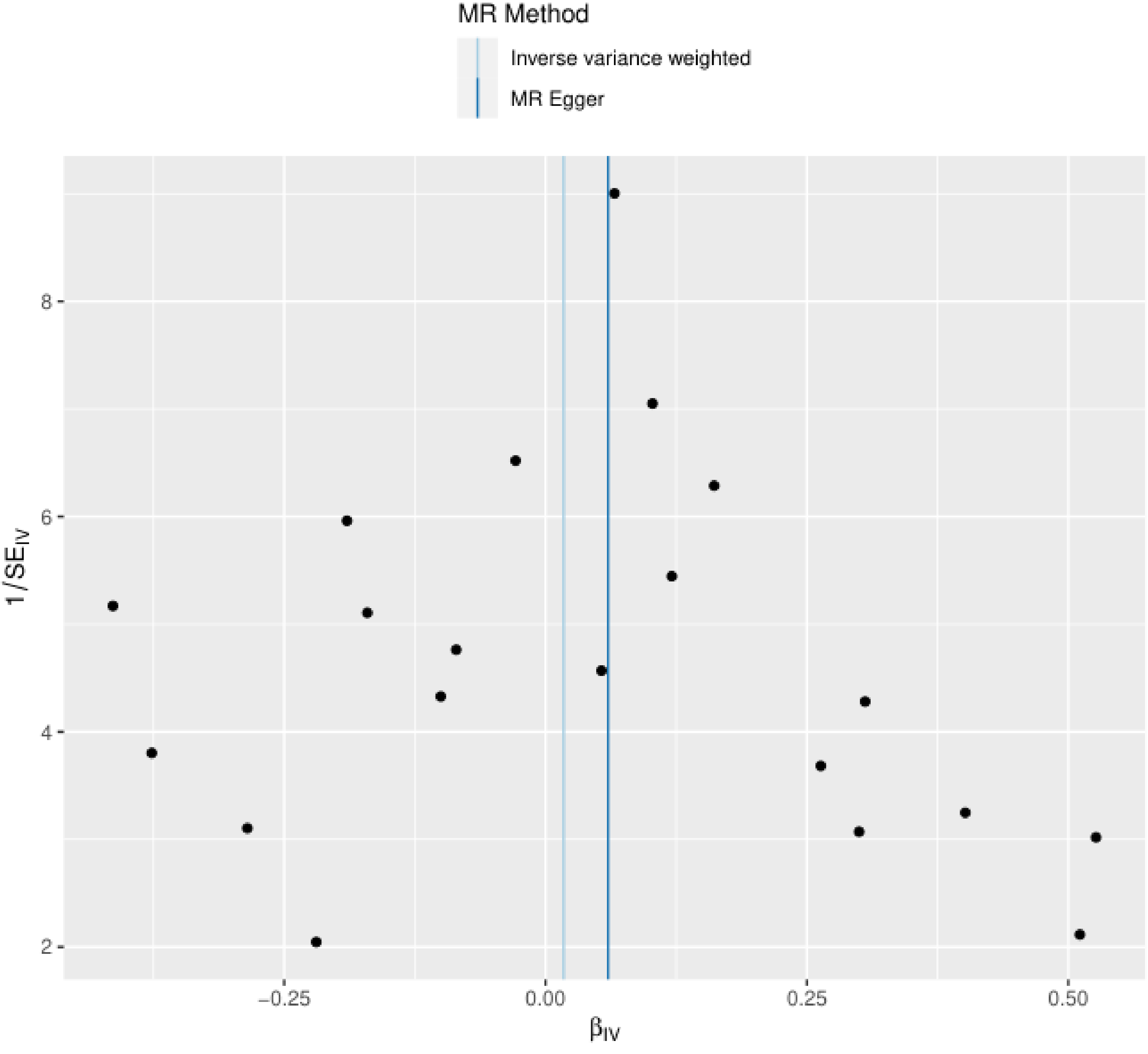

Funnel plot analyse of "CD24 on transitional" on 'Diabetic nephropathy'

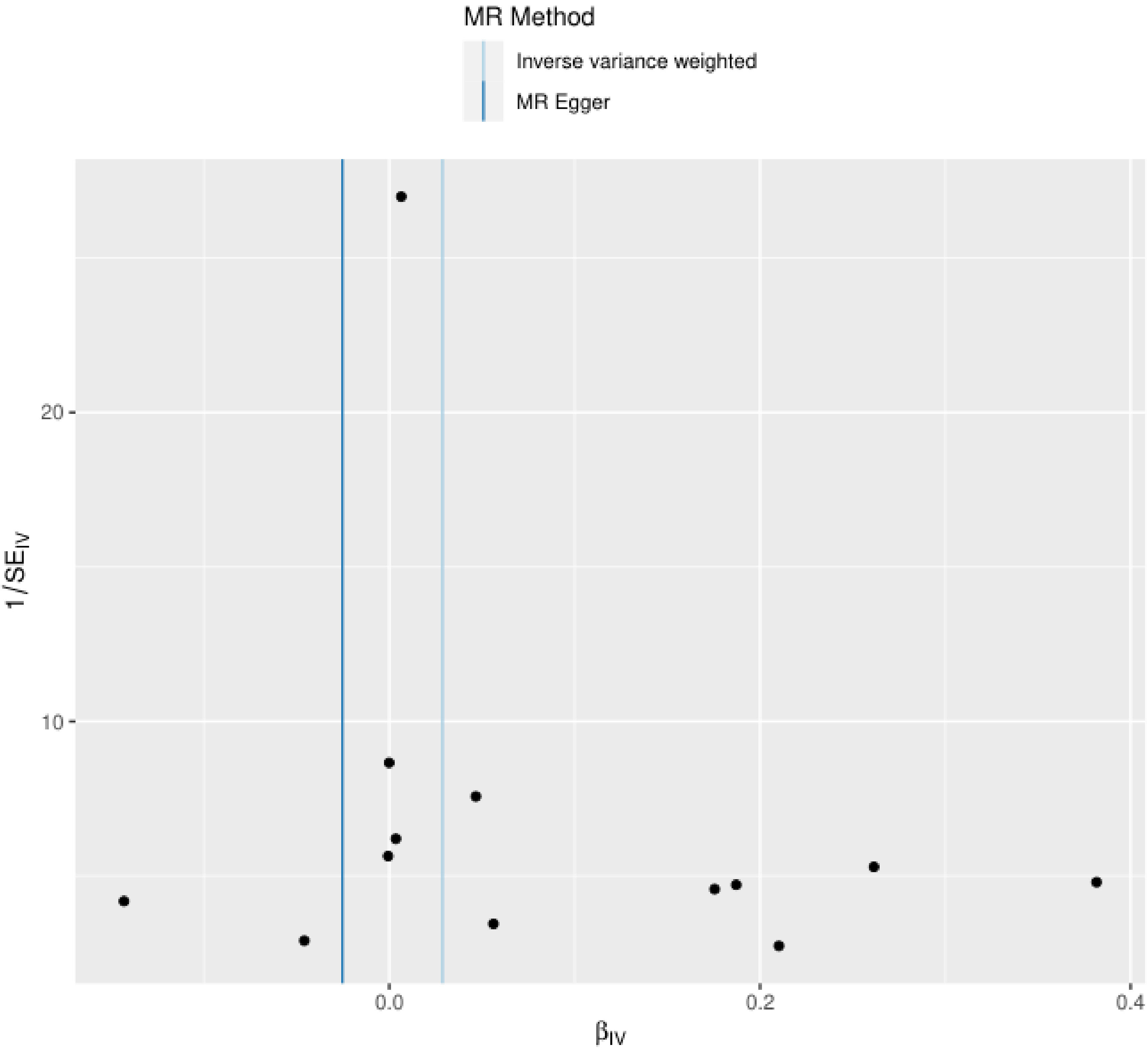

Funnel plot analyse of "CD62L- HLA DR++ monocyte %monocyte" on 'Diabetic nephropathy'

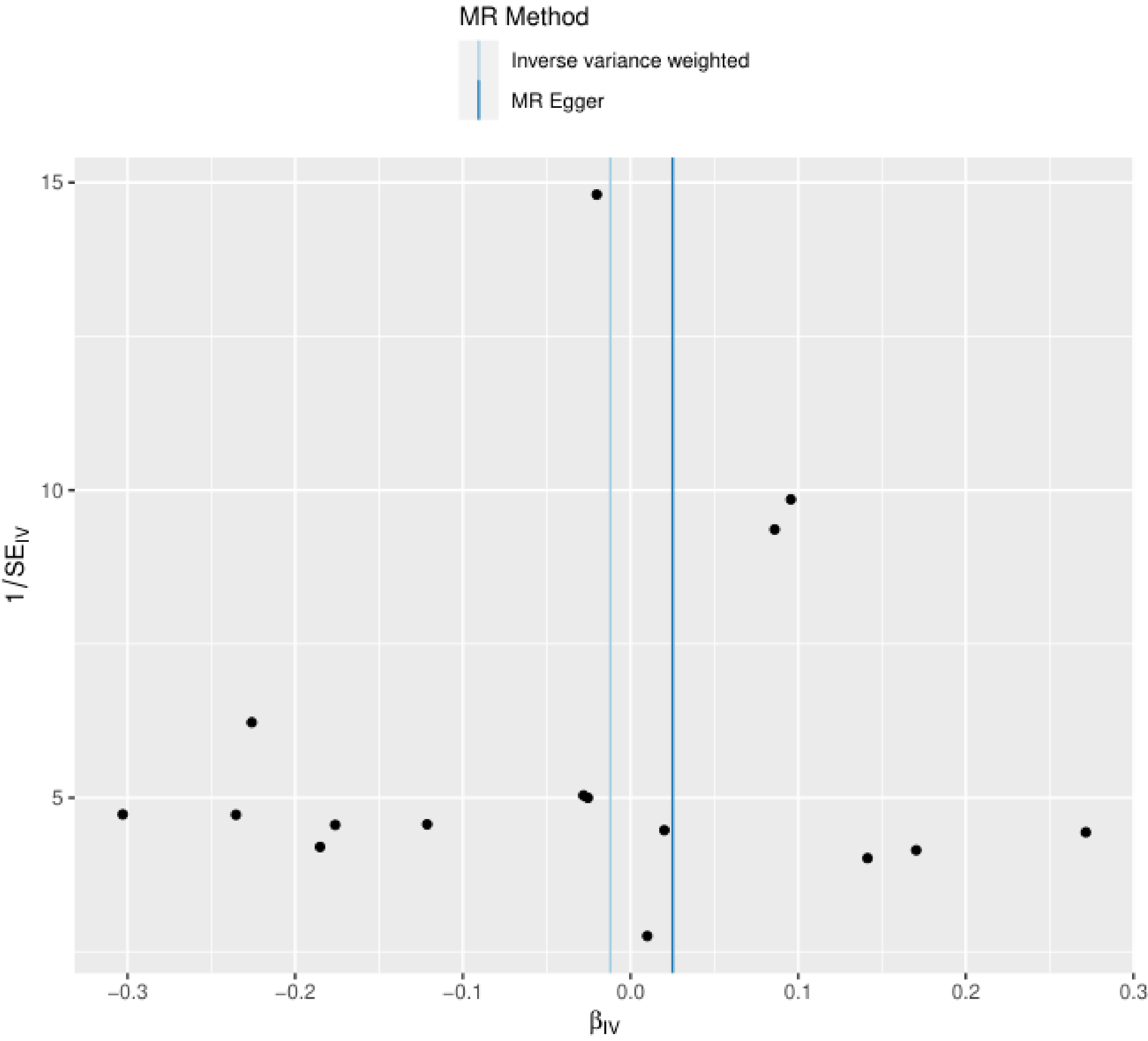

Funnel plot analyse of "CD4 Treg %T cell" on 'Diabetic nephropathy'

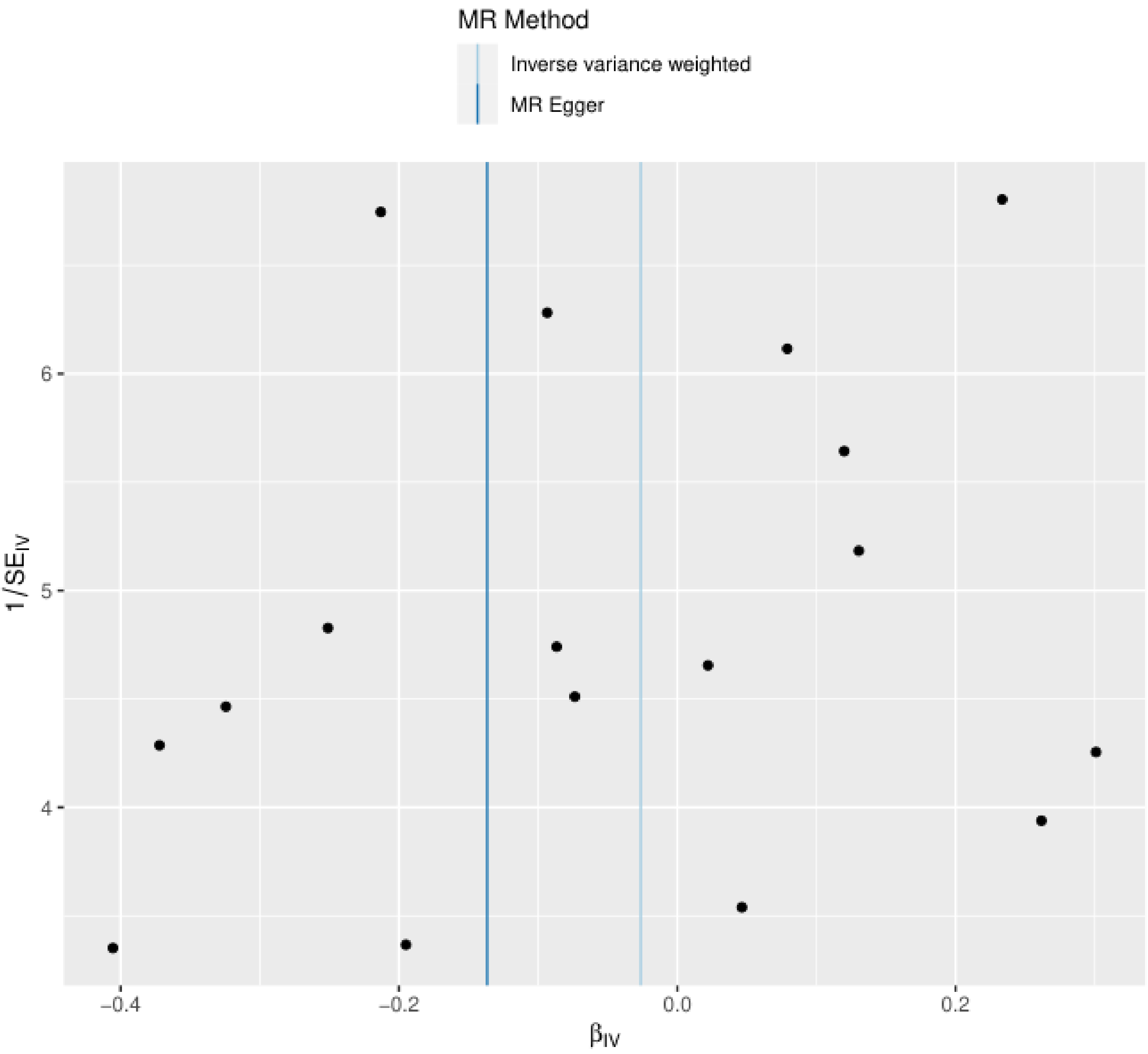

Funnel plot analyse of "CD14 on CD14+ CD16+ monocyte" on 'Diabetic nephropathy'

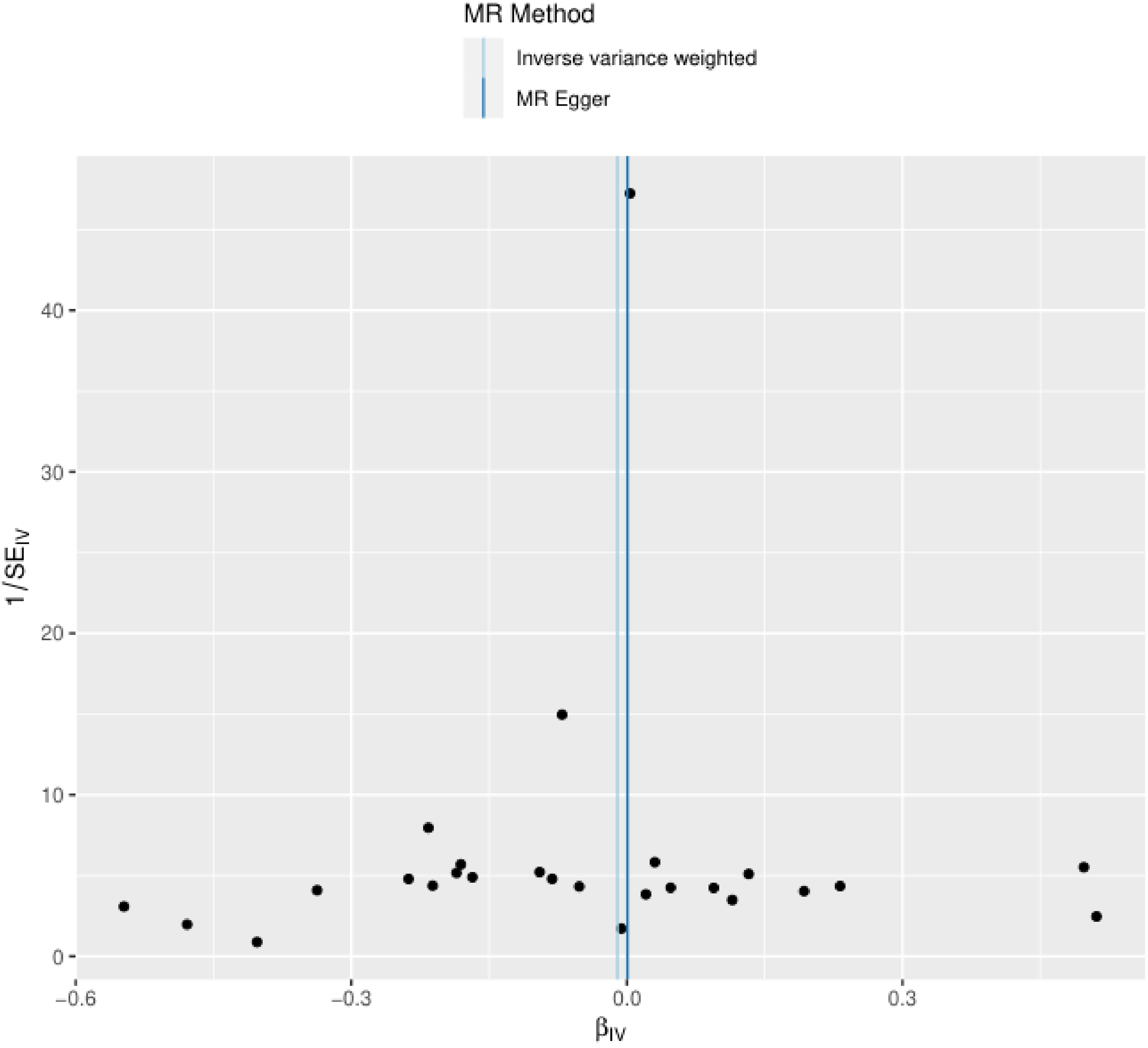

Funnel plot analyse of "Memory B cell %lymphocyte" on 'Diabetic nephropathy'

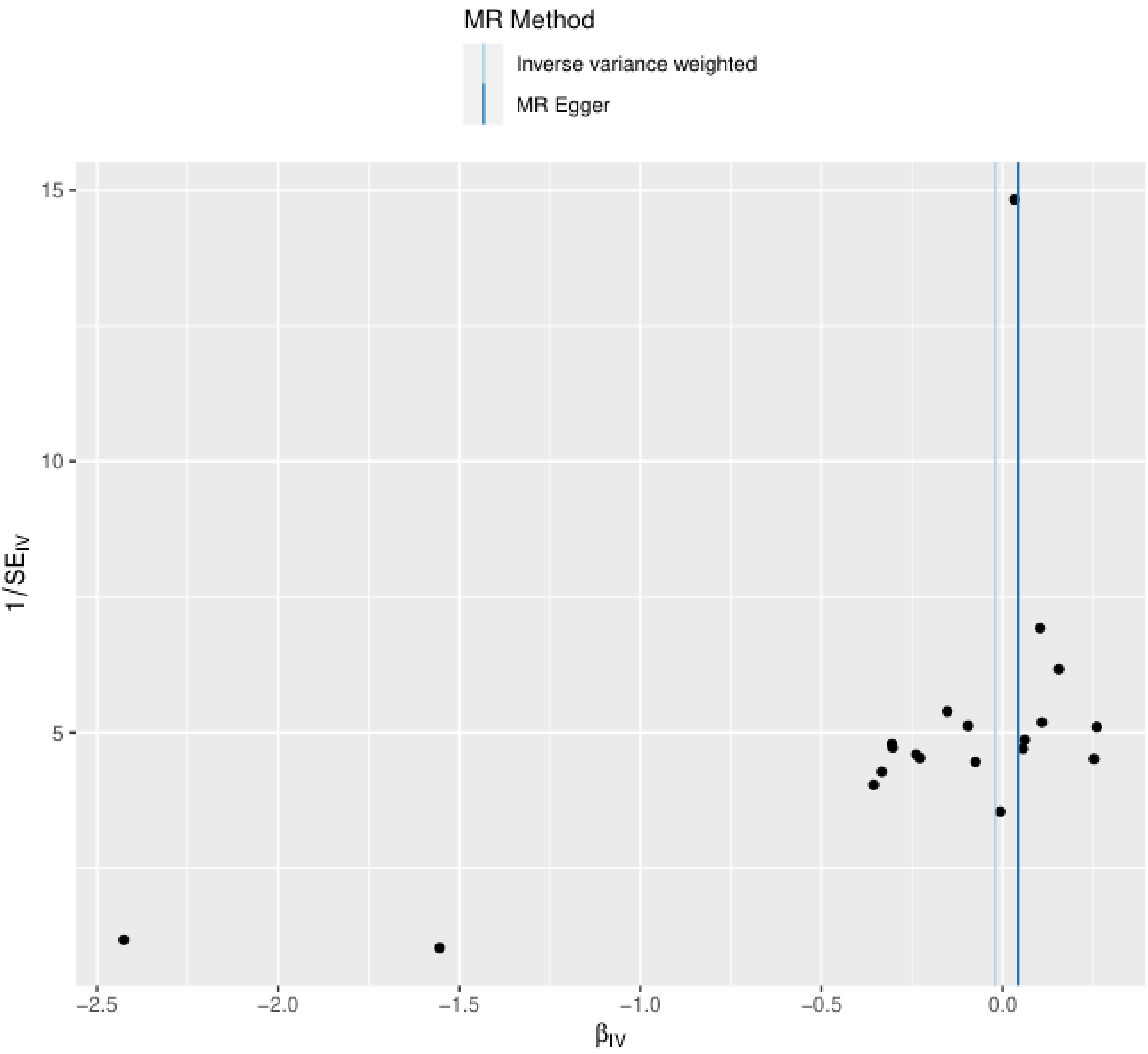

Funnel plot analyse of "CD64 on CD14- CD16+ monocyte " on 'Diabetic nephropathy'

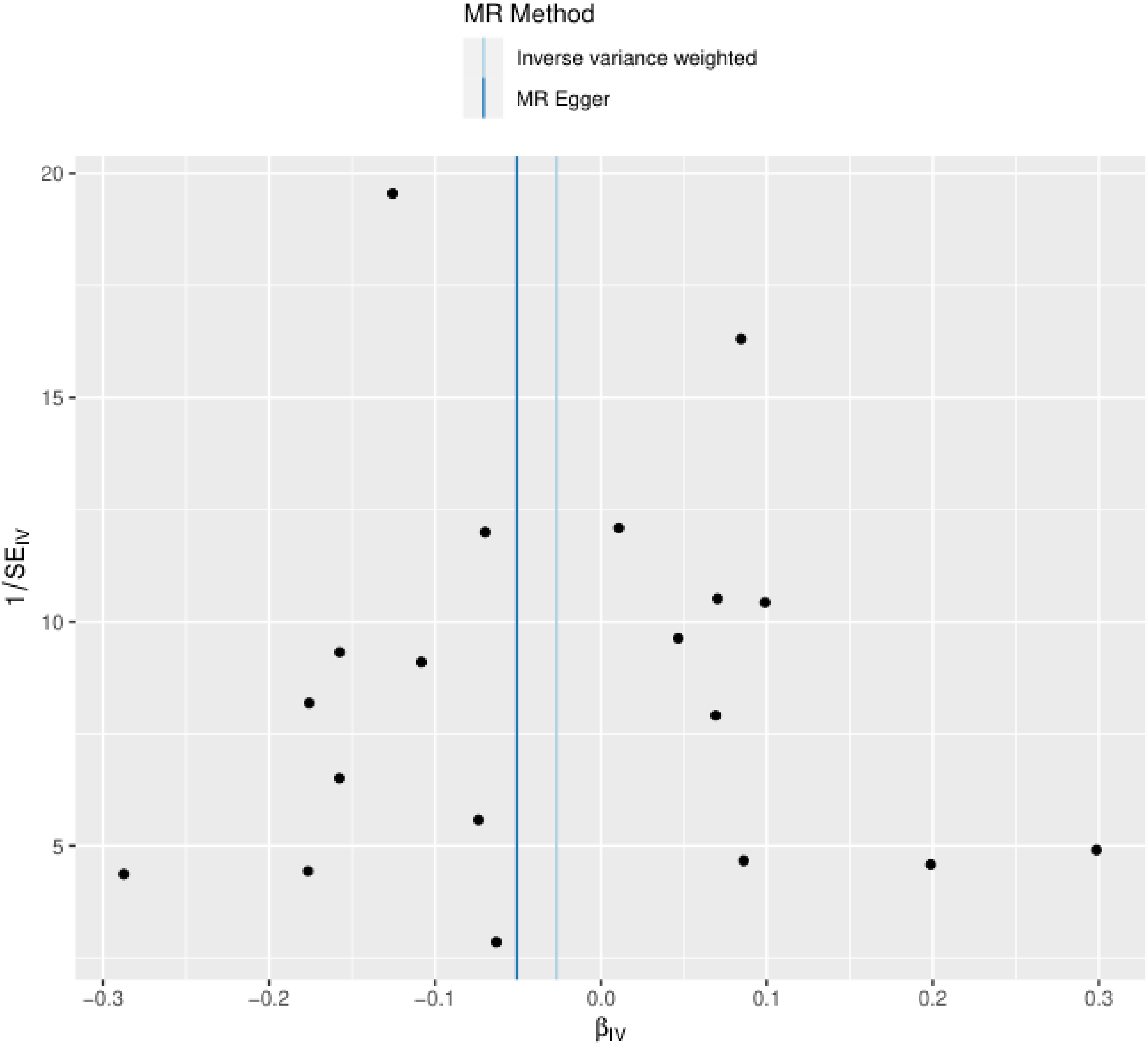

Funnel plot analyse of "CD28 on CD39+ activated Treg" on 'Diabetic nephropathy'

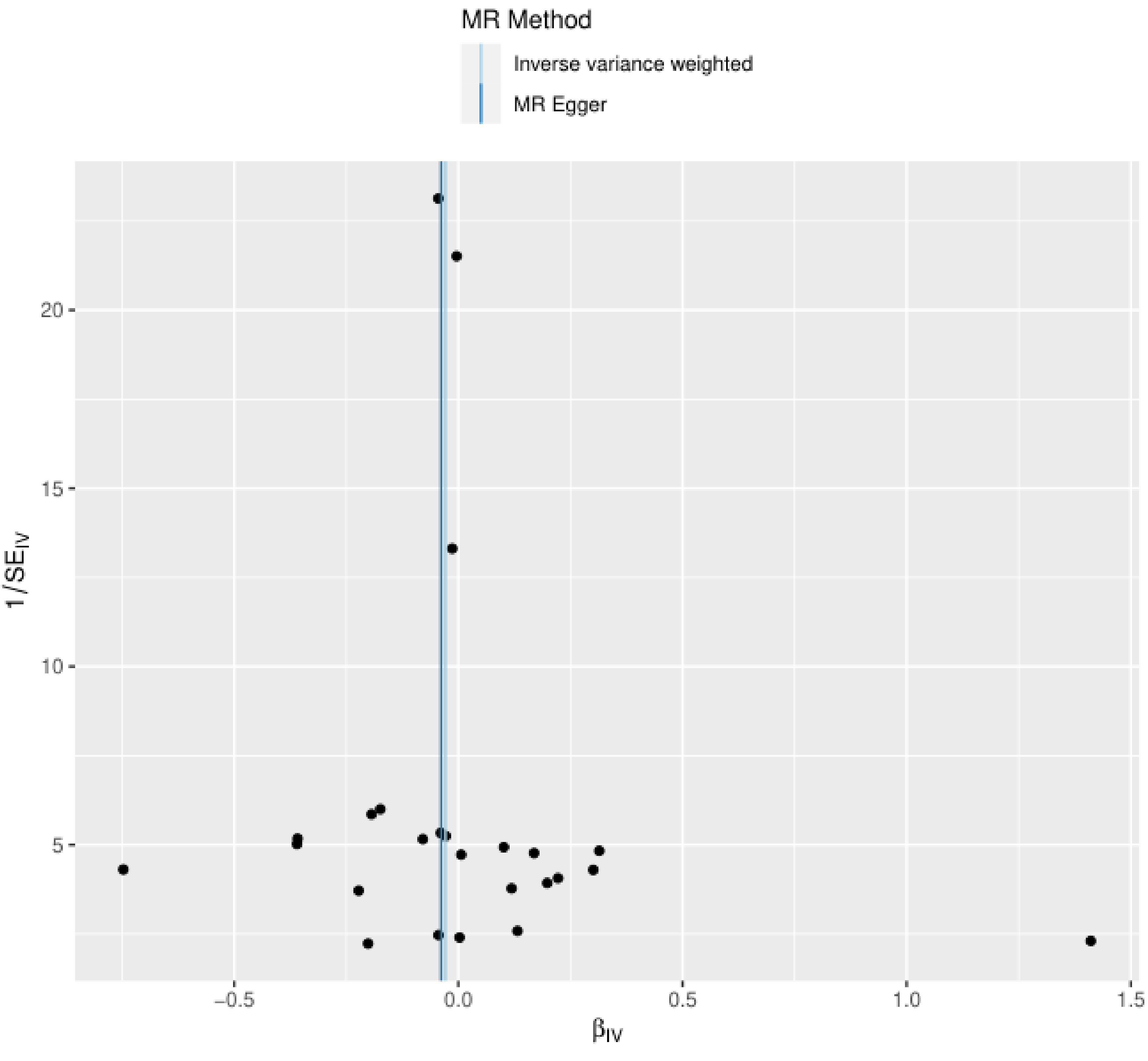

Funnel plot analyse of "CD24 on IgD+ CD38br" on 'Diabetic nephropathy'

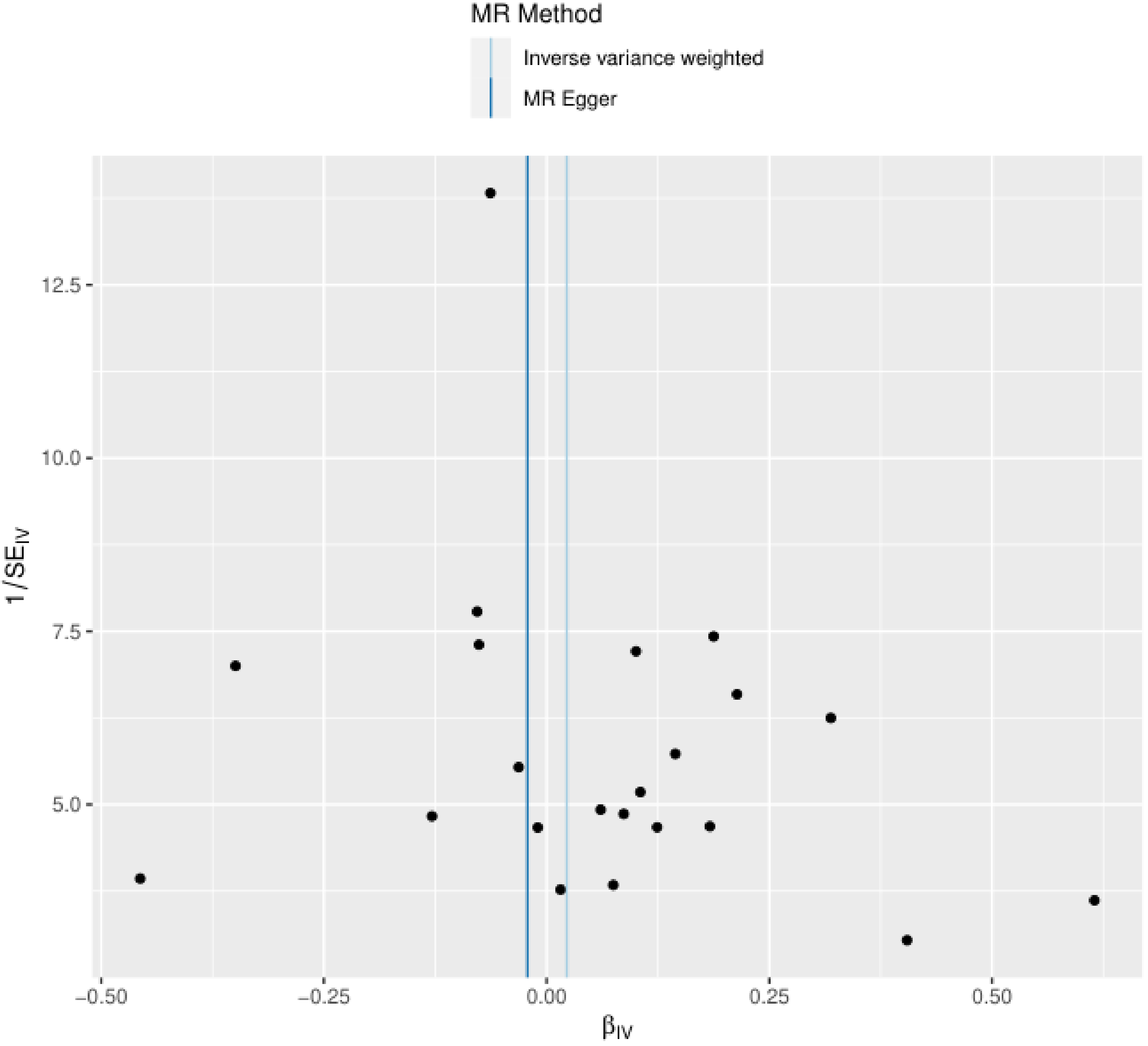

Funnel plot analyse of "SSC-A on HLA DR+ T cell" on 'Diabetic nephropathy'

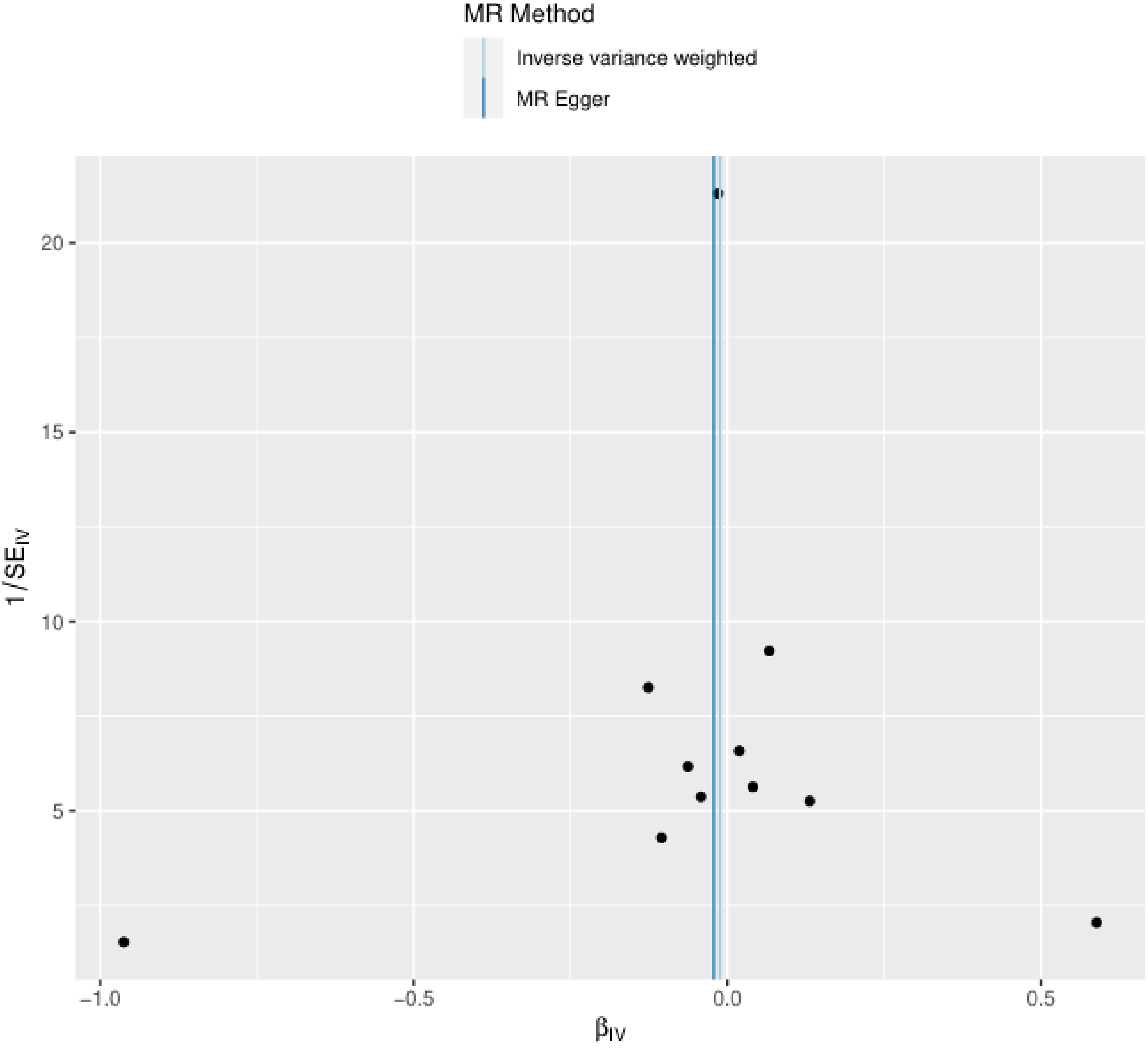

Funnel plot analyse of "CD3 on CD4 Treg " on 'Diabetic nephropathy'

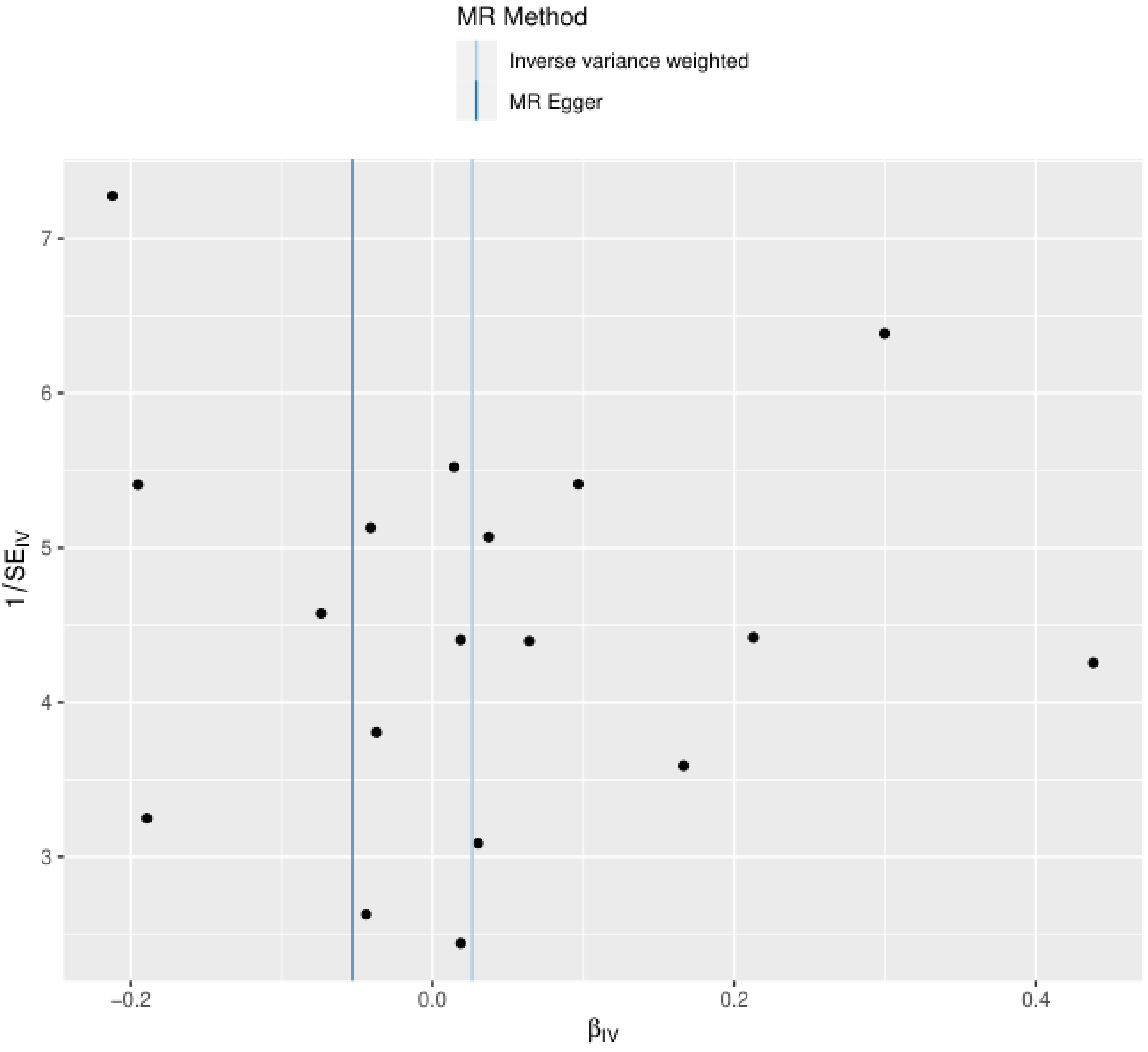

Funnel plot analyse of "IgD- CD38br %B cell" on 'Diabetic nephropathy'

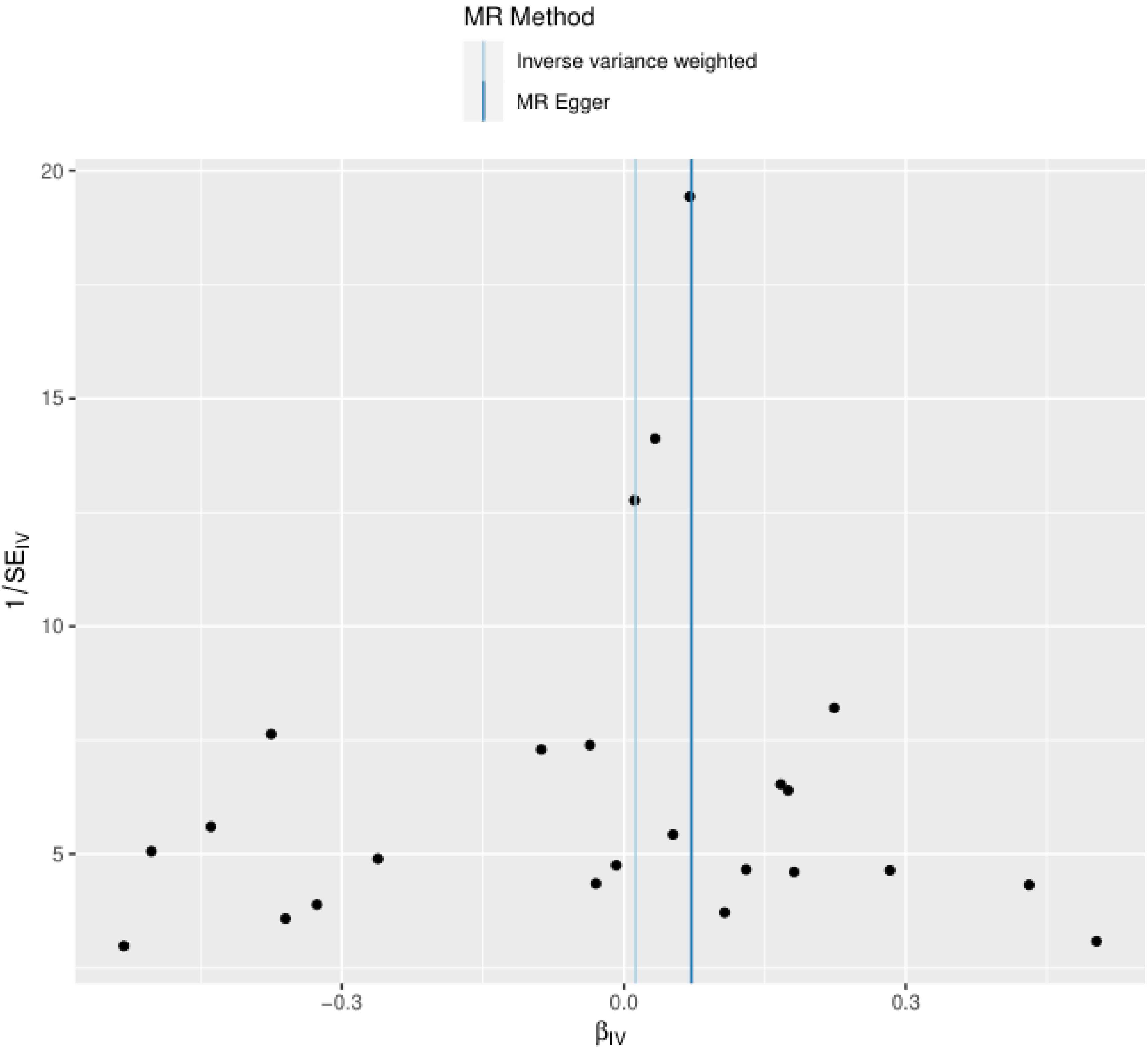

Funnel plot analyse of "Plasmacytoid DC %DC" on 'Diabetic nephropathy'

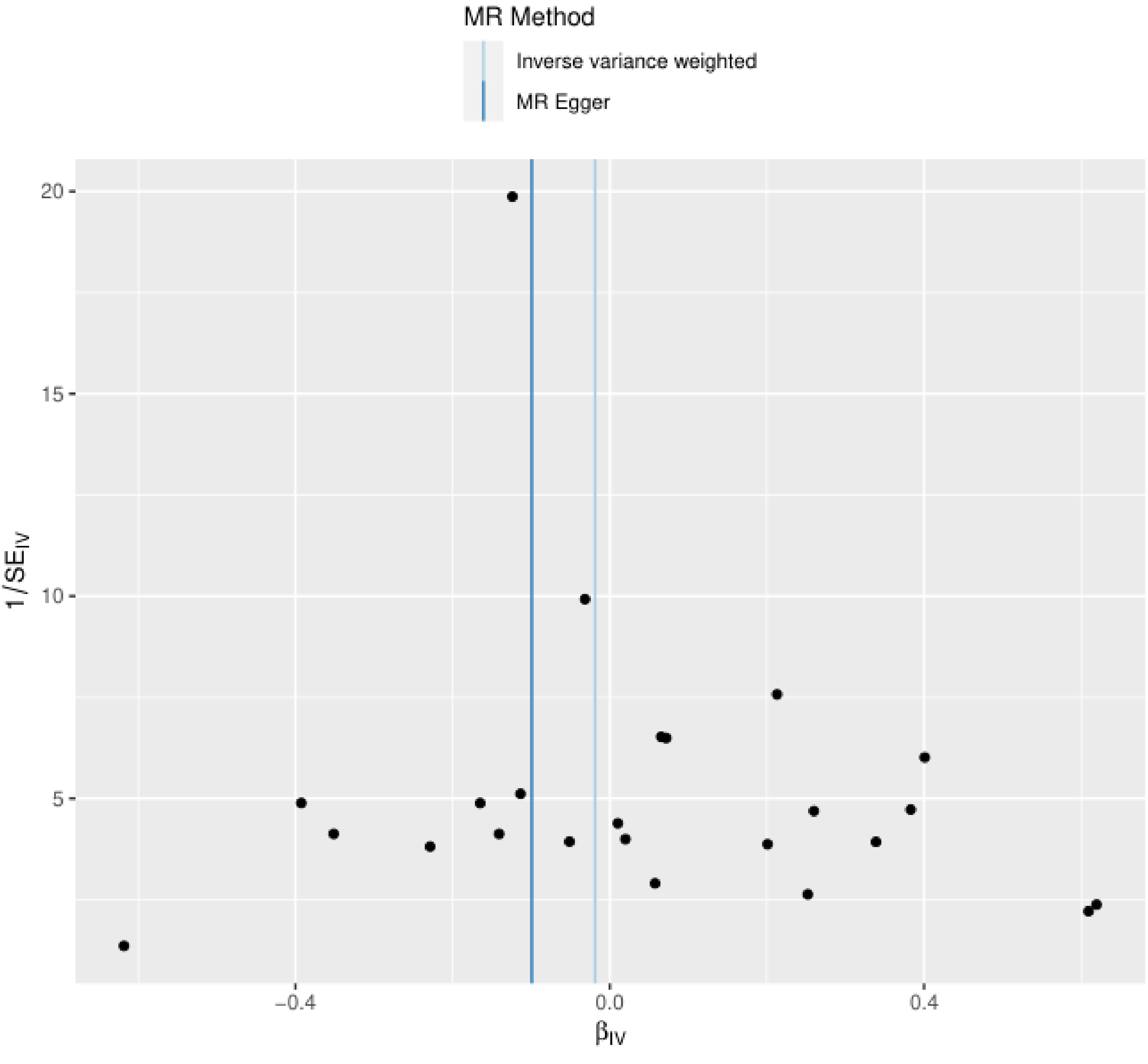

Funnel plot analyse of "CD28- DN (CD4-CD8-) %T cell" on 'Diabetic nephropathy'

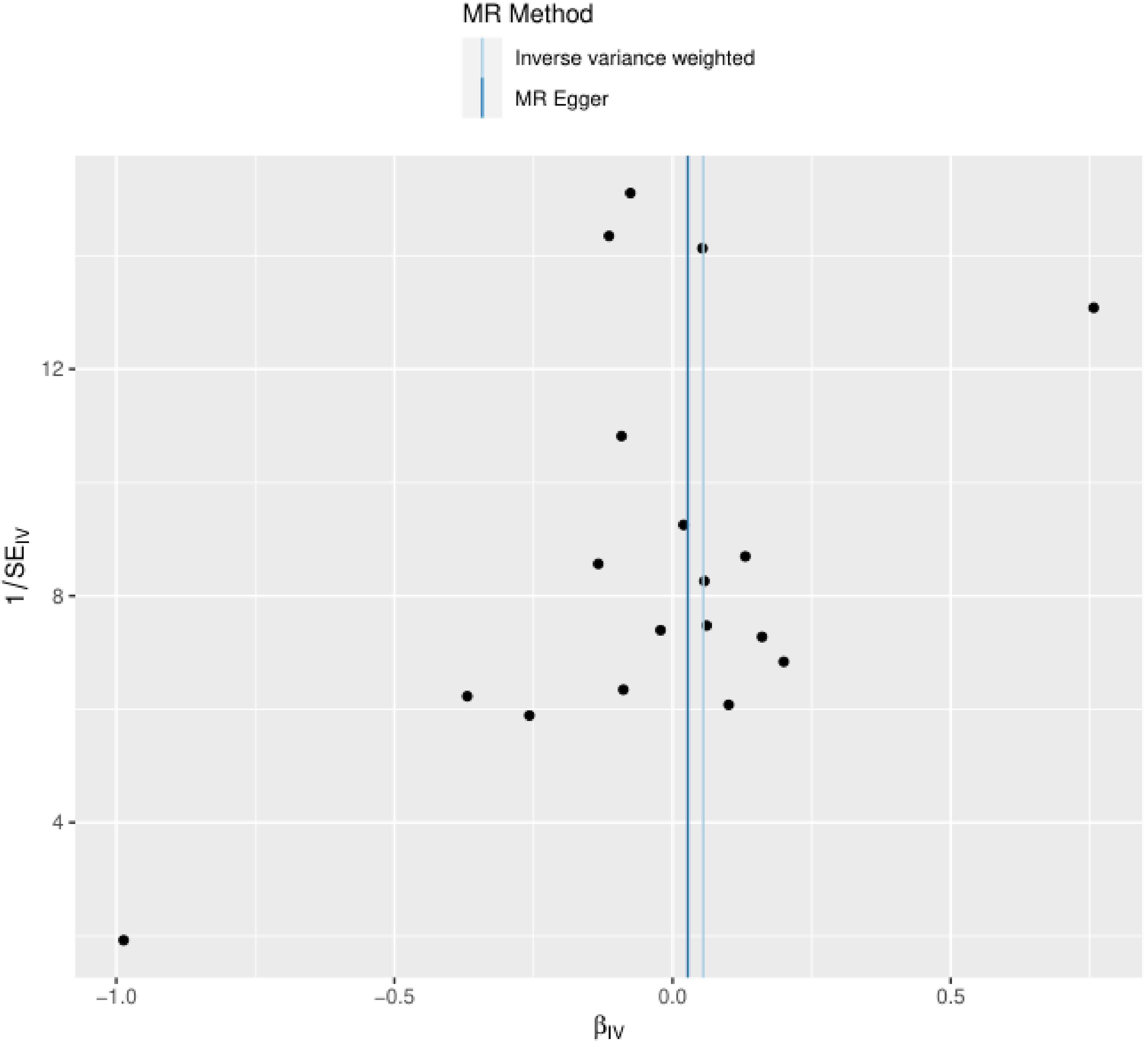

Funnel plot analyse of "HLA DR on CD33dim HLA DR+ CD11b- " on 'Diabetic nephropathy'

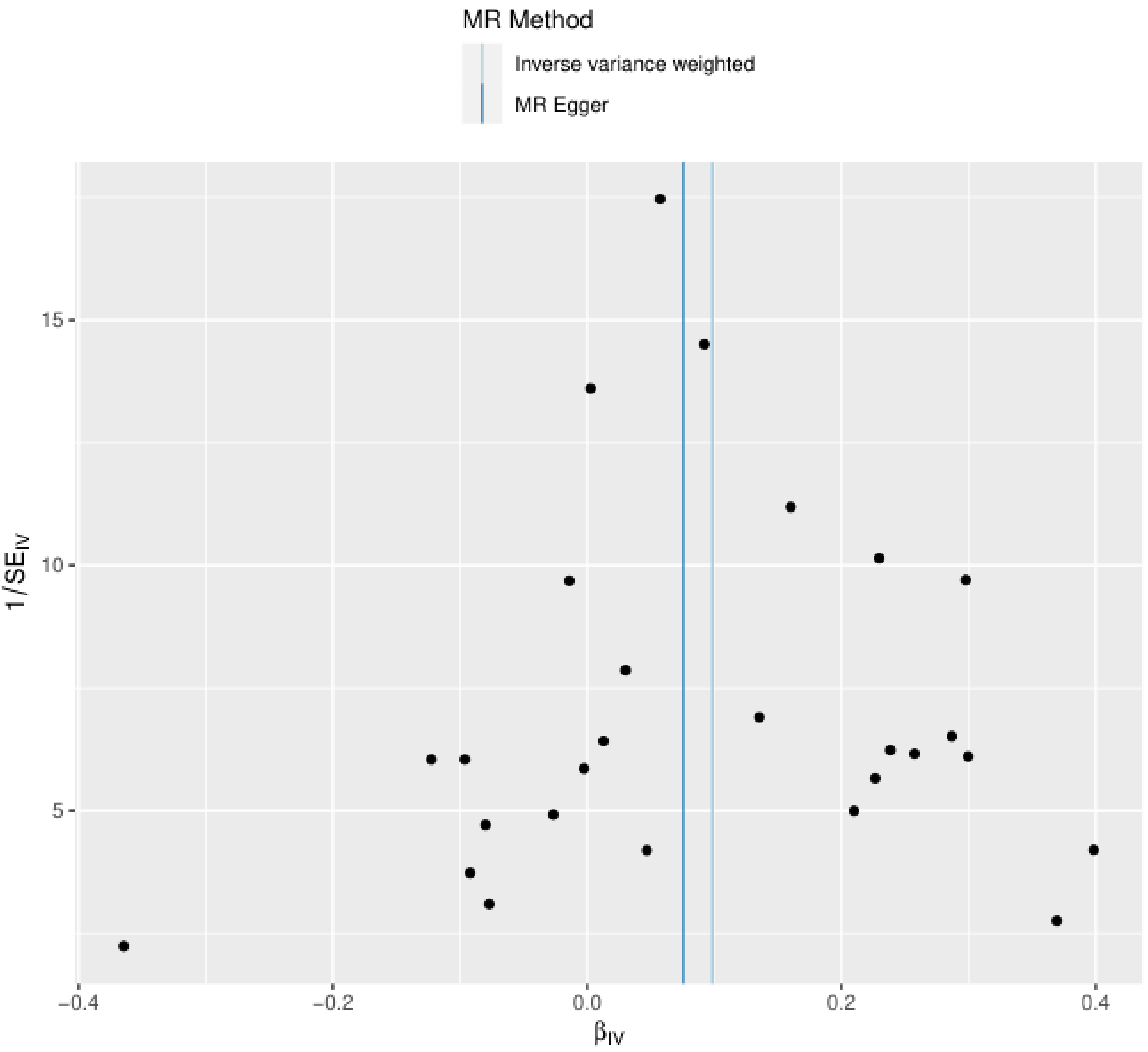

Funnel plot analyse of "CD33dim HLA DR+ CD11b+ AC" on 'Diabetic nephropathy'

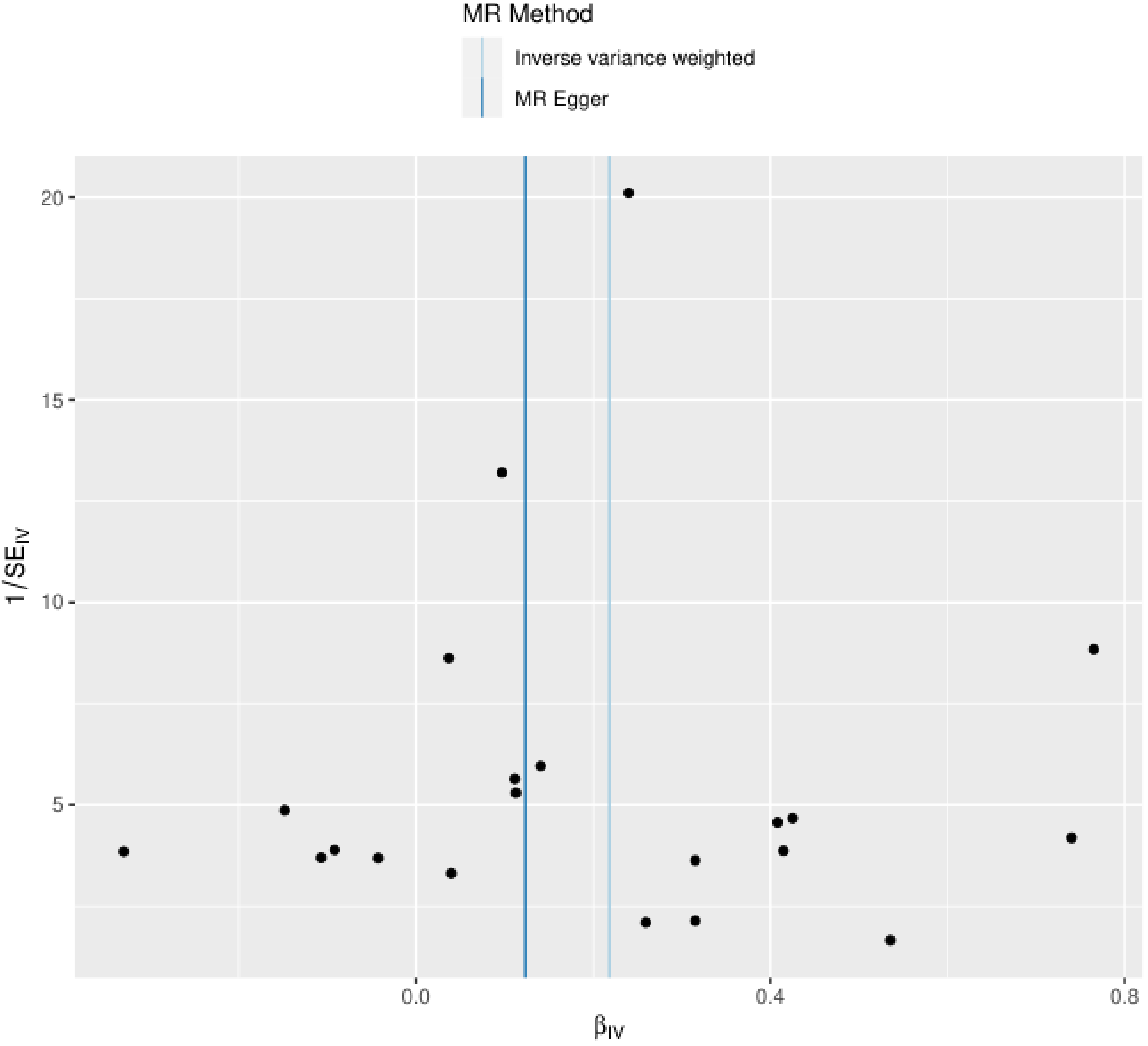

Funnel plot analyse of "HLA DR on CD14+ monocyte" on 'Diabetic nephropathy'

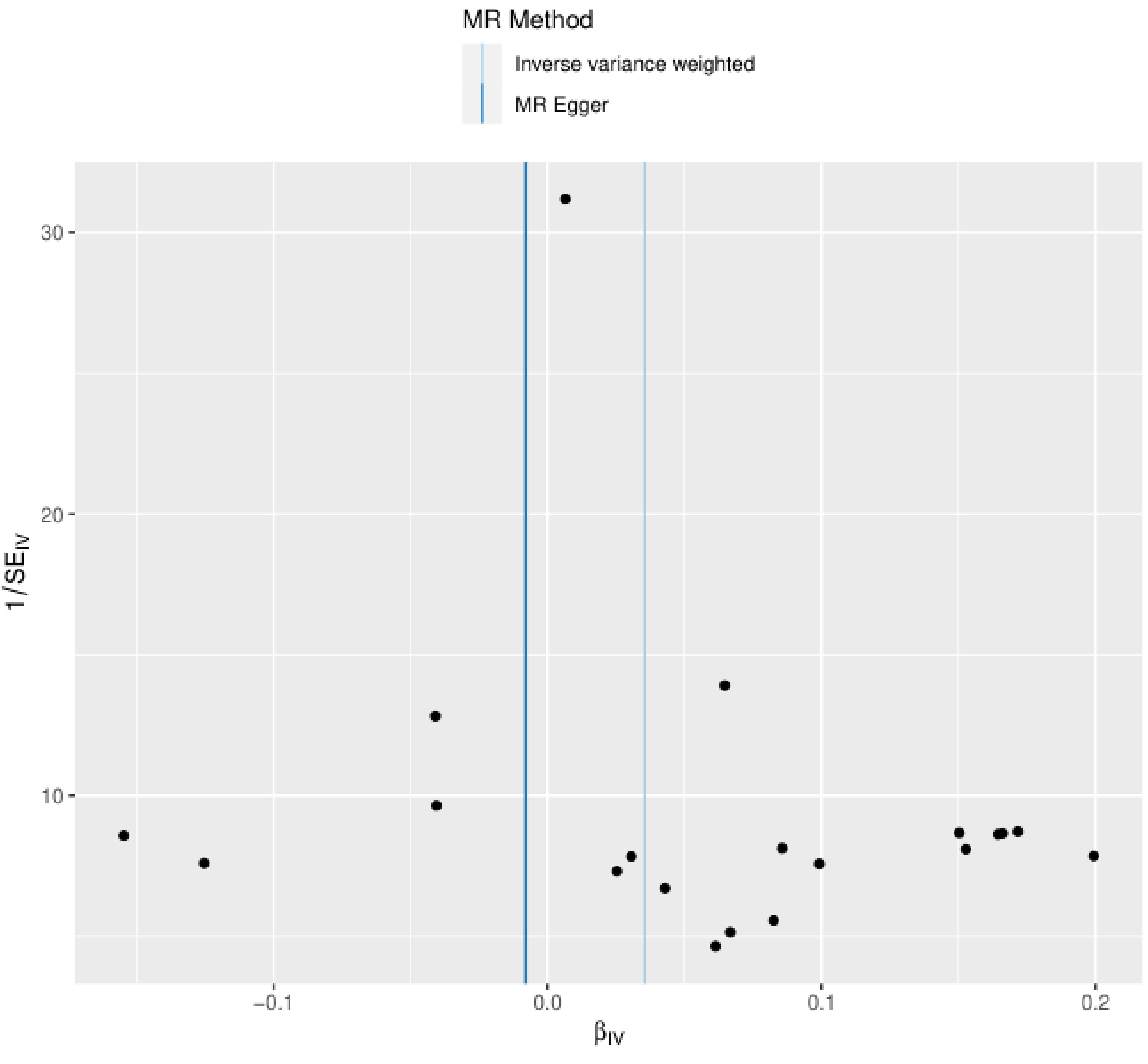

Funnel plot analyse of "CD33 on Gr MDSC " on 'Diabetic nephropathy'

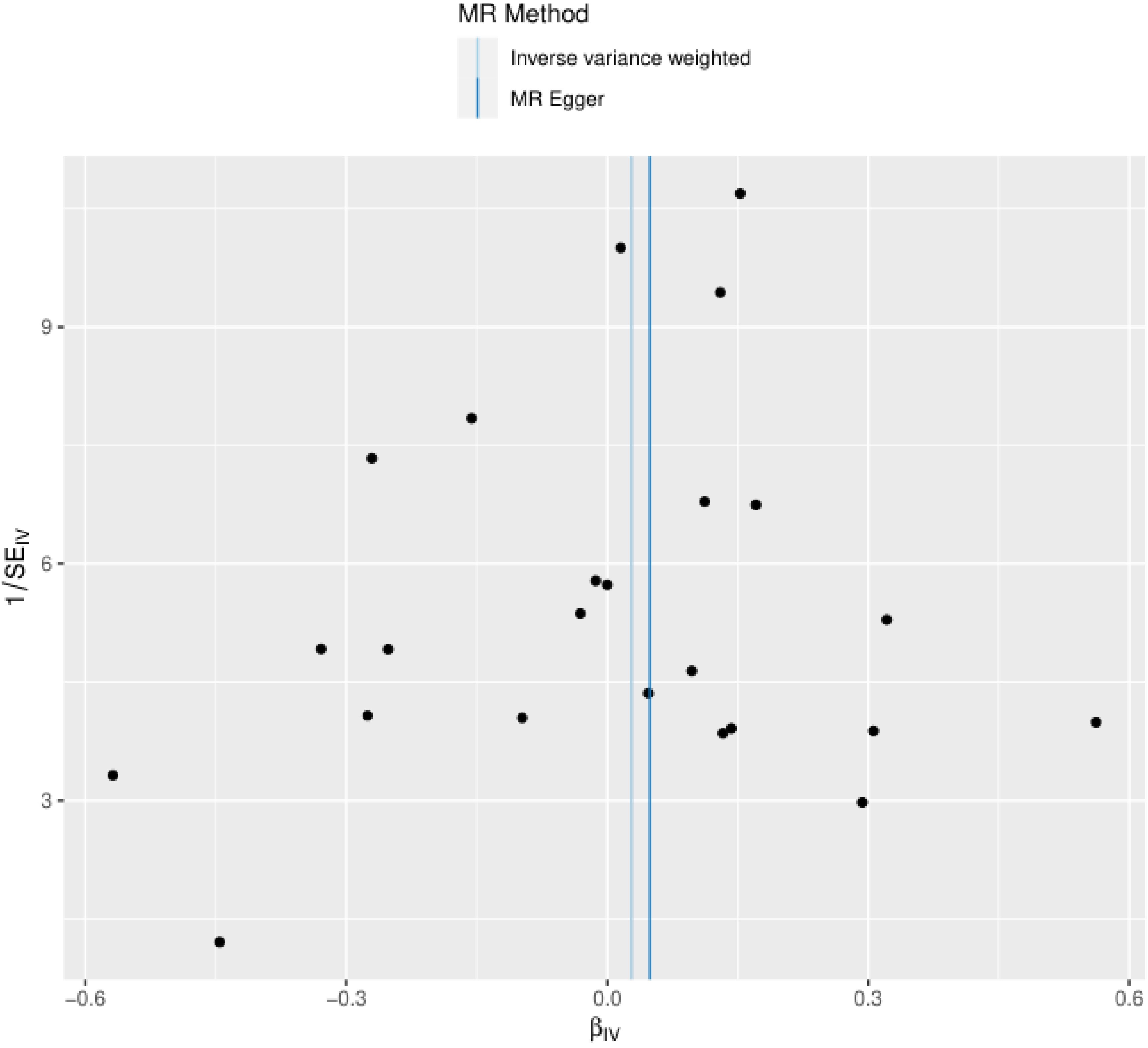

Funnel plot analyse of "CD39 on CD39+ CD8br " on 'Diabetic nephropathy'

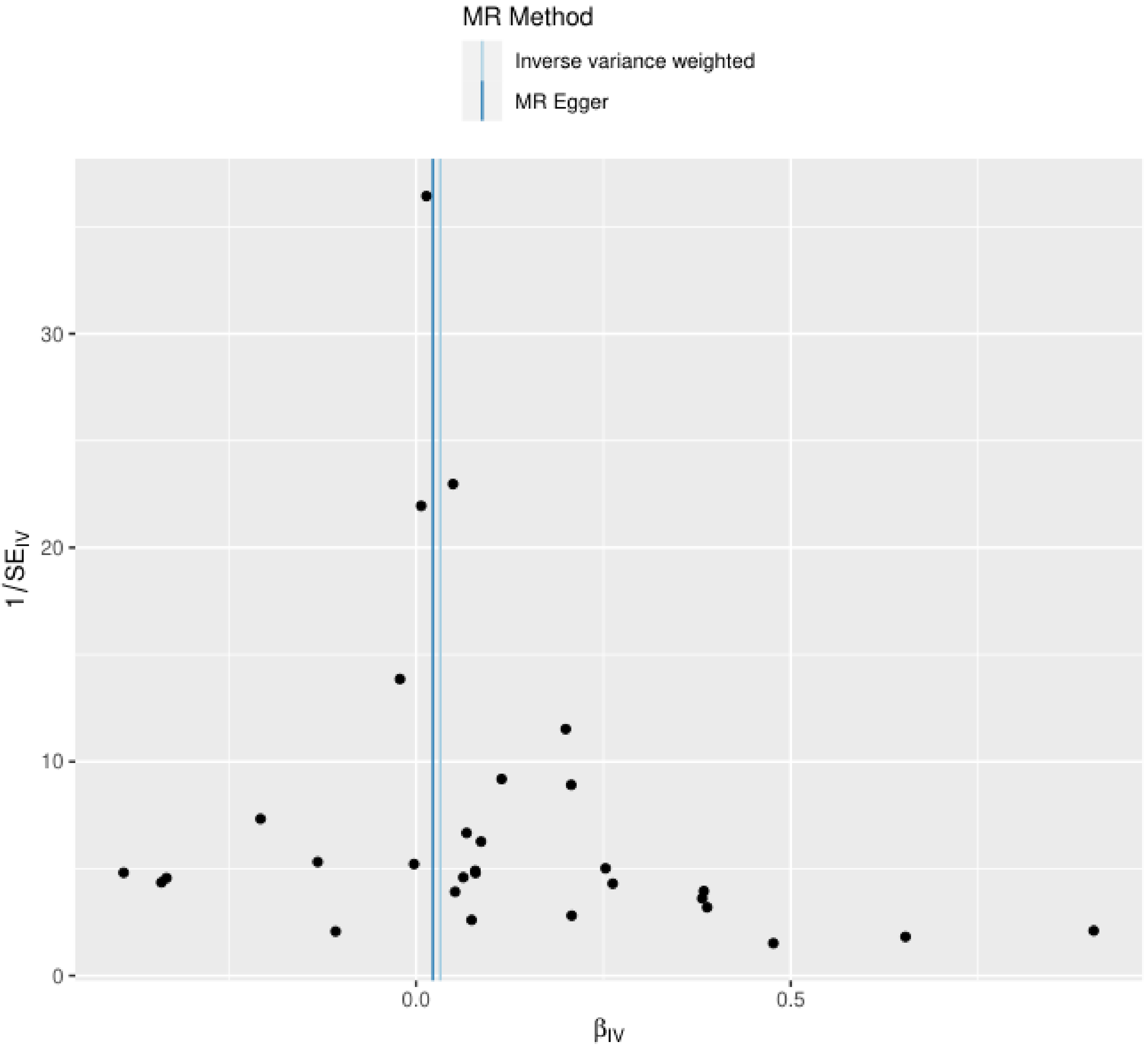

Funnel plot analyse of "CD25hi CD45RA- CD4 not Treg %T cell" on 'Diabetic nephropathy'

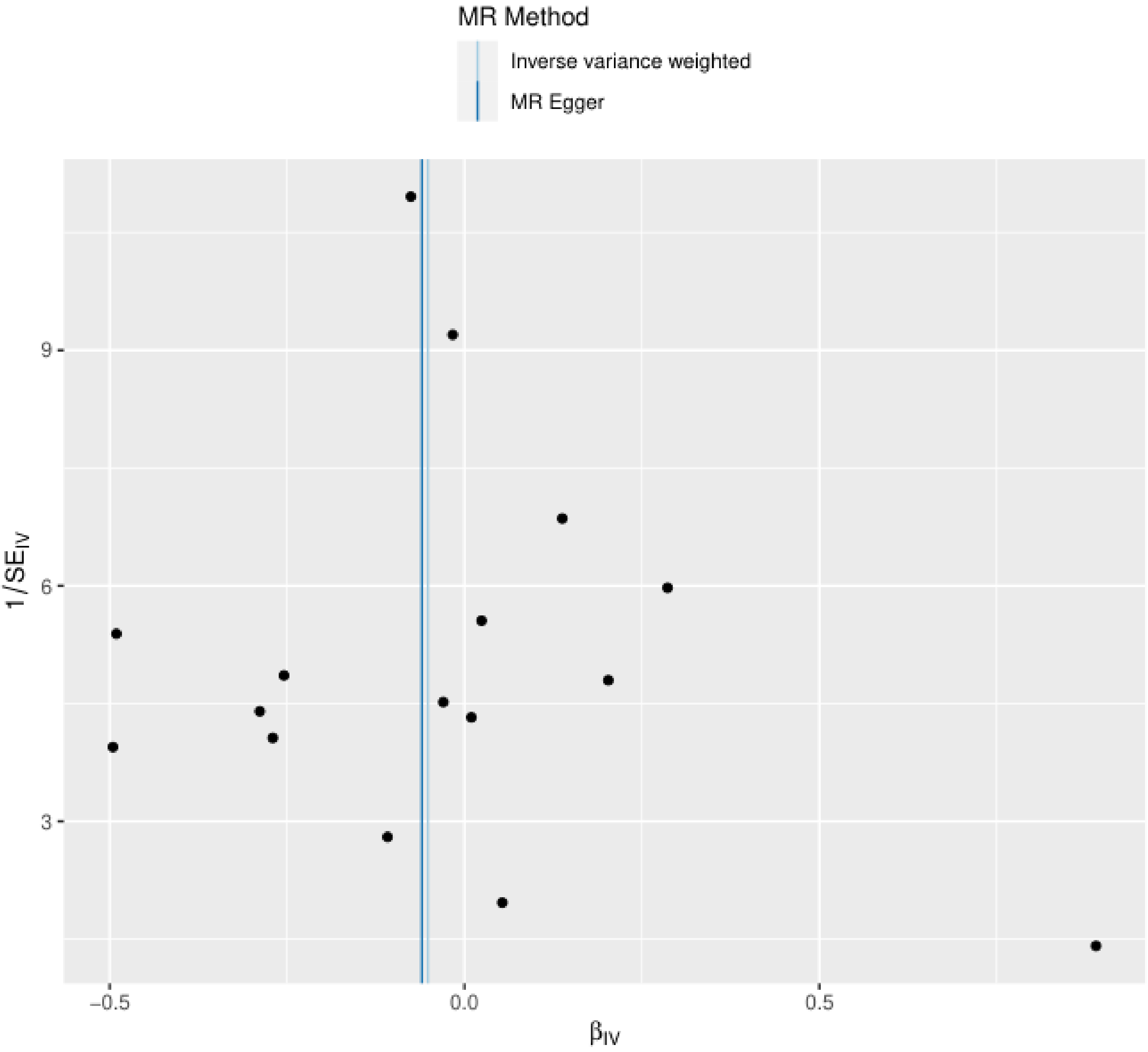

Funnel plot analyse of "CD8 on CD28+ CD45RA- CD8br " on 'Diabetic nephropathy'

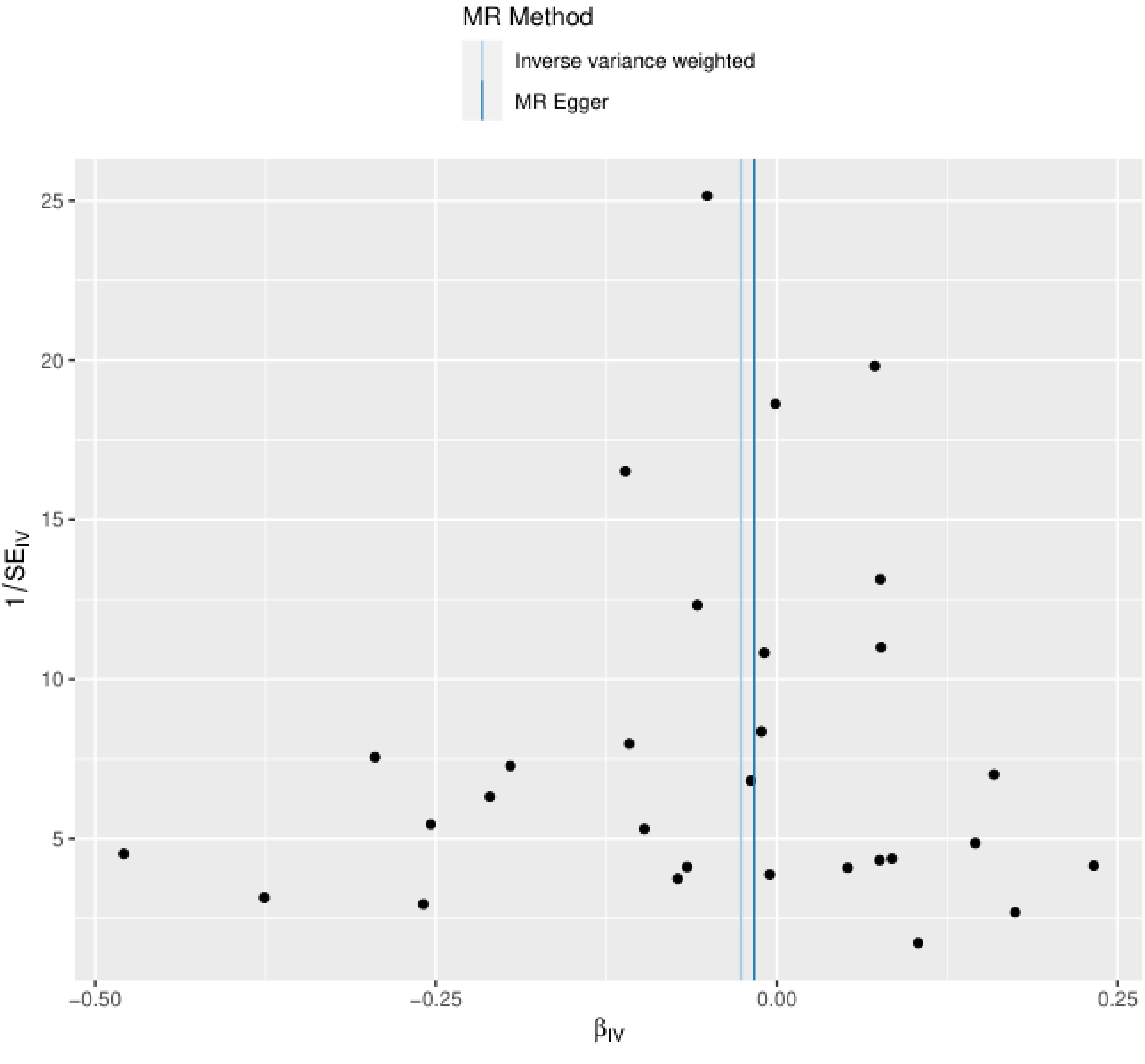

Funnel plot analyse of "Plasmacytoid DC AC" on 'Diabetic nephropathy'

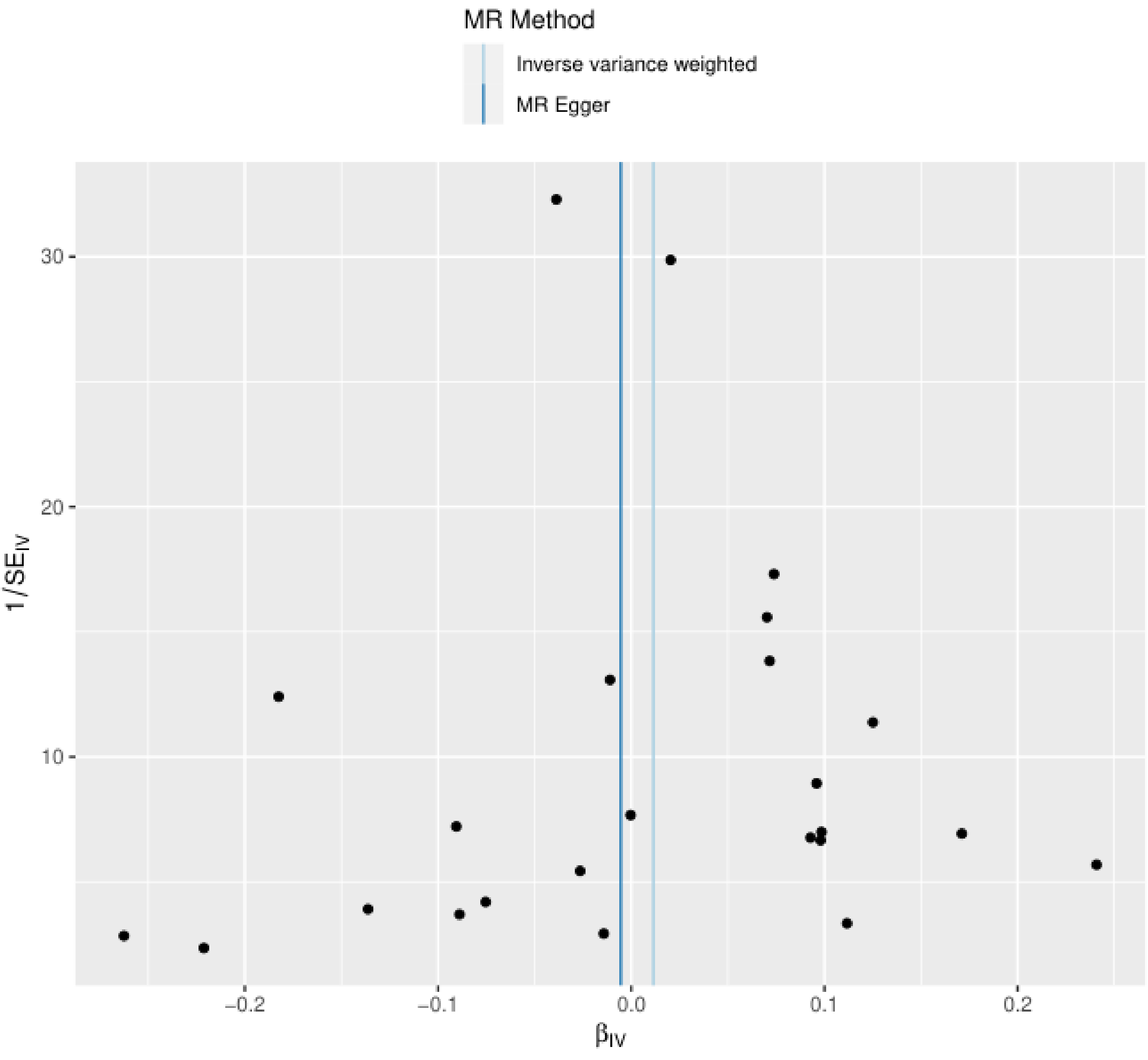

Funnel plot analyse of "CD25 on IgD+ CD38- unsw mem" on 'Diabetic nephropathy'

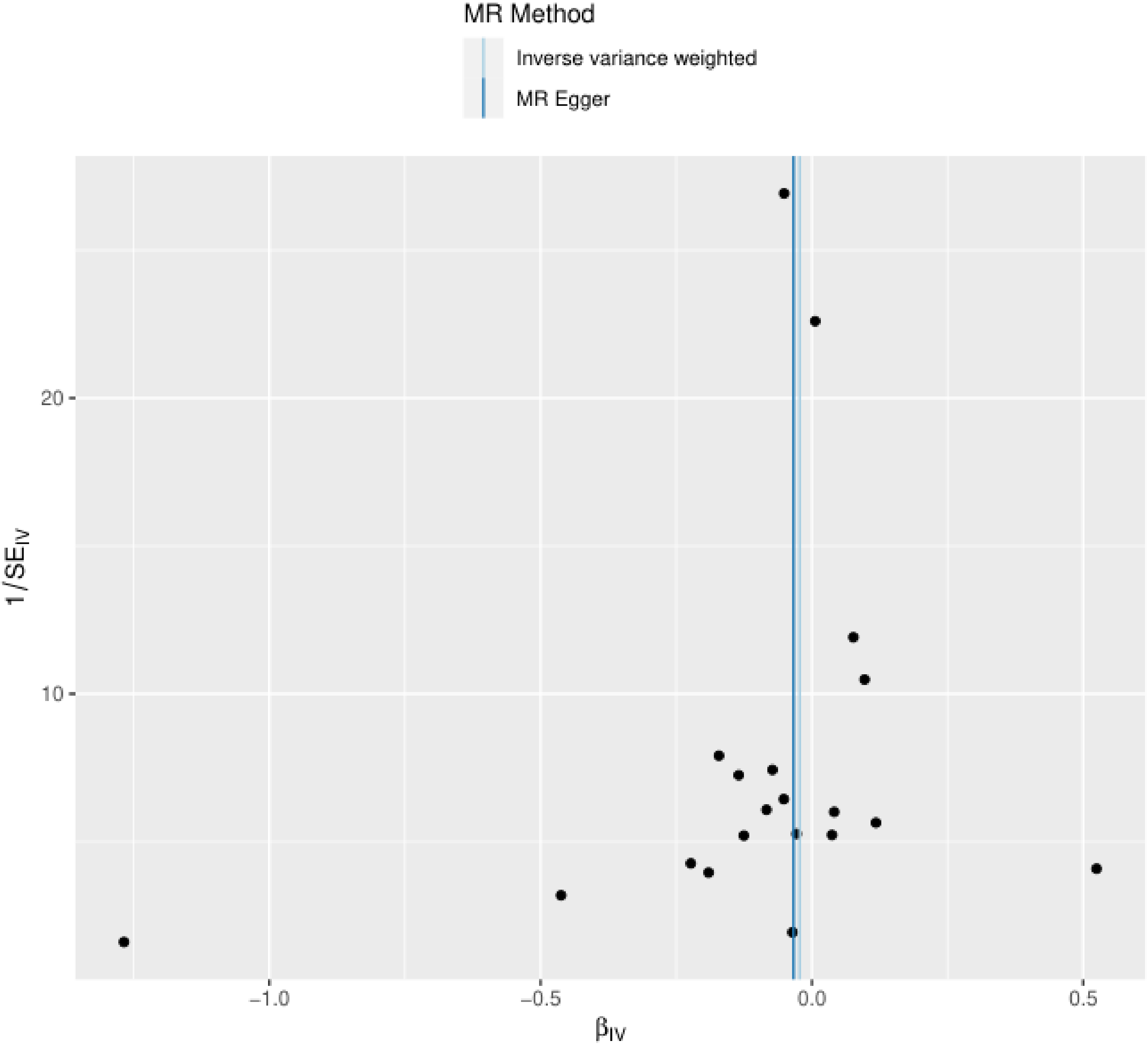

Funnel plot analyse of "IgD on IgD+ CD38dim" on 'Diabetic nephropathy'

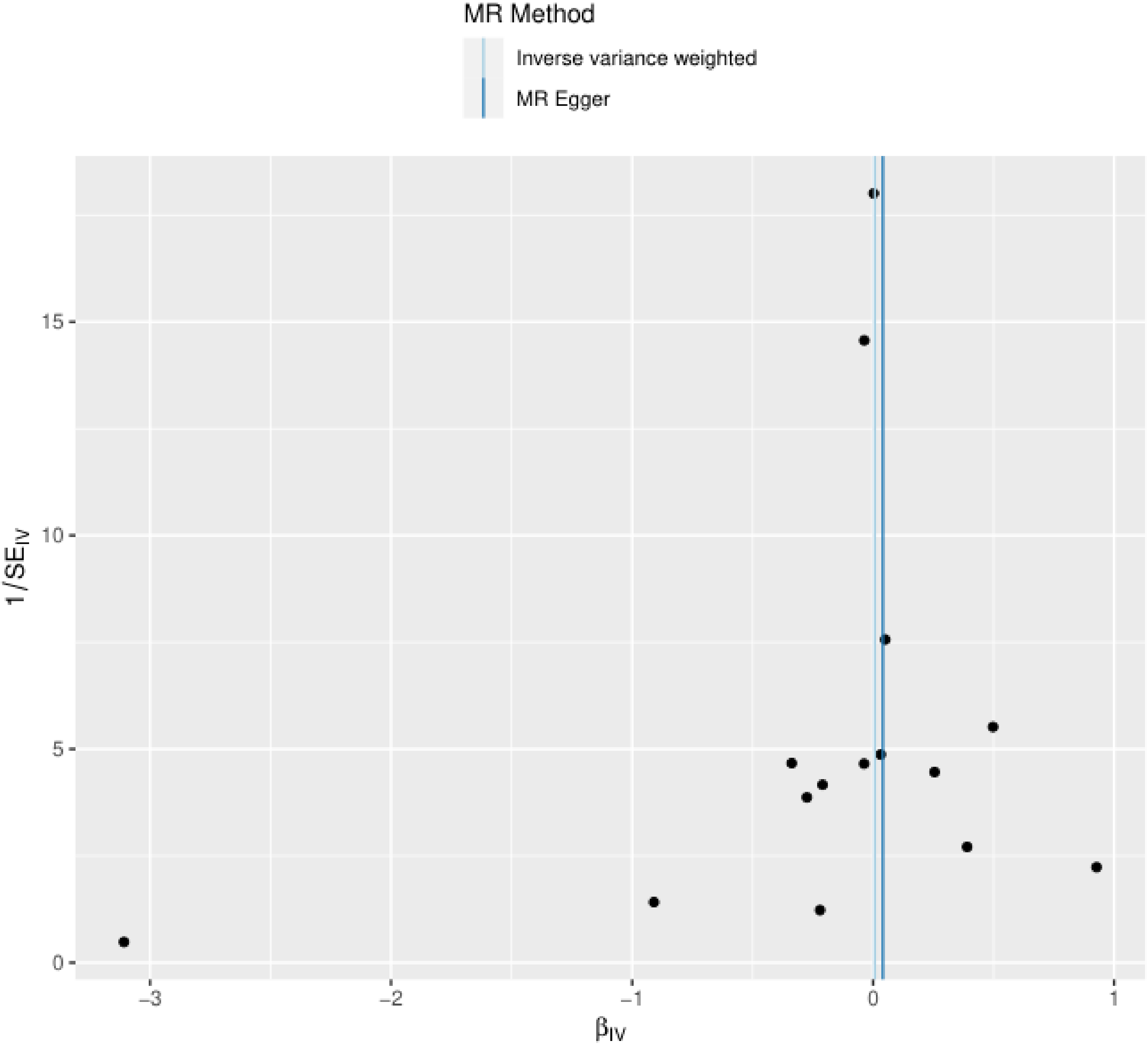

Funnel plot analyse of "IgD- CD38- AC" on 'Diabetic nephropathy'

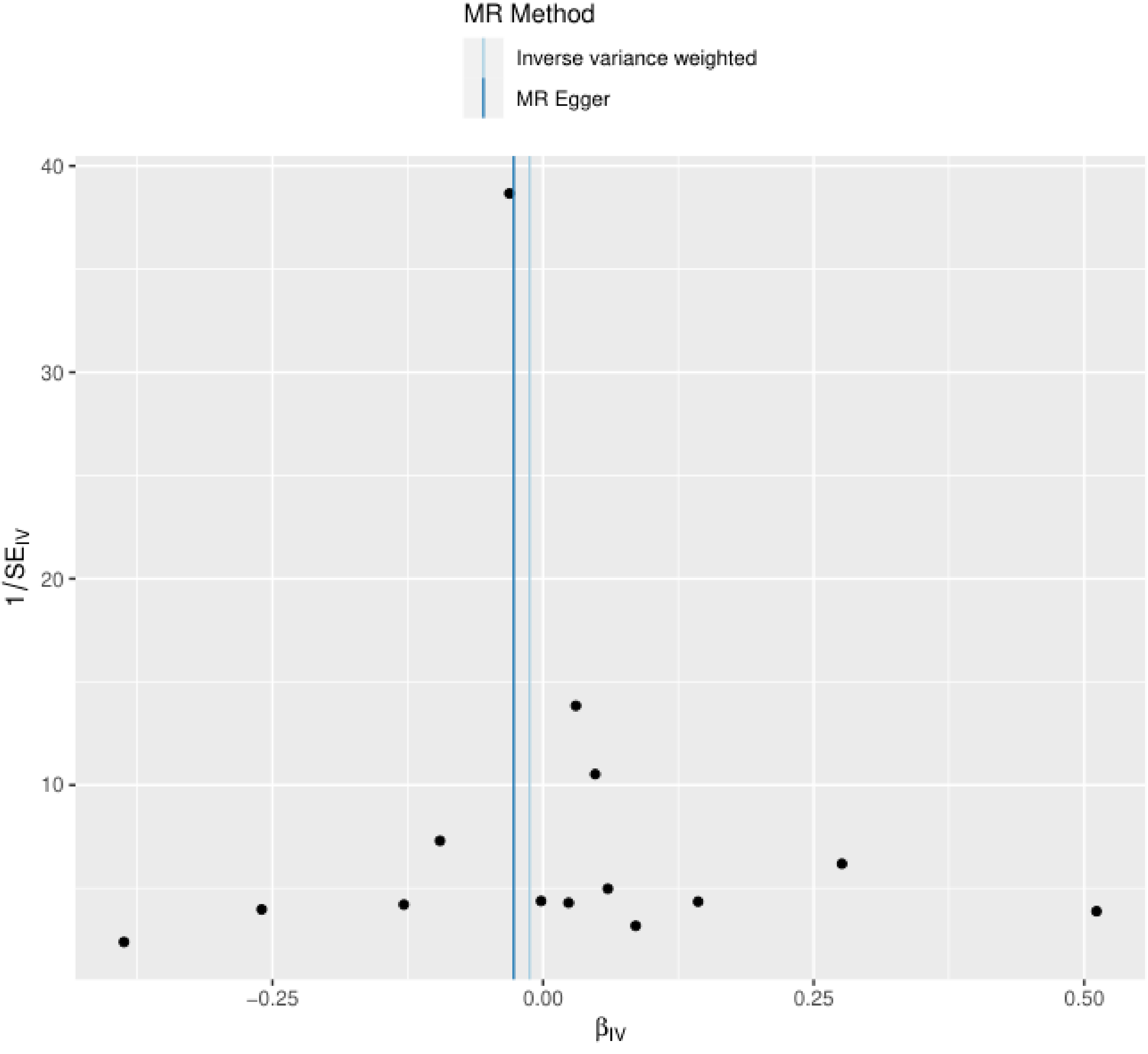

Funnel plot analyse of "CD20 on IgD- CD38br" on 'Diabetic nephropathy'

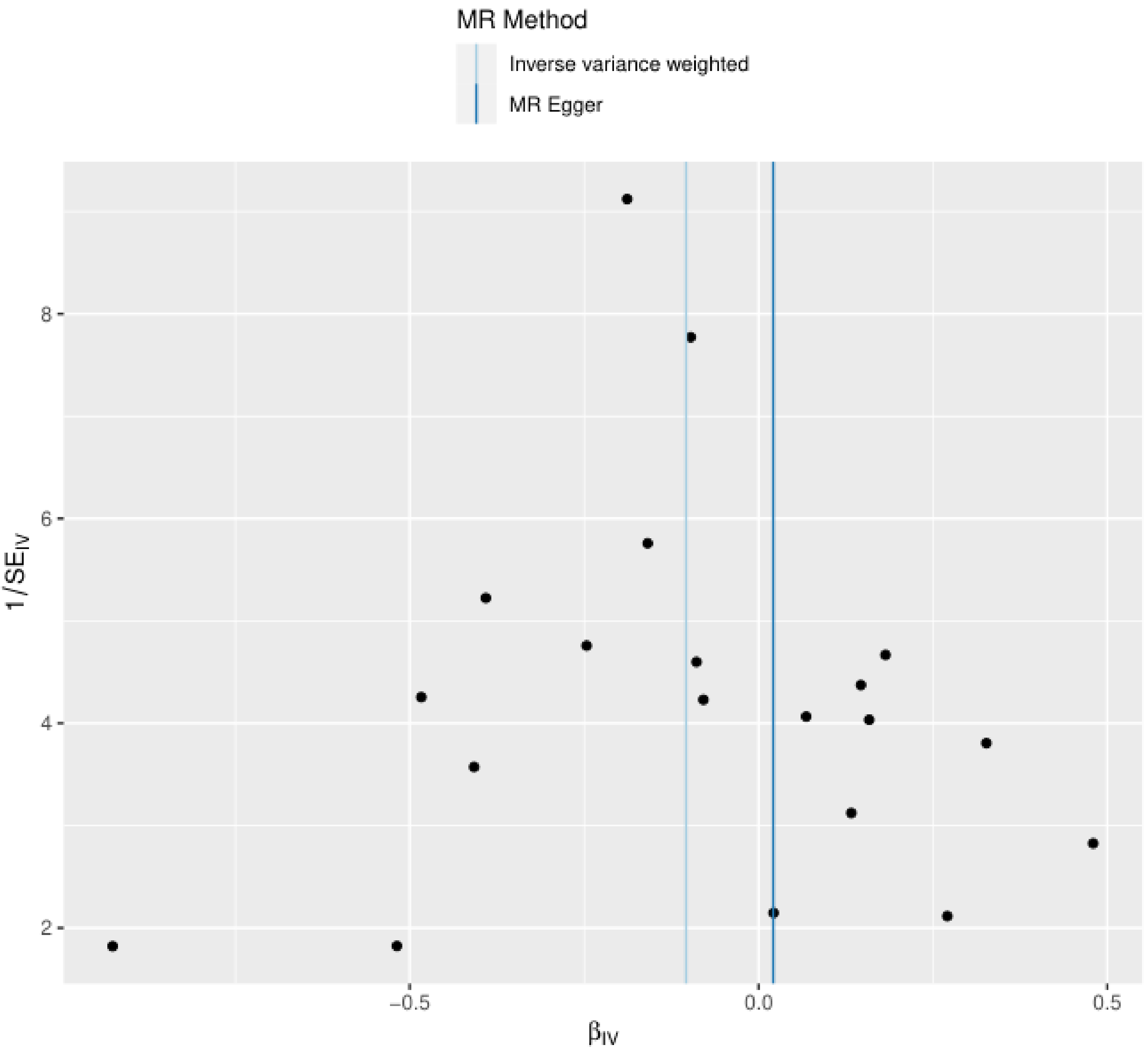

Funnel plot analyse of "IgD+ CD24+ %B cell" on 'Diabetic nephropathy'

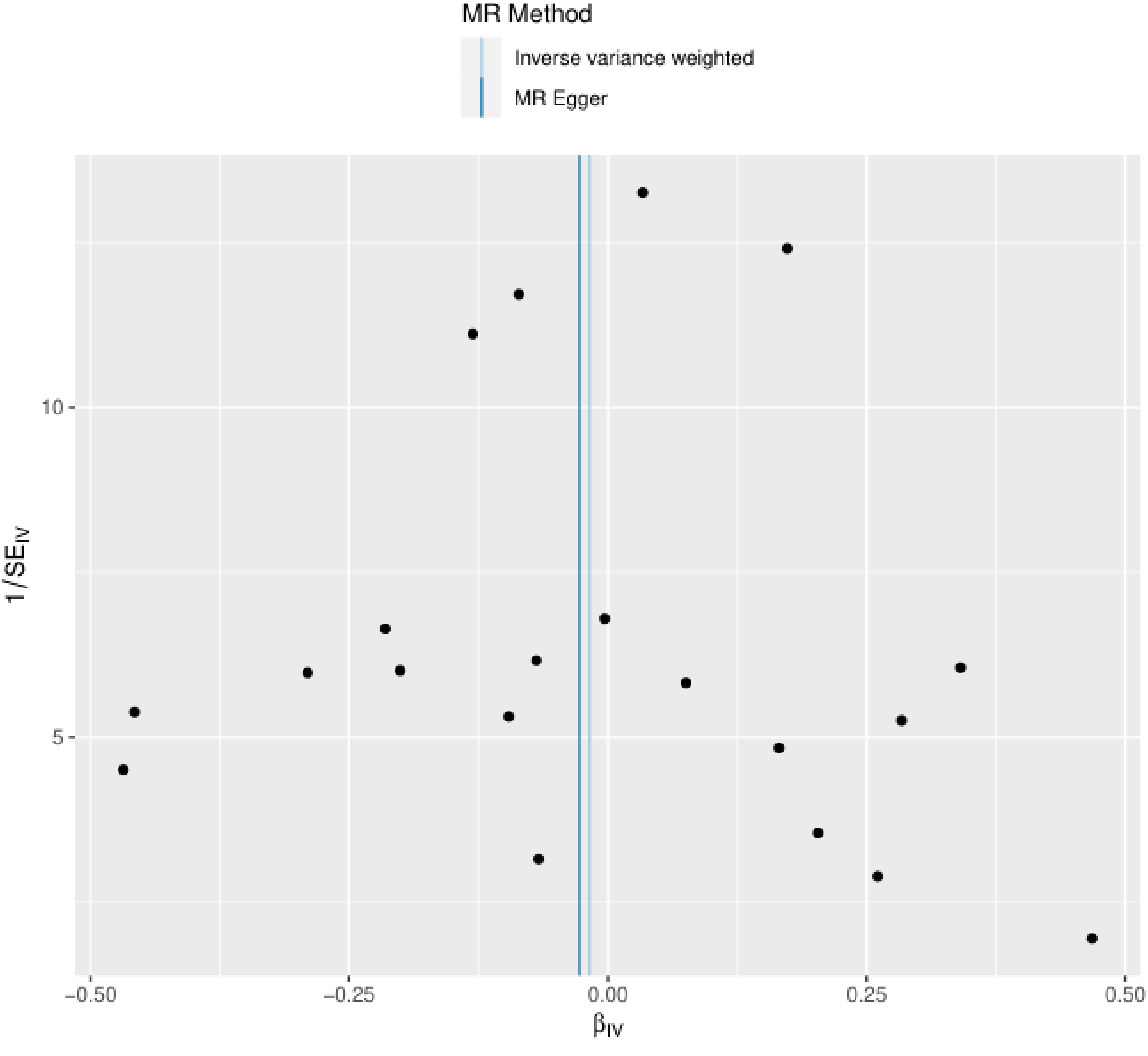

Funnel plot analyse of "PDL-1 on CD14- CD16+ monocyte" on 'Diabetic nephropathy'

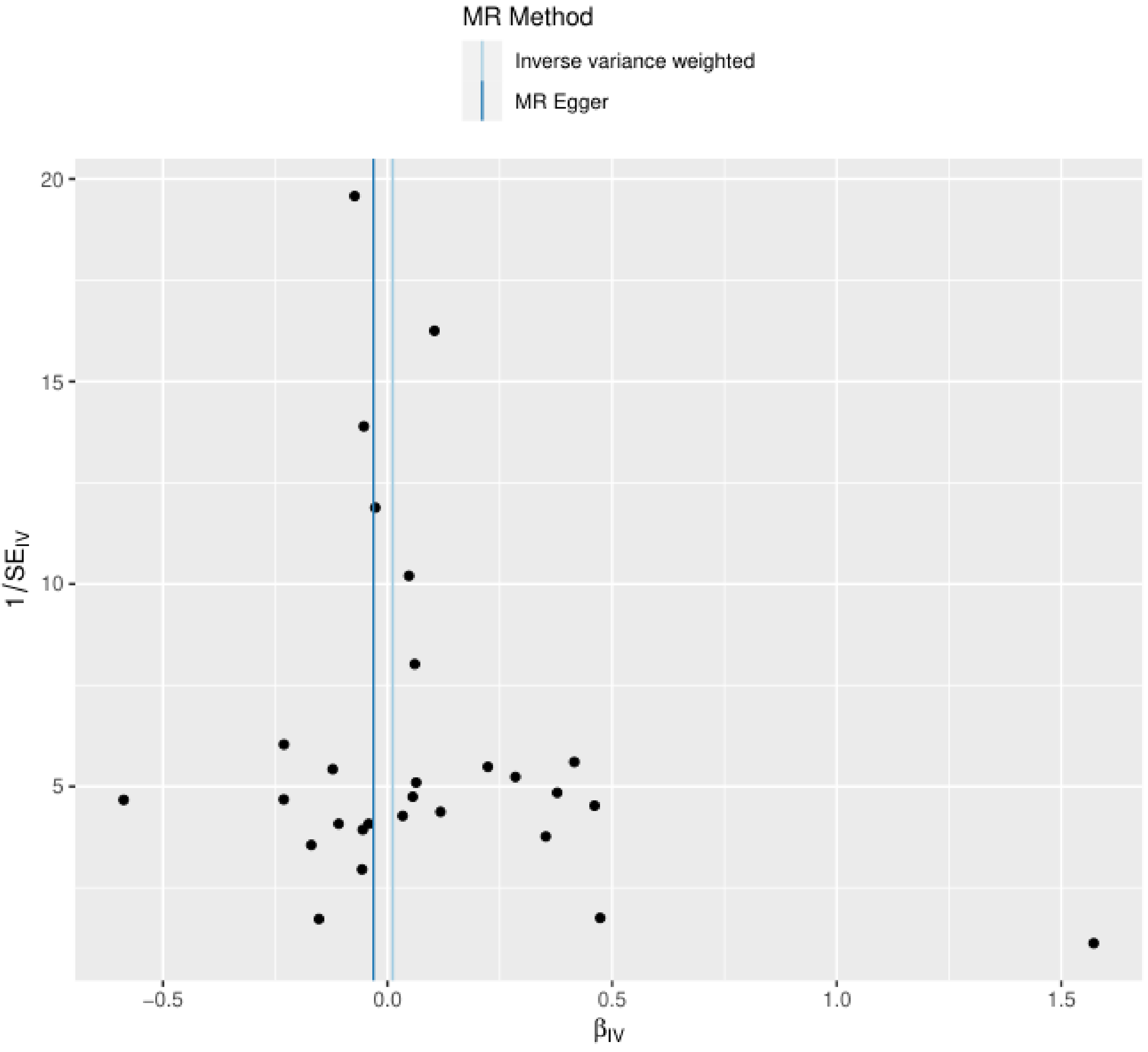

Funnel plot analyse of "PDL-1 on CD14+ CD16+ monocyte" on 'Diabetic nephropathy'

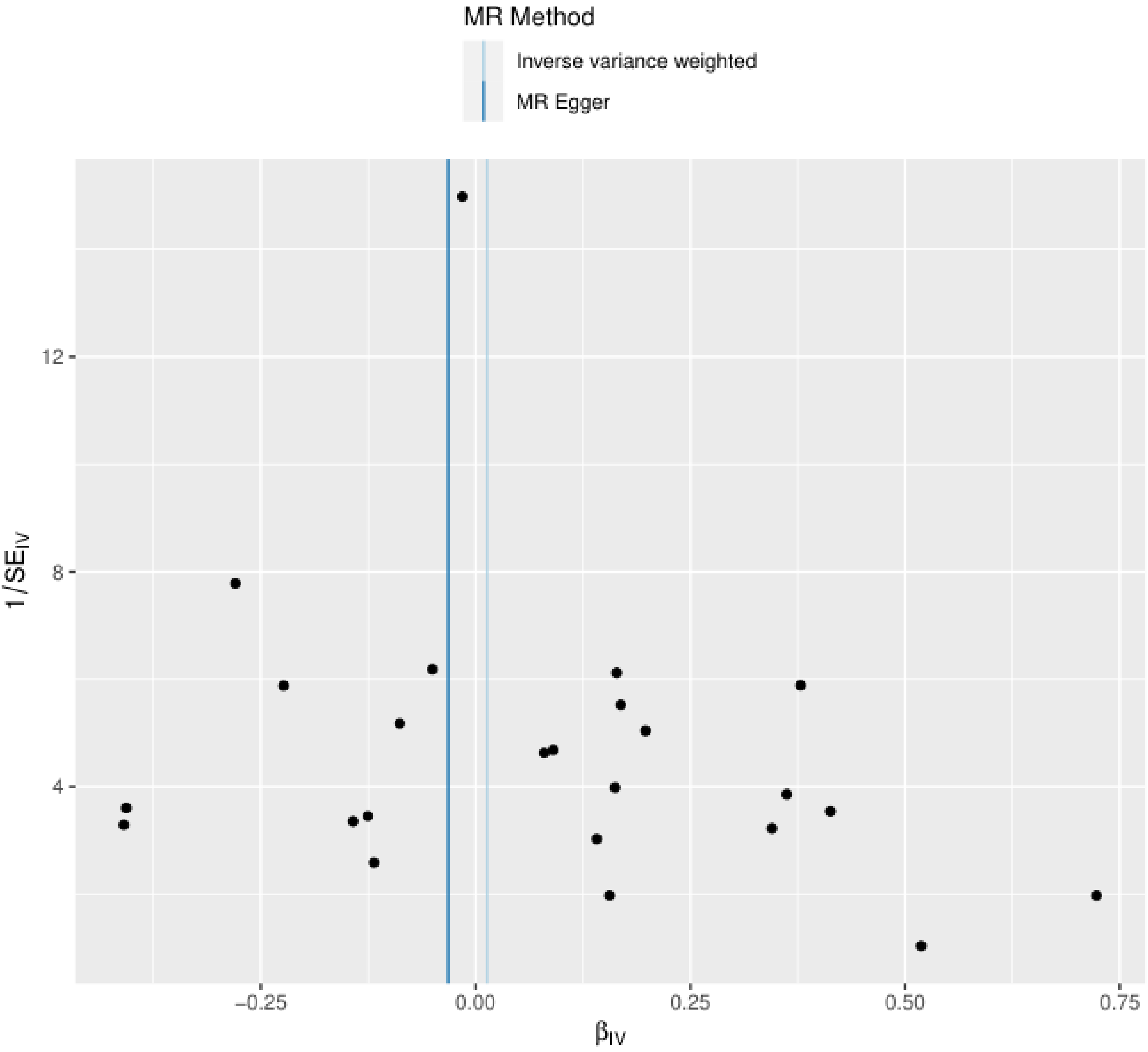

Funnel plot analyse of "CD28+ DN (CD4-CD8-) %T cell" on 'Diabetic nephropathy'

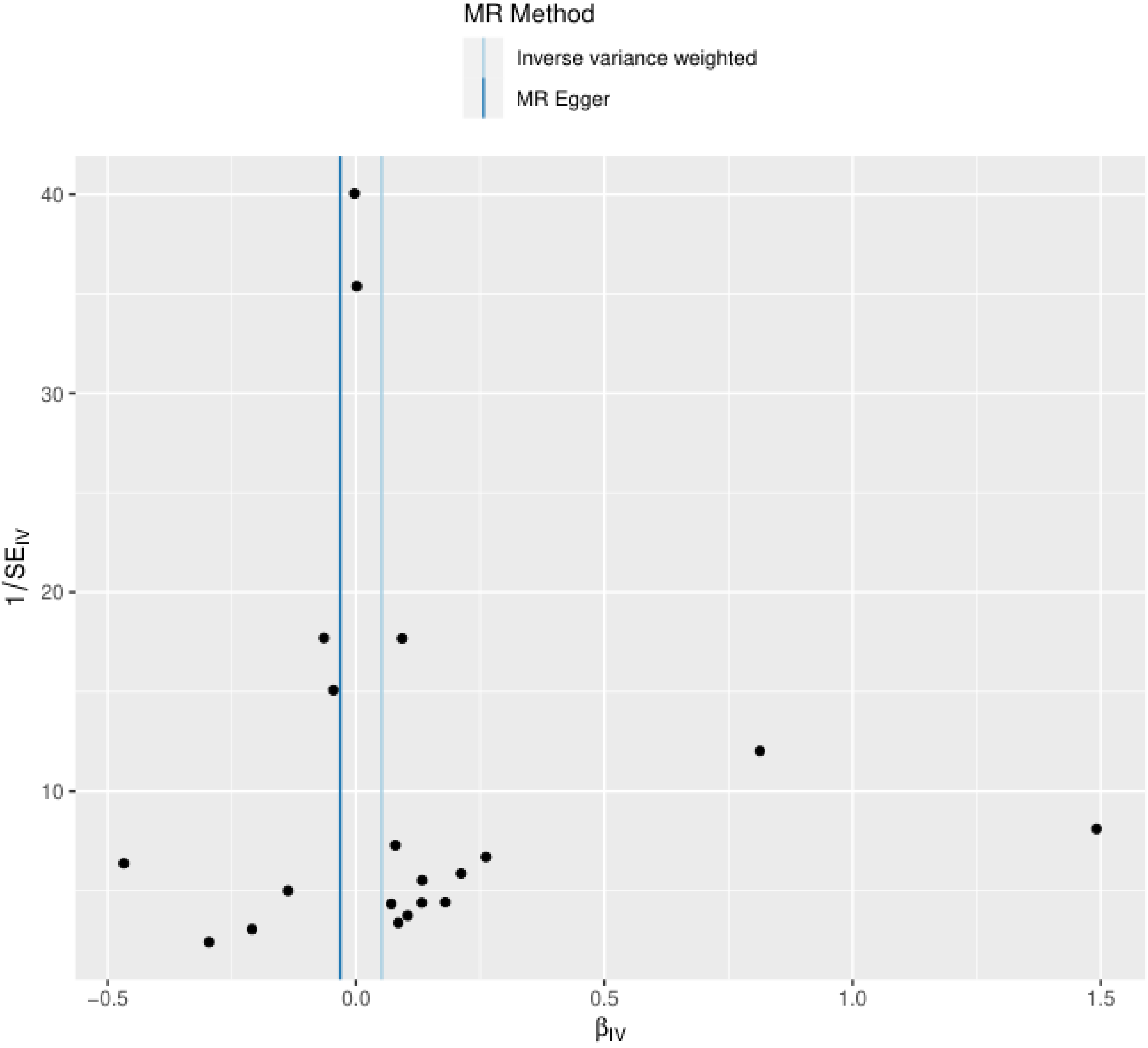

Funnel plot analyse of "HLA DR on CD14+ CD16+ monocyte" on 'Diabetic nephropathy'

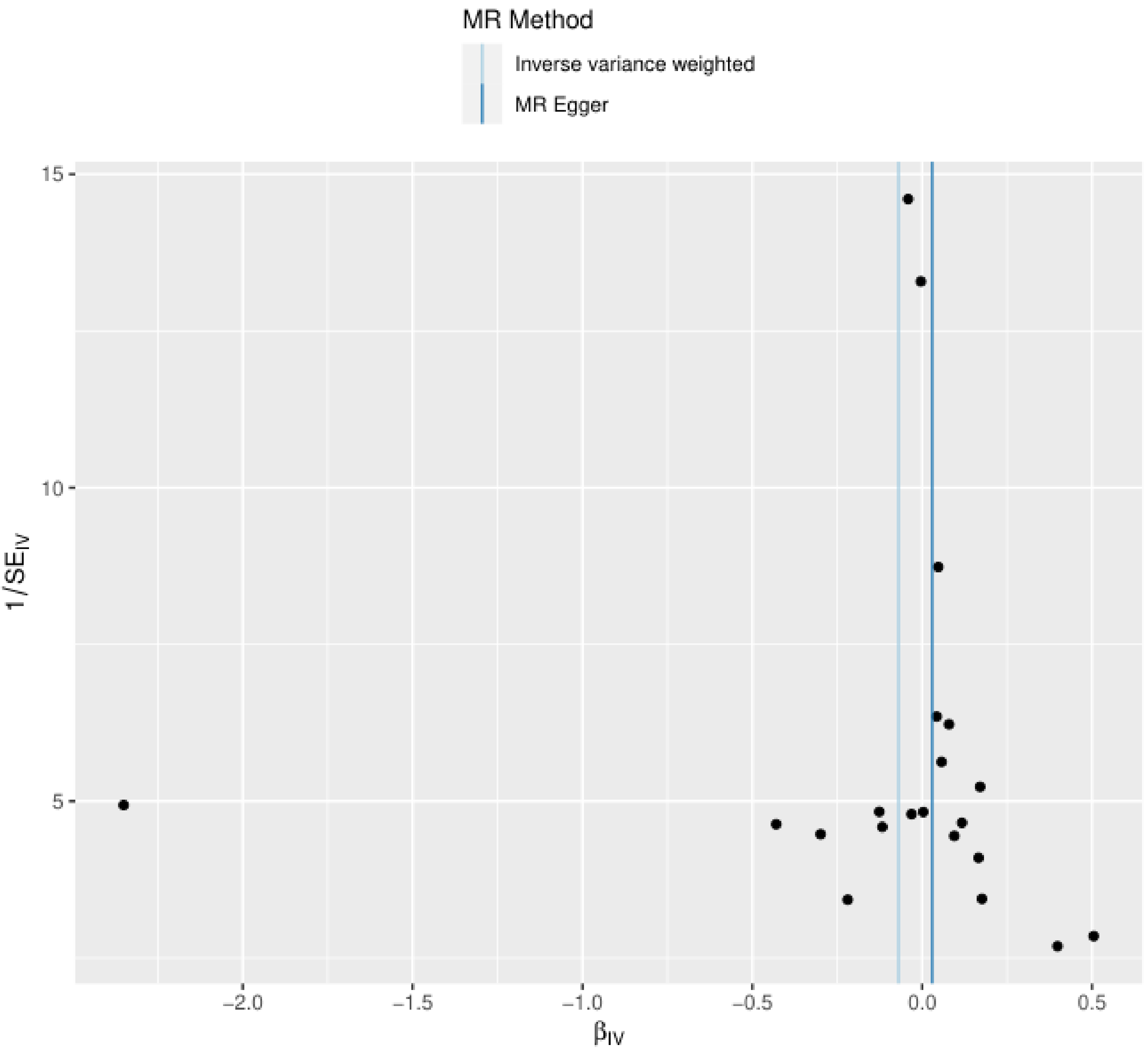

Funnel plot analyse of "SSC-A on HLA DR+ CD4+" on 'Diabetic nephropathy'

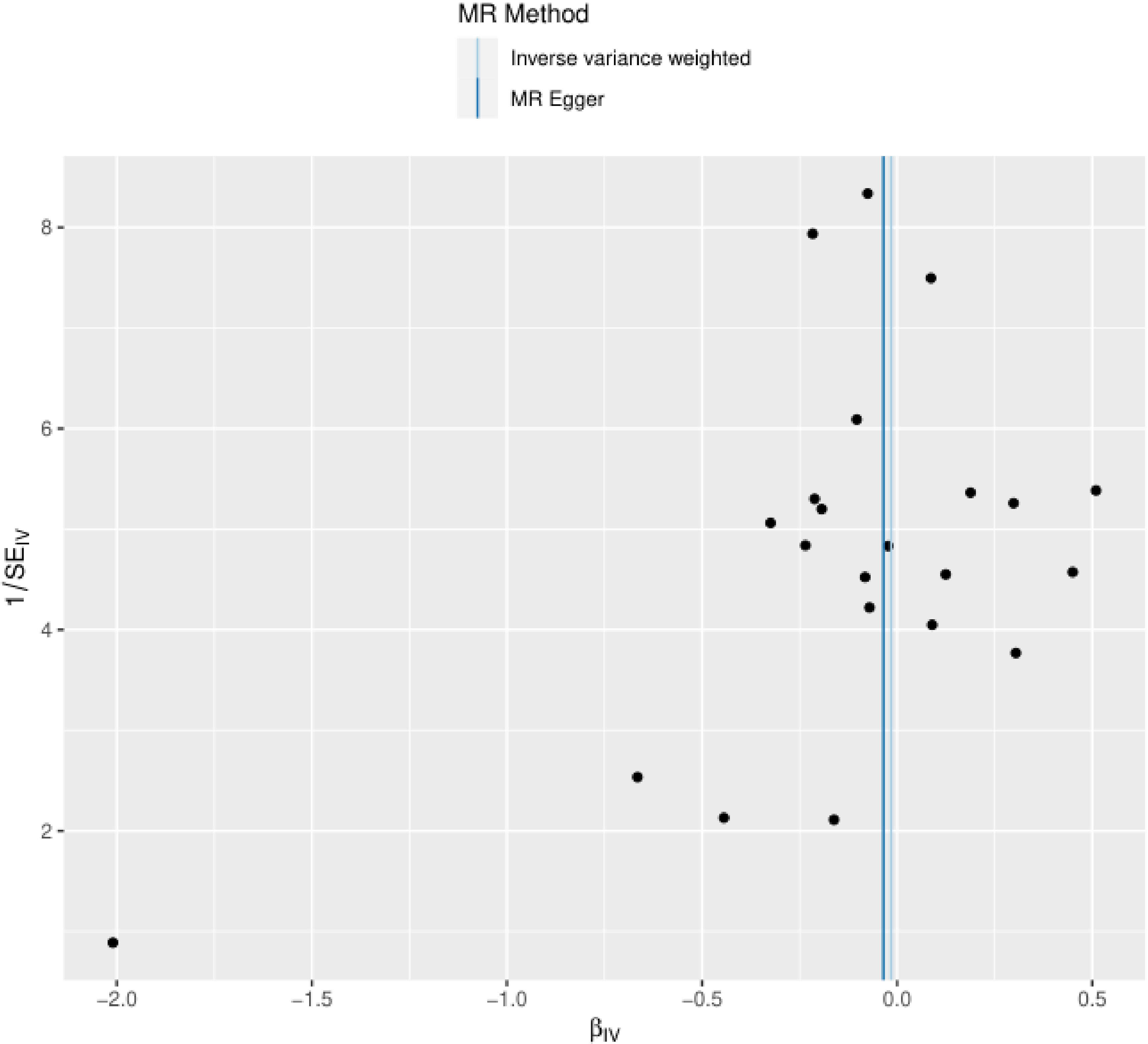

Funnel plot analyse of "Unsw Mem %lymphocyte" on 'Diabetic nephropathy'

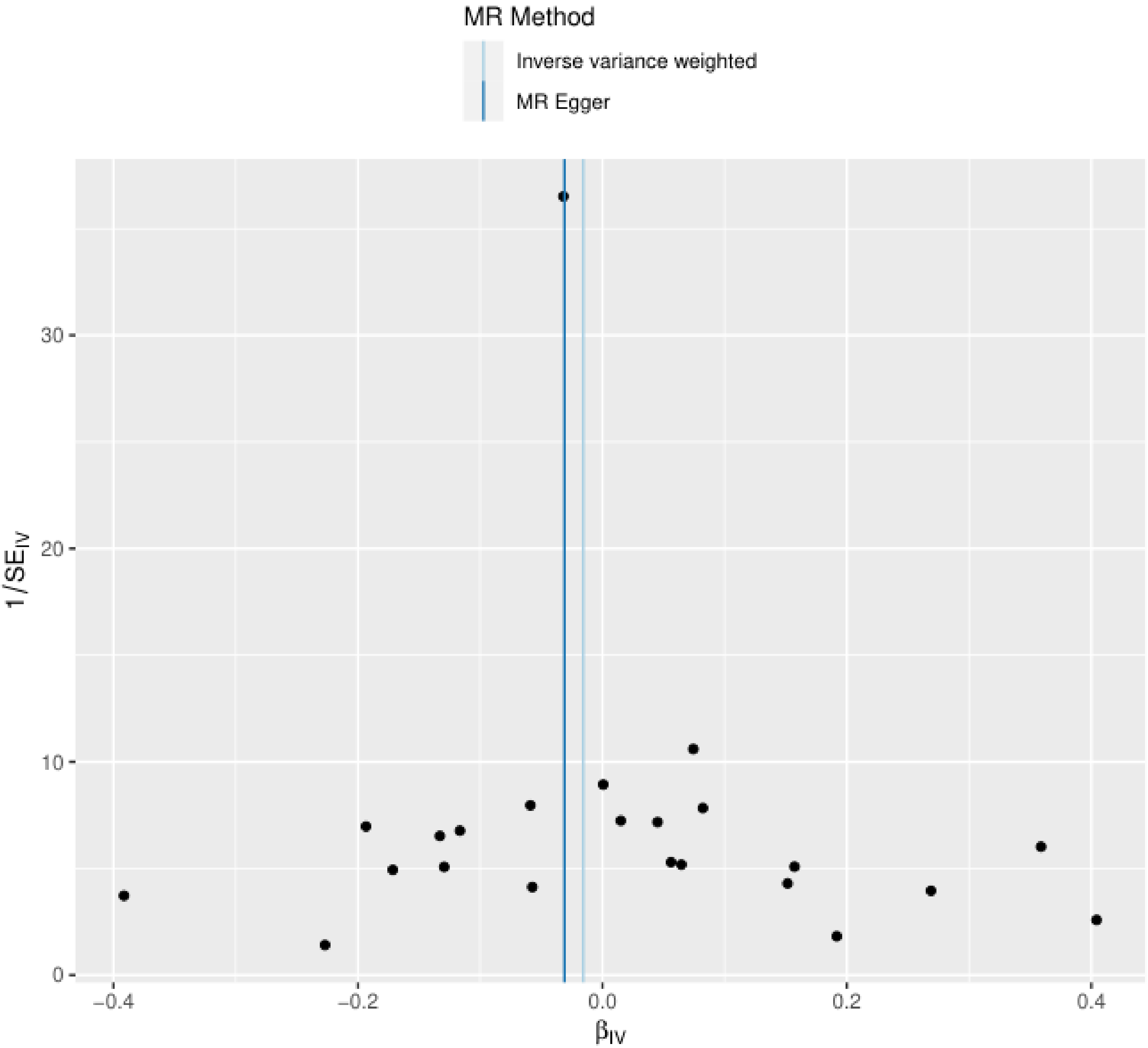

Funnel plot analyse of "CCR7 on naive CD4+" on 'Diabetic nephropathy'

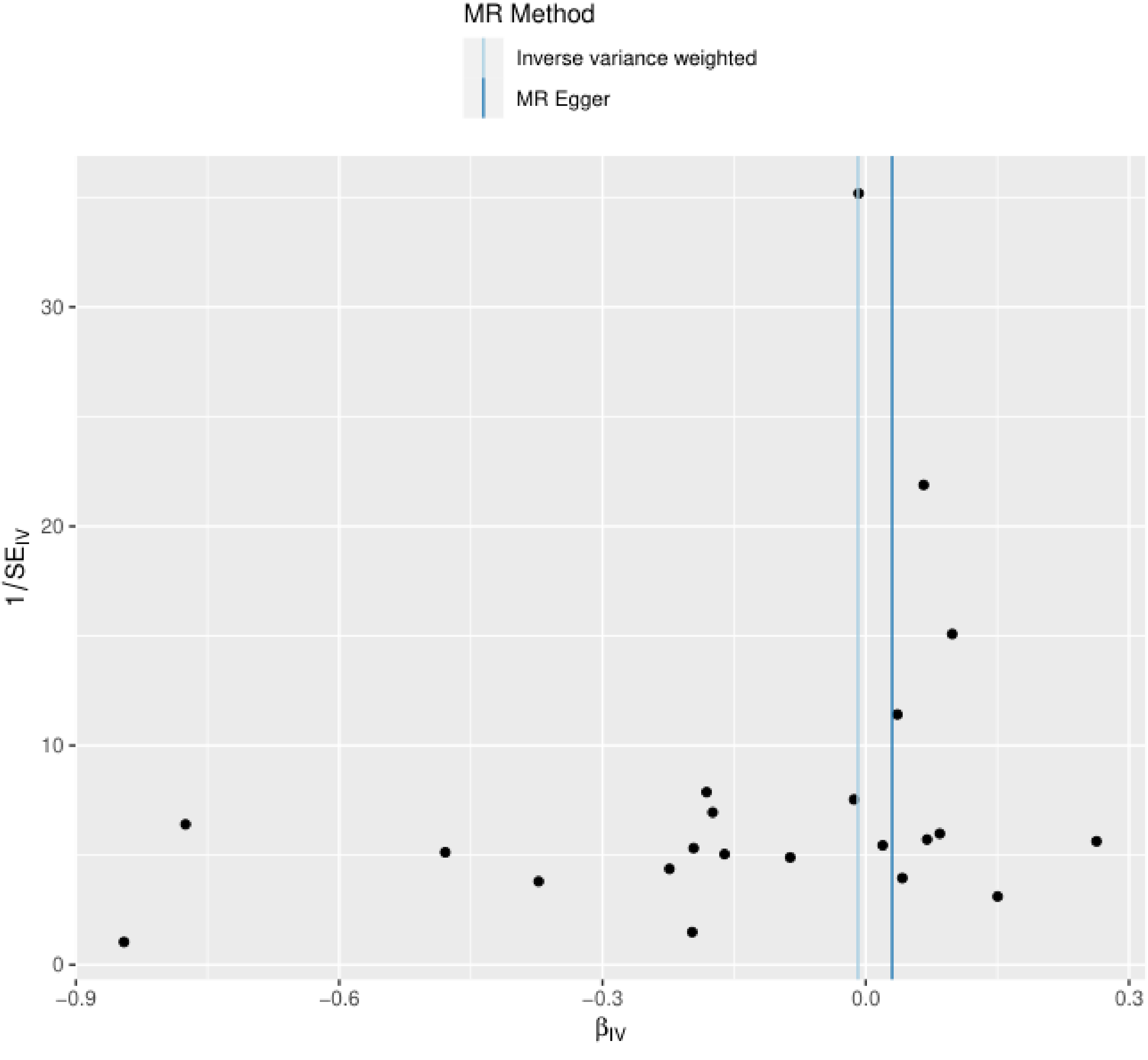

Funnel plot analyse of "CD4 on CD39+ secreting Treg " on 'Diabetic nephropathy'

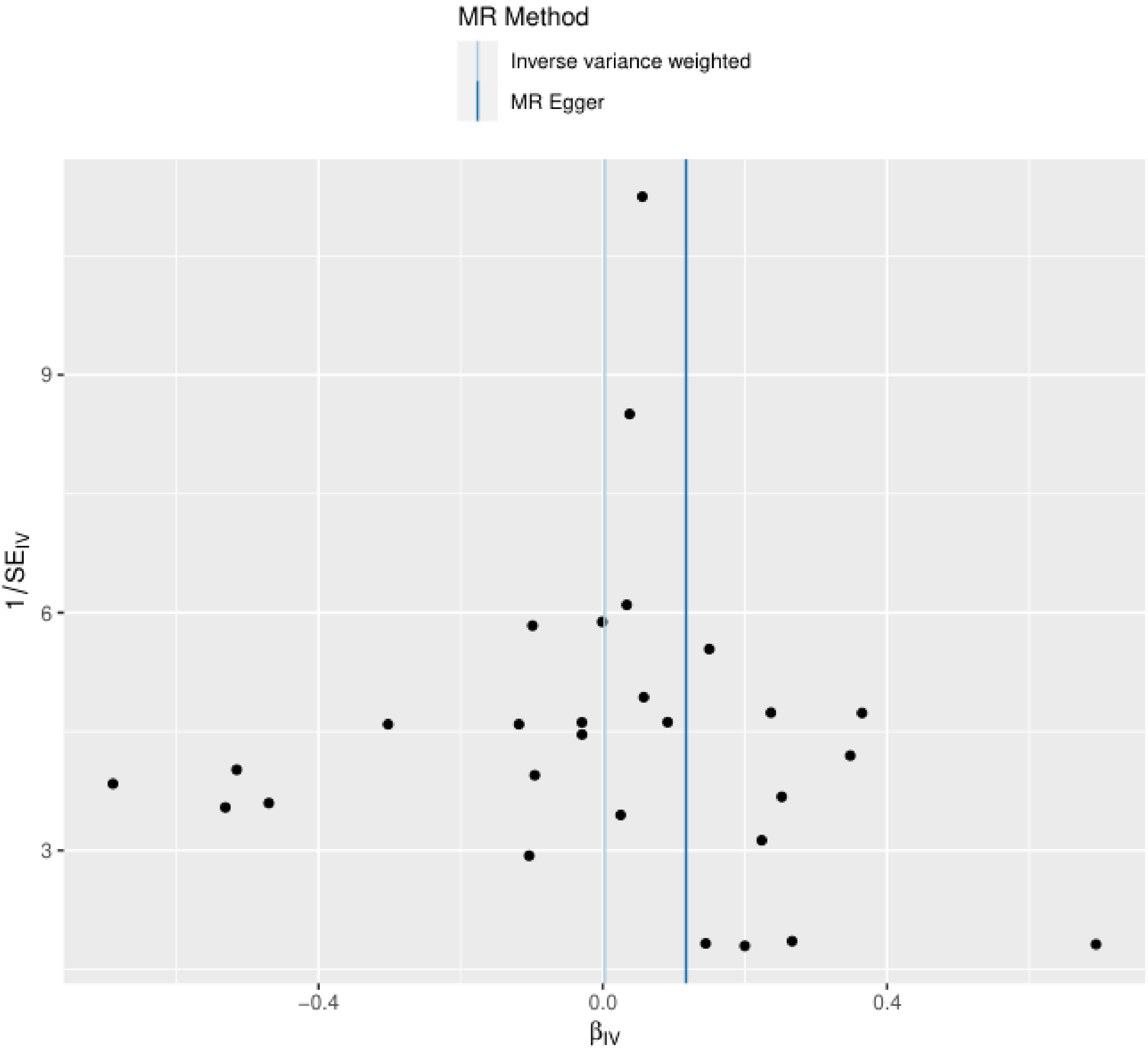

Funnel plot analyse of "CD8br NKT %T cell" on 'Diabetic nephropathy'

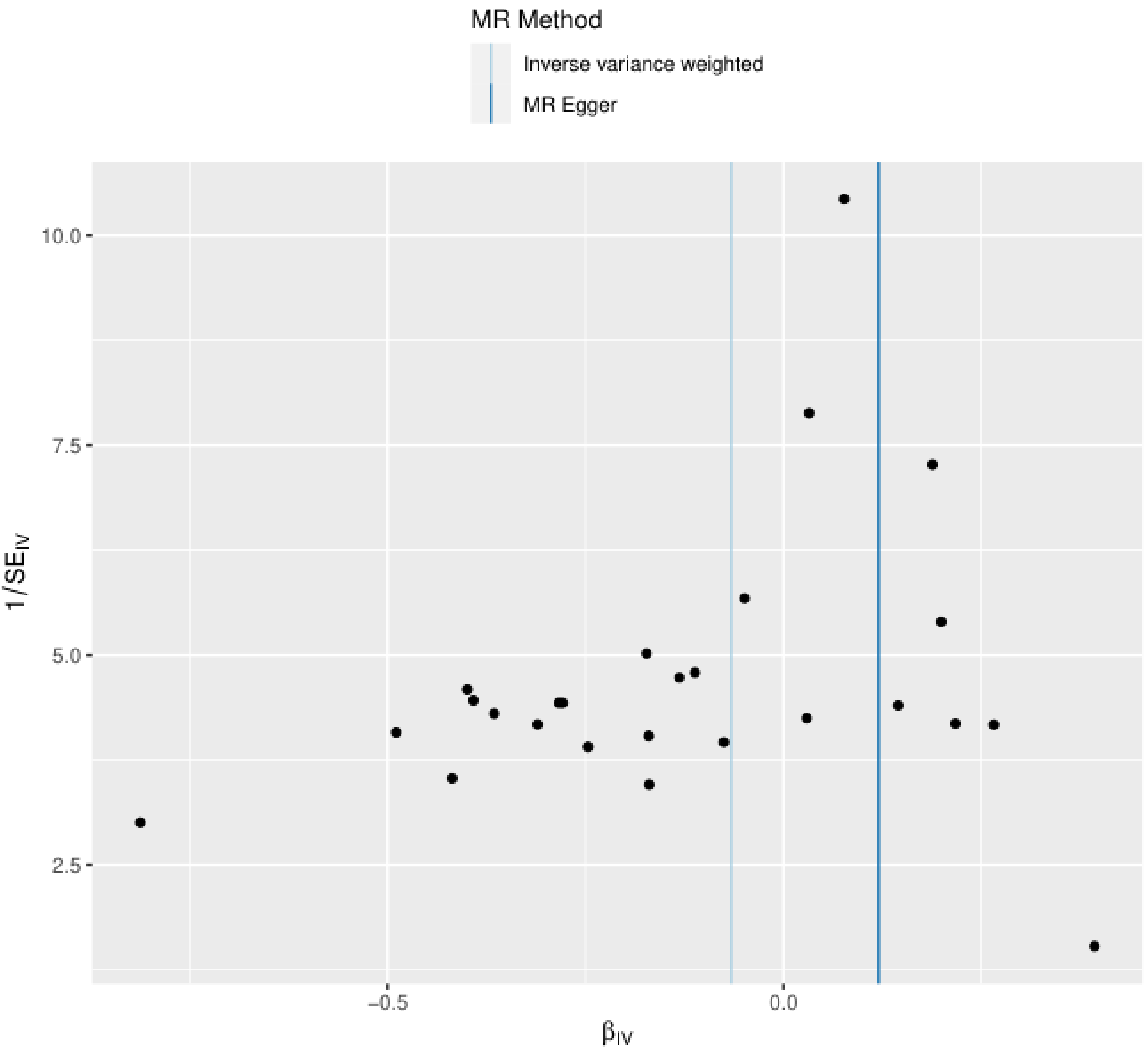

Funnel plot analyse of "CD25 on IgD- CD24-" on 'Diabetic nephropathy'

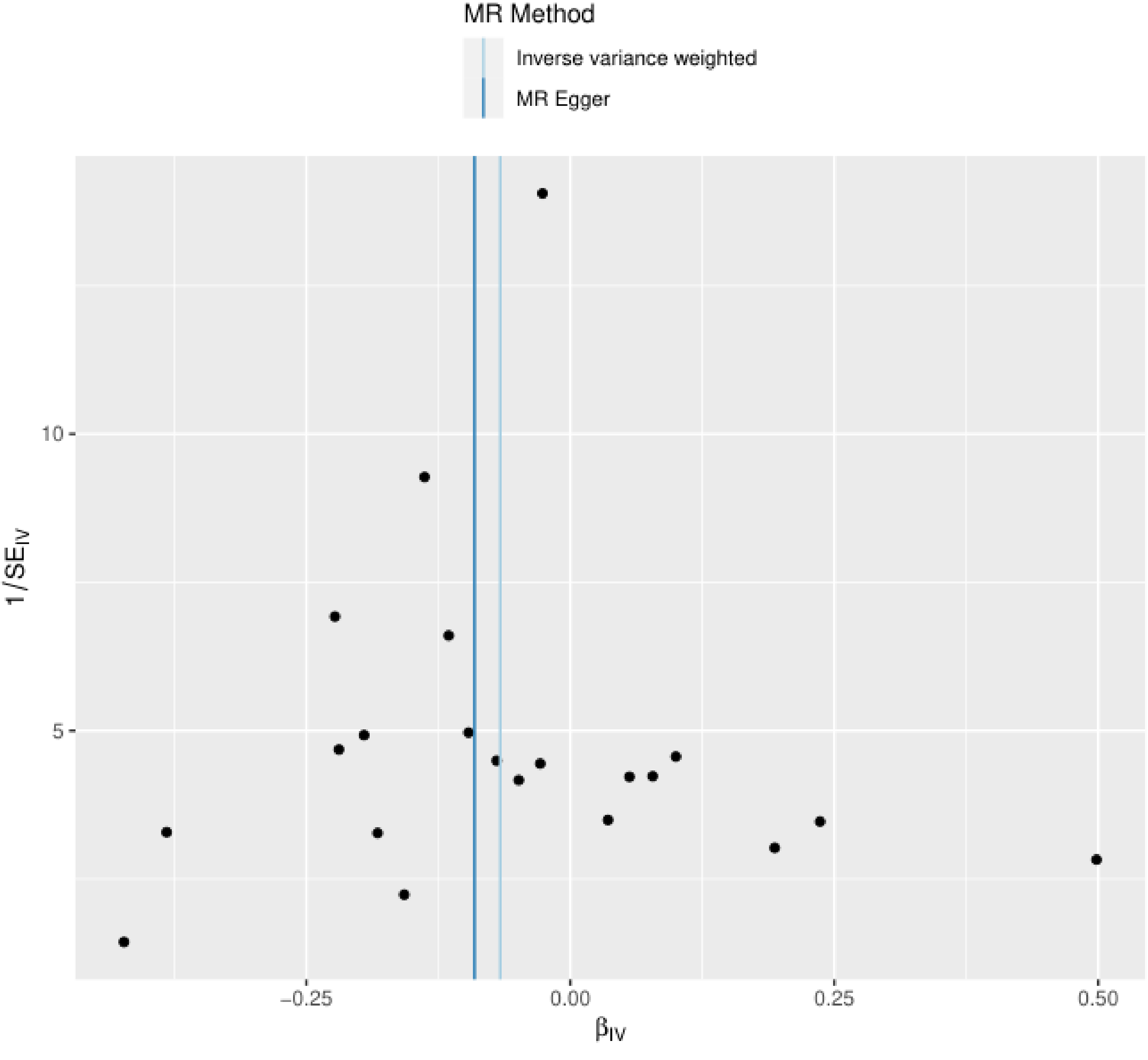

Funnel plot analyse of "DC AC" on 'Diabetic nephropathy'

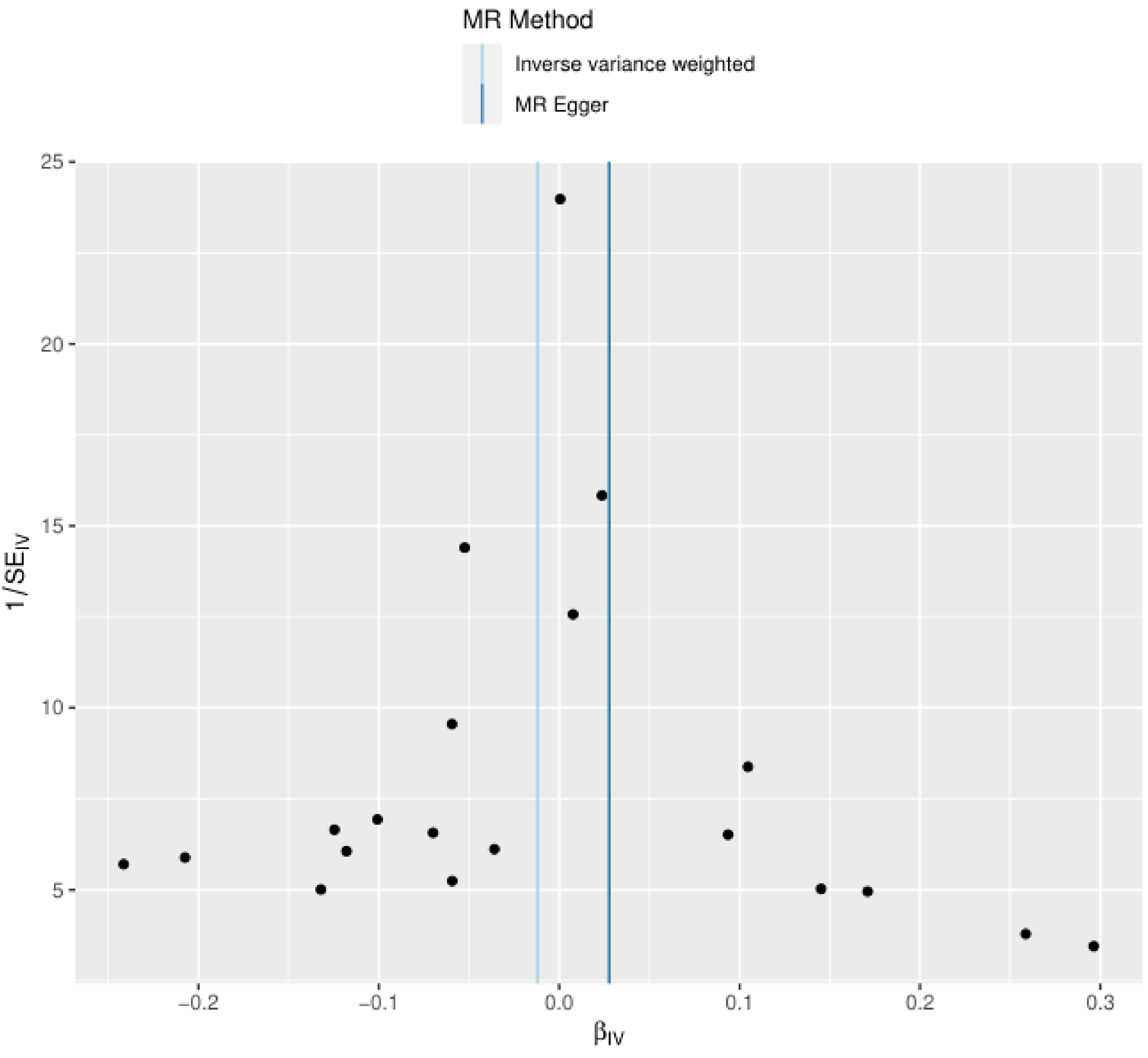

Funnel plot analyse of "Im MDSC %CD33dim HLA DR- CD66b-" on 'Diabetic nephropathy'

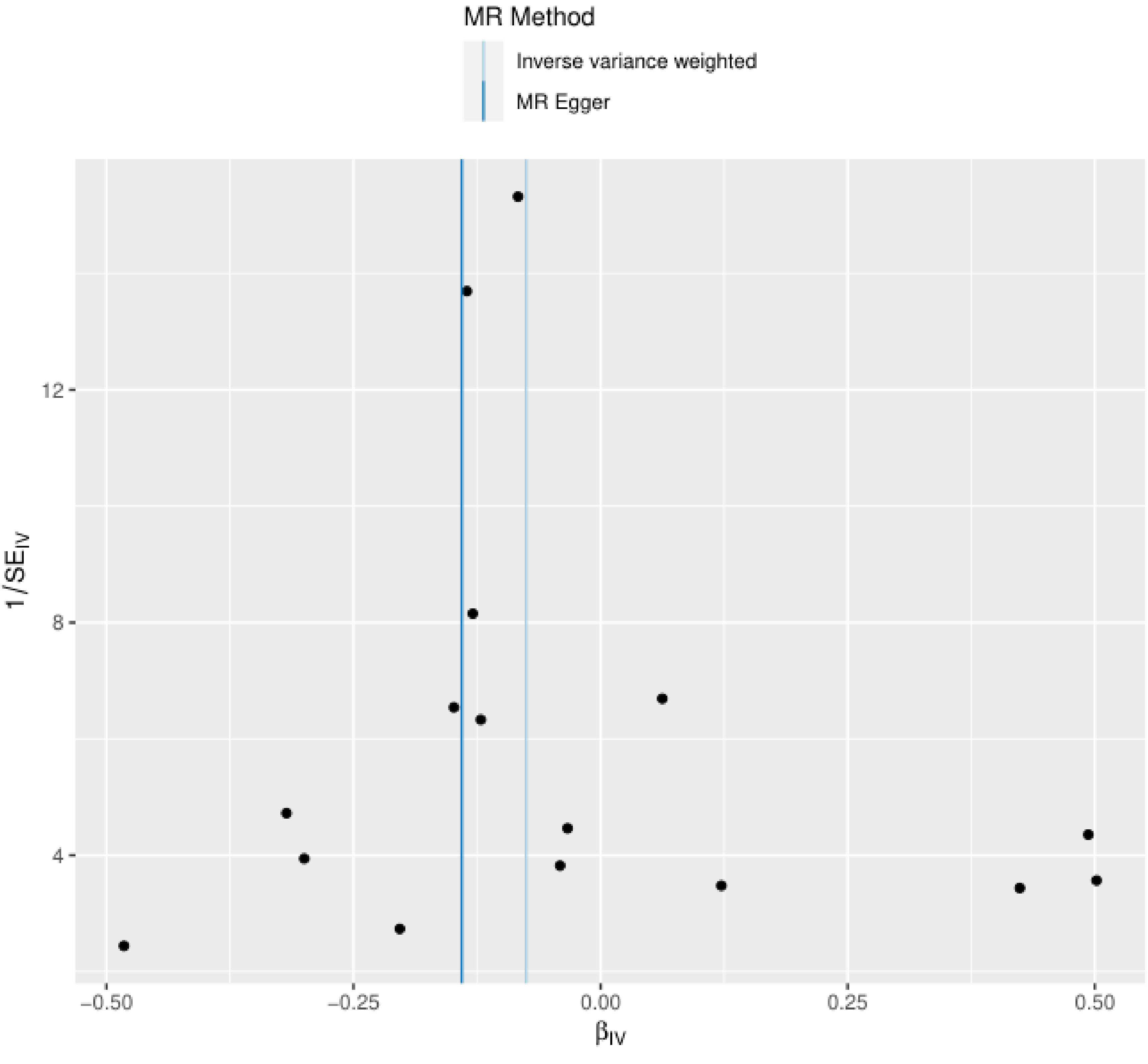

Funnel plot analyse of "CM DN (CD4-CD8-) %DN" on 'Diabetic nephropathy'

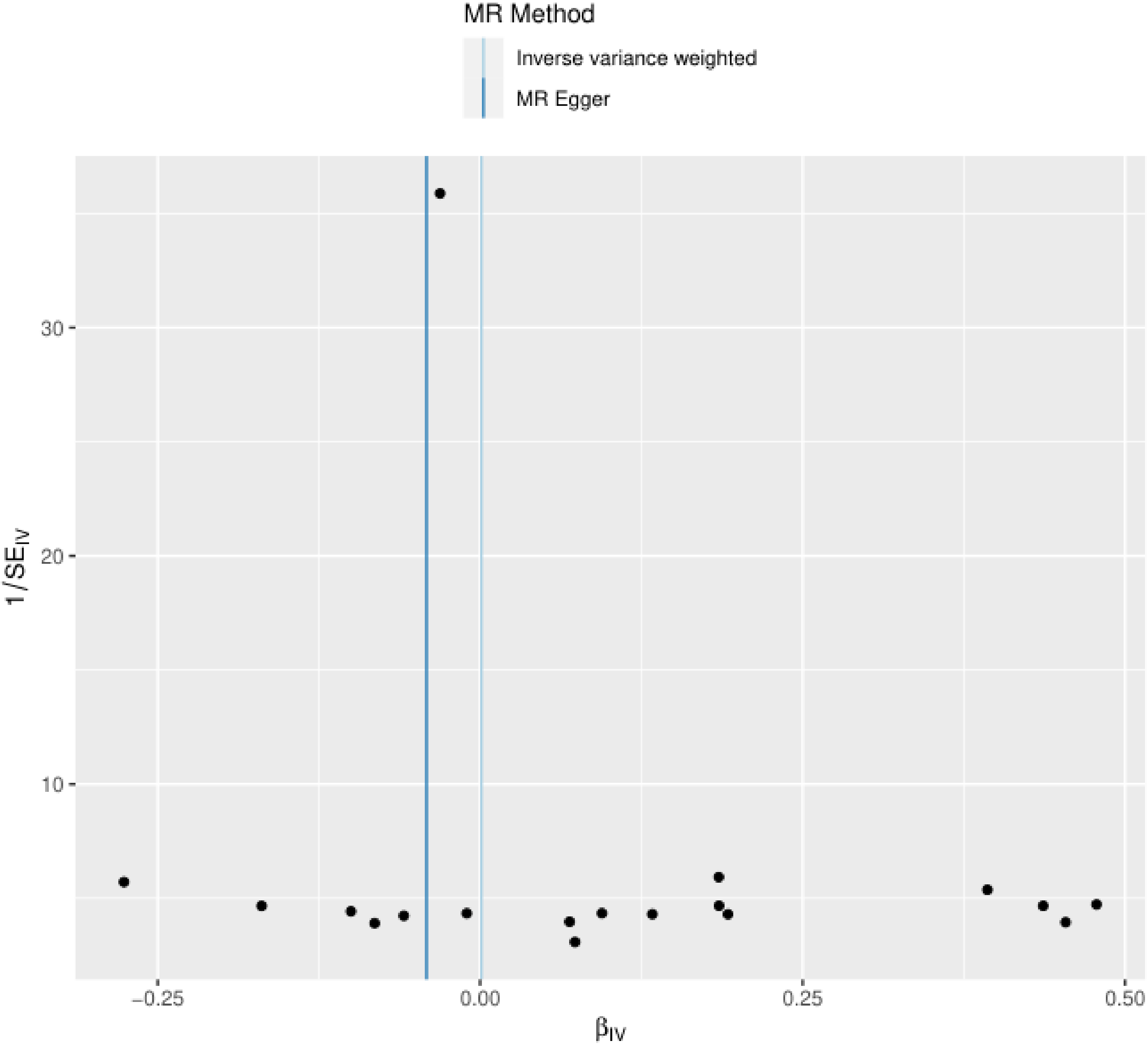

Funnel plot analyse of "TCRgd %T cell" on 'Diabetic nephropathy'

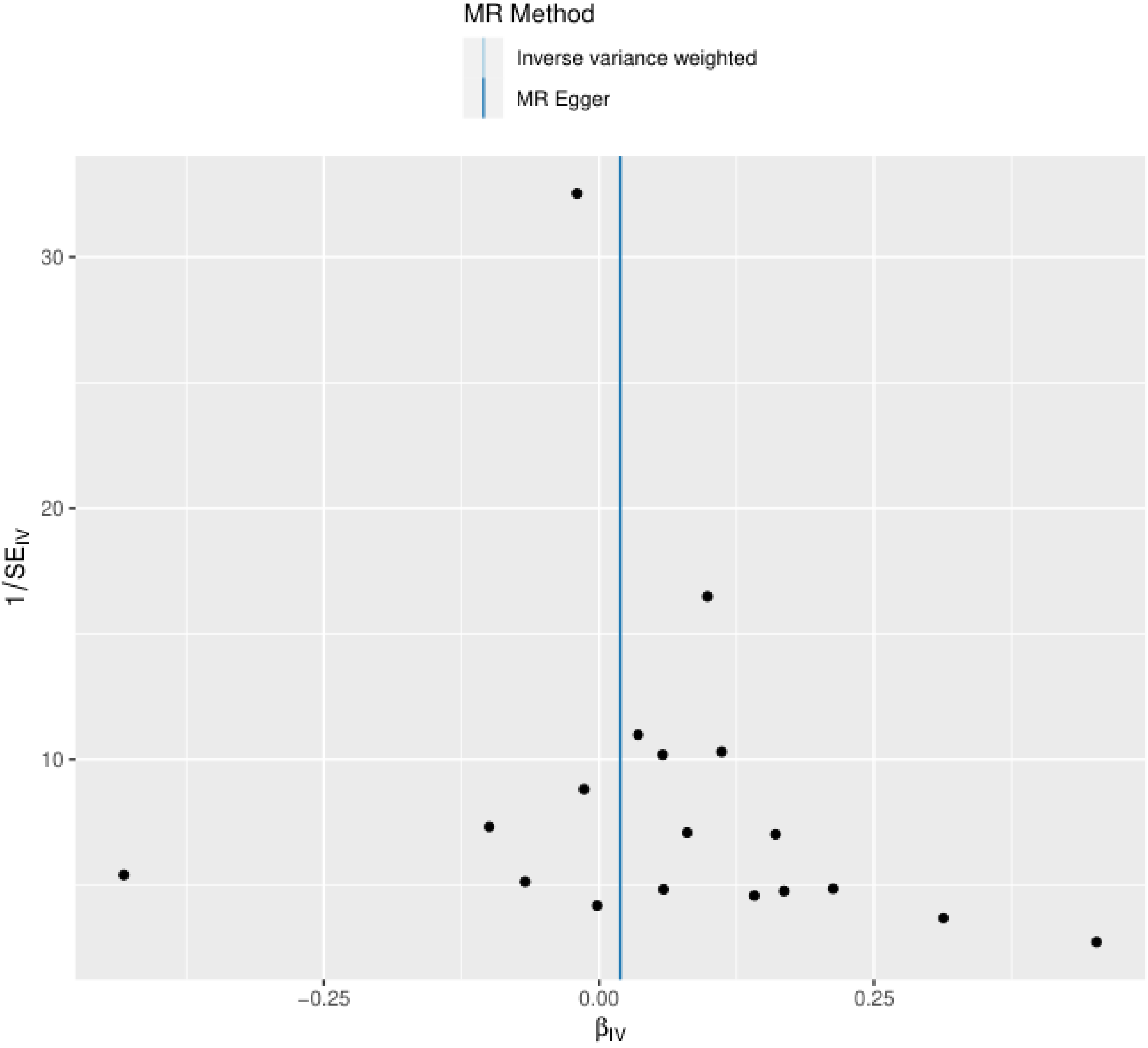

Funnel plot analyse of "BAFF-R on IgD+ CD24+" on 'Diabetic nephropathy'

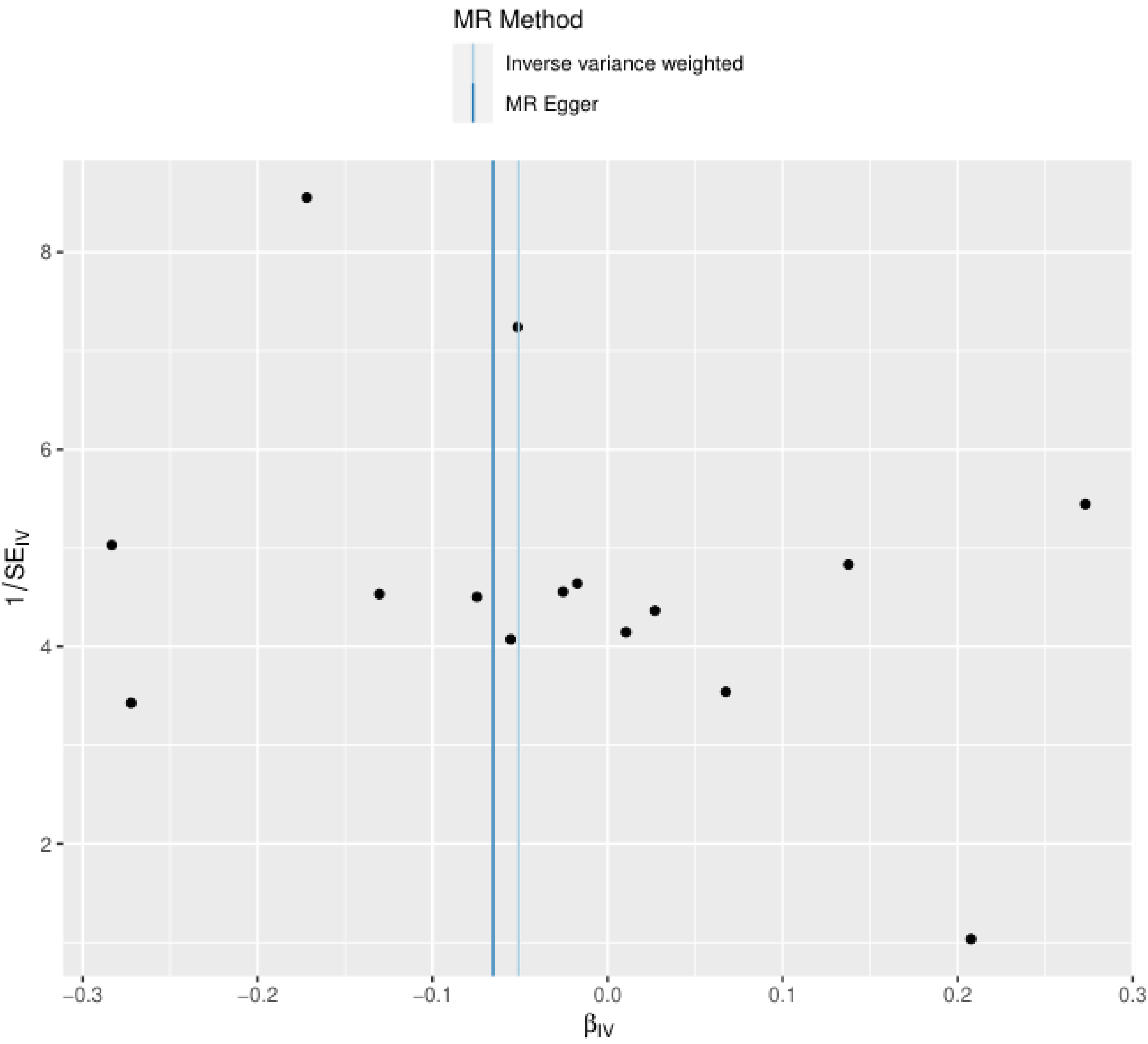

Funnel plot analyse of "CD25 on CD39+ resting Treg " on 'Diabetic nephropathy'

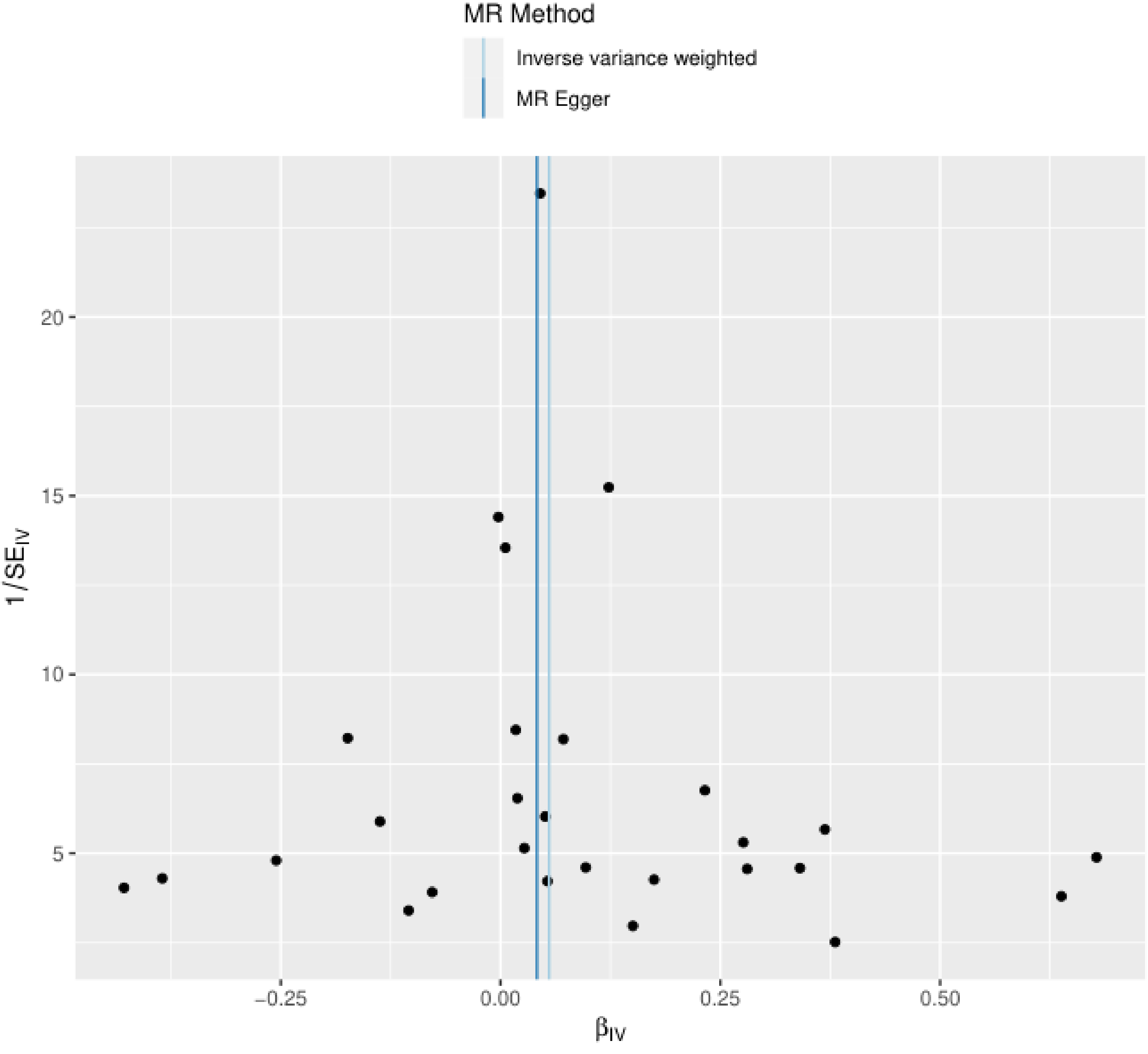

Funnel plot analyse of "CD4 on CD4 Treg" on 'Diabetic nephropathy'

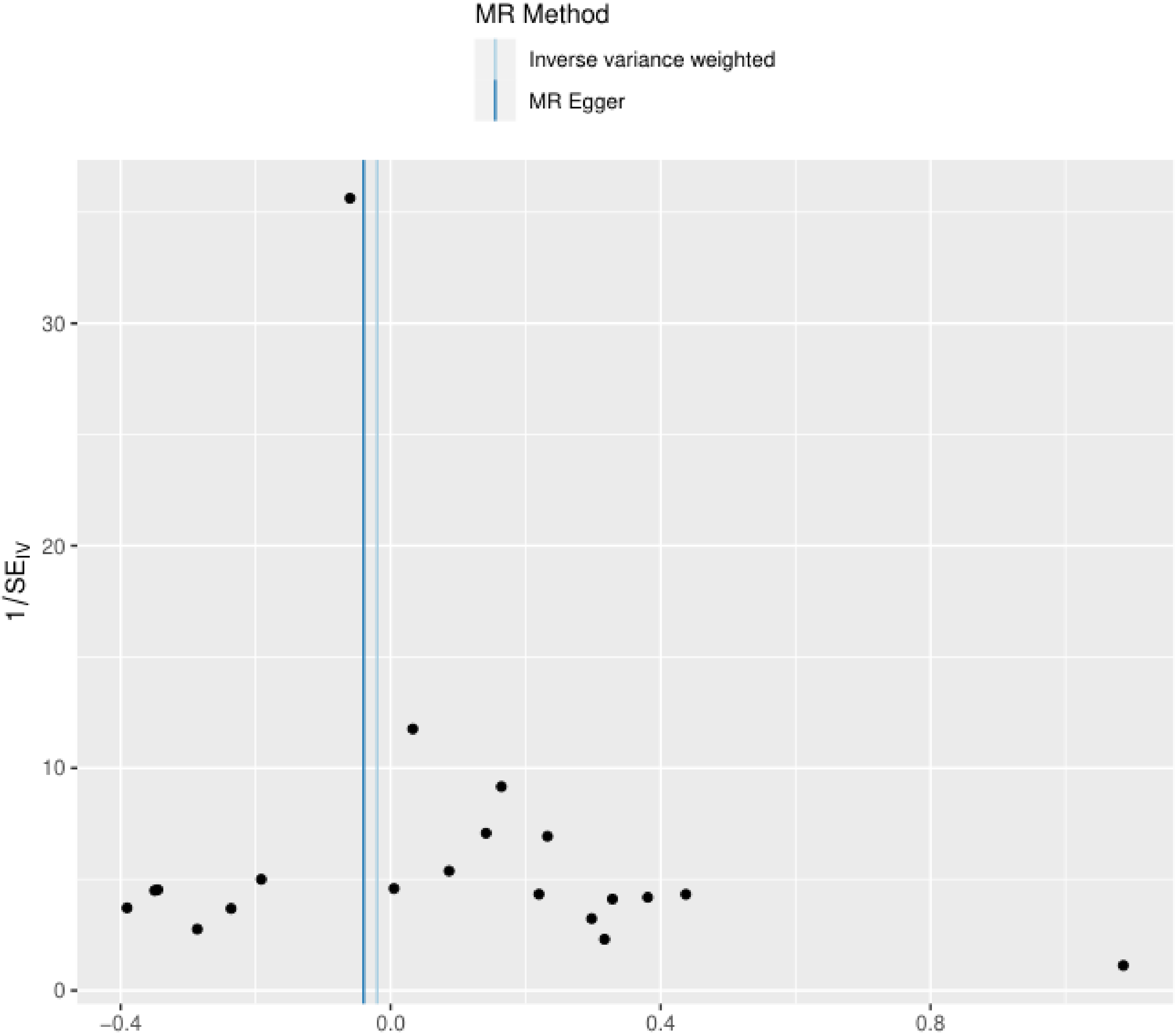

Funnel plot analyse of "DN (CD4-CD8-) %leukocyte" on 'Diabetic nephropathy'

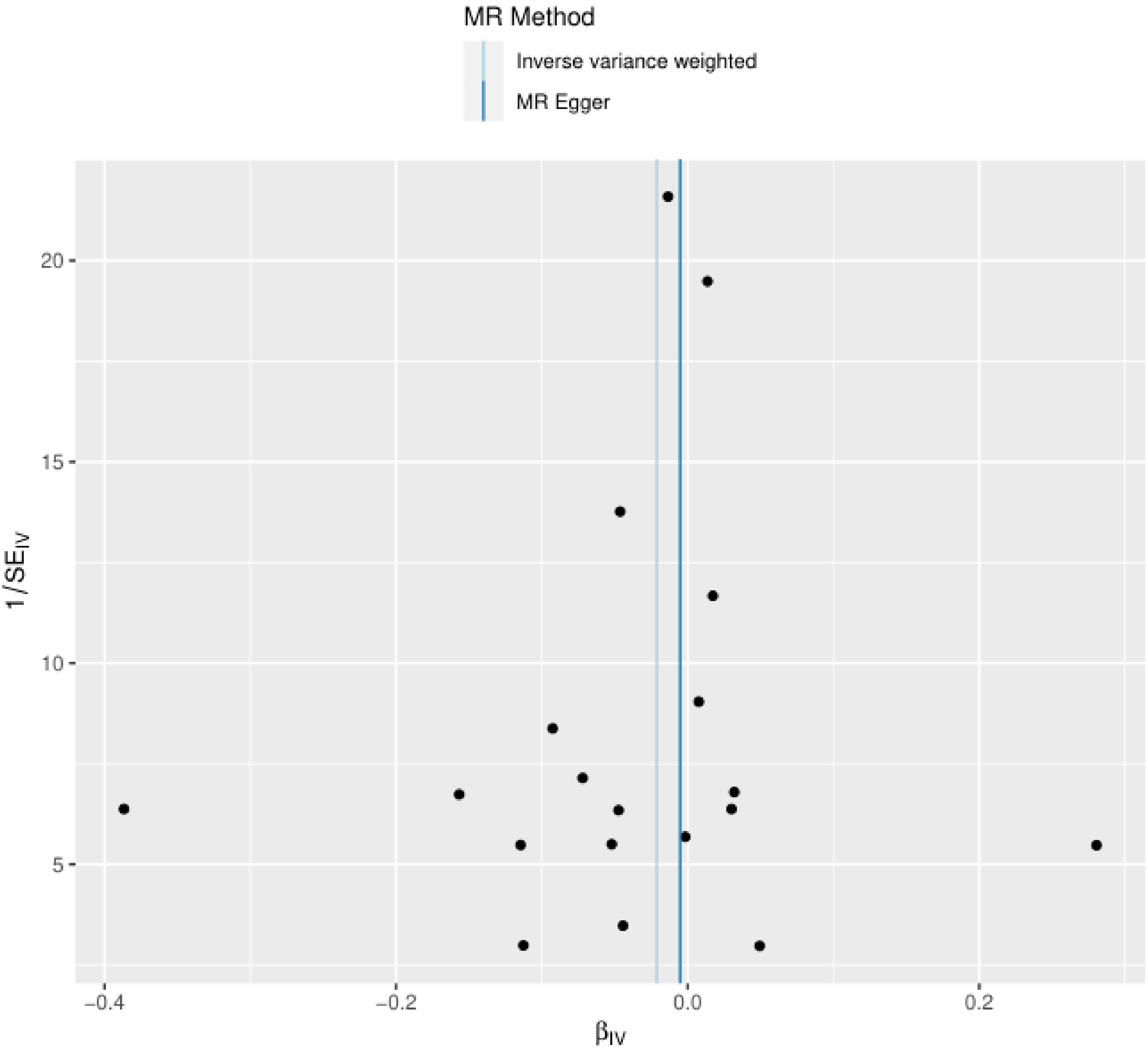

unnel plot analyse of "CD33br HLA DR+ CD14- %CD33br HLA DR+" on 'Diabetic nephropath

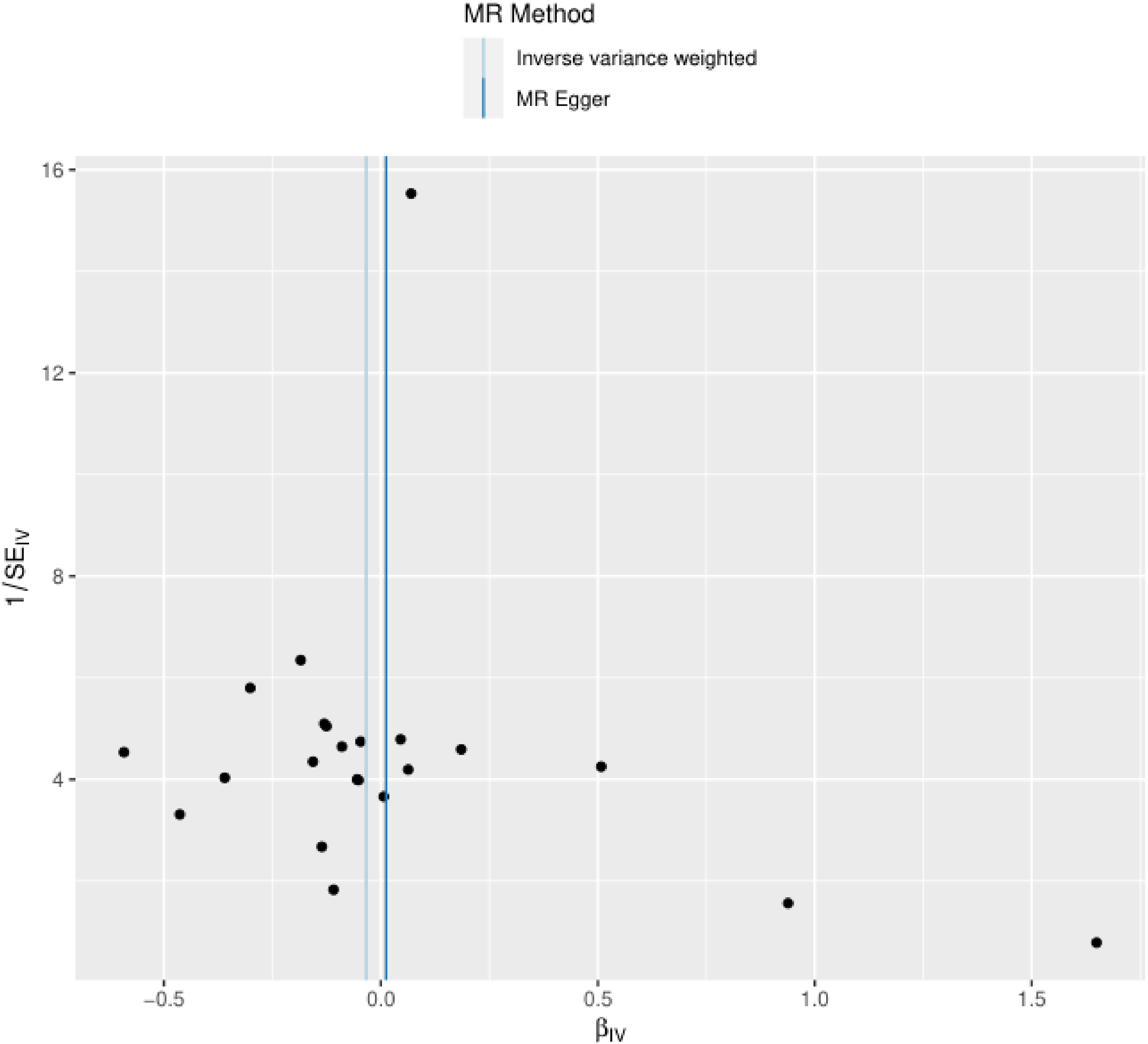

Funnel plot analyse of "FSC-A on NKT " on 'Diabetic nephropathy'

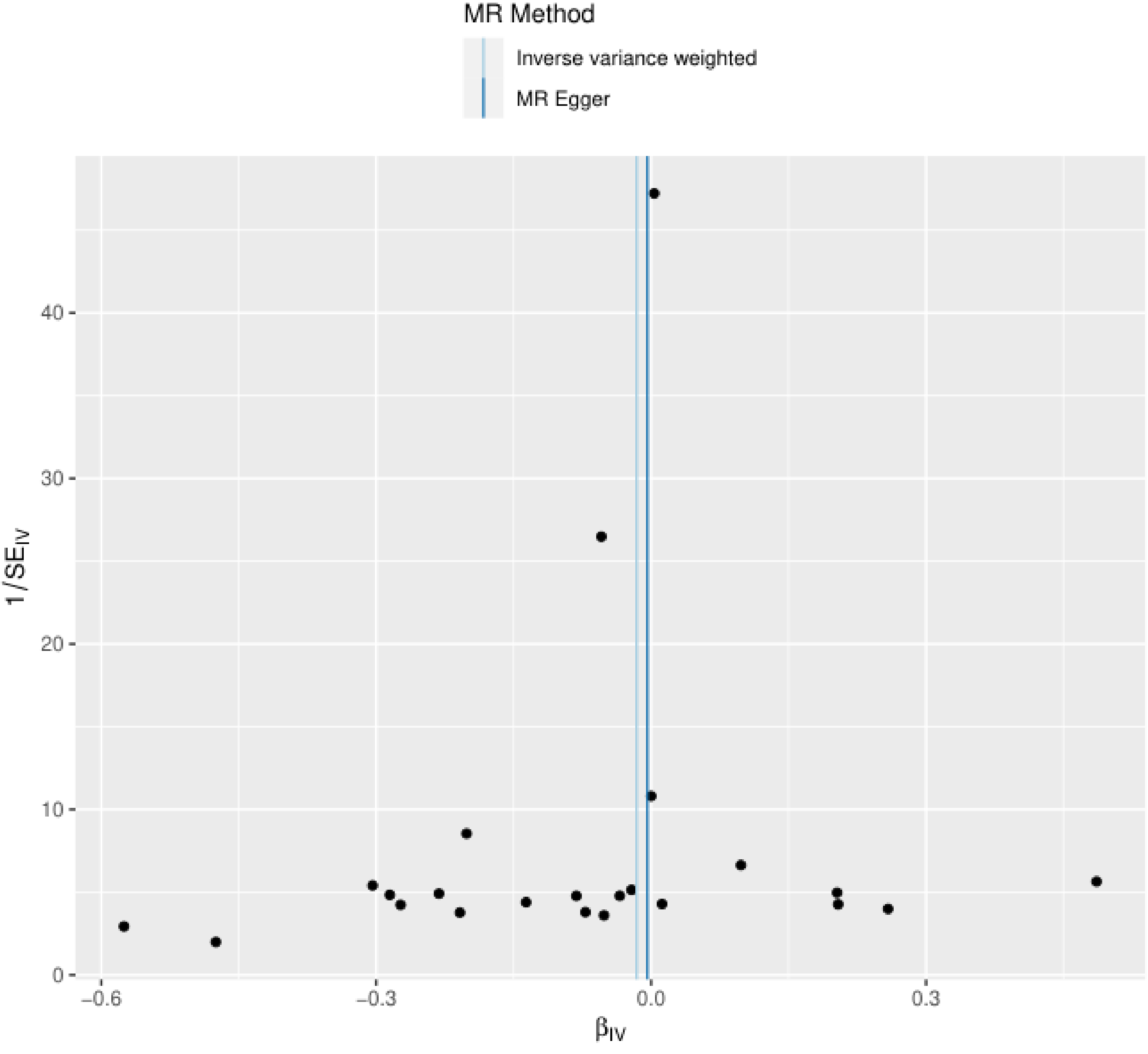

Funnel plot analyse of "CD24+ CD27+ %lymphocyte" on 'Diabetic nephropathy'

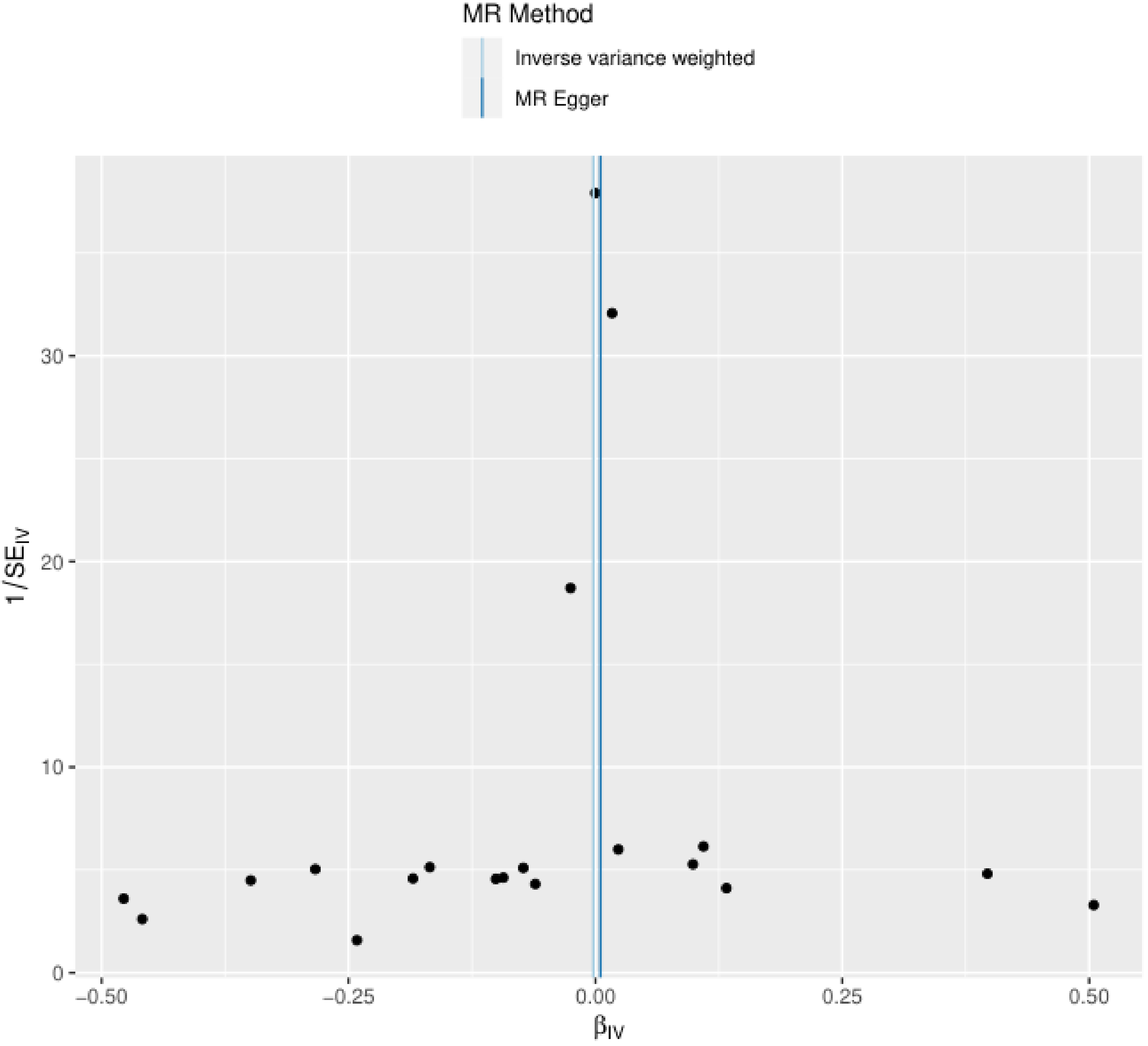

Funnel plot analyse of "SSC-A on NKT " on 'Diabetic nephropathy'

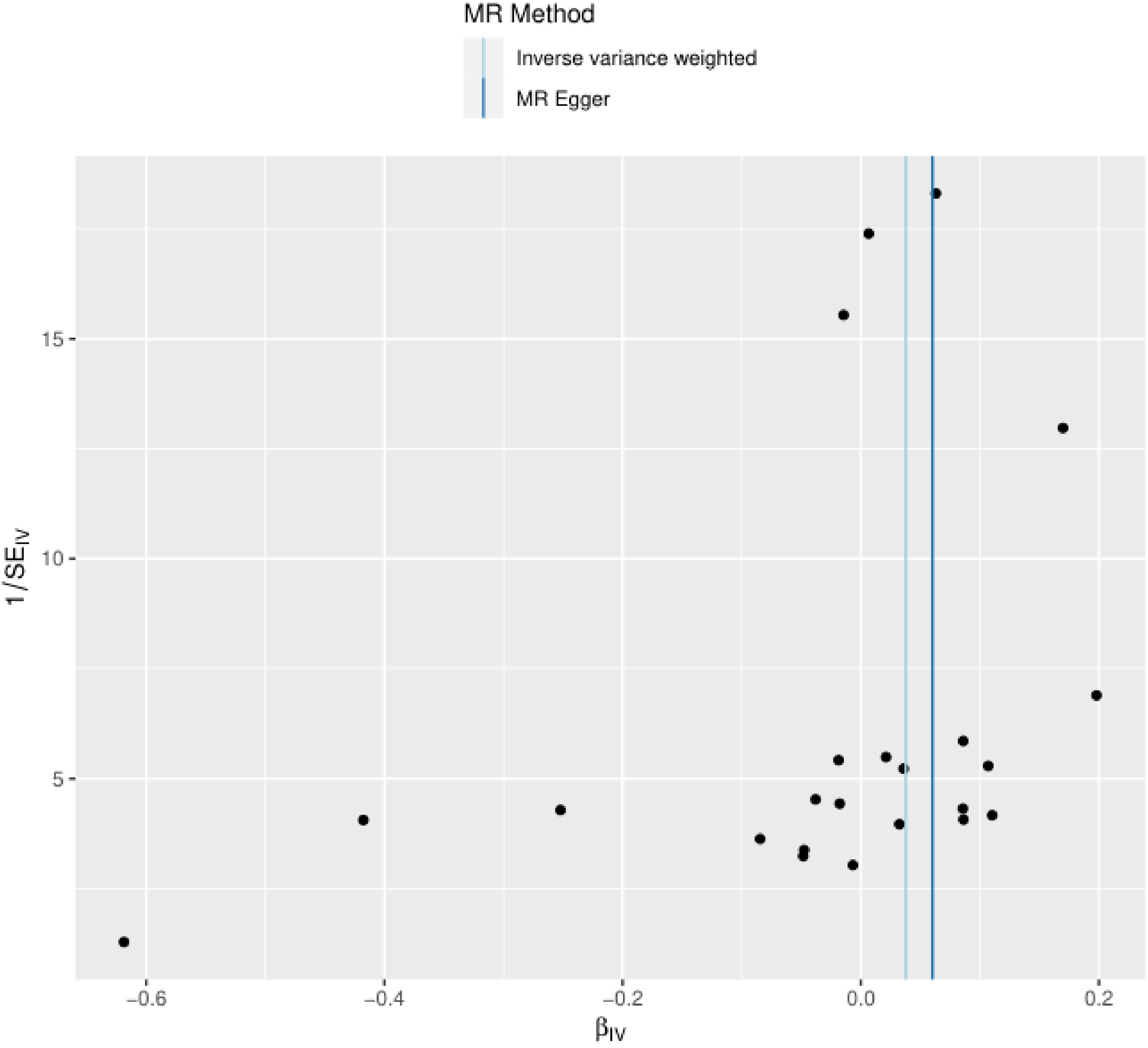

Funnel plot analyse of "CD11c+ CD62L- monocyte AC" on 'Diabetic nephropathy'

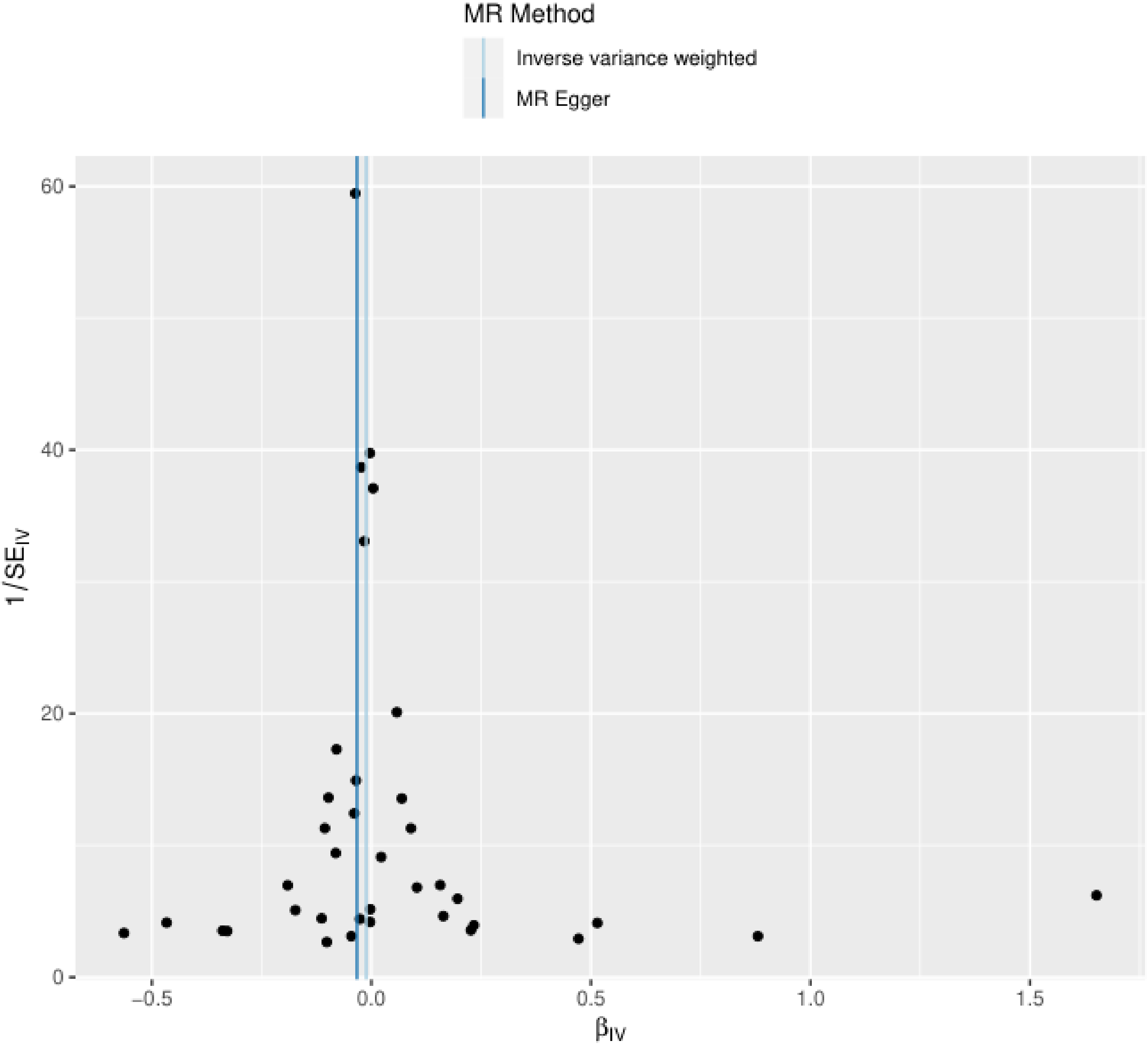

Funnel plot analyse of "Activated & secreting Treg %CD4 Treg" on 'Diabetic nephropathy'

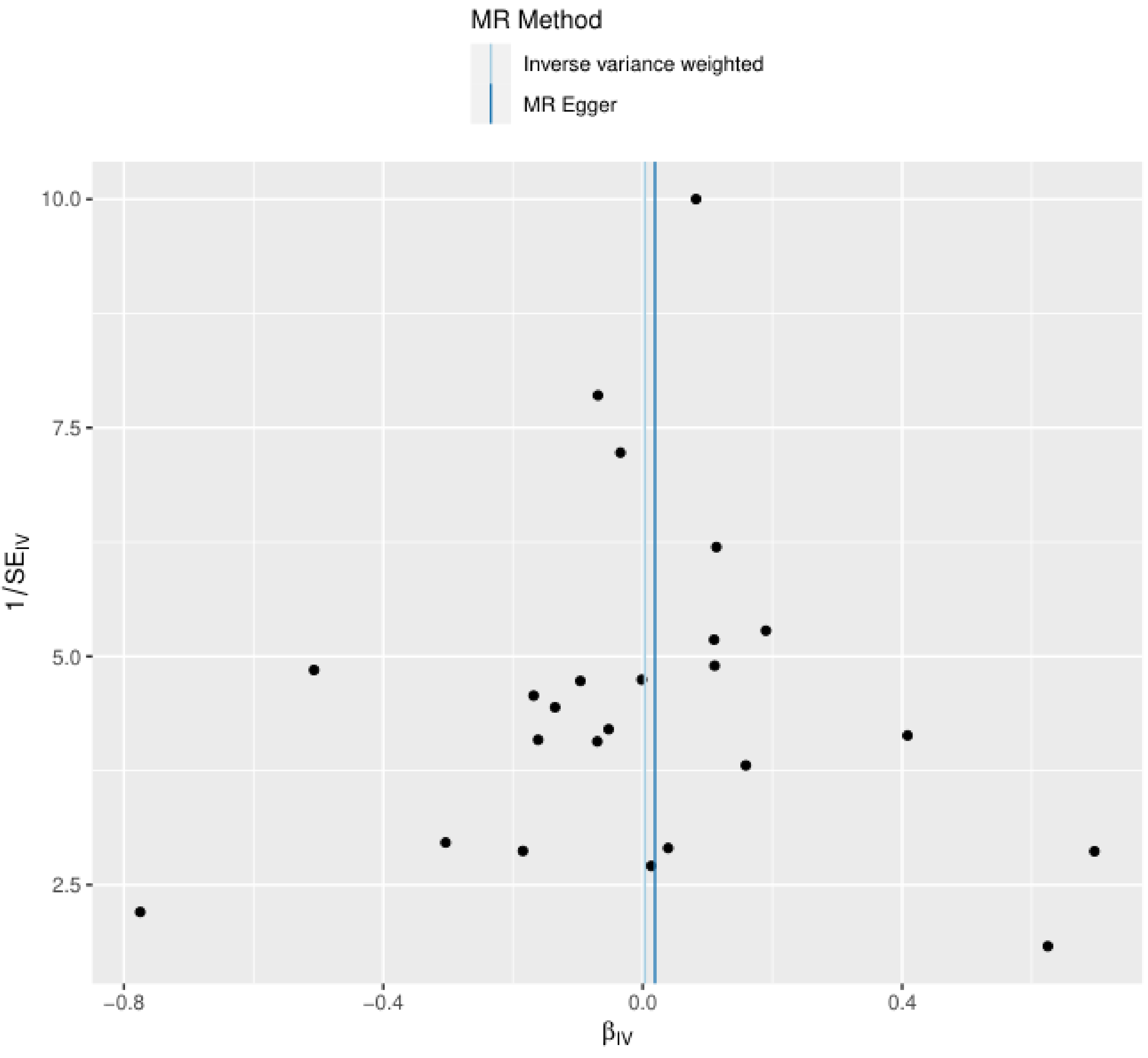

Funnel plot analyse of "CD16+ monocyte %monocyte" on 'Diabetic nephropathy'

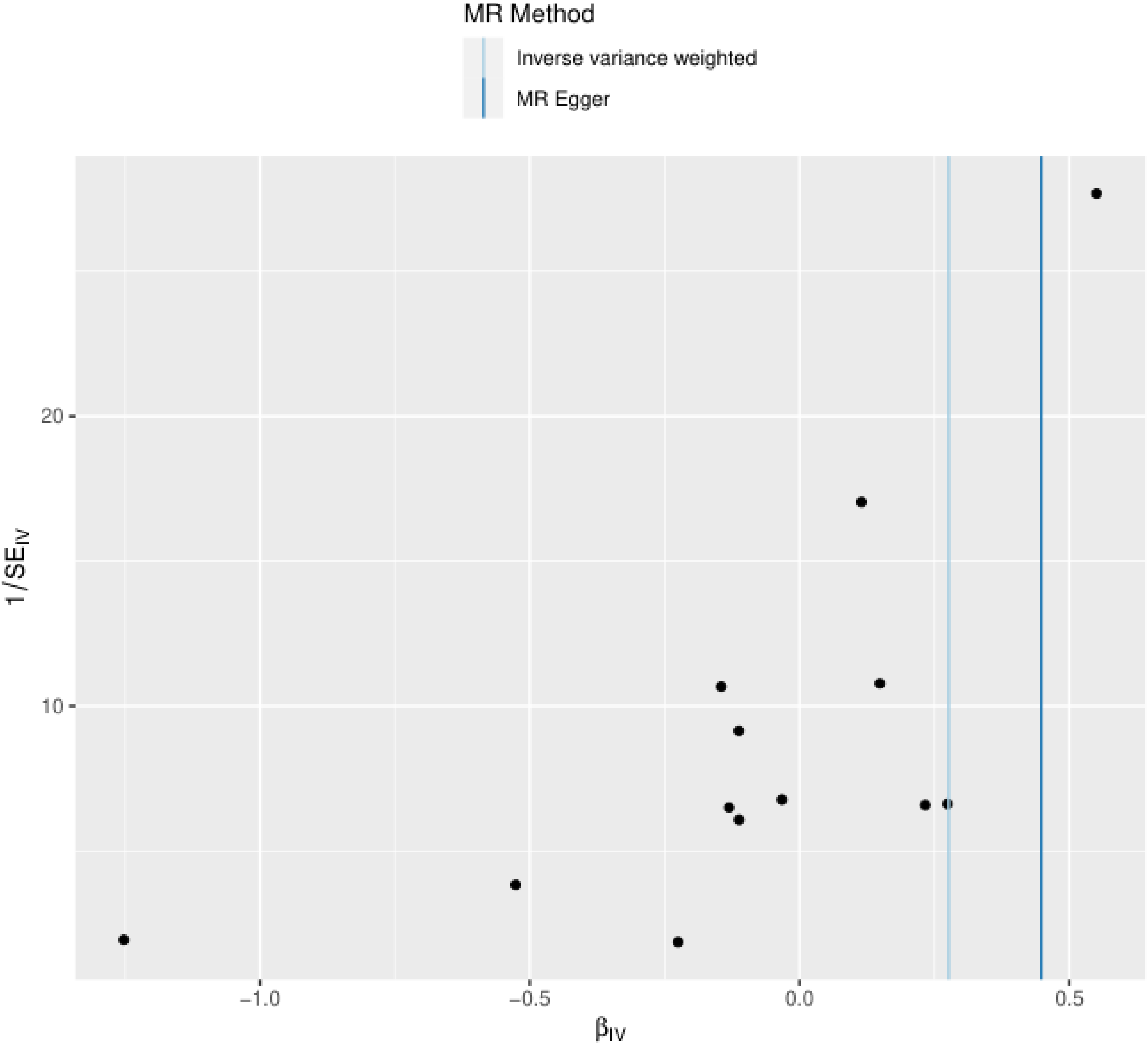

Funnel plot analyse of "HLA DR on CD33- HLA DR+ " on 'Diabetic nephropathy'

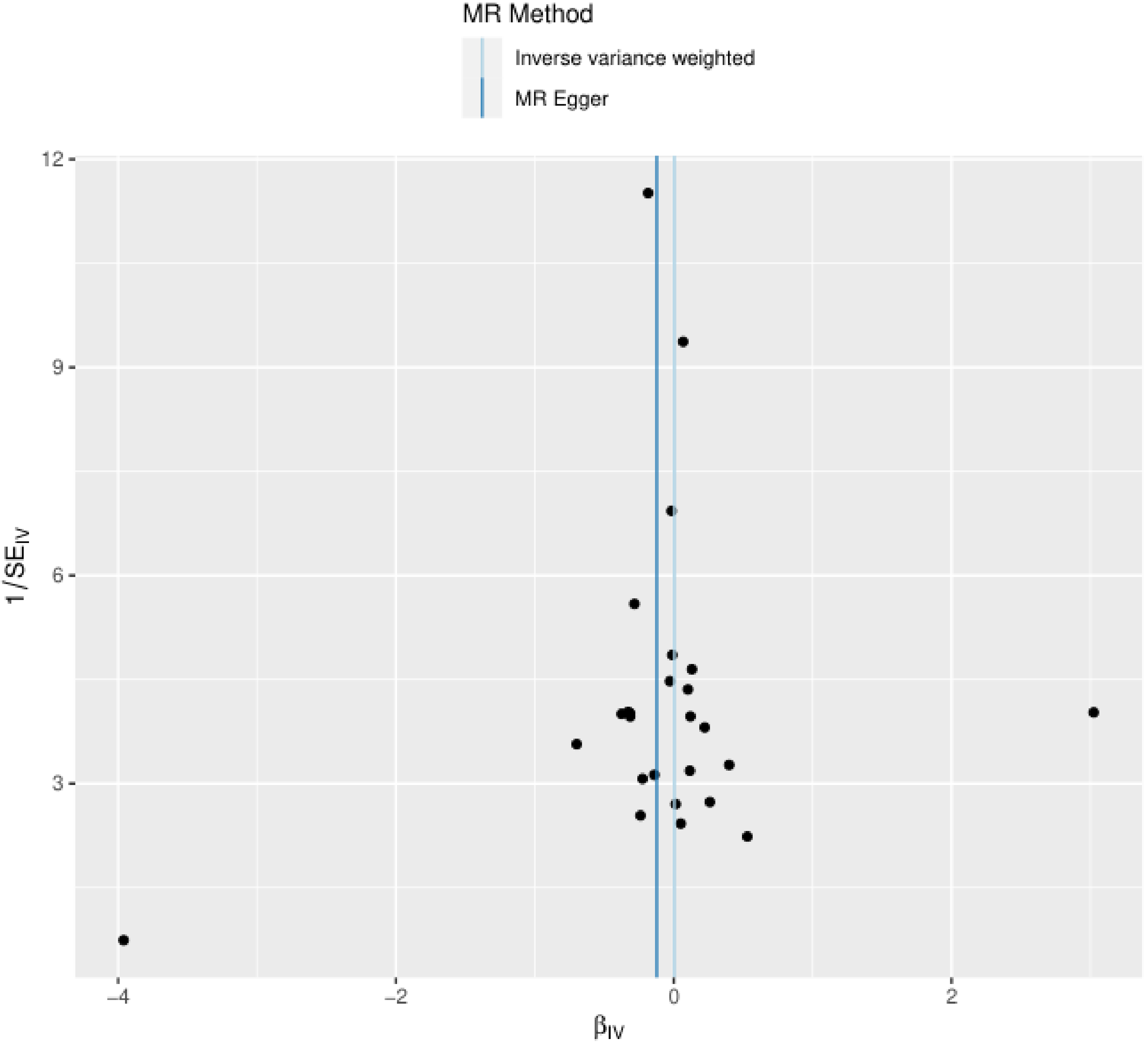

Funnel plot analyse of "HLA DR+ CD4+ %T cell" on 'Diabetic nephropathy'

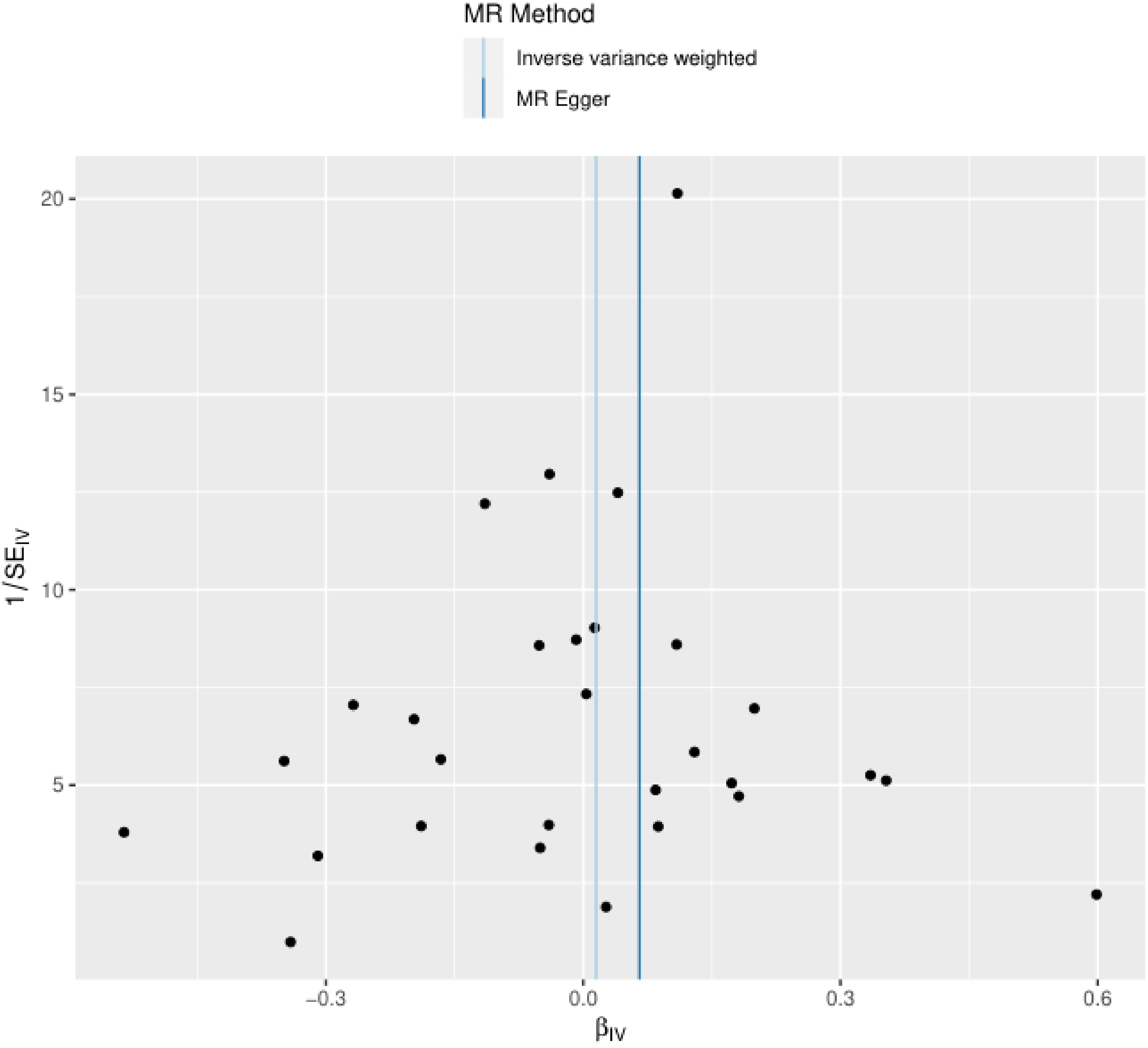

Funnel plot analyse of "CD16-CD56 on NK" on 'Diabetic nephropathy'

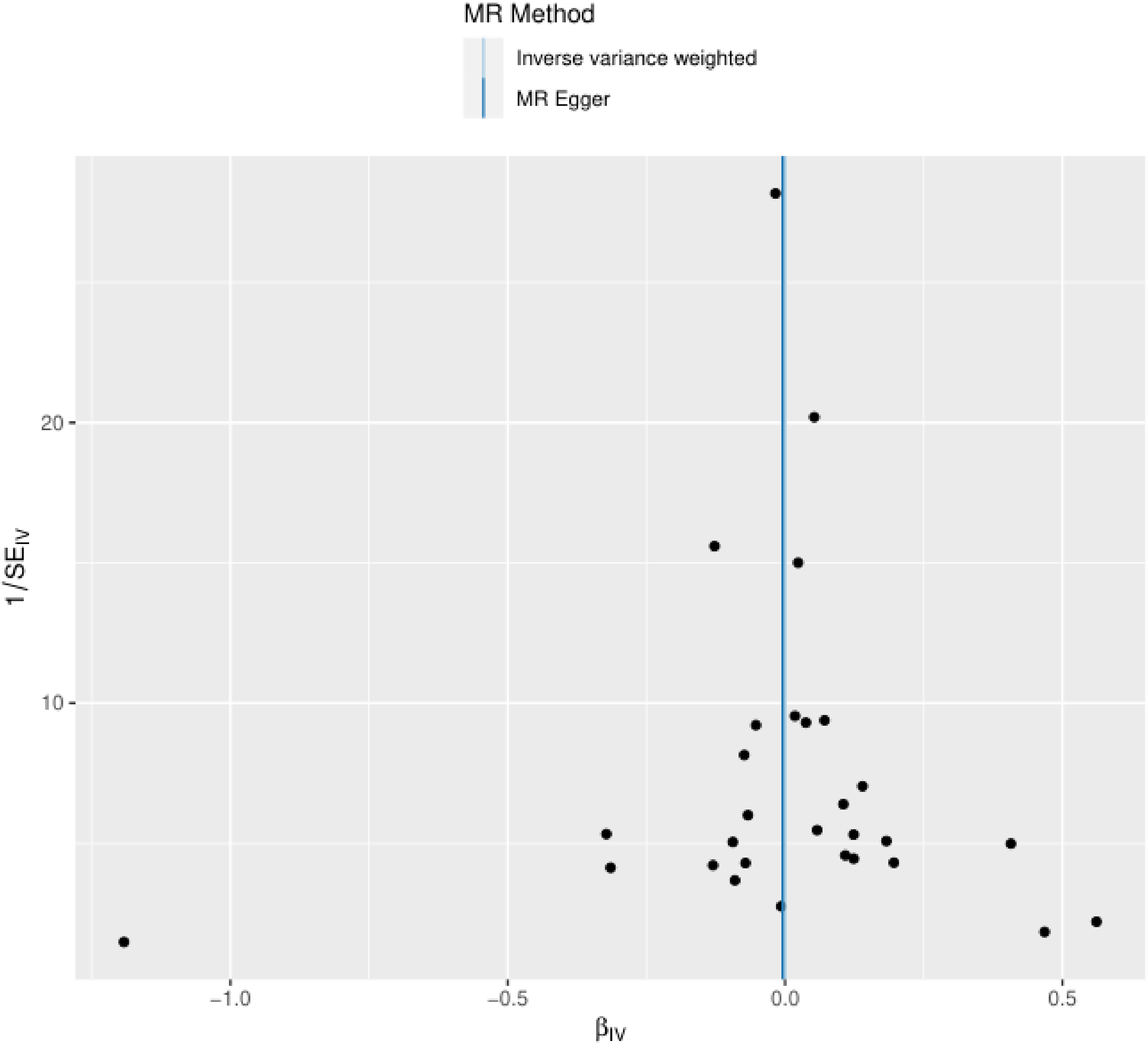

Funnel plot analyse of "CD39+ CD4+ AC" on 'Diabetic nephropathy'

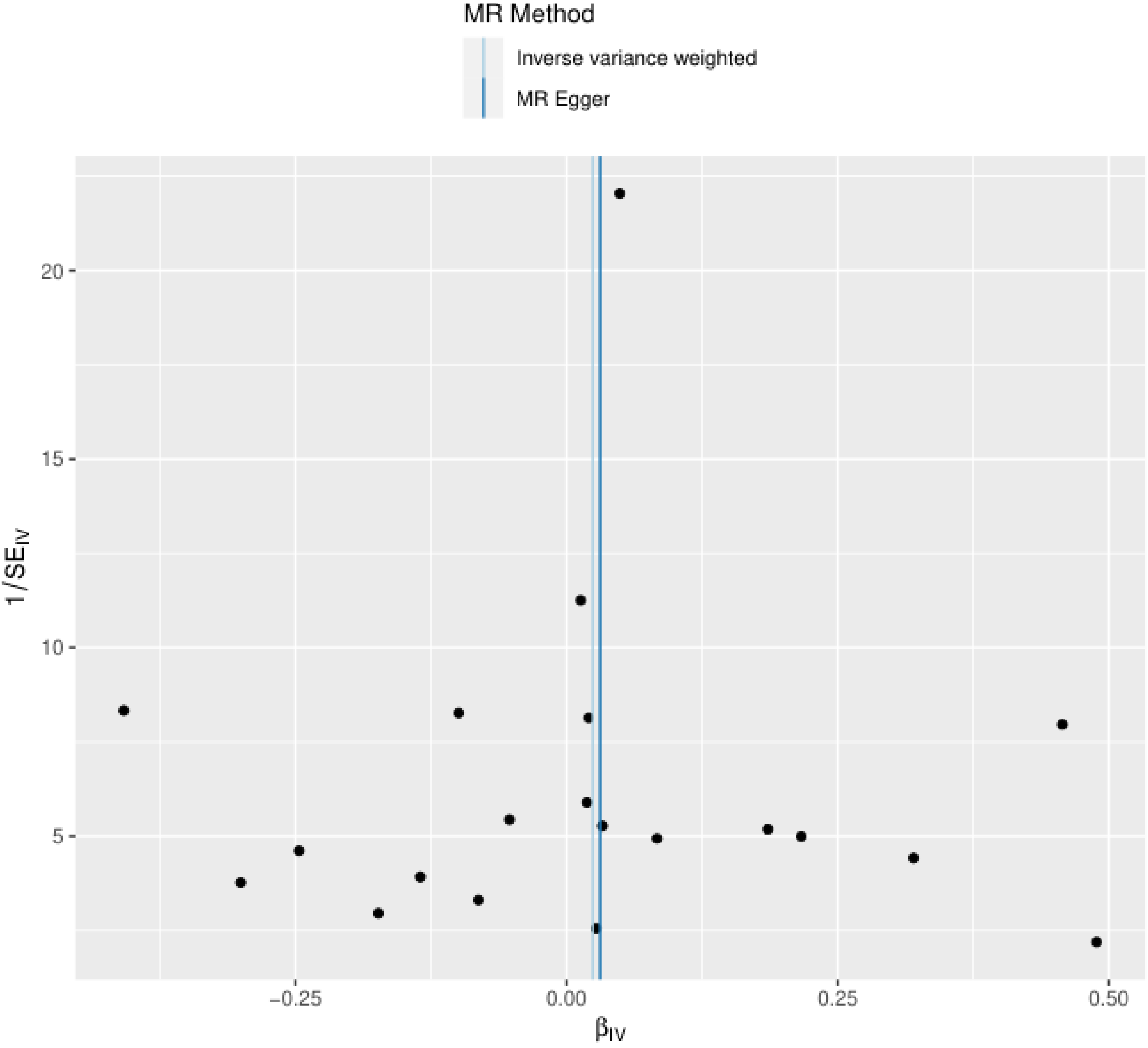

Funnel plot analyse of "CD4 on EM CD4+ " on 'Diabetic nephropathy'

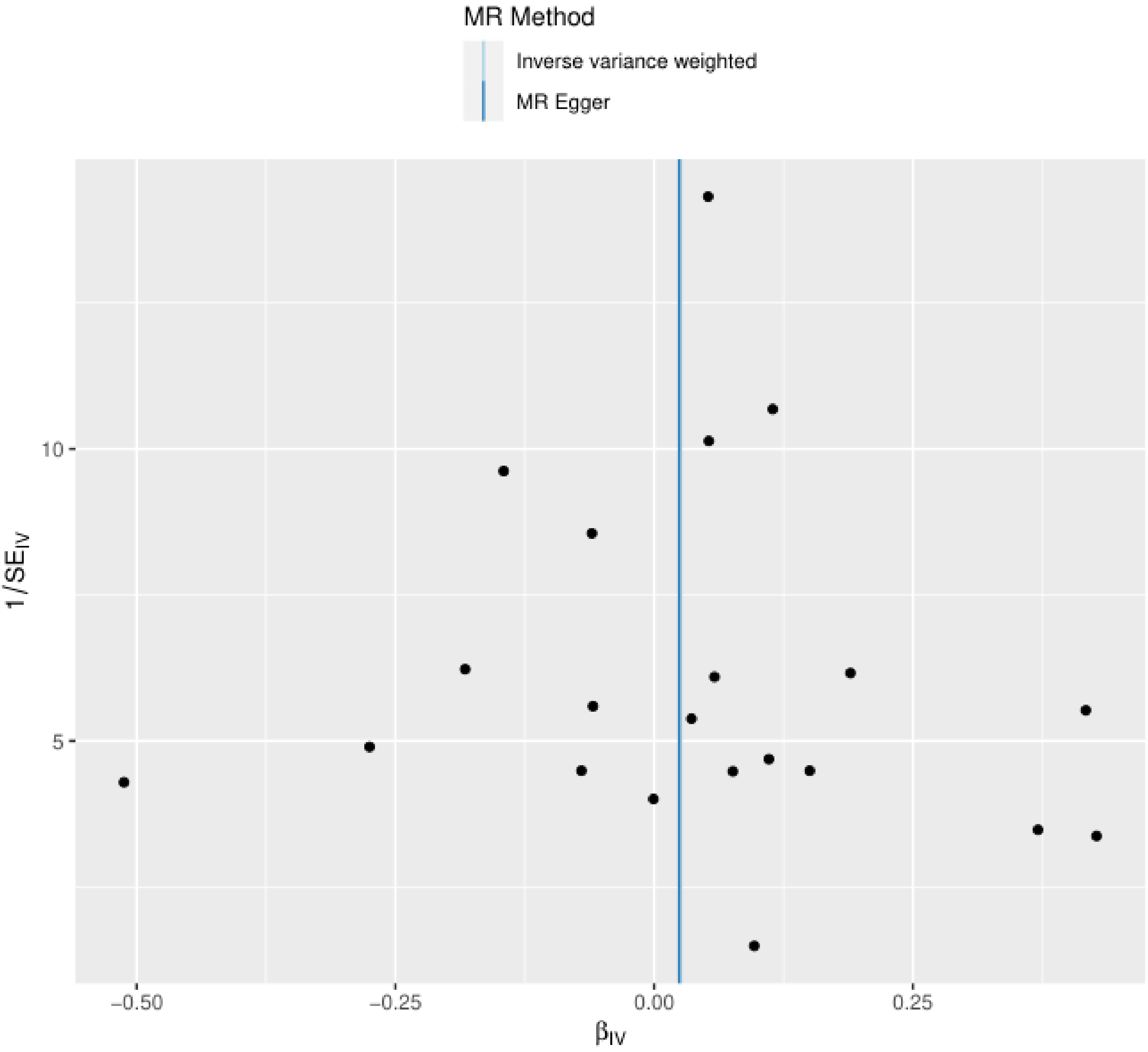

Funnel plot analyse of "CD80 on monocyte" on 'Diabetic nephropathy'

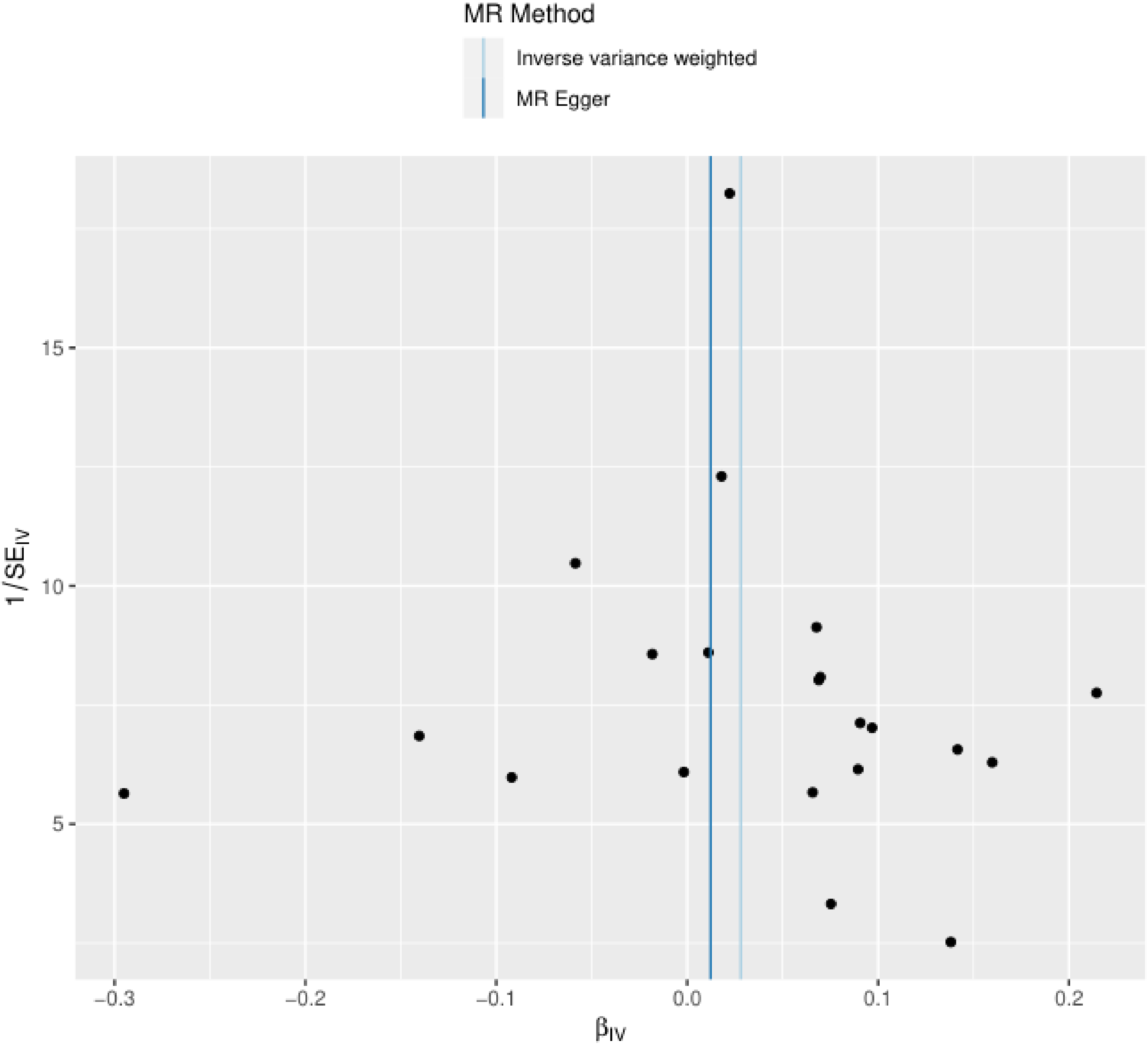

Funnel plot analyse of "CD66b on Gr MDSC" on 'Diabetic nephropathy'

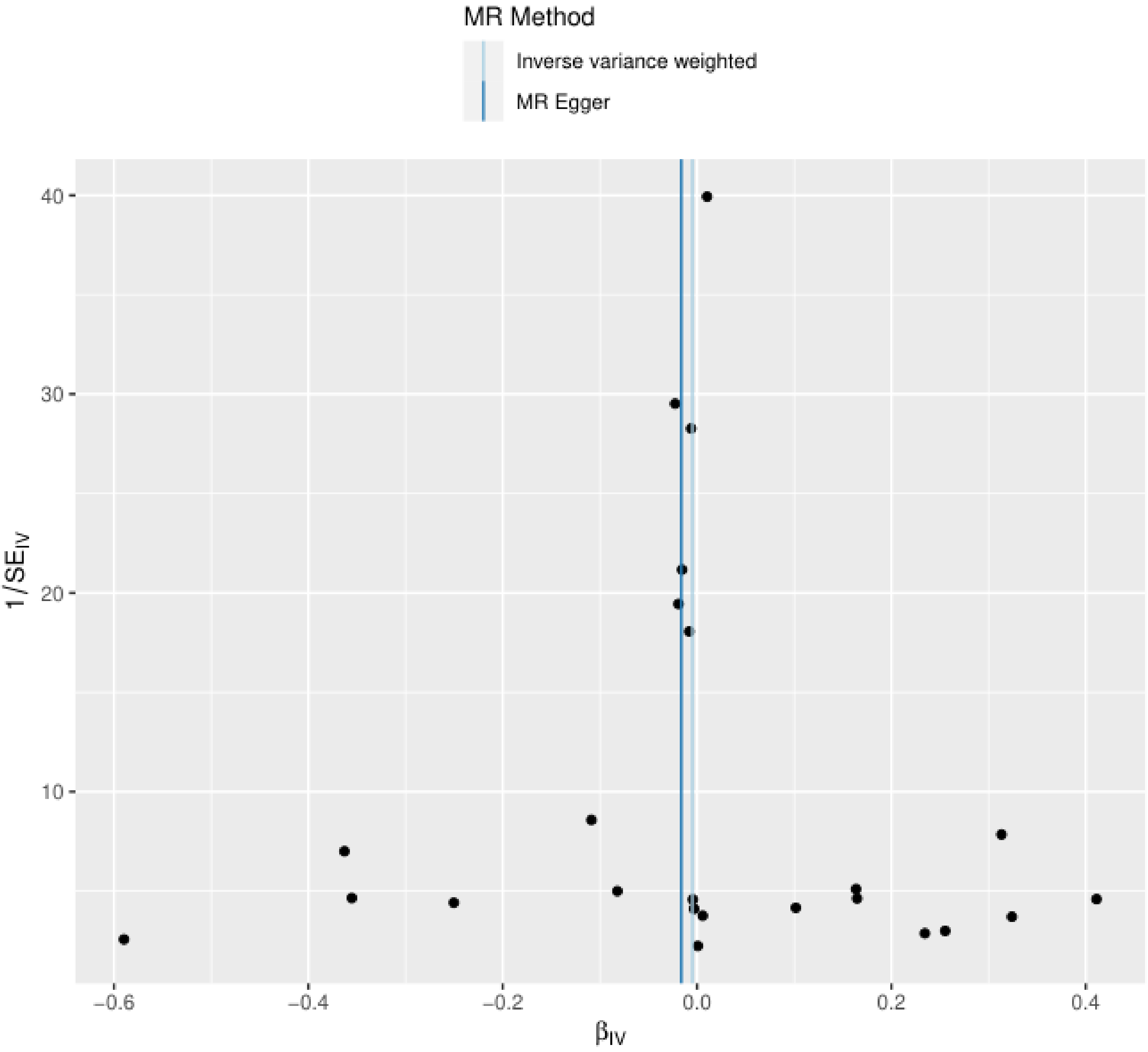

Funnel plot analyse of "CD86+ myeloid DC %DC" on 'Diabetic nephropathy'

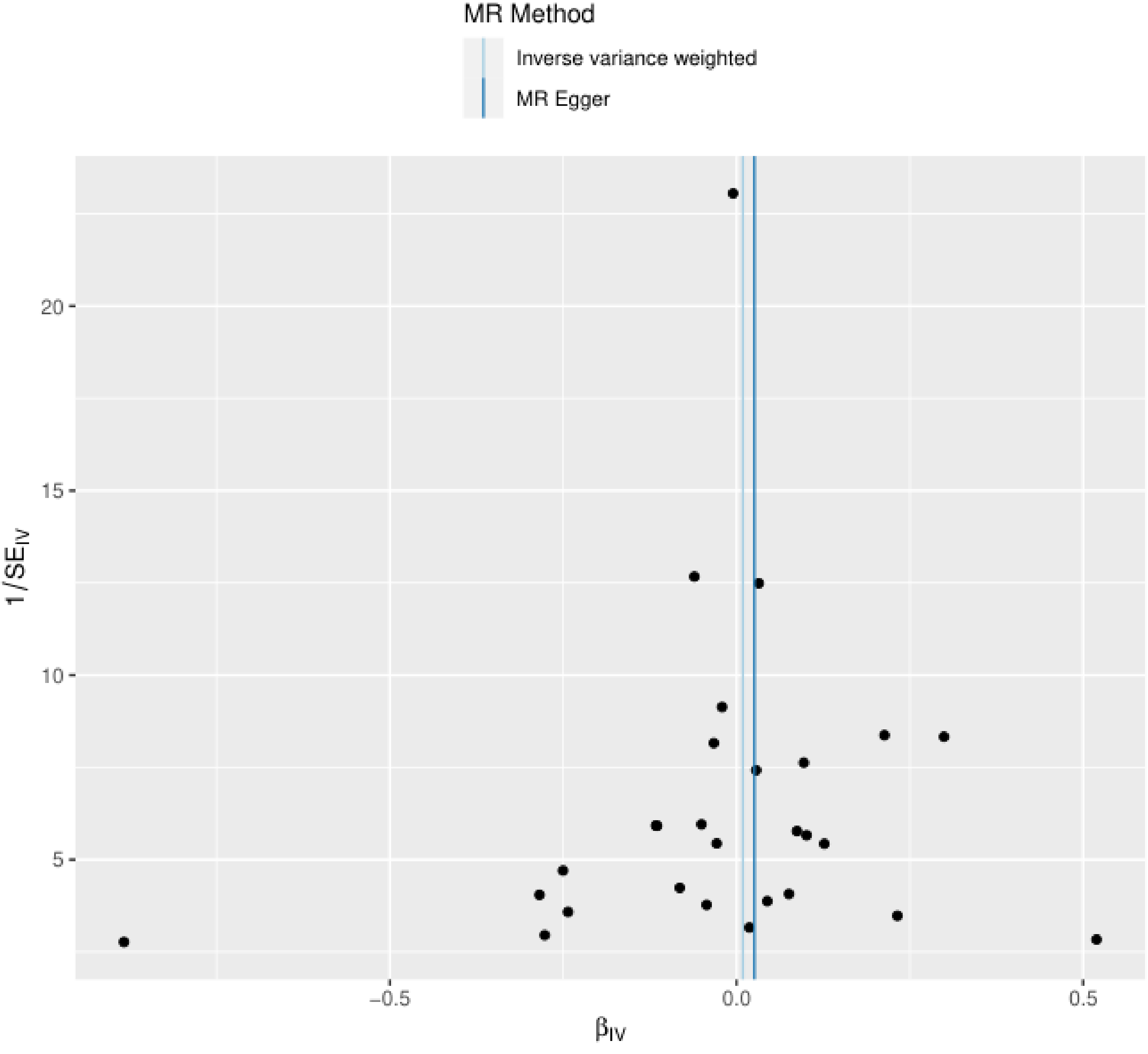

Funnel plot analyse of "CD28- CD8dim AC" on 'Diabetic nephropathy'

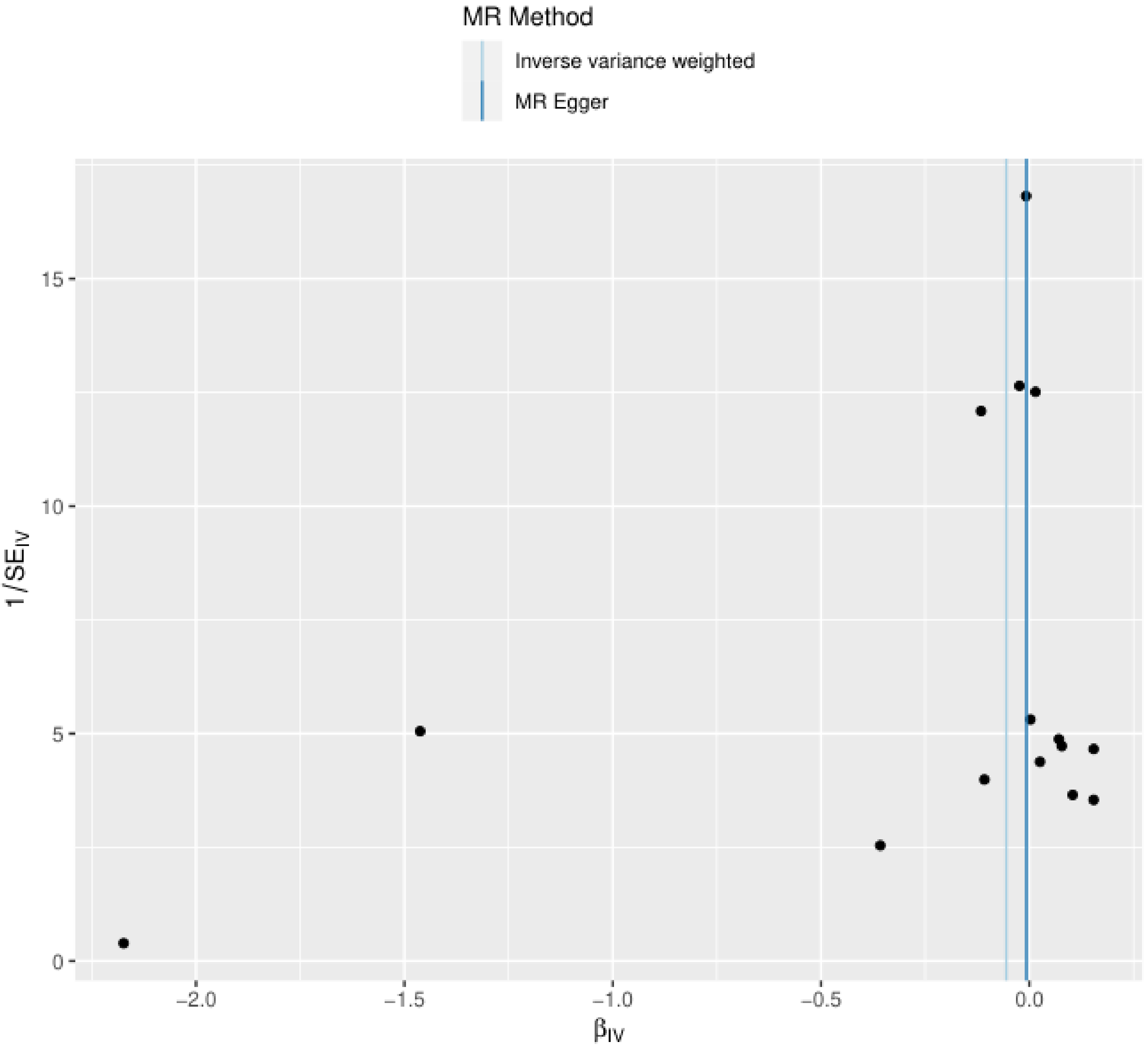

Funnel plot analyse of "CD45 on T cell" on 'Diabetic nephropathy'

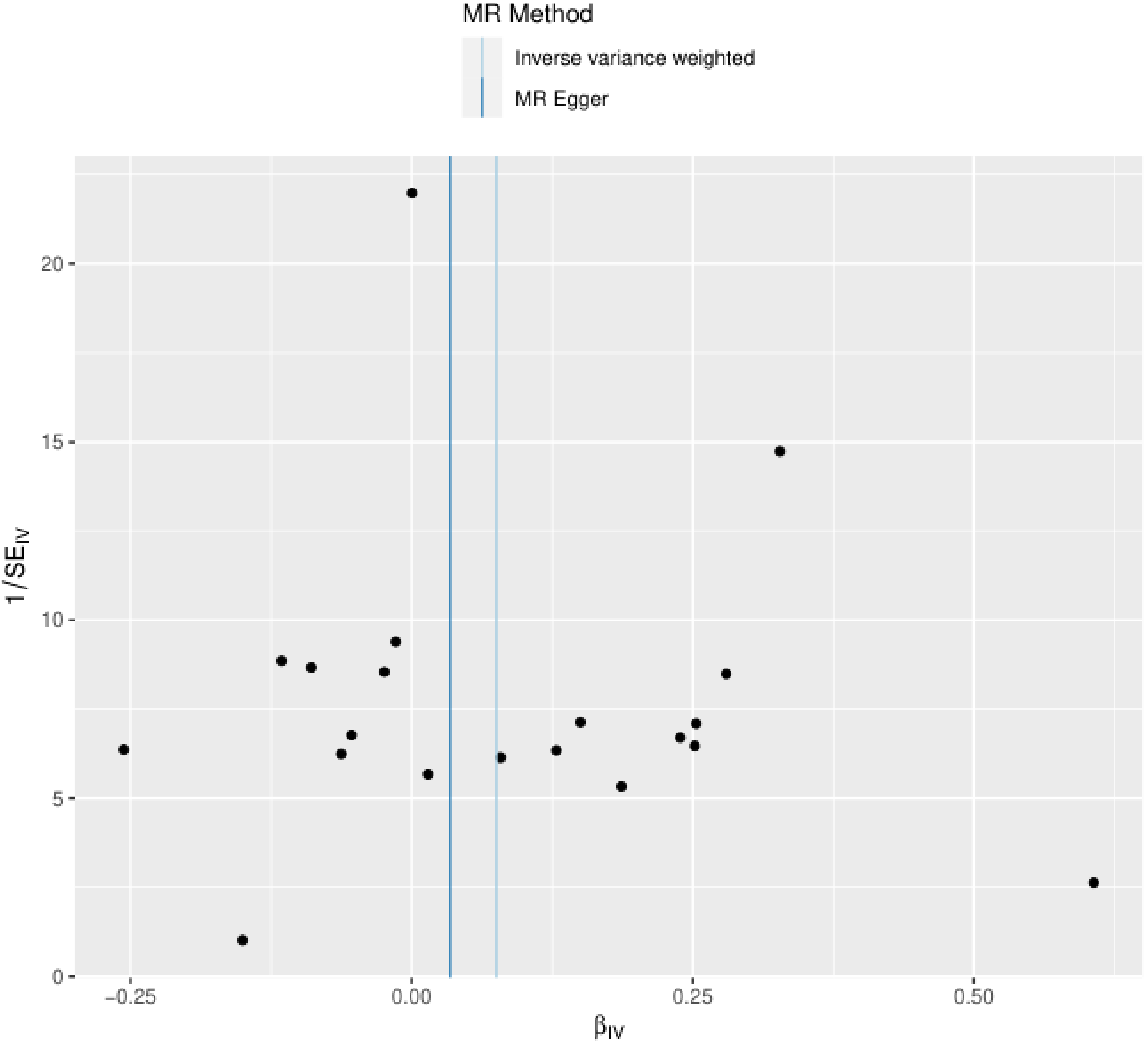

Funnel plot analyse of "HLA DR on CD33br HLA DR+ CD14dim " on 'Diabetic nephropathy'

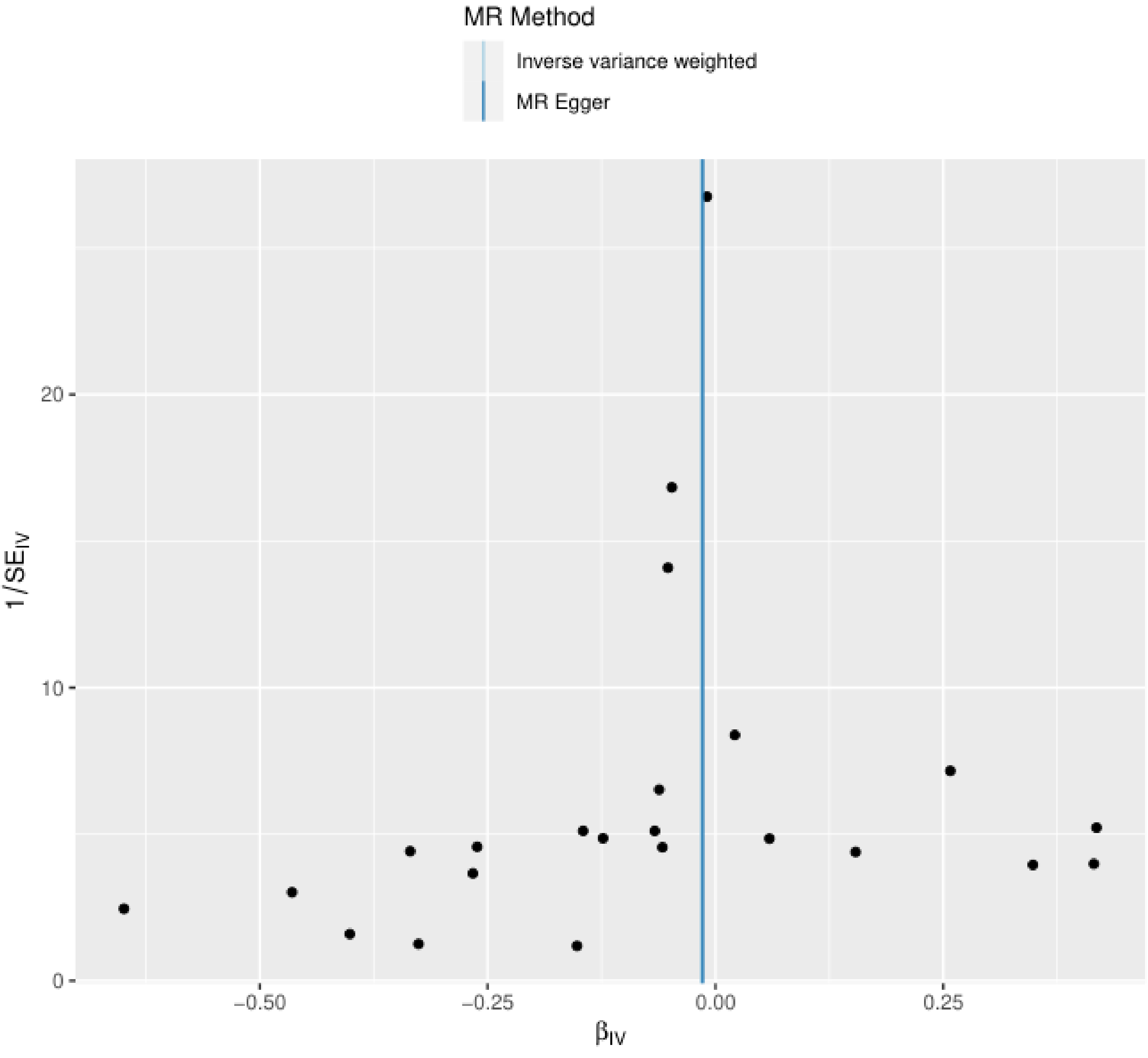

Funnel plot analyse of "CD25hi AC" on 'Diabetic nephropathy'

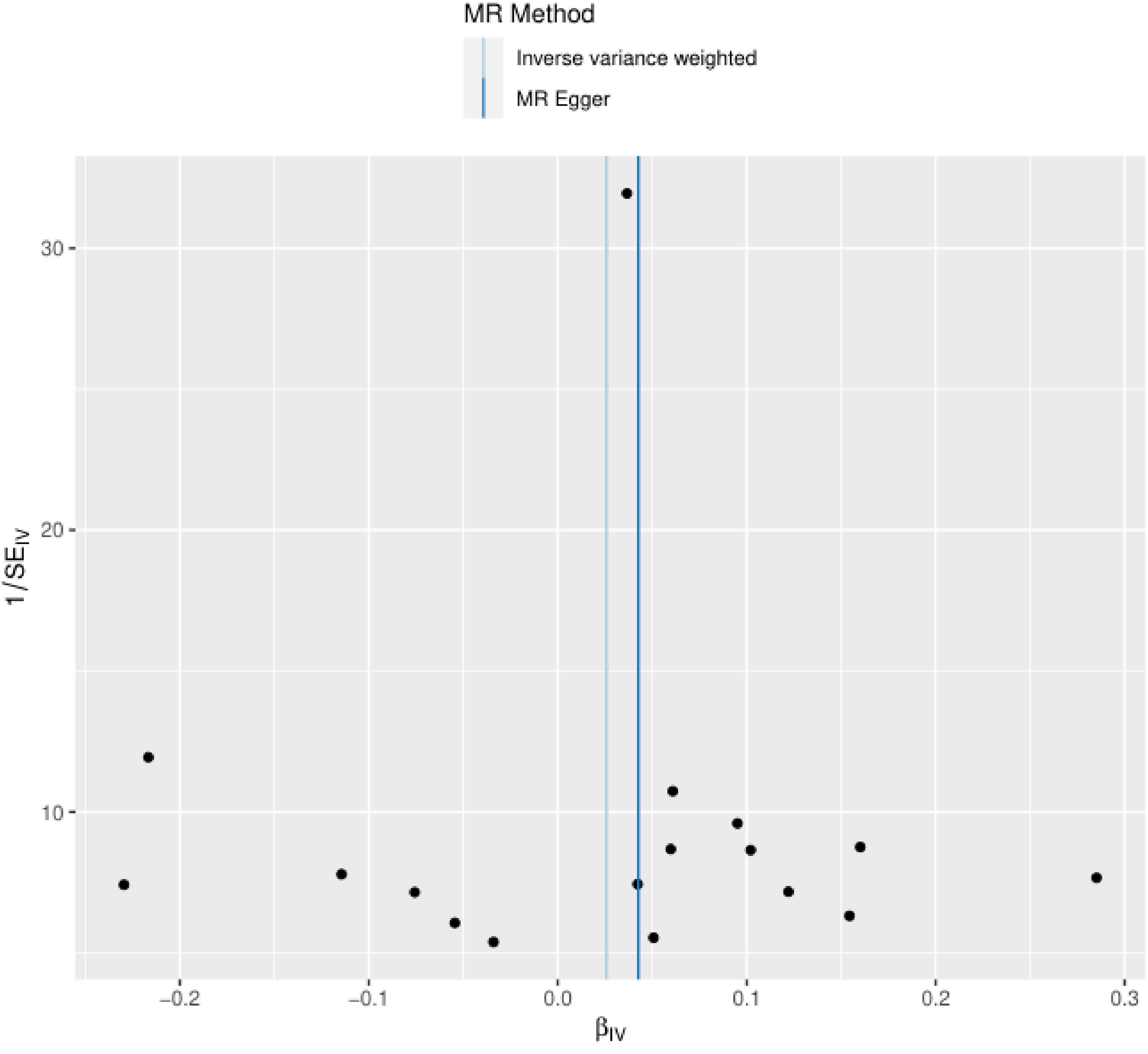

Funnel plot analyse of "HVEM on CM CD8br" on 'Diabetic nephropathy'

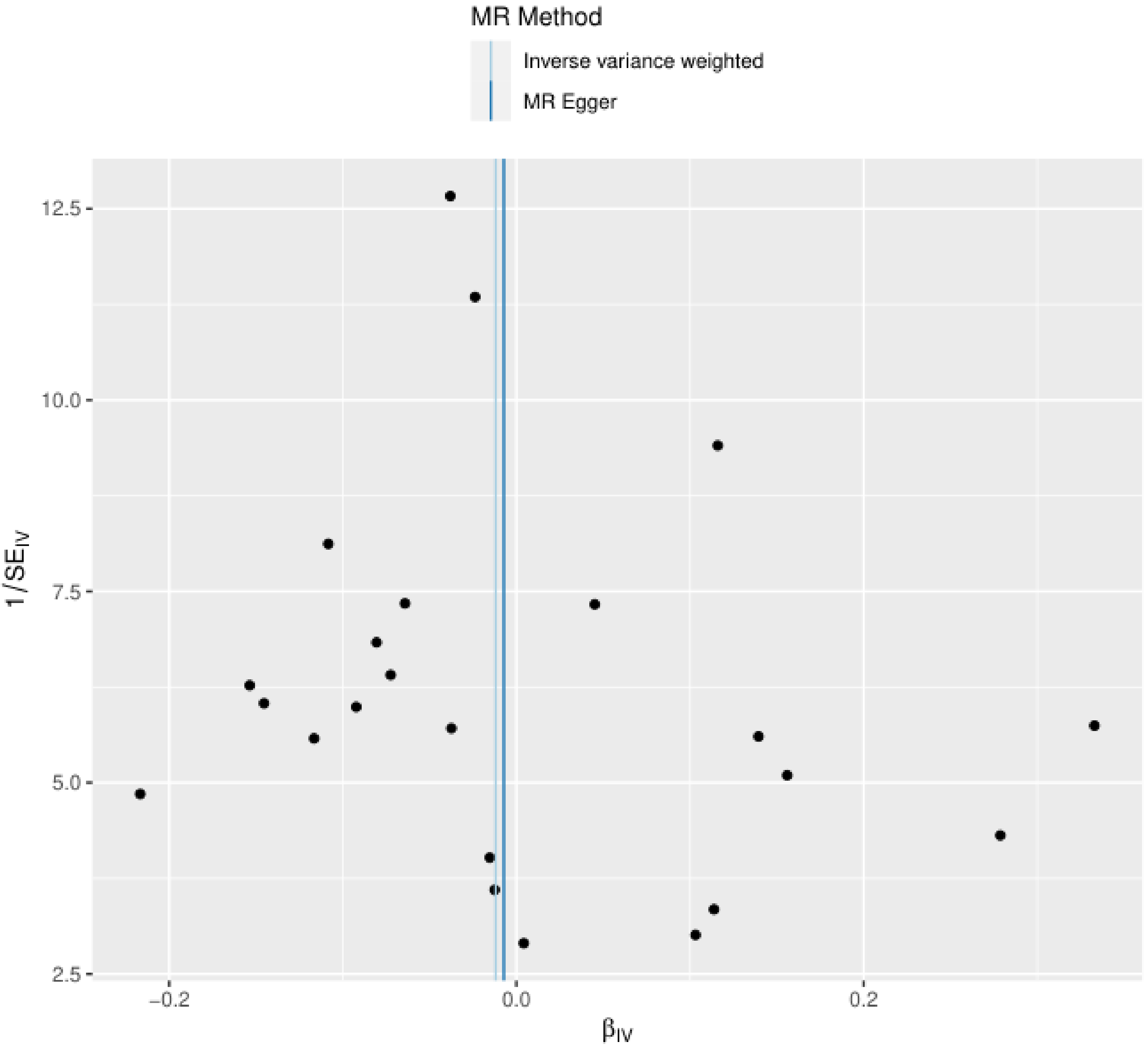

Funnel plot analyse of "CD62L- monocyte %monocyte" on 'Diabetic nephropathy'

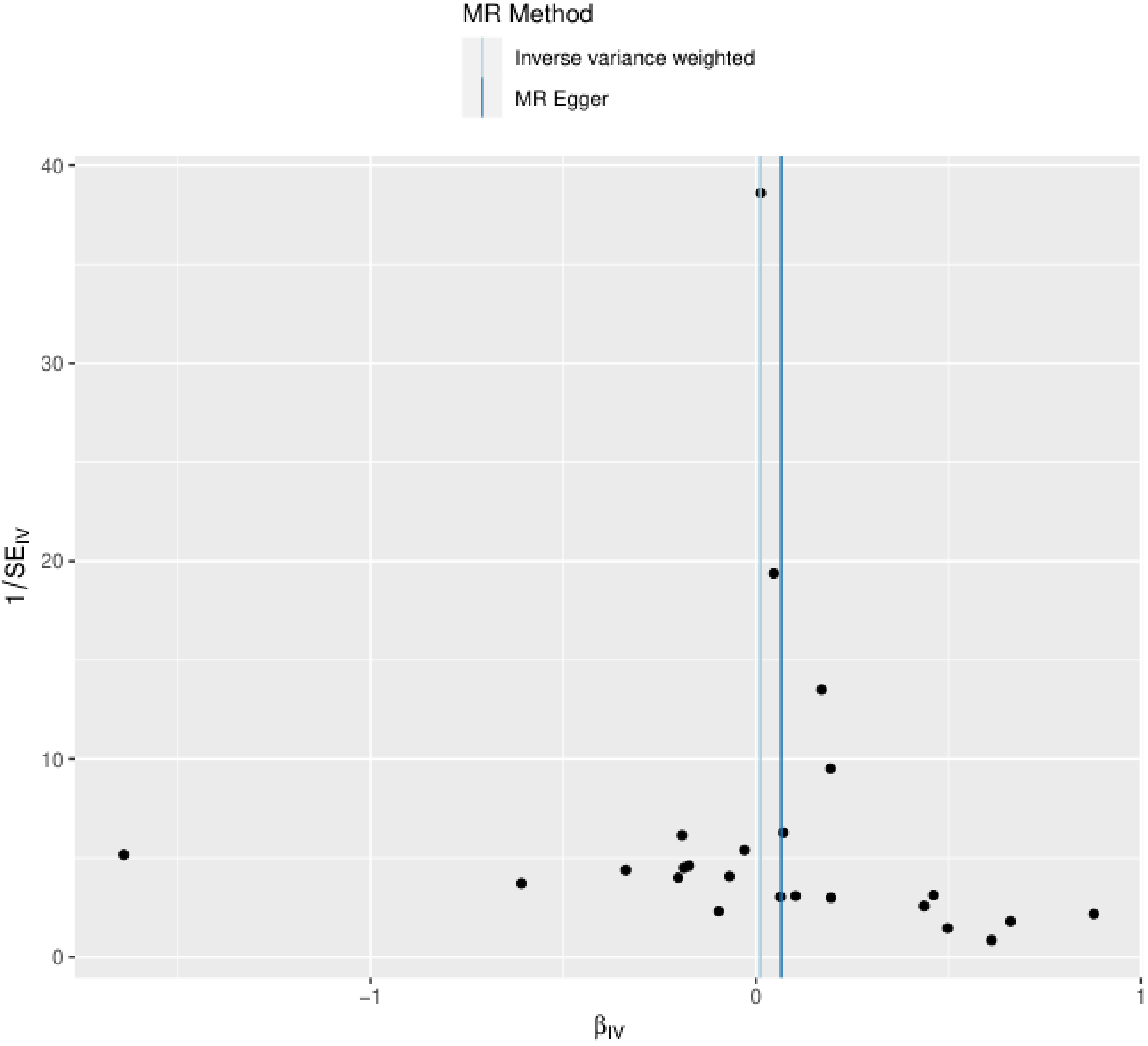

Funnel plot analyse of "CD25hi CD45RA- CD4 not Treg %CD4+" on 'Diabetic nephropathy'

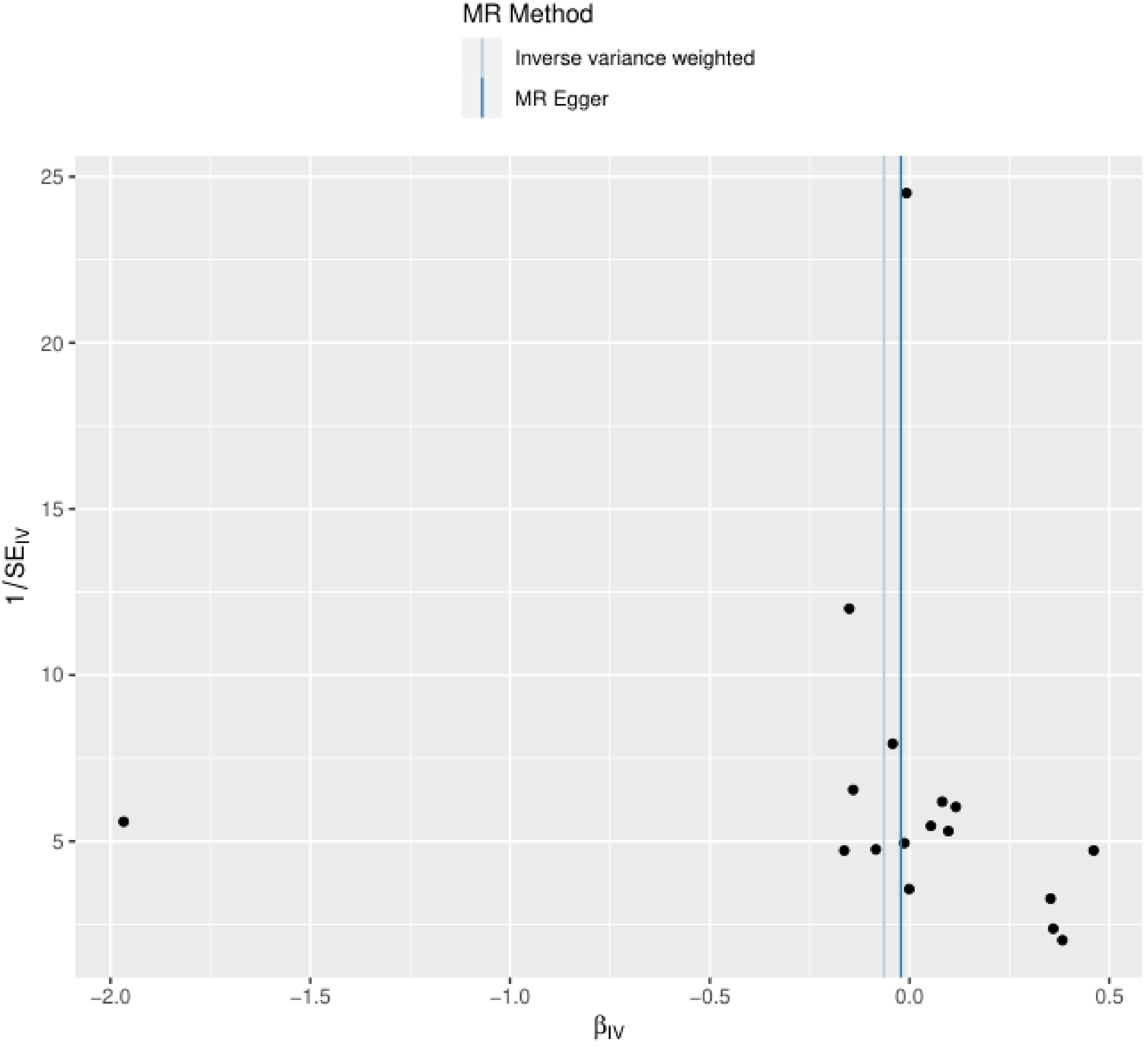

Funnel plot analyse of "CD45 on HLA DR+ CD4+" on 'Diabetic nephropathy'

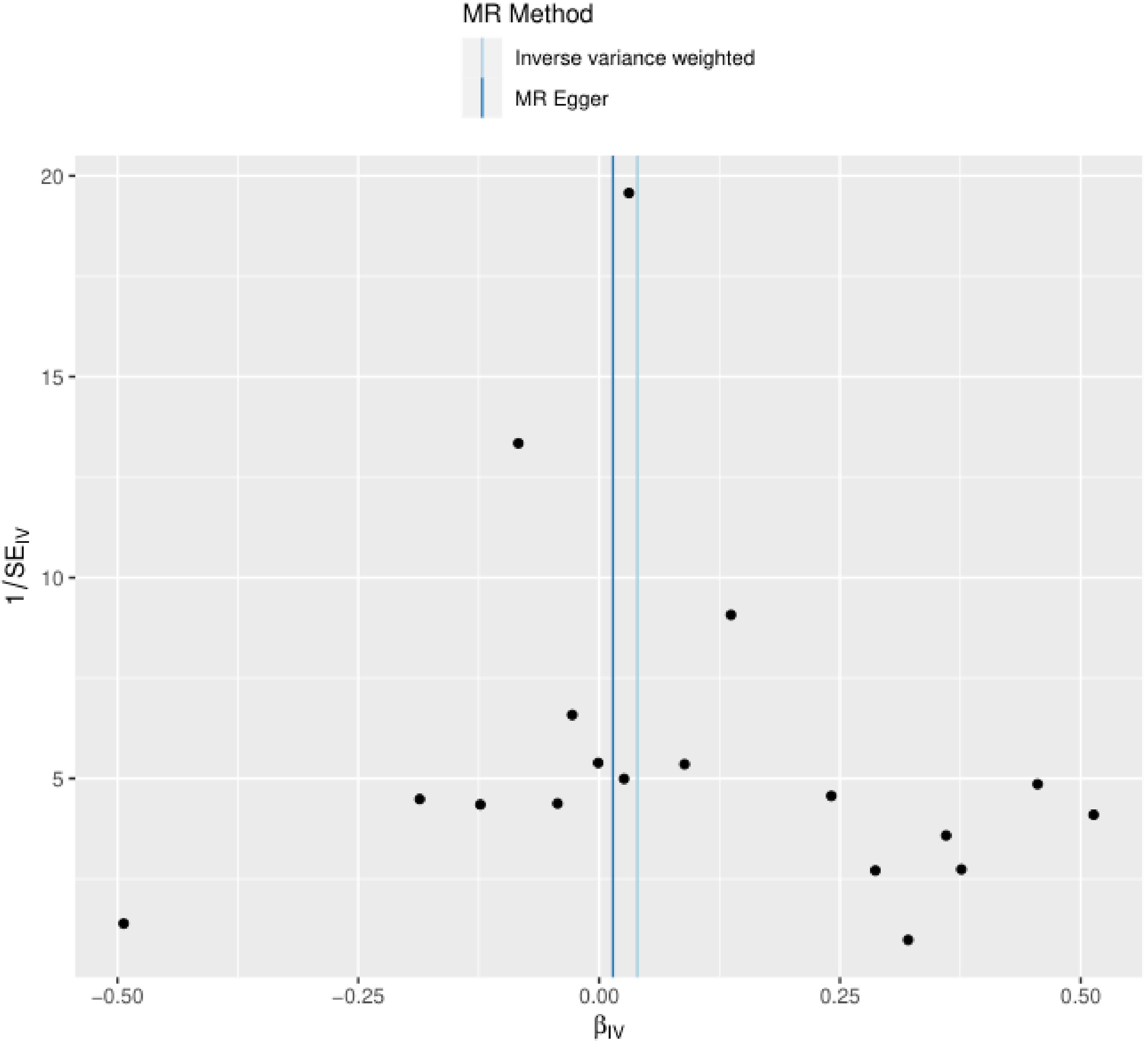

Funnel plot analyse of "TD CD8br %T cell" on 'Diabetic nephropathy'

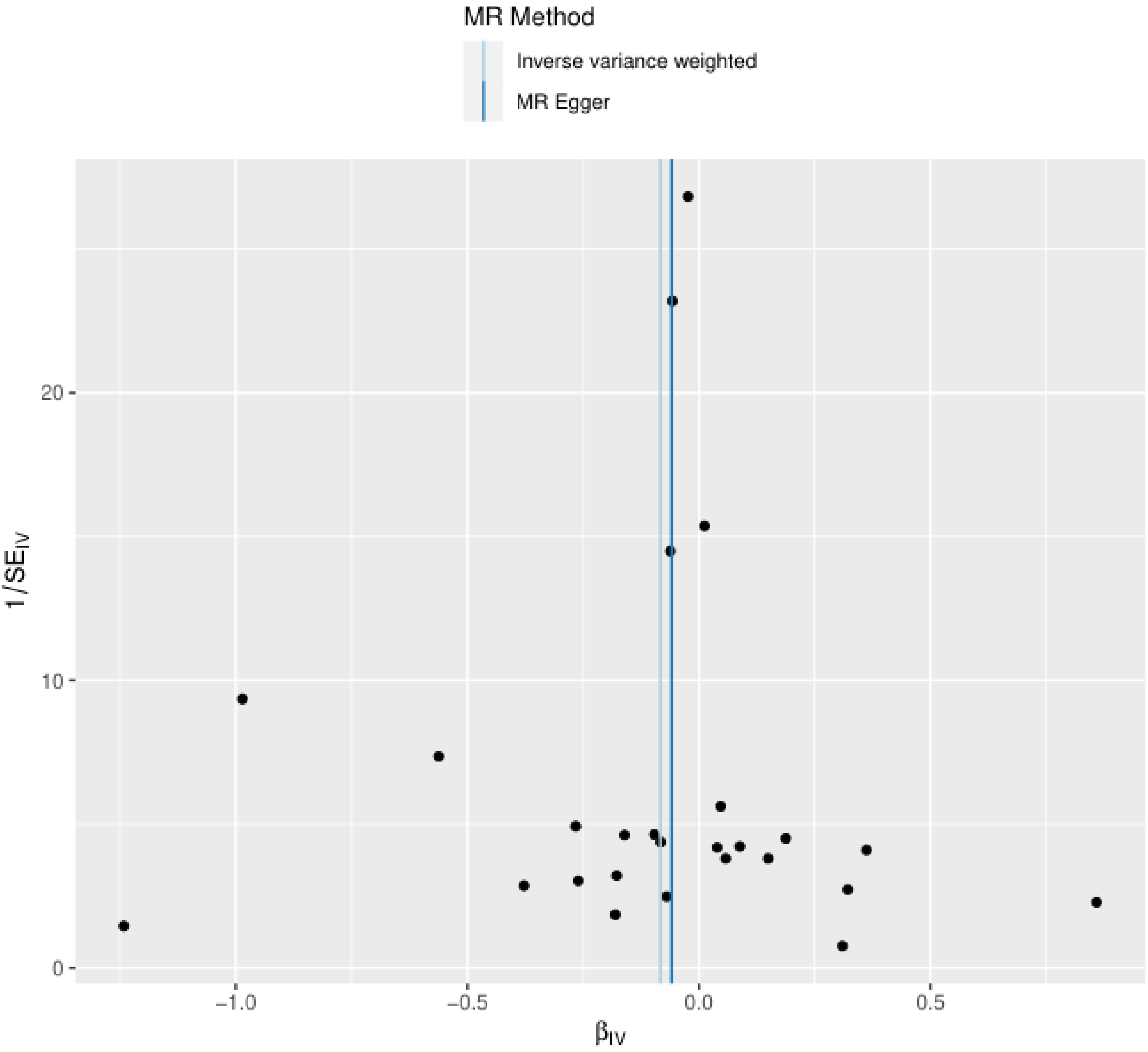

Funnel plot analyse of "TD CD4+ %T cell" on 'Diabetic nephropathy'

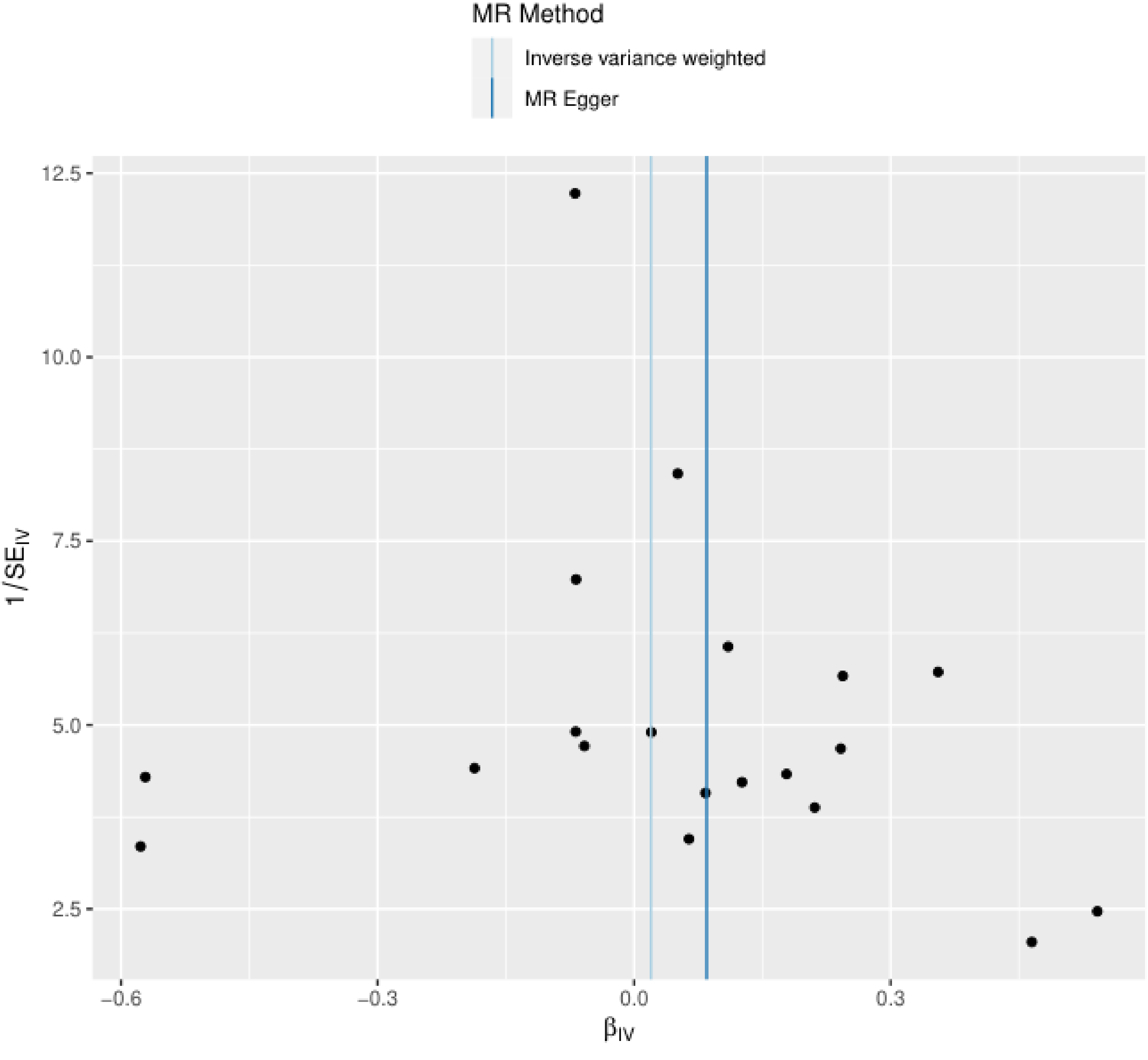

Funnel plot analyse of "CD19 on CD20- CD38- " on 'Diabetic nephropathy'

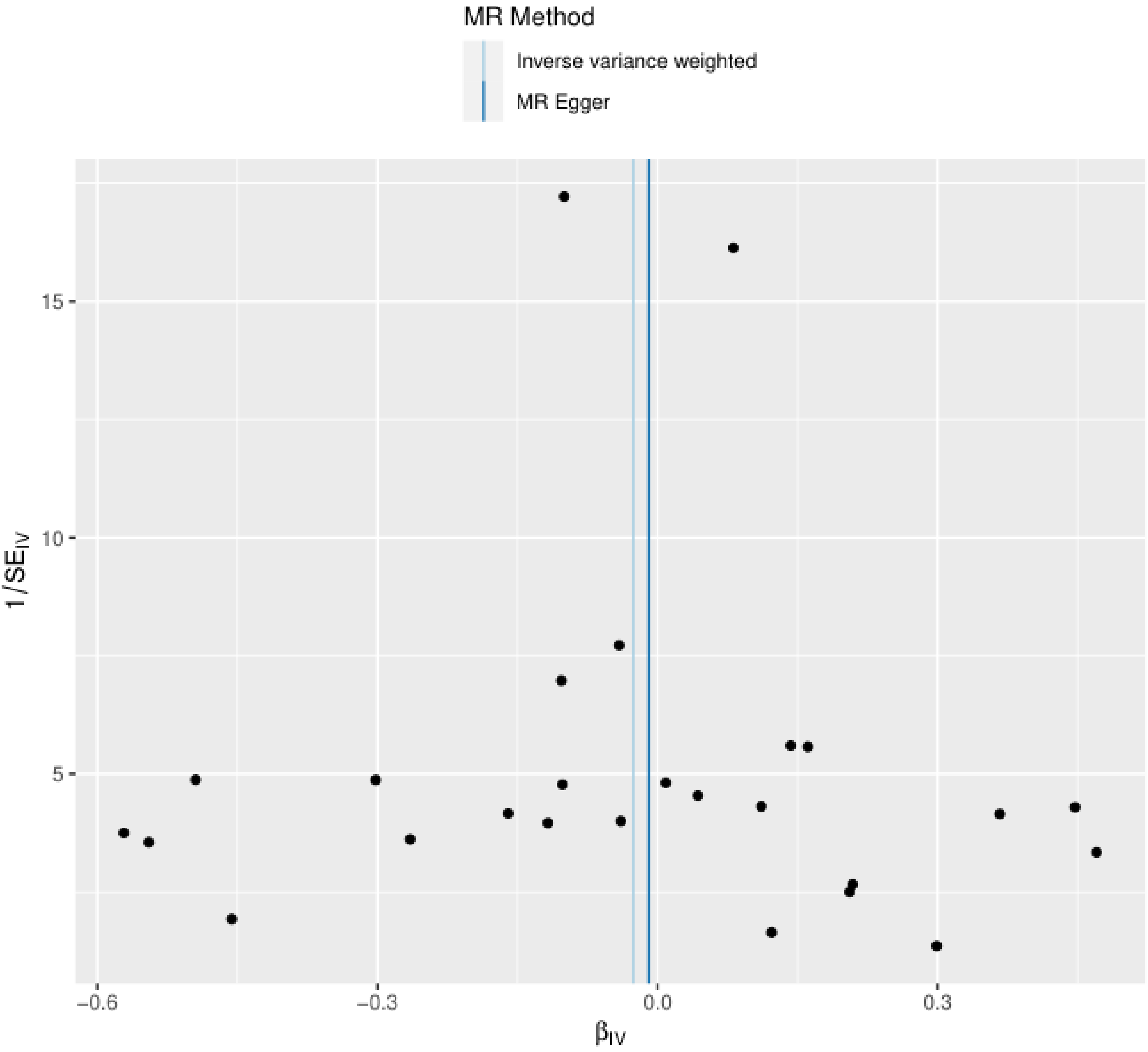

Funnel plot analyse of "CD20 on IgD- CD24-" on 'Diabetic nephropathy'

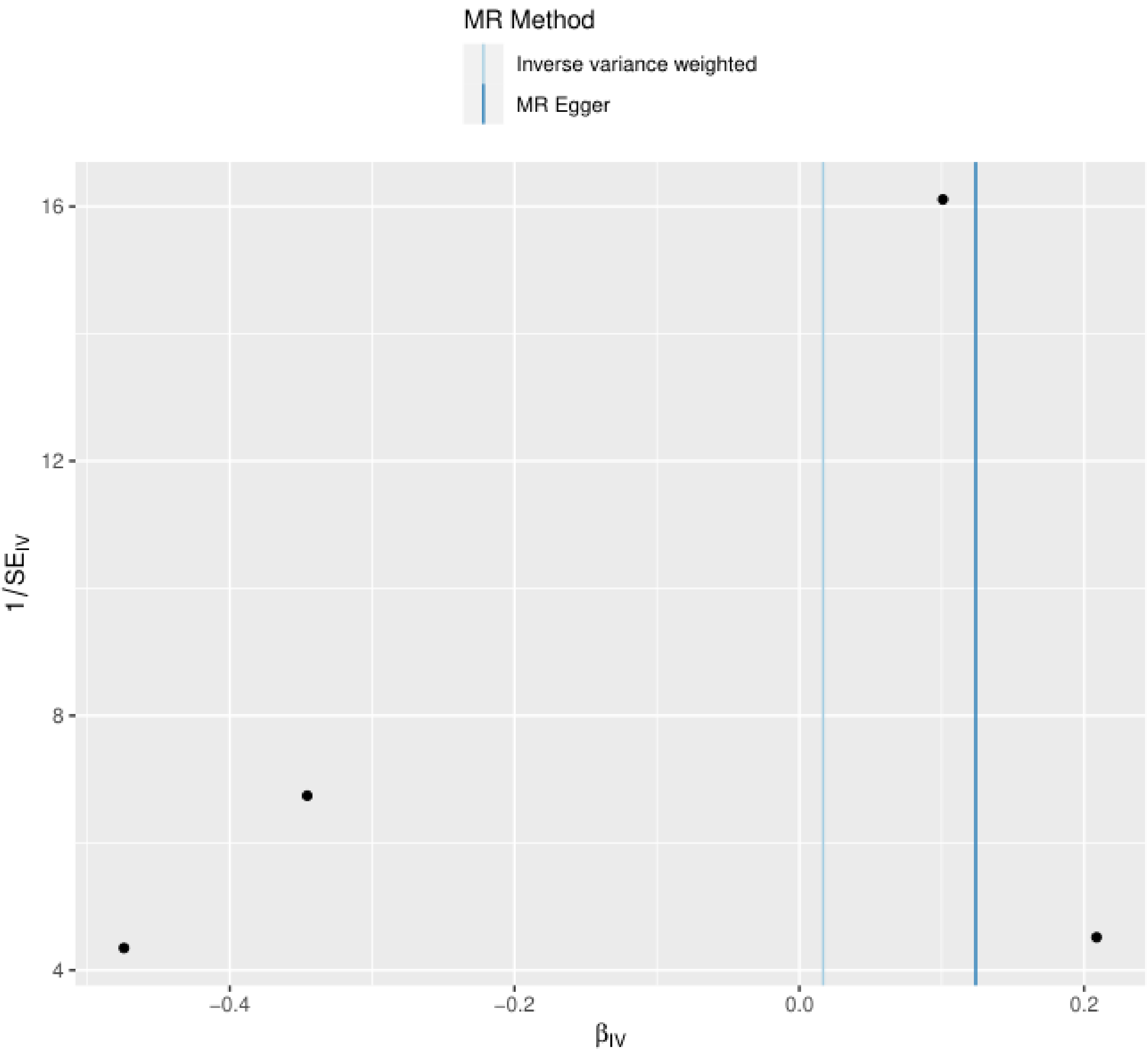

Funnel plot analyse of "BAFF-R on IgD- CD38dim" on 'Diabetic nephropathy'

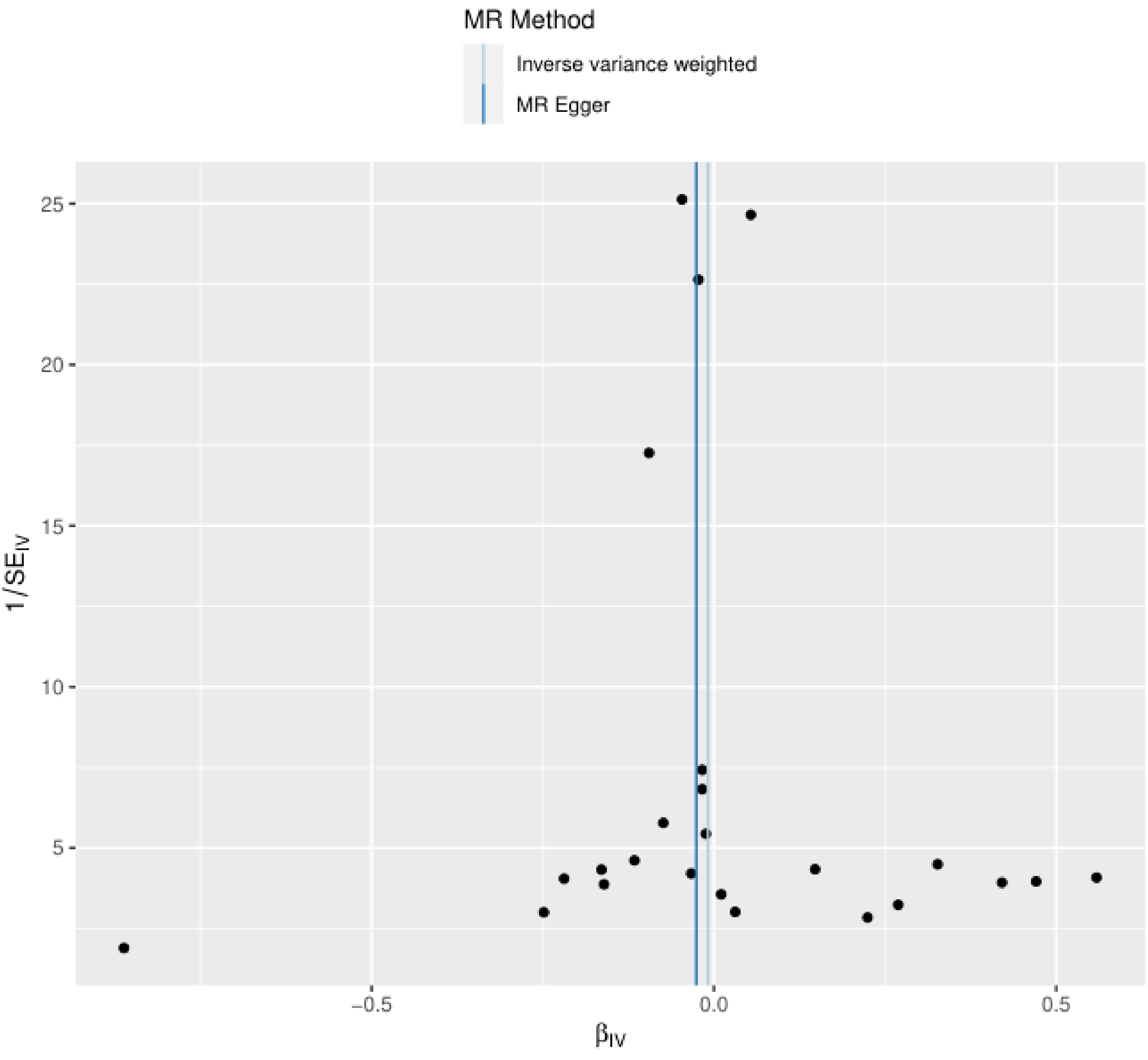

Funnel plot analyse of "PB/PC AC" on 'Diabetic nephropathy'

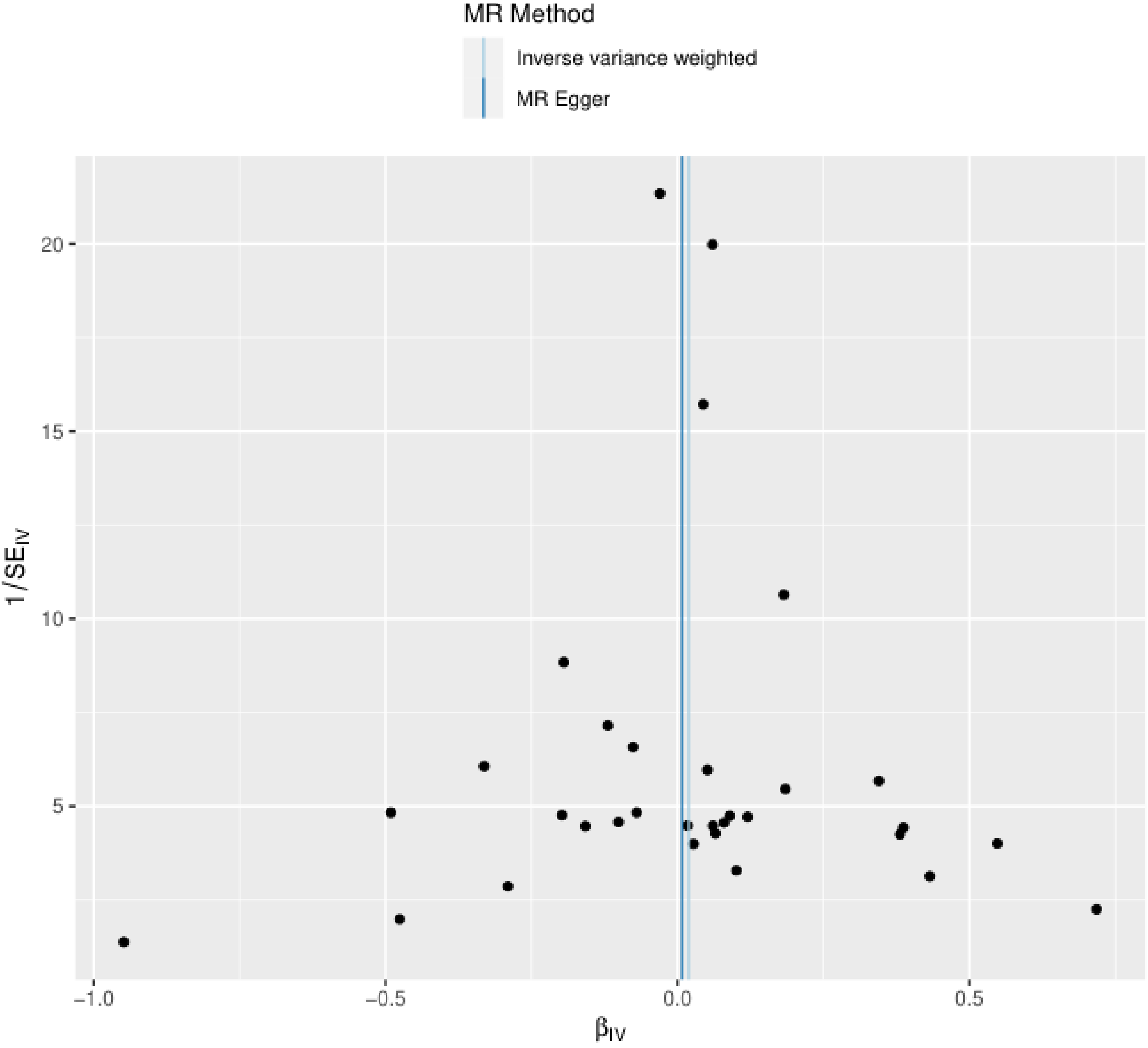

Funnel plot analyse of "Unsw mem %B cell" on 'Diabetic nephropathy'

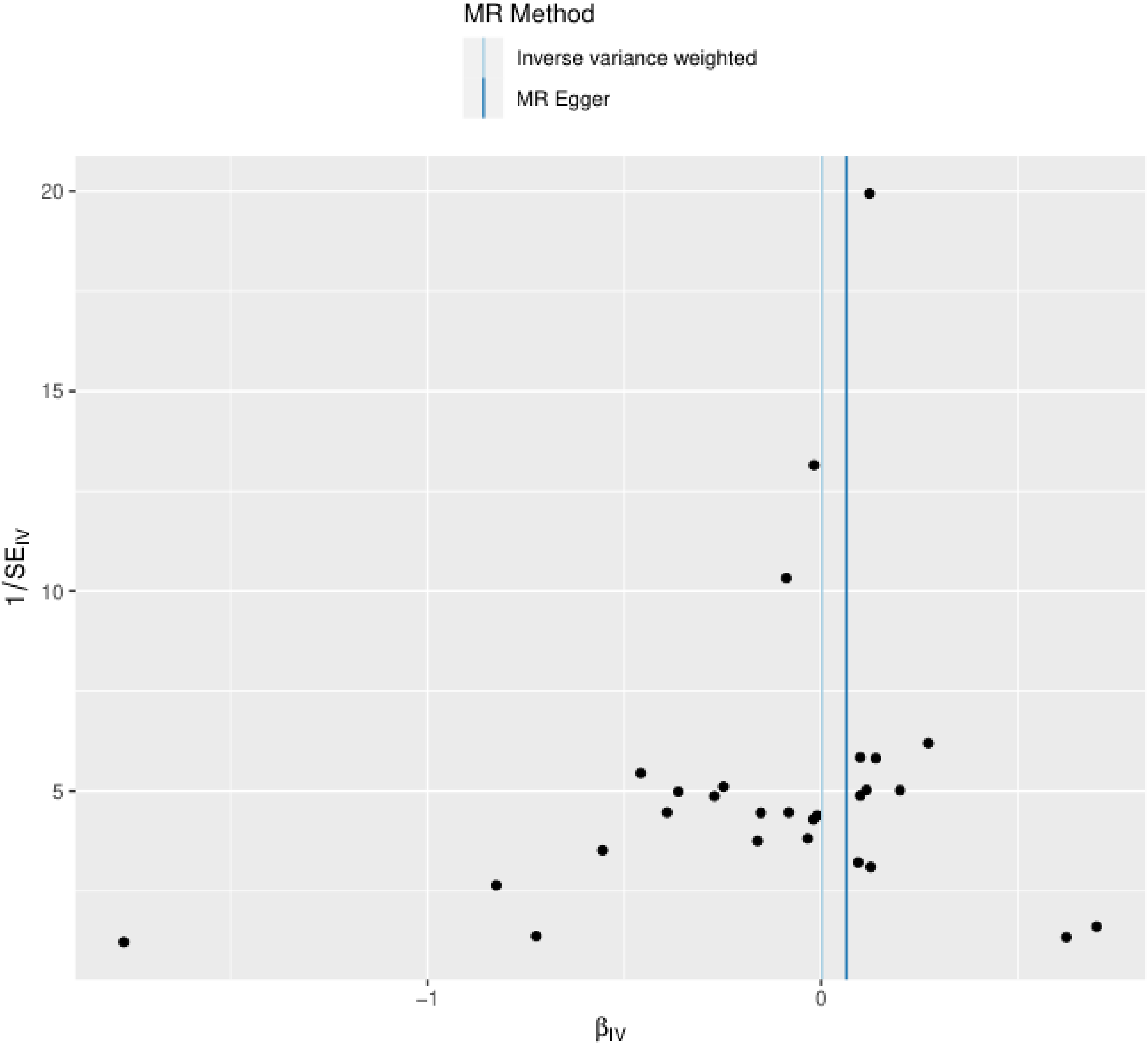

Funnel plot analyse of "CD28+ DN (CD4-CD8-) %DN" on 'Diabetic nephropathy'

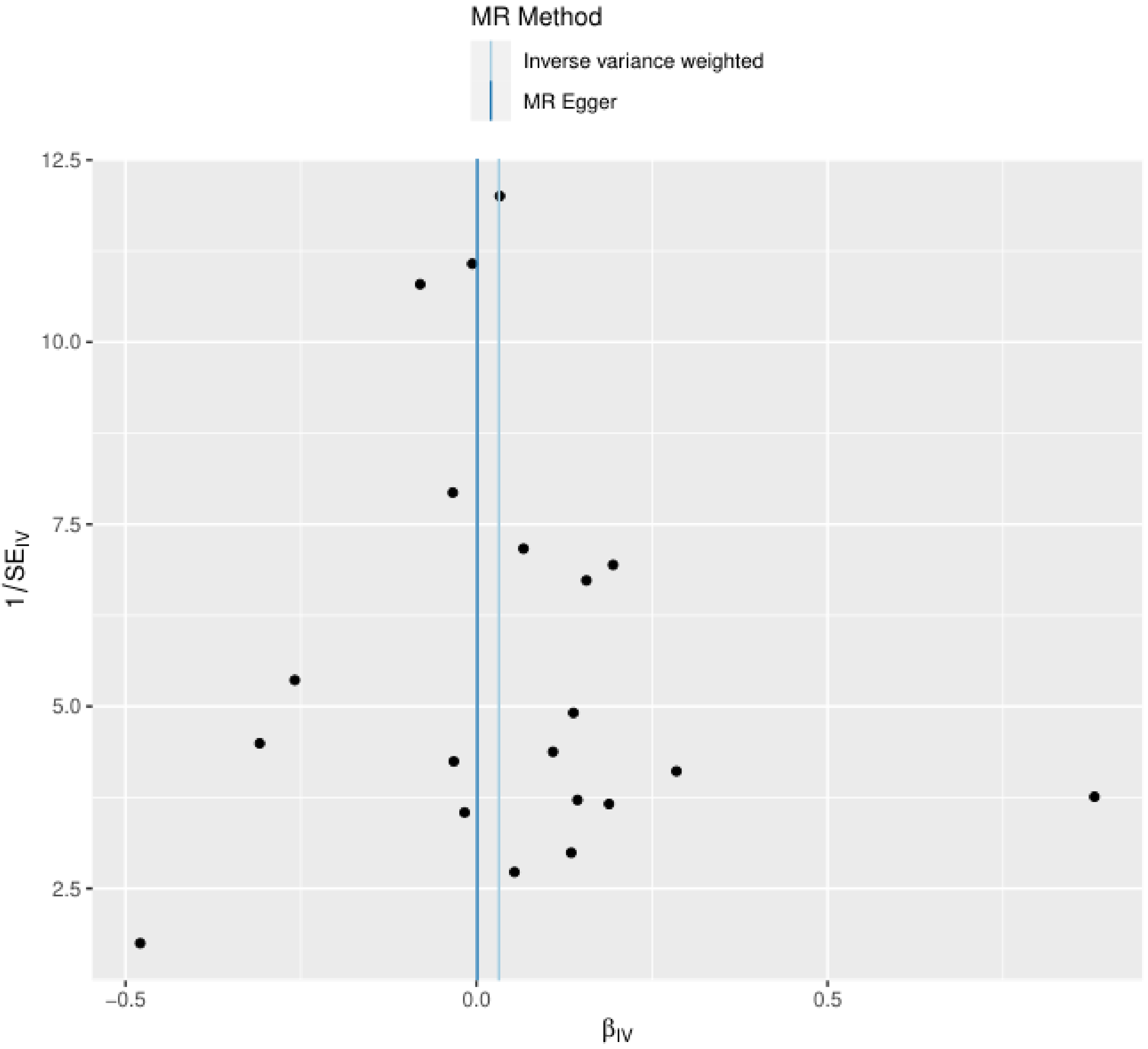

Funnel plot analyse of "CD8dim NKT %T cell" on 'Diabetic nephropathy'

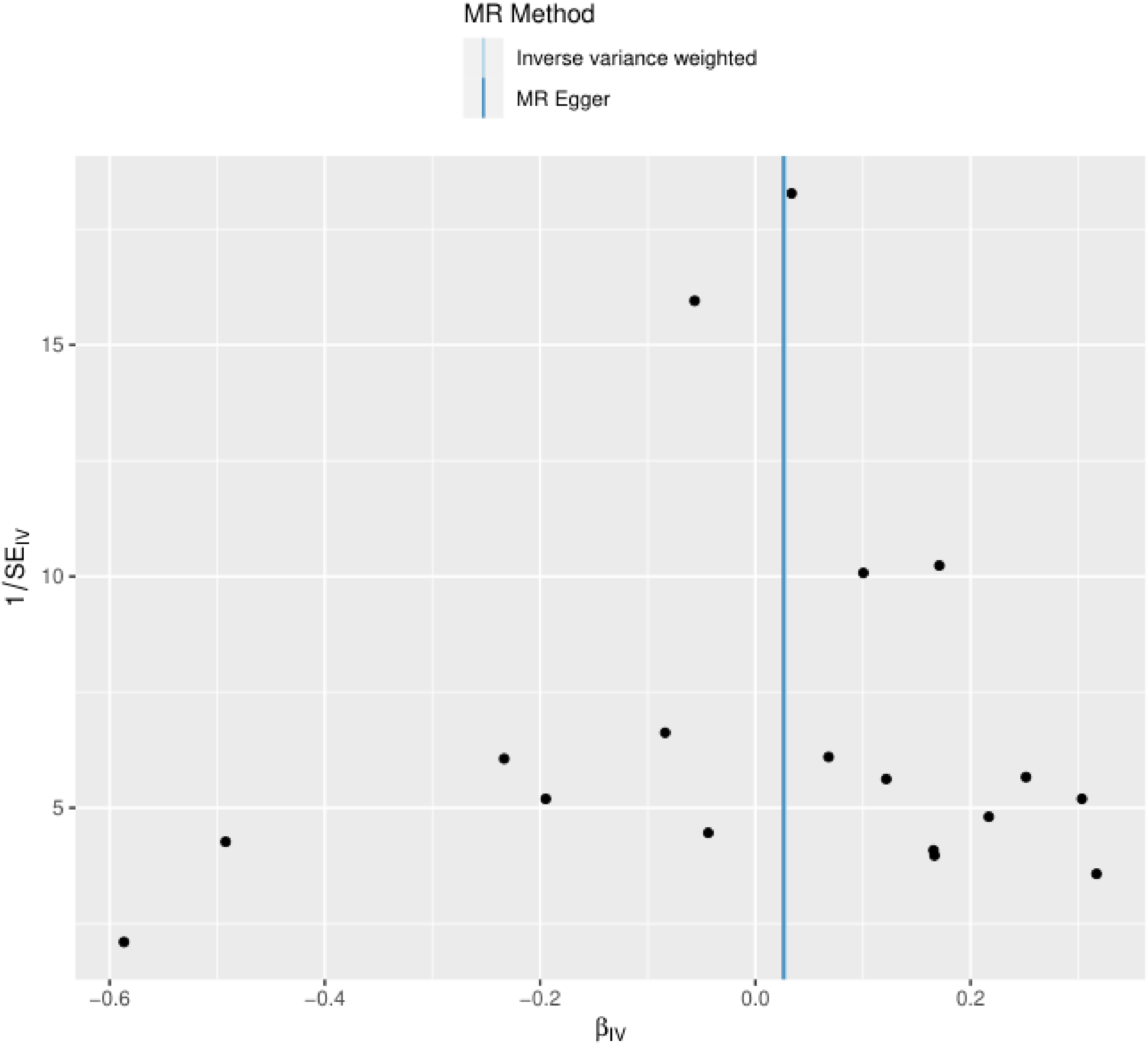

Funnel plot analyse of "IgD- CD24- %B cell" on 'Diabetic nephropathy'

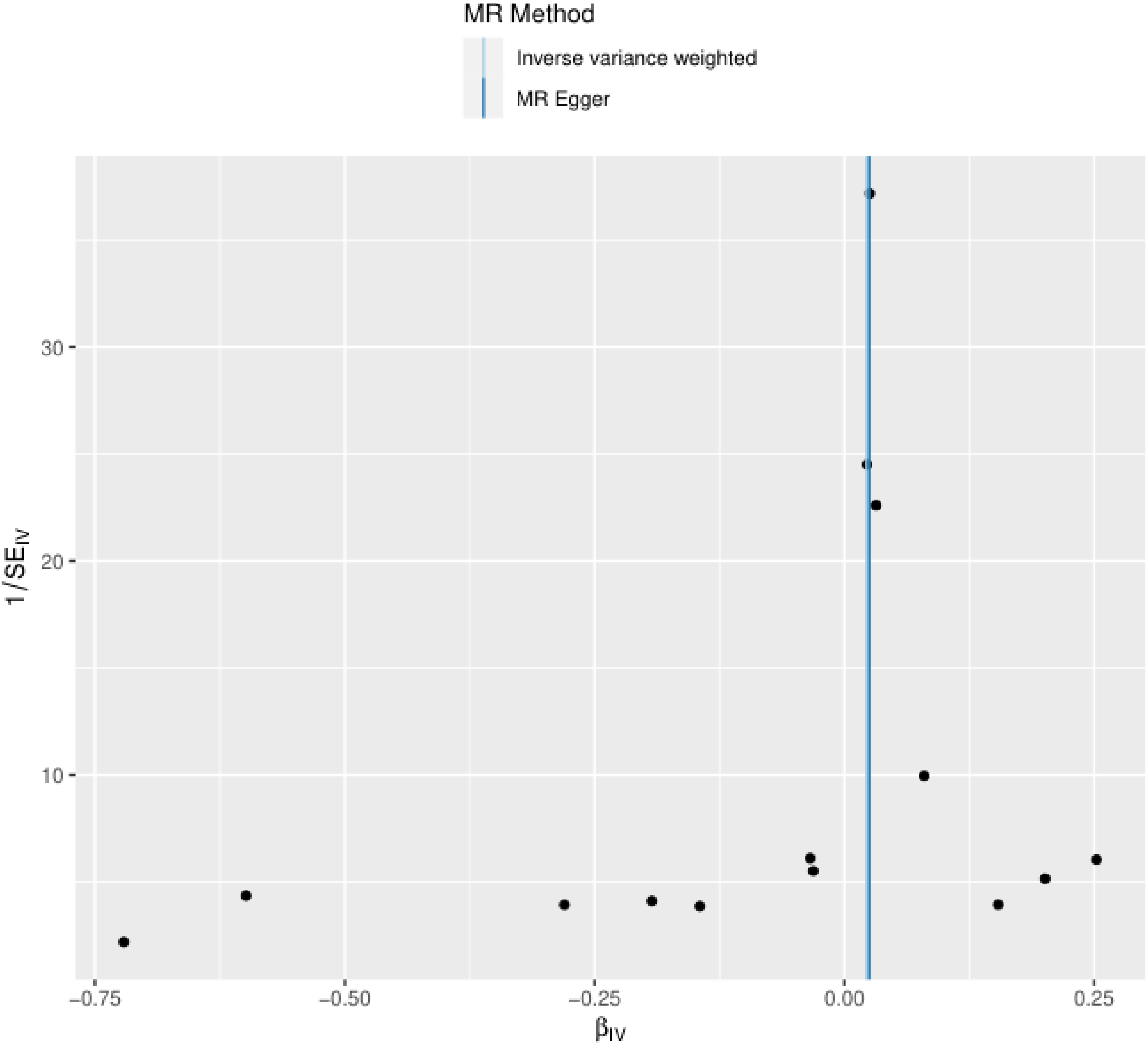

Funnel plot analyse of ''CD25 on secreting Treg '' on 'Diabetic nephropathy'

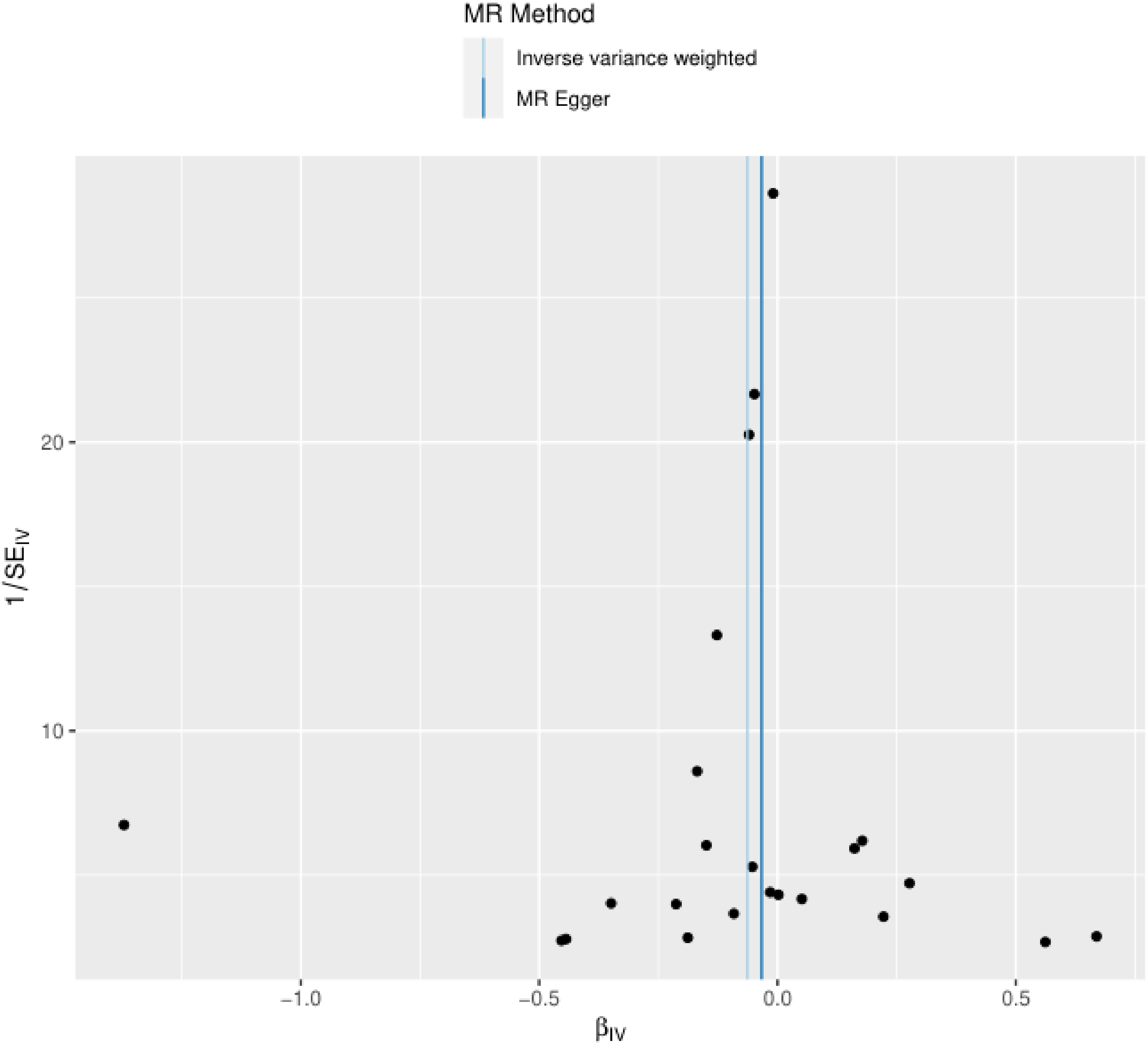

Funnel plot analyse of "FSC-A on CD4+" on 'Diabetic nephropathy'

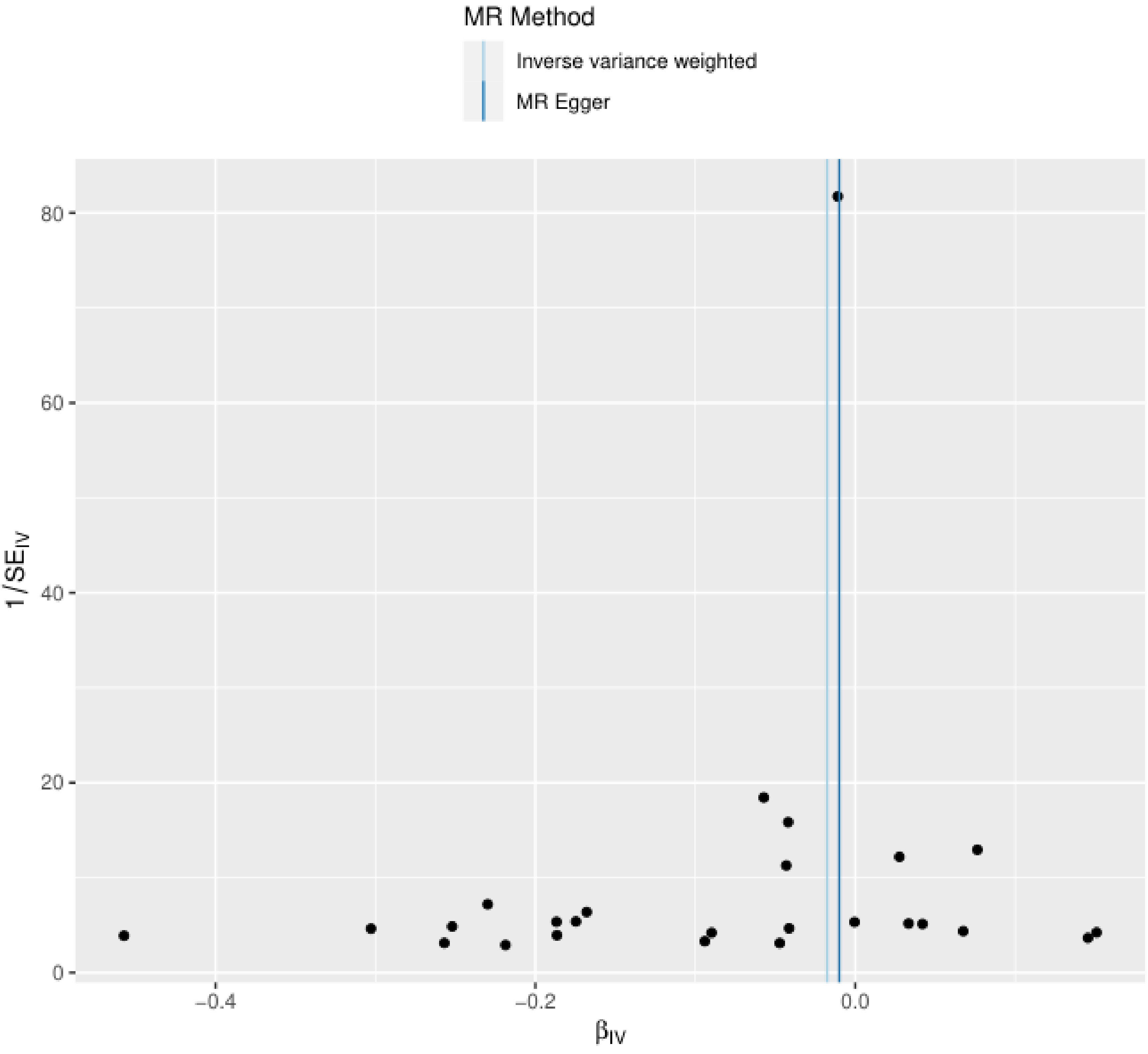

Funnel plot analyse of "CD25 on CD24+ CD27+" on 'Diabetic nephropathy'

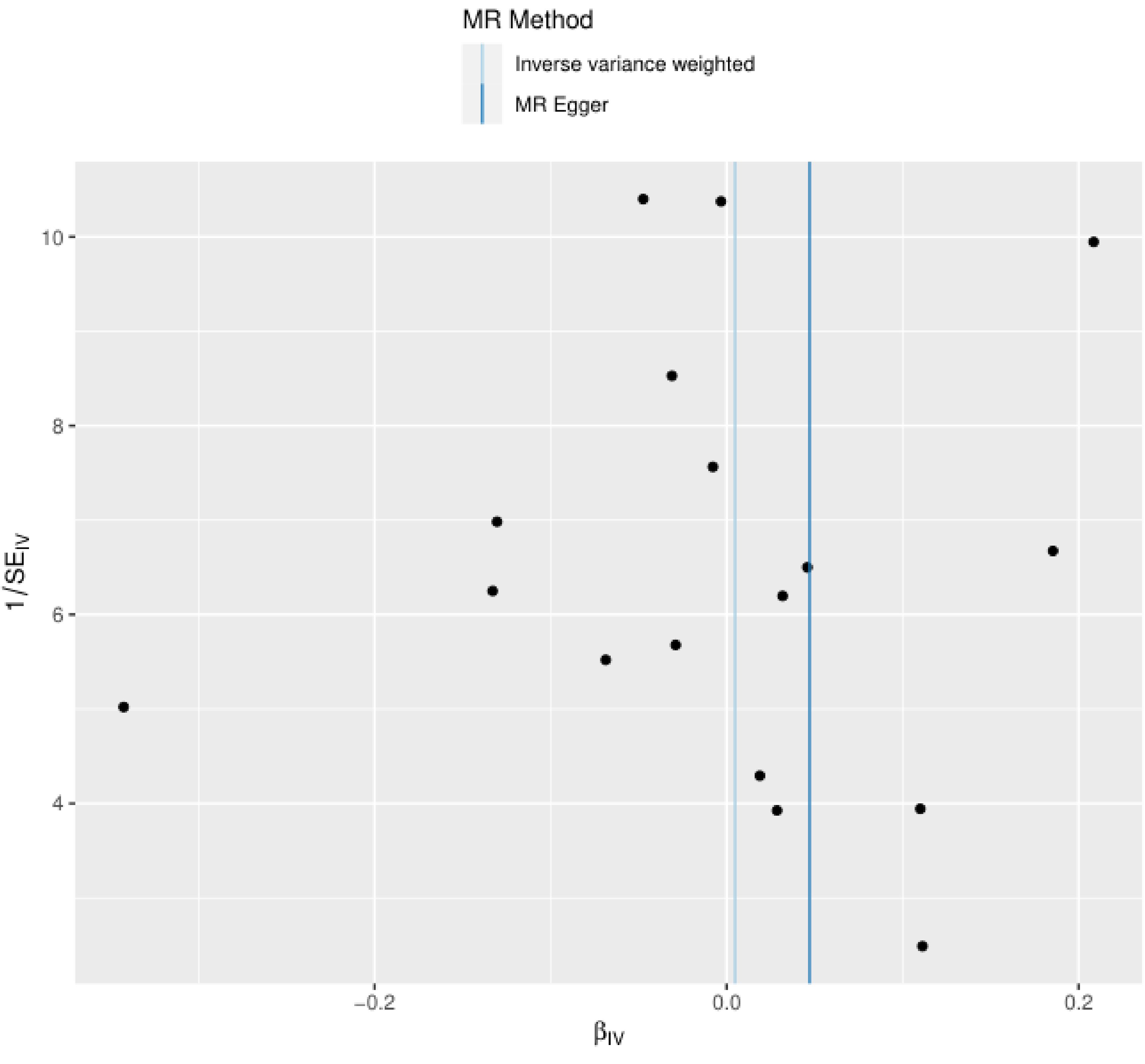

Funnel plot analyse of "CD14 on CD33br HLA DR+ CD14dim " on 'Diabetic nephropathy'

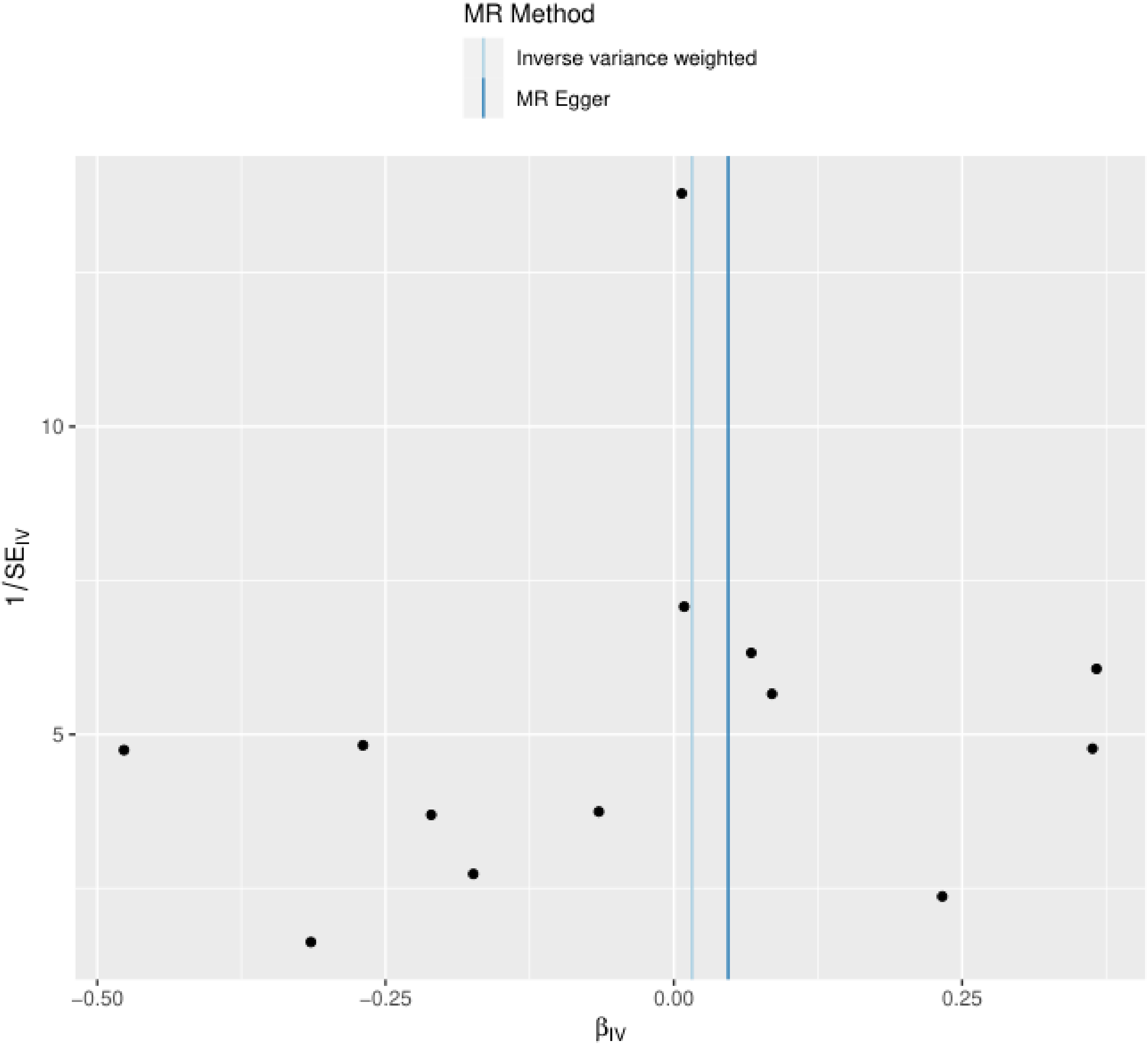

Funnel plot analyse of "CD45 on HLA DR+ NK" on 'Diabetic nephropathy'

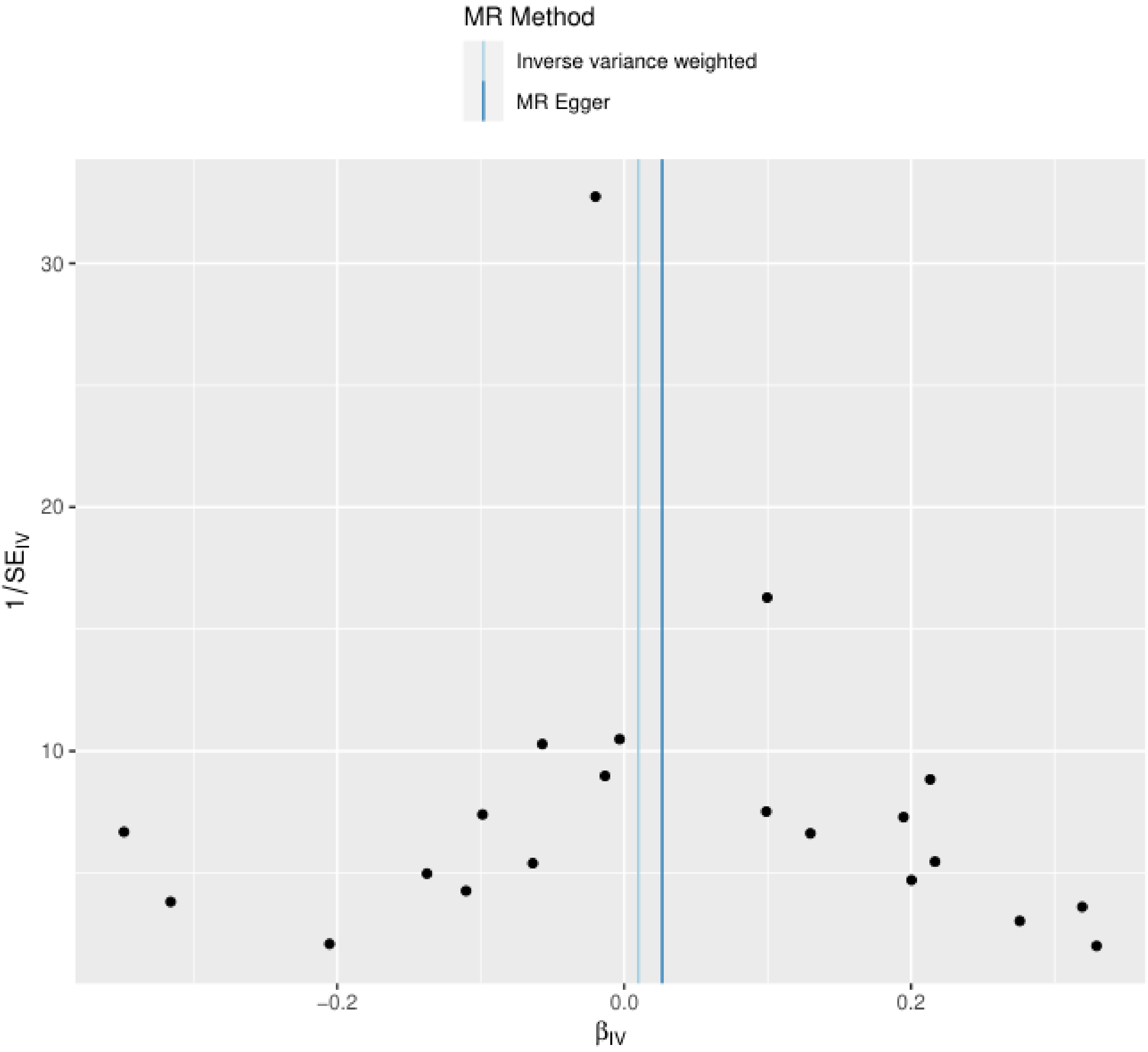

Funnel plot analyse of "BAFF-R on B cell" on 'Diabetic nephropathy'

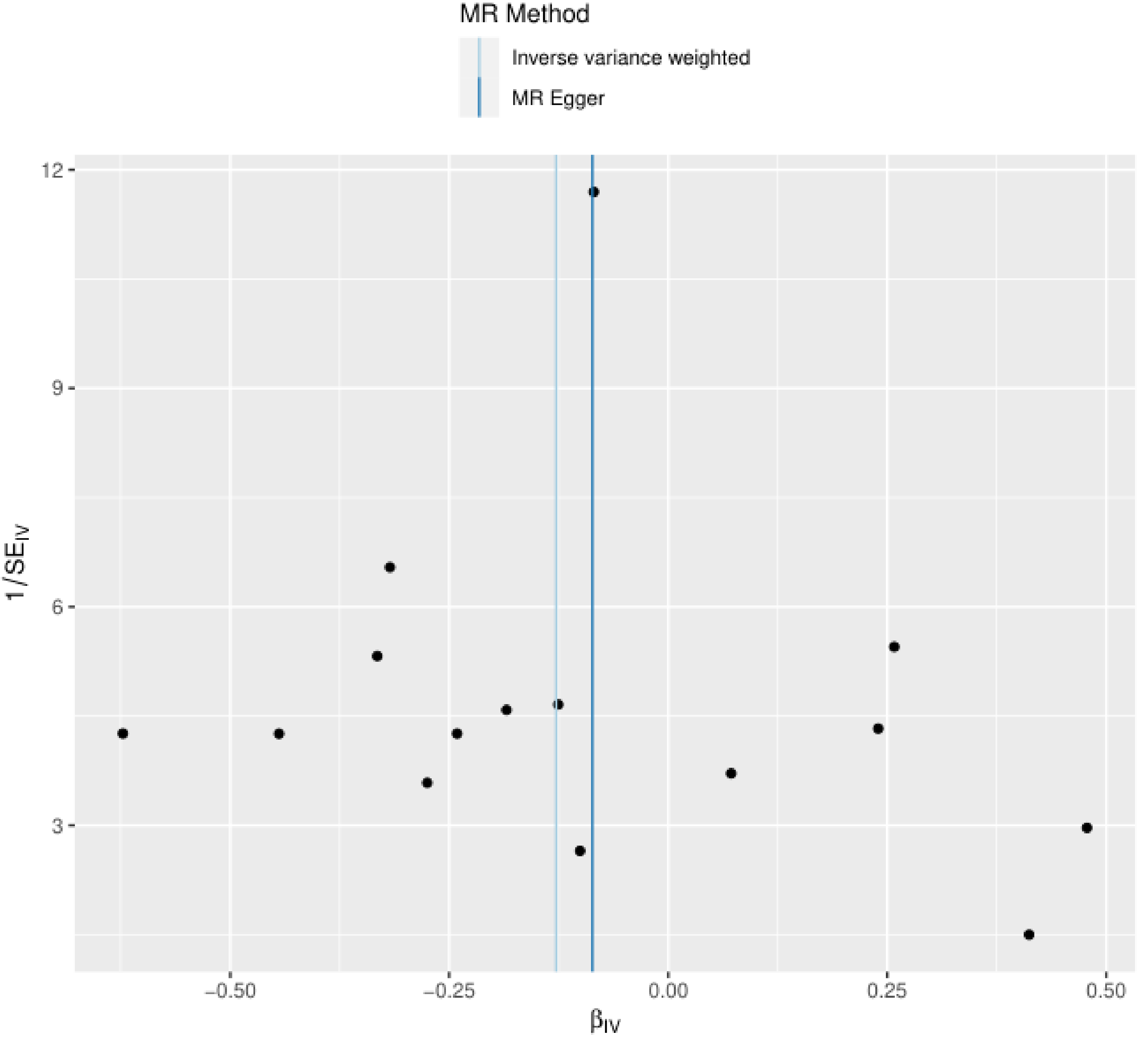

Funnel plot analyse of "CD4+ CD8dim %leukocyte" on 'Diabetic nephropathy'

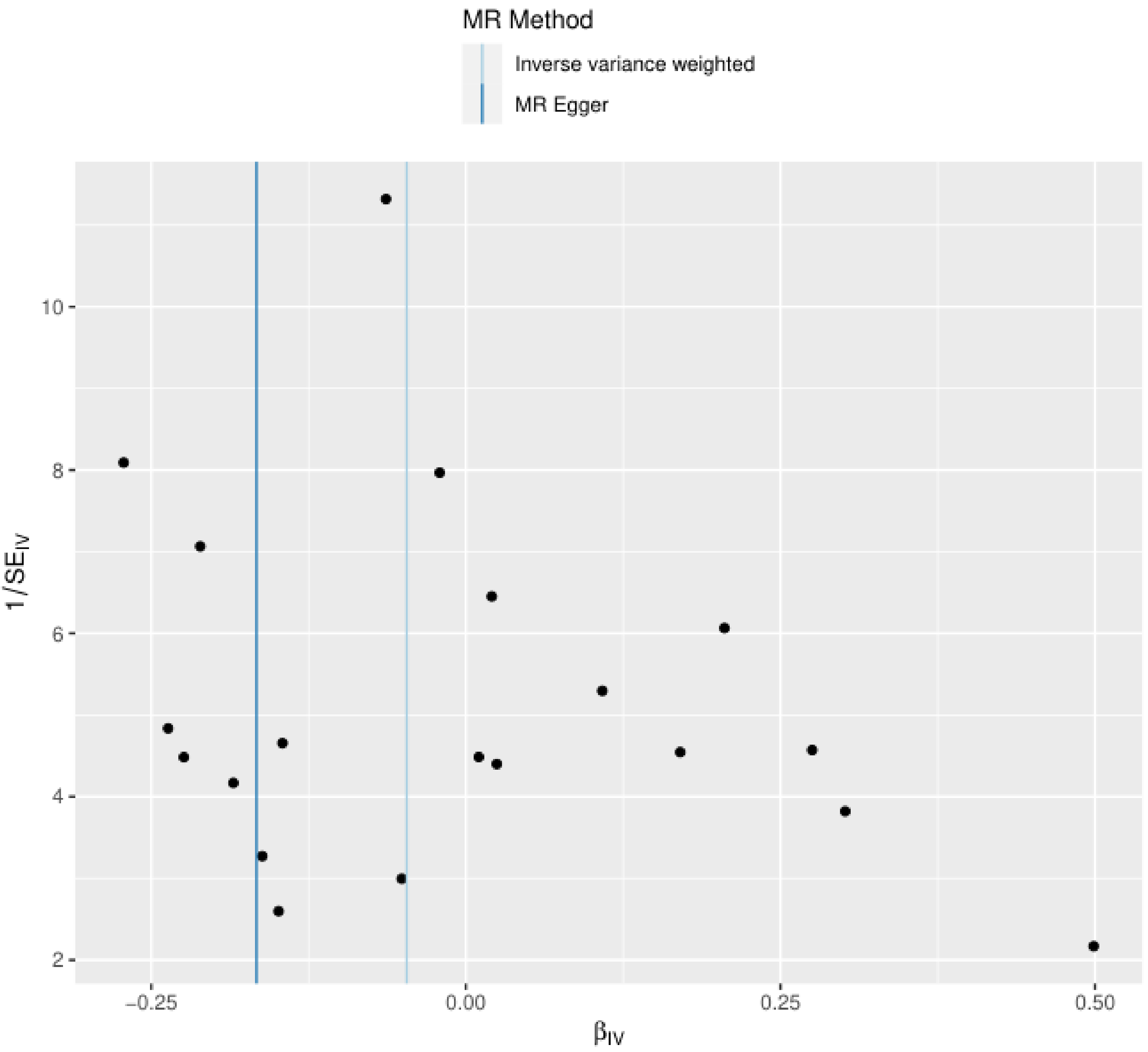

Funnel plot analyse of "HLA DR on HLA DR+ CD4+" on 'Diabetic nephropathy'

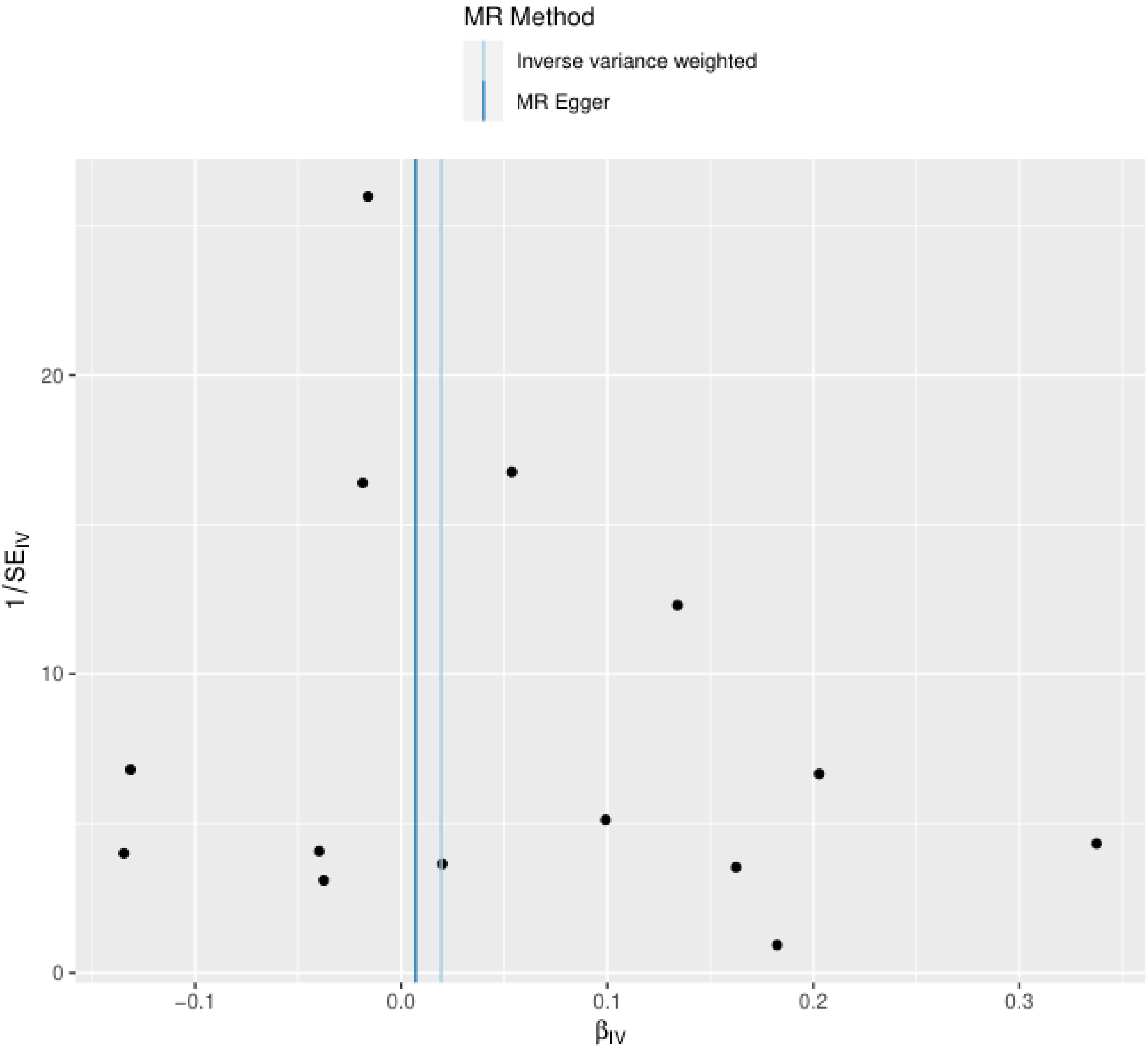

Funnel plot analyse of "EM CD8br %T cell" on 'Diabetic nephropathy'

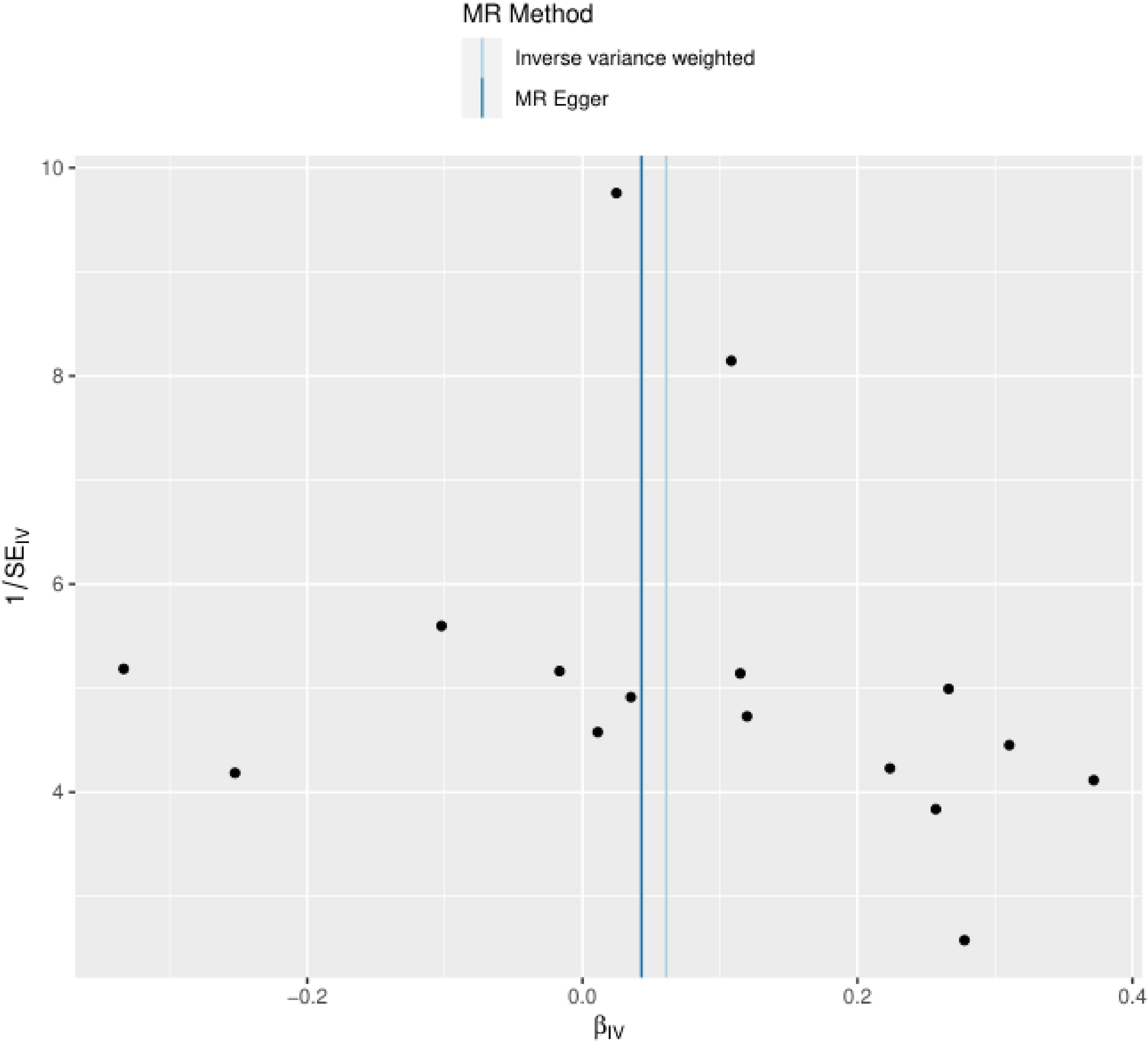

Funnel plot analyse of "CD3 on NKT " on 'Diabetic nephropathy'

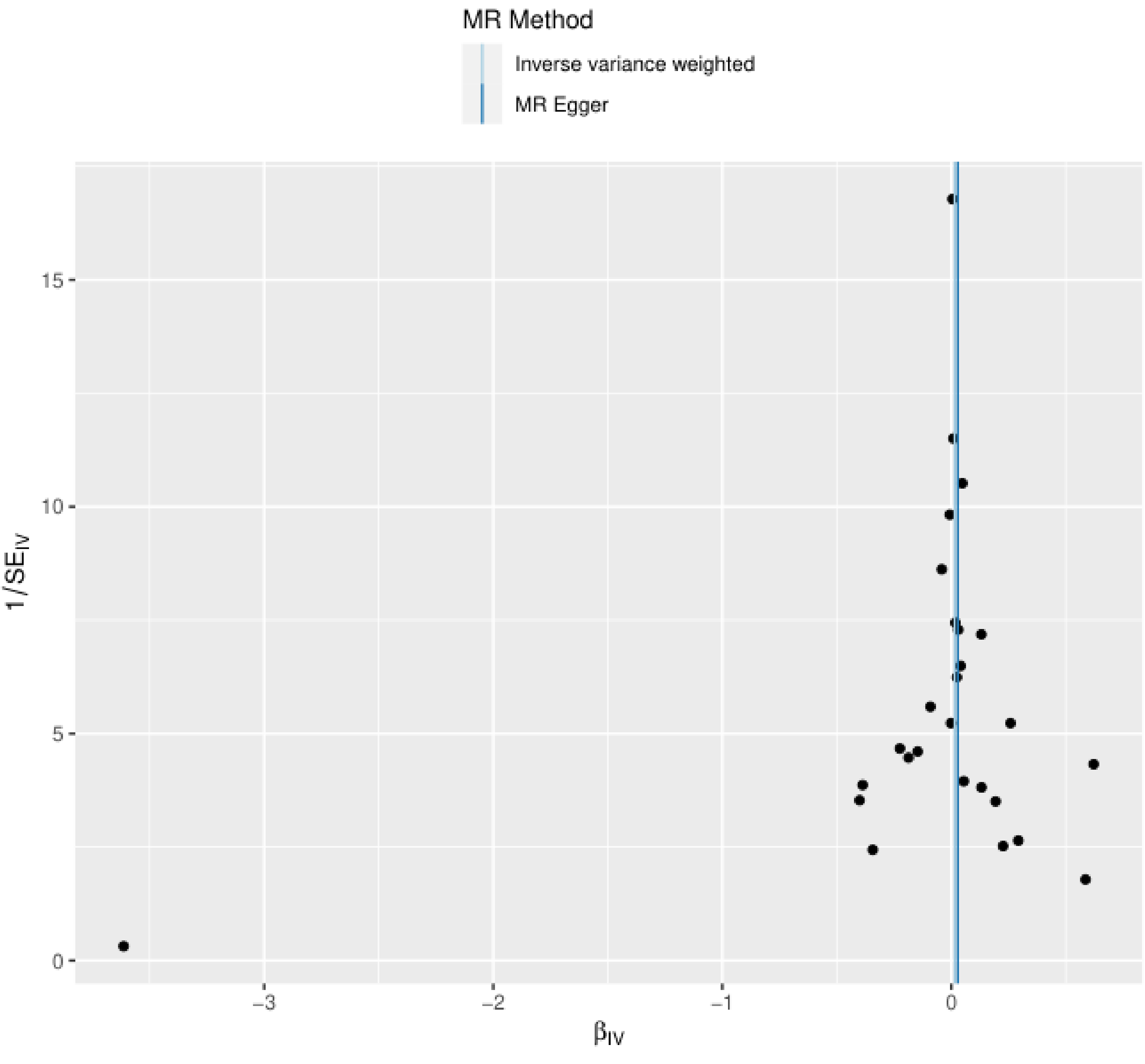

Funnel plot analyse of "CD16-CD56 on NKT" on 'Diabetic nephropathy'

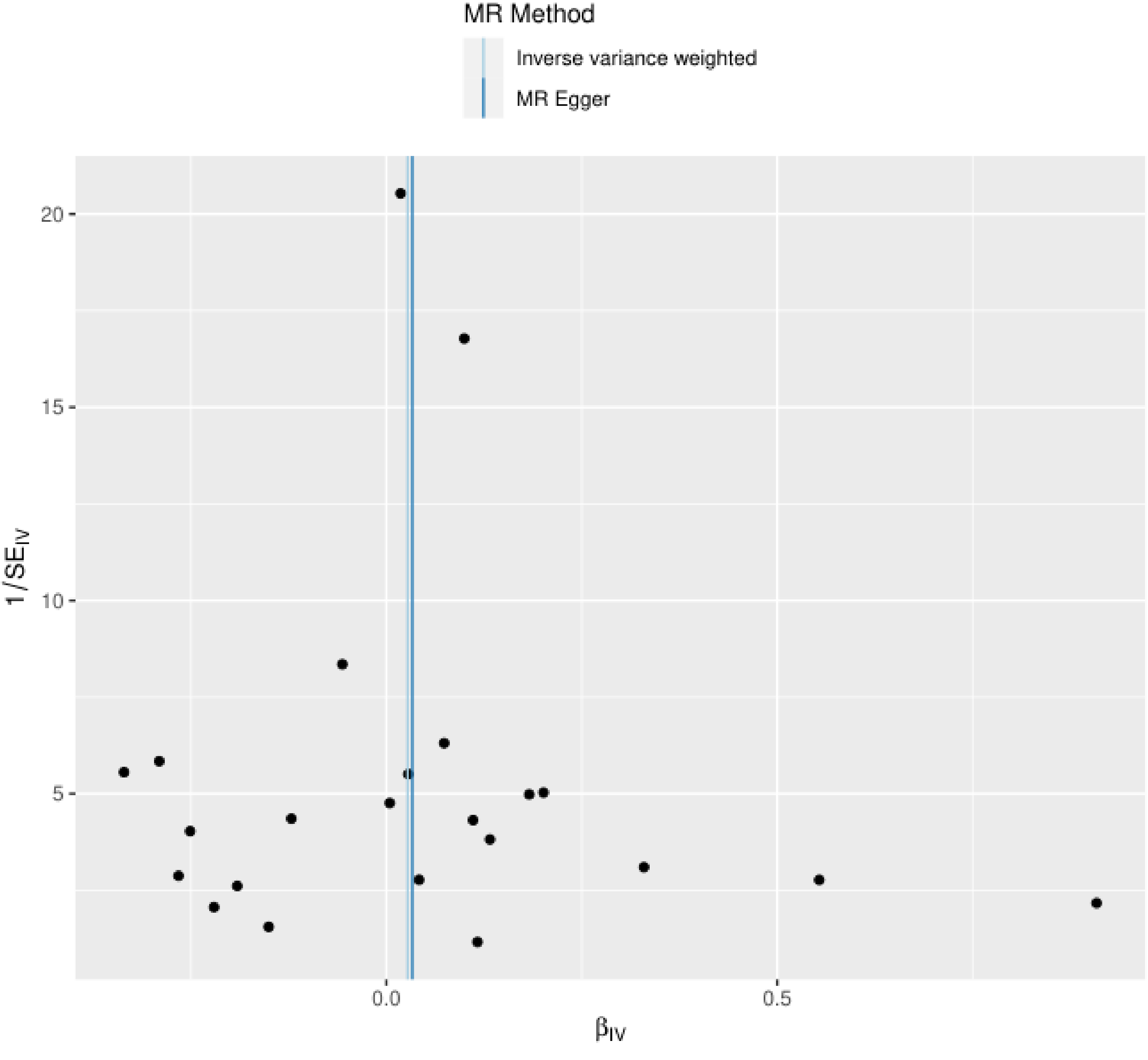

Funnel plot analyse of "CD16 on CD14+ CD16+ monocyte" on 'Diabetic nephropathy'

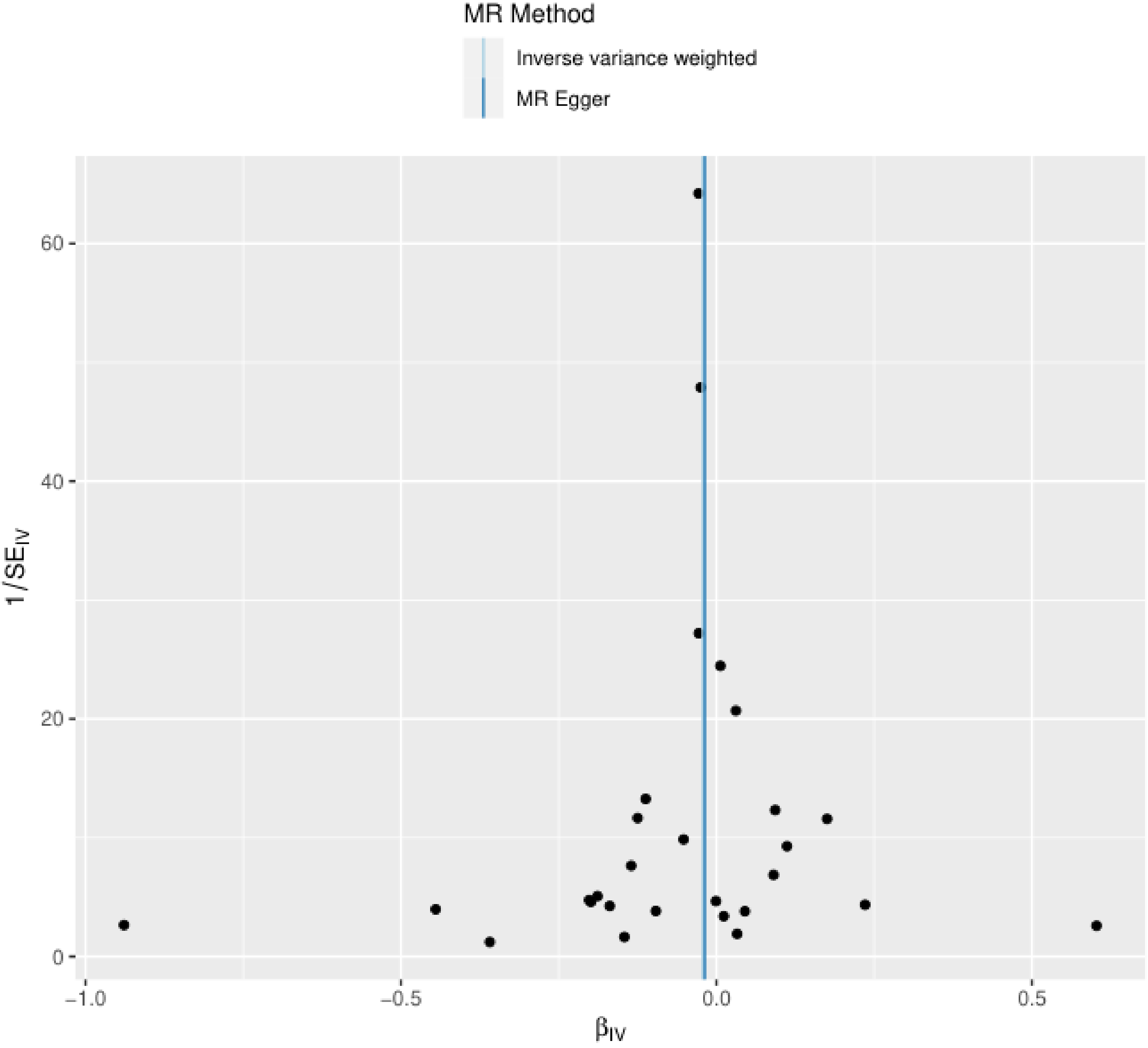

Funnel plot analyse of "CD28+ CD45RA- CD8dim %T cell" on 'Diabetic nephropathy'

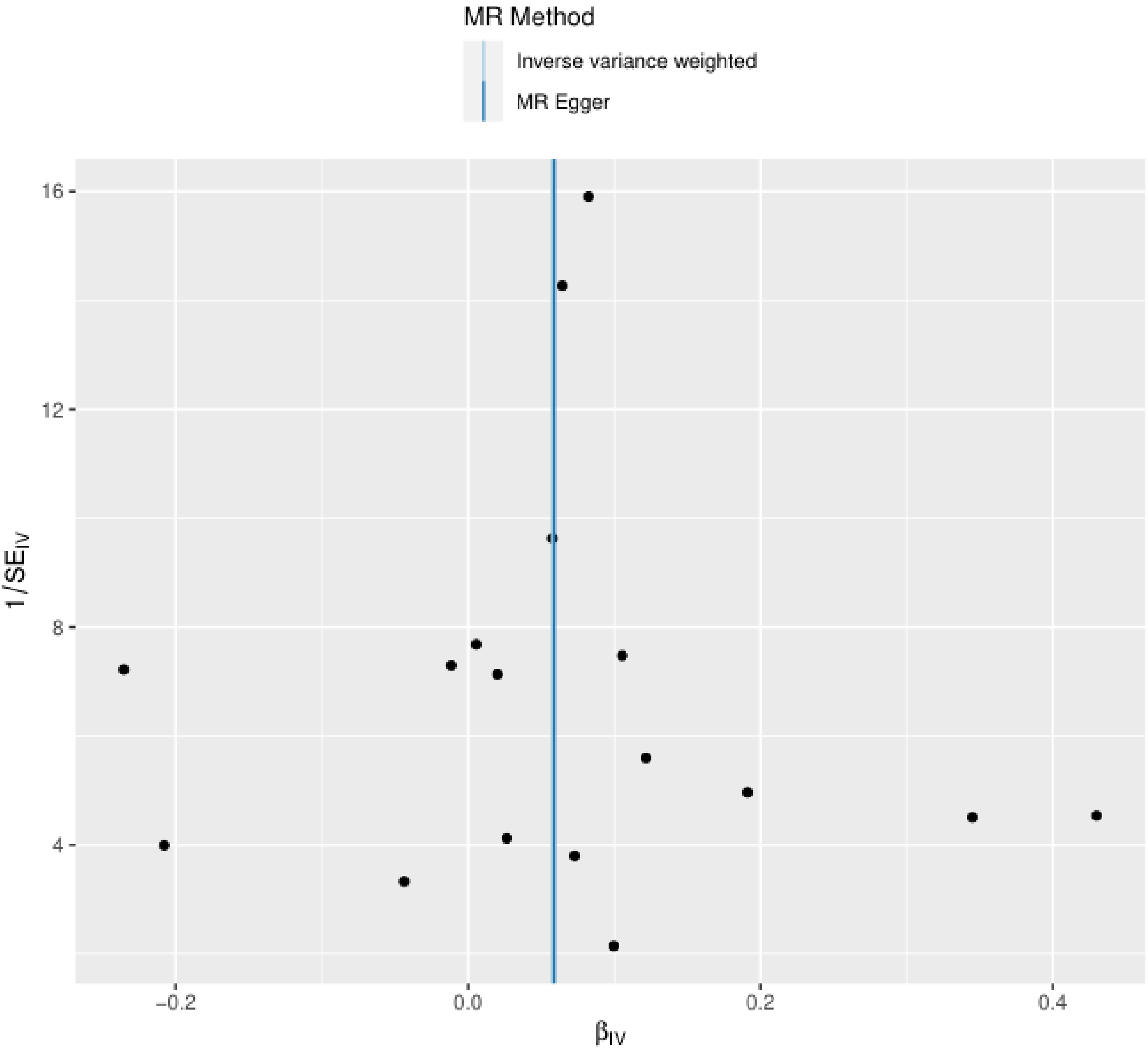

Funnel plot analyse of "CD38 on IgD+ CD38br" on 'Diabetic nephropathy'

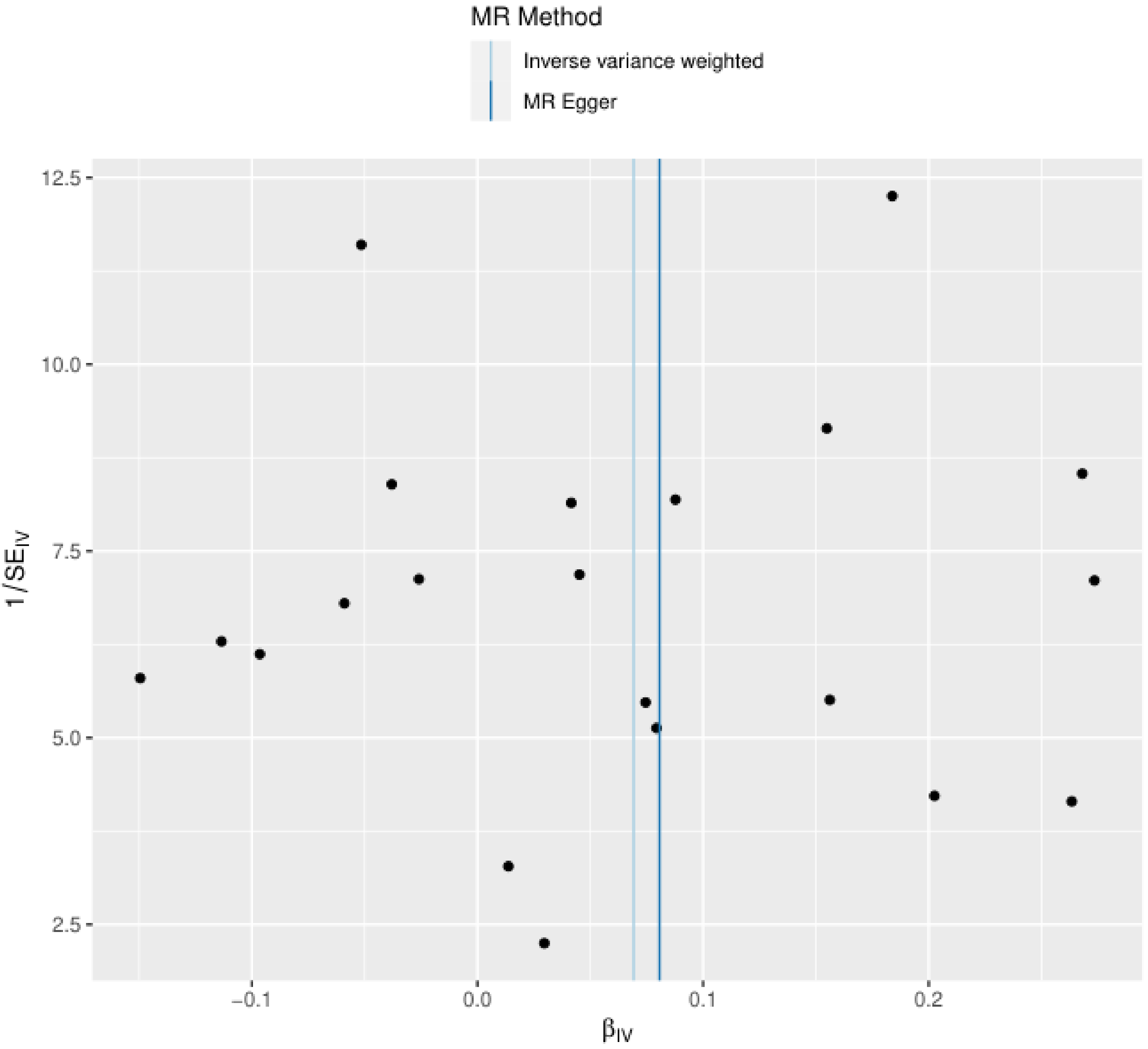

Funnel plot analyse of "CD33- HLA DR- AC" on 'Diabetic nephropathy'

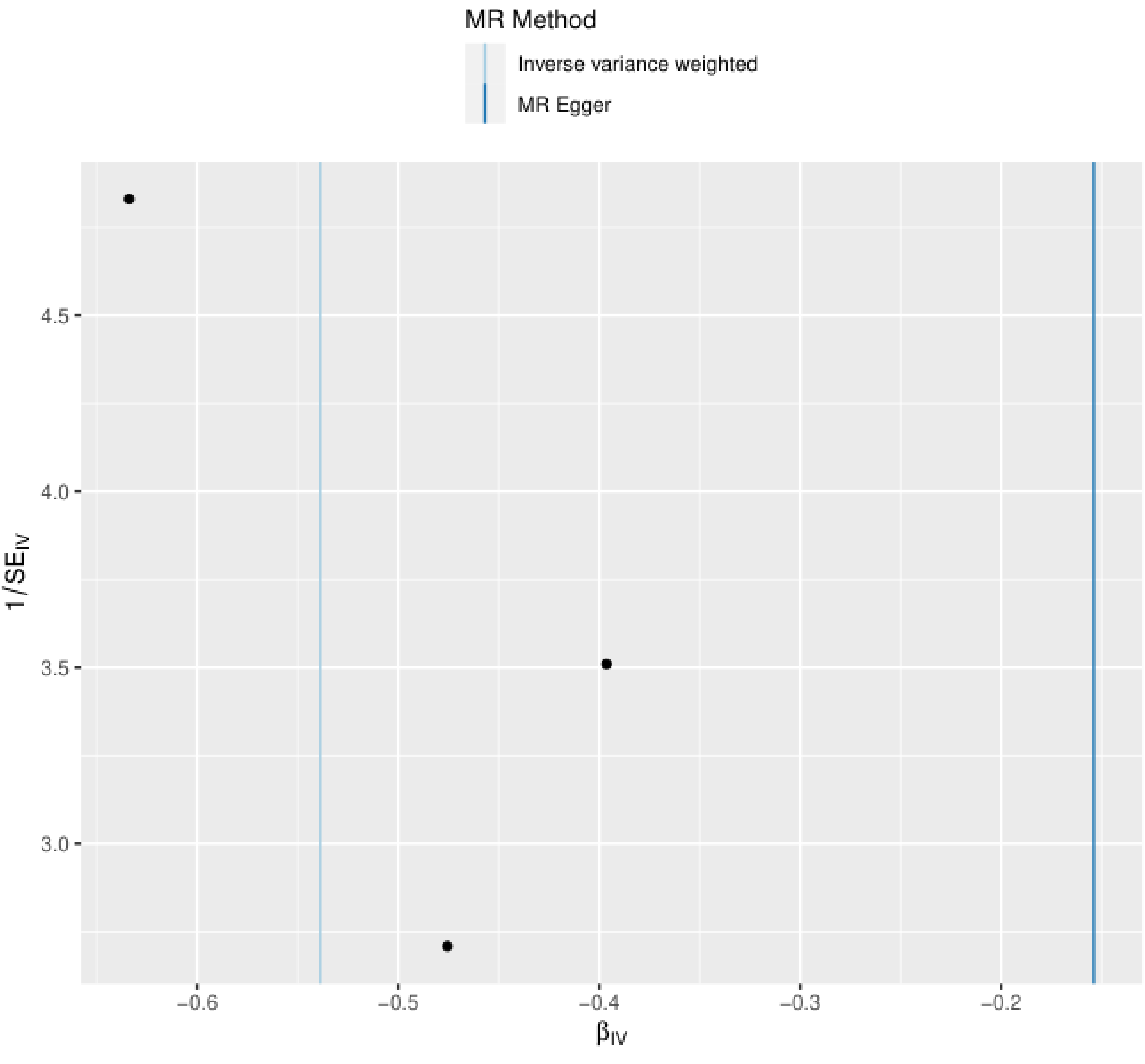

Funnel plot analyse of "CD28 on CD28+ DN (CD4-CD8-)" on 'Diabetic nephropathy'

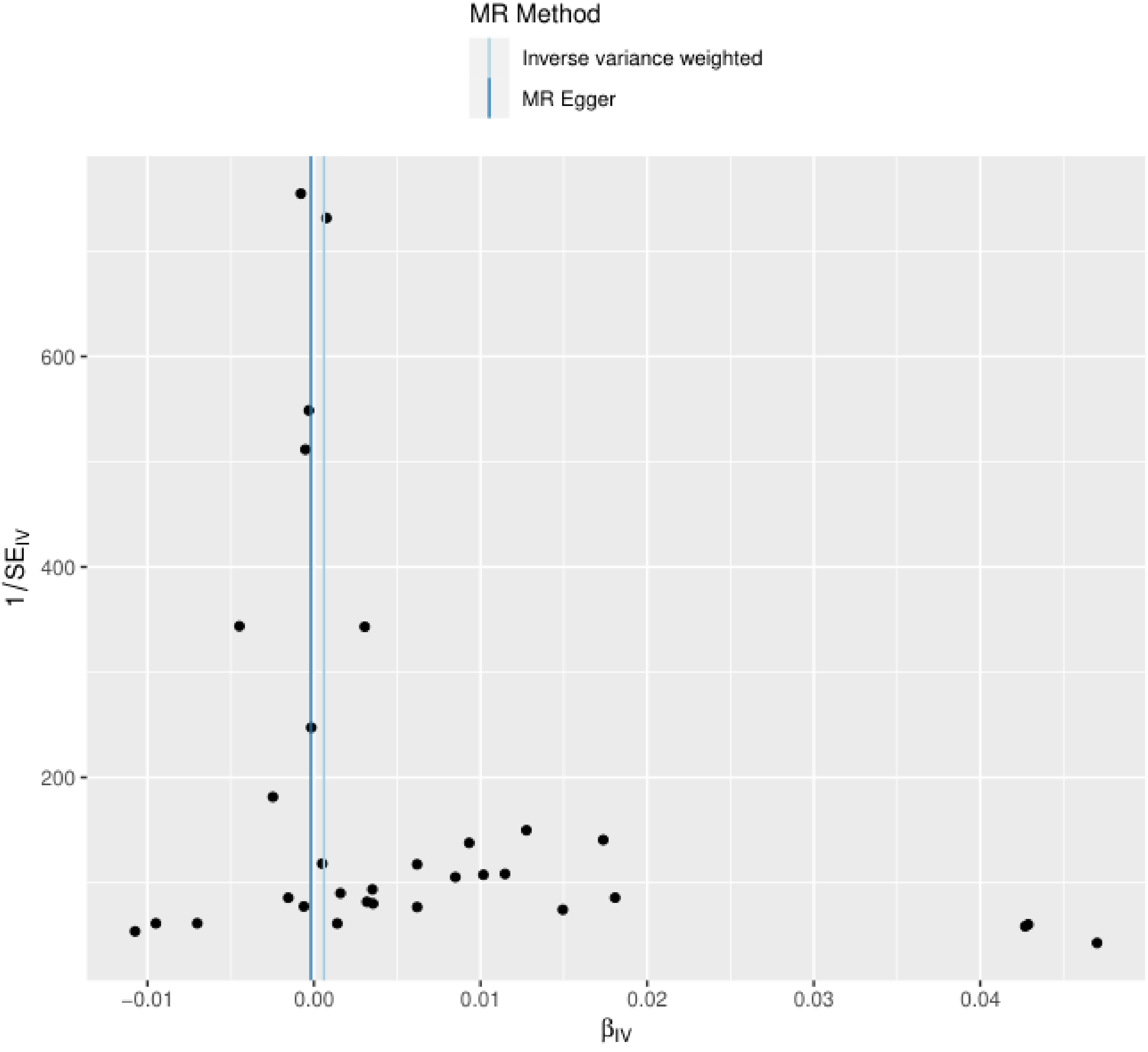

Funnel plot analyse of "CD45RA+ CD28- CD8br %CD8br" on 'Diabetic nephropathy'

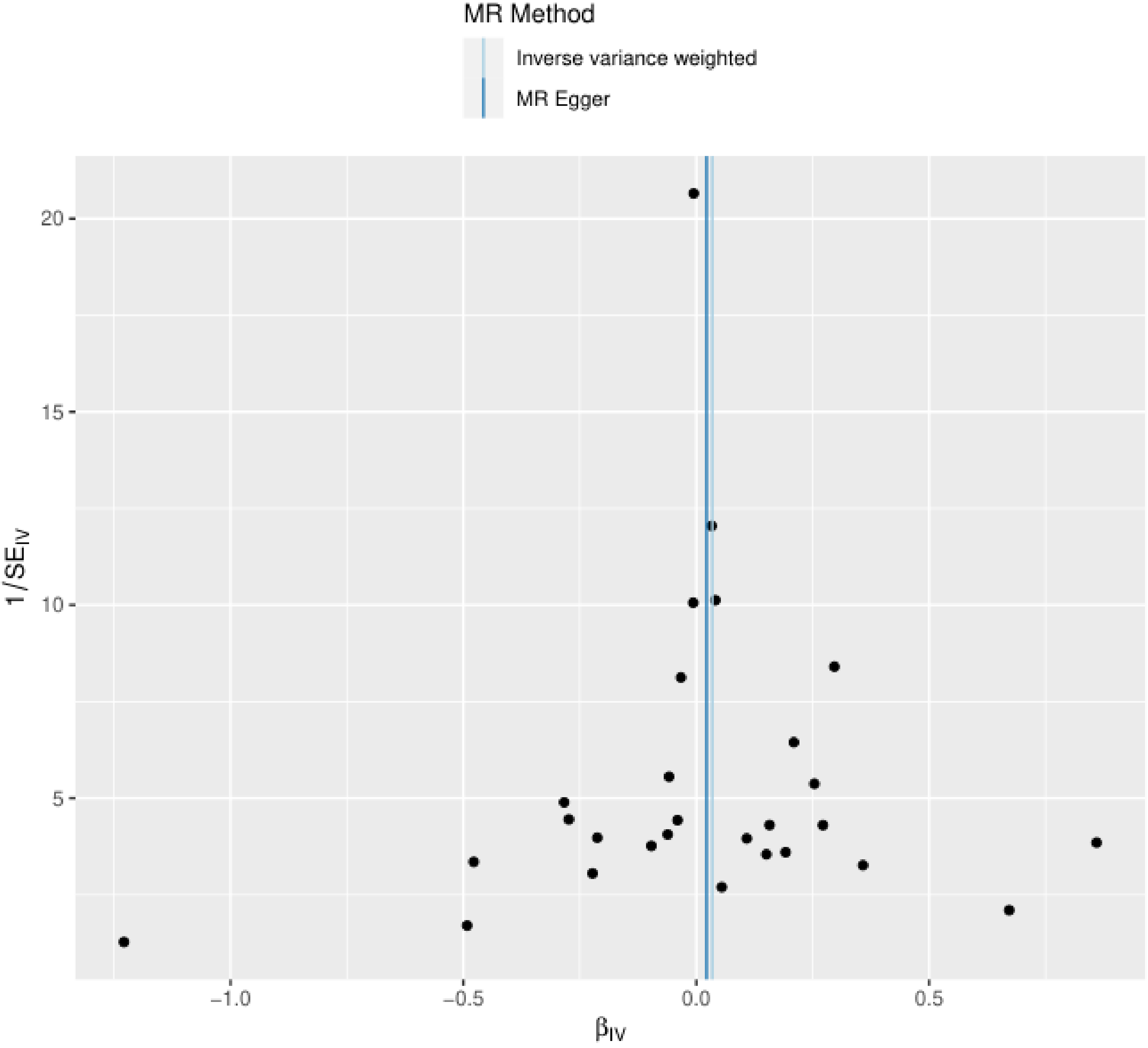

Funnel plot analyse of "CD8dim NKT AC" on 'Diabetic nephropathy'

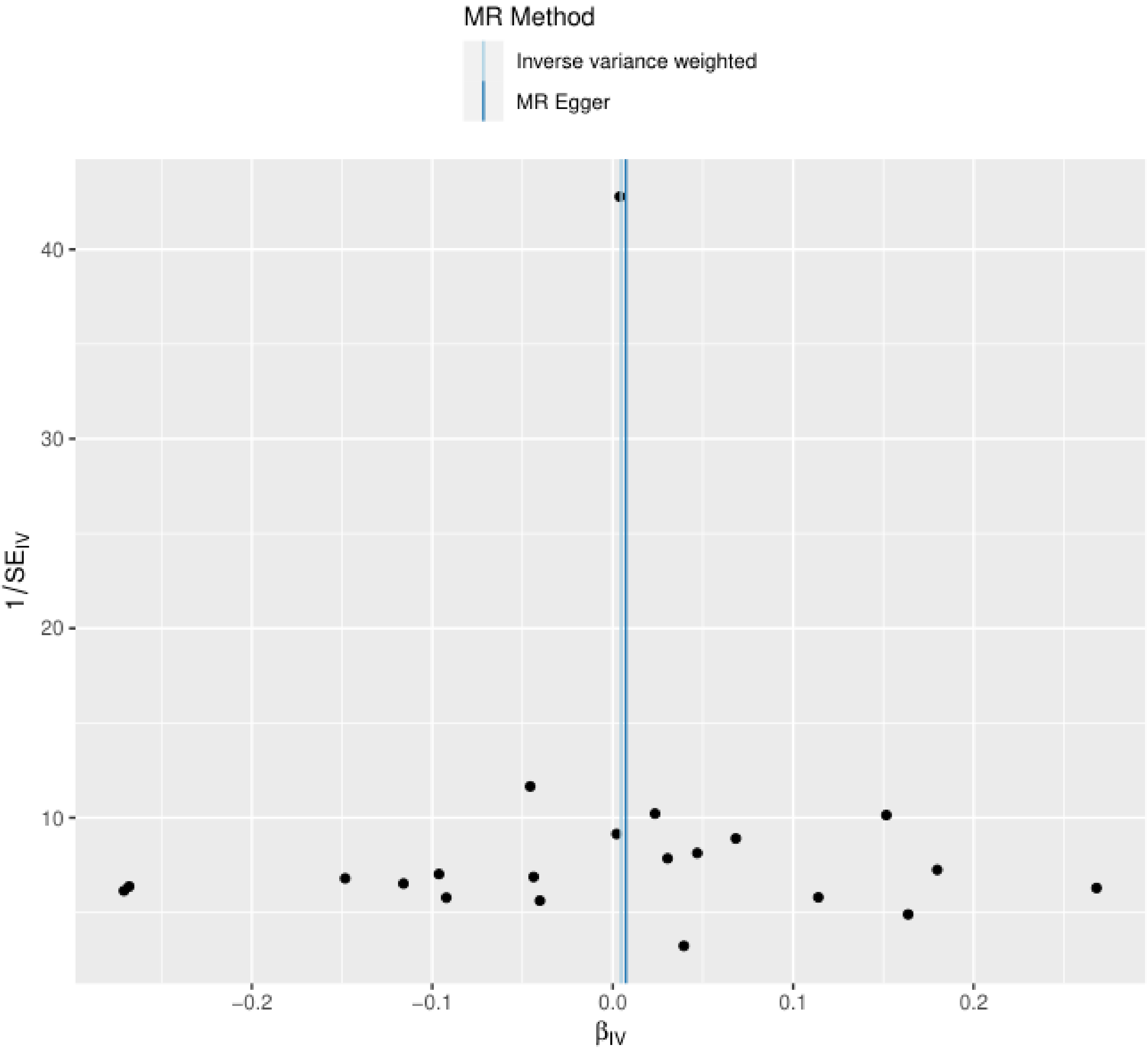

Funnel plot analyse of "CD33 on Mo MDSC " on 'Diabetic nephropathy'

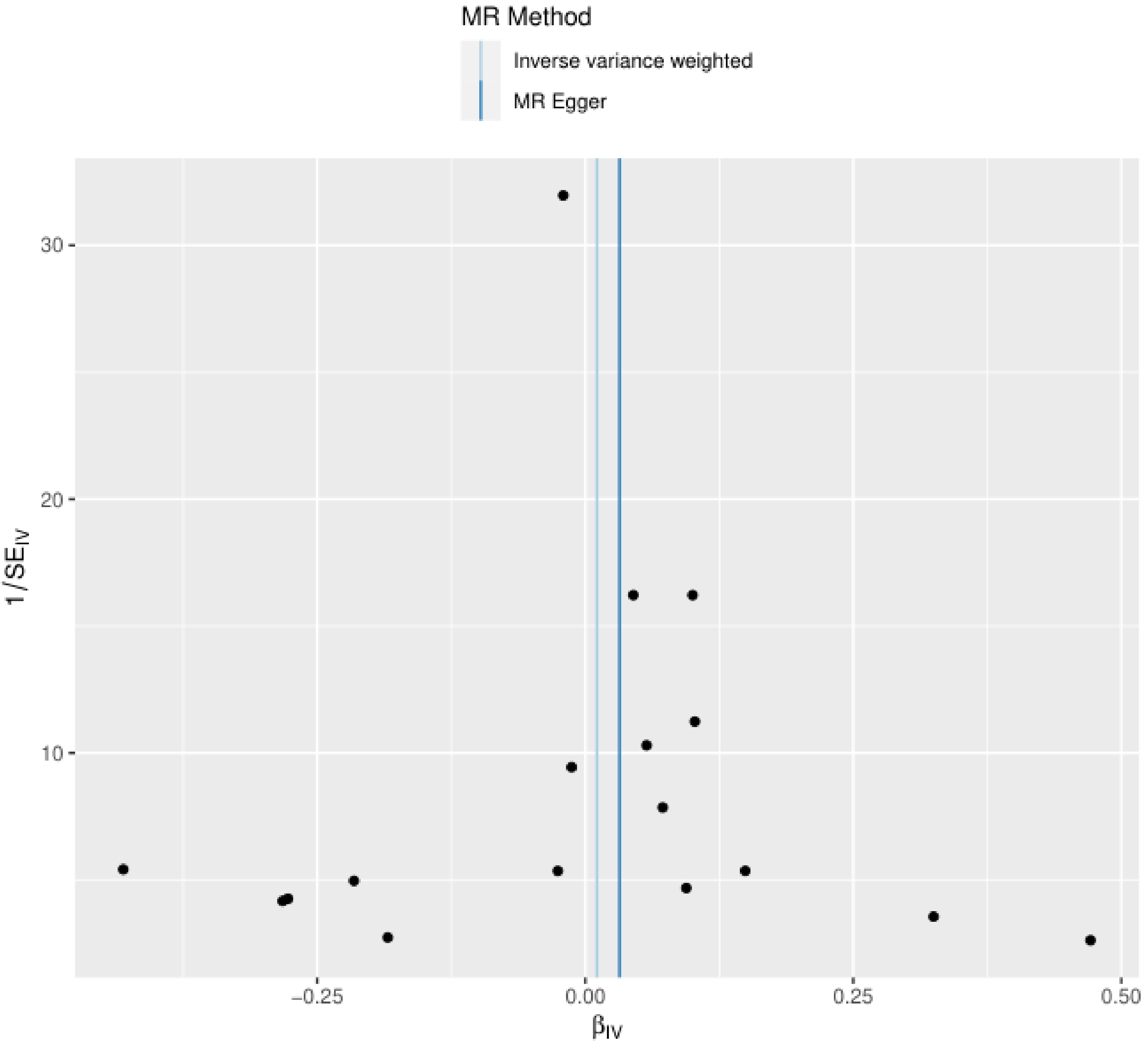

Funnel plot analyse of "BAFF-R on IgD- CD38-" on 'Diabetic nephropathy'

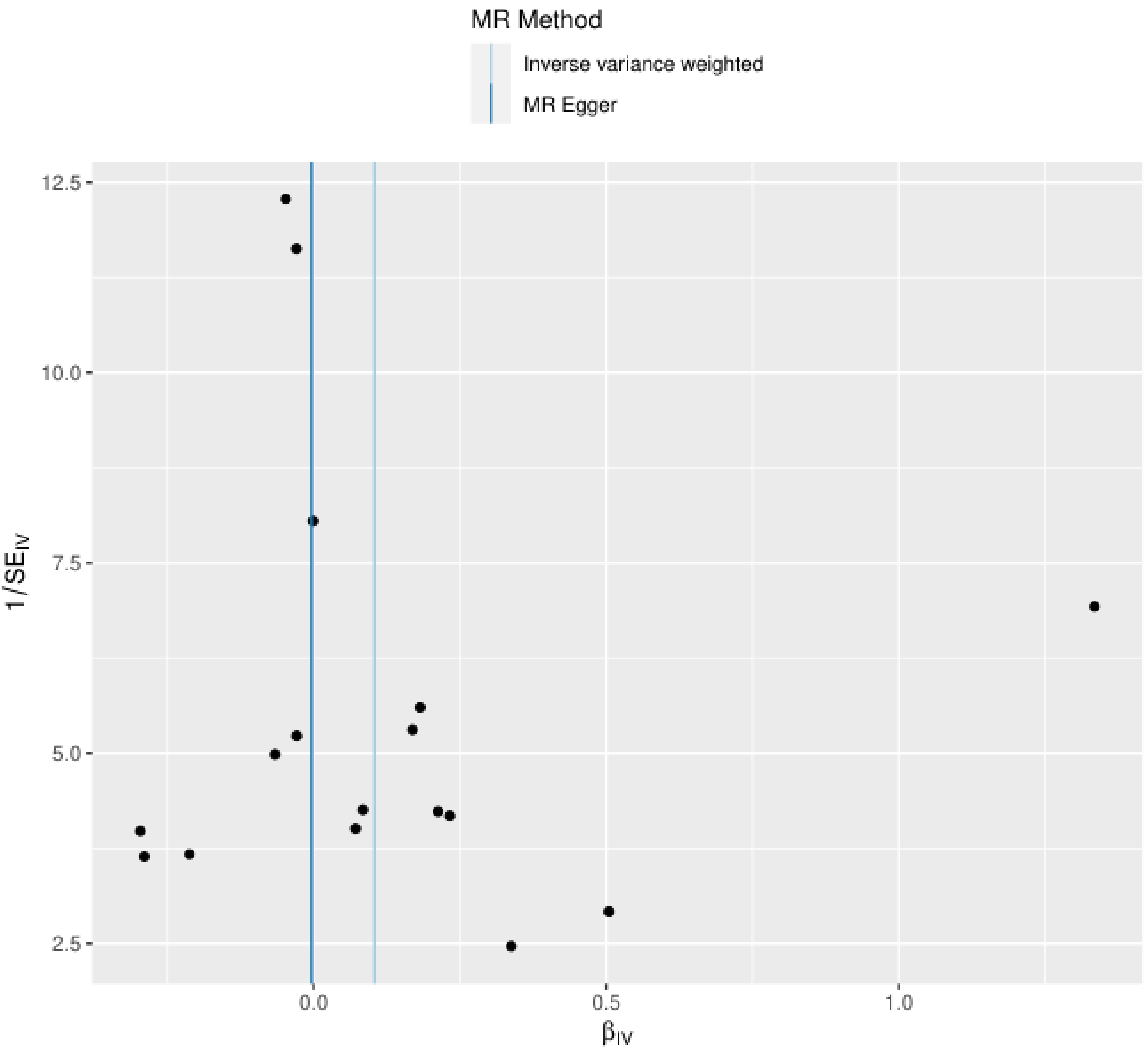

Funnel plot analyse of "T cell AC" on 'Diabetic nephropathy'

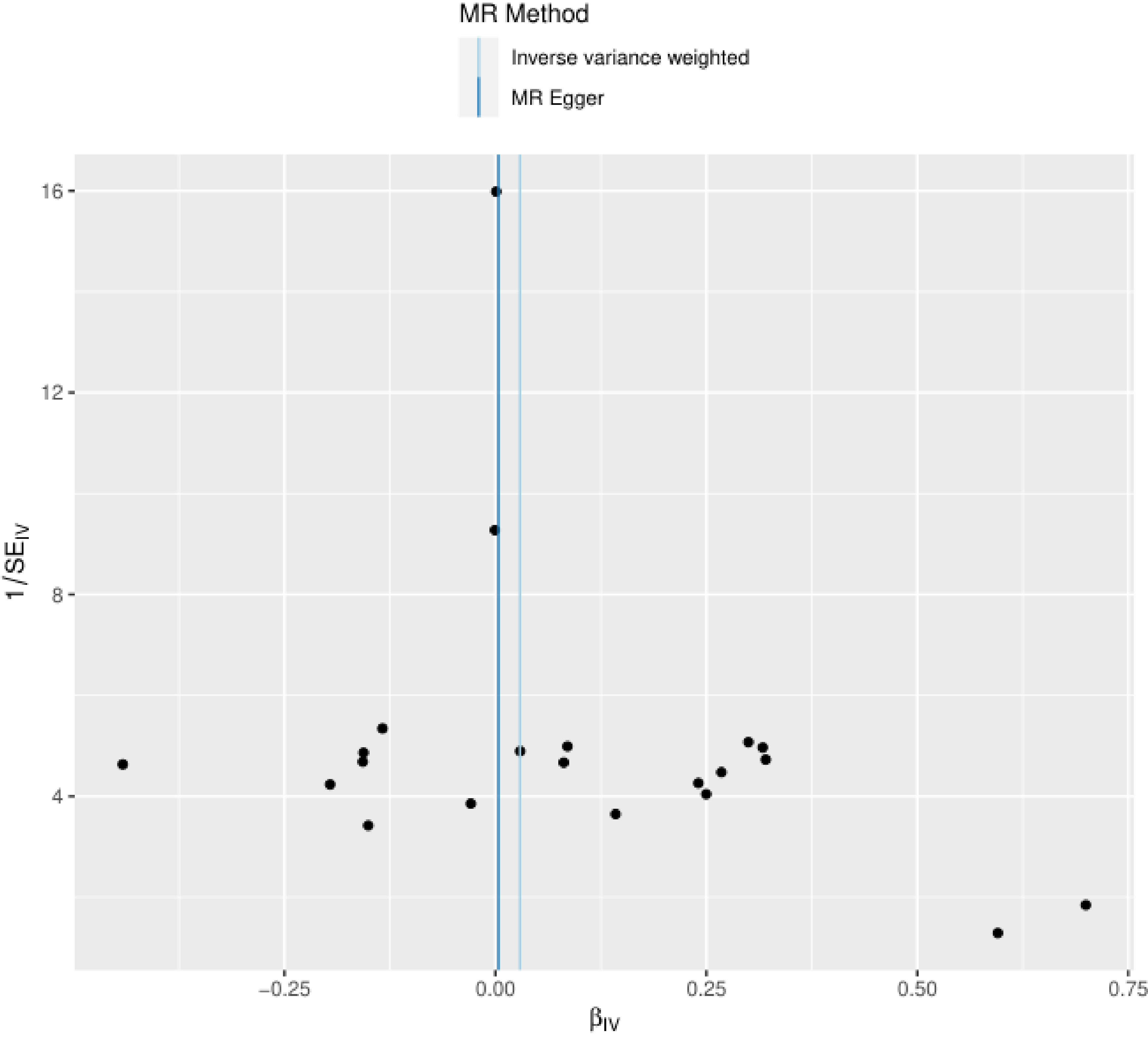

Funnel plot analyse of "TD DN (CD4-CD8-) AC" on 'Diabetic nephropathy'

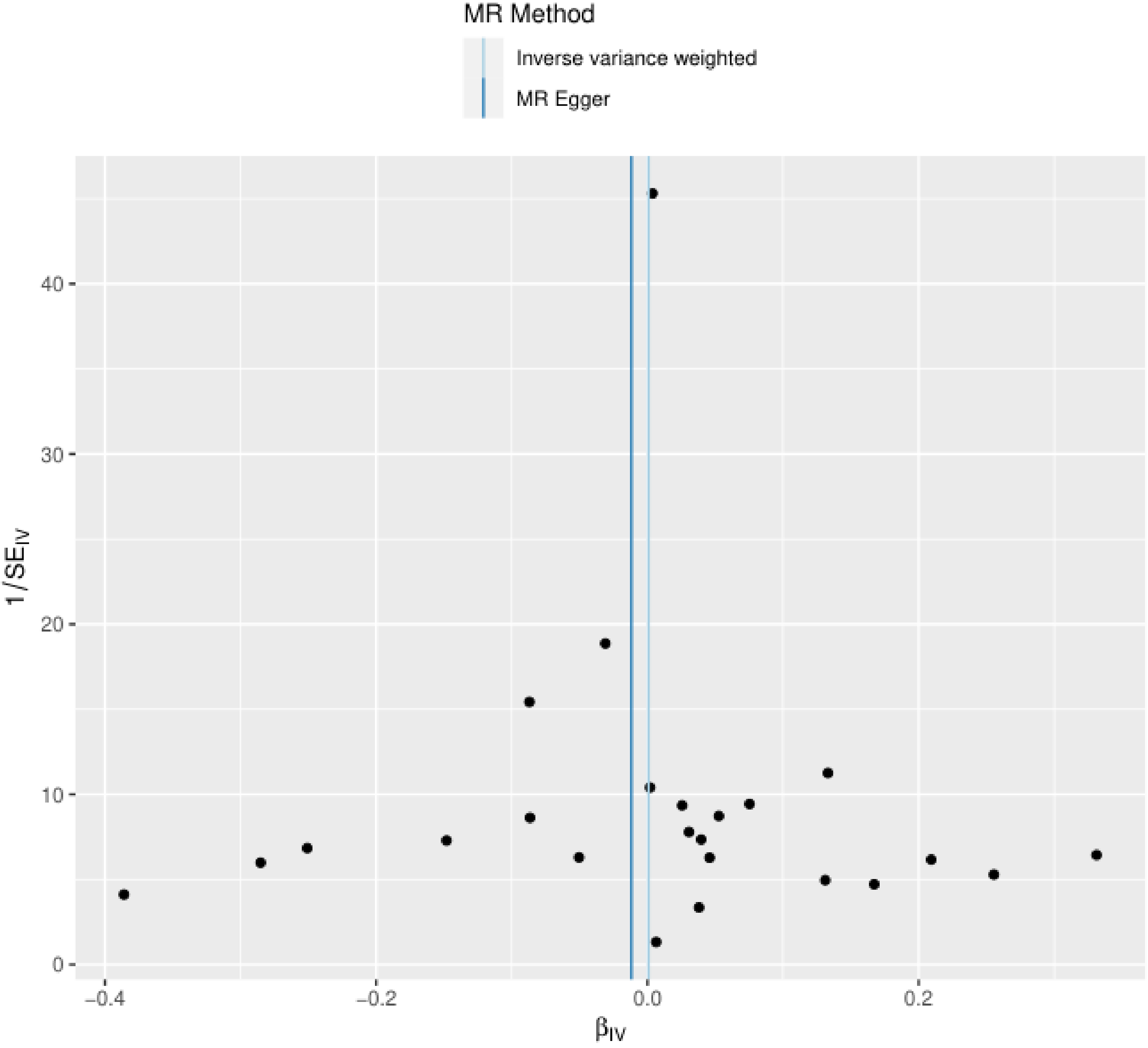

Funnel plot analyse of "CD33 on CD33br HLA DR+ " on 'Diabetic nephropathy'

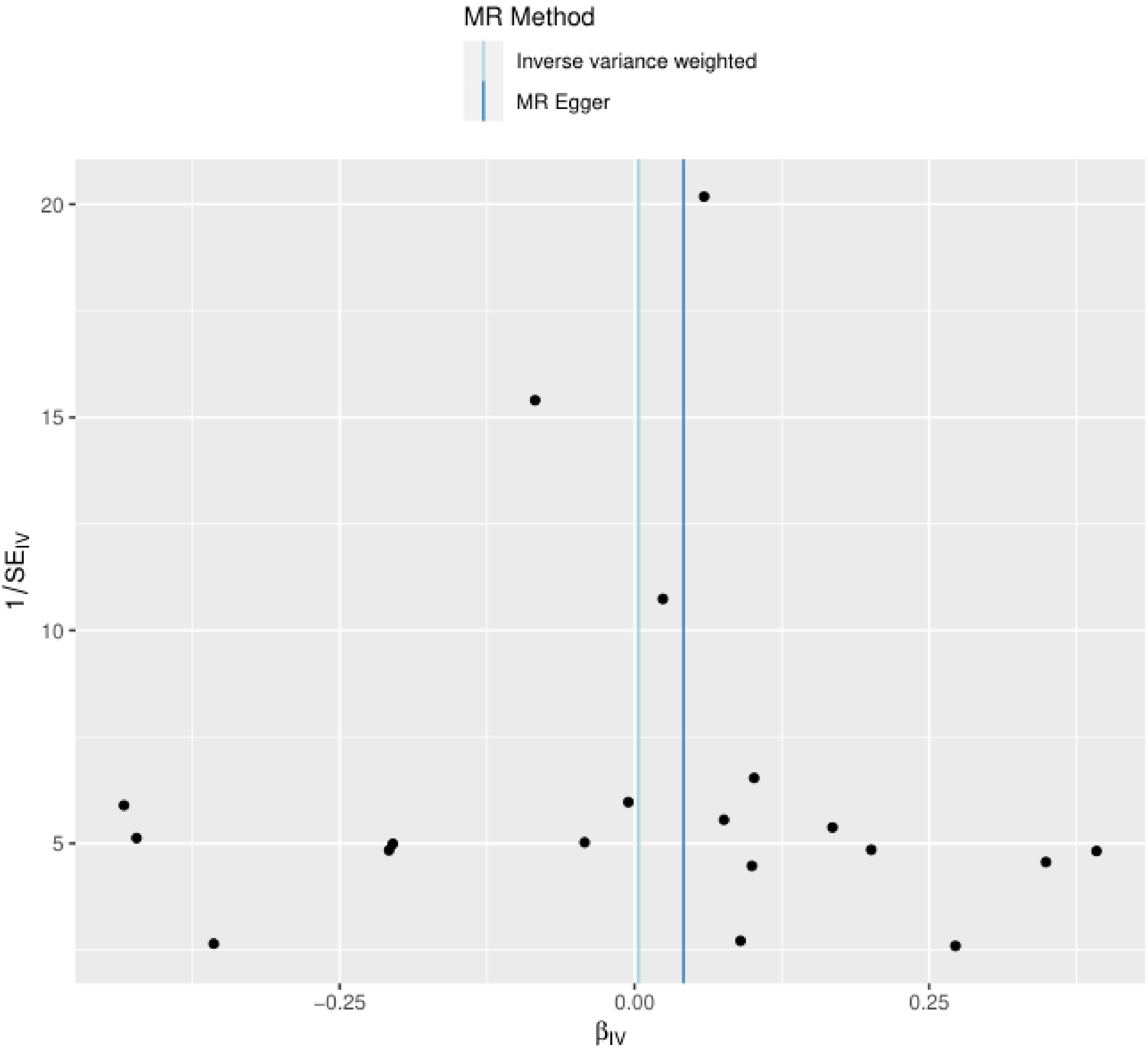

Funnel plot analyse of "CCR2 on plasmacytoid DC" on 'Diabetic nephropathy'

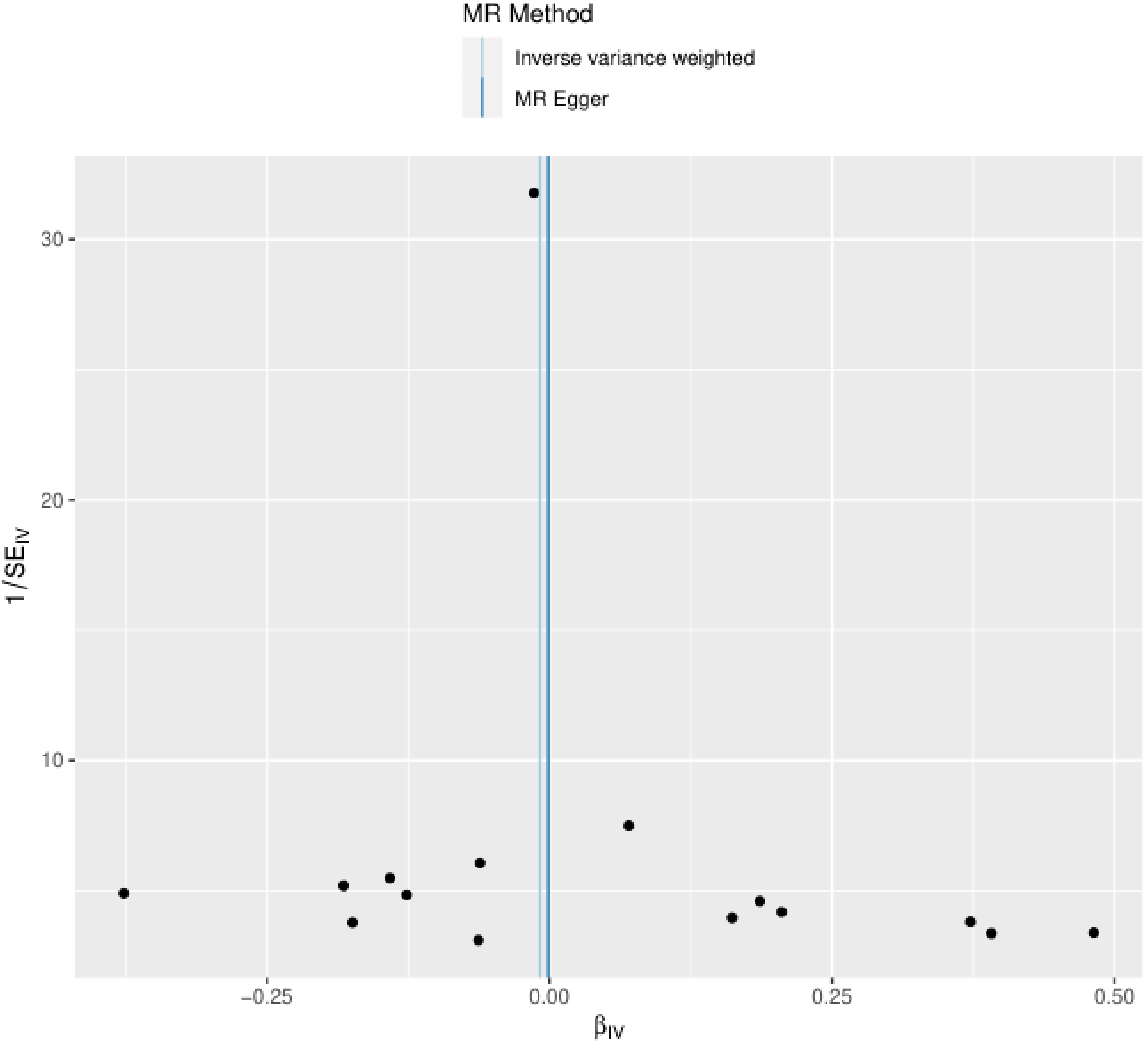

Funnel plot analyse of "CD11c+ HLA DR++ monocyte %monocyte" on 'Diabetic nephropathy'

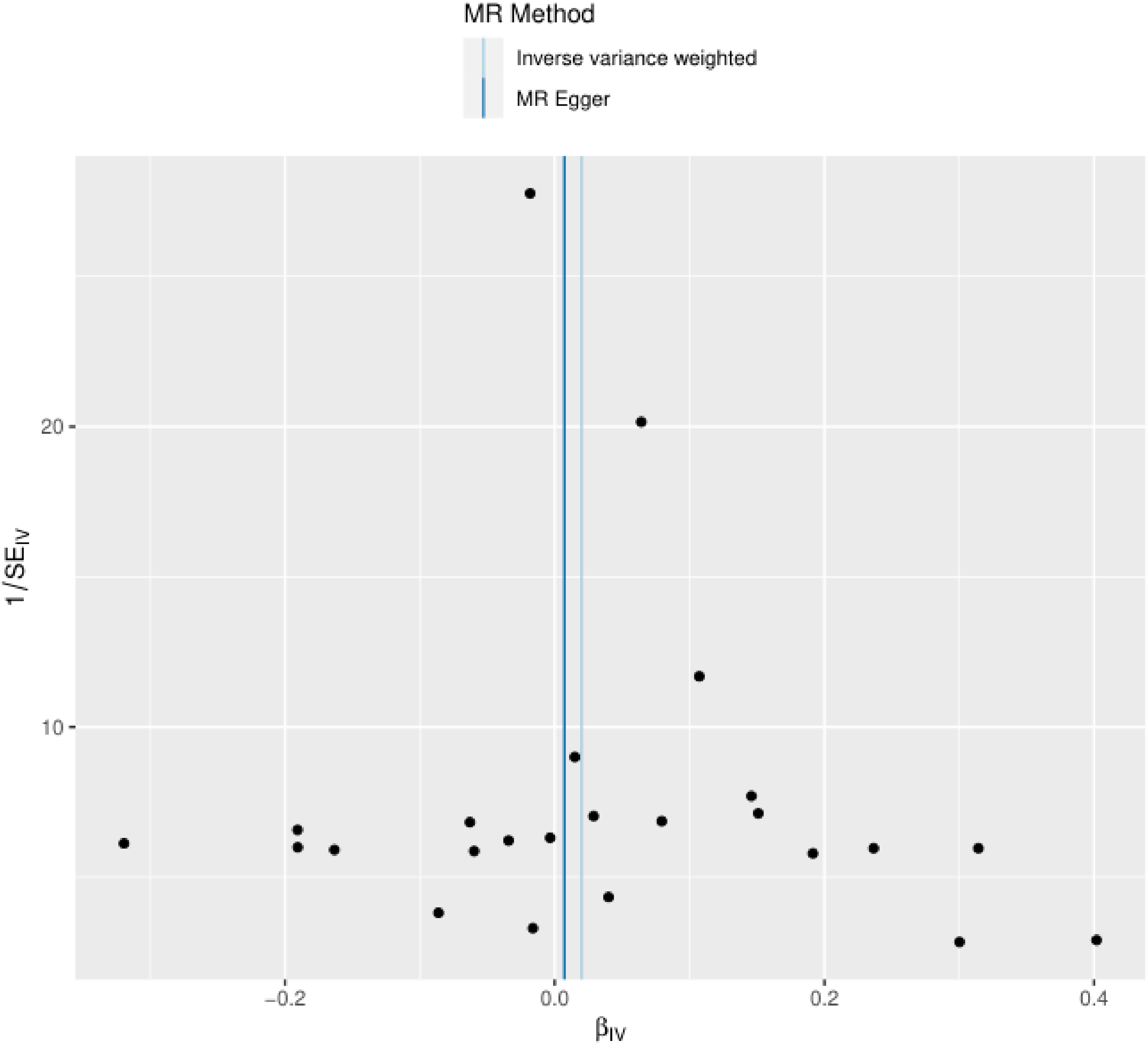

Funnel plot analyse of "CD25 on IgD+ CD38- naive" on 'Diabetic nephropathy'

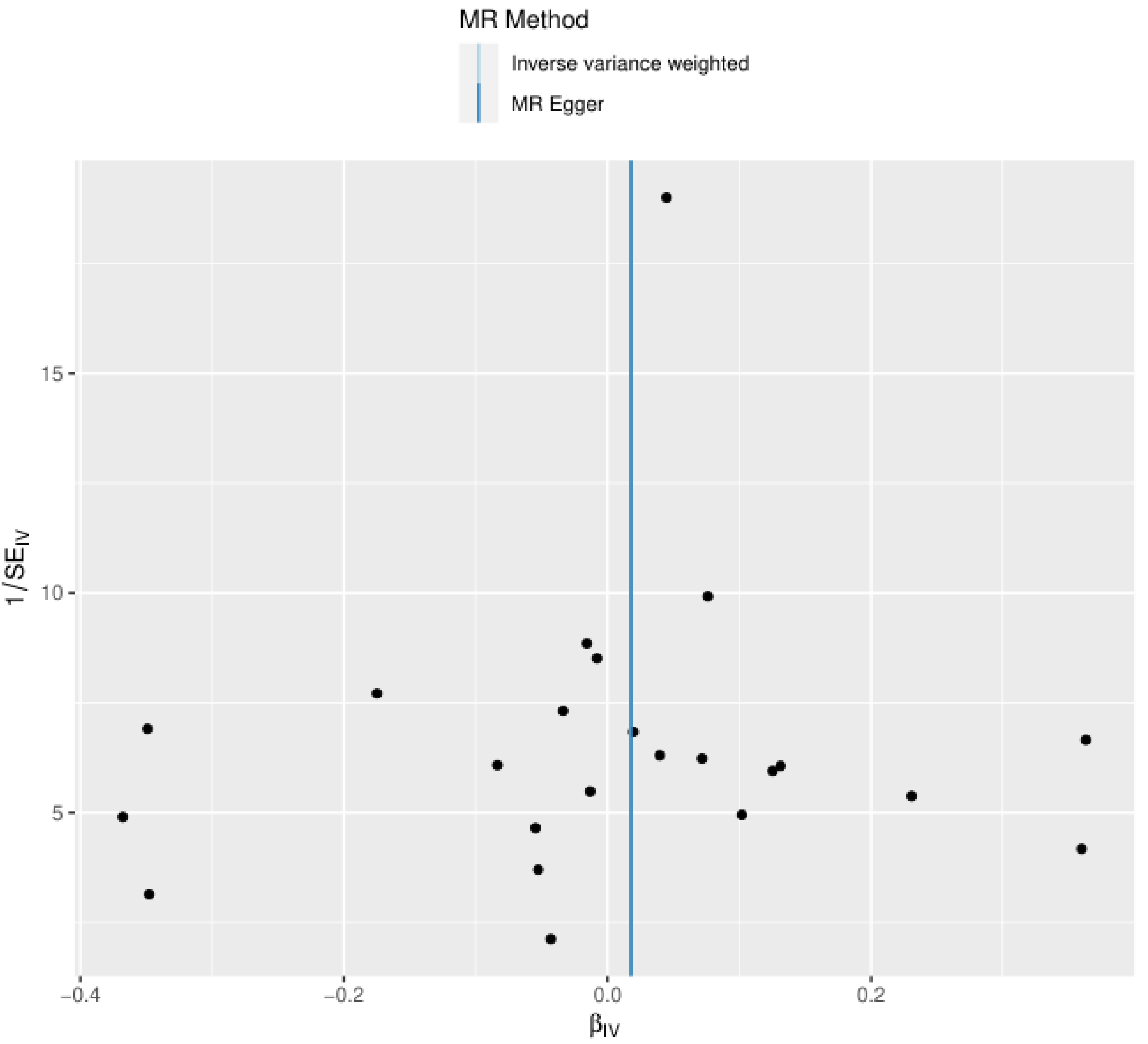

nnel plot analyse of "CD33br HLA DR+ CD14dim %CD33br HLA DR+" on 'Diabetic nephropat

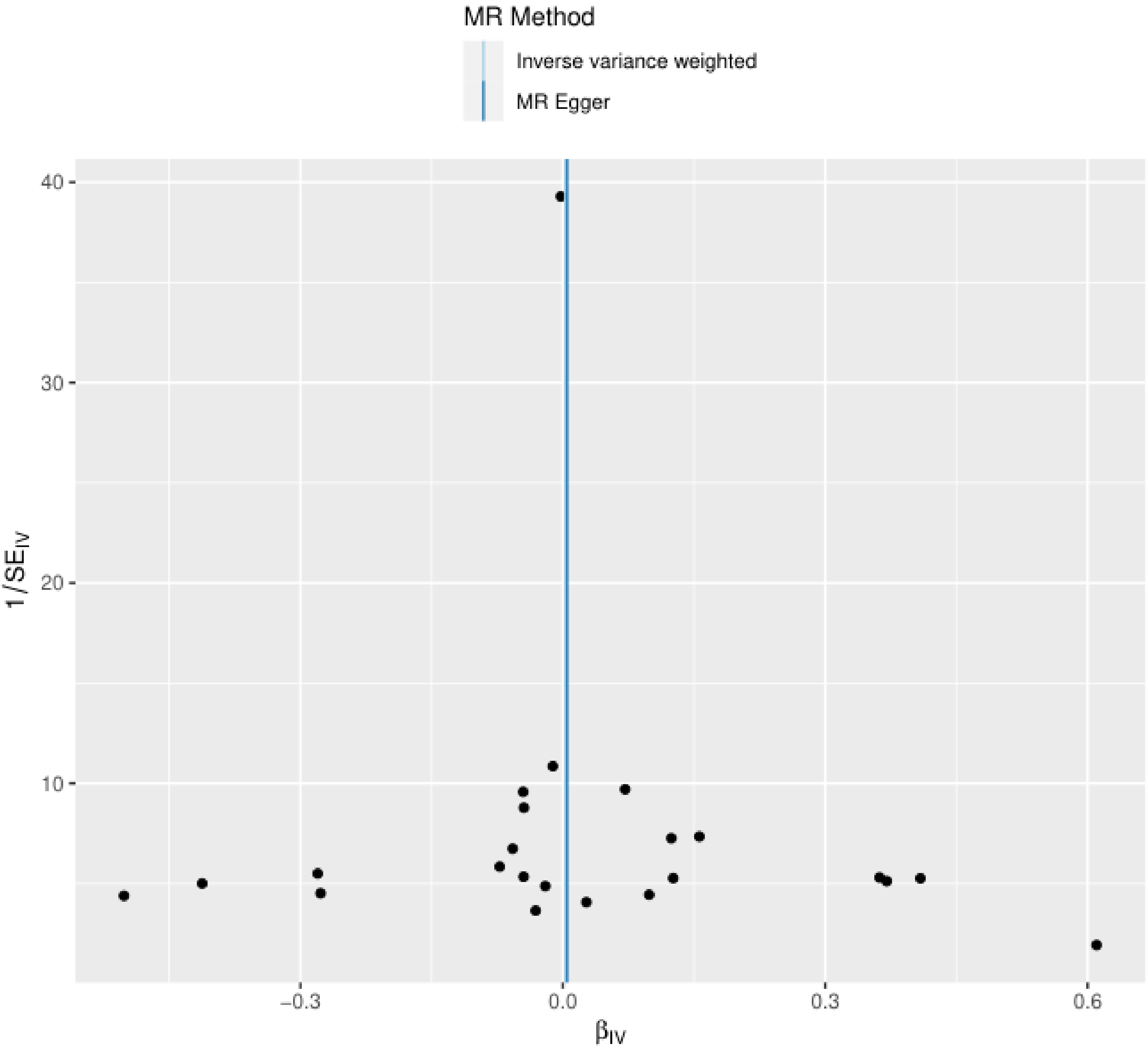

Funnel plot analyse of "CD45RA on naive CD8br" on 'Diabetic nephropathy'

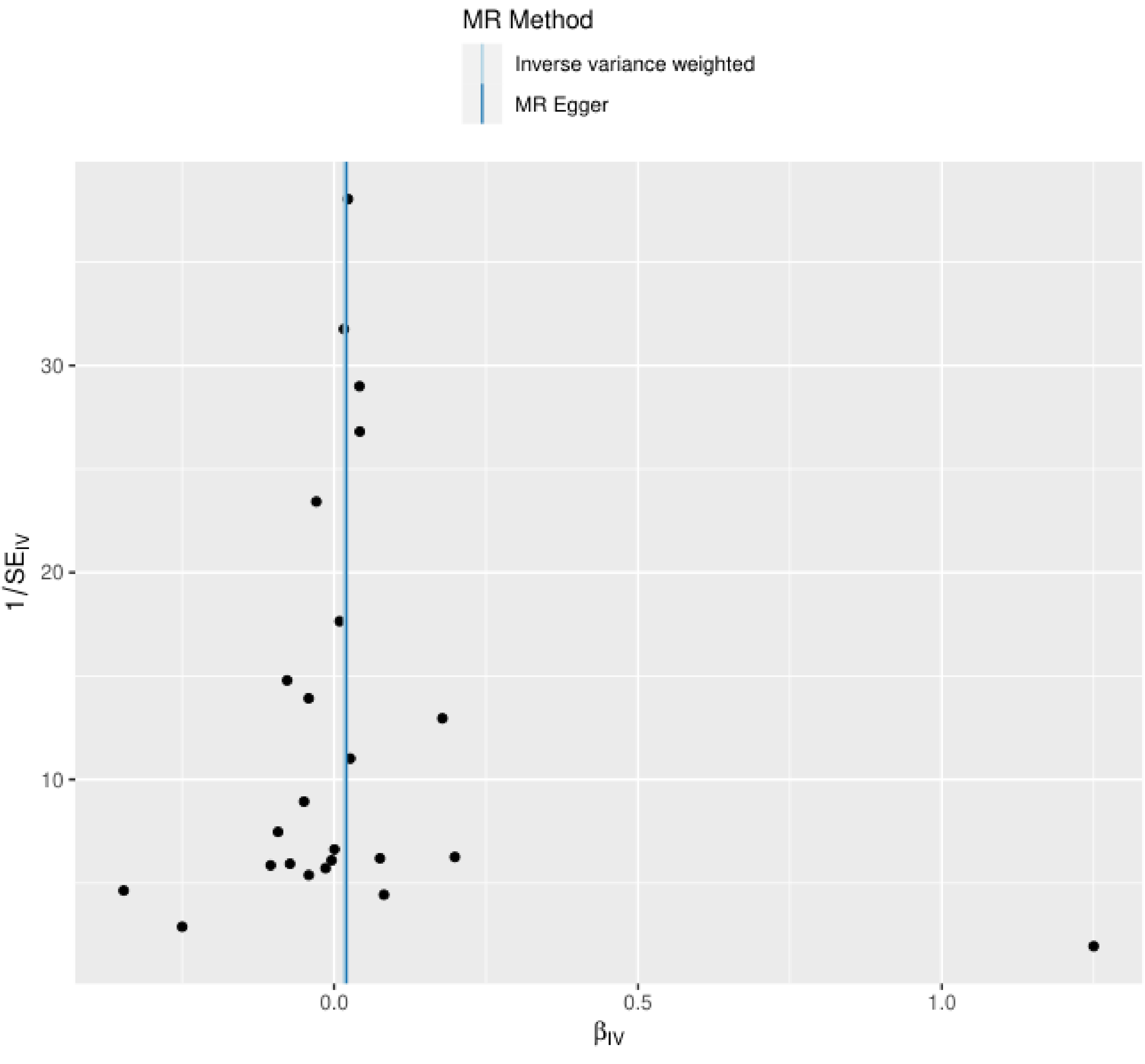

Funnel plot analyse of "CD33dim HLA DR- AC" on 'Diabetic nephropathy'

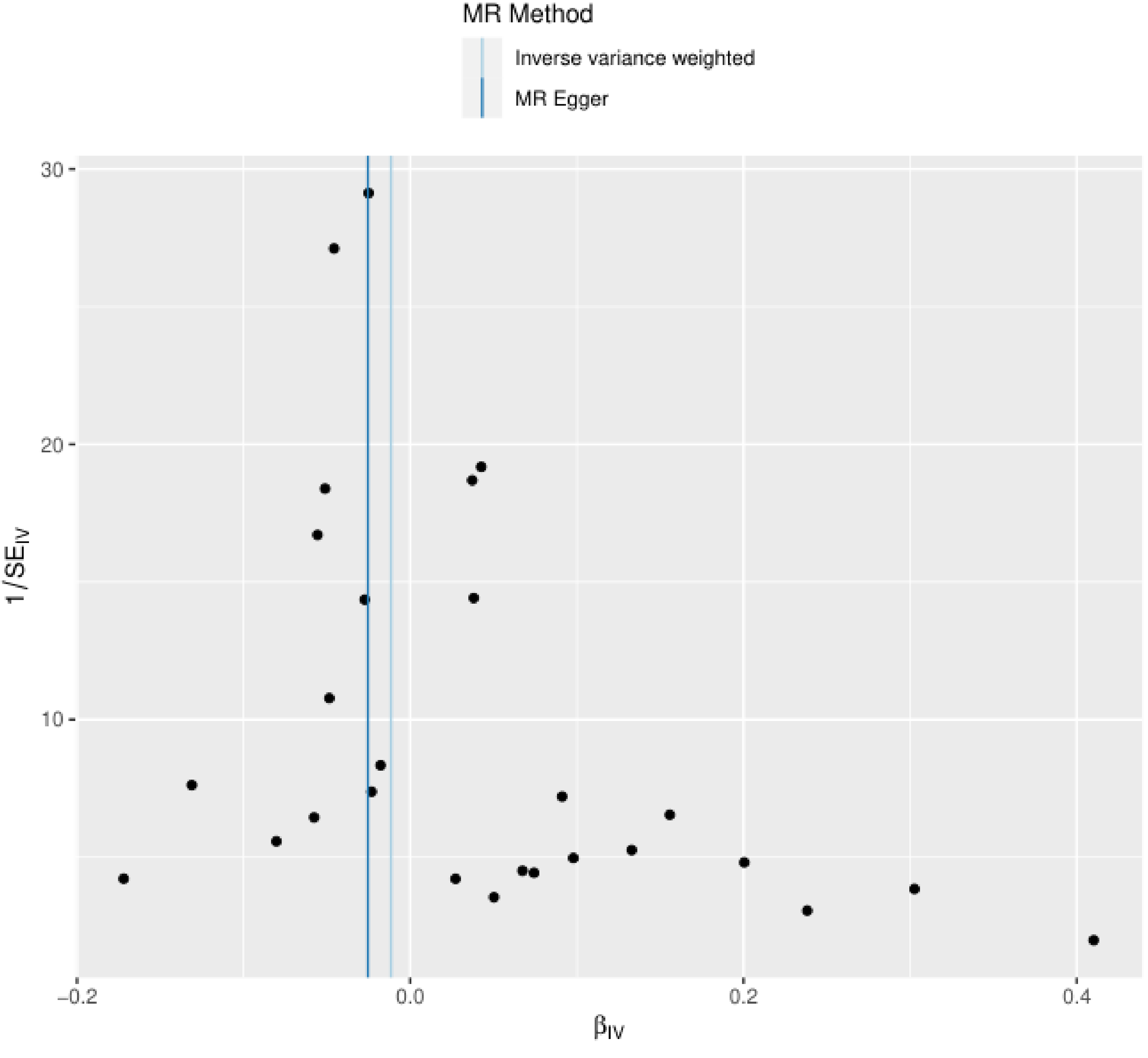

Funnel plot analyse of "CD25hi CD45RA+ CD4 not Treg %T cell" on 'Diabetic nephropathy'

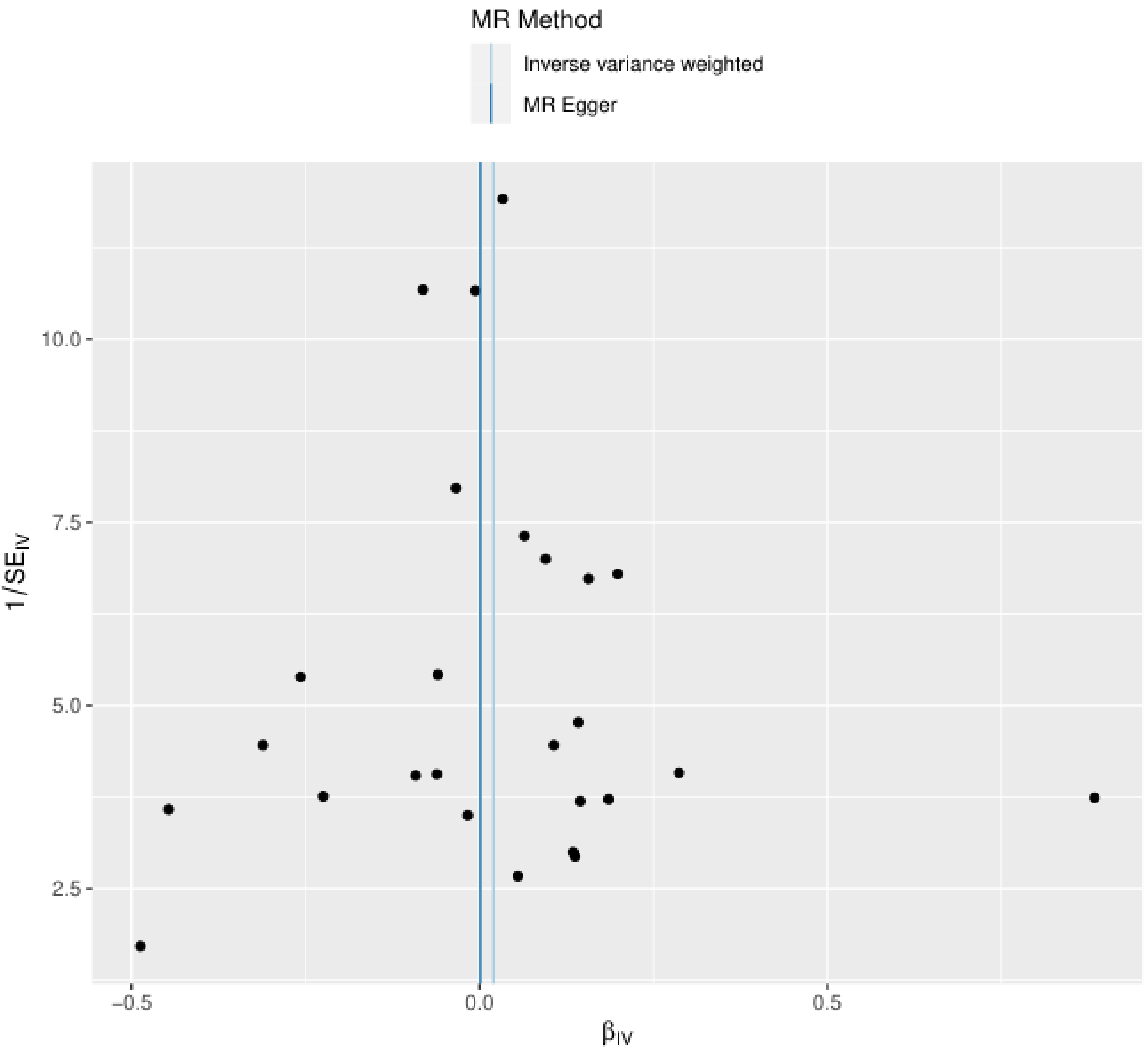

Funnel plot analyse of "CD8dim NKT %lymphocyte" on 'Diabetic nephropathy'

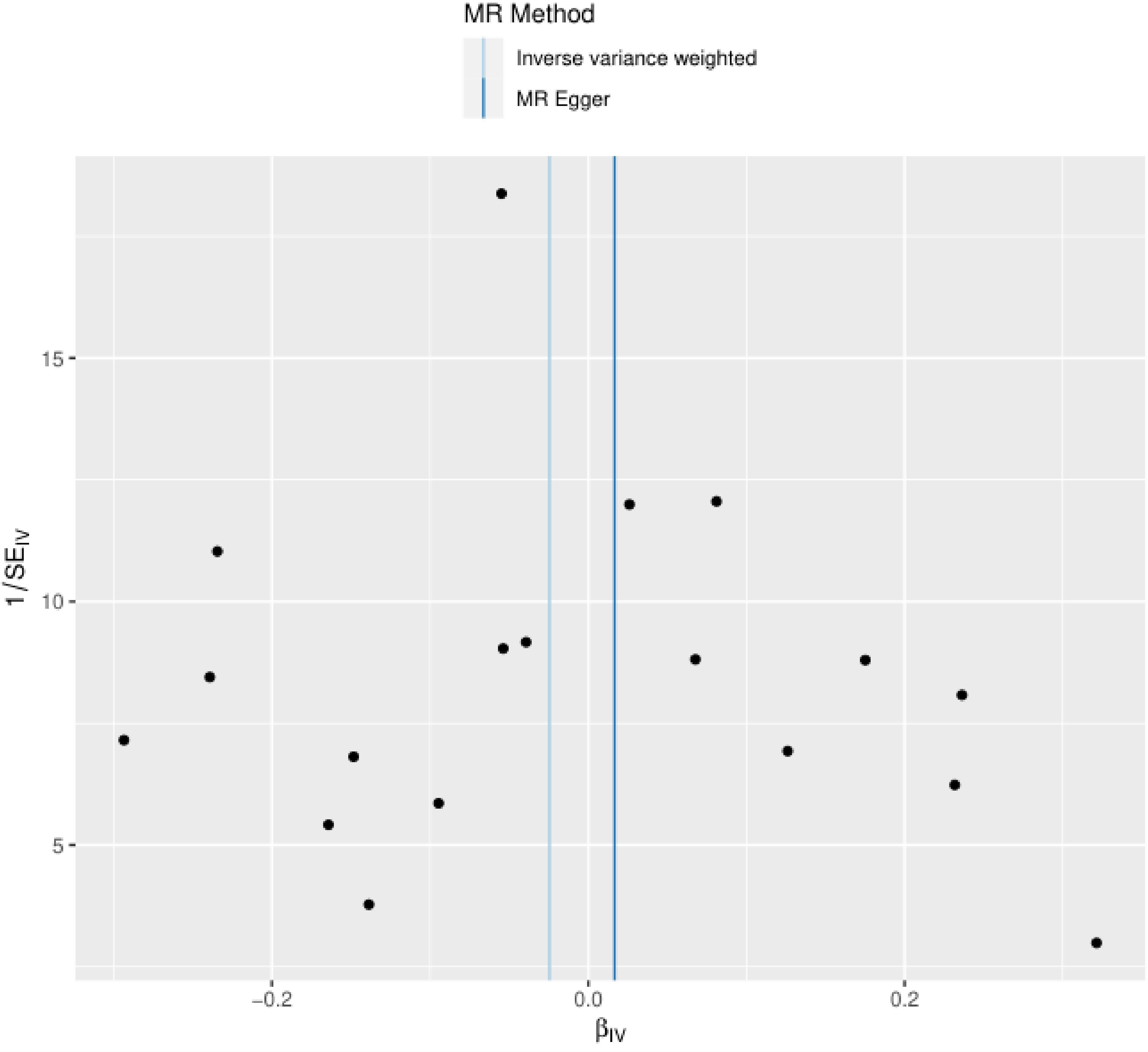

Funnel plot analyse of "HVEM on EM CD8br " on 'Diabetic nephropathy'

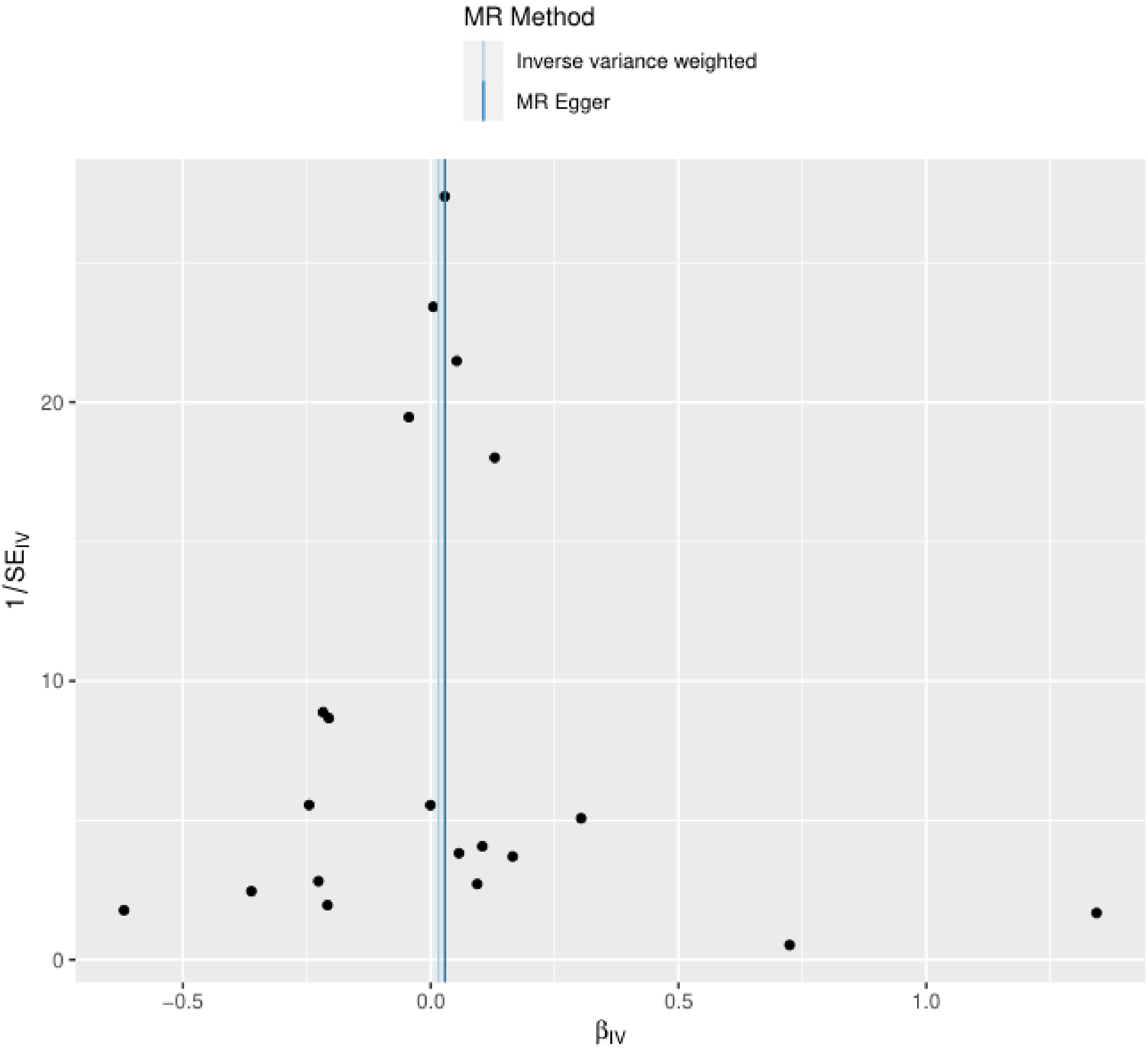

Funnel plot analyse of "CD40 on CD14+ CD16+ monocyte " on 'Diabetic nephropathy'

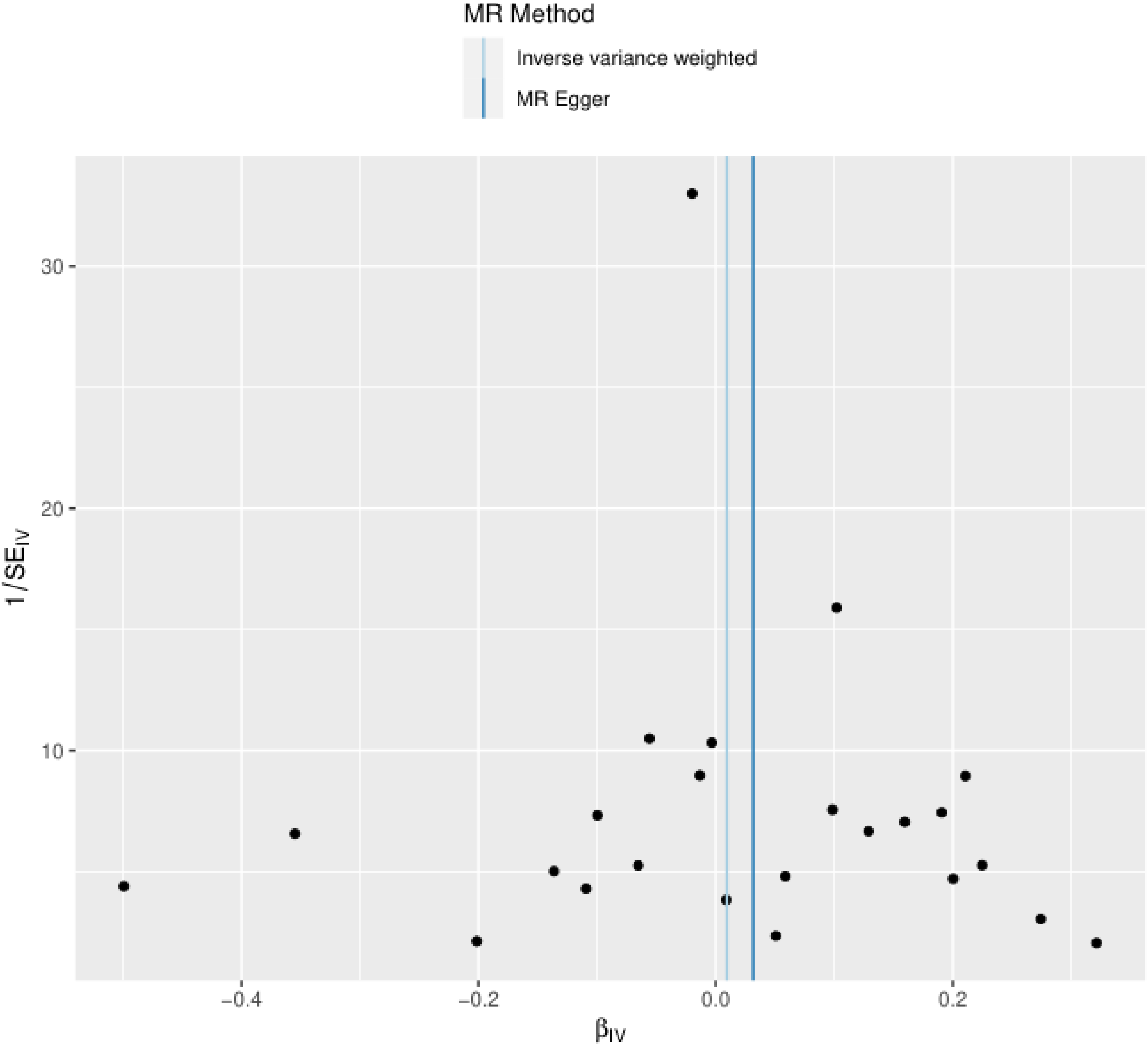

Funnel plot analyse of "BAFF-R on IgD+" on 'Diabetic nephropathy'

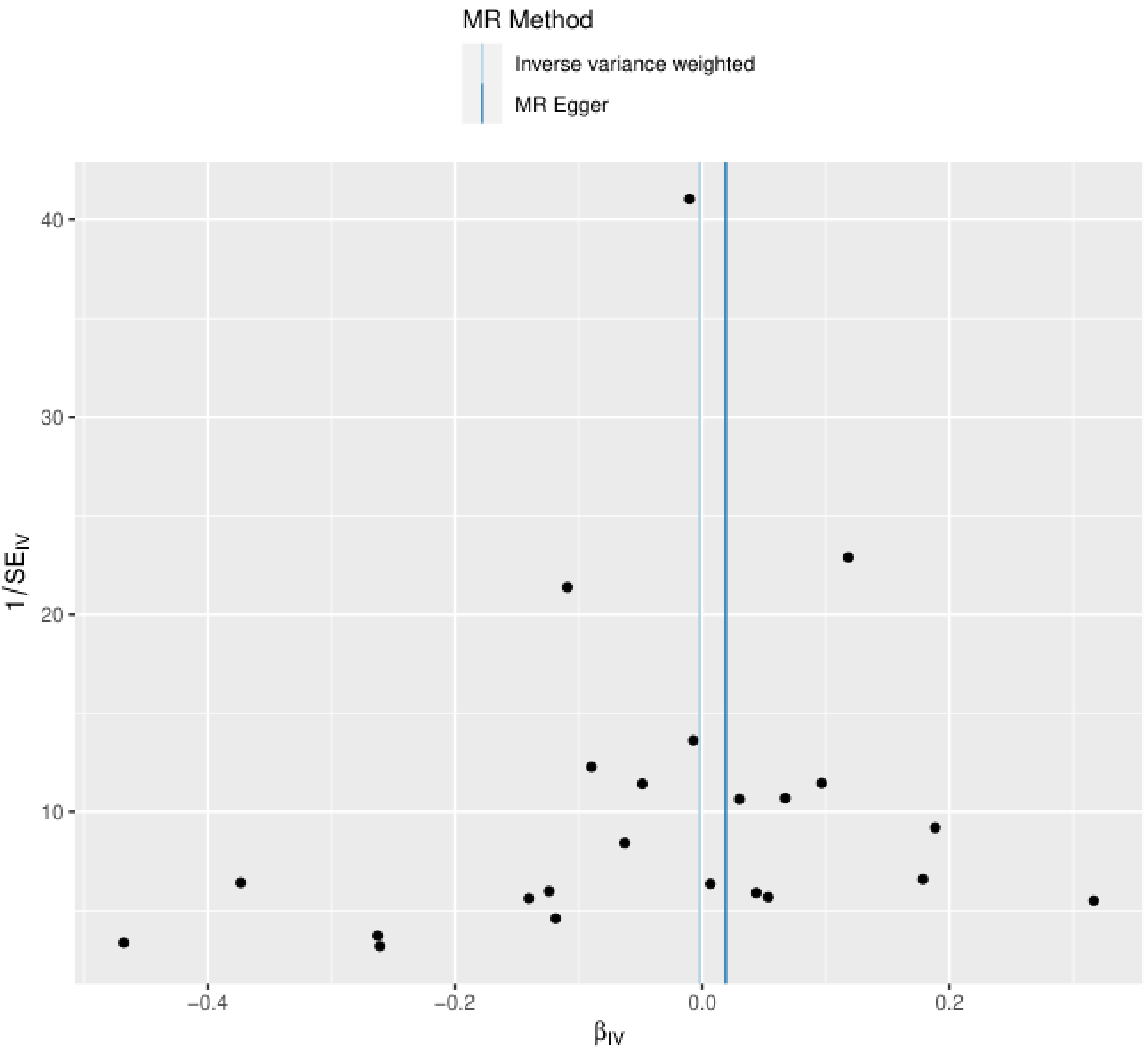

nel plot analyse of "CD33dim HLA DR+ CD11b- %CD33dim HLA DR+" on 'Diabetic nephropa

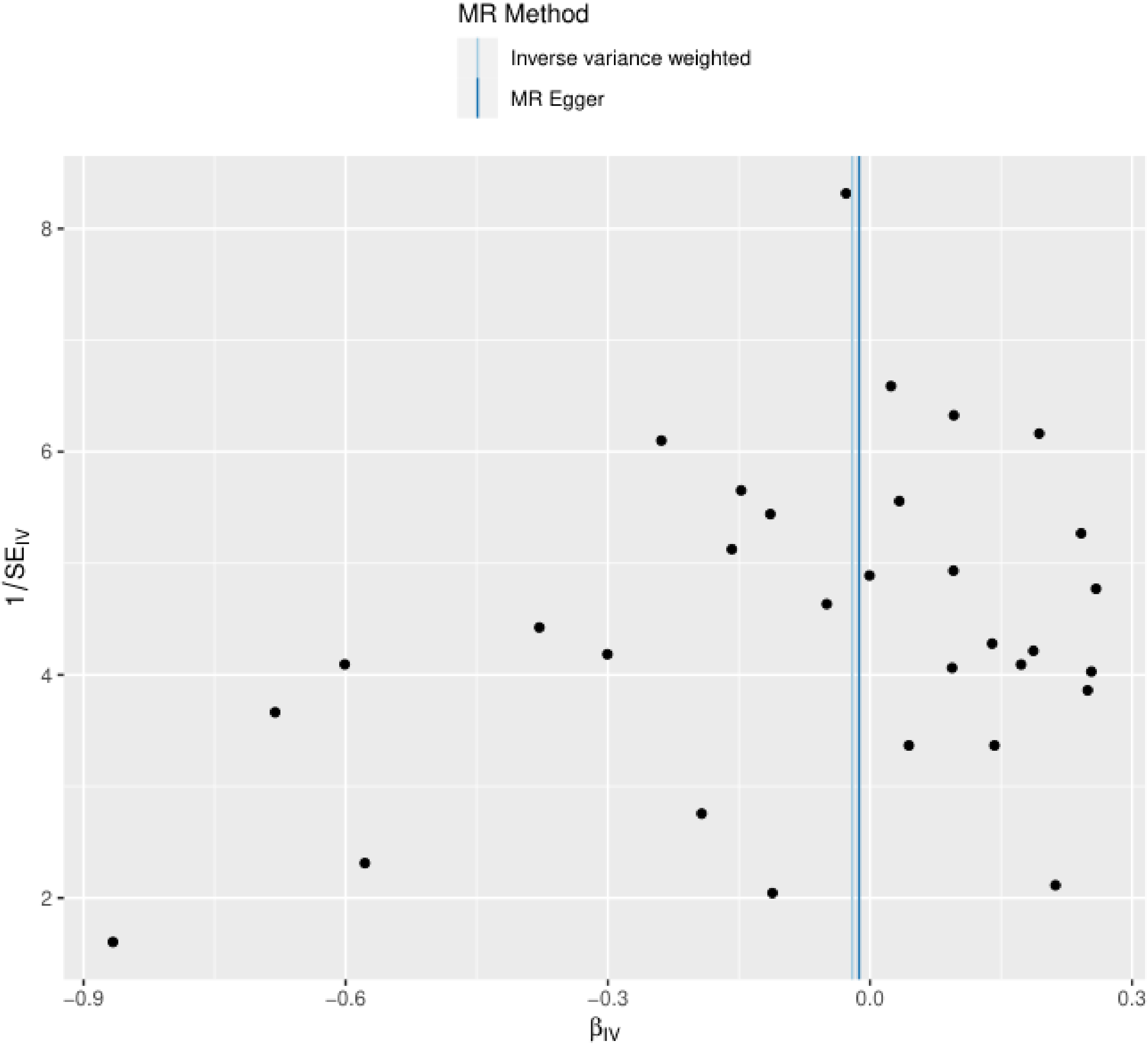

Funnel plot analyse of "CM CD8br %CD8br" on 'Diabetic nephropathy'

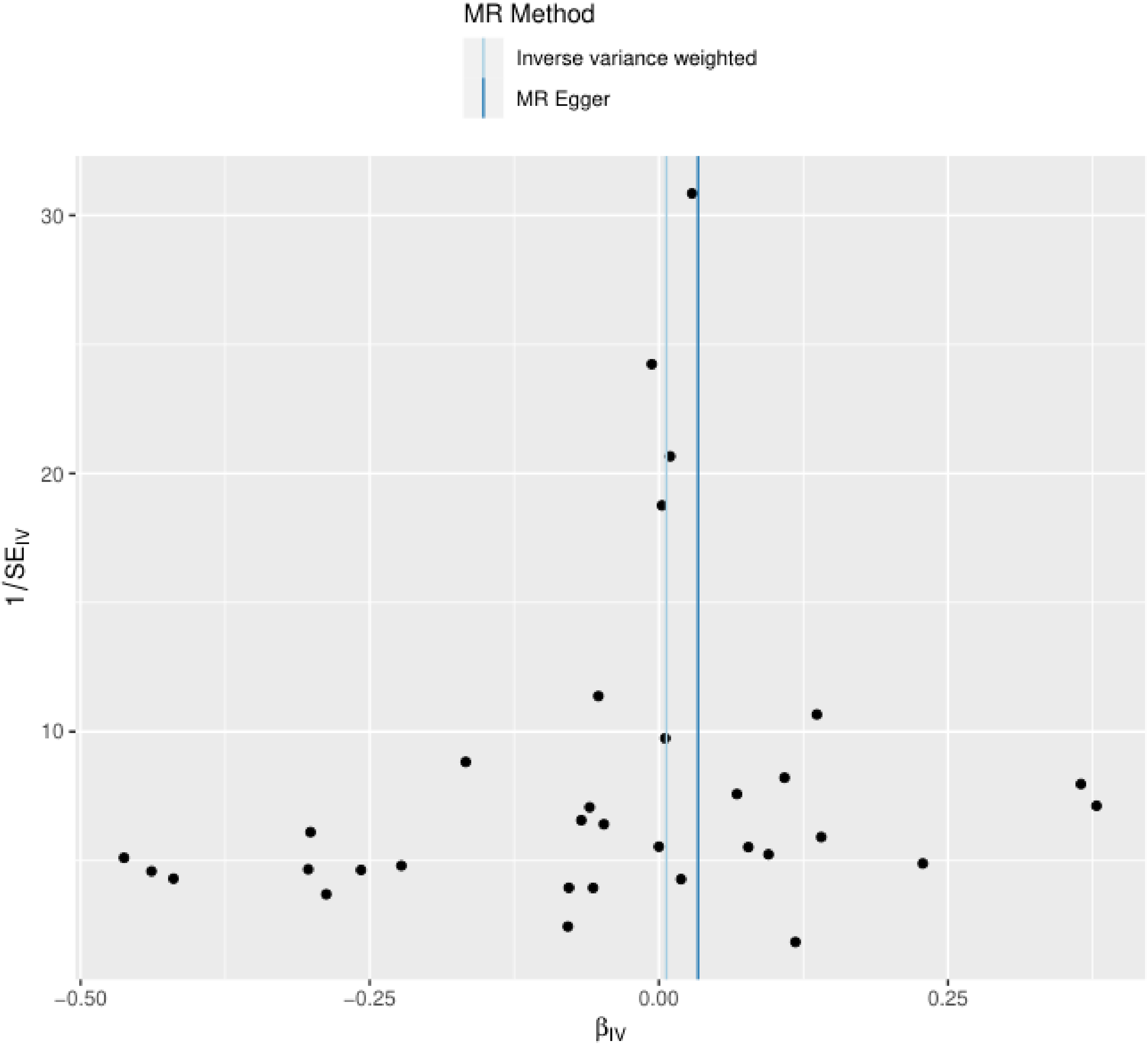

Funnel plot analyse of "CD4RA on TD CD4+ " on 'Diabetic nephropathy'

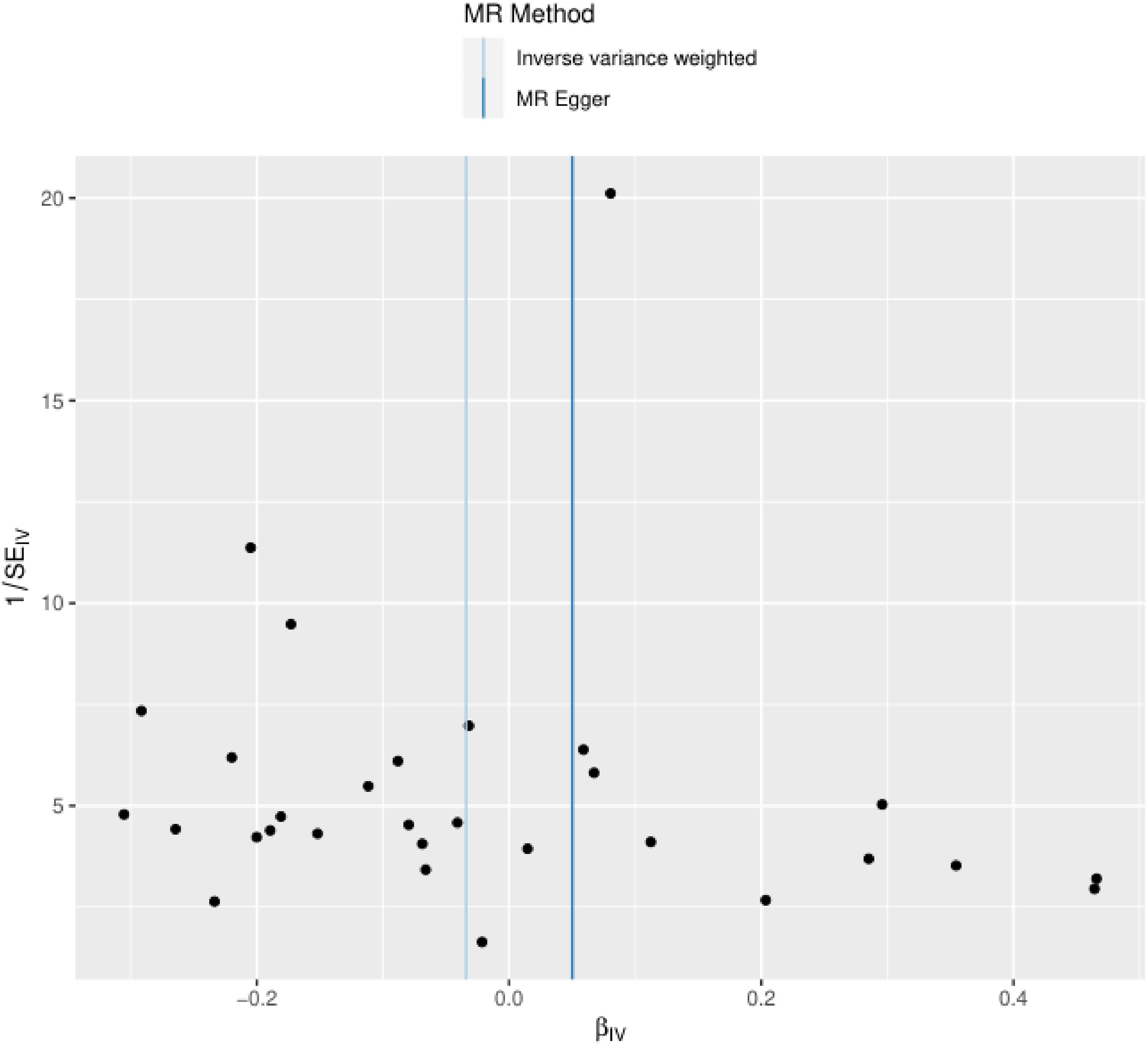

Funnel plot analyse of "CD27 on sw mem" on 'Diabetic nephropathy'

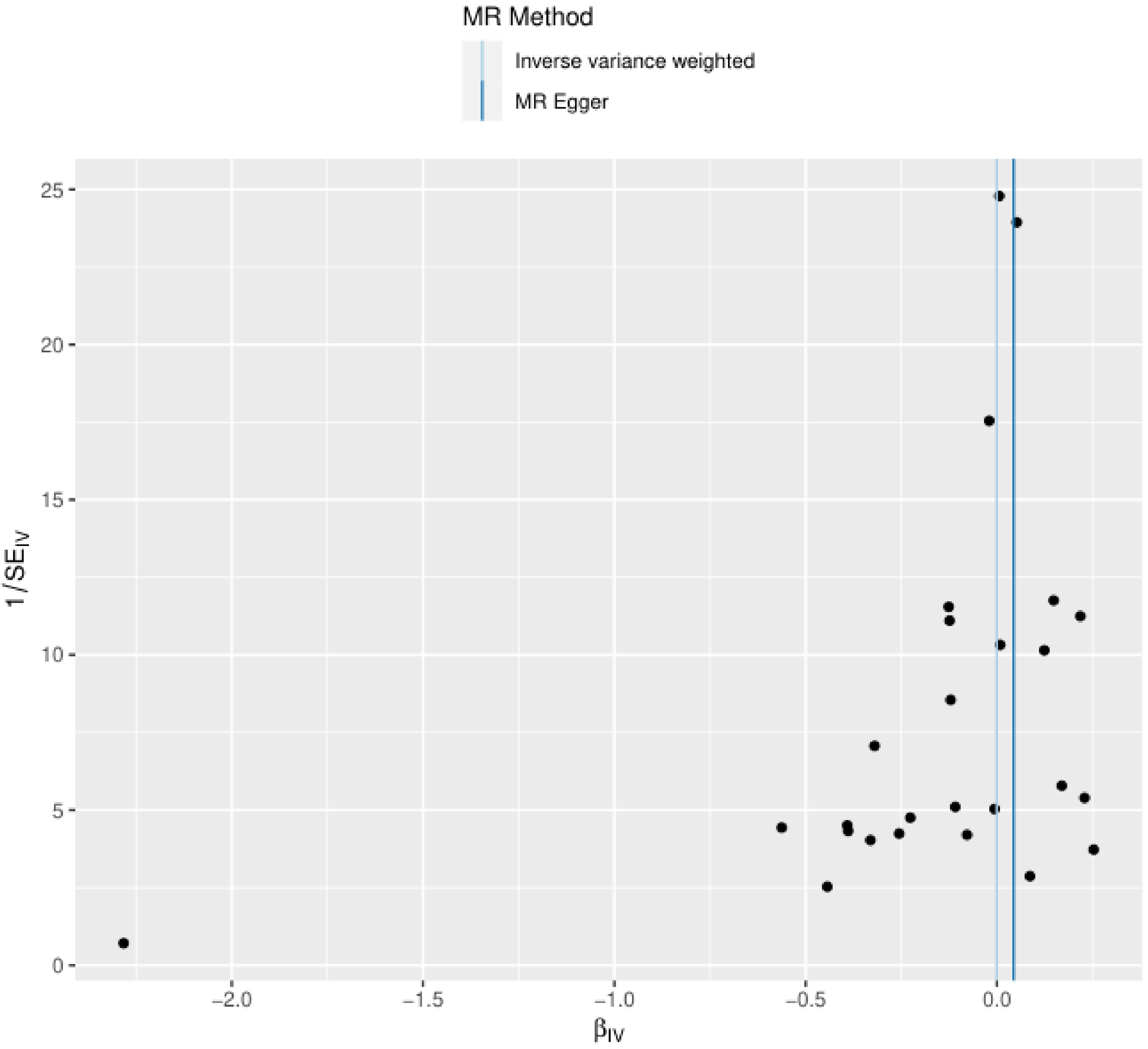

Funnel plot analyse of "CD28+ CD45RA- CD8dim %CD8dim" on 'Diabetic nephropathy'

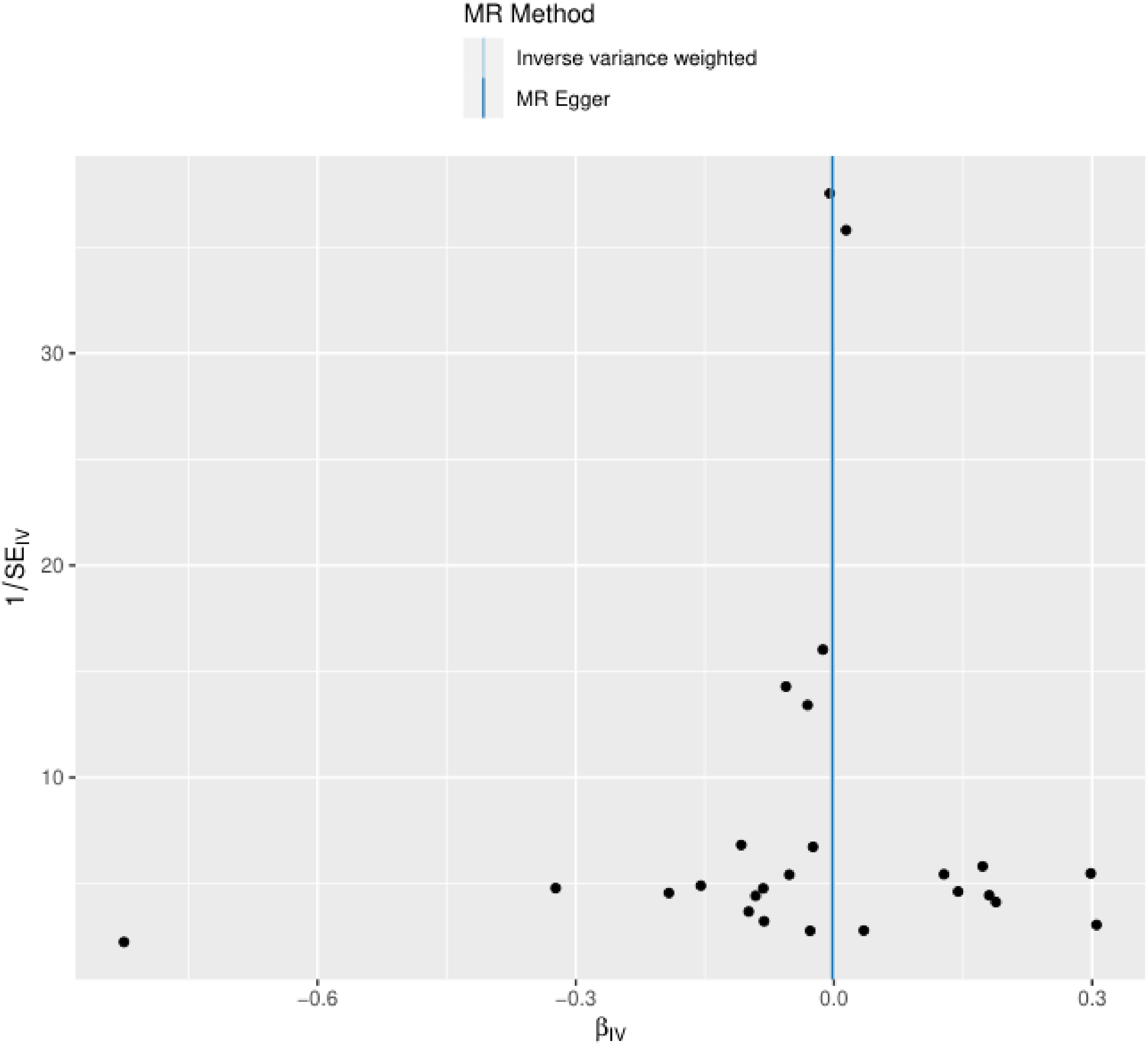

Funnel plot analyse of "CD19 on IgD+ CD38dim" on 'Diabetic nephropathy'

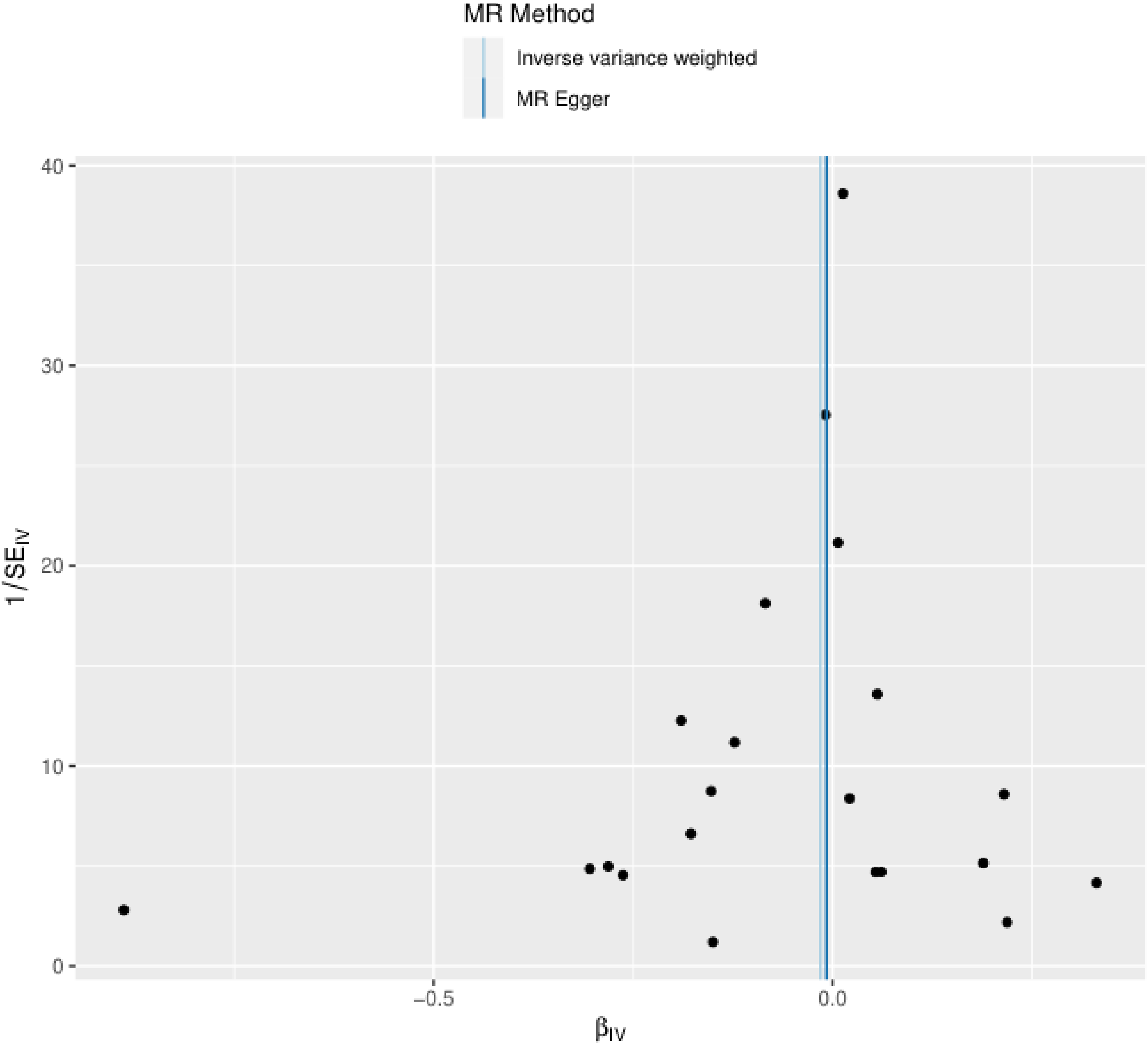

Funnel plot analyse of "CD25hi CD45RA- CD4 not Treg AC" on 'Diabetic nephropathy'

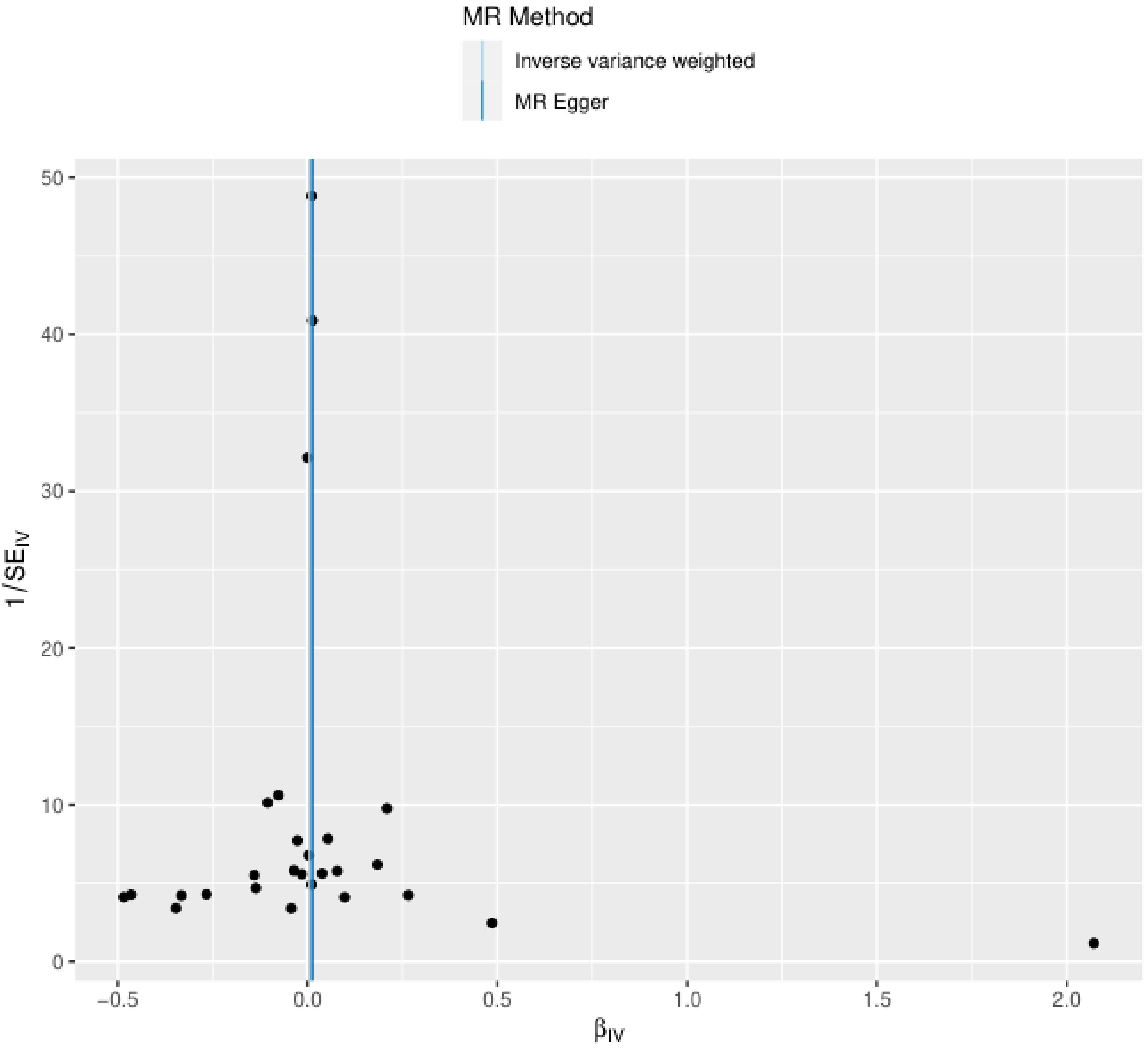

Funnel plot analyse of "CD25 on IgD+ CD38-" on 'Diabetic nephropathy'

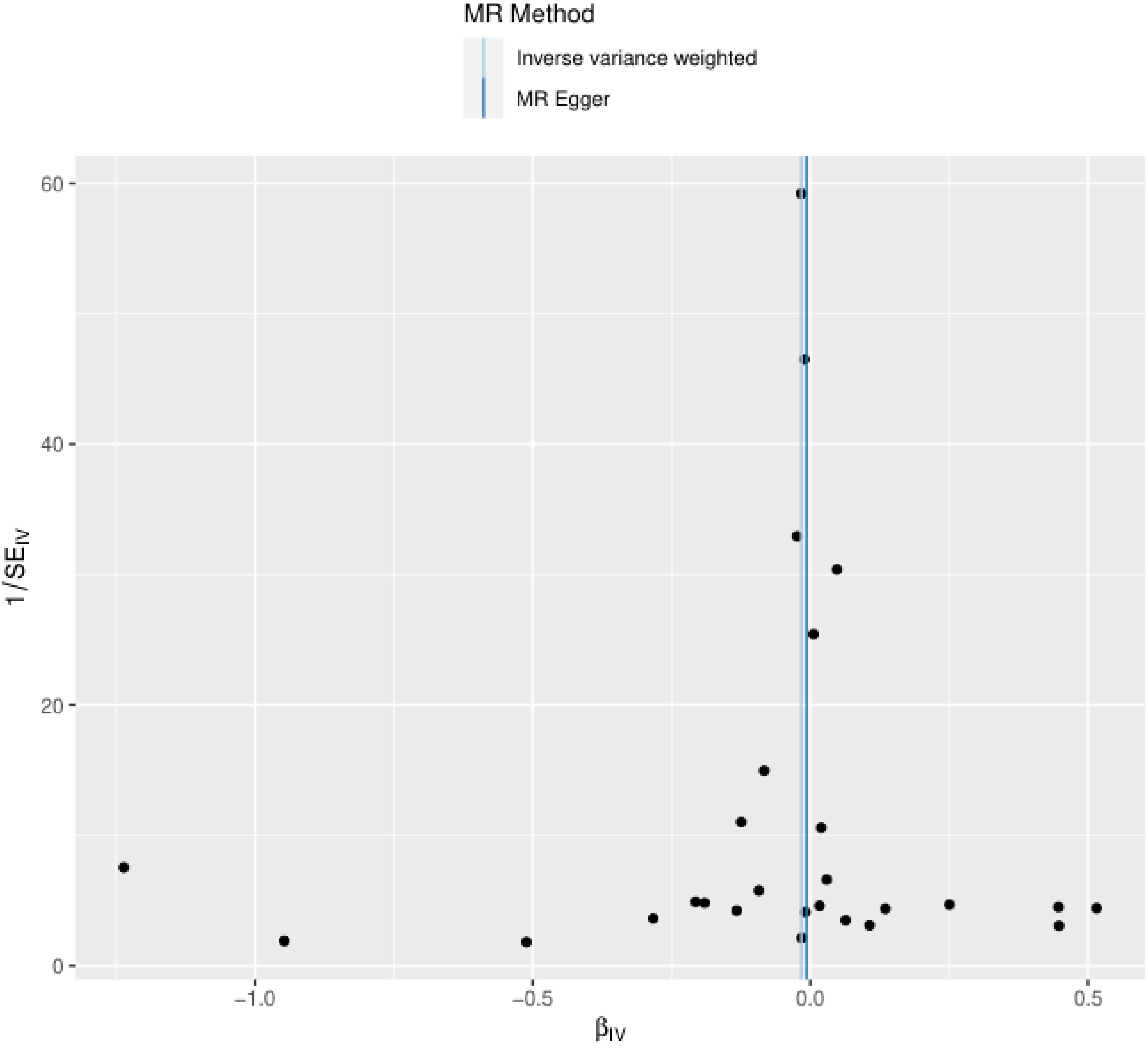

Funnel plot analyse of "CD28+ CD45RA- CD8br %CD8br" on 'Diabetic nephropathy'

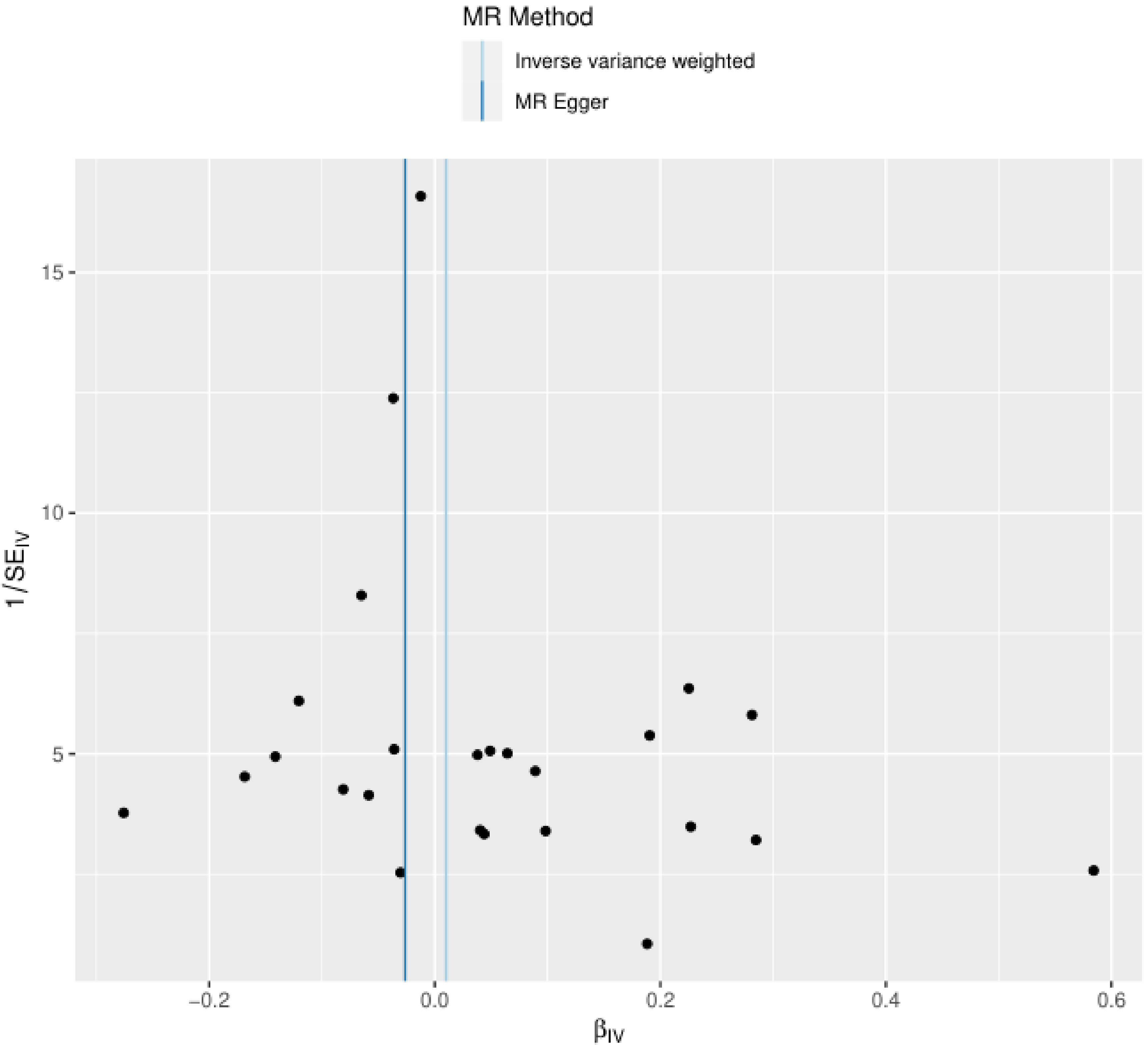

Funnel plot analyse of "CD19 on memory B cell" on 'Diabetic nephropathy'

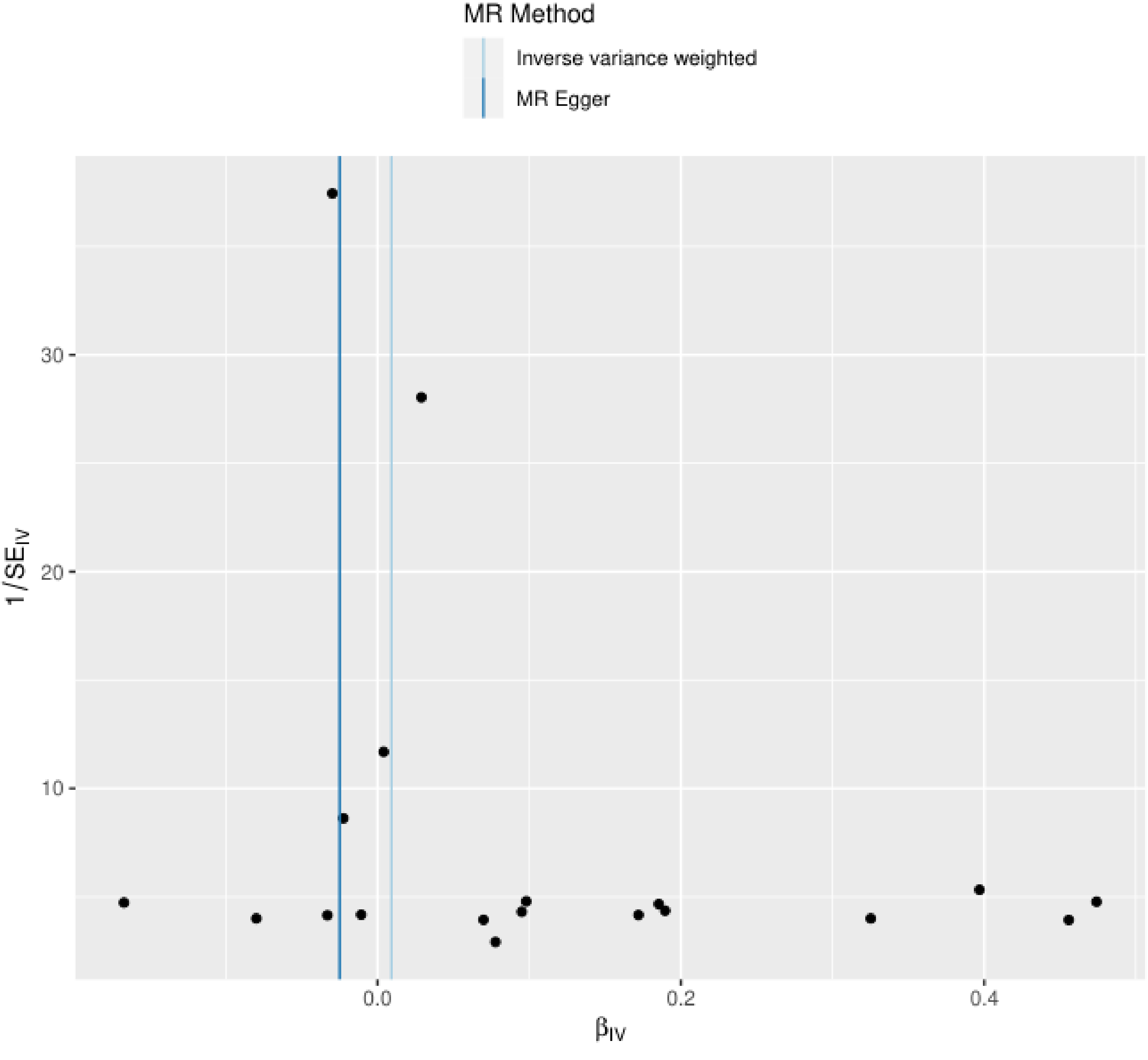

Funnel plot analyse of "TCRgd %lymphocyte" on 'Diabetic nephropathy'

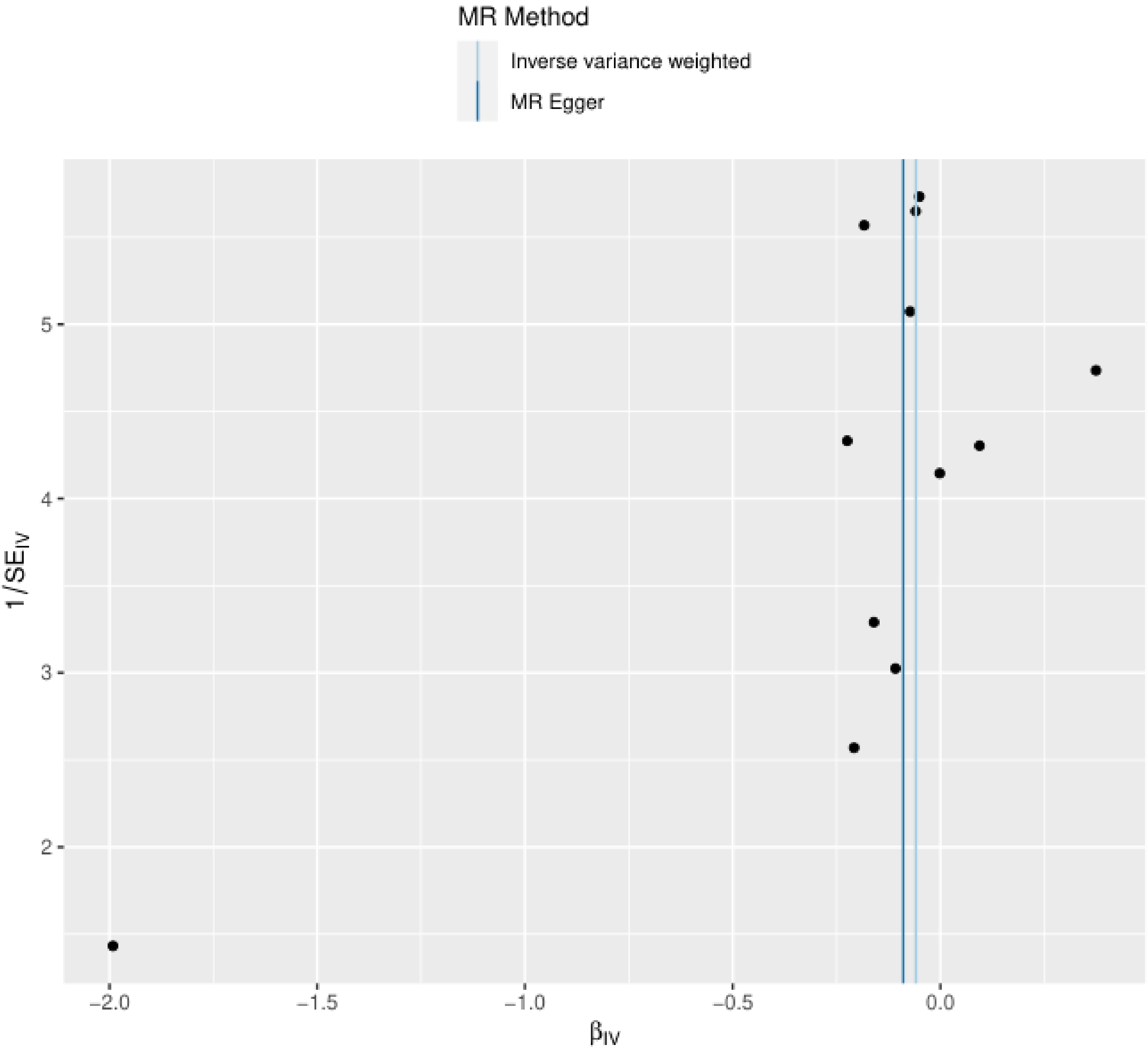

Funnel plot analyse of "CD64 on CD14+ CD16+ monocyte" on 'Diabetic nephropathy'

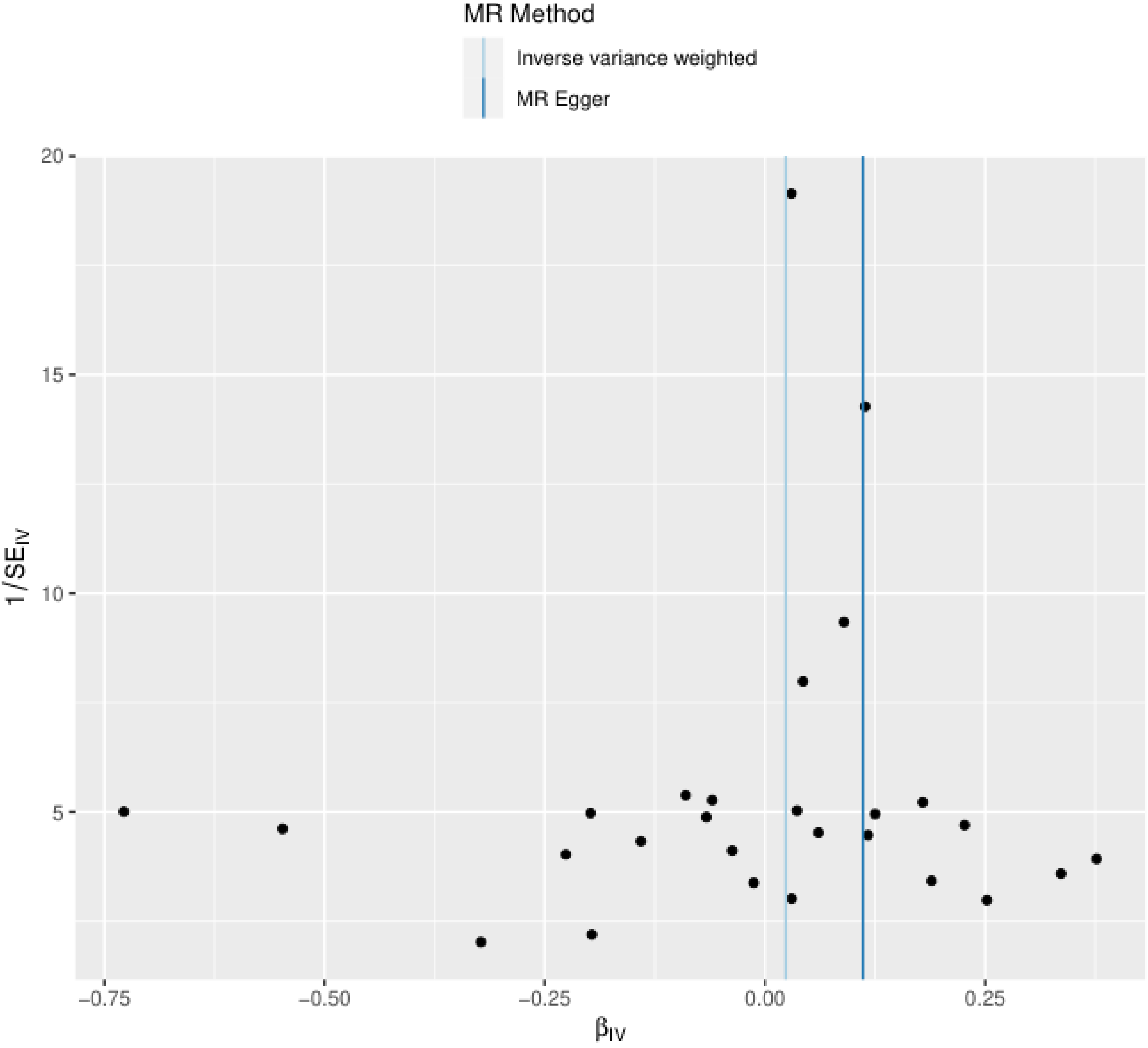

Funnel plot analyse of "CD45 on NK" on 'Diabetic nephropathy'

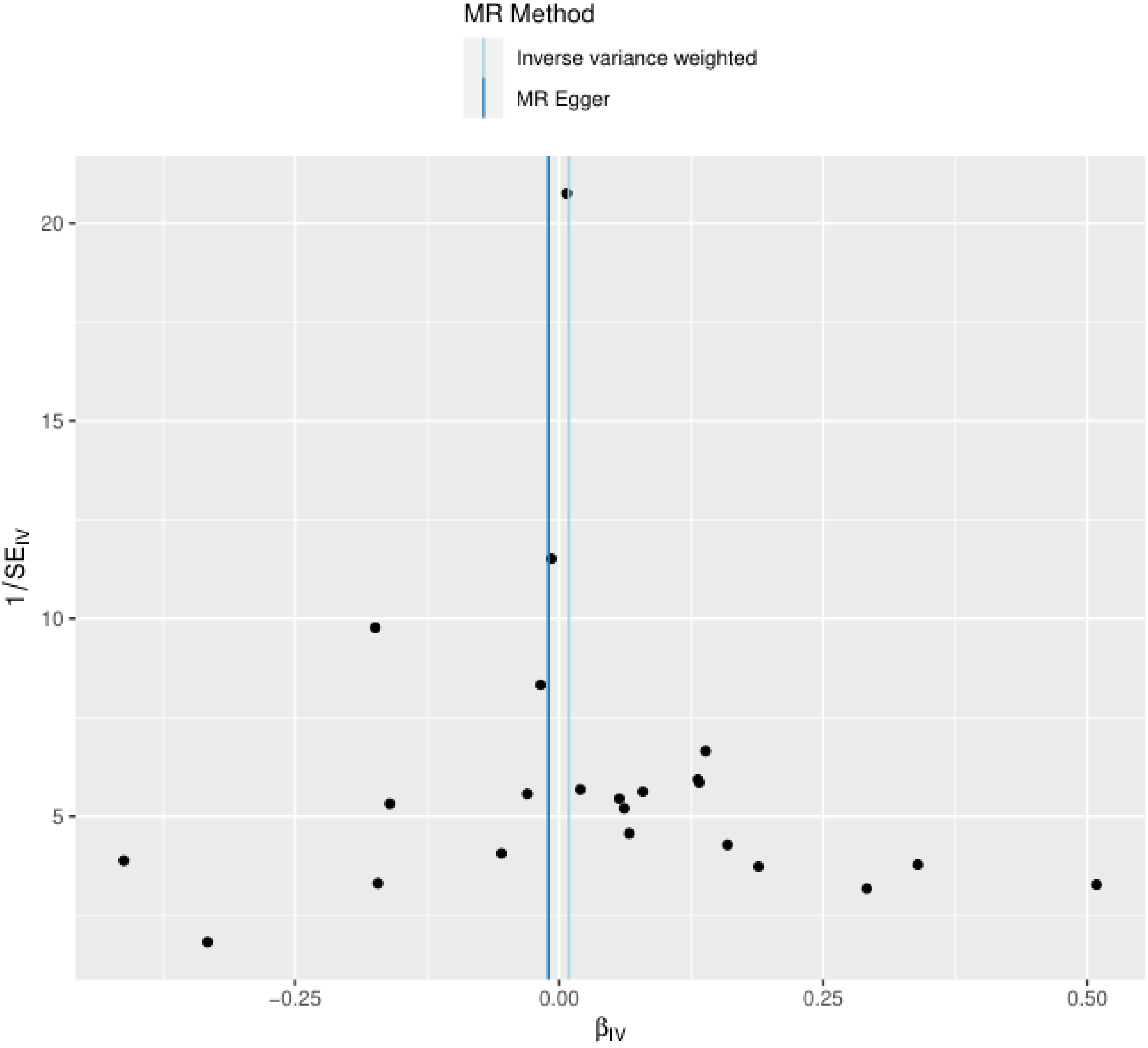

Funnel plot analyse of "CD38 on transitional" on 'Diabetic nephropathy'

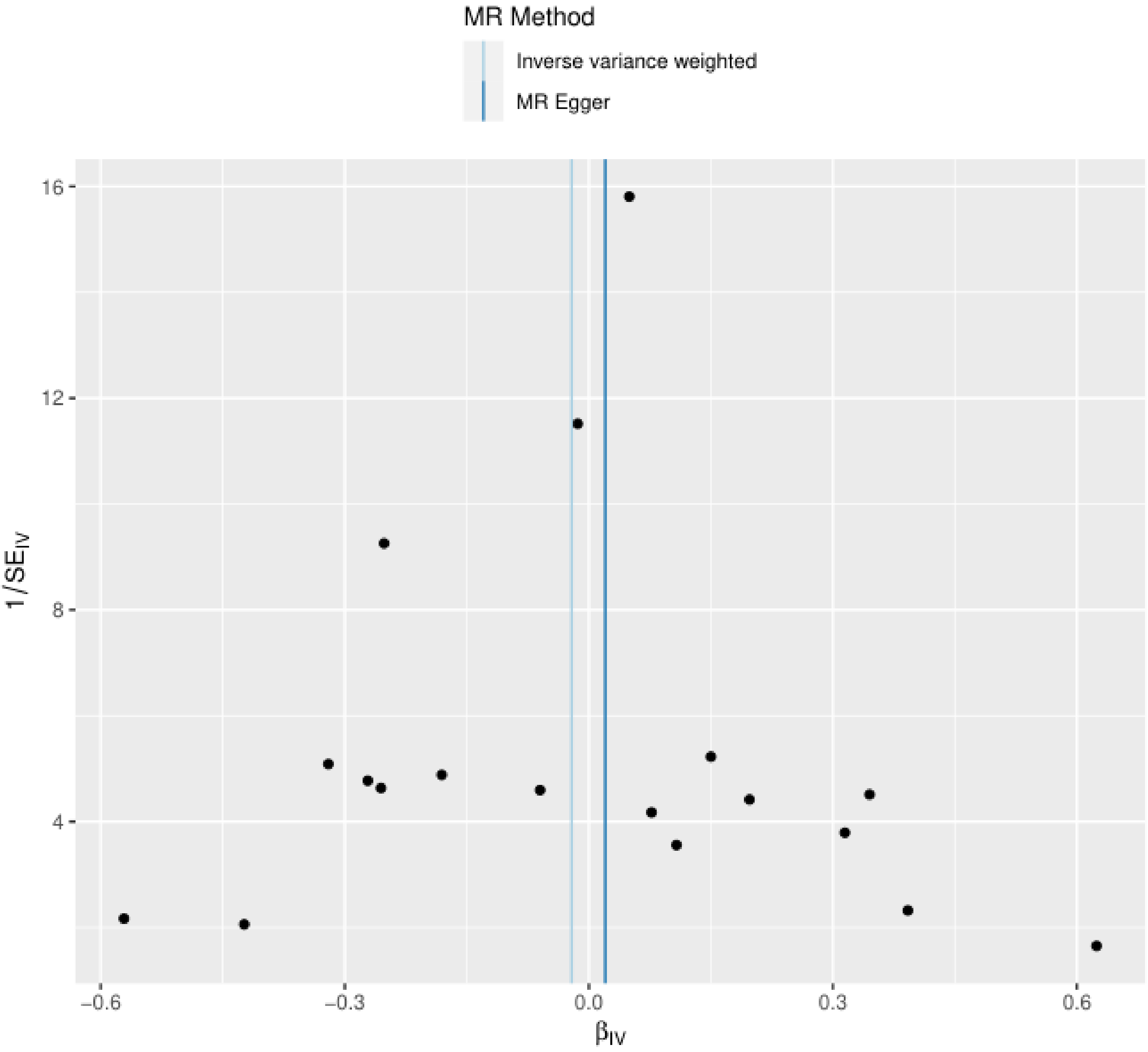

Funnel plot analyse of "CX3CR1 on CD14- CD16+ monocyte" on 'Diabetic nephropathy'

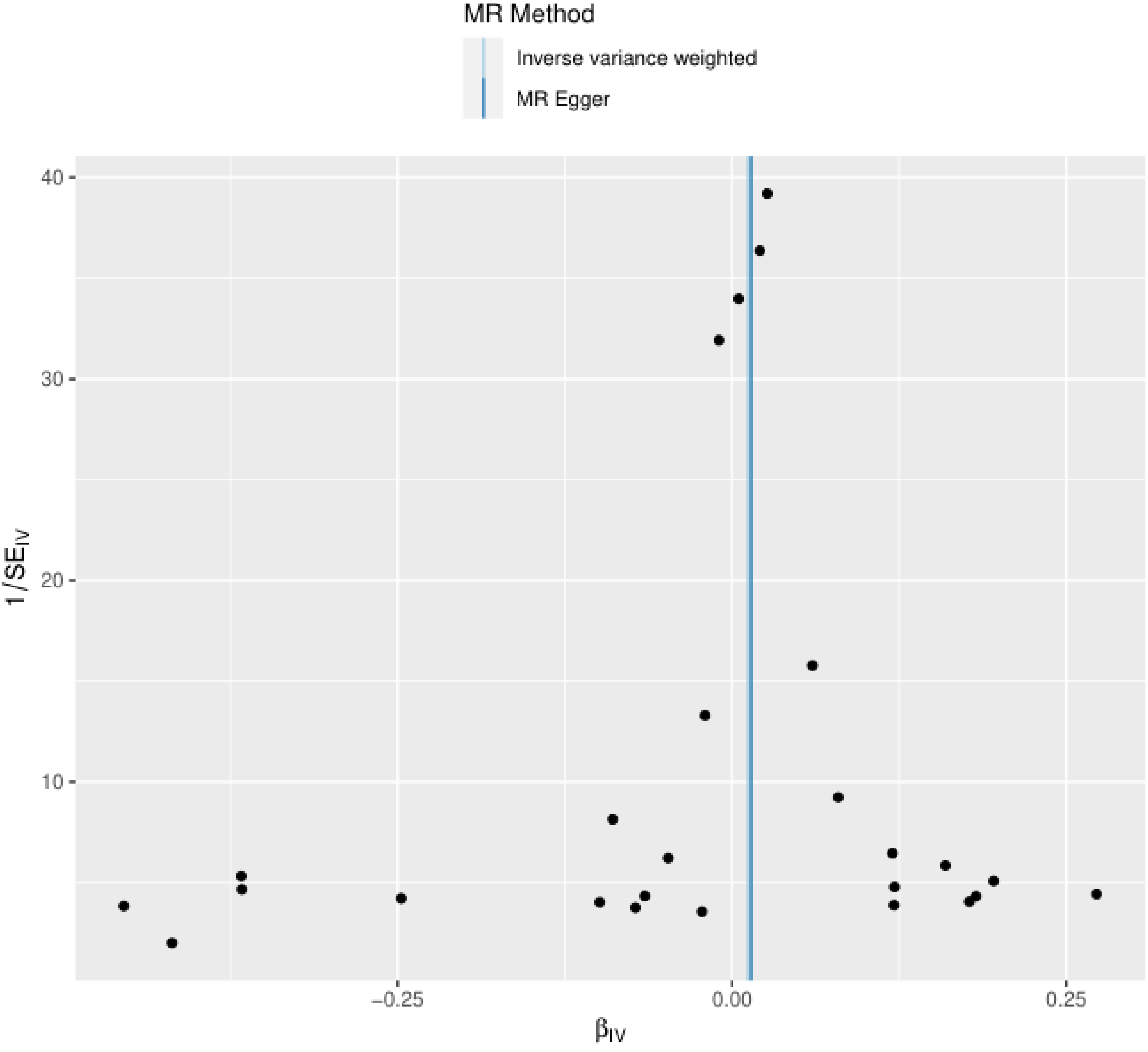

Funnel plot analyse of "CCR2 on CD14+ CD16- monocyte" on 'Diabetic nephropathy'

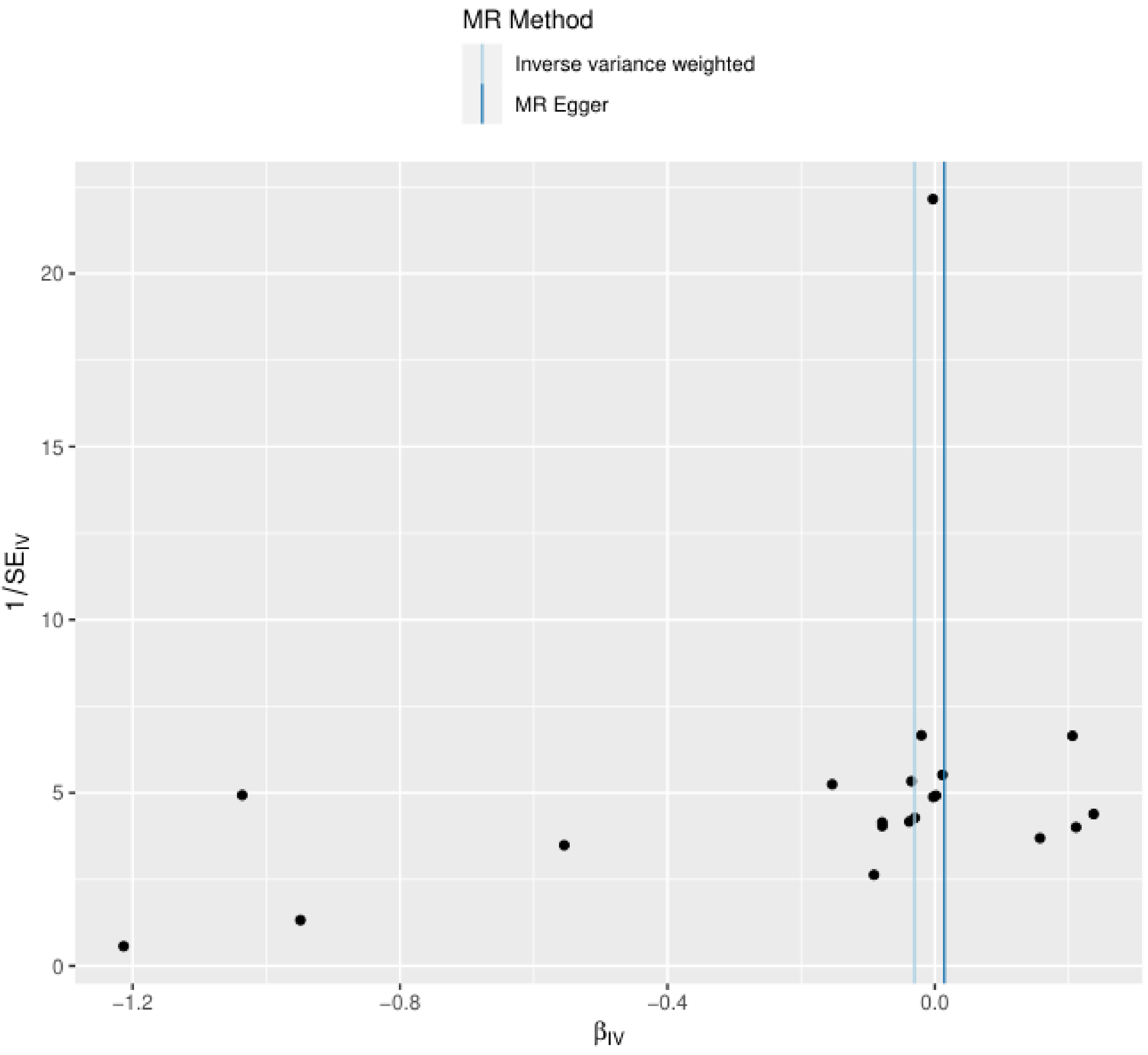

Funnel plot analyse of "CD25 on resting Treg " on 'Diabetic nephropathy'

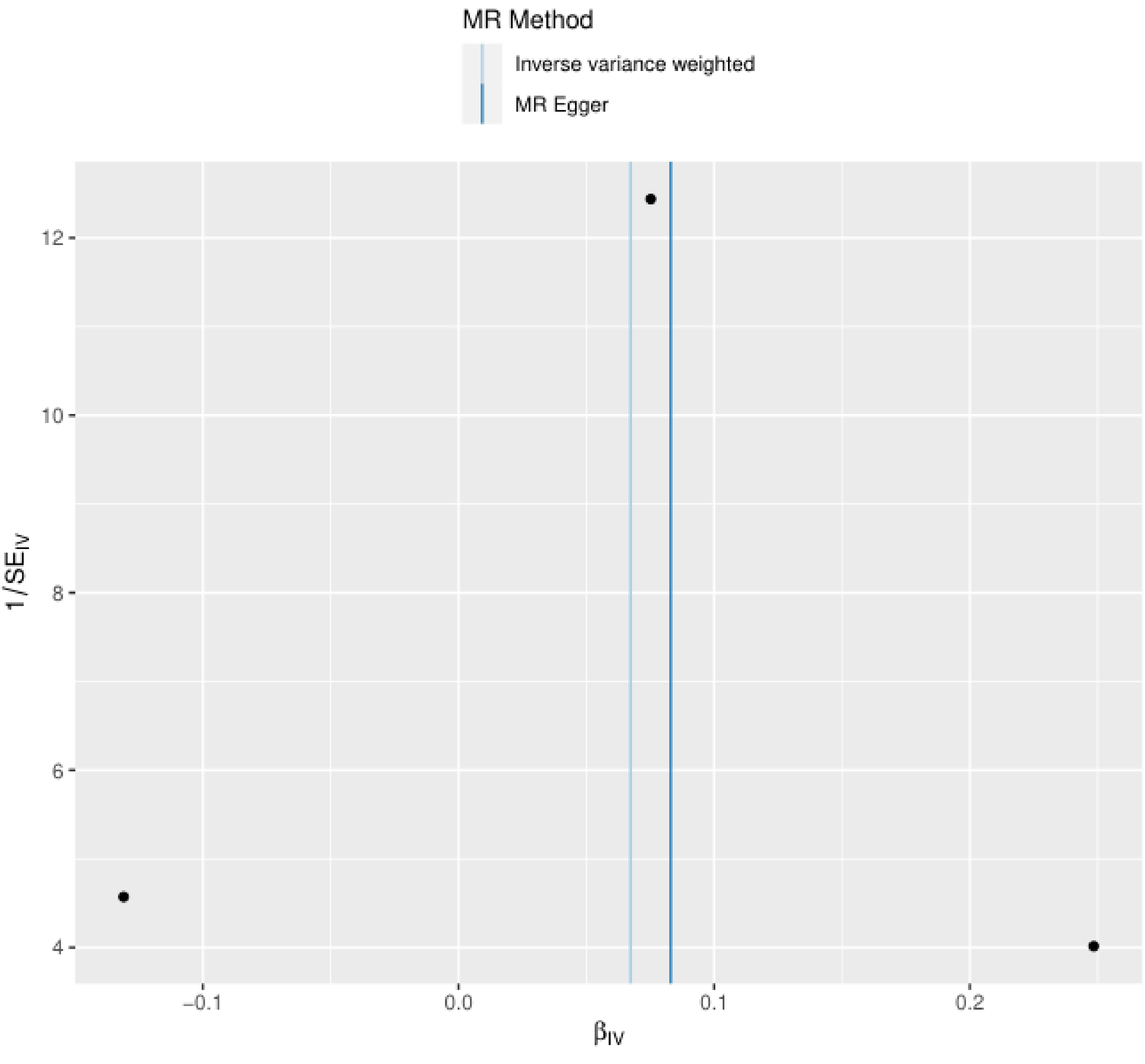

Funnel plot analyse of "CD24 on CD24+ CD27+" on 'Diabetic nephropathy'

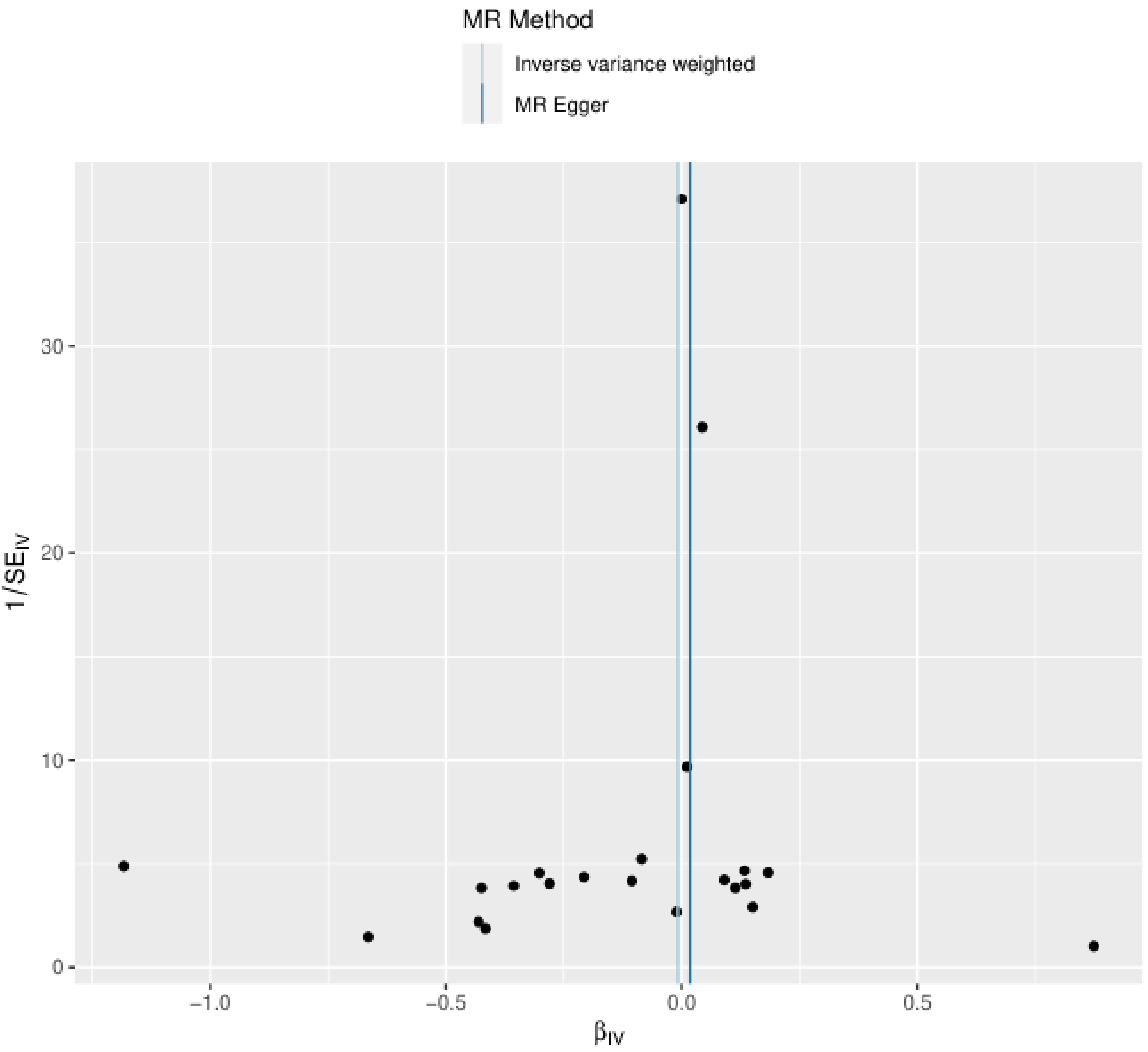

Funnel plot analyse of "IgD+ CD38- %B cell" on 'Diabetic nephropathy'

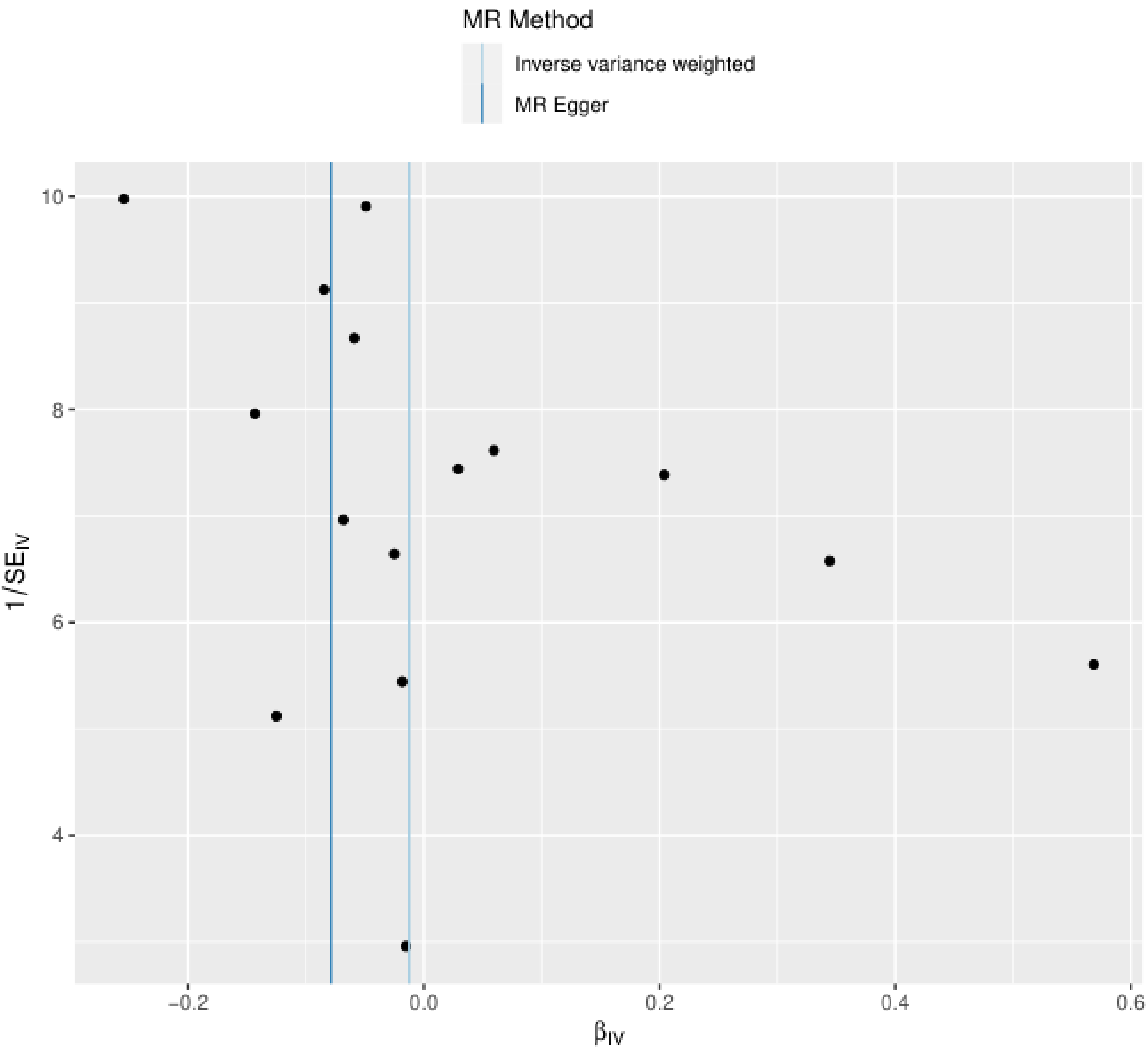

Funnel plot analyse of "HVEM on CD8br" on 'Diabetic nephropathy'

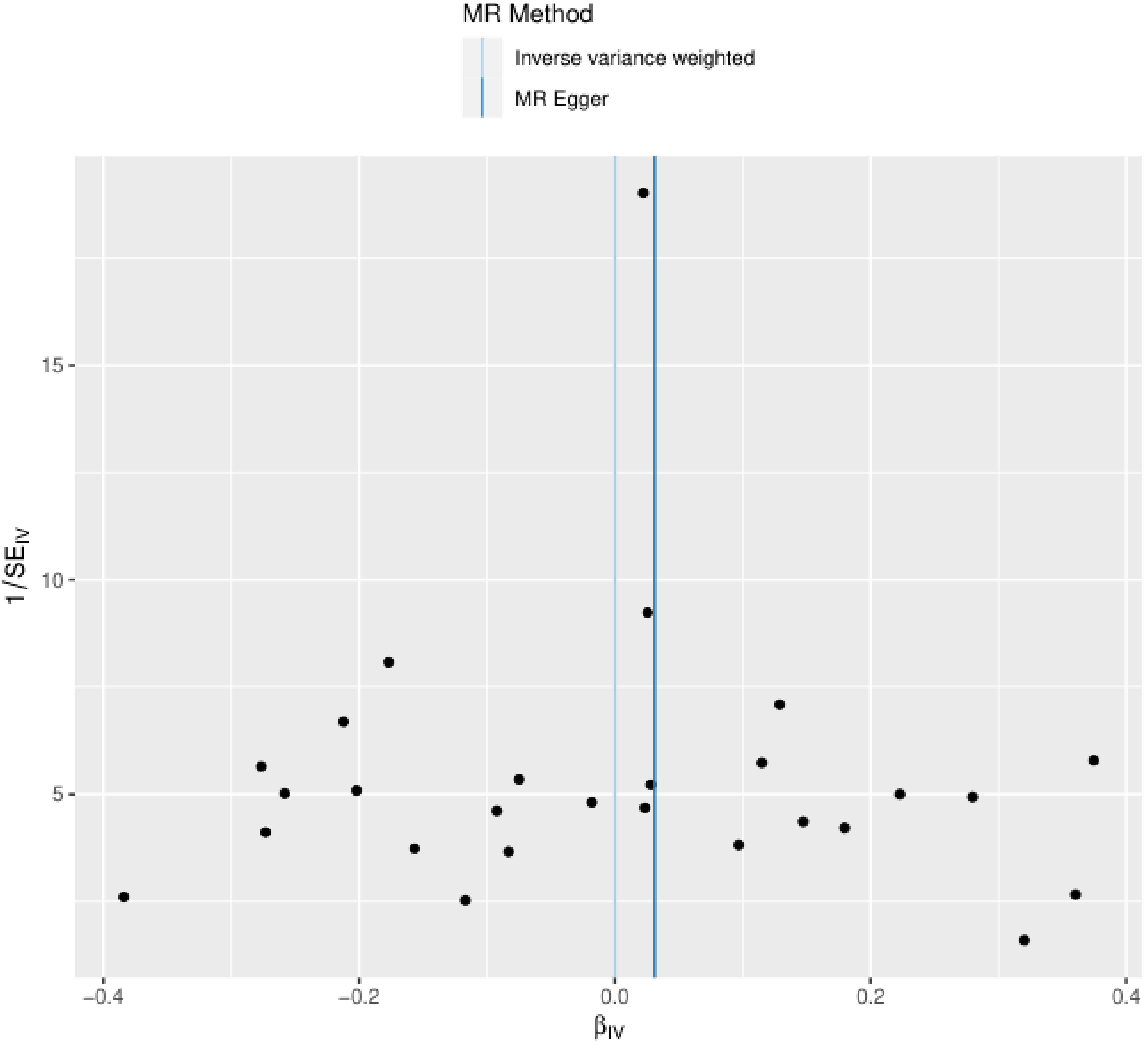

Funnel plot analyse of "CD4 on CD39+ CD4+ " on 'Diabetic nephropathy'

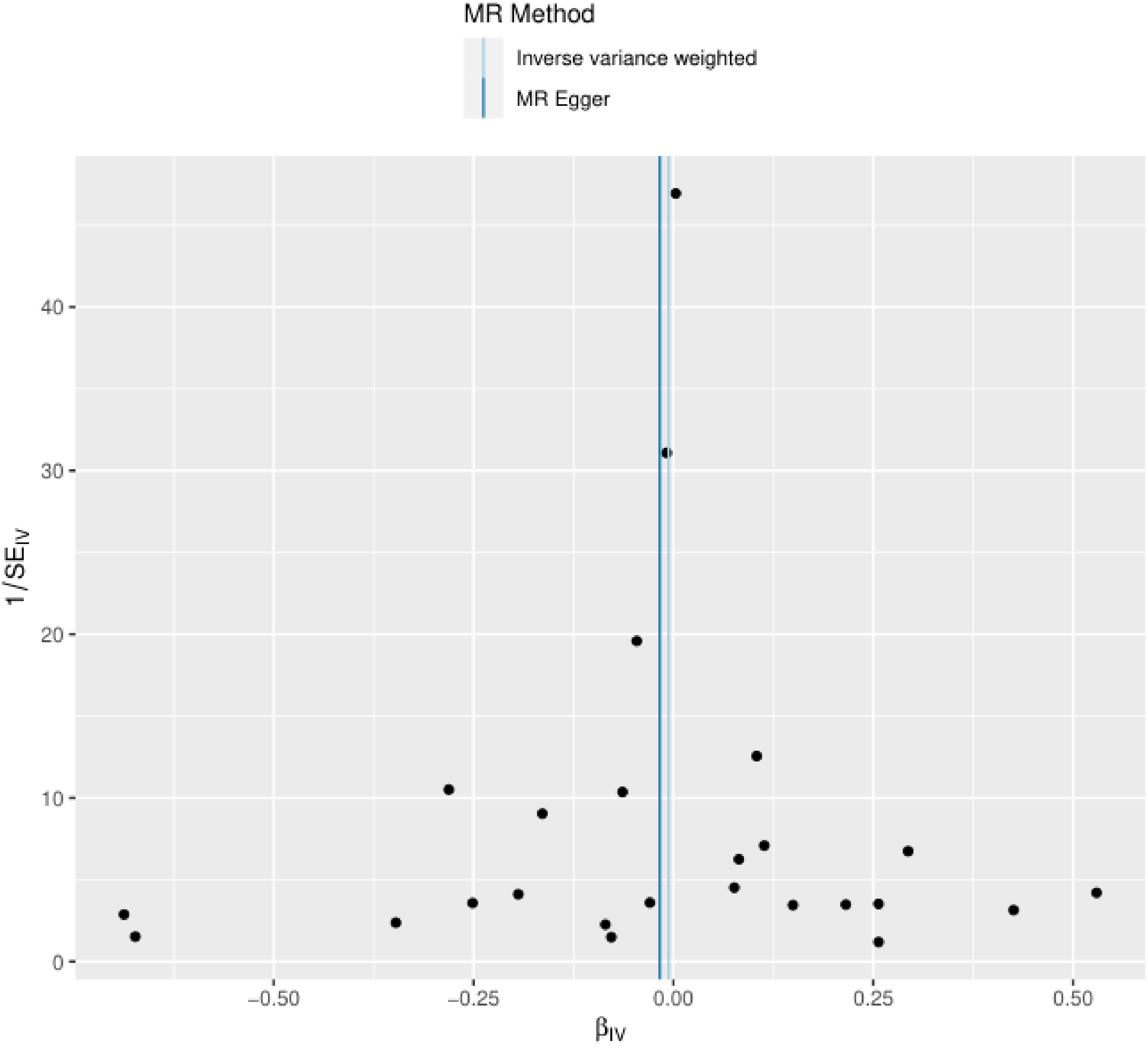

Funnel plot analyse of "CD45RA+ CD8br %CD8br" on 'Diabetic nephropathy'

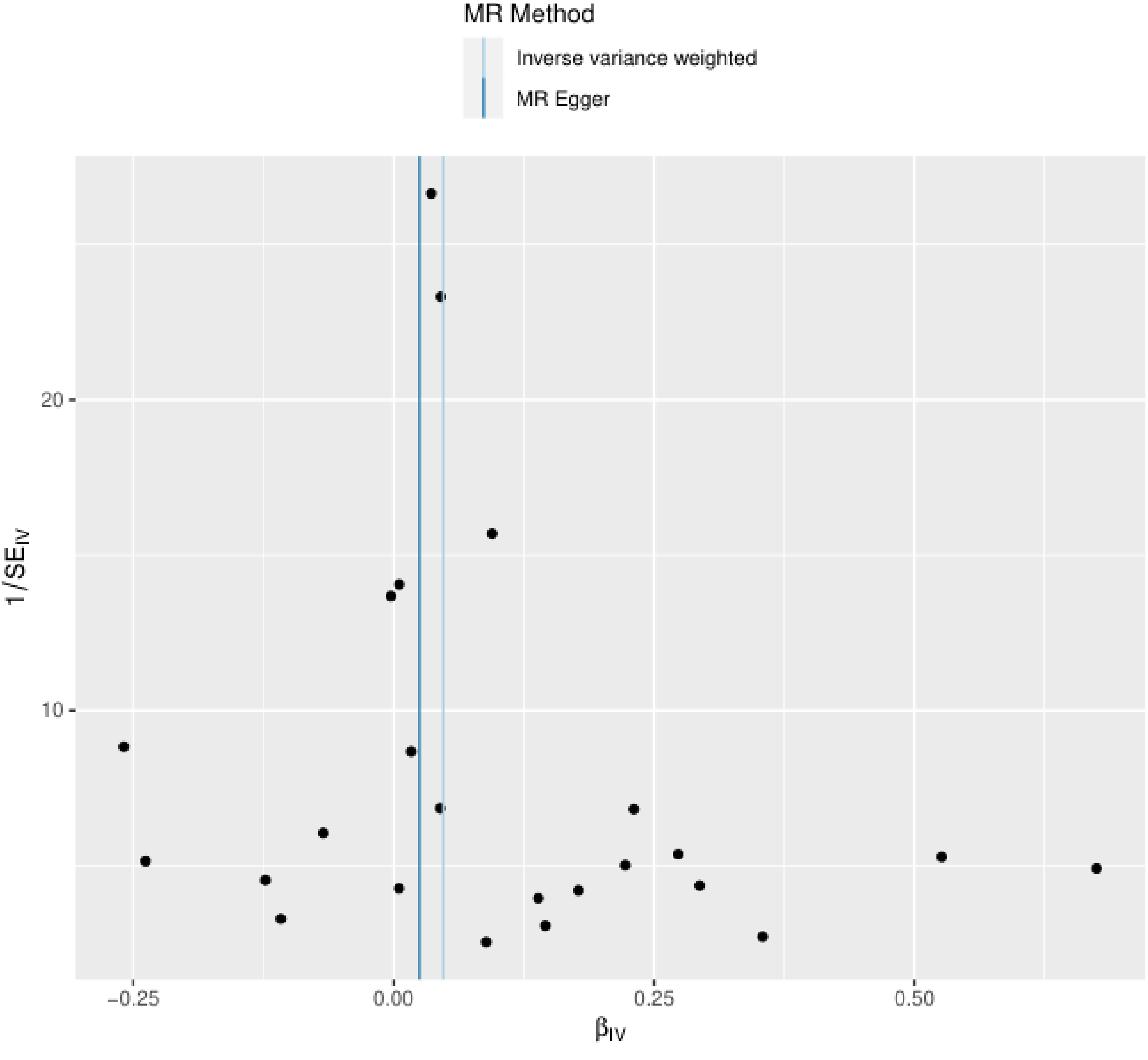

Funnel plot analyse of "CD4 on activated Treg " on 'Diabetic nephropathy'

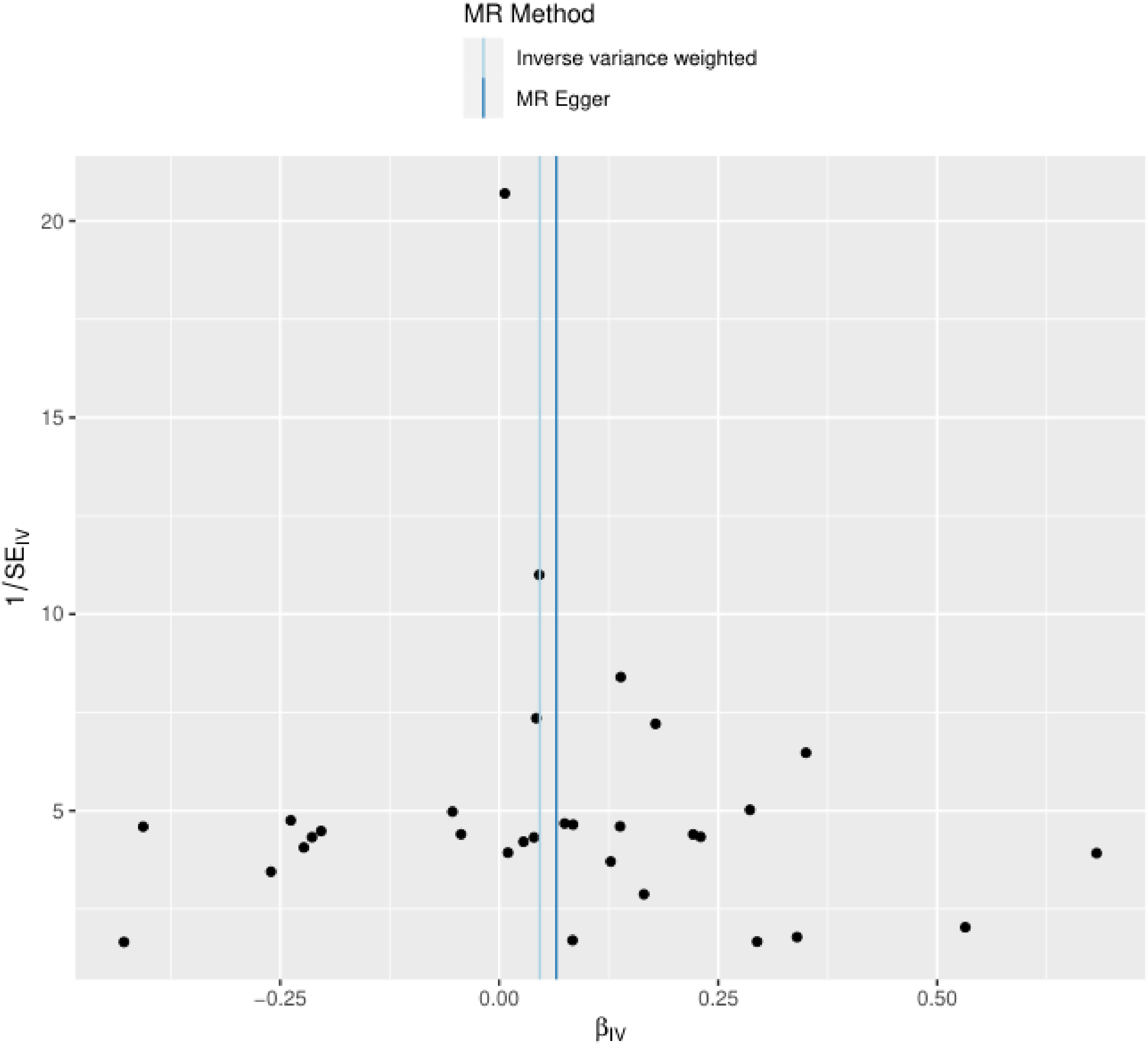

Funnel plot analyse of "IgD on transitional" on 'Diabetic nephropathy'

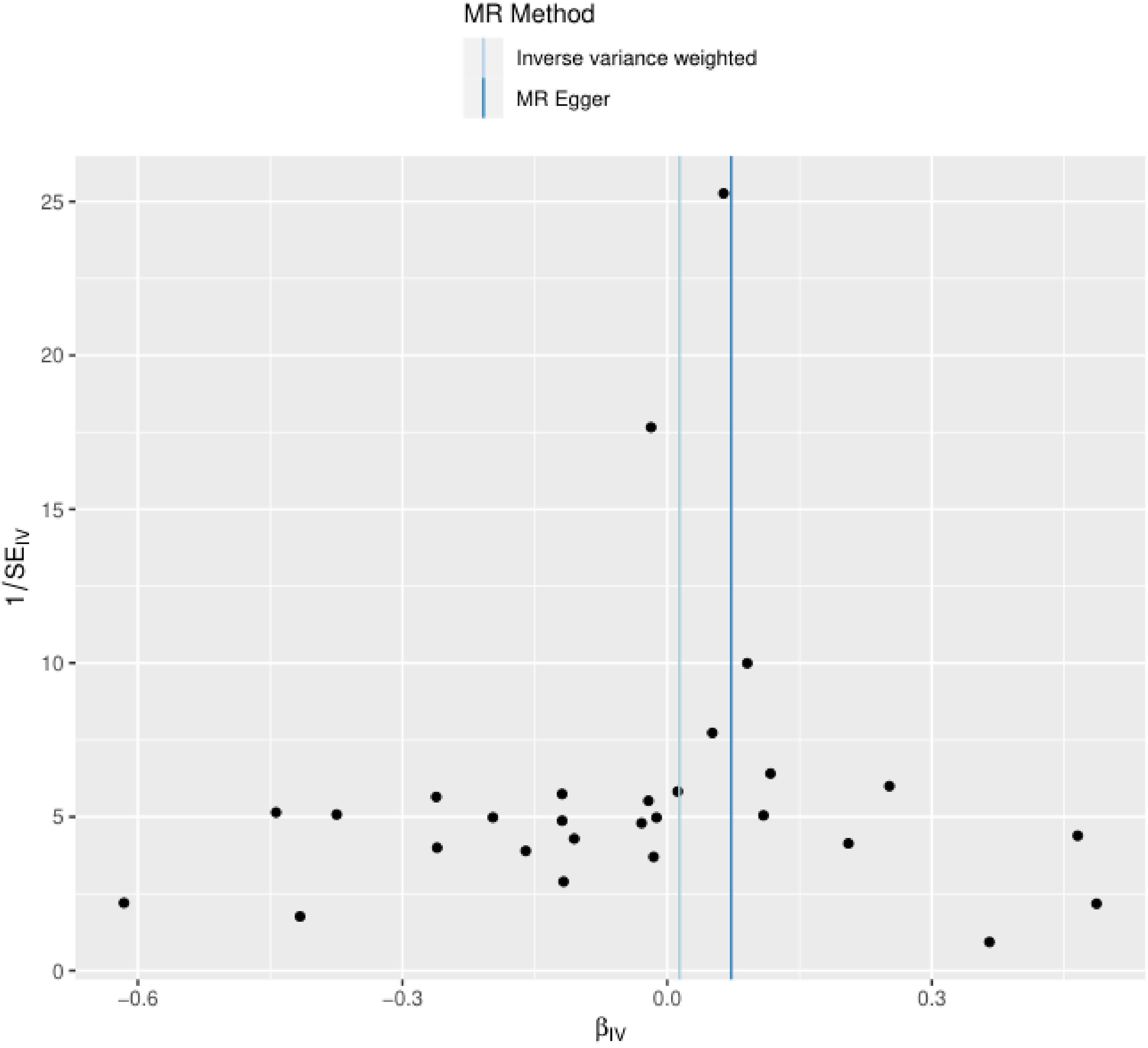

Funnel plot analyse of "CD3 on CD45RA- CD4+" on 'Diabetic nephropathy'

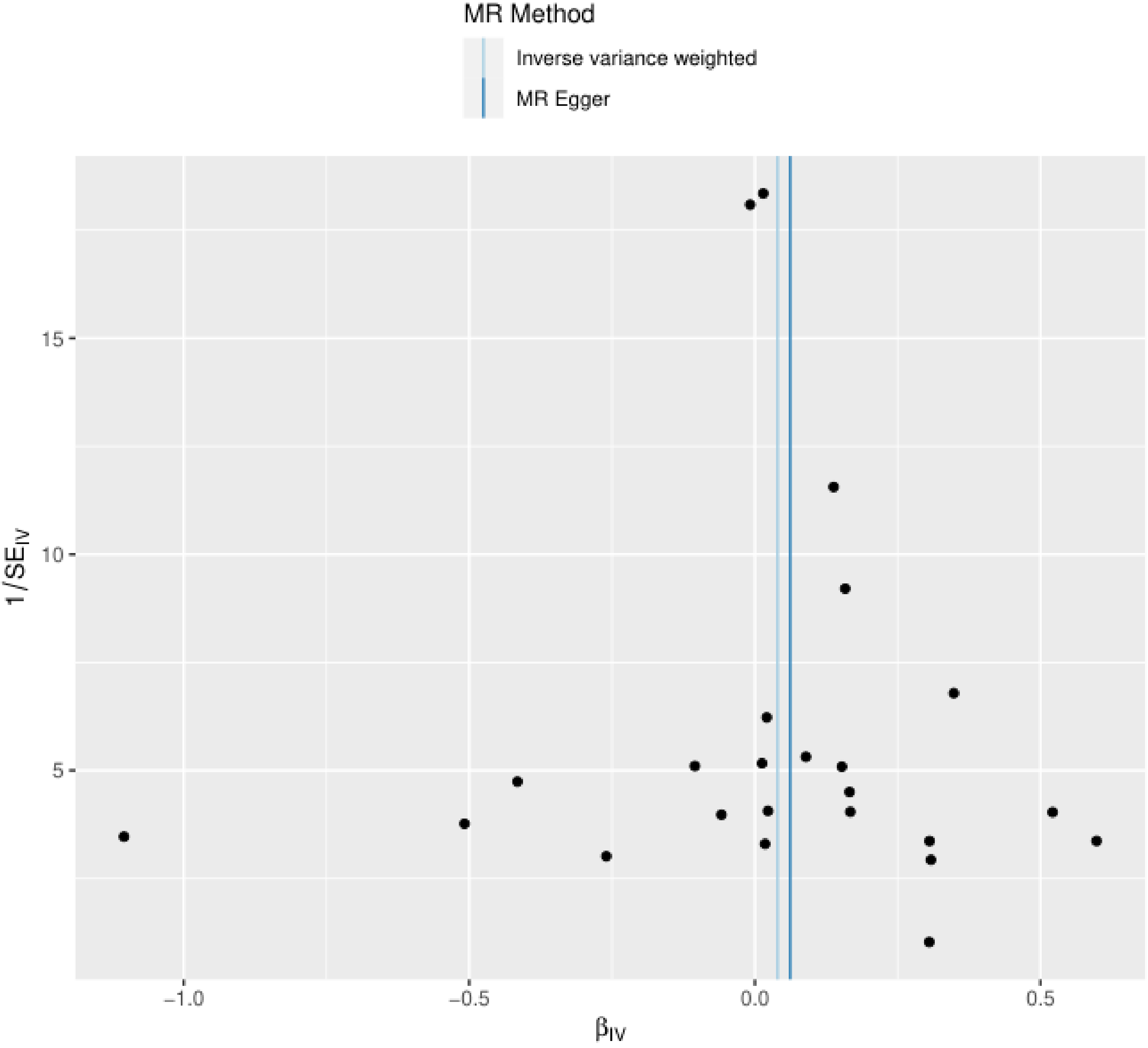

Funnel plot analyse of "TD CD8br %CD8br" on 'Diabetic nephropathy'

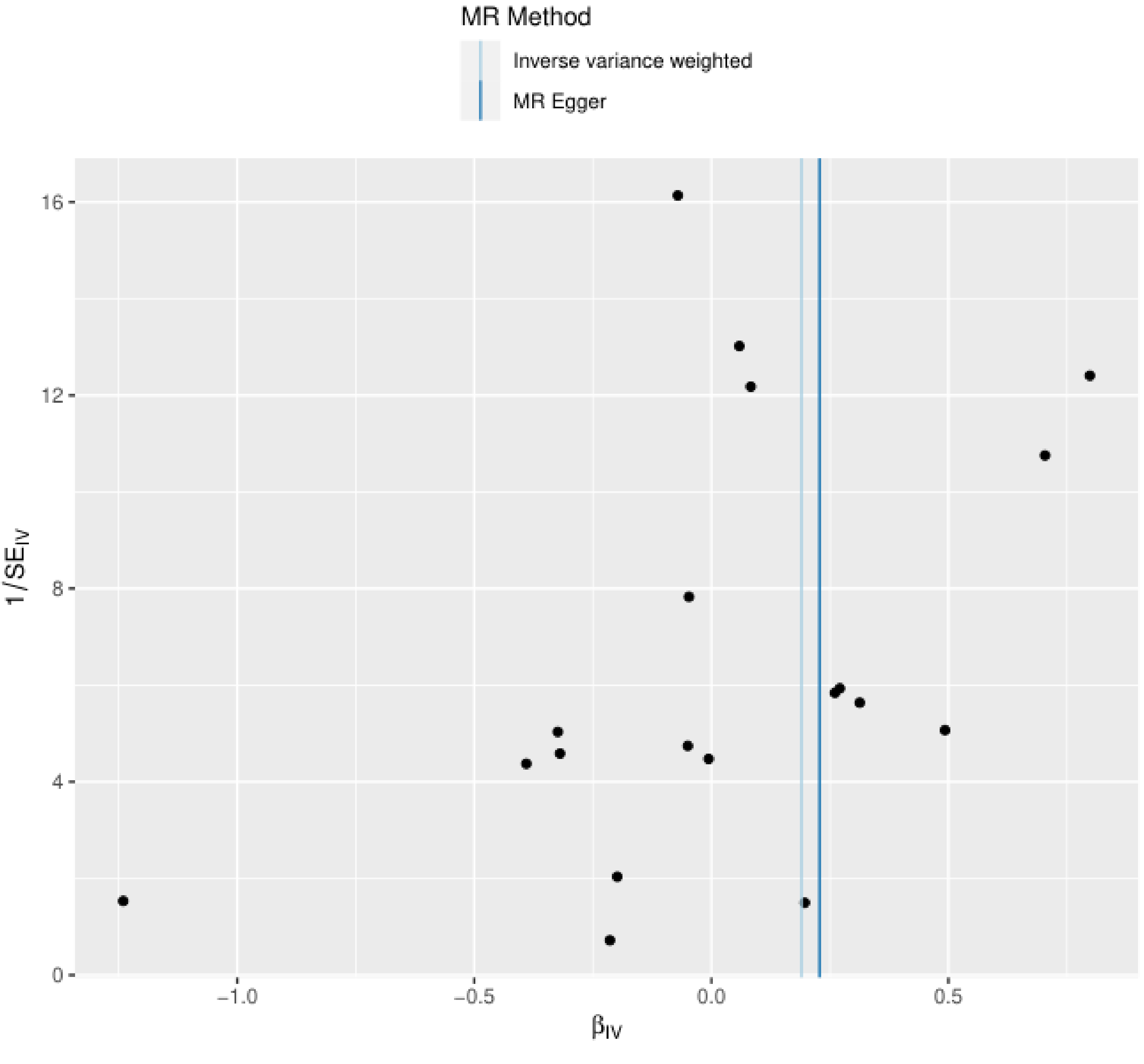

Funnel plot analyse of "HLA DR on CD14- CD16+ monocyte" on 'Diabetic nephropathy'

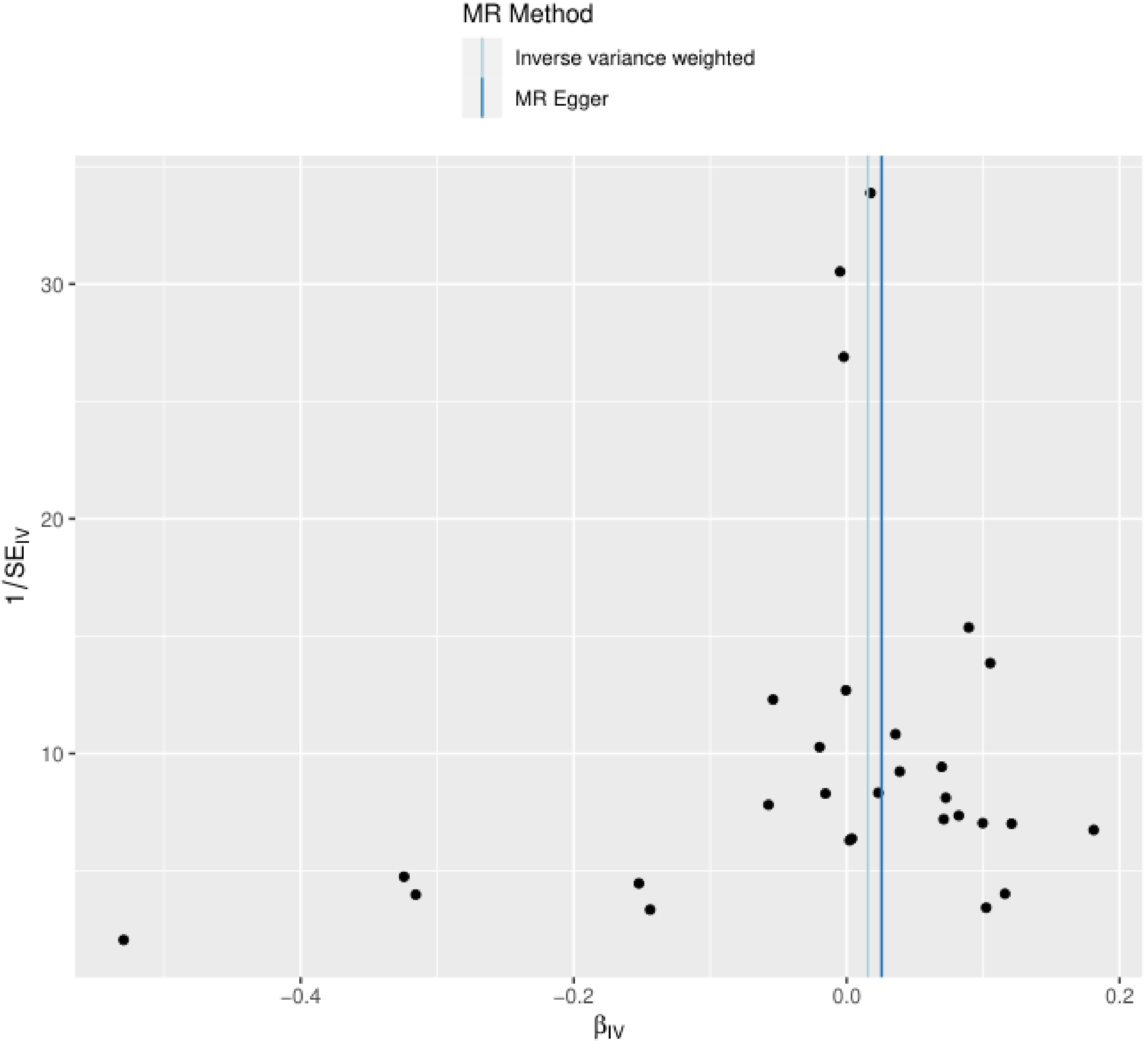

Funnel plot analyse of "CD45RA on resting Treg " on 'Diabetic nephropathy'

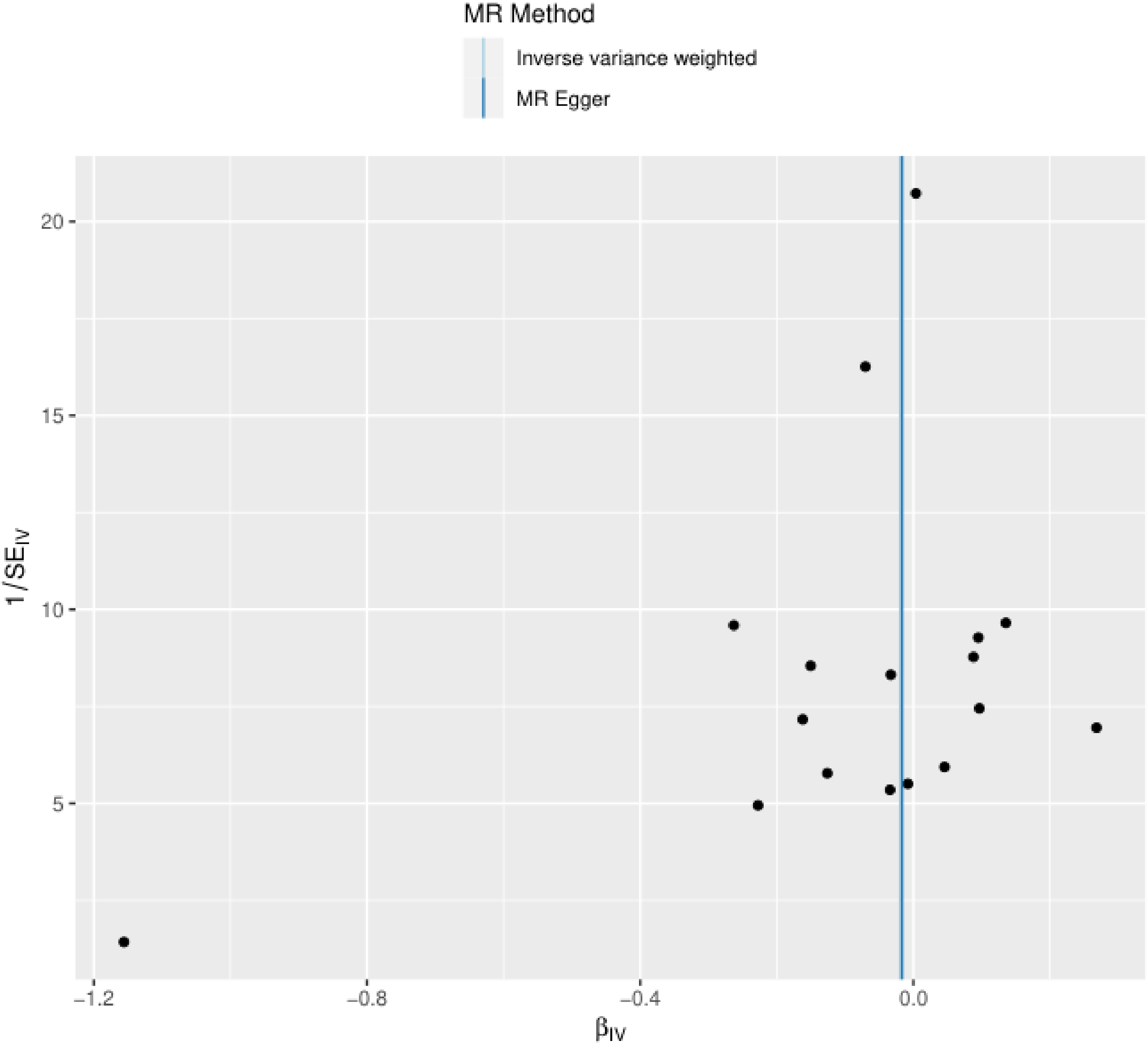

Funnel plot analyse of "HVEM on naive CD4+" on 'Diabetic nephropathy'

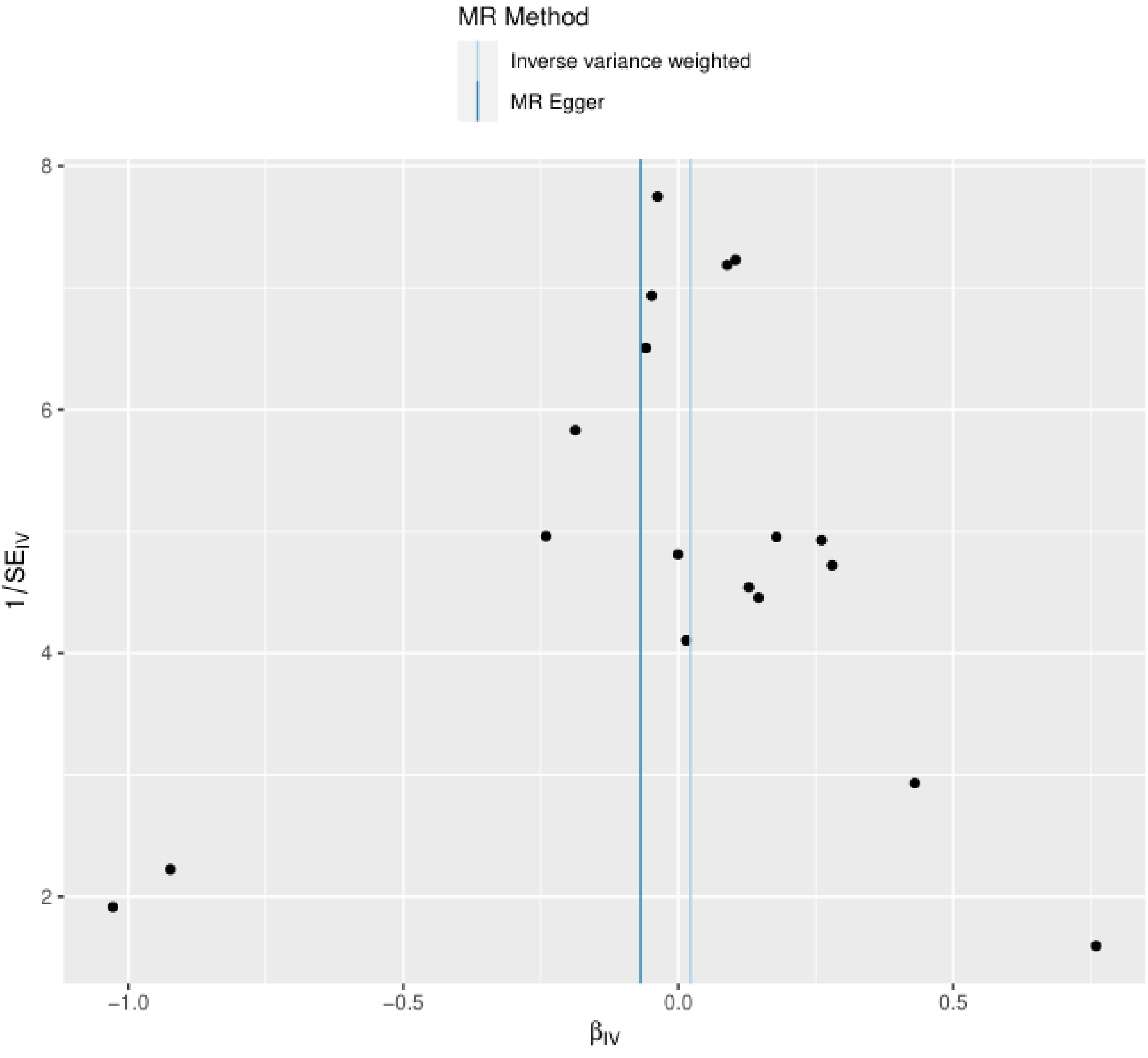

Funnel plot analyse of "CD127 on T cell" on 'Diabetic nephropathy'

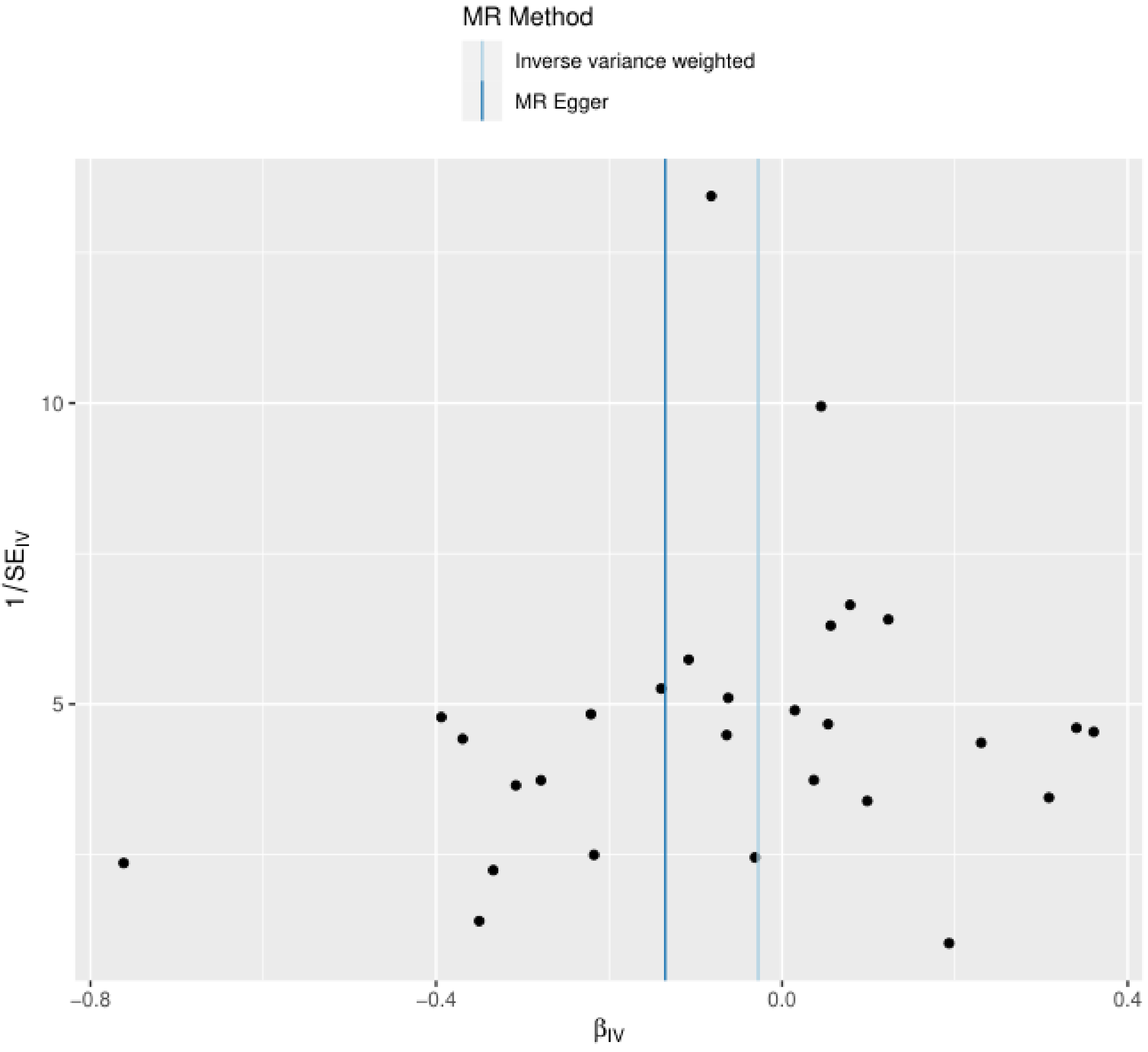

Funnel plot analyse of "CD19 on sw mem" on 'Diabetic nephropathy'

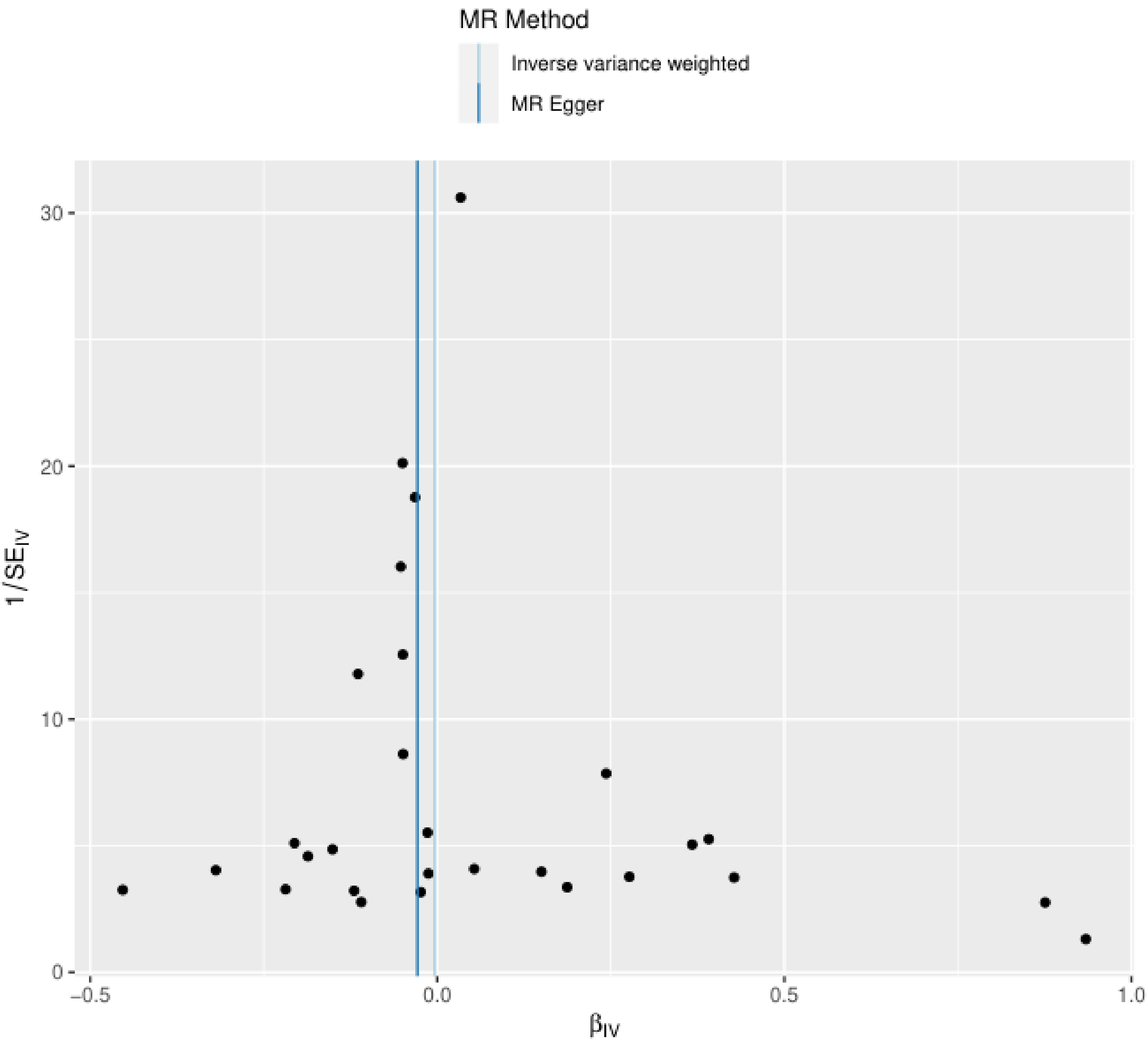

Funnel plot analyse of "CCR2 on CD14- CD16+ monocyte " on 'Diabetic nephropathy'

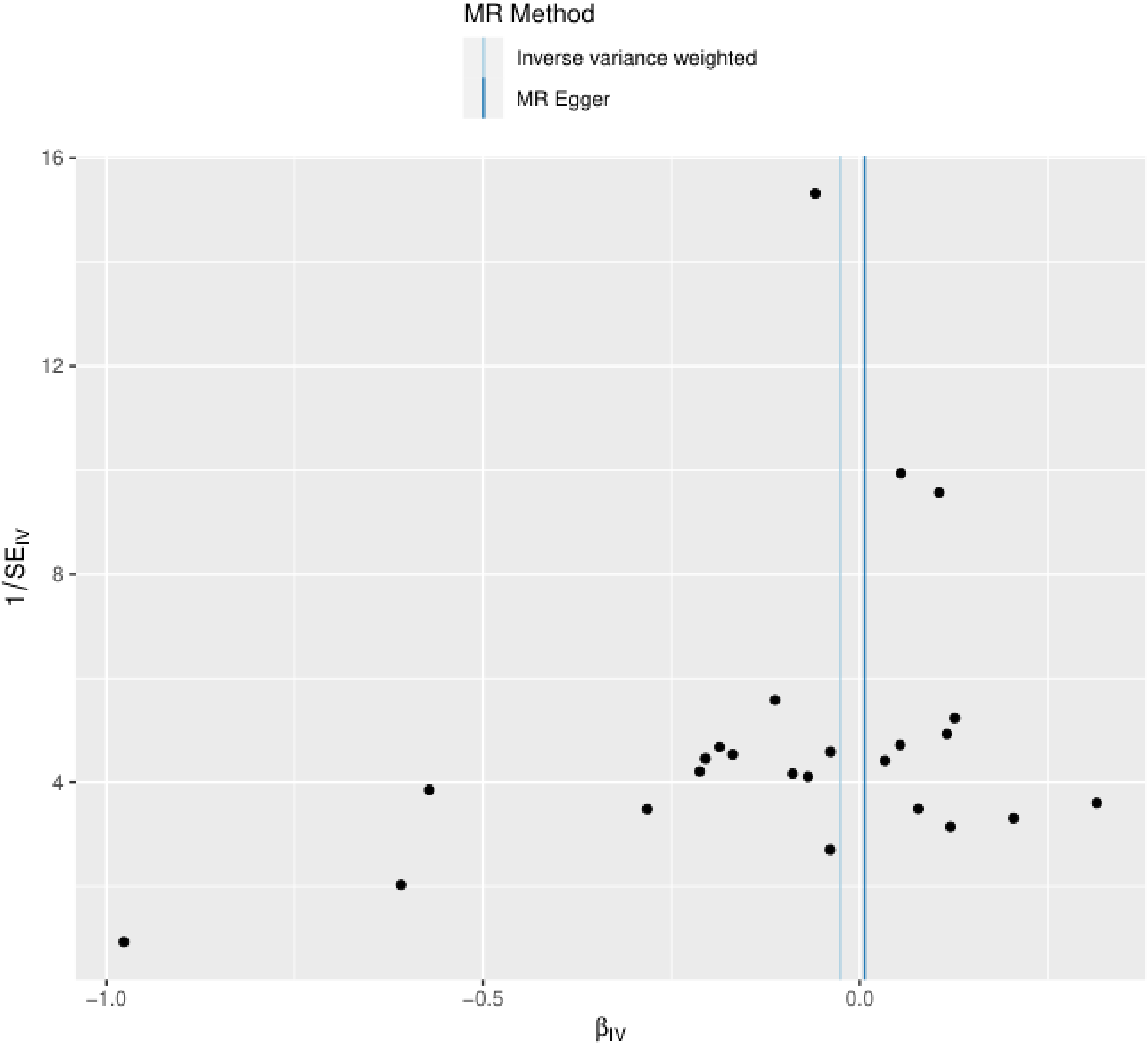

Funnel plot analyse of "IgD- CD24- %lymphocyte" on 'Diabetic nephropathy'

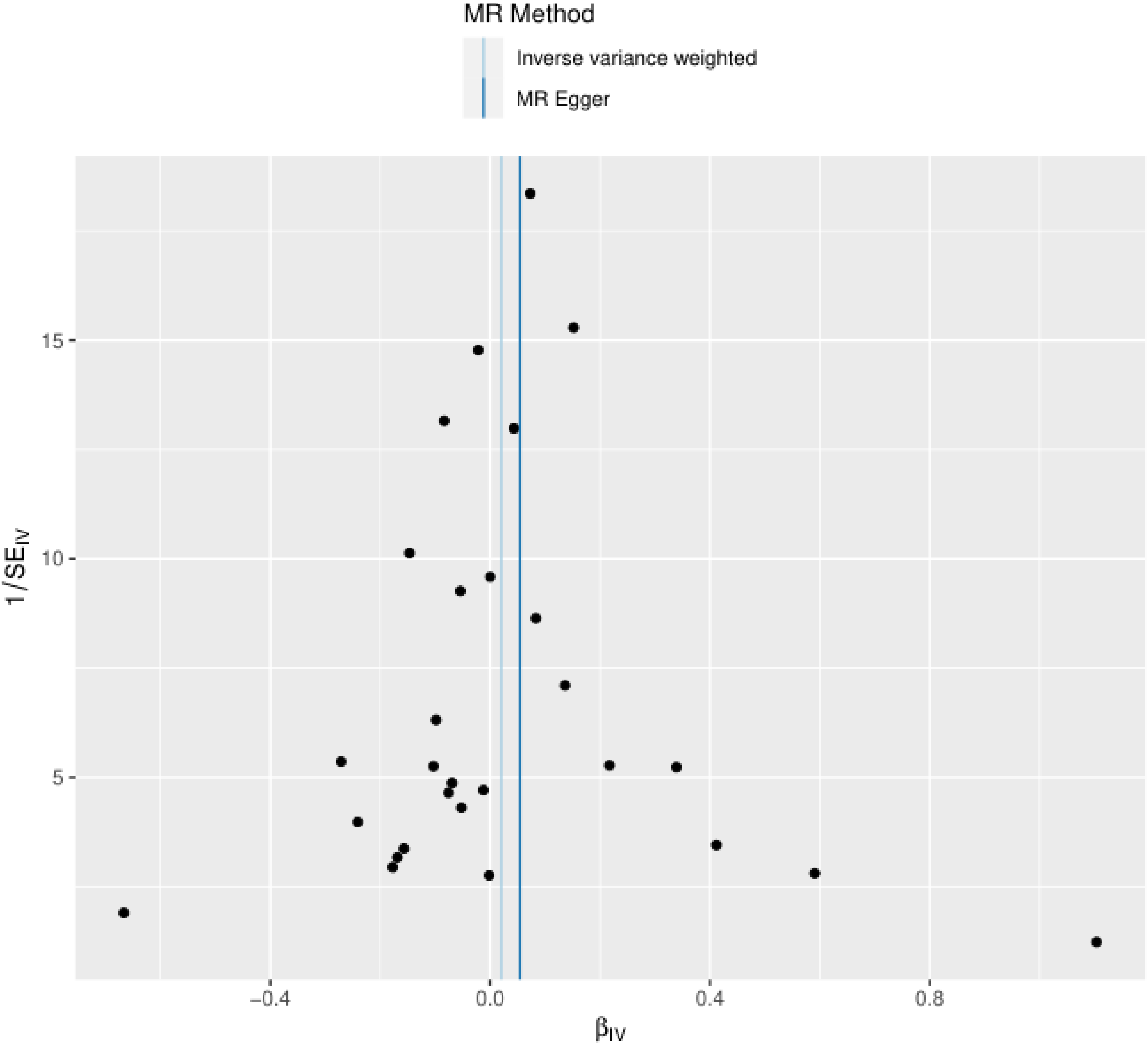

Funnel plot analyse of "CD64 on monocyte" on 'Diabetic nephropathy'

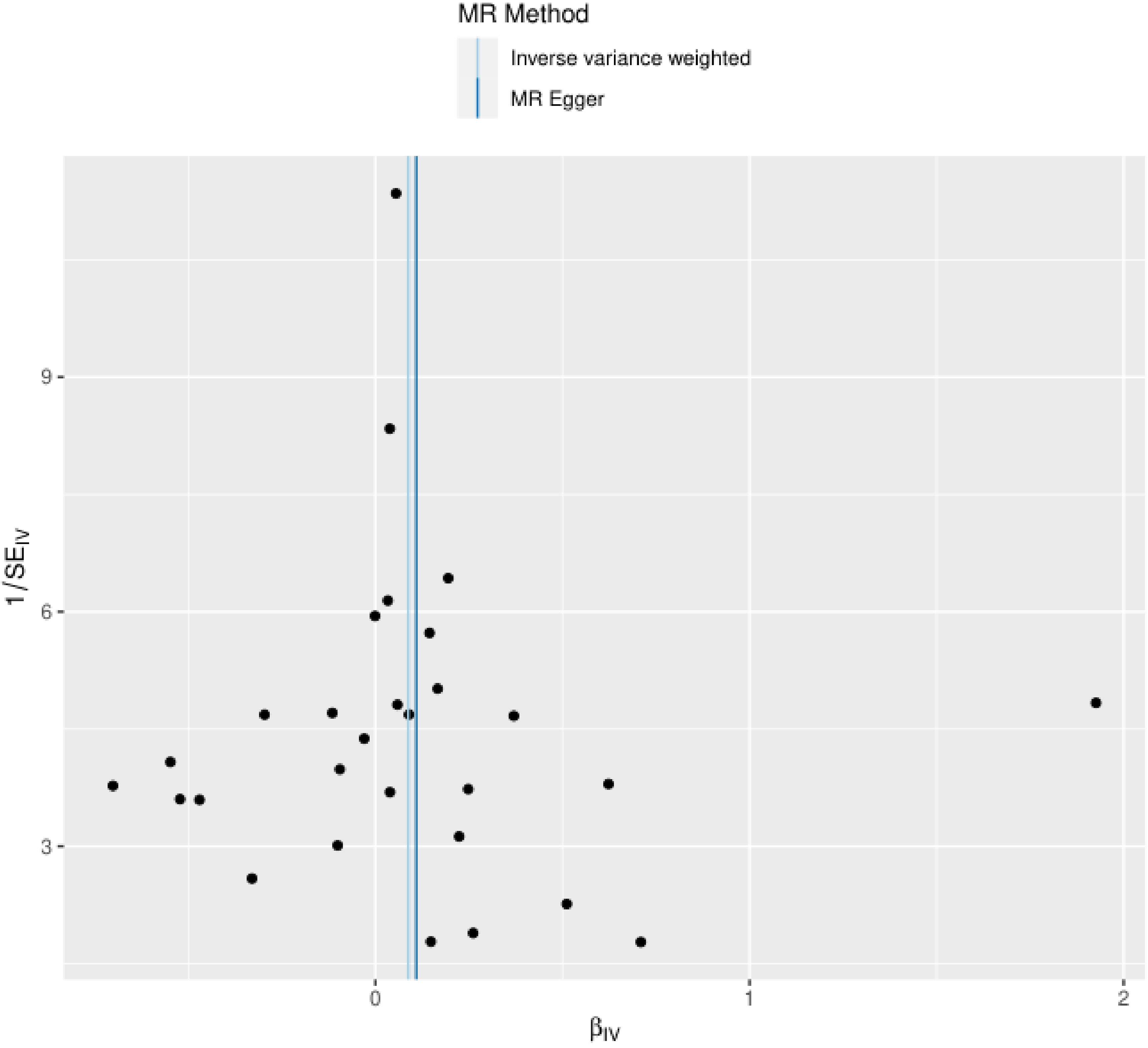

Funnel plot analyse of "CD8br NKT %lymphocyte" on 'Diabetic nephropathy'

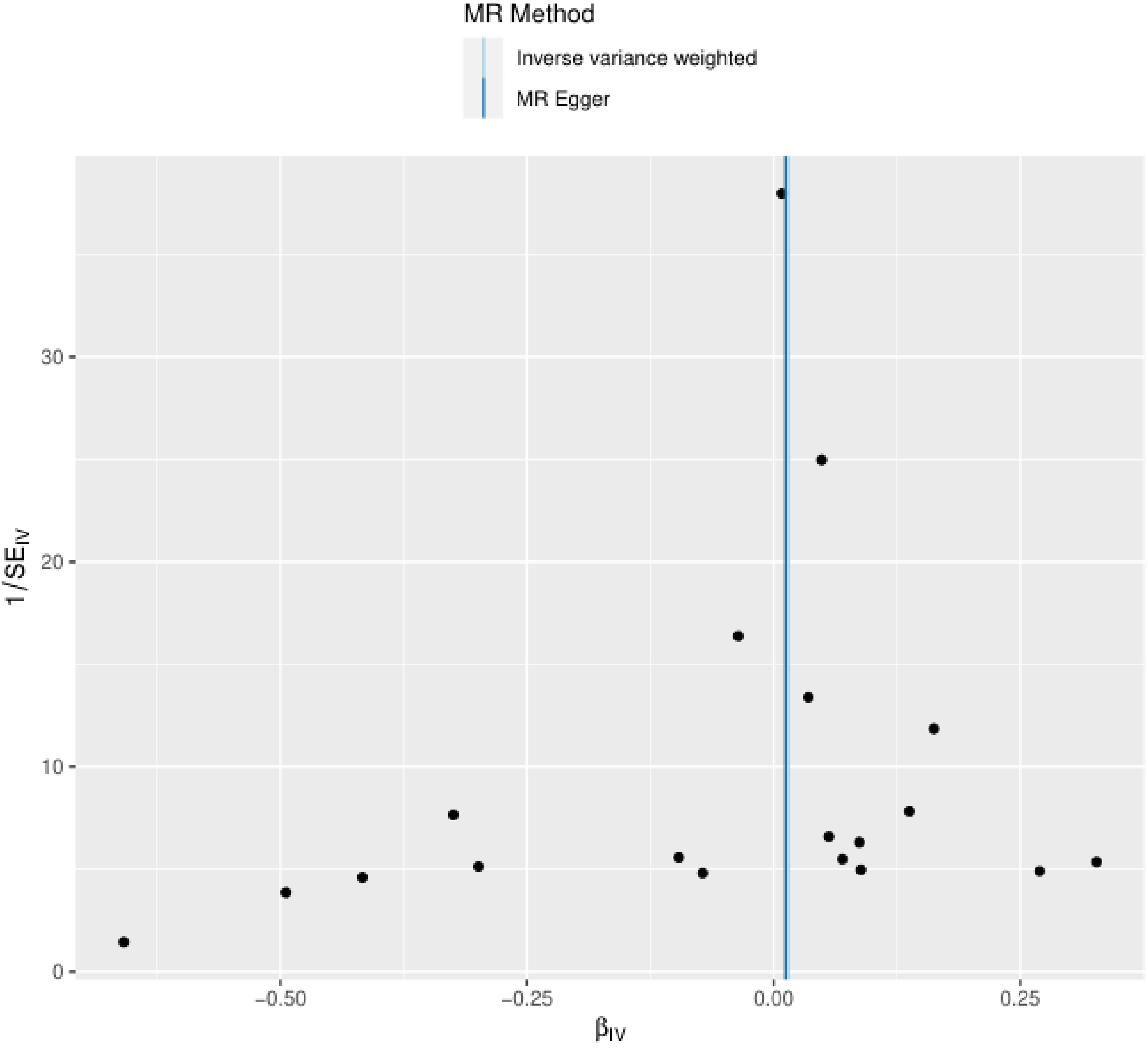

Funnel plot analyse of "FSC-A on myeloid DC " on 'Diabetic nephropathy'

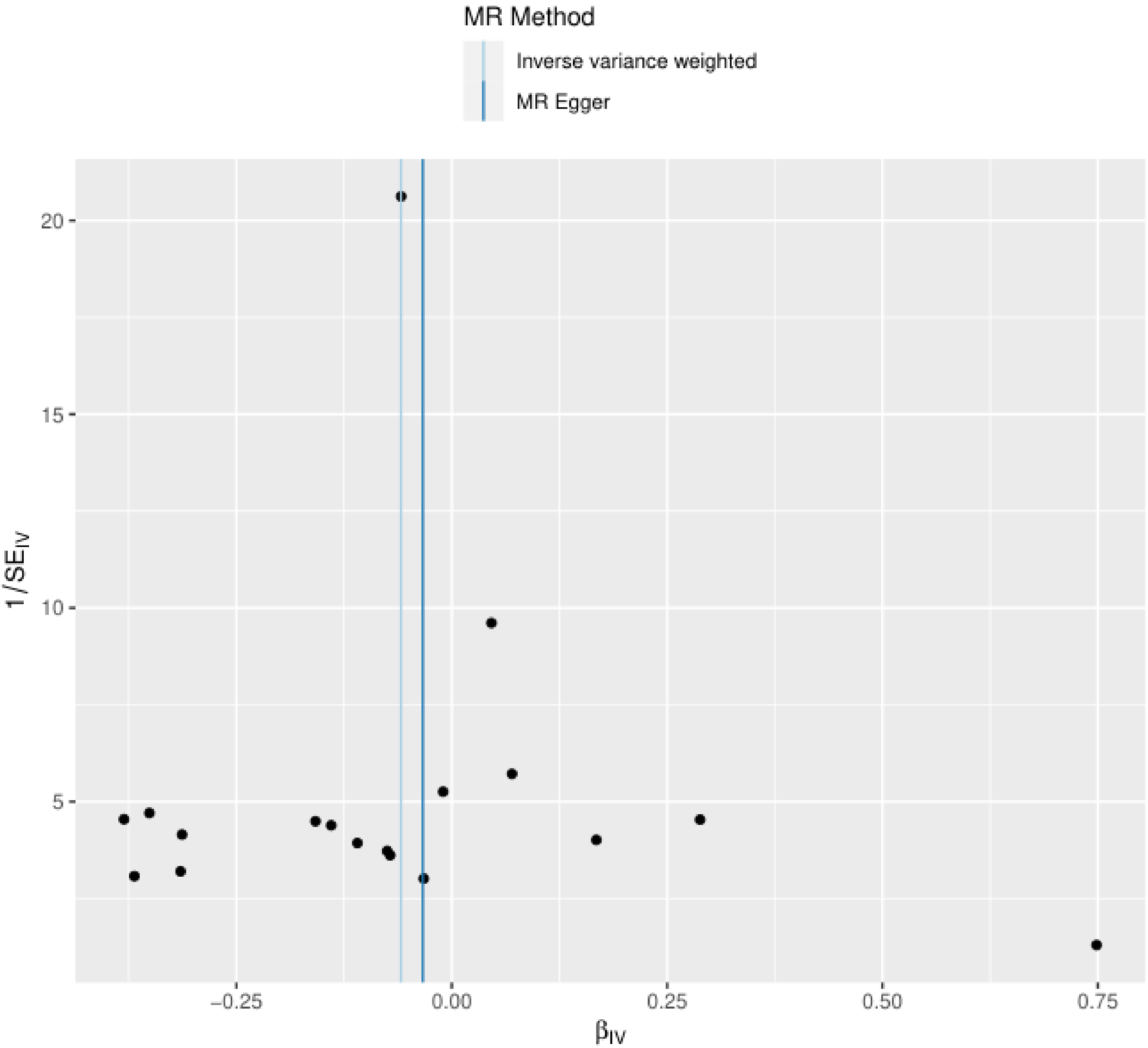

Funnel plot analyse of "FSC-A on CD8br" on 'Diabetic nephropathy'

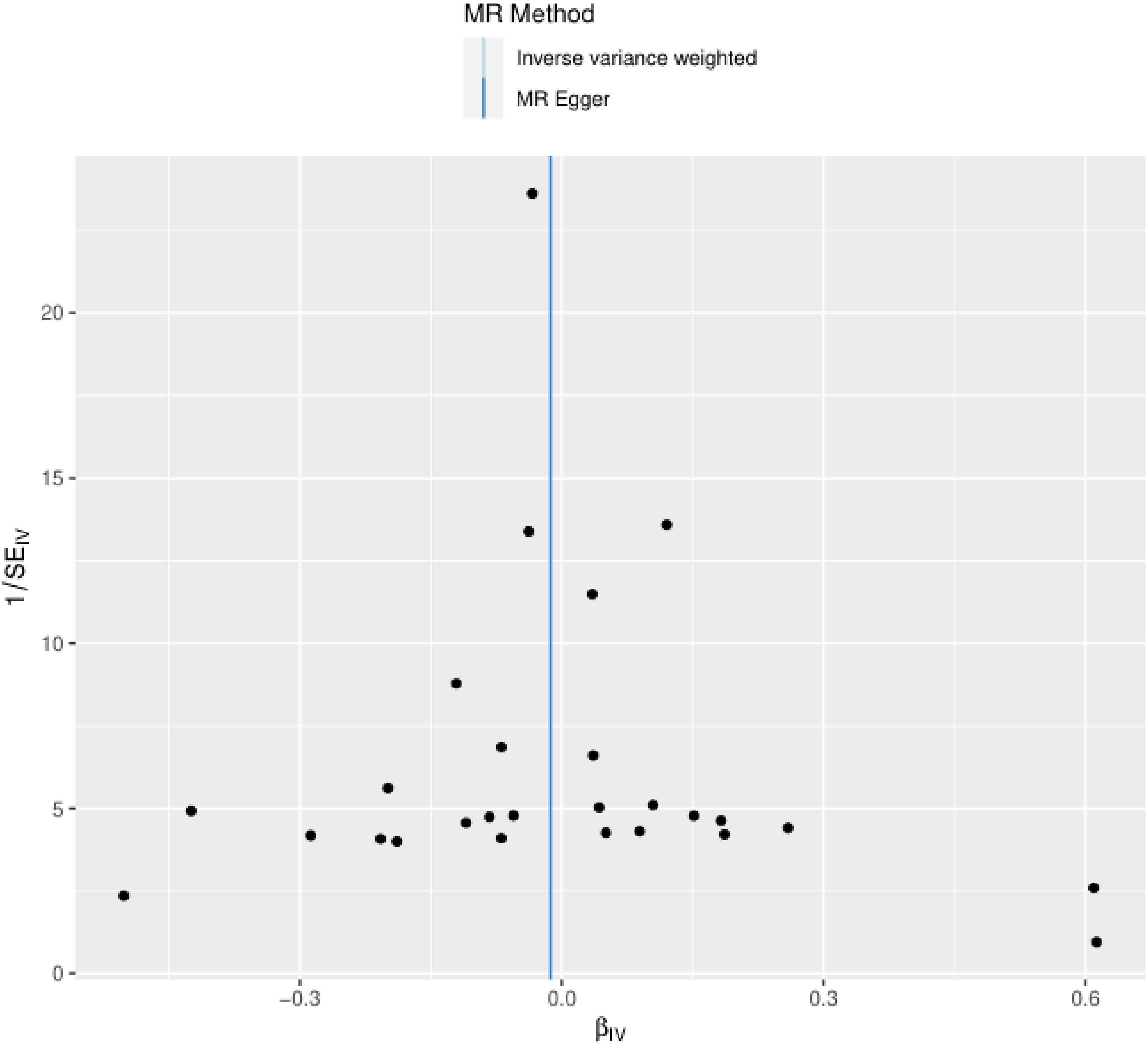

Funnel plot analyse of "CD3 on HLA DR+ T cell " on 'Diabetic nephropathy'

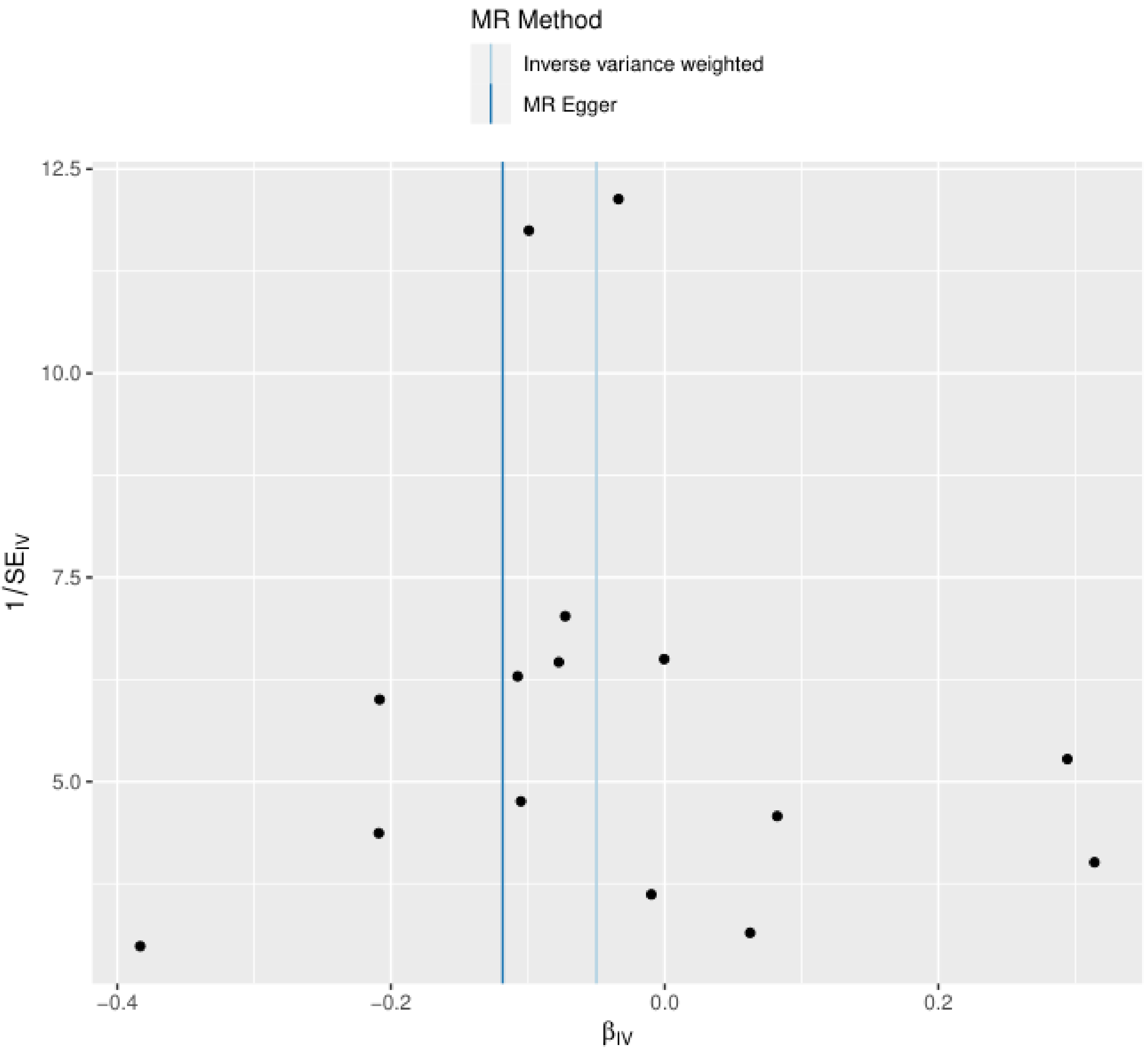

Funnel plot analyse of "CCR2 on CD62L+ myeloid DC" on 'Diabetic nephropathy'

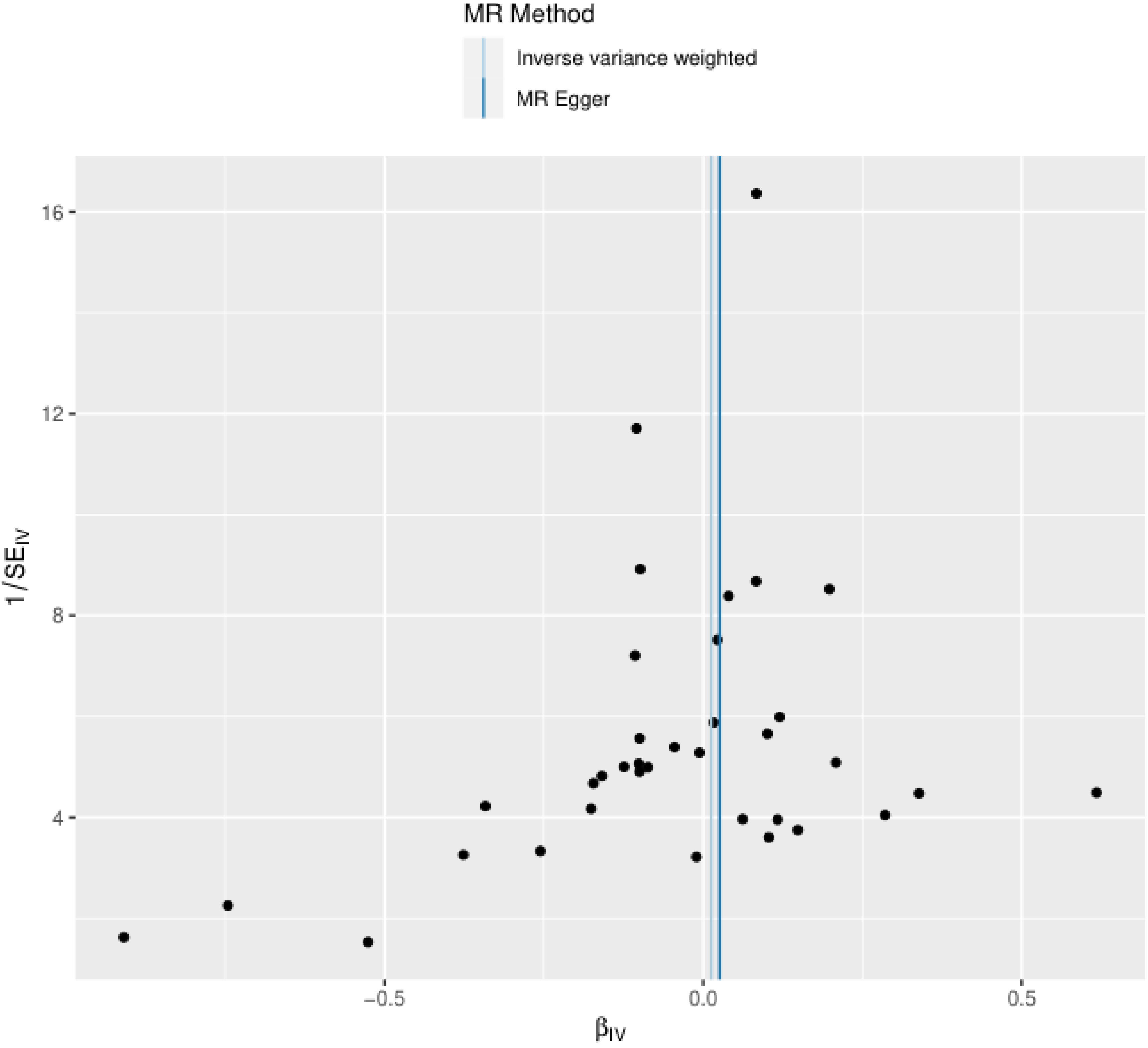

Funnel plot analyse of "CD3 on resting Treg " on 'Diabetic nephropathy'

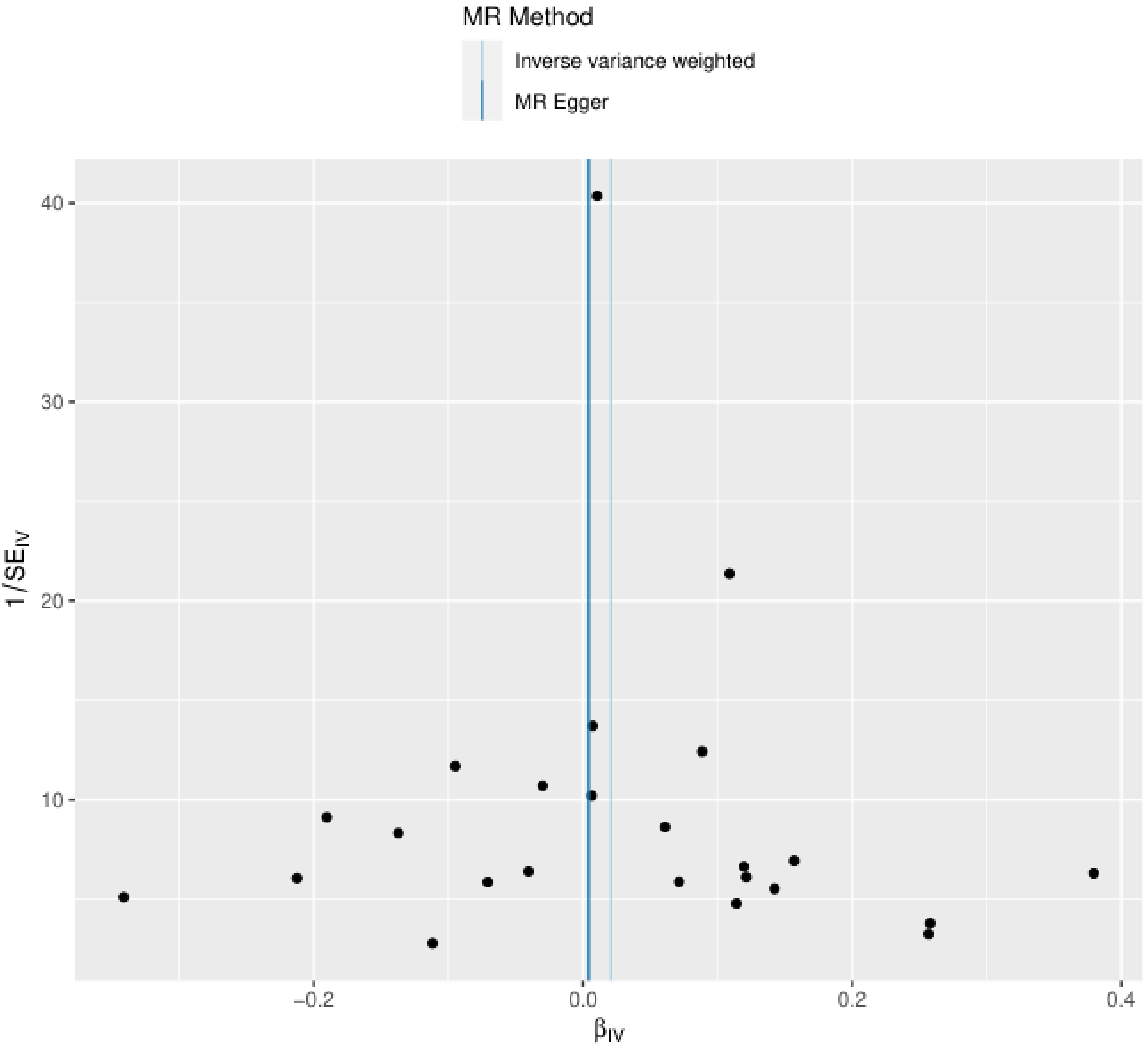

nel plot analyse of "CD33dim HLA DR+ CD11b+ %CD33dim HLA DR+" on 'Diabetic nephropa

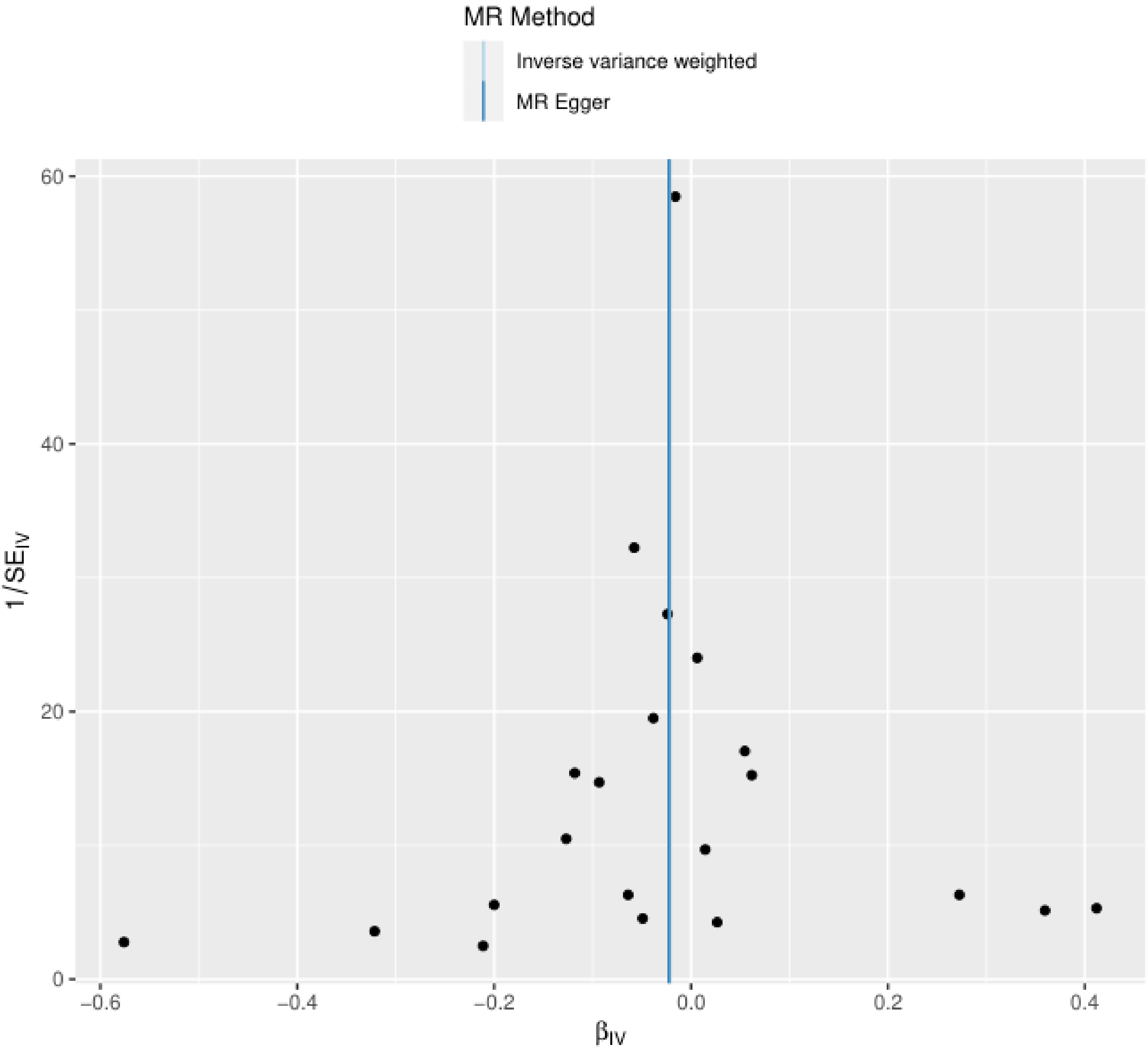

Funnel plot analyse of "Activated & secreting Treg AC" on 'Diabetic nephropathy'

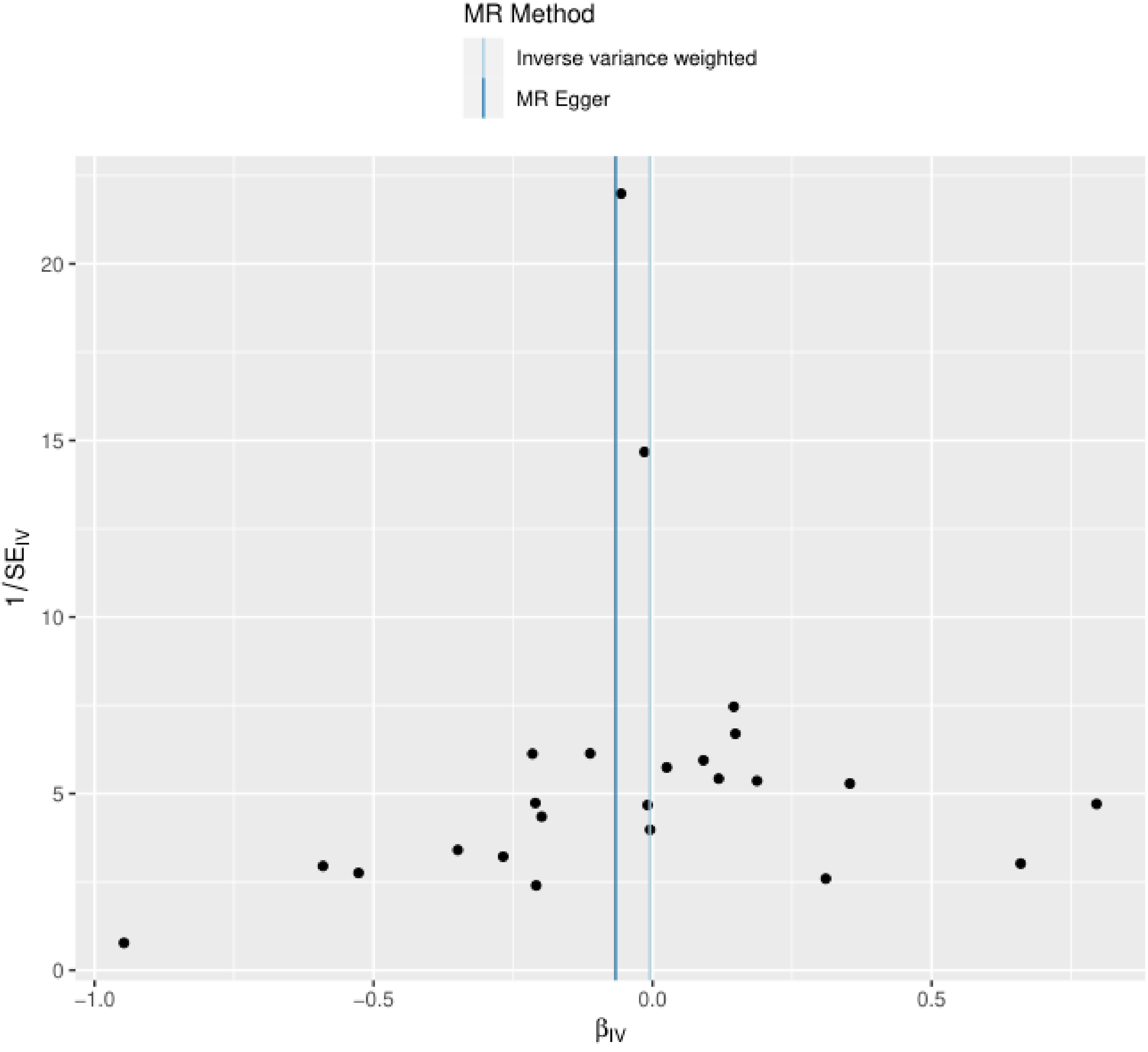

Funnel plot analyse of "FSC-A on CD14+ monocyte" on 'Diabetic nephropathy'

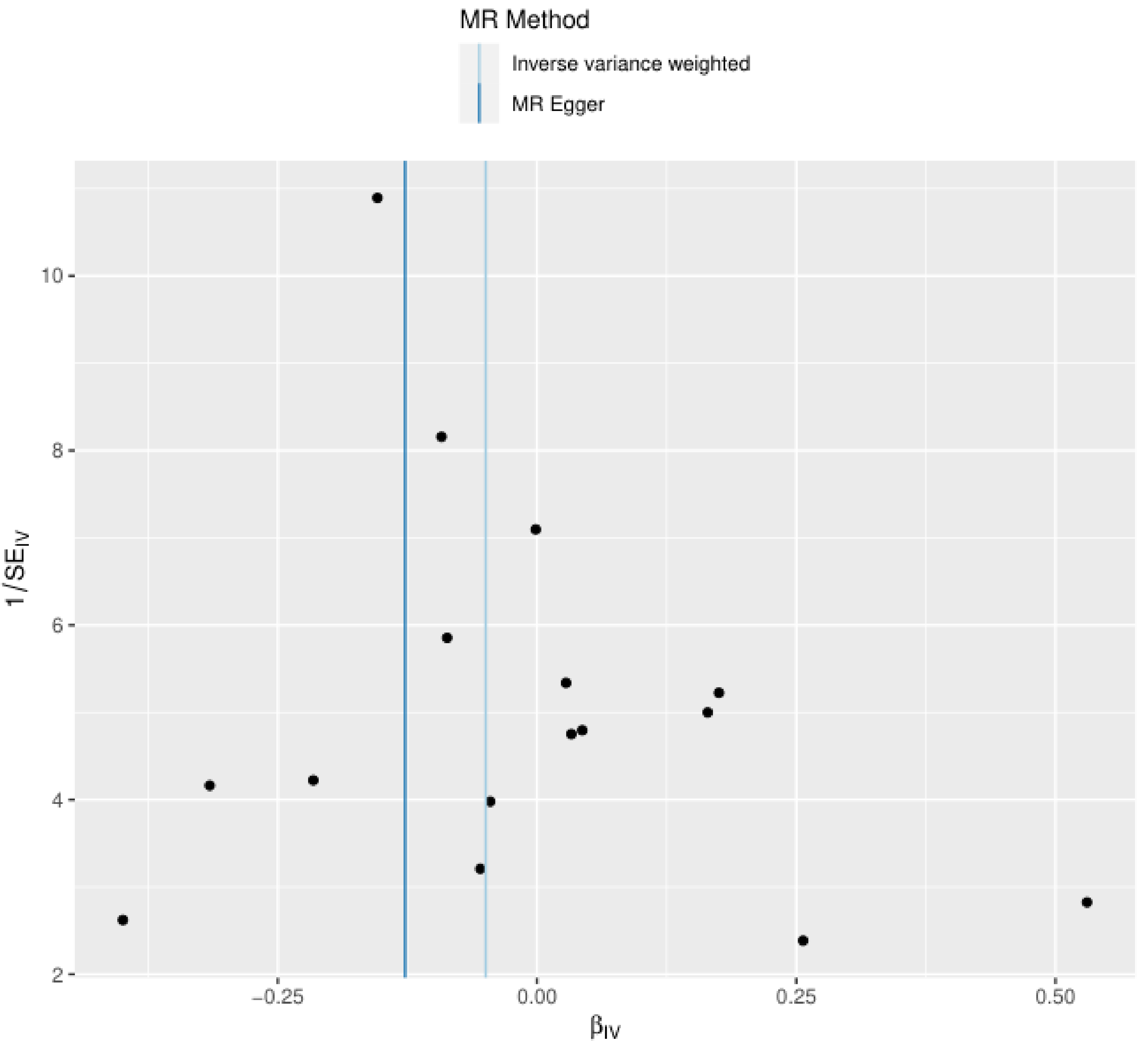

Funnel plot analyse of "IgD- CD27- %lymphocyte" on 'Diabetic nephropathy'

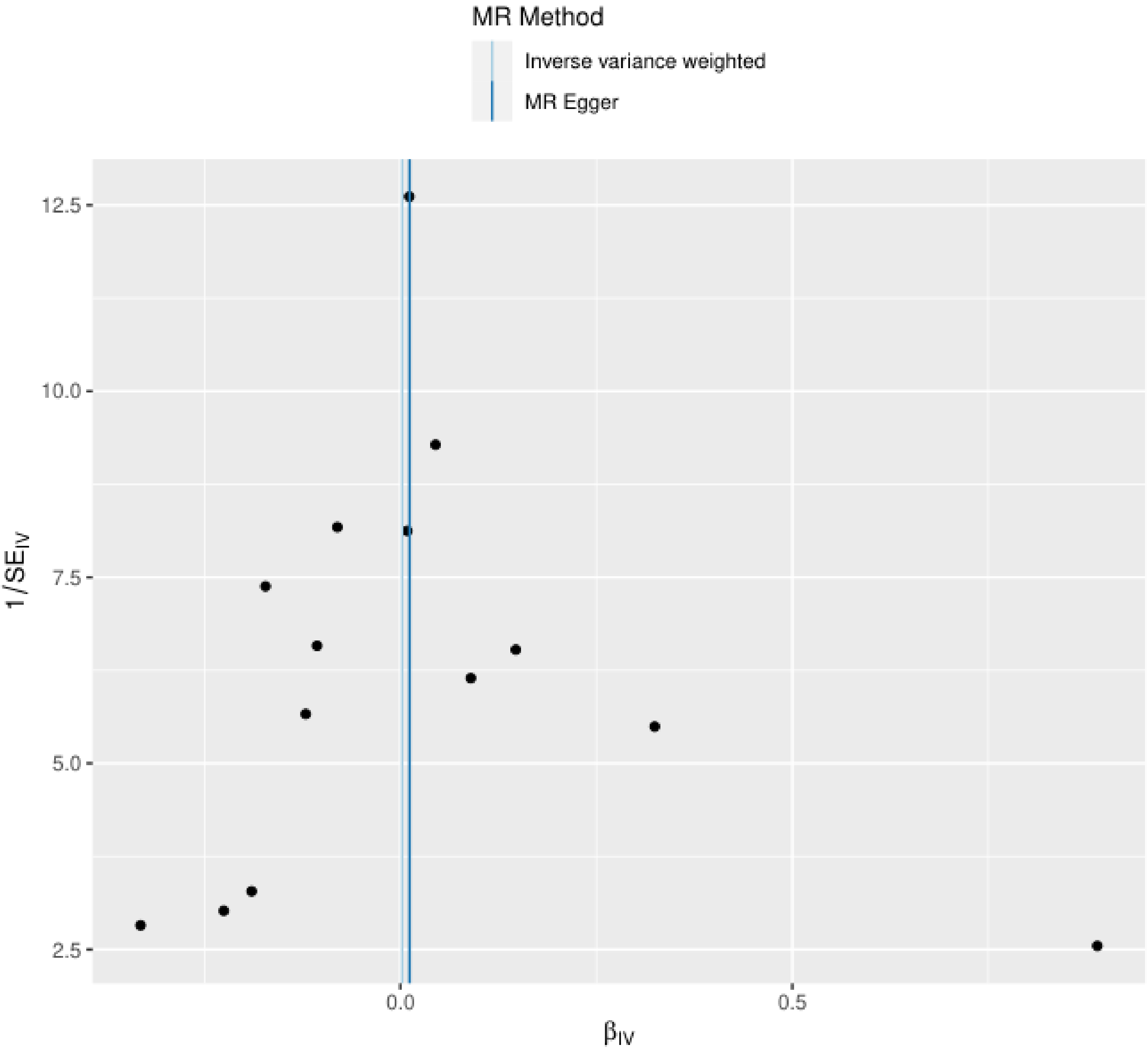

Funnel plot analyse of "CD45 on CD33- HLA DR- " on 'Diabetic nephropathy'

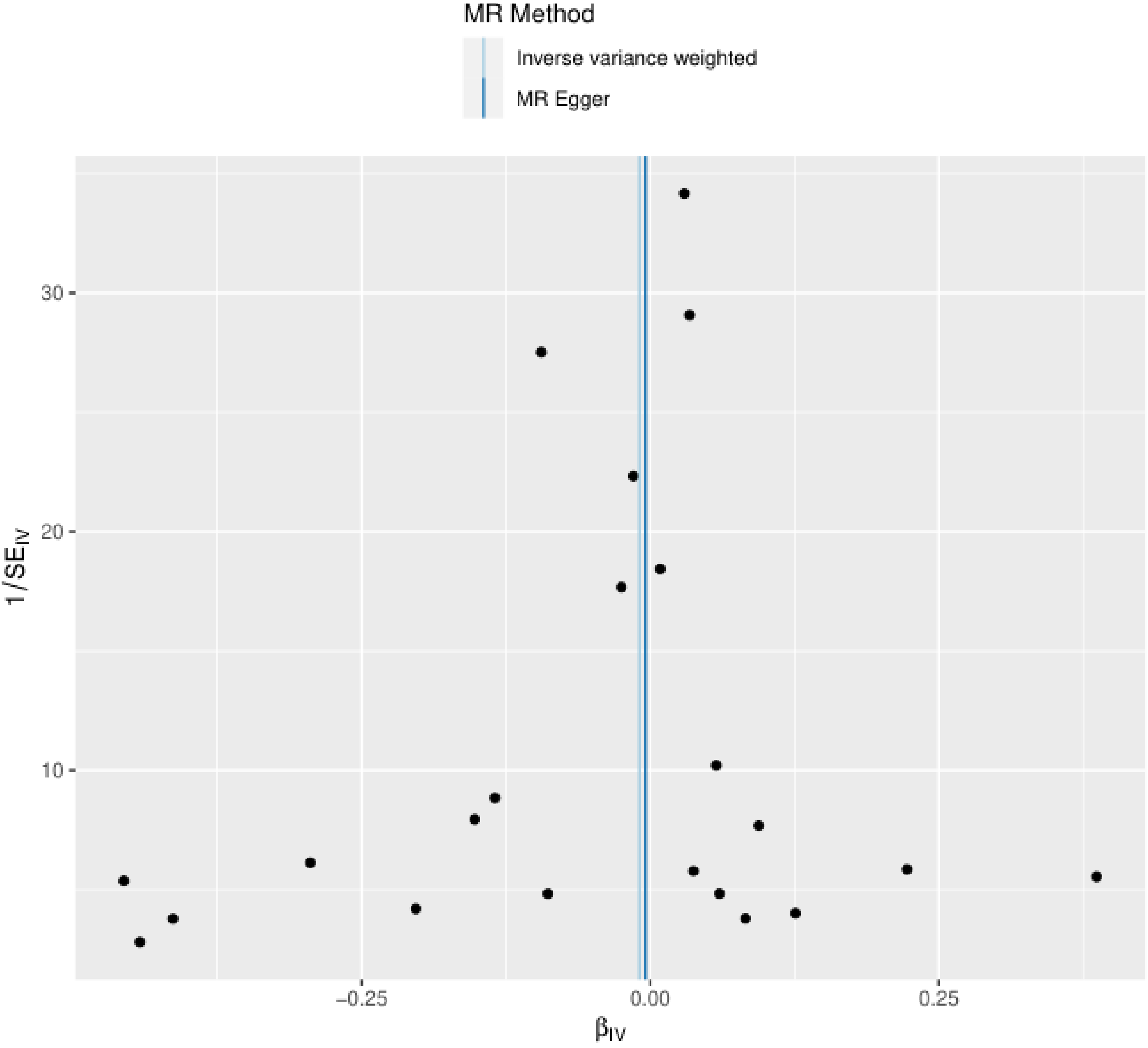

Funnel plot analyse of "CD39 on monocyte " on 'Diabetic nephropathy'

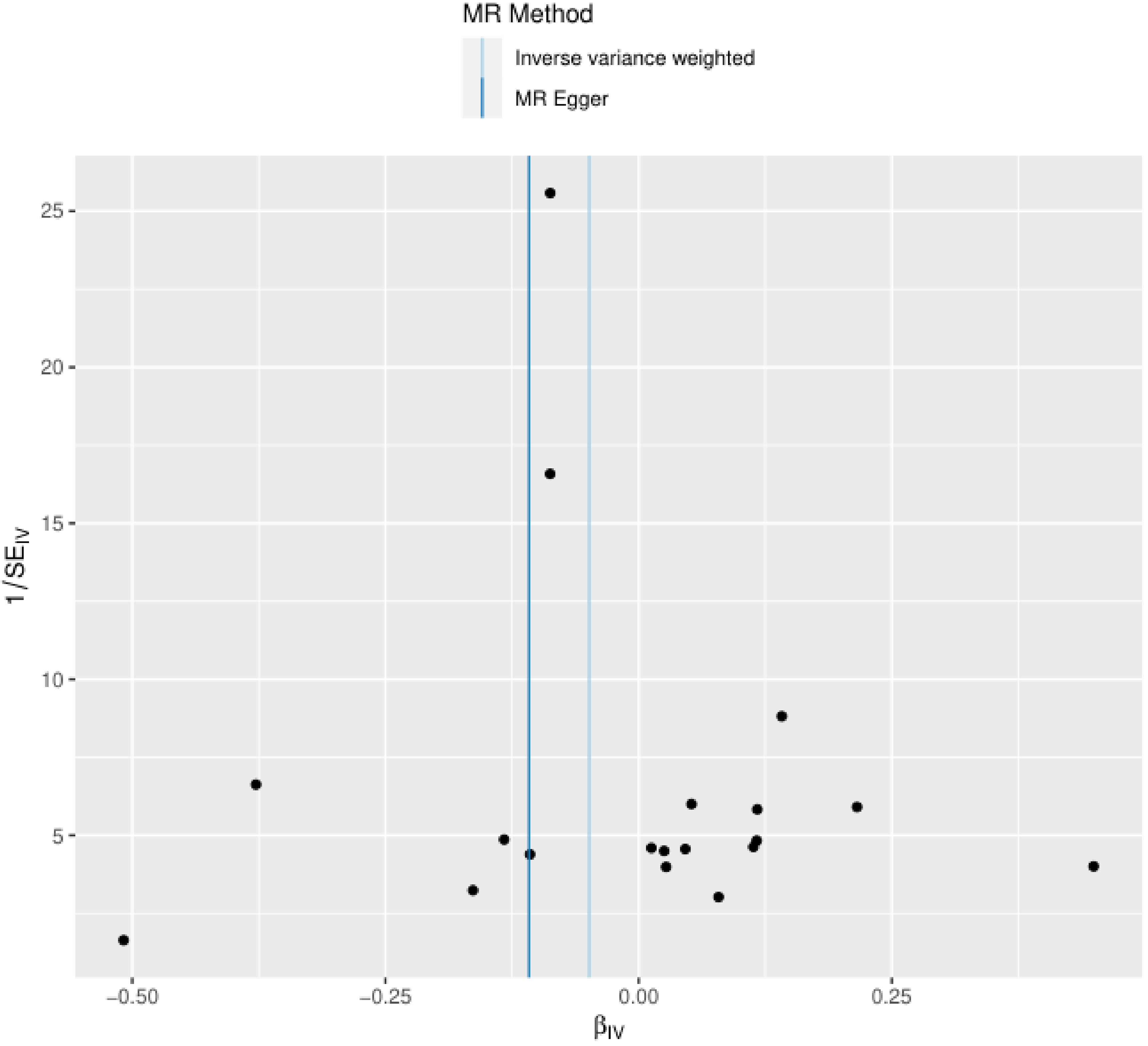

Funnel plot analyse of "HLA DR on HLA DR+ T cell" on 'Diabetic nephropathy'

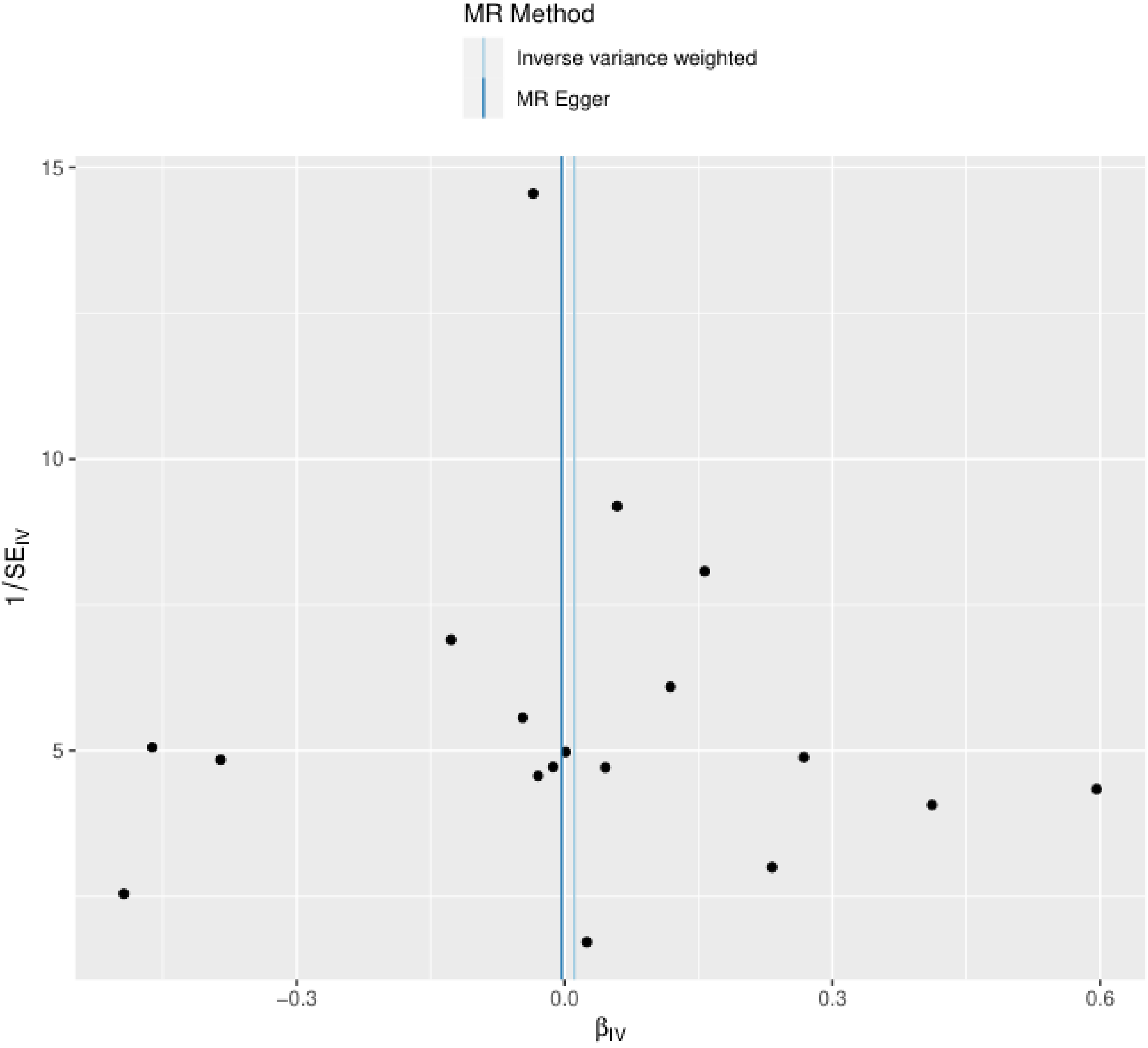

Funnel plot analyse of "CD3 on CM CD8br " on 'Diabetic nephropathy'

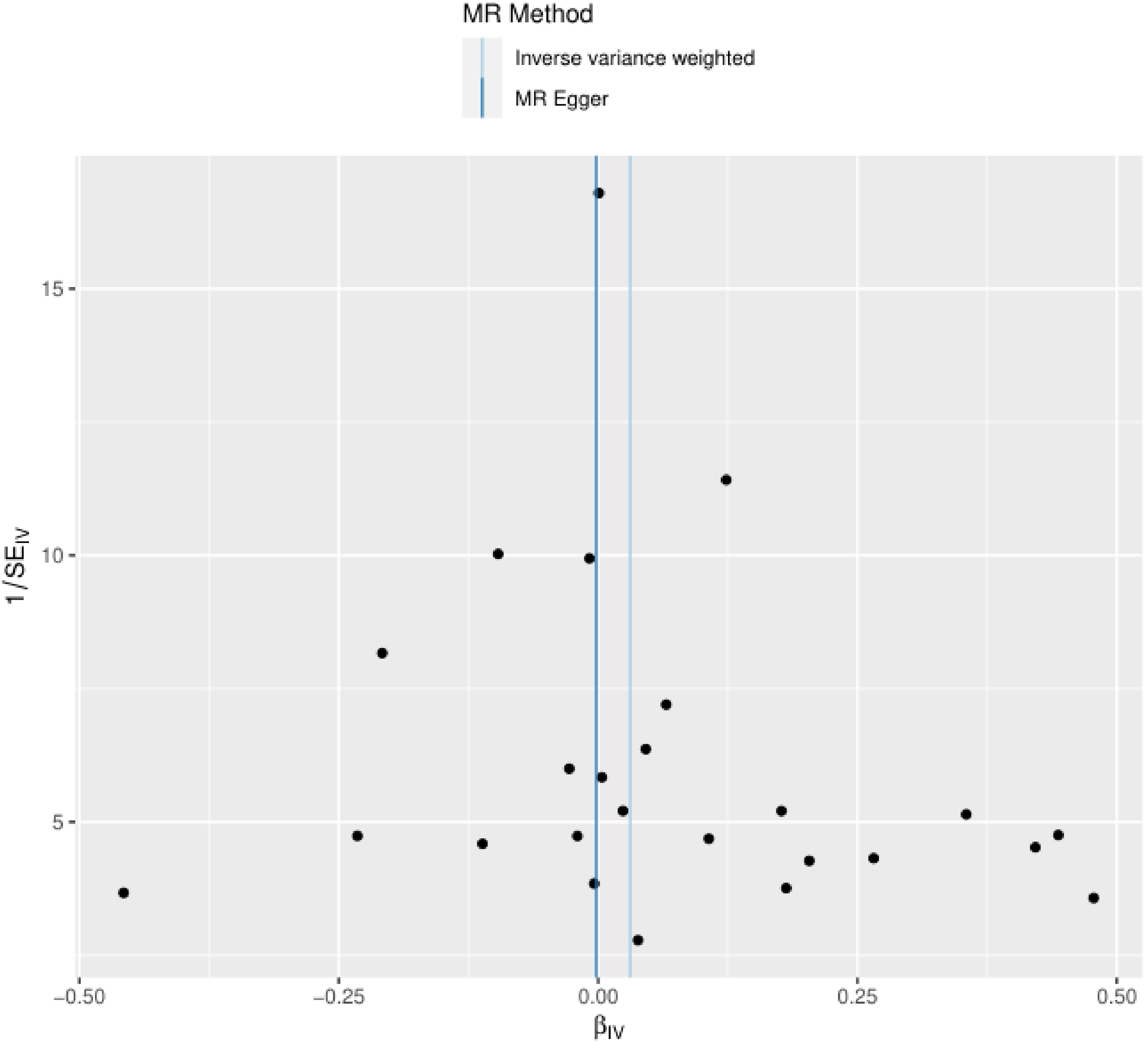

Funnel plot analyse of "CD38 on naive-mature B cell" on 'Diabetic nephropathy'

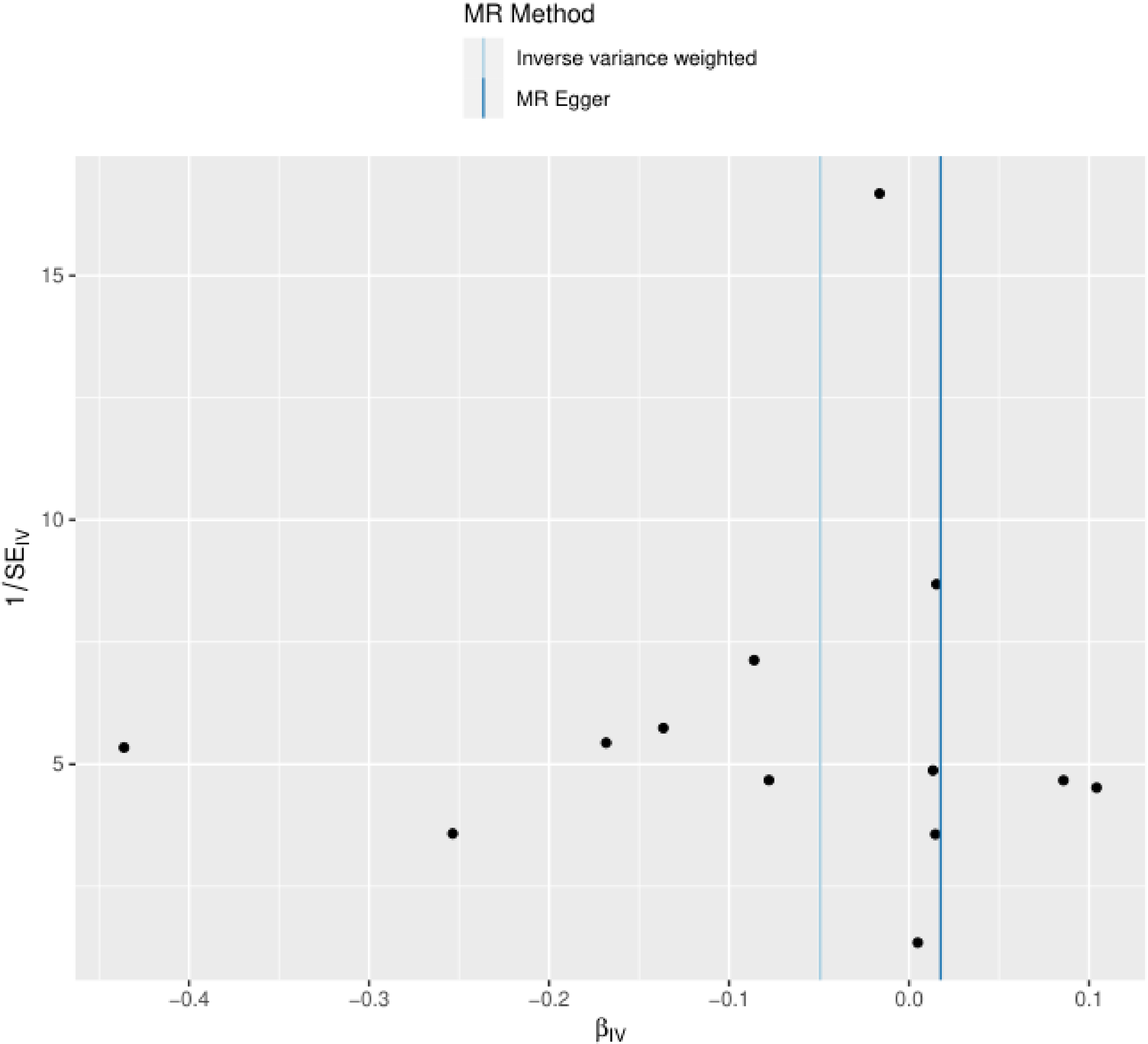

Funnel plot analyse of "CD4 on monocyte" on 'Diabetic nephropathy'

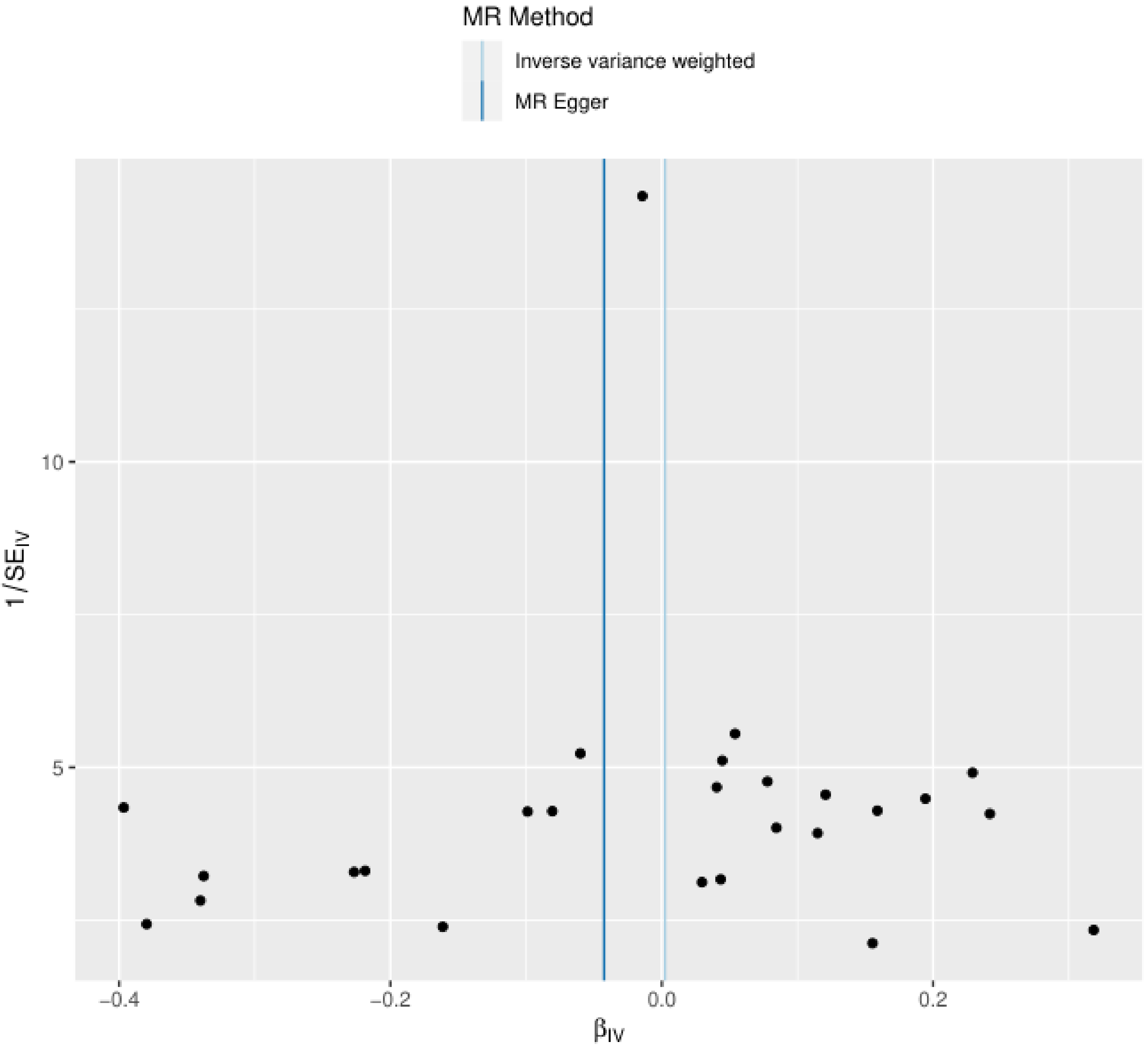

Funnel plot analyse of "CD19 on IgD- CD27-" on 'Diabetic nephropathy'

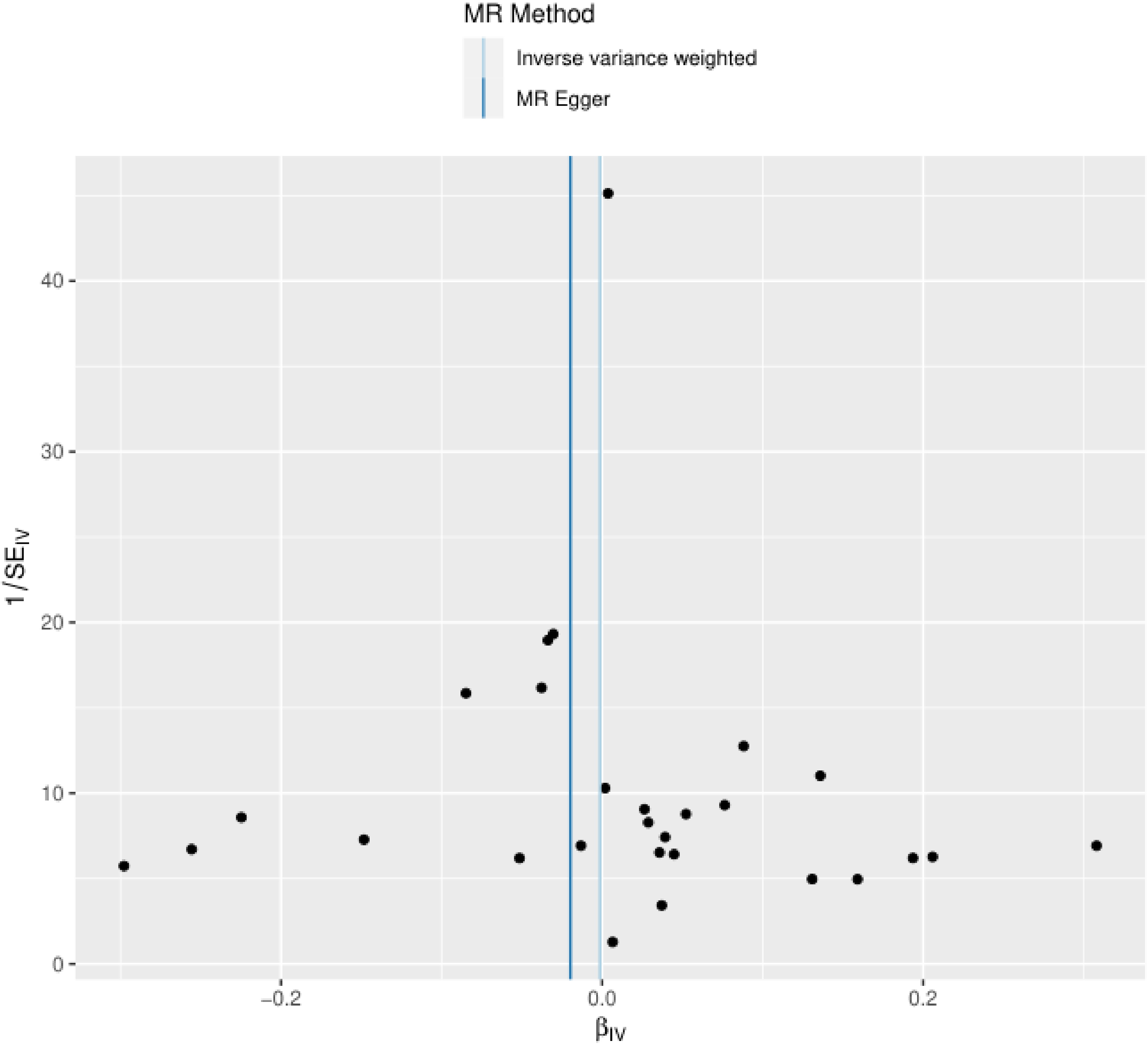

Funnel plot analyse of "CD33 on CD33br HLA DR+ CD14dim " on 'Diabetic nephropathy'

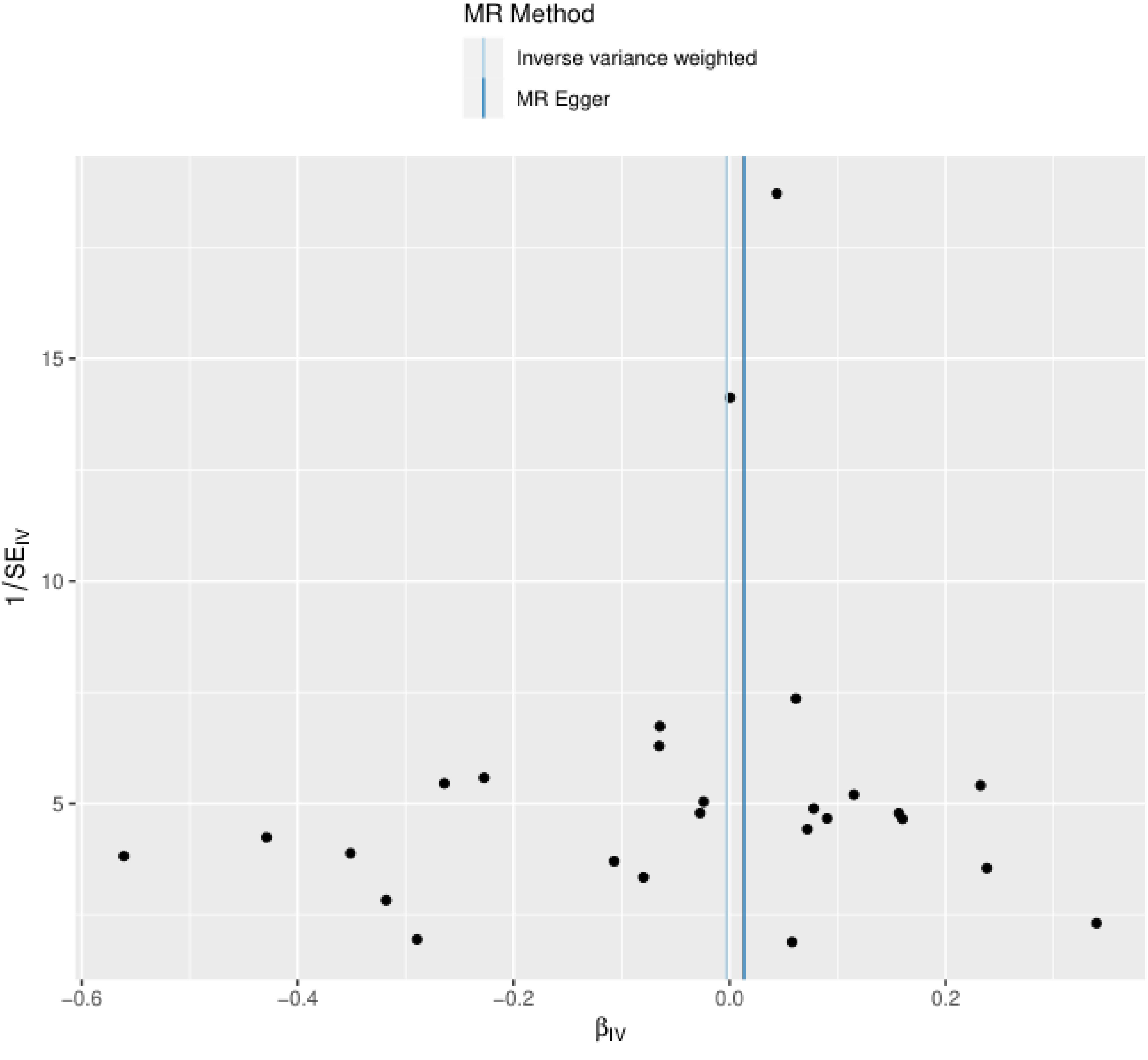

Funnel plot analyse of "Granulocyte %leukocyte" on 'Diabetic nephropathy'

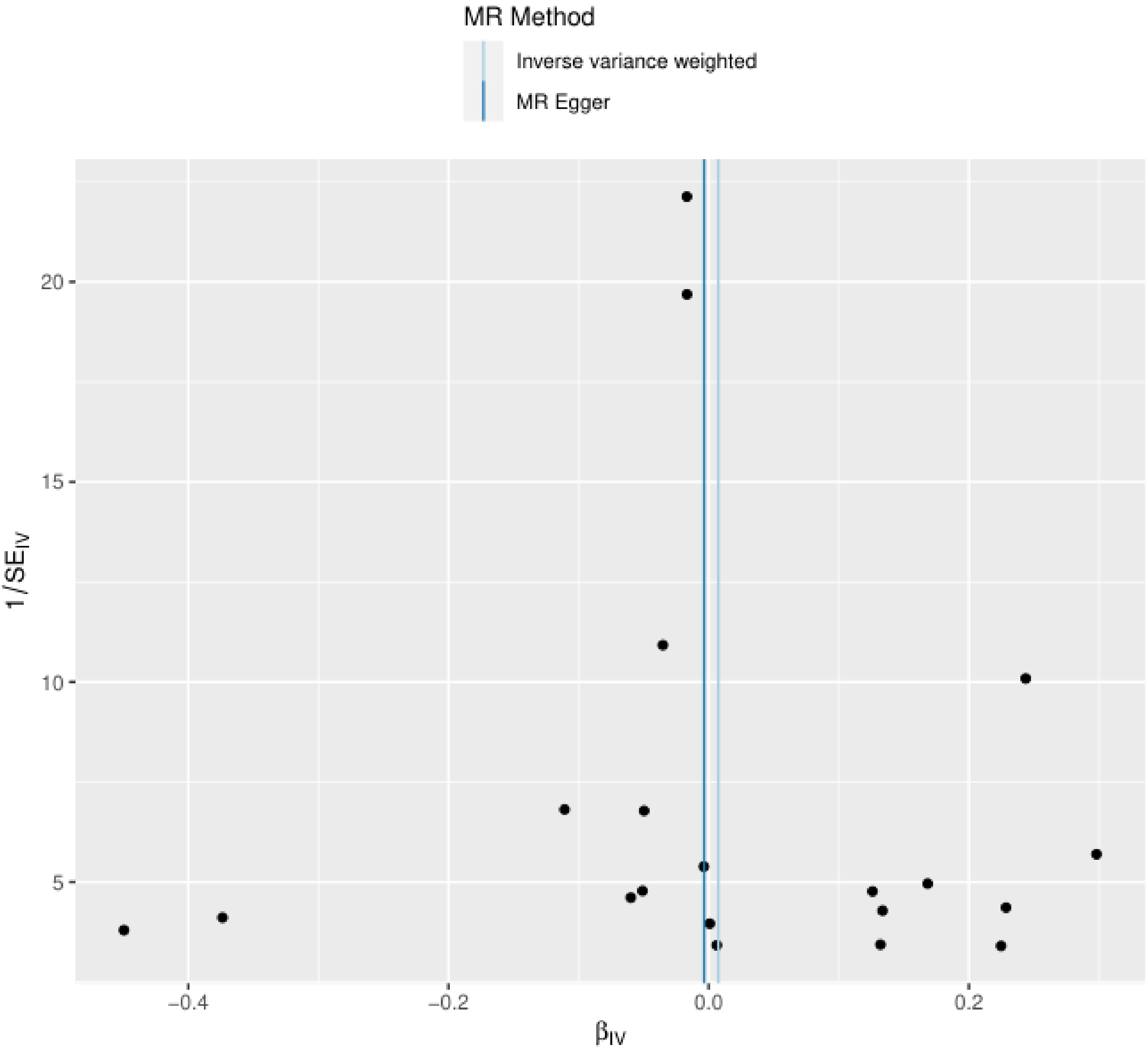

Funnel plot analyse of "CD62L- CD86+ myeloid DC AC" on 'Diabetic nephropathy'

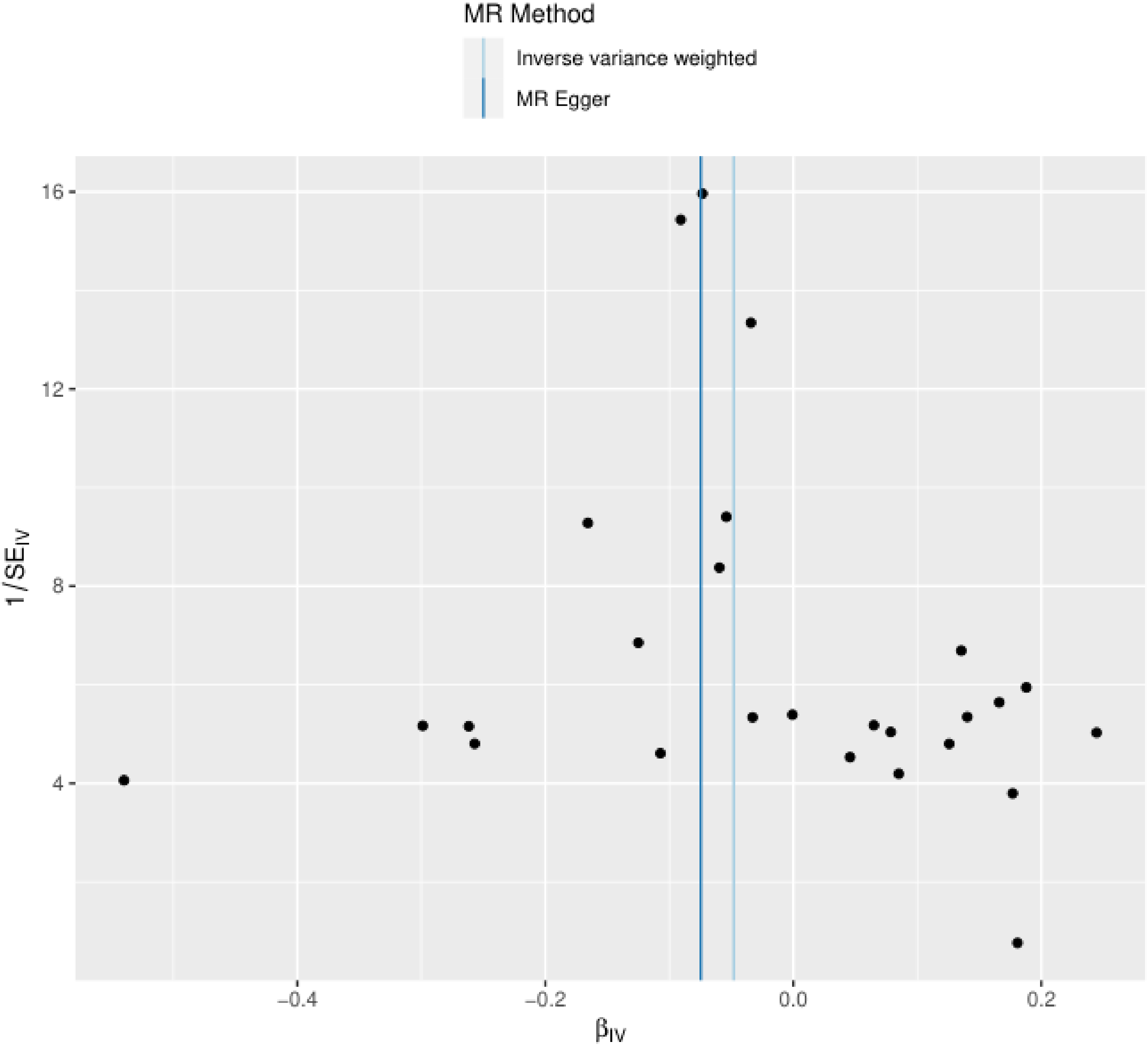

Funnel plot analyse of "CD3 on TD CD4+" on 'Diabetic nephropathy'

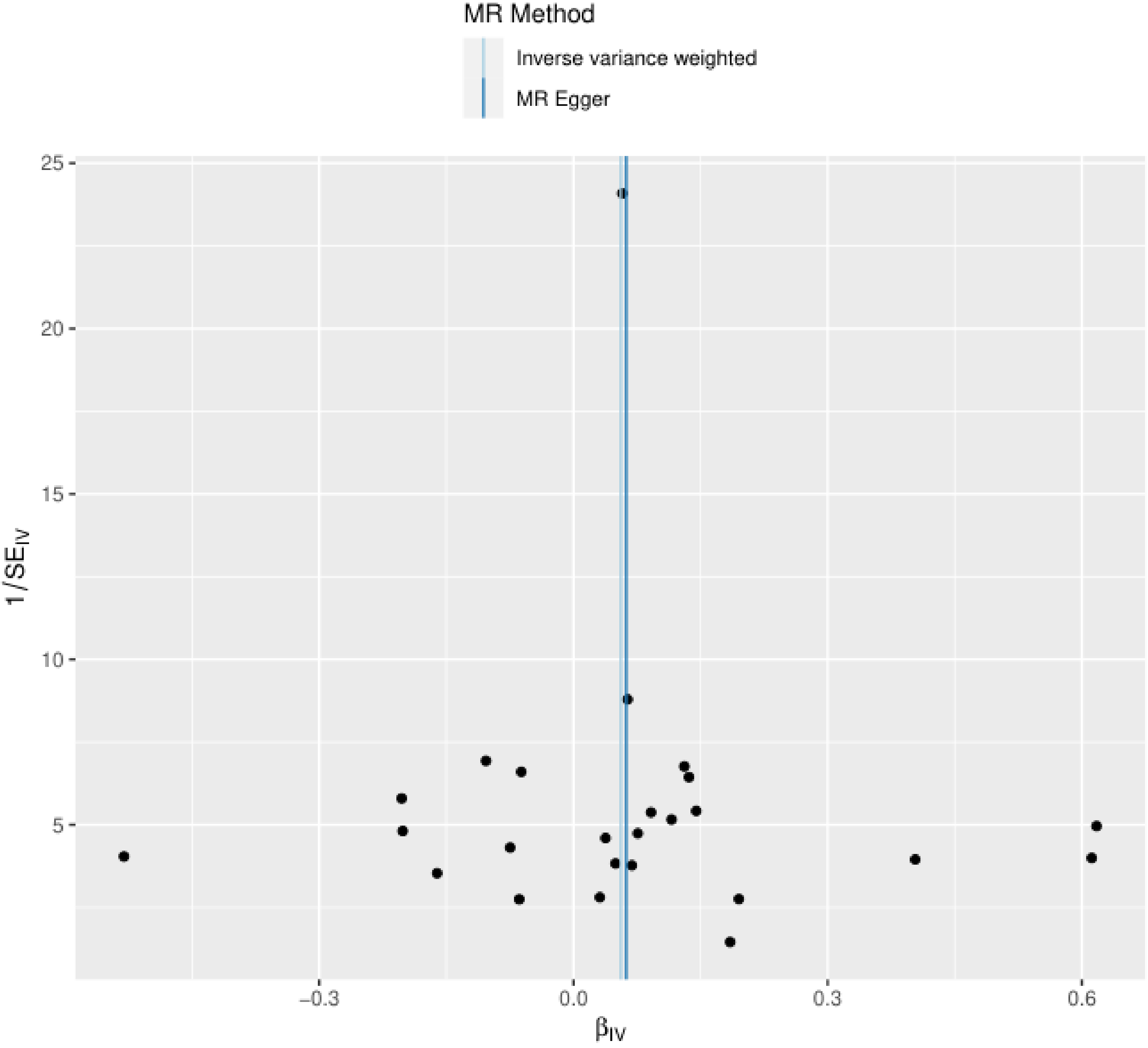

Funnel plot analyse of "CD20 on IgD+ CD38-" on 'Diabetic nephropathy'

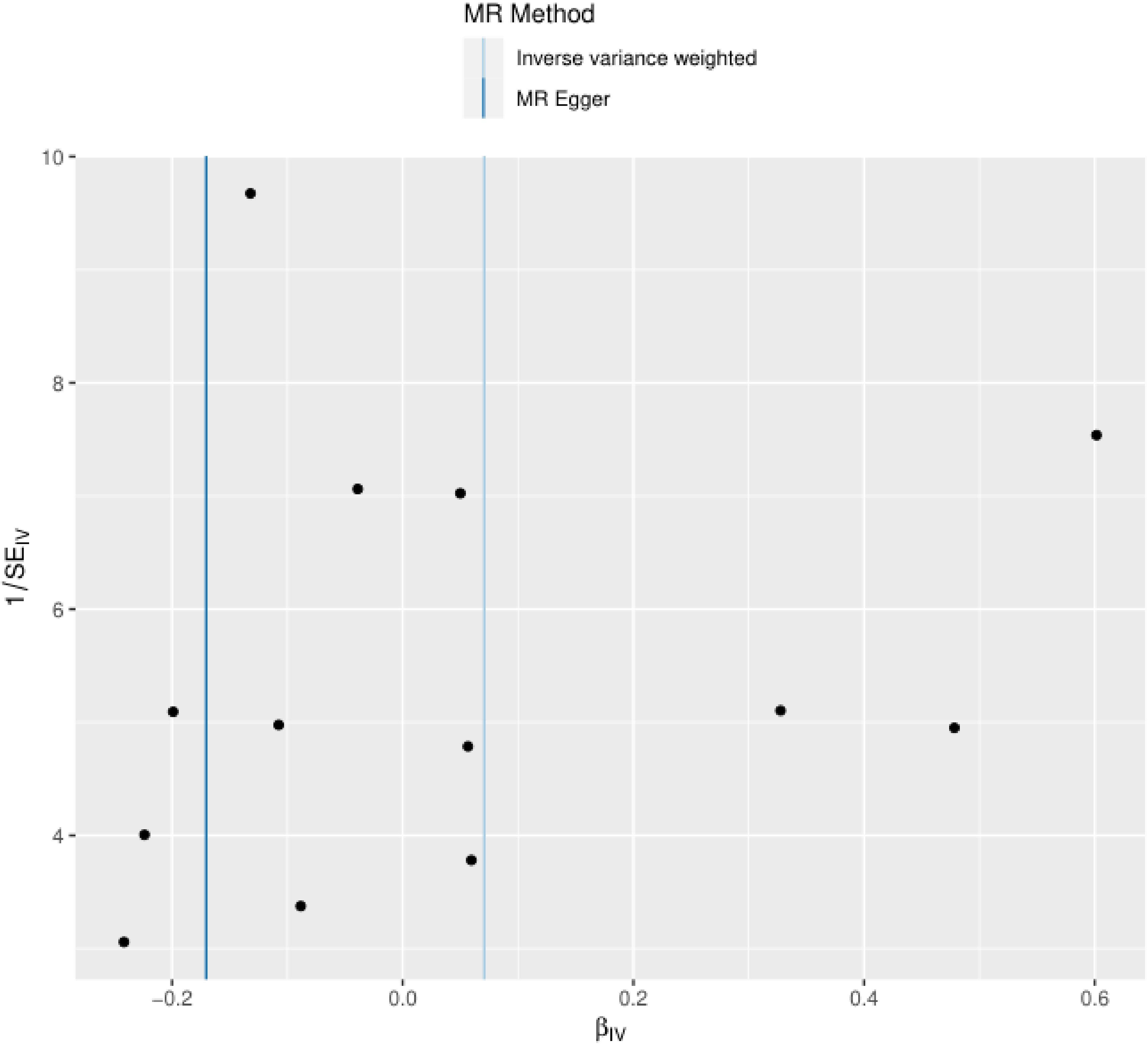

Funnel plot analyse of "CD8 on CM CD8br " on 'Diabetic nephropathy'

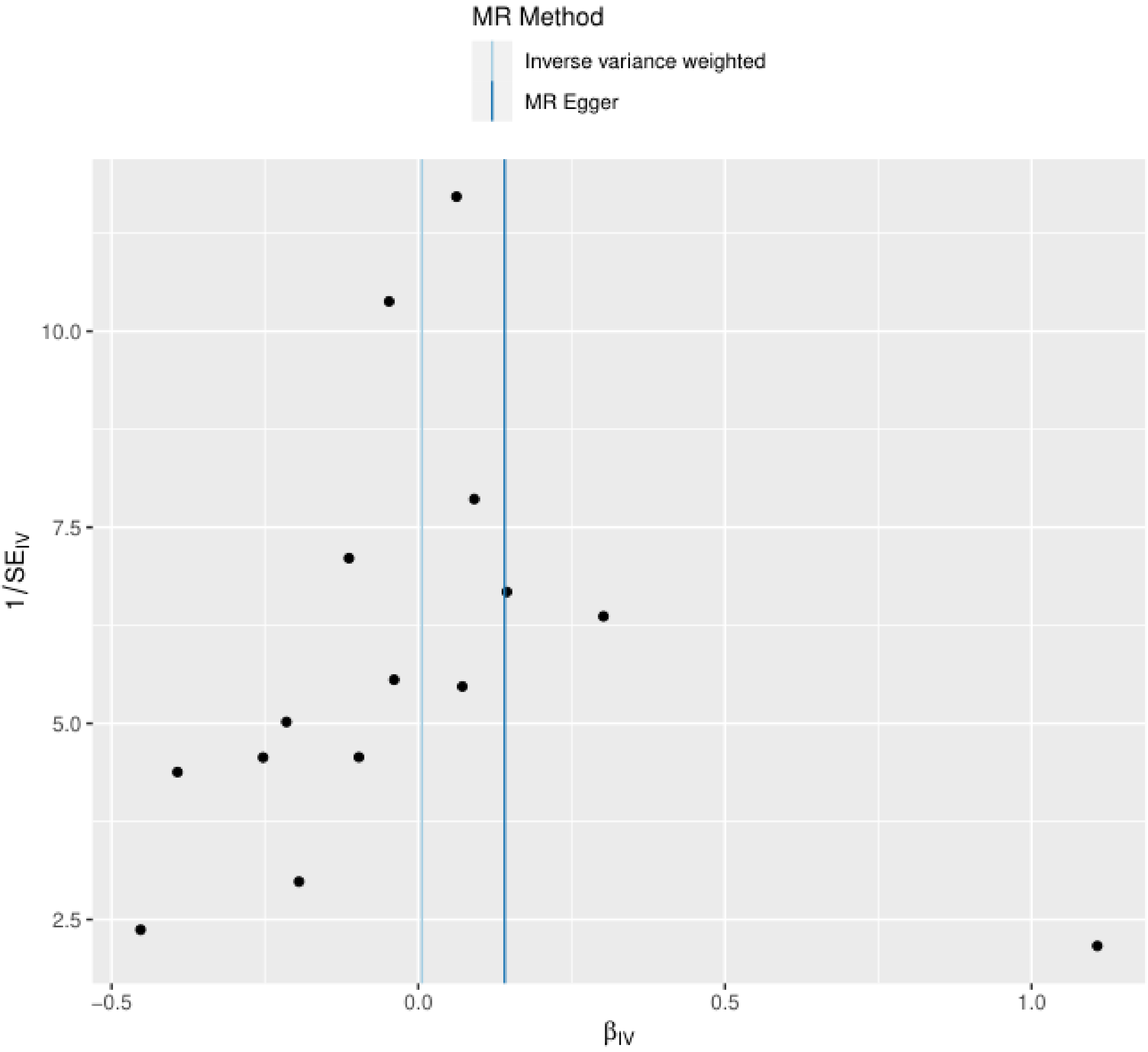

Funnel plot analyse of "CD62L on CD62L+ plasmacytoid DC " on 'Diabetic nephropathy'

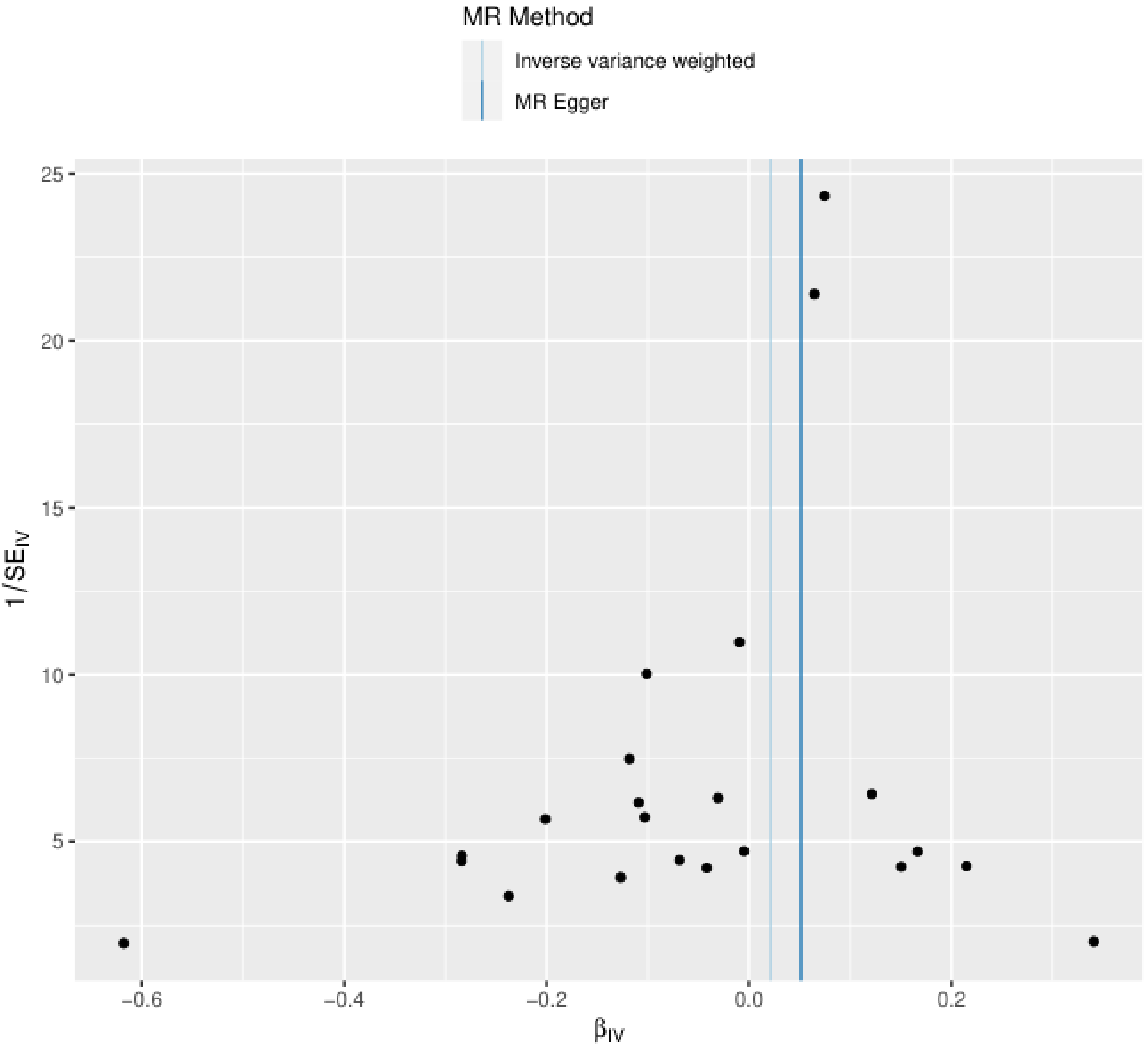

Funnel plot analyse of "CD25 on IgD- CD38-" on 'Diabetic nephropathy'

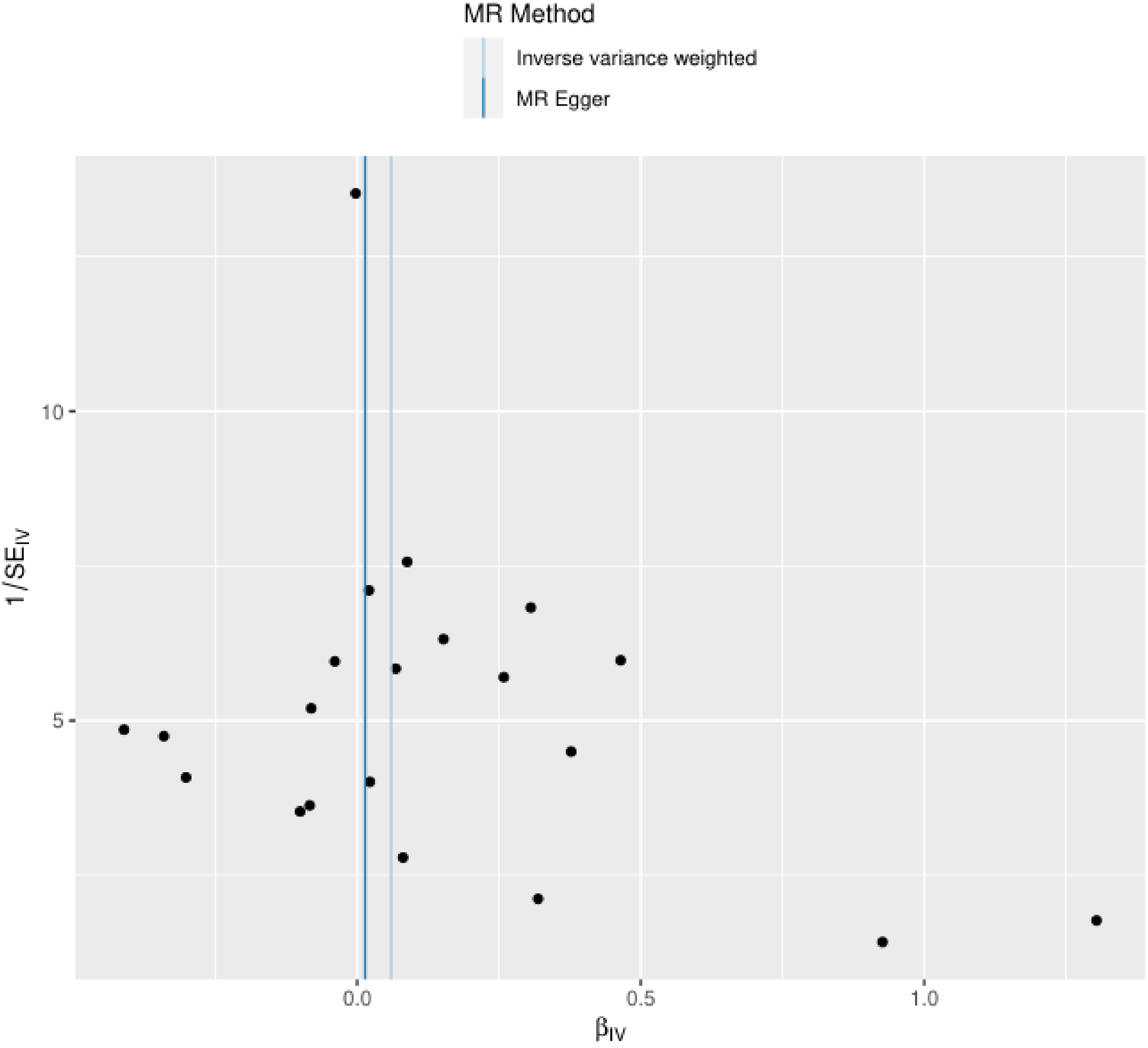

Funnel plot analyse of "CD4 on CD28+ CD4+ " on 'Diabetic nephropathy'

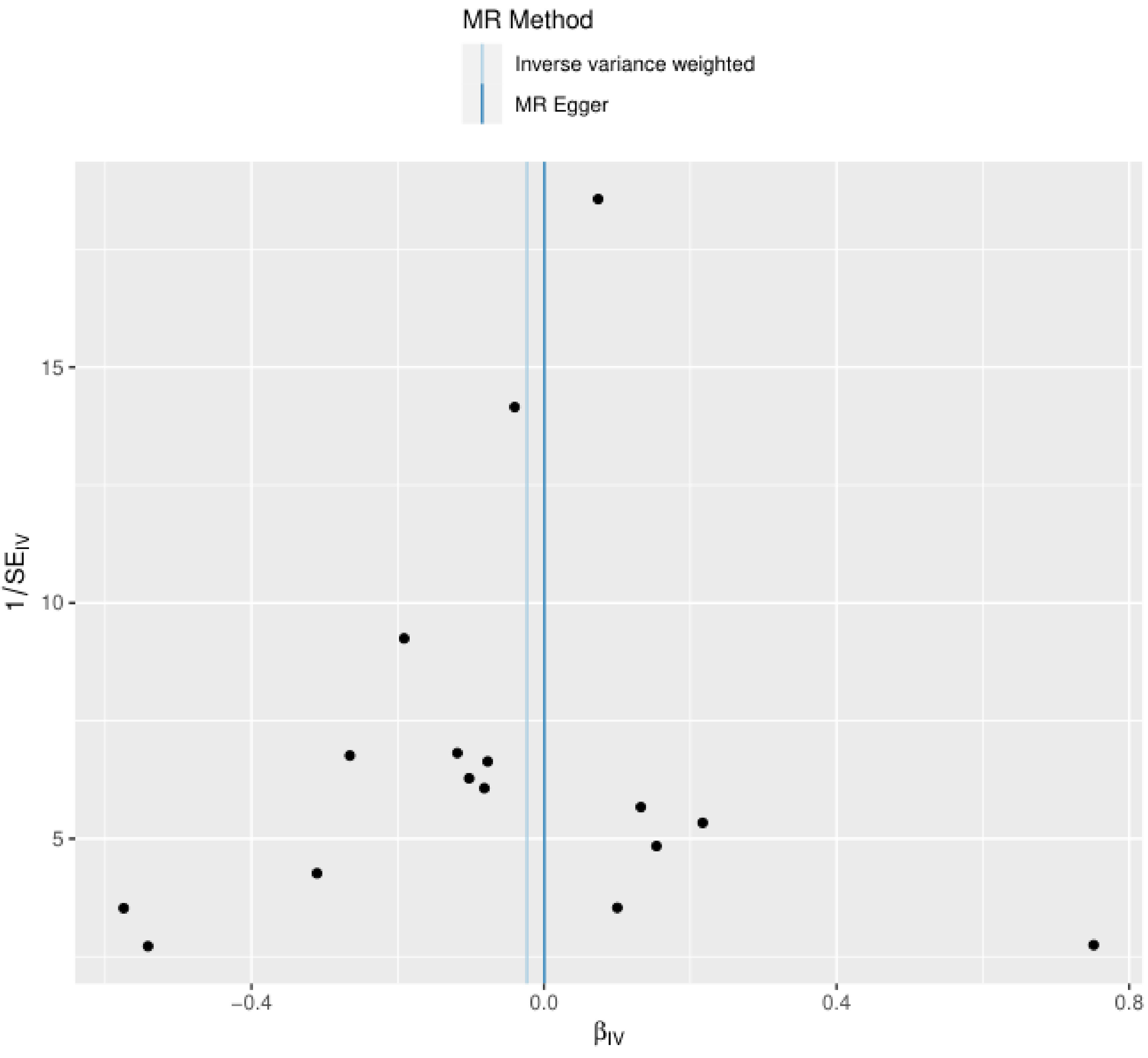

Funnel plot analyse of "CD86 on monocyte" on 'Diabetic nephropathy'

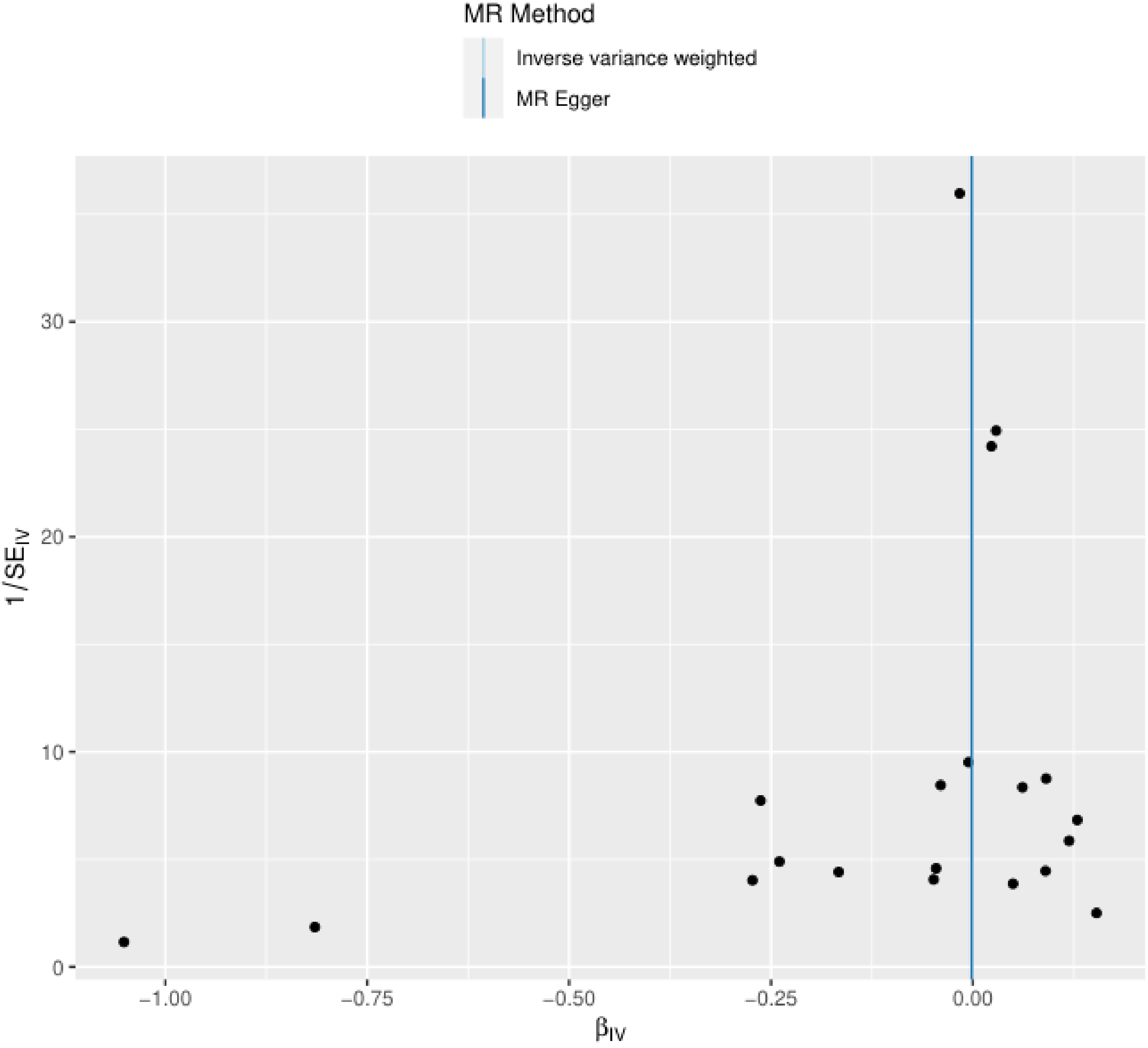

Funnel plot analyse of ''CD25 on activated & secreting Treg '' on 'Diabetic nephropathy'

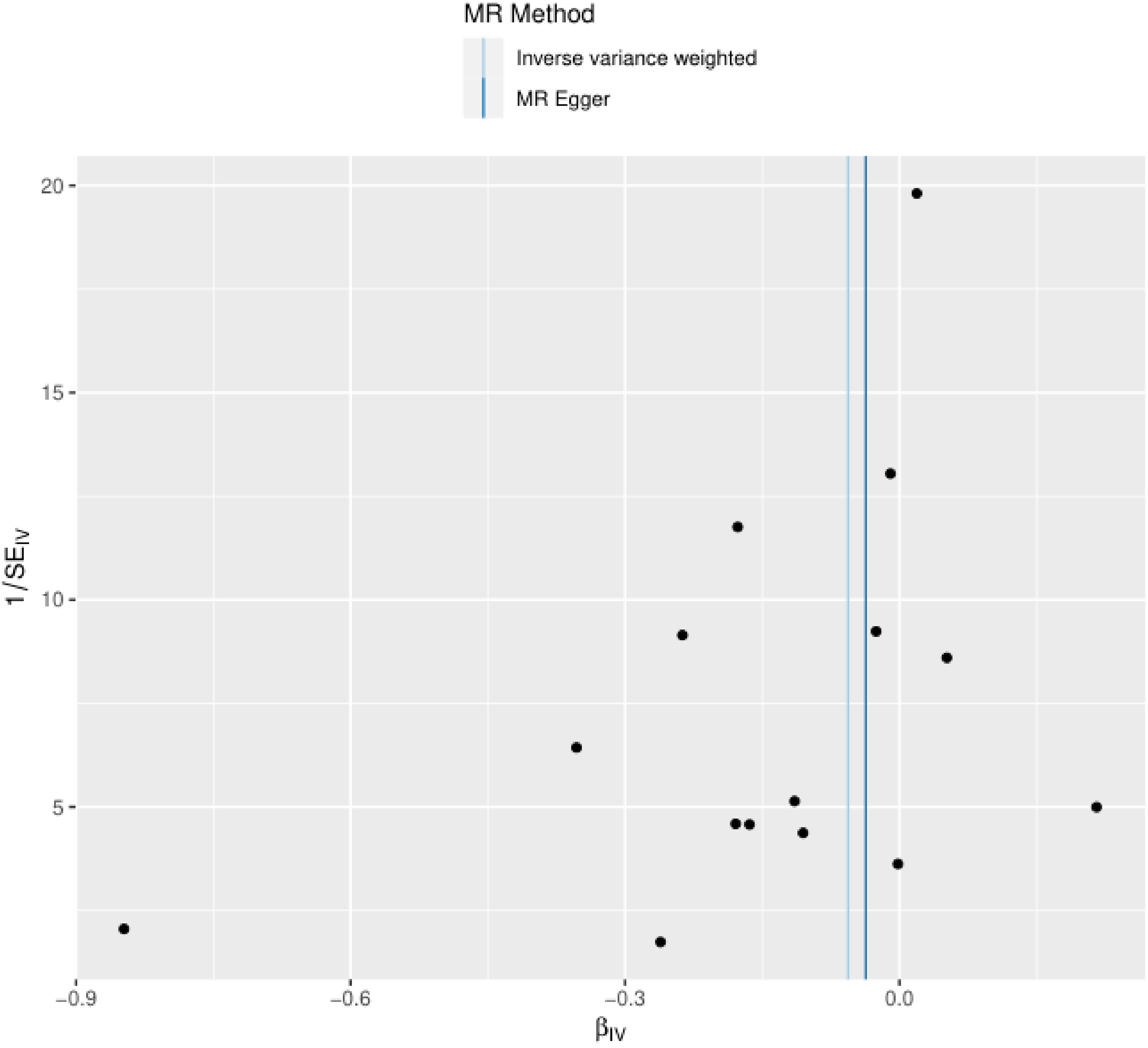

Funnel plot analyse of "CD3 on CD28+ DN (CD4-CD8-)" on 'Diabetic nephropathy'

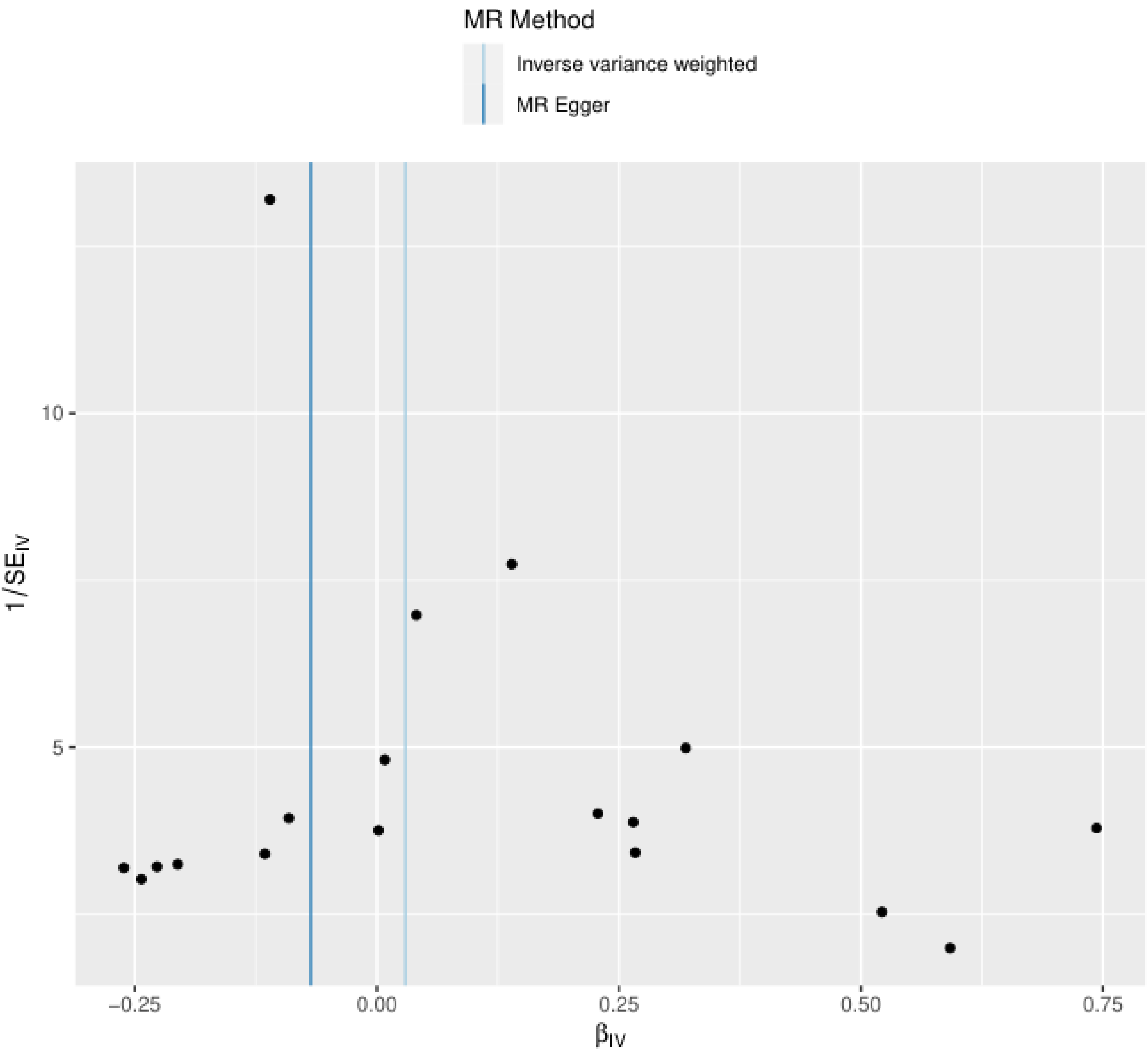

Funnel plot analyse of "Naive DN (CD4-CD8-) AC" on 'Diabetic nephropathy'

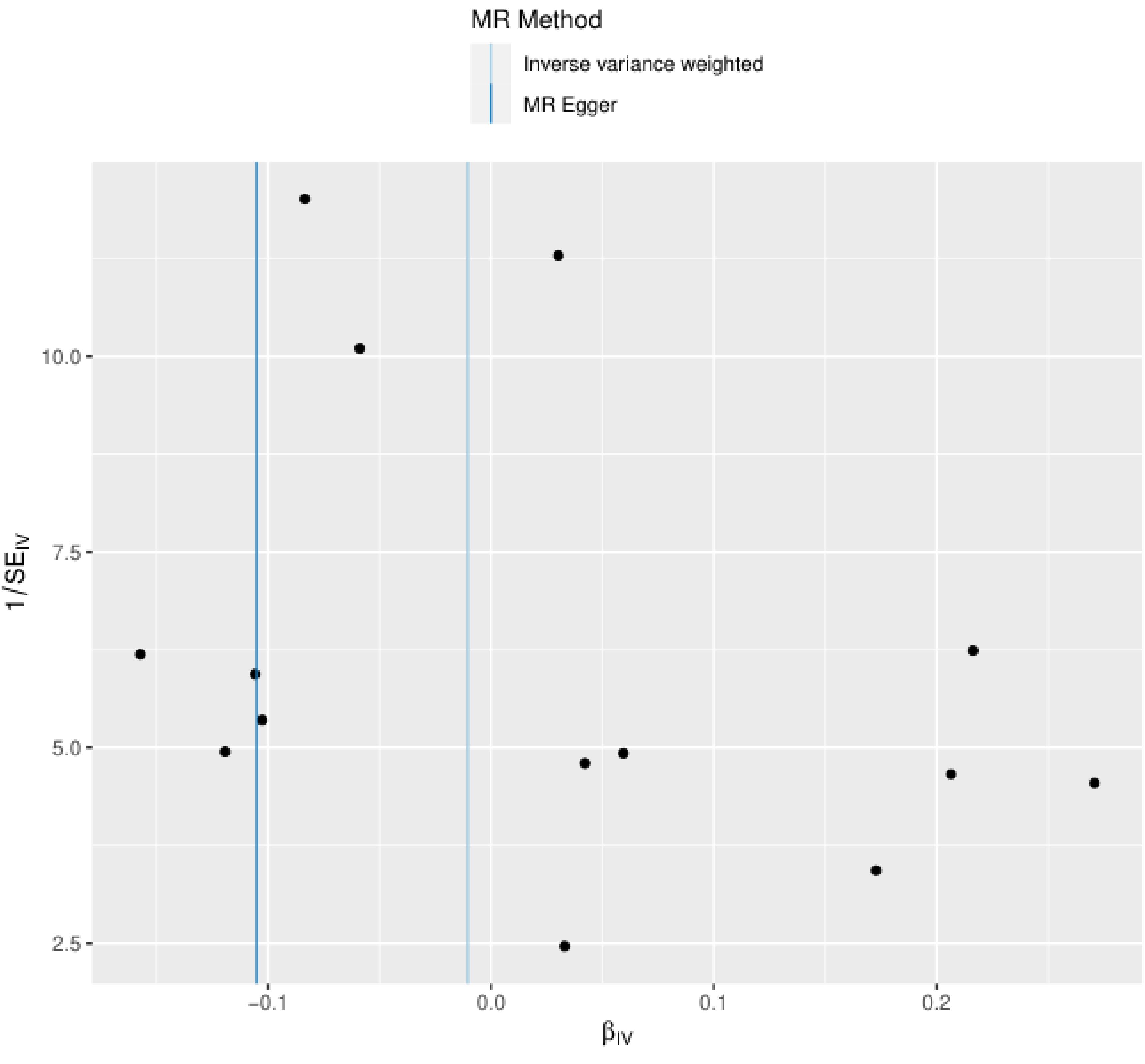

Funnel plot analyse of "CD28 on CD45RA- CD4 not Treg " on 'Diabetic nephropathy'

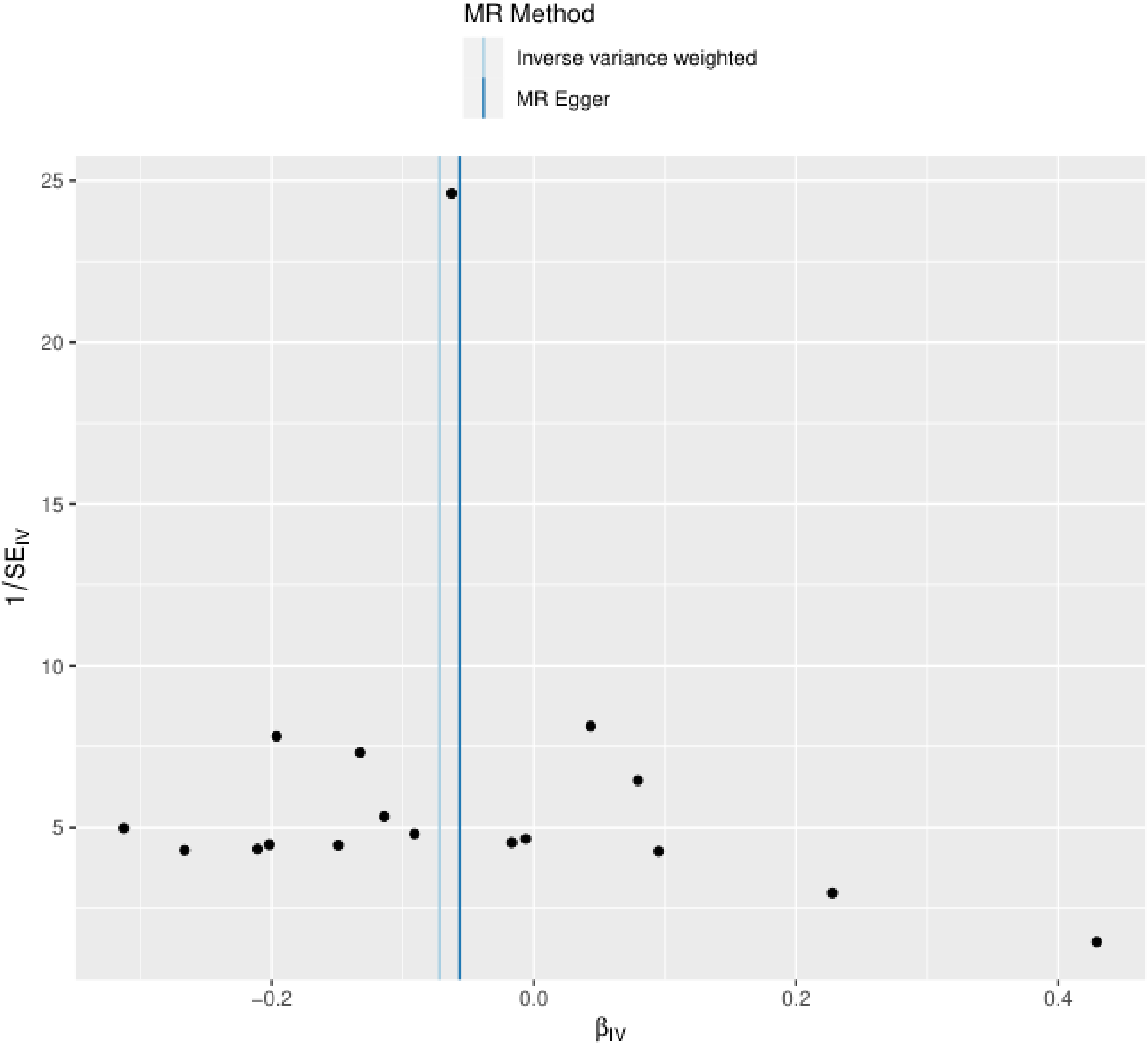

Funnel plot analyse of "FSC-A on HLA DR+ CD4+" on 'Diabetic nephropathy'

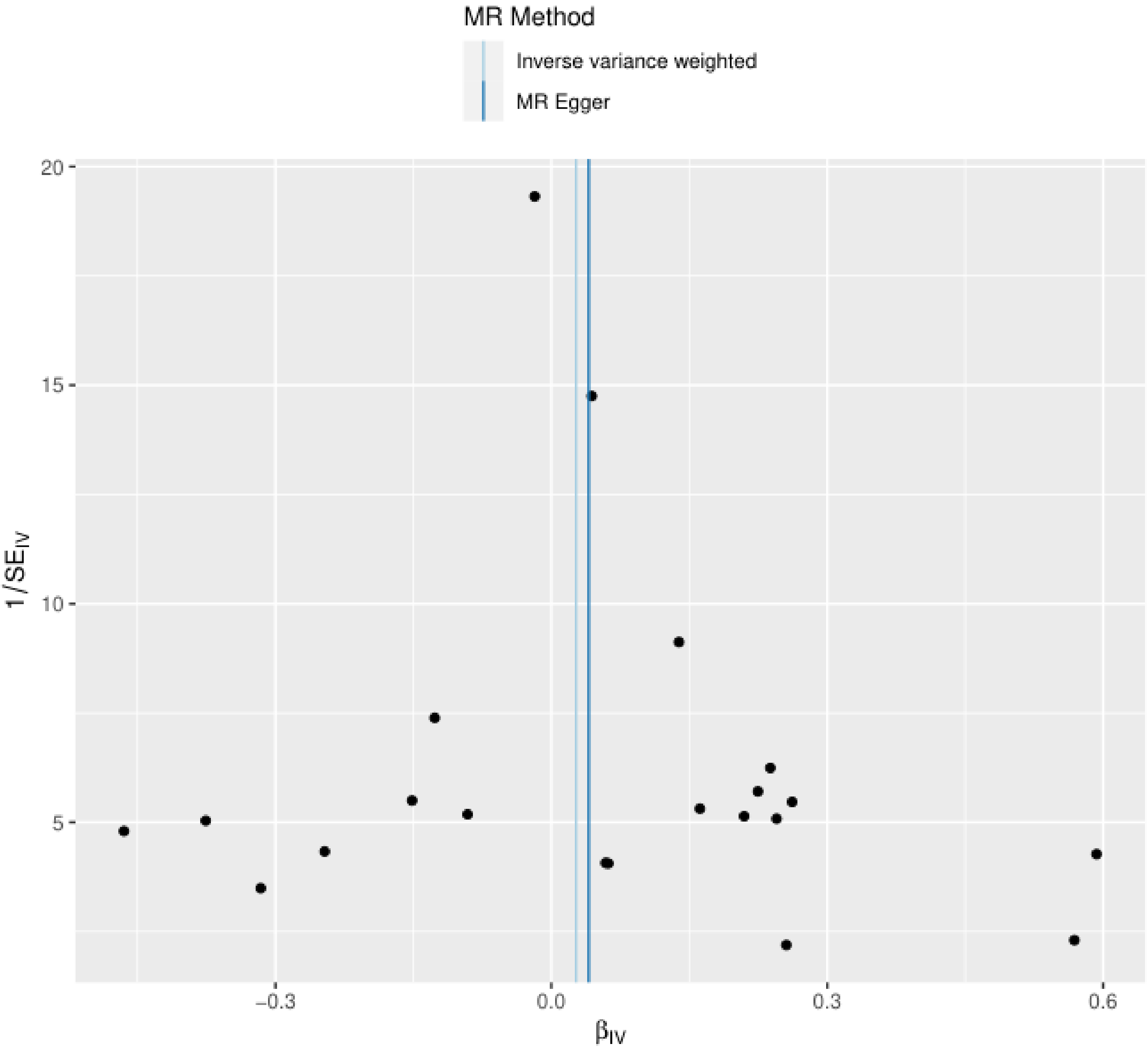

Funnel plot analyse of "CD86 on CD62L+ myeloid DC" on 'Diabetic nephropathy'

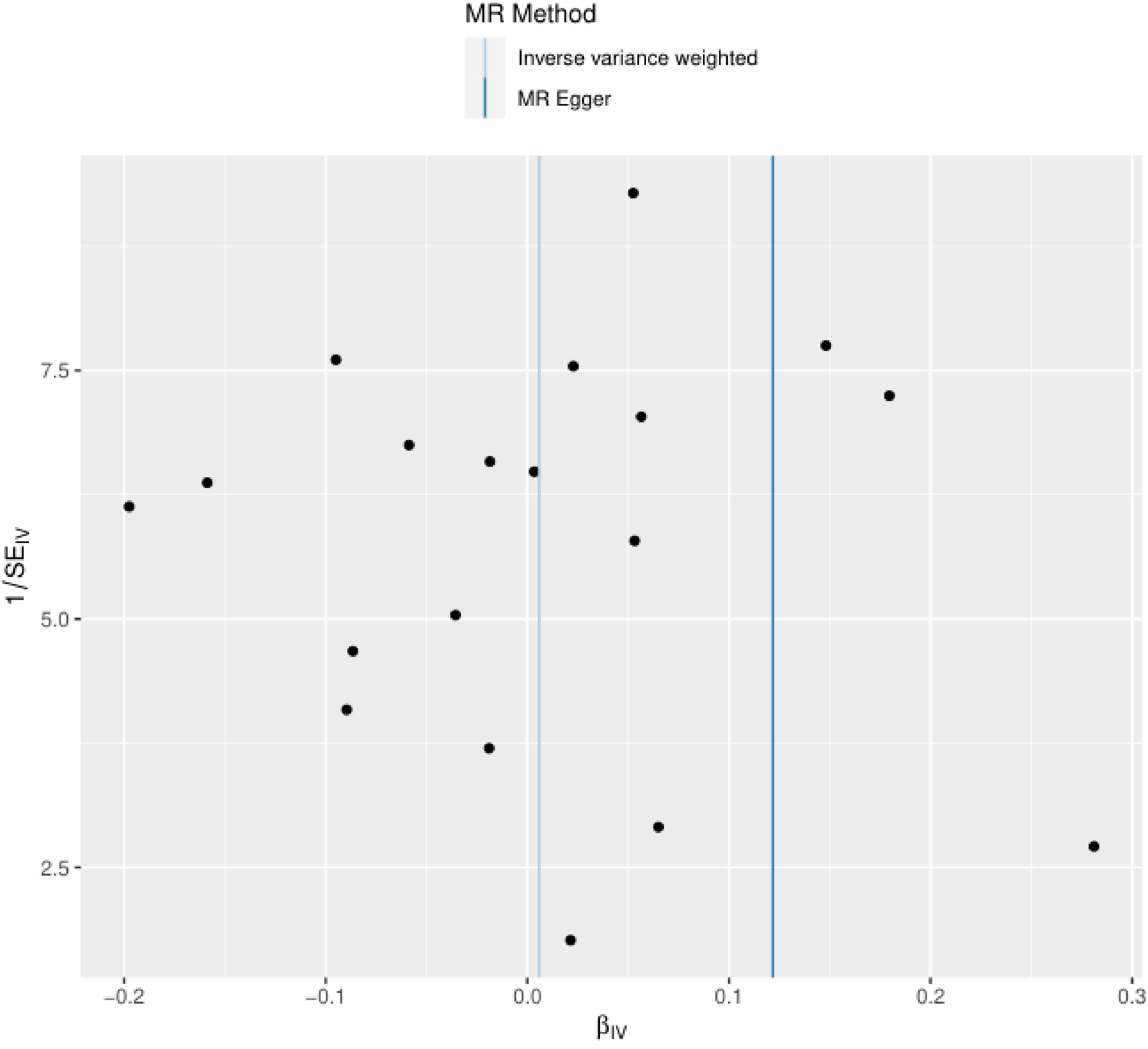

Funnel plot analyse of "CD20 on IgD+ CD38- unsw mem" on 'Diabetic nephropathy'

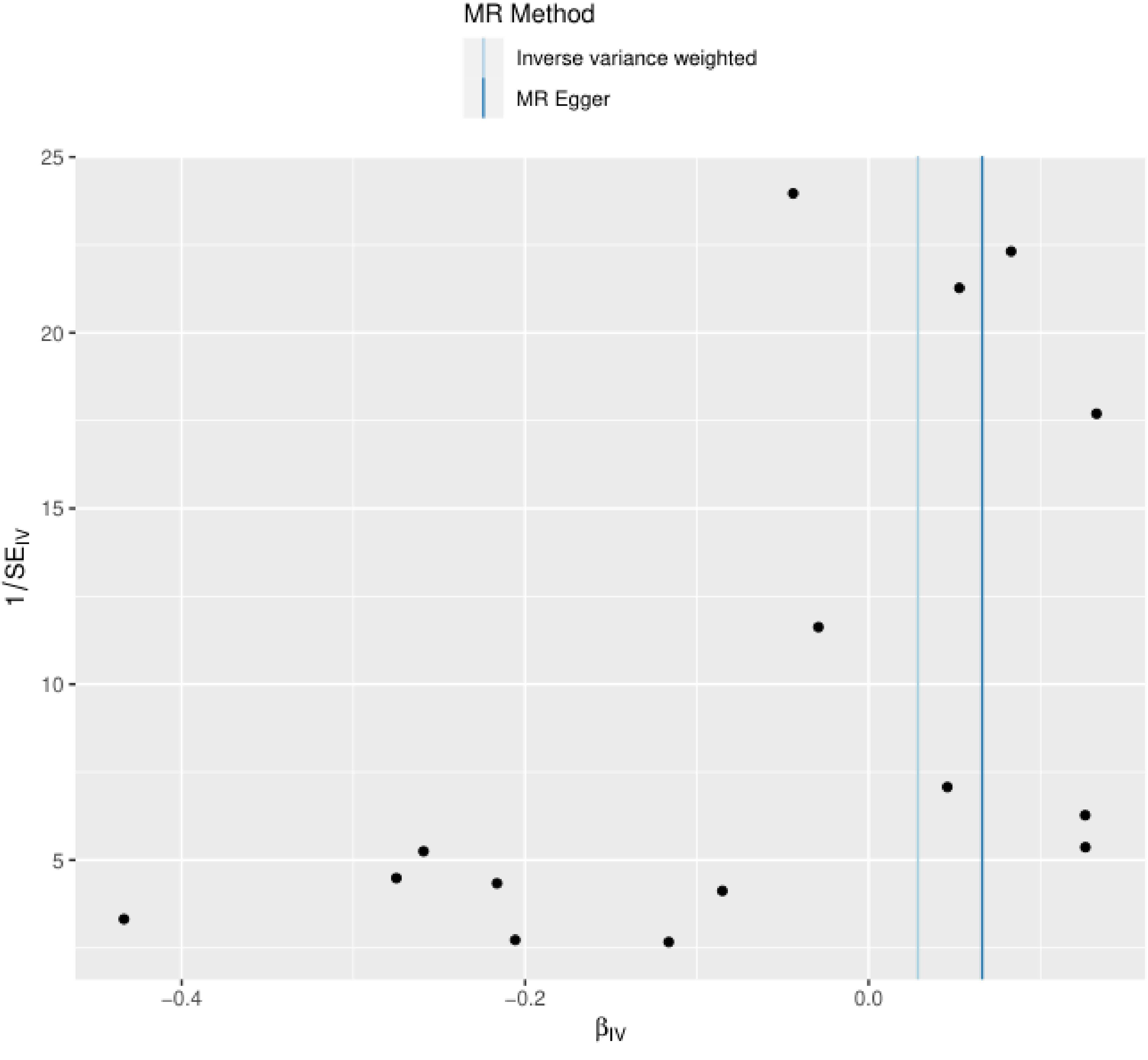

Funnel plot analyse of "FSC-A on HLA DR+ CD8br" on 'Diabetic nephropathy'

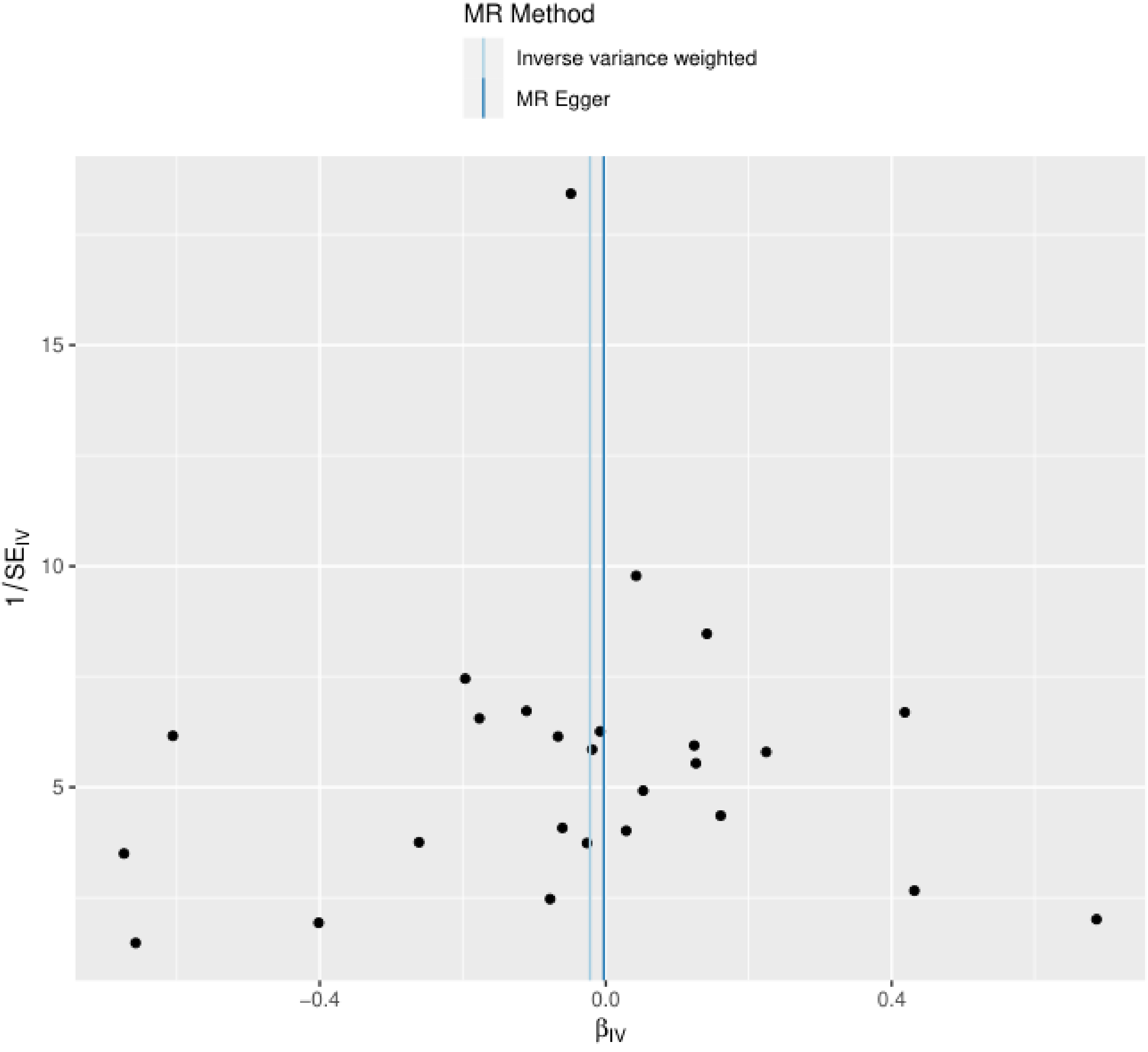

Funnel plot analyse of "Resting Treg AC" on 'Diabetic nephropathy'

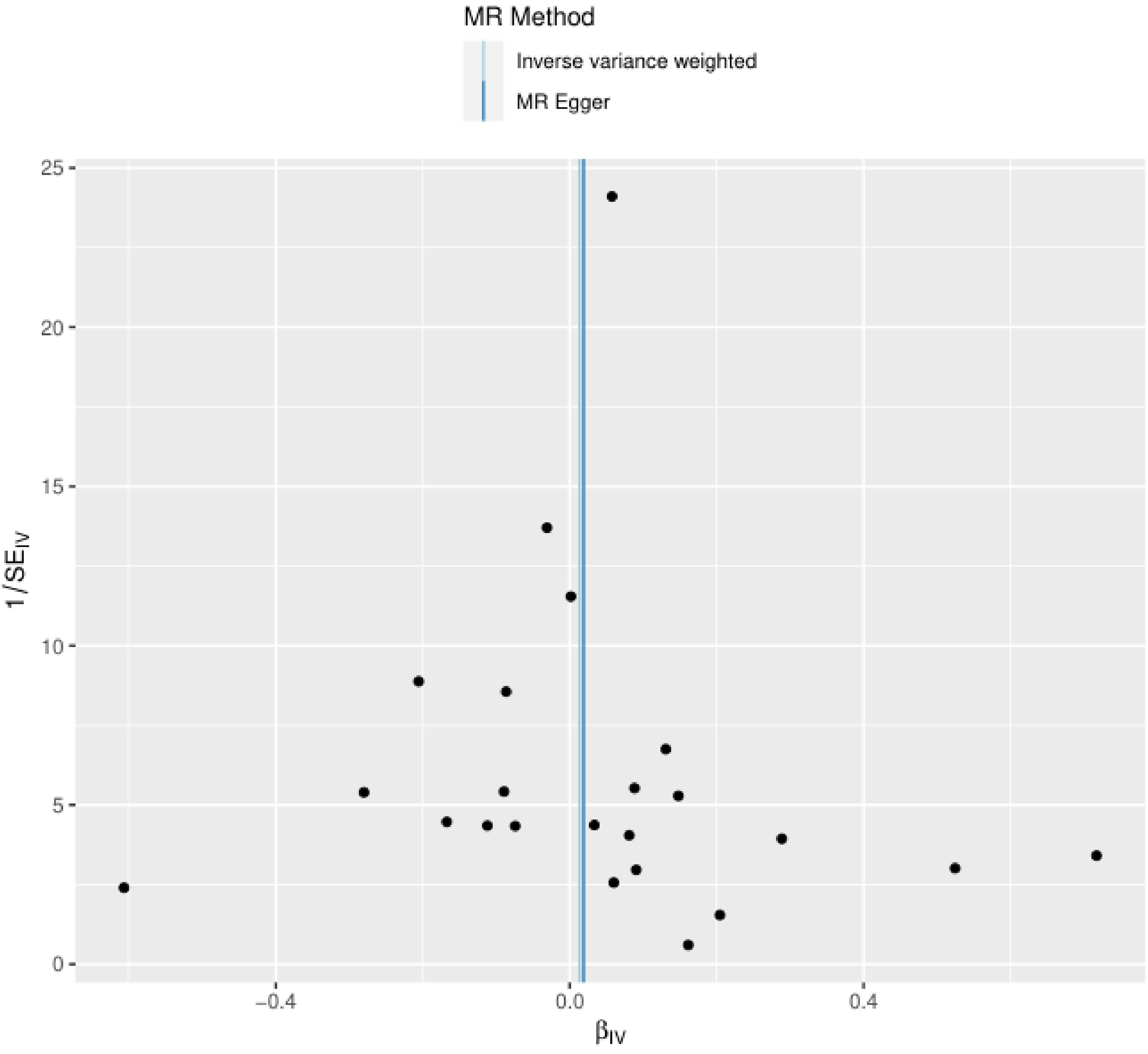

Funnel plot analyse of "CD20 on unsw mem" on 'Diabetic nephropathy'

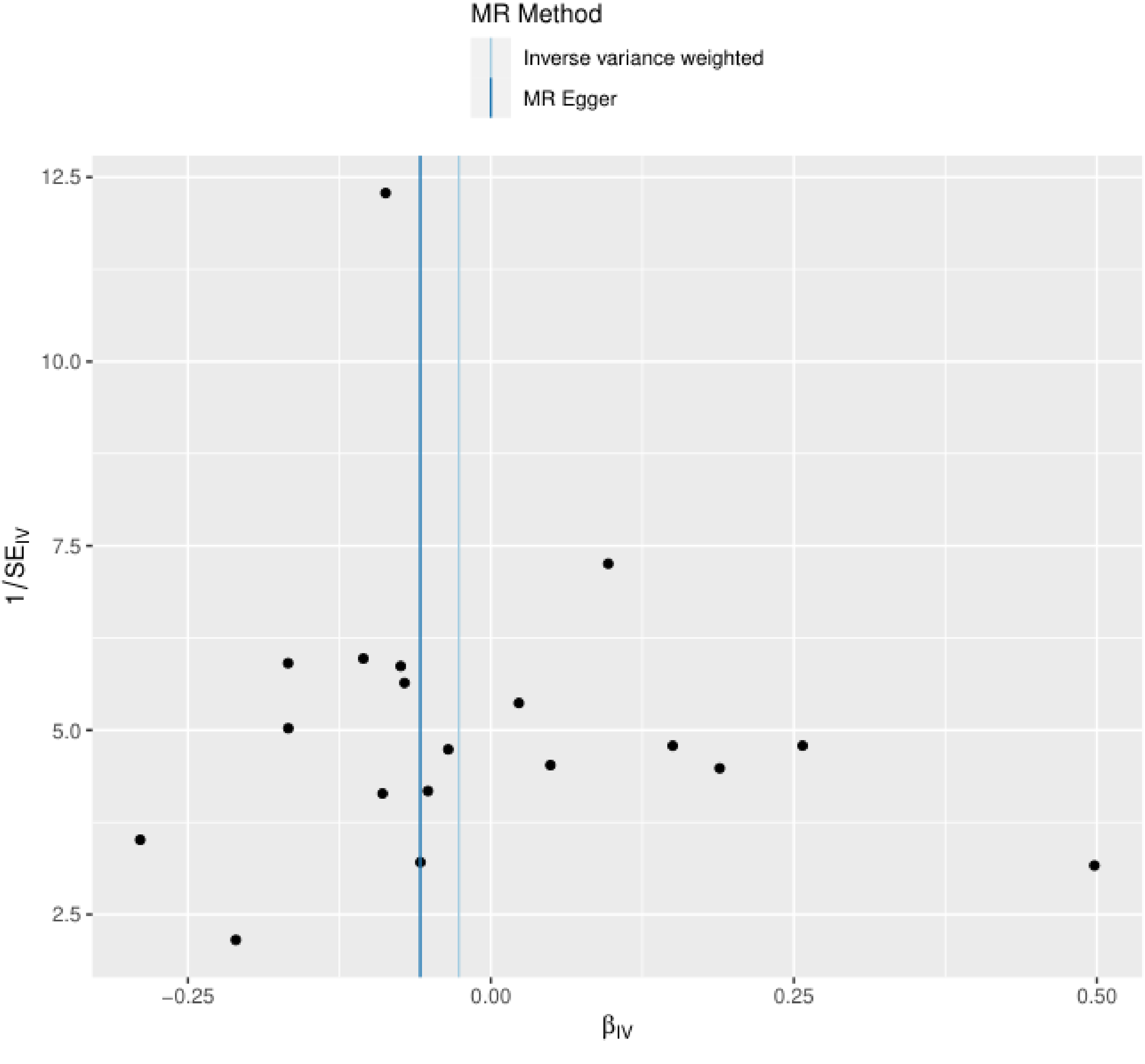

Funnel plot analyse of "CD28 on CD28+ CD45RA- CD8br " on 'Diabetic nephropathy'

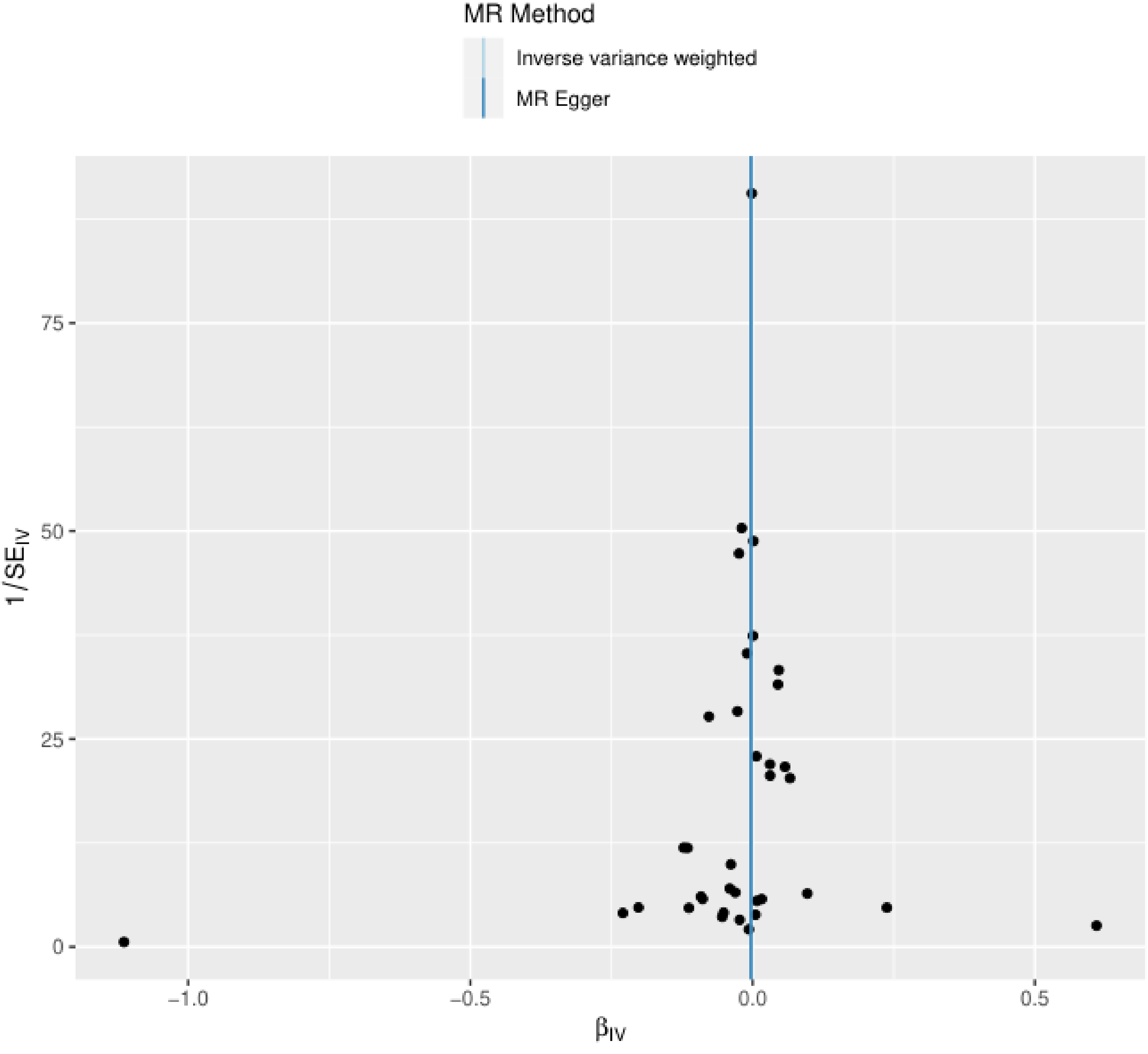

Funnel plot analyse of "CD28+ CD45RA- CD8dim AC" on 'Diabetic nephropathy'

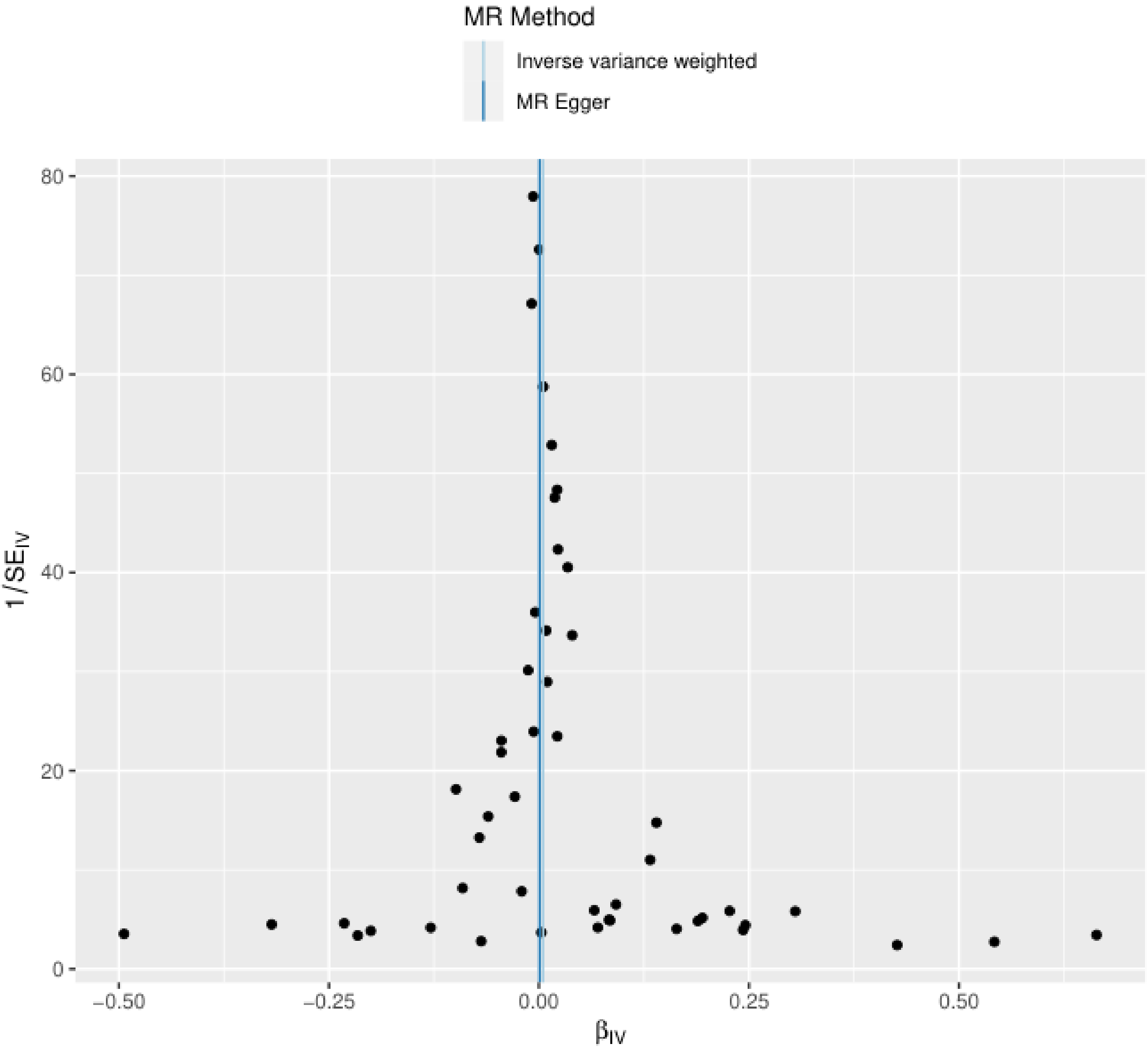

Funnel plot analyse of "CD28+ CD45RA+ CD8dim AC" on 'Diabetic nephropathy'

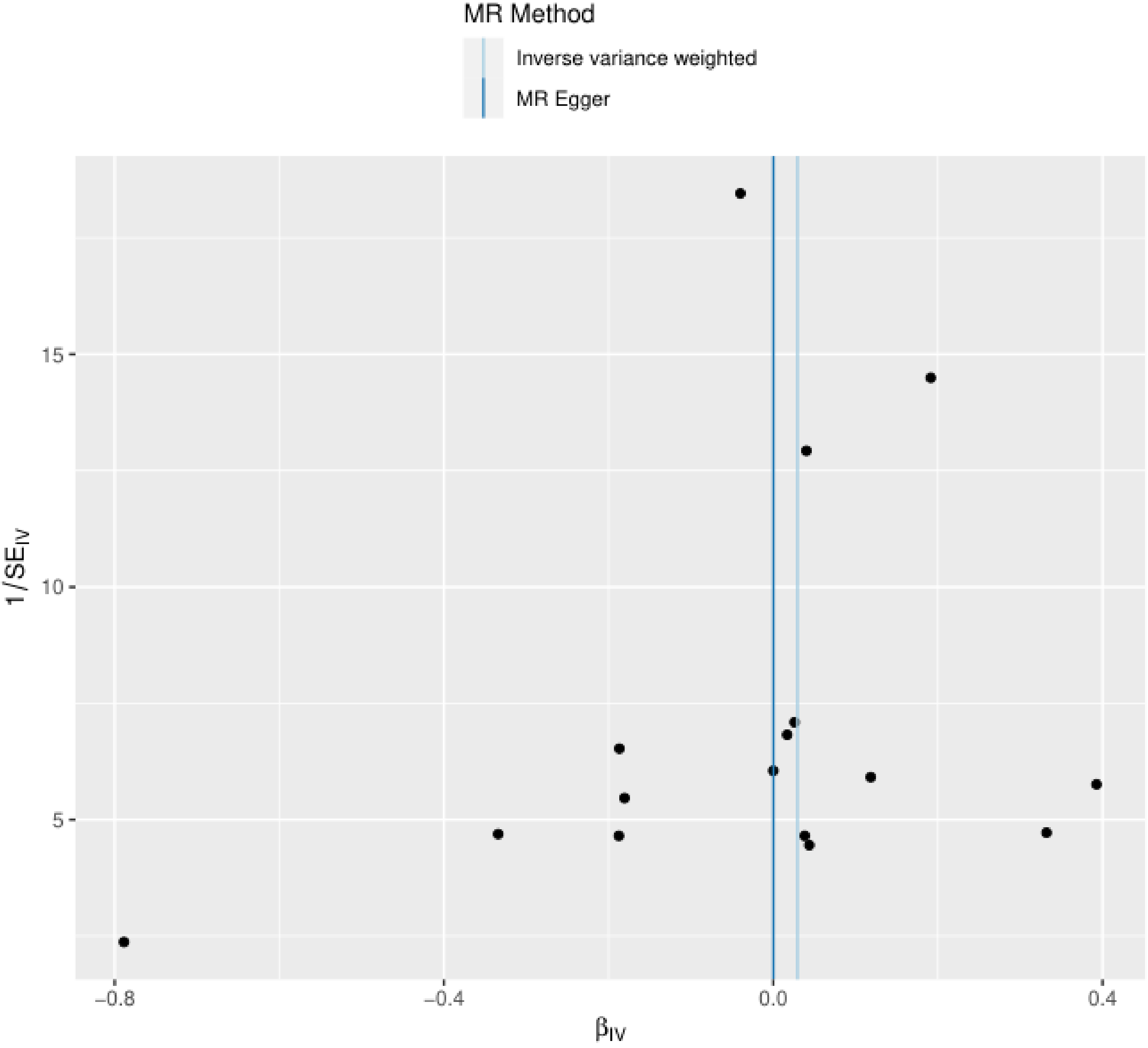

Funnel plot analyse of "CD28 on CD28+ CD45RA+ CD8br" on 'Diabetic nephropathy'

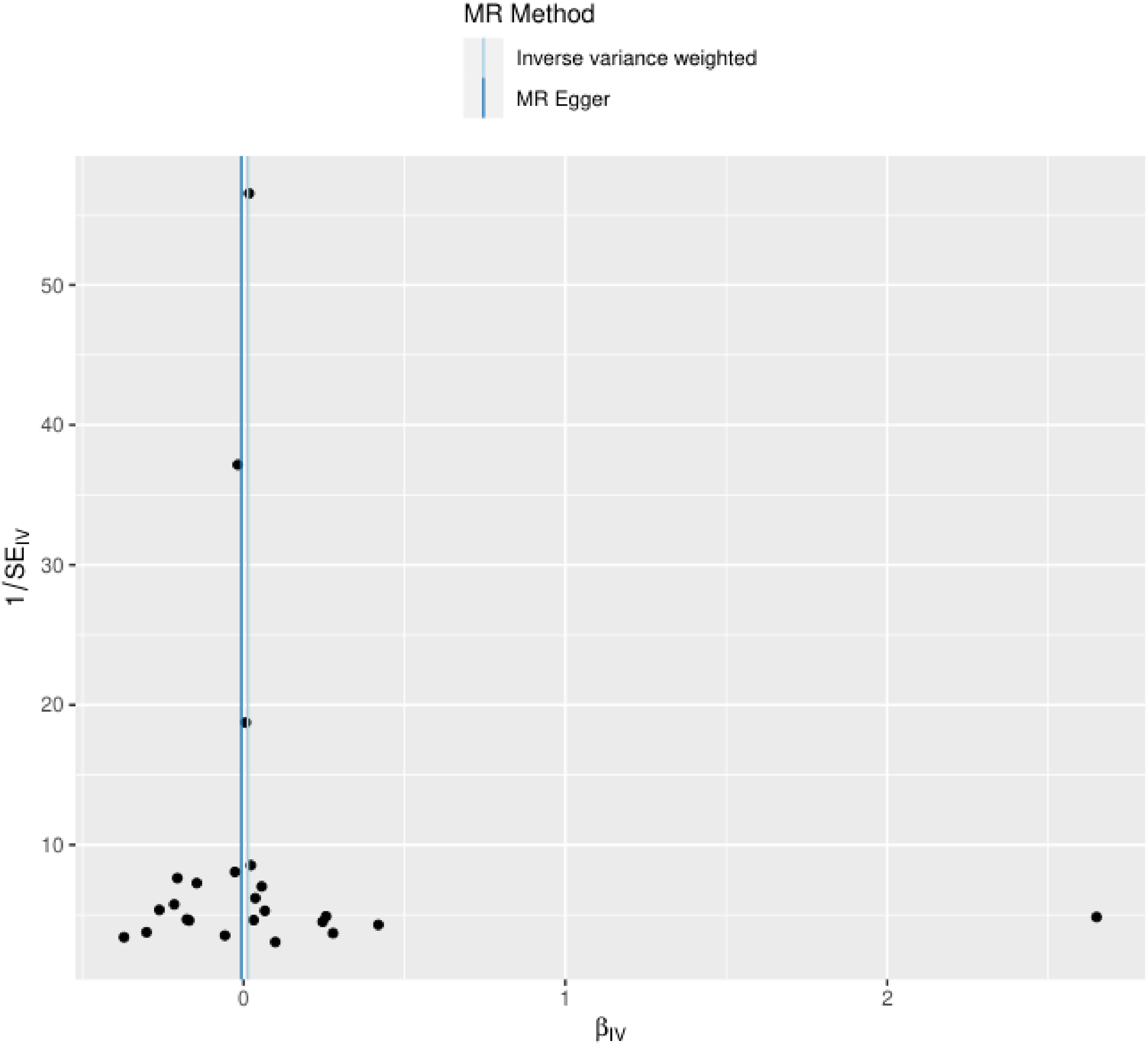

Funnel plot analyse of "CD20 on transitional" on 'Diabetic nephropathy'

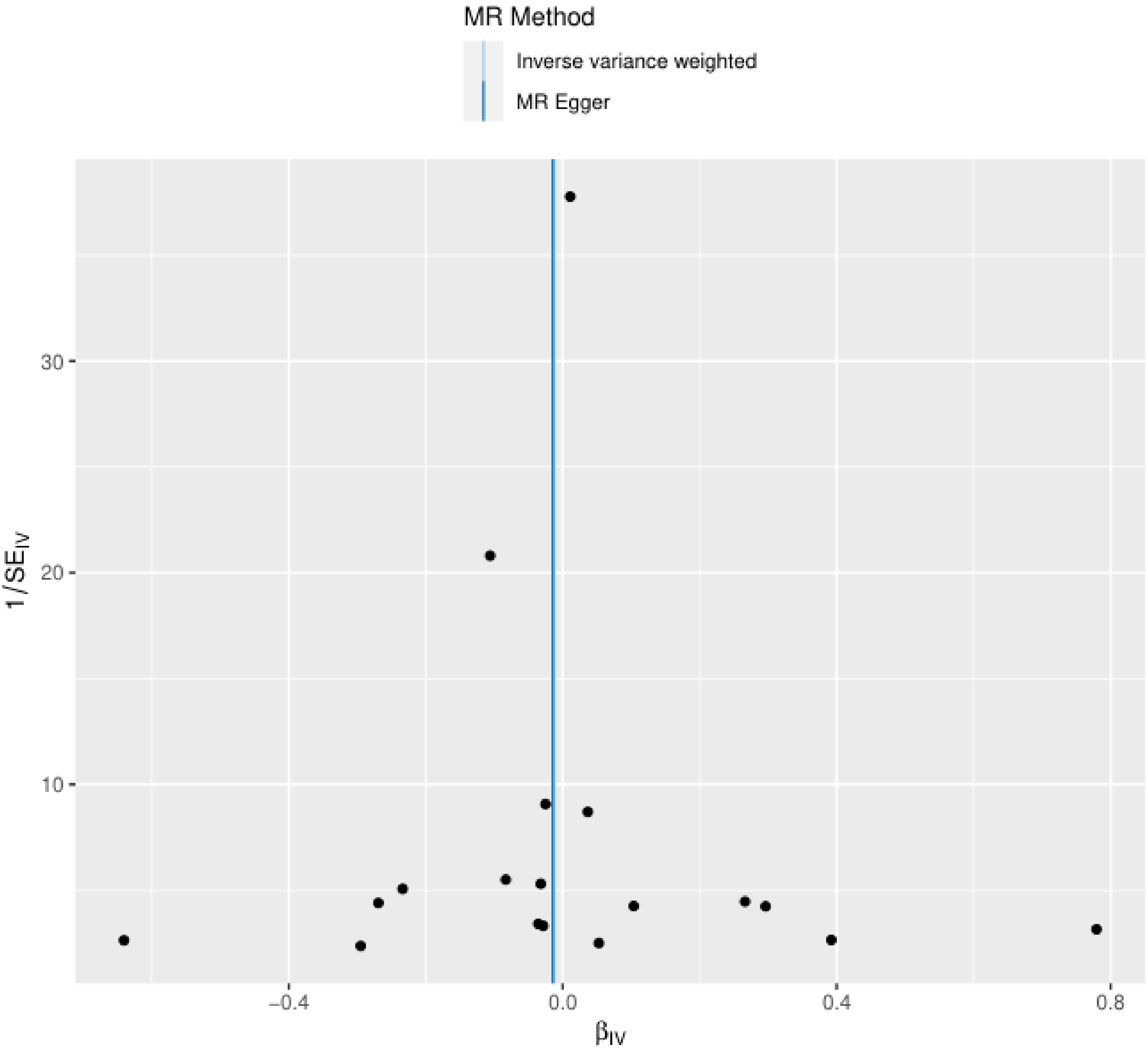

Funnel plot analyse of "IgD+ %Lymphocyte" on 'Diabetic nephropathy'

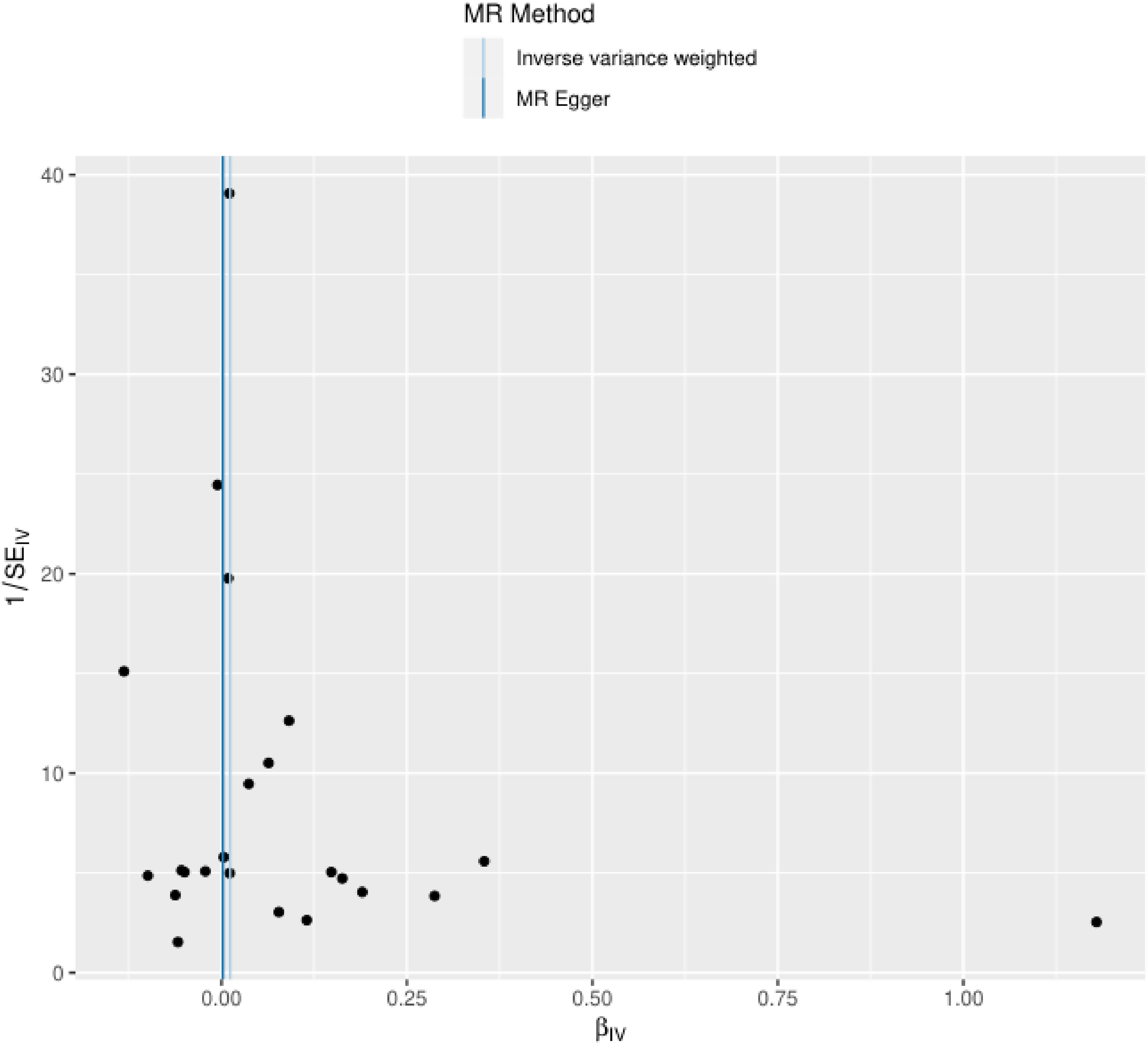

Funnel plot analyse of "CD39 on CD39+ secreting Treg " on 'Diabetic nephropathy'

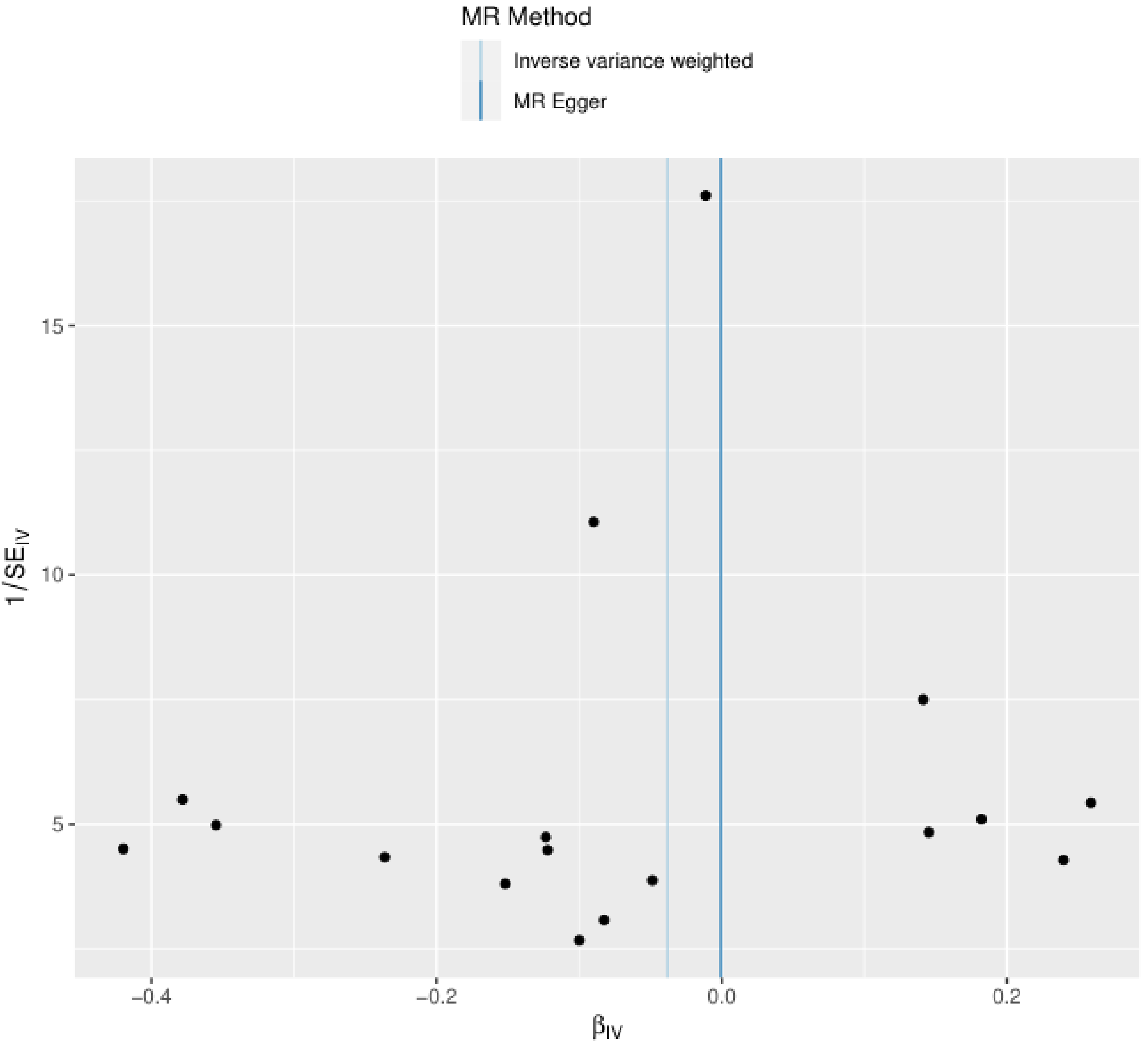

Funnel plot analyse of "CD4+ CD8dim AC" on 'Diabetic nephropathy'

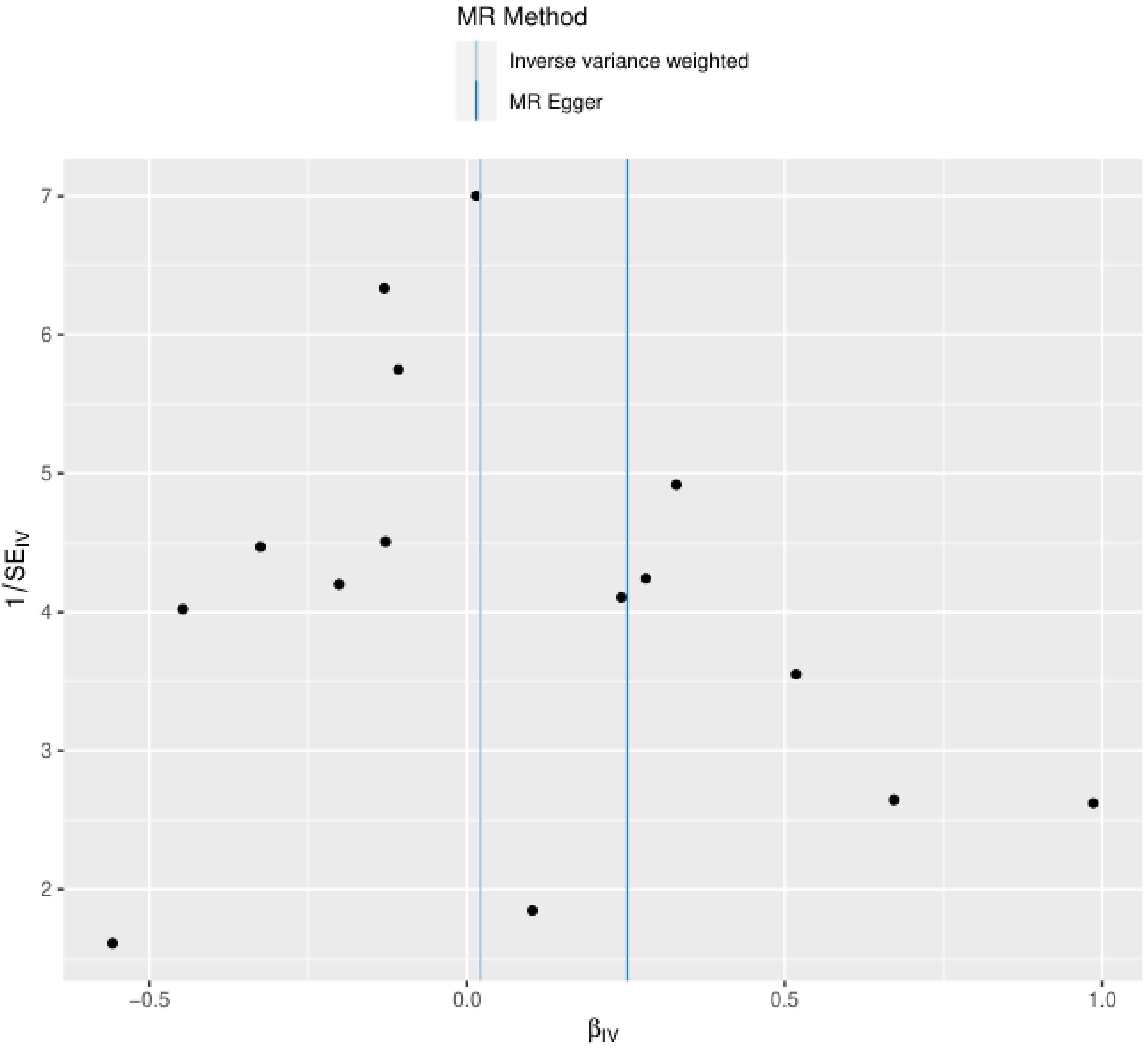

Funnel plot analyse of "CD27 on IgD- CD38br" on 'Diabetic nephropathy'

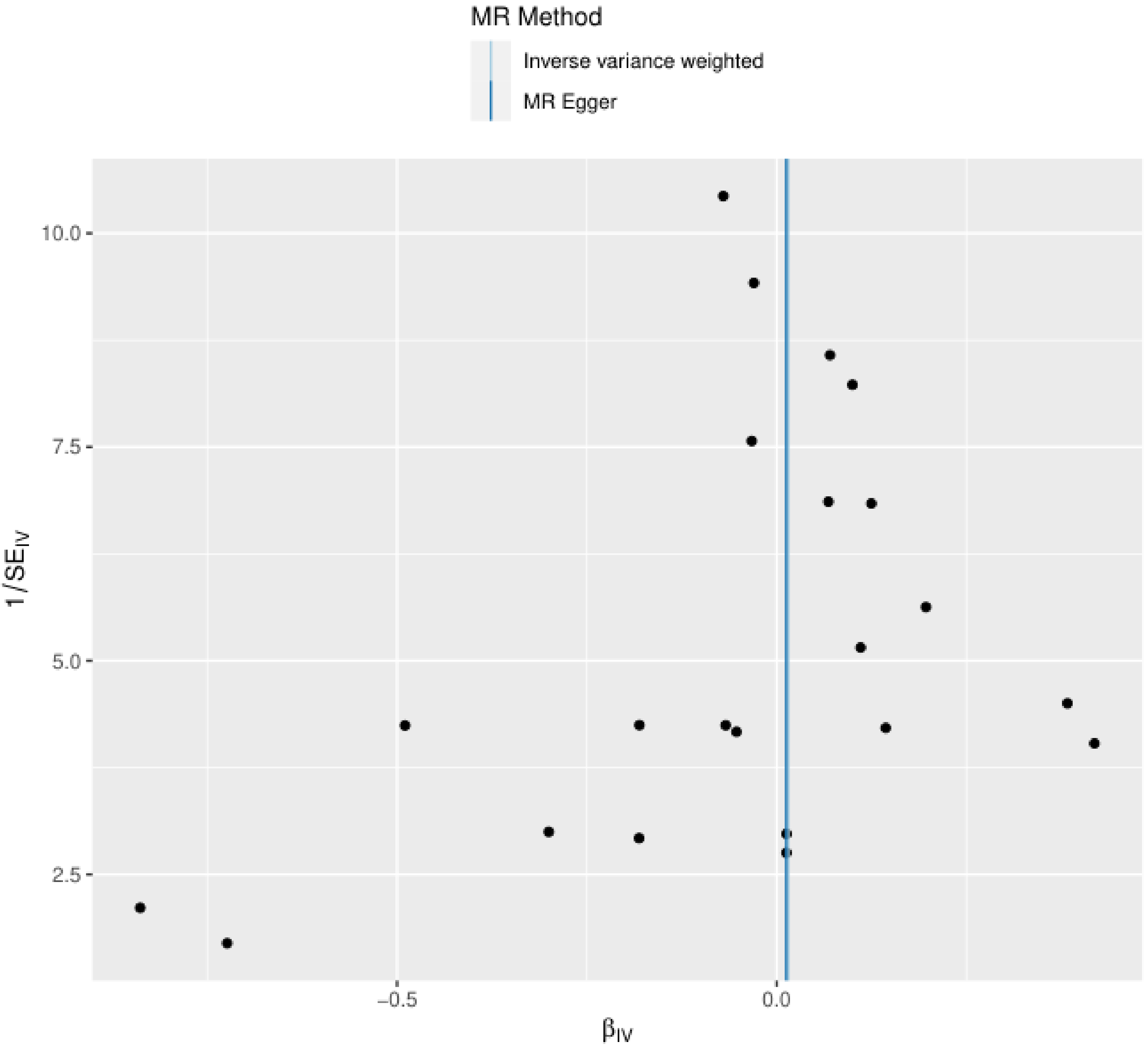

Funnel plot analyse of "CD14- CD16+ monocyte %monocyte" on 'Diabetic nephropathy'

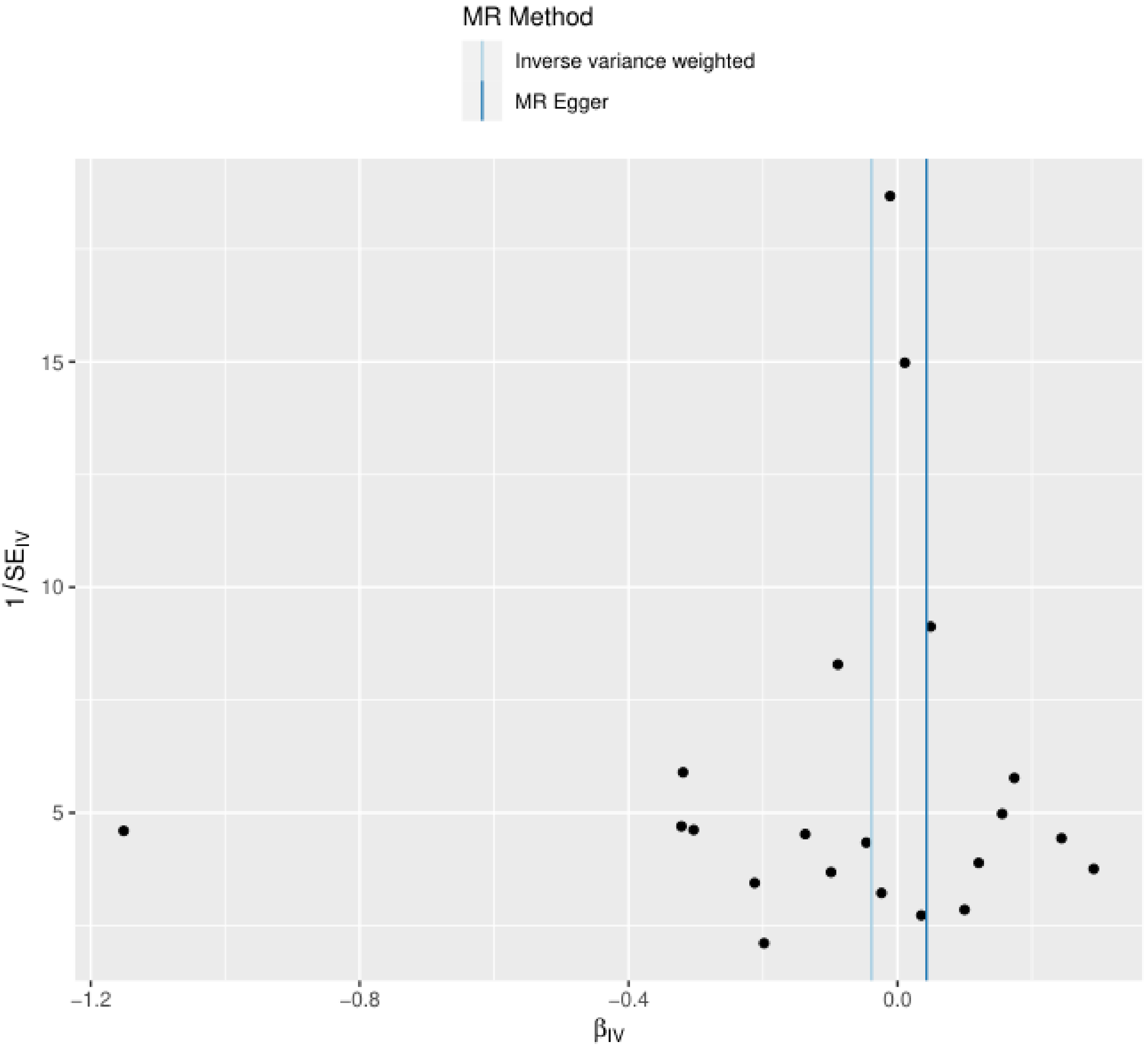

Funnel plot analyse of "CD19 on IgD+ CD38br" on 'Diabetic nephropathy'

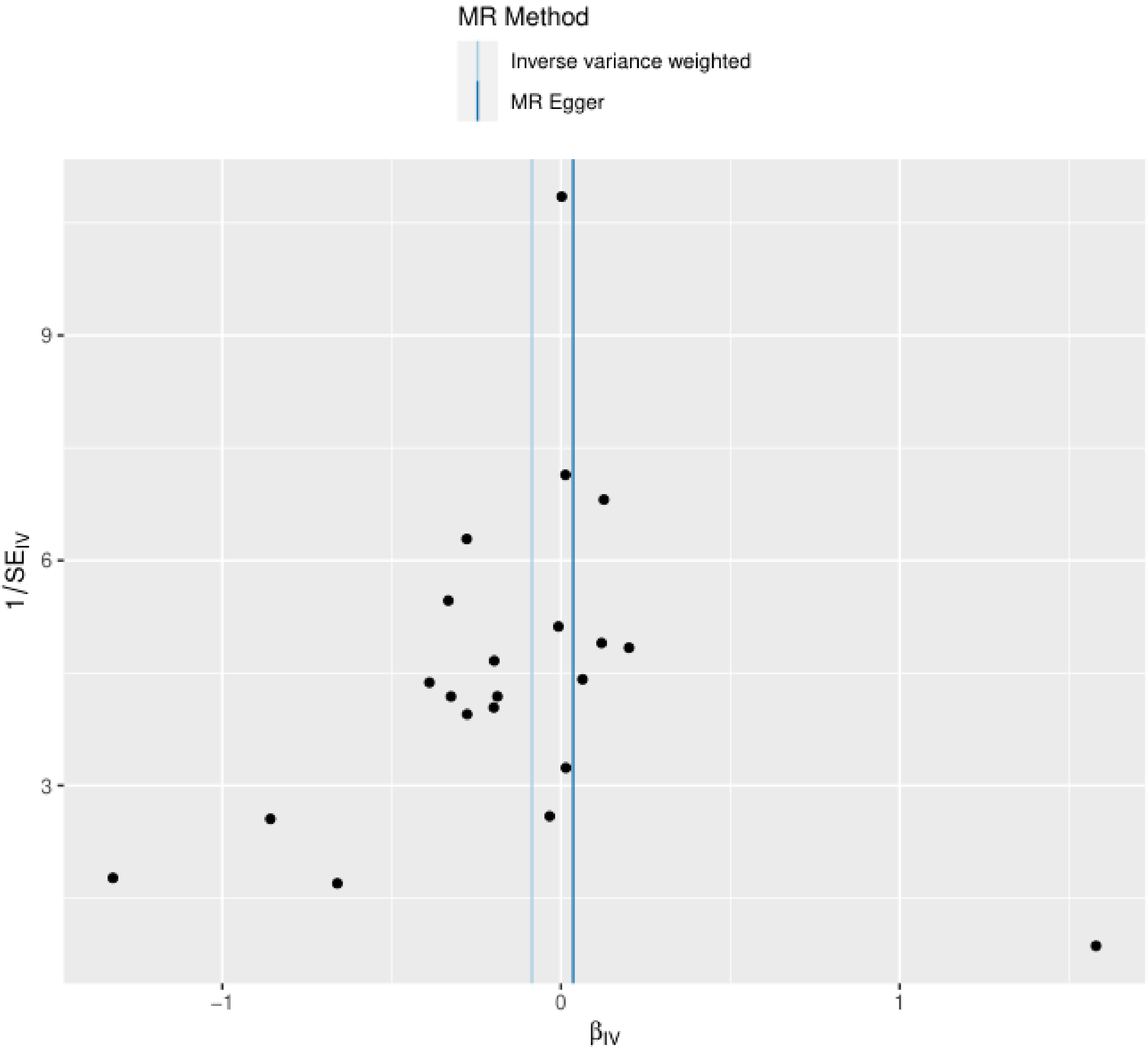

Funnel plot analyse of "DP (CD4+CD8+) %leukocyte" on 'Diabetic nephropathy'

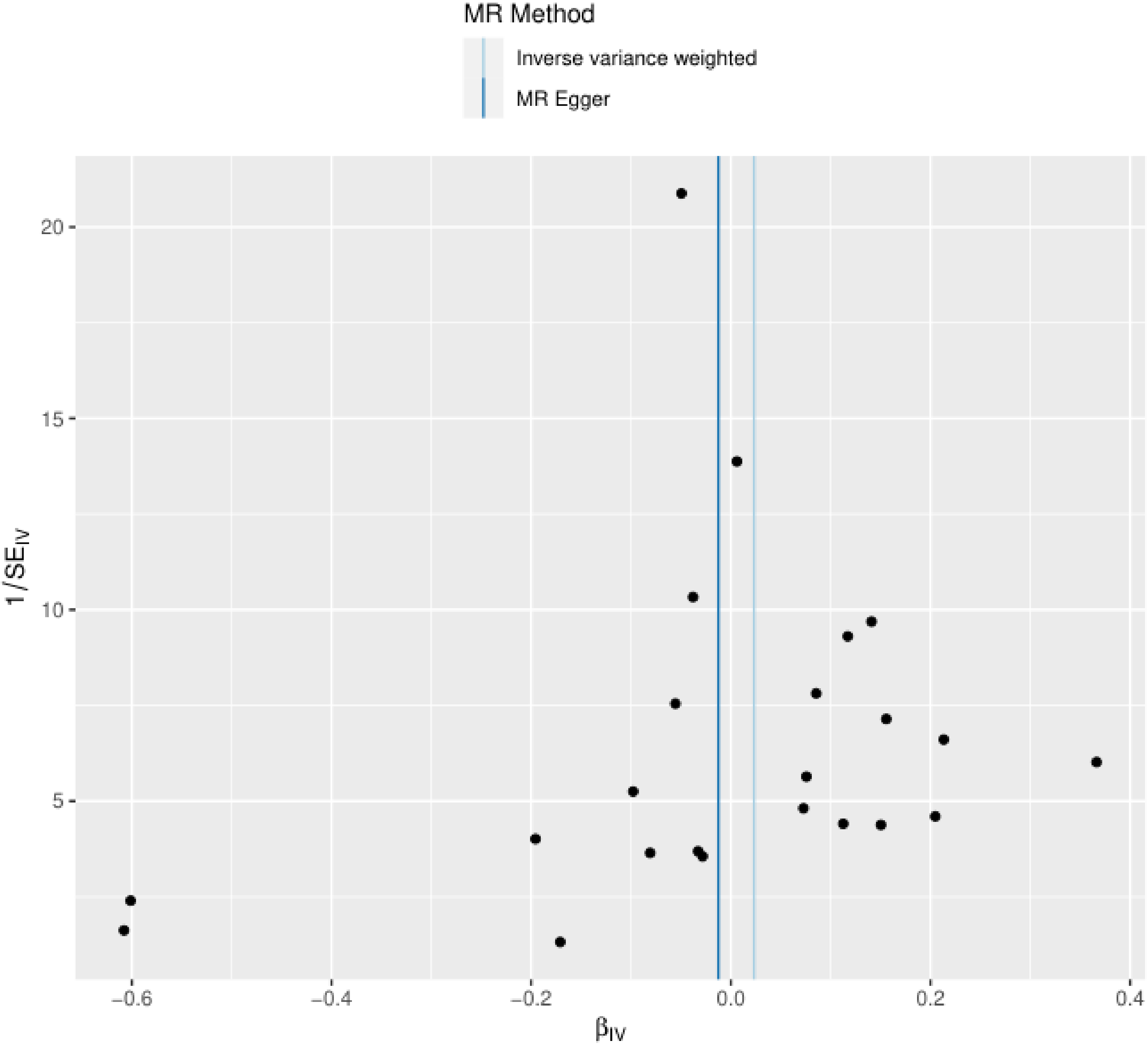

Funnel plot analyse of "CD39+ CD8br AC" on 'Diabetic nephropathy'

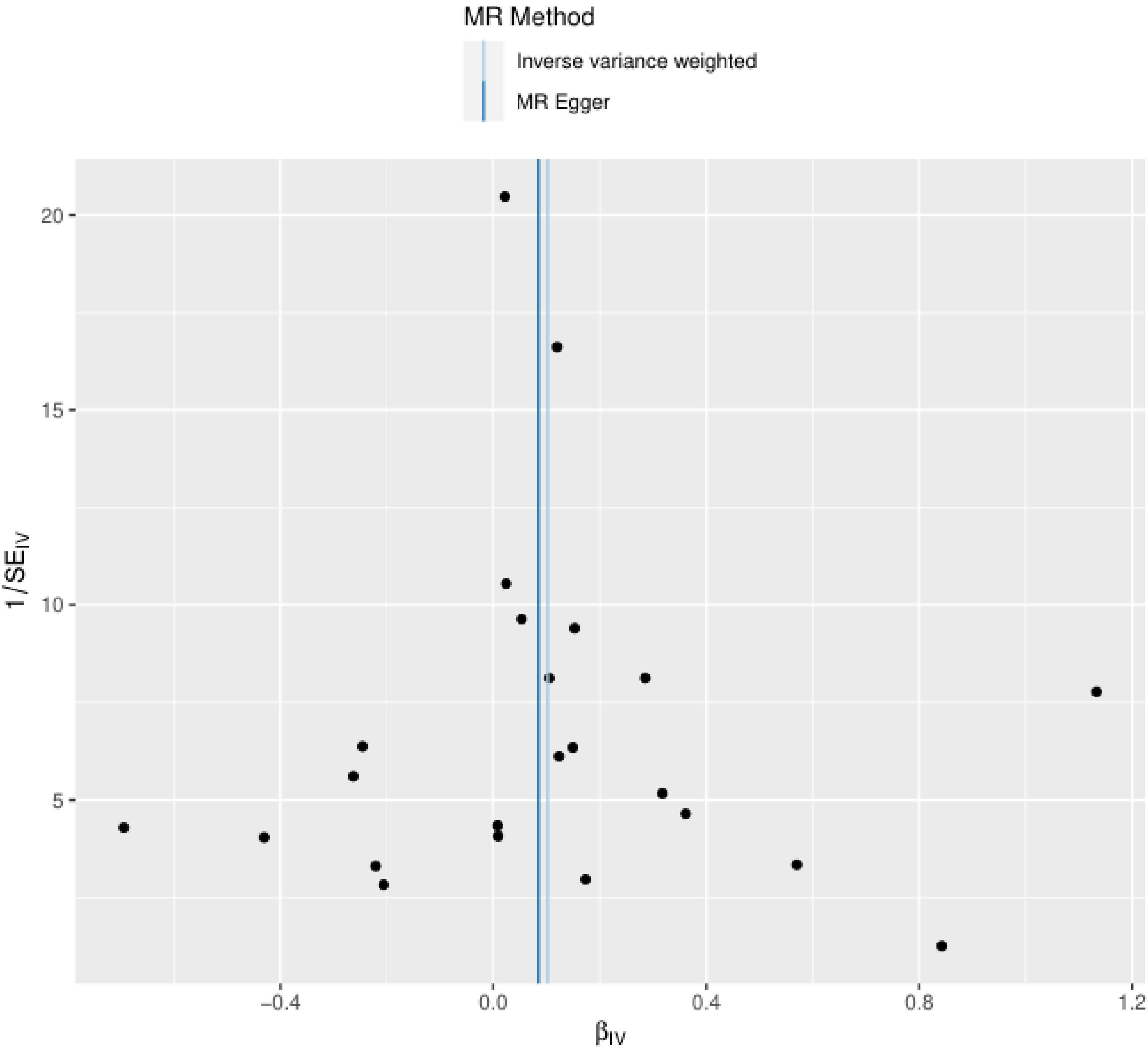

Funnel plot analyse of "HLA DR++ monocyte AC" on 'Diabetic nephropathy'

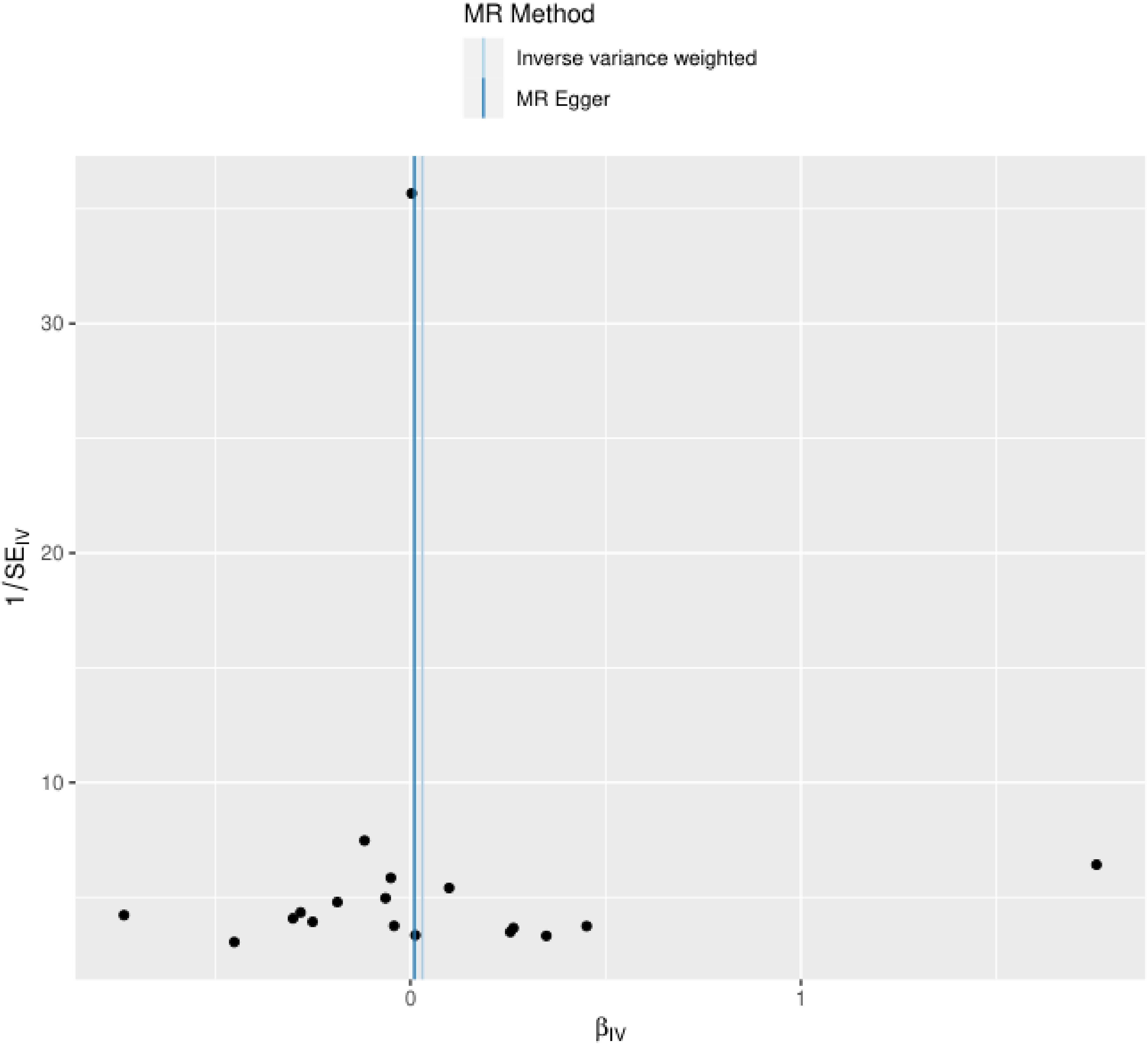

Funnel plot analyse of "CD20- %lymphocyte" on 'Diabetic nephropathy'

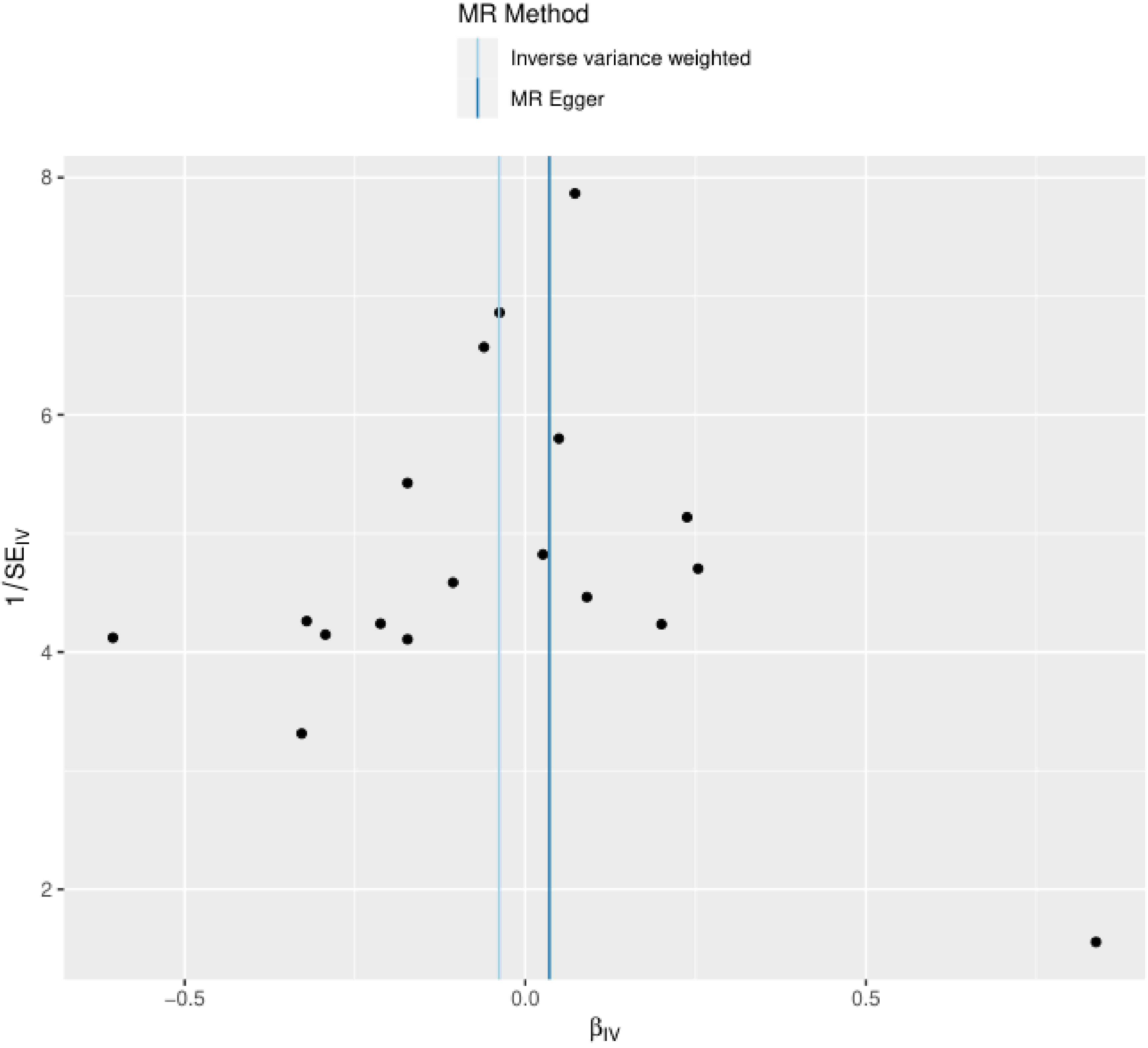

Funnel plot analyse of "CD3- lymphocyte AC" on 'Diabetic nephropathy'

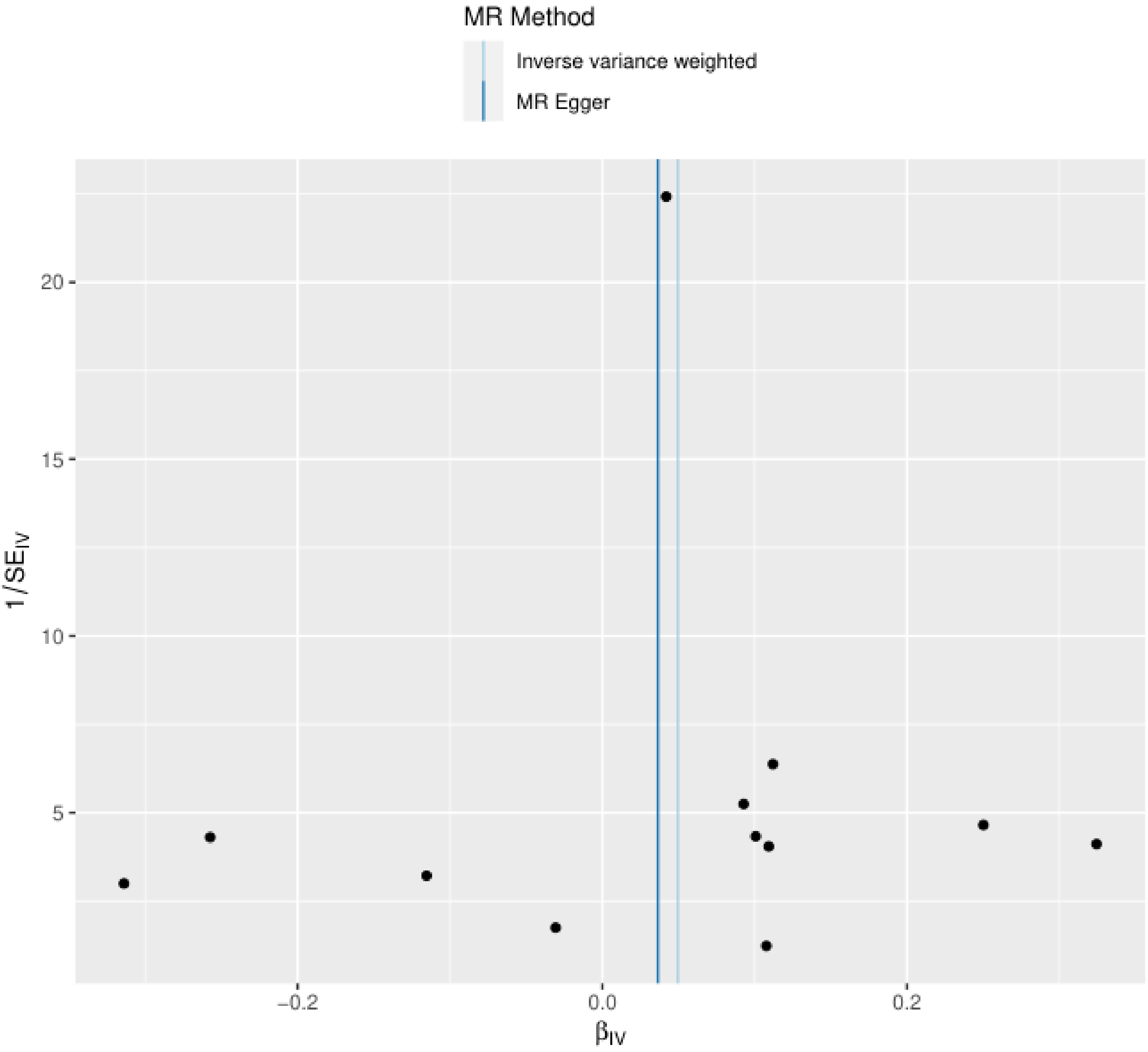

Funnel plot analyse of "CD25 on CD39+ activated Treg " on 'Diabetic nephropathy'

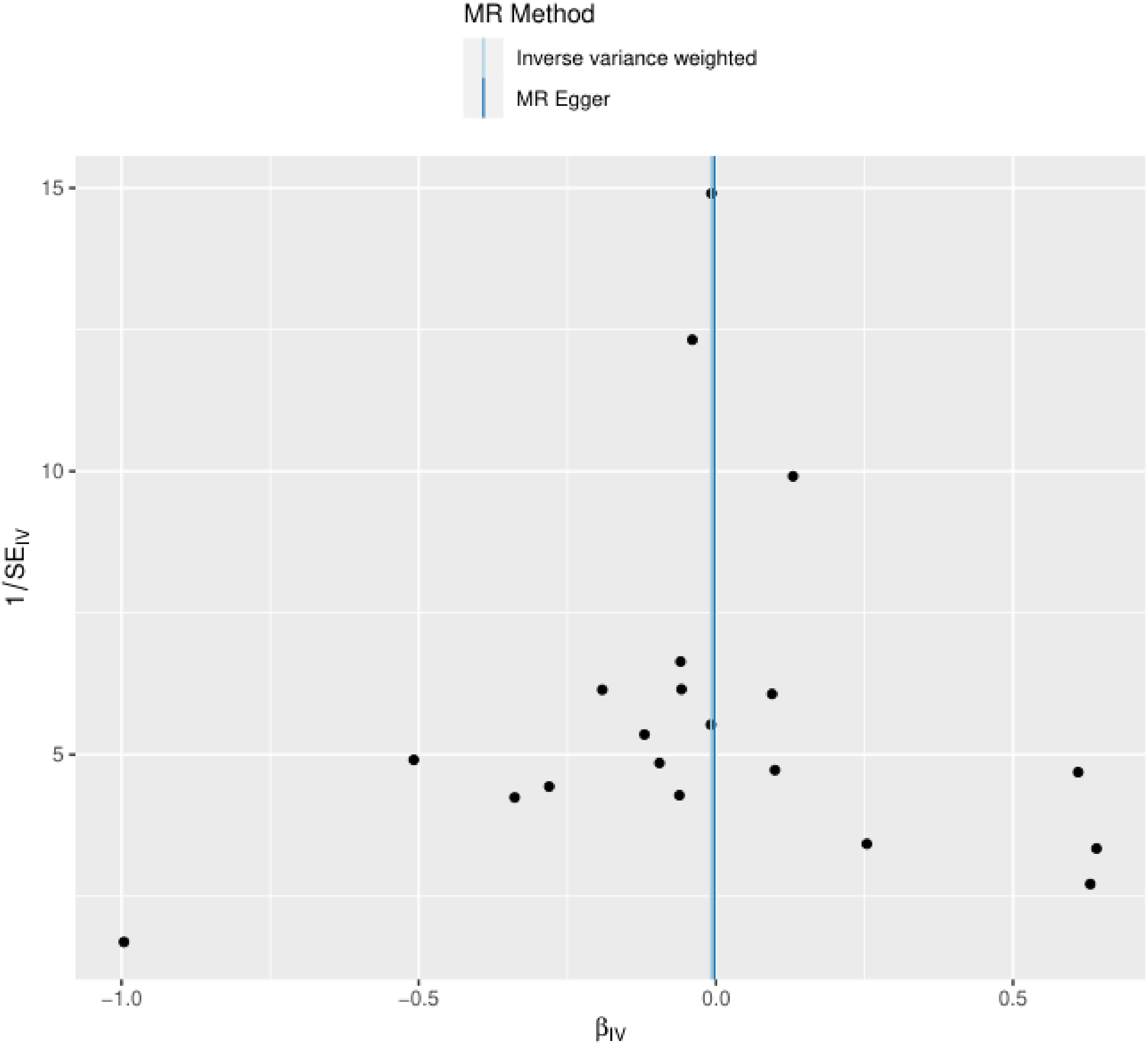

Funnel plot analyse of "CD3- lymphocyte %leukocyte" on 'Diabetic nephropathy'

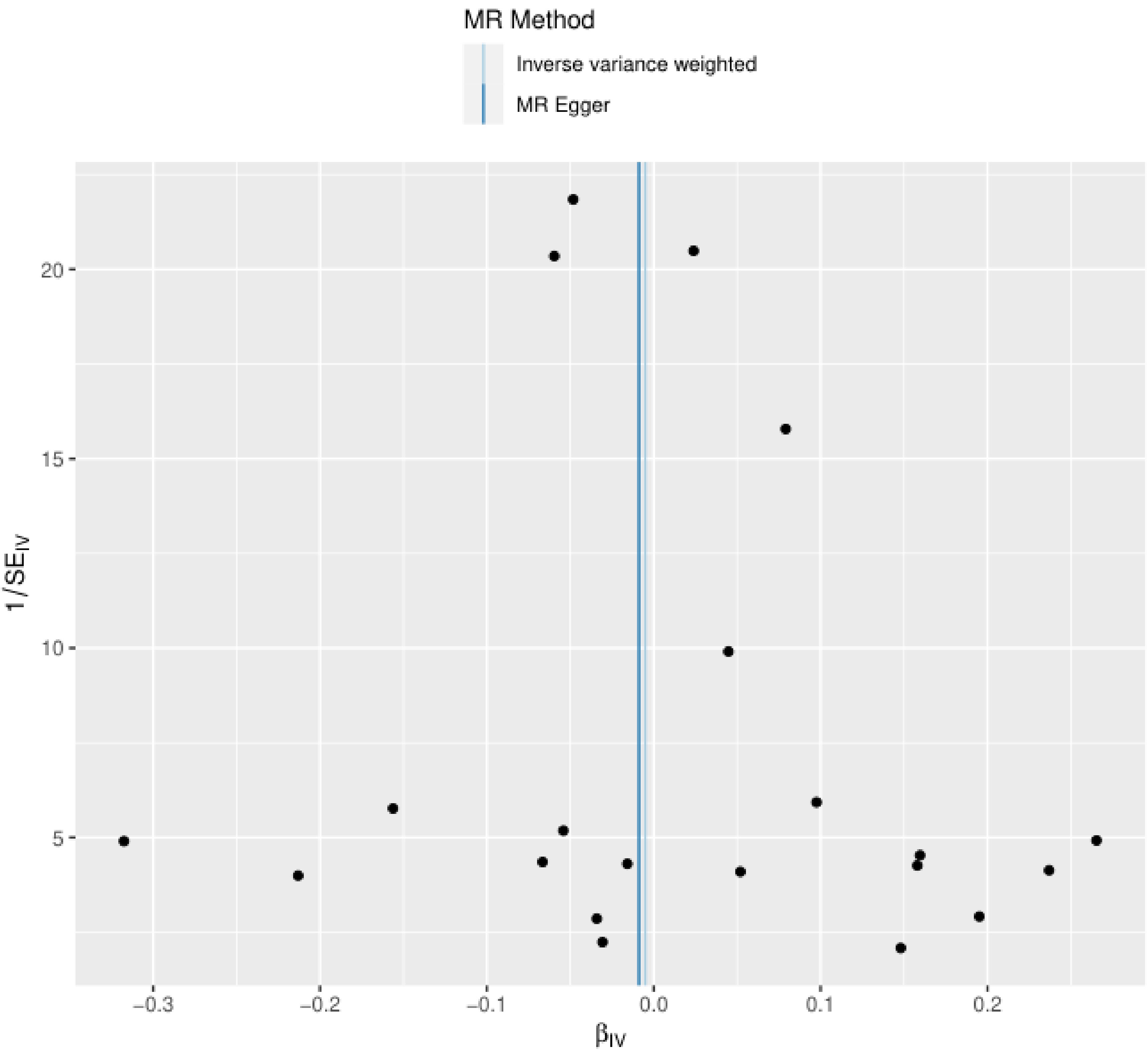

Funnel plot analyse of "FSC-A on T cell" on 'Diabetic nephropathy'

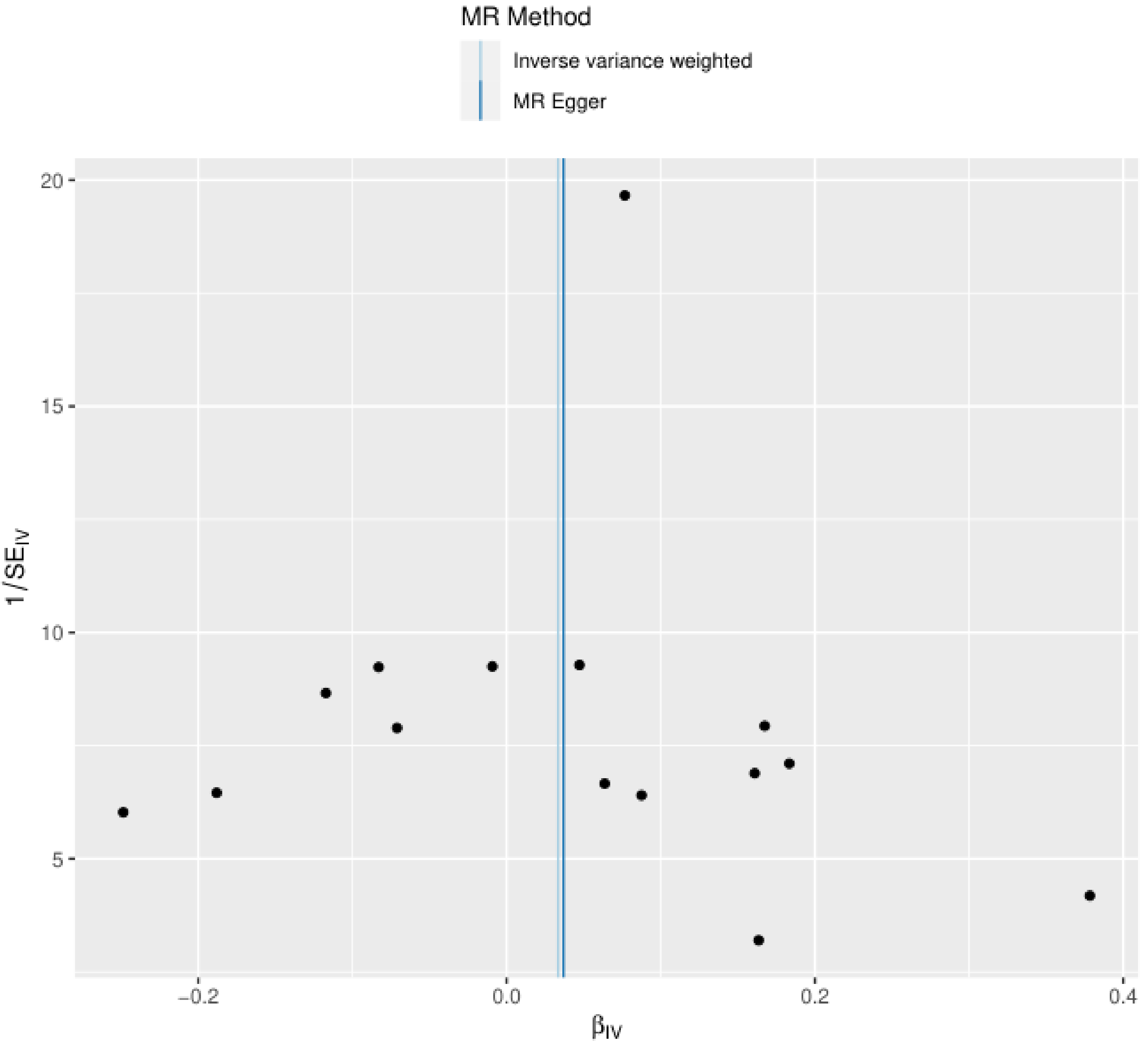

Funnel plot analyse of "CD45 on Mo MDSC " on 'Diabetic nephropathy'

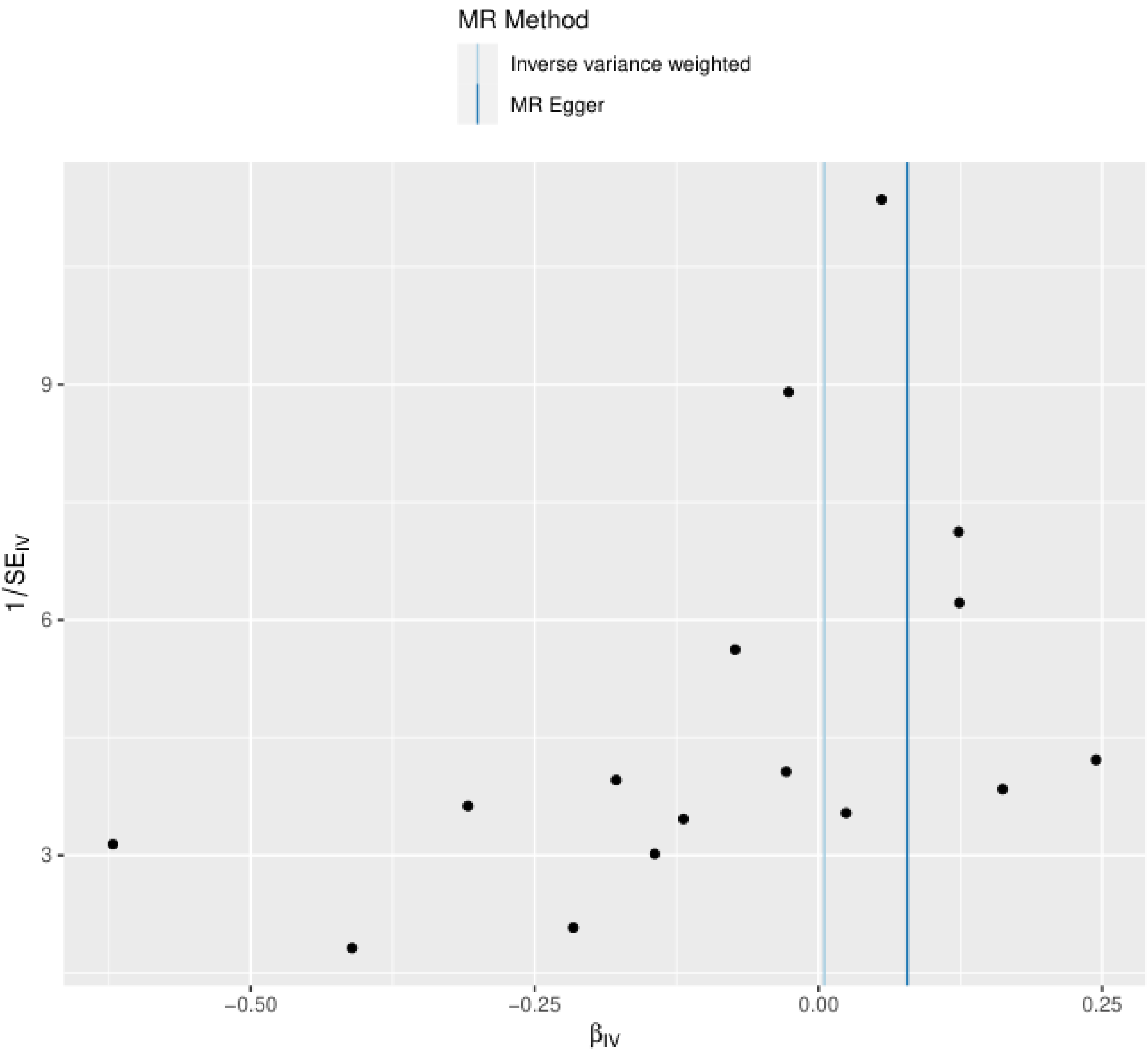

Funnel plot analyse of "CD14 on CD14+ CD16- monocyte" on 'Diabetic nephropathy'

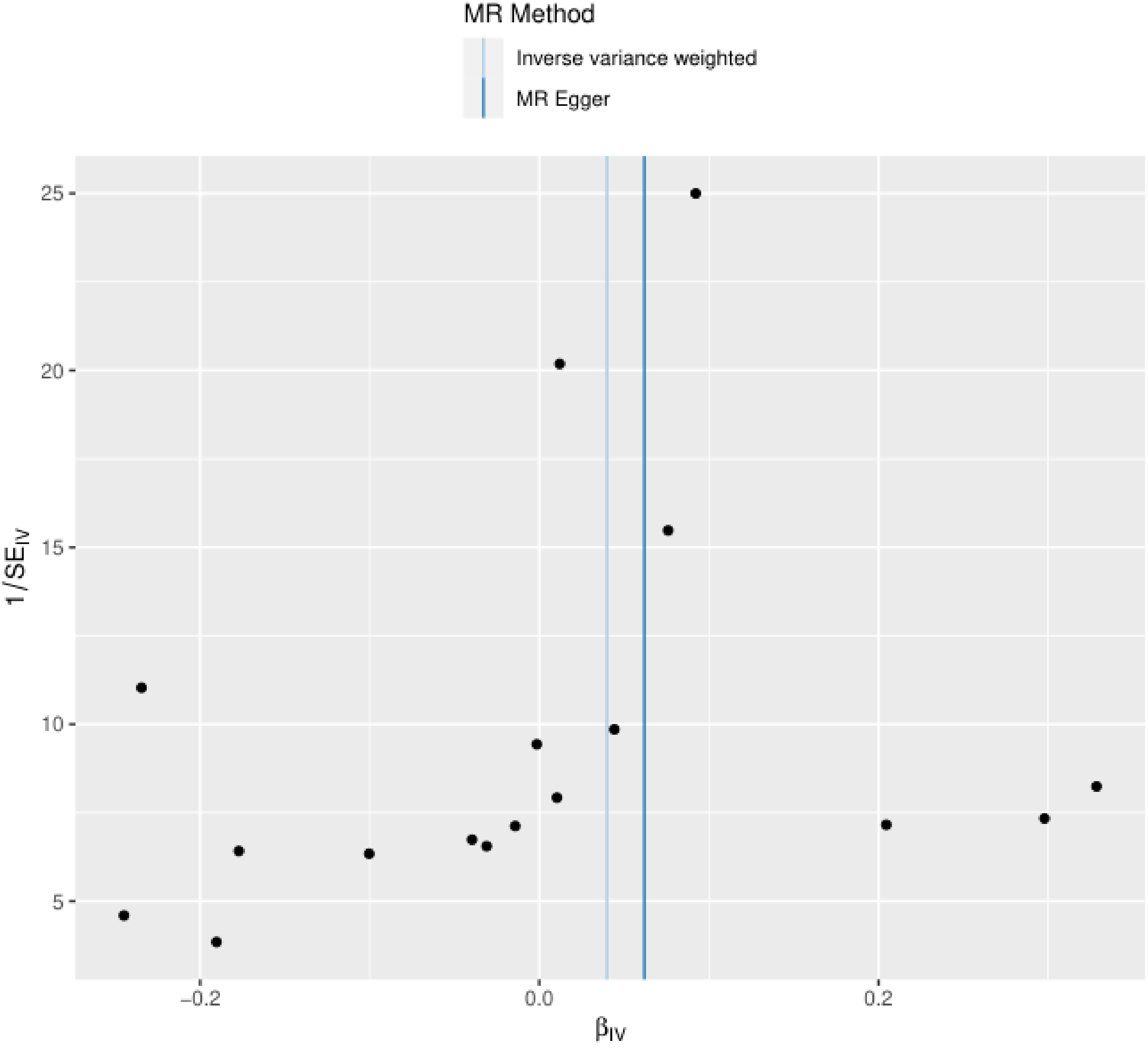

Funnel plot analyse of "HVEM on CM CD4+" on 'Diabetic nephropathy'

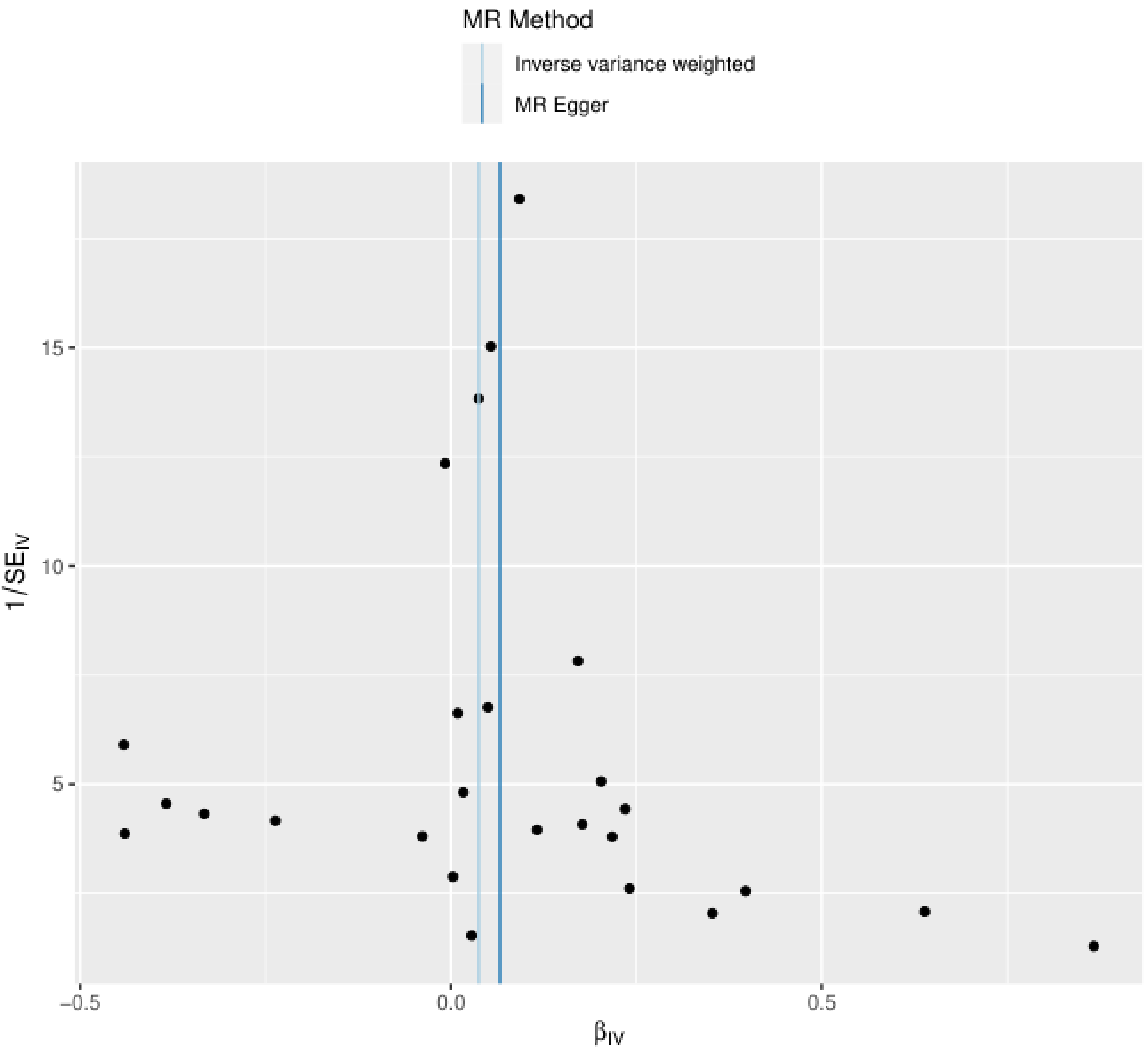

Funnel plot analyse of "IgD+ CD38br %B cell" on 'Diabetic nephropathy'

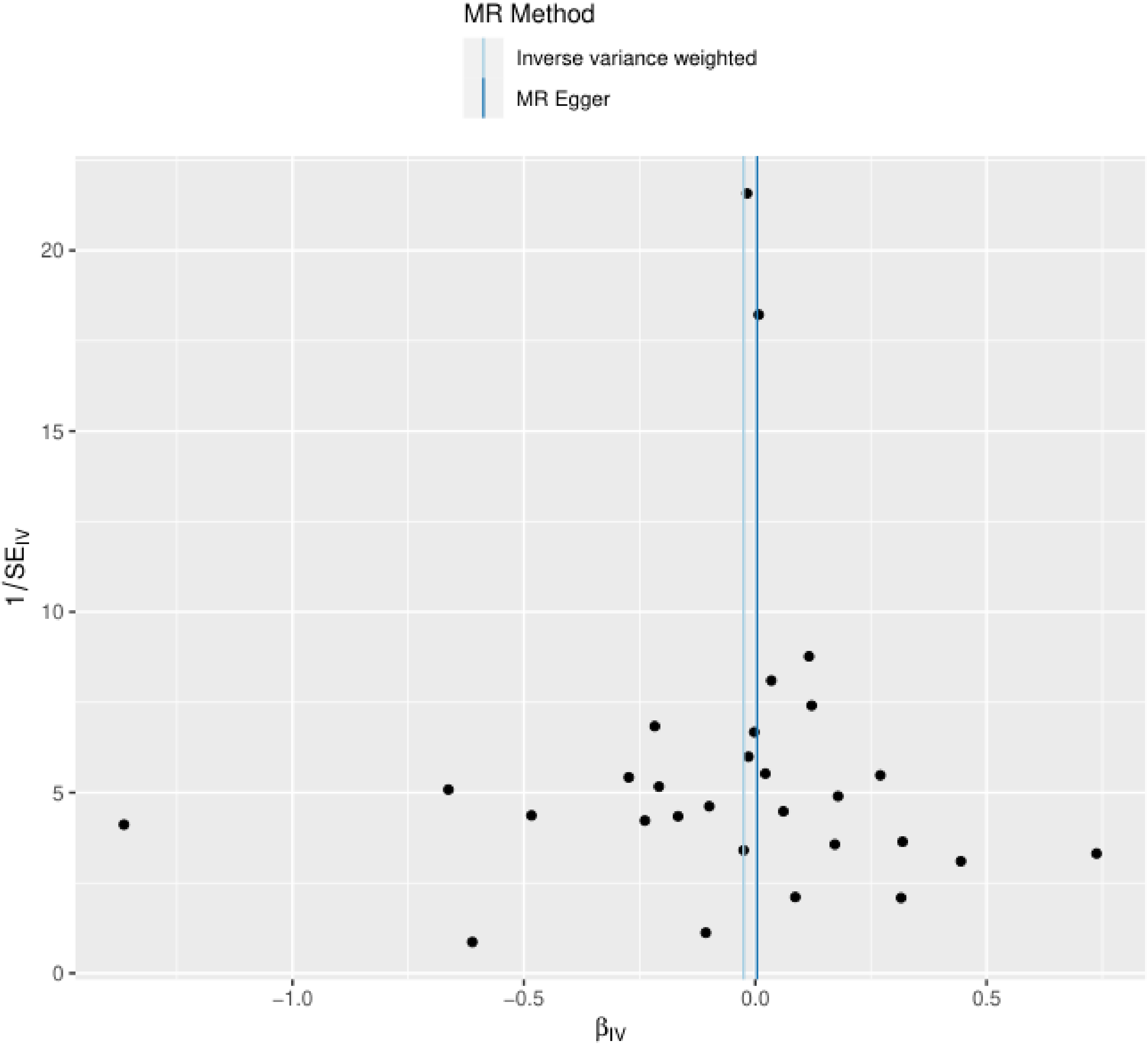

Funnel plot analyse of "IgD on IgD+ CD38-" on 'Diabetic nephropathy'

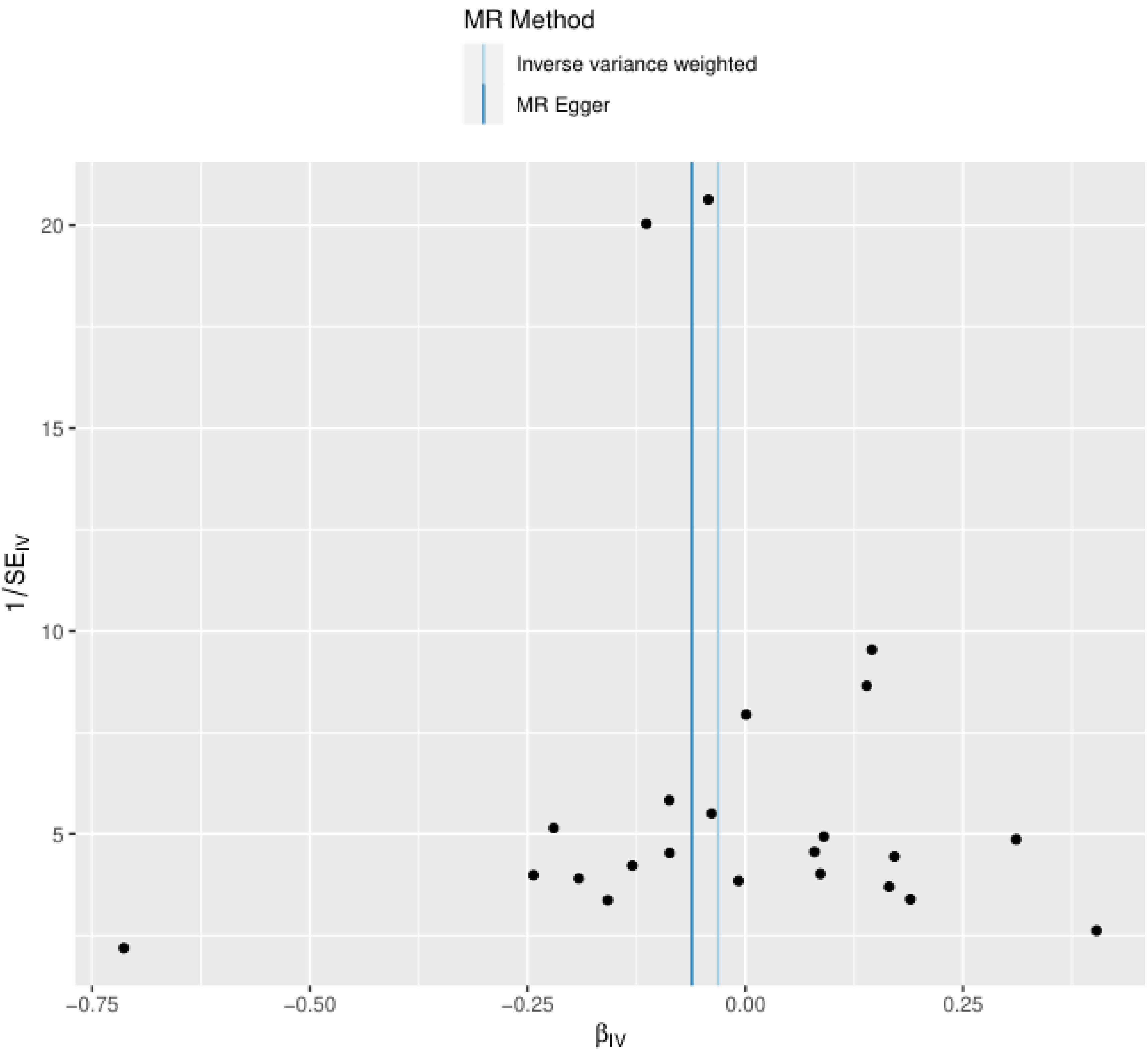

Funnel plot analyse of "CD20- CD38- %B cell" on 'Diabetic nephropathy'

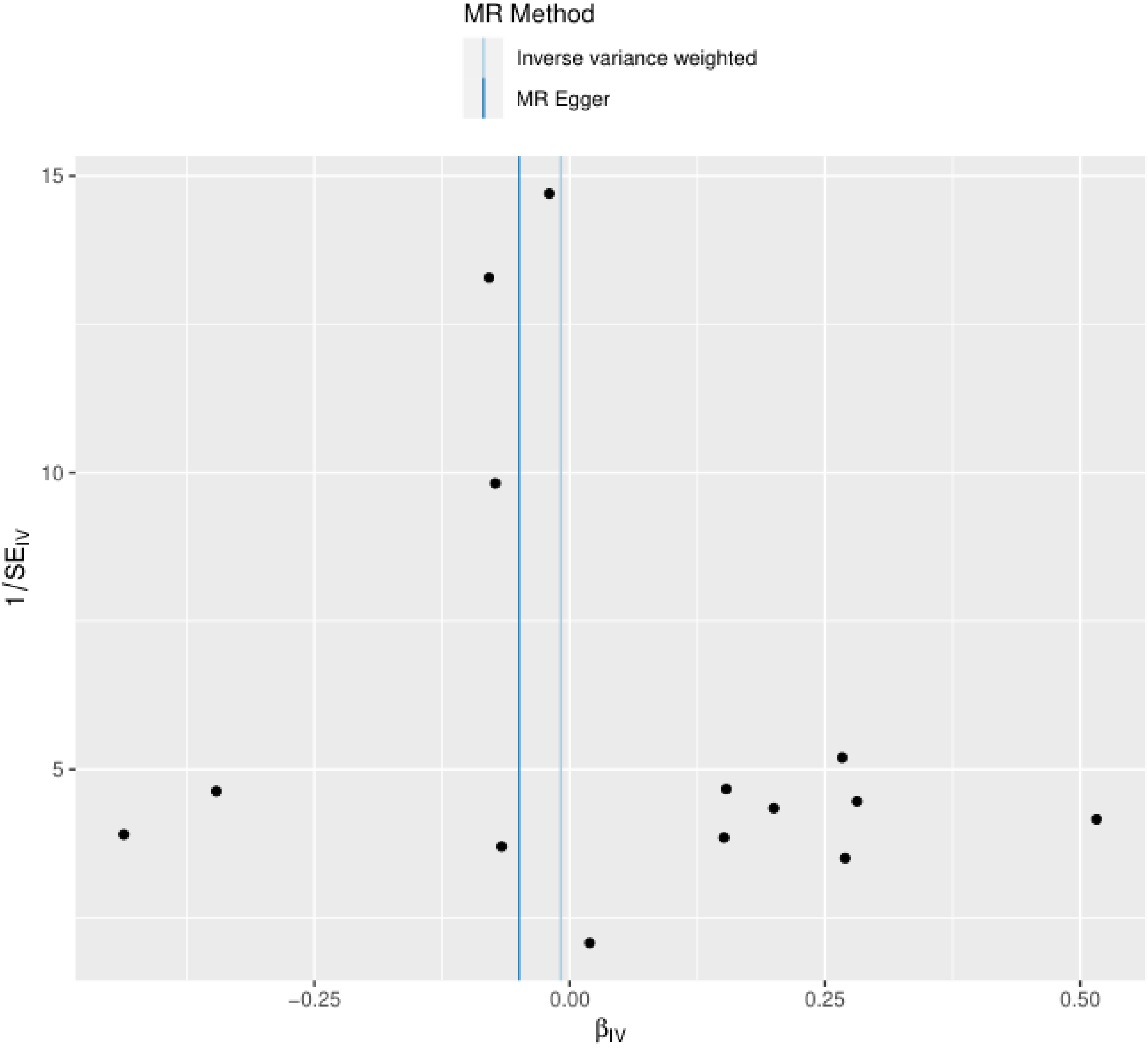

Funnel plot analyse of "CM DN (CD4-CD8-) %T cell" on 'Diabetic nephropathy'

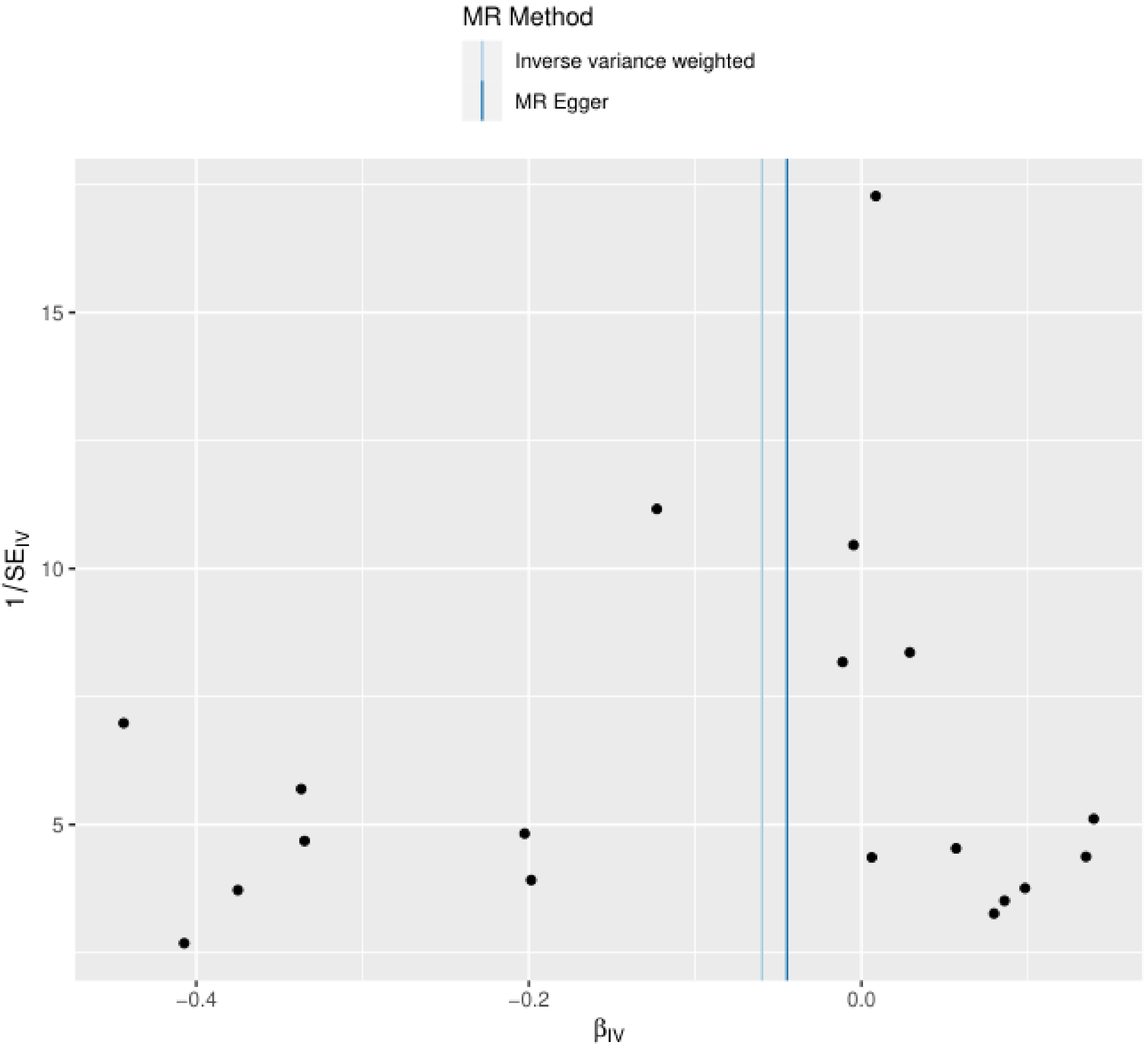

Funnel plot analyse of "Activated Treg %CD4" on 'Diabetic nephropathy'

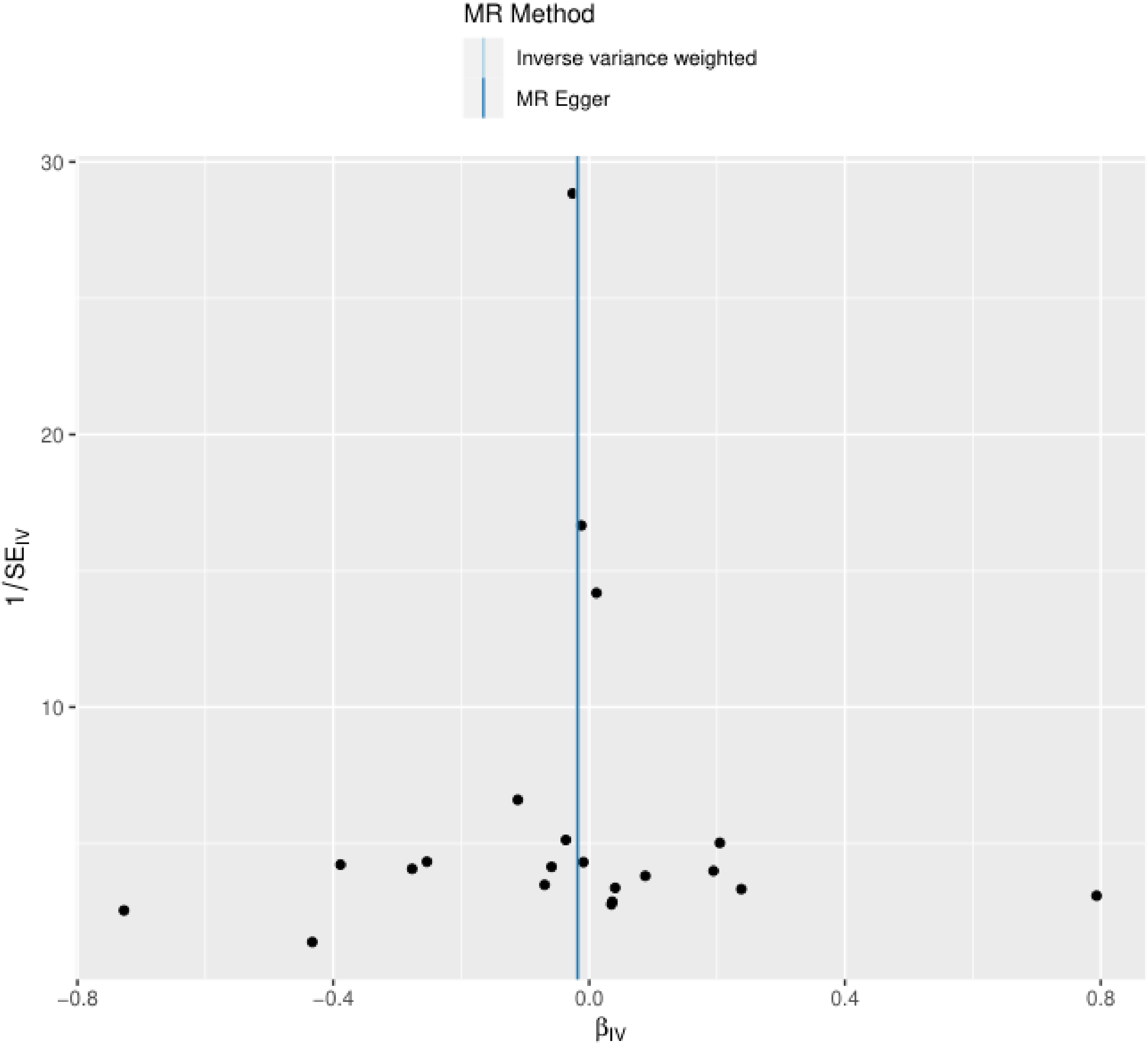

Funnel plot analyse of "CD19 on unsw mem" on 'Diabetic nephropathy'

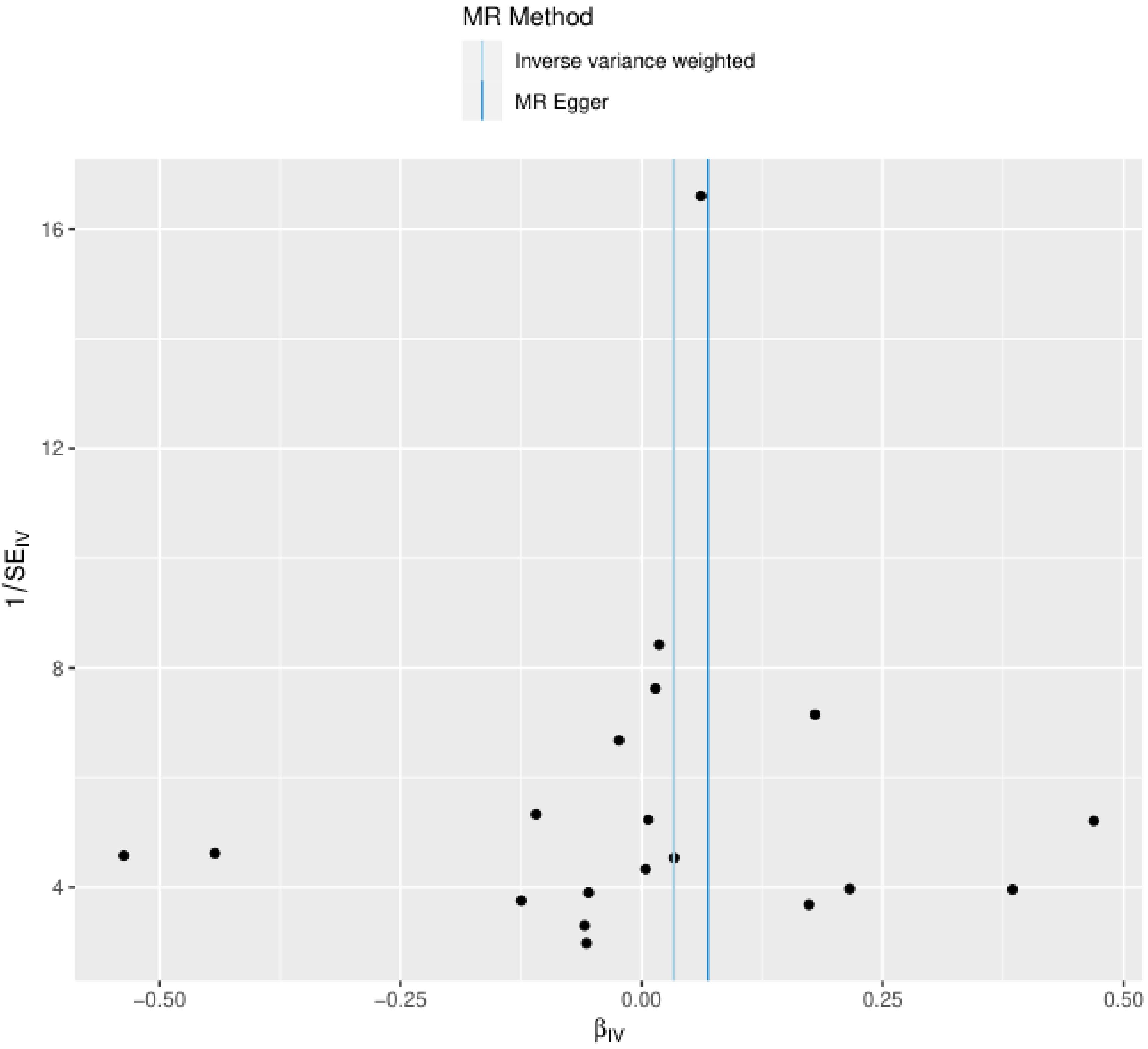

Funnel plot analyse of "CD11c+ monocyte %monocyte" on 'Diabetic nephropathy'

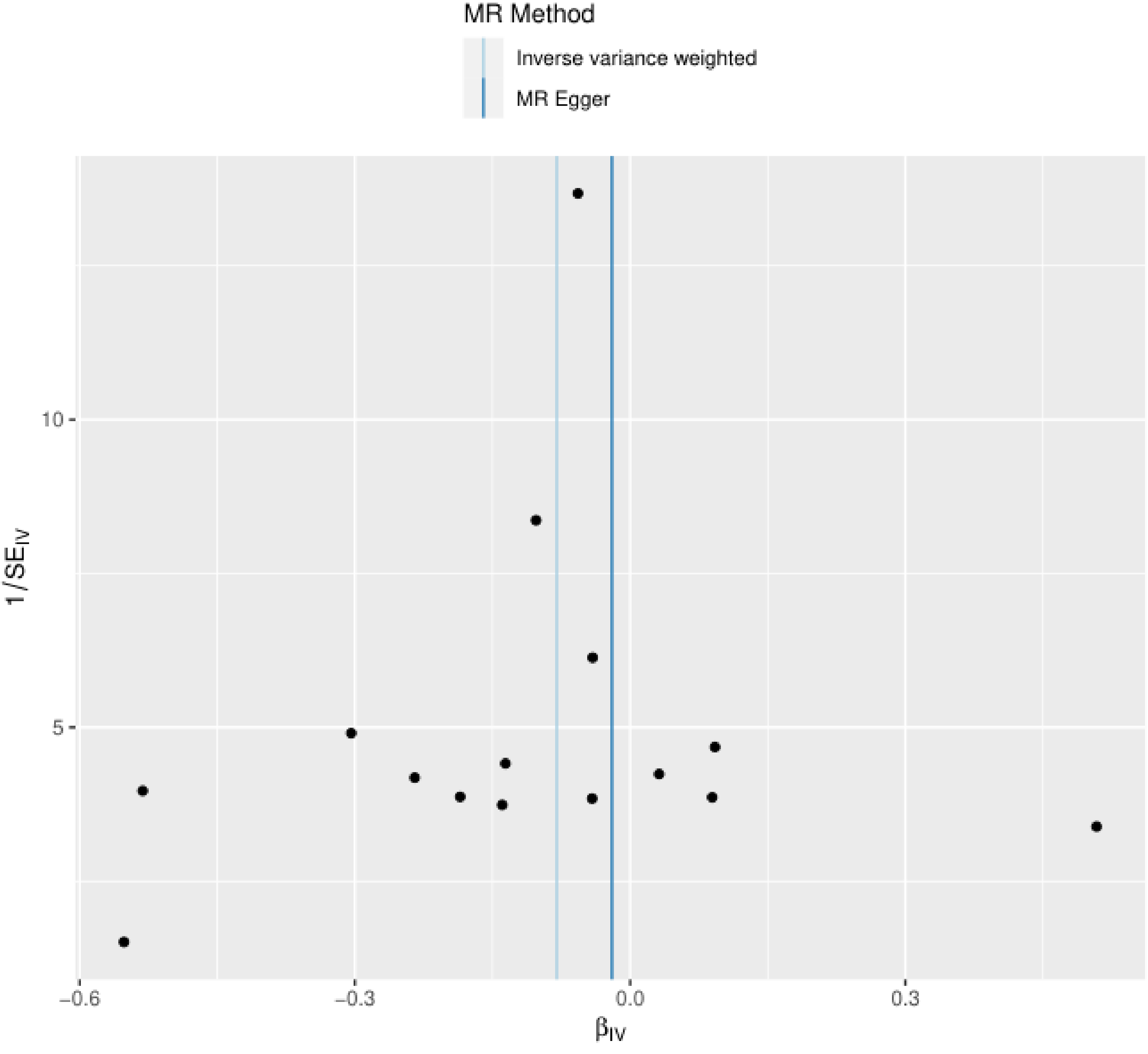

Funnel plot analyse of "CD4+ %leukocyte" on 'Diabetic nephropathy'

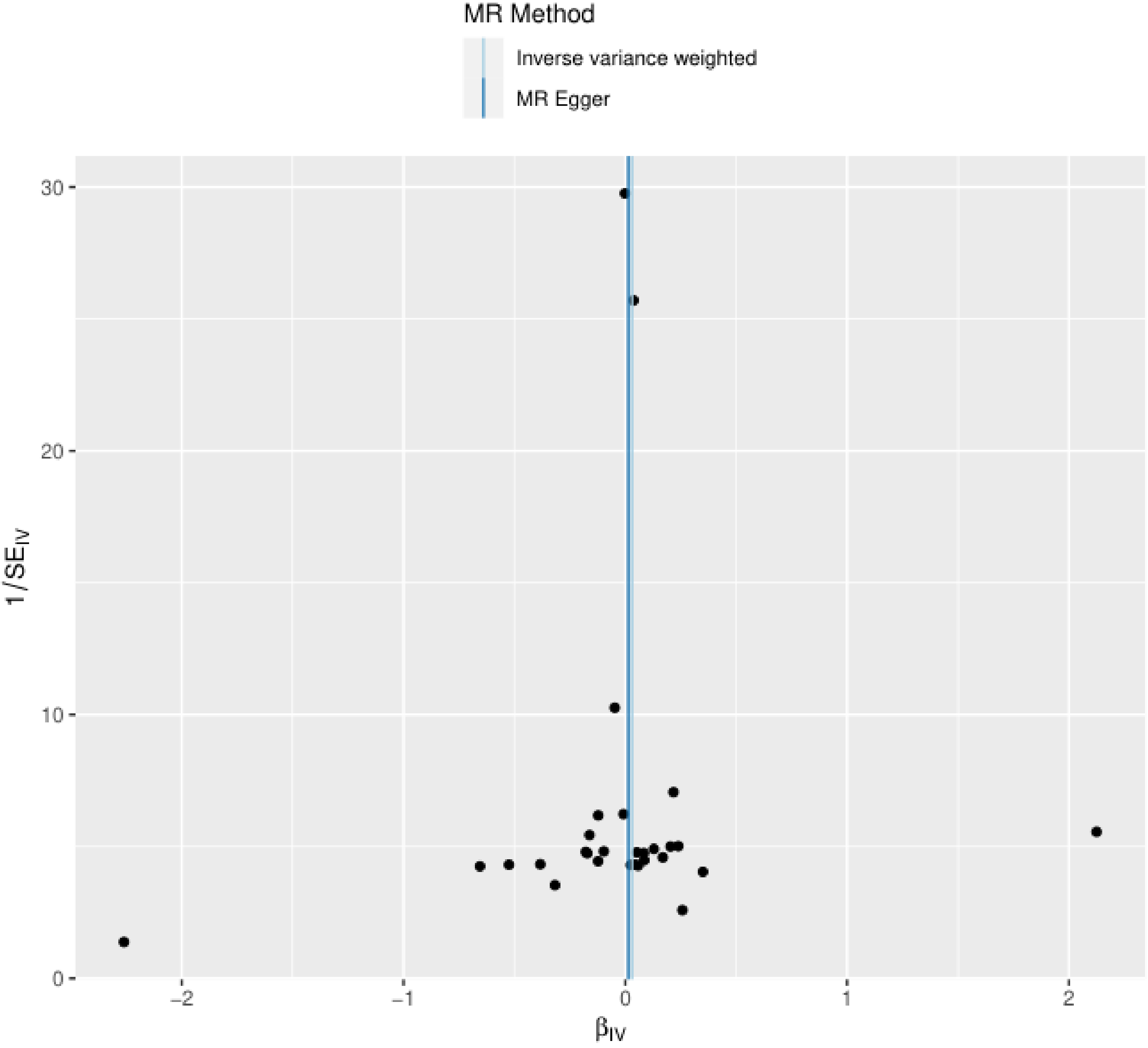

Funnel plot analyse of "CD28- CD127- CD25++ CD8br AC" on 'Diabetic nephropathy'

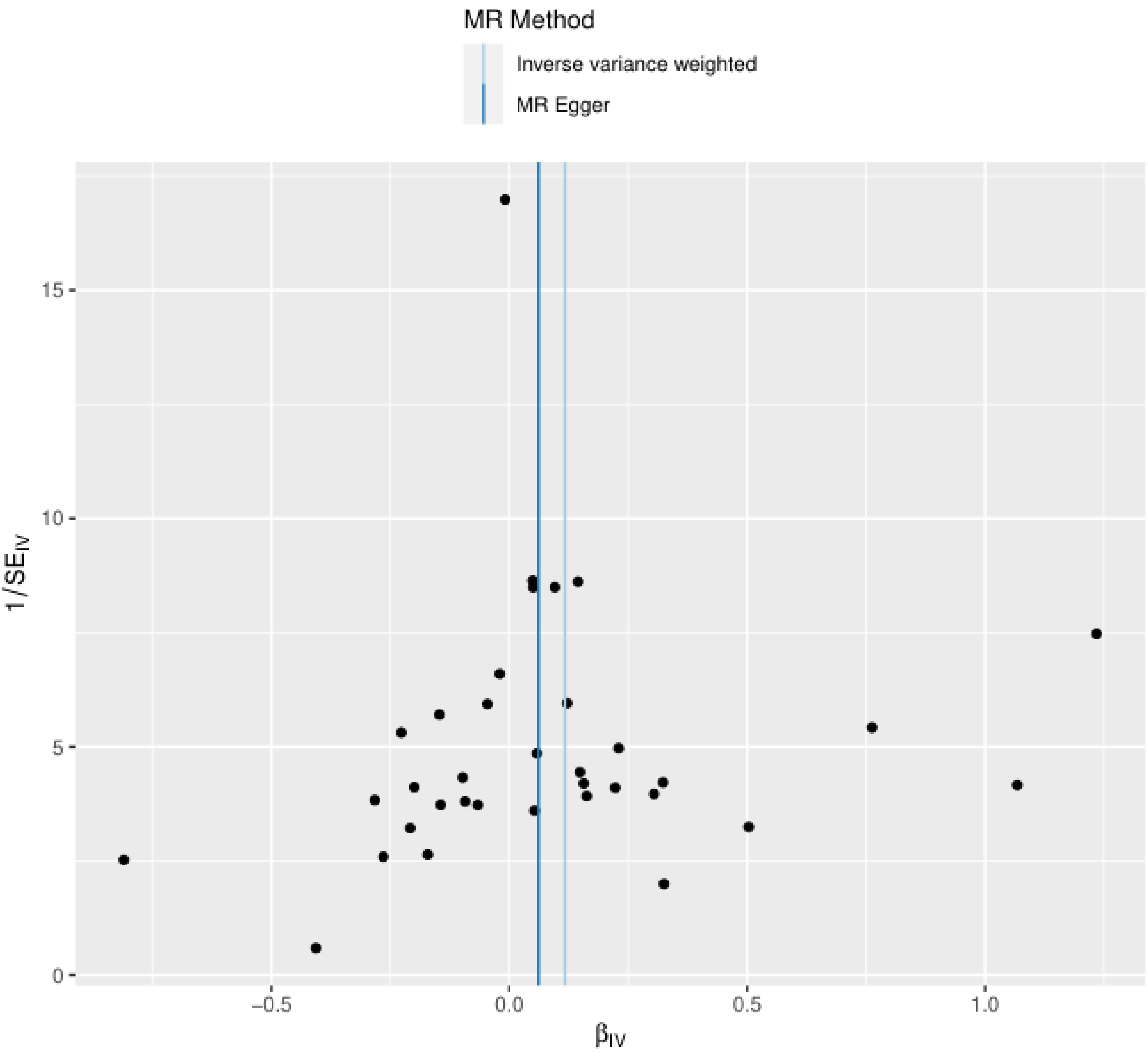

Funnel plot analyse of "Naive CD4+ %CD4+" on 'Diabetic nephropathy'

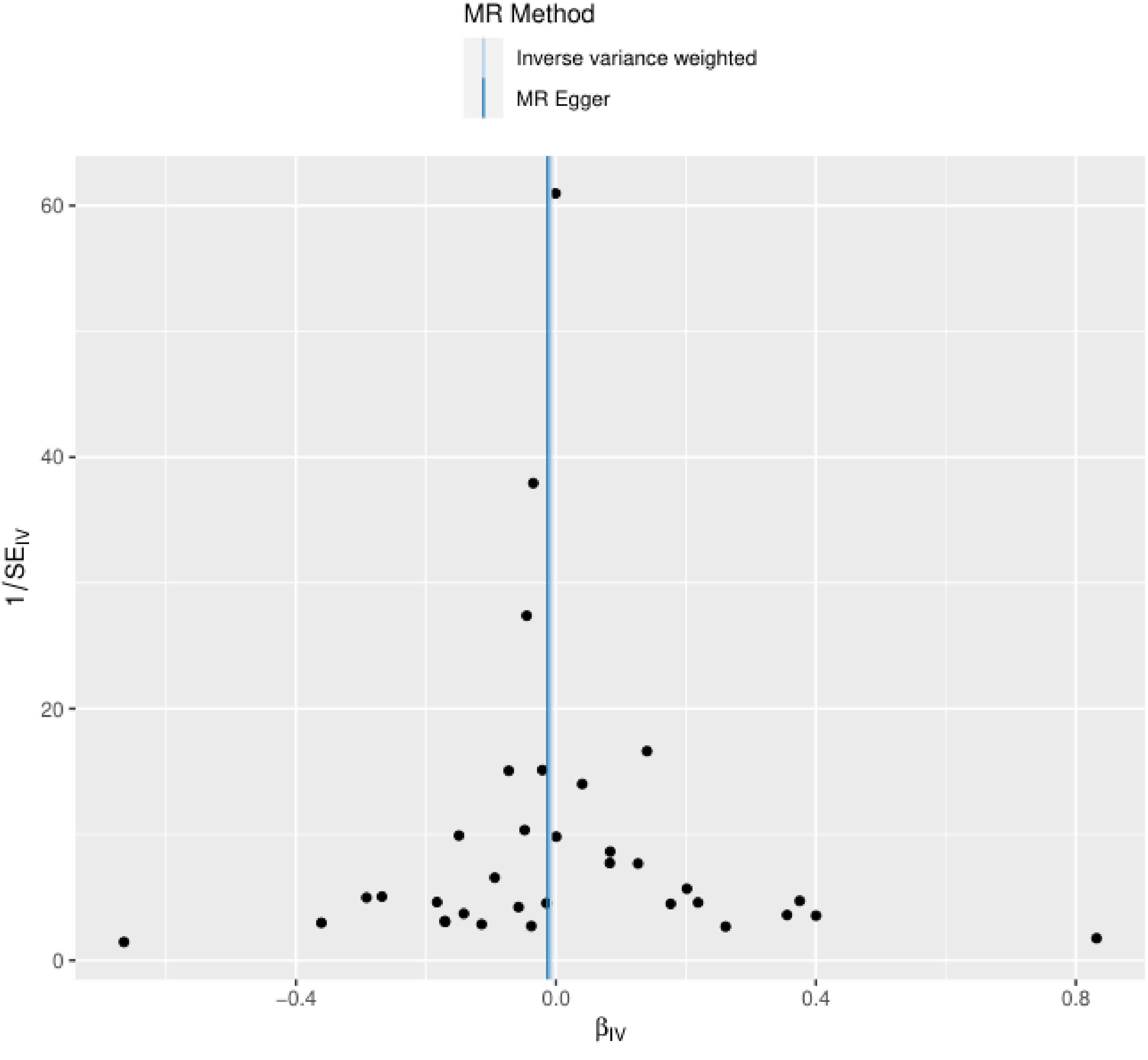

Funnel plot analyse of "CD64 on CD14+ CD16- monocyte " on 'Diabetic nephropathy'

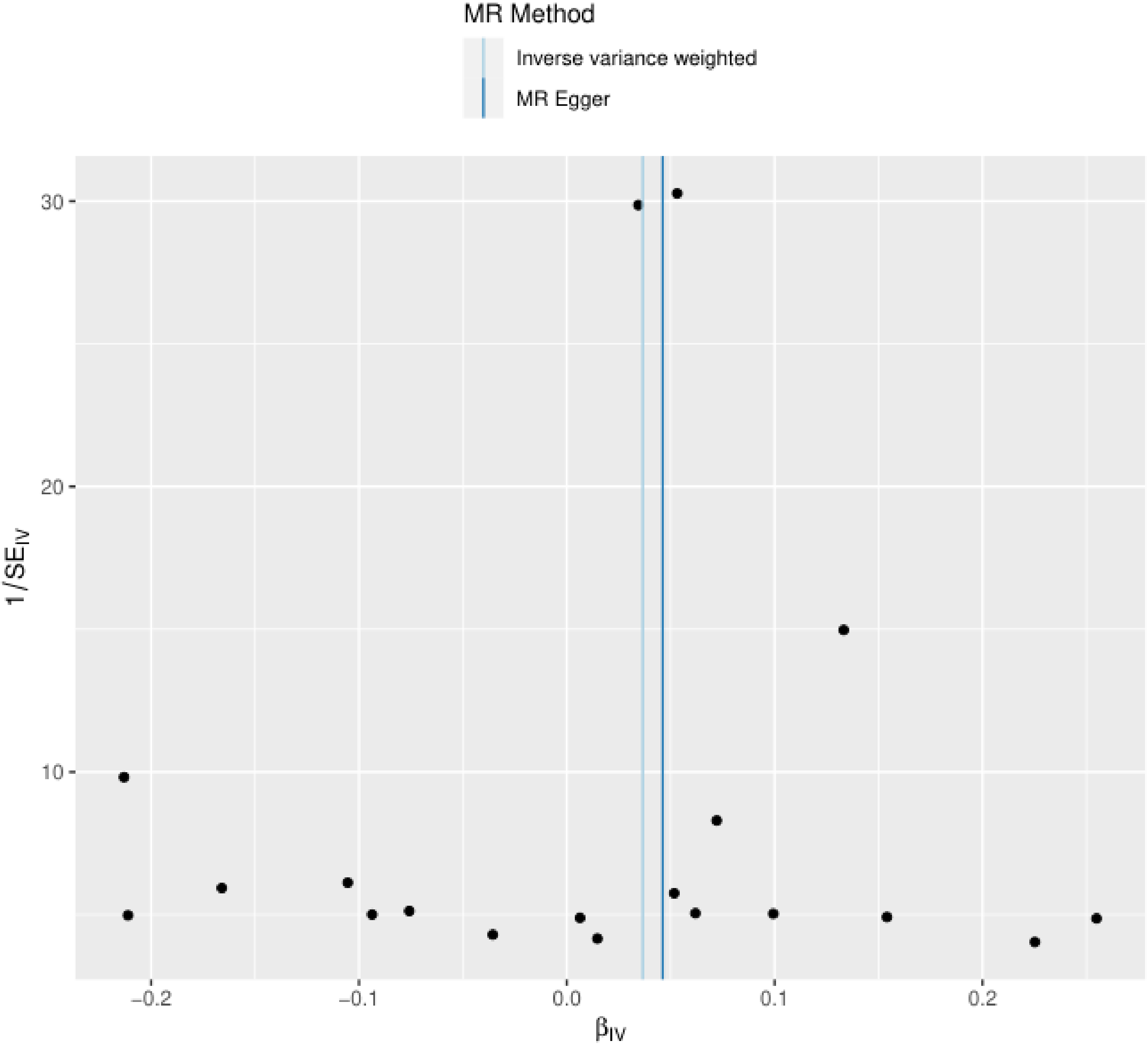

Funnel plot analyse of "CD28 on CD39+ CD8br " on 'Diabetic nephropathy'

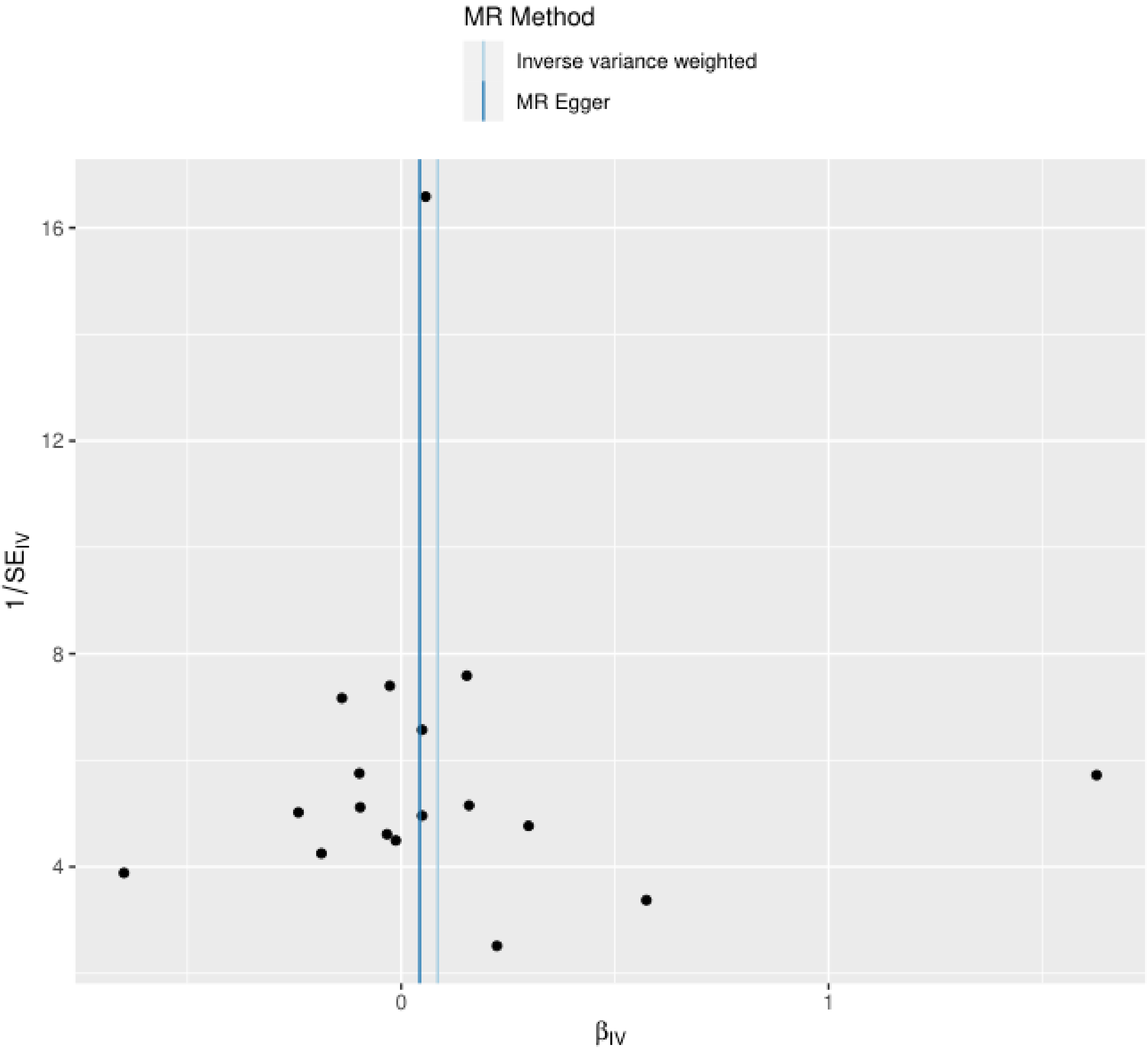

Funnel plot analyse of "CD8 on NKT" on 'Diabetic nephropathy'

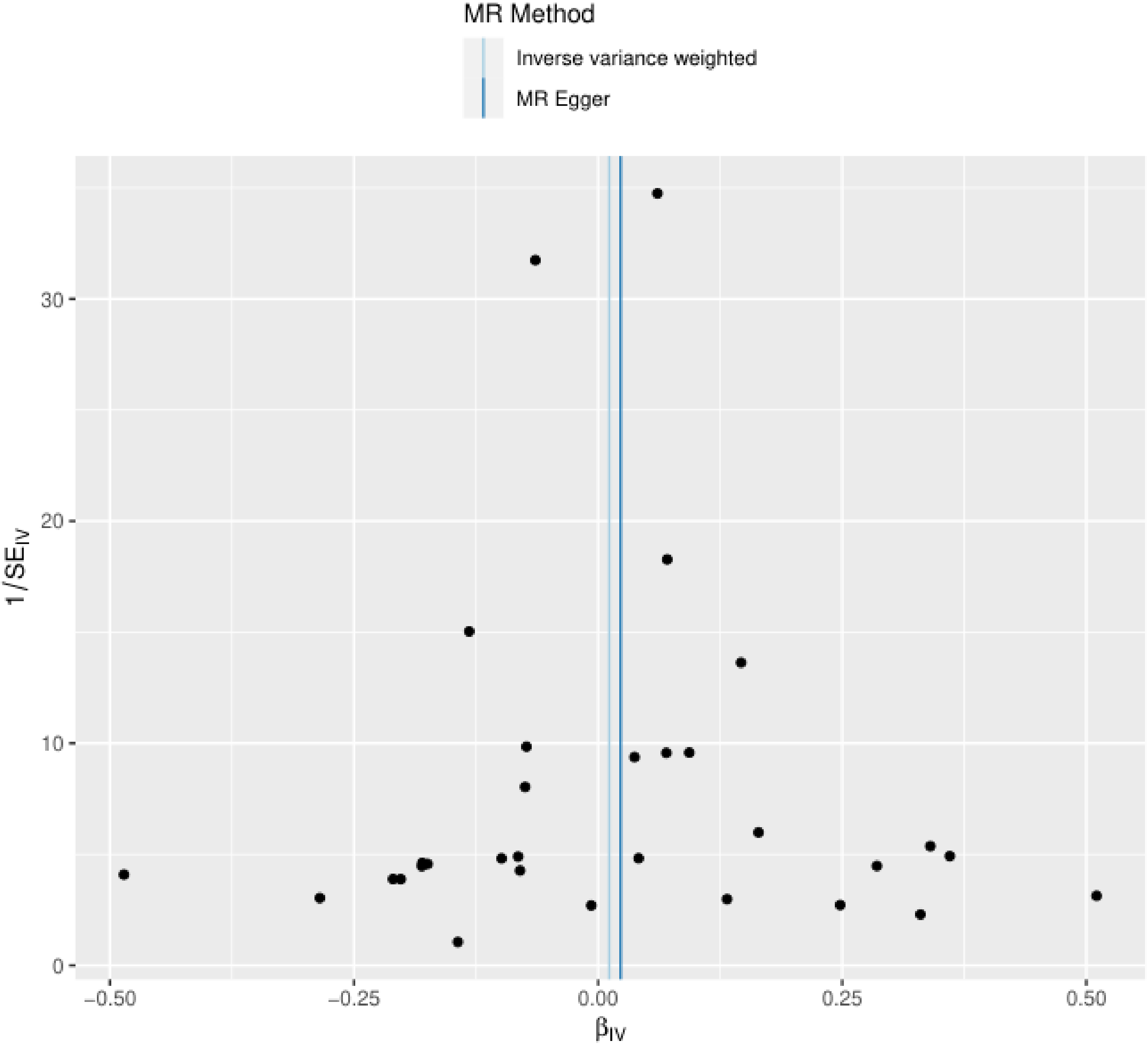

Funnel plot analyse of "CD39+ CD4+ %T cell" on 'Diabetic nephropathy'

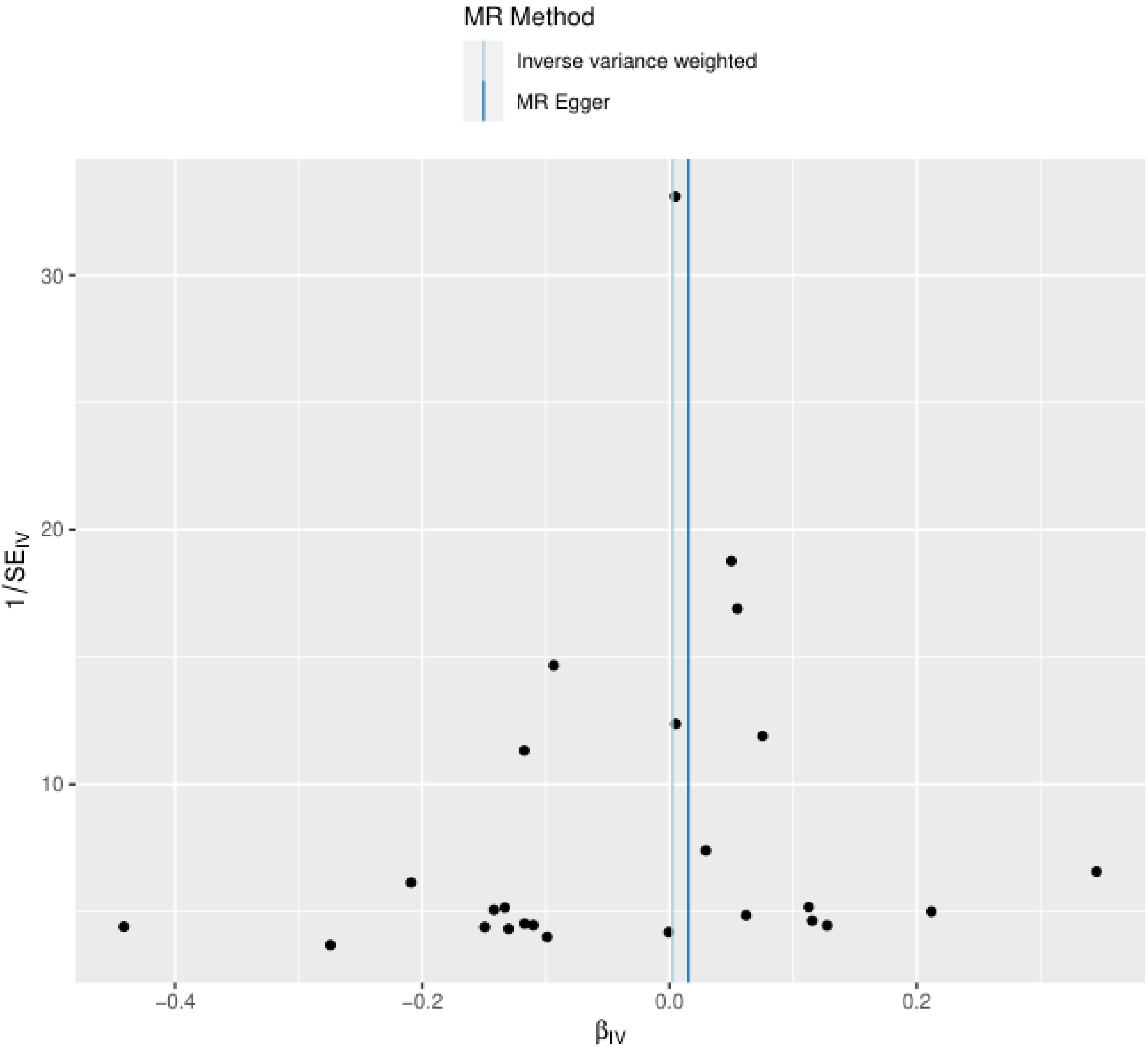

Funnel plot analyse of "Secreting Treg %CD4" on 'Diabetic nephropathy'

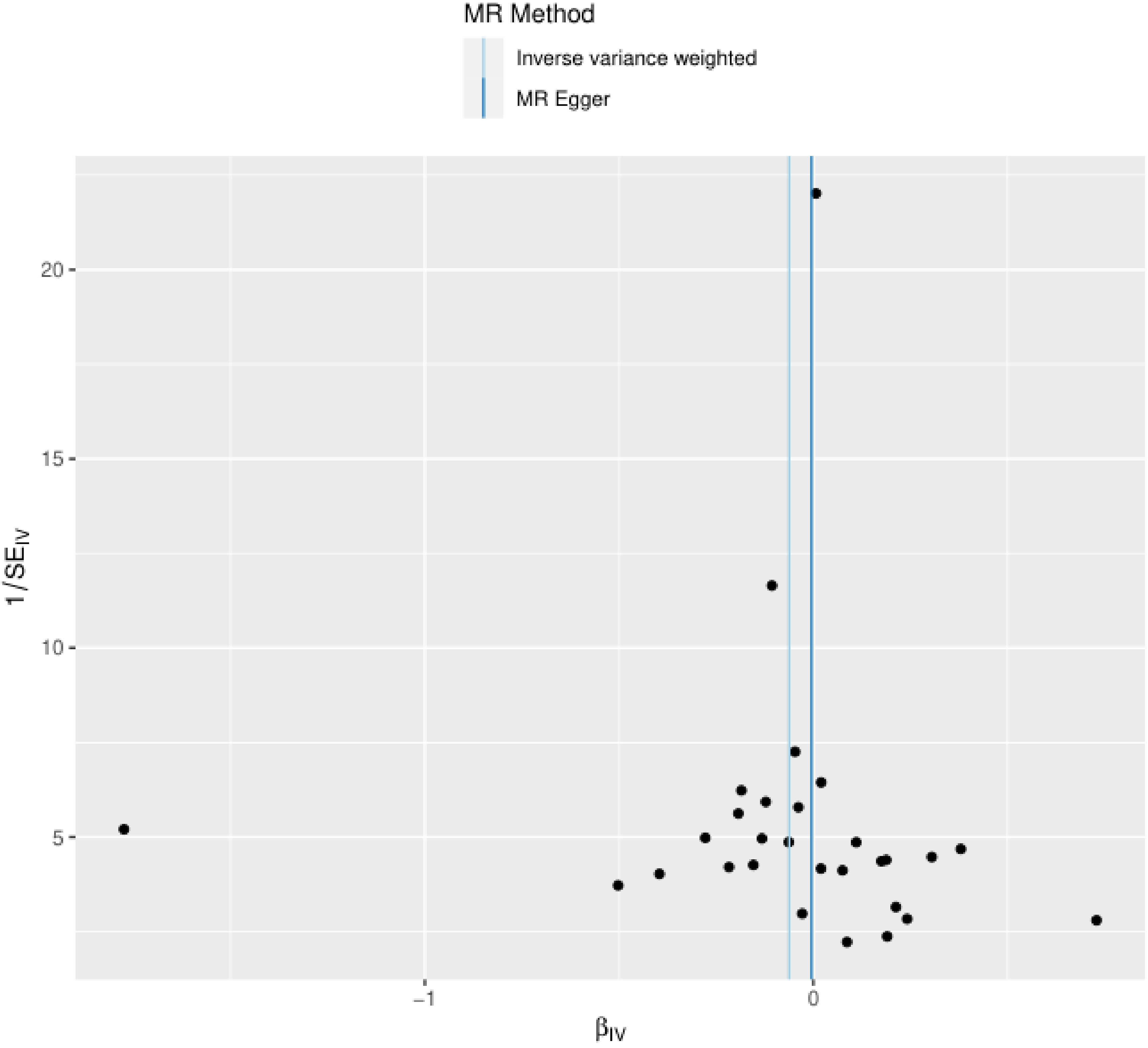

Funnel plot analyse of "CD45RA- CD4+ %CD4+" on 'Diabetic nephropathy'

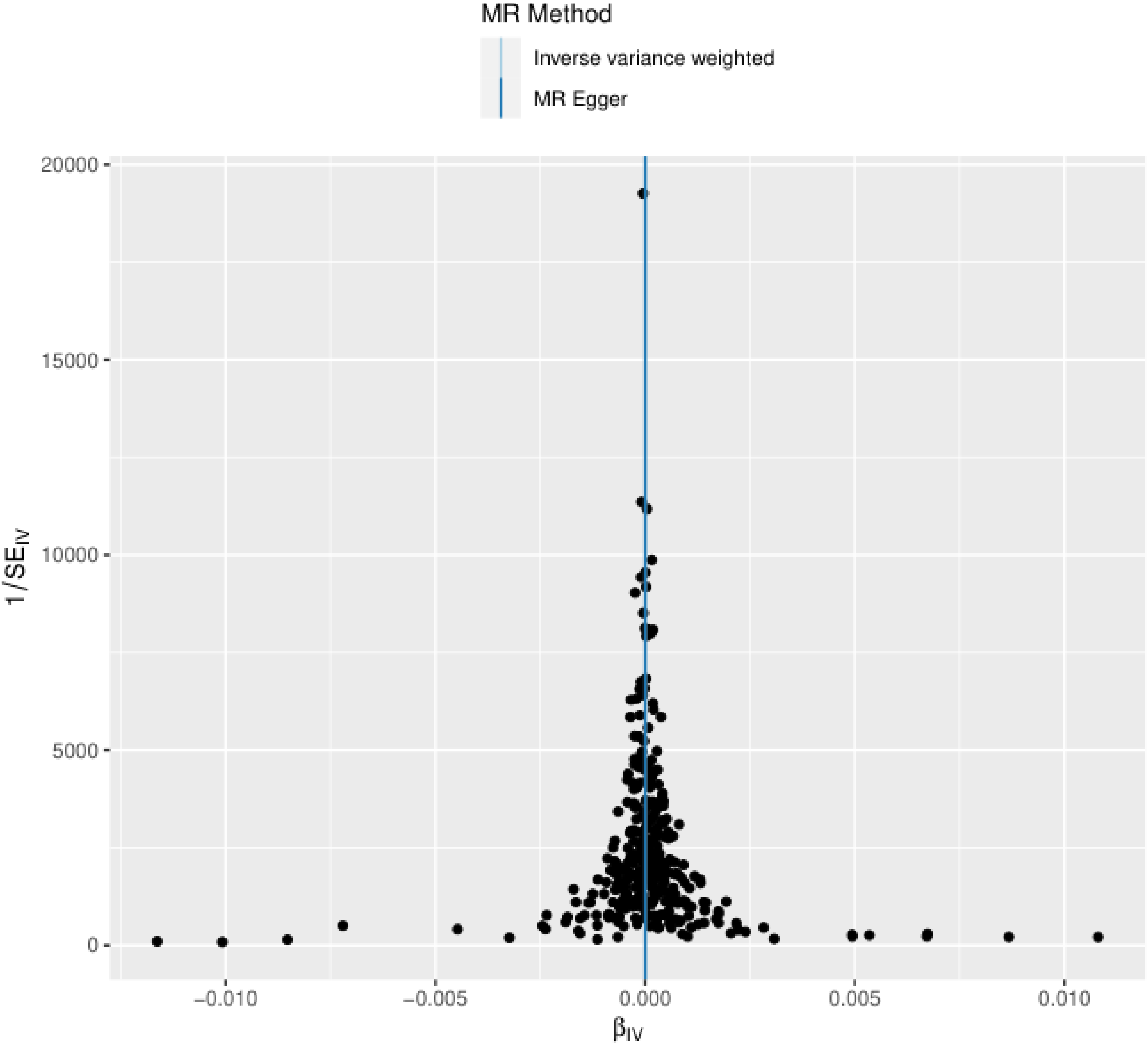

Funnel plot analyse of "CD45RA- CD28- CD8br AC" on 'Diabetic nephropathy'

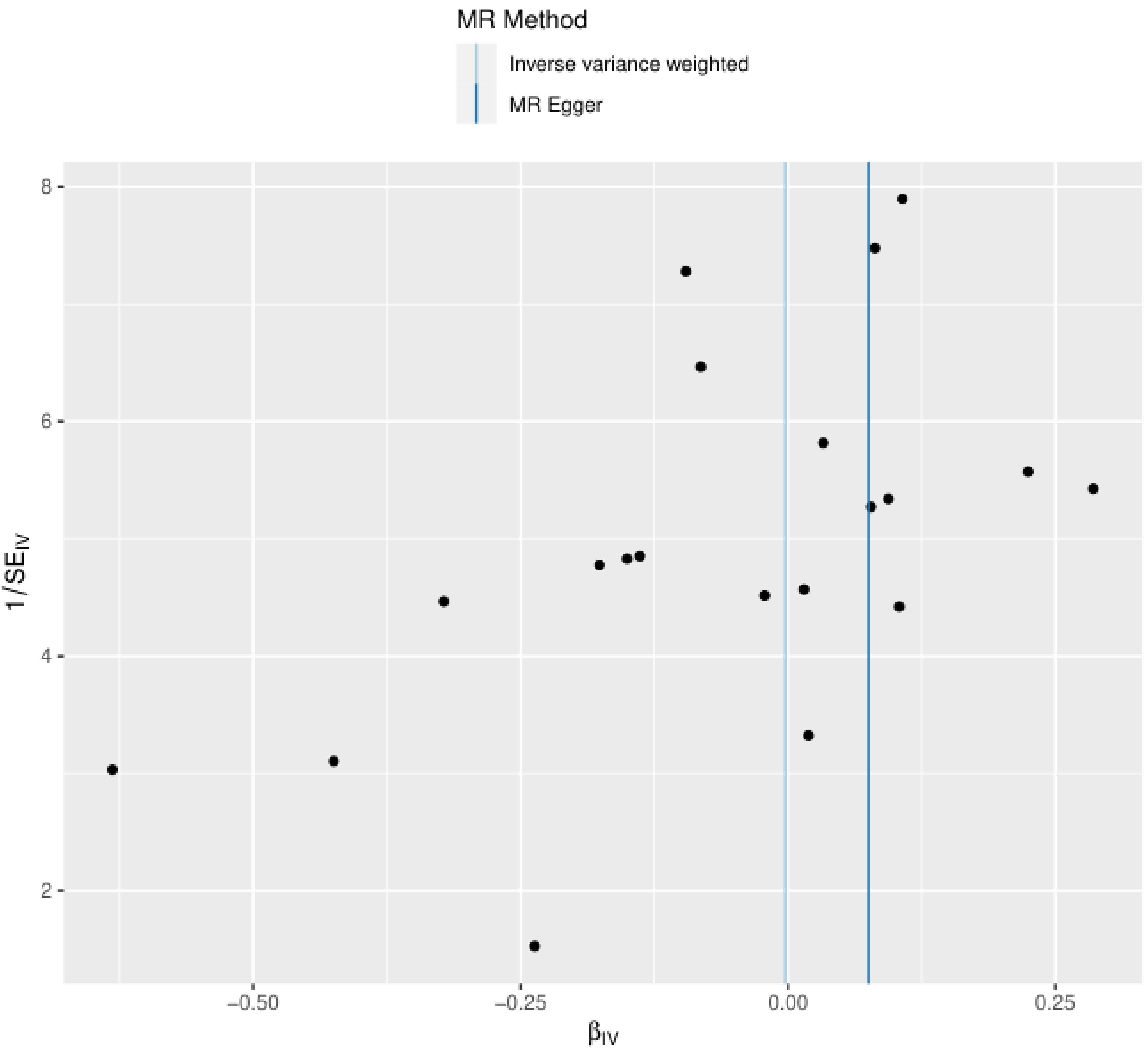

Funnel plot analyse of "HLA DR on HLA DR+ CD8br" on 'Diabetic nephropathy'

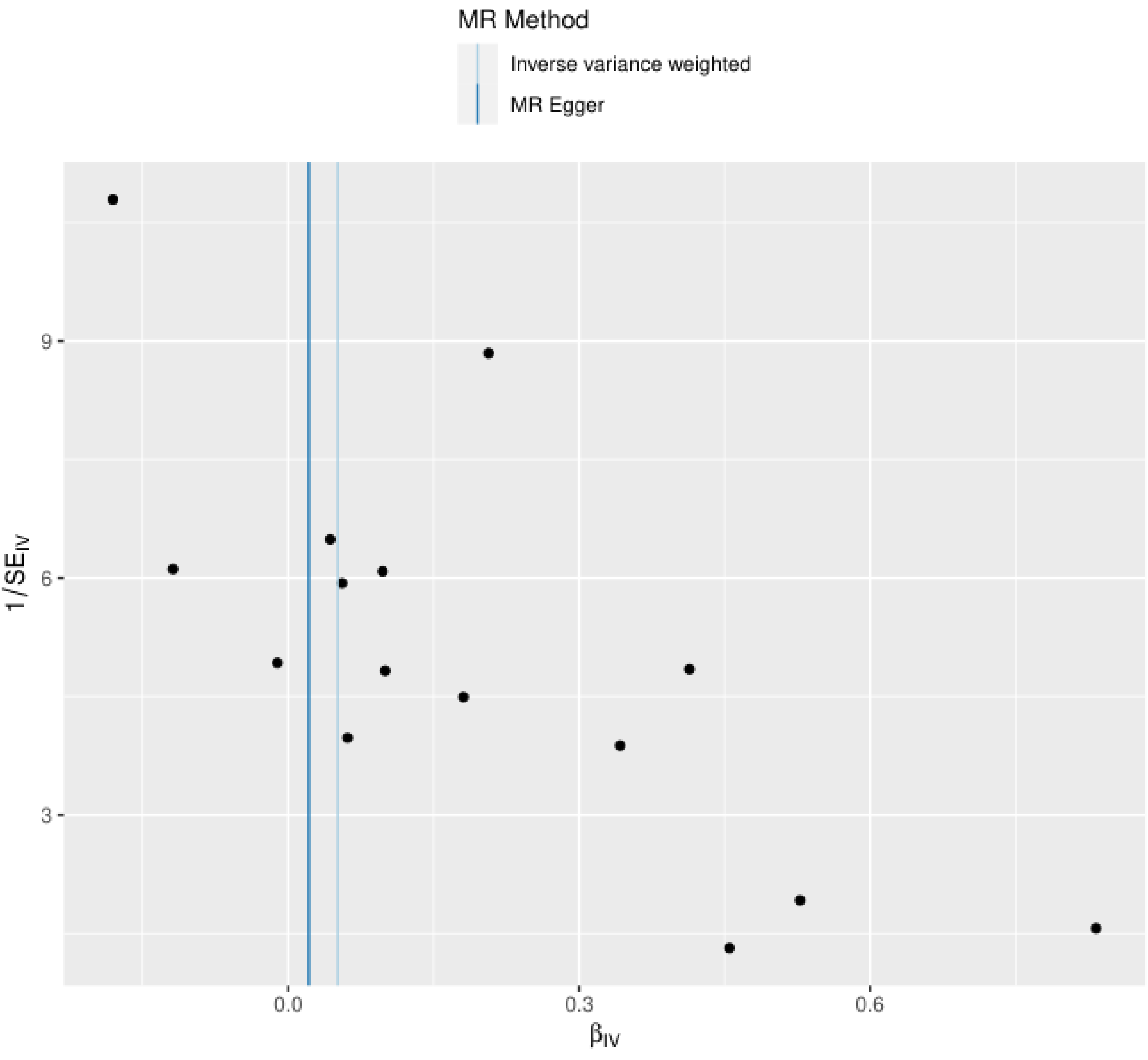

Funnel plot analyse of "PDL-1 on monocyte" on 'Diabetic nephropathy'

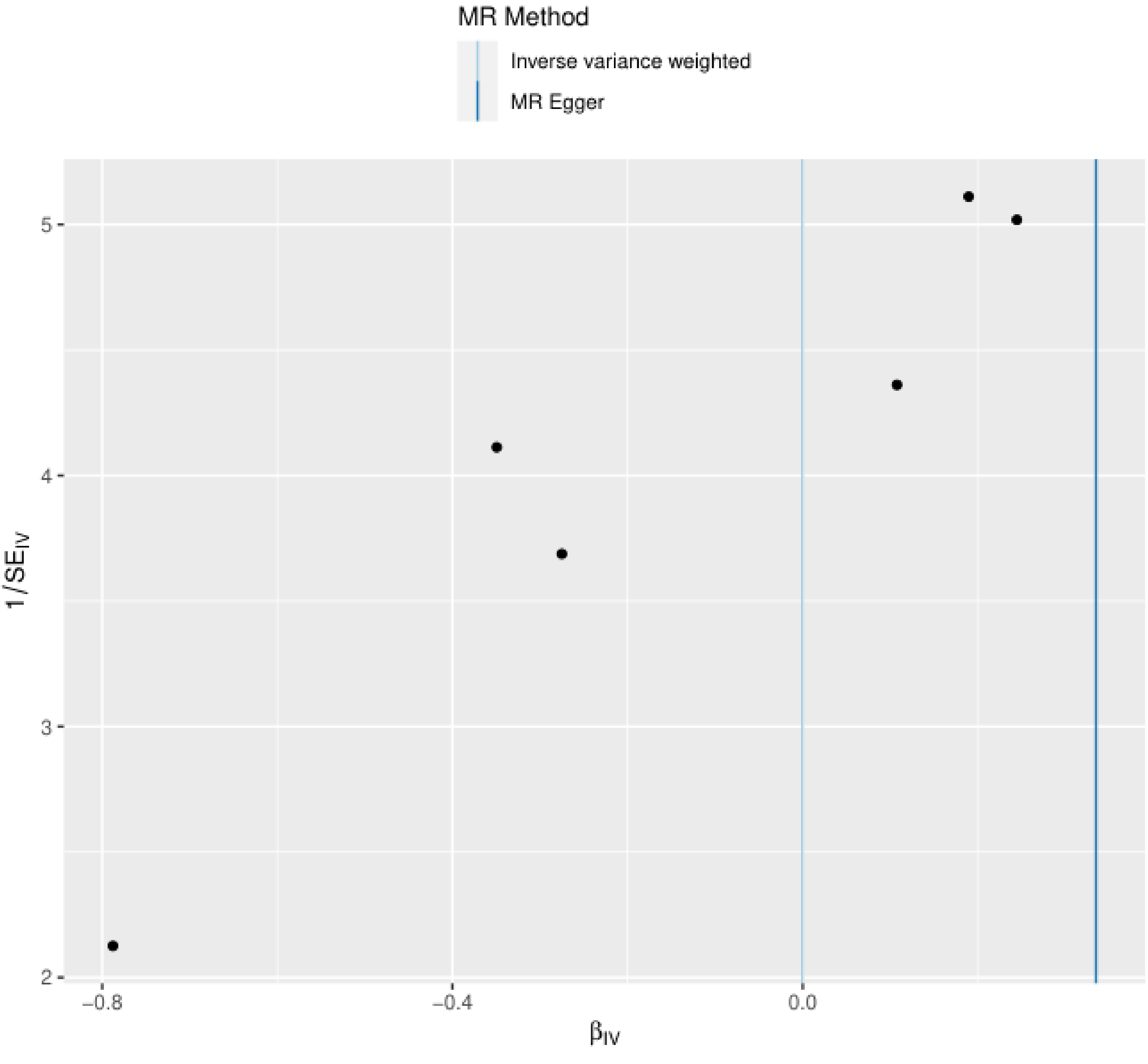

Funnel plot analyse of "CD3- lymphocyte %lymphocyte" on 'Diabetic nephropathy'

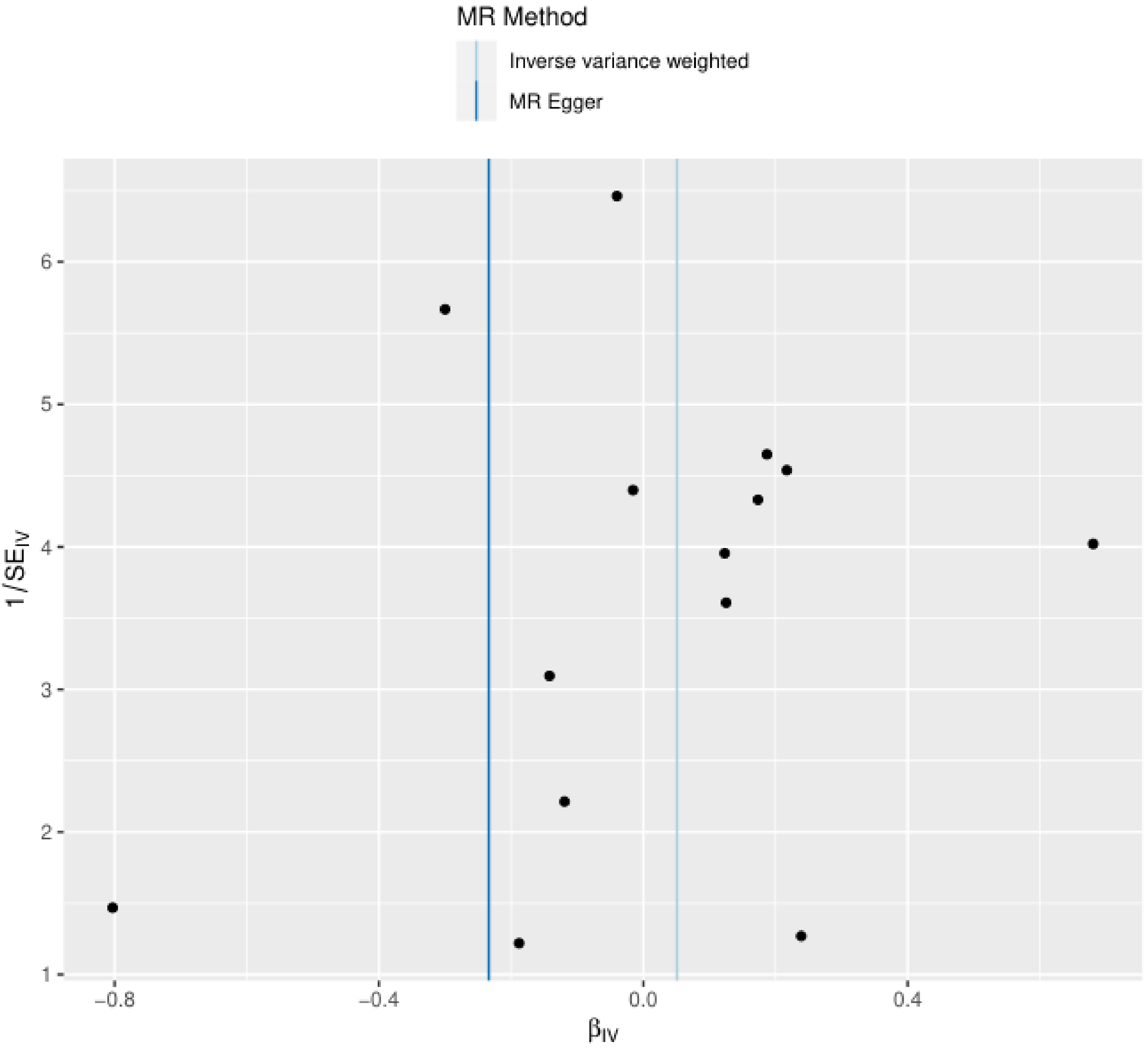

Funnel plot analyse of "CD38 on IgD+" on 'Diabetic nephropathy'

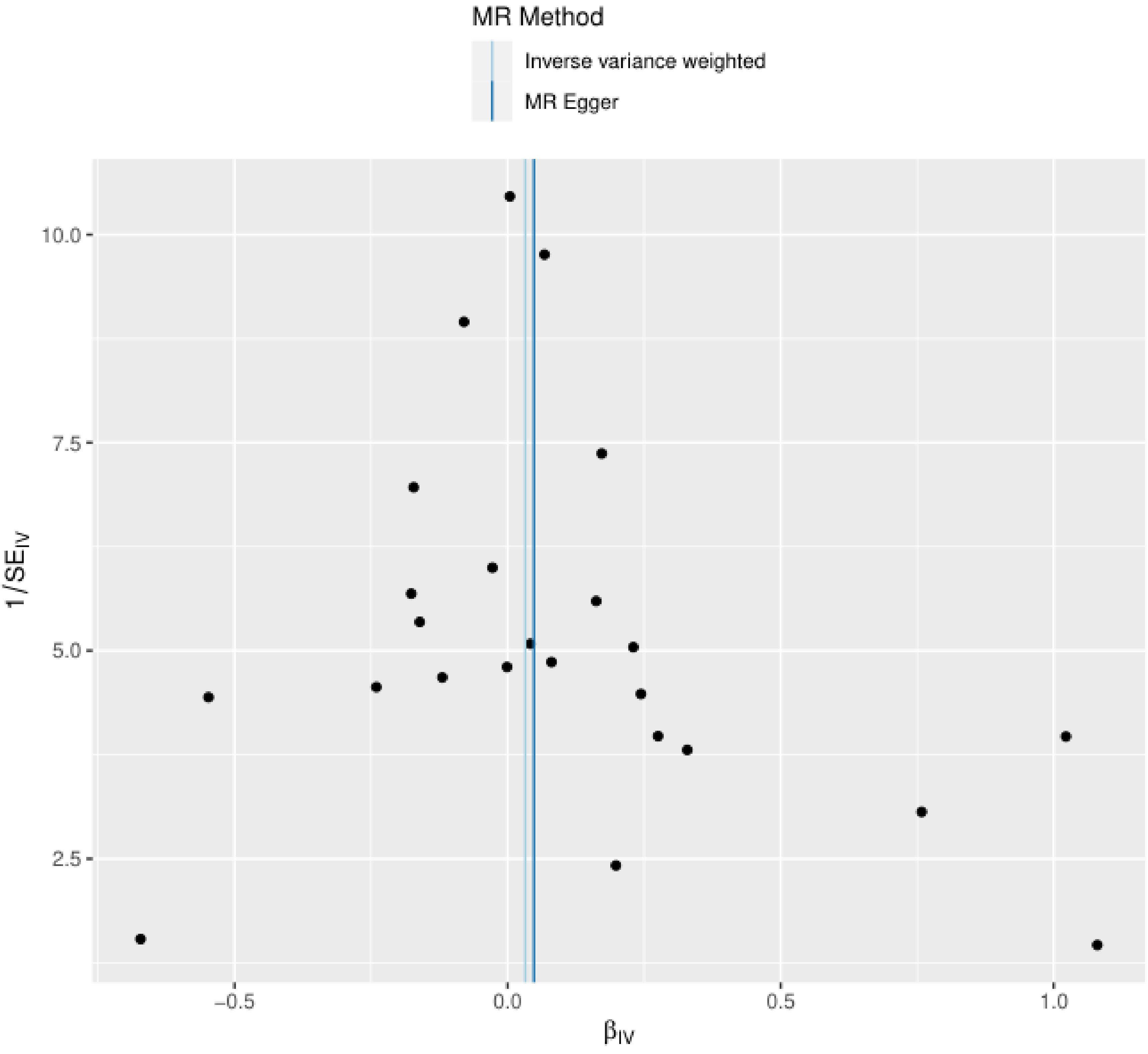

Funnel plot analyse of "CD8 on CD39+ CD8br " on 'Diabetic nephropathy'

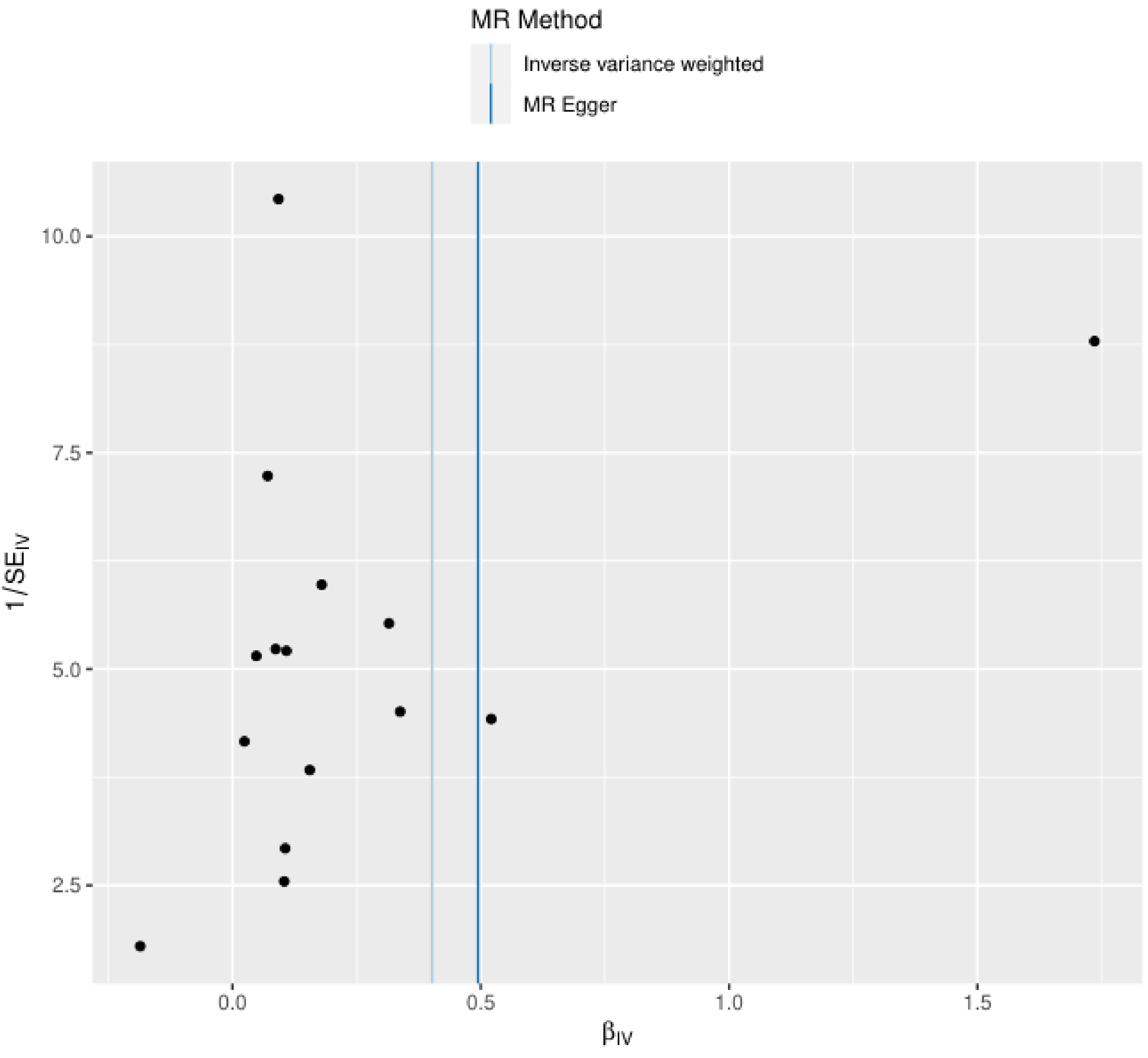

Funnel plot analyse of "CD64 on CD14- CD16- " on 'Diabetic nephropathy'

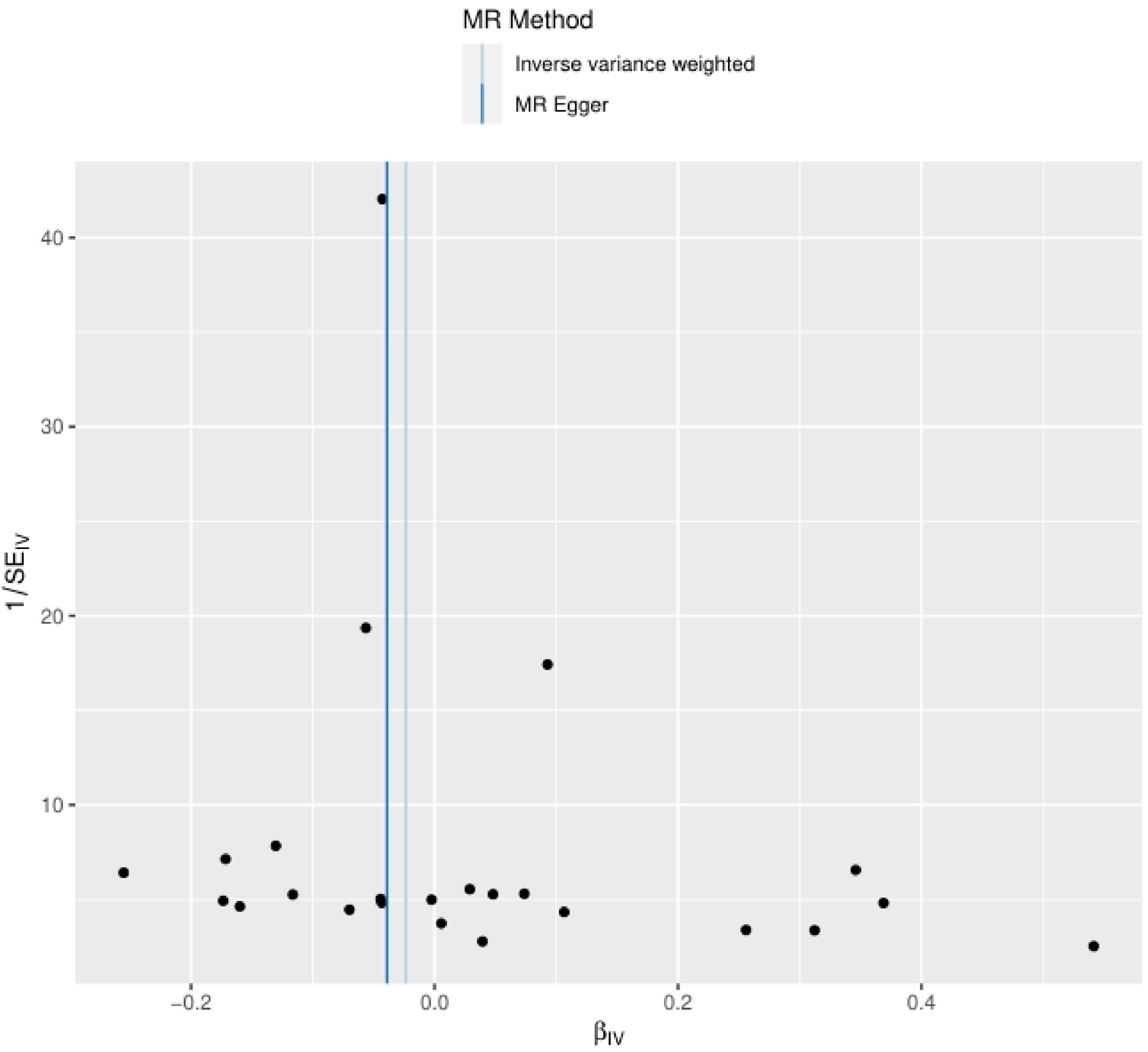

Funnel plot analyse of "CD27 on memory B cell" on 'Diabetic nephropathy'

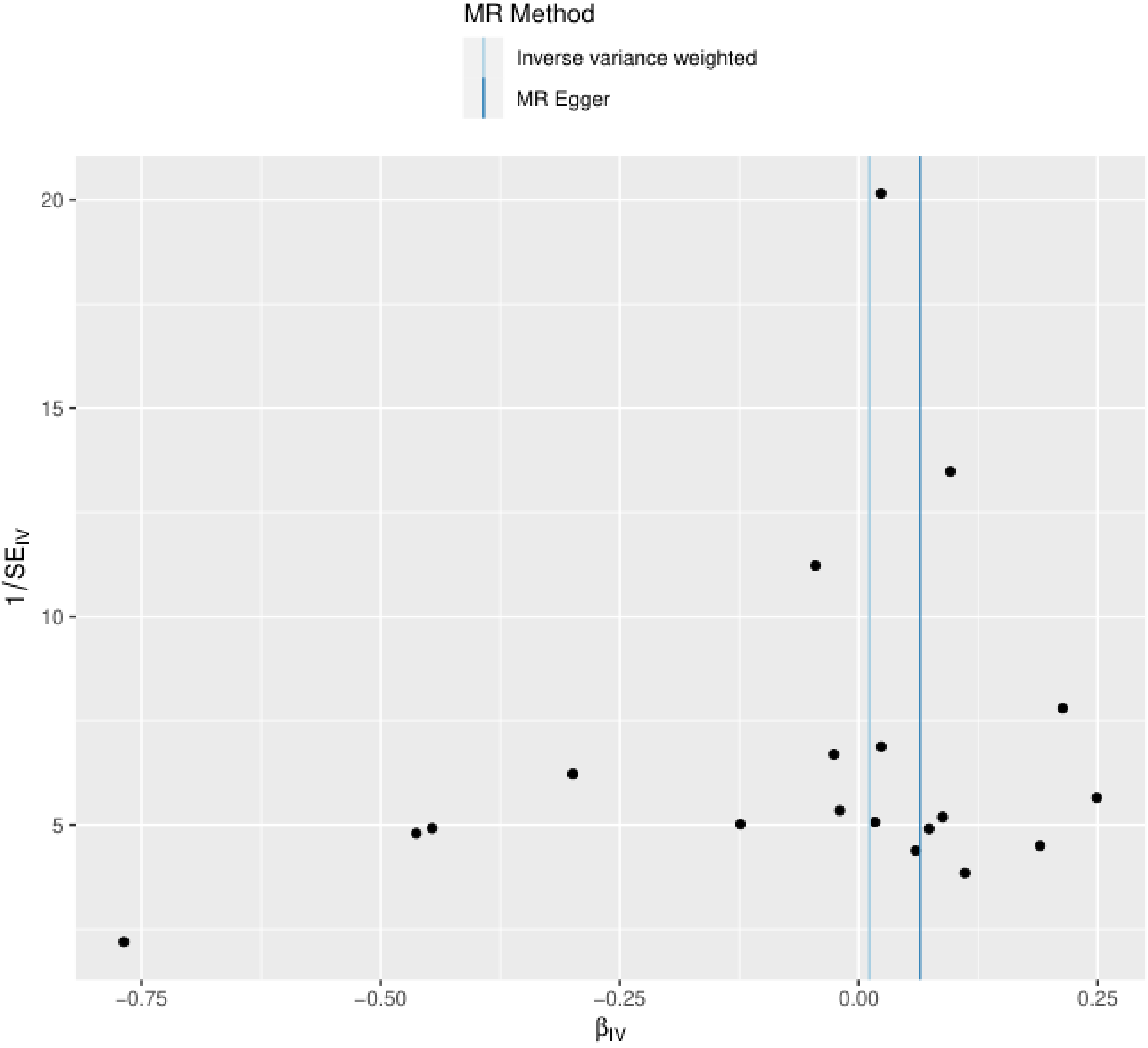

Funnel plot analyse of "CD3 on CD39+ resting Treg " on 'Diabetic nephropathy'

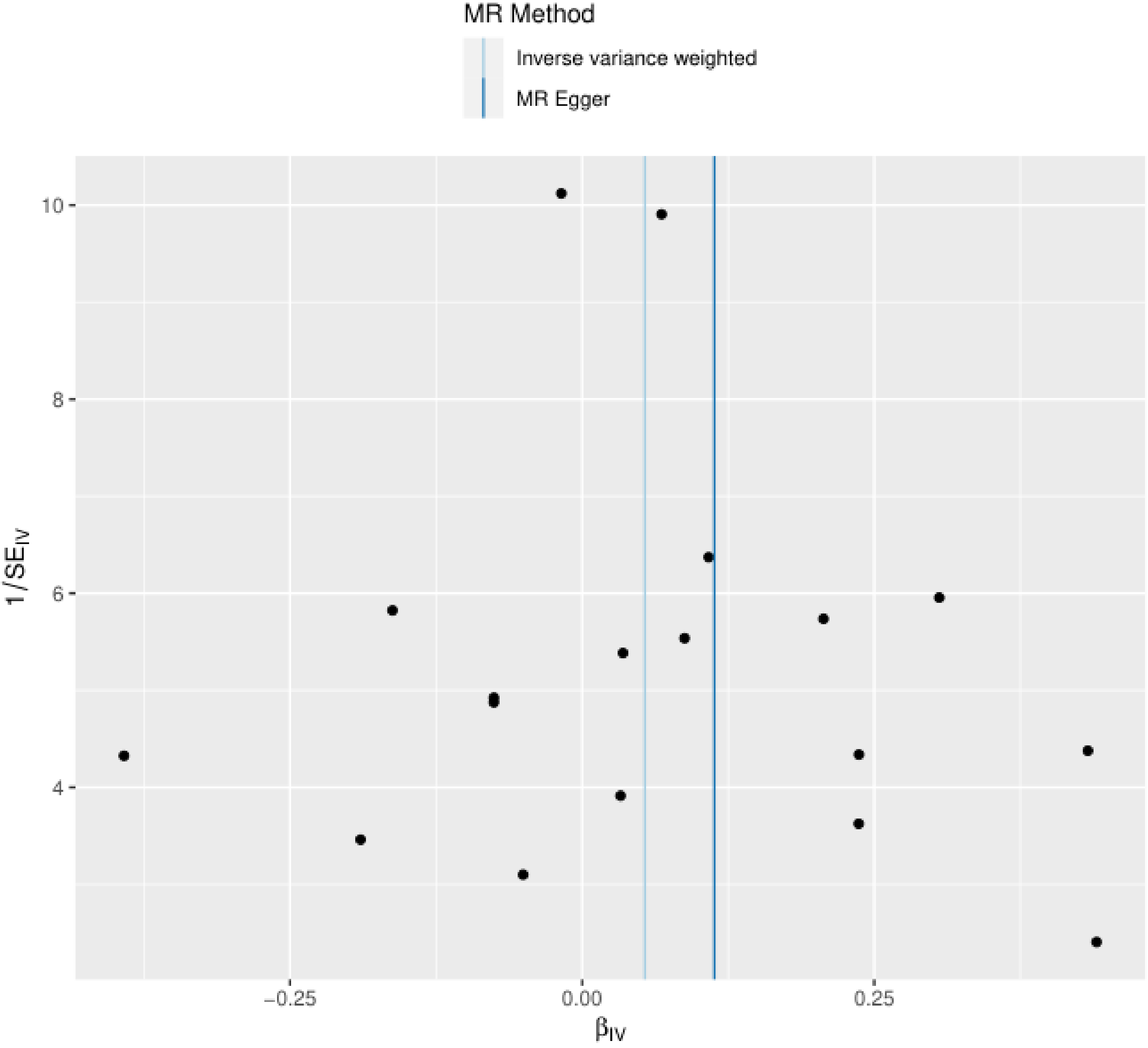

Funnel plot analyse of "CD11c+ HLA DR++ monocyte AC" on 'Diabetic nephropathy'

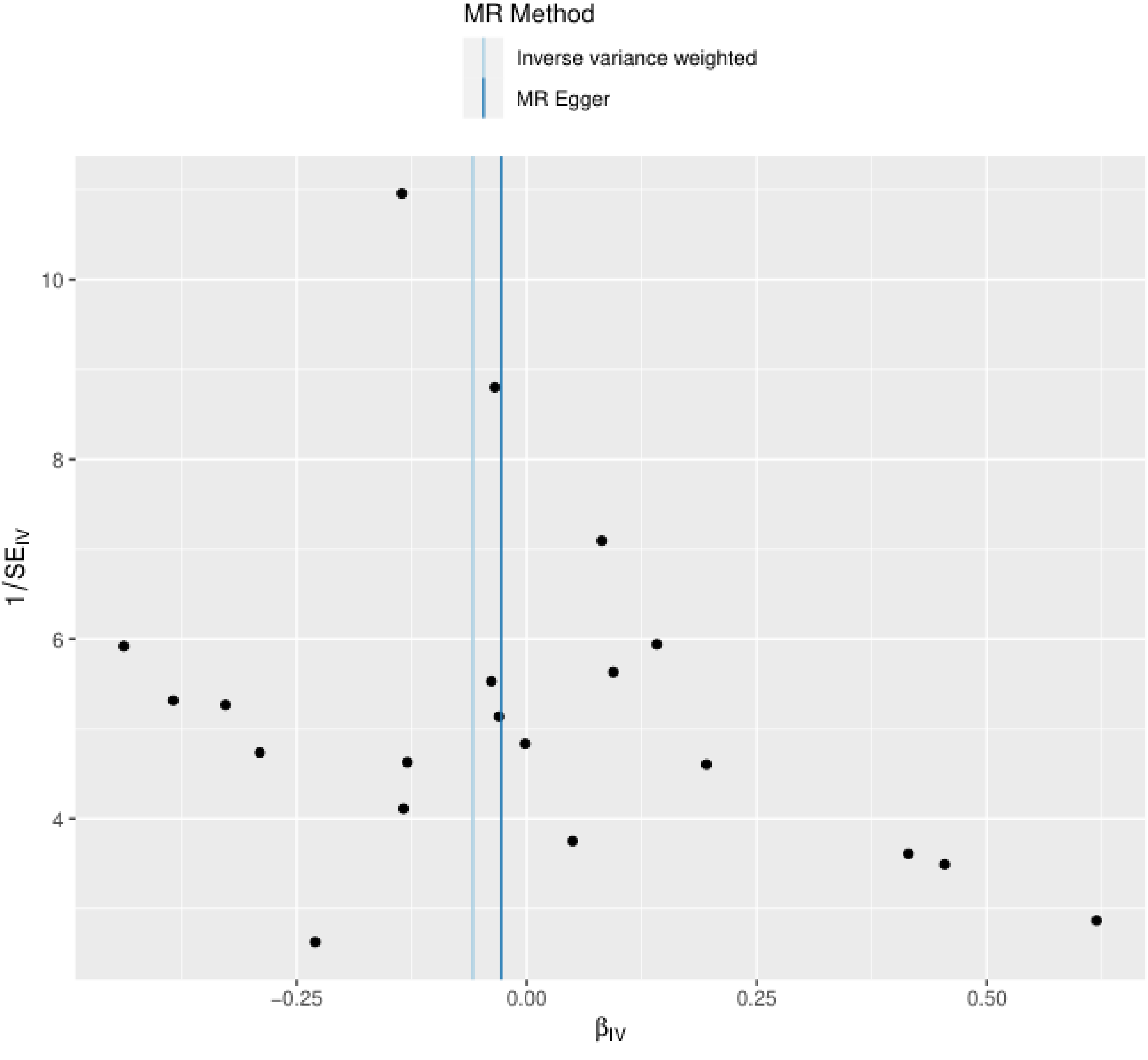

Funnel plot analyse of "CD86 on granulocyte" on 'Diabetic nephropathy'

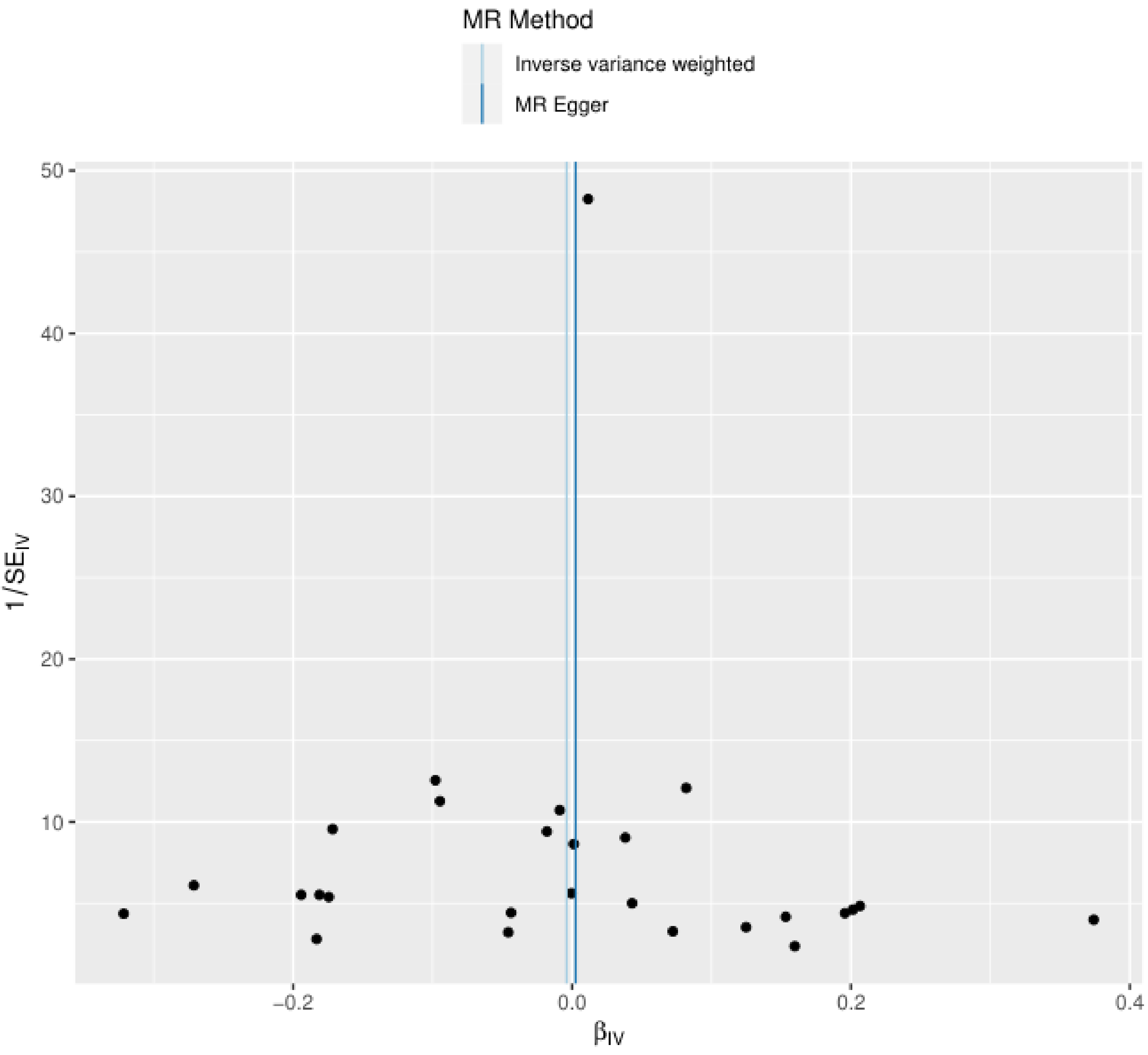

Funnel plot analyse of "CD25 on IgD+ CD24+" on 'Diabetic nephropathy'

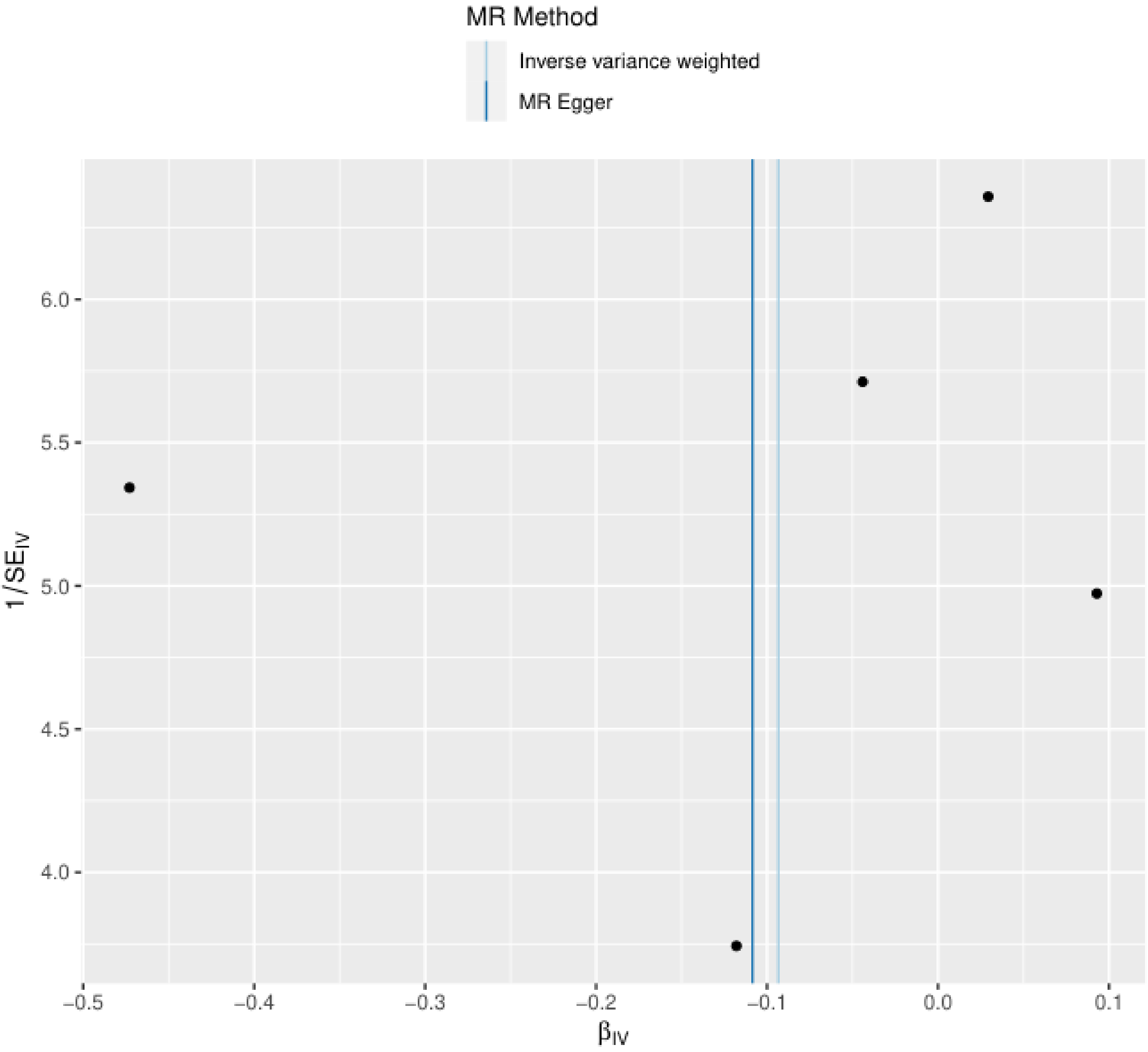

Funnel plot analyse of "CD25 on CD28+ CD4+ " on 'Diabetic nephropathy'

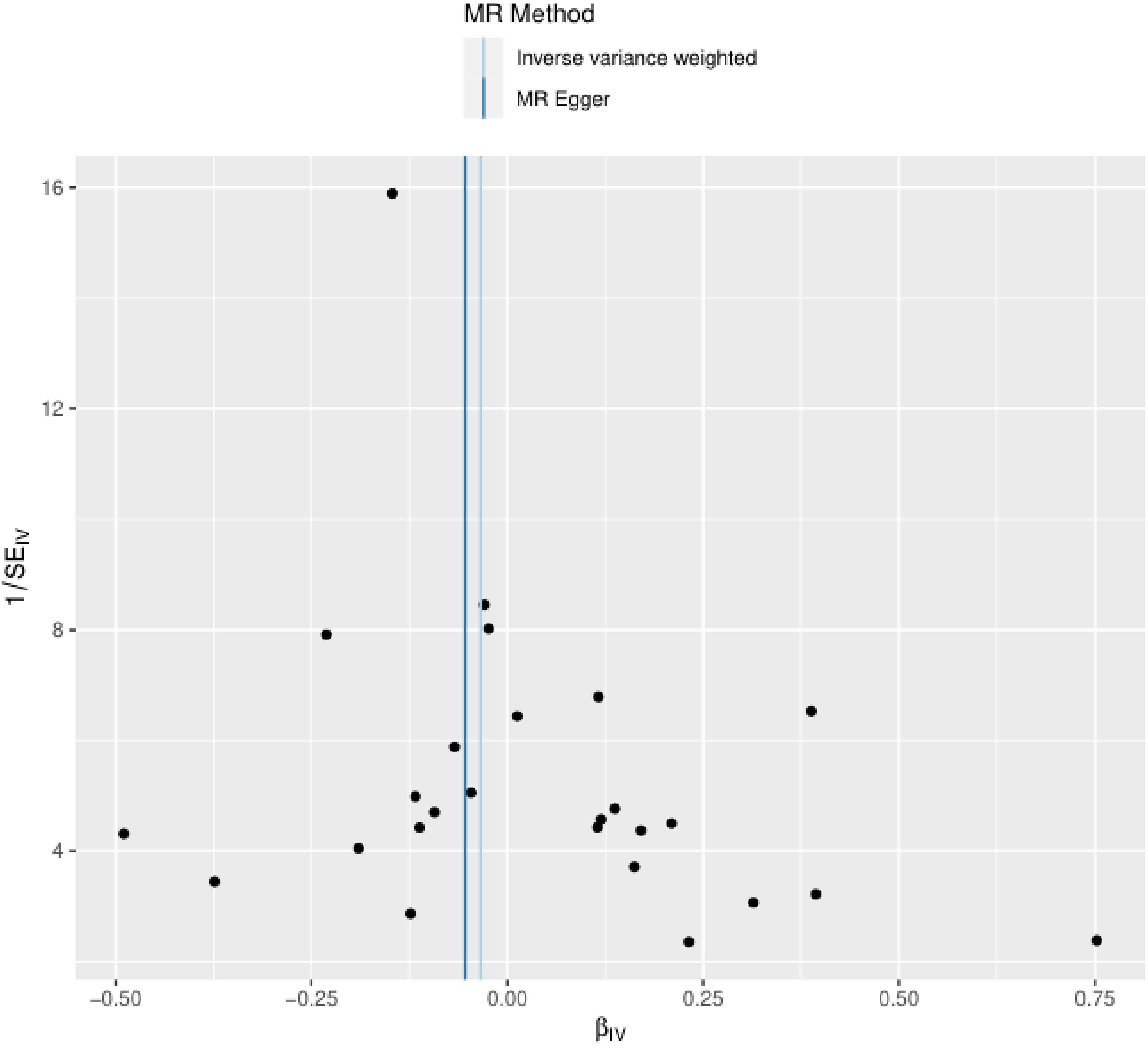

Funnel plot analyse of "CX3CR1 on monocyte" on 'Diabetic nephropathy'

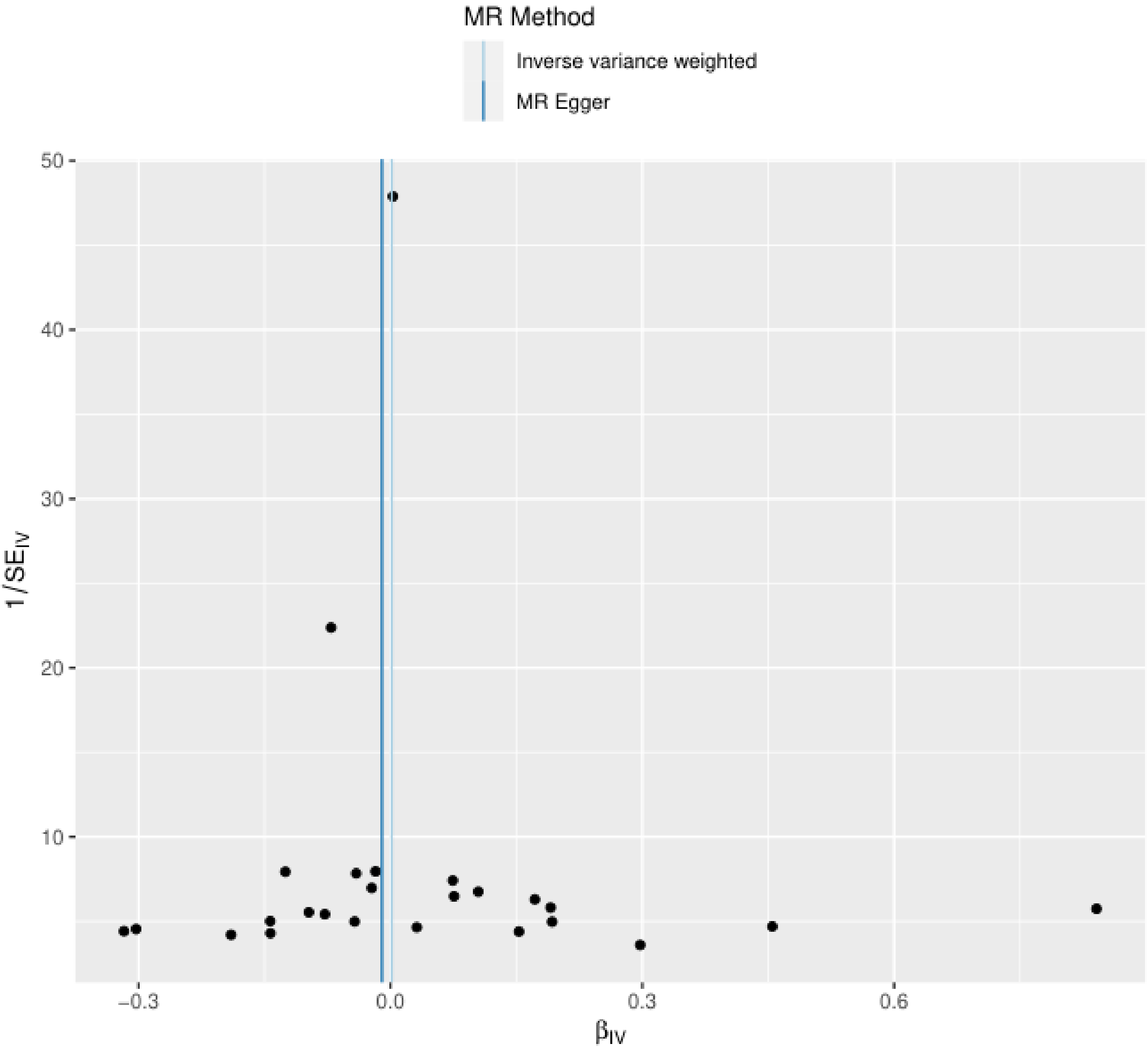

Funnel plot analyse of "CD20 on IgD+ CD24-" on 'Diabetic nephropathy'

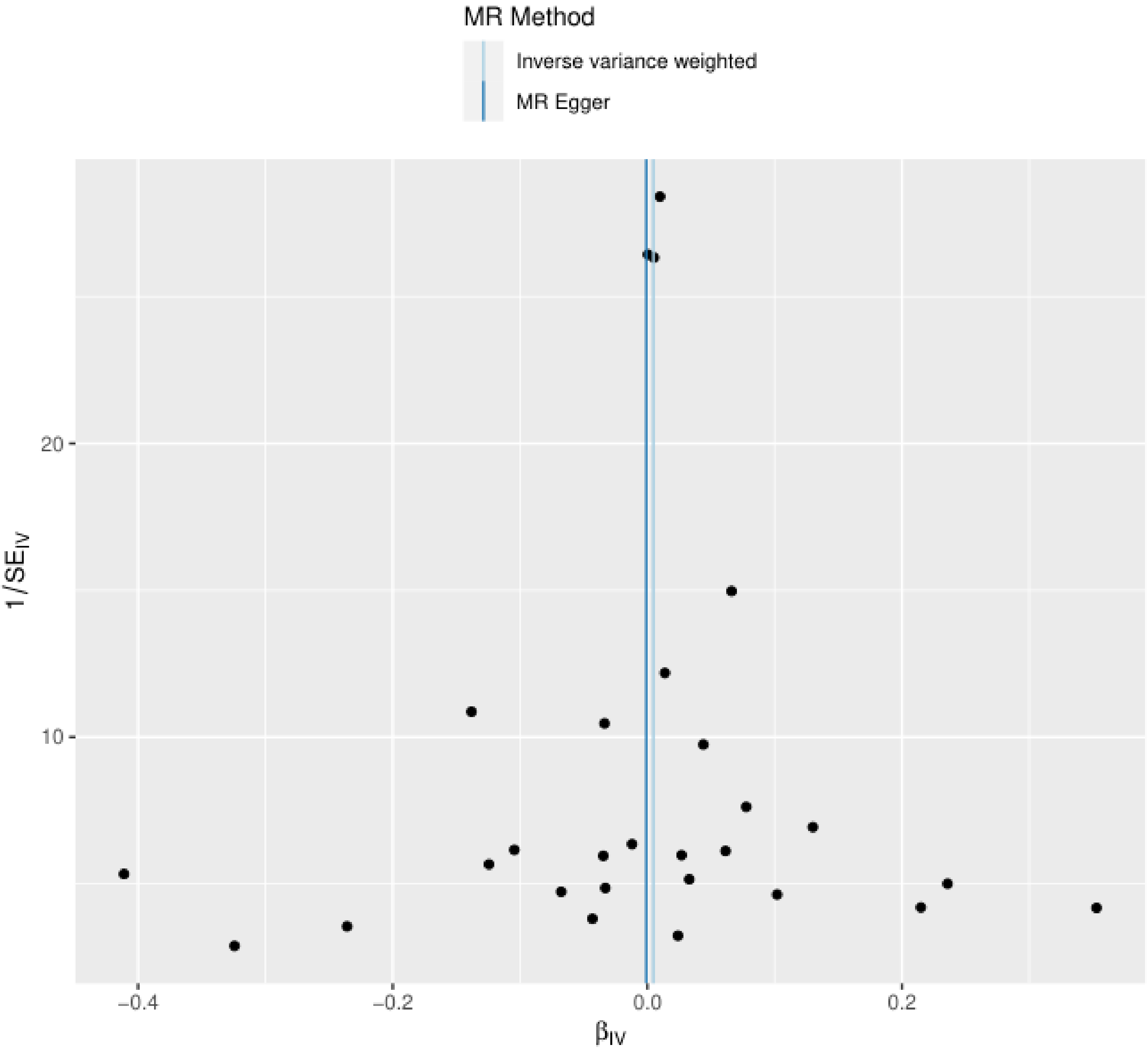

Funnel plot analyse of "CD33br HLA DR+ CD14dim AC" on 'Diabetic nephropathy'

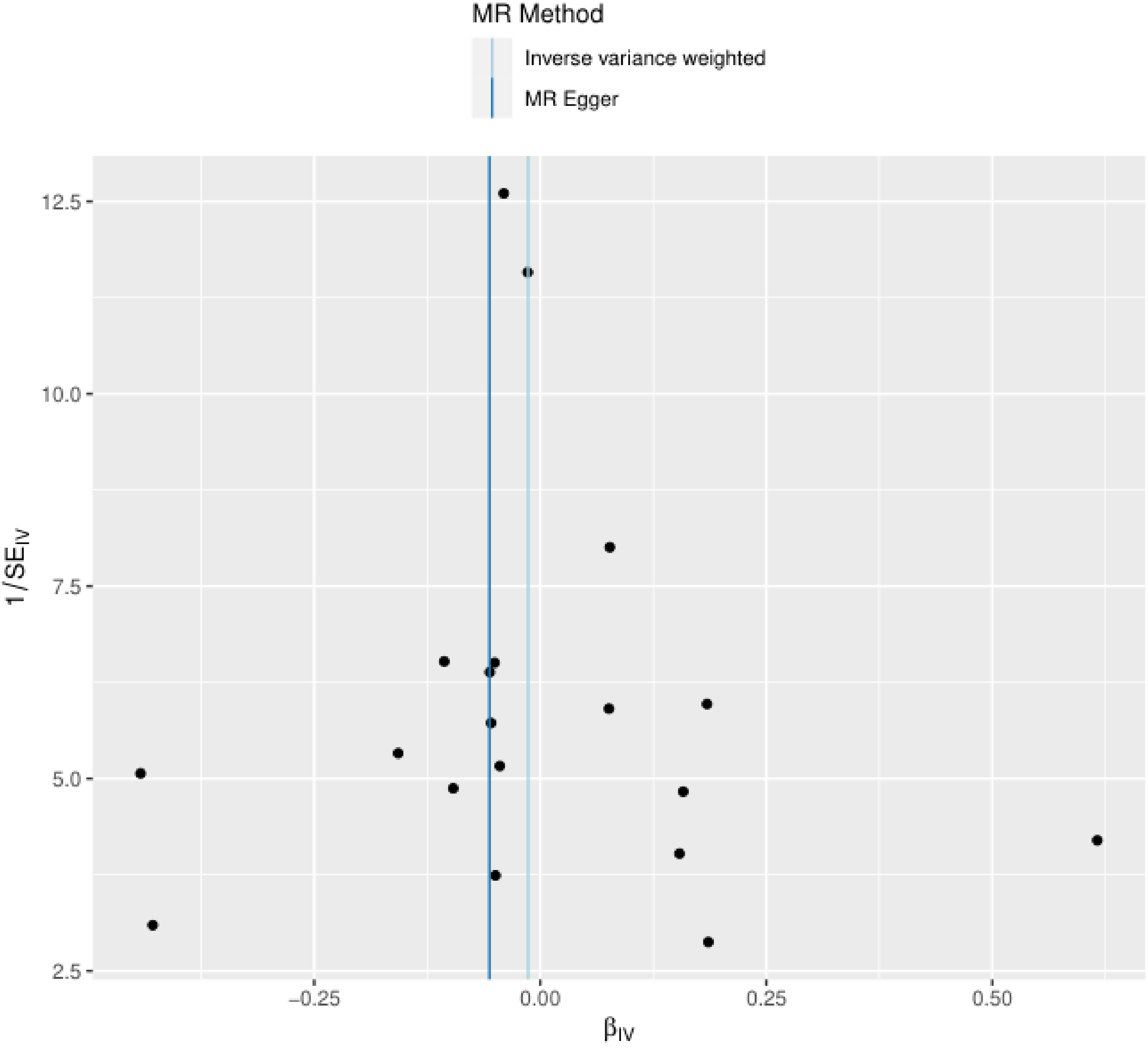

Funnel plot analyse of "CD3 on CD28+ CD45RA- CD8br " on 'Diabetic nephropathy'

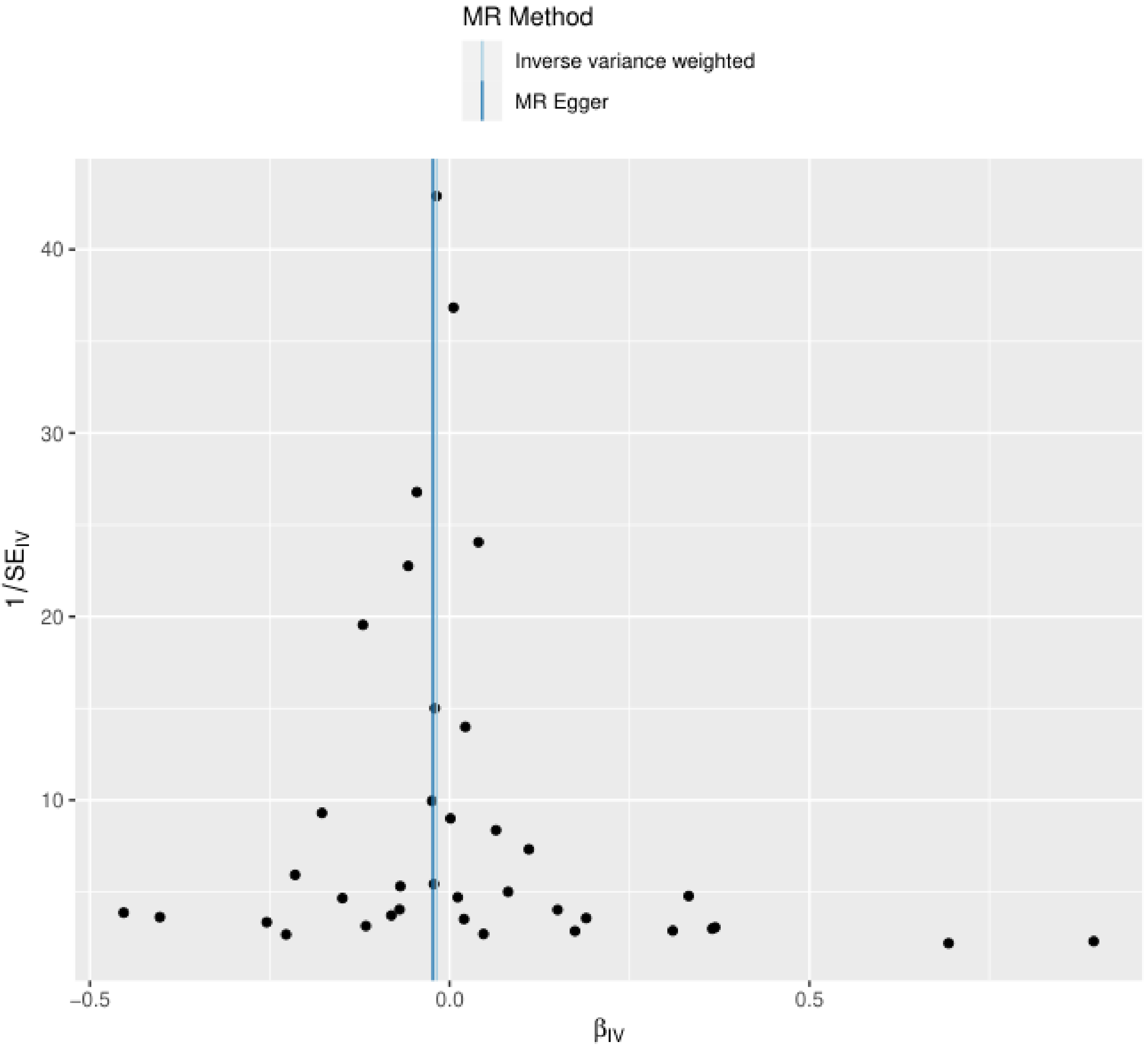

Funnel plot analyse of "Naive CD8br %CD8br" on 'Diabetic nephropathy'

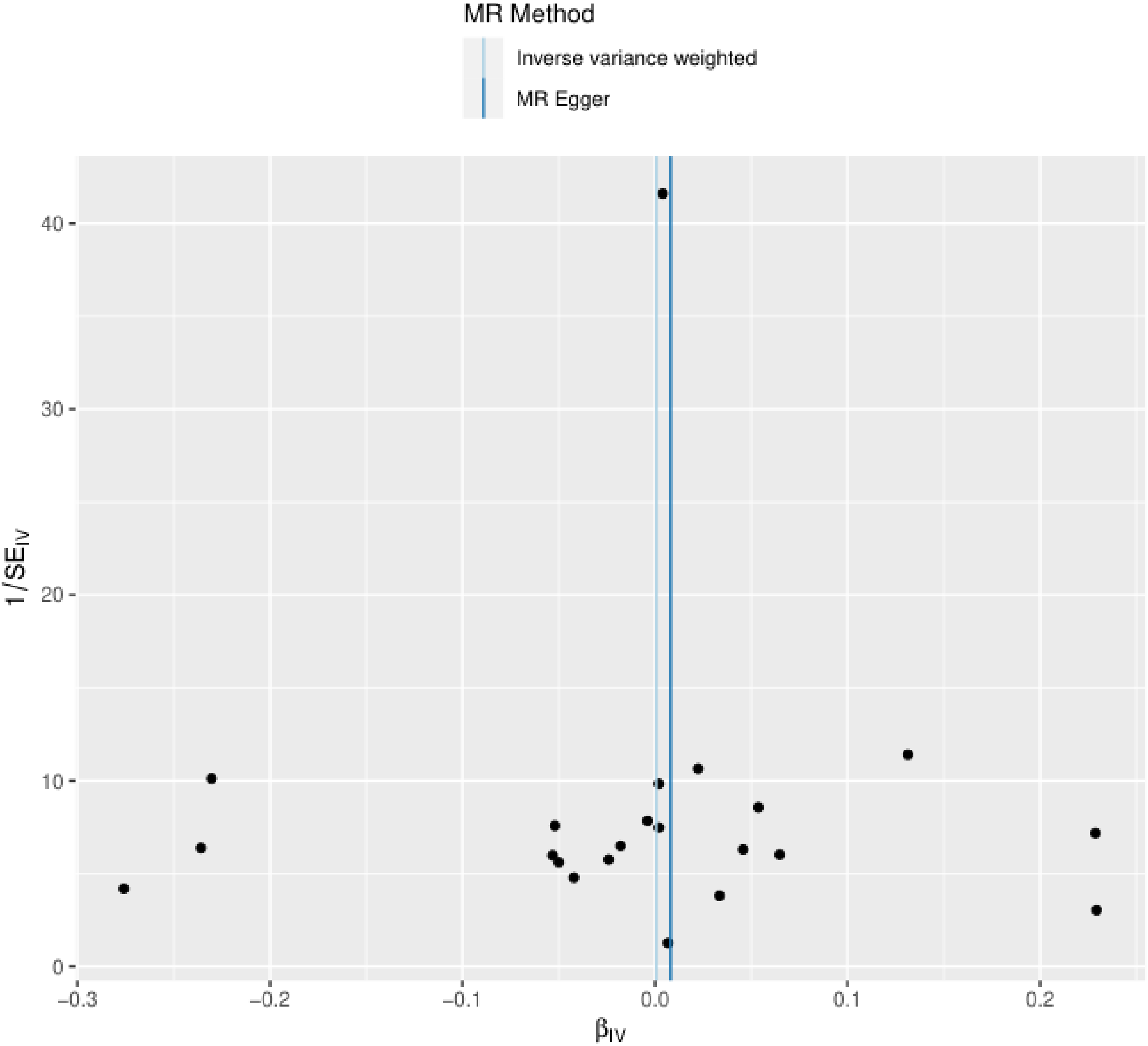

Funnel plot analyse of "CD33 on CD33dim HLA DR+ CD11b- " on 'Diabetic nephropathy'

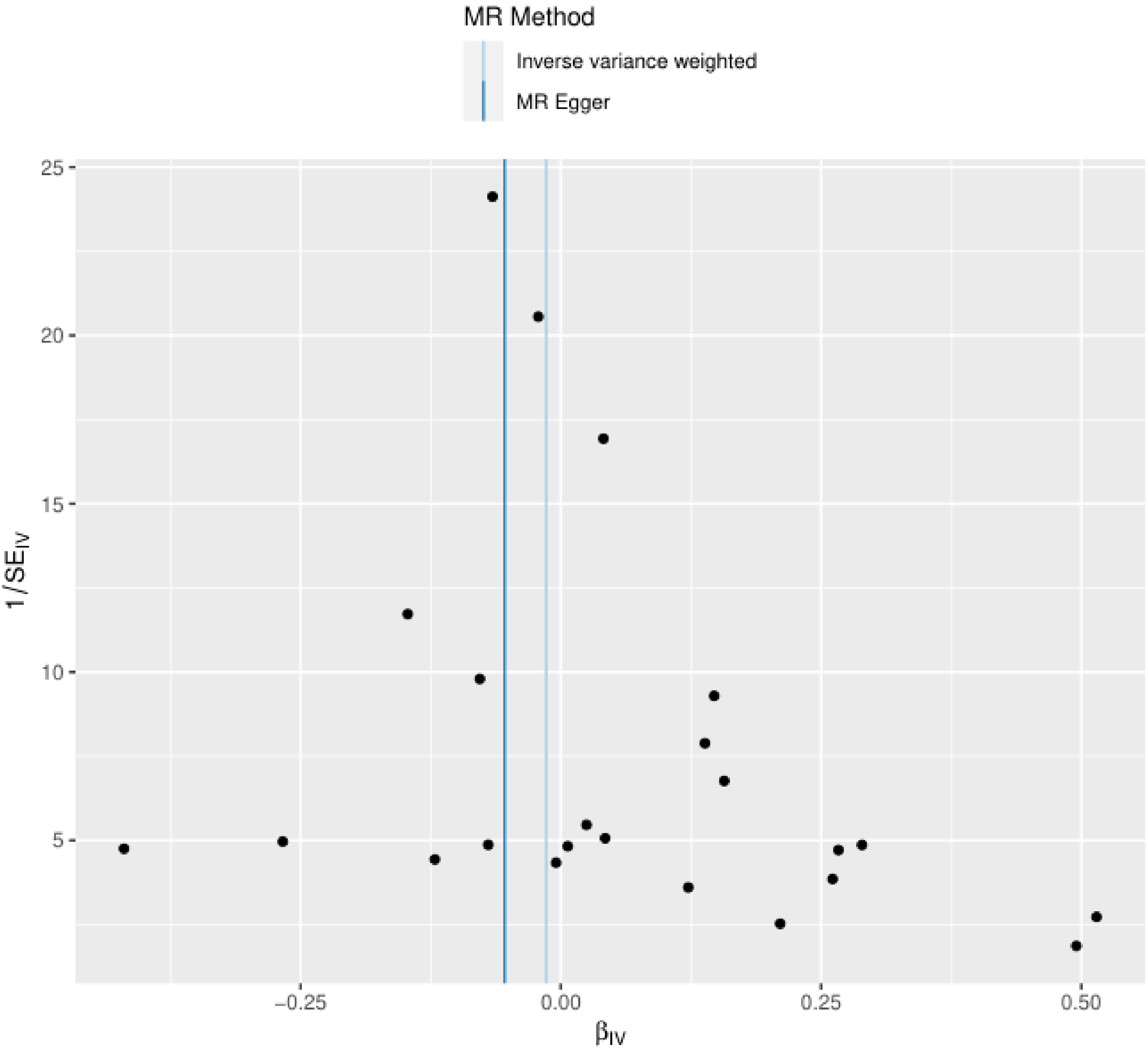

Funnel plot analyse of "CD25 on IgD+ CD38dim" on 'Diabetic nephropathy'

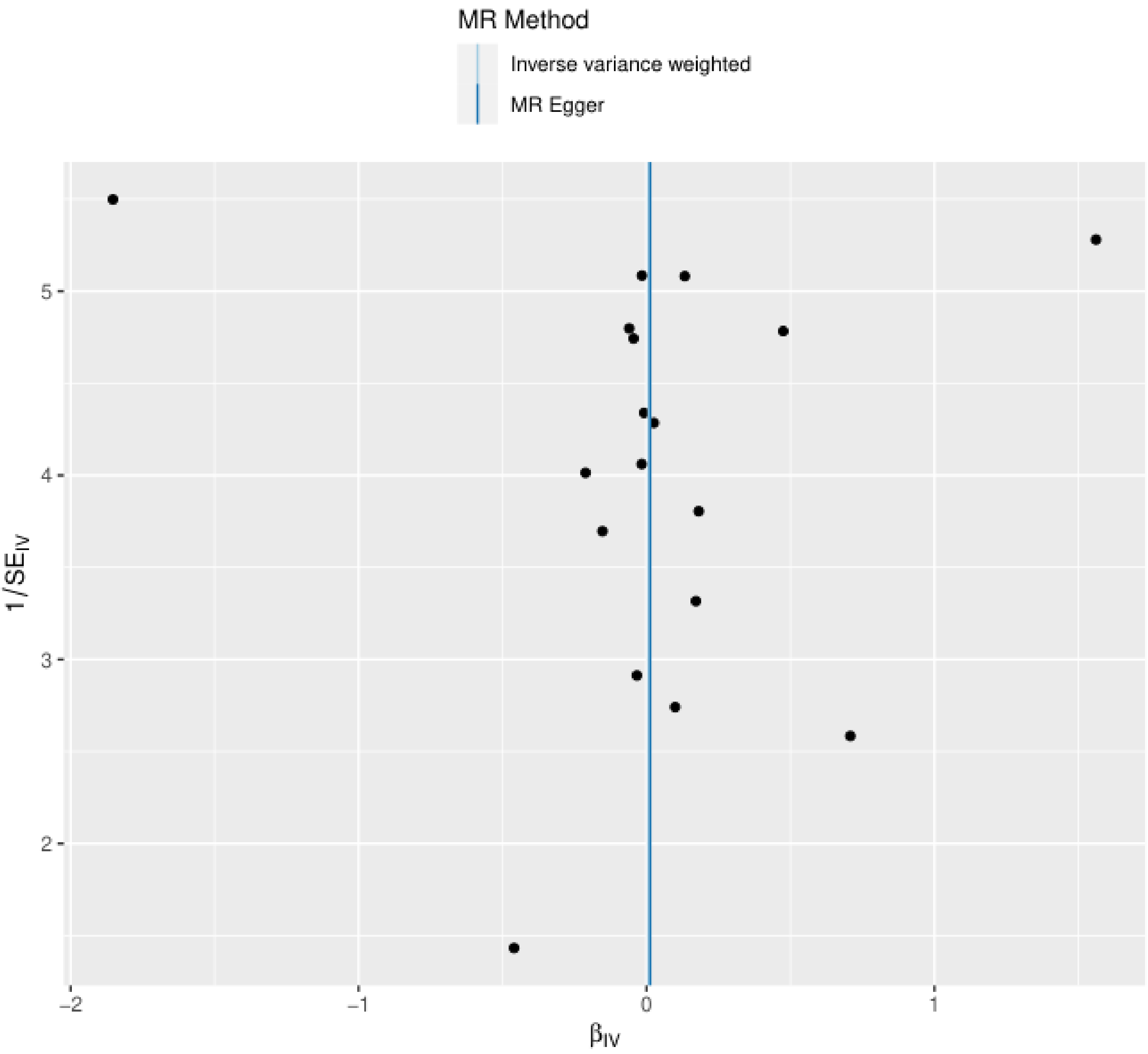

Funnel plot analyse of "SSC-A on lymphocyte" on 'Diabetic nephropathy'

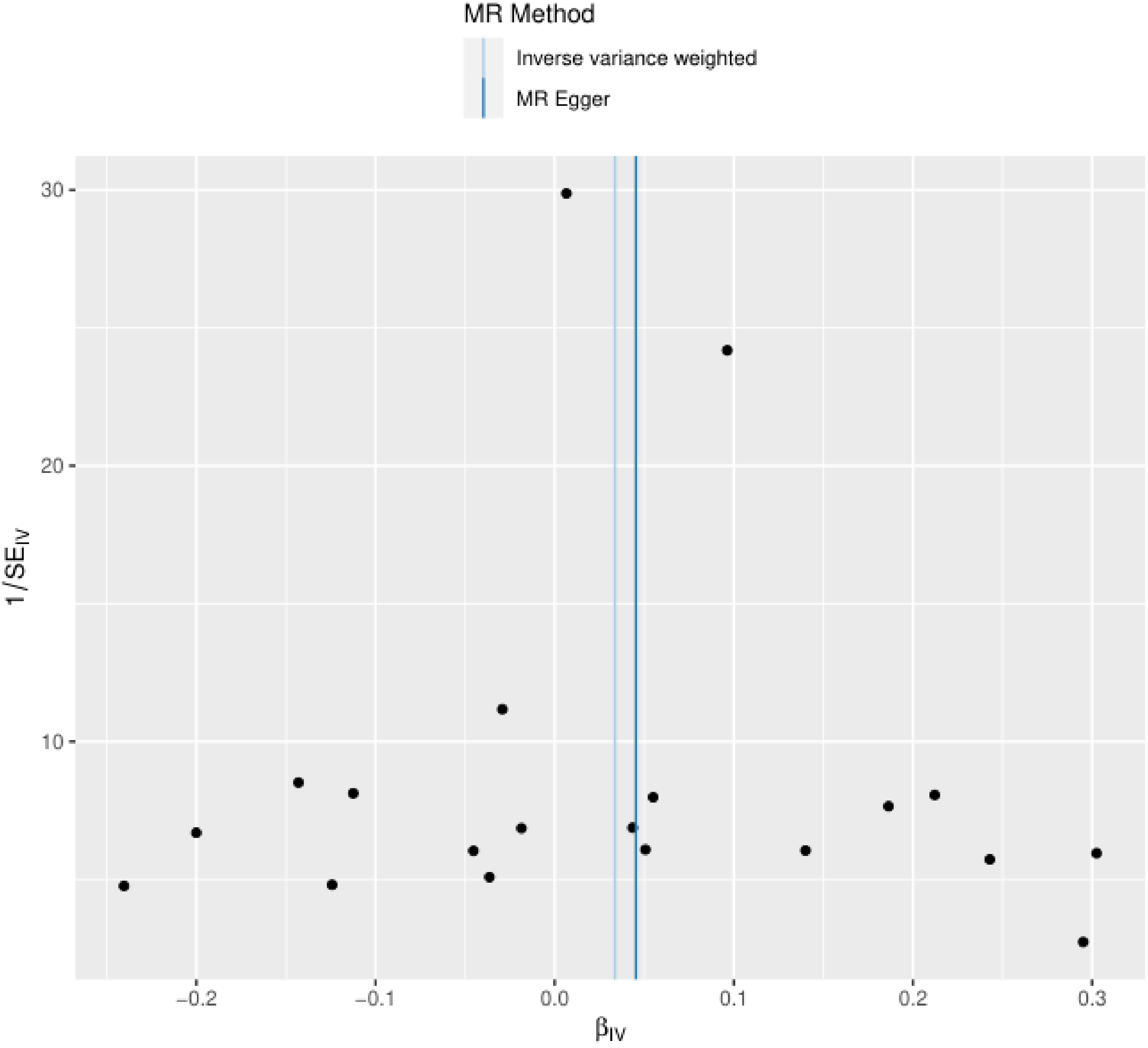

Funnel plot analyse of "CD11b on CD14+ monocyte" on 'Diabetic nephropathy'

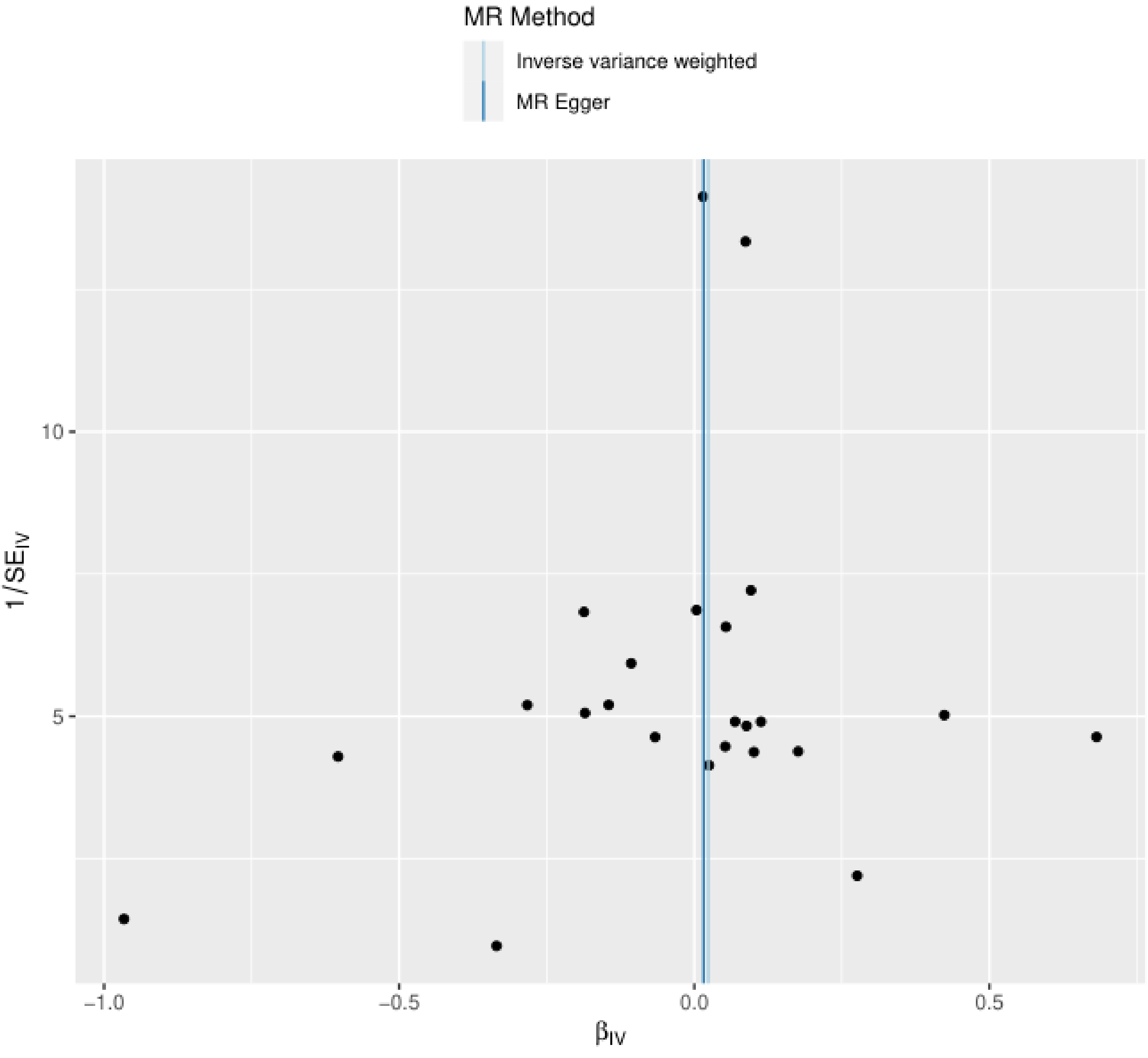

Funnel plot analyse of "CD8 on EM CD8br " on 'Diabetic nephropathy'

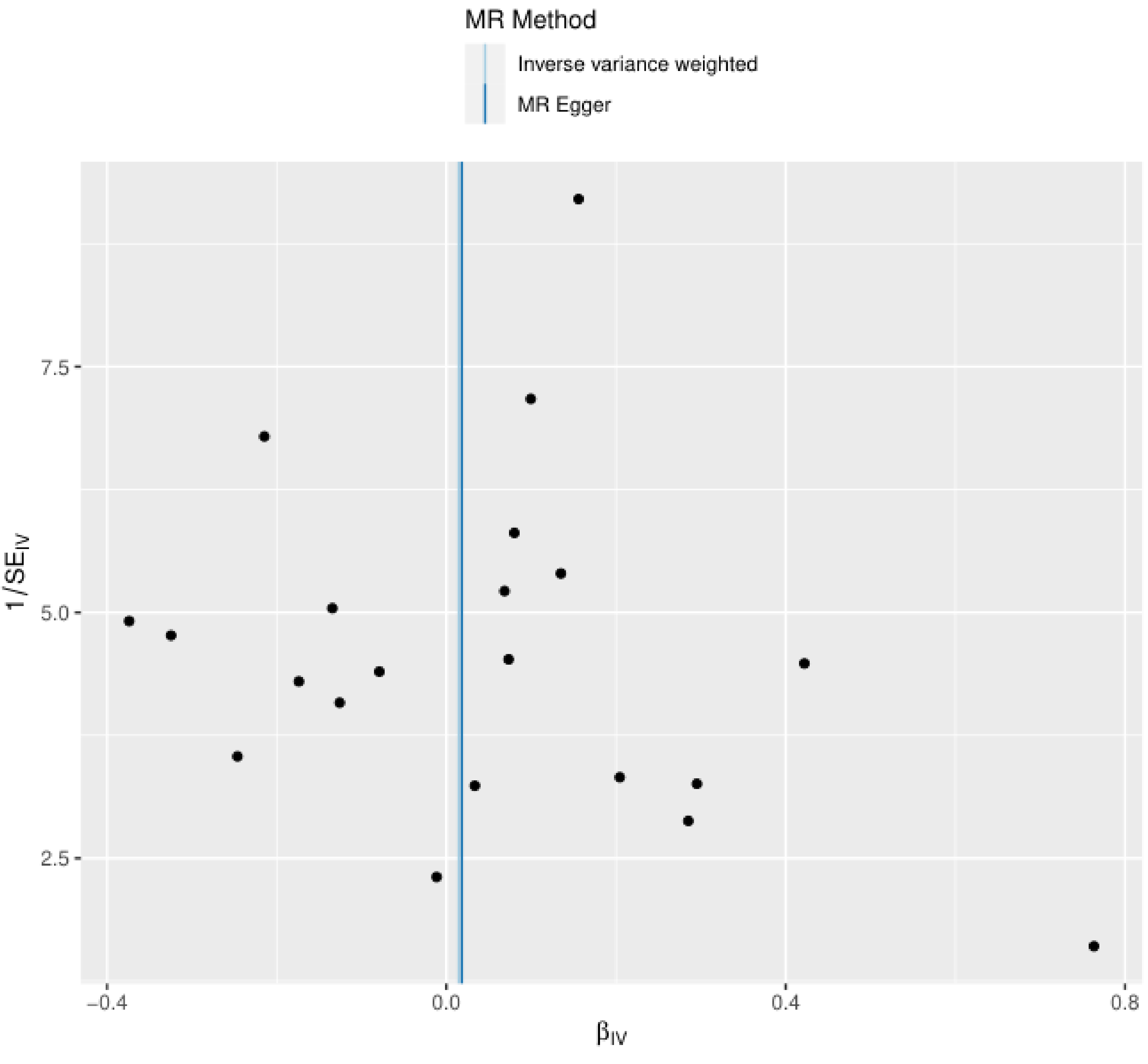

Funnel plot analyse of "EM DN (CD4-CD8-) AC" on 'Diabetic nephropathy'

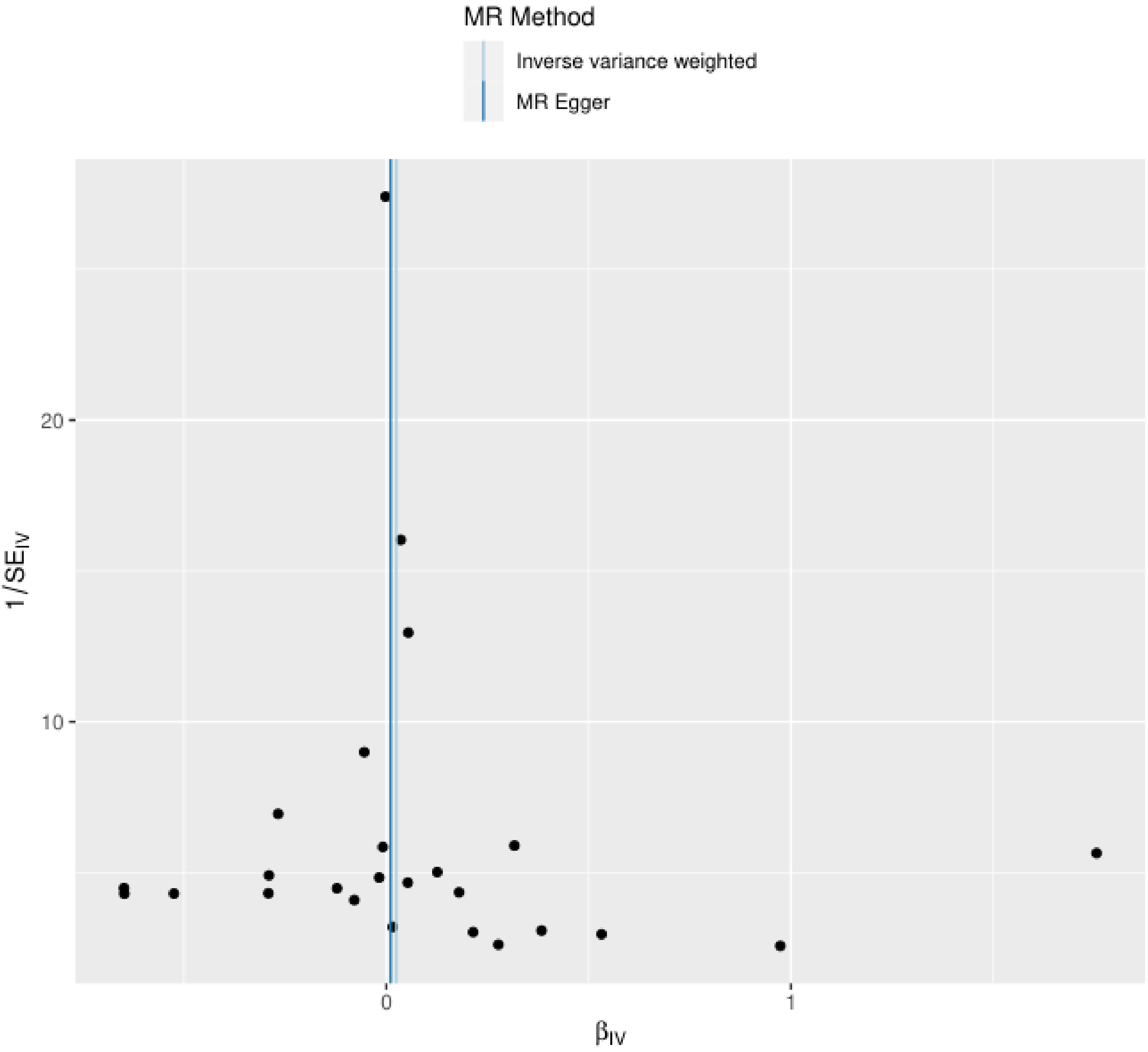

Funnel plot analyse of "CD28- CD25++ CD8br AC" on 'Diabetic nephropathy'

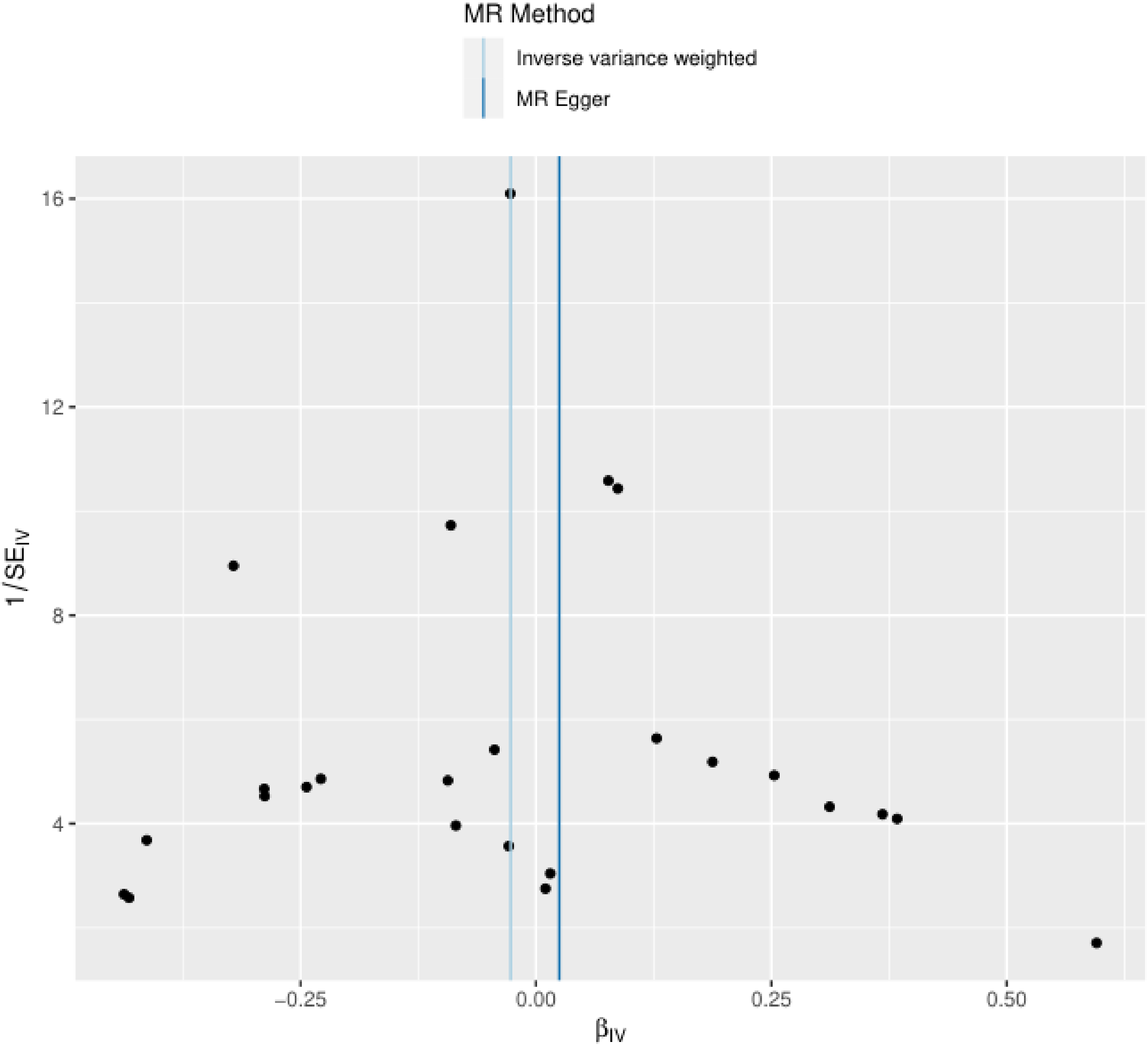

Funnel plot analyse of "CD3 on CD28+ CD4+" on 'Diabetic nephropathy'

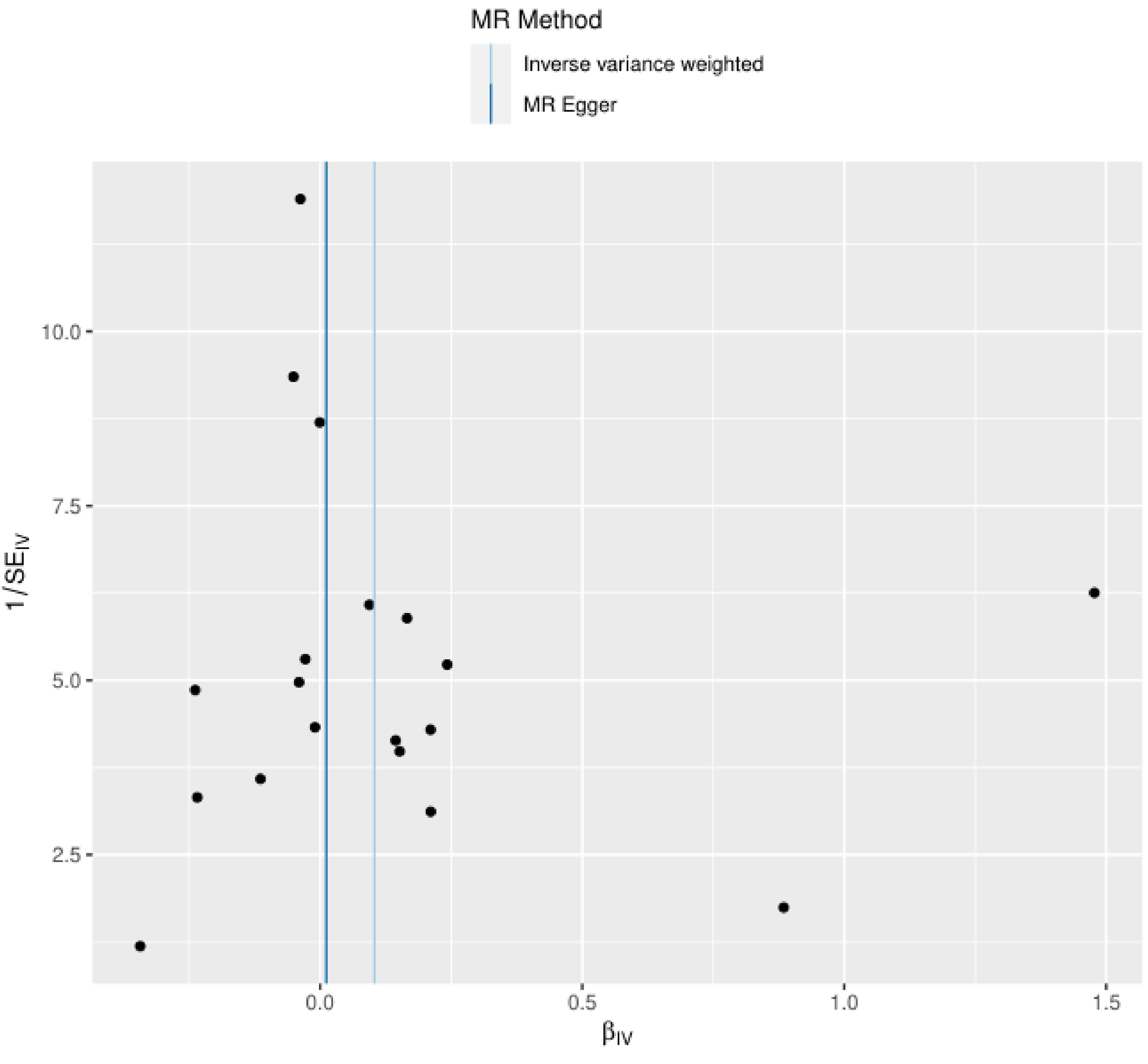

Funnel plot analyse of "Lymphocyte AC" on 'Diabetic nephropathy'

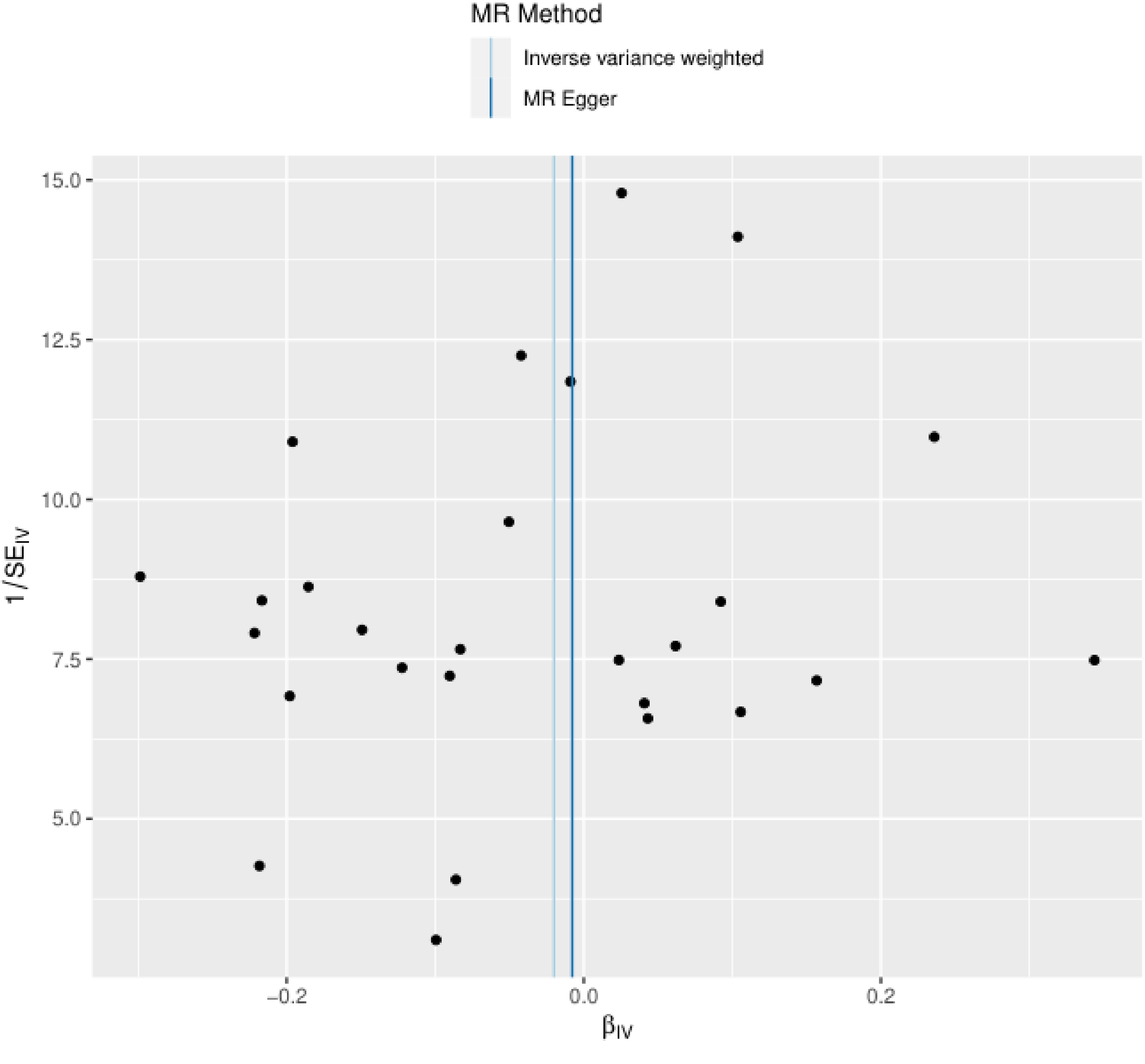

Funnel plot analyse of "HVEM on TD CD8br " on 'Diabetic nephropathy'

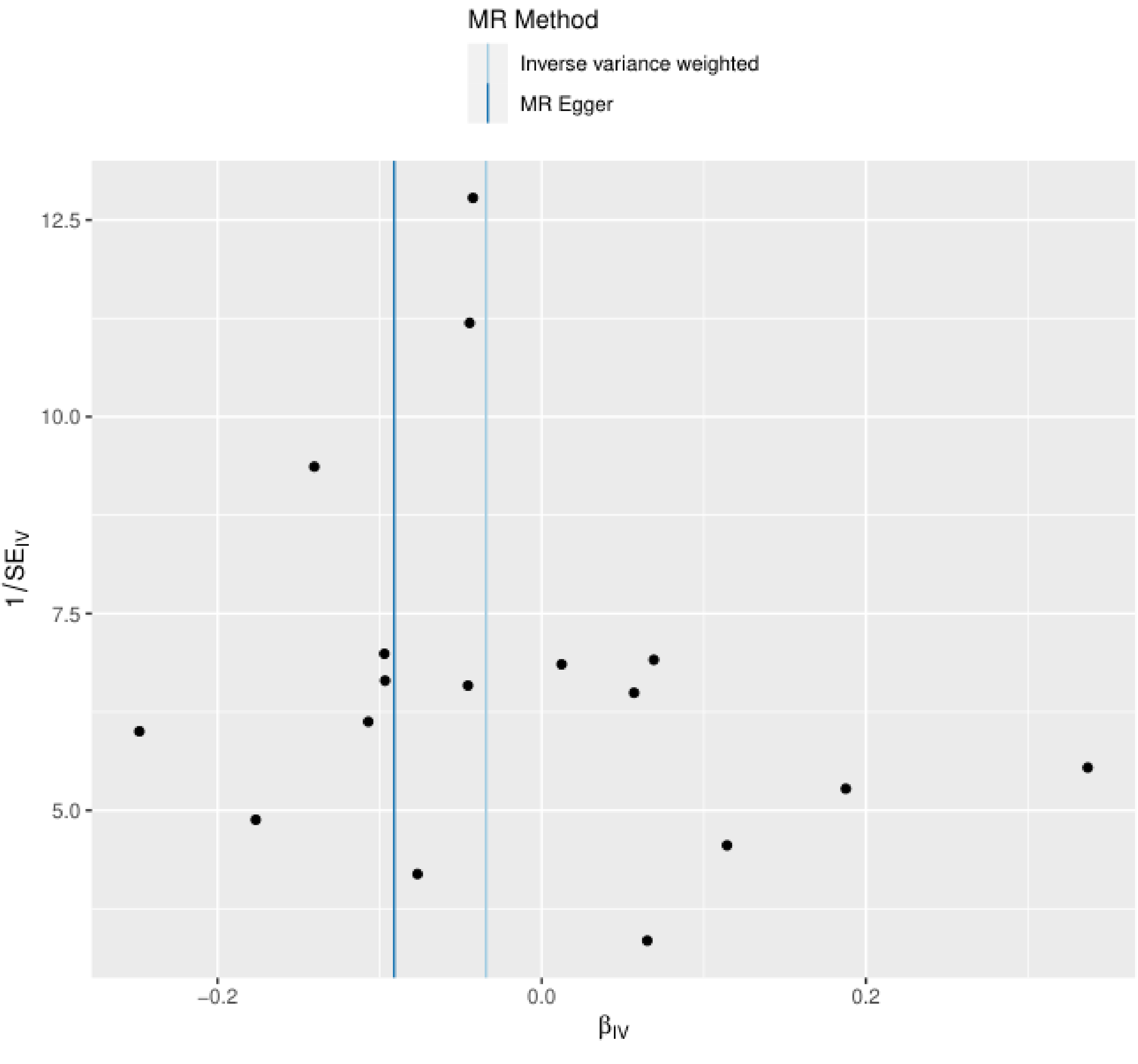

Funnel plot analyse of "CD66b++ myeloid cell AC" on 'Diabetic nephropathy'

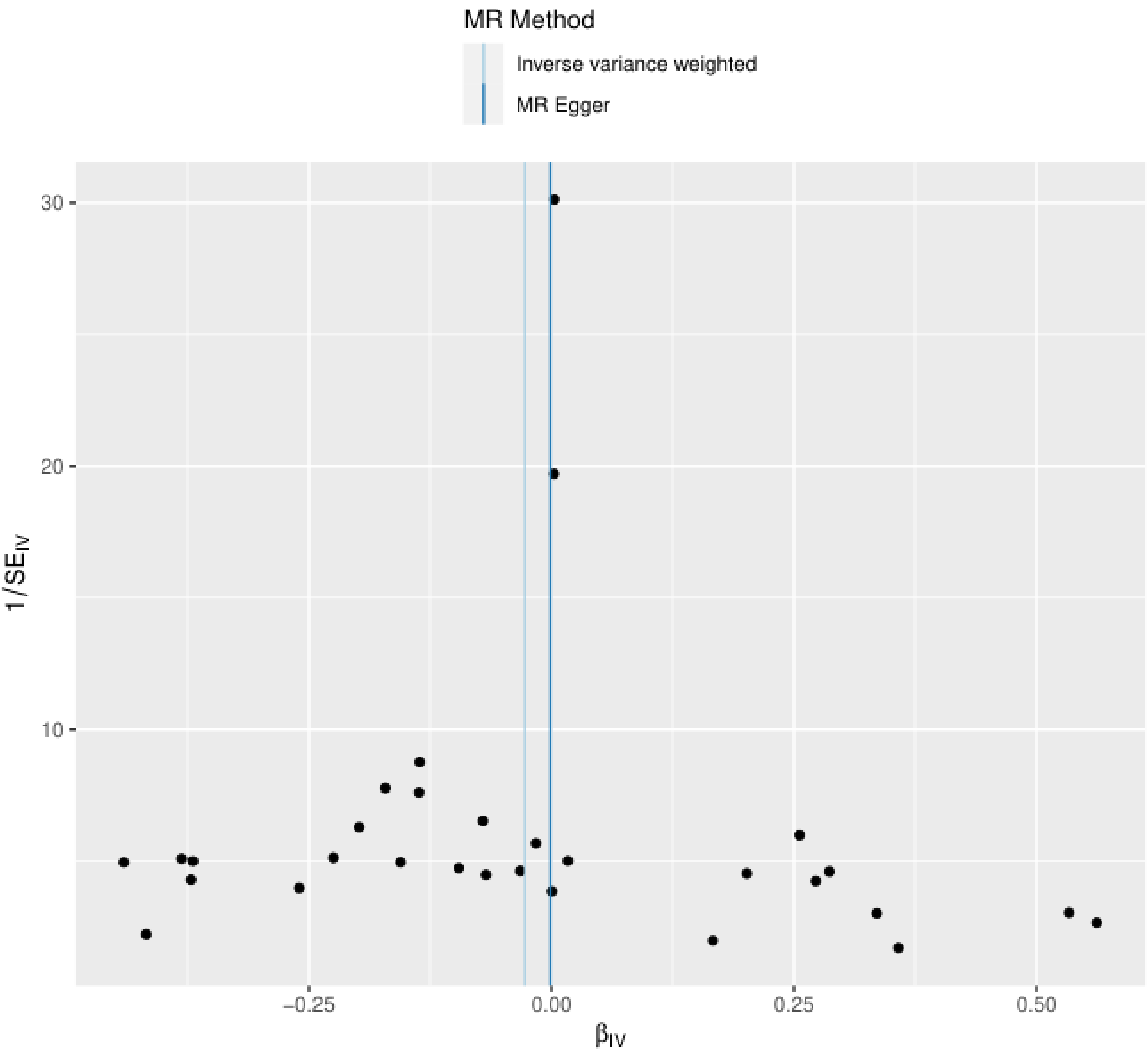

Funnel plot analyse of "CD11c on myeloid DC" on 'Diabetic nephropathy'

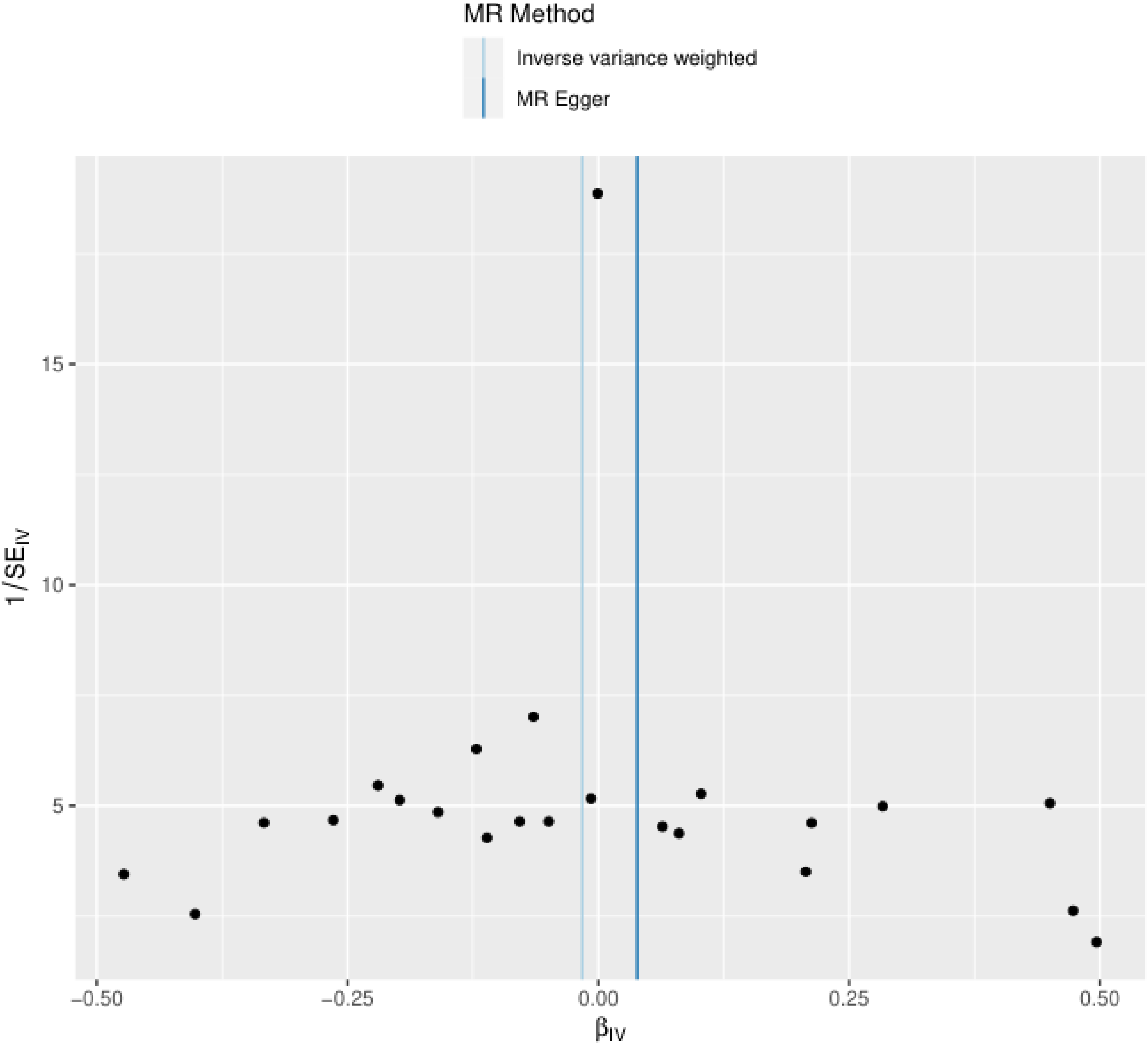

Funnel plot analyse of "CM CD4+ %T cell" on 'Diabetic nephropathy'

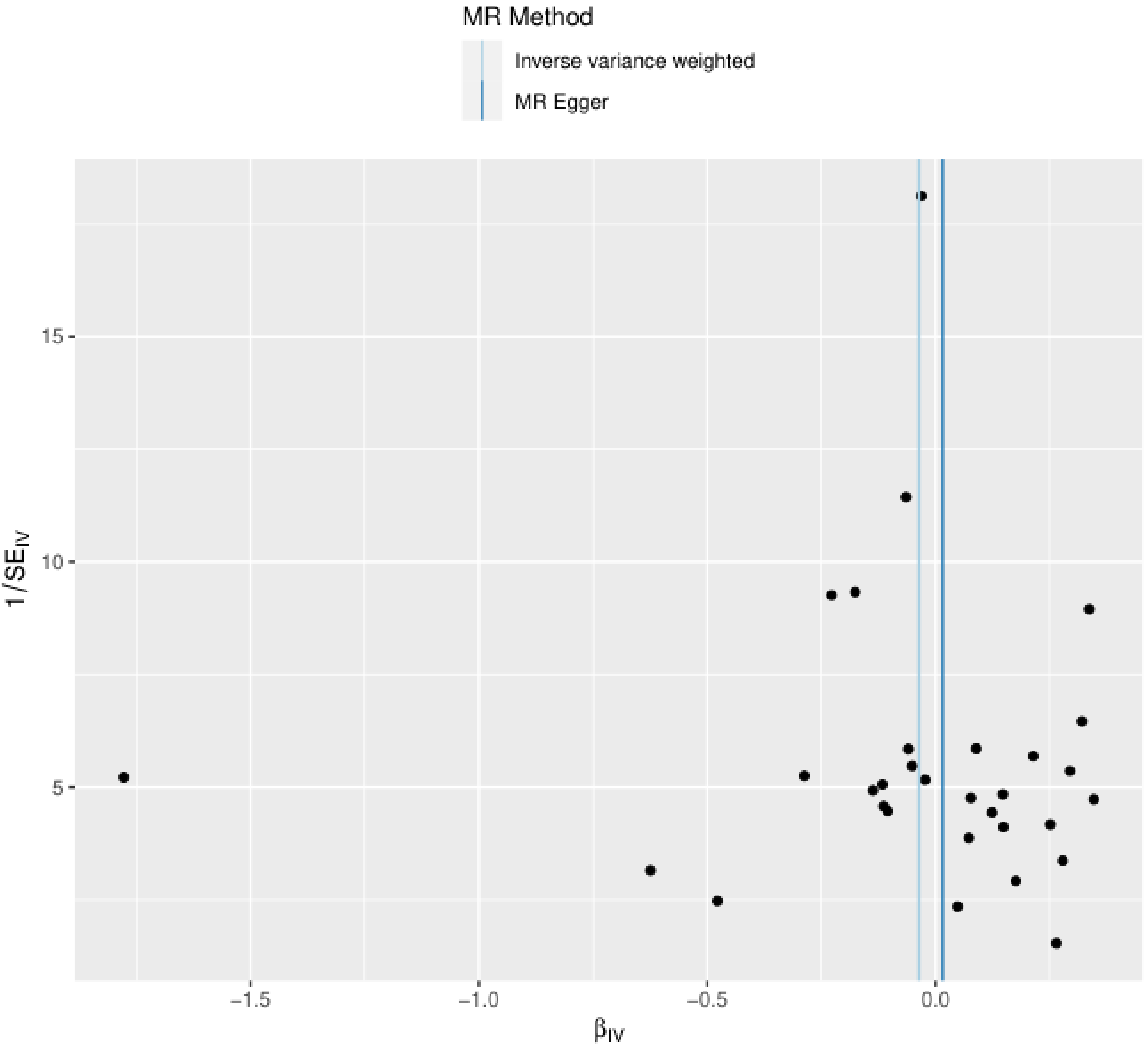

Funnel plot analyse of "CD20 on sw mem" on 'Diabetic nephropathy'

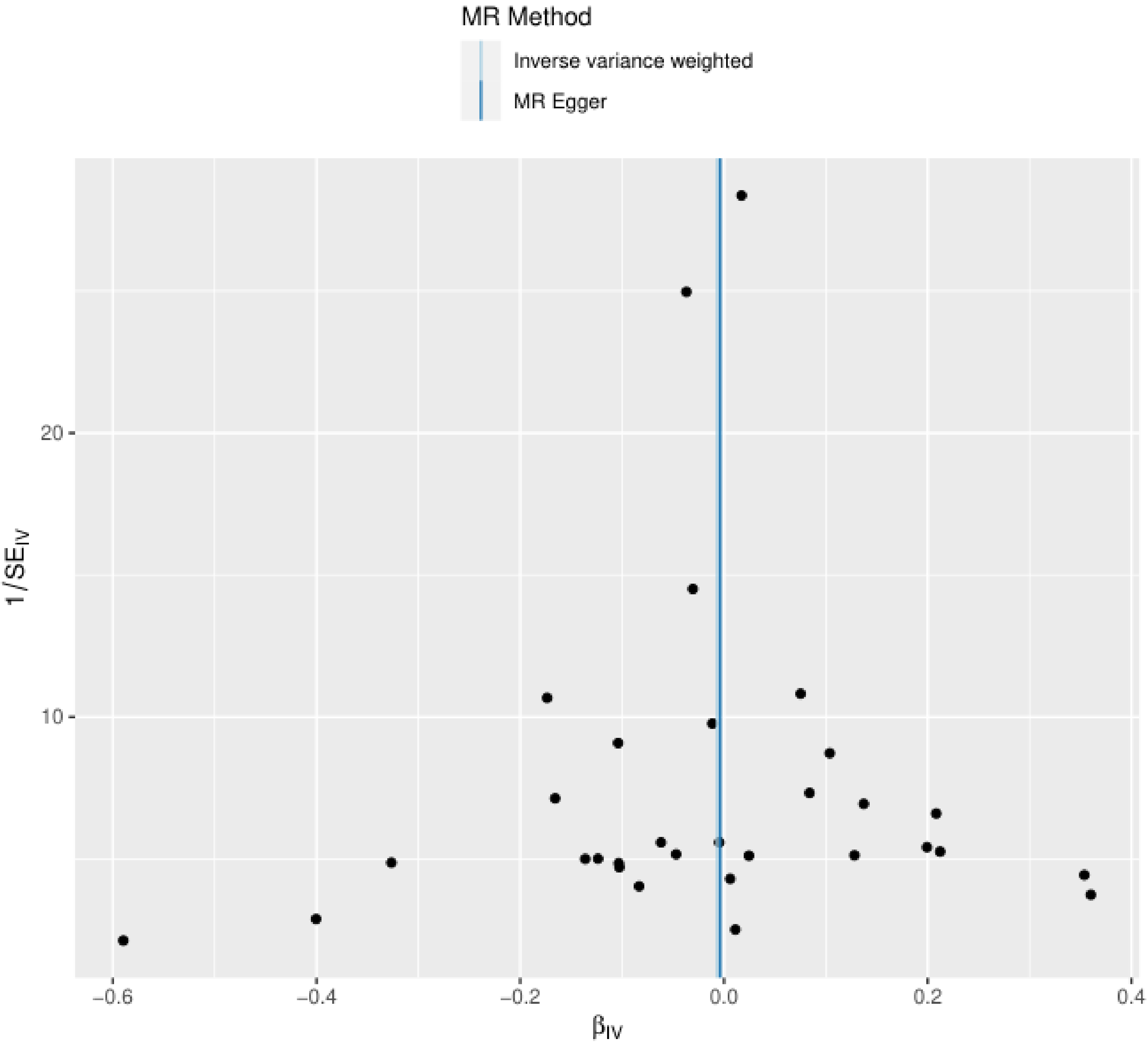

Funnel plot analyse of "CD3 on T cell" on 'Diabetic nephropathy'

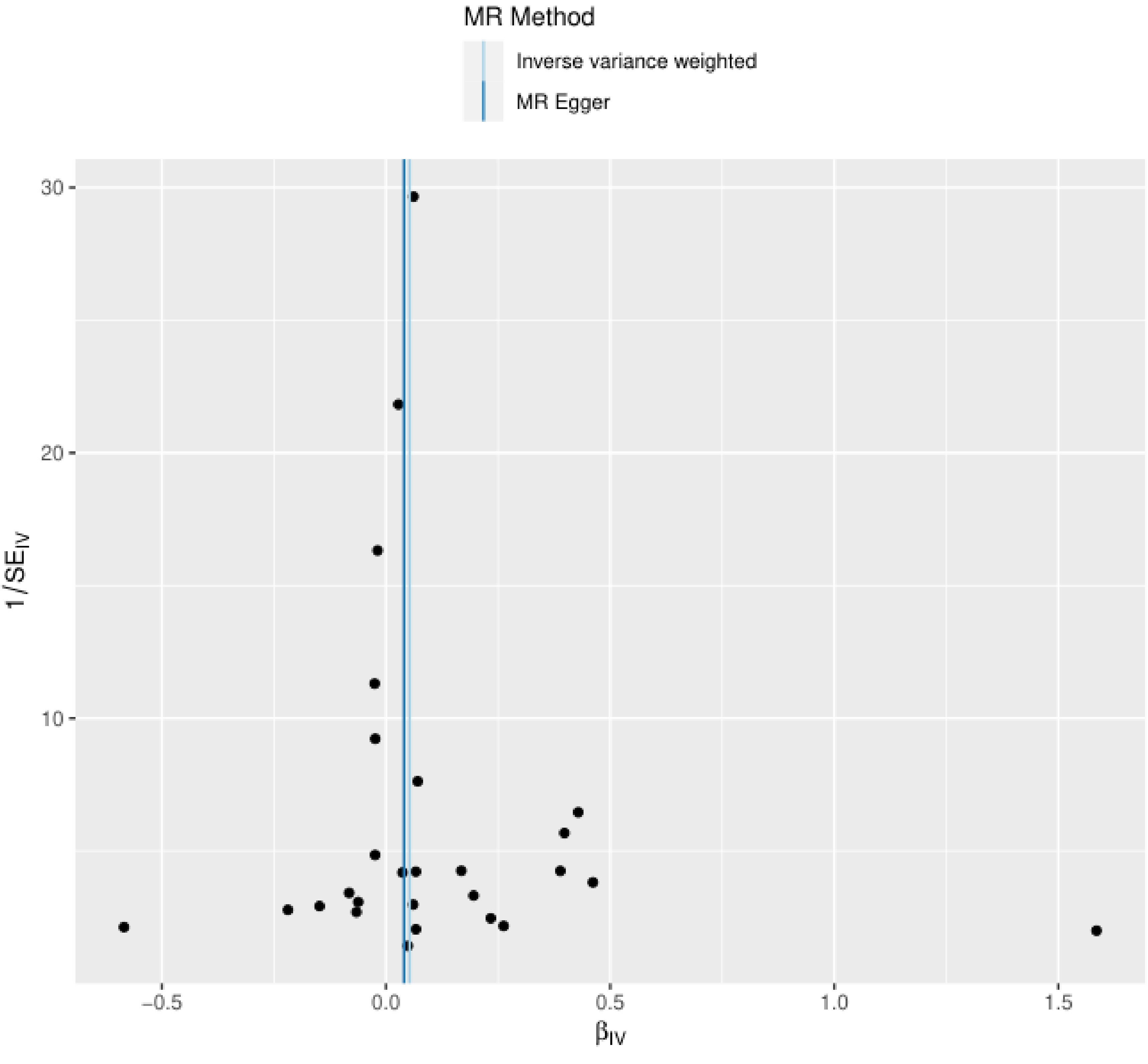

Funnel plot analyse of "Naive CD8br %T cell" on 'Diabetic nephropathy'

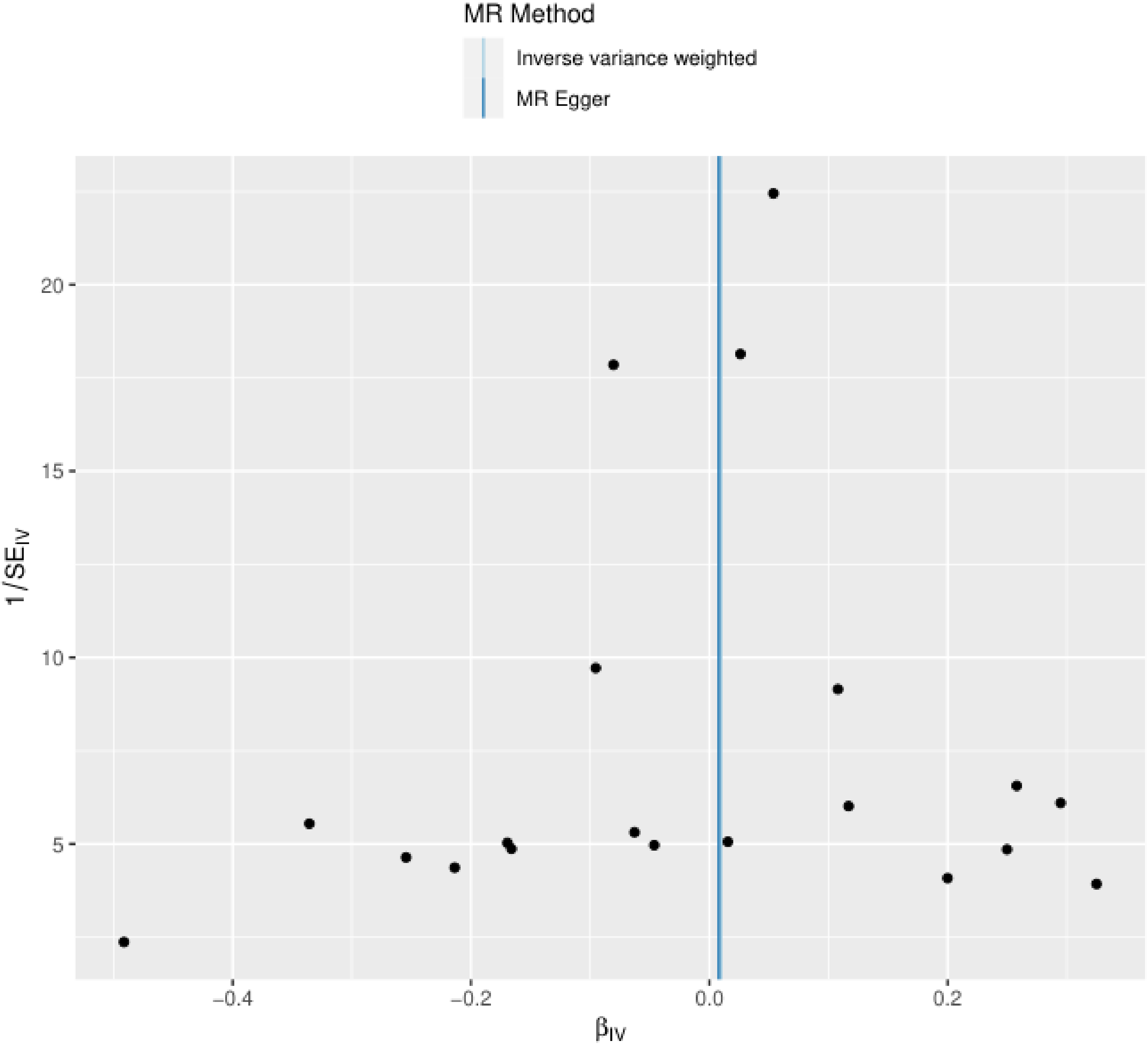

Funnel plot analyse of "CD80 on plasmacytoid DC" on 'Diabetic nephropathy'

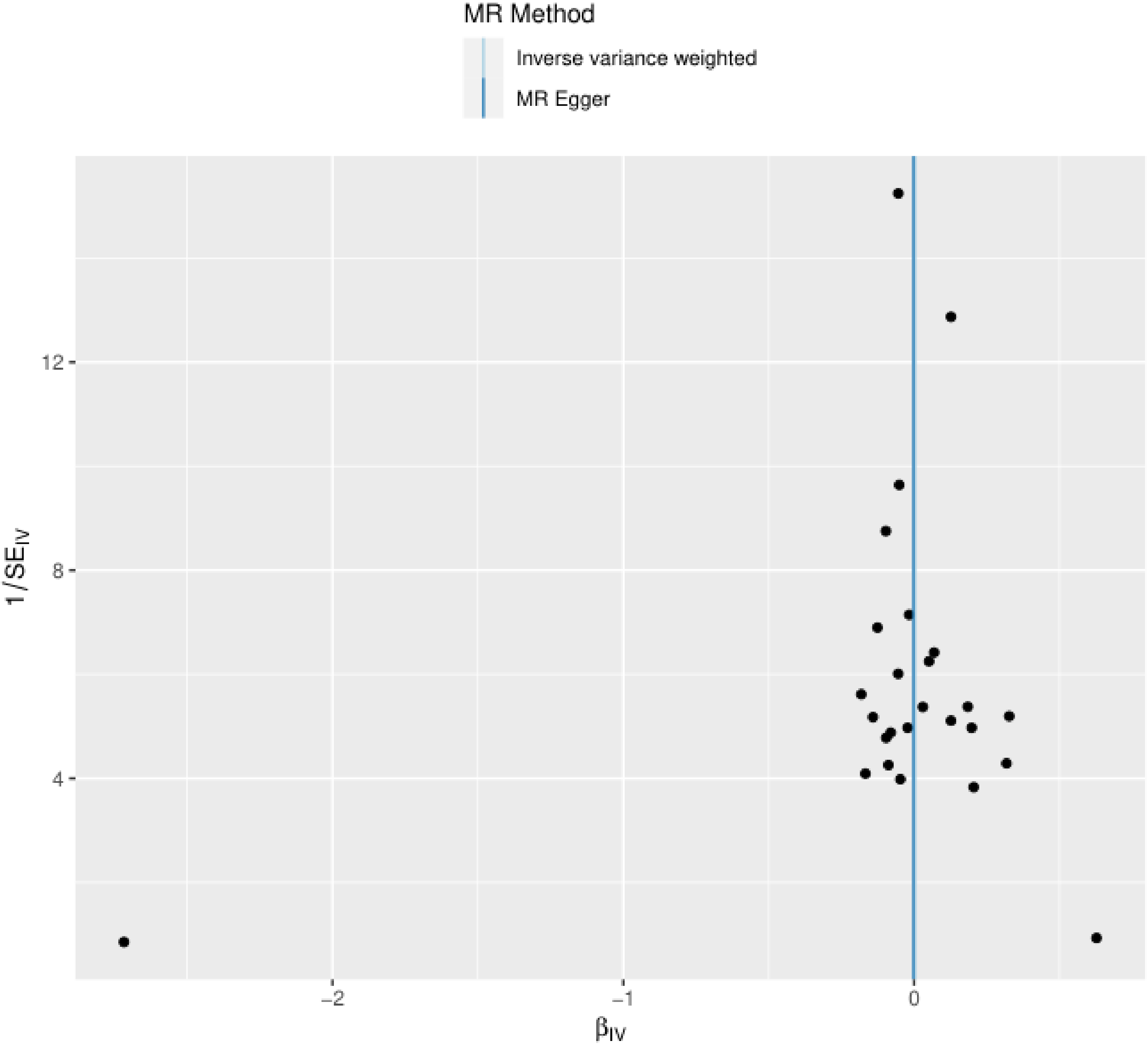

Funnel plot analyse of "CD3 on HLA DR+ CD8br" on 'Diabetic nephropathy'

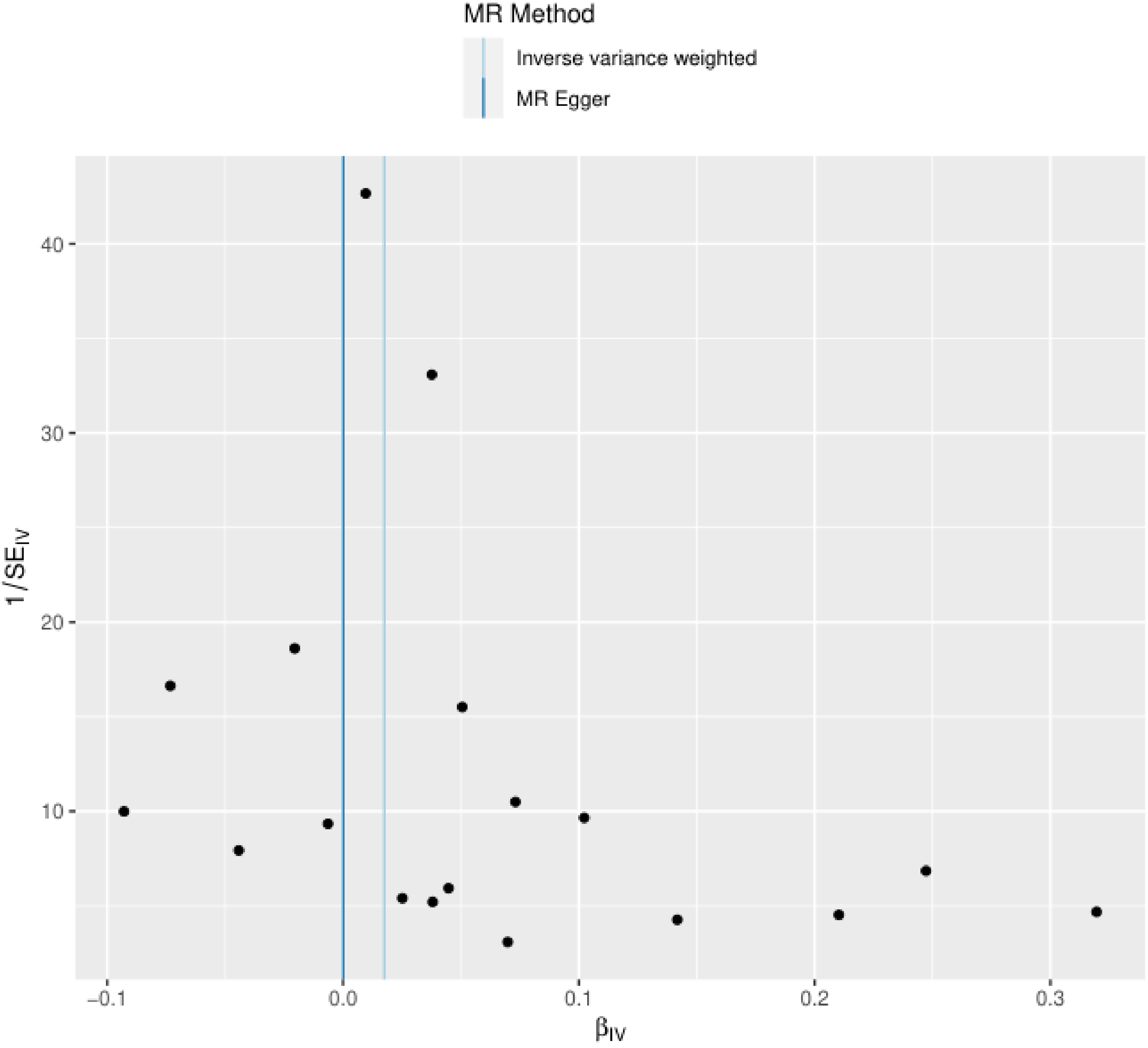

Funnel plot analyse of "CD123 on CD62L+ plasmacytoid DC" on 'Diabetic nephropathy'

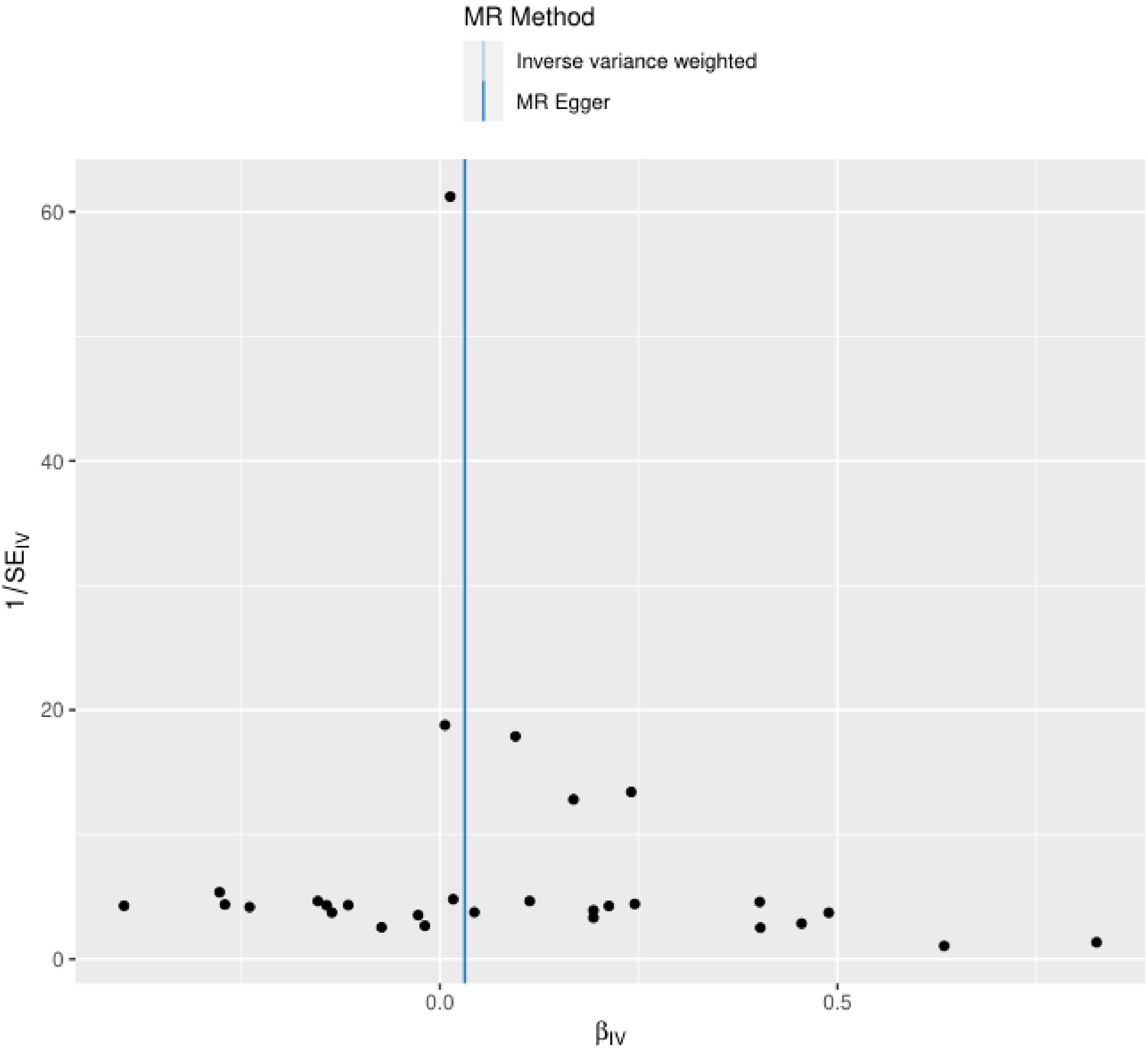

Funnel plot analyse of "IgD+ CD38br AC" on 'Diabetic nephropathy'

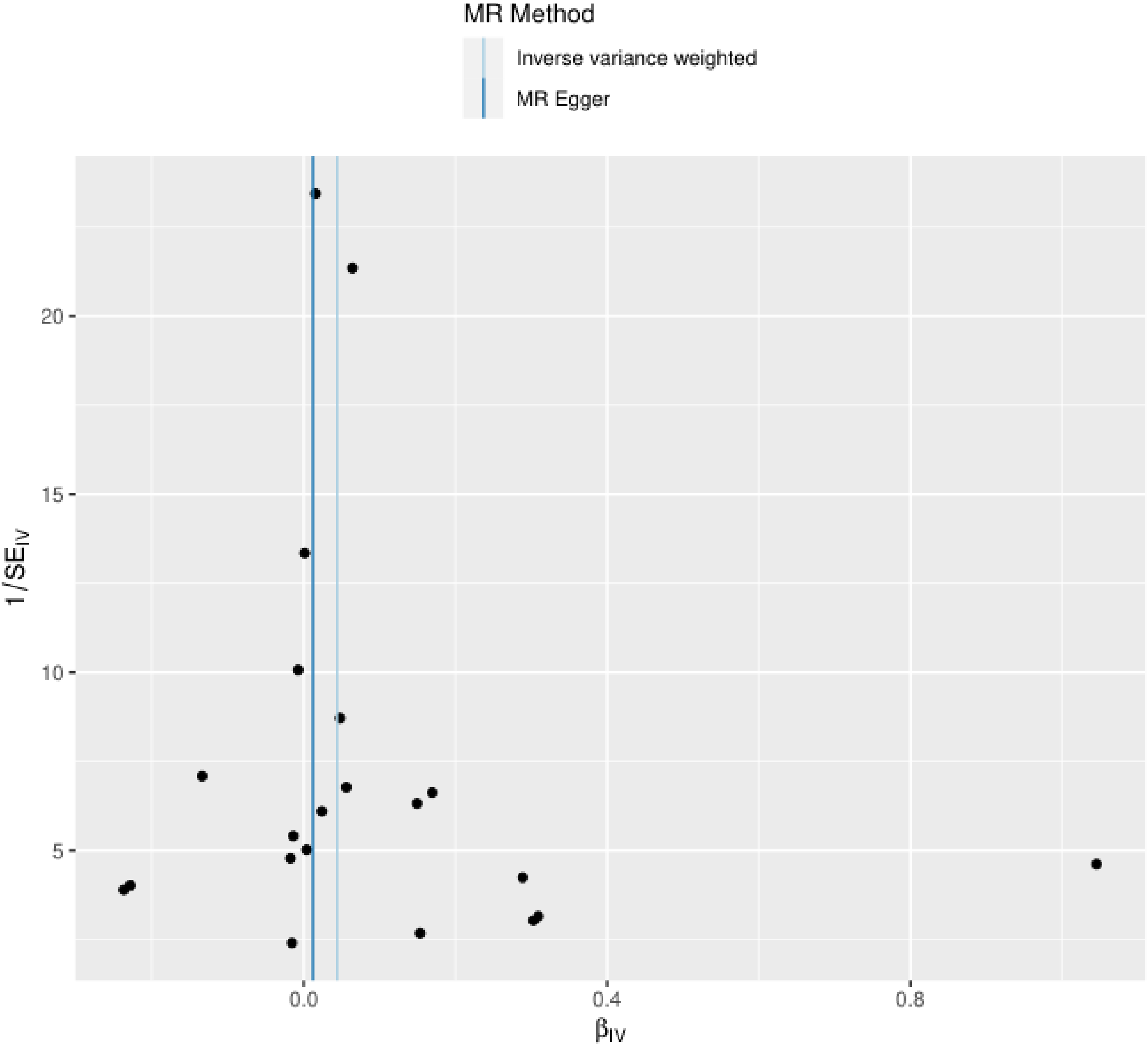

Funnel plot analyse of "CD24 on IgD+ CD38-" on 'Diabetic nephropathy'

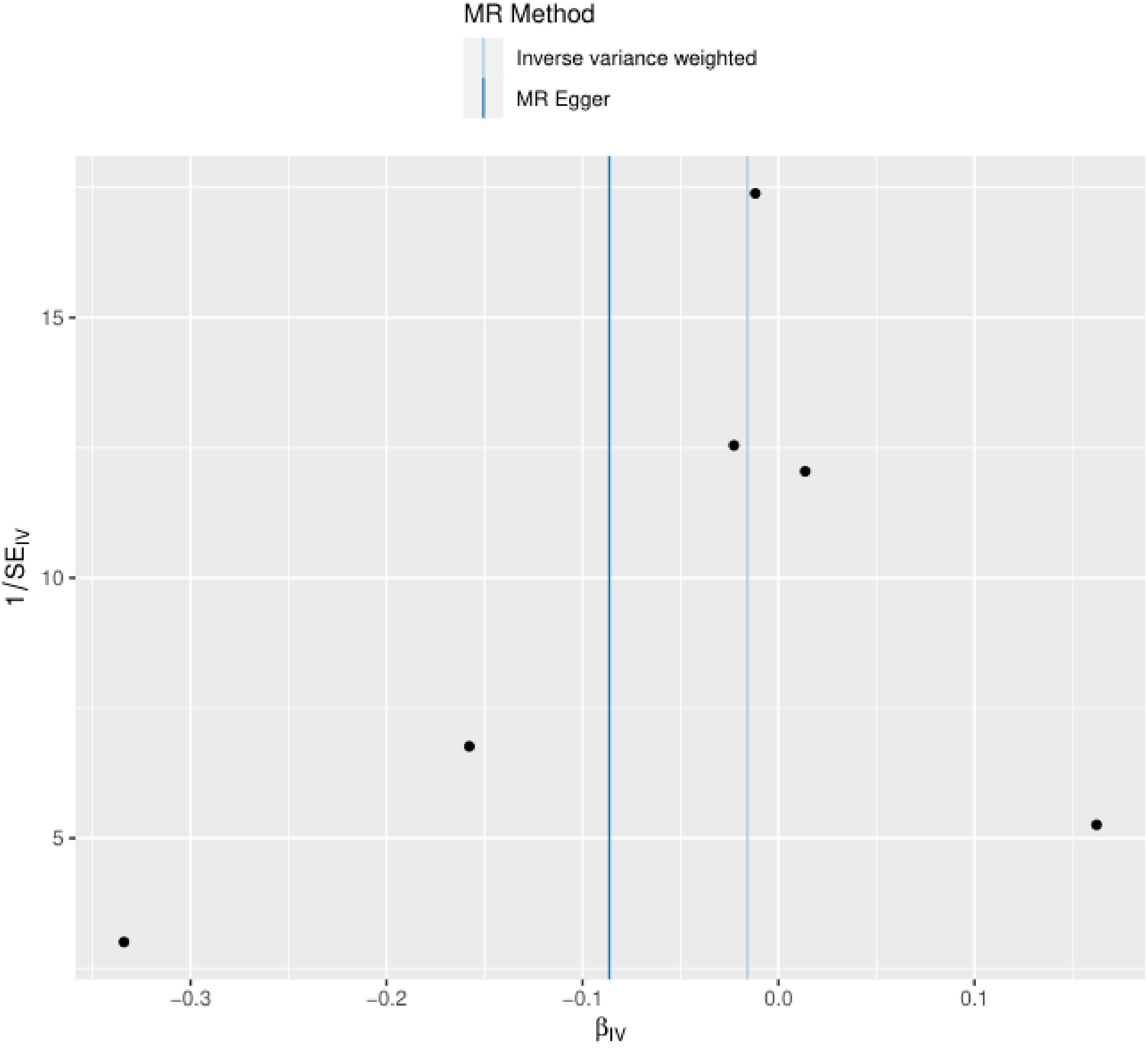

Funnel plot analyse of "HLA DR on HSC" on 'Diabetic nephropathy'

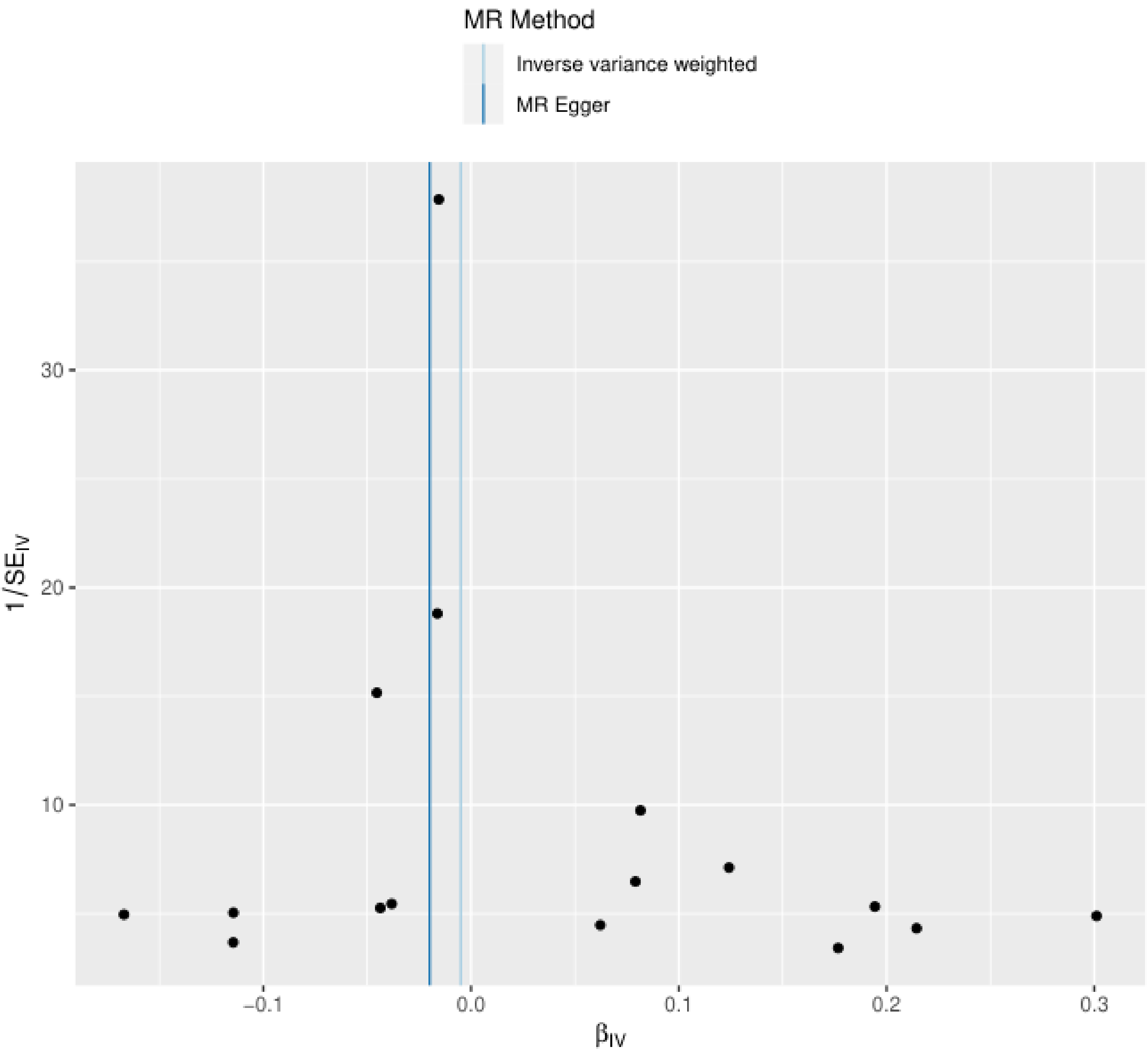

Funnel plot analyse of ''CD25 on CD39+ secreting Treg '' on 'Diabetic nephropathy'

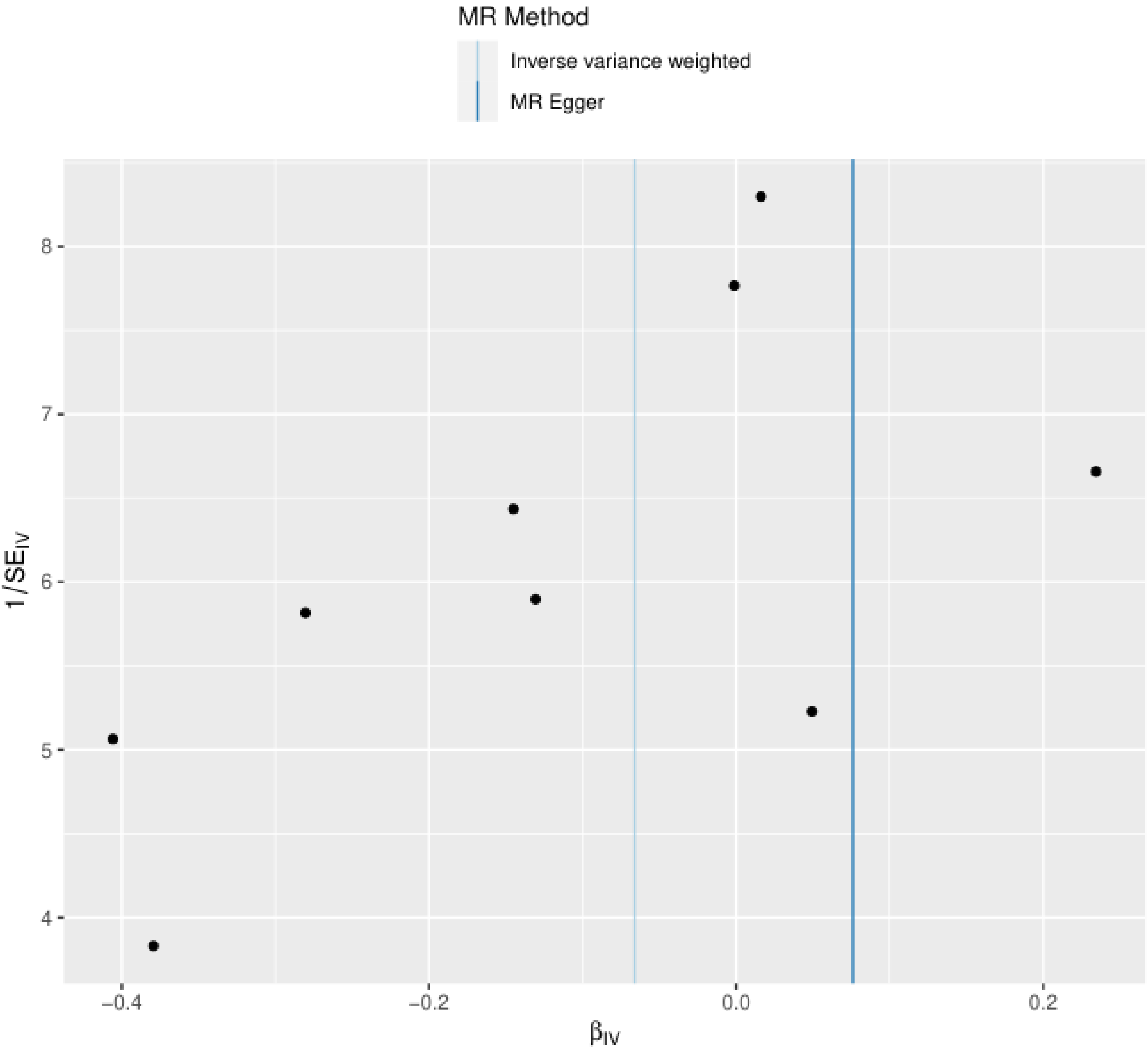

Funnel plot analyse of ''CD45 on CD33dim HLA DR+ CD11b- '' on 'Diabetic nephropathy'

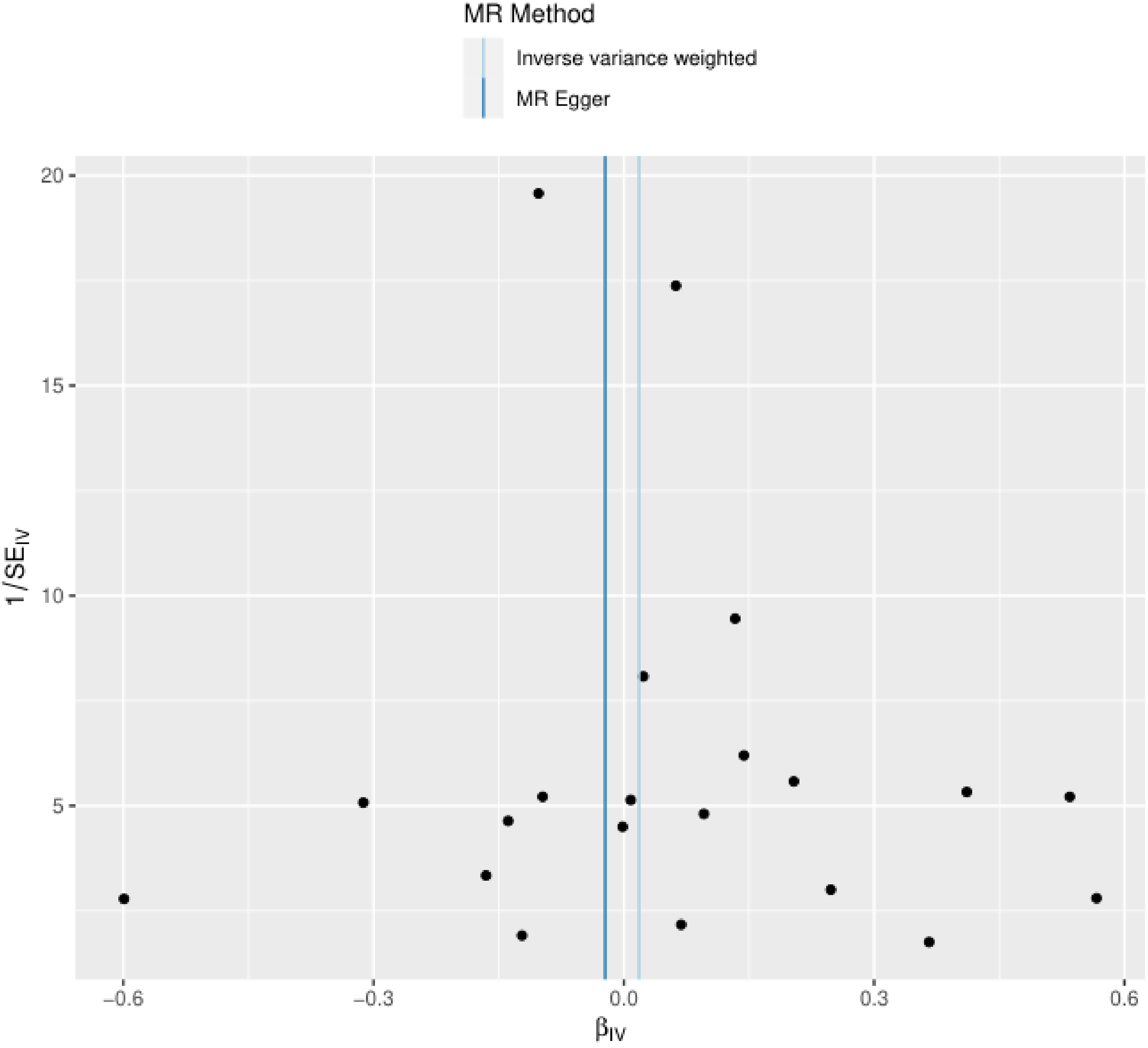

Funnel plot analyse of "FSC-A on granulocyte" on 'Diabetic nephropathy'

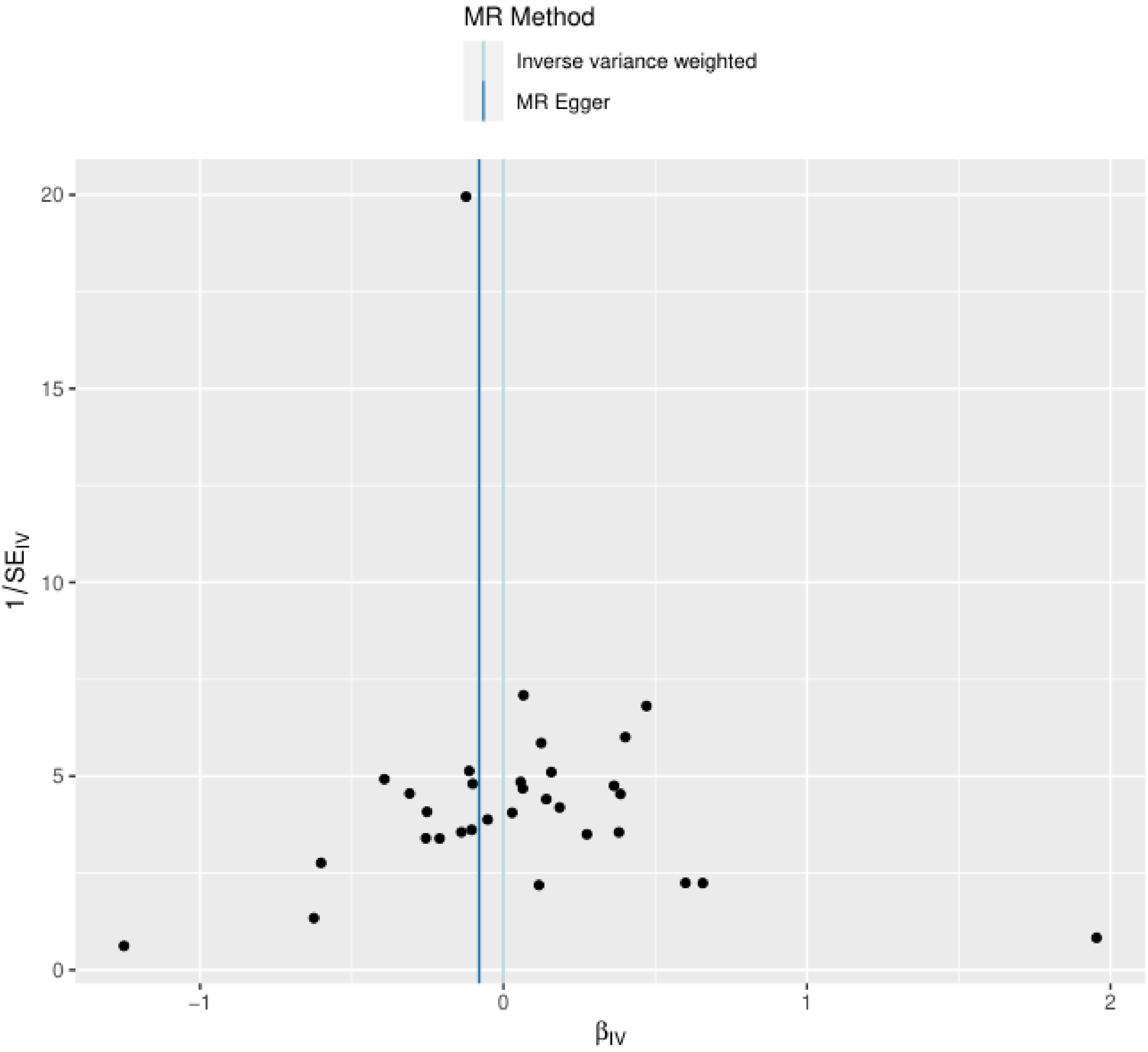

Funnel plot analyse of "CD28- DN (CD4-CD8-) AC" on 'Diabetic nephropathy'

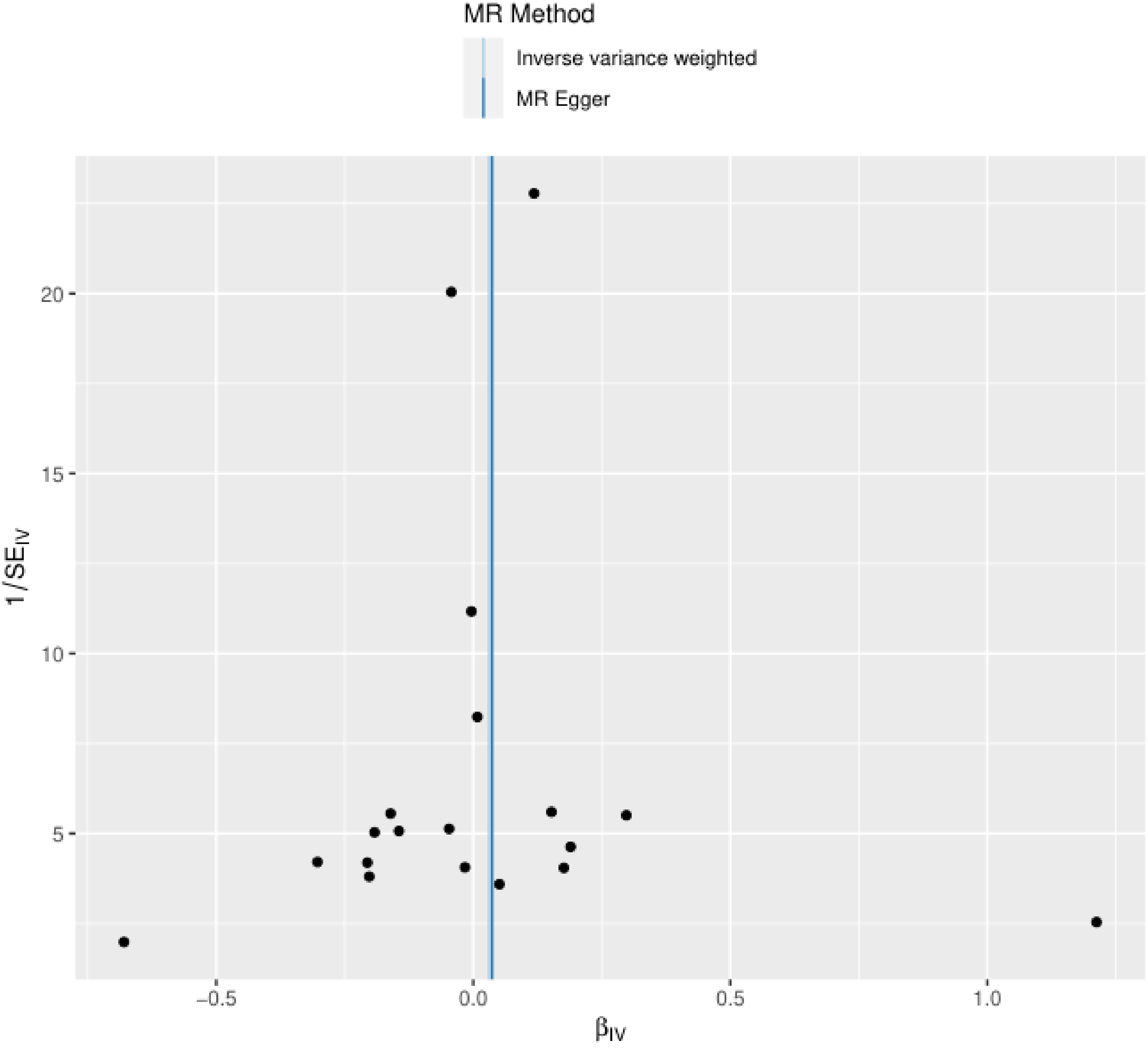

Funnel plot analyse of "CD45 on HLA DR+ CD8br" on 'Diabetic nephropathy'

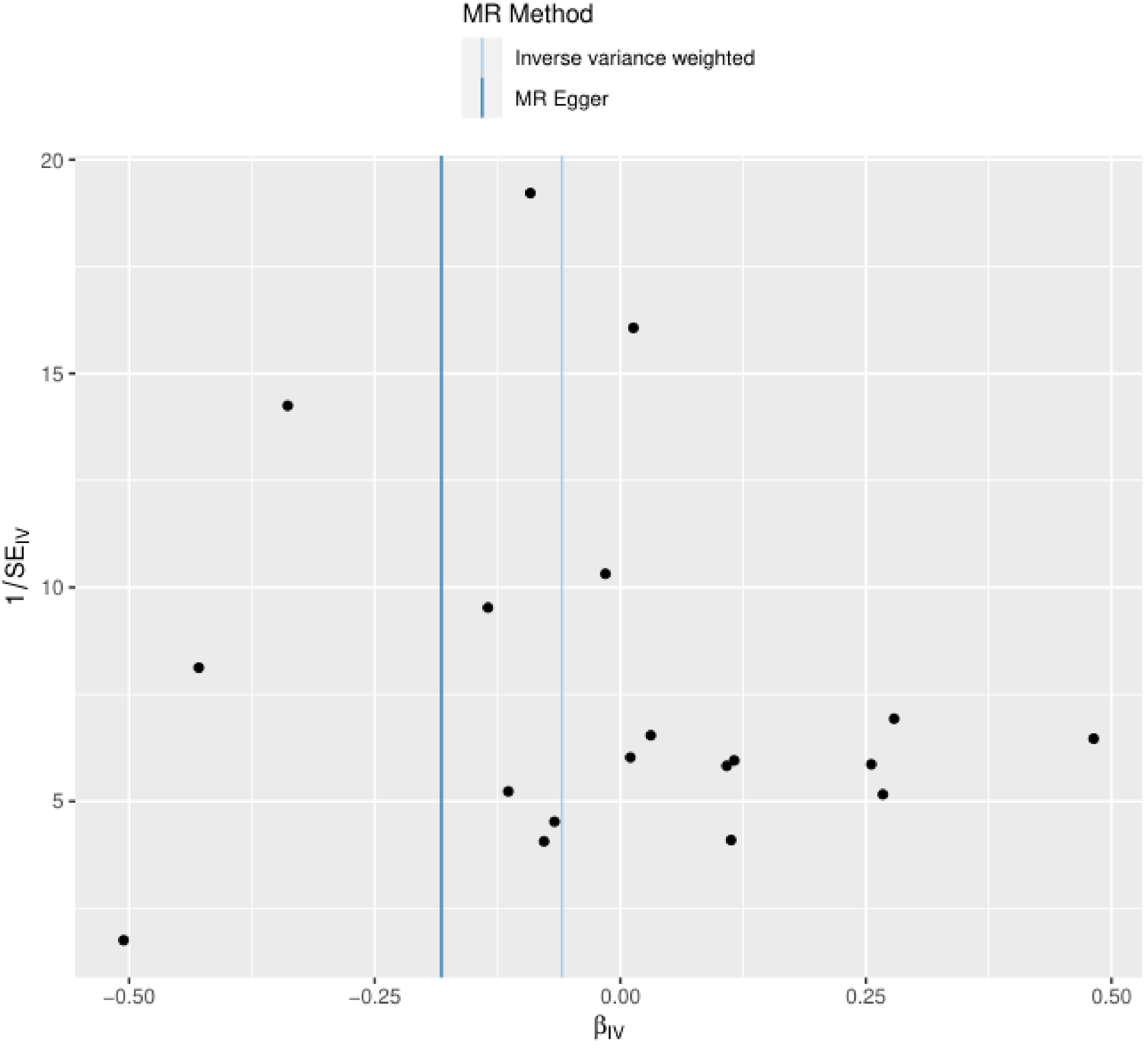

Funnel plot analyse of "Mo MDSC AC" on 'Diabetic nephropathy'

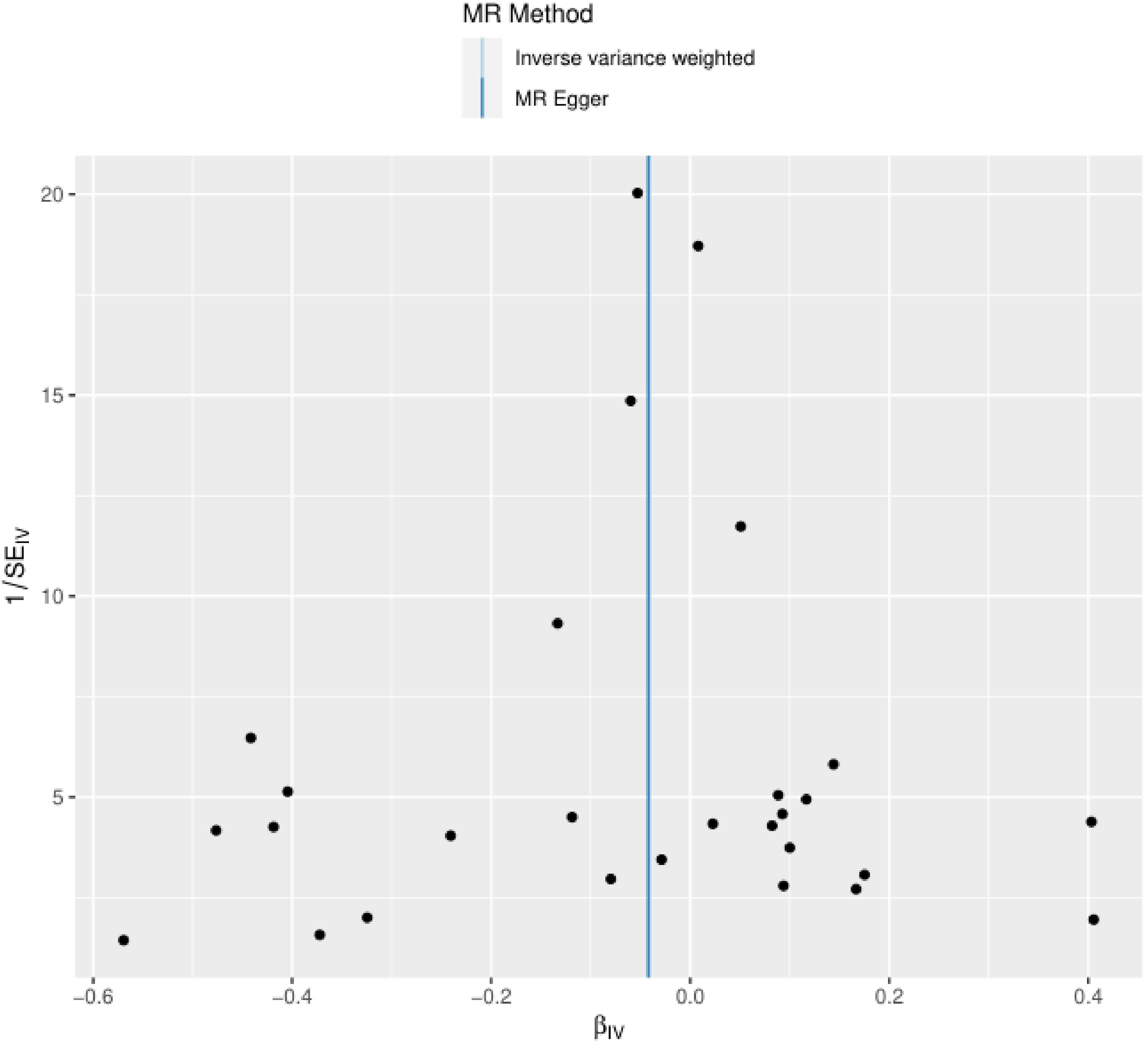

Funnel plot analyse of "EM CD4+ %CD4+" on 'Diabetic nephropathy'

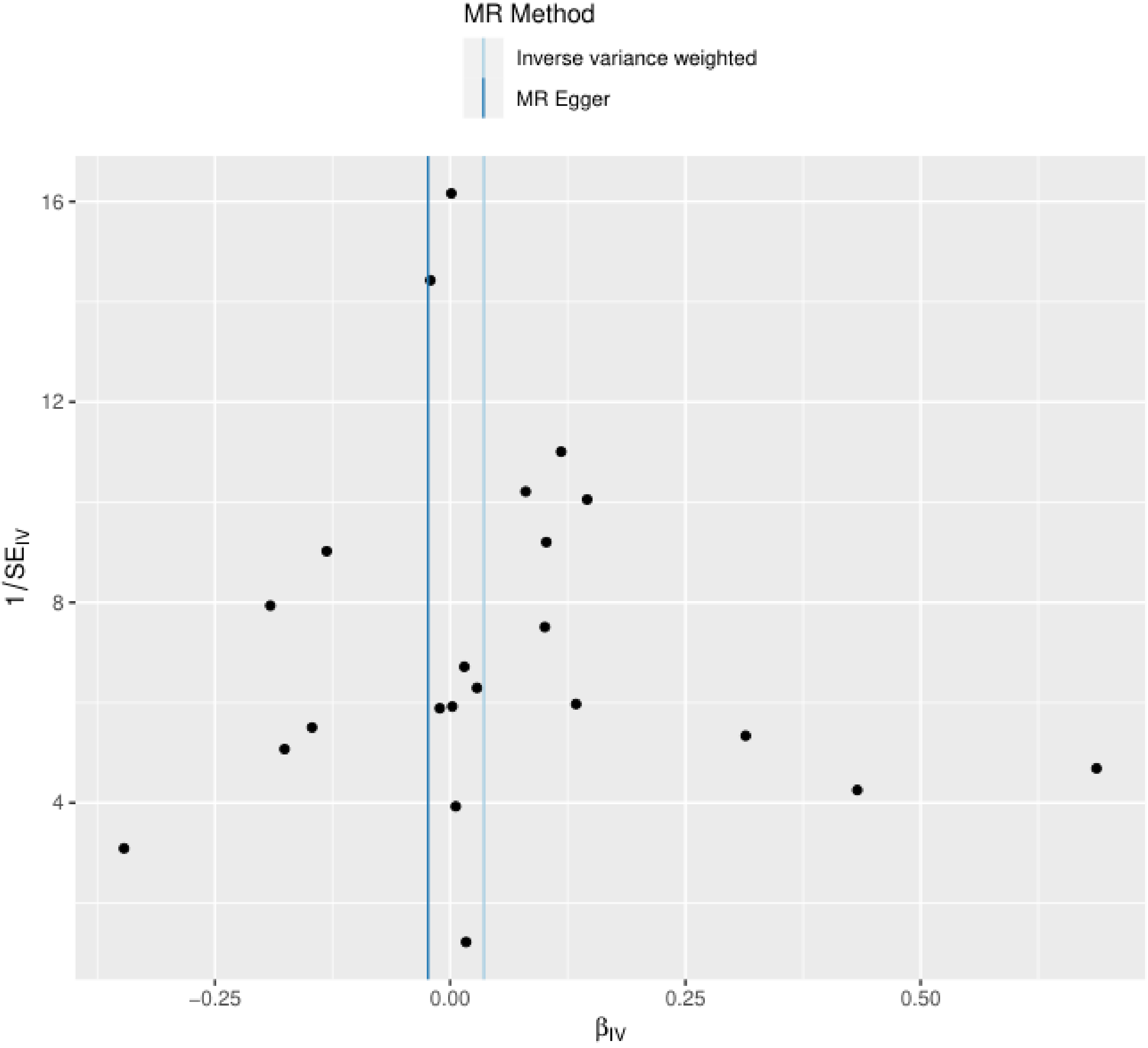

Funnel plot analyse of "CD45 on NKT" on 'Diabetic nephropathy'

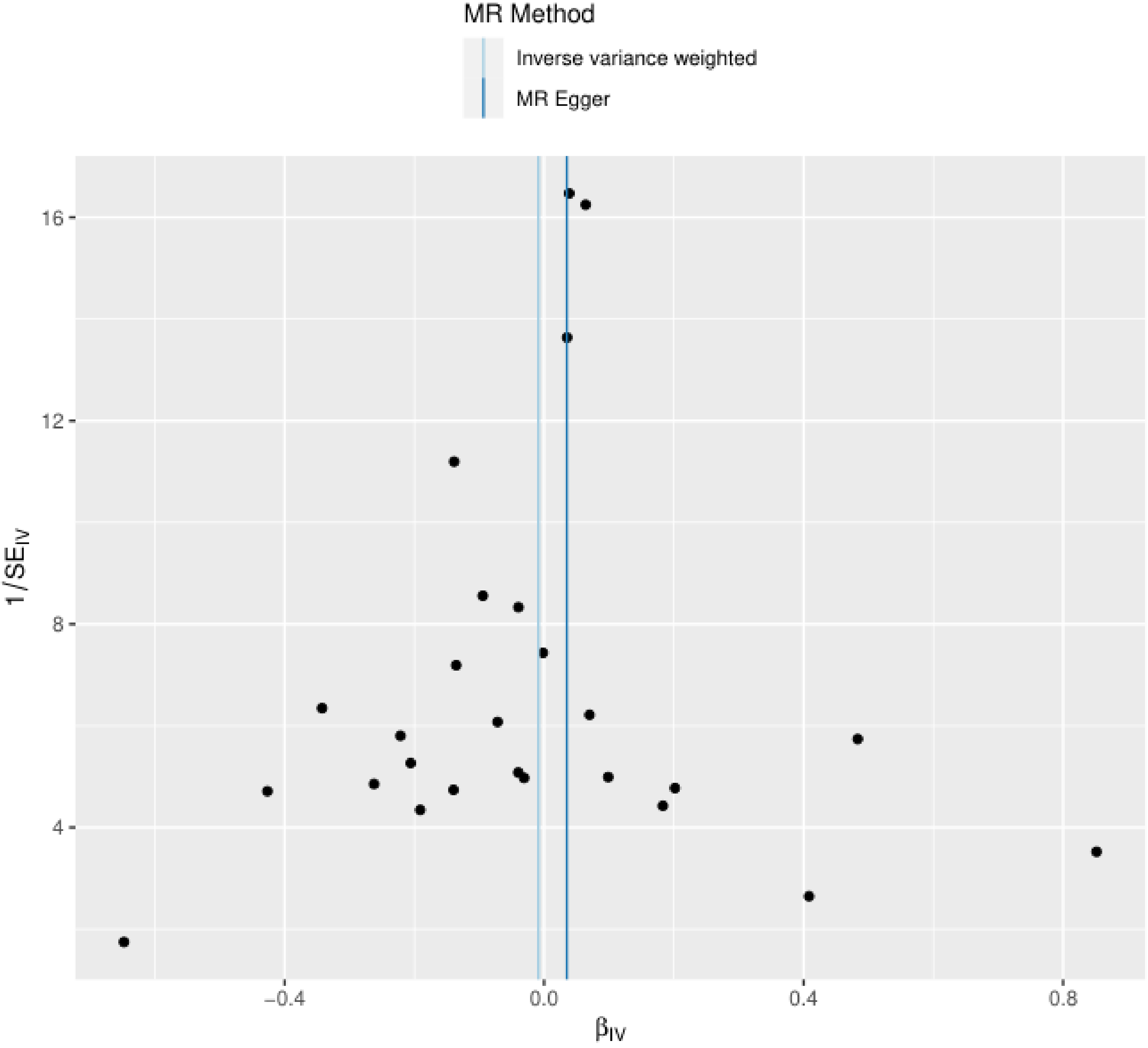

Funnel plot analyse of "CD28 on CD4+" on 'Diabetic nephropathy'

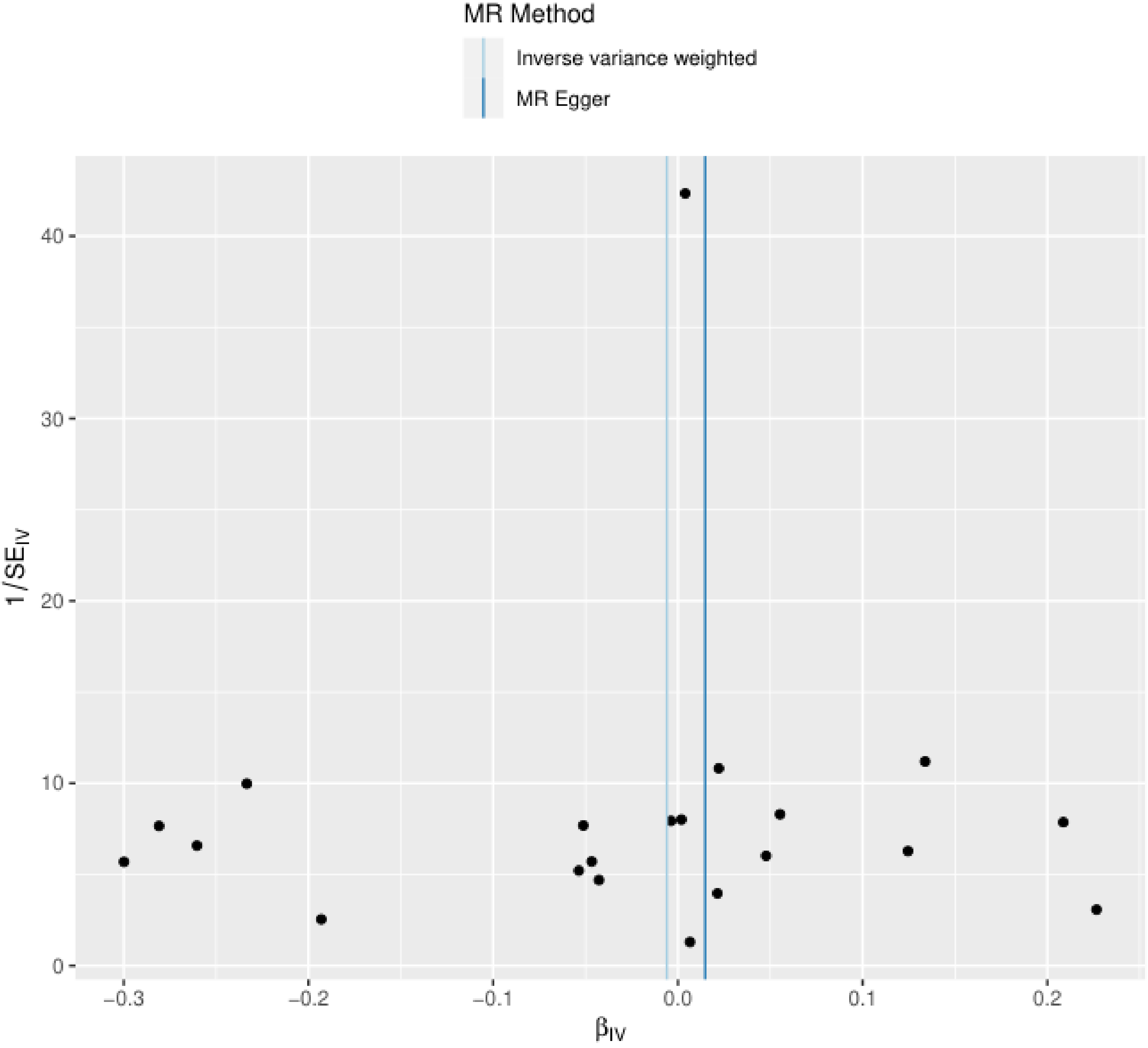

Funnel plot analyse of "CD33 on CD33dim HLA DR+ CD11b+ " on 'Diabetic nephropathy'

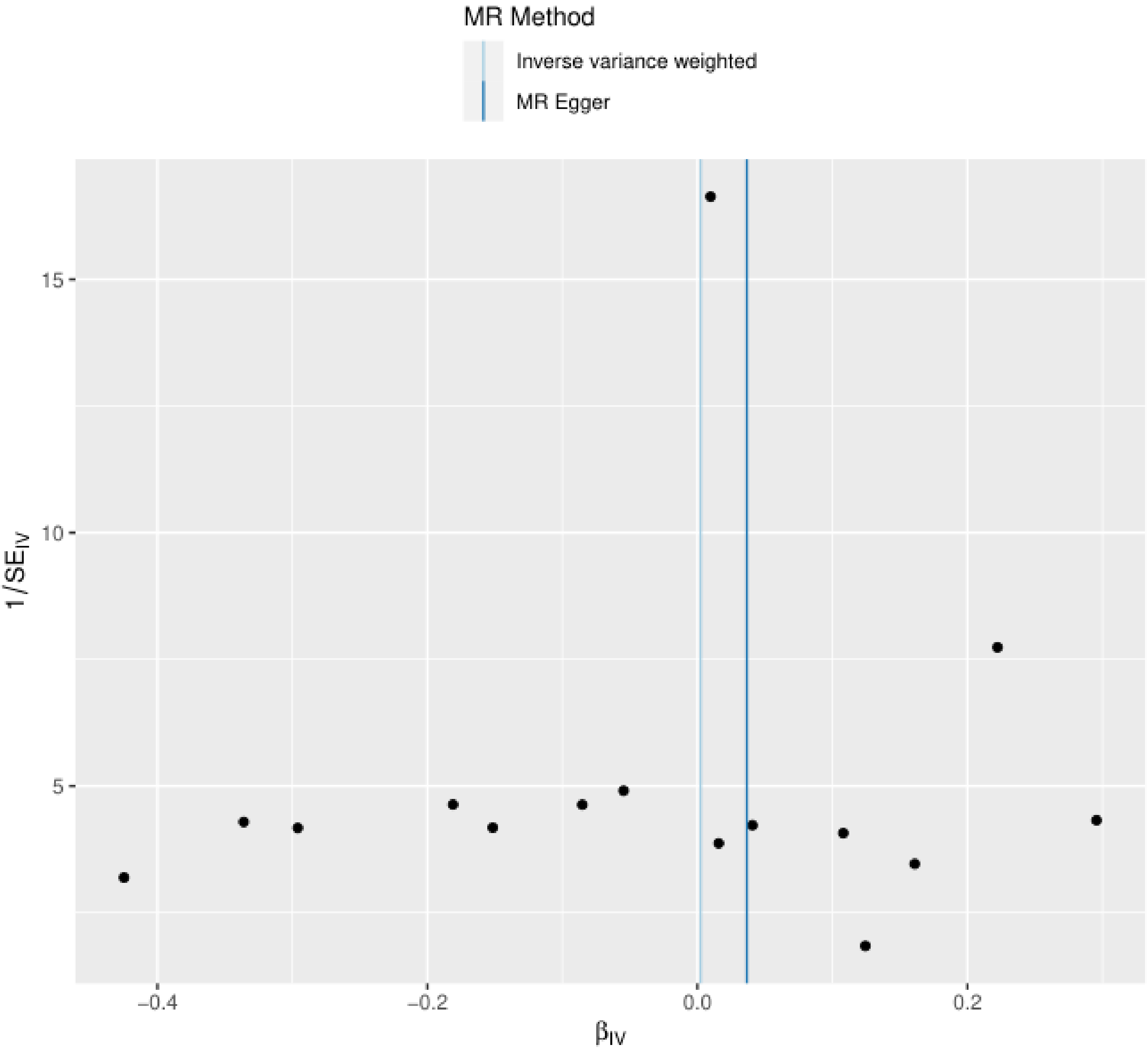

Funnel plot analyse of "IgD+ CD38dim %B cell" on 'Diabetic nephropathy'

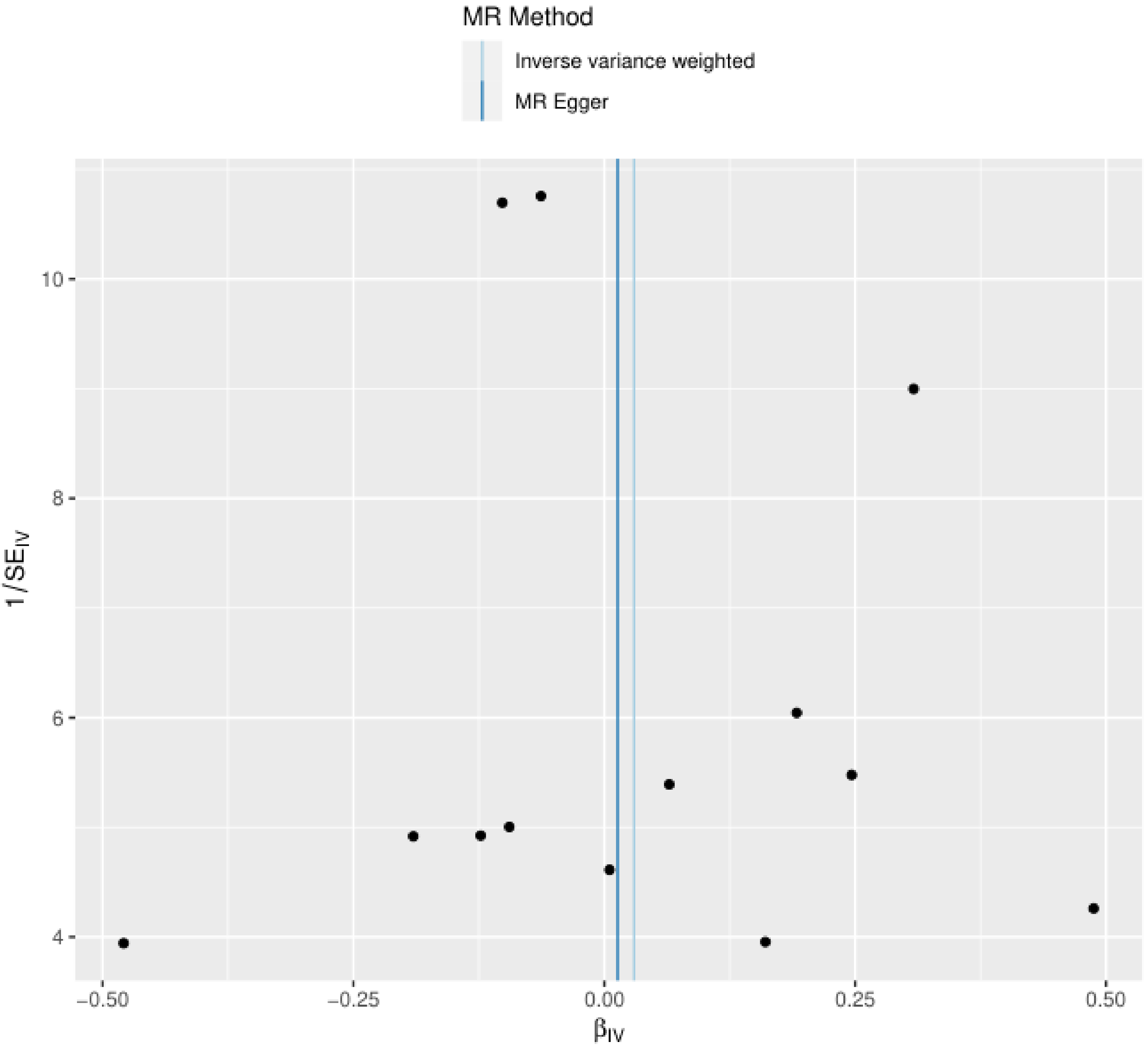

Funnel plot analyse of "CD28 on CD45RA+ CD4+" on 'Diabetic nephropathy'

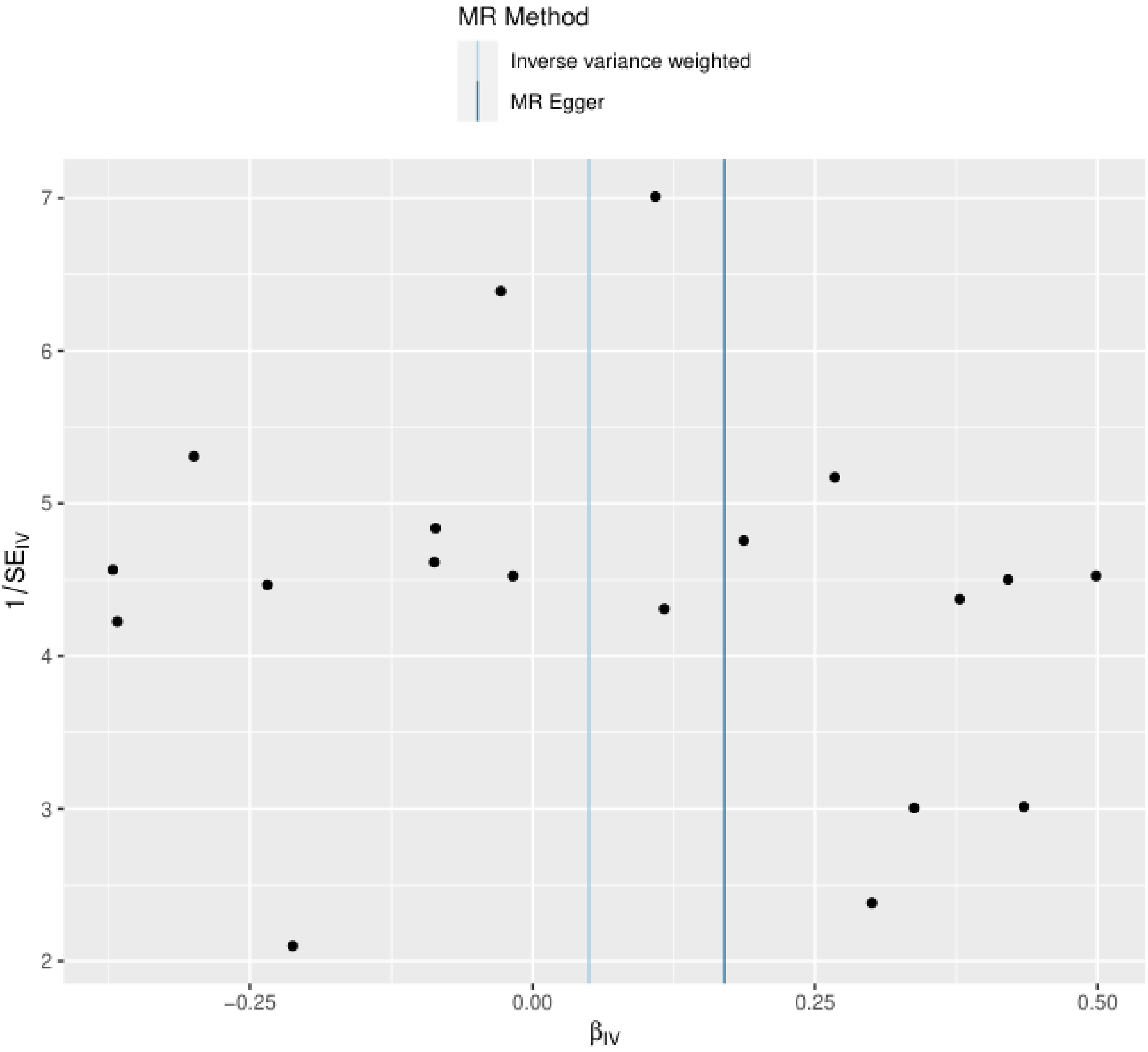

Funnel plot analyse of "CD62L- HLA DR++ monocyte AC" on 'Diabetic nephropathy'

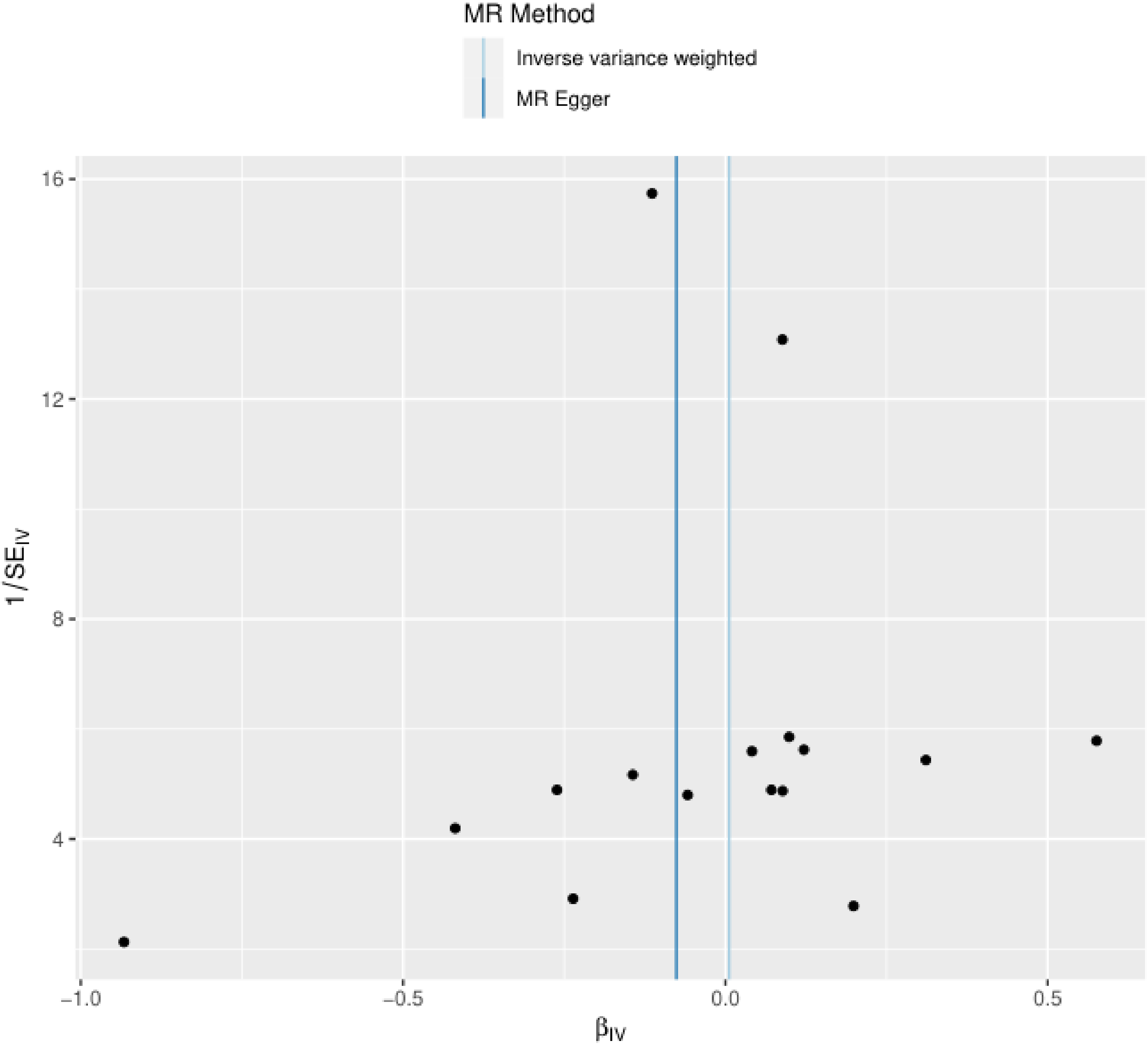

Funnel plot analyse of "CD8 on TD CD8br" on 'Diabetic nephropathy'

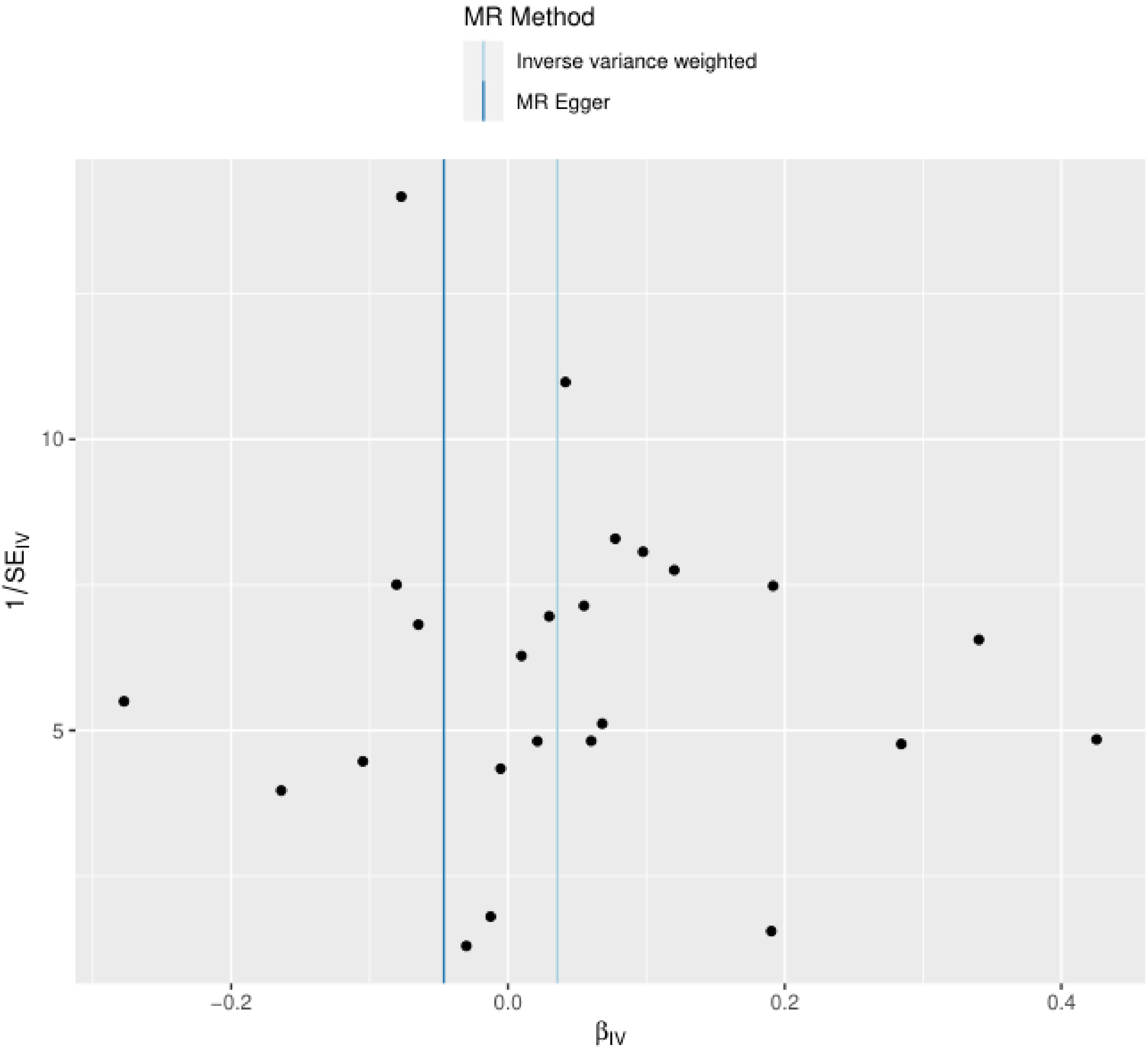

Funnel plot analyse of "CCR7 on naive CD8br" on 'Diabetic nephropathy'

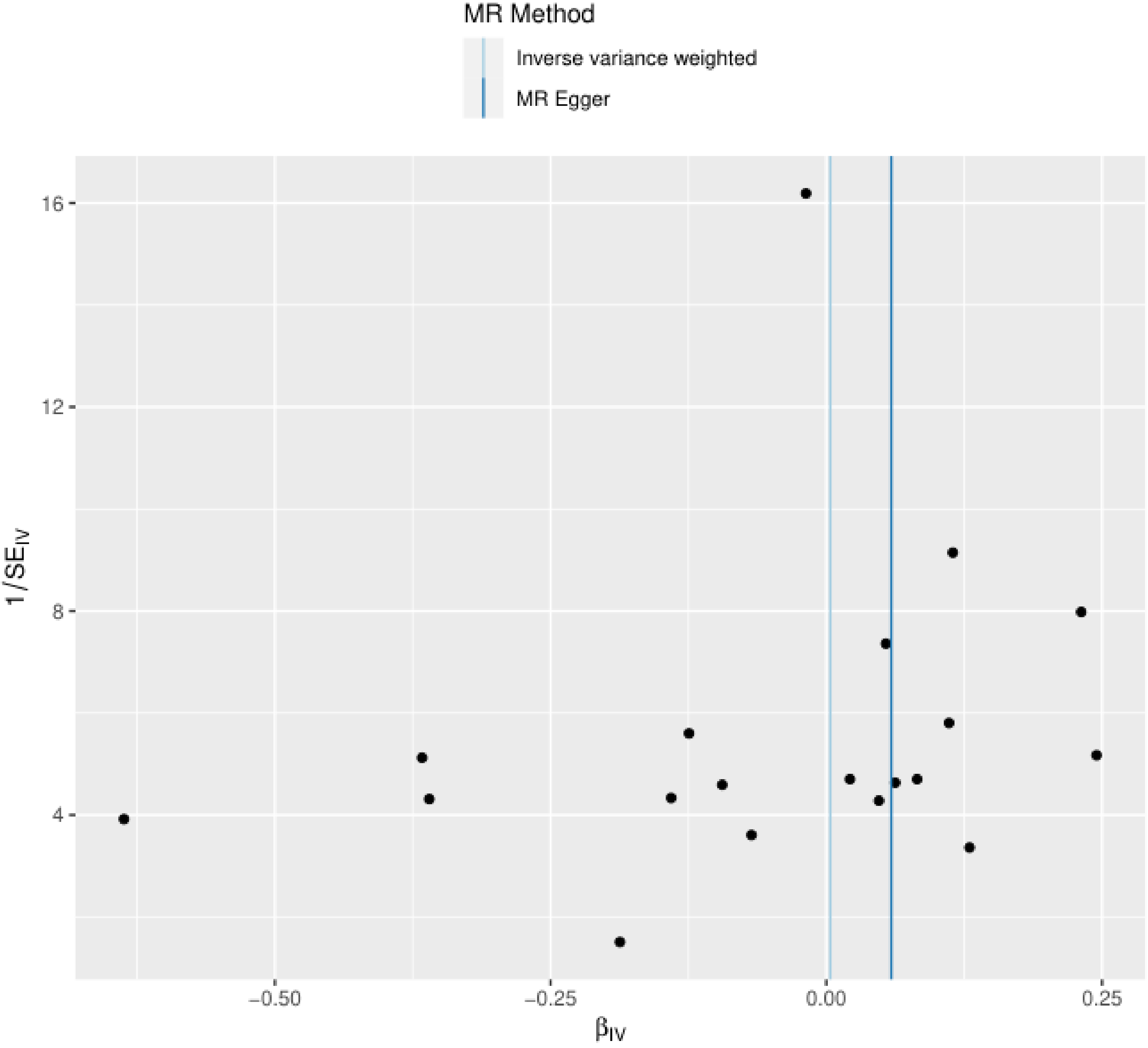

Funnel plot analyse of "CD25hi %T cell" on 'Diabetic nephropathy'

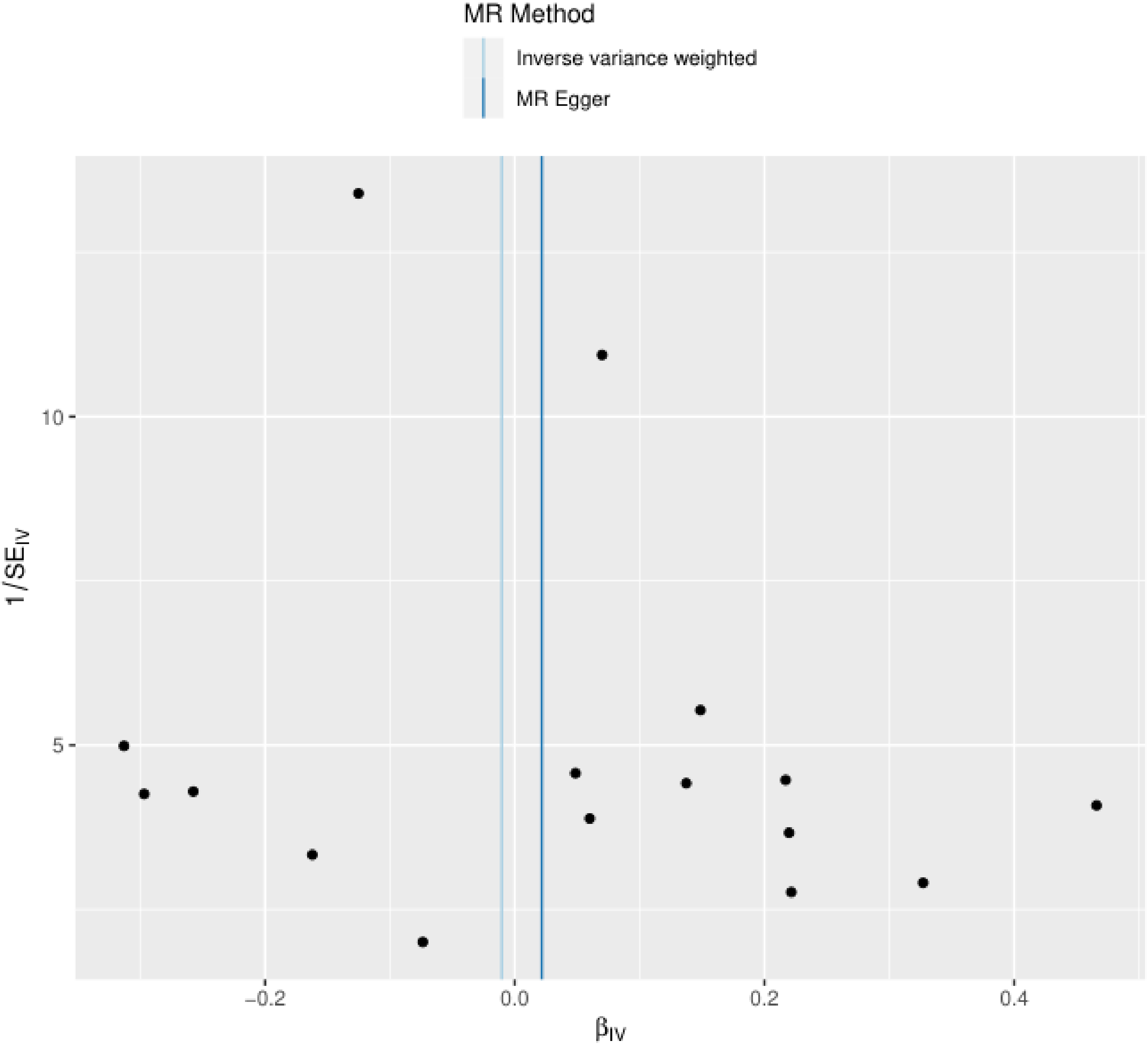

Funnel plot analyse of "IgD- CD27- %B cell" on 'Diabetic nephropathy'

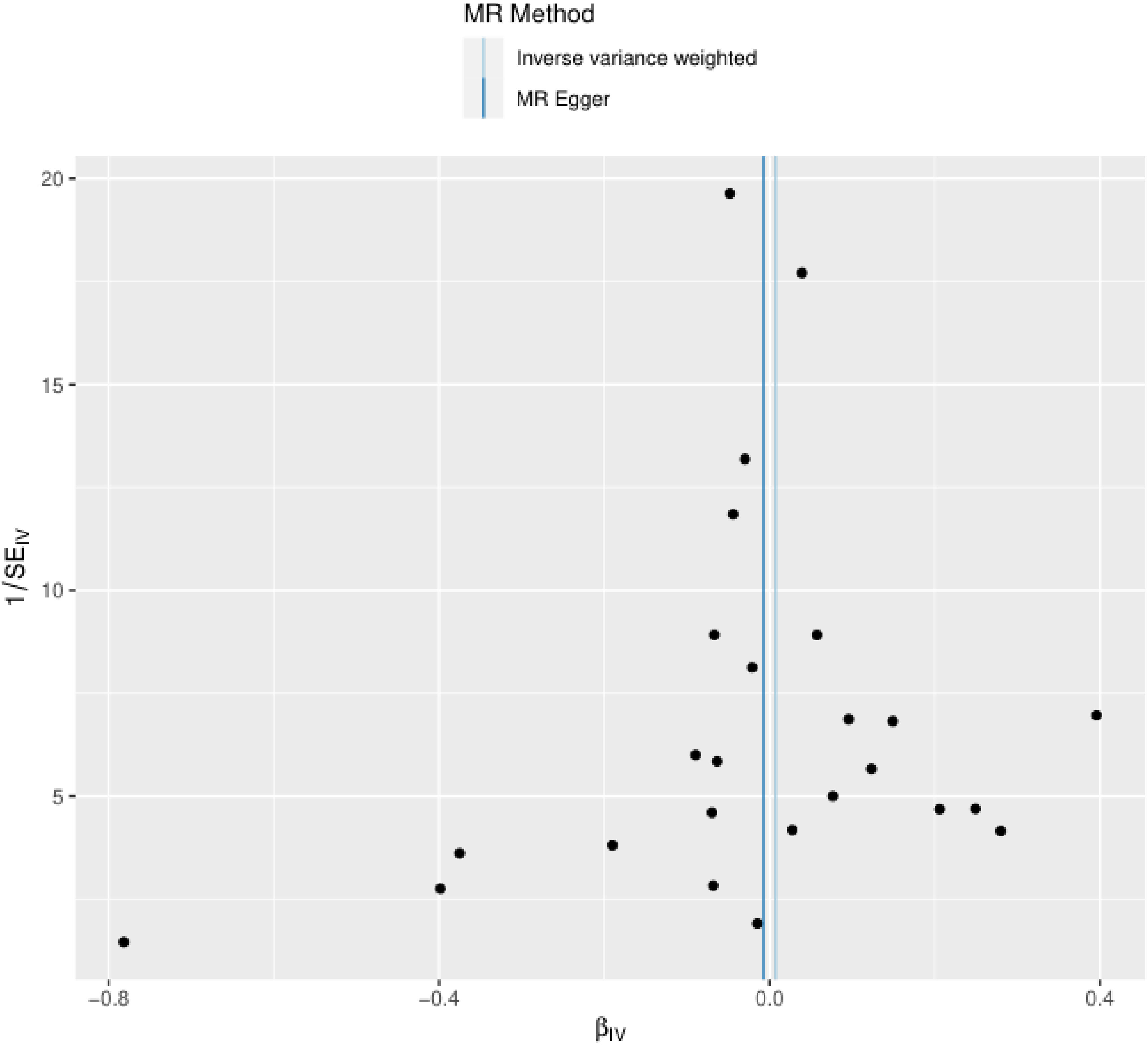

Funnel plot analyse of "CD25hi CD45RA+ CD4 not Treg AC" on 'Diabetic nephropathy'

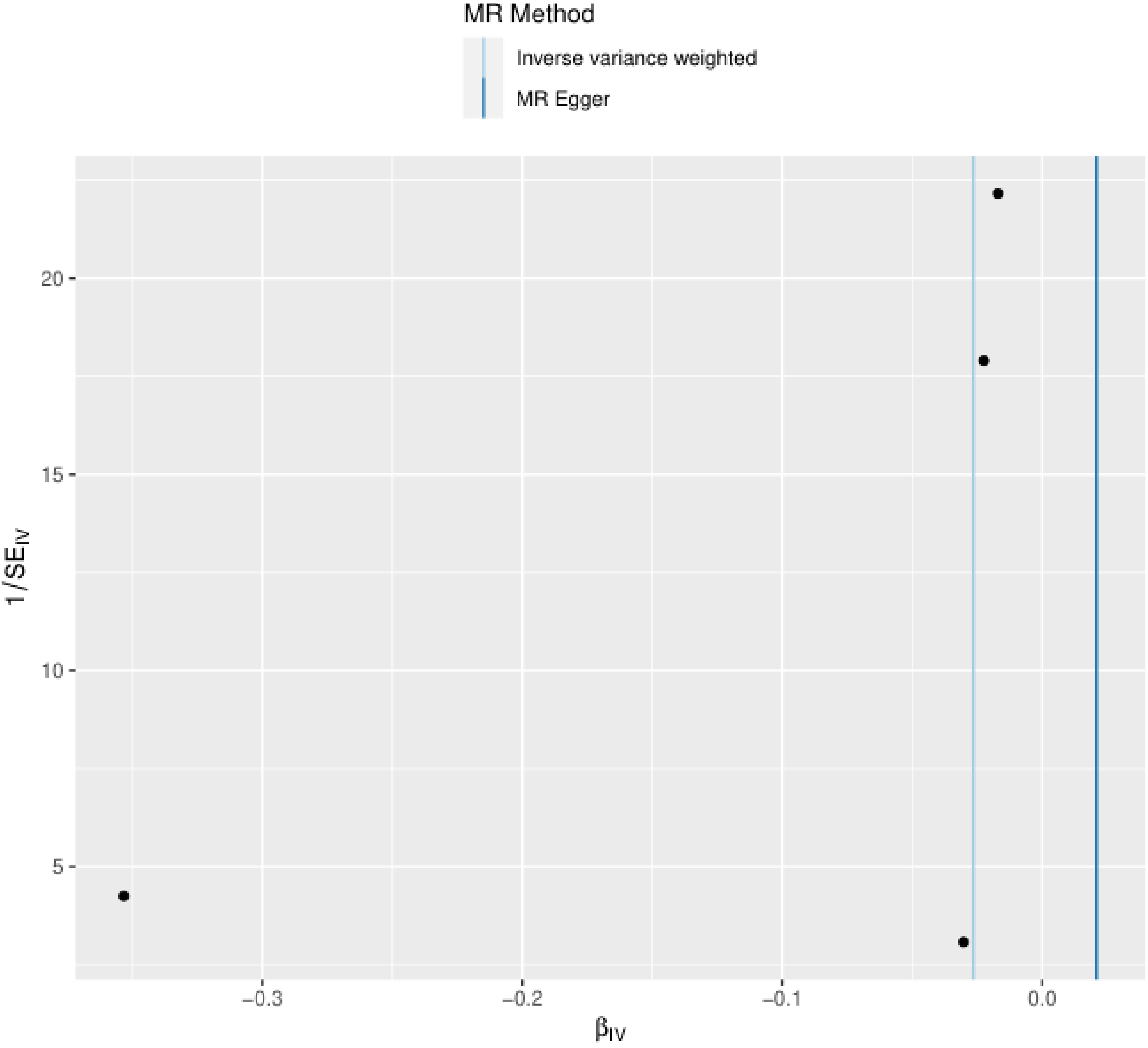

Funnel plot analyse of "CM DN (CD4-CD8-) AC" on 'Diabetic nephropathy'

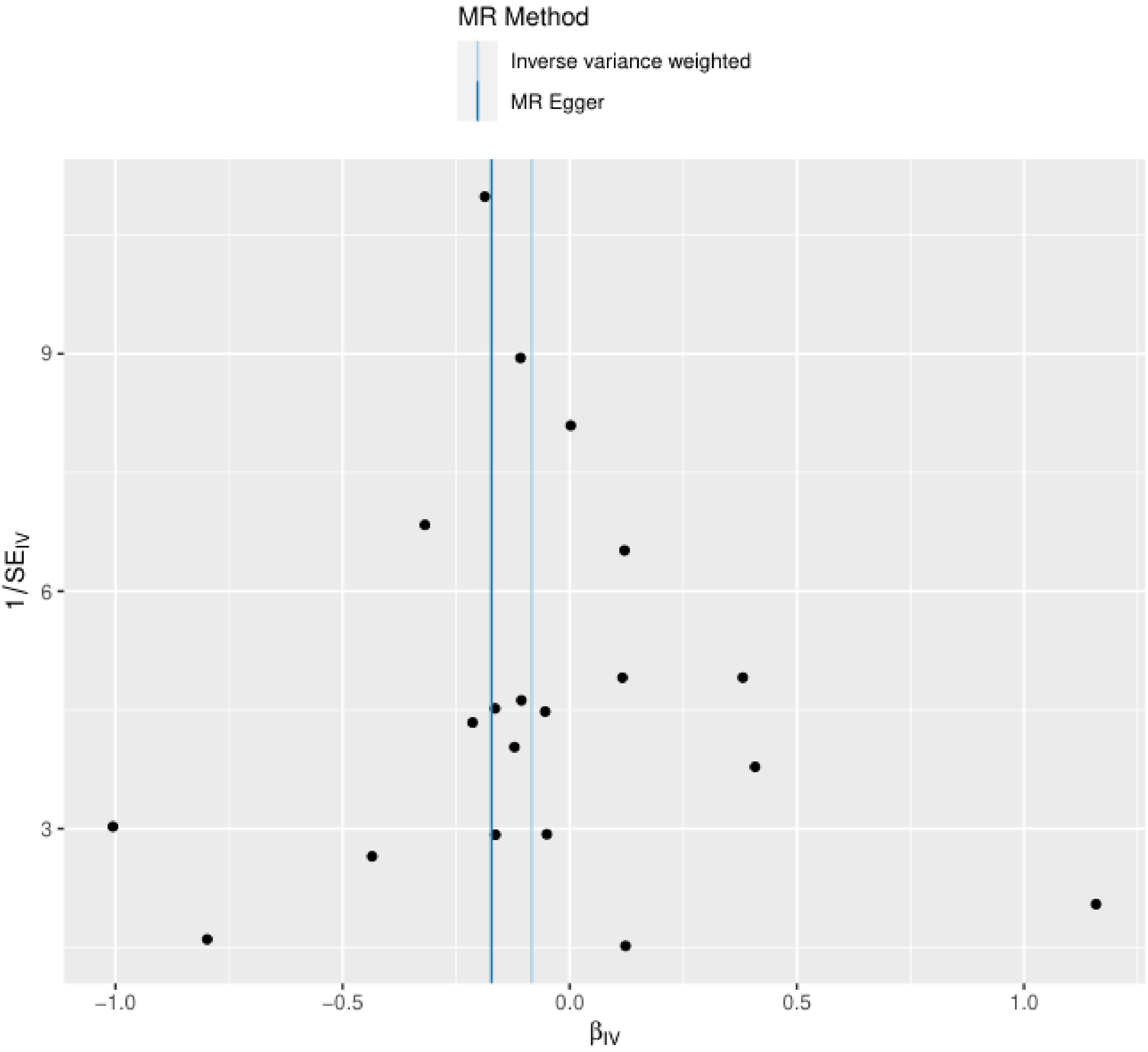

Funnel plot analyse of "CD86+ plasmacytoid DC AC" on 'Diabetic nephropathy'

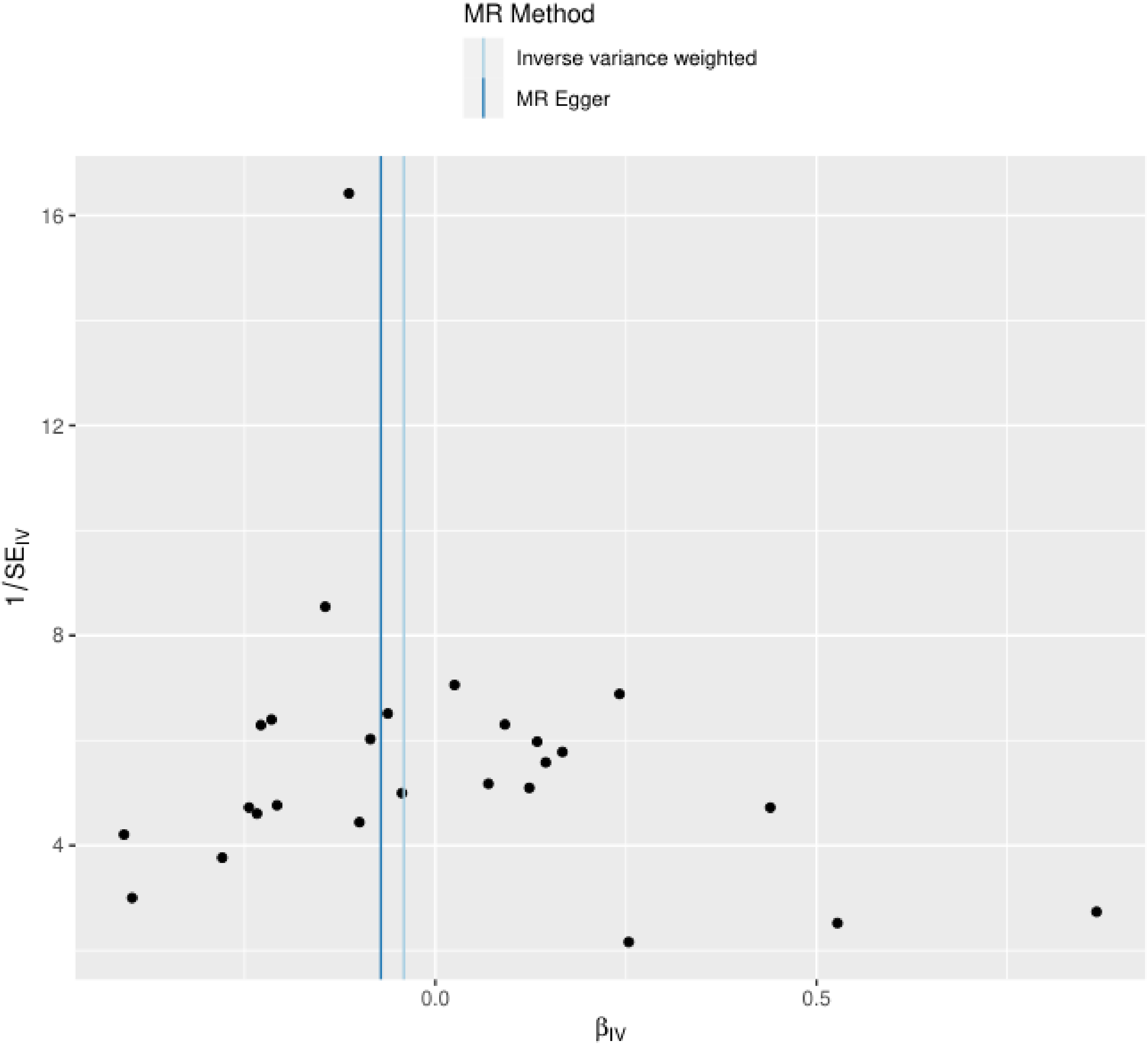

Funnel plot analyse of "CD62L on CD62L+ DC" on 'Diabetic nephropathy'

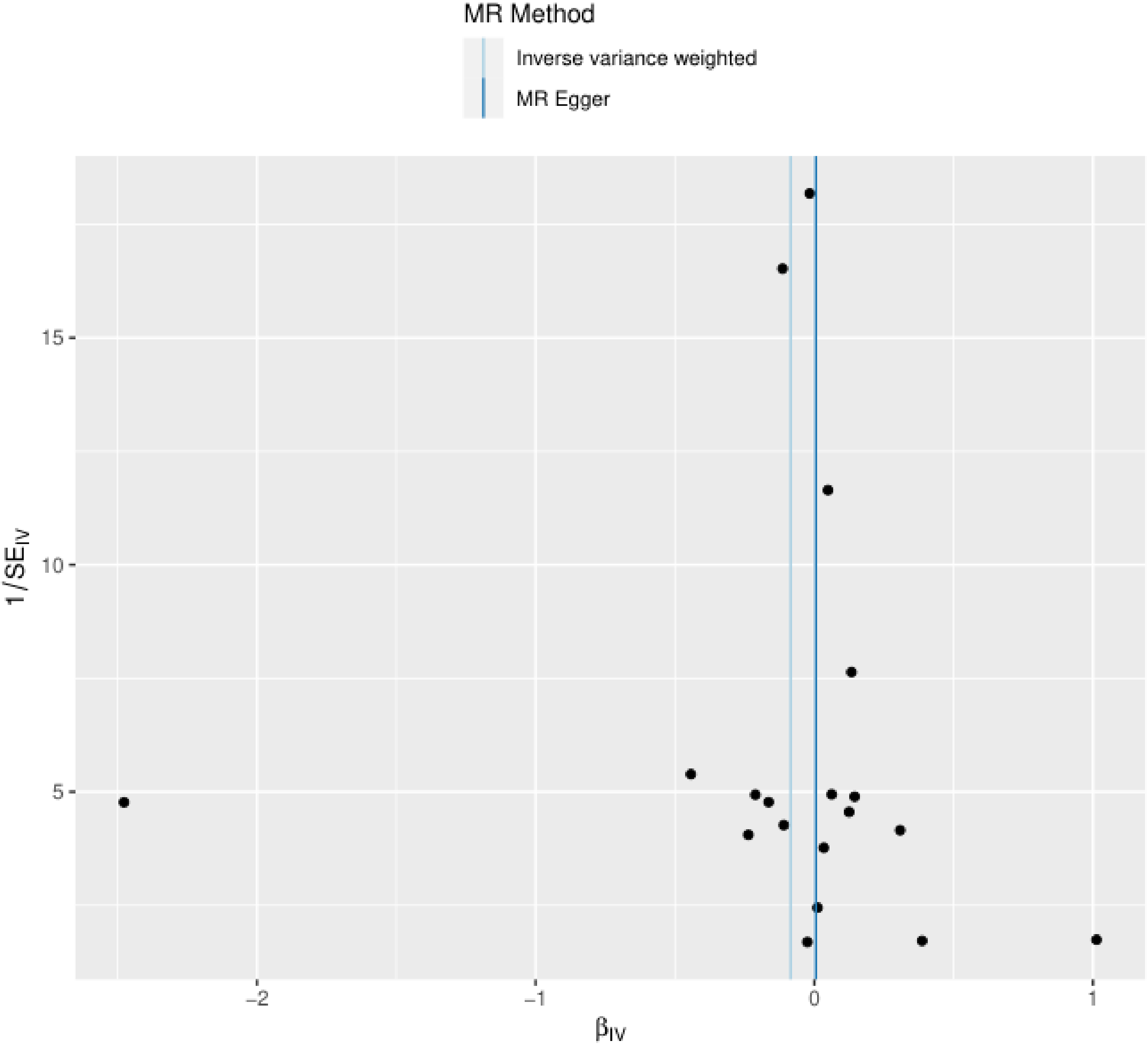

Funnel plot analyse of "CD4 Treg %CD4" on 'Diabetic nephropathy'

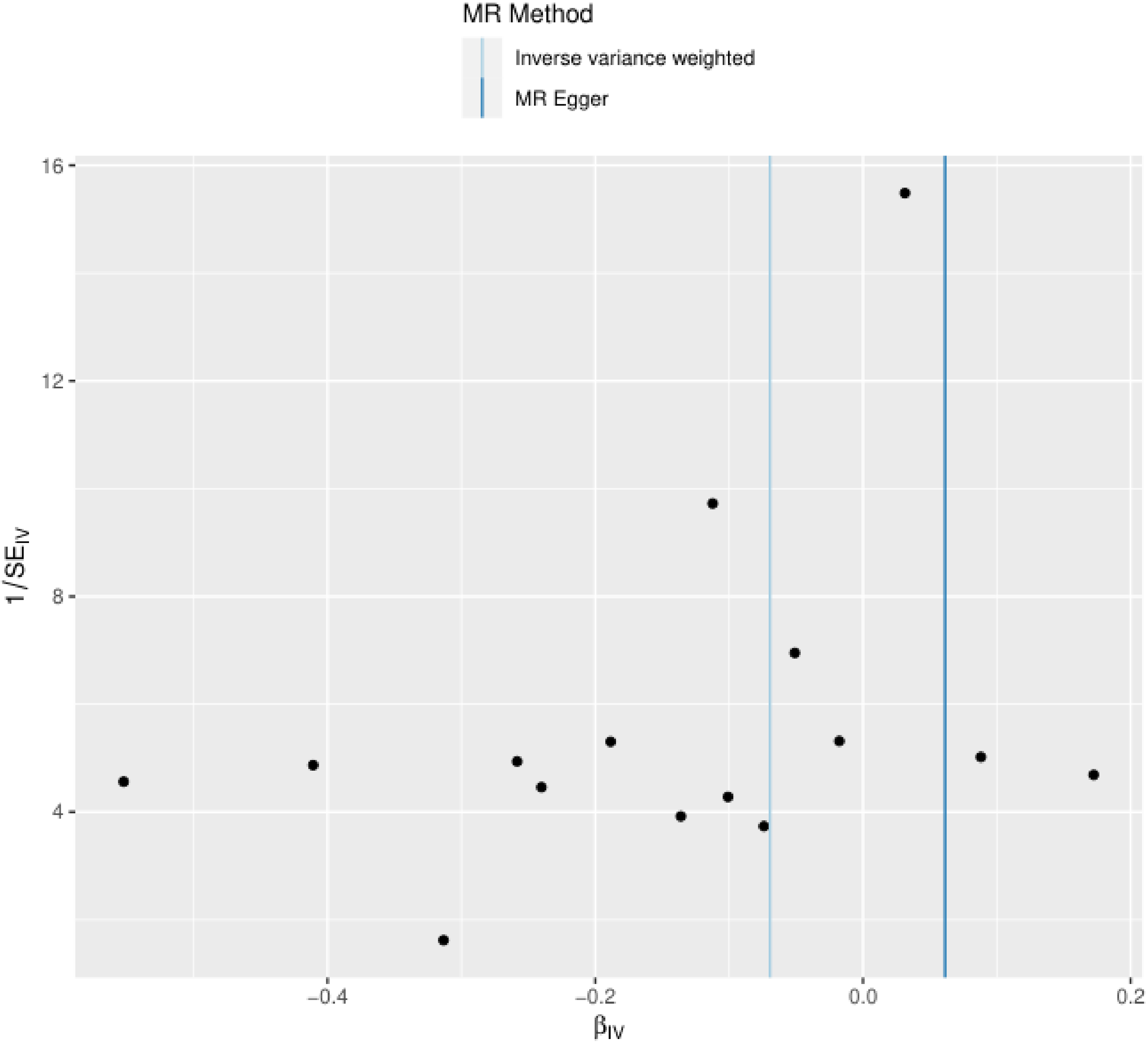

Funnel plot analyse of "CD127- CD8br AC" on 'Diabetic nephropathy'

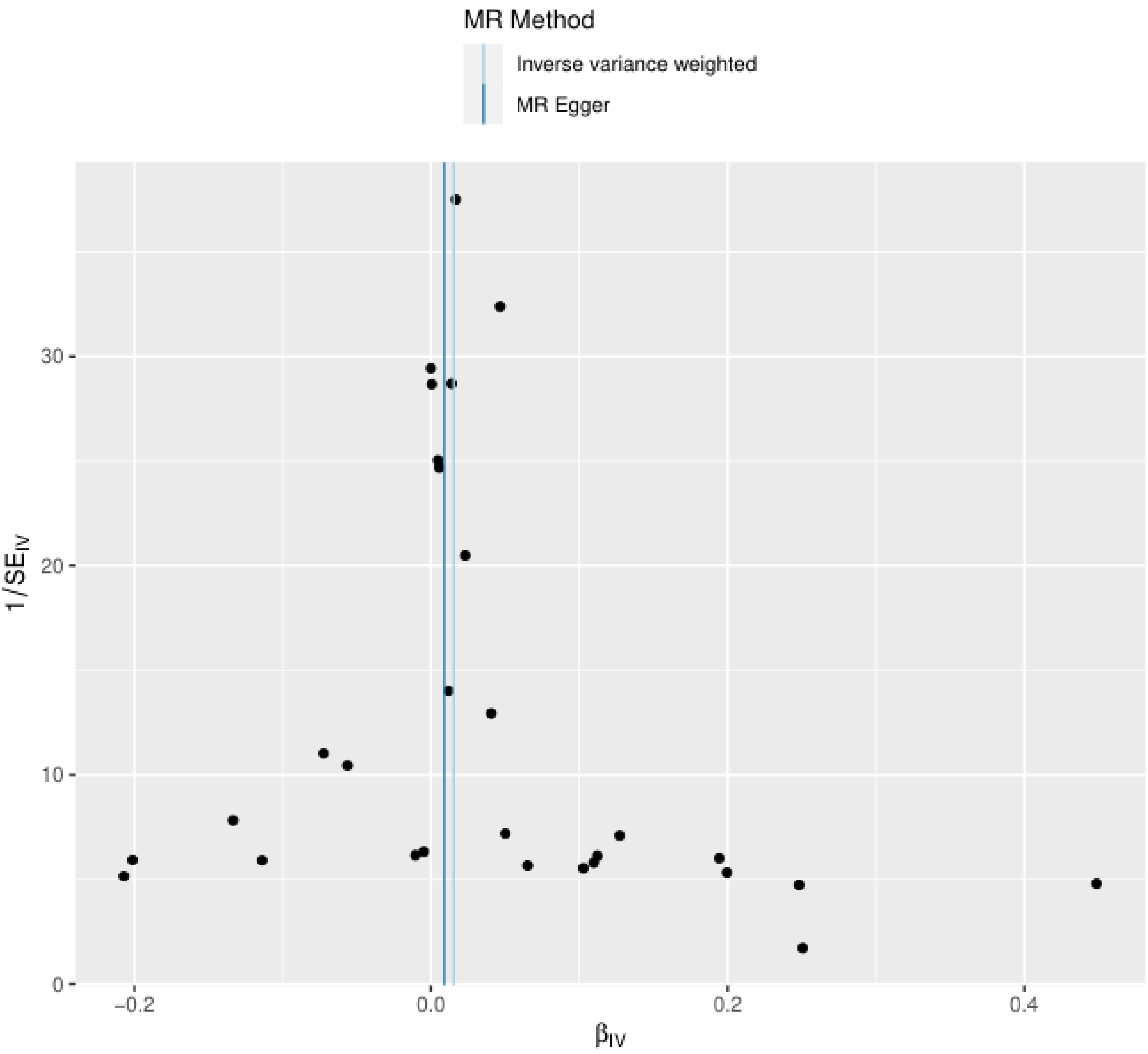

Funnel plot analyse of "CD33br HLA DR+ CD14- AC" on 'Diabetic nephropathy'

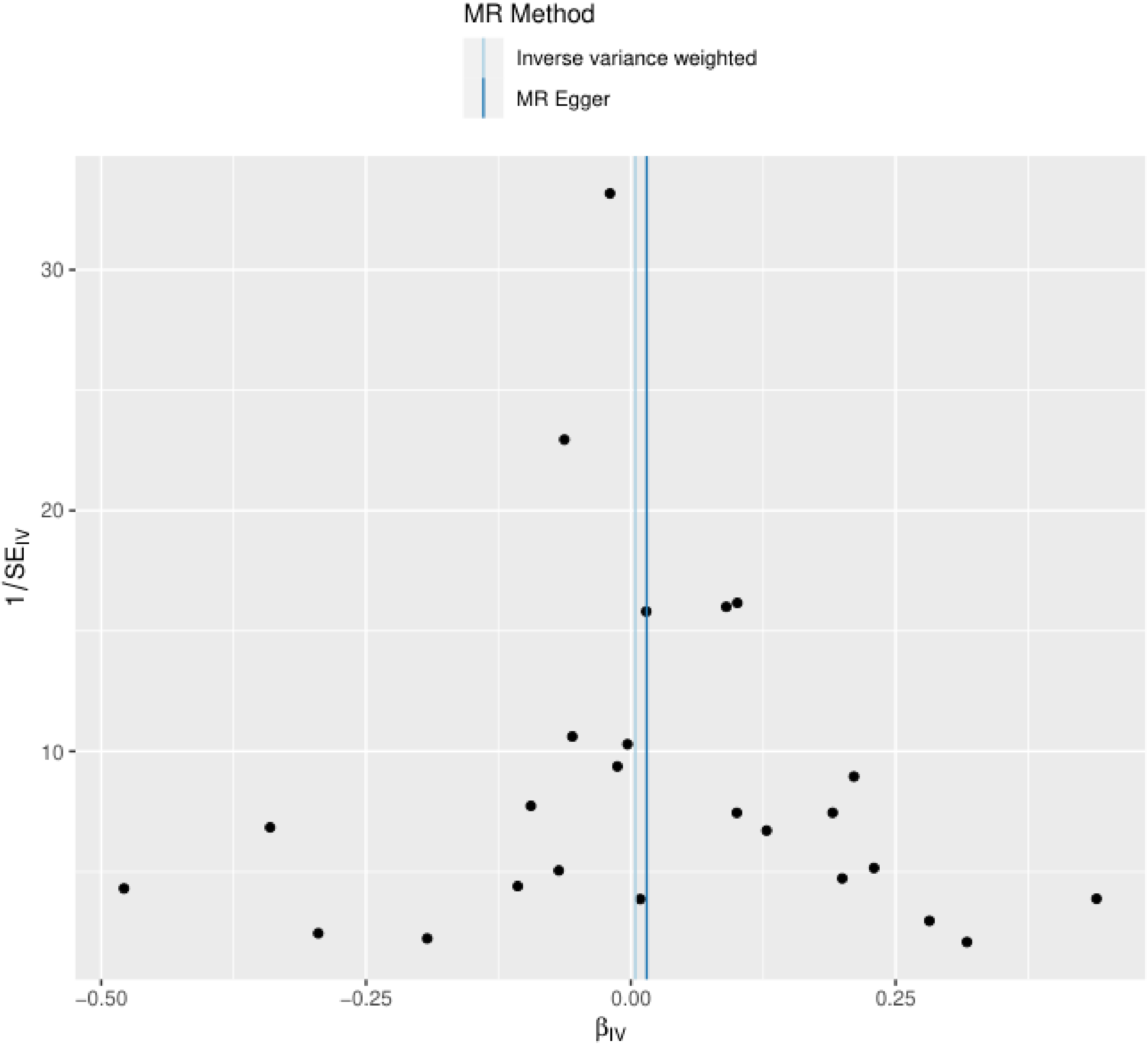

Funnel plot analyse of "BAFF-R on IgD+ CD38dim" on 'Diabetic nephropathy'

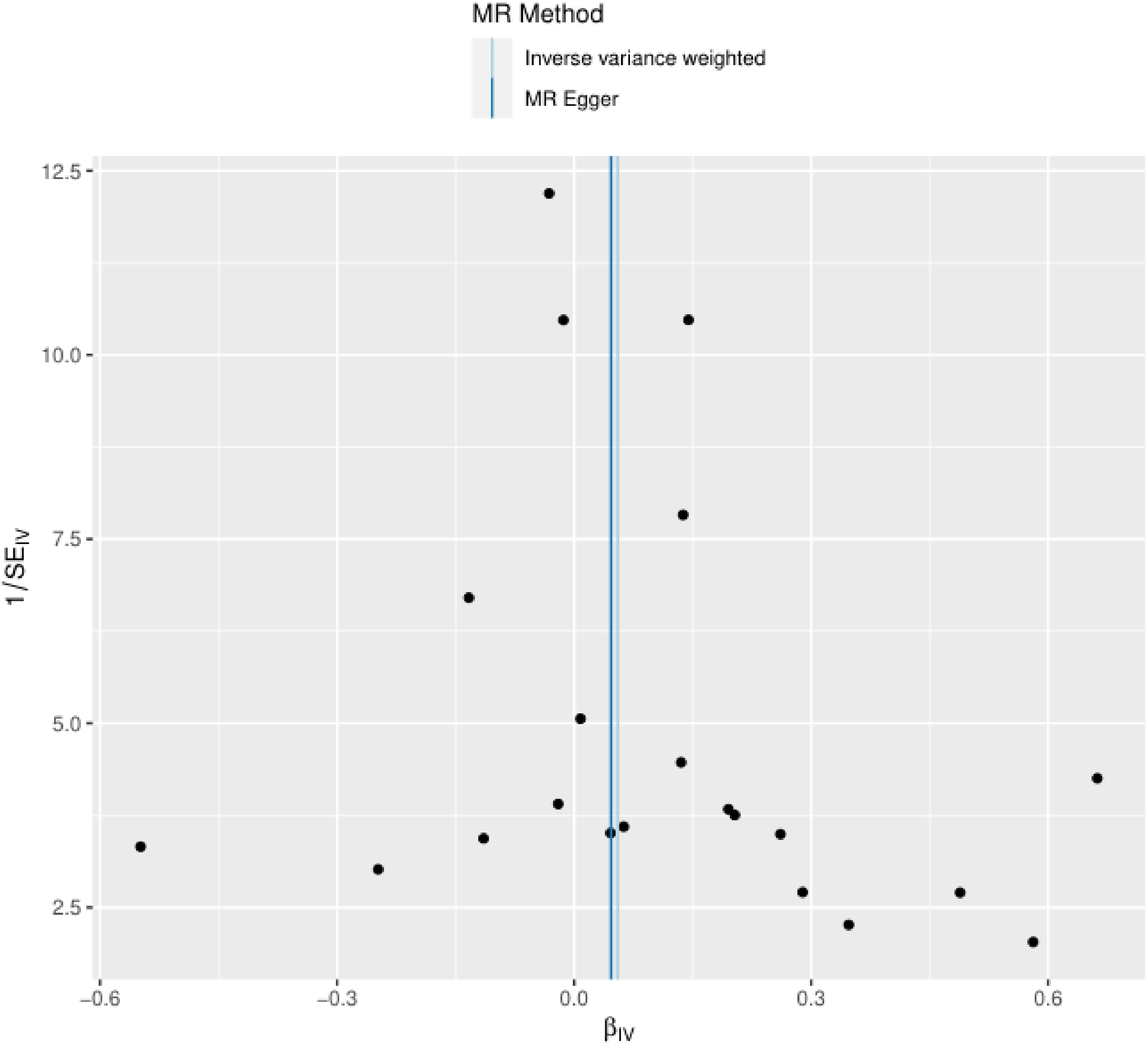

Funnel plot analyse of "Naive DN (CD4-CD8-) %T cell" on 'Diabetic nephropathy'

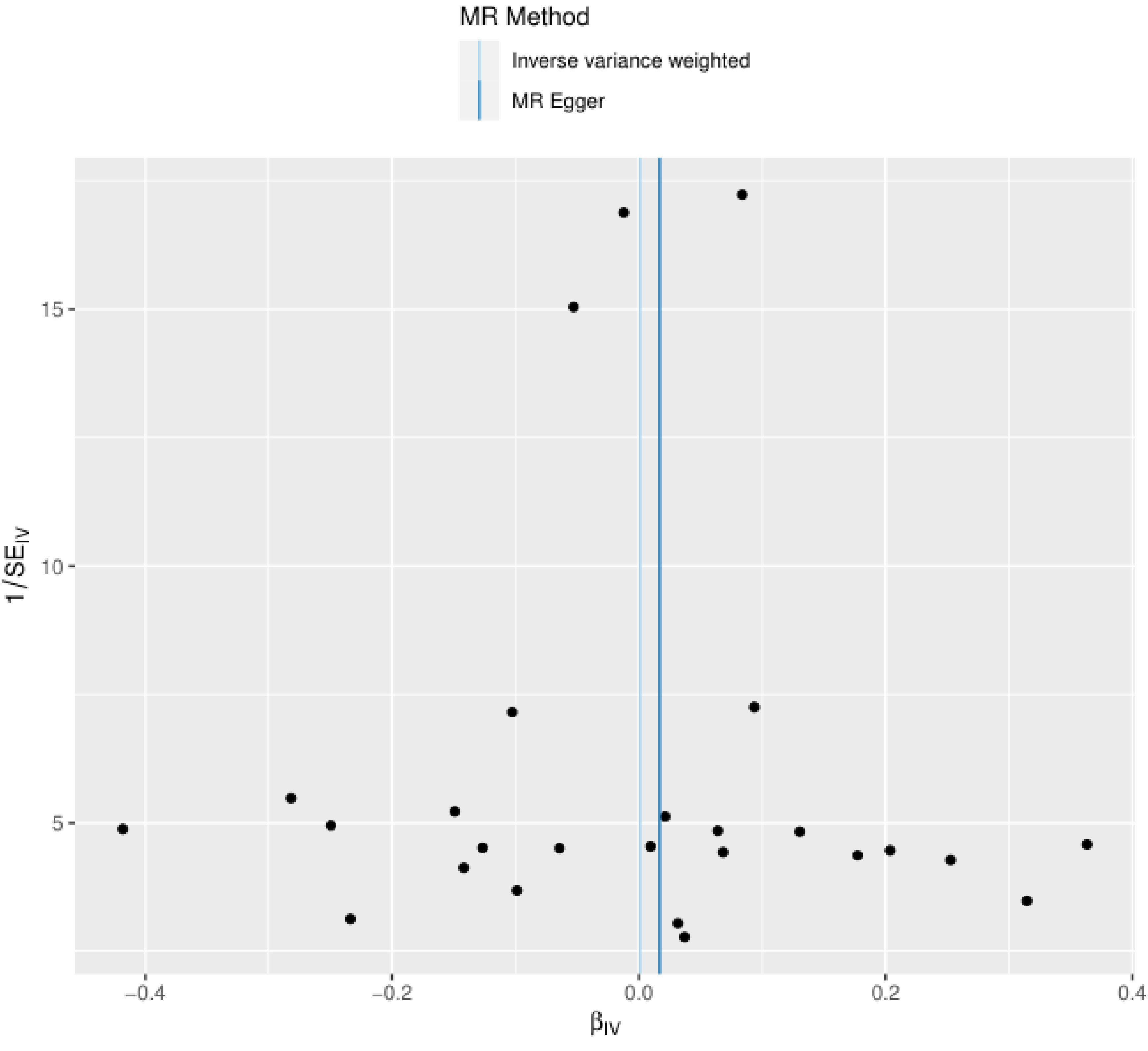

Funnel plot analyse of "CD19 on transitional" on 'Diabetic nephropathy'

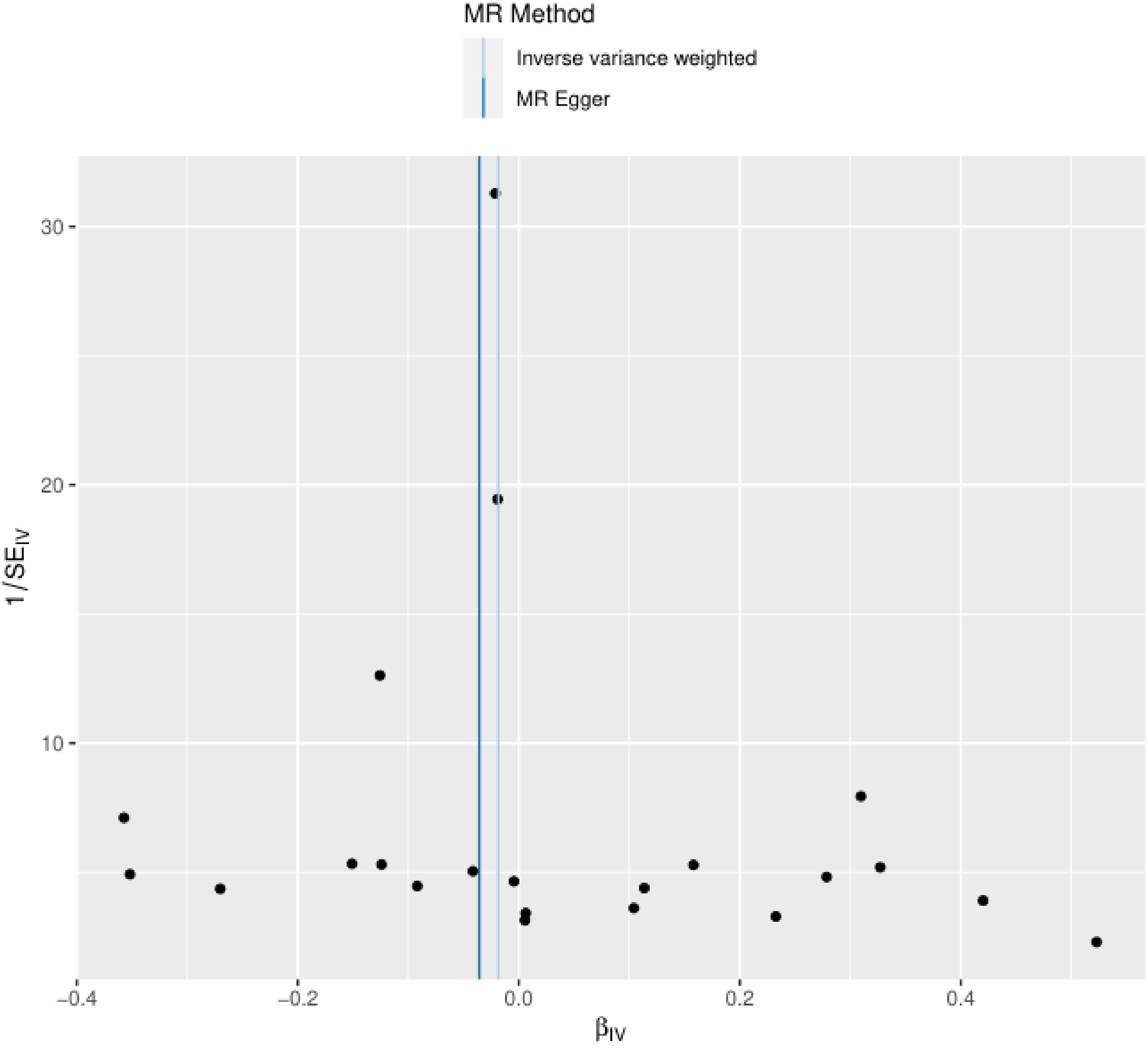

Funnel plot analyse of "CD86+ myeloid DC AC" on 'Diabetic nephropathy'

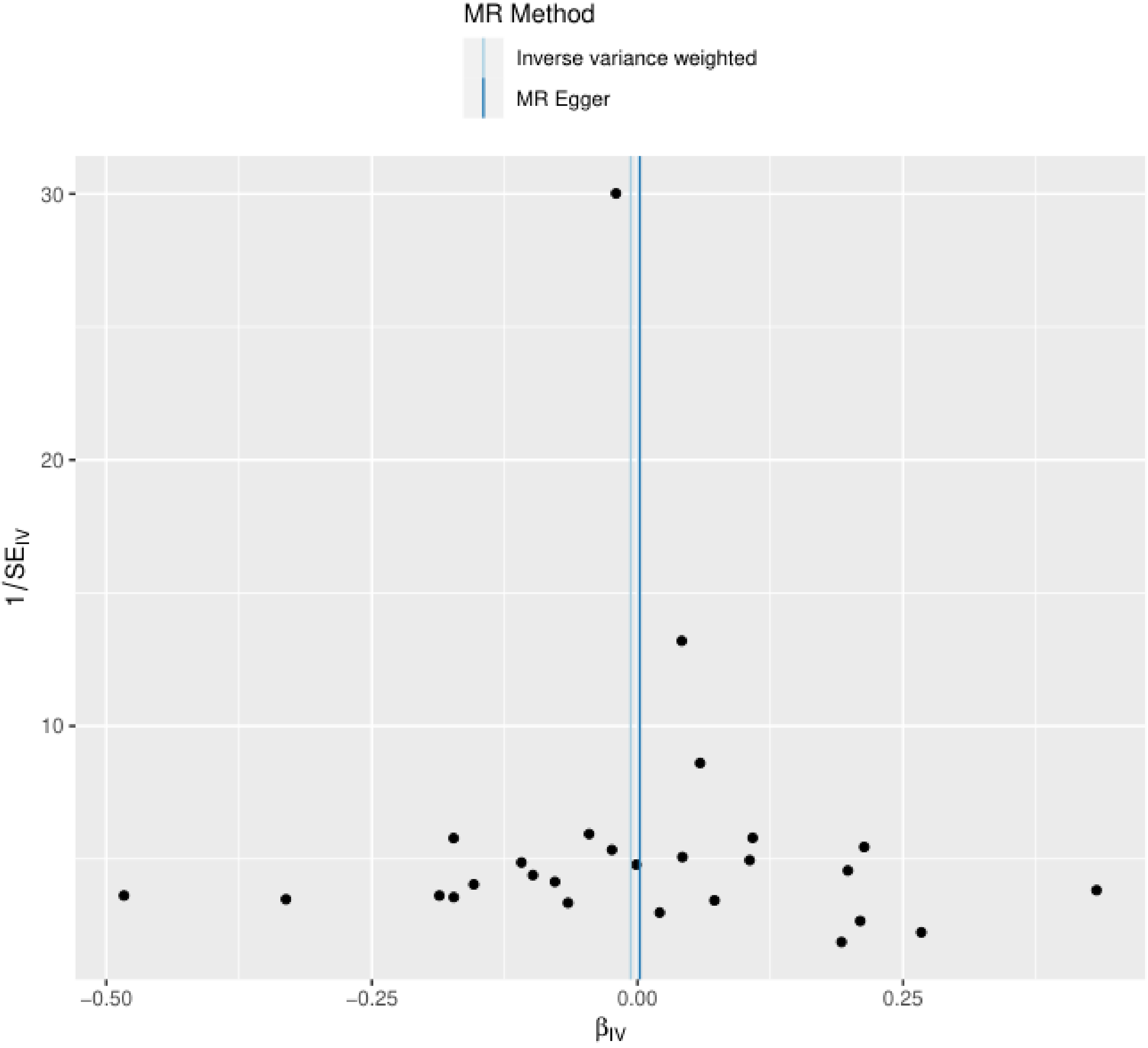

Funnel plot analyse of "PB/PC %B cell" on 'Diabetic nephropathy'

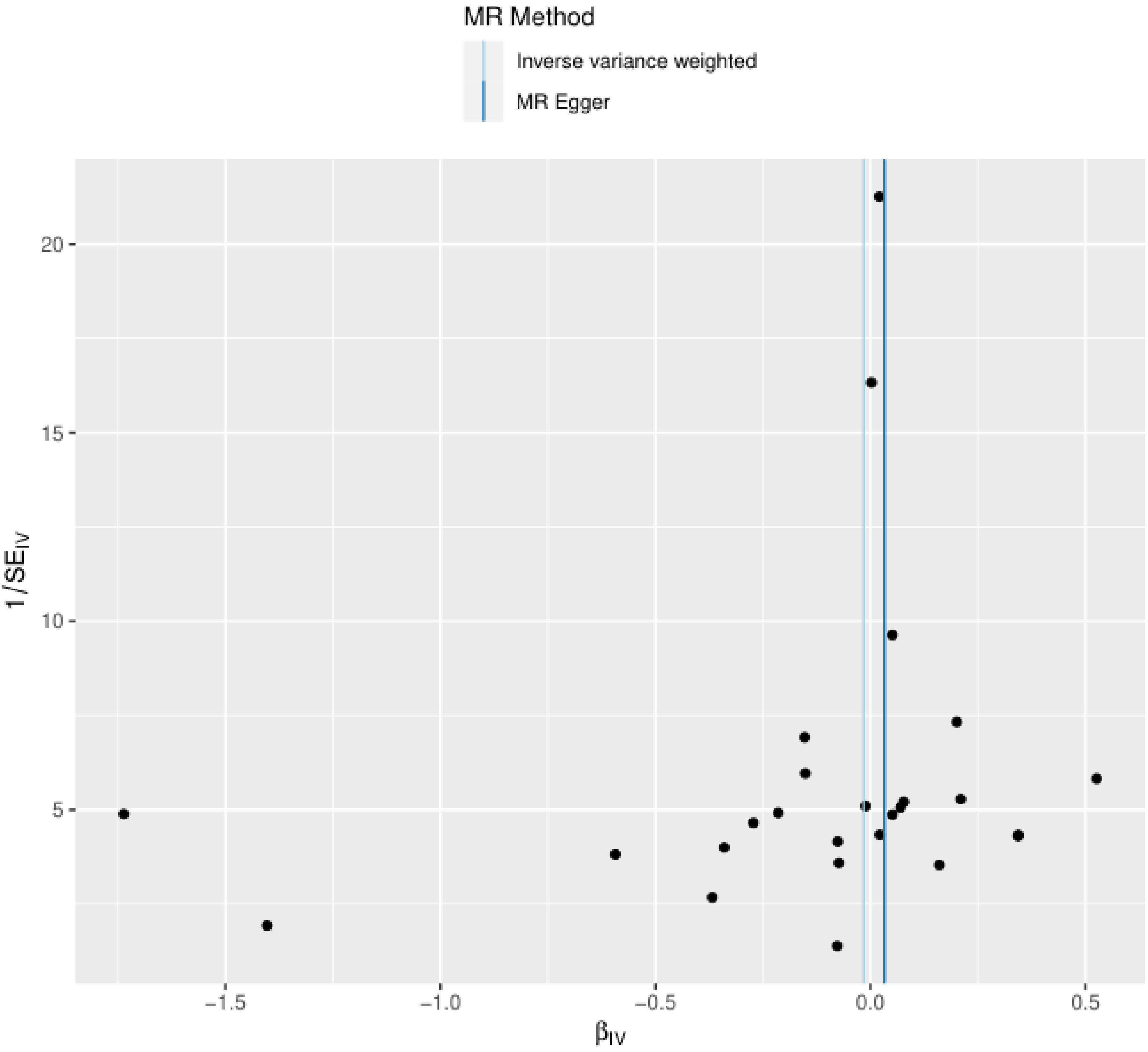

Funnel plot analyse of "CD20 on IgD+ CD38br" on 'Diabetic nephropathy'

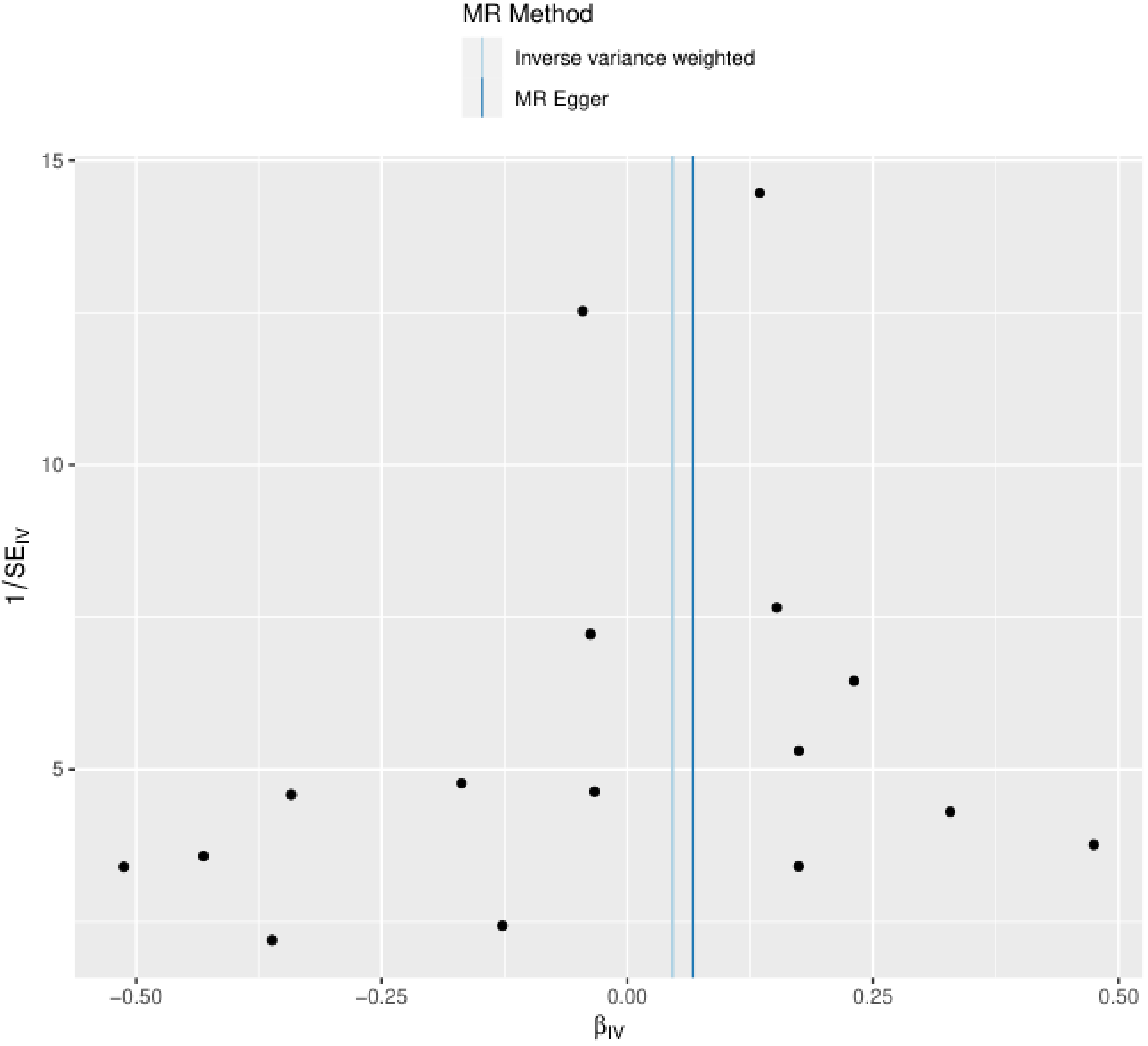

Funnel plot analyse of "CD45 on CD14+ monocyte" on 'Diabetic nephropathy'

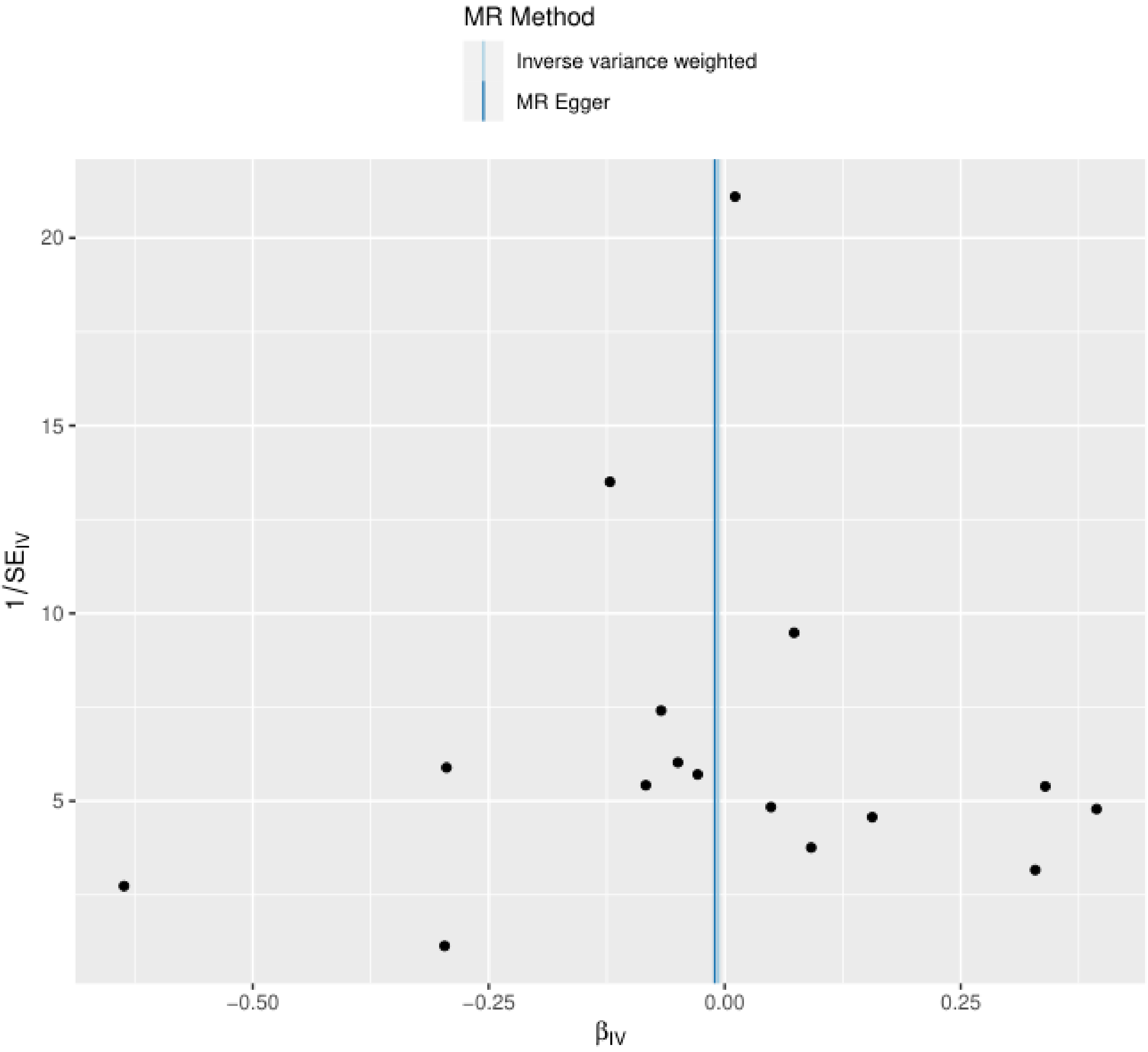

Funnel plot analyse of "CD45 on granulocyte" on 'Diabetic nephropathy'

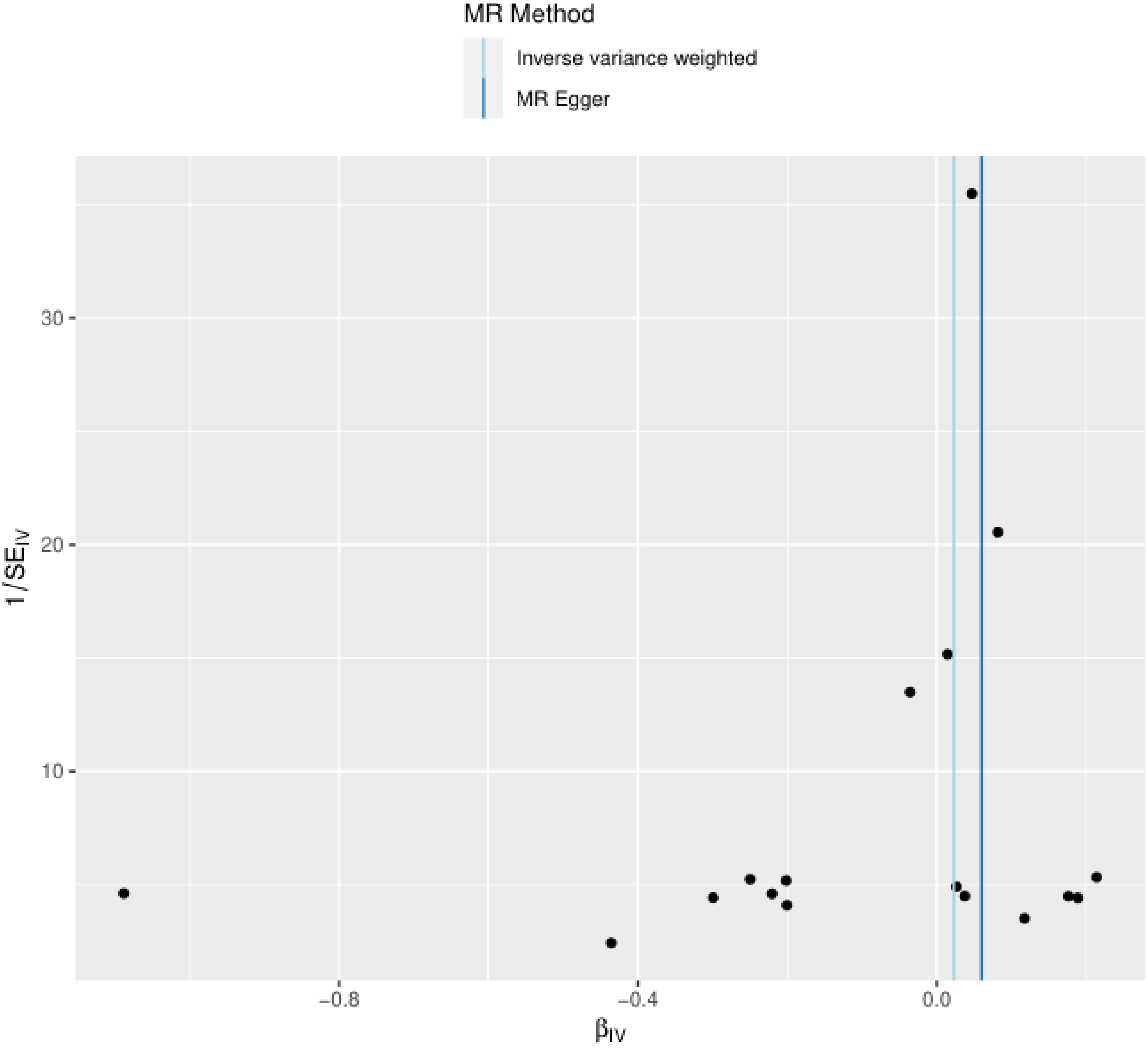

Funnel plot analyse of "CD45 on B cell" on 'Diabetic nephropathy'

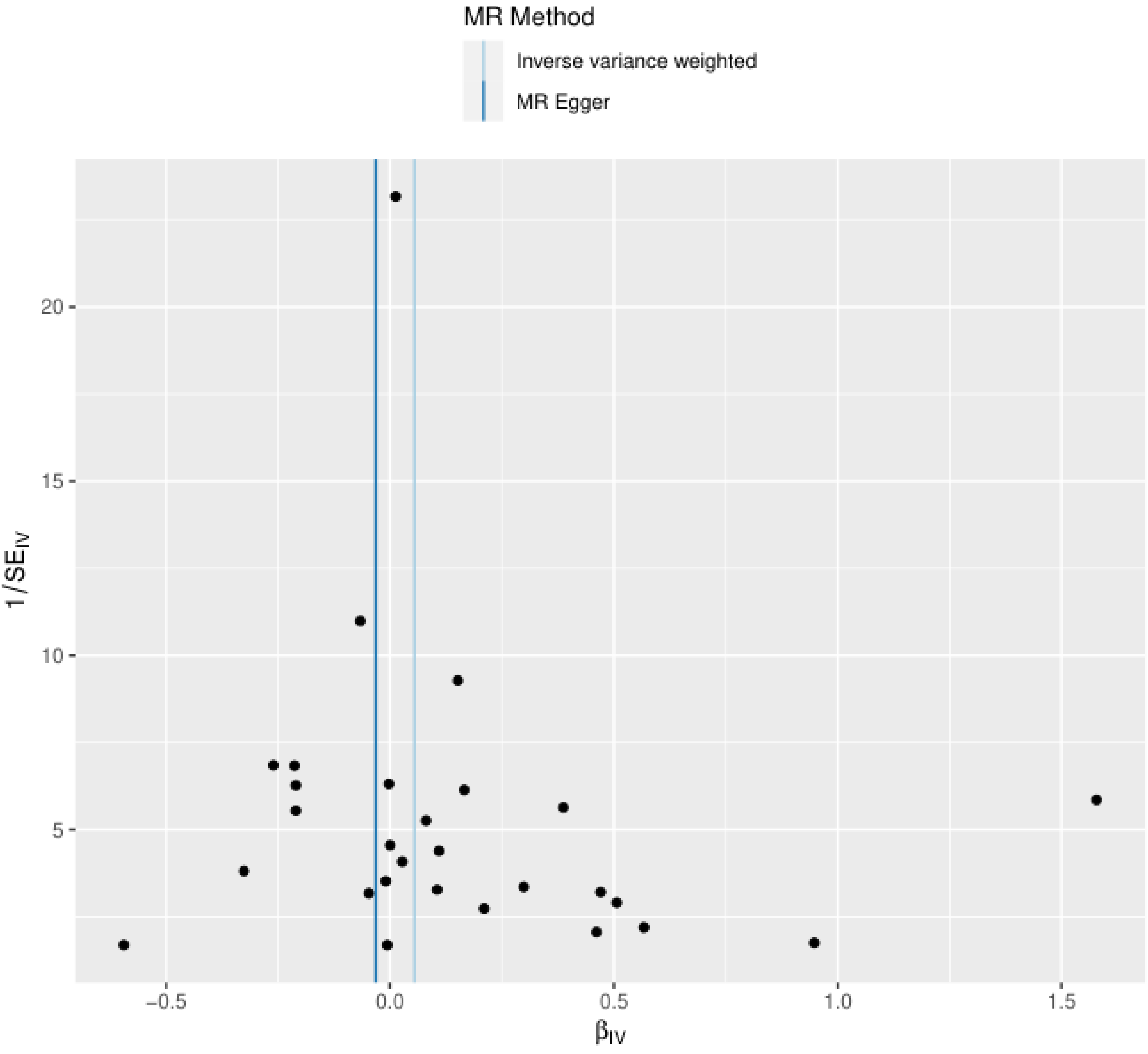

Funnel plot analyse of "CM CD4+ AC" on 'Diabetic nephropathy'

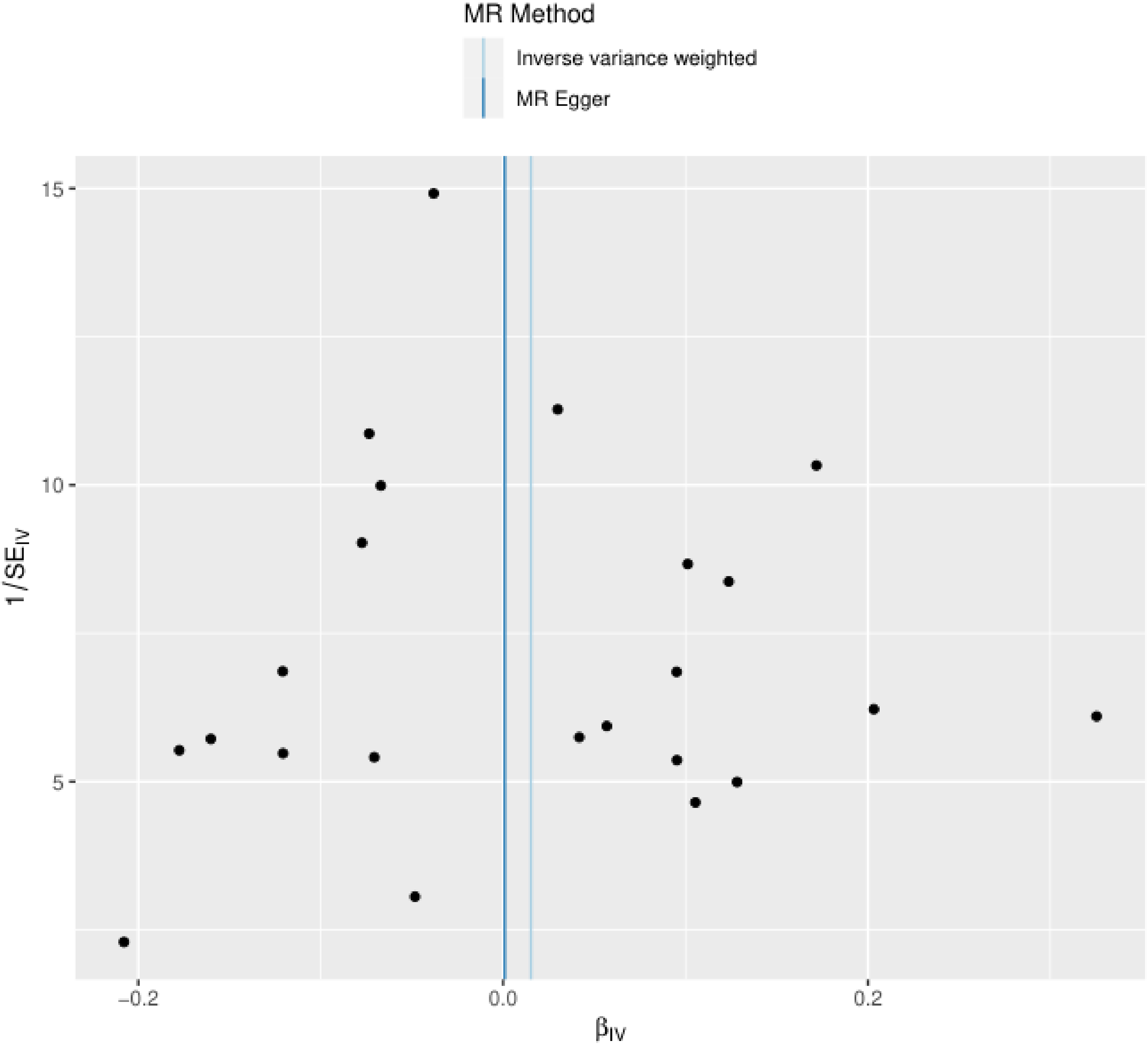

Funnel plot analyse of "Im MDSC AC" on 'Diabetic nephropathy'

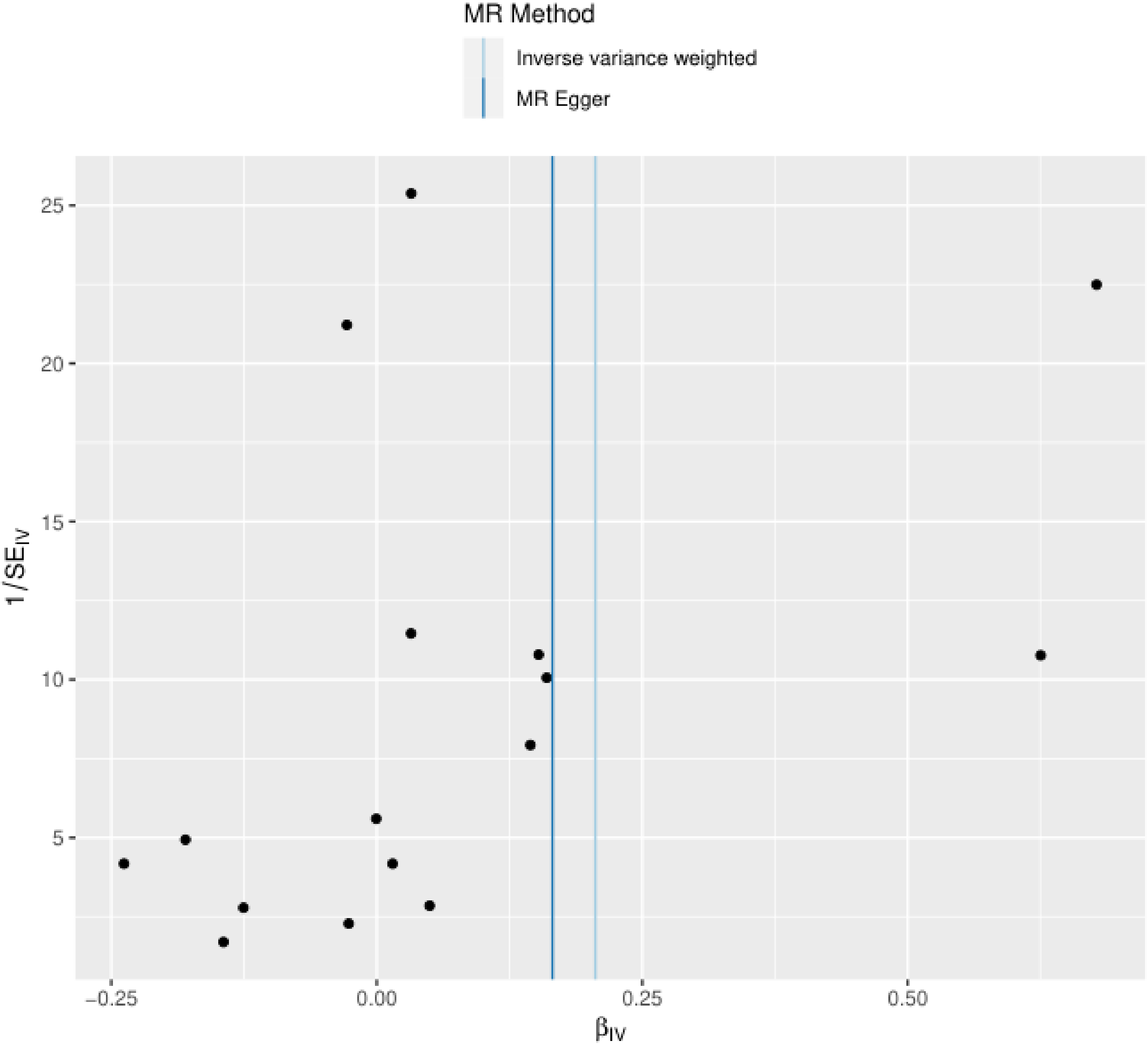

Funnel plot analyse of "HLA DR on myeloid DC" on 'Diabetic nephropathy'

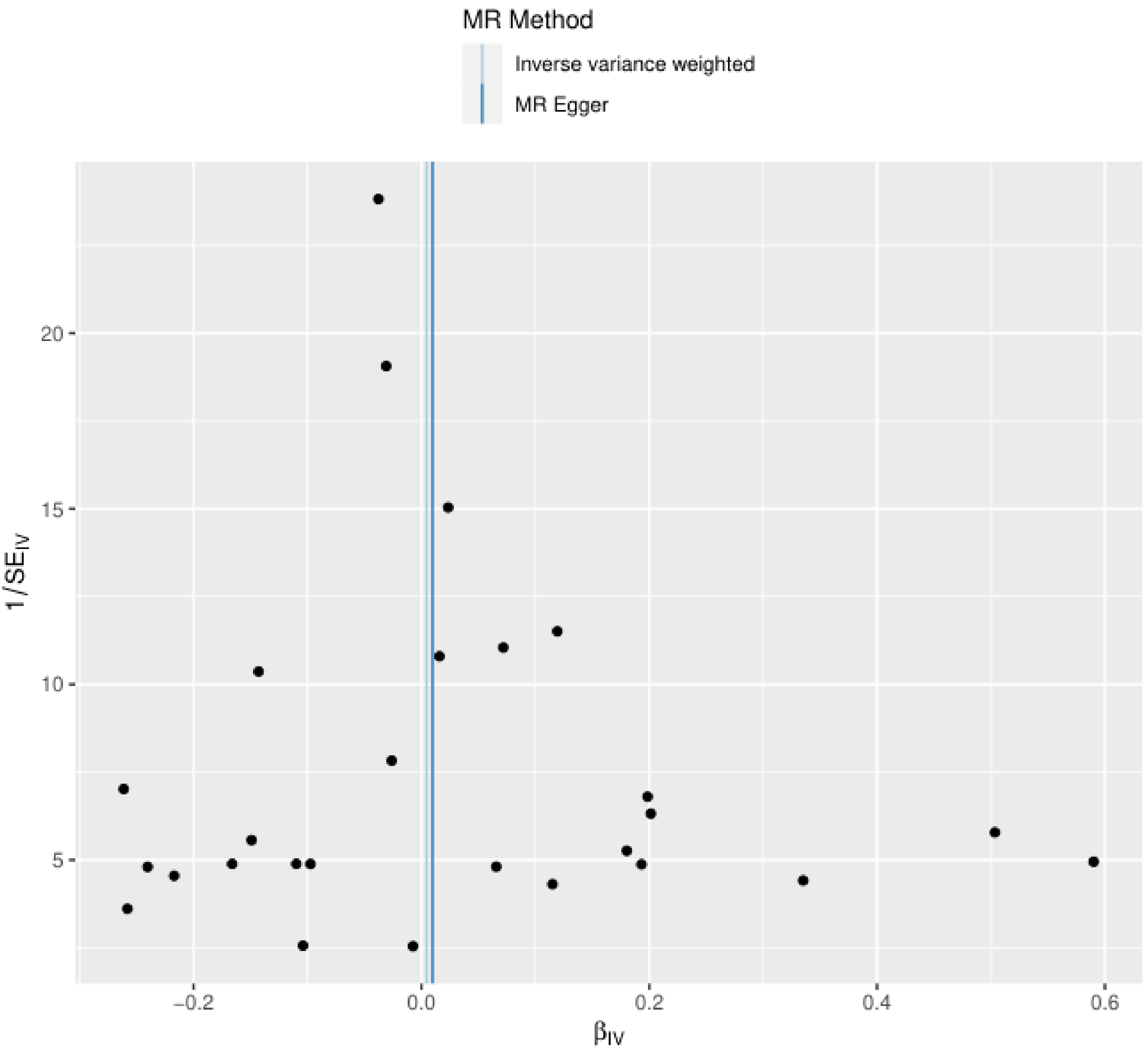

Funnel plot analyse of "CD39+ resting Treg AC" on 'Diabetic nephropathy'

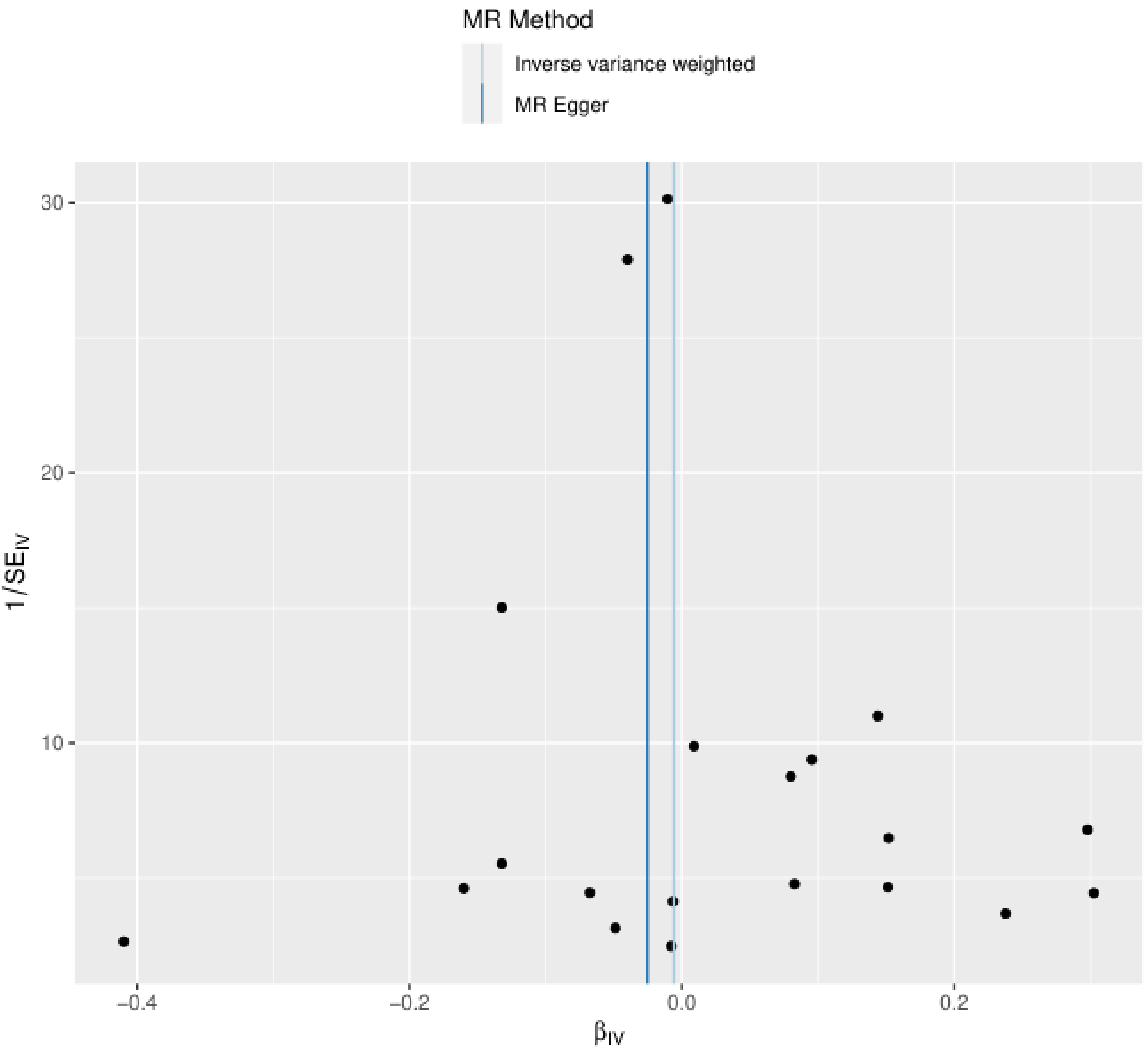

Funnel plot analyse of "CD39+ secreting Treg %CD4 Treg" on 'Diabetic nephropathy'

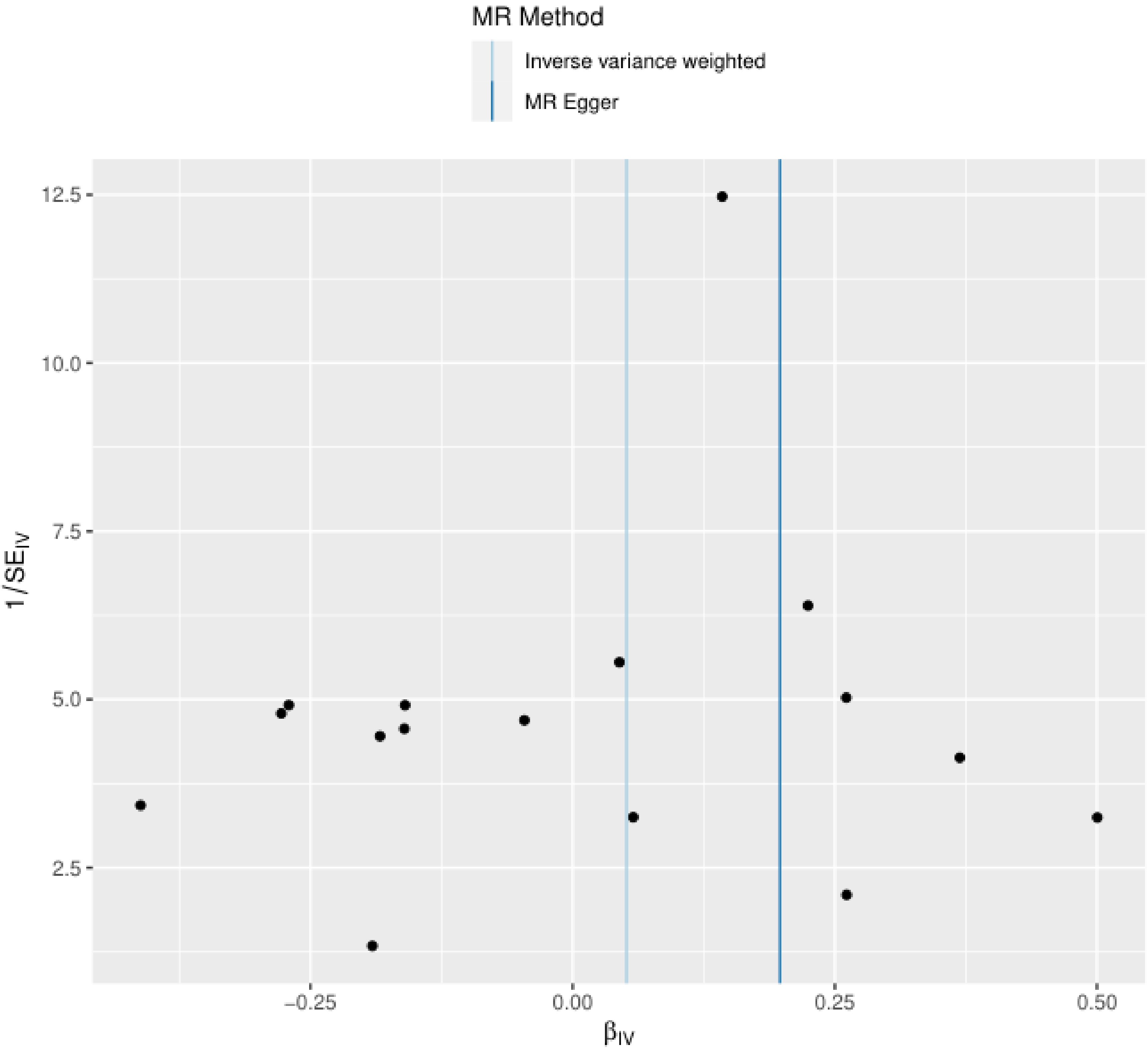

Funnel plot analyse of "CD62L on granulocyte" on 'Diabetic nephropathy'

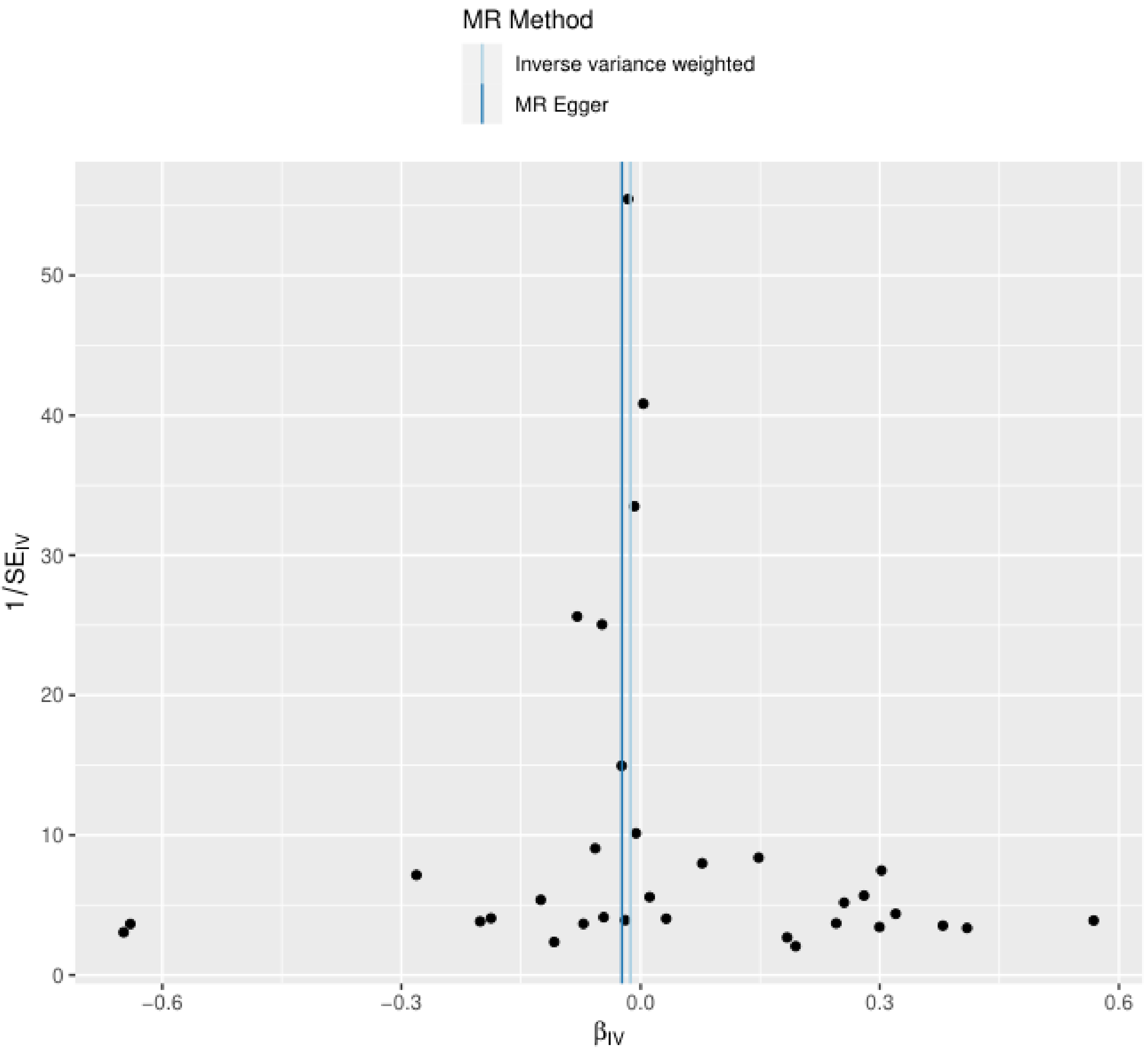

Funnel plot analyse of "CD45RA+ CD8br %T cell" on 'Diabetic nephropathy'

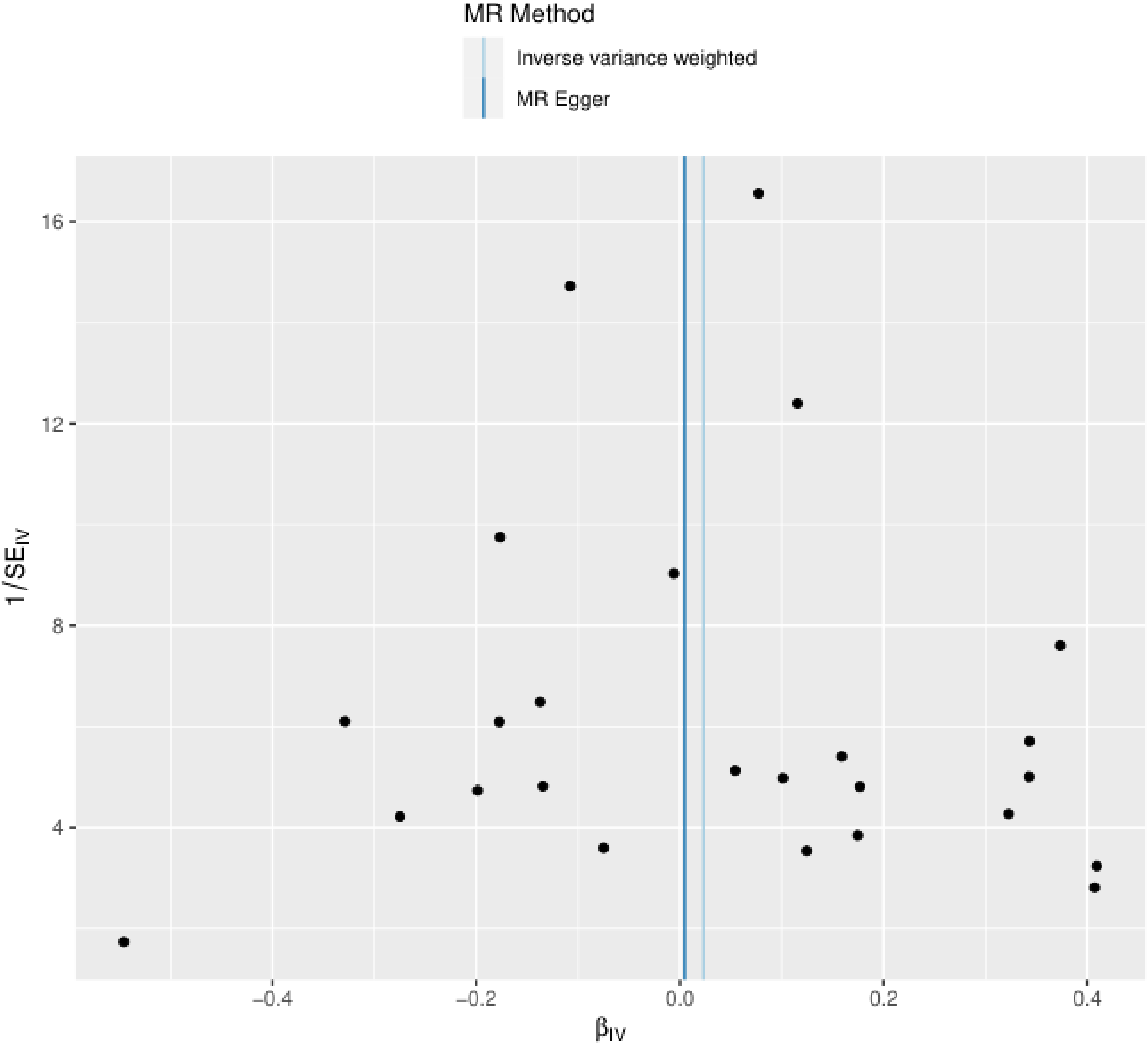

Funnel plot analyse of "CD24+ CD27+ %B cell" on 'Diabetic nephropathy'

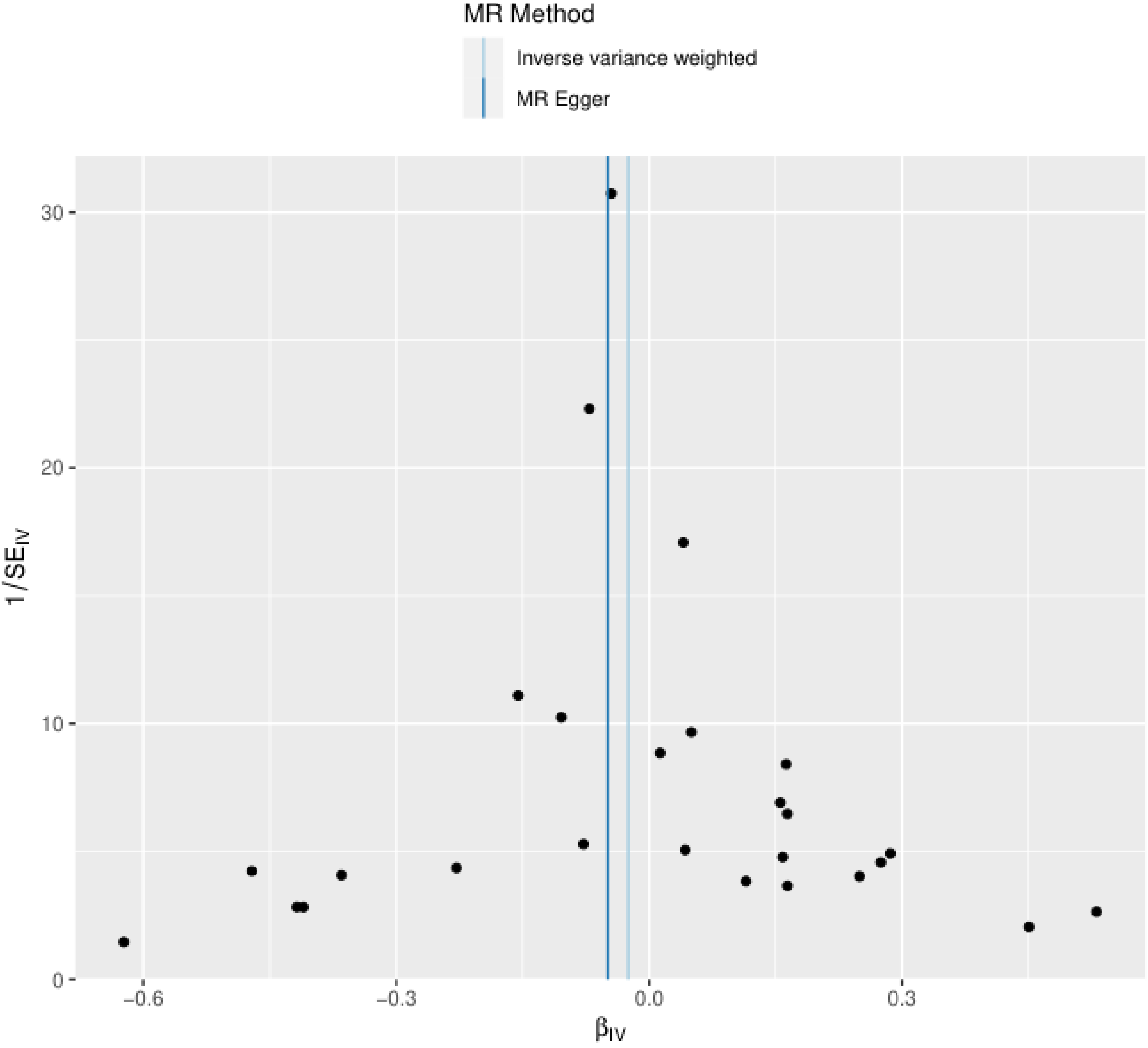

Funnel plot analyse of "CD25 on IgD+" on 'Diabetic nephropathy'

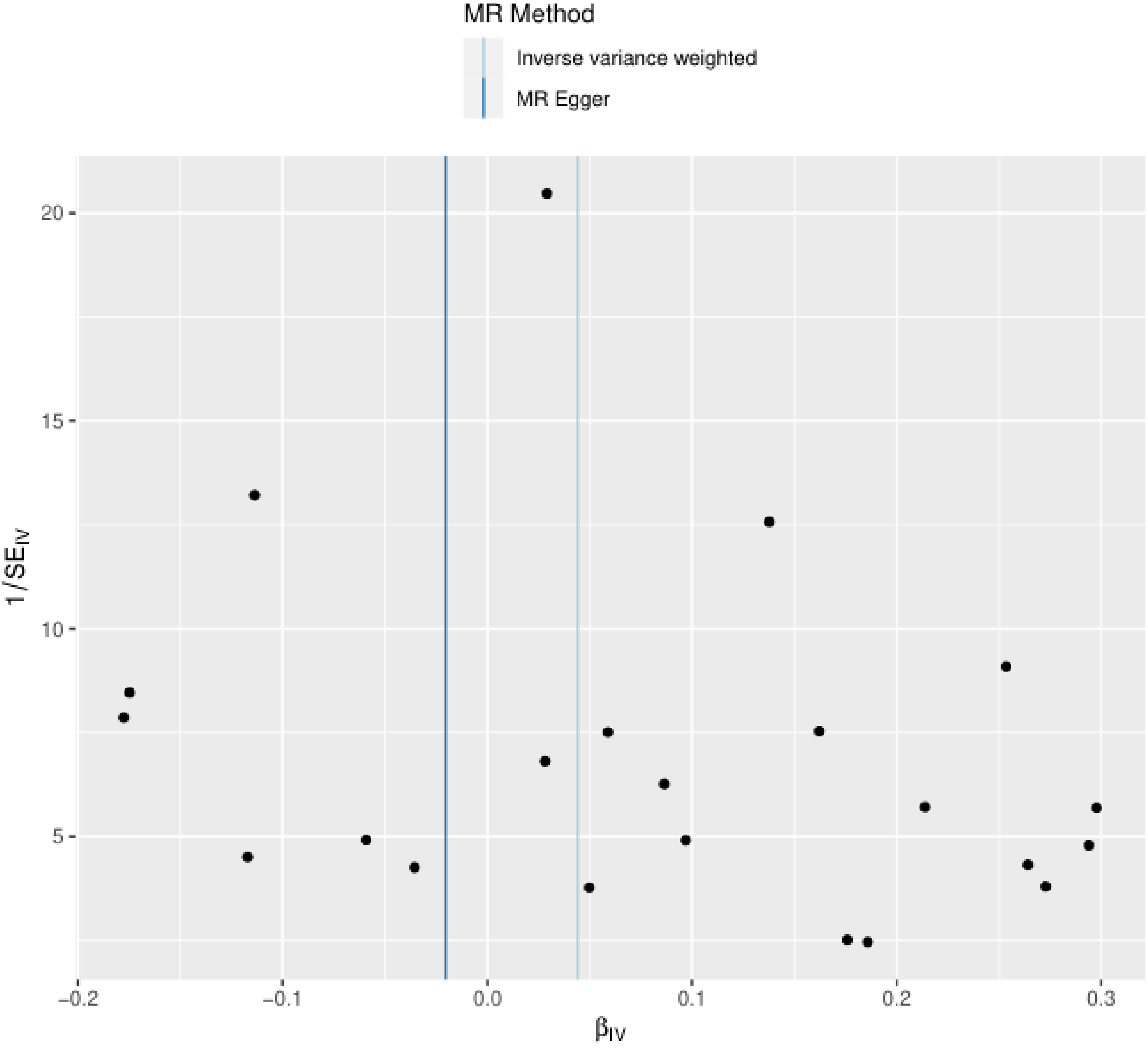

Funnel plot analyse of "SSC-A on monocyte" on 'Diabetic nephropathy'

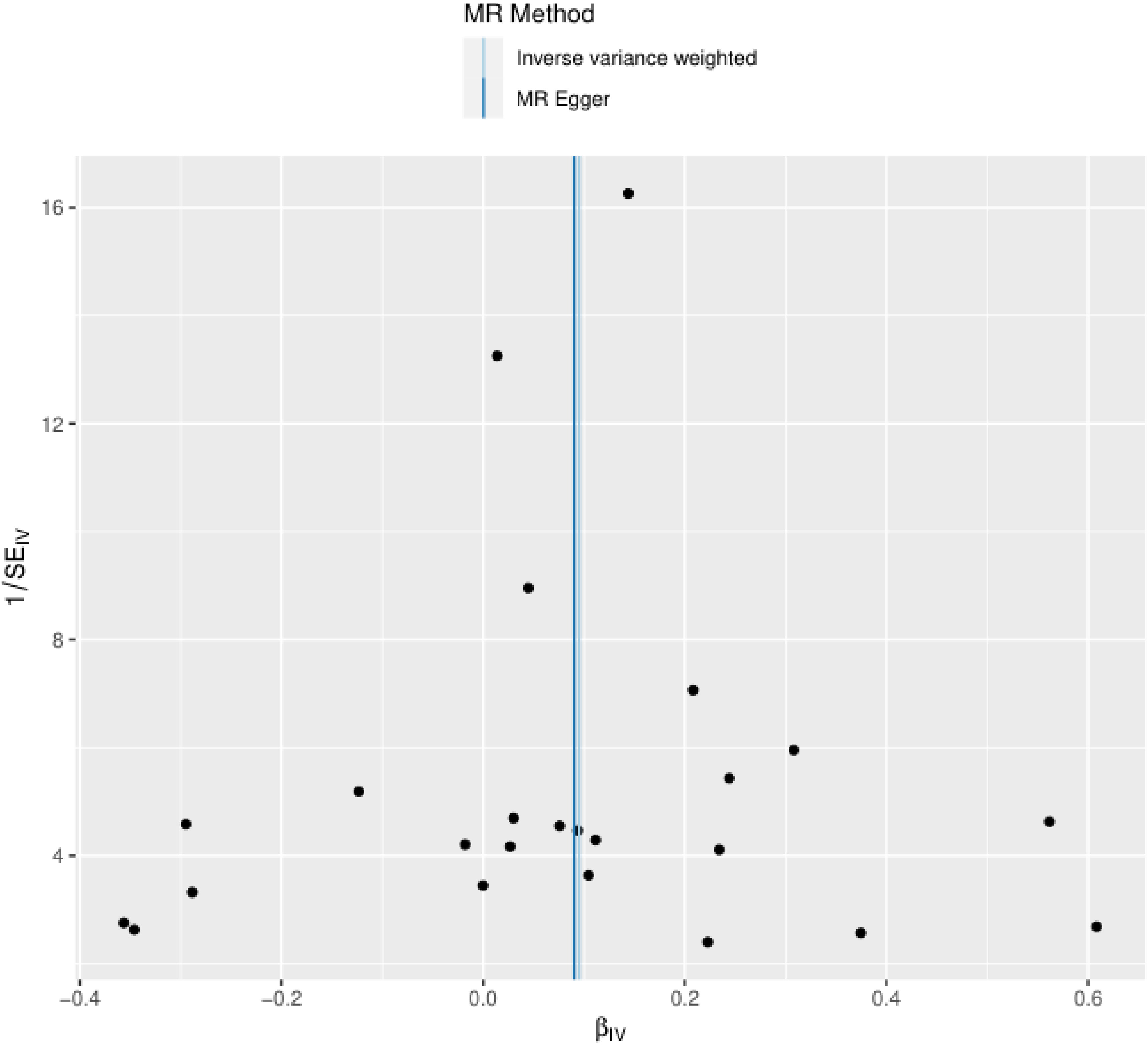

Funnel plot analyse of "CD45 on lymphocyte" on 'Diabetic nephropathy'

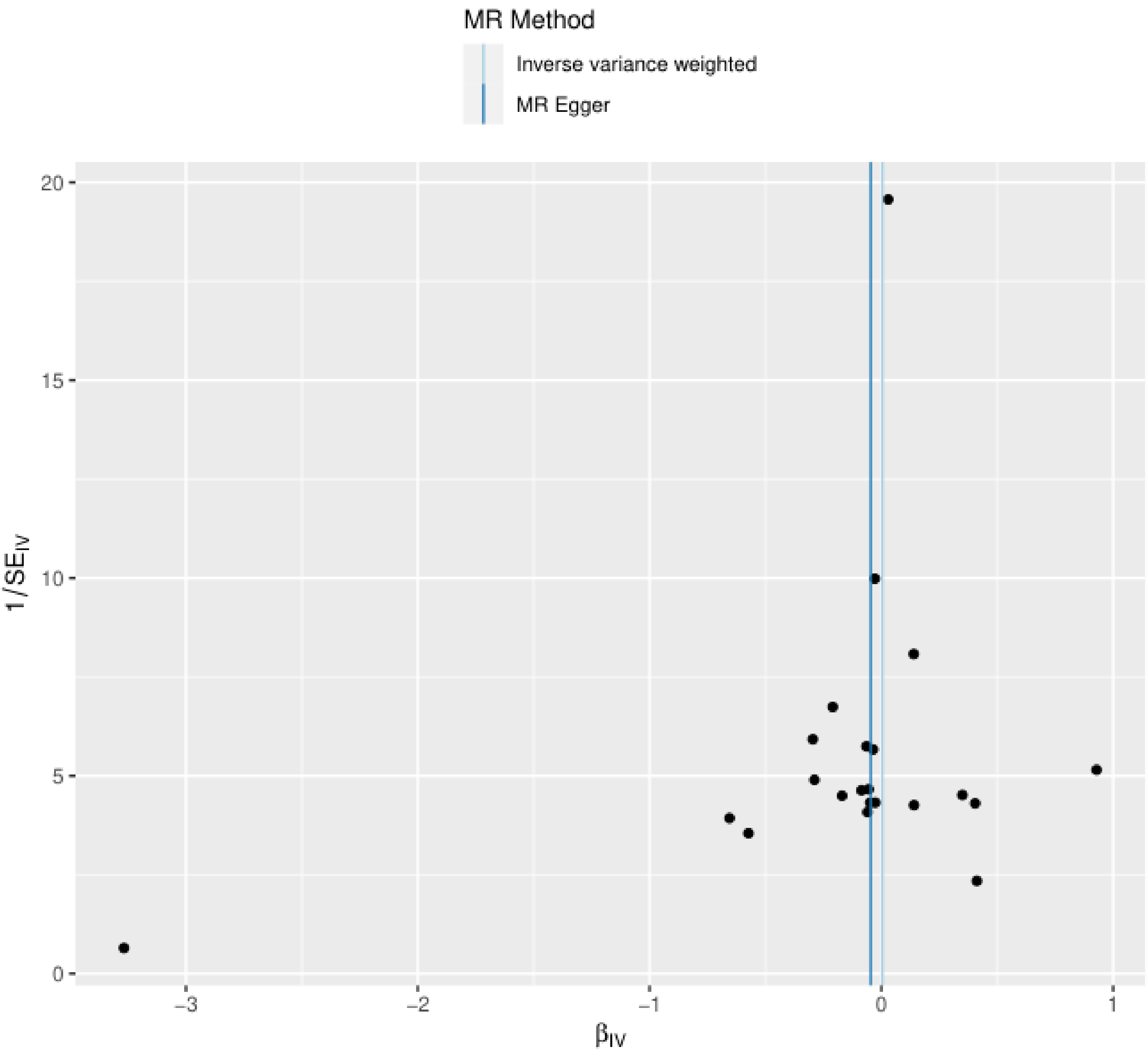

Funnel plot analyse of "CD8br %T cell" on 'Diabetic nephropathy'

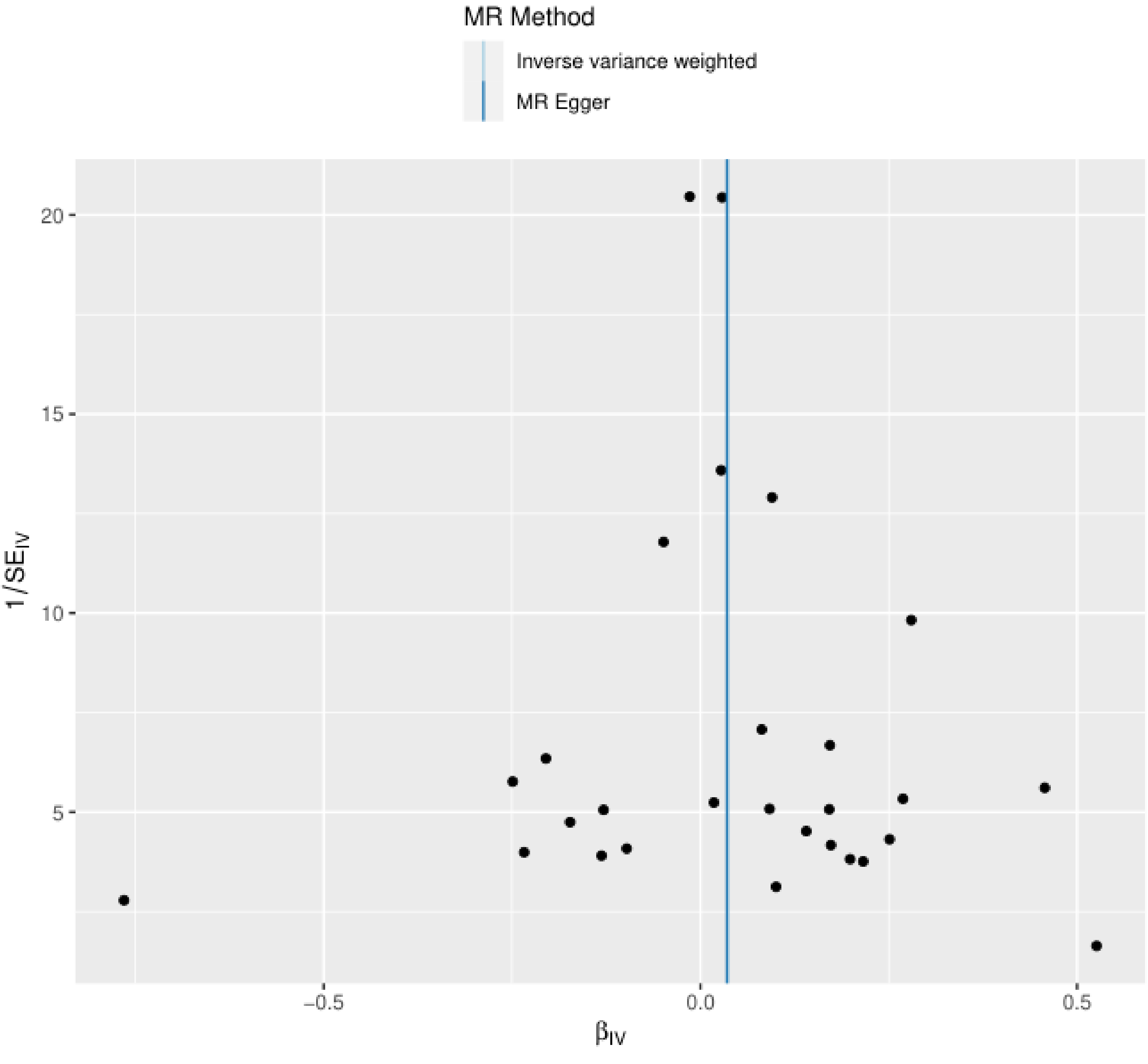

Funnel plot analyse of "SSC-A on HLA DR+ NK" on 'Diabetic nephropathy'

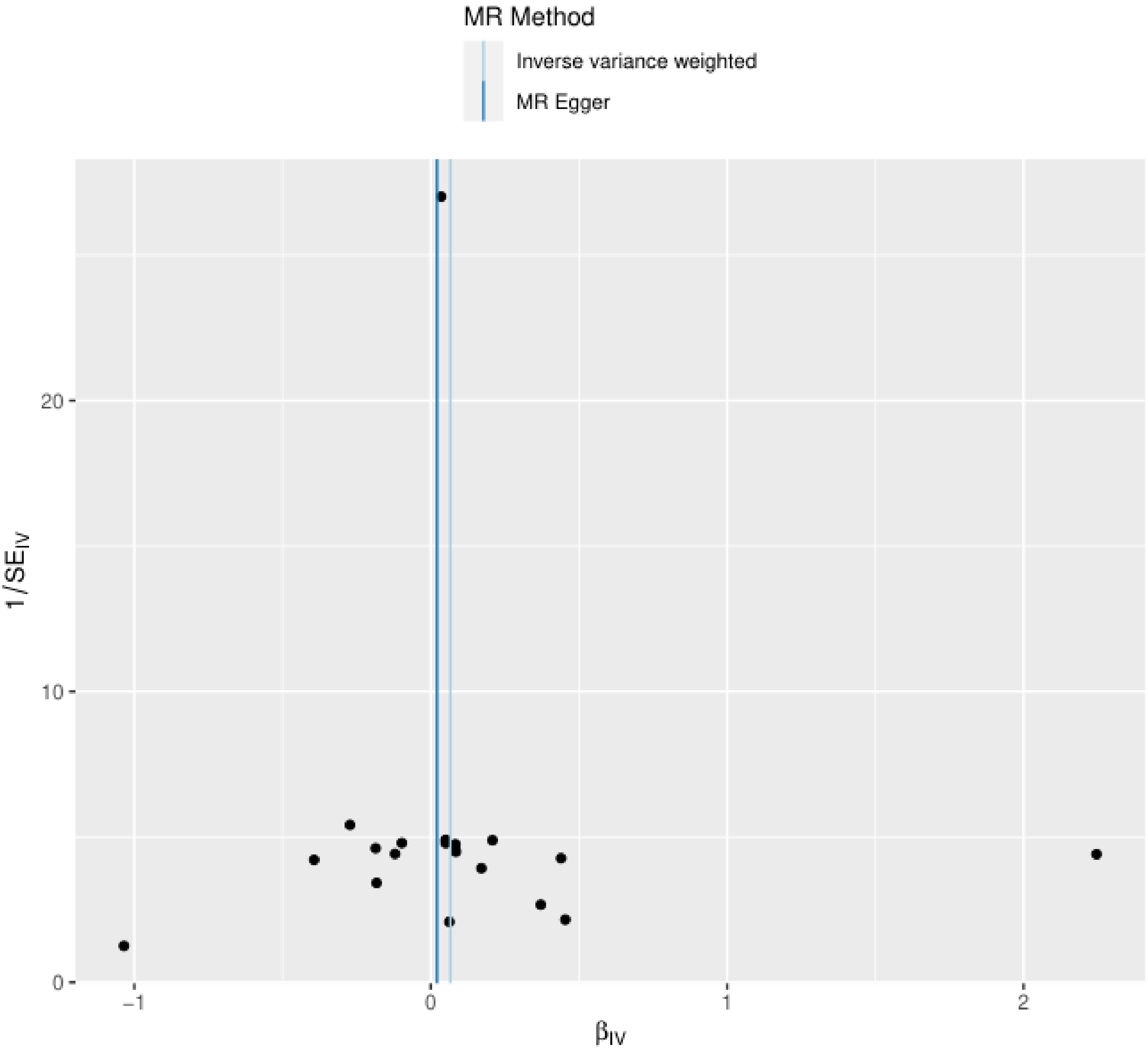

Funnel plot analyse of "CD28- CD127- CD25++ CD8br %T cell" on 'Diabetic nephropathy'

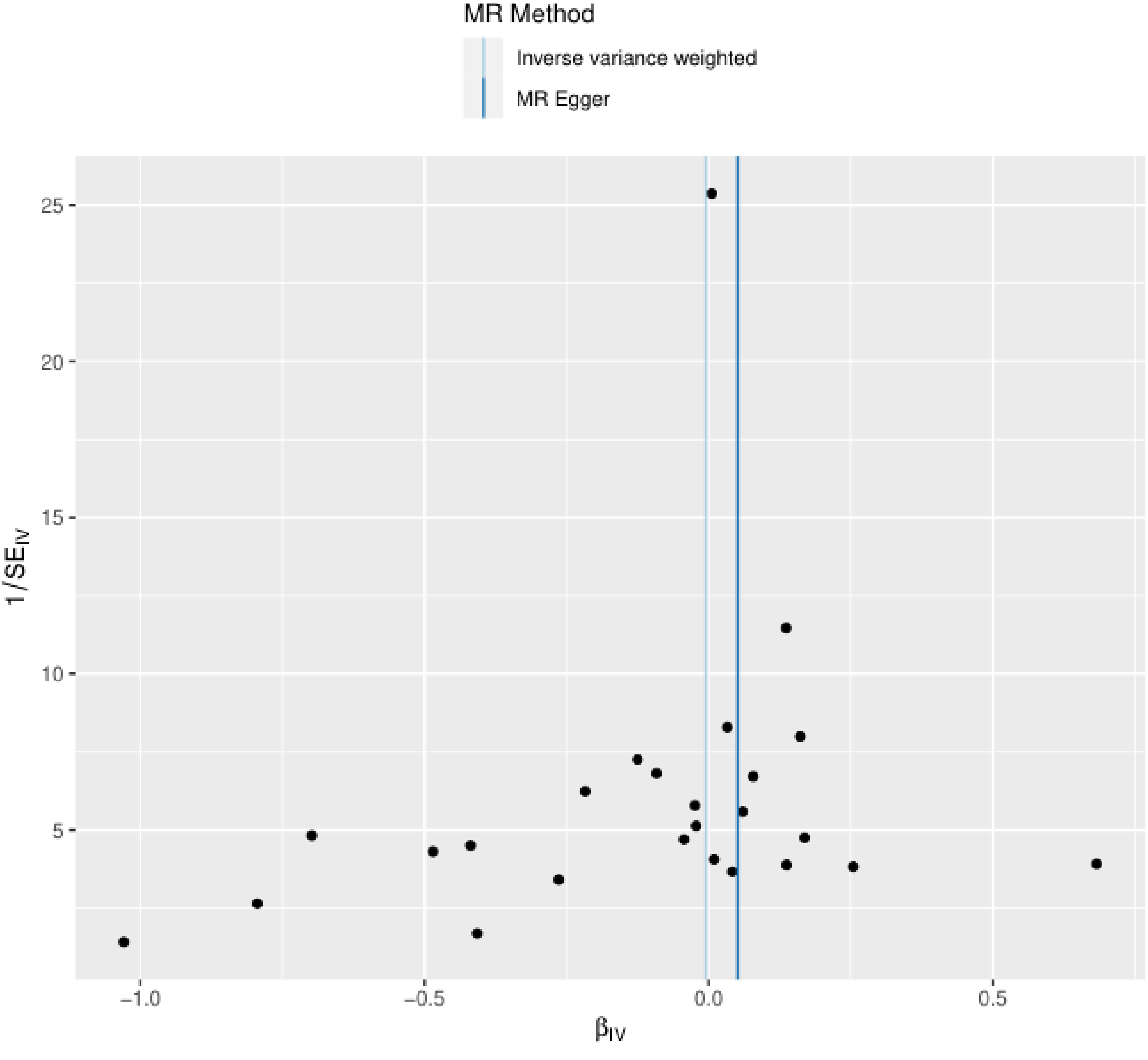

Funnel plot analyse of "IgD on IgD+ CD38br" on 'Diabetic nephropathy'

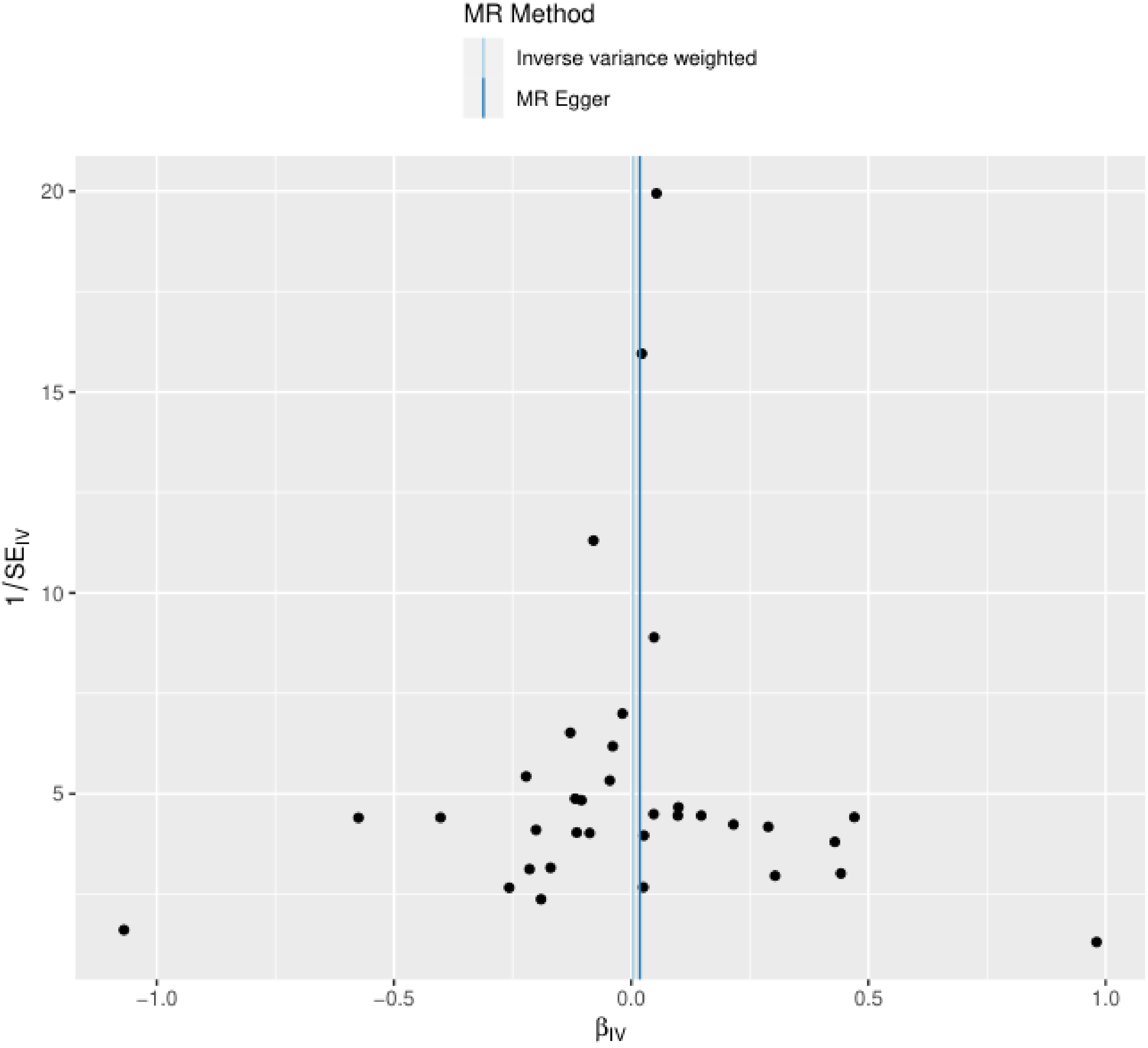

Funnel plot analyse of "Naive CD4+ %T cell" on 'Diabetic nephropathy'

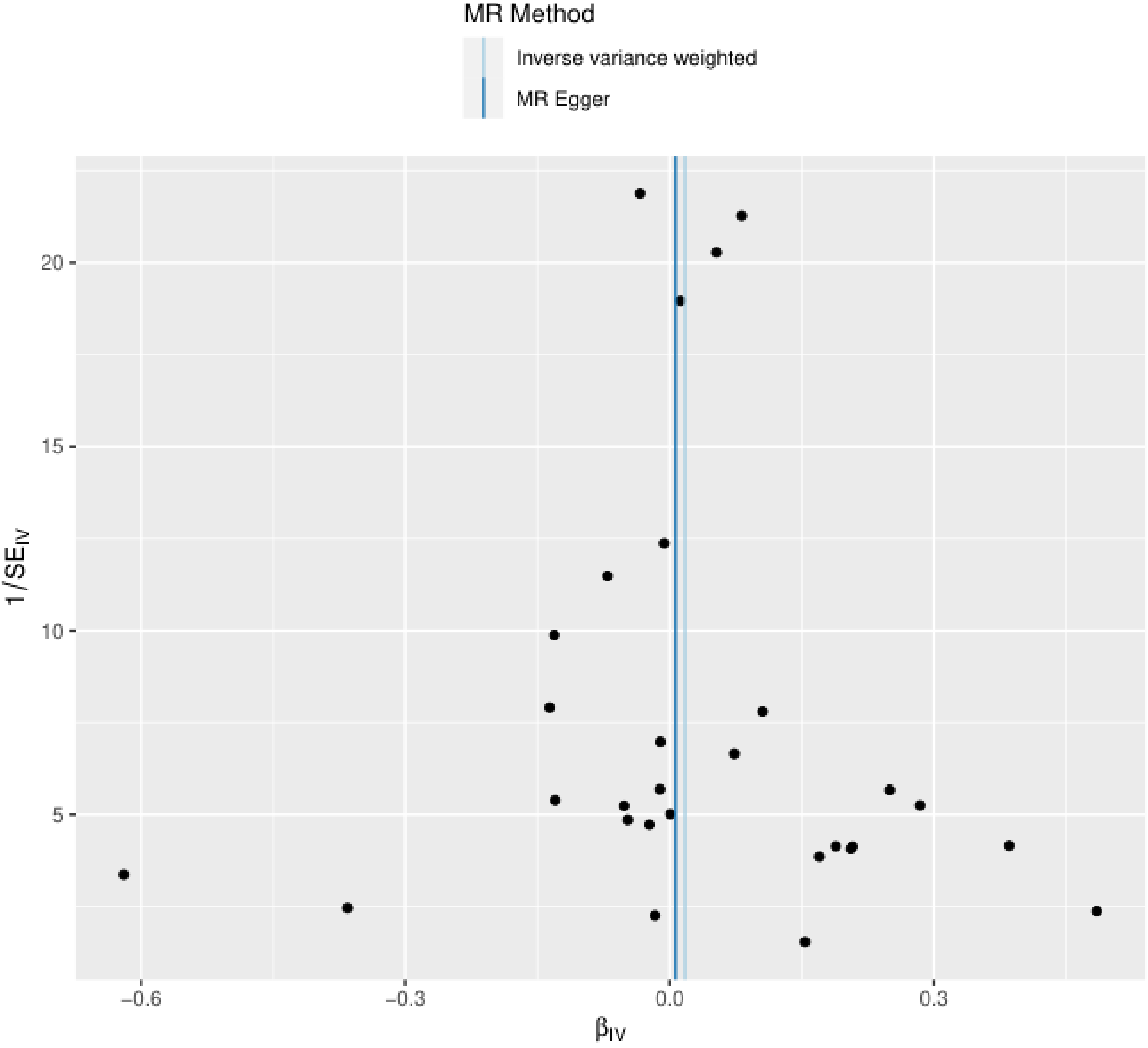

Funnel plot analyse of "CD24 on IgD- CD38-" on 'Diabetic nephropathy'

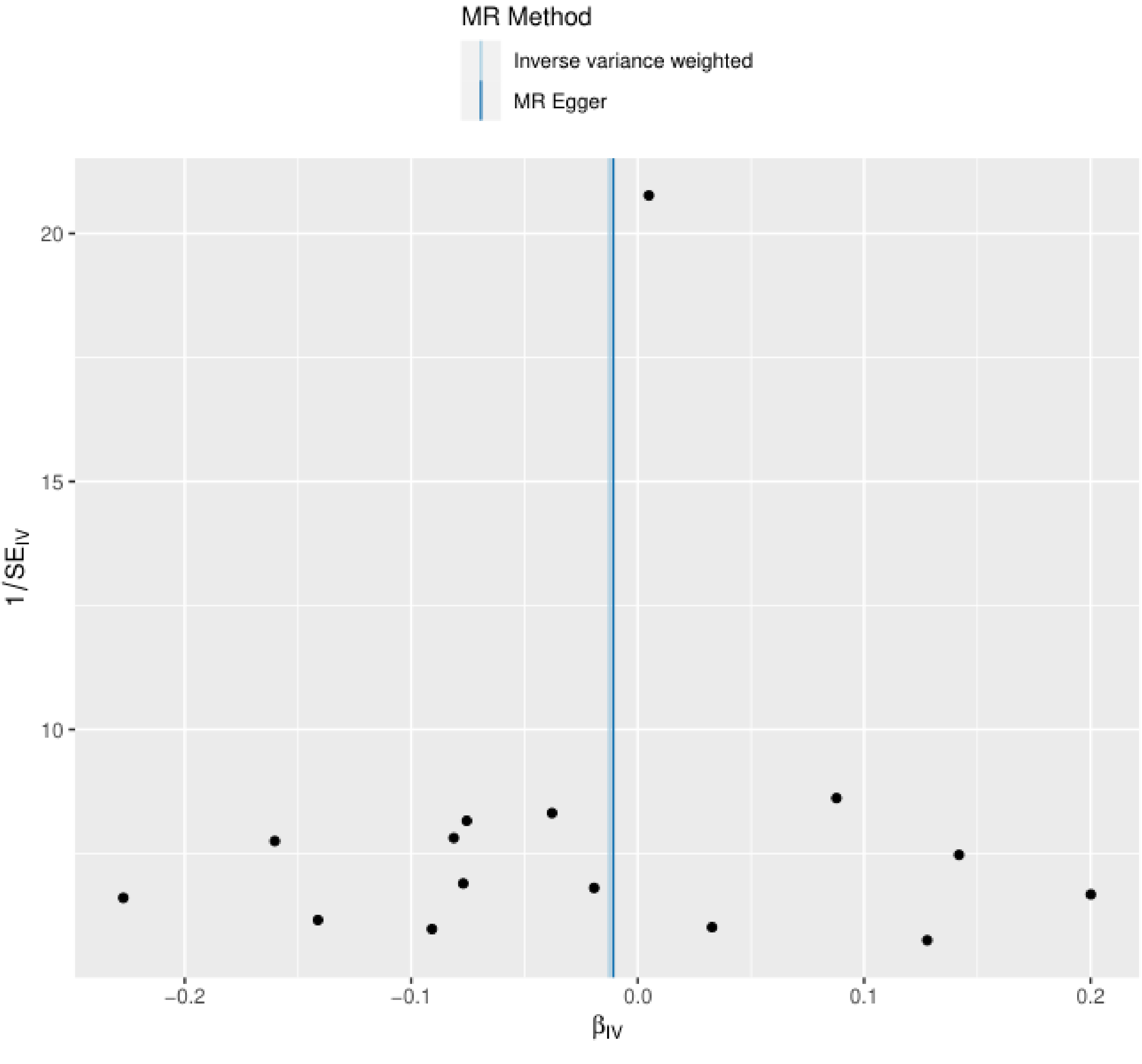

Funnel plot analyse of "CD45 on CD33br HLA DR+ CD14dim " on 'Diabetic nephropathy'

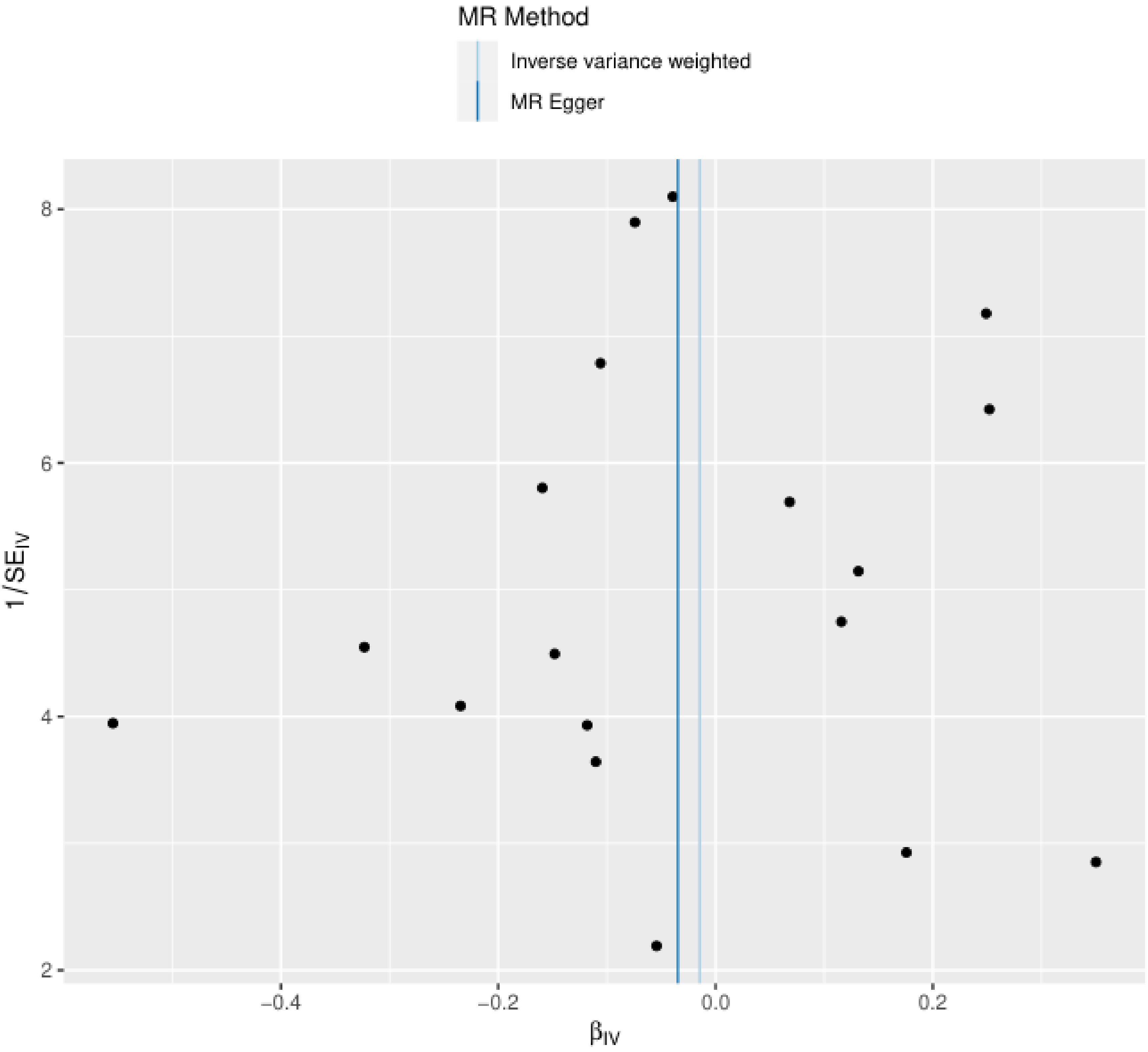

Funnel plot analyse of "CD20 on IgD- CD27-" on 'Diabetic nephropathy'

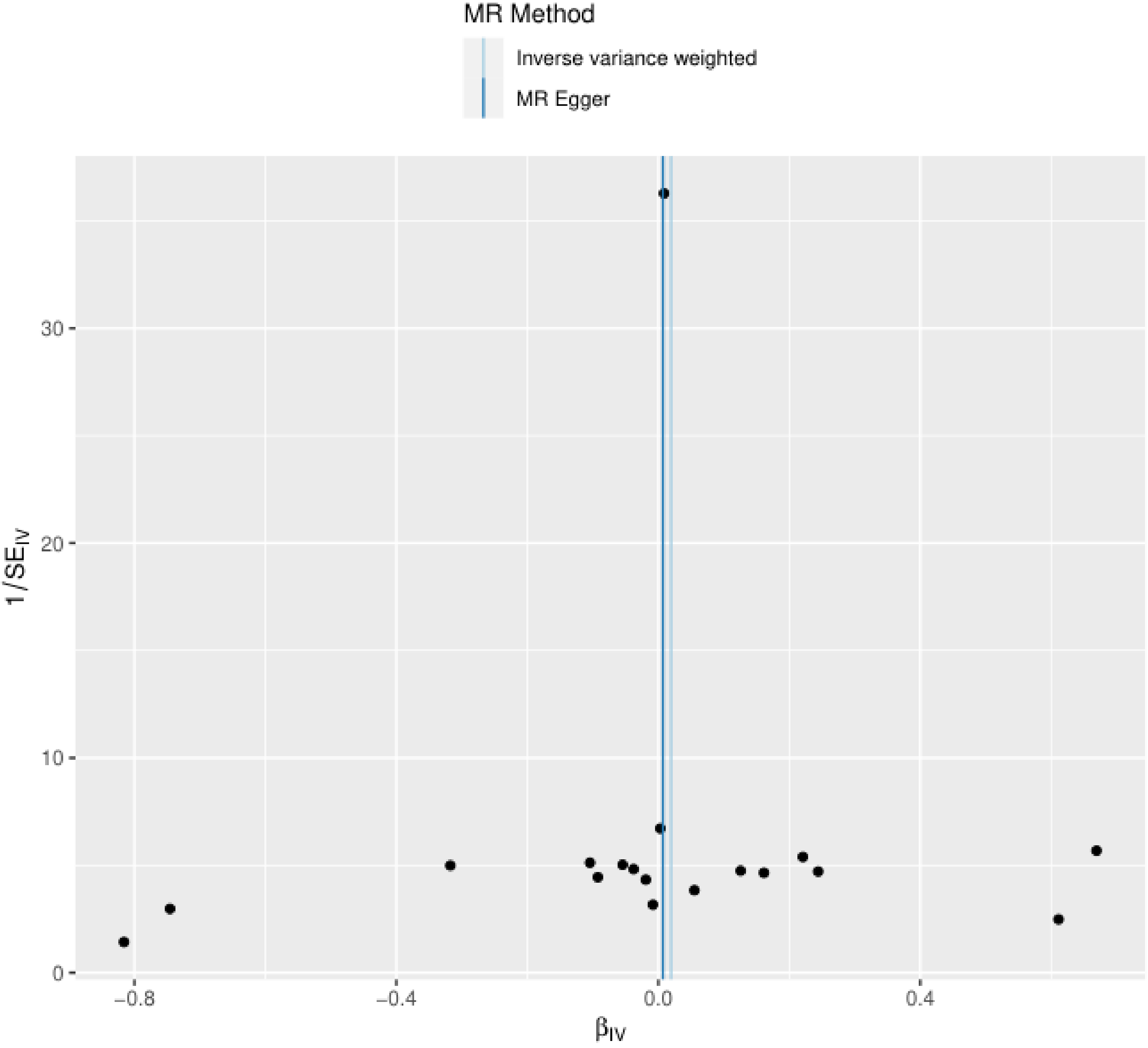

Funnel plot analyse of "FSC-A on monocyte" on 'Diabetic nephropathy'

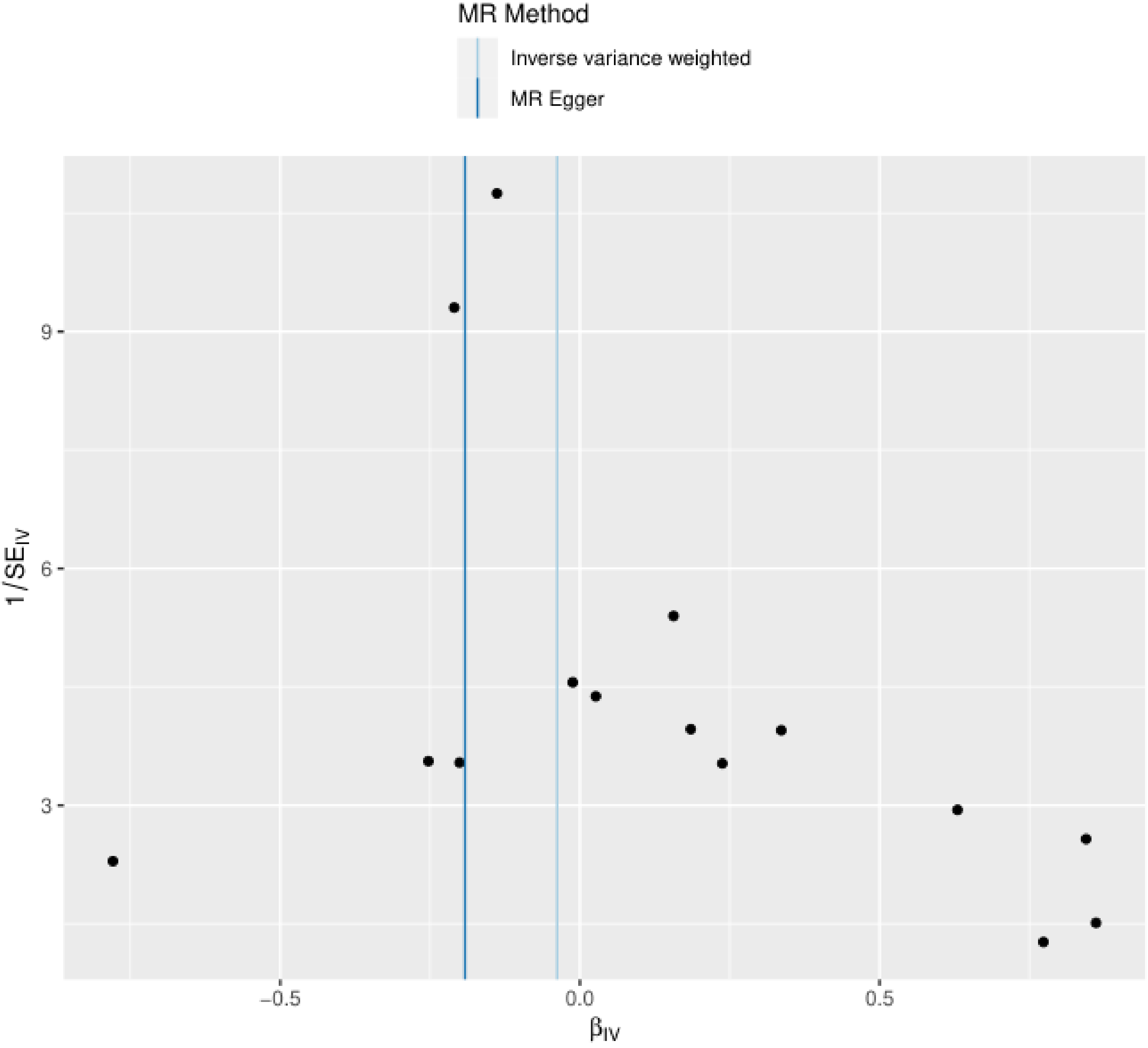

Funnel plot analyse of "PDL-1 on CD14+ CD16- monocyte" on 'Diabetic nephropathy'

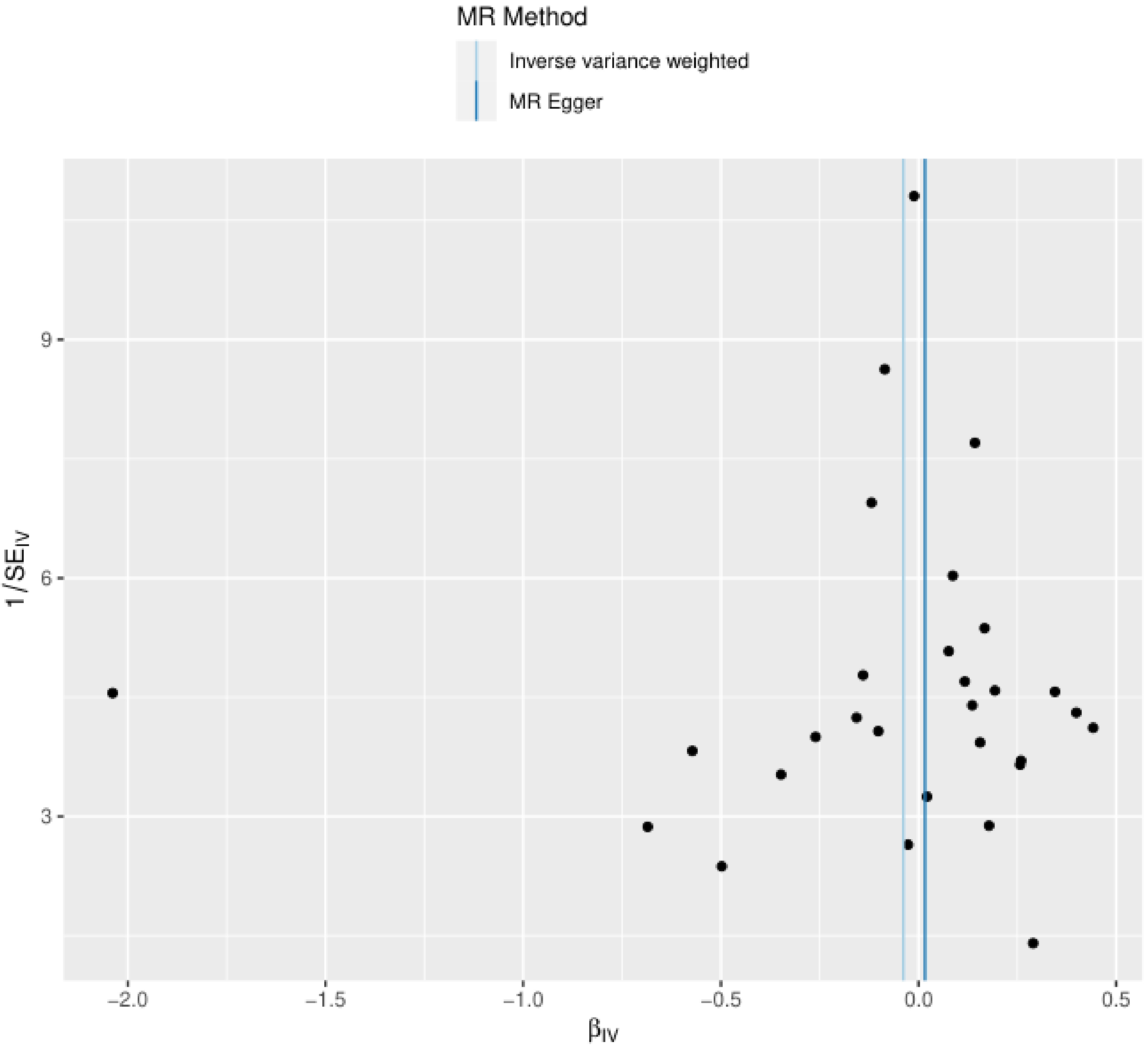

Funnel plot analyse of "CD20 on IgD- CD38-" on 'Diabetic nephropathy'

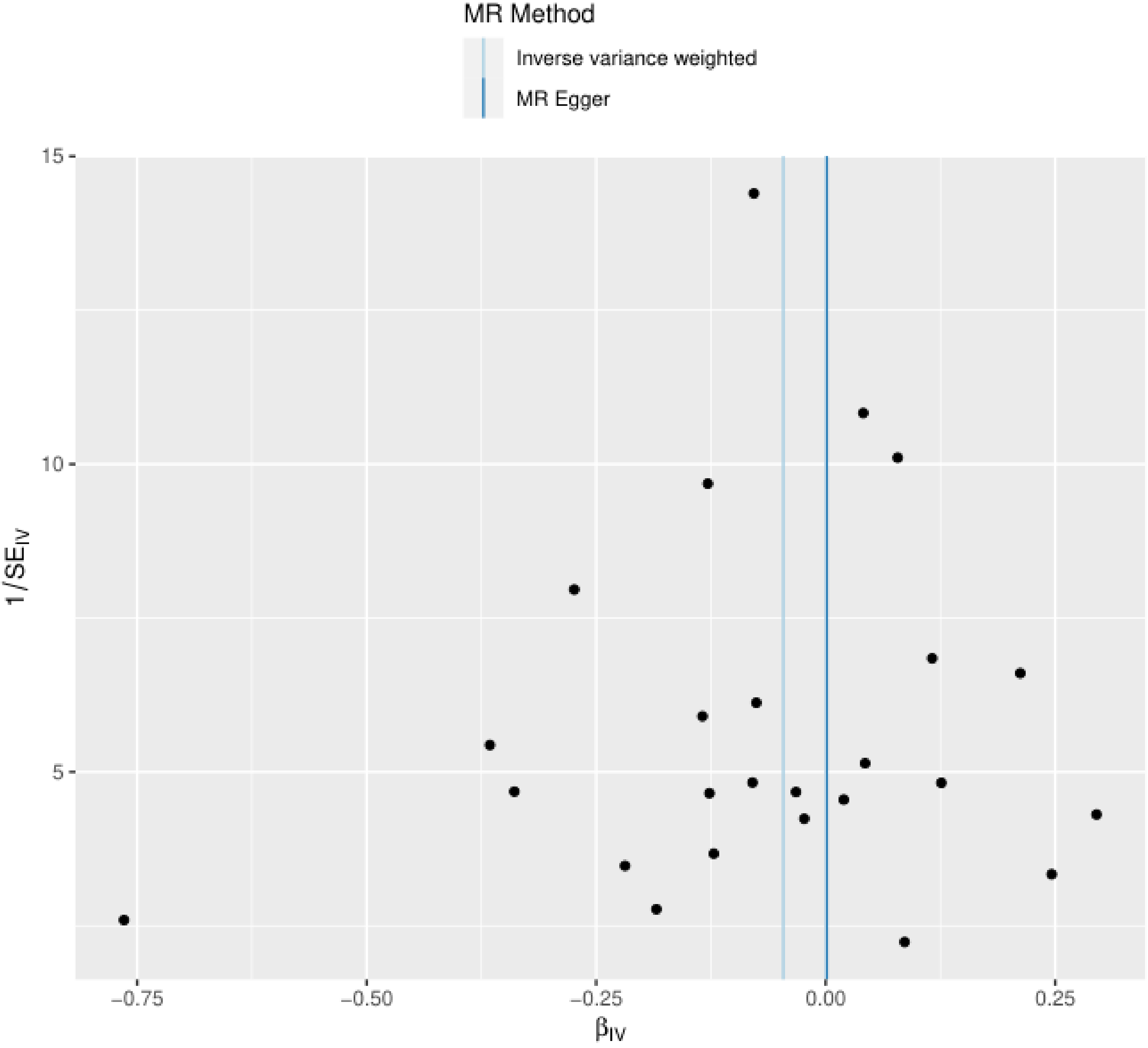

Funnel plot analyse of "CD4 on TD CD4+ " on 'Diabetic nephropathy'

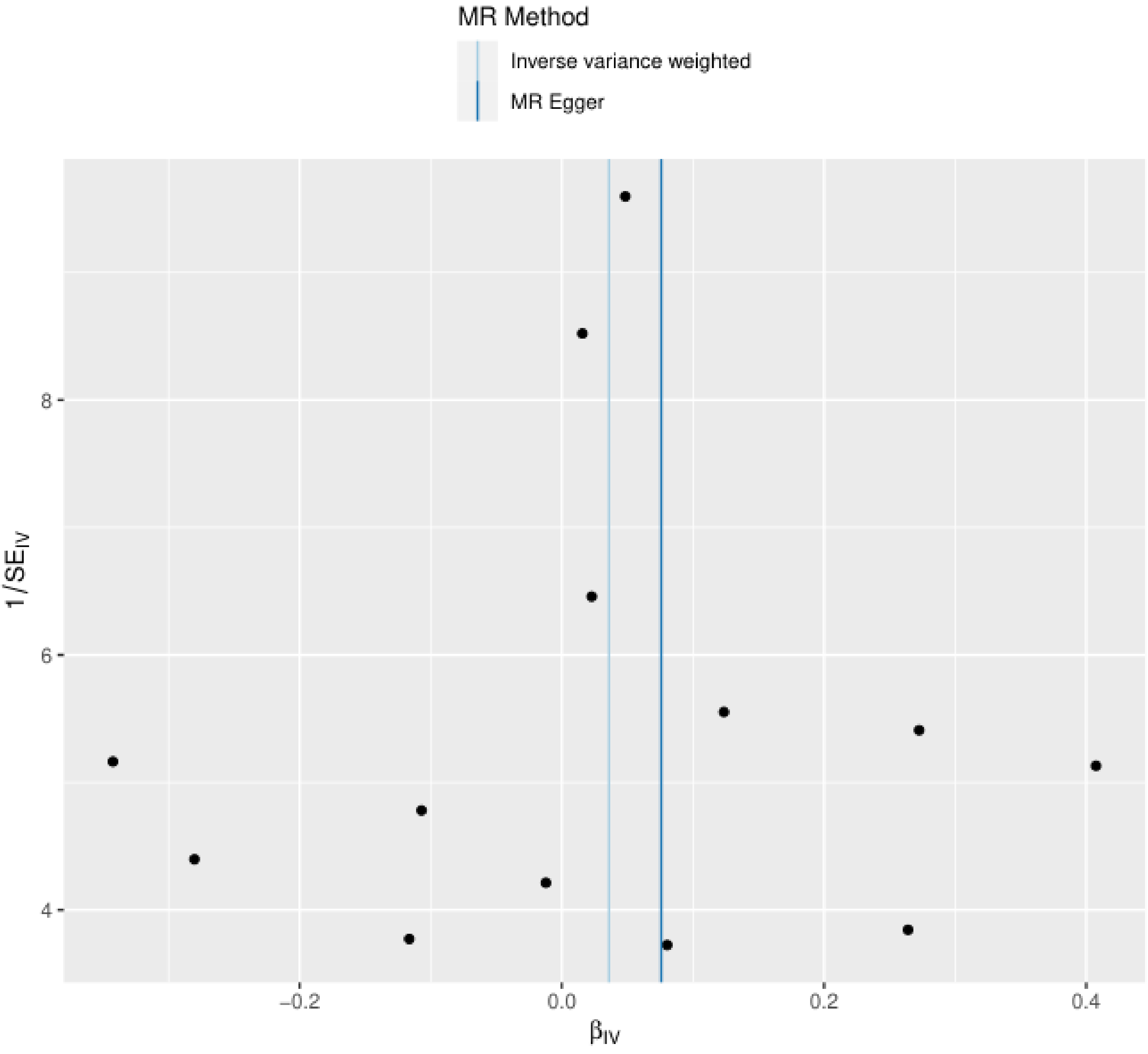

Funnel plot analyse of "CD4 on resting Treg " on 'Diabetic nephropathy'

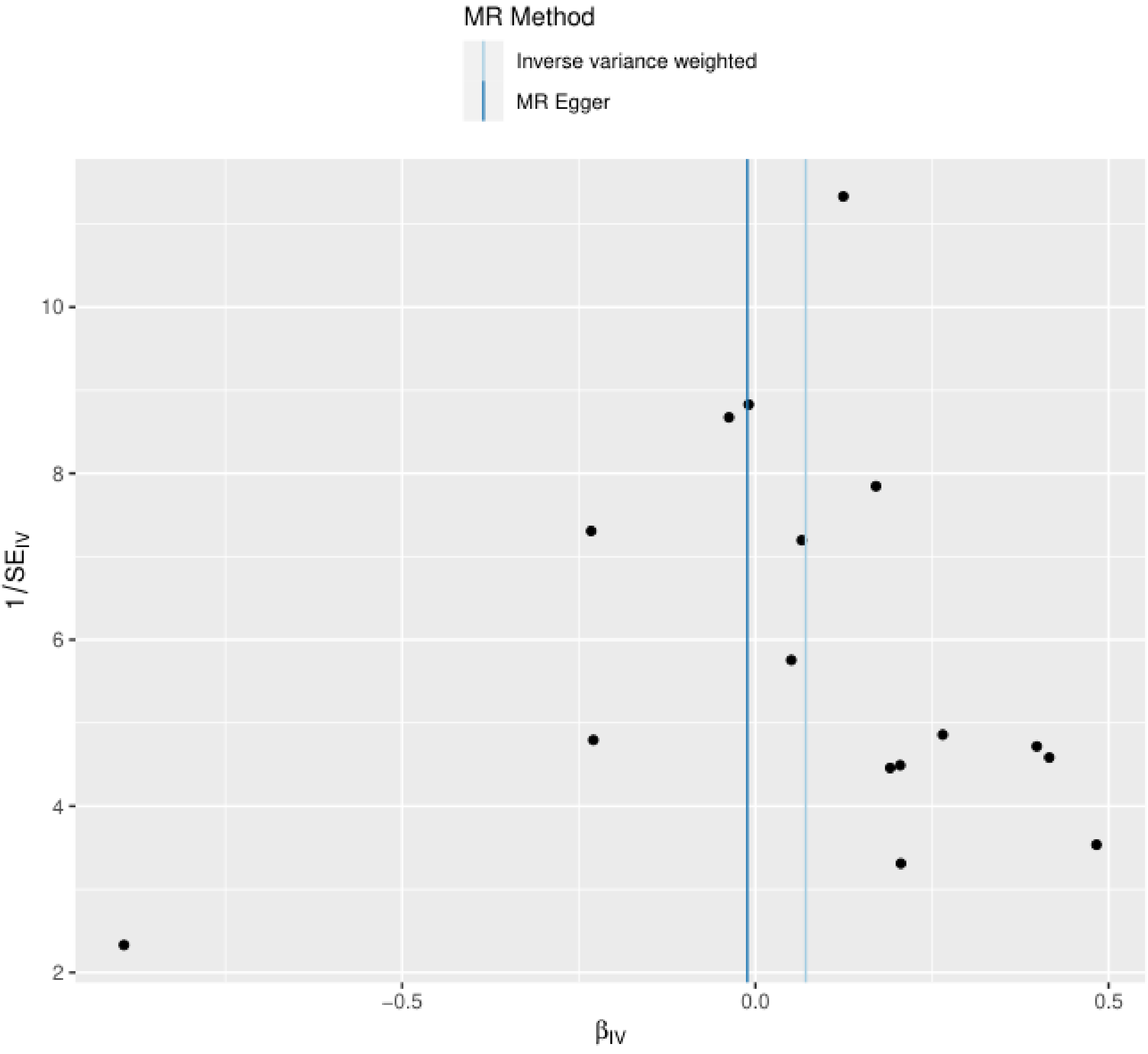

Funnel plot analyse of "CD38 on IgD+ CD24-" on 'Diabetic nephropathy'

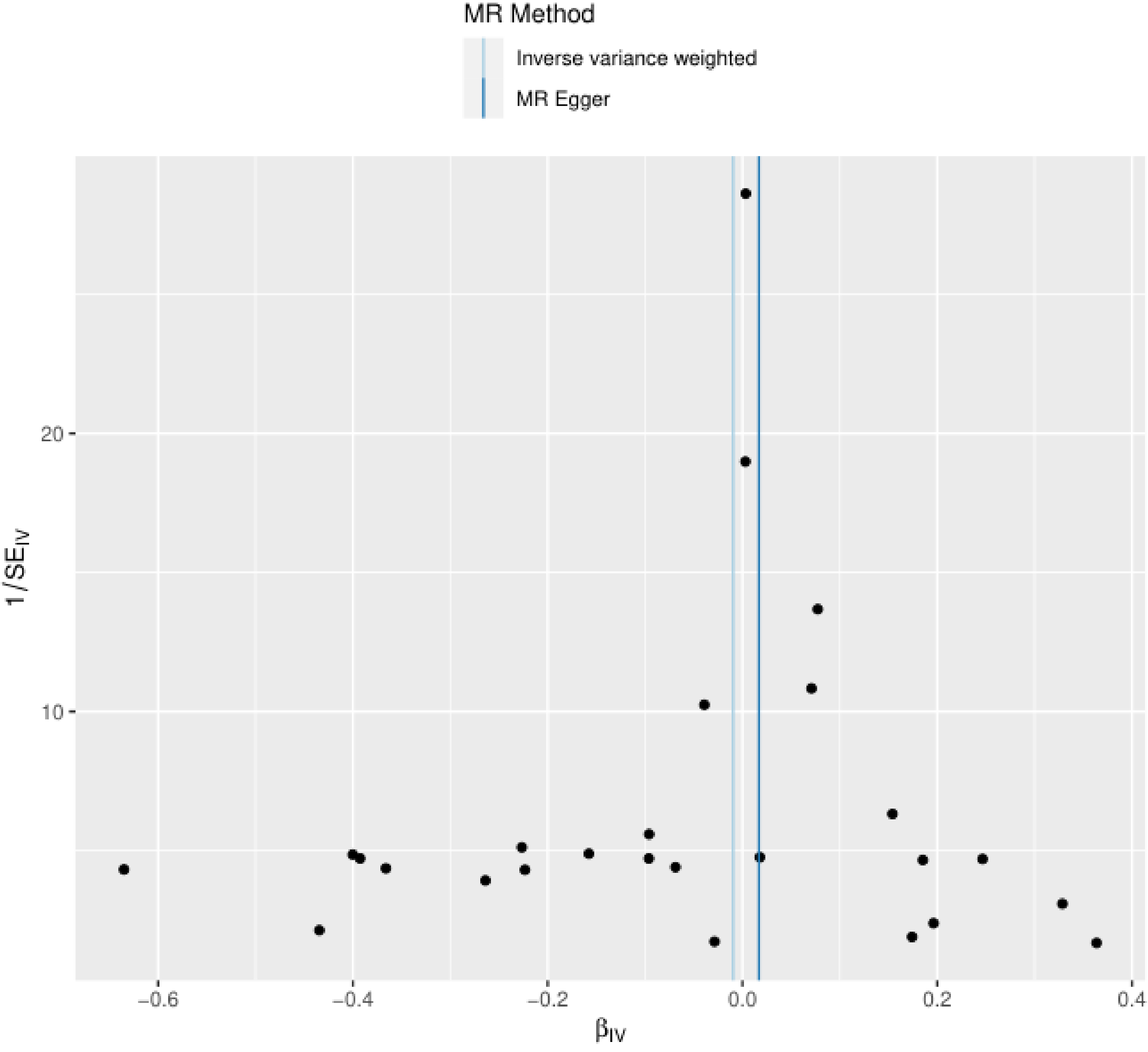

Funnel plot analyse of "CD11c on CD62L+ myeloid DC" on 'Diabetic nephropathy'

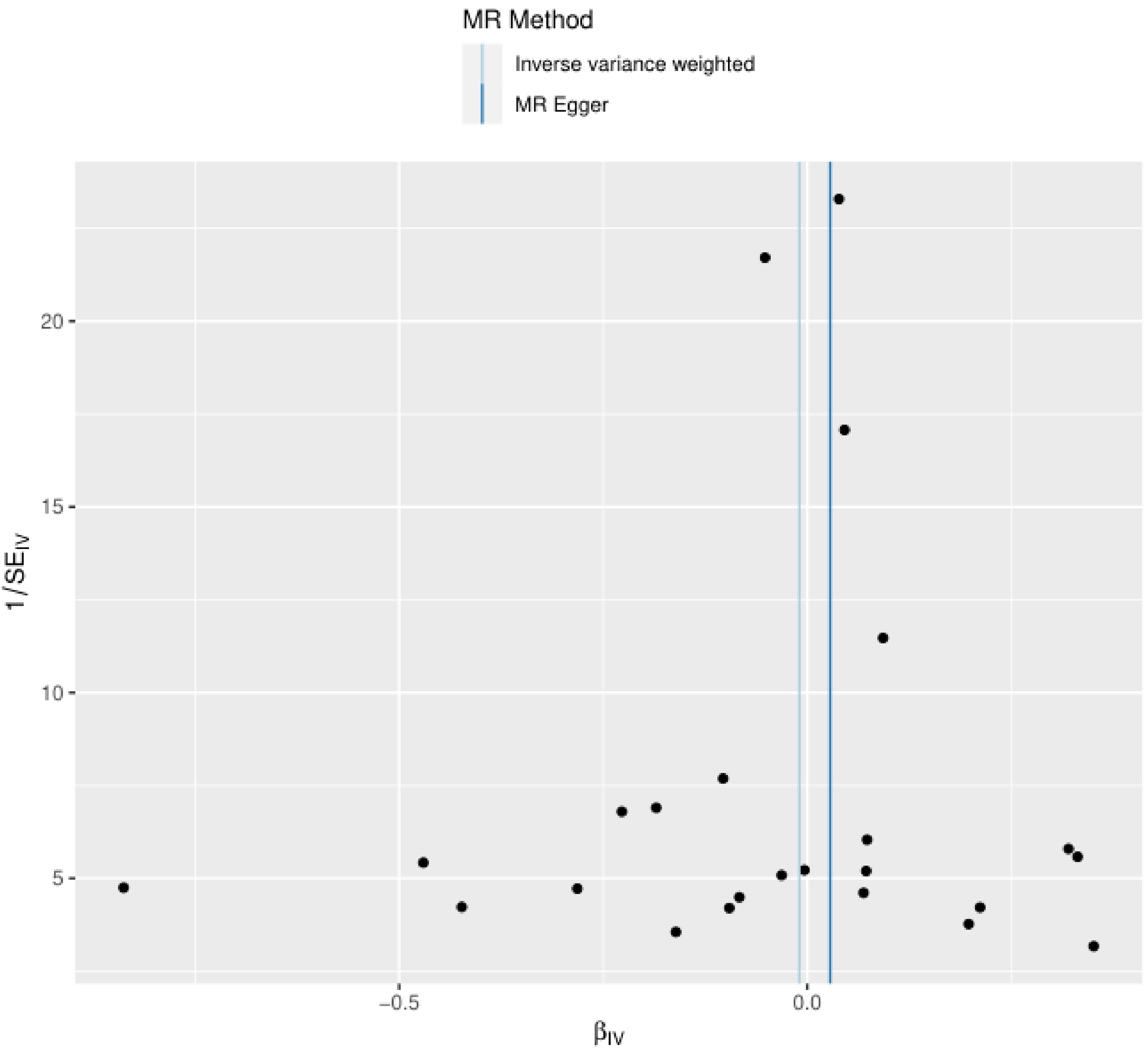

Funnel plot analyse of "CD8 on HLA DR+ CD8br" on 'Diabetic nephropathy'

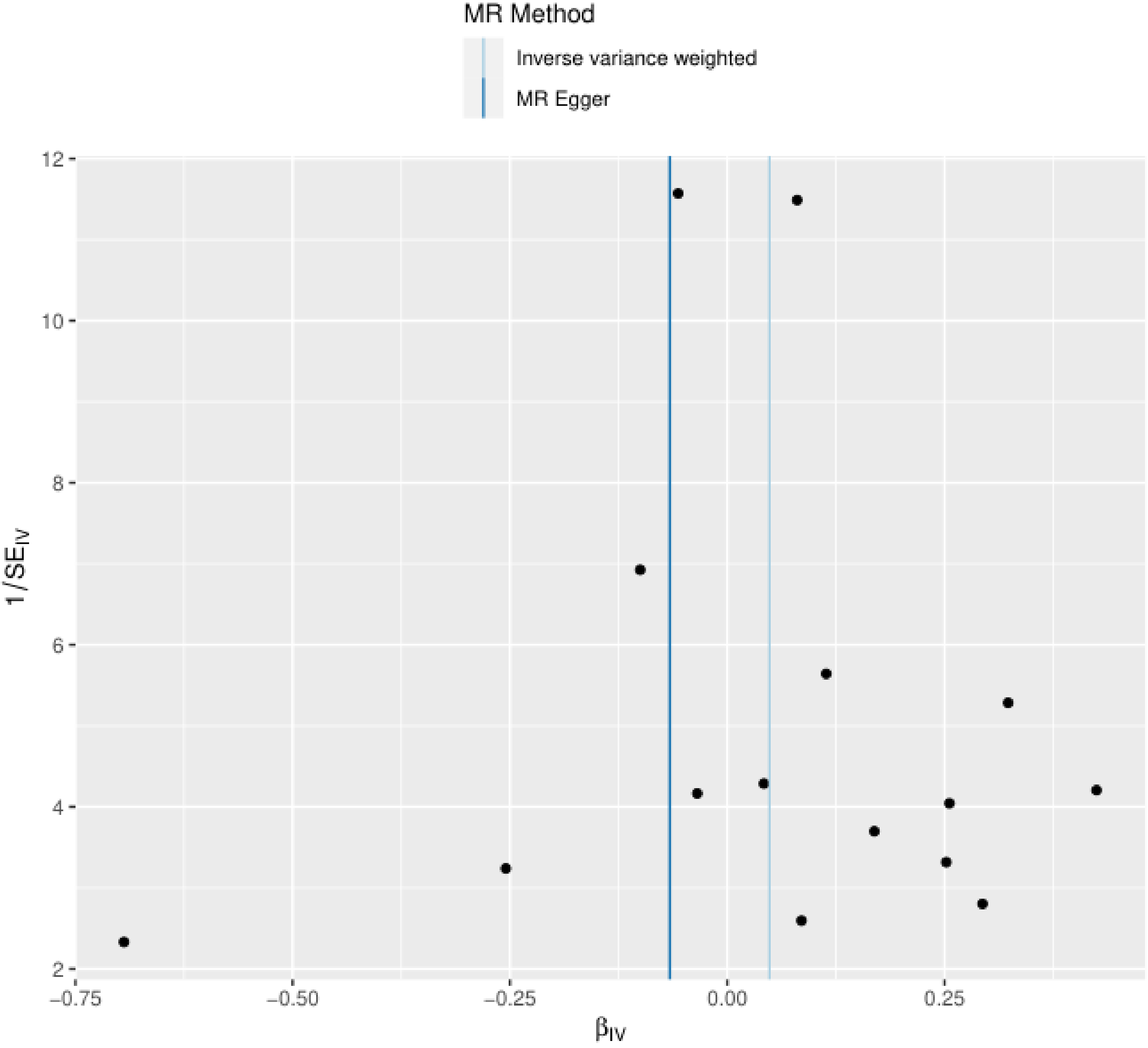

Funnel plot analyse of "BAFF-R on IgD- CD38br" on 'Diabetic nephropathy'

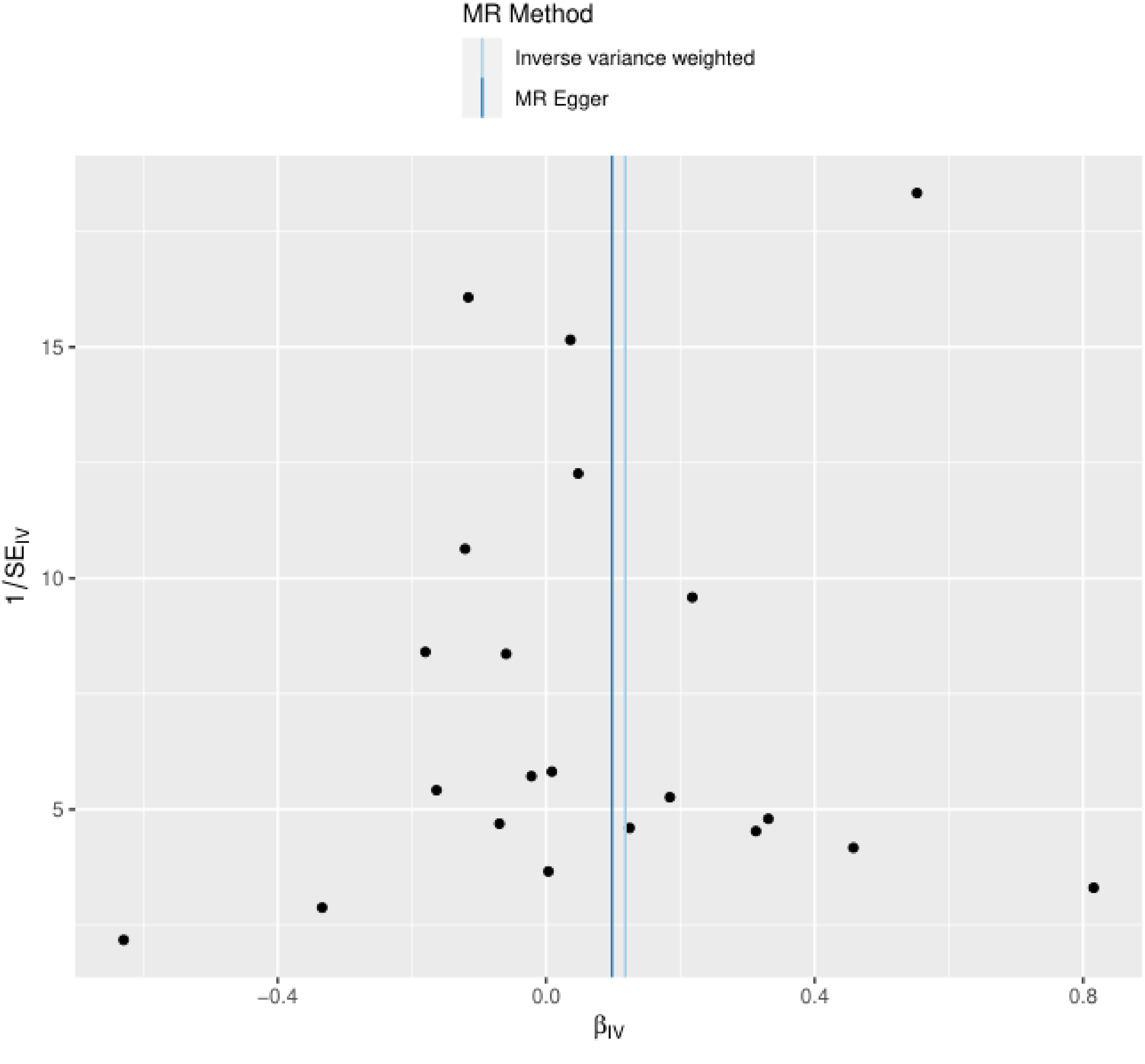

Funnel plot analyse of "HLA DR on B cell" on 'Diabetic nephropathy'

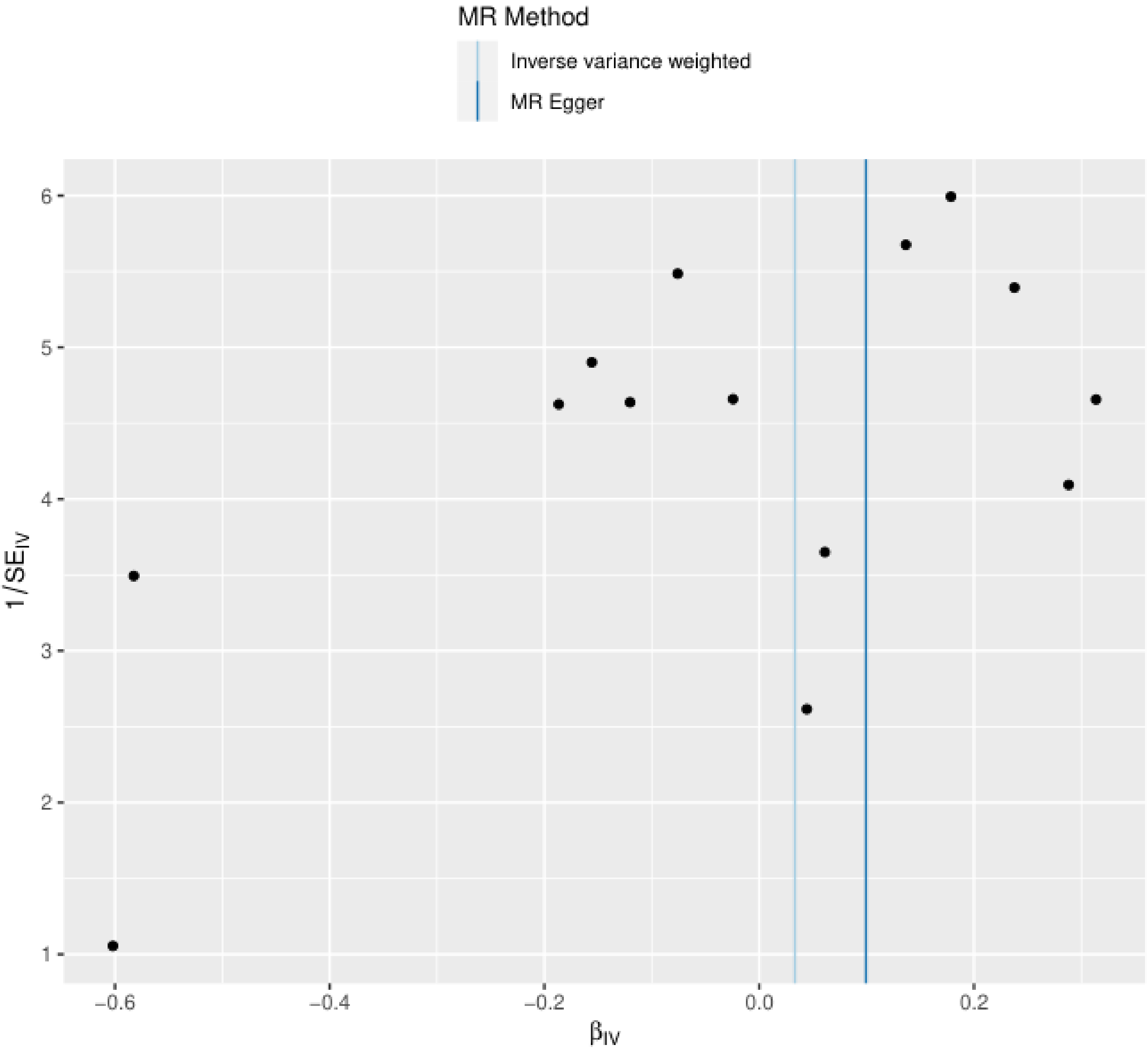

Funnel plot analyse of "CD8br and CD8dim %leukocyte" on 'Diabetic nephropathy'

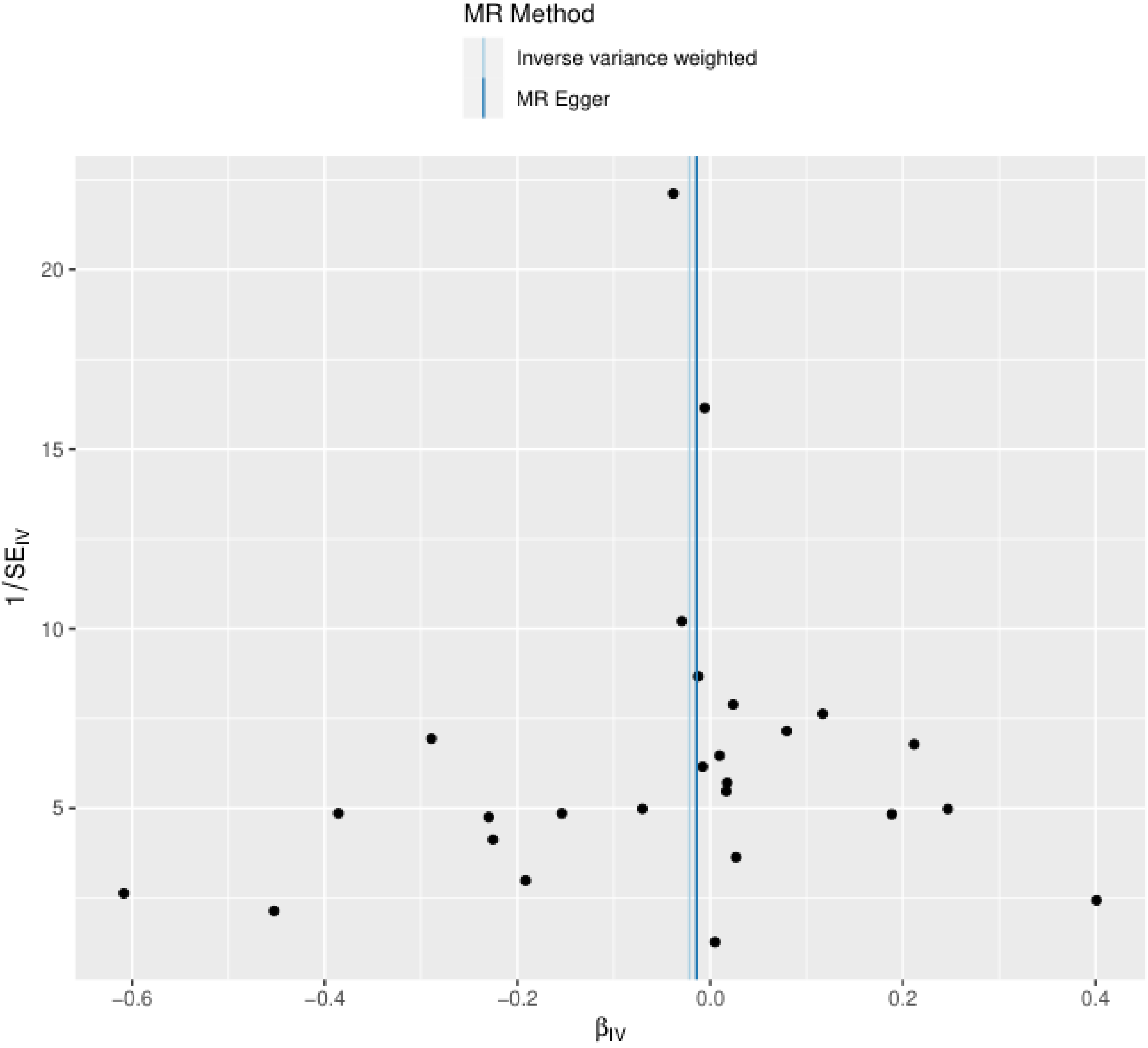

Funnel plot analyse of "SSC-A on granulocyte" on 'Diabetic nephropathy'

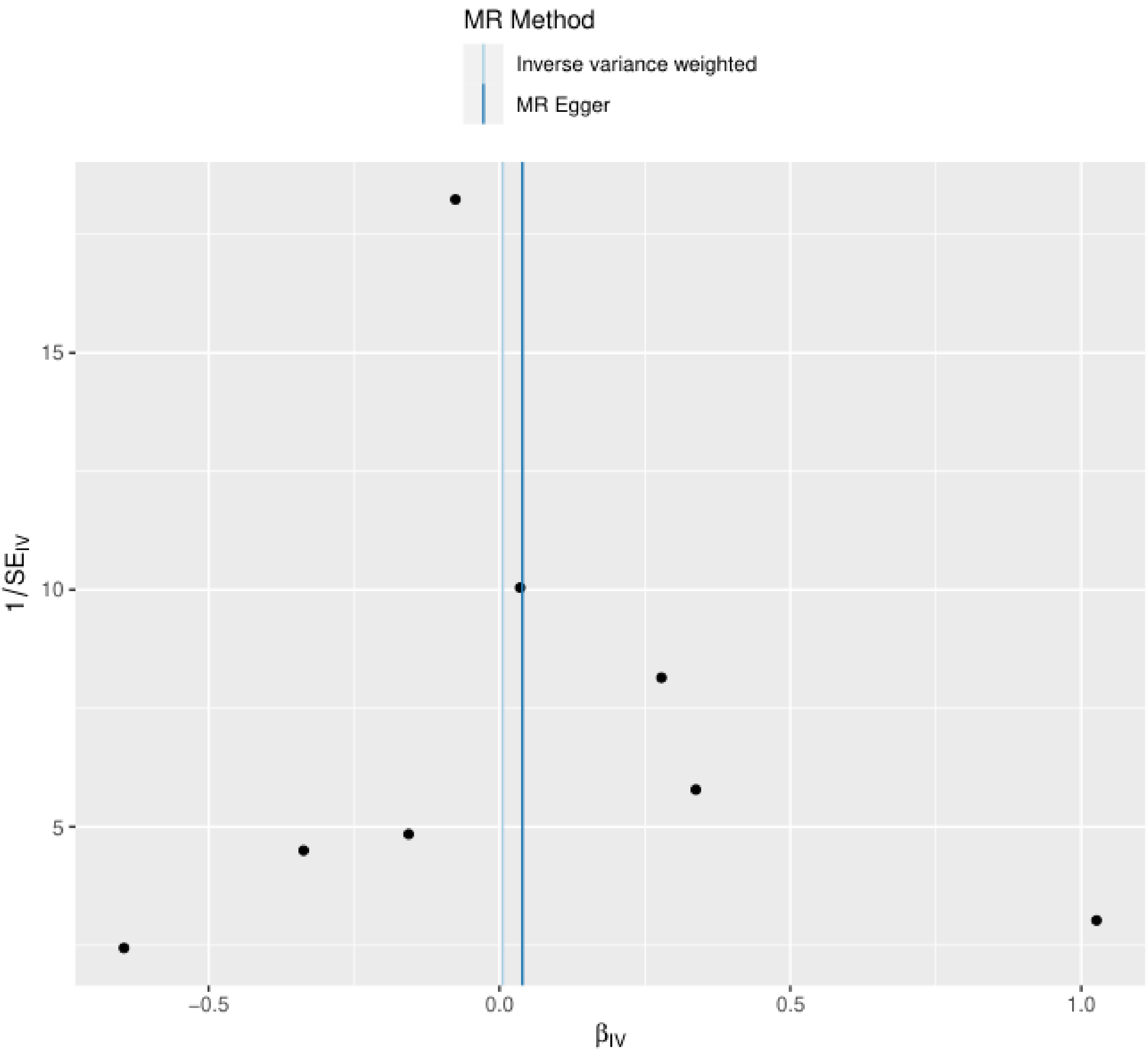

Funnel plot analyse of "CD45RA on CD39+ resting Treg " on 'Diabetic nephropathy'

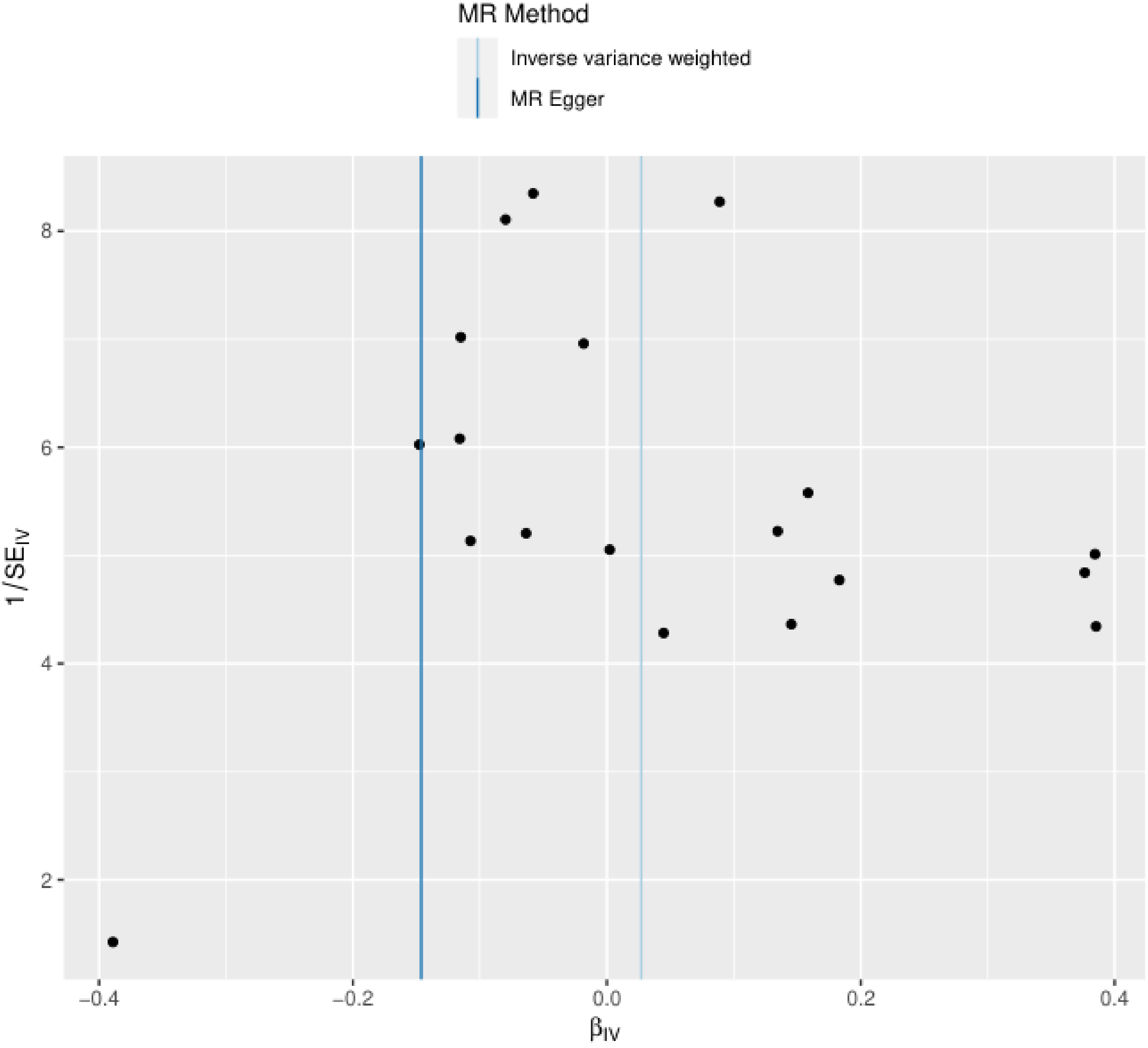

Funnel plot analyse of "CD3 on CD28- CD8br " on 'Diabetic nephropathy'

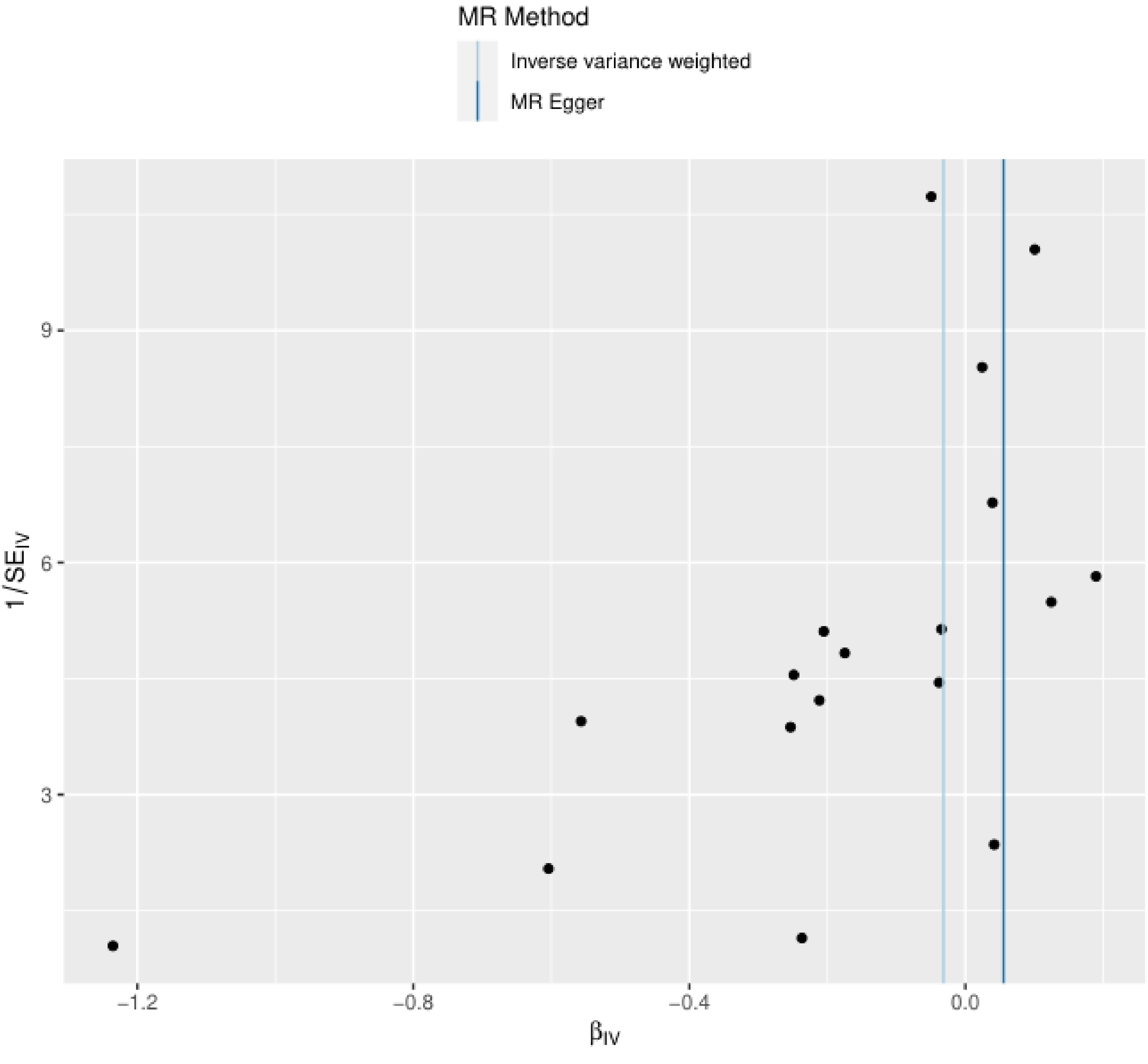

Funnel plot analyse of "IgD- CD24- AC" on 'Diabetic nephropathy'

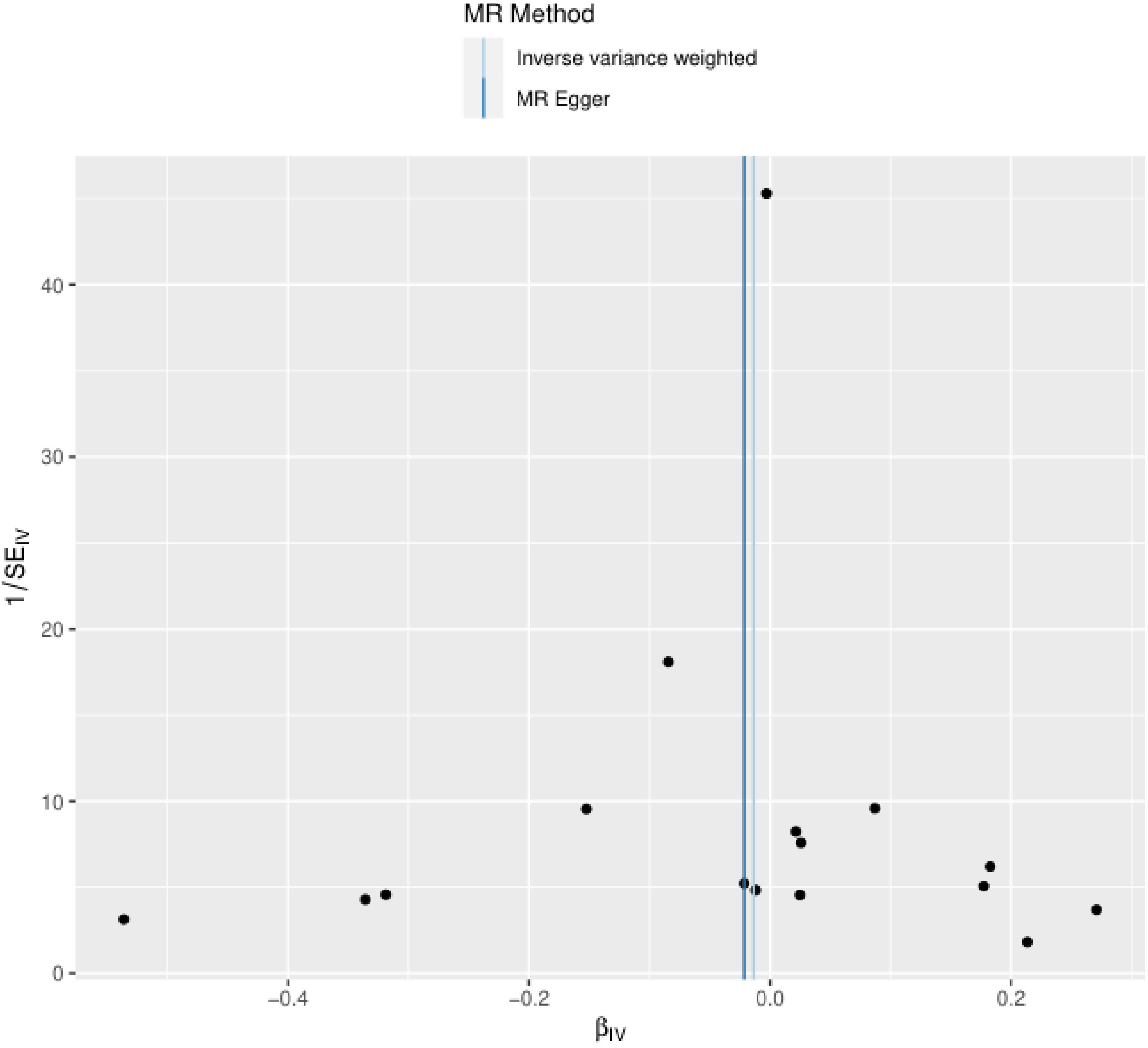

Funnel plot analyse of "CD45RA- CD4+ AC" on 'Diabetic nephropathy'

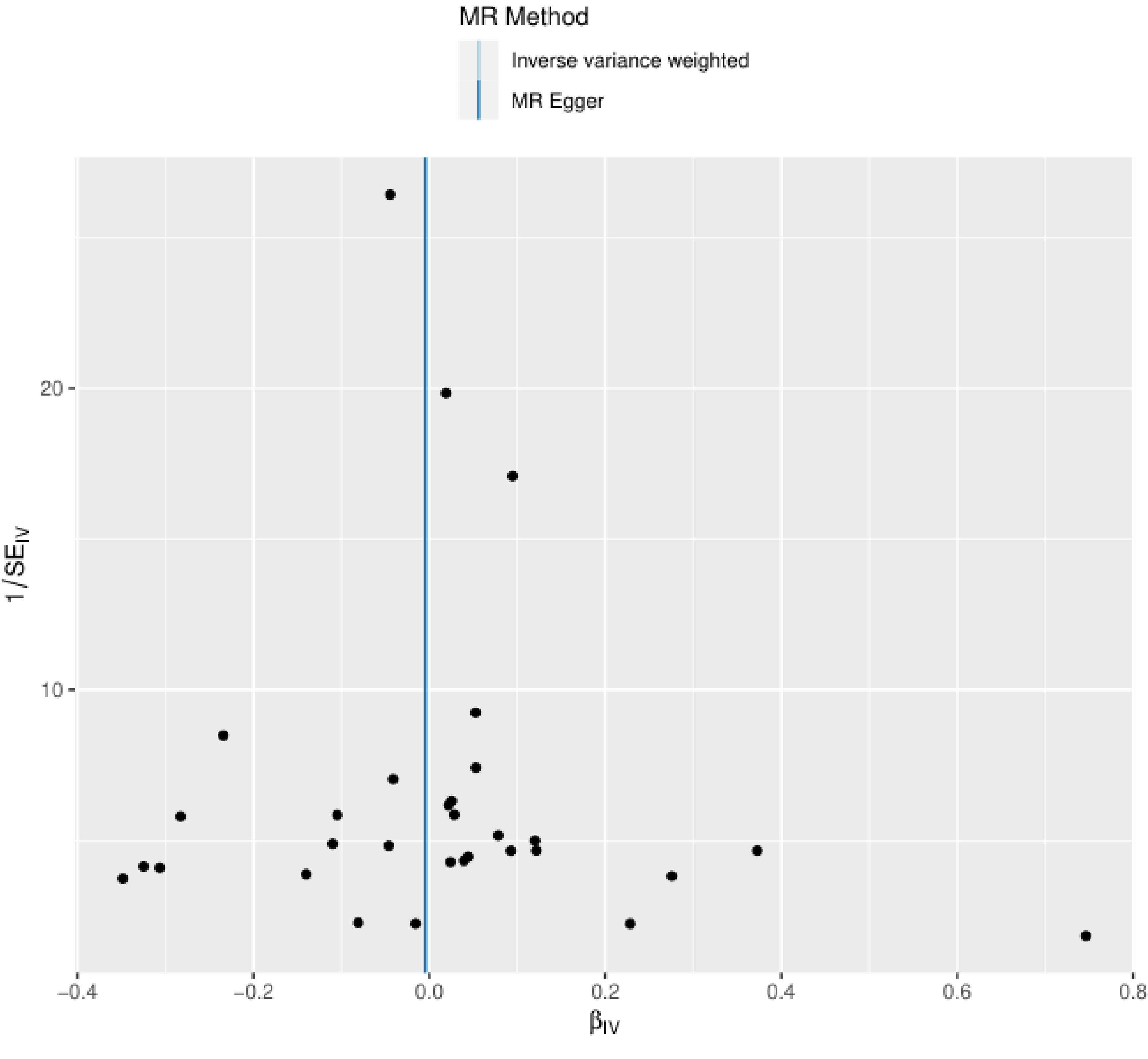

Funnel plot analyse of "CD27 on IgD+ CD24+" on 'Diabetic nephropathy'

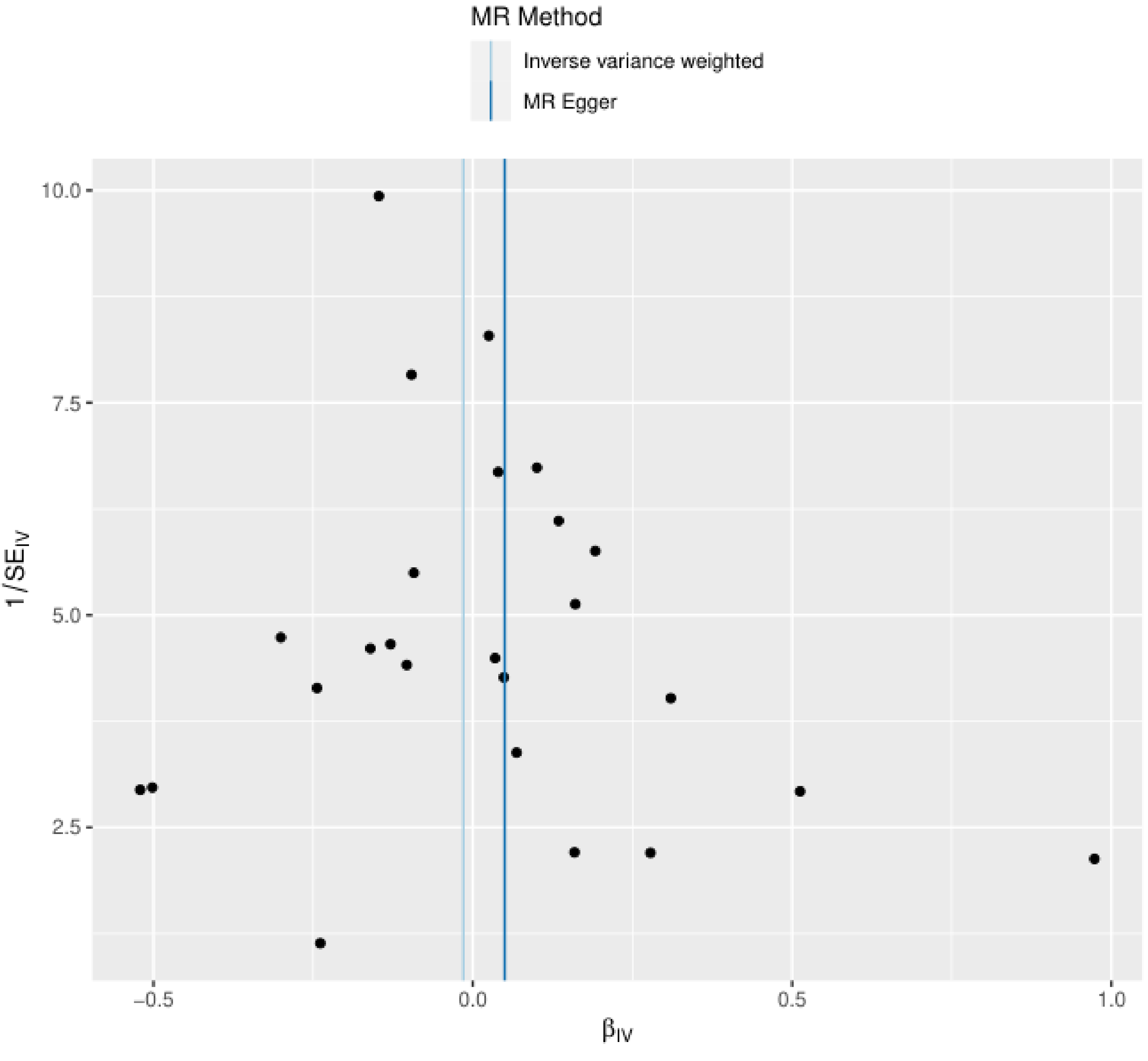

Funnel plot analyse of "IgD- CD27- AC" on 'Diabetic nephropathy'

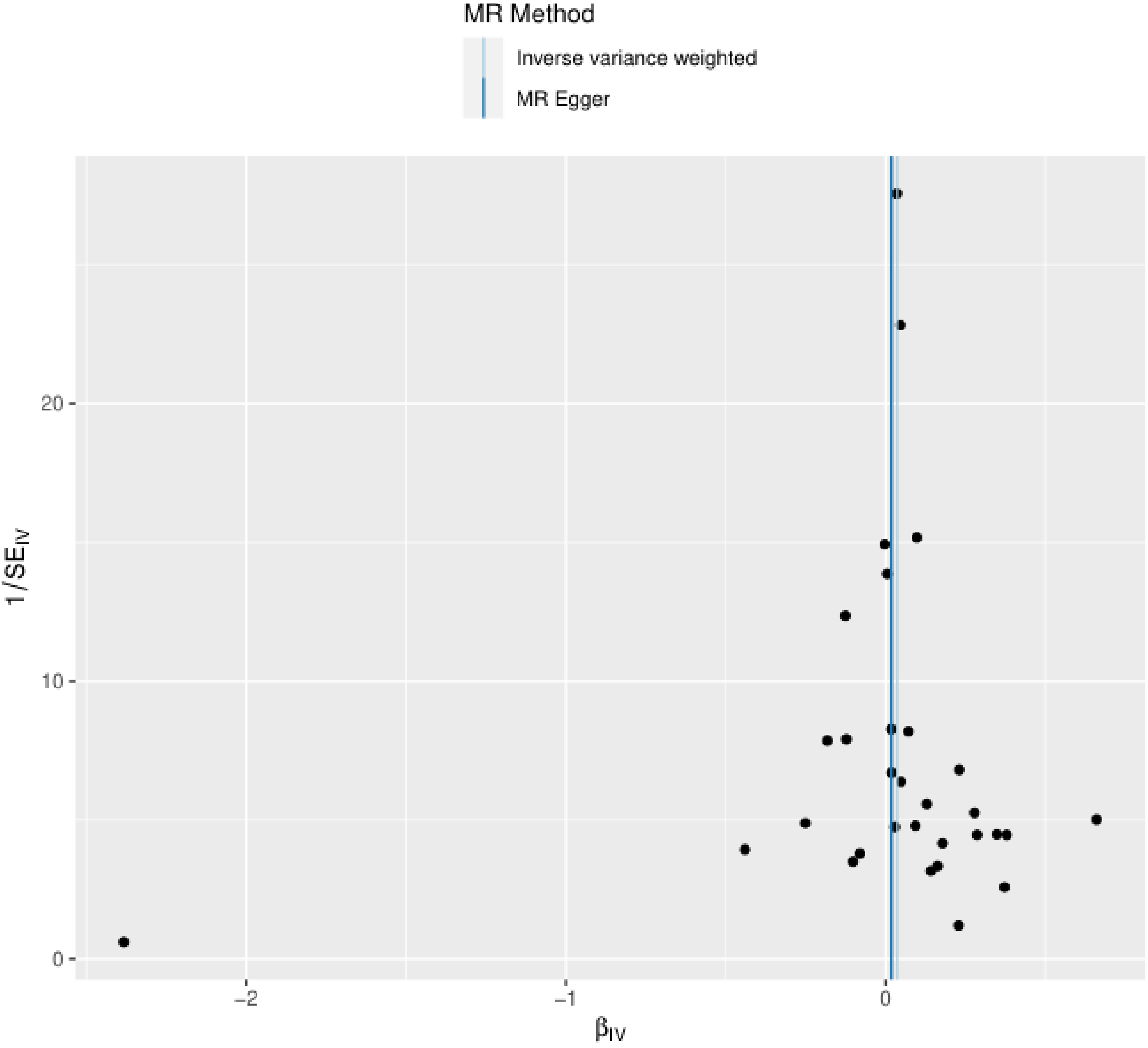

Funnel plot analyse of "CD4 on activated & secreting Treg " on 'Diabetic nephropathy'

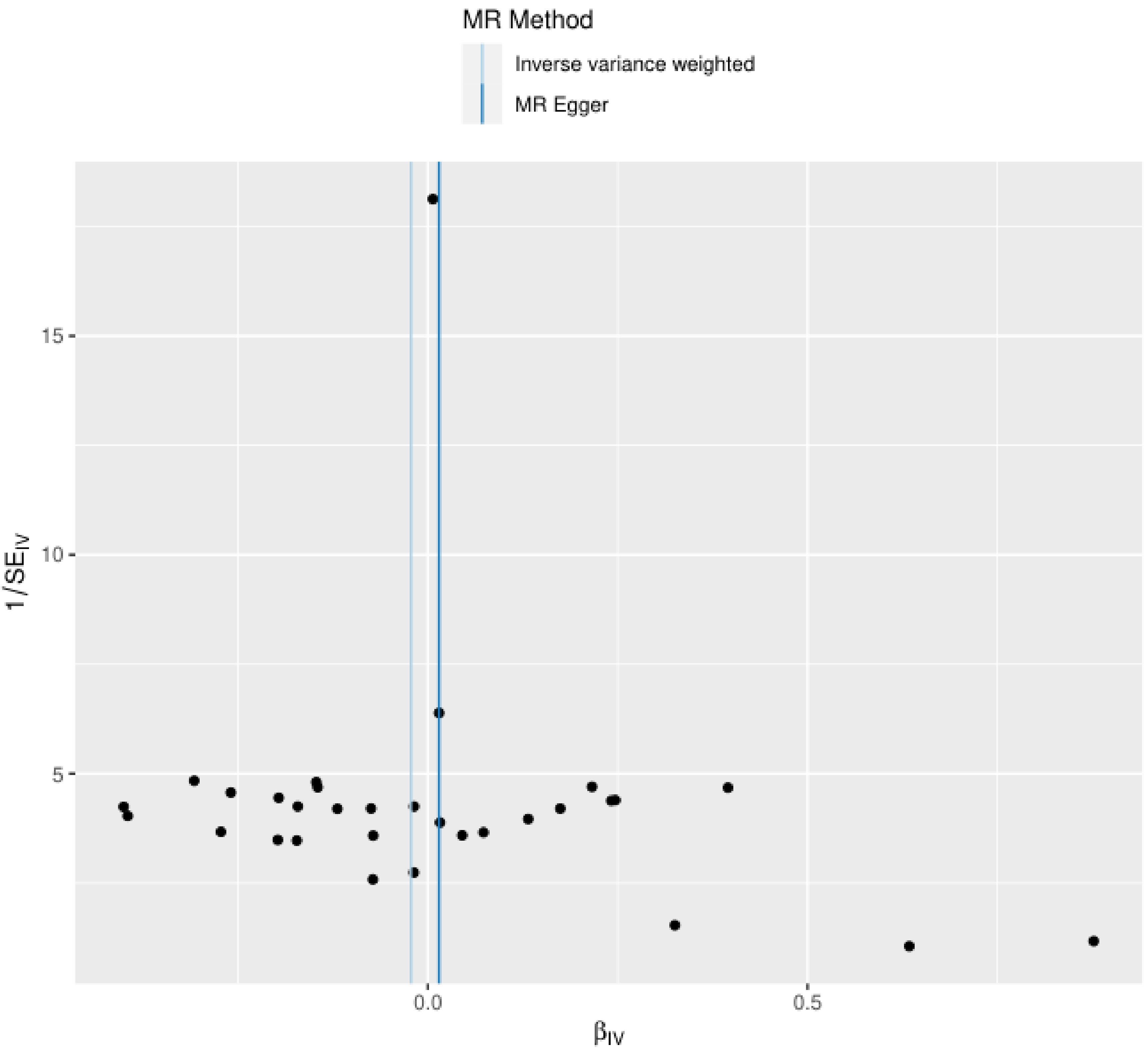

Funnel plot analyse of "Transitional AC" on 'Diabetic nephropathy'

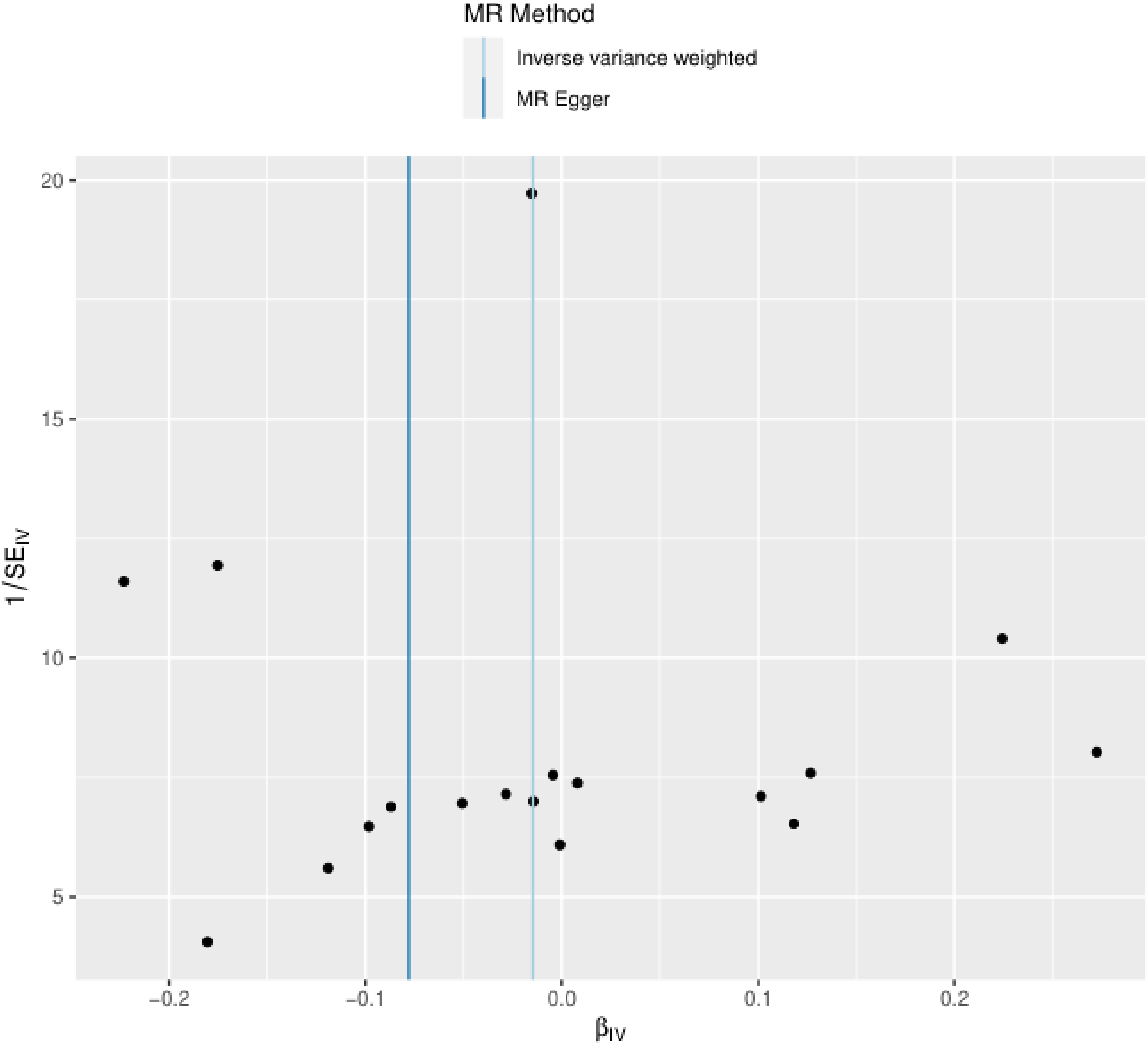

Funnel plot analyse of "HVEM on CD45RA- CD4+" on 'Diabetic nephropathy'

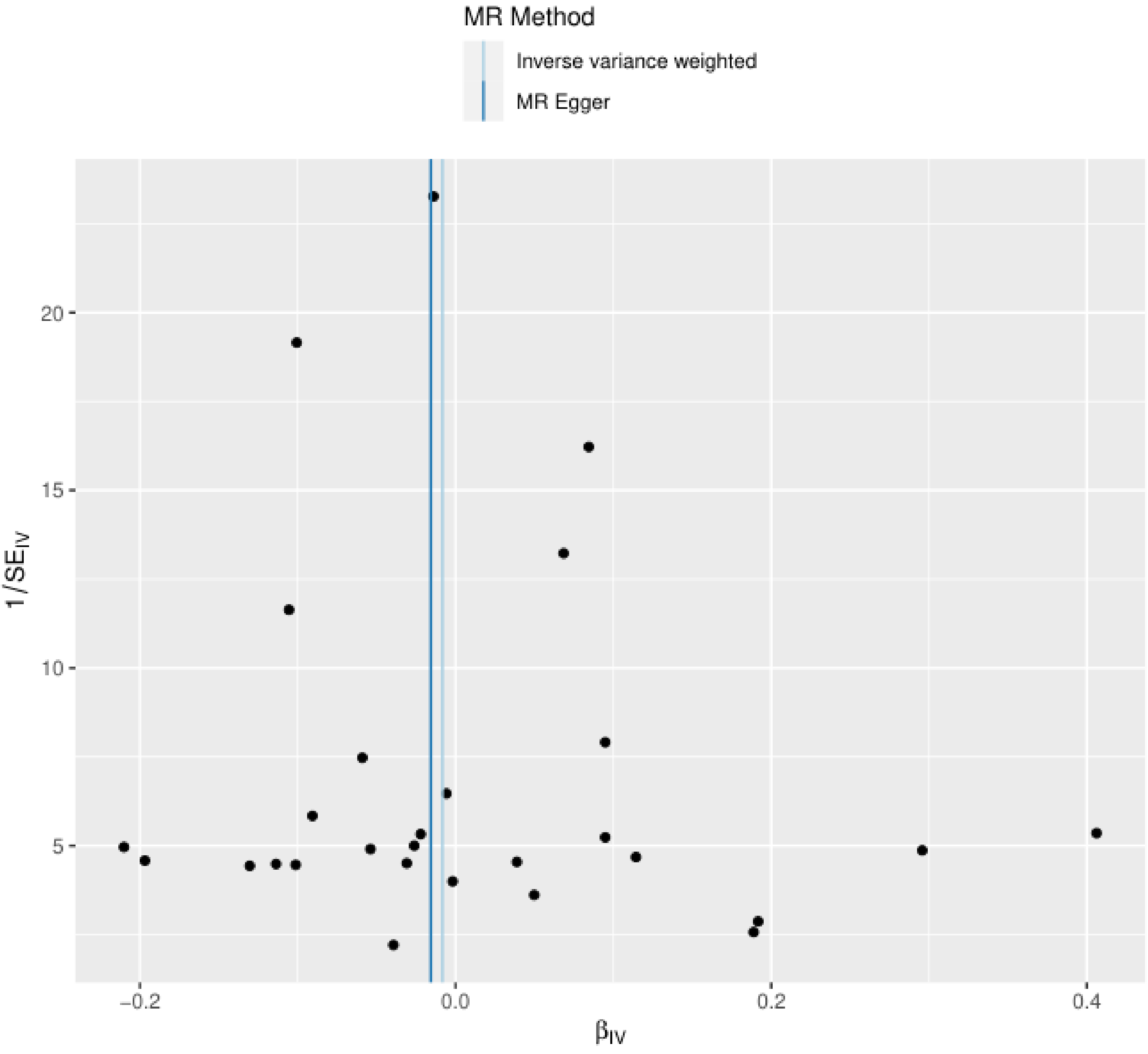

Funnel plot analyse of "CD3 on Naive CD4+" on 'Diabetic nephropathy'

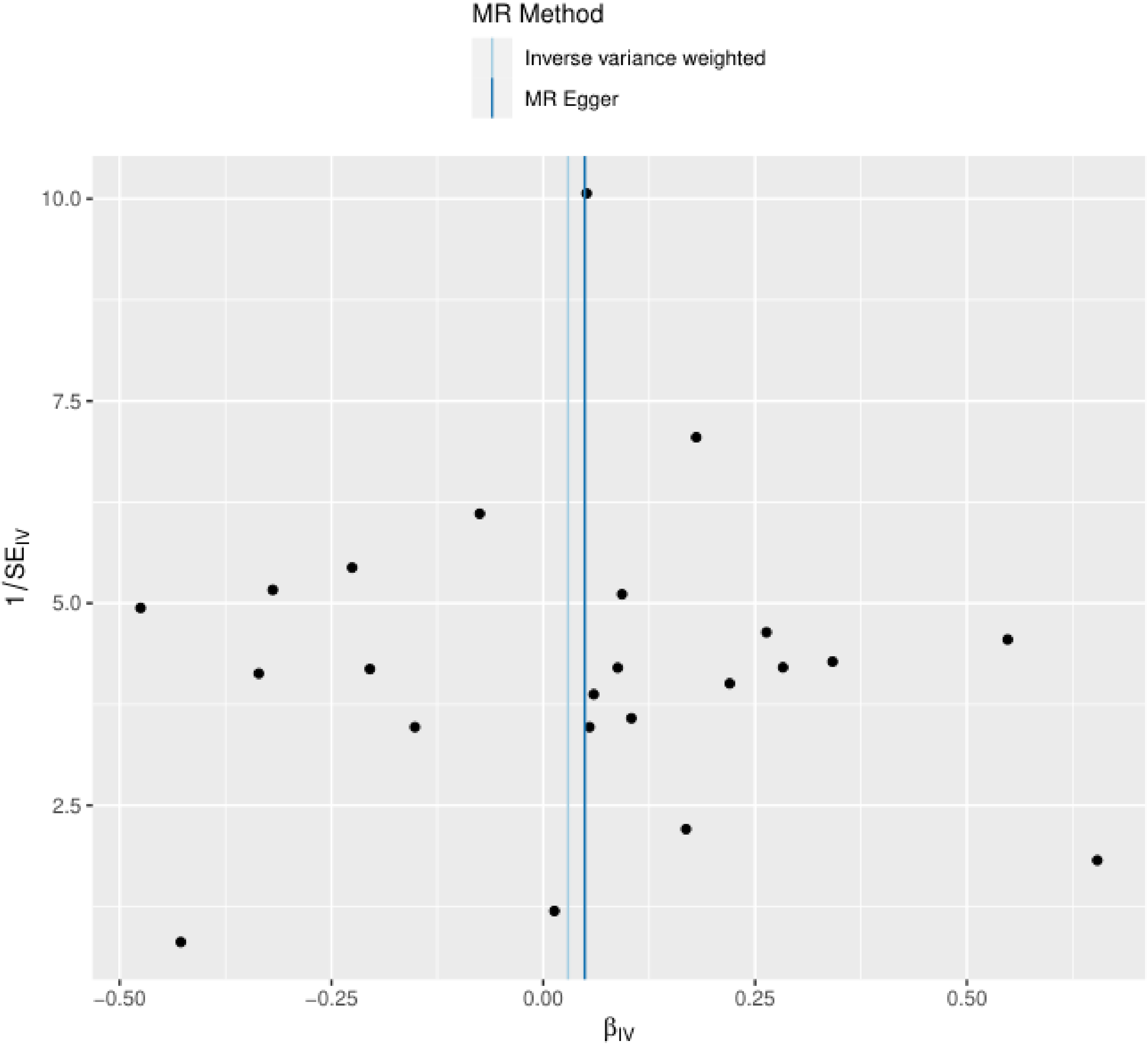

Funnel plot analyse of "CD28- CD8br %CD8br" on 'Diabetic nephropathy'

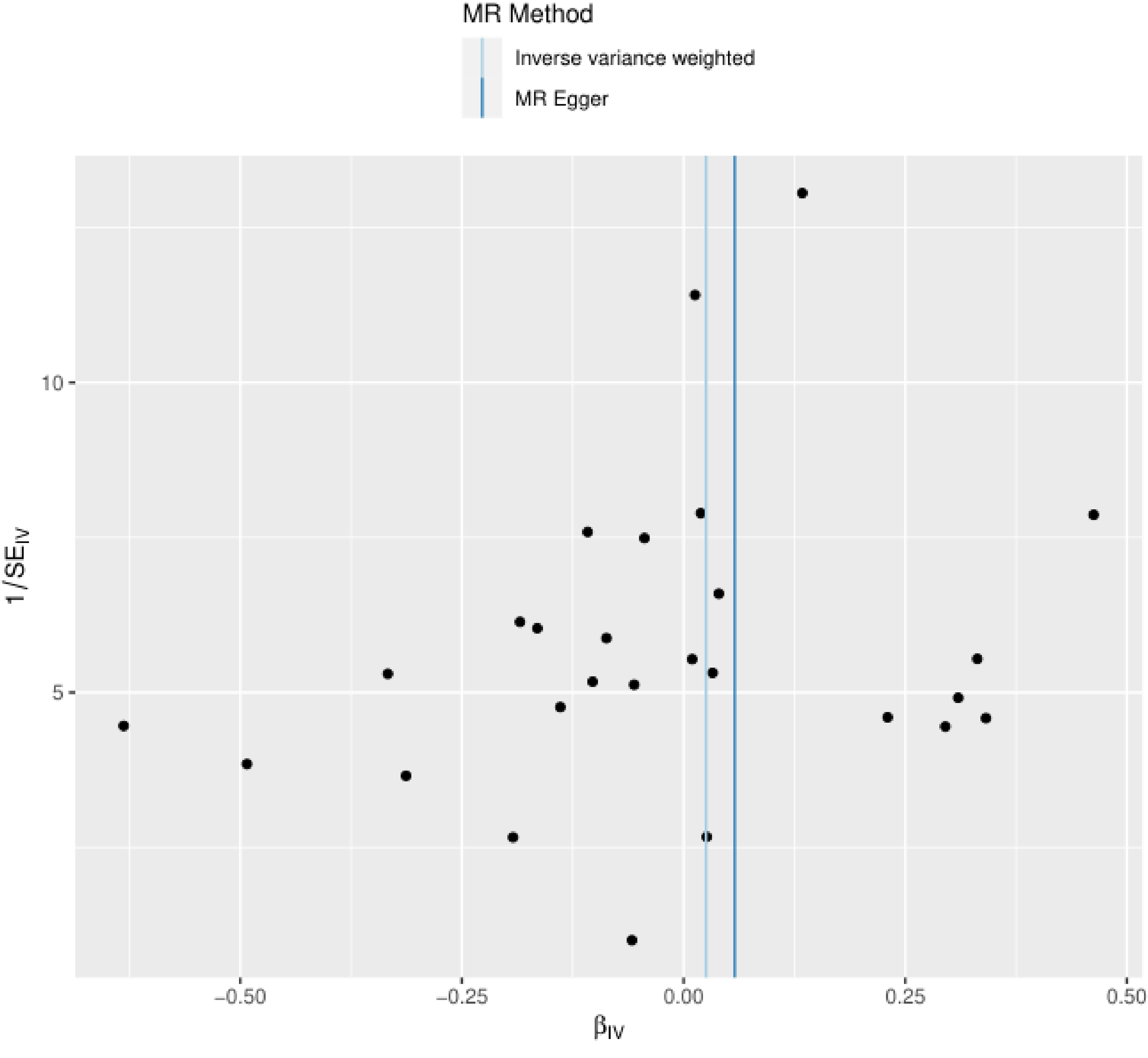

Funnel plot analyse of "CD4 on CD45RA+ CD4+ " on 'Diabetic nephropathy'

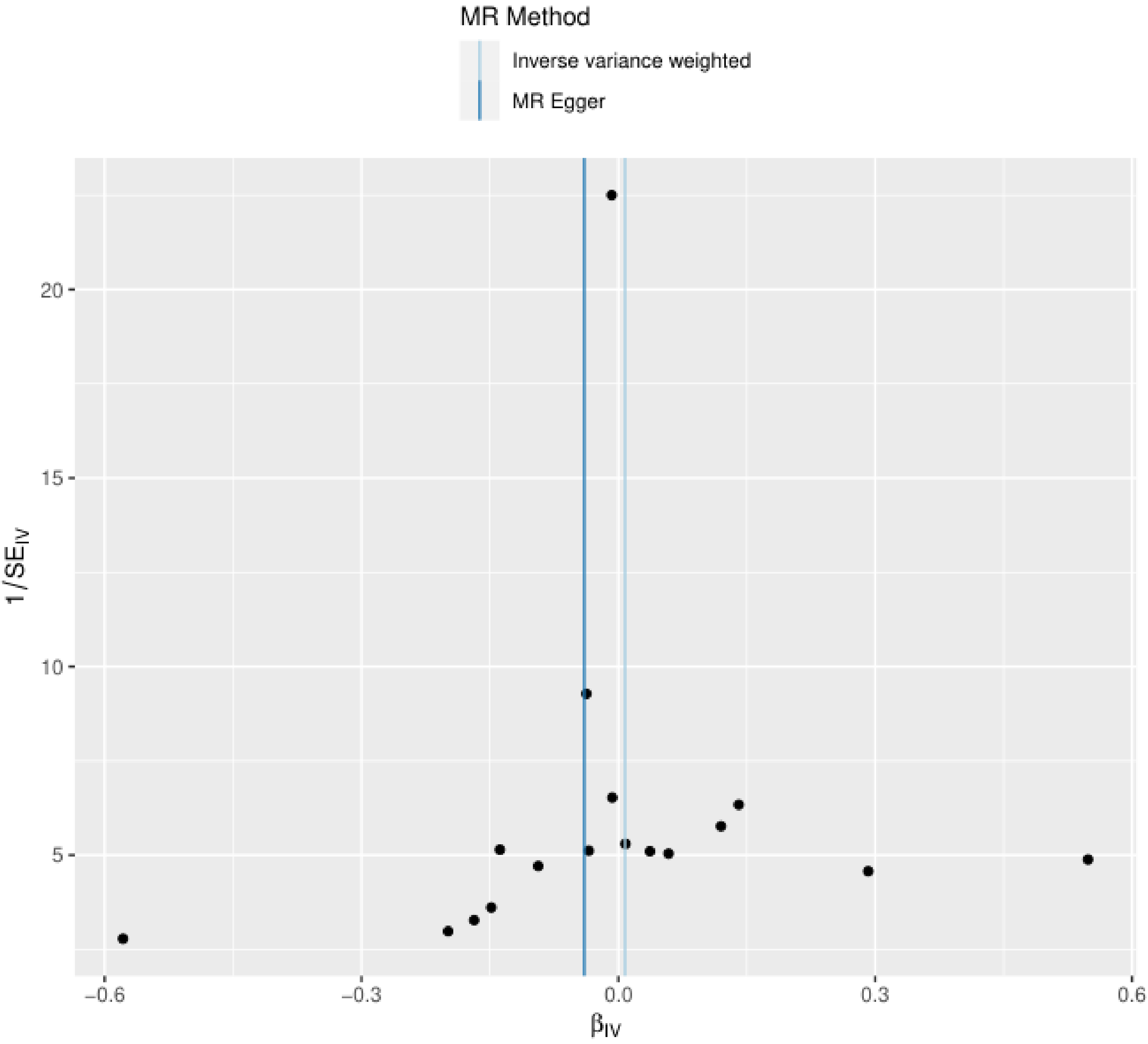

Funnel plot analyse of "CCR2 on granulocyte" on 'Diabetic nephropathy'

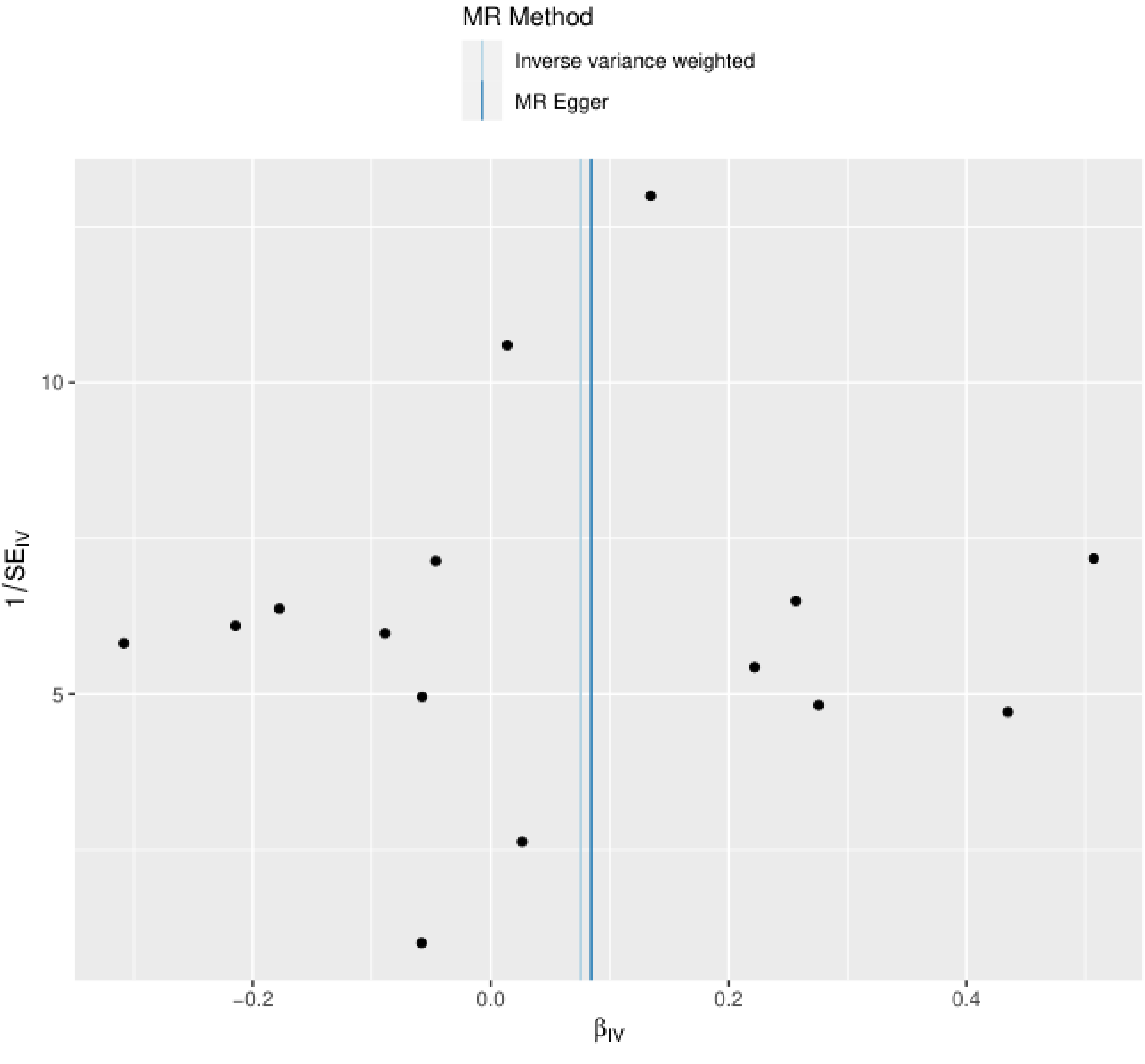

Funnel plot analyse of "CD4 on CM CD4 +" on 'Diabetic nephropathy'

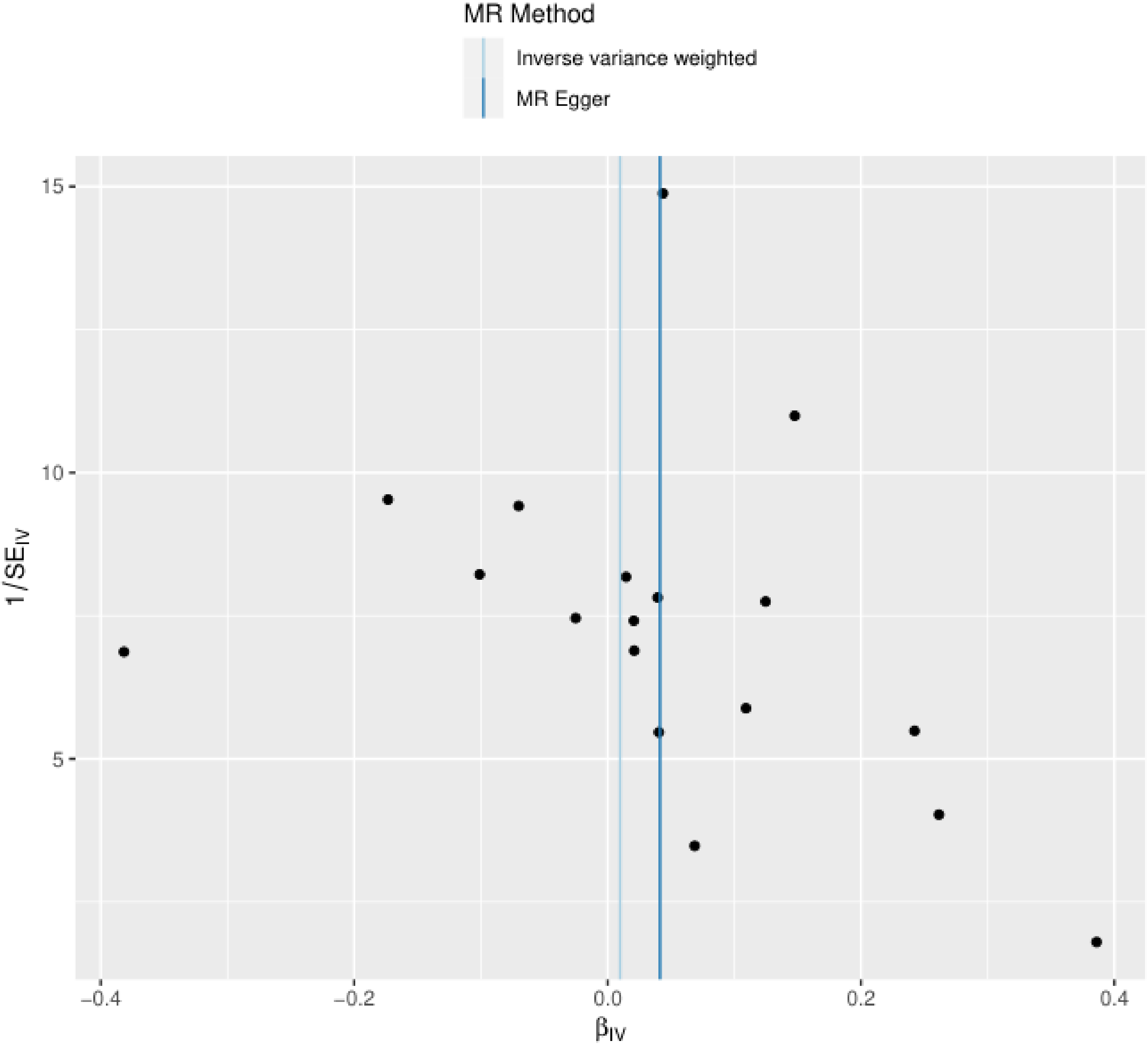

Funnel plot analyse of "CD45 on CD33dim HLA DR- " on 'Diabetic nephropathy'

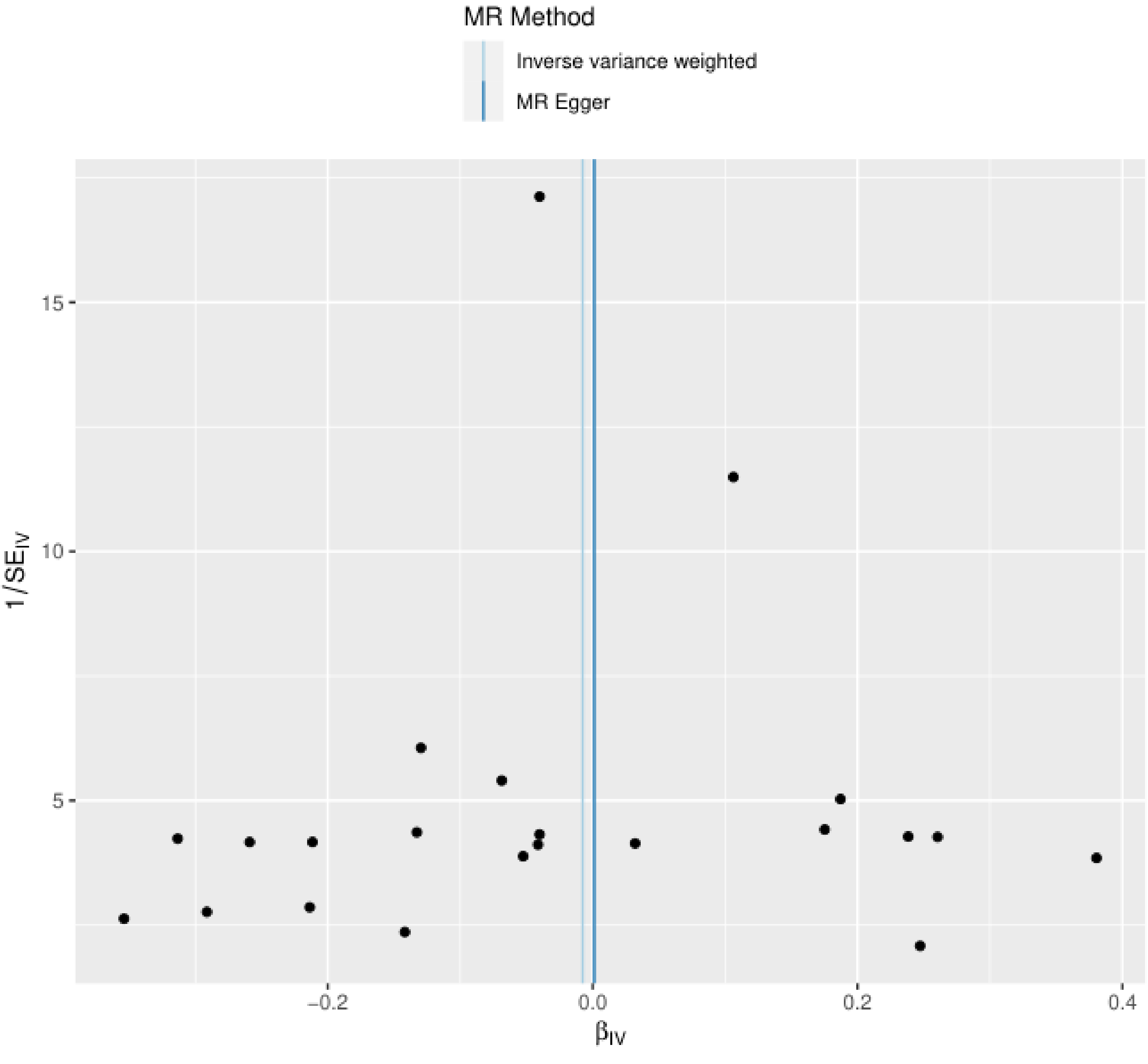

Funnel plot analyse of "CD19 on CD20- " on 'Diabetic nephropathy'

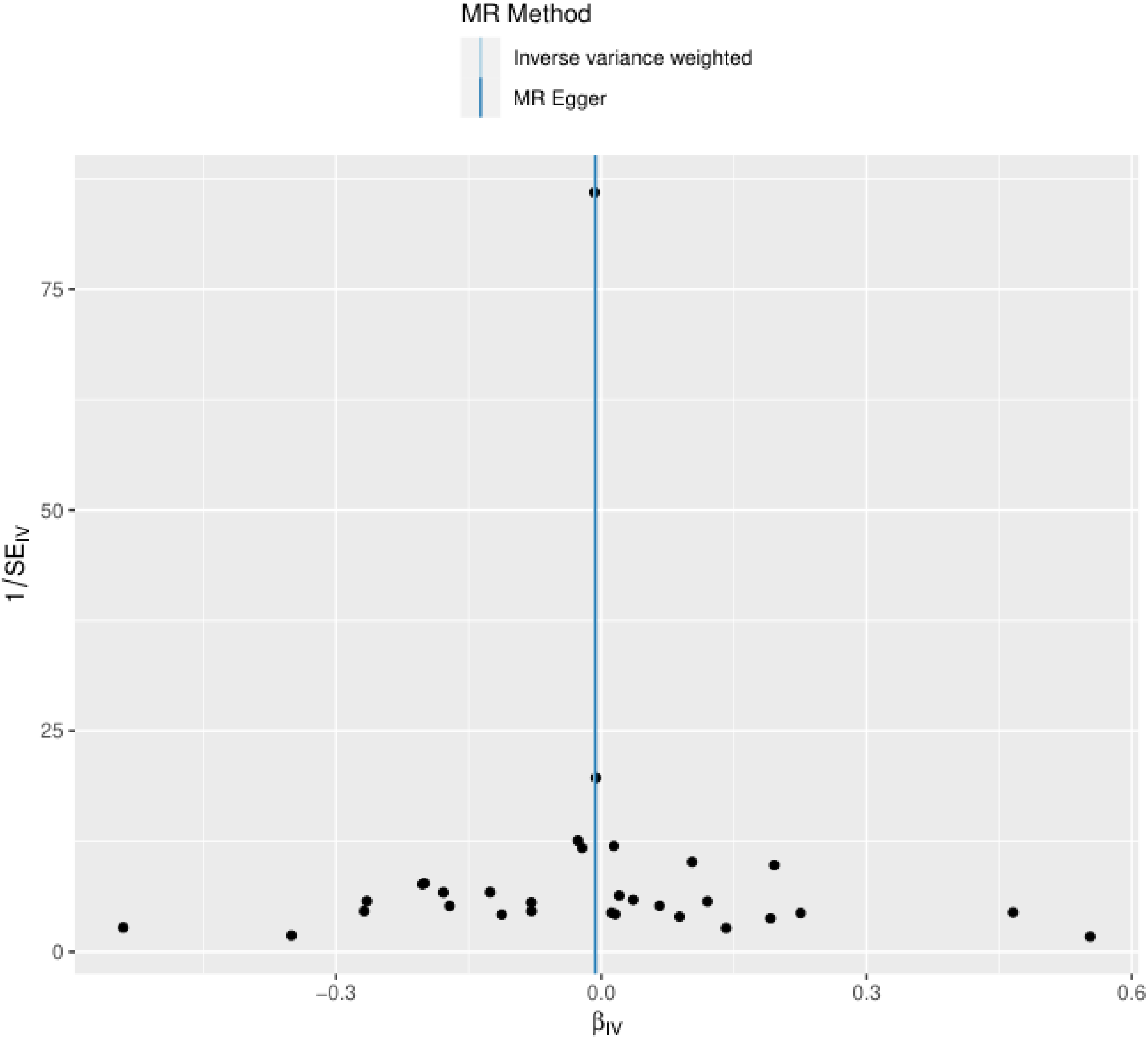

Funnel plot analyse of "CM CD4+ %CD4+" on 'Diabetic nephropathy'

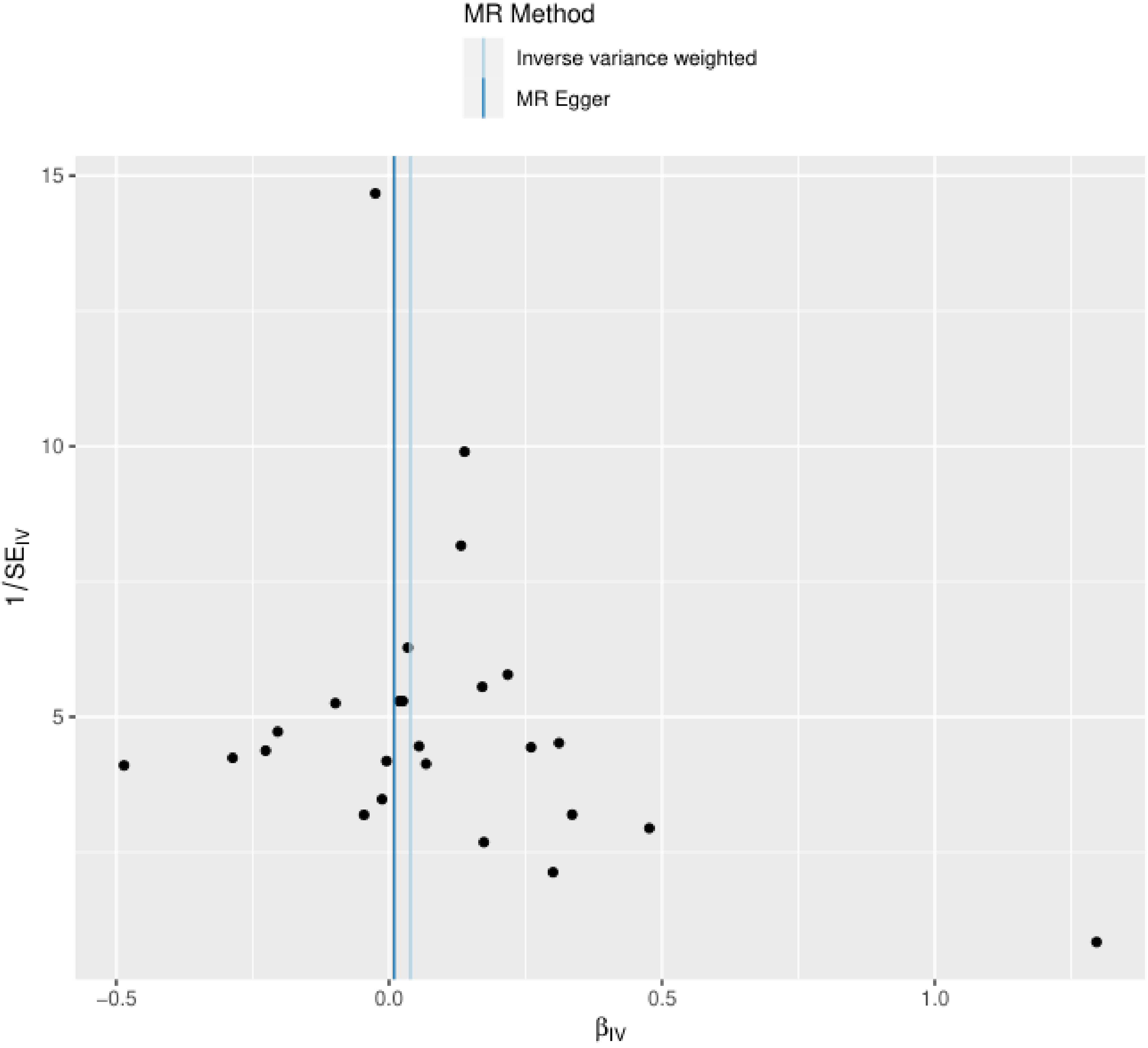

Funnel plot analyse of "CD25 on naive-mature B cell" on 'Diabetic nephropathy'

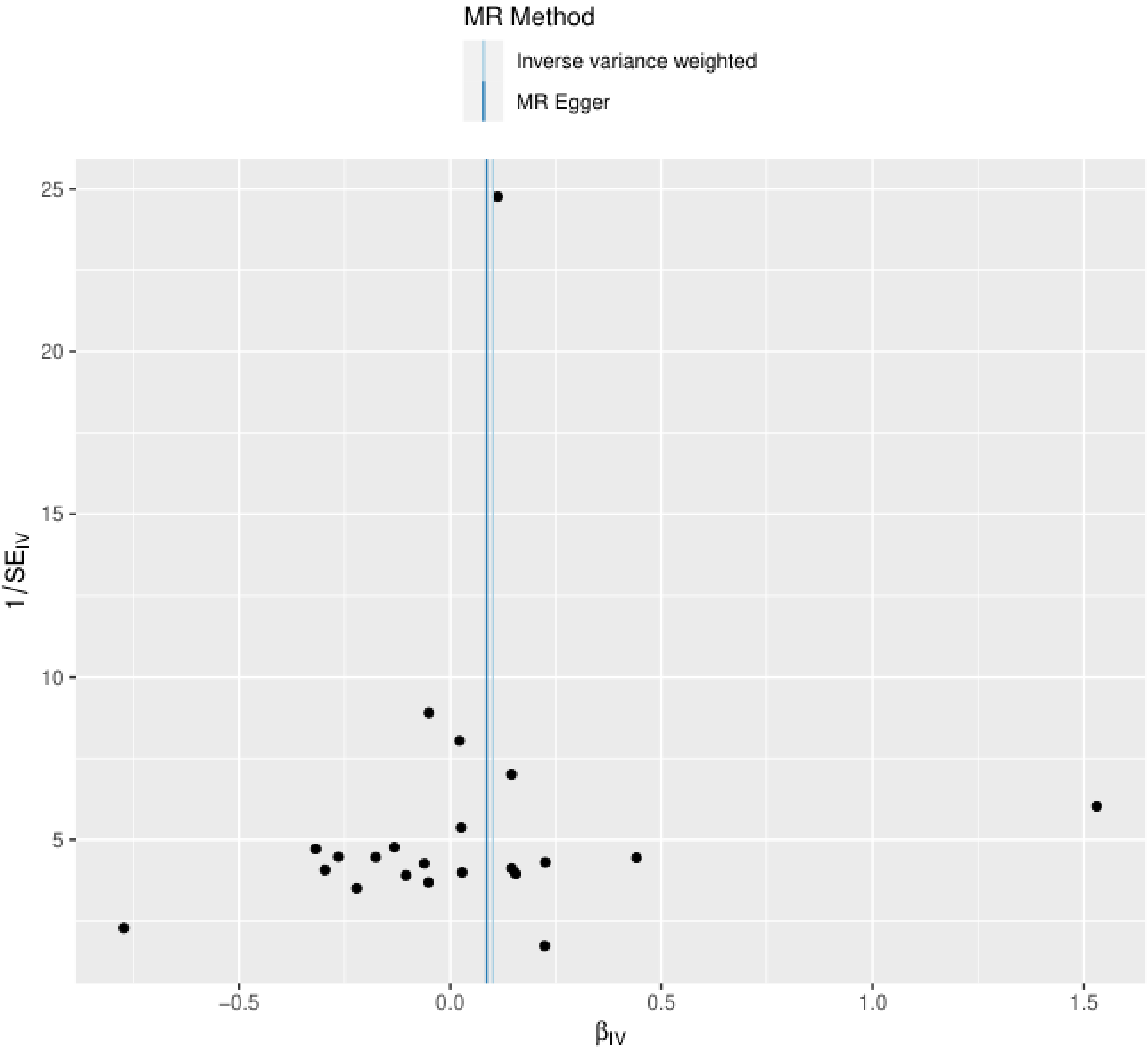

Funnel plot analyse of "CD4+ AC" on 'Diabetic nephropathy'

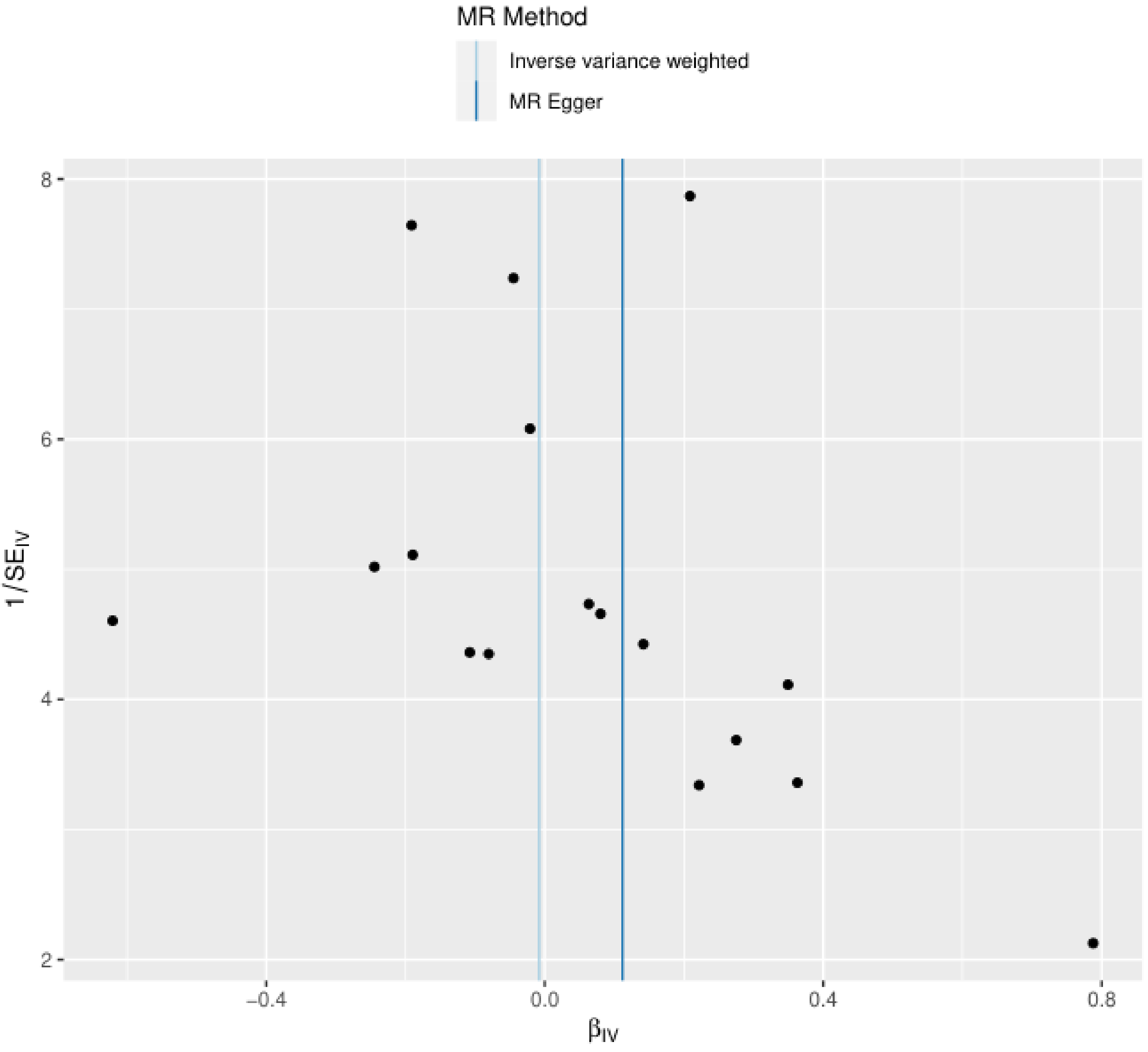

Funnel plot analyse of "T cell %lymphocyte" on 'Diabetic nephropathy'

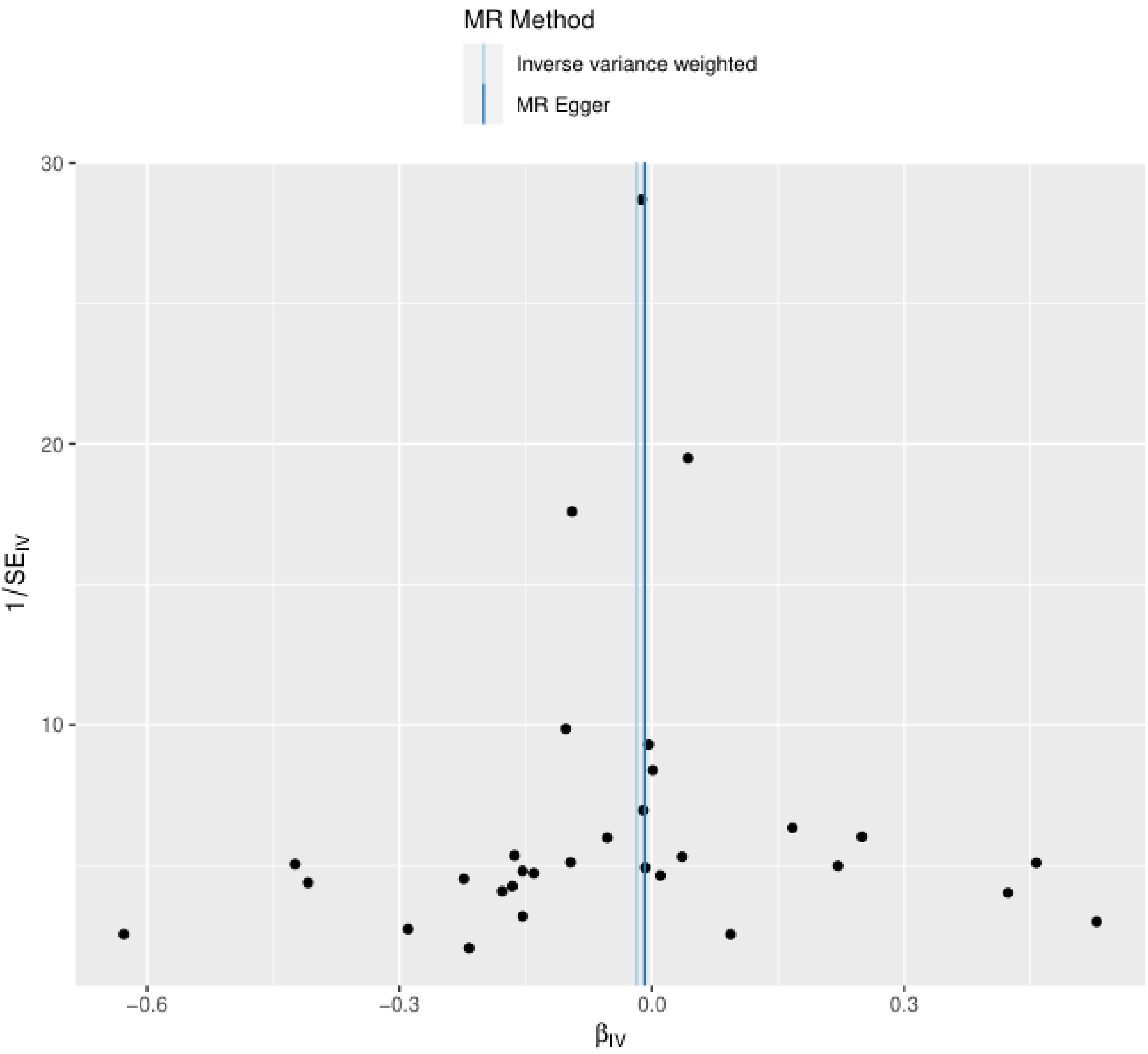

Funnel plot analyse of "CD24 on memory B cell" on 'Diabetic nephropathy'

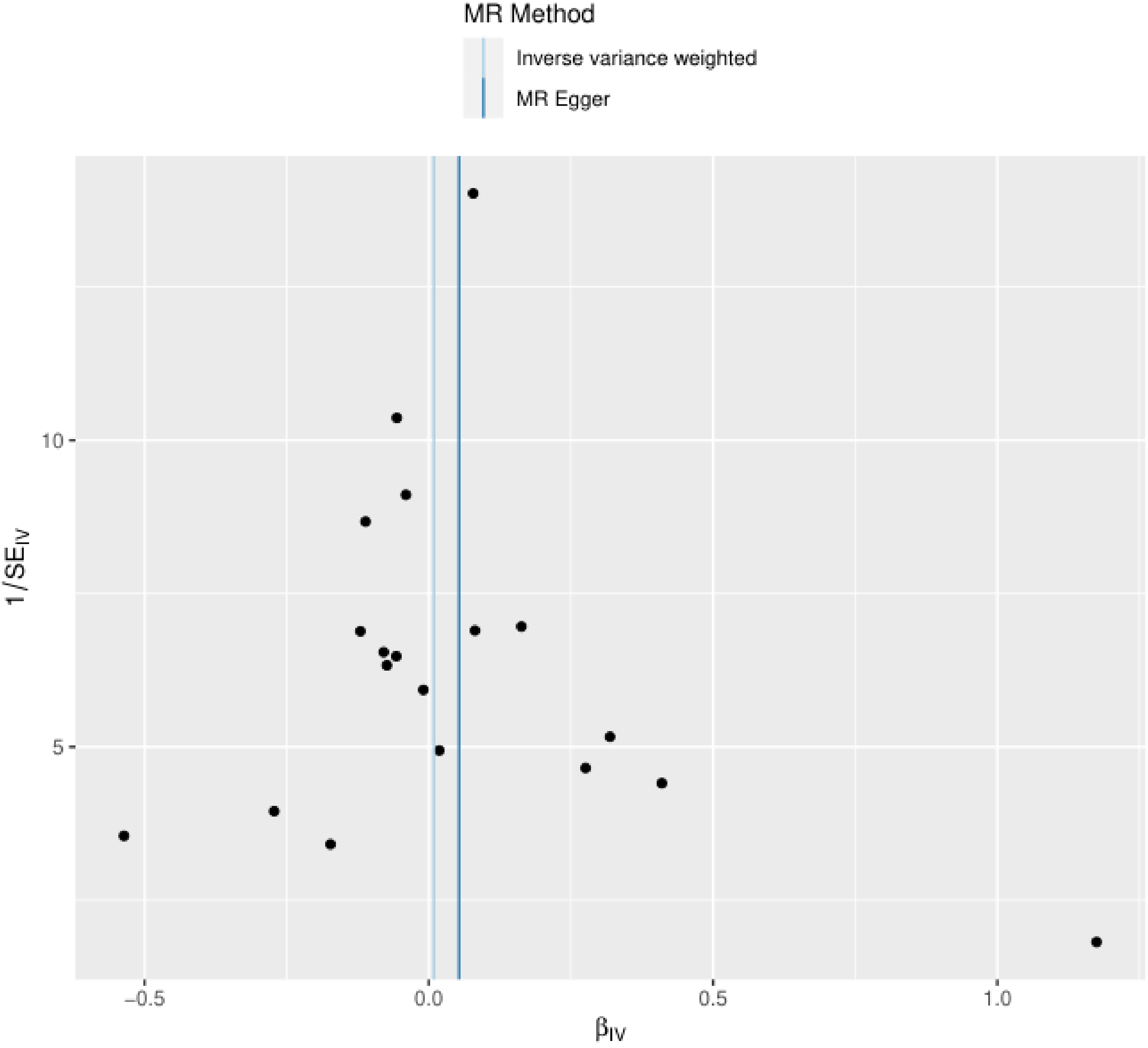

Funnel plot analyse of "CD11b on basophil" on 'Diabetic nephropathy'

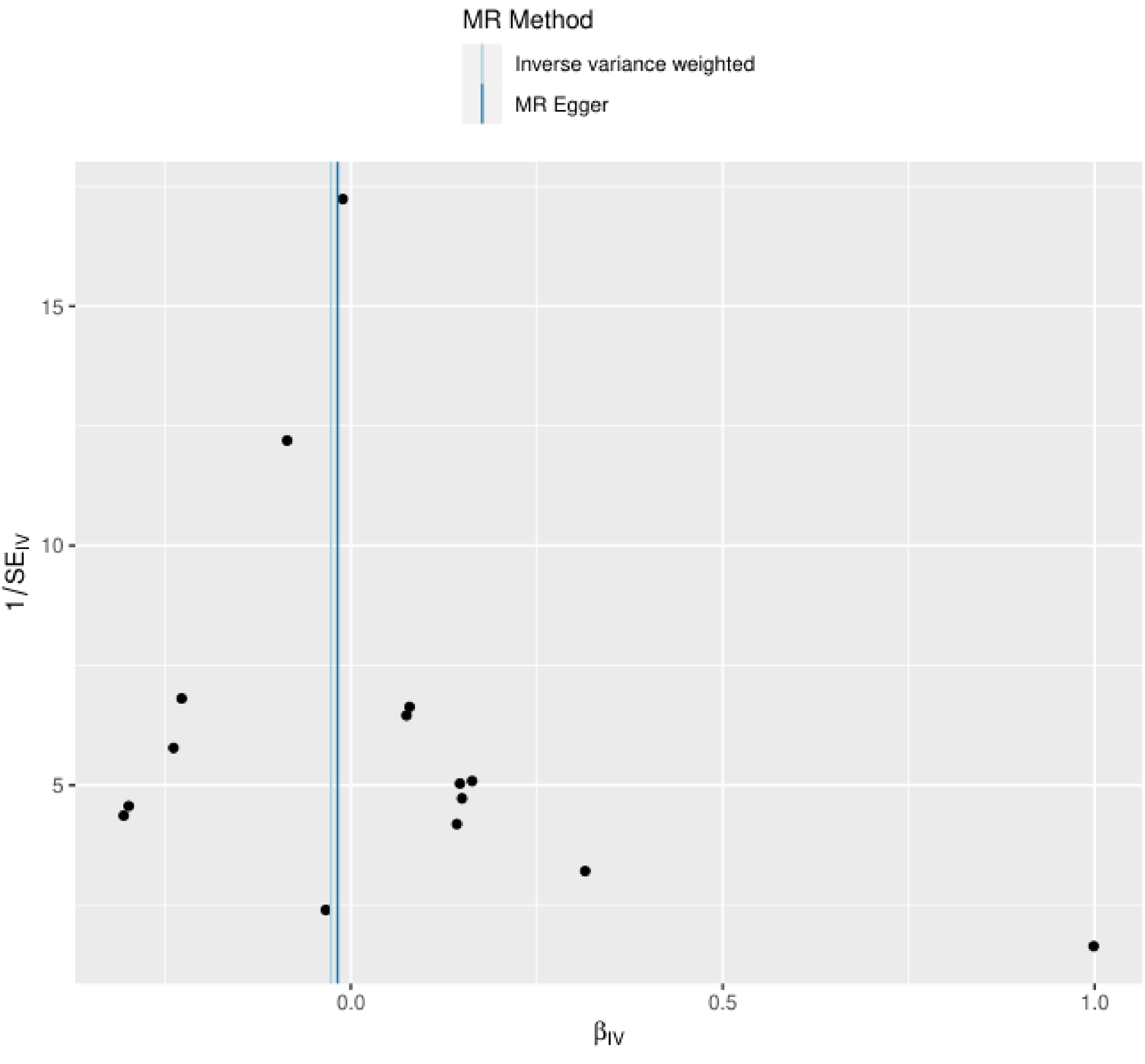

Funnel plot analyse of "CCR2 on CD14- CD16- " on 'Diabetic nephropathy'

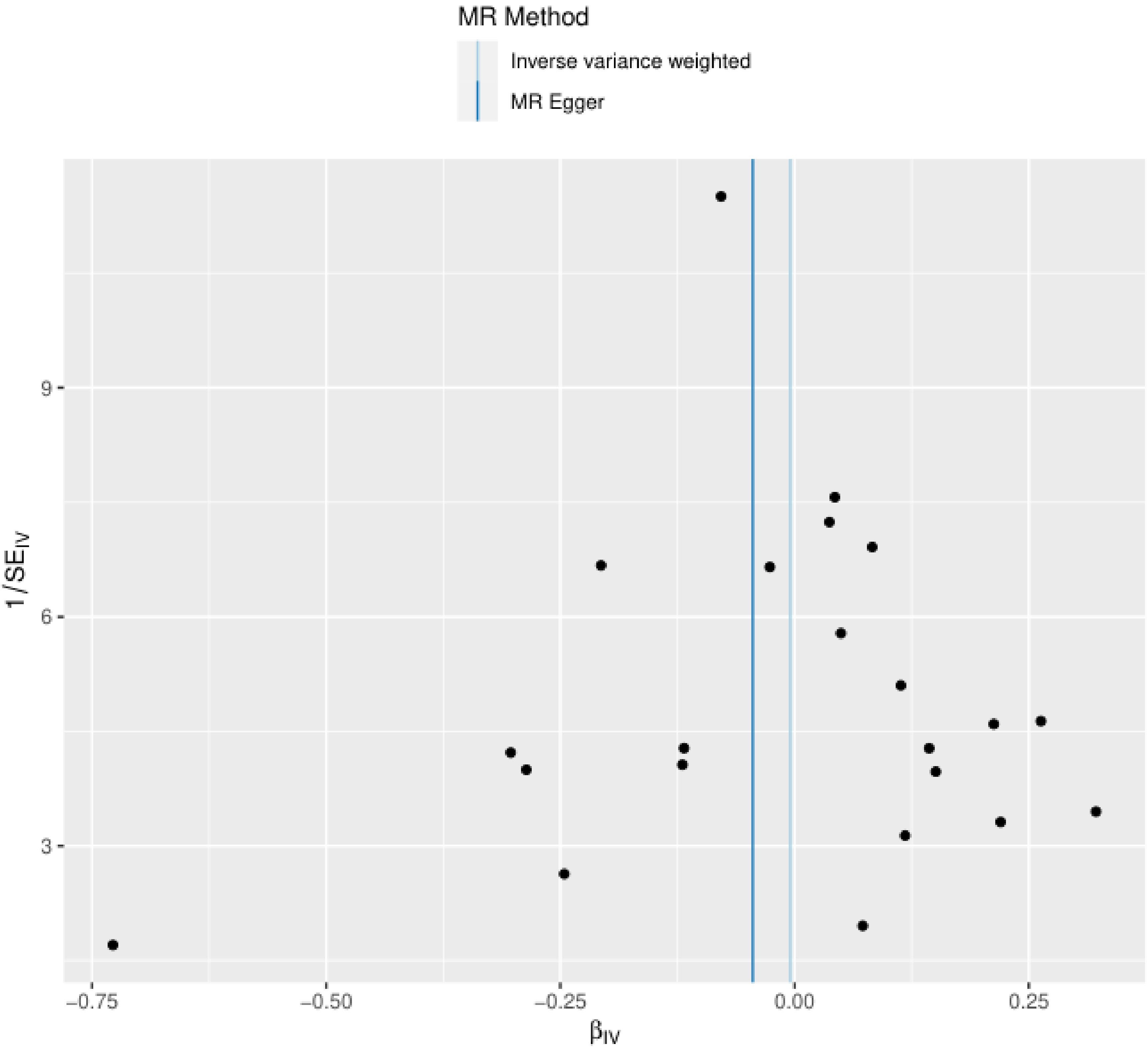

Funnel plot analyse of "NK AC" on 'Diabetic nephropathy'

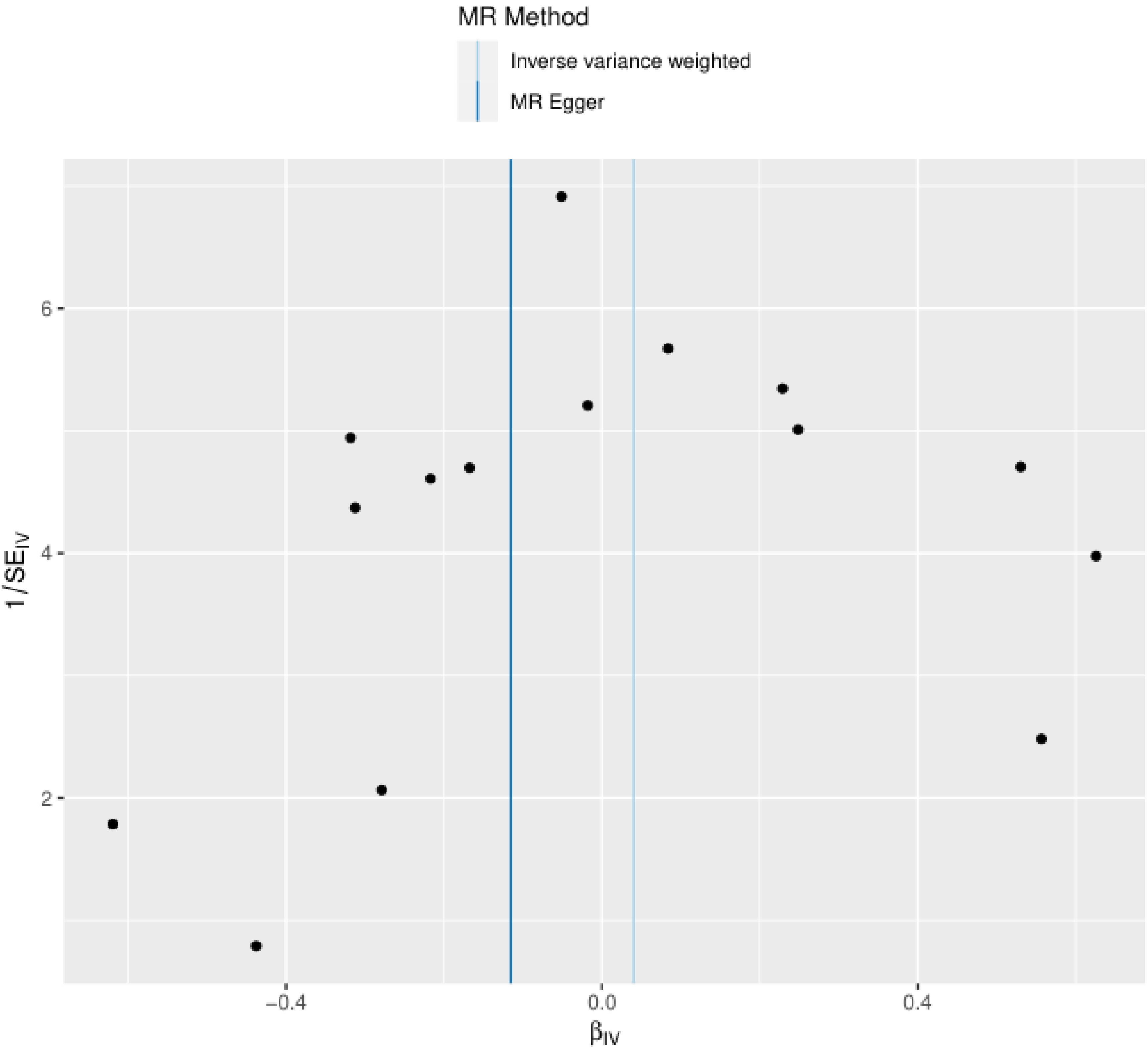

Funnel plot analyse of "CD28- CD8br AC" on 'Diabetic nephropathy'

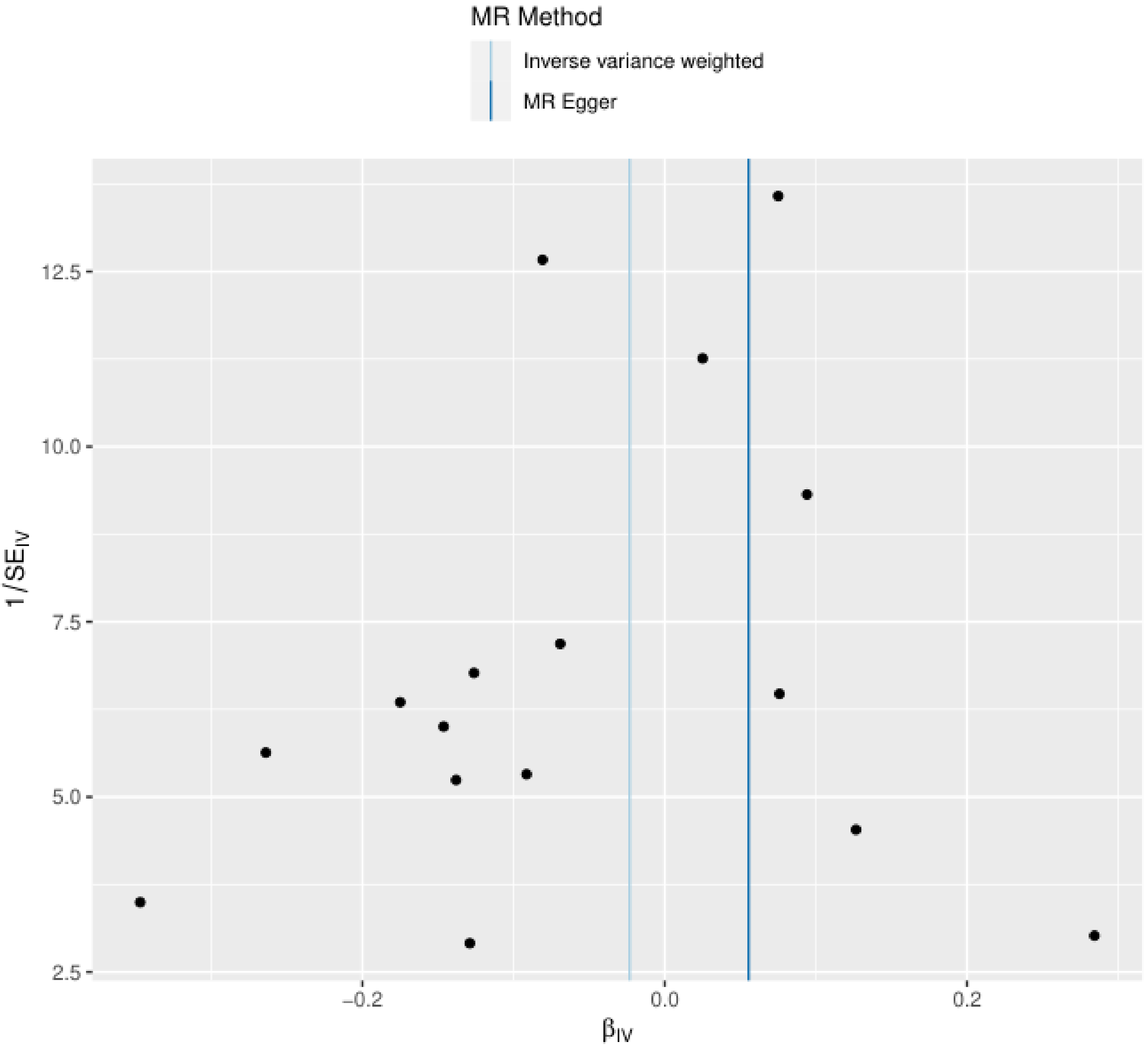

Funnel plot analyse of "CD20 on IgD+ CD38- naive" on 'Diabetic nephropathy'

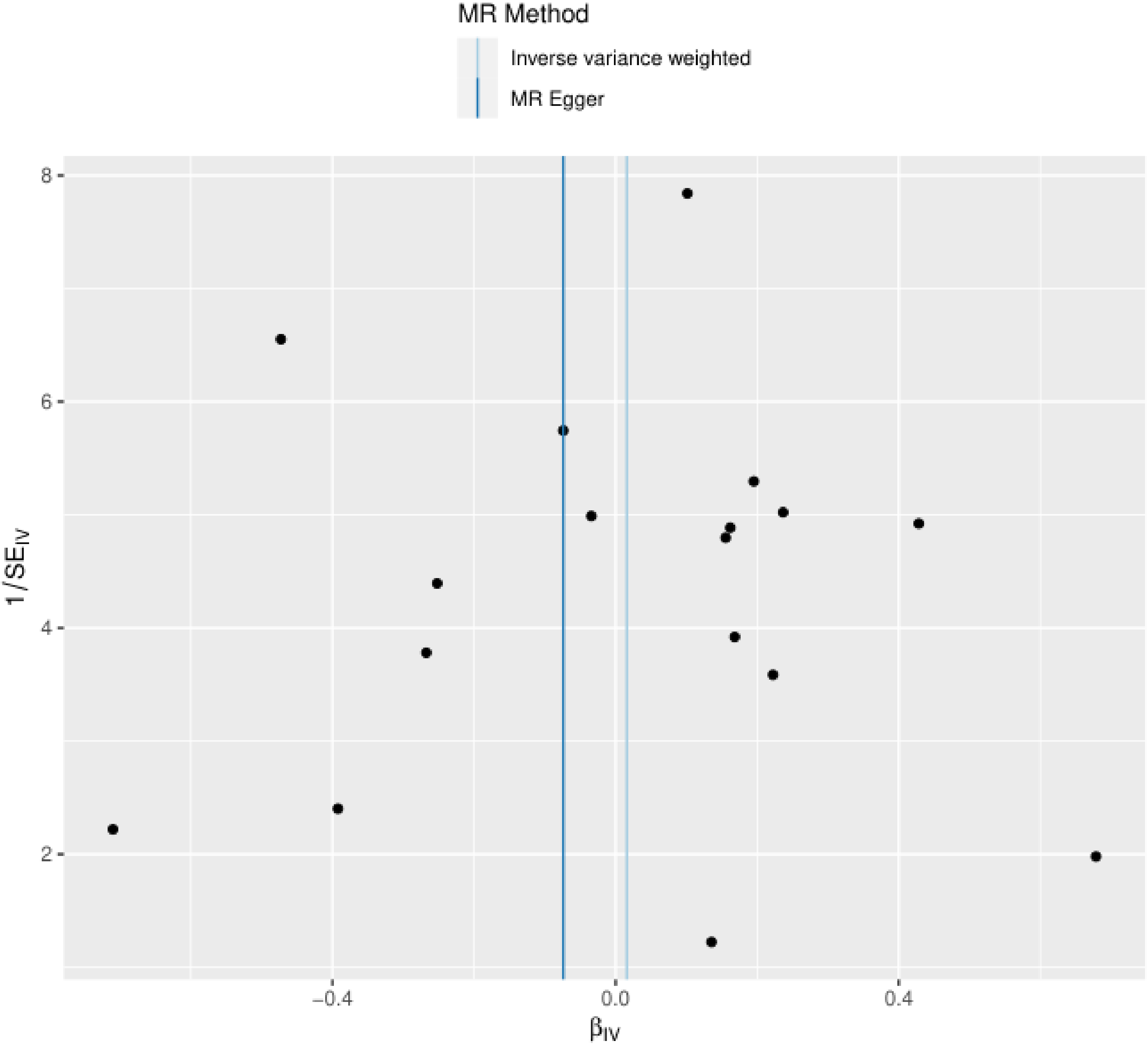

Funnel plot analyse of "CD25 on activated Treg " on 'Diabetic nephropathy'

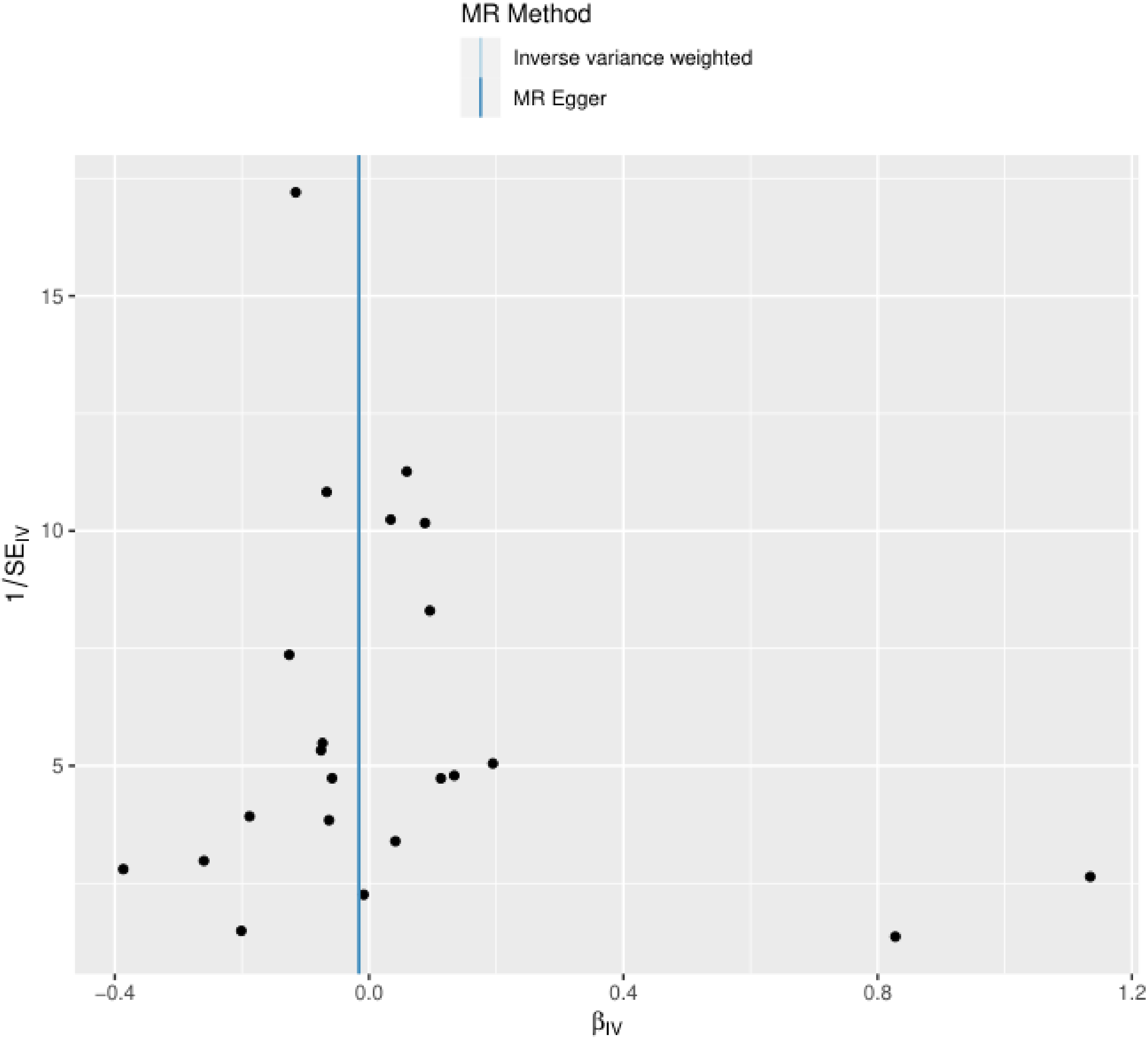

Funnel plot analyse of "CD39 on CD39+ activated Treg " on 'Diabetic nephropathy'

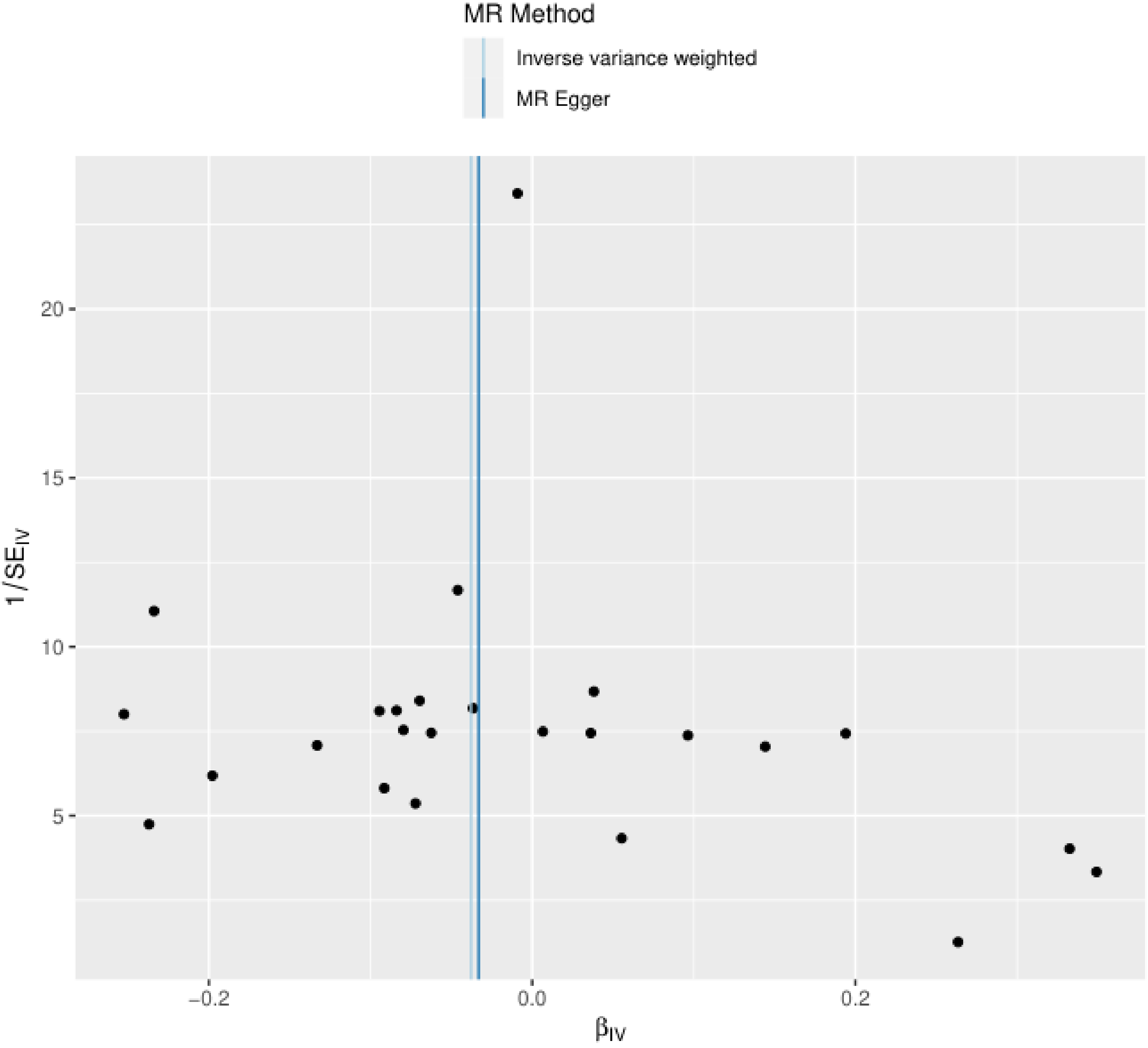

Funnel plot analyse of "HVEM on T cell" on 'Diabetic nephropathy'

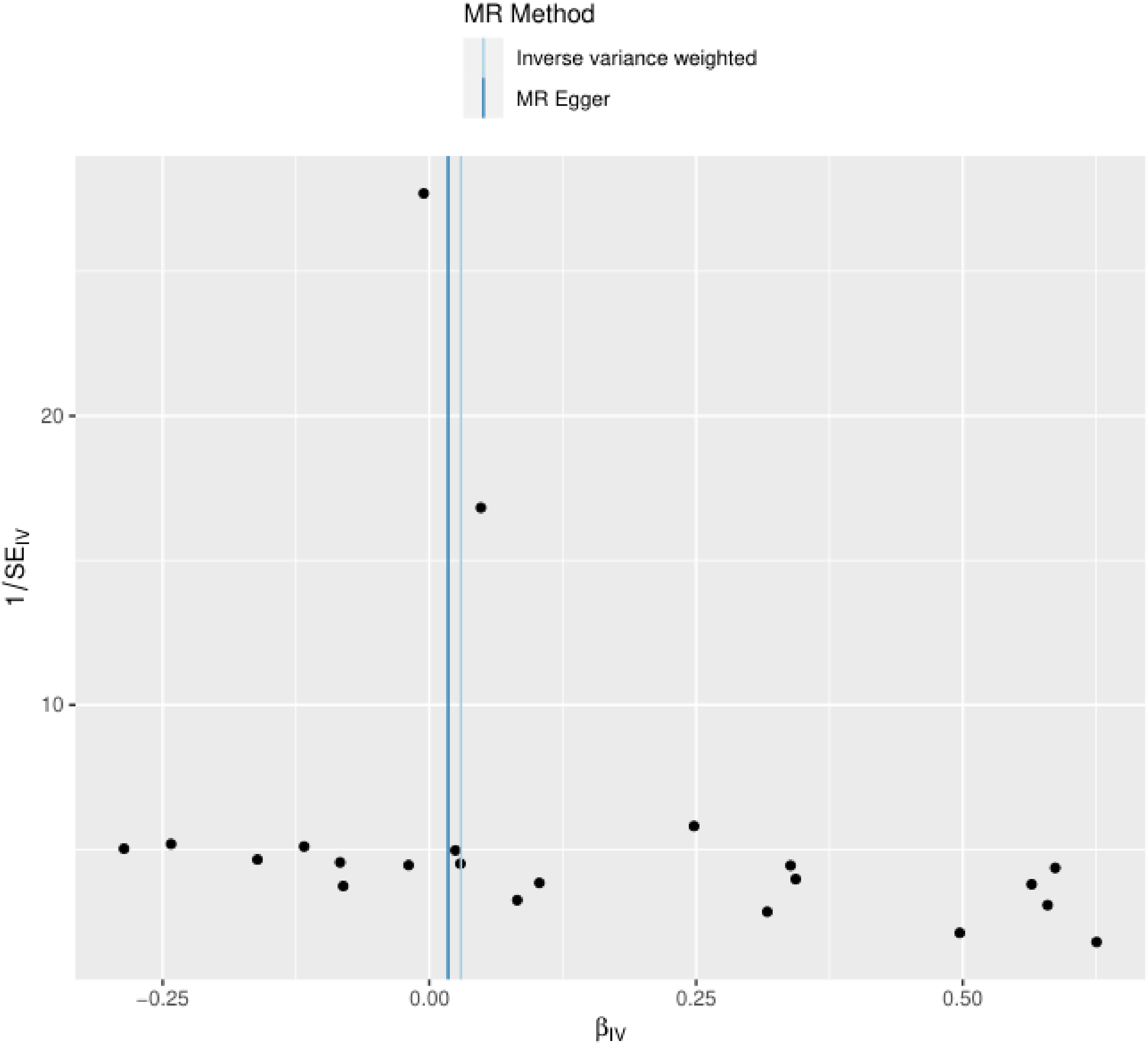

Funnel plot analyse of "Lymphocyte %leukocyte" on 'Diabetic nephropathy'

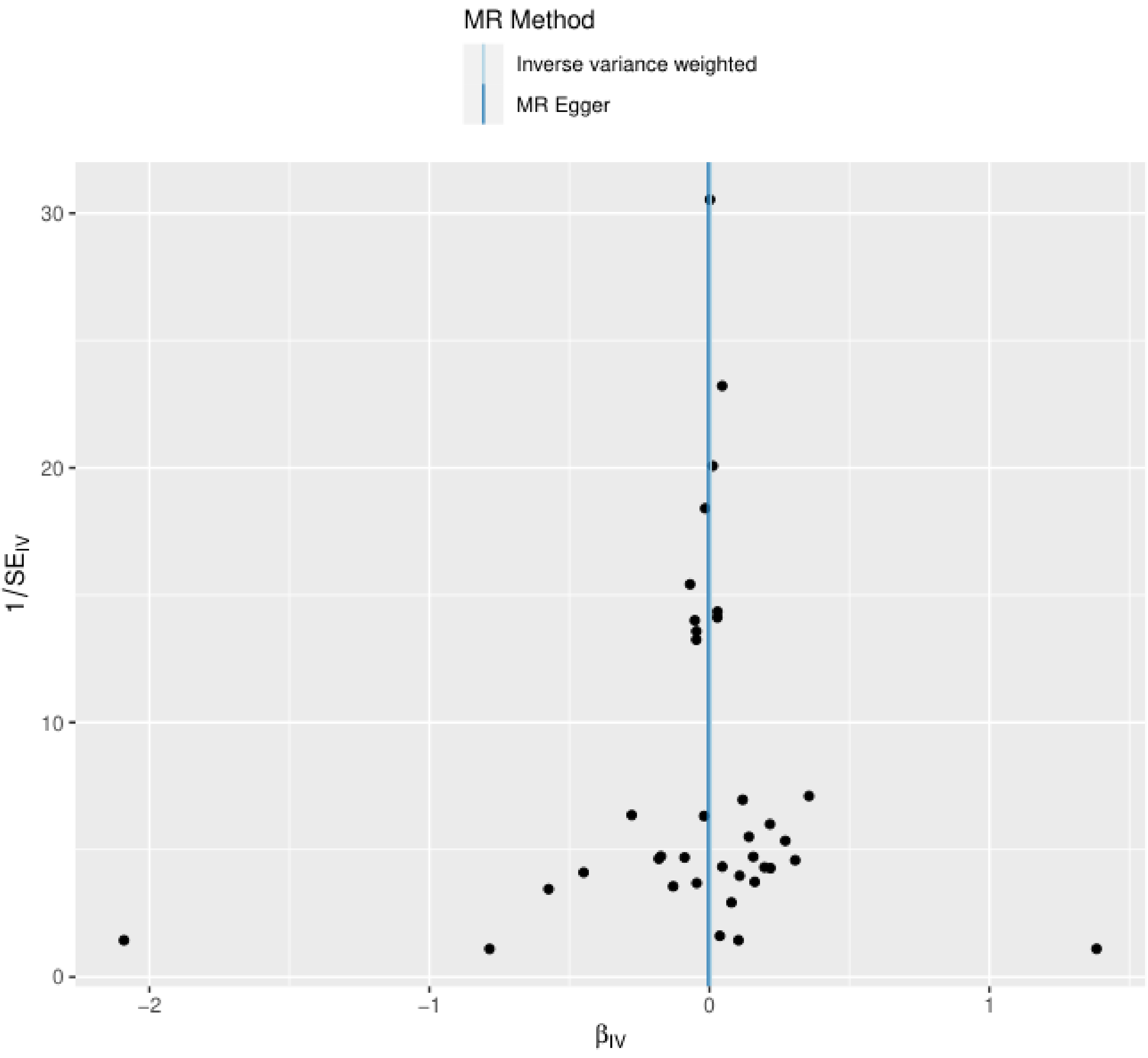

Funnel plot analyse of " CCR2 on CD14+ CD16+ monocyte" on 'Diabetic nephropathy'

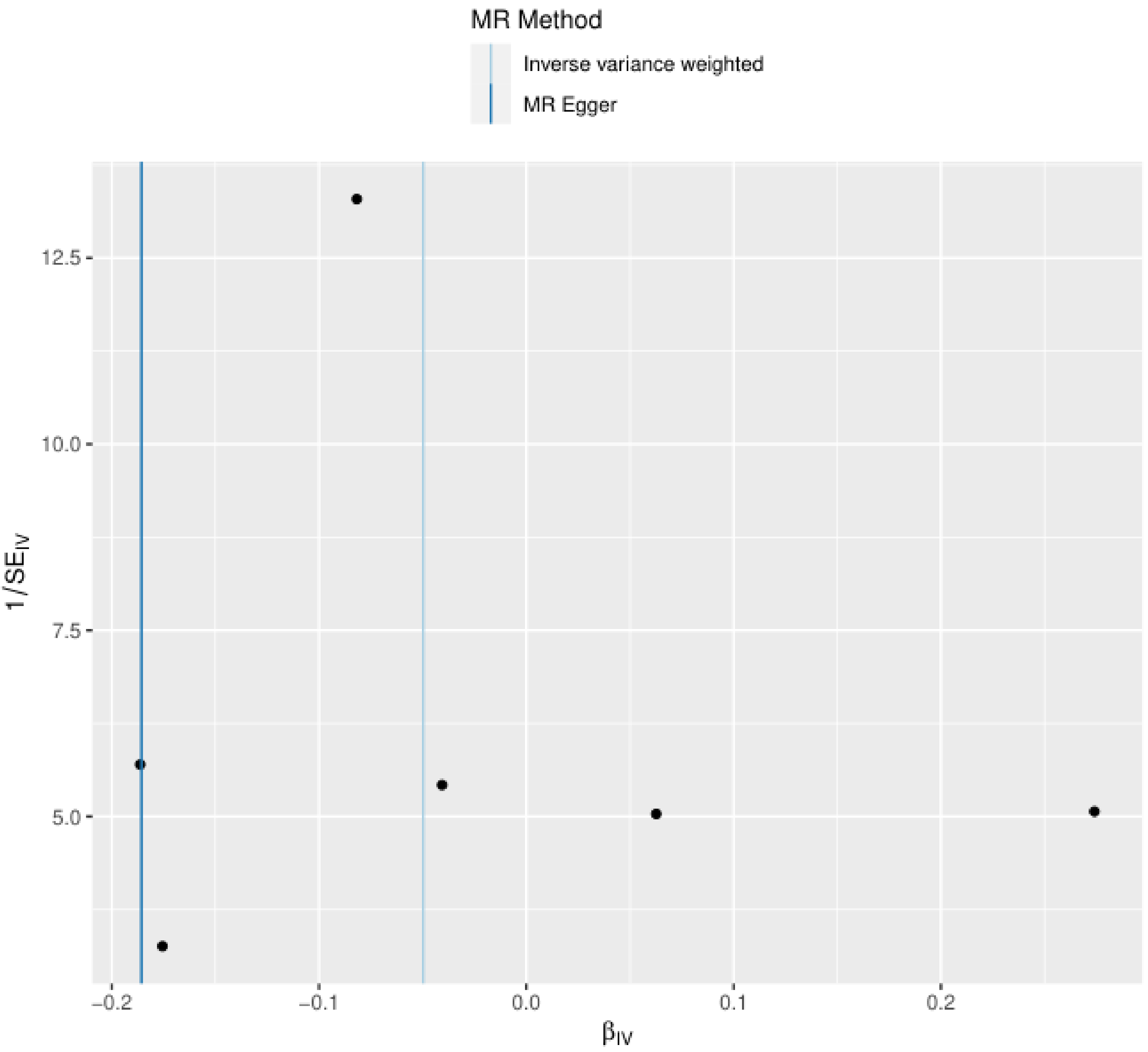

Funnel plot analyse of "CD28 on resting Treg " on 'Diabetic nephropathy'

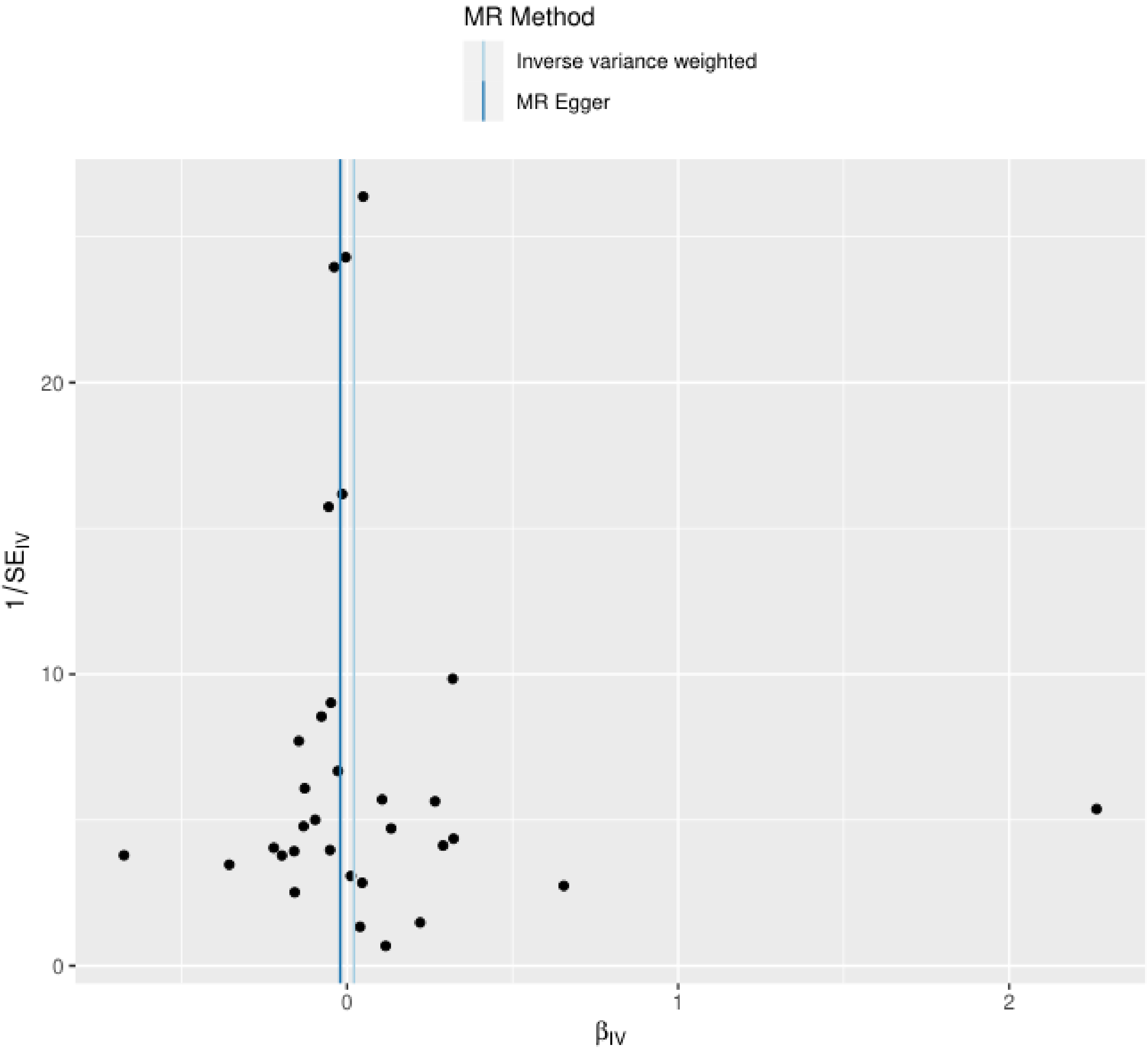

Funnel plot analyse of "HLA DR+ T cell%T cell" on 'Diabetic nephropathy'

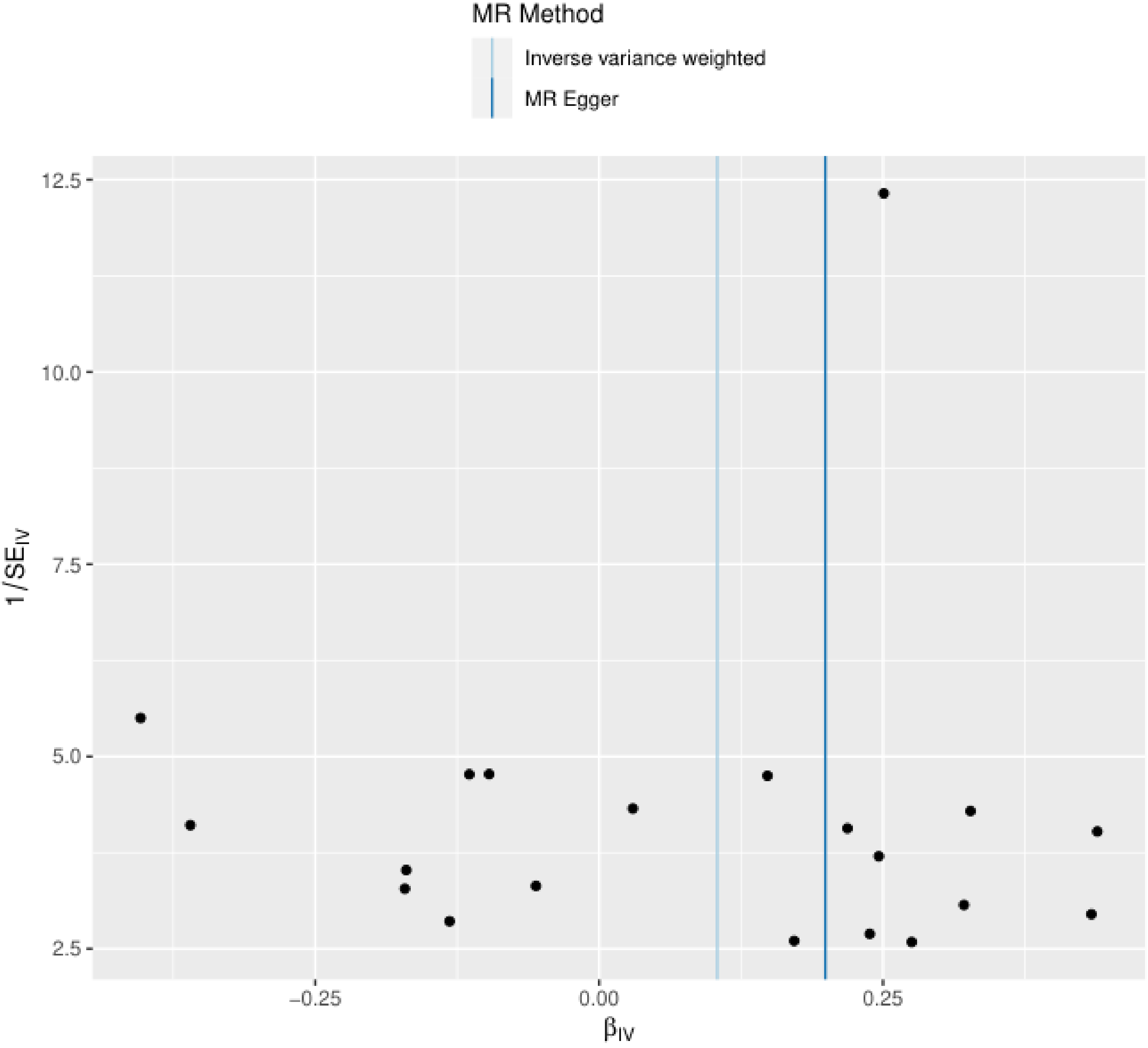

Funnel plot analyse of "CD25 on IgD- CD38br" on 'Diabetic nephropathy'

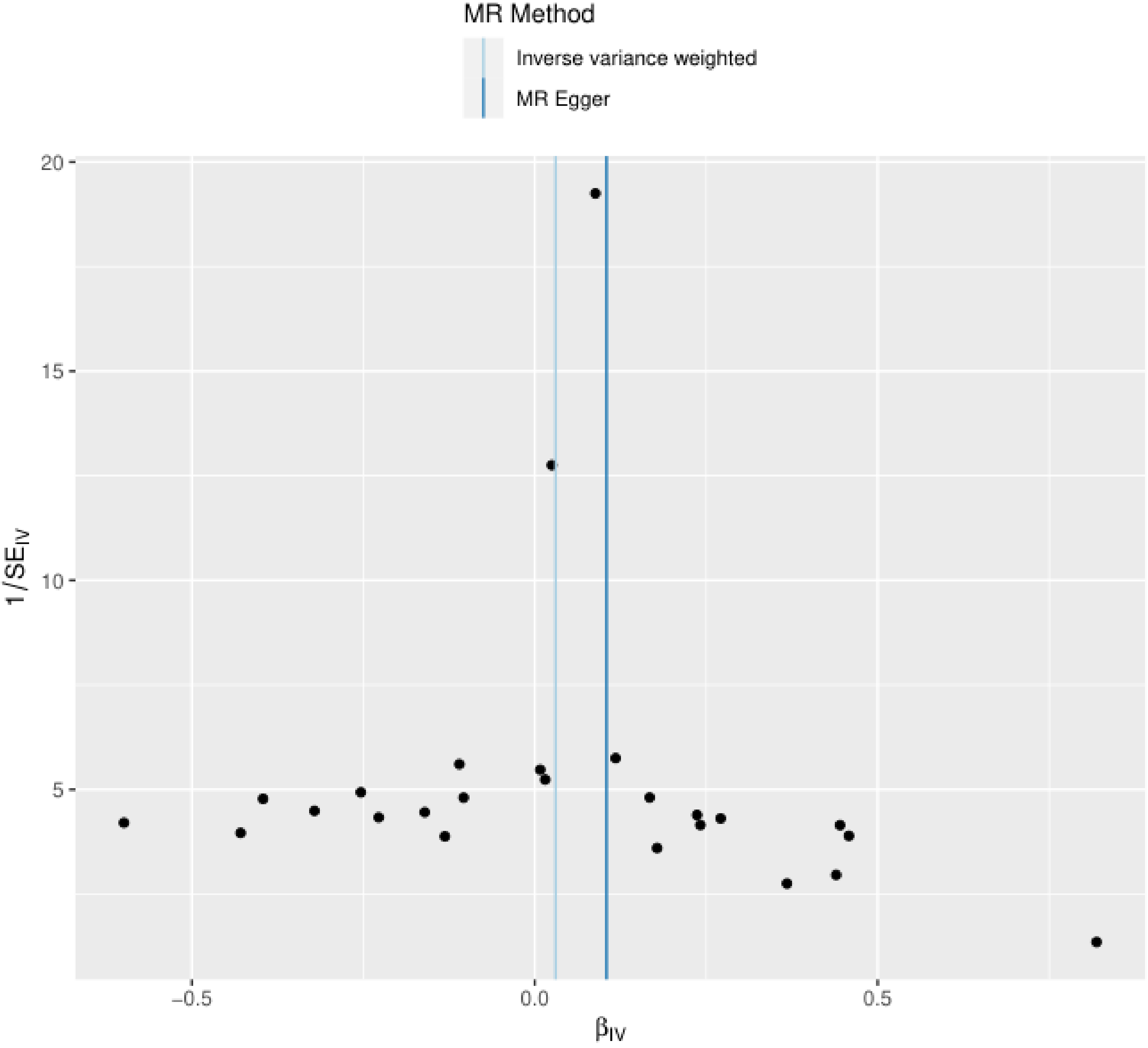

Funnel plot analyse of "IgD+ CD38br %lymphocyte" on 'Diabetic nephropathy'

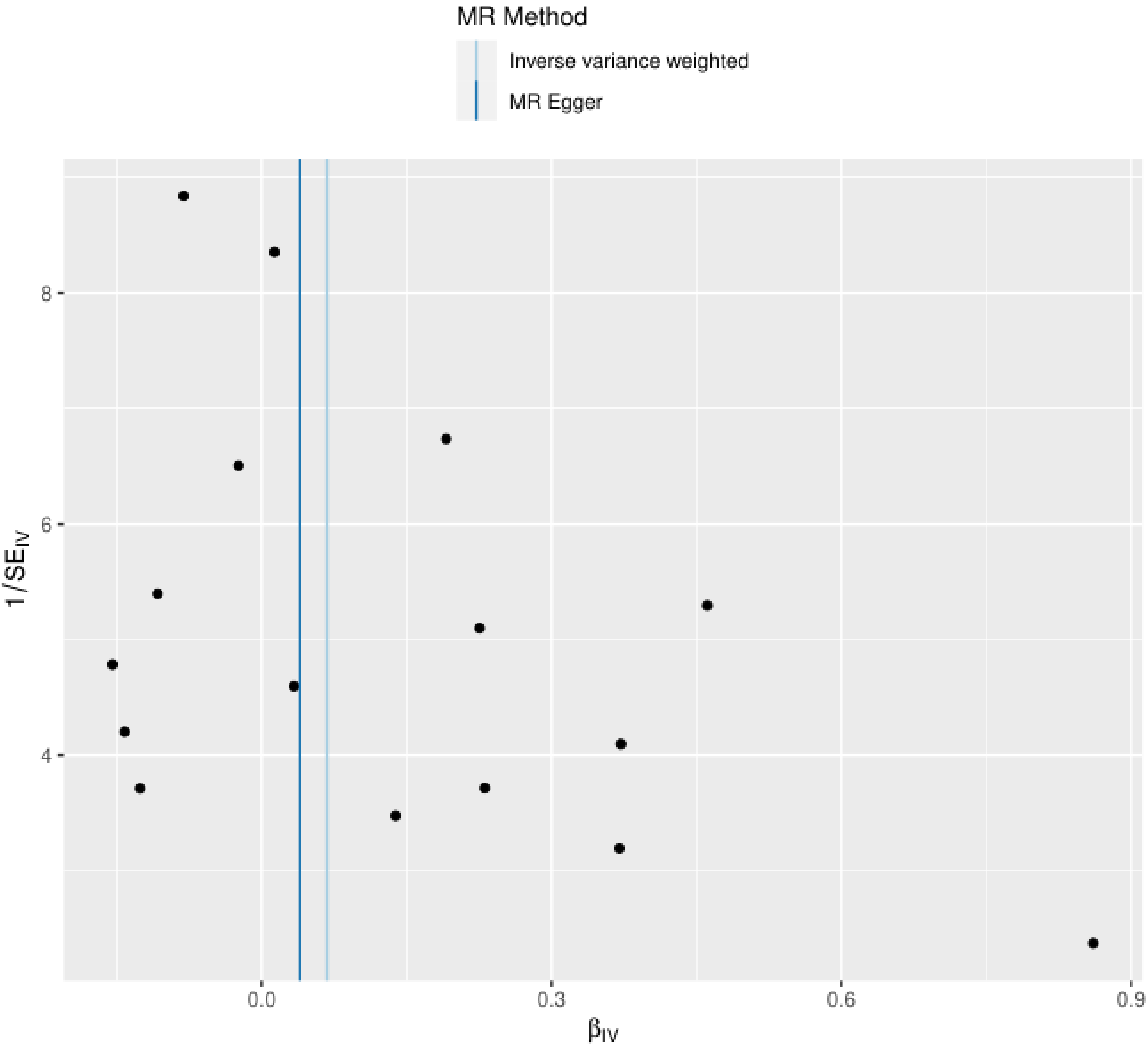

Funnel plot analyse of "CD11c+ CD62L- monocyte %monocyte" on 'Diabetic nephropathy'

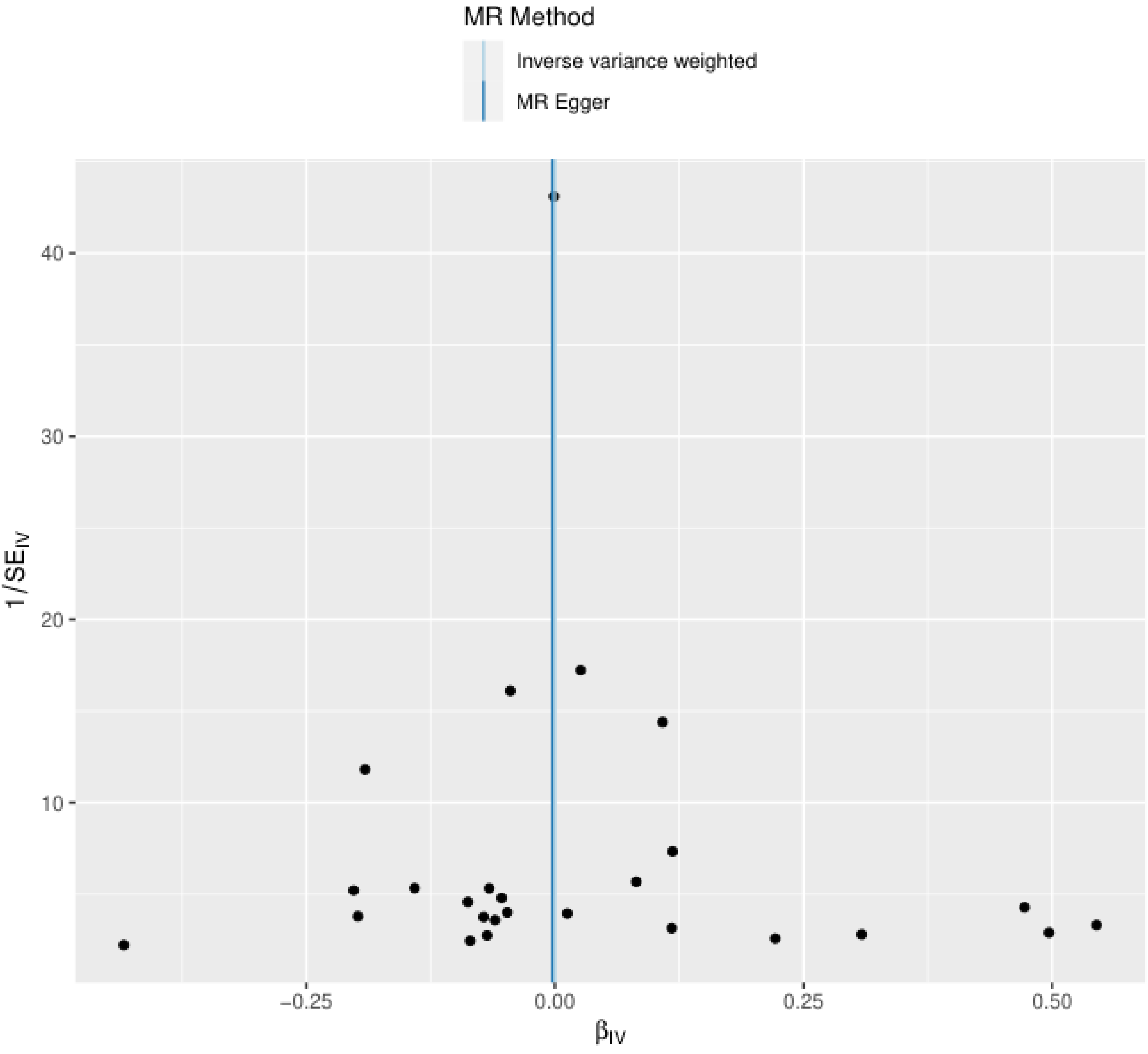

Funnel plot analyse of "Monocyte AC" on 'Diabetic nephropathy'

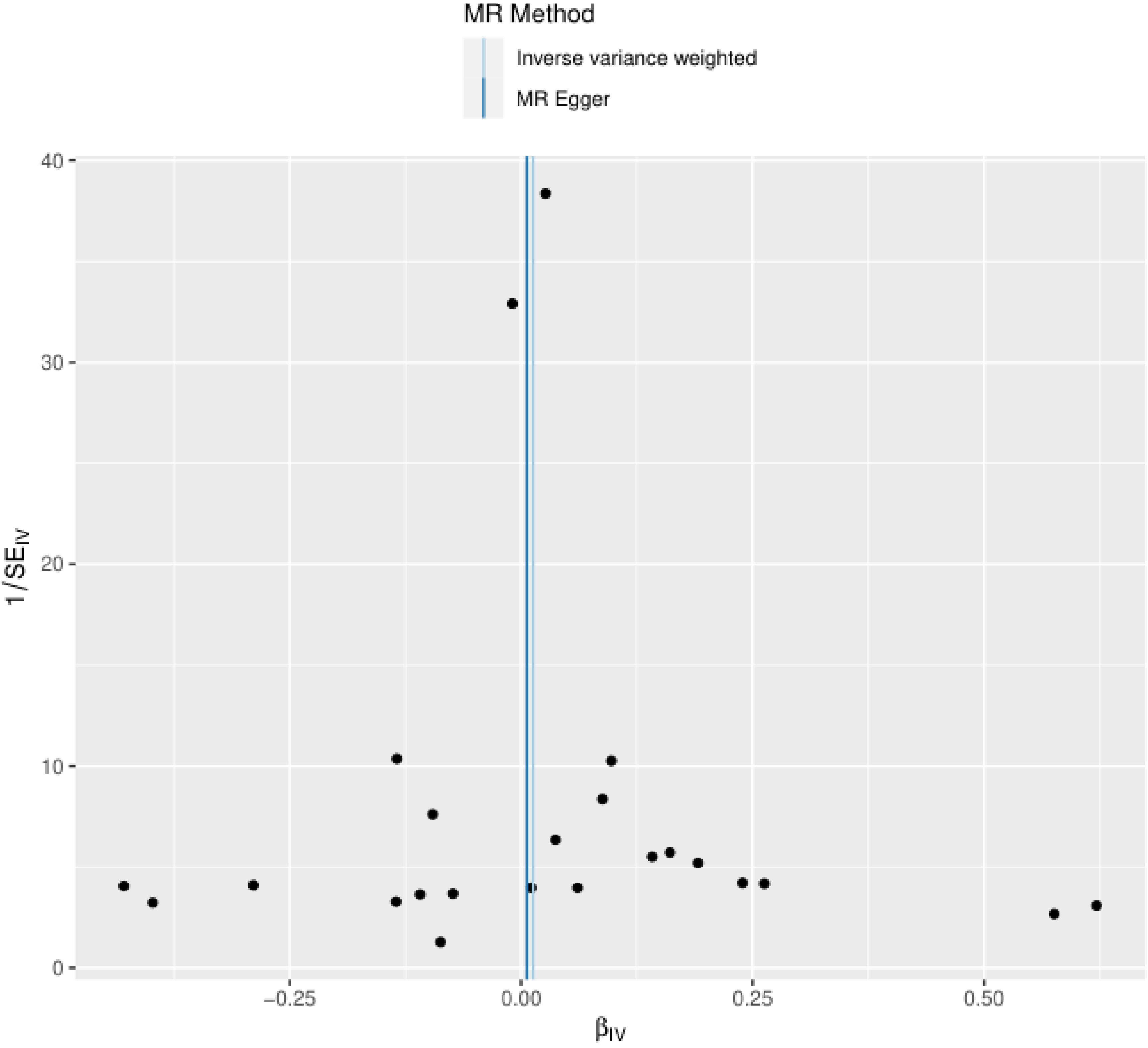

Funnel plot analyse of "CCR2 on monocyte" on 'Diabetic nephropathy'

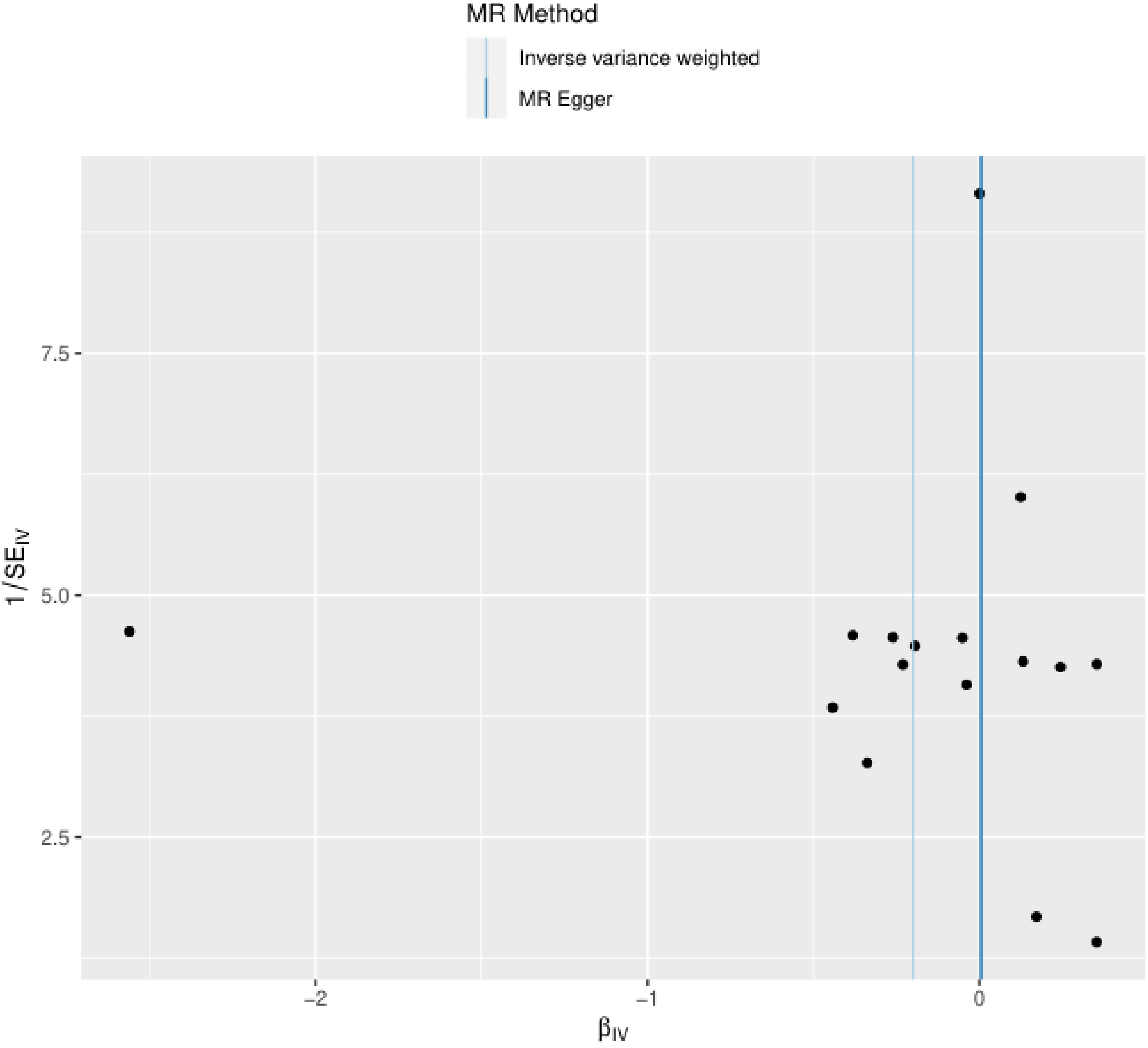

Funnel plot analyse of "Transitional %B cell" on 'Diabetic nephropathy'

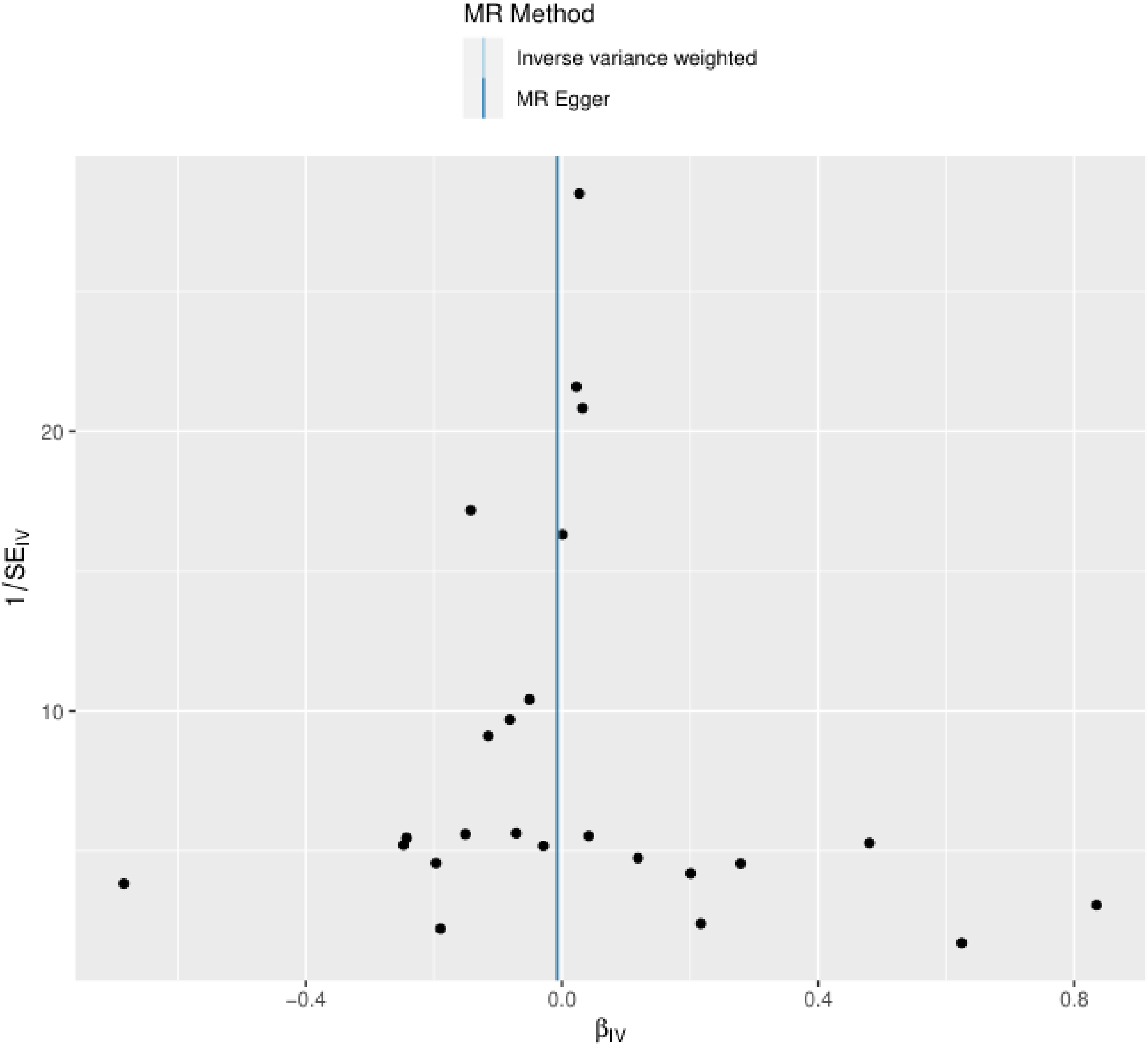

Funnel plot analyse of "CD28 on CD4 Treg " on 'Diabetic nephropathy'

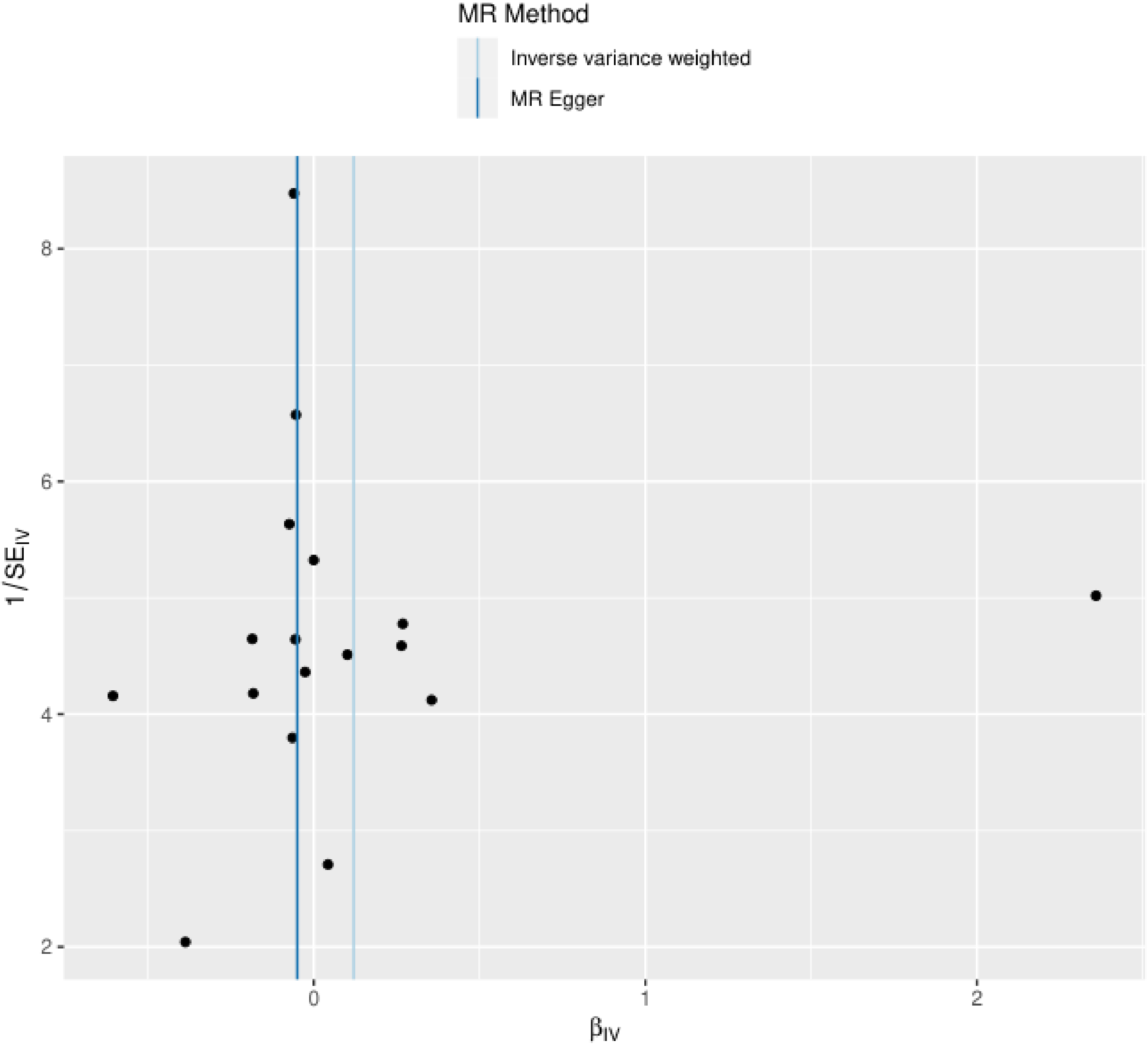

Funnel plot analyse of "CD8br %leukocyte" on 'Diabetic nephropathy'

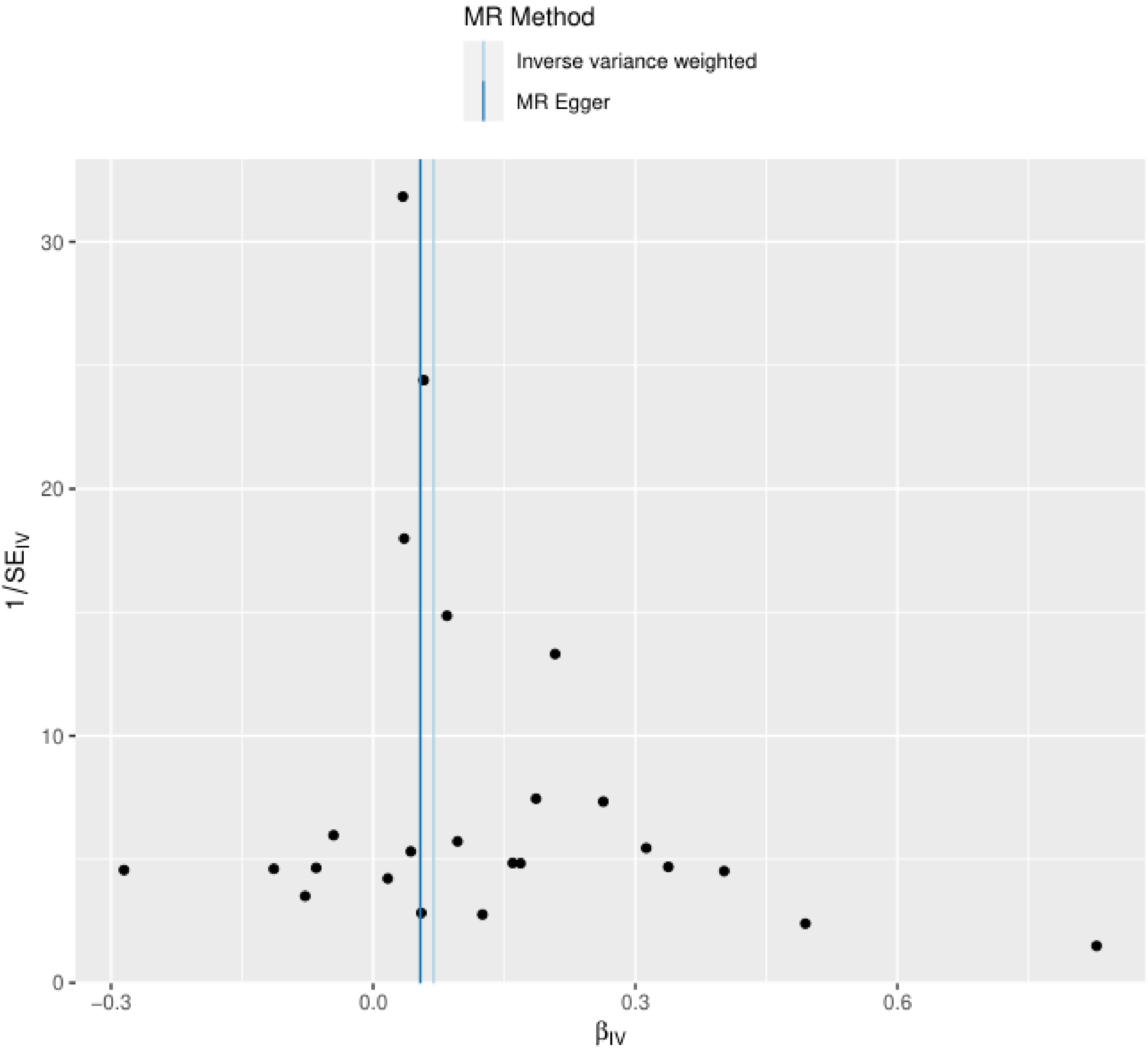

Funnel plot analyse of "CD16-CD56 on HLA DR+ NK" on 'Diabetic nephropathy'

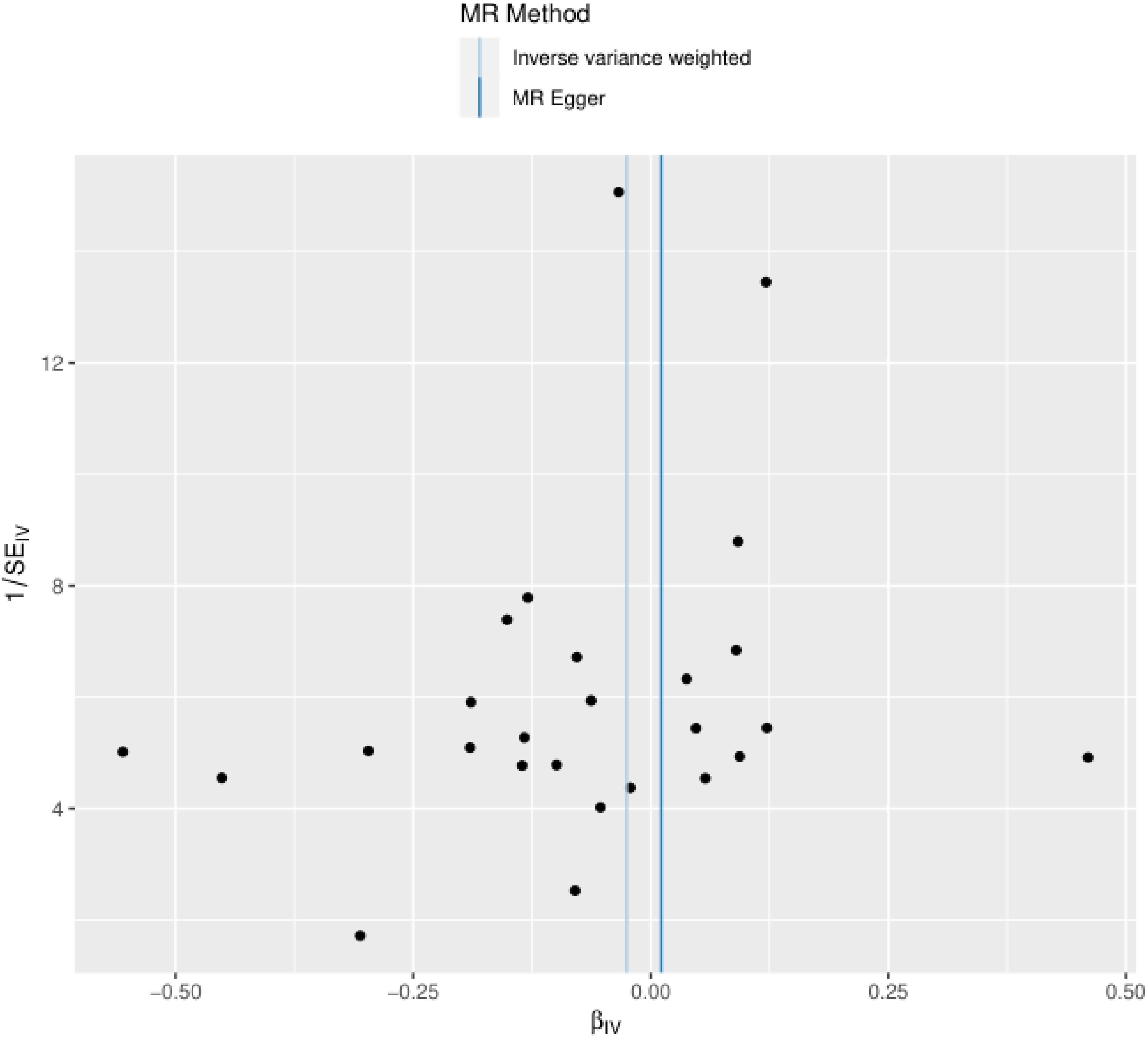

Funnel plot analyse of "CD3 on HLA DR+ CD4+" on 'Diabetic nephropathy'

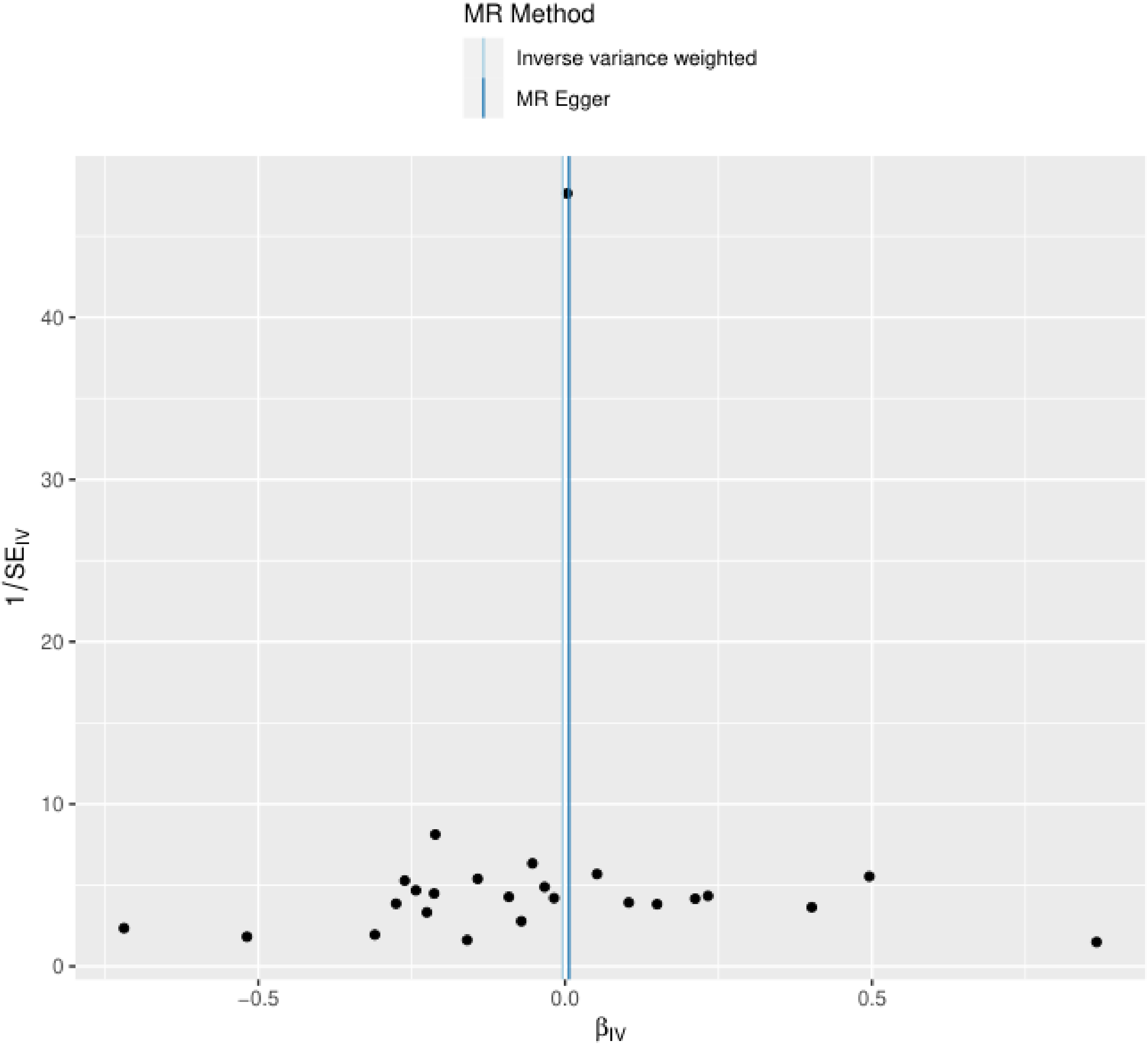

Funnel plot analyse of "CD24+ CD27+ AC" on 'Diabetic nephropathy'

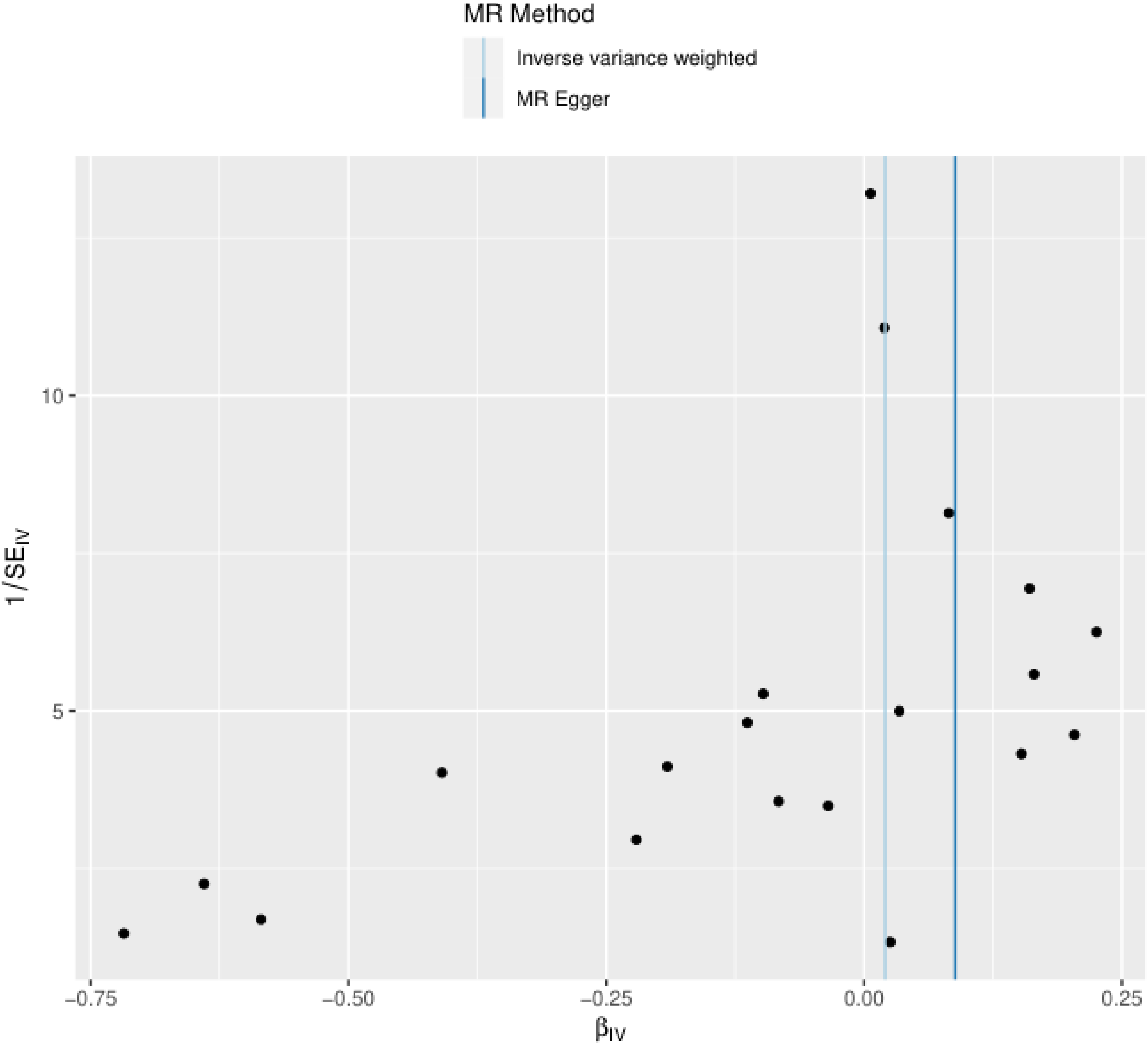

Funnel plot analyse of "CD39+ CD8br %T cell" on 'Diabetic nephropathy'

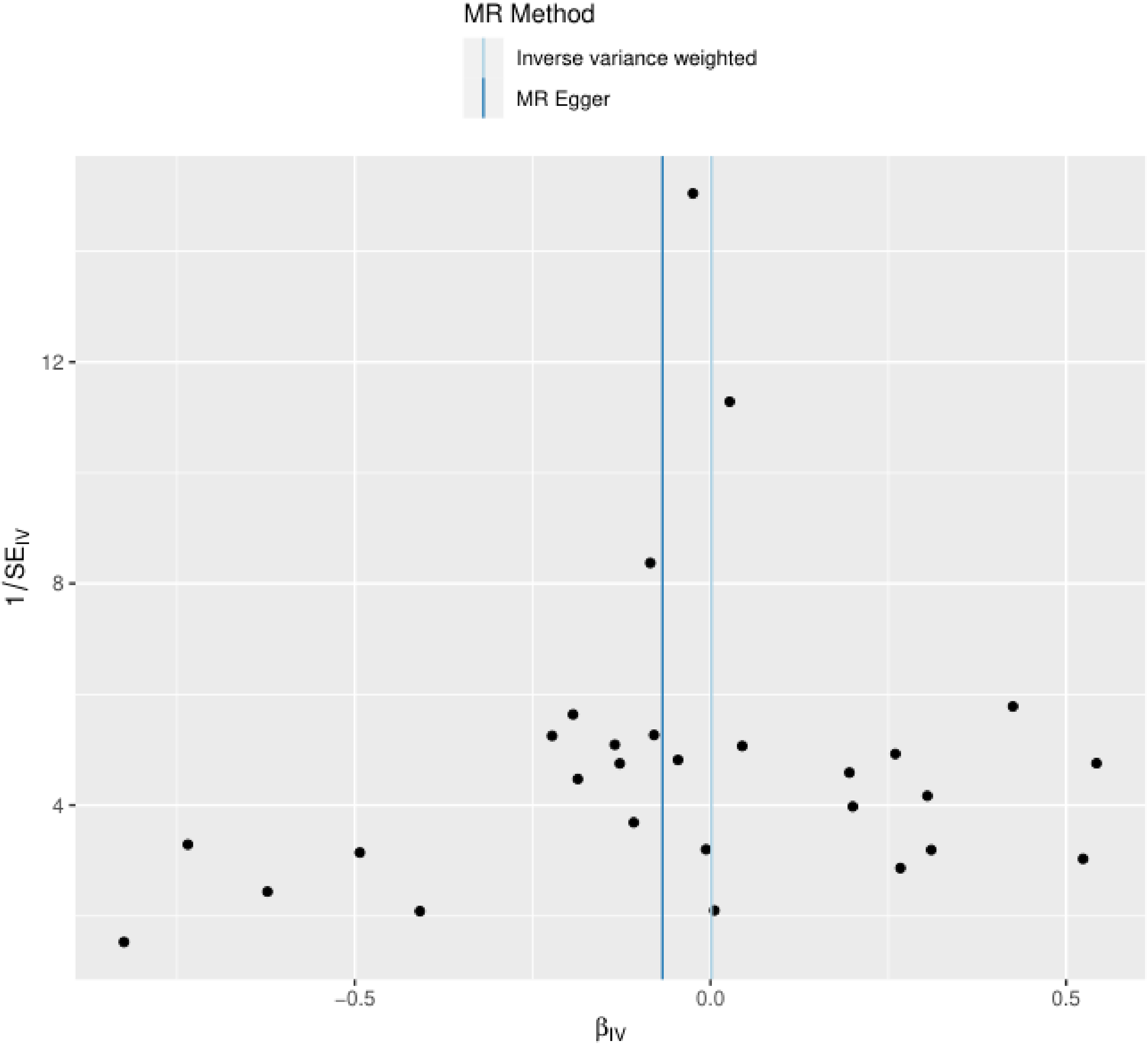

Funnel plot analyse of "Myeloid DC %DC" on 'Diabetic nephropathy'

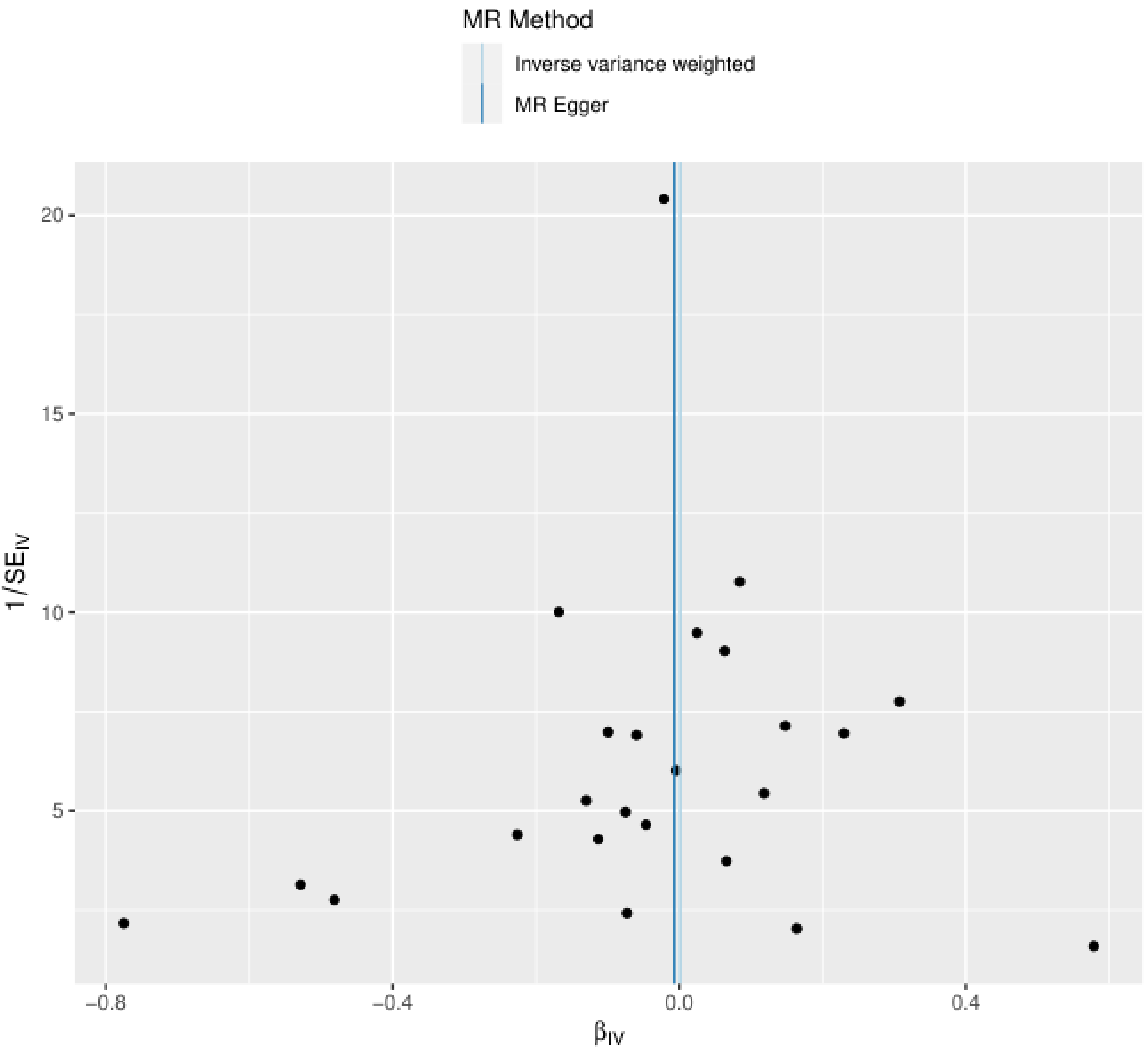

Funnel plot analyse of "CD3 on CD28+ CD45RA+ CD8br" on 'Diabetic nephropathy'

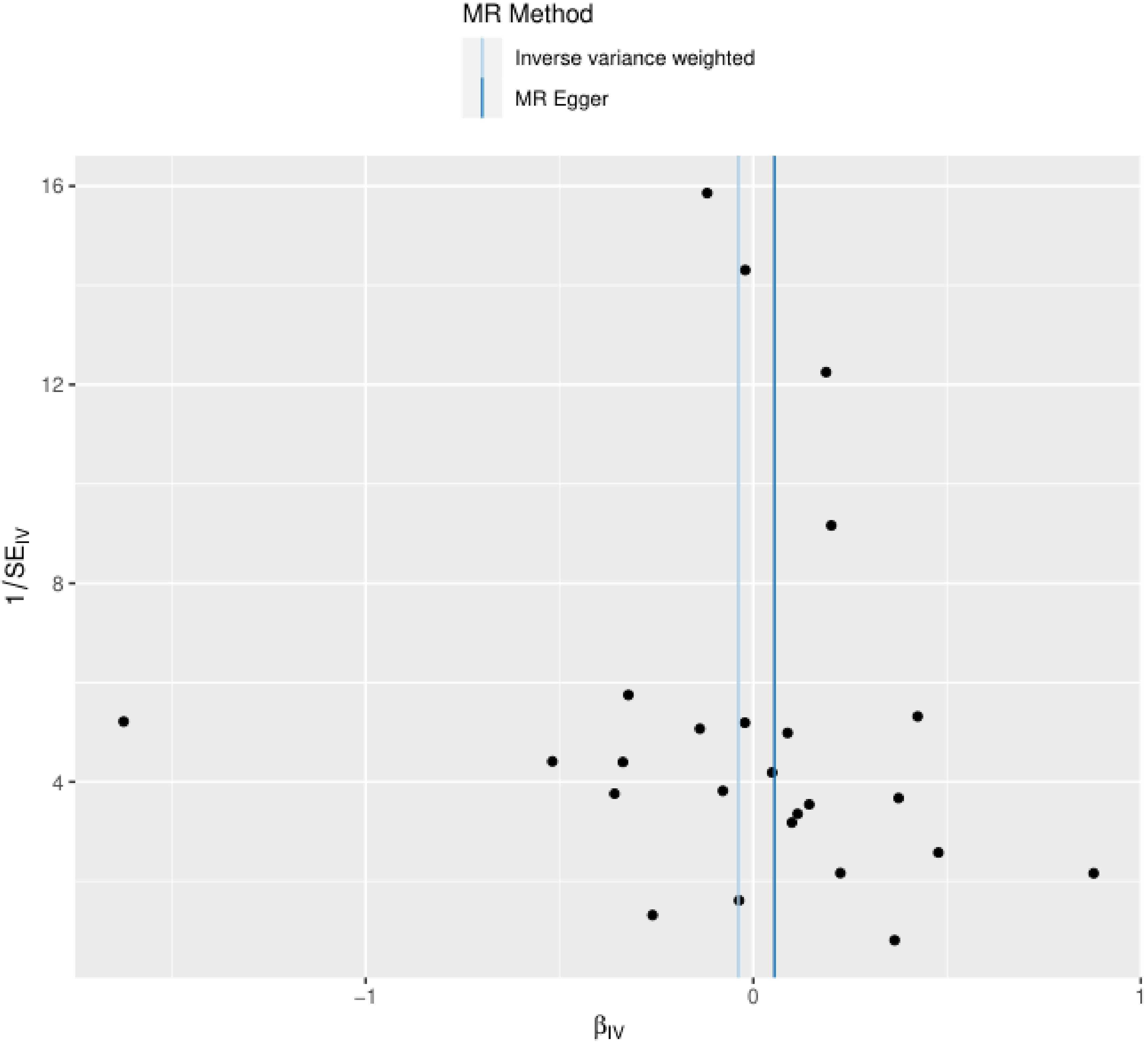

Funnel plot analyse of "CD25hi %CD4+" on 'Diabetic nephropathy'

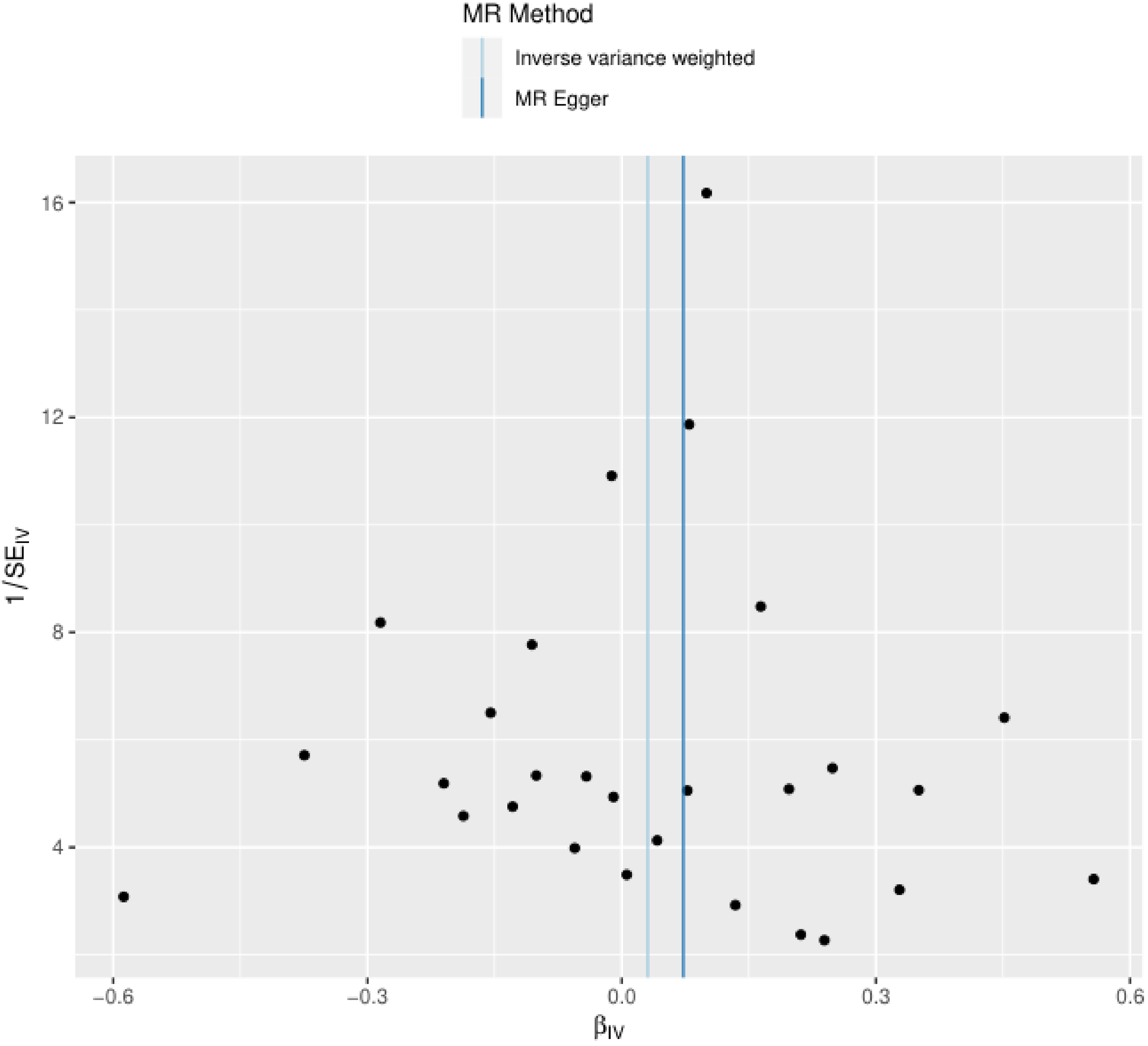

Funnel plot analyse of "CD27 on IgD- CD38-" on 'Diabetic nephropathy'

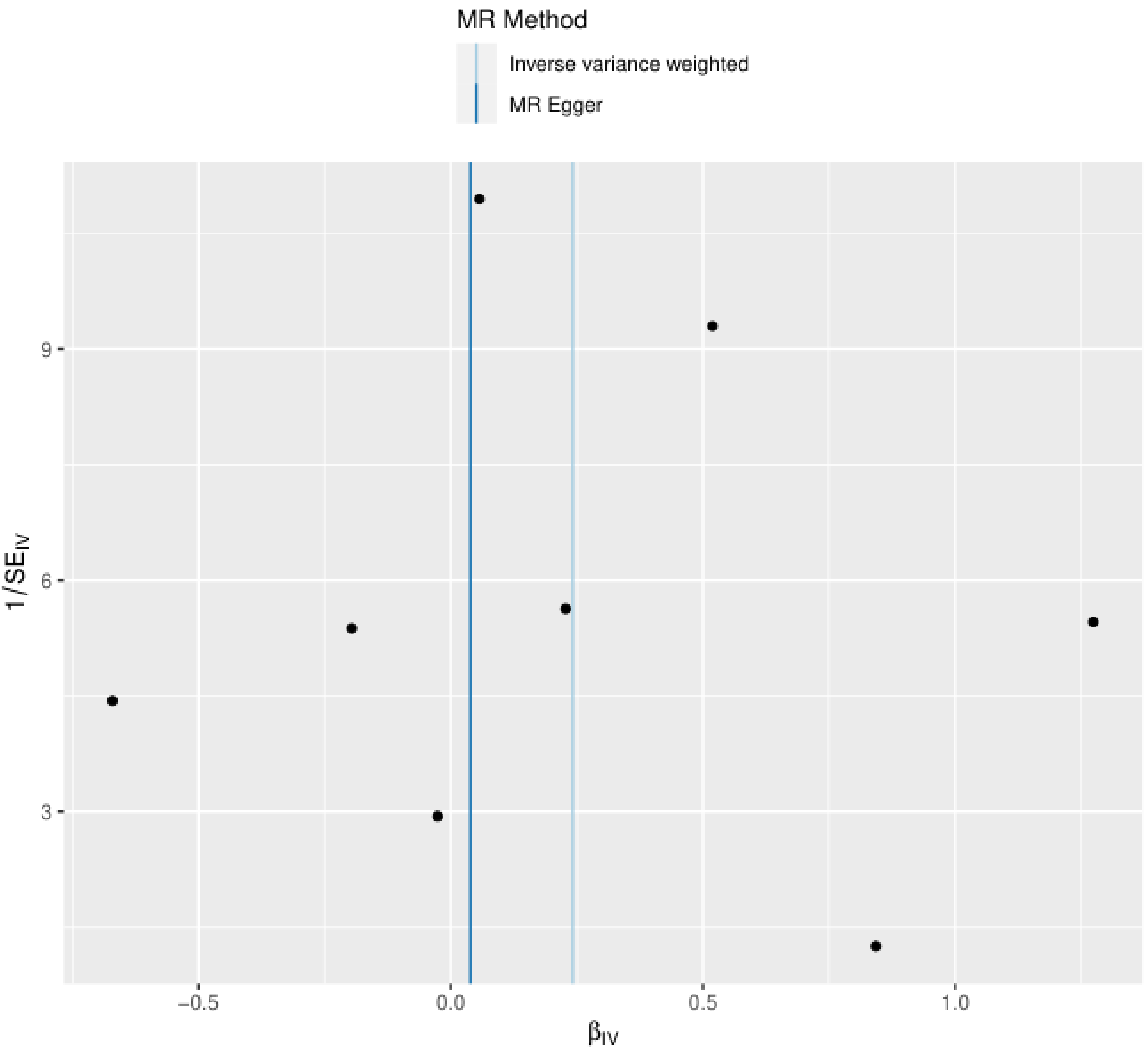

Funnel plot analyse of "HLA DR++ monocyte %leukocyte" on 'Diabetic nephropathy'

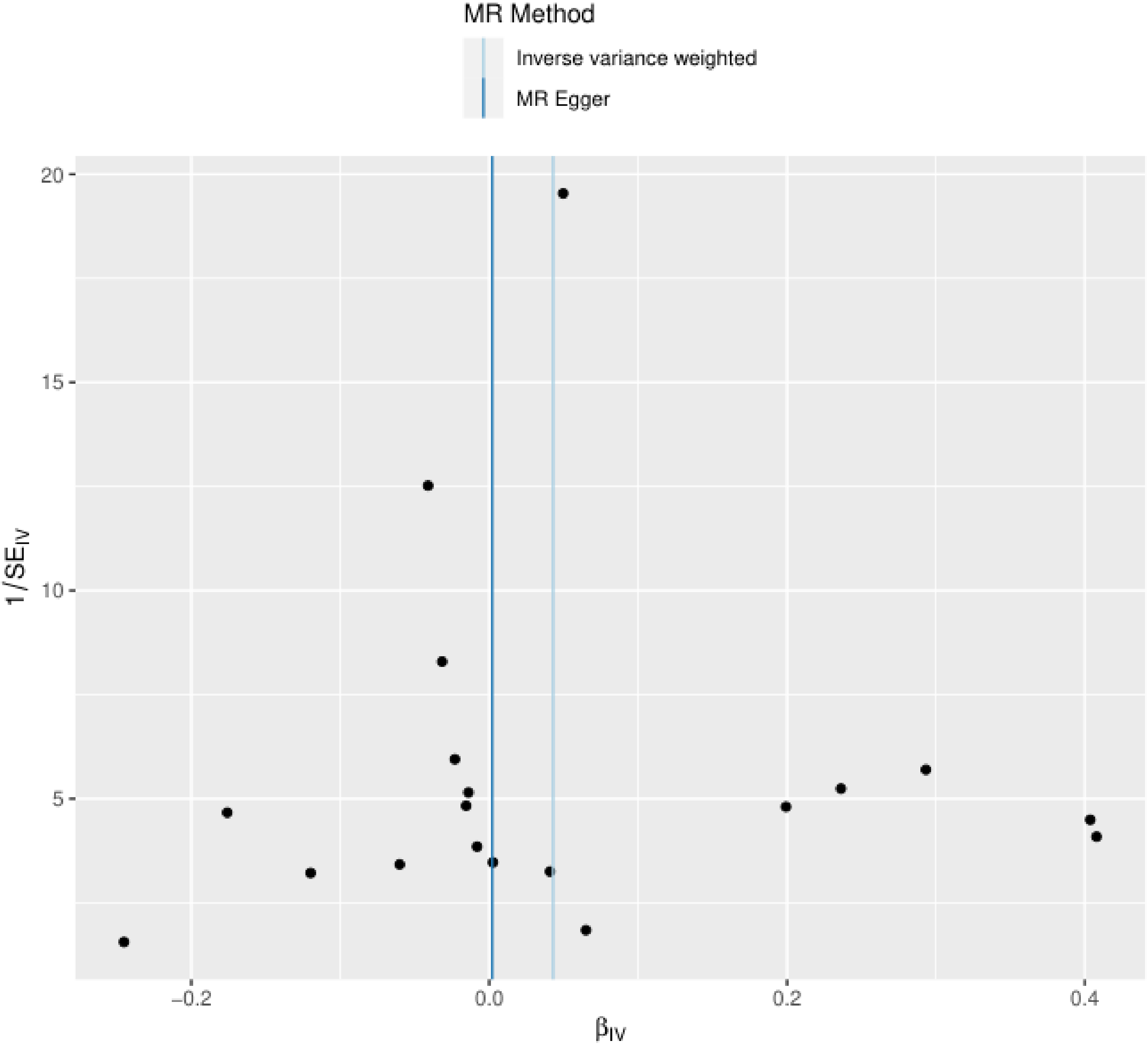

Funnel plot analyse of "FSC-A on NK" on 'Diabetic nephropathy'

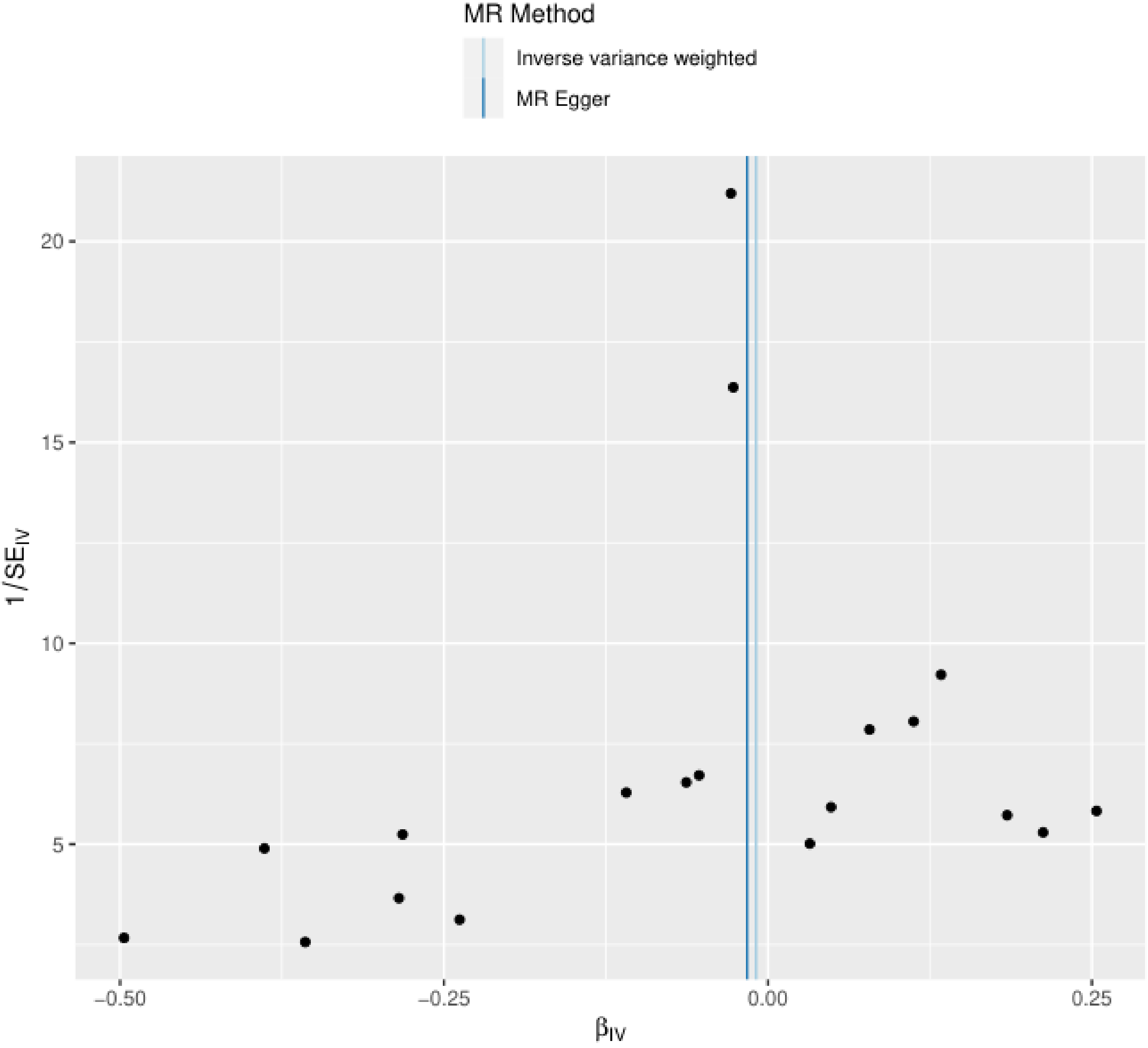

Funnel plot analyse of ''CD3 on EM CD4+ '' on 'Diabetic nephropathy'

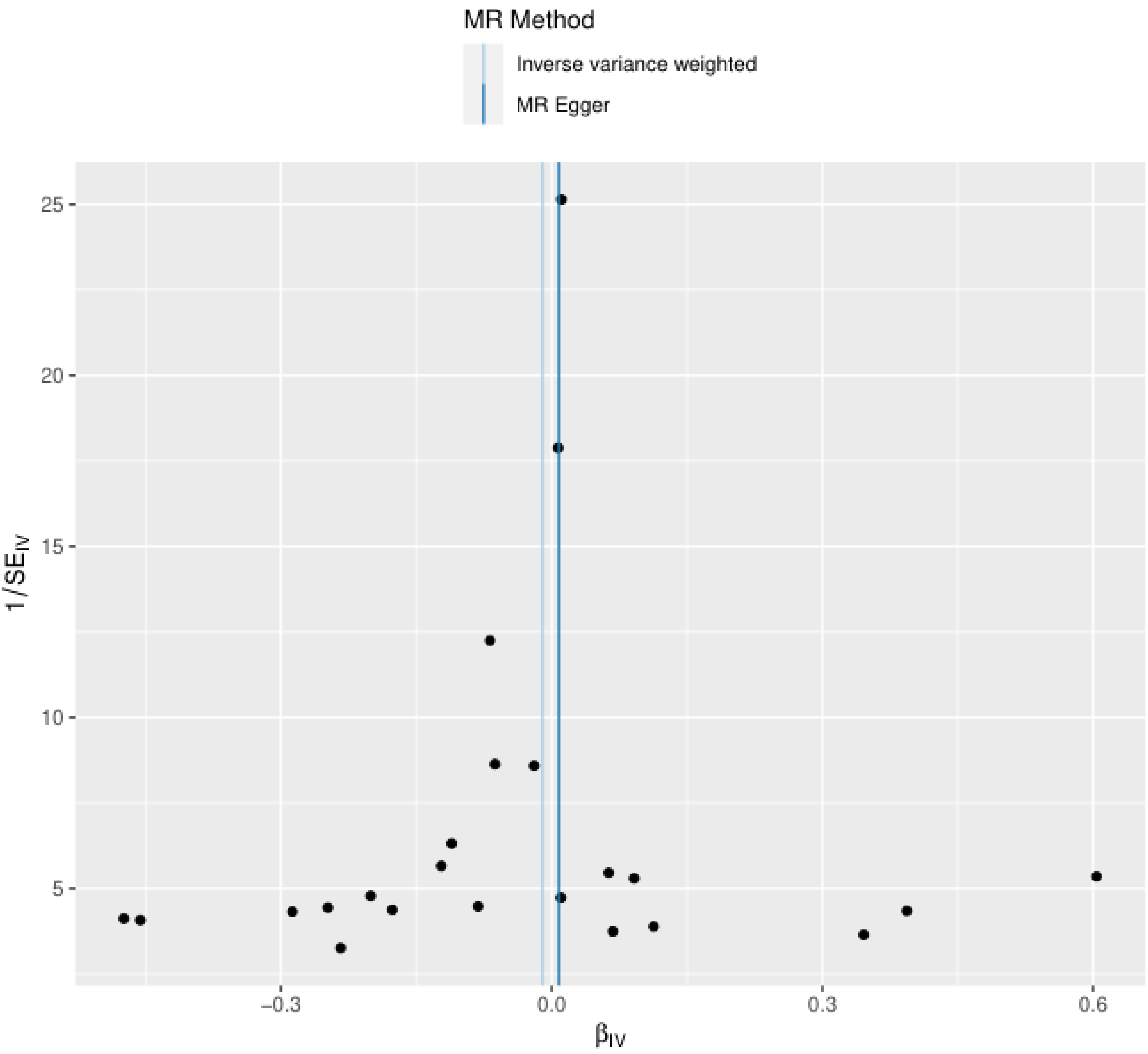

Funnel plot analyse of "IgD- CD38dim %lymphocyte" on 'Diabetic nephropathy'

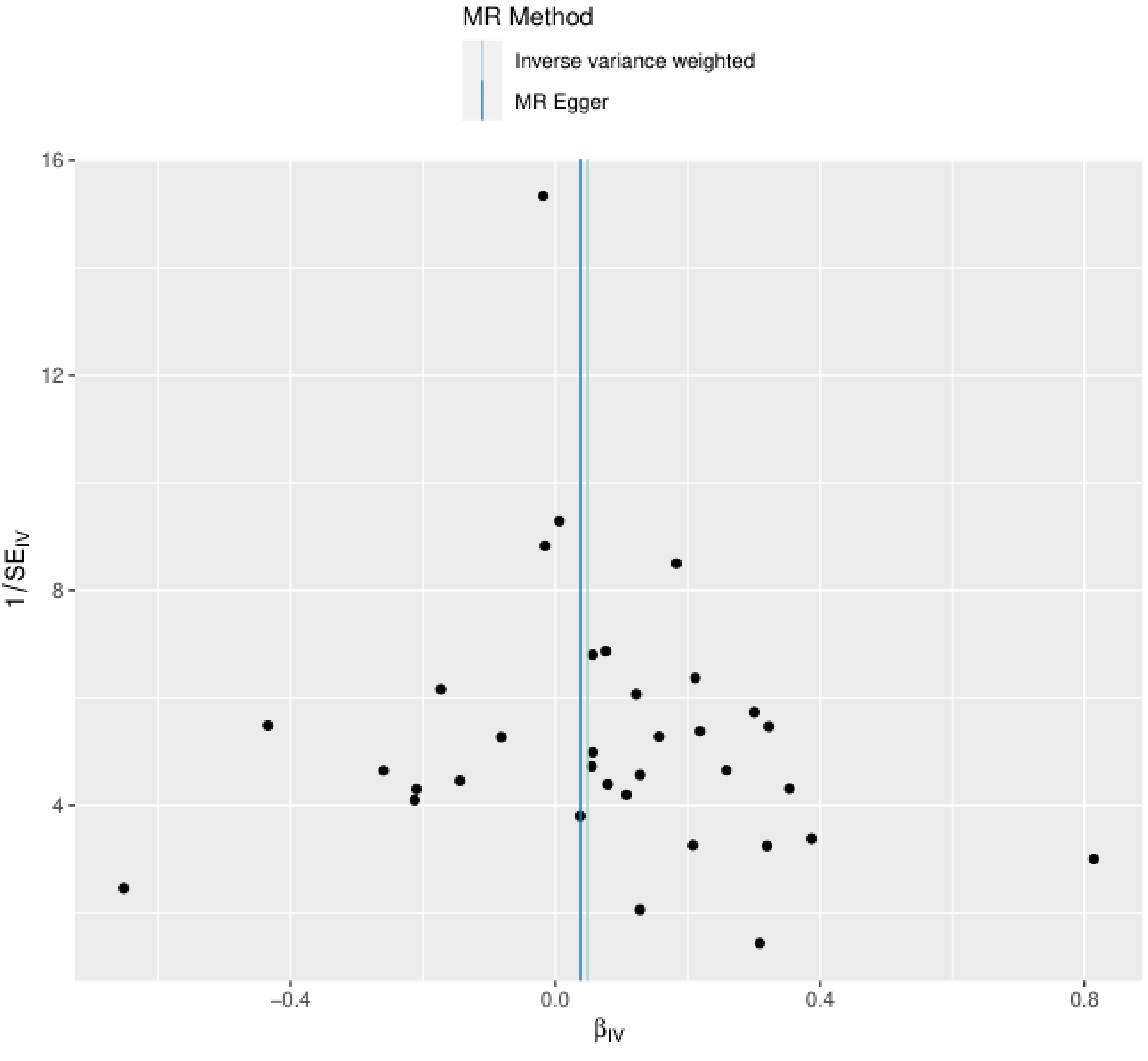

Funnel plot analyse of "NKT AC" on 'Diabetic nephropathy'

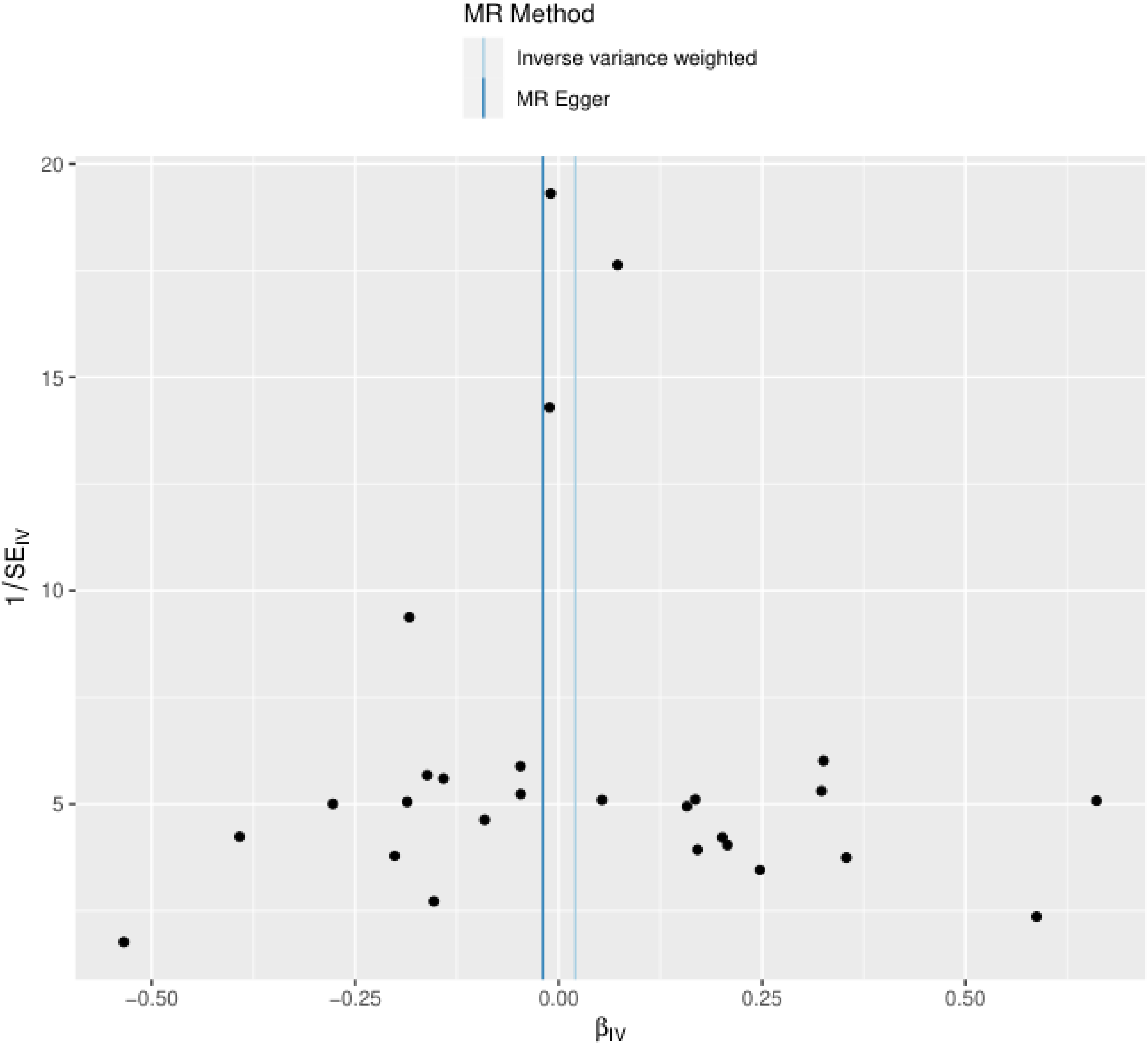

Funnel plot analyse of "Memory B cell %B cell" on 'Diabetic nephropathy'

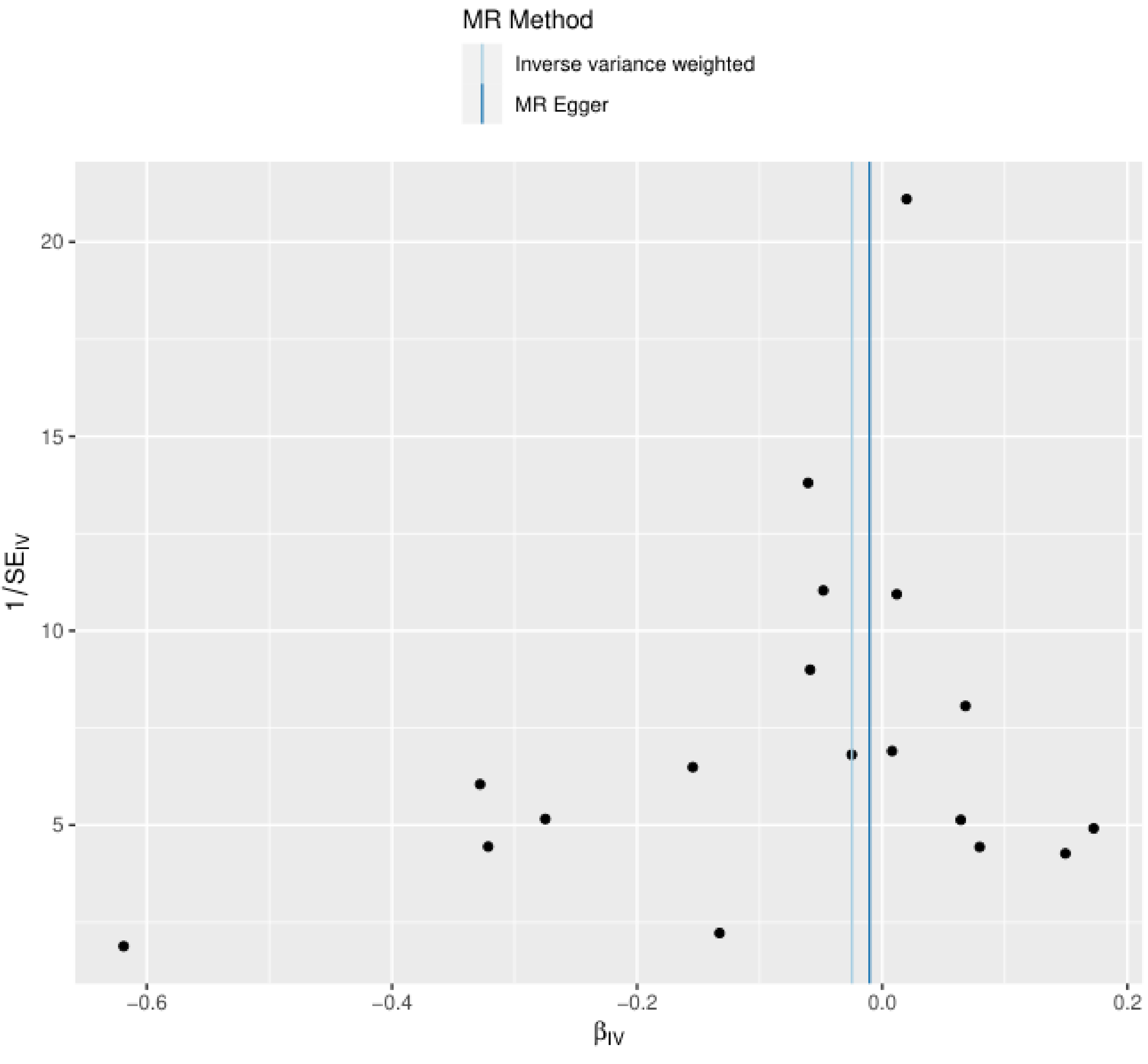

Funnel plot analyse of "CD28 on CD39+ CD4+ " on 'Diabetic nephropathy'

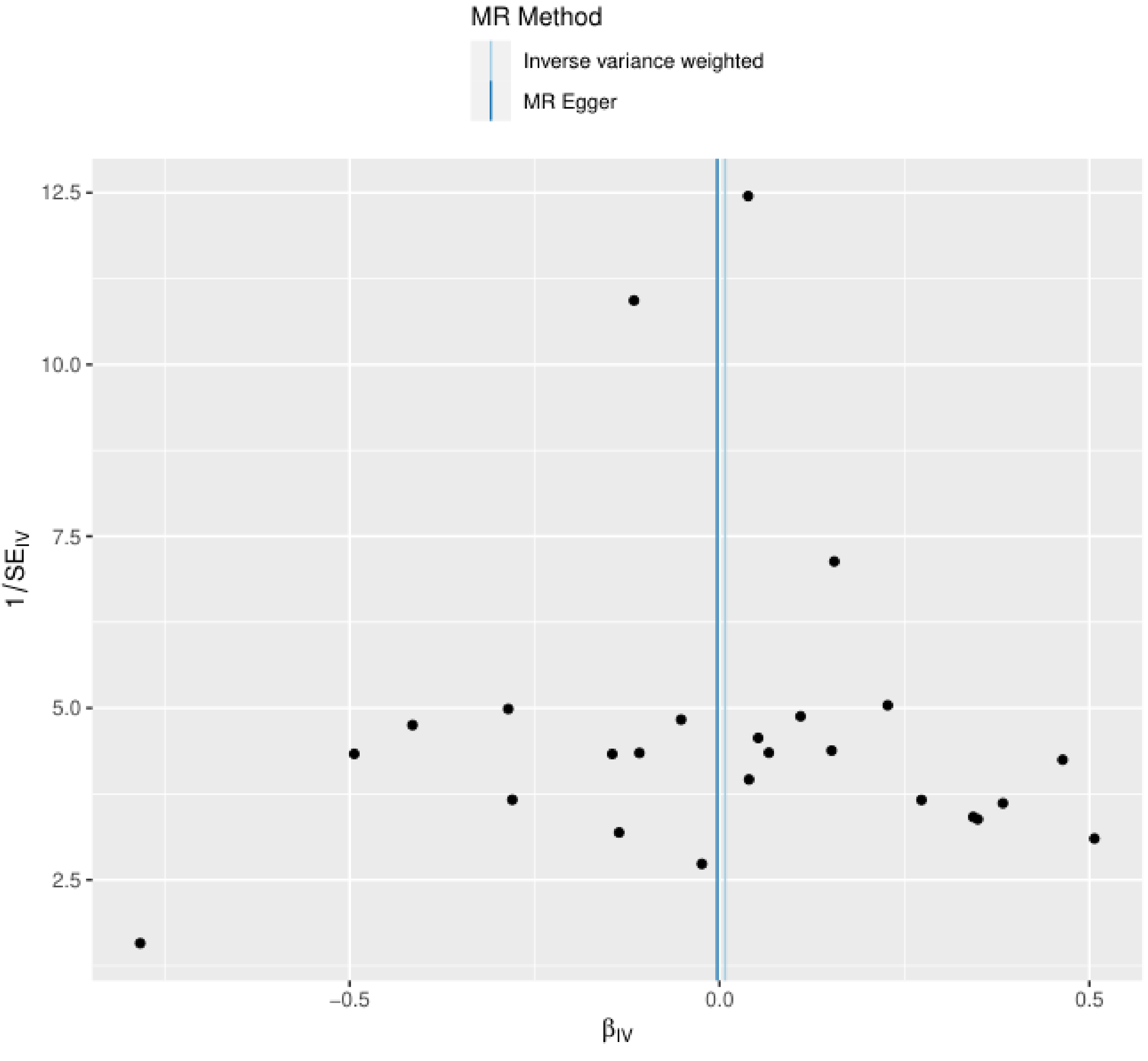

Funnel plot analyse of "CD28- CD25++ CD8br %CD8br" on 'Diabetic nephropathy'

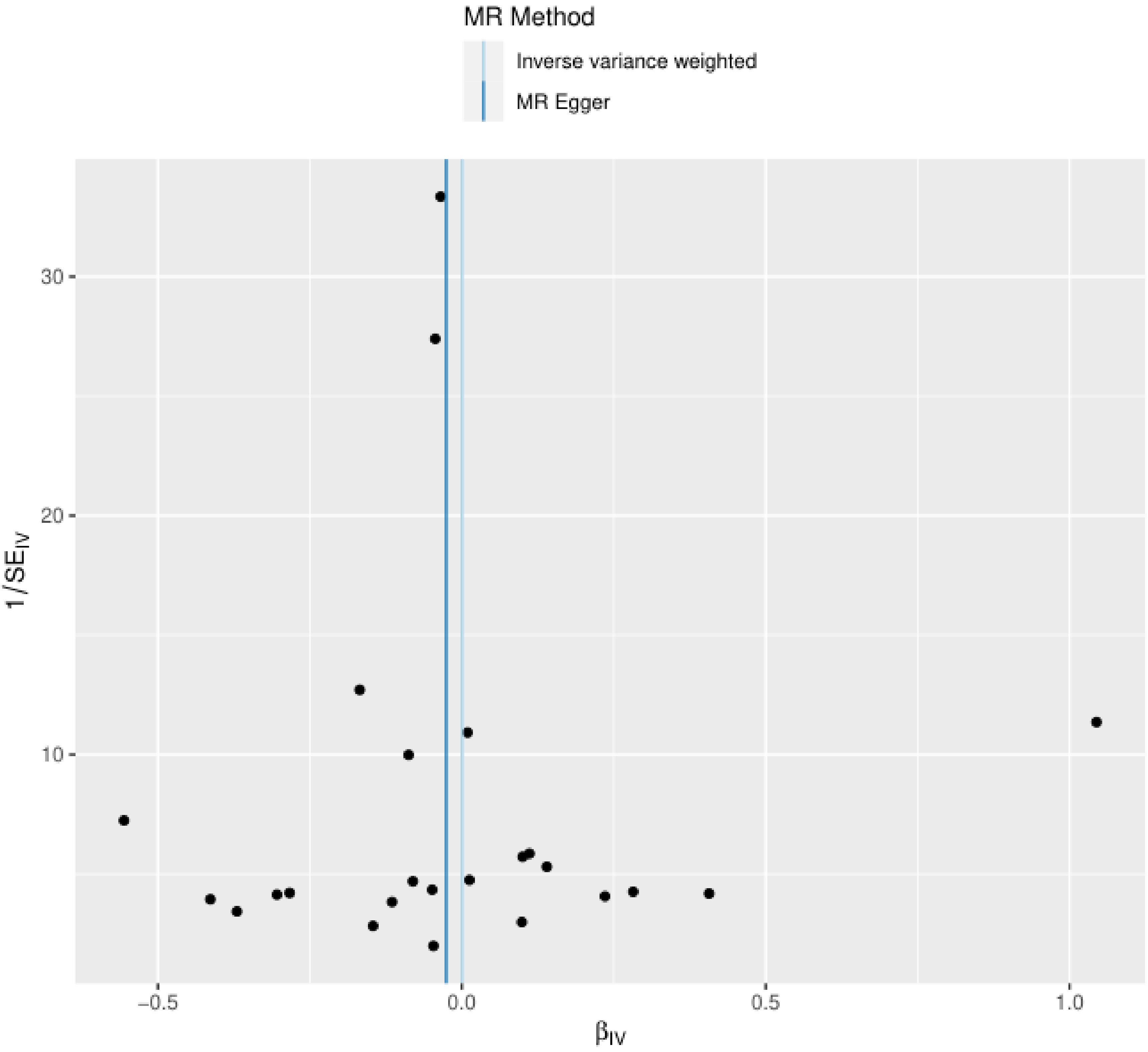

Funnel plot analyse of "HLA DR+ CD4+ AC" on 'Diabetic nephropathy'

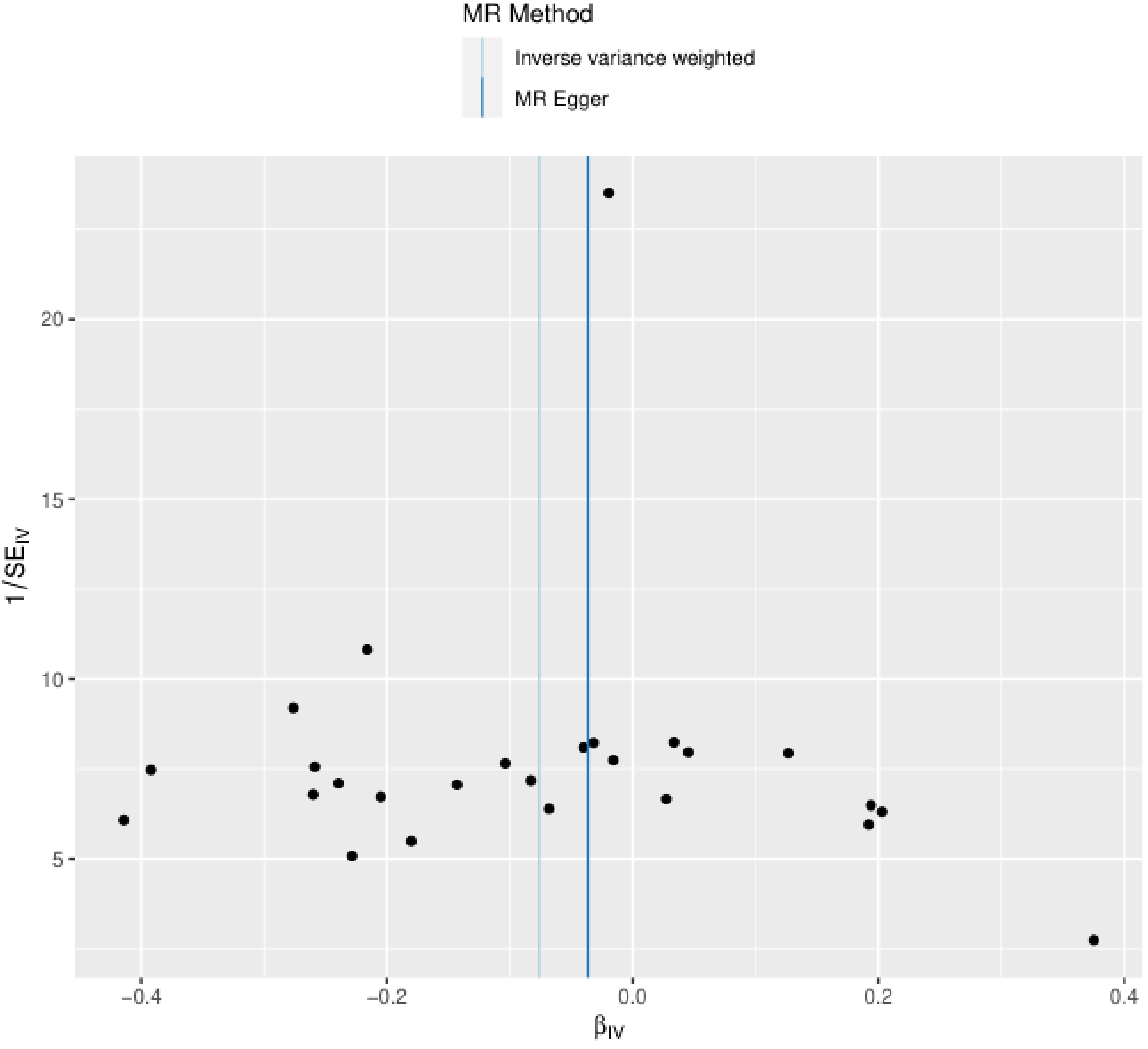

Funnel plot analyse of "HVEM on TD CD4+" on 'Diabetic nephropathy'

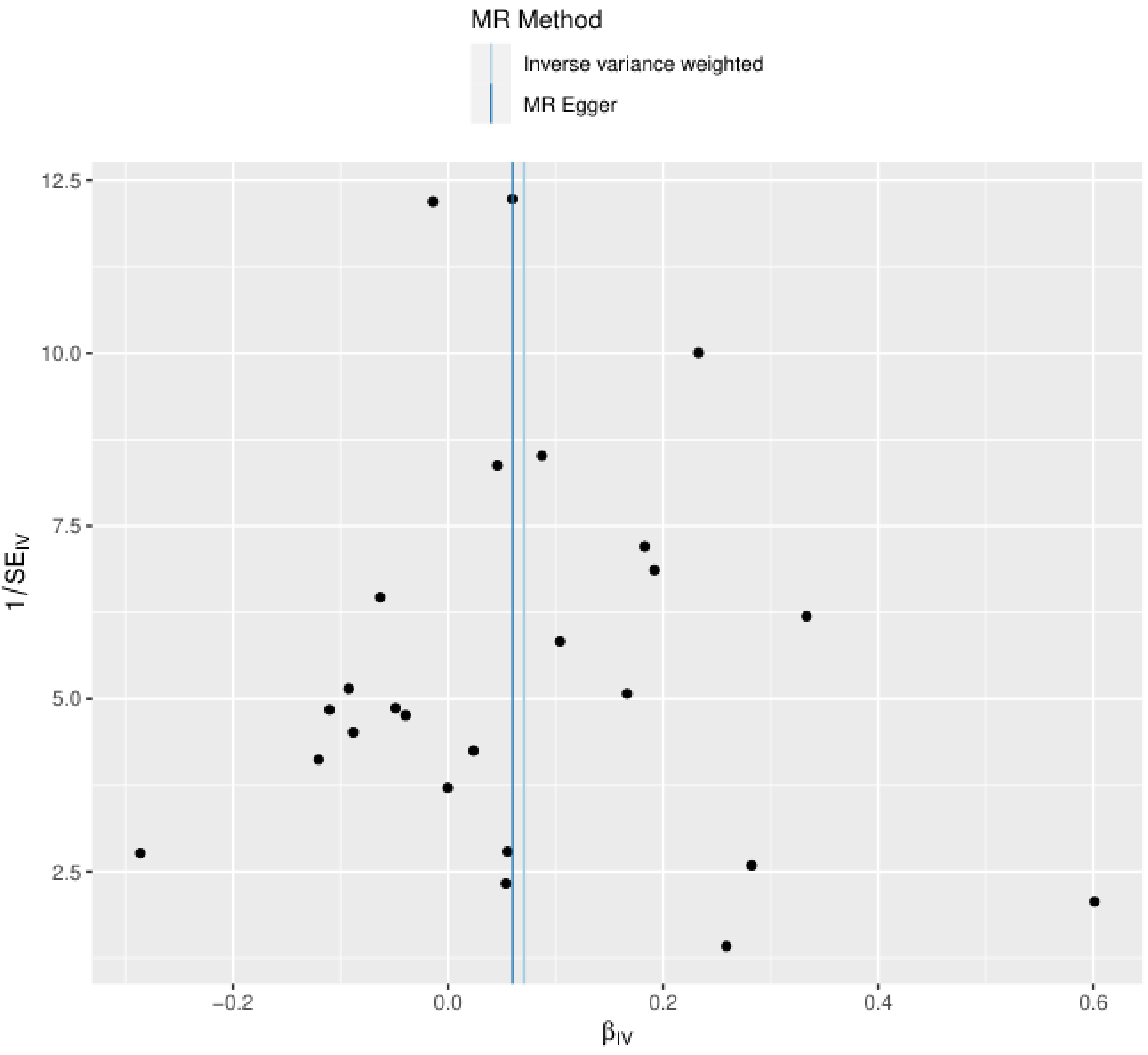

Funnel plot analyse of "CCR2 on monocyte " on 'Diabetic nephropathy'

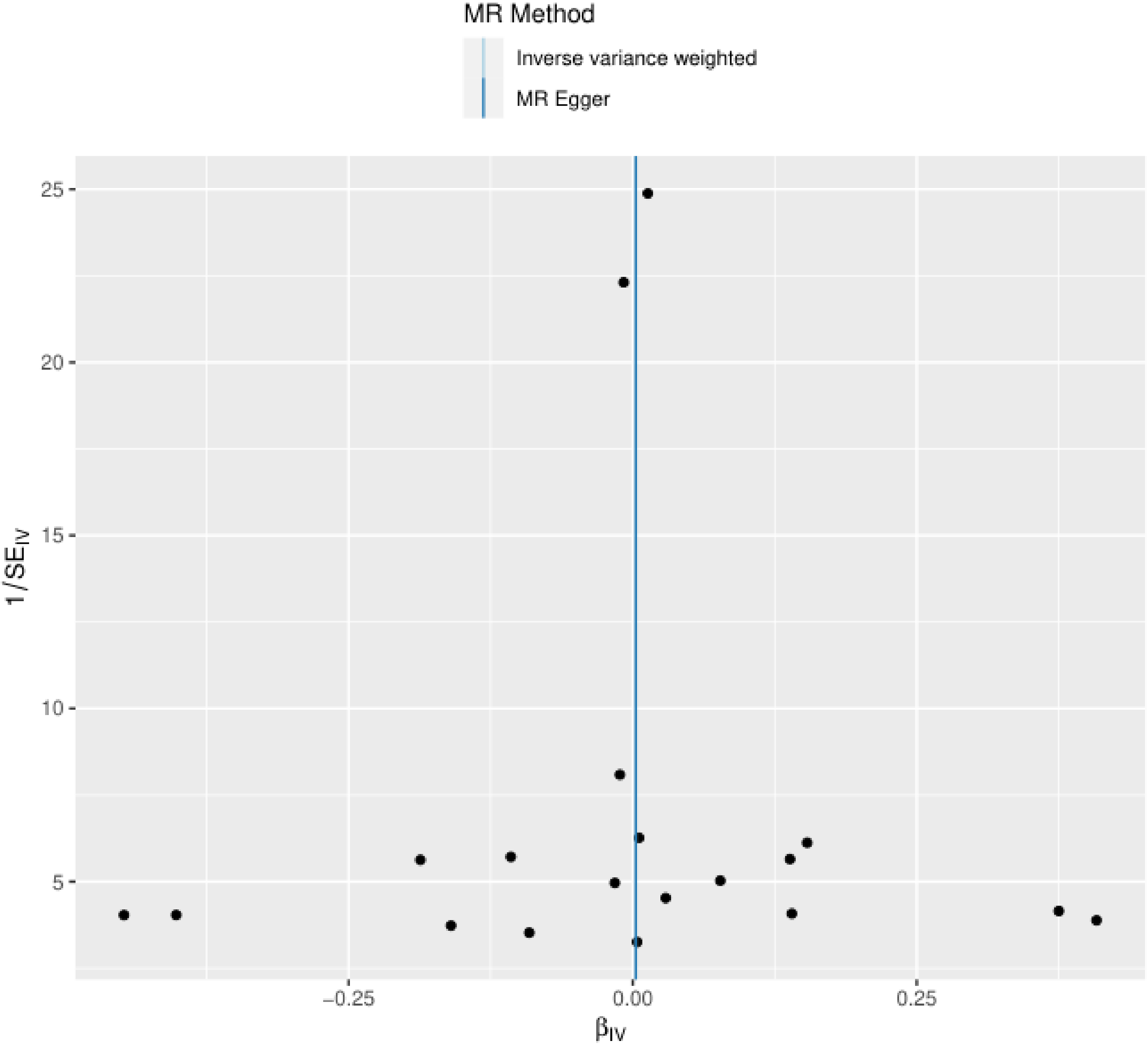

Funnel plot analyse of "CD62L- monocyte AC" on 'Diabetic nephropathy'

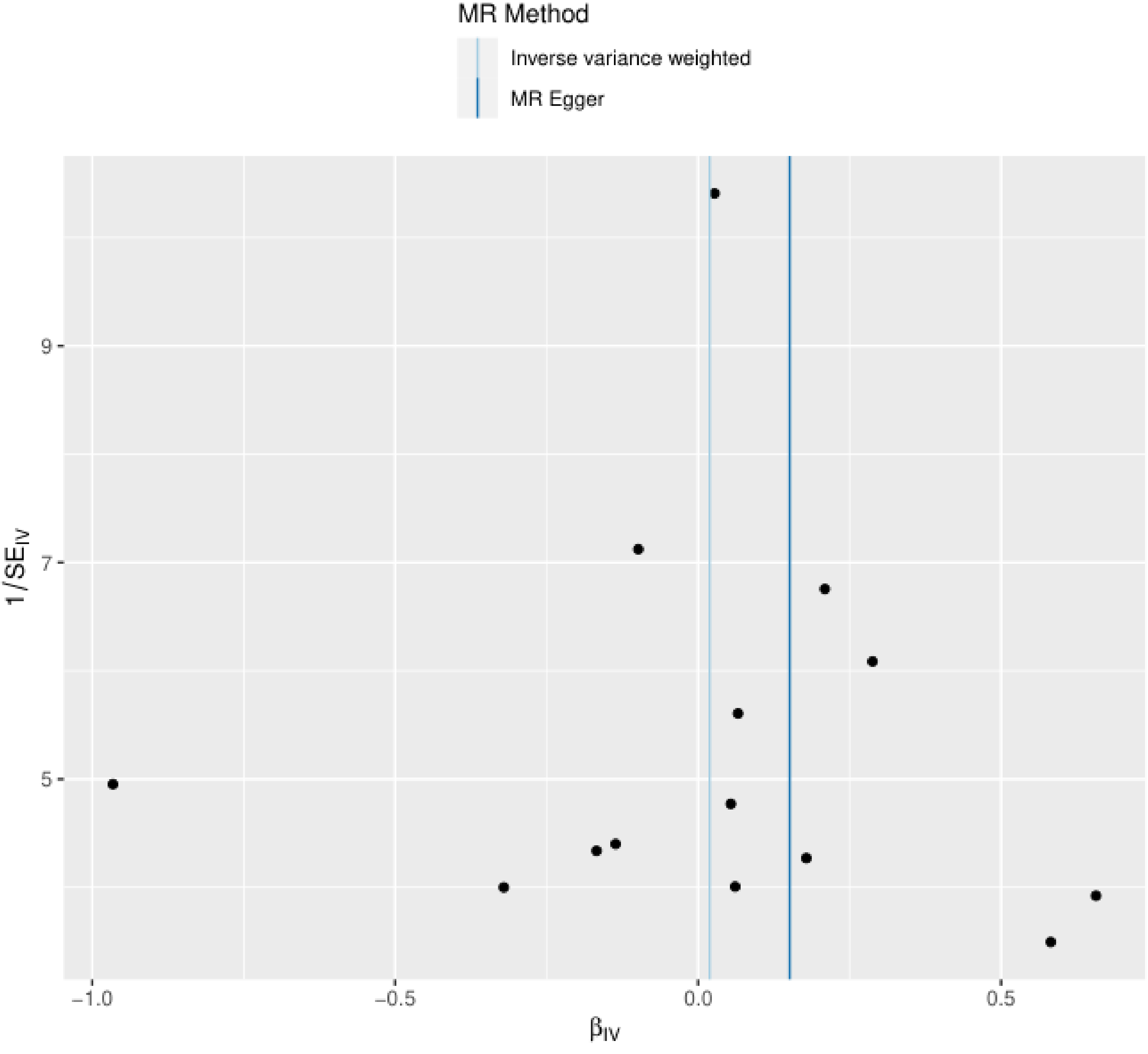

Funnel plot analyse of "CD4/CD8br" on 'Diabetic nephropathy'

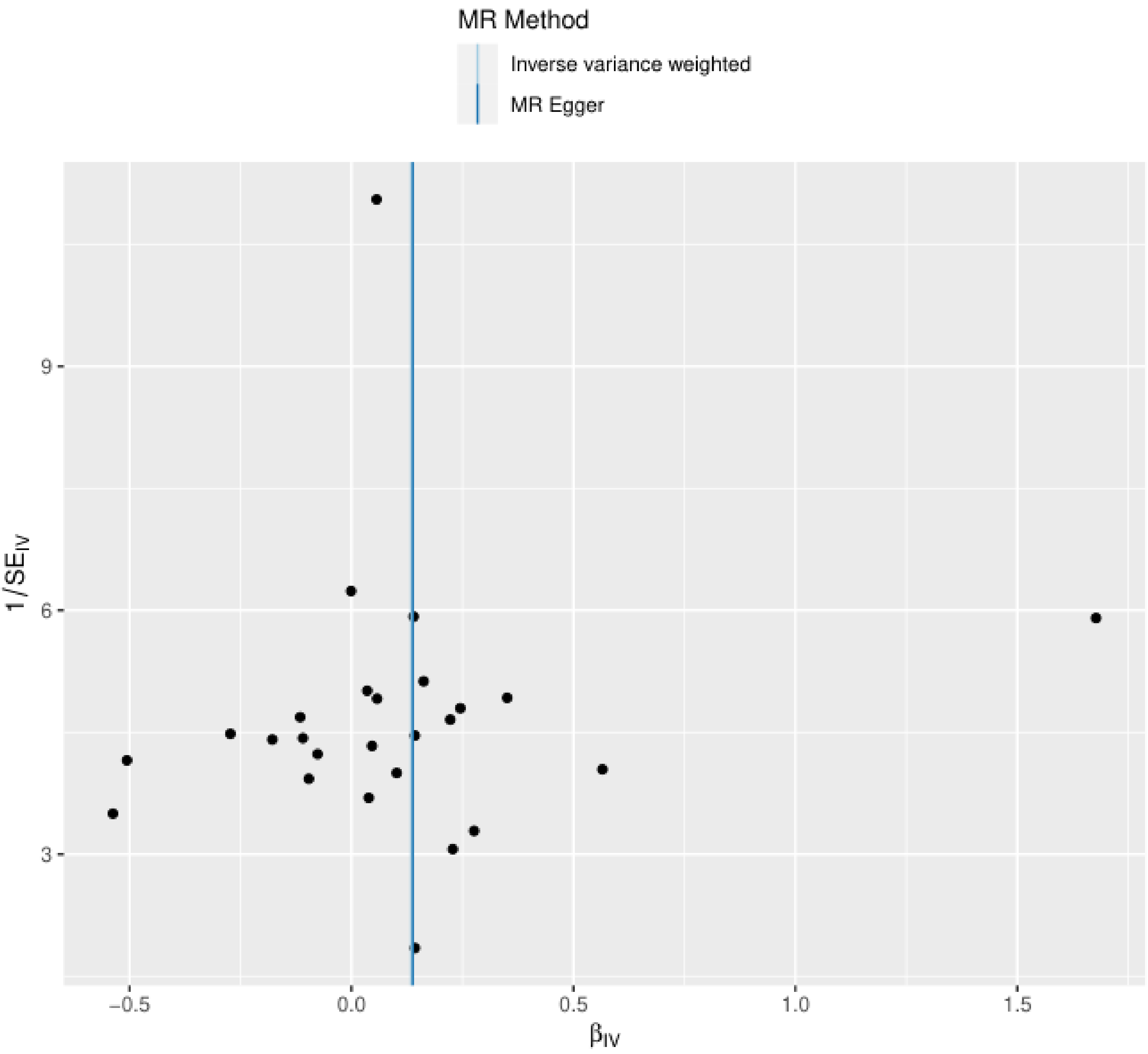

Funnel plot analyse of "CD8br NKT AC" on 'Diabetic nephropathy'

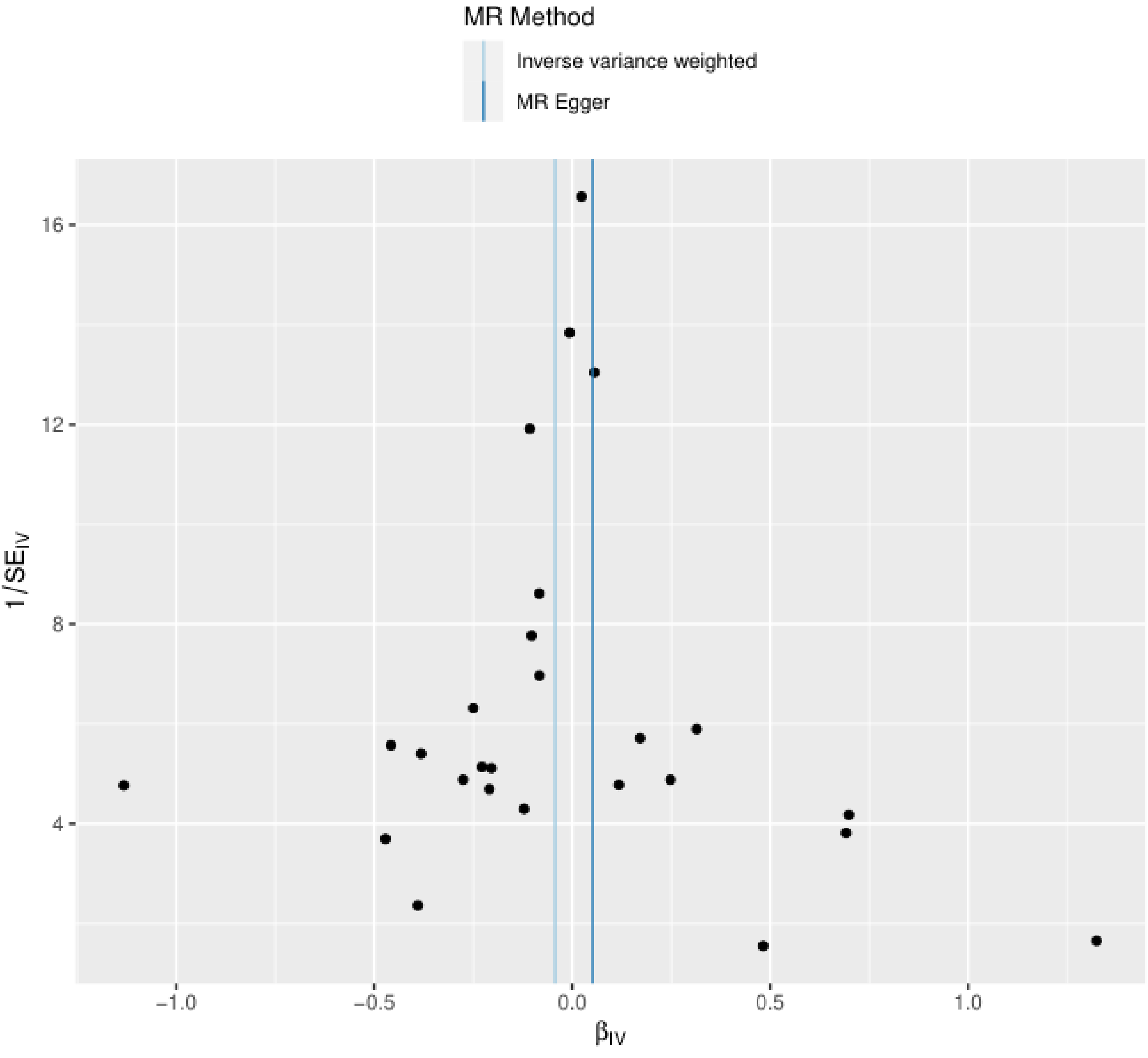

Funnel plot analyse of "CD8 on CD8br" on 'Diabetic nephropathy'

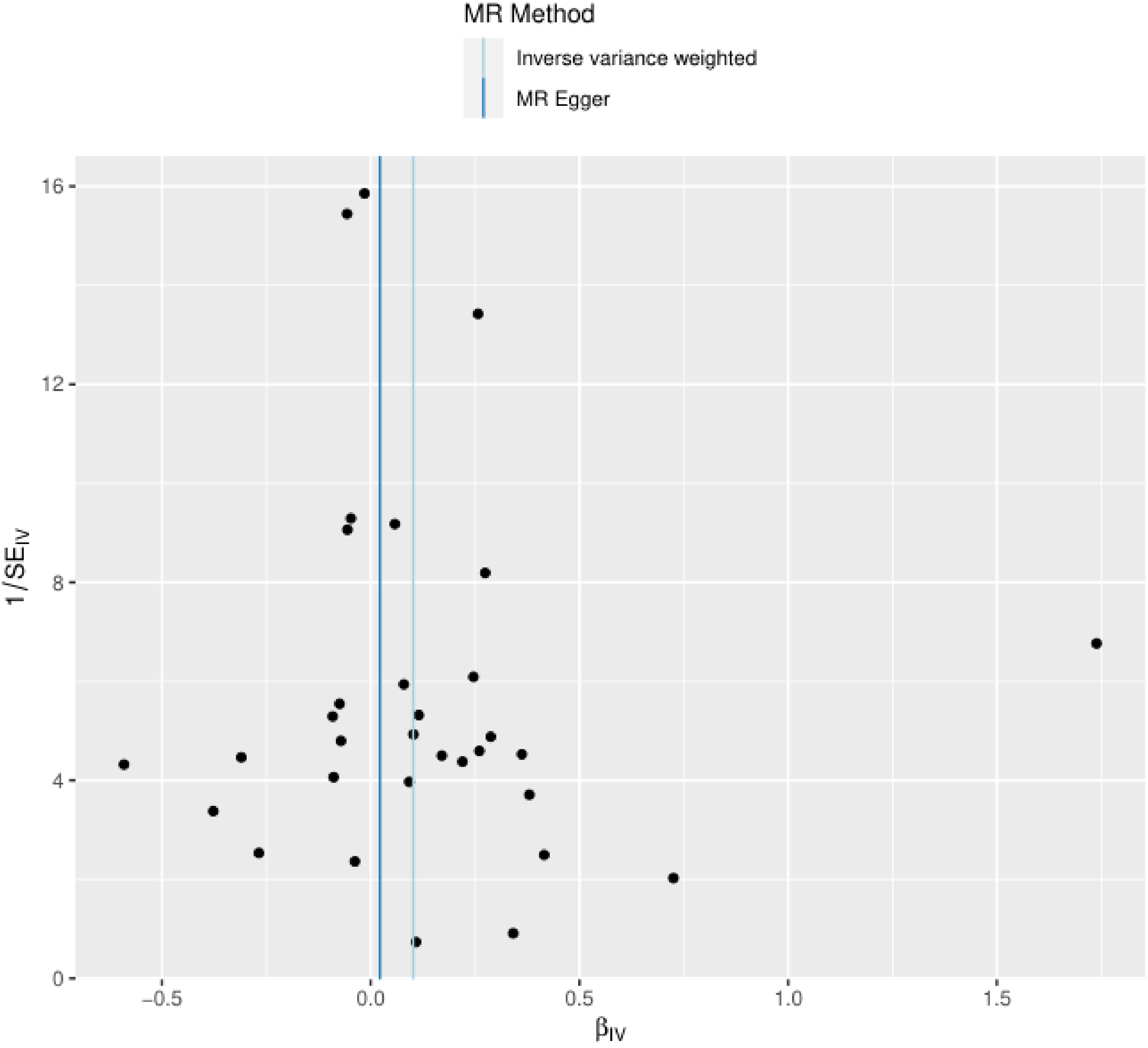

Funnel plot analyse of "HLA DR+ CD8br %lymphocyte" on 'Diabetic nephropathy'

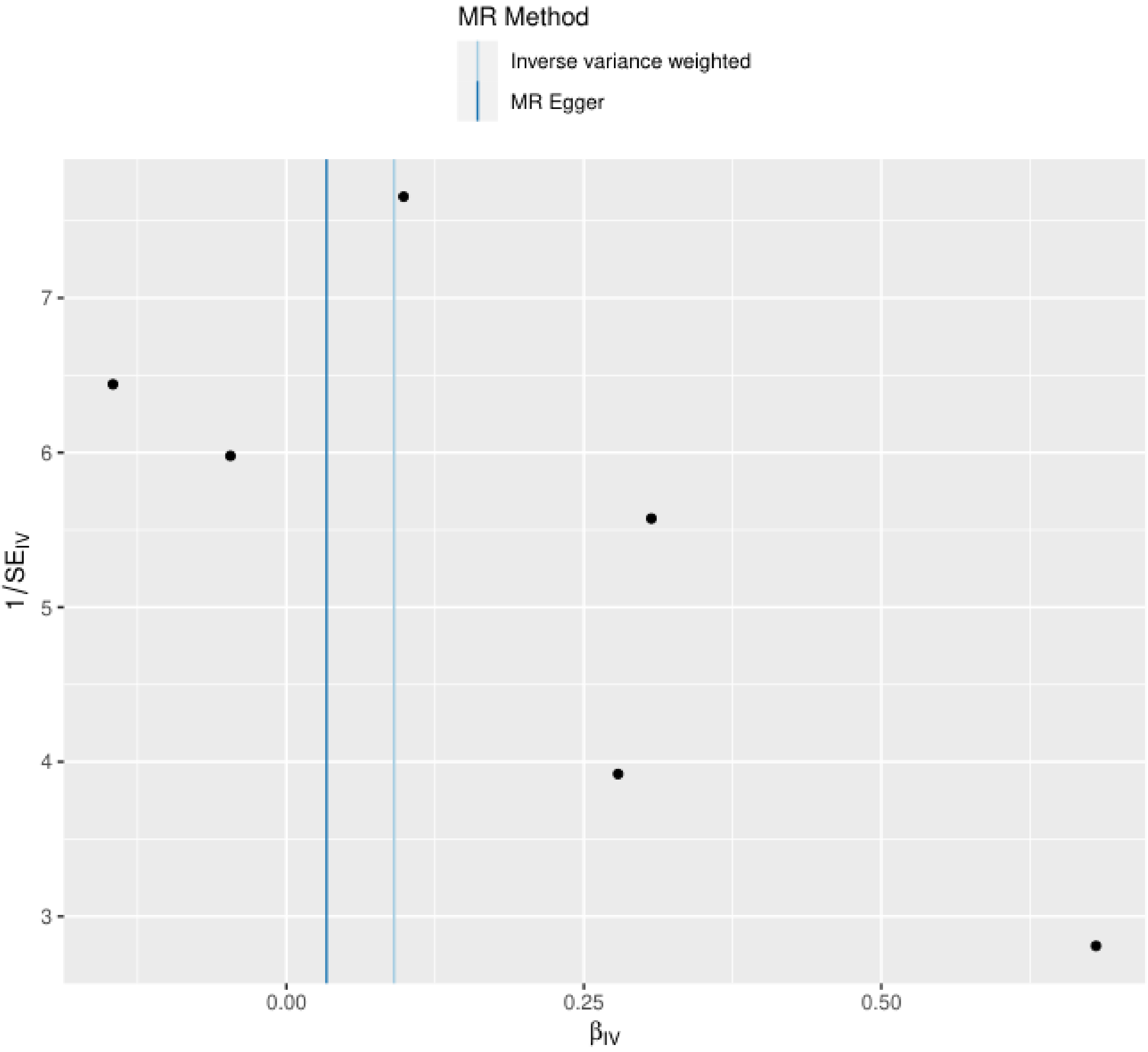

Funnel plot analyse of "CD24 on IgD- CD38dim" on 'Diabetic nephropathy'

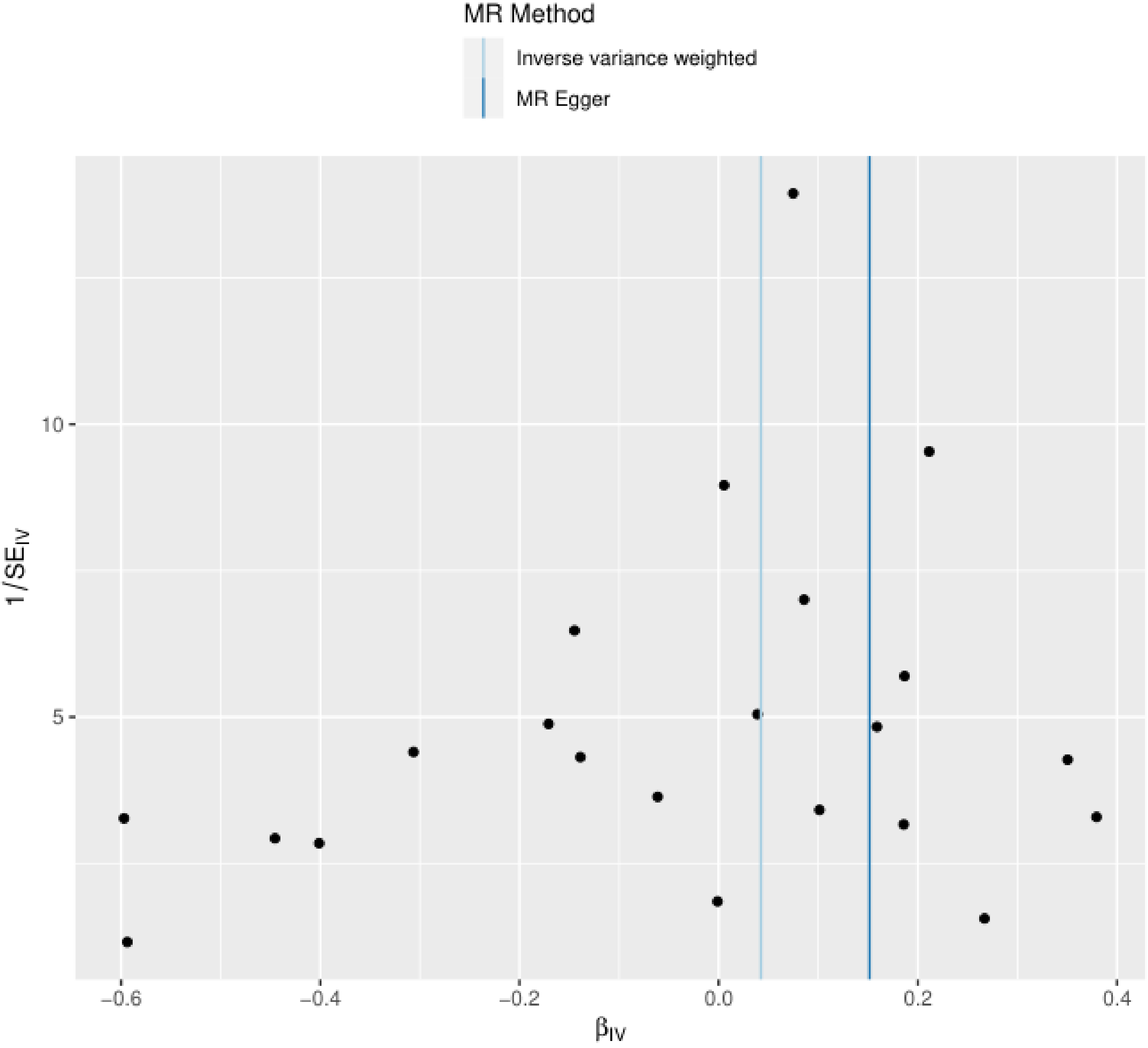

Funnel plot analyse of "CD4+ %T cell" on 'Diabetic nephropathy'

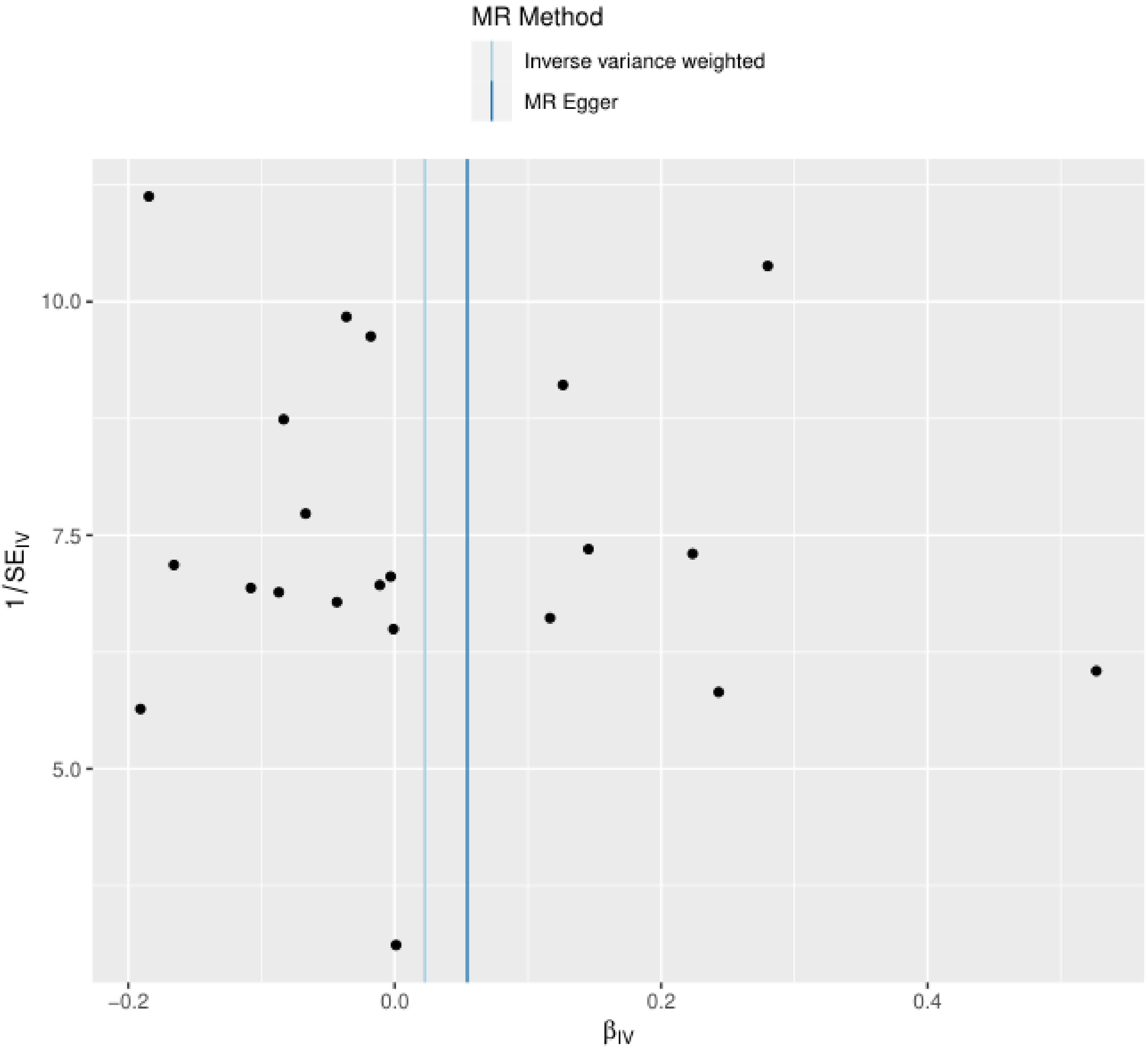

Funnel plot analyse of "HVEM on EM CD4+" on 'Diabetic nephropathy'

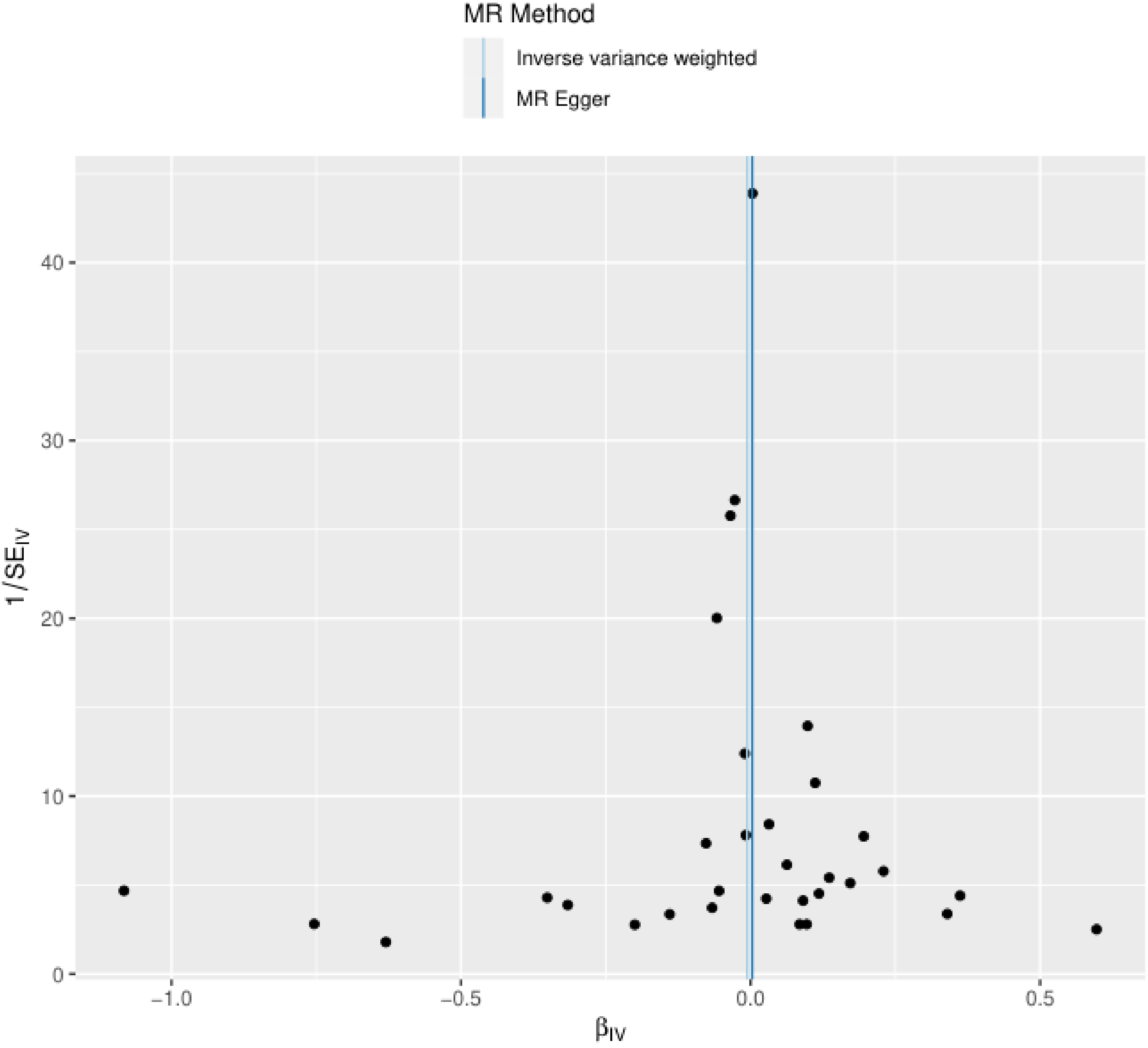

Funnel plot analyse of "Resting Treg %CD4 " on 'Diabetic nephropathy'

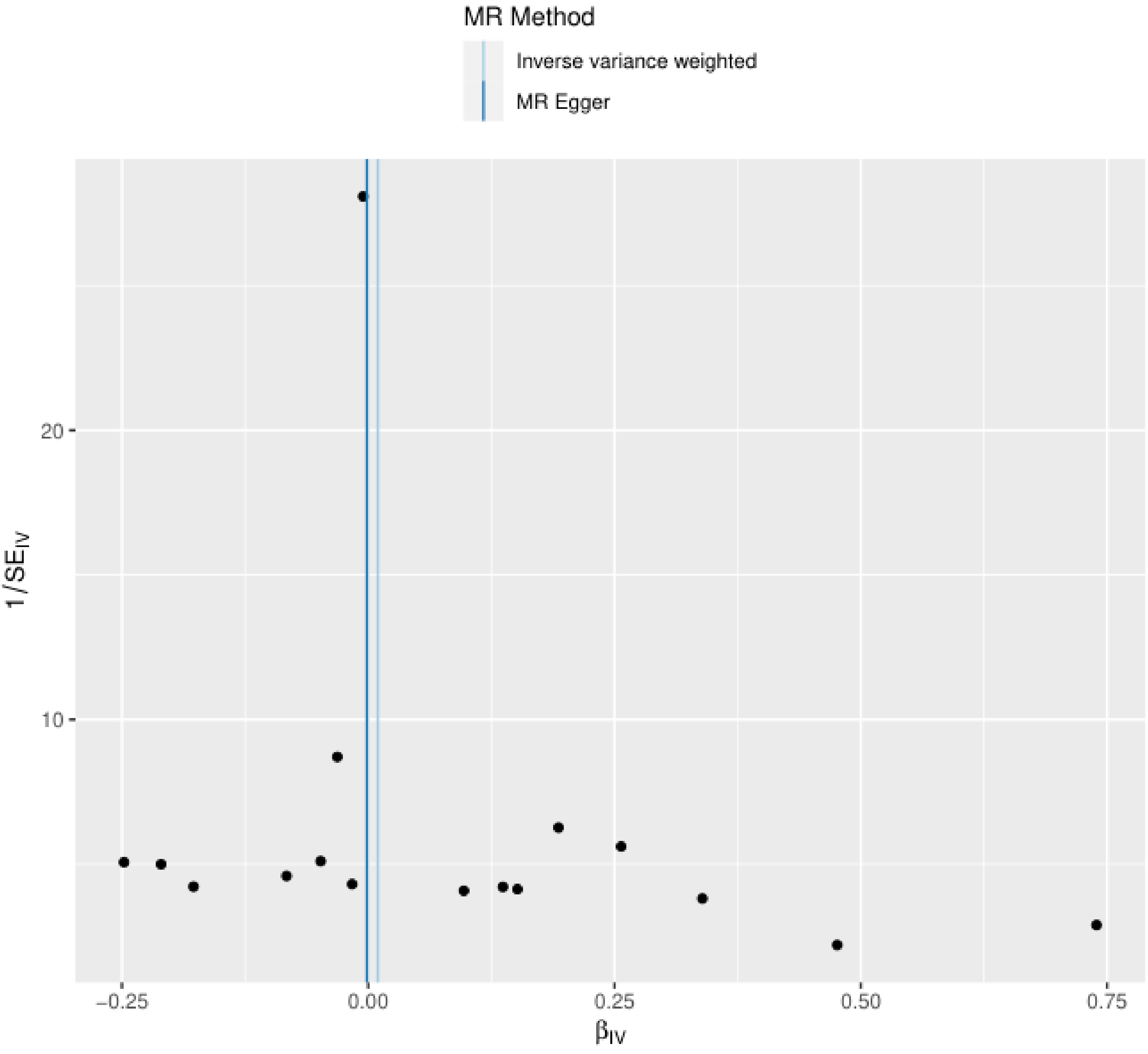

Funnel plot analyse of "T cell %leukocyte" on 'Diabetic nephropathy'

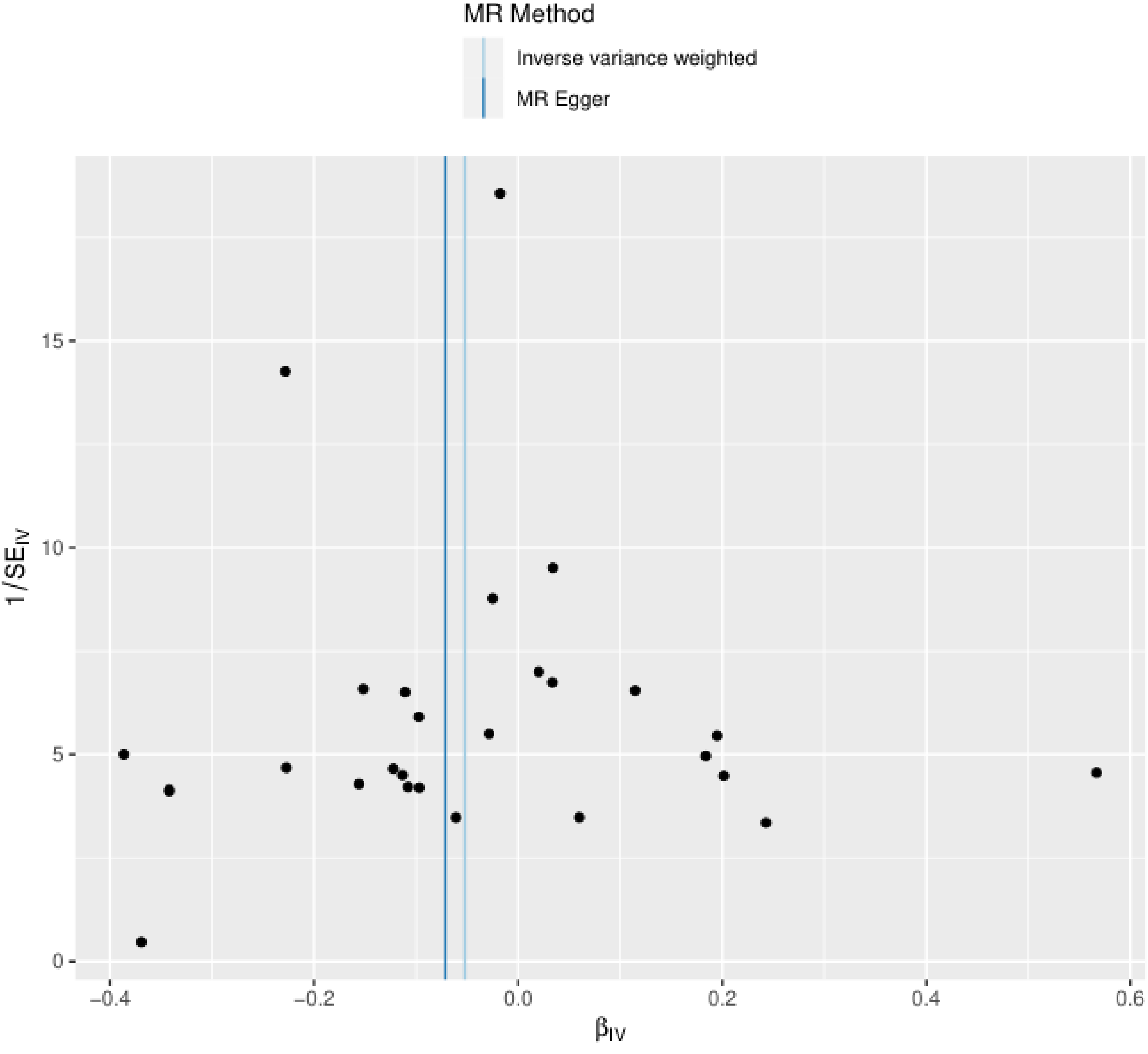

Funnel plot analyse of "CD3 on CD39+ secreting Treg " on 'Diabetic nephropathy'

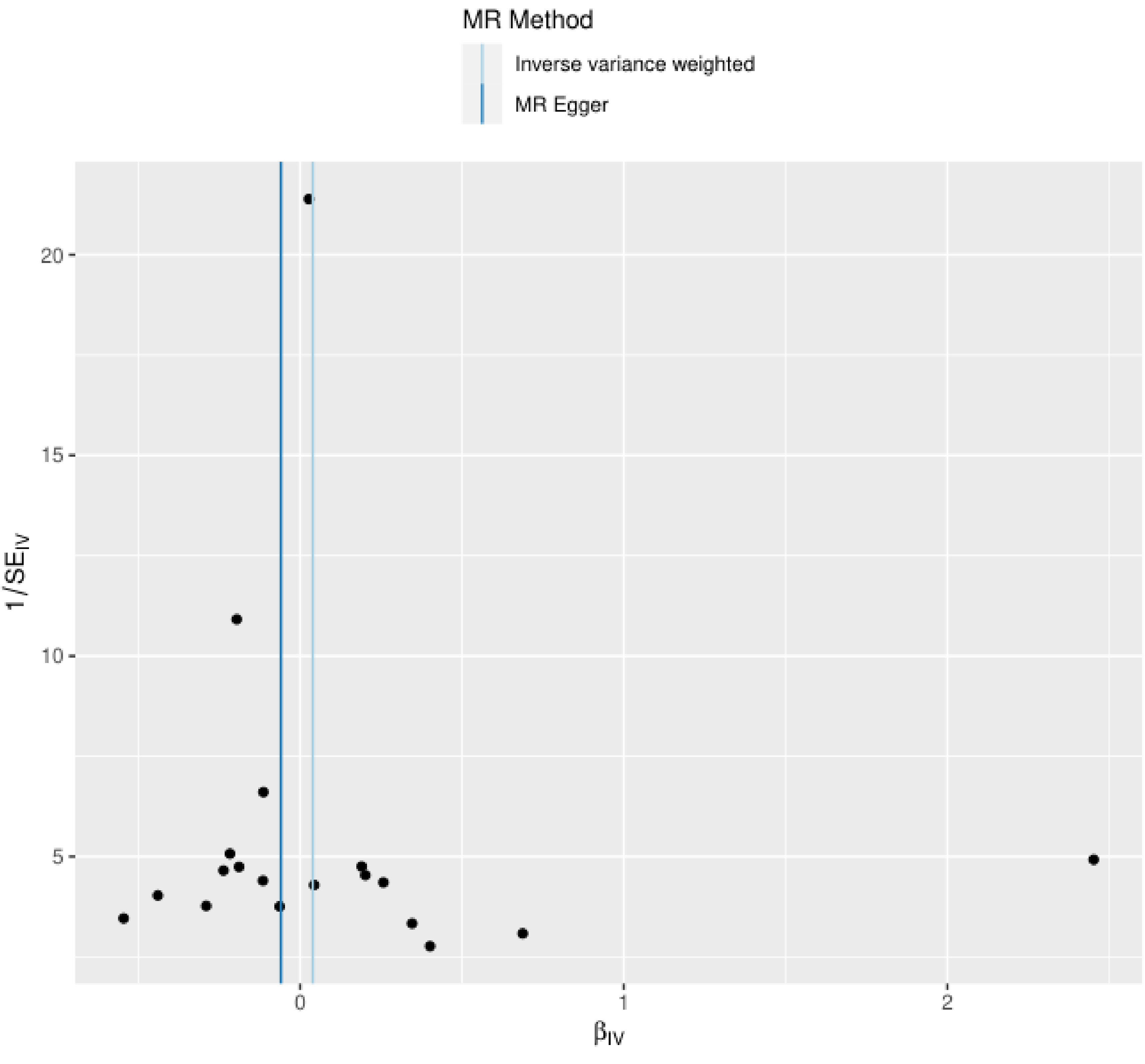

Funnel plot analyse of "CD20- %B cell" on 'Diabetic nephropathy'

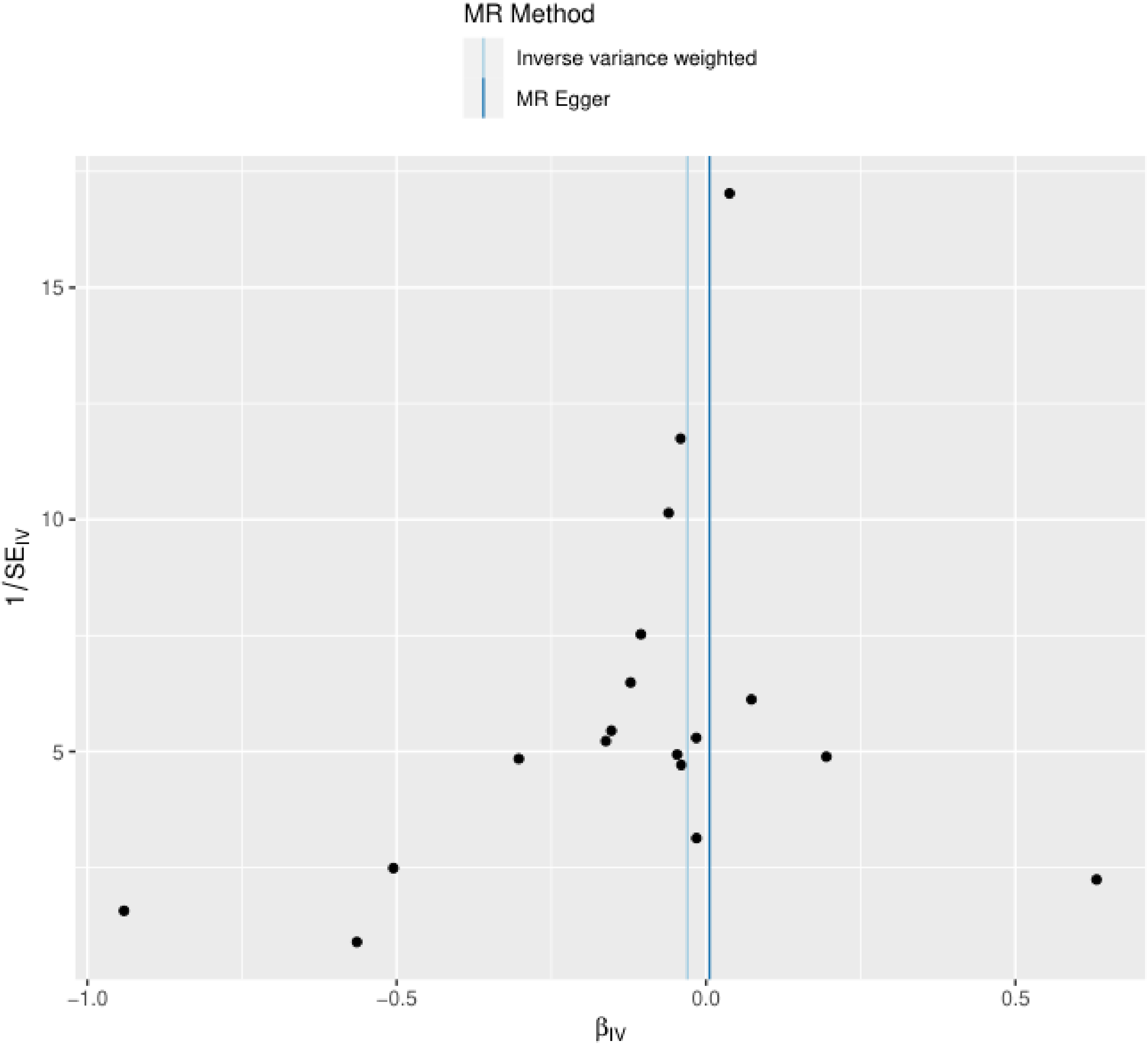

Funnel plot analyse of "CD3 on EM CD8br " on 'Diabetic nephropathy'

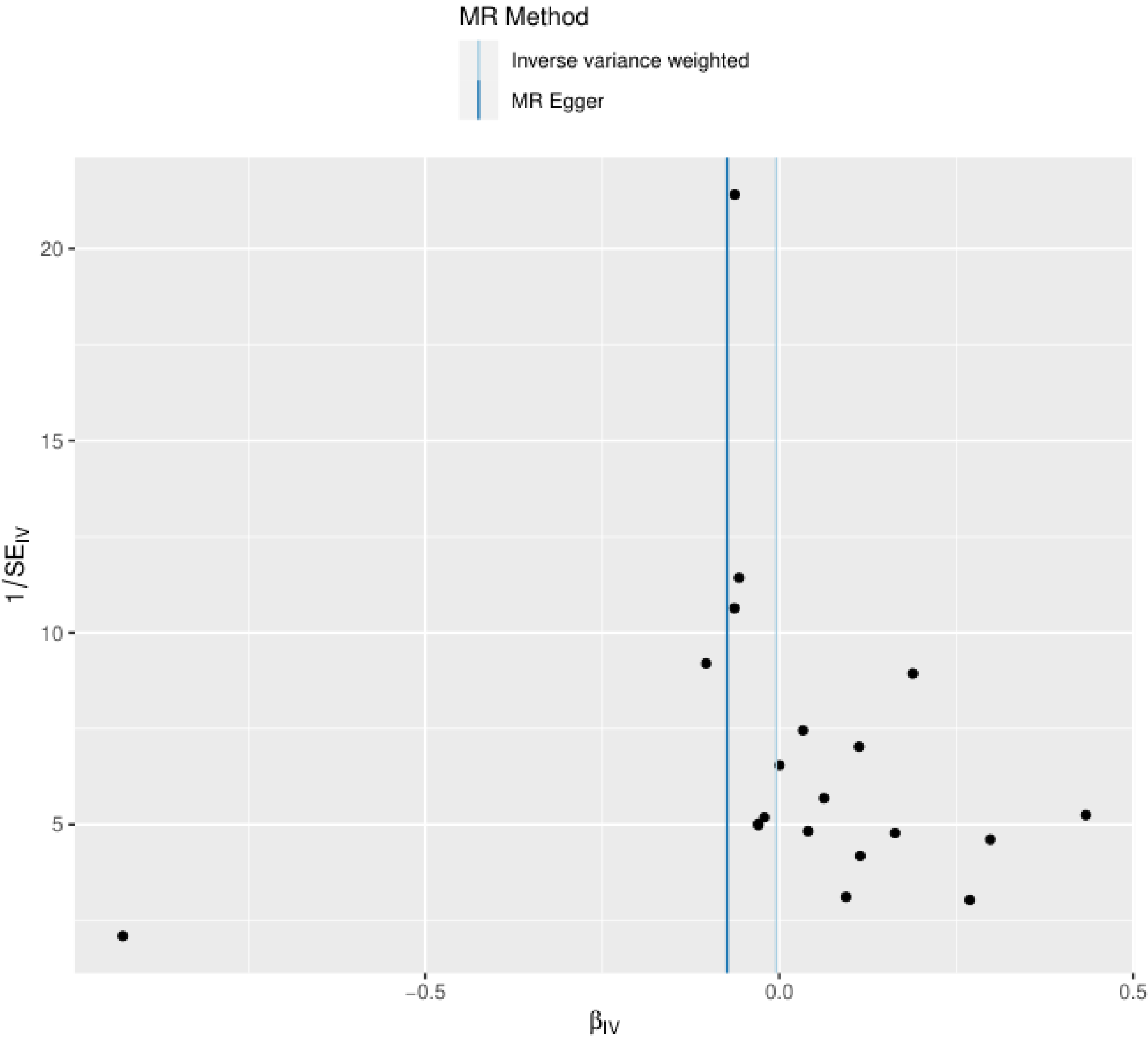

Funnel plot analyse of "CD127 on CD28+ CD4+" on 'Diabetic nephropathy'

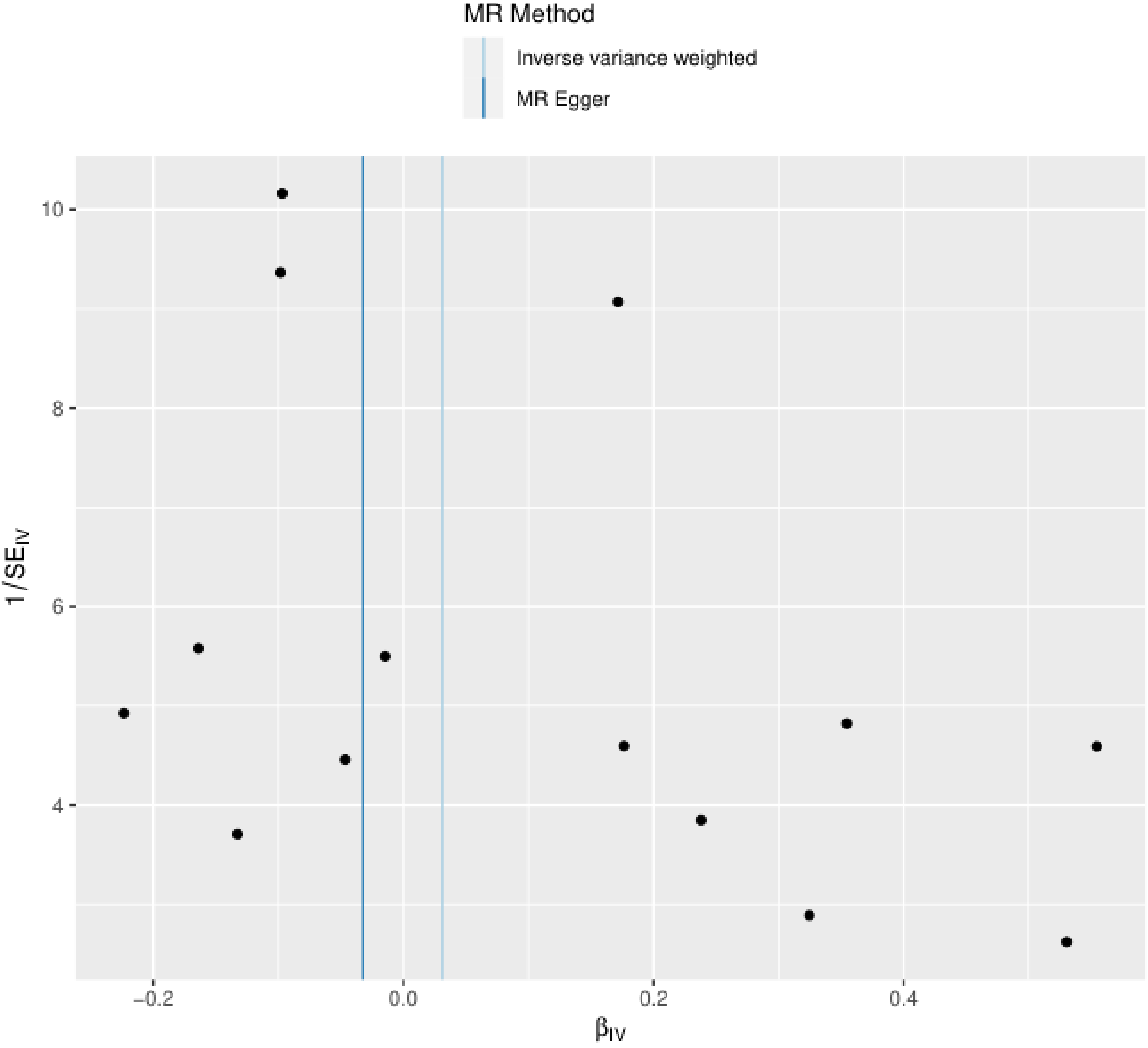

Funnel plot analyse of "Sw mem %B cell" on 'Diabetic nephropathy'

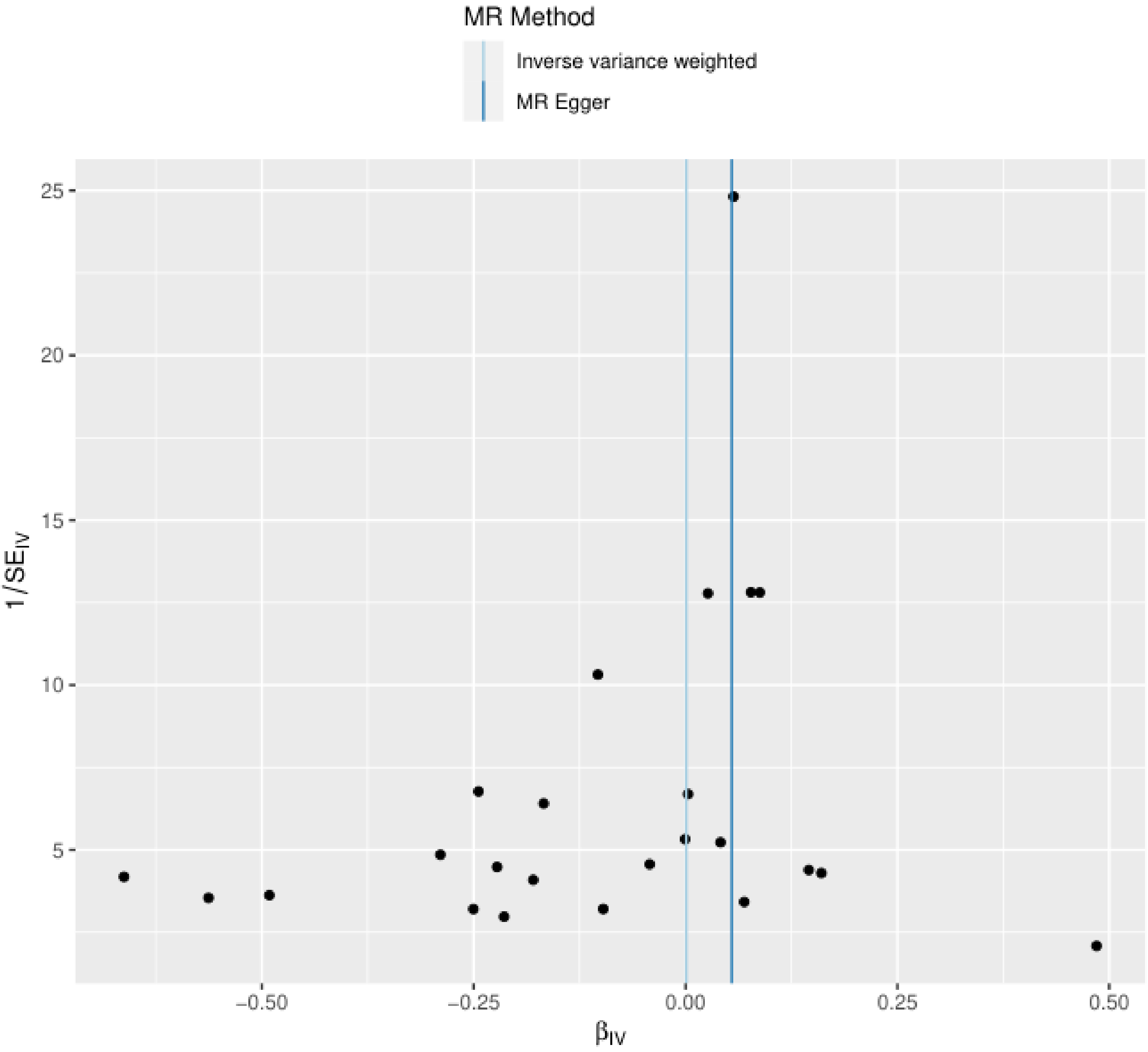

Funnel plot analyse of "CD25 on memory B cell" on 'Diabetic nephropathy'

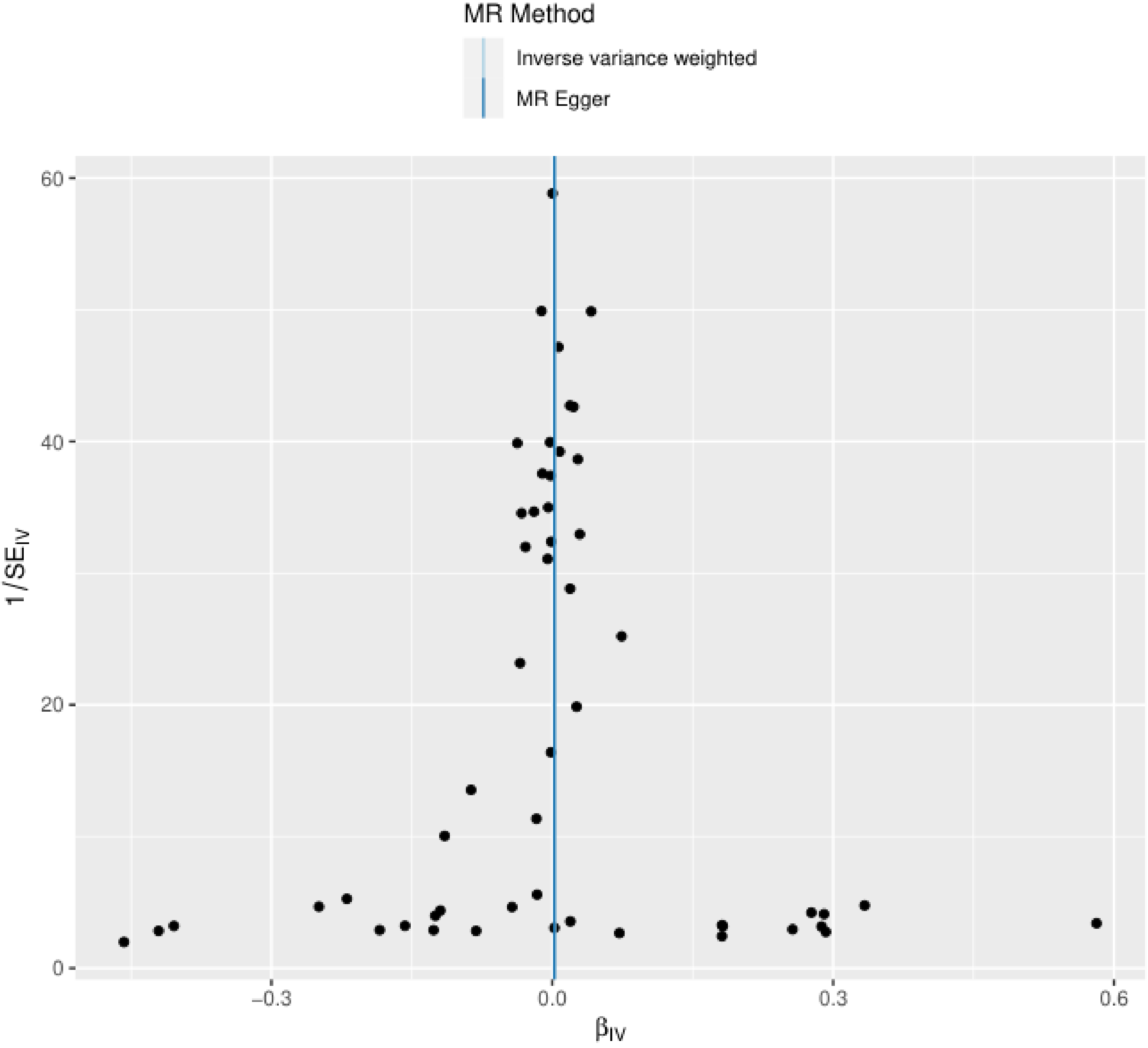

Funnel plot analyse of "CD28+ CD45RA+ CD8br AC" on 'Diabetic nephropathy'

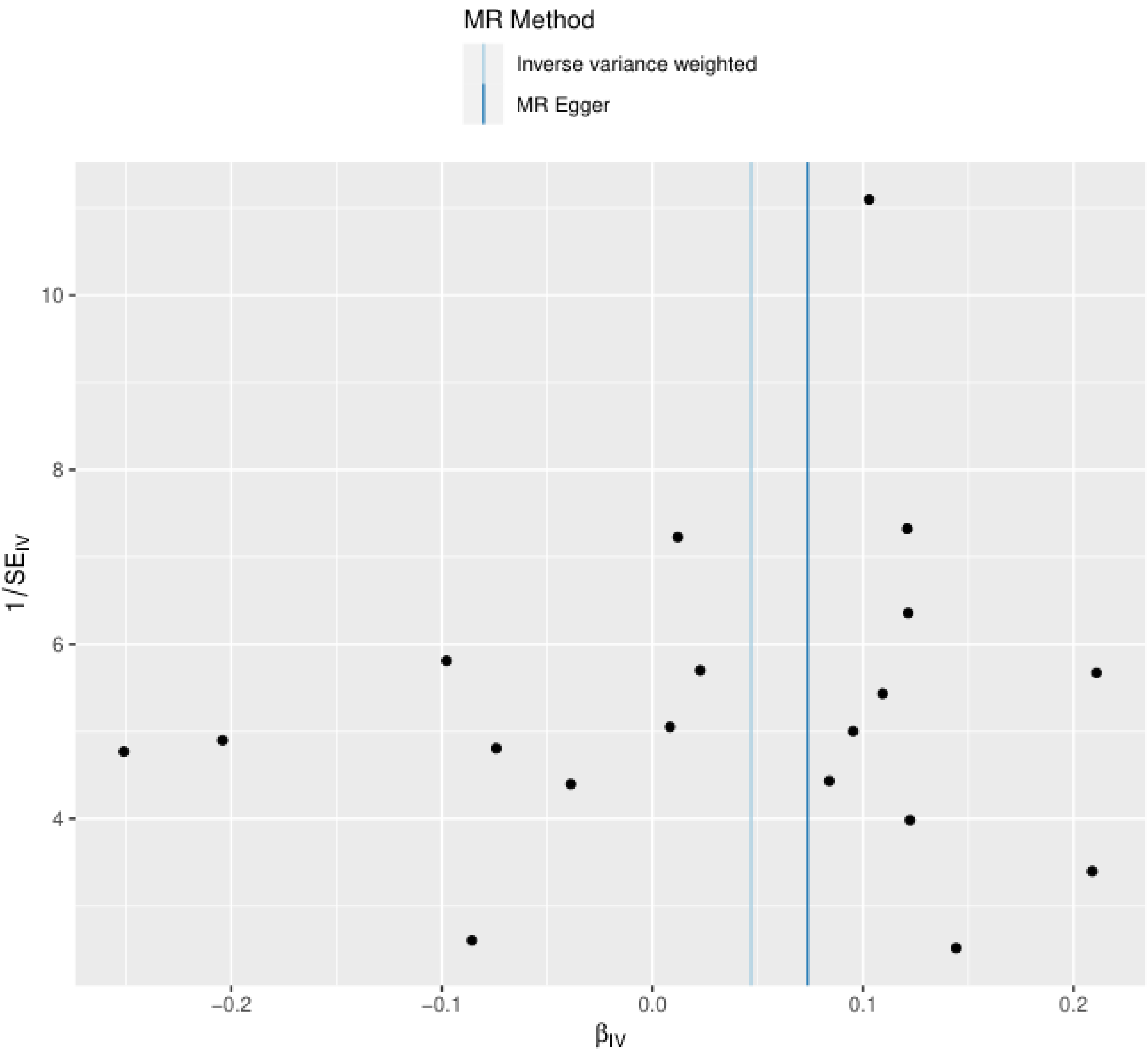

Funnel plot analyse of "CD127 on CD28+ CD45RA+ CD8br " on 'Diabetic nephropathy'

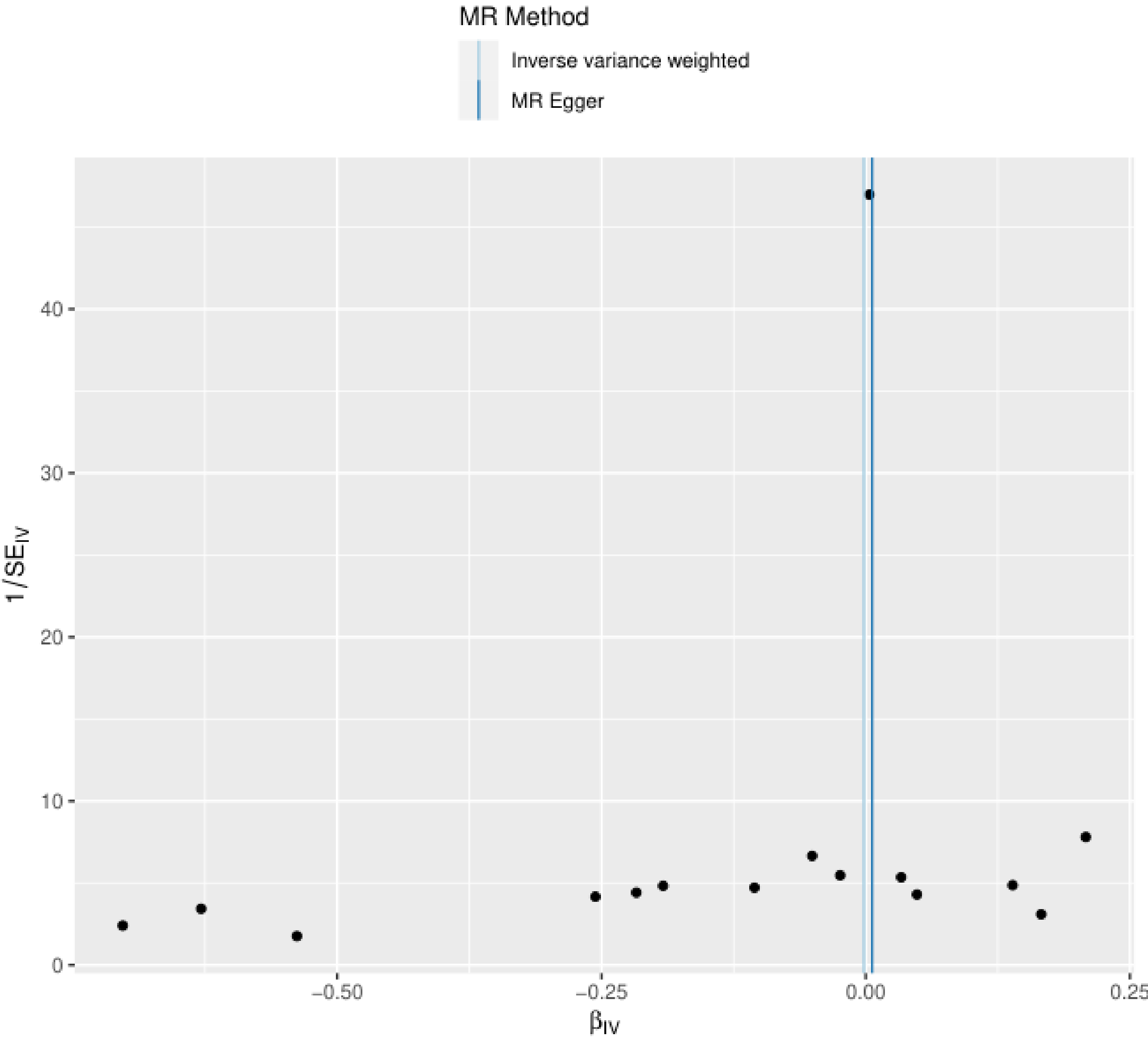

Funnel plot analyse of "Memory B cell AC" on 'Diabetic nephropathy'

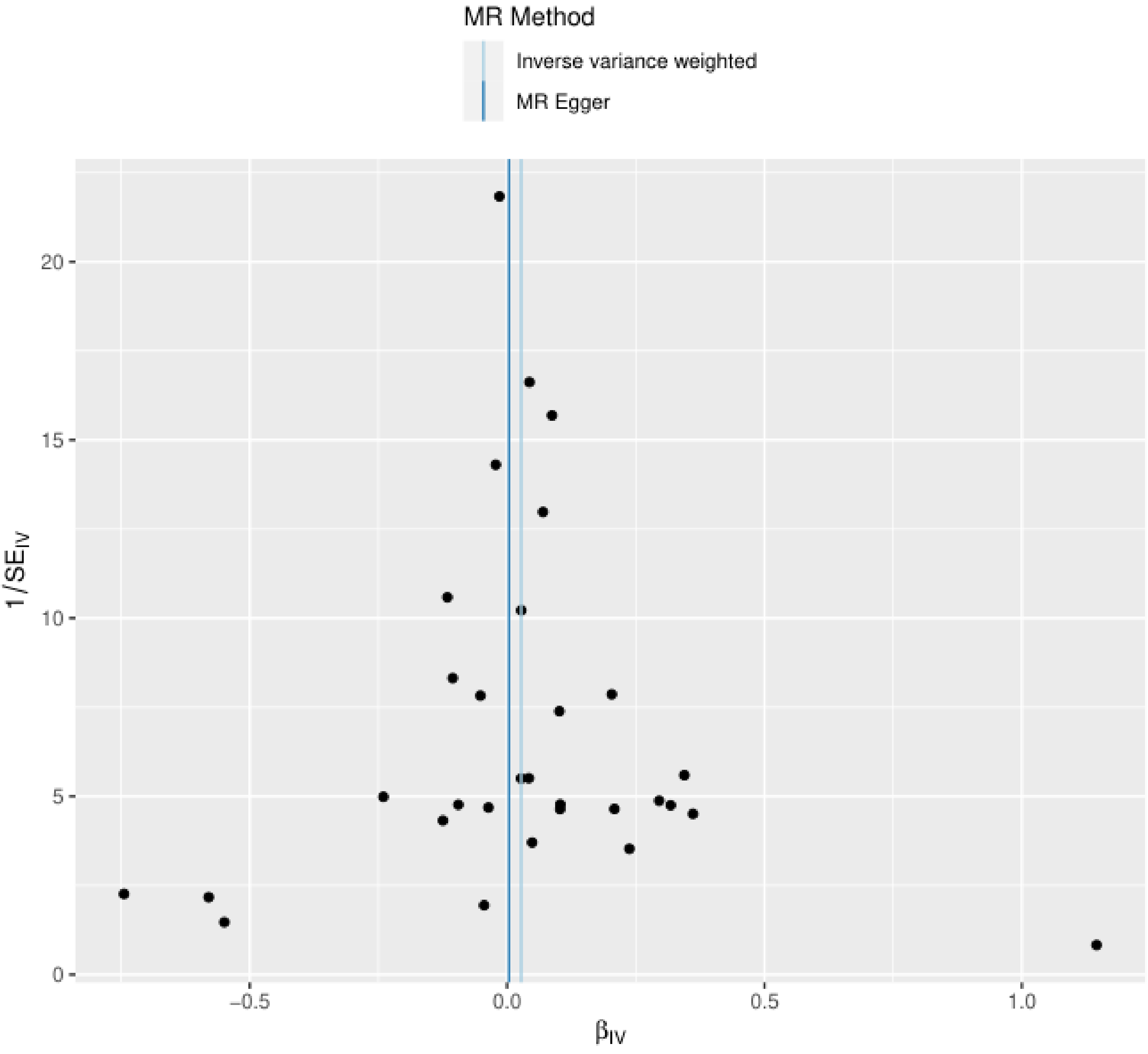

Funnel plot analyse of "CD3 on CD45RA+ CD4+" on 'Diabetic nephropathy'

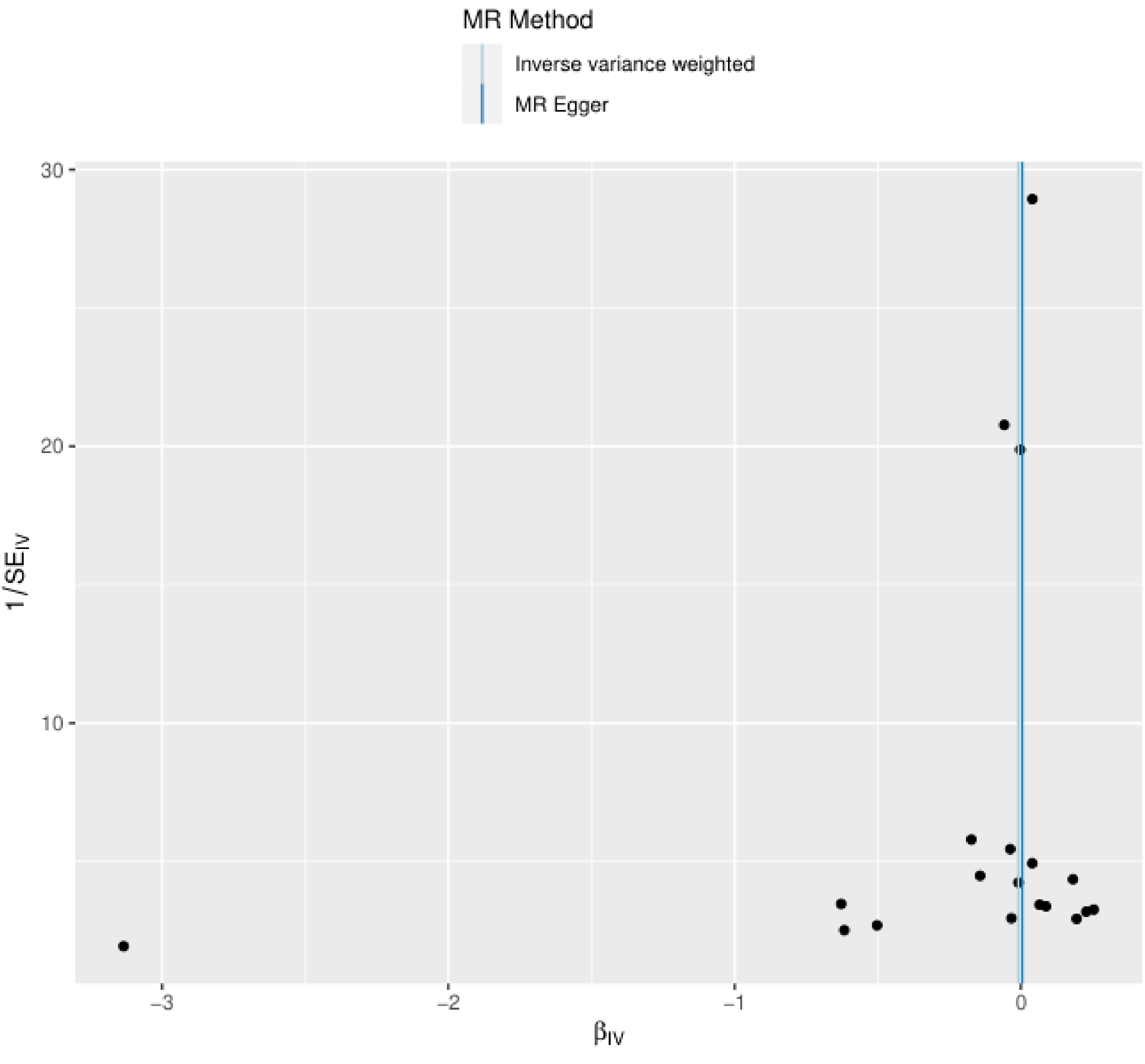

Funnel plot analyse of "SSC-A on CD8br" on 'Diabetic nephropathy'

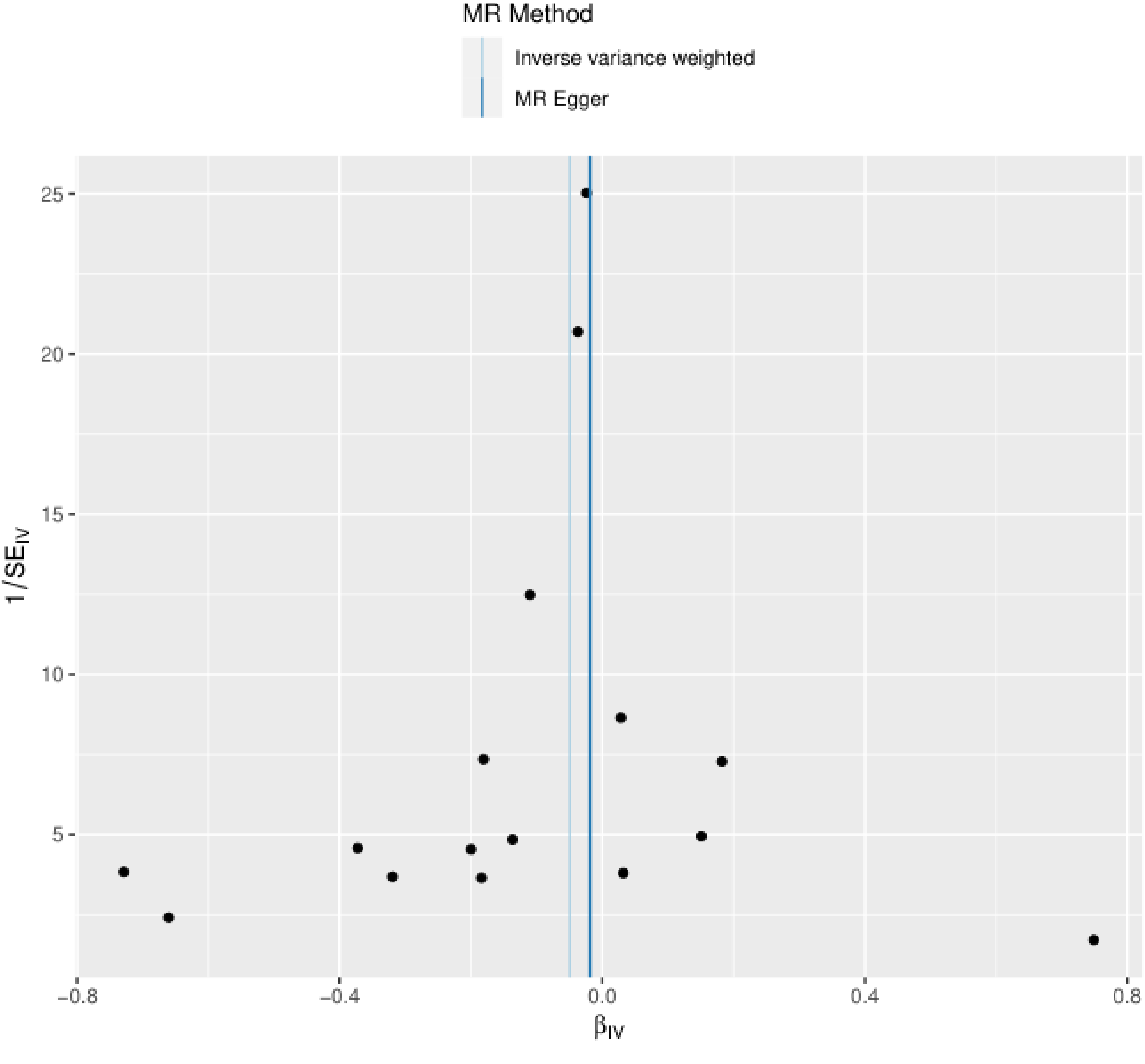

Funnel plot analyse of "Activated Treg AC" on 'Diabetic nephropathy'

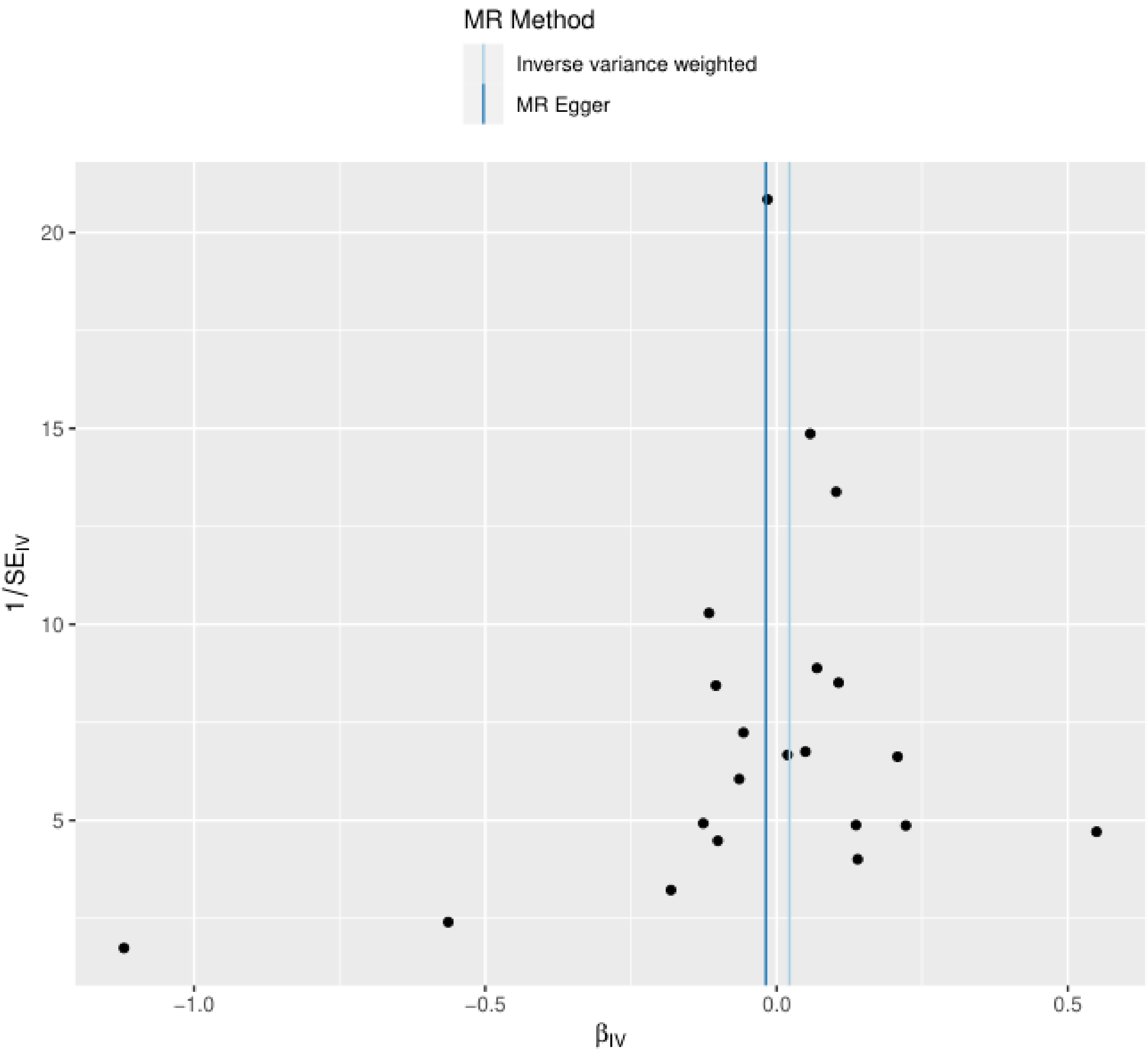

Funnel plot analyse of "CD3 on secreting Treg " on 'Diabetic nephropathy'

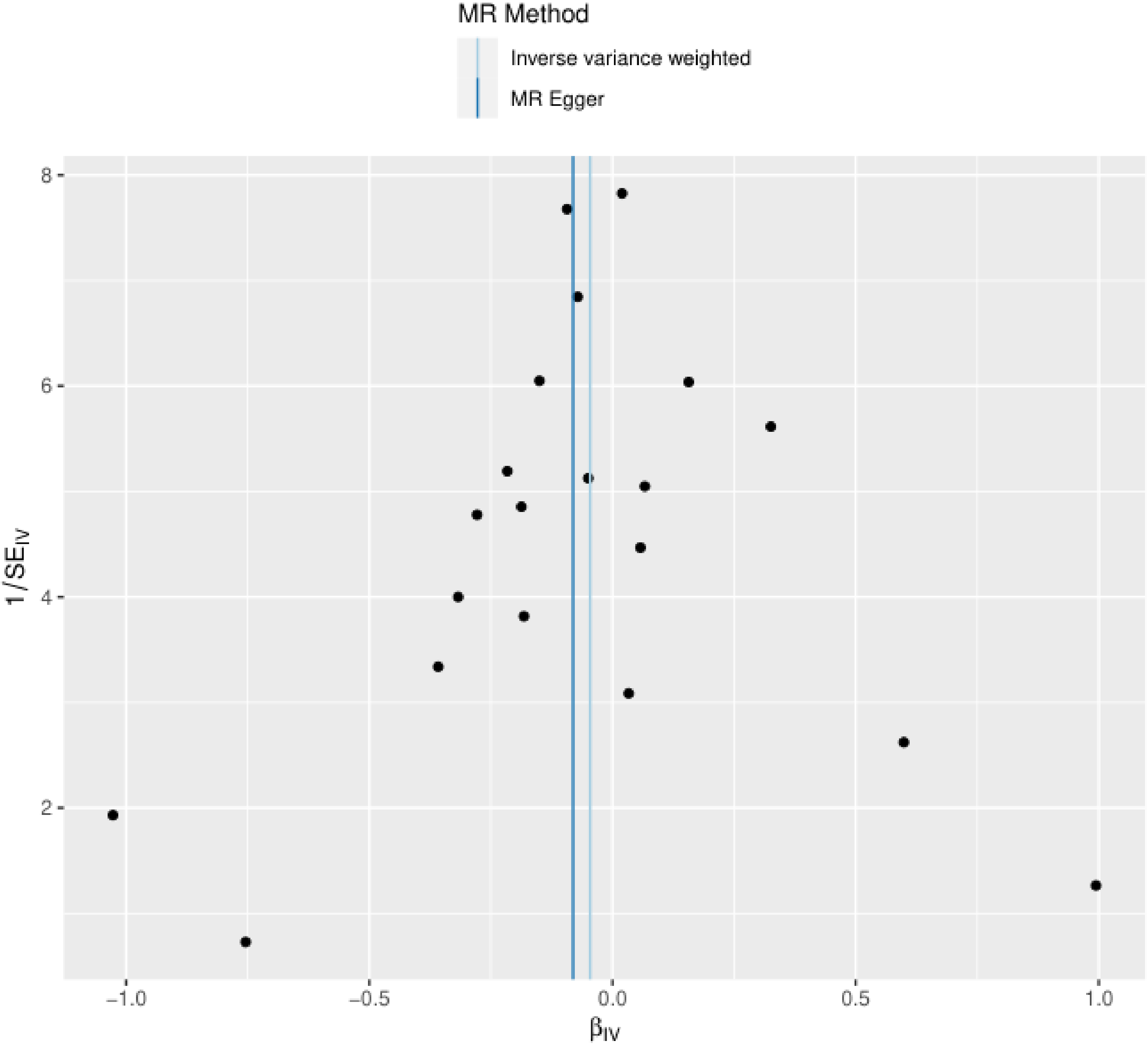

Funnel plot analyse of "CD8 on CD28- CD8br " on 'Diabetic nephropathy'

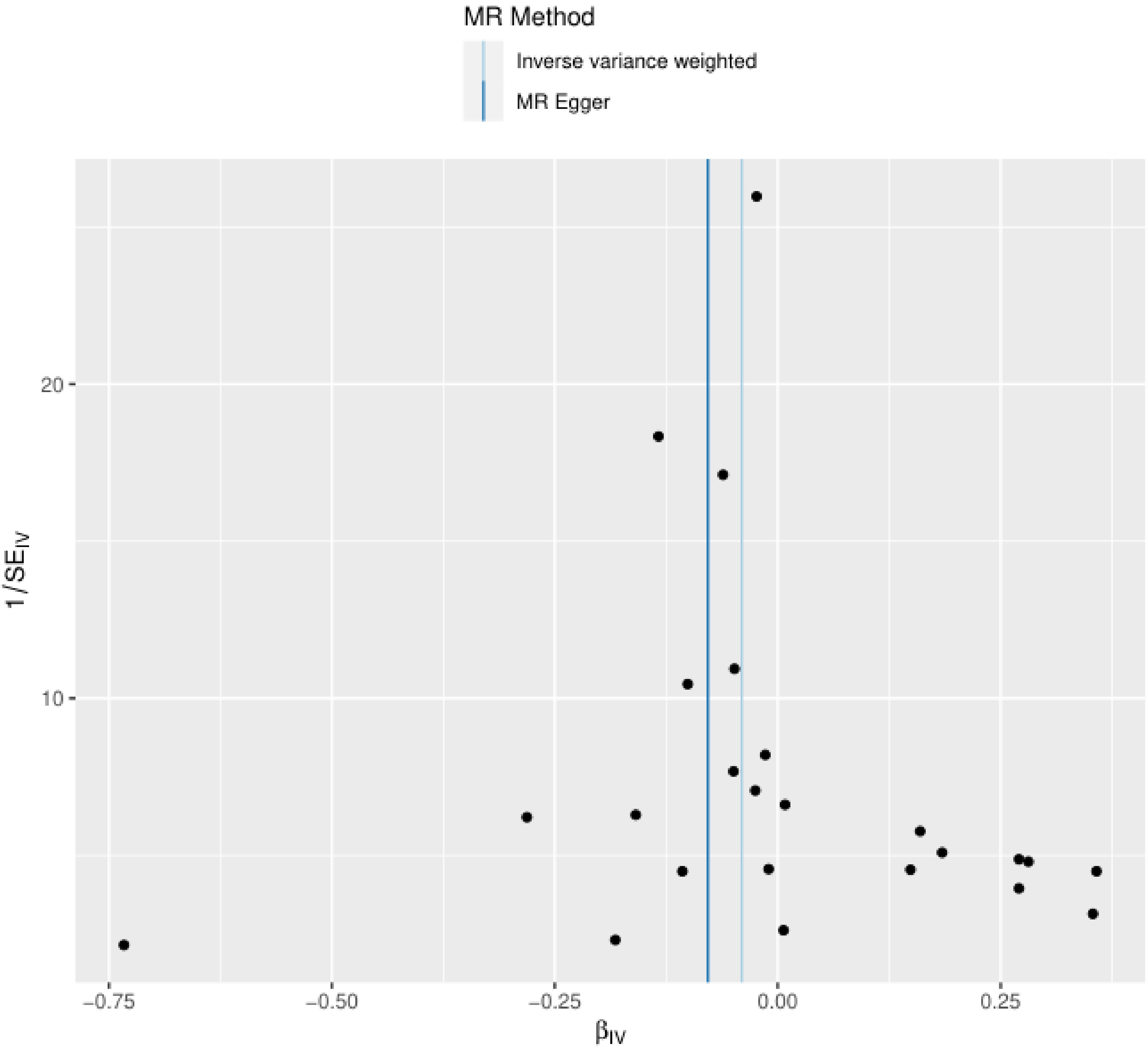

Funnel plot analyse of "CD28 on activated & secreting Treg" on 'Diabetic nephropathy'

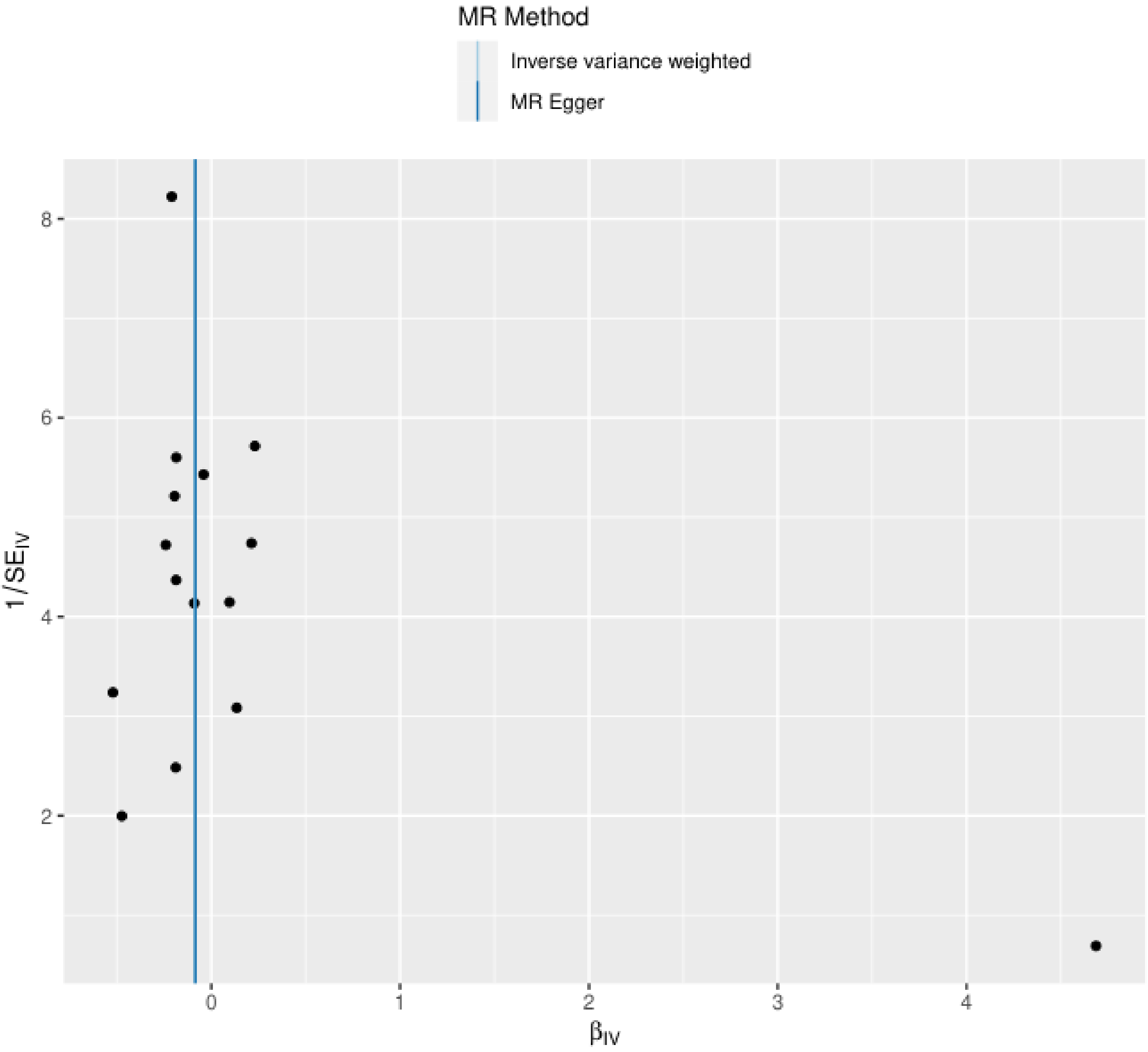

Funnel plot analyse of "IgD+ CD24+ %lymphocyte" on 'Diabetic nephropathy'

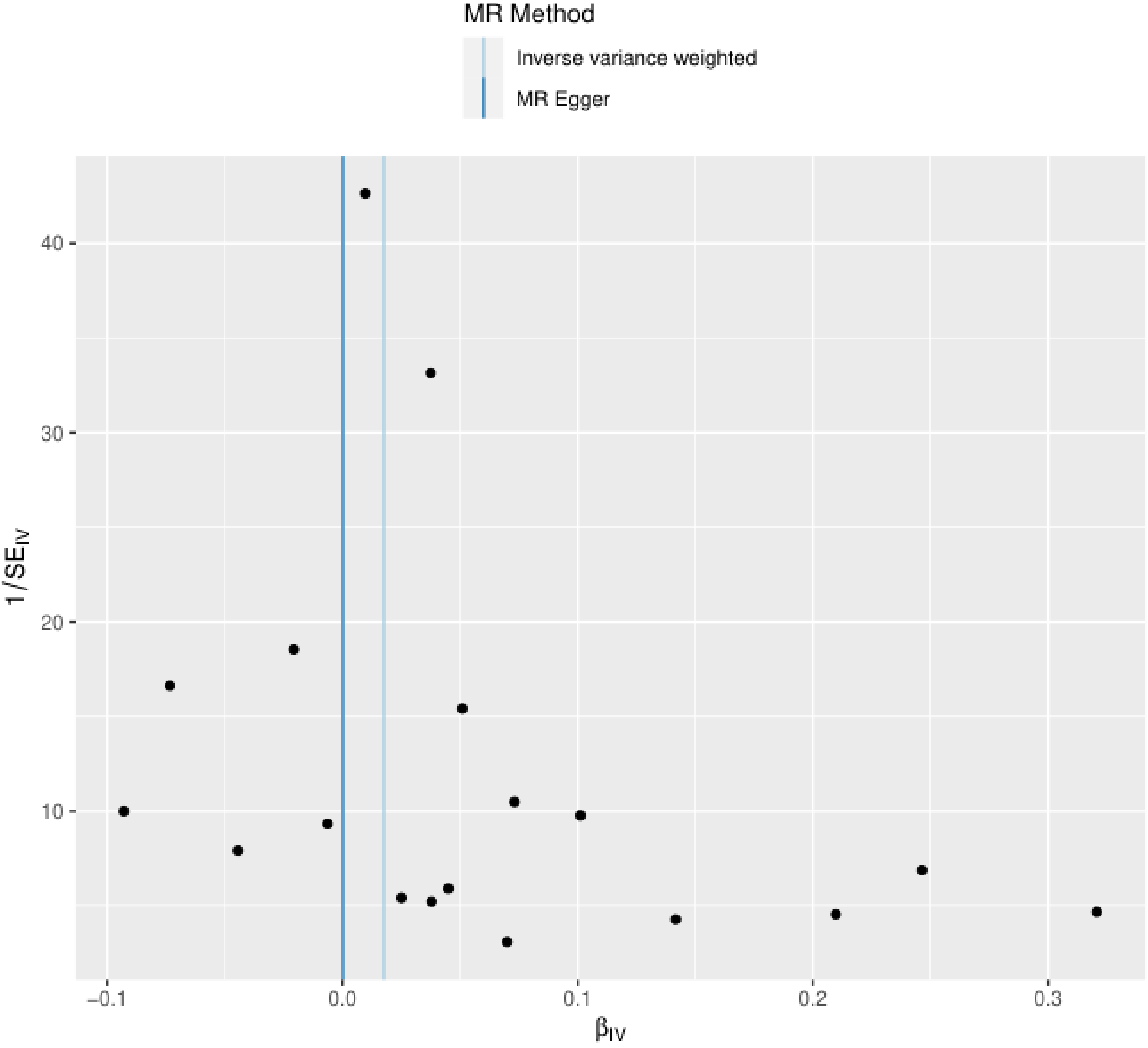

Funnel plot analyse of "CD123 on plasmacytoid DC" on 'Diabetic nephropathy'

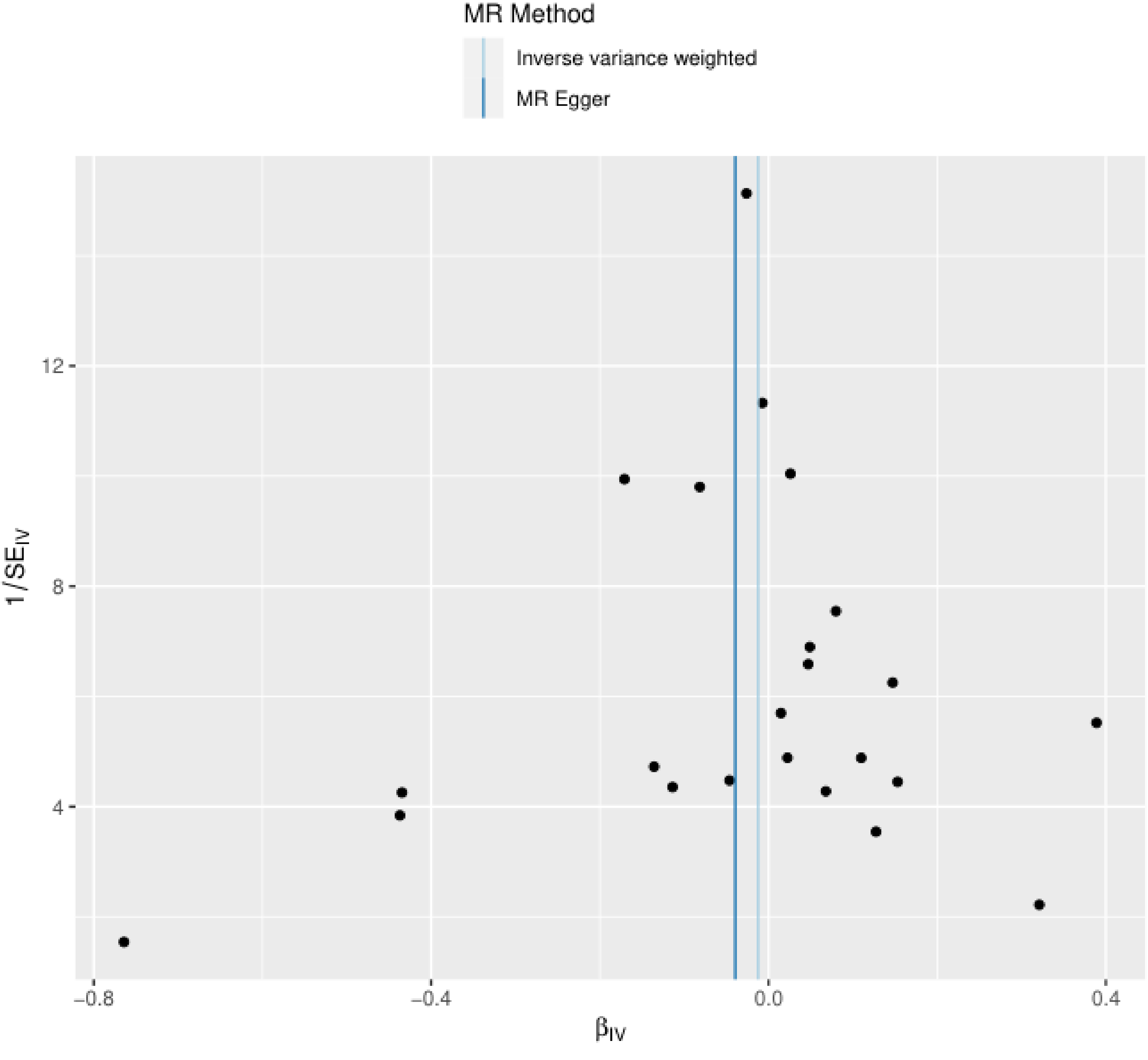

Funnel plot analyse of "CD38 on IgD+ CD38dim" on 'Diabetic nephropathy'

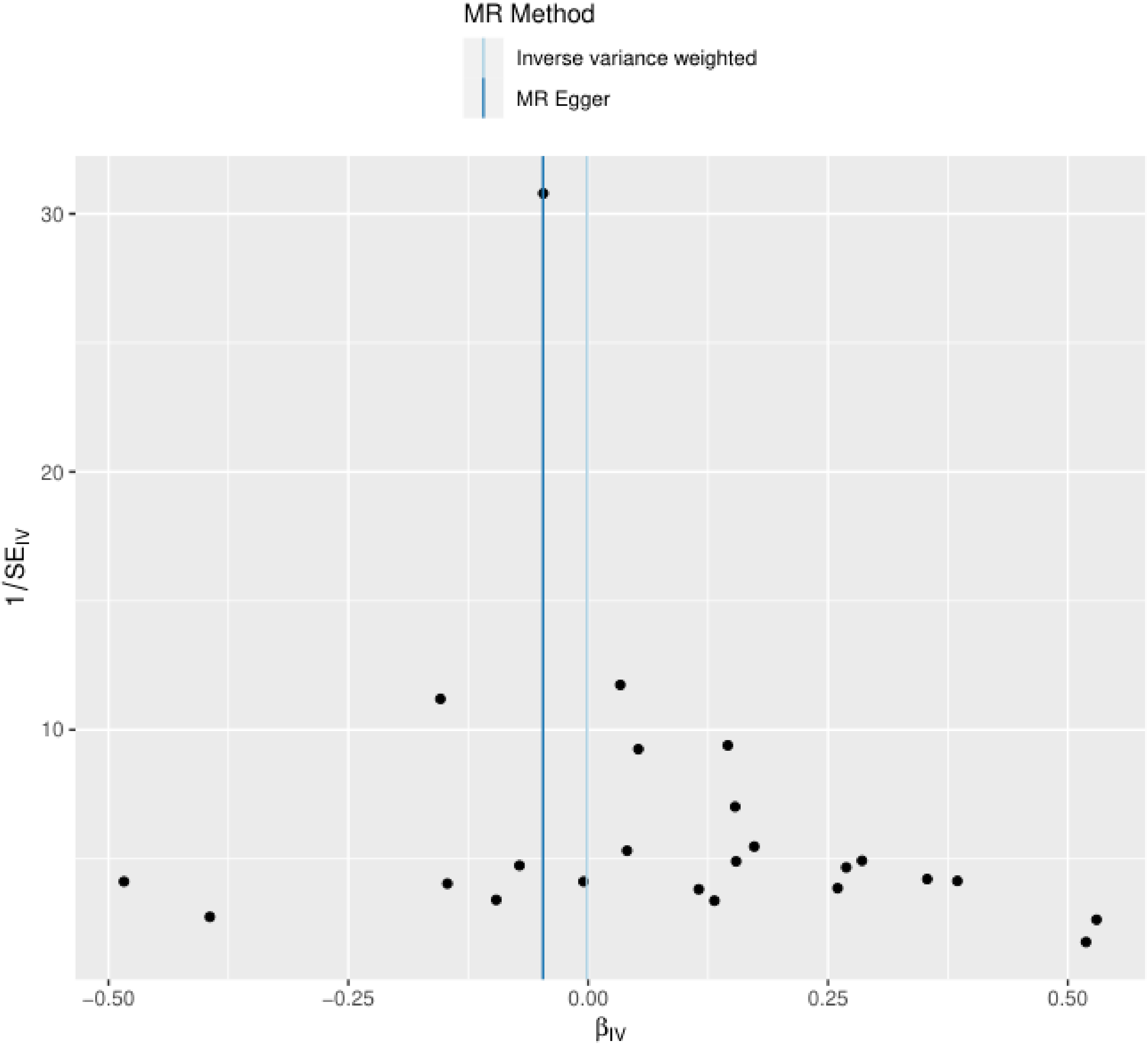

Funnel plot analyse of "CD25 on B cell" on 'Diabetic nephropathy'

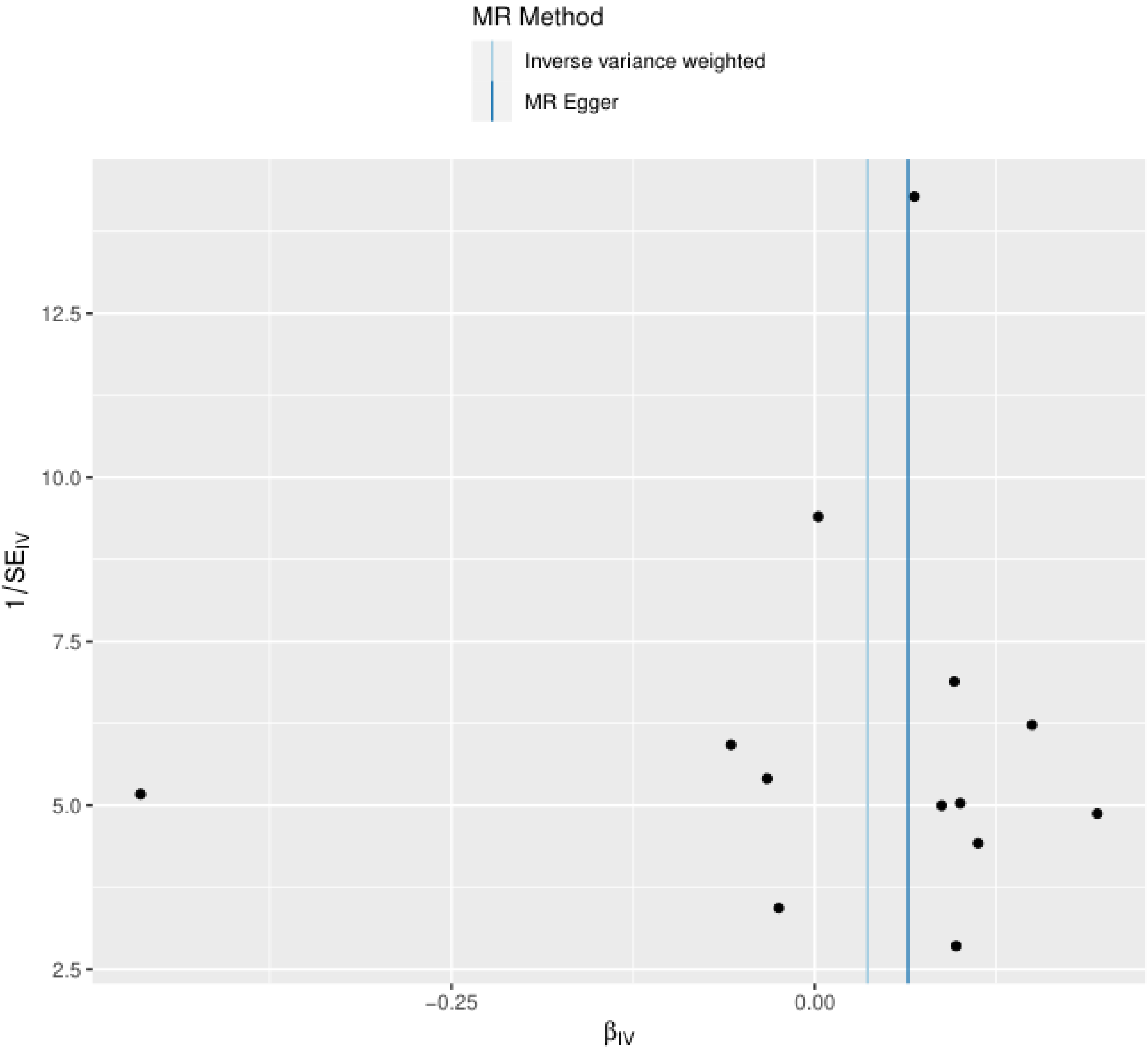

Funnel plot analyse of "SSC-A on B cell" on 'Diabetic nephropathy'

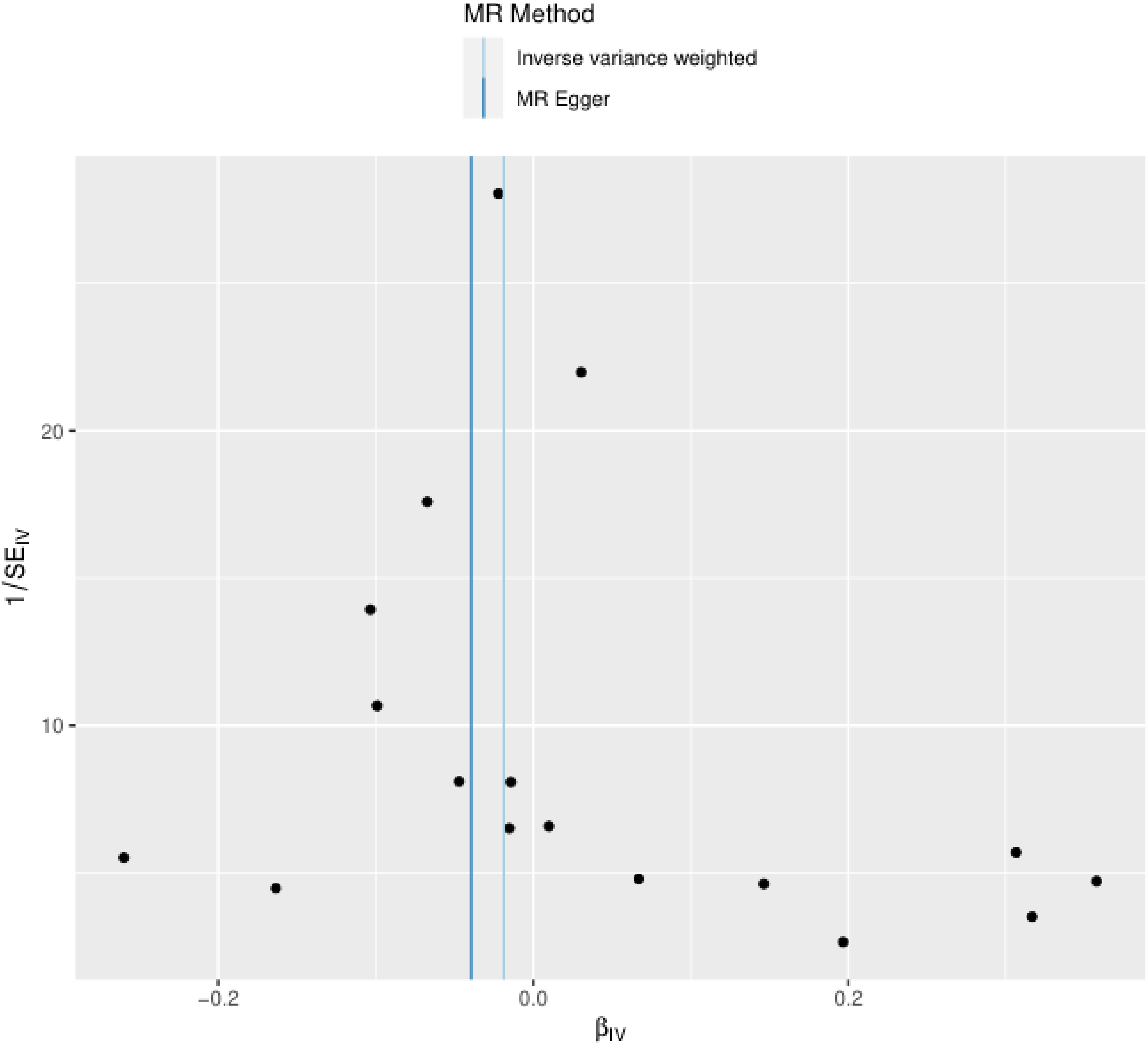

Funnel plot analyse of "CD28 on secreting Treg " on 'Diabetic nephropathy'

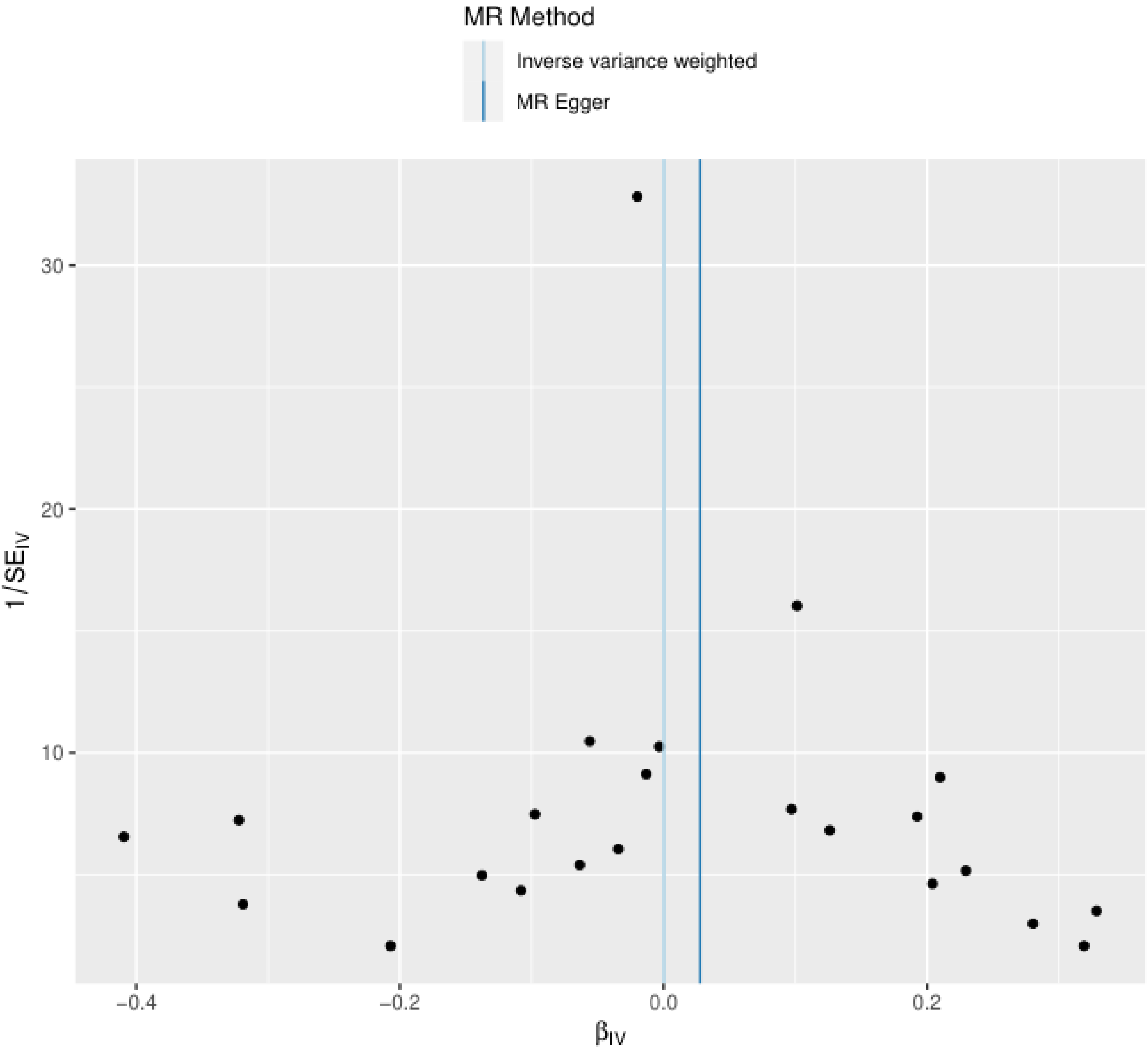

Funnel plot analyse of "BAFF-R on naive-mature B cell" on 'Diabetic nephropathy'

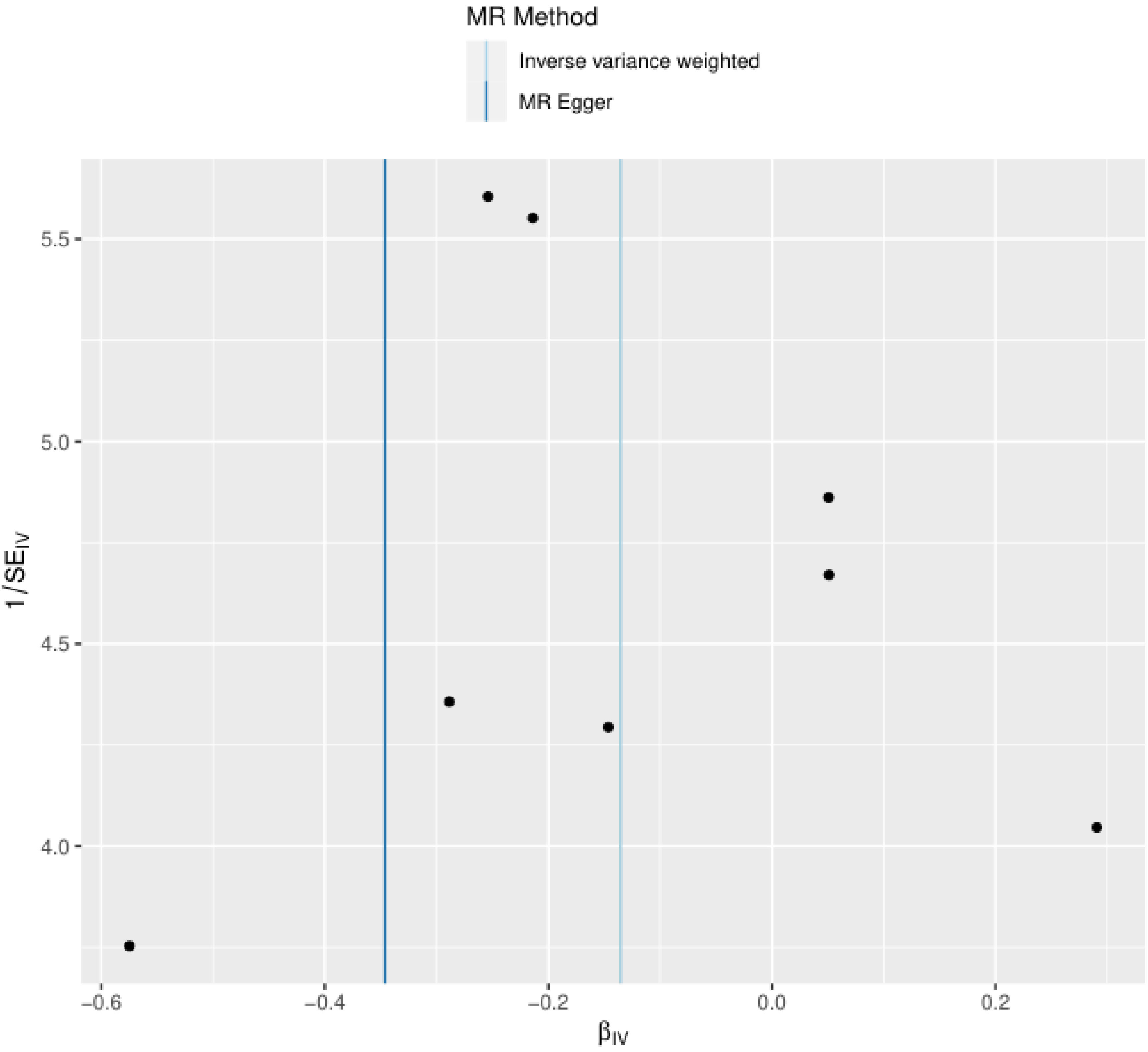

Funnel plot analyse of "CD28- CD25++ CD8br %T cell" on 'Diabetic nephropathy'

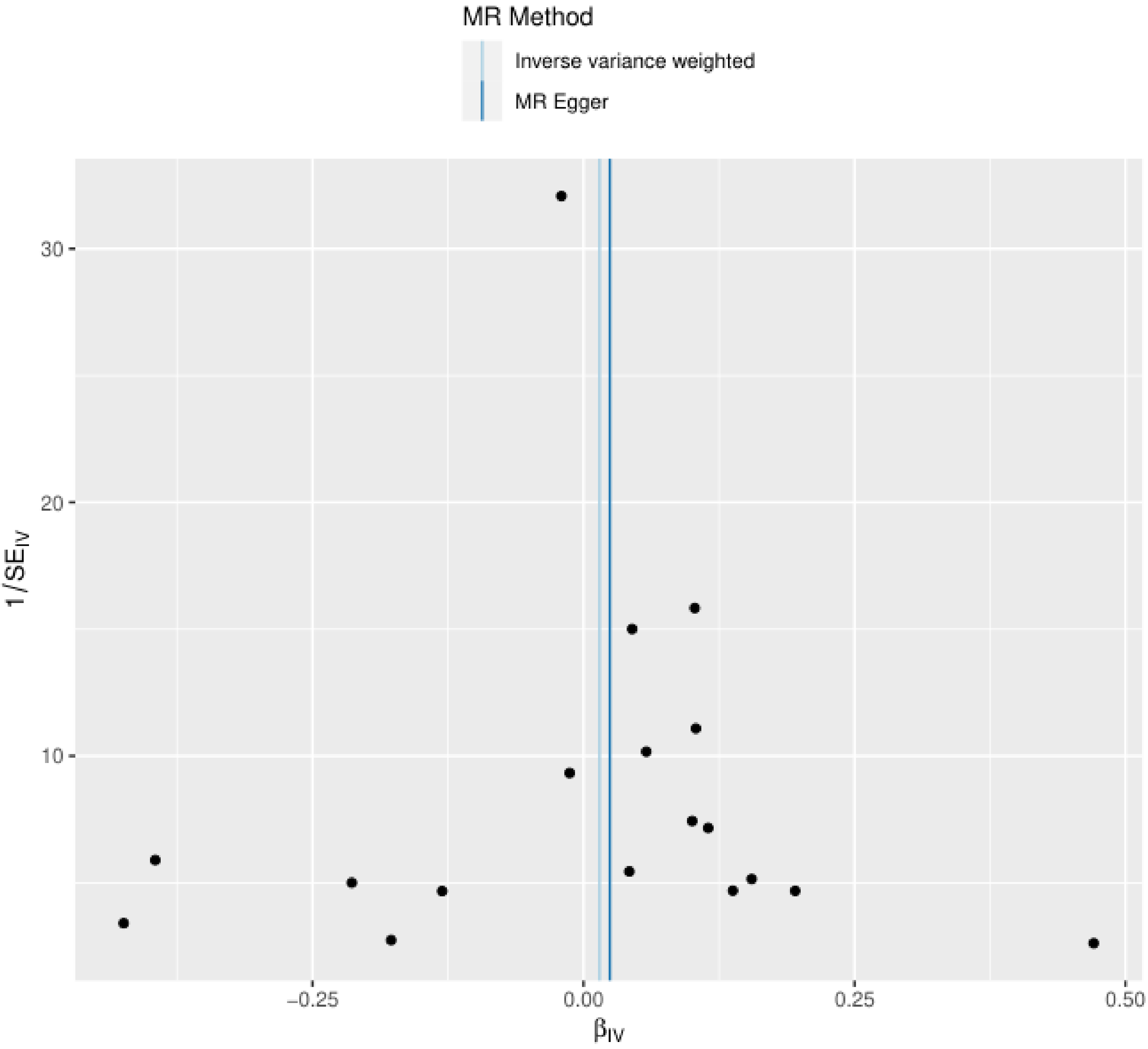

Funnel plot analyse of "BAFF-R on sw mem" on 'Diabetic nephropathy'

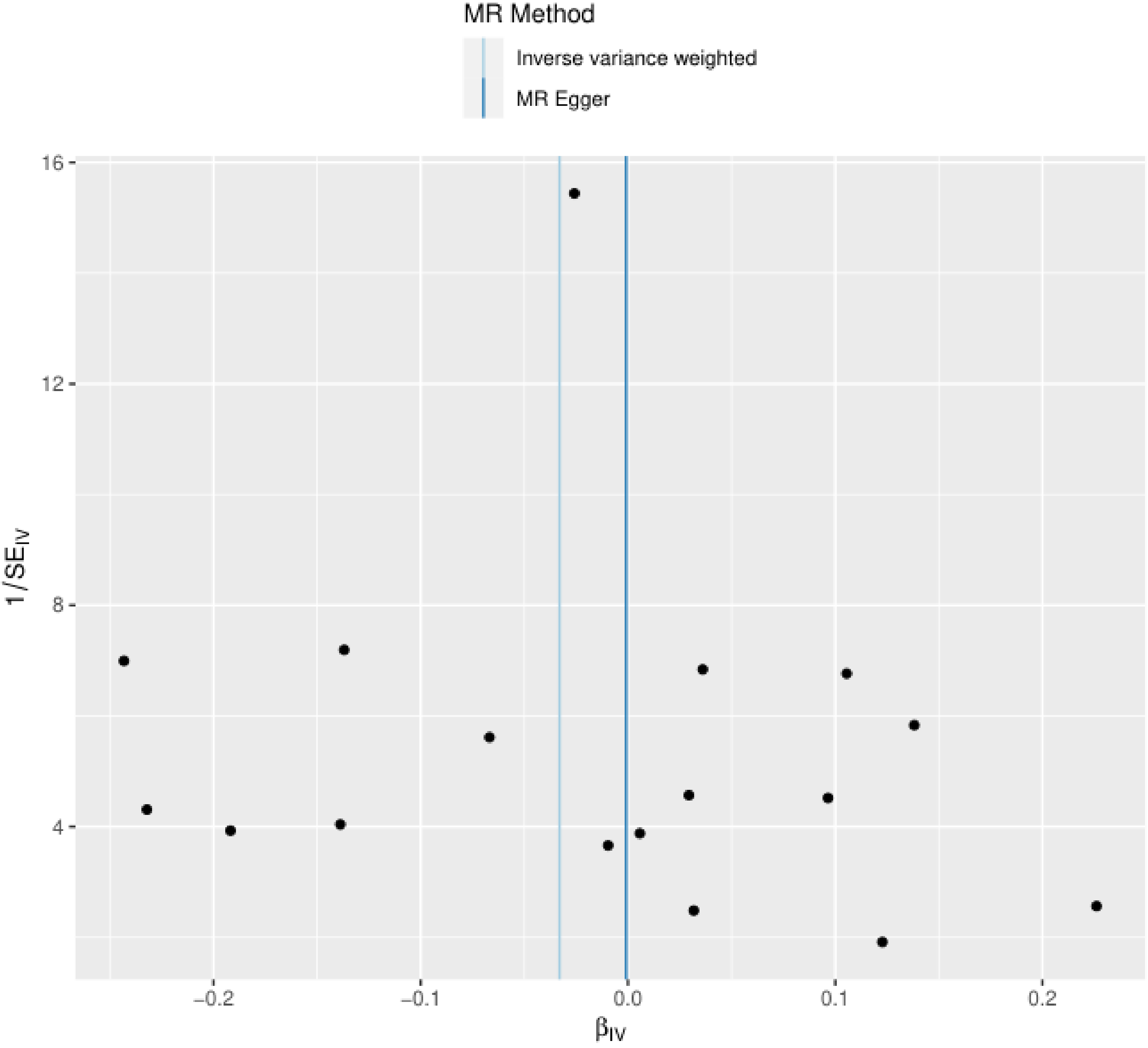

Funnel plot analyse of ''CD25 on CD39+ CD4 Treg '' on 'Diabetic nephropathy'

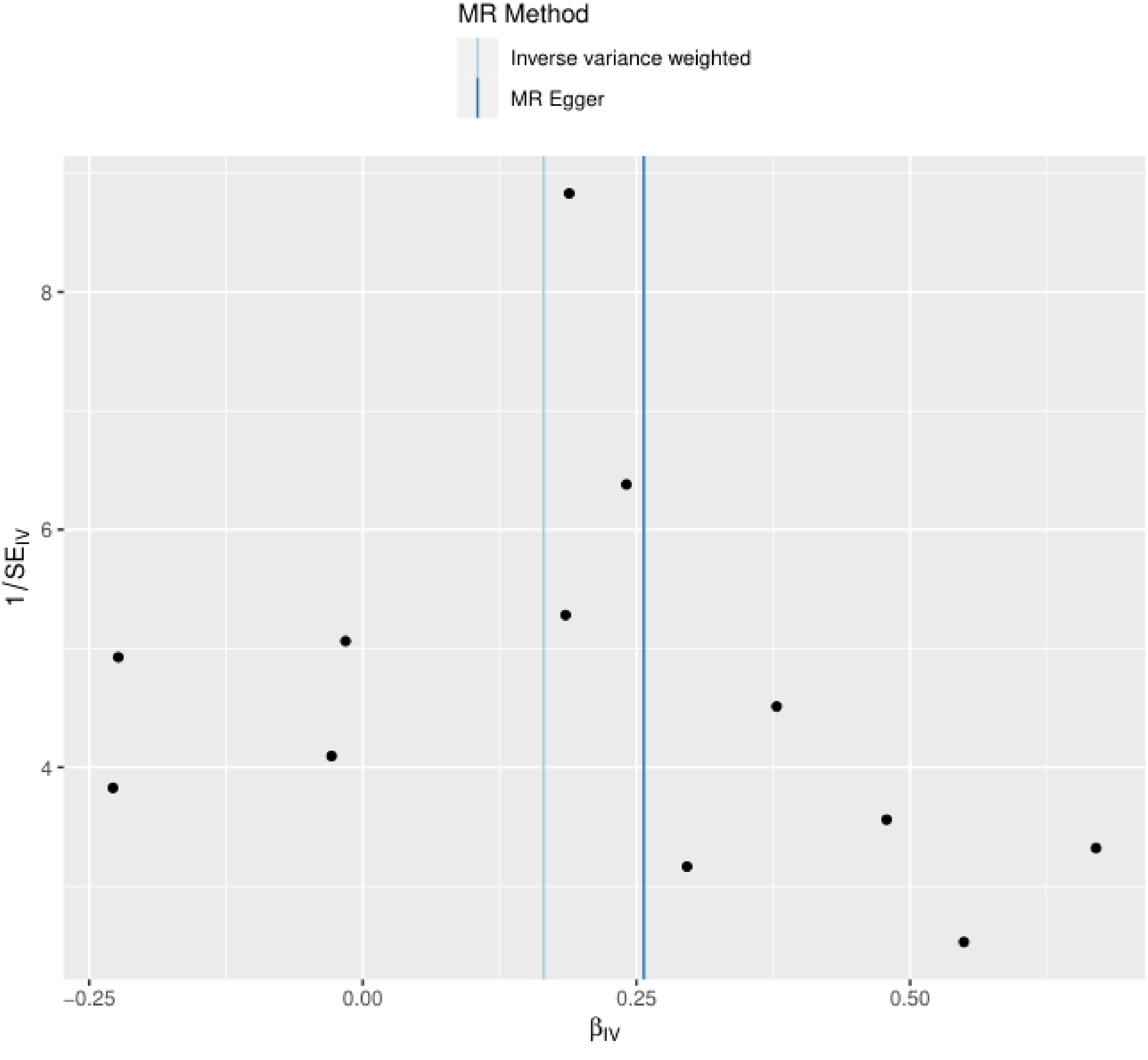

Funnel plot analyse of "IgD- CD38- %B cell" on 'Diabetic nephropathy'

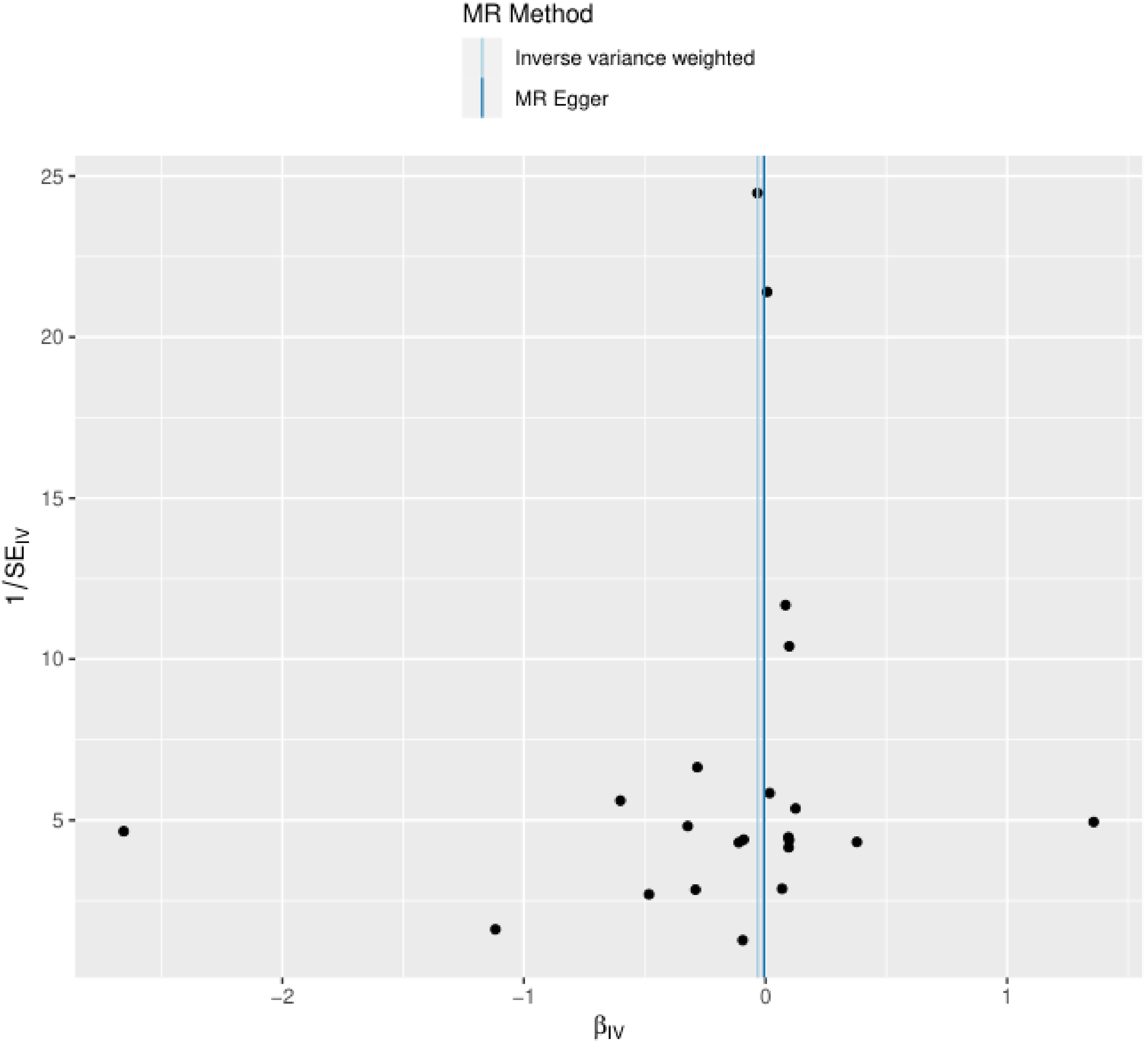

Funnel plot analyse of "IgD on unsw mem" on 'Diabetic nephropathy'

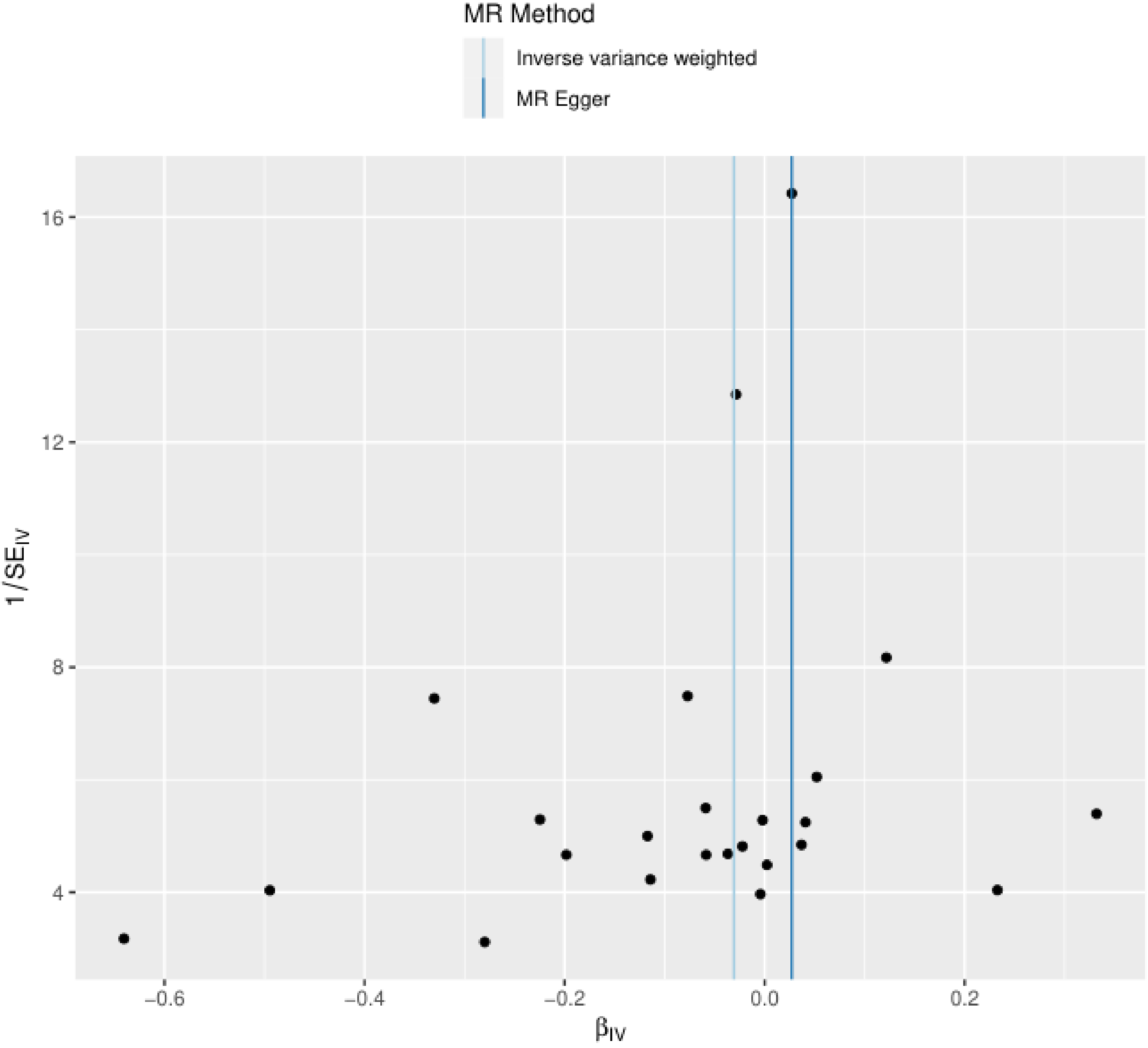

Funnel plot analyse of "HLA DR+ NK %NK" on 'Diabetic nephropathy'

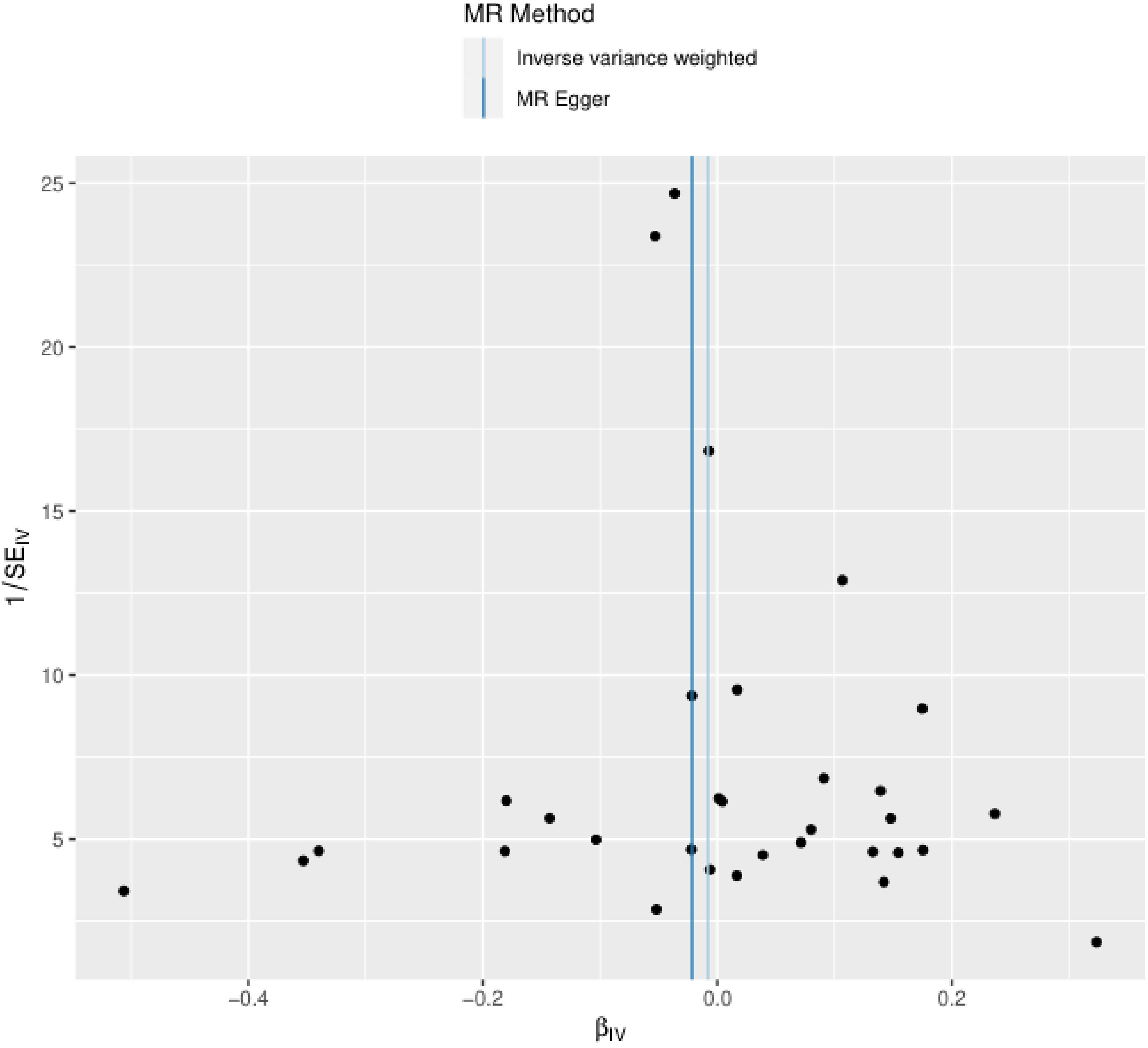

Funnel plot analyse of "CD45RA on naive CD4+ " on 'Diabetic nephropathy'

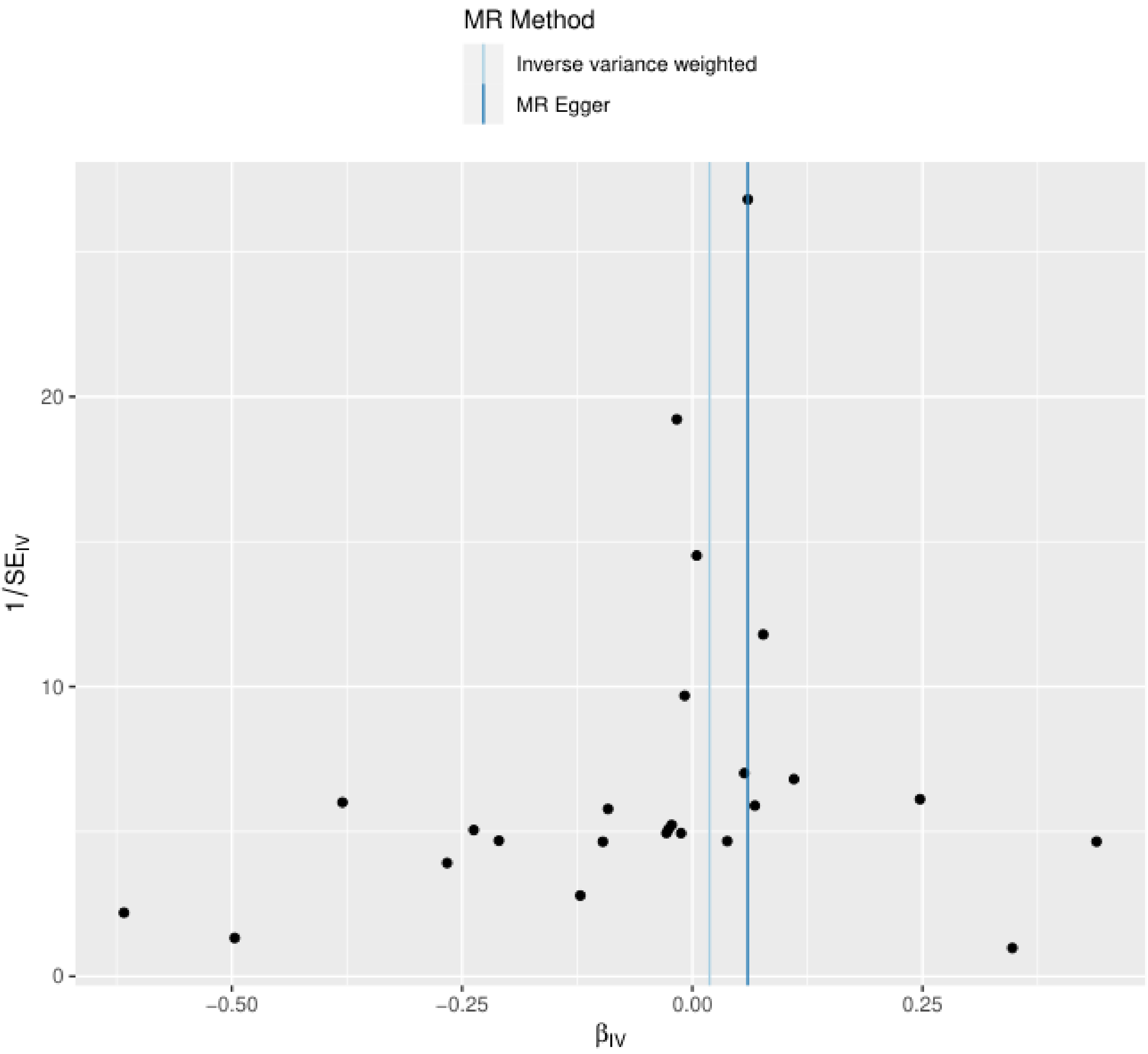

Funnel plot analyse of "CD3 on CM CD4+" on 'Diabetic nephropathy'

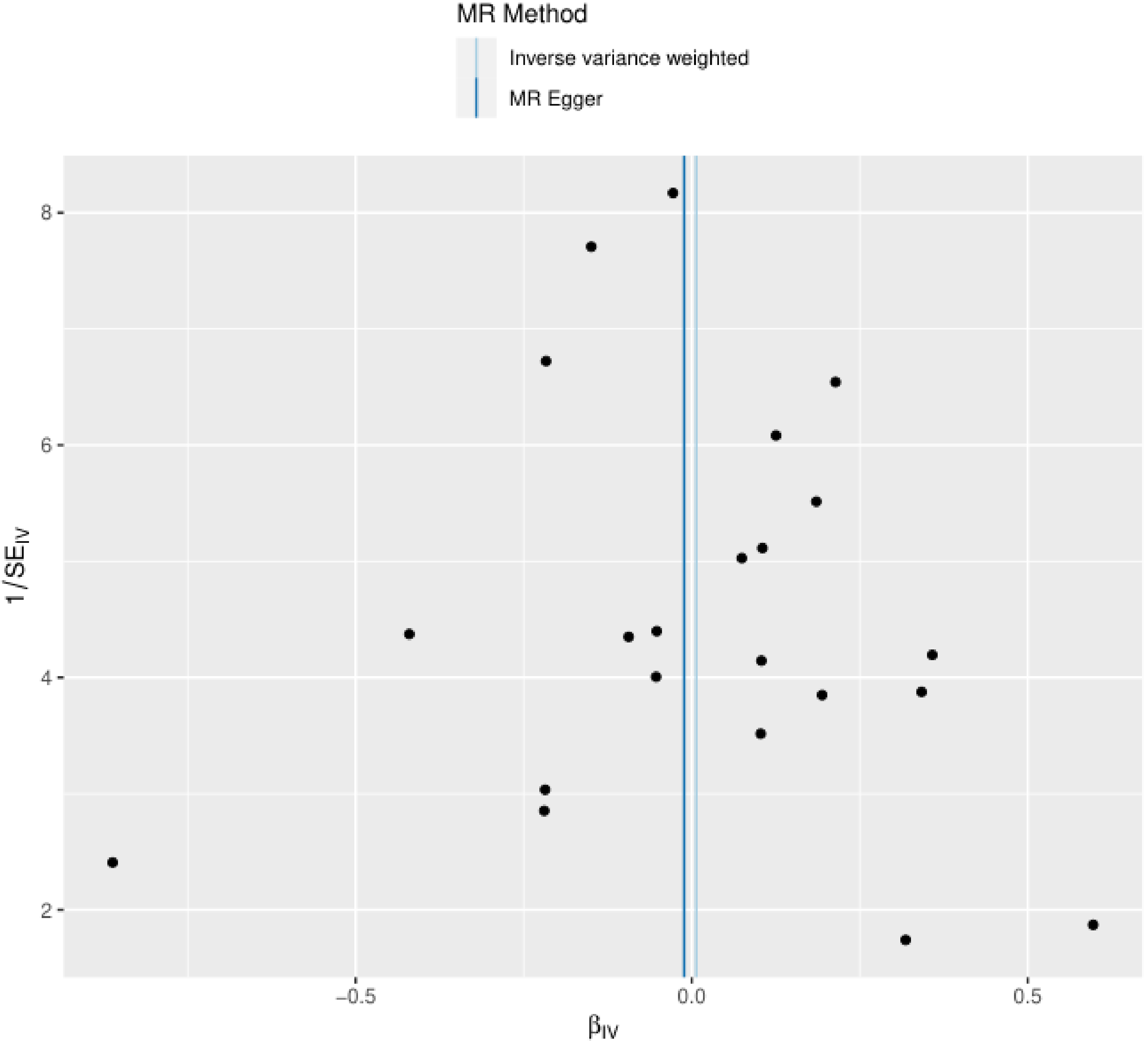

Funnel plot analyse of "CM CD8br %T cell" on 'Diabetic nephropathy'

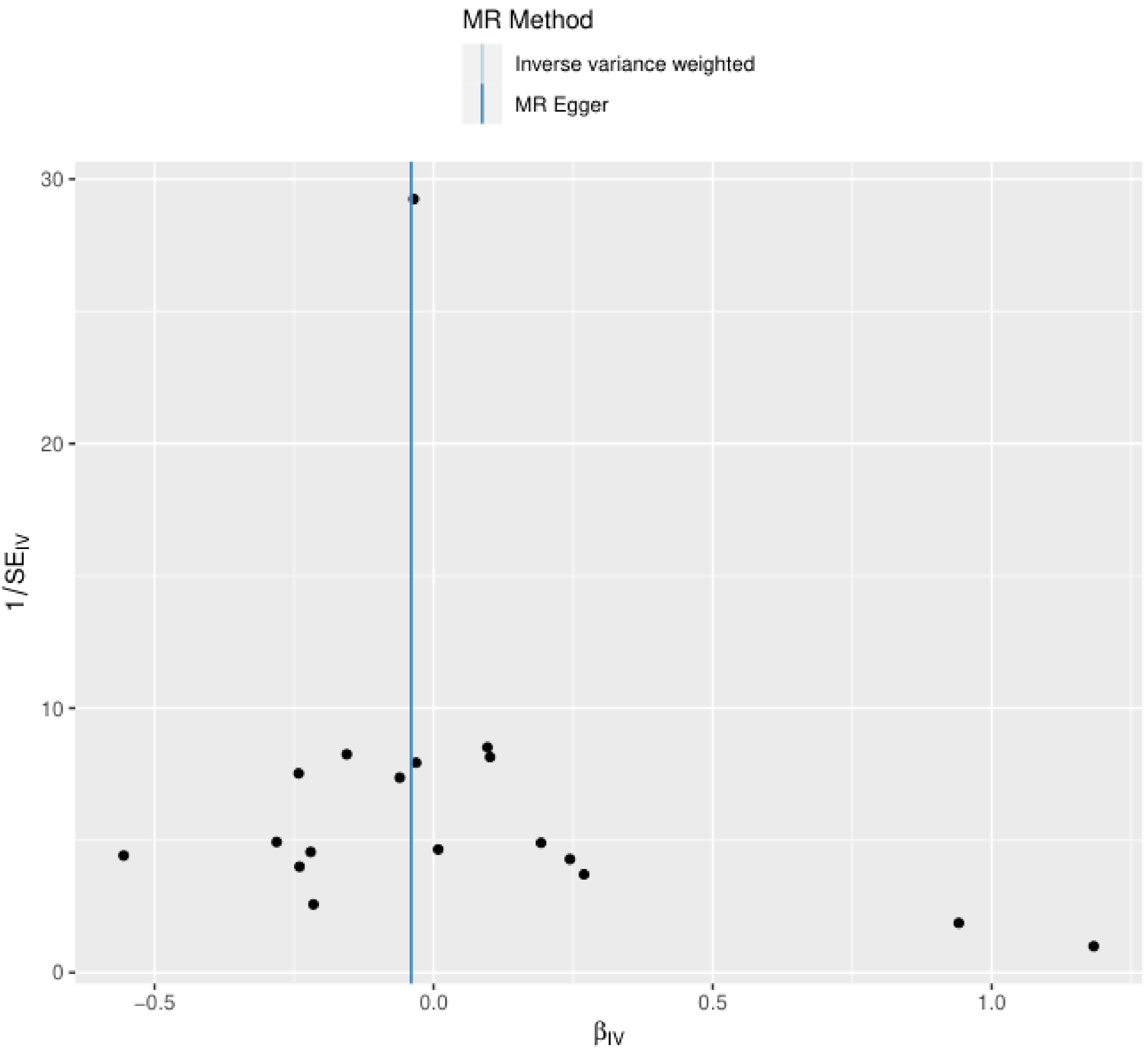

Funnel plot analyse of "CD14- CD16+ monocyte AC" on 'Diabetic nephropathy'

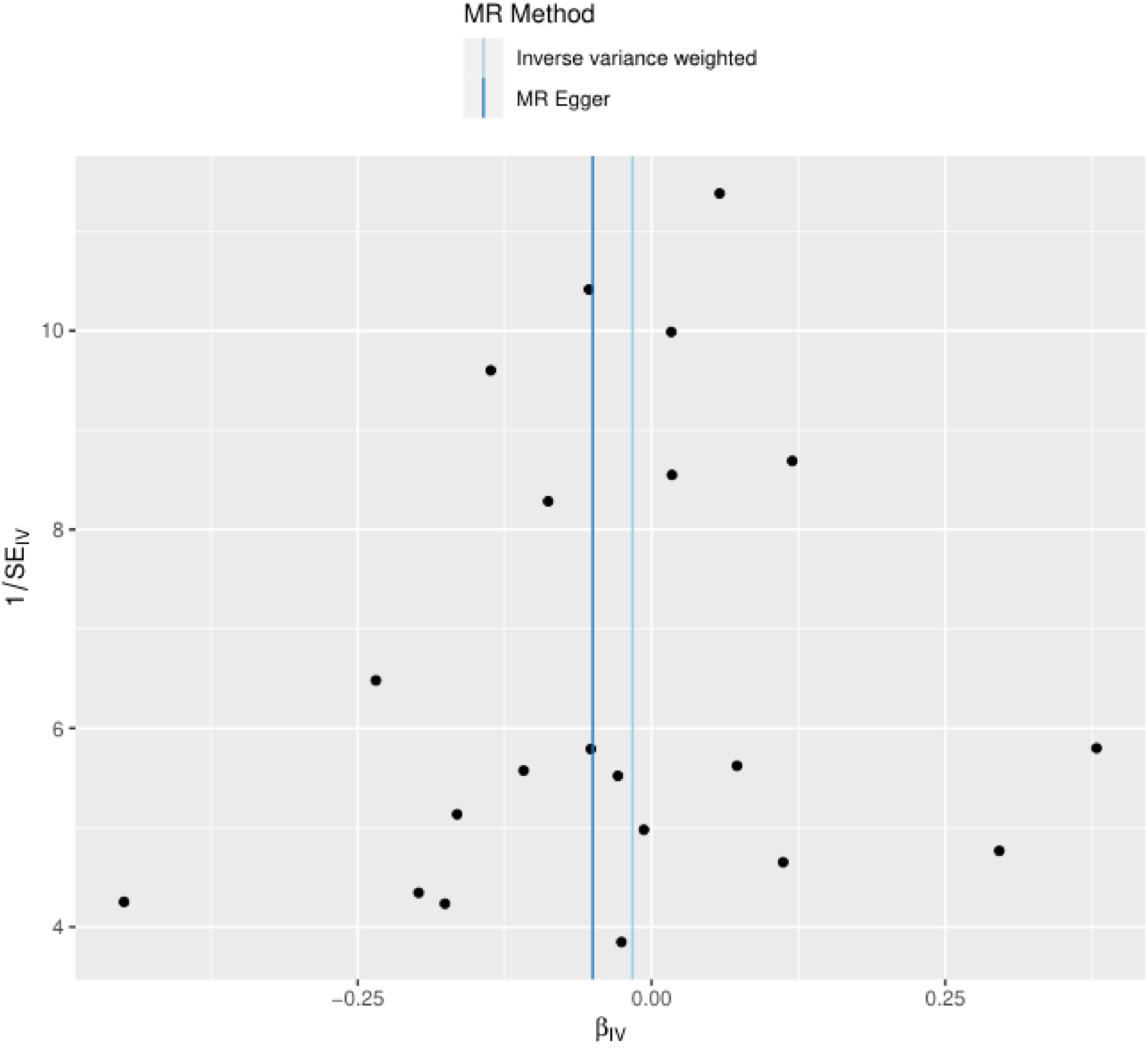

Funnel plot analyse of "Gr MDSC AC" on 'Diabetic nephropathy'

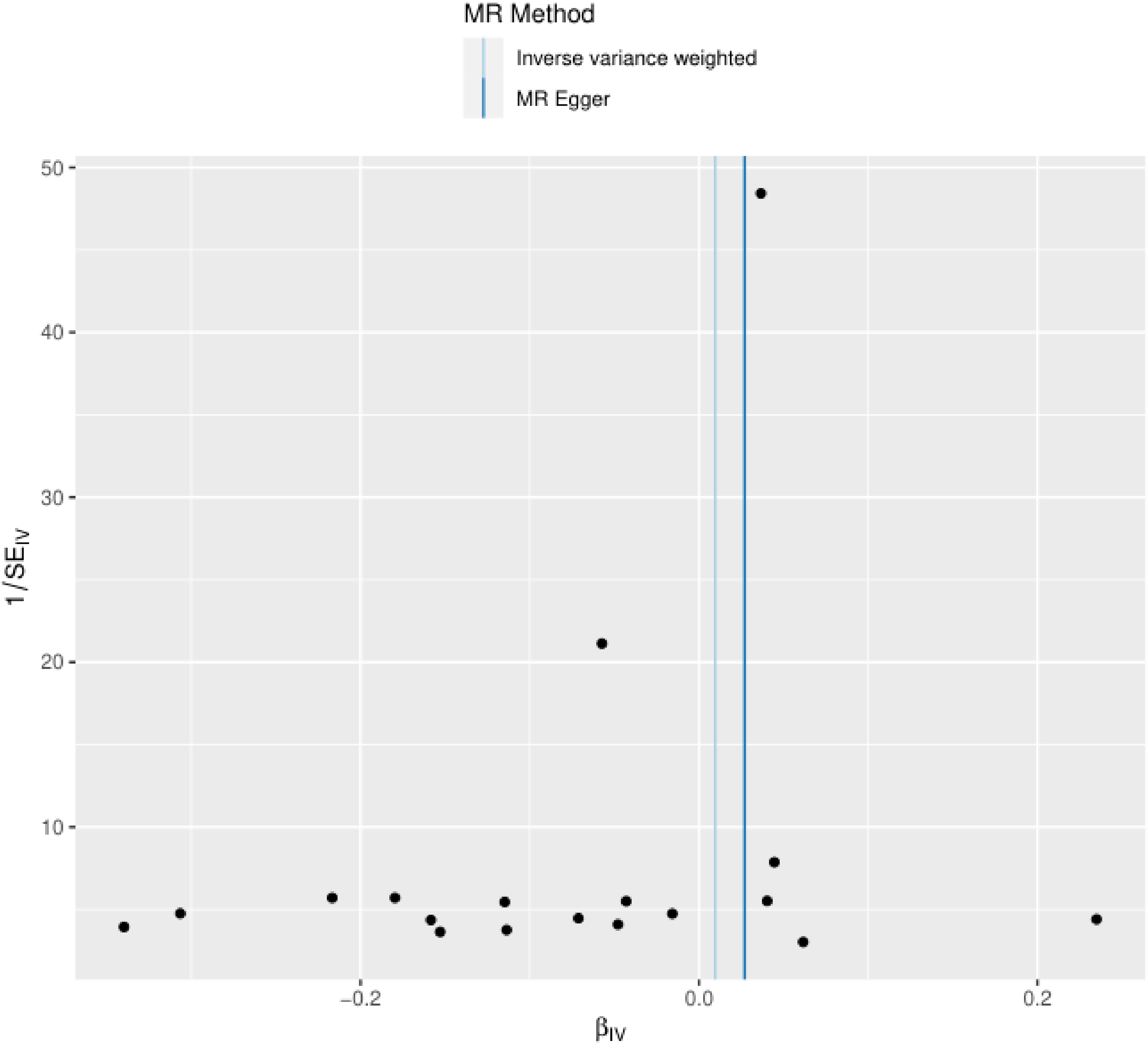

Funnel plot analyse of "FSC-A on HLA DR+ T cell" on 'Diabetic nephropathy'

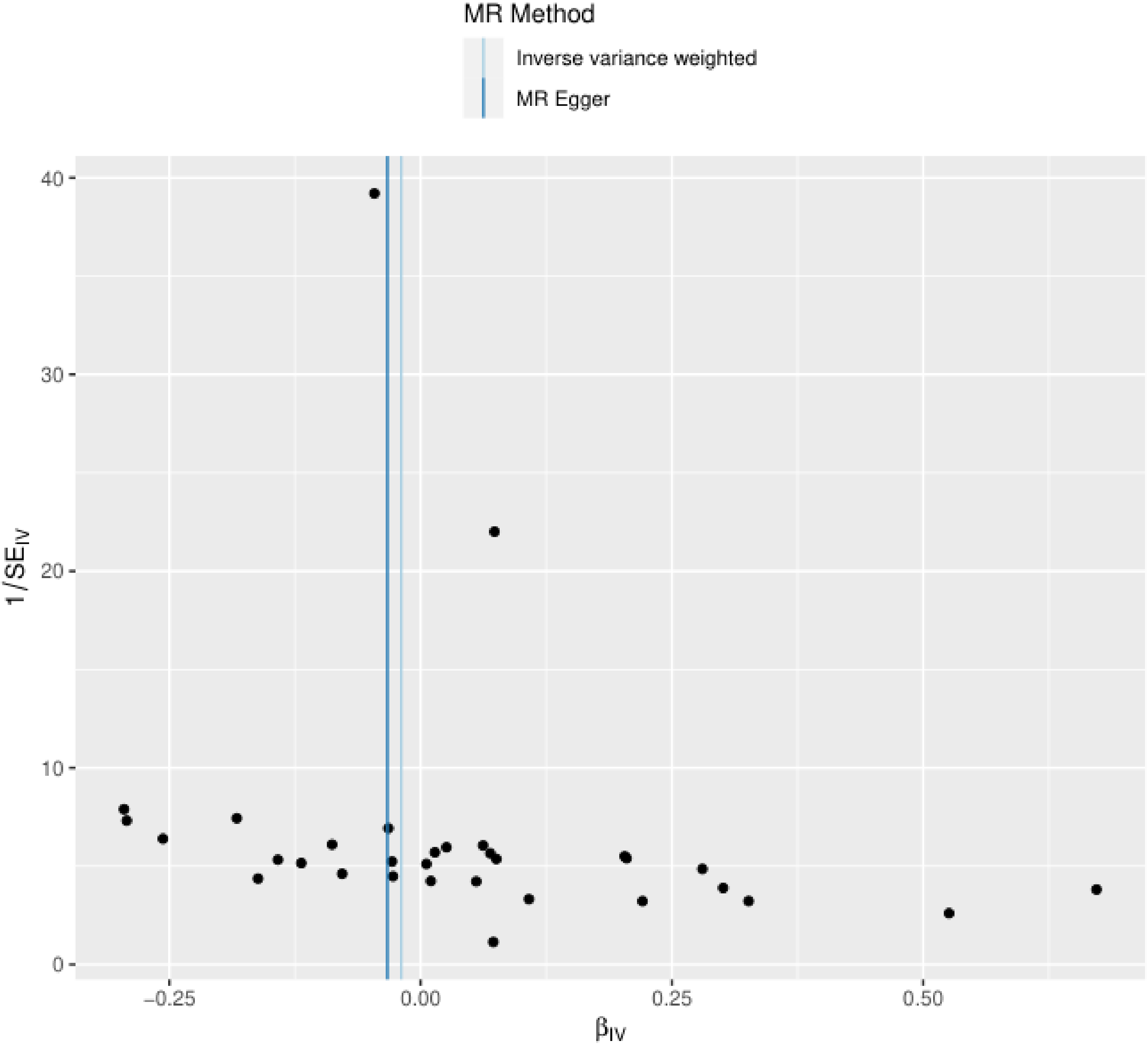

Funnel plot analyse of "CD27 on IgD- CD38dim" on 'Diabetic nephropathy'

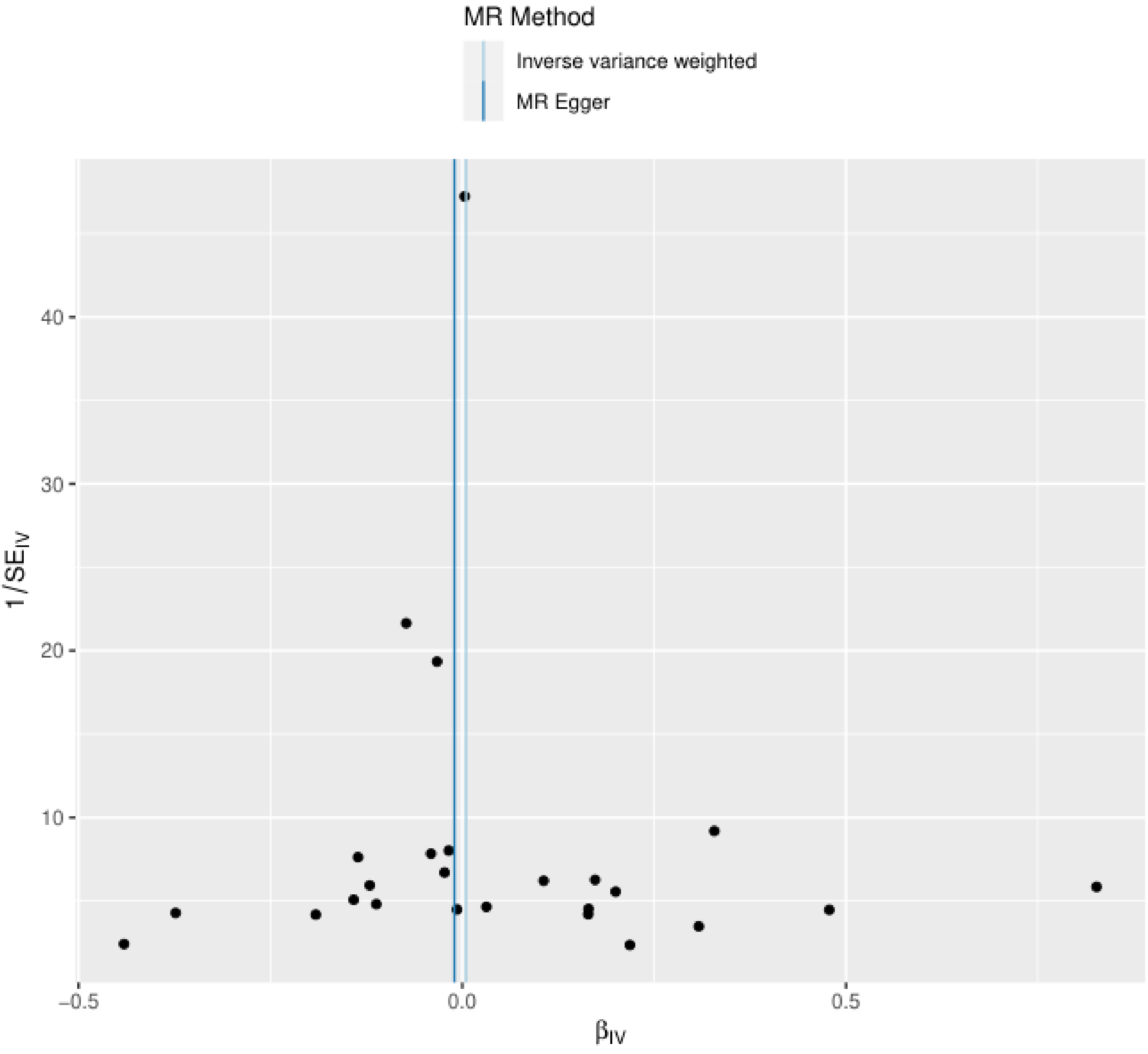

Funnel plot analyse of "CD20 on naive-mature B cell" on 'Diabetic nephropathy'

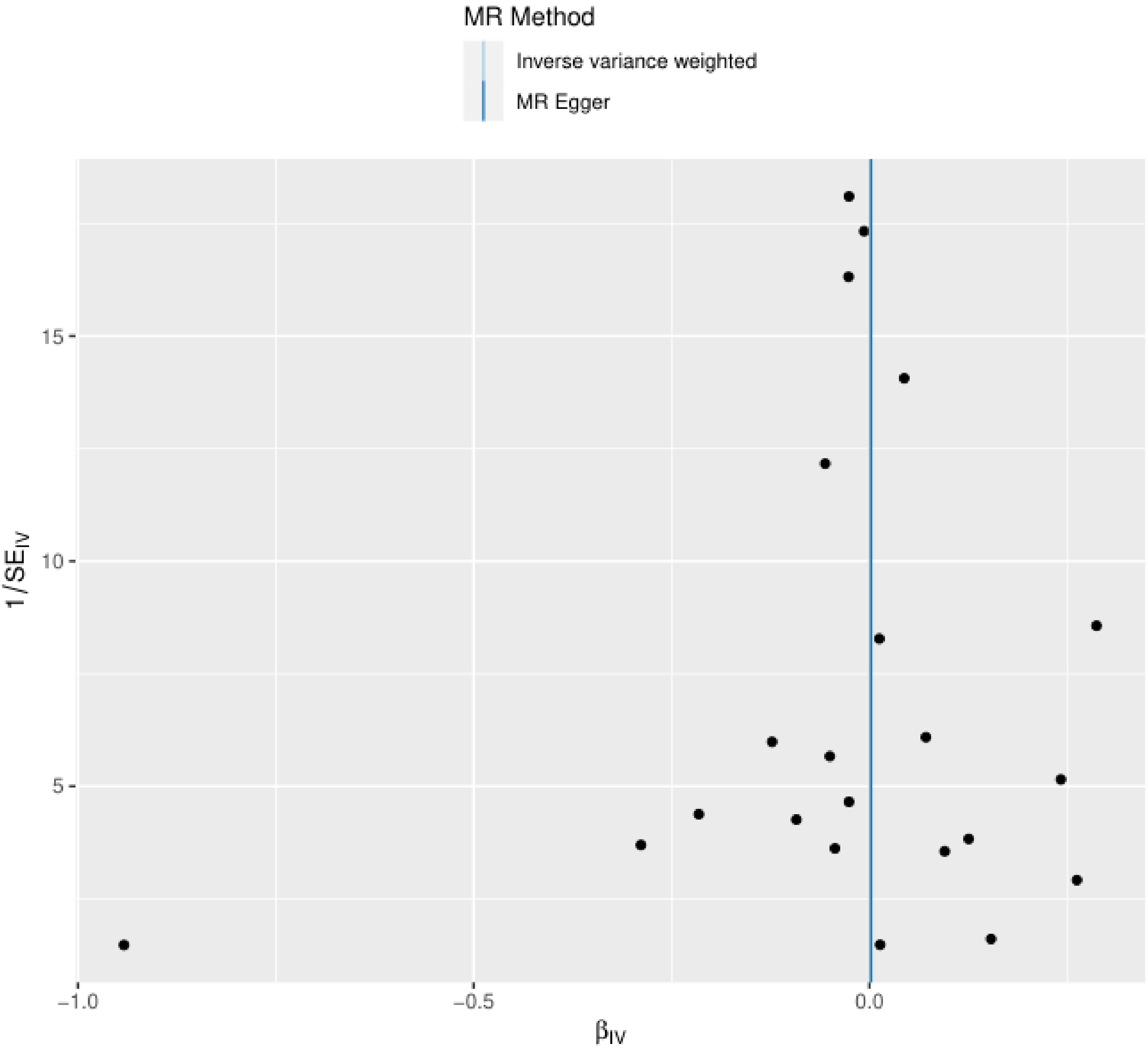

Funnel plot analyse of "CD62L- DC %DC" on 'Diabetic nephropathy'

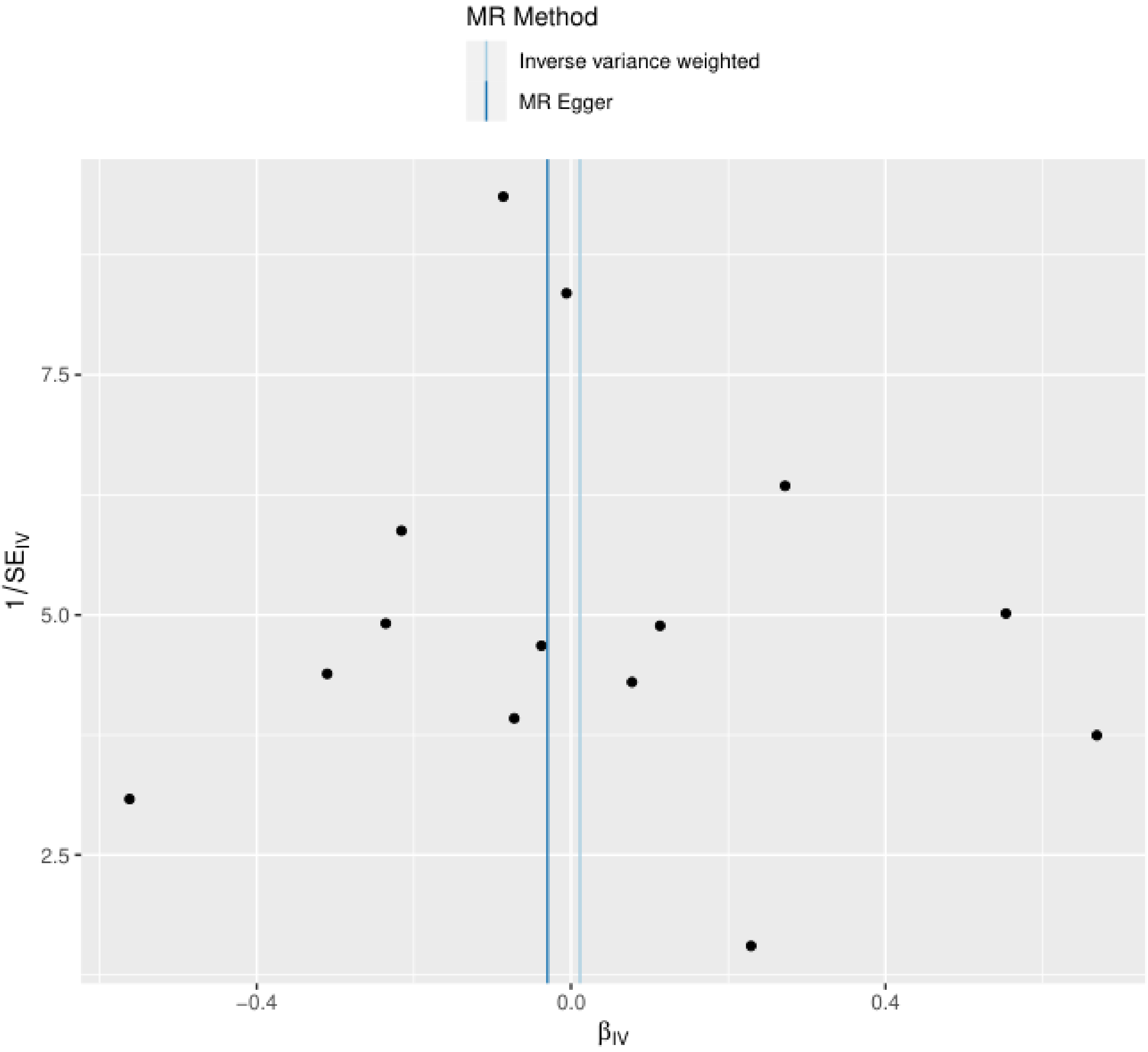

Funnel plot analyse of "CD45RA on TD CD8br" on 'Diabetic nephropathy'

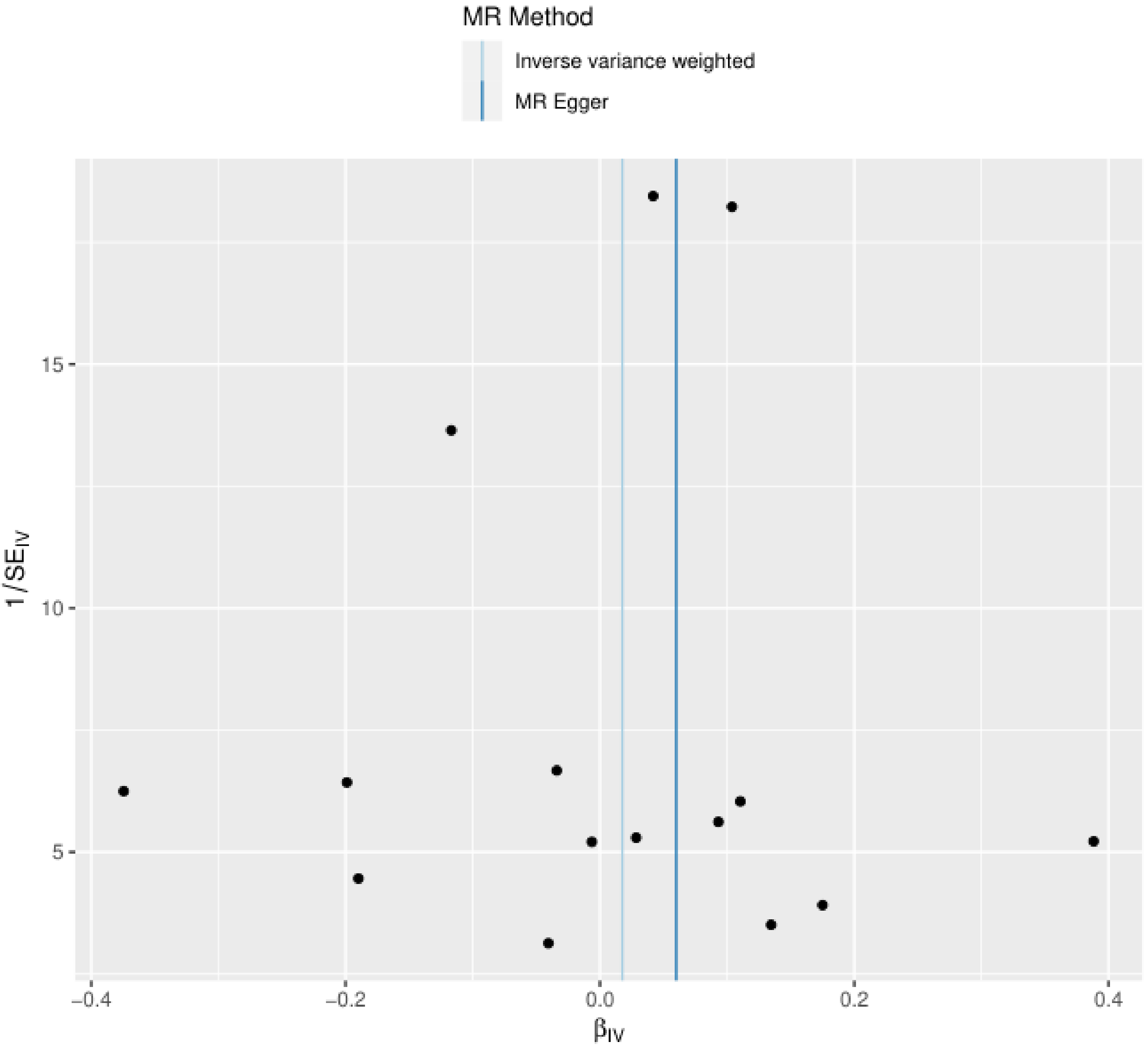

Funnel plot analyse of "CD45 on CD66b++ myelod cell " on 'Diabetic nephropathy'

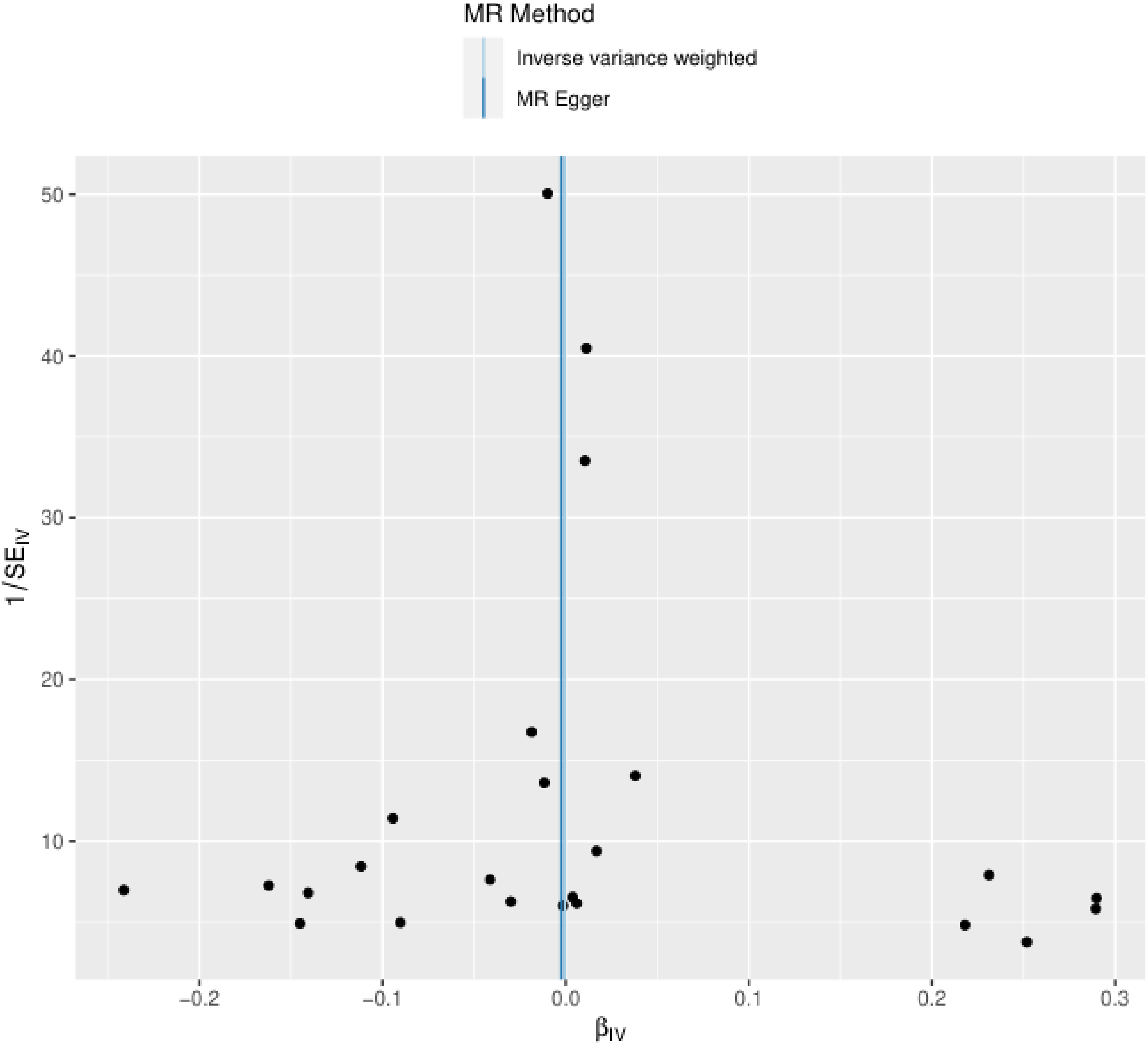

Funnel plot analyse of "CD45 on CD33- HLA DR+ " on 'Diabetic nephropathy'

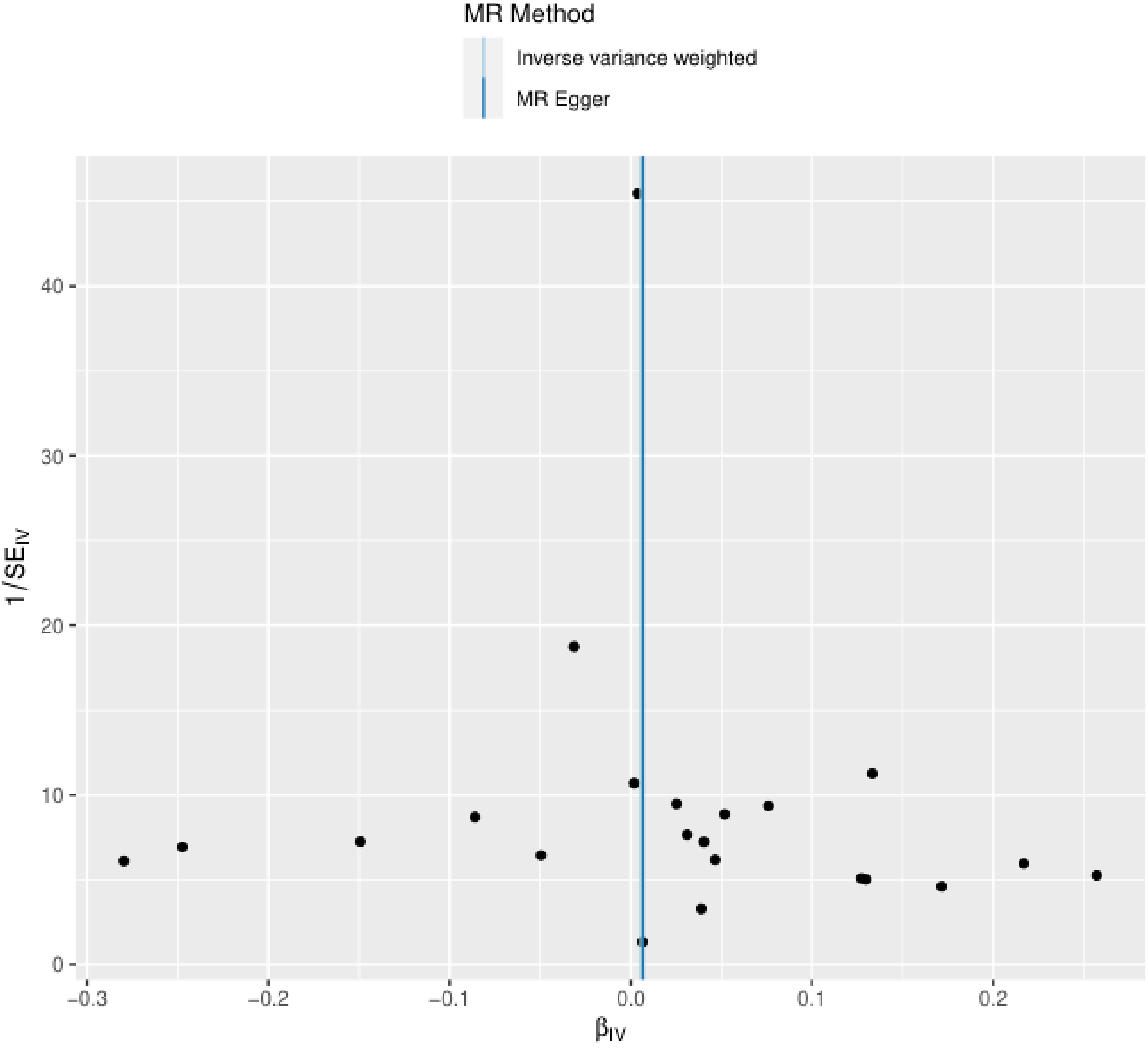

Funnel plot analyse of "CD33 on CD33br HLA DR+ CD14- " on 'Diabetic nephropathy'

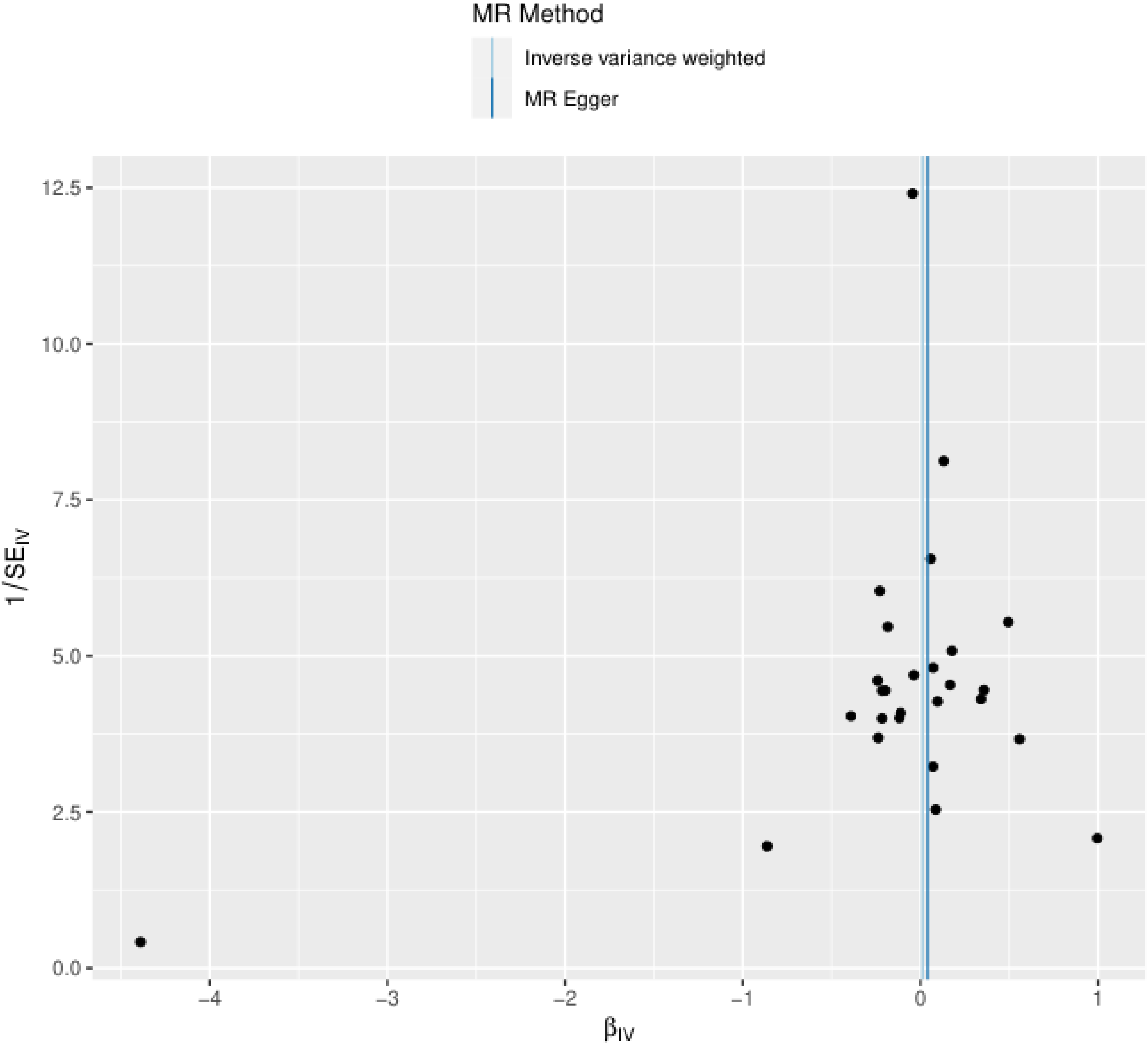

Funnel plot analyse of "IgD- CD38- %lymphocyte" on 'Diabetic nephropathy'

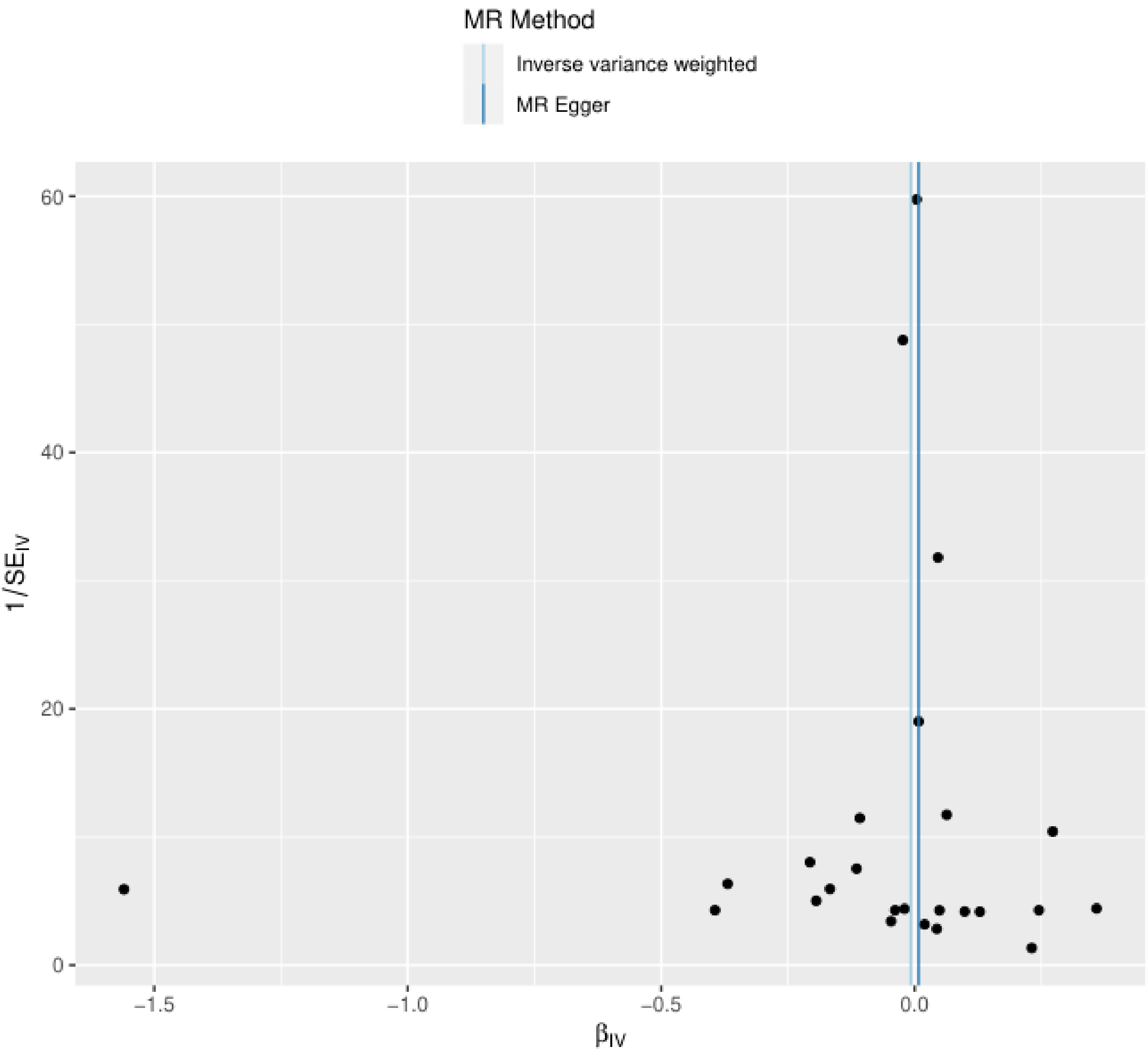

Funnel plot analyse of "CD45RA- CD4+ %T cell" on 'Diabetic nephropathy'

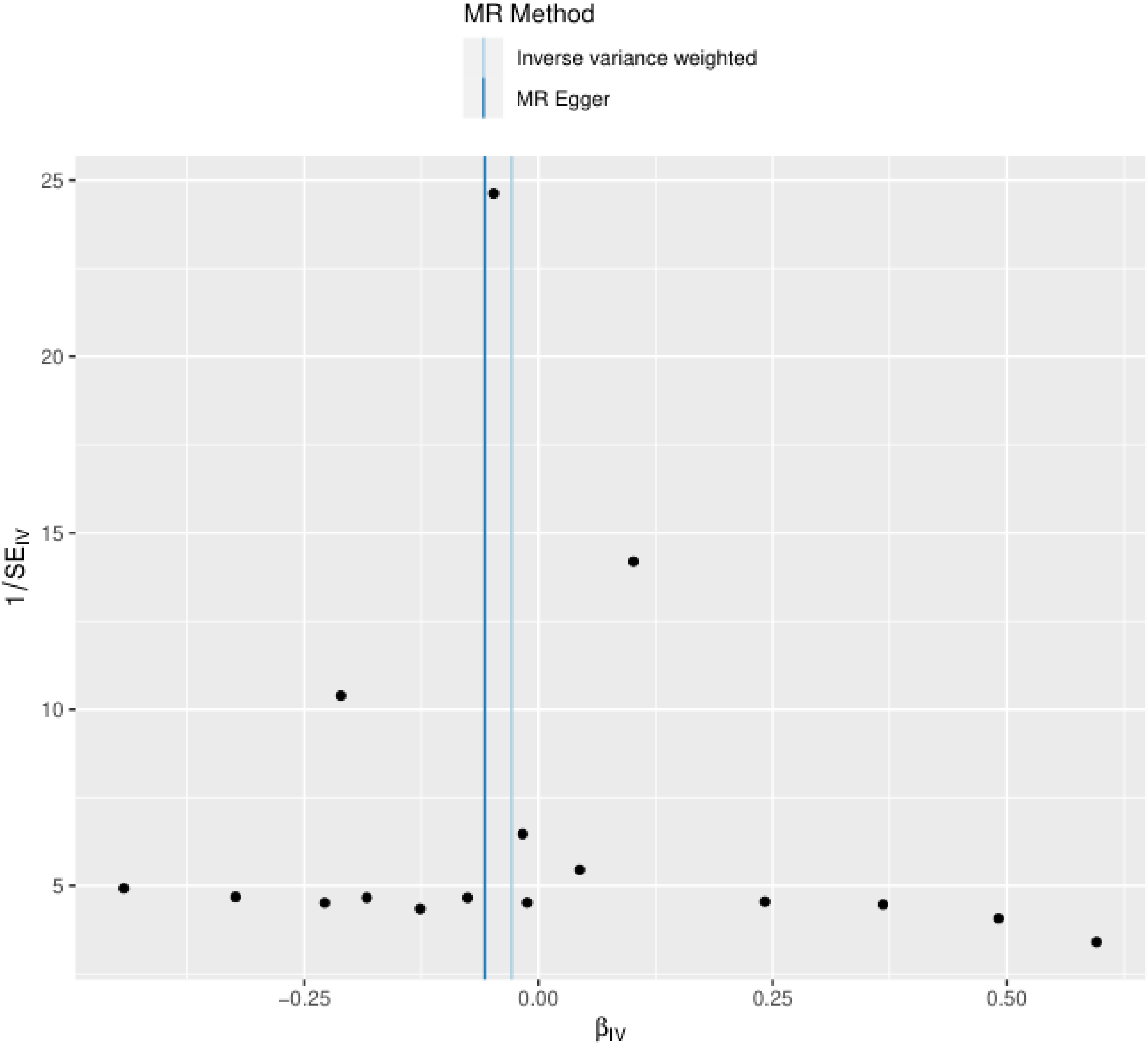

Funnel plot analyse of "CD27 on CD20-" on 'Diabetic nephropathy'

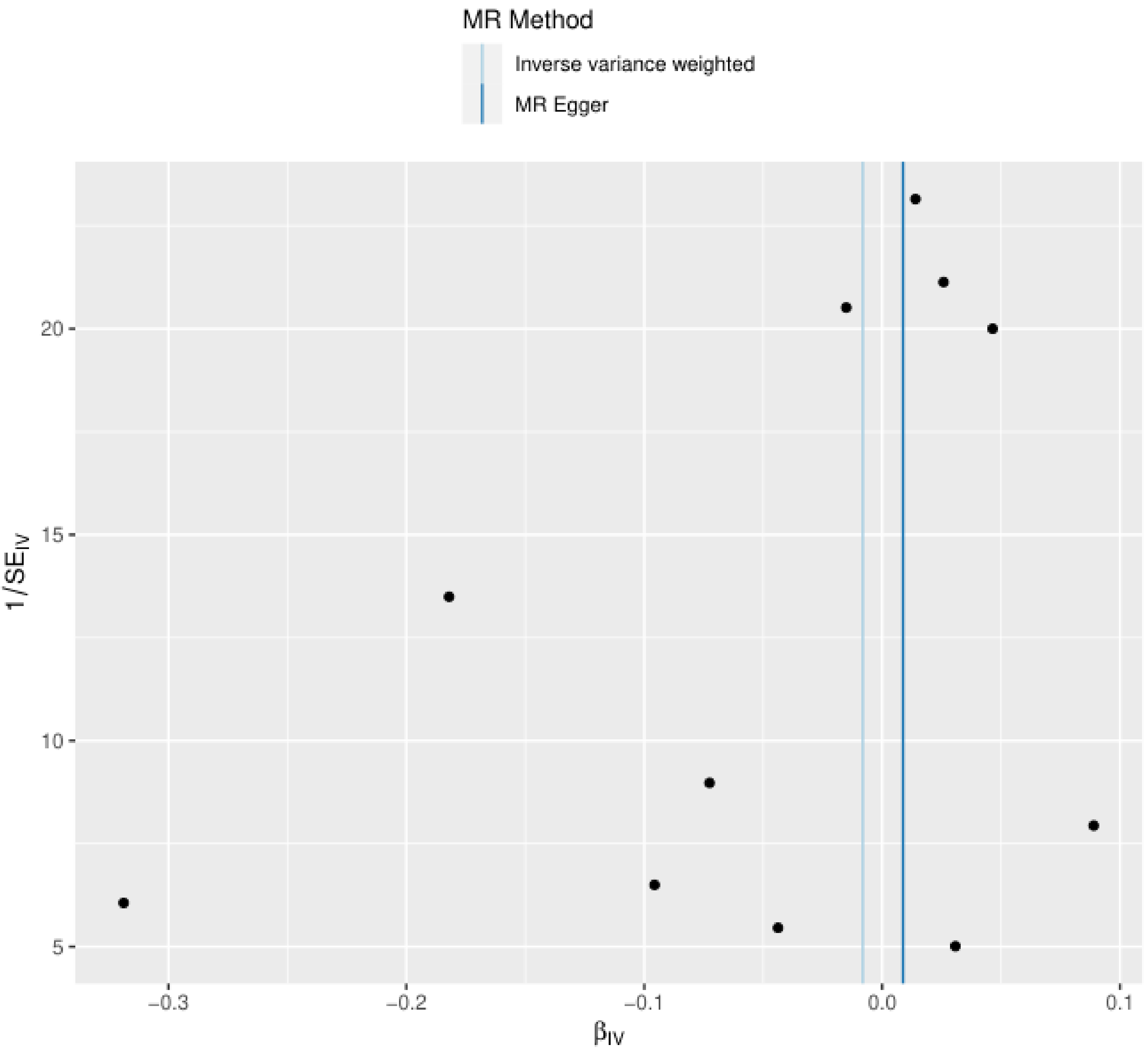

Funnel plot analyse of "CD45 on Im MDSC " on 'Diabetic nephropathy'

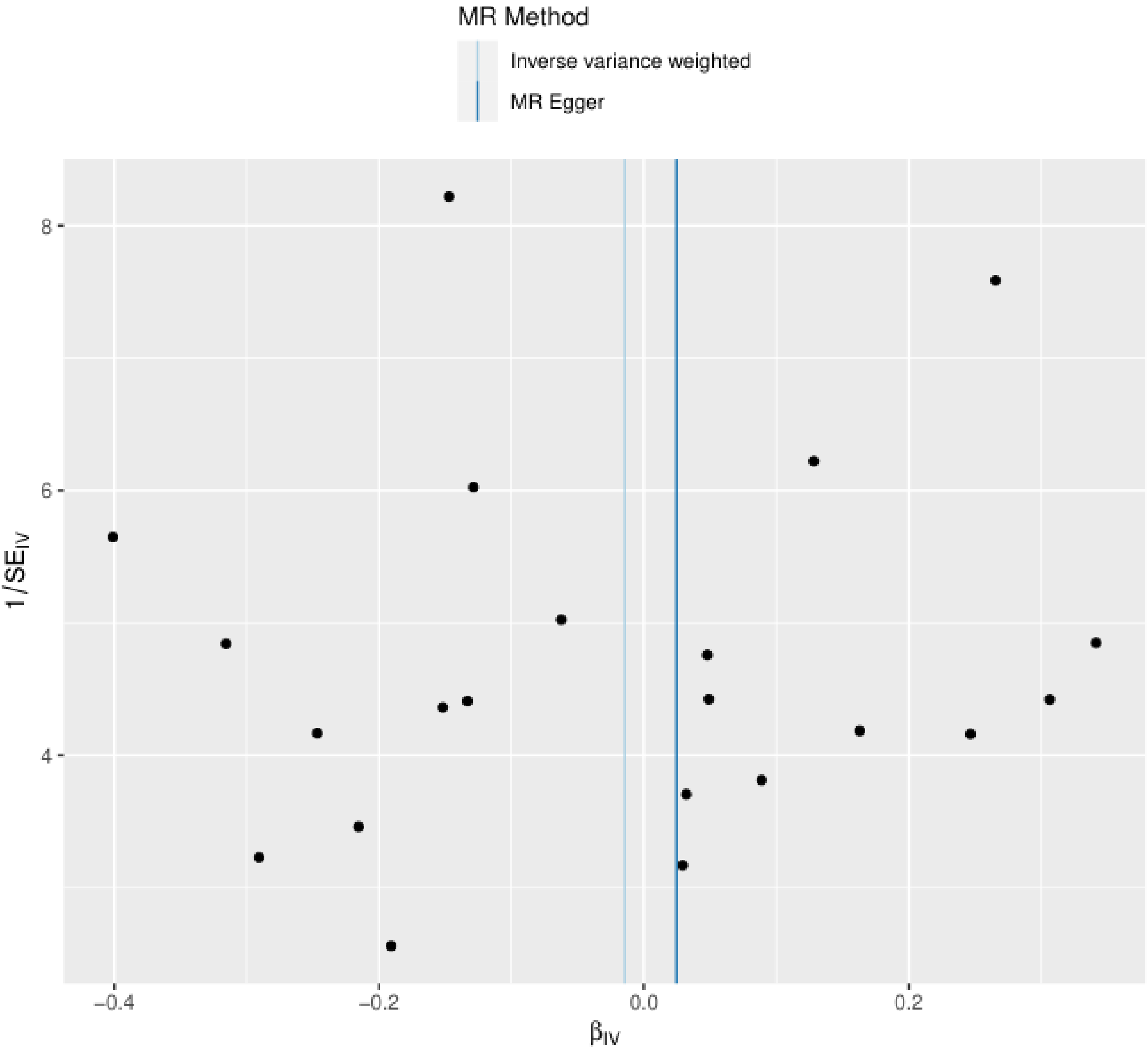

Funnel plot analyse of "CD19 on IgD- CD24-" on 'Diabetic nephropathy'

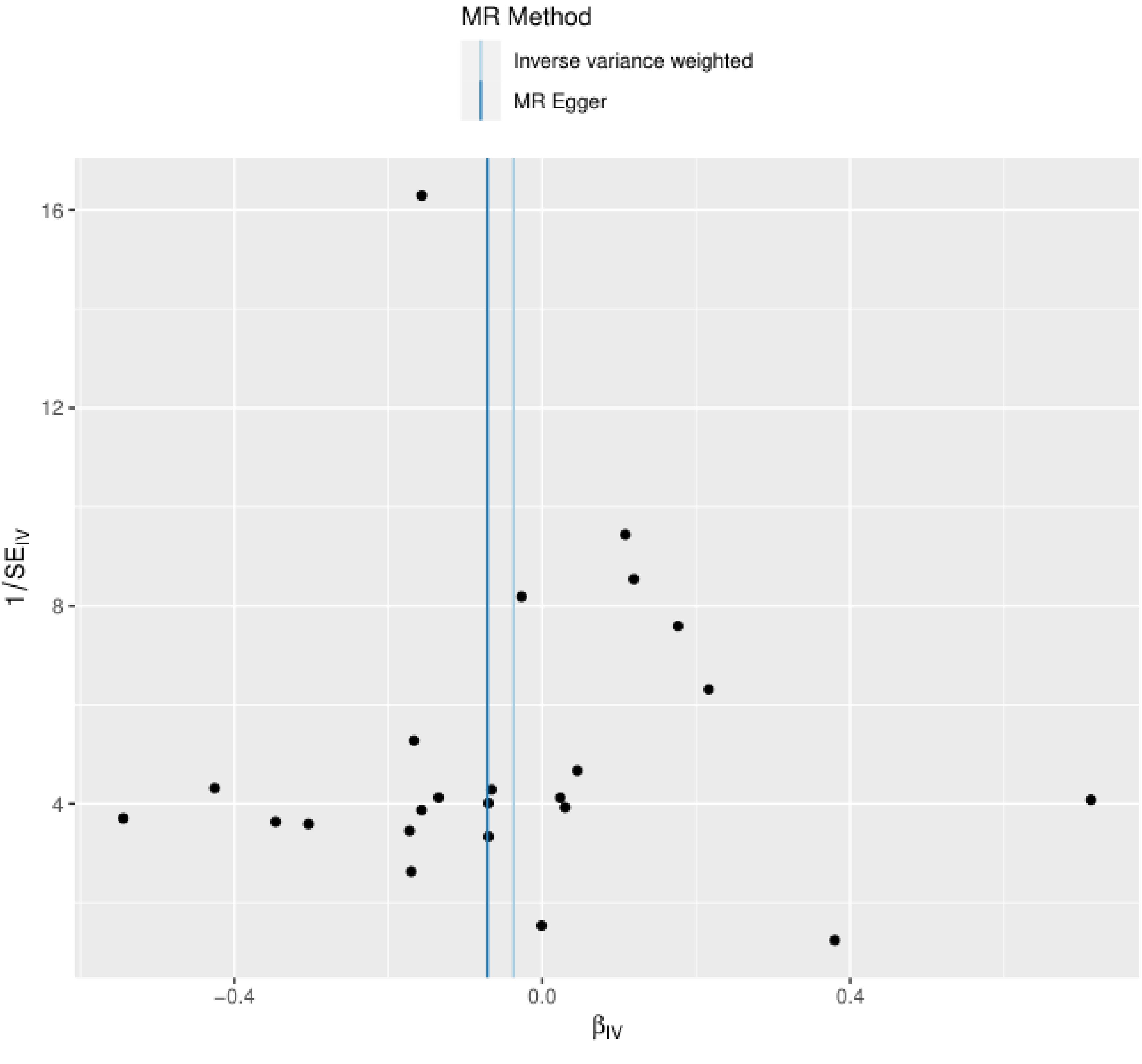

Funnel plot analyse of "CD62L- plasmacytoid DC AC" on 'Diabetic nephropathy'

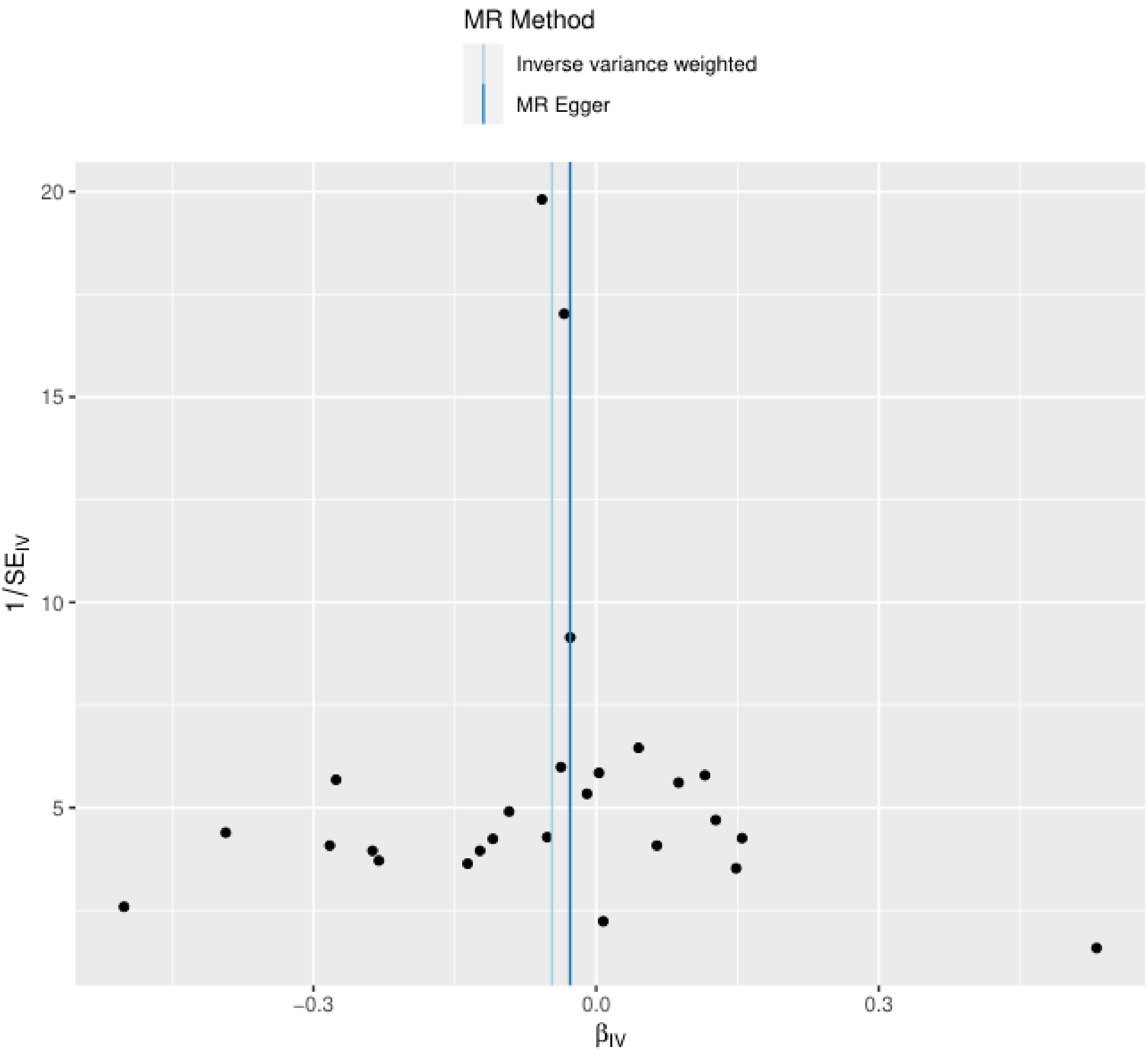

Funnel plot analyse of "PDL-1 on CD14- CD16- " on 'Diabetic nephropathy'

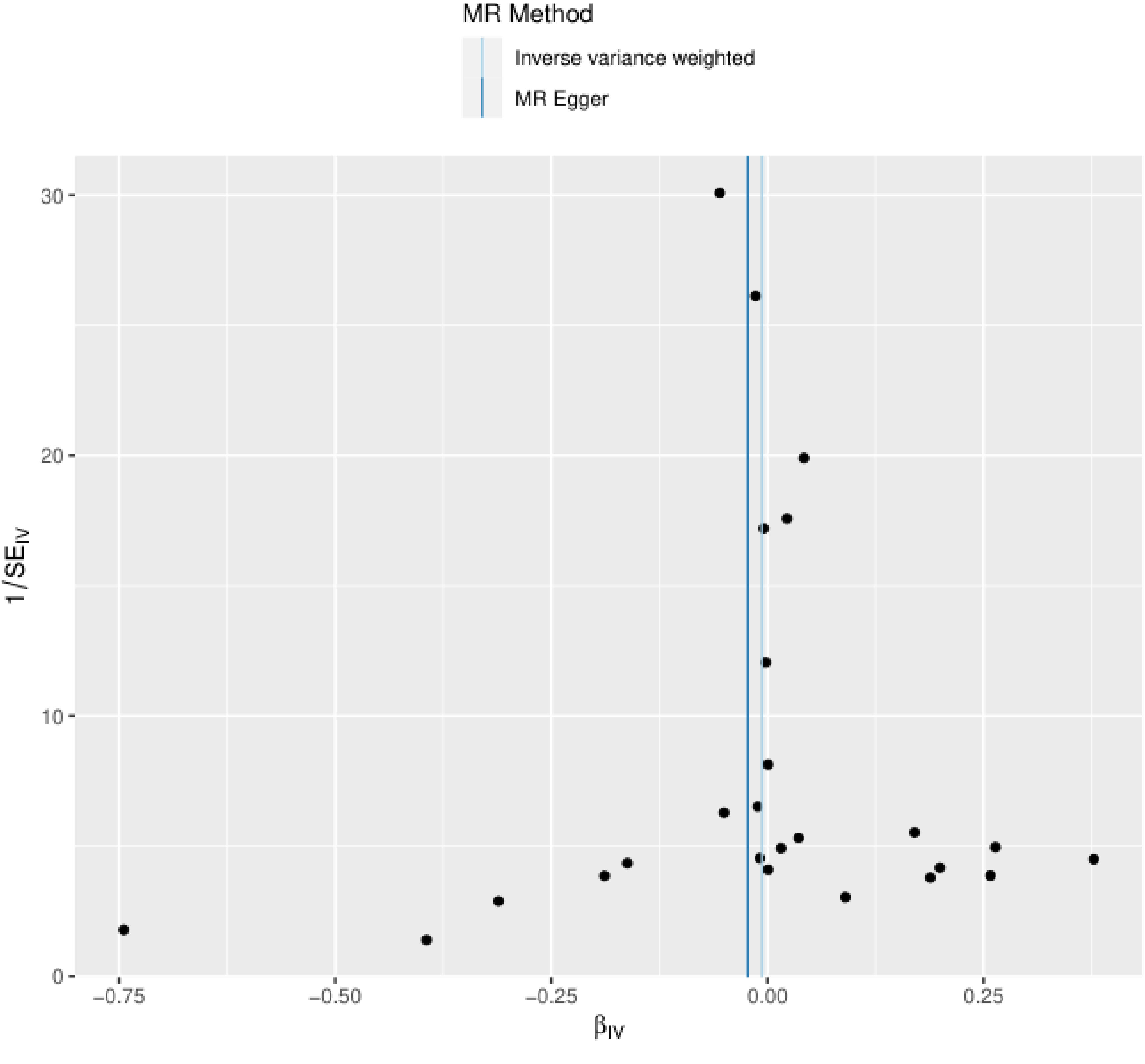

Funnel plot analyse of "CD24 on IgD+ CD24+" on 'Diabetic nephropathy'

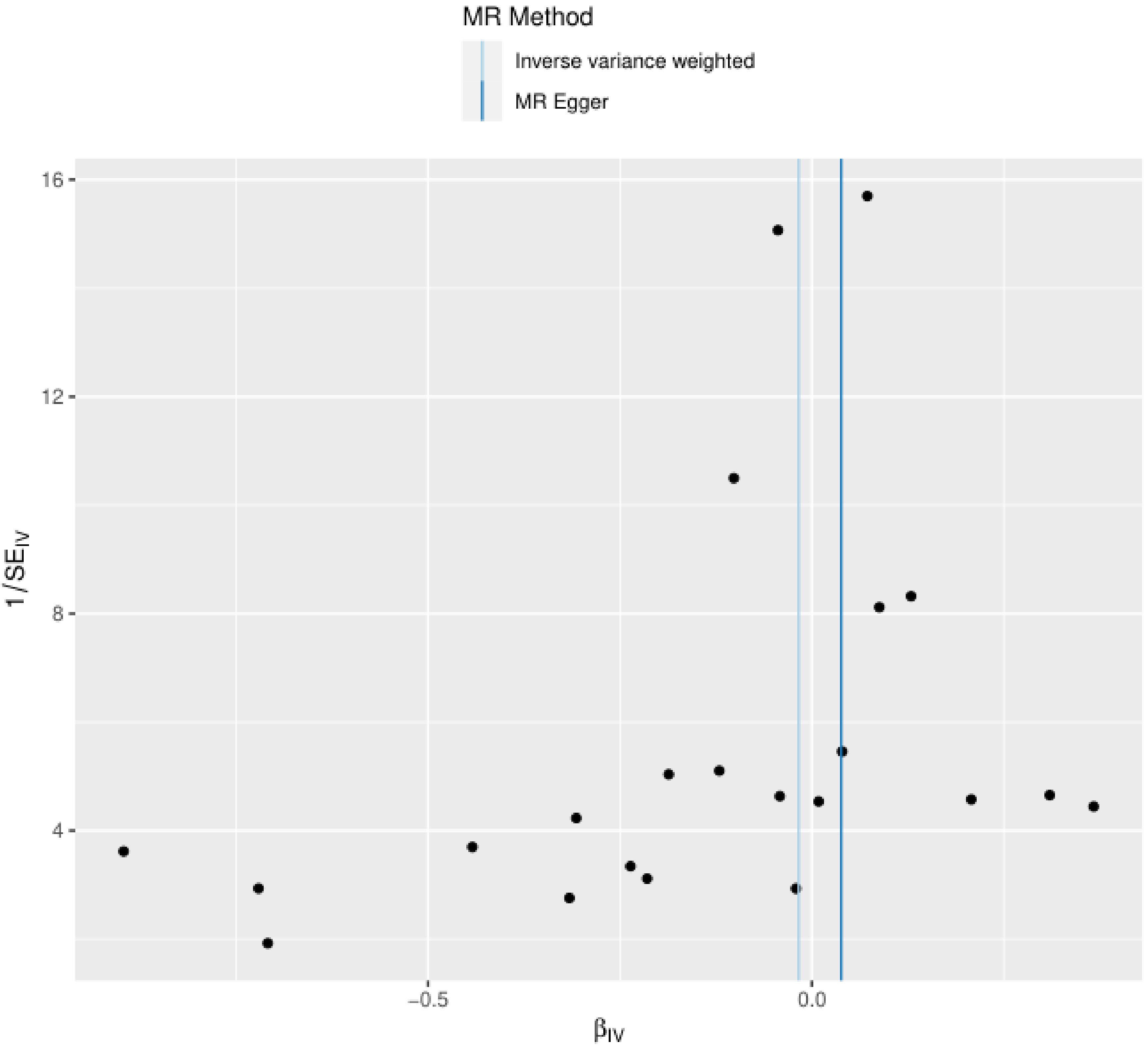

Funnel plot analyse of "CD25 on IgD- CD38dim" on 'Diabetic nephropathy'

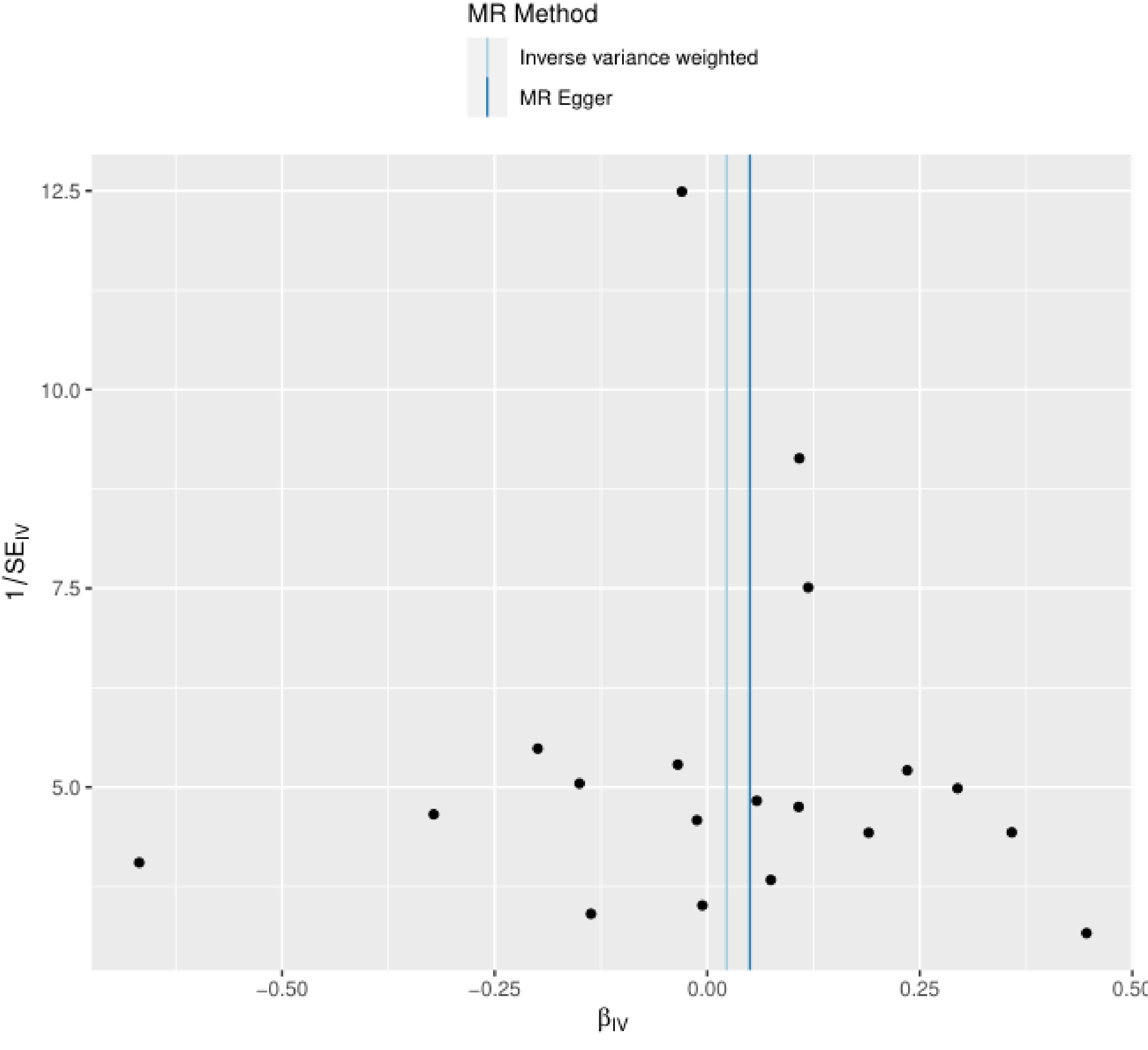

Funnel plot analyse of "CD86 on myeloid DC" on 'Diabetic nephropathy'

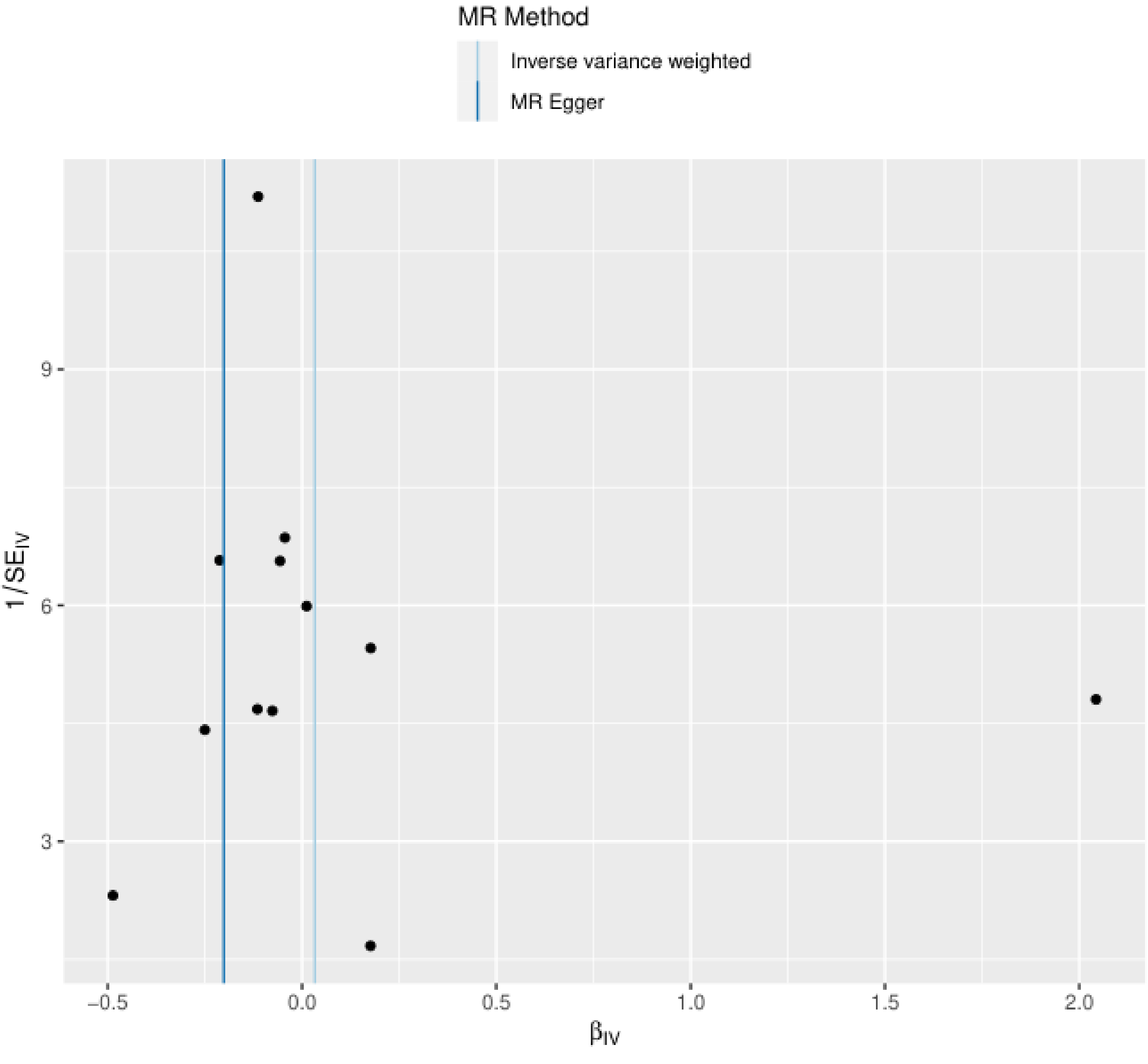

Funnel plot analyse of "TD CD4+ AC" on 'Diabetic nephropathy'

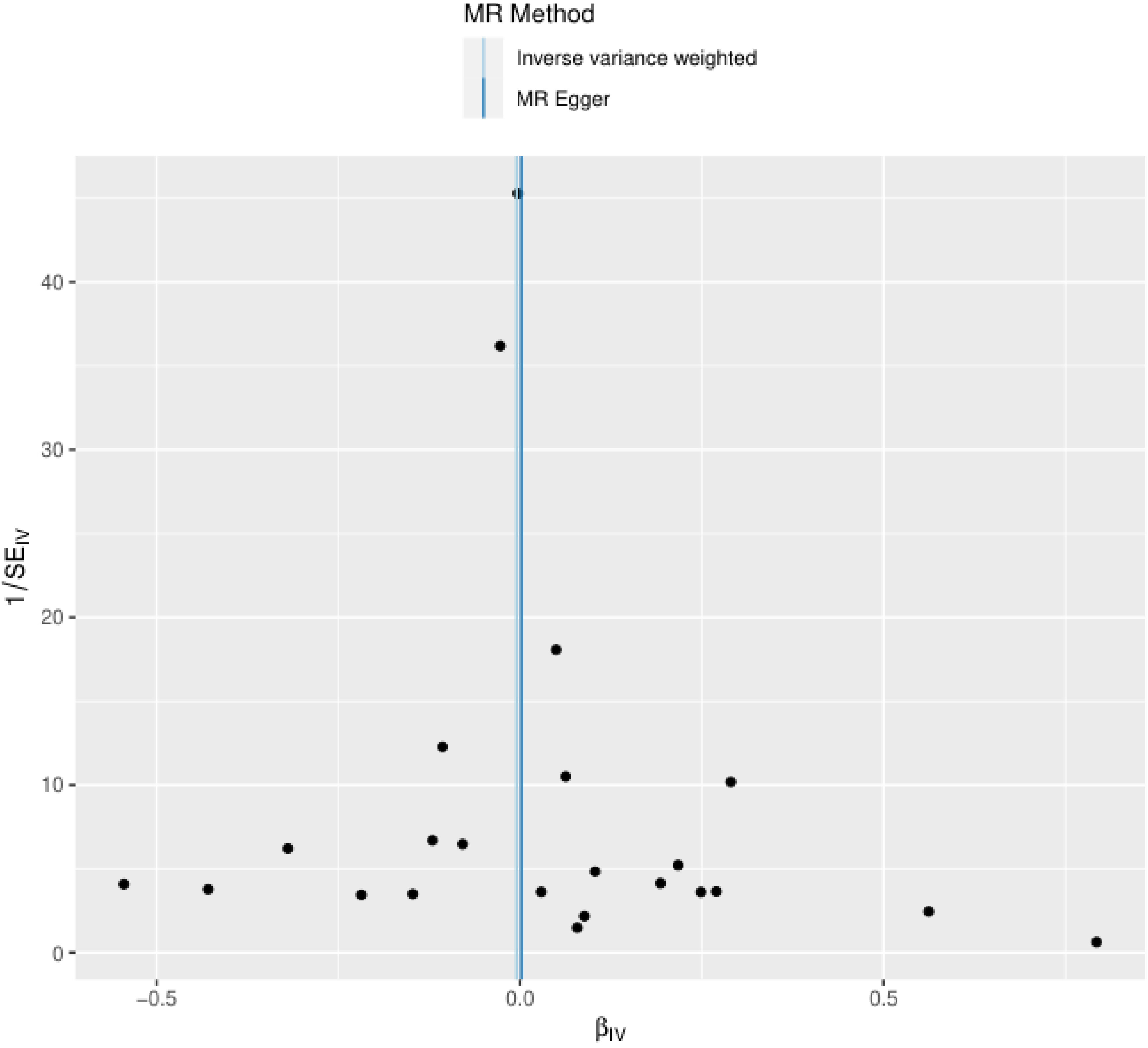

Funnel plot analyse of "EM CD8br %CD8br" on 'Diabetic nephropathy'

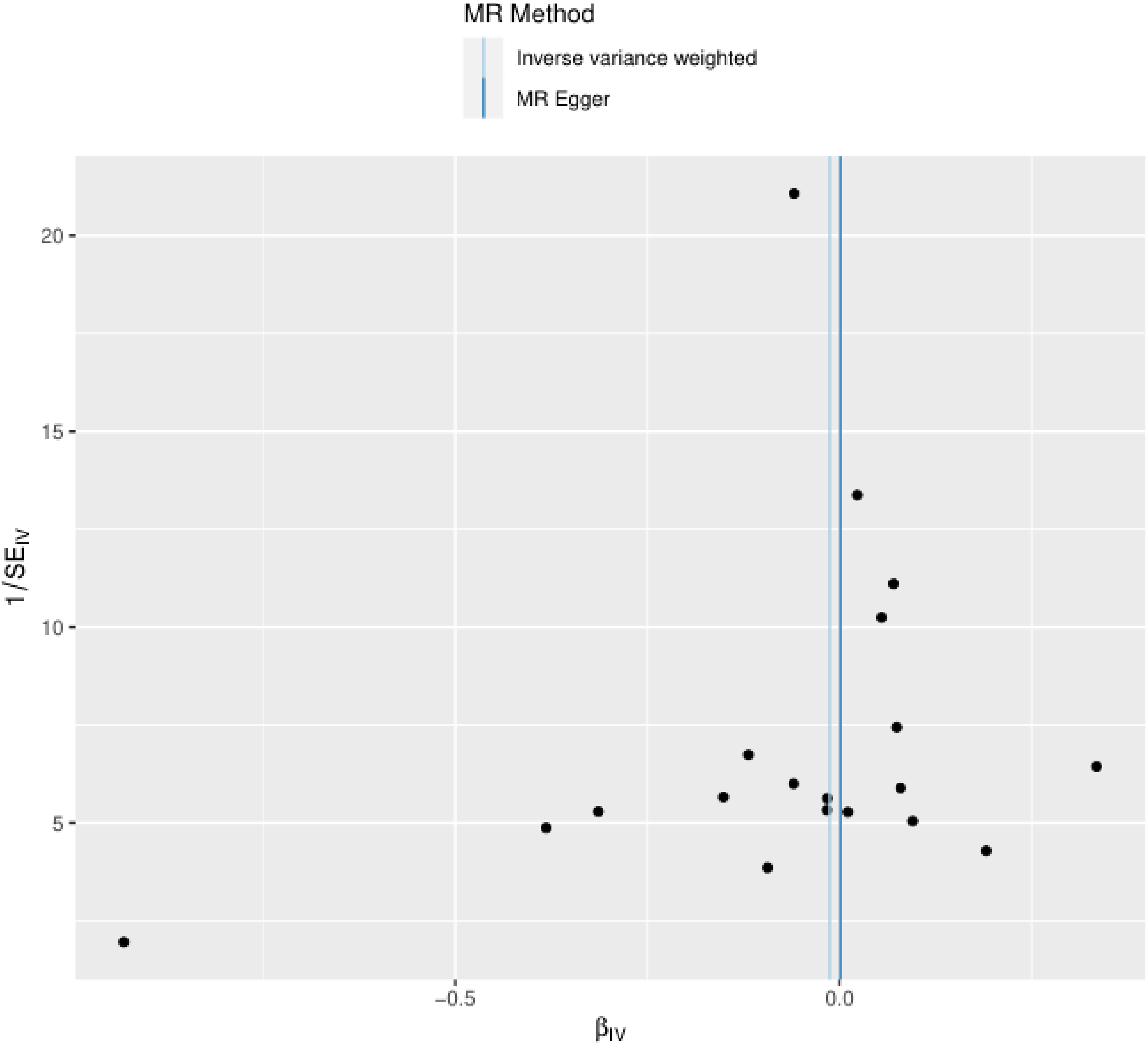

Funnel plot analyse of "HSC AC" on 'Diabetic nephropathy'

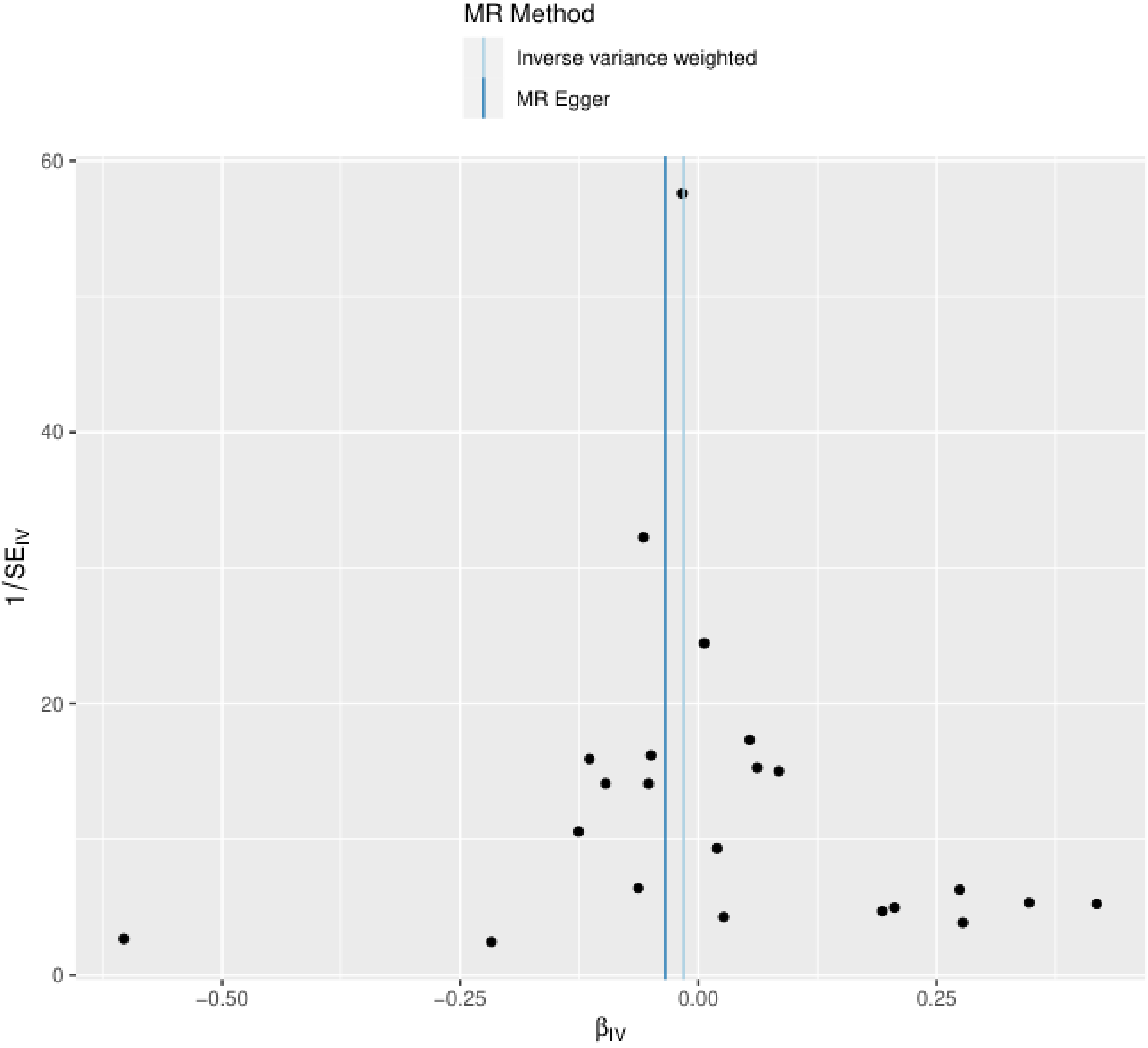

Funnel plot analyse of "Secreting Treg AC" on 'Diabetic nephropathy'

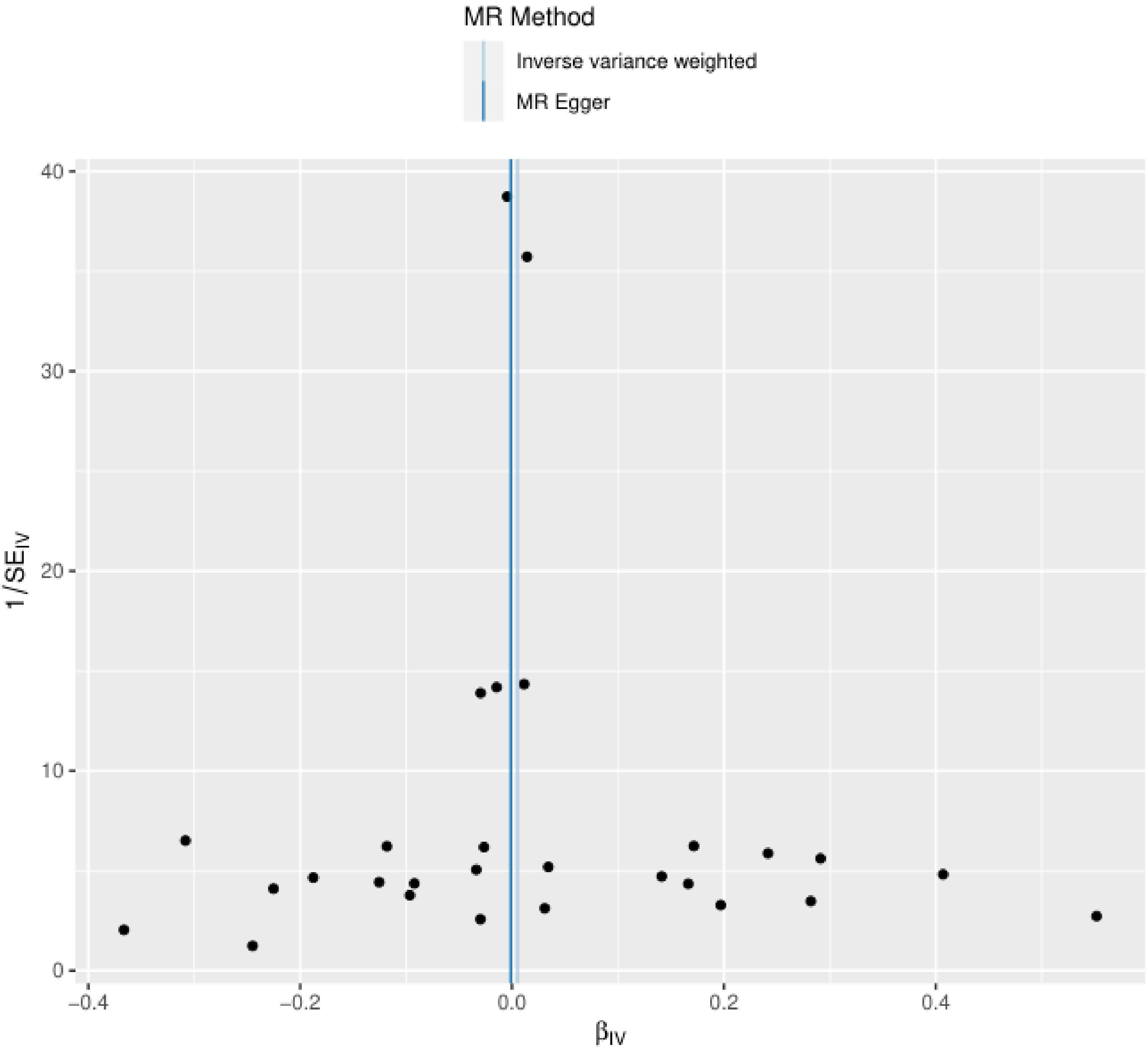

Funnel plot analyse of "CD19 on IgD+" on 'Diabetic nephropathy'

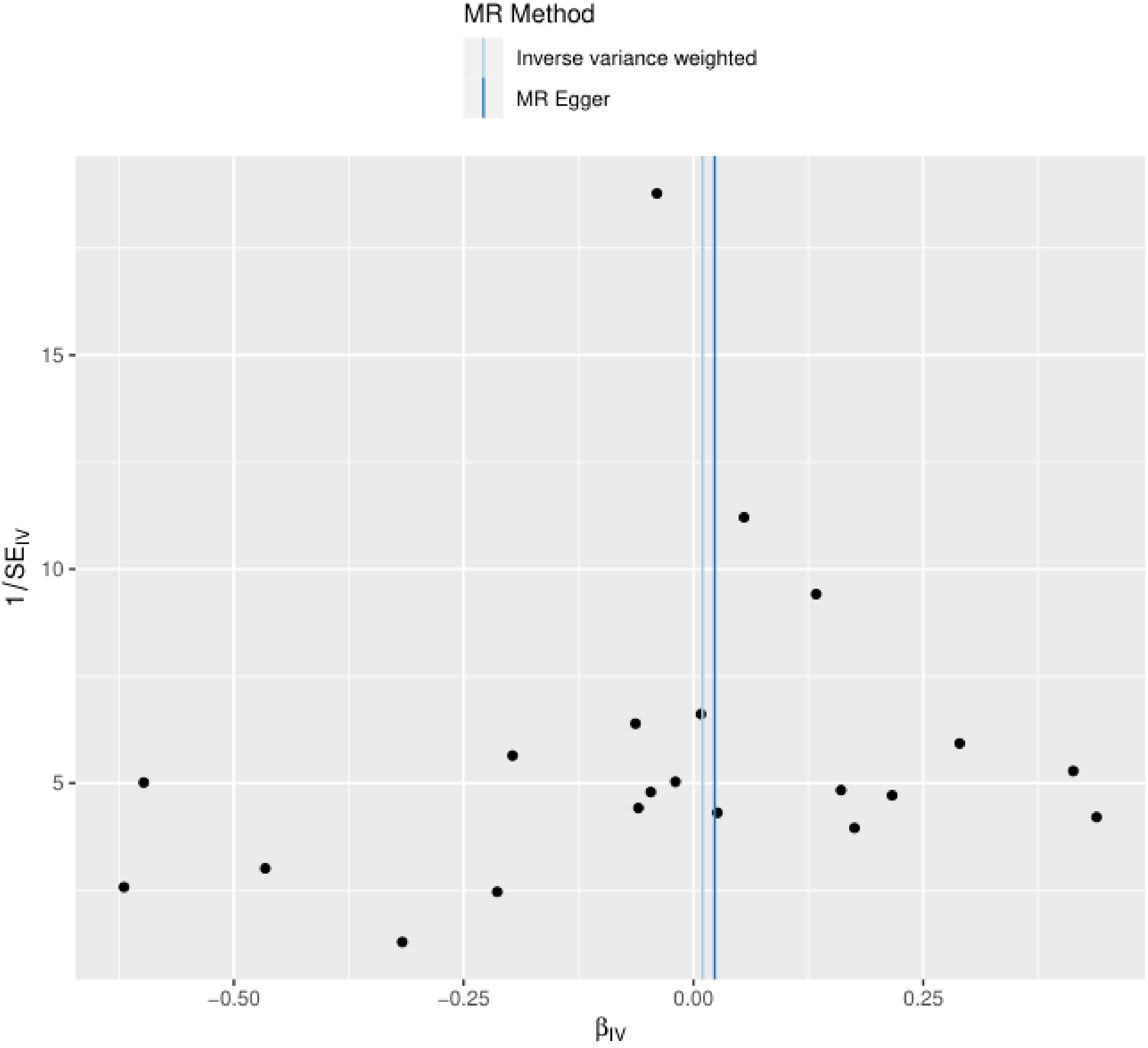

Funnel plot analyse of "CD4 Treg AC" on 'Diabetic nephropathy'

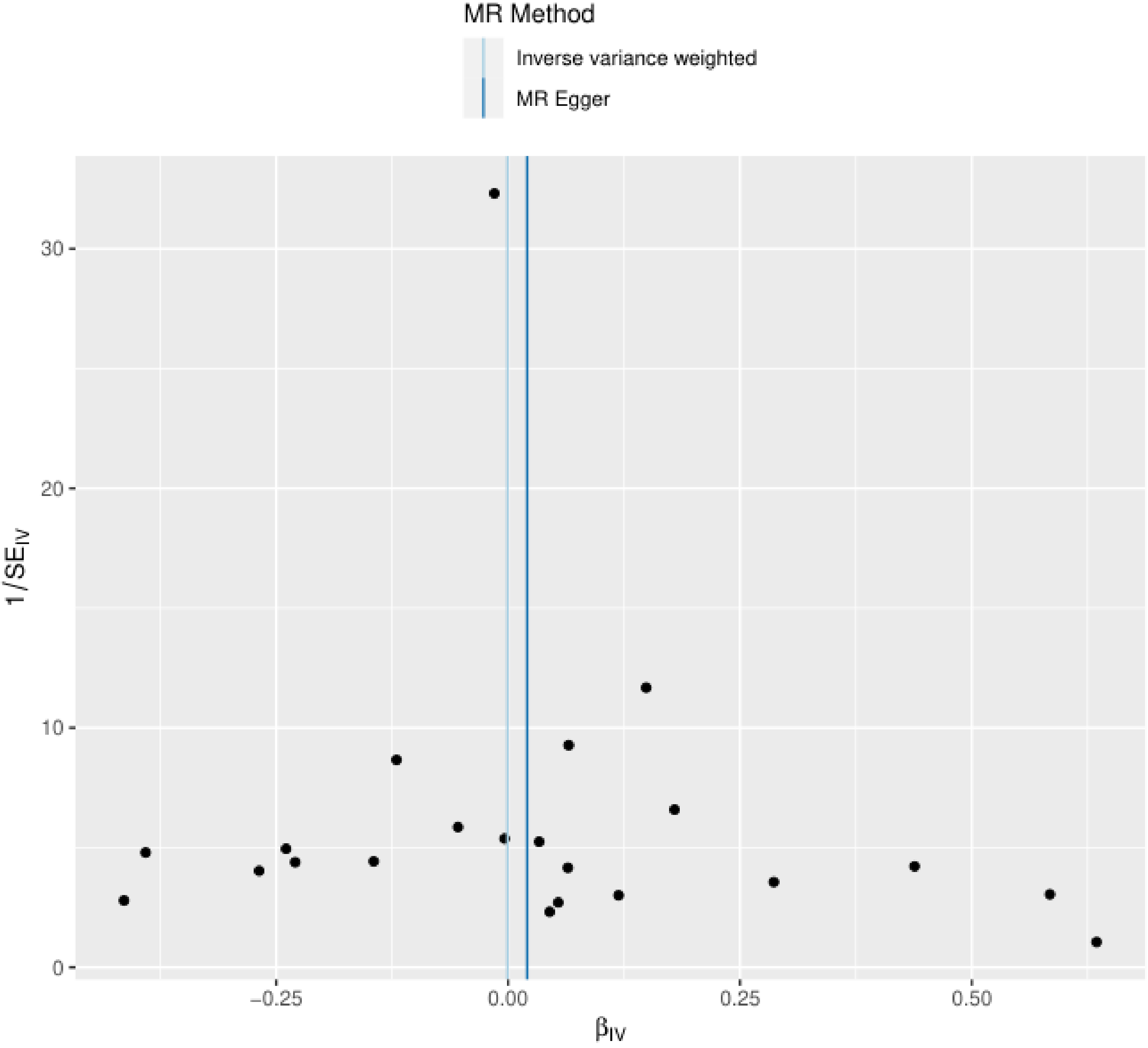

Funnel plot analyse of "Naive-mature B cell AC" on 'Diabetic nephropathy'

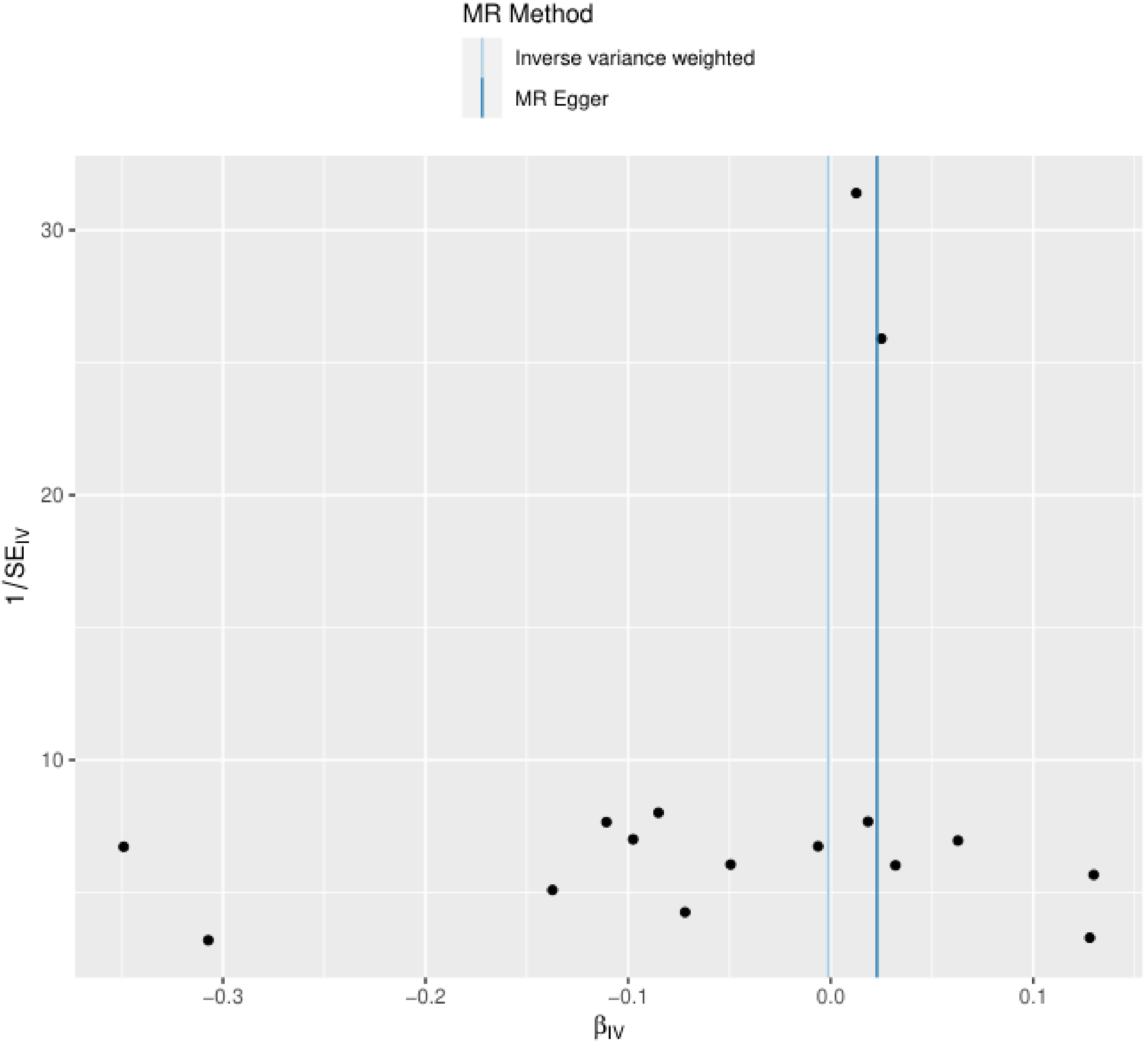

Funnel plot analyse of "CD45 on CD33br HLA DR+ " on 'Diabetic nephropathy'

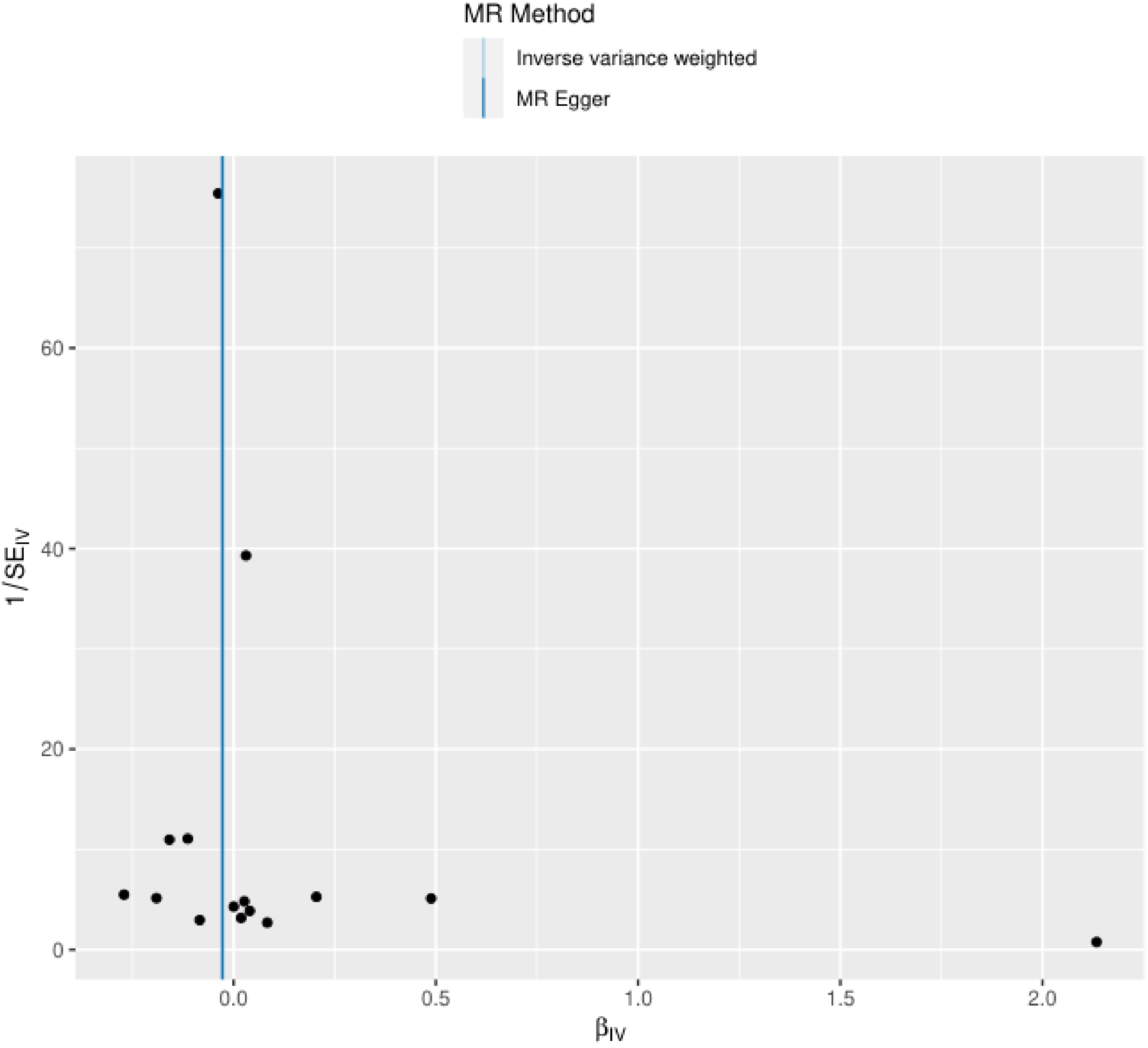

Funnel plot analyse of "CD28- CD8dim %CD8dim" on 'Diabetic nephropathy'

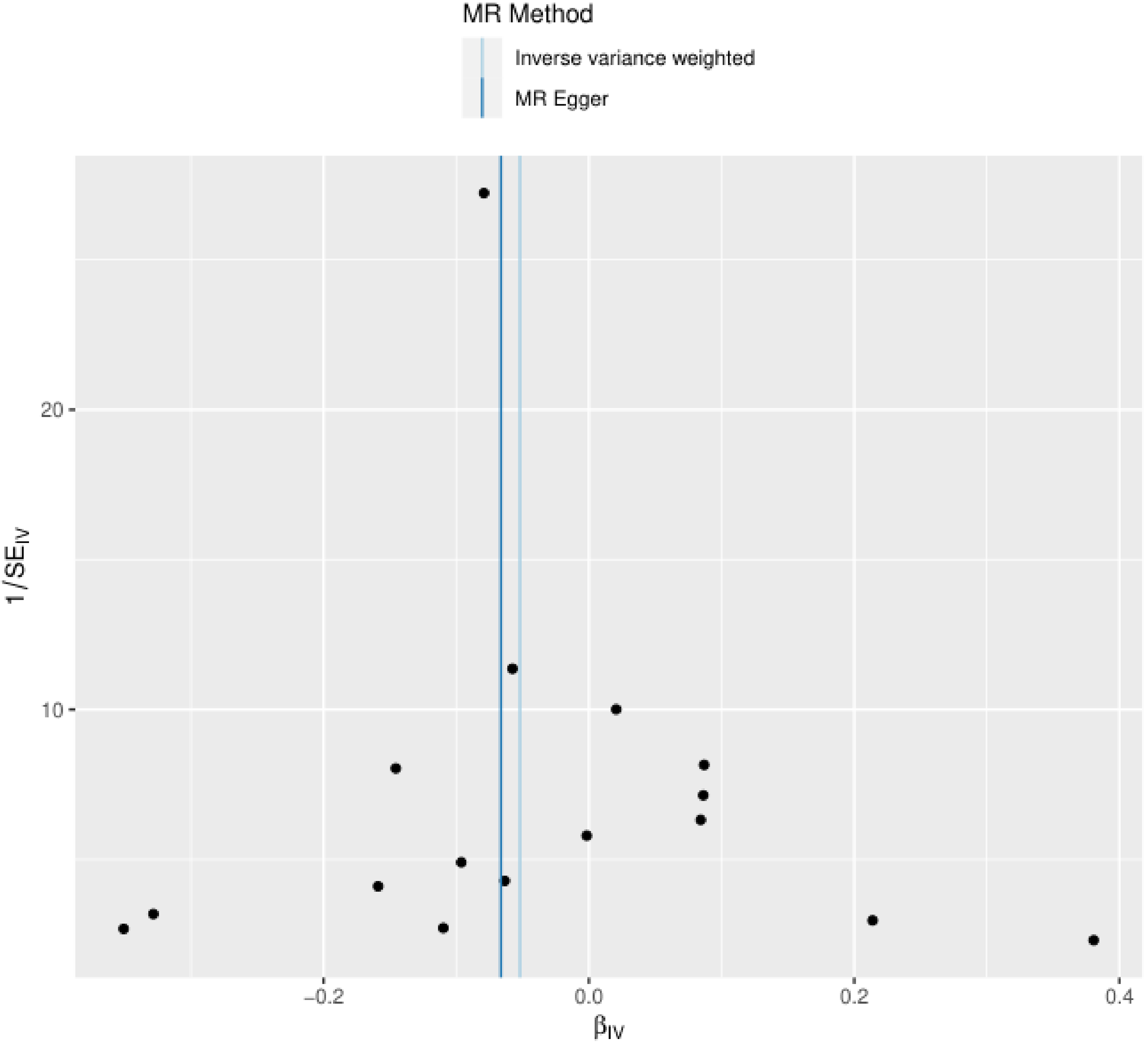

Funnel plot analyse of "BAFF-R on CD20- CD38-" on 'Diabetic nephropathy'

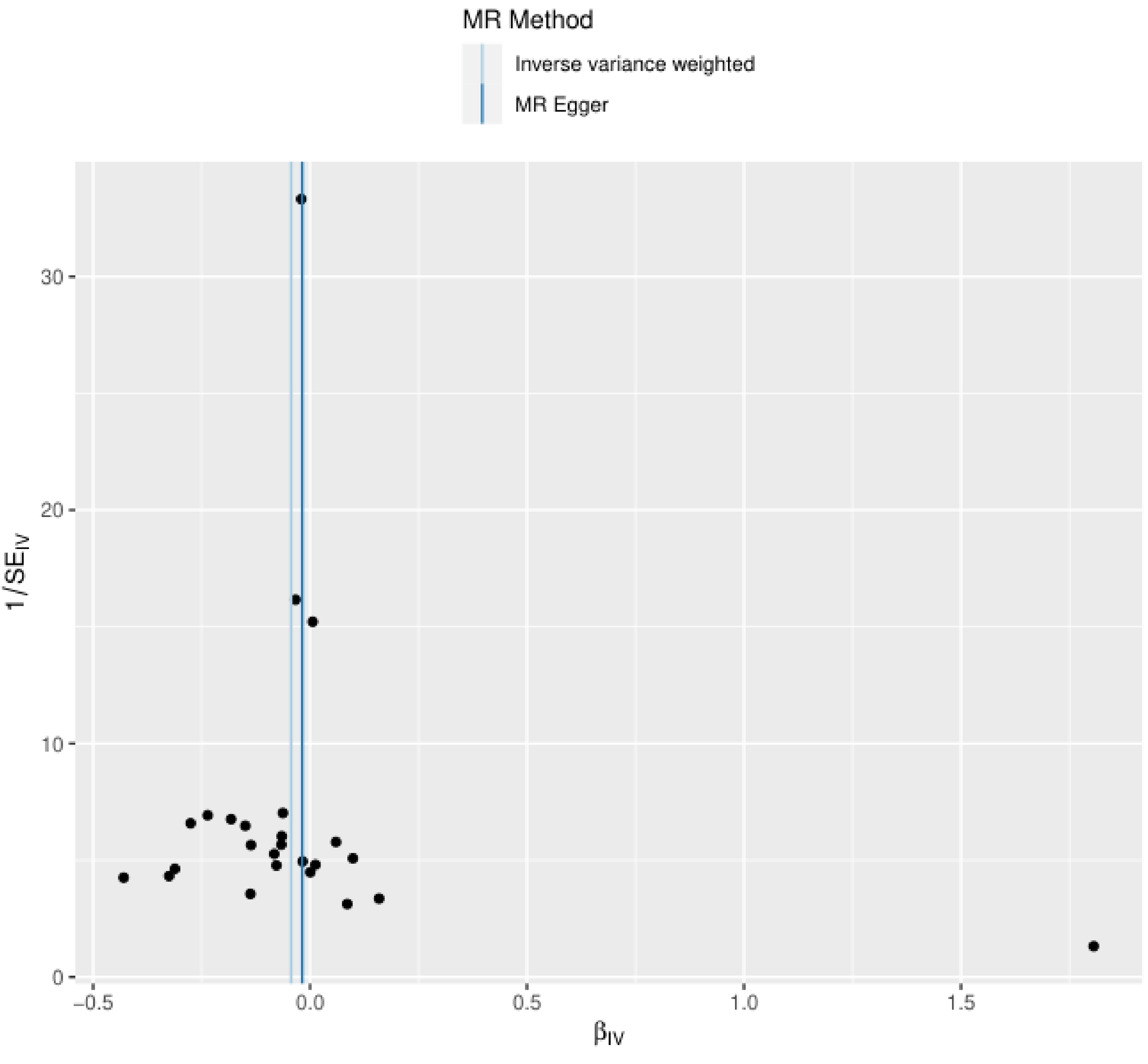

Funnel plot analyse of "CD62L on monocyte" on 'Diabetic nephropathy'

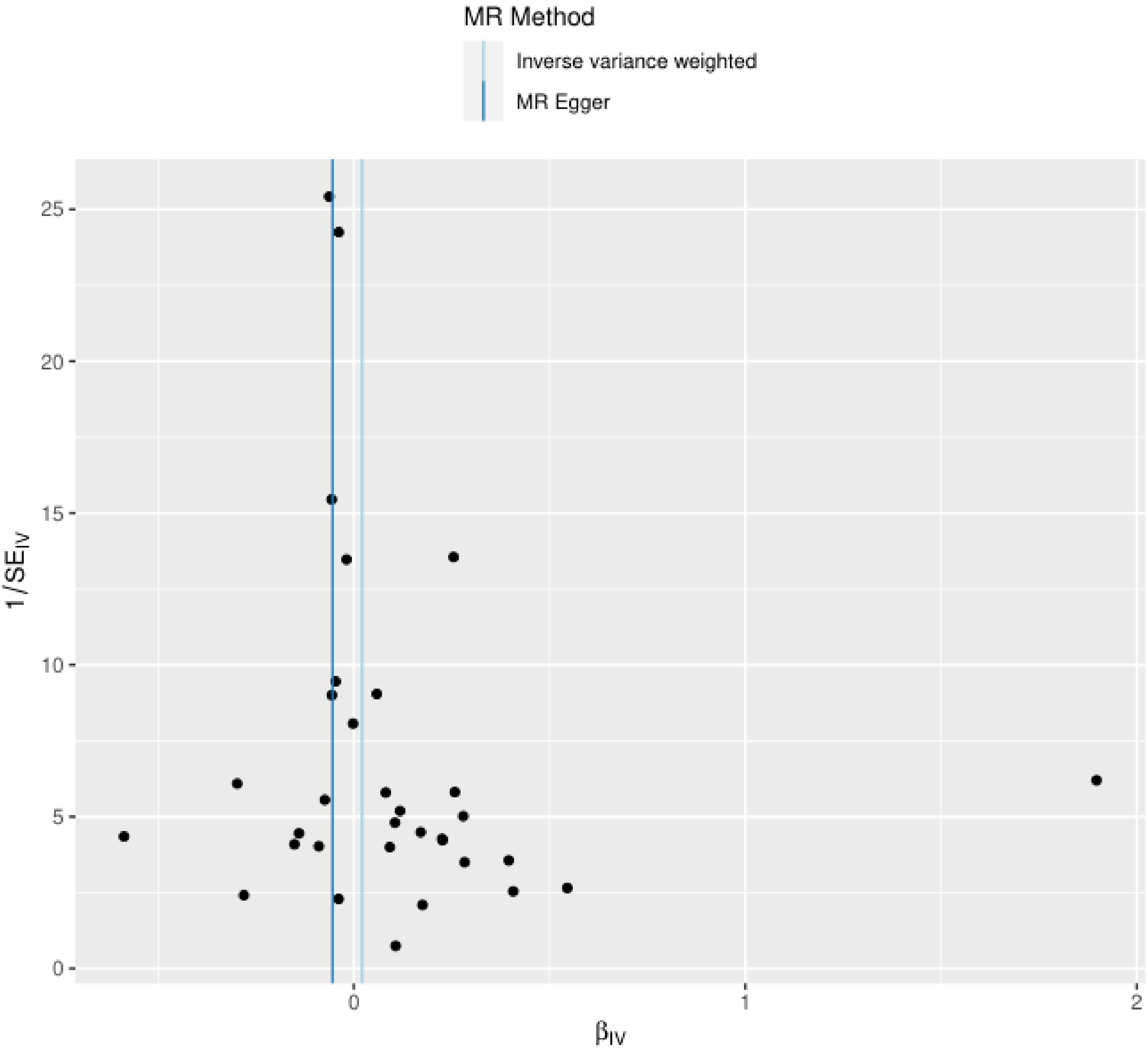

Funnel plot analyse of "HLA DR+ CD8br %T cell" on 'Diabetic nephropathy'

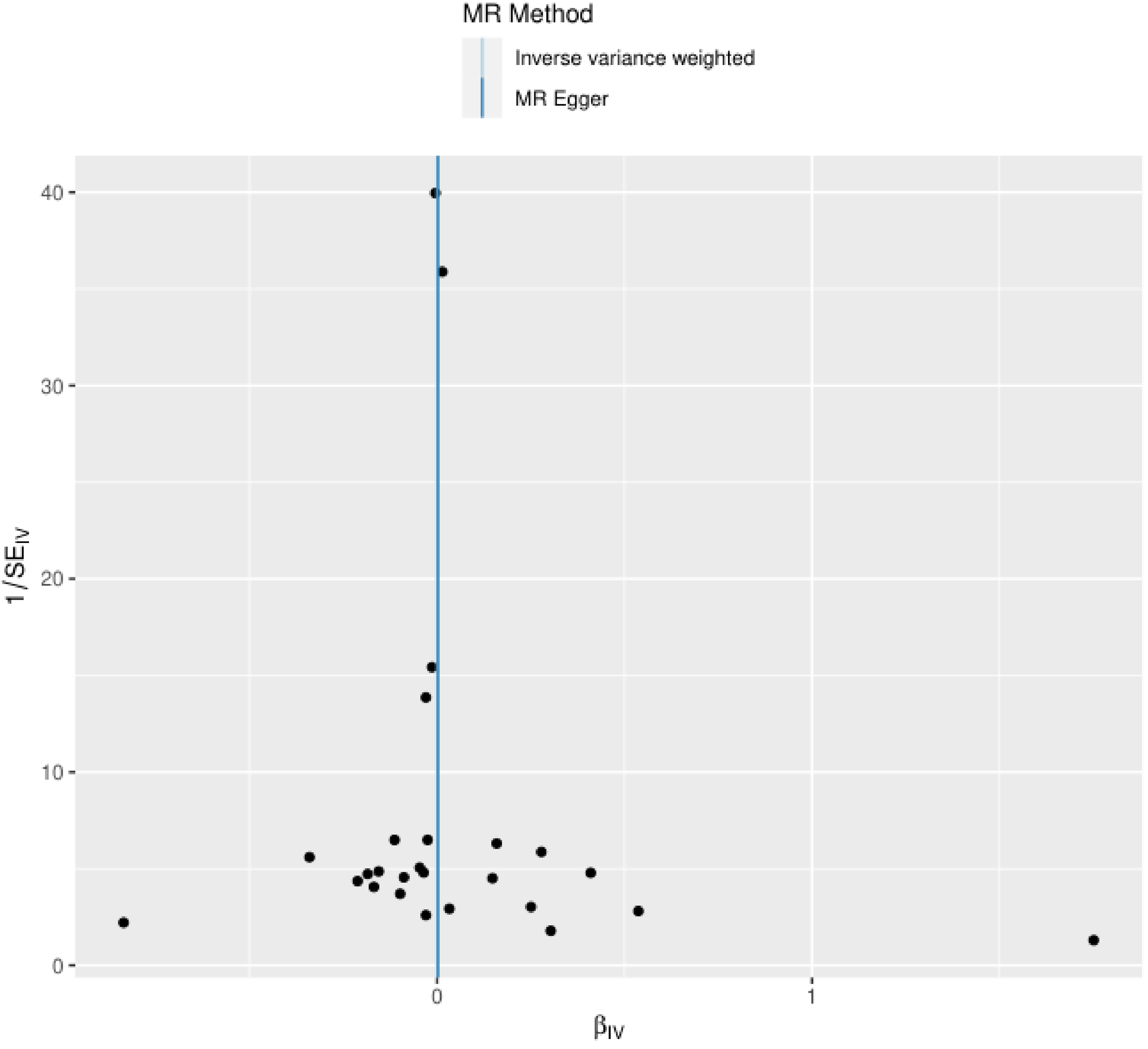

Funnel plot analyse of "CD19 on IgD+ CD24-" on 'Diabetic nephropathy'

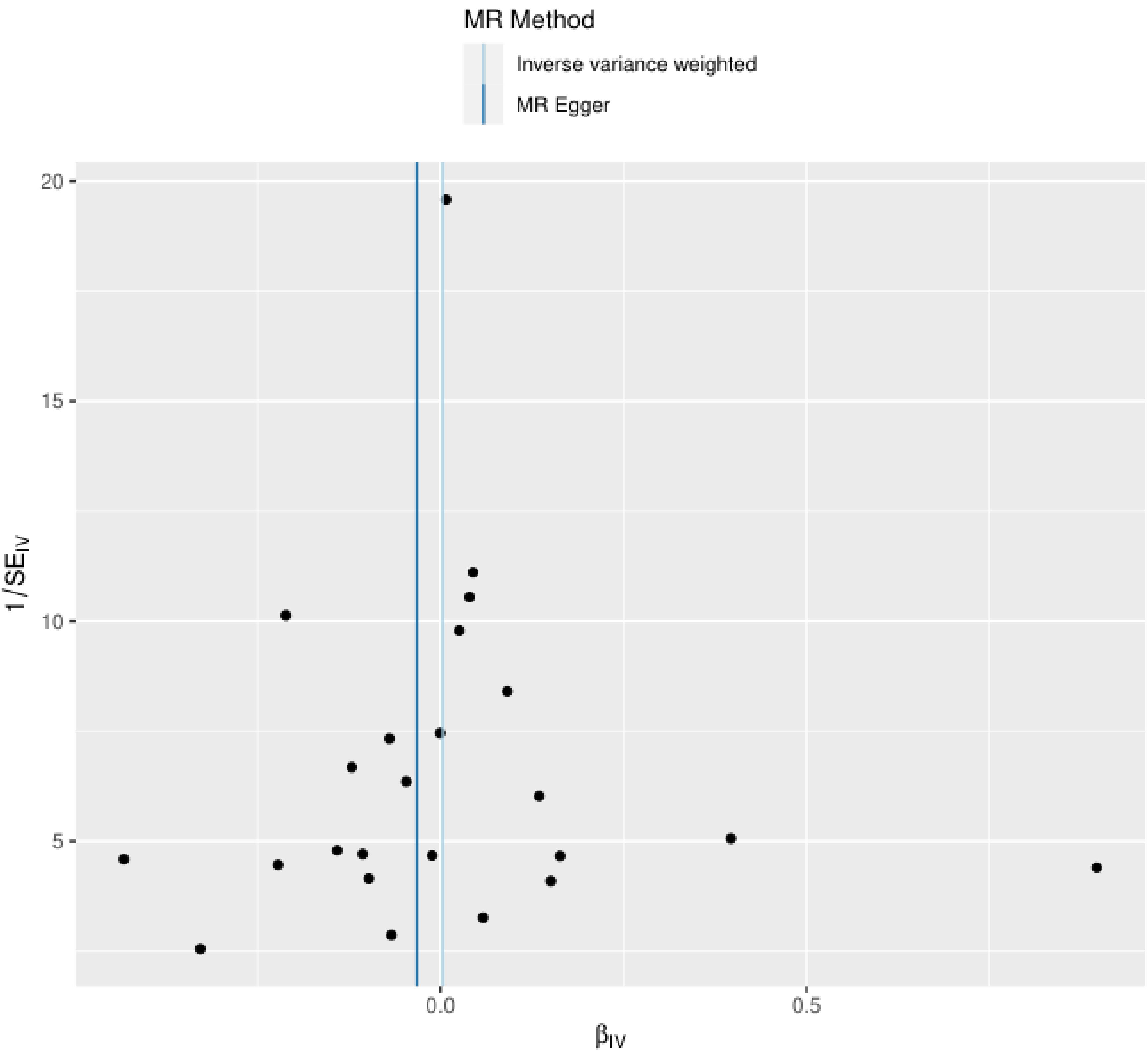

Funnel plot analyse of "CD8 on CD28+ CD45RA+ CD8br " on 'Diabetic nephropathy'

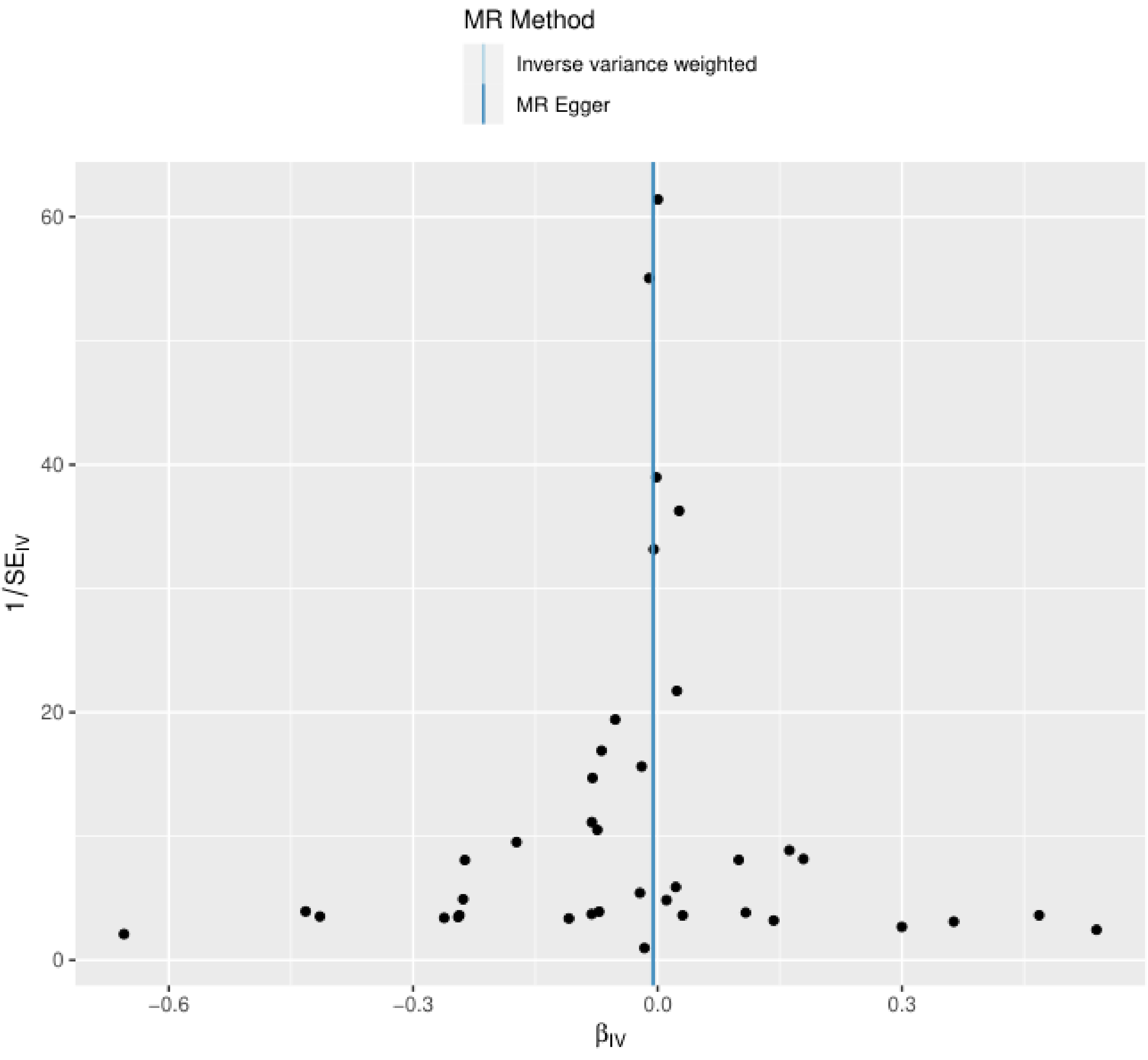

Funnel plot analyse of "CD28+ CD45RA+ CD8br %CD8br" on 'Diabetic nephropathy'

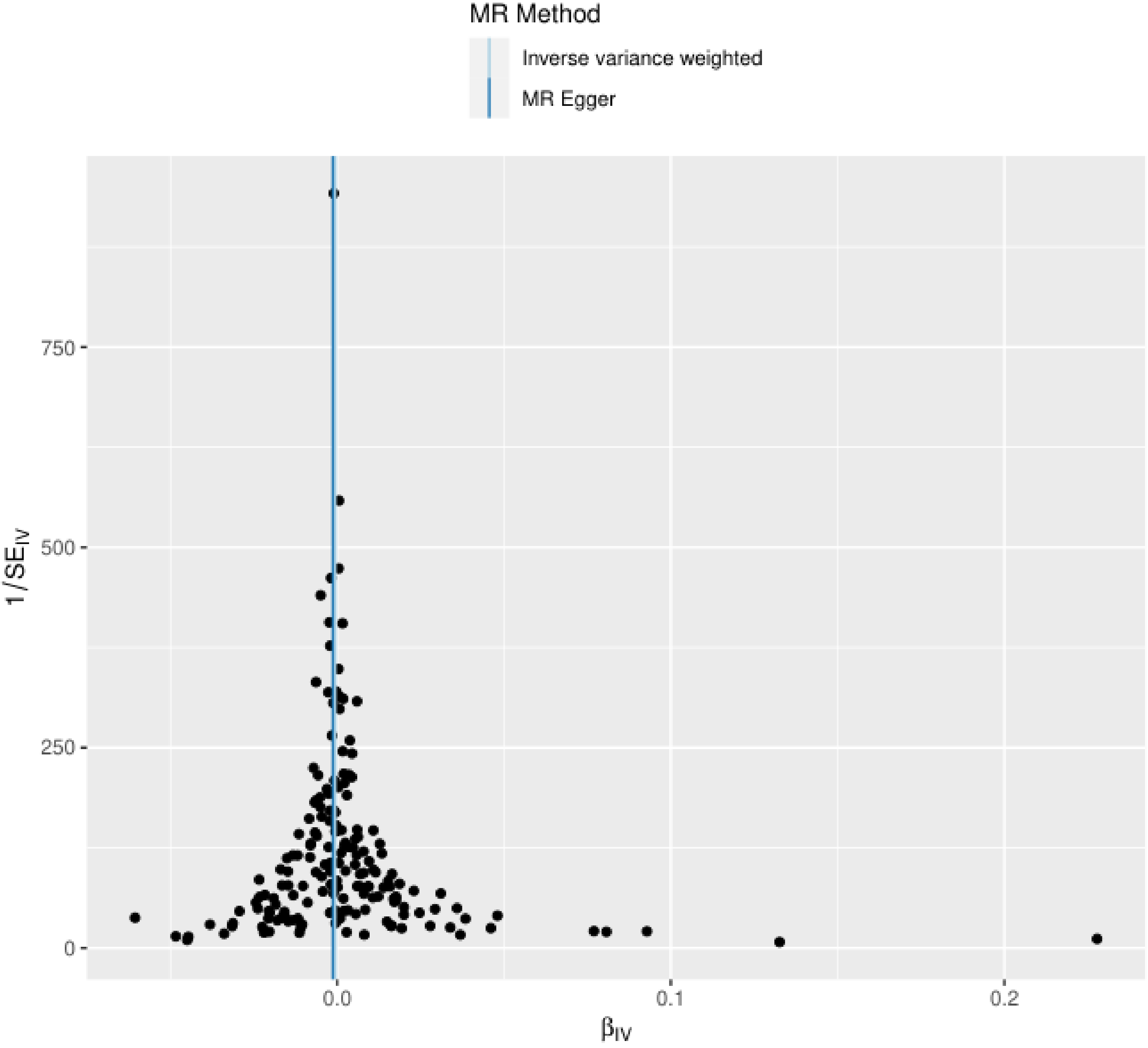

Funnel plot analyse of "CD45RA- CD28- CD8br %T cell" on 'Diabetic nephropathy'

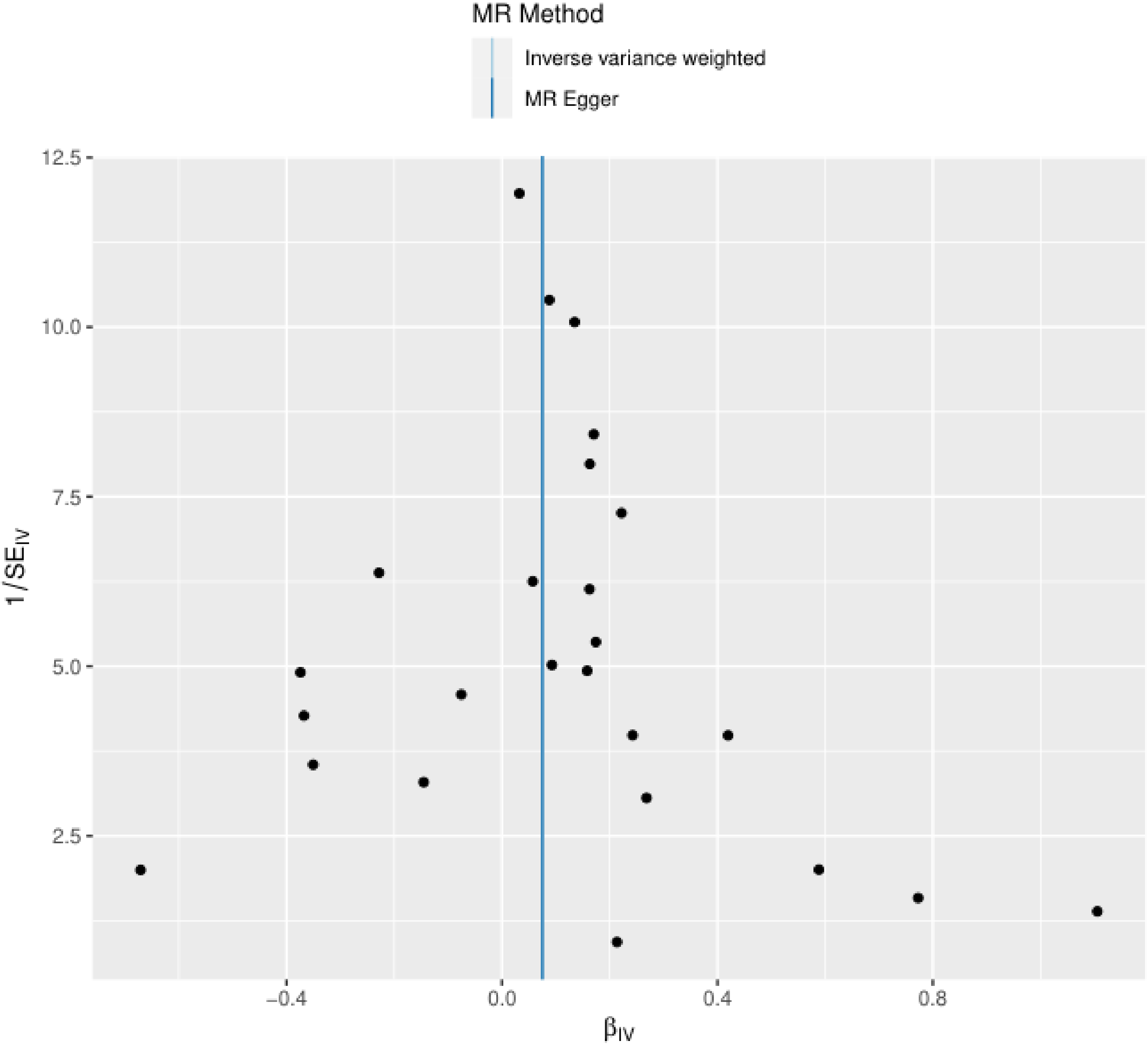

Funnel plot analyse of "EM DN (CD4-CD8-) %T cell" on 'Diabetic nephropathy'

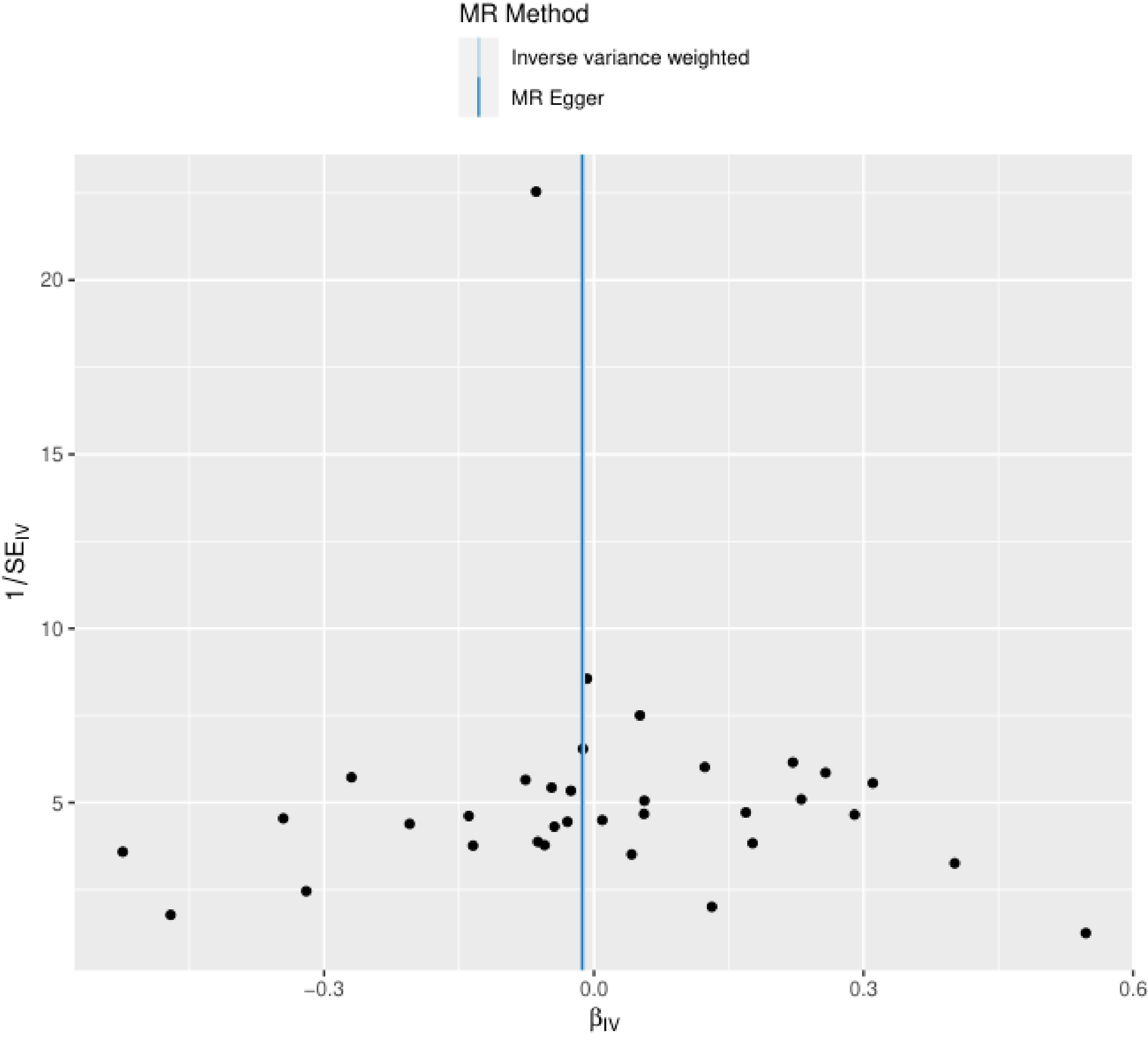

Funnel plot analyse of "NKT %lymphocyte" on 'Diabetic nephropathy'

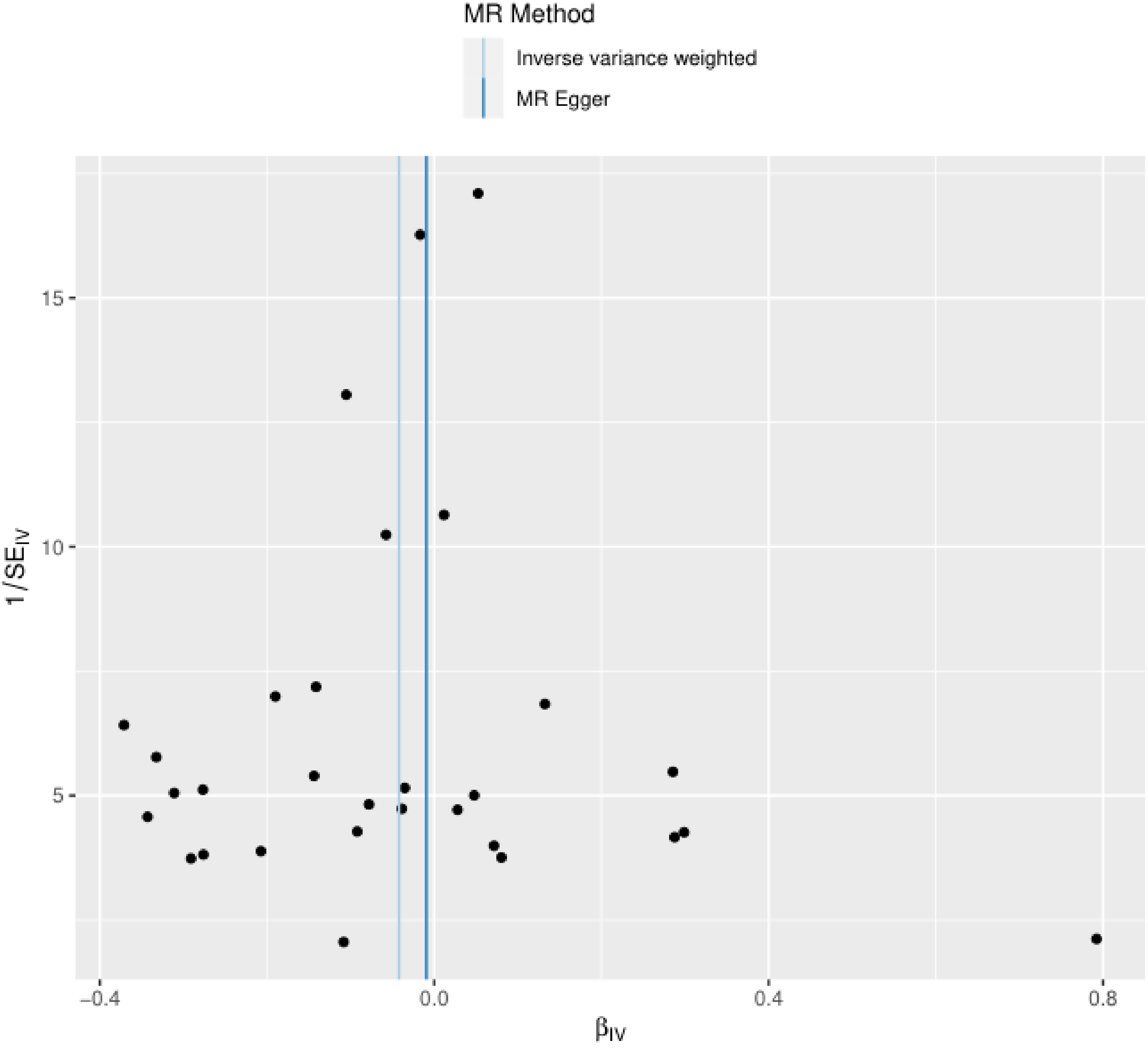

Funnel plot analyse of "Activated Treg %CD4 Treg" on 'Diabetic nephropathy'

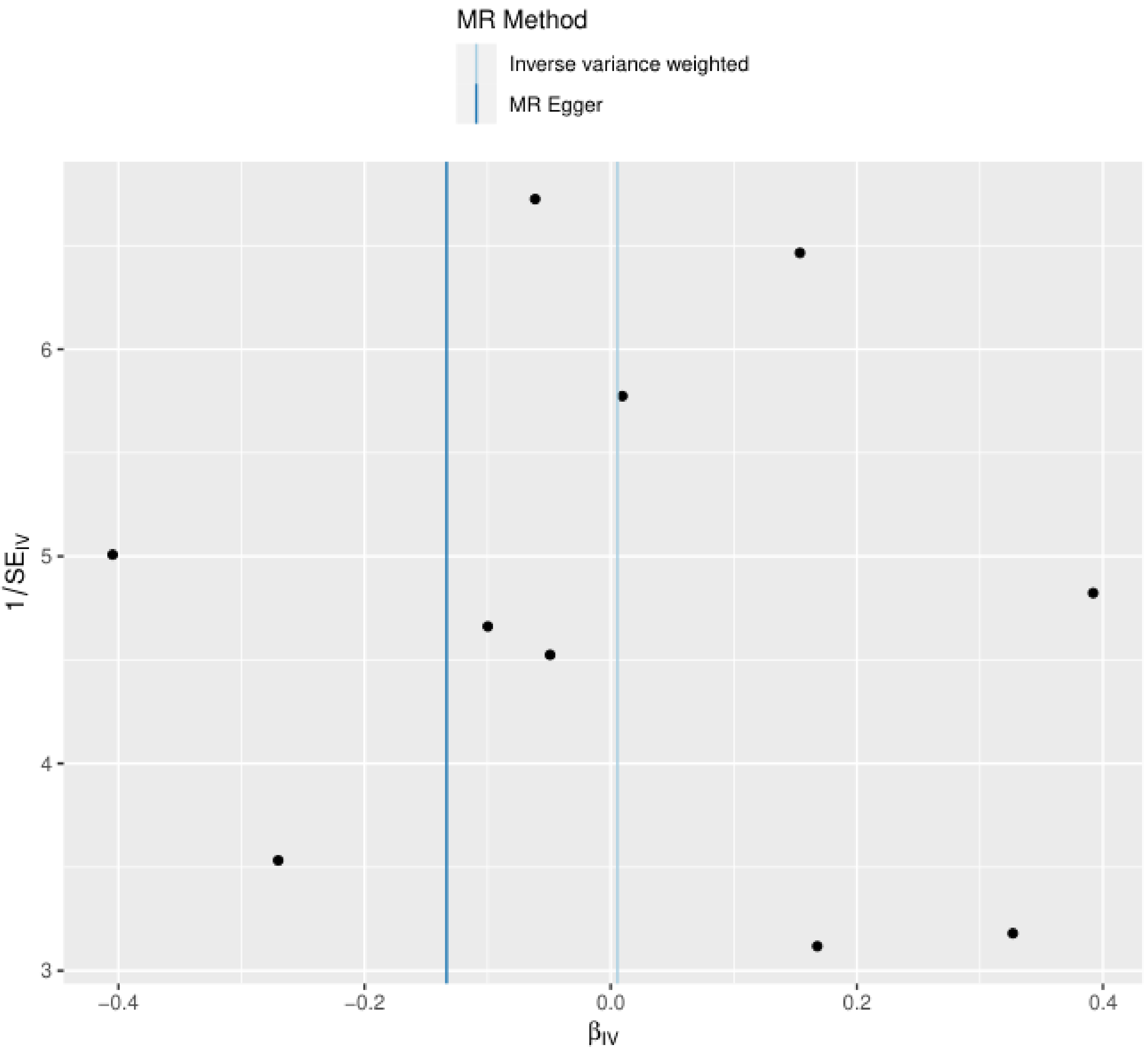

Funnel plot analyse of "DN (CD4-CD8-) NKT AC" on 'Diabetic nephropathy'

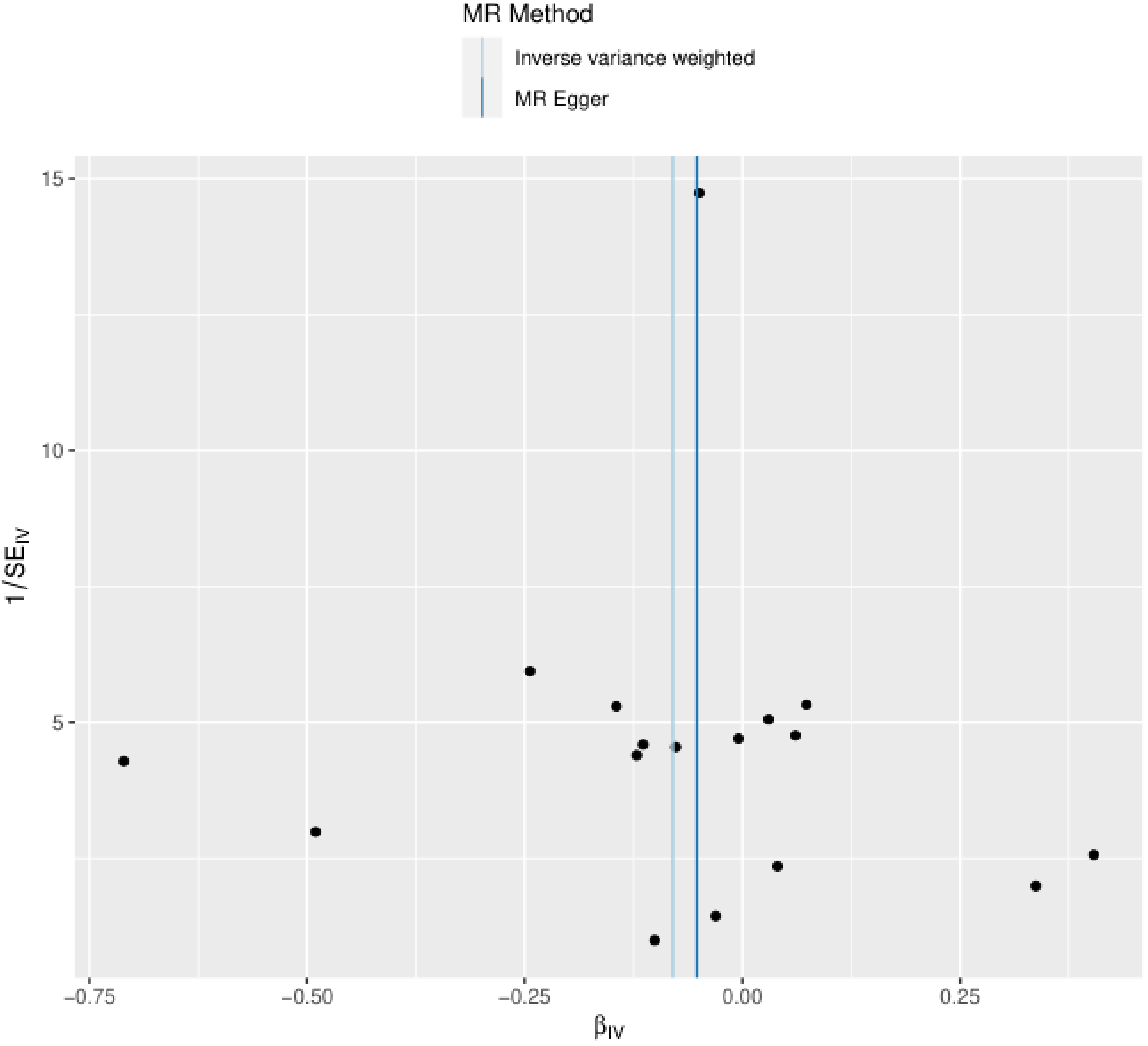

Funnel plot analyse of "CD8 on naive CD8br" on 'Diabetic nephropathy'

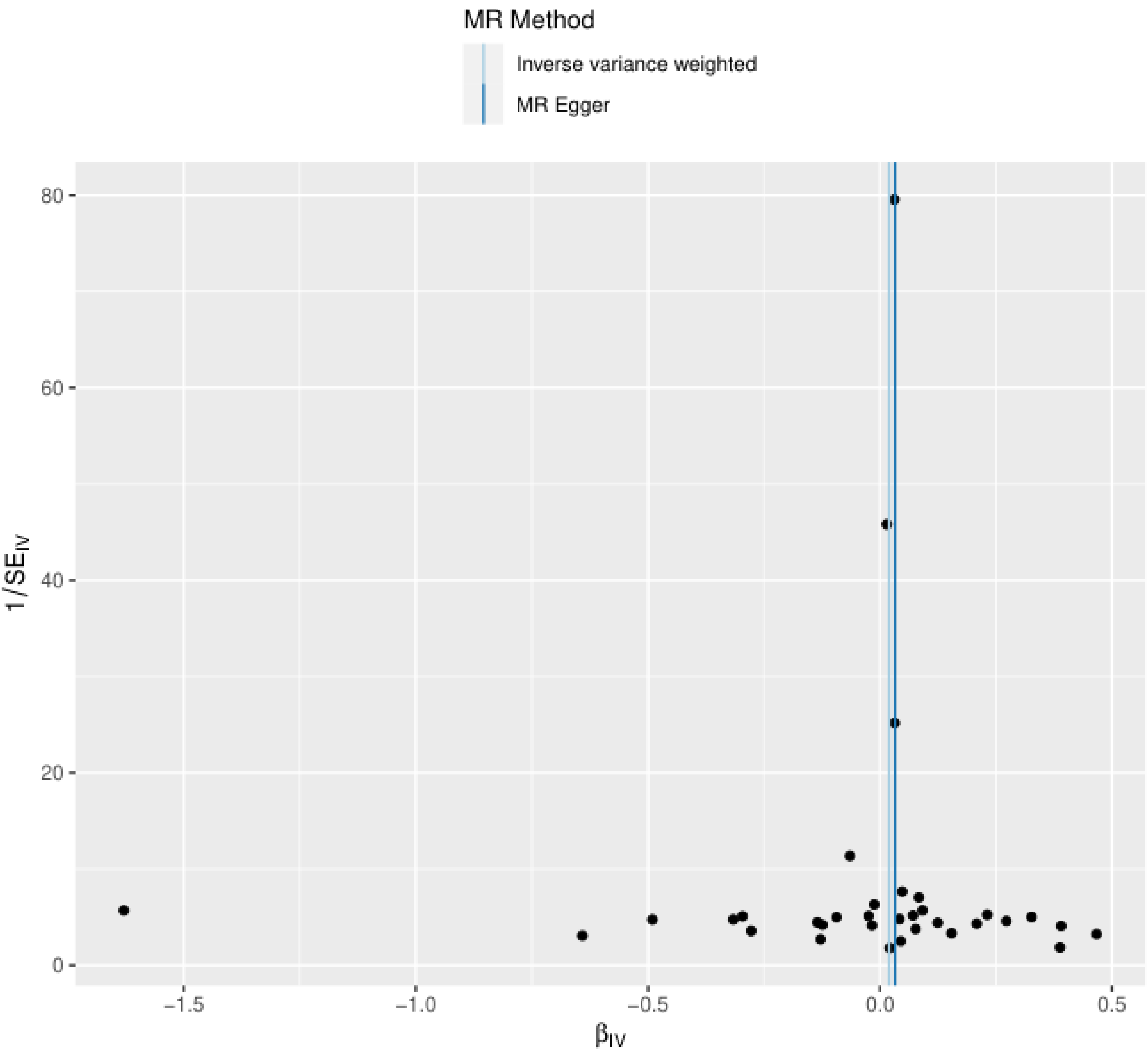

Funnel plot analyse of "CD20 on IgD- CD38dim" on 'Diabetic nephropathy'

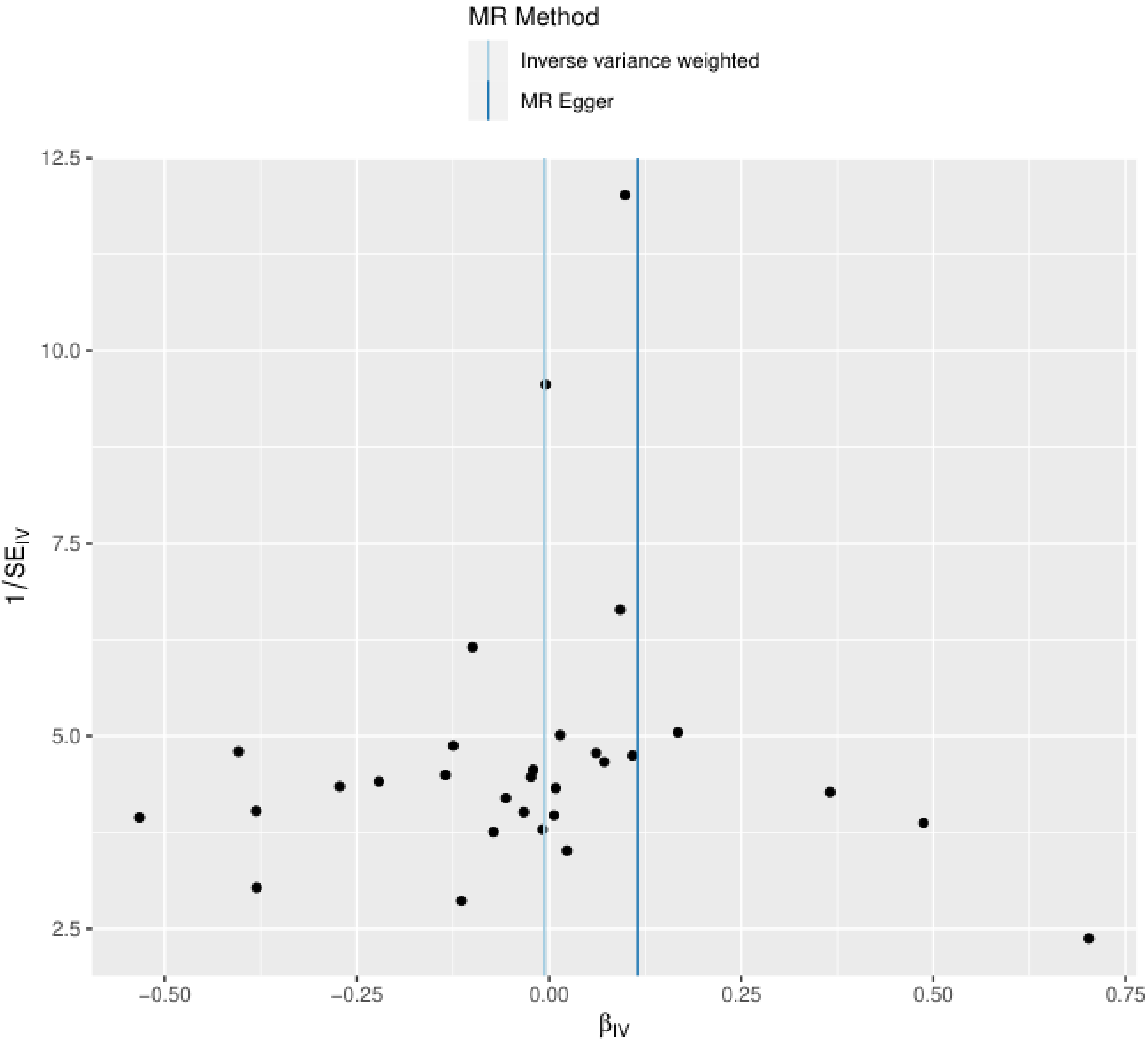

Funnel plot analyse of "B cell AC" on 'Diabetic nephropathy'

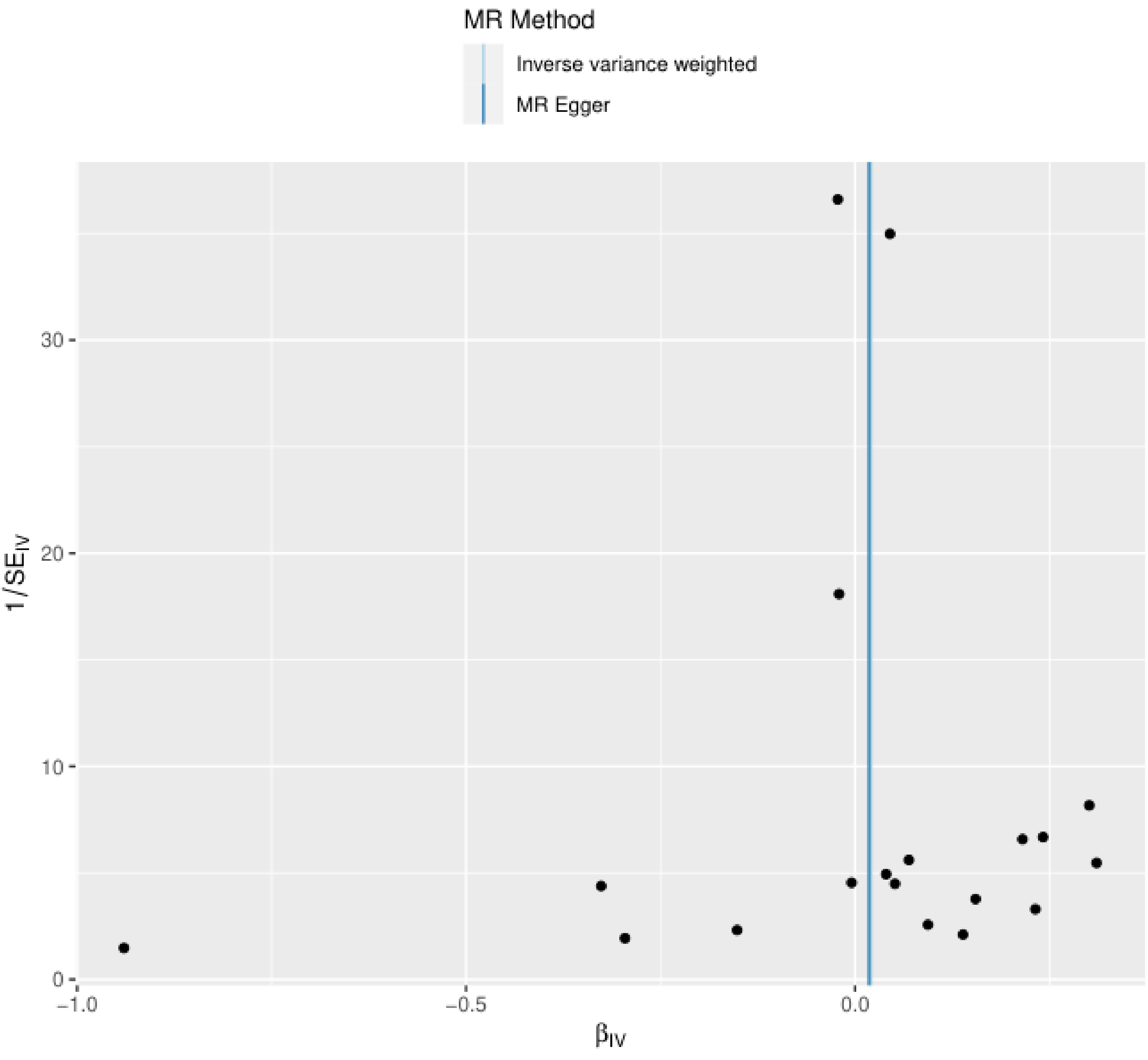

Funnel plot analyse of "CD62L- DC AC" on 'Diabetic nephropathy'

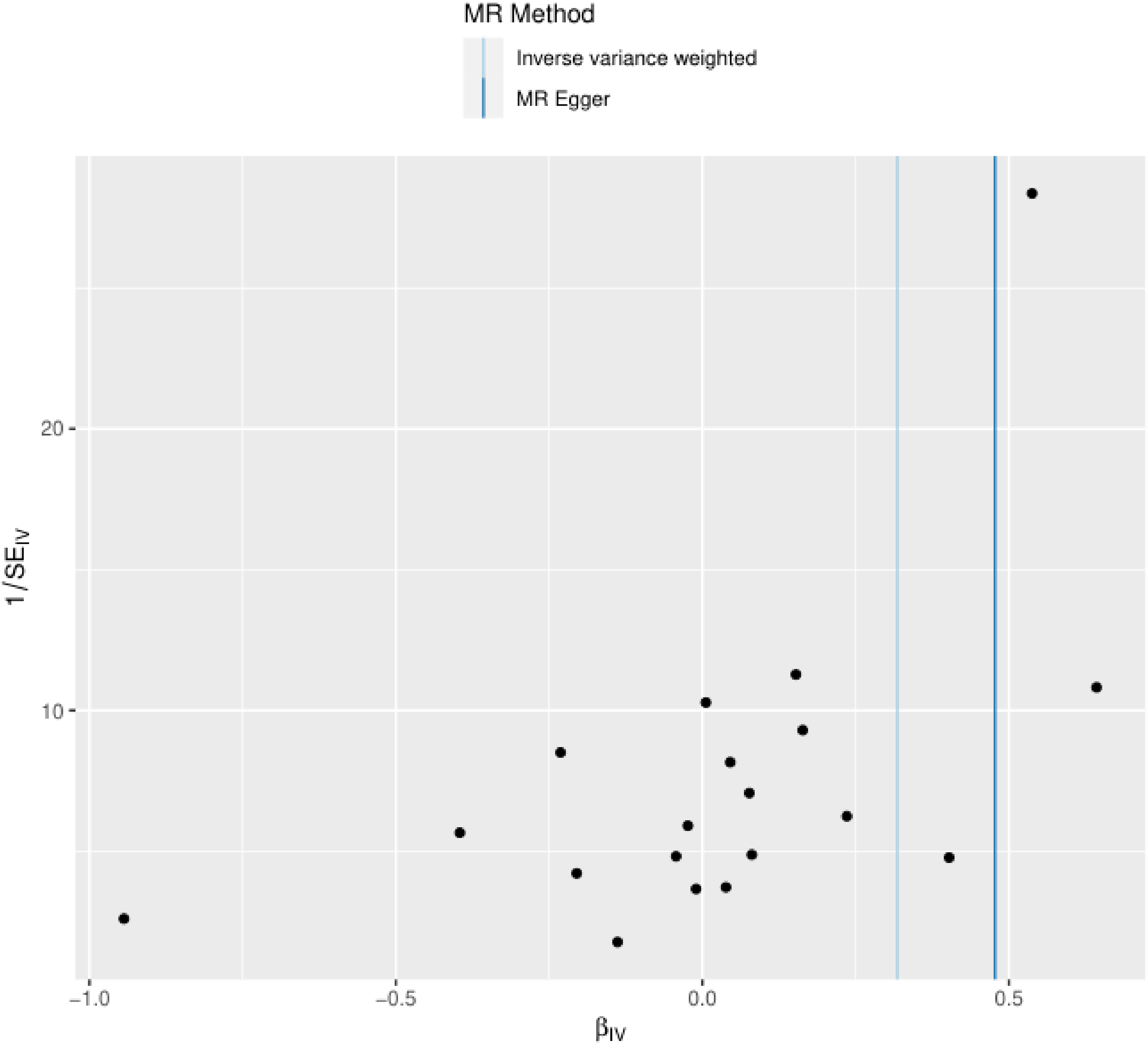

Funnel plot analyse of "HLA DR on DC" on 'Diabetic nephropathy'

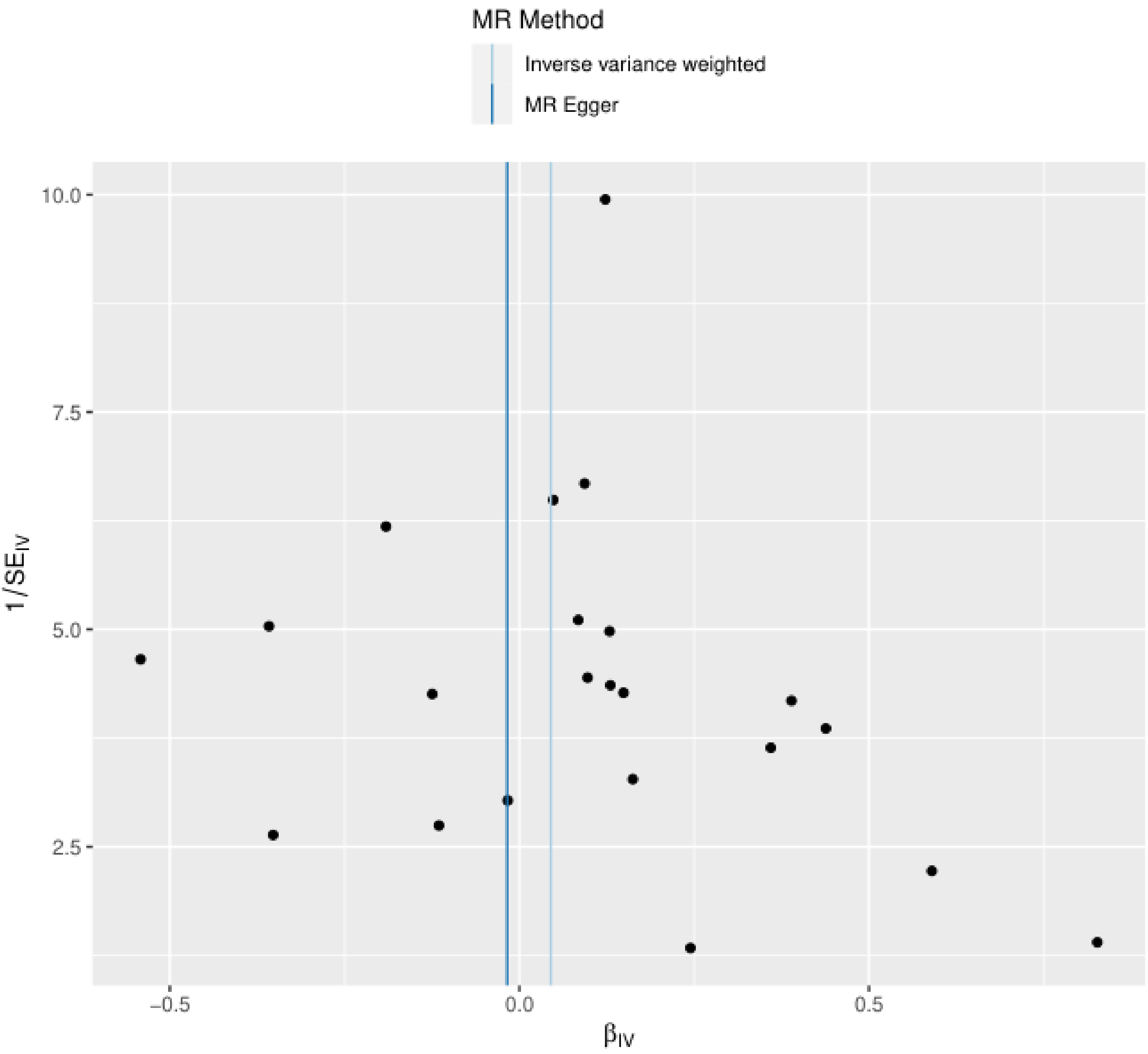

Funnel plot analyse of "CD19 on PB/PC" on 'Diabetic nephropathy'

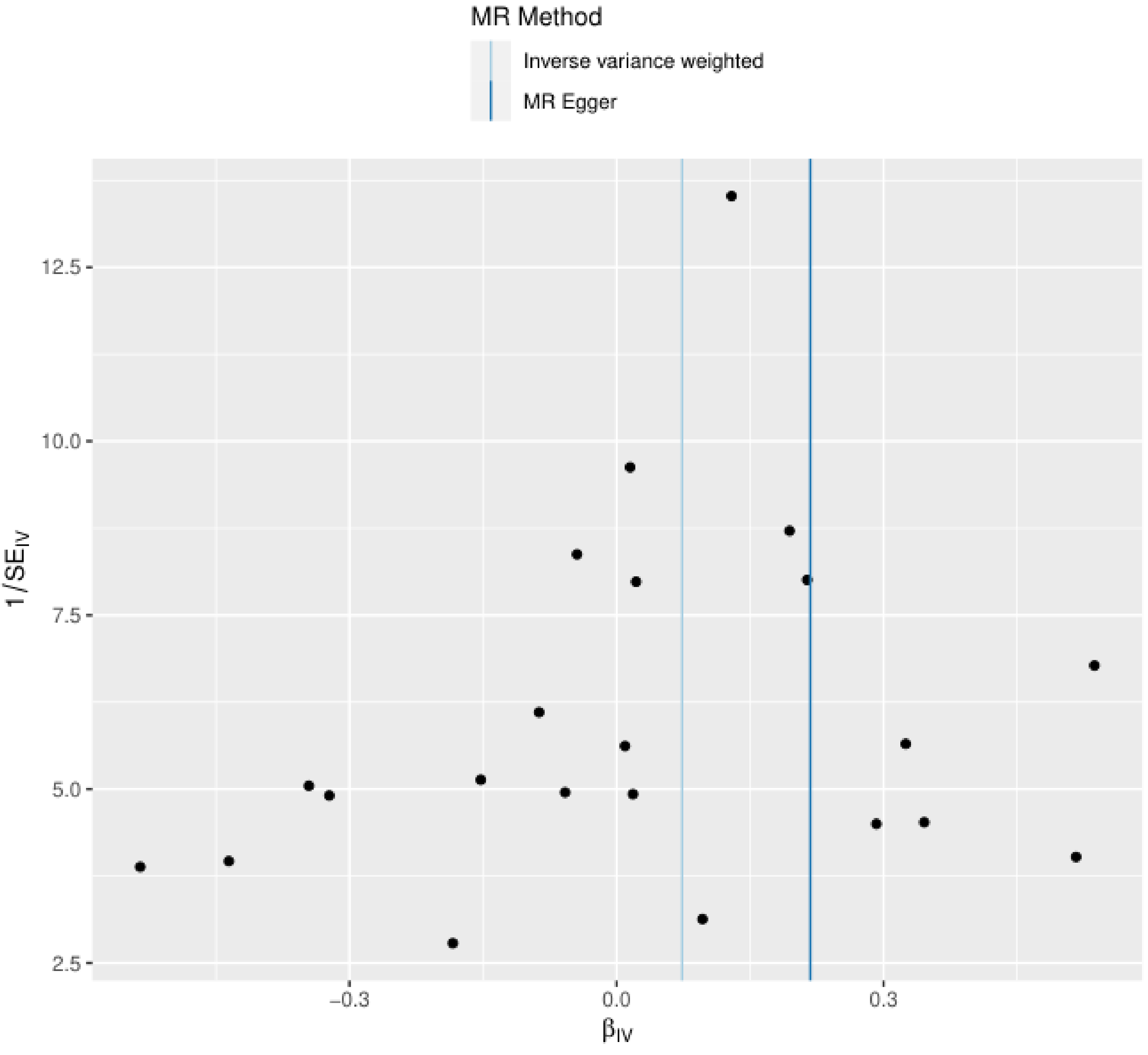

Funnel plot analyse of "CD4 on CD4+ " on 'Diabetic nephropathy'

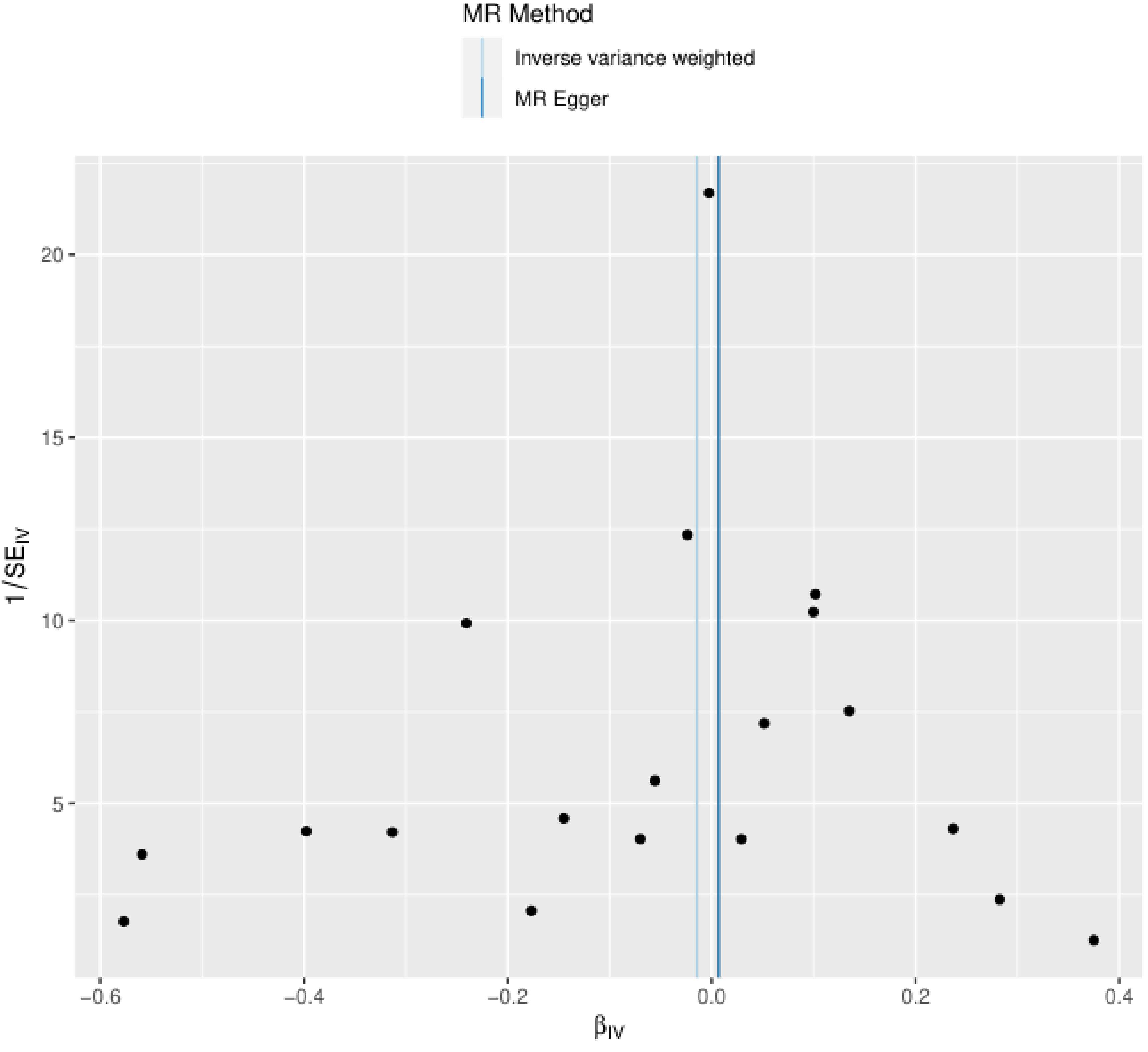

Funnel plot analyse of "CD62L- plasmacytoid DC %DC" on 'Diabetic nephropathy'

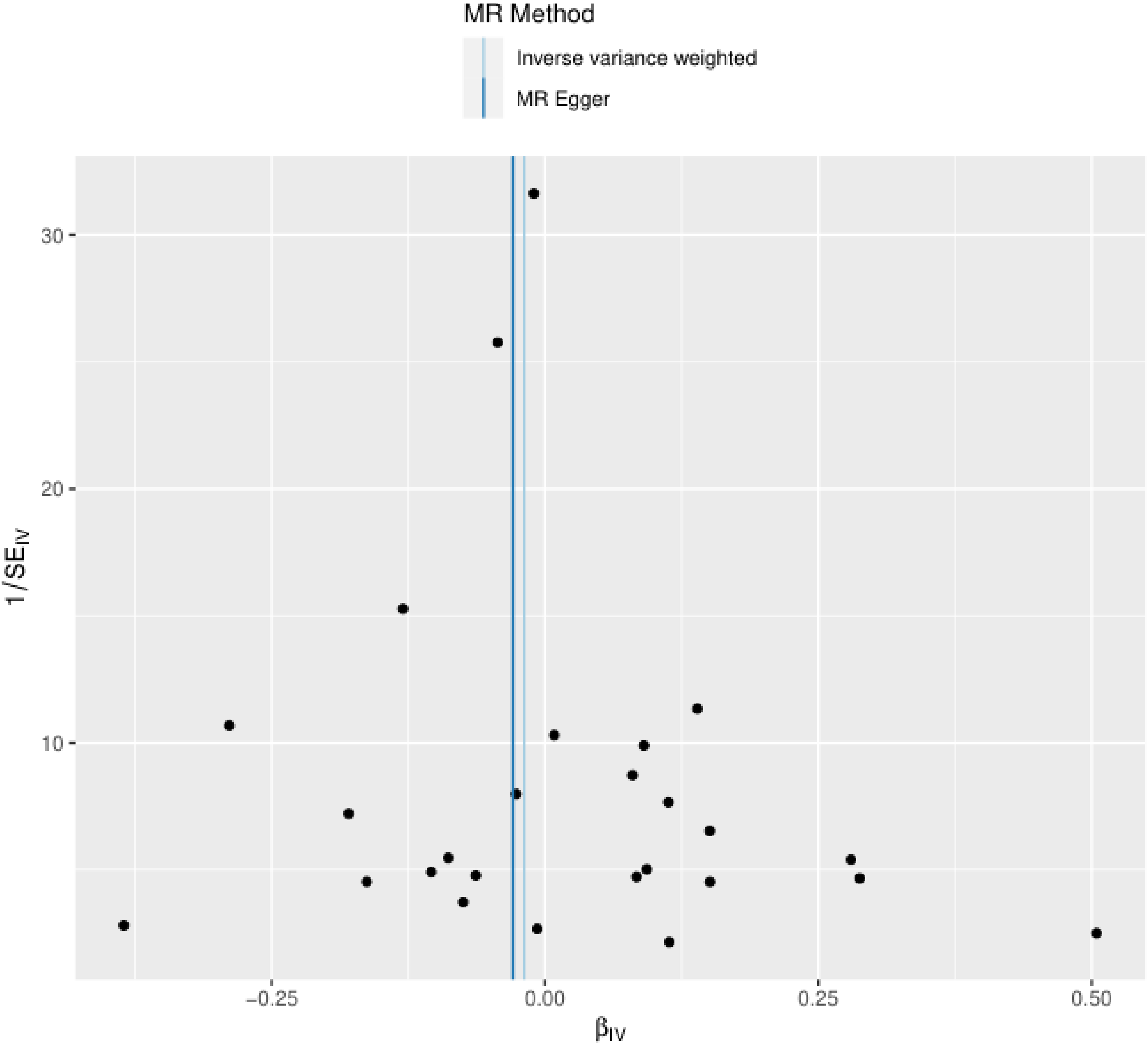

Funnel plot analyse of "CD39+ secreting Treg %secreting Treg" on 'Diabetic nephropathy'

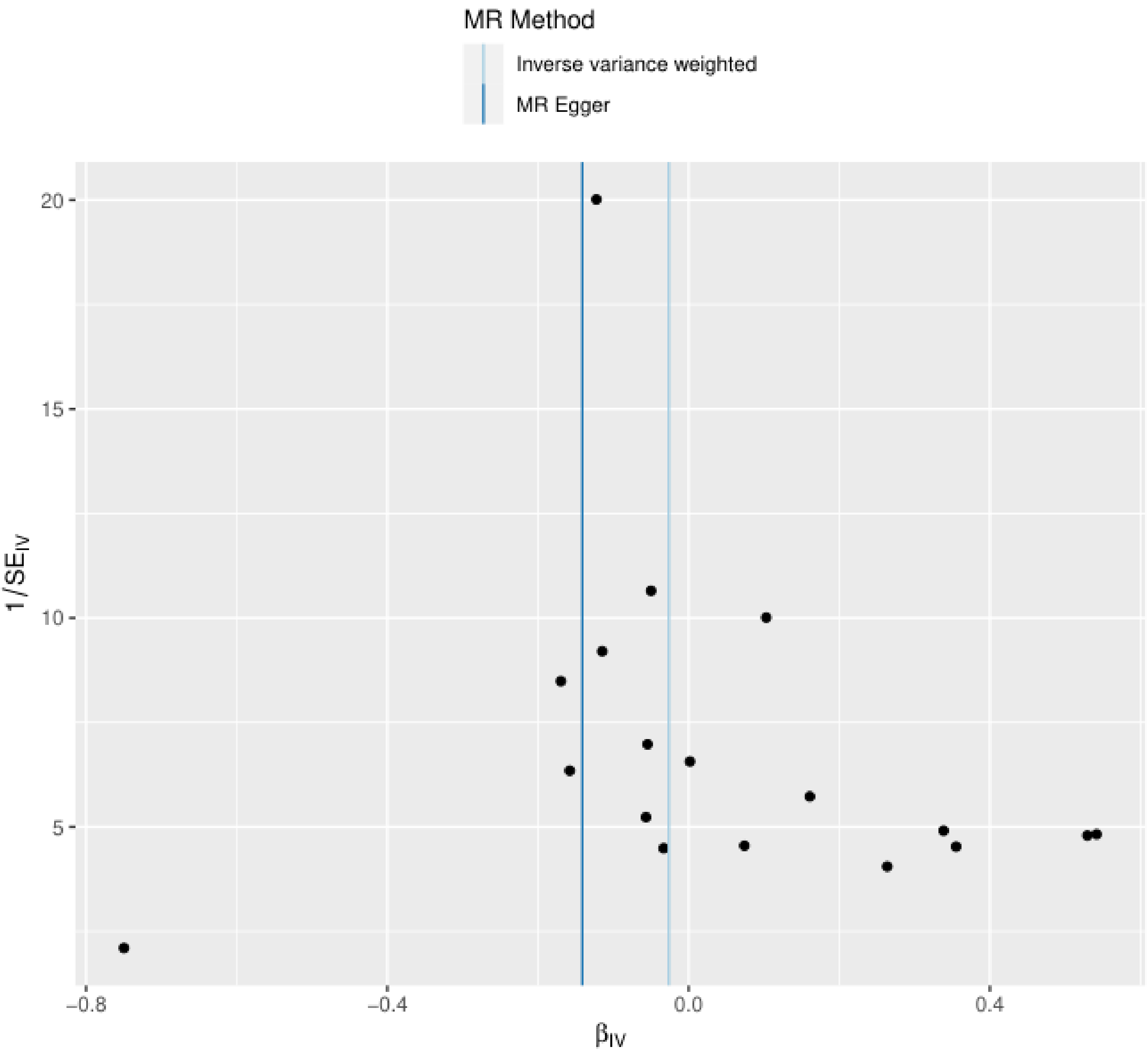

Funnel plot analyse of "CD28 on activated Treg" on 'Diabetic nephropathy'

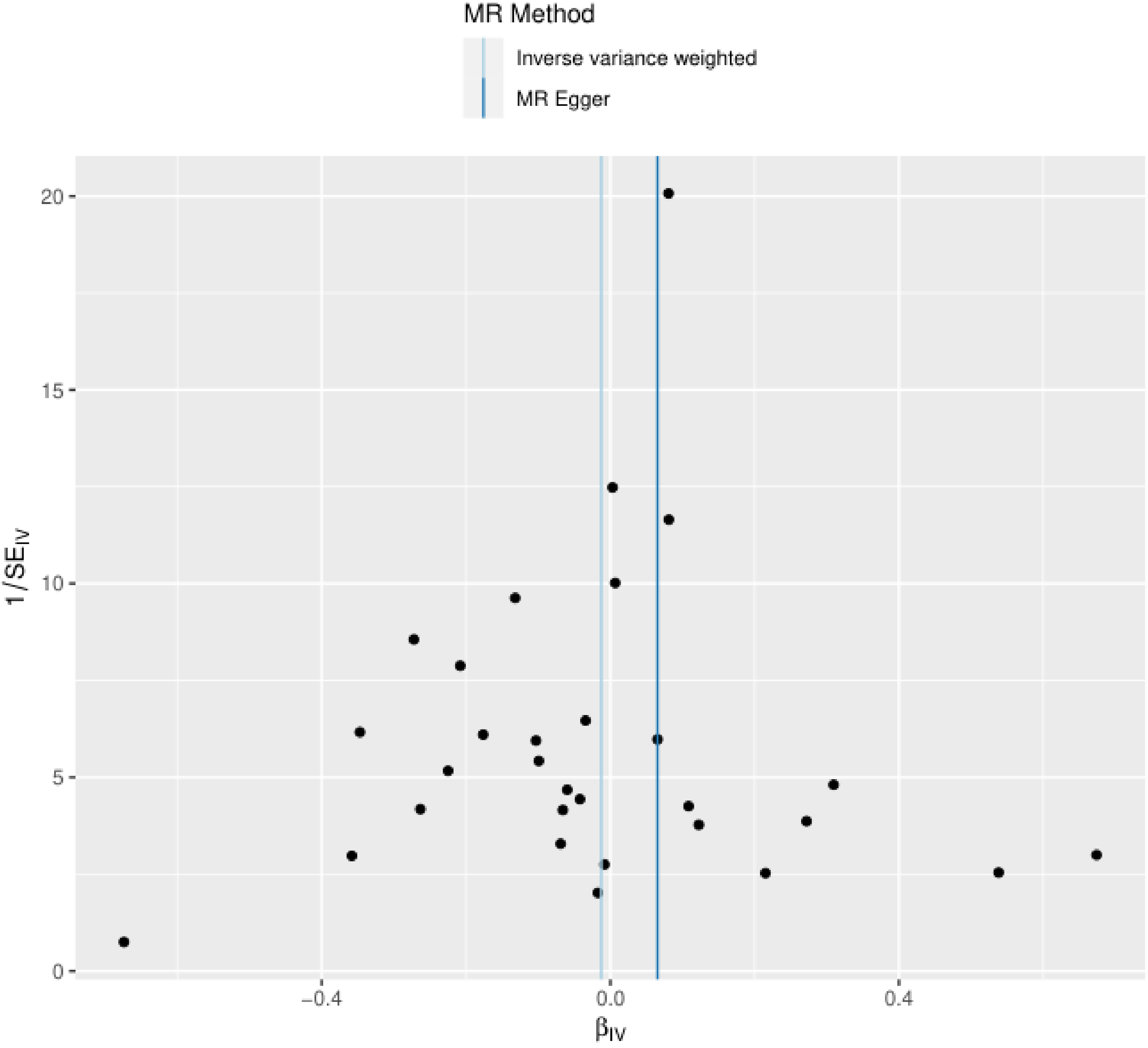

Funnel plot analyse of "CD27 on CD24+ CD27+" on 'Diabetic nephropathy'

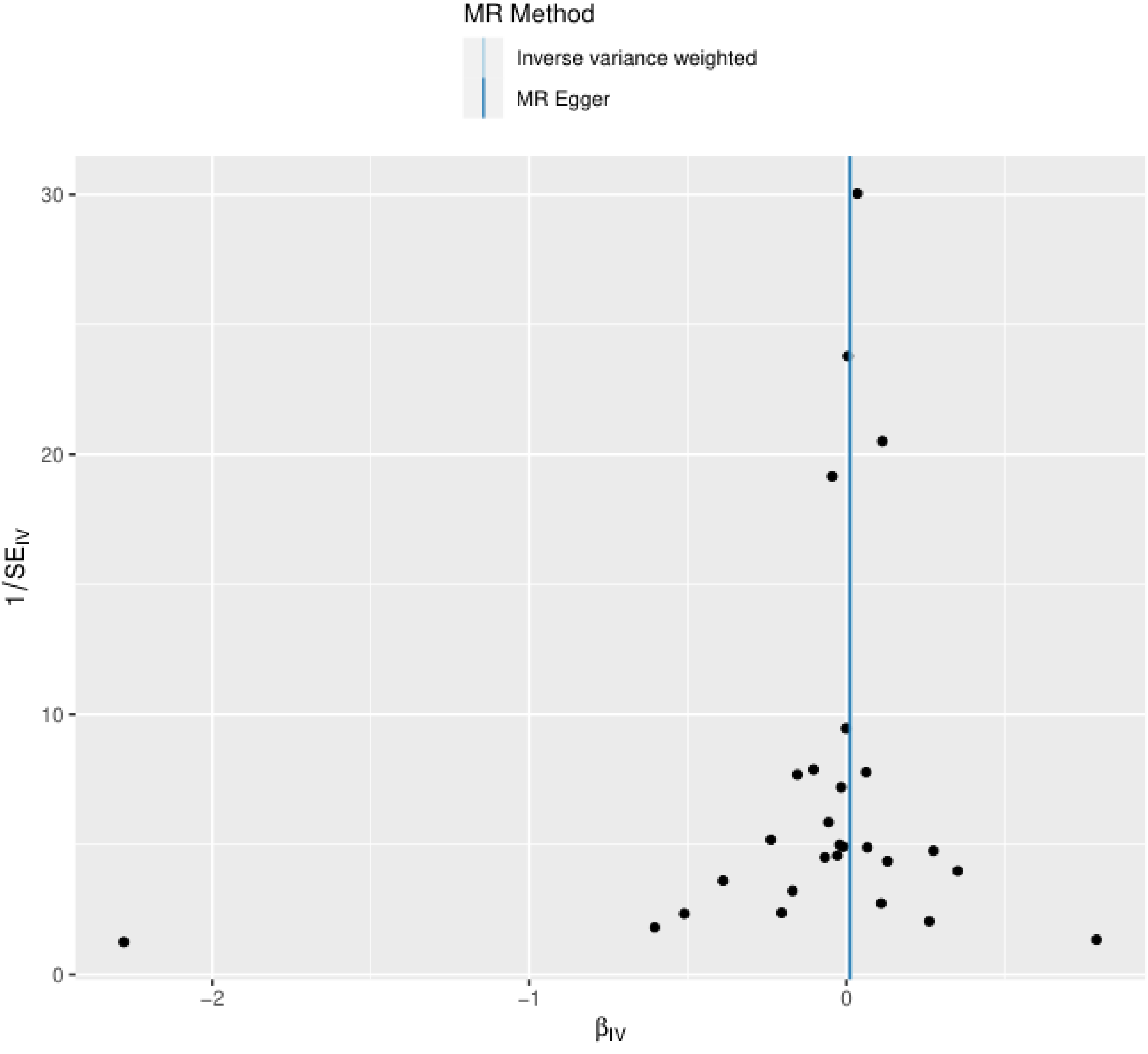

Funnel plot analyse of "CD40 on CD14- CD16+ monocyte " on 'Diabetic nephropathy'

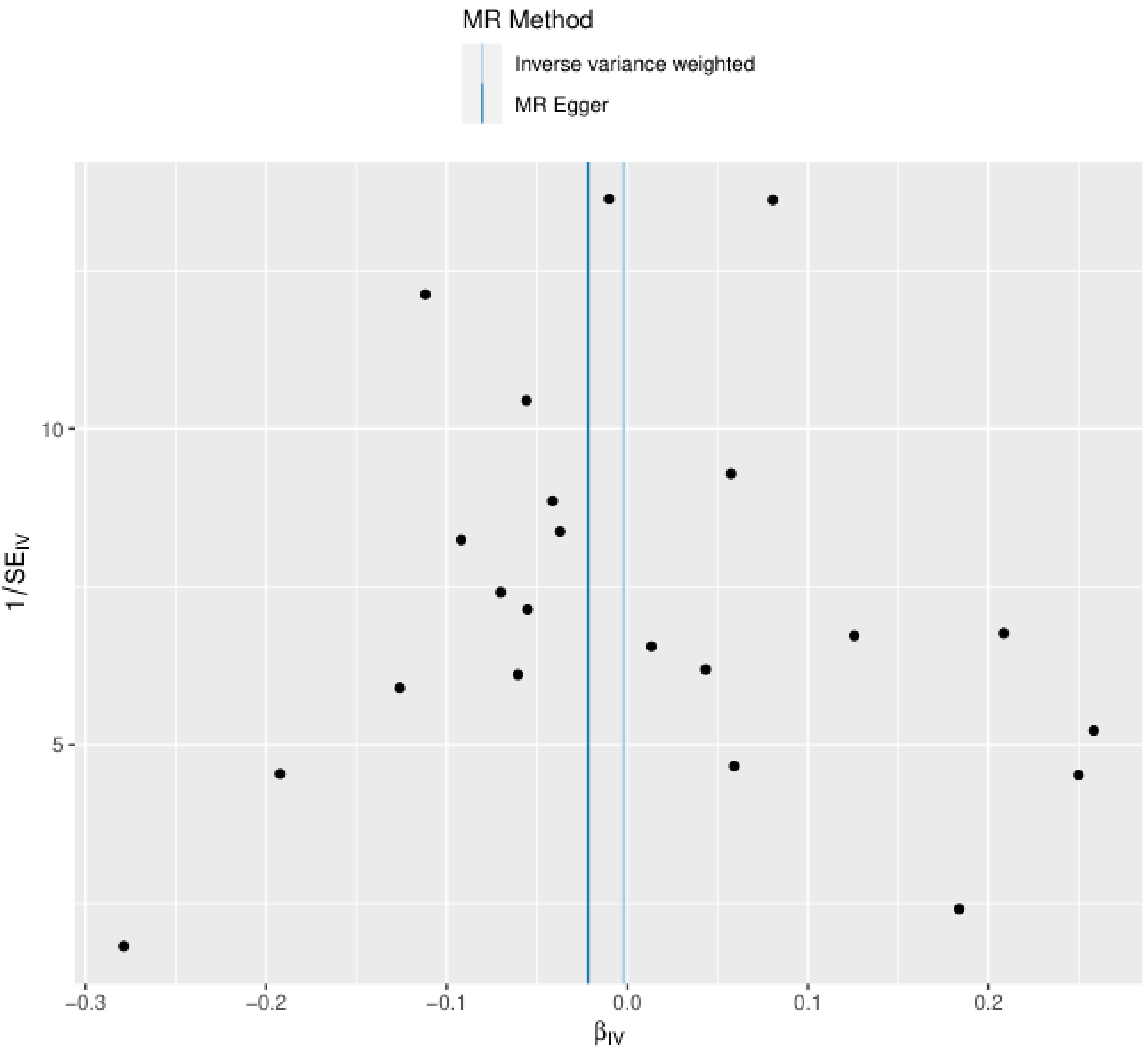

Funnel plot analyse of "CD11b on CD33dim HLA DR- " on 'Diabetic nephropathy'
