# Supplementary material for: Monocyte human leukocyte antigen-DR-mediated diabetic nephropathy progression is a promising therapeutic target
Source: Front Endocrinol (Lausanne). 2025 Dec 9;16:1733139. doi: 10.3389/fendo.2025.1733139 (PMC12722863; doi:10.3389/fendo.2025.1733139)

Supplementary Figure 5: Correlation analysis between MFI of HLA-DR on CD14+ and CD14+ CD16- monocyte with blood urea nitrogen, urinary protein excretion, fasting blood glucouse and serum albumin in patients with DN.

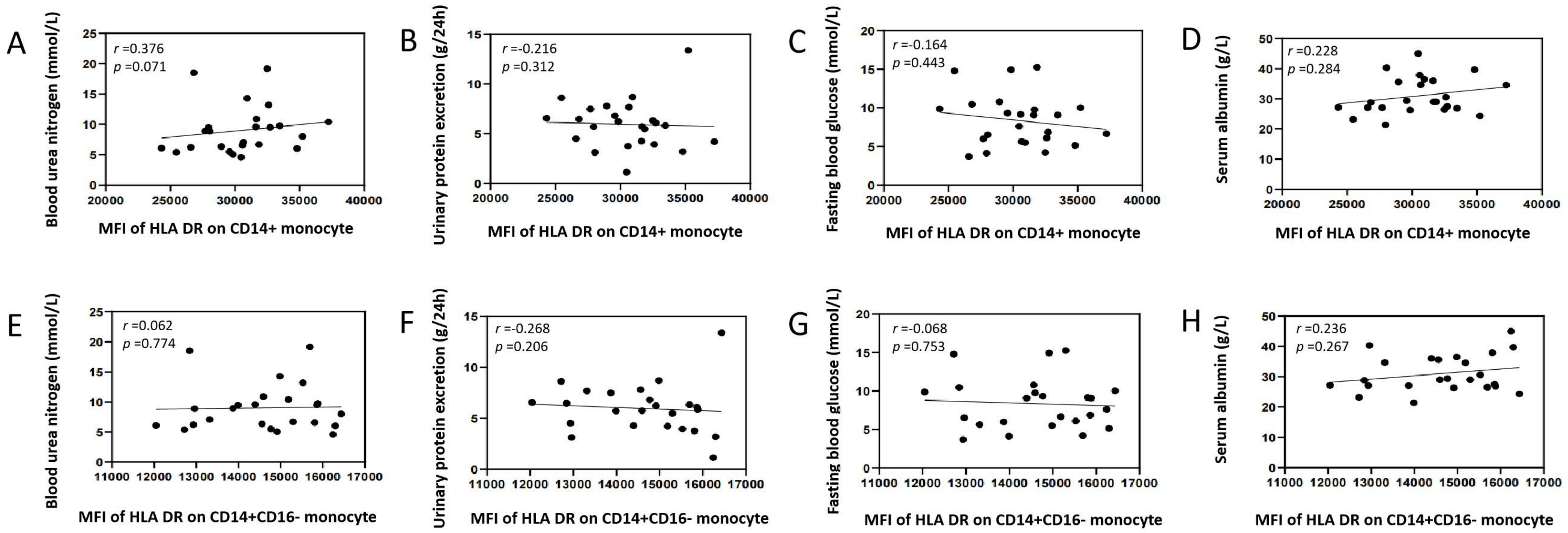

Supplement: Supplementary Figure 5 — Correlation analysis between MFI of HLA DR on CD14+ and CD14+ CD16− monocyte with blood urea nitrogen, urinary protein excretion, fasting blood glucose and serum albumin in patients with DN. MFI, median fluorescence intensities; HLA, human leukocyte antigen; DN, diabetic nephropathy. [file DataSheet5.pdf]
