# Supplementary material for: Monocyte human leukocyte antigen-DR-mediated diabetic nephropathy progression is a promising therapeutic target
Source: Front Endocrinol (Lausanne). 2025 Dec 9;16:1733139. doi: 10.3389/fendo.2025.1733139 (PMC12722863; doi:10.3389/fendo.2025.1733139)
Supplement: Supplementary Figure 7 — MFI of HLA DR on monocyte of different grades in renal pathology of DN patients. MFI, median fluorescence intensities; HLA, human leukocyte antigen; DN, diabetic nephropathy. [file DataSheet7.pdf]

Supplementary Figure 7: MFI of HLA DR on monocyte of different grades in renal pathology of DN patients.

A

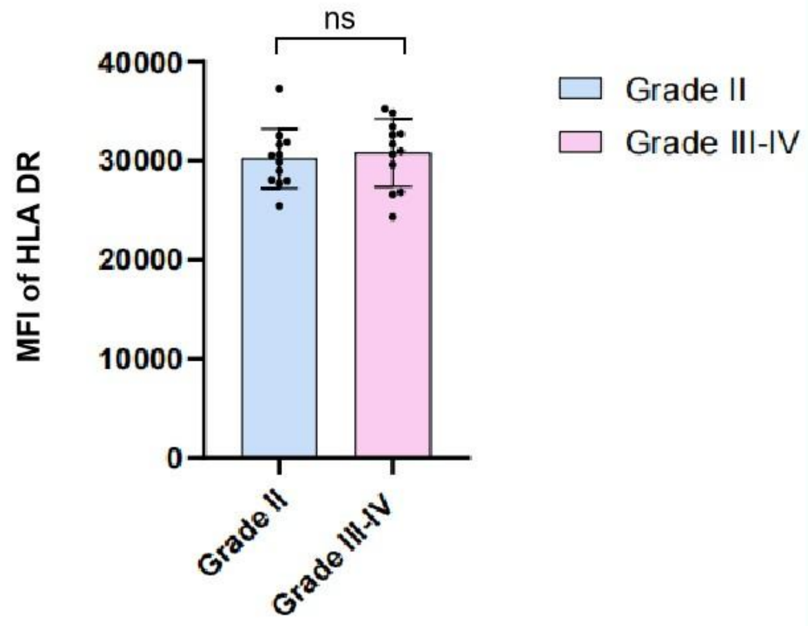

MFI of HLA DR on CD14+ monocyte

B

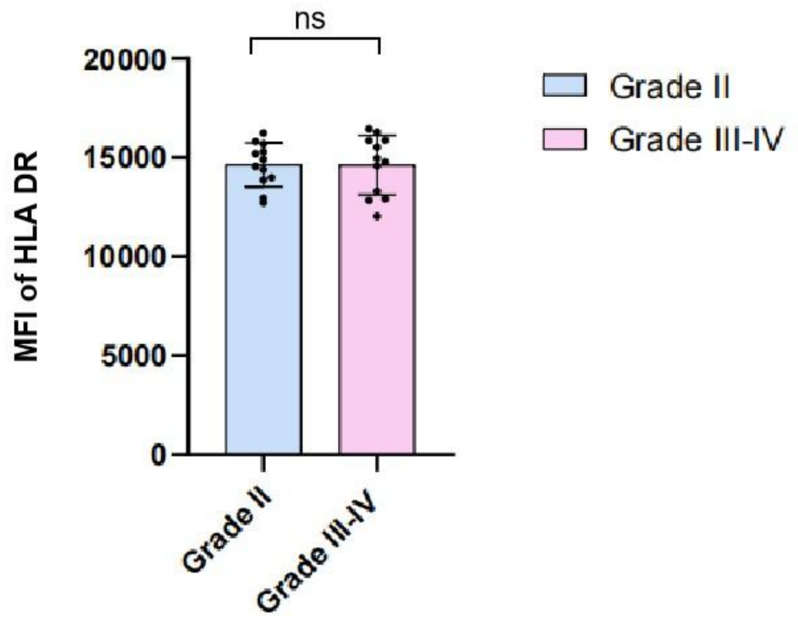

MFI of HLA DR on CD14+ CD16- monocyte
