# Supplementary material for: Monocyte human leukocyte antigen-DR-mediated diabetic nephropathy progression is a promising therapeutic target
Source: Front Endocrinol (Lausanne). 2025 Dec 9;16:1733139. doi: 10.3389/fendo.2025.1733139 (PMC12722863; doi:10.3389/fendo.2025.1733139)
Supplement: Supplementary Figure 1 — Leave one out analyses of all MR analyses of 731 immunophenotypes on DN. MR, mendelian randomization; DN, diabetic nephropathy. [file DataSheet1.pdf]

Supplementary Figure 1: Leave one out analyses of all MR analyses of 731 immunophenotypes on diabetic nephropathy

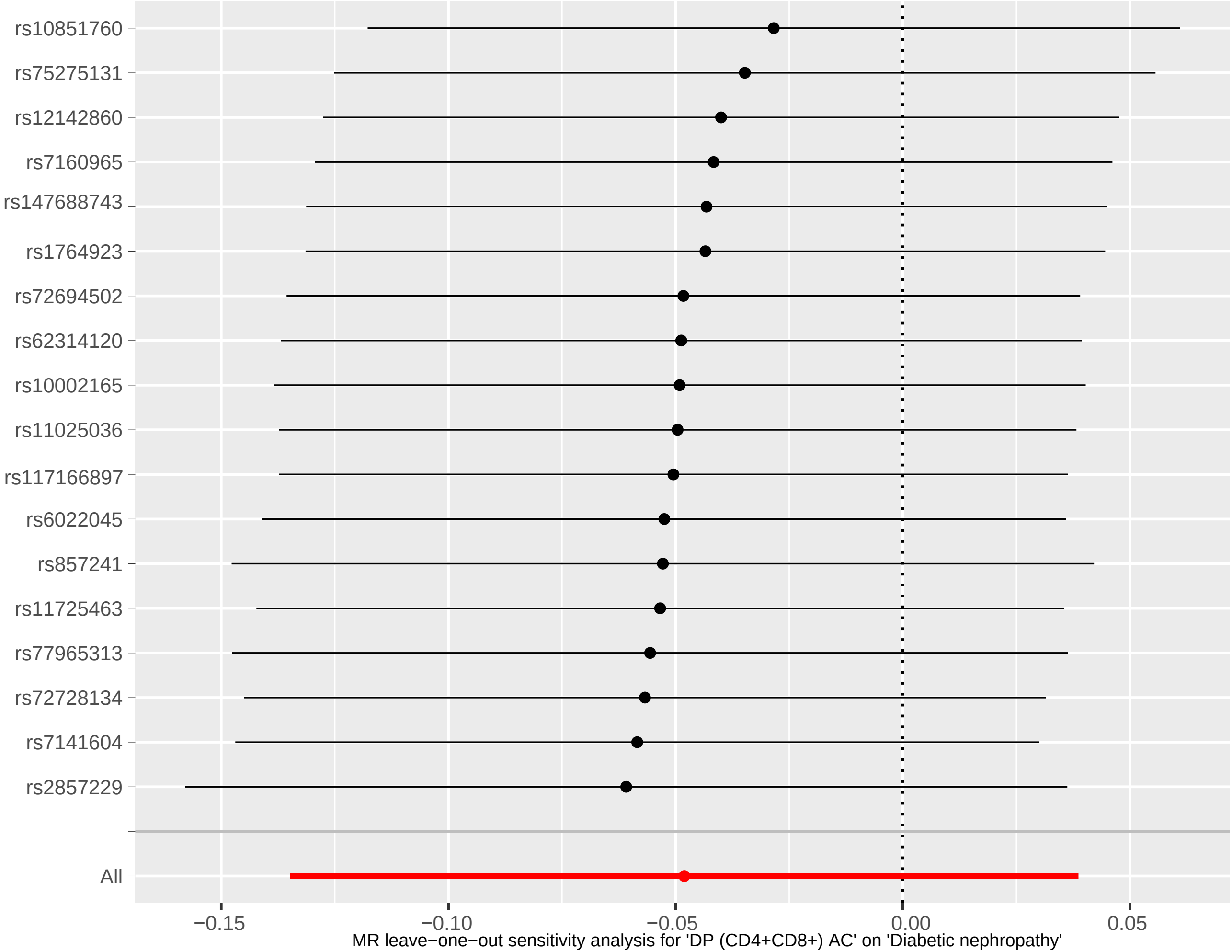

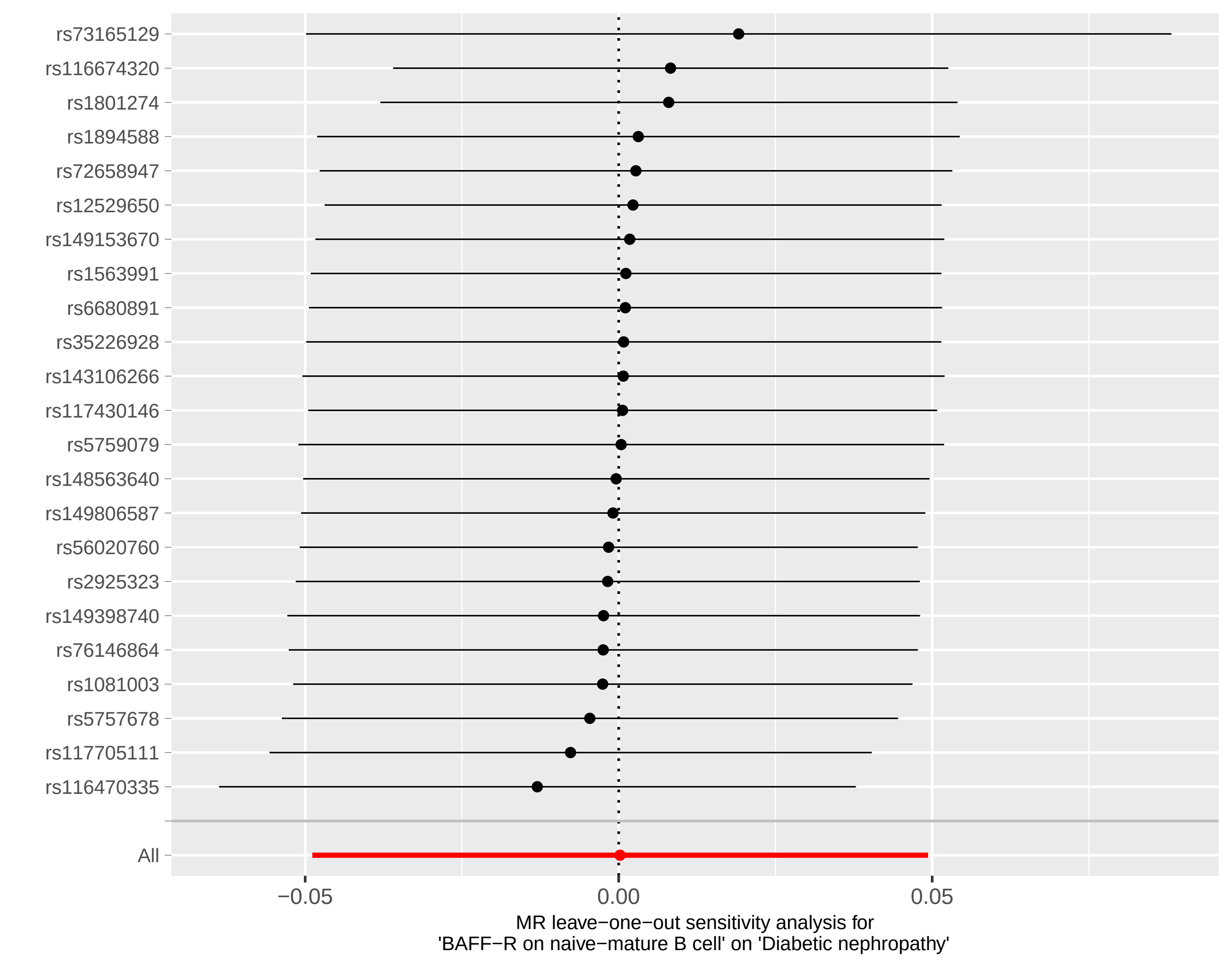

rs73165129
rs116674320
rs1801274
rs1894588
rs72658947
rs12529650
rs149153670
rs1563991
rs6680891
rs35226928
rs143106266
rs117430146
rs5759079
rs148563640
rs149806587
rs56020760
rs2925323
rs149398740
rs76146864
rs1081003
rs5757678
rs117705111
rs116470335
All
−0.05
0.00
0.05
MR leave−one−out sensitivity analysis for
'BAFF−R on naive−mature B cell' on 'Diabetic nephropathy'

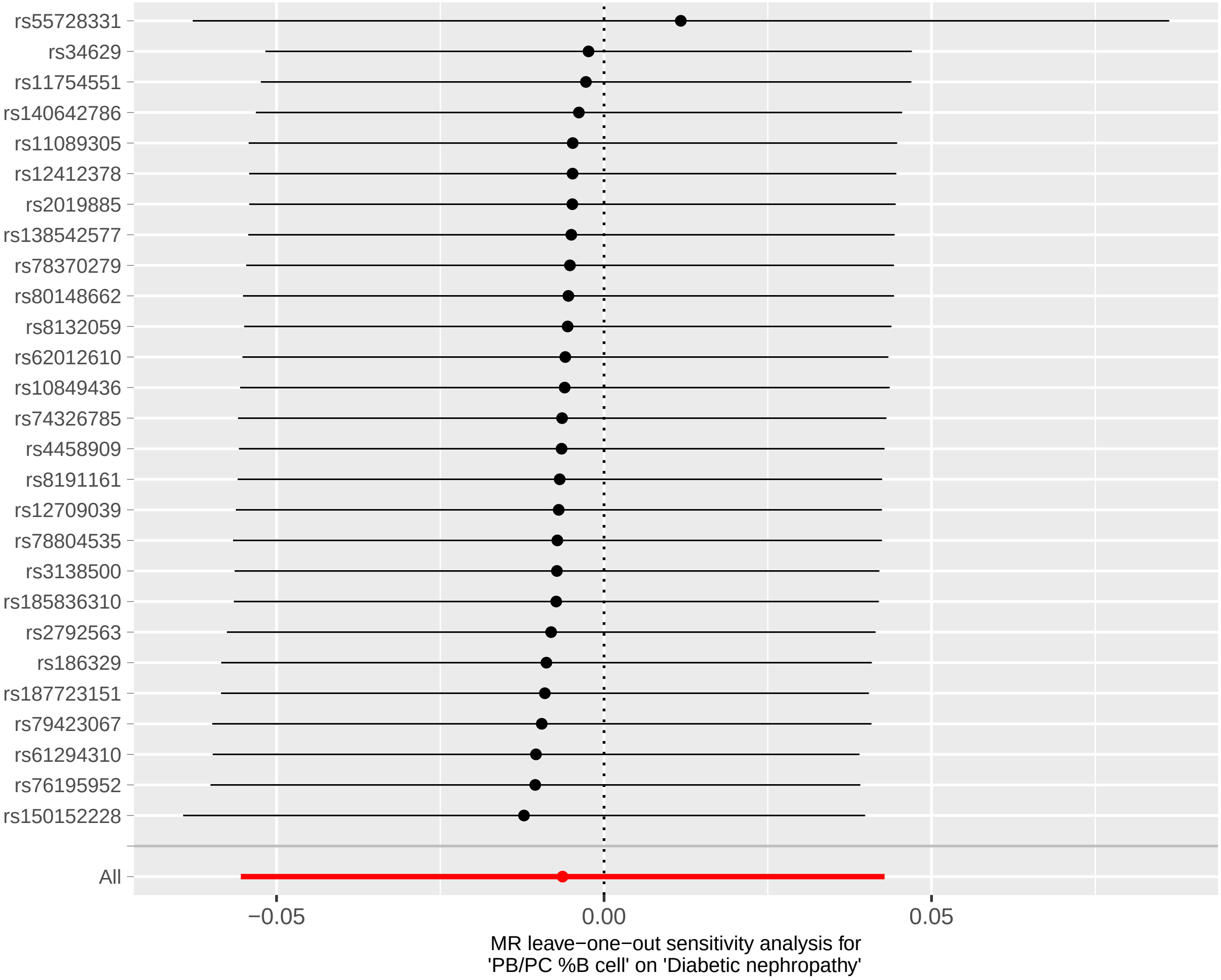
rs55728331
rs34629
rs11754551
rs140642786
rs11089305
rs12412378
rs2019885
rs138542577
rs78370279
rs80148662
rs8132059
rs62012610
rs10849436
rs74326785
rs4458909
rs8191161
rs12709039
rs78804535
rs3138500
rs185836310
rs2792563
rs186329
rs187723151
rs79423067
rs61294310
rs76195952
rs150152228
All
−0.05
0.00
0.05
MR leave−one−out sensitivity analysis for
'PB/PC %B cell' on 'Diabetic nephropathy'

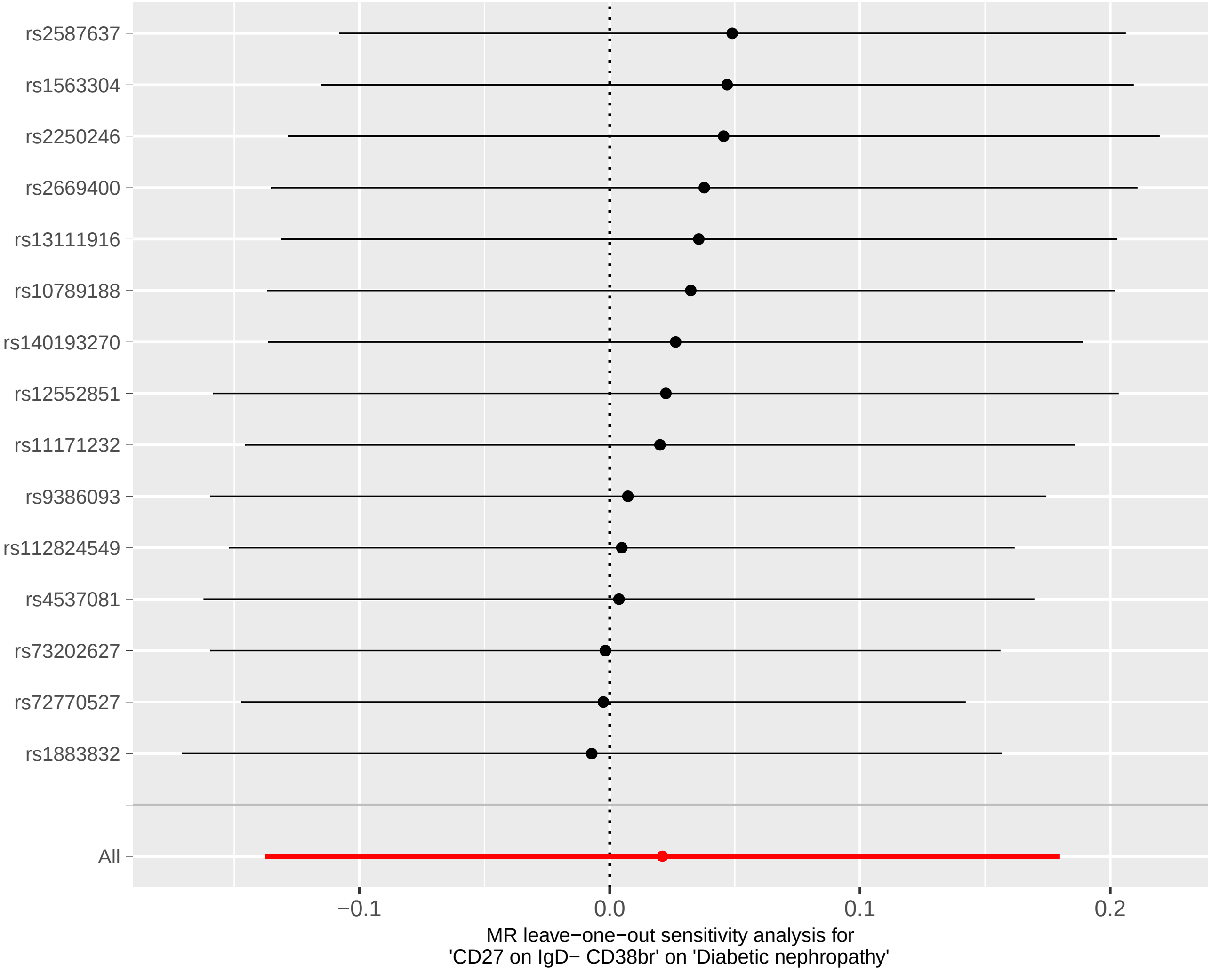
rs2587637
rs1563304
rs2250246
rs2669400
rs13111916
rs10789188
rs140193270
rs12552851
rs11171232
rs9386093
rs112824549
rs4537081
rs73202627
rs72770527
rs1883832
All
−0.1
0.0
0.1
0.2
MR leave−one−out sensitivity analysis for
'CD27 on IgD− CD38br' on 'Diabetic nephropathy'

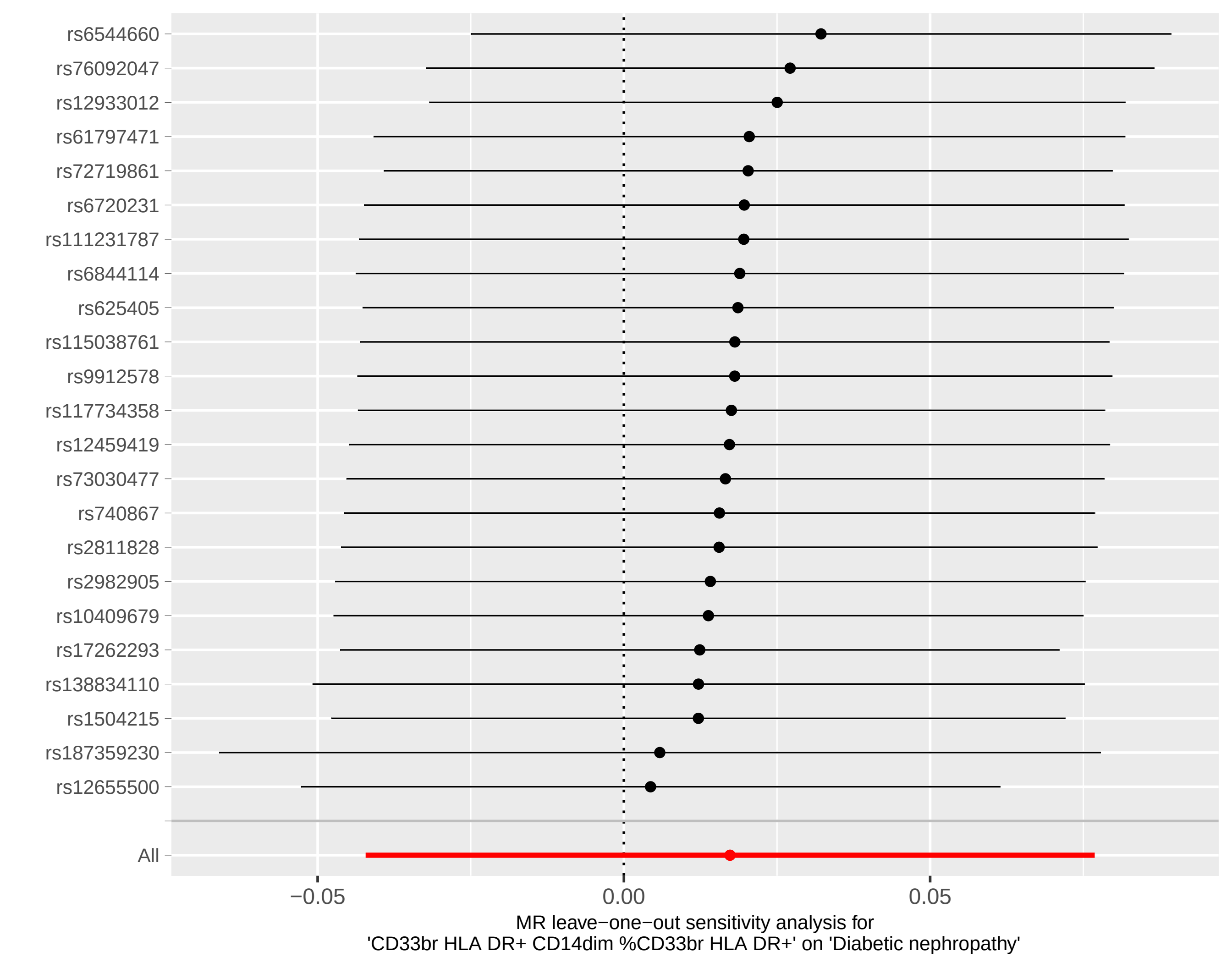
rs6544660
rs76092047
rs12933012
rs61797471
rs72719861
rs6720231
rs111231787
rs6844114
rs625405
rs115038761
rs9912578
rs117734358
rs12459419
rs73030477
rs740867
rs2811828
rs2982905
rs10409679
rs17262293
rs138834110
rs1504215
rs187359230
rs12655500
All
−0.05
0.00
0.05
MR leave−one−out sensitivity analysis for
'CD33br HLA DR+ CD14dim %CD33br HLA DR+' on 'Diabetic nephropathy'

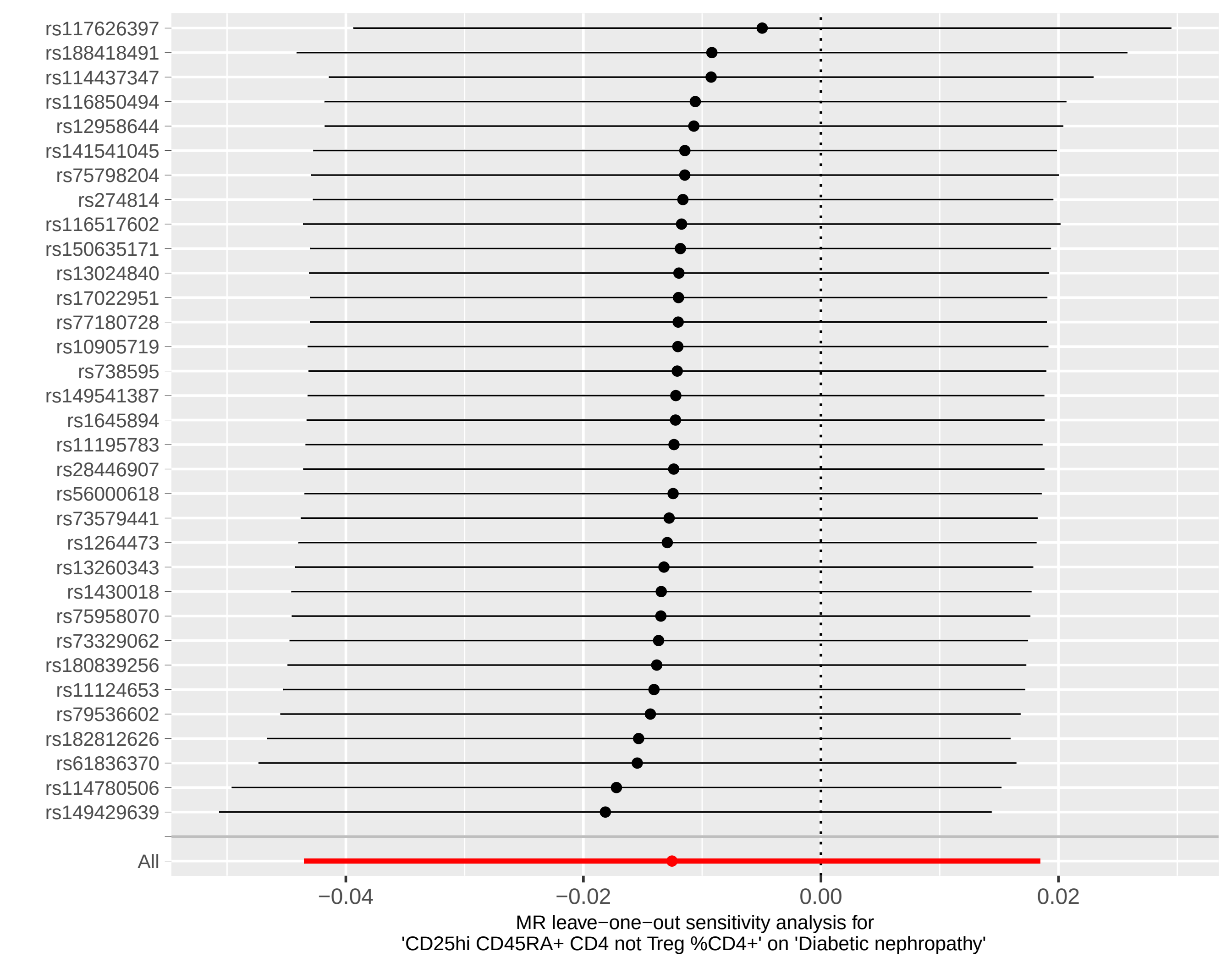
rs117626397
rs188418491
rs114437347
rs116850494
rs12958644
rs141541045
rs75798204
rs274814
rs116517602
rs150635171
rs13024840
rs17022951
rs77180728
rs10905719
rs738595
rs149541387
rs1645894
rs11195783
rs28446907
rs56000618
rs73579441
rs1264473
rs13260343
rs1430018
rs75958070
rs73329062
rs180839256
rs11124653
rs79536602
rs182812626
rs61836370
rs114780506
rs149429639
All
−0.04
−0.02
0.00
0.02
MR leave−one−out sensitivity analysis for
'CD25hi CD45RA+ CD4 not Treg %CD4+' on 'Diabetic nephropathy'

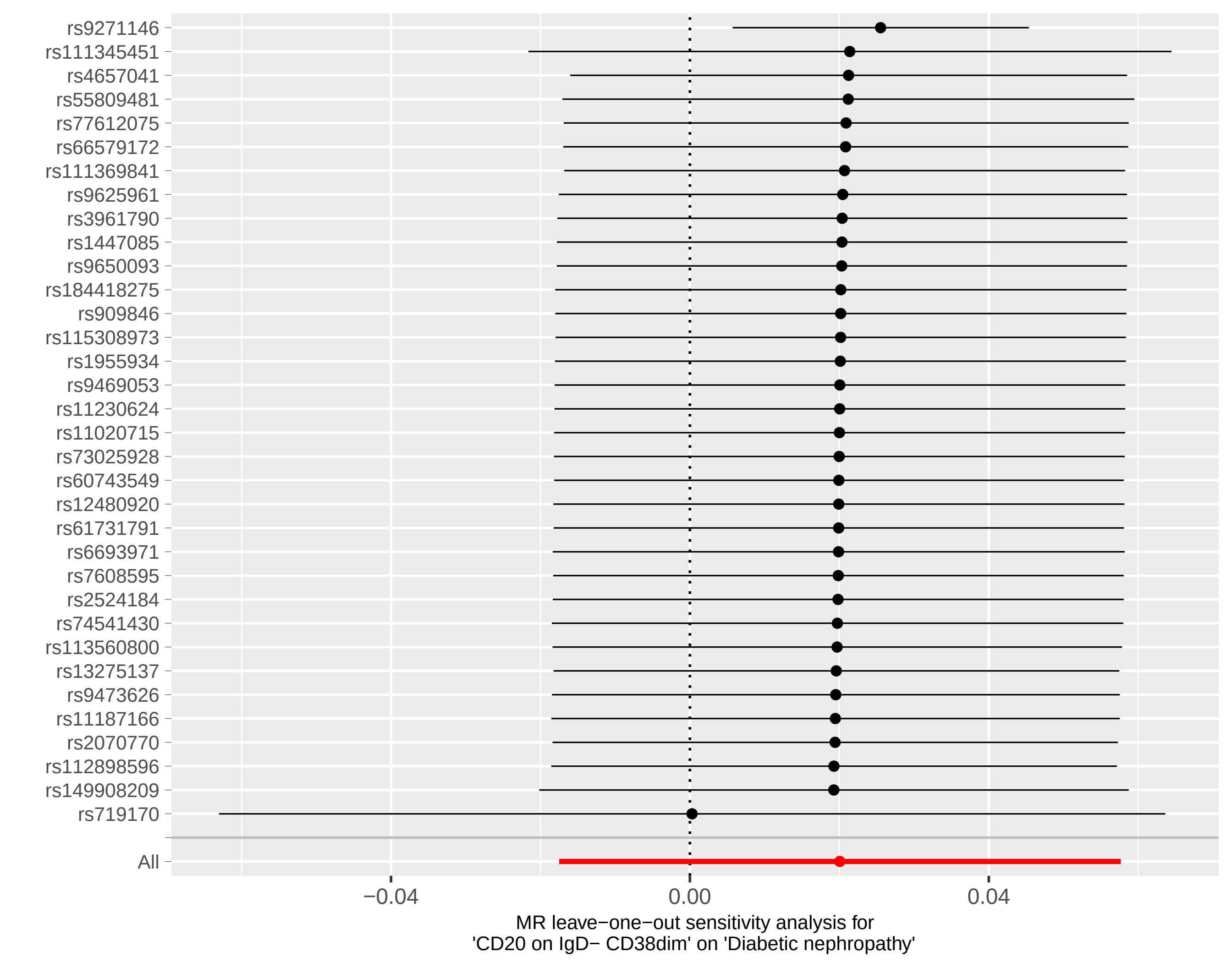
rs9271146
rs111345451
rs4657041
rs55809481
rs77612075
rs66579172
rs111369841
rs9625961
rs3961790
rs1447085
rs9650093
rs184418275
rs909846
rs115308973
rs1955934
rs9469053
rs11230624
rs11020715
rs73025928
rs60743549
rs12480920
rs61731791
rs6693971
rs7608595
rs2524184
rs74541430
rs113560800
rs13275137
rs9473626
rs11187166
rs2070770
rs112898596
rs149908209
rs719170
All
−0.04
0.00
0.04
MR leave−one−out sensitivity analysis for
'CD20 on IgD− CD38dim' on 'Diabetic nephropathy'

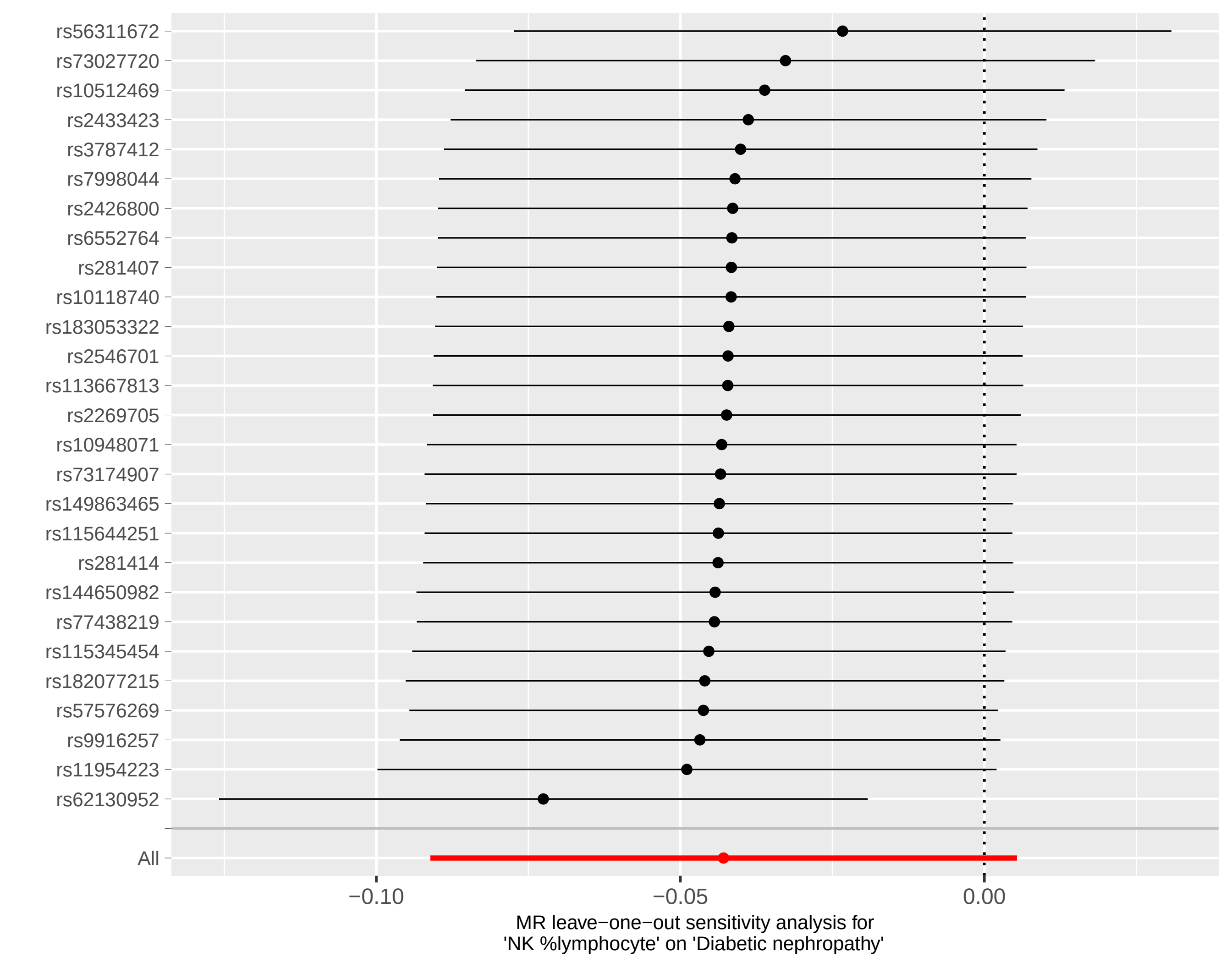
rs56311672
rs73027720
rs10512469
rs2433423
rs3787412
rs7998044
rs2426800
rs6552764
rs281407
rs10118740
rs183053322
rs2546701
rs113667813
rs2269705
rs10948071
rs73174907
rs149863465
rs115644251
rs281414
rs144650982
rs77438219
rs115345454
rs182077215
rs57576269
rs9916257
rs11954223
rs62130952
All
−0.10
−0.05
0.00
MR leave−one−out sensitivity analysis for
'NK %lymphocyte' on 'Diabetic nephropathy'

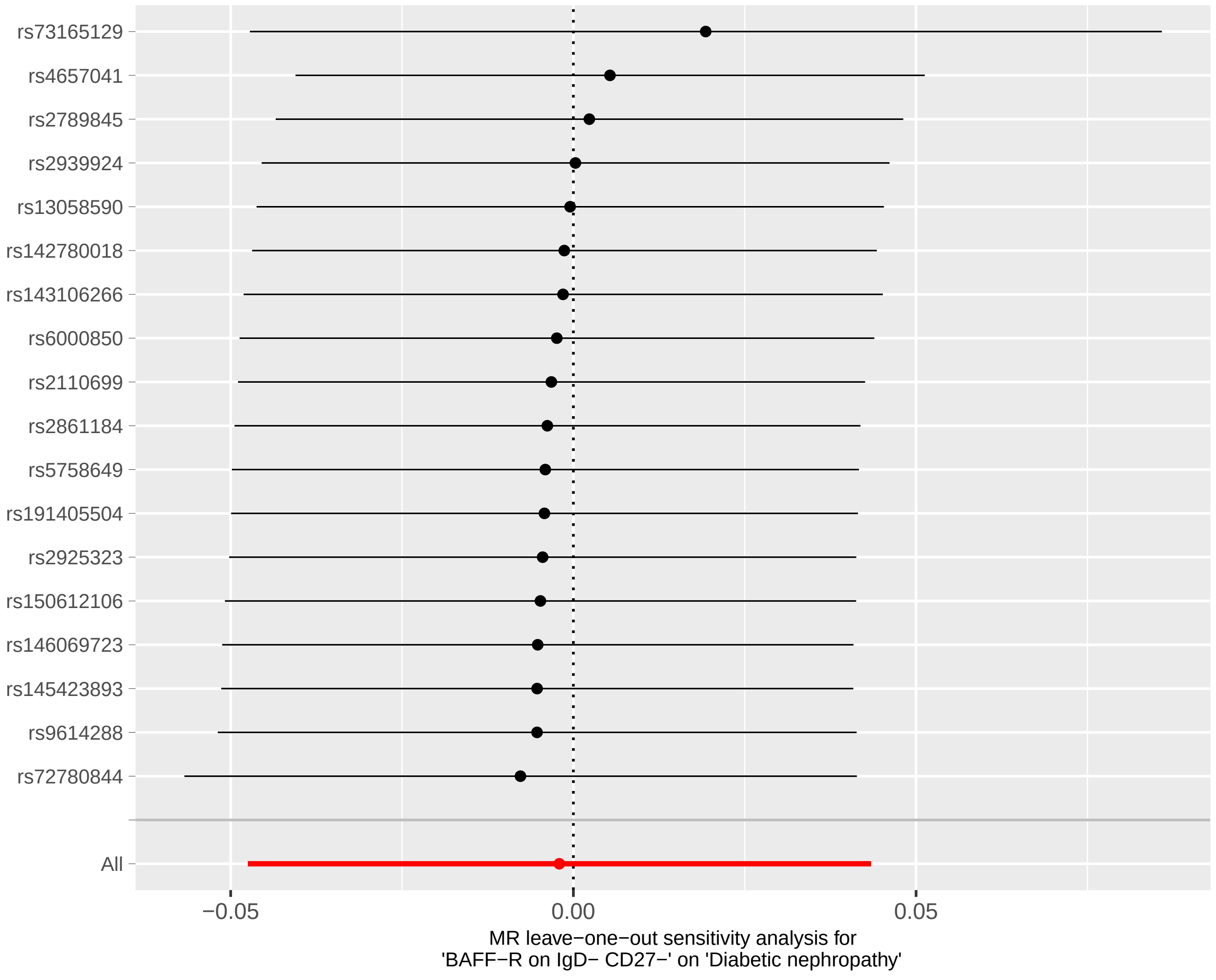
rs73165129
rs4657041
rs2789845
rs2939924
rs13058590
rs142780018
rs143106266
rs6000850
rs2110699
rs2861184
rs5758649
rs191405504
rs2925323
rs150612106
rs146069723
rs145423893
rs9614288
rs72780844
All
−0.05
0.00
0.05
MR leave−one−out sensitivity analysis for
'BAFF−R on IgD− CD27−' on 'Diabetic nephropathy'

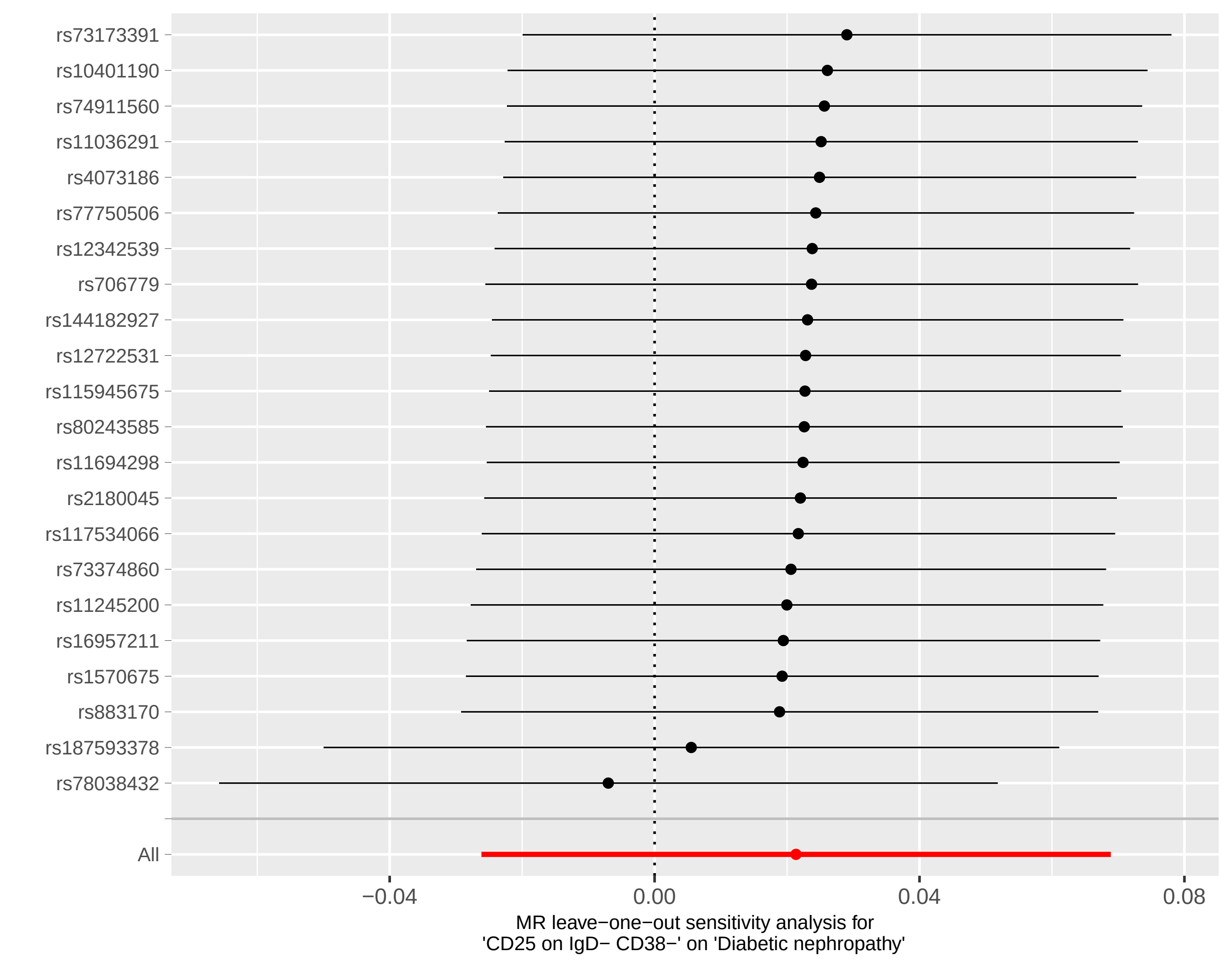
rs73173391
rs10401190
rs74911560
rs11036291
rs4073186
rs77750506
rs12342539
rs706779
rs144182927
rs12722531
rs115945675
rs80243585
rs11694298
rs2180045
rs117534066
rs73374860
rs11245200
rs16957211
rs1570675
rs883170
rs187593378
rs78038432
All
−0.04
0.00
0.04
0.08
MR leave−one−out sensitivity analysis for
'CD25 on IgD− CD38−' on 'Diabetic nephropathy'

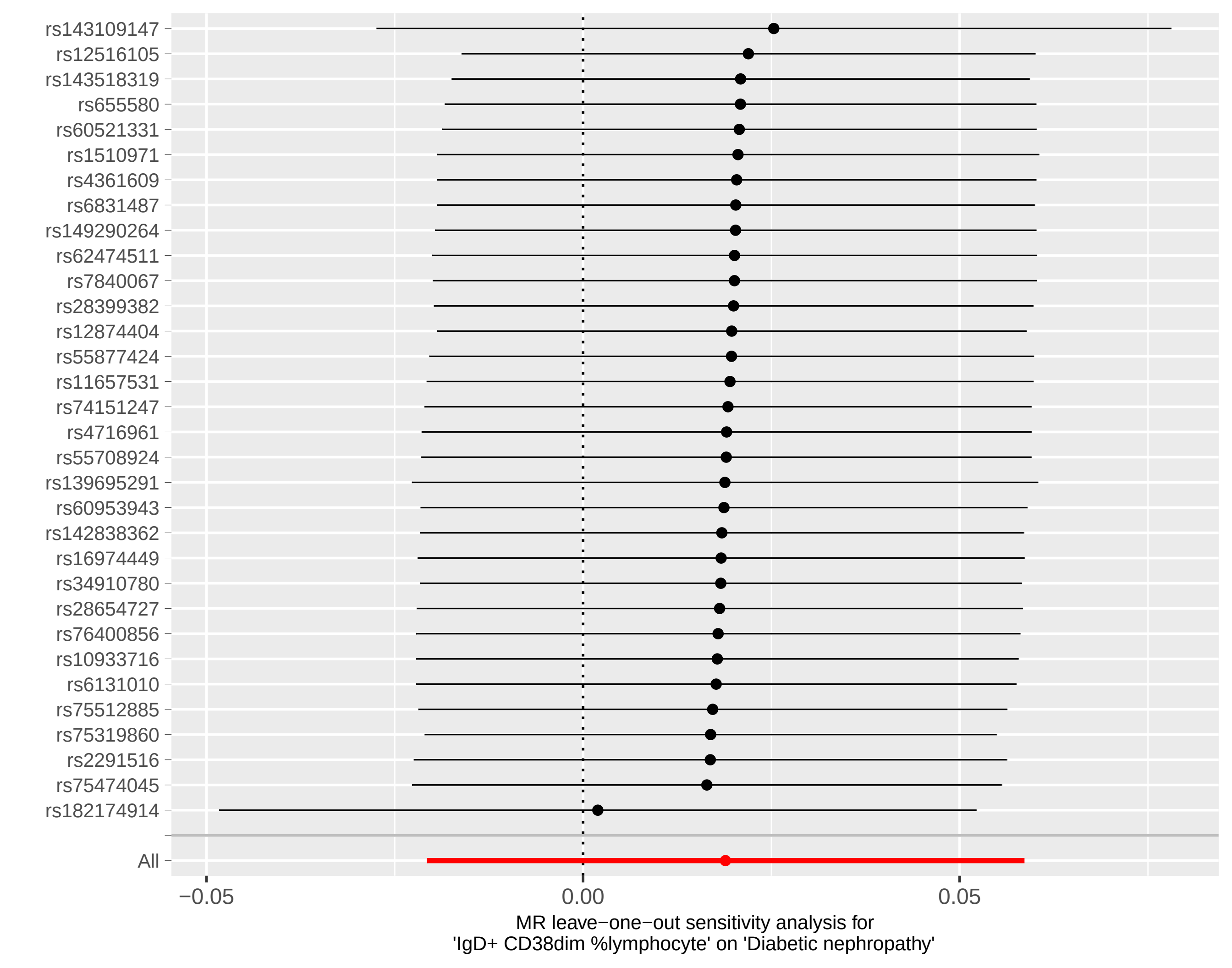
rs143109147
rs12516105
rs143518319
rs655580
rs60521331
rs1510971
rs4361609
rs6831487
rs149290264
rs62474511
rs7840067
rs28399382
rs12874404
rs55877424
rs11657531
rs74151247
rs4716961
rs55708924
rs139695291
rs60953943
rs142838362
rs16974449
rs34910780
rs28654727
rs76400856
rs10933716
rs6131010
rs75512885
rs75319860
rs2291516
rs75474045
rs182174914
All
−0.05
0.00
0.05
MR leave−one−out sensitivity analysis for
'IgD+ CD38dim %lymphocyte' on 'Diabetic nephropathy'

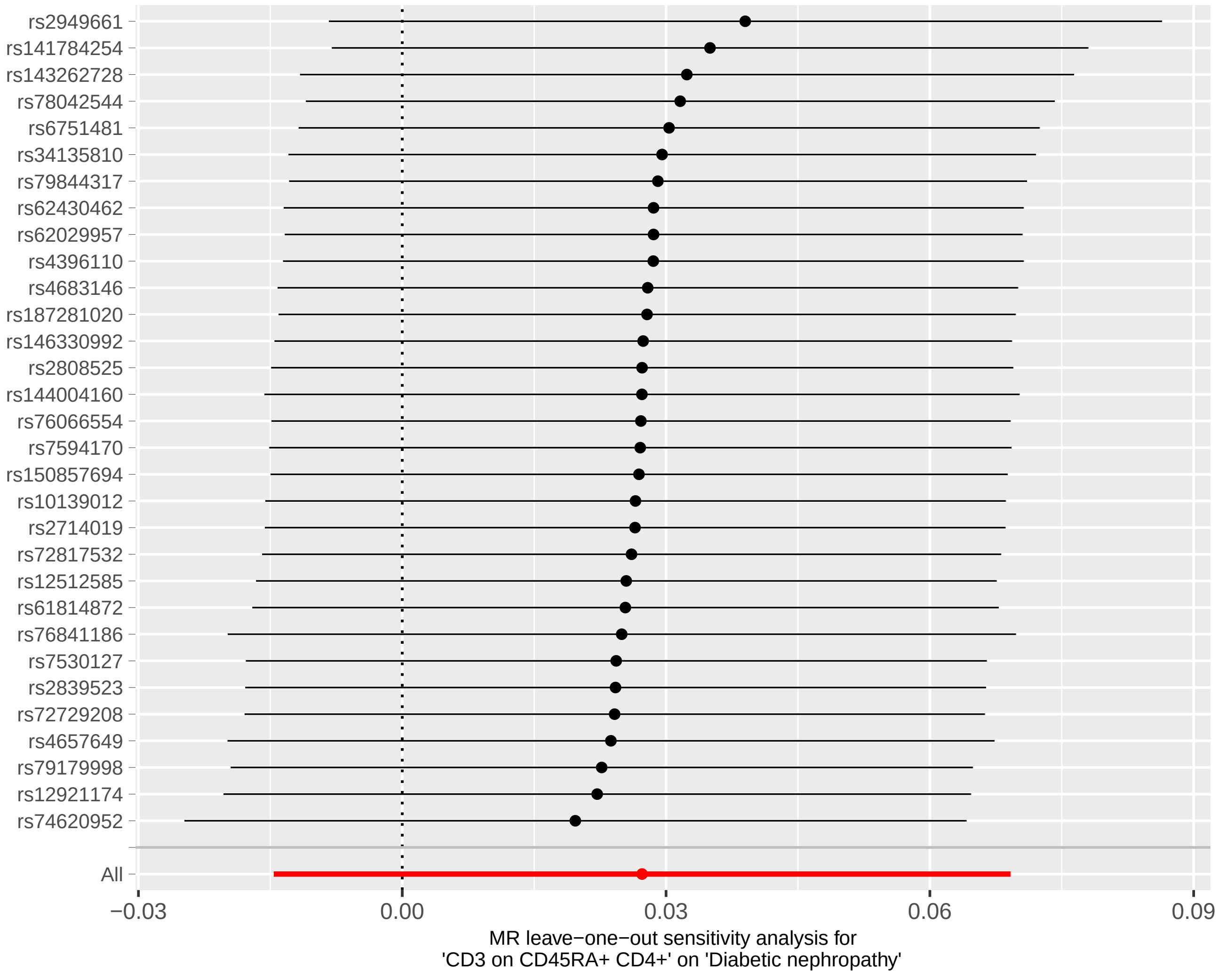
rs2949661
rs141784254
rs143262728
rs78042544
rs6751481
rs34135810
rs79844317
rs62430462
rs62029957
rs4396110
rs4683146
rs187281020
rs146330992
rs2808525
rs144004160
rs76066554
rs7594170
rs150857694
rs10139012
rs2714019
rs72817532
rs12512585
rs61814872
rs76841186
rs7530127
rs2839523
rs72729208
rs4657649
rs79179998
rs12921174
rs74620952
All
−0.03
0.00
0.03
0.06
0.09
MR leave−one−out sensitivity analysis for
'CD3 on CD45RA+ CD4+' on 'Diabetic nephropathy'

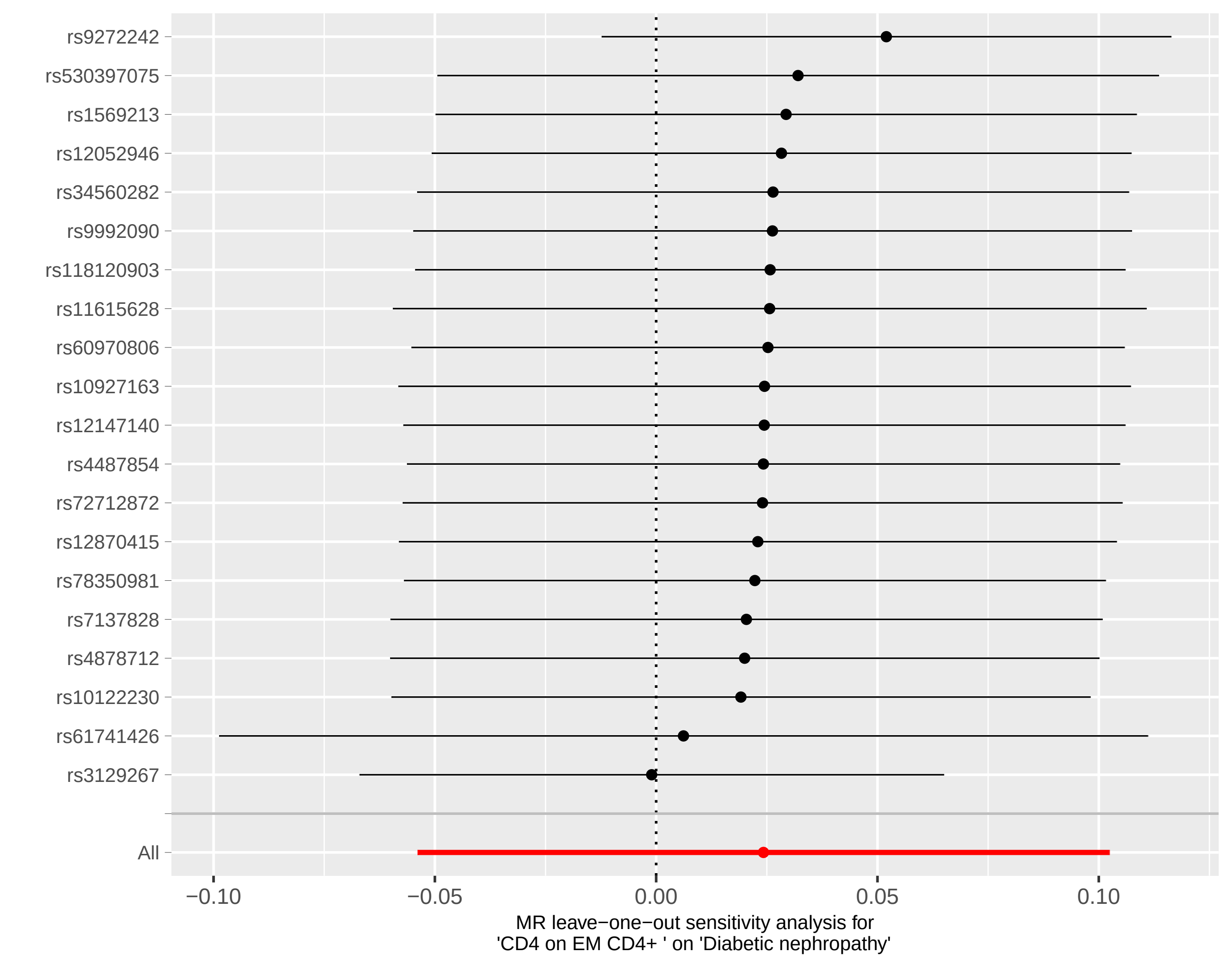
rs9272242
rs530397075
rs1569213
rs12052946
rs34560282
rs9992090
rs118120903
rs11615628
rs60970806
rs10927163
rs12147140
rs4487854
rs72712872
rs12870415
rs78350981
rs7137828
rs4878712
rs10122230
rs61741426
rs3129267
All
−0.10
−0.05
0.00
0.05
0.10
MR leave−one−out sensitivity analysis for
'CD4 on EM CD4+ ' on 'Diabetic nephropathy'

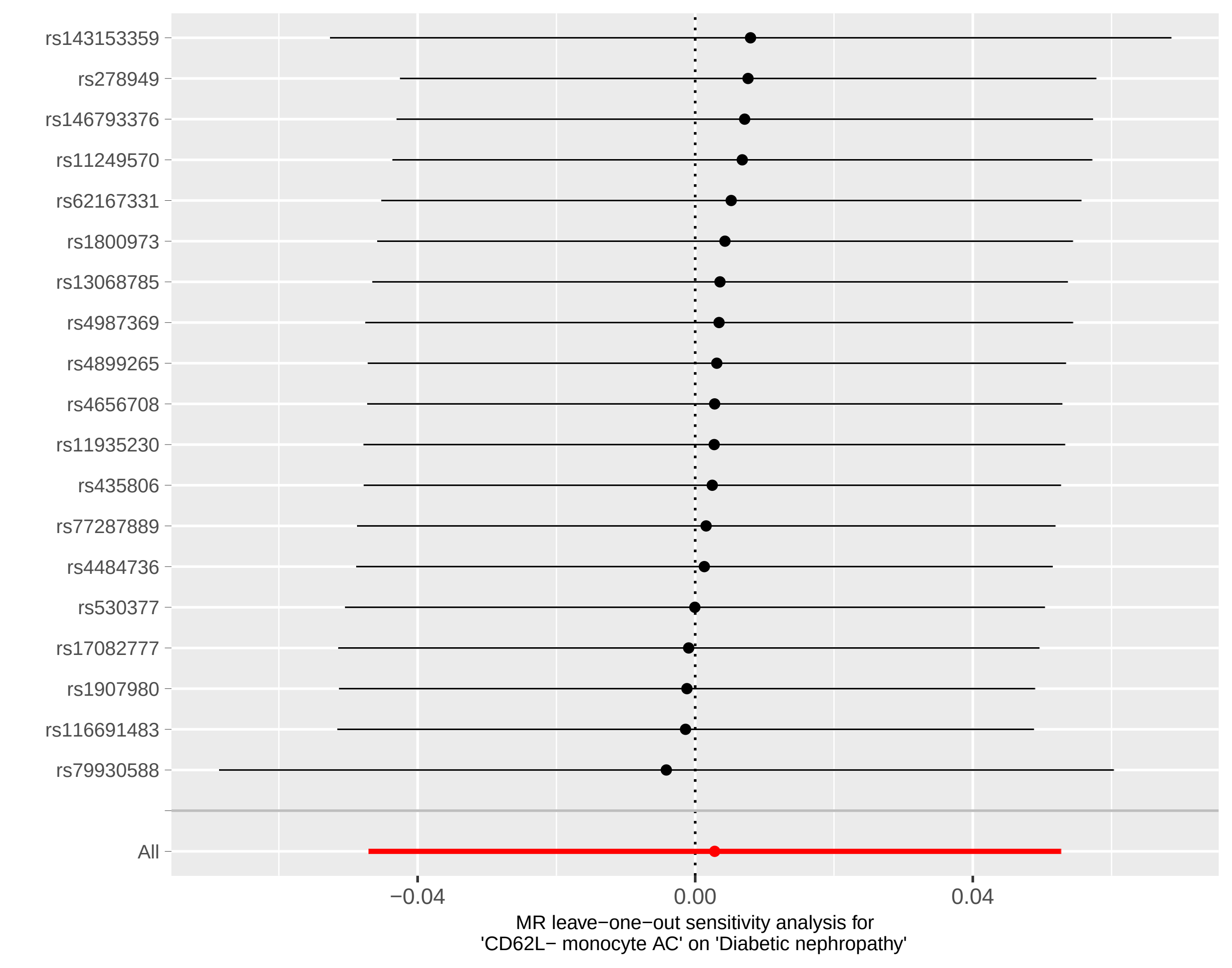
rs143153359
rs278949
rs146793376
rs11249570
rs62167331
rs1800973
rs13068785
rs4987369
rs4899265
rs4656708
rs11935230
rs435806
rs77287889
rs4484736
rs530377
rs17082777
rs1907980
rs116691483
rs79930588
All
−0.04
0.00
0.04
MR leave−one−out sensitivity analysis for
'CD62L− monocyte AC' on 'Diabetic nephropathy'

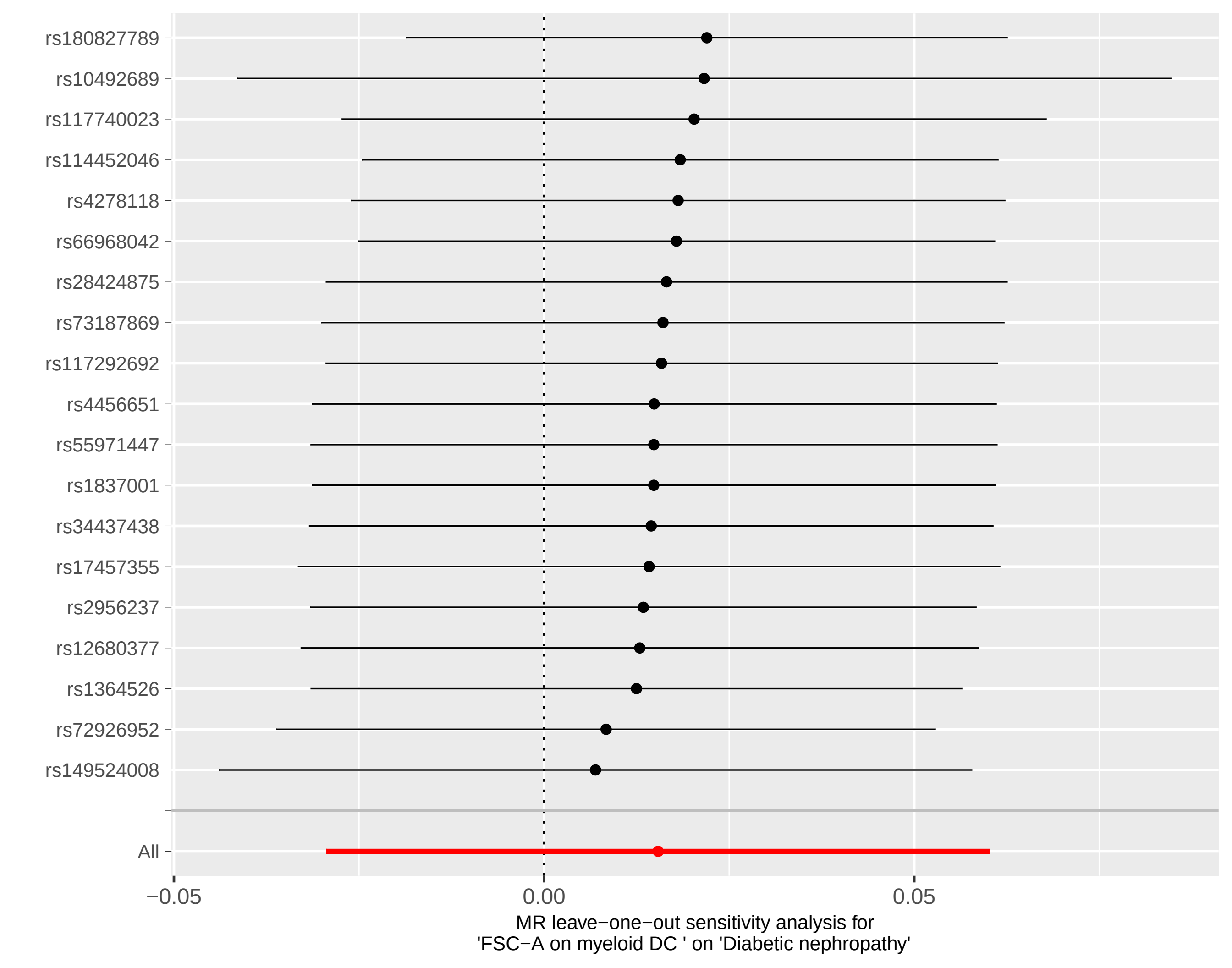
rs180827789
rs10492689
rs117740023
rs114452046
rs4278118
rs66968042
rs28424875
rs73187869
rs117292692
rs4456651
rs55971447
rs1837001
rs34437438
rs17457355
rs2956237
rs12680377
rs1364526
rs72926952
rs149524008
All
−0.05
0.00
0.05
MR leave−one−out sensitivity analysis for
'FSC−A on myeloid DC ' on 'Diabetic nephropathy'

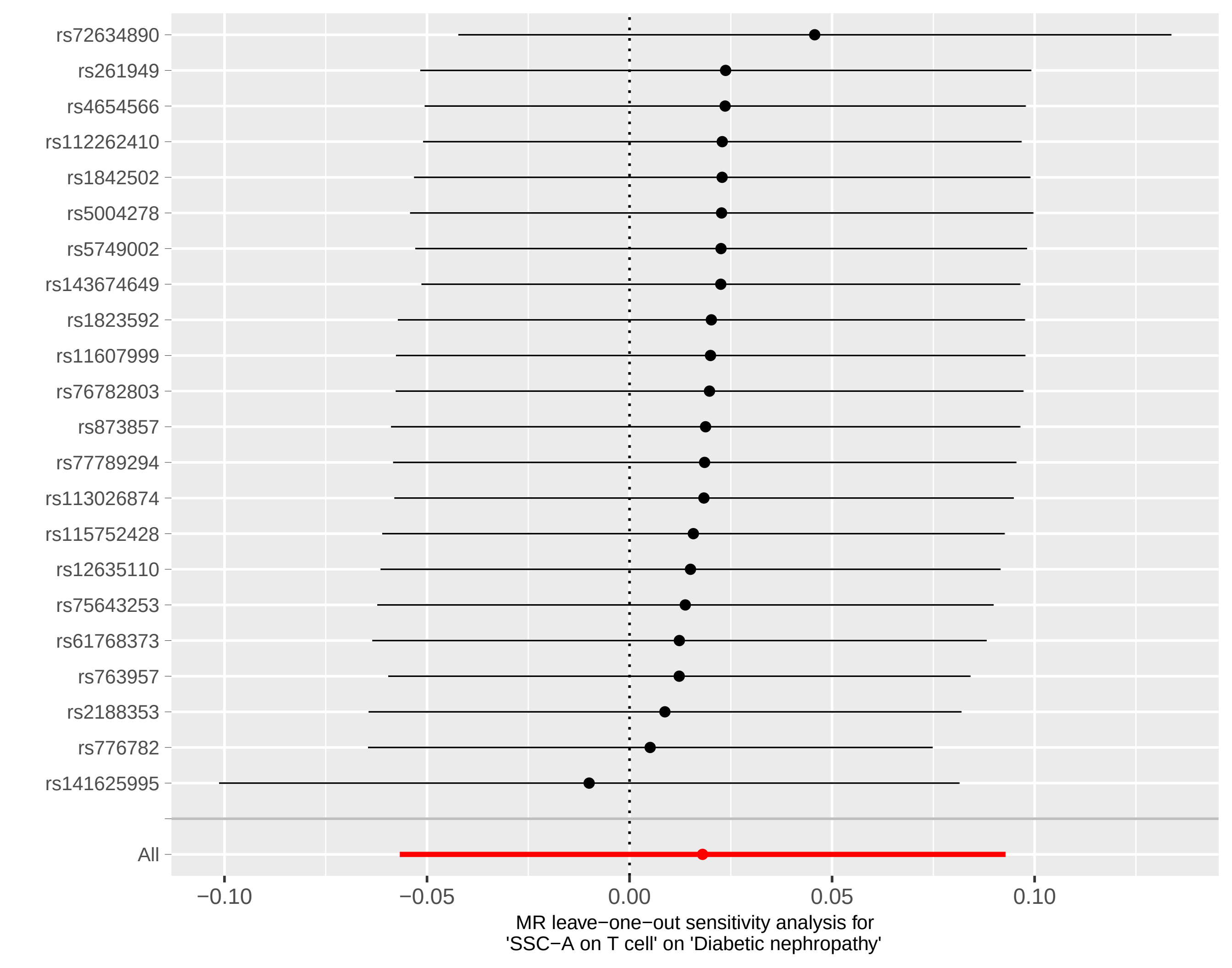
rs72634890
rs261949
rs4654566
rs112262410
rs1842502
rs5004278
rs5749002
rs143674649
rs1823592
rs11607999
rs76782803
rs873857
rs77789294
rs113026874
rs115752428
rs12635110
rs75643253
rs61768373
rs763957
rs2188353
rs776782
rs141625995
All
−0.10
−0.05
0.00
0.05
0.10
MR leave−one−out sensitivity analysis for
'SSC−A on T cell' on 'Diabetic nephropathy'

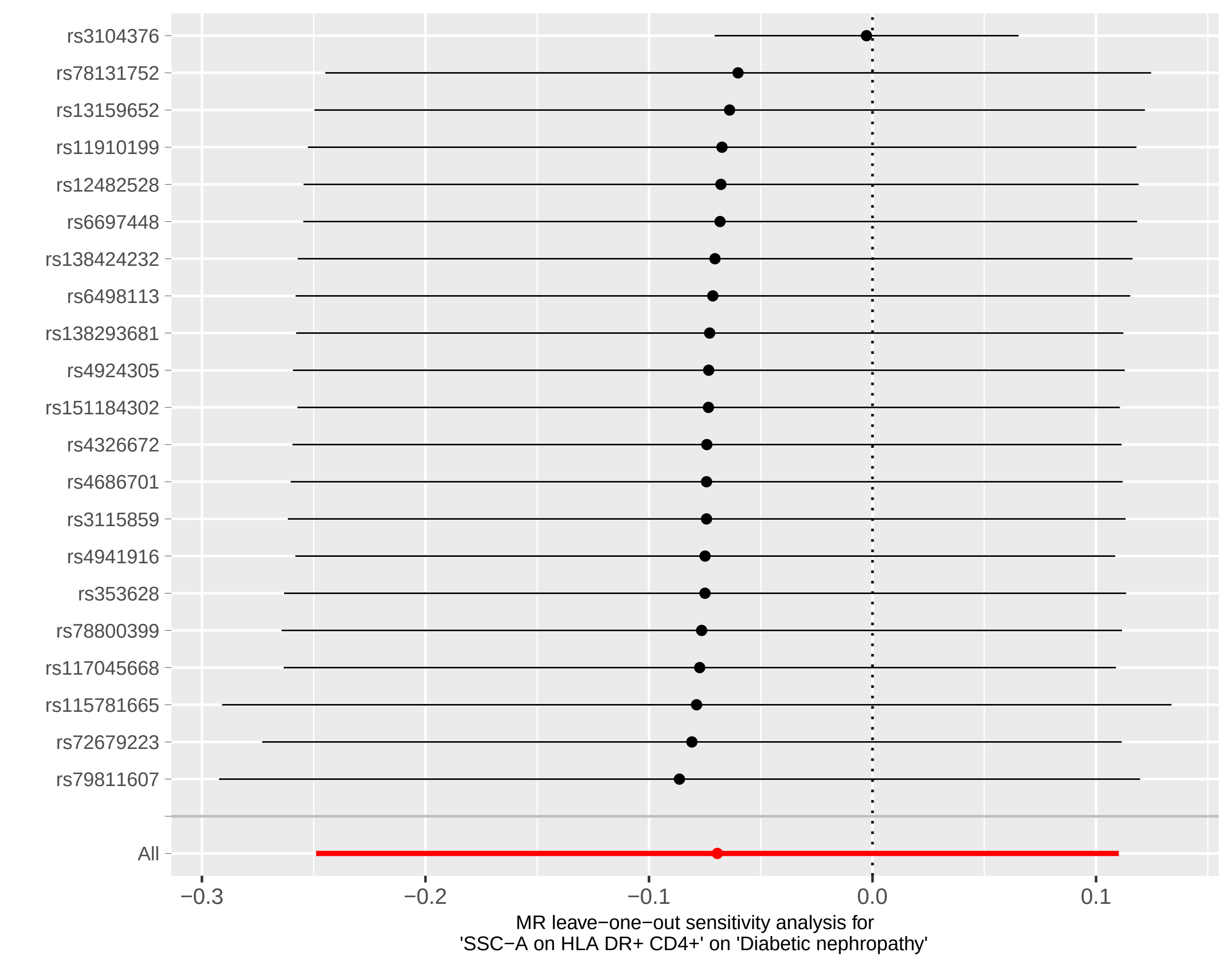

rs3104376
rs78131752
rs13159652
rs11910199
rs12482528
rs6697448
rs138424232
rs6498113
rs138293681
rs4924305
rs151184302
rs4326672
rs4686701
rs3115859
rs4941916
rs353628
rs78800399
rs117045668
rs115781665
rs72679223
rs79811607
All
−0.3
−0.2
−0.1
0.0
0.1
MR leave−one−out sensitivity analysis for
'SSC−A on HLA DR+ CD4+' on 'Diabetic nephropathy'

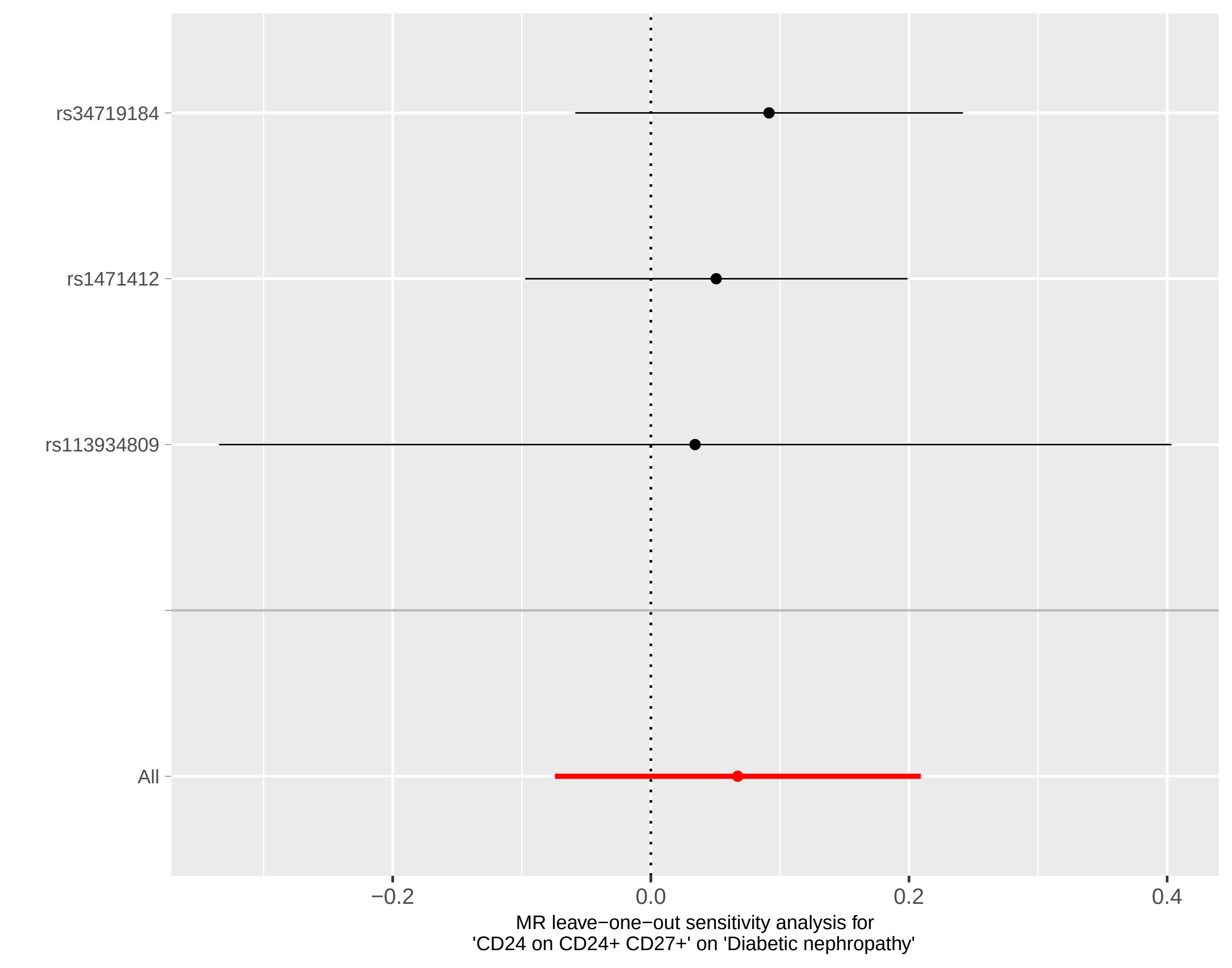

rs34719184
rs1471412
rs113934809
All
−0.2
0.0
0.2
0.4
MR leave−one−out sensitivity analysis for
'CD24 on CD24+ CD27+' on 'Diabetic nephropathy'

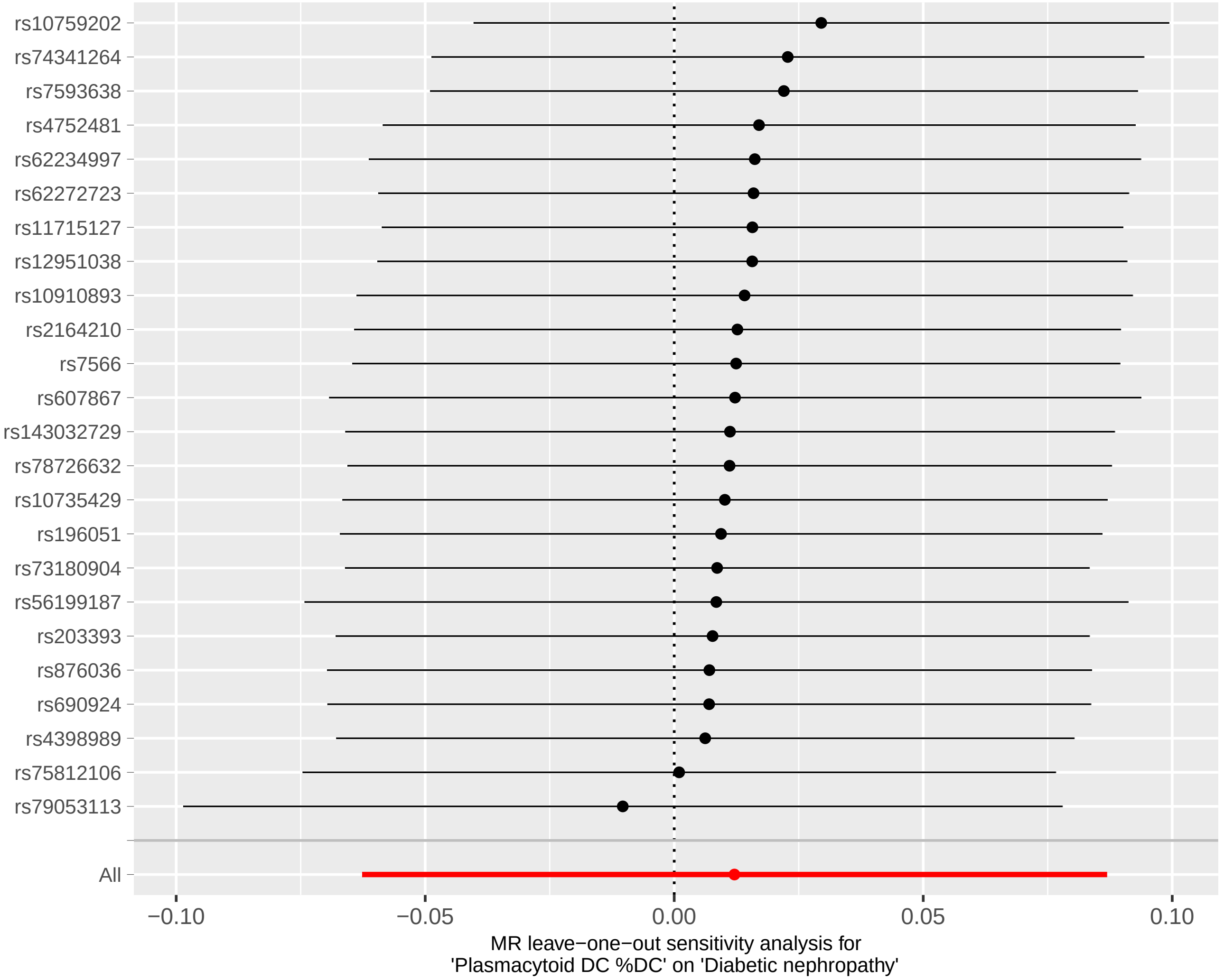
rs10759202
rs74341264
rs7593638
rs4752481
rs62234997
rs62272723
rs11715127
rs12951038
rs10910893
rs2164210
rs7566
rs607867
rs143032729
rs78726632
rs10735429
rs196051
rs73180904
rs56199187
rs203393
rs876036
rs690924
rs4398989
rs75812106
rs79053113
All
−0.10
−0.05
0.00
0.05
0.10
MR leave−one−out sensitivity analysis for
'Plasmacytoid DC %DC' on 'Diabetic nephropathy'

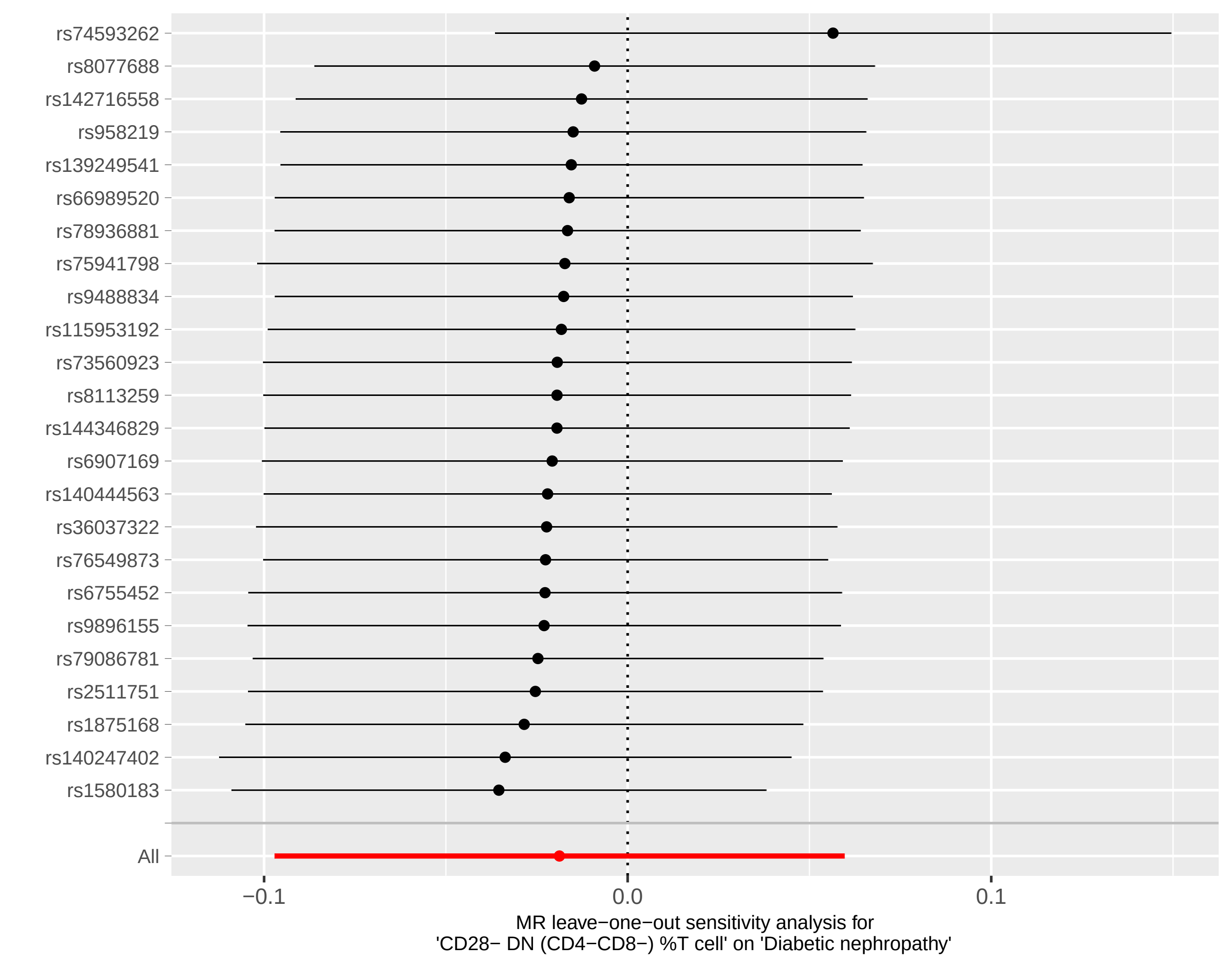
rs74593262
rs8077688
rs142716558
rs958219
rs139249541
rs66989520
rs78936881
rs75941798
rs9488834
rs115953192
rs73560923
rs8113259
rs144346829
rs6907169
rs140444563
rs36037322
rs76549873
rs6755452
rs9896155
rs79086781
rs2511751
rs1875168
rs140247402
rs1580183
All
−0.1
0.0
0.1
MR leave−one−out sensitivity analysis for
'CD28− DN (CD4−CD8−) %T cell' on 'Diabetic nephropathy'

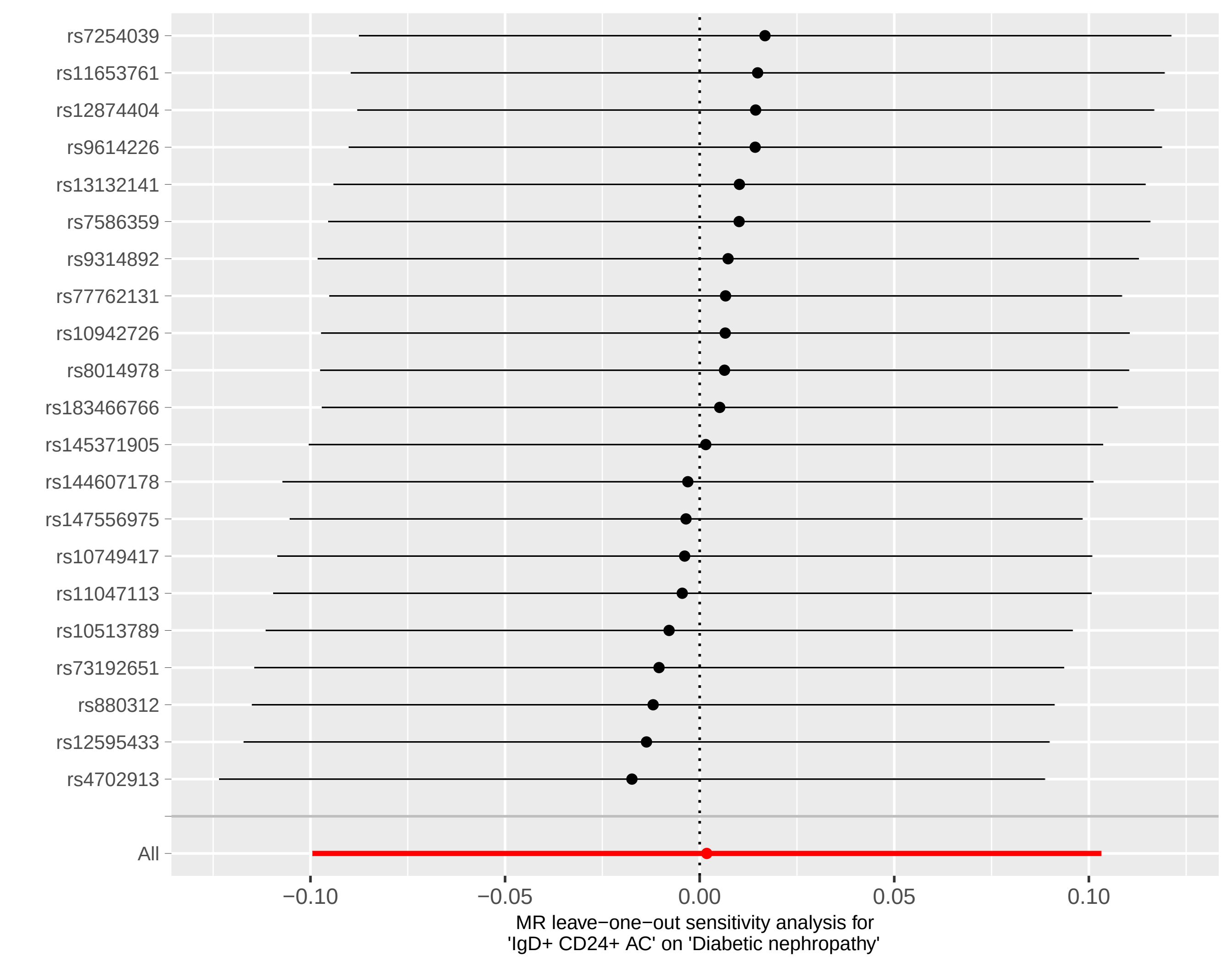
rs7254039
rs11653761
rs12874404
rs9614226
rs13132141
rs7586359
rs9314892
rs77762131
rs10942726
rs8014978
rs183466766
rs145371905
rs144607178
rs147556975
rs10749417
rs11047113
rs10513789
rs73192651
rs880312
rs12595433
rs4702913
All
−0.10
−0.05
0.00
0.05
0.10
MR leave−one−out sensitivity analysis for
'IgD+ CD24+ AC' on 'Diabetic nephropathy'

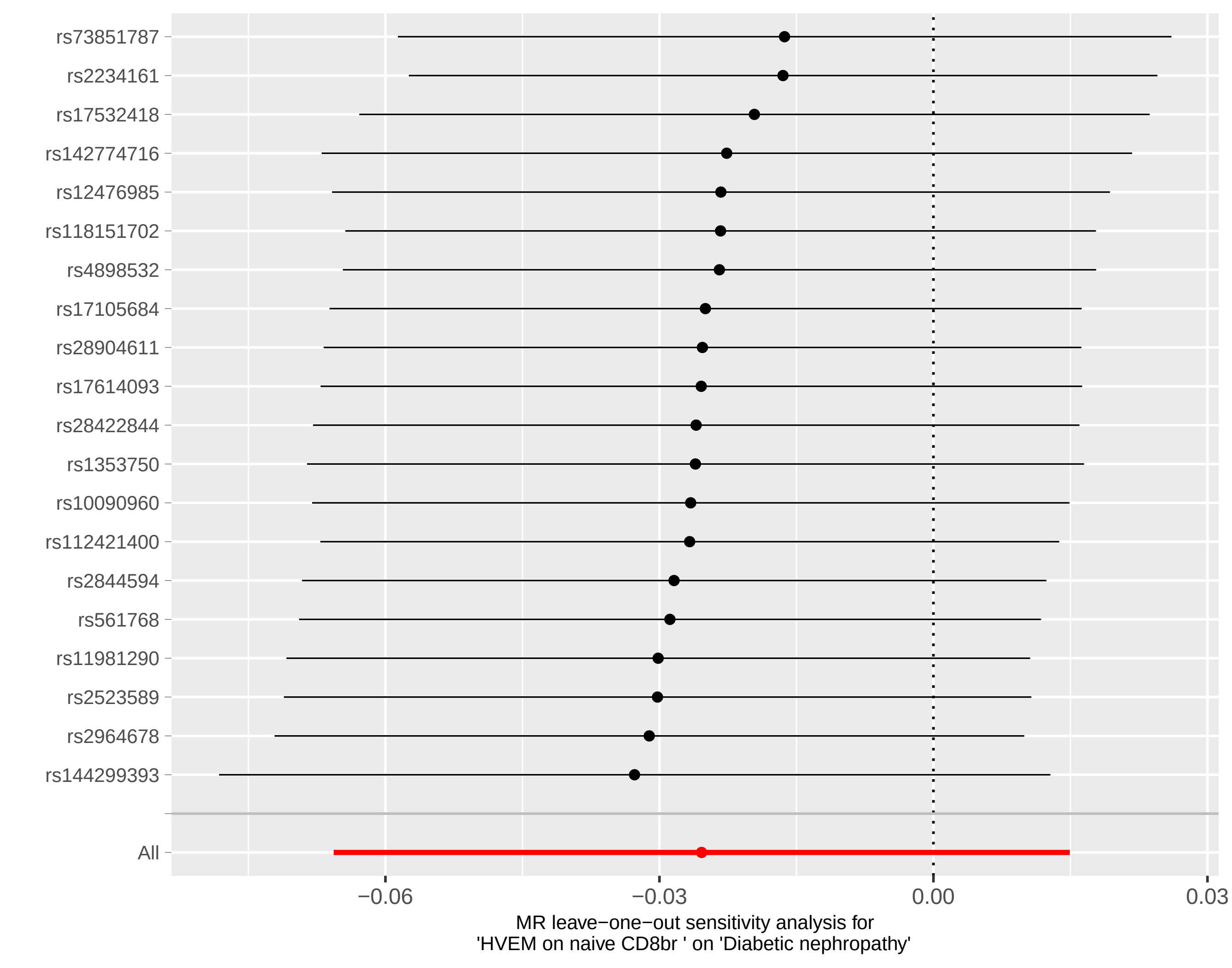
rs73851787
rs2234161
rs17532418
rs142774716
rs12476985
rs118151702
rs4898532
rs17105684
rs28904611
rs17614093
rs28422844
rs1353750
rs10090960
rs112421400
rs2844594
rs561768
rs11981290
rs2523589
rs2964678
rs144299393
All
−0.06
−0.03
0.00
0.03
MR leave−one−out sensitivity analysis for
'HVEM on naive CD8br ' on 'Diabetic nephropathy'

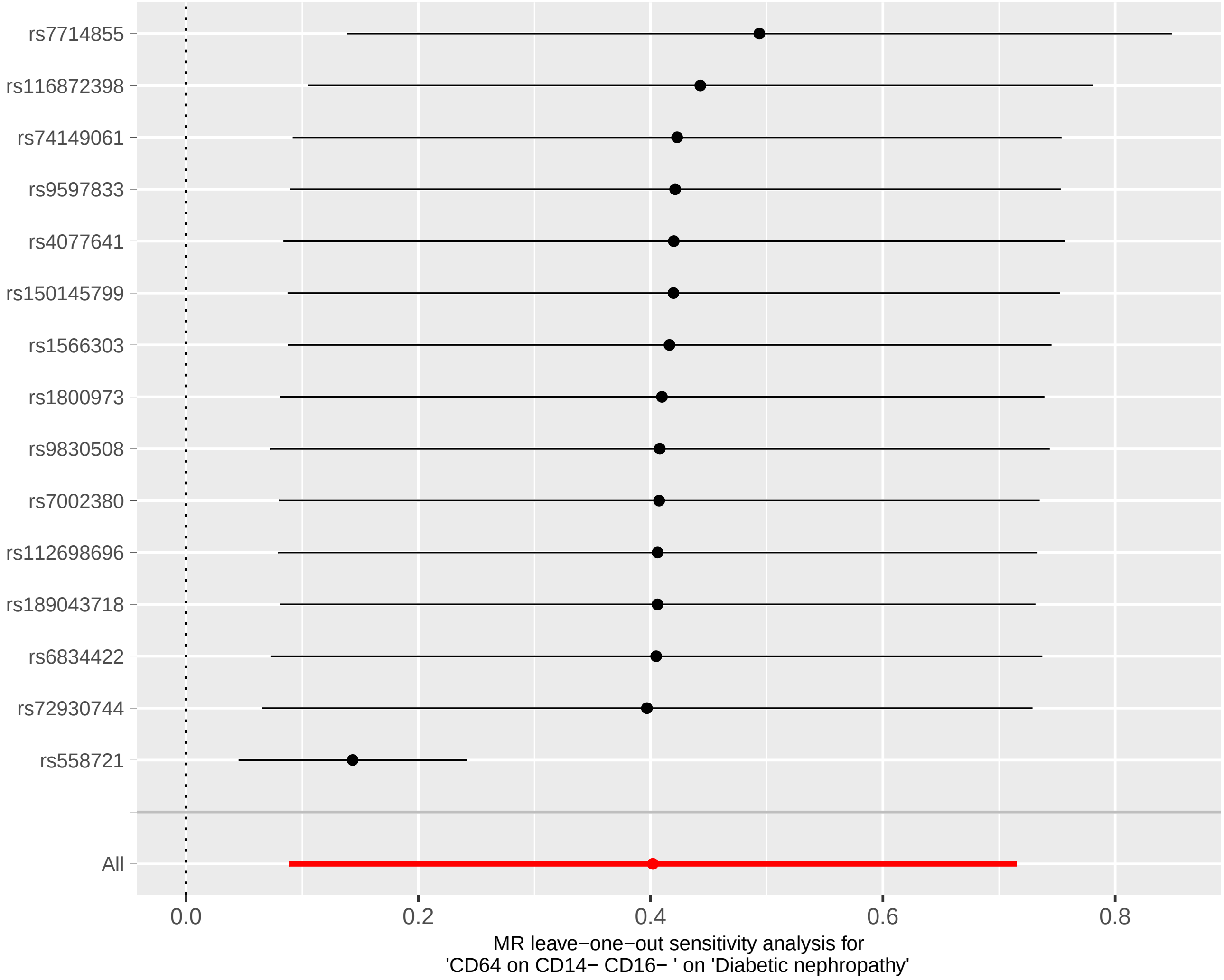
rs7714855
rs116872398
rs74149061
rs9597833
rs4077641
rs150145799
rs1566303
rs1800973
rs9830508
rs7002380
rs112698696
rs189043718
rs6834422
rs72930744
rs558721
All
0.0
0.2
0.4
0.6
0.8
MR leave−one−out sensitivity analysis for
'CD64 on CD14− CD16− ' on 'Diabetic nephropathy'

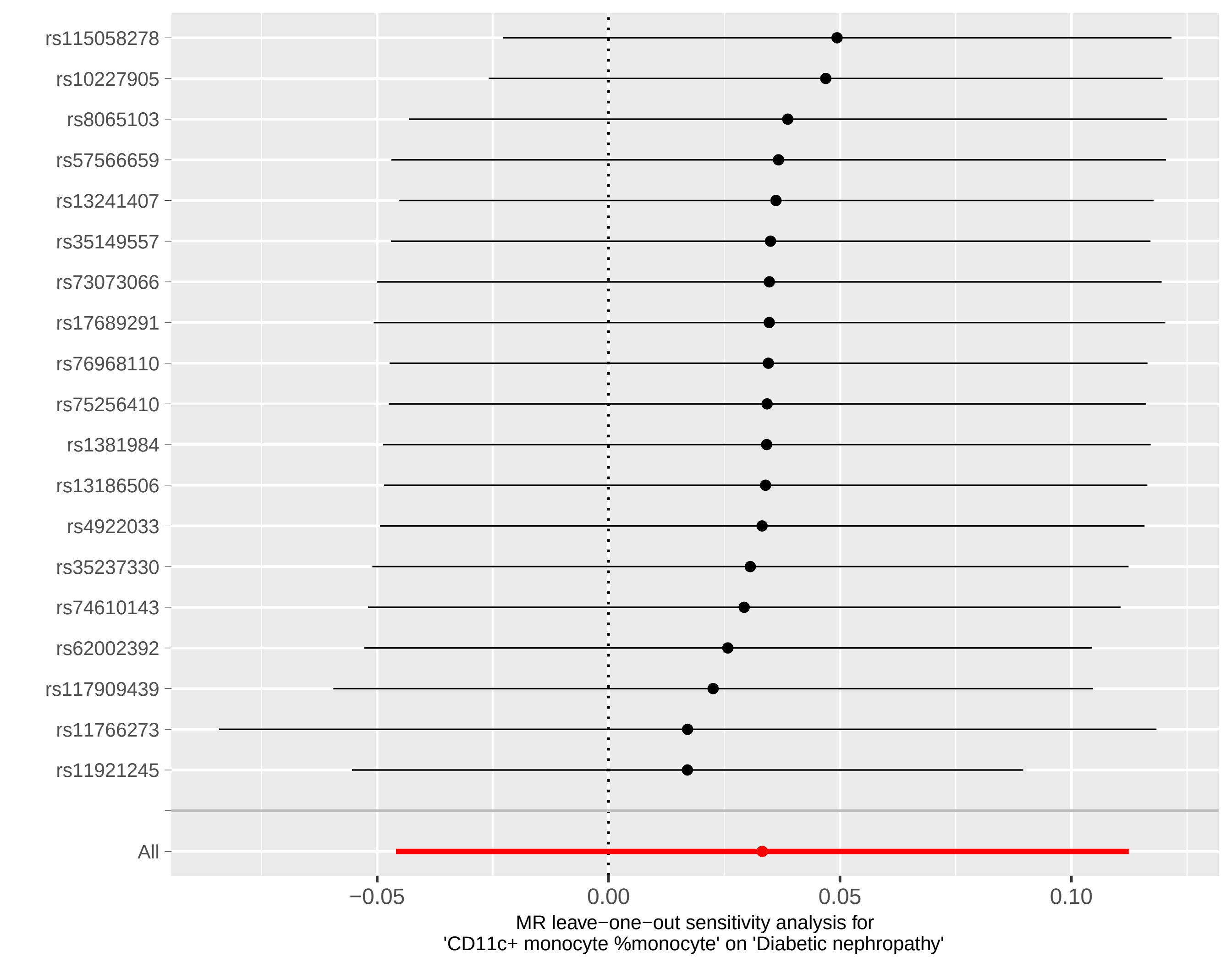
rs115058278
rs10227905
rs8065103
rs57566659
rs13241407
rs35149557
rs73073066
rs17689291
rs76968110
rs75256410
rs1381984
rs13186506
rs4922033
rs35237330
rs74610143
rs62002392
rs117909439
rs11766273
rs11921245
All
−0.05
0.00
0.05
0.10
MR leave−one−out sensitivity analysis for
'CD11c+ monocyte %monocyte' on 'Diabetic nephropathy'

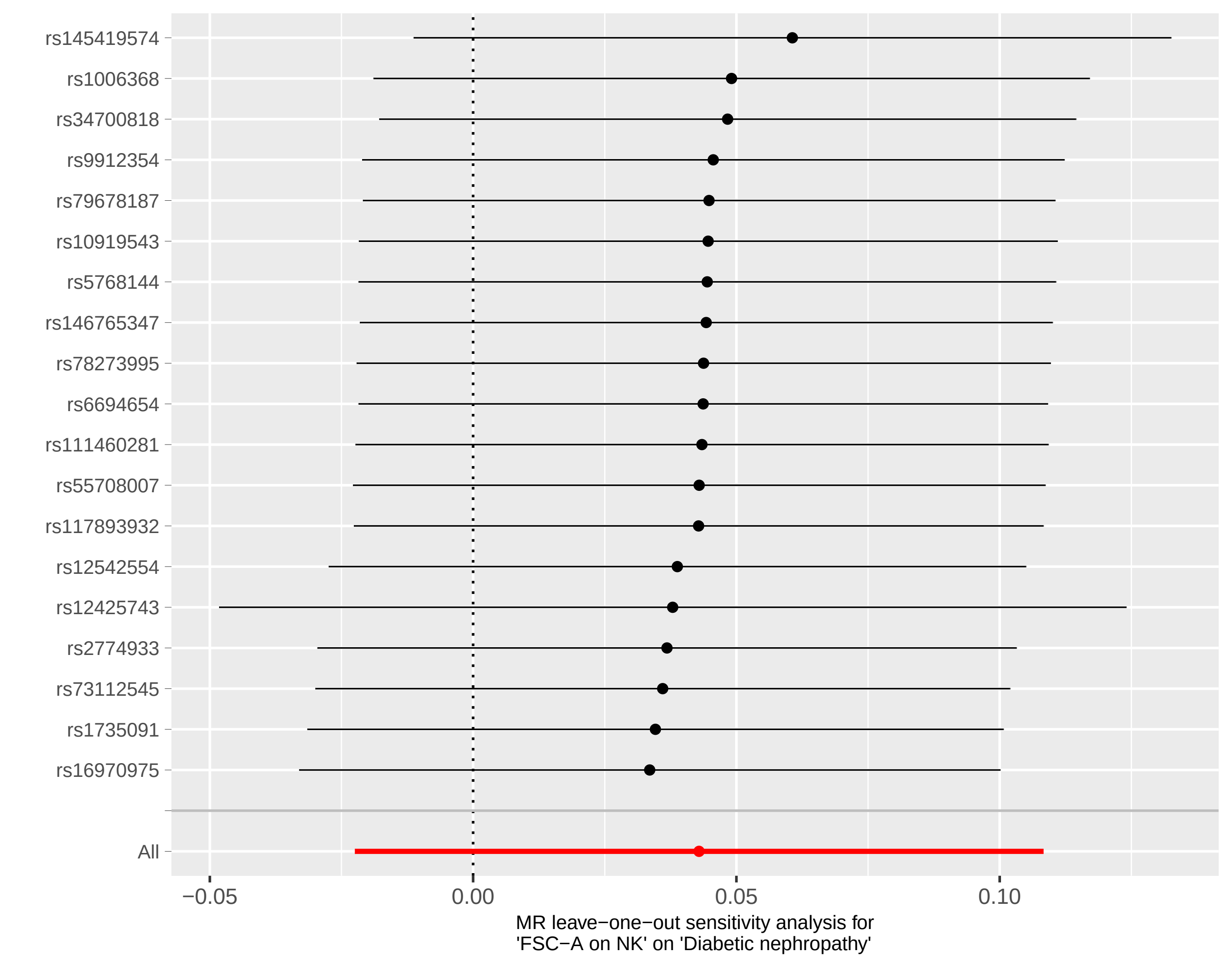
rs145419574
rs1006368
rs34700818
rs9912354
rs79678187
rs10919543
rs5768144
rs146765347
rs78273995
rs6694654
rs111460281
rs55708007
rs117893932
rs12542554
rs12425743
rs2774933
rs73112545
rs1735091
rs16970975
All
−0.05
0.00
0.05
0.10
MR leave−one−out sensitivity analysis for
'FSC−A on NK' on 'Diabetic nephropathy'

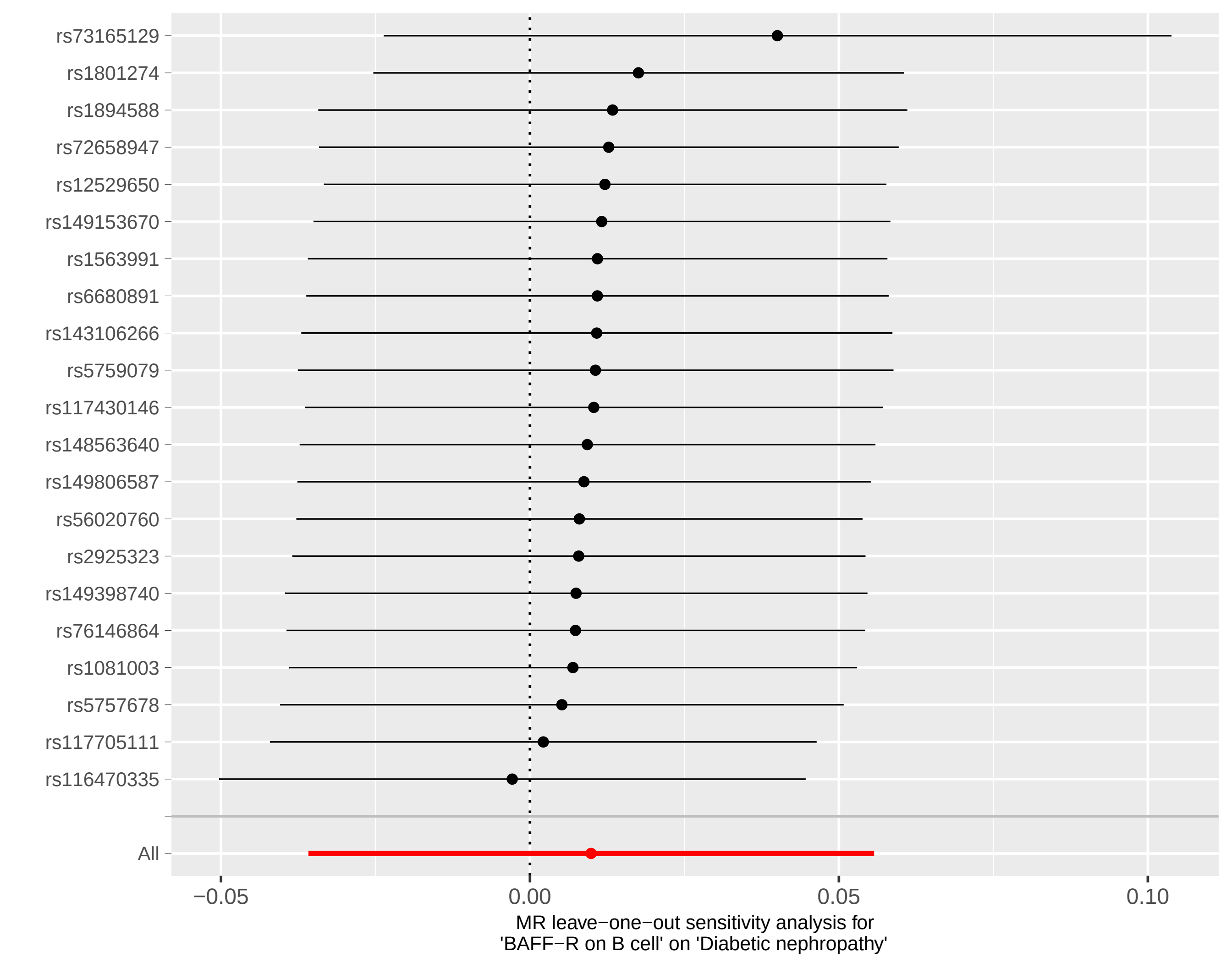

rs73165129
rs1801274
rs1894588
rs72658947
rs12529650
rs149153670
rs1563991
rs6680891
rs143106266
rs5759079
rs117430146
rs148563640
rs149806587
rs56020760
rs2925323
rs149398740
rs76146864
rs1081003
rs5757678
rs117705111
rs116470335
All
−0.05
0.00
0.05
0.10
MR leave−one−out sensitivity analysis for
'BAFF−R on B cell' on 'Diabetic nephropathy'

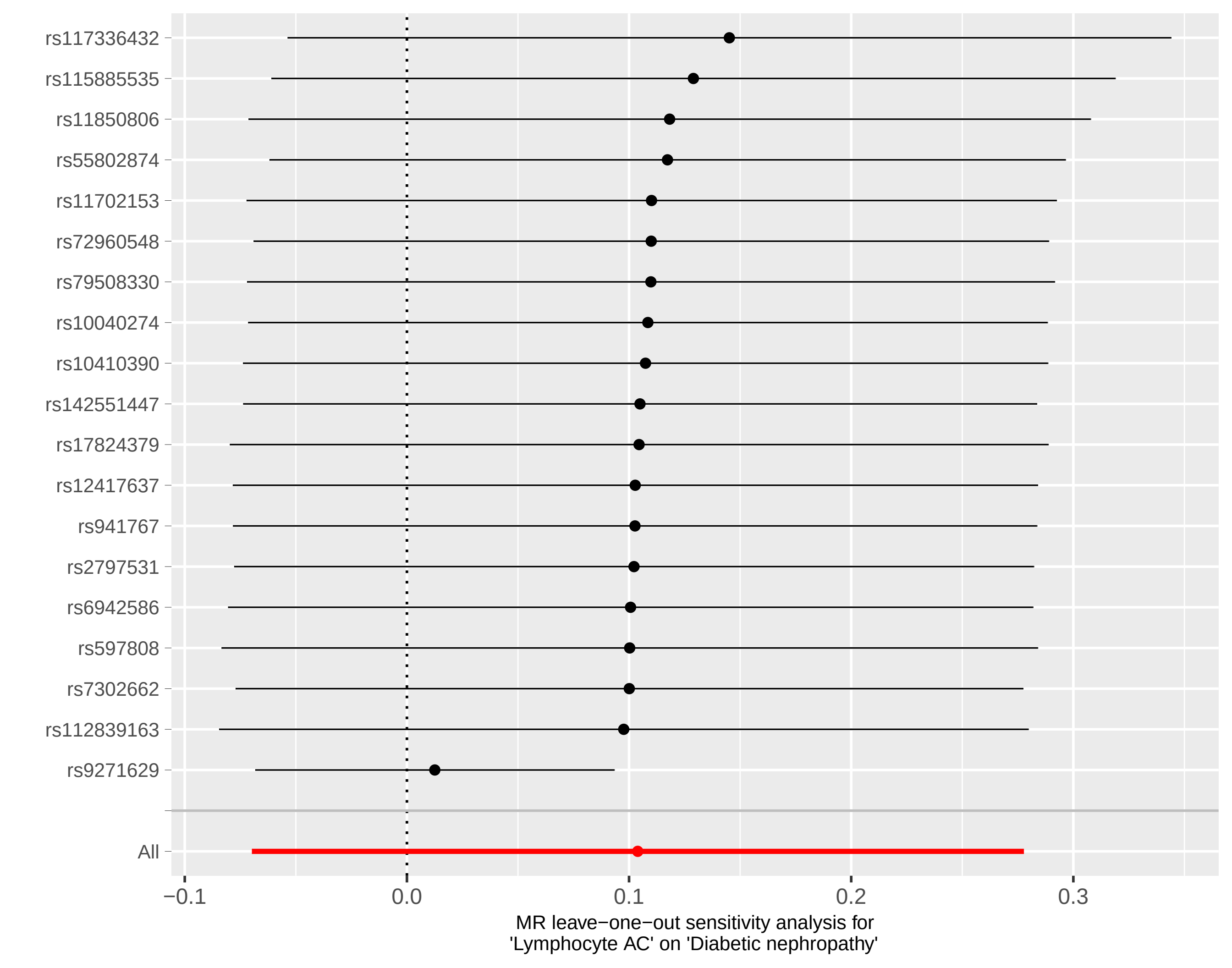
rs117336432
rs115885535
rs11850806
rs55802874
rs11702153
rs72960548
rs79508330
rs10040274
rs10410390
rs142551447
rs17824379
rs12417637
rs941767
rs2797531
rs6942586
rs597808
rs7302662
rs112839163
rs9271629
All
−0.1
0.0
0.1
0.2
0.3
MR leave−one−out sensitivity analysis for
'Lymphocyte AC' on 'Diabetic nephropathy'

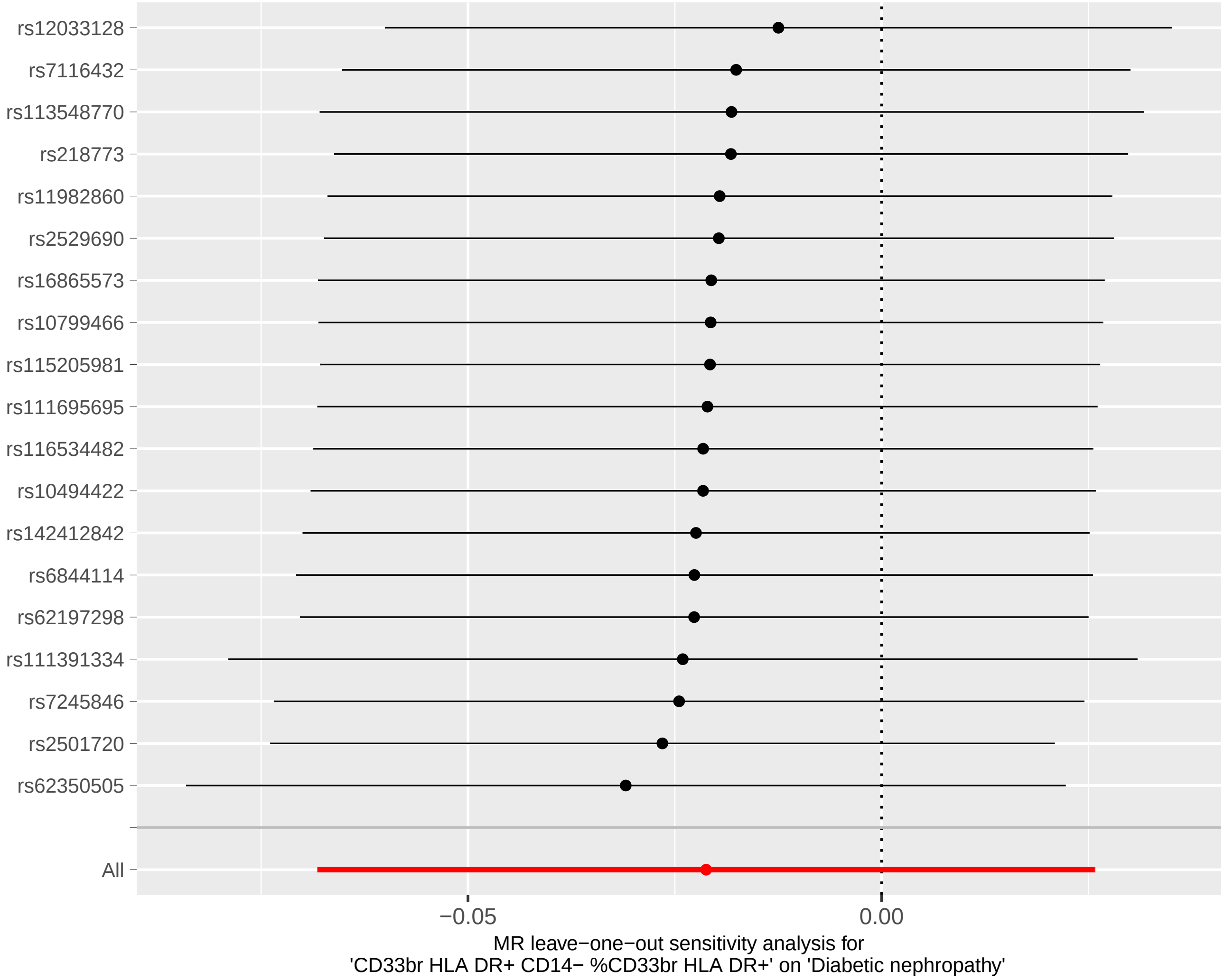
rs12033128
rs7116432
rs113548770
rs218773
rs11982860
rs2529690
rs16865573
rs10799466
rs115205981
rs111695695
rs116534482
rs10494422
rs142412842
rs6844114
rs62197298
rs111391334
rs7245846
rs2501720
rs62350505
All
−0.05
0.00
MR leave−one−out sensitivity analysis for
'CD33br HLA DR+ CD14− %CD33br HLA DR+' on 'Diabetic nephropathy'

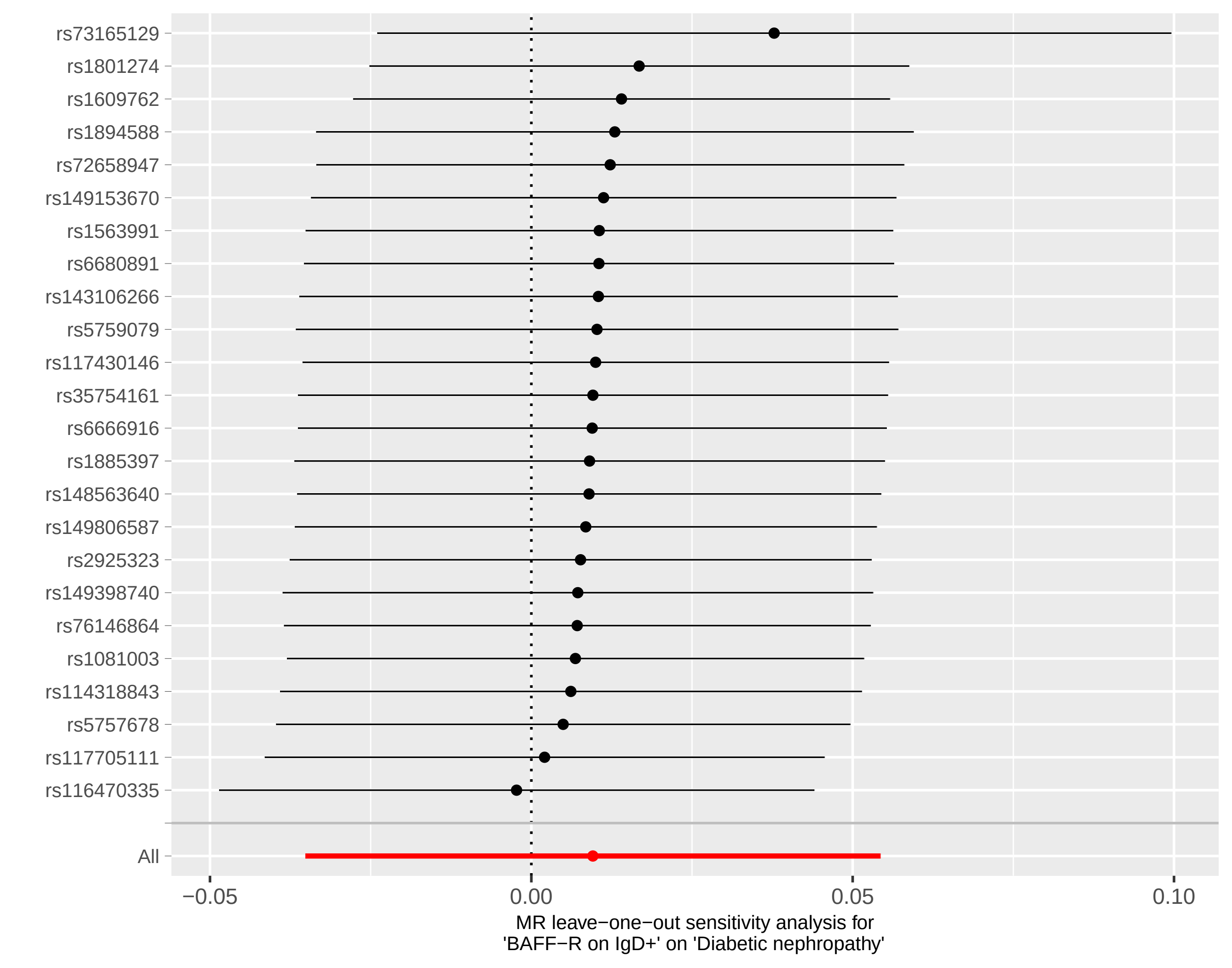
rs73165129
rs1801274
rs1609762
rs1894588
rs72658947
rs149153670
rs1563991
rs6680891
rs143106266
rs5759079
rs117430146
rs35754161
rs6666916
rs1885397
rs148563640
rs149806587
rs2925323
rs149398740
rs76146864
rs1081003
rs114318843
rs5757678
rs117705111
rs116470335
All
−0.05
0.00
0.05
0.10
MR leave−one−out sensitivity analysis for
'BAFF−R on IgD+' on 'Diabetic nephropathy'

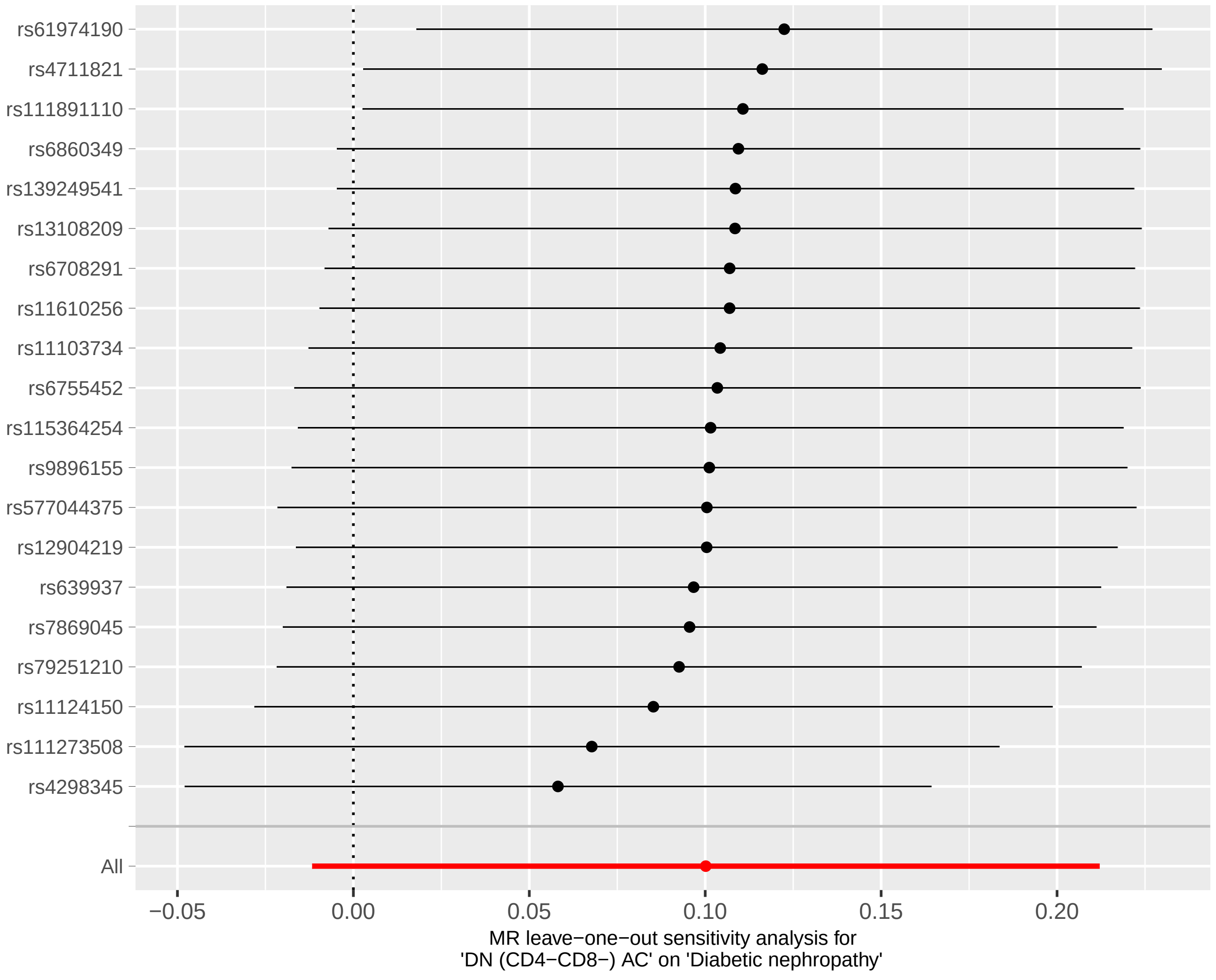
rs61974190
rs4711821
rs111891110
rs6860349
rs139249541
rs13108209
rs6708291
rs11610256
rs11103734
rs6755452
rs115364254
rs9896155
rs577044375
rs12904219
rs639937
rs7869045
rs79251210
rs11124150
rs111273508
rs4298345
All
−0.05
0.00
0.05
0.10
0.15
0.20
MR leave−one−out sensitivity analysis for
'DN (CD4−CD8−) AC' on 'Diabetic nephropathy'

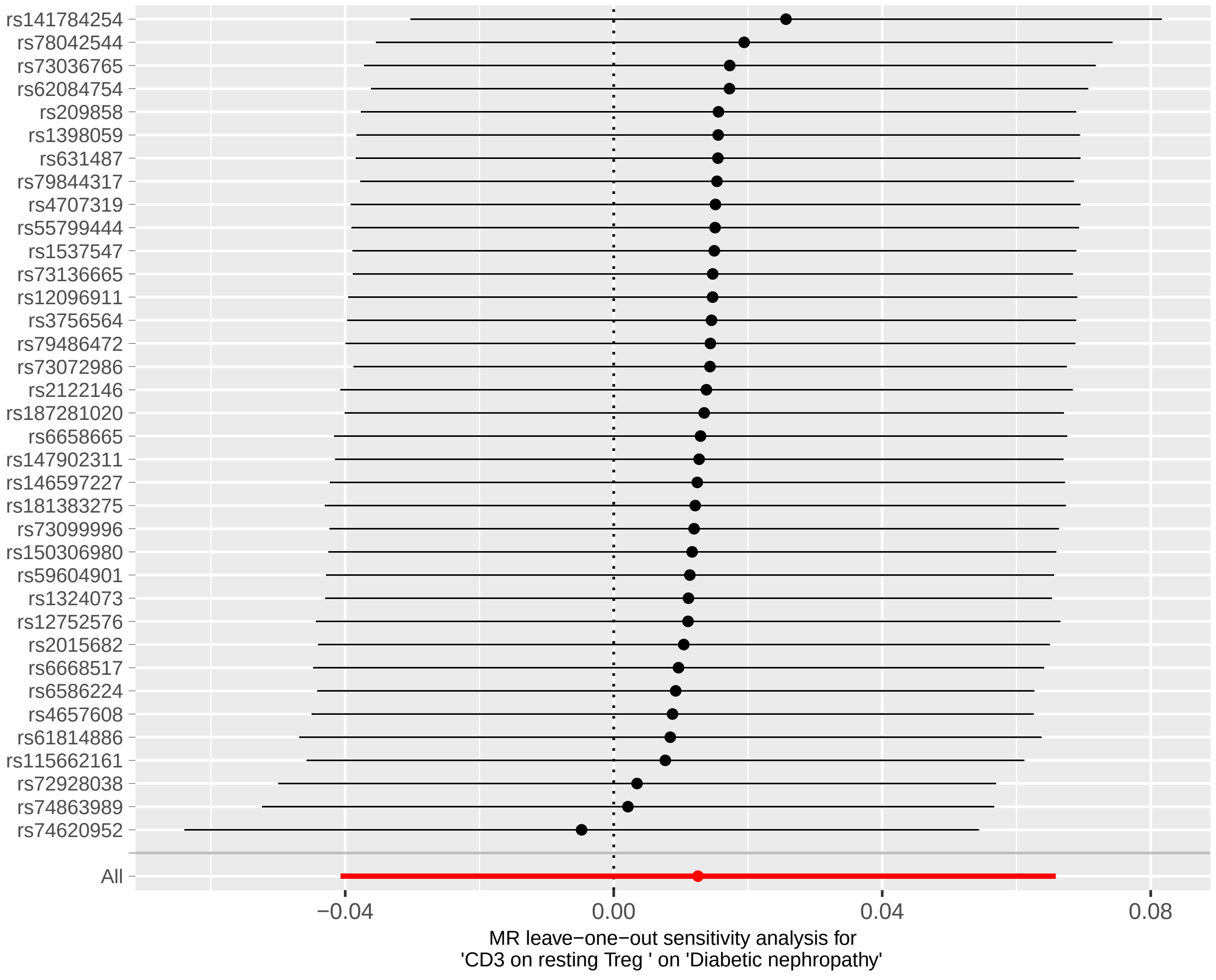
rs141784254
rs78042544
rs73036765
rs62084754
rs209858
rs1398059
rs631487
rs79844317
rs4707319
rs55799444
rs1537547
rs73136665
rs12096911
rs3756564
rs79486472
rs73072986
rs2122146
rs187281020
rs6658665
rs147902311
rs146597227
rs181383275
rs73099996
rs150306980
rs59604901
rs1324073
rs12752576
rs2015682
rs6668517
rs6586224
rs4657608
rs61814886
rs115662161
rs72928038
rs74863989
rs74620952
All
−0.04
0.00
0.04
0.08
MR leave−one−out sensitivity analysis for
'CD3 on resting Treg ' on 'Diabetic nephropathy'

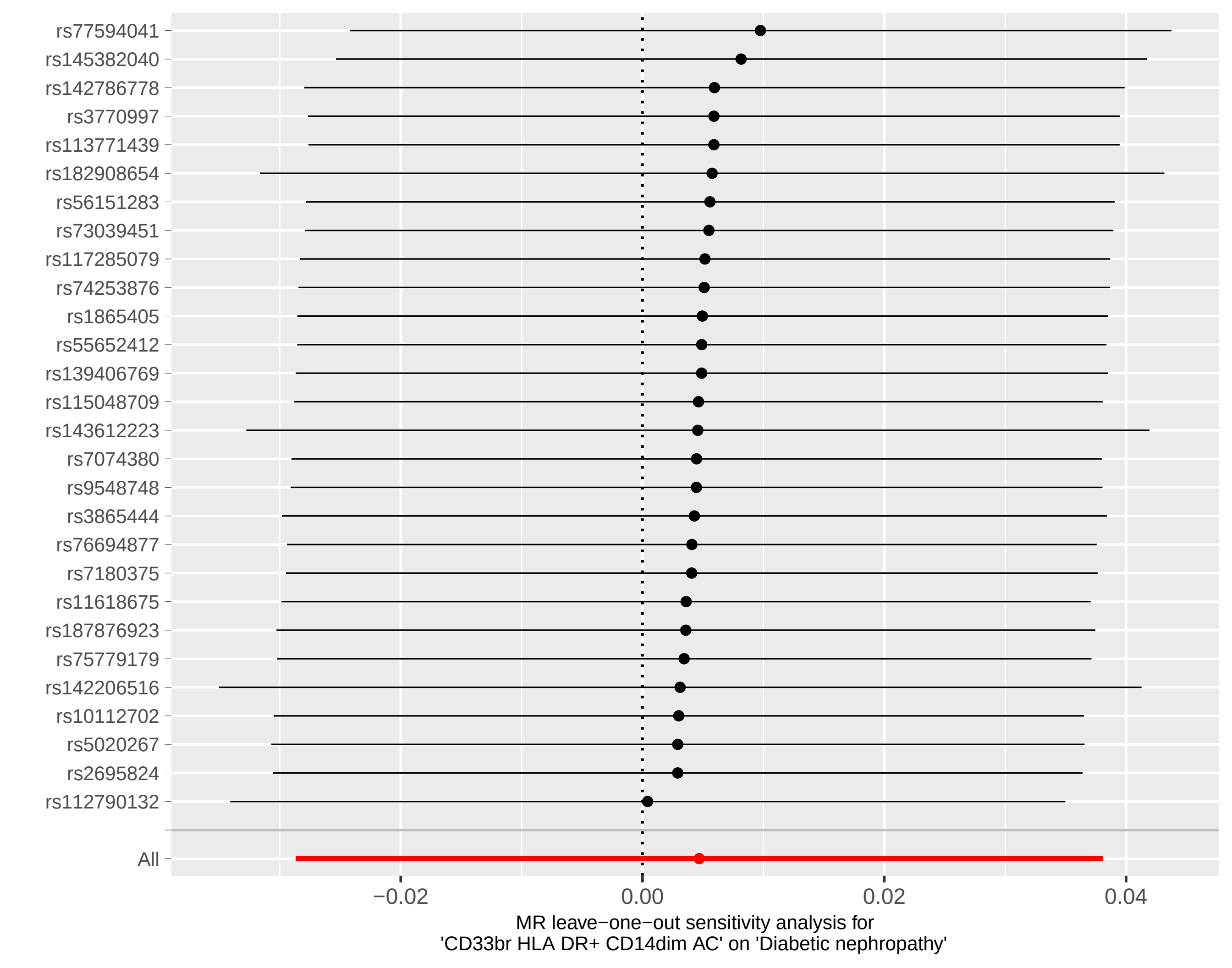
rs77594041
rs145382040
rs142786778
rs3770997
rs113771439
rs182908654
rs56151283
rs73039451
rs117285079
rs74253876
rs1865405
rs55652412
rs139406769
rs115048709
rs143612223
rs7074380
rs9548748
rs3865444
rs76694877
rs7180375
rs11618675
rs187876923
rs75779179
rs142206516
rs10112702
rs5020267
rs2695824
rs112790132
All
−0.02
0.00
0.02
0.04
MR leave−one−out sensitivity analysis for
'CD33br HLA DR+ CD14dim AC' on 'Diabetic nephropathy'

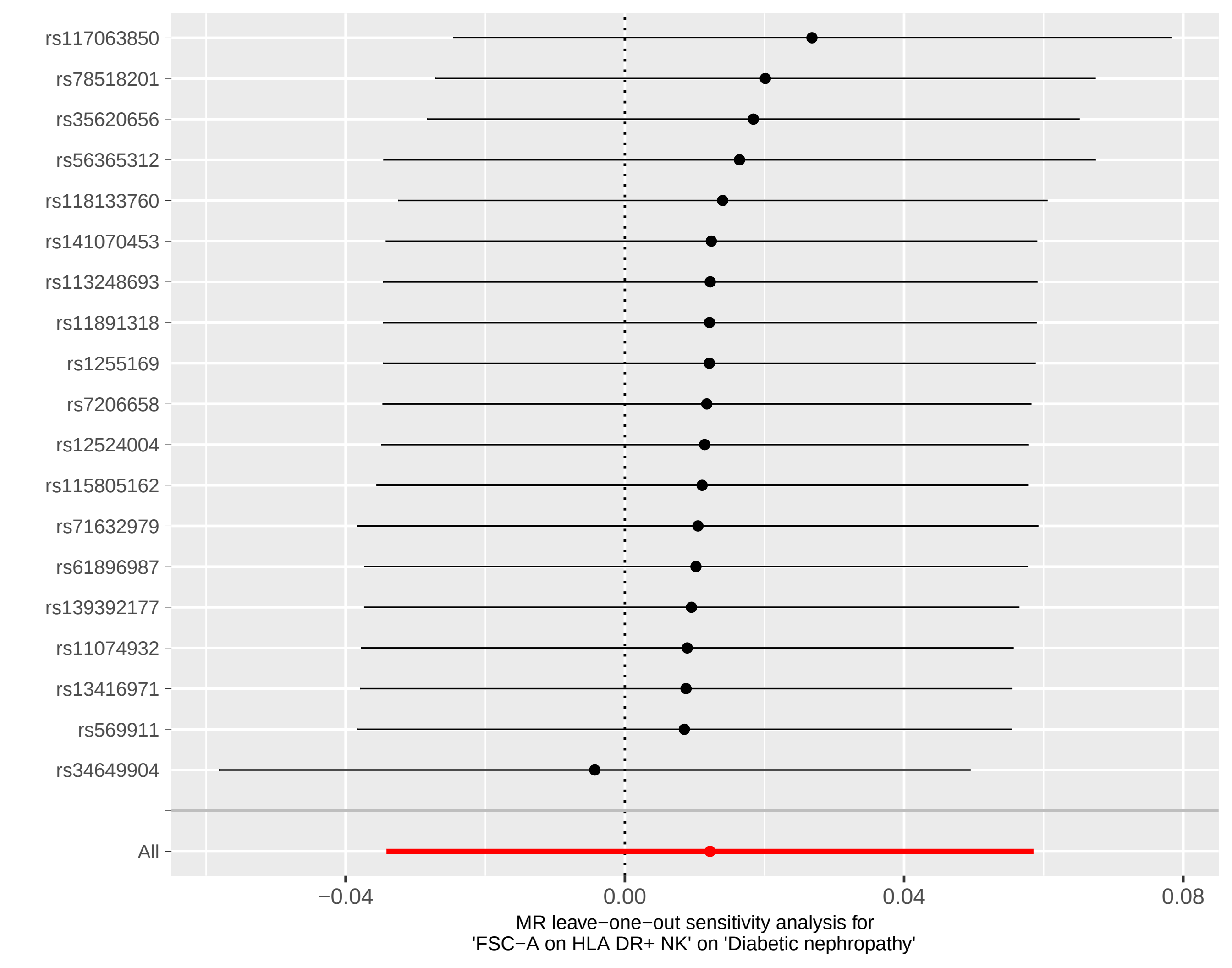
rs117063850
rs78518201
rs35620656
rs56365312
rs118133760
rs141070453
rs113248693
rs11891318
rs1255169
rs7206658
rs12524004
rs115805162
rs71632979
rs61896987
rs139392177
rs11074932
rs13416971
rs569911
rs34649904
All
−0.04
0.00
0.04
0.08
MR leave−one−out sensitivity analysis for
'FSC−A on HLA DR+ NK' on 'Diabetic nephropathy'

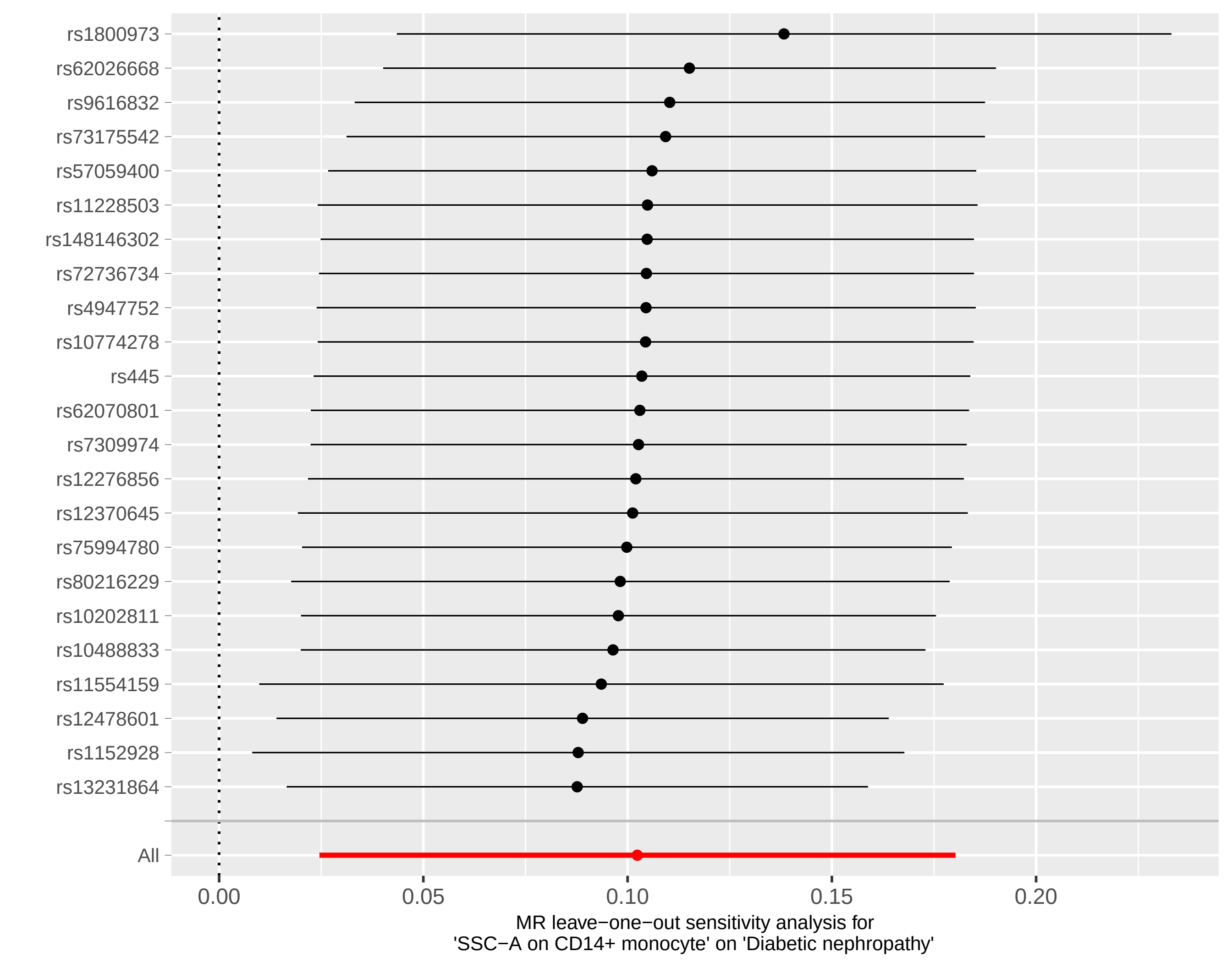
rs1800973
rs62026668
rs9616832
rs73175542
rs57059400
rs11228503
rs148146302
rs72736734
rs4947752
rs10774278
rs445
rs62070801
rs7309974
rs12276856
rs12370645
rs75994780
rs80216229
rs10202811
rs10488833
rs11554159
rs12478601
rs1152928
rs13231864
All
0.00
0.05
0.10
0.15
0.20
MR leave−one−out sensitivity analysis for
'SSC−A on CD14+ monocyte' on 'Diabetic nephropathy'

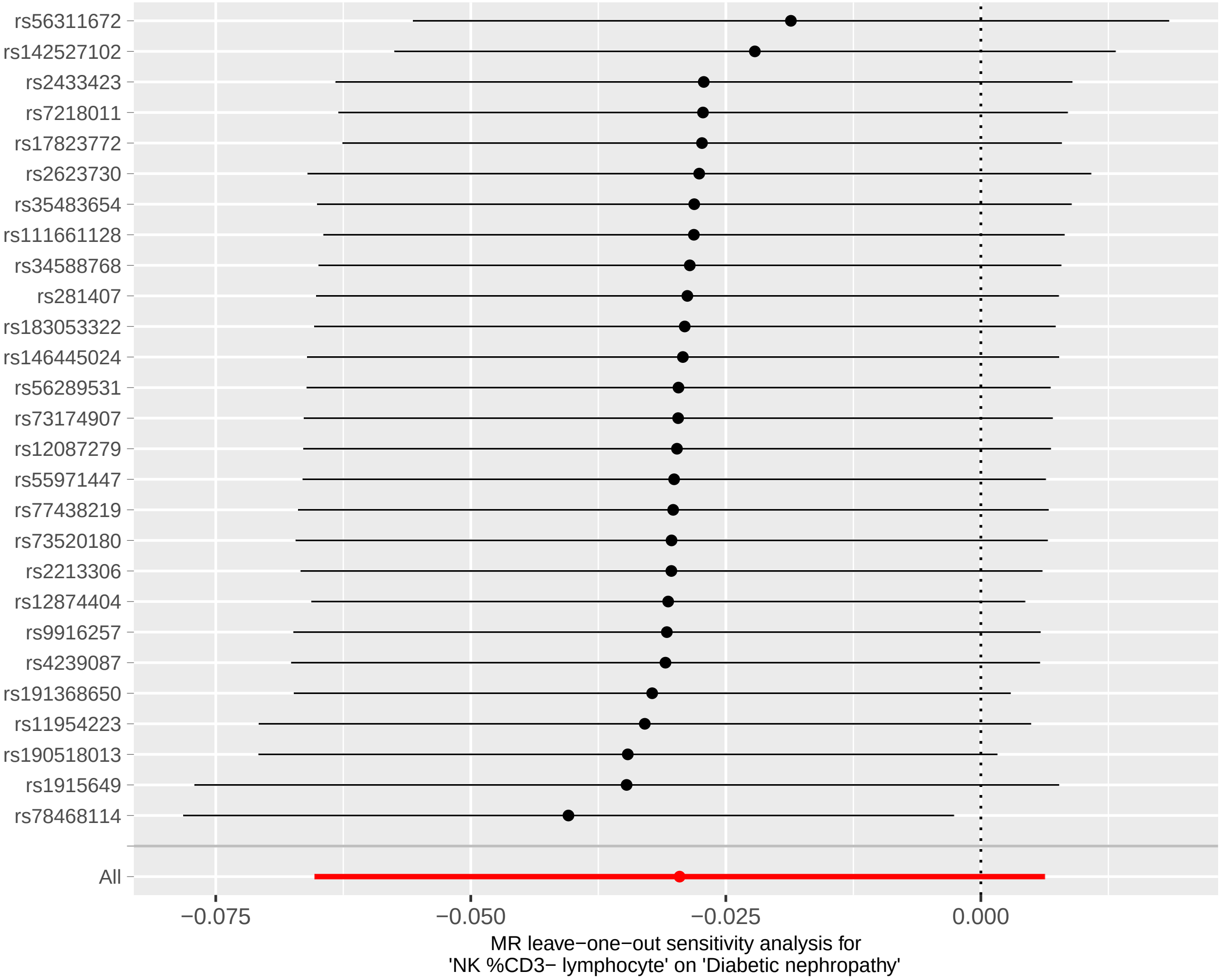
rs56311672
rs142527102
rs2433423
rs7218011
rs17823772
rs2623730
rs35483654
rs111661128
rs34588768
rs281407
rs183053322
rs146445024
rs56289531
rs73174907
rs12087279
rs55971447
rs77438219
rs73520180
rs2213306
rs12874404
rs9916257
rs4239087
rs191368650
rs11954223
rs190518013
rs1915649
rs78468114
All
−0.075
−0.050
−0.025
0.000
MR leave−one−out sensitivity analysis for
'NK %CD3− lymphocyte' on 'Diabetic nephropathy'

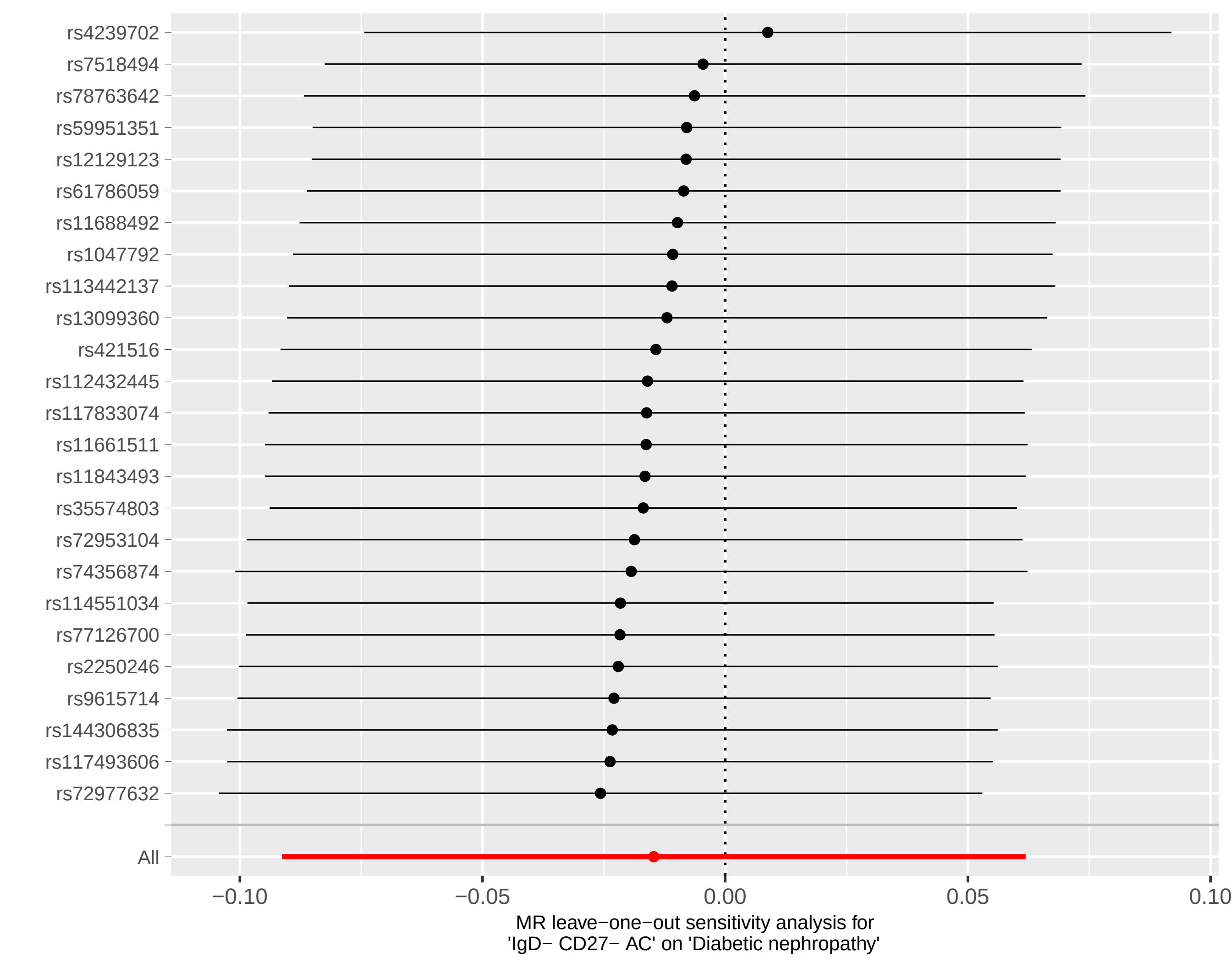
rs4239702
rs7518494
rs78763642
rs59951351
rs12129123
rs61786059
rs11688492
rs1047792
rs113442137
rs13099360
rs421516
rs112432445
rs117833074
rs11661511
rs11843493
rs35574803
rs72953104
rs74356874
rs114551034
rs77126700
rs2250246
rs9615714
rs144306835
rs117493606
rs72977632
All
−0.10
−0.05
0.00
0.05
0.10
MR leave−one−out sensitivity analysis for
'IgD− CD27− AC' on 'Diabetic nephropathy'

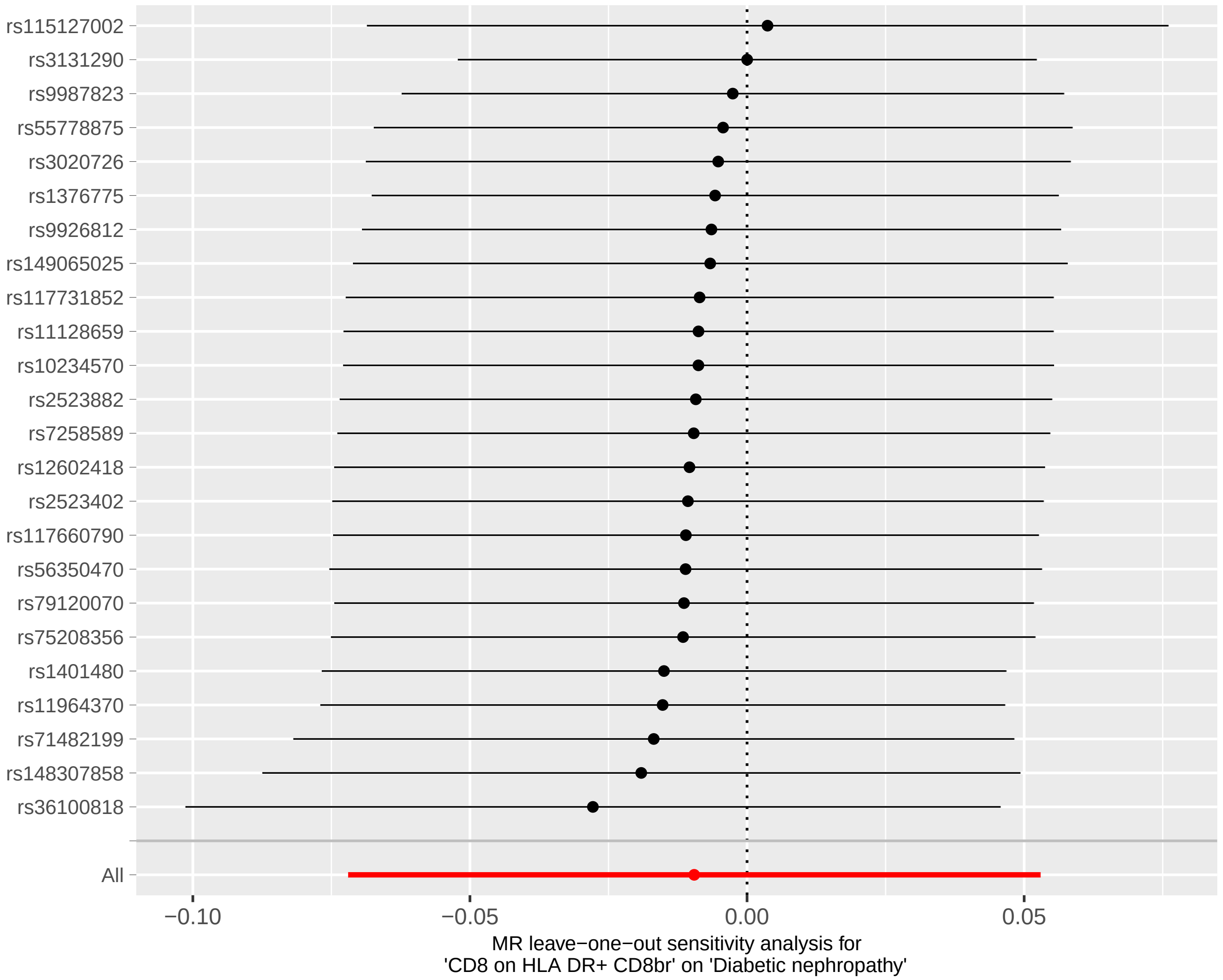
rs115127002
rs3131290
rs9987823
rs55778875
rs3020726
rs1376775
rs9926812
rs149065025
rs117731852
rs11128659
rs10234570
rs2523882
rs7258589
rs12602418
rs2523402
rs117660790
rs56350470
rs79120070
rs75208356
rs1401480
rs11964370
rs71482199
rs148307858
rs36100818
All
−0.10
−0.05
0.00
0.05
MR leave−one−out sensitivity analysis for
'CD8 on HLA DR+ CD8br' on 'Diabetic nephropathy'

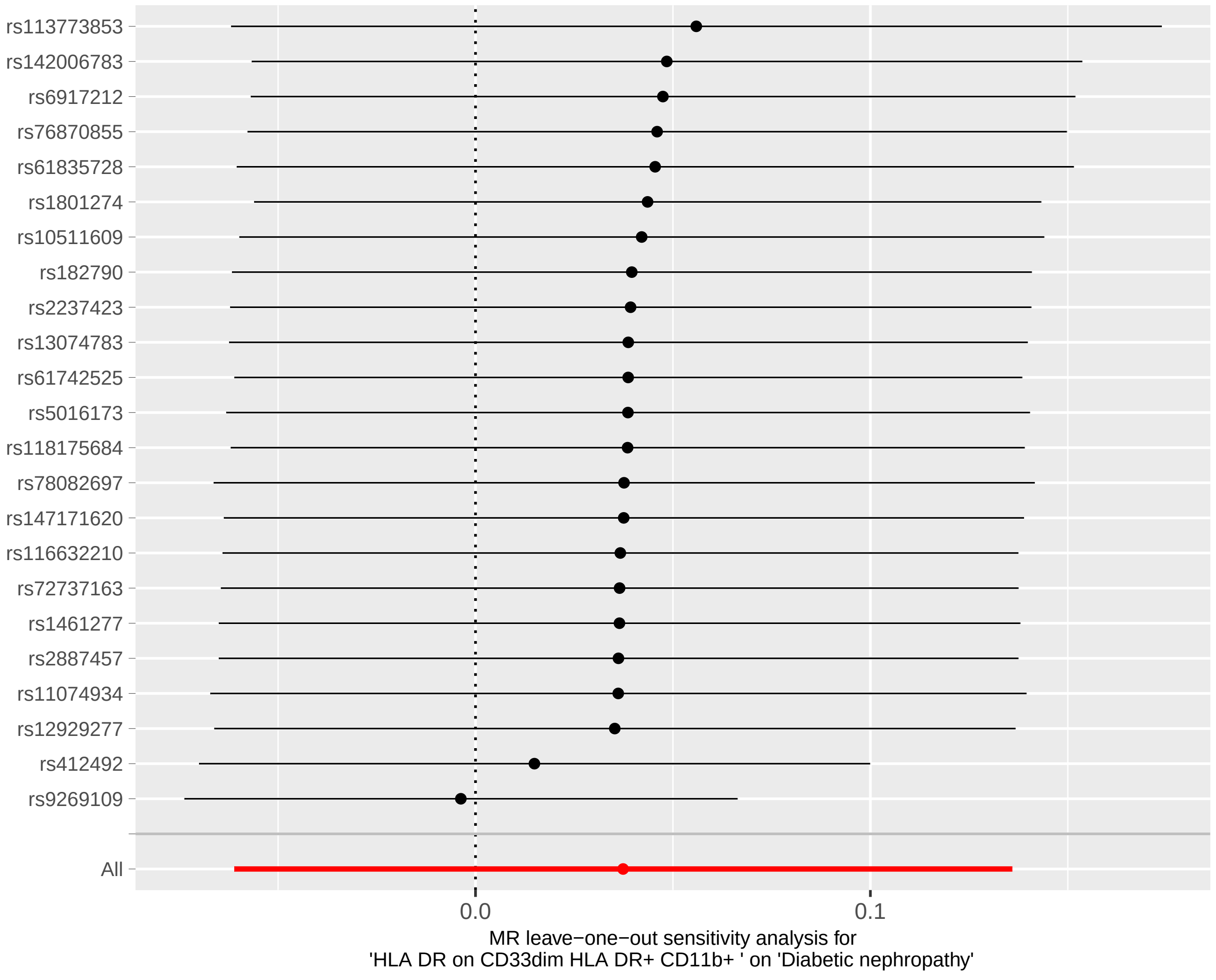
rs113773853
rs142006783
rs6917212
rs76870855
rs61835728
rs1801274
rs10511609
rs182790
rs2237423
rs13074783
rs61742525
rs5016173
rs118175684
rs78082697
rs147171620
rs116632210
rs72737163
rs1461277
rs2887457
rs11074934
rs12929277
rs412492
rs9269109
All
0.0
0.1
MR leave−one−out sensitivity analysis for
'HLA DR on CD33dim HLA DR+ CD11b+ ' on 'Diabetic nephropathy'

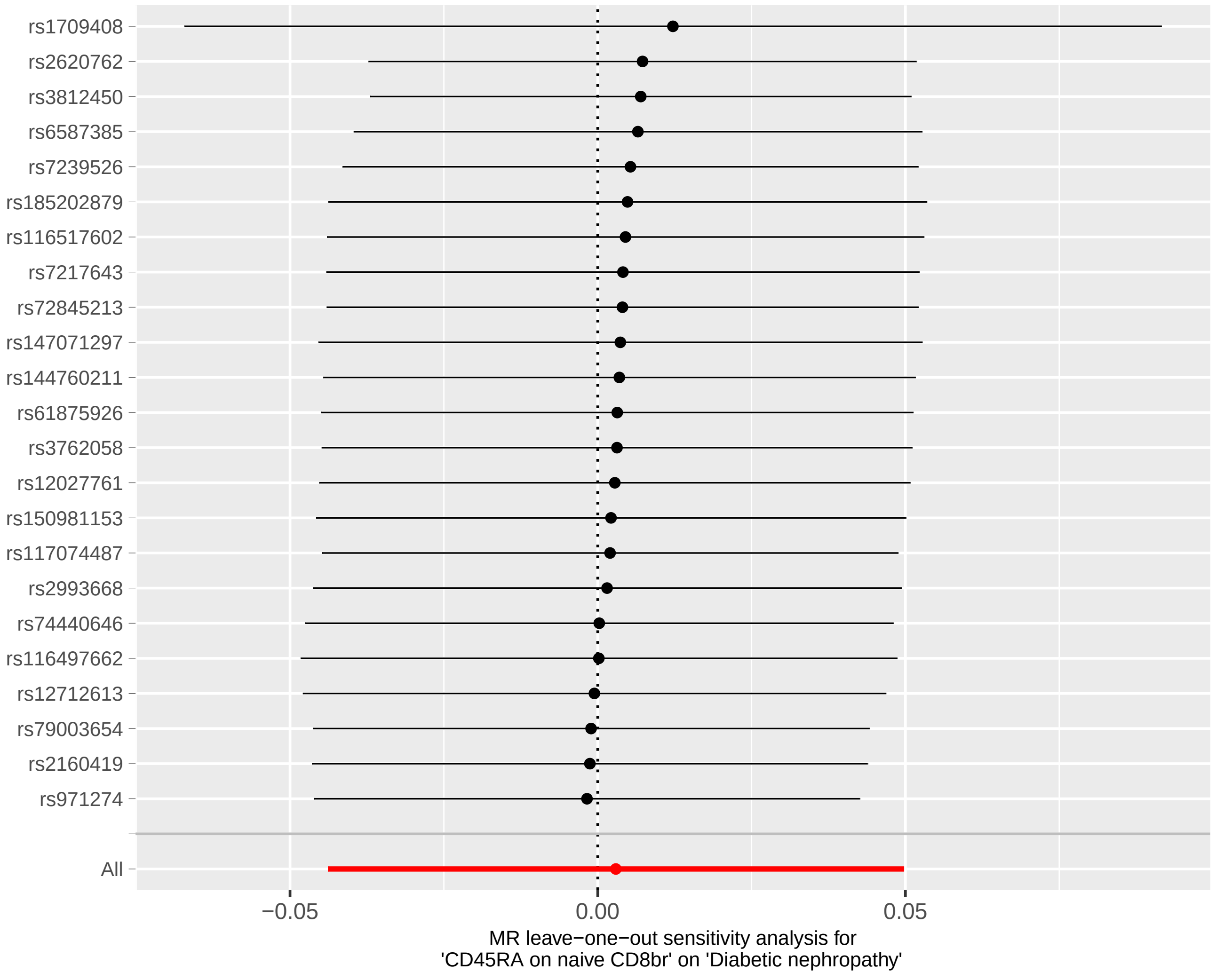
rs1709408
rs2620762
rs3812450
rs6587385
rs7239526
rs185202879
rs116517602
rs7217643
rs72845213
rs147071297
rs144760211
rs61875926
rs3762058
rs12027761
rs150981153
rs117074487
rs2993668
rs74440646
rs116497662
rs12712613
rs79003654
rs2160419
rs971274
All
−0.05
0.00
0.05
MR leave−one−out sensitivity analysis for
'CD45RA on naive CD8br' on 'Diabetic nephropathy'

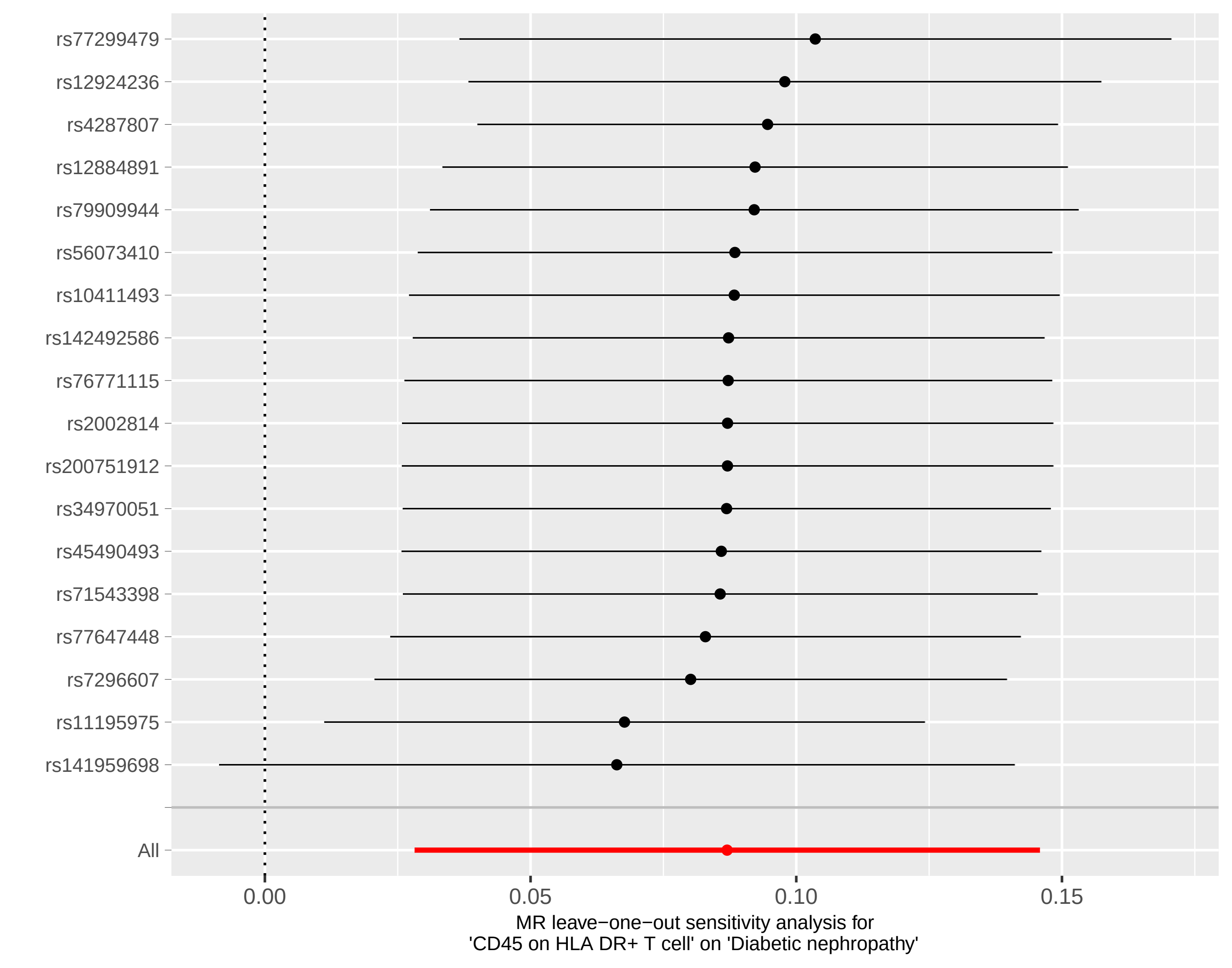
rs77299479
rs12924236
rs4287807
rs12884891
rs79909944
rs56073410
rs10411493
rs142492586
rs76771115
rs2002814
rs200751912
rs34970051
rs45490493
rs71543398
rs77647448
rs7296607
rs11195975
rs141959698
All
0.00
0.05
0.10
0.15
MR leave−one−out sensitivity analysis for
'CD45 on HLA DR+ T cell' on 'Diabetic nephropathy'

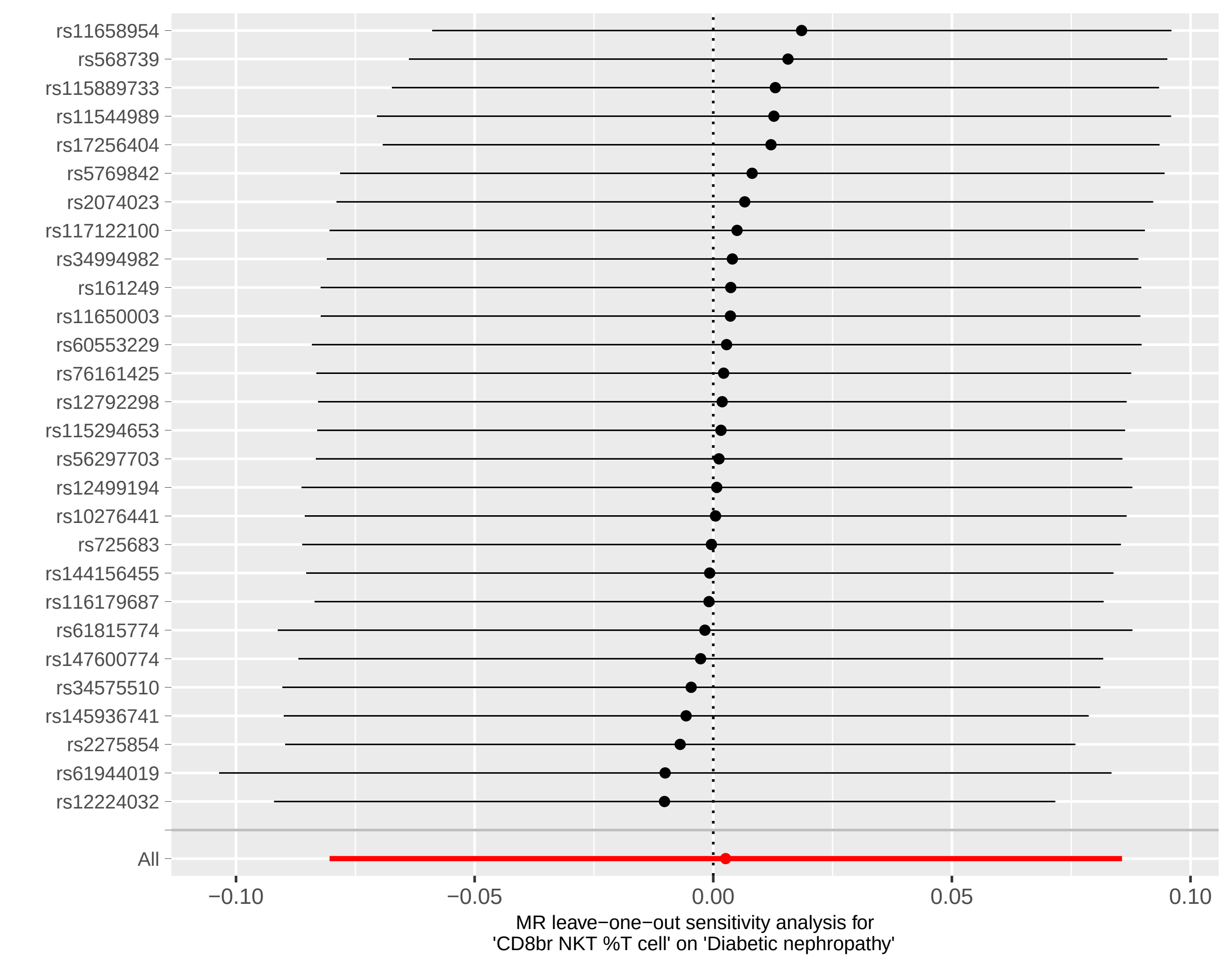
rs11658954
rs568739
rs115889733
rs11544989
rs17256404
rs5769842
rs2074023
rs117122100
rs34994982
rs161249
rs11650003
rs60553229
rs76161425
rs12792298
rs115294653
rs56297703
rs12499194
rs10276441
rs725683
rs144156455
rs116179687
rs61815774
rs147600774
rs34575510
rs145936741
rs2275854
rs61944019
rs12224032
All
−0.10
−0.05
0.00
0.05
0.10
MR leave−one−out sensitivity analysis for
'CD8br NKT %T cell' on 'Diabetic nephropathy'

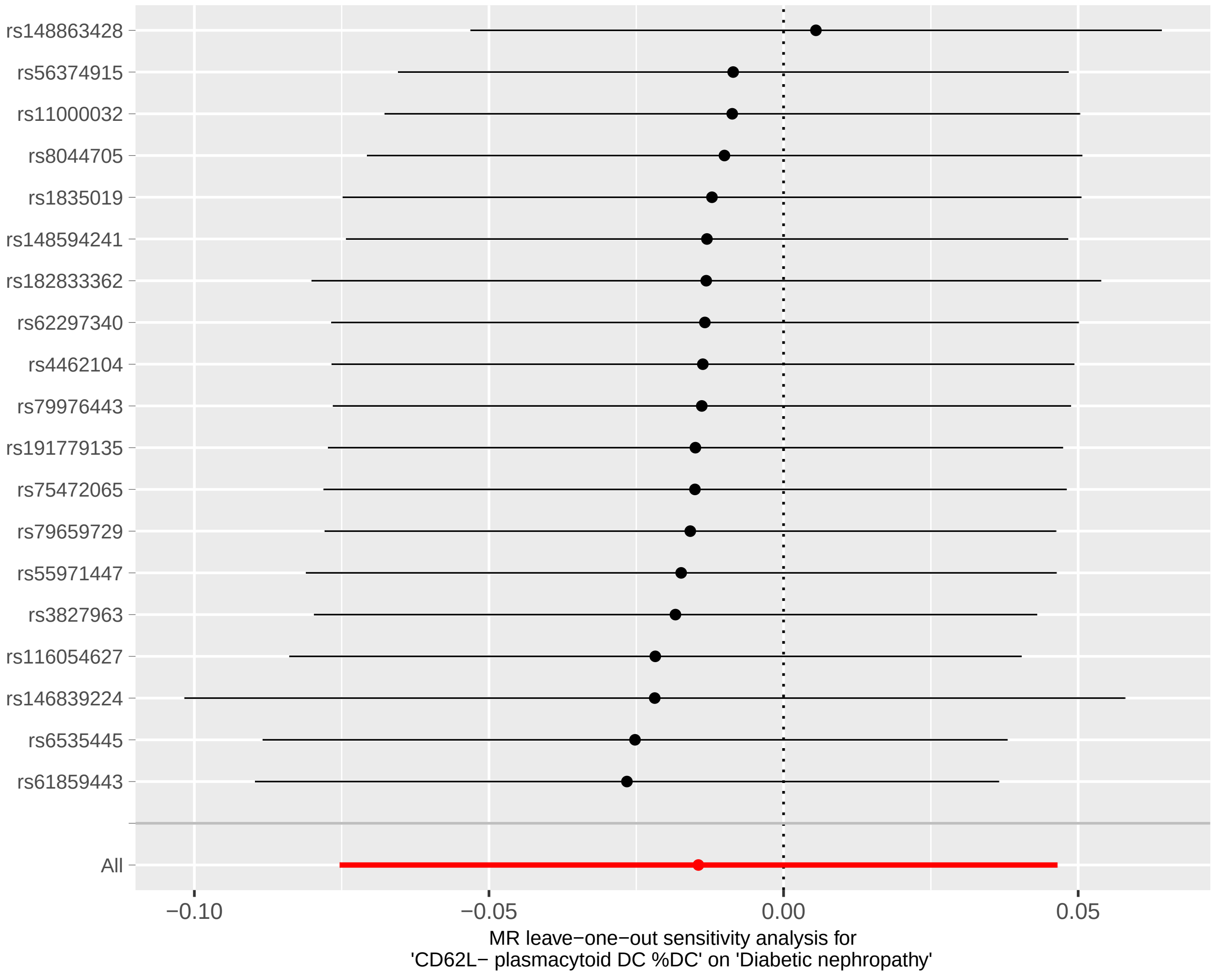

rs148863428
rs56374915
rs11000032
rs8044705
rs1835019
rs148594241
rs182833362
rs62297340
rs4462104
rs79976443
rs191779135
rs75472065
rs79659729
rs55971447
rs3827963
rs116054627
rs146839224
rs6535445
rs61859443
All
−0.10
−0.05
0.00
0.05
MR leave−one−out sensitivity analysis for
'CD62L− plasmacytoid DC %DC' on 'Diabetic nephropathy'

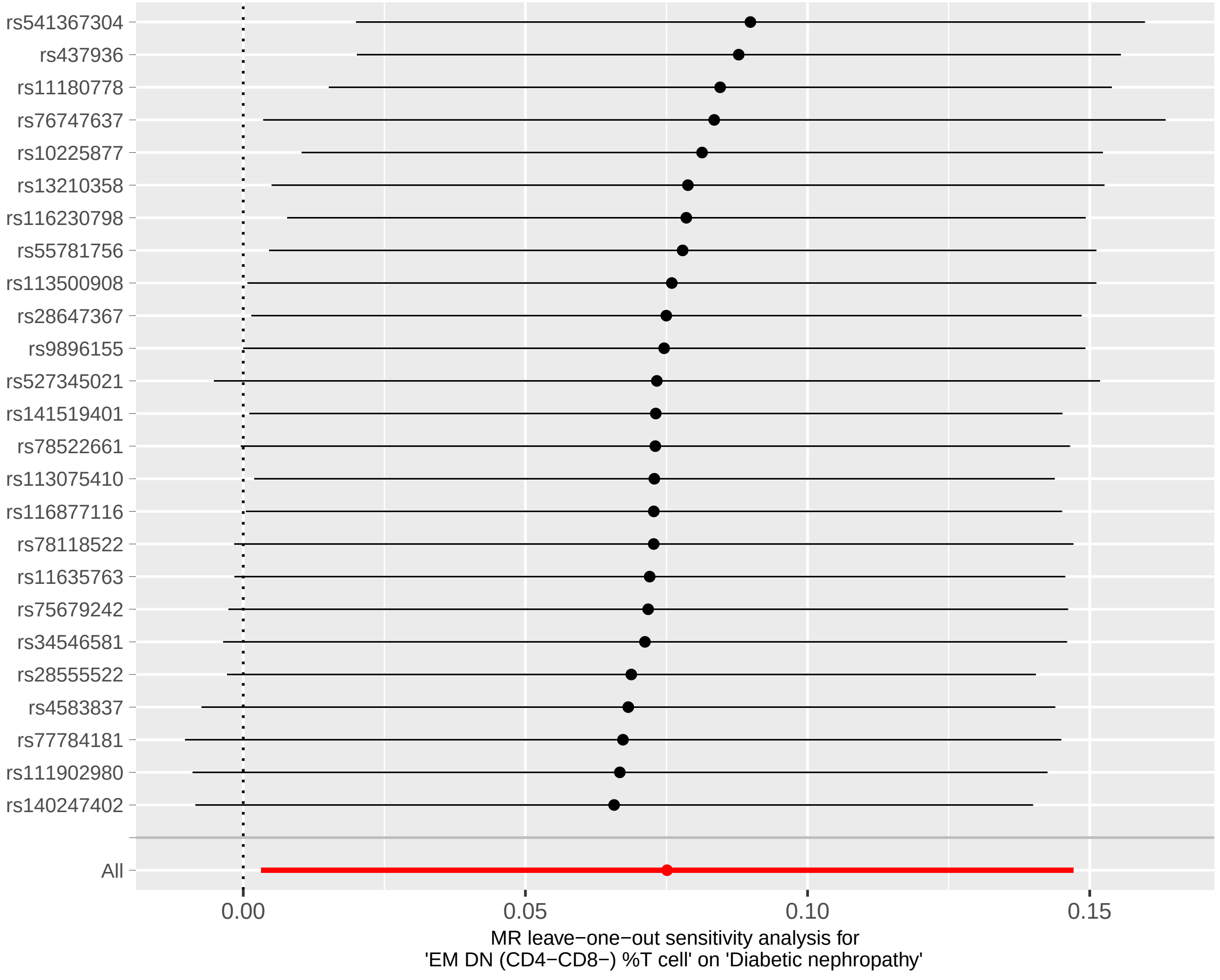

rs541367304
rs437936
rs11180778
rs76747637
rs10225877
rs13210358
rs116230798
rs55781756
rs113500908
rs28647367
rs9896155
rs527345021
rs141519401
rs78522661
rs113075410
rs116877116
rs78118522
rs11635763
rs75679242
rs34546581
rs28555522
rs4583837
rs77784181
rs111902980
rs140247402
All
0.00
0.05
0.10
0.15
MR leave−one−out sensitivity analysis for
'EM DN (CD4−CD8−) %T cell' on 'Diabetic nephropathy'

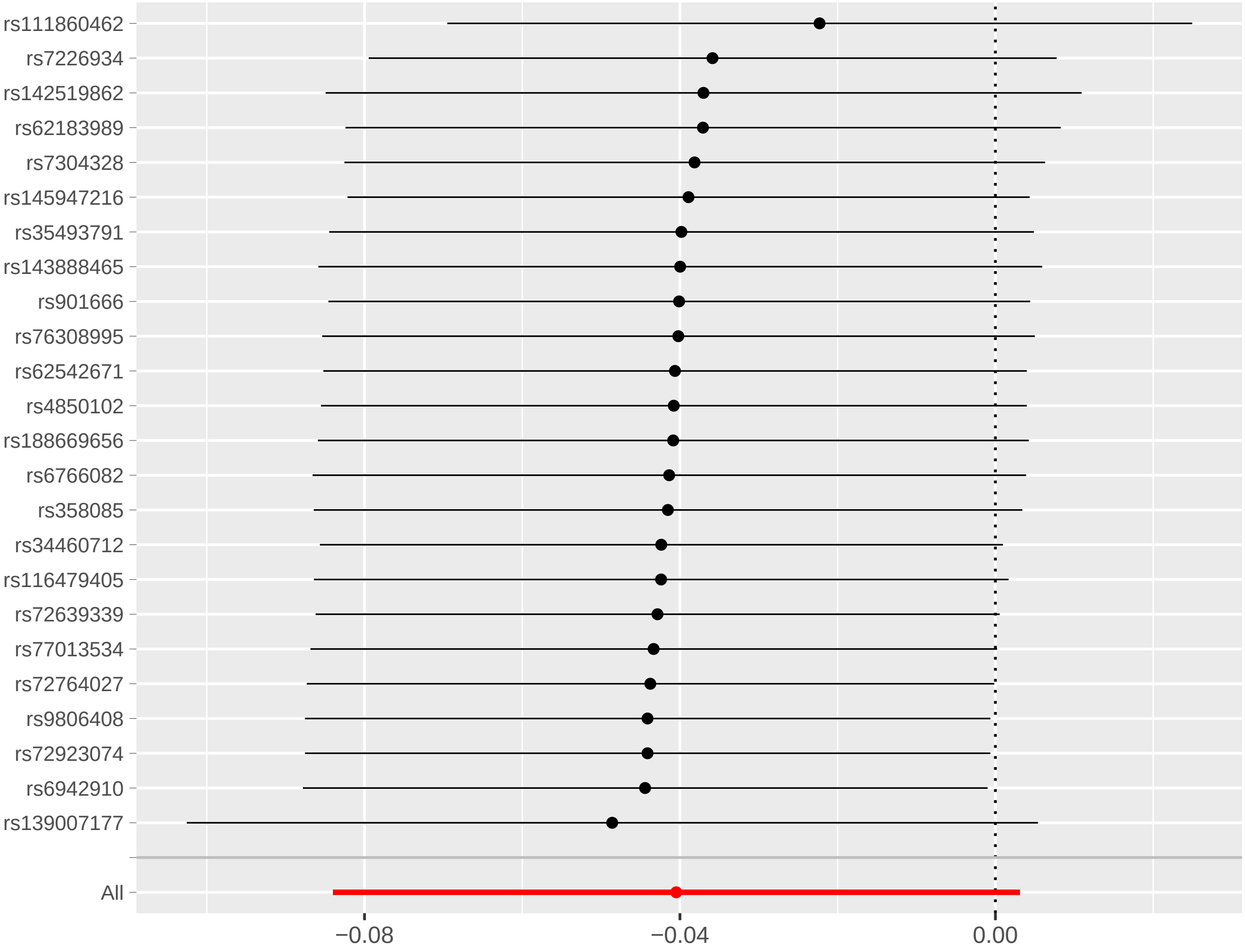

MR leave−one−out sensitivity analysis for
'CD28 on activated & secreting Treg' on 'Diabetic nephropathy'

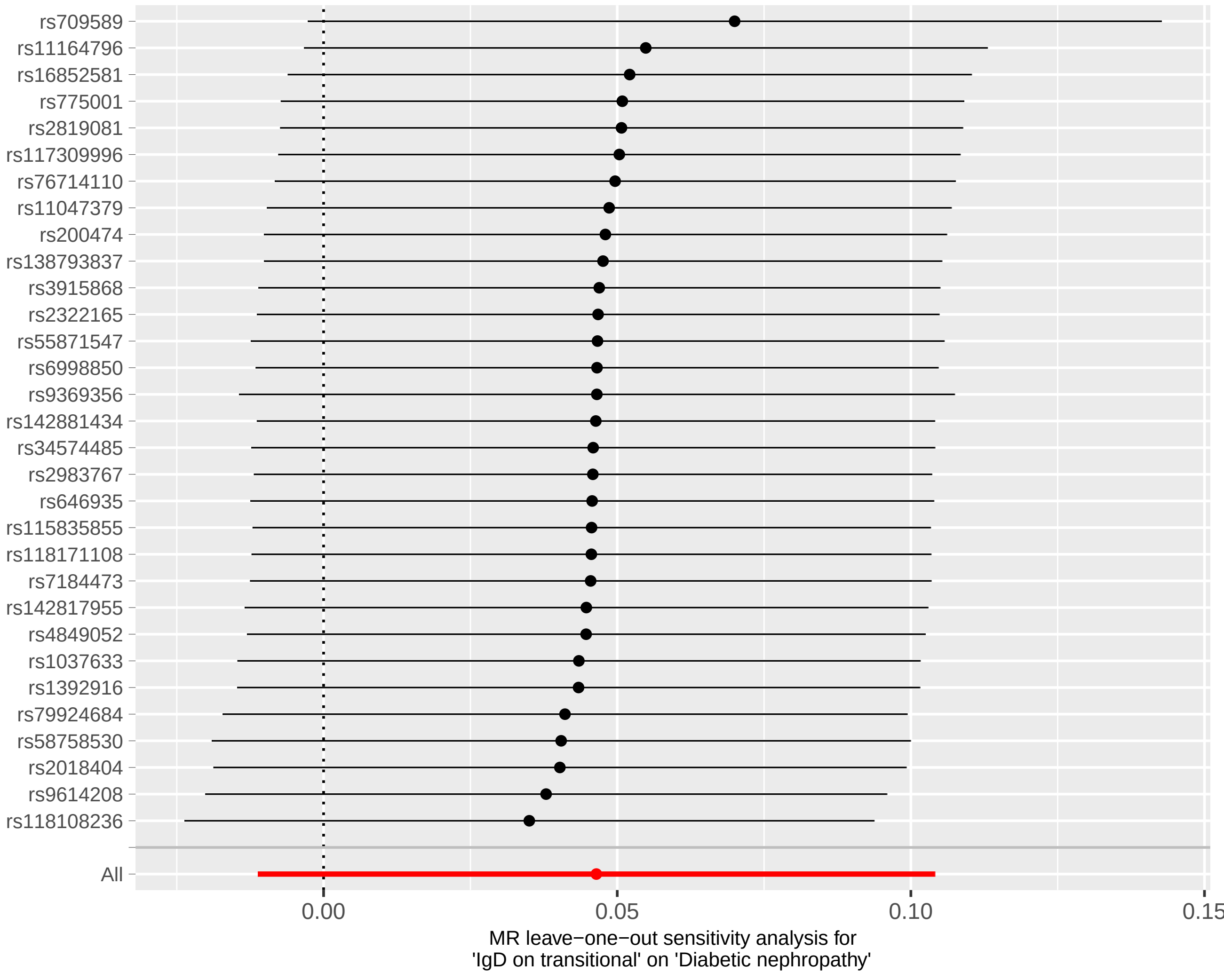

rs709589
rs11164796
rs16852581
rs775001
rs2819081
rs117309996
rs76714110
rs11047379
rs200474
rs138793837
rs3915868
rs2322165
rs55871547
rs6998850
rs9369356
rs142881434
rs34574485
rs2983767
rs646935
rs115835855
rs118171108
rs7184473
rs142817955
rs4849052
rs1037633
rs1392916
rs79924684
rs58758530
rs2018404
rs9614208
rs118108236
All
0.00
0.05
0.10
0.15
MR leave−one−out sensitivity analysis for
'IgD on transitional' on 'Diabetic nephropathy'

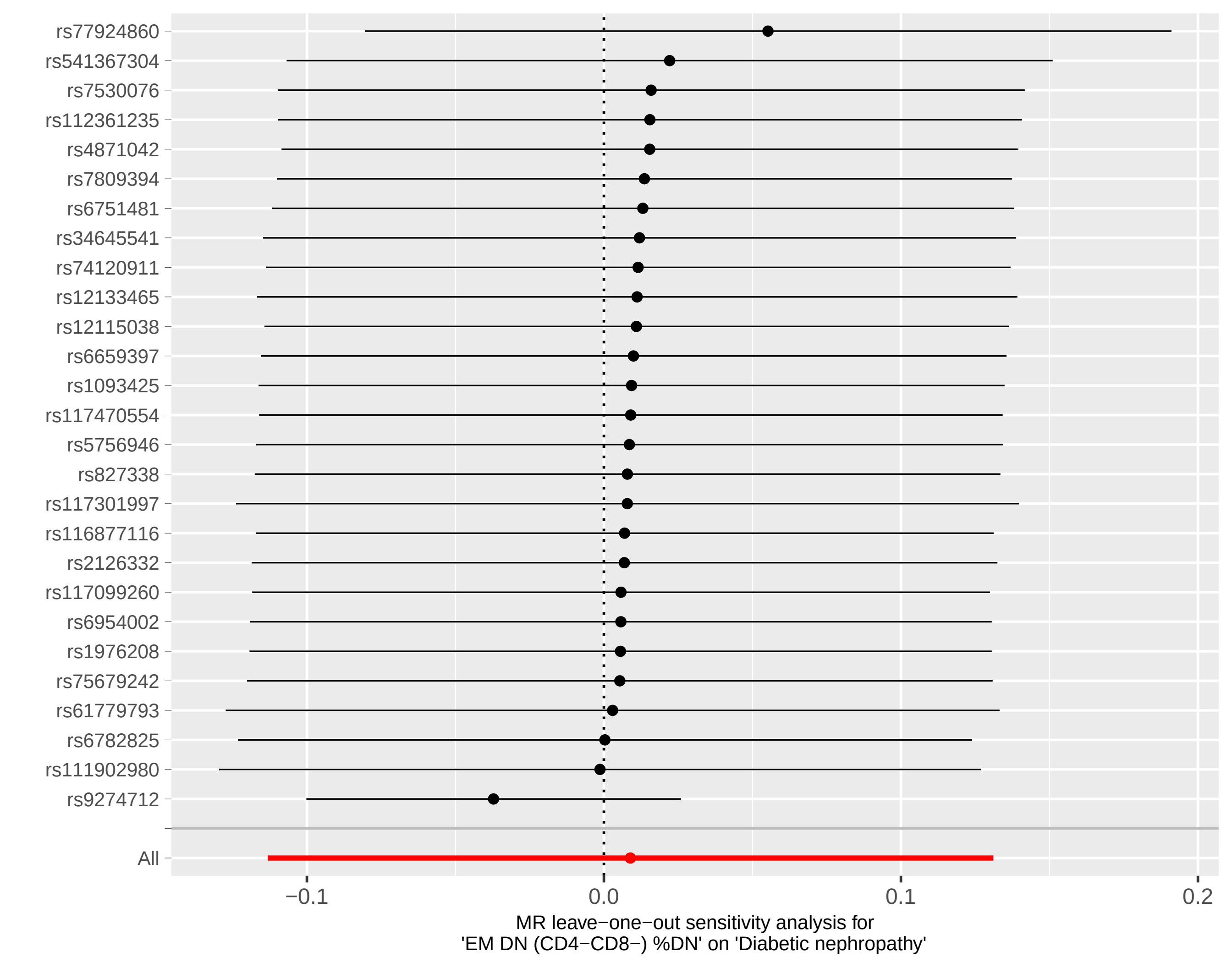
rs77924860
rs541367304
rs7530076
rs112361235
rs4871042
rs7809394
rs6751481
rs34645541
rs74120911
rs12133465
rs12115038
rs6659397
rs1093425
rs117470554
rs5756946
rs827338
rs117301997
rs116877116
rs2126332
rs117099260
rs6954002
rs1976208
rs75679242
rs61779793
rs6782825
rs111902980
rs9274712
All
−0.1
0.0
0.1
0.2
MR leave−one−out sensitivity analysis for
'EM DN (CD4−CD8−) %DN' on 'Diabetic nephropathy'

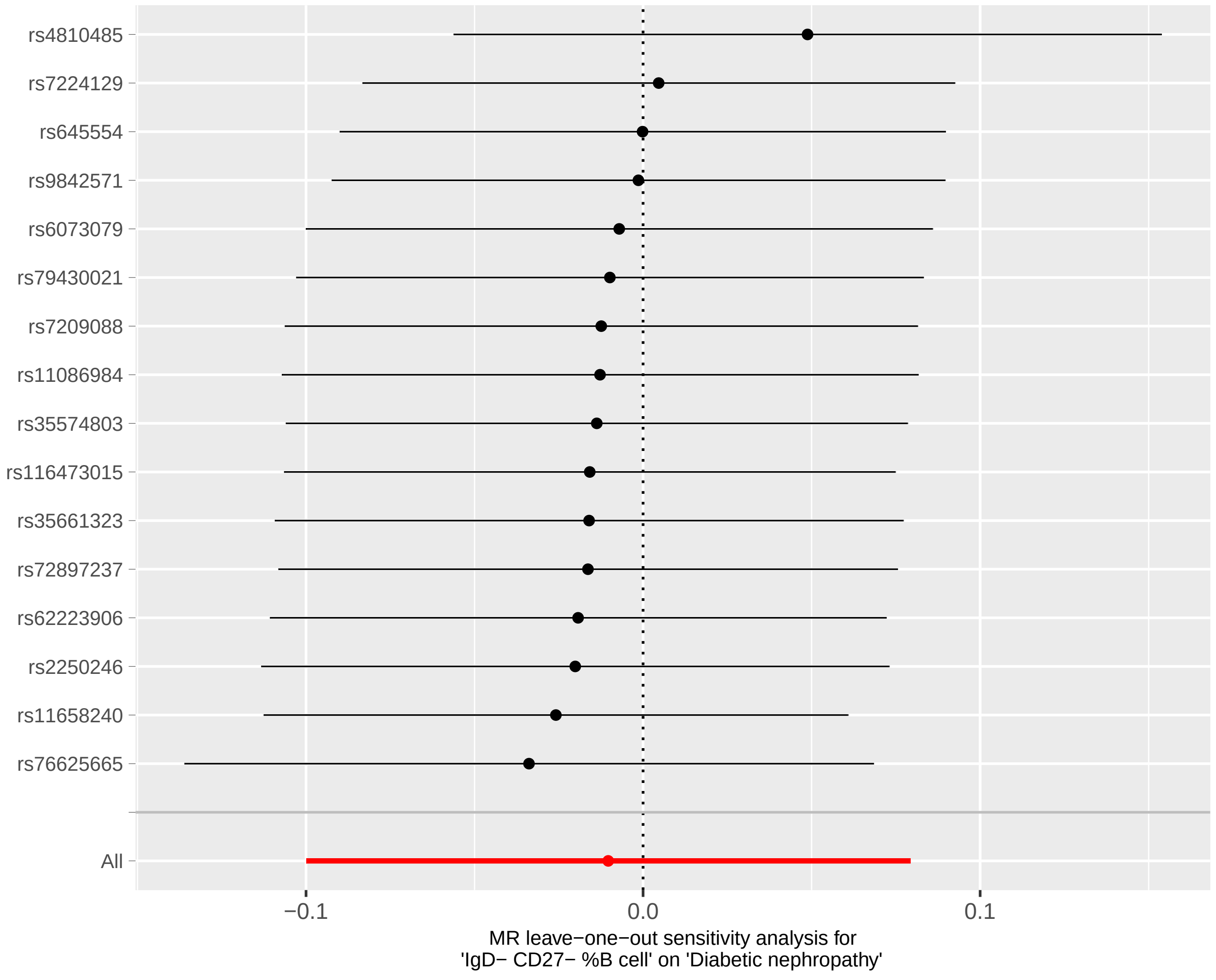
rs4810485
rs7224129
rs645554
rs9842571
rs6073079
rs79430021
rs7209088
rs11086984
rs35574803
rs116473015
rs35661323
rs72897237
rs62223906
rs2250246
rs11658240
rs76625665
All
−0.1
0.0
0.1
MR leave−one−out sensitivity analysis for
'IgD− CD27− %B cell' on 'Diabetic nephropathy'

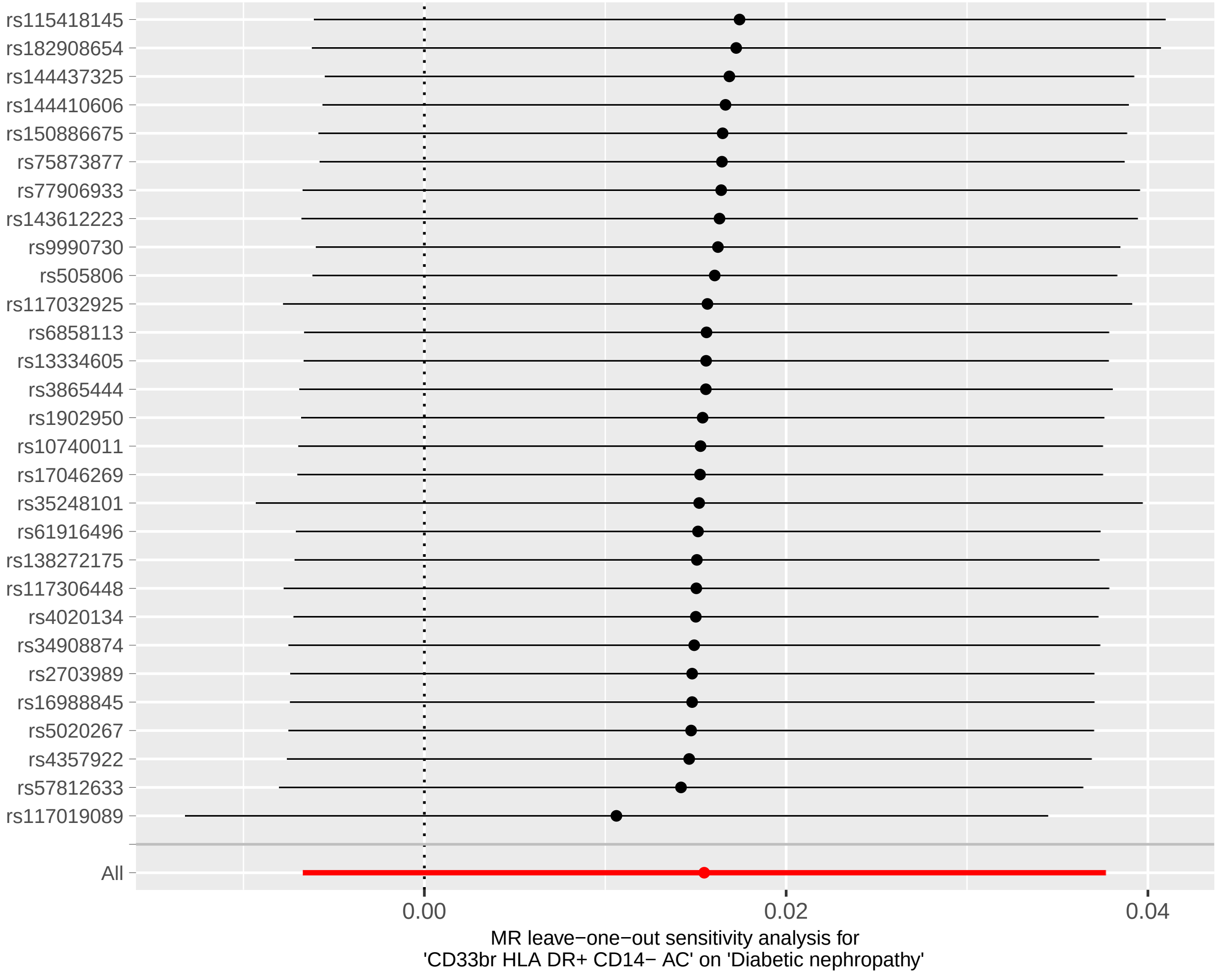
rs115418145
rs182908654
rs144437325
rs144410606
rs150886675
rs75873877
rs77906933
rs143612223
rs9990730
rs505806
rs117032925
rs6858113
rs13334605
rs3865444
rs1902950
rs10740011
rs17046269
rs35248101
rs61916496
rs138272175
rs117306448
rs4020134
rs34908874
rs2703989
rs16988845
rs5020267
rs4357922
rs57812633
rs117019089
All
0.00
0.02
0.04
MR leave−one−out sensitivity analysis for
'CD33br HLA DR+ CD14− AC' on 'Diabetic nephropathy'

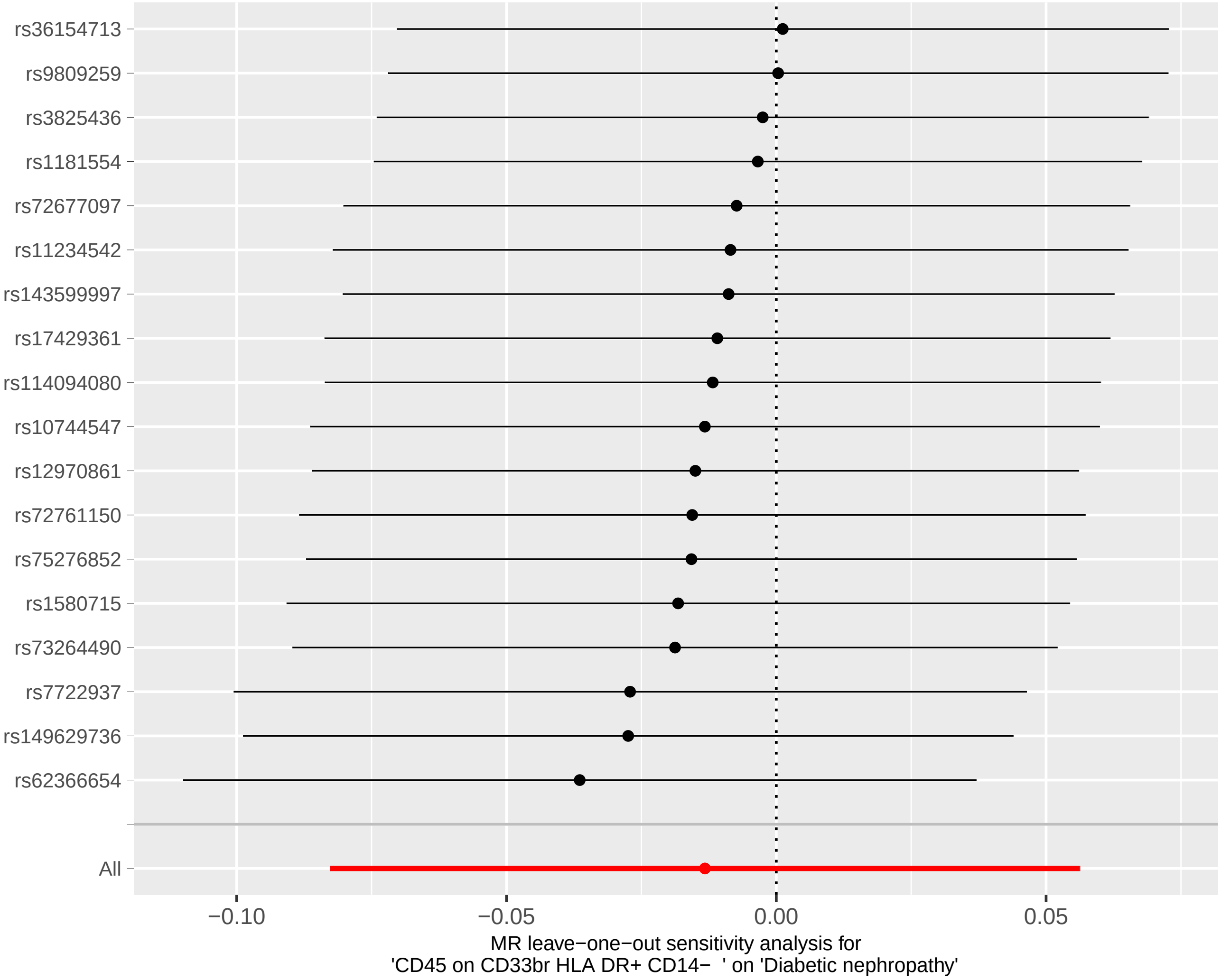
rs36154713
rs9809259
rs3825436
rs1181554
rs72677097
rs11234542
rs143599997
rs17429361
rs114094080
rs10744547
rs12970861
rs72761150
rs75276852
rs1580715
rs73264490
rs7722937
rs149629736
rs62366654
All
−0.10
−0.05
0.00
0.05
MR leave−one−out sensitivity analysis for
'CD45 on CD33br HLA DR+ CD14− ' on 'Diabetic nephropathy'

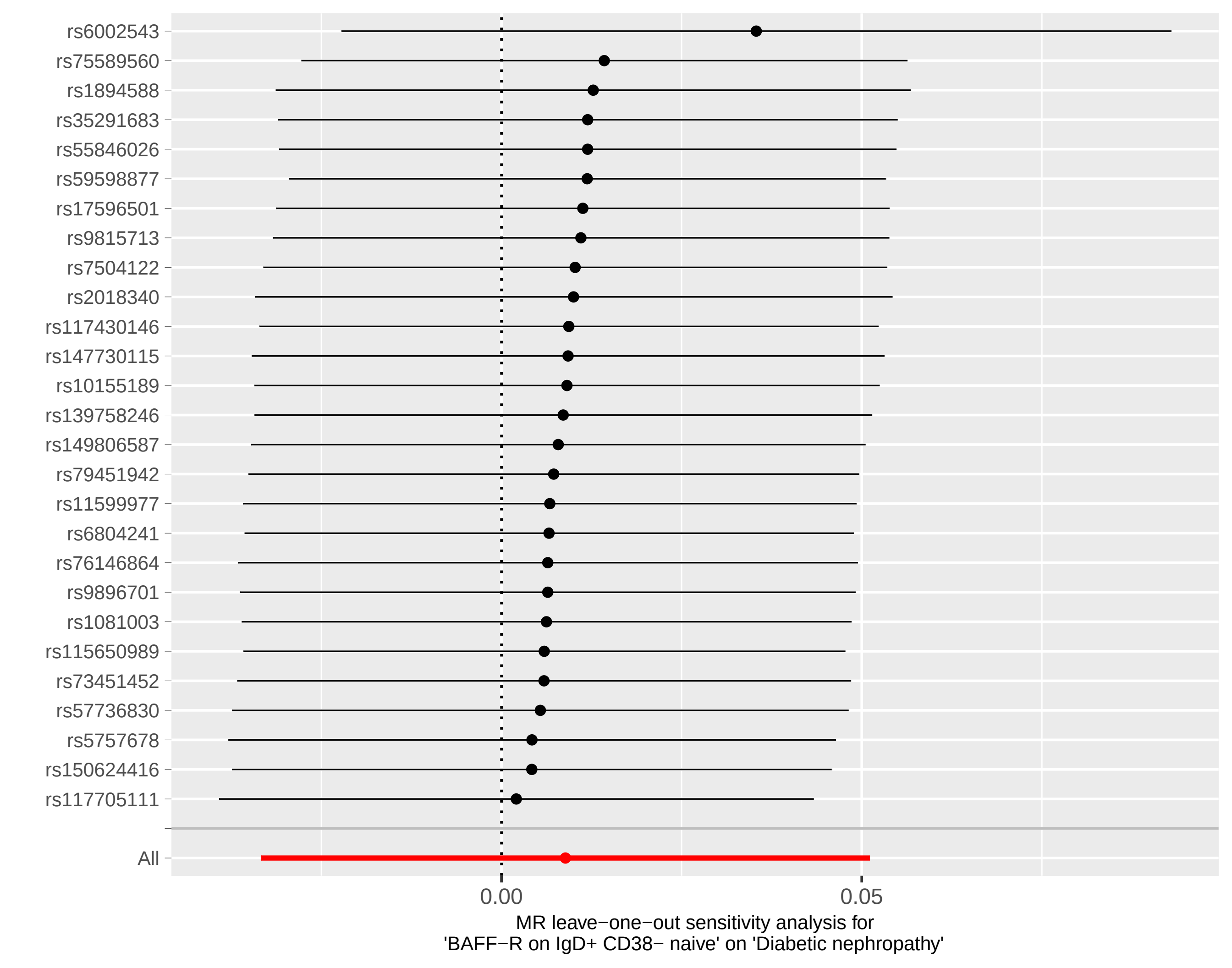
rs6002543
rs75589560
rs1894588
rs35291683
rs55846026
rs59598877
rs17596501
rs9815713
rs7504122
rs2018340
rs117430146
rs147730115
rs10155189
rs139758246
rs149806587
rs79451942
rs11599977
rs6804241
rs76146864
rs9896701
rs1081003
rs115650989
rs73451452
rs57736830
rs5757678
rs150624416
rs117705111
All
0.00
0.05
MR leave−one−out sensitivity analysis for
'BAFF−R on IgD+ CD38− naive' on 'Diabetic nephropathy'

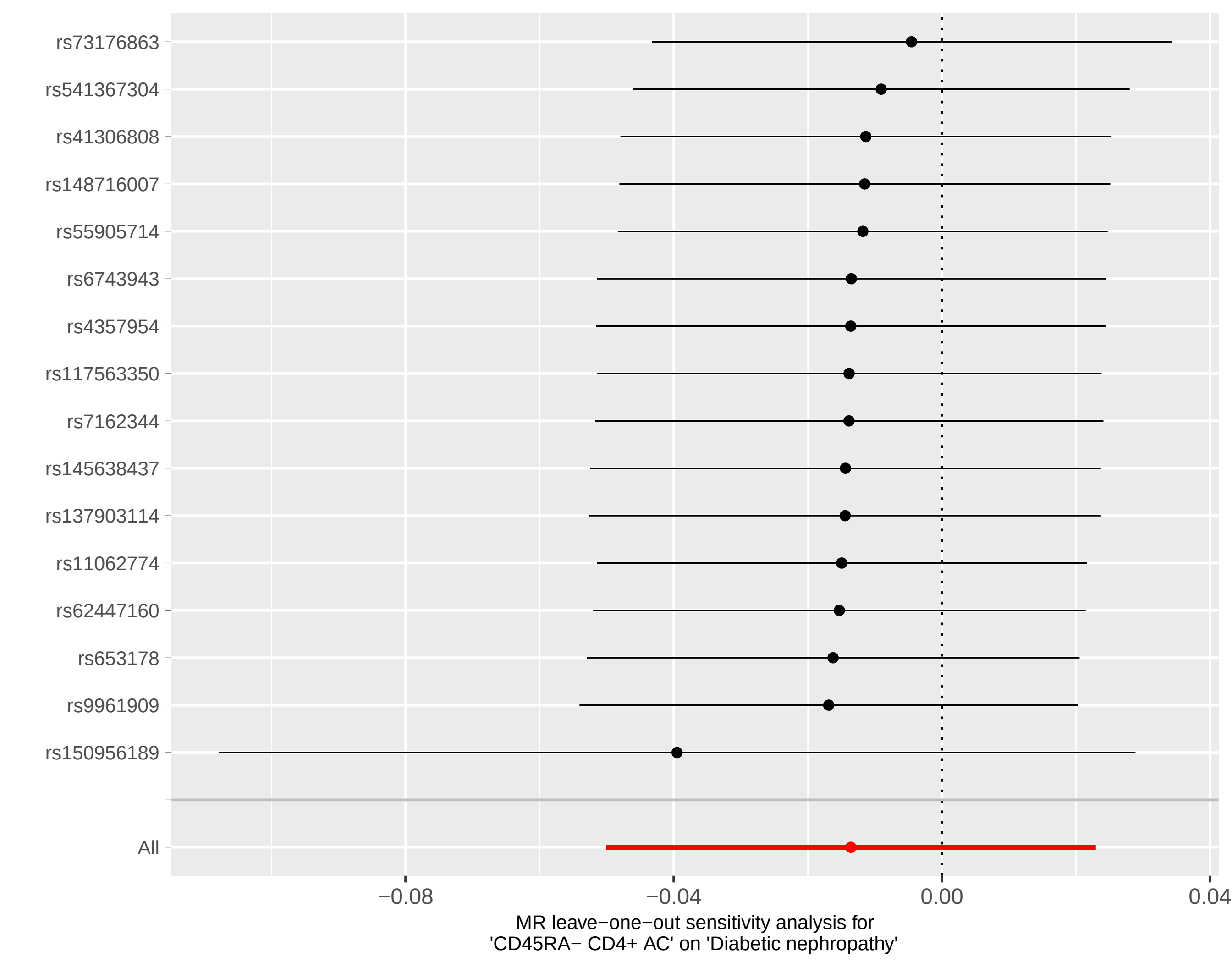
rs73176863
rs541367304
rs41306808
rs148716007
rs55905714
rs6743943
rs4357954
rs117563350
rs7162344
rs145638437
rs137903114
rs11062774
rs62447160
rs653178
rs9961909
rs150956189
All
−0.08
−0.04
0.00
0.04
MR leave−one−out sensitivity analysis for
'CD45RA− CD4+ AC' on 'Diabetic nephropathy'

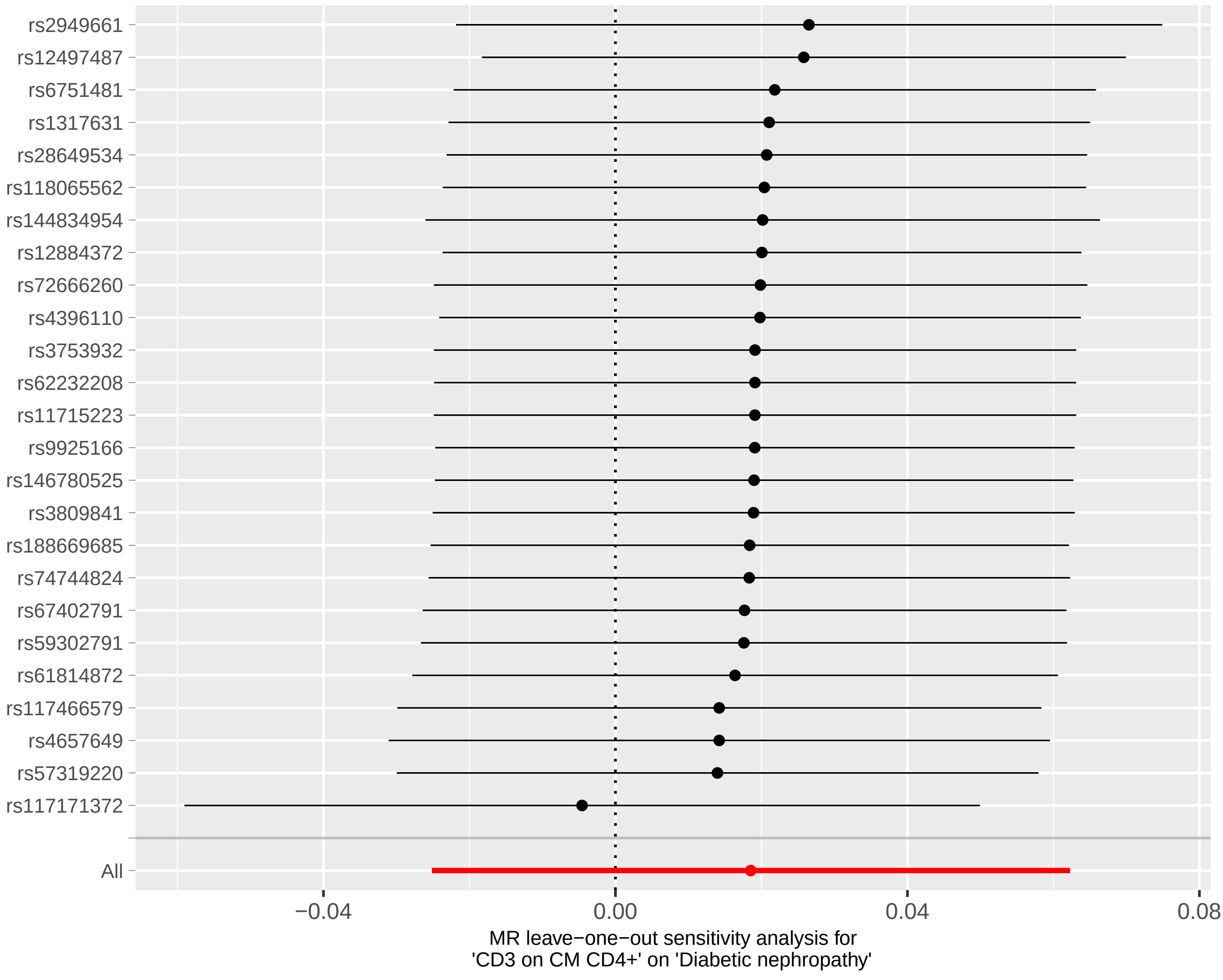
rs2949661
rs12497487
rs6751481
rs1317631
rs28649534
rs118065562
rs144834954
rs12884372
rs72666260
rs4396110
rs3753932
rs62232208
rs11715223
rs9925166
rs146780525
rs3809841
rs188669685
rs74744824
rs67402791
rs59302791
rs61814872
rs117466579
rs4657649
rs57319220
rs117171372
All
−0.04
0.00
0.04
0.08
MR leave−one−out sensitivity analysis for
'CD3 on CM CD4+' on 'Diabetic nephropathy'

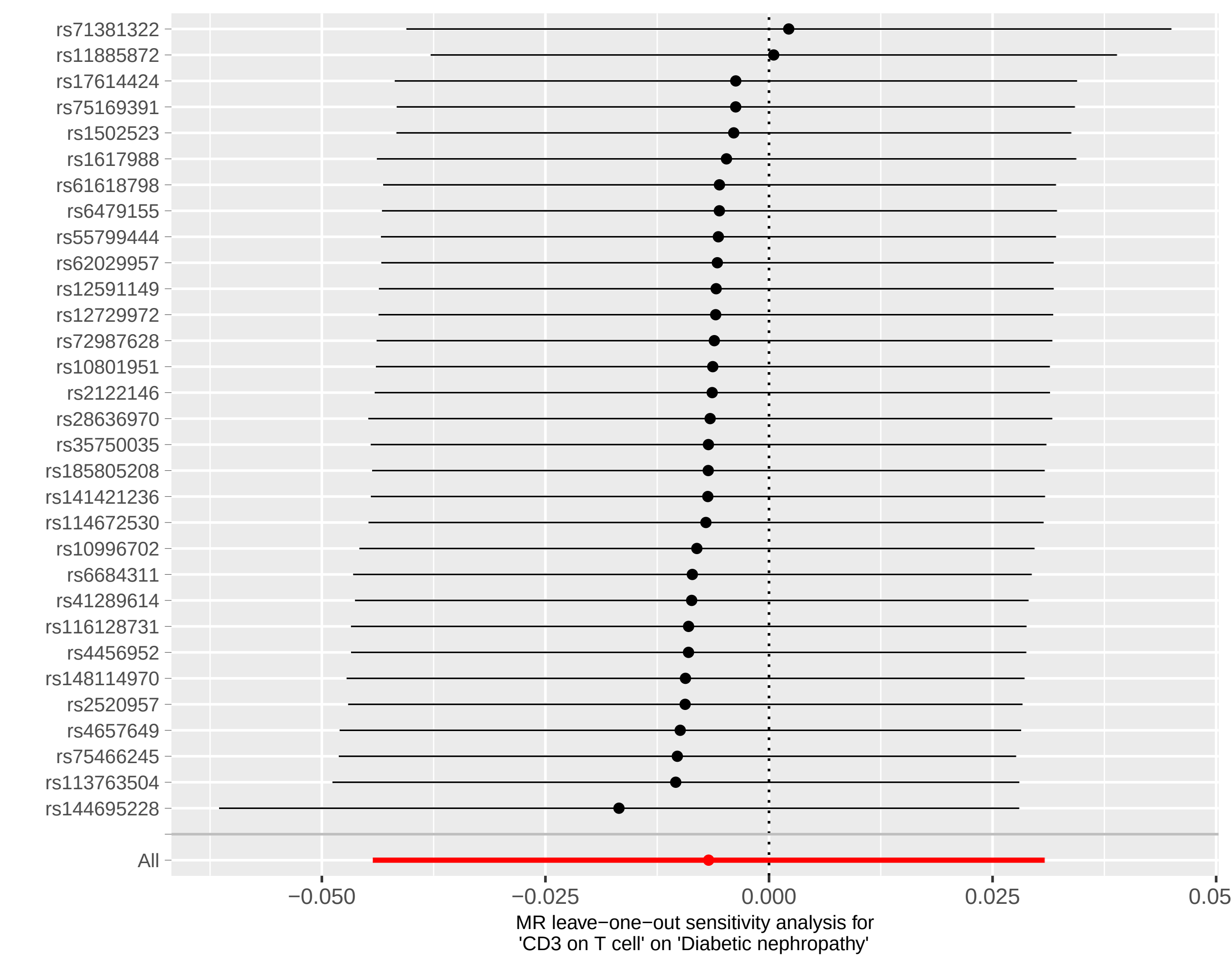

rs71381322
rs11885872
rs17614424
rs75169391
rs1502523
rs1617988
rs61618798
rs6479155
rs55799444
rs62029957
rs12591149
rs12729972
rs72987628
rs10801951
rs2122146
rs28636970
rs35750035
rs185805208
rs141421236
rs114672530
rs10996702
rs6684311
rs41289614
rs116128731
rs4456952
rs148114970
rs2520957
rs4657649
rs75466245
rs113763504
rs144695228
All
−0.050
−0.025
0.000
0.025
0.05
MR leave−one−out sensitivity analysis for
'CD3 on T cell' on 'Diabetic nephropathy'

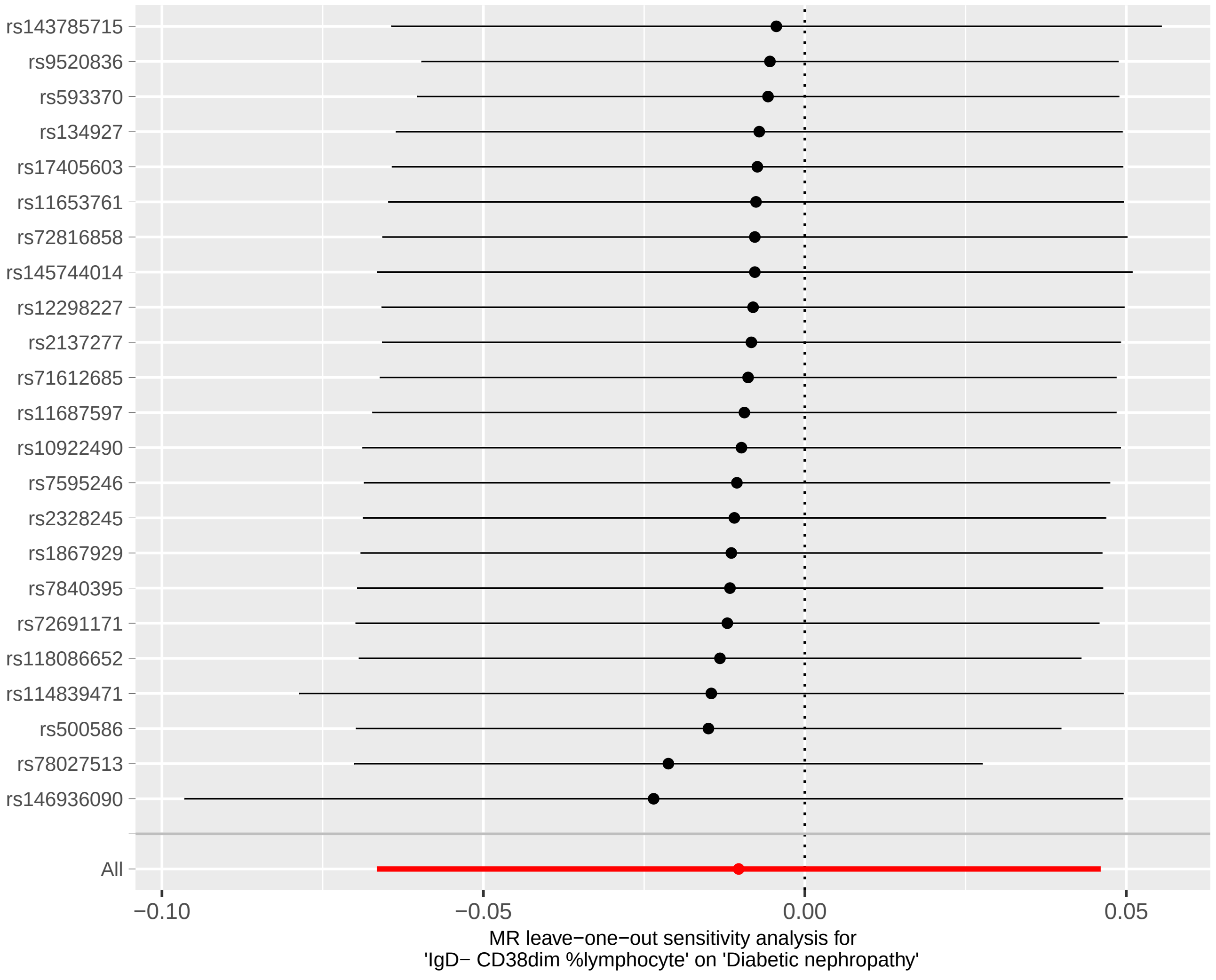

rs143785715
rs9520836
rs593370
rs134927
rs17405603
rs11653761
rs72816858
rs145744014
rs12298227
rs2137277
rs71612685
rs11687597
rs10922490
rs7595246
rs2328245
rs1867929
rs7840395
rs72691171
rs118086652
rs114839471
rs500586
rs78027513
rs146936090
All
−0.10
−0.05
0.00
0.05
MR leave−one−out sensitivity analysis for
'IgD− CD38dim %lymphocyte' on 'Diabetic nephropathy'

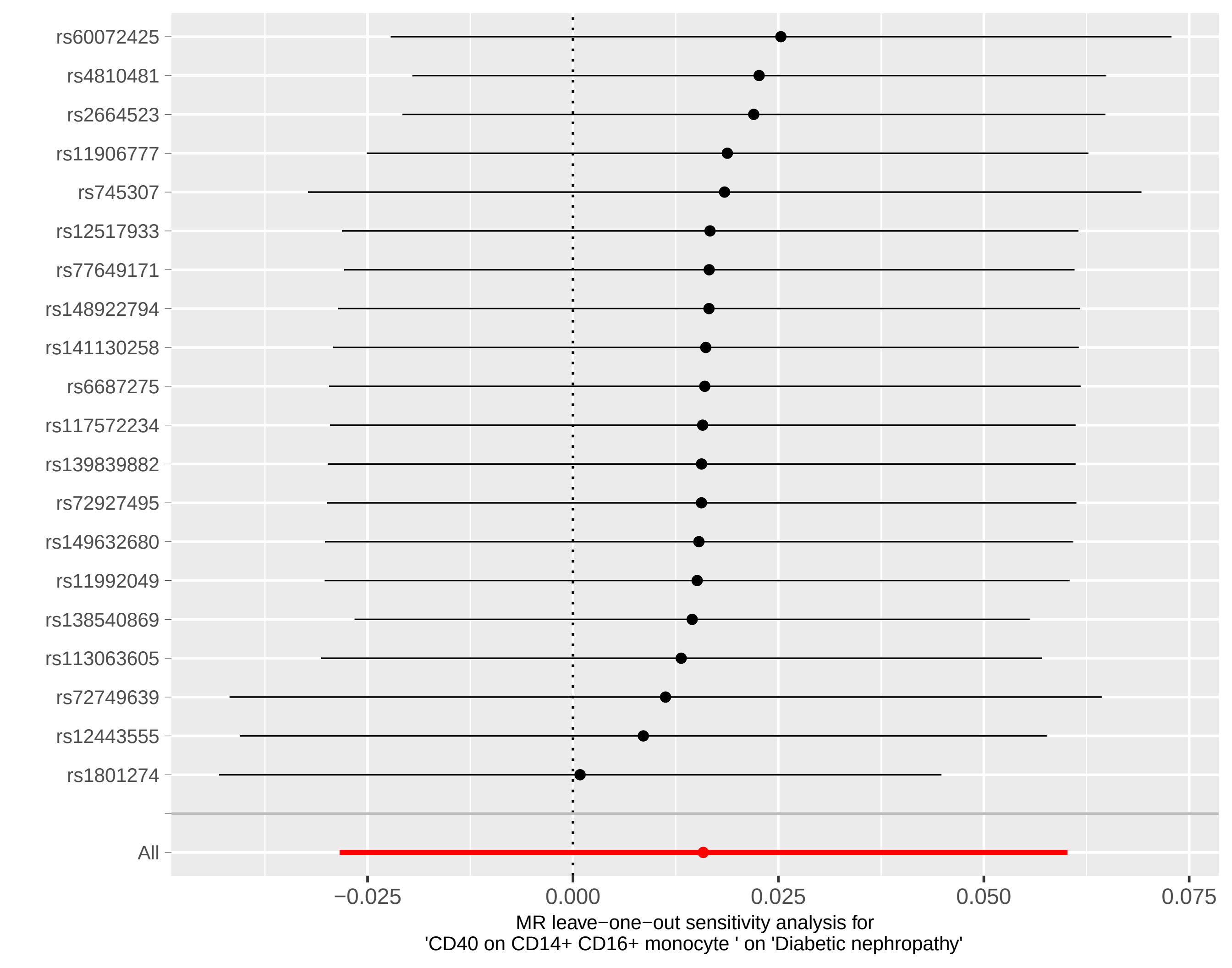
rs60072425
rs4810481
rs2664523
rs11906777
rs745307
rs12517933
rs77649171
rs148922794
rs141130258
rs6687275
rs117572234
rs139839882
rs72927495
rs149632680
rs11992049
rs138540869
rs113063605
rs72749639
rs12443555
rs1801274
All
−0.025
0.000
0.025
0.050
0.075
MR leave−one−out sensitivity analysis for
'CD40 on CD14+ CD16+ monocyte ' on 'Diabetic nephropathy'

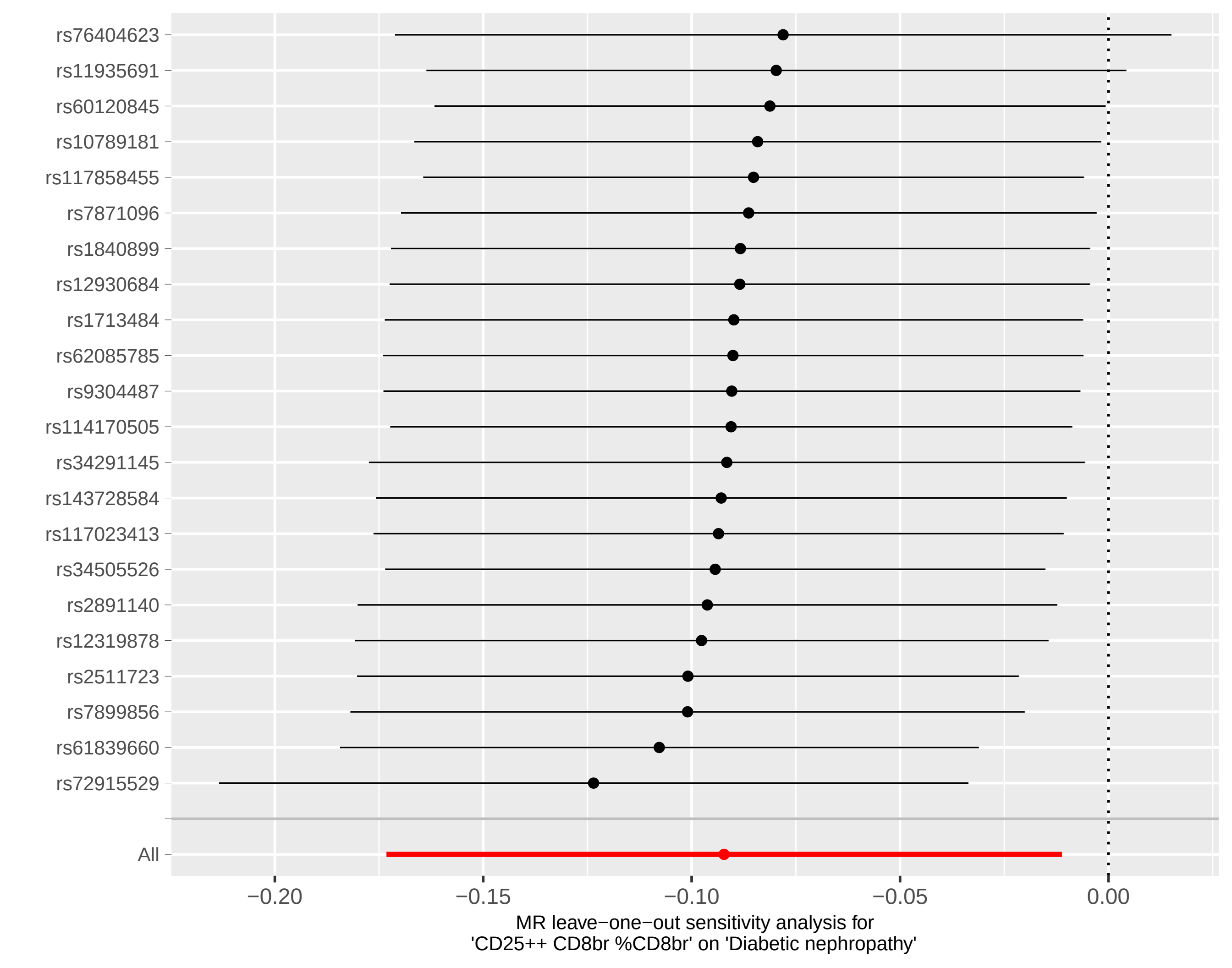

rs76404623
rs11935691
rs60120845
rs10789181
rs117858455
rs7871096
rs1840899
rs12930684
rs1713484
rs62085785
rs9304487
rs114170505
rs34291145
rs143728584
rs117023413
rs34505526
rs2891140
rs12319878
rs2511723
rs7899856
rs61839660
rs72915529
All
−0.20
−0.15
−0.10
−0.05
0.00
MR leave−one−out sensitivity analysis for
'CD25++ CD8br %CD8br' on 'Diabetic nephropathy'

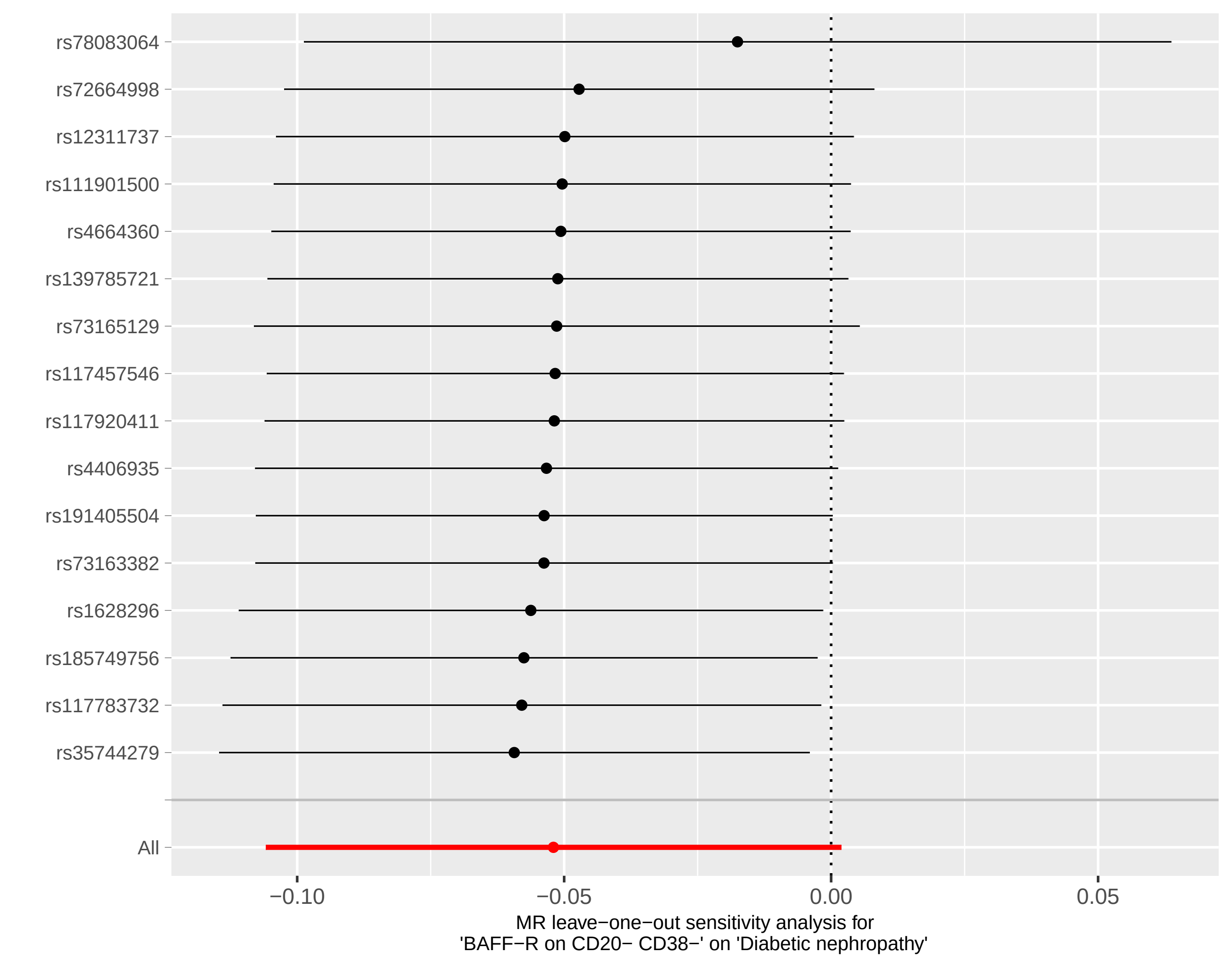
rs78083064
rs72664998
rs12311737
rs111901500
rs4664360
rs139785721
rs73165129
rs117457546
rs117920411
rs4406935
rs191405504
rs73163382
rs1628296
rs185749756
rs117783732
rs35744279
All
−0.10
−0.05
0.00
0.05
MR leave−one−out sensitivity analysis for
'BAFF−R on CD20− CD38−' on 'Diabetic nephropathy'

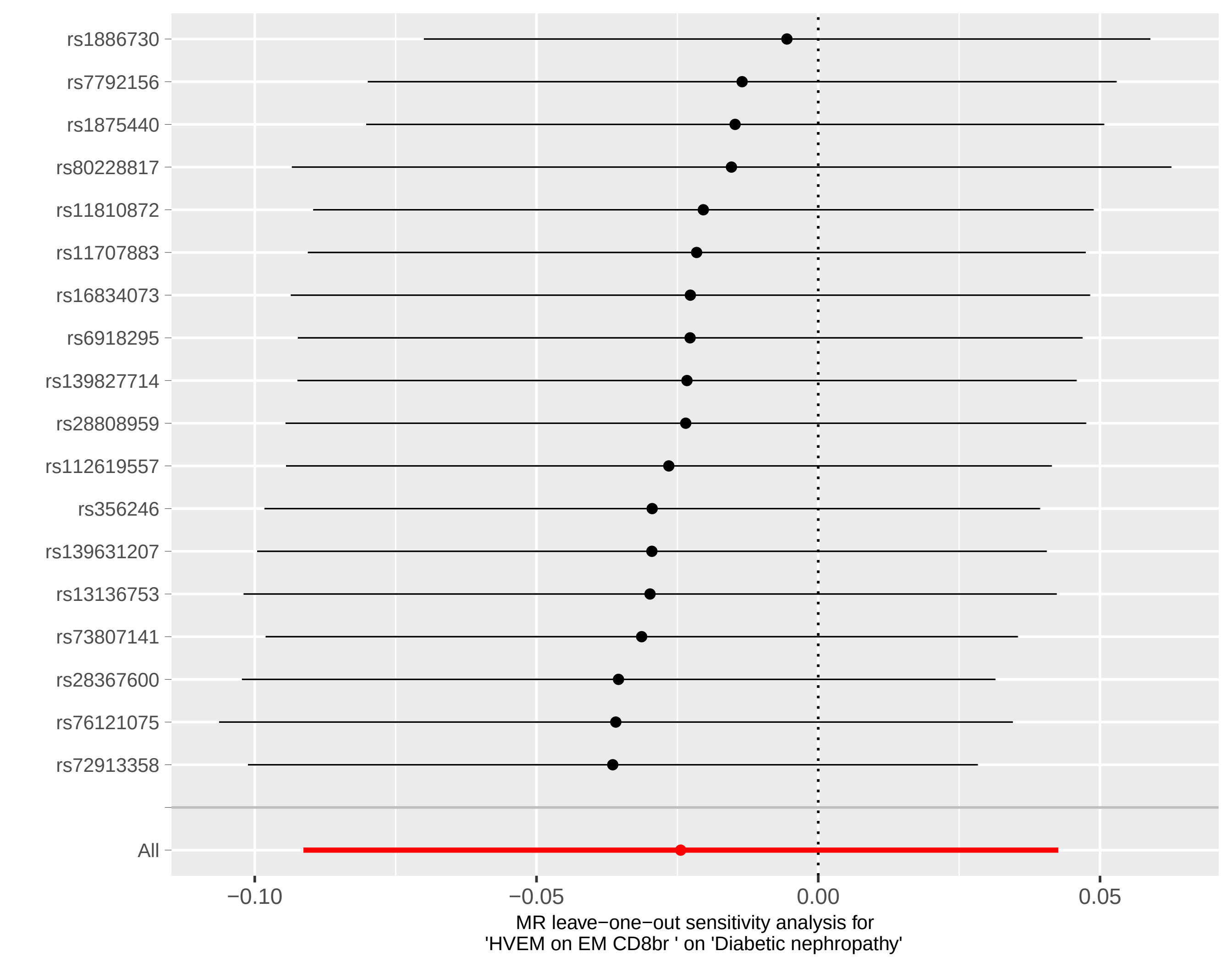
rs1886730
rs7792156
rs1875440
rs80228817
rs11810872
rs11707883
rs16834073
rs6918295
rs139827714
rs28808959
rs112619557
rs356246
rs139631207
rs13136753
rs73807141
rs28367600
rs76121075
rs72913358
All
−0.10
−0.05
0.00
0.05
MR leave−one−out sensitivity analysis for
'HVEM on EM CD8br ' on 'Diabetic nephropathy'

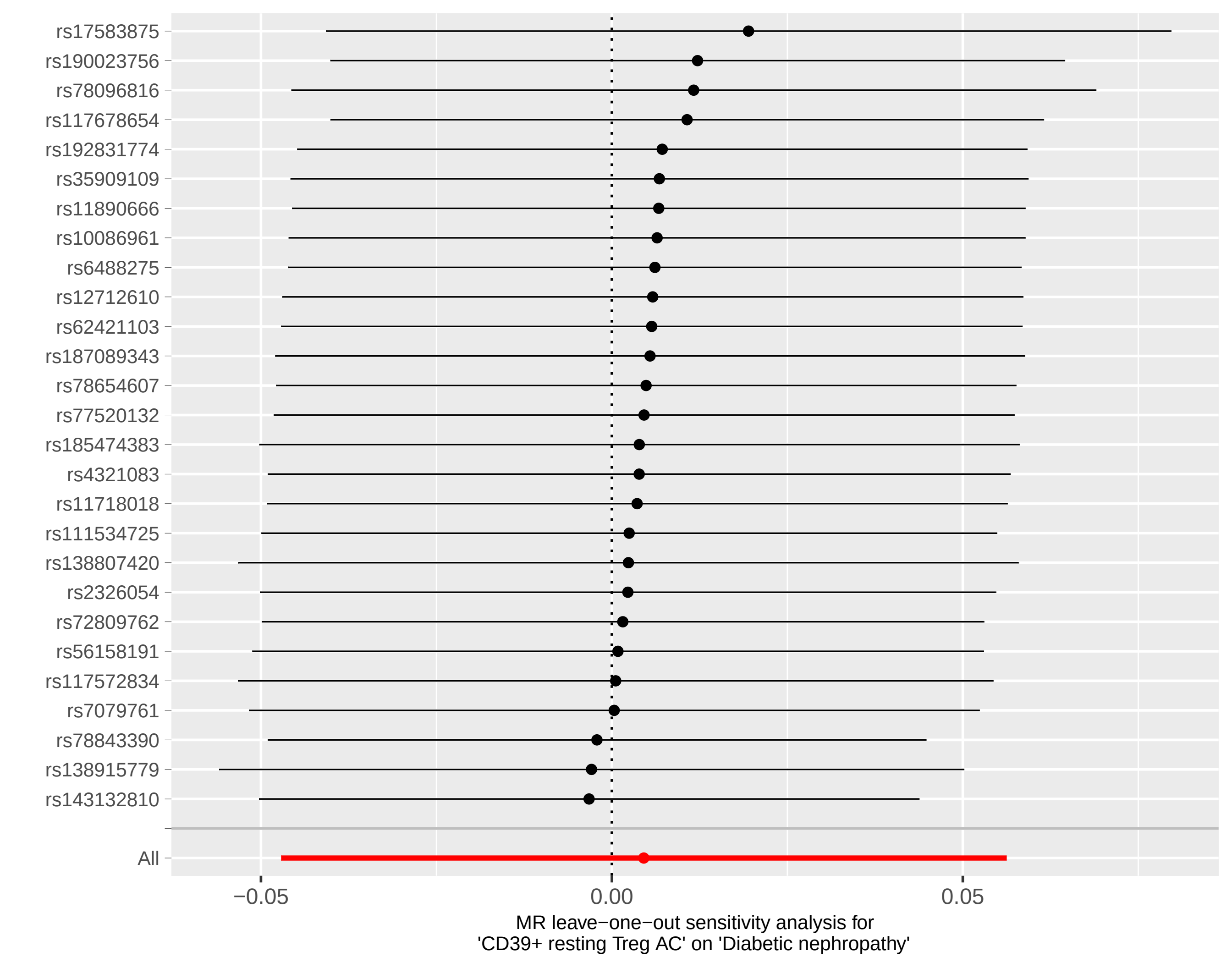
rs17583875
rs190023756
rs78096816
rs117678654
rs192831774
rs35909109
rs11890666
rs10086961
rs6488275
rs12712610
rs62421103
rs187089343
rs78654607
rs77520132
rs185474383
rs4321083
rs11718018
rs111534725
rs138807420
rs2326054
rs72809762
rs56158191
rs117572834
rs7079761
rs78843390
rs138915779
rs143132810
All
−0.05
0.00
0.05
MR leave−one−out sensitivity analysis for
'CD39+ resting Treg AC' on 'Diabetic nephropathy'

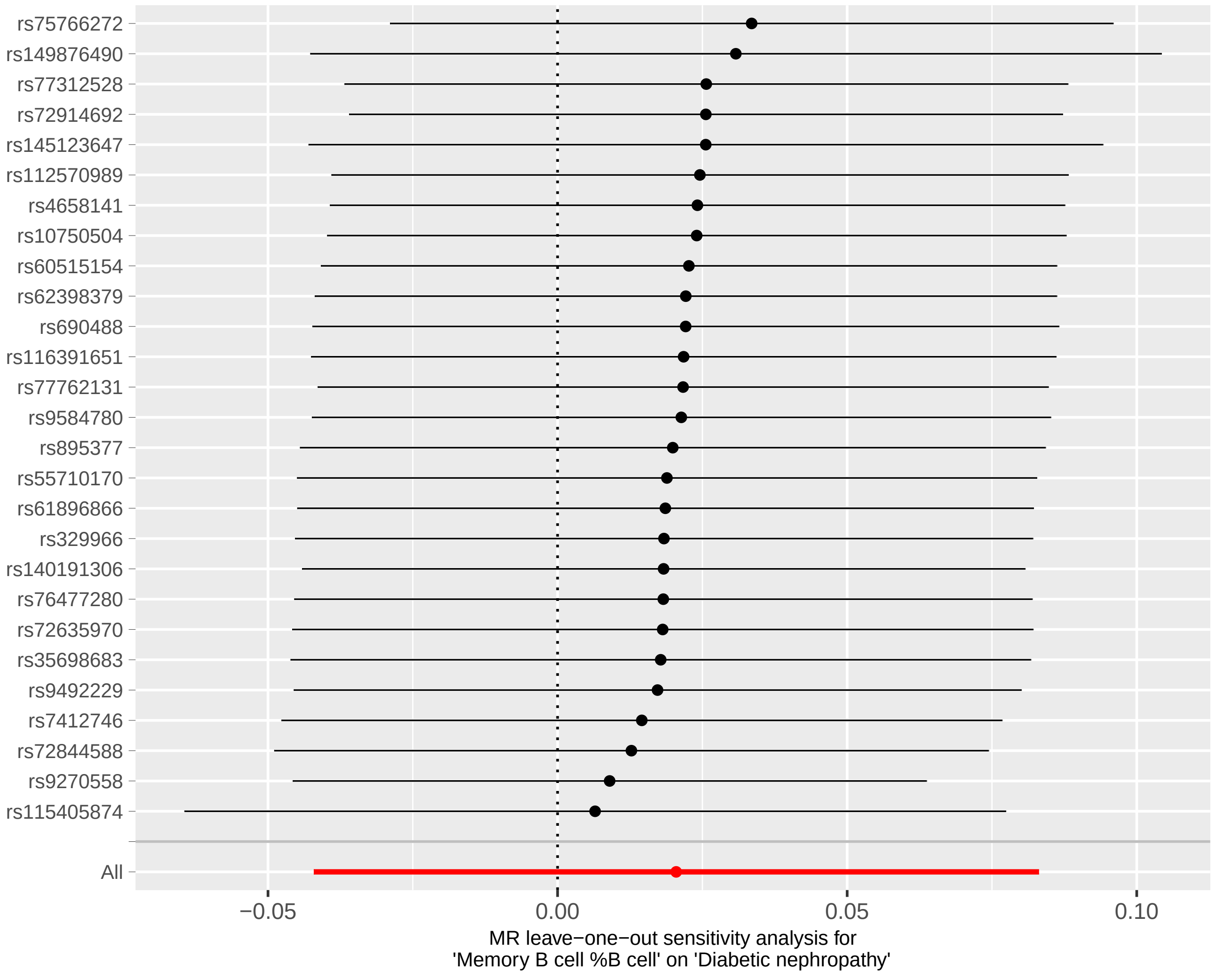
rs75766272
rs149876490
rs77312528
rs72914692
rs145123647
rs112570989
rs4658141
rs10750504
rs60515154
rs62398379
rs690488
rs116391651
rs77762131
rs9584780
rs895377
rs55710170
rs61896866
rs329966
rs140191306
rs76477280
rs72635970
rs35698683
rs9492229
rs7412746
rs72844588
rs9270558
rs115405874
All
−0.05
0.00
0.05
0.10
MR leave−one−out sensitivity analysis for
'Memory B cell %B cell' on 'Diabetic nephropathy'

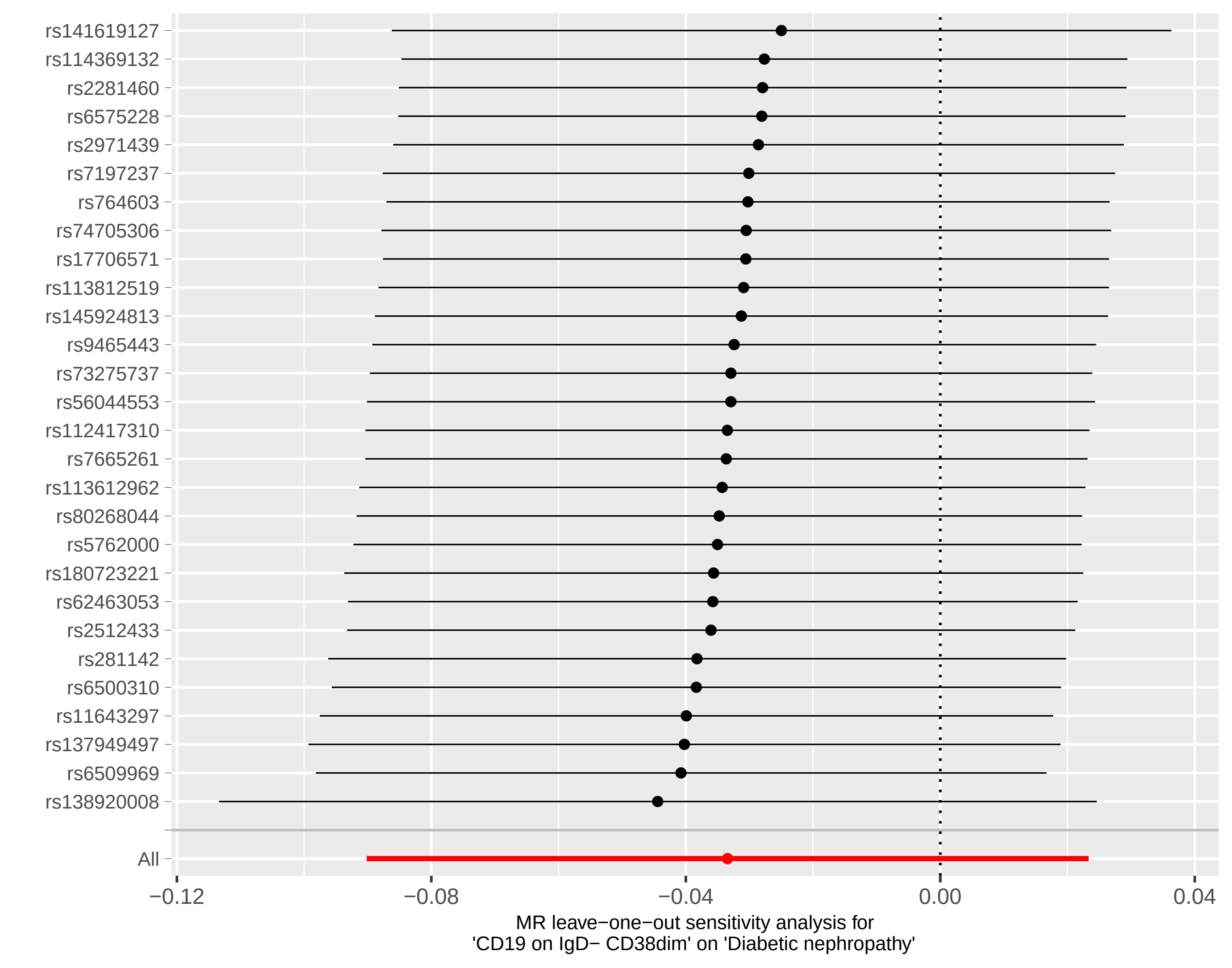
rs141619127
rs114369132
rs2281460
rs6575228
rs2971439
rs7197237
rs764603
rs74705306
rs17706571
rs113812519
rs145924813
rs9465443
rs73275737
rs56044553
rs112417310
rs7665261
rs113612962
rs80268044
rs5762000
rs180723221
rs62463053
rs2512433
rs281142
rs6500310
rs11643297
rs137949497
rs6509969
rs138920008
All
−0.12
−0.08
−0.04
0.00
0.04
MR leave−one−out sensitivity analysis for
'CD19 on IgD− CD38dim' on 'Diabetic nephropathy'

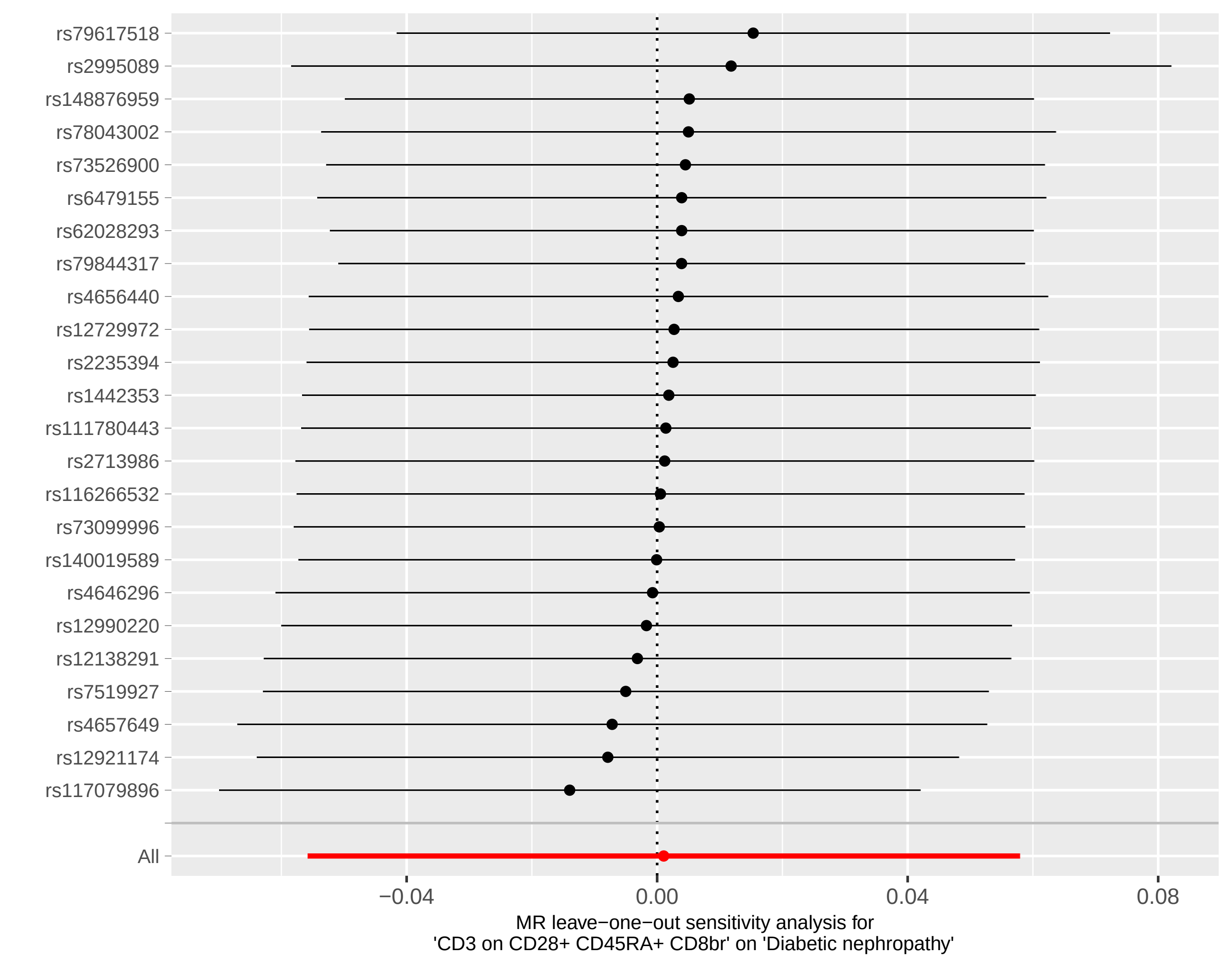

rs79617518
rs2995089
rs148876959
rs78043002
rs73526900
rs6479155
rs62028293
rs79844317
rs4656440
rs12729972
rs2235394
rs1442353
rs111780443
rs2713986
rs116266532
rs73099996
rs140019589
rs4646296
rs12990220
rs12138291
rs7519927
rs4657649
rs12921174
rs117079896
All
−0.04
0.00
0.04
0.08
MR leave−one−out sensitivity analysis for
'CD3 on CD28+ CD45RA+ CD8br' on 'Diabetic nephropathy'

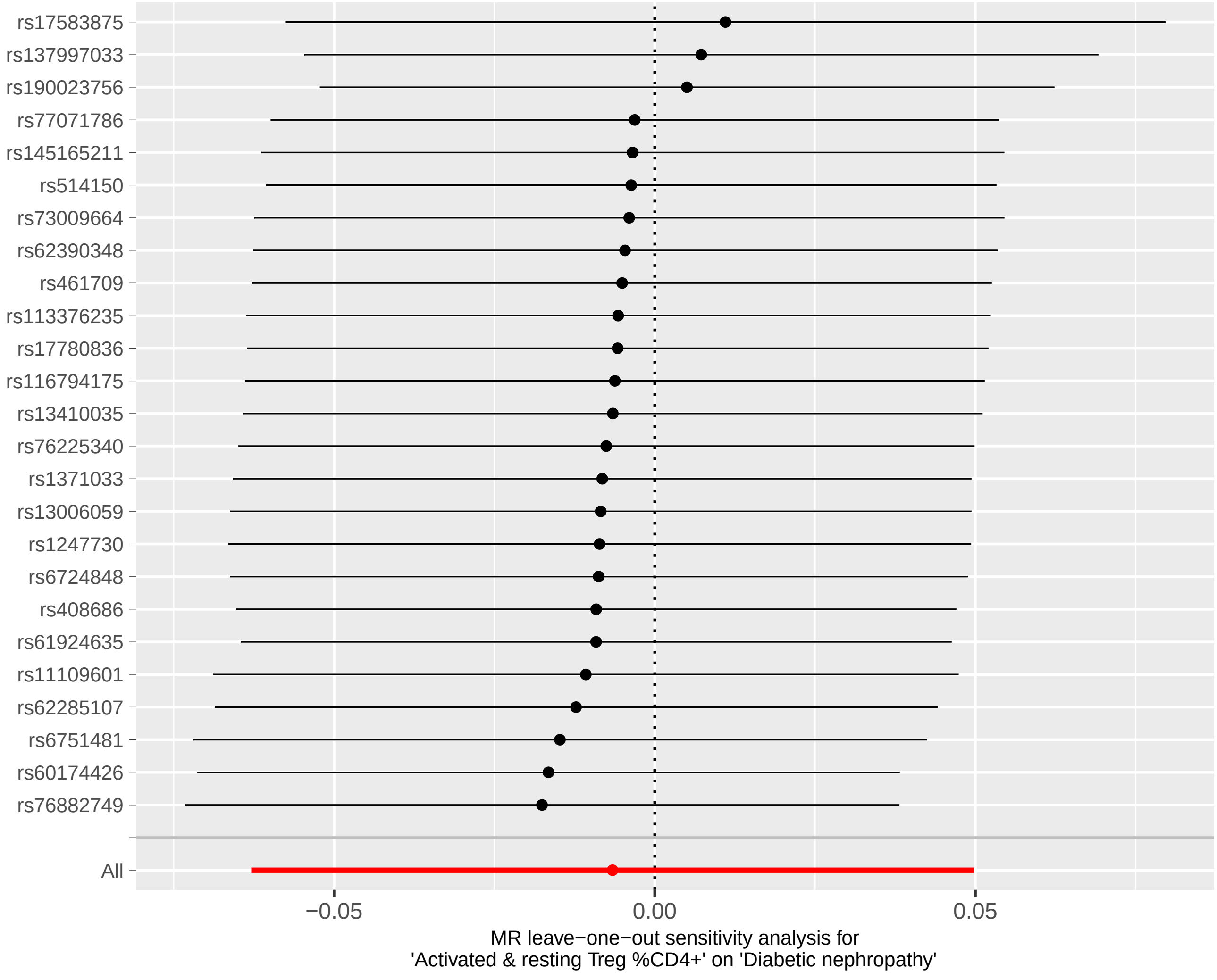

rs17583875
rs137997033
rs190023756
rs77071786
rs145165211
rs514150
rs73009664
rs62390348
rs461709
rs113376235
rs17780836
rs116794175
rs13410035
rs76225340
rs1371033
rs13006059
rs1247730
rs6724848
rs408686
rs61924635
rs11109601
rs62285107
rs6751481
rs60174426
rs76882749
All
−0.05
0.00
0.05
MR leave−one−out sensitivity analysis for
'Activated & resting Treg %CD4+' on 'Diabetic nephropathy'

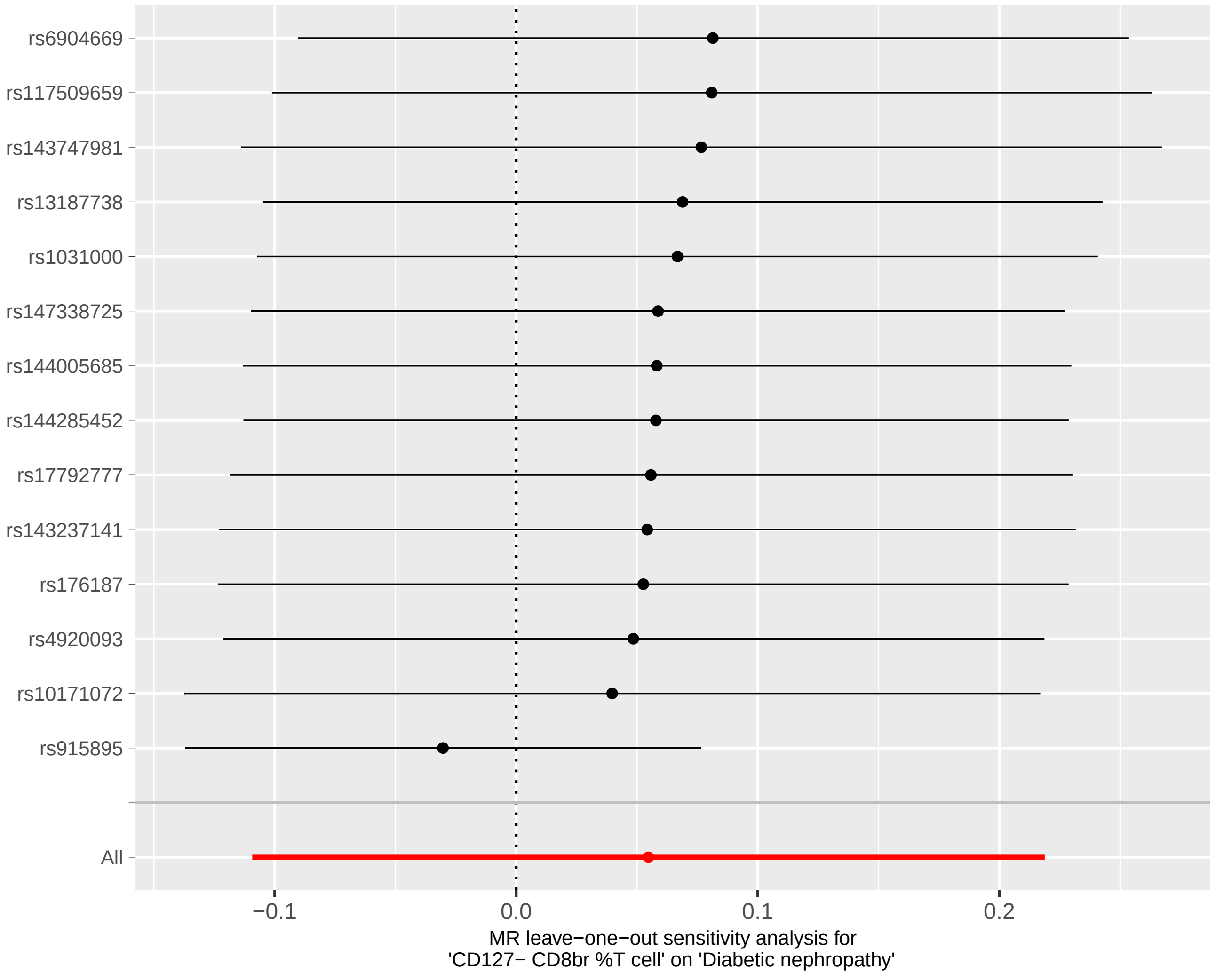
rs6904669
rs117509659
rs143747981
rs13187738
rs1031000
rs147338725
rs144005685
rs144285452
rs17792777
rs143237141
rs176187
rs4920093
rs10171072
rs915895
All
−0.1
0.0
0.1
0.2
MR leave−one−out sensitivity analysis for
'CD127− CD8br %T cell' on 'Diabetic nephropathy'

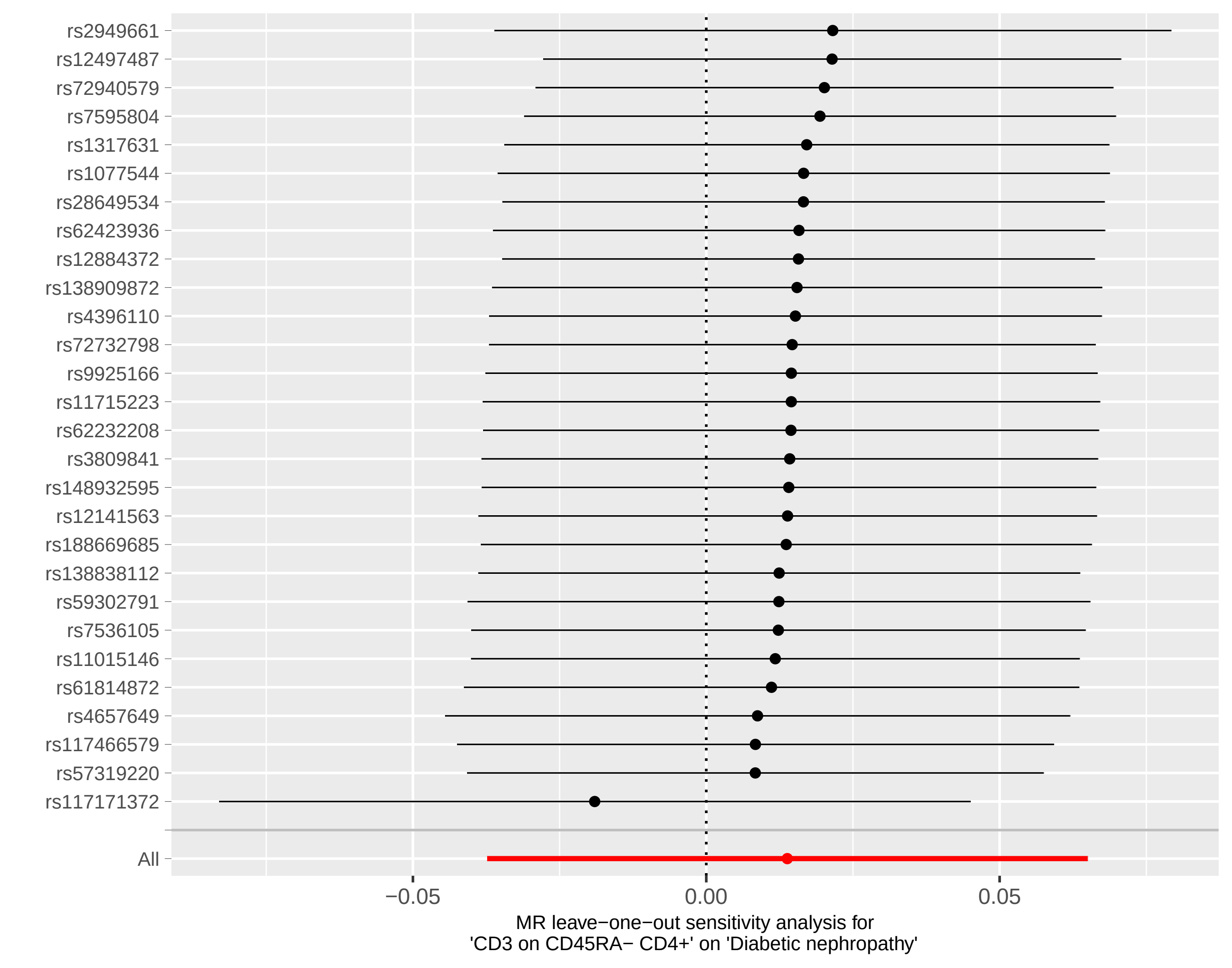

rs2949661
rs12497487
rs72940579
rs7595804
rs1317631
rs1077544
rs28649534
rs62423936
rs12884372
rs138909872
rs4396110
rs72732798
rs9925166
rs11715223
rs62232208
rs3809841
rs148932595
rs12141563
rs188669685
rs138838112
rs59302791
rs7536105
rs11015146
rs61814872
rs4657649
rs117466579
rs57319220
rs117171372
All
−0.05
0.00
0.05
MR leave−one−out sensitivity analysis for
'CD3 on CD45RA− CD4+' on 'Diabetic nephropathy'

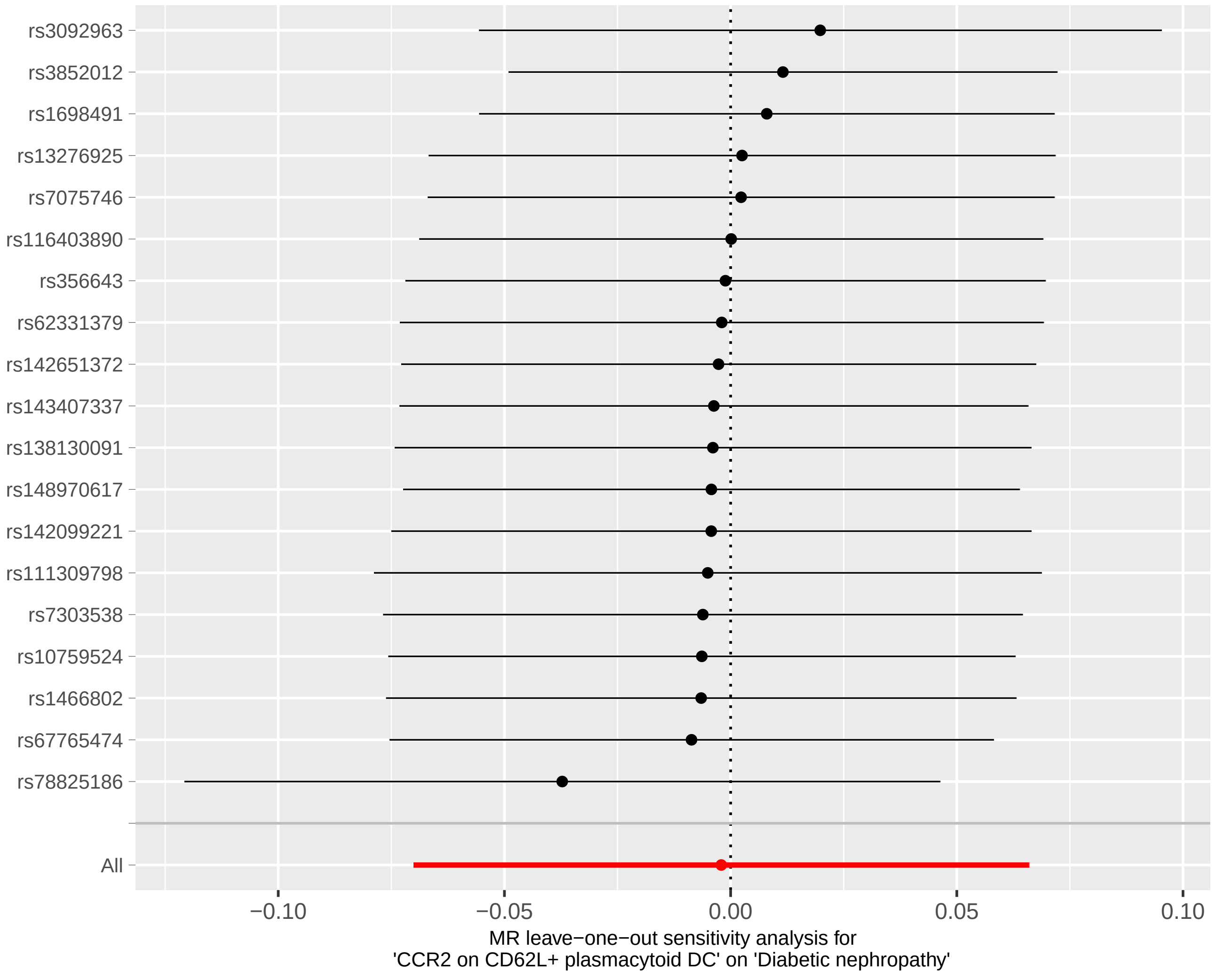

rs3092963
rs3852012
rs1698491
rs13276925
rs7075746
rs116403890
rs356643
rs62331379
rs142651372
rs143407337
rs138130091
rs148970617
rs142099221
rs111309798
rs7303538
rs10759524
rs1466802
rs67765474
rs78825186
All
−0.10
−0.05
0.00
0.05
0.10
MR leave−one−out sensitivity analysis for
'CCR2 on CD62L+ plasmacytoid DC' on 'Diabetic nephropathy'

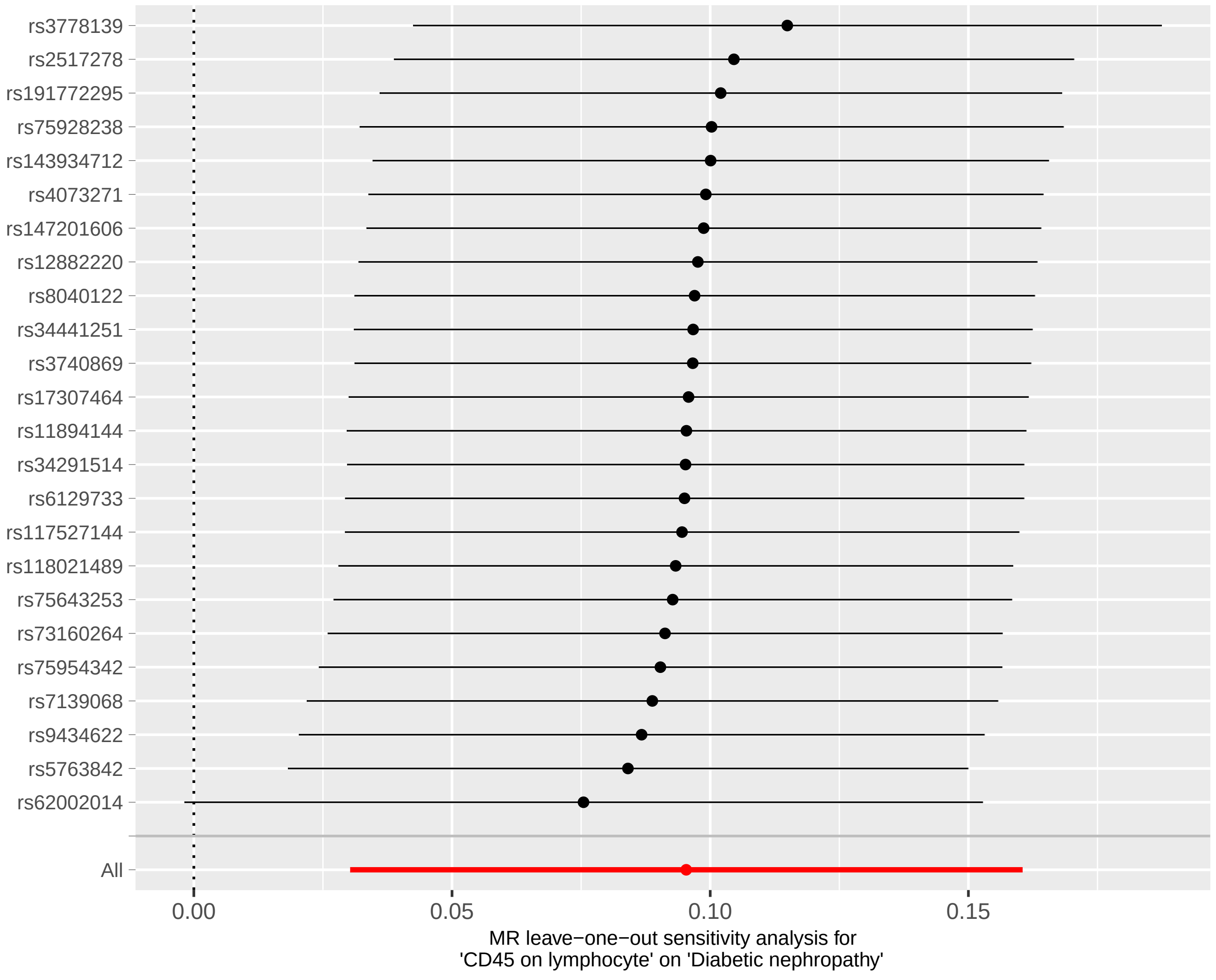

rs3778139
rs2517278
rs191772295
rs75928238
rs143934712
rs4073271
rs147201606
rs12882220
rs8040122
rs34441251
rs3740869
rs17307464
rs11894144
rs34291514
rs6129733
rs117527144
rs118021489
rs75643253
rs73160264
rs75954342
rs7139068
rs9434622
rs5763842
rs62002014
All
0.00
0.05
0.10
0.15
MR leave−one−out sensitivity analysis for
'CD45 on lymphocyte' on 'Diabetic nephropathy'

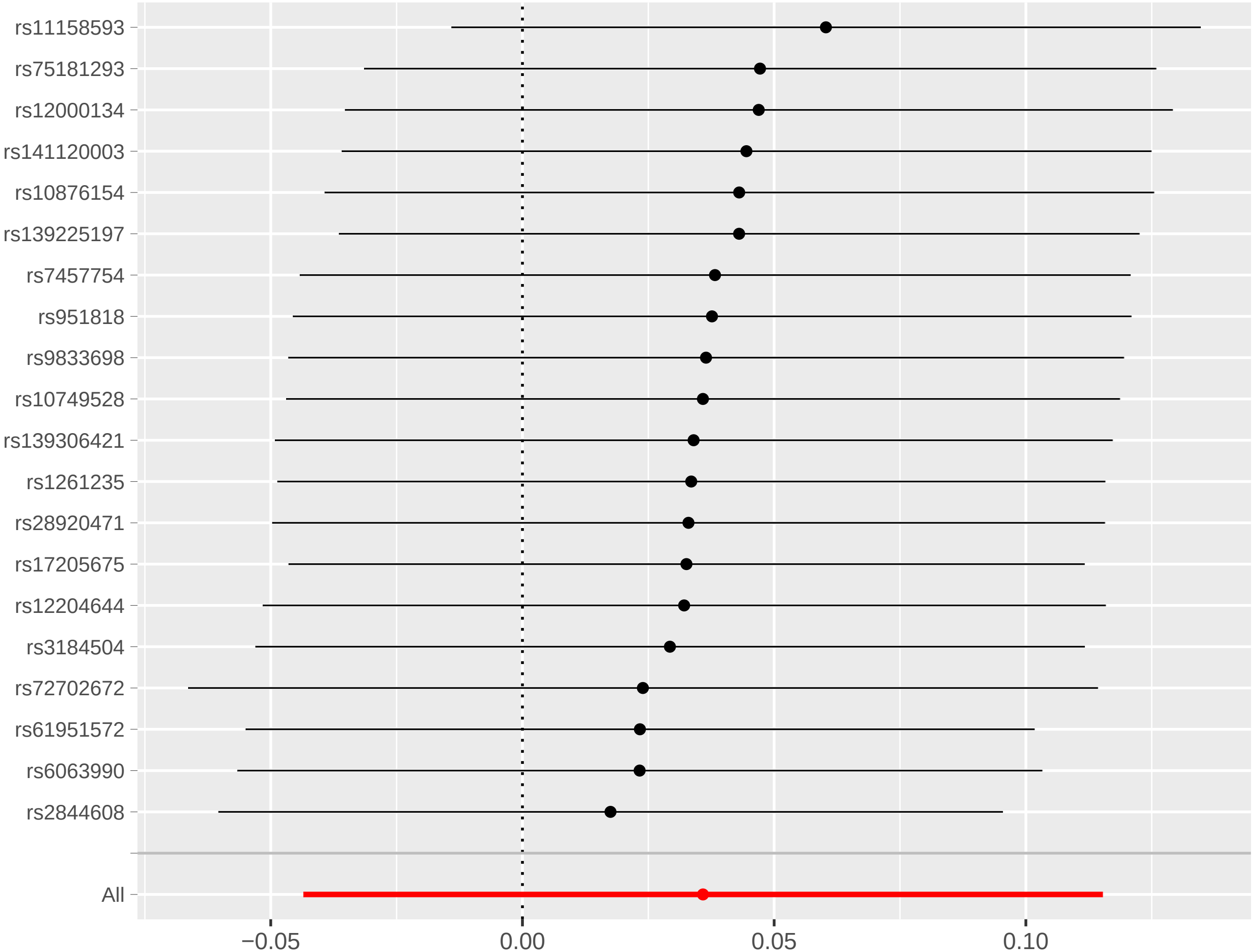

MR leave−one−out sensitivity analysis for
'CD4 on naive CD4+ ' on 'Diabetic nephropathy'

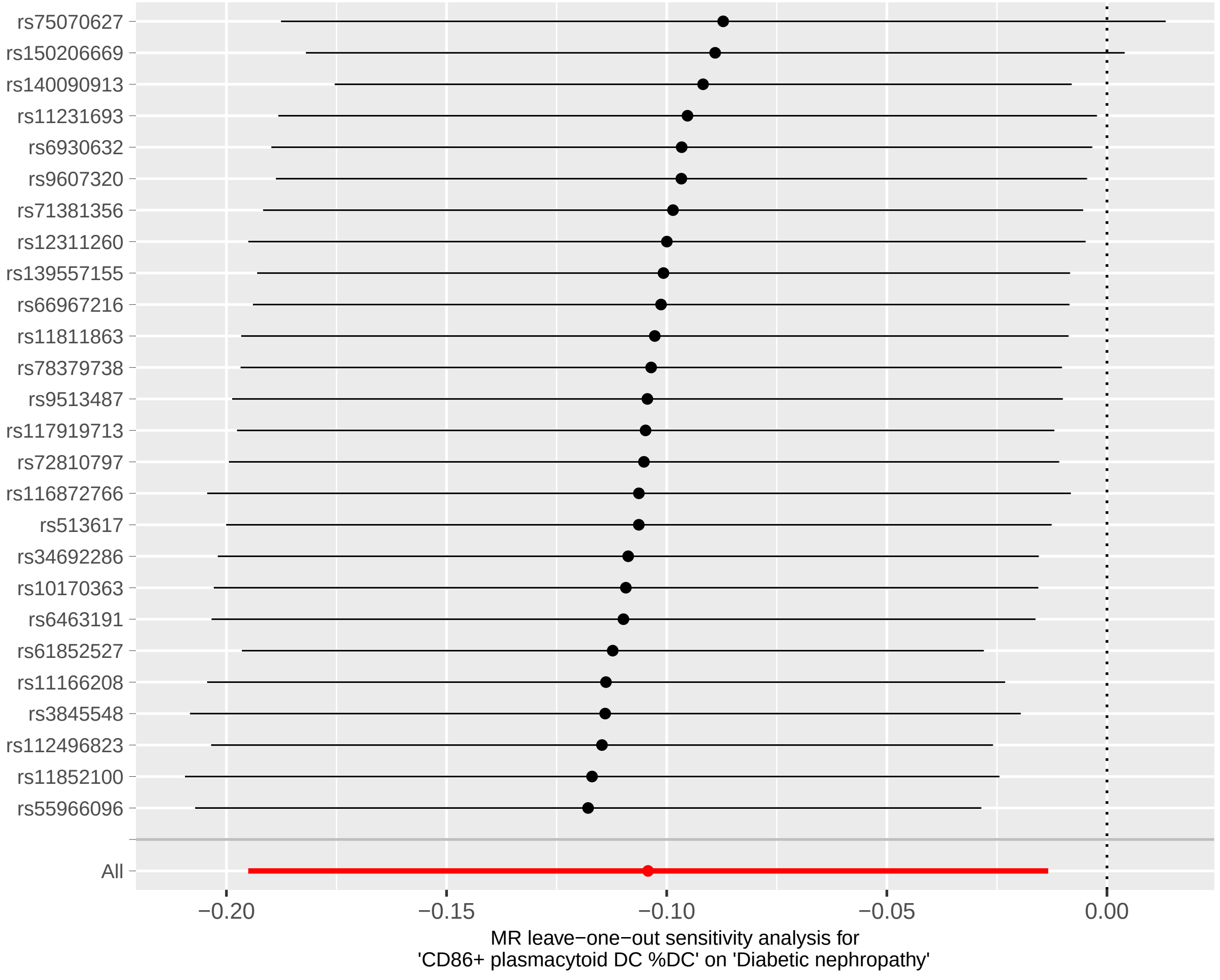

rs75070627
rs150206669
rs140090913
rs11231693
rs6930632
rs9607320
rs71381356
rs12311260
rs139557155
rs66967216
rs11811863
rs78379738
rs9513487
rs117919713
rs72810797
rs116872766
rs513617
rs34692286
rs10170363
rs6463191
rs61852527
rs11166208
rs3845548
rs112496823
rs11852100
rs55966096
All
−0.20
−0.15
−0.10
−0.05
0.00
MR leave−one−out sensitivity analysis for
'CD86+ plasmacytoid DC %DC' on 'Diabetic nephropathy'

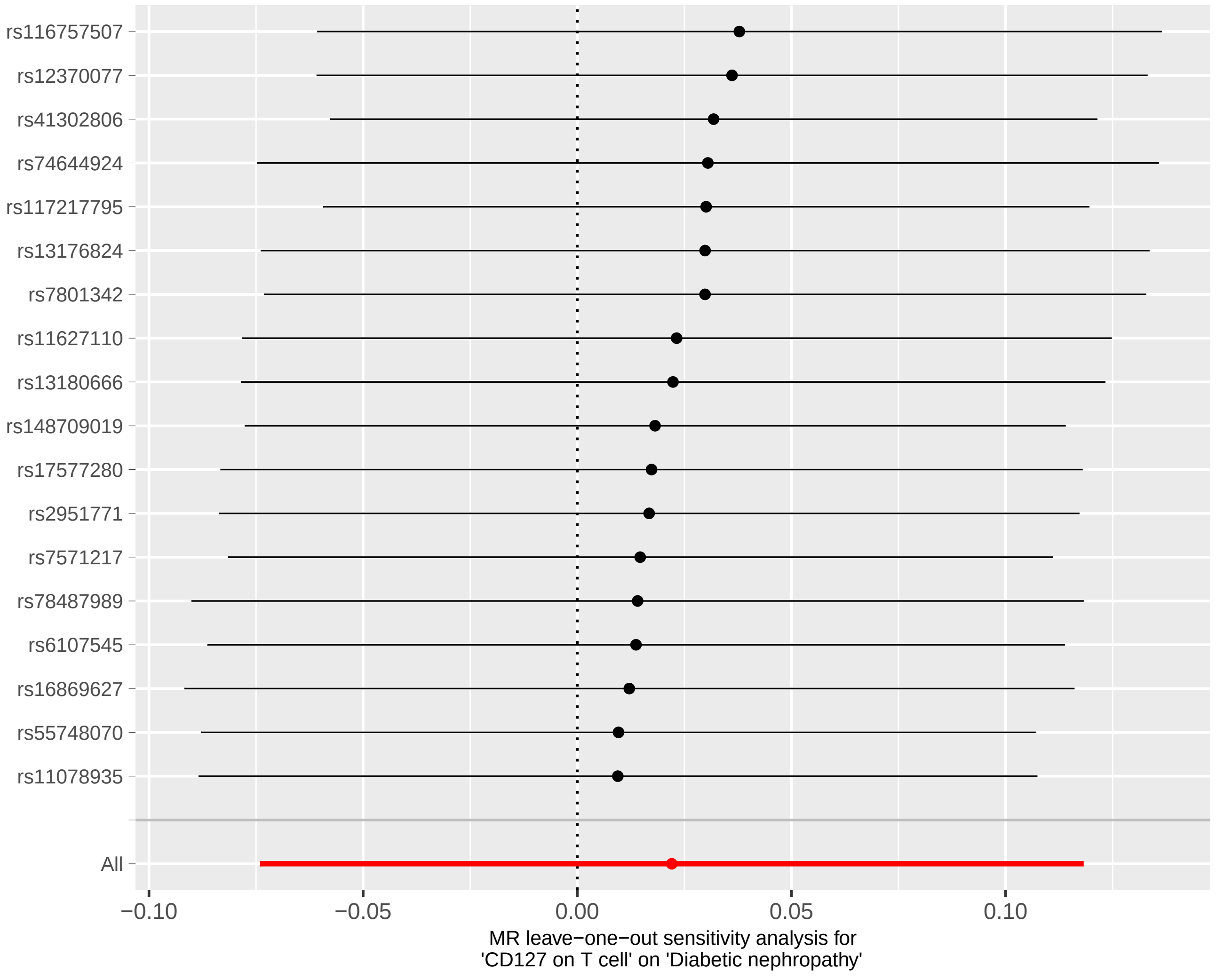

rs116757507
rs12370077
rs41302806
rs74644924
rs117217795
rs13176824
rs7801342
rs11627110
rs13180666
rs148709019
rs17577280
rs2951771
rs7571217
rs78487989
rs6107545
rs16869627
rs55748070
rs11078935
All
−0.10
−0.05
0.00
0.05
0.10
MR leave−one−out sensitivity analysis for
'CD127 on T cell' on 'Diabetic nephropathy'

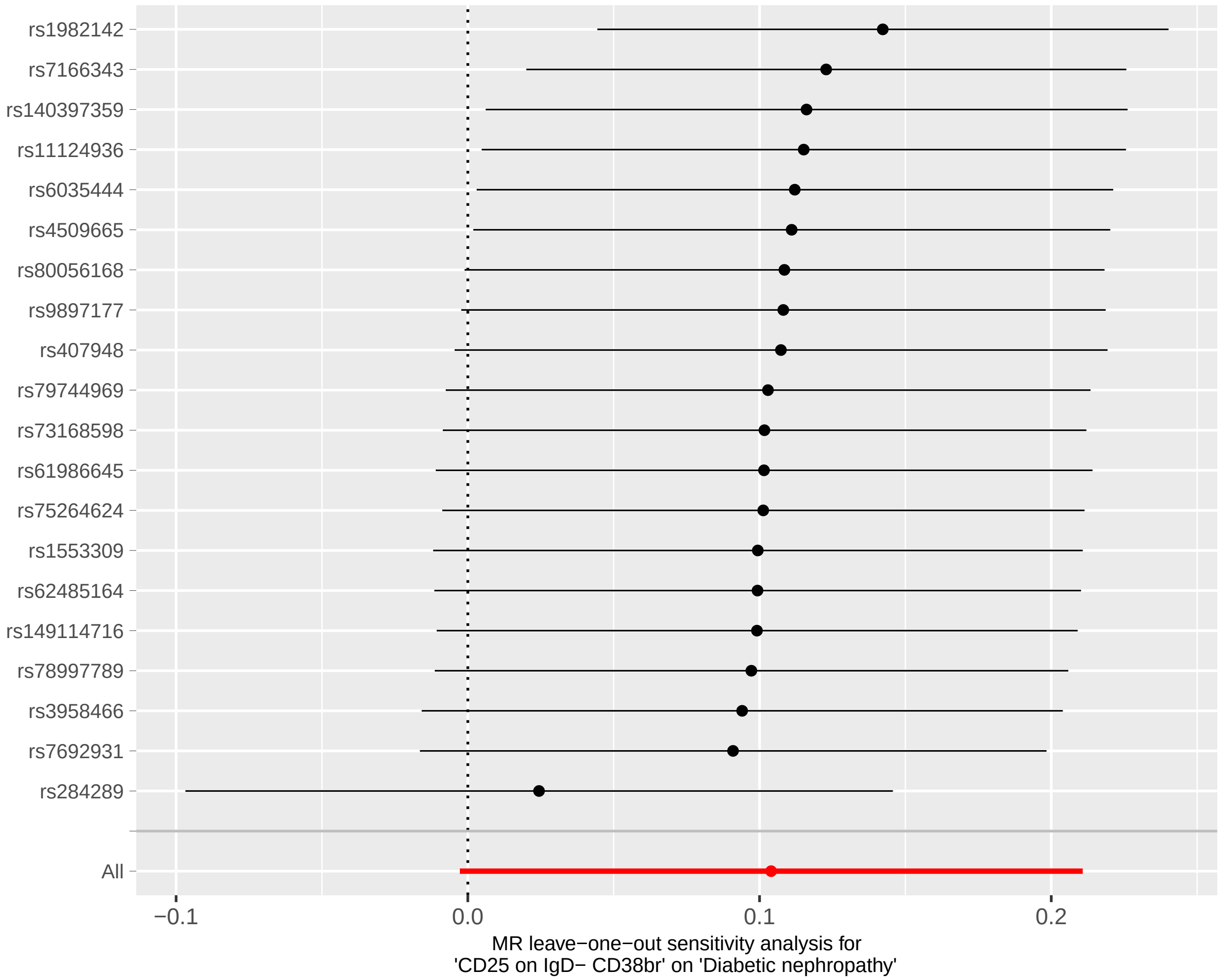
rs1982142
rs7166343
rs140397359
rs11124936
rs6035444
rs4509665
rs80056168
rs9897177
rs407948
rs79744969
rs73168598
rs61986645
rs75264624
rs1553309
rs62485164
rs149114716
rs78997789
rs3958466
rs7692931
rs284289
All
−0.1
0.0
0.1
0.2
MR leave−one−out sensitivity analysis for
'CD25 on IgD− CD38br' on 'Diabetic nephropathy'

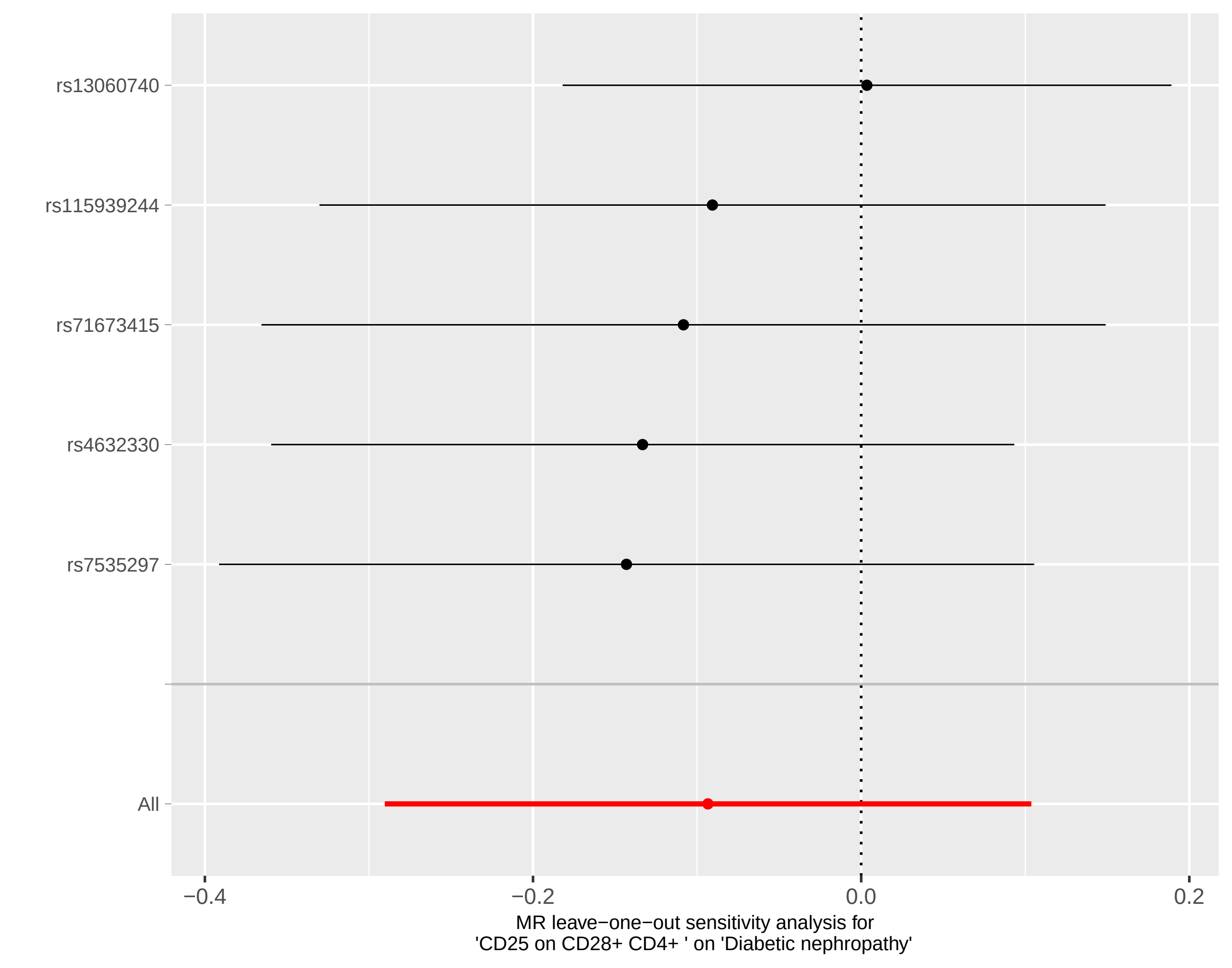
rs13060740
rs115939244
rs71673415
rs4632330
rs7535297
All
−0.4
−0.2
0.0
0.2
MR leave−one−out sensitivity analysis for
'CD25 on CD28+ CD4+ ' on 'Diabetic nephropathy'

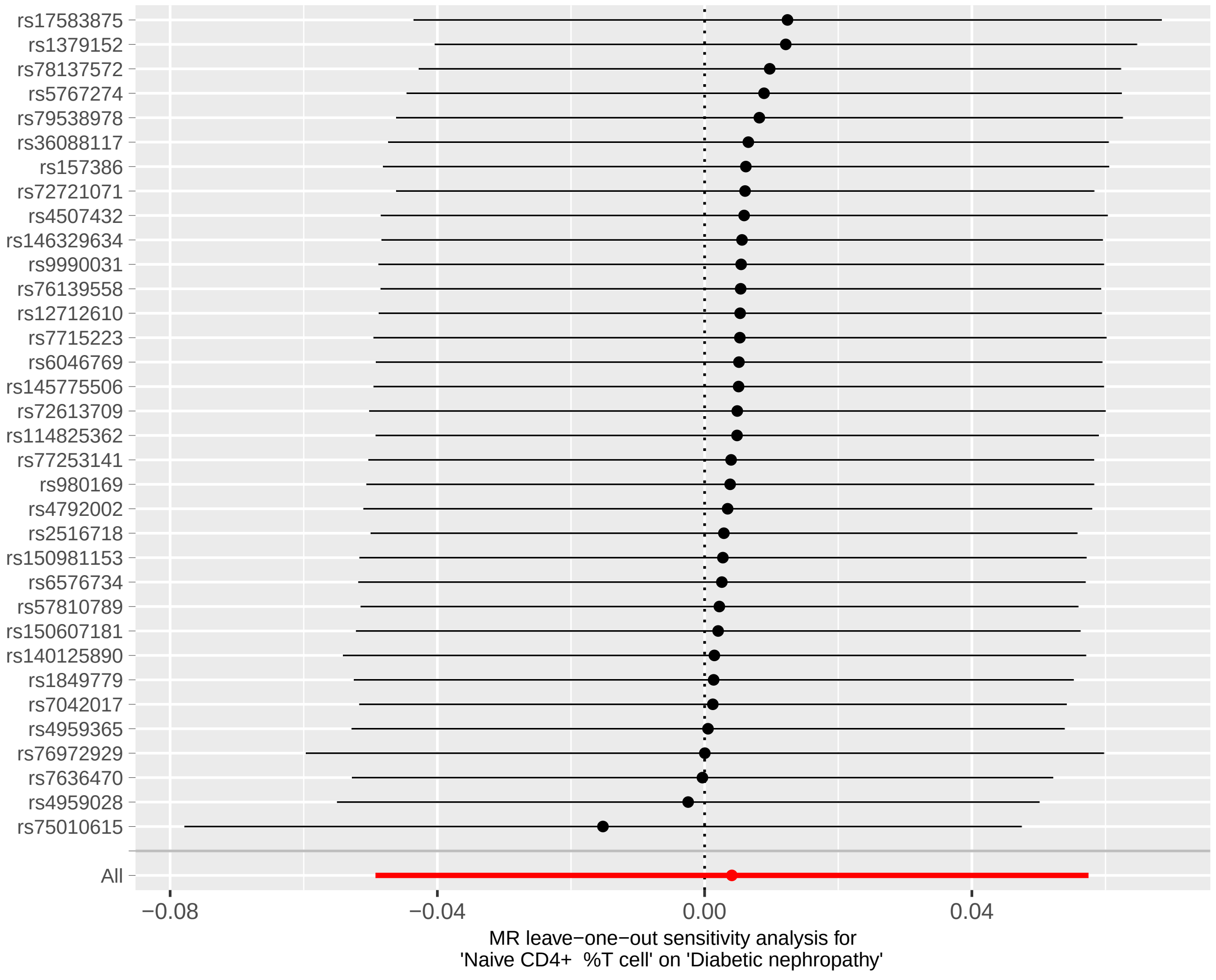

rs17583875
rs1379152
rs78137572
rs5767274
rs79538978
rs36088117
rs157386
rs72721071
rs4507432
rs146329634
rs9990031
rs76139558
rs12712610
rs7715223
rs6046769
rs145775506
rs72613709
rs114825362
rs77253141
rs980169
rs4792002
rs2516718
rs150981153
rs6576734
rs57810789
rs150607181
rs140125890
rs1849779
rs7042017
rs4959365
rs76972929
rs7636470
rs4959028
rs75010615
All
−0.08
−0.04
0.00
0.04
MR leave−one−out sensitivity analysis for
'Naive CD4+ %T cell' on 'Diabetic nephropathy'

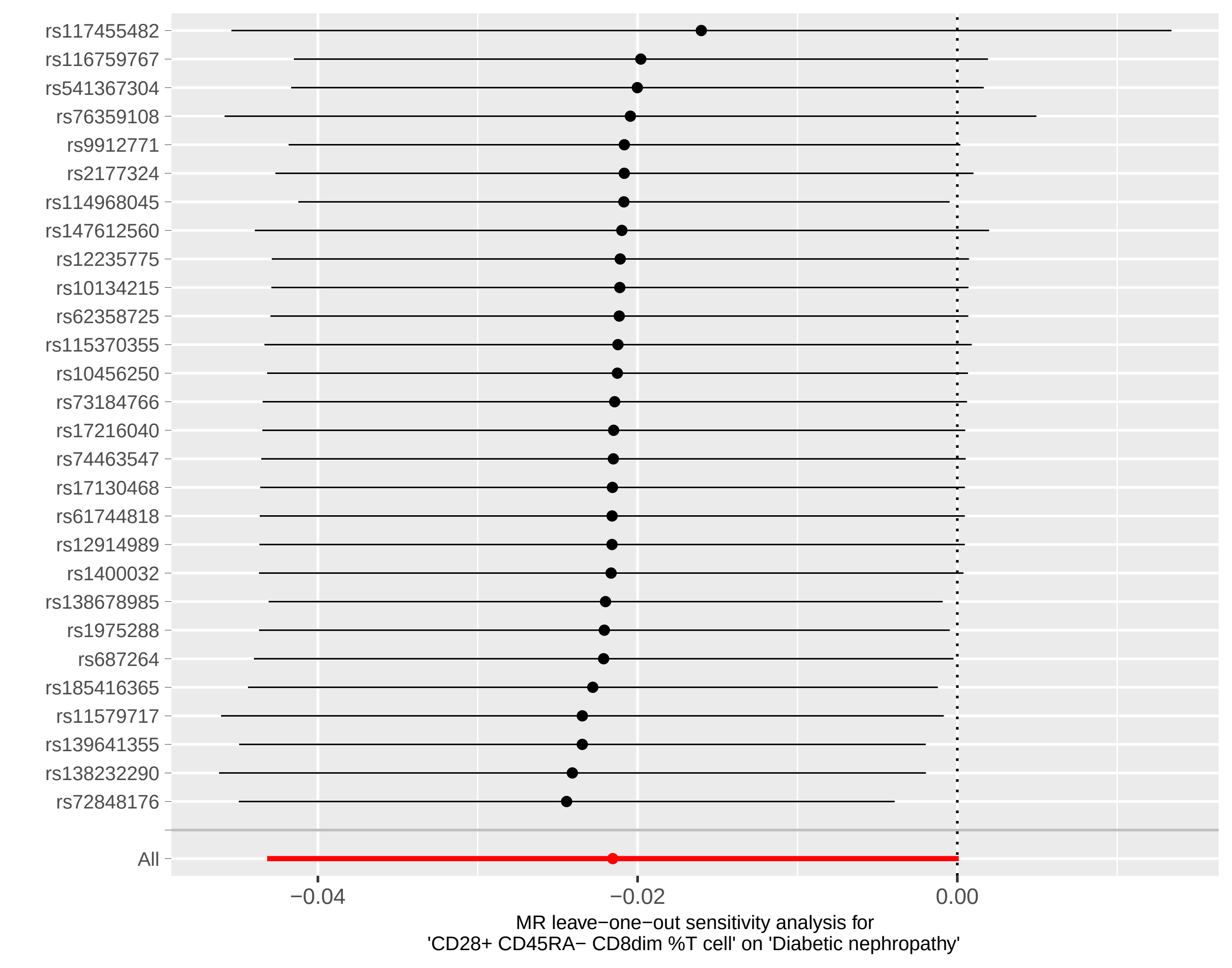
rs117455482
rs116759767
rs541367304
rs76359108
rs9912771
rs2177324
rs114968045
rs147612560
rs12235775
rs10134215
rs62358725
rs115370355
rs10456250
rs73184766
rs17216040
rs74463547
rs17130468
rs61744818
rs12914989
rs1400032
rs138678985
rs1975288
rs687264
rs185416365
rs11579717
rs139641355
rs138232290
rs72848176
All
−0.04
−0.02
0.00
MR leave−one−out sensitivity analysis for
'CD28+ CD45RA− CD8dim %T cell' on 'Diabetic nephropathy'

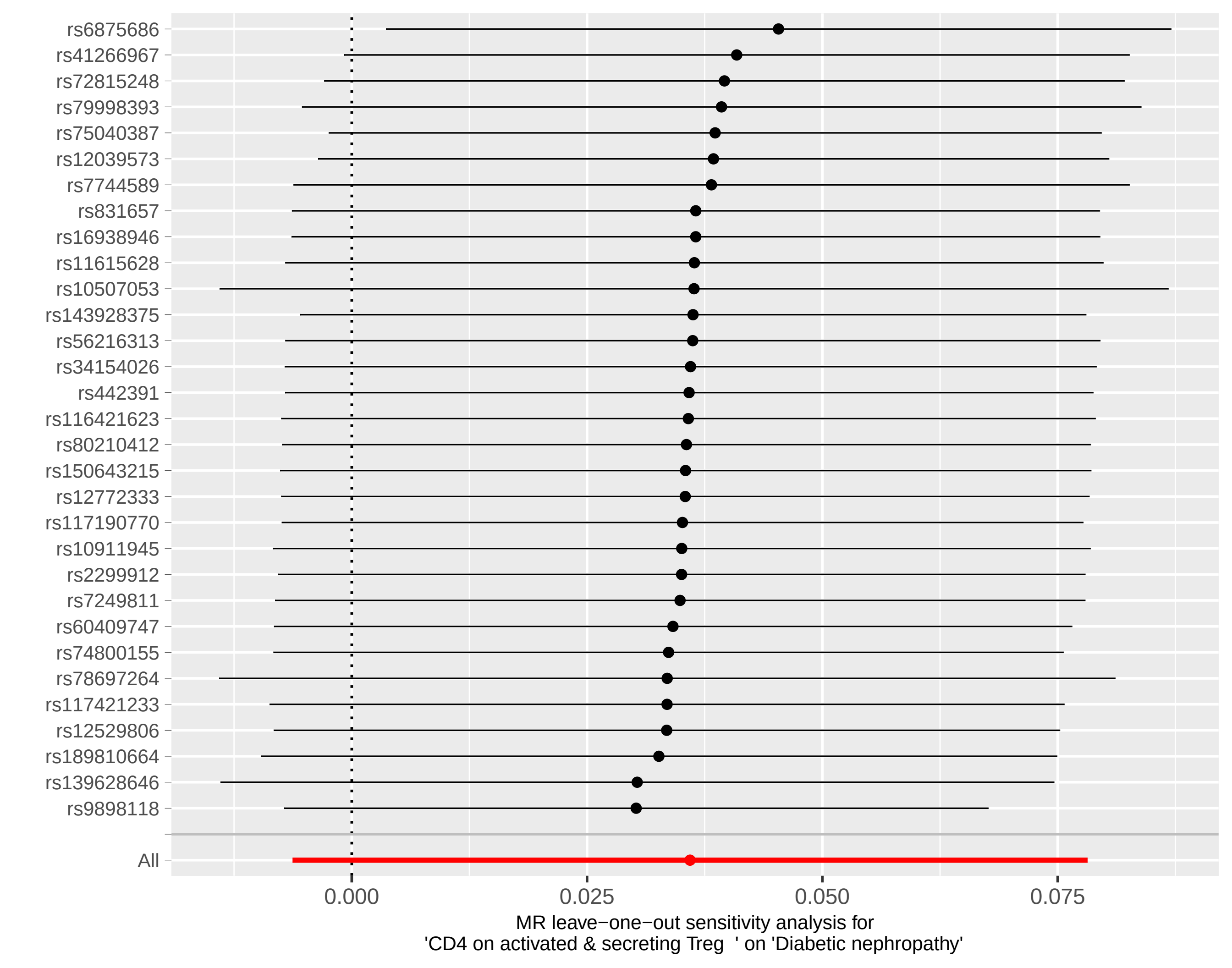
rs6875686
rs41266967
rs72815248
rs79998393
rs75040387
rs12039573
rs7744589
rs831657
rs16938946
rs11615628
rs10507053
rs143928375
rs56216313
rs34154026
rs442391
rs116421623
rs80210412
rs150643215
rs12772333
rs117190770
rs10911945
rs2299912
rs7249811
rs60409747
rs74800155
rs78697264
rs117421233
rs12529806
rs189810664
rs139628646
rs9898118
All
0.000
0.025
0.050
0.075
MR leave−one−out sensitivity analysis for
'CD4 on activated & secreting Treg ' on 'Diabetic nephropathy'

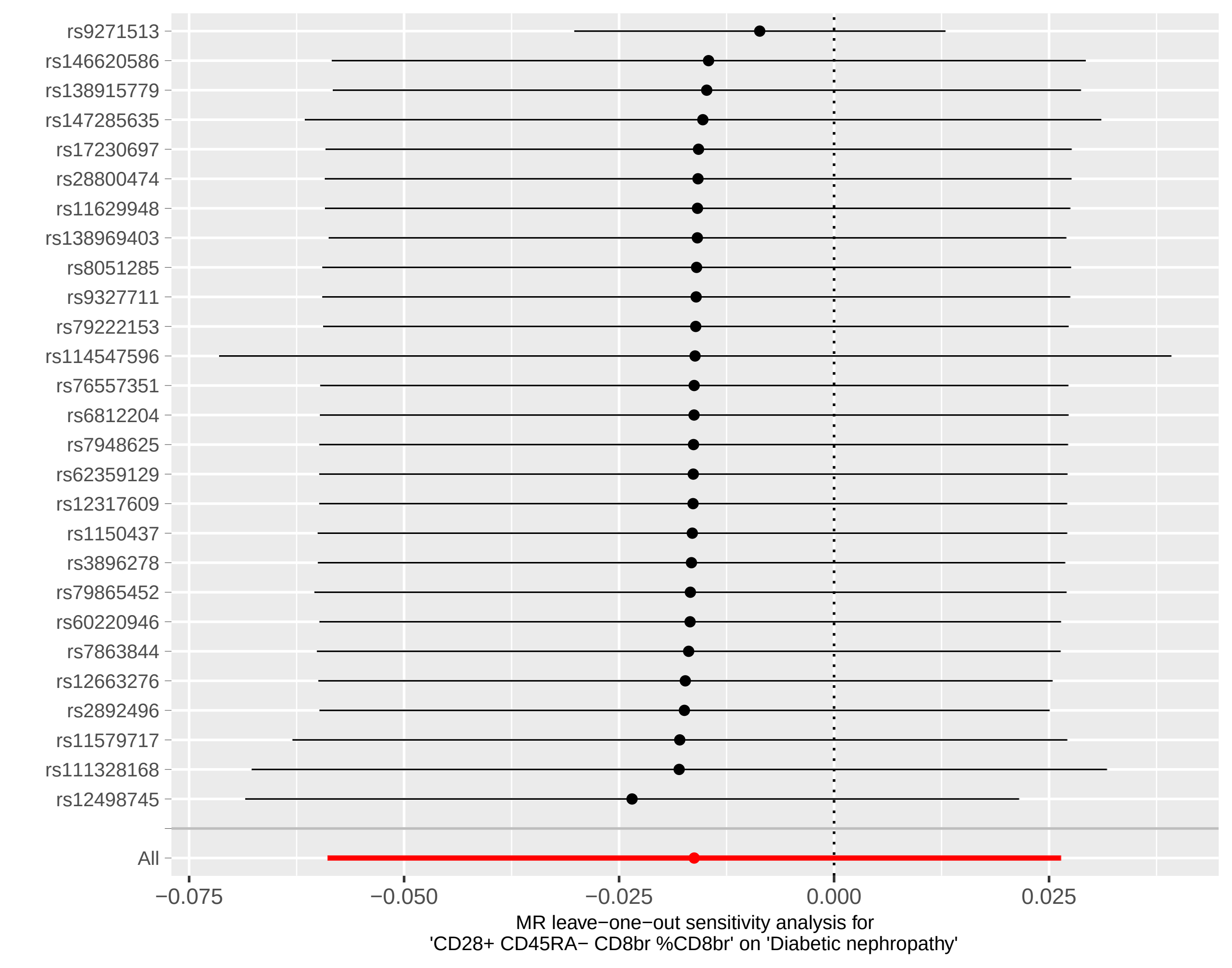
rs9271513
rs146620586
rs138915779
rs147285635
rs17230697
rs28800474
rs11629948
rs138969403
rs8051285
rs9327711
rs79222153
rs114547596
rs76557351
rs6812204
rs7948625
rs62359129
rs12317609
rs1150437
rs3896278
rs79865452
rs60220946
rs7863844
rs12663276
rs2892496
rs11579717
rs111328168
rs12498745
All
−0.075
−0.050
−0.025
0.000
0.025
MR leave−one−out sensitivity analysis for
'CD28+ CD45RA− CD8br %CD8br' on 'Diabetic nephropathy'

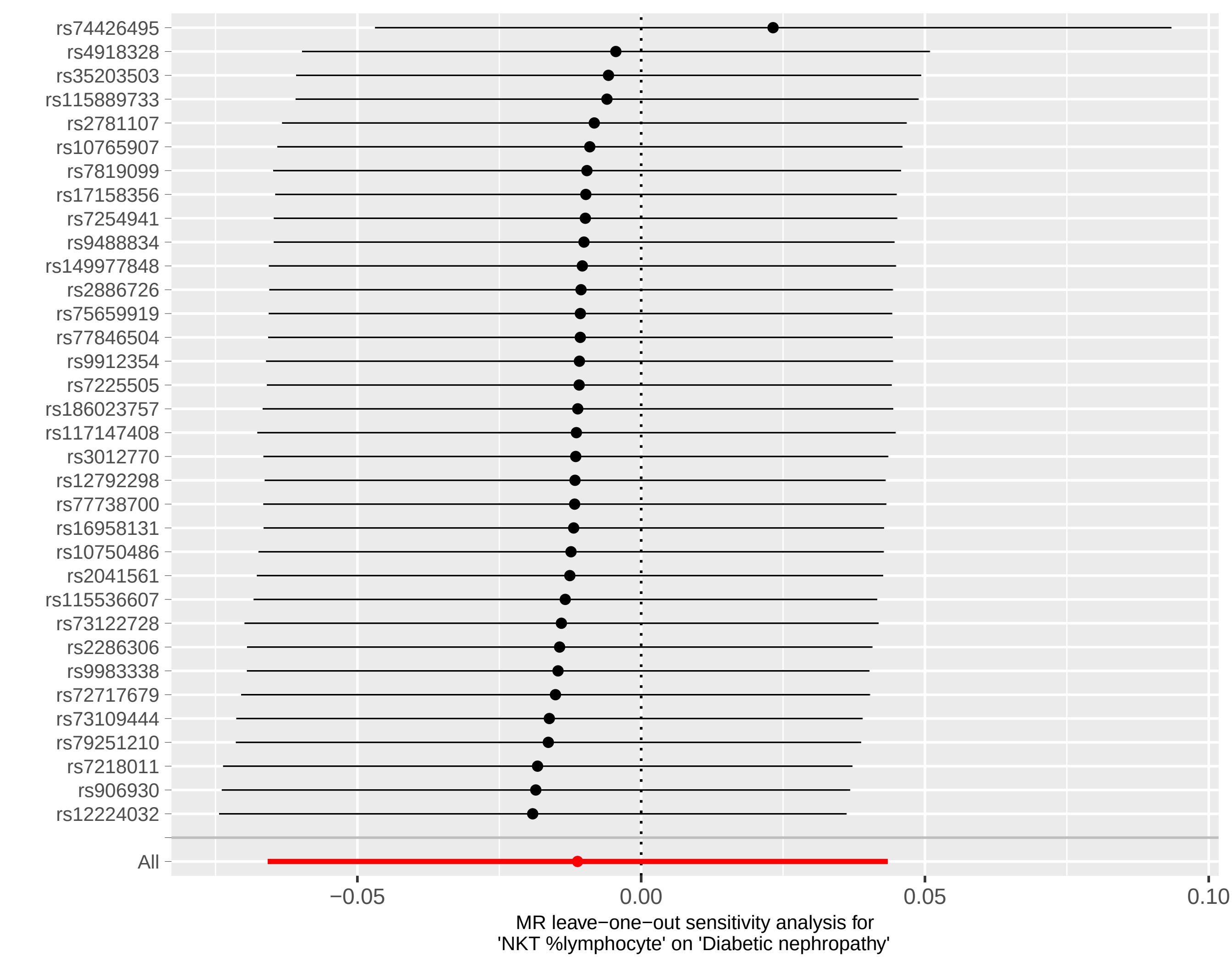
rs74426495
rs4918328
rs35203503
rs115889733
rs2781107
rs10765907
rs7819099
rs17158356
rs7254941
rs9488834
rs149977848
rs2886726
rs75659919
rs77846504
rs9912354
rs7225505
rs186023757
rs117147408
rs3012770
rs12792298
rs77738700
rs16958131
rs10750486
rs2041561
rs115536607
rs73122728
rs2286306
rs9983338
rs72717679
rs73109444
rs79251210
rs7218011
rs906930
rs12224032
All
−0.05
0.00
0.05
0.10
MR leave−one−out sensitivity analysis for
'NKT %lymphocyte' on 'Diabetic nephropathy'

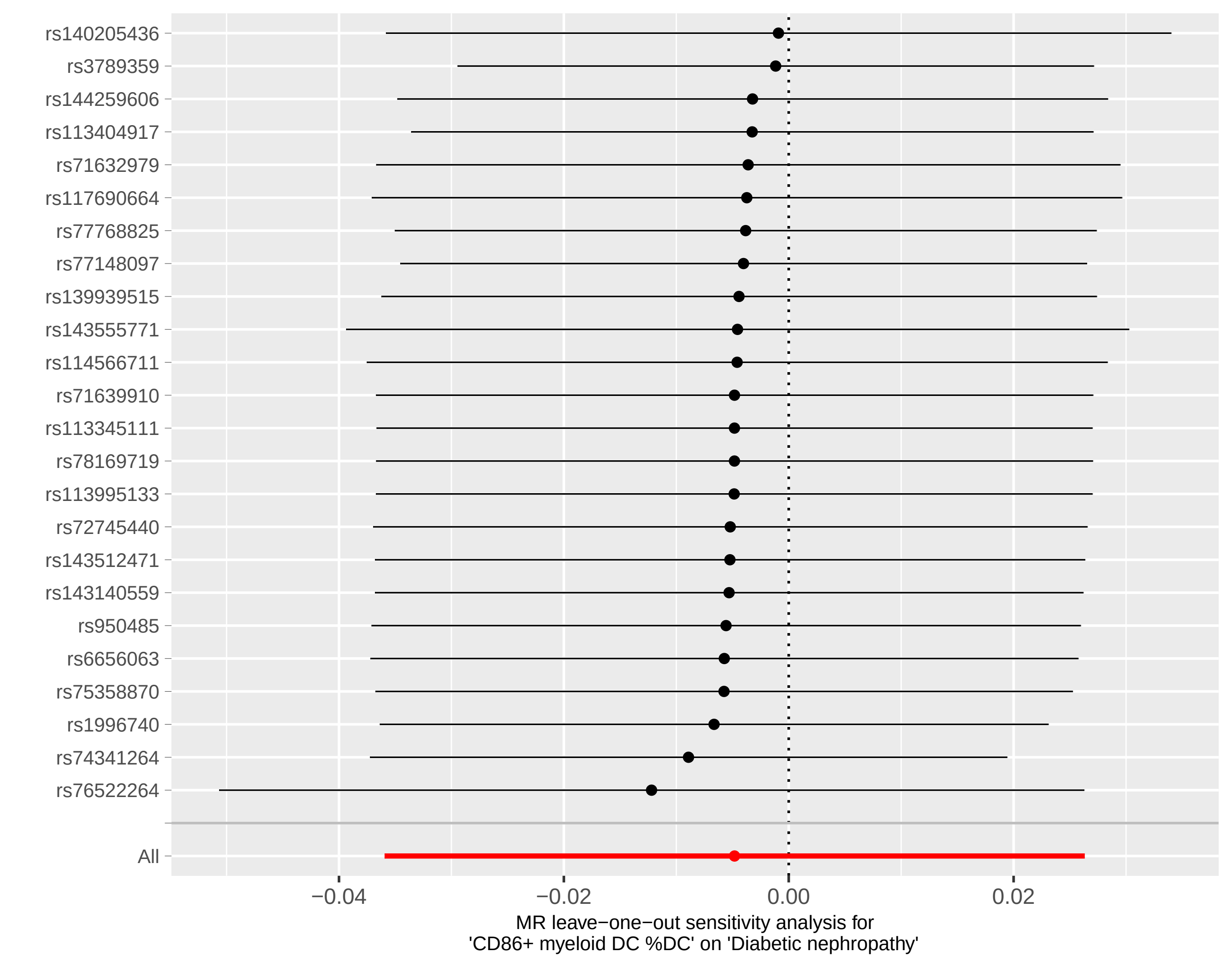
rs140205436
rs3789359
rs144259606
rs113404917
rs71632979
rs117690664
rs77768825
rs77148097
rs139939515
rs143555771
rs114566711
rs71639910
rs113345111
rs78169719
rs113995133
rs72745440
rs143512471
rs143140559
rs950485
rs6656063
rs75358870
rs1996740
rs74341264
rs76522264
All
−0.04
−0.02
0.00
0.02
MR leave−one−out sensitivity analysis for
'CD86+ myeloid DC %DC' on 'Diabetic nephropathy'

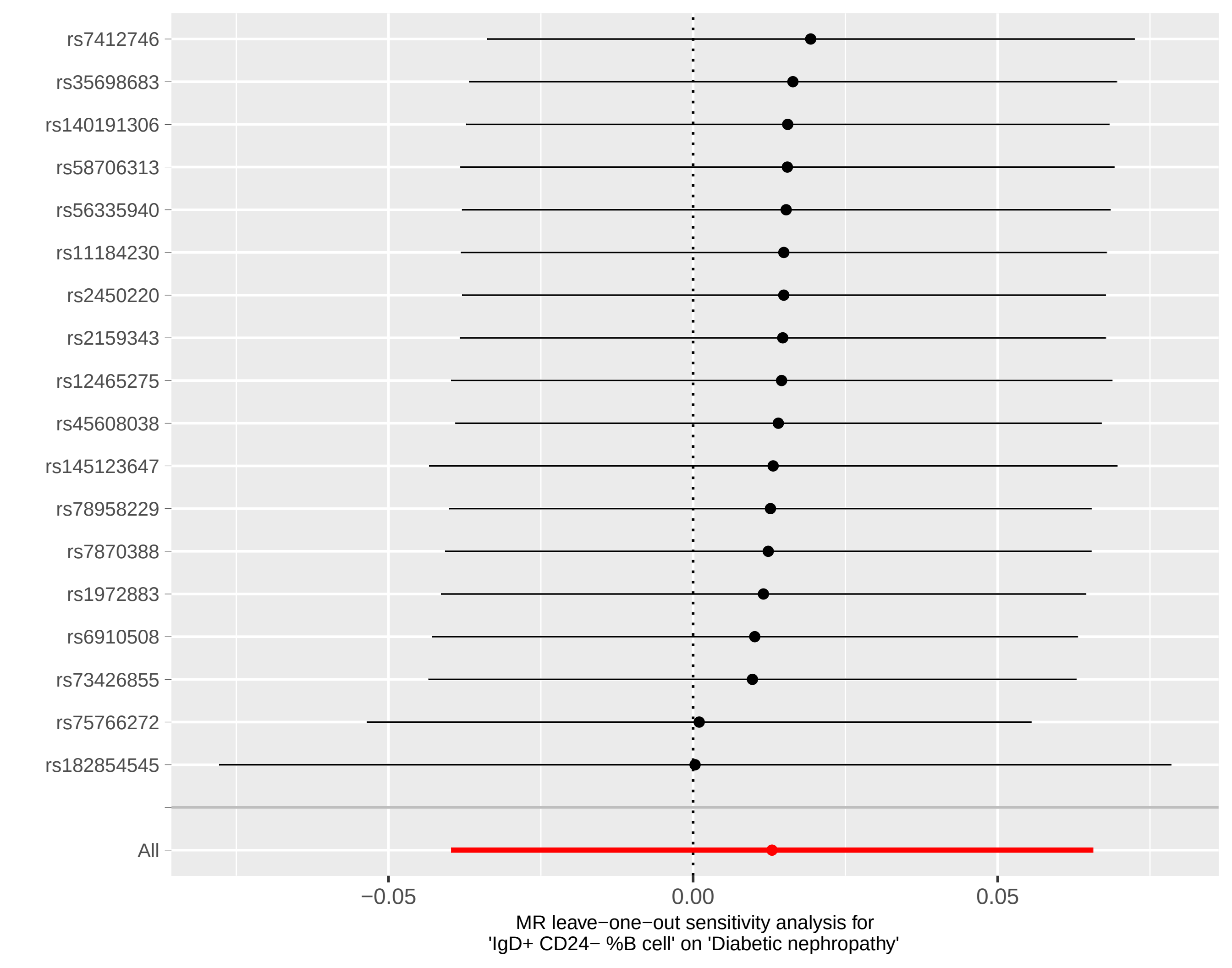
rs7412746
rs35698683
rs140191306
rs58706313
rs56335940
rs11184230
rs2450220
rs2159343
rs12465275
rs45608038
rs145123647
rs78958229
rs7870388
rs1972883
rs6910508
rs73426855
rs75766272
rs182854545
All
−0.05
0.00
0.05
MR leave−one−out sensitivity analysis for
'IgD+ CD24− %B cell' on 'Diabetic nephropathy'

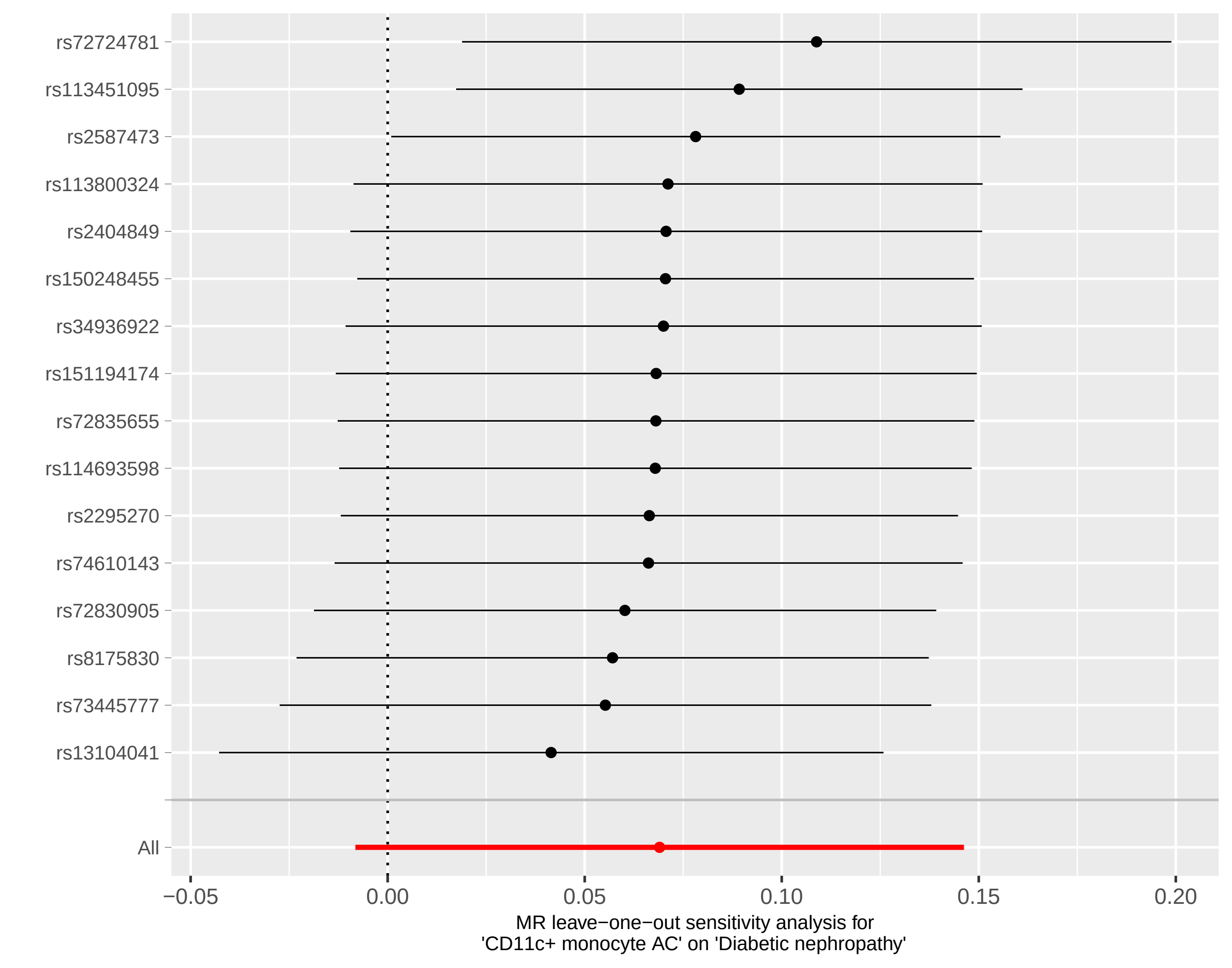

rs72724781
rs113451095
rs2587473
rs113800324
rs2404849
rs150248455
rs34936922
rs151194174
rs72835655
rs114693598
rs2295270
rs74610143
rs72830905
rs8175830
rs73445777
rs13104041
All
−0.05
0.00
0.05
0.10
0.15
0.20
MR leave−one−out sensitivity analysis for
'CD11c+ monocyte AC' on 'Diabetic nephropathy'

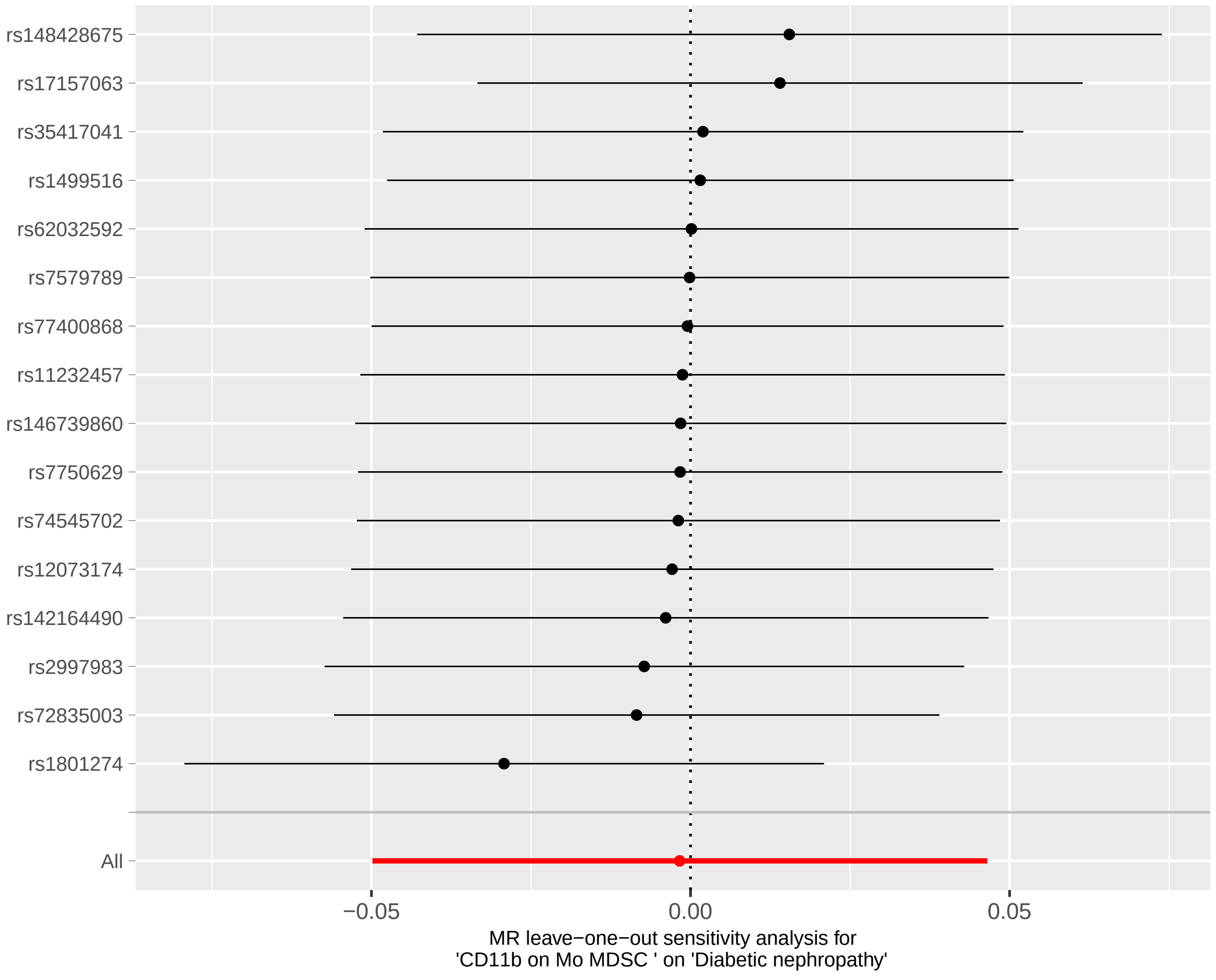
rs148428675
rs17157063
rs35417041
rs1499516
rs62032592
rs7579789
rs77400868
rs11232457
rs146739860
rs7750629
rs74545702
rs12073174
rs142164490
rs2997983
rs72835003
rs1801274
All
−0.05
0.00
0.05
MR leave−one−out sensitivity analysis for
'CD11b on Mo MDSC ' on 'Diabetic nephropathy'

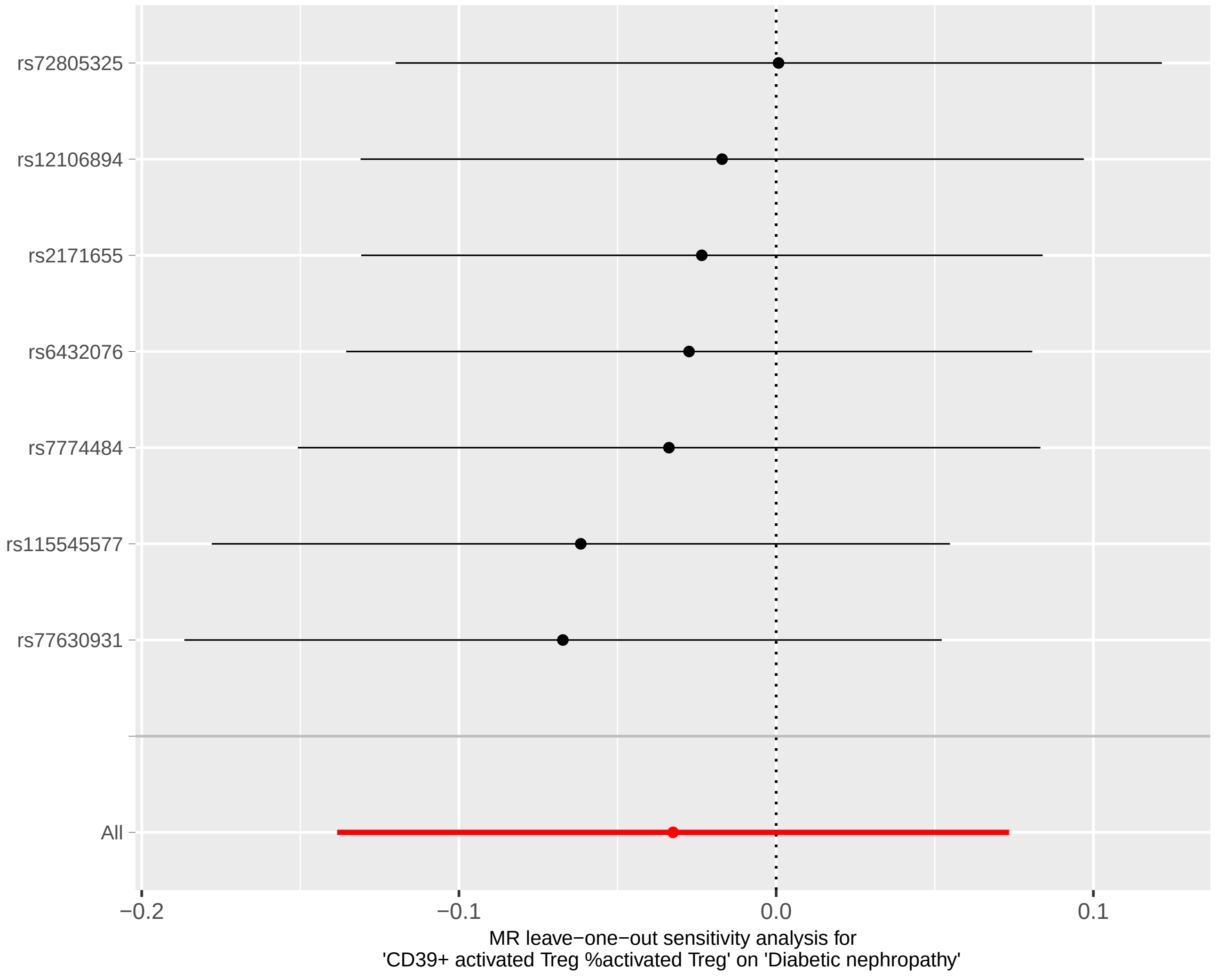

rs72805325
rs12106894
rs2171655
rs6432076
rs7774484
rs115545577
rs77630931
All
−0.2
−0.1
0.0
0.1
MR leave−one−out sensitivity analysis for
'CD39+ activated Treg %activated Treg' on 'Diabetic nephropathy'

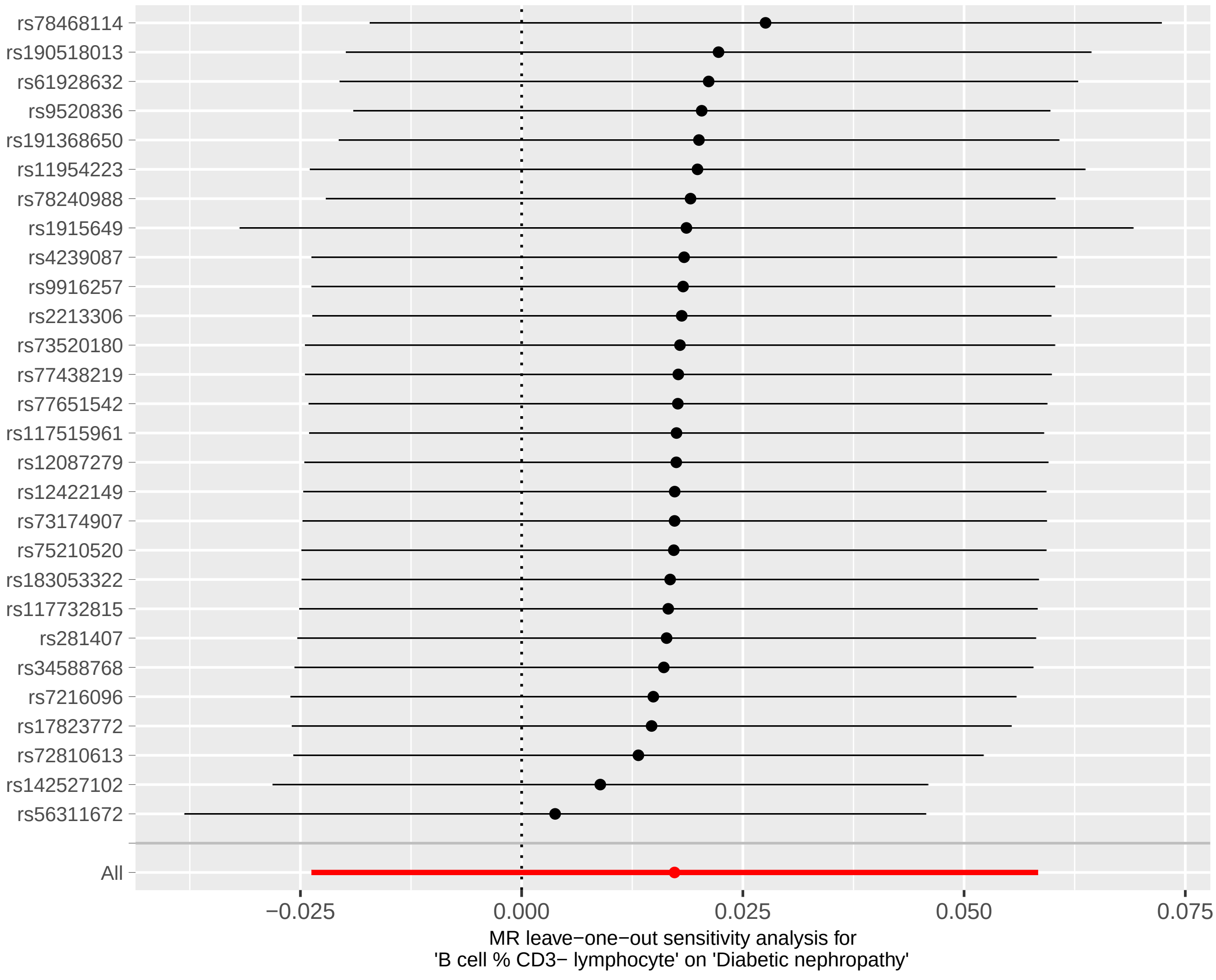
rs78468114
rs190518013
rs61928632
rs9520836
rs191368650
rs11954223
rs78240988
rs1915649
rs4239087
rs9916257
rs2213306
rs73520180
rs77438219
rs77651542
rs117515961
rs12087279
rs12422149
rs73174907
rs75210520
rs183053322
rs117732815
rs281407
rs34588768
rs7216096
rs17823772
rs72810613
rs142527102
rs56311672
All
−0.025
0.000
0.025
0.050
0.075
MR leave−one−out sensitivity analysis for
'B cell % CD3− lymphocyte' on 'Diabetic nephropathy'

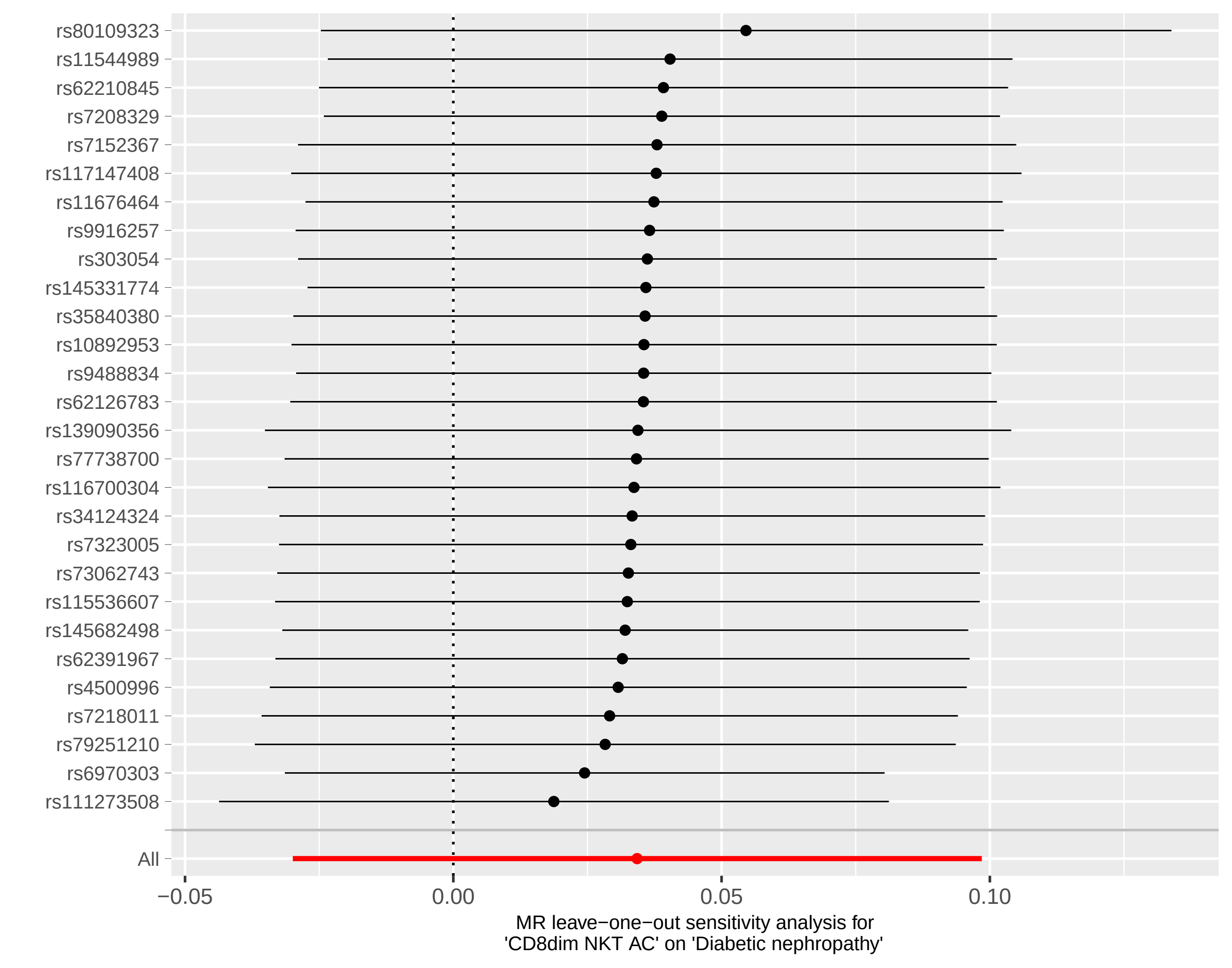
rs80109323
rs11544989
rs62210845
rs7208329
rs7152367
rs117147408
rs11676464
rs9916257
rs303054
rs145331774
rs35840380
rs10892953
rs9488834
rs62126783
rs139090356
rs77738700
rs116700304
rs34124324
rs7323005
rs73062743
rs115536607
rs145682498
rs62391967
rs4500996
rs7218011
rs79251210
rs6970303
rs111273508
All
−0.05
0.00
0.05
0.10
MR leave−one−out sensitivity analysis for
'CD8dim NKT AC' on 'Diabetic nephropathy'

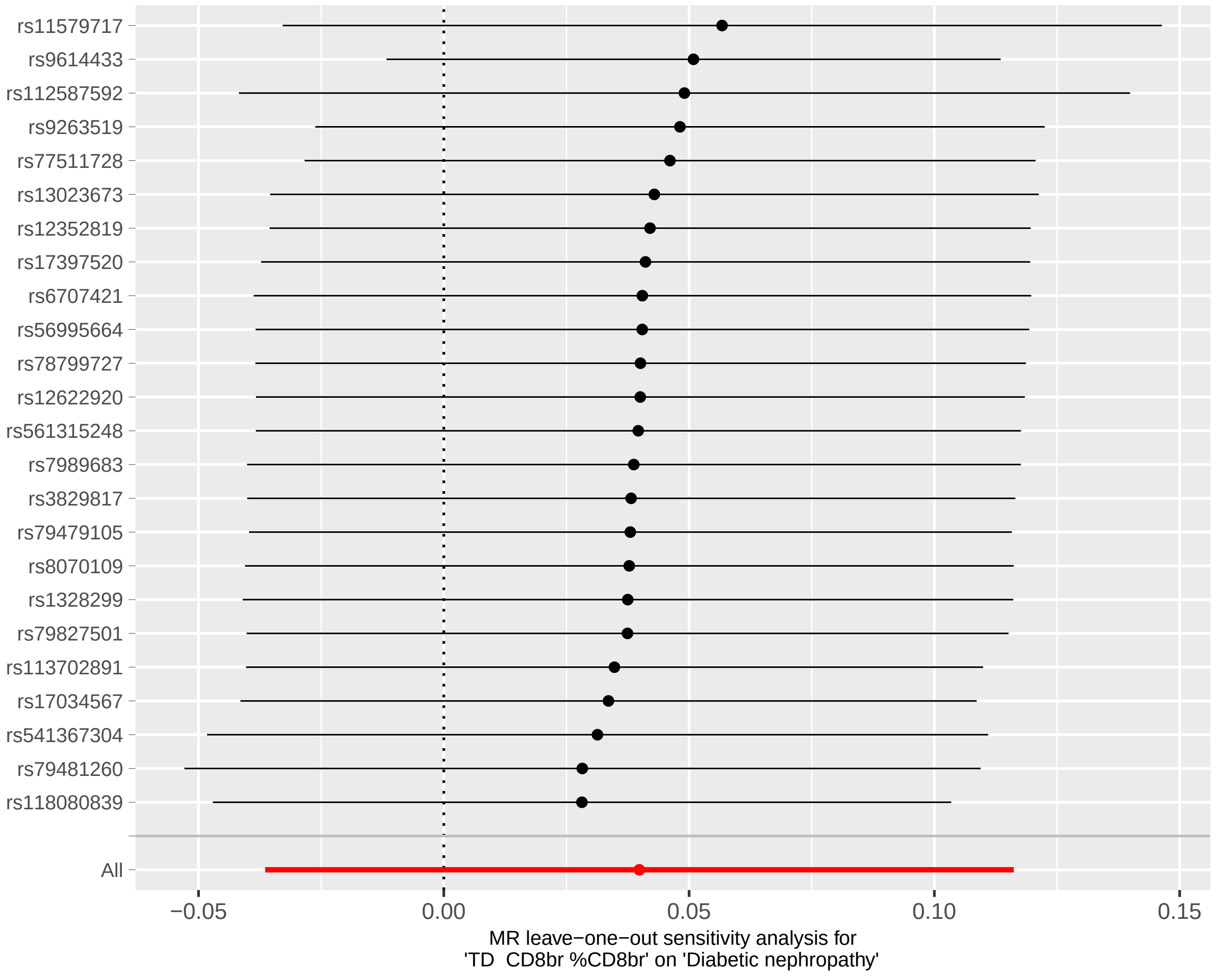
rs11579717
rs9614433
rs112587592
rs9263519
rs77511728
rs13023673
rs12352819
rs17397520
rs6707421
rs56995664
rs78799727
rs12622920
rs561315248
rs7989683
rs3829817
rs79479105
rs8070109
rs1328299
rs79827501
rs113702891
rs17034567
rs541367304
rs79481260
rs118080839
All
−0.05
0.00
0.05
0.10
0.15
MR leave−one−out sensitivity analysis for
'TD CD8br %CD8br' on 'Diabetic nephropathy'

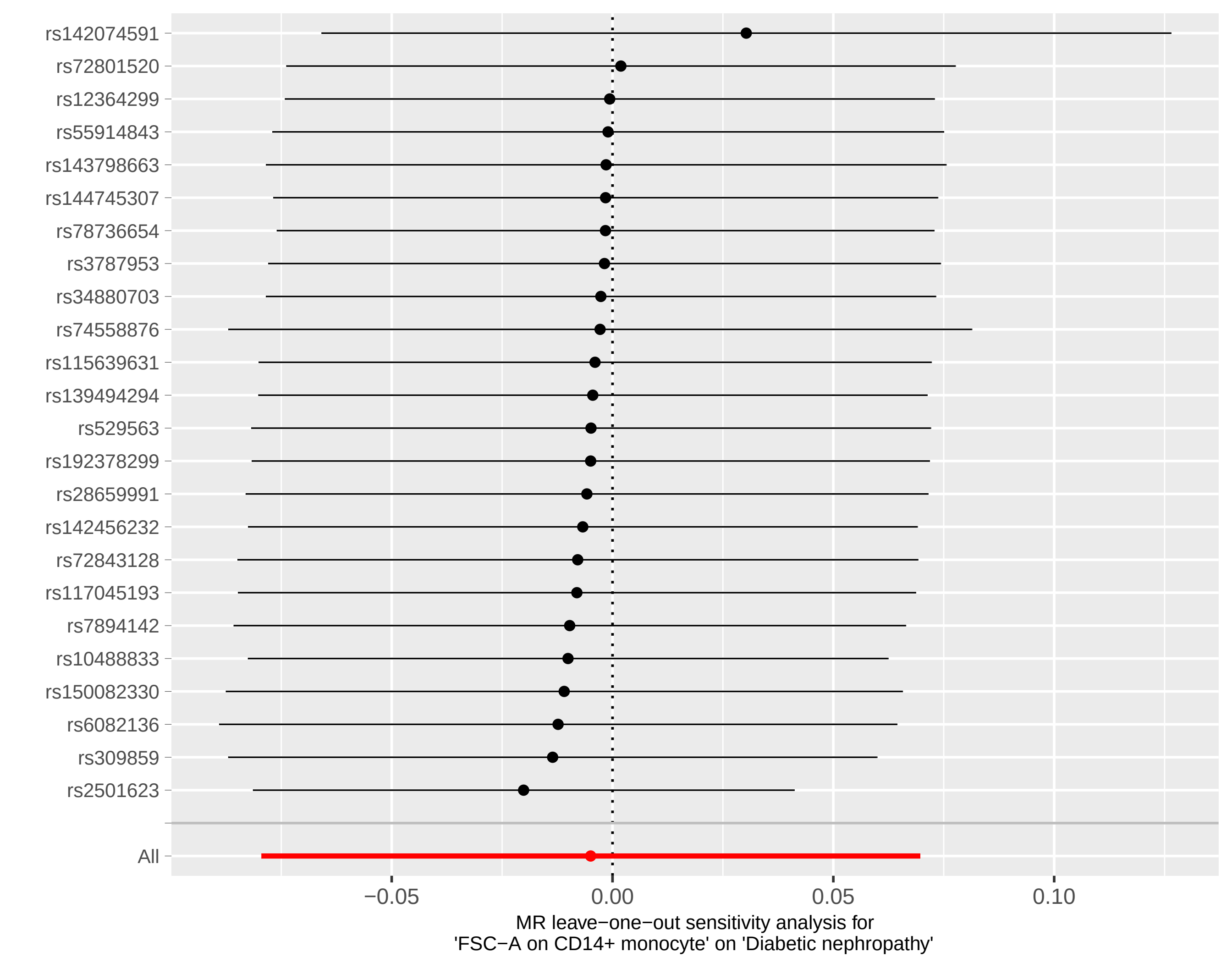
rs142074591
rs72801520
rs12364299
rs55914843
rs143798663
rs144745307
rs78736654
rs3787953
rs34880703
rs74558876
rs115639631
rs139494294
rs529563
rs192378299
rs28659991
rs142456232
rs72843128
rs117045193
rs7894142
rs10488833
rs150082330
rs6082136
rs309859
rs2501623
All
−0.05
0.00
0.05
0.10
MR leave−one−out sensitivity analysis for
'FSC−A on CD14+ monocyte' on 'Diabetic nephropathy'

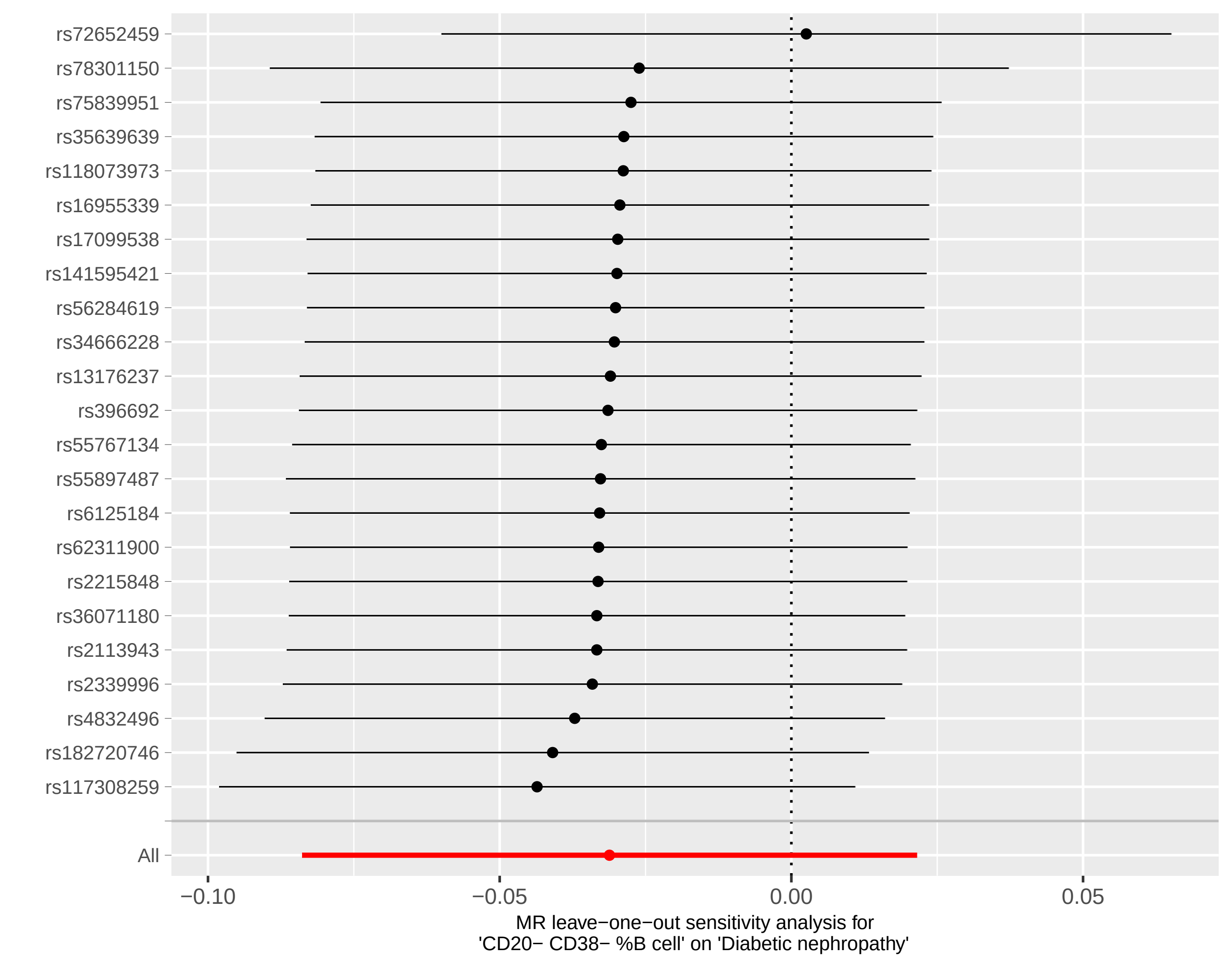
rs72652459
rs78301150
rs75839951
rs35639639
rs118073973
rs16955339
rs17099538
rs141595421
rs56284619
rs34666228
rs13176237
rs396692
rs55767134
rs55897487
rs6125184
rs62311900
rs2215848
rs36071180
rs2113943
rs2339996
rs4832496
rs182720746
rs117308259
All
−0.10
−0.05
0.00
0.05
MR leave−one−out sensitivity analysis for
'CD20− CD38− %B cell' on 'Diabetic nephropathy'

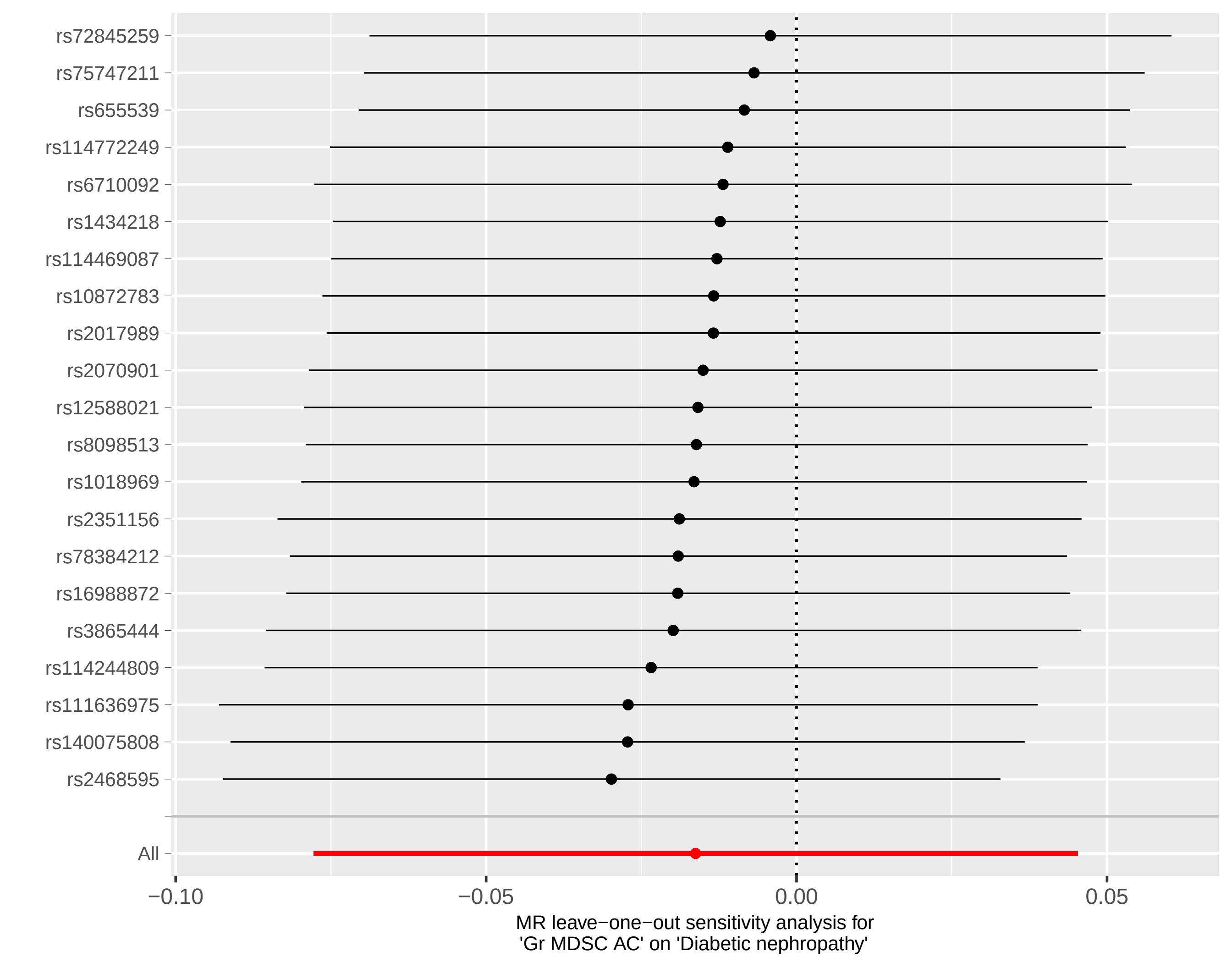
rs72845259
rs75747211
rs655539
rs114772249
rs6710092
rs1434218
rs114469087
rs10872783
rs2017989
rs2070901
rs12588021
rs8098513
rs1018969
rs2351156
rs78384212
rs16988872
rs3865444
rs114244809
rs111636975
rs140075808
rs2468595
All
−0.10
−0.05
0.00
0.05
MR leave−one−out sensitivity analysis for
'Gr MDSC AC' on 'Diabetic nephropathy'

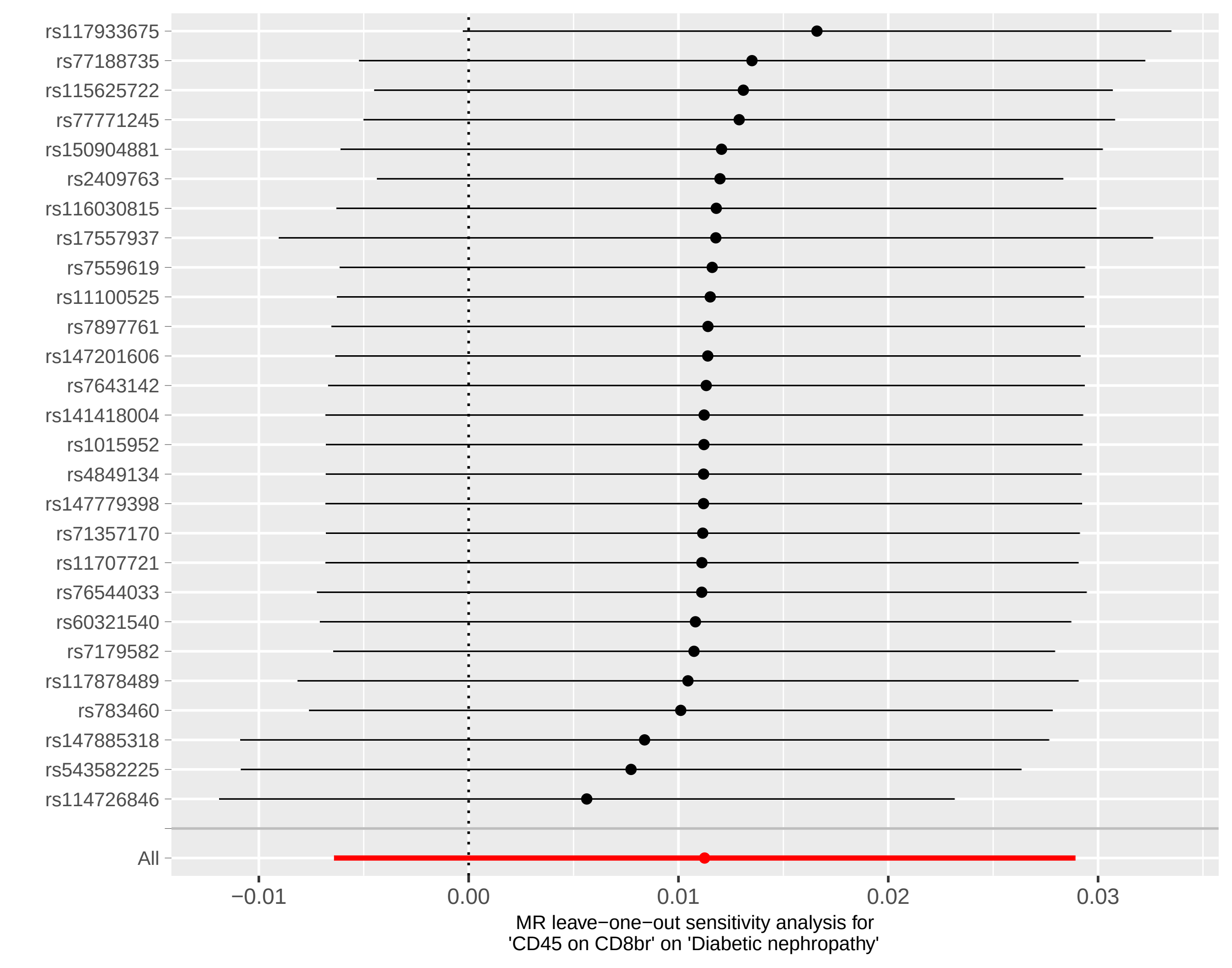

rs117933675
rs77188735
rs115625722
rs77771245
rs150904881
rs2409763
rs116030815
rs17557937
rs7559619
rs11100525
rs7897761
rs147201606
rs7643142
rs141418004
rs1015952
rs4849134
rs147779398
rs71357170
rs11707721
rs76544033
rs60321540
rs7179582
rs117878489
rs783460
rs147885318
rs543582225
rs114726846
All
−0.01
0.00
0.01
0.02
0.03
MR leave−one−out sensitivity analysis for
'CD45 on CD8br' on 'Diabetic nephropathy'

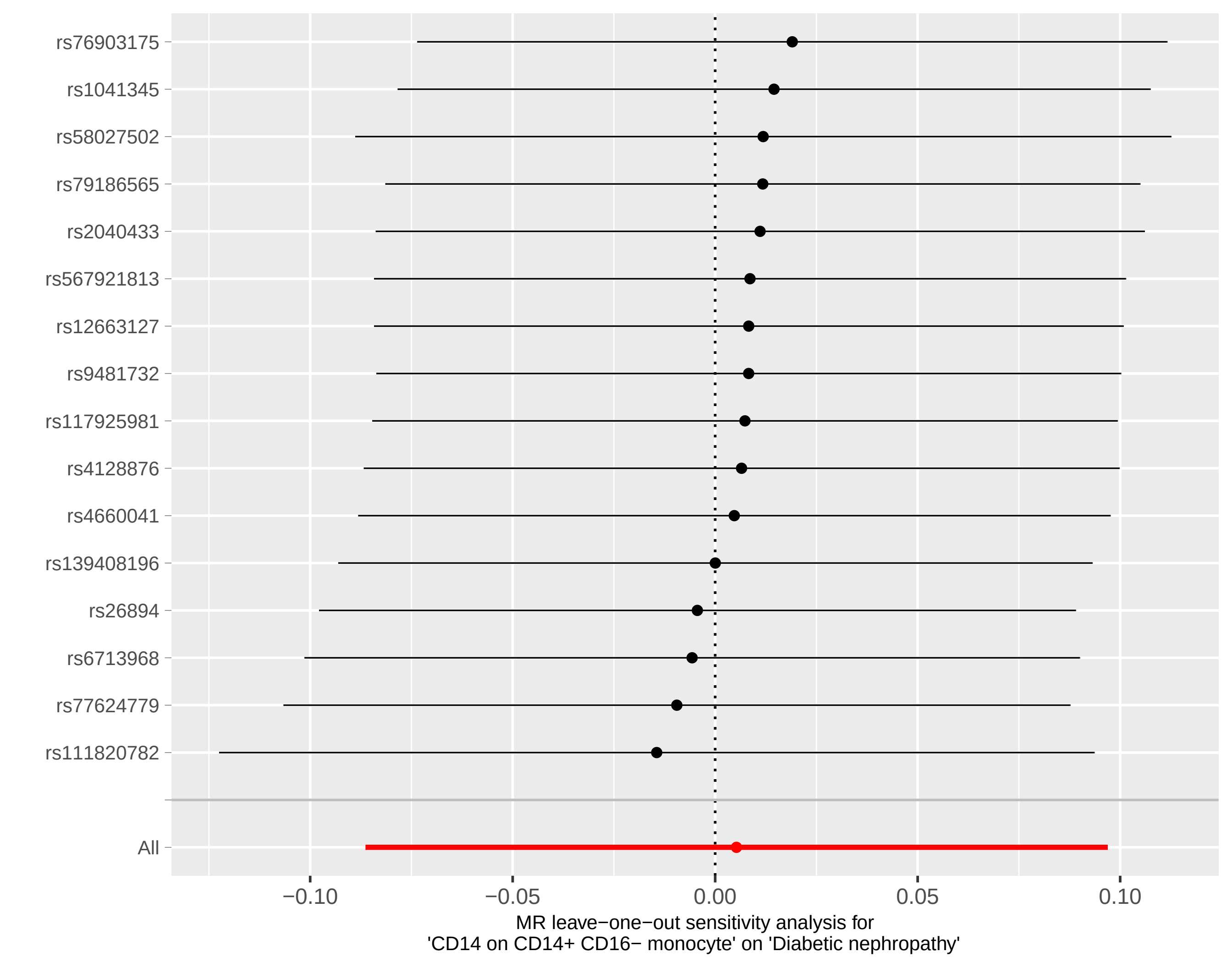
rs76903175
rs1041345
rs58027502
rs79186565
rs2040433
rs567921813
rs12663127
rs9481732
rs117925981
rs4128876
rs4660041
rs139408196
rs26894
rs6713968
rs77624779
rs111820782
All
−0.10
−0.05
0.00
0.05
0.10
MR leave−one−out sensitivity analysis for
'CD14 on CD14+ CD16− monocyte' on 'Diabetic nephropathy'

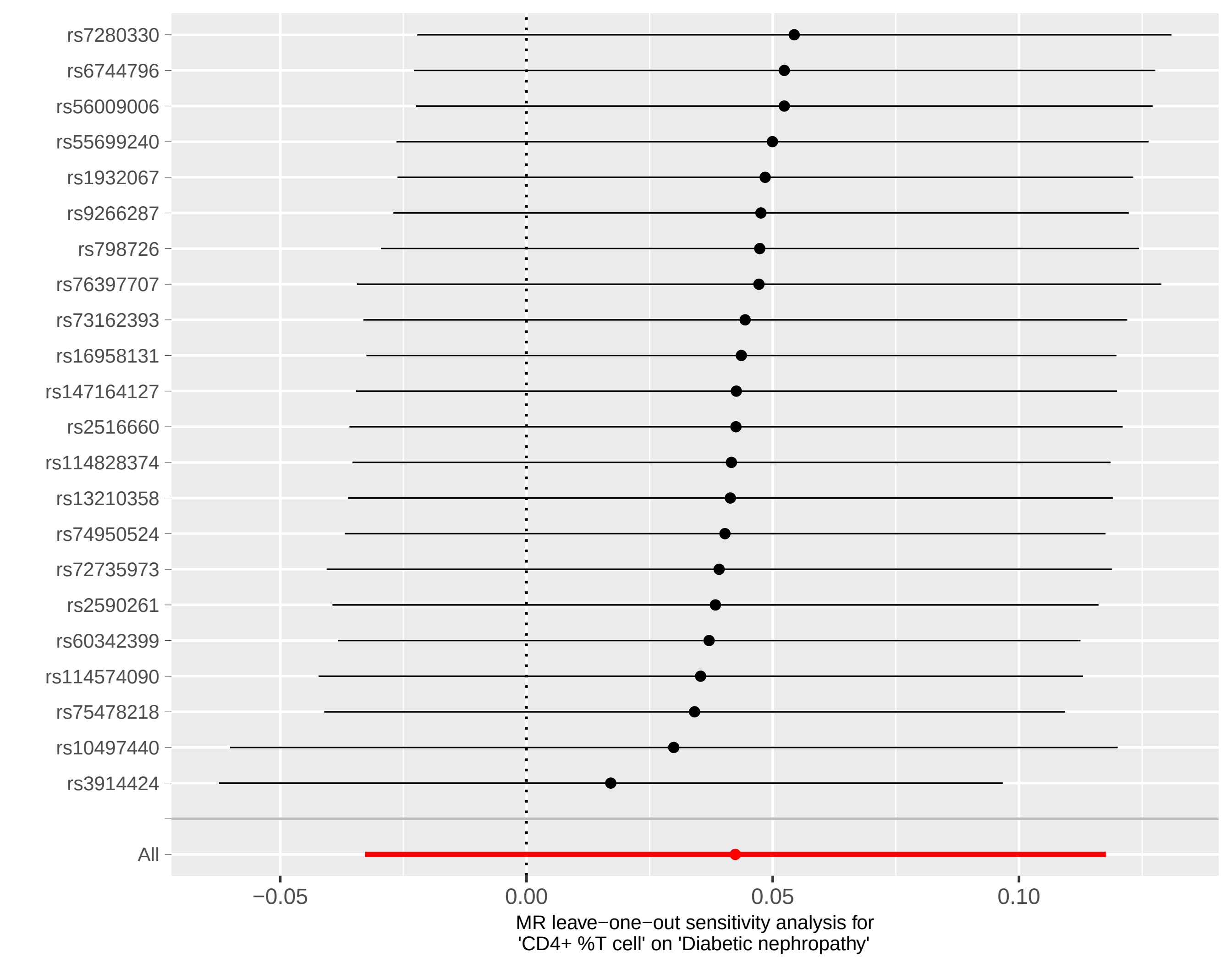

rs7280330
rs6744796
rs56009006
rs55699240
rs1932067
rs9266287
rs798726
rs76397707
rs73162393
rs16958131
rs147164127
rs2516660
rs114828374
rs13210358
rs74950524
rs72735973
rs2590261
rs60342399
rs114574090
rs75478218
rs10497440
rs3914424
All
−0.05
0.00
0.05
0.10
MR leave−one−out sensitivity analysis for
'CD4+ %T cell' on 'Diabetic nephropathy'

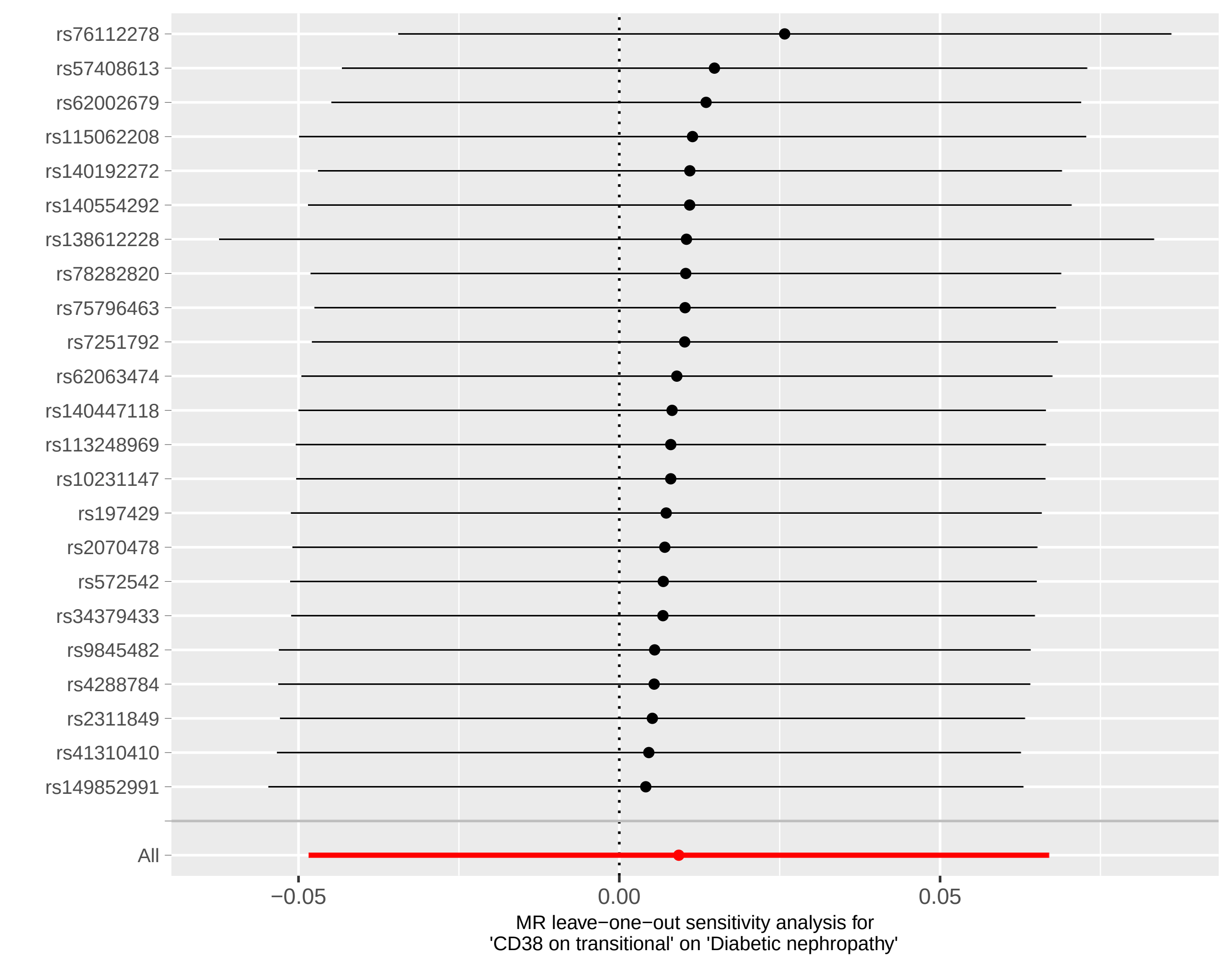
rs76112278
rs57408613
rs62002679
rs115062208
rs140192272
rs140554292
rs138612228
rs78282820
rs75796463
rs7251792
rs62063474
rs140447118
rs113248969
rs10231147
rs197429
rs2070478
rs572542
rs34379433
rs9845482
rs4288784
rs2311849
rs41310410
rs149852991
All
−0.05
0.00
0.05
MR leave−one−out sensitivity analysis for
'CD38 on transitional' on 'Diabetic nephropathy'

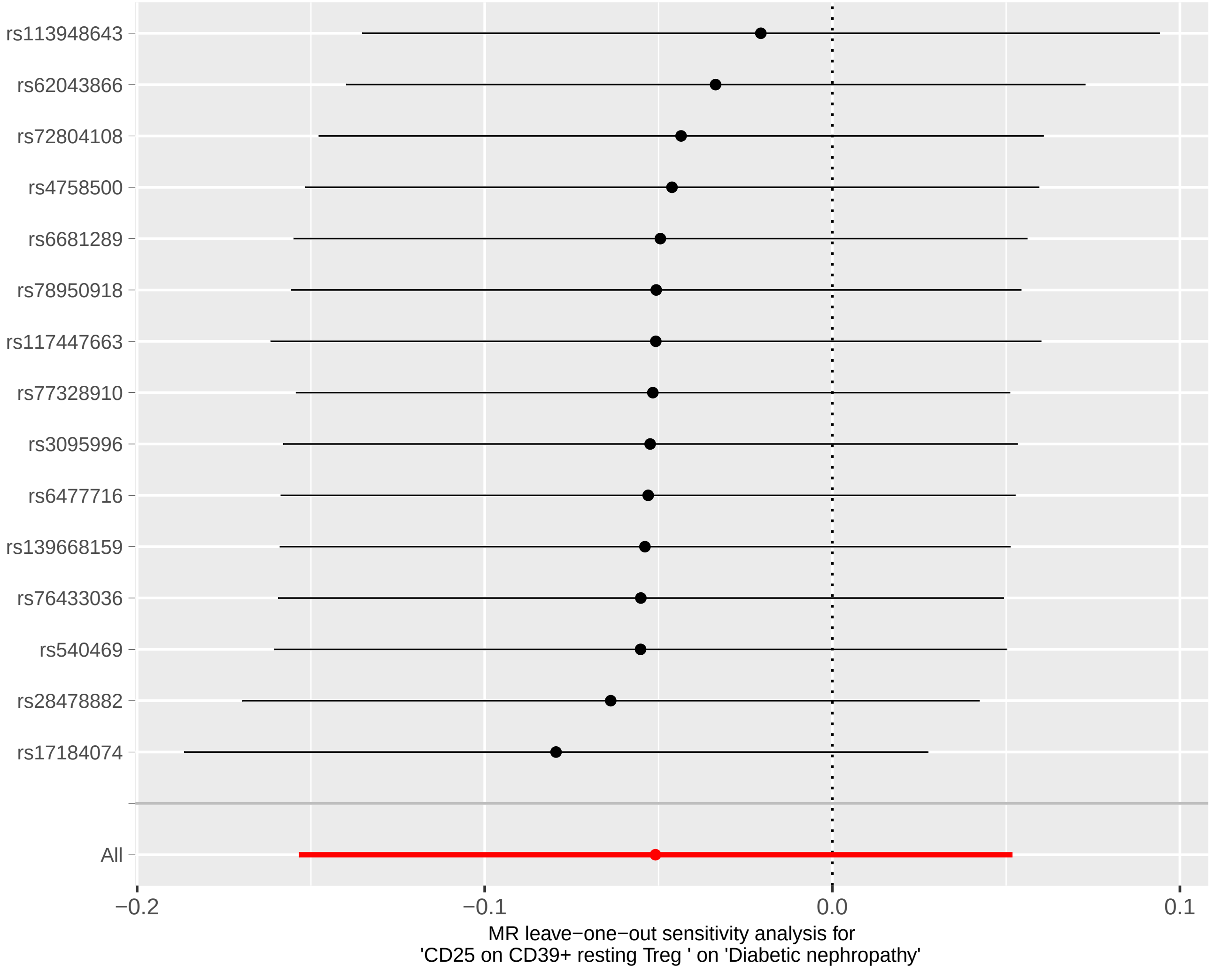
rs113948643
rs62043866
rs72804108
rs4758500
rs6681289
rs78950918
rs117447663
rs77328910
rs3095996
rs6477716
rs139668159
rs76433036
rs540469
rs28478882
rs17184074
All
−0.2
−0.1
0.0
0.1
MR leave−one−out sensitivity analysis for
'CD25 on CD39+ resting Treg ' on 'Diabetic nephropathy'

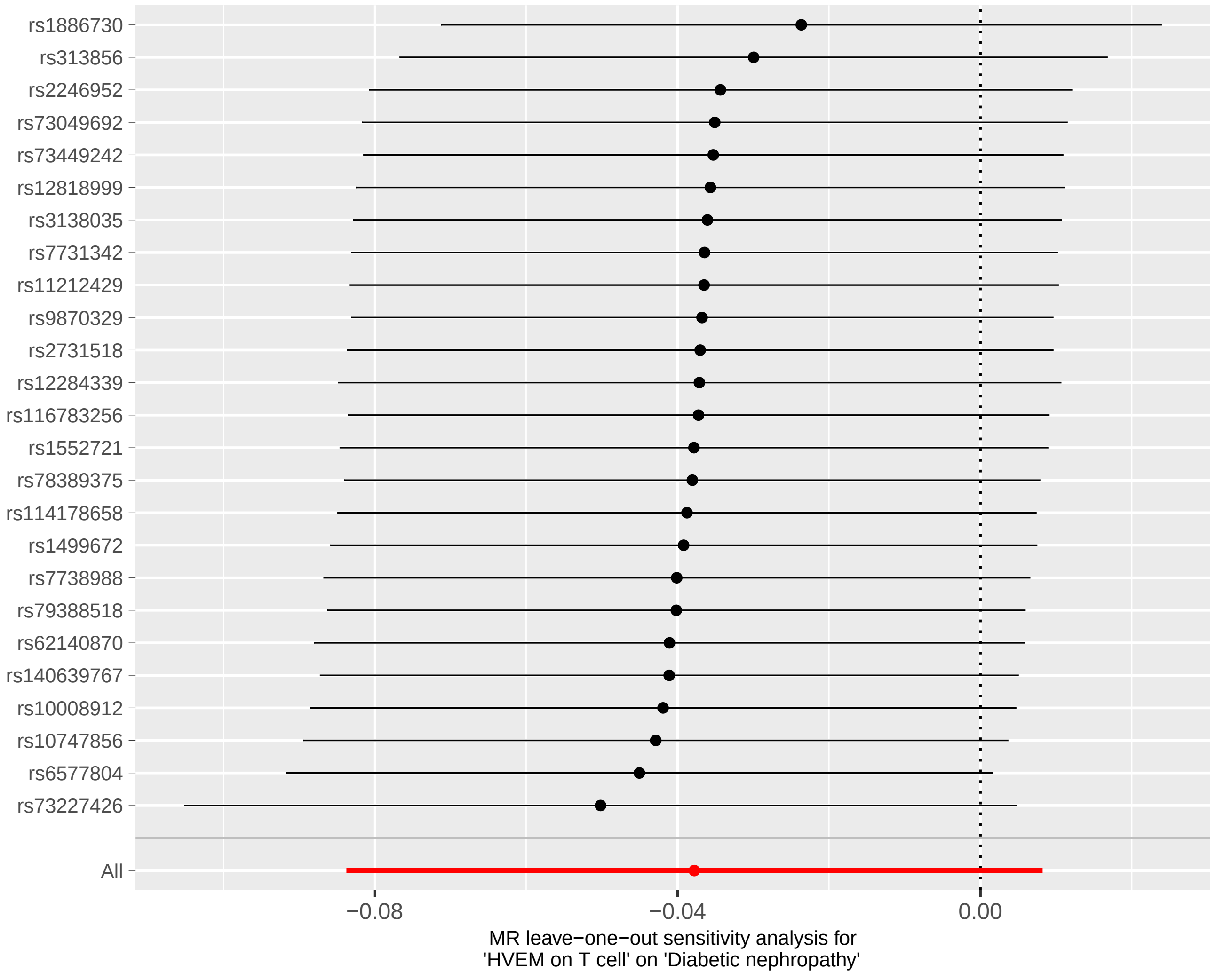
rs1886730
rs313856
rs2246952
rs73049692
rs73449242
rs12818999
rs3138035
rs7731342
rs11212429
rs9870329
rs2731518
rs12284339
rs116783256
rs1552721
rs78389375
rs114178658
rs1499672
rs7738988
rs79388518
rs62140870
rs140639767
rs10008912
rs10747856
rs6577804
rs73227426
All
−0.08
−0.04
0.00
MR leave−one−out sensitivity analysis for
'HVEM on T cell' on 'Diabetic nephropathy'

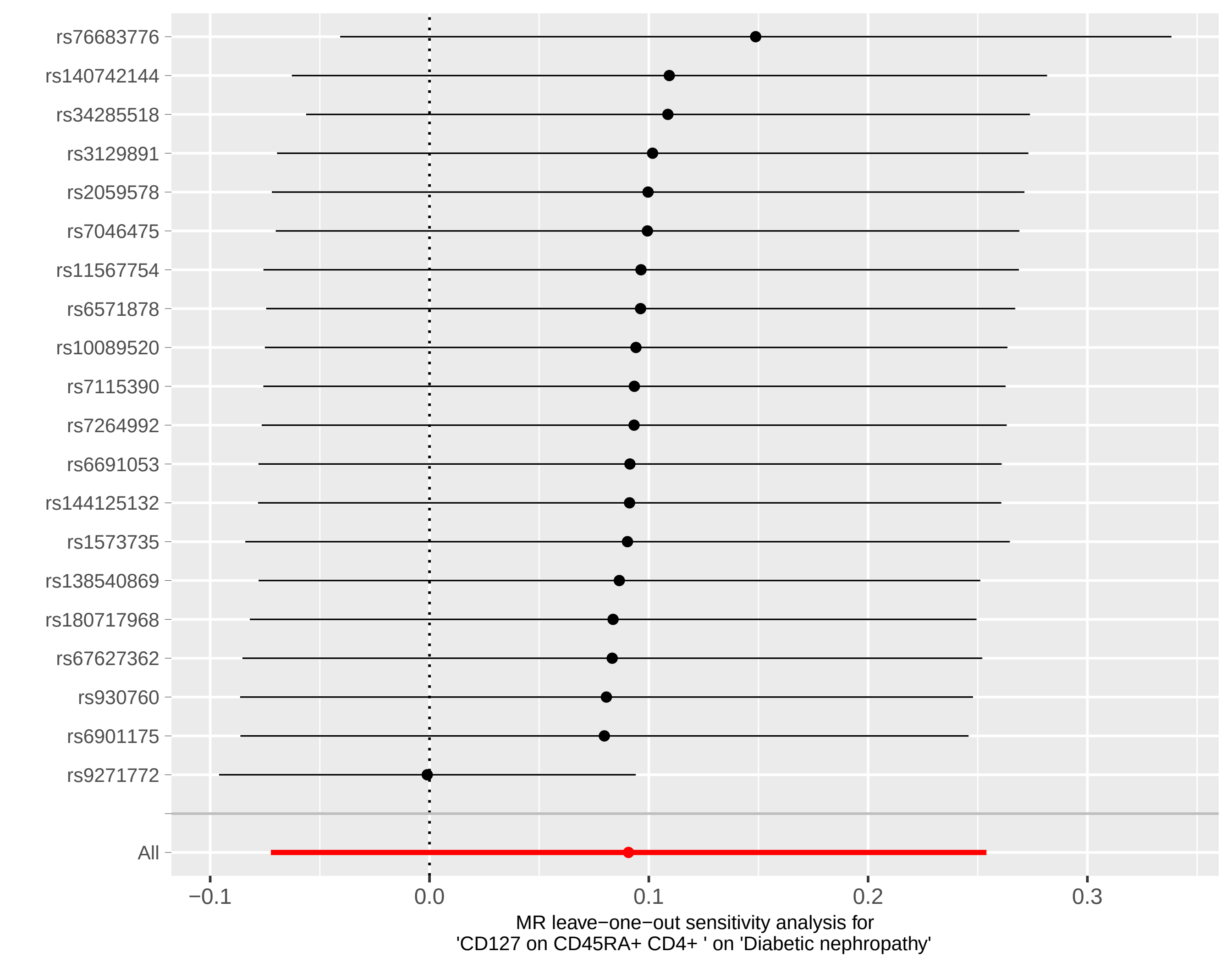
rs76683776
rs140742144
rs34285518
rs3129891
rs2059578
rs7046475
rs11567754
rs6571878
rs10089520
rs7115390
rs7264992
rs6691053
rs144125132
rs1573735
rs138540869
rs180717968
rs67627362
rs930760
rs6901175
rs9271772
All
−0.1
0.0
0.1
0.2
0.3
MR leave−one−out sensitivity analysis for
'CD127 on CD45RA+ CD4+ ' on 'Diabetic nephropathy'

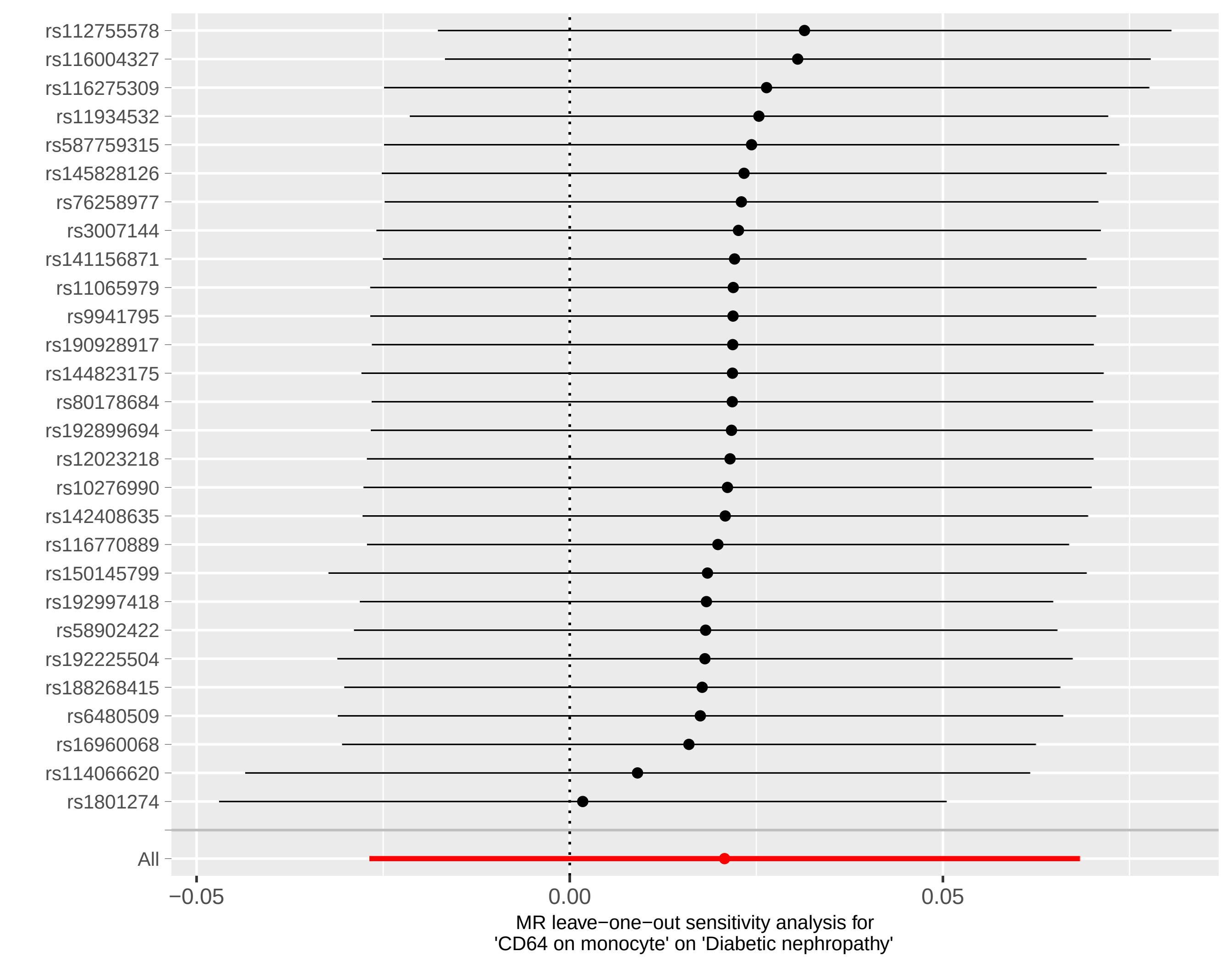
rs112755578
rs116004327
rs116275309
rs11934532
rs587759315
rs145828126
rs76258977
rs3007144
rs141156871
rs11065979
rs9941795
rs190928917
rs144823175
rs80178684
rs192899694
rs12023218
rs10276990
rs142408635
rs116770889
rs150145799
rs192997418
rs58902422
rs192225504
rs188268415
rs6480509
rs16960068
rs114066620
rs1801274
All
−0.05
0.00
0.05
MR leave−one−out sensitivity analysis for
'CD64 on monocyte' on 'Diabetic nephropathy'

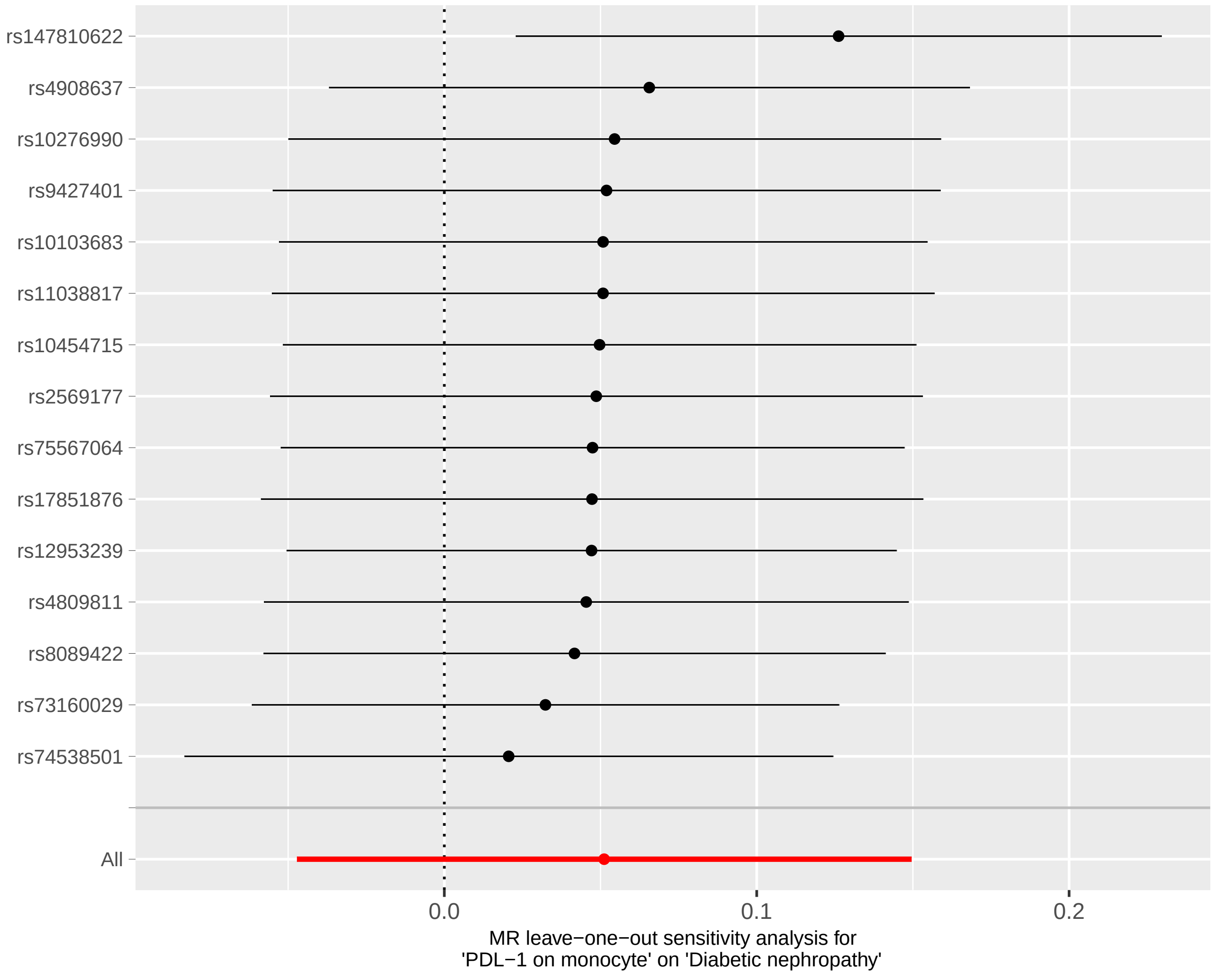
rs147810622
rs4908637
rs10276990
rs9427401
rs10103683
rs11038817
rs10454715
rs2569177
rs75567064
rs17851876
rs12953239
rs4809811
rs8089422
rs73160029
rs74538501
All
0.0
0.1
0.2
MR leave−one−out sensitivity analysis for
'PDL−1 on monocyte' on 'Diabetic nephropathy'

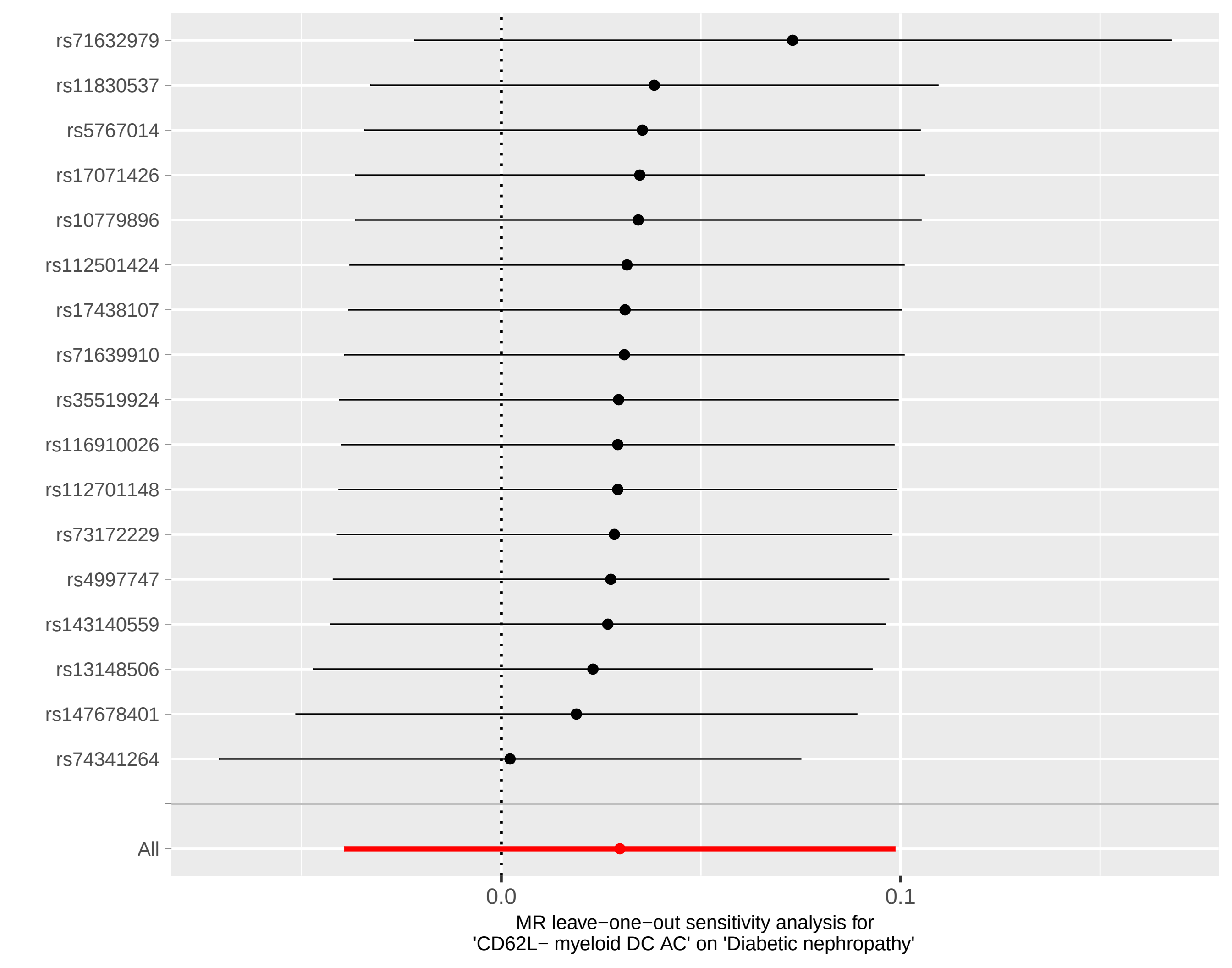
rs71632979
rs11830537
rs5767014
rs17071426
rs10779896
rs112501424
rs17438107
rs71639910
rs35519924
rs116910026
rs112701148
rs73172229
rs4997747
rs143140559
rs13148506
rs147678401
rs74341264
All
0.0
0.1
MR leave−one−out sensitivity analysis for
'CD62L− myeloid DC AC' on 'Diabetic nephropathy'

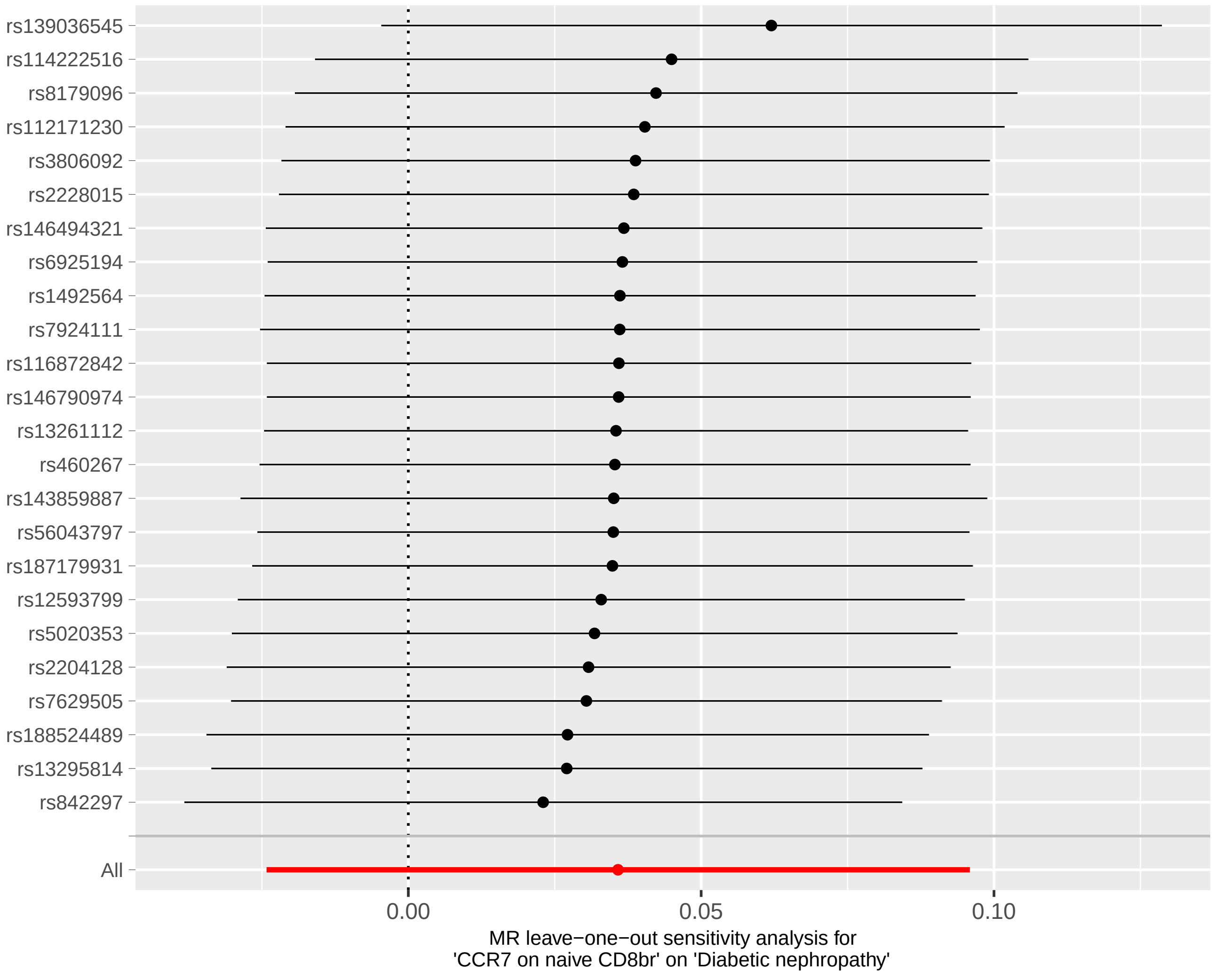
rs139036545
rs114222516
rs8179096
rs112171230
rs3806092
rs2228015
rs146494321
rs6925194
rs1492564
rs7924111
rs116872842
rs146790974
rs13261112
rs460267
rs143859887
rs56043797
rs187179931
rs12593799
rs5020353
rs2204128
rs7629505
rs188524489
rs13295814
rs842297
All
0.00
0.05
0.10
MR leave−one−out sensitivity analysis for
'CCR7 on naive CD8br' on 'Diabetic nephropathy'

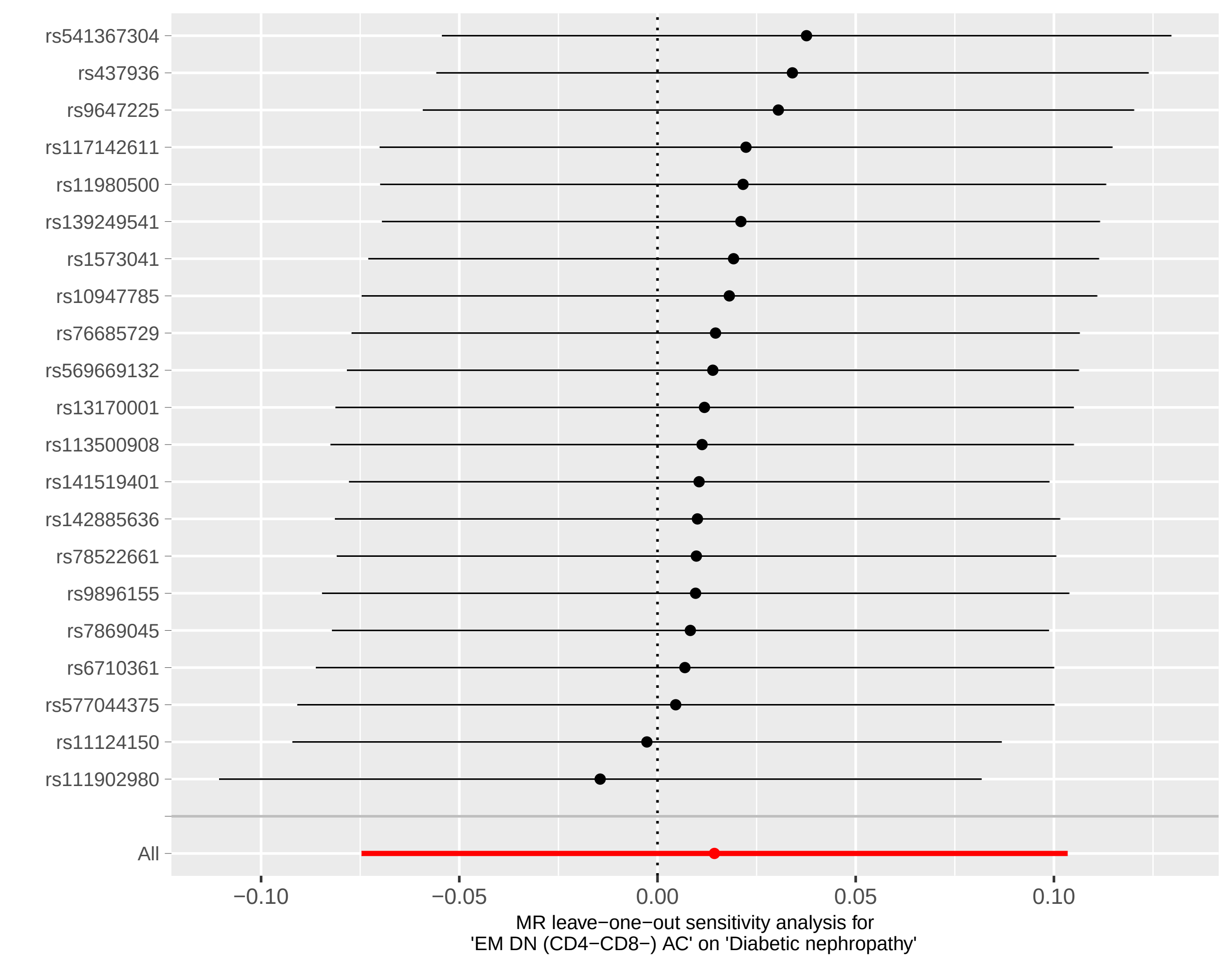

rs541367304
rs437936
rs9647225
rs117142611
rs11980500
rs139249541
rs1573041
rs10947785
rs76685729
rs569669132
rs13170001
rs113500908
rs141519401
rs142885636
rs78522661
rs9896155
rs7869045
rs6710361
rs577044375
rs11124150
rs111902980
All
−0.10
−0.05
0.00
0.05
0.10
MR leave−one−out sensitivity analysis for
'EM DN (CD4−CD8−) AC' on 'Diabetic nephropathy'

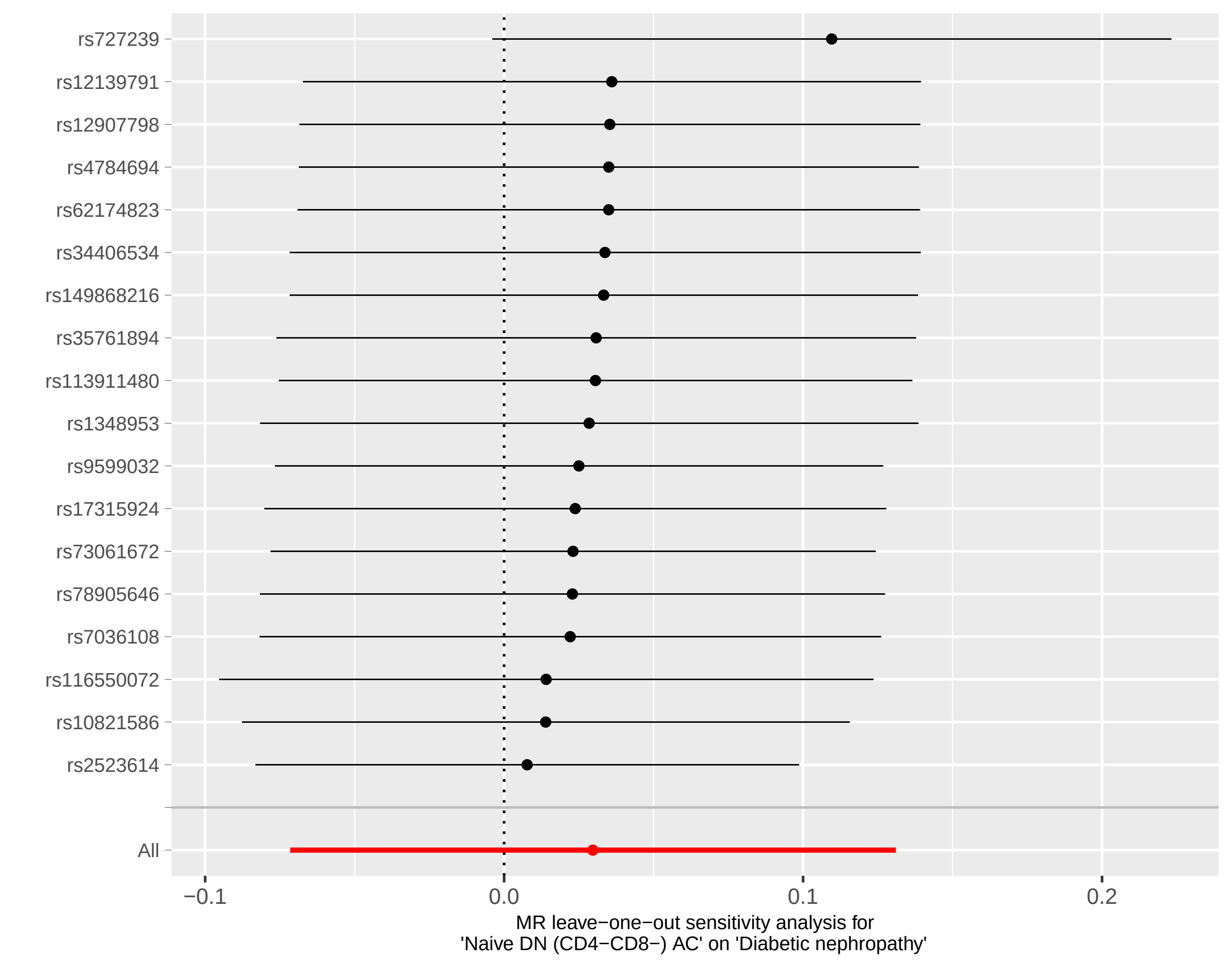

rs727239
rs12139791
rs12907798
rs4784694
rs62174823
rs34406534
rs149868216
rs35761894
rs113911480
rs1348953
rs9599032
rs17315924
rs73061672
rs78905646
rs7036108
rs116550072
rs10821586
rs2523614
All
−0.1
0.0
0.1
0.2
MR leave−one−out sensitivity analysis for
'Naive DN (CD4−CD8−) AC' on 'Diabetic nephropathy'

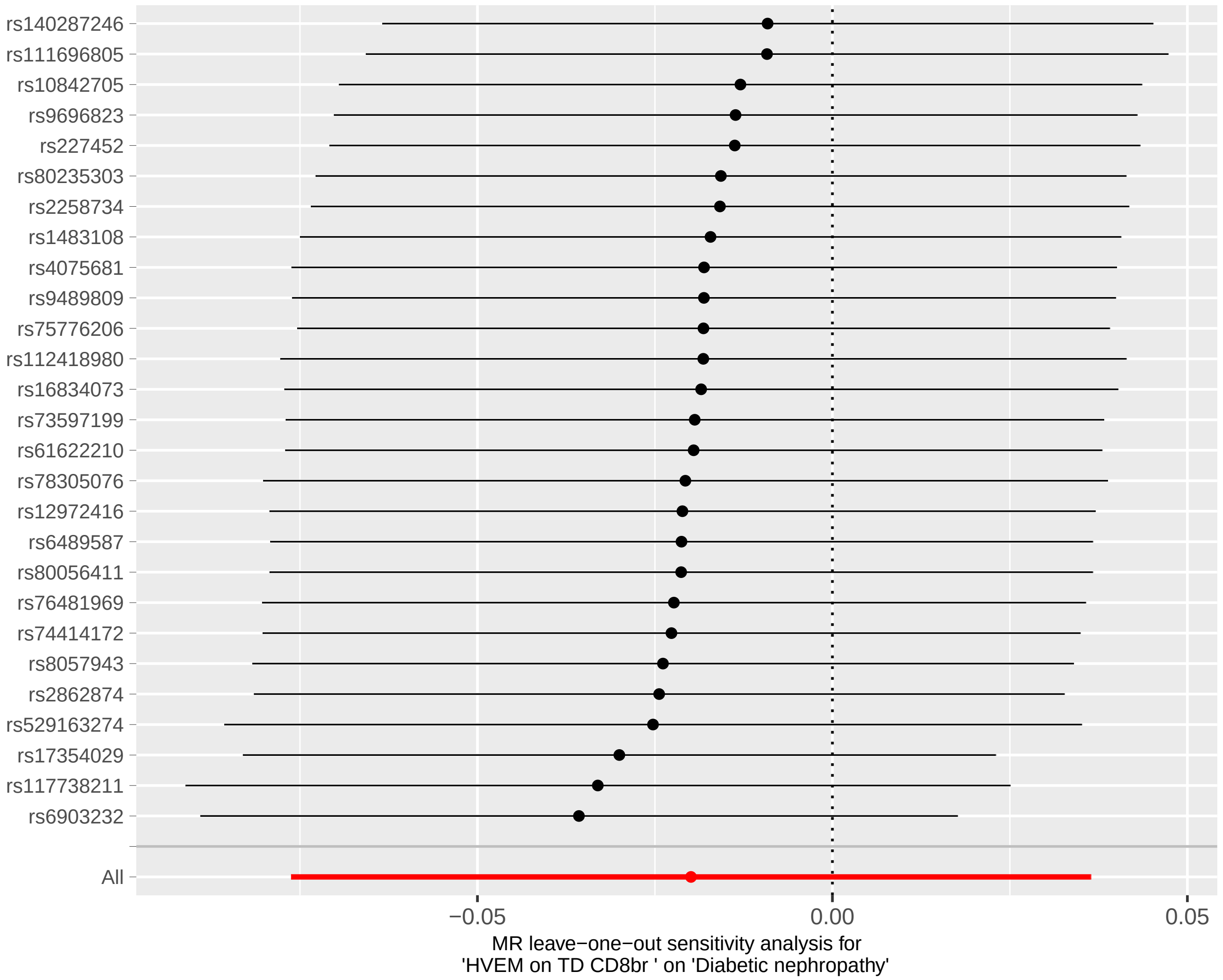
rs140287246
rs111696805
rs10842705
rs9696823
rs227452
rs80235303
rs2258734
rs1483108
rs4075681
rs9489809
rs75776206
rs112418980
rs16834073
rs73597199
rs61622210
rs78305076
rs12972416
rs6489587
rs80056411
rs76481969
rs74414172
rs8057943
rs2862874
rs529163274
rs17354029
rs117738211
rs6903232
All
−0.05
0.00
0.05
MR leave−one−out sensitivity analysis for
'HVEM on TD CD8br ' on 'Diabetic nephropathy'

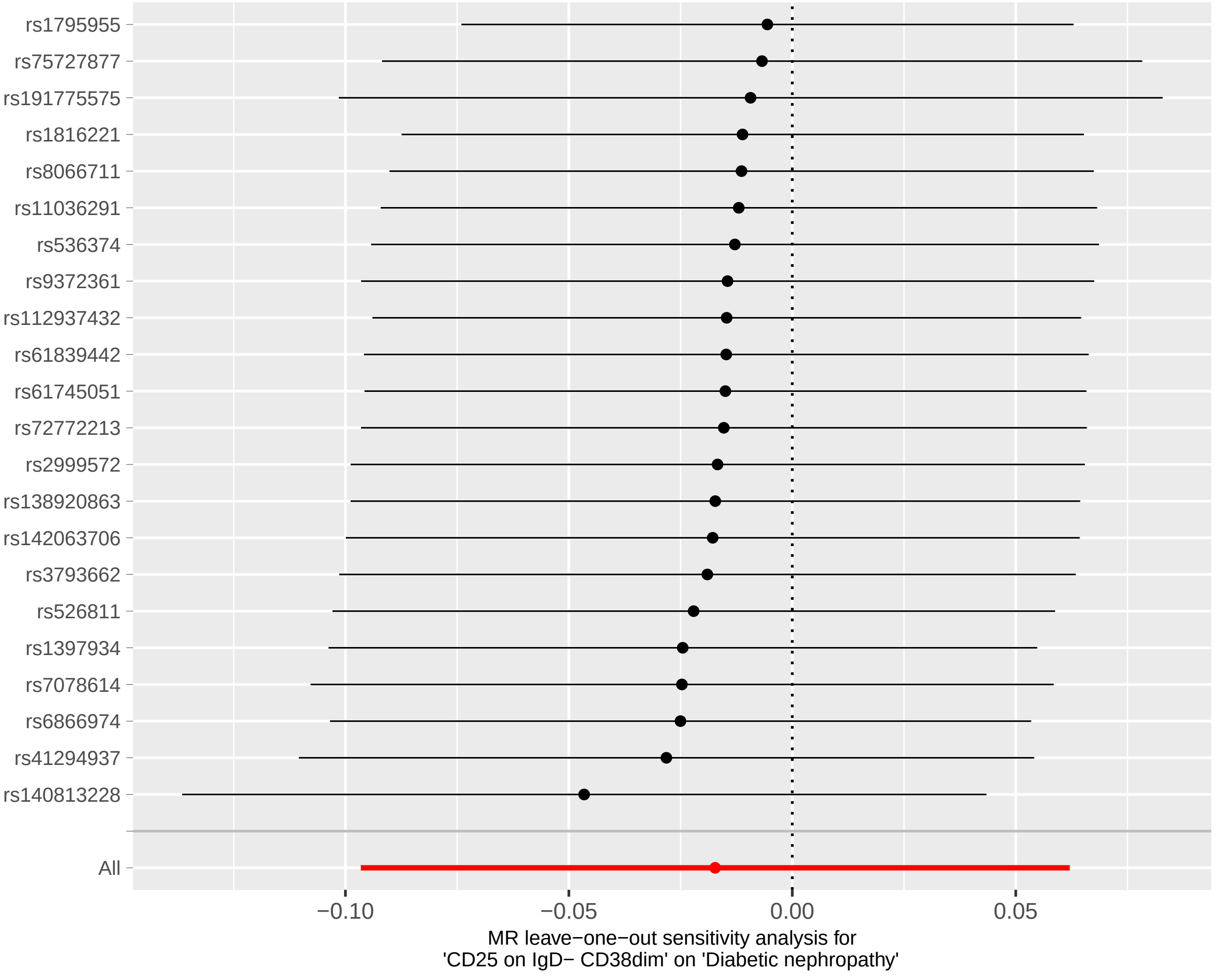
rs1795955
rs75727877
rs191775575
rs1816221
rs8066711
rs11036291
rs536374
rs9372361
rs112937432
rs61839442
rs61745051
rs72772213
rs2999572
rs138920863
rs142063706
rs3793662
rs526811
rs1397934
rs7078614
rs6866974
rs41294937
rs140813228
All
−0.10
−0.05
0.00
0.05
MR leave−one−out sensitivity analysis for
'CD25 on IgD− CD38dim' on 'Diabetic nephropathy'

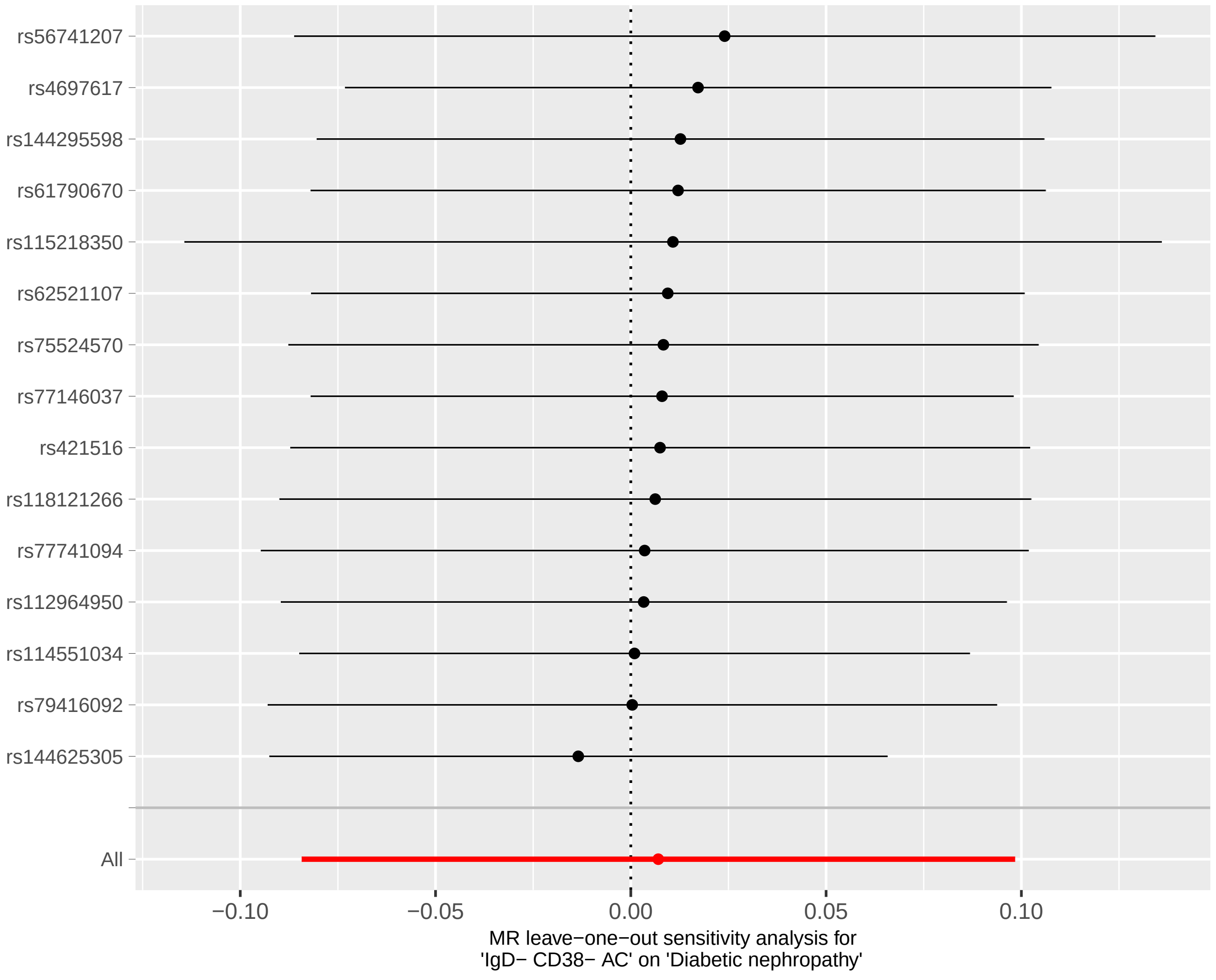
rs56741207
rs4697617
rs144295598
rs61790670
rs115218350
rs62521107
rs75524570
rs77146037
rs421516
rs118121266
rs77741094
rs112964950
rs114551034
rs79416092
rs144625305
All
−0.10
−0.05
0.00
0.05
0.10
MR leave−one−out sensitivity analysis for
'IgD− CD38− AC' on 'Diabetic nephropathy'

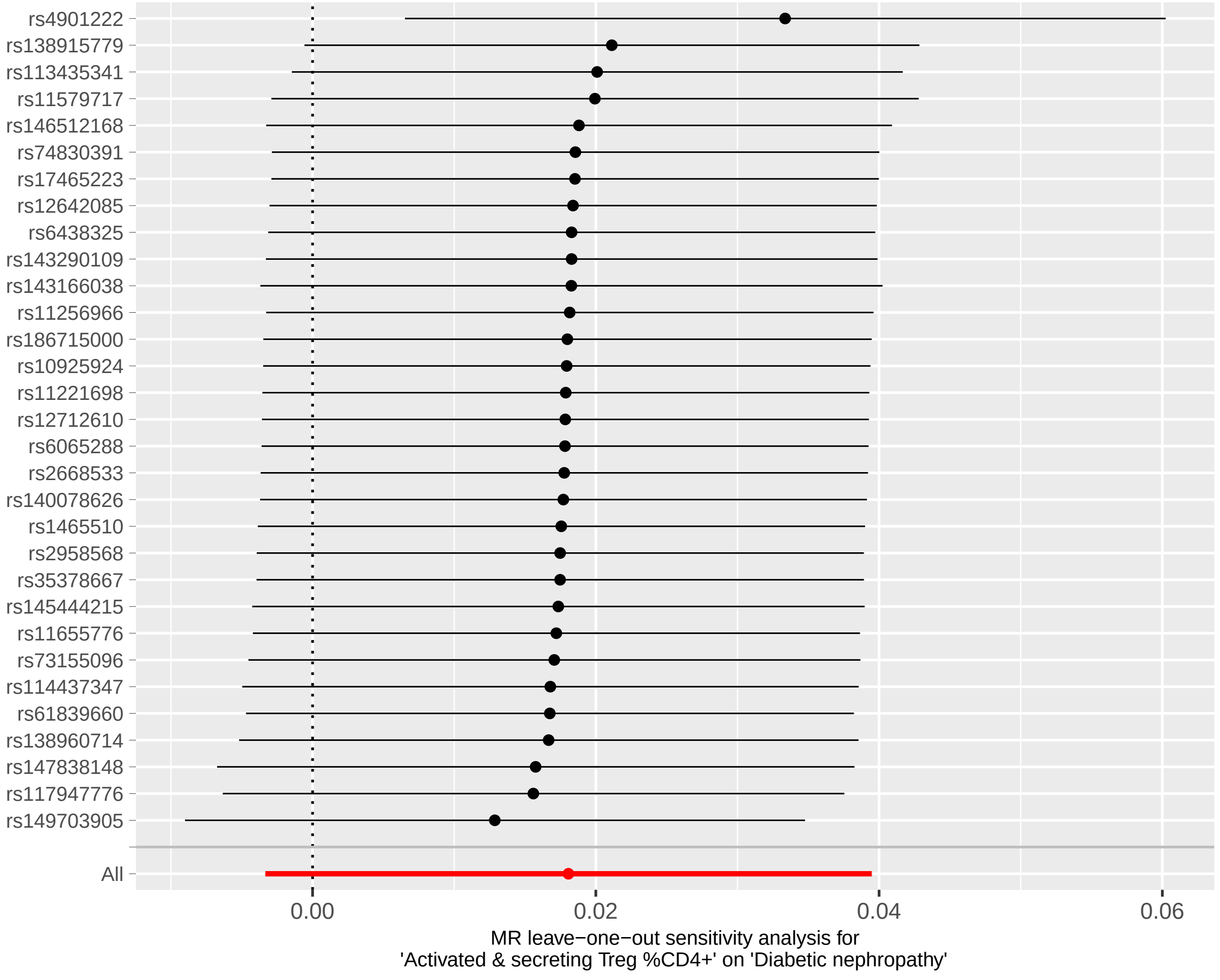
rs4901222
rs138915779
rs113435341
rs11579717
rs146512168
rs74830391
rs17465223
rs12642085
rs6438325
rs143290109
rs143166038
rs11256966
rs186715000
rs10925924
rs11221698
rs12712610
rs6065288
rs2668533
rs140078626
rs1465510
rs2958568
rs35378667
rs145444215
rs11655776
rs73155096
rs114437347
rs61839660
rs138960714
rs147838148
rs117947776
rs149703905
All
0.00
0.02
0.04
0.06
MR leave−one−out sensitivity analysis for
'Activated & secreting Treg %CD4+' on 'Diabetic nephropathy'

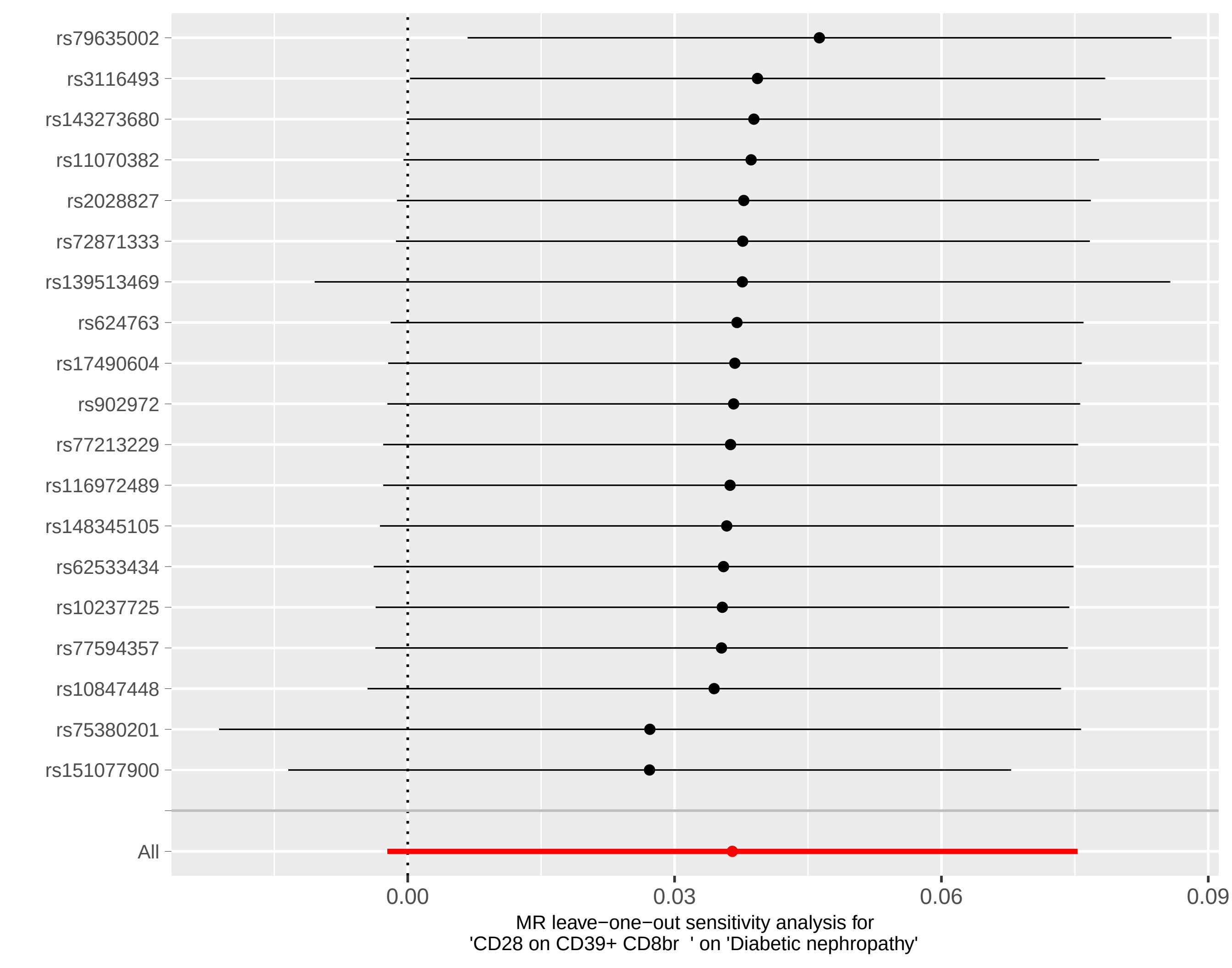
rs79635002
rs3116493
rs143273680
rs11070382
rs2028827
rs72871333
rs139513469
rs624763
rs17490604
rs902972
rs77213229
rs116972489
rs148345105
rs62533434
rs10237725
rs77594357
rs10847448
rs75380201
rs151077900
All
0.00
0.03
0.06
0.09
MR leave−one−out sensitivity analysis for
'CD28 on CD39+ CD8br ' on 'Diabetic nephropathy'

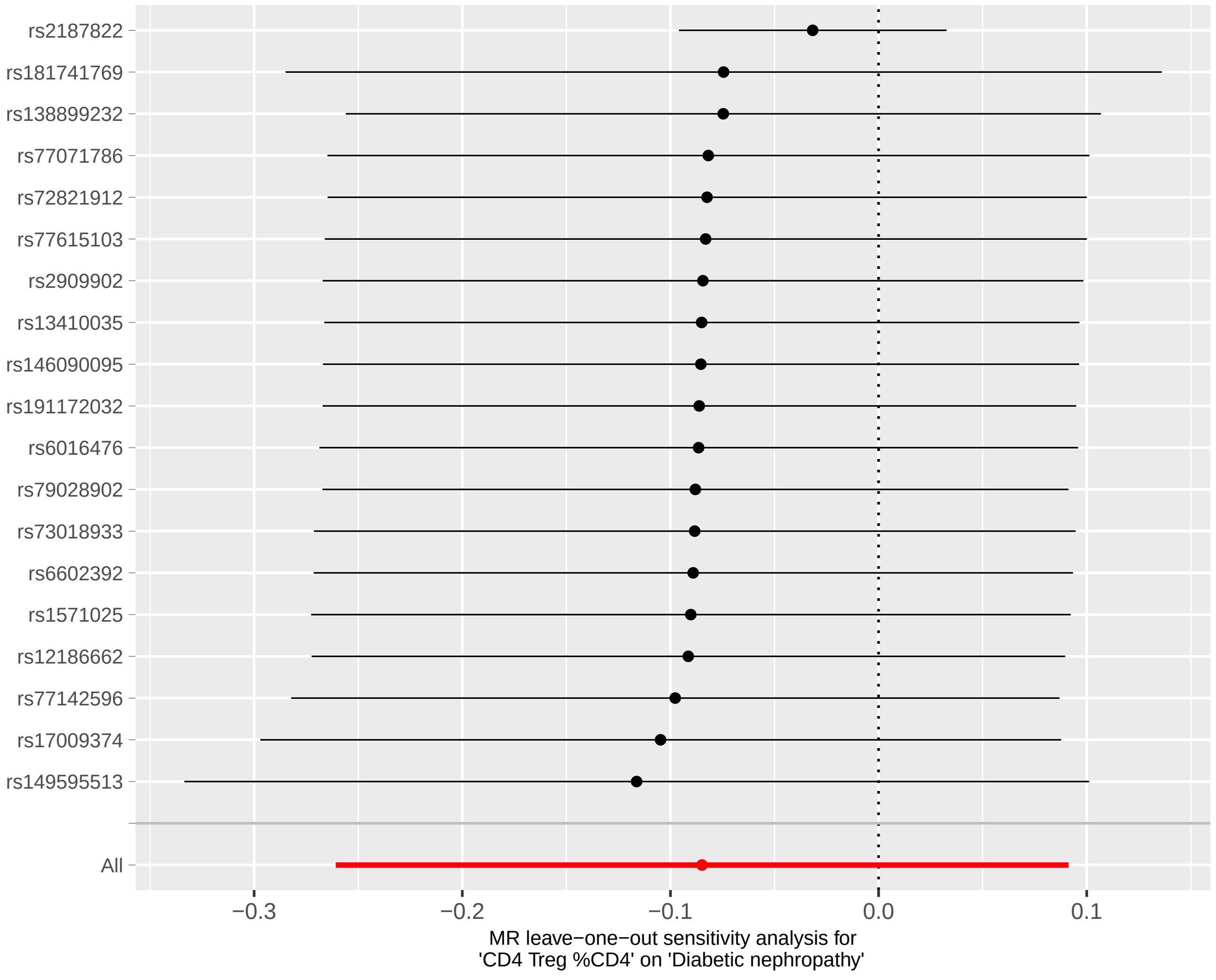
rs2187822
rs181741769
rs138899232
rs77071786
rs72821912
rs77615103
rs2909902
rs13410035
rs146090095
rs191172032
rs6016476
rs79028902
rs73018933
rs6602392
rs1571025
rs12186662
rs77142596
rs17009374
rs149595513
All
−0.3
−0.2
−0.1
0.0
0.1
MR leave−one−out sensitivity analysis for
'CD4 Treg %CD4' on 'Diabetic nephropathy'

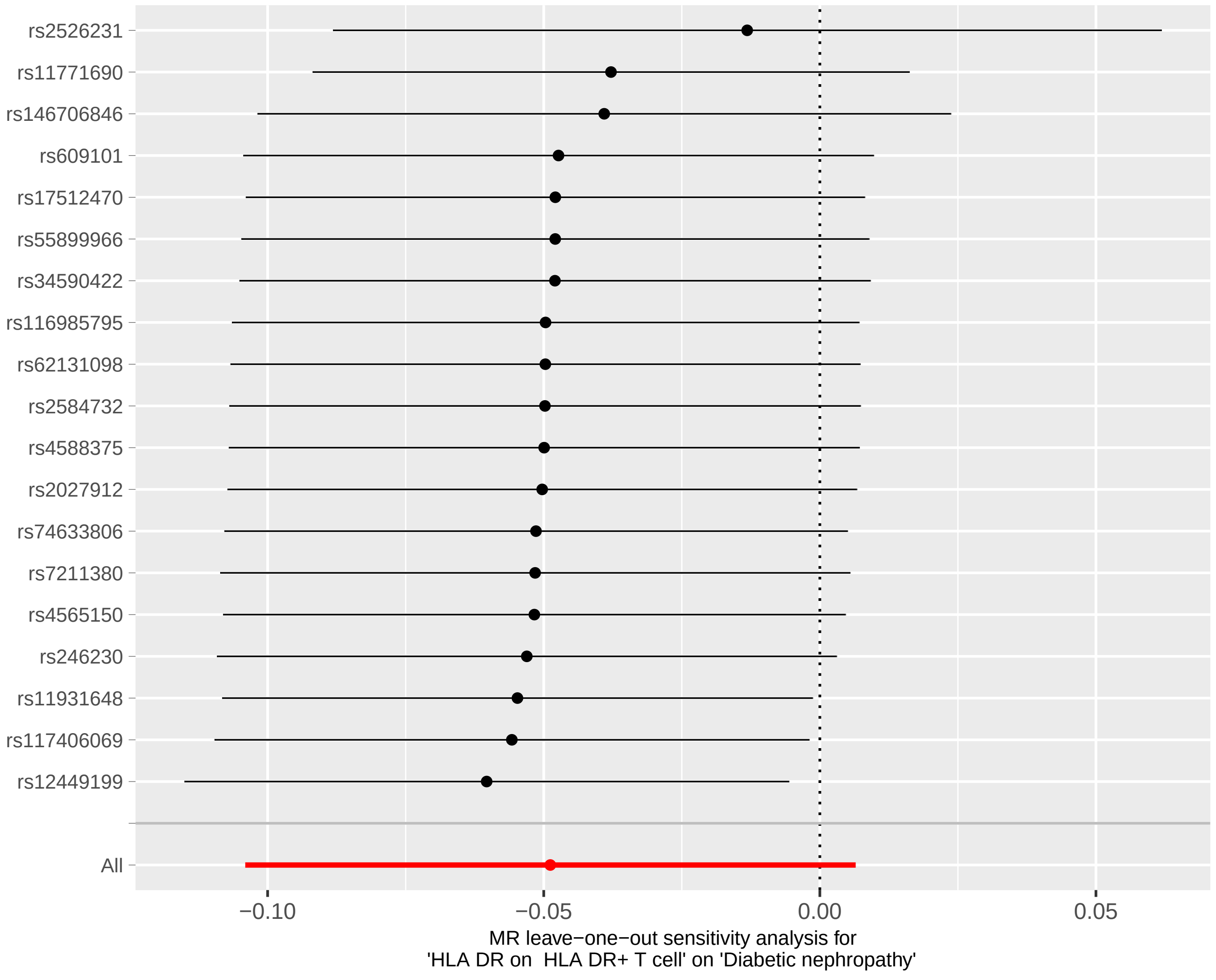
rs2526231
rs11771690
rs146706846
rs609101
rs17512470
rs55899966
rs34590422
rs116985795
rs62131098
rs2584732
rs4588375
rs2027912
rs74633806
rs7211380
rs4565150
rs246230
rs11931648
rs117406069
rs12449199
All
−0.10
−0.05
0.00
0.05
MR leave−one−out sensitivity analysis for
'HLA DR on HLA DR+ T cell' on 'Diabetic nephropathy'

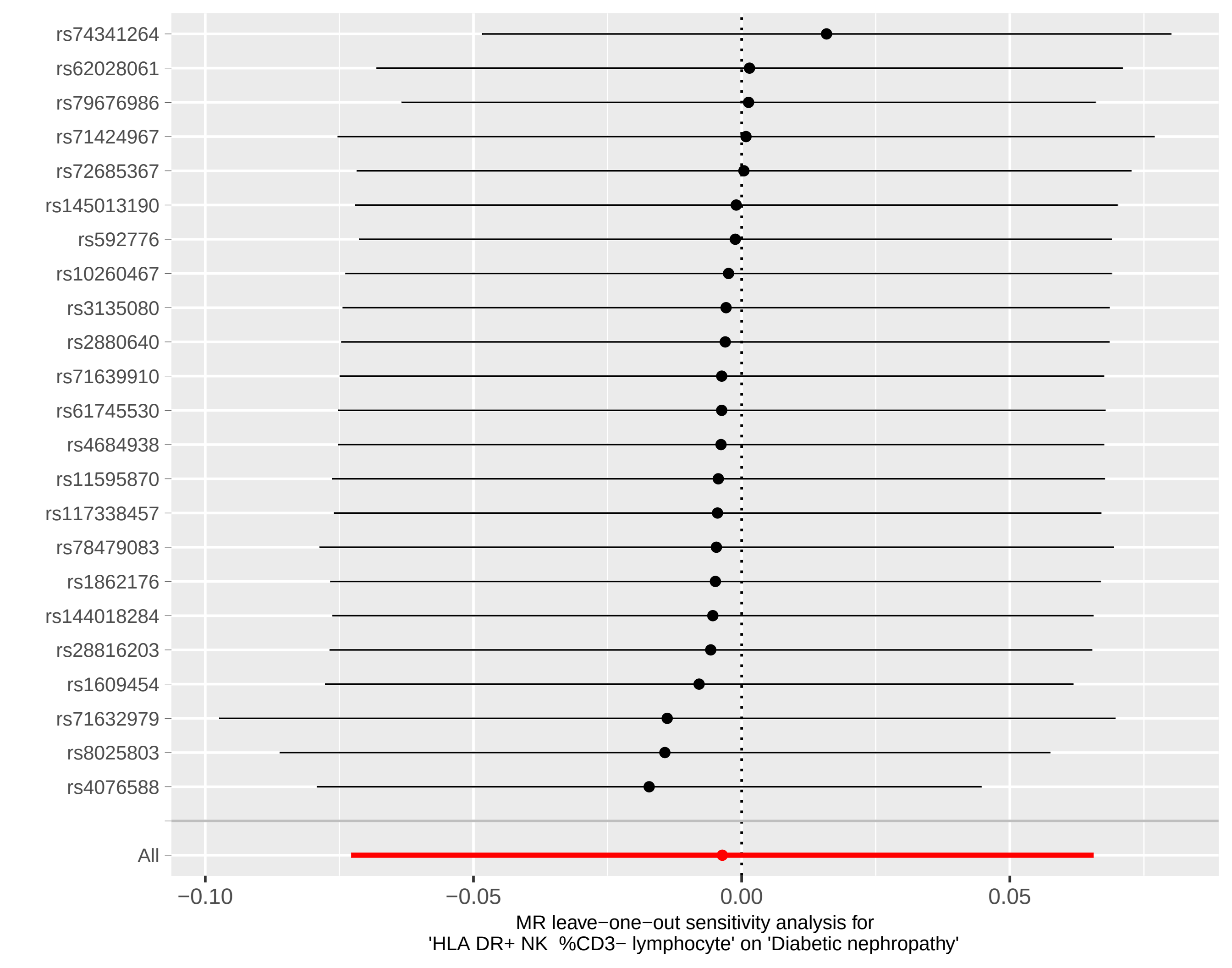
rs74341264
rs62028061
rs79676986
rs71424967
rs72685367
rs145013190
rs592776
rs10260467
rs3135080
rs2880640
rs71639910
rs61745530
rs4684938
rs11595870
rs117338457
rs78479083
rs1862176
rs144018284
rs28816203
rs1609454
rs71632979
rs8025803
rs4076588
All
−0.10
−0.05
0.00
0.05
MR leave−one−out sensitivity analysis for
'HLA DR+ NK %CD3− lymphocyte' on 'Diabetic nephropathy'

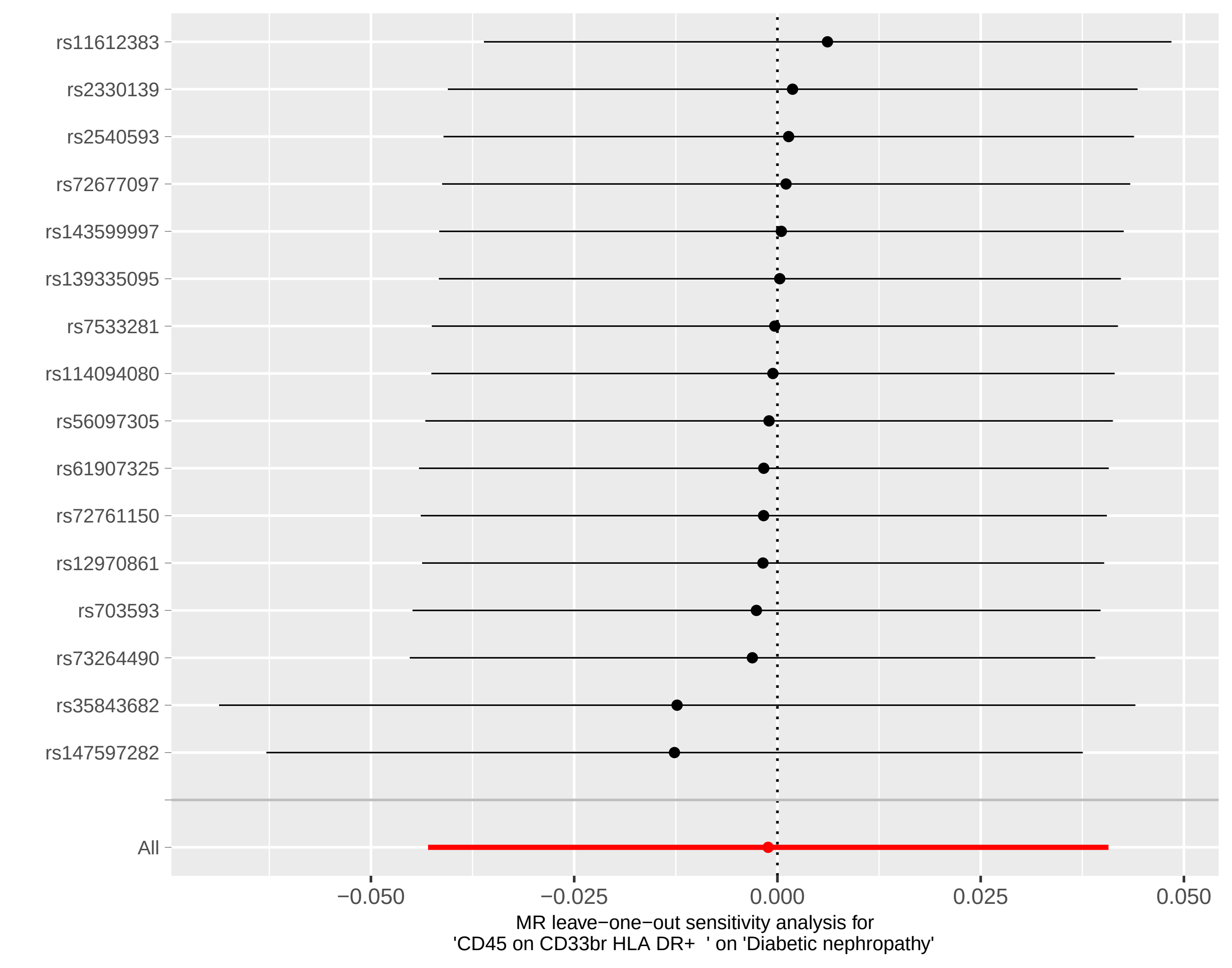
rs11612383
rs2330139
rs2540593
rs72677097
rs143599997
rs139335095
rs7533281
rs114094080
rs56097305
rs61907325
rs72761150
rs12970861
rs703593
rs73264490
rs35843682
rs147597282
All
−0.050
−0.025
0.000
0.025
0.050
MR leave−one−out sensitivity analysis for
'CD45 on CD33br HLA DR+ ' on 'Diabetic nephropathy'

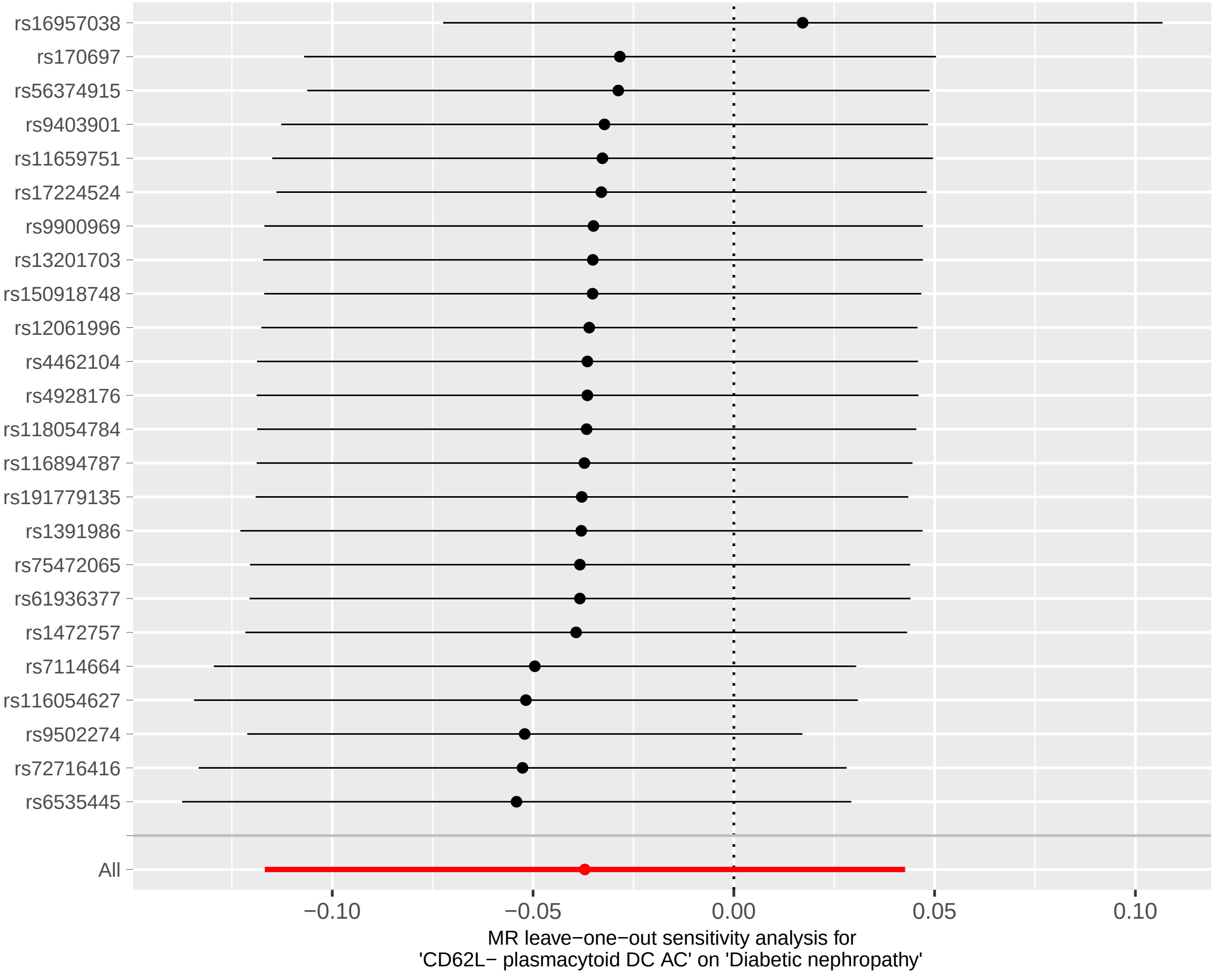

rs16957038
rs170697
rs56374915
rs9403901
rs11659751
rs17224524
rs9900969
rs13201703
rs150918748
rs12061996
rs4462104
rs4928176
rs118054784
rs116894787
rs191779135
rs1391986
rs75472065
rs61936377
rs1472757
rs7114664
rs116054627
rs9502274
rs72716416
rs6535445
All
−0.10
−0.05
0.00
0.05
0.10
MR leave−one−out sensitivity analysis for
'CD62L− plasmacytoid DC AC' on 'Diabetic nephropathy'

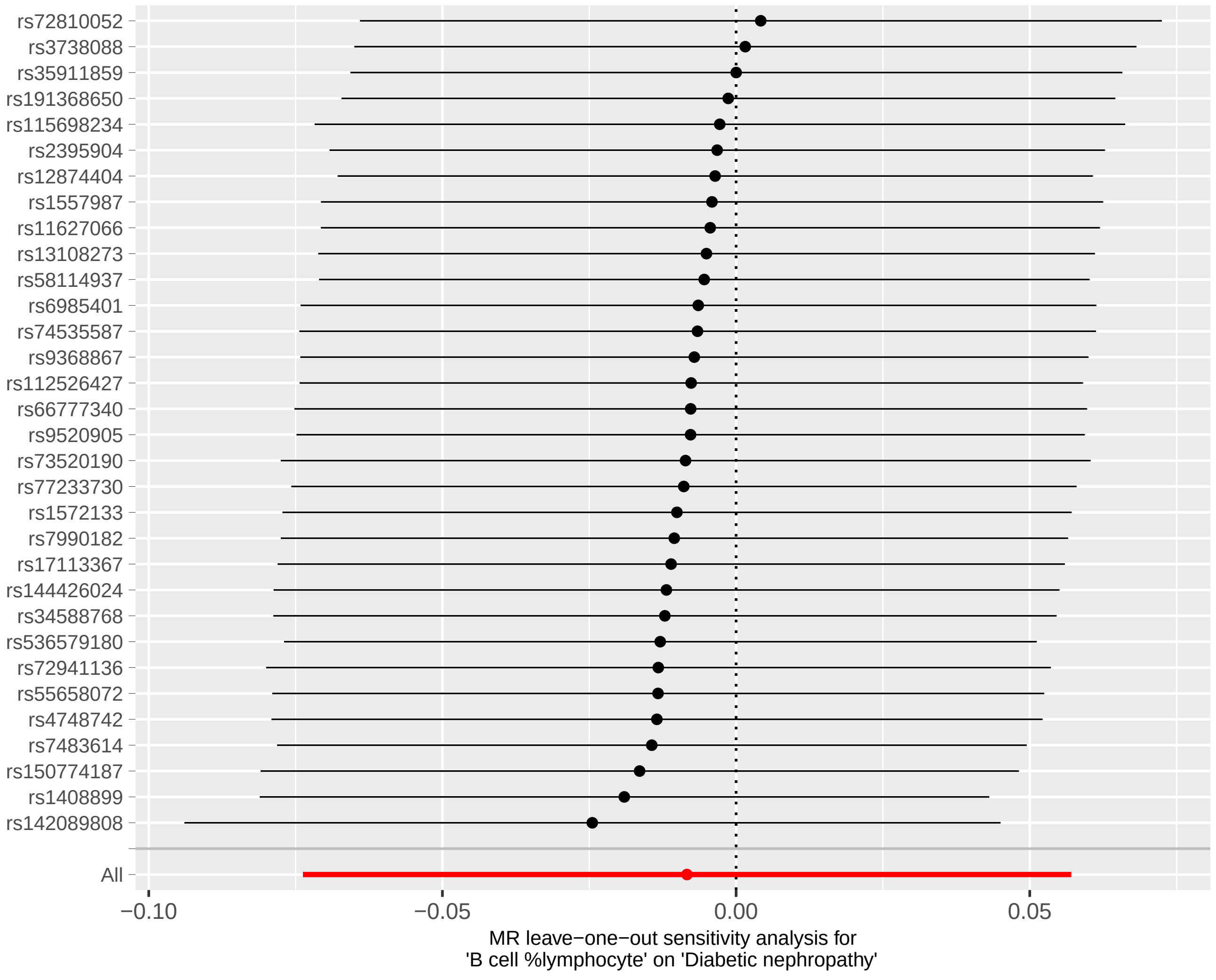
rs72810052
rs3738088
rs35911859
rs191368650
rs115698234
rs2395904
rs12874404
rs1557987
rs11627066
rs13108273
rs58114937
rs6985401
rs74535587
rs9368867
rs112526427
rs66777340
rs9520905
rs73520190
rs77233730
rs1572133
rs7990182
rs17113367
rs144426024
rs34588768
rs536579180
rs72941136
rs55658072
rs4748742
rs7483614
rs150774187
rs1408899
rs142089808
All
−0.10
−0.05
0.00
0.05
MR leave−one−out sensitivity analysis for
'B cell %lymphocyte' on 'Diabetic nephropathy'

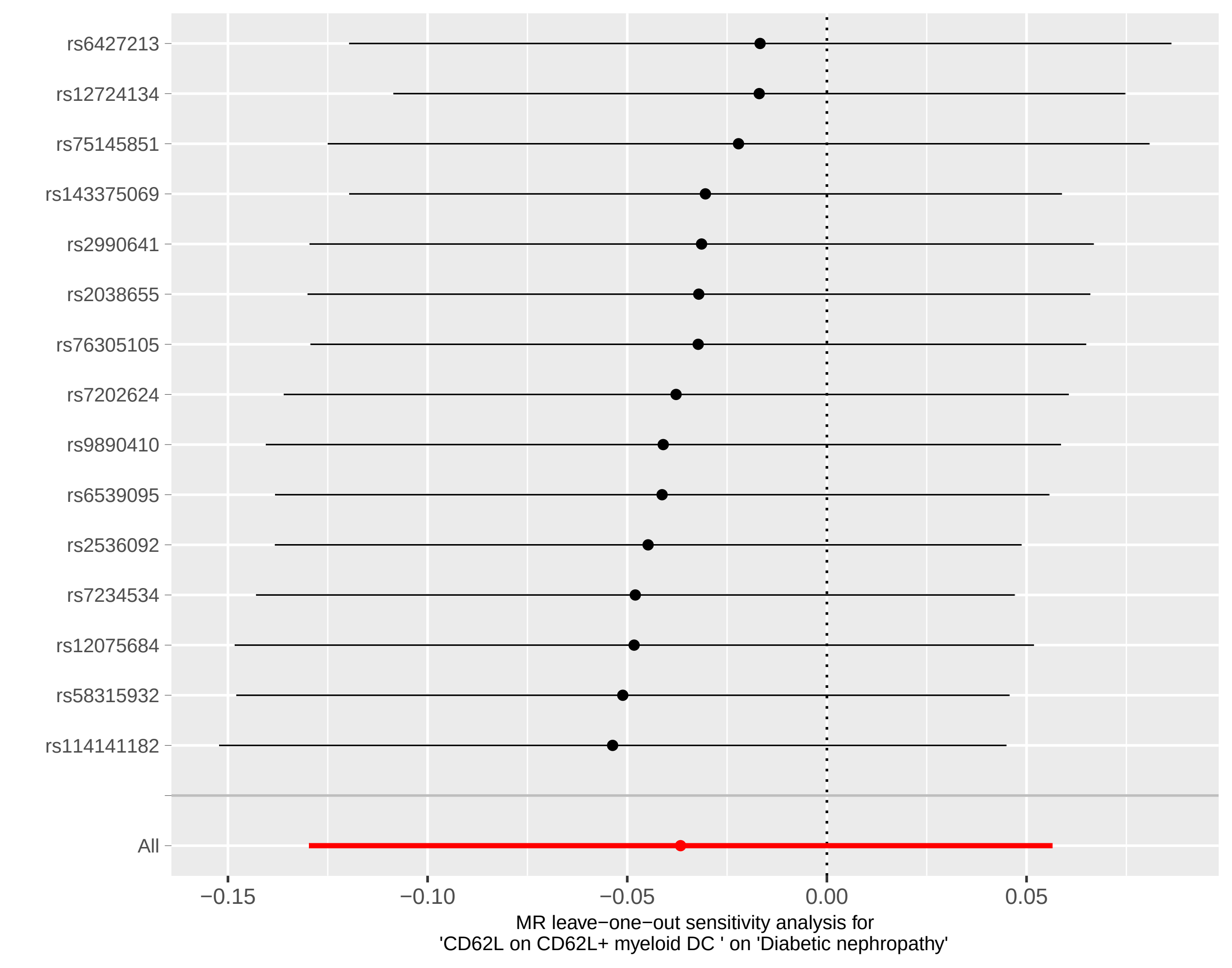
rs6427213
rs12724134
rs75145851
rs143375069
rs2990641
rs2038655
rs76305105
rs7202624
rs9890410
rs6539095
rs2536092
rs7234534
rs12075684
rs58315932
rs114141182
All
−0.15
−0.10
−0.05
0.00
0.05
MR leave−one−out sensitivity analysis for
'CD62L on CD62L+ myeloid DC ' on 'Diabetic nephropathy'

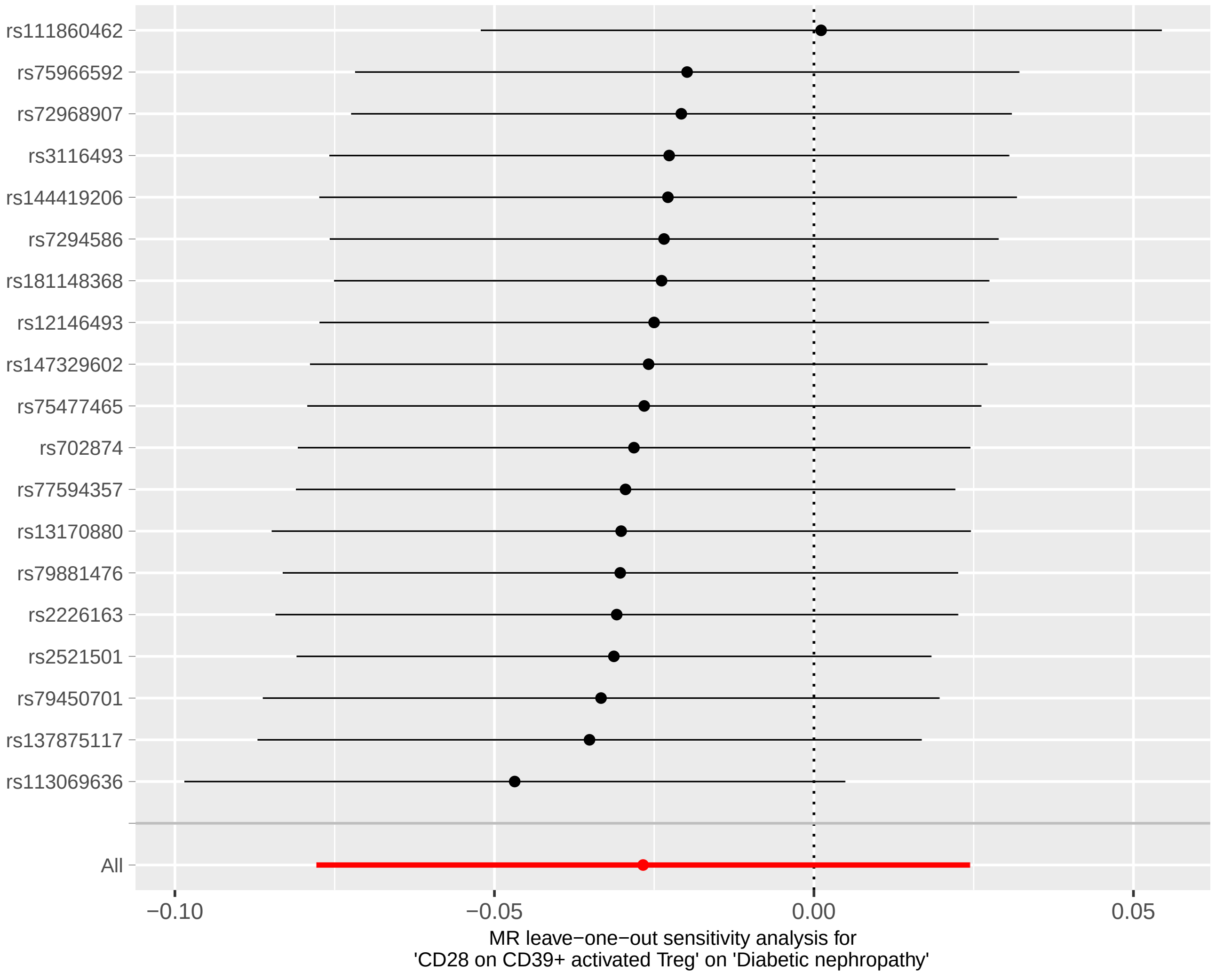
rs111860462
rs75966592
rs72968907
rs3116493
rs144419206
rs7294586
rs181148368
rs12146493
rs147329602
rs75477465
rs702874
rs77594357
rs13170880
rs79881476
rs2226163
rs2521501
rs79450701
rs137875117
rs113069636
All
−0.10
−0.05
0.00
0.05
MR leave−one−out sensitivity analysis for
'CD28 on CD39+ activated Treg' on 'Diabetic nephropathy'

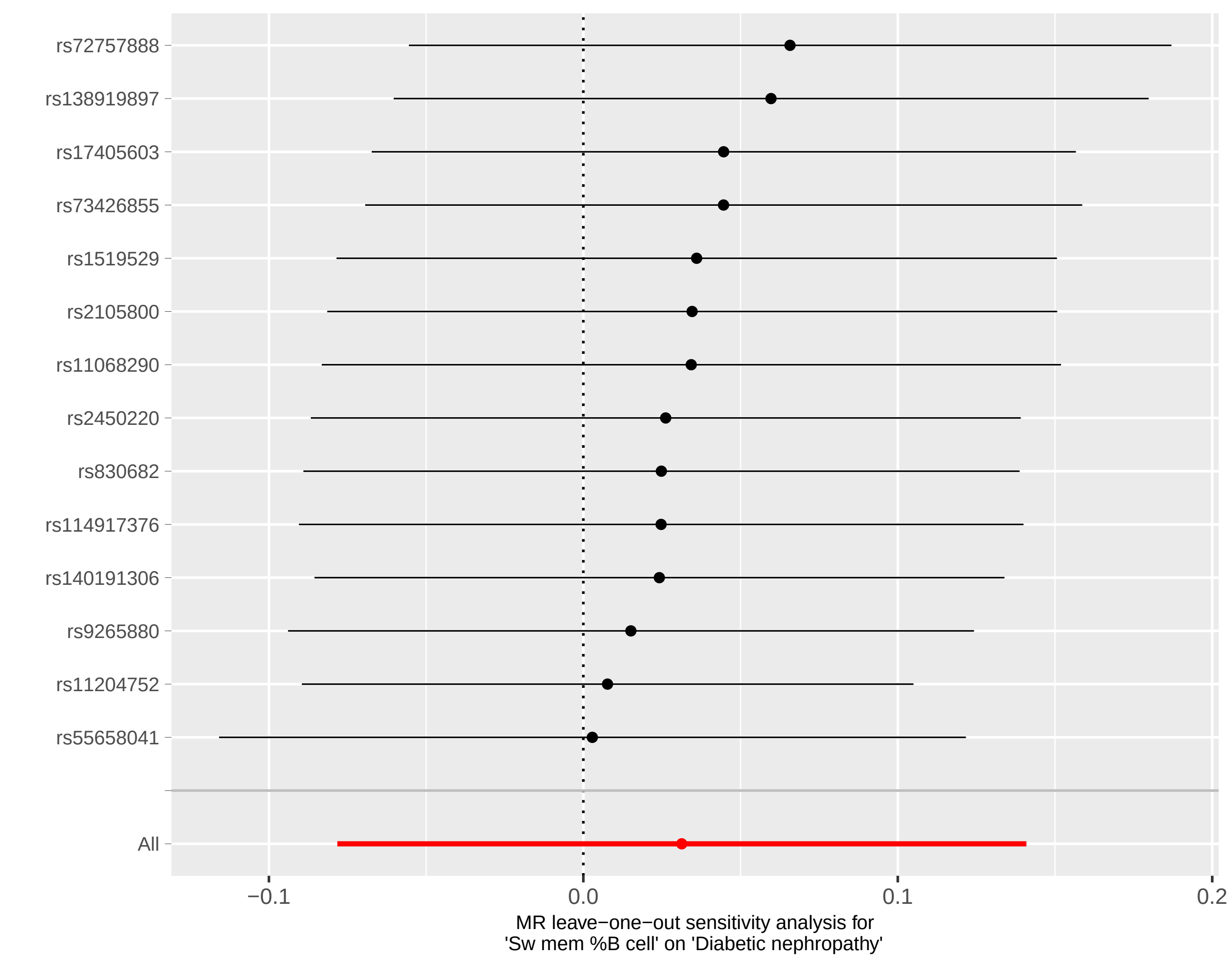
rs72757888
rs138919897
rs17405603
rs73426855
rs1519529
rs2105800
rs11068290
rs2450220
rs830682
rs114917376
rs140191306
rs9265880
rs11204752
rs55658041
All
−0.1
0.0
0.1
0.2
MR leave−one−out sensitivity analysis for
'Sw mem %B cell' on 'Diabetic nephropathy'

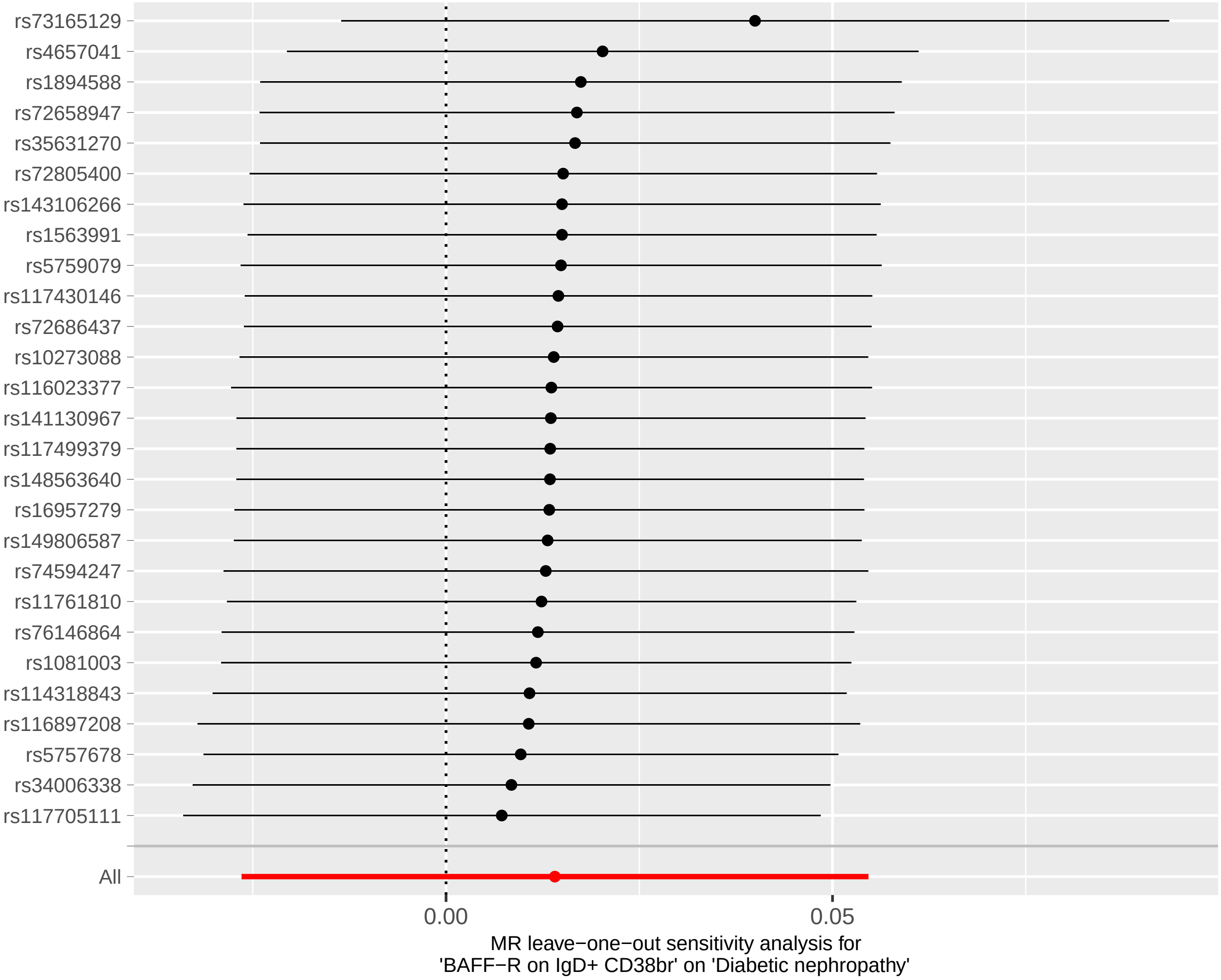
rs73165129
rs4657041
rs1894588
rs72658947
rs35631270
rs72805400
rs143106266
rs1563991
rs5759079
rs117430146
rs72686437
rs10273088
rs116023377
rs141130967
rs117499379
rs148563640
rs16957279
rs149806587
rs74594247
rs11761810
rs76146864
rs1081003
rs114318843
rs116897208
rs5757678
rs34006338
rs117705111
All
0.00
0.05
MR leave−one−out sensitivity analysis for
'BAFF−R on IgD+ CD38br' on 'Diabetic nephropathy'

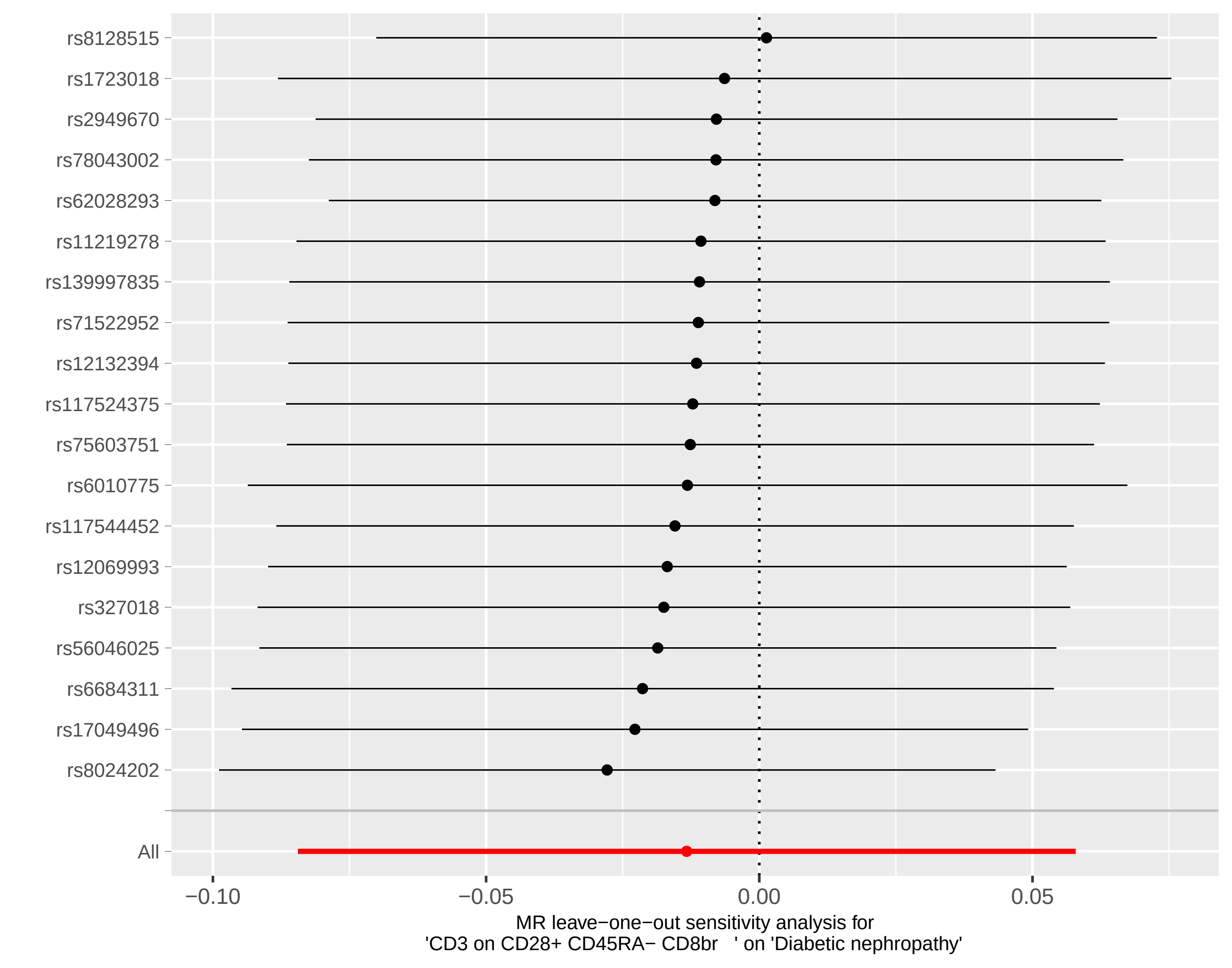
rs8128515
rs1723018
rs2949670
rs78043002
rs62028293
rs11219278
rs139997835
rs71522952
rs12132394
rs117524375
rs75603751
rs6010775
rs117544452
rs12069993
rs327018
rs56046025
rs6684311
rs17049496
rs8024202
All
−0.10
−0.05
0.00
0.05
MR leave−one−out sensitivity analysis for
'CD3 on CD28+ CD45RA− CD8br ' on 'Diabetic nephropathy'

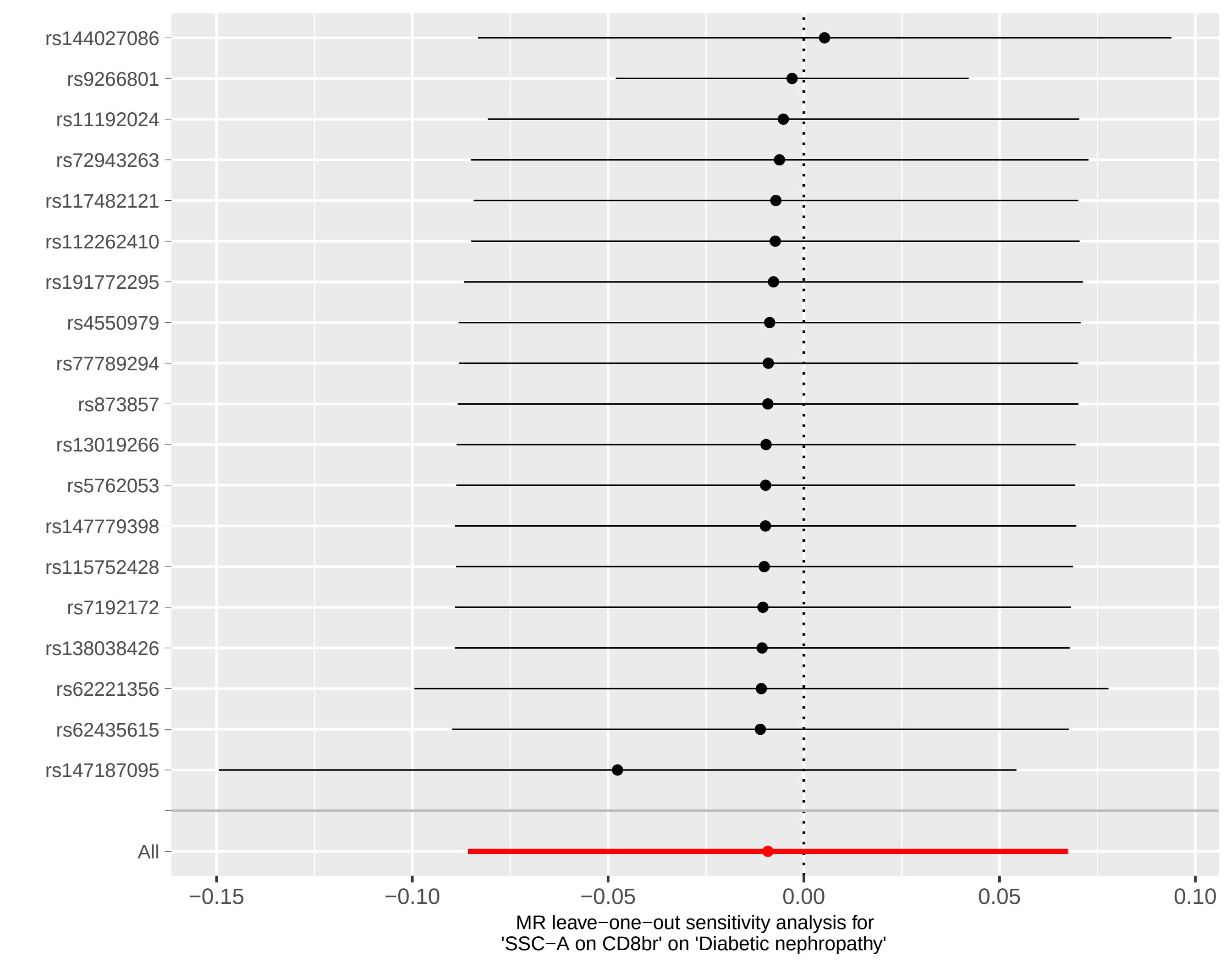
rs144027086
rs9266801
rs11192024
rs72943263
rs117482121
rs112262410
rs191772295
rs4550979
rs77789294
rs873857
rs13019266
rs5762053
rs147779398
rs115752428
rs7192172
rs138038426
rs62221356
rs62435615
rs147187095
All
−0.15
−0.10
−0.05
0.00
0.05
0.10
MR leave−one−out sensitivity analysis for
'SSC−A on CD8br' on 'Diabetic nephropathy'

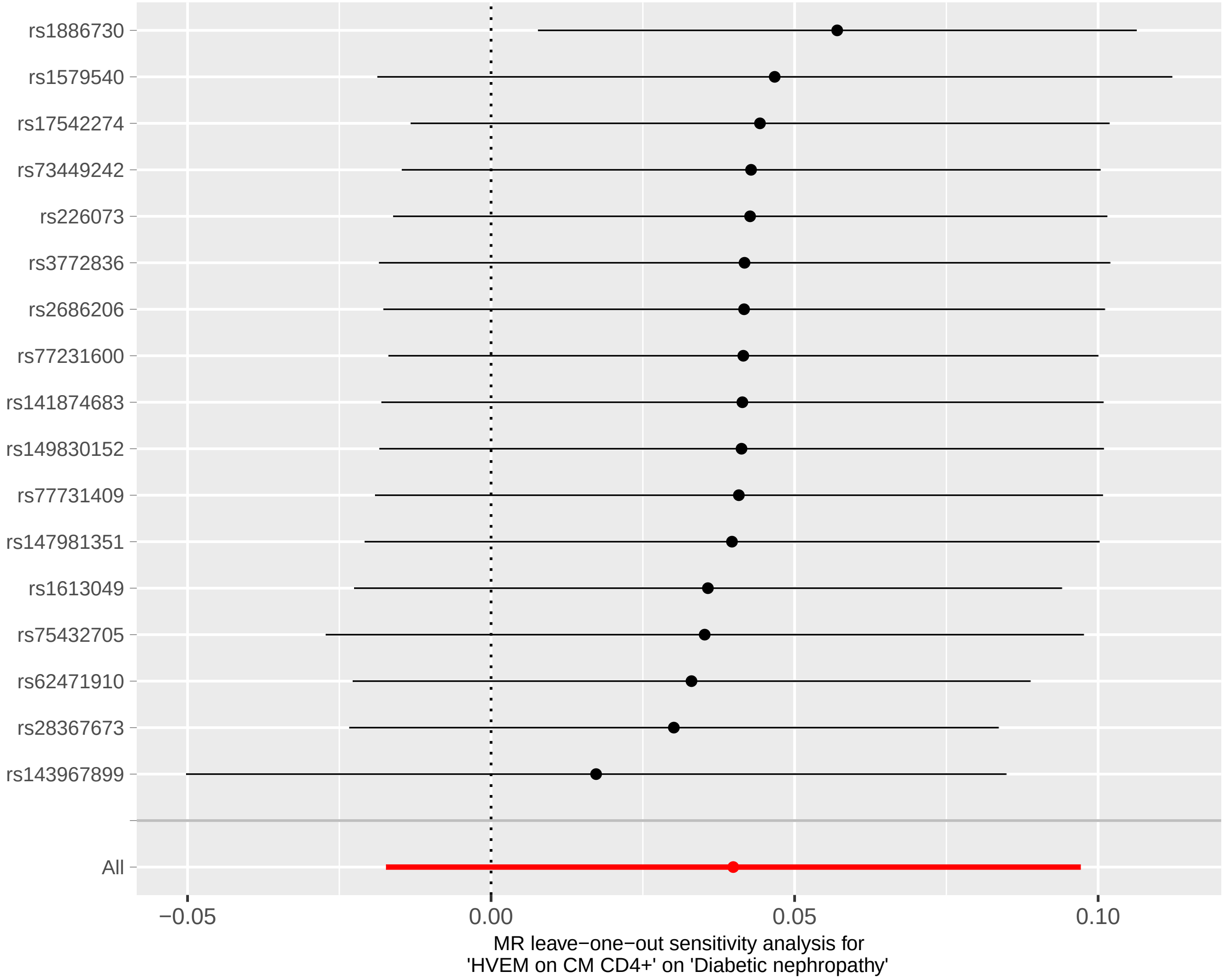
rs1886730
rs1579540
rs17542274
rs73449242
rs226073
rs3772836
rs2686206
rs77231600
rs141874683
rs149830152
rs77731409
rs147981351
rs1613049
rs75432705
rs62471910
rs28367673
rs143967899
All
−0.05
0.00
0.05
0.10
MR leave−one−out sensitivity analysis for
'HVEM on CM CD4+' on 'Diabetic nephropathy'

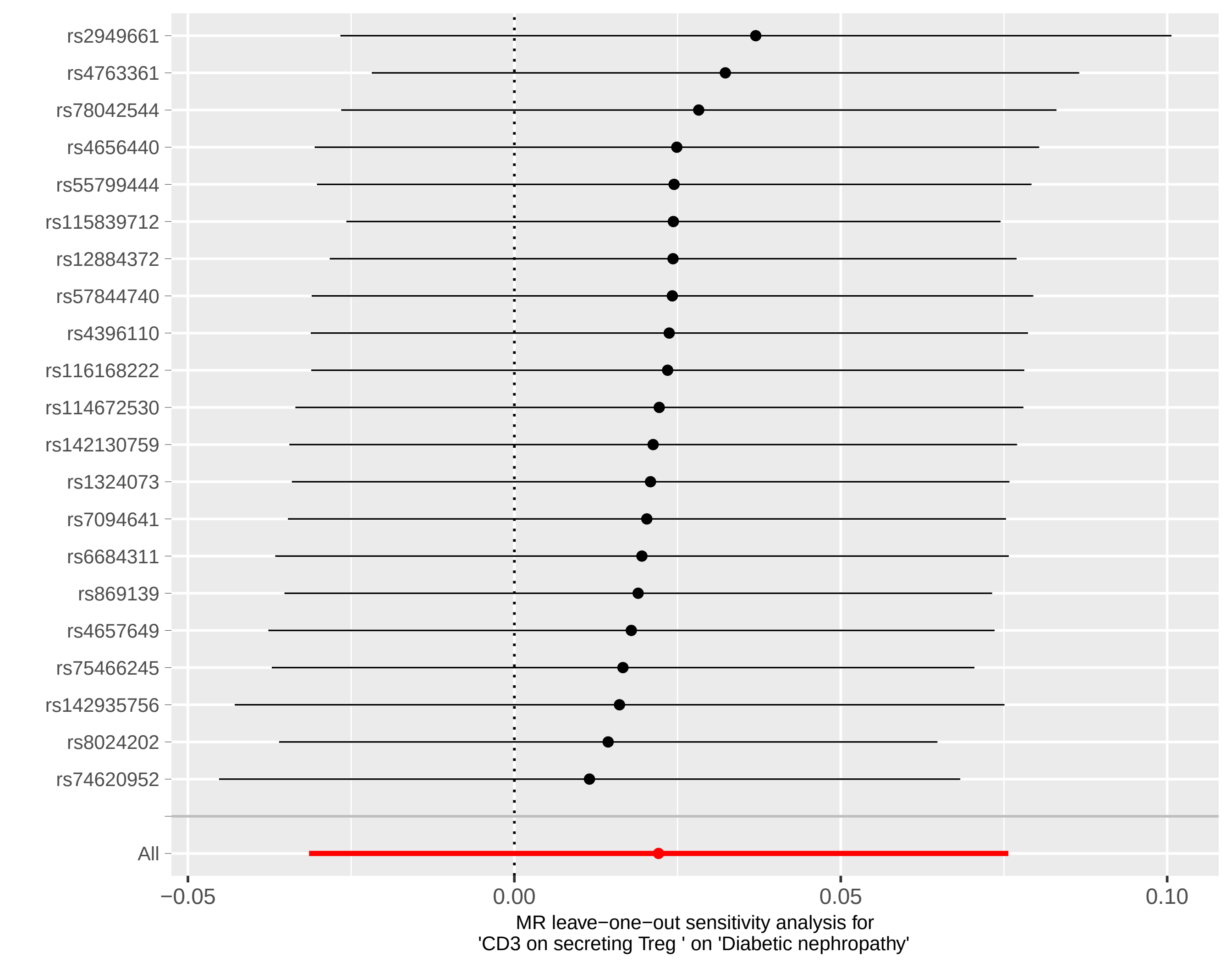
rs2949661
rs4763361
rs78042544
rs4656440
rs55799444
rs115839712
rs12884372
rs57844740
rs4396110
rs116168222
rs114672530
rs142130759
rs1324073
rs7094641
rs6684311
rs869139
rs4657649
rs75466245
rs142935756
rs8024202
rs74620952
All
−0.05
0.00
0.05
0.10
MR leave−one−out sensitivity analysis for
'CD3 on secreting Treg ' on 'Diabetic nephropathy'

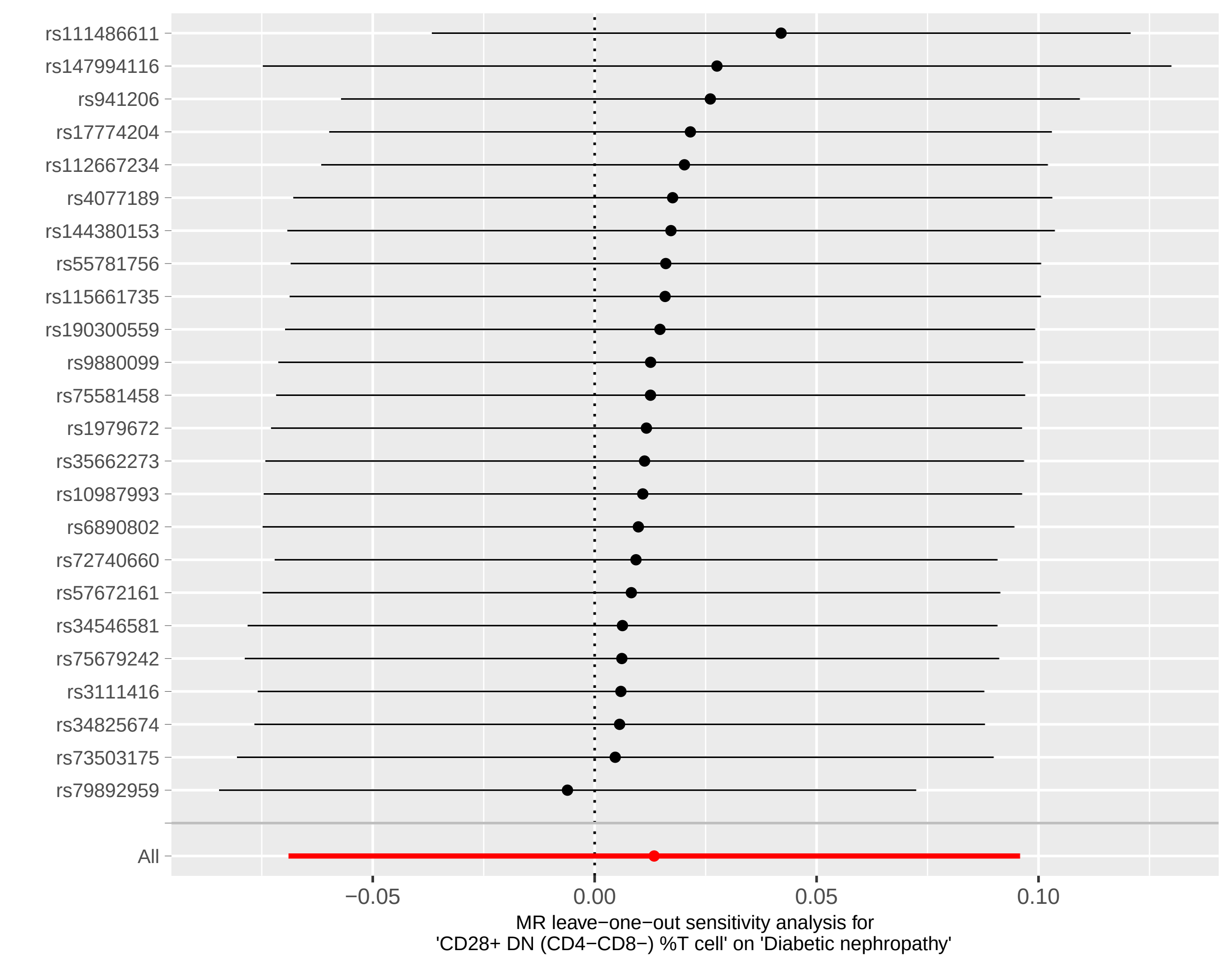
rs111486611
rs147994116
rs941206
rs17774204
rs112667234
rs4077189
rs144380153
rs55781756
rs115661735
rs190300559
rs9880099
rs75581458
rs1979672
rs35662273
rs10987993
rs6890802
rs72740660
rs57672161
rs34546581
rs75679242
rs3111416
rs34825674
rs73503175
rs79892959
All
−0.05
0.00
0.05
0.10
MR leave−one−out sensitivity analysis for
'CD28+ DN (CD4−CD8−) %T cell' on 'Diabetic nephropathy'

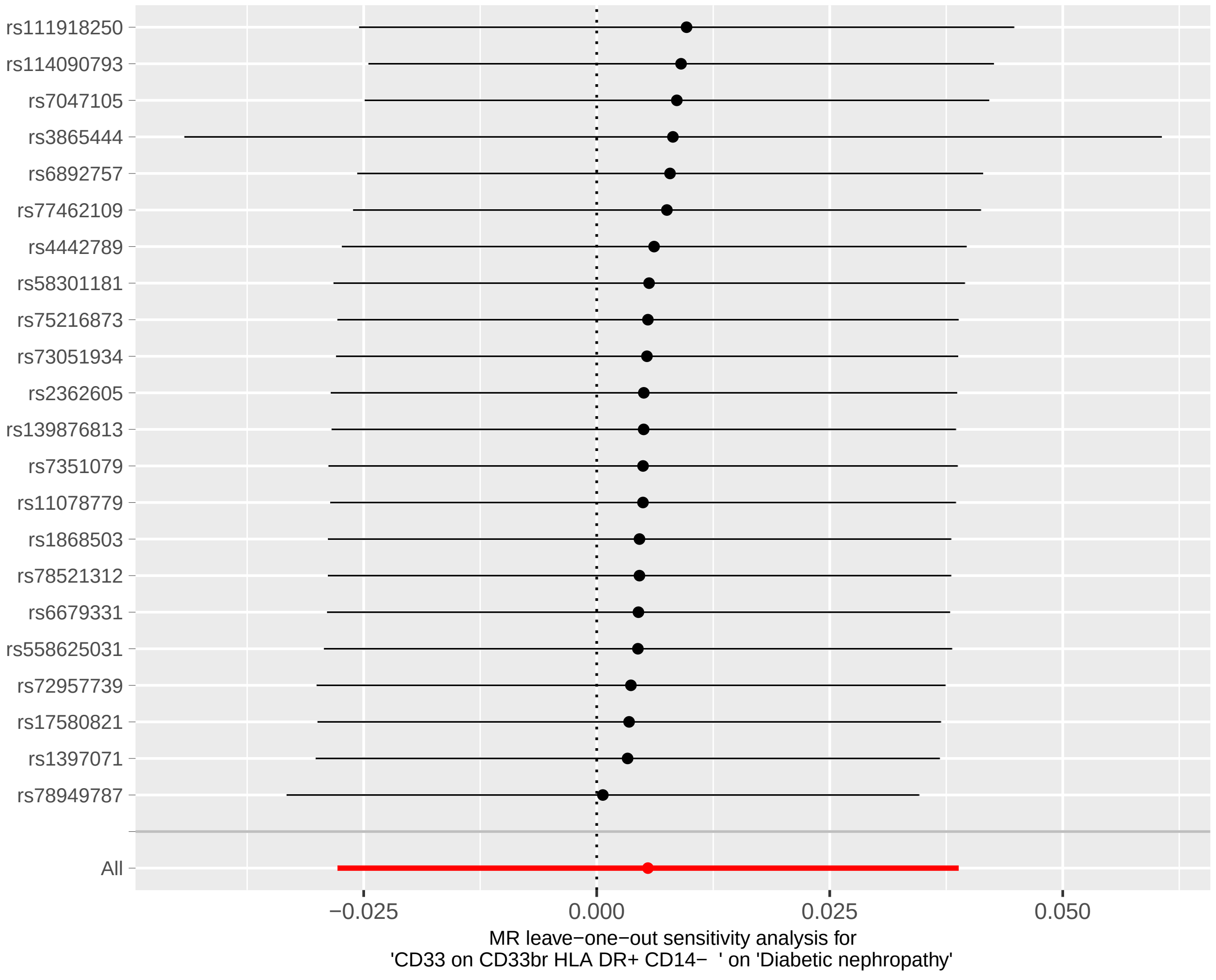
rs111918250
rs114090793
rs7047105
rs3865444
rs6892757
rs77462109
rs4442789
rs58301181
rs75216873
rs73051934
rs2362605
rs139876813
rs7351079
rs11078779
rs1868503
rs78521312
rs6679331
rs558625031
rs72957739
rs17580821
rs1397071
rs78949787
All
−0.025
0.000
0.025
0.050
MR leave−one−out sensitivity analysis for
'CD33 on CD33br HLA DR+ CD14− ' on 'Diabetic nephropathy'

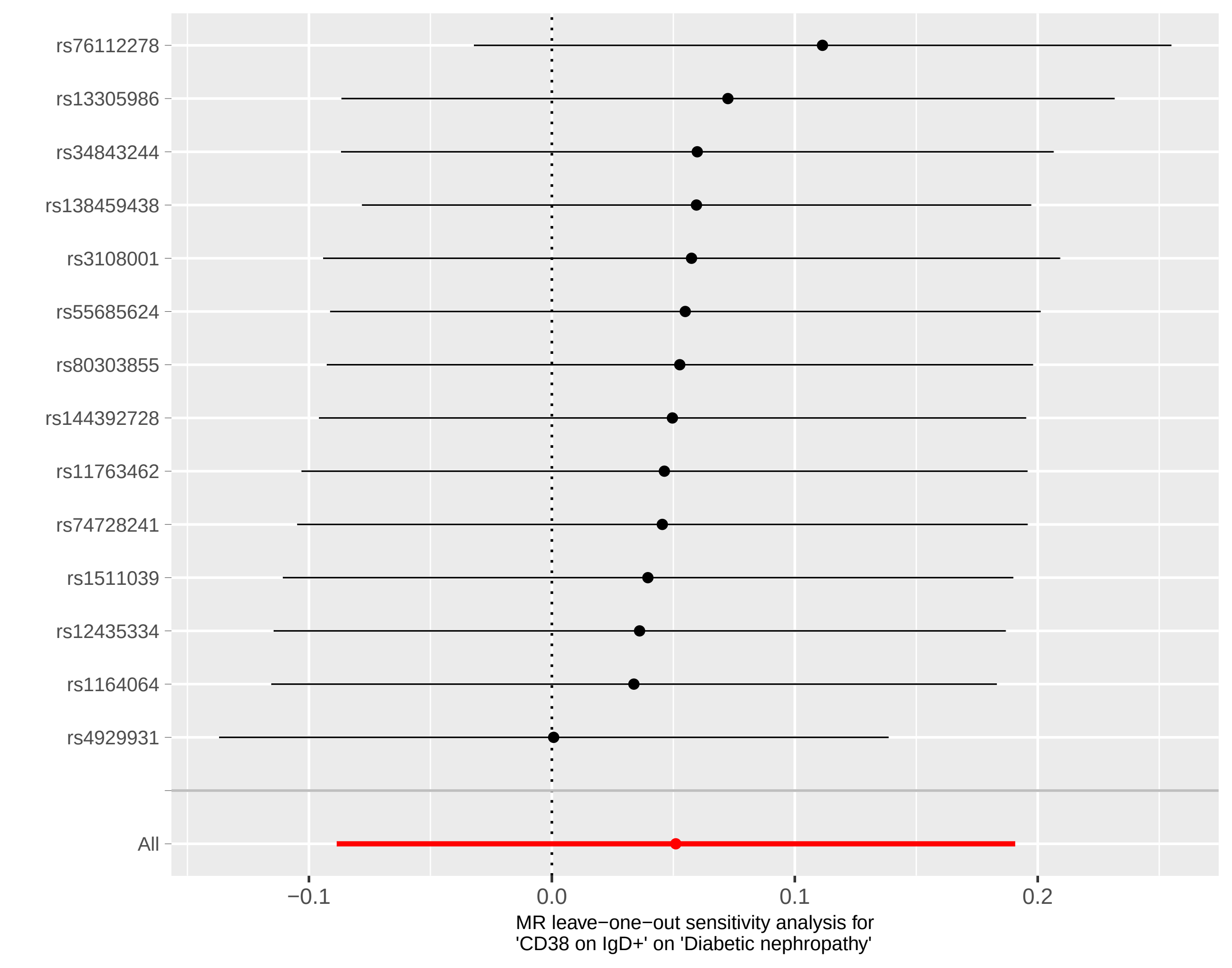
rs76112278
rs13305986
rs34843244
rs138459438
rs3108001
rs55685624
rs80303855
rs144392728
rs11763462
rs74728241
rs1511039
rs12435334
rs1164064
rs4929931
All
−0.1
0.0
0.1
0.2
MR leave−one−out sensitivity analysis for
'CD38 on IgD+' on 'Diabetic nephropathy'

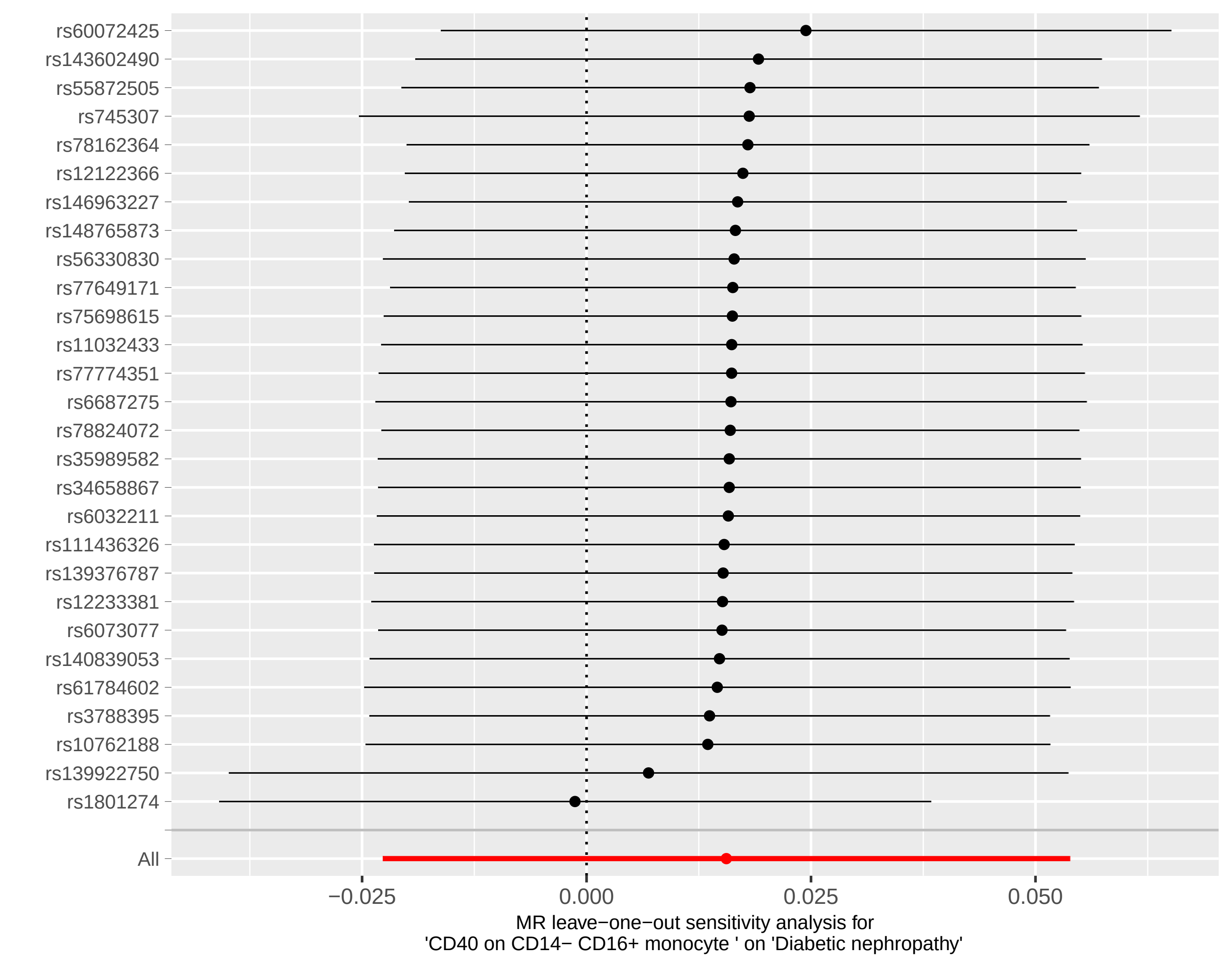

rs60072425
rs143602490
rs55872505
rs745307
rs78162364
rs12122366
rs146963227
rs148765873
rs56330830
rs77649171
rs75698615
rs11032433
rs77774351
rs6687275
rs78824072
rs35989582
rs34658867
rs6032211
rs111436326
rs139376787
rs12233381
rs6073077
rs140839053
rs61784602
rs3788395
rs10762188
rs139922750
rs1801274
All
−0.025
0.000
0.025
0.050
MR leave−one−out sensitivity analysis for
'CD40 on CD14− CD16+ monocyte ' on 'Diabetic nephropathy'

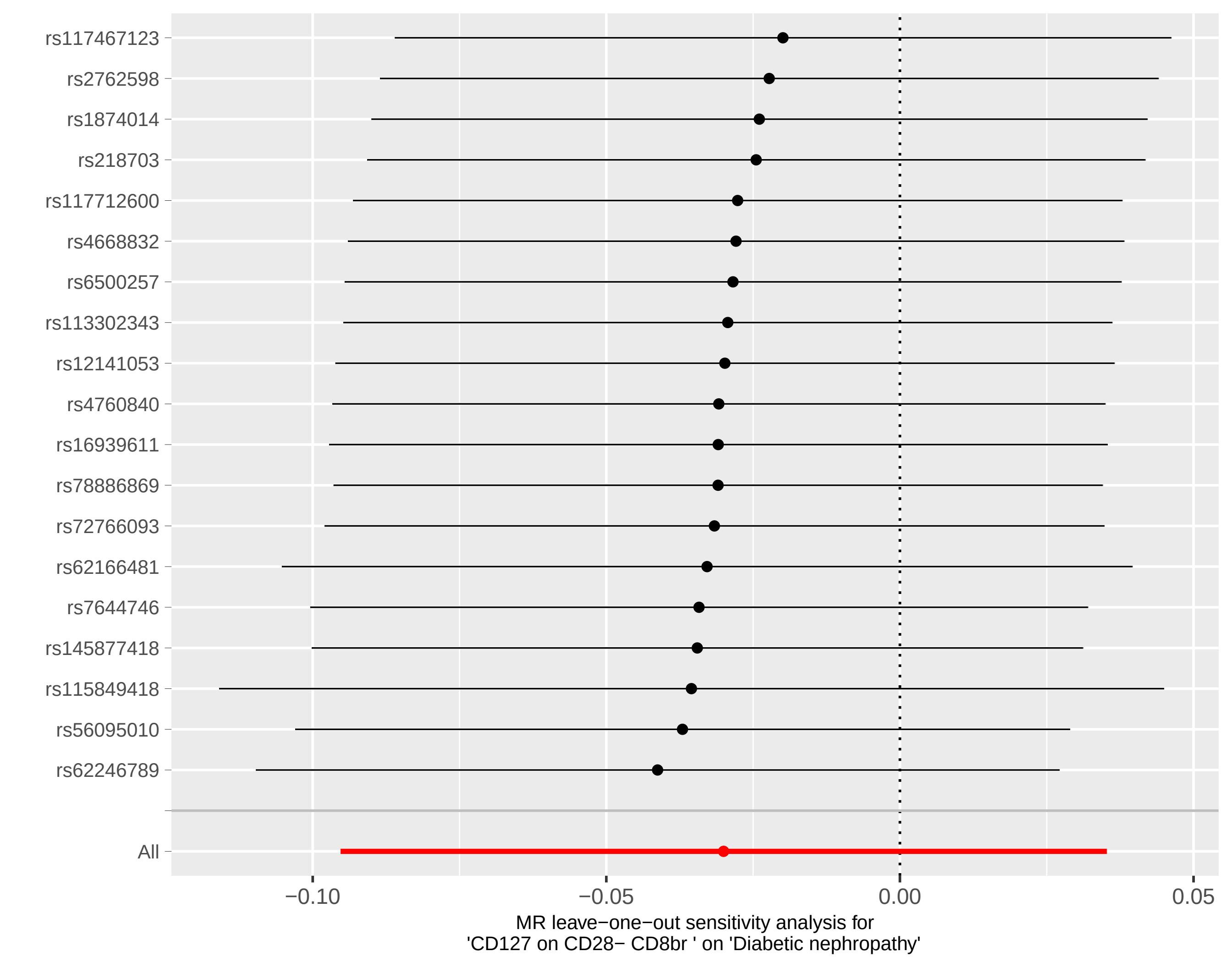
rs117467123
rs2762598
rs1874014
rs218703
rs117712600
rs4668832
rs6500257
rs113302343
rs12141053
rs4760840
rs16939611
rs78886869
rs72766093
rs62166481
rs7644746
rs145877418
rs115849418
rs56095010
rs62246789
All
−0.10
−0.05
0.00
0.05
MR leave−one−out sensitivity analysis for
'CD127 on CD28− CD8br ' on 'Diabetic nephropathy'

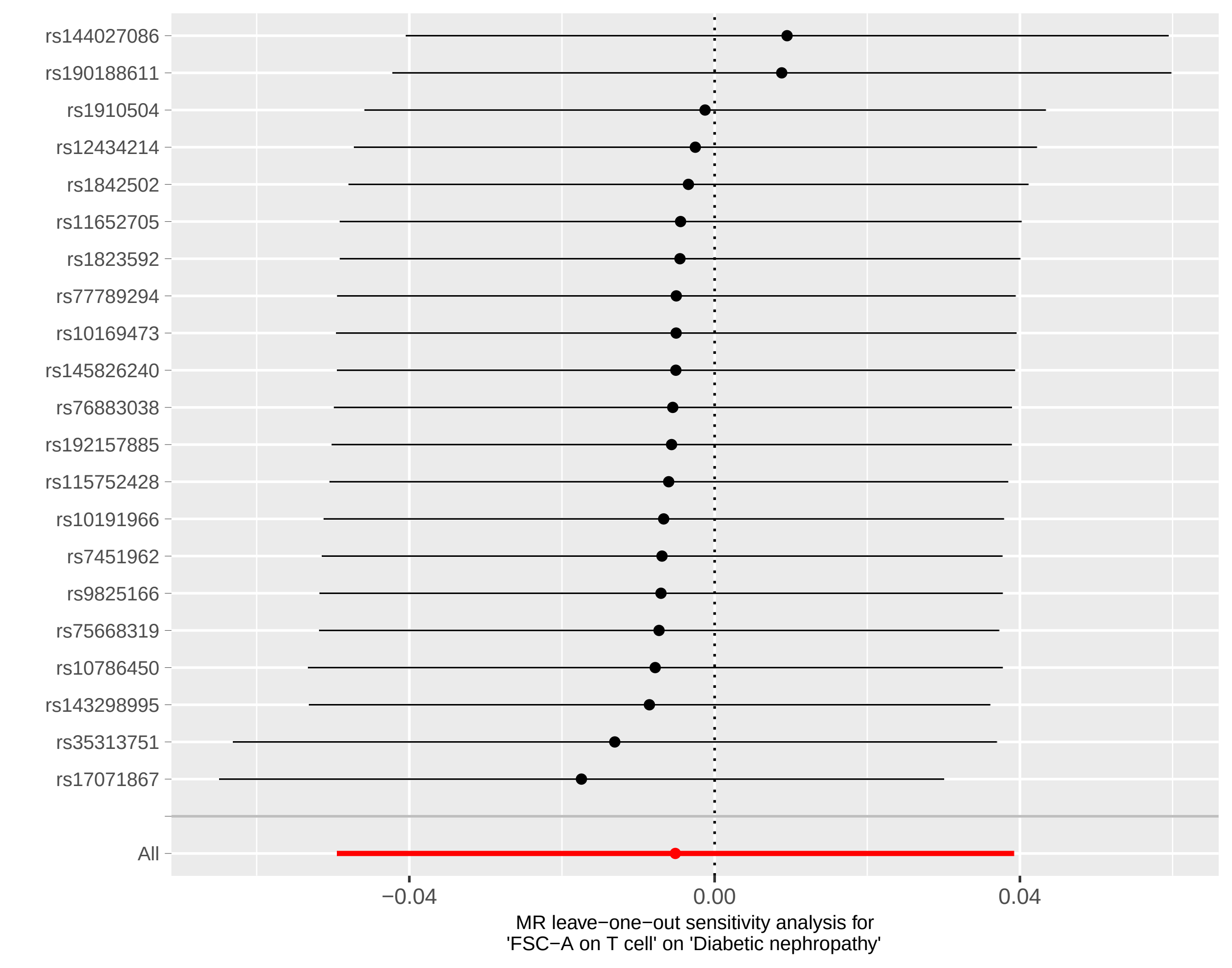
rs144027086
rs190188611
rs1910504
rs12434214
rs1842502
rs11652705
rs1823592
rs77789294
rs10169473
rs145826240
rs76883038
rs192157885
rs115752428
rs10191966
rs7451962
rs9825166
rs75668319
rs10786450
rs143298995
rs35313751
rs17071867
All
−0.04
0.00
0.04
MR leave−one−out sensitivity analysis for
'FSC−A on T cell' on 'Diabetic nephropathy'

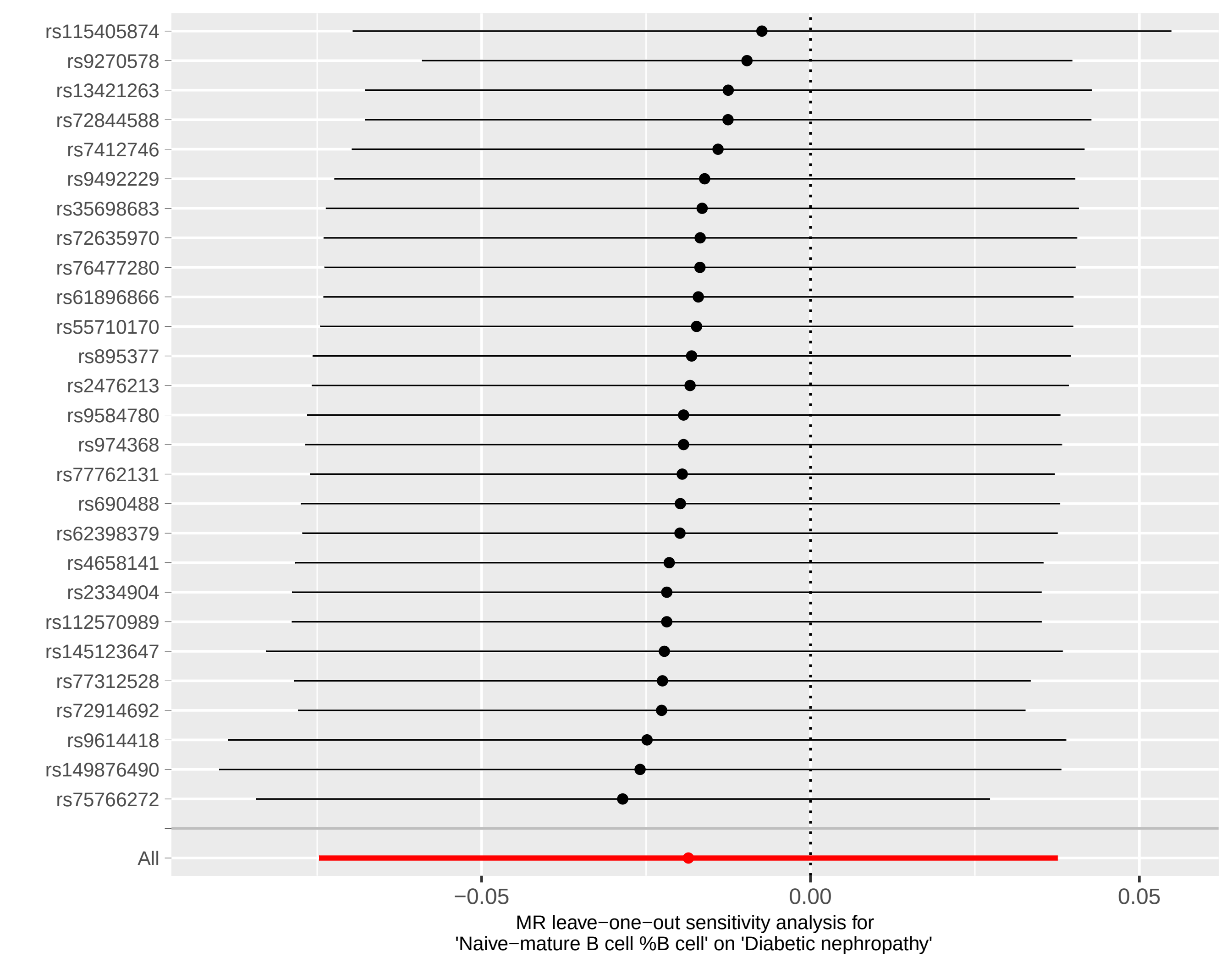
rs115405874
rs9270578
rs13421263
rs72844588
rs7412746
rs9492229
rs35698683
rs72635970
rs76477280
rs61896866
rs55710170
rs895377
rs2476213
rs9584780
rs974368
rs77762131
rs690488
rs62398379
rs4658141
rs2334904
rs112570989
rs145123647
rs77312528
rs72914692
rs9614418
rs149876490
rs75766272
All
−0.05
0.00
0.05
MR leave−one−out sensitivity analysis for
'Naive−mature B cell %B cell' on 'Diabetic nephropathy'

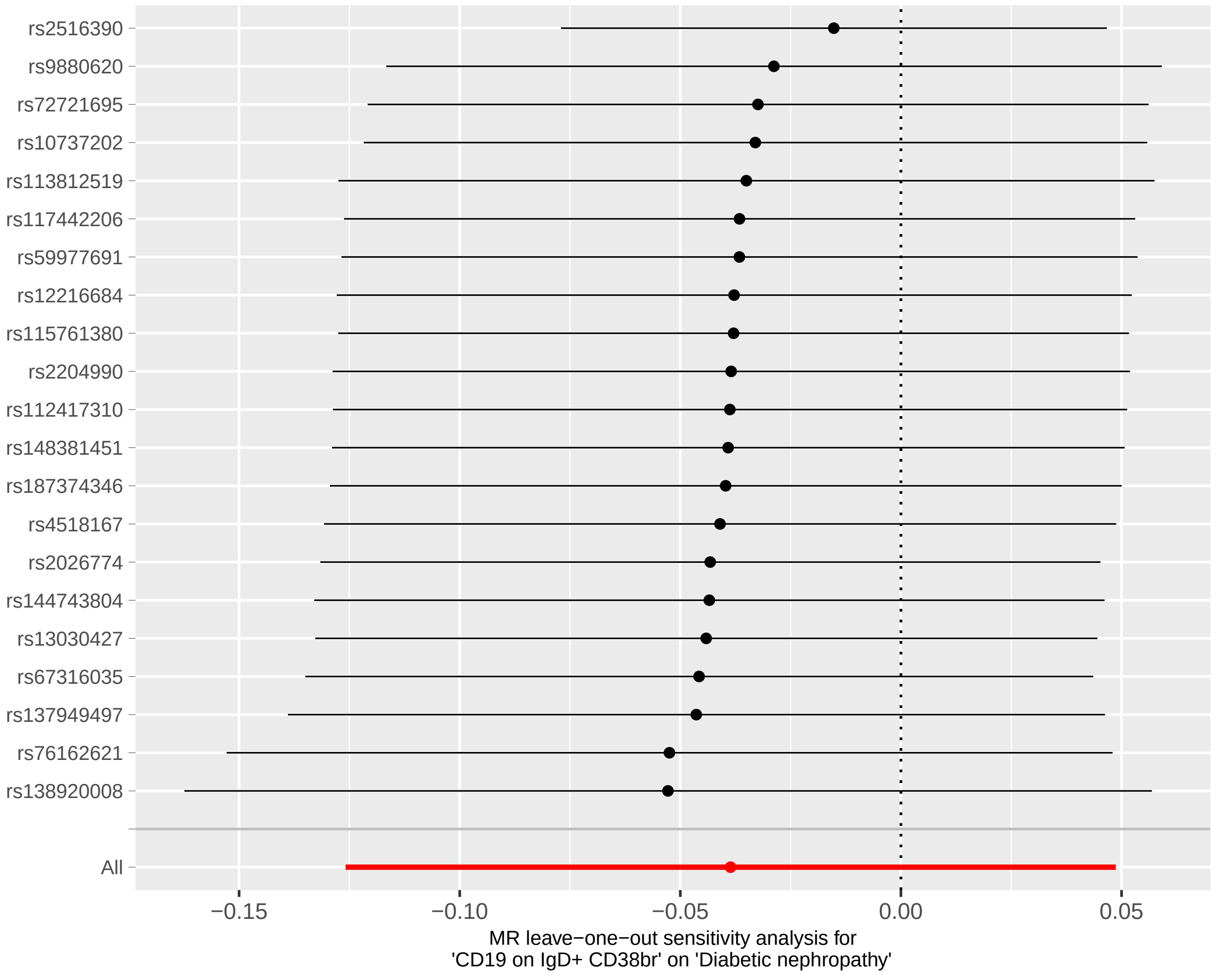

rs2516390
rs9880620
rs72721695
rs10737202
rs113812519
rs117442206
rs59977691
rs12216684
rs115761380
rs2204990
rs112417310
rs148381451
rs187374346
rs4518167
rs2026774
rs144743804
rs13030427
rs67316035
rs137949497
rs76162621
rs138920008
All
−0.15
−0.10
−0.05
0.00
0.05
MR leave−one−out sensitivity analysis for
'CD19 on IgD+ CD38br' on 'Diabetic nephropathy'

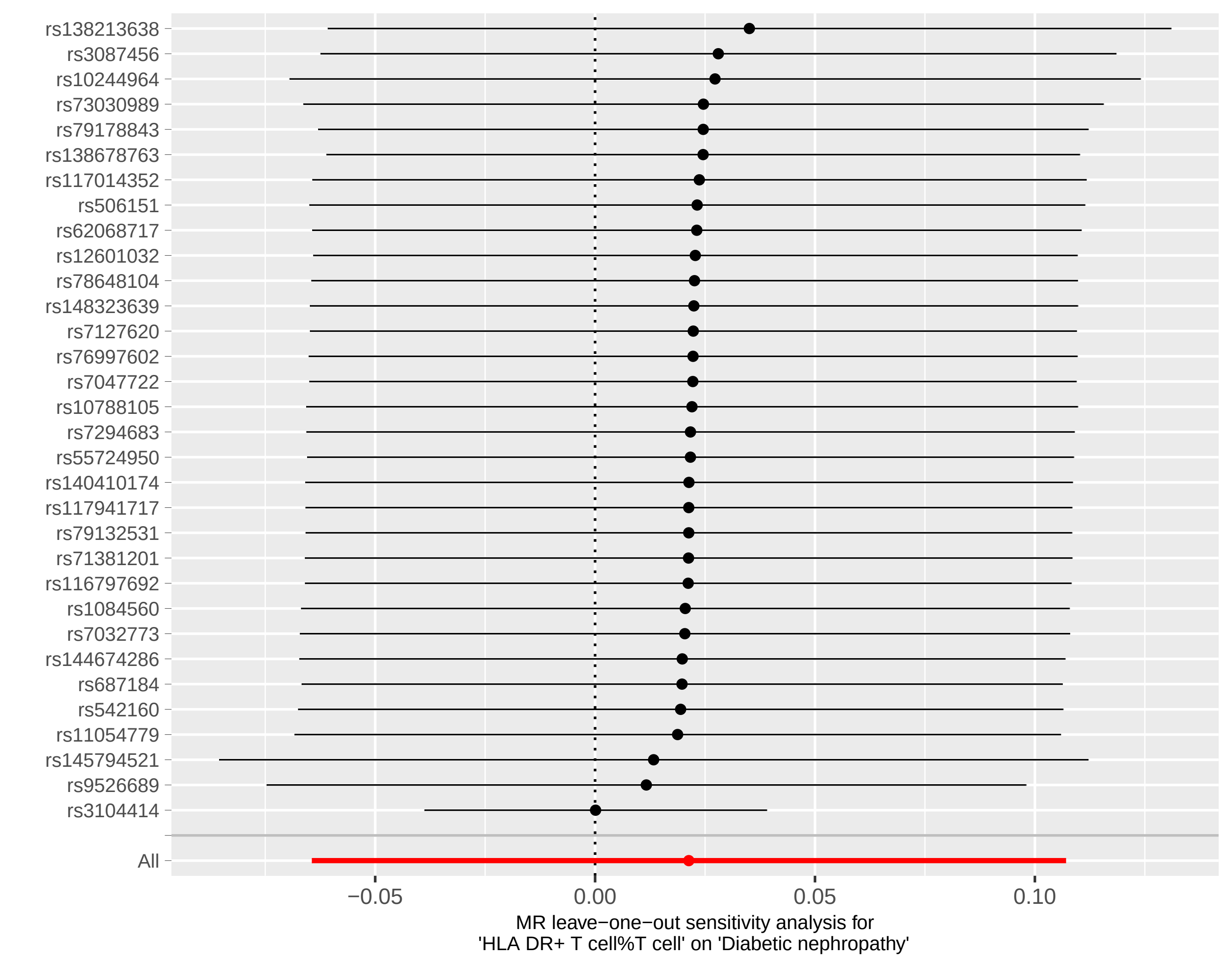
rs138213638
rs3087456
rs10244964
rs73030989
rs79178843
rs138678763
rs117014352
rs506151
rs62068717
rs12601032
rs78648104
rs148323639
rs7127620
rs76997602
rs7047722
rs10788105
rs7294683
rs55724950
rs140410174
rs117941717
rs79132531
rs71381201
rs116797692
rs1084560
rs7032773
rs144674286
rs687184
rs542160
rs11054779
rs145794521
rs9526689
rs3104414
All
−0.05
0.00
0.05
0.10
MR leave−one−out sensitivity analysis for
'HLA DR+ T cell%T cell' on 'Diabetic nephropathy'

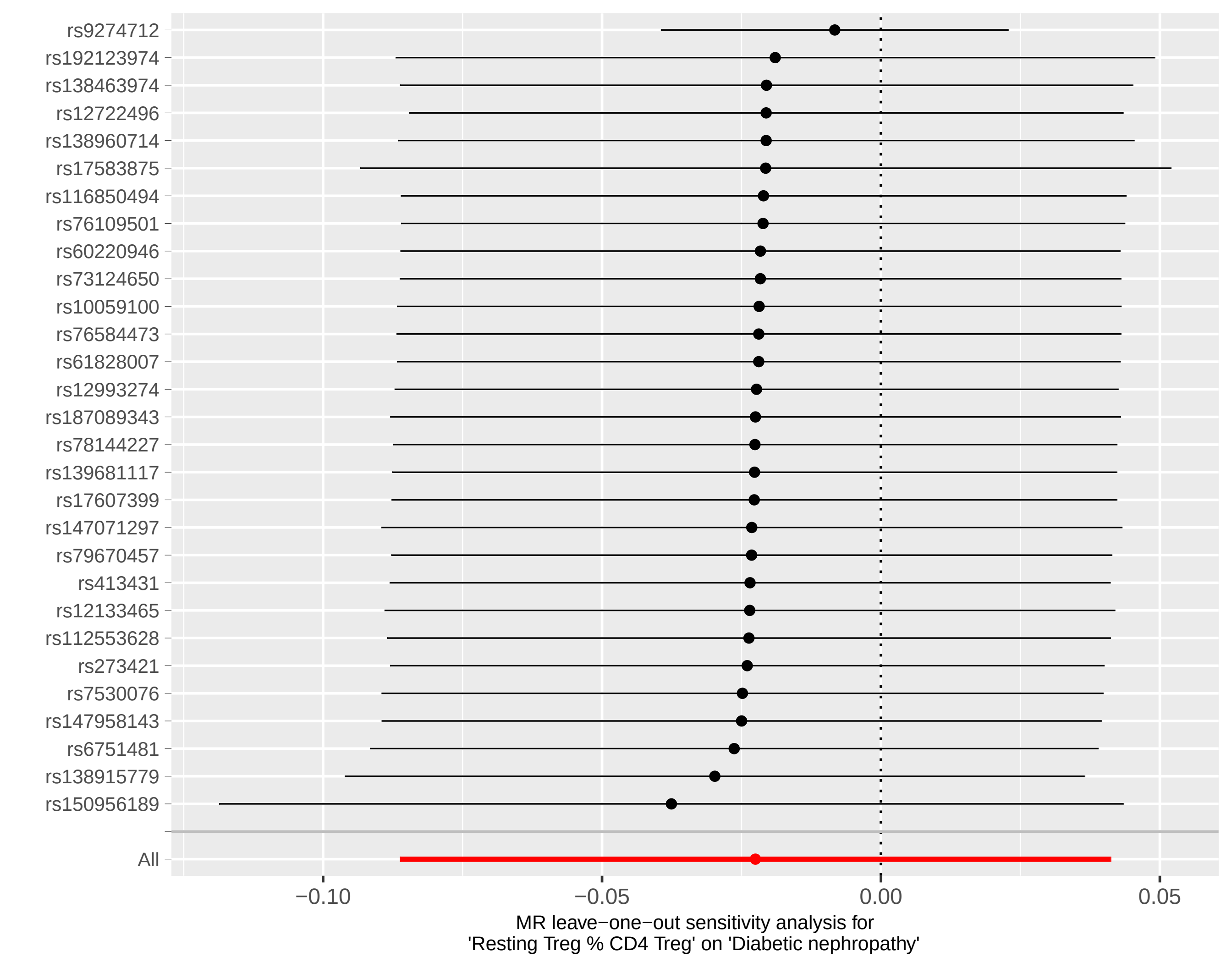

rs9274712
rs192123974
rs138463974
rs12722496
rs138960714
rs17583875
rs116850494
rs76109501
rs60220946
rs73124650
rs10059100
rs76584473
rs61828007
rs12993274
rs187089343
rs78144227
rs139681117
rs17607399
rs147071297
rs79670457
rs413431
rs12133465
rs112553628
rs273421
rs7530076
rs147958143
rs6751481
rs138915779
rs150956189
All
−0.10
−0.05
0.00
0.05
MR leave−one−out sensitivity analysis for
'Resting Treg % CD4 Treg' on 'Diabetic nephropathy'

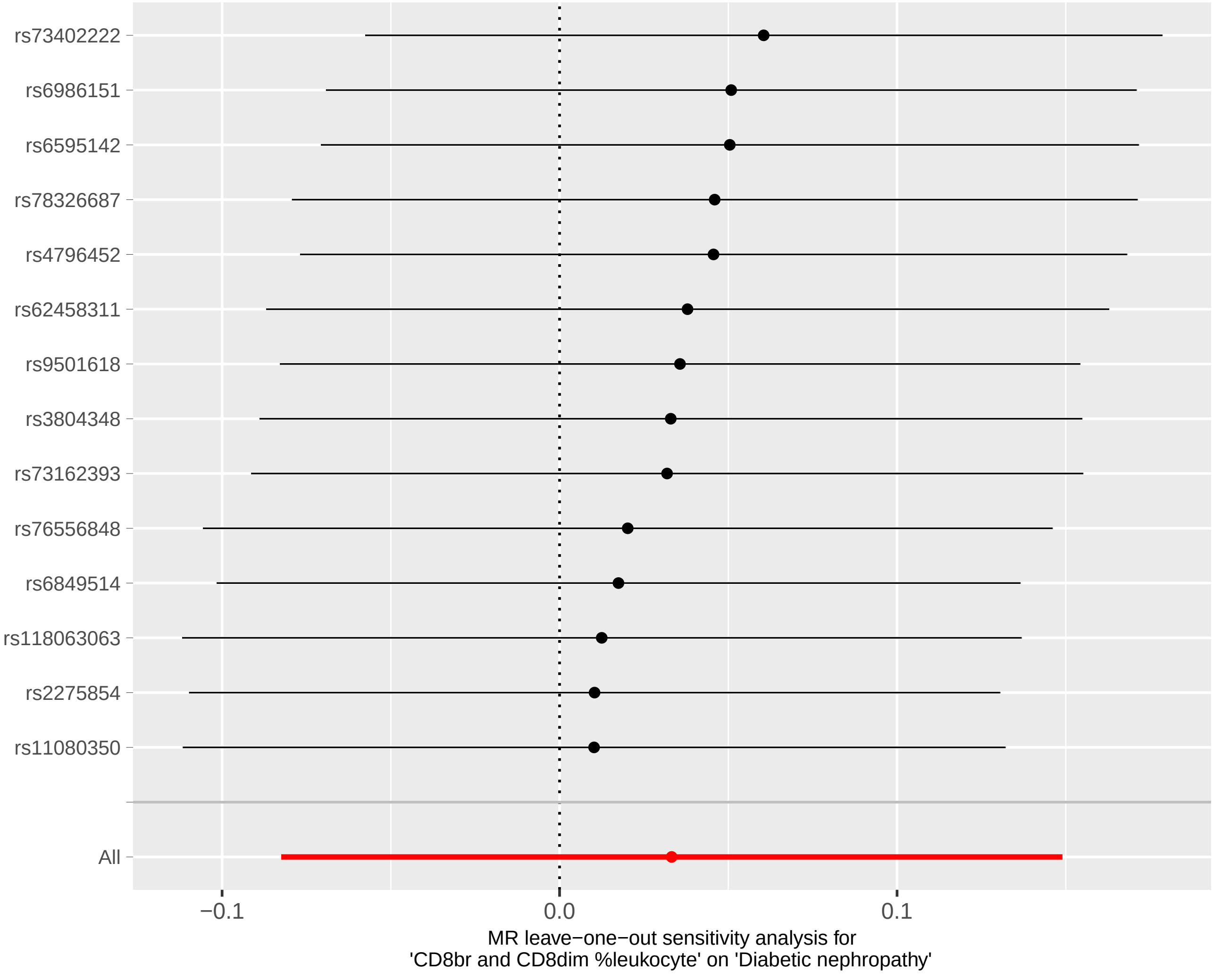

rs73402222
rs6986151
rs6595142
rs78326687
rs4796452
rs62458311
rs9501618
rs3804348
rs73162393
rs76556848
rs6849514
rs118063063
rs2275854
rs11080350
All
−0.1
0.0
0.1
MR leave−one−out sensitivity analysis for
'CD8br and CD8dim %leukocyte' on 'Diabetic nephropathy'

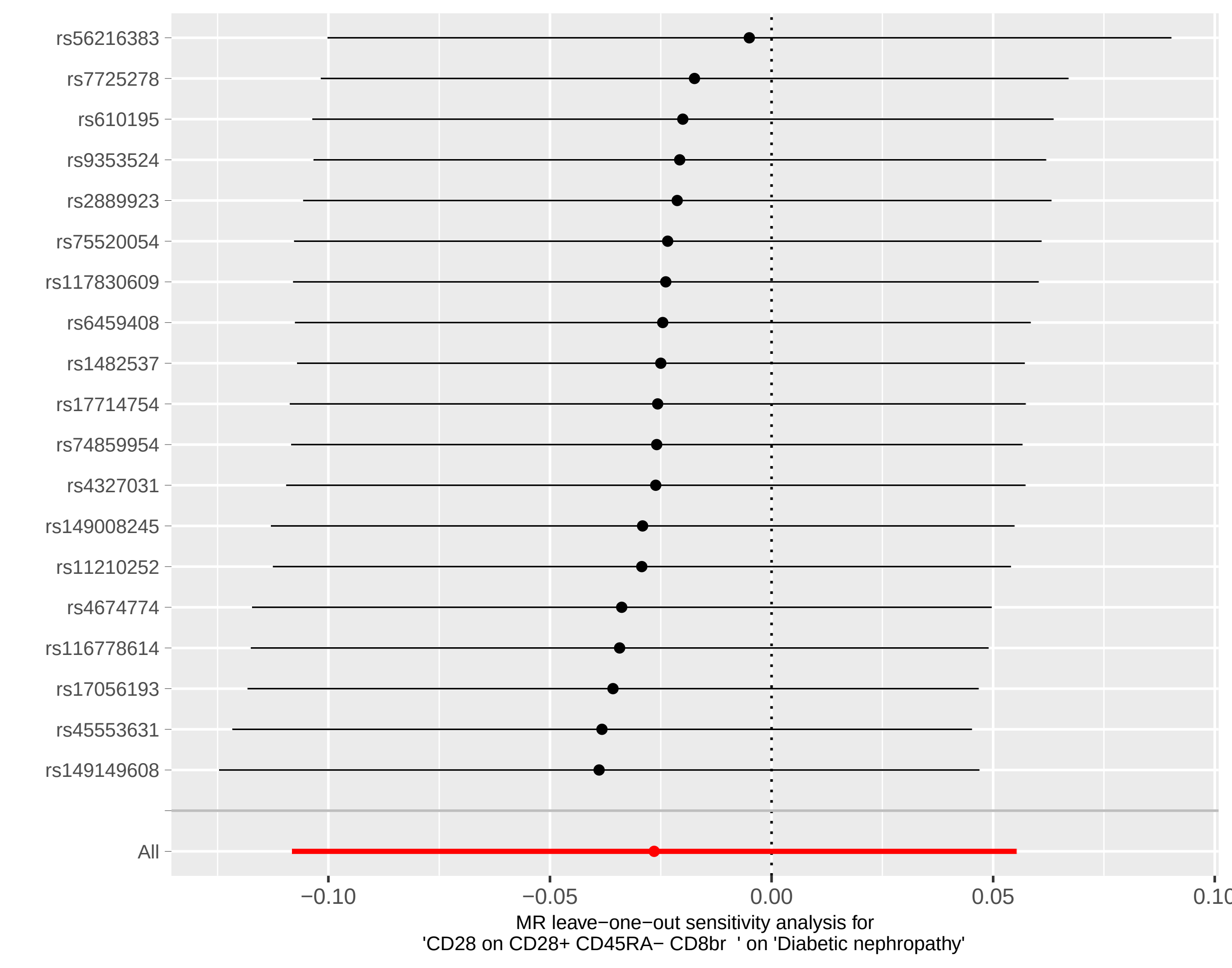
rs56216383
rs7725278
rs610195
rs9353524
rs2889923
rs75520054
rs117830609
rs6459408
rs1482537
rs17714754
rs74859954
rs4327031
rs149008245
rs11210252
rs4674774
rs116778614
rs17056193
rs45553631
rs149149608
All
−0.10
−0.05
0.00
0.05
0.10
MR leave−one−out sensitivity analysis for
'CD28 on CD28+ CD45RA− CD8br ' on 'Diabetic nephropathy'

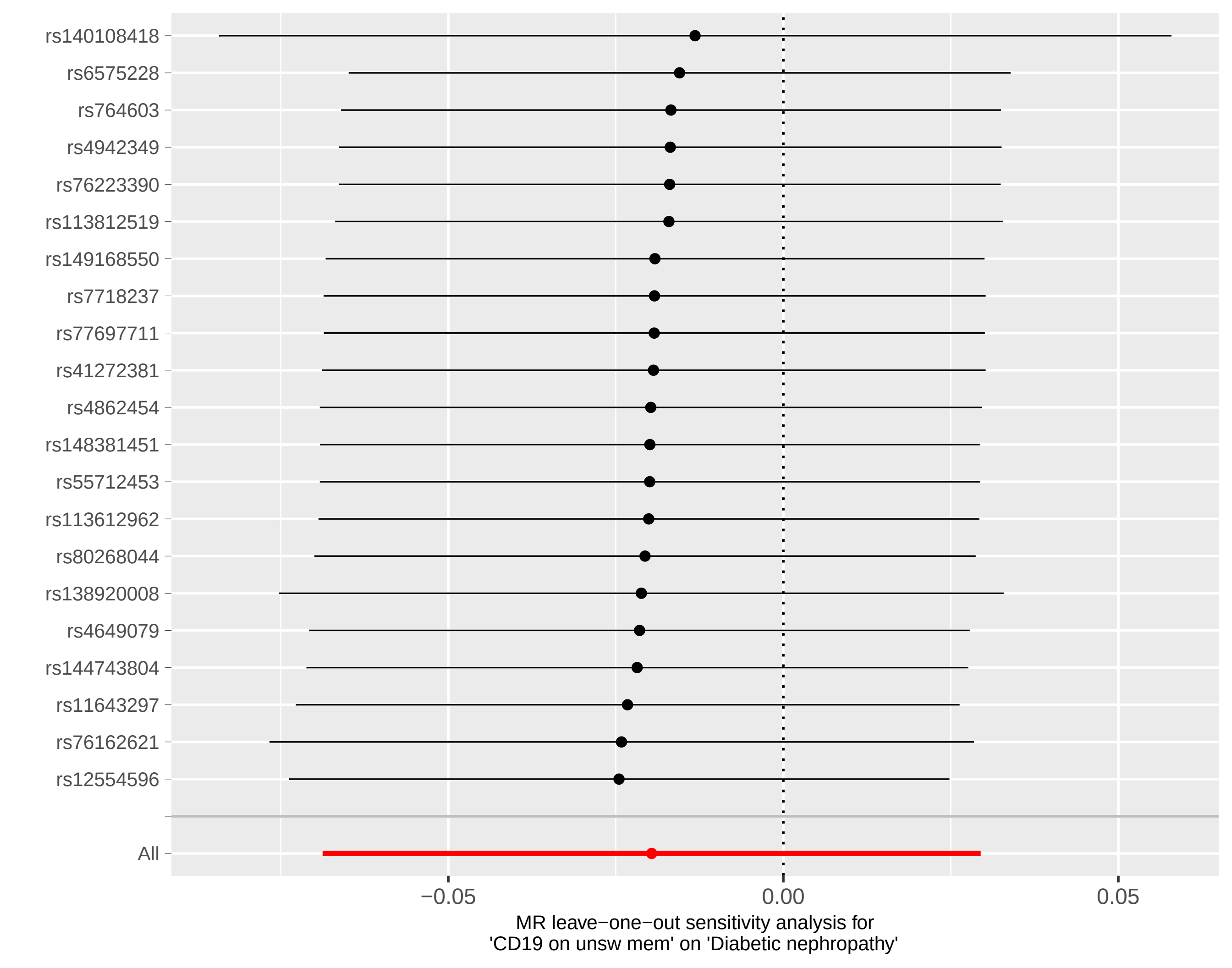
rs140108418
rs6575228
rs764603
rs4942349
rs76223390
rs113812519
rs149168550
rs7718237
rs77697711
rs41272381
rs4862454
rs148381451
rs55712453
rs113612962
rs80268044
rs138920008
rs4649079
rs144743804
rs11643297
rs76162621
rs12554596
All
−0.05
0.00
0.05
MR leave−one−out sensitivity analysis for
'CD19 on unsw mem' on 'Diabetic nephropathy'

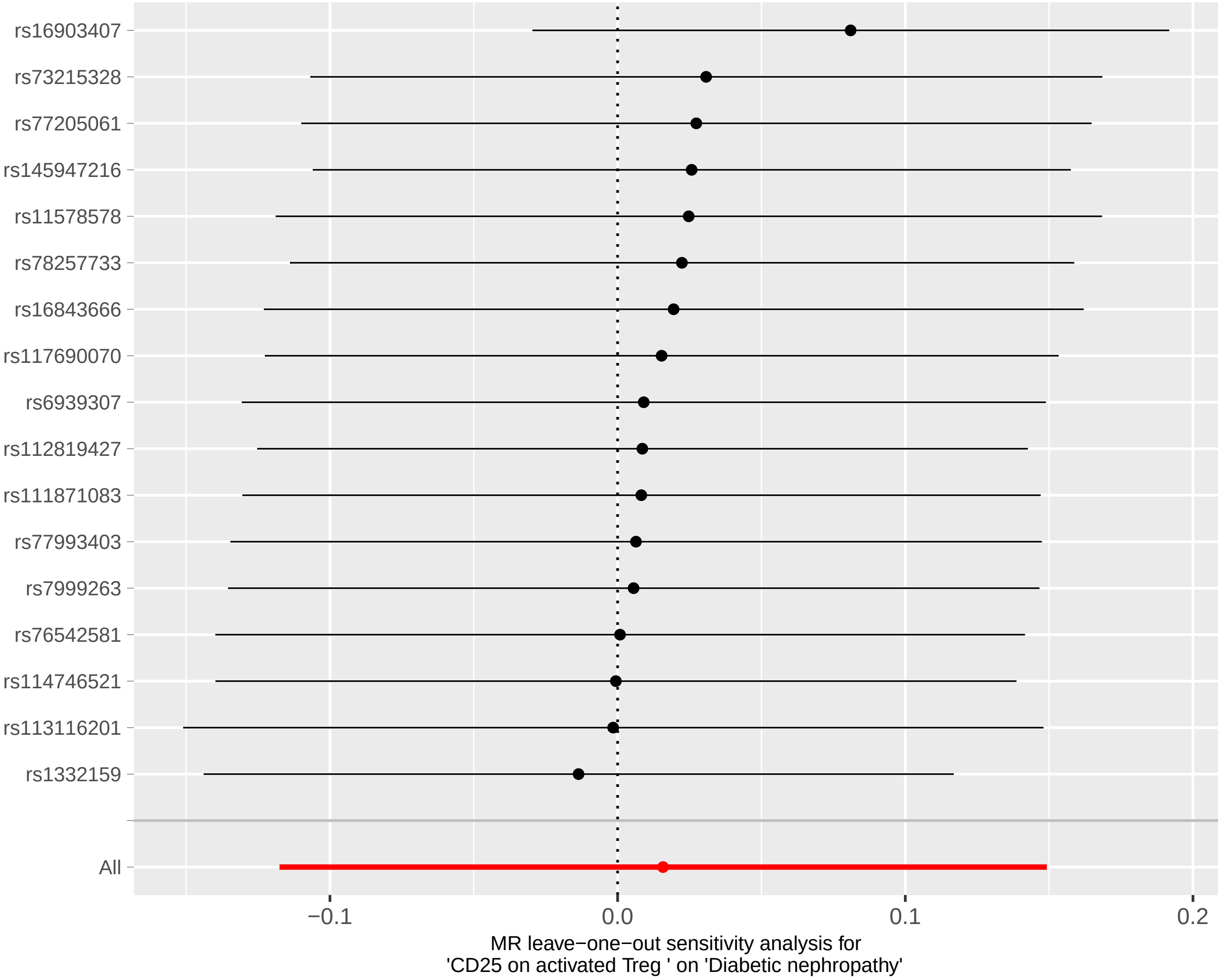
rs16903407
rs73215328
rs77205061
rs145947216
rs11578578
rs78257733
rs16843666
rs117690070
rs6939307
rs112819427
rs111871083
rs77993403
rs7999263
rs76542581
rs114746521
rs113116201
rs1332159
All
−0.1
0.0
0.1
0.2
MR leave−one−out sensitivity analysis for
'CD25 on activated Treg ' on 'Diabetic nephropathy'

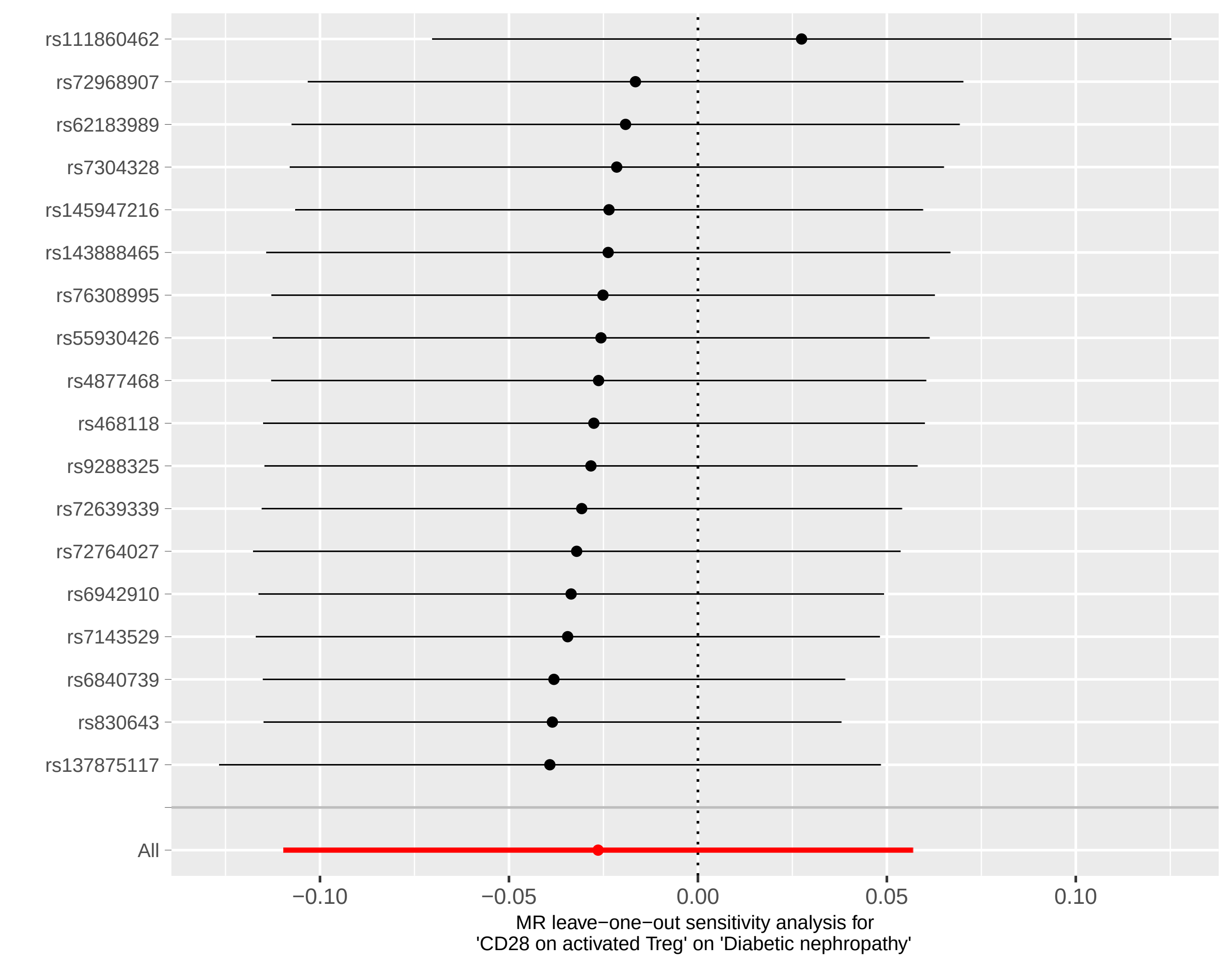
rs111860462
rs72968907
rs62183989
rs7304328
rs145947216
rs143888465
rs76308995
rs55930426
rs4877468
rs468118
rs9288325
rs72639339
rs72764027
rs6942910
rs7143529
rs6840739
rs830643
rs137875117
All
−0.10
−0.05
0.00
0.05
0.10
MR leave−one−out sensitivity analysis for
'CD28 on activated Treg' on 'Diabetic nephropathy'

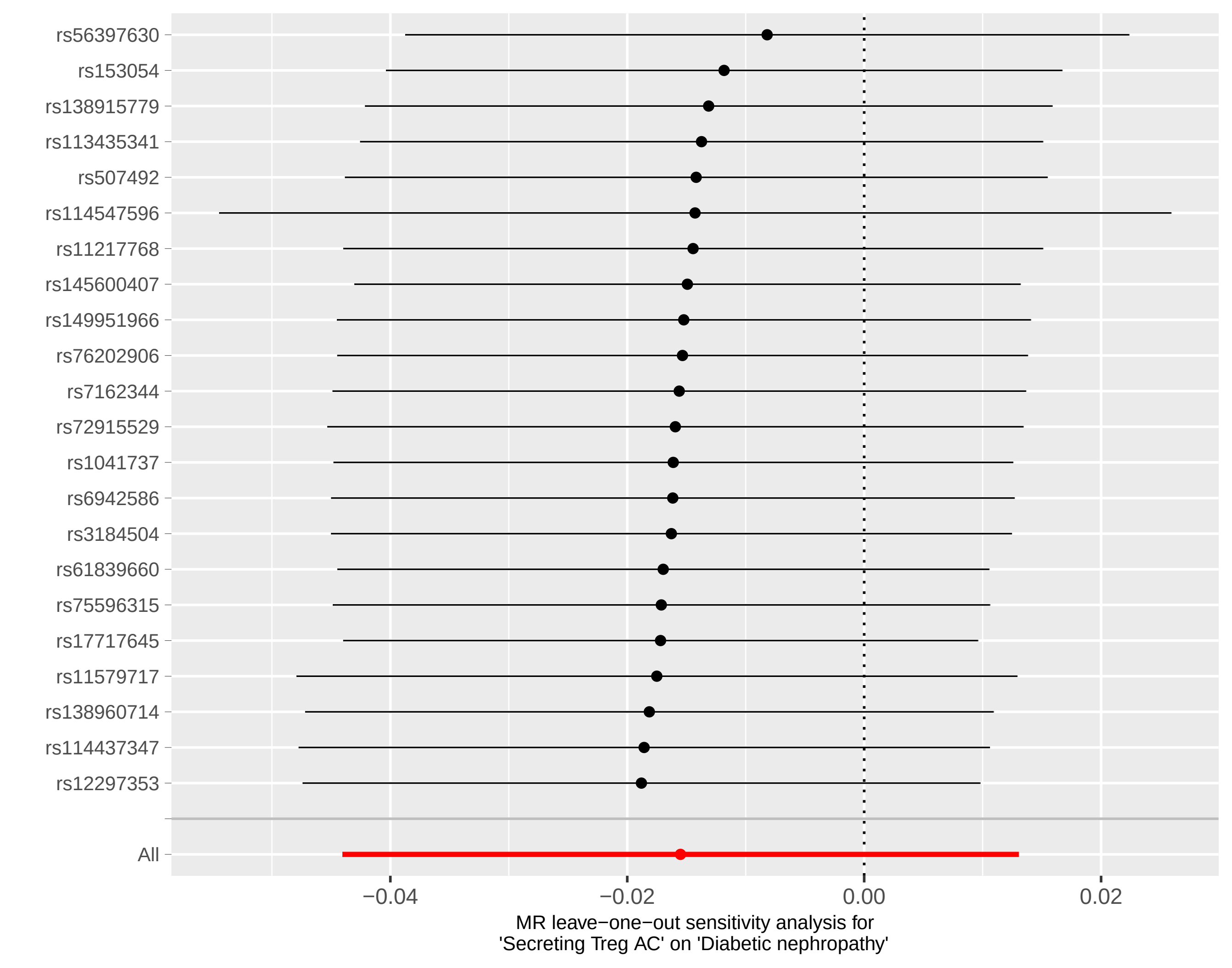
rs56397630
rs153054
rs138915779
rs113435341
rs507492
rs114547596
rs11217768
rs145600407
rs149951966
rs76202906
rs7162344
rs72915529
rs1041737
rs6942586
rs3184504
rs61839660
rs75596315
rs17717645
rs11579717
rs138960714
rs114437347
rs12297353
All
−0.04
−0.02
0.00
0.02
MR leave−one−out sensitivity analysis for
'Secreting Treg AC' on 'Diabetic nephropathy'

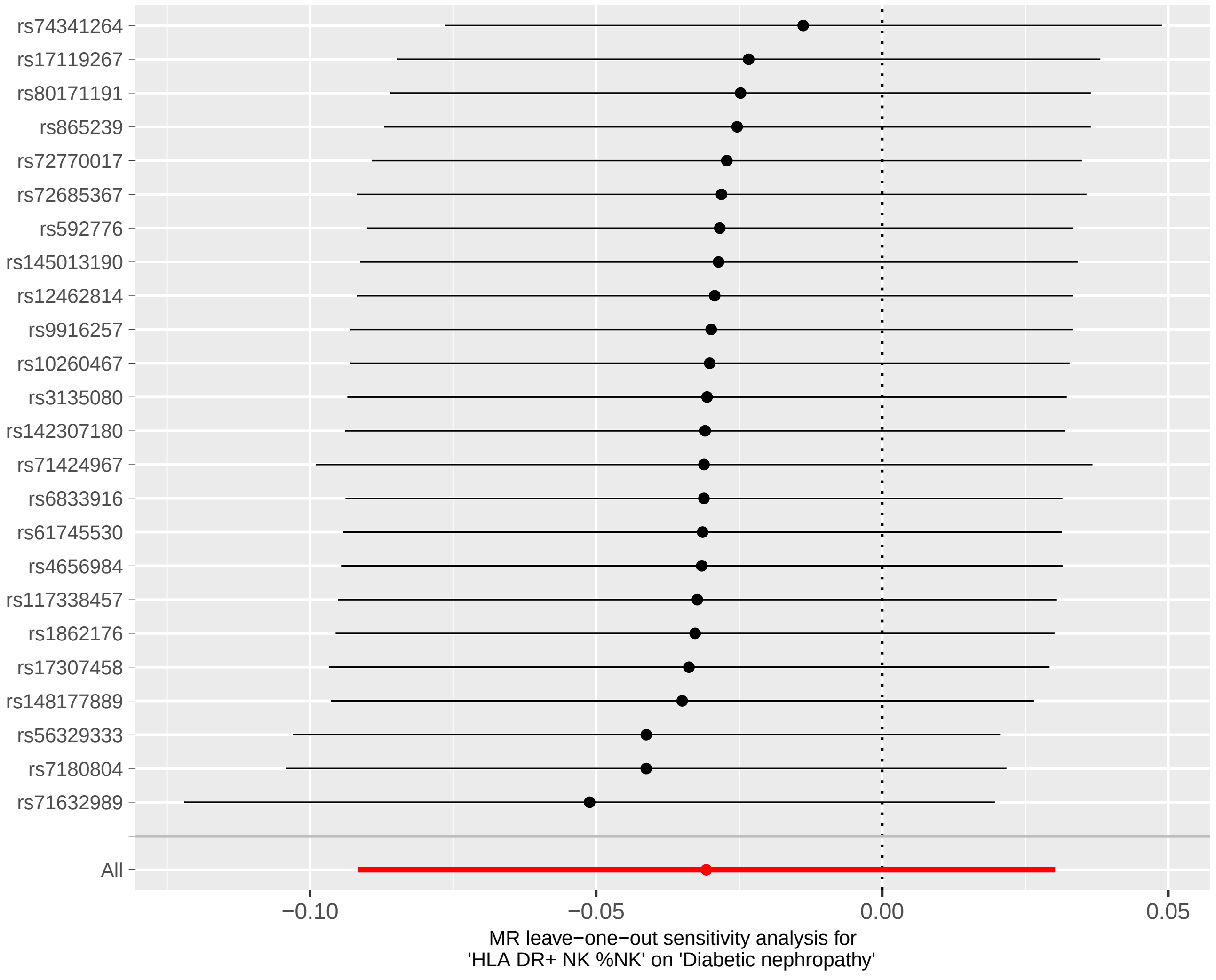

rs74341264
rs17119267
rs80171191
rs865239
rs72770017
rs72685367
rs592776
rs145013190
rs12462814
rs9916257
rs10260467
rs3135080
rs142307180
rs71424967
rs6833916
rs61745530
rs4656984
rs117338457
rs1862176
rs17307458
rs148177889
rs56329333
rs7180804
rs71632989
All
−0.10
−0.05
0.00
0.05
MR leave−one−out sensitivity analysis for
'HLA DR+ NK %NK' on 'Diabetic nephropathy'

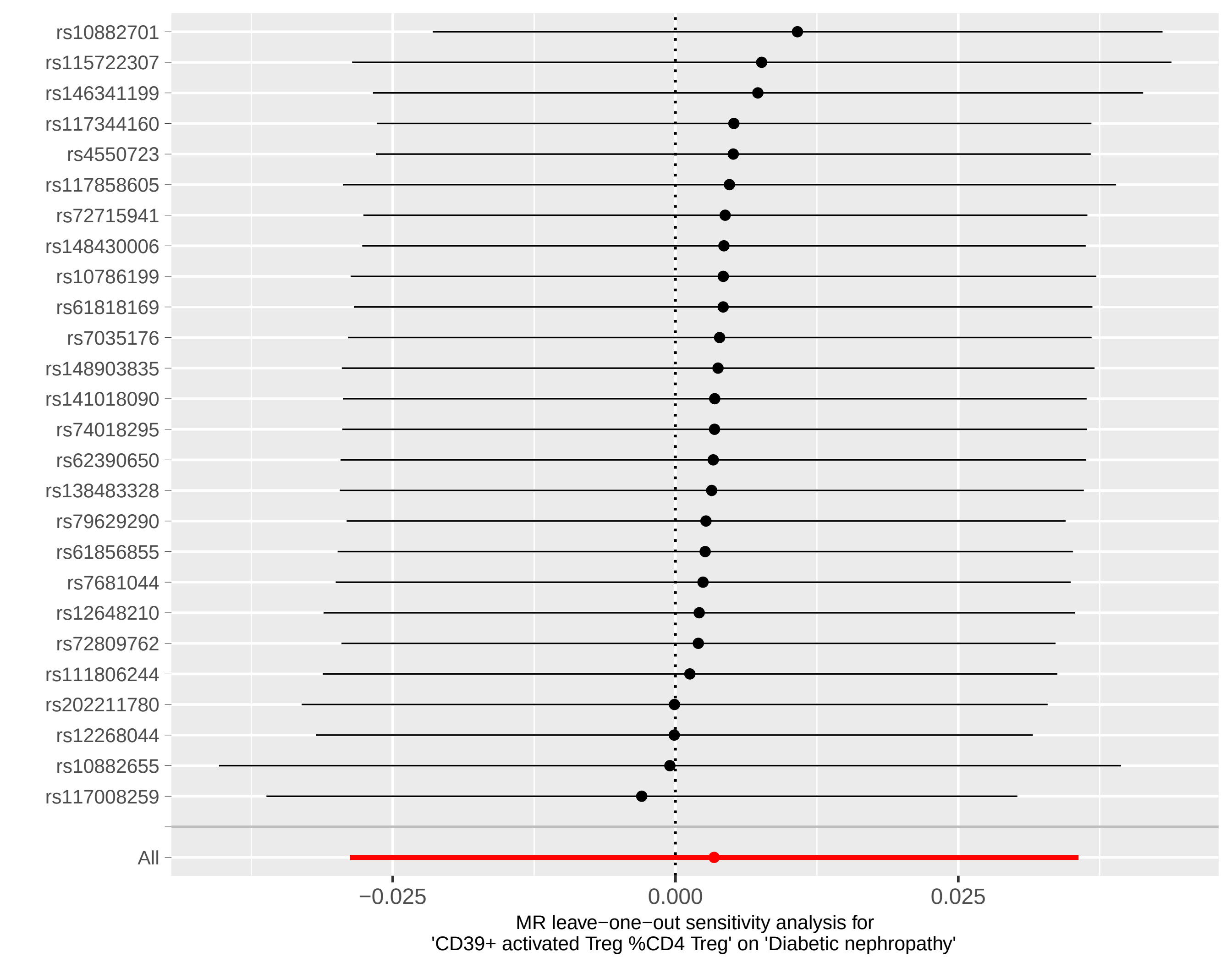
rs10882701
rs115722307
rs146341199
rs117344160
rs4550723
rs117858605
rs72715941
rs148430006
rs10786199
rs61818169
rs7035176
rs148903835
rs141018090
rs74018295
rs62390650
rs138483328
rs79629290
rs61856855
rs7681044
rs12648210
rs72809762
rs111806244
rs202211780
rs12268044
rs10882655
rs117008259
All
−0.025
0.000
0.025
MR leave−one−out sensitivity analysis for
'CD39+ activated Treg %CD4 Treg' on 'Diabetic nephropathy'

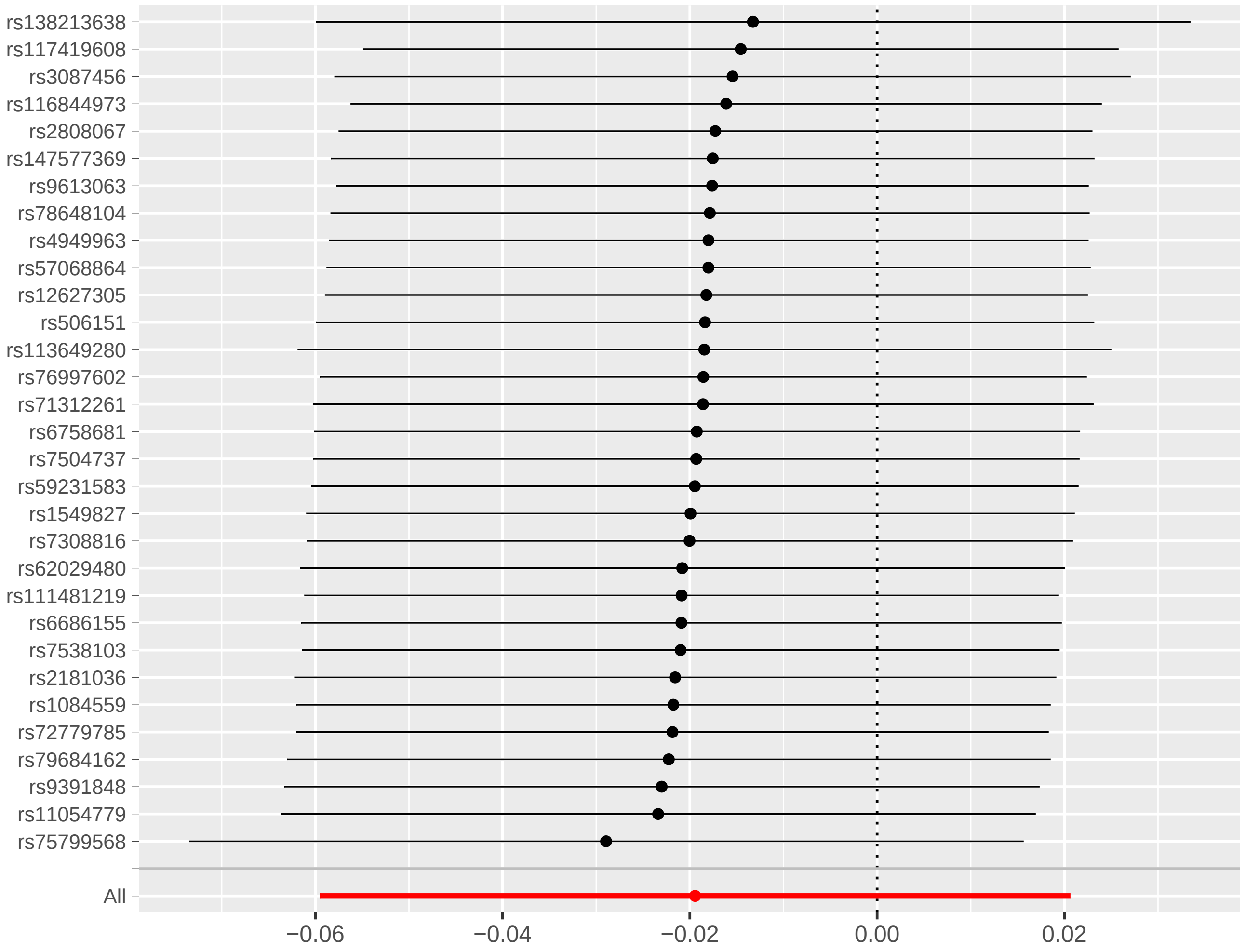

MR leave−one−out sensitivity analysis for
'HLA DR+ T cell AC' on 'Diabetic nephropathy'

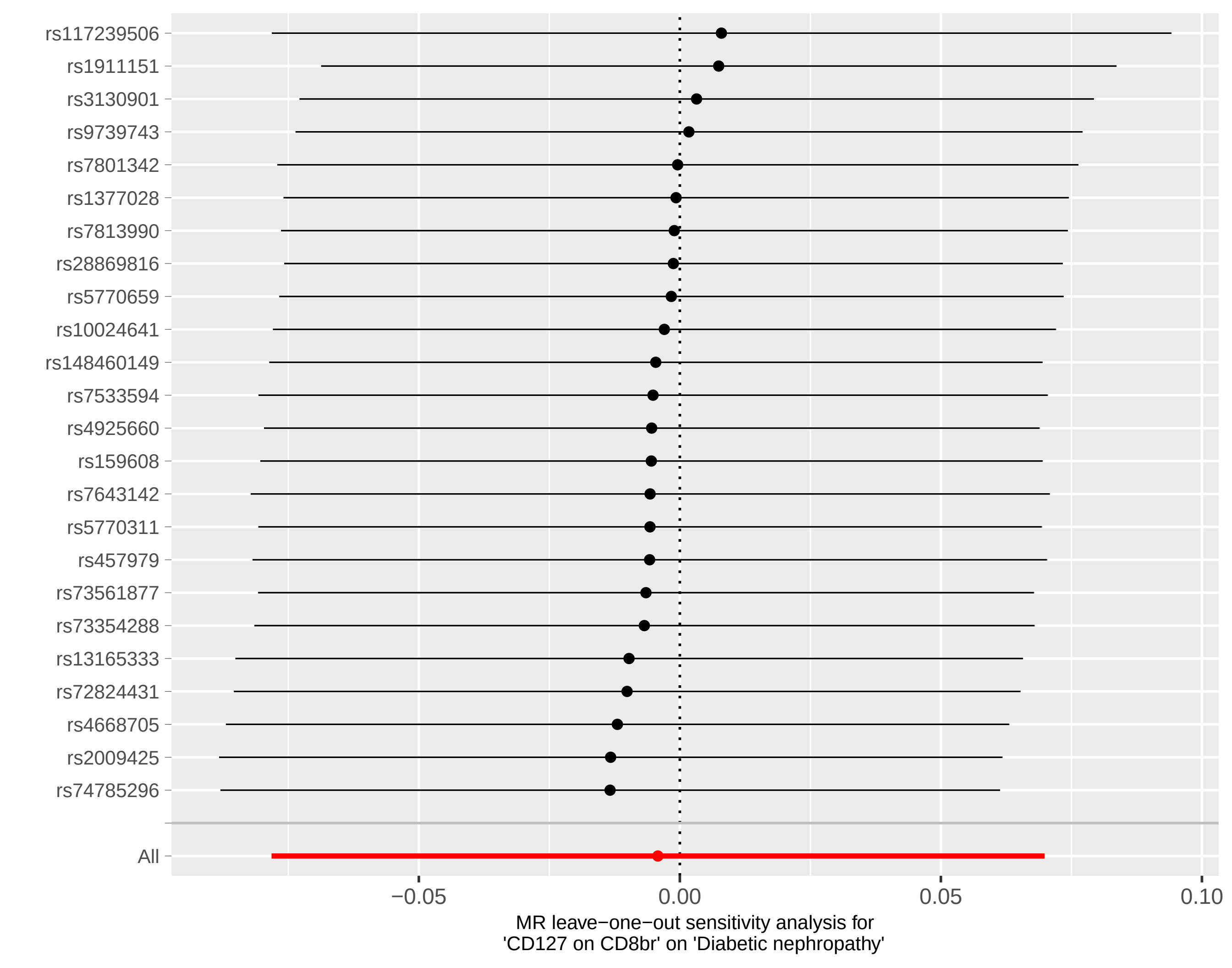
rs117239506
rs1911151
rs3130901
rs9739743
rs7801342
rs1377028
rs7813990
rs28869816
rs5770659
rs10024641
rs148460149
rs7533594
rs4925660
rs159608
rs7643142
rs5770311
rs457979
rs73561877
rs73354288
rs13165333
rs72824431
rs4668705
rs2009425
rs74785296
All
−0.05
0.00
0.05
0.10
MR leave−one−out sensitivity analysis for
'CD127 on CD8br' on 'Diabetic nephropathy'

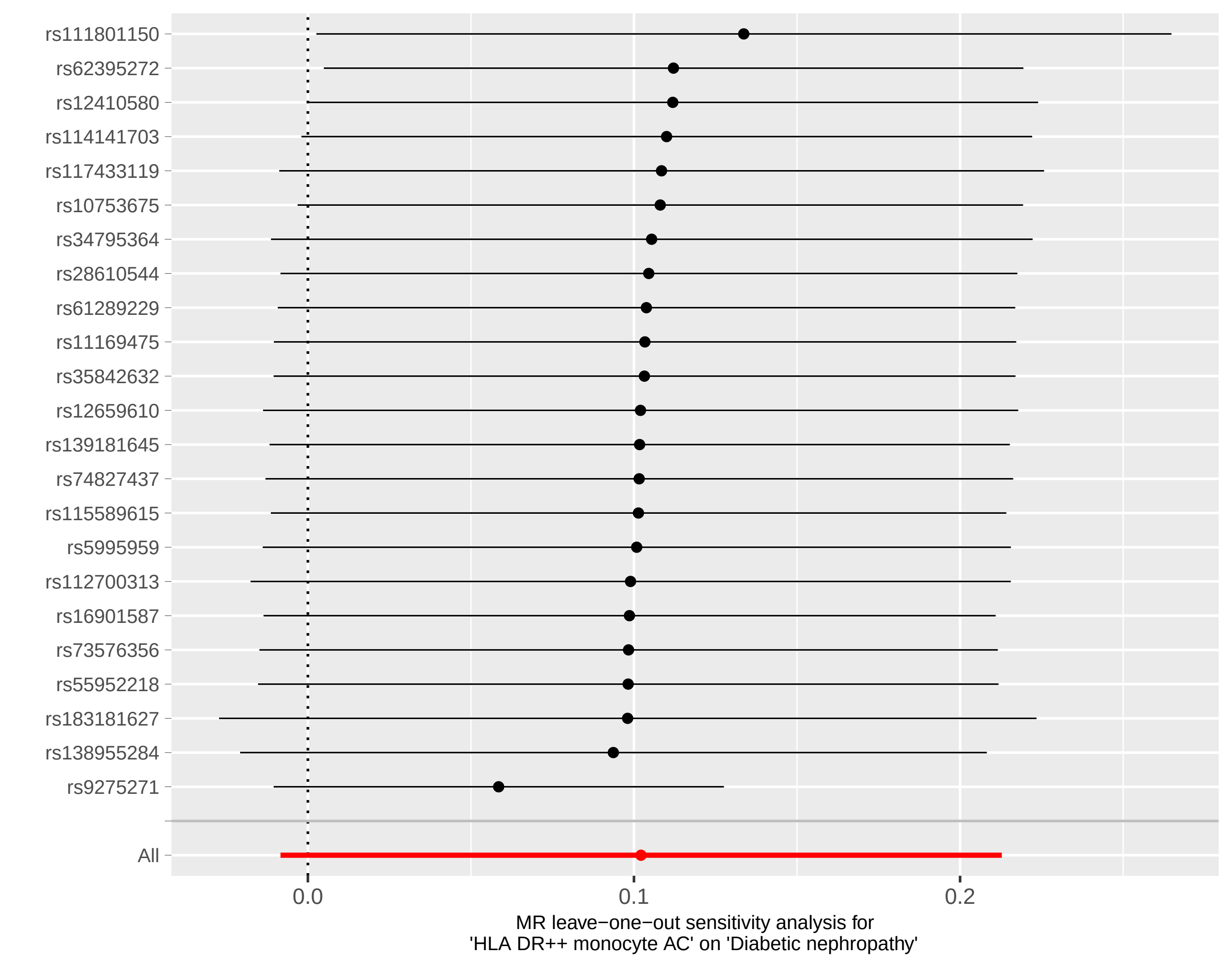

rs111801150
rs62395272
rs12410580
rs114141703
rs117433119
rs10753675
rs34795364
rs28610544
rs61289229
rs11169475
rs35842632
rs12659610
rs139181645
rs74827437
rs115589615
rs5995959
rs112700313
rs16901587
rs73576356
rs55952218
rs183181627
rs138955284
rs9275271
All
0.0
0.1
0.2
MR leave−one−out sensitivity analysis for
'HLA DR++ monocyte AC' on 'Diabetic nephropathy'

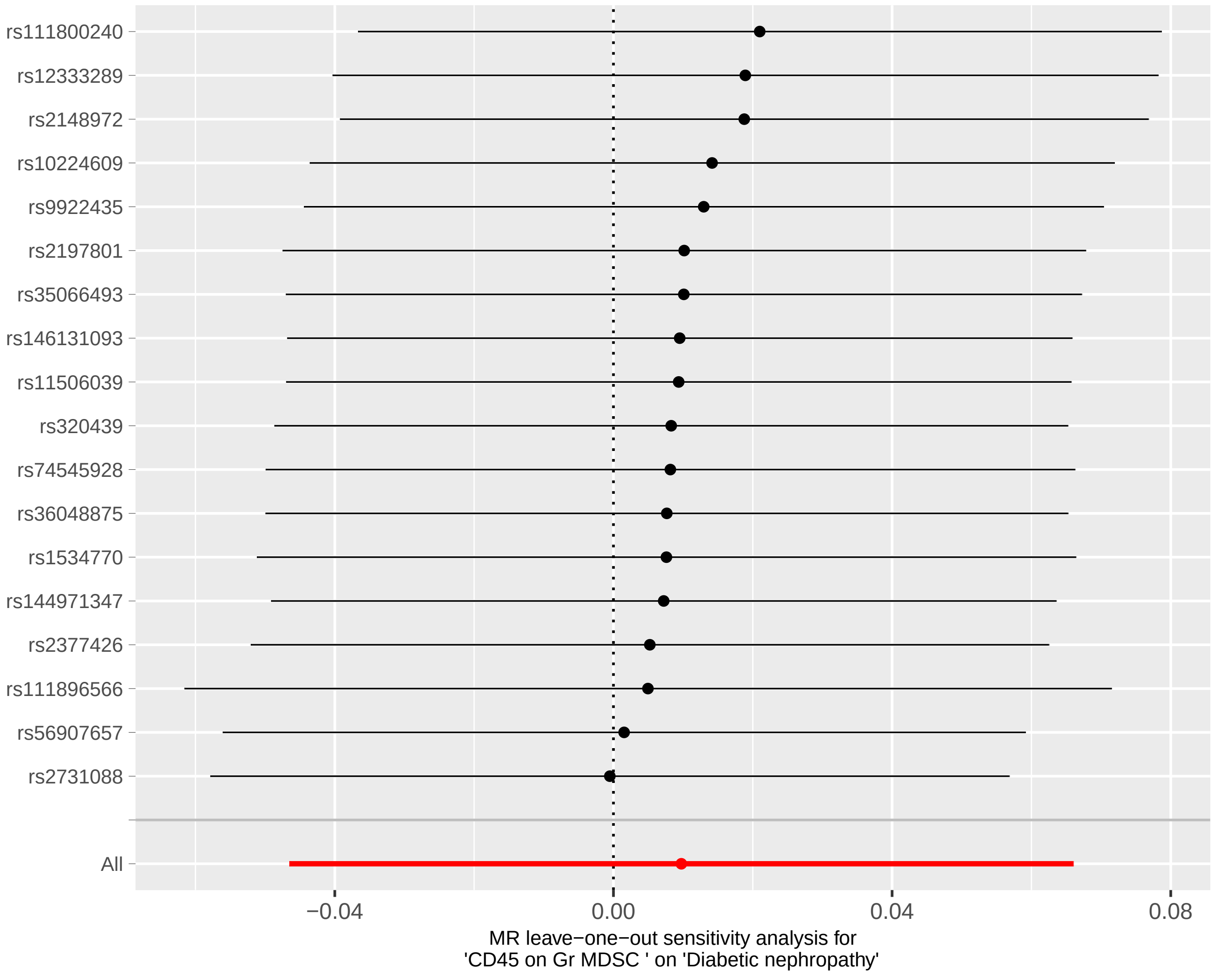

rs111800240
rs12333289
rs2148972
rs10224609
rs9922435
rs2197801
rs35066493
rs146131093
rs11506039
rs320439
rs74545928
rs36048875
rs1534770
rs144971347
rs2377426
rs111896566
rs56907657
rs2731088
All
−0.04
0.00
0.04
0.08
MR leave−one−out sensitivity analysis for
'CD45 on Gr MDSC ' on 'Diabetic nephropathy'

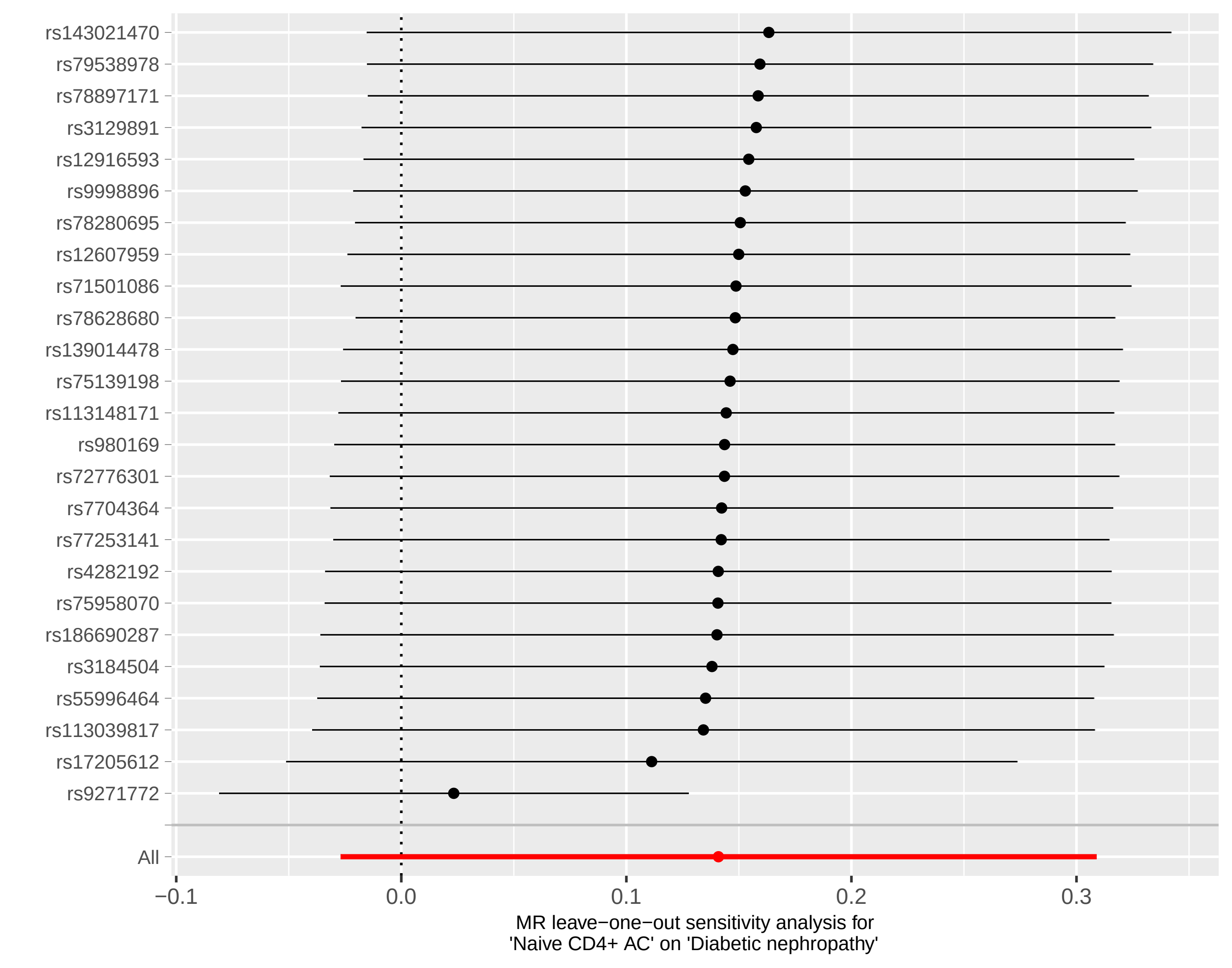
rs143021470
rs79538978
rs78897171
rs3129891
rs12916593
rs9998896
rs78280695
rs12607959
rs71501086
rs78628680
rs139014478
rs75139198
rs113148171
rs980169
rs72776301
rs7704364
rs77253141
rs4282192
rs75958070
rs186690287
rs3184504
rs55996464
rs113039817
rs17205612
rs9271772
All
−0.1
0.0
0.1
0.2
0.3
MR leave−one−out sensitivity analysis for
'Naive CD4+ AC' on 'Diabetic nephropathy'

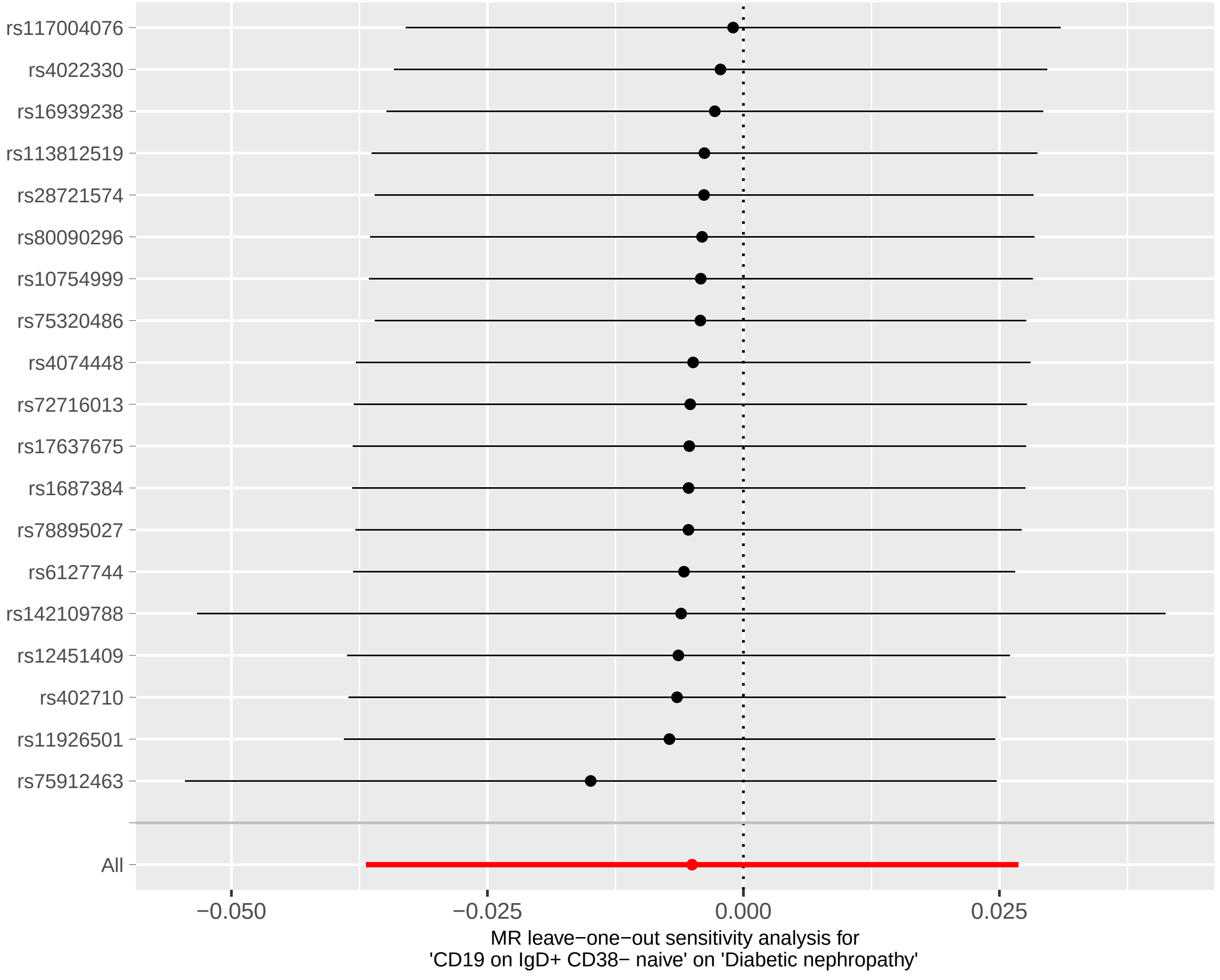
rs117004076
rs4022330
rs16939238
rs113812519
rs28721574
rs80090296
rs10754999
rs75320486
rs4074448
rs72716013
rs17637675
rs1687384
rs78895027
rs6127744
rs142109788
rs12451409
rs402710
rs11926501
rs75912463
All
−0.050
−0.025
0.000
0.025
MR leave−one−out sensitivity analysis for
'CD19 on IgD+ CD38− naive' on 'Diabetic nephropathy'

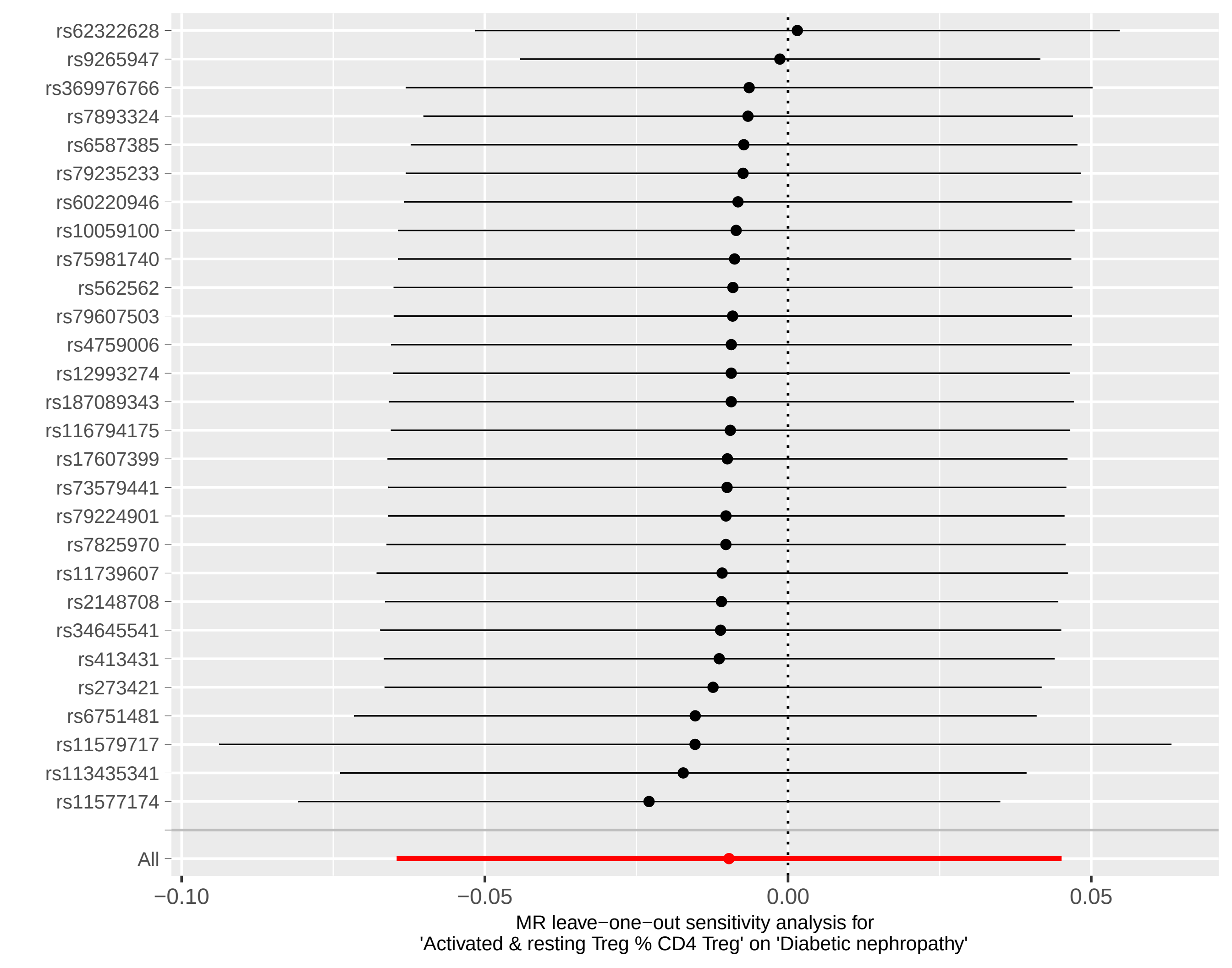

rs62322628
rs9265947
rs369976766
rs7893324
rs6587385
rs79235233
rs60220946
rs10059100
rs75981740
rs562562
rs79607503
rs4759006
rs12993274
rs187089343
rs116794175
rs17607399
rs73579441
rs79224901
rs7825970
rs11739607
rs2148708
rs34645541
rs413431
rs273421
rs6751481
rs11579717
rs113435341
rs11577174
All
−0.10
−0.05
0.00
0.05
MR leave−one−out sensitivity analysis for
'Activated & resting Treg % CD4 Treg' on 'Diabetic nephropathy'

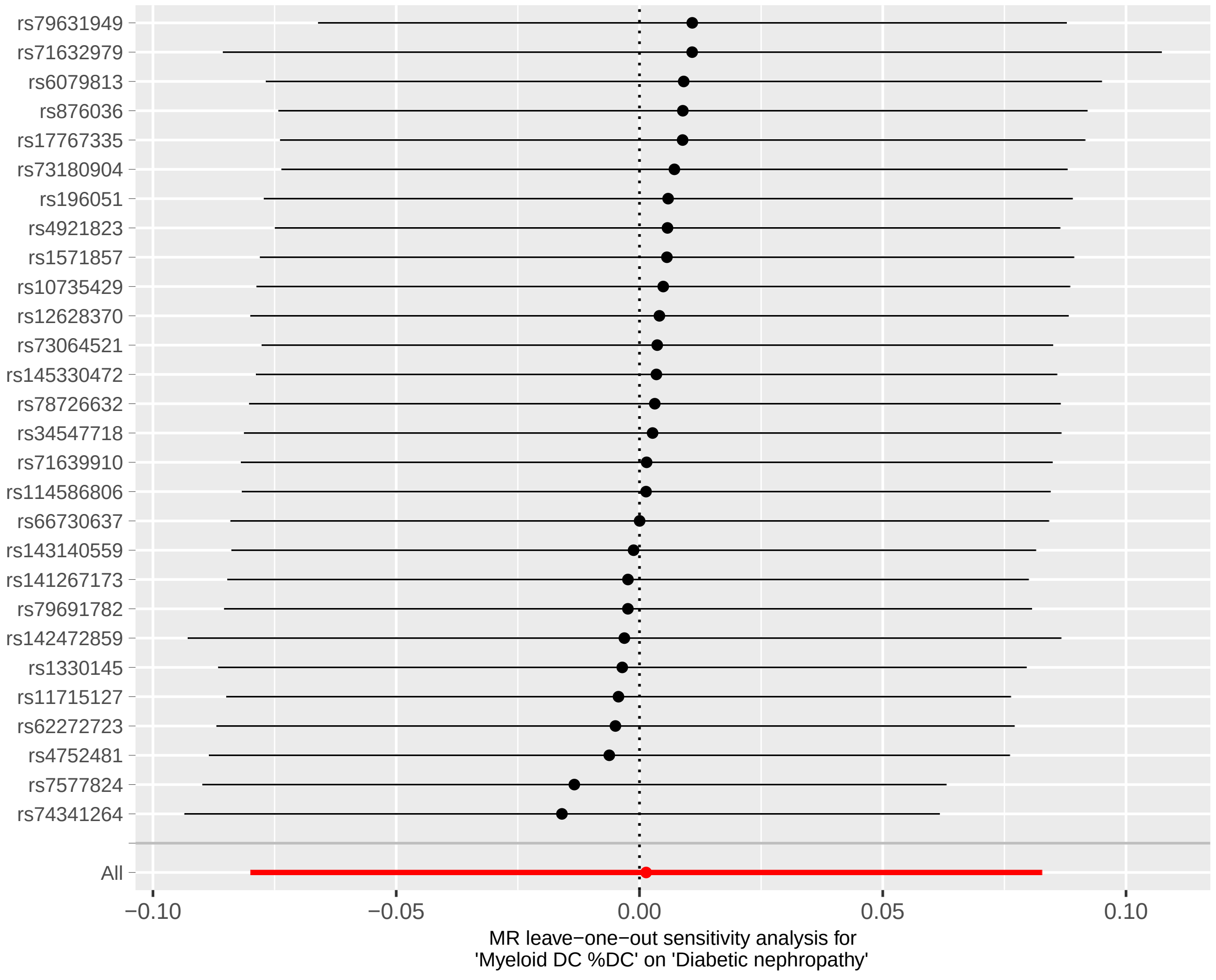

rs79631949
rs71632979
rs6079813
rs876036
rs17767335
rs73180904
rs196051
rs4921823
rs1571857
rs10735429
rs12628370
rs73064521
rs145330472
rs78726632
rs34547718
rs71639910
rs114586806
rs66730637
rs143140559
rs141267173
rs79691782
rs142472859
rs1330145
rs11715127
rs62272723
rs4752481
rs7577824
rs74341264
All
−0.10
−0.05
0.00
0.05
0.10
MR leave−one−out sensitivity analysis for
'Myeloid DC %DC' on 'Diabetic nephropathy'

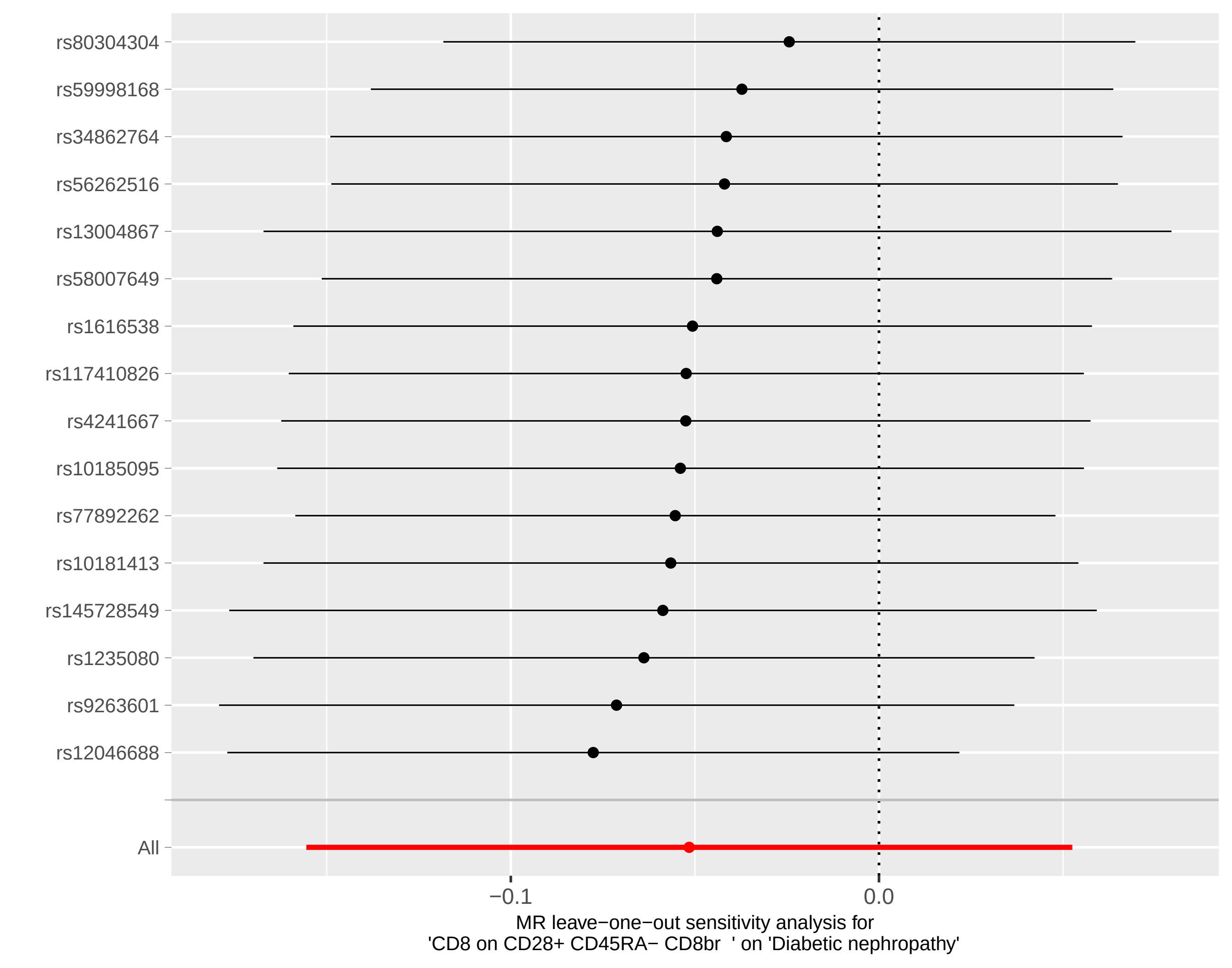
rs80304304
rs59998168
rs34862764
rs56262516
rs13004867
rs58007649
rs1616538
rs117410826
rs4241667
rs10185095
rs77892262
rs10181413
rs145728549
rs1235080
rs9263601
rs12046688
All
−0.1
0.0
MR leave−one−out sensitivity analysis for
'CD8 on CD28+ CD45RA− CD8br ' on 'Diabetic nephropathy'

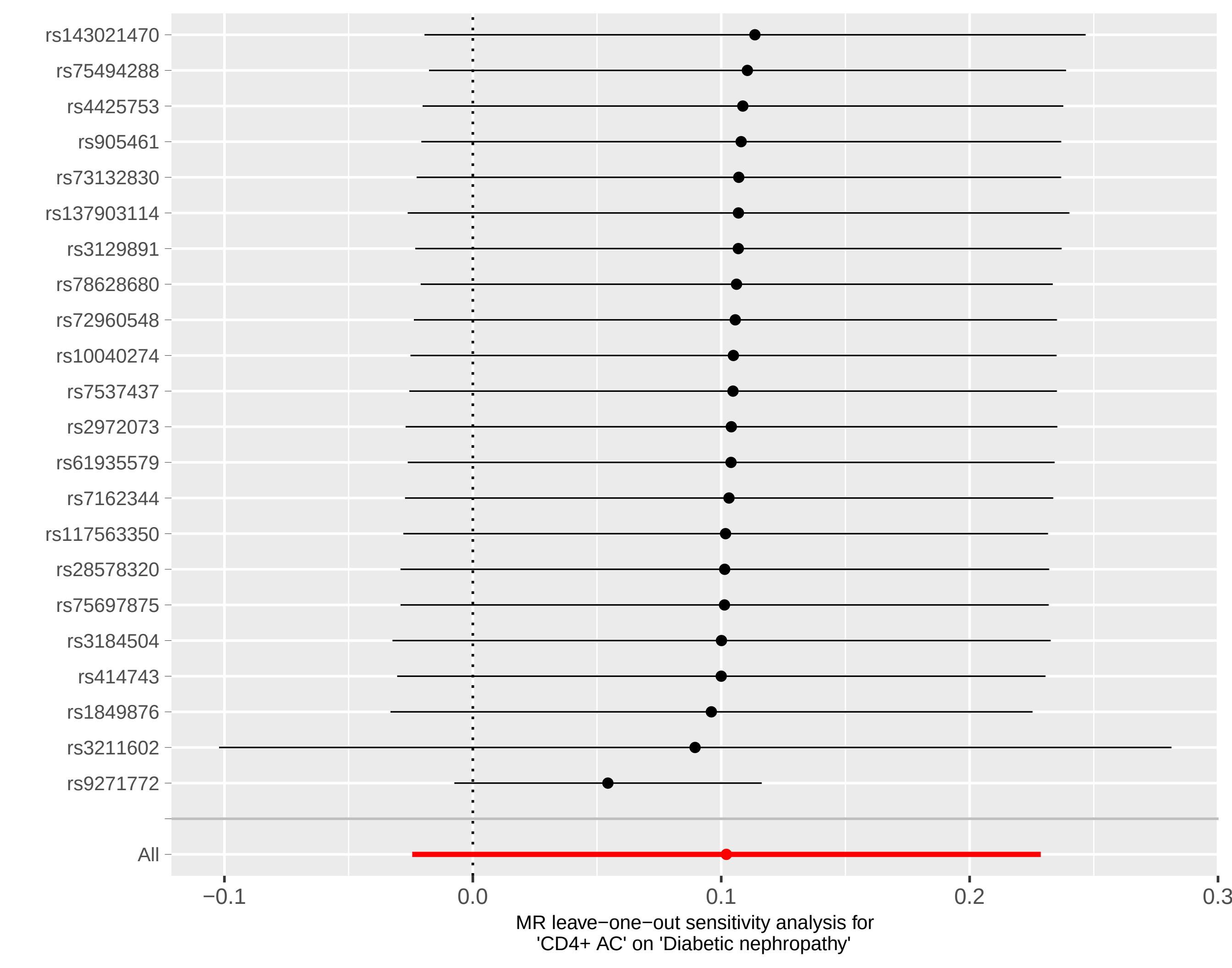

rs143021470
rs75494288
rs4425753
rs905461
rs73132830
rs137903114
rs3129891
rs78628680
rs72960548
rs10040274
rs7537437
rs2972073
rs61935579
rs7162344
rs117563350
rs28578320
rs75697875
rs3184504
rs414743
rs1849876
rs3211602
rs9271772
All
−0.1
0.0
0.1
0.2
0.3
MR leave−one−out sensitivity analysis for
'CD4+ AC' on 'Diabetic nephropathy'

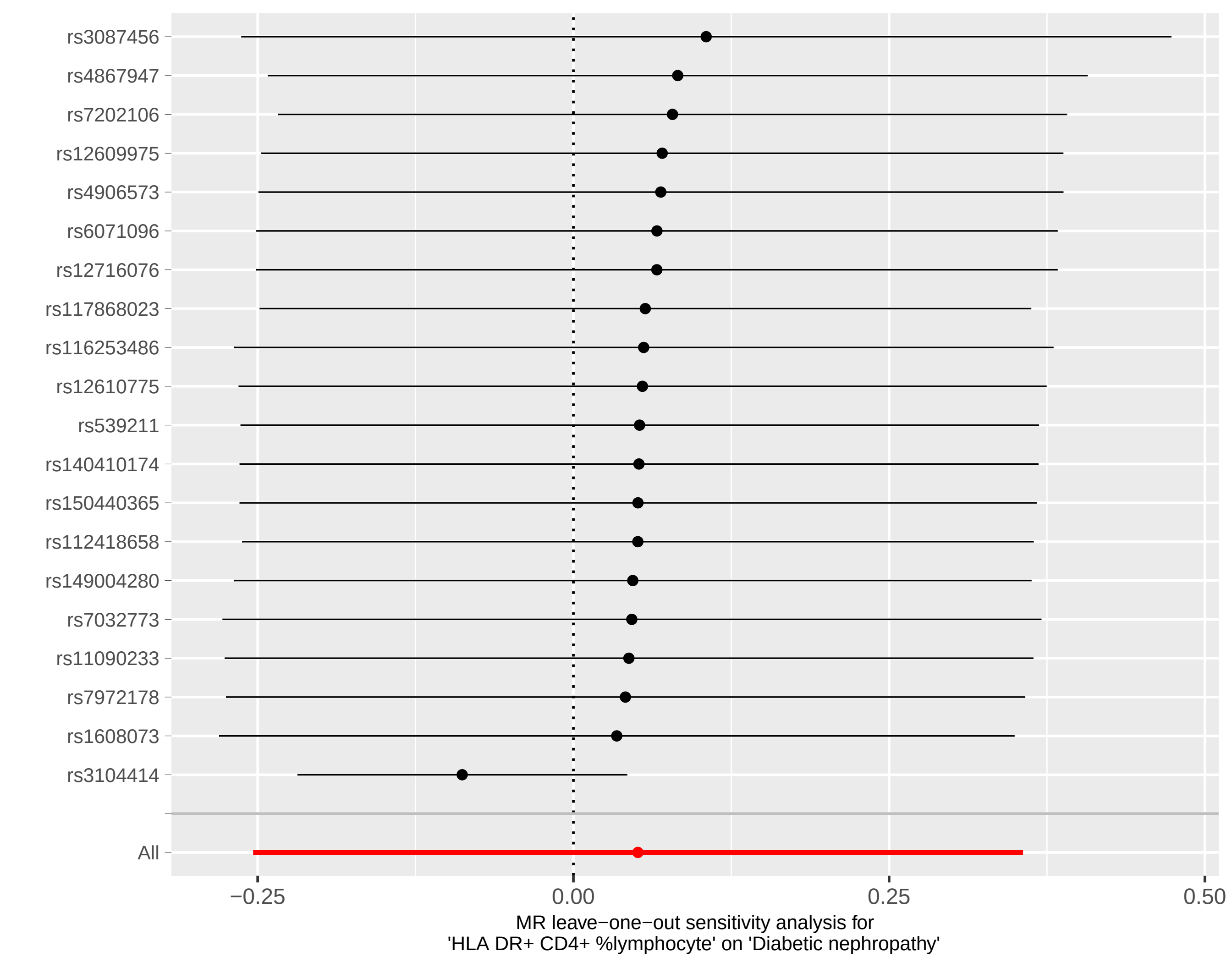
rs3087456
rs4867947
rs7202106
rs12609975
rs4906573
rs6071096
rs12716076
rs117868023
rs116253486
rs12610775
rs539211
rs140410174
rs150440365
rs112418658
rs149004280
rs7032773
rs11090233
rs7972178
rs1608073
rs3104414
All
−0.25
0.00
0.25
0.50
MR leave−one−out sensitivity analysis for
'HLA DR+ CD4+ %lymphocyte' on 'Diabetic nephropathy'

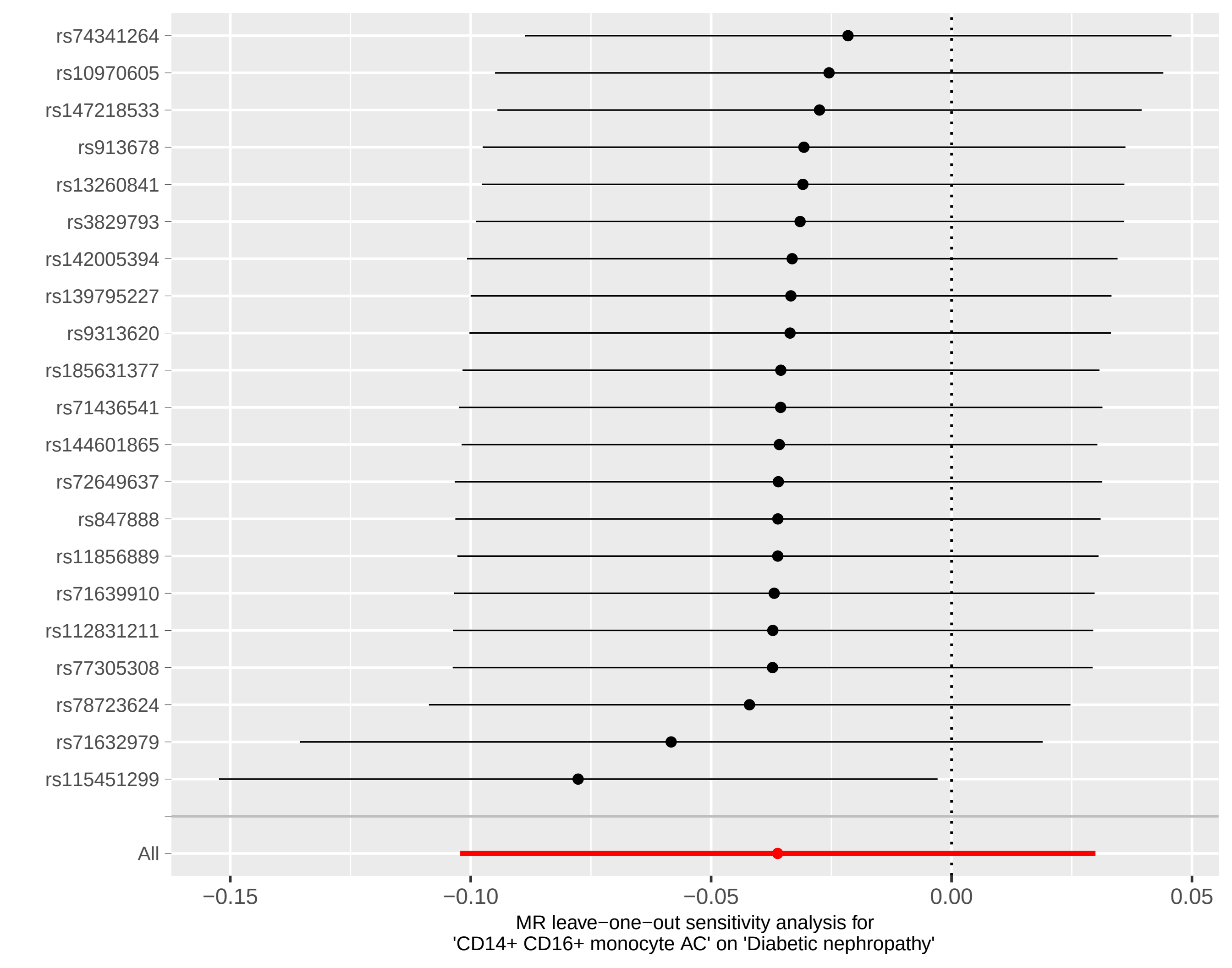
rs74341264
rs10970605
rs147218533
rs913678
rs13260841
rs3829793
rs142005394
rs139795227
rs9313620
rs185631377
rs71436541
rs144601865
rs72649637
rs847888
rs11856889
rs71639910
rs112831211
rs77305308
rs78723624
rs71632979
rs115451299
All
−0.15
−0.10
−0.05
0.00
0.05
MR leave−one−out sensitivity analysis for
'CD14+ CD16+ monocyte AC' on 'Diabetic nephropathy'

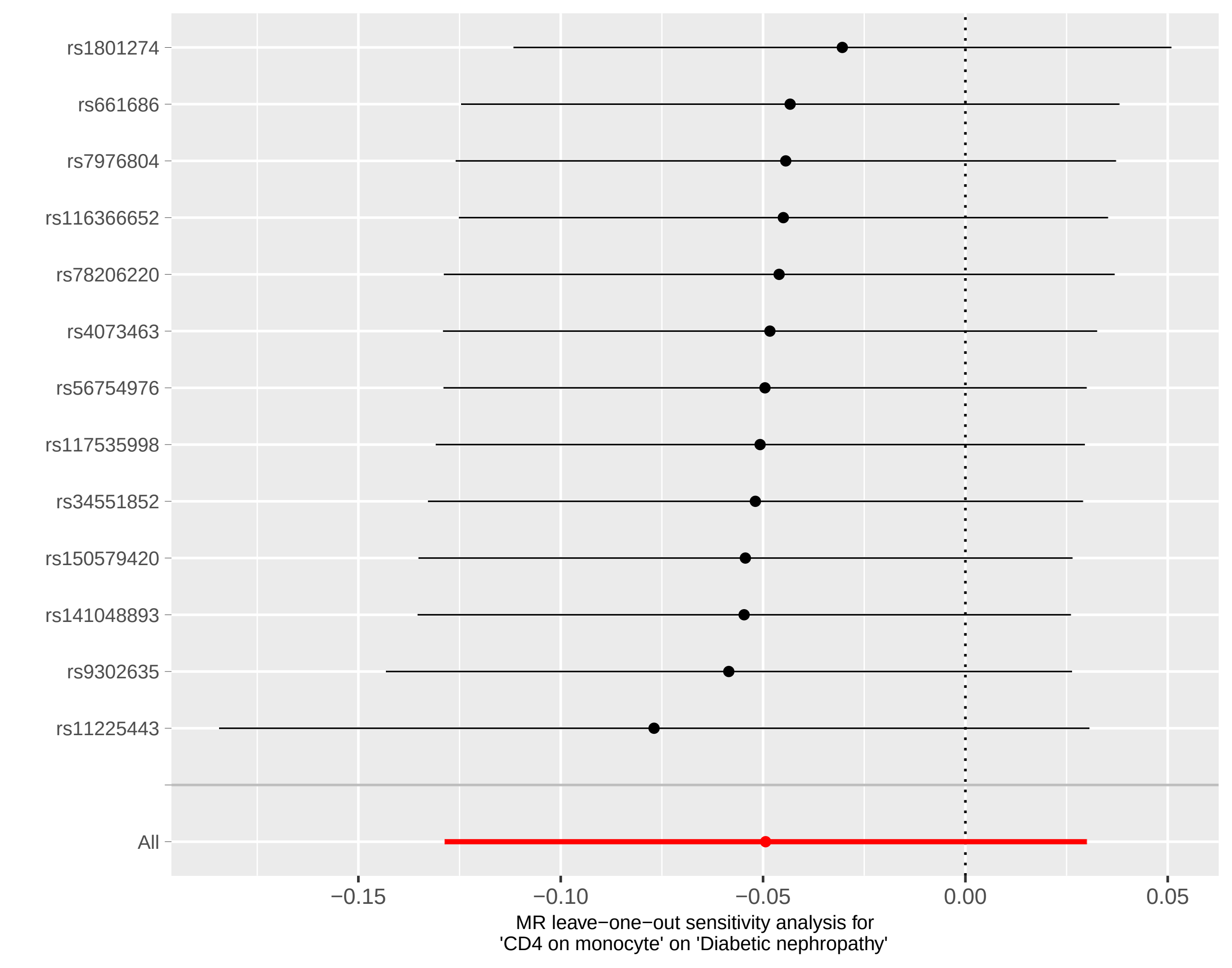
rs1801274
rs661686
rs7976804
rs116366652
rs78206220
rs4073463
rs56754976
rs117535998
rs34551852
rs150579420
rs141048893
rs9302635
rs11225443
All
−0.15
−0.10
−0.05
0.00
0.05
MR leave−one−out sensitivity analysis for
'CD4 on monocyte' on 'Diabetic nephropathy'

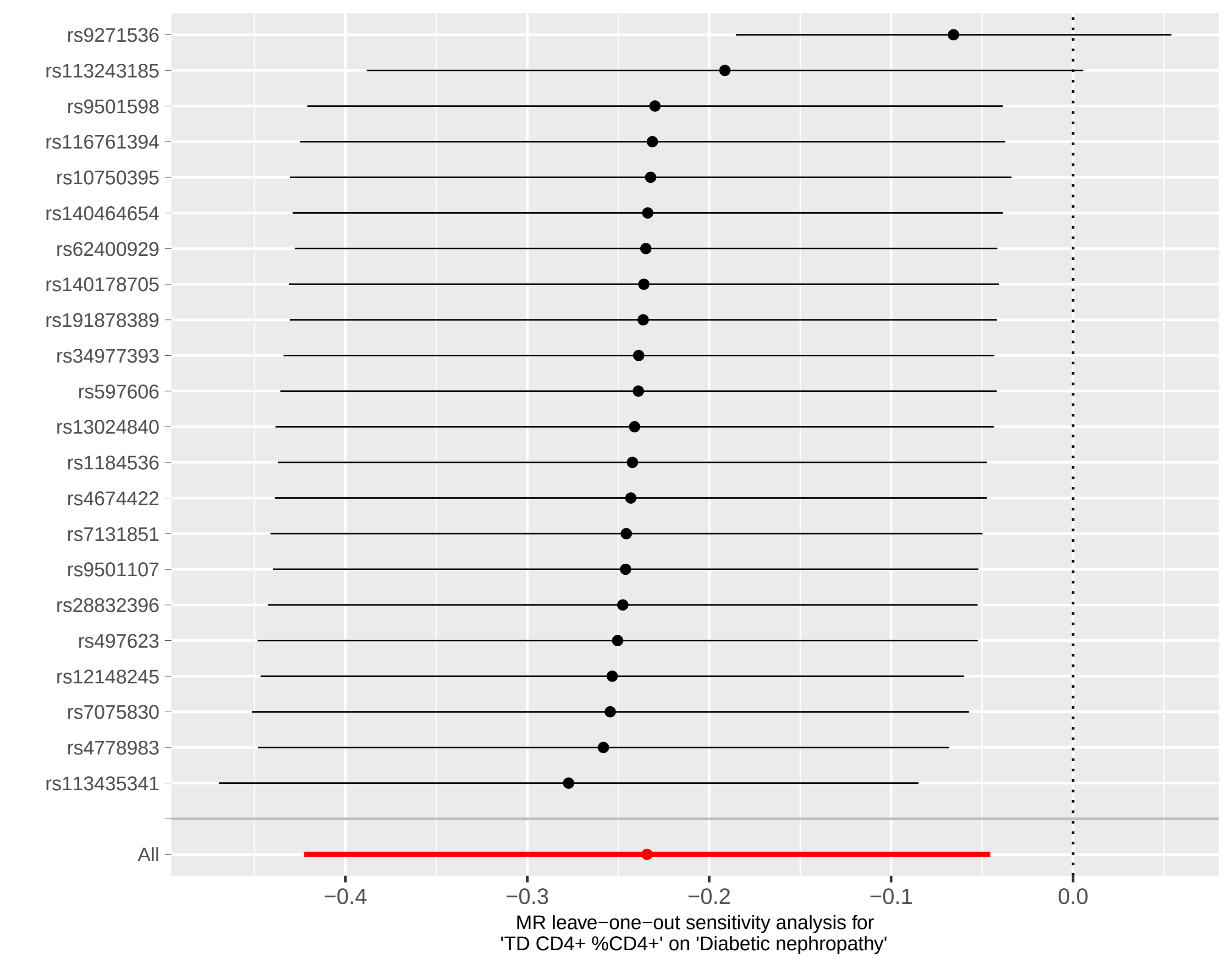
rs9271536
rs113243185
rs9501598
rs116761394
rs10750395
rs140464654
rs62400929
rs140178705
rs191878389
rs34977393
rs597606
rs13024840
rs1184536
rs4674422
rs7131851
rs9501107
rs28832396
rs497623
rs12148245
rs7075830
rs4778983
rs113435341
All
−0.4
−0.3
−0.2
−0.1
0.0
MR leave−one−out sensitivity analysis for
'TD CD4+ %CD4+' on 'Diabetic nephropathy'

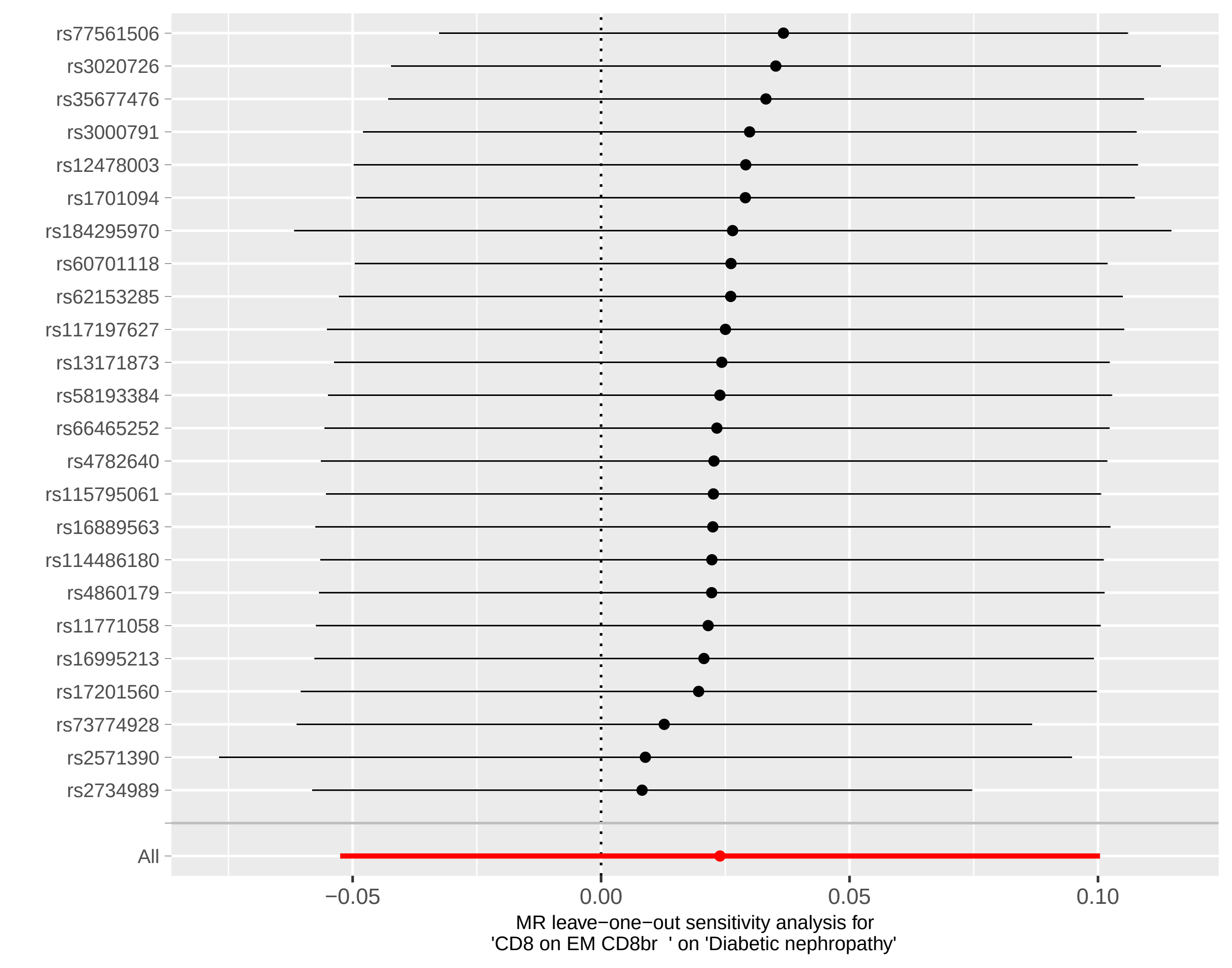
rs77561506
rs3020726
rs35677476
rs3000791
rs12478003
rs1701094
rs184295970
rs60701118
rs62153285
rs117197627
rs13171873
rs58193384
rs66465252
rs4782640
rs115795061
rs16889563
rs114486180
rs4860179
rs11771058
rs16995213
rs17201560
rs73774928
rs2571390
rs2734989
All
−0.05
0.00
0.05
0.10
MR leave−one−out sensitivity analysis for
'CD8 on EM CD8br ' on 'Diabetic nephropathy'

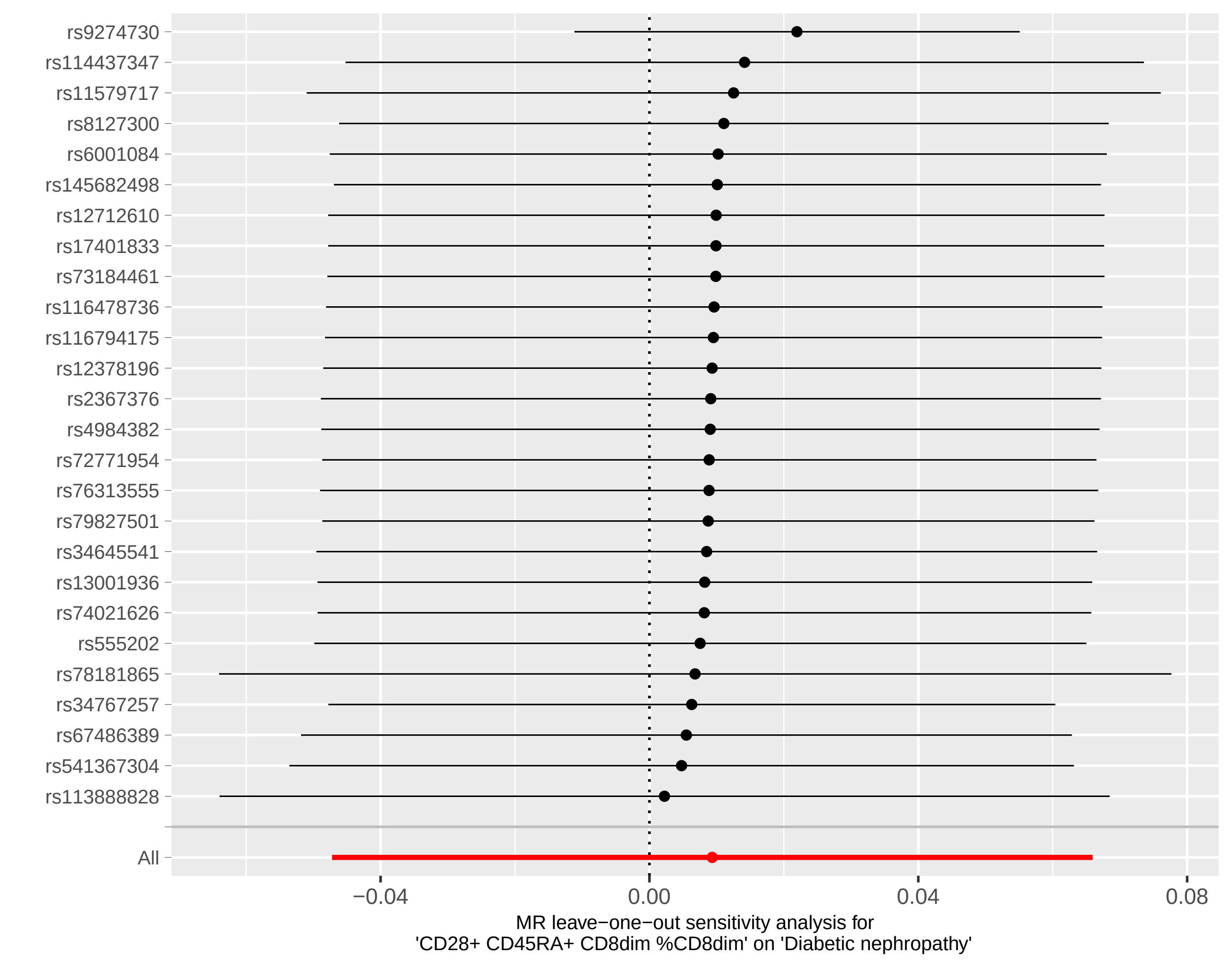
rs9274730
rs114437347
rs11579717
rs8127300
rs6001084
rs145682498
rs12712610
rs17401833
rs73184461
rs116478736
rs116794175
rs12378196
rs2367376
rs4984382
rs72771954
rs76313555
rs79827501
rs34645541
rs13001936
rs74021626
rs555202
rs78181865
rs34767257
rs67486389
rs541367304
rs113888828
All
−0.04
0.00
0.04
0.08
MR leave−one−out sensitivity analysis for
'CD28+ CD45RA+ CD8dim %CD8dim' on 'Diabetic nephropathy'

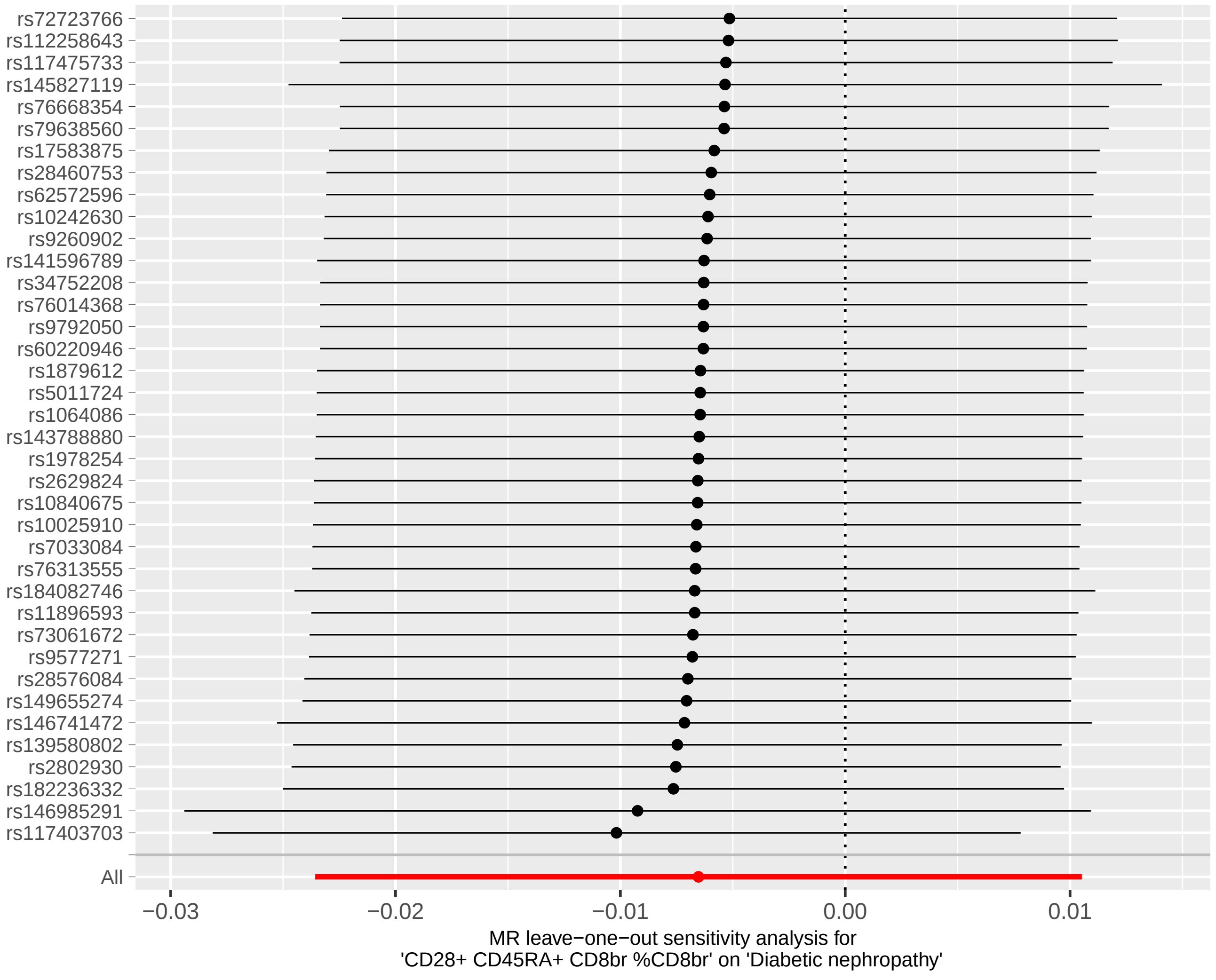

rs72723766
rs112258643
rs117475733
rs145827119
rs76668354
rs79638560
rs17583875
rs28460753
rs62572596
rs10242630
rs9260902
rs141596789
rs34752208
rs76014368
rs9792050
rs60220946
rs1879612
rs5011724
rs1064086
rs143788880
rs1978254
rs2629824
rs10840675
rs10025910
rs7033084
rs76313555
rs184082746
rs11896593
rs73061672
rs9577271
rs28576084
rs149655274
rs146741472
rs139580802
rs2802930
rs182236332
rs146985291
rs117403703
All
−0.03
−0.02
−0.01
0.00
0.01
MR leave−one−out sensitivity analysis for
'CD28+ CD45RA+ CD8br %CD8br' on 'Diabetic nephropathy'

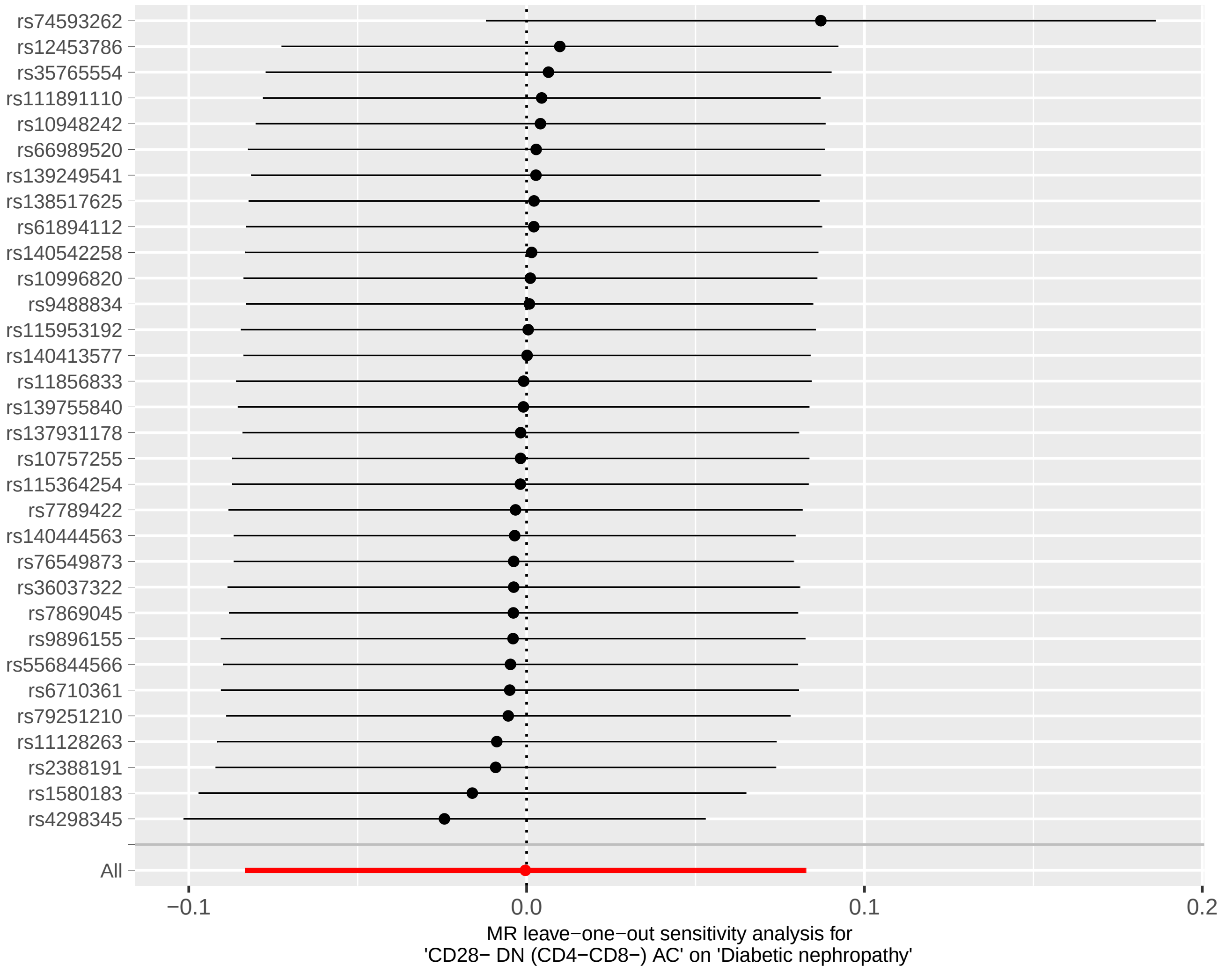
rs74593262
rs12453786
rs35765554
rs111891110
rs10948242
rs66989520
rs139249541
rs138517625
rs61894112
rs140542258
rs10996820
rs9488834
rs115953192
rs140413577
rs11856833
rs139755840
rs137931178
rs10757255
rs115364254
rs7789422
rs140444563
rs76549873
rs36037322
rs7869045
rs9896155
rs556844566
rs6710361
rs79251210
rs11128263
rs2388191
rs1580183
rs4298345
All
−0.1
0.0
0.1
0.2
MR leave−one−out sensitivity analysis for
'CD28− DN (CD4−CD8−) AC' on 'Diabetic nephropathy'

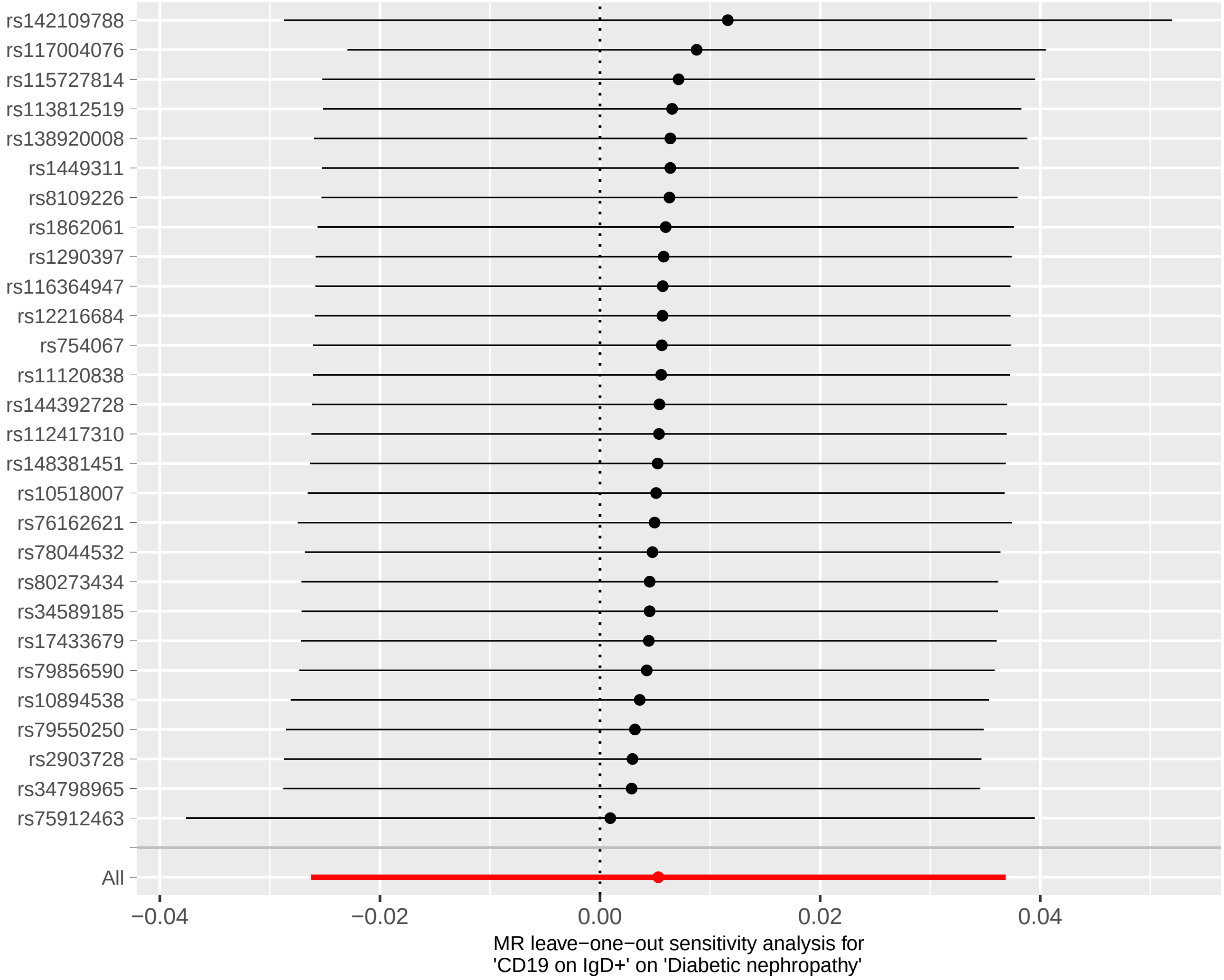

rs142109788
rs117004076
rs115727814
rs113812519
rs138920008
rs1449311
rs8109226
rs1862061
rs1290397
rs116364947
rs12216684
rs754067
rs11120838
rs144392728
rs112417310
rs148381451
rs10518007
rs76162621
rs78044532
rs80273434
rs34589185
rs17433679
rs79856590
rs10894538
rs79550250
rs2903728
rs34798965
rs75912463
All
−0.04
−0.02
0.00
0.02
0.04
MR leave−one−out sensitivity analysis for
'CD19 on IgD+' on 'Diabetic nephropathy'

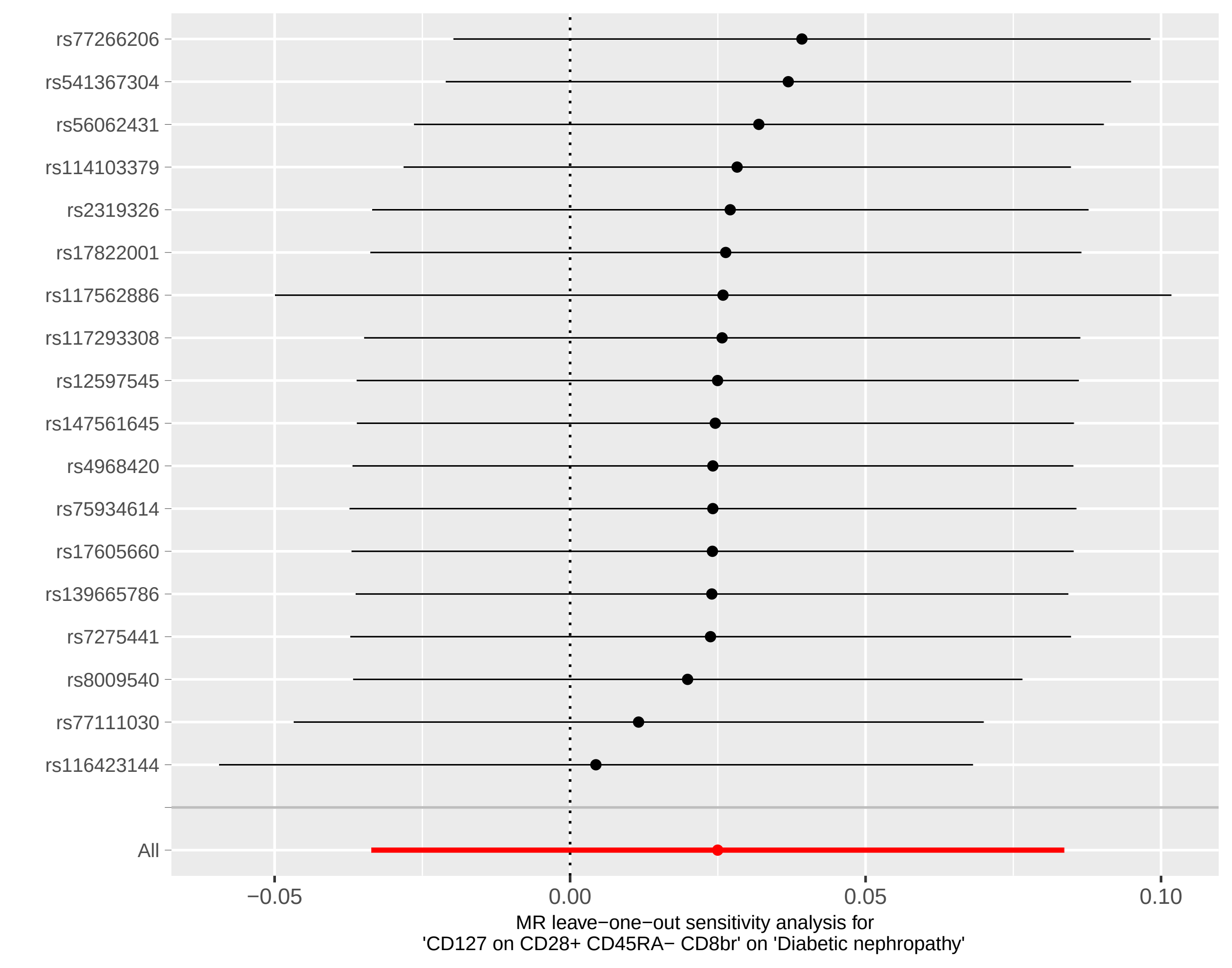

rs77266206
rs541367304
rs56062431
rs114103379
rs2319326
rs17822001
rs117562886
rs117293308
rs12597545
rs147561645
rs4968420
rs75934614
rs17605660
rs139665786
rs7275441
rs8009540
rs77111030
rs116423144
All
−0.05
0.00
0.05
0.10
MR leave−one−out sensitivity analysis for
'CD127 on CD28+ CD45RA− CD8br' on 'Diabetic nephropathy'

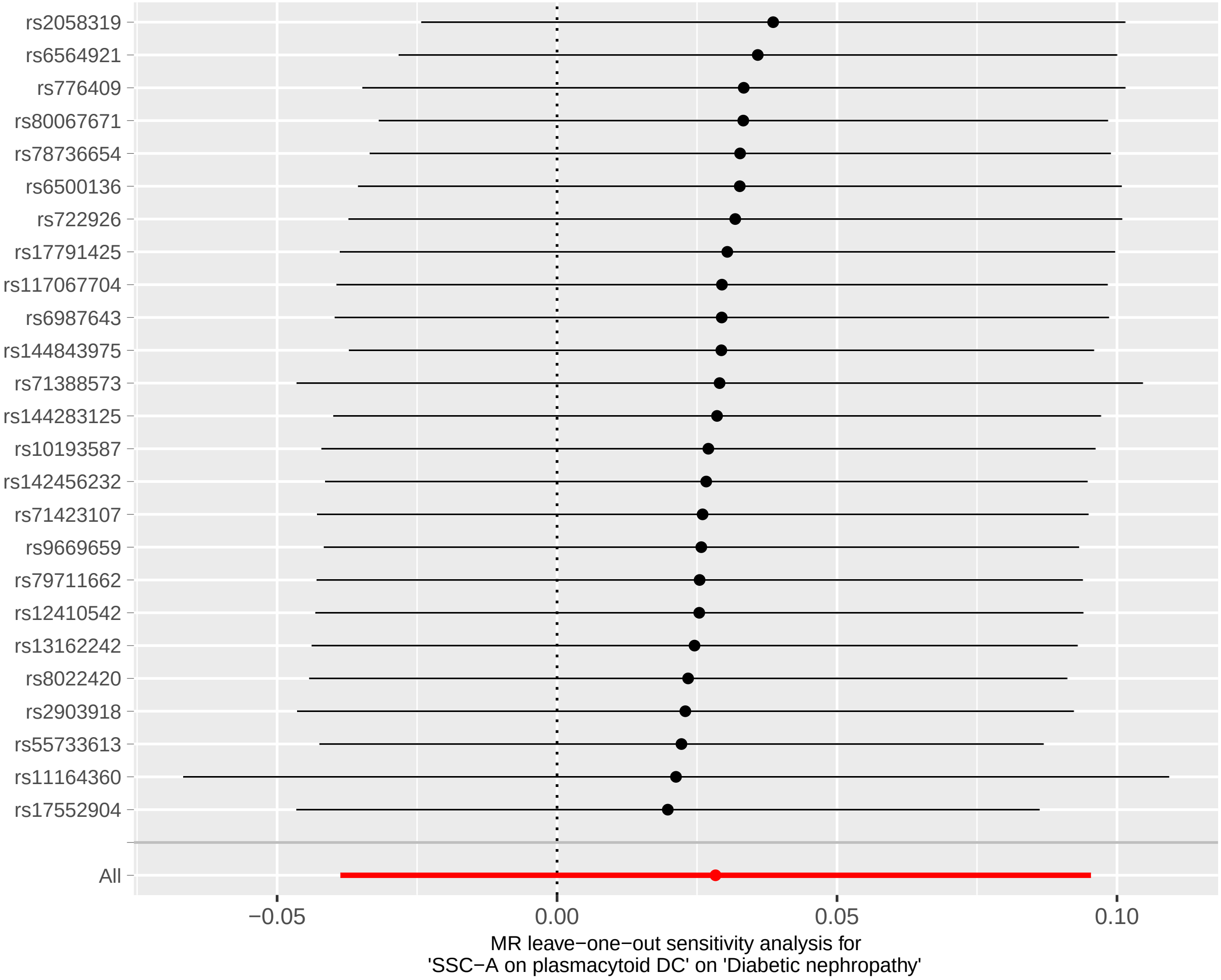
rs2058319
rs6564921
rs776409
rs80067671
rs78736654
rs6500136
rs722926
rs17791425
rs117067704
rs6987643
rs144843975
rs71388573
rs144283125
rs10193587
rs142456232
rs71423107
rs9669659
rs79711662
rs12410542
rs13162242
rs8022420
rs2903918
rs55733613
rs11164360
rs17552904
All
−0.05
0.00
0.05
0.10
MR leave−one−out sensitivity analysis for
'SSC−A on plasmacytoid DC' on 'Diabetic nephropathy'

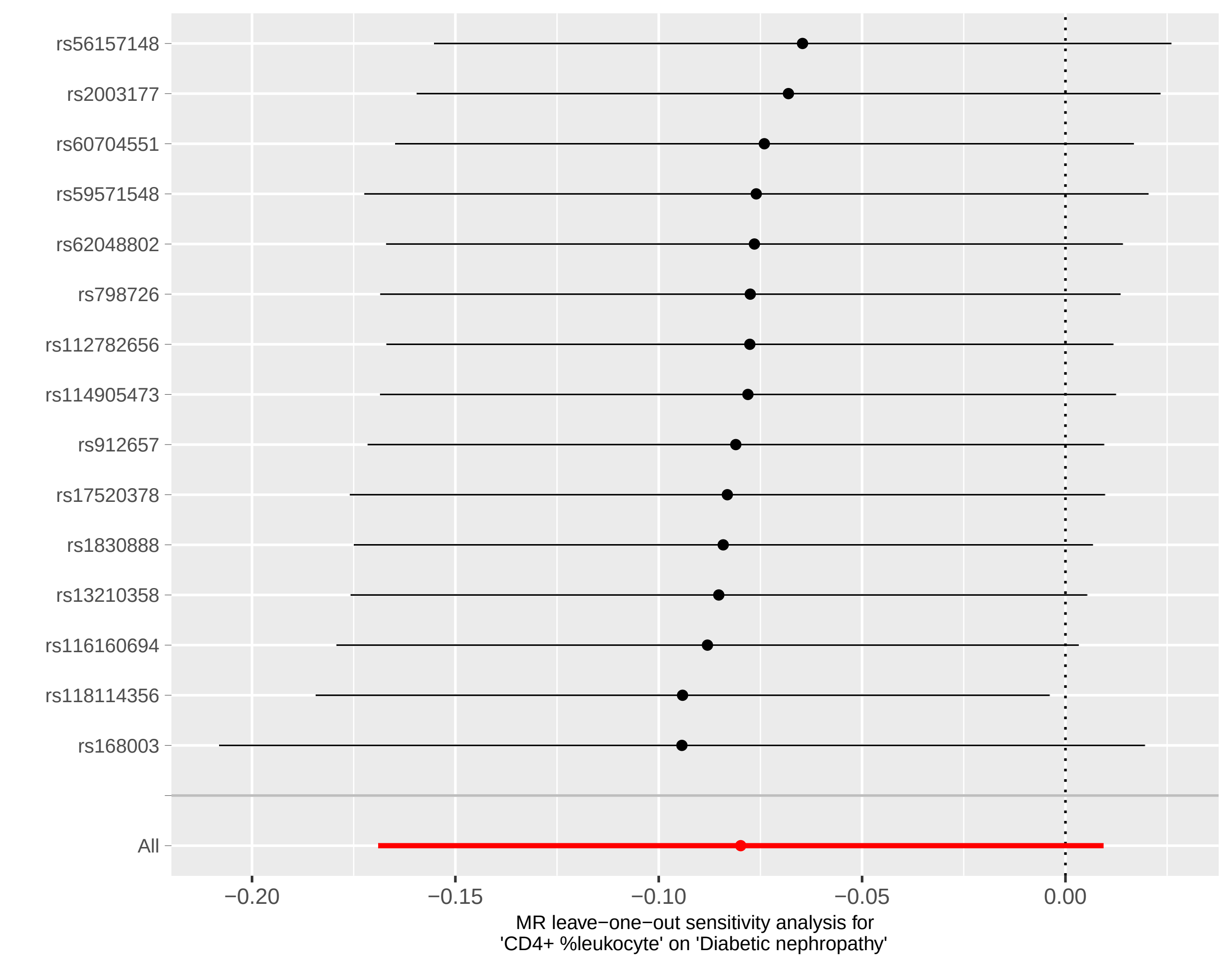
rs56157148
rs2003177
rs60704551
rs59571548
rs62048802
rs798726
rs112782656
rs114905473
rs912657
rs17520378
rs1830888
rs13210358
rs116160694
rs118114356
rs168003
All
−0.20
−0.15
−0.10
−0.05
0.00
MR leave−one−out sensitivity analysis for
'CD4+ %leukocyte' on 'Diabetic nephropathy'

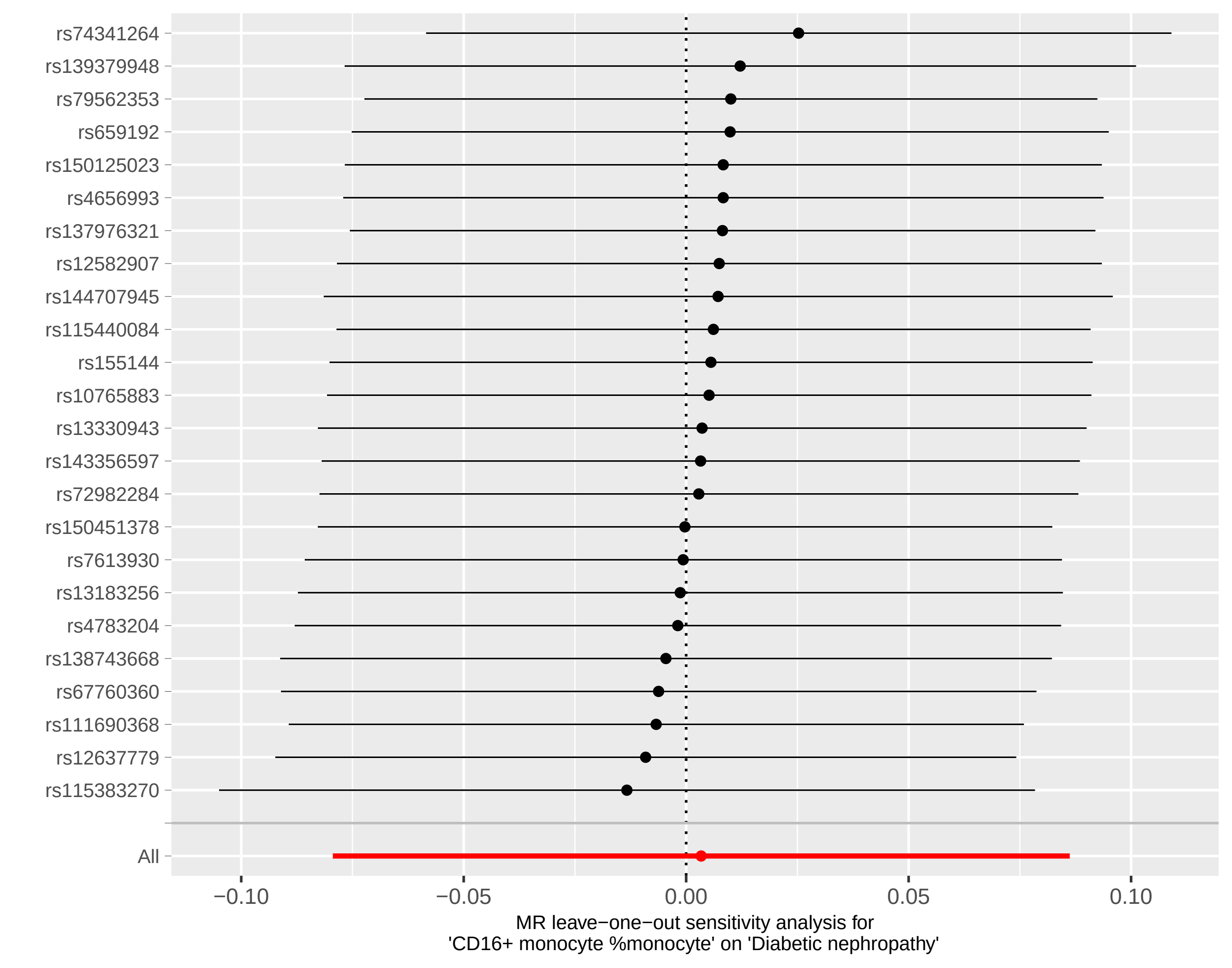
rs74341264
rs139379948
rs79562353
rs659192
rs150125023
rs4656993
rs137976321
rs12582907
rs144707945
rs115440084
rs155144
rs10765883
rs13330943
rs143356597
rs72982284
rs150451378
rs7613930
rs13183256
rs4783204
rs138743668
rs67760360
rs111690368
rs12637779
rs115383270
All
−0.10
−0.05
0.00
0.05
0.10
MR leave−one−out sensitivity analysis for
'CD16+ monocyte %monocyte' on 'Diabetic nephropathy'

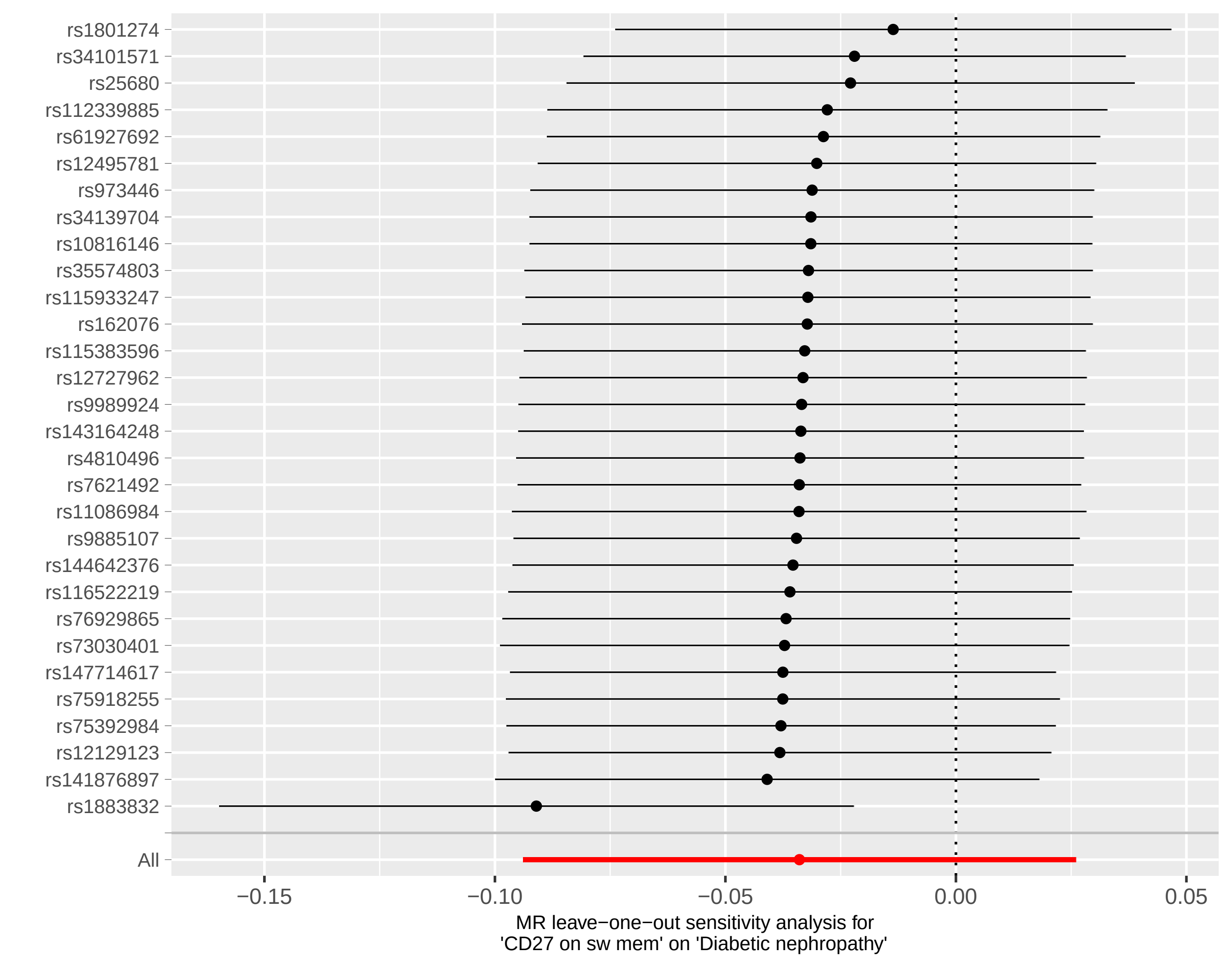

rs1801274
rs34101571
rs25680
rs112339885
rs61927692
rs12495781
rs973446
rs34139704
rs10816146
rs35574803
rs115933247
rs162076
rs115383596
rs12727962
rs9989924
rs143164248
rs4810496
rs7621492
rs11086984
rs9885107
rs144642376
rs116522219
rs76929865
rs73030401
rs147714617
rs75918255
rs75392984
rs12129123
rs141876897
rs1883832
All
−0.15
−0.10
−0.05
0.00
0.05
MR leave−one−out sensitivity analysis for
'CD27 on sw mem' on 'Diabetic nephropathy'

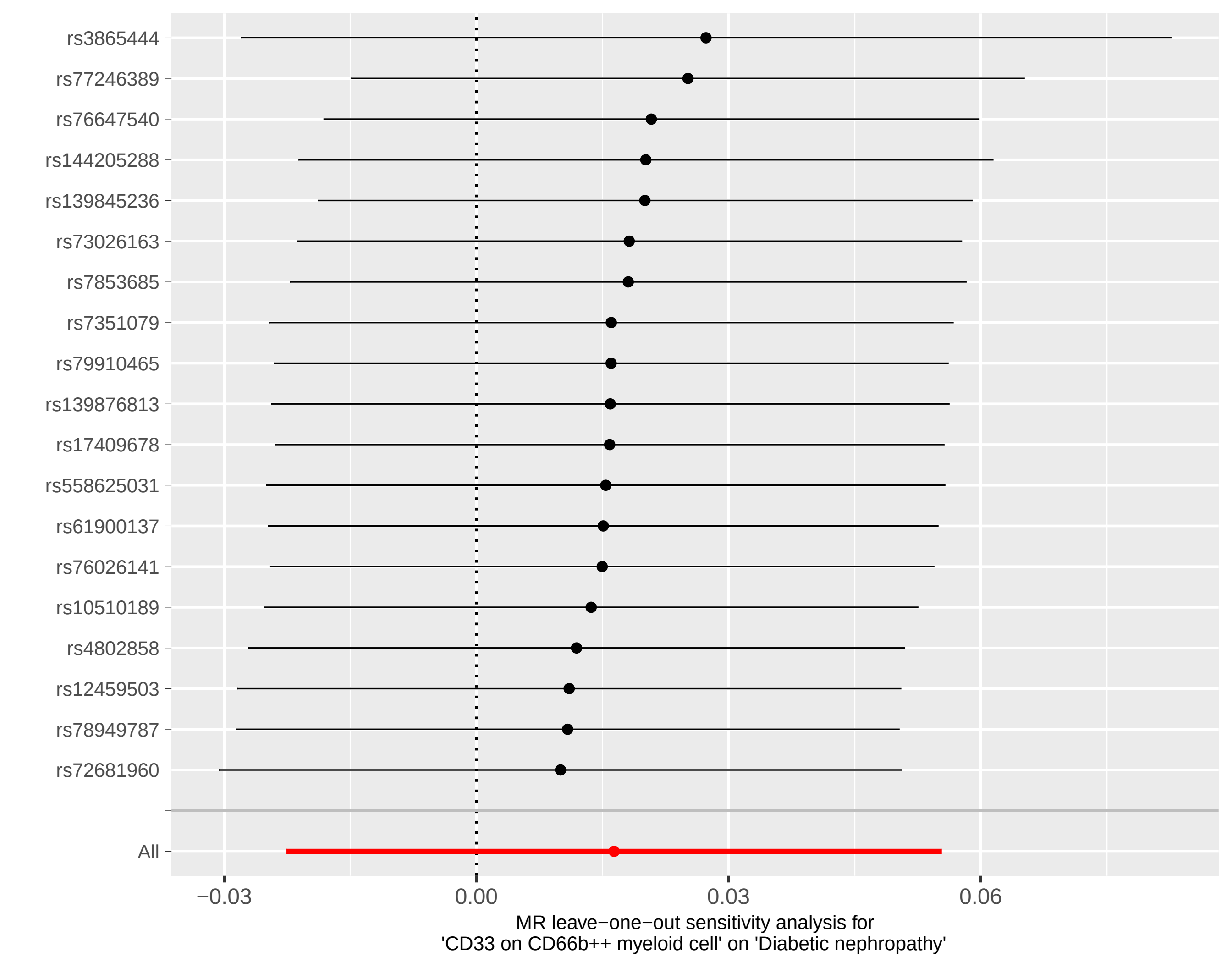
rs3865444
rs77246389
rs76647540
rs144205288
rs139845236
rs73026163
rs7853685
rs7351079
rs79910465
rs139876813
rs17409678
rs558625031
rs61900137
rs76026141
rs10510189
rs4802858
rs12459503
rs78949787
rs72681960
All
−0.03
0.00
0.03
0.06
MR leave−one−out sensitivity analysis for
'CD33 on CD66b++ myeloid cell' on 'Diabetic nephropathy'

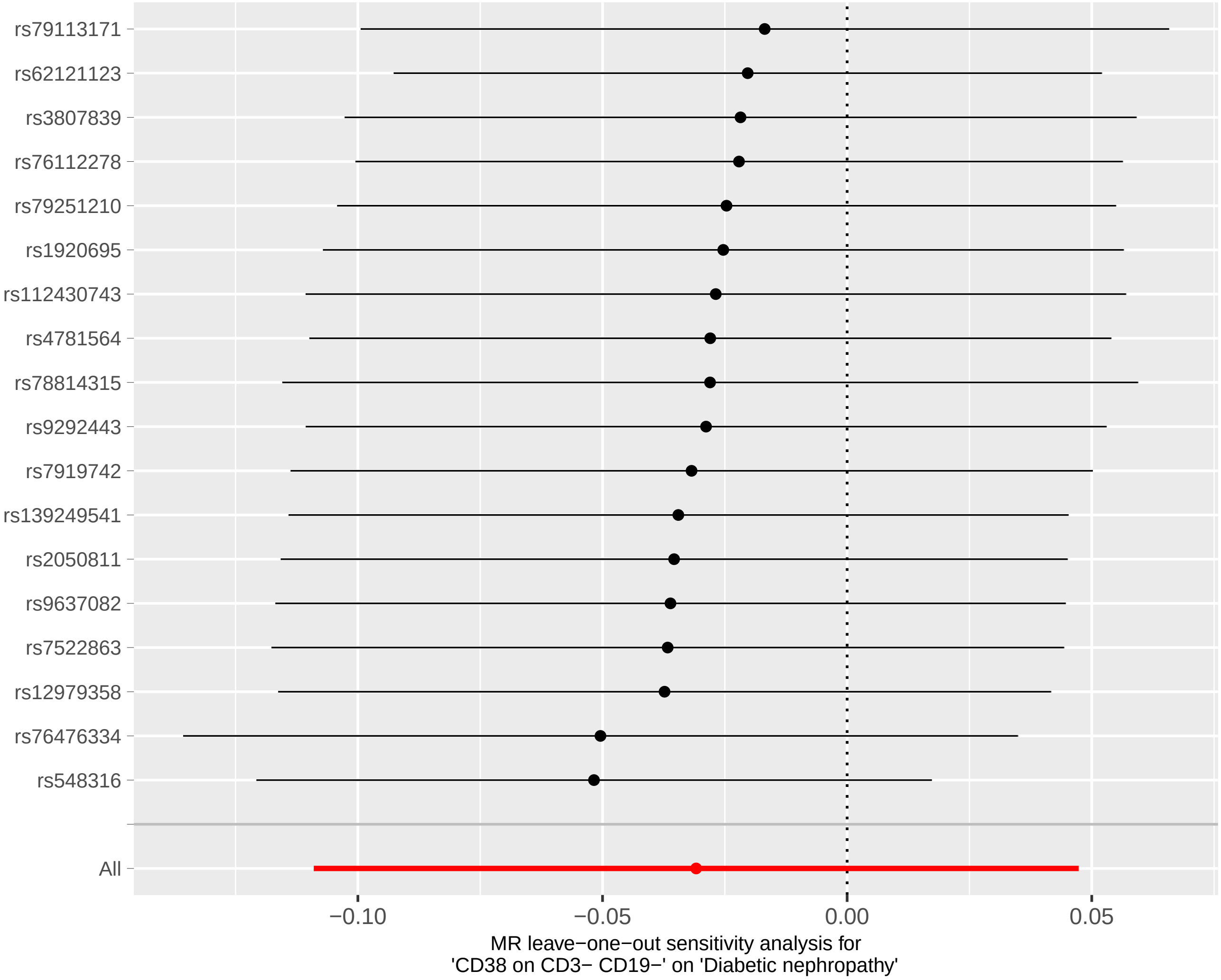

rs79113171
rs62121123
rs3807839
rs76112278
rs79251210
rs1920695
rs112430743
rs4781564
rs78814315
rs9292443
rs7919742
rs139249541
rs2050811
rs9637082
rs7522863
rs12979358
rs76476334
rs548316
All
−0.10
−0.05
0.00
0.05
MR leave−one−out sensitivity analysis for
'CD38 on CD3− CD19−' on 'Diabetic nephropathy'

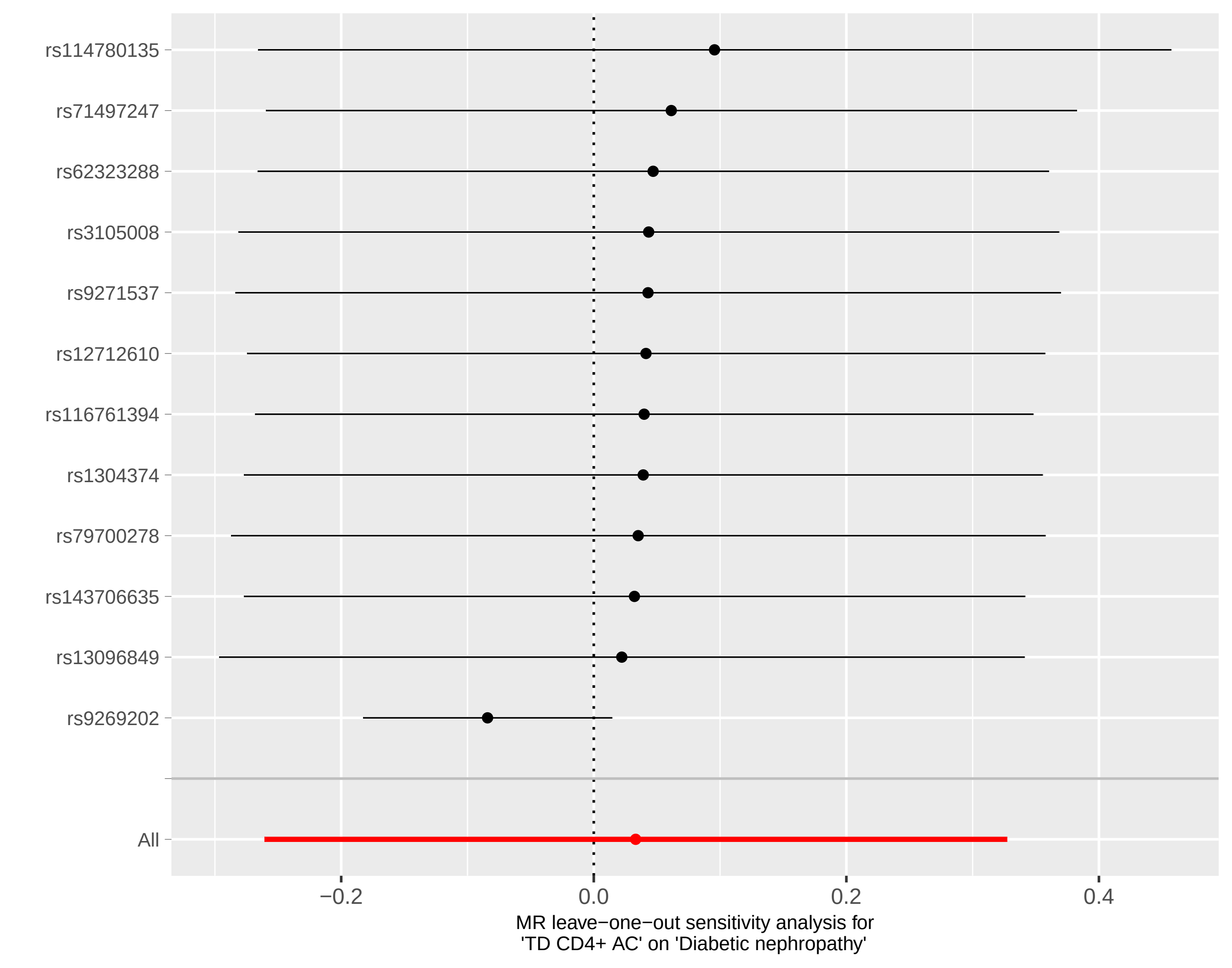
rs114780135
rs71497247
rs62323288
rs3105008
rs9271537
rs12712610
rs116761394
rs1304374
rs79700278
rs143706635
rs13096849
rs9269202
All
−0.2
0.0
0.2
0.4
MR leave−one−out sensitivity analysis for
'TD CD4+ AC' on 'Diabetic nephropathy'

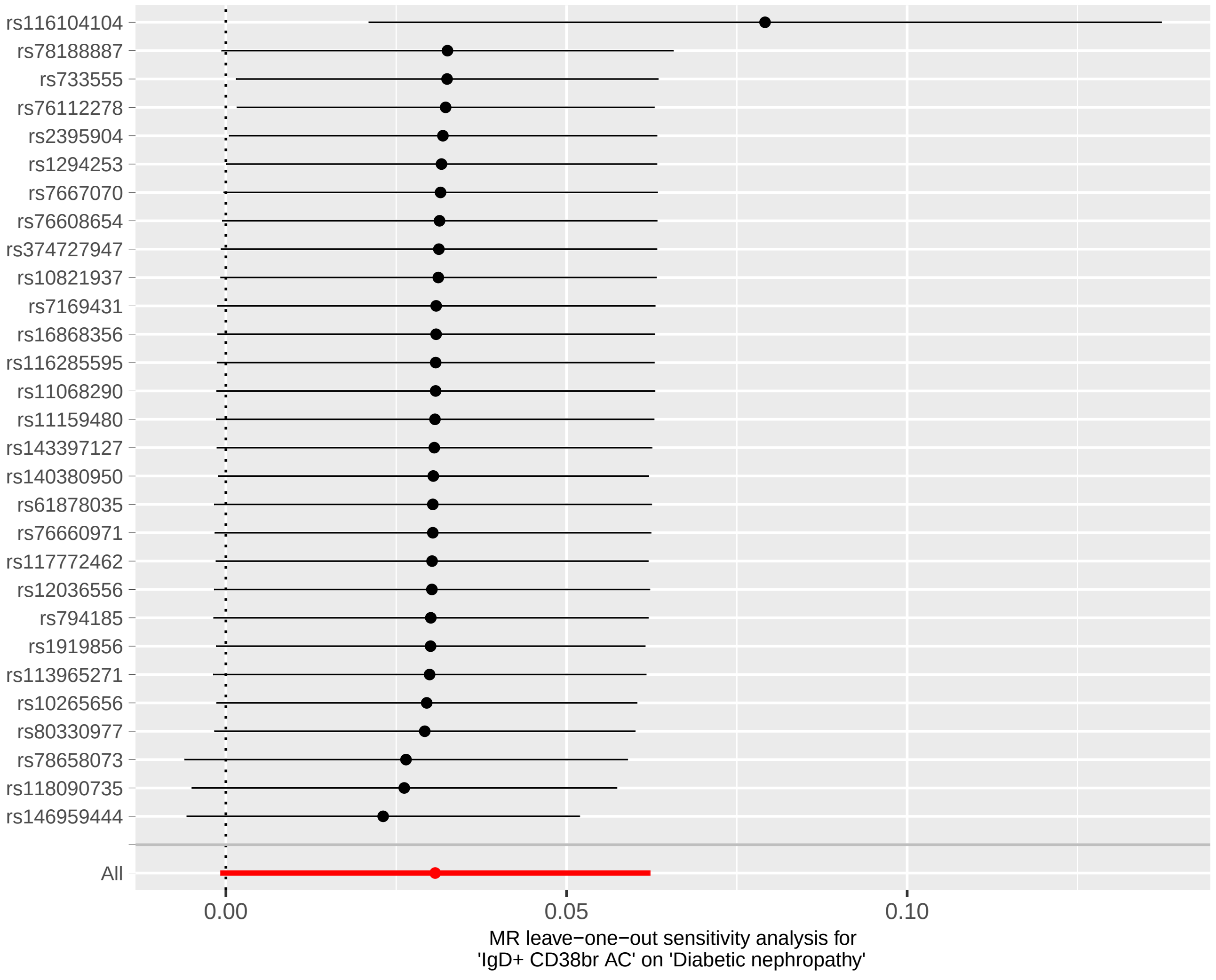
rs116104104
rs78188887
rs733555
rs76112278
rs2395904
rs1294253
rs7667070
rs76608654
rs374727947
rs10821937
rs7169431
rs16868356
rs116285595
rs11068290
rs11159480
rs143397127
rs140380950
rs61878035
rs76660971
rs117772462
rs12036556
rs794185
rs1919856
rs113965271
rs10265656
rs80330977
rs78658073
rs118090735
rs146959444
All
0.00
0.05
0.10
MR leave−one−out sensitivity analysis for
'IgD+ CD38br AC' on 'Diabetic nephropathy'

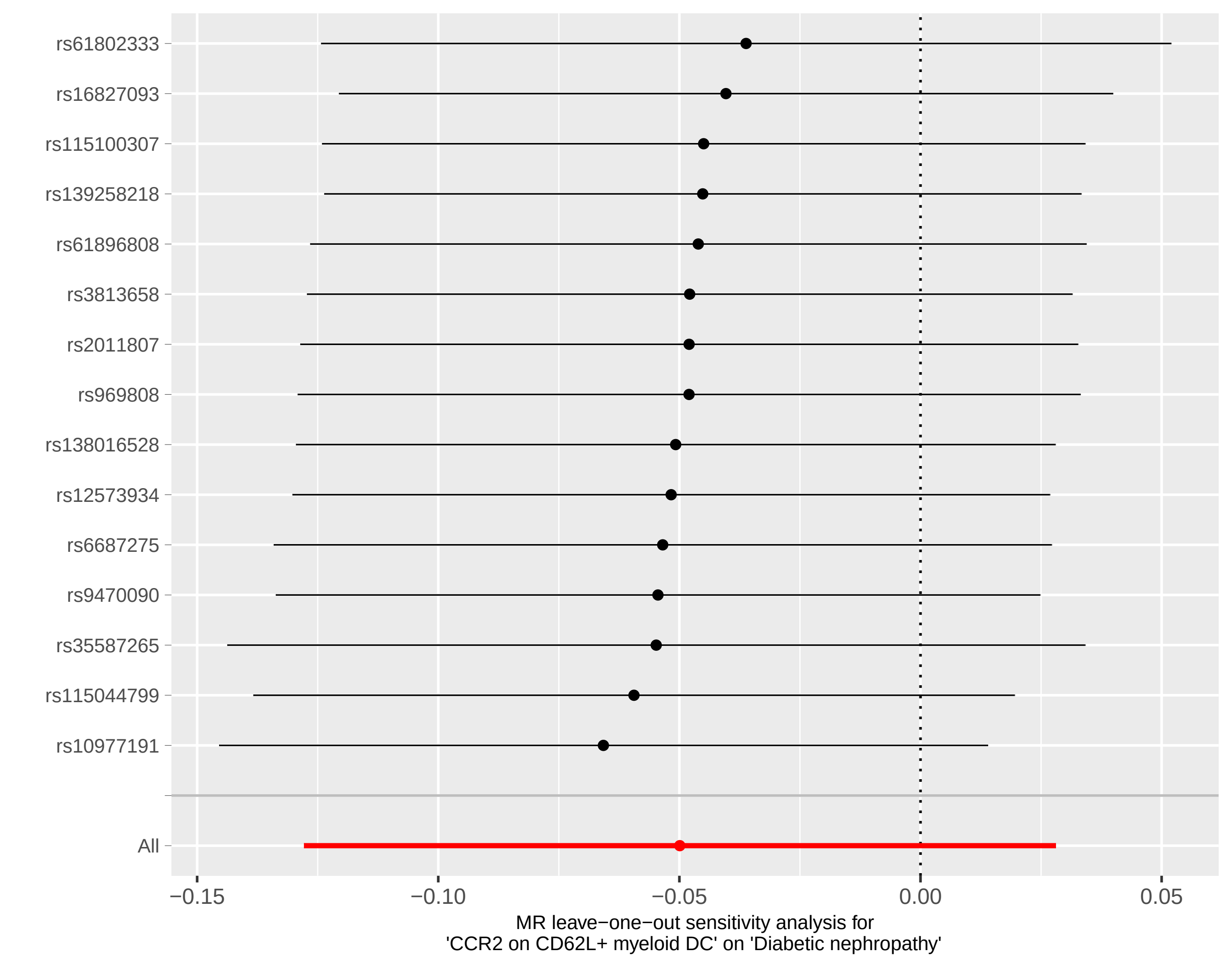
rs61802333
rs16827093
rs115100307
rs139258218
rs61896808
rs3813658
rs2011807
rs969808
rs138016528
rs12573934
rs6687275
rs9470090
rs35587265
rs115044799
rs10977191
All
−0.15
−0.10
−0.05
0.00
0.05
MR leave−one−out sensitivity analysis for
'CCR2 on CD62L+ myeloid DC' on 'Diabetic nephropathy'

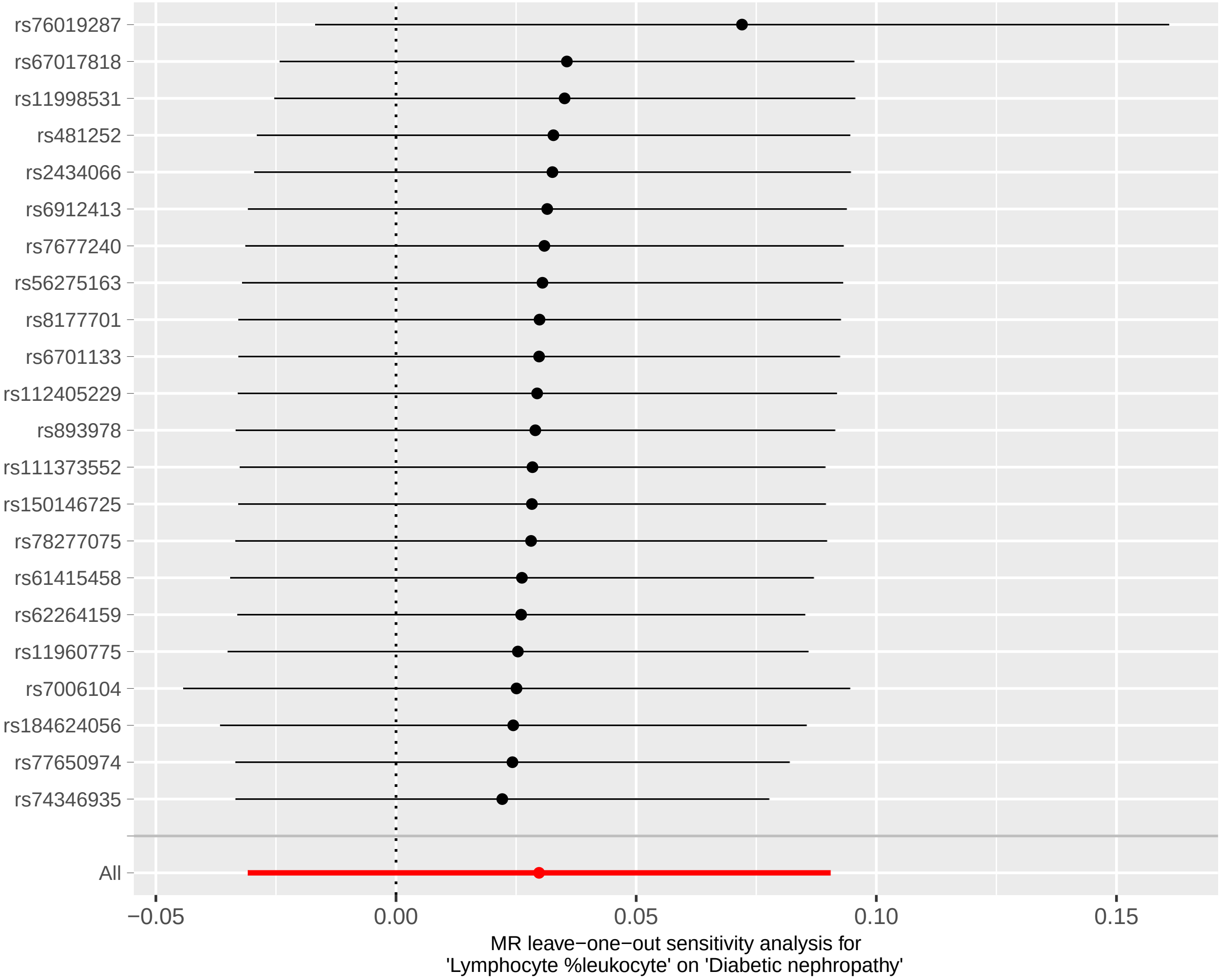

rs76019287
rs67017818
rs11998531
rs481252
rs2434066
rs6912413
rs7677240
rs56275163
rs8177701
rs6701133
rs112405229
rs893978
rs111373552
rs150146725
rs78277075
rs61415458
rs62264159
rs11960775
rs7006104
rs184624056
rs77650974
rs74346935
All
−0.05
0.00
0.05
0.10
0.15
MR leave−one−out sensitivity analysis for
'Lymphocyte %leukocyte' on 'Diabetic nephropathy'

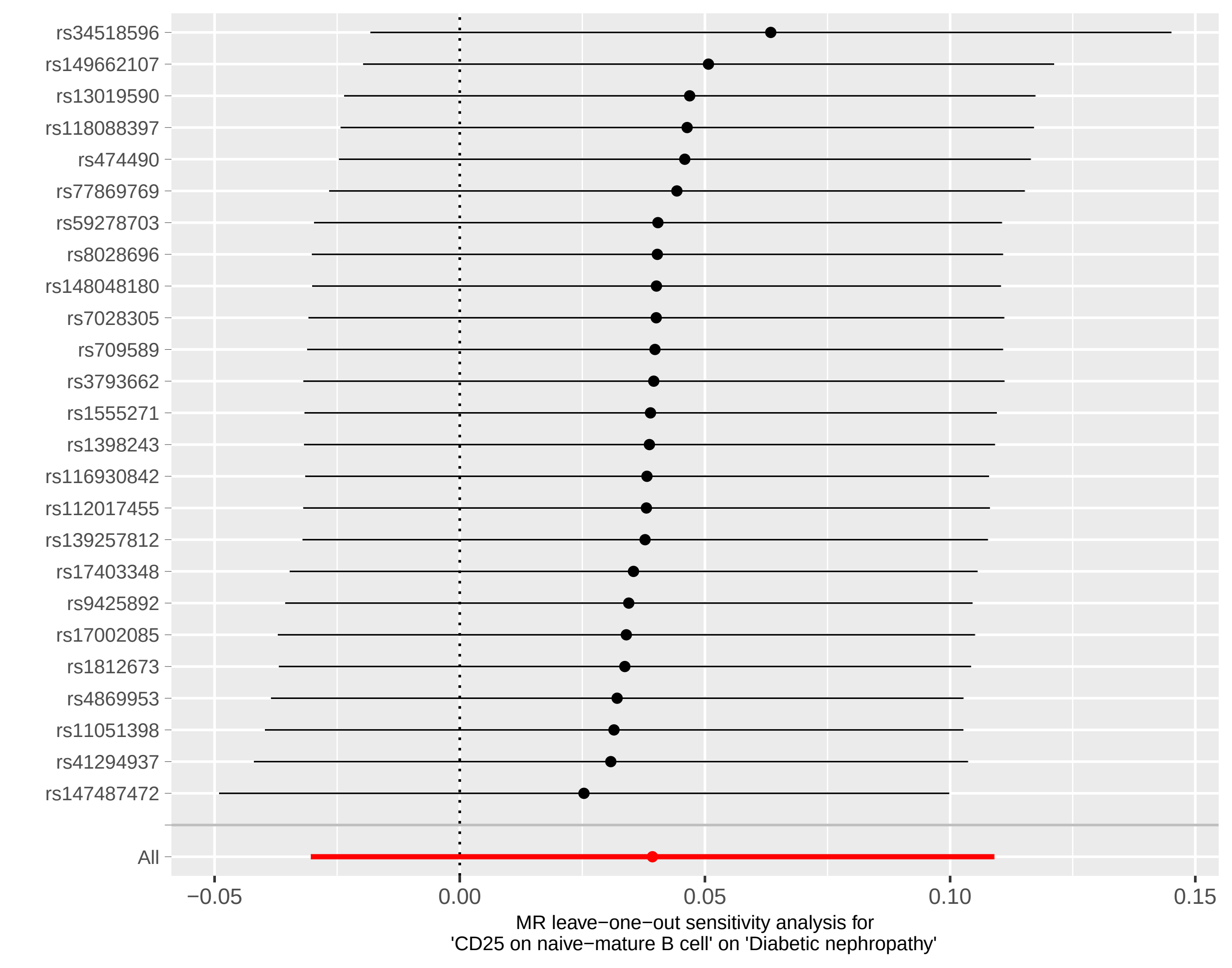
rs34518596
rs149662107
rs13019590
rs118088397
rs474490
rs77869769
rs59278703
rs8028696
rs148048180
rs7028305
rs709589
rs3793662
rs1555271
rs1398243
rs116930842
rs112017455
rs139257812
rs17403348
rs9425892
rs17002085
rs1812673
rs4869953
rs11051398
rs41294937
rs147487472
All
−0.05
0.00
0.05
0.10
0.15
MR leave−one−out sensitivity analysis for
'CD25 on naive−mature B cell' on 'Diabetic nephropathy'

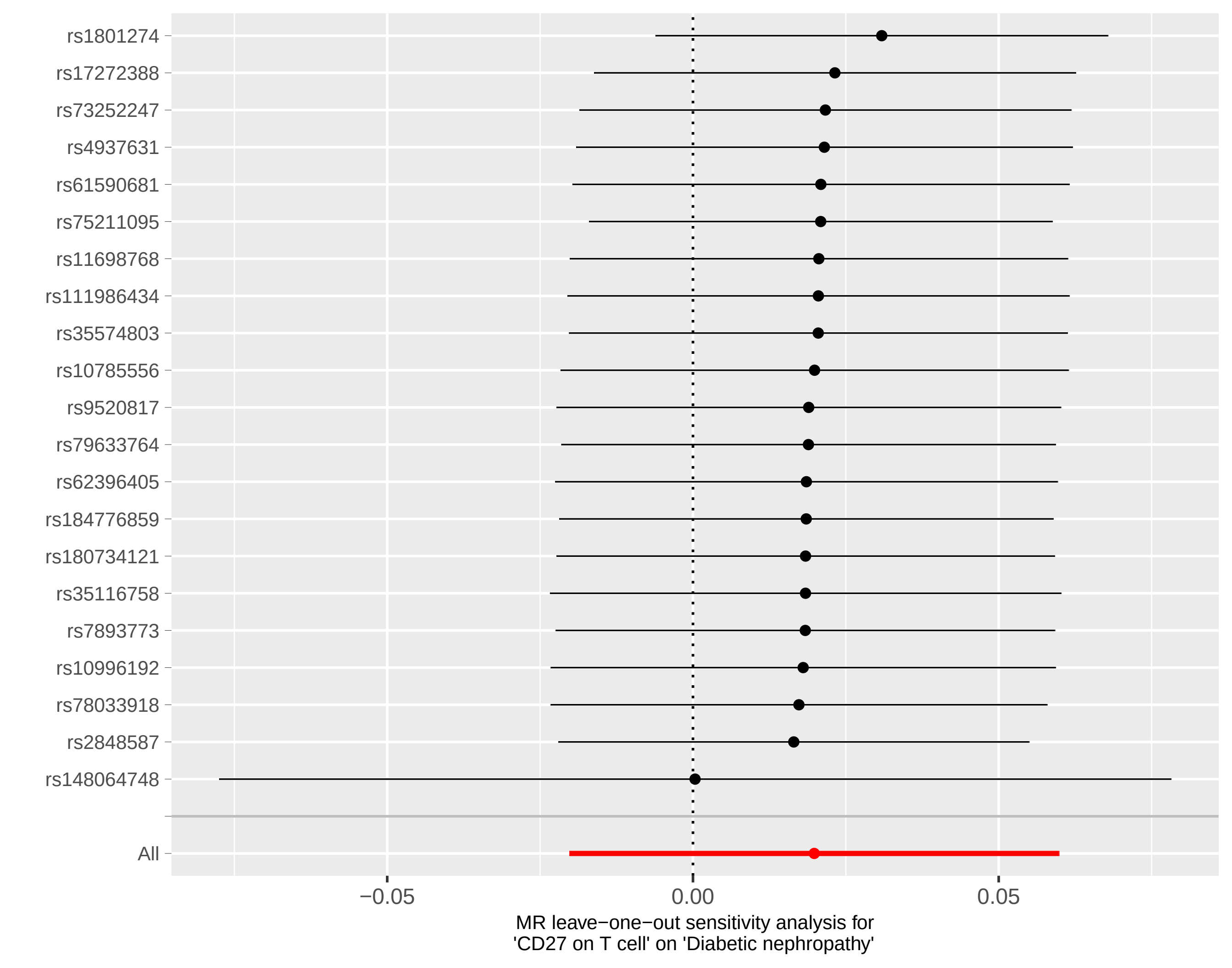
rs1801274
rs17272388
rs73252247
rs4937631
rs61590681
rs75211095
rs11698768
rs111986434
rs35574803
rs10785556
rs9520817
rs79633764
rs62396405
rs184776859
rs180734121
rs35116758
rs7893773
rs10996192
rs78033918
rs2848587
rs148064748
All
−0.05
0.00
0.05
MR leave−one−out sensitivity analysis for
'CD27 on T cell' on 'Diabetic nephropathy'

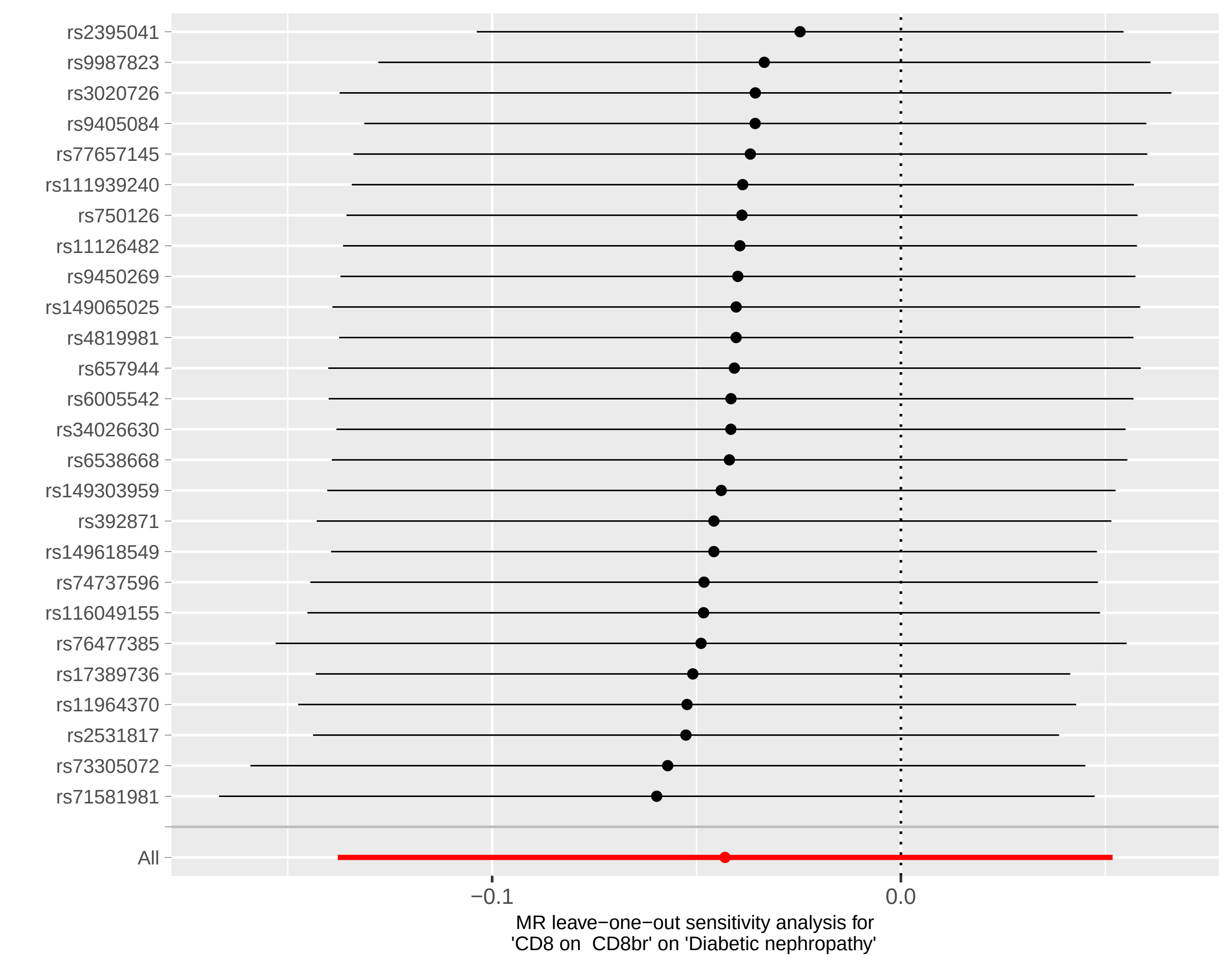
rs2395041
rs9987823
rs3020726
rs9405084
rs77657145
rs111939240
rs750126
rs11126482
rs9450269
rs149065025
rs4819981
rs657944
rs6005542
rs34026630
rs6538668
rs149303959
rs392871
rs149618549
rs74737596
rs116049155
rs76477385
rs17389736
rs11964370
rs2531817
rs73305072
rs71581981
All
−0.1
0.0
MR leave−one−out sensitivity analysis for
'CD8 on CD8br' on 'Diabetic nephropathy'

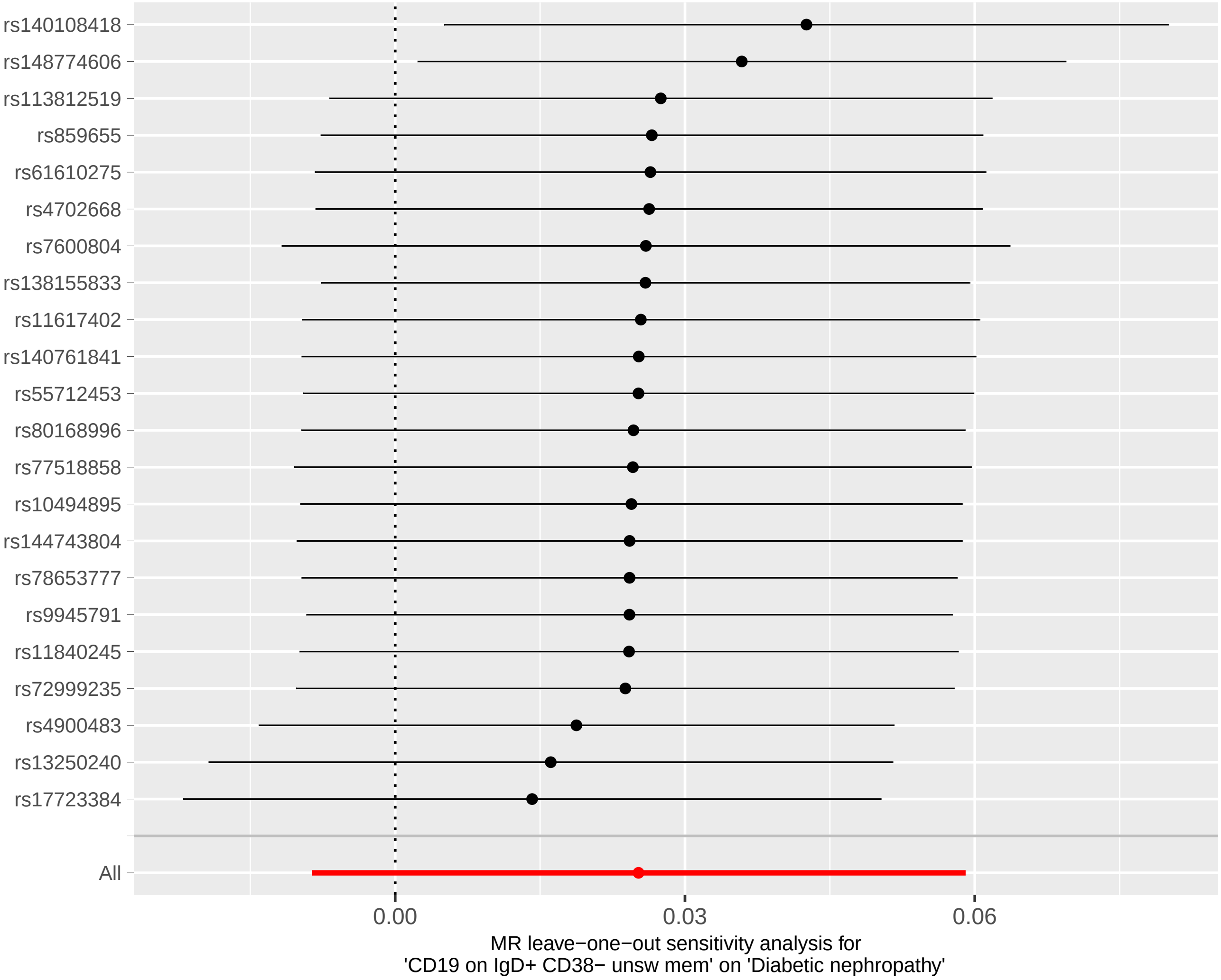
rs140108418
rs148774606
rs113812519
rs859655
rs61610275
rs4702668
rs7600804
rs138155833
rs11617402
rs140761841
rs55712453
rs80168996
rs77518858
rs10494895
rs144743804
rs78653777
rs9945791
rs11840245
rs72999235
rs4900483
rs13250240
rs17723384
All
0.00
0.03
0.06
MR leave−one−out sensitivity analysis for
'CD19 on IgD+ CD38− unsw mem' on 'Diabetic nephropathy'

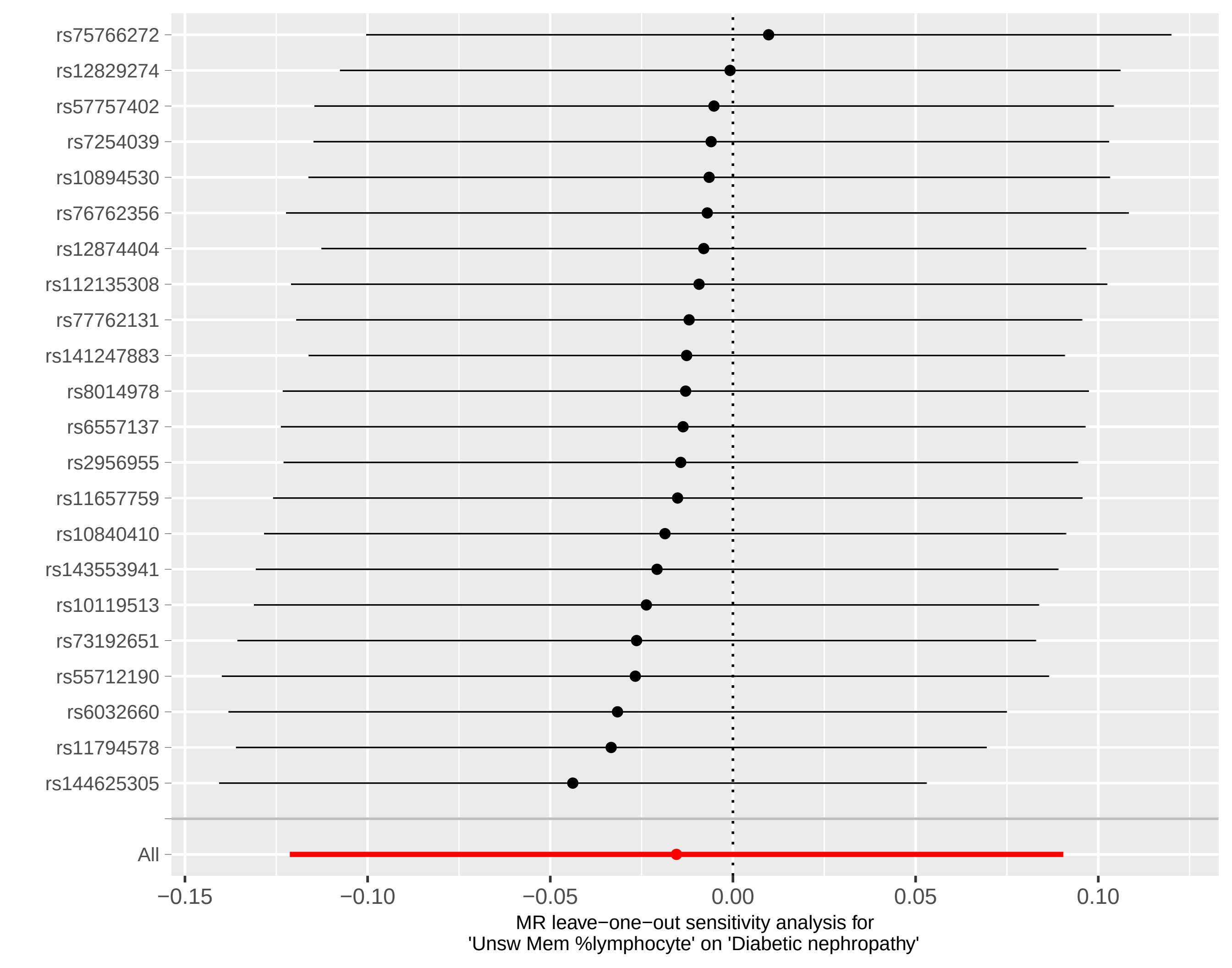
rs75766272
rs12829274
rs57757402
rs7254039
rs10894530
rs76762356
rs12874404
rs112135308
rs77762131
rs141247883
rs8014978
rs6557137
rs2956955
rs11657759
rs10840410
rs143553941
rs10119513
rs73192651
rs55712190
rs6032660
rs11794578
rs144625305
All
−0.15
−0.10
−0.05
0.00
0.05
0.10
MR leave−one−out sensitivity analysis for
'Unsw Mem %lymphocyte' on 'Diabetic nephropathy'

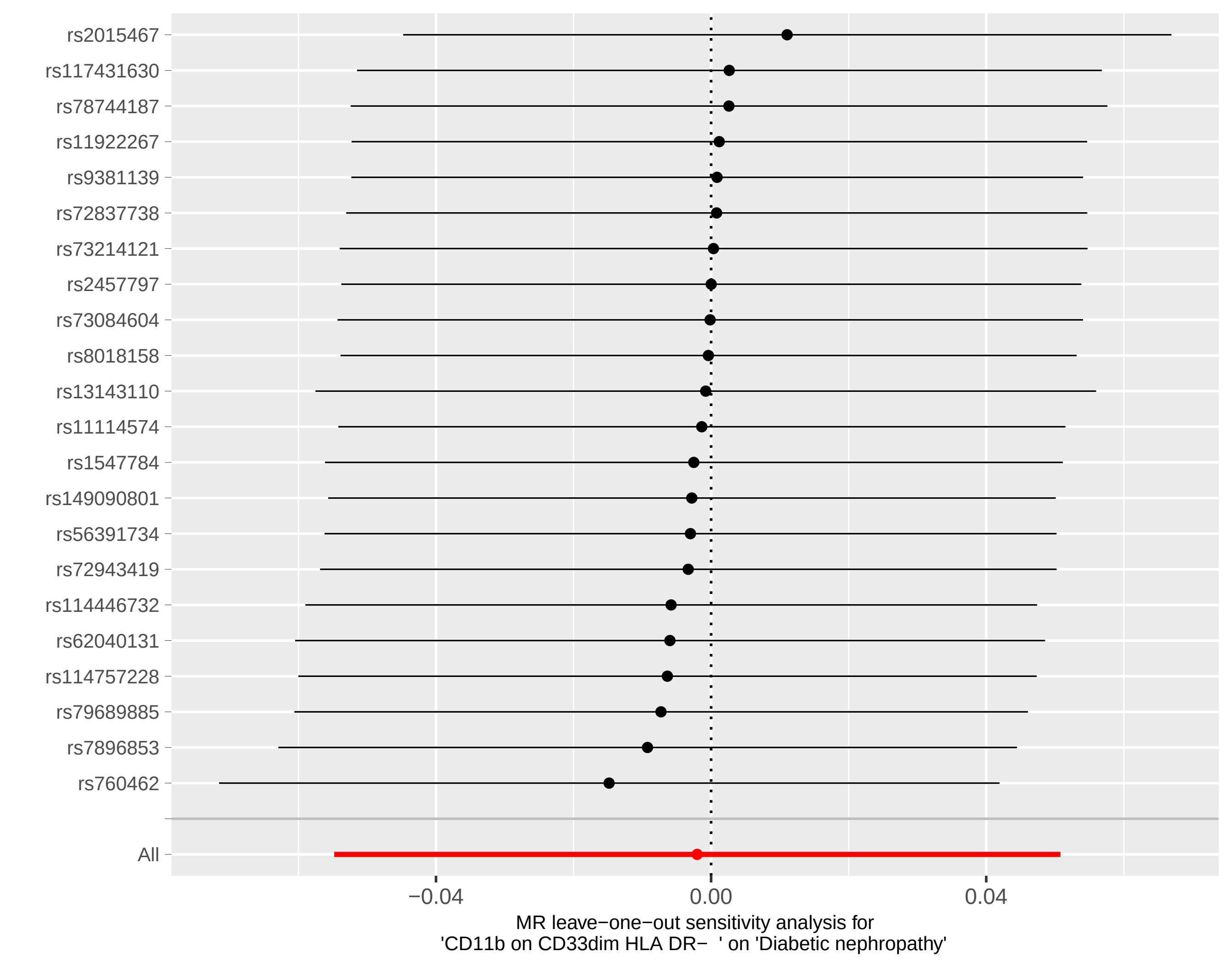
rs2015467
rs117431630
rs78744187
rs11922267
rs9381139
rs72837738
rs73214121
rs2457797
rs73084604
rs8018158
rs13143110
rs11114574
rs1547784
rs149090801
rs56391734
rs72943419
rs114446732
rs62040131
rs114757228
rs79689885
rs7896853
rs760462
All
−0.04
0.00
0.04
MR leave−one−out sensitivity analysis for
'CD11b on CD33dim HLA DR− ' on 'Diabetic nephropathy'

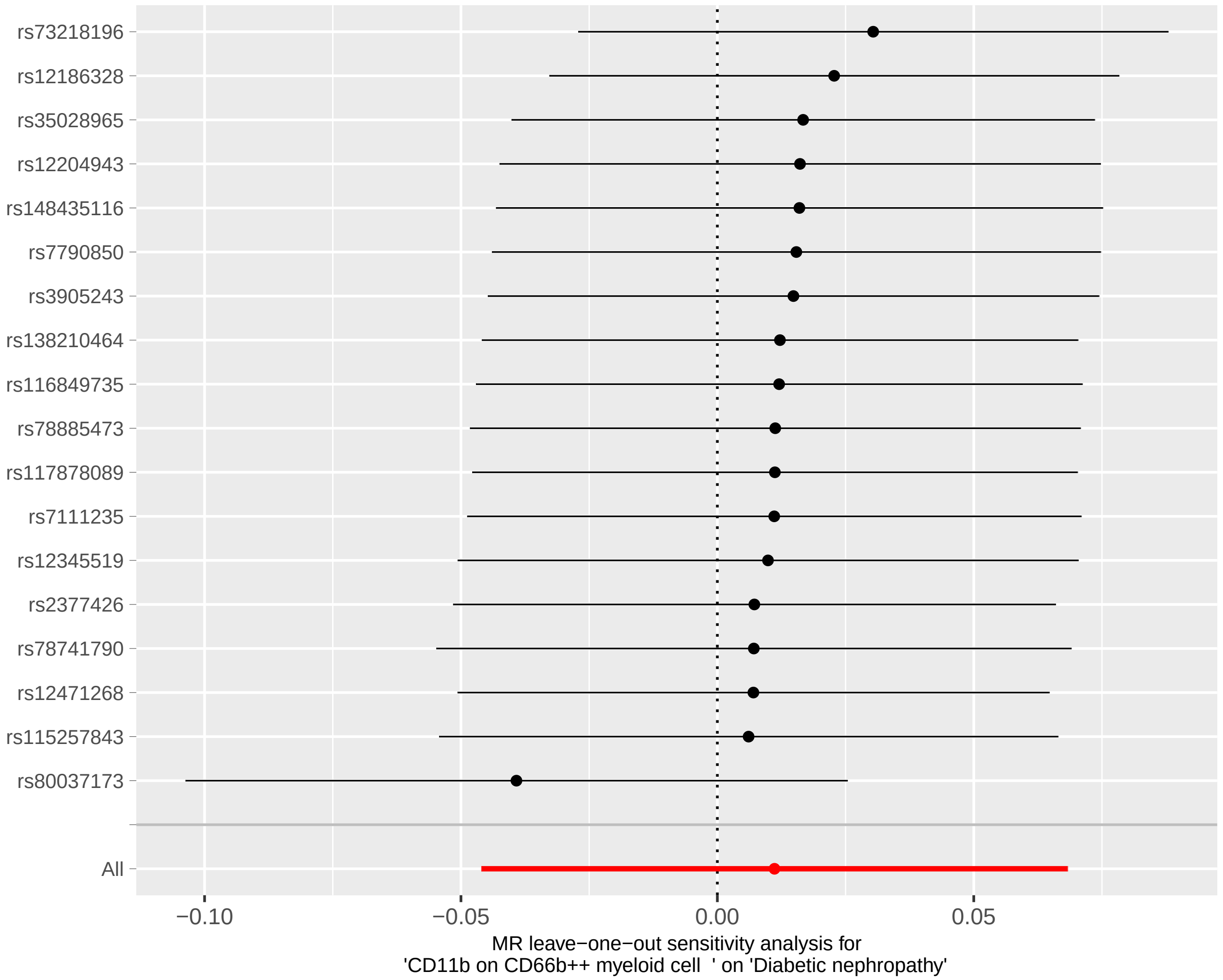
rs73218196
rs12186328
rs35028965
rs12204943
rs148435116
rs7790850
rs3905243
rs138210464
rs116849735
rs78885473
rs117878089
rs7111235
rs12345519
rs2377426
rs78741790
rs12471268
rs115257843
rs80037173
All
−0.10
−0.05
0.00
0.05
MR leave−one−out sensitivity analysis for
'CD11b on CD66b++ myeloid cell ' on 'Diabetic nephropathy'

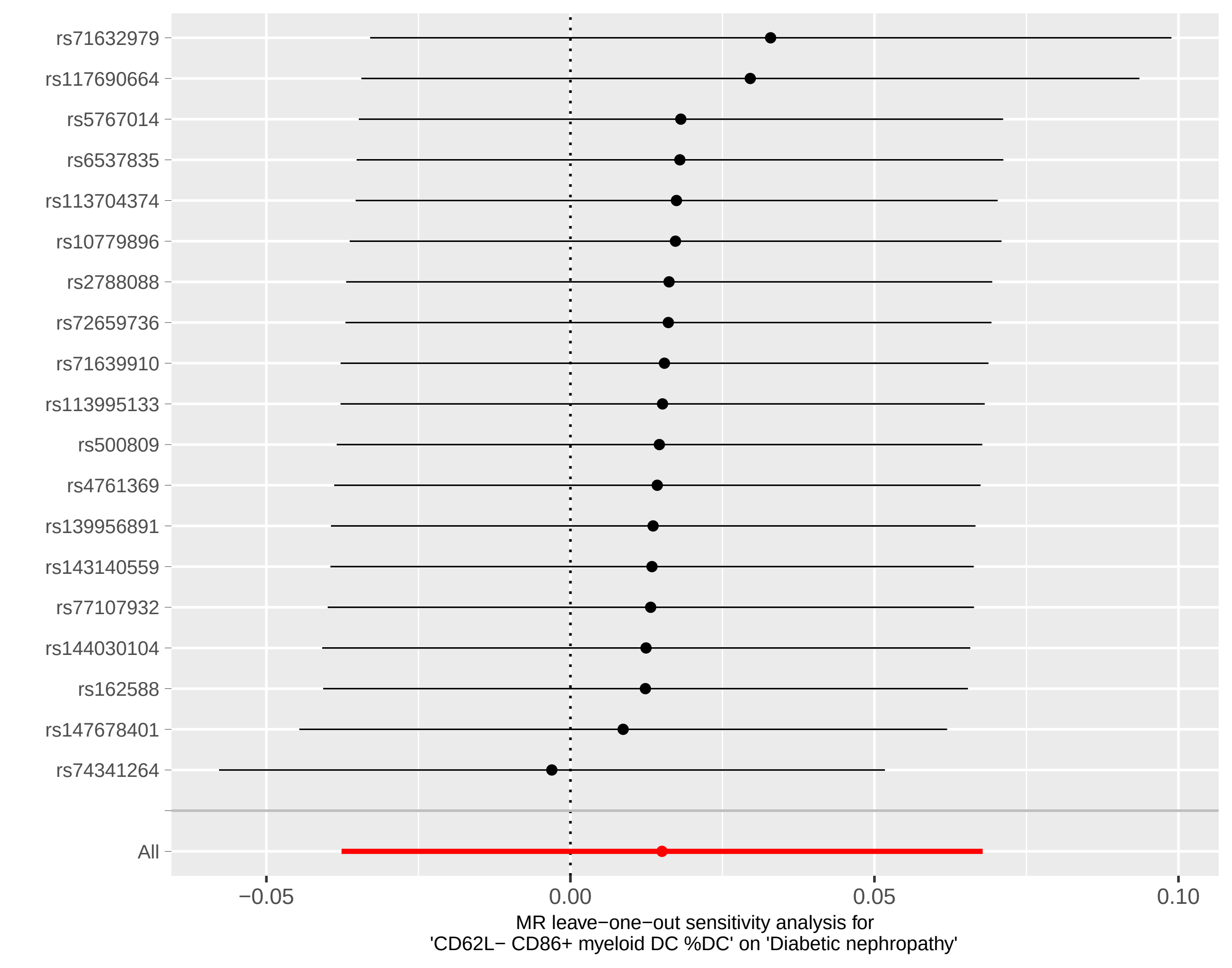
rs71632979
rs117690664
rs5767014
rs6537835
rs113704374
rs10779896
rs2788088
rs72659736
rs71639910
rs113995133
rs500809
rs4761369
rs139956891
rs143140559
rs77107932
rs144030104
rs162588
rs147678401
rs74341264
All
−0.05
0.00
0.05
0.10
MR leave−one−out sensitivity analysis for
'CD62L− CD86+ myeloid DC %DC' on 'Diabetic nephropathy'

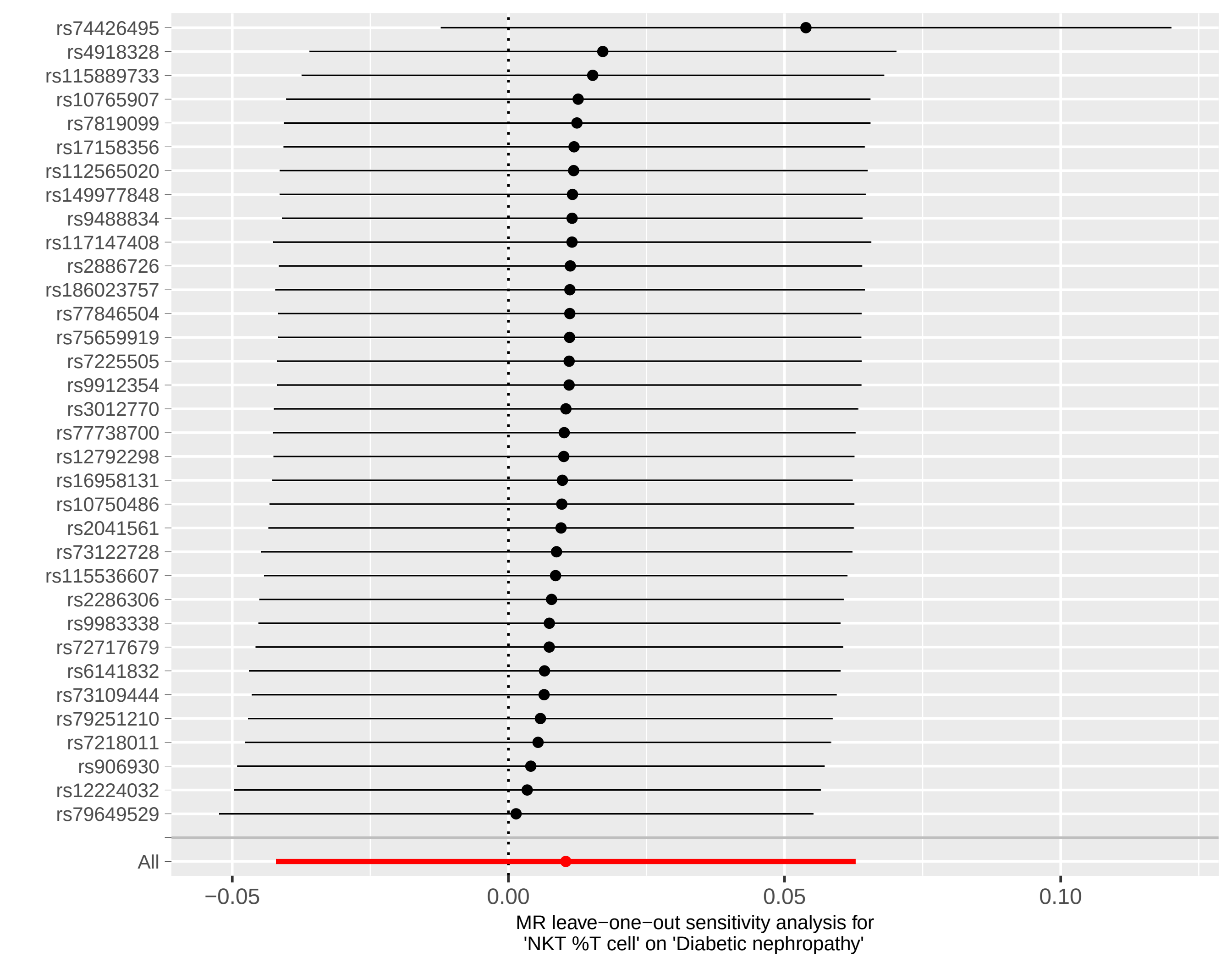

rs74426495
rs4918328
rs115889733
rs10765907
rs7819099
rs17158356
rs112565020
rs149977848
rs9488834
rs117147408
rs2886726
rs186023757
rs77846504
rs75659919
rs7225505
rs9912354
rs3012770
rs77738700
rs12792298
rs16958131
rs10750486
rs2041561
rs73122728
rs115536607
rs2286306
rs9983338
rs72717679
rs6141832
rs73109444
rs79251210
rs7218011
rs906930
rs12224032
rs79649529
All
−0.05
0.00
0.05
0.10
MR leave−one−out sensitivity analysis for
'NKT %T cell' on 'Diabetic nephropathy'

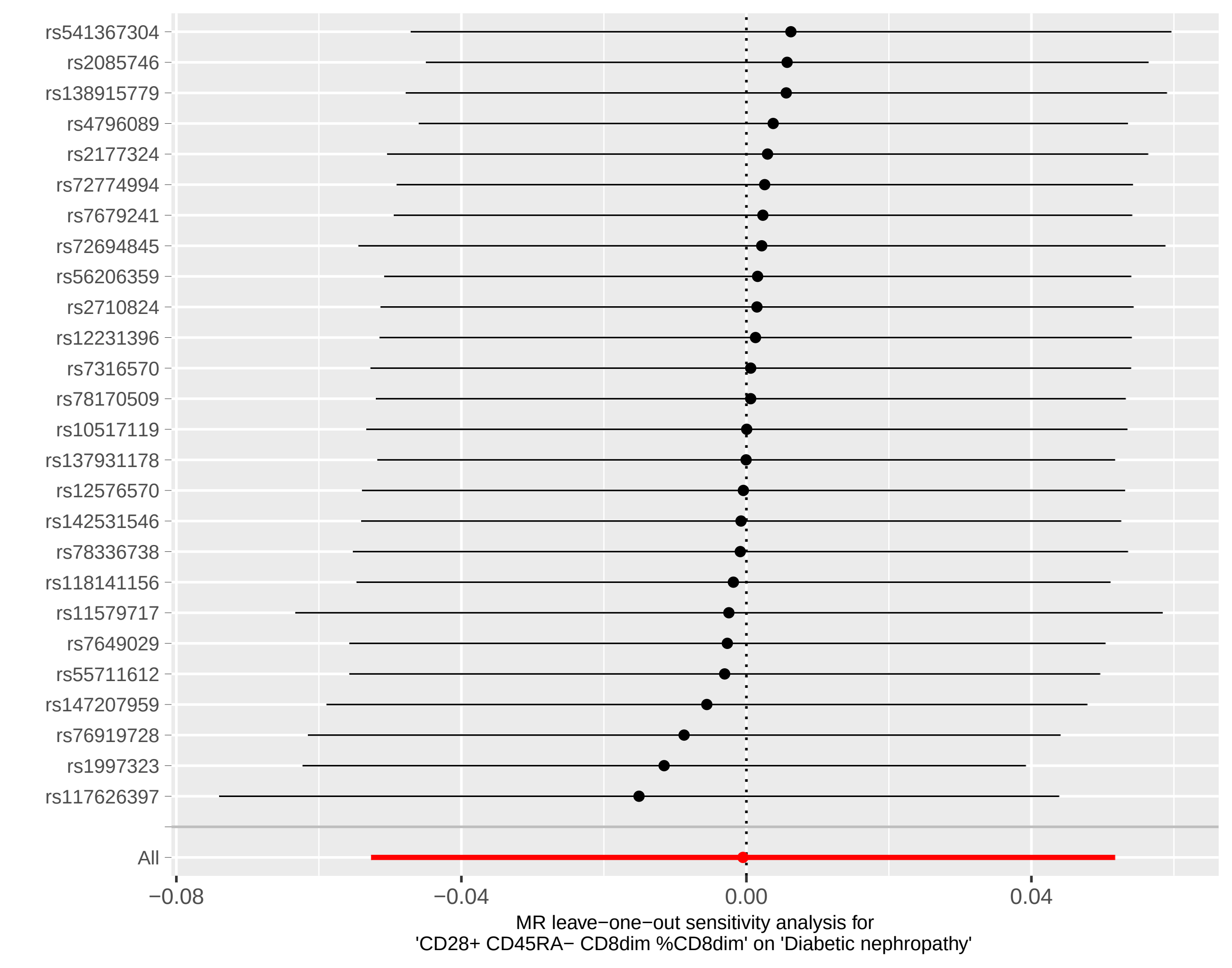
rs541367304
rs2085746
rs138915779
rs4796089
rs2177324
rs72774994
rs7679241
rs72694845
rs56206359
rs2710824
rs12231396
rs7316570
rs78170509
rs10517119
rs137931178
rs12576570
rs142531546
rs78336738
rs118141156
rs11579717
rs7649029
rs55711612
rs147207959
rs76919728
rs1997323
rs117626397
All
−0.08
−0.04
0.00
0.04
MR leave−one−out sensitivity analysis for
'CD28+ CD45RA− CD8dim %CD8dim' on 'Diabetic nephropathy'

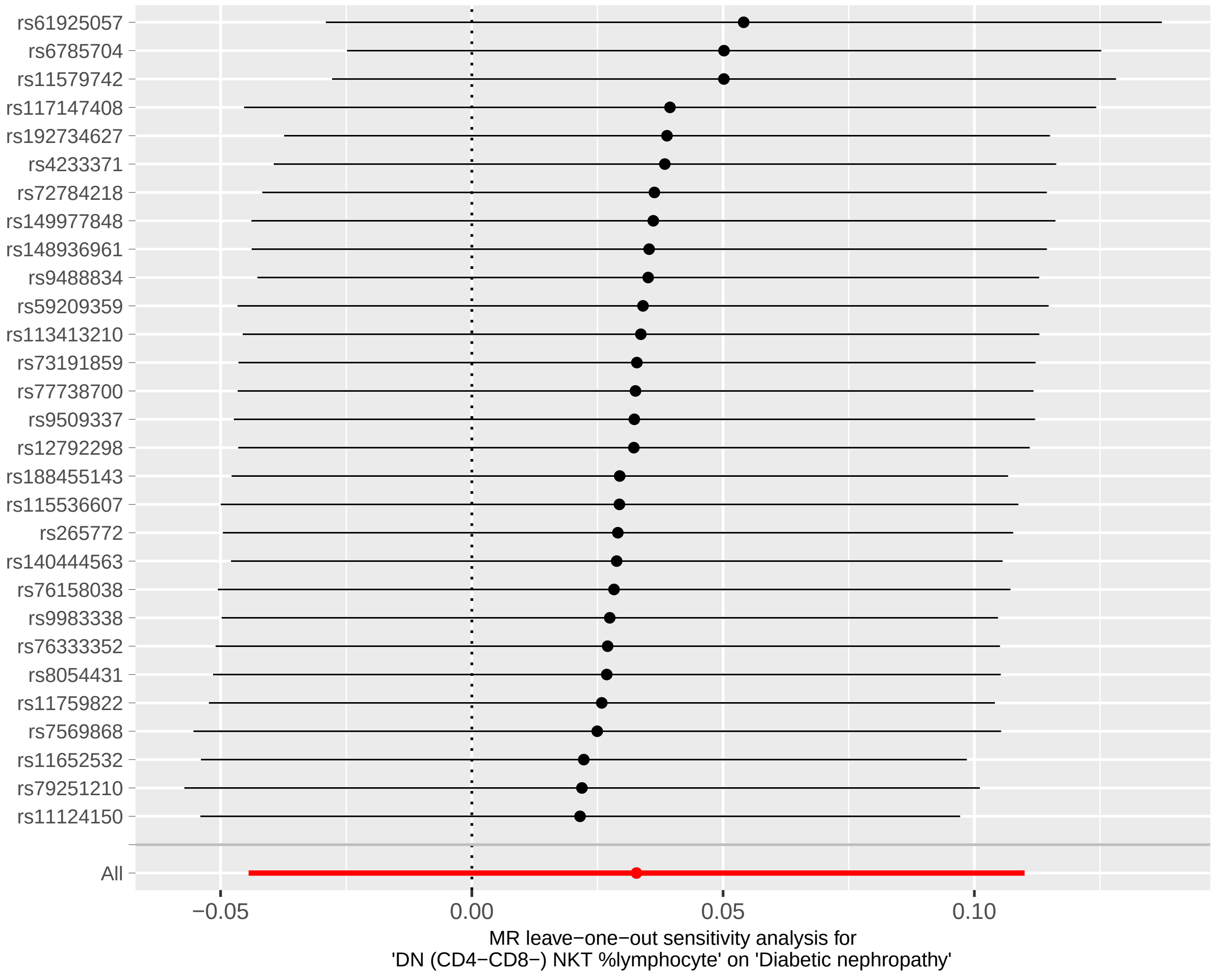
rs61925057
rs6785704
rs11579742
rs117147408
rs192734627
rs4233371
rs72784218
rs149977848
rs148936961
rs9488834
rs59209359
rs113413210
rs73191859
rs77738700
rs9509337
rs12792298
rs188455143
rs115536607
rs265772
rs140444563
rs76158038
rs9983338
rs76333352
rs8054431
rs11759822
rs7569868
rs11652532
rs79251210
rs11124150
All
−0.05
0.00
0.05
0.10
MR leave−one−out sensitivity analysis for
'DN (CD4−CD8−) NKT %lymphocyte' on 'Diabetic nephropathy'

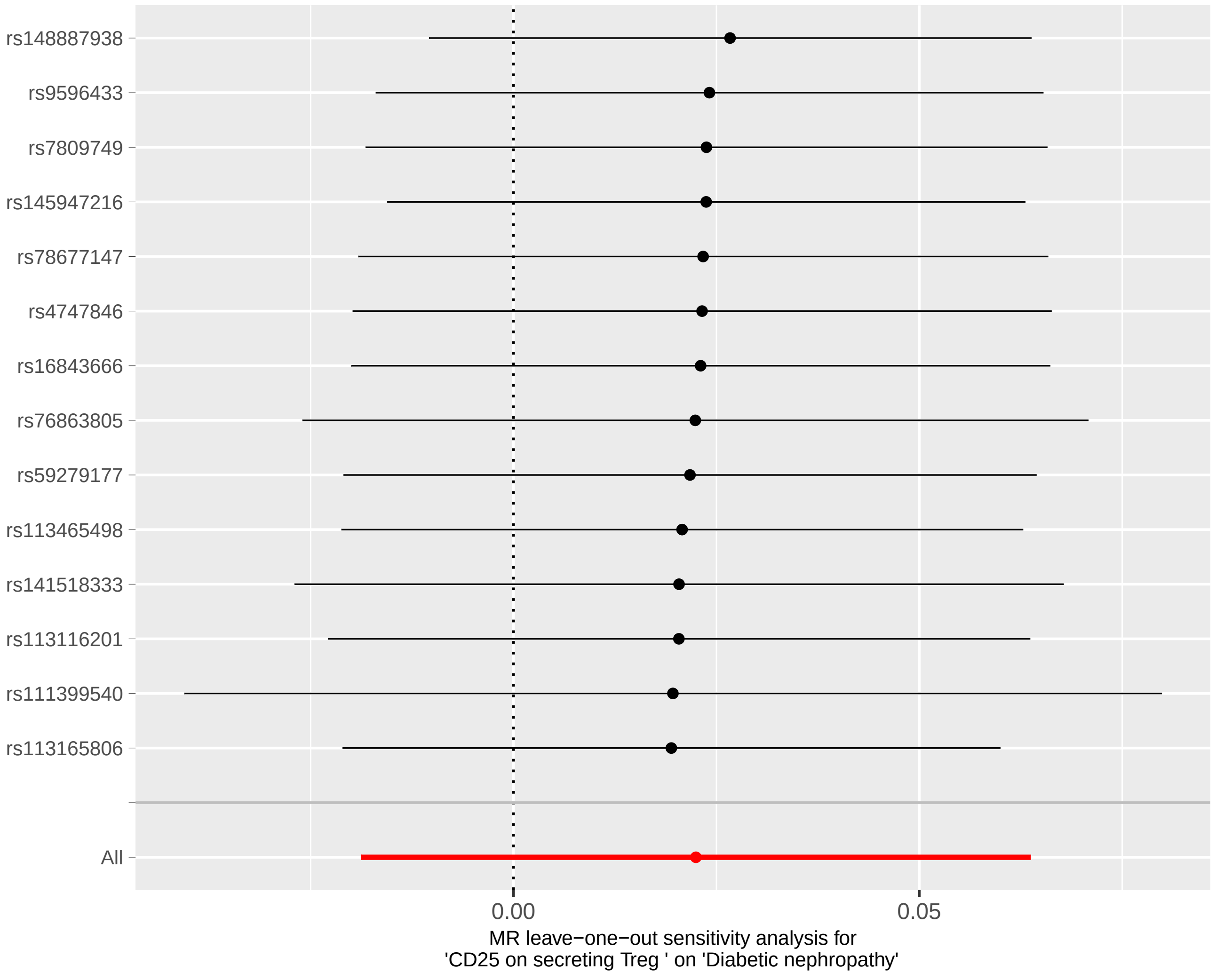
rs148887938
rs9596433
rs7809749
rs145947216
rs78677147
rs4747846
rs16843666
rs76863805
rs59279177
rs113465498
rs141518333
rs113116201
rs111399540
rs113165806
All
0.00
0.05
MR leave−one−out sensitivity analysis for
'CD25 on secreting Treg ' on 'Diabetic nephropathy'

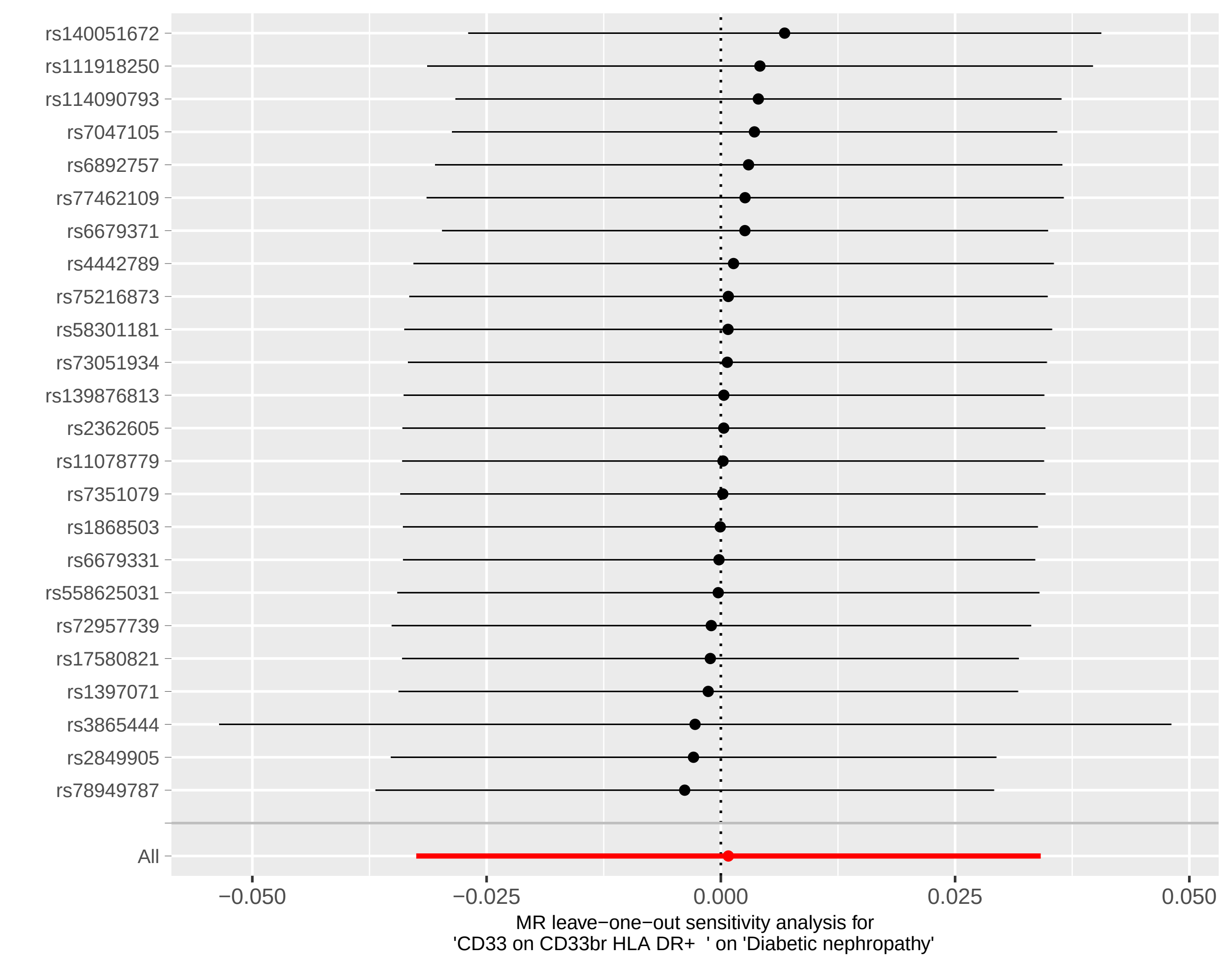
rs140051672
rs111918250
rs114090793
rs7047105
rs6892757
rs77462109
rs6679371
rs4442789
rs75216873
rs58301181
rs73051934
rs139876813
rs2362605
rs11078779
rs7351079
rs1868503
rs6679331
rs558625031
rs72957739
rs17580821
rs1397071
rs3865444
rs2849905
rs78949787
All
−0.050
−0.025
0.000
0.025
0.050
MR leave−one−out sensitivity analysis for
'CD33 on CD33br HLA DR+ ' on 'Diabetic nephropathy'

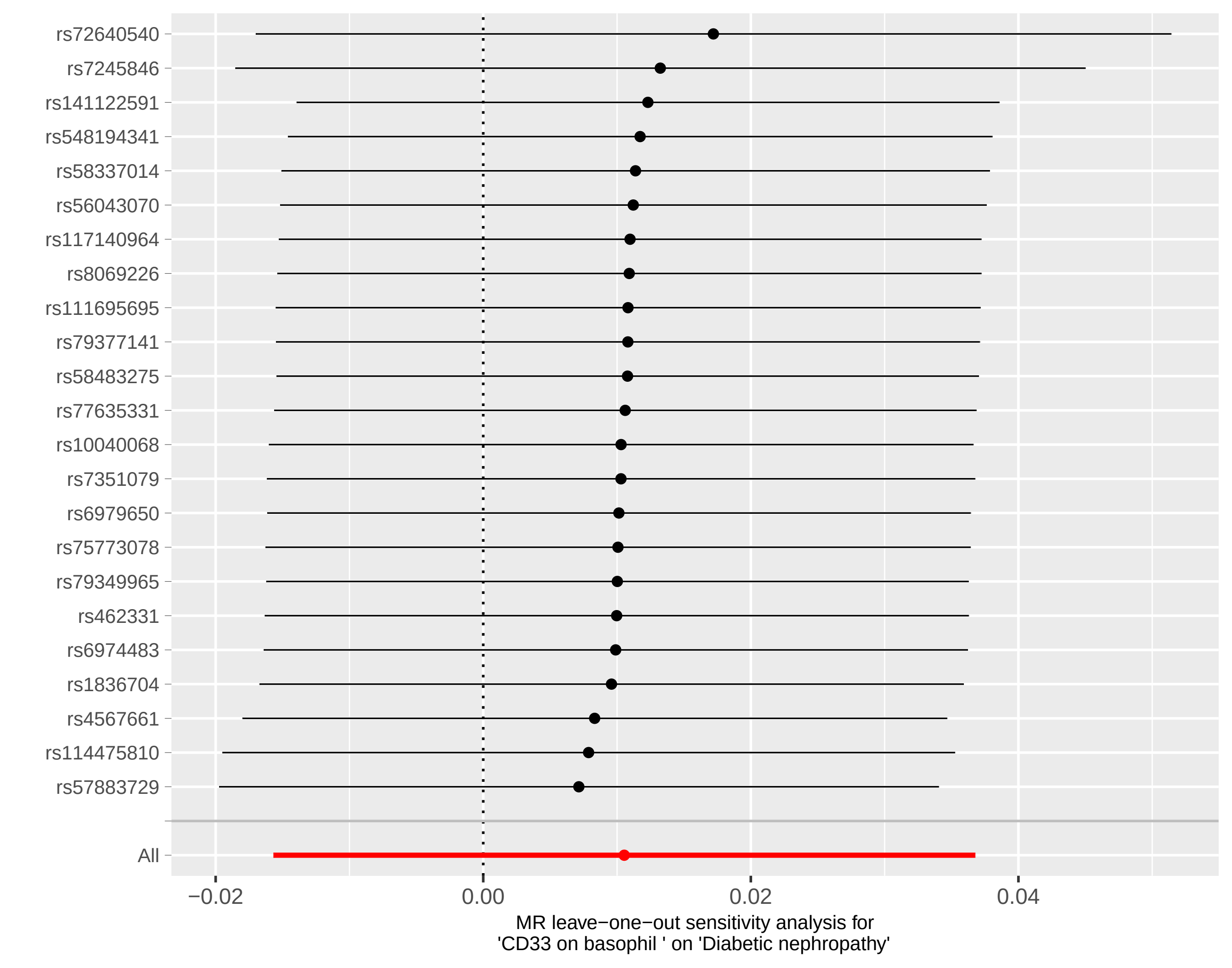
rs72640540
rs7245846
rs141122591
rs548194341
rs58337014
rs56043070
rs117140964
rs8069226
rs111695695
rs79377141
rs58483275
rs77635331
rs10040068
rs7351079
rs6979650
rs75773078
rs79349965
rs462331
rs6974483
rs1836704
rs4567661
rs114475810
rs57883729
All
−0.02
0.00
0.02
0.04
MR leave−one−out sensitivity analysis for
'CD33 on basophil ' on 'Diabetic nephropathy'

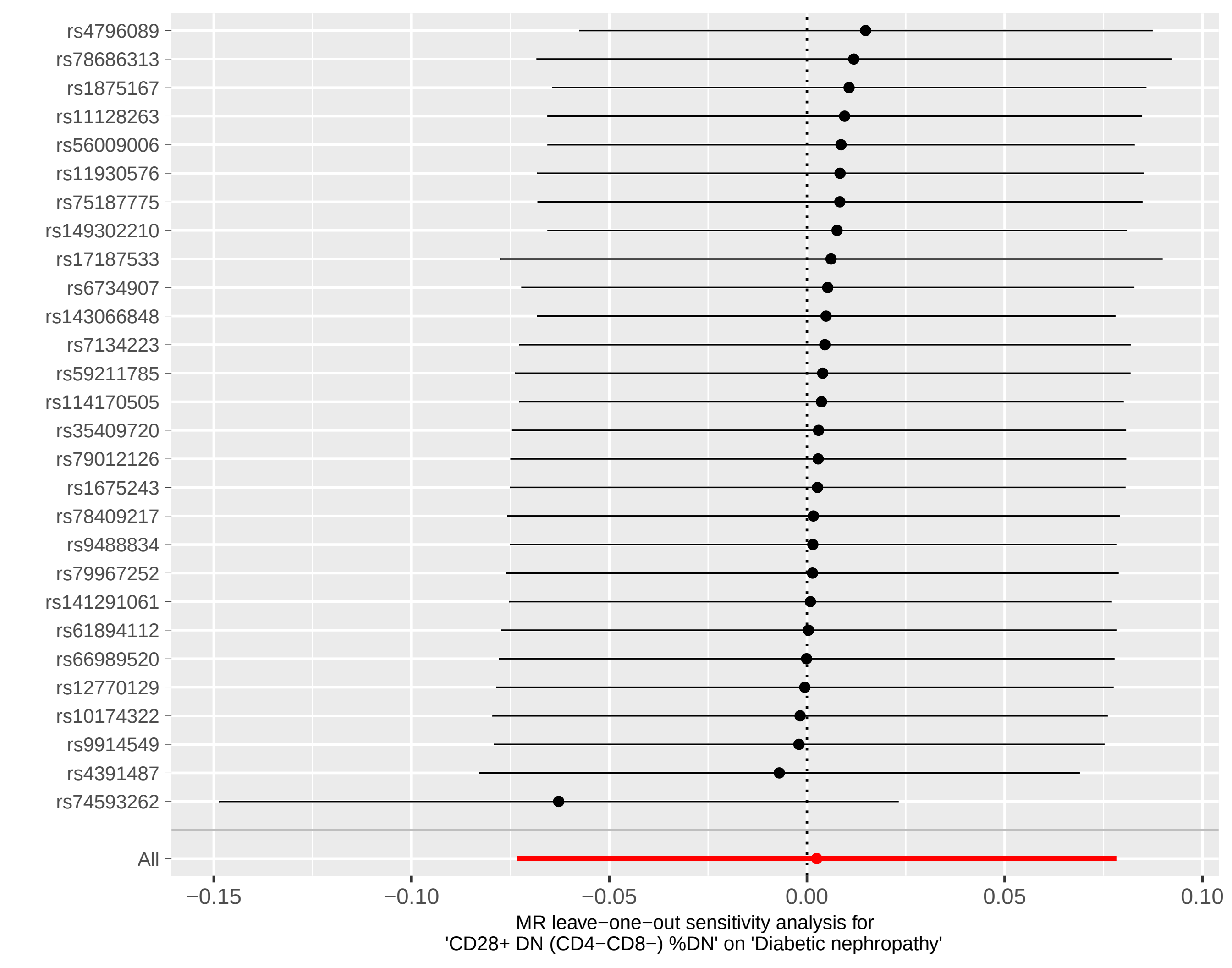
rs4796089
rs78686313
rs1875167
rs11128263
rs56009006
rs11930576
rs75187775
rs149302210
rs17187533
rs6734907
rs143066848
rs7134223
rs59211785
rs114170505
rs35409720
rs79012126
rs1675243
rs78409217
rs9488834
rs79967252
rs141291061
rs61894112
rs66989520
rs12770129
rs10174322
rs9914549
rs4391487
rs74593262
All
−0.15
−0.10
−0.05
0.00
0.05
0.10
MR leave−one−out sensitivity analysis for
'CD28+ DN (CD4−CD8−) %DN' on 'Diabetic nephropathy'

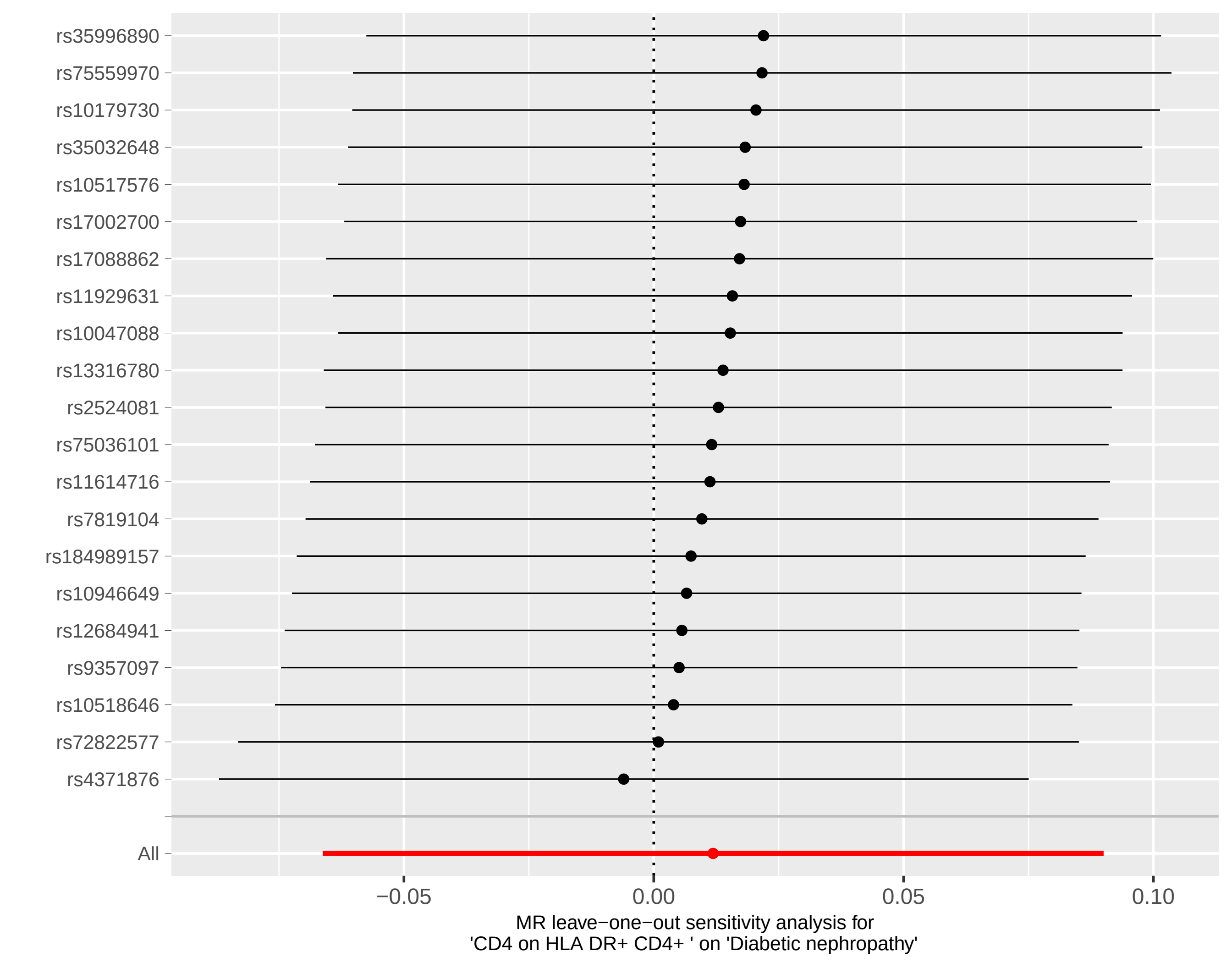
rs35996890
rs75559970
rs10179730
rs35032648
rs10517576
rs17002700
rs17088862
rs11929631
rs10047088
rs13316780
rs2524081
rs75036101
rs11614716
rs7819104
rs184989157
rs10946649
rs12684941
rs9357097
rs10518646
rs72822577
rs4371876
All
−0.05
0.00
0.05
0.10
MR leave−one−out sensitivity analysis for
'CD4 on HLA DR+ CD4+ ' on 'Diabetic nephropathy'

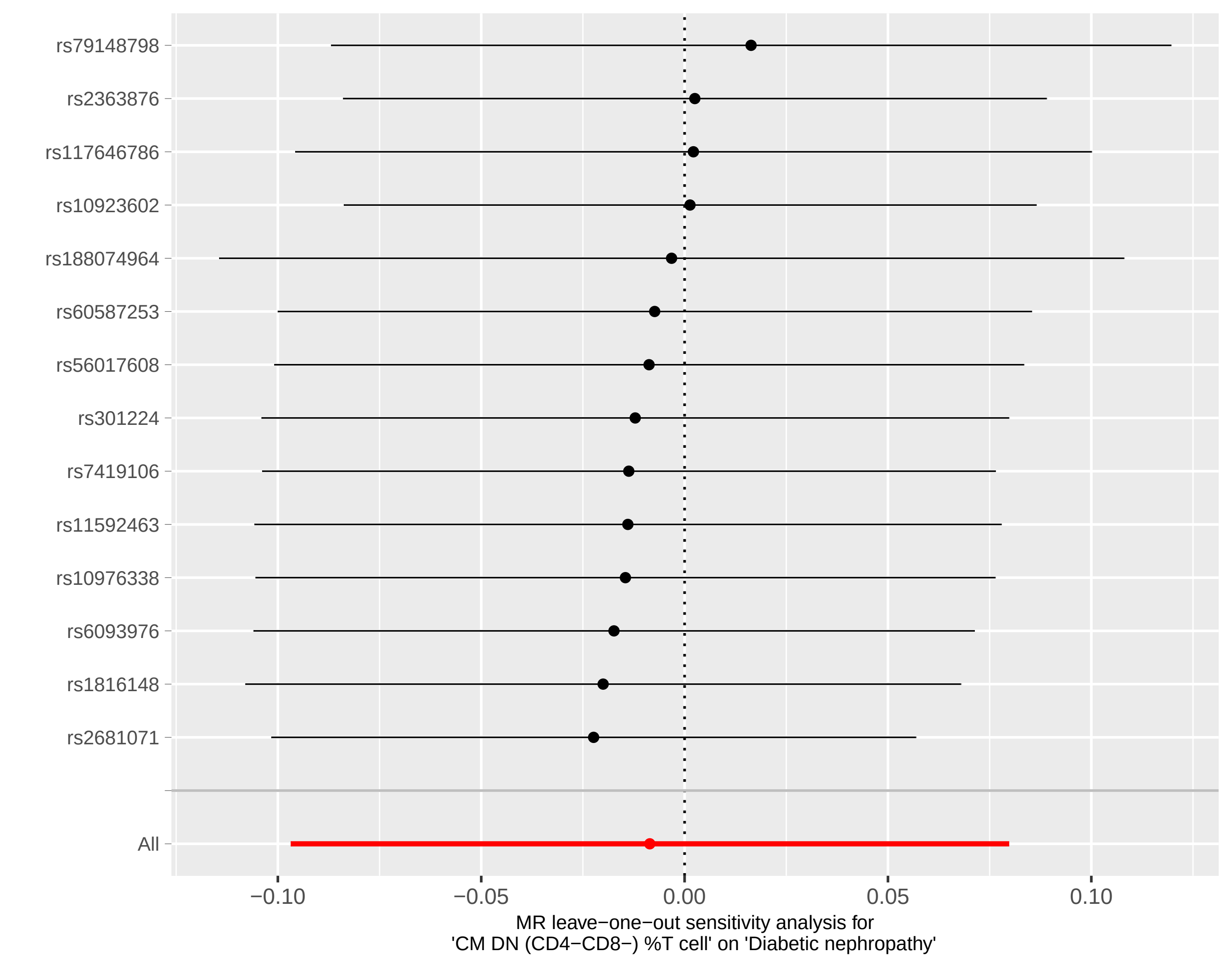
rs79148798
rs2363876
rs117646786
rs10923602
rs188074964
rs60587253
rs56017608
rs301224
rs7419106
rs11592463
rs10976338
rs6093976
rs1816148
rs2681071
All
−0.10
−0.05
0.00
0.05
0.10
MR leave−one−out sensitivity analysis for
'CM DN (CD4−CD8−) %T cell' on 'Diabetic nephropathy'

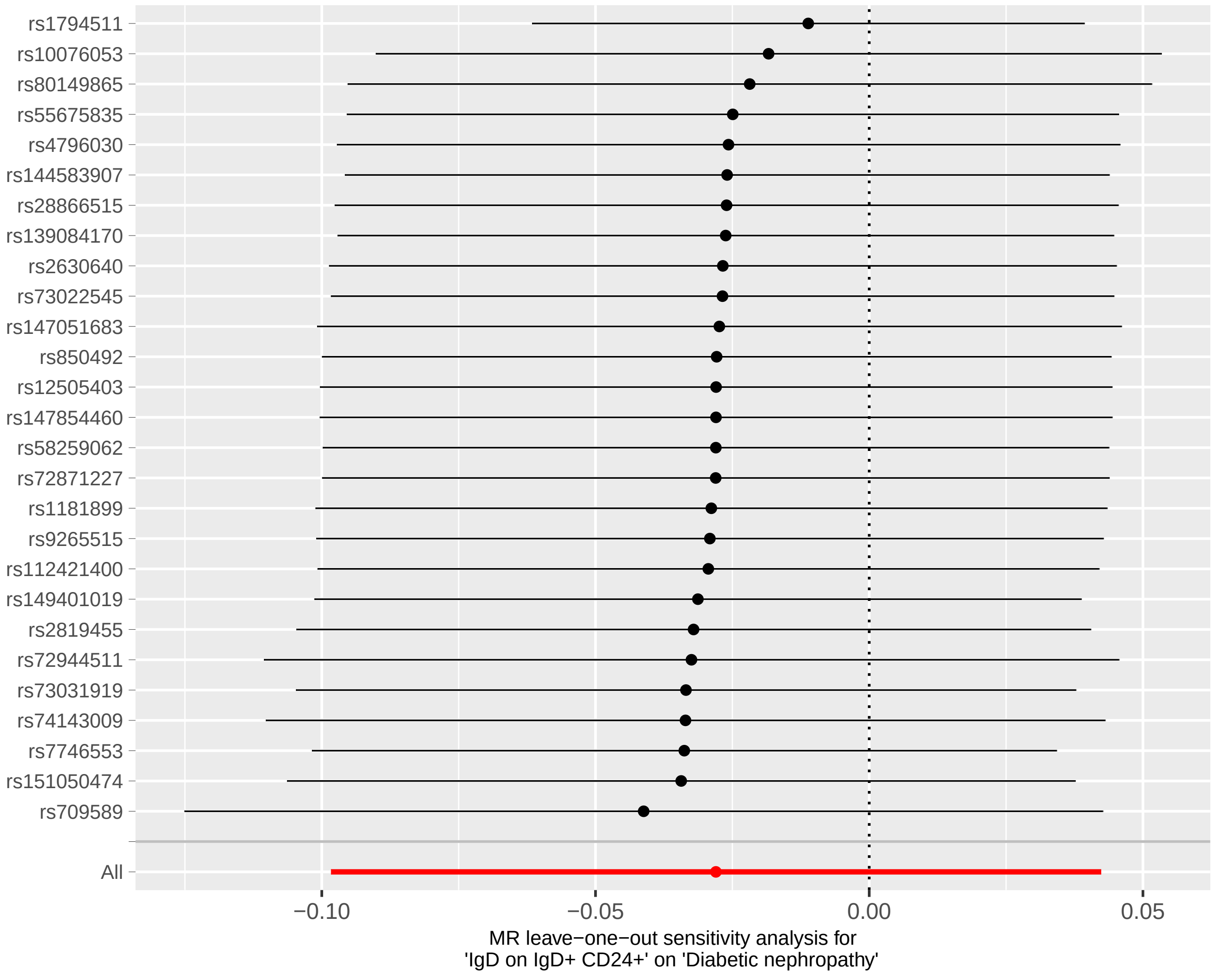

rs1794511
rs10076053
rs80149865
rs55675835
rs4796030
rs144583907
rs28866515
rs139084170
rs2630640
rs73022545
rs147051683
rs850492
rs12505403
rs147854460
rs58259062
rs72871227
rs1181899
rs9265515
rs112421400
rs149401019
rs2819455
rs72944511
rs73031919
rs74143009
rs7746553
rs151050474
rs709589
All
−0.10
−0.05
0.00
0.05
MR leave−one−out sensitivity analysis for
'IgD on IgD+ CD24+' on 'Diabetic nephropathy'

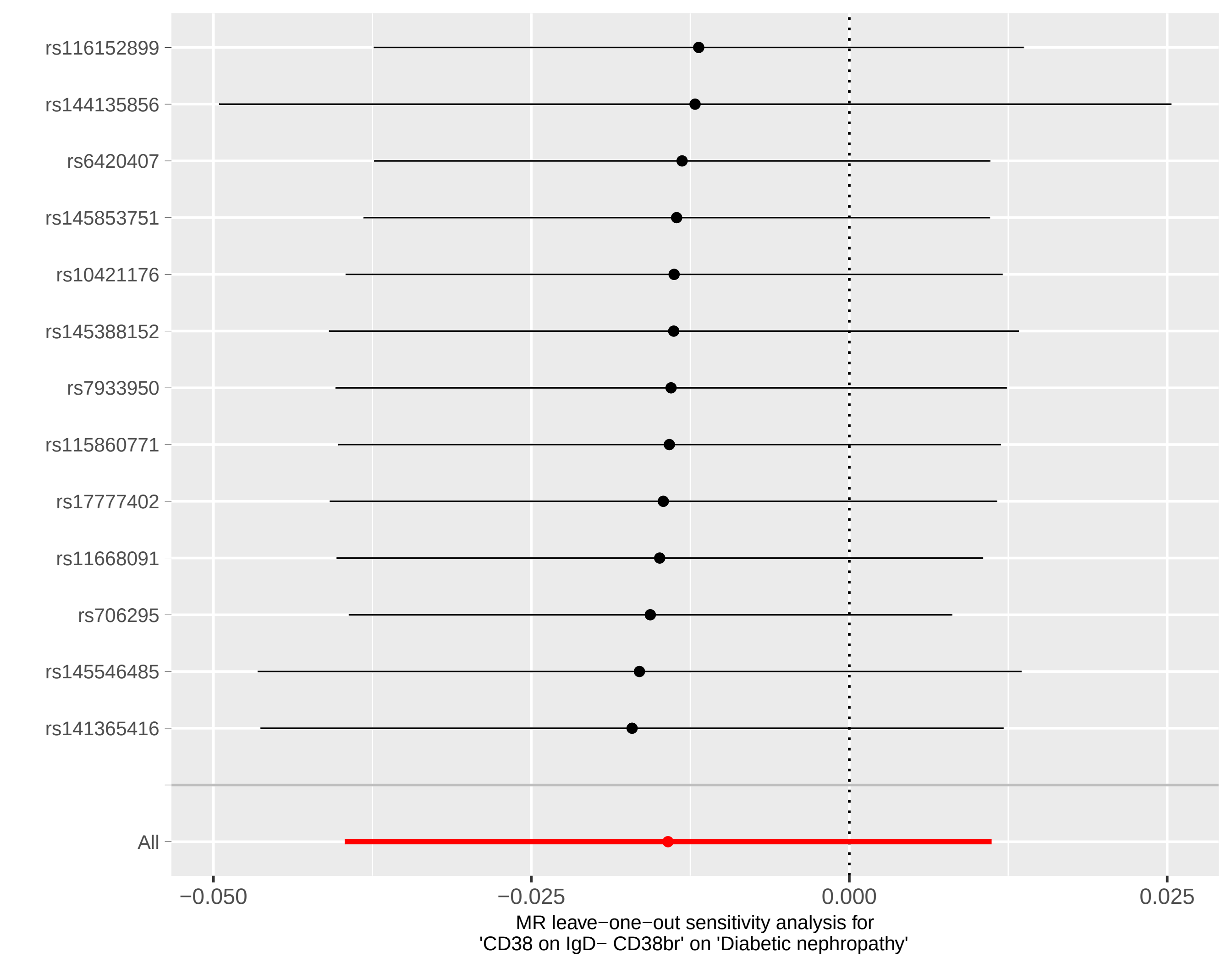
rs116152899
rs144135856
rs6420407
rs145853751
rs10421176
rs145388152
rs7933950
rs115860771
rs17777402
rs11668091
rs706295
rs145546485
rs141365416
All
−0.050
−0.025
0.000
0.025
MR leave−one−out sensitivity analysis for
'CD38 on IgD− CD38br' on 'Diabetic nephropathy'

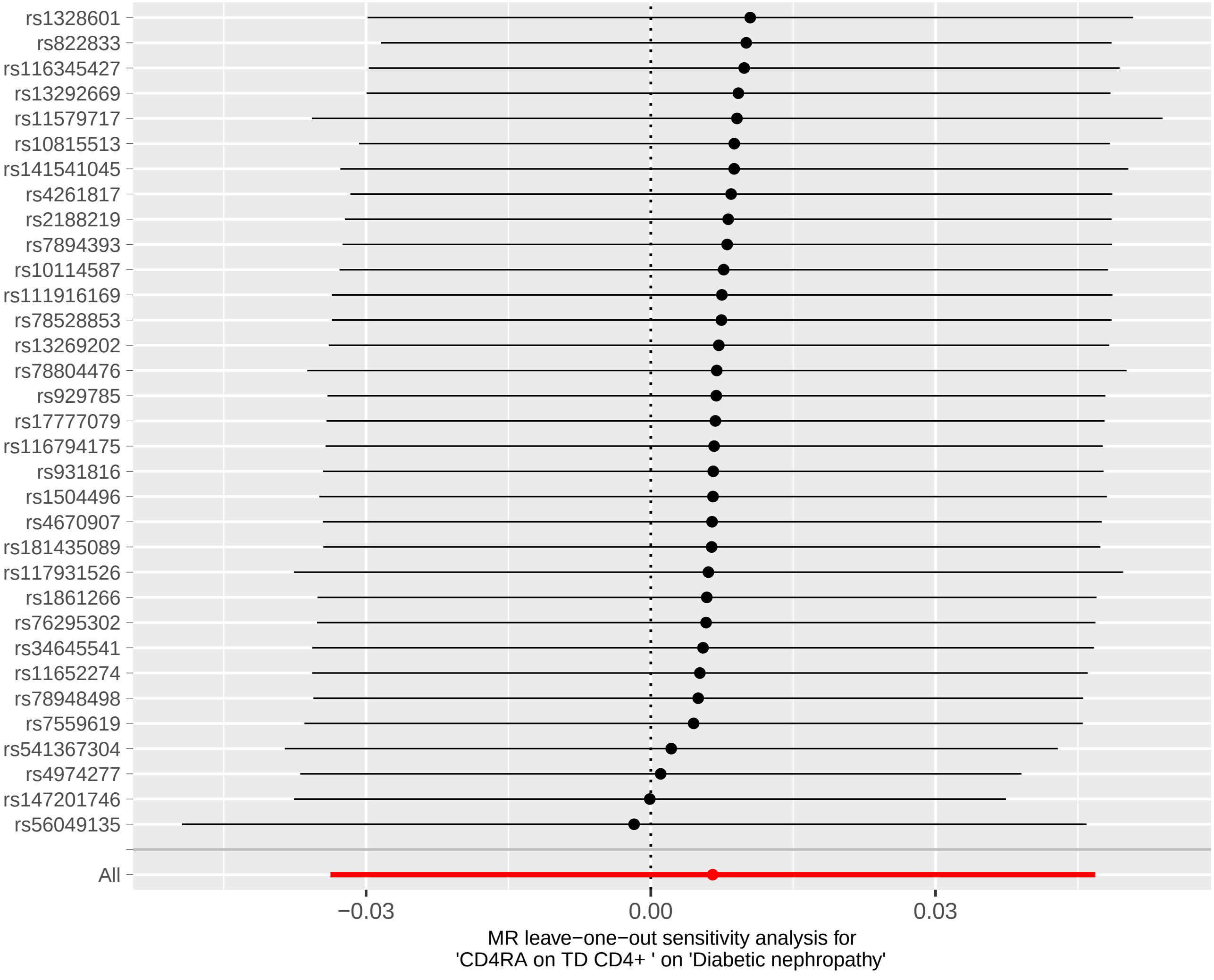
rs1328601
rs822833
rs116345427
rs13292669
rs11579717
rs10815513
rs141541045
rs4261817
rs2188219
rs7894393
rs10114587
rs111916169
rs78528853
rs13269202
rs78804476
rs929785
rs17777079
rs116794175
rs931816
rs1504496
rs4670907
rs181435089
rs117931526
rs1861266
rs76295302
rs34645541
rs11652274
rs78948498
rs7559619
rs541367304
rs4974277
rs147201746
rs56049135
All
−0.03
0.00
0.03
MR leave−one−out sensitivity analysis for
'CD4RA on TD CD4+ ' on 'Diabetic nephropathy'

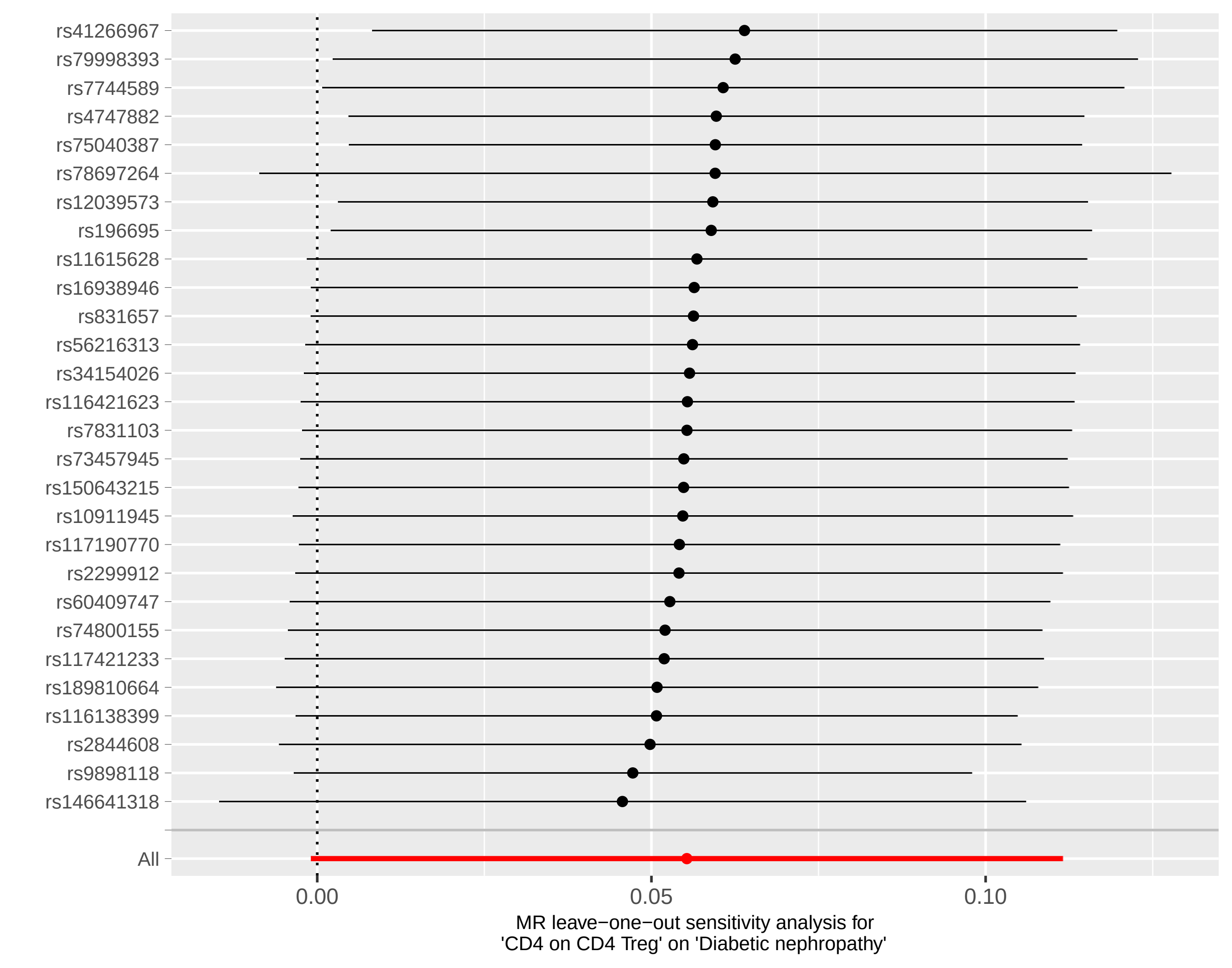
rs41266967
rs79998393
rs7744589
rs4747882
rs75040387
rs78697264
rs12039573
rs196695
rs11615628
rs16938946
rs831657
rs56216313
rs34154026
rs116421623
rs7831103
rs73457945
rs150643215
rs10911945
rs117190770
rs2299912
rs60409747
rs74800155
rs117421233
rs189810664
rs116138399
rs2844608
rs9898118
rs146641318
All
0.00
0.05
0.10
MR leave−one−out sensitivity analysis for
'CD4 on CD4 Treg' on 'Diabetic nephropathy'

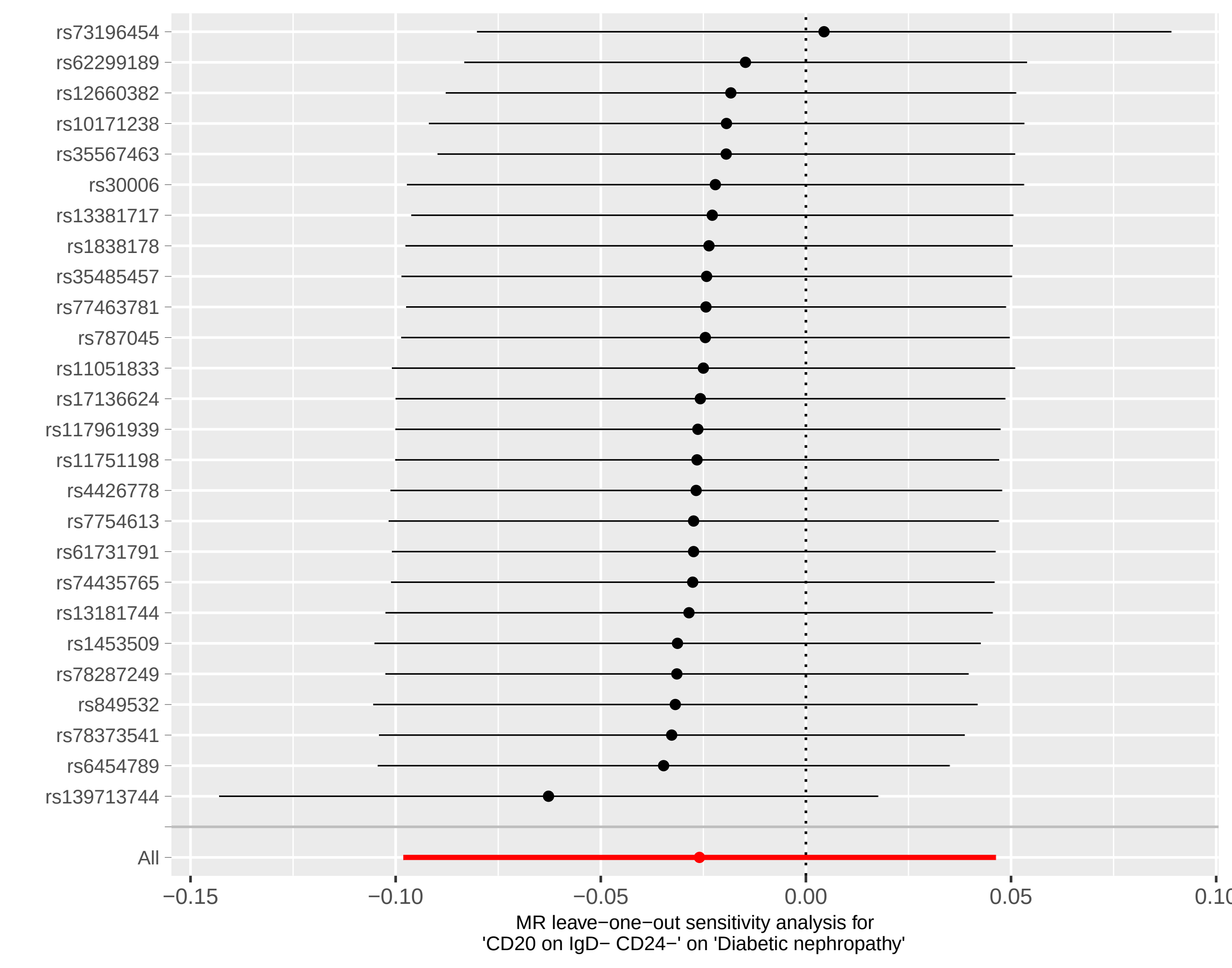
rs73196454
rs62299189
rs12660382
rs10171238
rs35567463
rs30006
rs13381717
rs1838178
rs35485457
rs77463781
rs787045
rs11051833
rs17136624
rs117961939
rs11751198
rs4426778
rs7754613
rs61731791
rs74435765
rs13181744
rs1453509
rs78287249
rs849532
rs78373541
rs6454789
rs139713744
All
−0.15
−0.10
−0.05
0.00
0.05
MR leave−one−out sensitivity analysis for
'CD20 on IgD− CD24−' on 'Diabetic nephropathy'

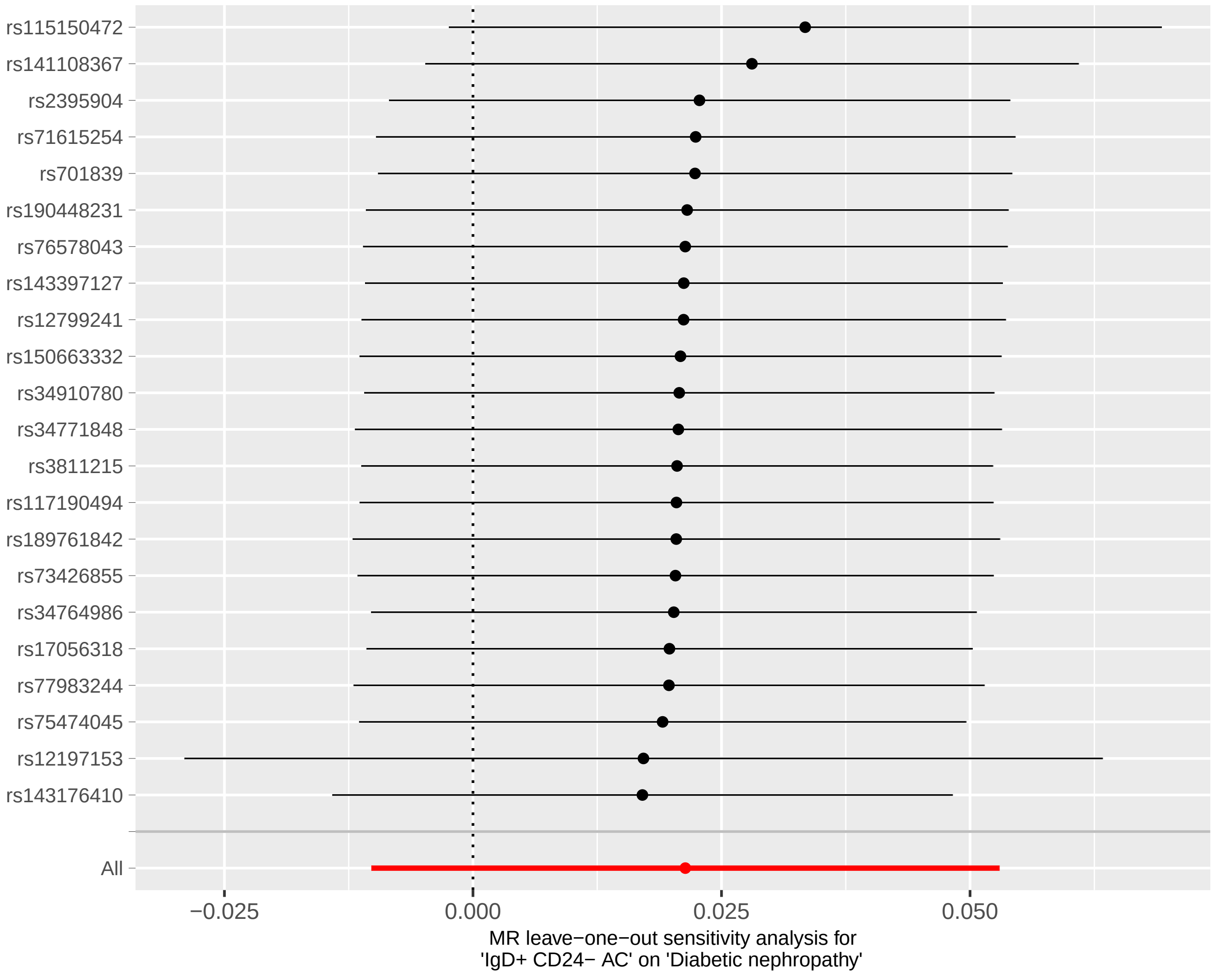
rs115150472
rs141108367
rs2395904
rs71615254
rs701839
rs190448231
rs76578043
rs143397127
rs12799241
rs150663332
rs34910780
rs34771848
rs3811215
rs117190494
rs189761842
rs73426855
rs34764986
rs17056318
rs77983244
rs75474045
rs12197153
rs143176410
All
−0.025
0.000
0.025
0.050
MR leave−one−out sensitivity analysis for
'IgD+ CD24− AC' on 'Diabetic nephropathy'

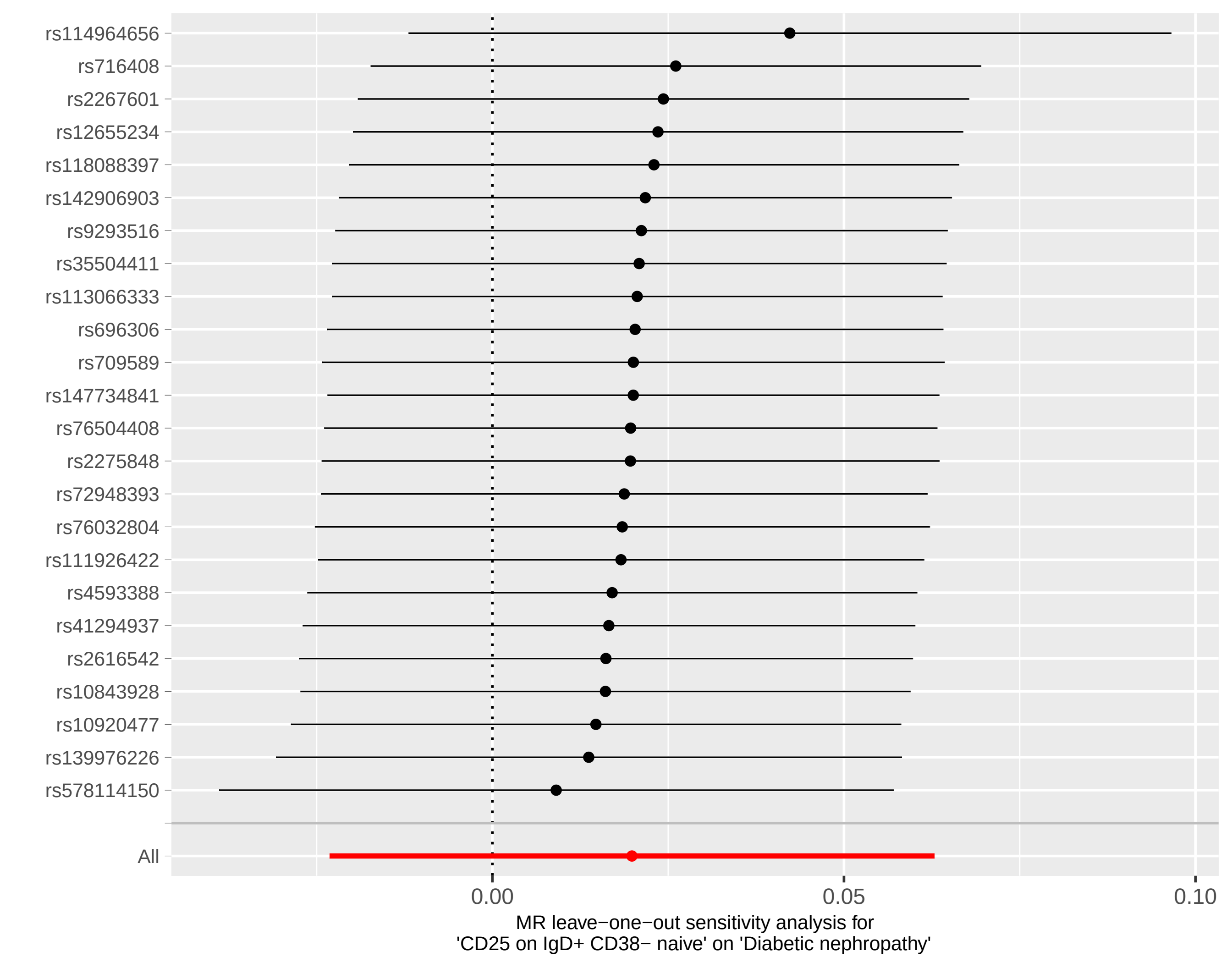
rs114964656
rs716408
rs2267601
rs12655234
rs118088397
rs142906903
rs9293516
rs35504411
rs113066333
rs696306
rs709589
rs147734841
rs76504408
rs2275848
rs72948393
rs76032804
rs111926422
rs4593388
rs41294937
rs2616542
rs10843928
rs10920477
rs139976226
rs578114150
All
0.00
0.05
0.10
MR leave−one−out sensitivity analysis for
'CD25 on IgD+ CD38− naive' on 'Diabetic nephropathy'

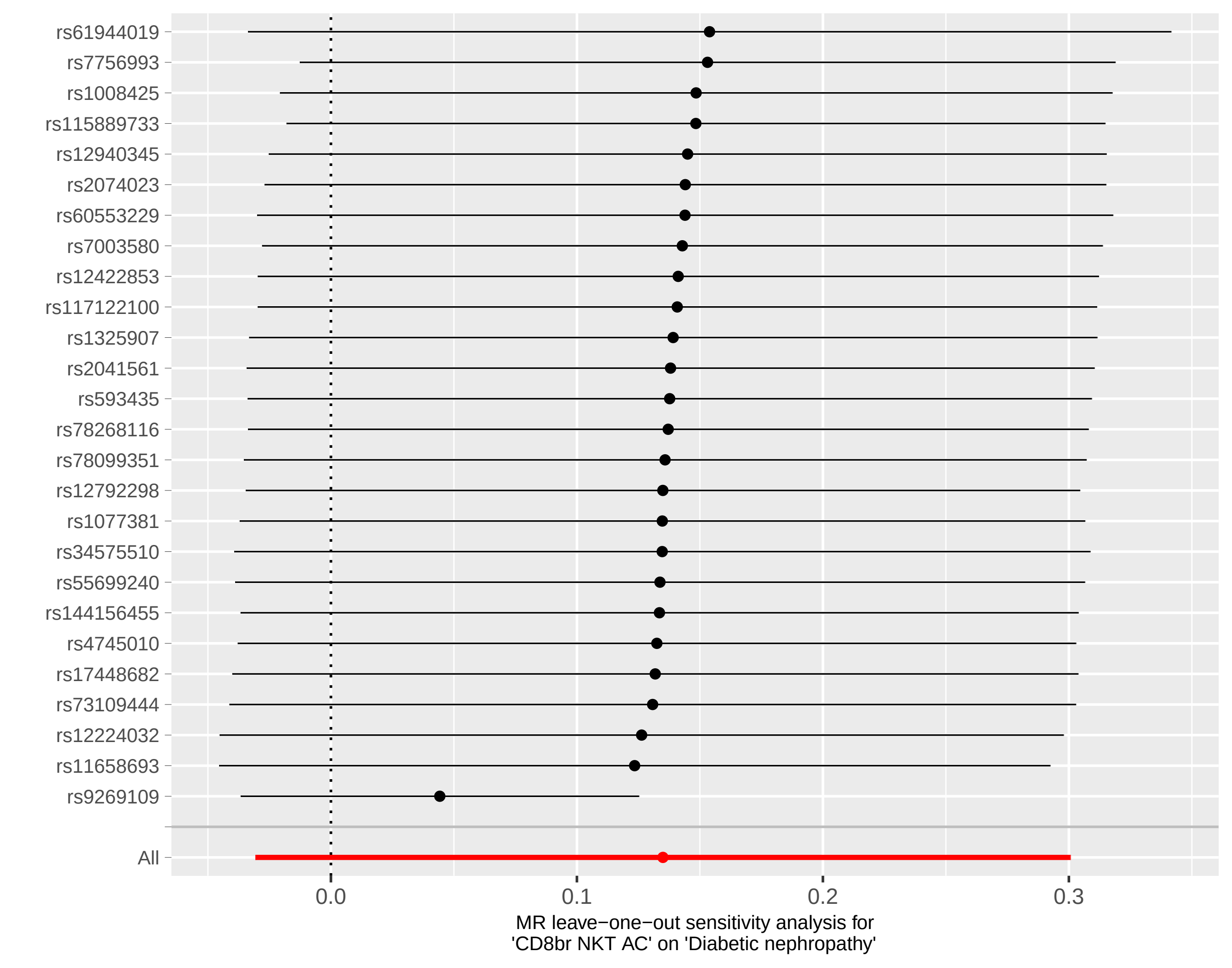
rs61944019
rs7756993
rs1008425
rs115889733
rs12940345
rs2074023
rs60553229
rs7003580
rs12422853
rs117122100
rs1325907
rs2041561
rs593435
rs78268116
rs78099351
rs12792298
rs1077381
rs34575510
rs55699240
rs144156455
rs4745010
rs17448682
rs73109444
rs12224032
rs11658693
rs9269109
All
0.0
0.1
0.2
0.3
MR leave−one−out sensitivity analysis for
'CD8br NKT AC' on 'Diabetic nephropathy'

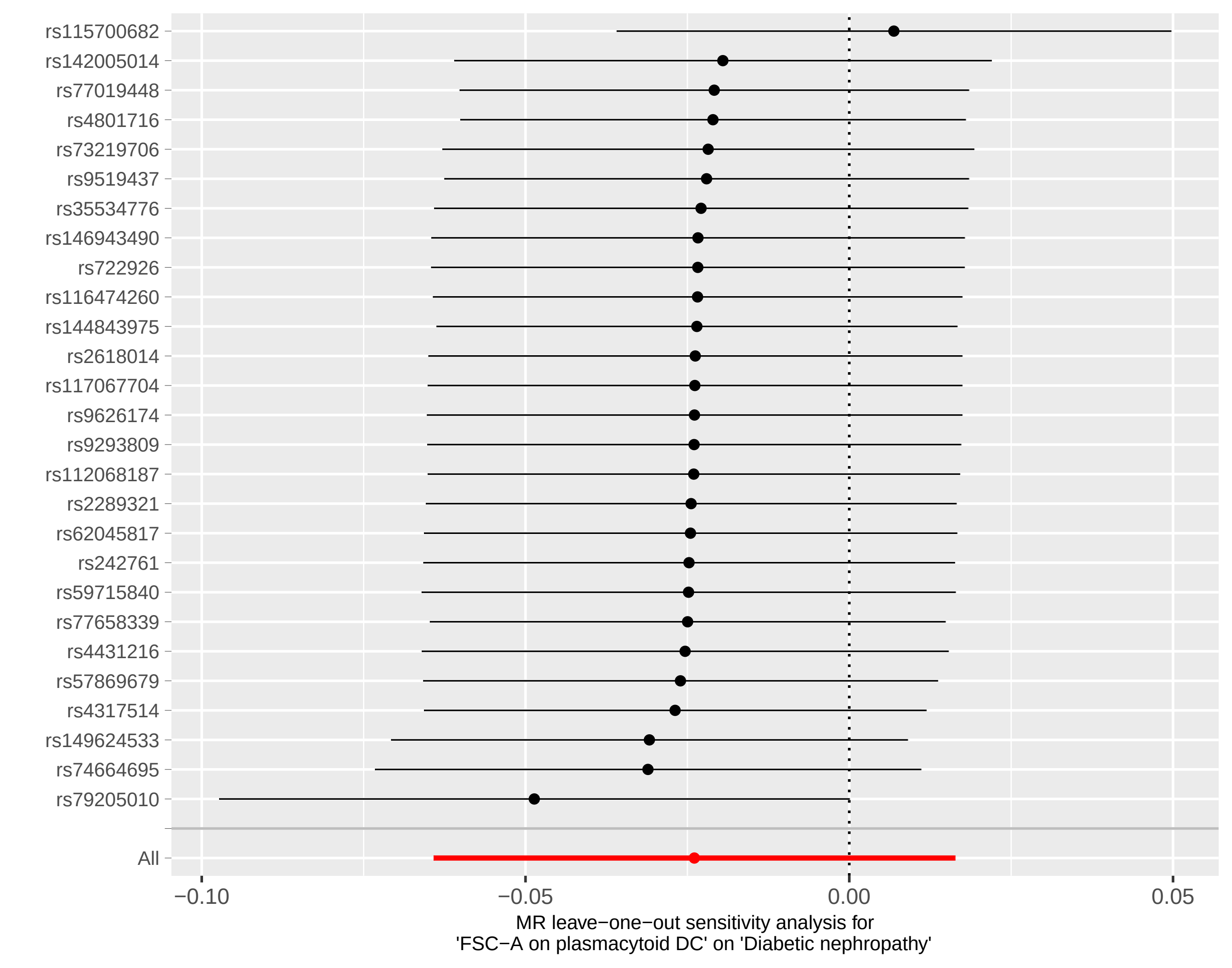
rs115700682
rs142005014
rs77019448
rs4801716
rs73219706
rs9519437
rs35534776
rs146943490
rs722926
rs116474260
rs144843975
rs2618014
rs117067704
rs9626174
rs9293809
rs112068187
rs2289321
rs62045817
rs242761
rs59715840
rs77658339
rs4431216
rs57869679
rs4317514
rs149624533
rs74664695
rs79205010
All
−0.10
−0.05
0.00
0.05
MR leave−one−out sensitivity analysis for
'FSC−A on plasmacytoid DC' on 'Diabetic nephropathy'

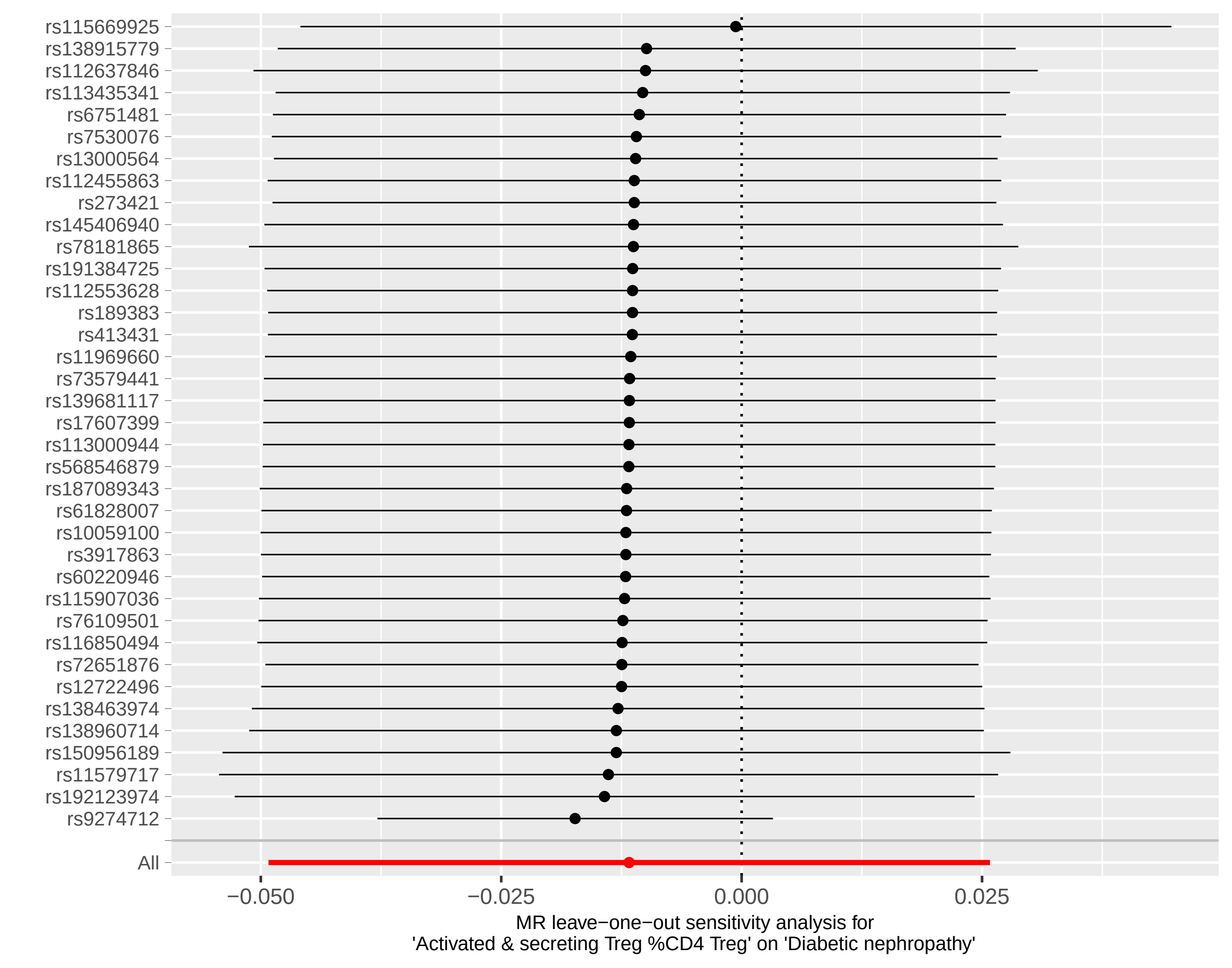
rs115669925
rs138915779
rs112637846
rs113435341
rs6751481
rs7530076
rs13000564
rs112455863
rs273421
rs145406940
rs78181865
rs191384725
rs112553628
rs189383
rs413431
rs11969660
rs73579441
rs139681117
rs17607399
rs113000944
rs568546879
rs187089343
rs61828007
rs10059100
rs3917863
rs60220946
rs115907036
rs76109501
rs116850494
rs72651876
rs12722496
rs138463974
rs138960714
rs150956189
rs11579717
rs192123974
rs9274712
All
−0.050
−0.025
0.000
0.025
MR leave−one−out sensitivity analysis for
'Activated & secreting Treg %CD4 Treg' on 'Diabetic nephropathy'

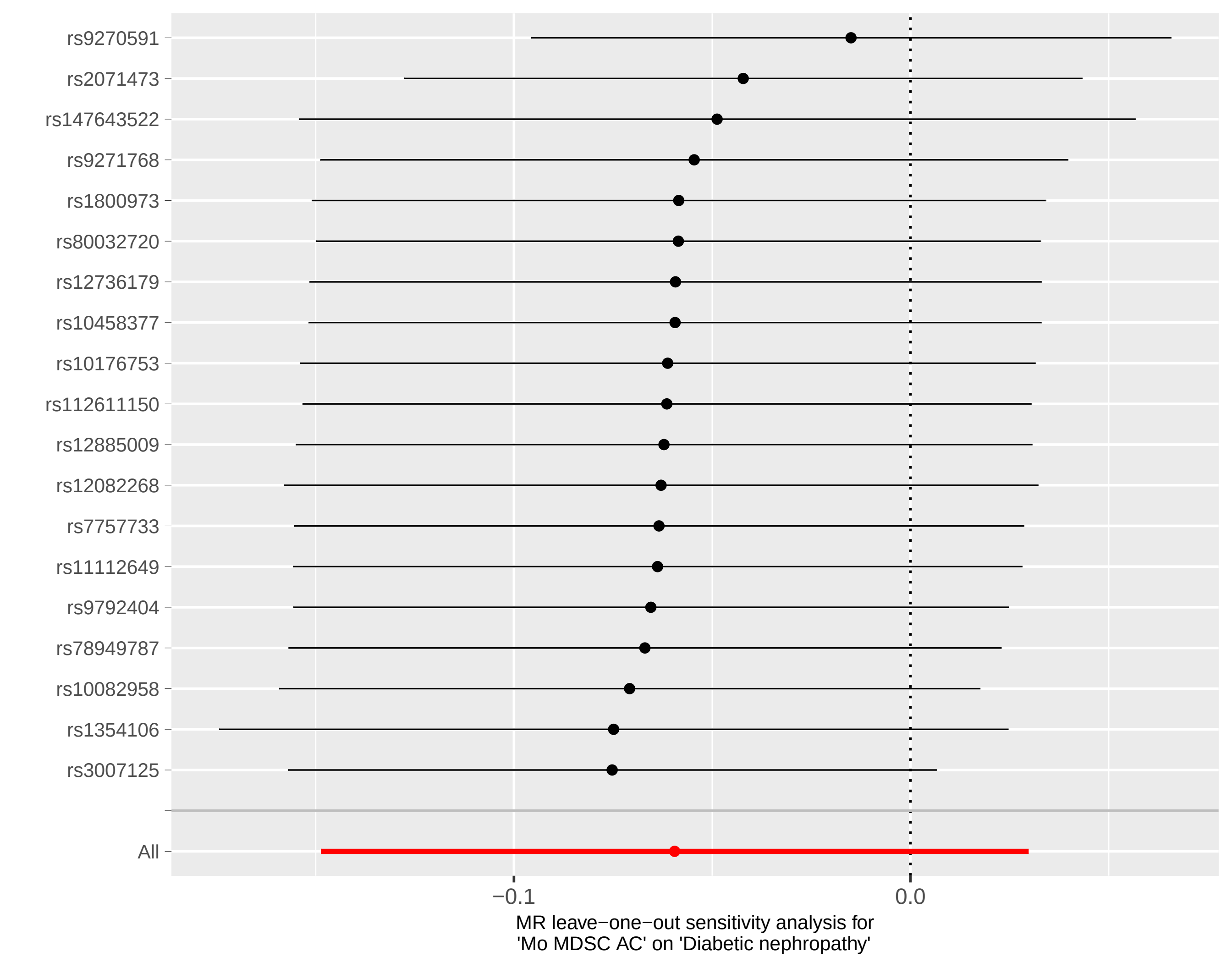

rs9270591
rs2071473
rs147643522
rs9271768
rs1800973
rs80032720
rs12736179
rs10458377
rs10176753
rs112611150
rs12885009
rs12082268
rs7757733
rs11112649
rs9792404
rs78949787
rs10082958
rs1354106
rs3007125
All
−0.1
0.0
MR leave−one−out sensitivity analysis for
'Mo MDSC AC' on 'Diabetic nephropathy'

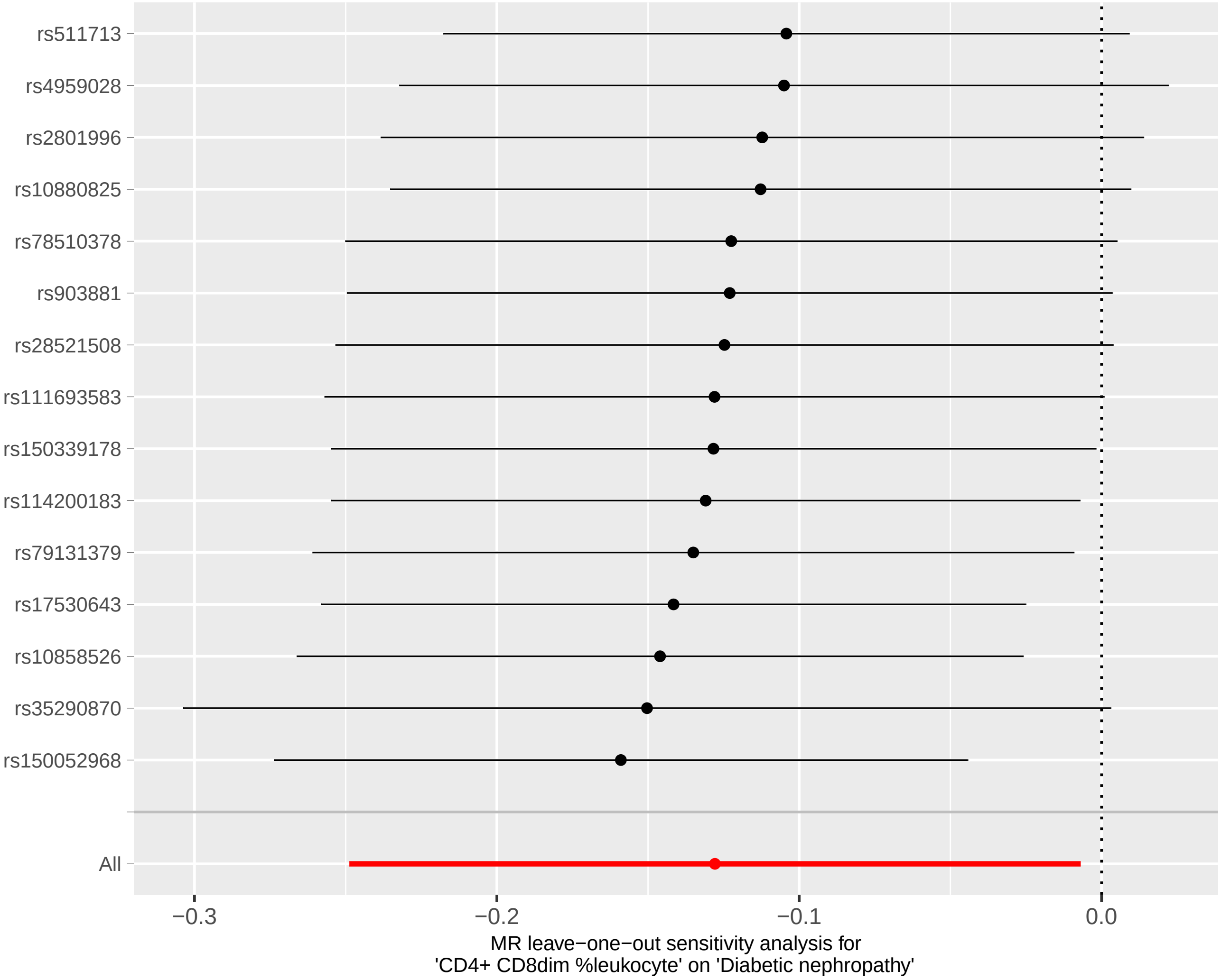
rs511713
rs4959028
rs2801996
rs10880825
rs78510378
rs903881
rs28521508
rs111693583
rs150339178
rs114200183
rs79131379
rs17530643
rs10858526
rs35290870
rs150052968
All
−0.3
−0.2
−0.1
0.0
MR leave−one−out sensitivity analysis for
'CD4+ CD8dim %leukocyte' on 'Diabetic nephropathy'

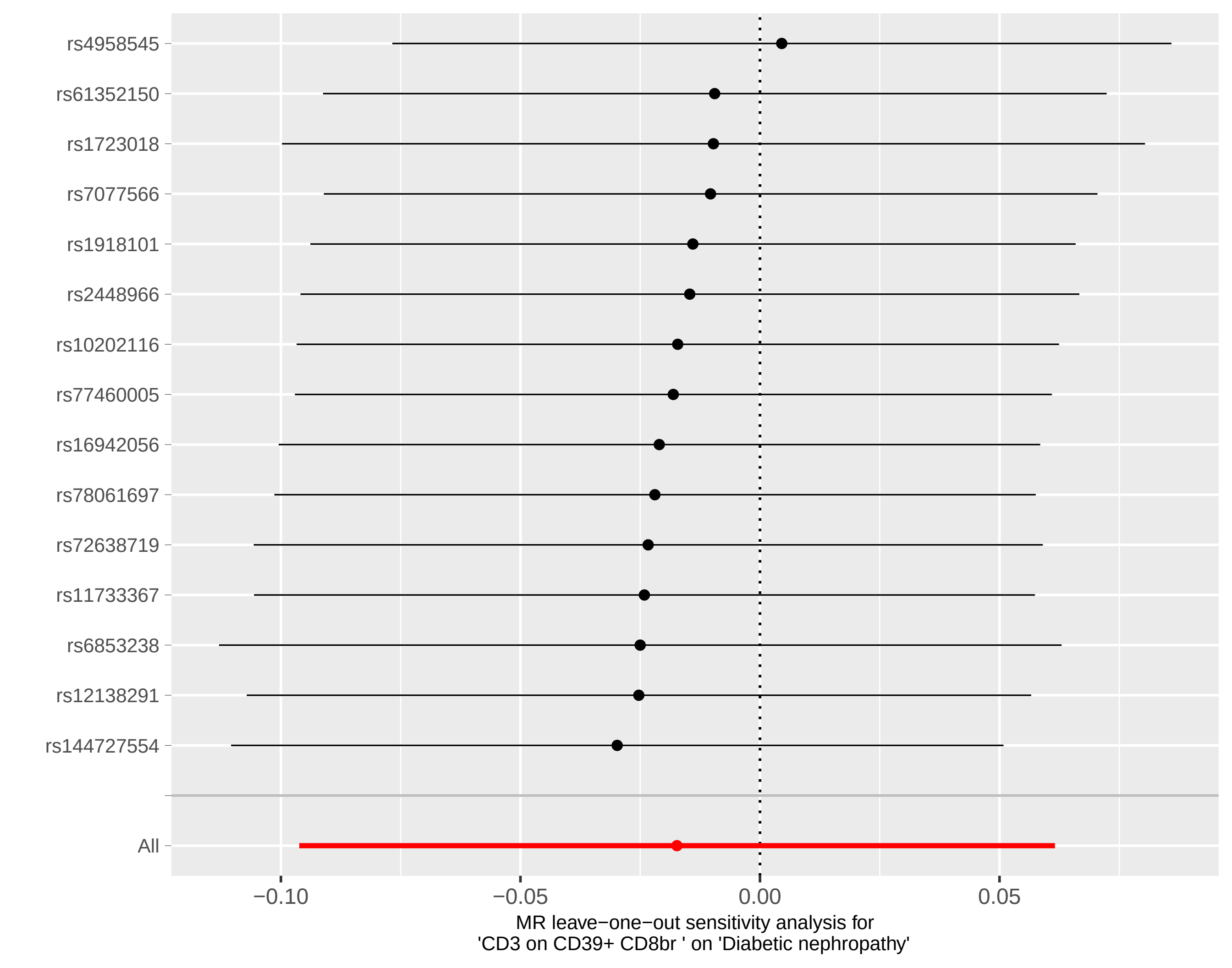
rs4958545
rs61352150
rs1723018
rs7077566
rs1918101
rs2448966
rs10202116
rs77460005
rs16942056
rs78061697
rs72638719
rs11733367
rs6853238
rs12138291
rs144727554
All
−0.10
−0.05
0.00
0.05
MR leave−one−out sensitivity analysis for
'CD3 on CD39+ CD8br ' on 'Diabetic nephropathy'

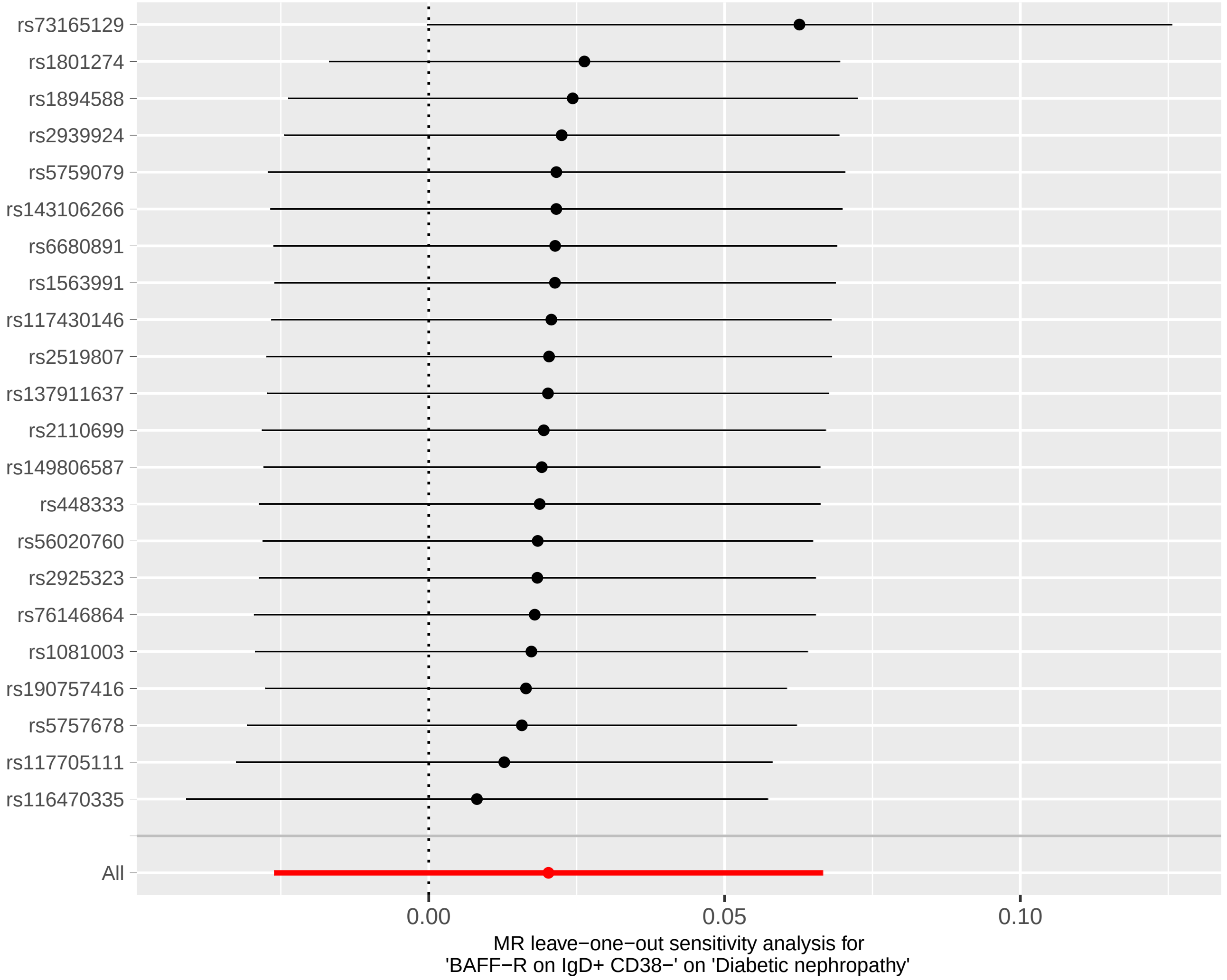
rs73165129
rs1801274
rs1894588
rs2939924
rs5759079
rs143106266
rs6680891
rs1563991
rs117430146
rs2519807
rs137911637
rs2110699
rs149806587
rs448333
rs56020760
rs2925323
rs76146864
rs1081003
rs190757416
rs5757678
rs117705111
rs116470335
All
0.00
0.05
0.10
MR leave−one−out sensitivity analysis for
'BAFF−R on IgD+ CD38−' on 'Diabetic nephropathy'

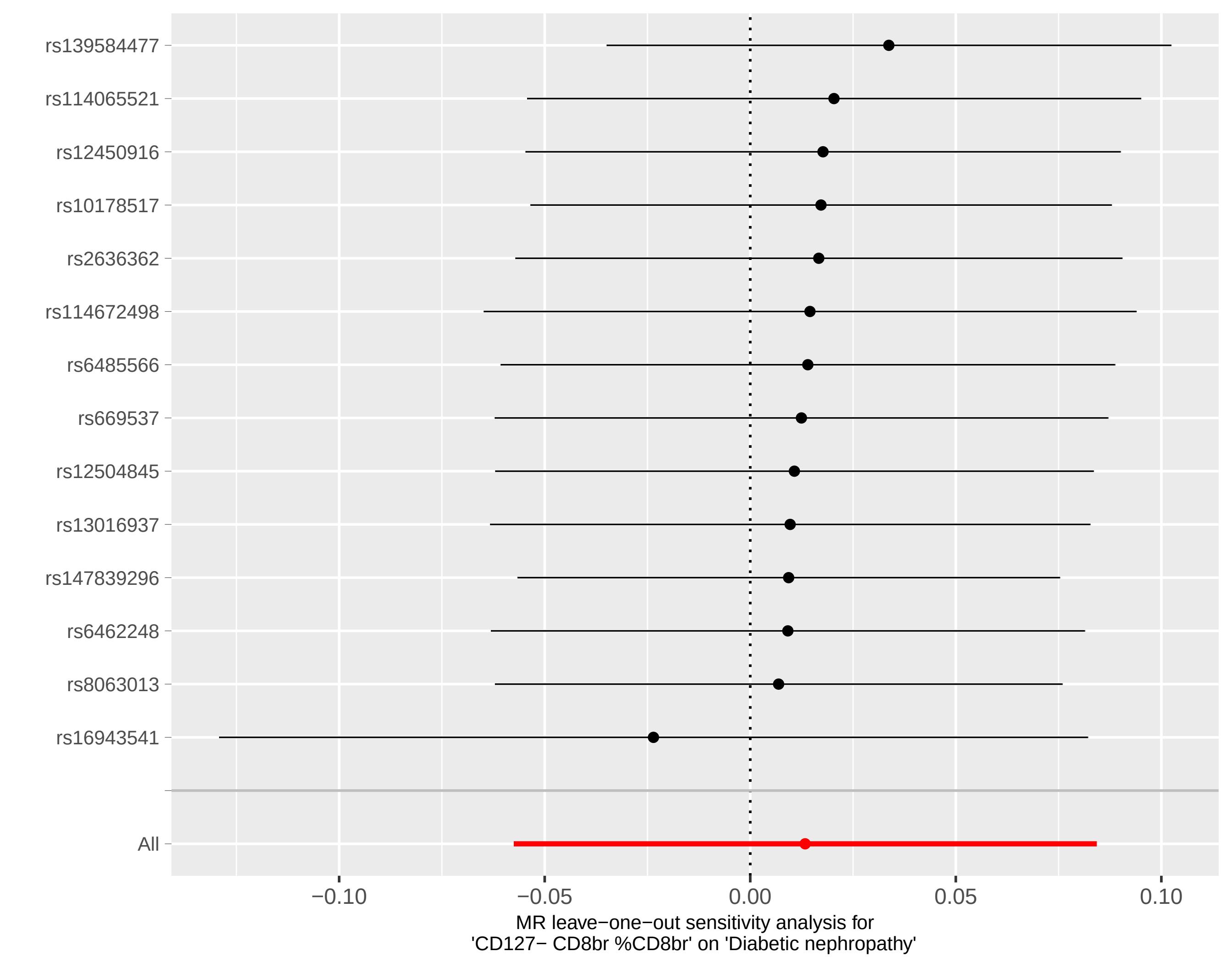
rs139584477
rs114065521
rs12450916
rs10178517
rs2636362
rs114672498
rs6485566
rs669537
rs12504845
rs13016937
rs147839296
rs6462248
rs8063013
rs16943541
All
−0.10
−0.05
0.00
0.05
0.10
MR leave−one−out sensitivity analysis for
'CD127− CD8br %CD8br' on 'Diabetic nephropathy'

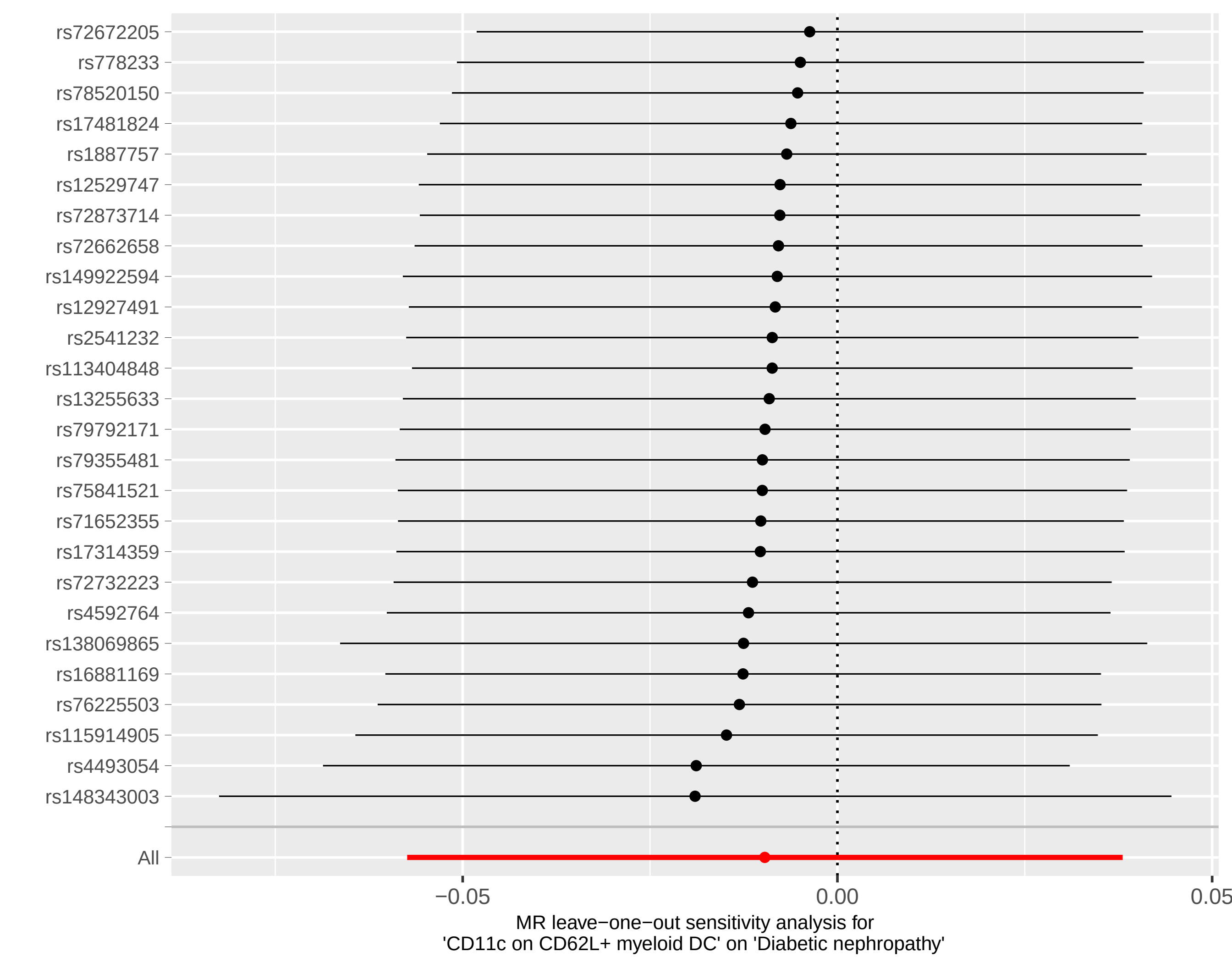
rs72672205
rs778233
rs78520150
rs17481824
rs1887757
rs12529747
rs72873714
rs72662658
rs149922594
rs12927491
rs2541232
rs113404848
rs13255633
rs79792171
rs79355481
rs75841521
rs71652355
rs17314359
rs72732223
rs4592764
rs138069865
rs16881169
rs76225503
rs115914905
rs4493054
rs148343003
All
−0.05
0.00
0.05
MR leave−one−out sensitivity analysis for
'CD11c on CD62L+ myeloid DC' on 'Diabetic nephropathy'

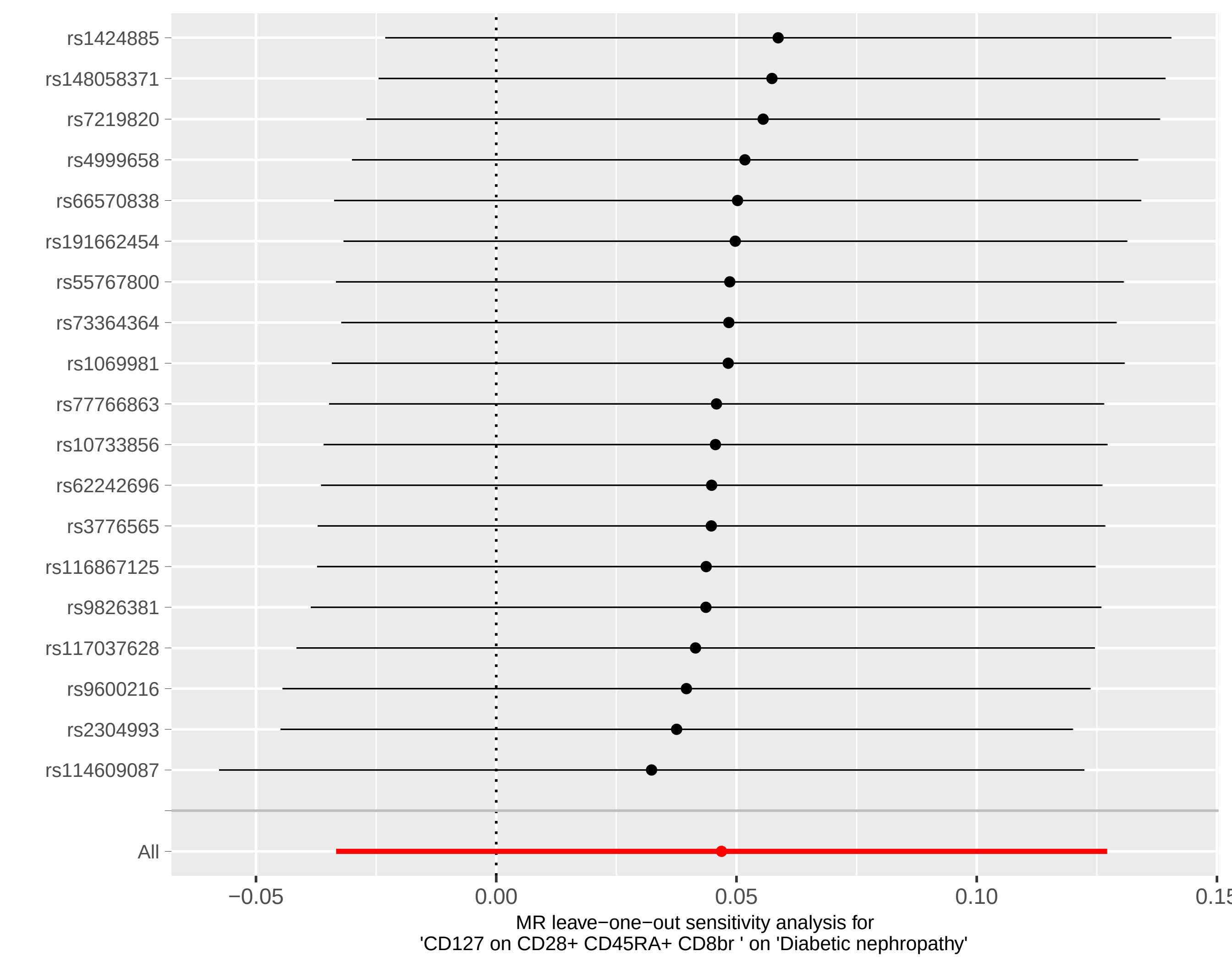
rs1424885
rs148058371
rs7219820
rs4999658
rs66570838
rs191662454
rs55767800
rs73364364
rs1069981
rs77766863
rs10733856
rs62242696
rs3776565
rs116867125
rs9826381
rs117037628
rs9600216
rs2304993
rs114609087
All
−0.05
0.00
0.05
0.10
MR leave−one−out sensitivity analysis for
'CD127 on CD28+ CD45RA+ CD8br ' on 'Diabetic nephropathy'

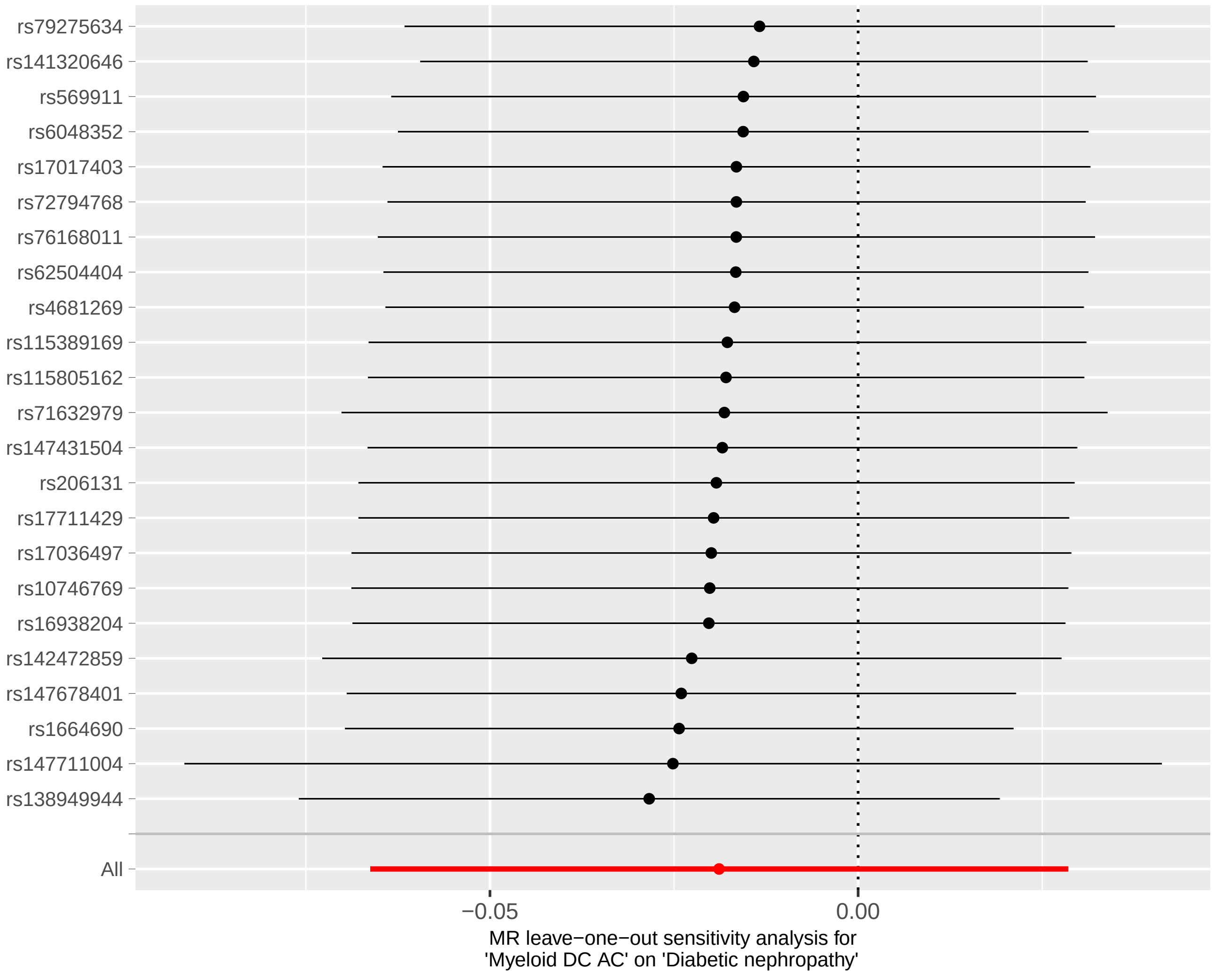

rs79275634
rs141320646
rs569911
rs6048352
rs17017403
rs72794768
rs76168011
rs62504404
rs4681269
rs115389169
rs115805162
rs71632979
rs147431504
rs206131
rs17711429
rs17036497
rs10746769
rs16938204
rs142472859
rs147678401
rs1664690
rs147711004
rs138949944
All
−0.05
0.00
MR leave−one−out sensitivity analysis for
'Myeloid DC AC' on 'Diabetic nephropathy'

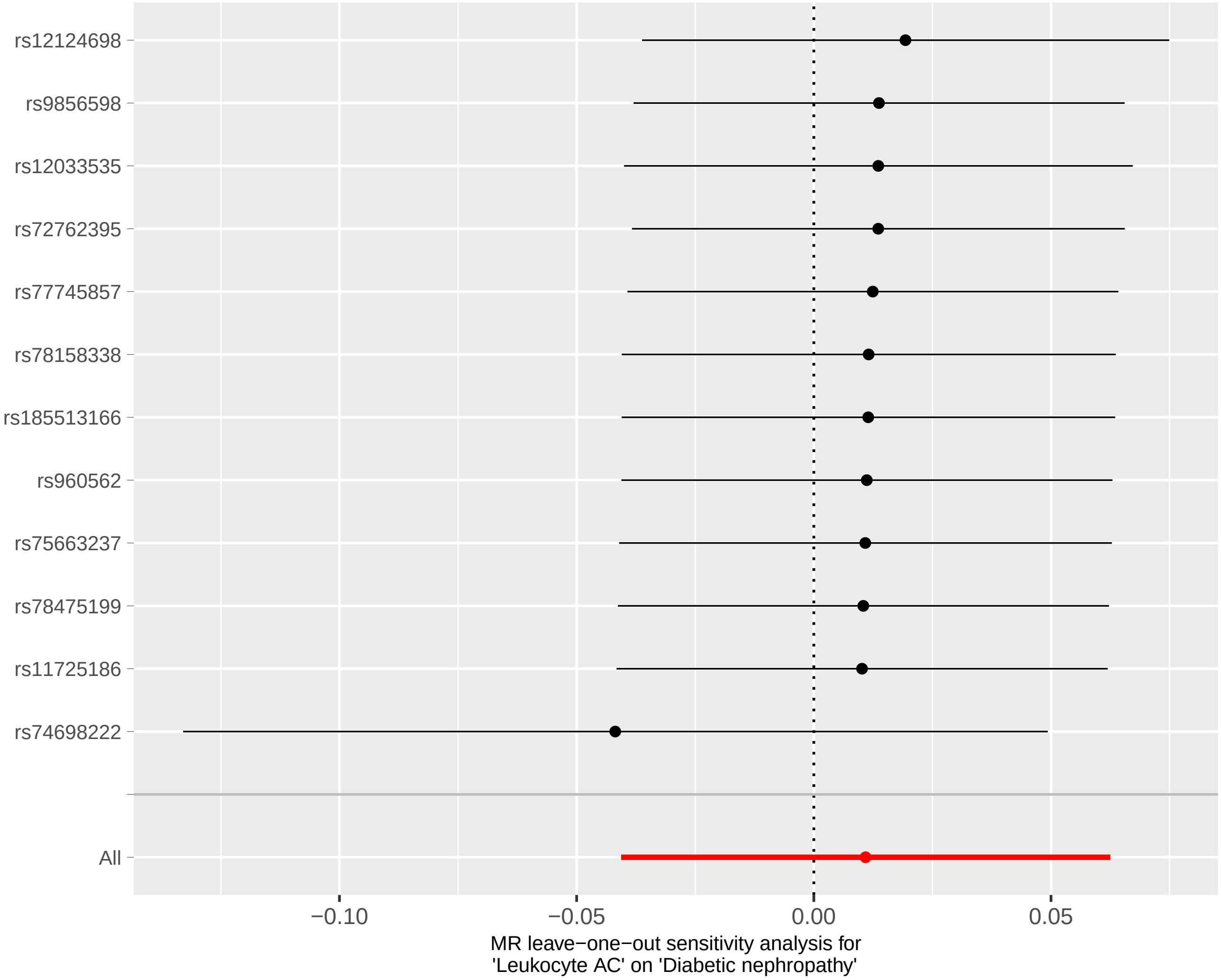
rs12124698
rs9856598
rs12033535
rs72762395
rs77745857
rs78158338
rs185513166
rs960562
rs75663237
rs78475199
rs11725186
rs74698222
All
−0.10
−0.05
0.00
0.05
MR leave−one−out sensitivity analysis for
'Leukocyte AC' on 'Diabetic nephropathy'

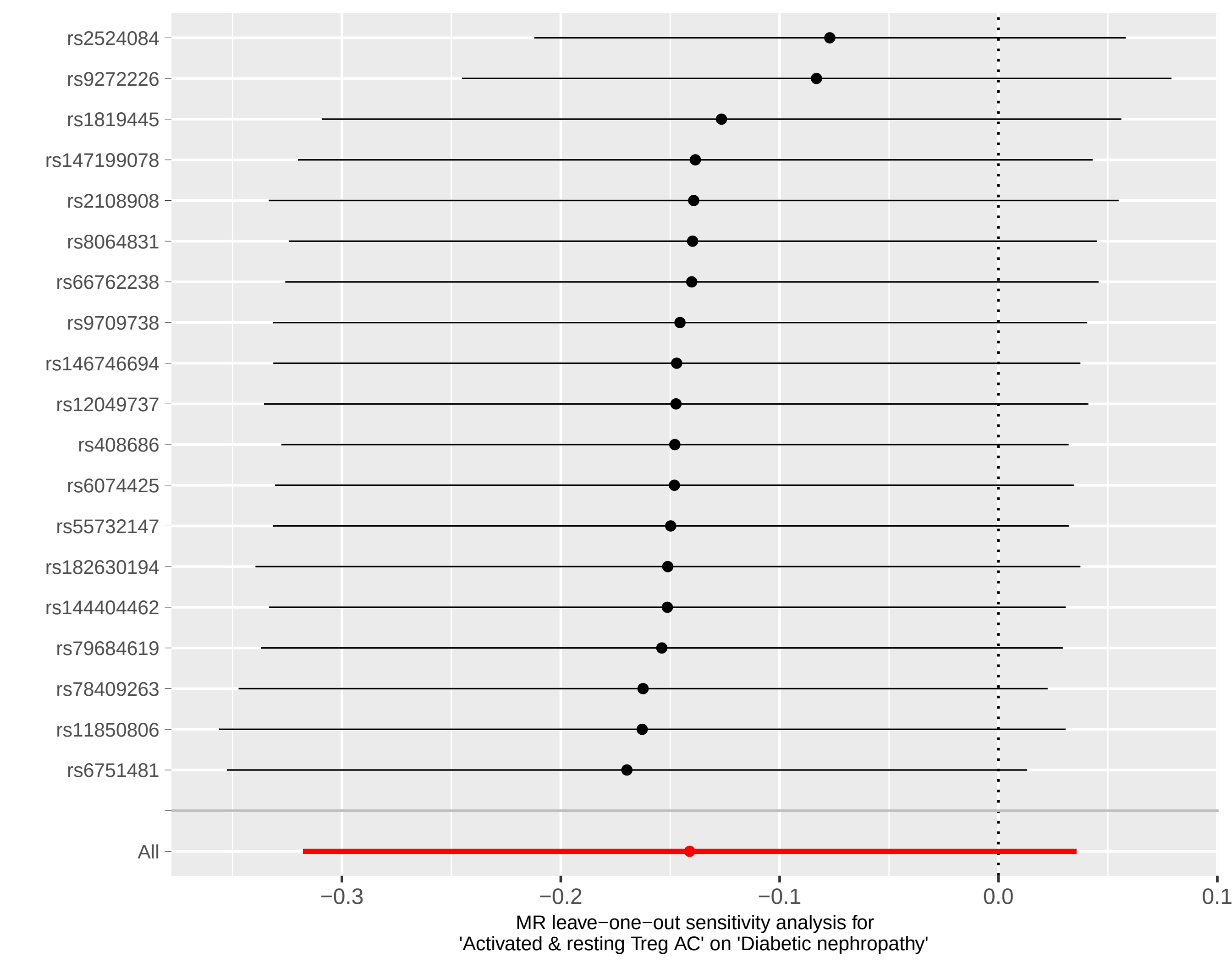
rs2524084
rs9272226
rs1819445
rs147199078
rs2108908
rs8064831
rs66762238
rs9709738
rs146746694
rs12049737
rs408686
rs6074425
rs55732147
rs182630194
rs144404462
rs79684619
rs78409263
rs11850806
rs6751481
All
−0.3
−0.2
−0.1
0.0
0.1
MR leave−one−out sensitivity analysis for
'Activated & resting Treg AC' on 'Diabetic nephropathy'

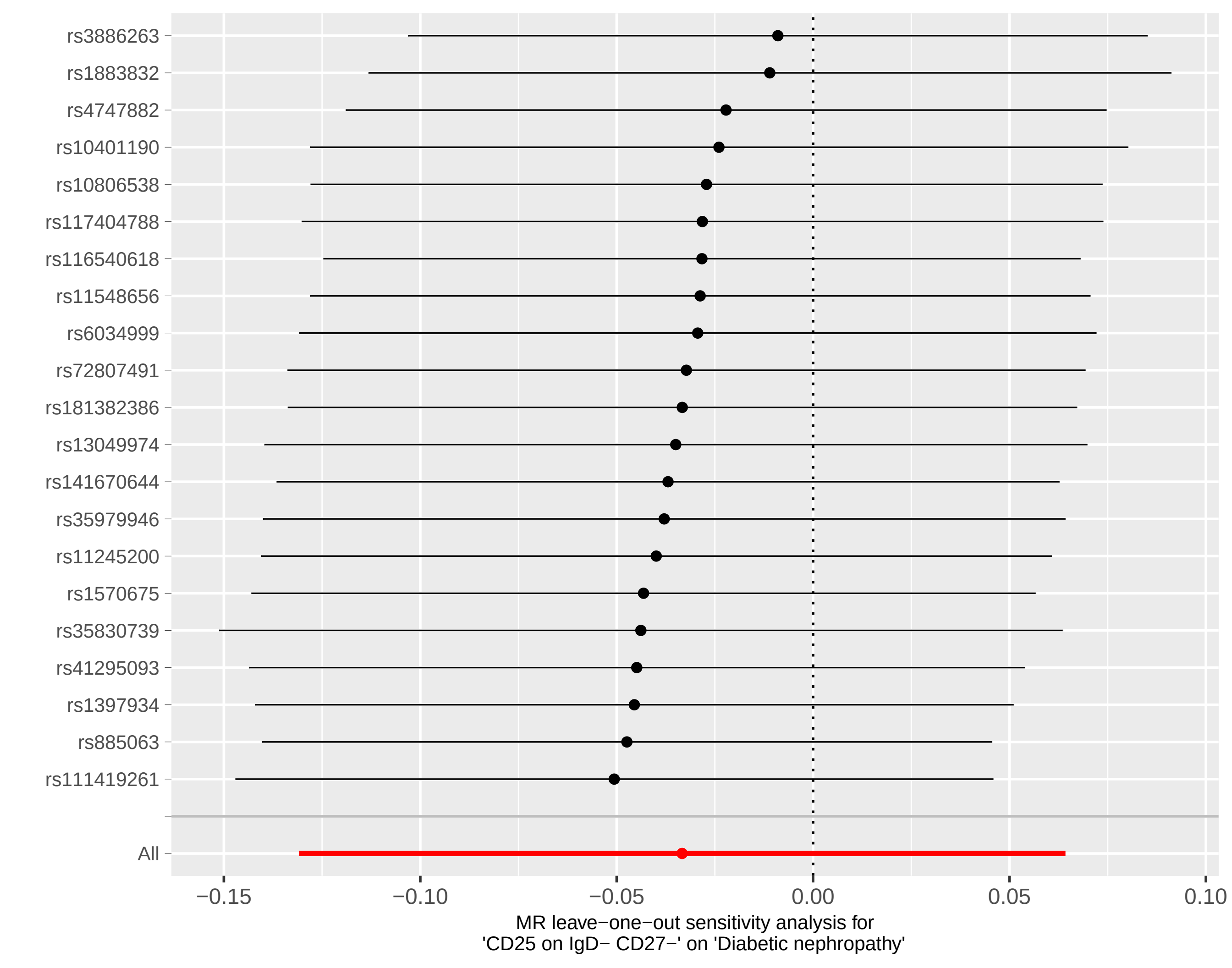
rs3886263
rs1883832
rs4747882
rs10401190
rs10806538
rs117404788
rs116540618
rs11548656
rs6034999
rs72807491
rs181382386
rs13049974
rs141670644
rs35979946
rs11245200
rs1570675
rs35830739
rs41295093
rs1397934
rs885063
rs111419261
All
−0.15
−0.10
−0.05
0.00
0.05
0.10
MR leave−one−out sensitivity analysis for
'CD25 on IgD− CD27−' on 'Diabetic nephropathy'

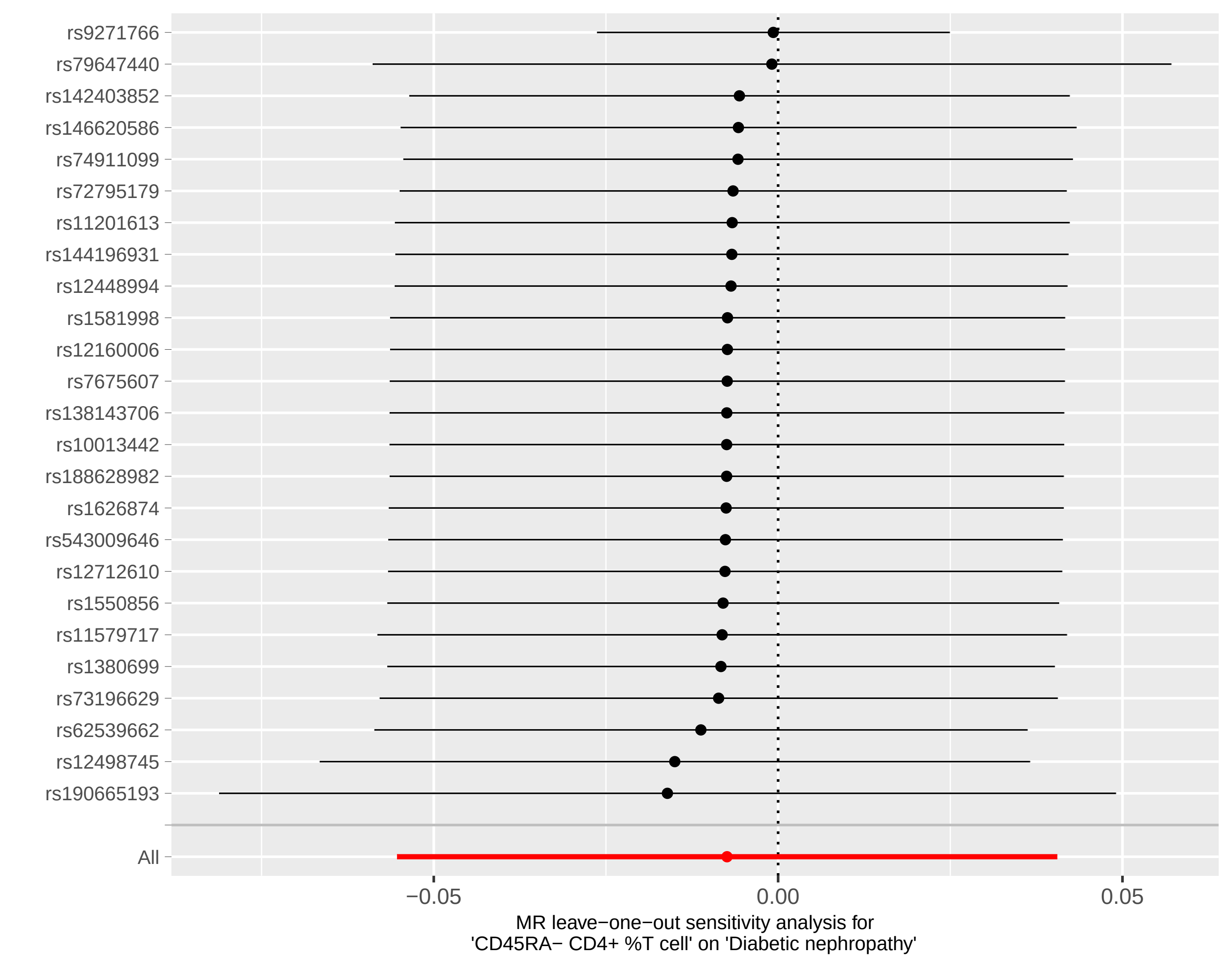
rs9271766
rs79647440
rs142403852
rs146620586
rs74911099
rs72795179
rs11201613
rs144196931
rs12448994
rs1581998
rs12160006
rs7675607
rs138143706
rs10013442
rs188628982
rs1626874
rs543009646
rs12712610
rs1550856
rs11579717
rs1380699
rs73196629
rs62539662
rs12498745
rs190665193
All
−0.05
0.00
0.05
MR leave−one−out sensitivity analysis for
'CD45RA− CD4+ %T cell' on 'Diabetic nephropathy'

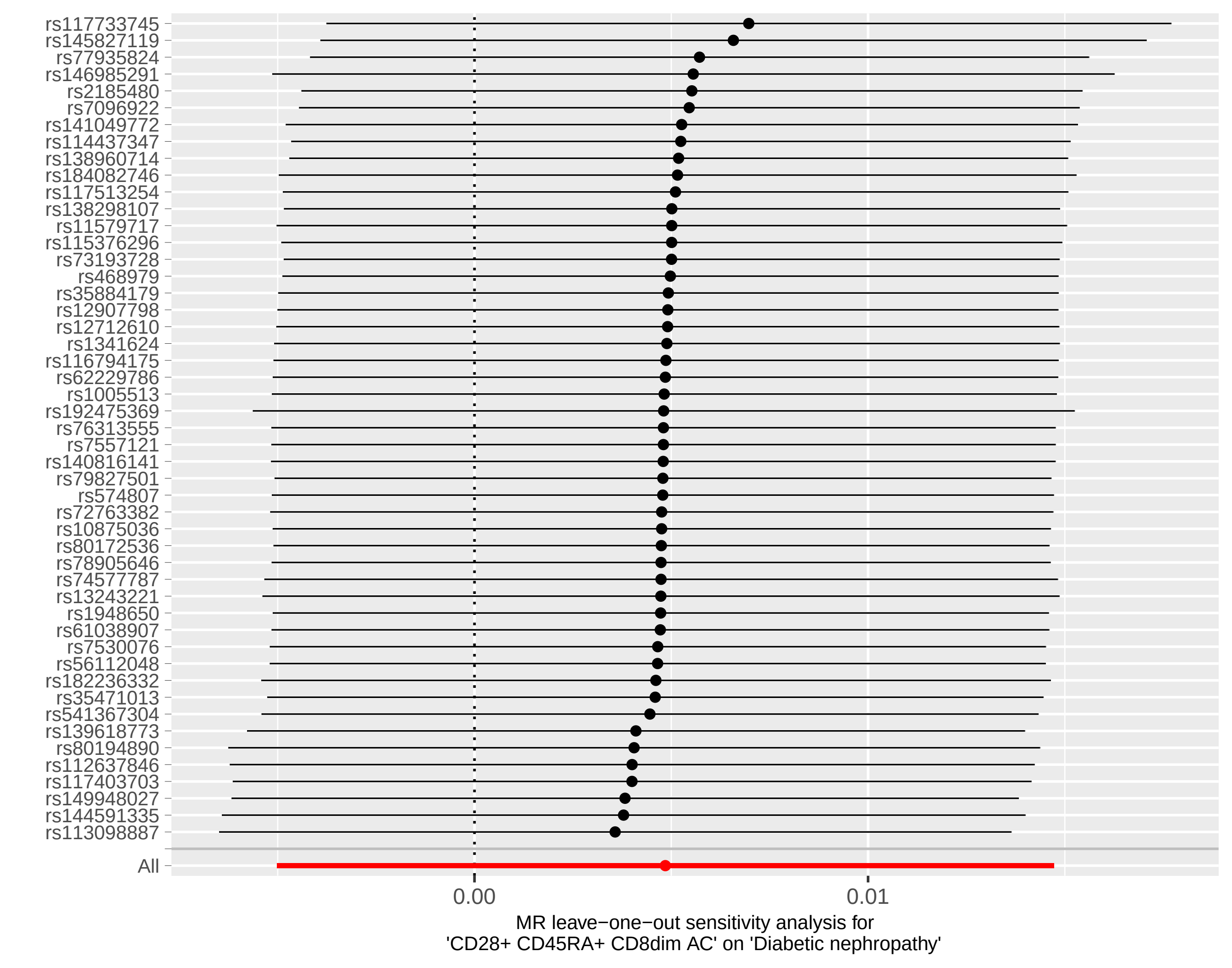

rs117733745
rs145827119
rs77935824
rs146985291
rs2185480
rs7096922
rs141049772
rs114437347
rs138960714
rs184082746
rs117513254
rs138298107
rs11579717
rs115376296
rs73193728
rs468979
rs35884179
rs12907798
rs12712610
rs1341624
rs116794175
rs62229786
rs1005513
rs192475369
rs76313555
rs7557121
rs140816141
rs79827501
rs574807
rs72763382
rs10875036
rs80172536
rs78905646
rs74577787
rs13243221
rs1948650
rs61038907
rs7530076
rs56112048
rs182236332
rs35471013
rs541367304
rs139618773
rs80194890
rs112637846
rs117403703
rs149948027
rs144591335
rs113098887
All
0.00
0.01
MR leave−one−out sensitivity analysis for
'CD28+ CD45RA+ CD8dim AC' on 'Diabetic nephropathy'

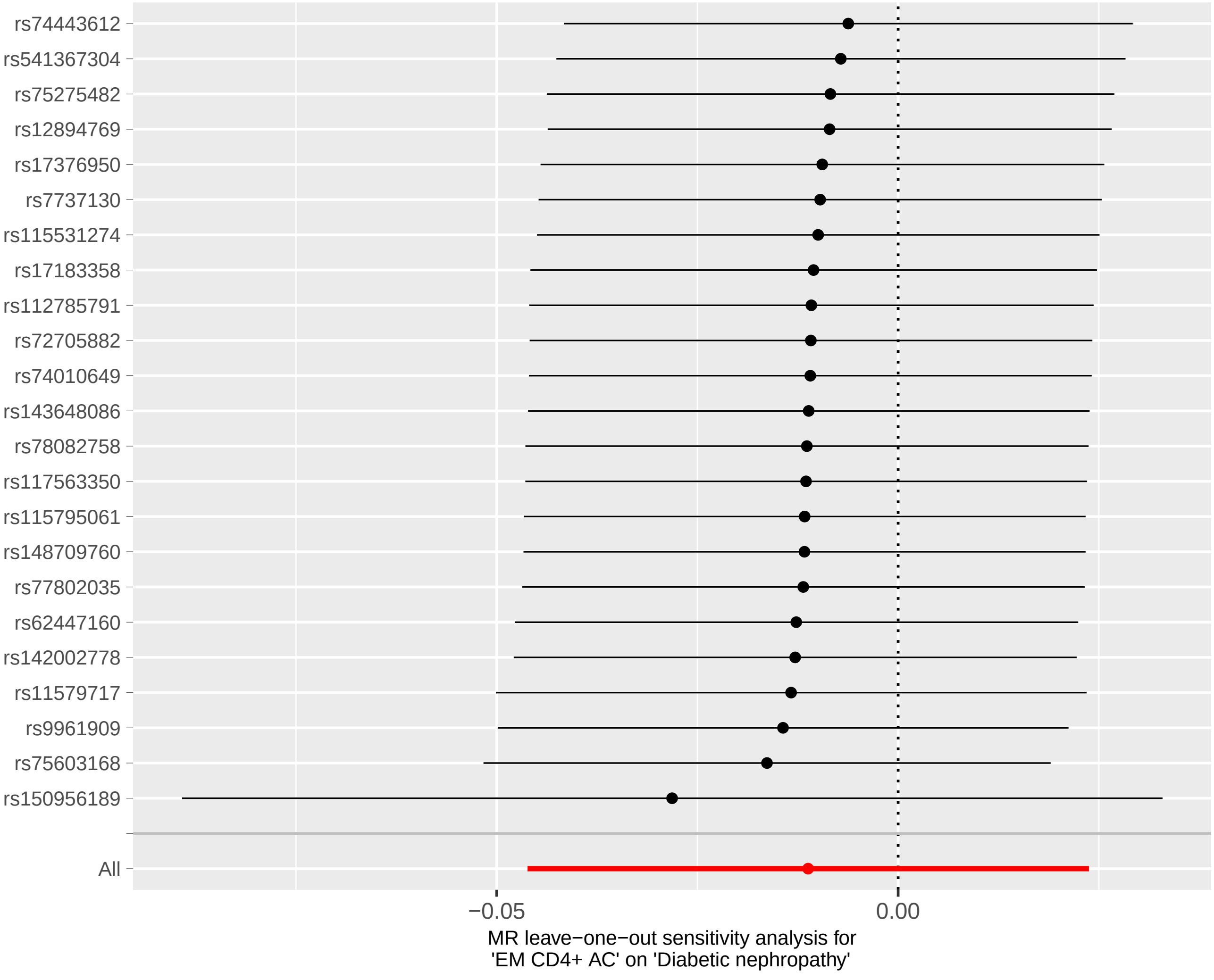
rs74443612
rs541367304
rs75275482
rs12894769
rs17376950
rs7737130
rs115531274
rs17183358
rs112785791
rs72705882
rs74010649
rs143648086
rs78082758
rs117563350
rs115795061
rs148709760
rs77802035
rs62447160
rs142002778
rs11579717
rs9961909
rs75603168
rs150956189
All
−0.05
0.00
MR leave−one−out sensitivity analysis for
'EM CD4+ AC' on 'Diabetic nephropathy'

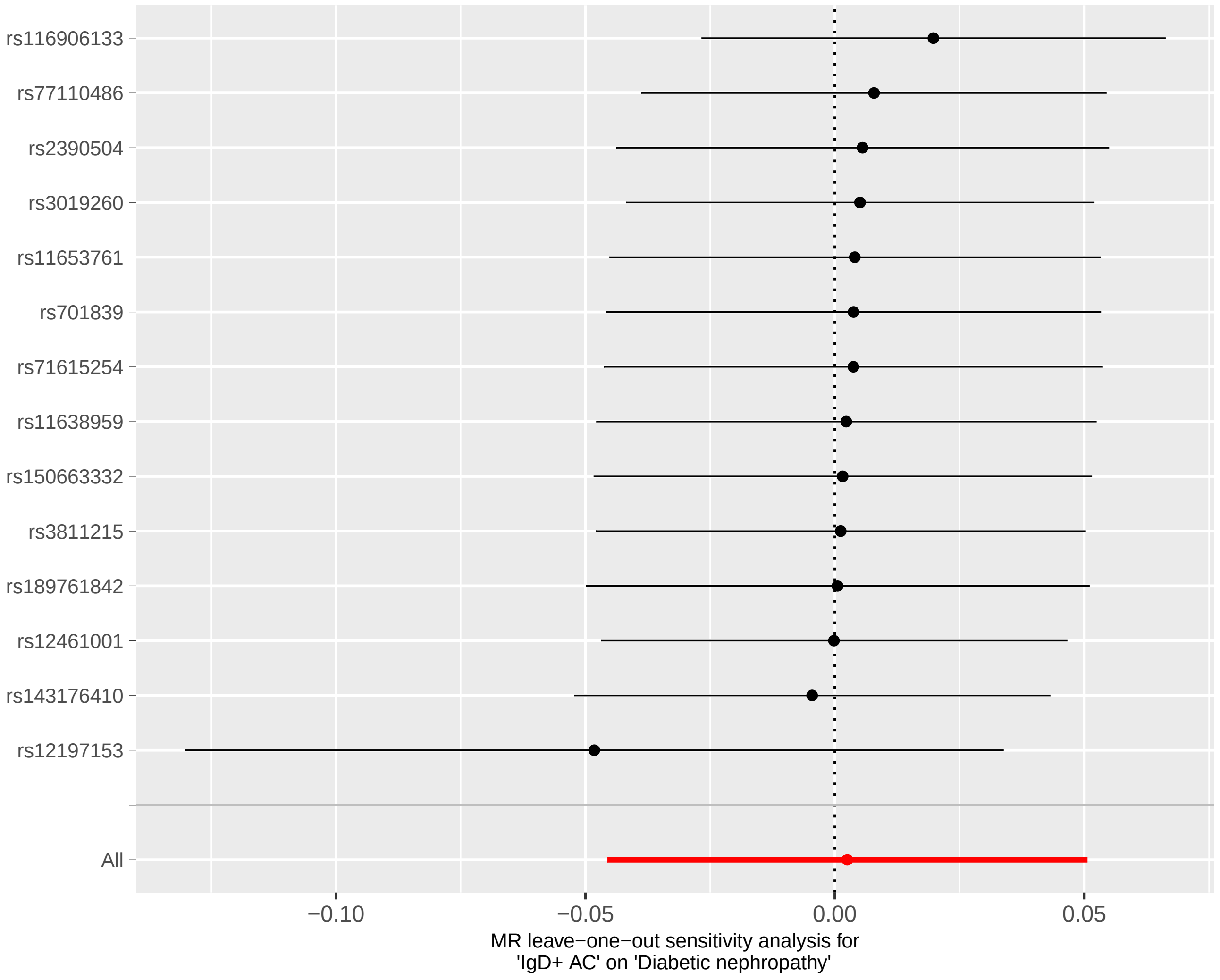
rs116906133
rs77110486
rs2390504
rs3019260
rs11653761
rs701839
rs71615254
rs11638959
rs150663332
rs3811215
rs189761842
rs12461001
rs143176410
rs12197153
All
−0.10
−0.05
0.00
0.05
MR leave−one−out sensitivity analysis for
'IgD+ AC' on 'Diabetic nephropathy'

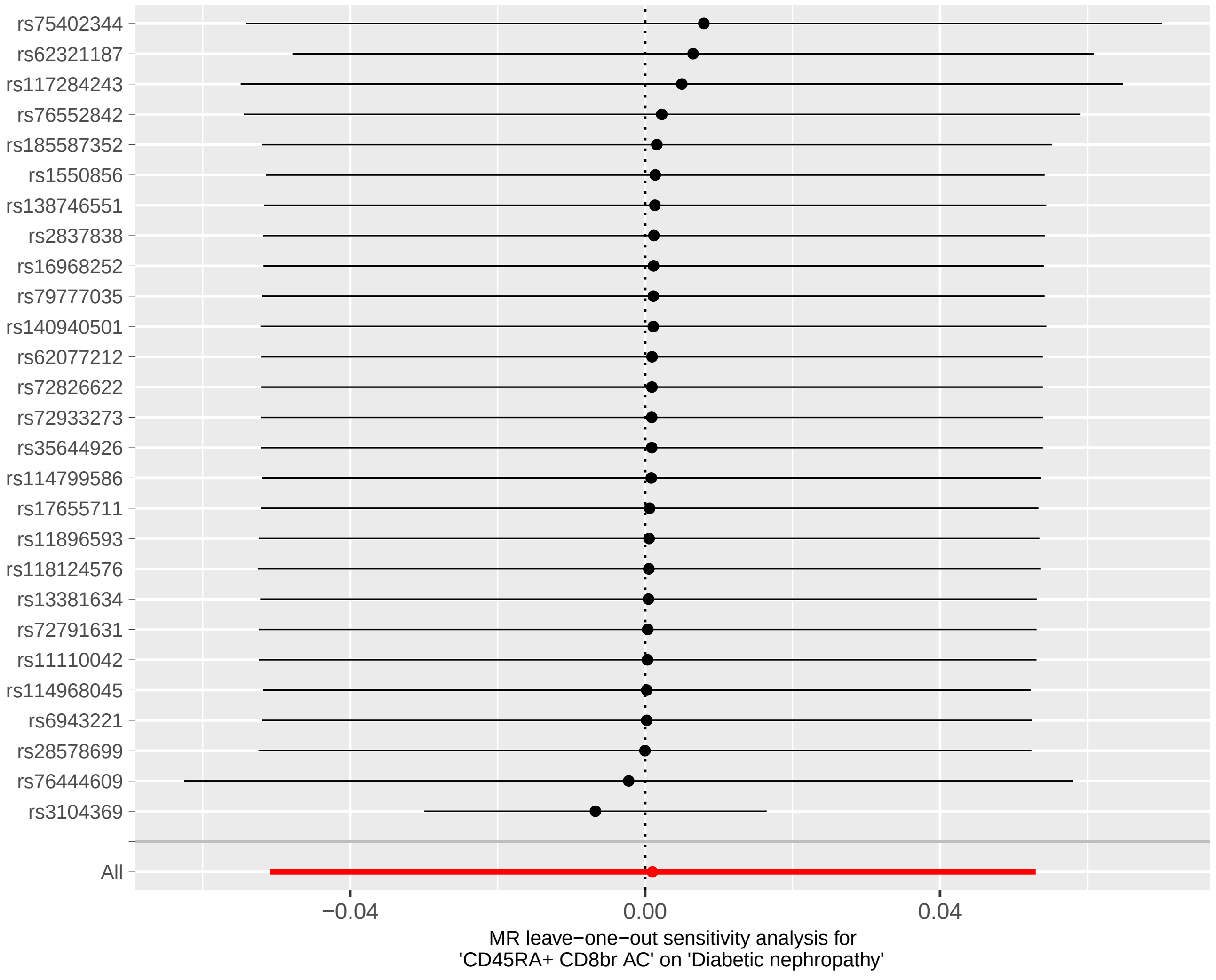
rs75402344
rs62321187
rs117284243
rs76552842
rs185587352
rs1550856
rs138746551
rs2837838
rs16968252
rs79777035
rs140940501
rs62077212
rs72826622
rs72933273
rs35644926
rs114799586
rs17655711
rs11896593
rs118124576
rs13381634
rs72791631
rs11110042
rs114968045
rs6943221
rs28578699
rs76444609
rs3104369
All
−0.04
0.00
0.04
MR leave−one−out sensitivity analysis for
'CD45RA+ CD8br AC' on 'Diabetic nephropathy'

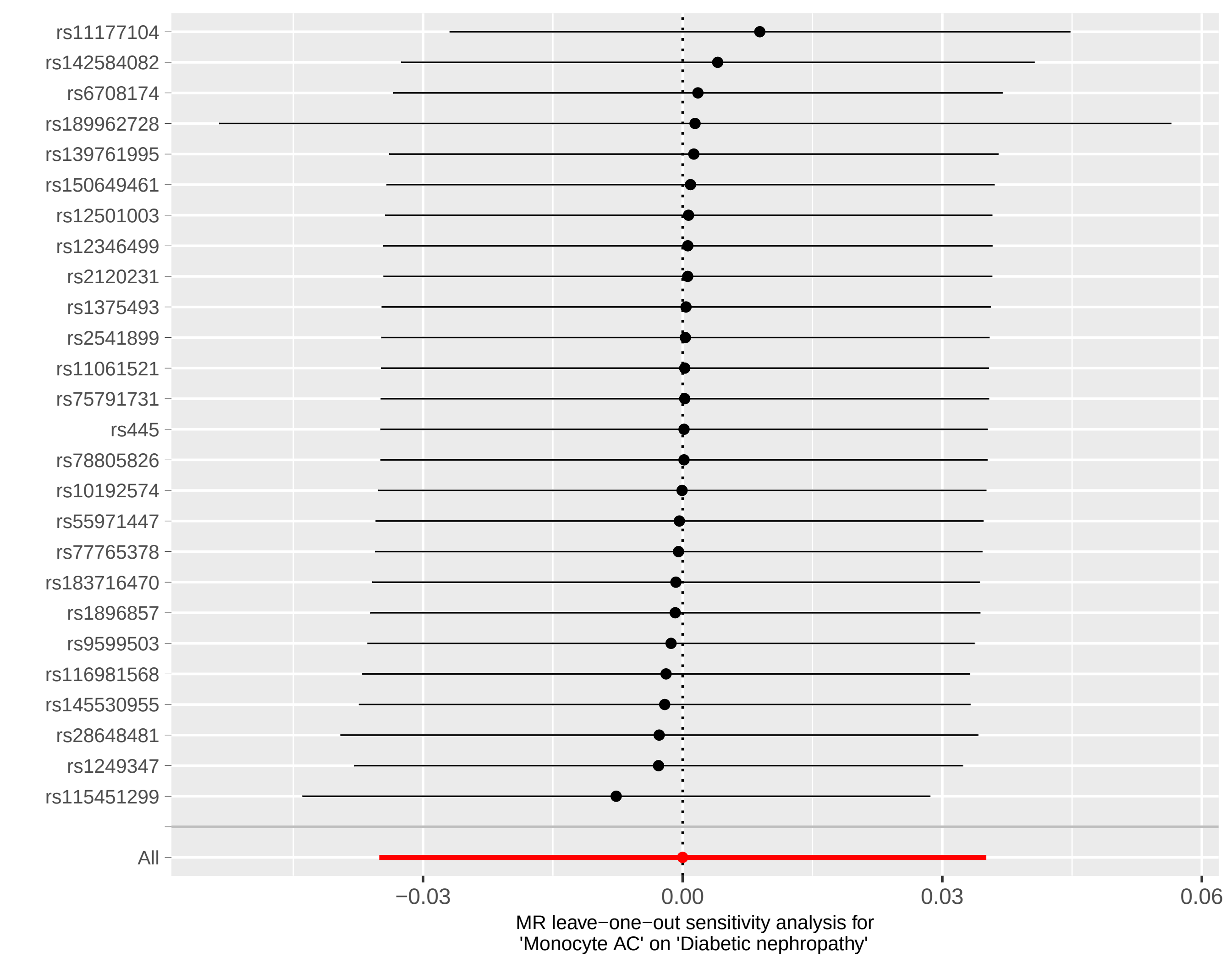

rs11177104
rs142584082
rs6708174
rs189962728
rs139761995
rs150649461
rs12501003
rs12346499
rs2120231
rs1375493
rs2541899
rs11061521
rs75791731
rs445
rs78805826
rs10192574
rs55971447
rs77765378
rs183716470
rs1896857
rs9599503
rs116981568
rs145530955
rs28648481
rs1249347
rs115451299
All
−0.03
0.00
0.03
0.06
MR leave−one−out sensitivity analysis for
'Monocyte AC' on 'Diabetic nephropathy'

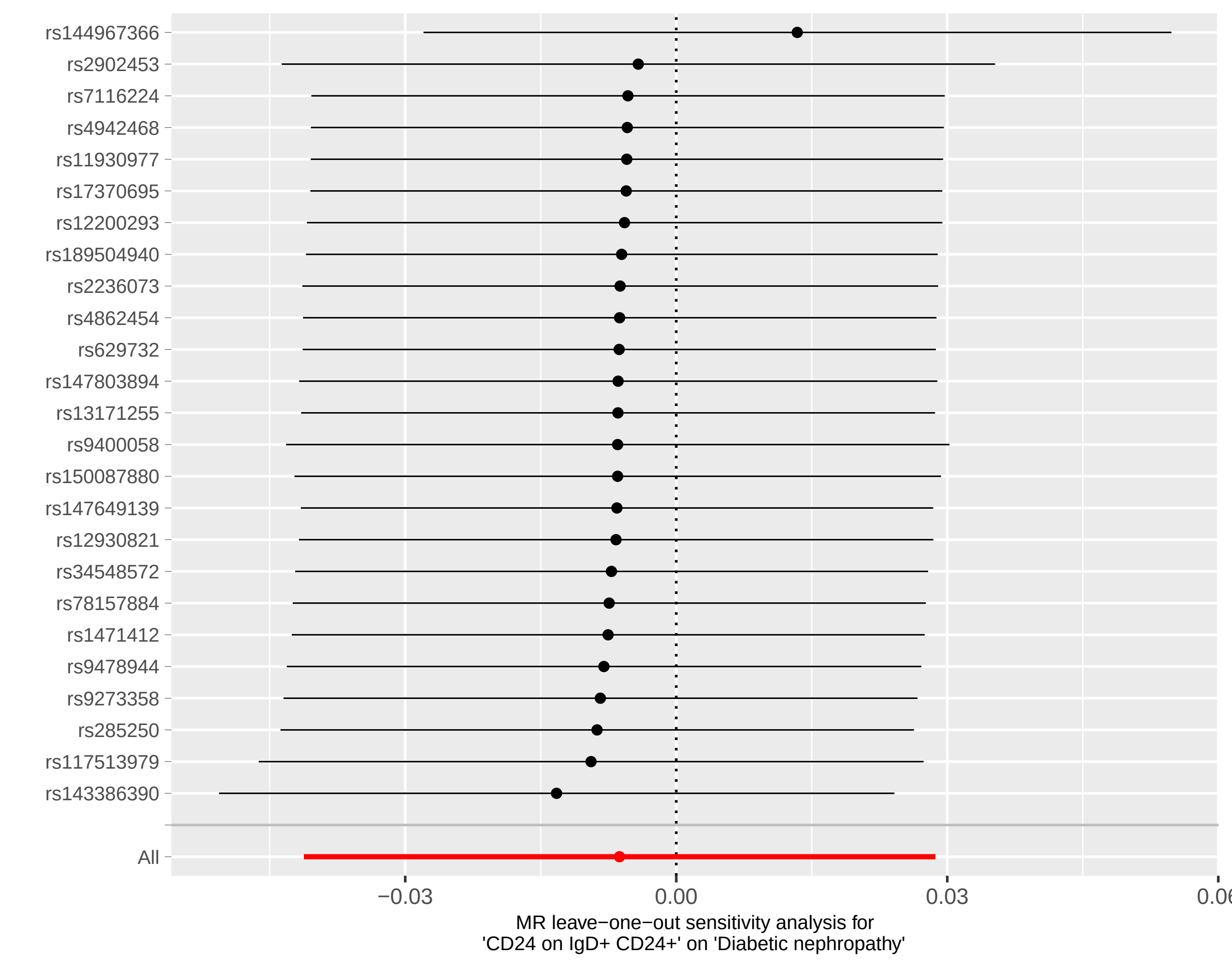
rs144967366
rs2902453
rs7116224
rs4942468
rs11930977
rs17370695
rs12200293
rs189504940
rs2236073
rs4862454
rs629732
rs147803894
rs13171255
rs9400058
rs150087880
rs147649139
rs12930821
rs34548572
rs78157884
rs1471412
rs9478944
rs9273358
rs285250
rs117513979
rs143386390
All
−0.03
0.00
0.03
0.06
MR leave−one−out sensitivity analysis for
'CD24 on IgD+ CD24+' on 'Diabetic nephropathy'

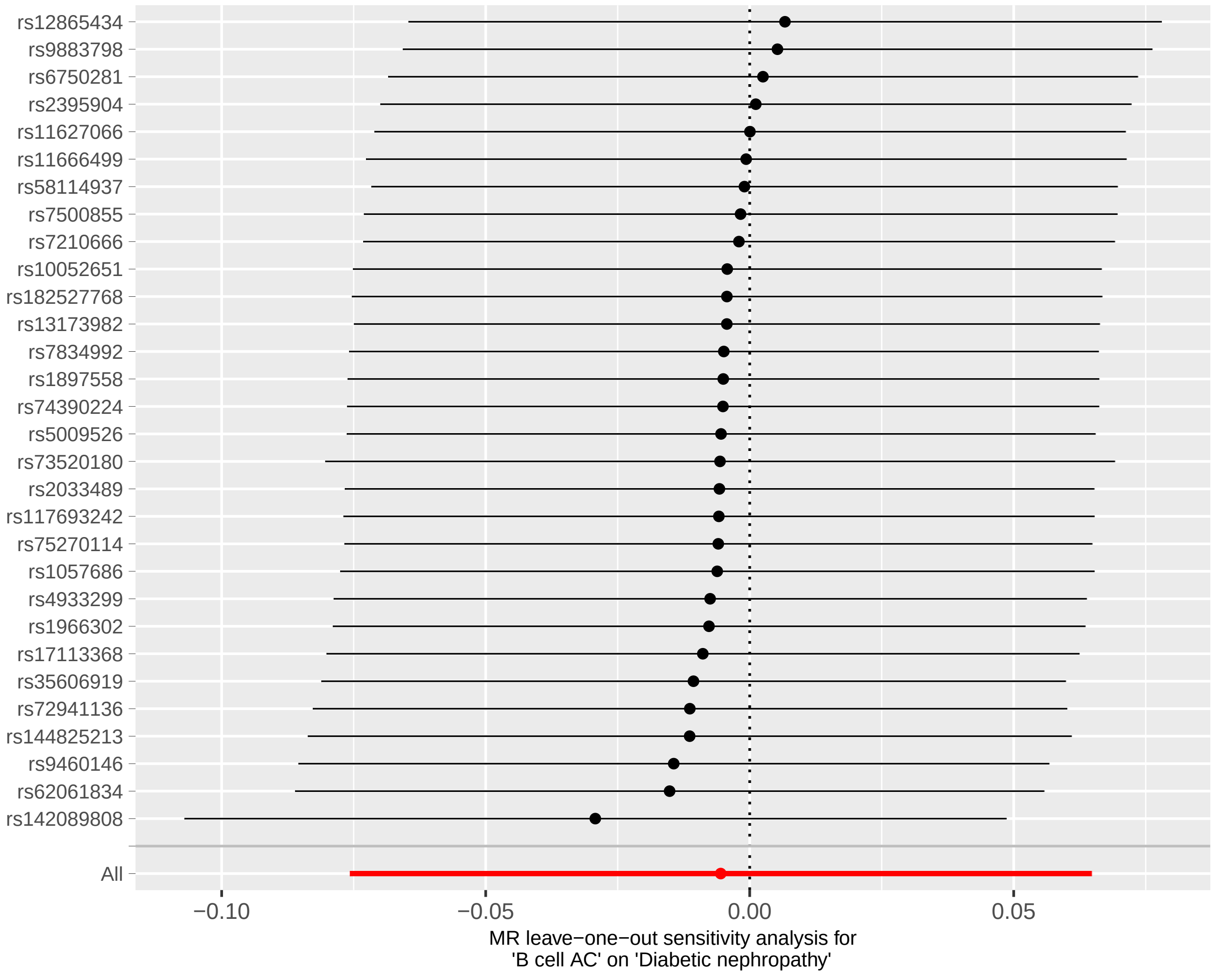
rs12865434
rs9883798
rs6750281
rs2395904
rs11627066
rs11666499
rs58114937
rs7500855
rs7210666
rs10052651
rs182527768
rs13173982
rs7834992
rs1897558
rs74390224
rs5009526
rs73520180
rs2033489
rs117693242
rs75270114
rs1057686
rs4933299
rs1966302
rs17113368
rs35606919
rs72941136
rs144825213
rs9460146
rs62061834
rs142089808
All
−0.10
−0.05
0.00
0.05
MR leave−one−out sensitivity analysis for
'B cell AC' on 'Diabetic nephropathy'

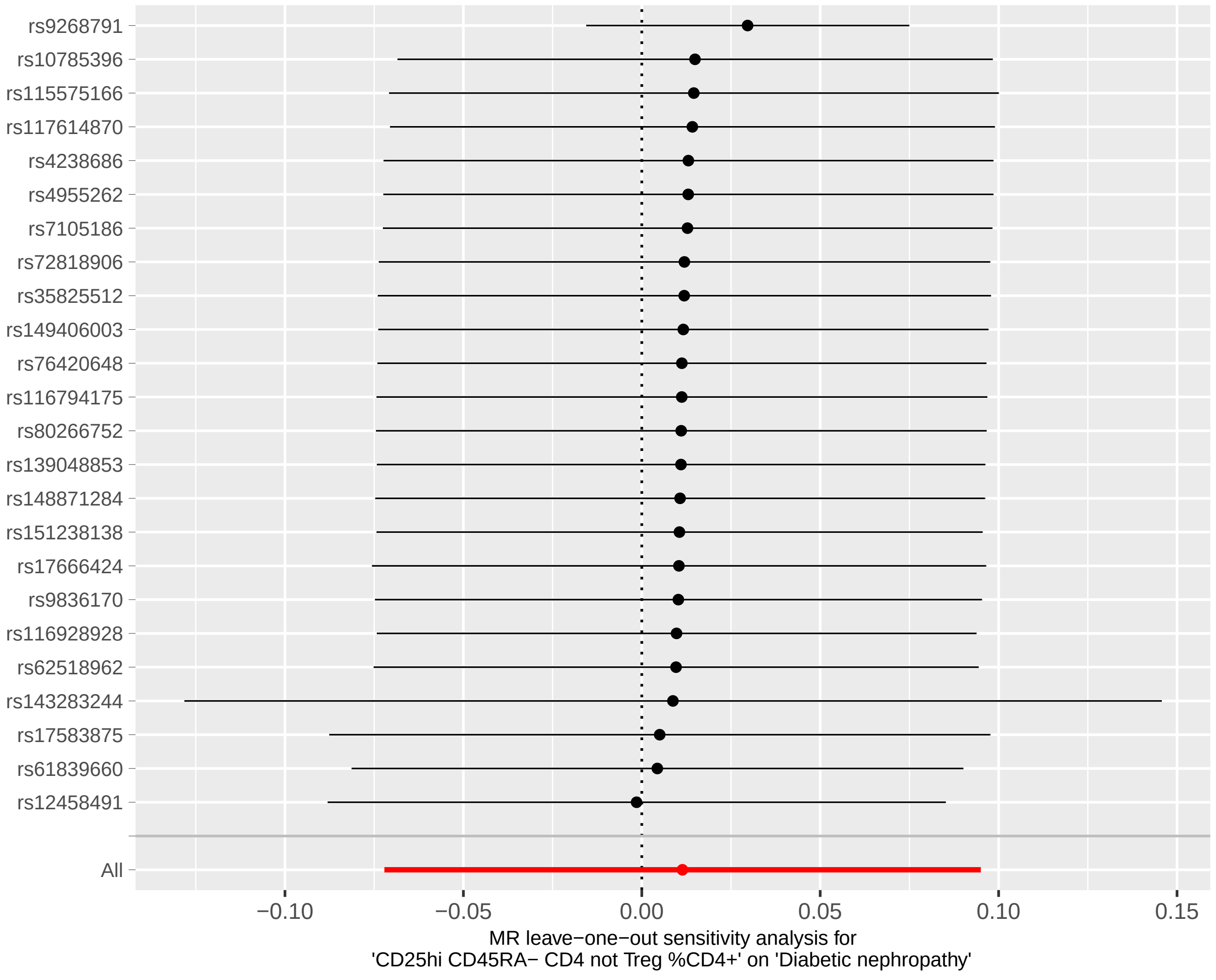
rs9268791
rs10785396
rs115575166
rs117614870
rs4238686
rs4955262
rs7105186
rs72818906
rs35825512
rs149406003
rs76420648
rs116794175
rs80266752
rs139048853
rs148871284
rs151238138
rs17666424
rs9836170
rs116928928
rs62518962
rs143283244
rs17583875
rs61839660
rs12458491
All
−0.10
−0.05
0.00
0.05
0.10
0.15
MR leave−one−out sensitivity analysis for
'CD25hi CD45RA− CD4 not Treg %CD4+' on 'Diabetic nephropathy'

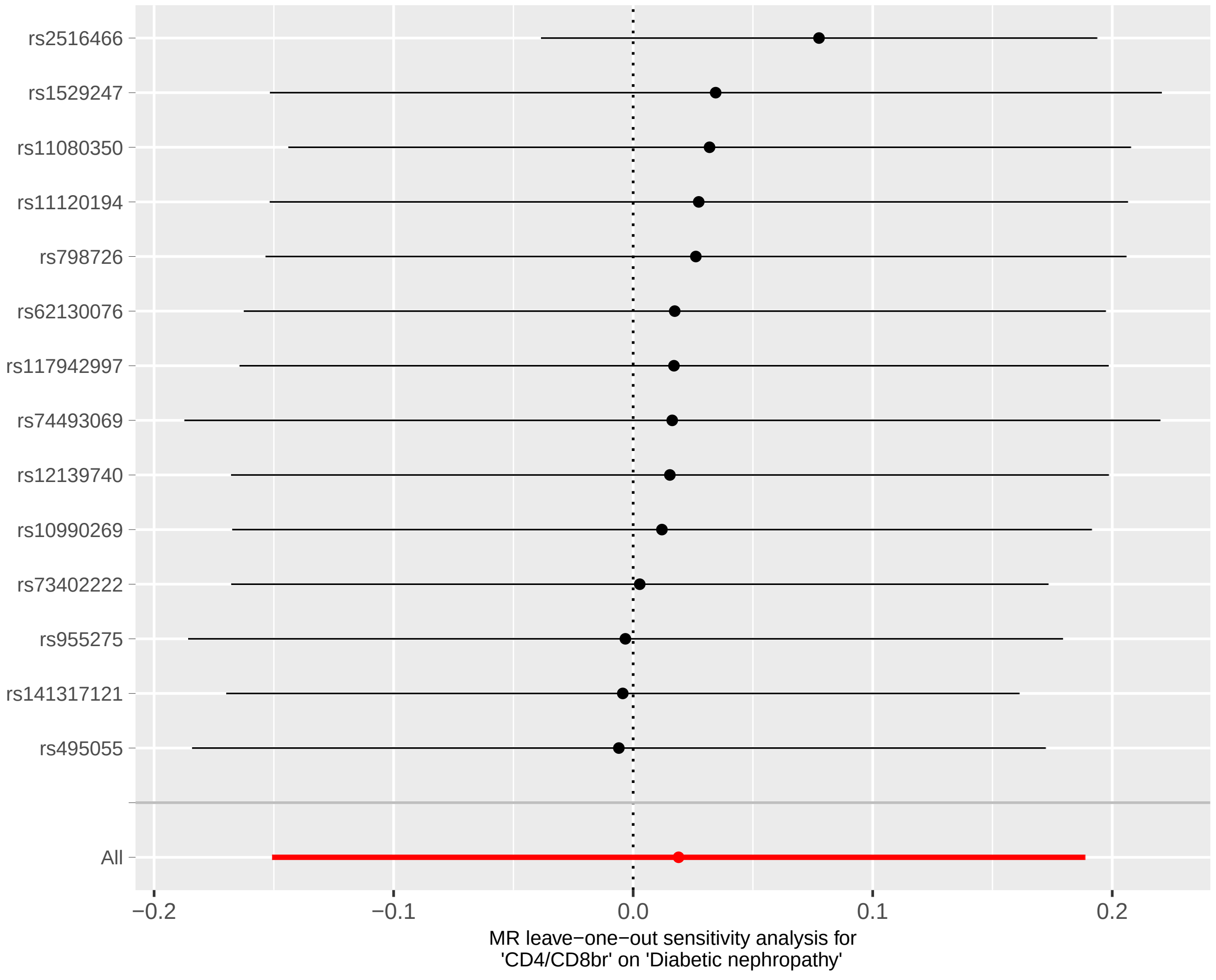
rs2516466
rs1529247
rs11080350
rs11120194
rs798726
rs62130076
rs117942997
rs74493069
rs12139740
rs10990269
rs73402222
rs955275
rs141317121
rs495055
All
−0.2
−0.1
0.0
0.1
0.2
MR leave−one−out sensitivity analysis for
'CD4/CD8br' on 'Diabetic nephropathy'

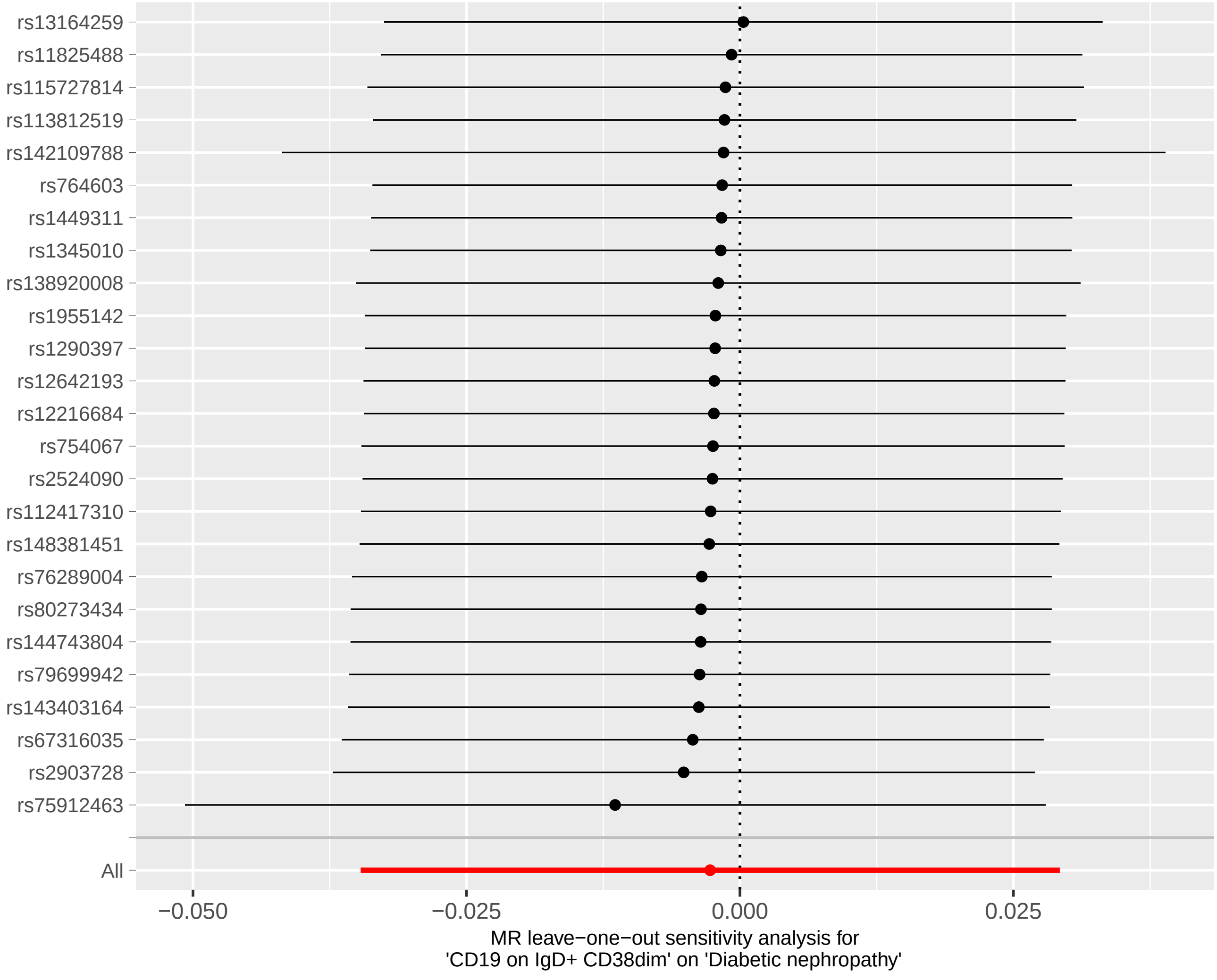
rs13164259
rs11825488
rs115727814
rs113812519
rs142109788
rs764603
rs1449311
rs1345010
rs138920008
rs1955142
rs1290397
rs12642193
rs12216684
rs754067
rs2524090
rs112417310
rs148381451
rs76289004
rs80273434
rs144743804
rs79699942
rs143403164
rs67316035
rs2903728
rs75912463
All
−0.050
−0.025
0.000
0.025
MR leave−one−out sensitivity analysis for
'CD19 on IgD+ CD38dim' on 'Diabetic nephropathy'

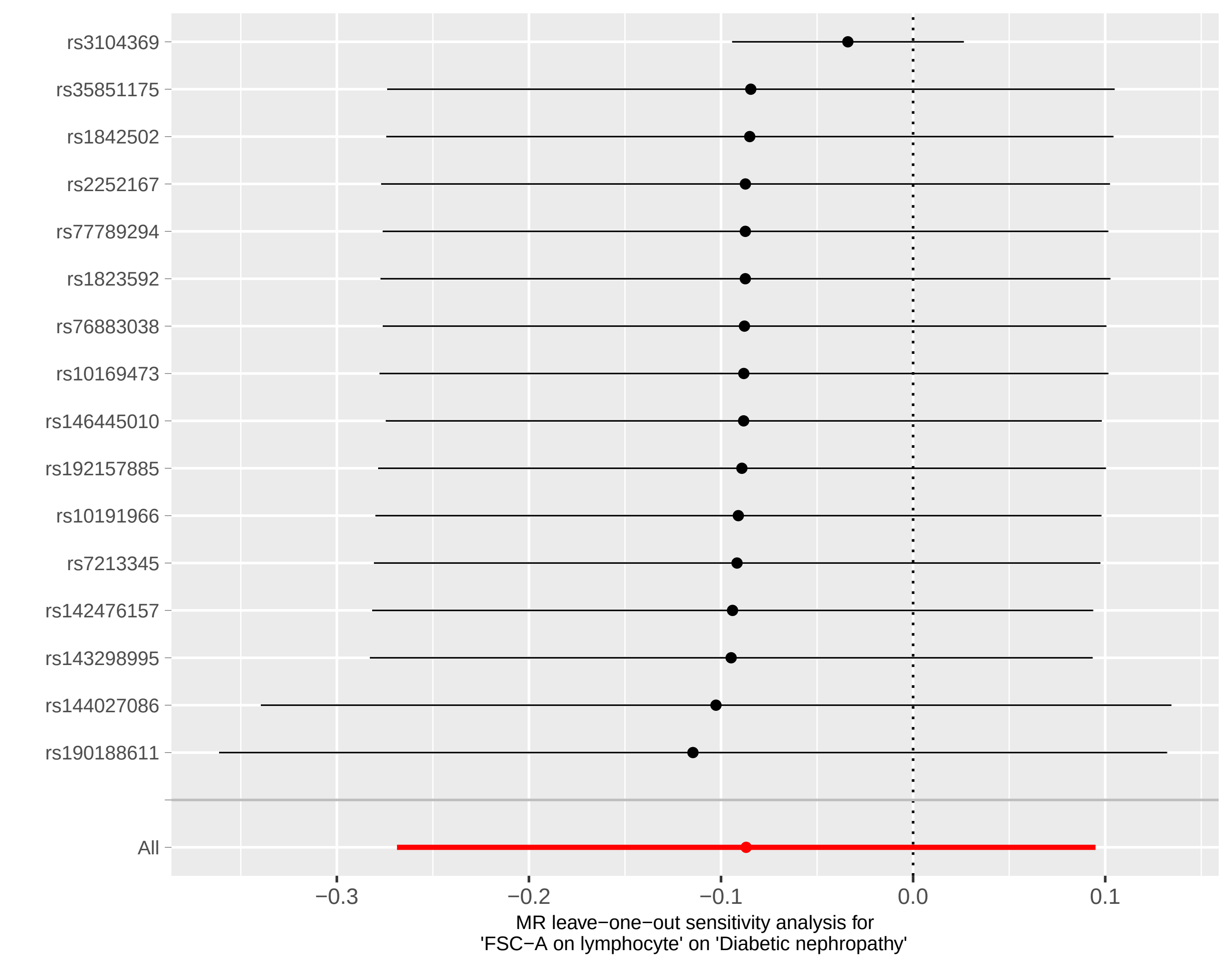
rs3104369
rs35851175
rs1842502
rs2252167
rs77789294
rs1823592
rs76883038
rs10169473
rs146445010
rs192157885
rs10191966
rs7213345
rs142476157
rs143298995
rs144027086
rs190188611
All
−0.3
−0.2
−0.1
0.0
0.1
MR leave−one−out sensitivity analysis for
'FSC−A on lymphocyte' on 'Diabetic nephropathy'

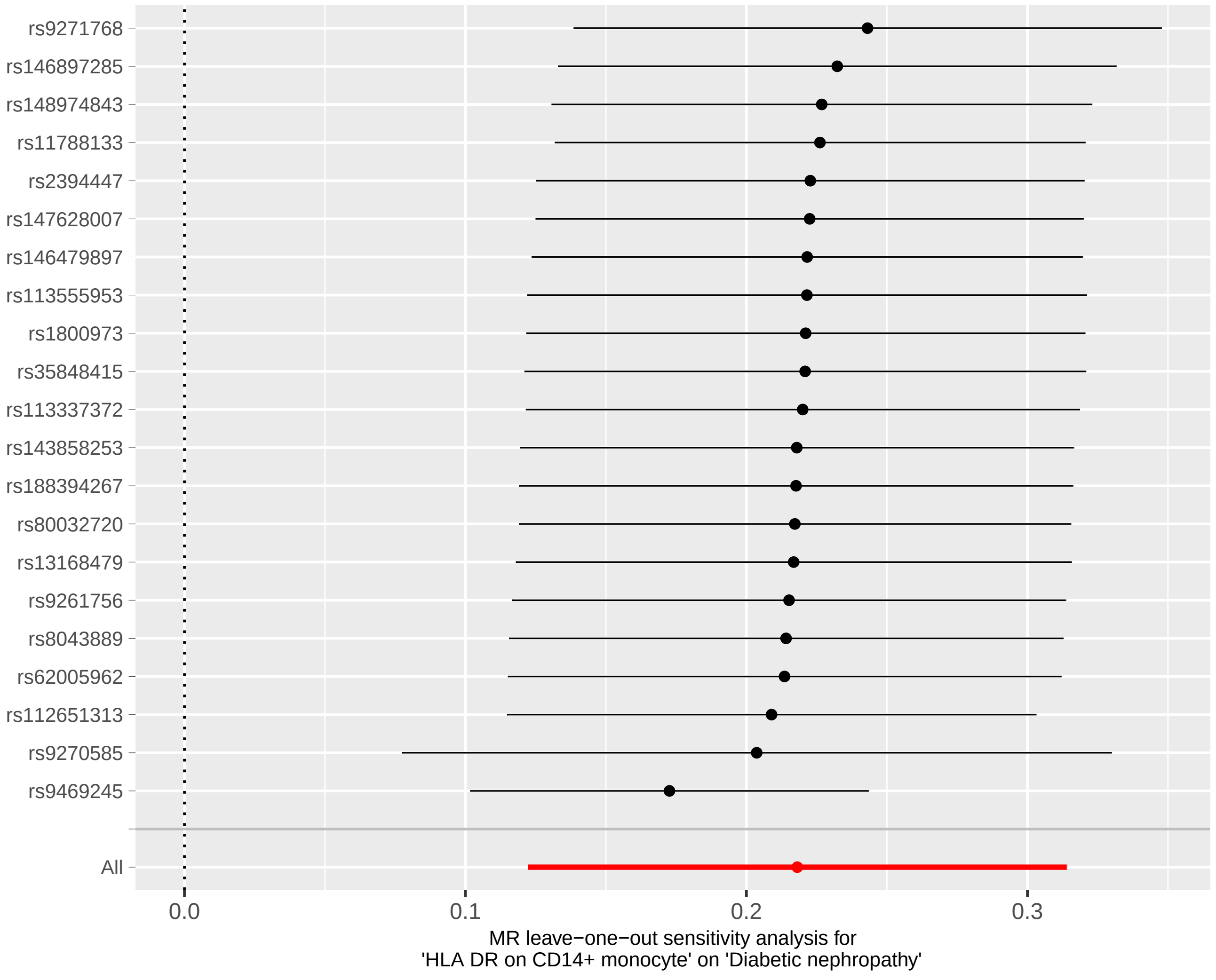

rs9271768
rs146897285
rs148974843
rs11788133
rs2394447
rs147628007
rs146479897
rs113555953
rs1800973
rs35848415
rs113337372
rs143858253
rs188394267
rs80032720
rs13168479
rs9261756
rs8043889
rs62005962
rs112651313
rs9270585
rs9469245
All
0.0
0.1
0.2
0.3
MR leave−one−out sensitivity analysis for
'HLA DR on CD14+ monocyte' on 'Diabetic nephropathy'

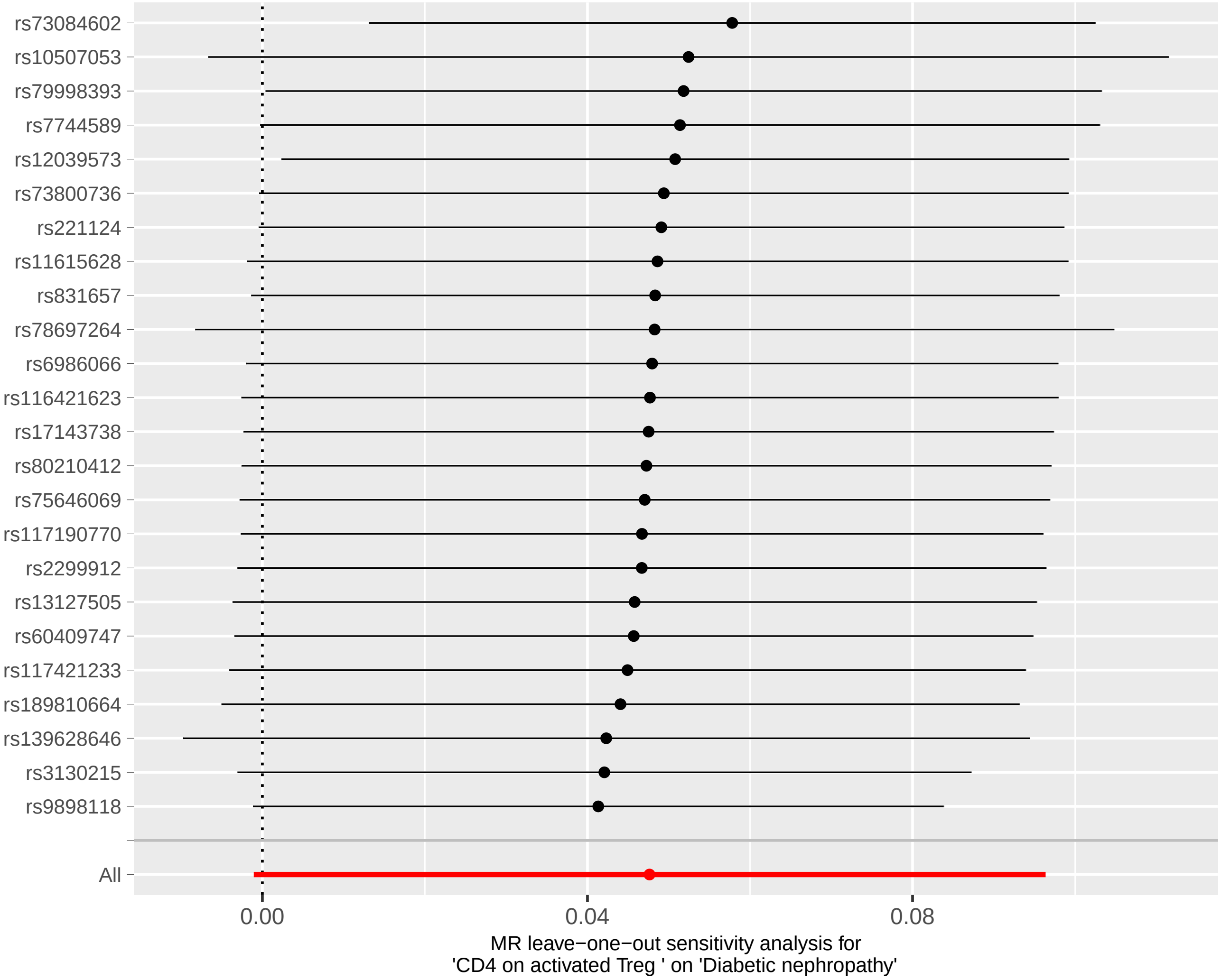
rs73084602
rs10507053
rs79998393
rs7744589
rs12039573
rs73800736
rs221124
rs11615628
rs831657
rs78697264
rs6986066
rs116421623
rs17143738
rs80210412
rs75646069
rs117190770
rs2299912
rs13127505
rs60409747
rs117421233
rs189810664
rs139628646
rs3130215
rs9898118
All
0.00
0.04
0.08
MR leave−one−out sensitivity analysis for
'CD4 on activated Treg ' on 'Diabetic nephropathy'

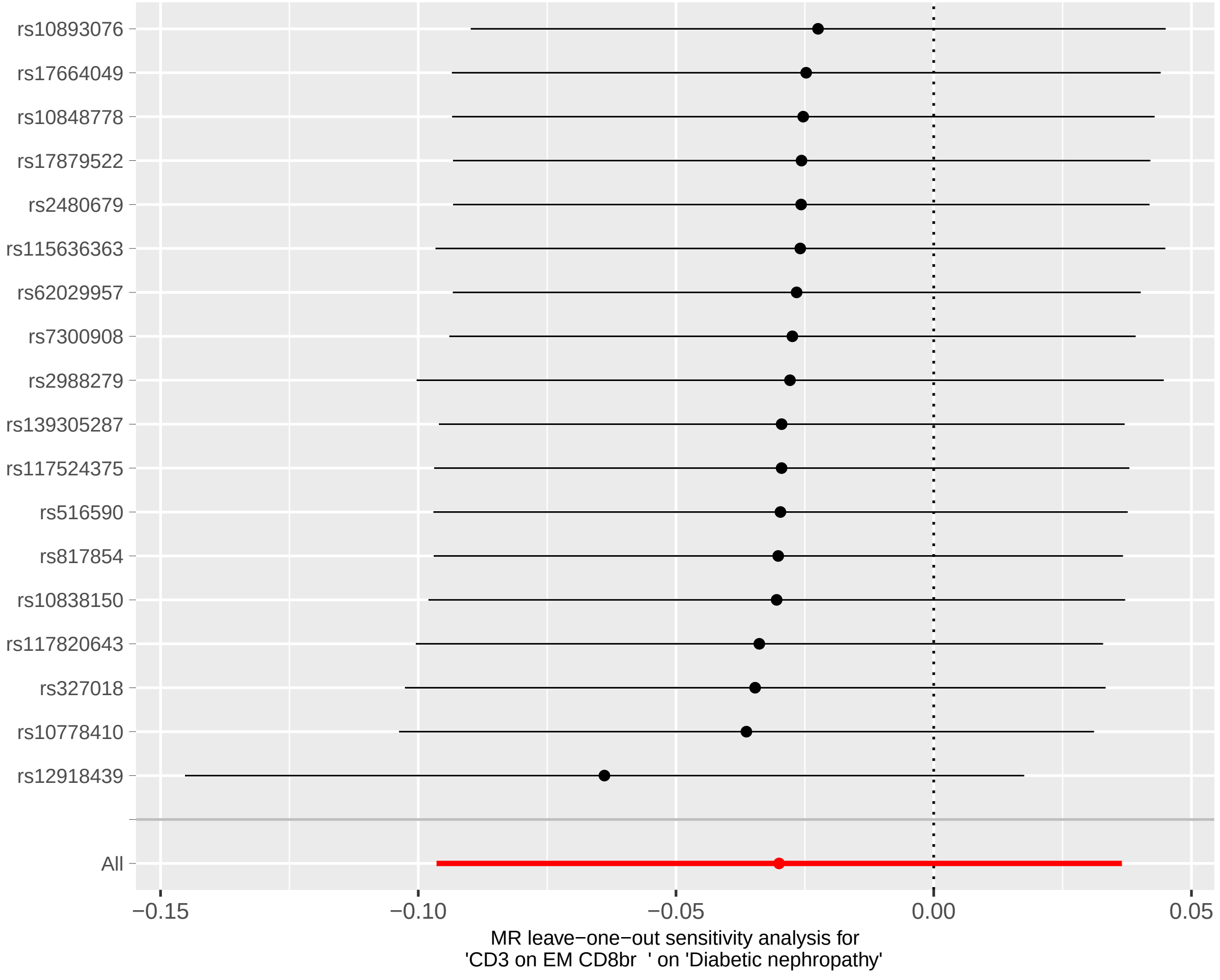

rs10893076
rs17664049
rs10848778
rs17879522
rs2480679
rs115636363
rs62029957
rs7300908
rs2988279
rs139305287
rs117524375
rs516590
rs817854
rs10838150
rs117820643
rs327018
rs10778410
rs12918439
All
−0.15
−0.10
−0.05
0.00
0.05
MR leave−one−out sensitivity analysis for
'CD3 on EM CD8br ' on 'Diabetic nephropathy'

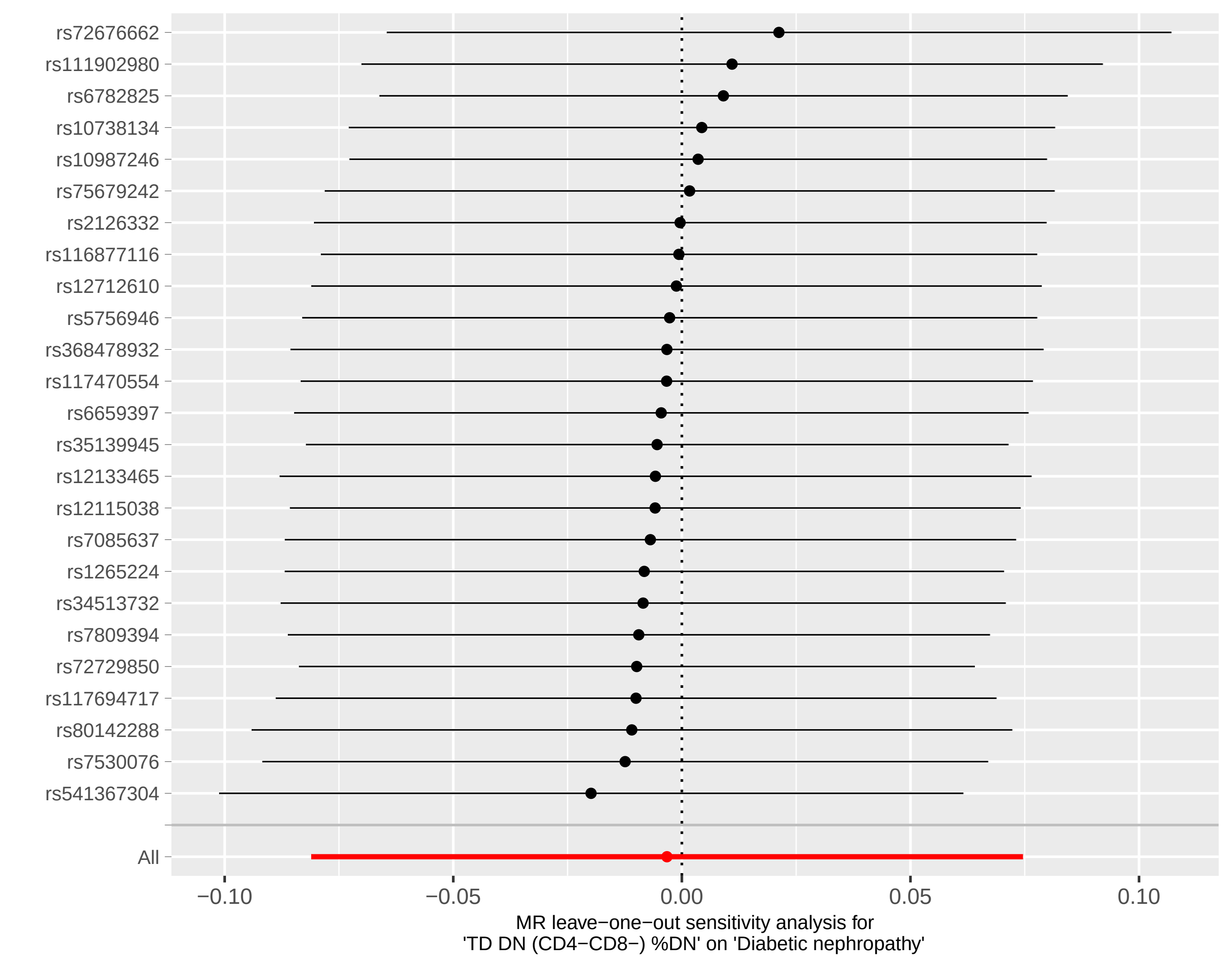
rs72676662
rs111902980
rs6782825
rs10738134
rs10987246
rs75679242
rs2126332
rs116877116
rs12712610
rs5756946
rs368478932
rs117470554
rs6659397
rs35139945
rs12133465
rs12115038
rs7085637
rs1265224
rs34513732
rs7809394
rs72729850
rs117694717
rs80142288
rs7530076
rs541367304
All
−0.10
−0.05
0.00
0.05
0.10
MR leave−one−out sensitivity analysis for
'TD DN (CD4−CD8−) %DN' on 'Diabetic nephropathy'

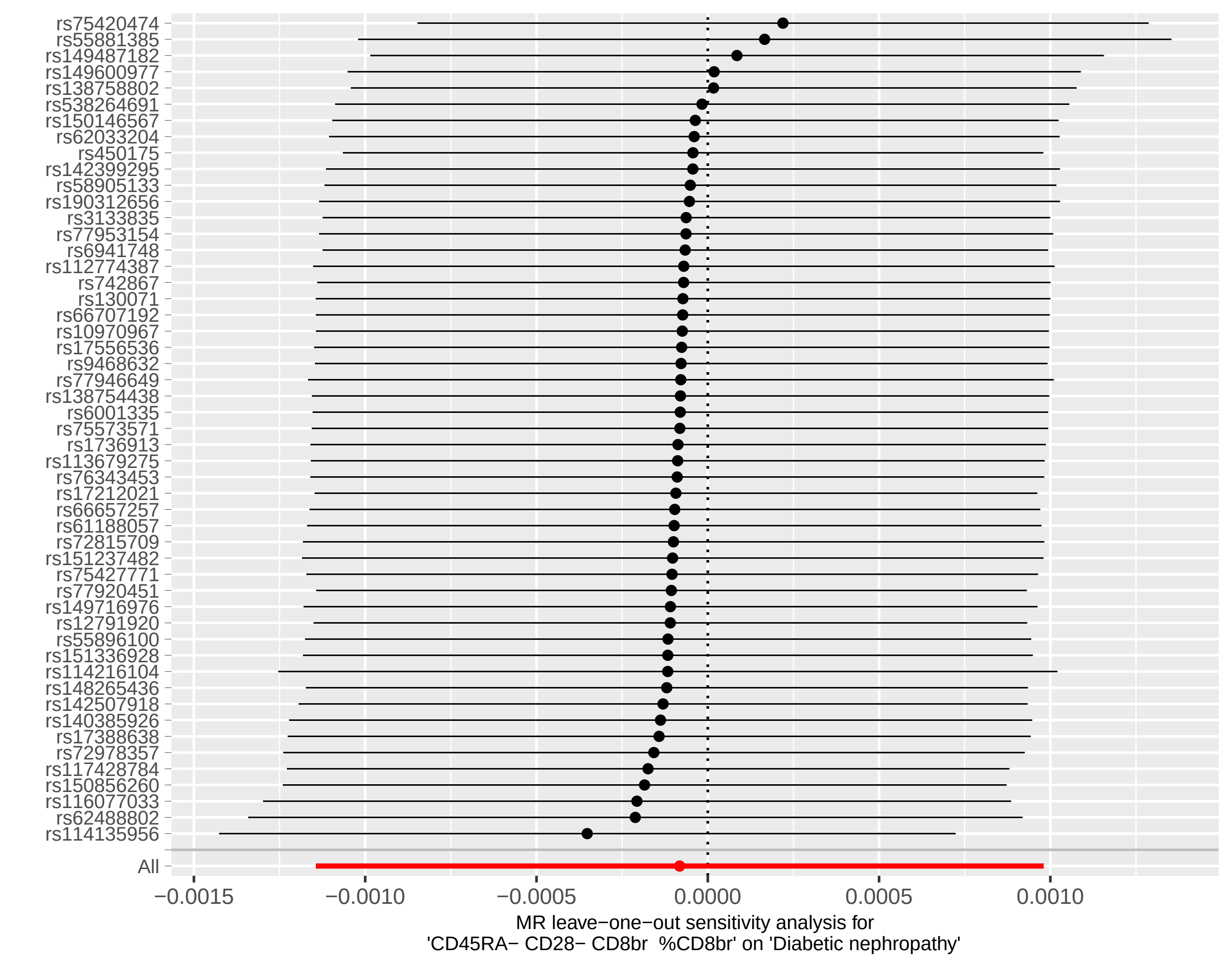

rs75420474
rs55881385
rs149487182
rs149600977
rs138758802
rs538264691
rs150146567
rs62033204
rs450175
rs142399295
rs58905133
rs190312656
rs3133835
rs77953154
rs6941748
rs112774387
rs742867
rs130071
rs66707192
rs10970967
rs17556536
rs9468632
rs77946649
rs138754438
rs6001335
rs75573571
rs1736913
rs113679275
rs76343453
rs17212021
rs66657257
rs61188057
rs72815709
rs151237482
rs75427771
rs77920451
rs149716976
rs12791920
rs55896100
rs151336928
rs114216104
rs148265436
rs142507918
rs140385926
rs17388638
rs72978357
rs117428784
rs150856260
rs116077033
rs62488802
rs114135956
All
−0.0015
−0.0010
−0.0005
0.0000
0.0005
0.0010
MR leave−one−out sensitivity analysis for
'CD45RA− CD28− CD8br %CD8br' on 'Diabetic nephropathy'

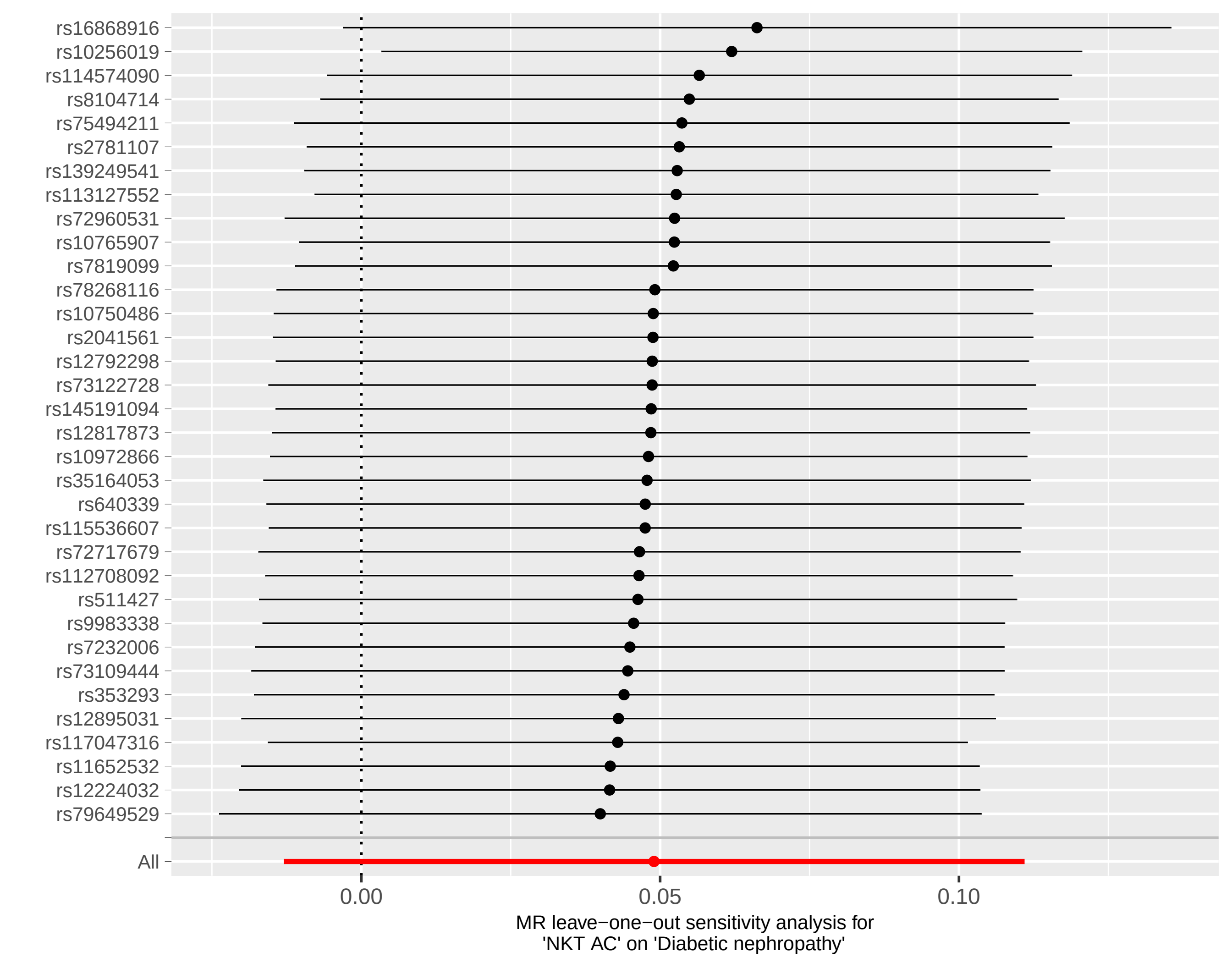
rs16868916
rs10256019
rs114574090
rs8104714
rs75494211
rs2781107
rs139249541
rs113127552
rs72960531
rs10765907
rs7819099
rs78268116
rs10750486
rs2041561
rs12792298
rs73122728
rs145191094
rs12817873
rs10972866
rs35164053
rs640339
rs115536607
rs72717679
rs112708092
rs511427
rs9983338
rs7232006
rs73109444
rs353293
rs12895031
rs117047316
rs11652532
rs12224032
rs79649529
All
0.00
0.05
0.10
MR leave−one−out sensitivity analysis for
'NKT AC' on 'Diabetic nephropathy'

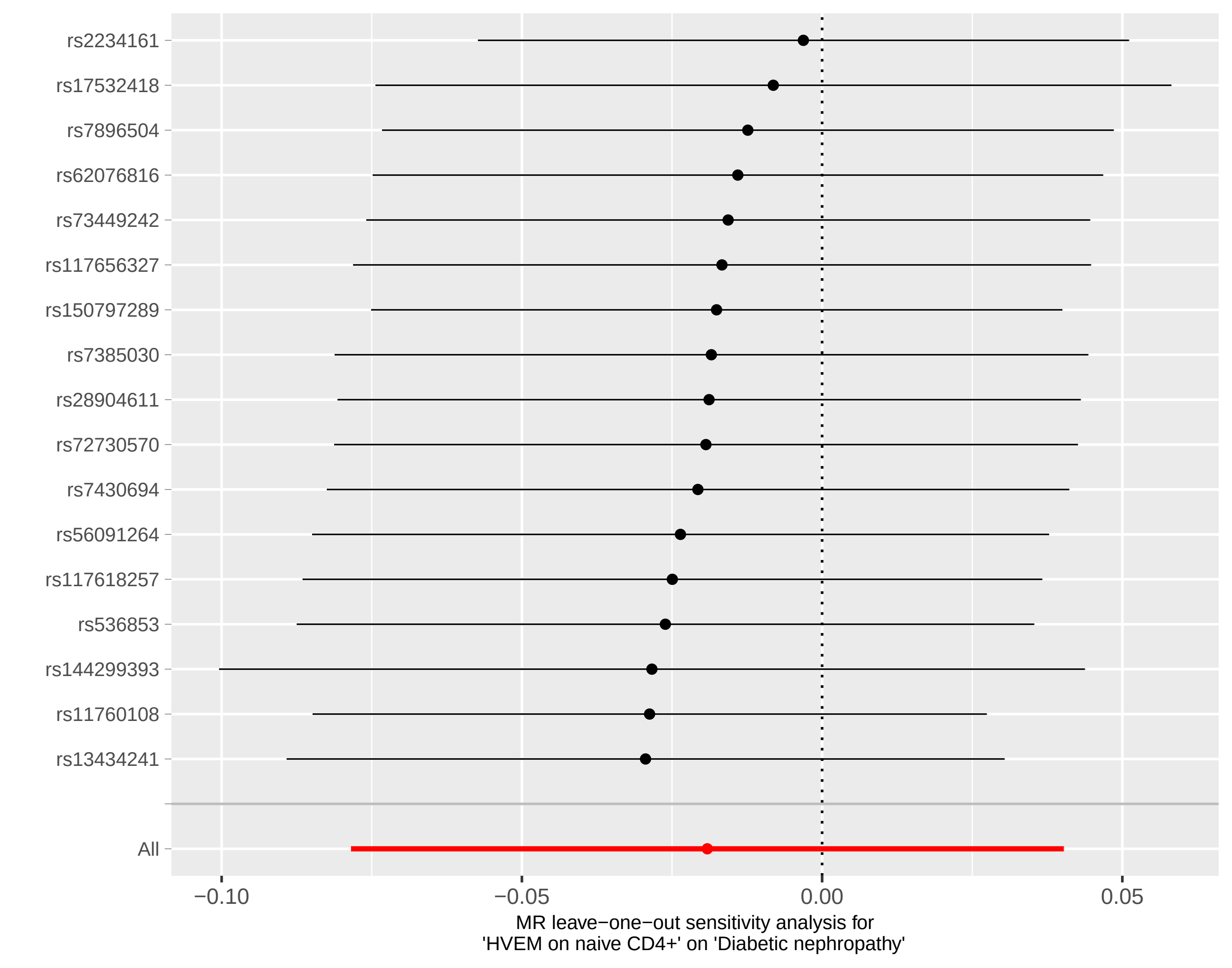
rs2234161
rs17532418
rs7896504
rs62076816
rs73449242
rs117656327
rs150797289
rs7385030
rs28904611
rs72730570
rs7430694
rs56091264
rs117618257
rs536853
rs144299393
rs11760108
rs13434241
All
−0.10
−0.05
0.00
0.05
MR leave−one−out sensitivity analysis for
'HVEM on naive CD4+' on 'Diabetic nephropathy'

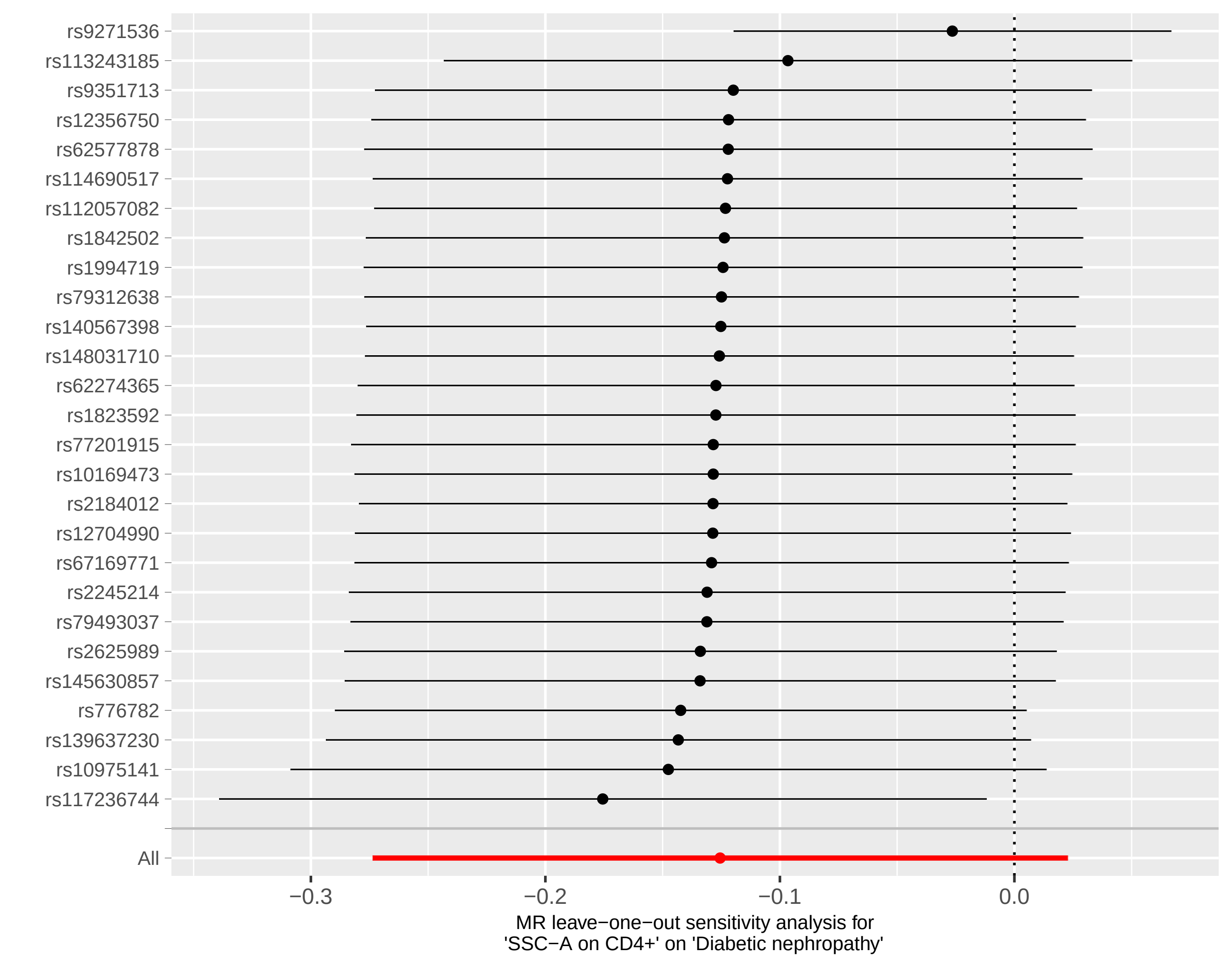
rs9271536
rs113243185
rs9351713
rs12356750
rs62577878
rs114690517
rs112057082
rs1842502
rs1994719
rs79312638
rs140567398
rs148031710
rs62274365
rs1823592
rs77201915
rs10169473
rs2184012
rs12704990
rs67169771
rs2245214
rs79493037
rs2625989
rs145630857
rs776782
rs139637230
rs10975141
rs117236744
All
−0.3
−0.2
−0.1
0.0
MR leave−one−out sensitivity analysis for
'SSC−A on CD4+' on 'Diabetic nephropathy'

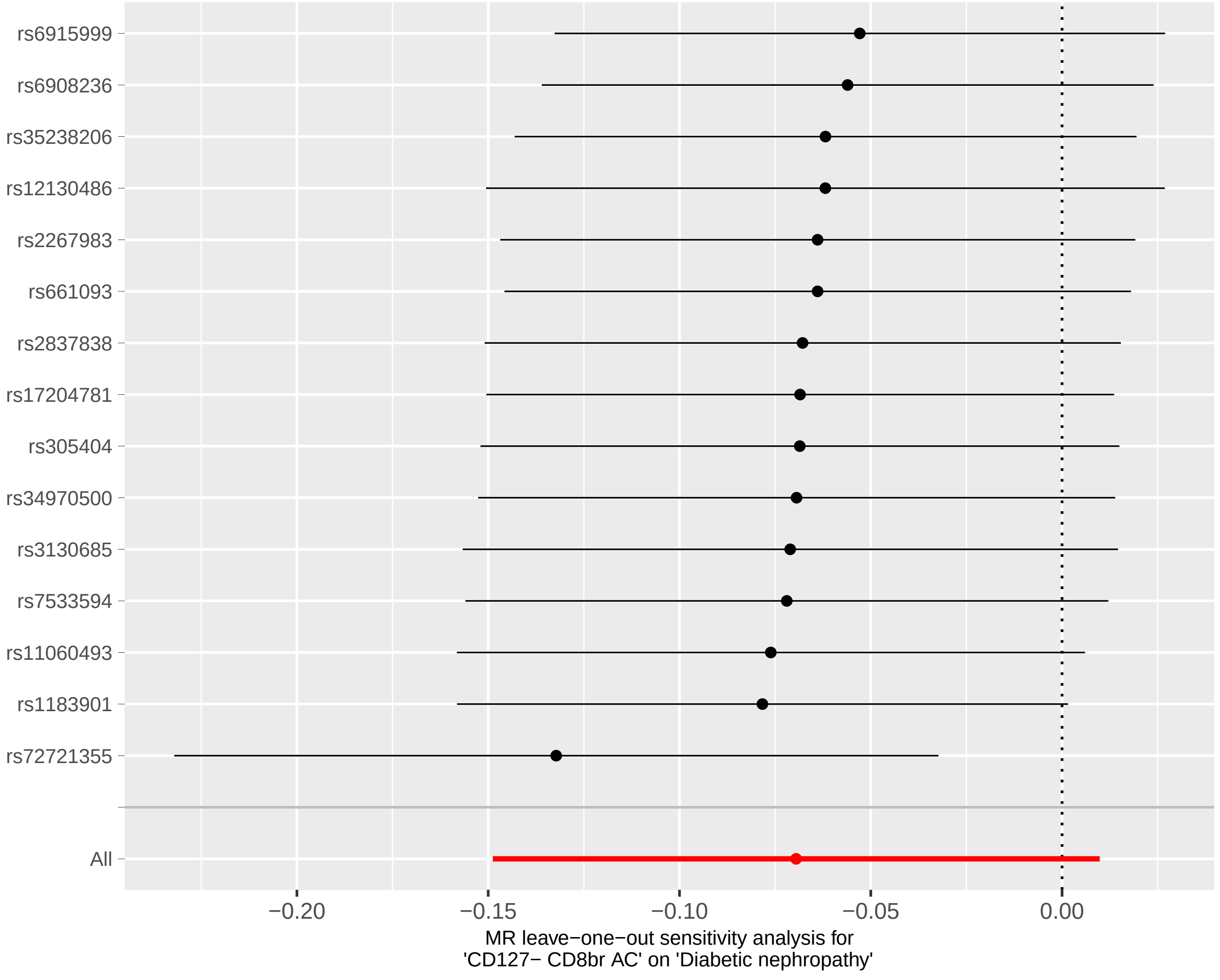
rs6915999
rs6908236
rs35238206
rs12130486
rs2267983
rs661093
rs2837838
rs17204781
rs305404
rs34970500
rs3130685
rs7533594
rs11060493
rs1183901
rs72721355
All
−0.20
−0.15
−0.10
−0.05
0.00
MR leave−one−out sensitivity analysis for
'CD127− CD8br AC' on 'Diabetic nephropathy'

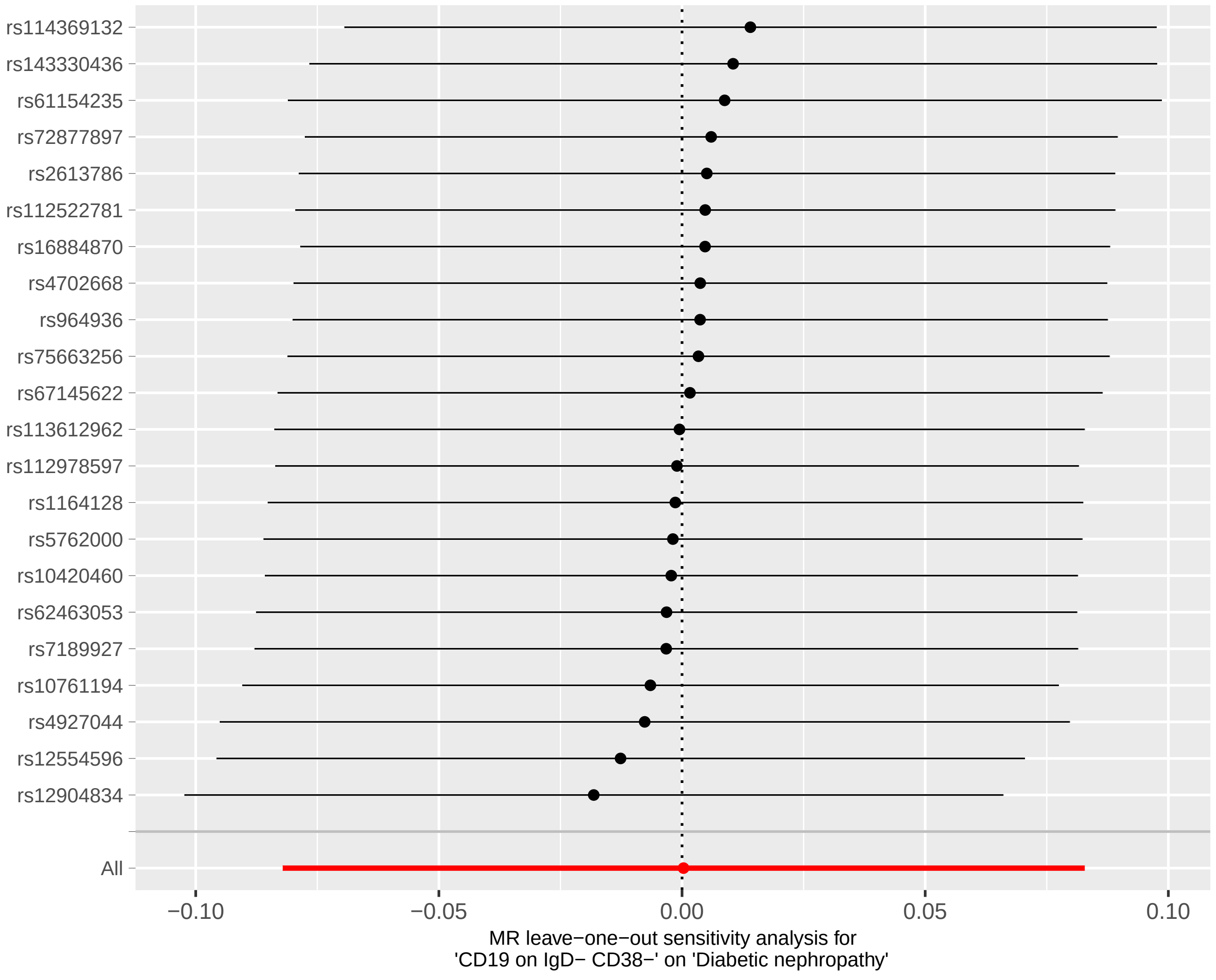
rs114369132
rs143330436
rs61154235
rs72877897
rs2613786
rs112522781
rs16884870
rs4702668
rs964936
rs75663256
rs67145622
rs113612962
rs112978597
rs1164128
rs5762000
rs10420460
rs62463053
rs7189927
rs10761194
rs4927044
rs12554596
rs12904834
All
−0.10
−0.05
0.00
0.05
0.10
MR leave−one−out sensitivity analysis for
'CD19 on IgD− CD38−' on 'Diabetic nephropathy'

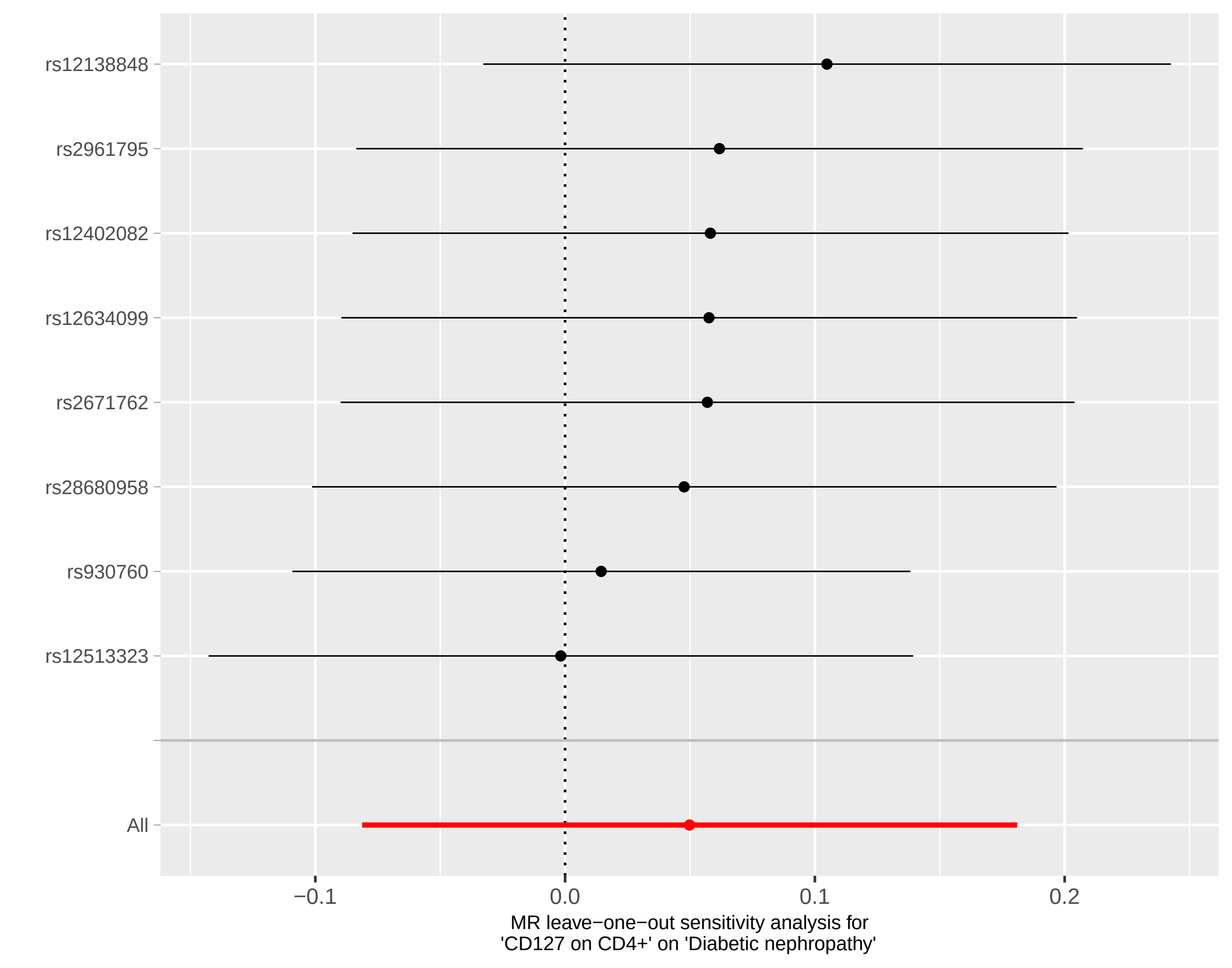
rs12138848
rs2961795
rs12402082
rs12634099
rs2671762
rs28680958
rs930760
rs12513323
All
−0.1
0.0
0.1
0.2
MR leave−one−out sensitivity analysis for
'CD127 on CD4+' on 'Diabetic nephropathy'

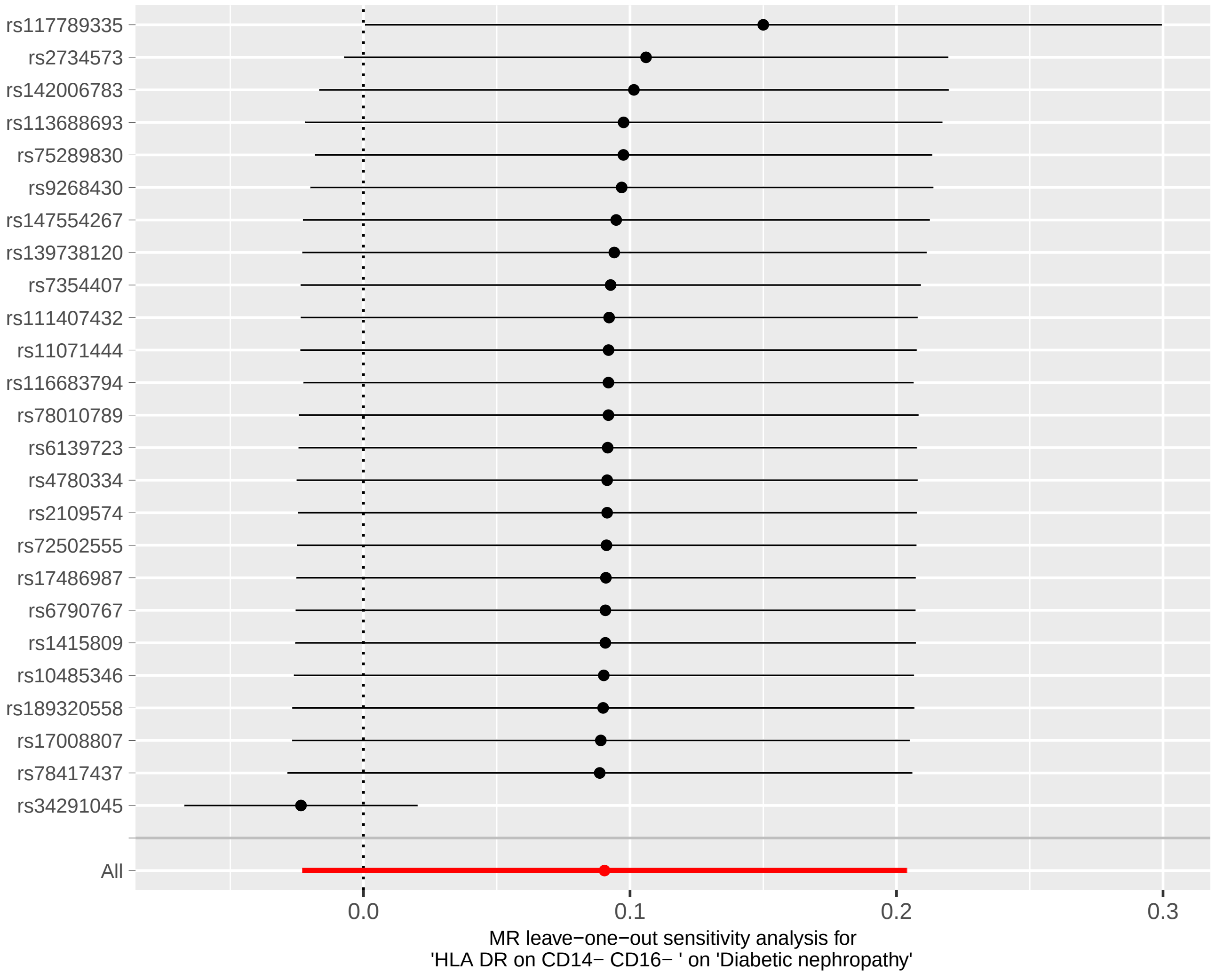
rs117789335
rs2734573
rs142006783
rs113688693
rs75289830
rs9268430
rs147554267
rs139738120
rs7354407
rs111407432
rs11071444
rs116683794
rs78010789
rs6139723
rs4780334
rs2109574
rs72502555
rs17486987
rs6790767
rs1415809
rs10485346
rs189320558
rs17008807
rs78417437
rs34291045
All
0.0
0.1
0.2
0.3
MR leave−one−out sensitivity analysis for
'HLA DR on CD14− CD16− ' on 'Diabetic nephropathy'

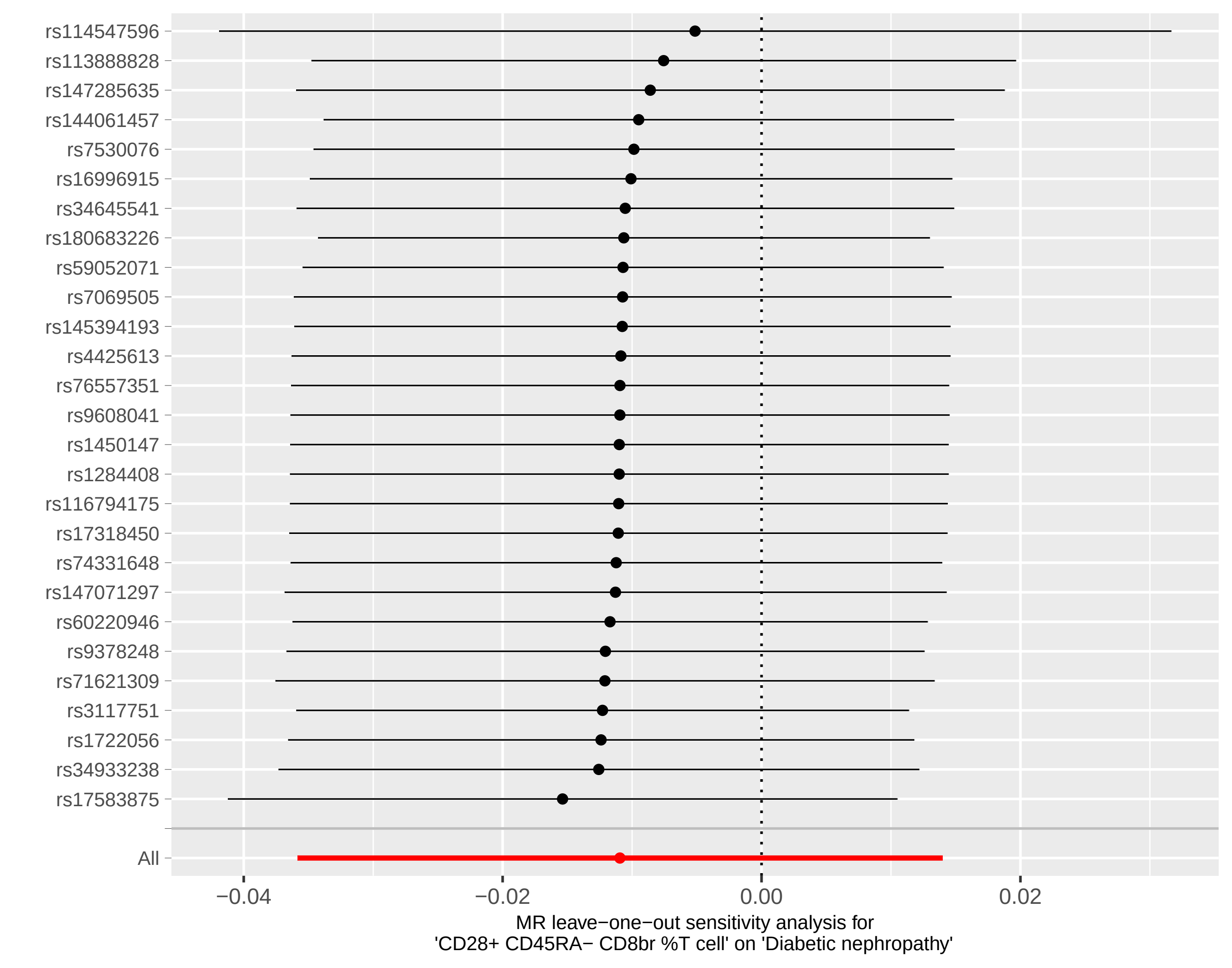
rs114547596
rs113888828
rs147285635
rs144061457
rs7530076
rs16996915
rs34645541
rs180683226
rs59052071
rs7069505
rs145394193
rs4425613
rs76557351
rs9608041
rs1450147
rs1284408
rs116794175
rs17318450
rs74331648
rs147071297
rs60220946
rs9378248
rs71621309
rs3117751
rs1722056
rs34933238
rs17583875
All
−0.04
−0.02
0.00
0.02
MR leave−one−out sensitivity analysis for
'CD28+ CD45RA− CD8br %T cell' on 'Diabetic nephropathy'

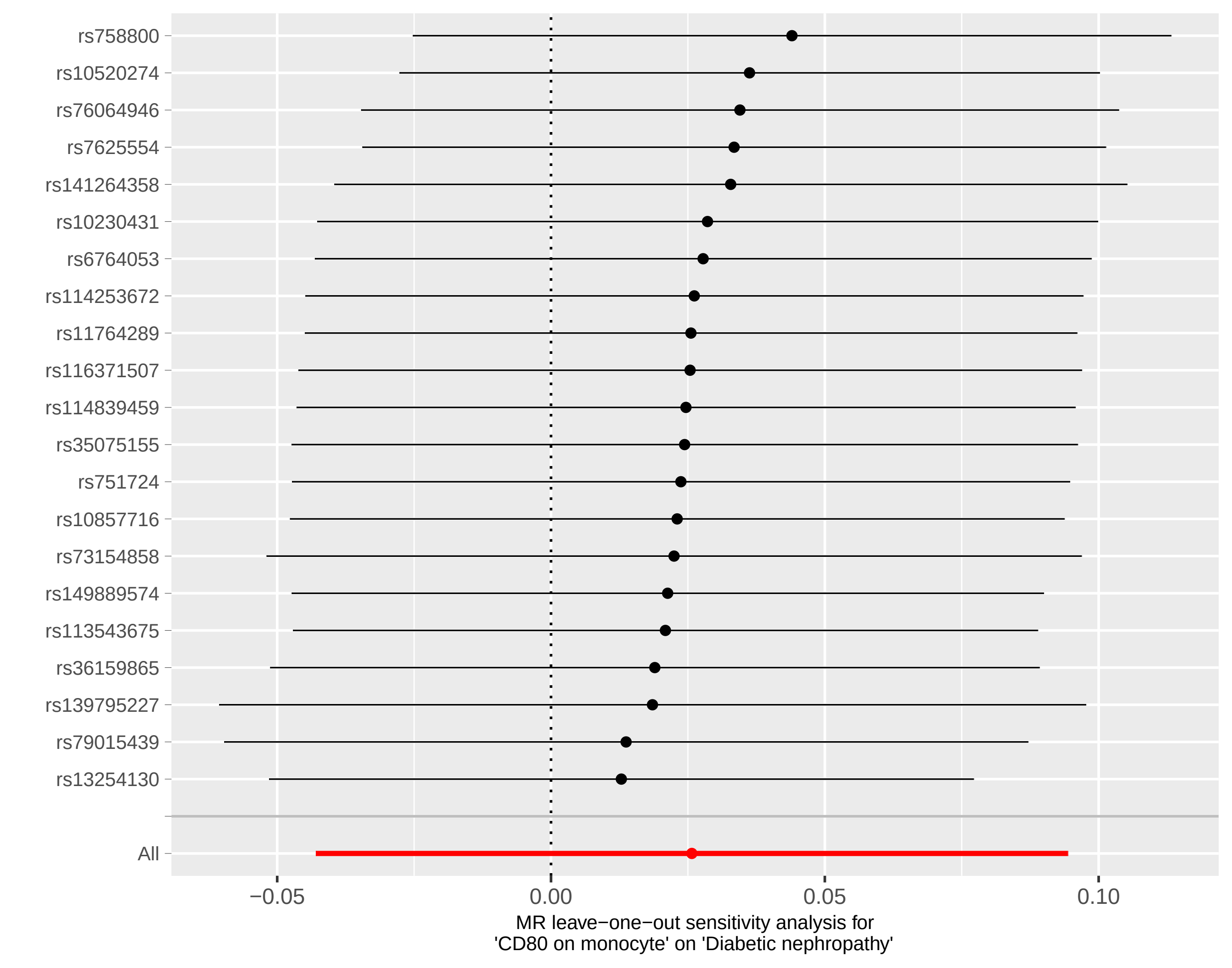
rs758800
rs10520274
rs76064946
rs7625554
rs141264358
rs10230431
rs6764053
rs114253672
rs11764289
rs116371507
rs114839459
rs35075155
rs751724
rs10857716
rs73154858
rs149889574
rs113543675
rs36159865
rs139795227
rs79015439
rs13254130
All
−0.05
0.00
0.05
0.10
MR leave−one−out sensitivity analysis for
'CD80 on monocyte' on 'Diabetic nephropathy'

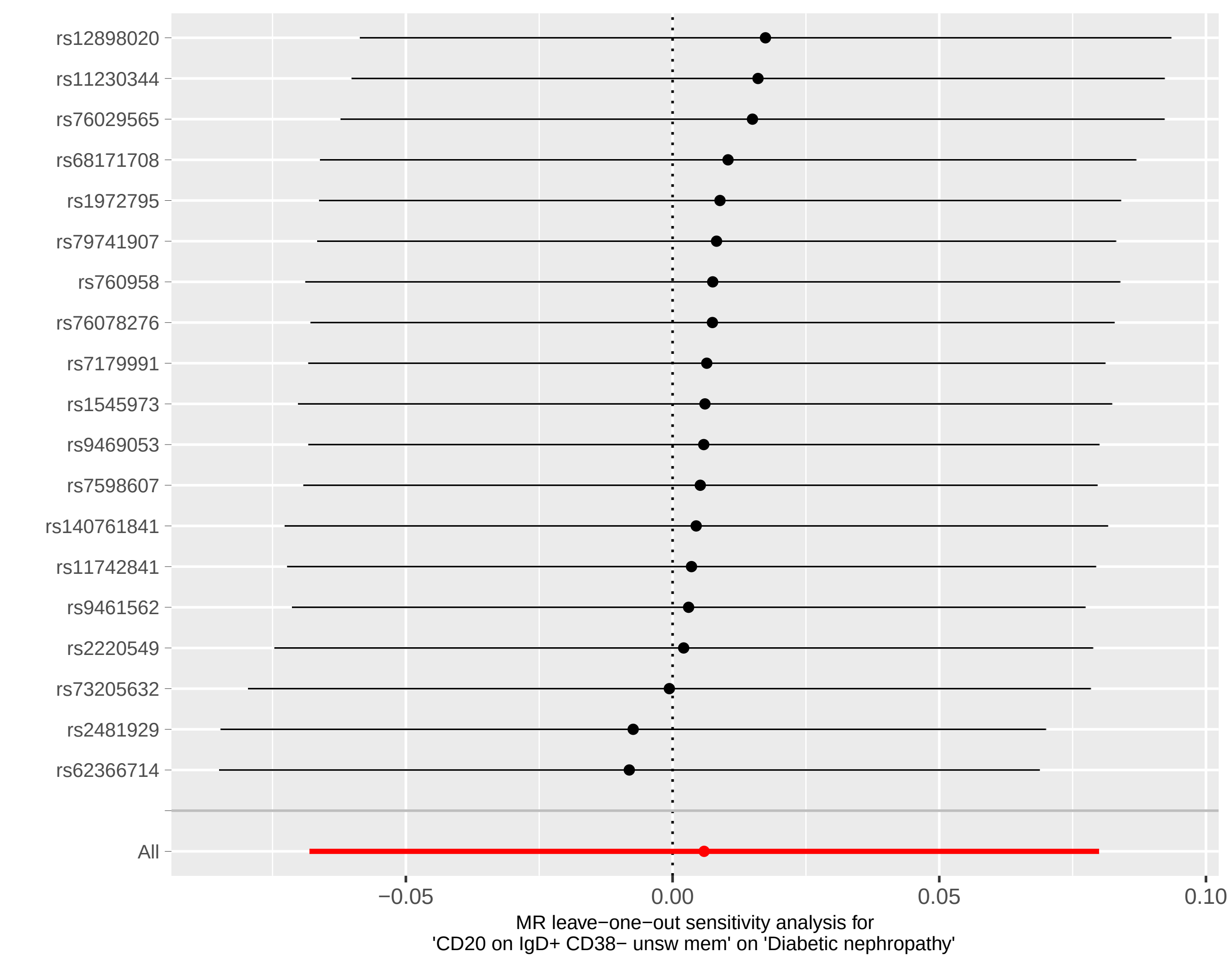
rs12898020
rs11230344
rs76029565
rs68171708
rs1972795
rs79741907
rs760958
rs76078276
rs7179991
rs1545973
rs9469053
rs7598607
rs140761841
rs11742841
rs9461562
rs2220549
rs73205632
rs2481929
rs62366714
All
−0.05
0.00
0.05
0.10
MR leave−one−out sensitivity analysis for
'CD20 on IgD+ CD38− unsw mem' on 'Diabetic nephropathy'

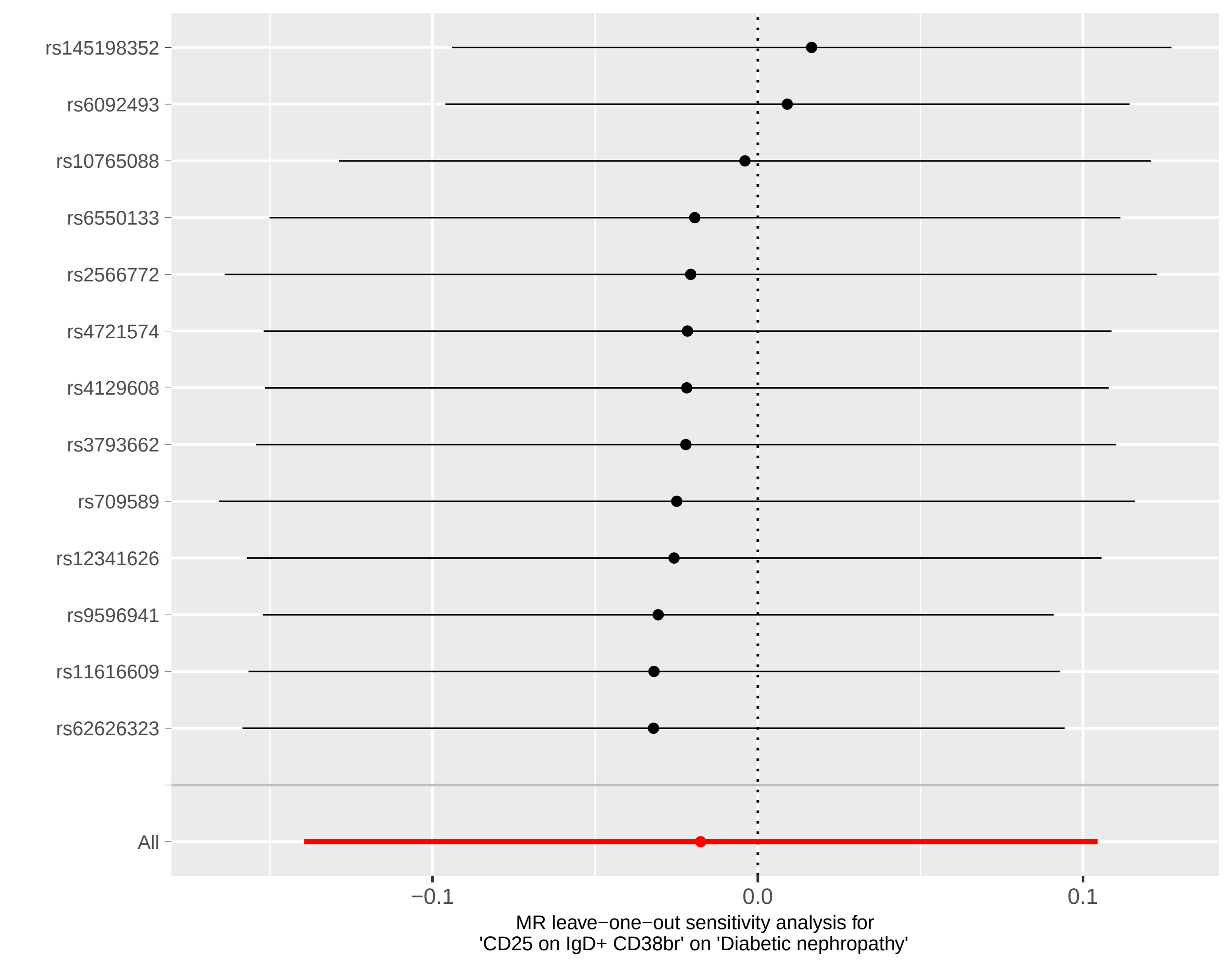

rs145198352
rs6092493
rs10765088
rs6550133
rs2566772
rs4721574
rs4129608
rs3793662
rs709589
rs12341626
rs9596941
rs11616609
rs62626323
All
−0.1
0.0
0.1
MR leave−one−out sensitivity analysis for
'CD25 on IgD+ CD38br' on 'Diabetic nephropathy'

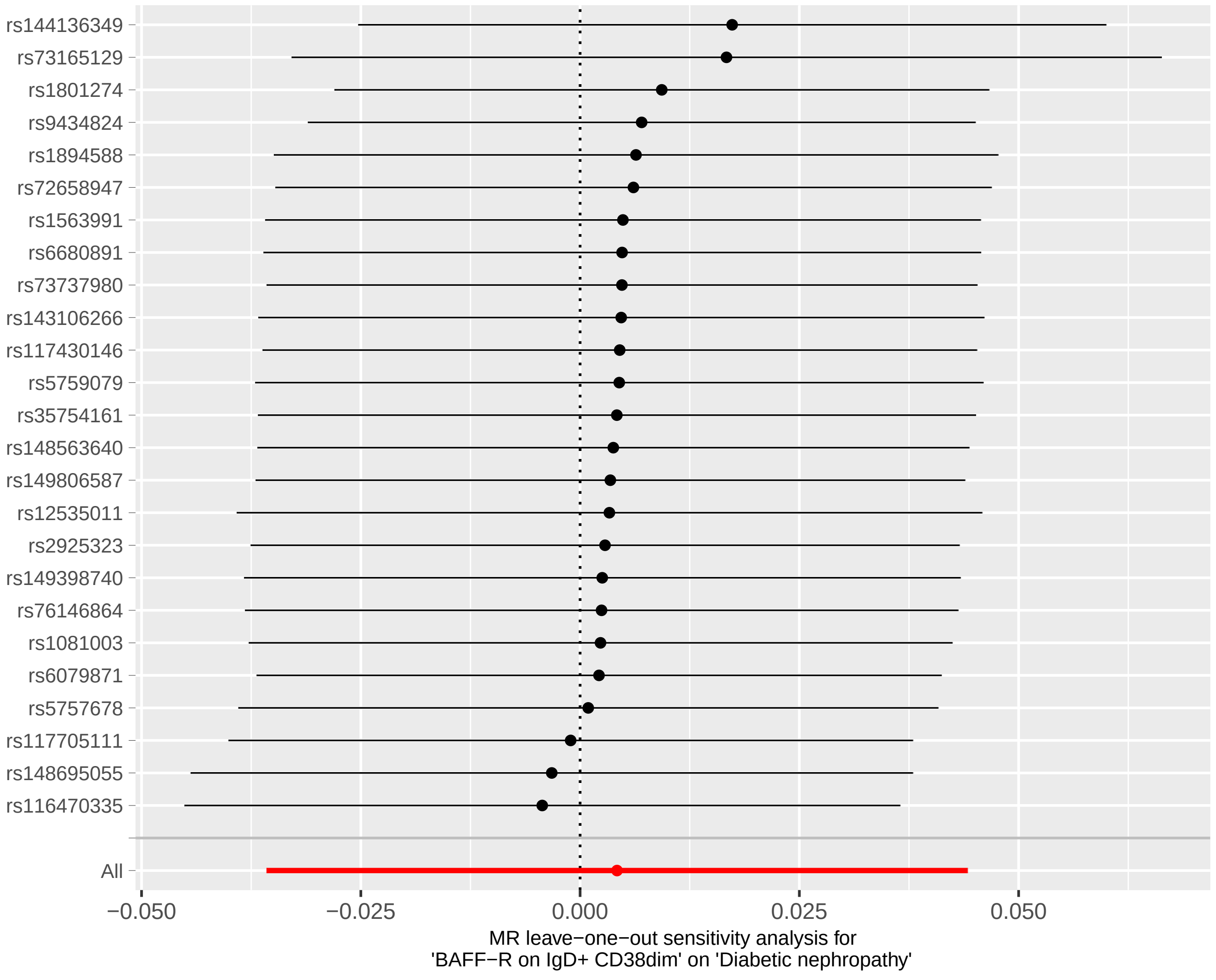

rs144136349
rs73165129
rs1801274
rs9434824
rs1894588
rs72658947
rs1563991
rs6680891
rs73737980
rs143106266
rs117430146
rs5759079
rs35754161
rs148563640
rs149806587
rs12535011
rs2925323
rs149398740
rs76146864
rs1081003
rs6079871
rs5757678
rs117705111
rs148695055
rs116470335
All
−0.050
−0.025
0.000
0.025
0.050
MR leave−one−out sensitivity analysis for
'BAFF−R on IgD+ CD38dim' on 'Diabetic nephropathy'

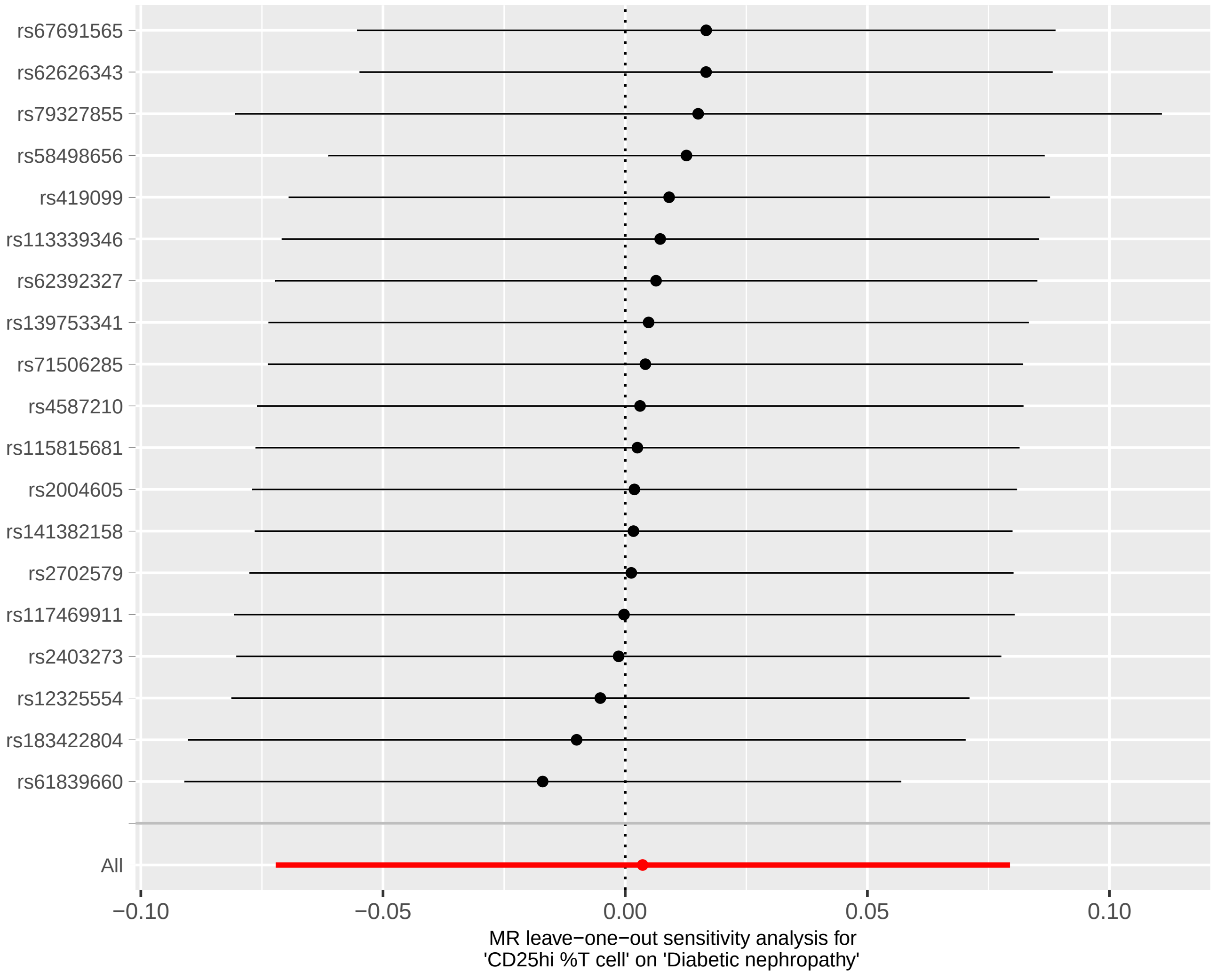
rs67691565
rs62626343
rs79327855
rs58498656
rs419099
rs113339346
rs62392327
rs139753341
rs71506285
rs4587210
rs115815681
rs2004605
rs141382158
rs2702579
rs117469911
rs2403273
rs12325554
rs183422804
rs61839660
All
−0.10
−0.05
0.00
0.05
0.10
MR leave−one−out sensitivity analysis for
'CD25hi %T cell' on 'Diabetic nephropathy'

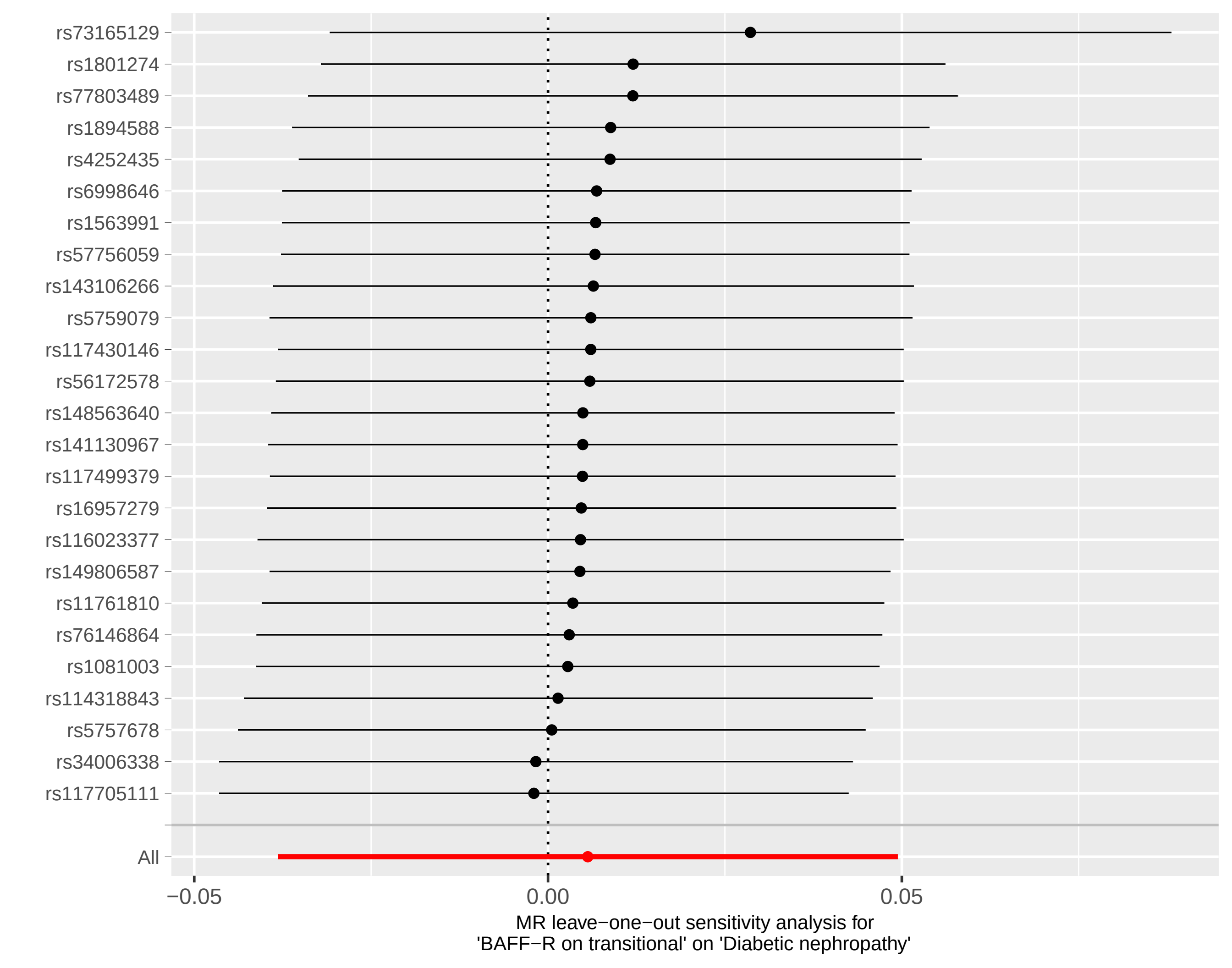
rs73165129
rs1801274
rs77803489
rs1894588
rs4252435
rs6998646
rs1563991
rs57756059
rs143106266
rs5759079
rs117430146
rs56172578
rs148563640
rs141130967
rs117499379
rs16957279
rs116023377
rs149806587
rs11761810
rs76146864
rs1081003
rs114318843
rs5757678
rs34006338
rs117705111
All
−0.05
0.00
0.05
MR leave−one−out sensitivity analysis for
'BAFF−R on transitional' on 'Diabetic nephropathy'

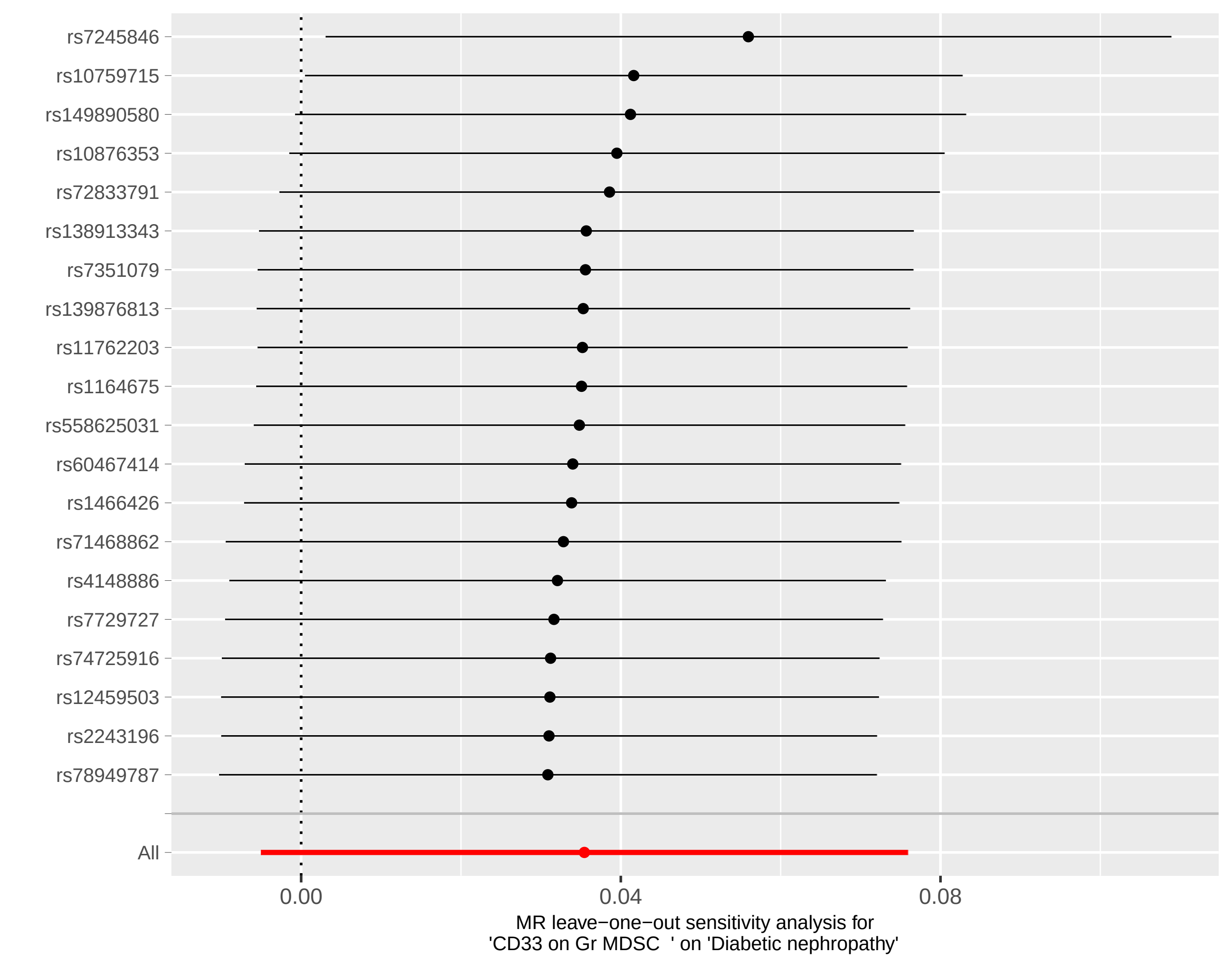
rs7245846
rs10759715
rs149890580
rs10876353
rs72833791
rs138913343
rs7351079
rs139876813
rs11762203
rs1164675
rs558625031
rs60467414
rs1466426
rs71468862
rs4148886
rs7729727
rs74725916
rs12459503
rs2243196
rs78949787
All
0.00
0.04
0.08
MR leave−one−out sensitivity analysis for
'CD33 on Gr MDSC ' on 'Diabetic nephropathy'

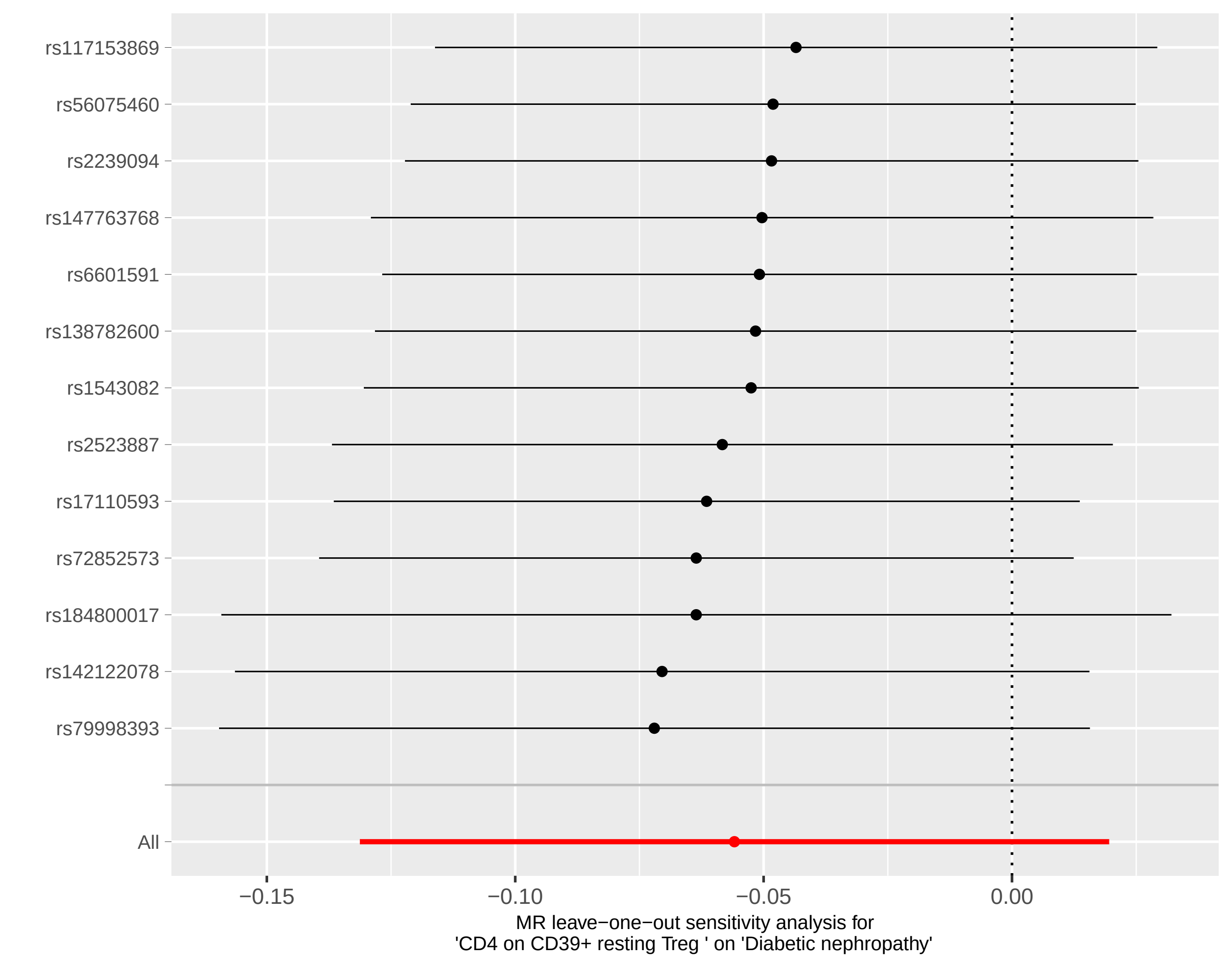

rs117153869
rs56075460
rs2239094
rs147763768
rs6601591
rs138782600
rs1543082
rs2523887
rs17110593
rs72852573
rs184800017
rs142122078
rs79998393
All
−0.15
−0.10
−0.05
0.00
MR leave−one−out sensitivity analysis for
'CD4 on CD39+ resting Treg ' on 'Diabetic nephropathy'

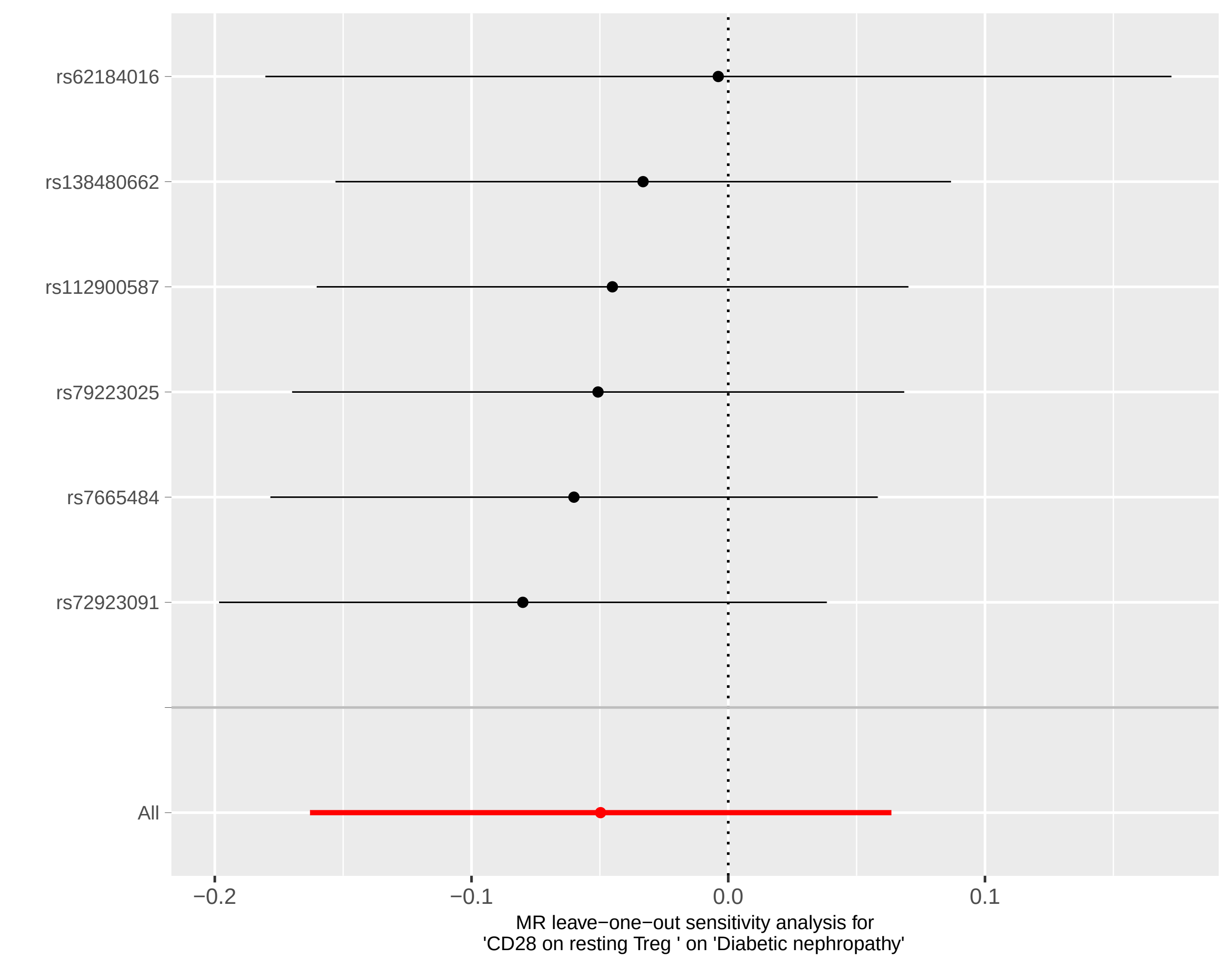
rs62184016
rs138480662
rs112900587
rs79223025
rs7665484
rs72923091
All
−0.2
−0.1
0.0
0.1
MR leave−one−out sensitivity analysis for
'CD28 on resting Treg ' on 'Diabetic nephropathy'

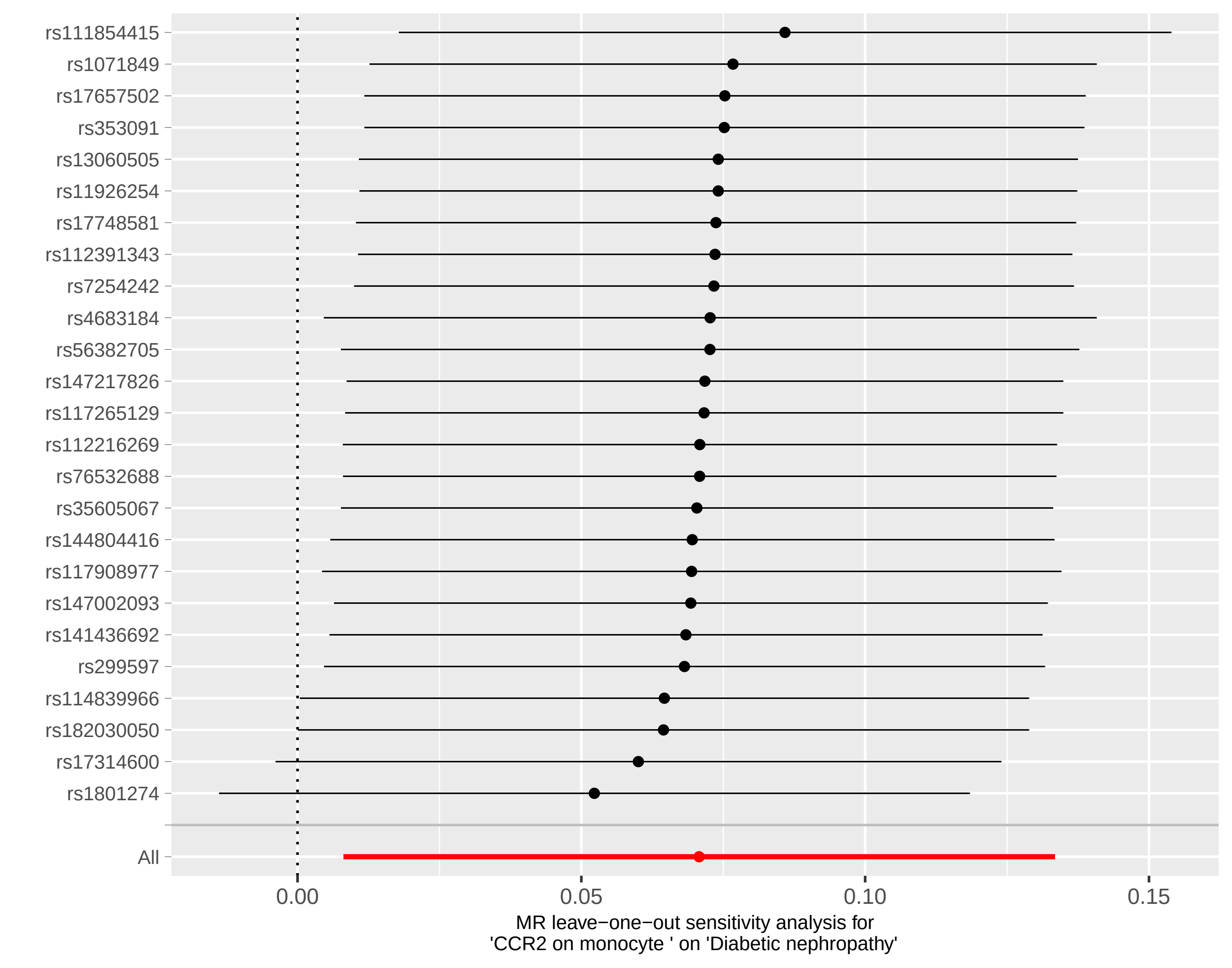
rs111854415
rs1071849
rs17657502
rs353091
rs13060505
rs11926254
rs17748581
rs112391343
rs7254242
rs4683184
rs56382705
rs147217826
rs117265129
rs112216269
rs76532688
rs35605067
rs144804416
rs117908977
rs147002093
rs141436692
rs299597
rs114839966
rs182030050
rs17314600
rs1801274
All
0.00
0.05
0.10
0.15
MR leave−one−out sensitivity analysis for
'CCR2 on monocyte ' on 'Diabetic nephropathy'

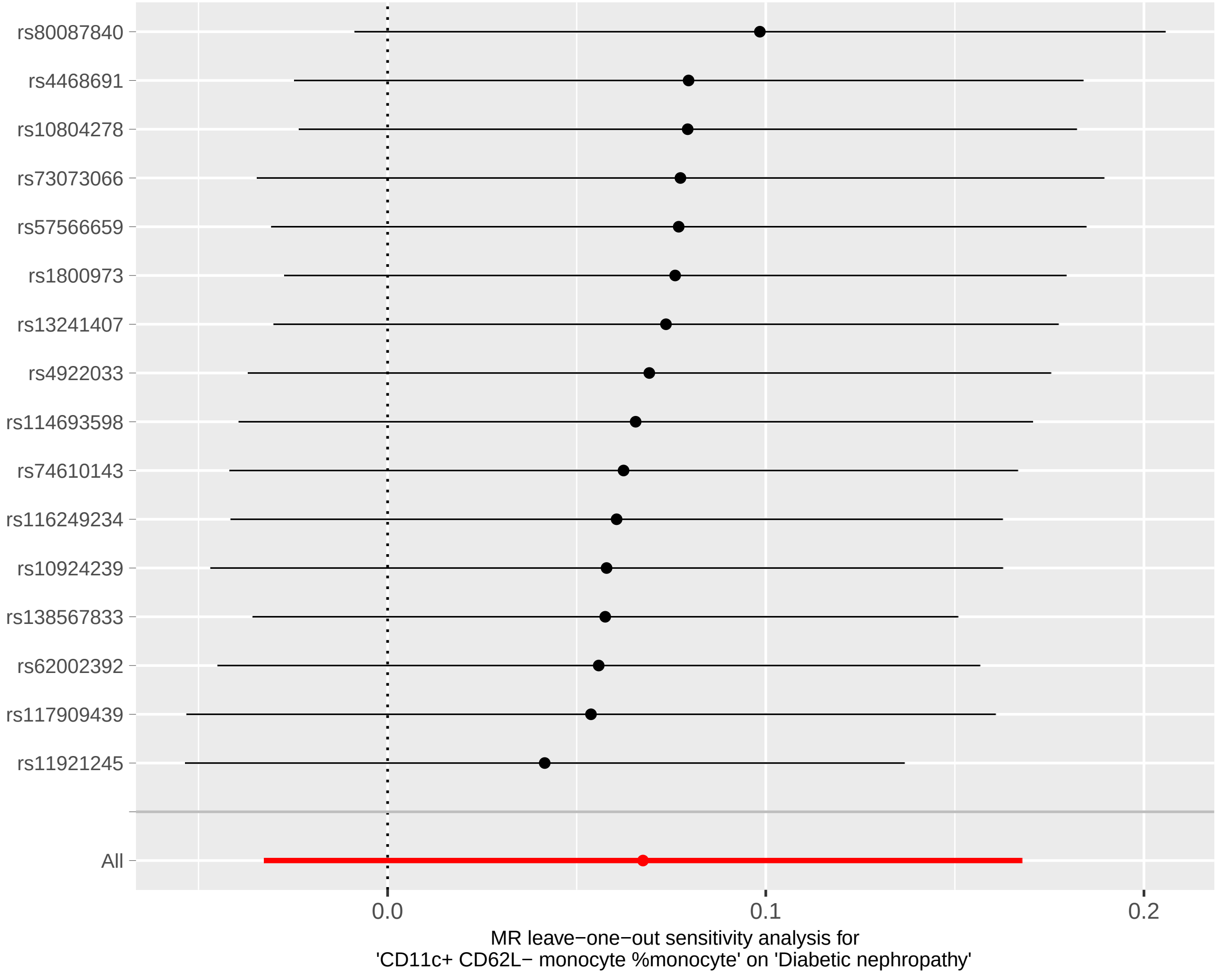
rs80087840
rs4468691
rs10804278
rs73073066
rs57566659
rs1800973
rs13241407
rs4922033
rs114693598
rs74610143
rs116249234
rs10924239
rs138567833
rs62002392
rs117909439
rs11921245
All
0.0
0.1
0.2
MR leave−one−out sensitivity analysis for
'CD11c+ CD62L− monocyte %monocyte' on 'Diabetic nephropathy'

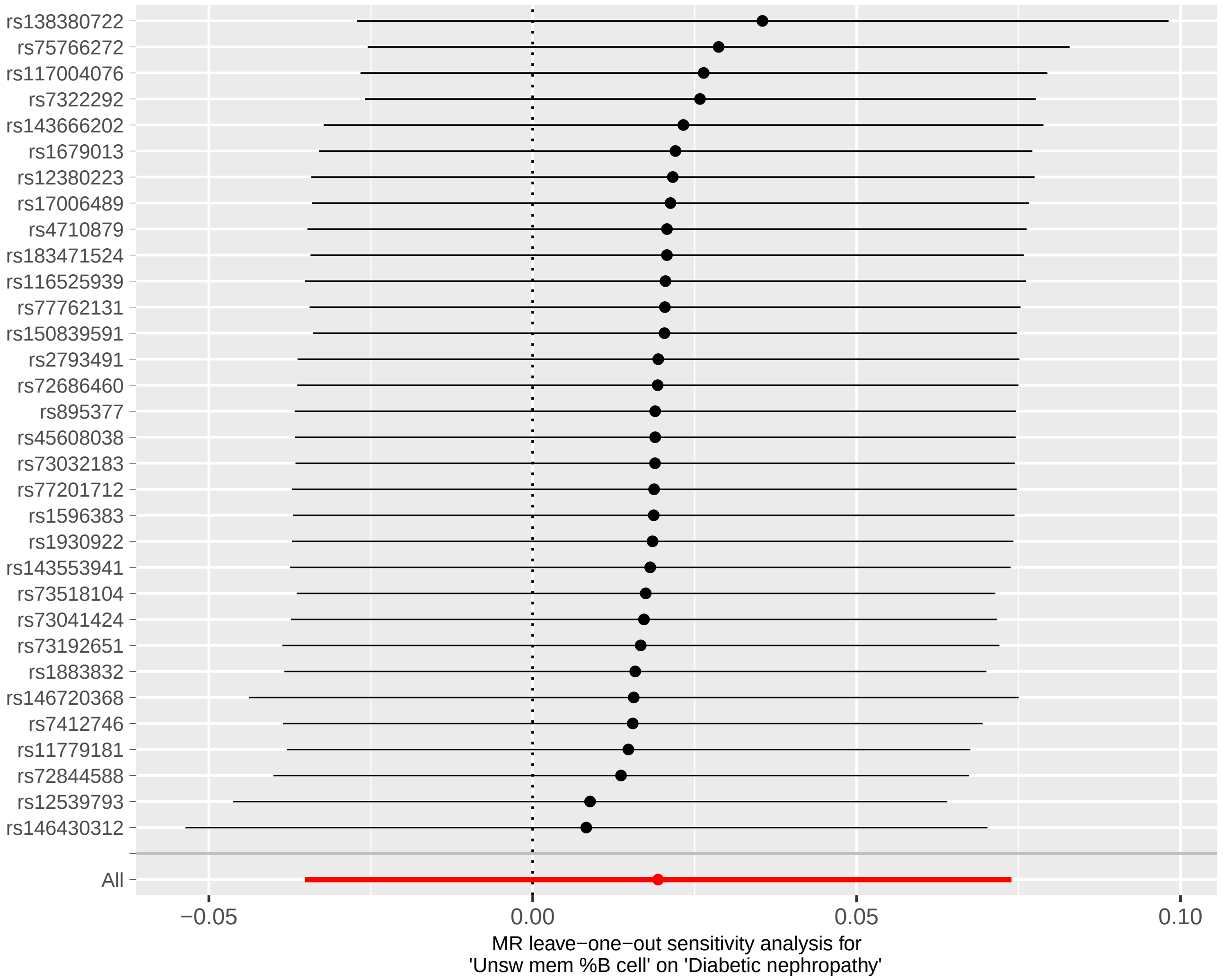

rs138380722
rs75766272
rs117004076
rs7322292
rs143666202
rs1679013
rs12380223
rs17006489
rs4710879
rs183471524
rs116525939
rs77762131
rs150839591
rs2793491
rs72686460
rs895377
rs45608038
rs73032183
rs77201712
rs1596383
rs1930922
rs143553941
rs73518104
rs73041424
rs73192651
rs1883832
rs146720368
rs7412746
rs11779181
rs72844588
rs12539793
rs146430312
All
−0.05
0.00
0.05
0.10
MR leave−one−out sensitivity analysis for
'Unsw mem %B cell' on 'Diabetic nephropathy'

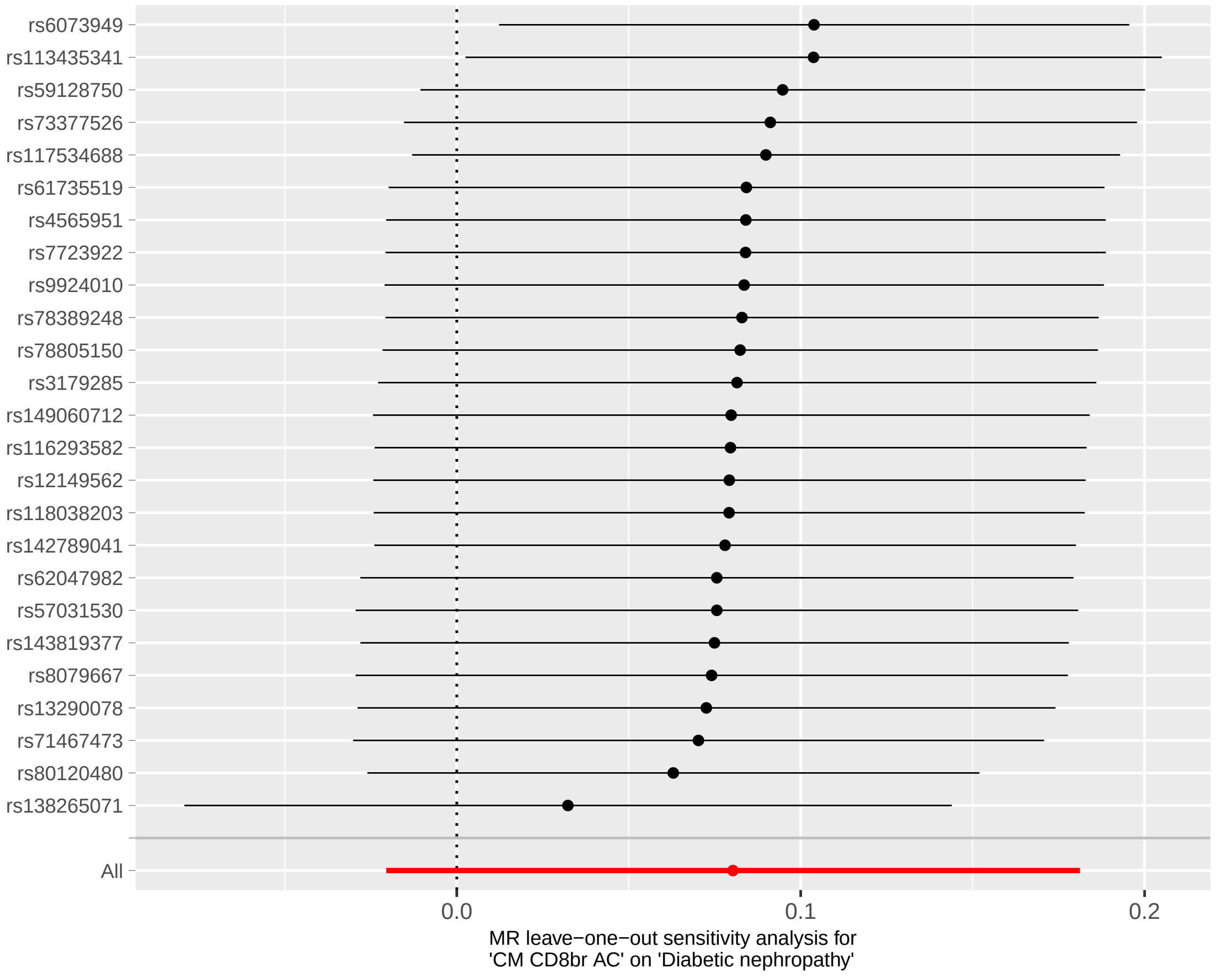
rs6073949
rs113435341
rs59128750
rs73377526
rs117534688
rs61735519
rs4565951
rs7723922
rs9924010
rs78389248
rs78805150
rs3179285
rs149060712
rs116293582
rs12149562
rs118038203
rs142789041
rs62047982
rs57031530
rs143819377
rs8079667
rs13290078
rs71467473
rs80120480
rs138265071
All
0.0
0.1
0.2
MR leave−one−out sensitivity analysis for
'CM CD8br AC' on 'Diabetic nephropathy'

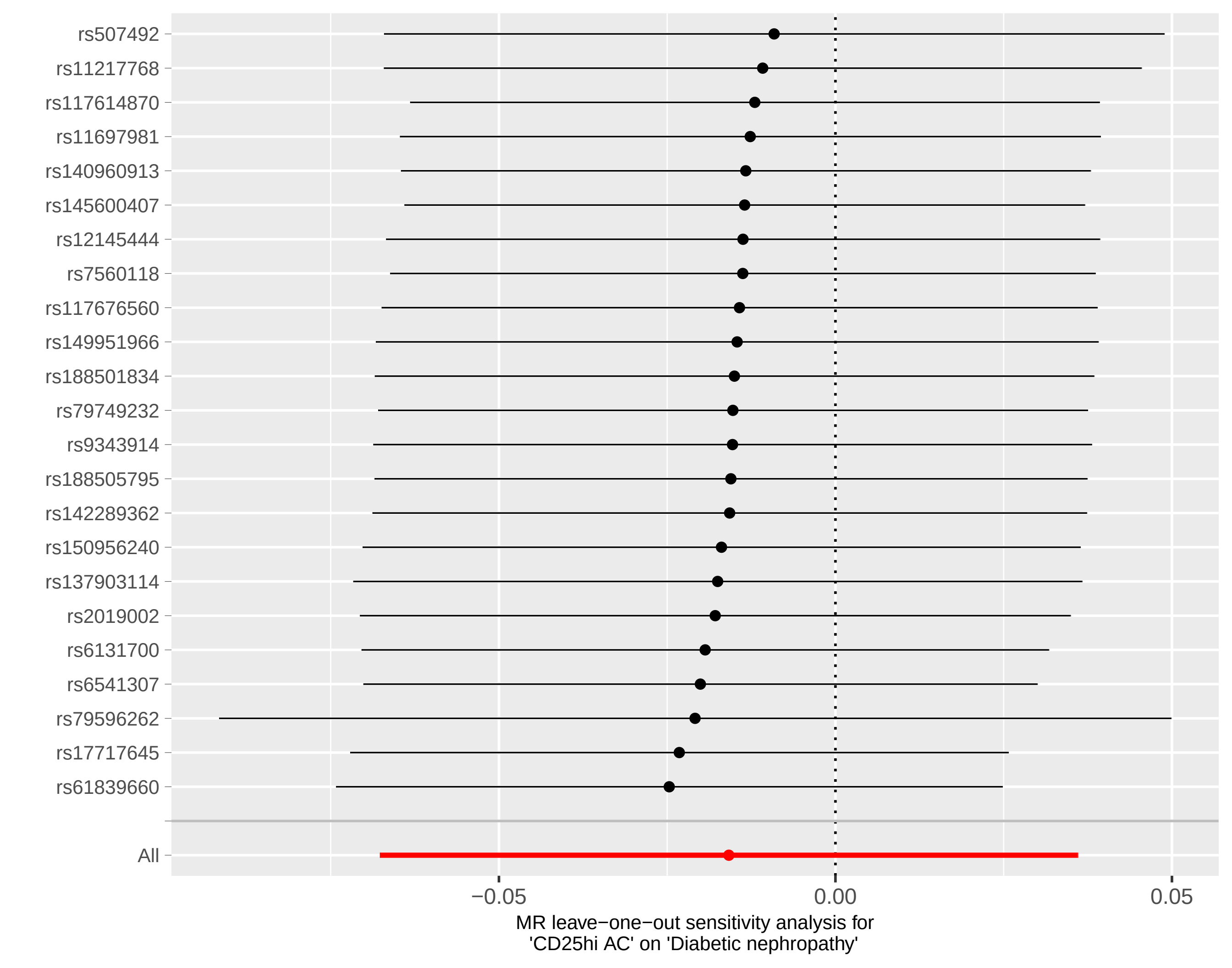
rs507492
rs11217768
rs117614870
rs11697981
rs140960913
rs145600407
rs12145444
rs7560118
rs117676560
rs149951966
rs188501834
rs79749232
rs9343914
rs188505795
rs142289362
rs150956240
rs137903114
rs2019002
rs6131700
rs6541307
rs79596262
rs17717645
rs61839660
All
−0.05
0.00
0.05
MR leave−one−out sensitivity analysis for
'CD25hi AC' on 'Diabetic nephropathy'

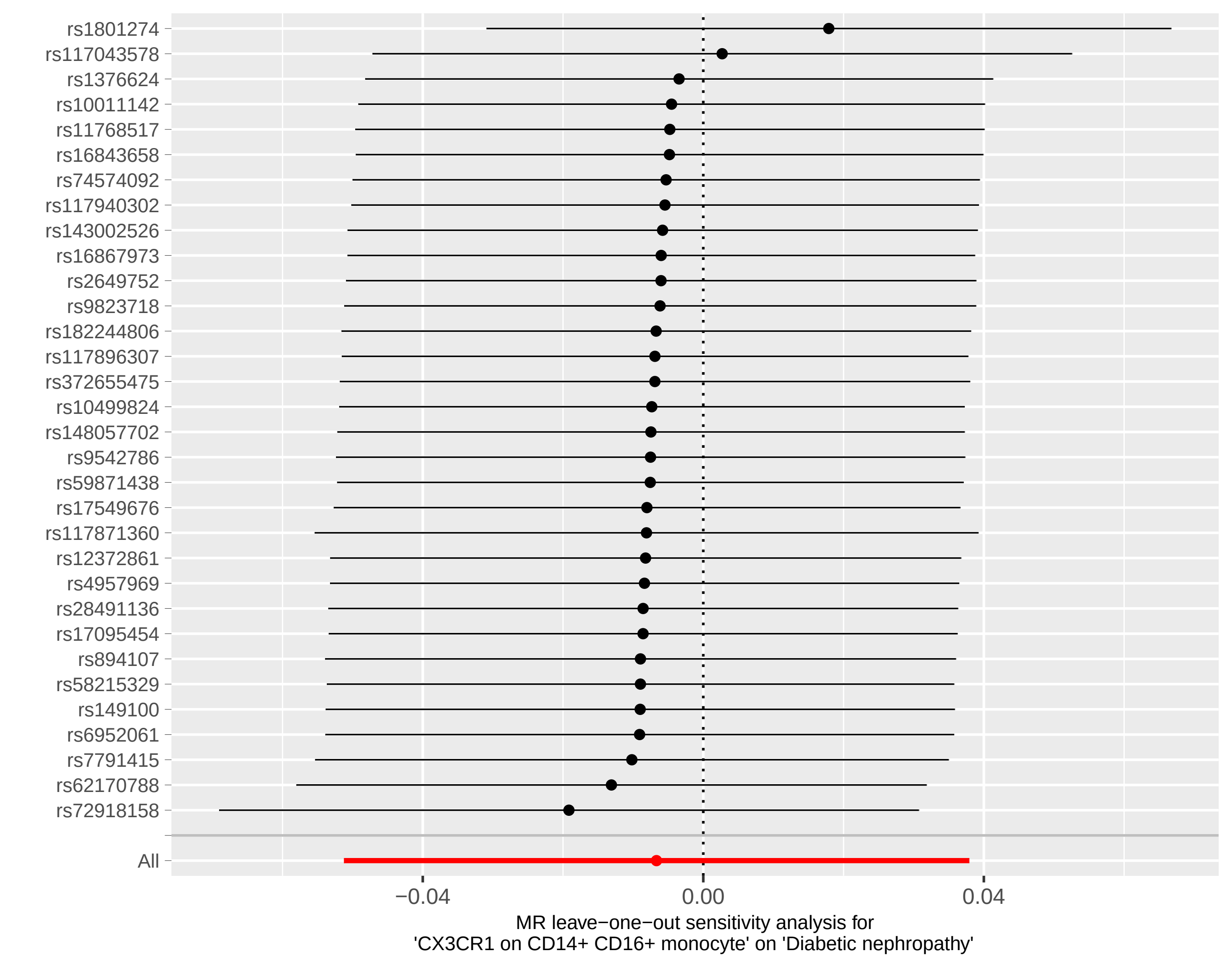
rs1801274
rs117043578
rs1376624
rs10011142
rs11768517
rs16843658
rs74574092
rs117940302
rs143002526
rs16867973
rs2649752
rs9823718
rs182244806
rs117896307
rs372655475
rs10499824
rs148057702
rs9542786
rs59871438
rs17549676
rs117871360
rs12372861
rs4957969
rs28491136
rs17095454
rs894107
rs58215329
rs149100
rs6952061
rs7791415
rs62170788
rs72918158
All
−0.04
0.00
0.04
MR leave−one−out sensitivity analysis for
'CX3CR1 on CD14+ CD16+ monocyte' on 'Diabetic nephropathy'

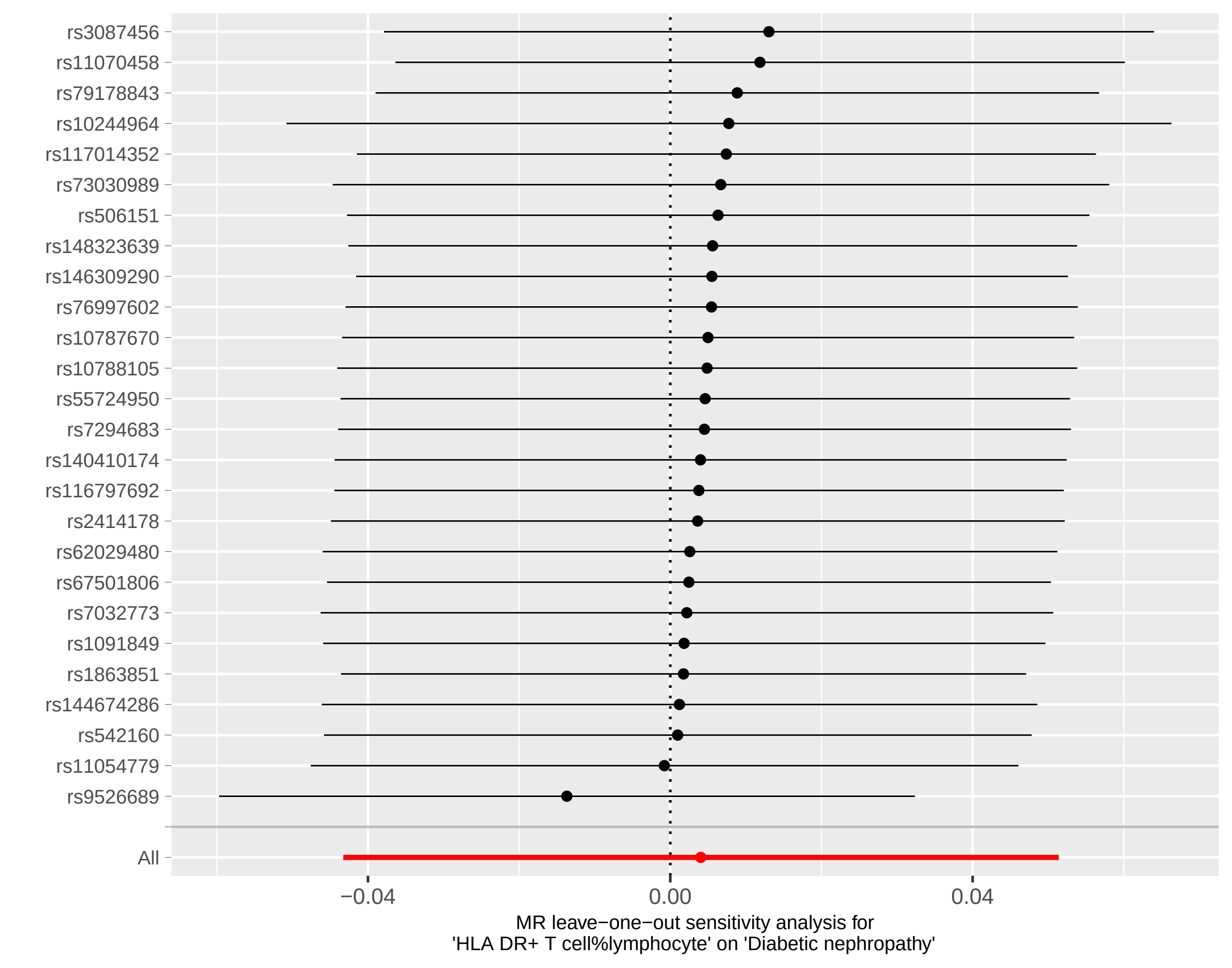
rs3087456
rs11070458
rs79178843
rs10244964
rs117014352
rs73030989
rs506151
rs148323639
rs146309290
rs76997602
rs10787670
rs10788105
rs55724950
rs7294683
rs140410174
rs116797692
rs2414178
rs62029480
rs67501806
rs7032773
rs1091849
rs1863851
rs144674286
rs542160
rs11054779
rs9526689
All
−0.04
0.00
0.04
MR leave−one−out sensitivity analysis for
'HLA DR+ T cell%lymphocyte' on 'Diabetic nephropathy'

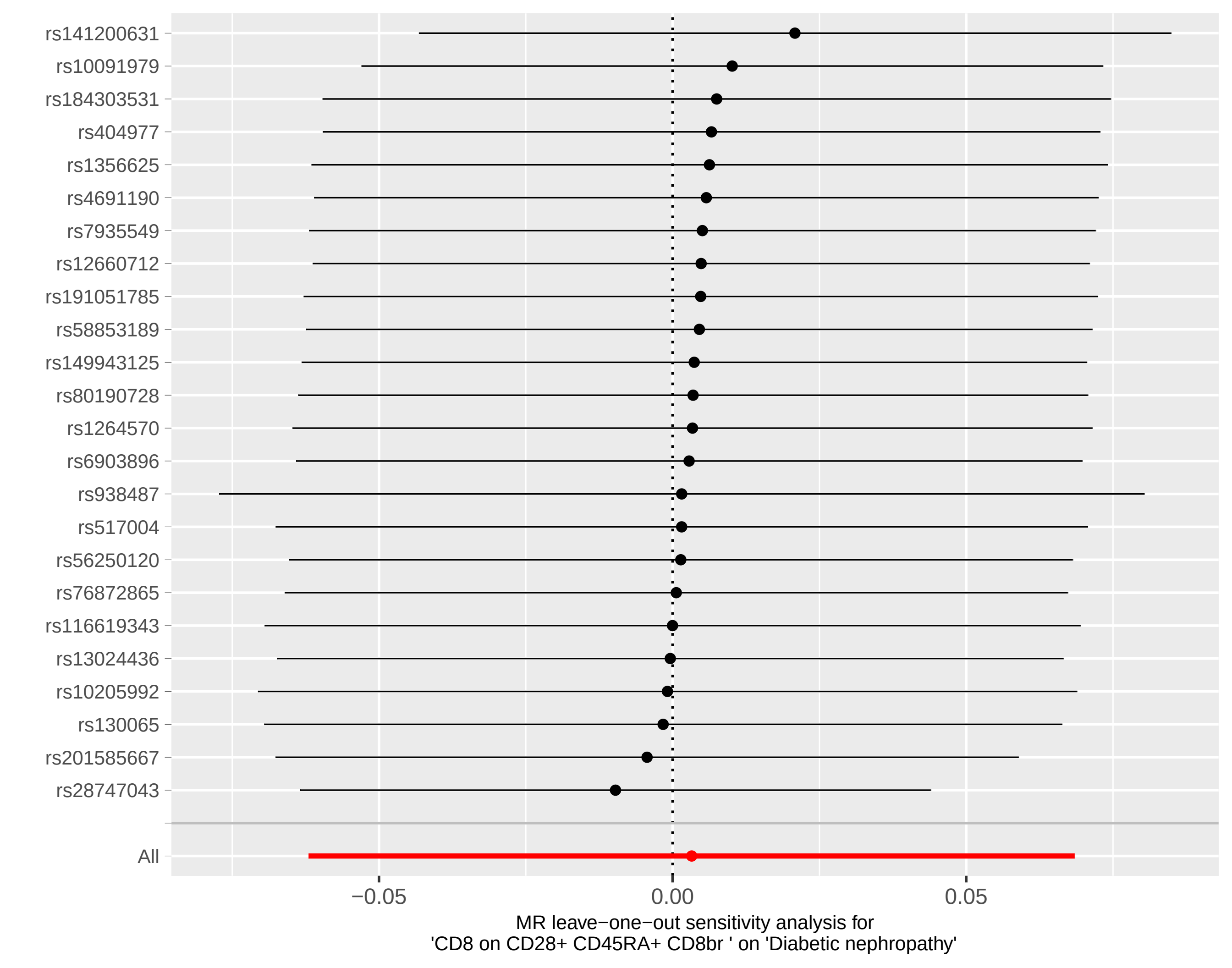
rs141200631
rs10091979
rs184303531
rs404977
rs1356625
rs4691190
rs7935549
rs12660712
rs191051785
rs58853189
rs149943125
rs80190728
rs1264570
rs6903896
rs938487
rs517004
rs56250120
rs76872865
rs116619343
rs13024436
rs10205992
rs130065
rs201585667
rs28747043
All
−0.05
0.00
0.05
MR leave−one−out sensitivity analysis for
'CD8 on CD28+ CD45RA+ CD8br ' on 'Diabetic nephropathy'

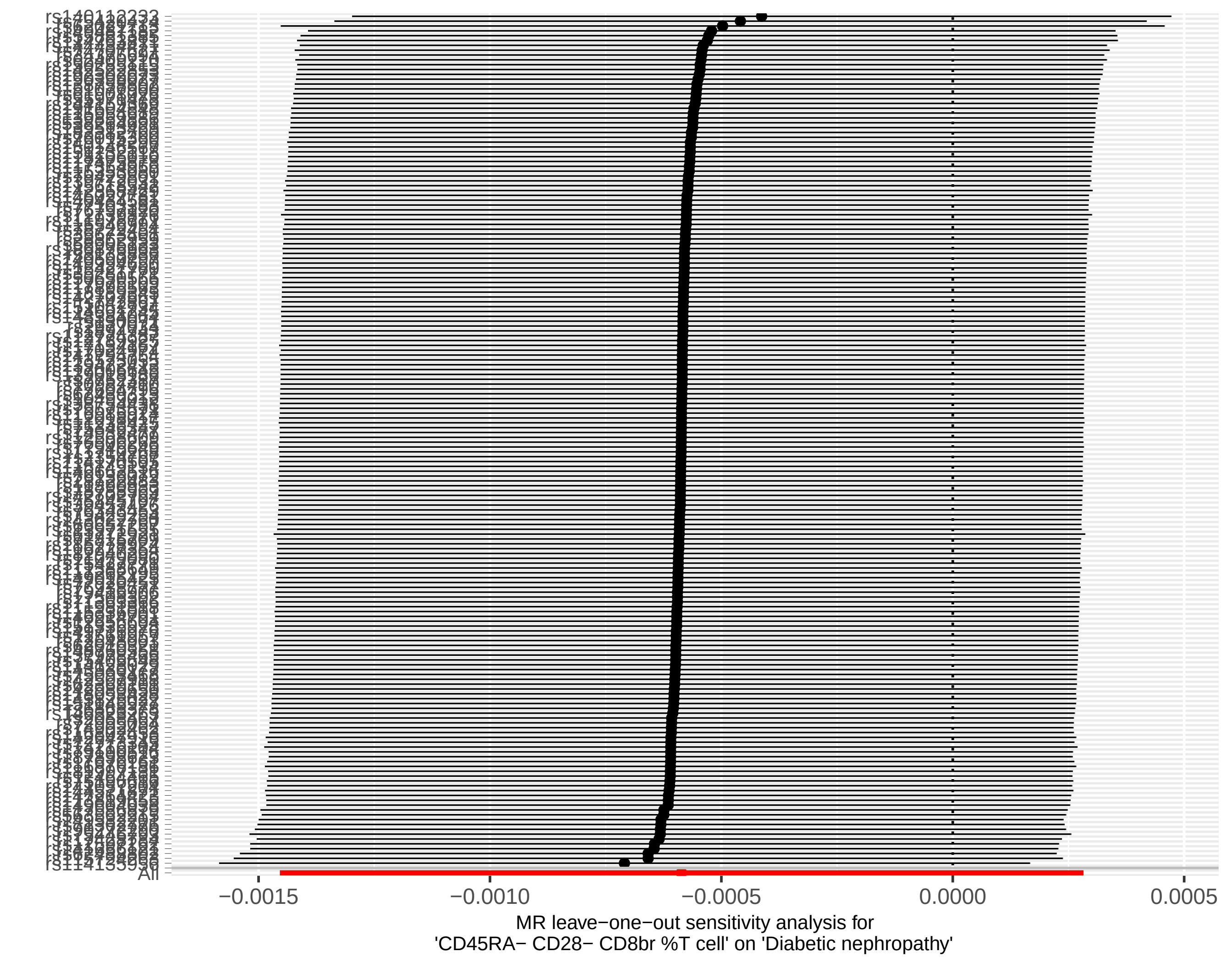
All
−0.0015
−0.0010
−0.0005
0.0000
0.0005
MR leave−one−out sensitivity analysis for
'CD45RA− CD28− CD8br %T cell' on 'Diabetic nephropathy'

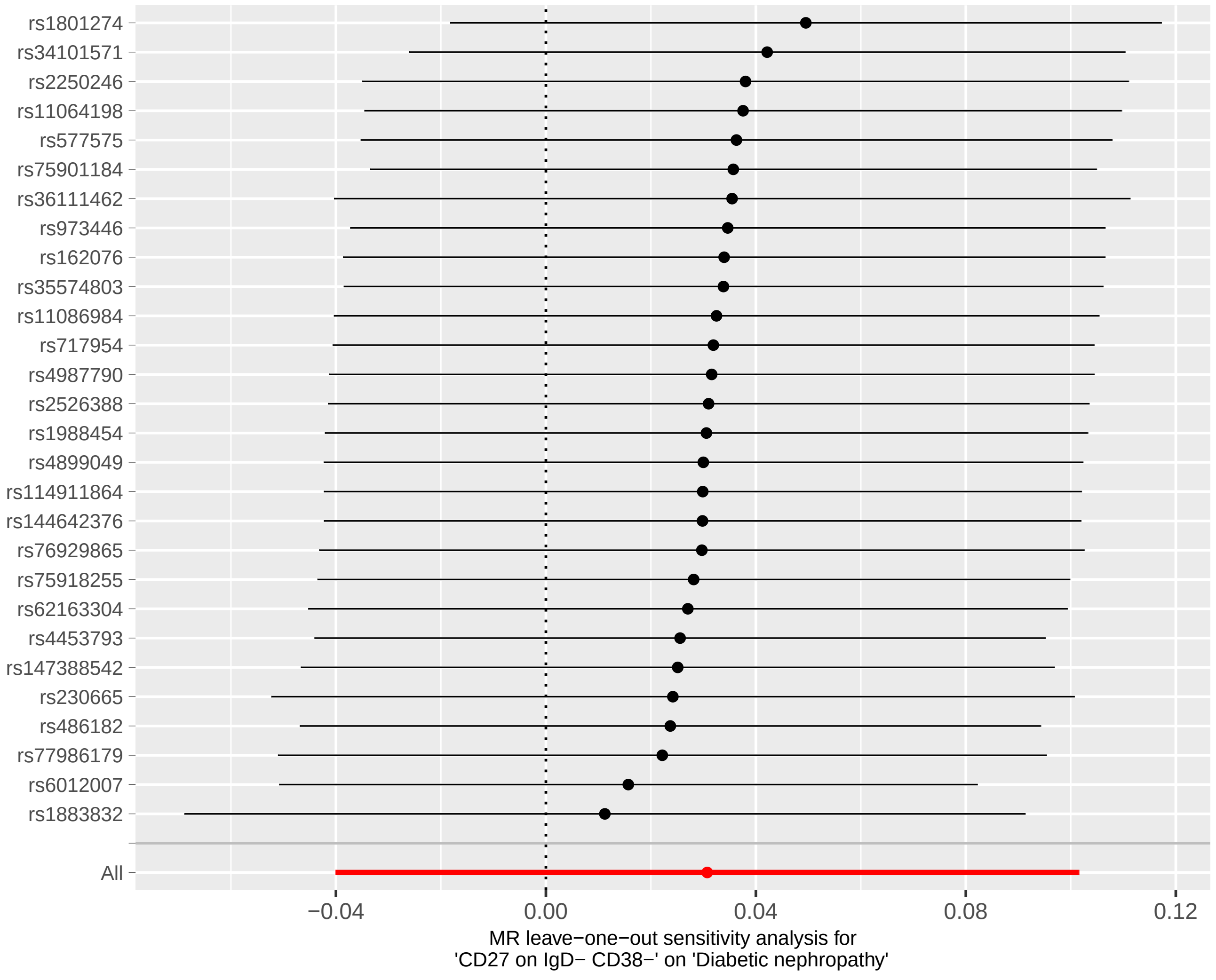

rs1801274
rs34101571
rs2250246
rs11064198
rs577575
rs75901184
rs36111462
rs973446
rs162076
rs35574803
rs11086984
rs717954
rs4987790
rs2526388
rs1988454
rs4899049
rs114911864
rs144642376
rs76929865
rs75918255
rs62163304
rs4453793
rs147388542
rs230665
rs486182
rs77986179
rs6012007
rs1883832
All
−0.04
0.00
0.04
0.08
0.12
MR leave−one−out sensitivity analysis for
'CD27 on IgD− CD38−' on 'Diabetic nephropathy'

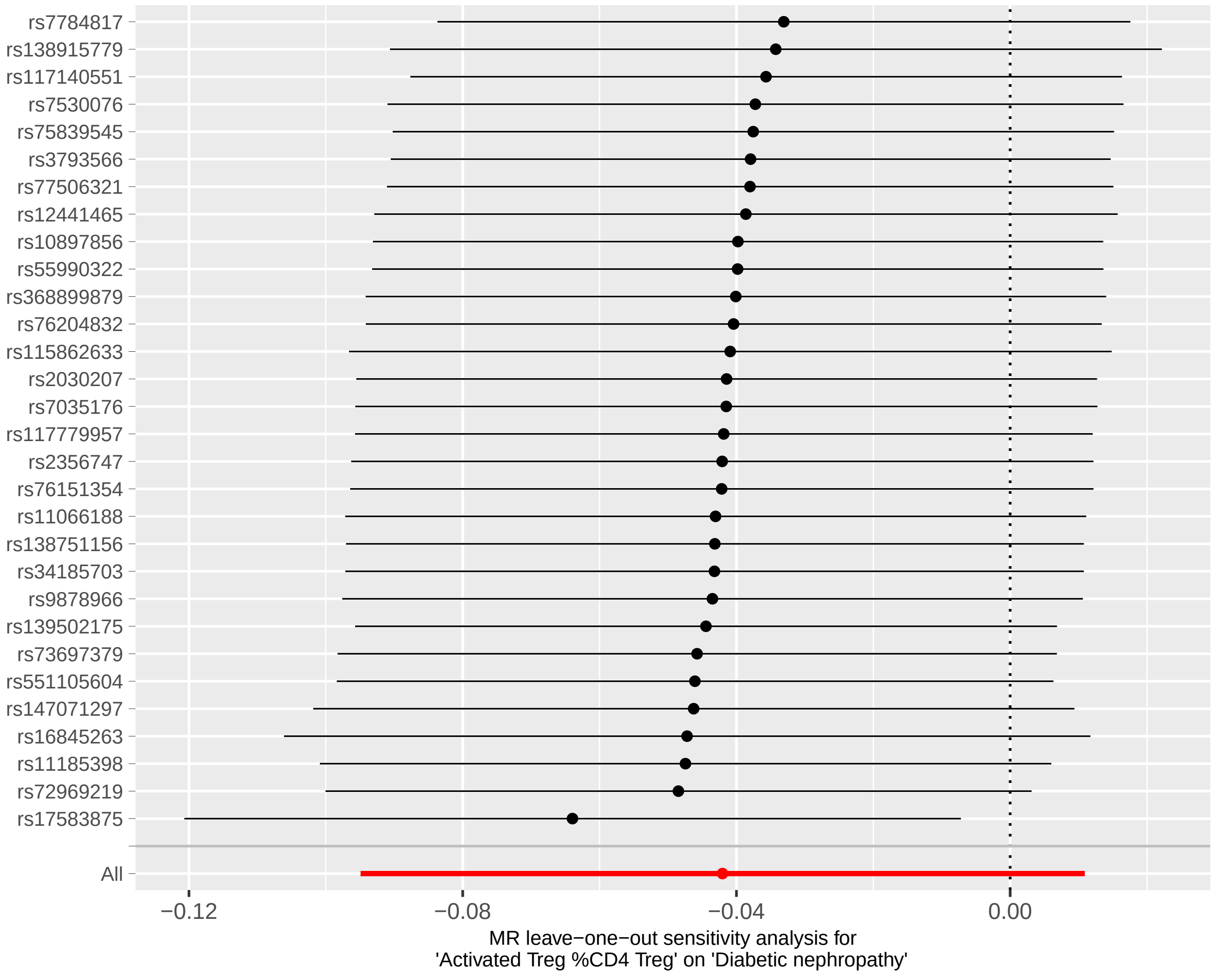
rs7784817
rs138915779
rs117140551
rs7530076
rs75839545
rs3793566
rs77506321
rs12441465
rs10897856
rs55990322
rs368899879
rs76204832
rs115862633
rs2030207
rs7035176
rs117779957
rs2356747
rs76151354
rs11066188
rs138751156
rs34185703
rs9878966
rs139502175
rs73697379
rs551105604
rs147071297
rs16845263
rs11185398
rs72969219
rs17583875
All
−0.12
−0.08
−0.04
0.00
MR leave−one−out sensitivity analysis for
'Activated Treg %CD4 Treg' on 'Diabetic nephropathy'

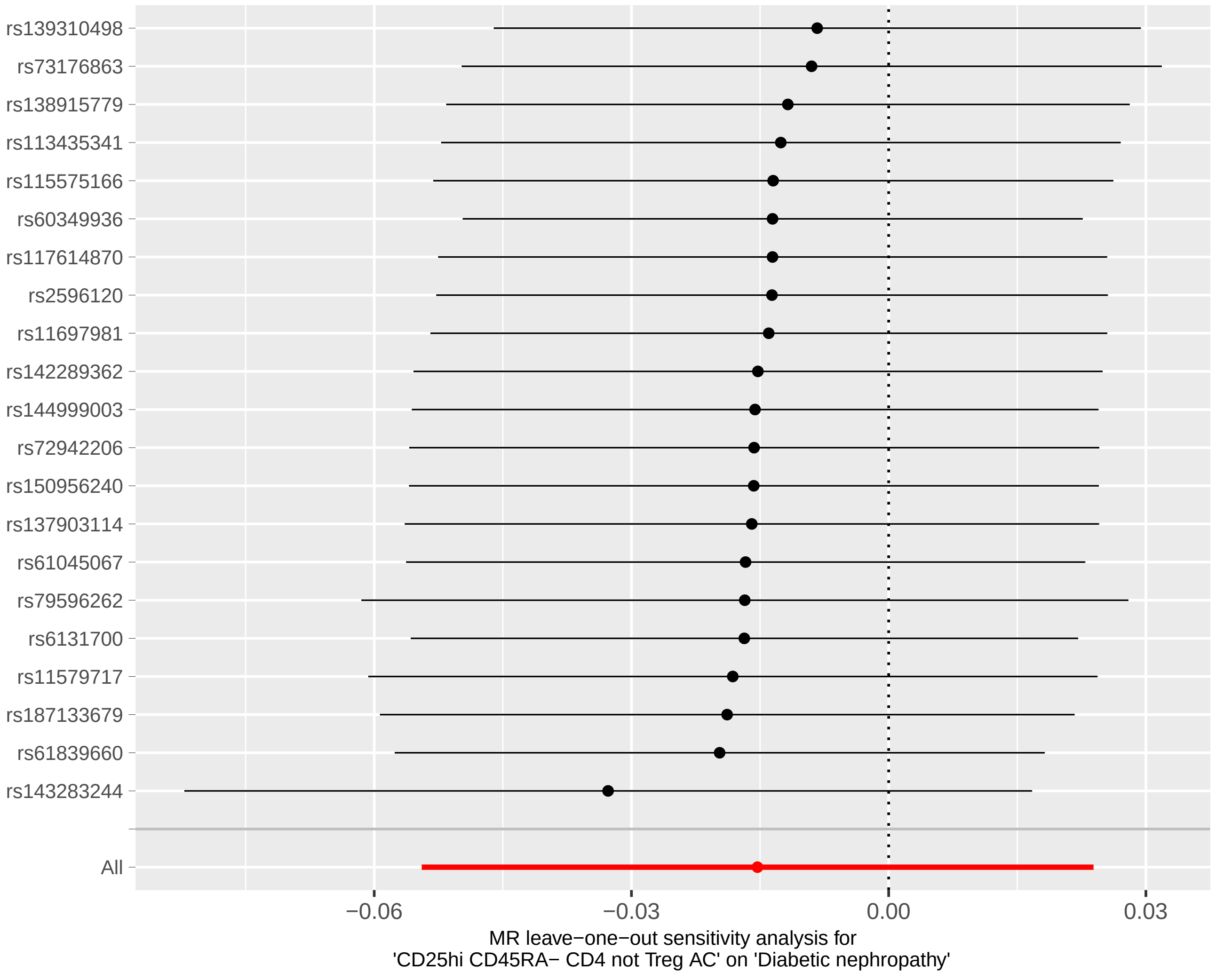
rs139310498
rs73176863
rs138915779
rs113435341
rs115575166
rs60349936
rs117614870
rs2596120
rs11697981
rs142289362
rs144999003
rs72942206
rs150956240
rs137903114
rs61045067
rs79596262
rs6131700
rs11579717
rs187133679
rs61839660
rs143283244
All
−0.06
−0.03
0.00
0.03
MR leave−one−out sensitivity analysis for
'CD25hi CD45RA− CD4 not Treg AC' on 'Diabetic nephropathy'

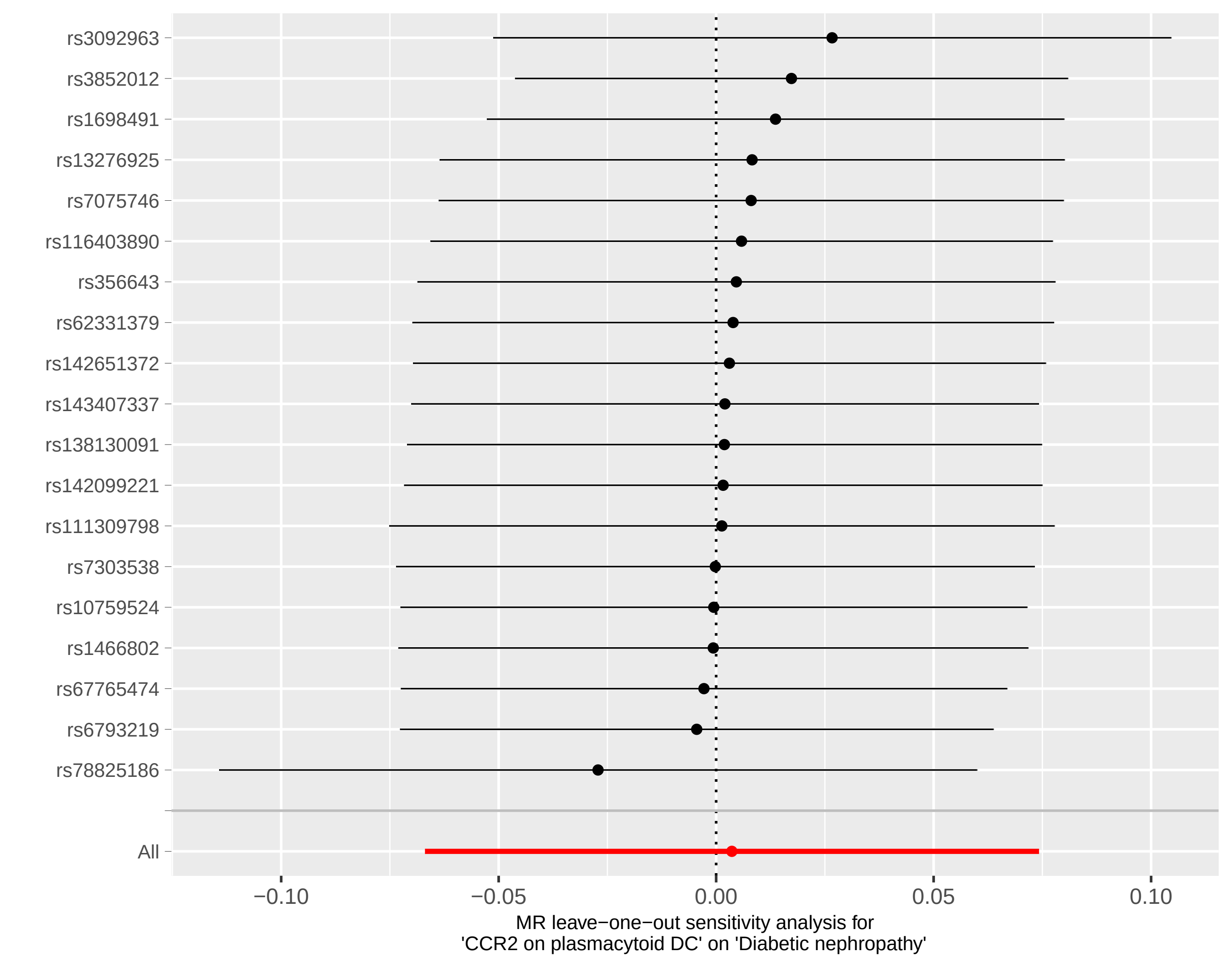

rs3092963
rs3852012
rs1698491
rs13276925
rs7075746
rs116403890
rs356643
rs62331379
rs142651372
rs143407337
rs138130091
rs142099221
rs111309798
rs7303538
rs10759524
rs1466802
rs67765474
rs6793219
rs78825186
All
−0.10
−0.05
0.00
0.05
0.10
MR leave−one−out sensitivity analysis for
'CCR2 on plasmacytoid DC' on 'Diabetic nephropathy'

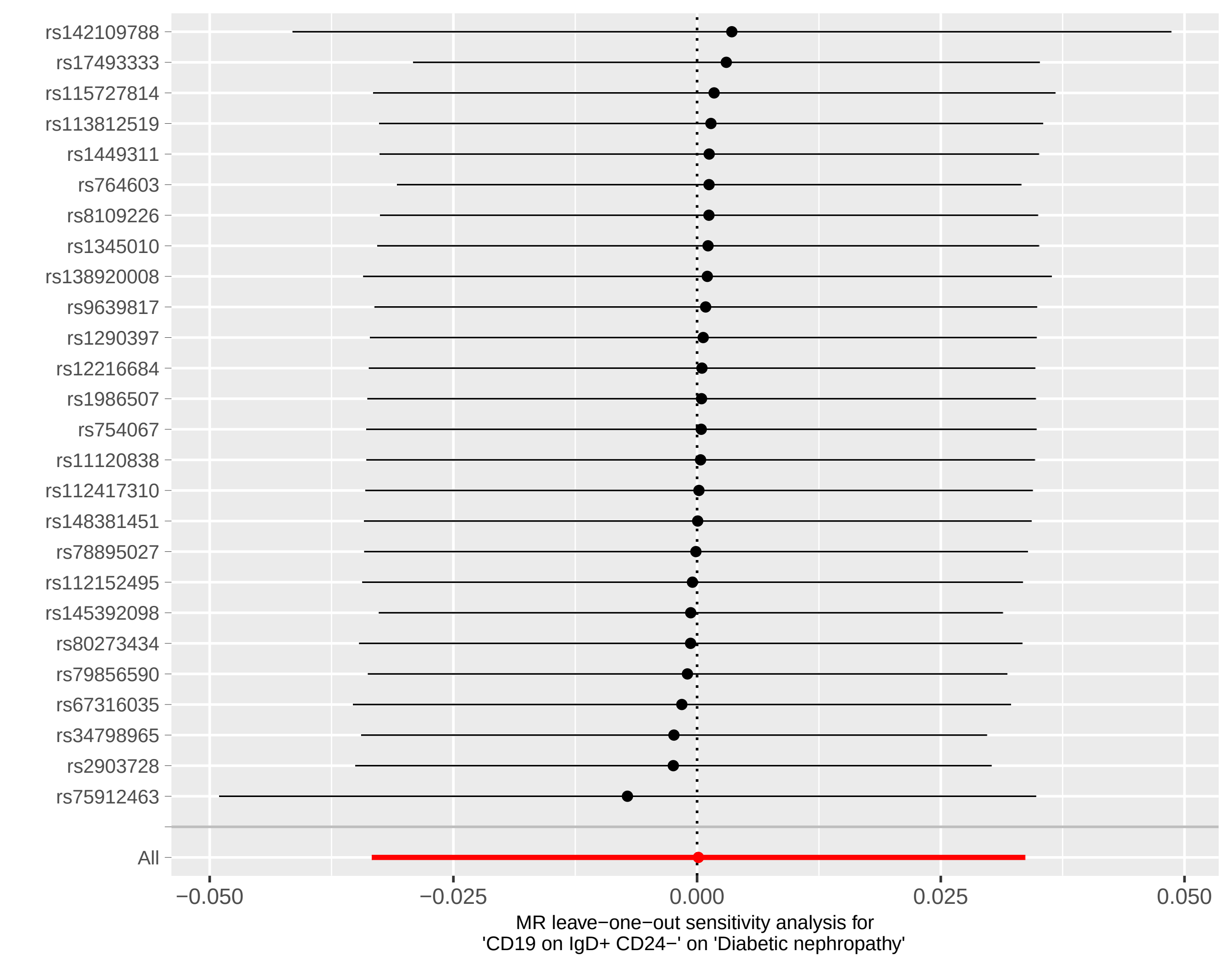
rs142109788
rs17493333
rs115727814
rs113812519
rs1449311
rs764603
rs8109226
rs1345010
rs138920008
rs9639817
rs1290397
rs12216684
rs1986507
rs754067
rs11120838
rs112417310
rs148381451
rs78895027
rs112152495
rs145392098
rs80273434
rs79856590
rs67316035
rs34798965
rs2903728
rs75912463
All
−0.050
−0.025
0.000
0.025
0.050
MR leave−one−out sensitivity analysis for
'CD19 on IgD+ CD24−' on 'Diabetic nephropathy'

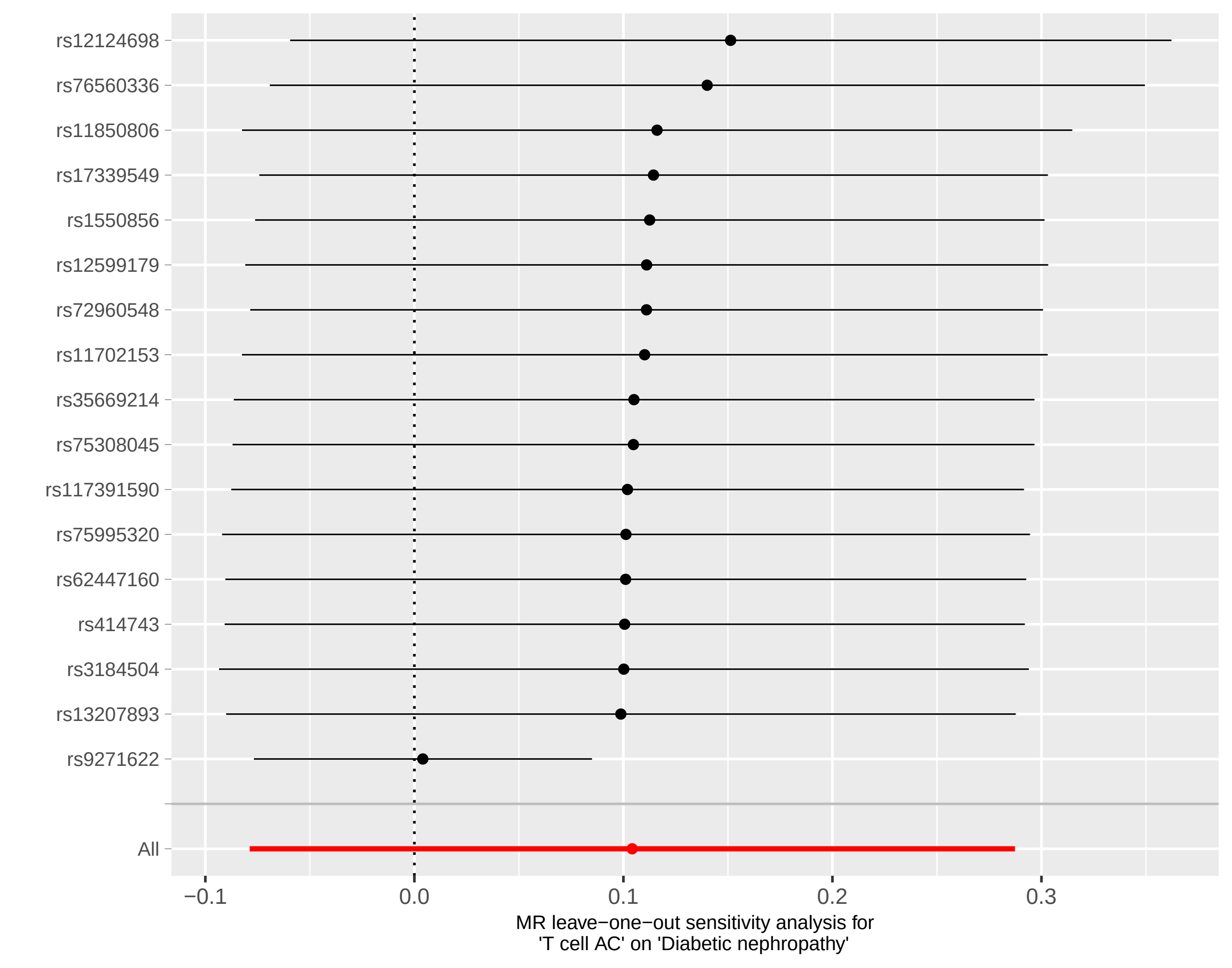
rs12124698
rs76560336
rs11850806
rs17339549
rs1550856
rs12599179
rs72960548
rs11702153
rs35669214
rs75308045
rs117391590
rs75995320
rs62447160
rs414743
rs3184504
rs13207893
rs9271622
All
−0.1
0.0
0.1
0.2
0.3
MR leave−one−out sensitivity analysis for
'T cell AC' on 'Diabetic nephropathy'

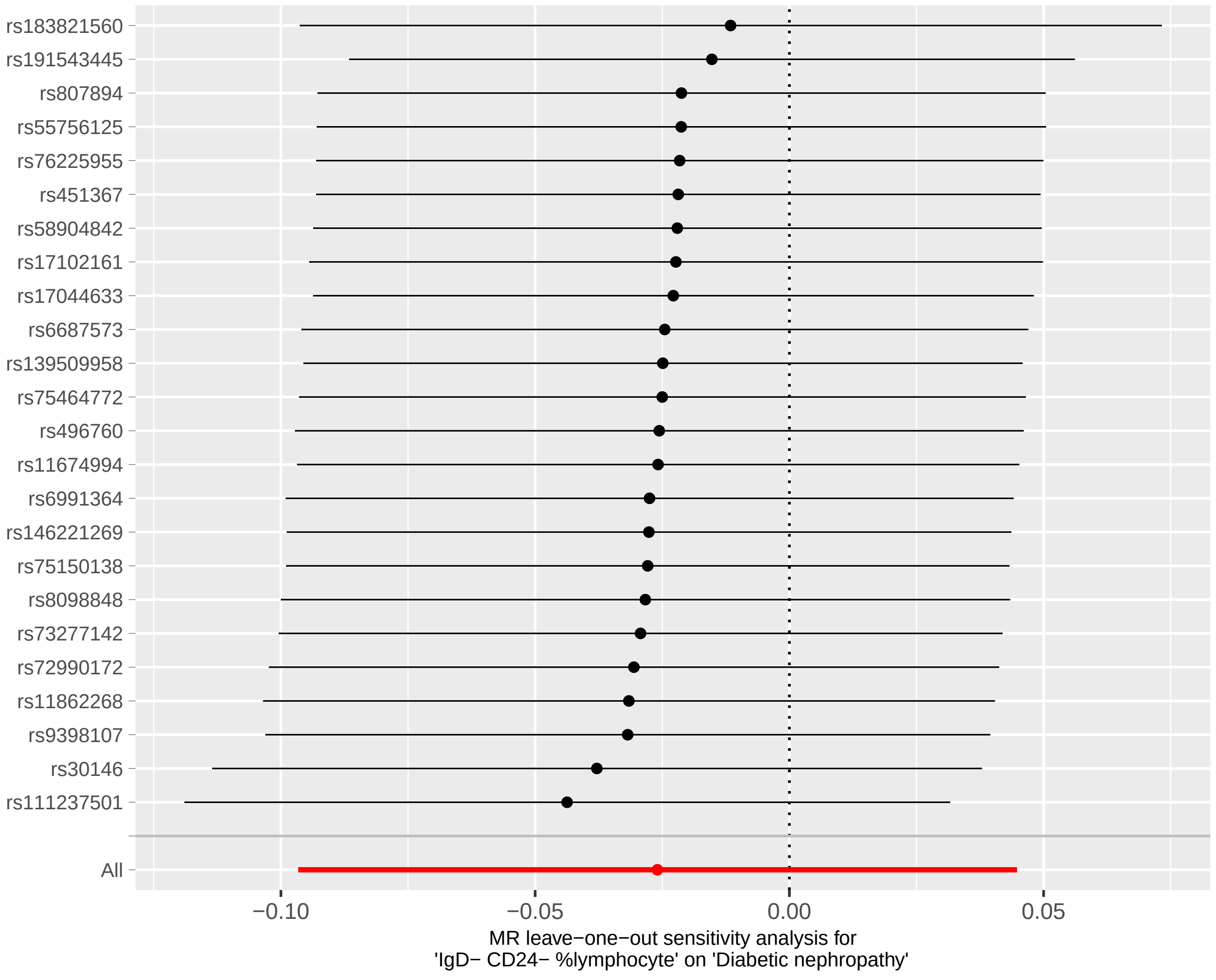
rs183821560
rs191543445
rs807894
rs55756125
rs76225955
rs451367
rs58904842
rs17102161
rs17044633
rs6687573
rs139509958
rs75464772
rs496760
rs11674994
rs6991364
rs146221269
rs75150138
rs8098848
rs73277142
rs72990172
rs11862268
rs9398107
rs30146
rs111237501
All
−0.10
−0.05
0.00
0.05
MR leave−one−out sensitivity analysis for
'IgD− CD24− %lymphocyte' on 'Diabetic nephropathy'

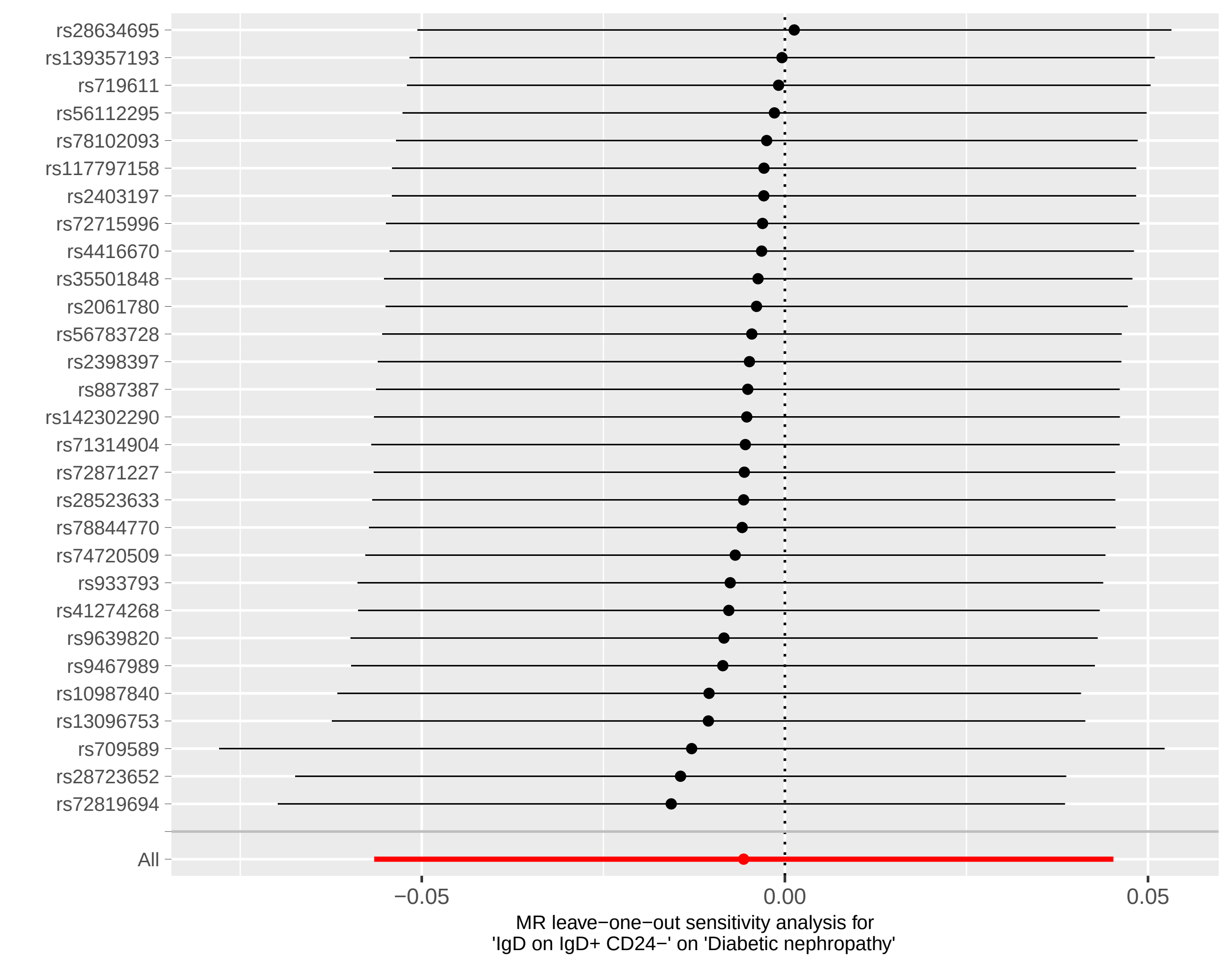
rs28634695
rs139357193
rs719611
rs56112295
rs78102093
rs117797158
rs2403197
rs72715996
rs4416670
rs35501848
rs2061780
rs56783728
rs2398397
rs887387
rs142302290
rs71314904
rs72871227
rs28523633
rs78844770
rs74720509
rs933793
rs41274268
rs9639820
rs9467989
rs10987840
rs13096753
rs709589
rs28723652
rs72819694
All
−0.05
0.00
0.05
MR leave−one−out sensitivity analysis for
'IgD on IgD+ CD24−' on 'Diabetic nephropathy'

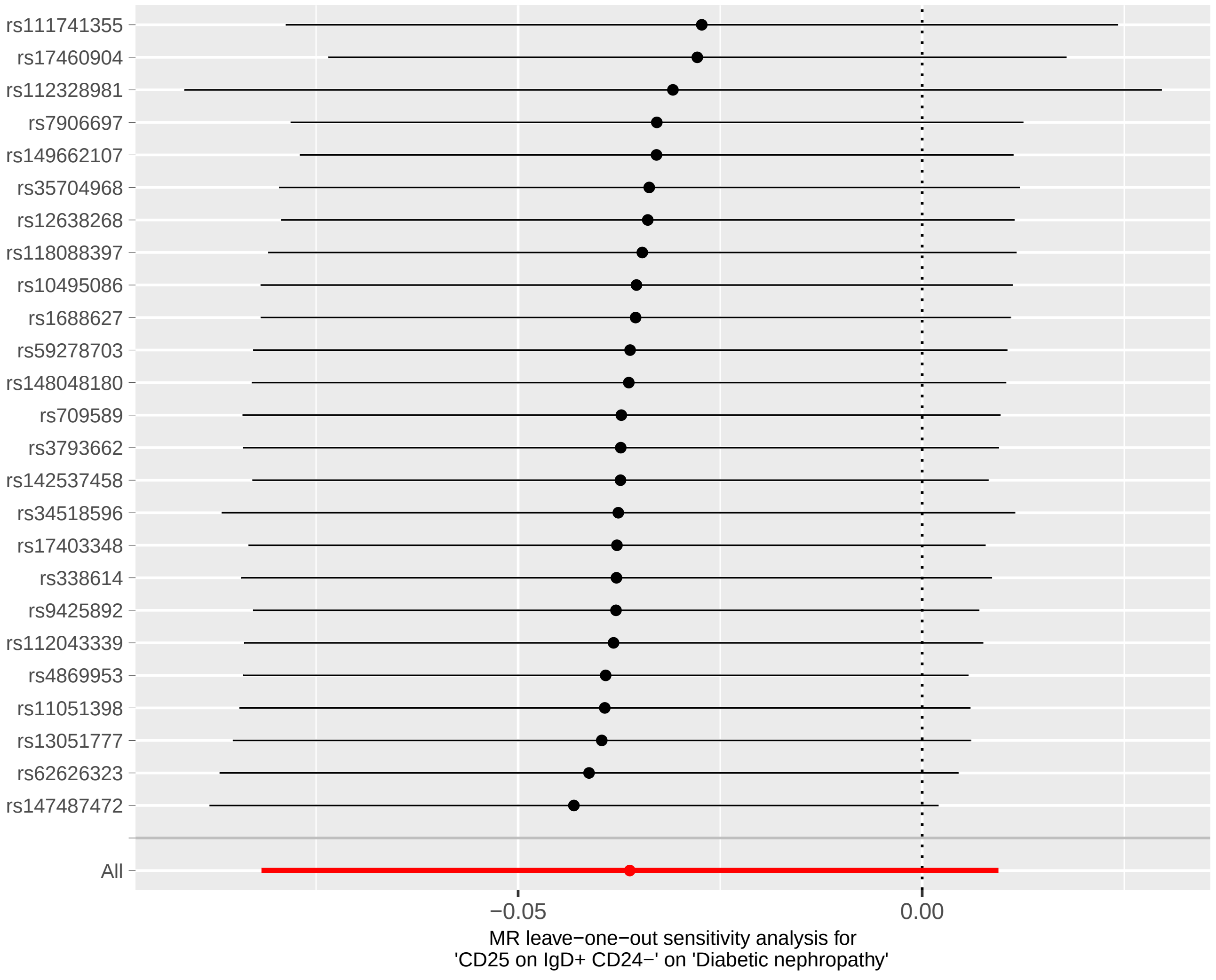
rs111741355
rs17460904
rs112328981
rs7906697
rs149662107
rs35704968
rs12638268
rs118088397
rs10495086
rs1688627
rs59278703
rs148048180
rs709589
rs3793662
rs142537458
rs34518596
rs17403348
rs338614
rs9425892
rs112043339
rs4869953
rs11051398
rs13051777
rs62626323
rs147487472
All
−0.05
0.00
MR leave−one−out sensitivity analysis for
'CD25 on IgD+ CD24−' on 'Diabetic nephropathy'

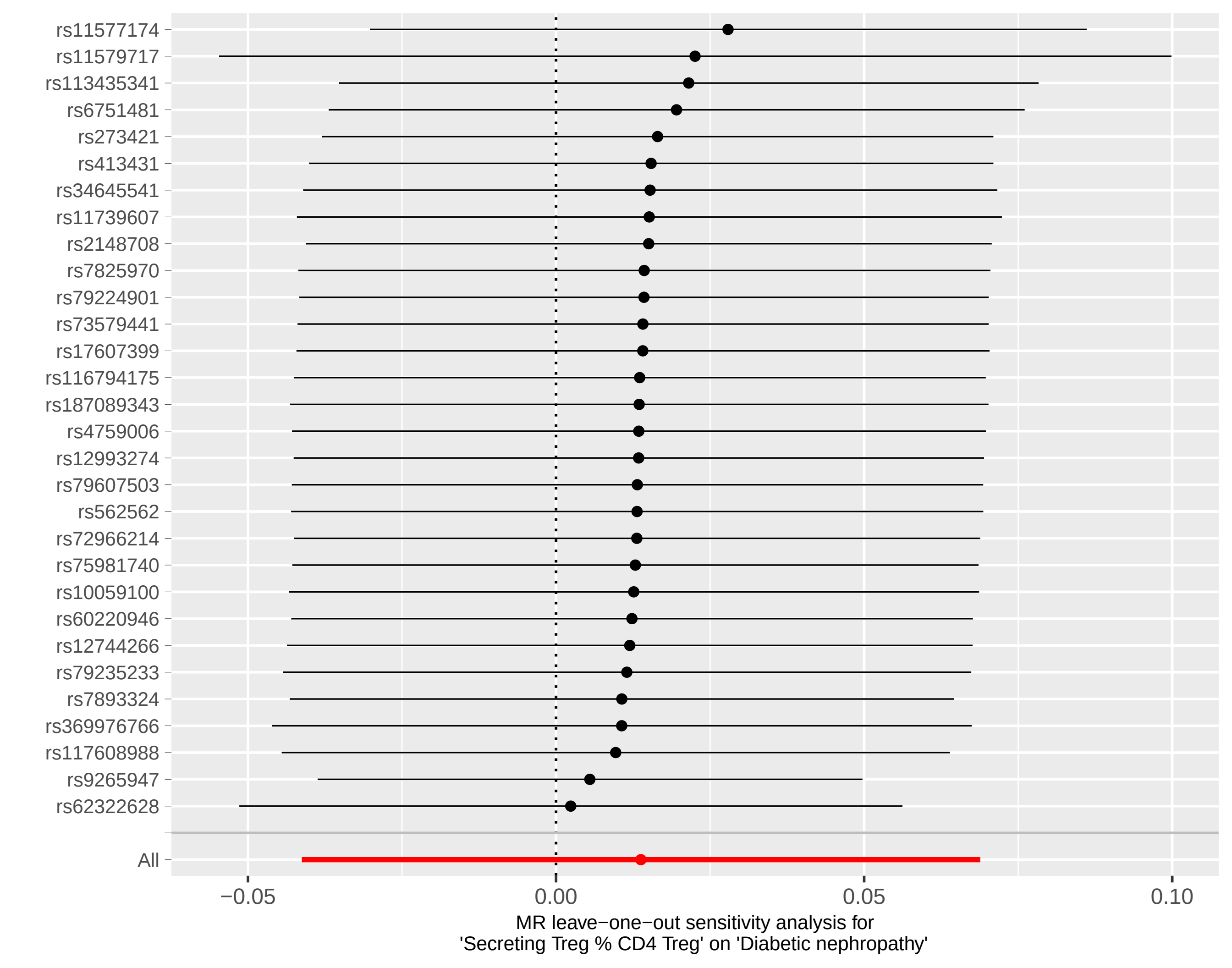
rs11577174
rs11579717
rs113435341
rs6751481
rs273421
rs413431
rs34645541
rs11739607
rs2148708
rs7825970
rs79224901
rs73579441
rs17607399
rs116794175
rs187089343
rs4759006
rs12993274
rs79607503
rs562562
rs72966214
rs75981740
rs10059100
rs60220946
rs12744266
rs79235233
rs7893324
rs369976766
rs117608988
rs9265947
rs62322628
All
−0.05
0.00
0.05
0.10
MR leave−one−out sensitivity analysis for
'Secreting Treg % CD4 Treg' on 'Diabetic nephropathy'

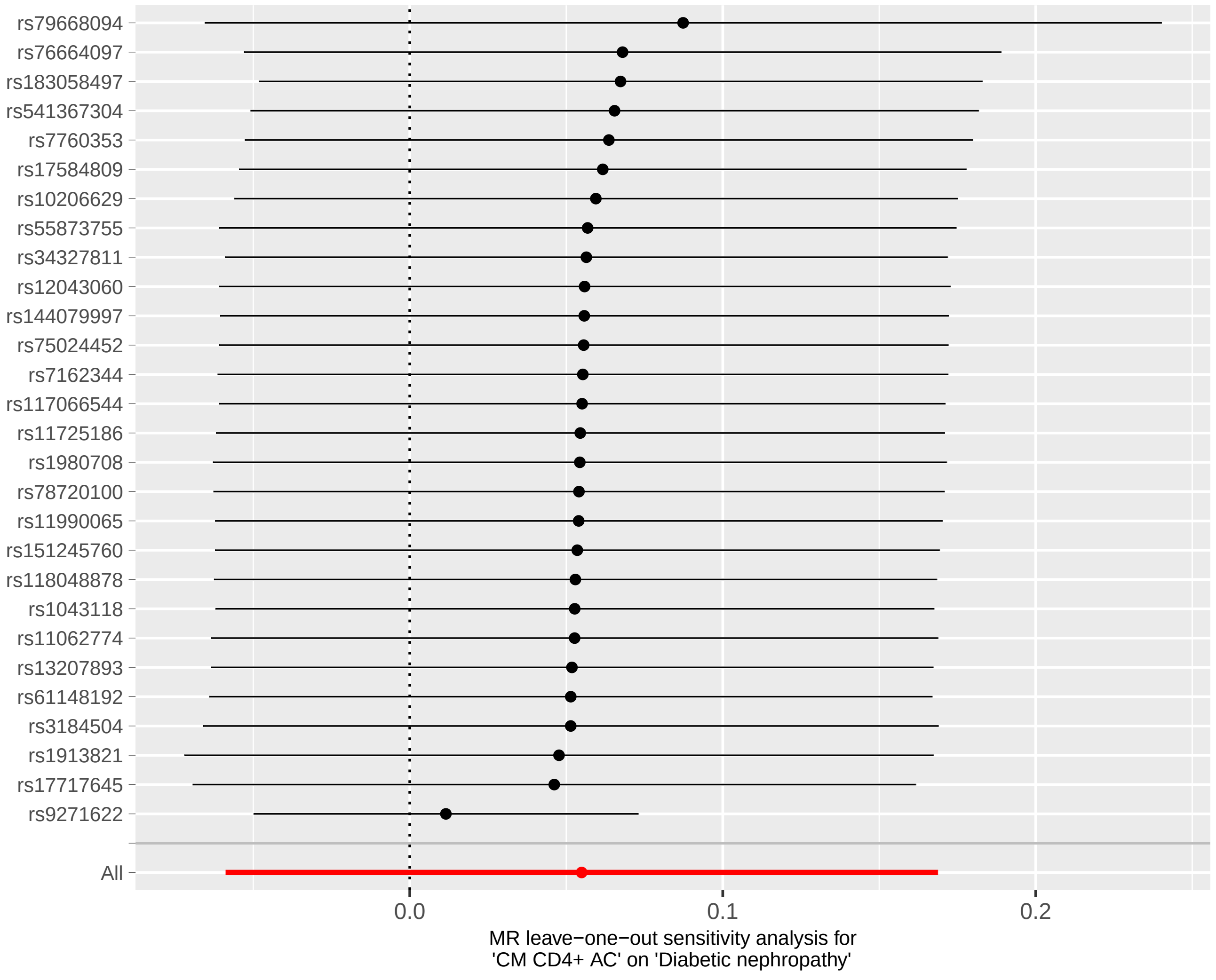
rs79668094
rs76664097
rs183058497
rs541367304
rs7760353
rs17584809
rs10206629
rs55873755
rs34327811
rs12043060
rs144079997
rs75024452
rs7162344
rs117066544
rs11725186
rs1980708
rs78720100
rs11990065
rs151245760
rs118048878
rs1043118
rs11062774
rs13207893
rs61148192
rs3184504
rs1913821
rs17717645
rs9271622
All
0.0
0.1
0.2
MR leave−one−out sensitivity analysis for
'CM CD4+ AC' on 'Diabetic nephropathy'

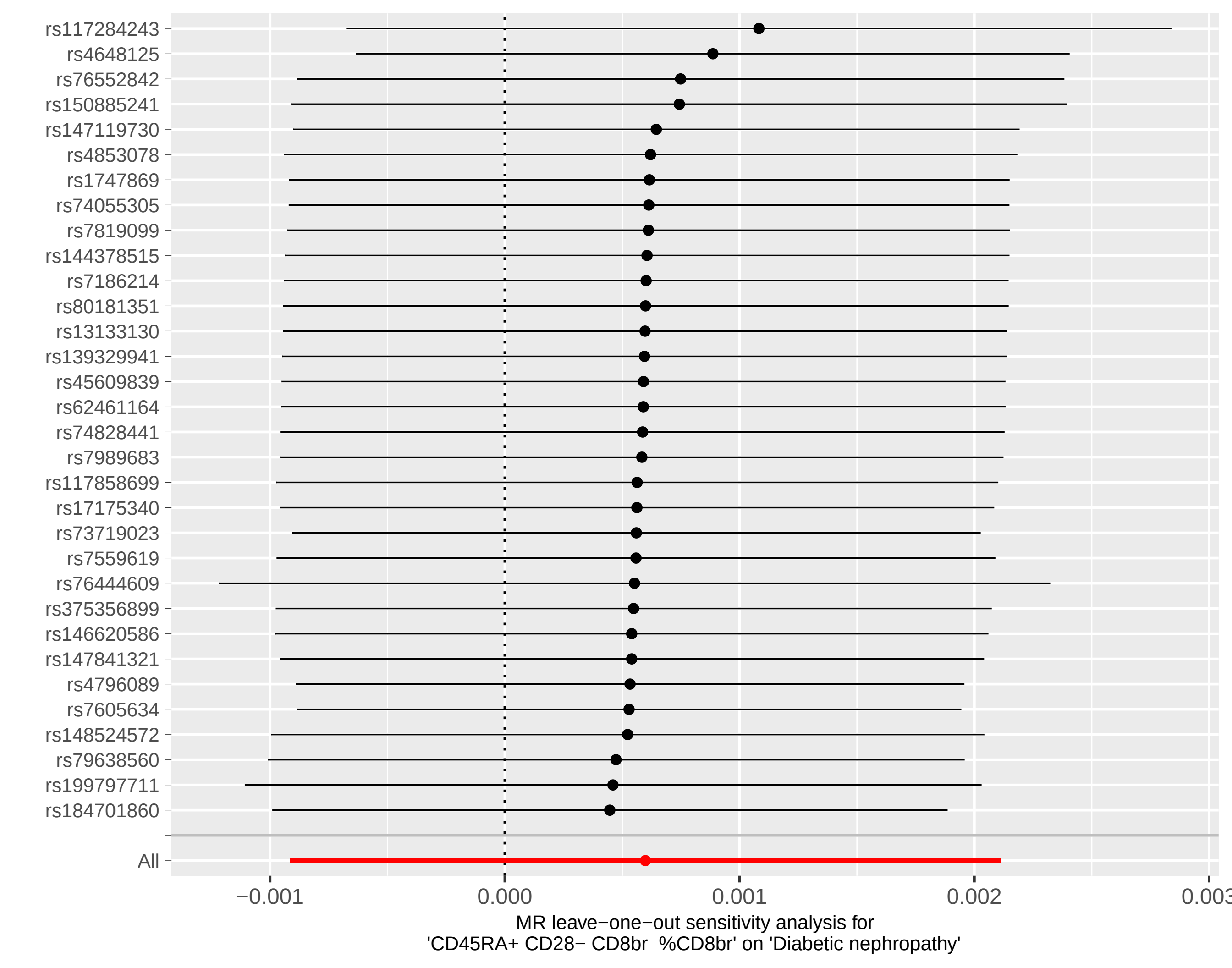
rs117284243
rs4648125
rs76552842
rs150885241
rs147119730
rs4853078
rs1747869
rs74055305
rs7819099
rs144378515
rs7186214
rs80181351
rs13133130
rs139329941
rs45609839
rs62461164
rs74828441
rs7989683
rs117858699
rs17175340
rs73719023
rs7559619
rs76444609
rs375356899
rs146620586
rs147841321
rs4796089
rs7605634
rs148524572
rs79638560
rs199797711
rs184701860
All
−0.001
0.000
0.001
0.002
0.003
MR leave−one−out sensitivity analysis for
'CD45RA+ CD28− CD8br %CD8br' on 'Diabetic nephropathy'

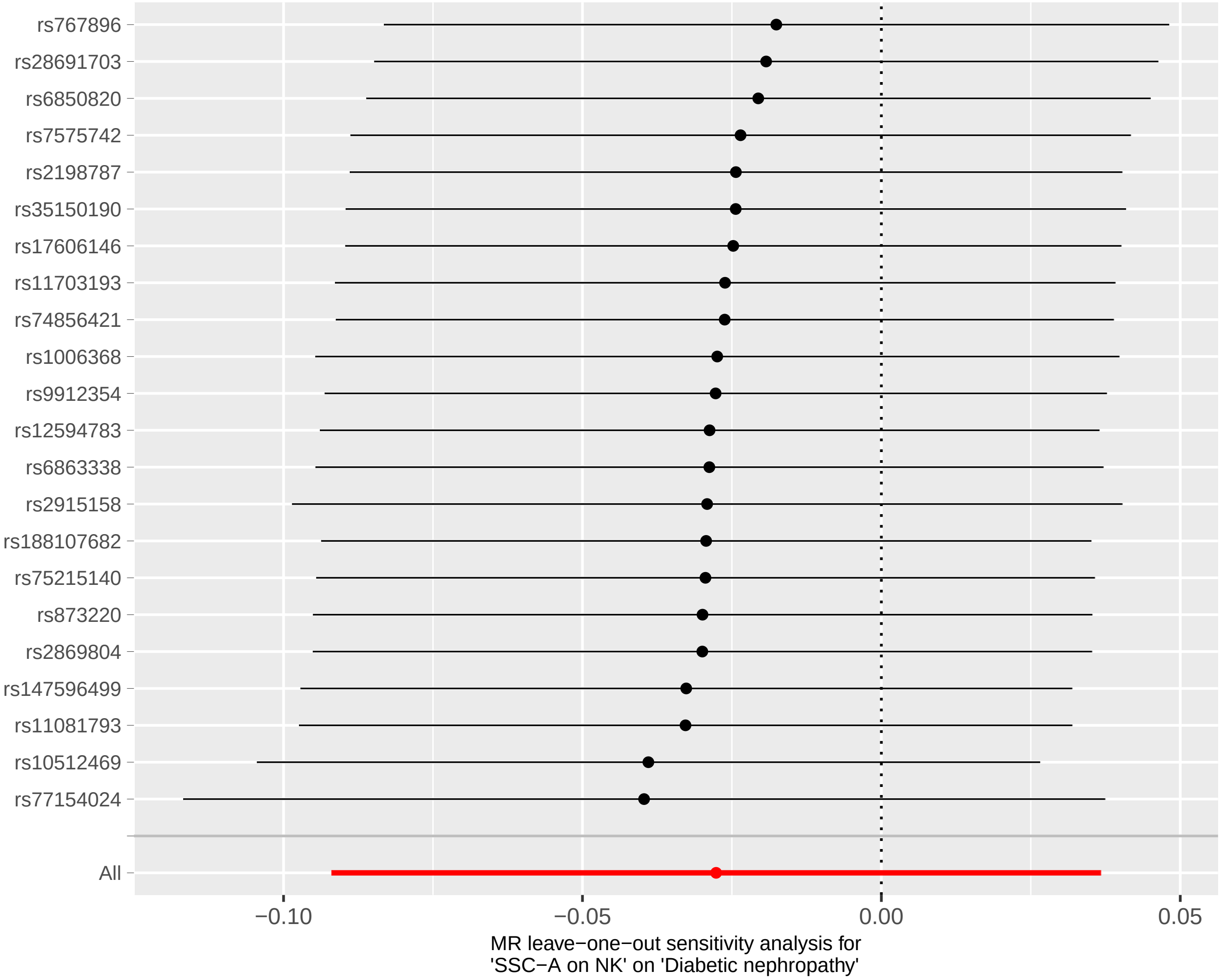

rs767896
rs28691703
rs6850820
rs7575742
rs2198787
rs35150190
rs17606146
rs11703193
rs74856421
rs1006368
rs9912354
rs12594783
rs6863338
rs2915158
rs188107682
rs75215140
rs873220
rs2869804
rs147596499
rs11081793
rs10512469
rs77154024
All
−0.10
−0.05
0.00
0.05
MR leave−one−out sensitivity analysis for
'SSC−A on NK' on 'Diabetic nephropathy'

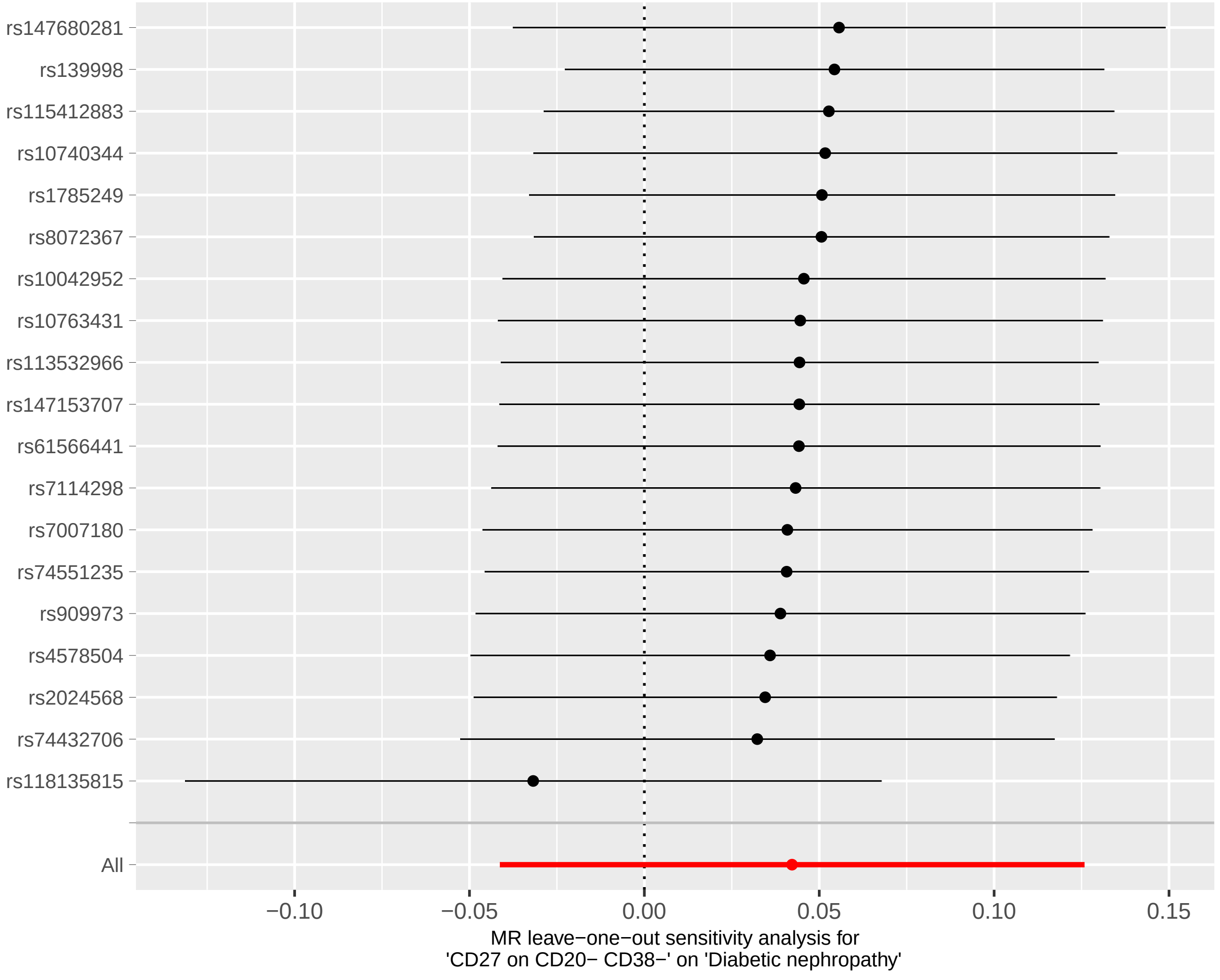
rs147680281
rs139998
rs115412883
rs10740344
rs1785249
rs8072367
rs10042952
rs10763431
rs113532966
rs147153707
rs61566441
rs7114298
rs7007180
rs74551235
rs909973
rs4578504
rs2024568
rs74432706
rs118135815
All
−0.10
−0.05
0.00
0.05
0.10
0.15
MR leave−one−out sensitivity analysis for
'CD27 on CD20− CD38−' on 'Diabetic nephropathy'

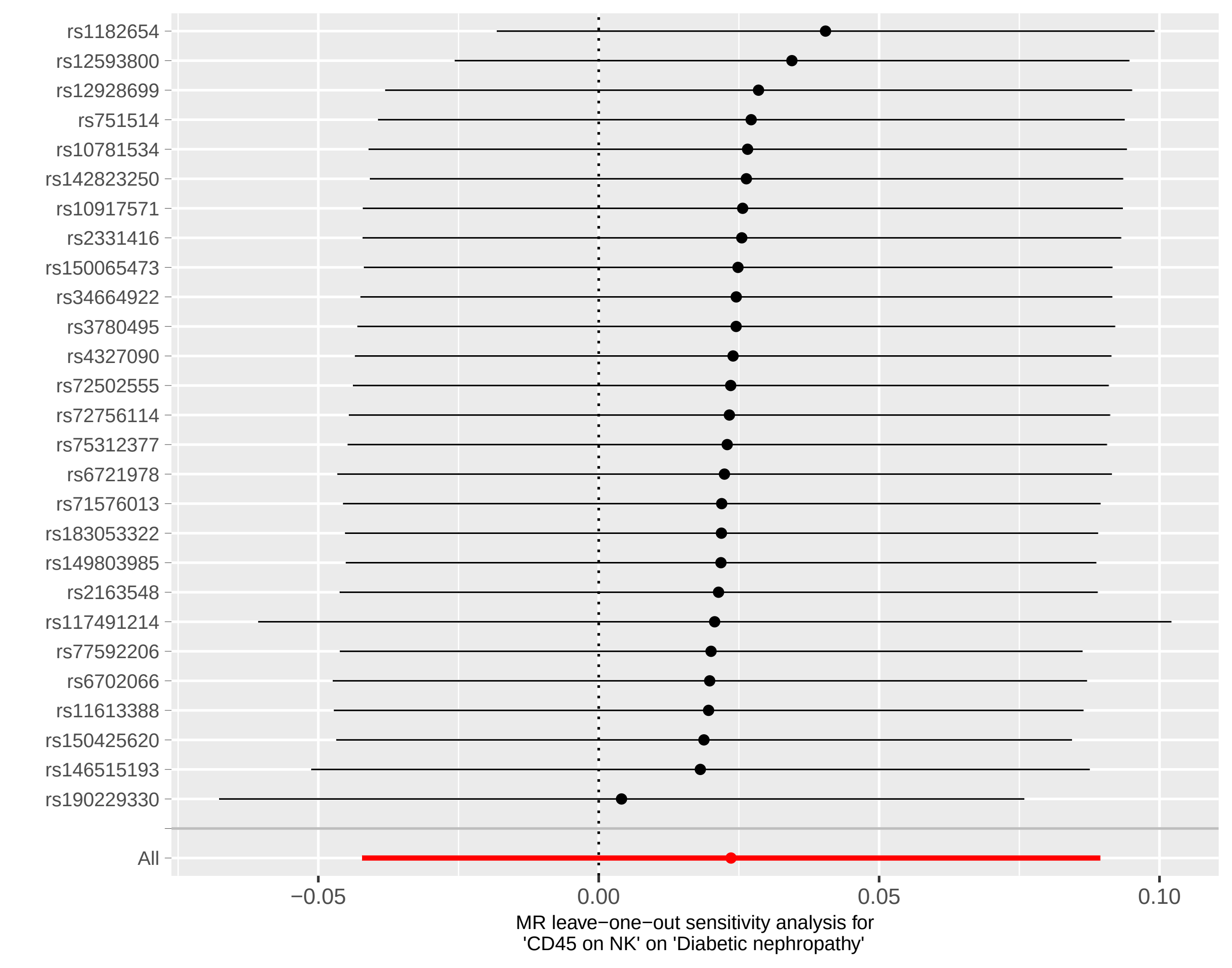

rs1182654
rs12593800
rs12928699
rs751514
rs10781534
rs142823250
rs10917571
rs2331416
rs150065473
rs34664922
rs3780495
rs4327090
rs72502555
rs72756114
rs75312377
rs6721978
rs71576013
rs183053322
rs149803985
rs2163548
rs117491214
rs77592206
rs6702066
rs11613388
rs150425620
rs146515193
rs190229330
All
−0.05
0.00
0.05
0.10
MR leave−one−out sensitivity analysis for
'CD45 on NK' on 'Diabetic nephropathy'

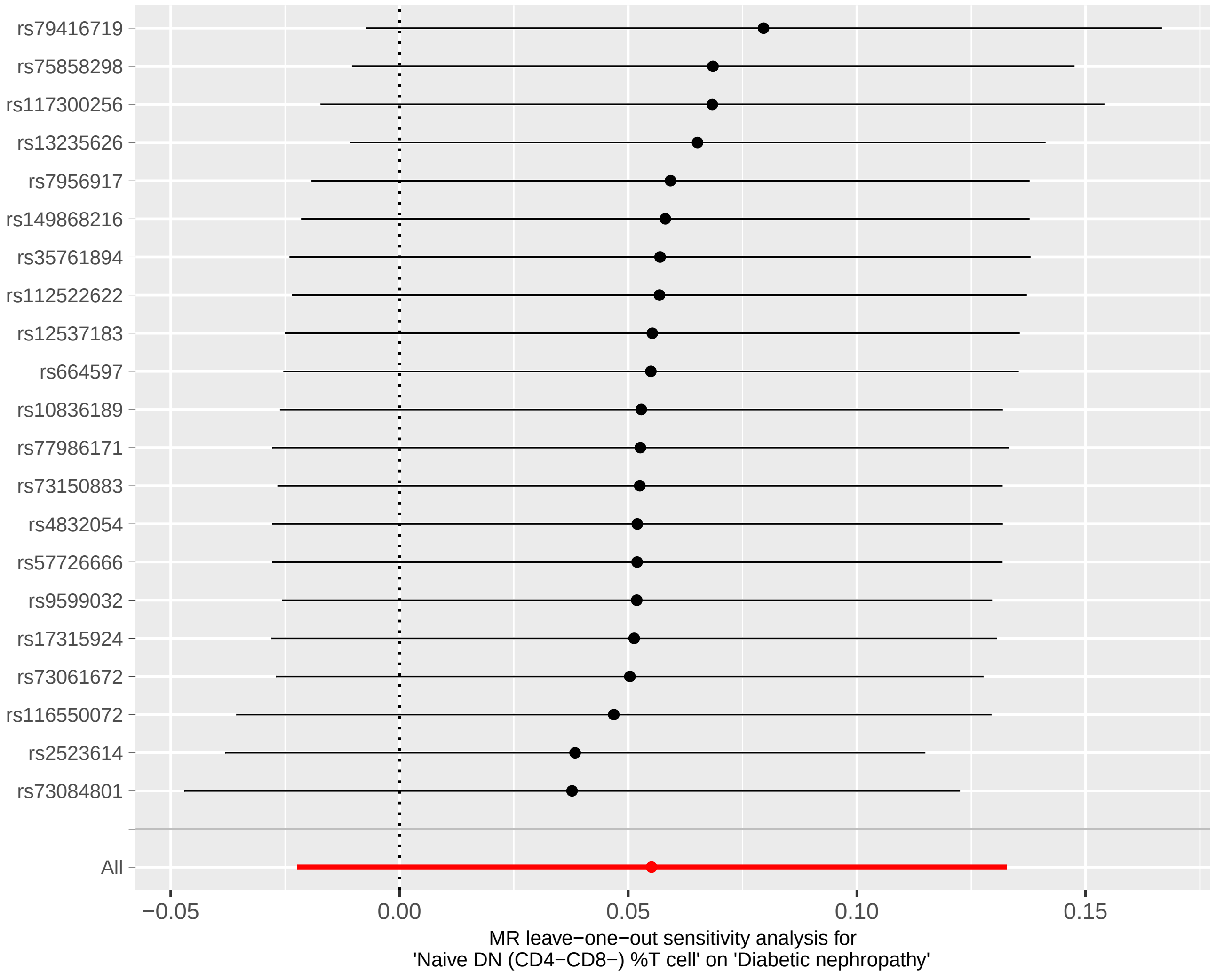

rs79416719
rs75858298
rs117300256
rs13235626
rs7956917
rs149868216
rs35761894
rs112522622
rs12537183
rs664597
rs10836189
rs77986171
rs73150883
rs4832054
rs57726666
rs9599032
rs17315924
rs73061672
rs116550072
rs2523614
rs73084801
All
−0.05
0.00
0.05
0.10
0.15
MR leave−one−out sensitivity analysis for
'Naive DN (CD4−CD8−) %T cell' on 'Diabetic nephropathy'

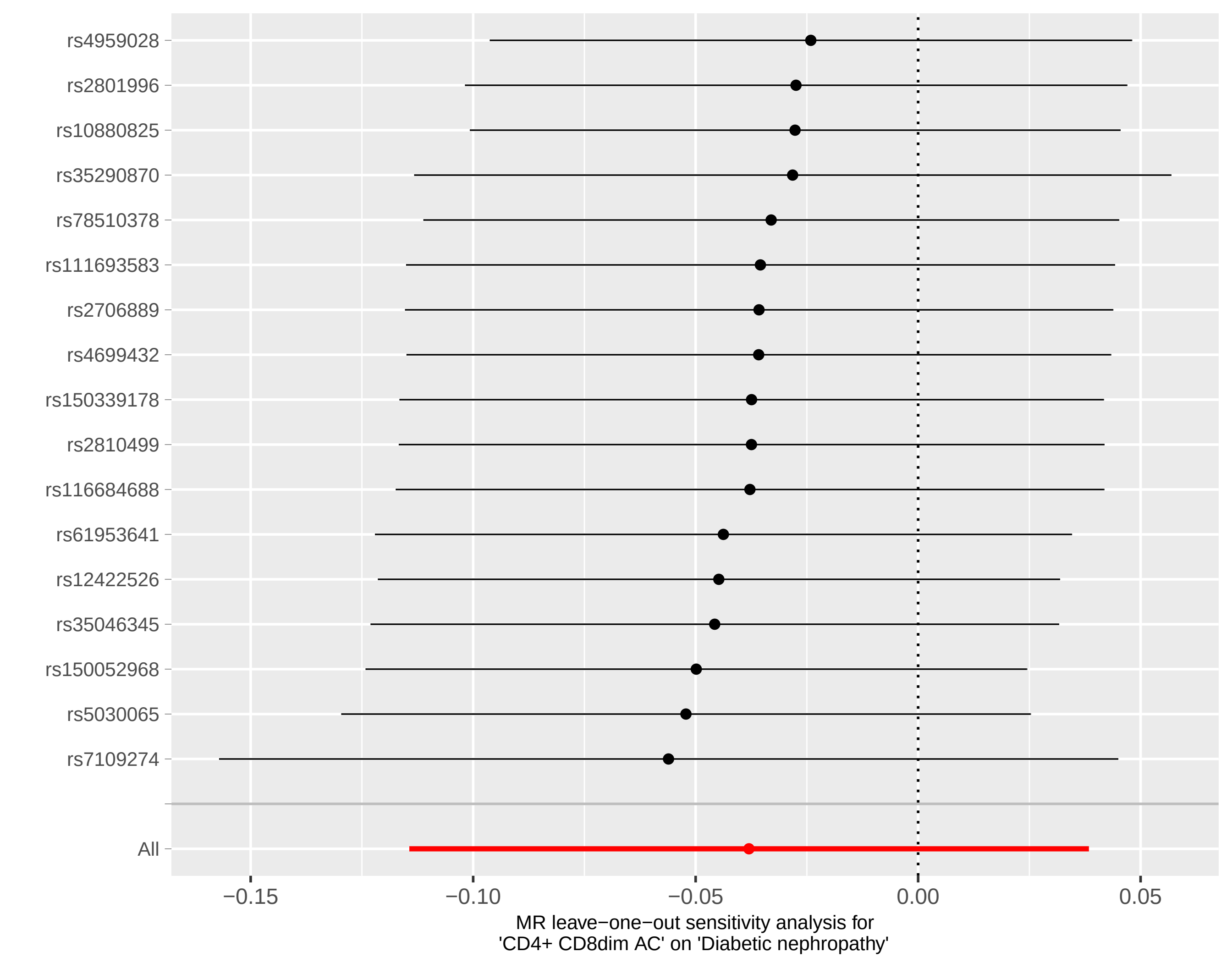

rs4959028
rs2801996
rs10880825
rs35290870
rs78510378
rs111693583
rs2706889
rs4699432
rs150339178
rs2810499
rs116684688
rs61953641
rs12422526
rs35046345
rs150052968
rs5030065
rs7109274
All
−0.15
−0.10
−0.05
0.00
0.05
MR leave−one−out sensitivity analysis for
'CD4+ CD8dim AC' on 'Diabetic nephropathy'

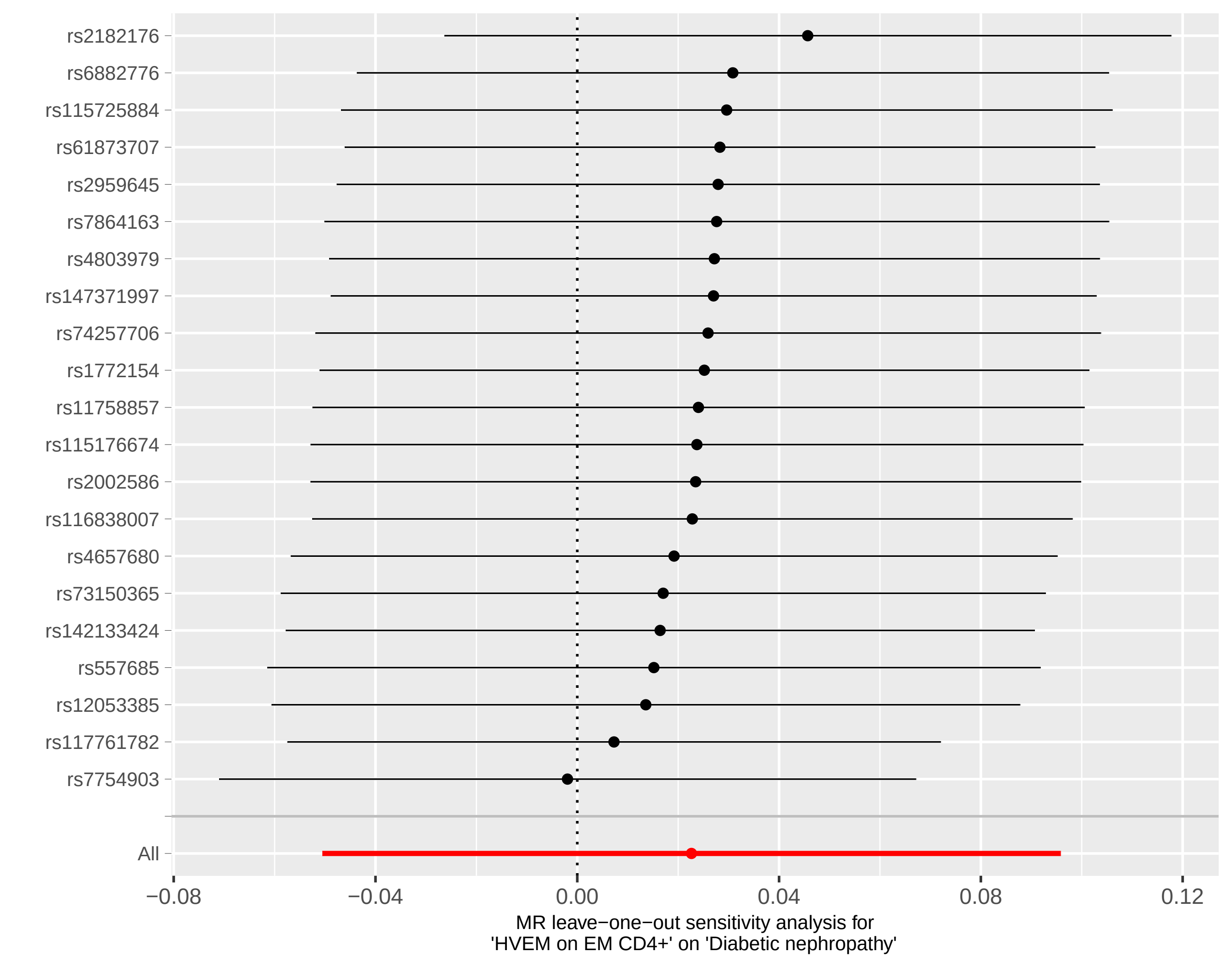
rs2182176
rs6882776
rs115725884
rs61873707
rs2959645
rs7864163
rs4803979
rs147371997
rs74257706
rs1772154
rs11758857
rs115176674
rs2002586
rs116838007
rs4657680
rs73150365
rs142133424
rs557685
rs12053385
rs117761782
rs7754903
All
−0.08
−0.04
0.00
0.04
0.08
0.12
MR leave−one−out sensitivity analysis for
'HVEM on EM CD4+' on 'Diabetic nephropathy'

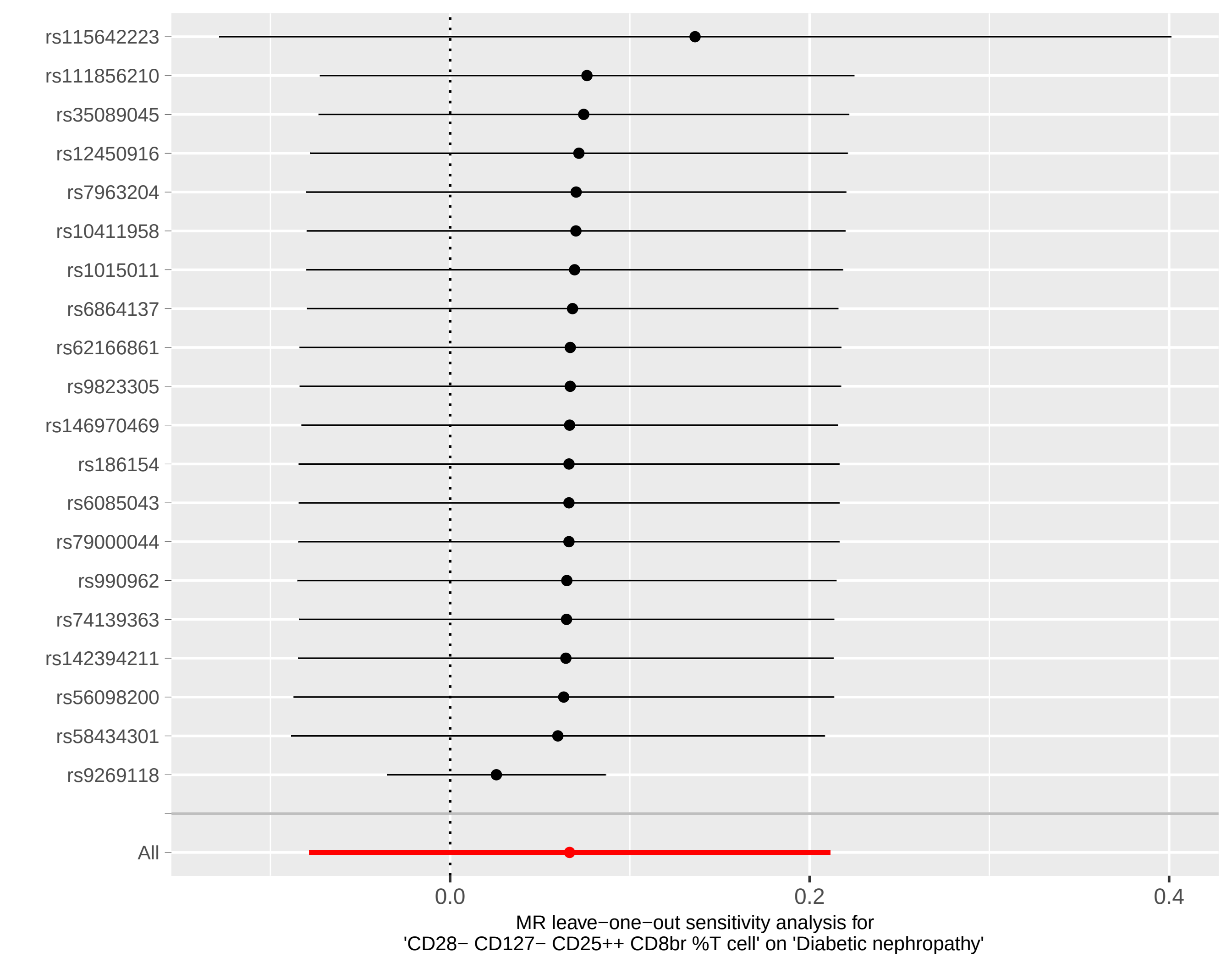
rs115642223
rs111856210
rs35089045
rs12450916
rs7963204
rs10411958
rs1015011
rs6864137
rs62166861
rs9823305
rs146970469
rs186154
rs6085043
rs79000044
rs990962
rs74139363
rs142394211
rs56098200
rs58434301
rs9269118
All
0.0
0.2
0.4
MR leave−one−out sensitivity analysis for
'CD28− CD127− CD25++ CD8br %T cell' on 'Diabetic nephropathy'

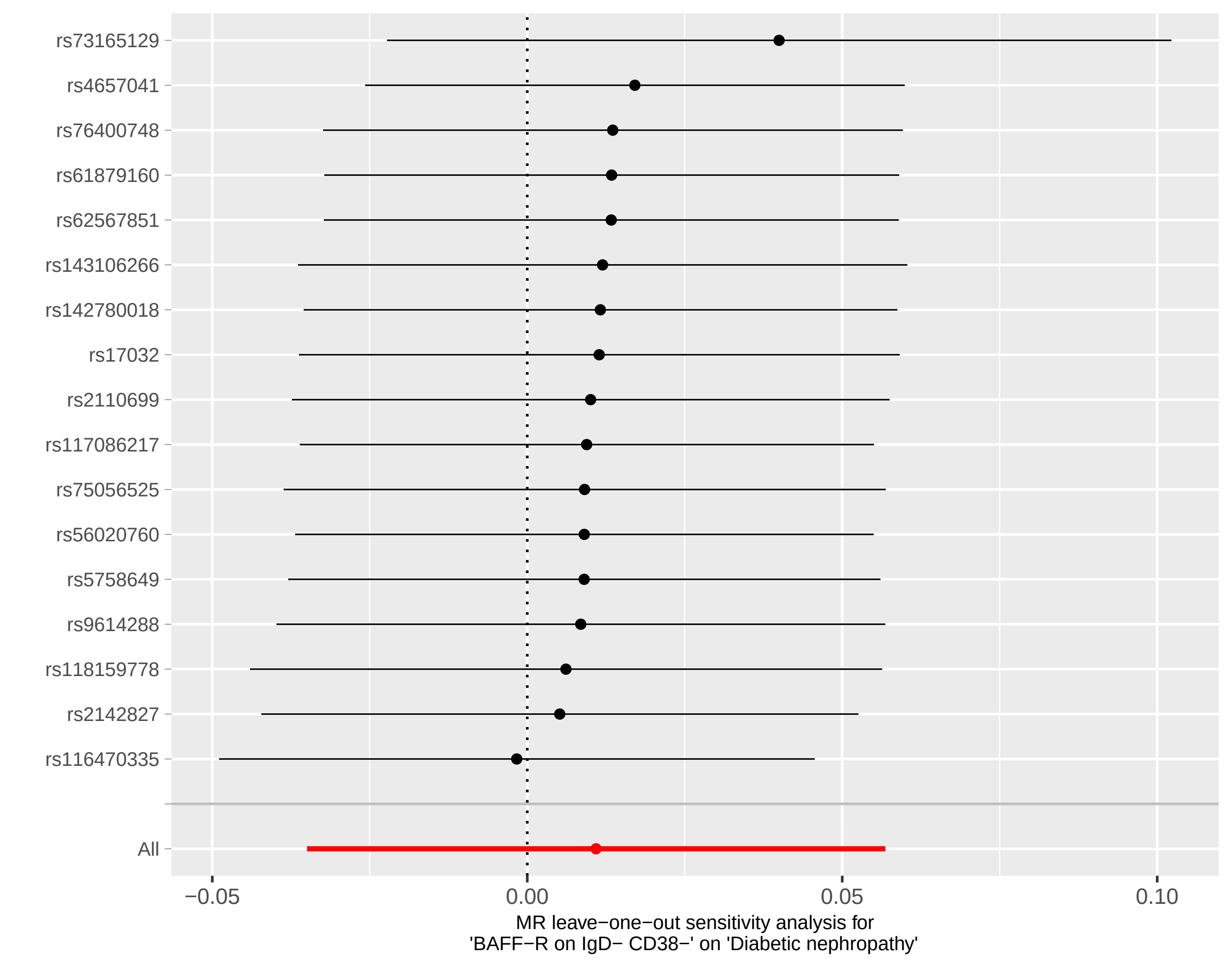

rs73165129
rs4657041
rs76400748
rs61879160
rs62567851
rs143106266
rs142780018
rs17032
rs2110699
rs117086217
rs75056525
rs56020760
rs5758649
rs9614288
rs118159778
rs2142827
rs116470335
All
−0.05
0.00
0.05
0.10
MR leave−one−out sensitivity analysis for
'BAFF−R on IgD− CD38−' on 'Diabetic nephropathy'

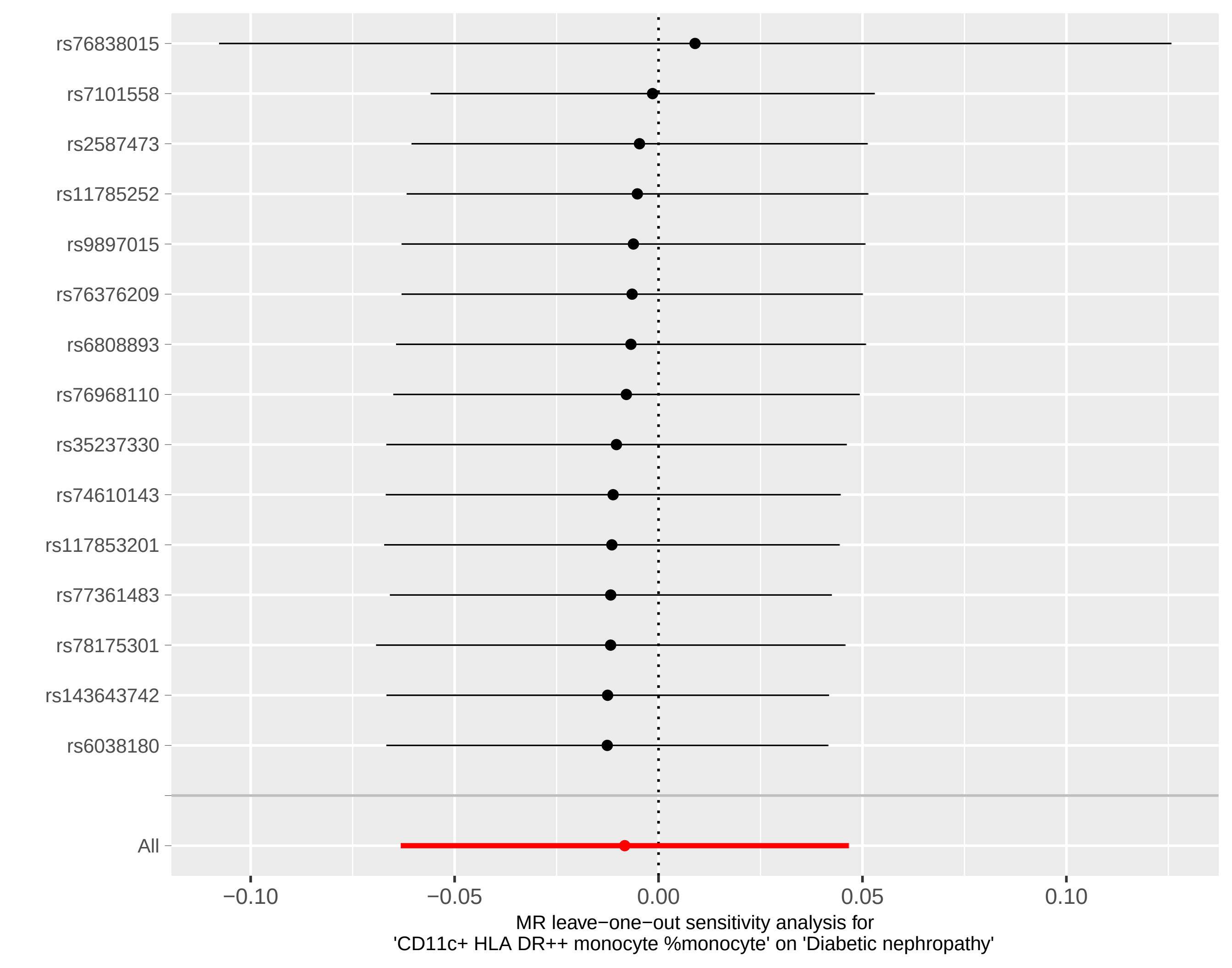
rs76838015
rs7101558
rs2587473
rs11785252
rs9897015
rs76376209
rs6808893
rs76968110
rs35237330
rs74610143
rs117853201
rs77361483
rs78175301
rs143643742
rs6038180
All
−0.10
−0.05
0.00
0.05
0.10
MR leave−one−out sensitivity analysis for
'CD11c+ HLA DR++ monocyte %monocyte' on 'Diabetic nephropathy'

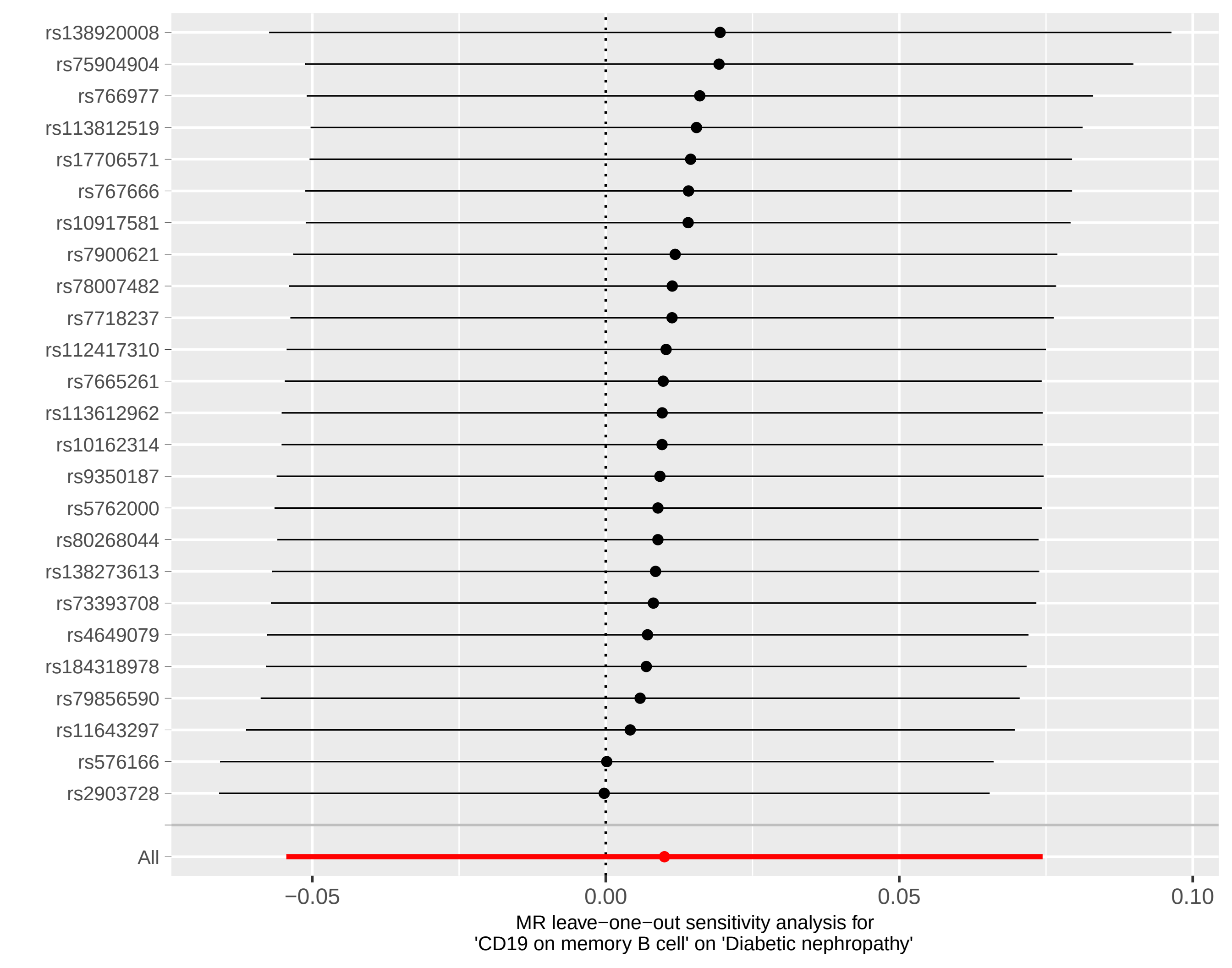
rs138920008
rs75904904
rs766977
rs113812519
rs17706571
rs767666
rs10917581
rs7900621
rs78007482
rs7718237
rs112417310
rs7665261
rs113612962
rs10162314
rs9350187
rs5762000
rs80268044
rs138273613
rs73393708
rs4649079
rs184318978
rs79856590
rs11643297
rs576166
rs2903728
All
−0.05
0.00
0.05
0.10
MR leave−one−out sensitivity analysis for
'CD19 on memory B cell' on 'Diabetic nephropathy'

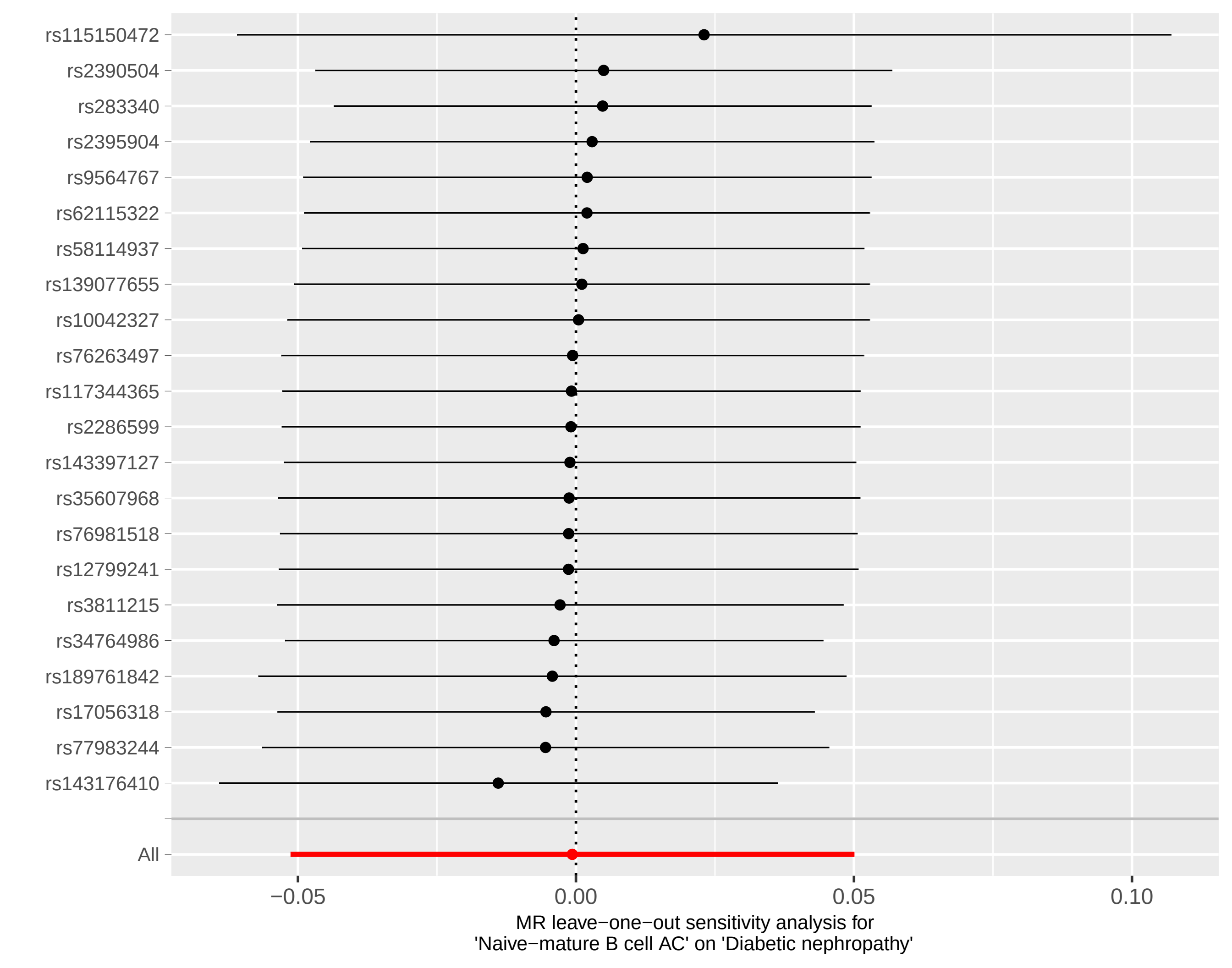
rs115150472
rs2390504
rs283340
rs2395904
rs9564767
rs62115322
rs58114937
rs139077655
rs10042327
rs76263497
rs117344365
rs2286599
rs143397127
rs35607968
rs76981518
rs12799241
rs3811215
rs34764986
rs189761842
rs17056318
rs77983244
rs143176410
All
−0.05
0.00
0.05
0.10
MR leave−one−out sensitivity analysis for
'Naive−mature B cell AC' on 'Diabetic nephropathy'

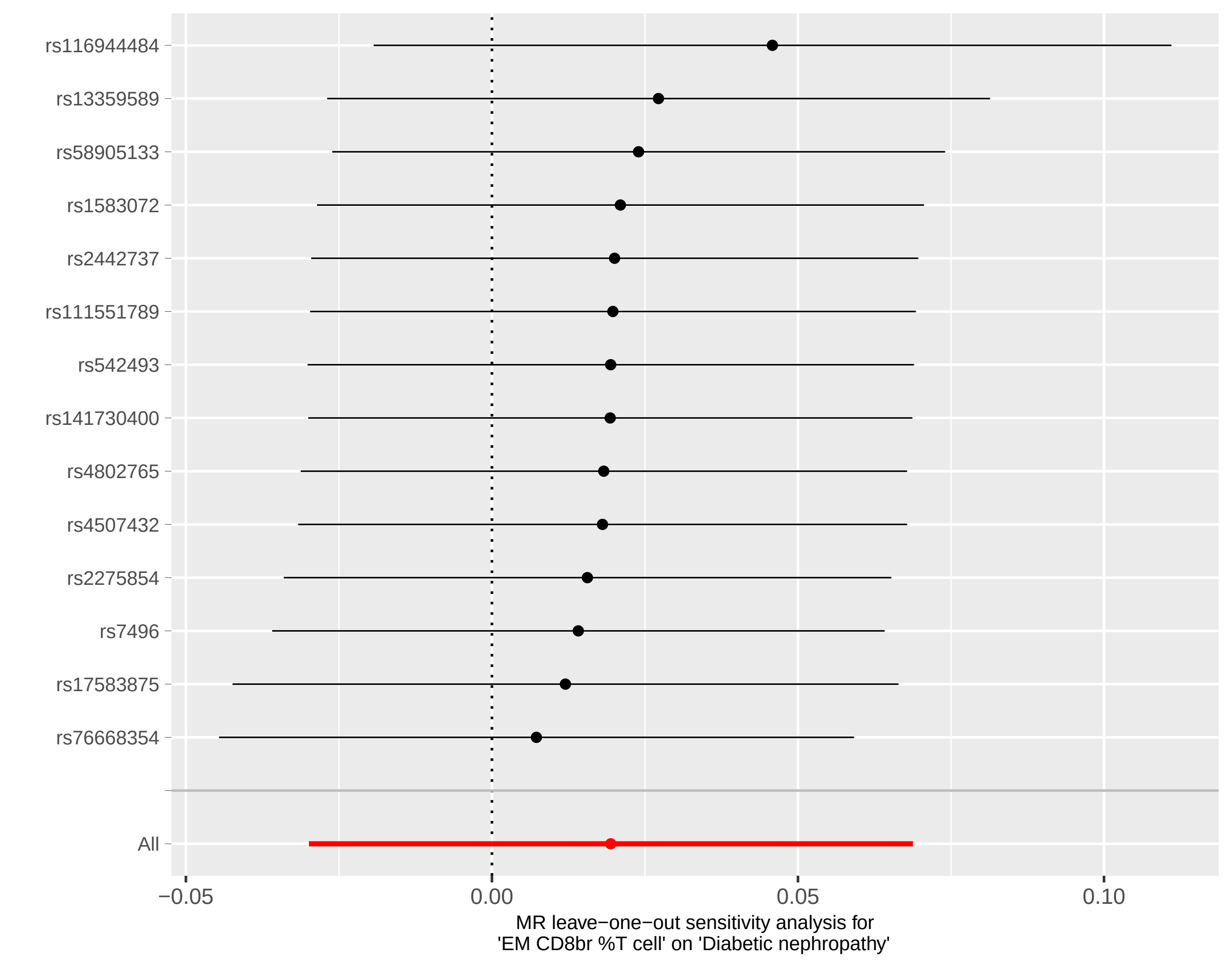
rs116944484
rs13359589
rs58905133
rs1583072
rs2442737
rs111551789
rs542493
rs141730400
rs4802765
rs4507432
rs2275854
rs7496
rs17583875
rs76668354
All
−0.05
0.00
0.05
0.10
MR leave−one−out sensitivity analysis for
'EM CD8br %T cell' on 'Diabetic nephropathy'

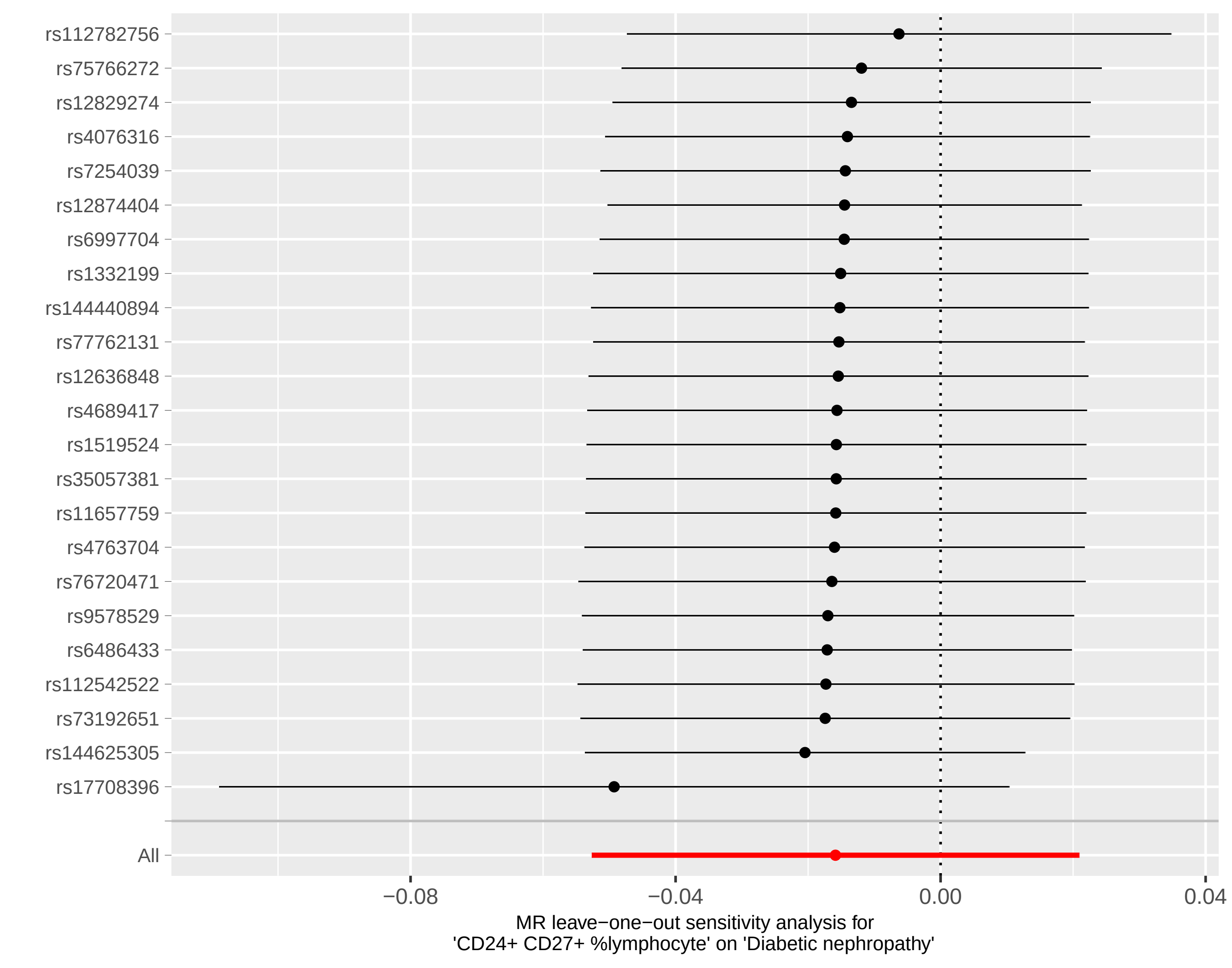
rs112782756
rs75766272
rs12829274
rs4076316
rs7254039
rs12874404
rs6997704
rs1332199
rs144440894
rs77762131
rs12636848
rs4689417
rs1519524
rs35057381
rs11657759
rs4763704
rs76720471
rs9578529
rs6486433
rs112542522
rs73192651
rs144625305
rs17708396
All
−0.08
−0.04
0.00
0.04
MR leave−one−out sensitivity analysis for
'CD24+ CD27+ %lymphocyte' on 'Diabetic nephropathy'

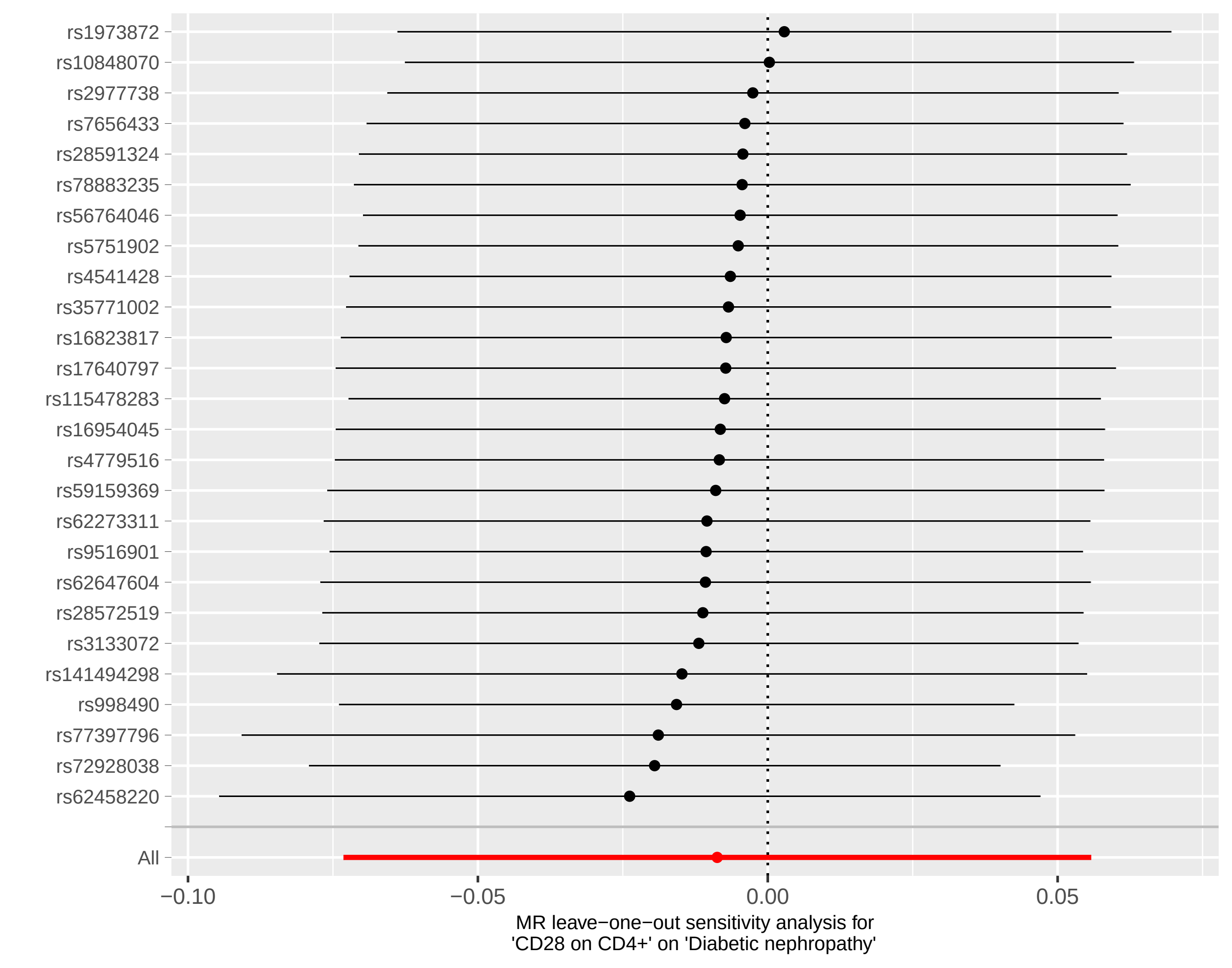
rs1973872
rs10848070
rs2977738
rs7656433
rs28591324
rs78883235
rs56764046
rs5751902
rs4541428
rs35771002
rs16823817
rs17640797
rs115478283
rs16954045
rs4779516
rs59159369
rs62273311
rs9516901
rs62647604
rs28572519
rs3133072
rs141494298
rs998490
rs77397796
rs72928038
rs62458220
All
−0.10
−0.05
0.00
0.05
MR leave−one−out sensitivity analysis for
'CD28 on CD4+' on 'Diabetic nephropathy'

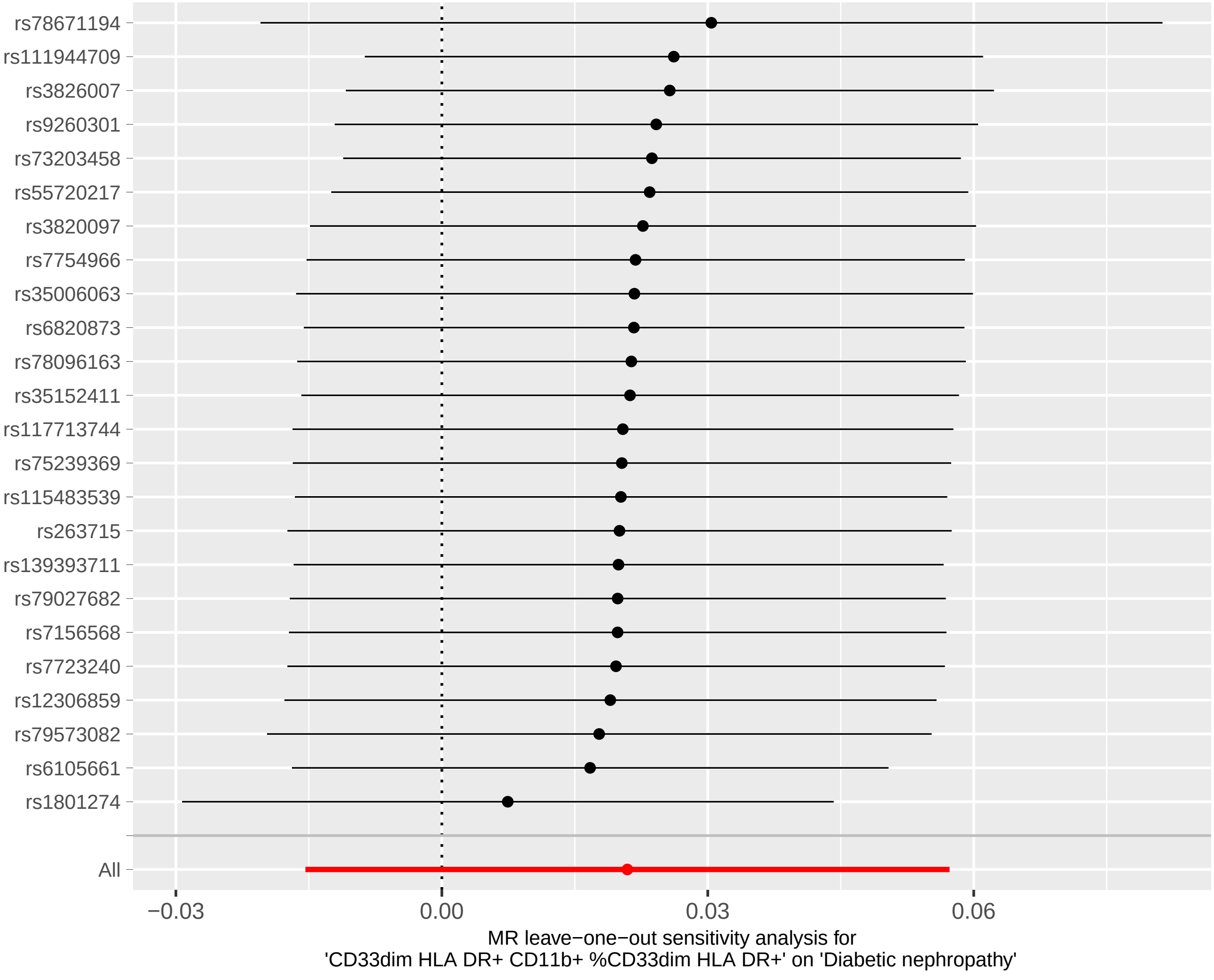
rs78671194
rs111944709
rs3826007
rs9260301
rs73203458
rs55720217
rs3820097
rs7754966
rs35006063
rs6820873
rs78096163
rs35152411
rs117713744
rs75239369
rs115483539
rs263715
rs139393711
rs79027682
rs7156568
rs7723240
rs12306859
rs79573082
rs6105661
rs1801274
All
−0.03
0.00
0.03
0.06
MR leave−one−out sensitivity analysis for
'CD33dim HLA DR+ CD11b+ %CD33dim HLA DR+' on 'Diabetic nephropathy'

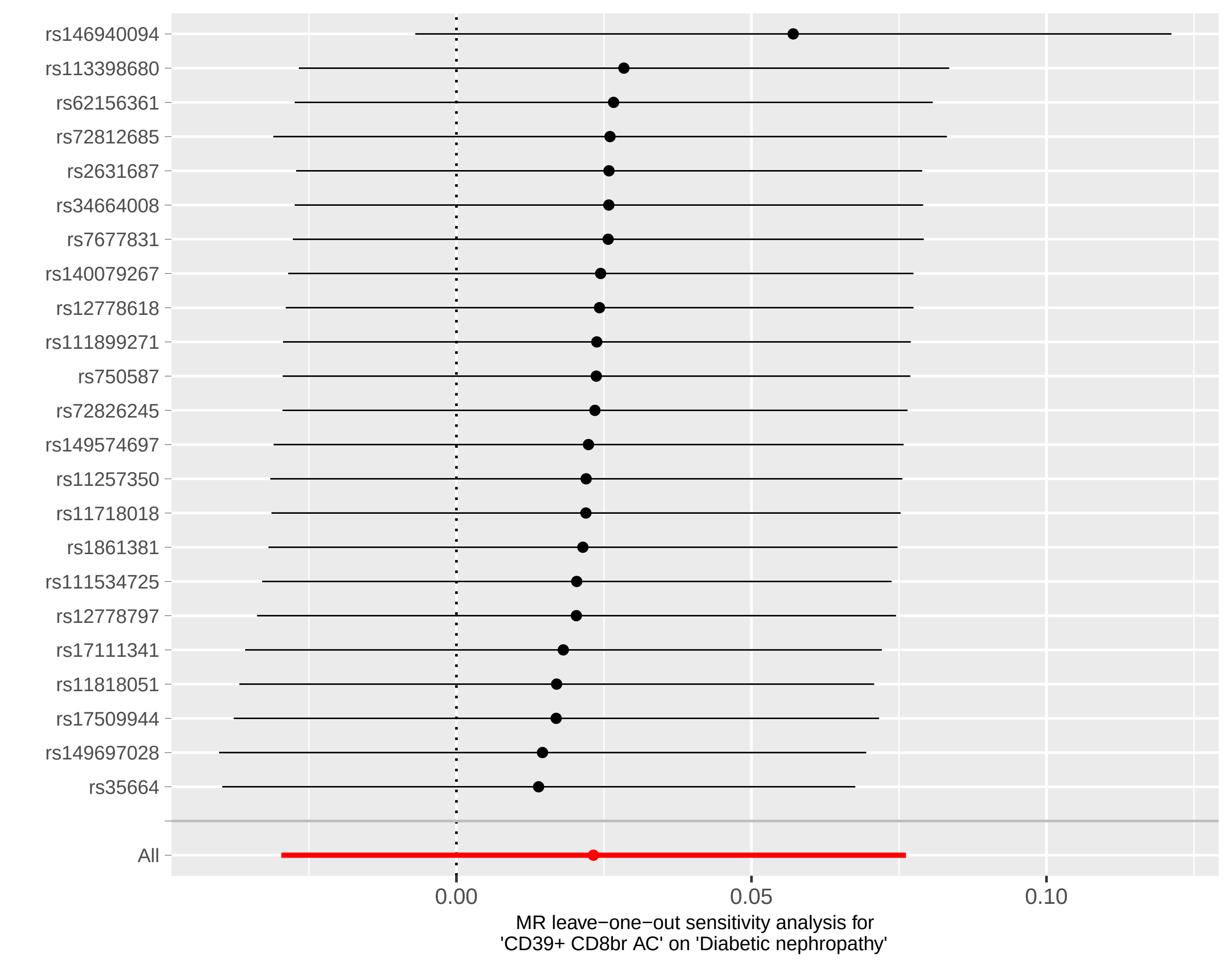
rs146940094
rs113398680
rs62156361
rs72812685
rs2631687
rs34664008
rs7677831
rs140079267
rs12778618
rs111899271
rs750587
rs72826245
rs149574697
rs11257350
rs11718018
rs1861381
rs111534725
rs12778797
rs17111341
rs11818051
rs17509944
rs149697028
rs35664
All
0.00
0.05
0.10
MR leave−one−out sensitivity analysis for
'CD39+ CD8br AC' on 'Diabetic nephropathy'

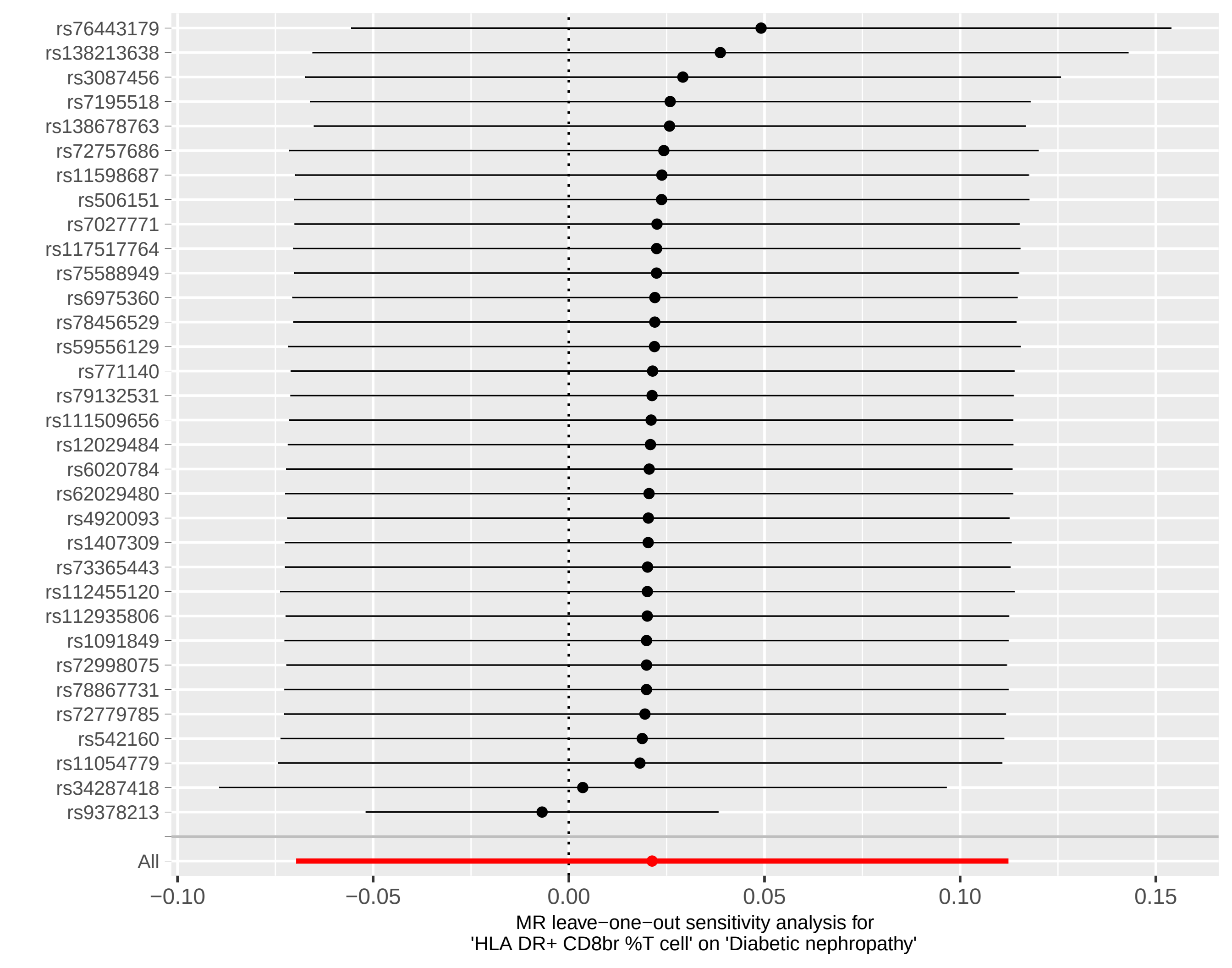
rs76443179
rs138213638
rs3087456
rs7195518
rs138678763
rs72757686
rs11598687
rs506151
rs7027771
rs117517764
rs75588949
rs6975360
rs78456529
rs59556129
rs771140
rs79132531
rs111509656
rs12029484
rs6020784
rs62029480
rs4920093
rs1407309
rs73365443
rs112455120
rs112935806
rs1091849
rs72998075
rs78867731
rs72779785
rs542160
rs11054779
rs34287418
rs9378213
All
−0.10
−0.05
0.00
0.05
0.10
0.15
MR leave−one−out sensitivity analysis for
'HLA DR+ CD8br %T cell' on 'Diabetic nephropathy'

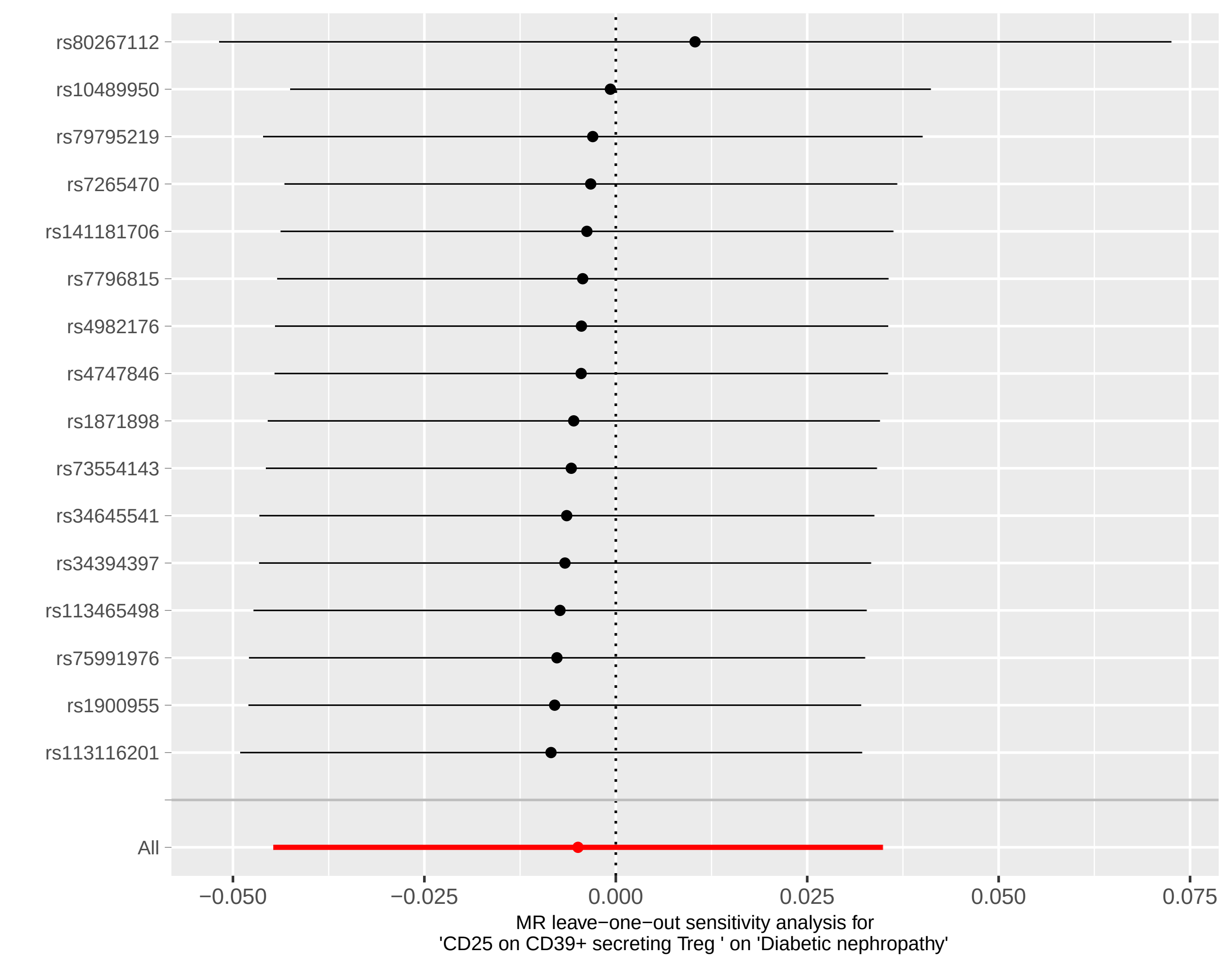
rs80267112
rs10489950
rs79795219
rs7265470
rs141181706
rs7796815
rs4982176
rs4747846
rs1871898
rs73554143
rs34645541
rs34394397
rs113465498
rs75991976
rs1900955
rs113116201
All
−0.050
−0.025
0.000
0.025
0.050
0.075
MR leave−one−out sensitivity analysis for
'CD25 on CD39+ secreting Treg ' on 'Diabetic nephropathy'

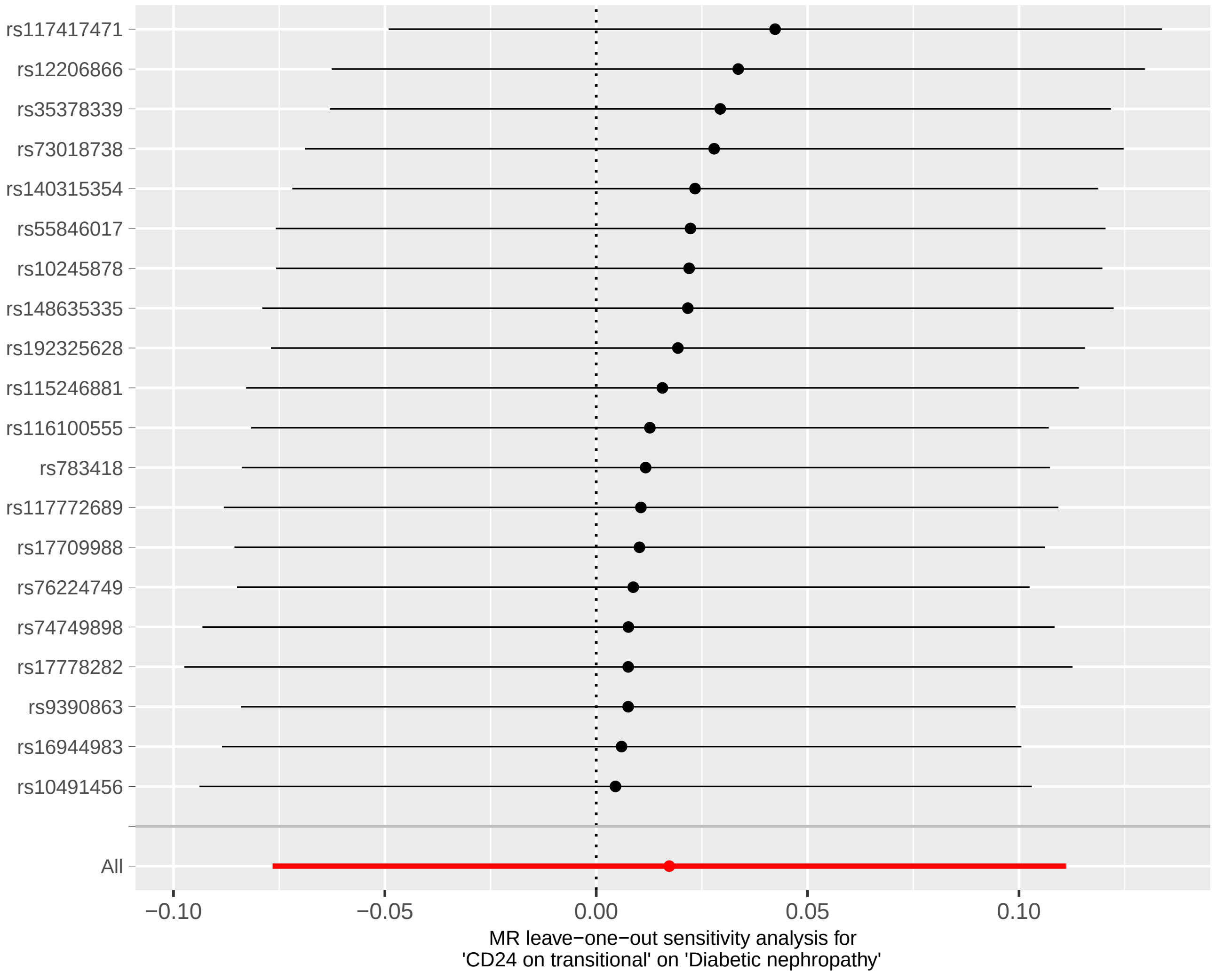
rs117417471
rs12206866
rs35378339
rs73018738
rs140315354
rs55846017
rs10245878
rs148635335
rs192325628
rs115246881
rs116100555
rs783418
rs117772689
rs17709988
rs76224749
rs74749898
rs17778282
rs9390863
rs16944983
rs10491456
All
−0.10
−0.05
0.00
0.05
0.10
MR leave−one−out sensitivity analysis for
'CD24 on transitional' on 'Diabetic nephropathy'

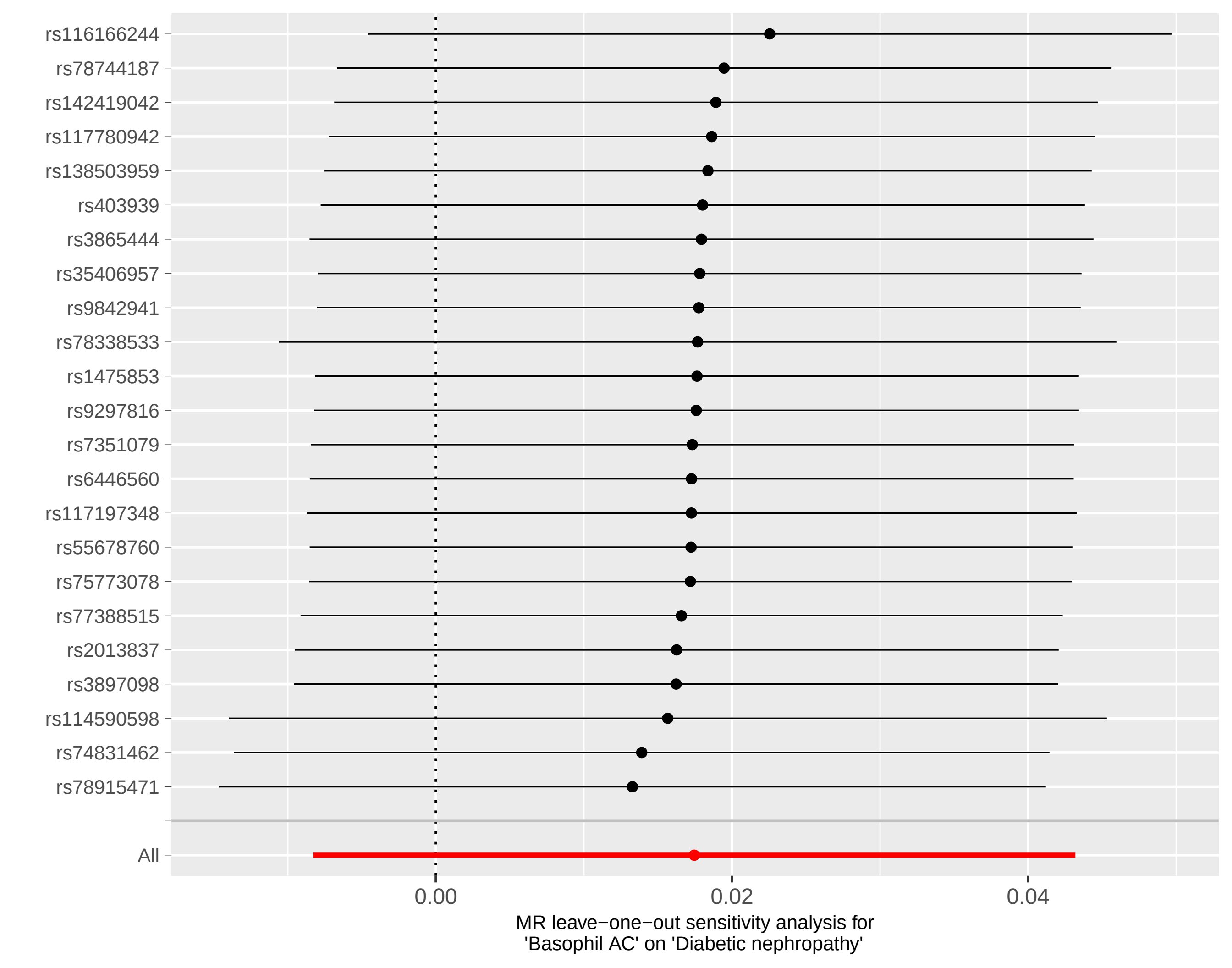

rs116166244
rs78744187
rs142419042
rs117780942
rs138503959
rs403939
rs3865444
rs35406957
rs9842941
rs78338533
rs1475853
rs9297816
rs7351079
rs6446560
rs117197348
rs55678760
rs75773078
rs77388515
rs2013837
rs3897098
rs114590598
rs74831462
rs78915471
All
0.00
0.02
0.04
MR leave−one−out sensitivity analysis for
'Basophil AC' on 'Diabetic nephropathy'

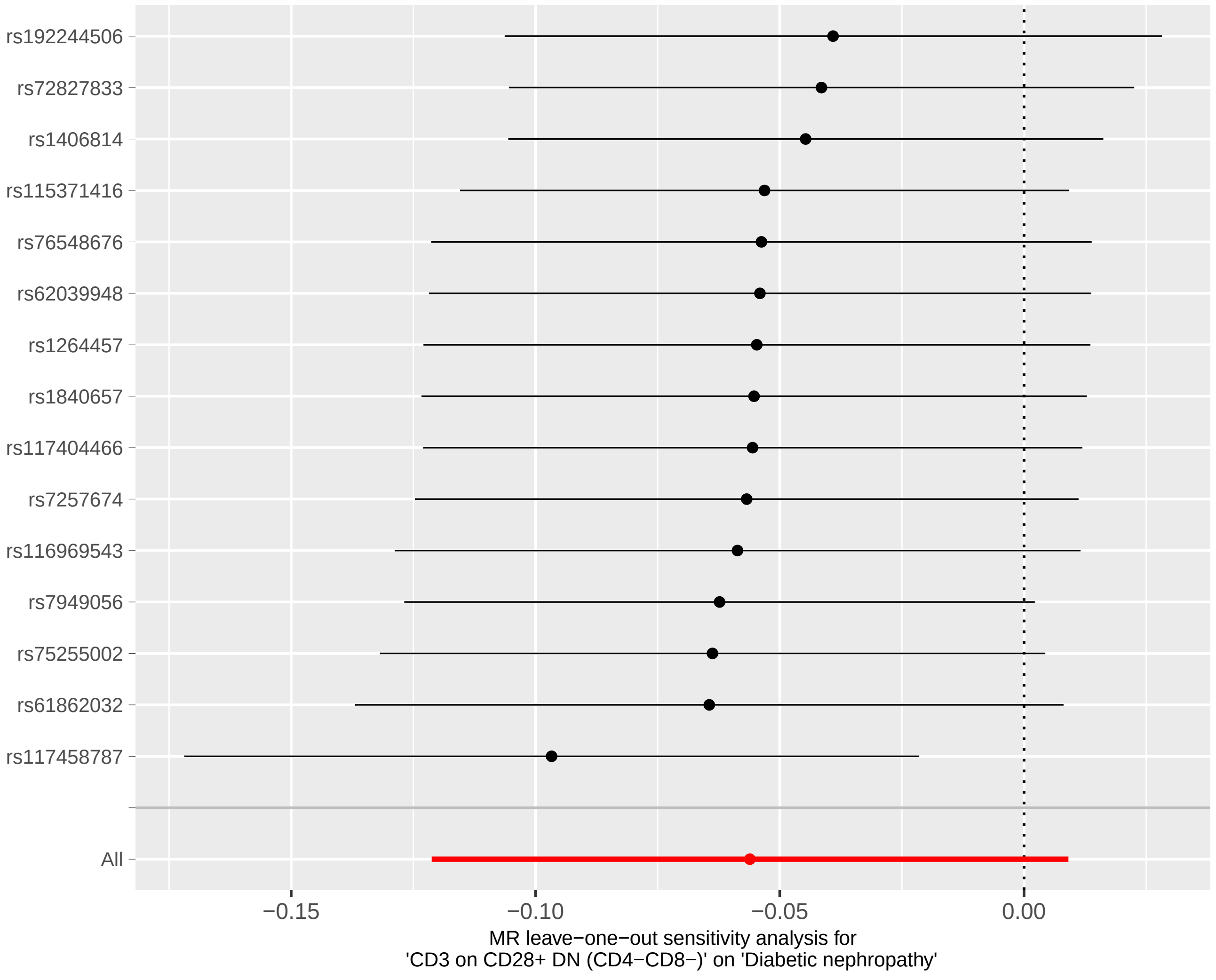
rs192244506
rs72827833
rs1406814
rs115371416
rs76548676
rs62039948
rs1264457
rs1840657
rs117404466
rs7257674
rs116969543
rs7949056
rs75255002
rs61862032
rs117458787
All
−0.15
−0.10
−0.05
0.00
MR leave−one−out sensitivity analysis for
'CD3 on CD28+ DN (CD4−CD8−)' on 'Diabetic nephropathy'

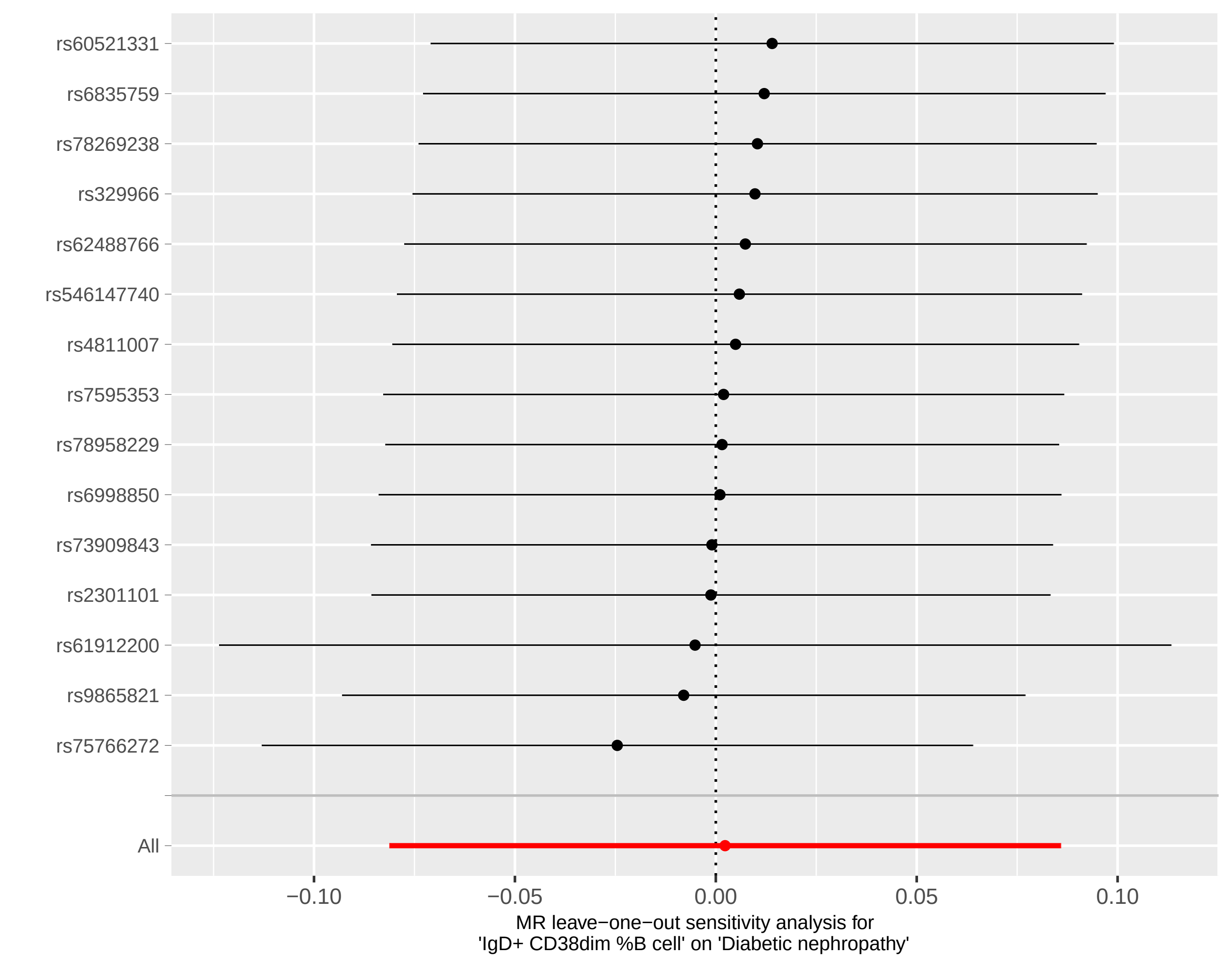

rs60521331
rs6835759
rs78269238
rs329966
rs62488766
rs546147740
rs4811007
rs7595353
rs78958229
rs6998850
rs73909843
rs2301101
rs61912200
rs9865821
rs75766272
All
−0.10
−0.05
0.00
0.05
0.10
MR leave−one−out sensitivity analysis for
'IgD+ CD38dim %B cell' on 'Diabetic nephropathy'

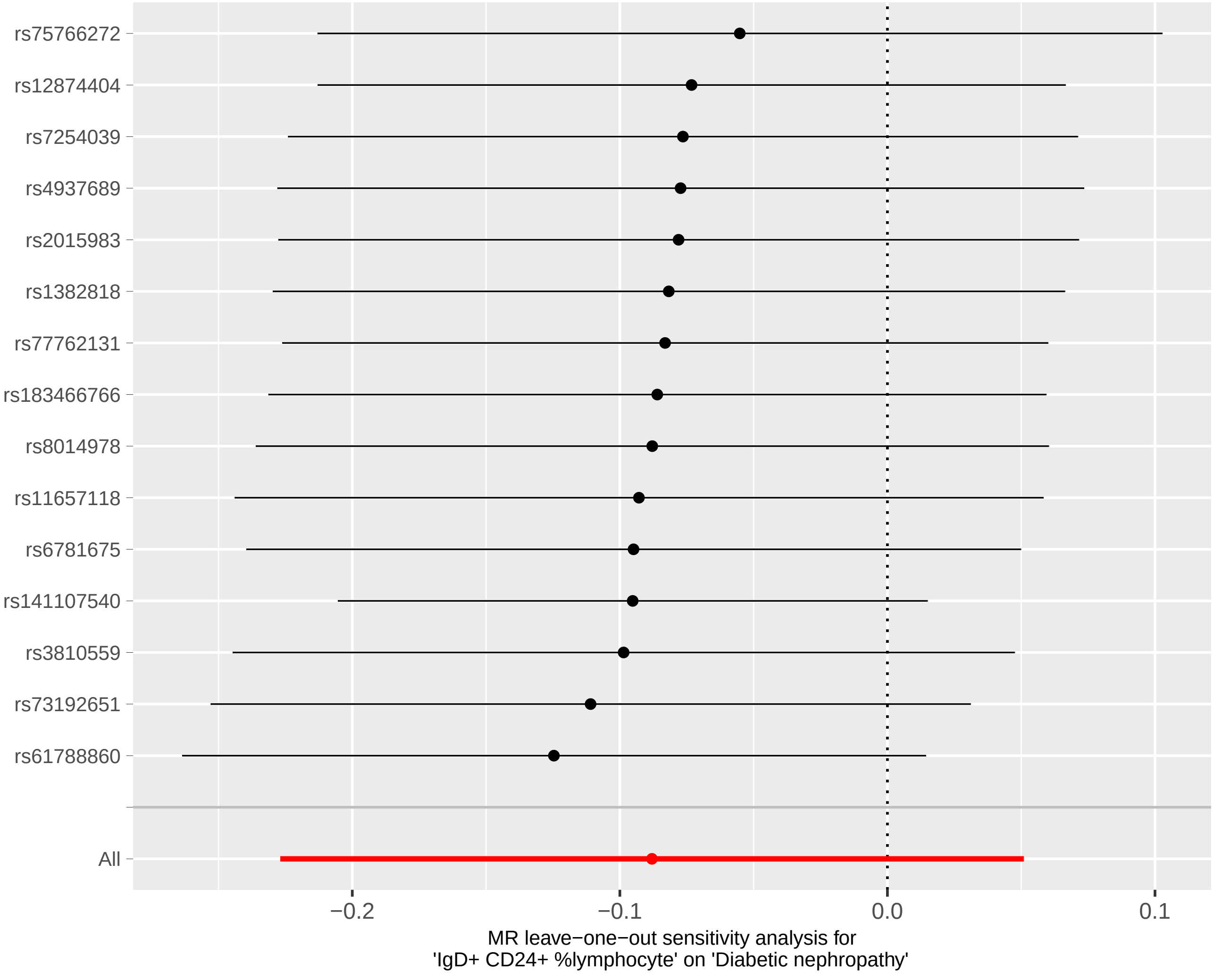
rs75766272
rs12874404
rs7254039
rs4937689
rs2015983
rs1382818
rs77762131
rs183466766
rs8014978
rs11657118
rs6781675
rs141107540
rs3810559
rs73192651
rs61788860
All
−0.2
−0.1
0.0
0.1
MR leave−one−out sensitivity analysis for
'IgD+ CD24+ %lymphocyte' on 'Diabetic nephropathy'

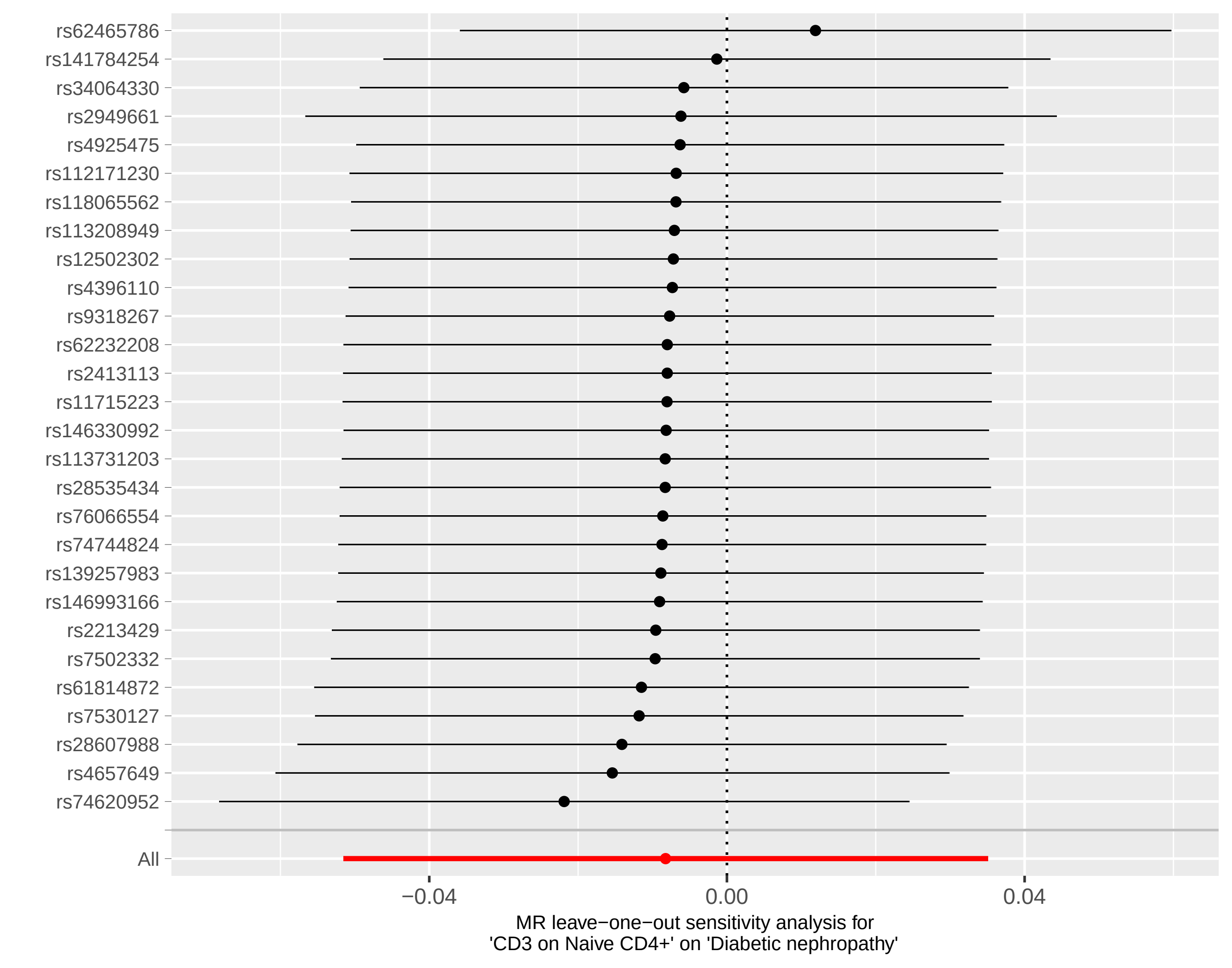

rs62465786
rs141784254
rs34064330
rs2949661
rs4925475
rs112171230
rs118065562
rs113208949
rs12502302
rs4396110
rs9318267
rs62232208
rs2413113
rs11715223
rs146330992
rs113731203
rs28535434
rs76066554
rs74744824
rs139257983
rs146993166
rs2213429
rs7502332
rs61814872
rs7530127
rs28607988
rs4657649
rs74620952
All
−0.04
0.00
0.04
MR leave−one−out sensitivity analysis for
'CD3 on Naive CD4+' on 'Diabetic nephropathy'

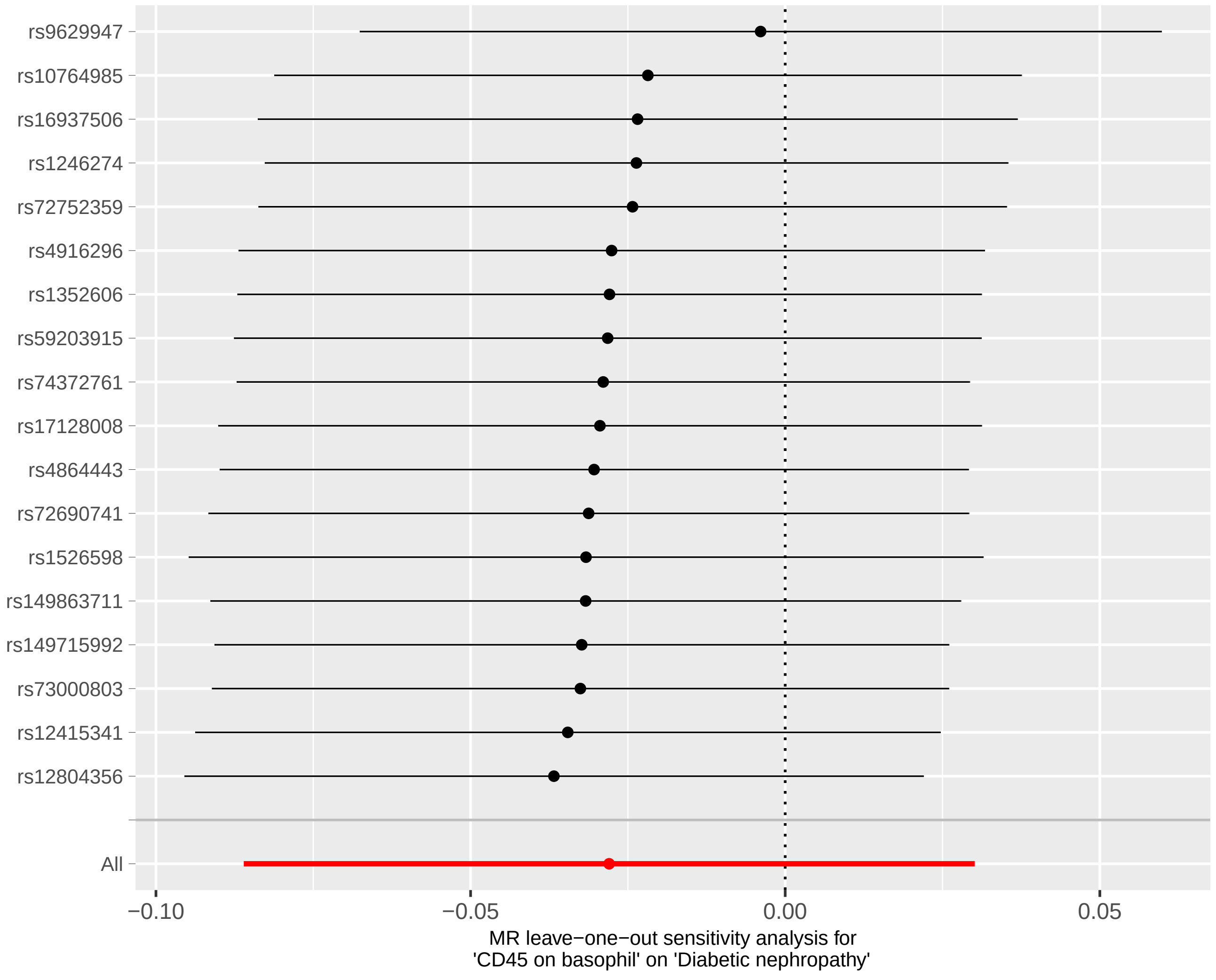
rs9629947
rs10764985
rs16937506
rs1246274
rs72752359
rs4916296
rs1352606
rs59203915
rs74372761
rs17128008
rs4864443
rs72690741
rs1526598
rs149863711
rs149715992
rs73000803
rs12415341
rs12804356
All
−0.10
−0.05
0.00
0.05
MR leave−one−out sensitivity analysis for
'CD45 on basophil' on 'Diabetic nephropathy'

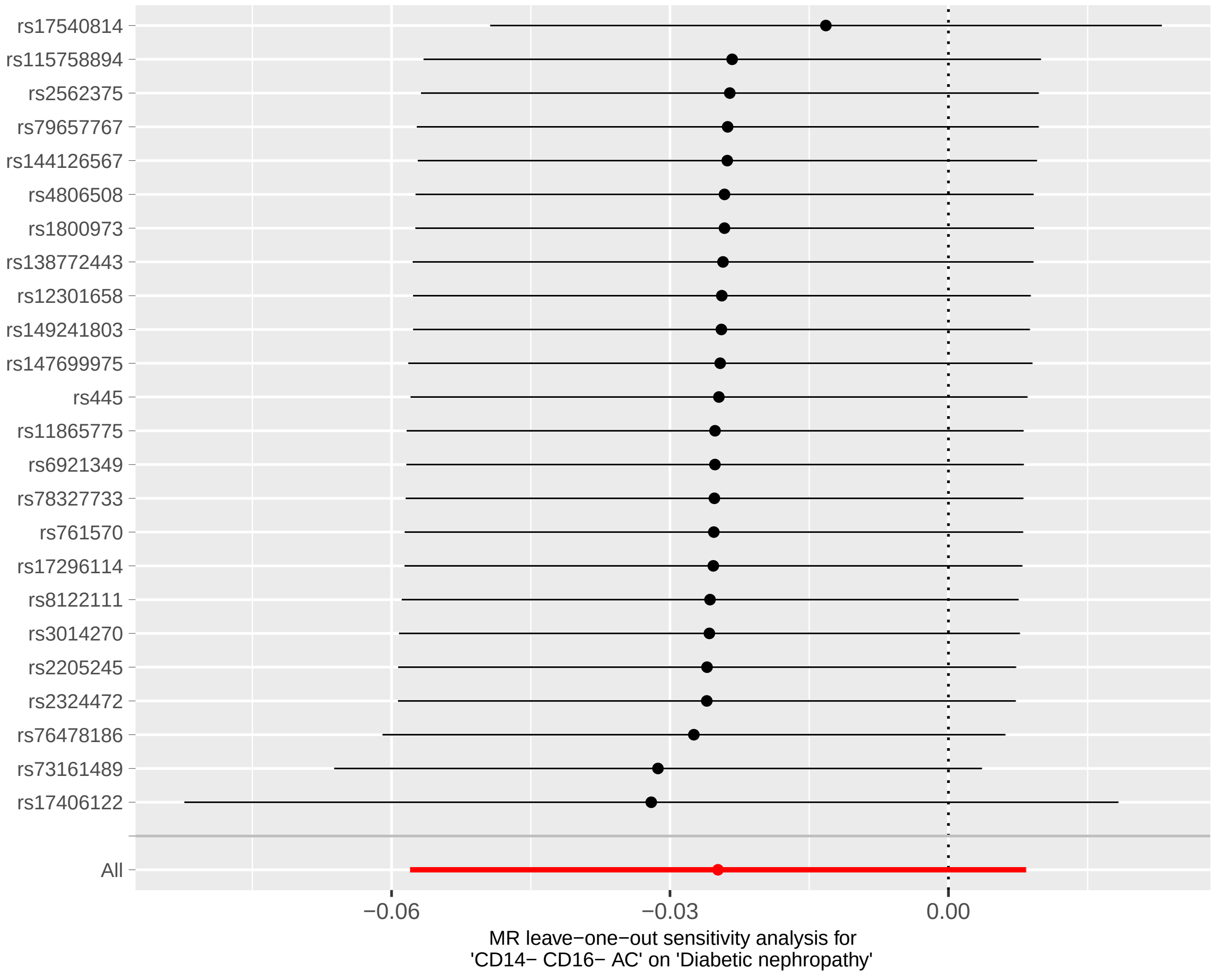
rs17540814
rs115758894
rs2562375
rs79657767
rs144126567
rs4806508
rs1800973
rs138772443
rs12301658
rs149241803
rs147699975
rs445
rs11865775
rs6921349
rs78327733
rs761570
rs17296114
rs8122111
rs3014270
rs2205245
rs2324472
rs76478186
rs73161489
rs17406122
All
−0.06
−0.03
0.00
MR leave−one−out sensitivity analysis for
'CD14− CD16− AC' on 'Diabetic nephropathy'

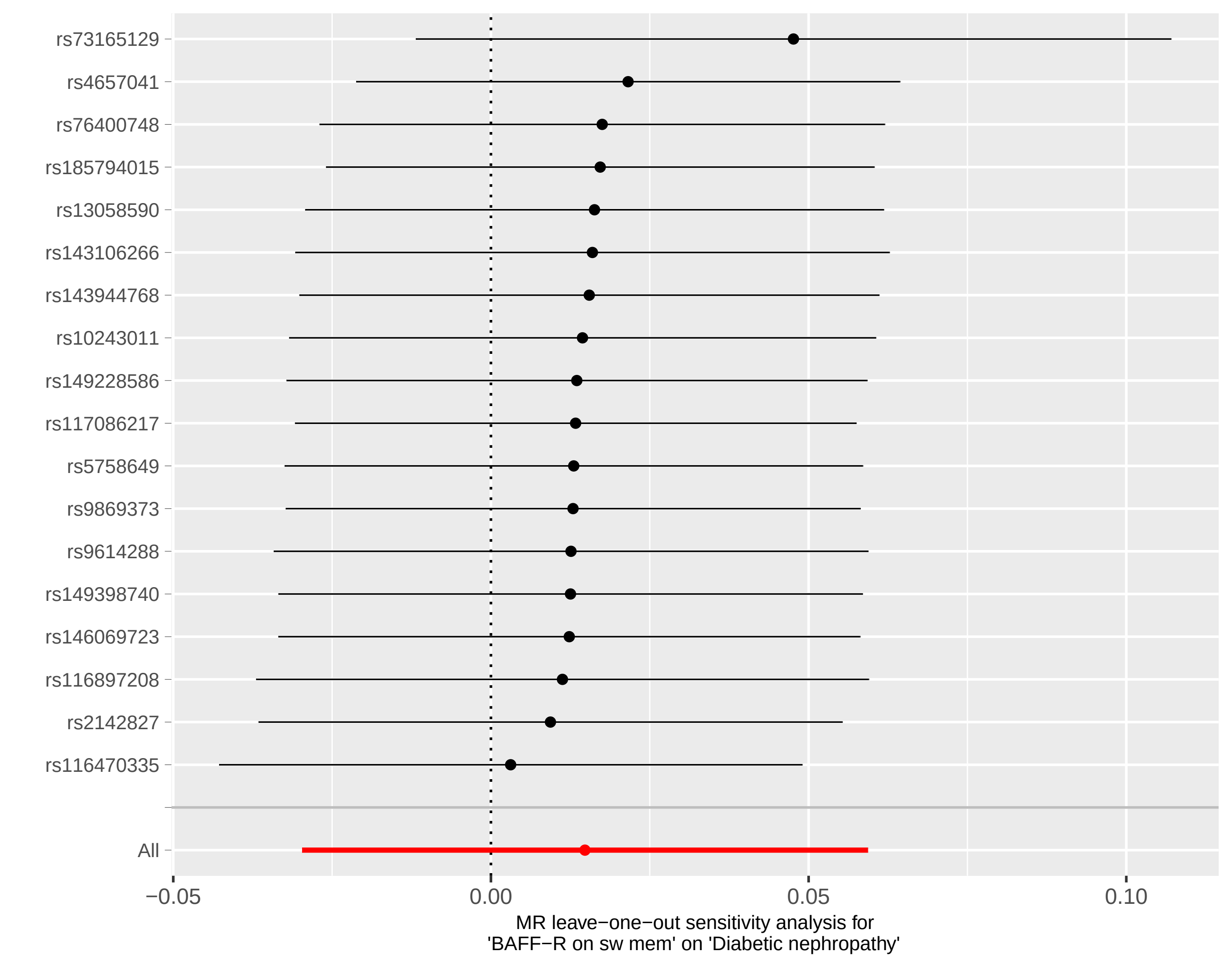
rs73165129
rs4657041
rs76400748
rs185794015
rs13058590
rs143106266
rs143944768
rs10243011
rs149228586
rs117086217
rs5758649
rs9869373
rs9614288
rs149398740
rs146069723
rs116897208
rs2142827
rs116470335
All
−0.05
0.00
0.05
0.10
MR leave−one−out sensitivity analysis for
'BAFF−R on sw mem' on 'Diabetic nephropathy'

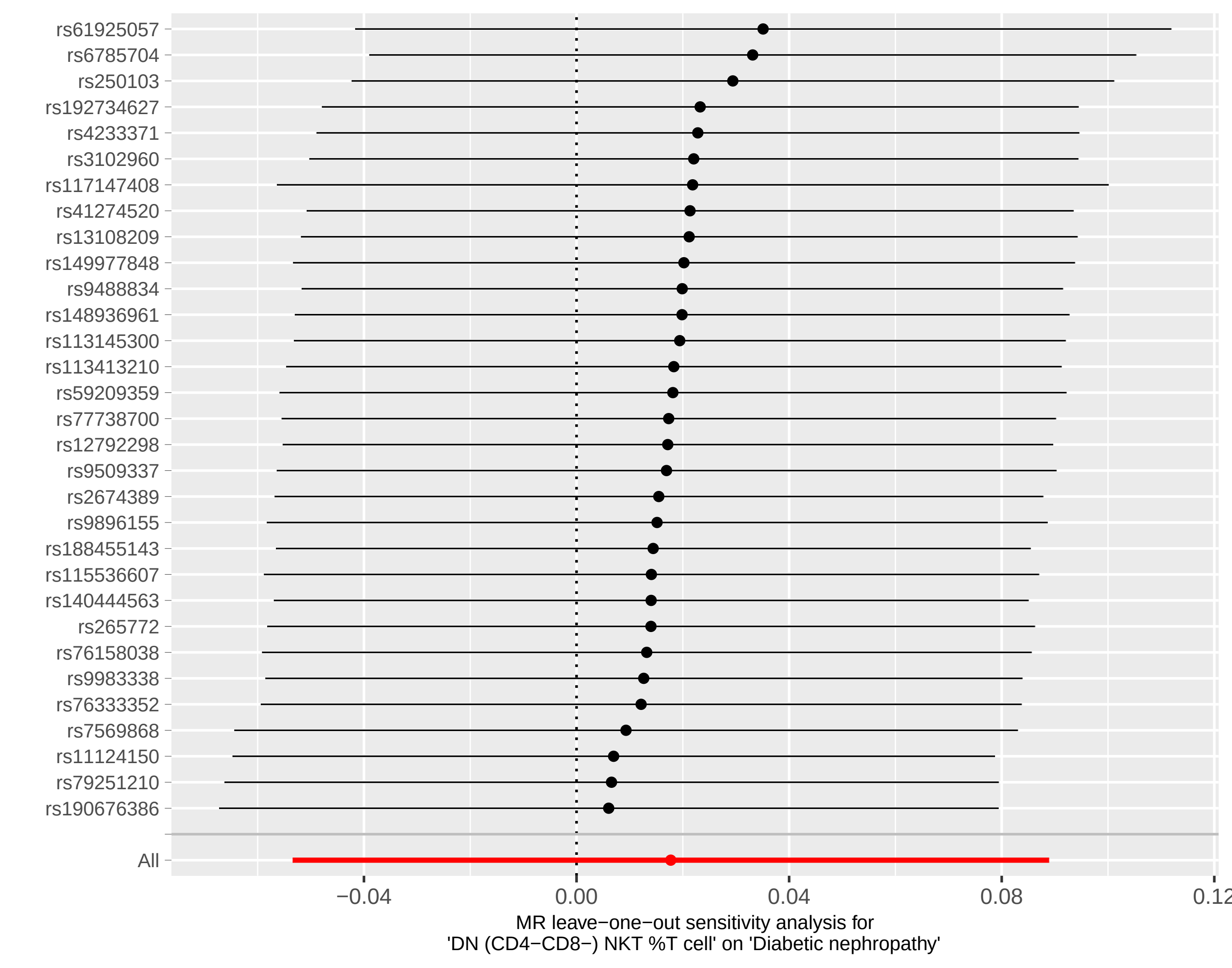
rs61925057
rs6785704
rs250103
rs192734627
rs4233371
rs3102960
rs117147408
rs41274520
rs13108209
rs149977848
rs9488834
rs148936961
rs113145300
rs113413210
rs59209359
rs77738700
rs12792298
rs9509337
rs2674389
rs9896155
rs188455143
rs115536607
rs140444563
rs265772
rs76158038
rs9983338
rs76333352
rs7569868
rs11124150
rs79251210
rs190676386
All
−0.04
0.00
0.04
0.08
0.12
MR leave−one−out sensitivity analysis for
'DN (CD4−CD8−) NKT %T cell' on 'Diabetic nephropathy'

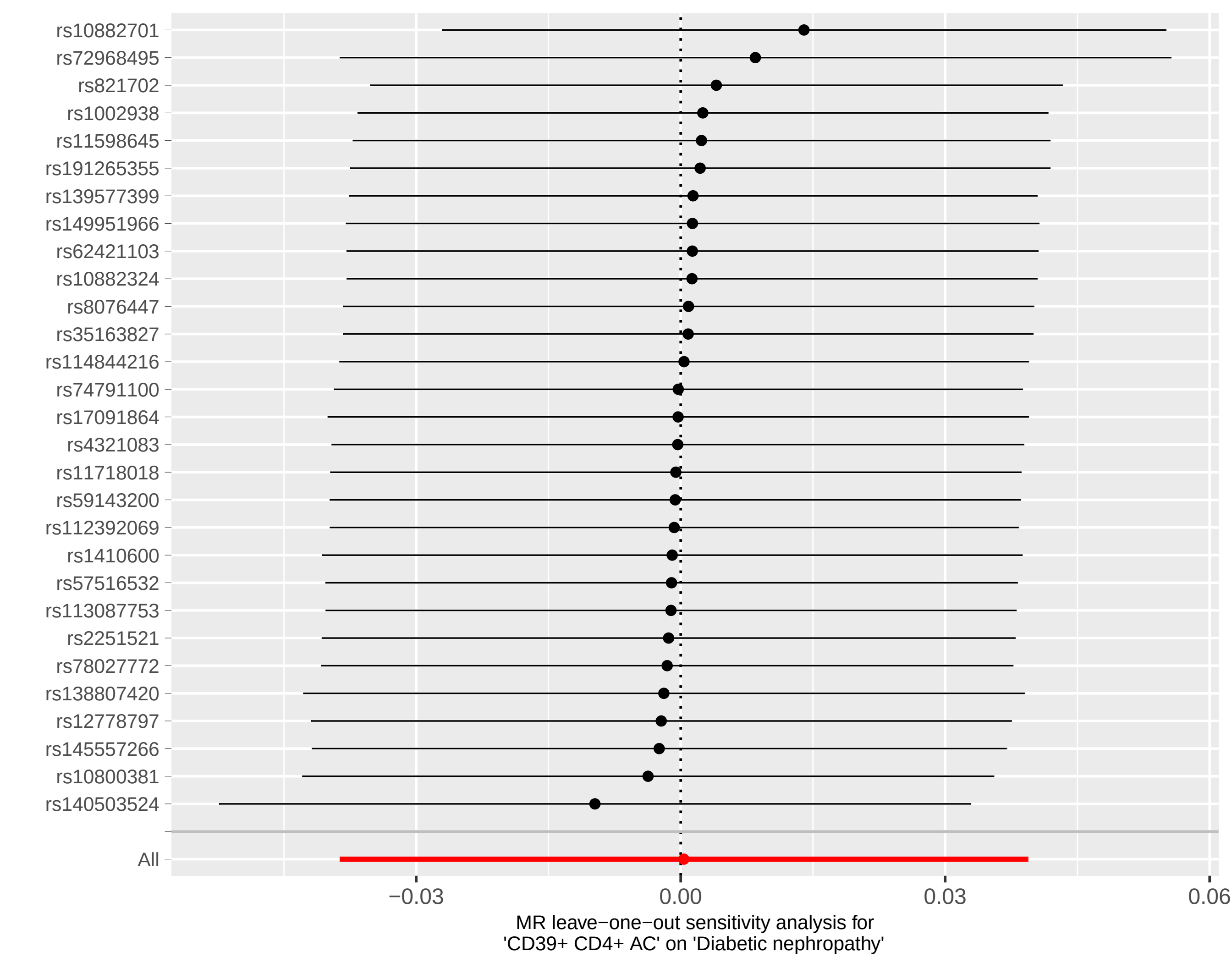
rs10882701
rs72968495
rs821702
rs1002938
rs11598645
rs191265355
rs139577399
rs149951966
rs62421103
rs10882324
rs8076447
rs35163827
rs114844216
rs74791100
rs17091864
rs4321083
rs11718018
rs59143200
rs112392069
rs1410600
rs57516532
rs113087753
rs2251521
rs78027772
rs138807420
rs12778797
rs145557266
rs10800381
rs140503524
All
−0.03
0.00
0.03
0.06
MR leave−one−out sensitivity analysis for
'CD39+ CD4+ AC' on 'Diabetic nephropathy'

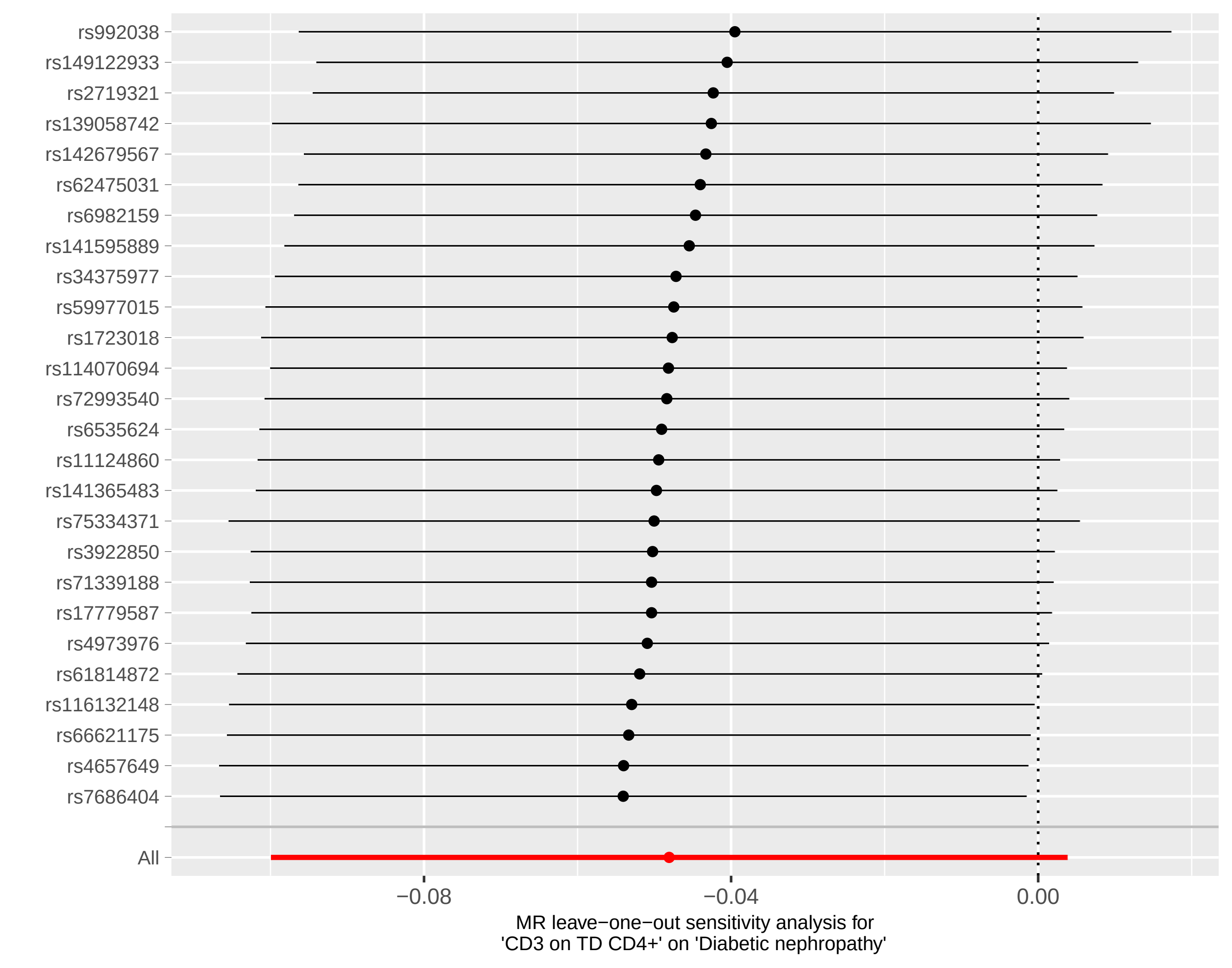
rs992038
rs149122933
rs2719321
rs139058742
rs142679567
rs62475031
rs6982159
rs141595889
rs34375977
rs59977015
rs1723018
rs114070694
rs72993540
rs6535624
rs11124860
rs141365483
rs75334371
rs3922850
rs71339188
rs17779587
rs4973976
rs61814872
rs116132148
rs66621175
rs4657649
rs7686404
All
−0.08
−0.04
0.00
MR leave−one−out sensitivity analysis for
'CD3 on TD CD4+' on 'Diabetic nephropathy'

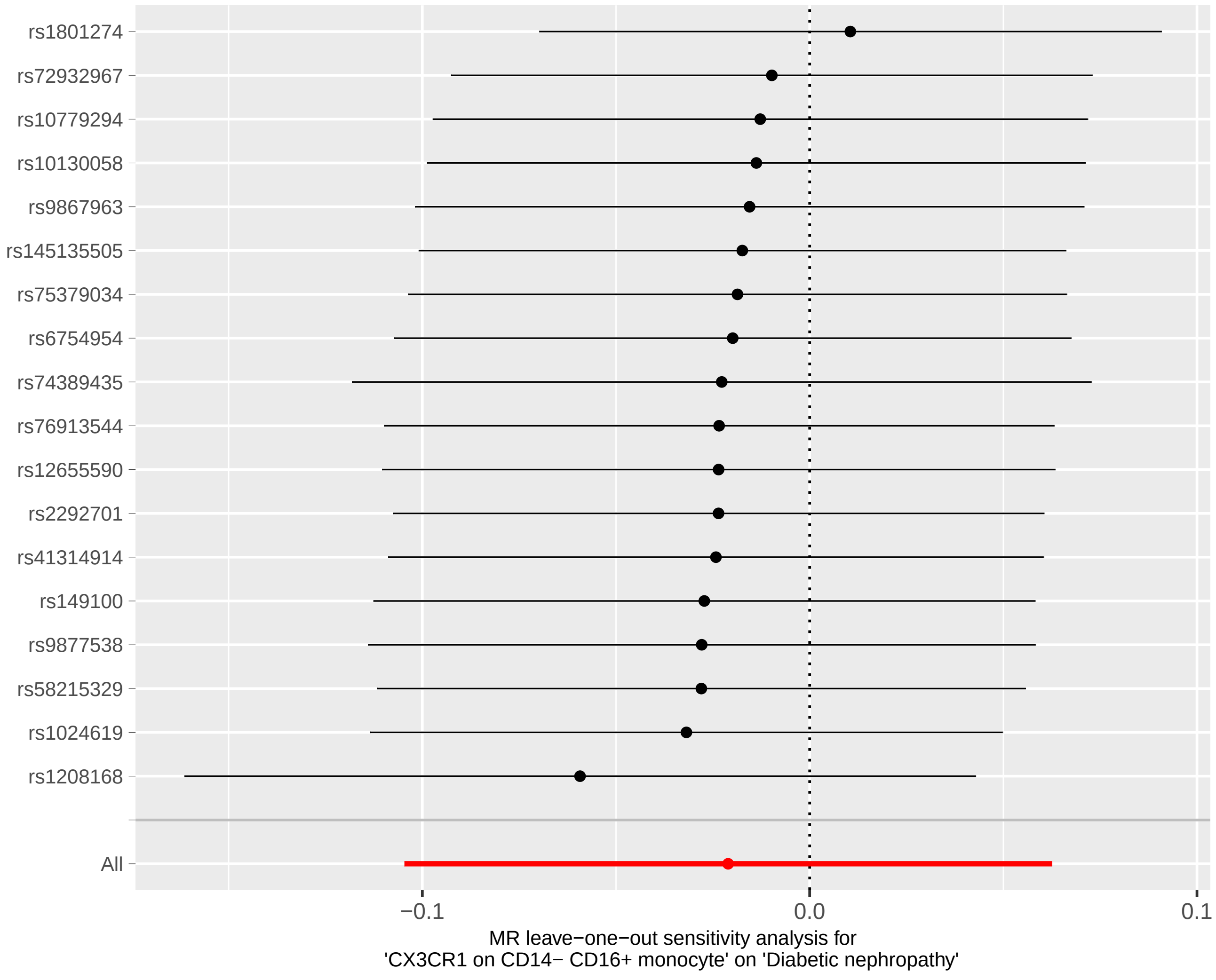

rs1801274
rs72932967
rs10779294
rs10130058
rs9867963
rs145135505
rs75379034
rs6754954
rs74389435
rs76913544
rs12655590
rs2292701
rs41314914
rs149100
rs9877538
rs58215329
rs1024619
rs1208168
All
−0.1
0.0
0.1
MR leave−one−out sensitivity analysis for
'CX3CR1 on CD14− CD16+ monocyte' on 'Diabetic nephropathy'

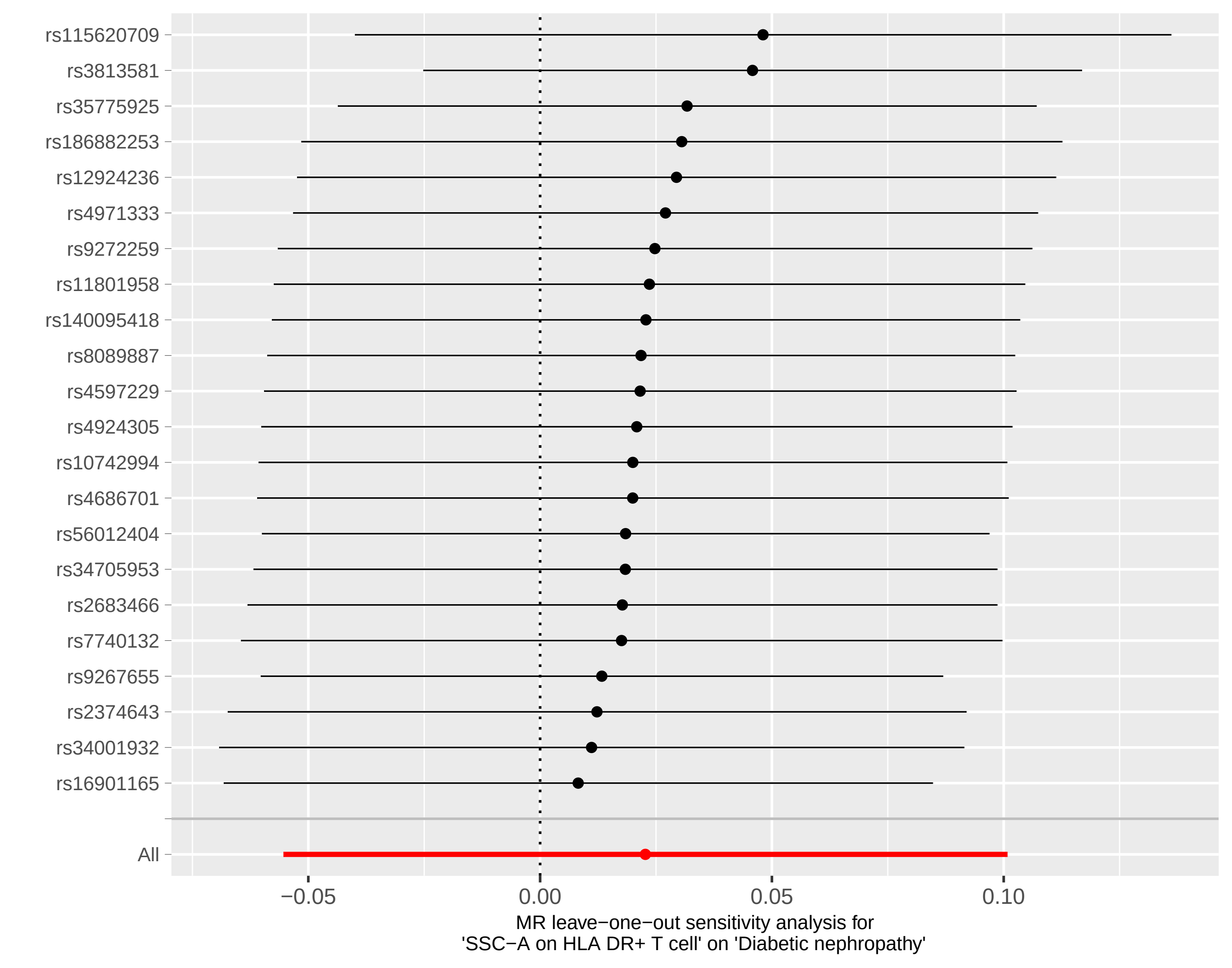

rs115620709
rs3813581
rs35775925
rs186882253
rs12924236
rs4971333
rs9272259
rs11801958
rs140095418
rs8089887
rs4597229
rs4924305
rs10742994
rs4686701
rs56012404
rs34705953
rs2683466
rs7740132
rs9267655
rs2374643
rs34001932
rs16901165
All
−0.05
0.00
0.05
0.10
MR leave−one−out sensitivity analysis for
'SSC−A on HLA DR+ T cell' on 'Diabetic nephropathy'

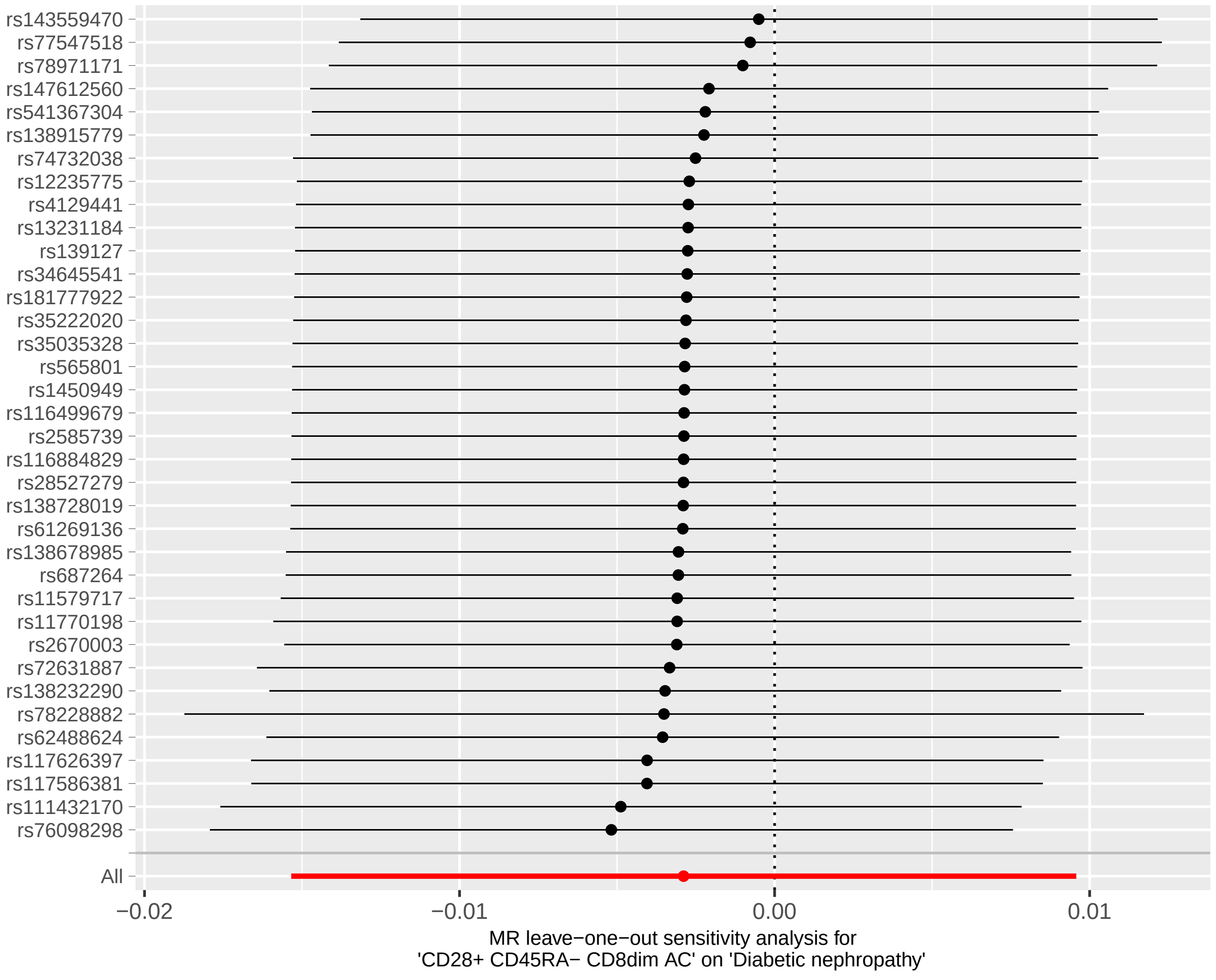
rs143559470
rs77547518
rs78971171
rs147612560
rs541367304
rs138915779
rs74732038
rs12235775
rs4129441
rs13231184
rs139127
rs34645541
rs181777922
rs35222020
rs35035328
rs565801
rs1450949
rs116499679
rs2585739
rs116884829
rs28527279
rs138728019
rs61269136
rs138678985
rs687264
rs11579717
rs11770198
rs2670003
rs72631887
rs138232290
rs78228882
rs62488624
rs117626397
rs117586381
rs111432170
rs76098298
All
−0.02
−0.01
0.00
0.01
MR leave−one−out sensitivity analysis for
'CD28+ CD45RA− CD8dim AC' on 'Diabetic nephropathy'

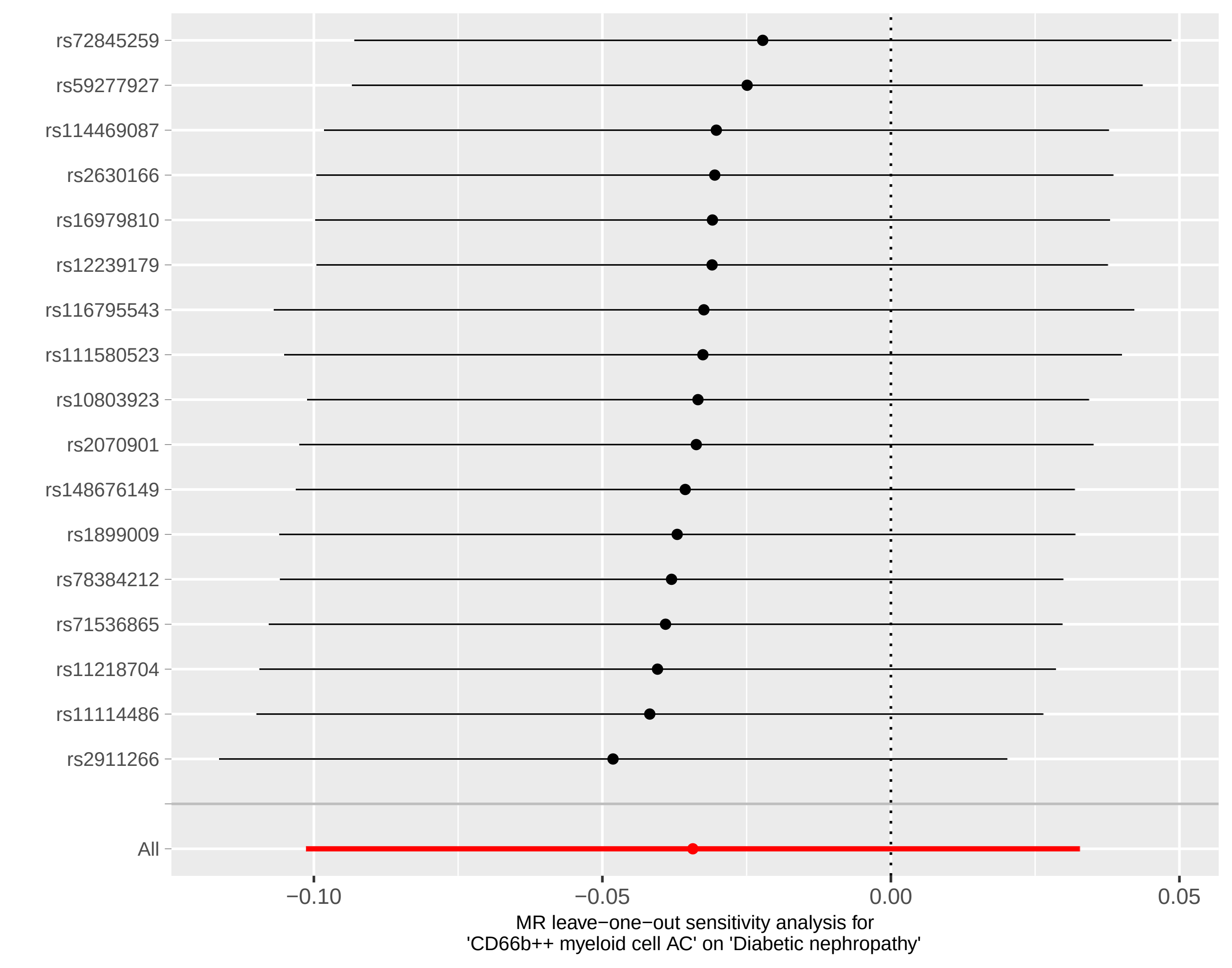
rs72845259
rs59277927
rs114469087
rs2630166
rs16979810
rs12239179
rs116795543
rs111580523
rs10803923
rs2070901
rs148676149
rs1899009
rs78384212
rs71536865
rs11218704
rs11114486
rs2911266
All
−0.10
−0.05
0.00
0.05
MR leave−one−out sensitivity analysis for
'CD66b++ myeloid cell AC' on 'Diabetic nephropathy'

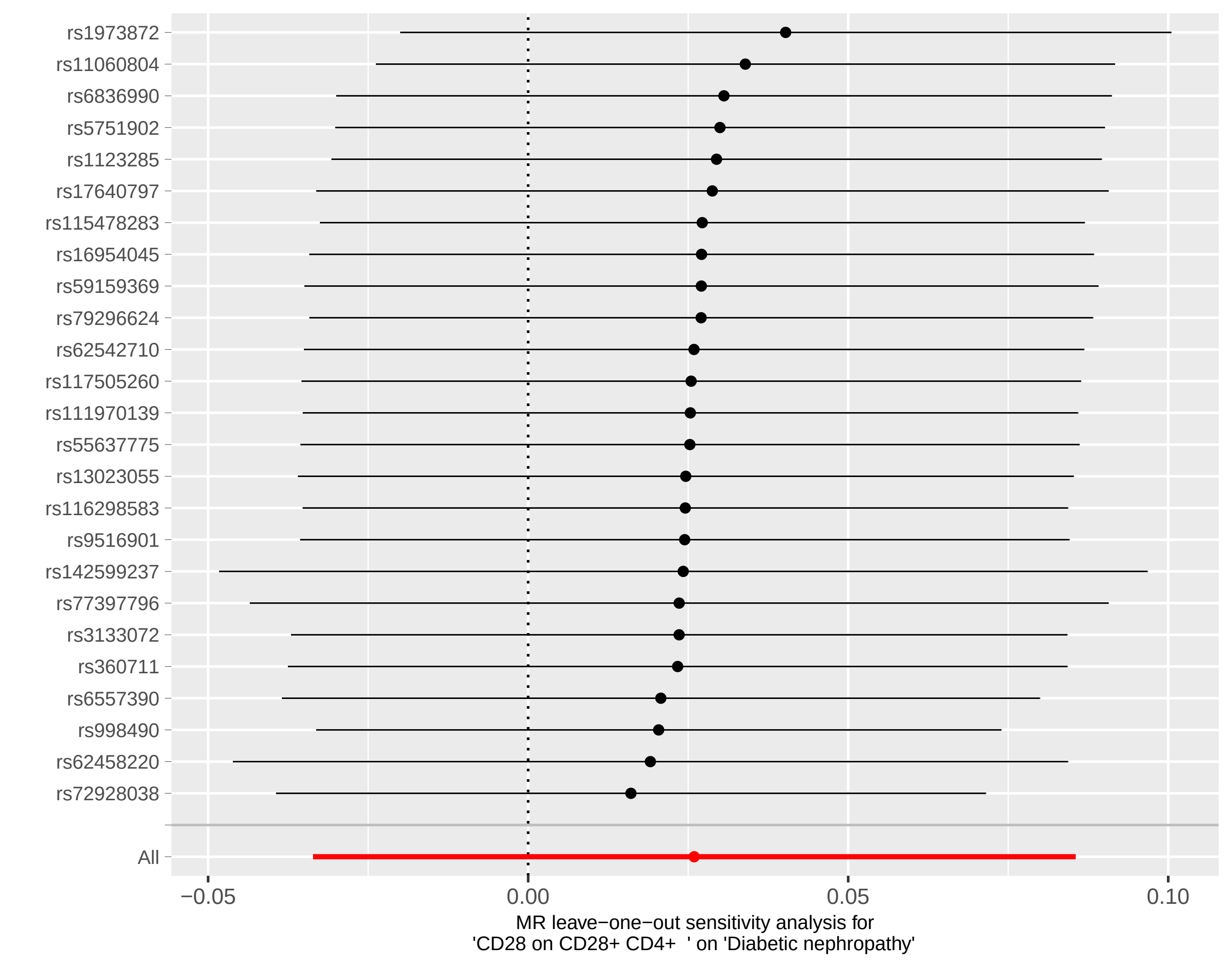
rs1973872
rs11060804
rs6836990
rs5751902
rs1123285
rs17640797
rs115478283
rs16954045
rs59159369
rs79296624
rs62542710
rs117505260
rs111970139
rs55637775
rs13023055
rs116298583
rs9516901
rs142599237
rs77397796
rs3133072
rs360711
rs6557390
rs998490
rs62458220
rs72928038
All
−0.05
0.00
0.05
0.10
MR leave−one−out sensitivity analysis for
'CD28 on CD28+ CD4+ ' on 'Diabetic nephropathy'

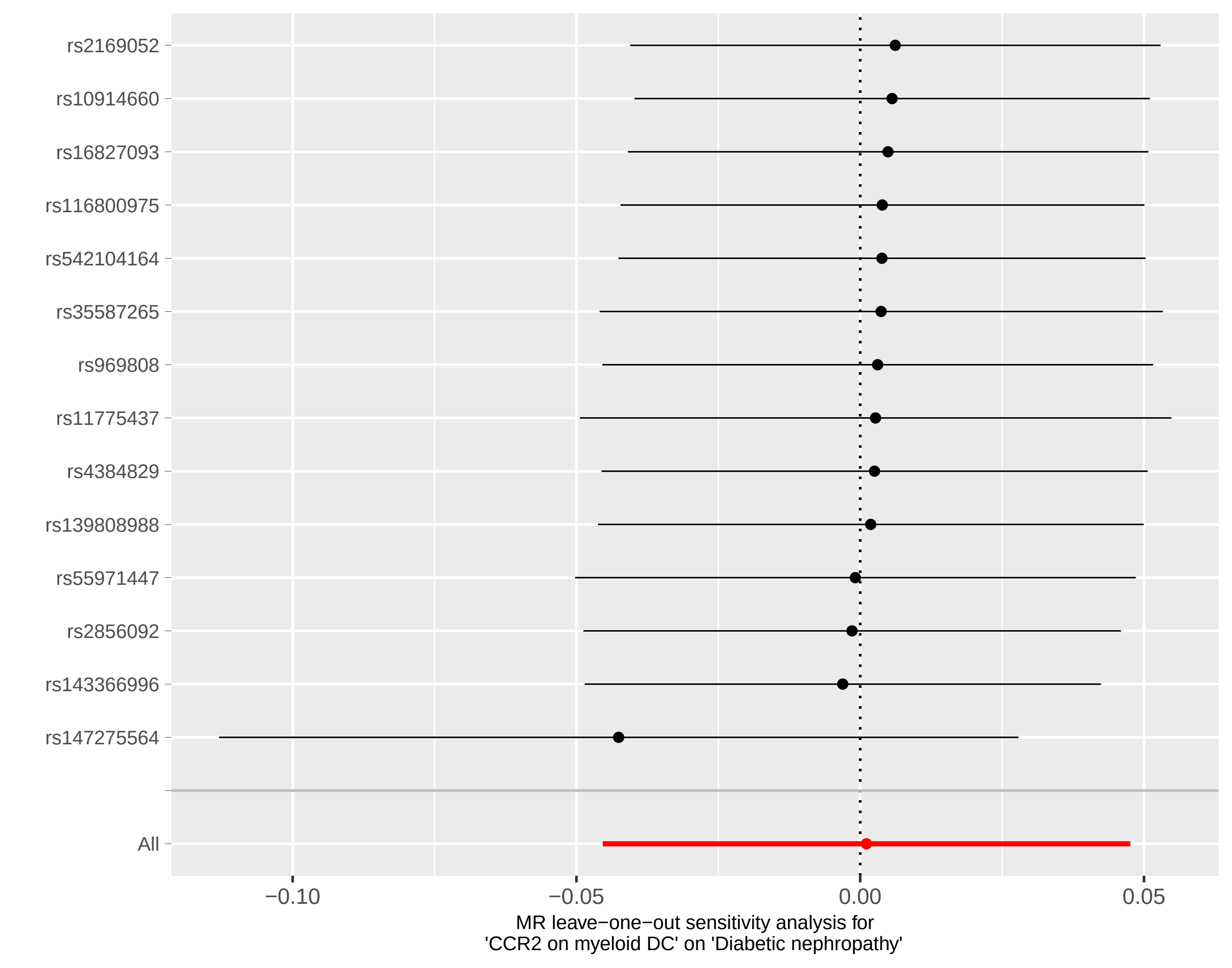
rs2169052
rs10914660
rs16827093
rs116800975
rs542104164
rs35587265
rs969808
rs11775437
rs4384829
rs139808988
rs55971447
rs2856092
rs143366996
rs147275564
All
−0.10
−0.05
0.00
0.05
MR leave−one−out sensitivity analysis for
'CCR2 on myeloid DC' on 'Diabetic nephropathy'

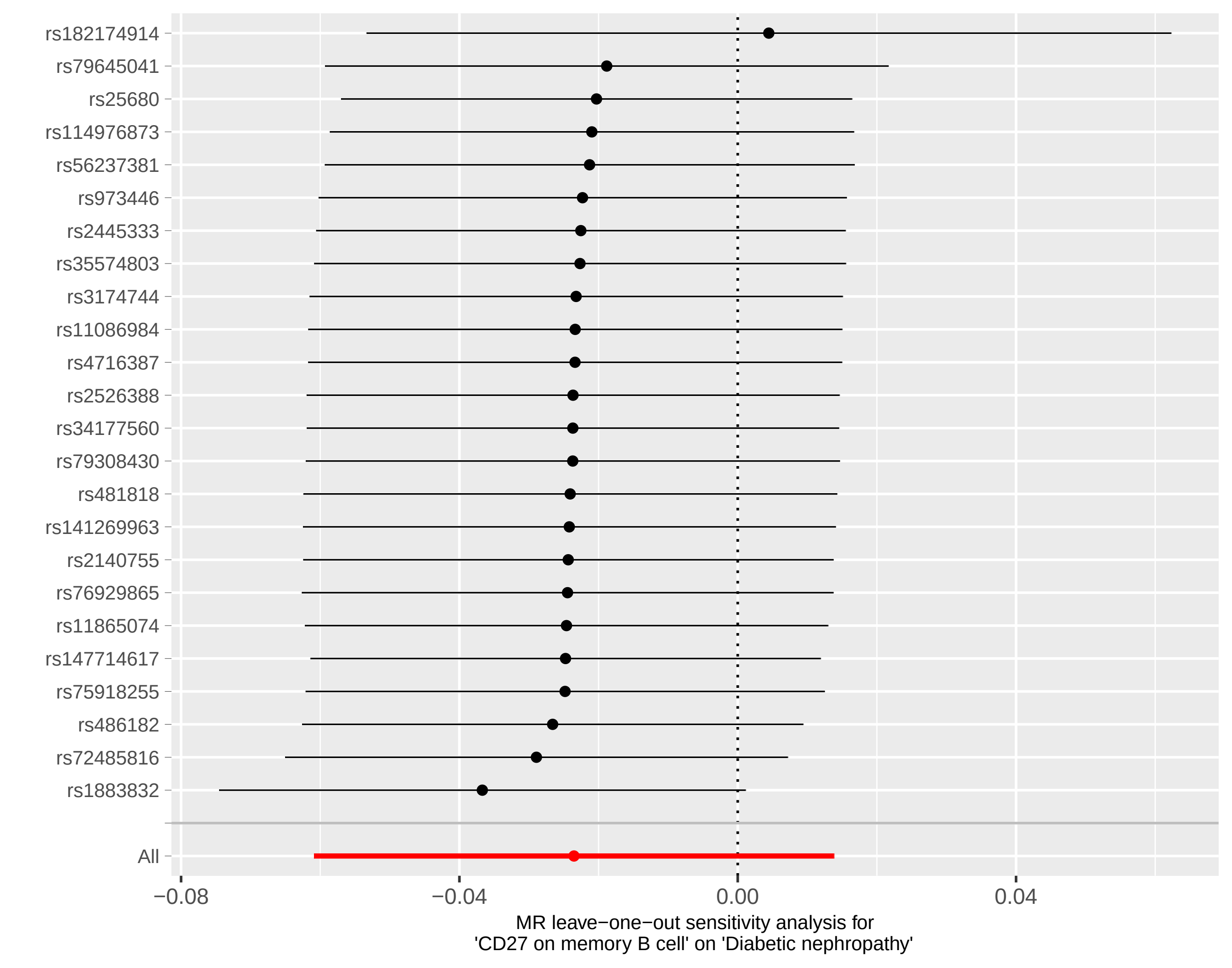
rs182174914
rs79645041
rs25680
rs114976873
rs56237381
rs973446
rs2445333
rs35574803
rs3174744
rs11086984
rs4716387
rs2526388
rs34177560
rs79308430
rs481818
rs141269963
rs2140755
rs76929865
rs11865074
rs147714617
rs75918255
rs486182
rs72485816
rs1883832
All
−0.08
−0.04
0.00
0.04
MR leave−one−out sensitivity analysis for
'CD27 on memory B cell' on 'Diabetic nephropathy'

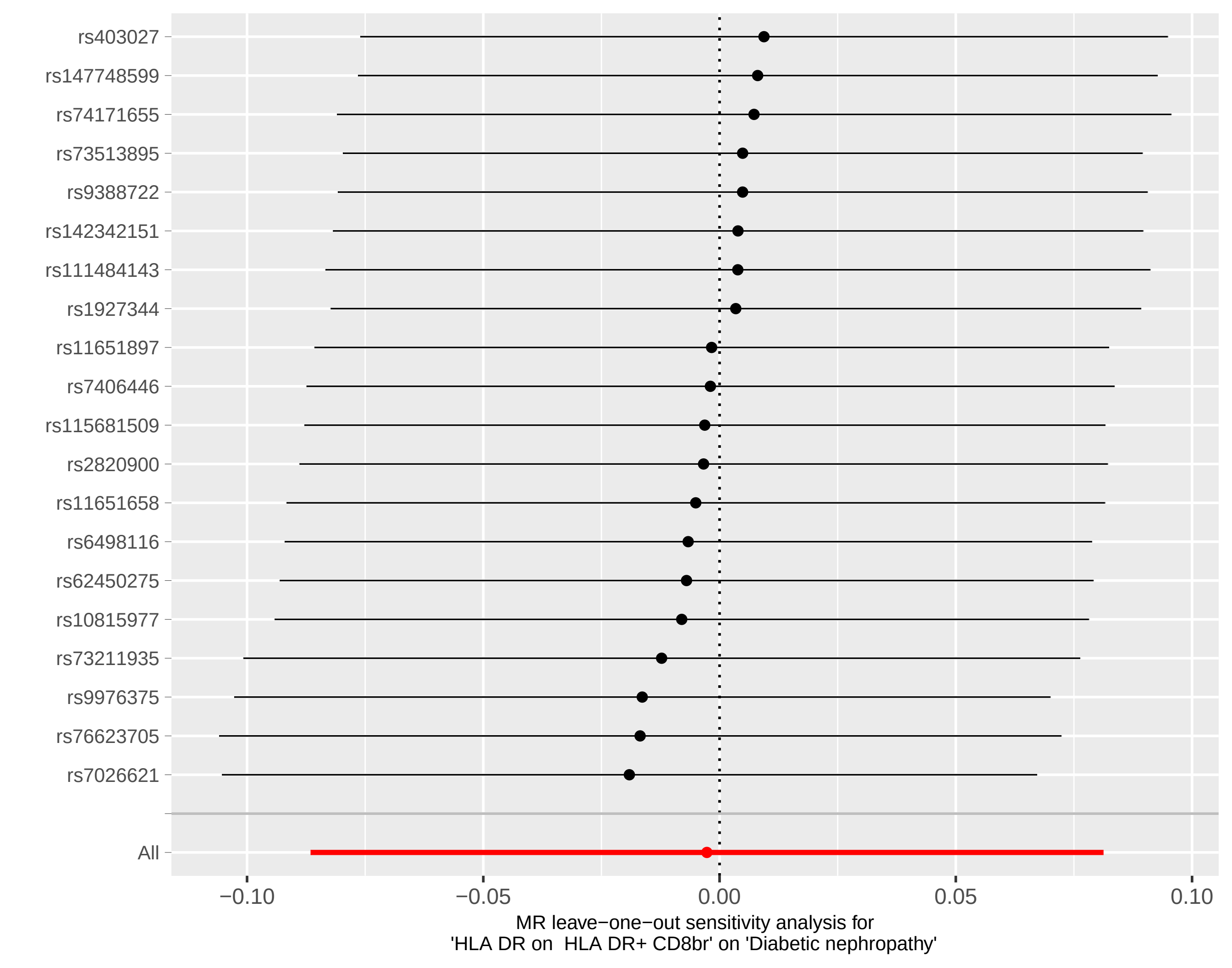
rs403027
rs147748599
rs74171655
rs73513895
rs9388722
rs142342151
rs111484143
rs1927344
rs11651897
rs7406446
rs115681509
rs2820900
rs11651658
rs6498116
rs62450275
rs10815977
rs73211935
rs9976375
rs76623705
rs7026621
All
−0.10
−0.05
0.00
0.05
0.10
MR leave−one−out sensitivity analysis for
'HLA DR on HLA DR+ CD8br' on 'Diabetic nephropathy'

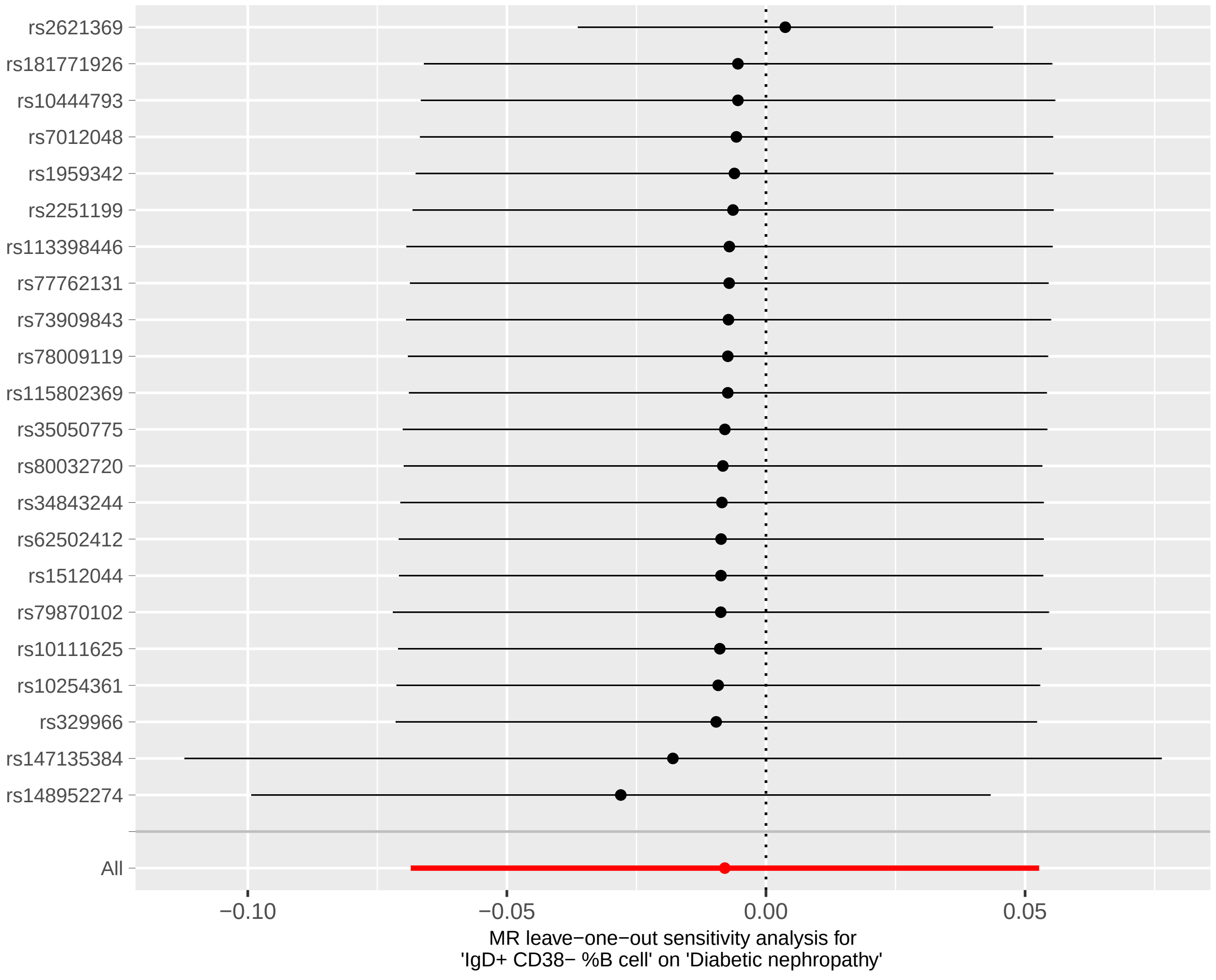
rs2621369
rs181771926
rs10444793
rs7012048
rs1959342
rs2251199
rs113398446
rs77762131
rs73909843
rs78009119
rs115802369
rs35050775
rs80032720
rs34843244
rs62502412
rs1512044
rs79870102
rs10111625
rs10254361
rs329966
rs147135384
rs148952274
All
−0.10
−0.05
0.00
0.05
MR leave−one−out sensitivity analysis for
'IgD+ CD38− %B cell' on 'Diabetic nephropathy'

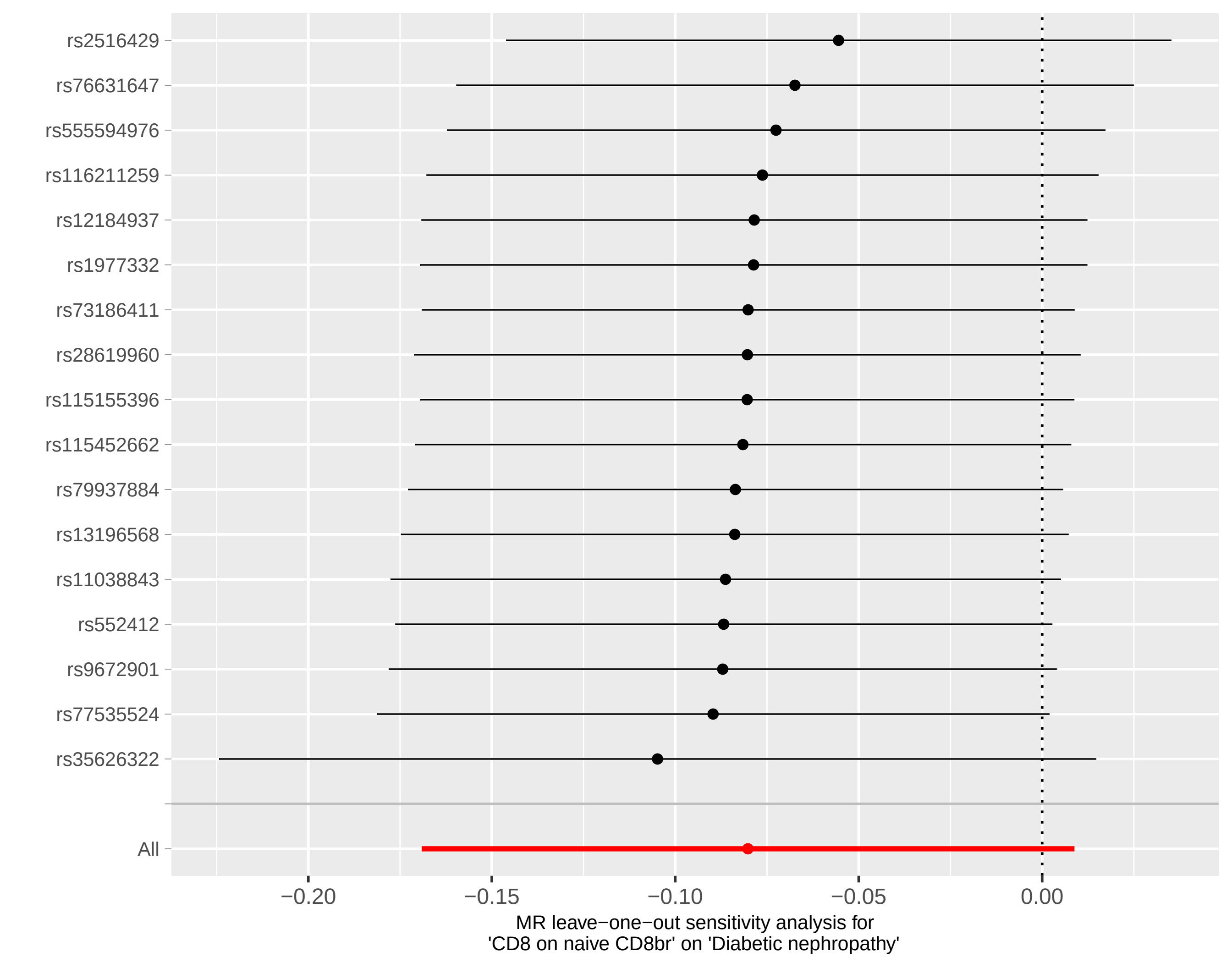

rs2516429
rs76631647
rs555594976
rs116211259
rs12184937
rs1977332
rs73186411
rs28619960
rs115155396
rs115452662
rs79937884
rs13196568
rs11038843
rs552412
rs9672901
rs77535524
rs35626322
All
−0.20
−0.15
−0.10
−0.05
0.00
MR leave−one−out sensitivity analysis for
'CD8 on naive CD8br' on 'Diabetic nephropathy'

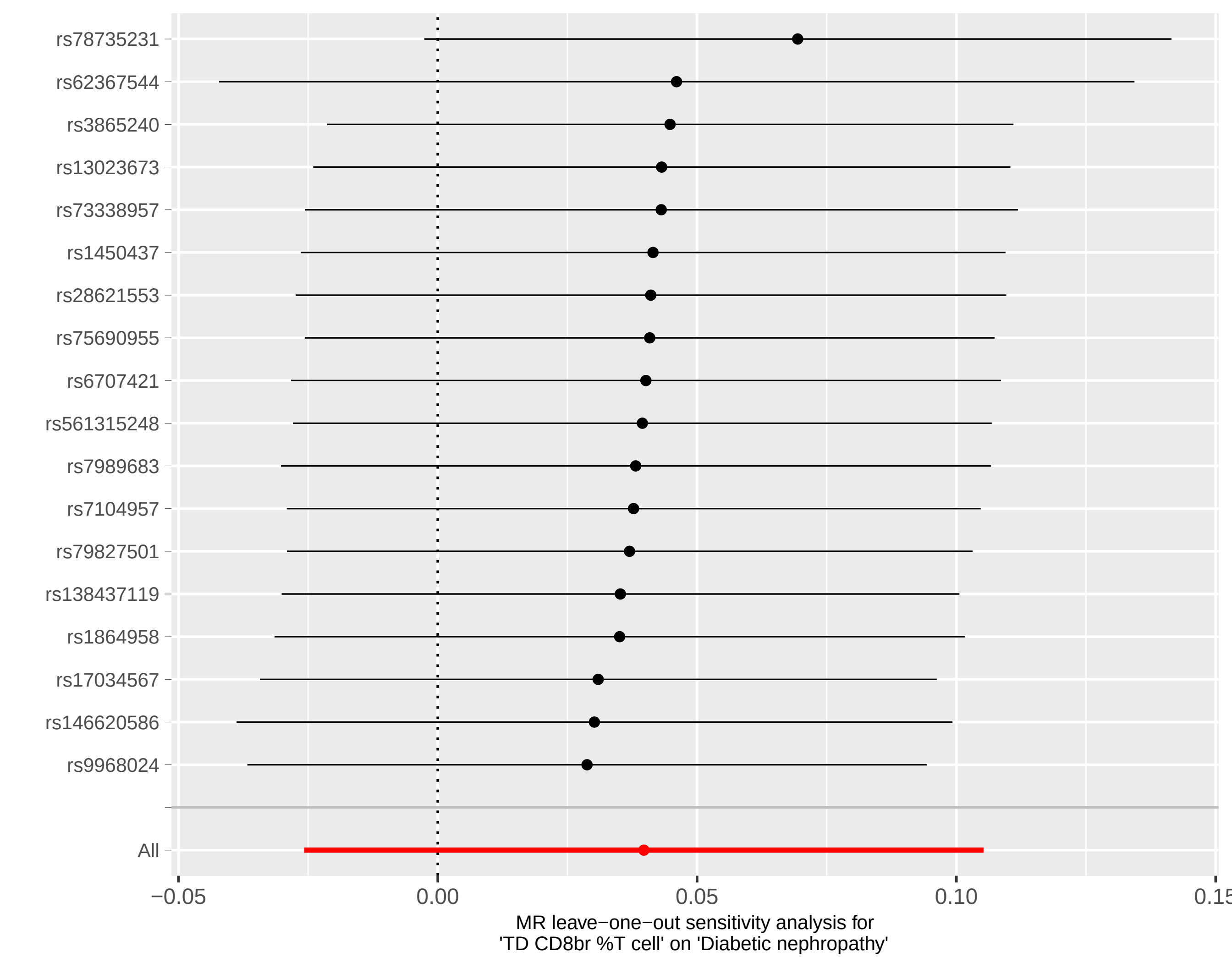
rs78735231
rs62367544
rs3865240
rs13023673
rs73338957
rs1450437
rs28621553
rs75690955
rs6707421
rs561315248
rs7989683
rs7104957
rs79827501
rs138437119
rs1864958
rs17034567
rs146620586
rs9968024
All
−0.05
0.00
0.05
0.10
0.15
MR leave−one−out sensitivity analysis for
'TD CD8br %T cell' on 'Diabetic nephropathy'

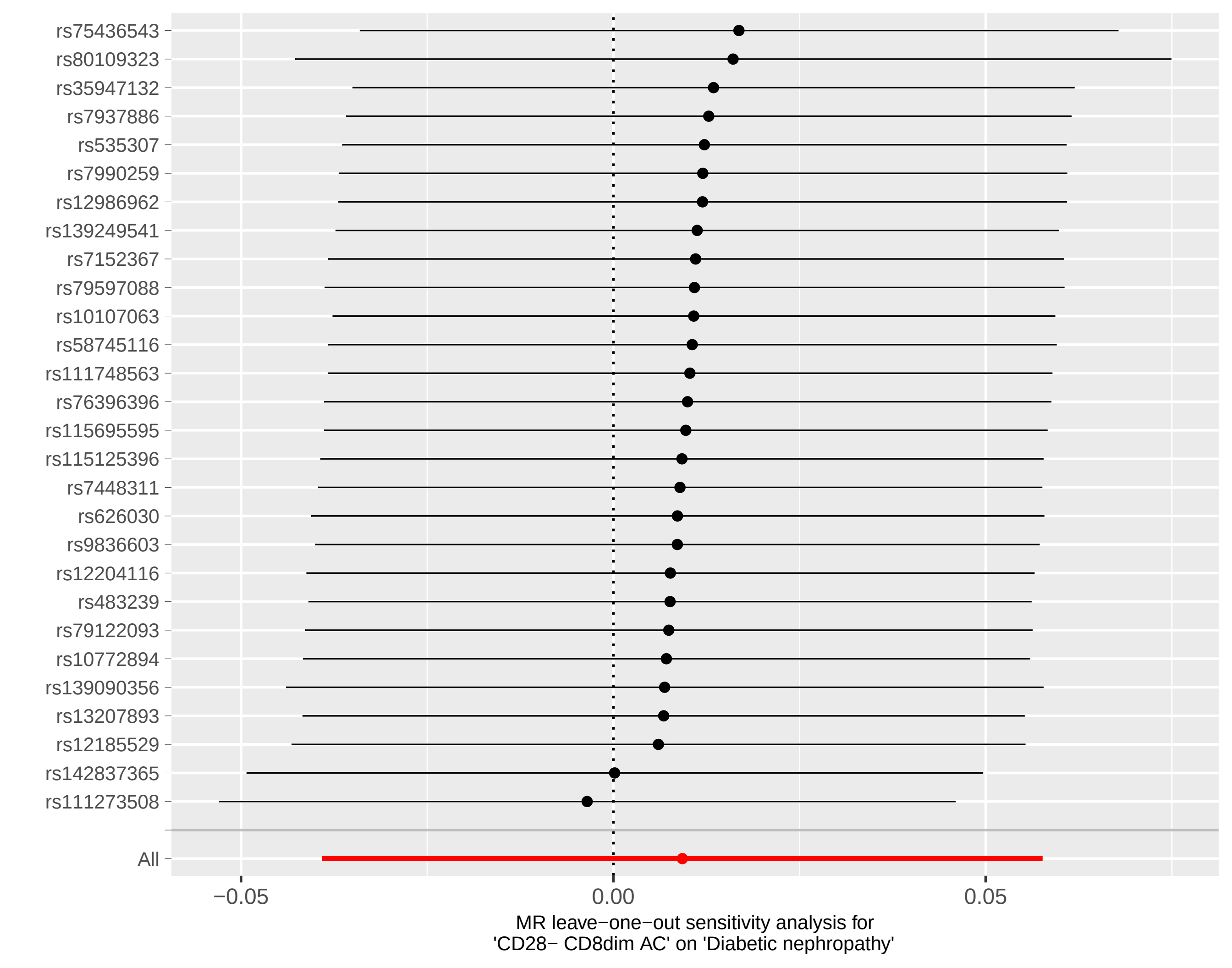
rs75436543
rs80109323
rs35947132
rs7937886
rs535307
rs7990259
rs12986962
rs139249541
rs7152367
rs79597088
rs10107063
rs58745116
rs111748563
rs76396396
rs115695595
rs115125396
rs7448311
rs626030
rs9836603
rs12204116
rs483239
rs79122093
rs10772894
rs139090356
rs13207893
rs12185529
rs142837365
rs111273508
All
−0.05
0.00
0.05
MR leave−one−out sensitivity analysis for
'CD28− CD8dim AC' on 'Diabetic nephropathy'

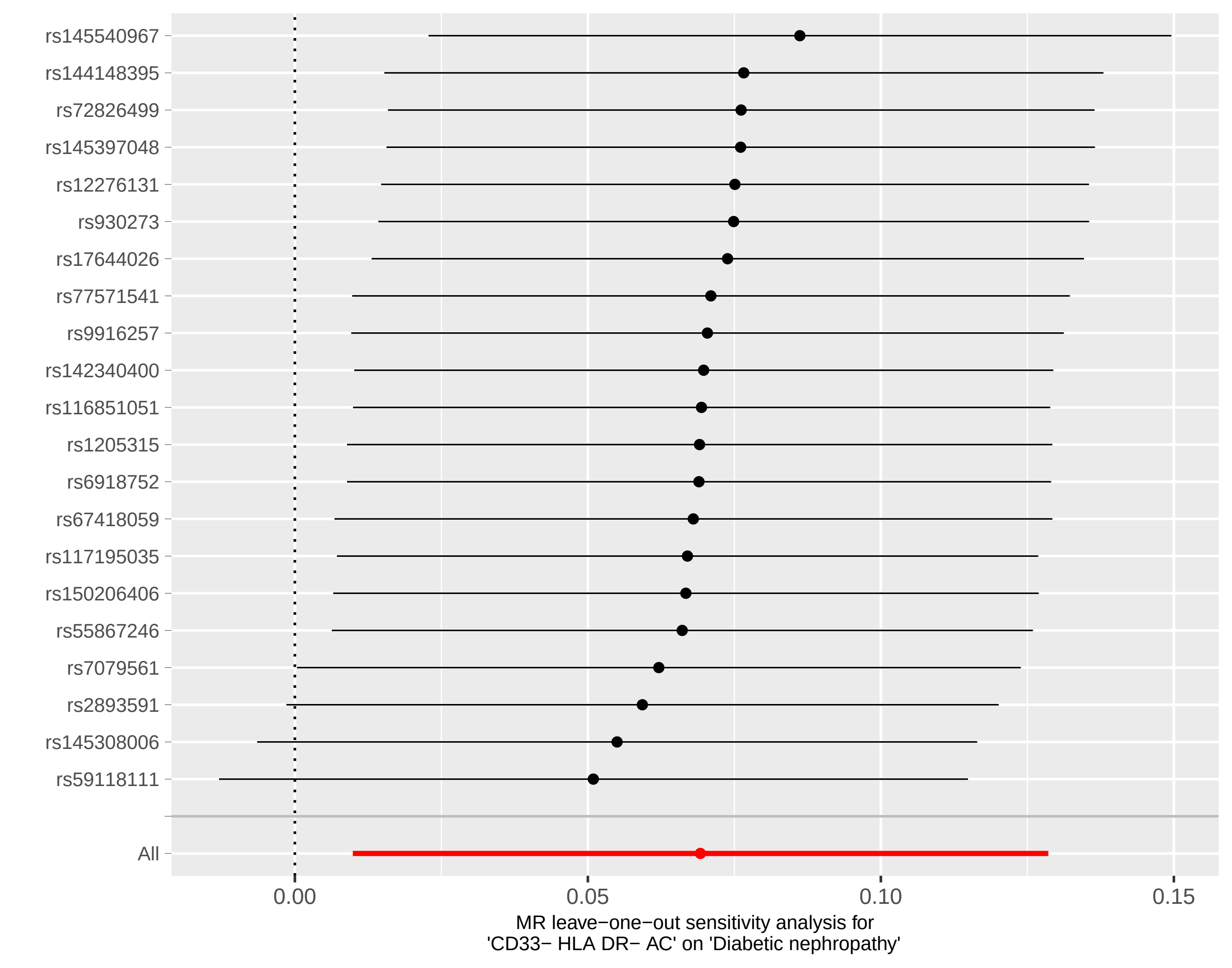
rs145540967
rs144148395
rs72826499
rs145397048
rs12276131
rs930273
rs17644026
rs77571541
rs9916257
rs142340400
rs116851051
rs1205315
rs6918752
rs67418059
rs117195035
rs150206406
rs55867246
rs7079561
rs2893591
rs145308006
rs59118111
All
0.00
0.05
0.10
0.15
MR leave−one−out sensitivity analysis for
'CD33− HLA DR− AC' on 'Diabetic nephropathy'

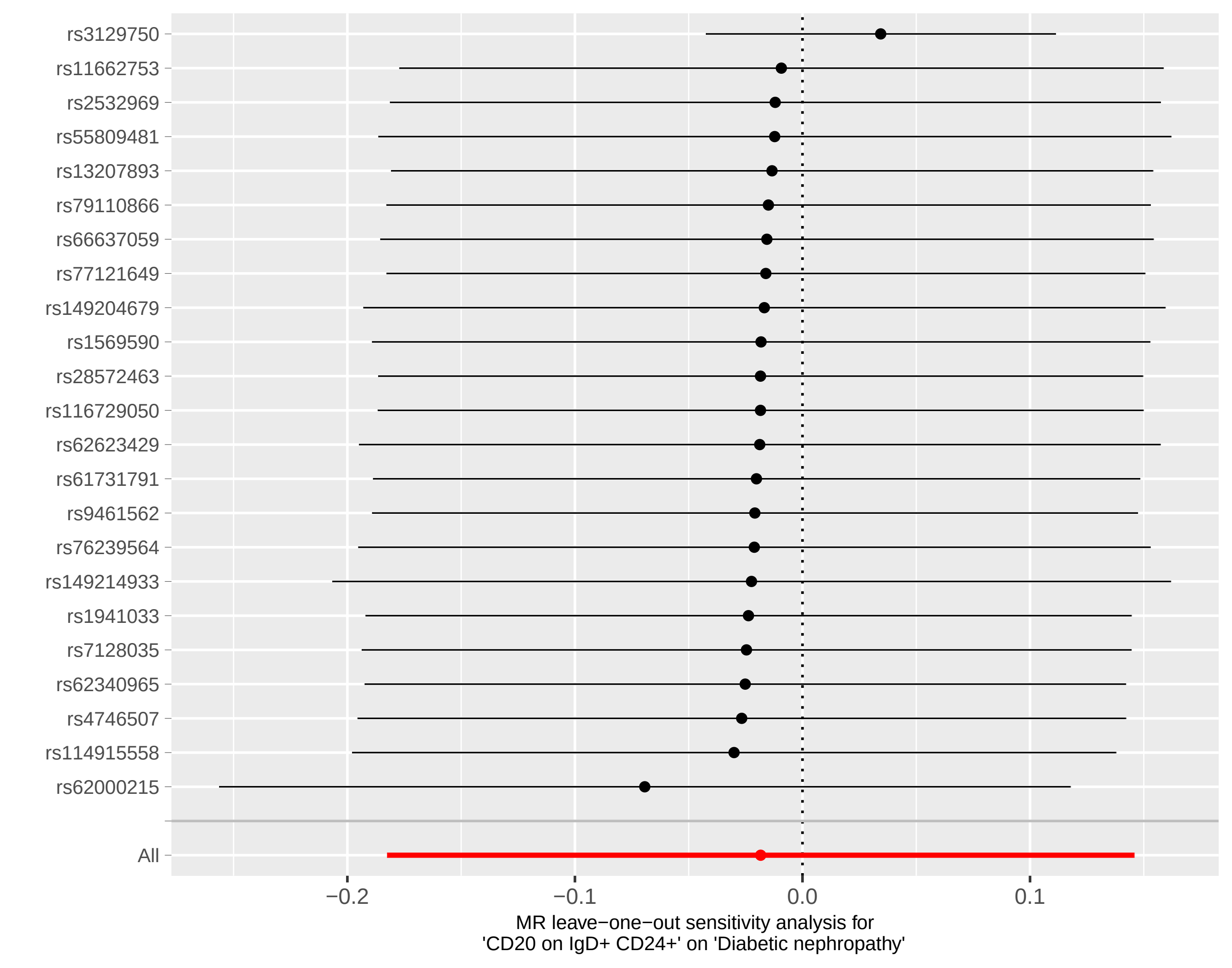
rs3129750
rs11662753
rs2532969
rs55809481
rs13207893
rs79110866
rs66637059
rs77121649
rs149204679
rs1569590
rs28572463
rs116729050
rs62623429
rs61731791
rs9461562
rs76239564
rs149214933
rs1941033
rs7128035
rs62340965
rs4746507
rs114915558
rs62000215
All
−0.2
−0.1
0.0
0.1
MR leave−one−out sensitivity analysis for
'CD20 on IgD+ CD24+' on 'Diabetic nephropathy'

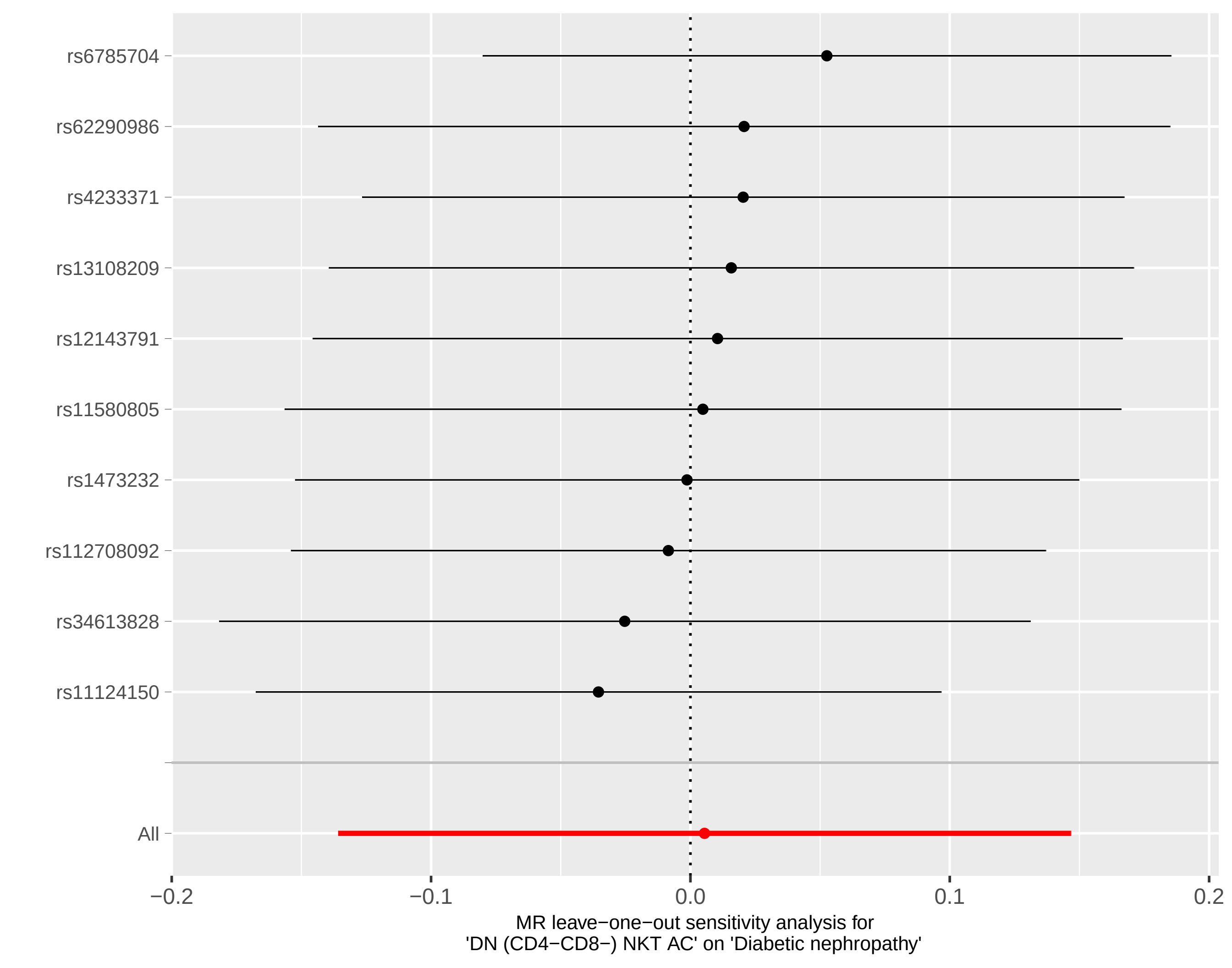

rs6785704
rs62290986
rs4233371
rs13108209
rs12143791
rs11580805
rs1473232
rs112708092
rs34613828
rs11124150
All
−0.2
−0.1
0.0
0.1
0.2
MR leave−one−out sensitivity analysis for
'DN (CD4−CD8−) NKT AC' on 'Diabetic nephropathy'

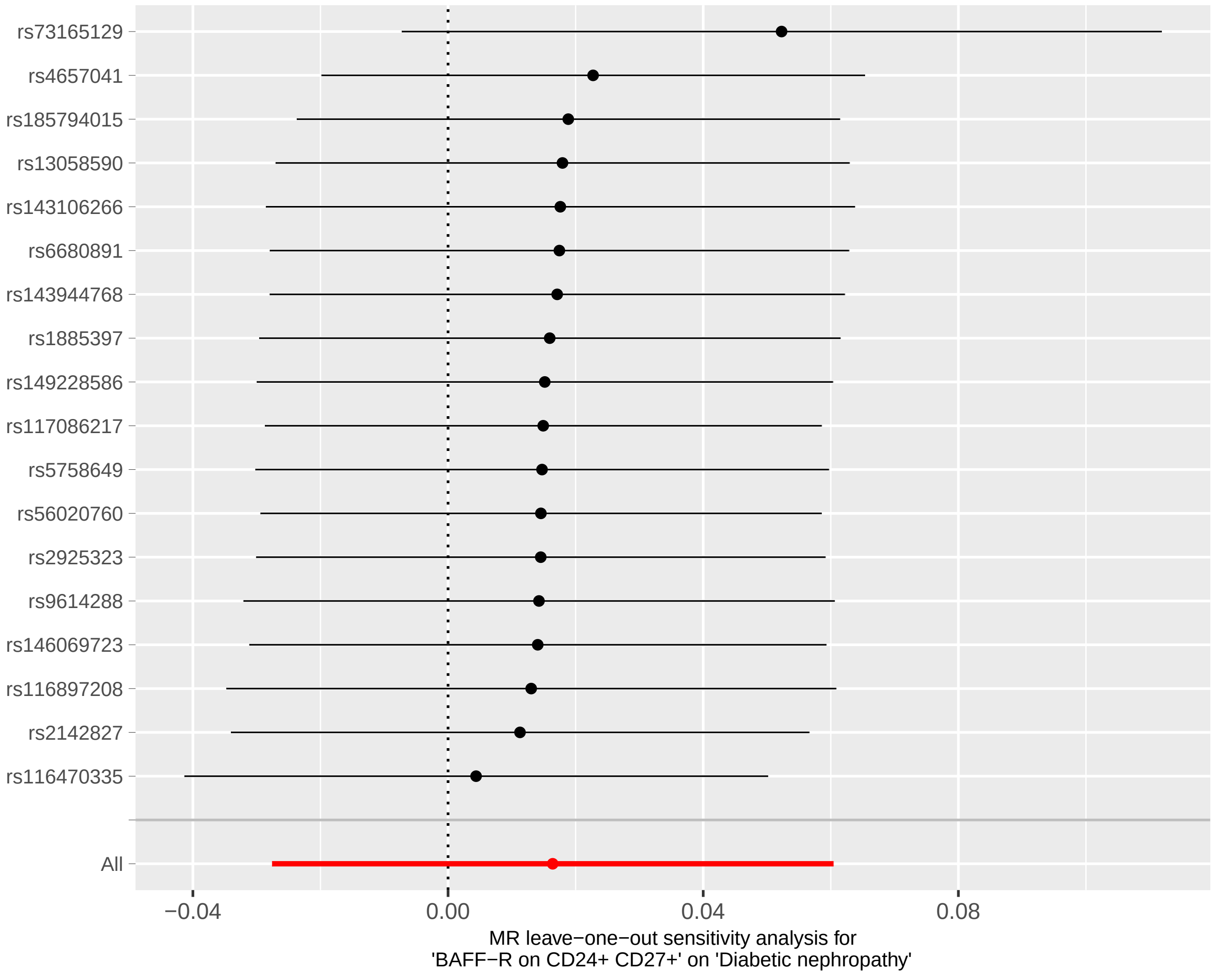
rs73165129
rs4657041
rs185794015
rs13058590
rs143106266
rs6680891
rs143944768
rs1885397
rs149228586
rs117086217
rs5758649
rs56020760
rs2925323
rs9614288
rs146069723
rs116897208
rs2142827
rs116470335
All
−0.04
0.00
0.04
0.08
MR leave−one−out sensitivity analysis for
'BAFF−R on CD24+ CD27+' on 'Diabetic nephropathy'

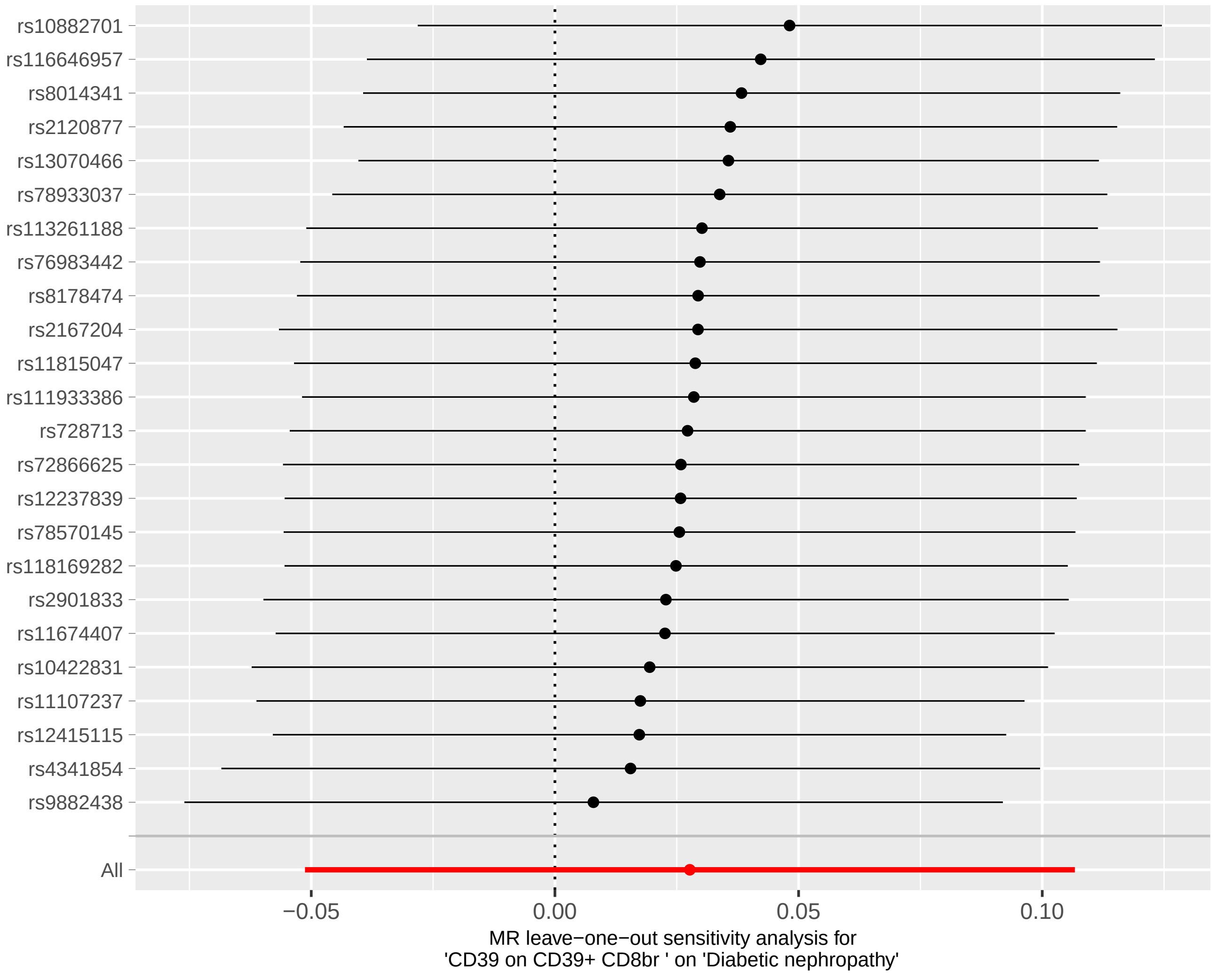
rs10882701
rs116646957
rs8014341
rs2120877
rs13070466
rs78933037
rs113261188
rs76983442
rs8178474
rs2167204
rs11815047
rs111933386
rs728713
rs72866625
rs12237839
rs78570145
rs118169282
rs2901833
rs11674407
rs10422831
rs11107237
rs12415115
rs4341854
rs9882438
All
−0.05
0.00
0.05
0.10
MR leave−one−out sensitivity analysis for
'CD39 on CD39+ CD8br ' on 'Diabetic nephropathy'

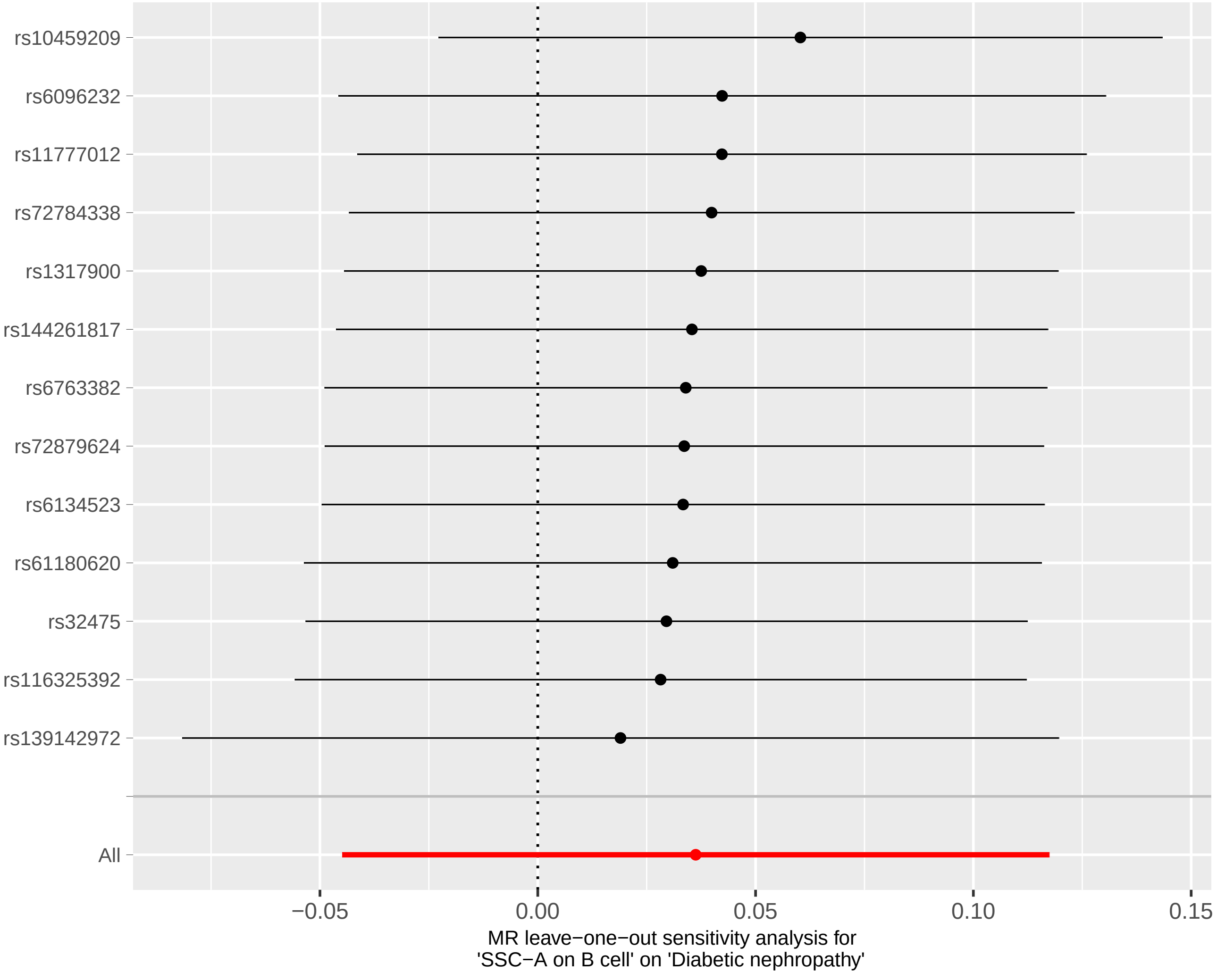
rs10459209
rs6096232
rs11777012
rs72784338
rs1317900
rs144261817
rs6763382
rs72879624
rs6134523
rs61180620
rs32475
rs116325392
rs139142972
All
−0.05
0.00
0.05
0.10
0.15
MR leave−one−out sensitivity analysis for
'SSC−A on B cell' on 'Diabetic nephropathy'

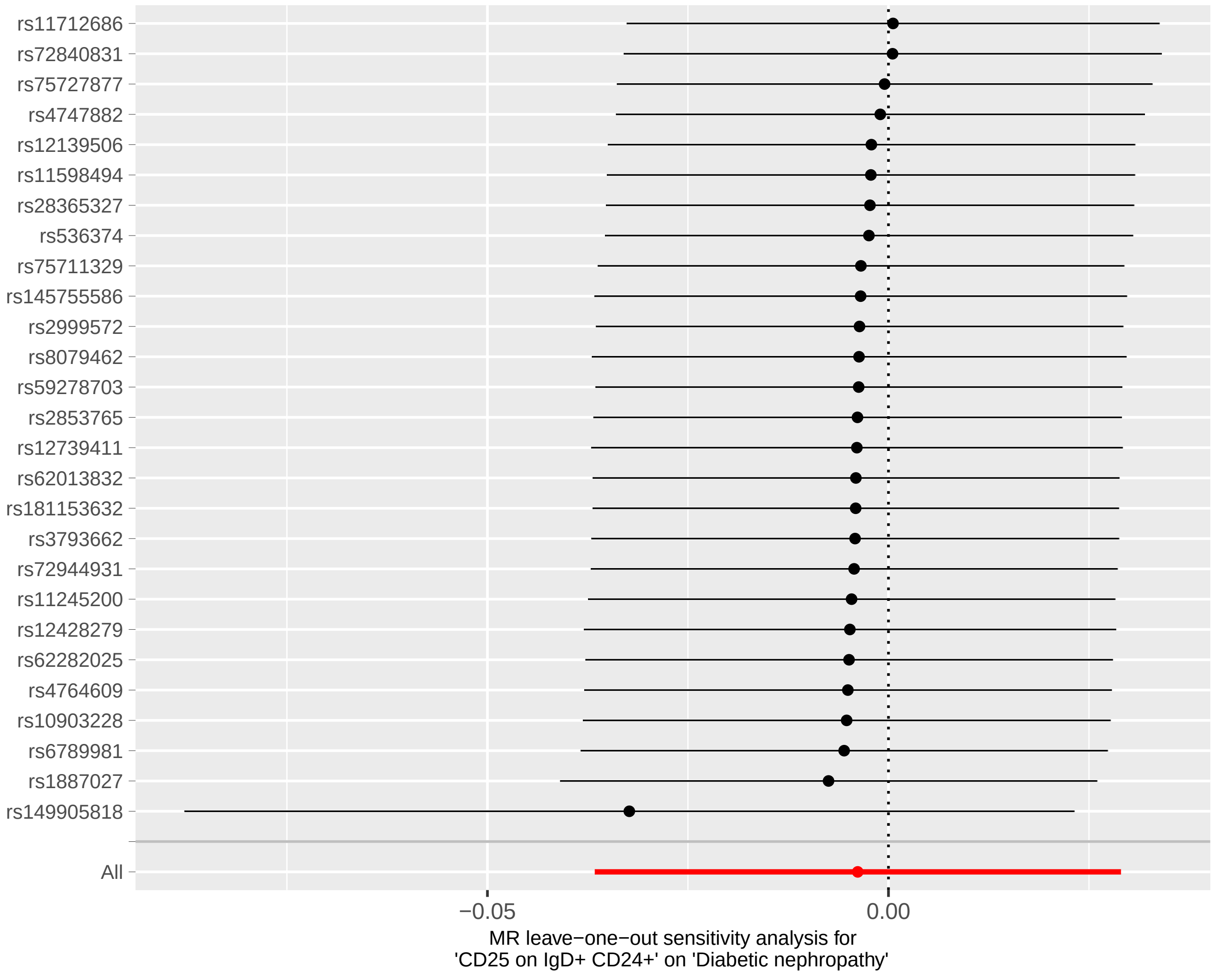
rs11712686
rs72840831
rs75727877
rs4747882
rs12139506
rs11598494
rs28365327
rs536374
rs75711329
rs145755586
rs2999572
rs8079462
rs59278703
rs2853765
rs12739411
rs62013832
rs181153632
rs3793662
rs72944931
rs11245200
rs12428279
rs62282025
rs4764609
rs10903228
rs6789981
rs1887027
rs149905818
All
−0.05
0.00
MR leave−one−out sensitivity analysis for
'CD25 on IgD+ CD24+' on 'Diabetic nephropathy'

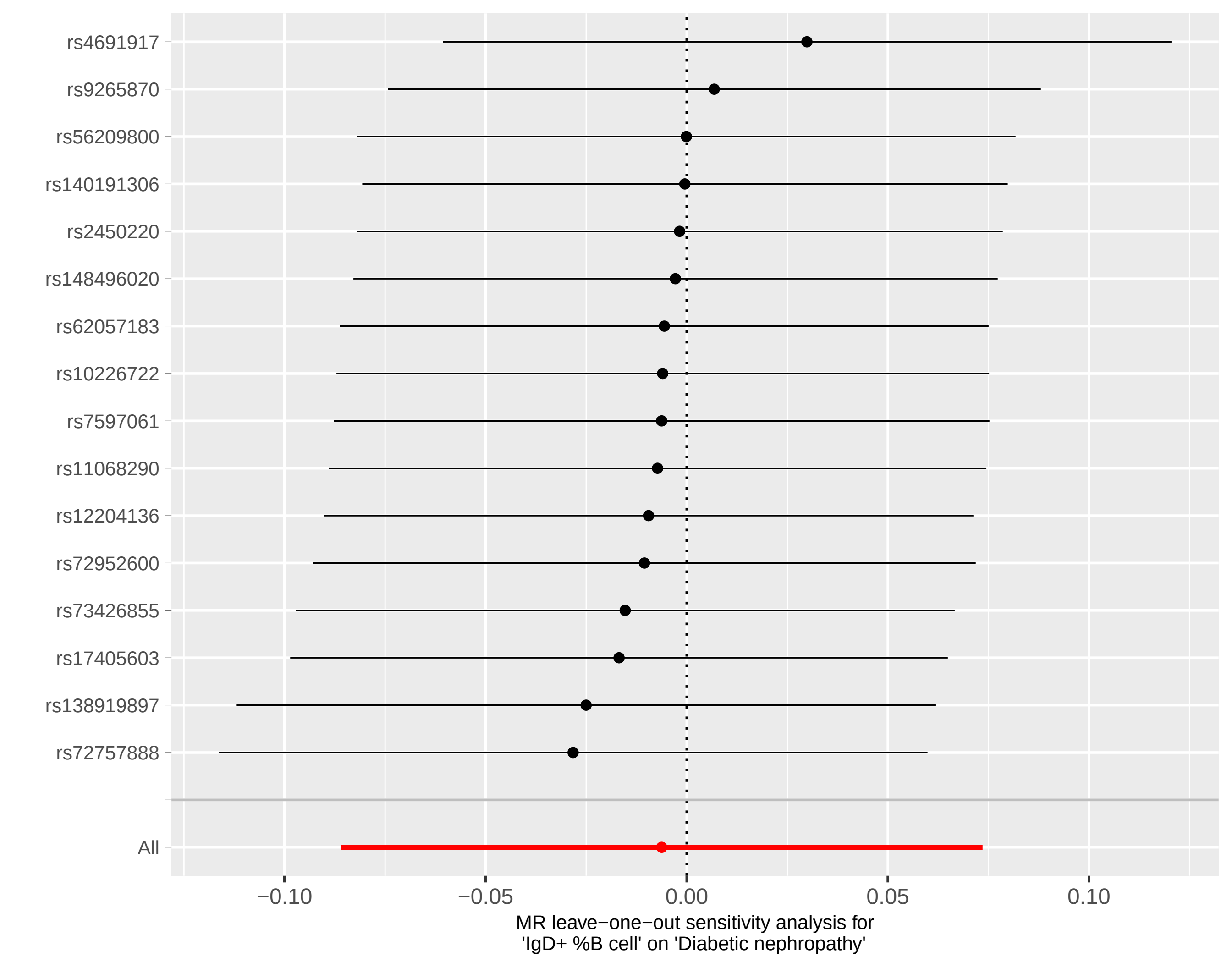

rs4691917
rs9265870
rs56209800
rs140191306
rs2450220
rs148496020
rs62057183
rs10226722
rs7597061
rs11068290
rs12204136
rs72952600
rs73426855
rs17405603
rs138919897
rs72757888
All
−0.10
−0.05
0.00
0.05
0.10
MR leave−one−out sensitivity analysis for
'IgD+ %B cell' on 'Diabetic nephropathy'

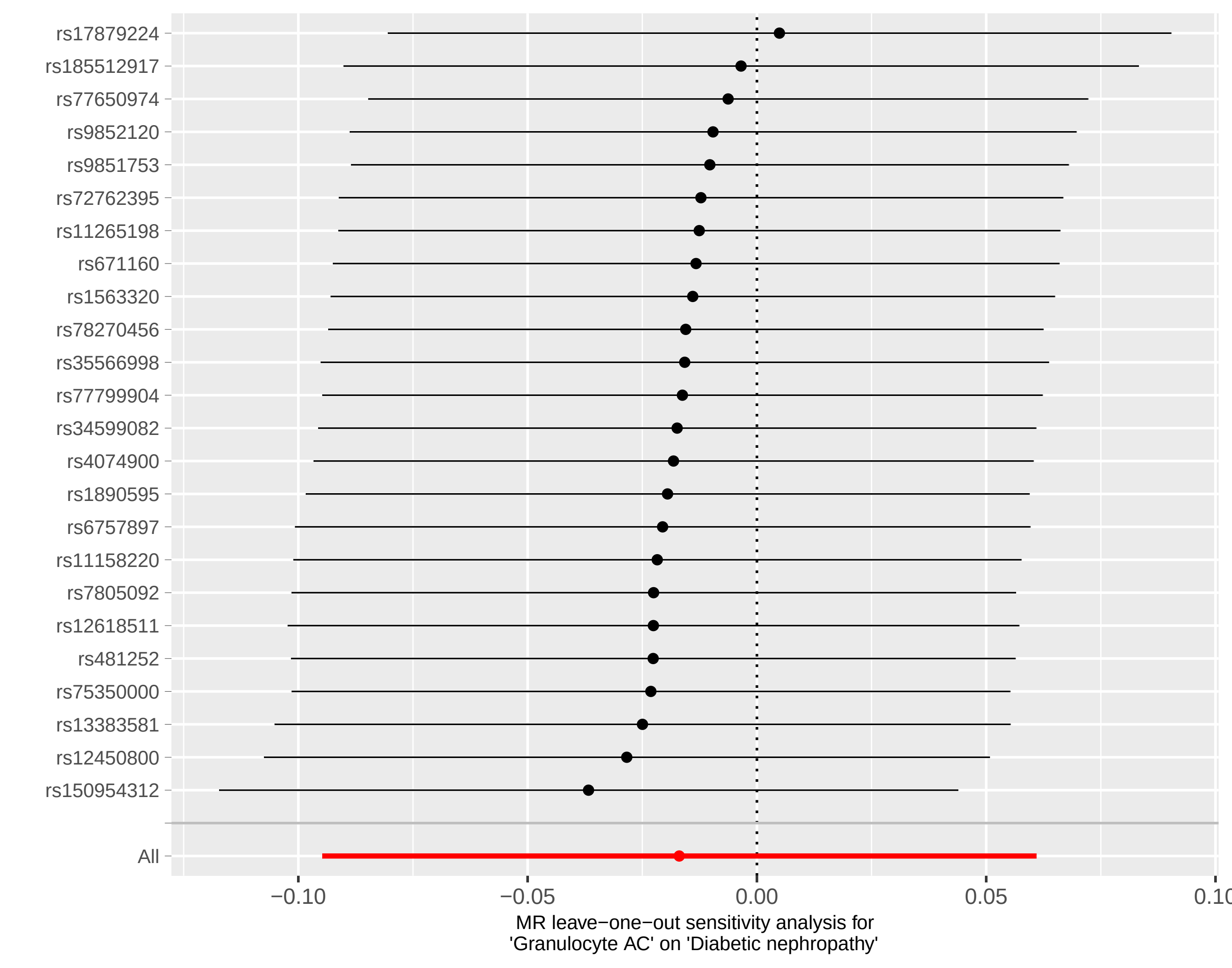
rs17879224
rs185512917
rs77650974
rs9852120
rs9851753
rs72762395
rs11265198
rs671160
rs1563320
rs78270456
rs35566998
rs77799904
rs34599082
rs4074900
rs1890595
rs6757897
rs11158220
rs7805092
rs12618511
rs481252
rs75350000
rs13383581
rs12450800
rs150954312
All
−0.10
−0.05
0.00
0.05
0.10
MR leave−one−out sensitivity analysis for
'Granulocyte AC' on 'Diabetic nephropathy'

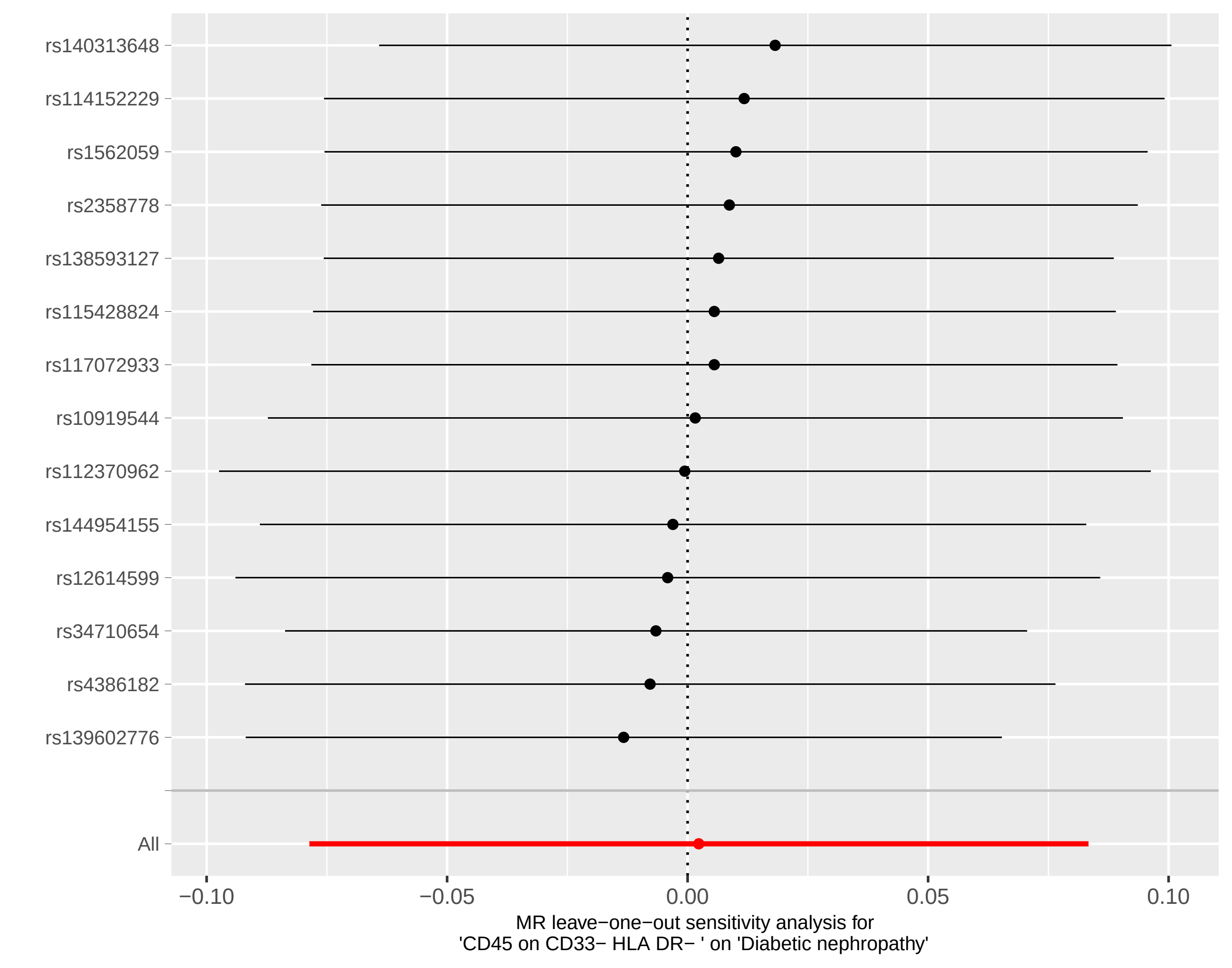
rs140313648
rs114152229
rs1562059
rs2358778
rs138593127
rs115428824
rs117072933
rs10919544
rs112370962
rs144954155
rs12614599
rs34710654
rs4386182
rs139602776
All
−0.10
−0.05
0.00
0.05
0.10
MR leave−one−out sensitivity analysis for
'CD45 on CD33− HLA DR− ' on 'Diabetic nephropathy'

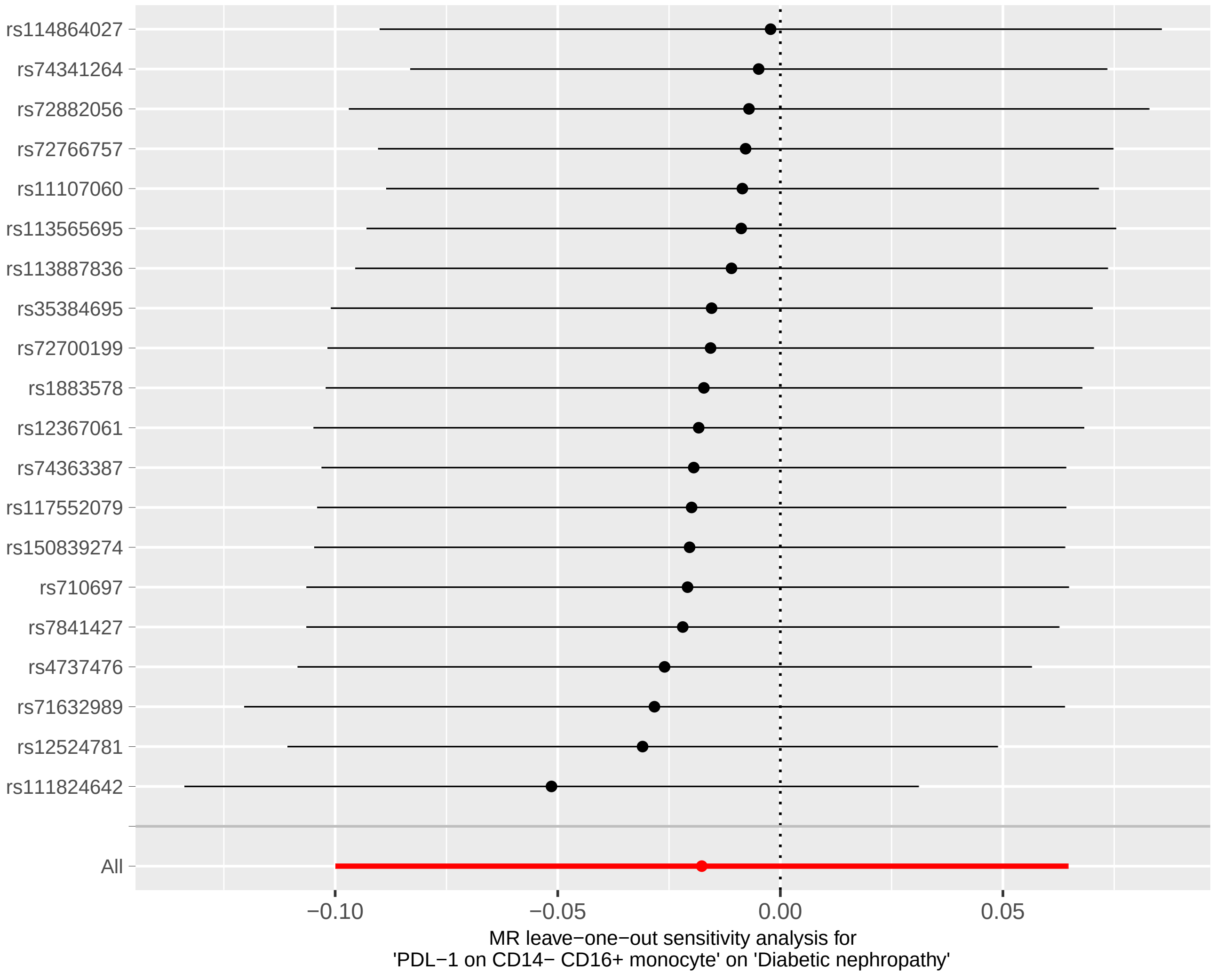
rs114864027
rs74341264
rs72882056
rs72766757
rs11107060
rs113565695
rs113887836
rs35384695
rs72700199
rs1883578
rs12367061
rs74363387
rs117552079
rs150839274
rs710697
rs7841427
rs4737476
rs71632989
rs12524781
rs111824642
All
−0.10
−0.05
0.00
0.05
MR leave−one−out sensitivity analysis for
'PDL−1 on CD14− CD16+ monocyte' on 'Diabetic nephropathy'

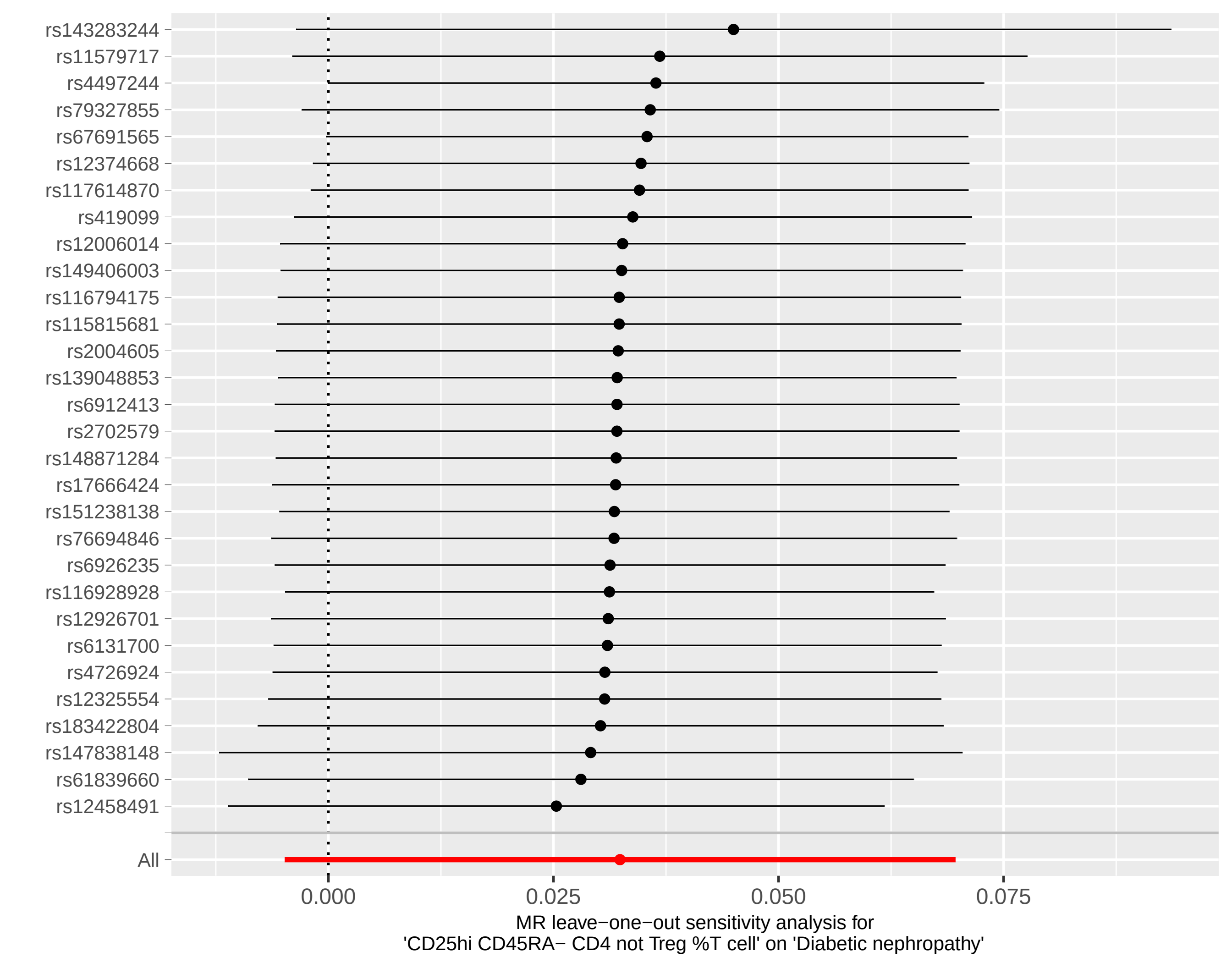

rs143283244
rs11579717
rs4497244
rs79327855
rs67691565
rs12374668
rs117614870
rs419099
rs12006014
rs149406003
rs116794175
rs115815681
rs2004605
rs139048853
rs6912413
rs2702579
rs148871284
rs17666424
rs151238138
rs76694846
rs6926235
rs116928928
rs12926701
rs6131700
rs4726924
rs12325554
rs183422804
rs147838148
rs61839660
rs12458491
All
0.000
0.025
0.050
0.075
MR leave−one−out sensitivity analysis for
'CD25hi CD45RA− CD4 not Treg %T cell' on 'Diabetic nephropathy'

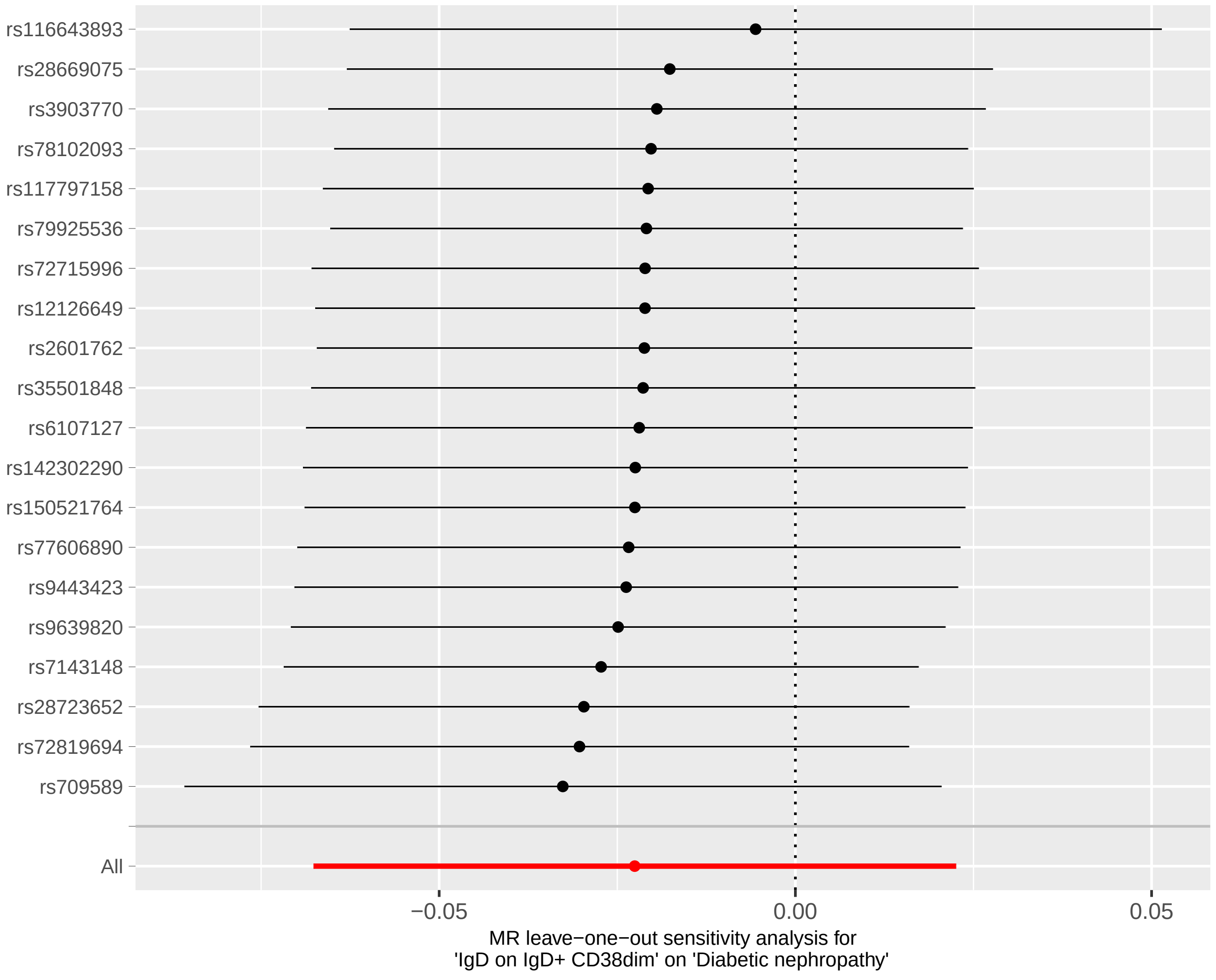
rs116643893
rs28669075
rs3903770
rs78102093
rs117797158
rs79925536
rs72715996
rs12126649
rs2601762
rs35501848
rs6107127
rs142302290
rs150521764
rs77606890
rs9443423
rs9639820
rs7143148
rs28723652
rs72819694
rs709589
All
−0.05
0.00
0.05
MR leave−one−out sensitivity analysis for
'IgD on IgD+ CD38dim' on 'Diabetic nephropathy'

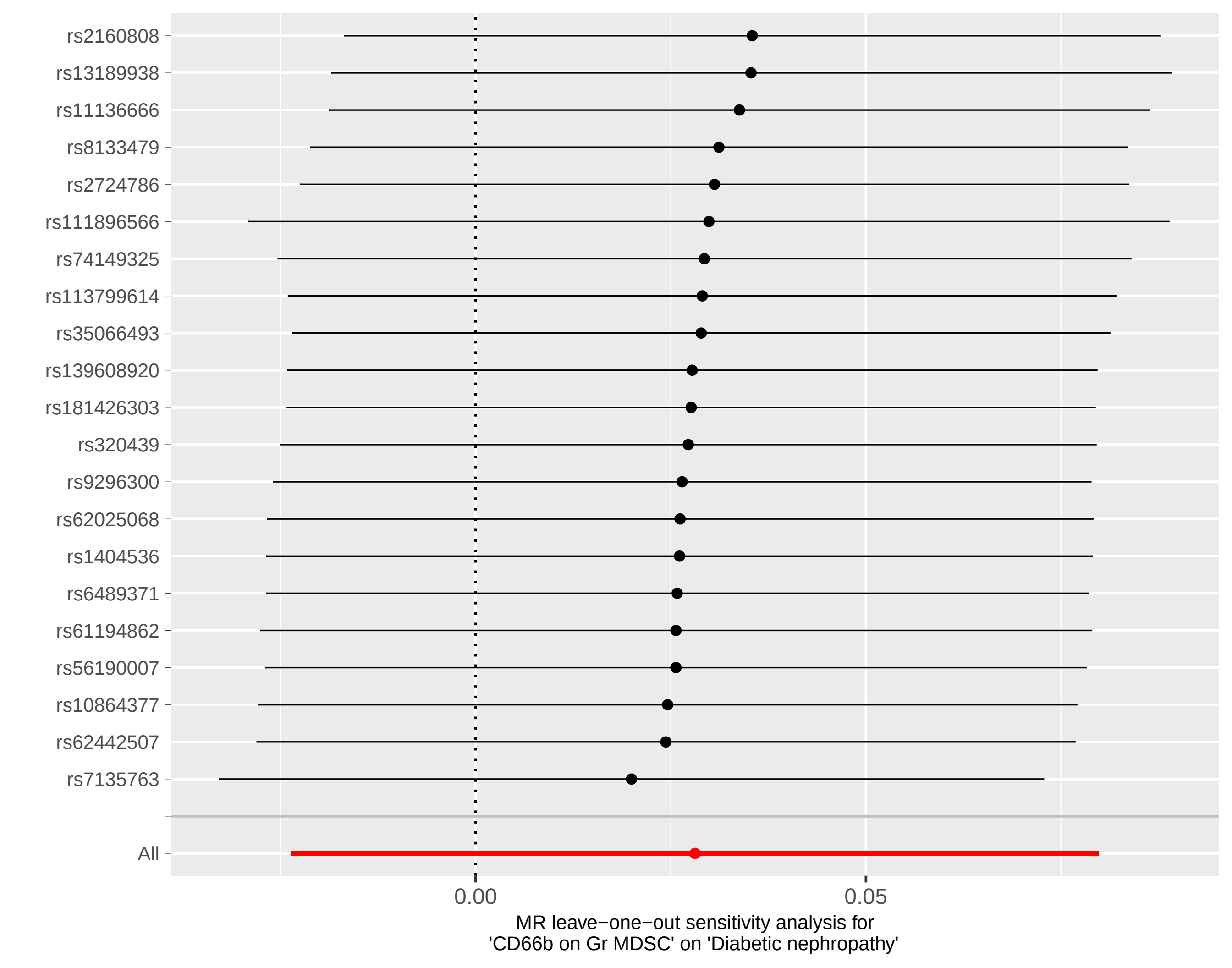
rs2160808
rs13189938
rs11136666
rs8133479
rs2724786
rs111896566
rs74149325
rs113799614
rs35066493
rs139608920
rs181426303
rs320439
rs9296300
rs62025068
rs1404536
rs6489371
rs61194862
rs56190007
rs10864377
rs62442507
rs7135763
All
0.00
0.05
MR leave−one−out sensitivity analysis for
'CD66b on Gr MDSC' on 'Diabetic nephropathy'

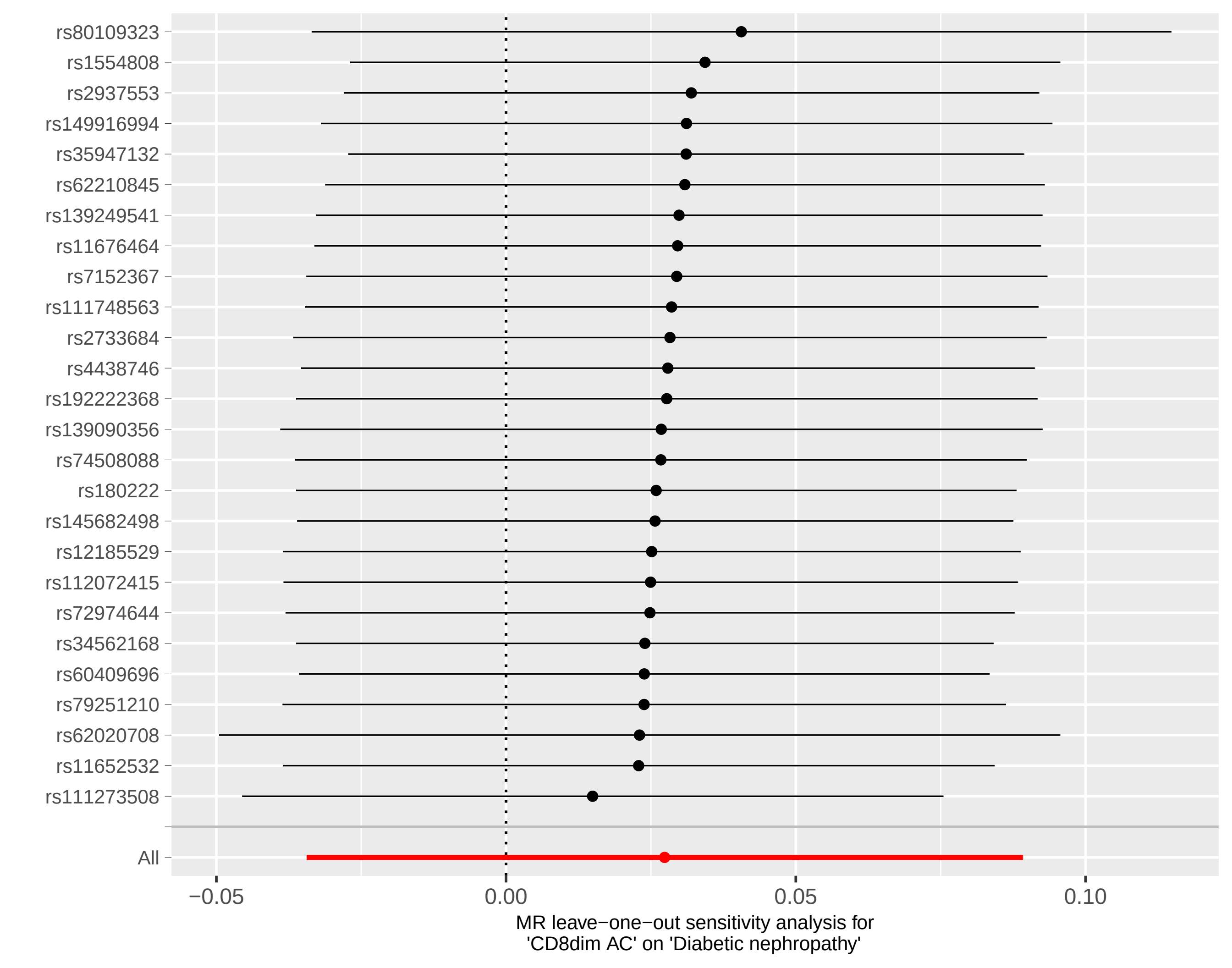

rs80109323
rs1554808
rs2937553
rs149916994
rs35947132
rs62210845
rs139249541
rs11676464
rs7152367
rs111748563
rs2733684
rs4438746
rs192222368
rs139090356
rs74508088
rs180222
rs145682498
rs12185529
rs112072415
rs72974644
rs34562168
rs60409696
rs79251210
rs62020708
rs11652532
rs111273508
All
−0.05
0.00
0.05
0.10
MR leave−one−out sensitivity analysis for
'CD8dim AC' on 'Diabetic nephropathy'

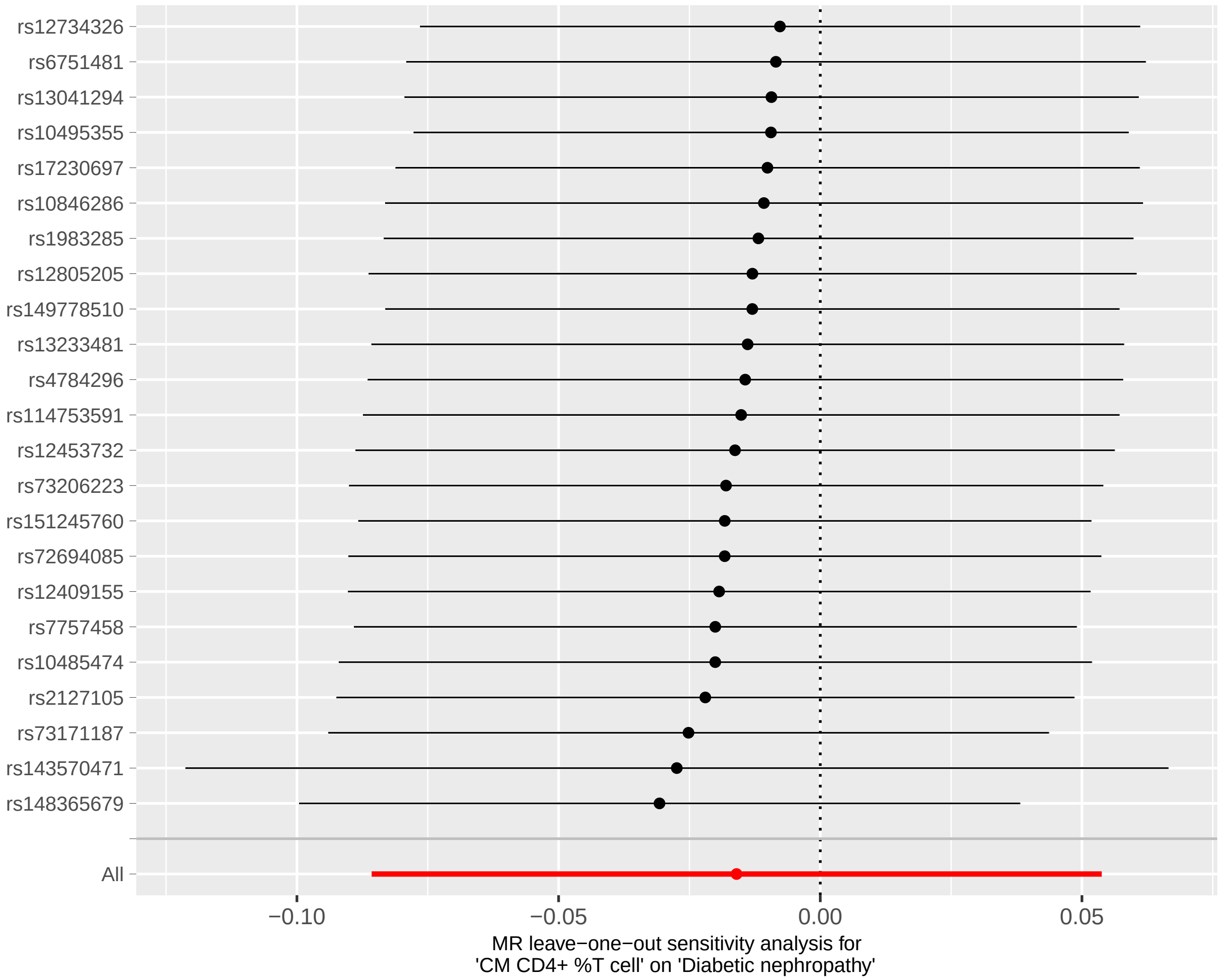
rs12734326
rs6751481
rs13041294
rs10495355
rs17230697
rs10846286
rs1983285
rs12805205
rs149778510
rs13233481
rs4784296
rs114753591
rs12453732
rs73206223
rs151245760
rs72694085
rs12409155
rs7757458
rs10485474
rs2127105
rs73171187
rs143570471
rs148365679
All
−0.10
−0.05
0.00
0.05
MR leave−one−out sensitivity analysis for
'CM CD4+ %T cell' on 'Diabetic nephropathy'

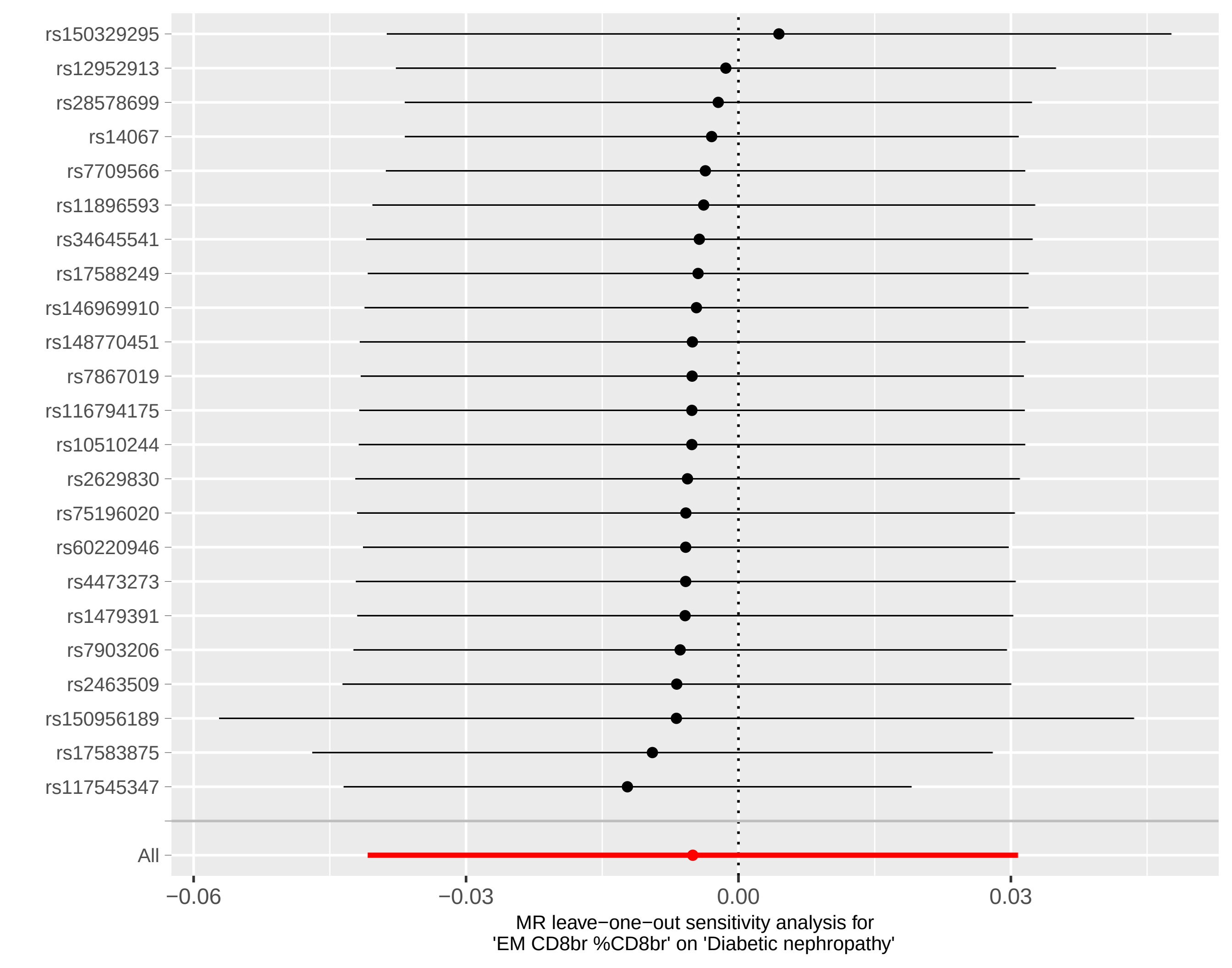
rs150329295
rs12952913
rs28578699
rs14067
rs7709566
rs11896593
rs34645541
rs17588249
rs146969910
rs148770451
rs7867019
rs116794175
rs10510244
rs2629830
rs75196020
rs60220946
rs4473273
rs1479391
rs7903206
rs2463509
rs150956189
rs17583875
rs117545347
All
−0.06
−0.03
0.00
0.03
MR leave−one−out sensitivity analysis for
'EM CD8br %CD8br' on 'Diabetic nephropathy'

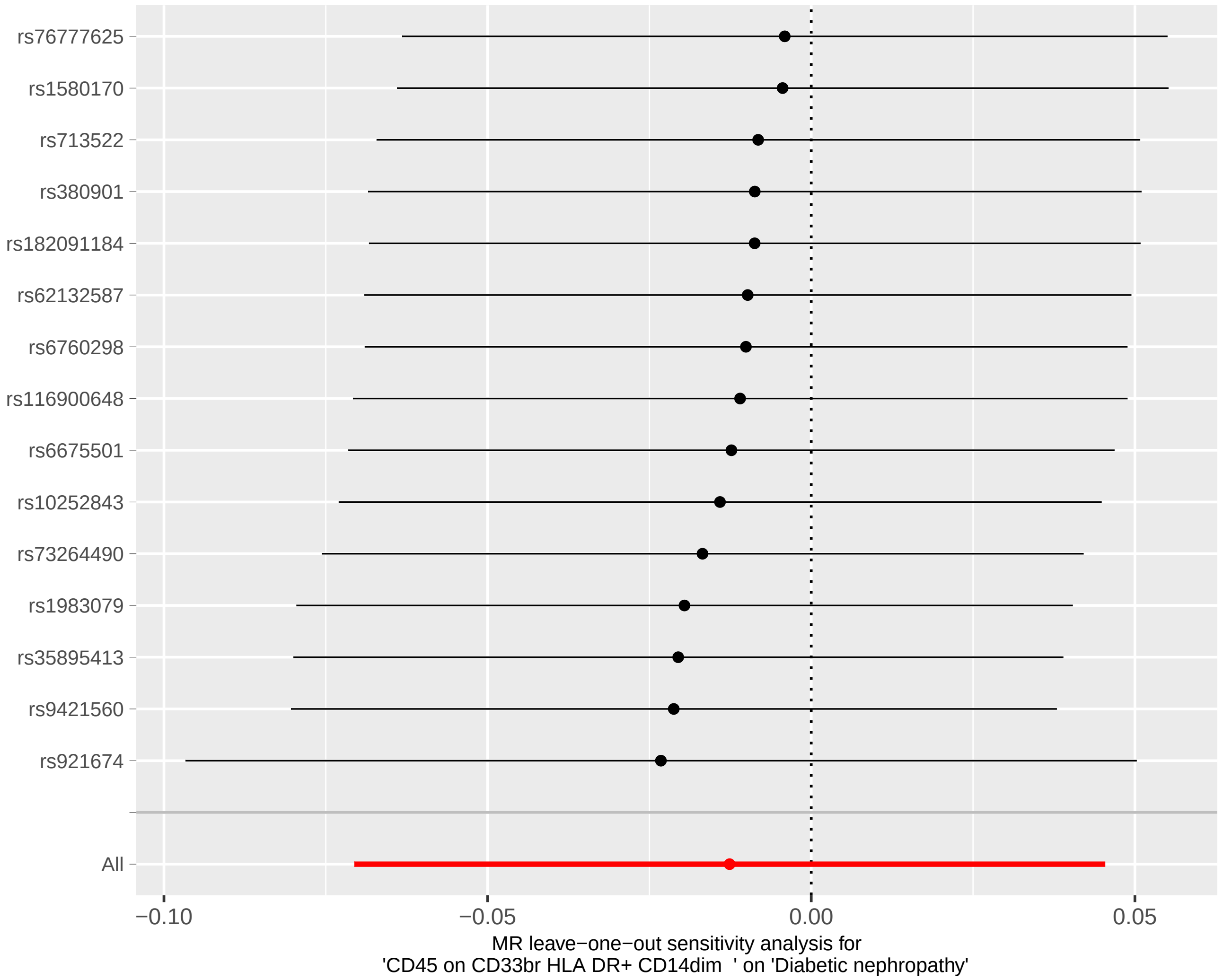
rs76777625
rs1580170
rs713522
rs380901
rs182091184
rs62132587
rs6760298
rs116900648
rs6675501
rs10252843
rs73264490
rs1983079
rs35895413
rs9421560
rs921674
All
−0.10
−0.05
0.00
0.05
MR leave−one−out sensitivity analysis for
'CD45 on CD33br HLA DR+ CD14dim ' on 'Diabetic nephropathy'

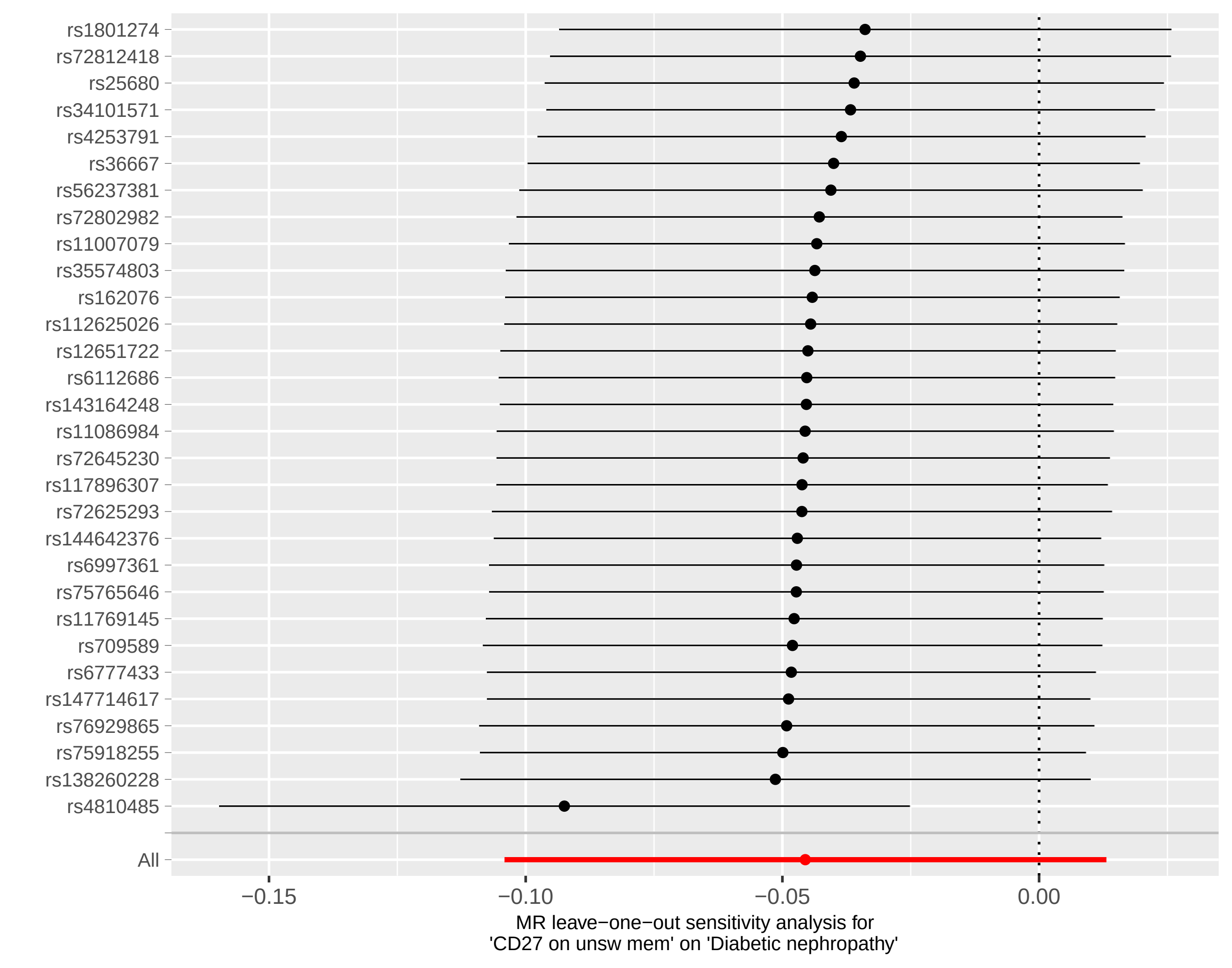
rs1801274
rs72812418
rs25680
rs34101571
rs4253791
rs36667
rs56237381
rs72802982
rs11007079
rs35574803
rs162076
rs112625026
rs12651722
rs6112686
rs143164248
rs11086984
rs72645230
rs117896307
rs72625293
rs144642376
rs6997361
rs75765646
rs11769145
rs709589
rs6777433
rs147714617
rs76929865
rs75918255
rs138260228
rs4810485
All
−0.15
−0.10
−0.05
0.00
MR leave−one−out sensitivity analysis for
'CD27 on unsw mem' on 'Diabetic nephropathy'

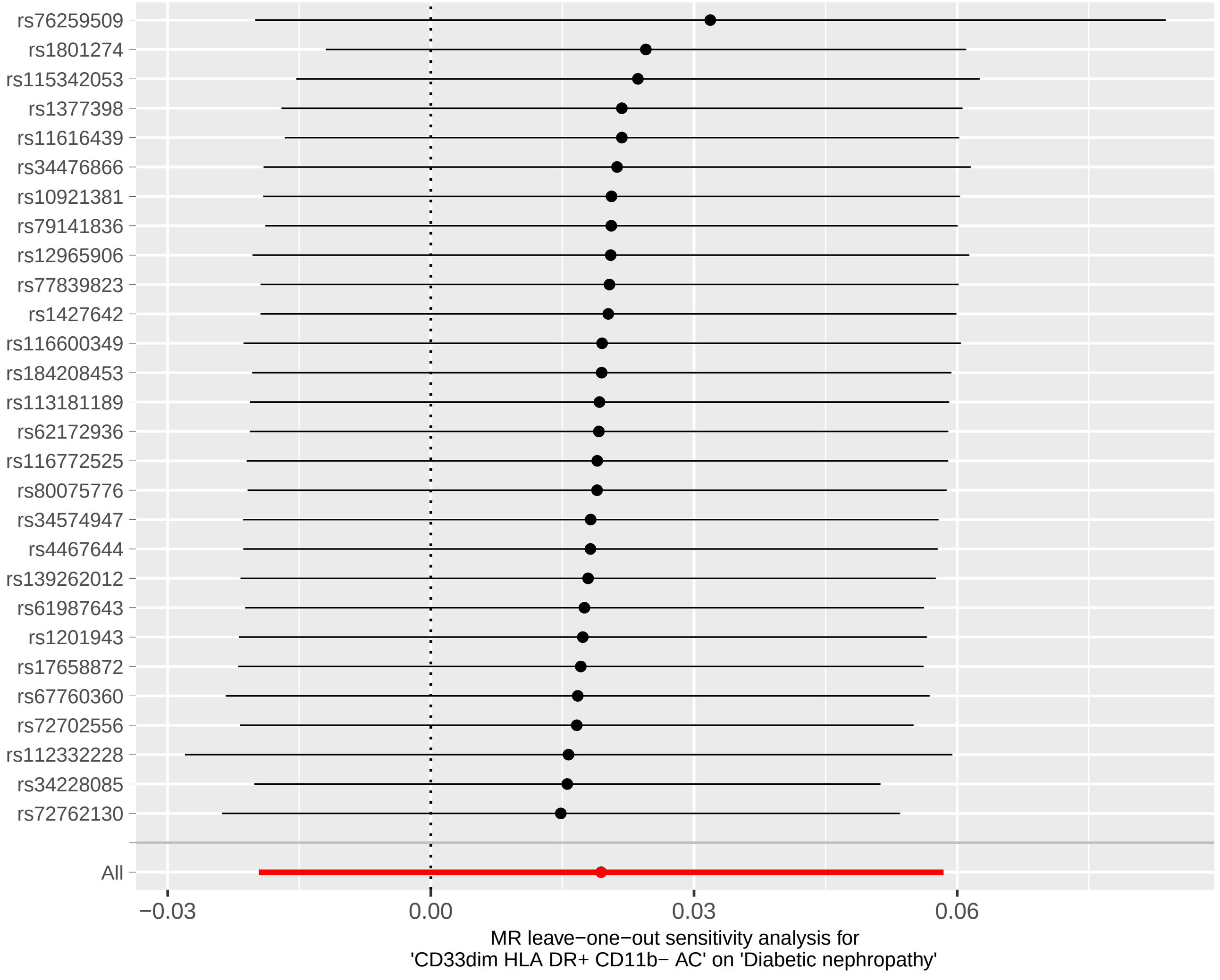

rs76259509
rs1801274
rs115342053
rs1377398
rs11616439
rs34476866
rs10921381
rs79141836
rs12965906
rs77839823
rs1427642
rs116600349
rs184208453
rs113181189
rs62172936
rs116772525
rs80075776
rs34574947
rs4467644
rs139262012
rs61987643
rs1201943
rs17658872
rs67760360
rs72702556
rs112332228
rs34228085
rs72762130
All
−0.03
0.00
0.03
0.06
MR leave−one−out sensitivity analysis for
'CD33dim HLA DR+ CD11b− AC' on 'Diabetic nephropathy'

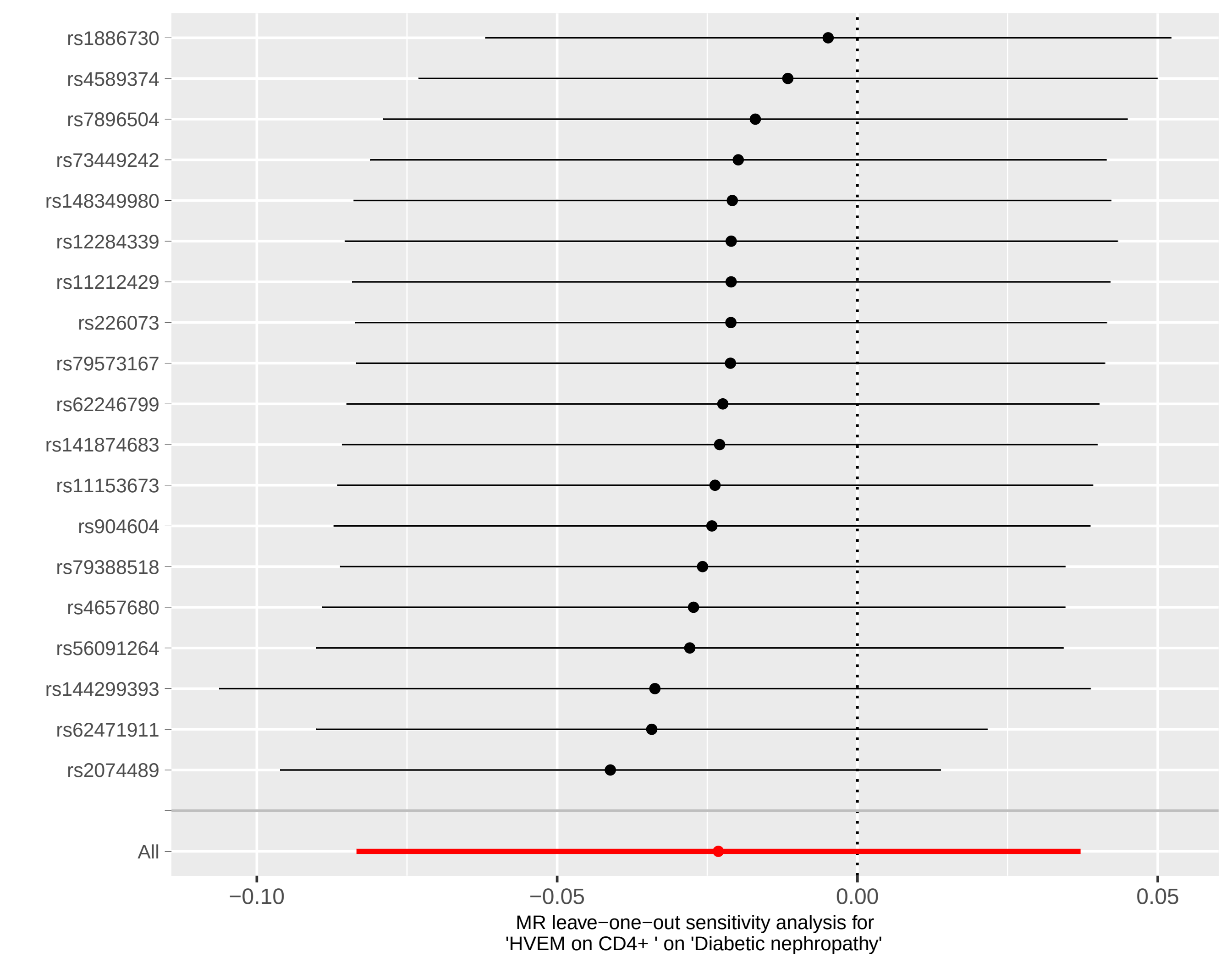
rs1886730
rs4589374
rs7896504
rs73449242
rs148349980
rs12284339
rs11212429
rs226073
rs79573167
rs62246799
rs141874683
rs11153673
rs904604
rs79388518
rs4657680
rs56091264
rs144299393
rs62471911
rs2074489
All
−0.10
−0.05
0.00
0.05
MR leave−one−out sensitivity analysis for
'HVEM on CD4+ ' on 'Diabetic nephropathy'

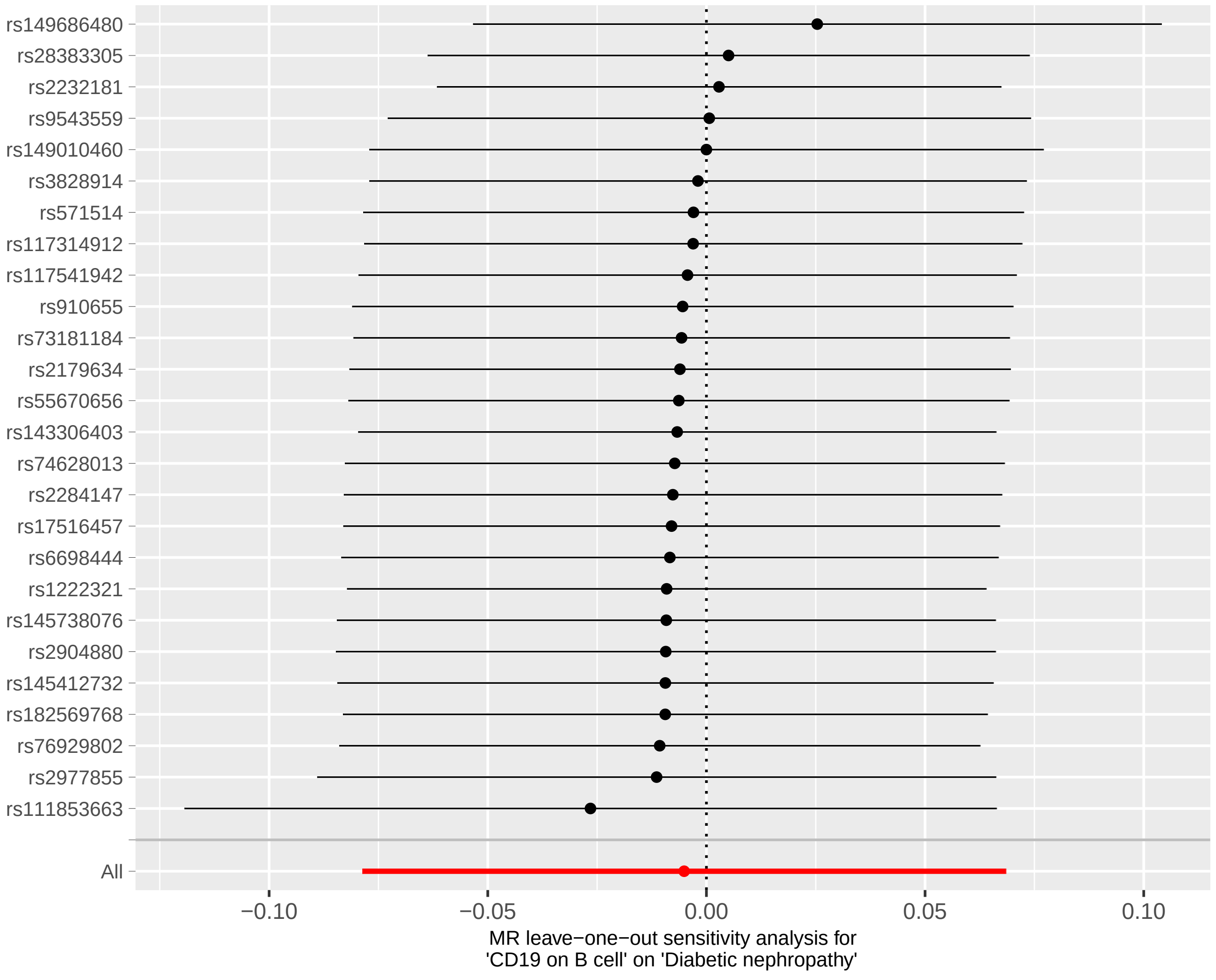
rs149686480
rs28383305
rs2232181
rs9543559
rs149010460
rs3828914
rs571514
rs117314912
rs117541942
rs910655
rs73181184
rs2179634
rs55670656
rs143306403
rs74628013
rs2284147
rs17516457
rs6698444
rs1222321
rs145738076
rs2904880
rs145412732
rs182569768
rs76929802
rs2977855
rs111853663
All
−0.10
−0.05
0.00
0.05
0.10
MR leave−one−out sensitivity analysis for
'CD19 on B cell' on 'Diabetic nephropathy'

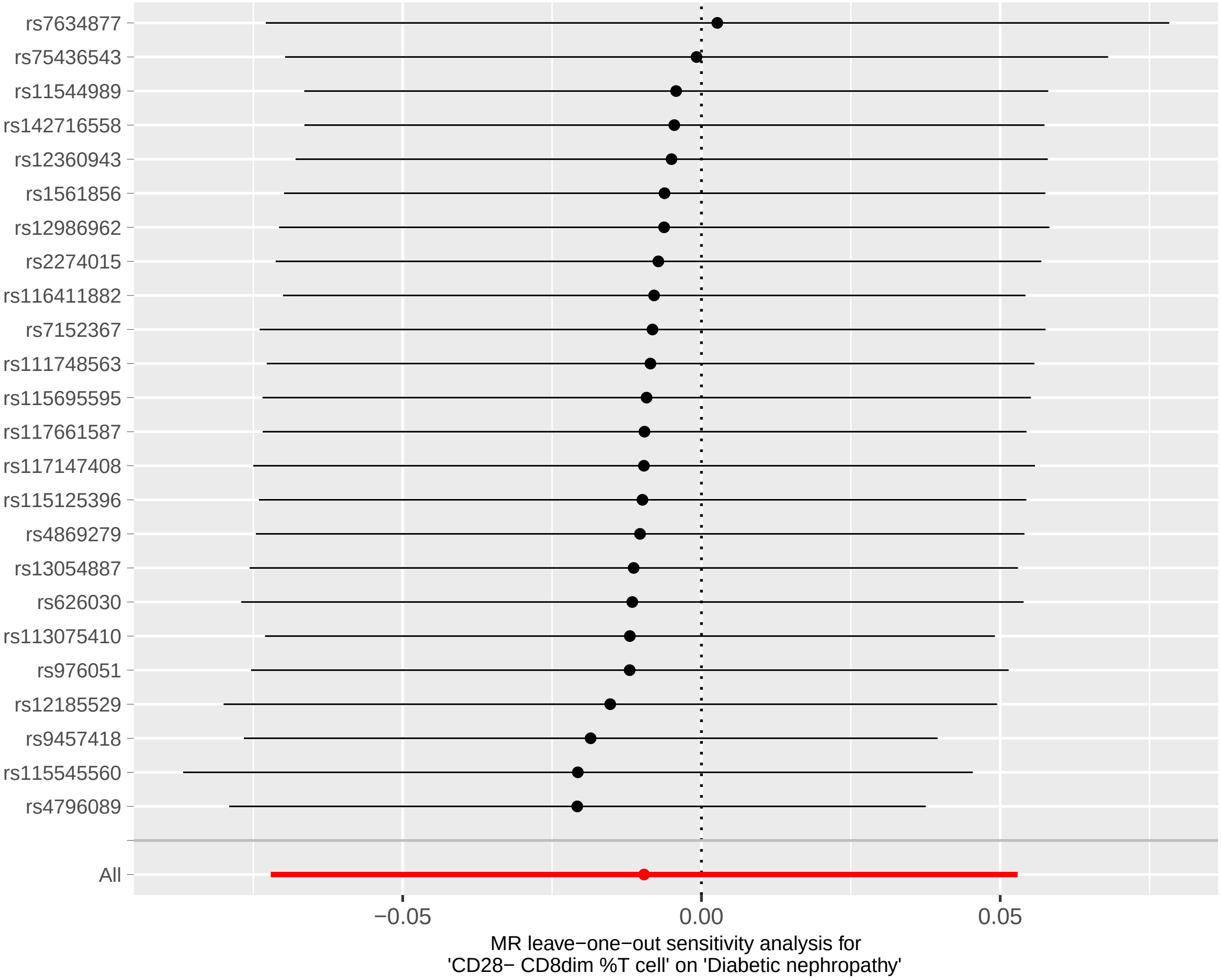

rs7634877
rs75436543
rs11544989
rs142716558
rs12360943
rs1561856
rs12986962
rs2274015
rs116411882
rs7152367
rs111748563
rs115695595
rs117661587
rs117147408
rs115125396
rs4869279
rs13054887
rs626030
rs113075410
rs976051
rs12185529
rs9457418
rs115545560
rs4796089
All
−0.05
0.00
0.05
MR leave−one−out sensitivity analysis for
'CD28− CD8dim %T cell' on 'Diabetic nephropathy'

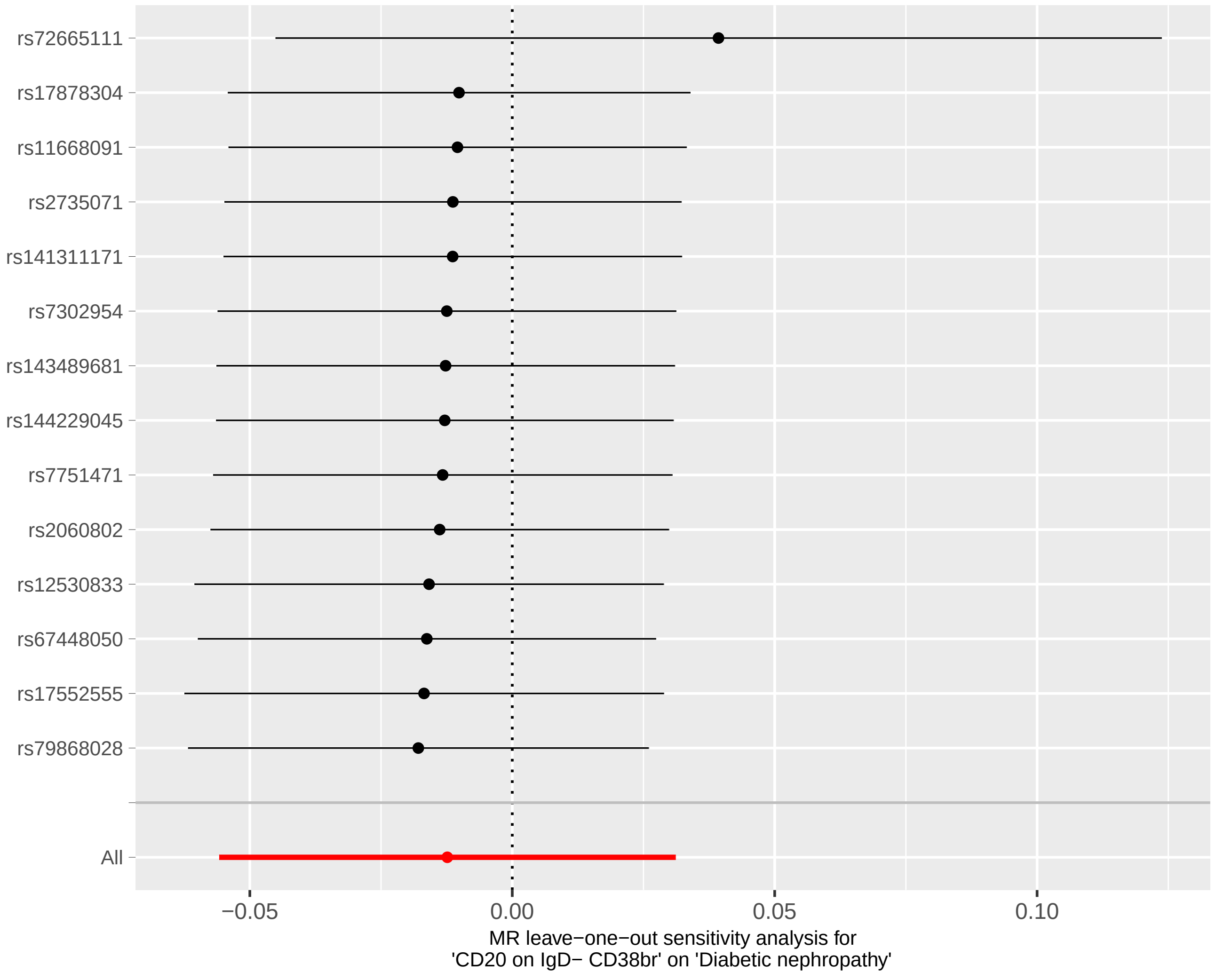
rs72665111
rs17878304
rs11668091
rs2735071
rs141311171
rs7302954
rs143489681
rs144229045
rs7751471
rs2060802
rs12530833
rs67448050
rs17552555
rs79868028
All
−0.05
0.00
0.05
0.10
MR leave−one−out sensitivity analysis for
'CD20 on IgD− CD38br' on 'Diabetic nephropathy'

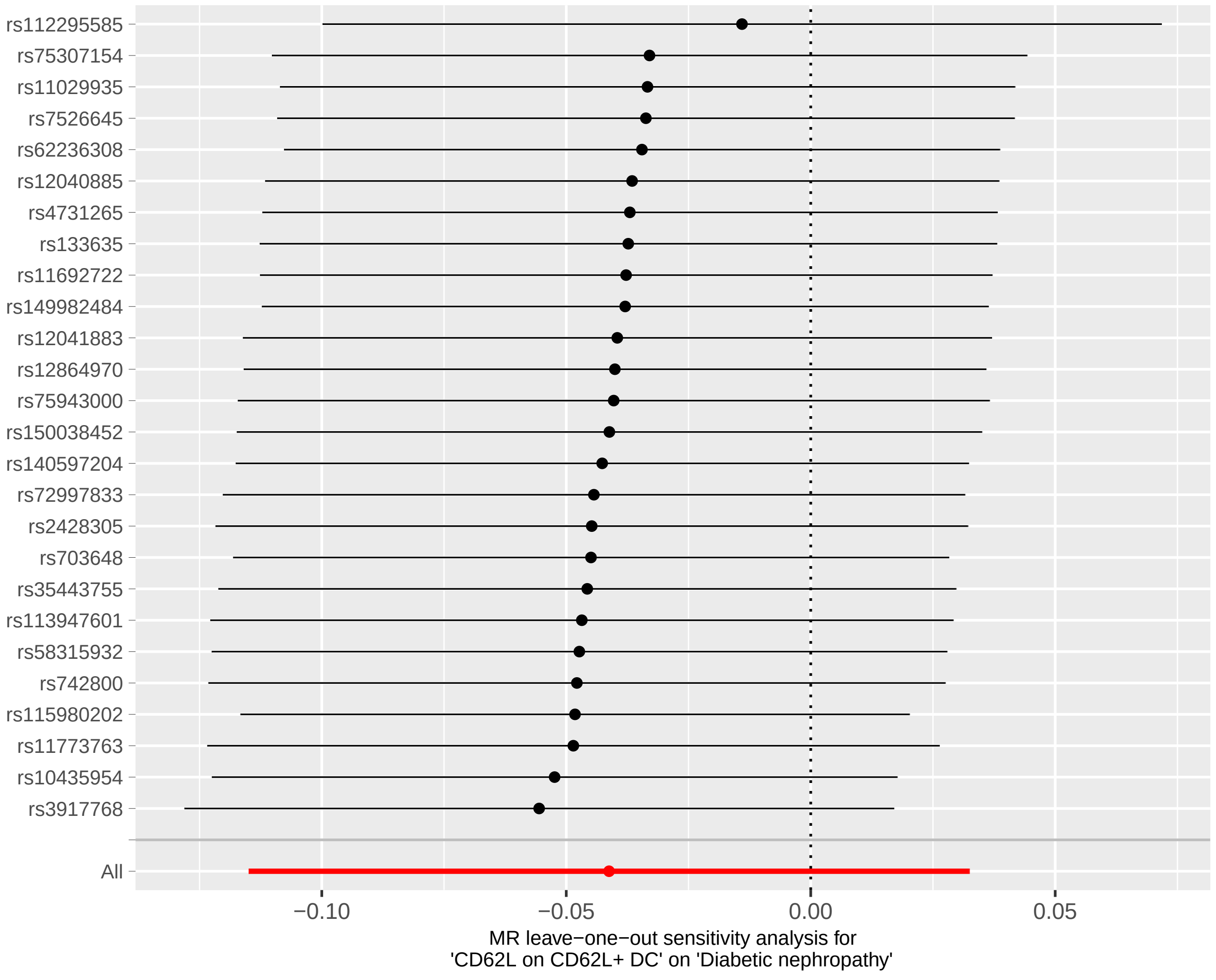
rs112295585
rs75307154
rs11029935
rs7526645
rs62236308
rs12040885
rs4731265
rs133635
rs11692722
rs149982484
rs12041883
rs12864970
rs75943000
rs150038452
rs140597204
rs72997833
rs2428305
rs703648
rs35443755
rs113947601
rs58315932
rs742800
rs115980202
rs11773763
rs10435954
rs3917768
All
−0.10
−0.05
0.00
0.05
MR leave−one−out sensitivity analysis for
'CD62L on CD62L+ DC' on 'Diabetic nephropathy'

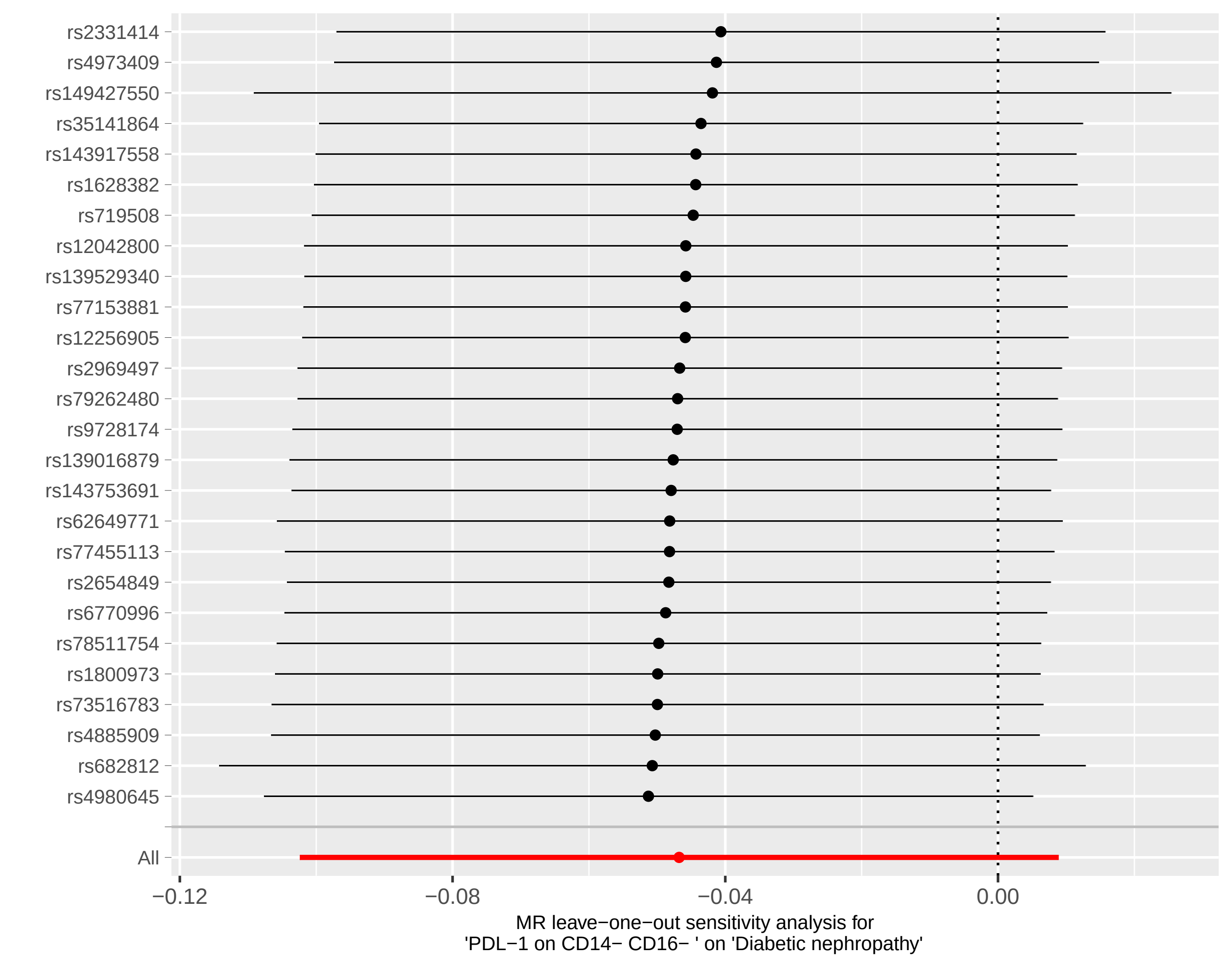
rs2331414
rs4973409
rs149427550
rs35141864
rs143917558
rs1628382
rs719508
rs12042800
rs139529340
rs77153881
rs12256905
rs2969497
rs79262480
rs9728174
rs139016879
rs143753691
rs62649771
rs77455113
rs2654849
rs6770996
rs78511754
rs1800973
rs73516783
rs4885909
rs682812
rs4980645
All
−0.12
−0.08
−0.04
0.00
MR leave−one−out sensitivity analysis for
'PDL−1 on CD14− CD16− ' on 'Diabetic nephropathy'

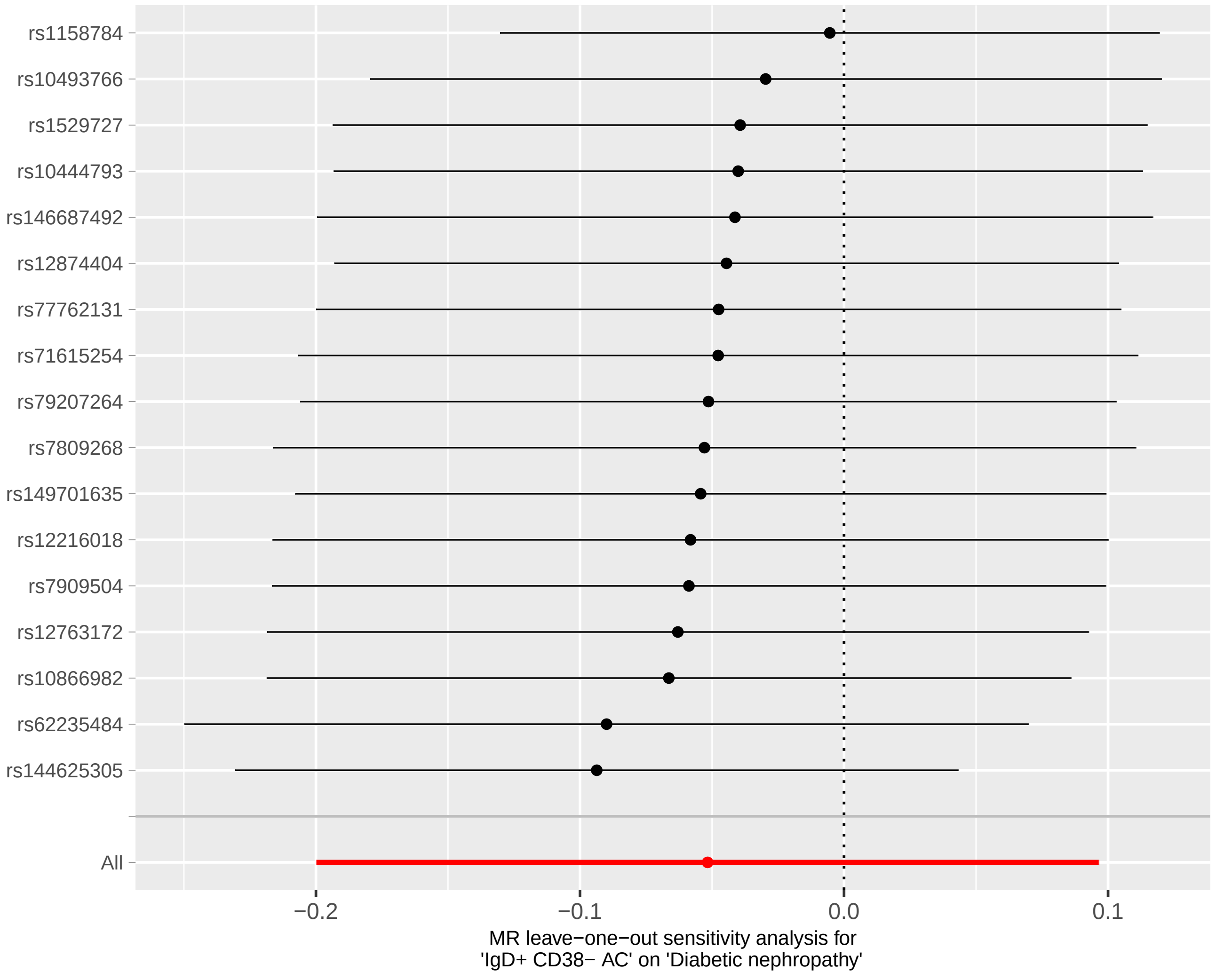

rs1158784
rs10493766
rs1529727
rs10444793
rs146687492
rs12874404
rs77762131
rs71615254
rs79207264
rs7809268
rs149701635
rs12216018
rs7909504
rs12763172
rs10866982
rs62235484
rs144625305
All
−0.2
−0.1
0.0
0.1
MR leave−one−out sensitivity analysis for
'IgD+ CD38− AC' on 'Diabetic nephropathy'

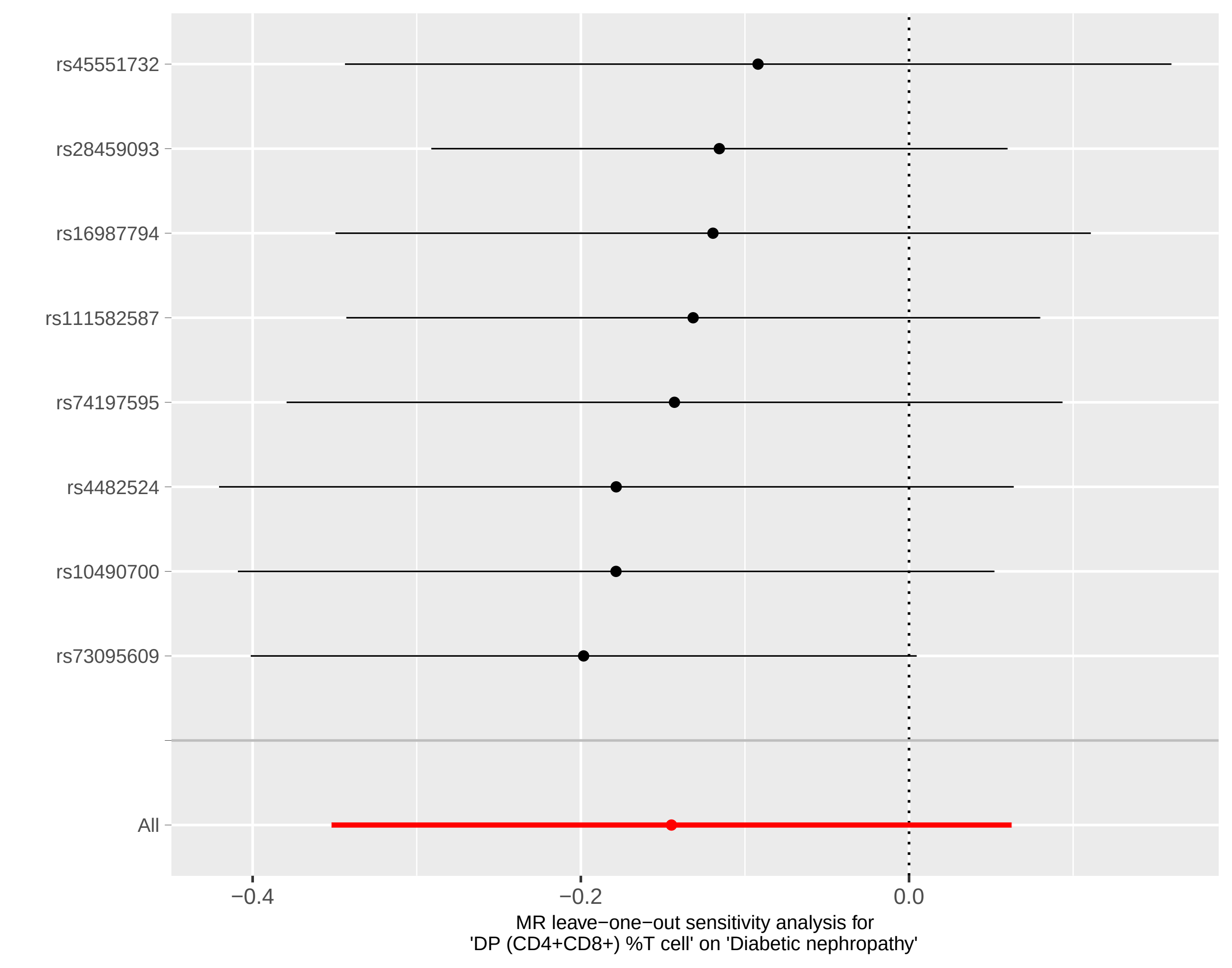
rs45551732
rs28459093
rs16987794
rs111582587
rs74197595
rs4482524
rs10490700
rs73095609
All
−0.4
−0.2
0.0
MR leave−one−out sensitivity analysis for
'DP (CD4+CD8+) %T cell' on 'Diabetic nephropathy'

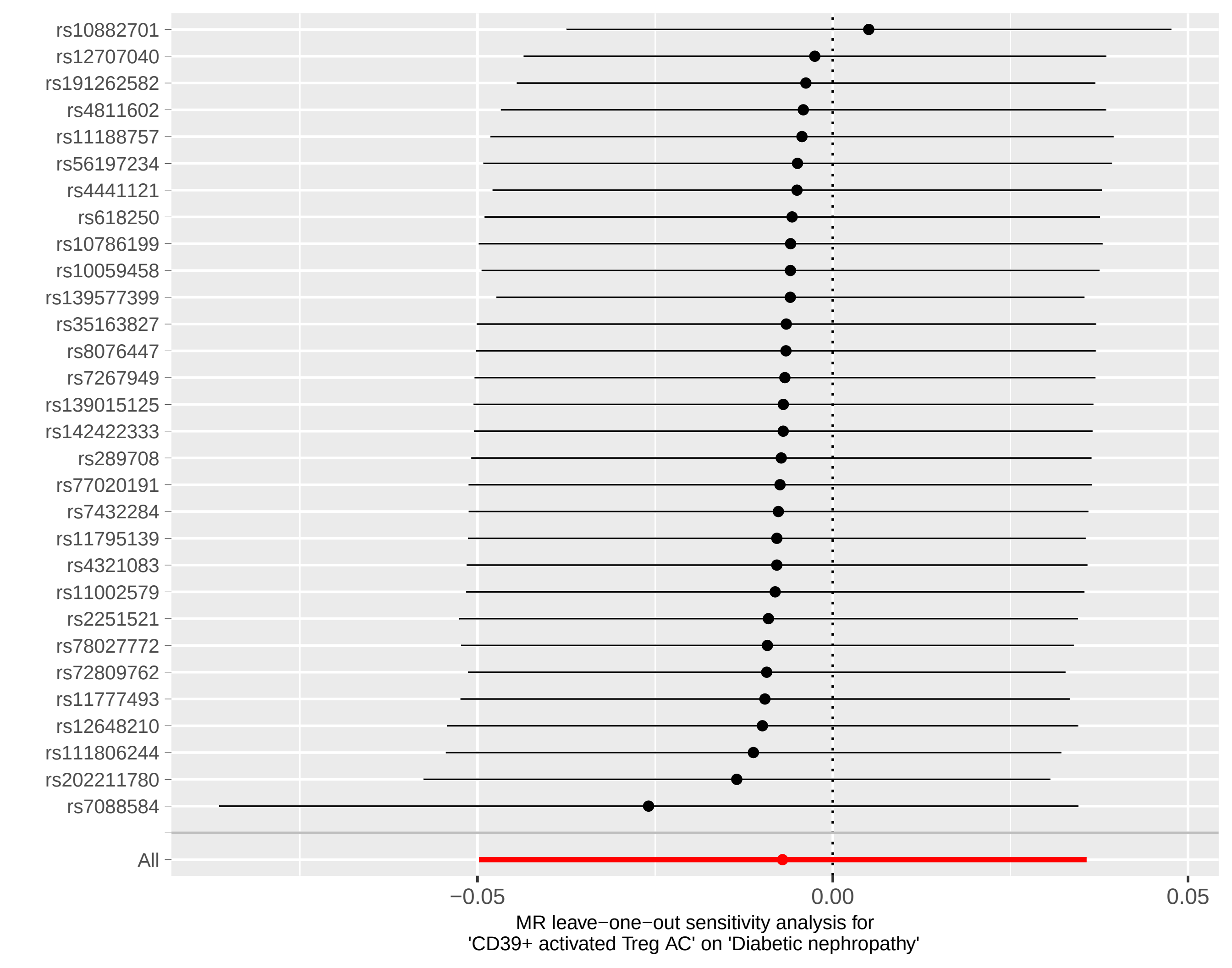
rs10882701
rs12707040
rs191262582
rs4811602
rs11188757
rs56197234
rs4441121
rs618250
rs10786199
rs10059458
rs139577399
rs35163827
rs8076447
rs7267949
rs139015125
rs142422333
rs289708
rs77020191
rs7432284
rs11795139
rs4321083
rs11002579
rs2251521
rs78027772
rs72809762
rs11777493
rs12648210
rs111806244
rs202211780
rs7088584
All
−0.05
0.00
0.05
MR leave−one−out sensitivity analysis for
'CD39+ activated Treg AC' on 'Diabetic nephropathy'

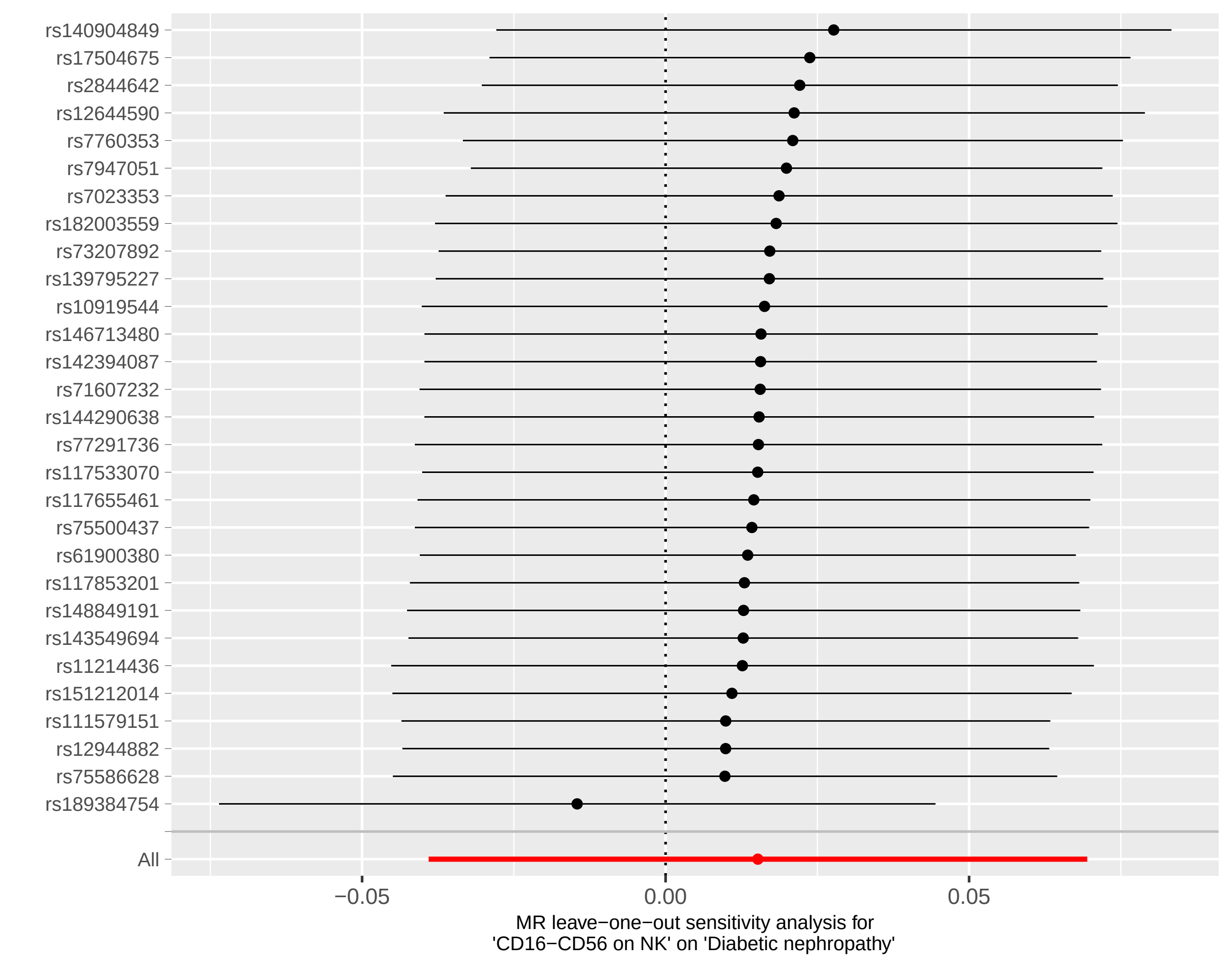
rs140904849
rs17504675
rs2844642
rs12644590
rs7760353
rs7947051
rs7023353
rs182003559
rs73207892
rs139795227
rs10919544
rs146713480
rs142394087
rs71607232
rs144290638
rs77291736
rs117533070
rs117655461
rs75500437
rs61900380
rs117853201
rs148849191
rs143549694
rs11214436
rs151212014
rs111579151
rs12944882
rs75586628
rs189384754
All
−0.05
0.00
0.05
MR leave−one−out sensitivity analysis for
'CD16−CD56 on NK' on 'Diabetic nephropathy'

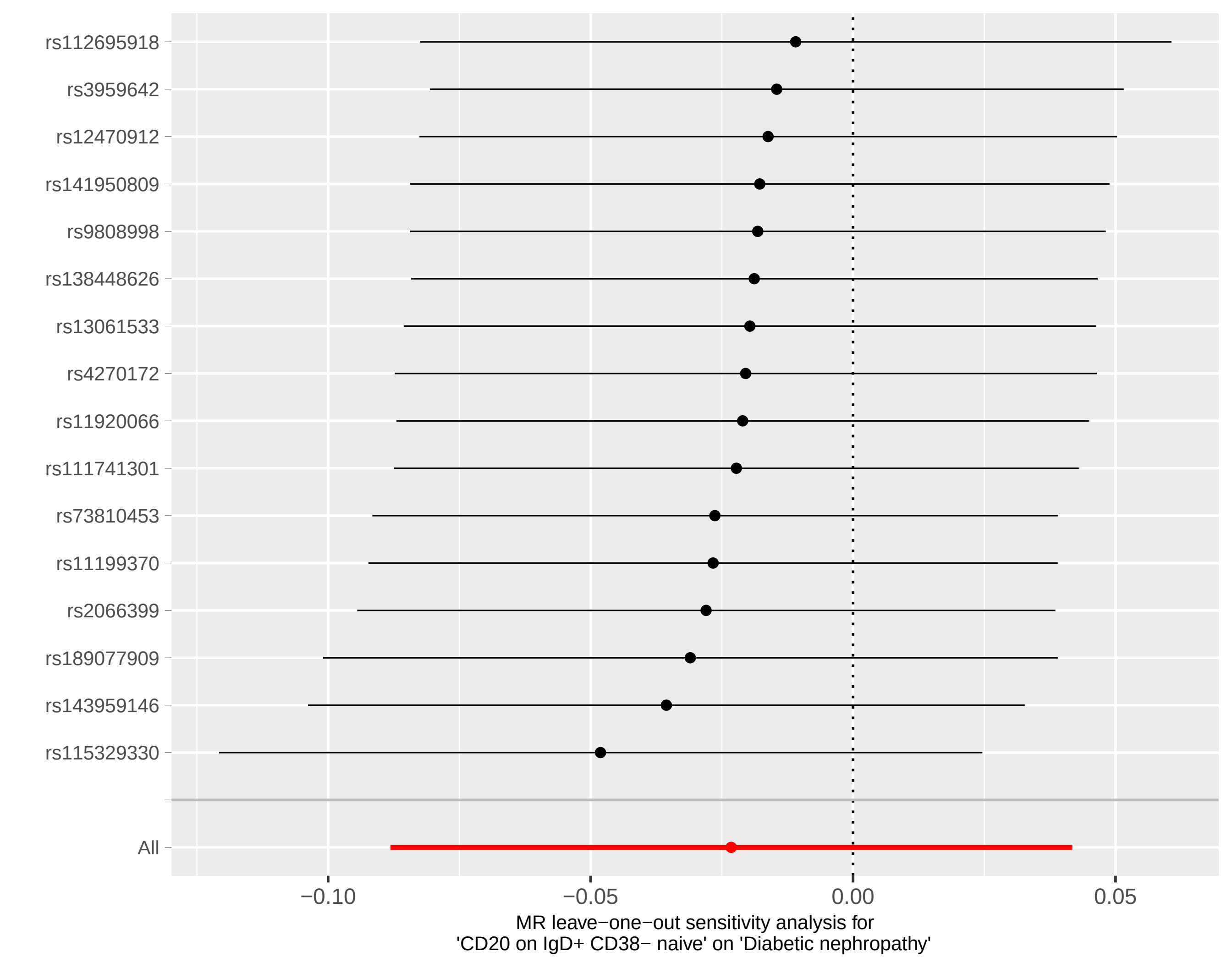
rs112695918
rs3959642
rs12470912
rs141950809
rs9808998
rs138448626
rs13061533
rs4270172
rs11920066
rs111741301
rs73810453
rs11199370
rs2066399
rs189077909
rs143959146
rs115329330
All
−0.10
−0.05
0.00
0.05
MR leave−one−out sensitivity analysis for
'CD20 on IgD+ CD38− naive' on 'Diabetic nephropathy'

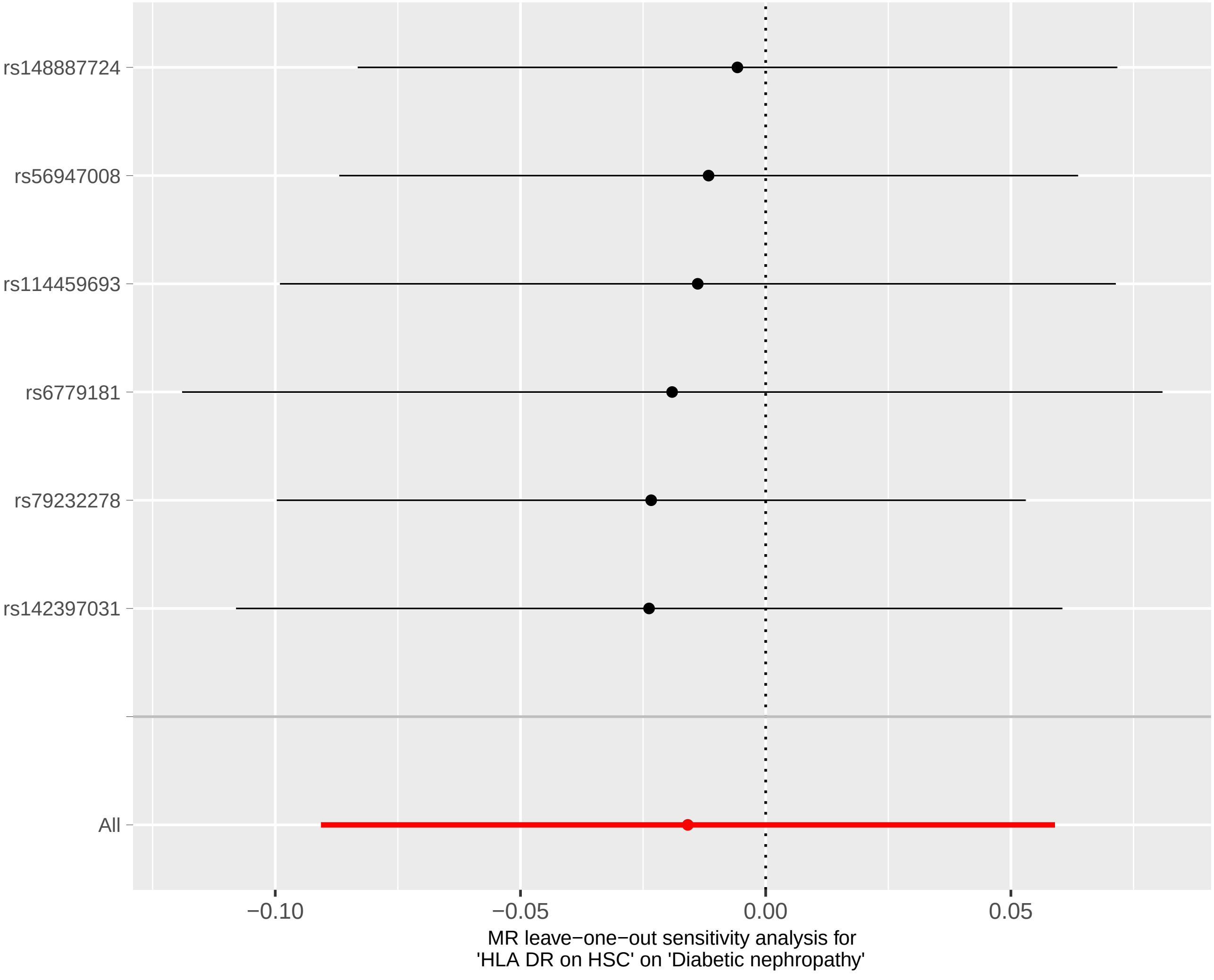

rs148887724
rs56947008
rs114459693
rs6779181
rs79232278
rs142397031
All
−0.10
−0.05
0.00
0.05
MR leave−one−out sensitivity analysis for
'HLA DR on HSC' on 'Diabetic nephropathy'

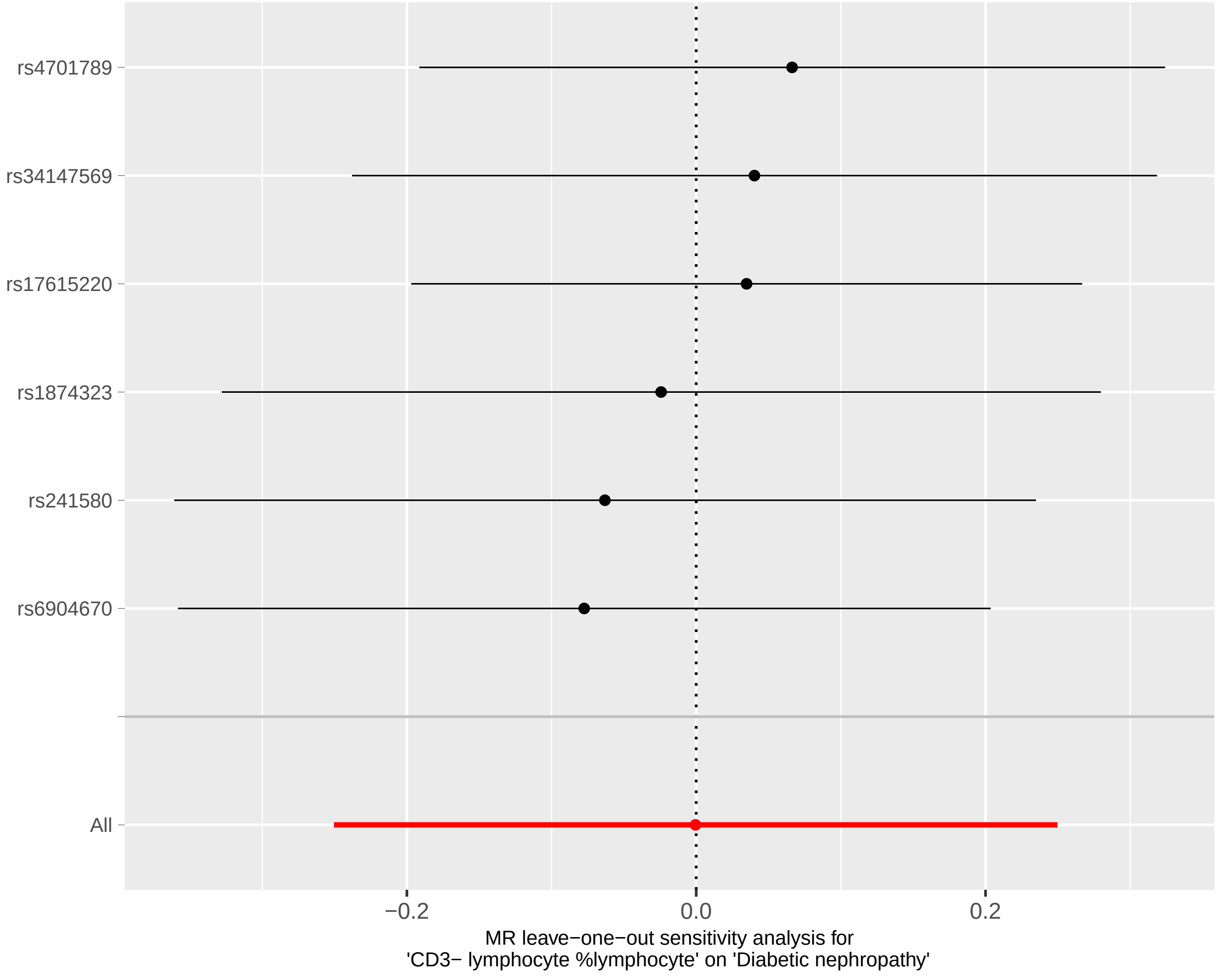
rs4701789
rs34147569
rs17615220
rs1874323
rs241580
rs6904670
All
−0.2
0.0
0.2
MR leave−one−out sensitivity analysis for
'CD3− lymphocyte %lymphocyte' on 'Diabetic nephropathy'

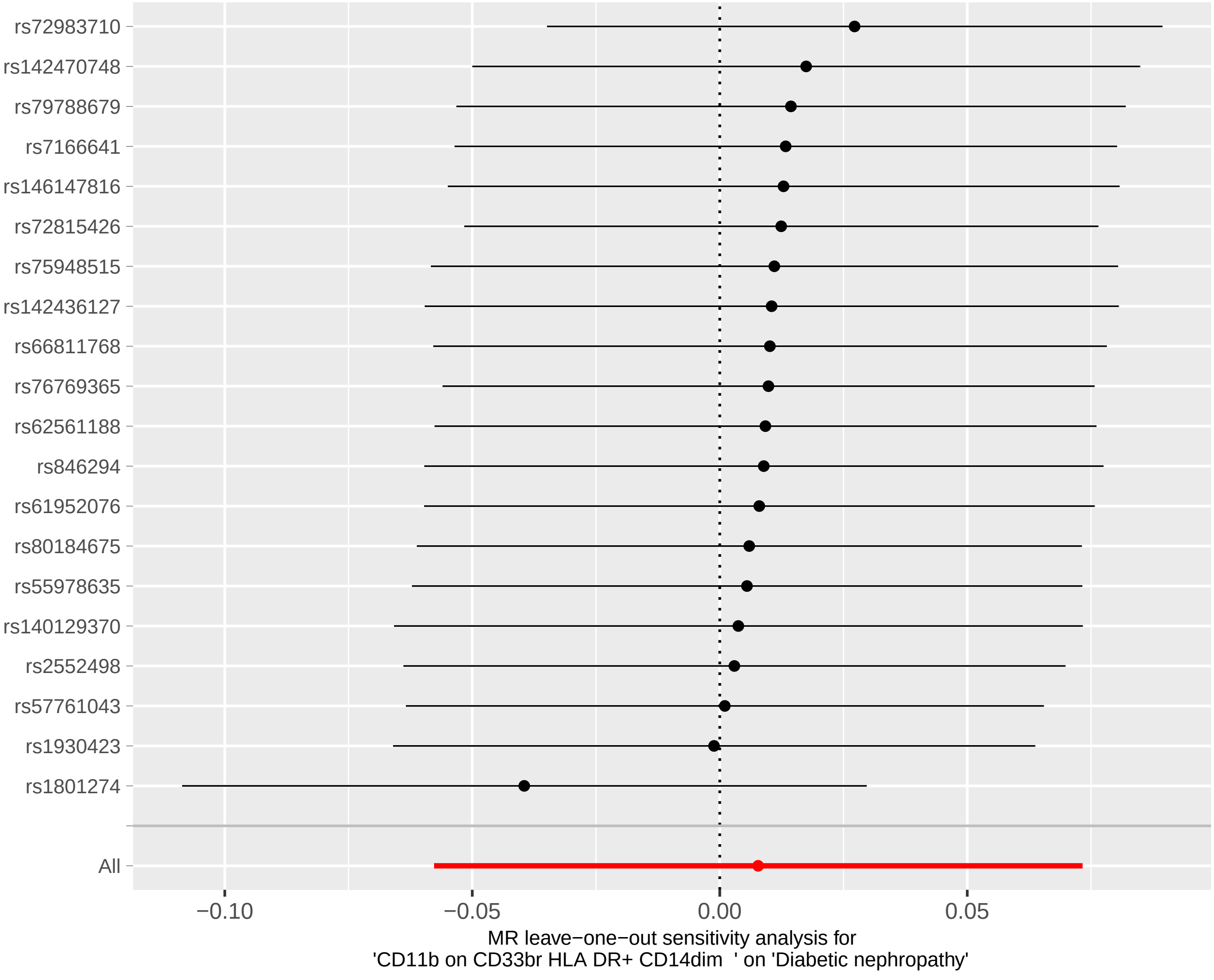
rs72983710
rs142470748
rs79788679
rs7166641
rs146147816
rs72815426
rs75948515
rs142436127
rs66811768
rs76769365
rs62561188
rs846294
rs61952076
rs80184675
rs55978635
rs140129370
rs2552498
rs57761043
rs1930423
rs1801274
All
−0.10
−0.05
0.00
0.05
MR leave−one−out sensitivity analysis for
'CD11b on CD33br HLA DR+ CD14dim ' on 'Diabetic nephropathy'

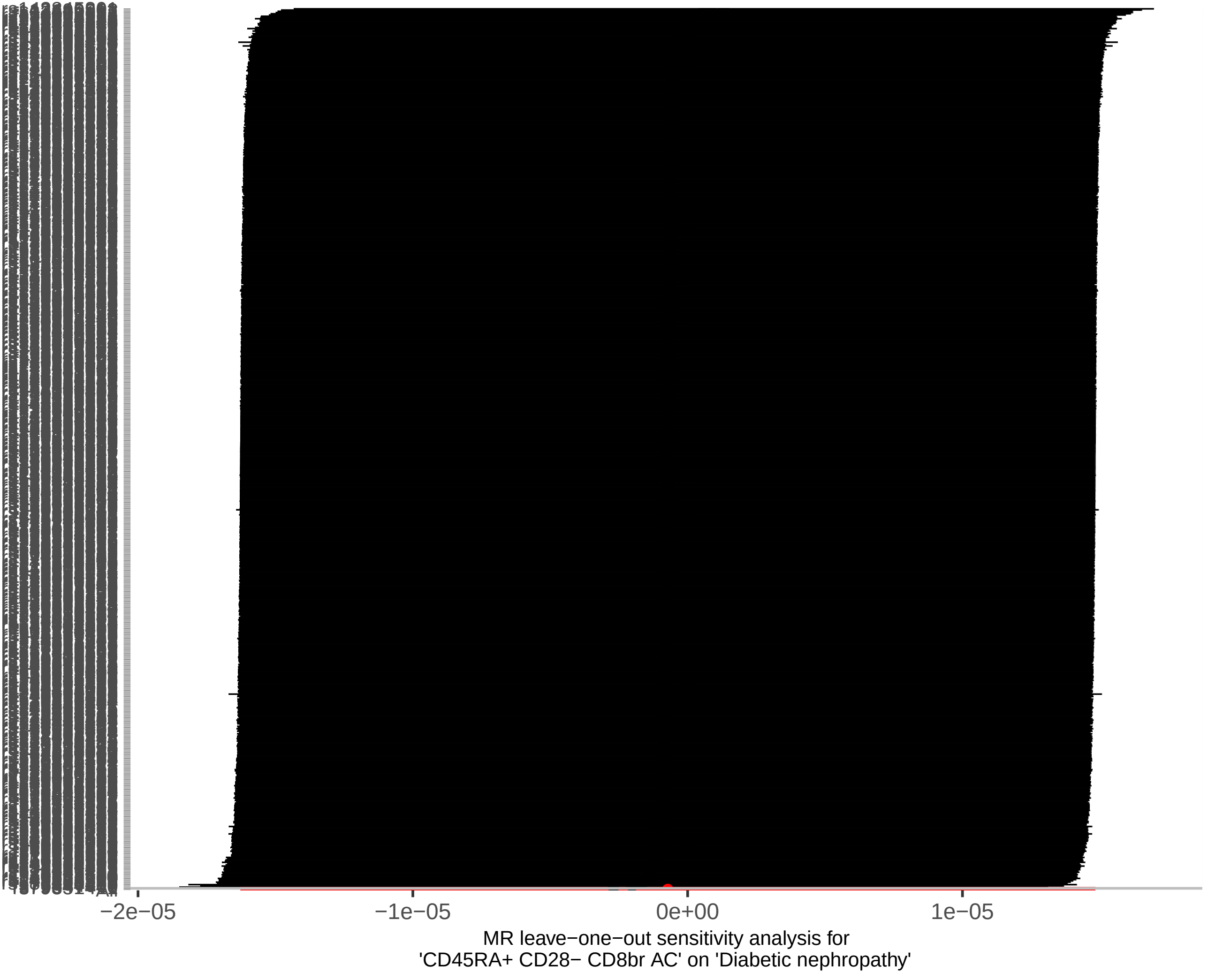

−2e−05
−1e−05
0e+00
1e−05
MR leave−one−out sensitivity analysis for
'CD45RA+ CD28− CD8br AC' on 'Diabetic nephropathy'

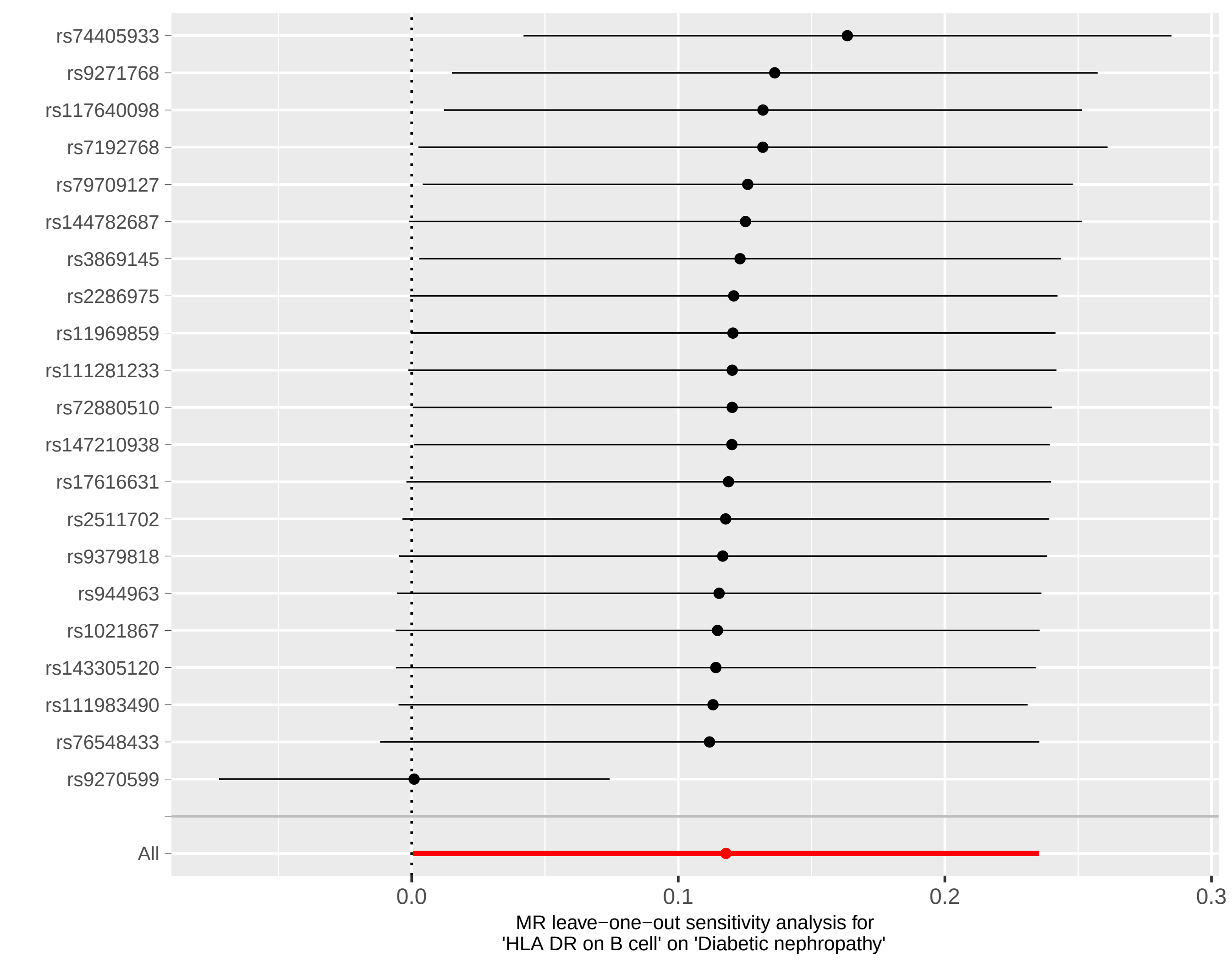

rs74405933
rs9271768
rs117640098
rs7192768
rs79709127
rs144782687
rs3869145
rs2286975
rs11969859
rs111281233
rs72880510
rs147210938
rs17616631
rs2511702
rs9379818
rs944963
rs1021867
rs143305120
rs111983490
rs76548433
rs9270599
All
0.0
0.1
0.2
0.3
MR leave−one−out sensitivity analysis for
'HLA DR on B cell' on 'Diabetic nephropathy'

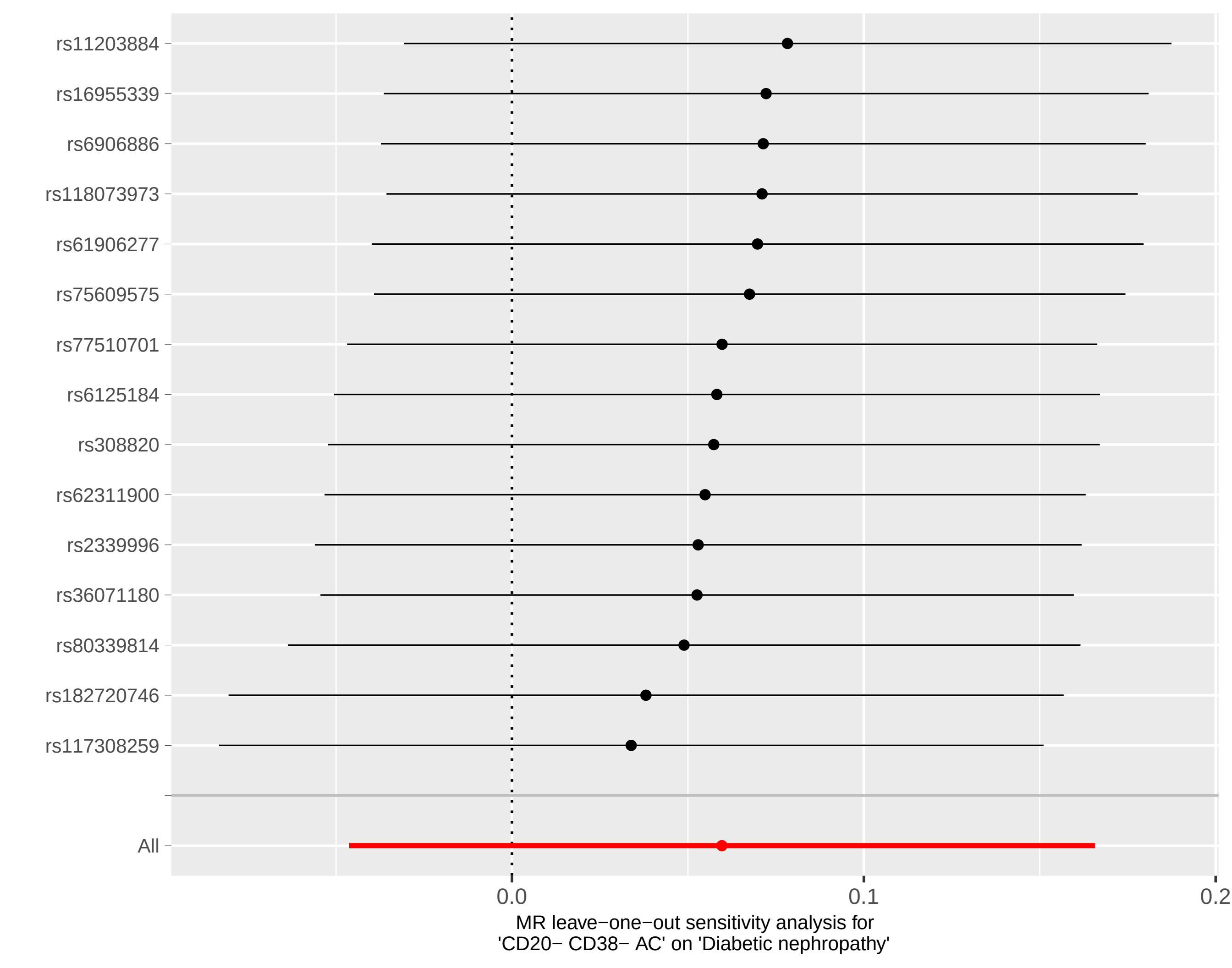
rs11203884
rs16955339
rs6906886
rs118073973
rs61906277
rs75609575
rs77510701
rs6125184
rs308820
rs62311900
rs2339996
rs36071180
rs80339814
rs182720746
rs117308259
All
0.0
0.1
0.2
MR leave−one−out sensitivity analysis for
'CD20− CD38− AC' on 'Diabetic nephropathy'

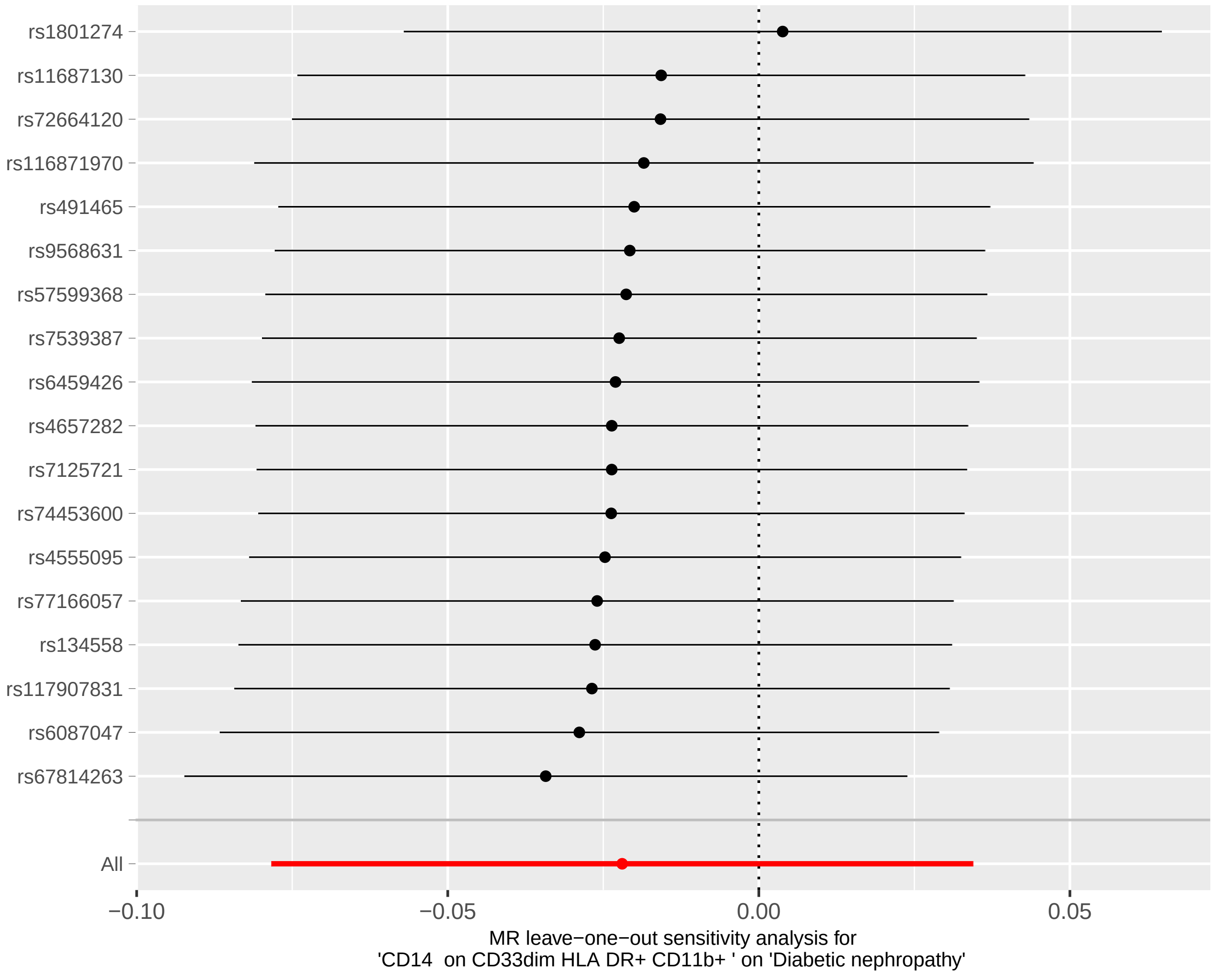

rs1801274
rs11687130
rs72664120
rs116871970
rs491465
rs9568631
rs57599368
rs7539387
rs6459426
rs4657282
rs7125721
rs74453600
rs4555095
rs77166057
rs134558
rs117907831
rs6087047
rs67814263
All
−0.10
−0.05
0.00
0.05
MR leave−one−out sensitivity analysis for
'CD14 on CD33dim HLA DR+ CD11b+ ' on 'Diabetic nephropathy'

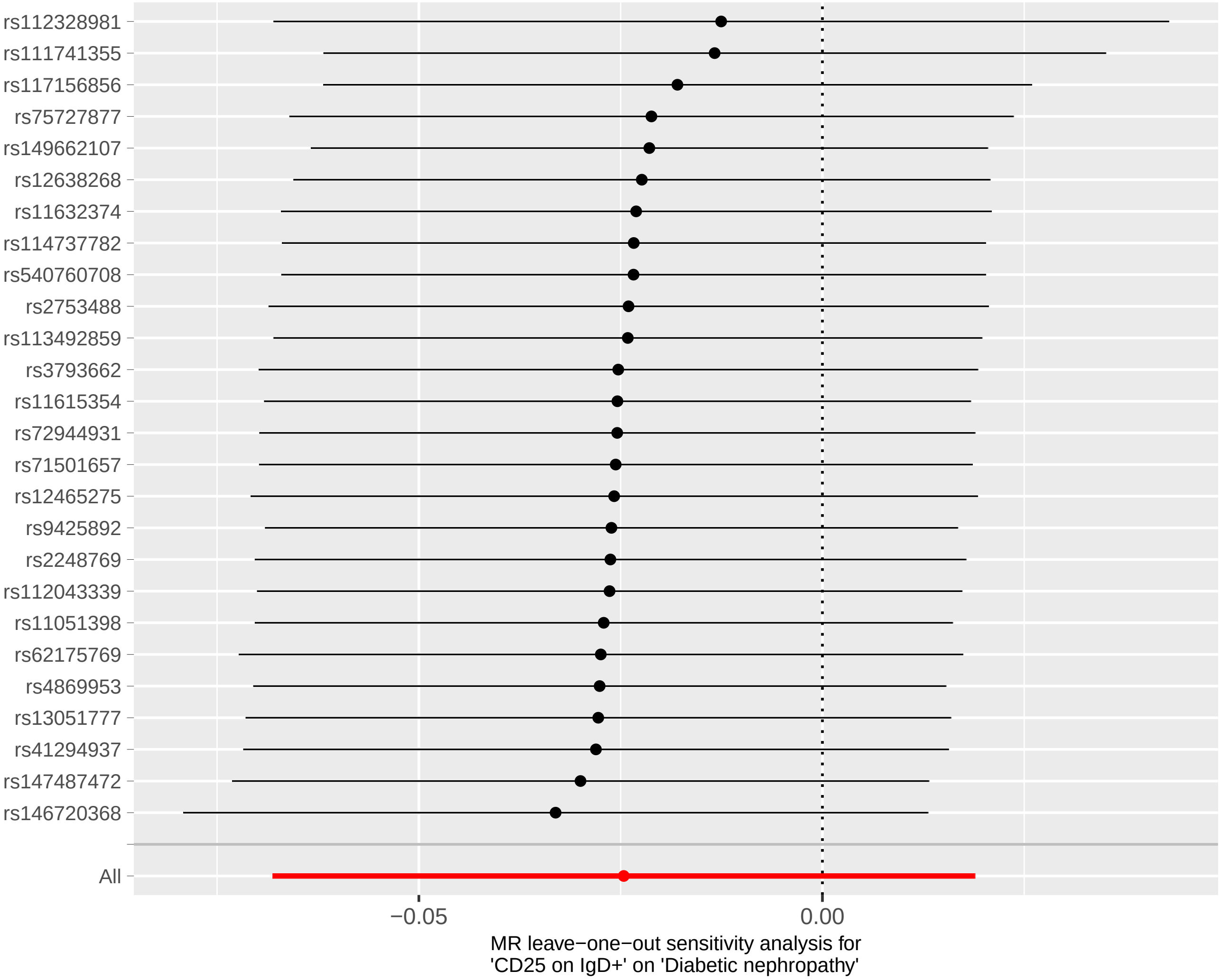
rs112328981
rs111741355
rs117156856
rs75727877
rs149662107
rs12638268
rs11632374
rs114737782
rs540760708
rs2753488
rs113492859
rs3793662
rs11615354
rs72944931
rs71501657
rs12465275
rs9425892
rs2248769
rs112043339
rs11051398
rs62175769
rs4869953
rs13051777
rs41294937
rs147487472
rs146720368
All
−0.05
0.00
MR leave−one−out sensitivity analysis for
'CD25 on IgD+' on 'Diabetic nephropathy'

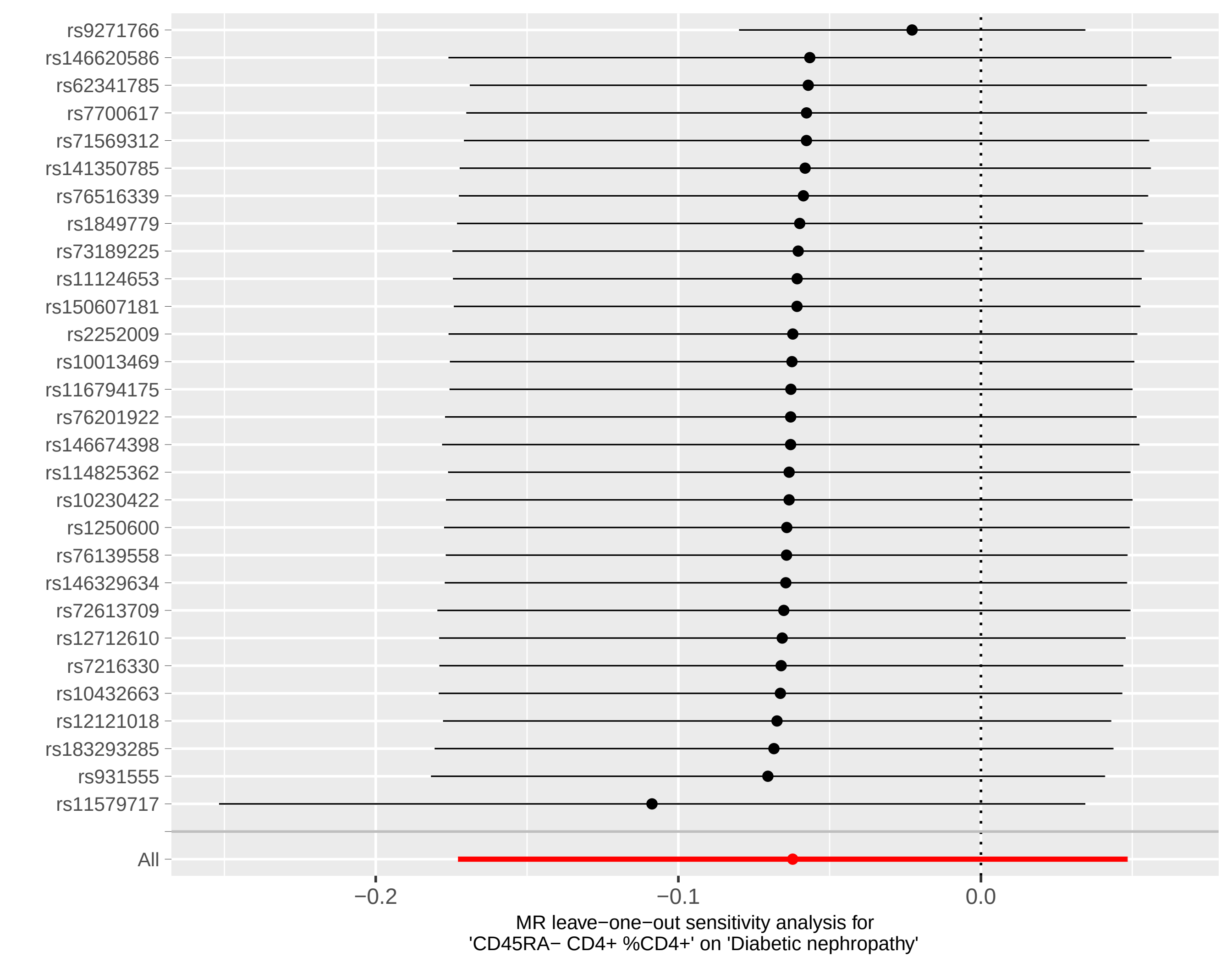
rs9271766
rs146620586
rs62341785
rs7700617
rs71569312
rs141350785
rs76516339
rs1849779
rs73189225
rs11124653
rs150607181
rs2252009
rs10013469
rs116794175
rs76201922
rs146674398
rs114825362
rs10230422
rs1250600
rs76139558
rs146329634
rs72613709
rs12712610
rs7216330
rs10432663
rs12121018
rs183293285
rs931555
rs11579717
All
−0.2
−0.1
0.0
MR leave−one−out sensitivity analysis for
'CD45RA− CD4+ %CD4+' on 'Diabetic nephropathy'

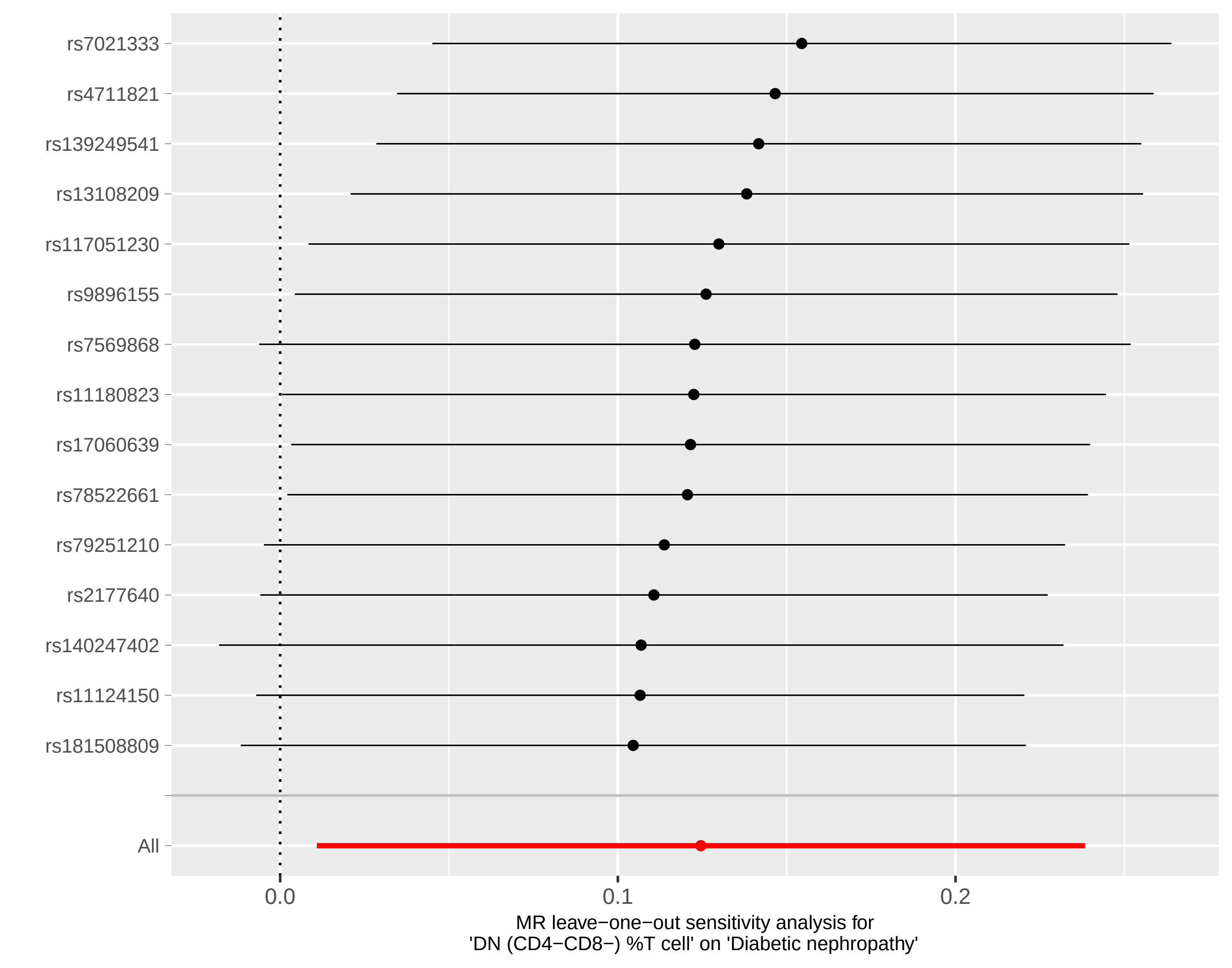
rs7021333
rs4711821
rs139249541
rs13108209
rs117051230
rs9896155
rs7569868
rs11180823
rs17060639
rs78522661
rs79251210
rs2177640
rs140247402
rs11124150
rs181508809
All
0.0
0.1
0.2
MR leave−one−out sensitivity analysis for
'DN (CD4−CD8−) %T cell' on 'Diabetic nephropathy'

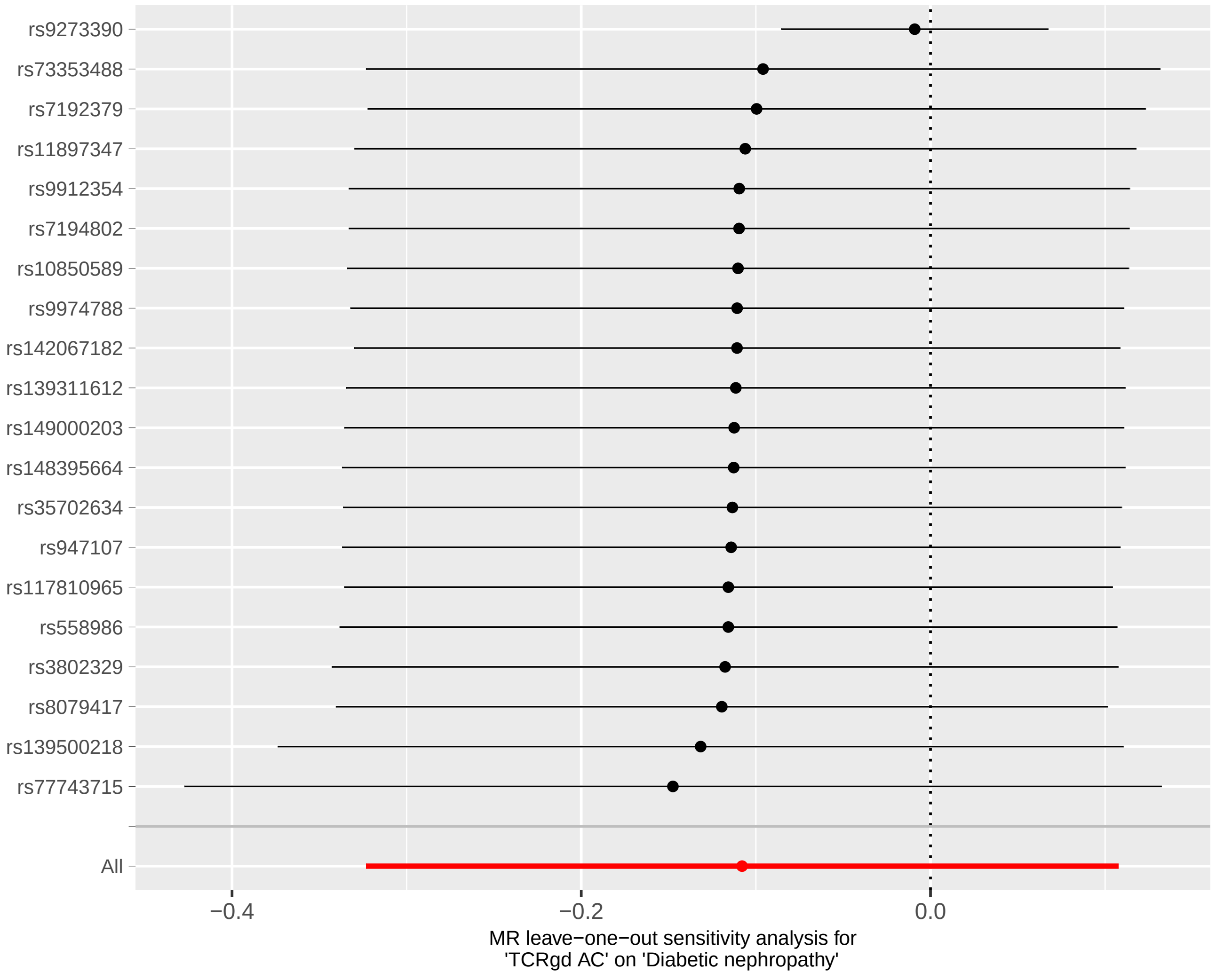
rs9273390
rs73353488
rs7192379
rs11897347
rs9912354
rs7194802
rs10850589
rs9974788
rs142067182
rs139311612
rs149000203
rs148395664
rs35702634
rs947107
rs117810965
rs558986
rs3802329
rs8079417
rs139500218
rs77743715
All
−0.4
−0.2
0.0
MR leave−one−out sensitivity analysis for
'TCRgd AC' on 'Diabetic nephropathy'

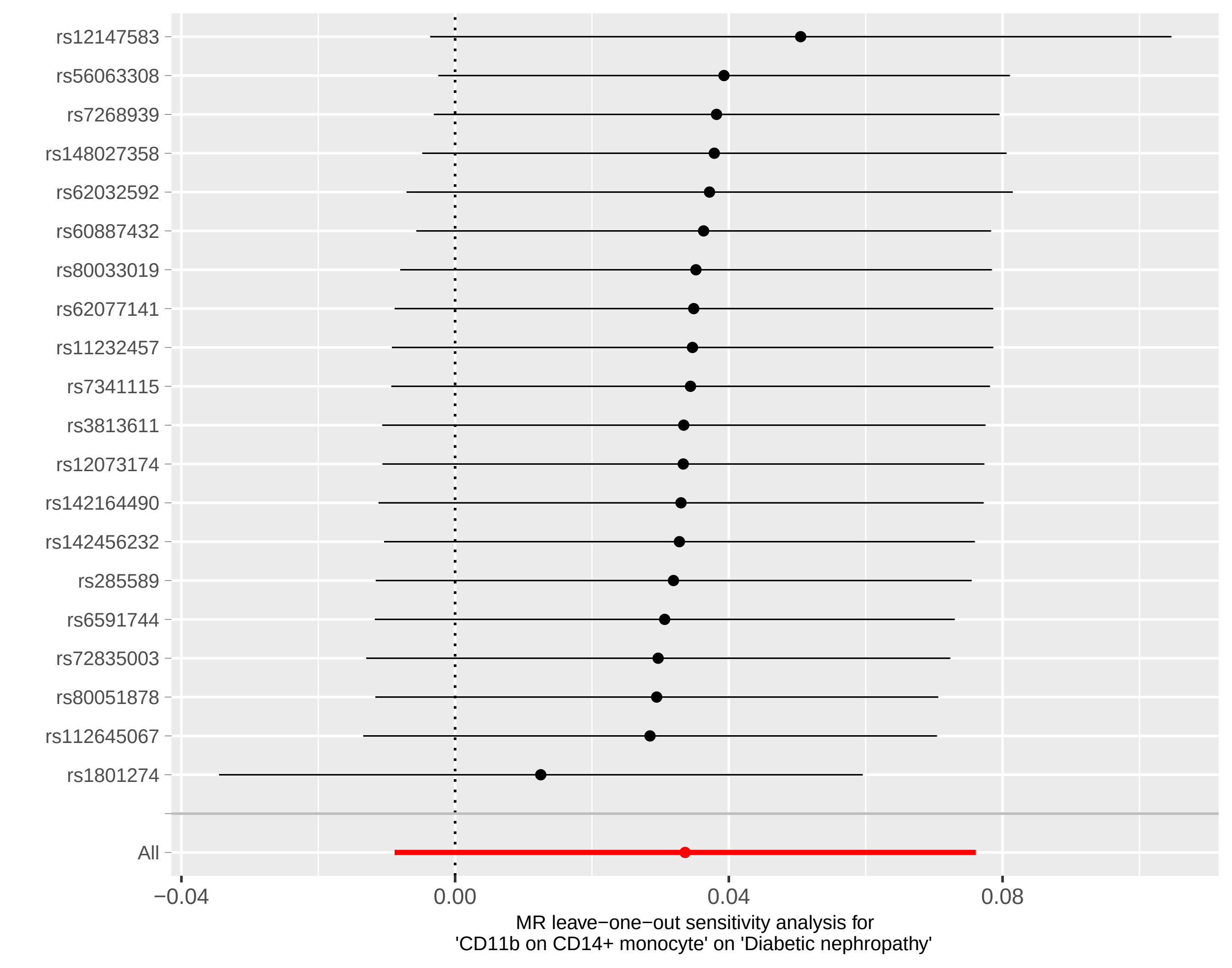

rs12147583
rs56063308
rs7268939
rs148027358
rs62032592
rs60887432
rs80033019
rs62077141
rs11232457
rs7341115
rs3813611
rs12073174
rs142164490
rs142456232
rs285589
rs6591744
rs72835003
rs80051878
rs112645067
rs1801274
All
−0.04
0.00
0.04
0.08
MR leave−one−out sensitivity analysis for
'CD11b on CD14+ monocyte' on 'Diabetic nephropathy'

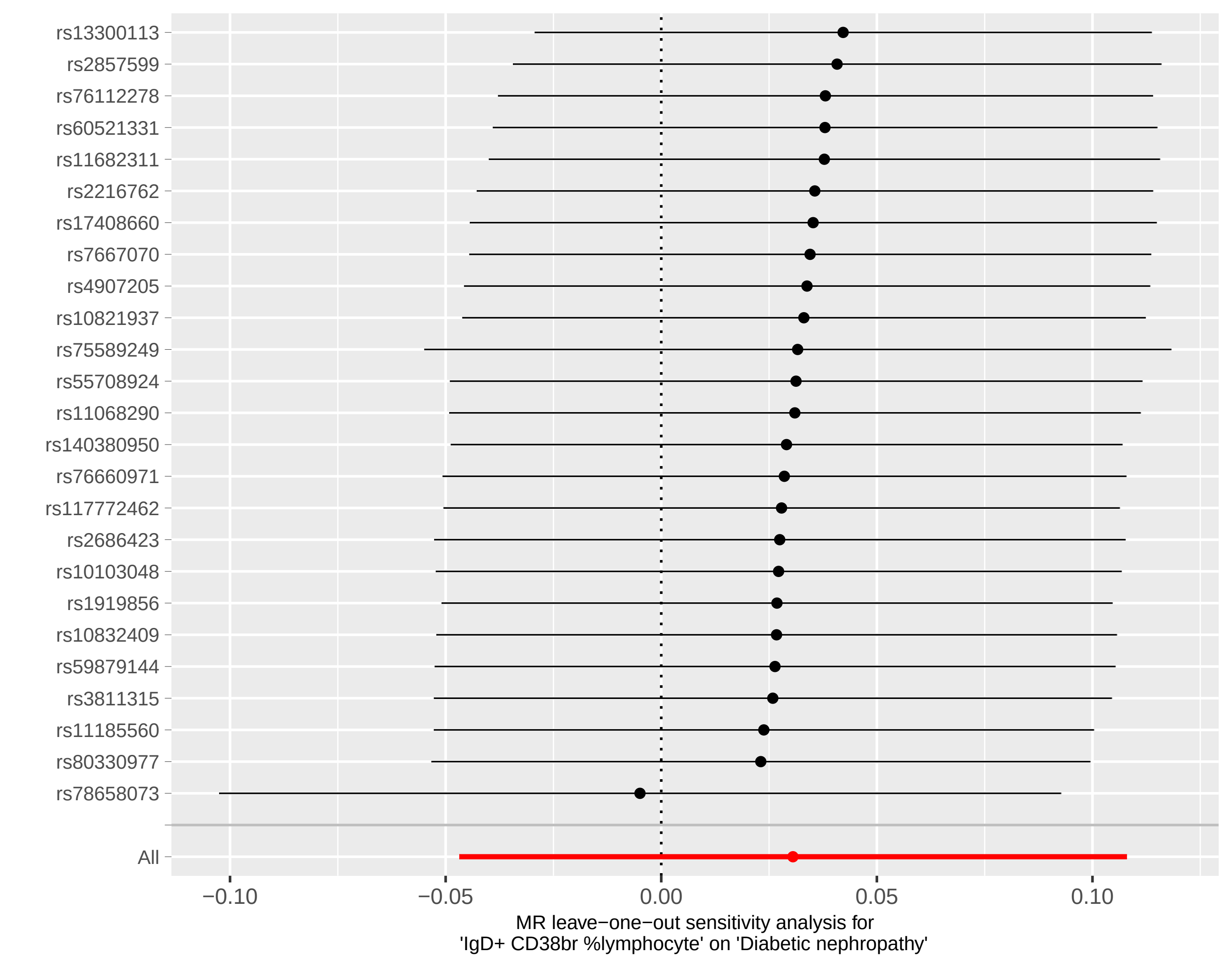

rs13300113
rs2857599
rs76112278
rs60521331
rs11682311
rs2216762
rs17408660
rs7667070
rs4907205
rs10821937
rs75589249
rs55708924
rs11068290
rs140380950
rs76660971
rs117772462
rs2686423
rs10103048
rs1919856
rs10832409
rs59879144
rs3811315
rs11185560
rs80330977
rs78658073
All
−0.10
−0.05
0.00
0.05
0.10
MR leave−one−out sensitivity analysis for
'IgD+ CD38br %lymphocyte' on 'Diabetic nephropathy'

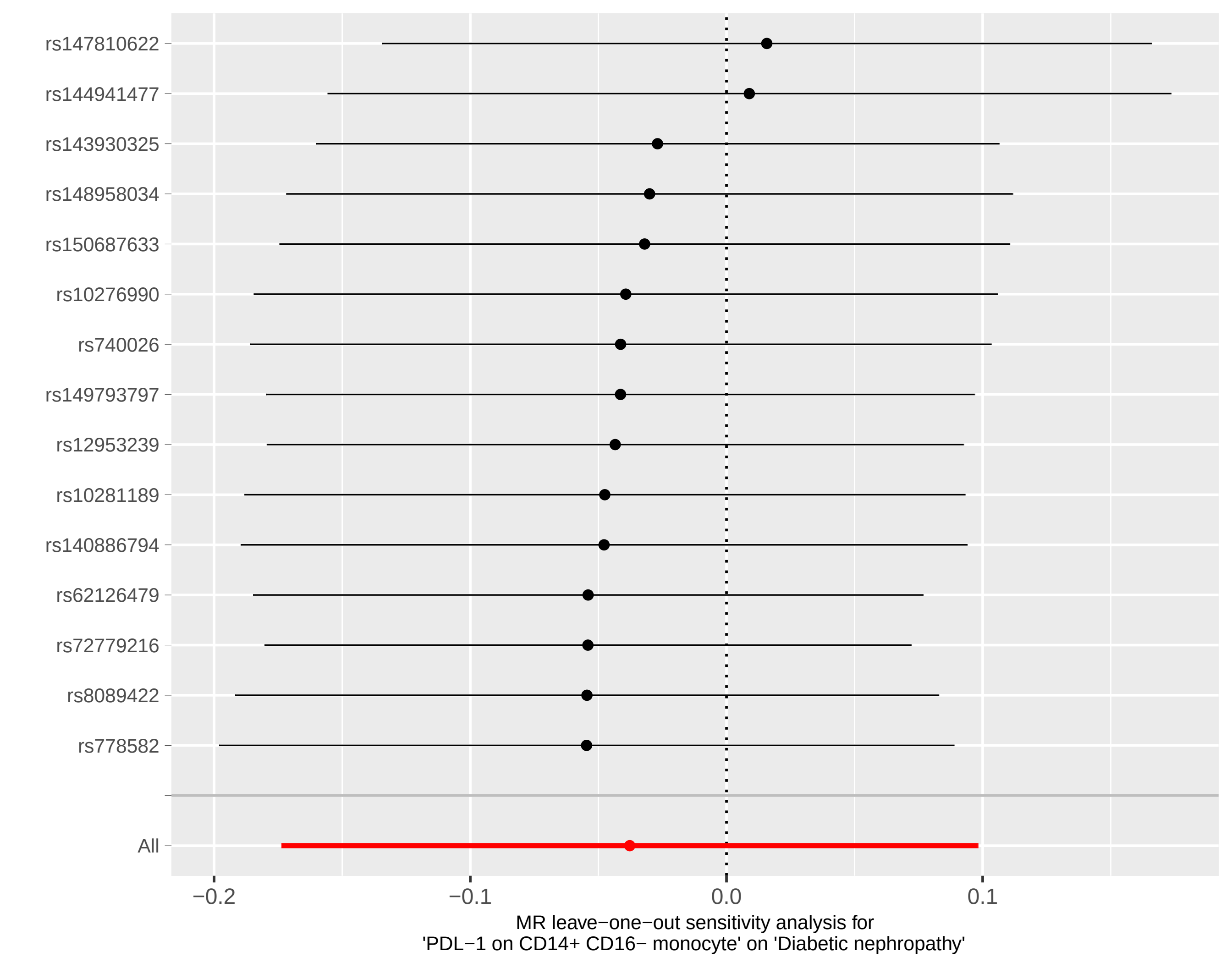
rs147810622
rs144941477
rs143930325
rs148958034
rs150687633
rs10276990
rs740026
rs149793797
rs12953239
rs10281189
rs140886794
rs62126479
rs72779216
rs8089422
rs778582
All
−0.2
−0.1
0.0
0.1
MR leave−one−out sensitivity analysis for
'PDL−1 on CD14+ CD16− monocyte' on 'Diabetic nephropathy'

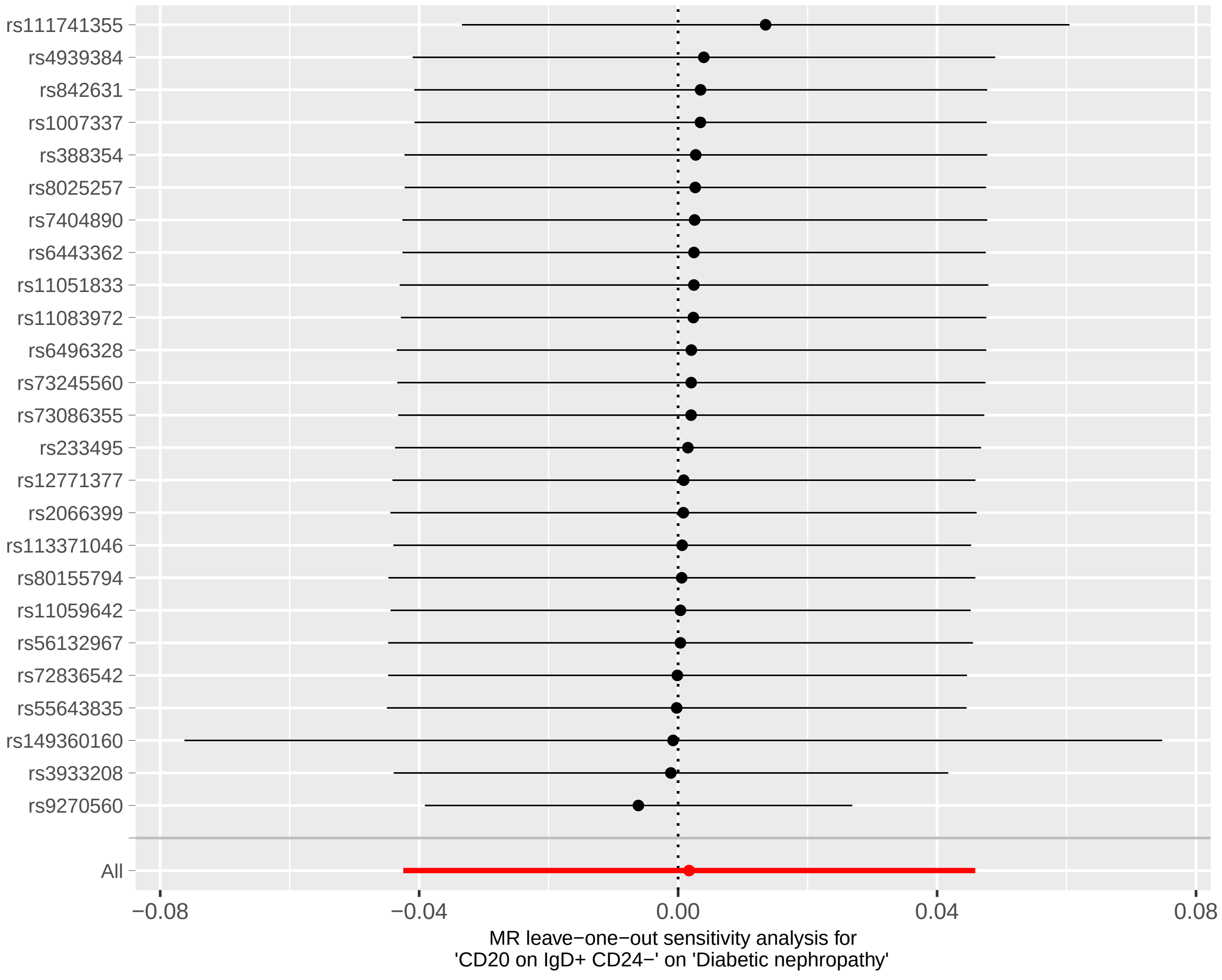
rs111741355
rs4939384
rs842631
rs1007337
rs388354
rs8025257
rs7404890
rs6443362
rs11051833
rs11083972
rs6496328
rs73245560
rs73086355
rs233495
rs12771377
rs2066399
rs113371046
rs80155794
rs11059642
rs56132967
rs72836542
rs55643835
rs149360160
rs3933208
rs9270560
All
−0.08
−0.04
0.00
0.04
0.08
MR leave−one−out sensitivity analysis for
'CD20 on IgD+ CD24−' on 'Diabetic nephropathy'

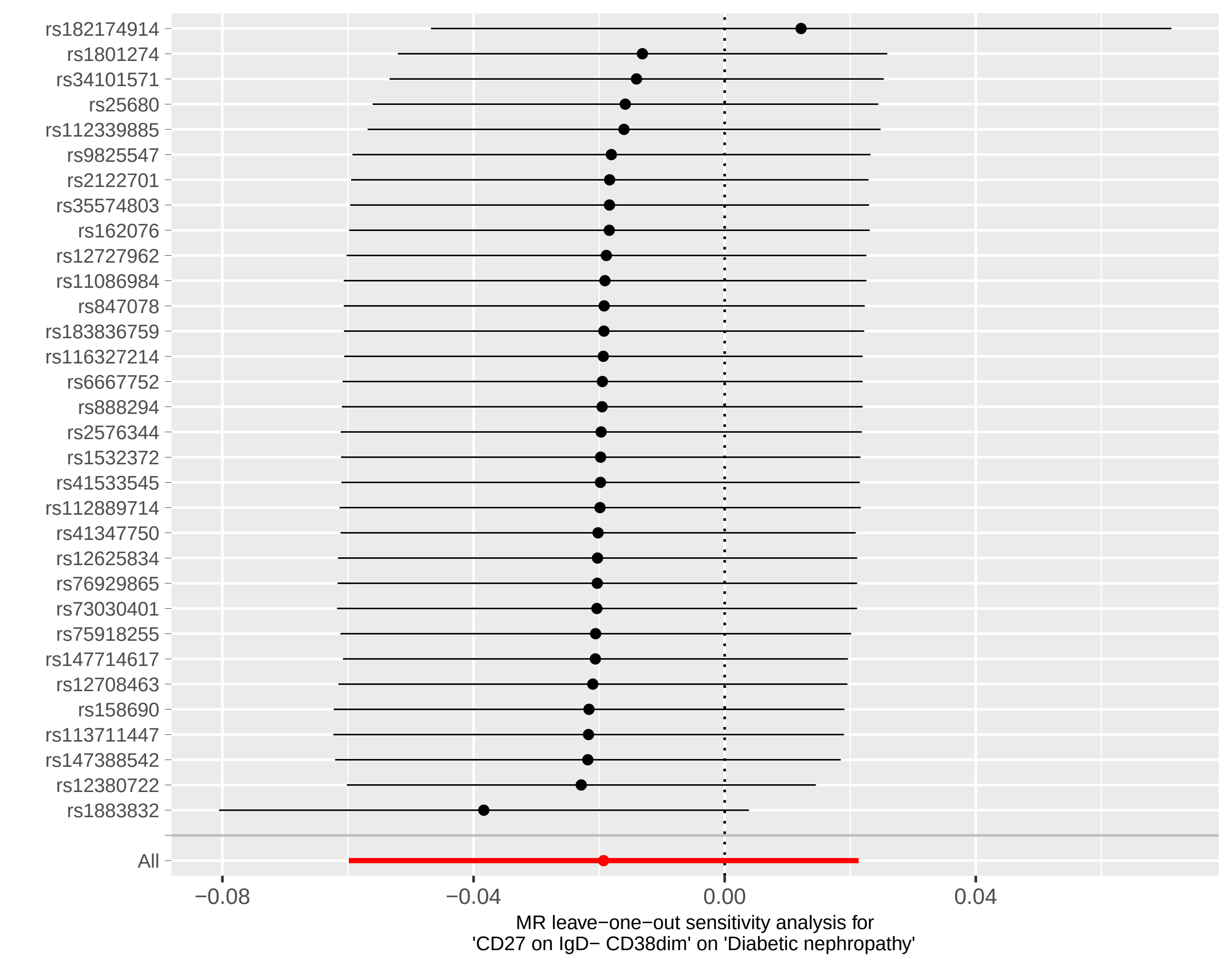
rs182174914
rs1801274
rs34101571
rs25680
rs112339885
rs9825547
rs2122701
rs35574803
rs162076
rs12727962
rs11086984
rs847078
rs183836759
rs116327214
rs6667752
rs888294
rs2576344
rs1532372
rs41533545
rs112889714
rs41347750
rs12625834
rs76929865
rs73030401
rs75918255
rs147714617
rs12708463
rs158690
rs113711447
rs147388542
rs12380722
rs1883832
All
−0.08
−0.04
0.00
0.04
MR leave−one−out sensitivity analysis for
'CD27 on IgD− CD38dim' on 'Diabetic nephropathy'

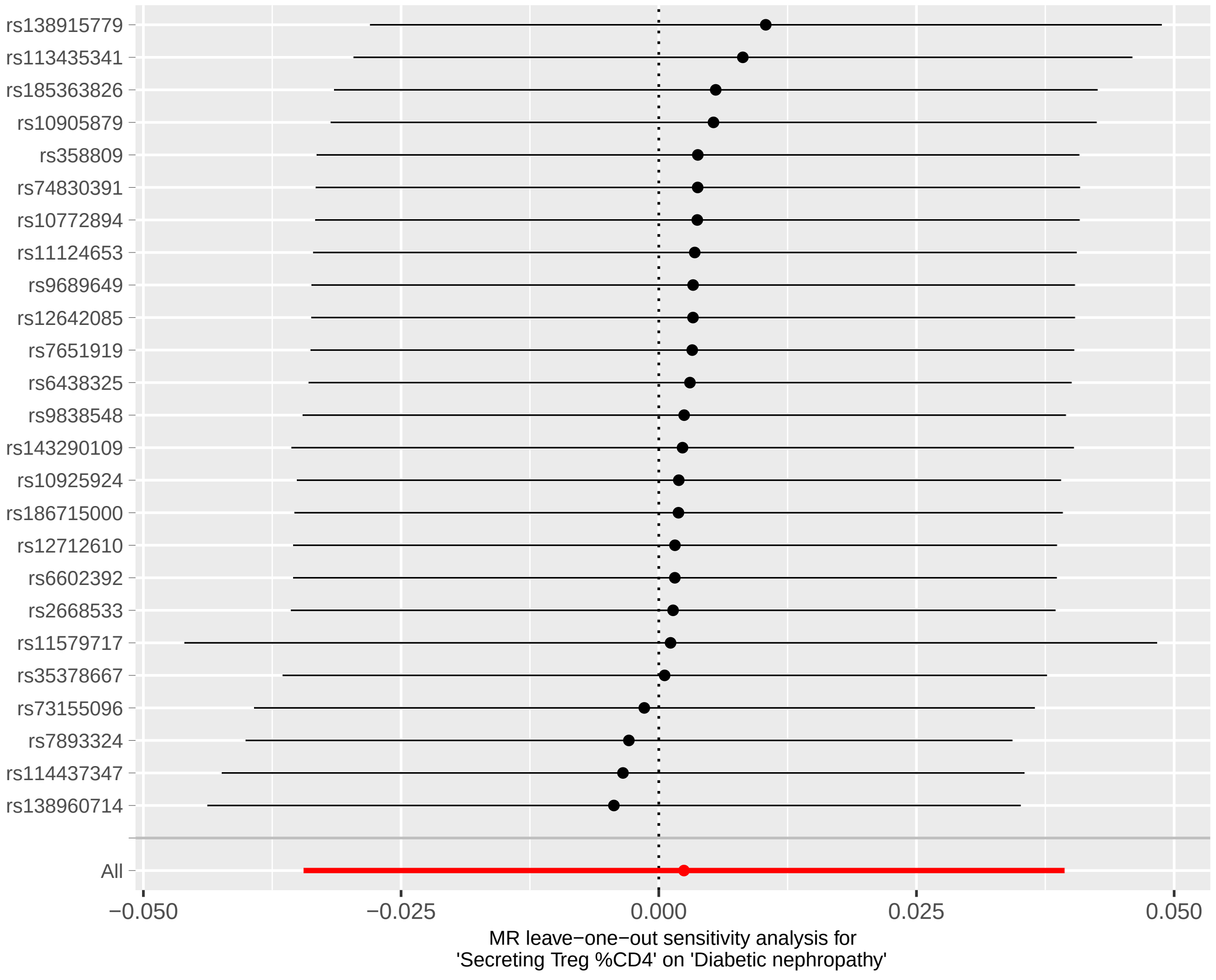
rs138915779
rs113435341
rs185363826
rs10905879
rs358809
rs74830391
rs10772894
rs11124653
rs9689649
rs12642085
rs7651919
rs6438325
rs9838548
rs143290109
rs10925924
rs186715000
rs12712610
rs6602392
rs2668533
rs11579717
rs35378667
rs73155096
rs7893324
rs114437347
rs138960714
All
−0.050
−0.025
0.000
0.025
0.050
MR leave−one−out sensitivity analysis for
'Secreting Treg %CD4' on 'Diabetic nephropathy'

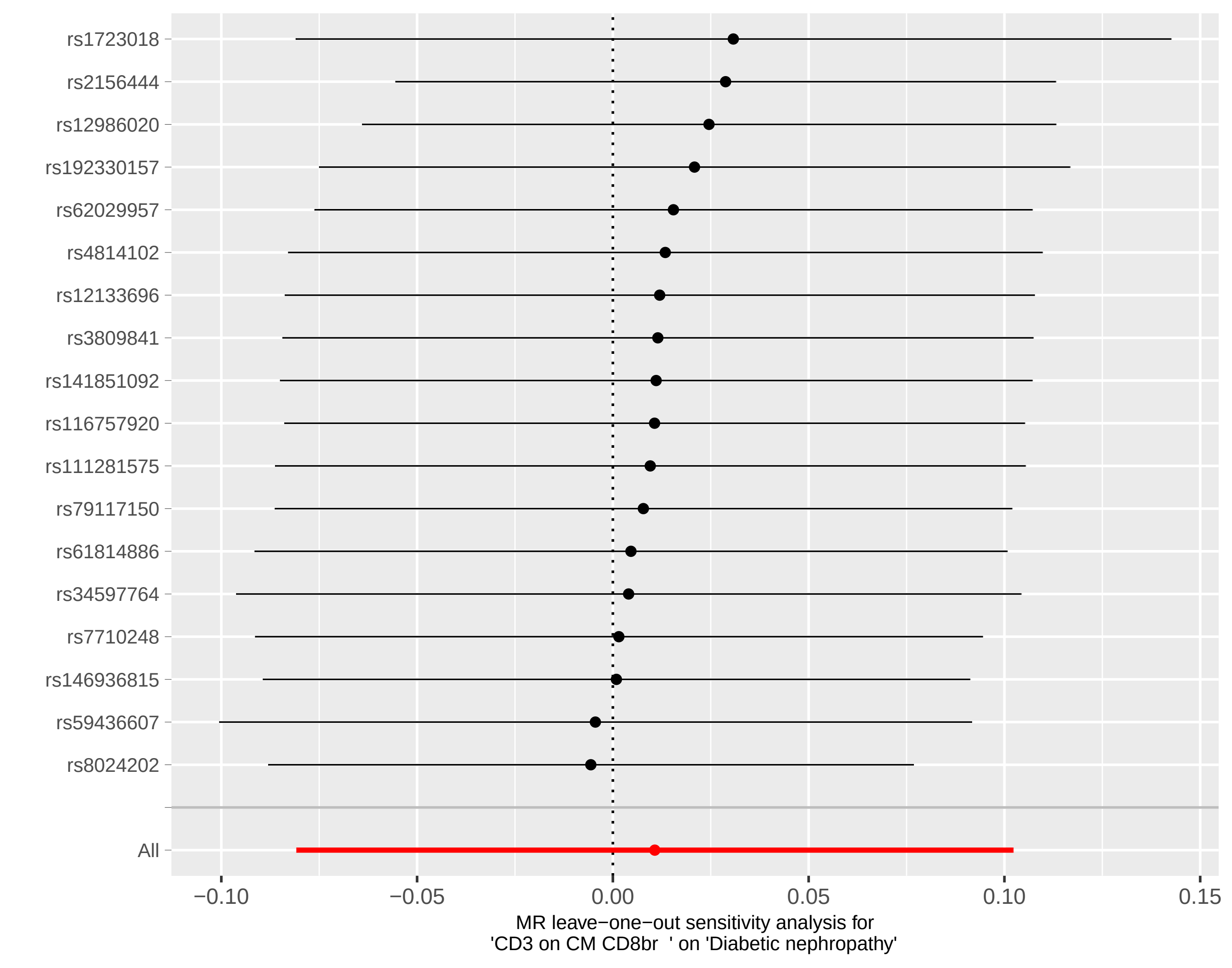

rs1723018
rs2156444
rs12986020
rs192330157
rs62029957
rs4814102
rs12133696
rs3809841
rs141851092
rs116757920
rs111281575
rs79117150
rs61814886
rs34597764
rs7710248
rs146936815
rs59436607
rs8024202
All
−0.10
−0.05
0.00
0.05
0.10
0.15
MR leave−one−out sensitivity analysis for
'CD3 on CM CD8br ' on 'Diabetic nephropathy'

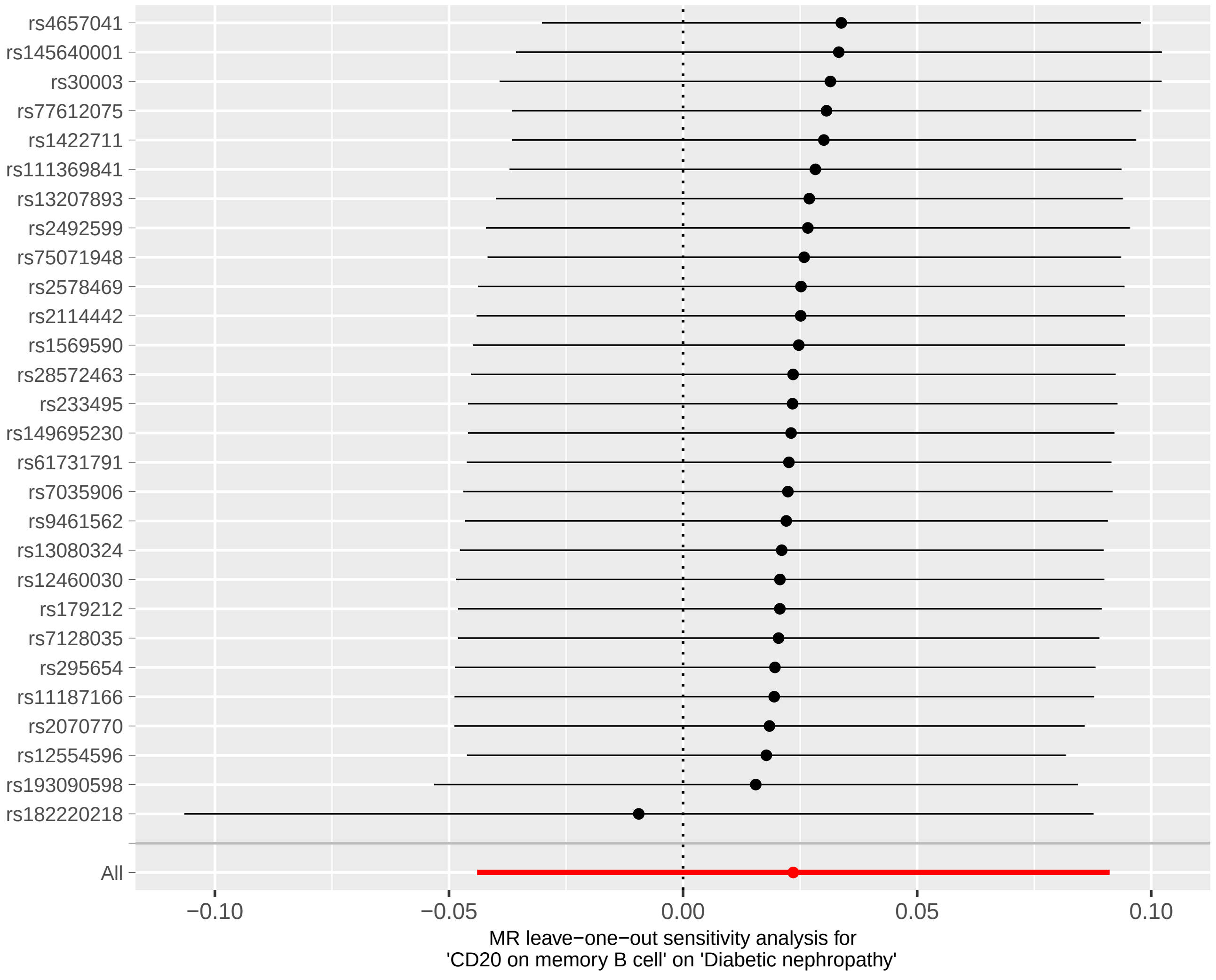
rs4657041
rs145640001
rs30003
rs77612075
rs1422711
rs111369841
rs13207893
rs2492599
rs75071948
rs2578469
rs2114442
rs1569590
rs28572463
rs233495
rs149695230
rs61731791
rs7035906
rs9461562
rs13080324
rs12460030
rs179212
rs7128035
rs295654
rs11187166
rs2070770
rs12554596
rs193090598
rs182220218
All
−0.10
−0.05
0.00
0.05
0.10
MR leave−one−out sensitivity analysis for
'CD20 on memory B cell' on 'Diabetic nephropathy'

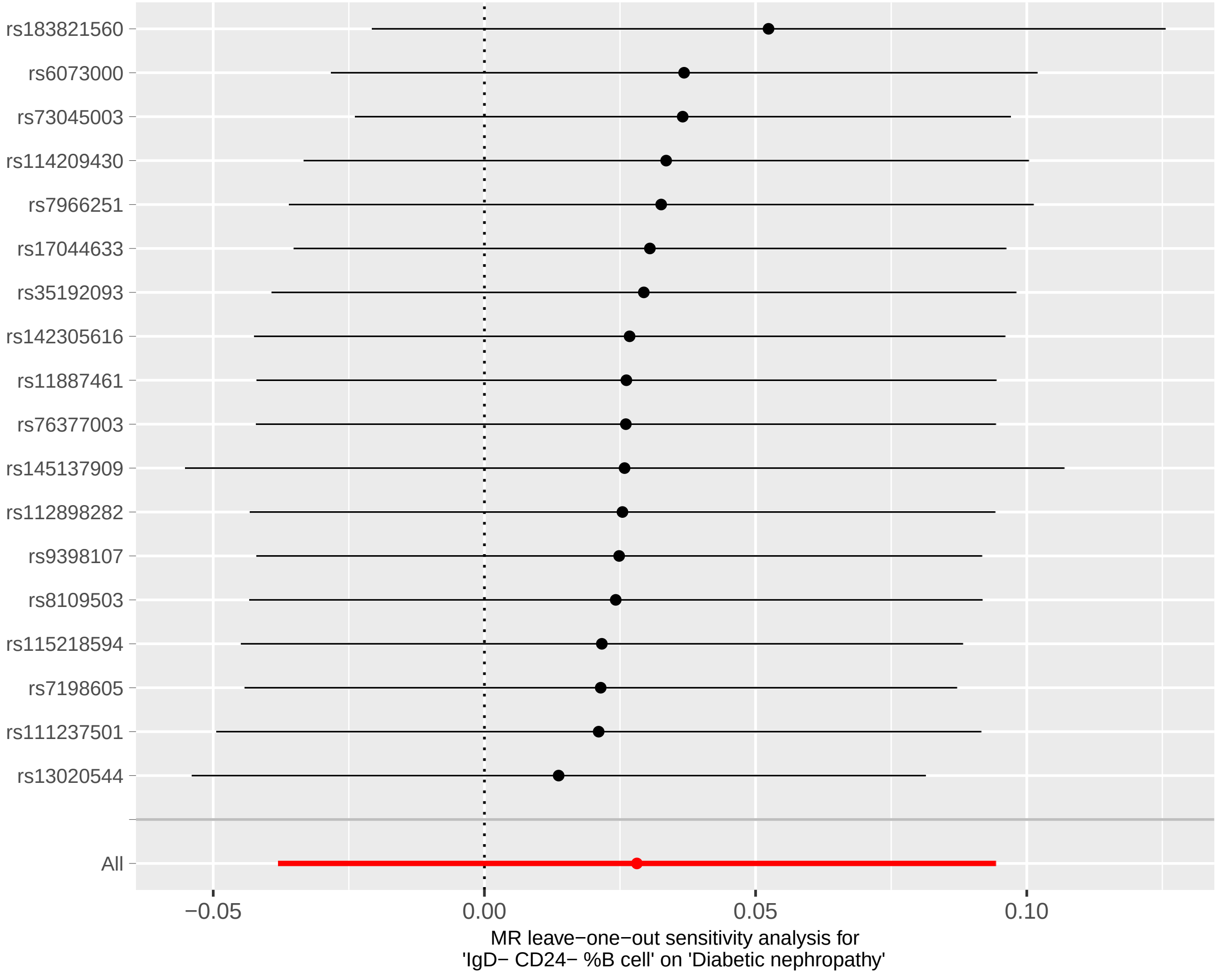
rs183821560
rs6073000
rs73045003
rs114209430
rs7966251
rs17044633
rs35192093
rs142305616
rs11887461
rs76377003
rs145137909
rs112898282
rs9398107
rs8109503
rs115218594
rs7198605
rs111237501
rs13020544
All
−0.05
0.00
0.05
0.10
MR leave−one−out sensitivity analysis for
'IgD− CD24− %B cell' on 'Diabetic nephropathy'

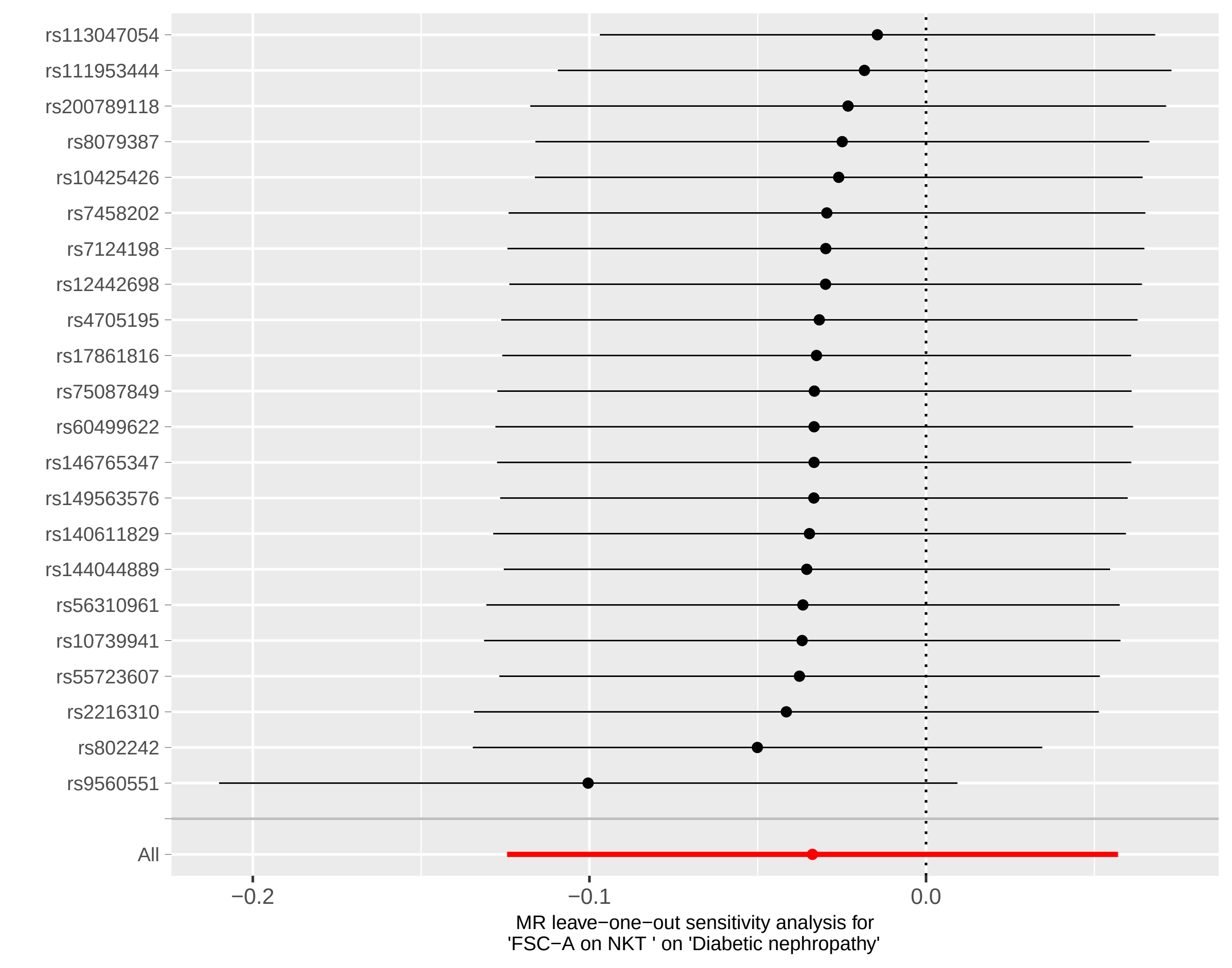

rs113047054
rs111953444
rs200789118
rs8079387
rs10425426
rs7458202
rs7124198
rs12442698
rs4705195
rs17861816
rs75087849
rs60499622
rs146765347
rs149563576
rs140611829
rs144044889
rs56310961
rs10739941
rs55723607
rs2216310
rs802242
rs9560551
All
−0.2
−0.1
0.0
MR leave−one−out sensitivity analysis for
'FSC−A on NKT ' on 'Diabetic nephropathy'

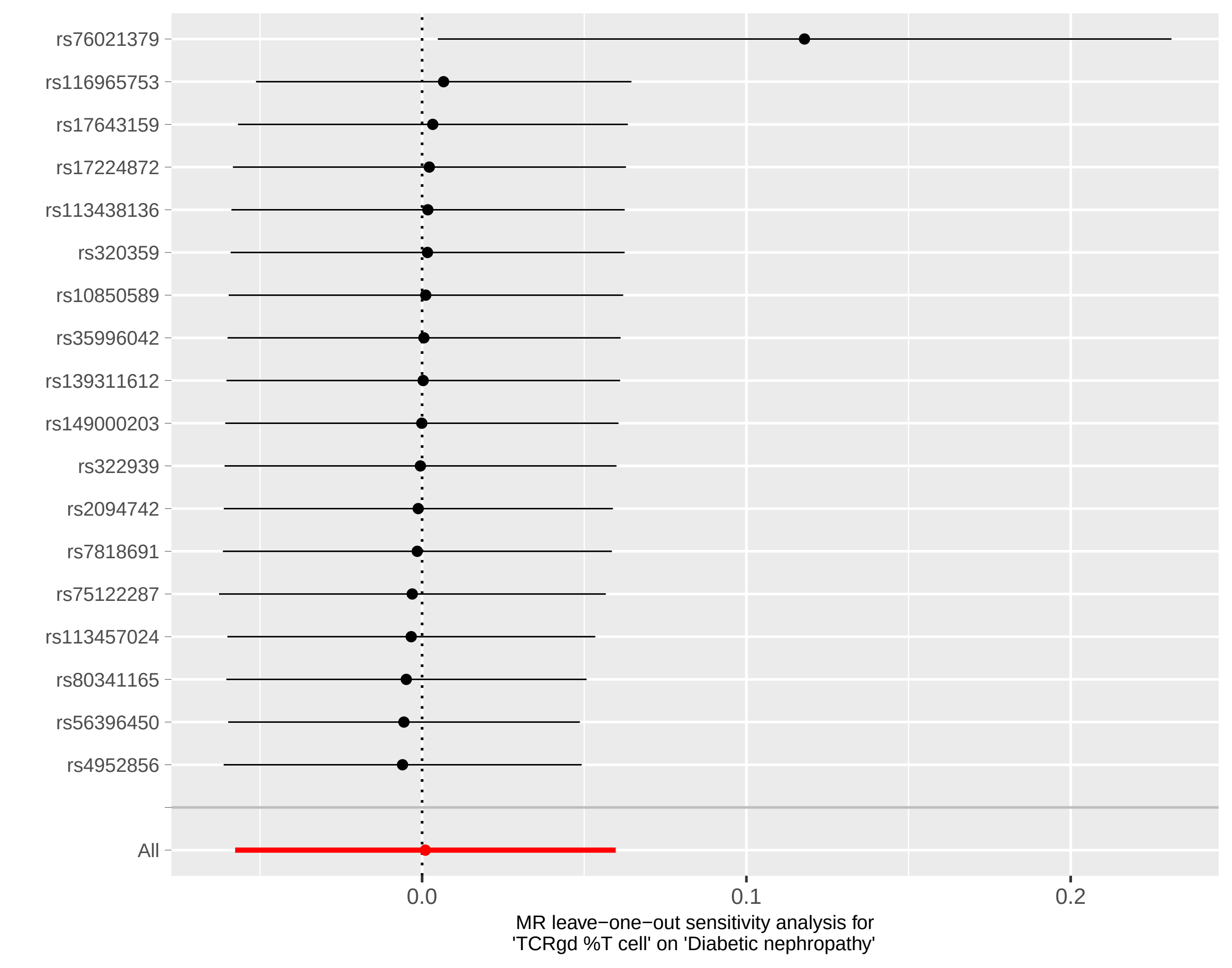

rs76021379
rs116965753
rs17643159
rs17224872
rs113438136
rs320359
rs10850589
rs35996042
rs139311612
rs149000203
rs322939
rs2094742
rs7818691
rs75122287
rs113457024
rs80341165
rs56396450
rs4952856
All
0.0
0.1
0.2
MR leave−one−out sensitivity analysis for
'TCRgd %T cell' on 'Diabetic nephropathy'

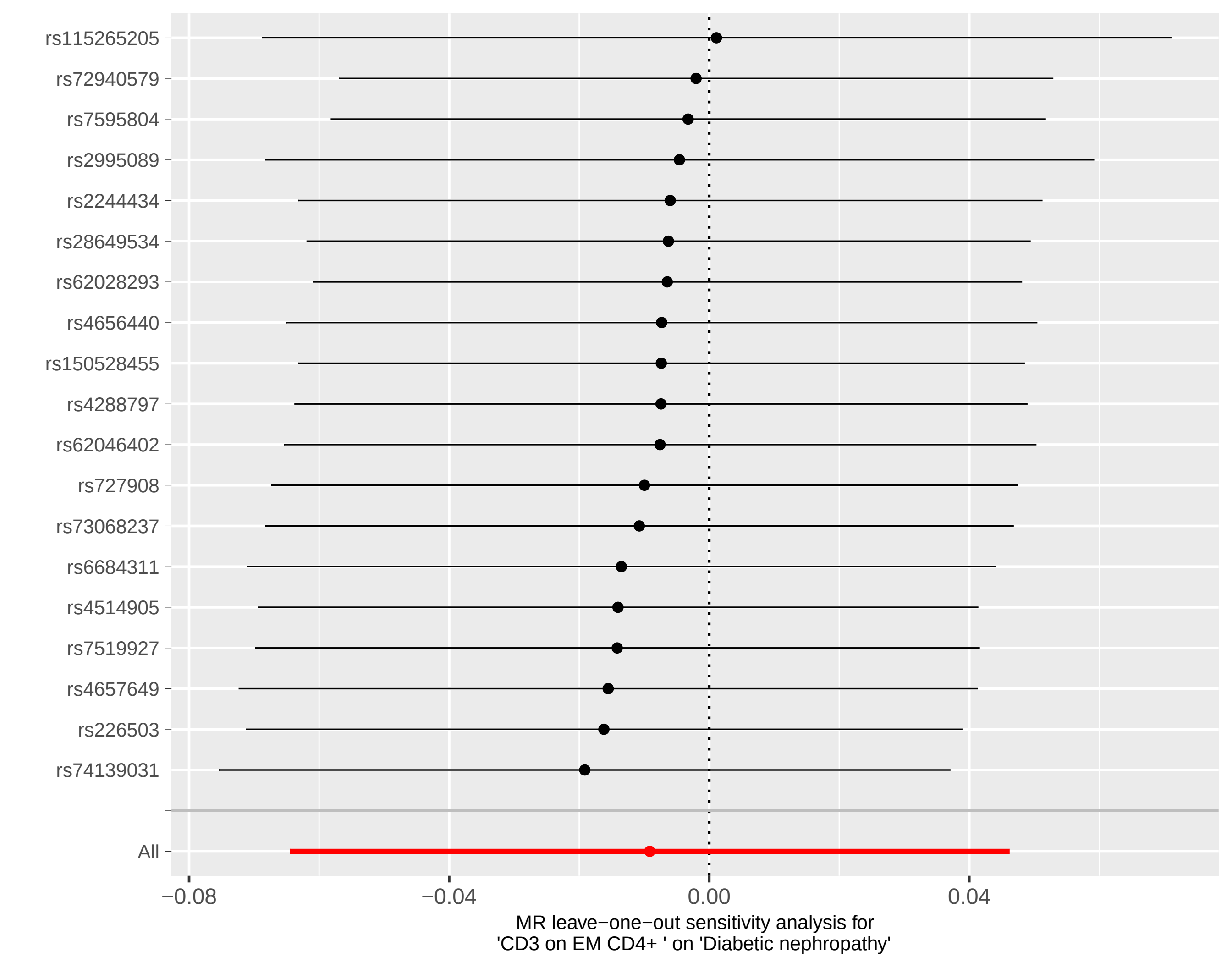

rs115265205
rs72940579
rs7595804
rs2995089
rs2244434
rs28649534
rs62028293
rs4656440
rs150528455
rs4288797
rs62046402
rs727908
rs73068237
rs6684311
rs4514905
rs7519927
rs4657649
rs226503
rs74139031
All
−0.08
−0.04
0.00
0.04
MR leave−one−out sensitivity analysis for
'CD3 on EM CD4+ ' on 'Diabetic nephropathy'

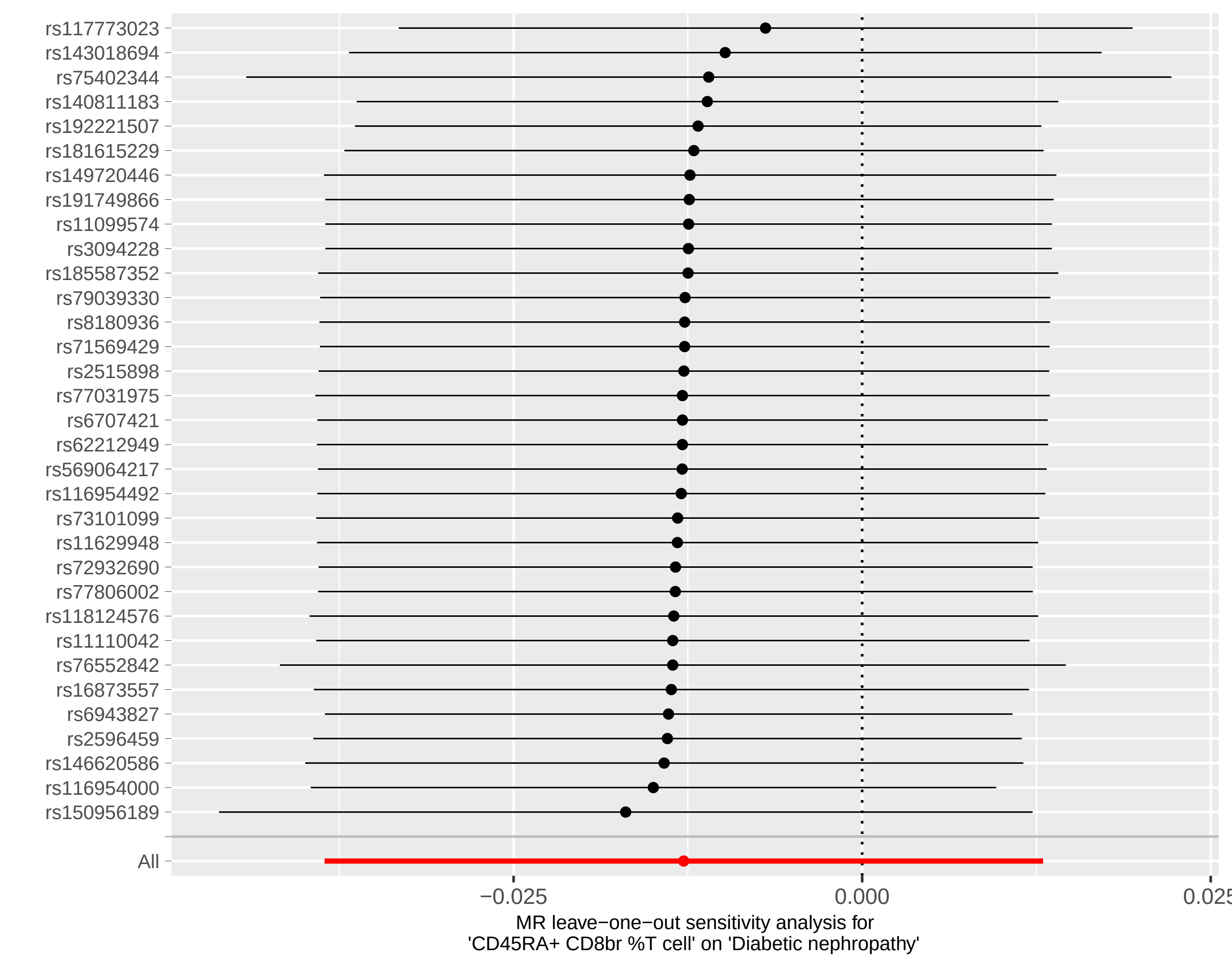

rs117773023
rs143018694
rs75402344
rs140811183
rs192221507
rs181615229
rs149720446
rs191749866
rs11099574
rs3094228
rs185587352
rs79039330
rs8180936
rs71569429
rs2515898
rs77031975
rs6707421
rs62212949
rs569064217
rs116954492
rs73101099
rs11629948
rs72932690
rs77806002
rs118124576
rs11110042
rs76552842
rs16873557
rs6943827
rs2596459
rs146620586
rs116954000
rs150956189
All
−0.025
0.000
0.025
MR leave−one−out sensitivity analysis for
'CD45RA+ CD8br %T cell' on 'Diabetic nephropathy'

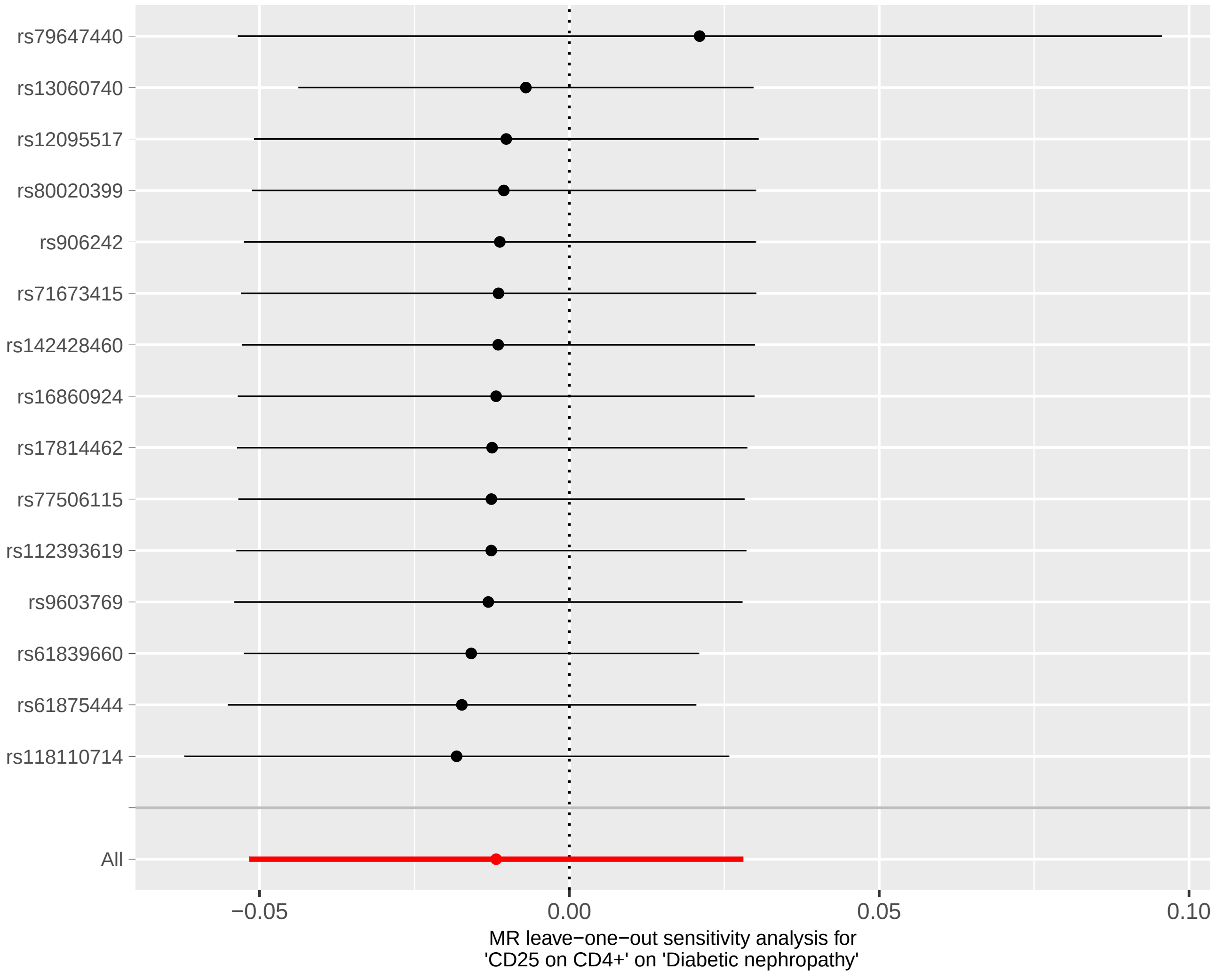
rs79647440
rs13060740
rs12095517
rs80020399
rs906242
rs71673415
rs142428460
rs16860924
rs17814462
rs77506115
rs112393619
rs9603769
rs61839660
rs61875444
rs118110714
All
−0.05
0.00
0.05
0.10
MR leave−one−out sensitivity analysis for
'CD25 on CD4+' on 'Diabetic nephropathy'

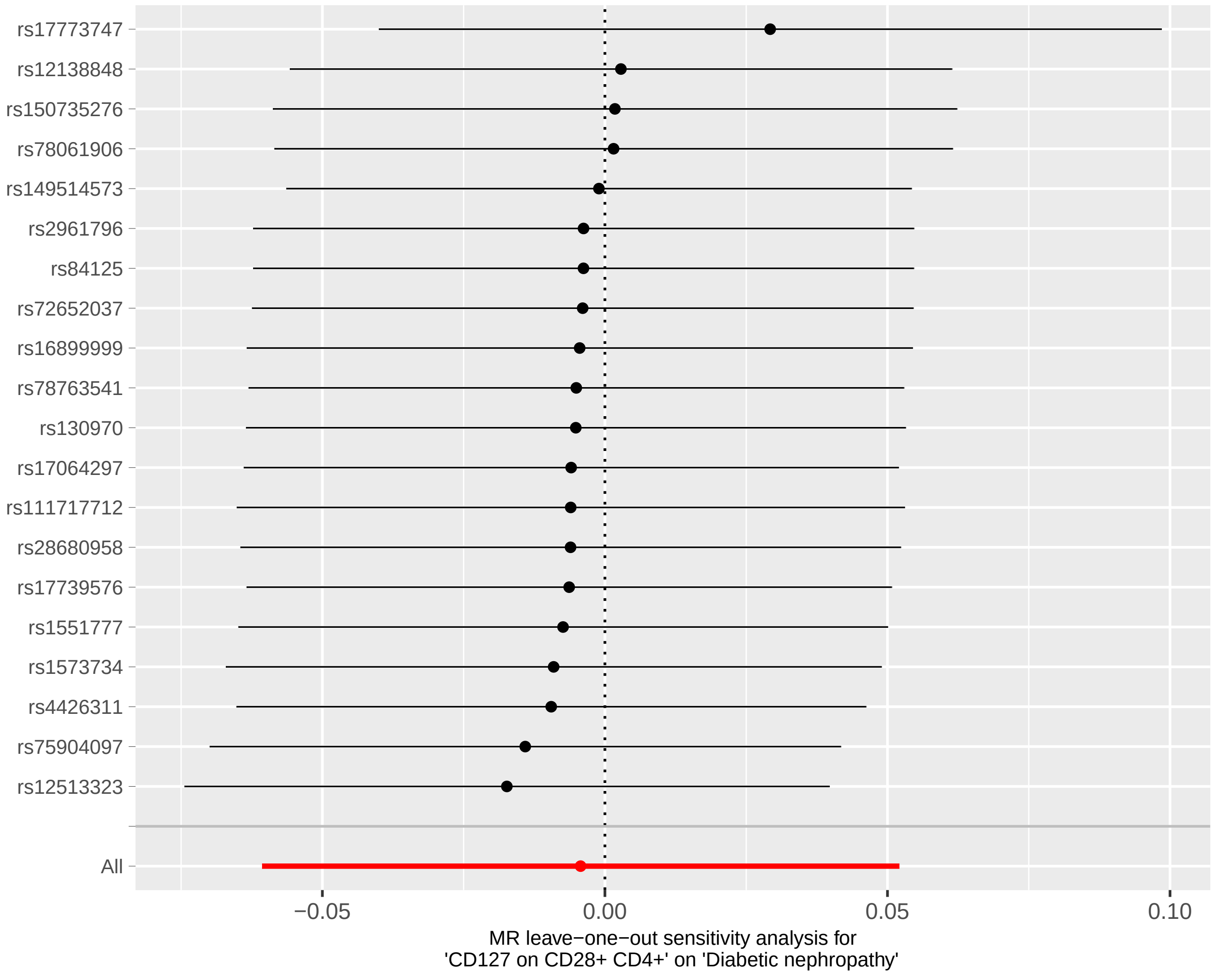
rs17773747
rs12138848
rs150735276
rs78061906
rs149514573
rs2961796
rs84125
rs72652037
rs16899999
rs78763541
rs130970
rs17064297
rs111717712
rs28680958
rs17739576
rs1551777
rs1573734
rs4426311
rs75904097
rs12513323
All
−0.05
0.00
0.05
0.10
MR leave−one−out sensitivity analysis for
'CD127 on CD28+ CD4+' on 'Diabetic nephropathy'

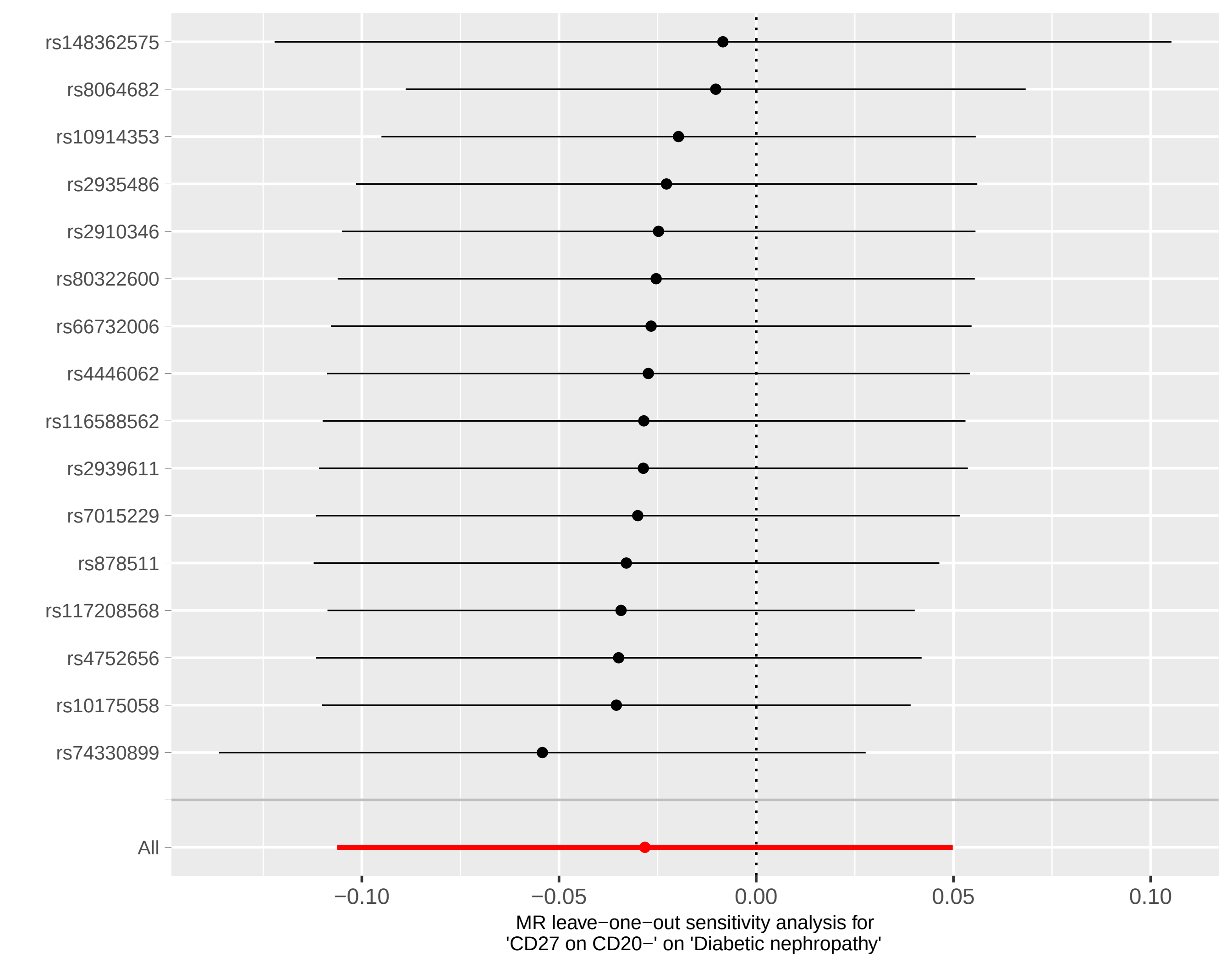

rs148362575
rs8064682
rs10914353
rs2935486
rs2910346
rs80322600
rs66732006
rs4446062
rs116588562
rs2939611
rs7015229
rs878511
rs117208568
rs4752656
rs10175058
rs74330899
All
−0.10
−0.05
0.00
0.05
0.10
MR leave−one−out sensitivity analysis for
'CD27 on CD20−' on 'Diabetic nephropathy'

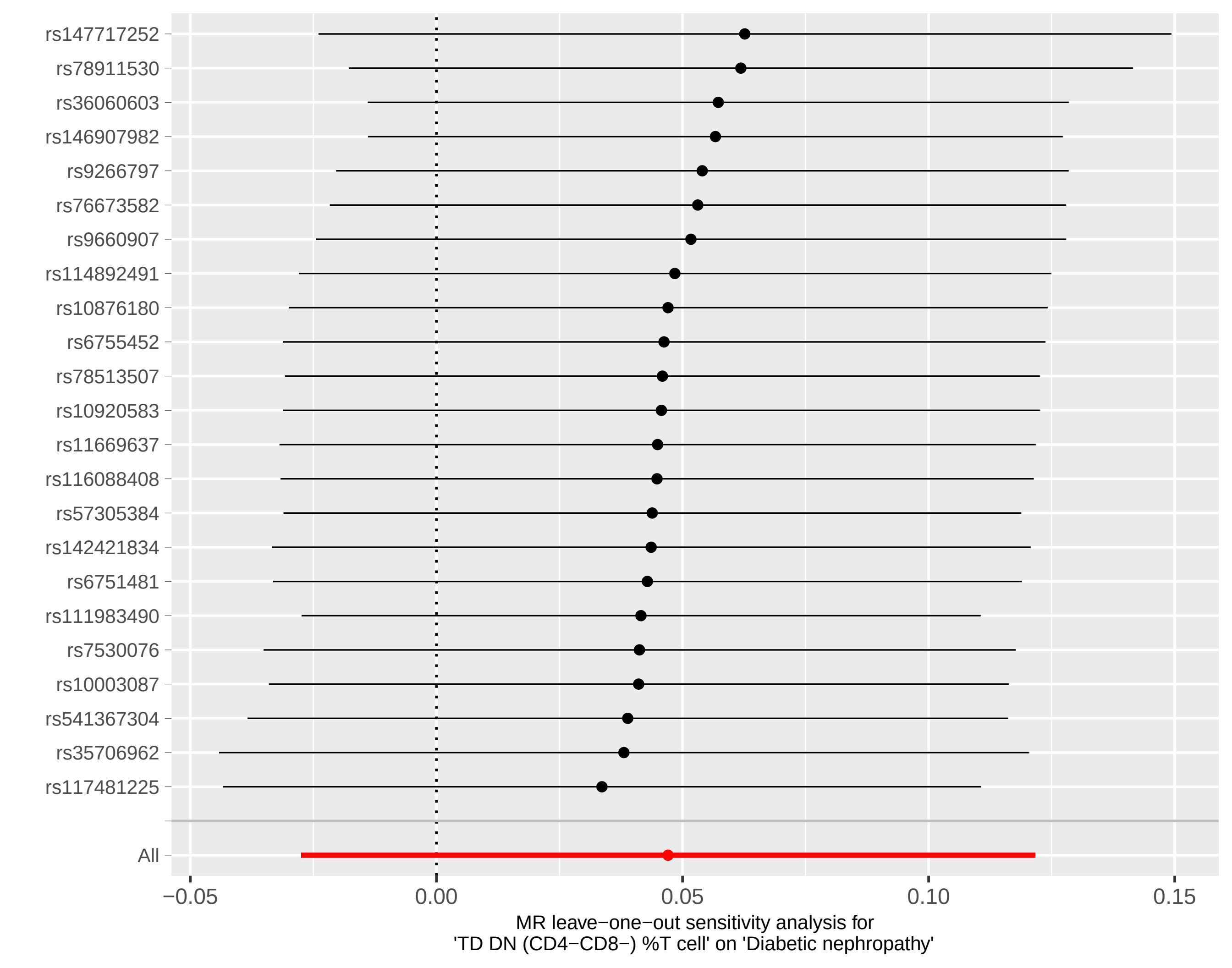
rs147717252
rs78911530
rs36060603
rs146907982
rs9266797
rs76673582
rs9660907
rs114892491
rs10876180
rs6755452
rs78513507
rs10920583
rs11669637
rs116088408
rs57305384
rs142421834
rs6751481
rs111983490
rs7530076
rs10003087
rs541367304
rs35706962
rs117481225
All
−0.05
0.00
0.05
0.10
0.15
MR leave−one−out sensitivity analysis for
'TD DN (CD4−CD8−) %T cell' on 'Diabetic nephropathy'

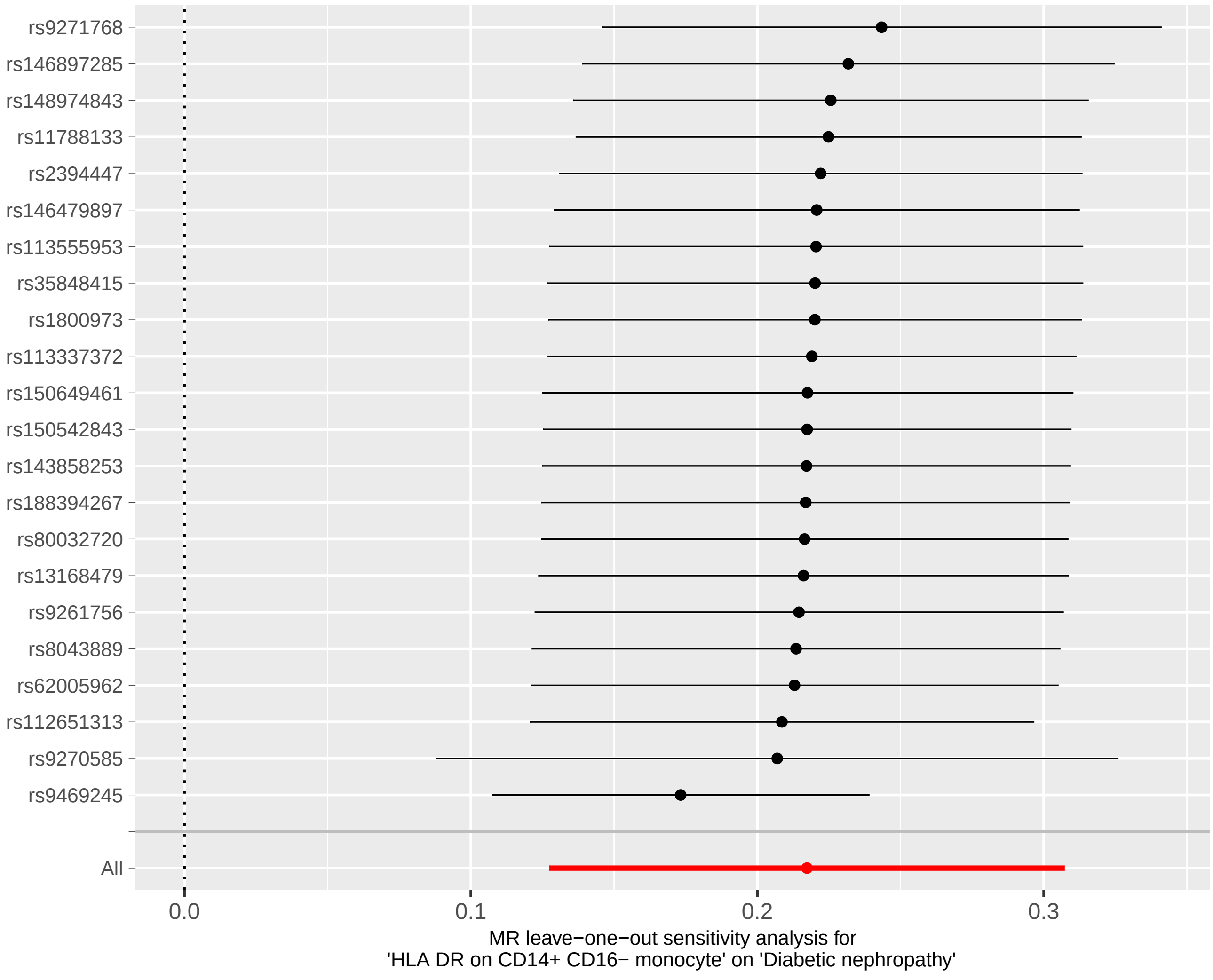
rs9271768
rs146897285
rs148974843
rs11788133
rs2394447
rs146479897
rs113555953
rs35848415
rs1800973
rs113337372
rs150649461
rs150542843
rs143858253
rs188394267
rs80032720
rs13168479
rs9261756
rs8043889
rs62005962
rs112651313
rs9270585
rs9469245
All
0.0
0.1
0.2
0.3
MR leave−one−out sensitivity analysis for
'HLA DR on CD14+ CD16− monocyte' on 'Diabetic nephropathy'

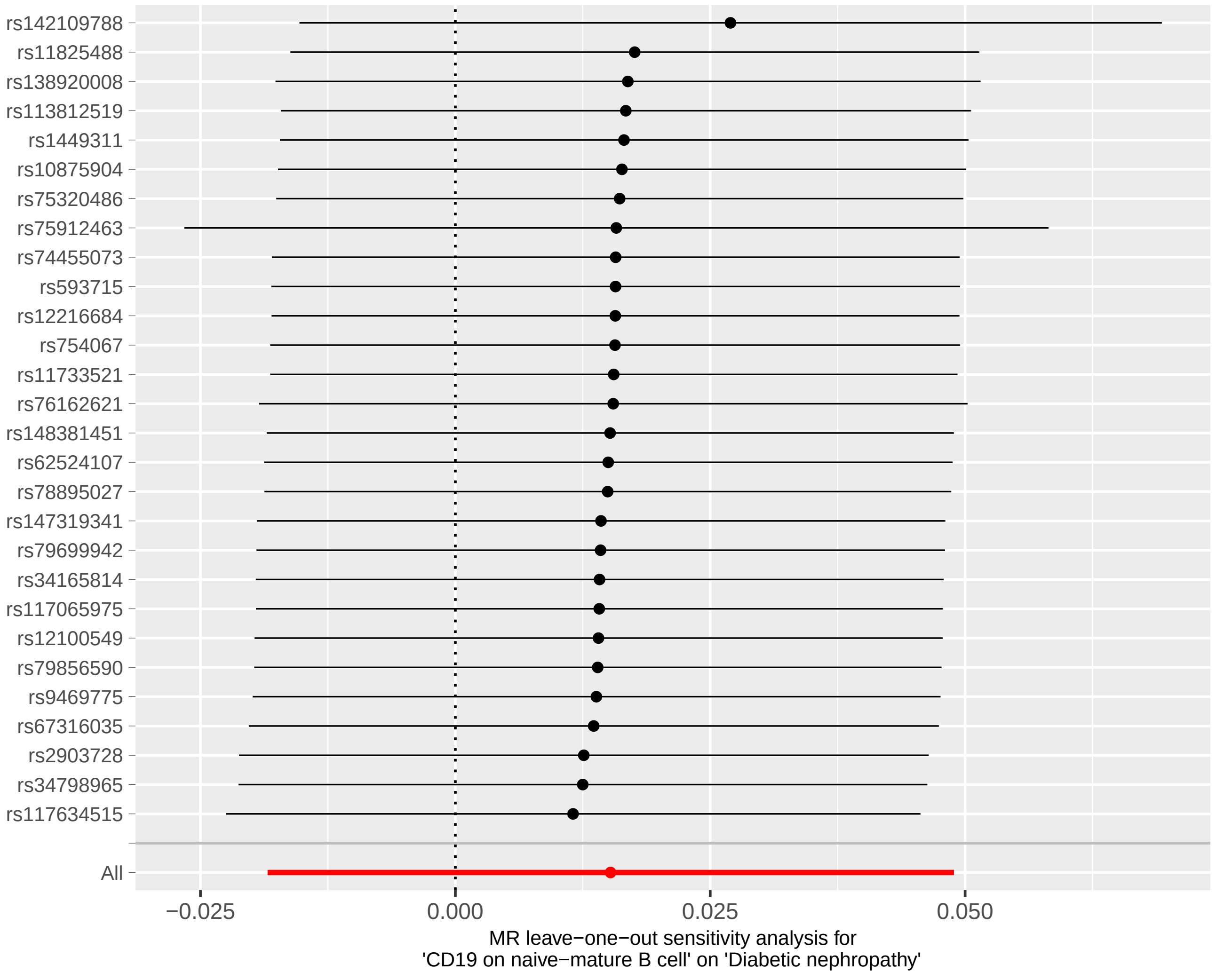
rs142109788
rs11825488
rs138920008
rs113812519
rs1449311
rs10875904
rs75320486
rs75912463
rs74455073
rs593715
rs12216684
rs754067
rs11733521
rs76162621
rs148381451
rs62524107
rs78895027
rs147319341
rs79699942
rs34165814
rs117065975
rs12100549
rs79856590
rs9469775
rs67316035
rs2903728
rs34798965
rs117634515
All
−0.025
0.000
0.025
0.050
MR leave−one−out sensitivity analysis for
'CD19 on naive−mature B cell' on 'Diabetic nephropathy'

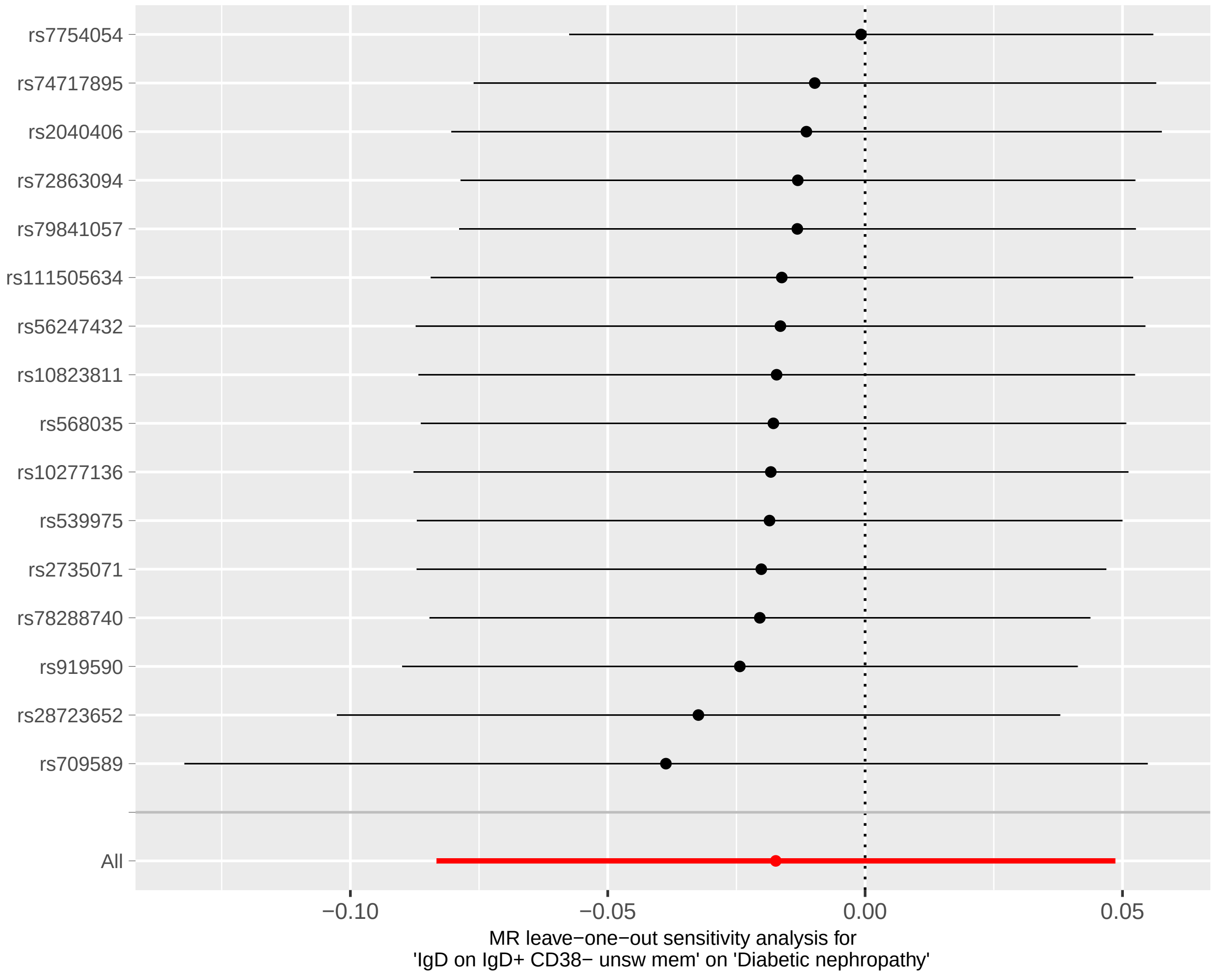

rs7754054
rs74717895
rs2040406
rs72863094
rs79841057
rs111505634
rs56247432
rs10823811
rs568035
rs10277136
rs539975
rs2735071
rs78288740
rs919590
rs28723652
rs709589
All
−0.10
−0.05
0.00
0.05
MR leave−one−out sensitivity analysis for
'IgD on IgD+ CD38− unsw mem' on 'Diabetic nephropathy'

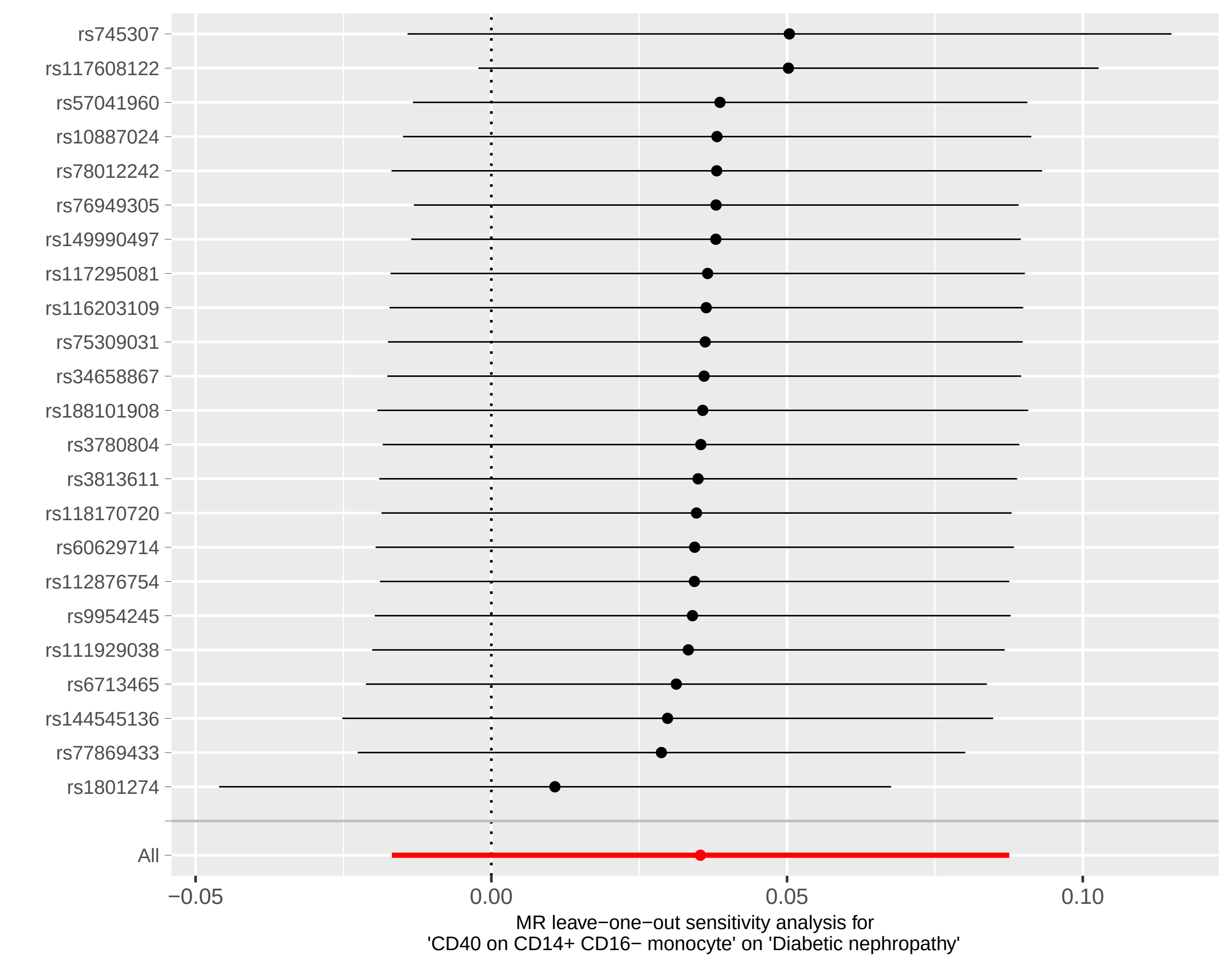
rs745307
rs117608122
rs57041960
rs10887024
rs78012242
rs76949305
rs149990497
rs117295081
rs116203109
rs75309031
rs34658867
rs188101908
rs3780804
rs3813611
rs118170720
rs60629714
rs112876754
rs9954245
rs111929038
rs6713465
rs144545136
rs77869433
rs1801274
All
−0.05
0.00
0.05
0.10
MR leave−one−out sensitivity analysis for
'CD40 on CD14+ CD16− monocyte' on 'Diabetic nephropathy'

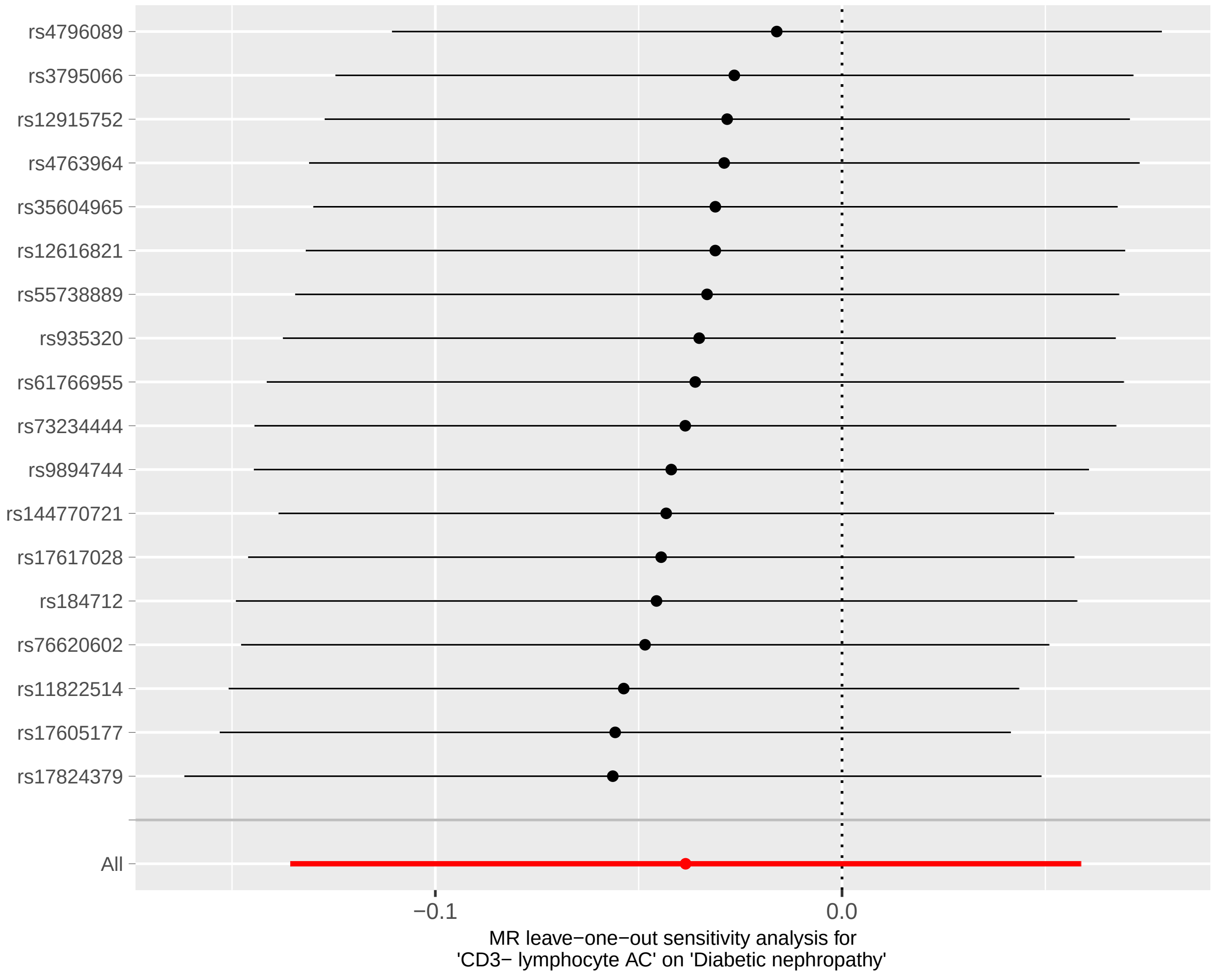
rs4796089
rs3795066
rs12915752
rs4763964
rs35604965
rs12616821
rs55738889
rs935320
rs61766955
rs73234444
rs9894744
rs144770721
rs17617028
rs184712
rs76620602
rs11822514
rs17605177
rs17824379
All
−0.1
0.0
MR leave−one−out sensitivity analysis for
'CD3− lymphocyte AC' on 'Diabetic nephropathy'

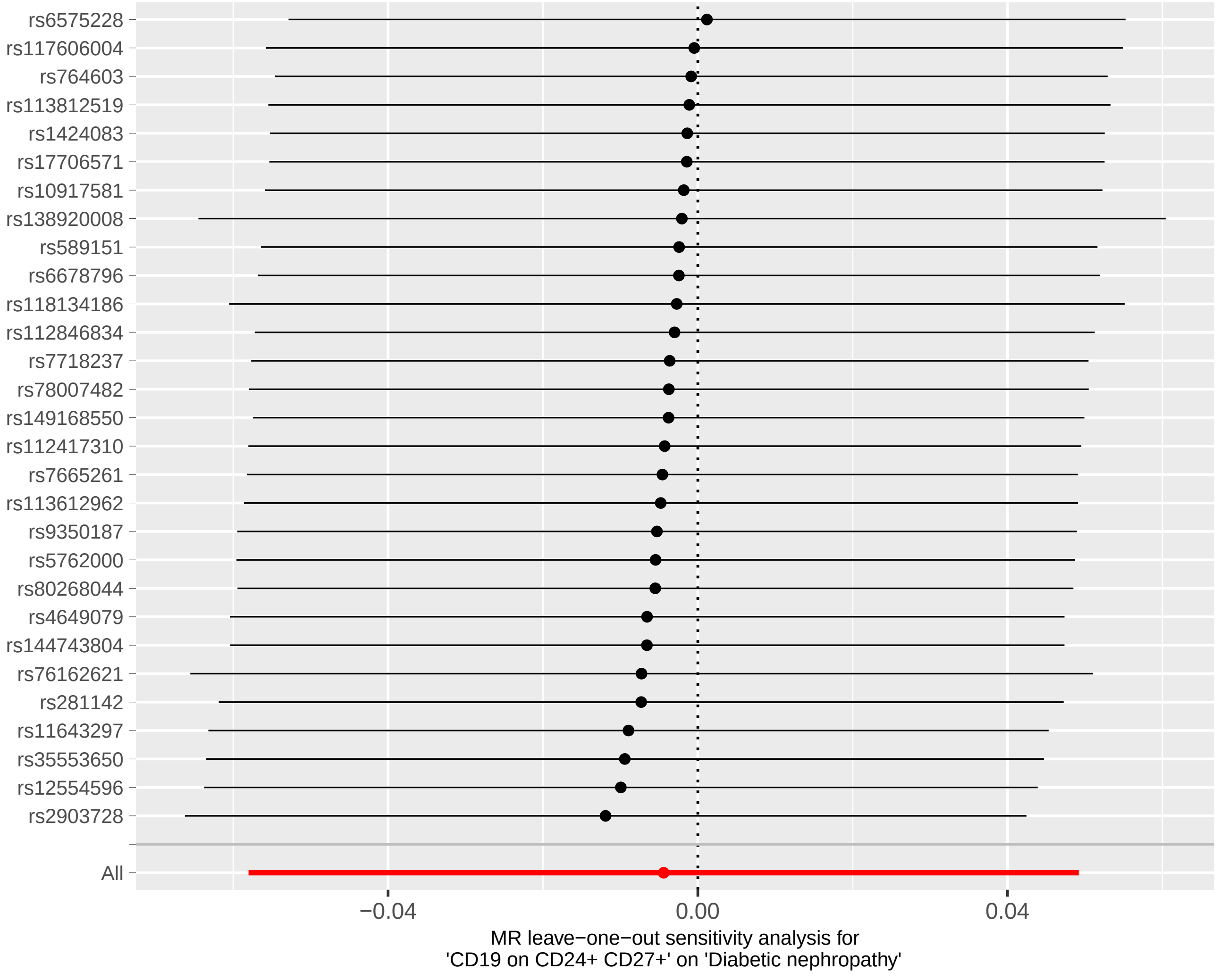
rs6575228
rs117606004
rs764603
rs113812519
rs1424083
rs17706571
rs10917581
rs138920008
rs589151
rs6678796
rs118134186
rs112846834
rs7718237
rs78007482
rs149168550
rs112417310
rs7665261
rs113612962
rs9350187
rs5762000
rs80268044
rs4649079
rs144743804
rs76162621
rs281142
rs11643297
rs35553650
rs12554596
rs2903728
All
−0.04
0.00
0.04
MR leave−one−out sensitivity analysis for
'CD19 on CD24+ CD27+' on 'Diabetic nephropathy'

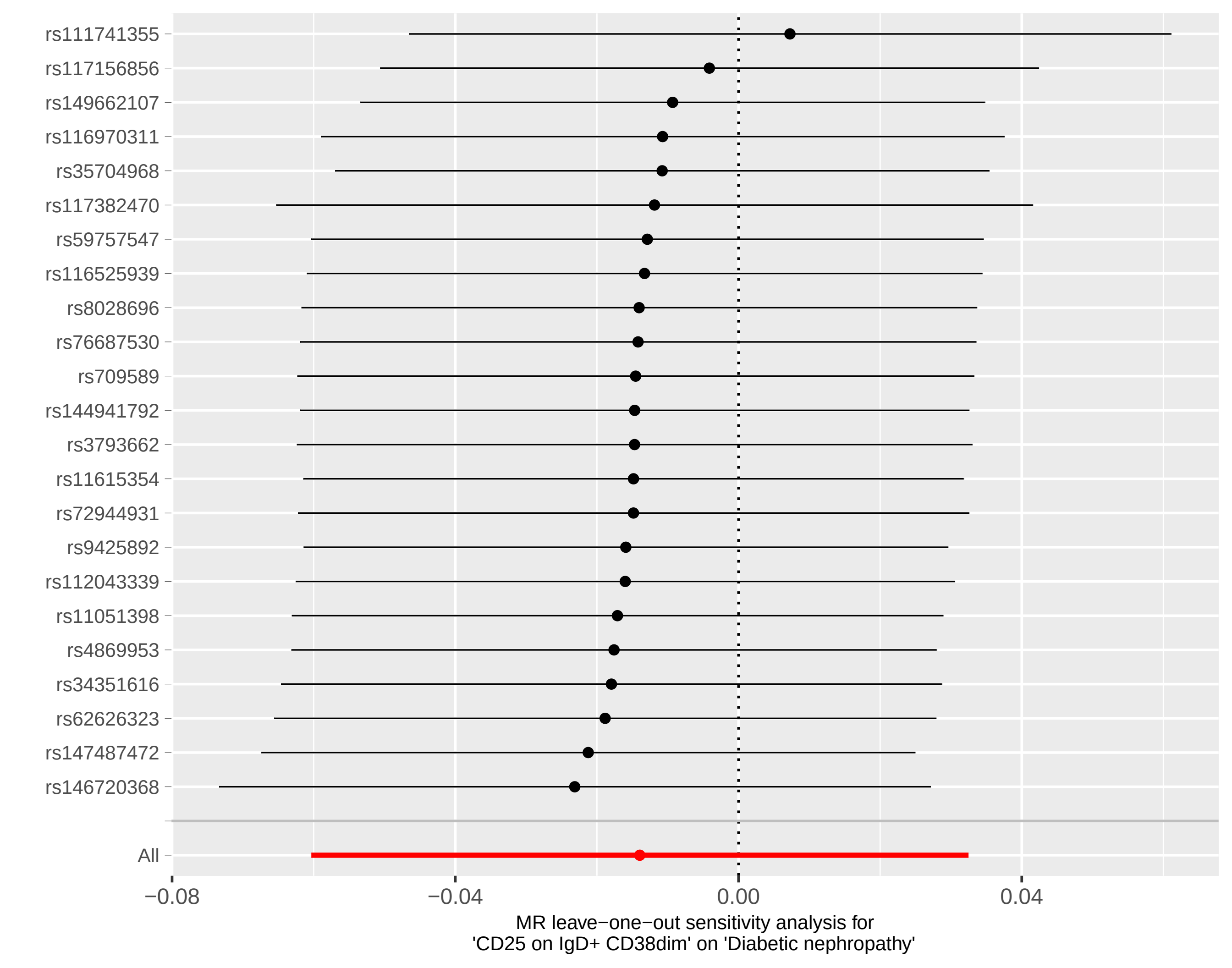

rs111741355
rs117156856
rs149662107
rs116970311
rs35704968
rs117382470
rs59757547
rs116525939
rs8028696
rs76687530
rs709589
rs144941792
rs3793662
rs11615354
rs72944931
rs9425892
rs112043339
rs11051398
rs4869953
rs34351616
rs62626323
rs147487472
rs146720368
All
−0.08
−0.04
0.00
0.04
MR leave−one−out sensitivity analysis for
'CD25 on IgD+ CD38dim' on 'Diabetic nephropathy'

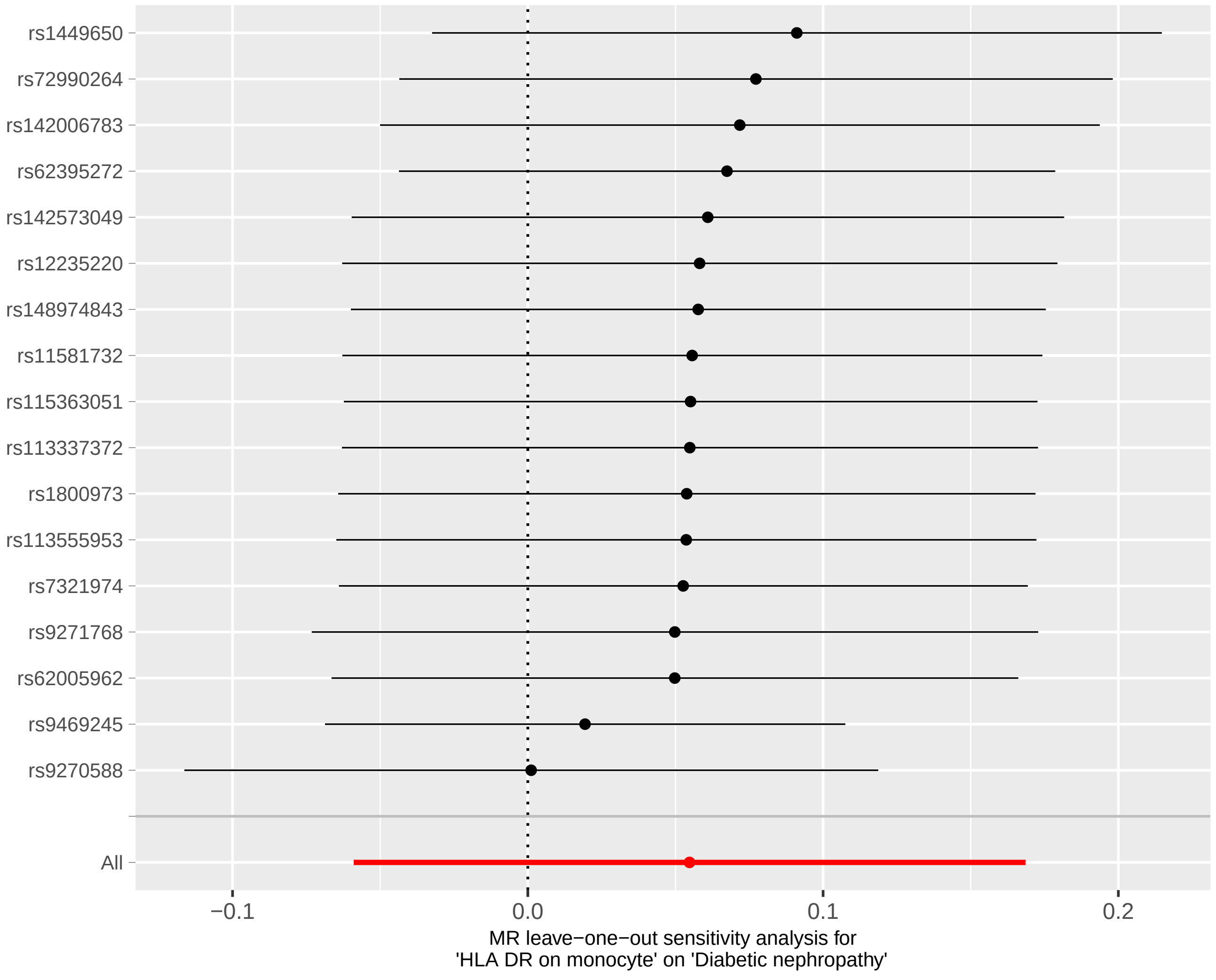
rs1449650
rs72990264
rs142006783
rs62395272
rs142573049
rs12235220
rs148974843
rs11581732
rs115363051
rs113337372
rs1800973
rs113555953
rs7321974
rs9271768
rs62005962
rs9469245
rs9270588
All
−0.1
0.0
0.1
0.2
MR leave−one−out sensitivity analysis for
'HLA DR on monocyte' on 'Diabetic nephropathy'

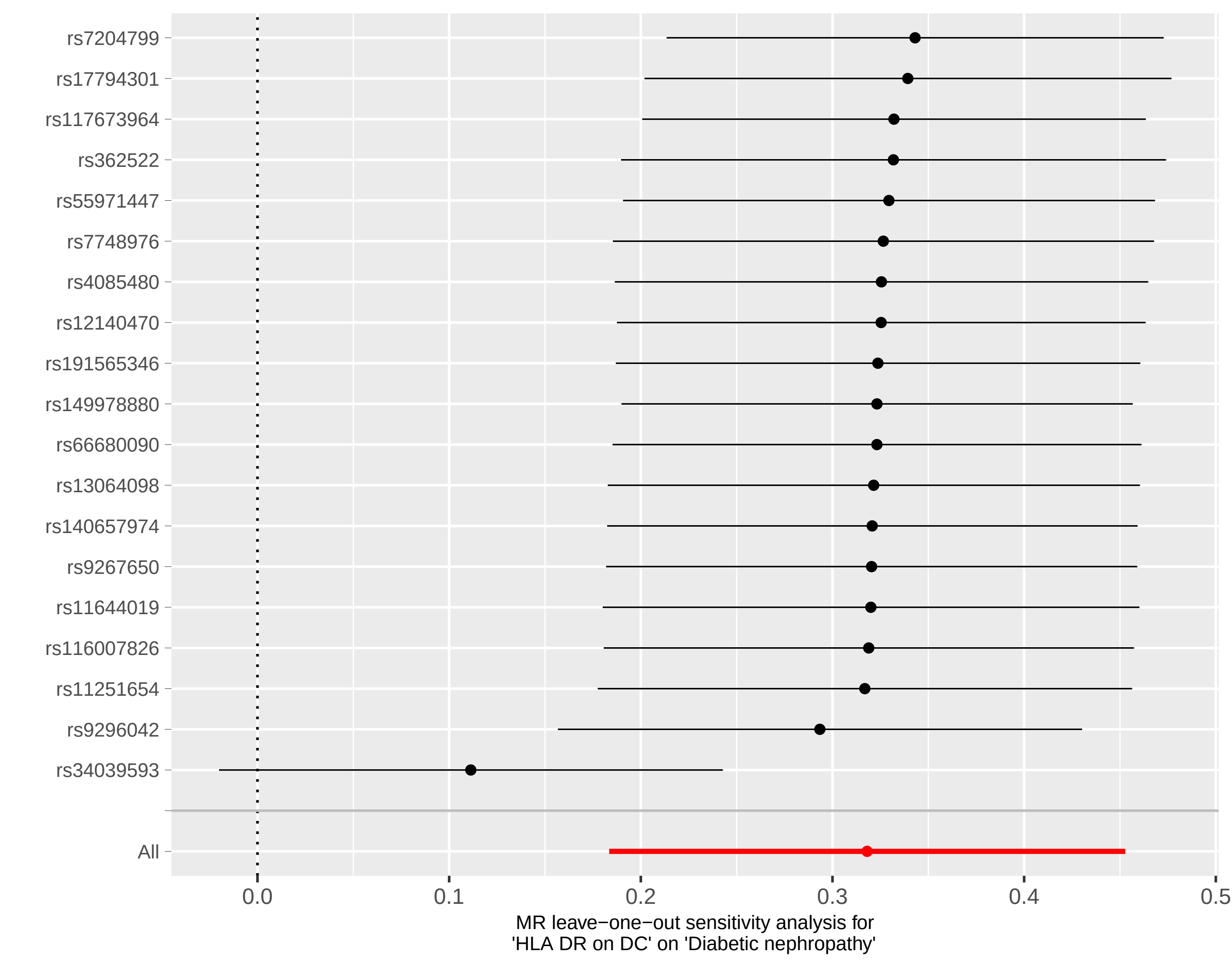
rs7204799
rs17794301
rs117673964
rs362522
rs55971447
rs7748976
rs4085480
rs12140470
rs191565346
rs149978880
rs66680090
rs13064098
rs140657974
rs9267650
rs11644019
rs116007826
rs11251654
rs9296042
rs34039593
All
0.0
0.1
0.2
0.3
0.4
0.5
MR leave−one−out sensitivity analysis for
'HLA DR on DC' on 'Diabetic nephropathy'

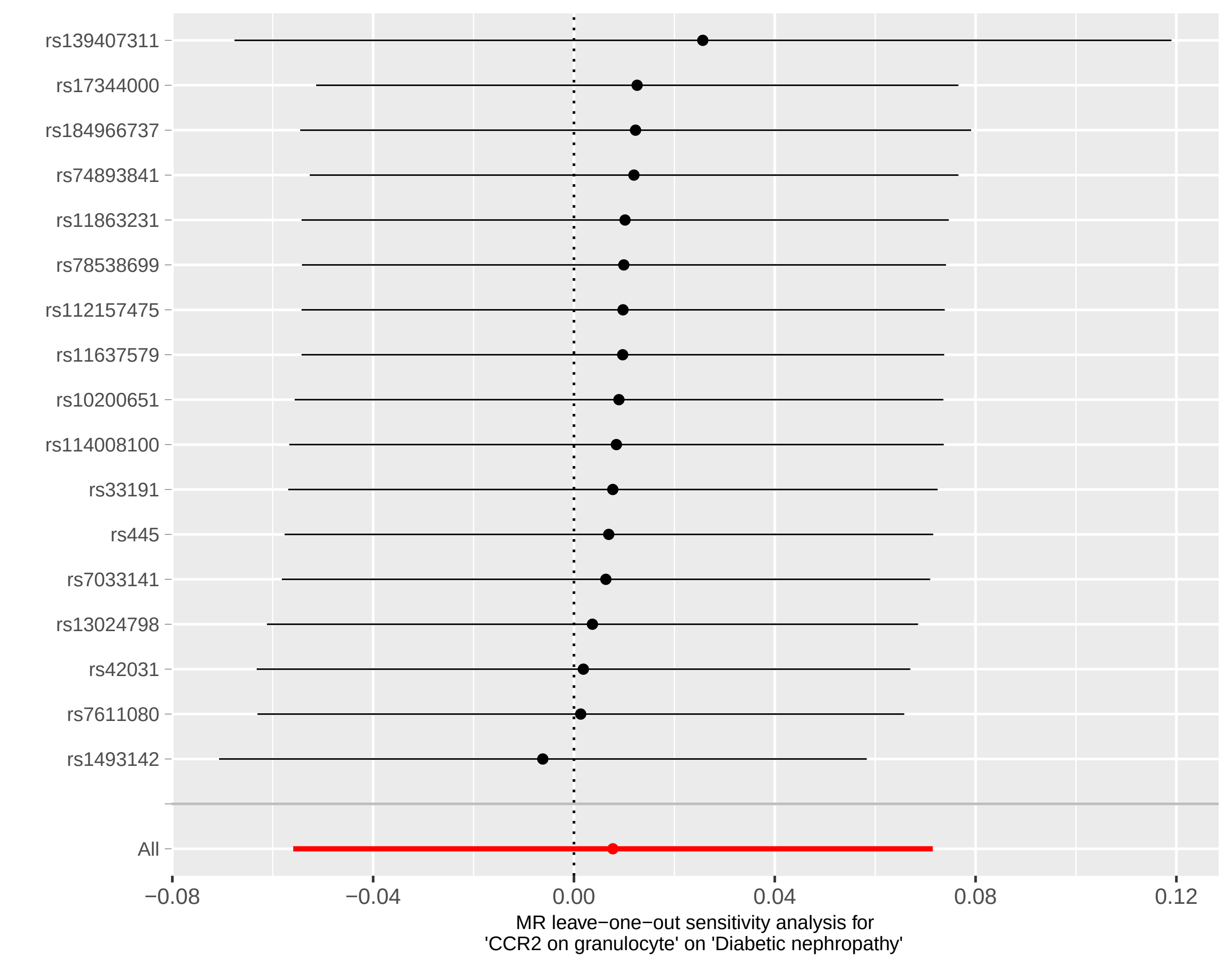
rs139407311
rs17344000
rs184966737
rs74893841
rs11863231
rs78538699
rs112157475
rs11637579
rs10200651
rs114008100
rs33191
rs445
rs7033141
rs13024798
rs42031
rs7611080
rs1493142
All
−0.08
−0.04
0.00
0.04
0.08
0.12
MR leave−one−out sensitivity analysis for
'CCR2 on granulocyte' on 'Diabetic nephropathy'

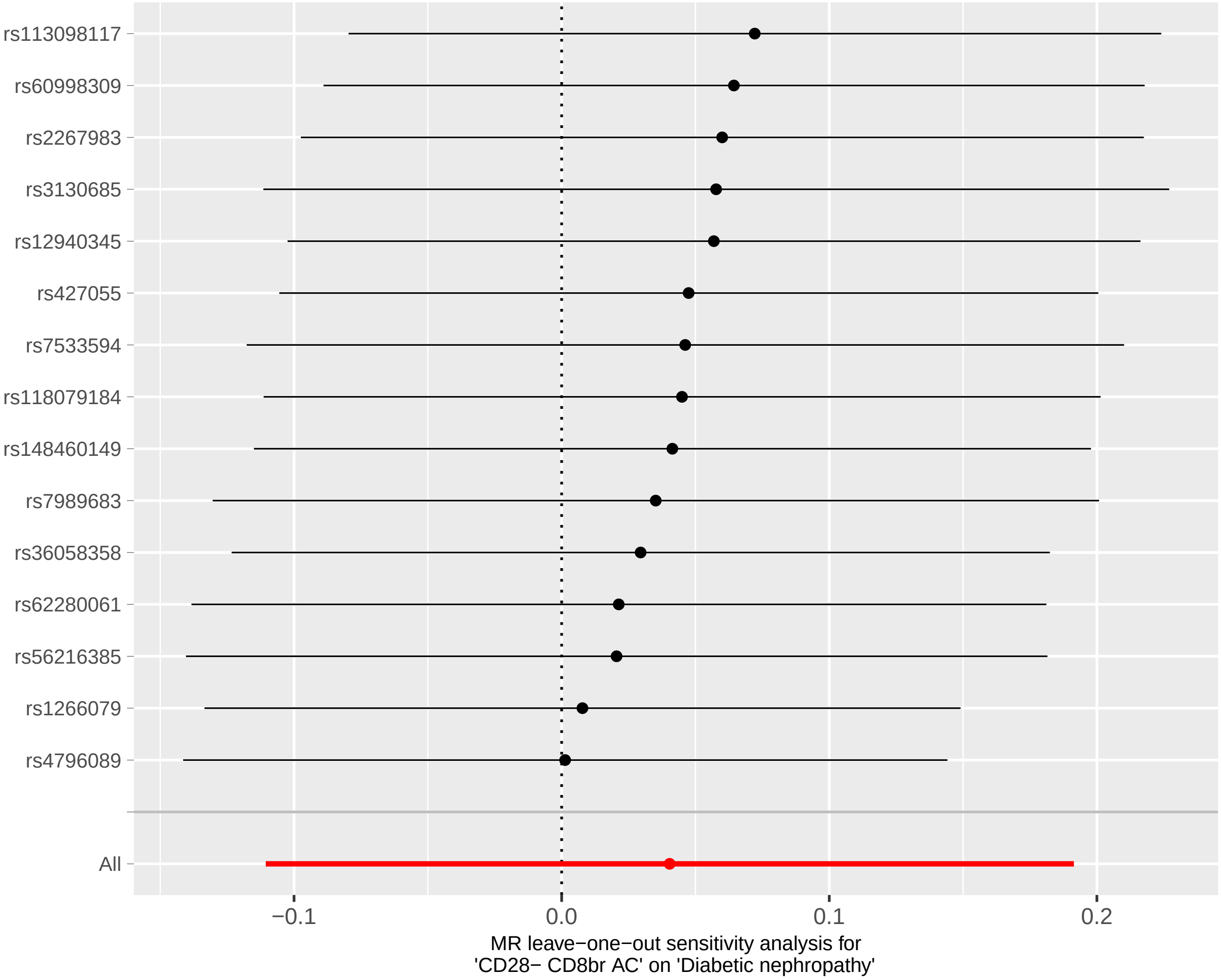
rs113098117
rs60998309
rs2267983
rs3130685
rs12940345
rs427055
rs7533594
rs118079184
rs148460149
rs7989683
rs36058358
rs62280061
rs56216385
rs1266079
rs4796089
All
−0.1
0.0
0.1
0.2
MR leave−one−out sensitivity analysis for
'CD28− CD8br AC' on 'Diabetic nephropathy'

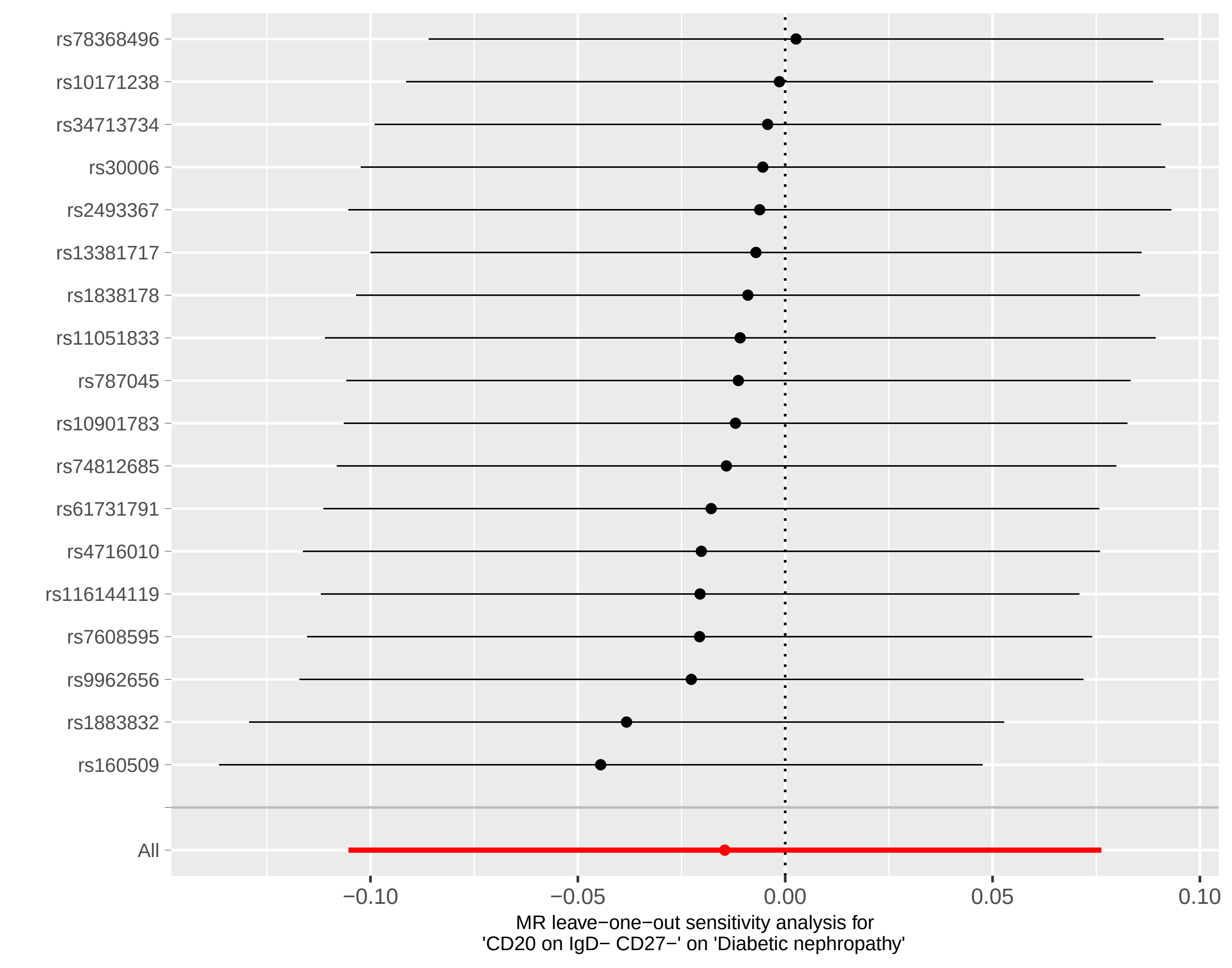
rs78368496
rs10171238
rs34713734
rs30006
rs2493367
rs13381717
rs1838178
rs11051833
rs787045
rs10901783
rs74812685
rs61731791
rs4716010
rs116144119
rs7608595
rs9962656
rs1883832
rs160509
All
−0.10
−0.05
0.00
0.05
0.10
MR leave−one−out sensitivity analysis for
'CD20 on IgD− CD27−' on 'Diabetic nephropathy'

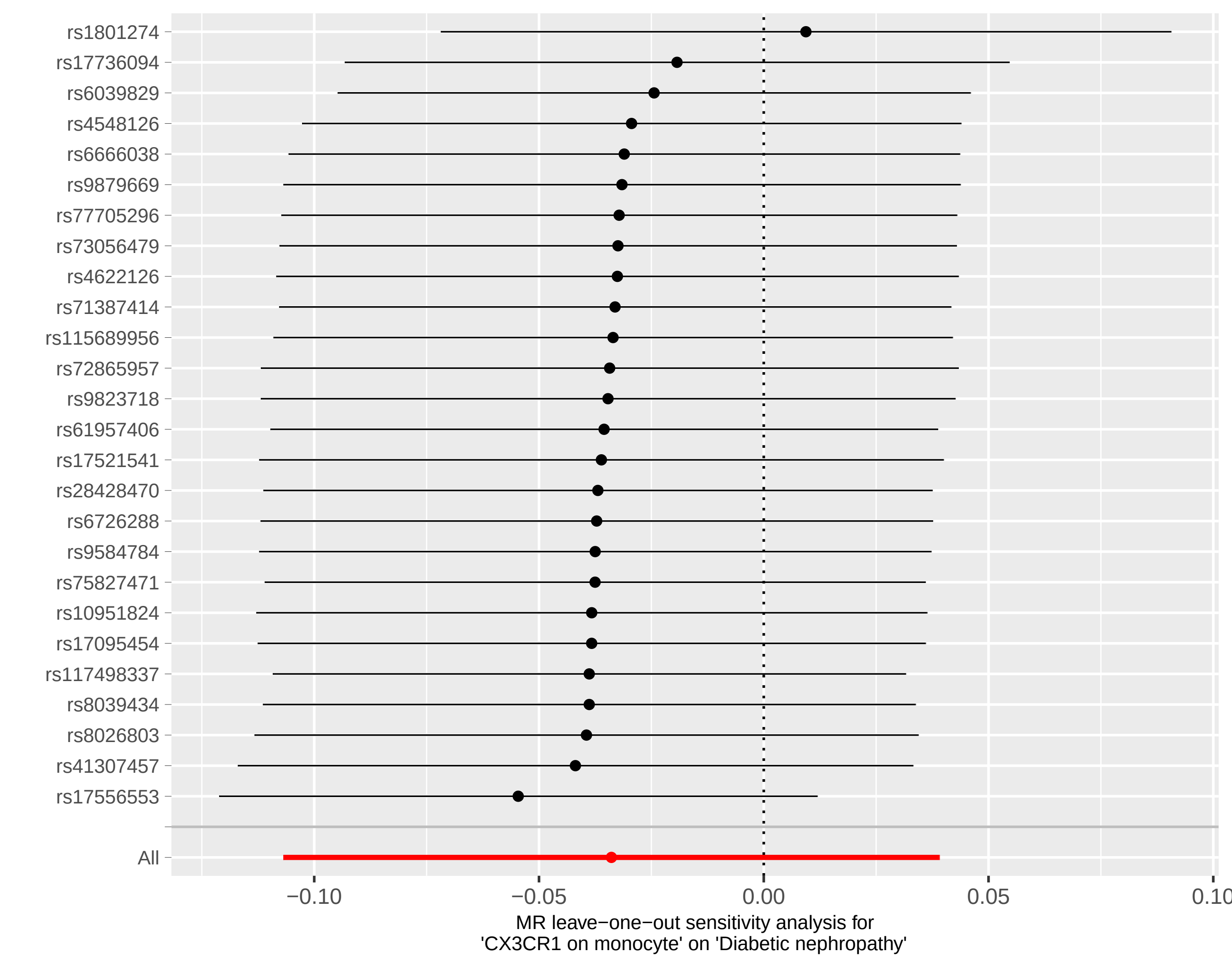
rs1801274
rs17736094
rs6039829
rs4548126
rs6666038
rs9879669
rs77705296
rs73056479
rs4622126
rs71387414
rs115689956
rs72865957
rs9823718
rs61957406
rs17521541
rs28428470
rs6726288
rs9584784
rs75827471
rs10951824
rs17095454
rs117498337
rs8039434
rs8026803
rs41307457
rs17556553
All
−0.10
−0.05
0.00
0.05
0.10
MR leave−one−out sensitivity analysis for
'CX3CR1 on monocyte' on 'Diabetic nephropathy'

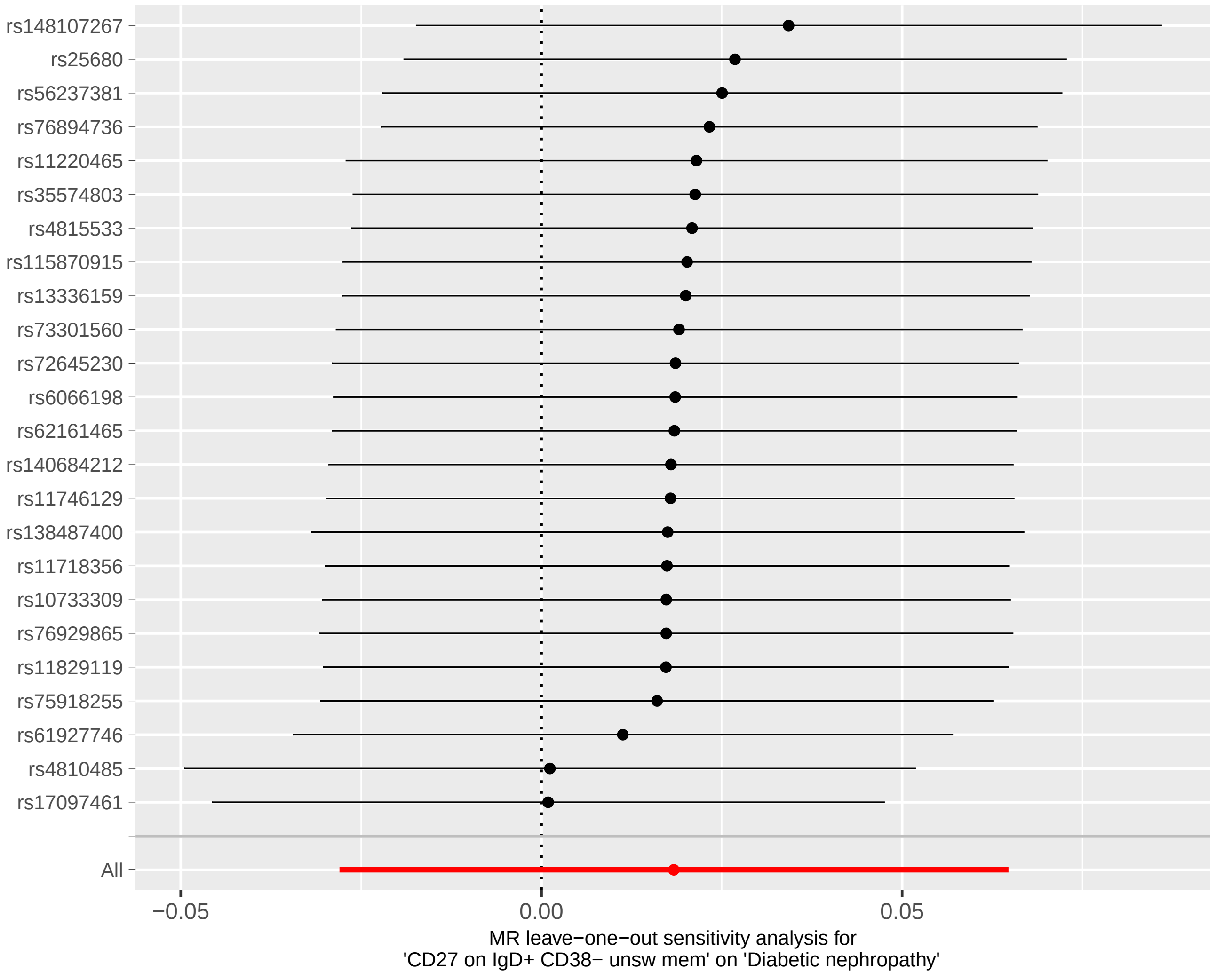

rs148107267
rs25680
rs56237381
rs76894736
rs11220465
rs35574803
rs4815533
rs115870915
rs13336159
rs73301560
rs72645230
rs6066198
rs62161465
rs140684212
rs11746129
rs138487400
rs11718356
rs10733309
rs76929865
rs11829119
rs75918255
rs61927746
rs4810485
rs17097461
All
−0.05
0.00
0.05
MR leave−one−out sensitivity analysis for
'CD27 on IgD+ CD38− unsw mem' on 'Diabetic nephropathy'

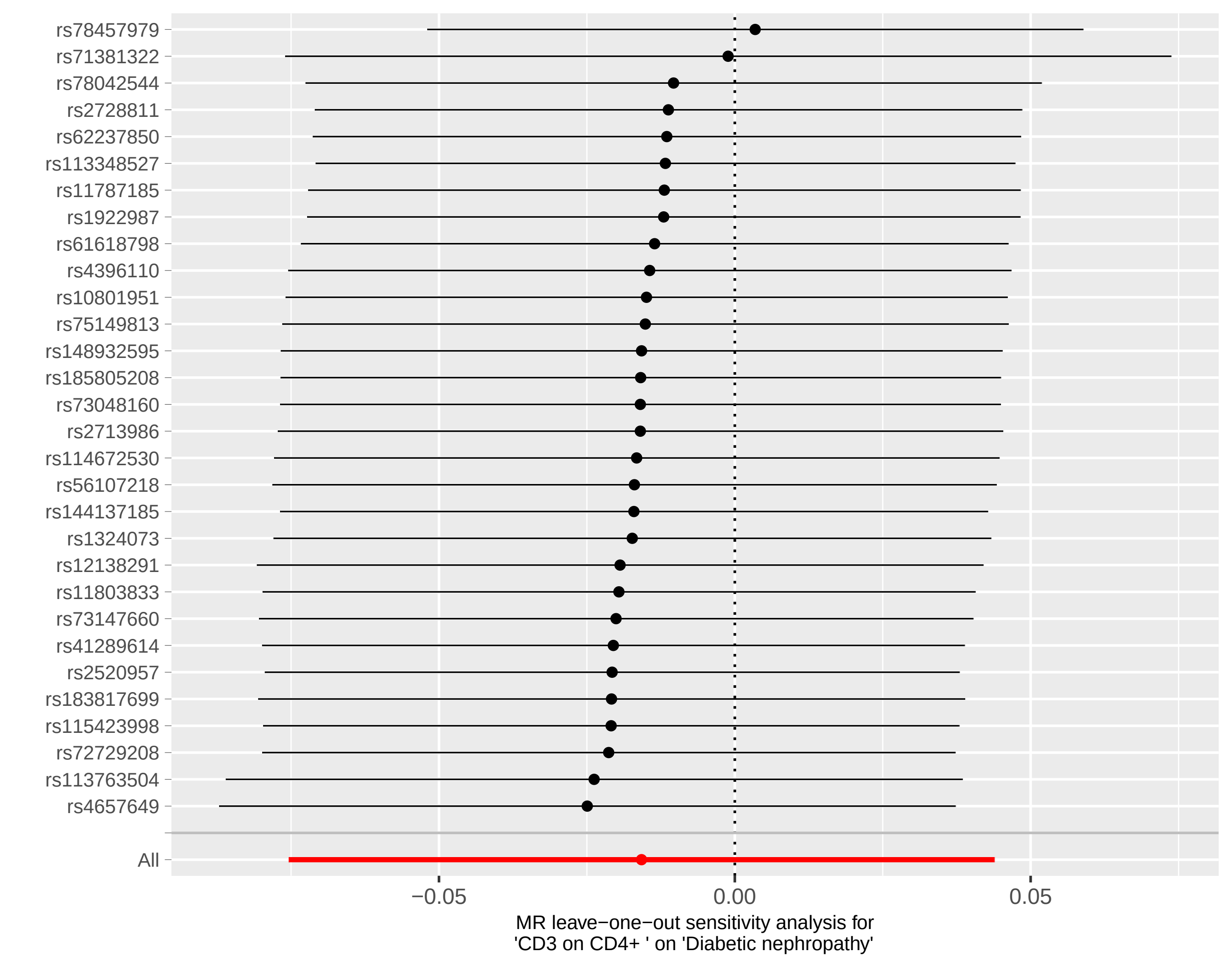
rs78457979
rs71381322
rs78042544
rs2728811
rs62237850
rs113348527
rs11787185
rs1922987
rs61618798
rs4396110
rs10801951
rs75149813
rs148932595
rs185805208
rs73048160
rs2713986
rs114672530
rs56107218
rs144137185
rs1324073
rs12138291
rs11803833
rs73147660
rs41289614
rs2520957
rs183817699
rs115423998
rs72729208
rs113763504
rs4657649
All
−0.05
0.00
0.05
MR leave−one−out sensitivity analysis for
'CD3 on CD4+ ' on 'Diabetic nephropathy'

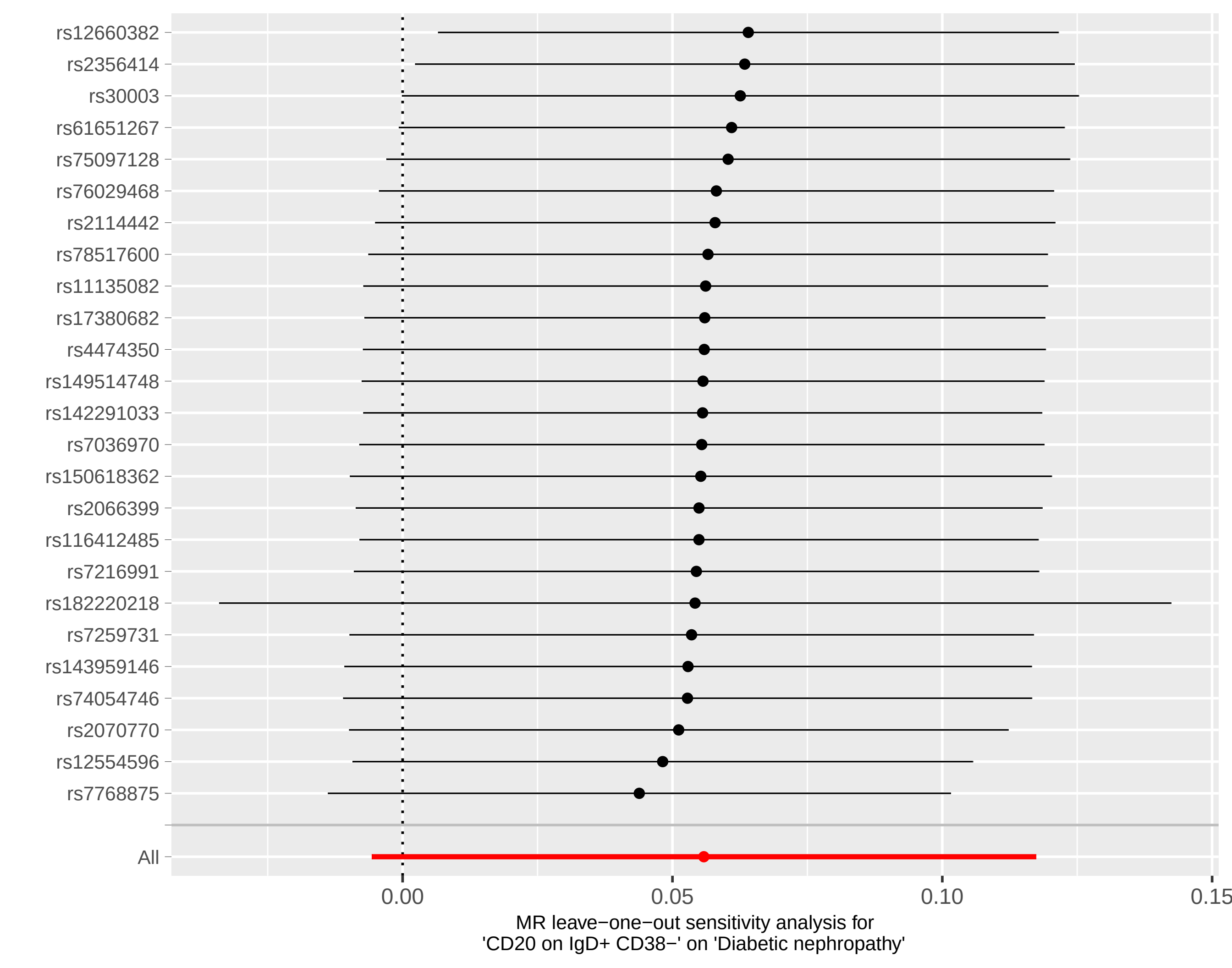

rs12660382
rs2356414
rs30003
rs61651267
rs75097128
rs76029468
rs2114442
rs78517600
rs11135082
rs17380682
rs4474350
rs149514748
rs142291033
rs7036970
rs150618362
rs2066399
rs116412485
rs7216991
rs182220218
rs7259731
rs143959146
rs74054746
rs2070770
rs12554596
rs7768875
All
0.00
0.05
0.10
0.15
MR leave−one−out sensitivity analysis for
'CD20 on IgD+ CD38−' on 'Diabetic nephropathy'

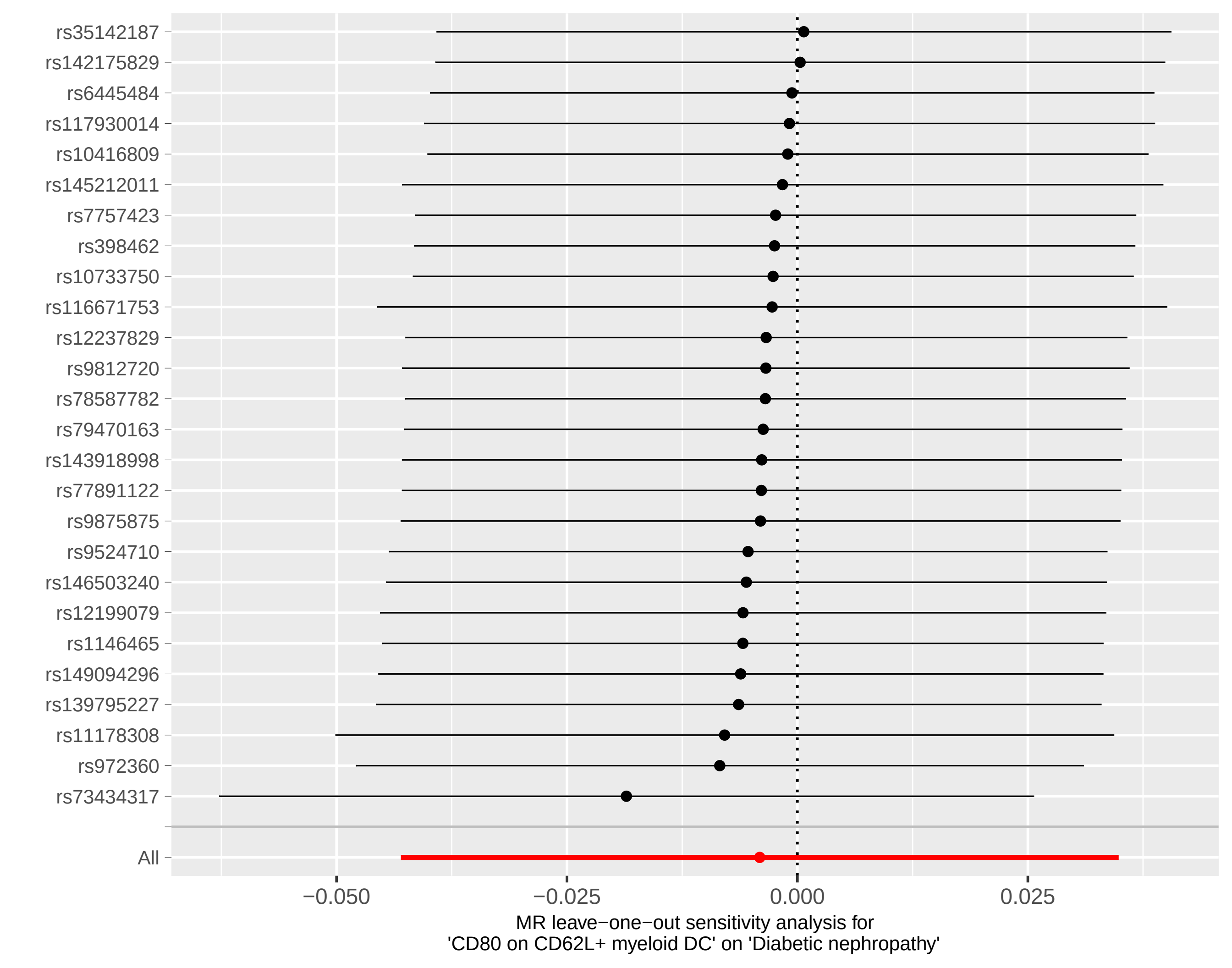
rs35142187
rs142175829
rs6445484
rs117930014
rs10416809
rs145212011
rs7757423
rs398462
rs10733750
rs116671753
rs12237829
rs9812720
rs78587782
rs79470163
rs143918998
rs77891122
rs9875875
rs9524710
rs146503240
rs12199079
rs1146465
rs149094296
rs139795227
rs11178308
rs972360
rs73434317
All
−0.050
−0.025
0.000
0.025
MR leave−one−out sensitivity analysis for
'CD80 on CD62L+ myeloid DC' on 'Diabetic nephropathy'

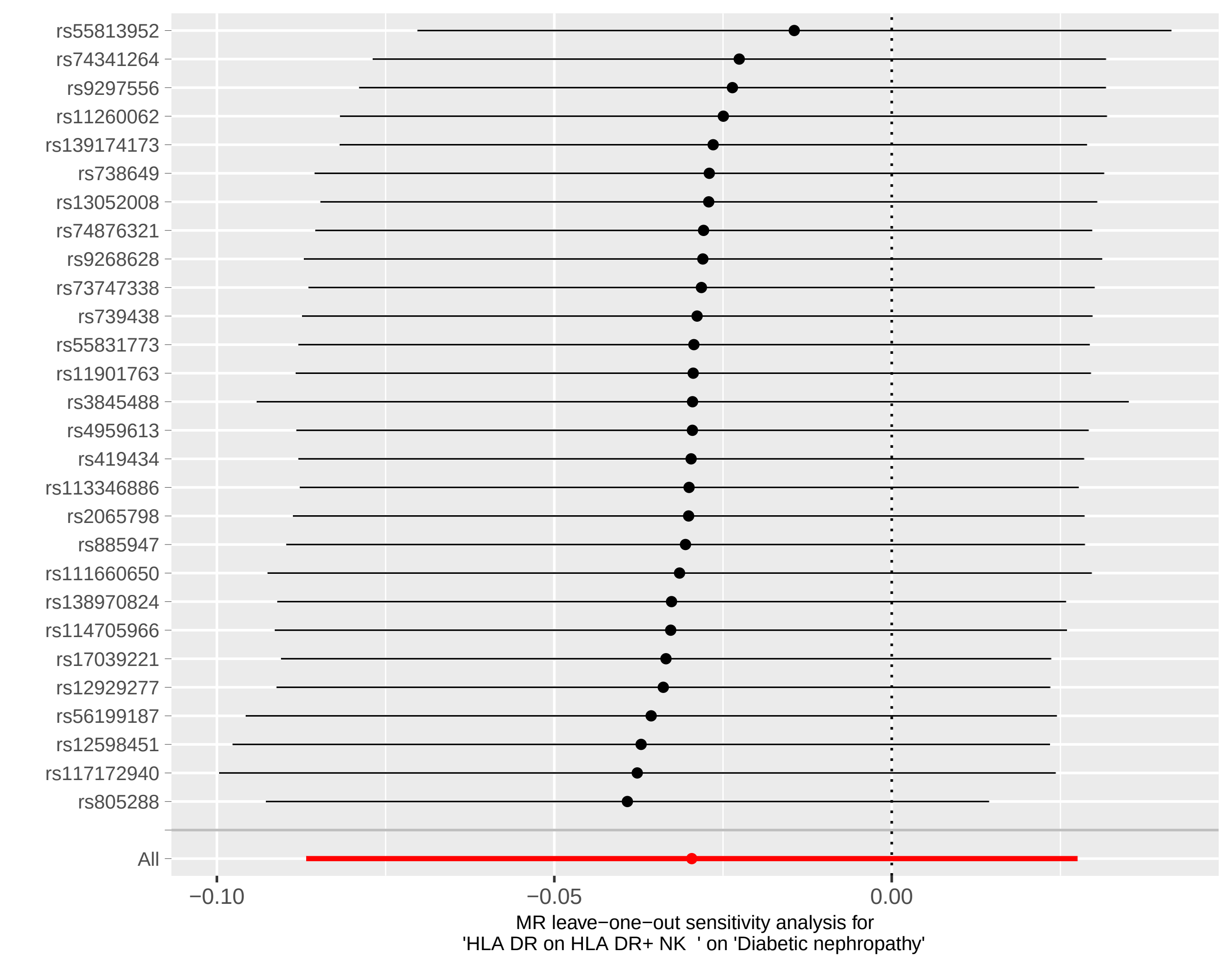
rs55813952
rs74341264
rs9297556
rs11260062
rs139174173
rs738649
rs13052008
rs74876321
rs9268628
rs73747338
rs739438
rs55831773
rs11901763
rs3845488
rs4959613
rs419434
rs113346886
rs2065798
rs885947
rs111660650
rs138970824
rs114705966
rs17039221
rs12929277
rs56199187
rs12598451
rs117172940
rs805288
All
−0.10
−0.05
0.00
MR leave−one−out sensitivity analysis for
'HLA DR on HLA DR+ NK ' on 'Diabetic nephropathy'

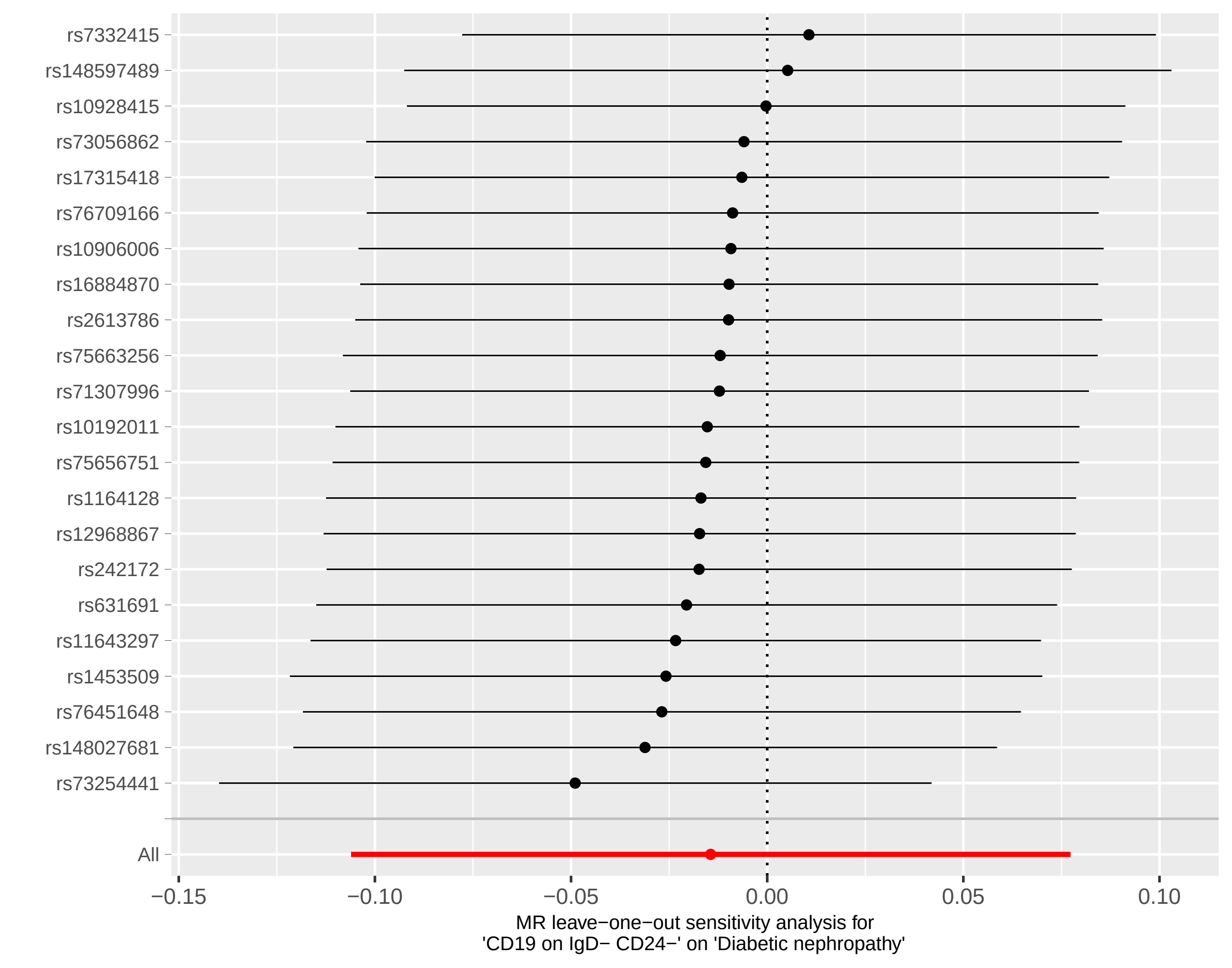
rs7332415
rs148597489
rs10928415
rs73056862
rs17315418
rs76709166
rs10906006
rs16884870
rs2613786
rs75663256
rs71307996
rs10192011
rs75656751
rs1164128
rs12968867
rs242172
rs631691
rs11643297
rs1453509
rs76451648
rs148027681
rs73254441
All
−0.15
−0.10
−0.05
0.00
0.05
0.10
MR leave−one−out sensitivity analysis for
'CD19 on IgD− CD24−' on 'Diabetic nephropathy'

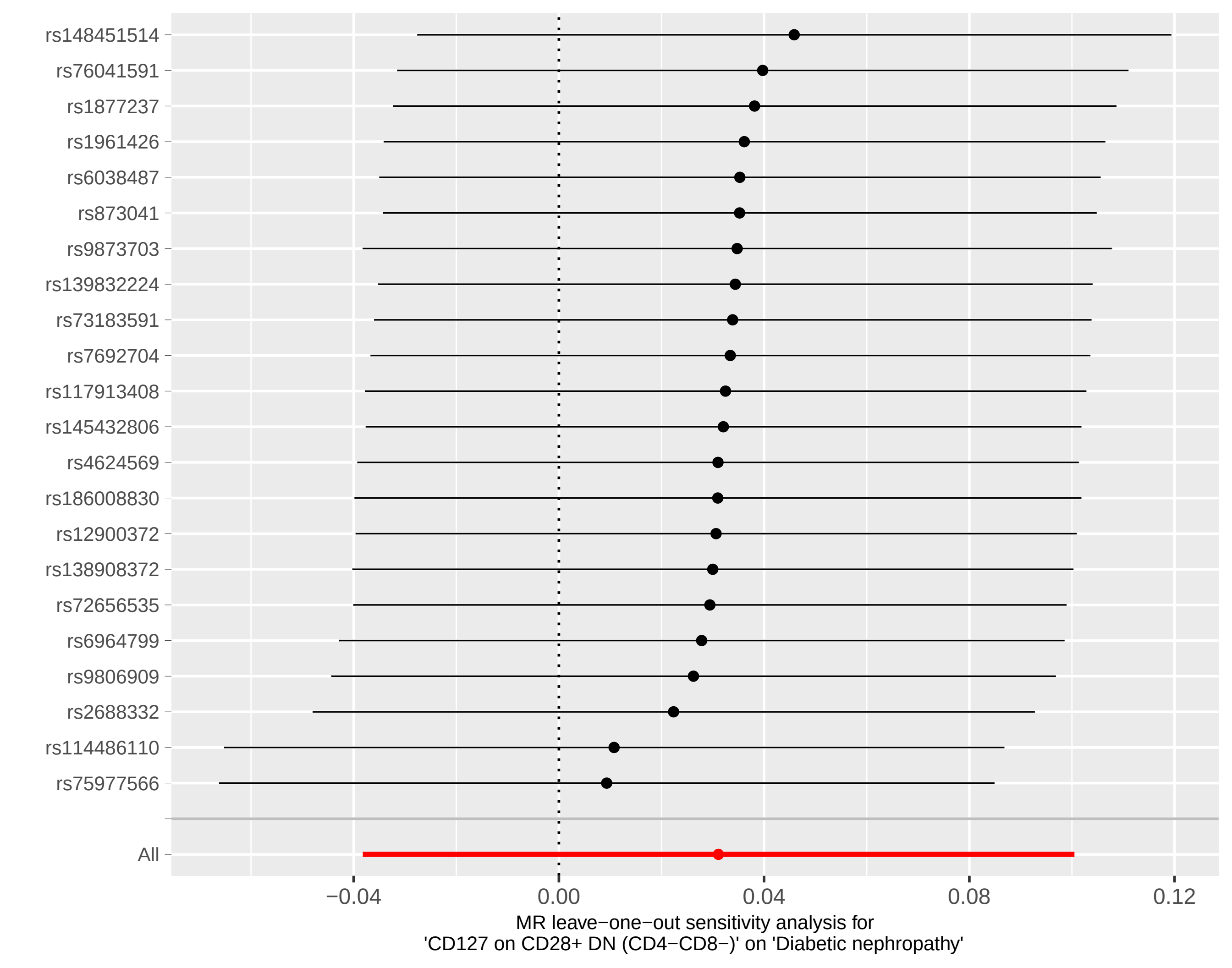
rs148451514
rs76041591
rs1877237
rs1961426
rs6038487
rs873041
rs9873703
rs139832224
rs73183591
rs7692704
rs117913408
rs145432806
rs4624569
rs186008830
rs12900372
rs138908372
rs72656535
rs6964799
rs9806909
rs2688332
rs114486110
rs75977566
All
−0.04
0.00
0.04
0.08
0.12
MR leave−one−out sensitivity analysis for
'CD127 on CD28+ DN (CD4−CD8−)' on 'Diabetic nephropathy'

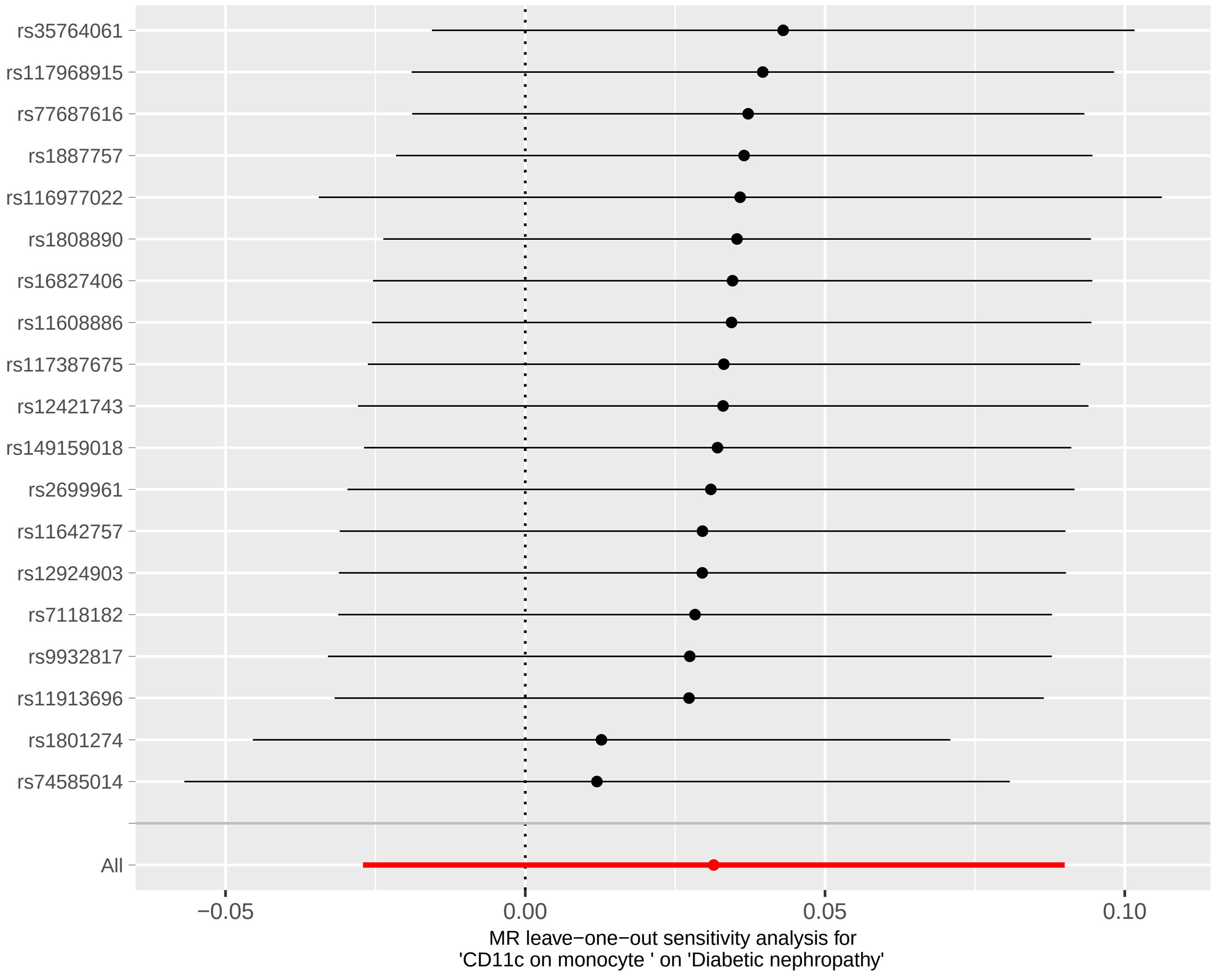

rs35764061
rs117968915
rs77687616
rs1887757
rs116977022
rs1808890
rs16827406
rs11608886
rs117387675
rs12421743
rs149159018
rs2699961
rs11642757
rs12924903
rs7118182
rs9932817
rs11913696
rs1801274
rs74585014
All
−0.05
0.00
0.05
0.10
MR leave−one−out sensitivity analysis for
'CD11c on monocyte ' on 'Diabetic nephropathy'

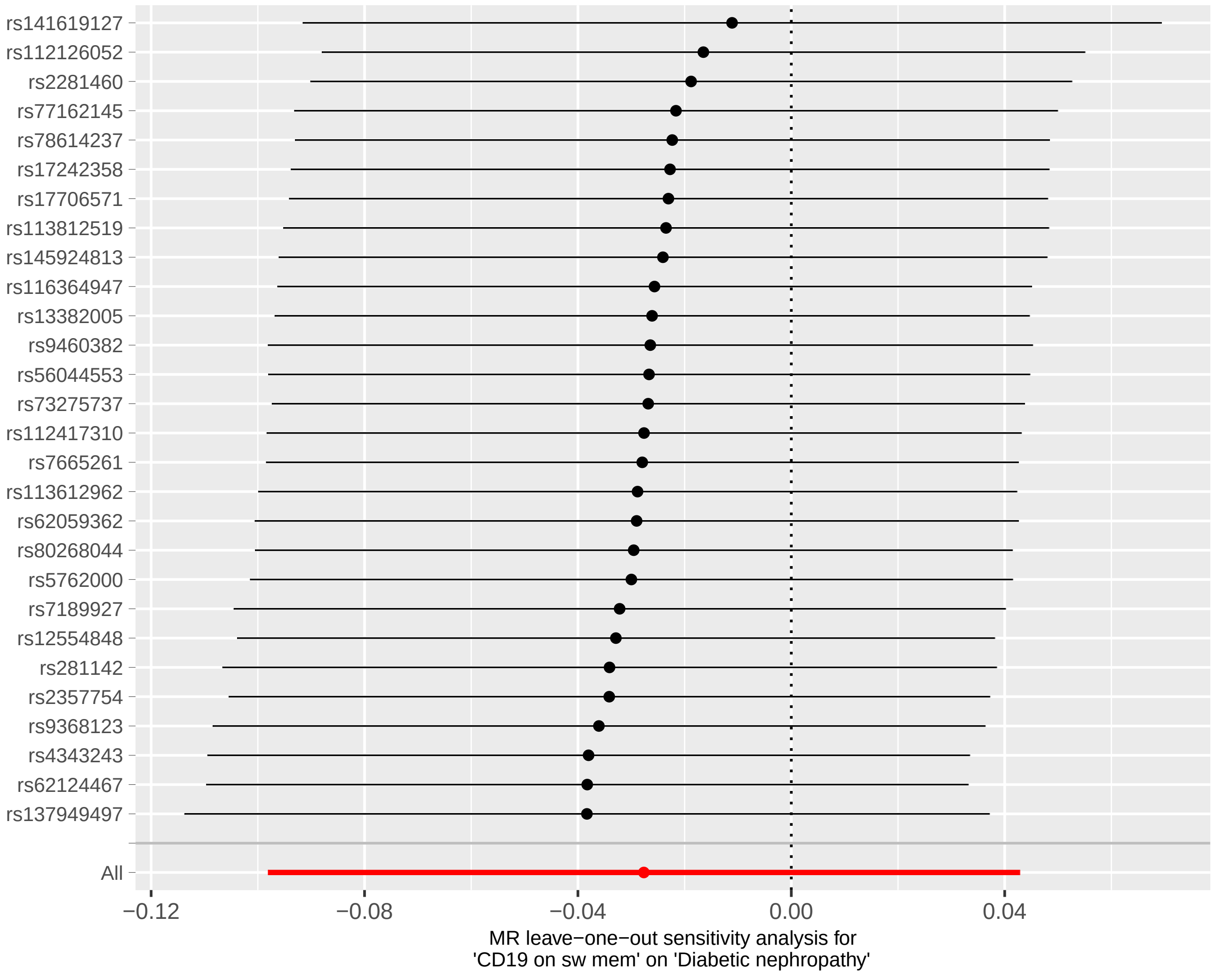

rs141619127
rs112126052
rs2281460
rs77162145
rs78614237
rs17242358
rs17706571
rs113812519
rs145924813
rs116364947
rs13382005
rs9460382
rs56044553
rs73275737
rs112417310
rs7665261
rs113612962
rs62059362
rs80268044
rs5762000
rs7189927
rs12554848
rs281142
rs2357754
rs9368123
rs4343243
rs62124467
rs137949497
All
−0.12
−0.08
−0.04
0.00
0.04
MR leave−one−out sensitivity analysis for
'CD19 on sw mem' on 'Diabetic nephropathy'

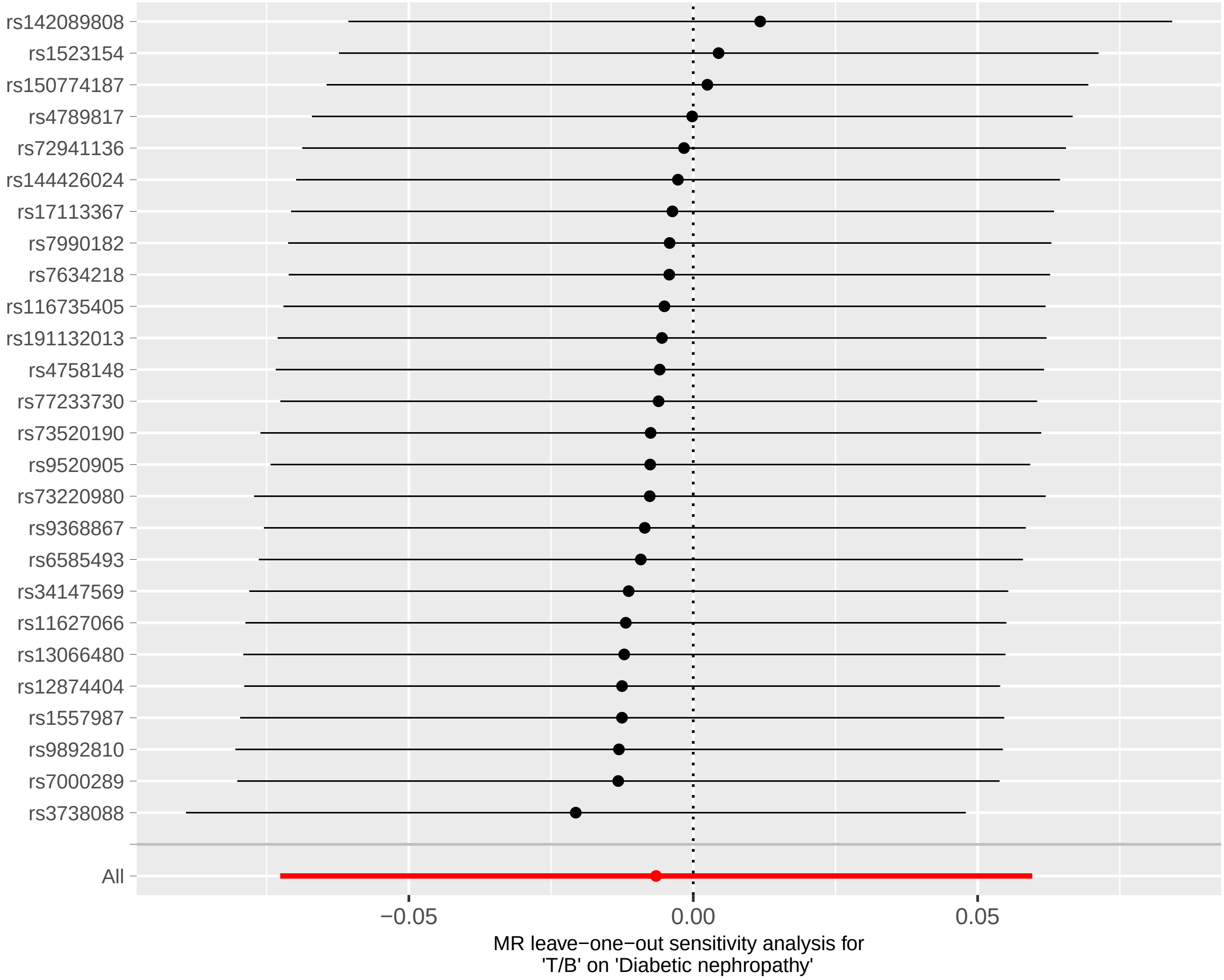

rs142089808
rs1523154
rs150774187
rs4789817
rs72941136
rs144426024
rs17113367
rs7990182
rs7634218
rs116735405
rs191132013
rs4758148
rs77233730
rs73520190
rs9520905
rs73220980
rs9368867
rs6585493
rs34147569
rs11627066
rs13066480
rs12874404
rs1557987
rs9892810
rs7000289
rs3738088
All
−0.05
0.00
0.05
MR leave−one−out sensitivity analysis for
'T/B' on 'Diabetic nephropathy'

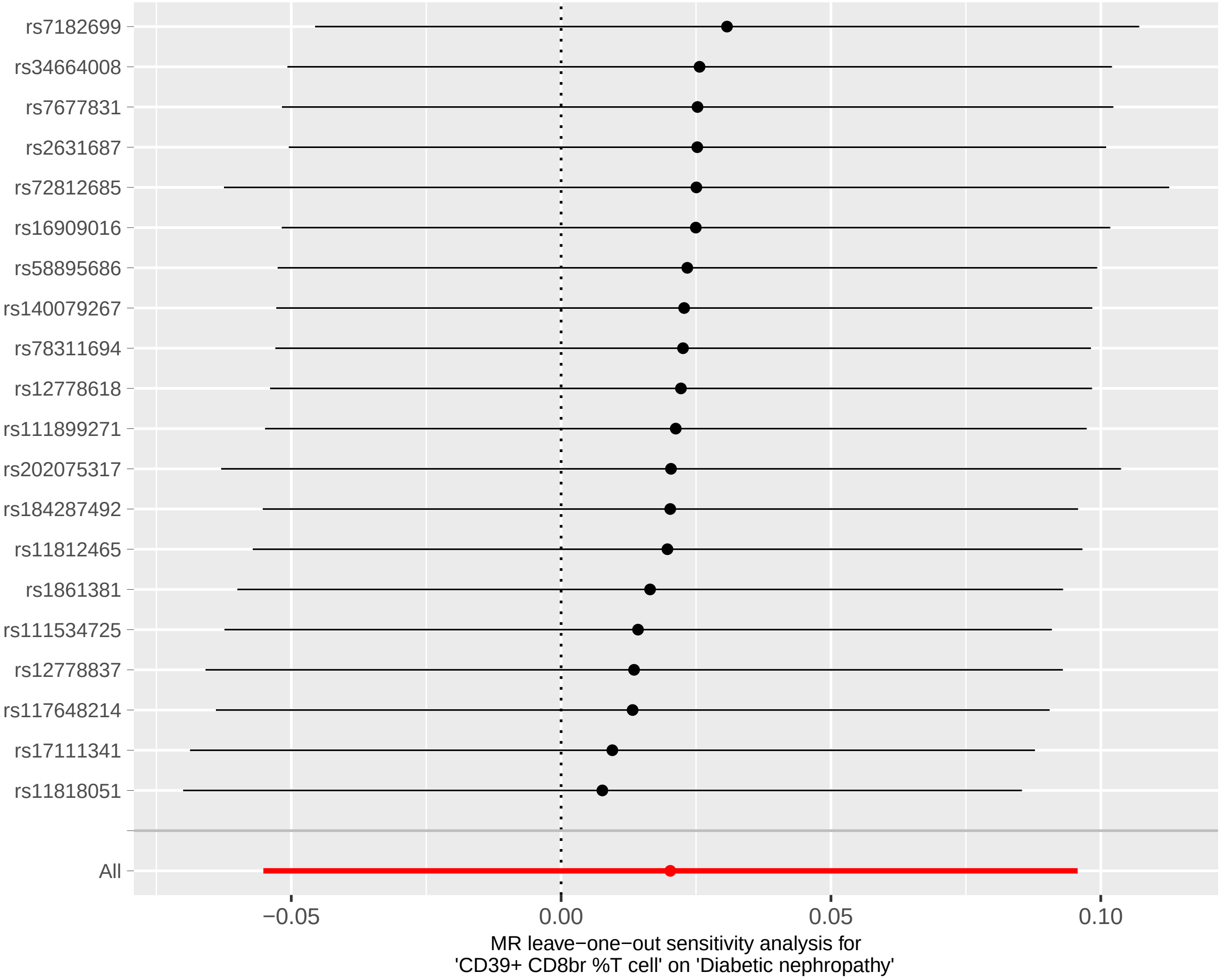

rs7182699
rs34664008
rs7677831
rs2631687
rs72812685
rs16909016
rs58895686
rs140079267
rs78311694
rs12778618
rs111899271
rs202075317
rs184287492
rs11812465
rs1861381
rs111534725
rs12778837
rs117648214
rs17111341
rs11818051
All
−0.05
0.00
0.05
0.10
MR leave−one−out sensitivity analysis for
'CD39+ CD8br %T cell' on 'Diabetic nephropathy'

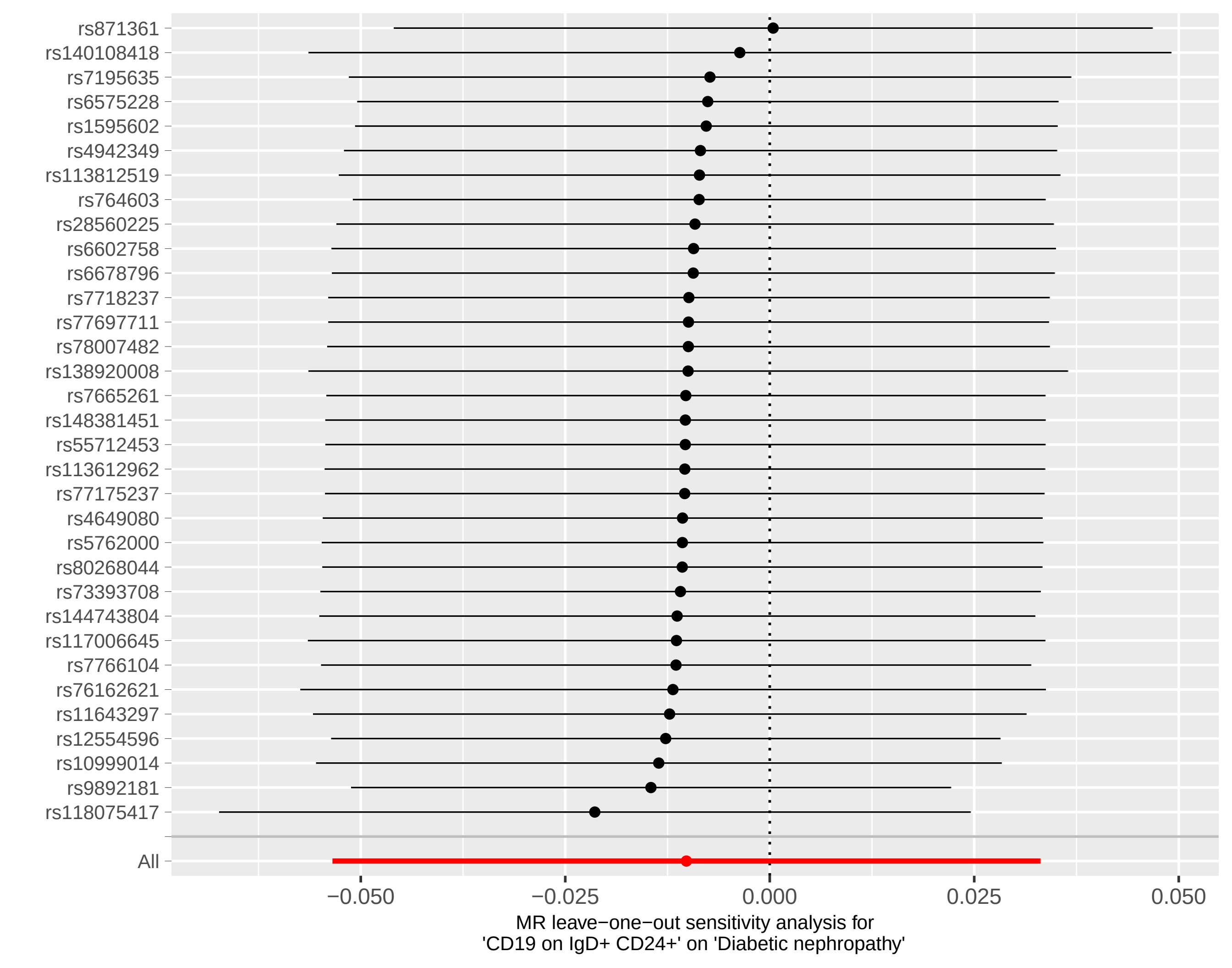
rs871361
rs140108418
rs7195635
rs6575228
rs1595602
rs4942349
rs113812519
rs764603
rs28560225
rs6602758
rs6678796
rs7718237
rs77697711
rs78007482
rs138920008
rs7665261
rs148381451
rs55712453
rs113612962
rs77175237
rs4649080
rs5762000
rs80268044
rs73393708
rs144743804
rs117006645
rs7766104
rs76162621
rs11643297
rs12554596
rs10999014
rs9892181
rs118075417
All
−0.050
−0.025
0.000
0.025
0.050
MR leave−one−out sensitivity analysis for
'CD19 on IgD+ CD24+' on 'Diabetic nephropathy'

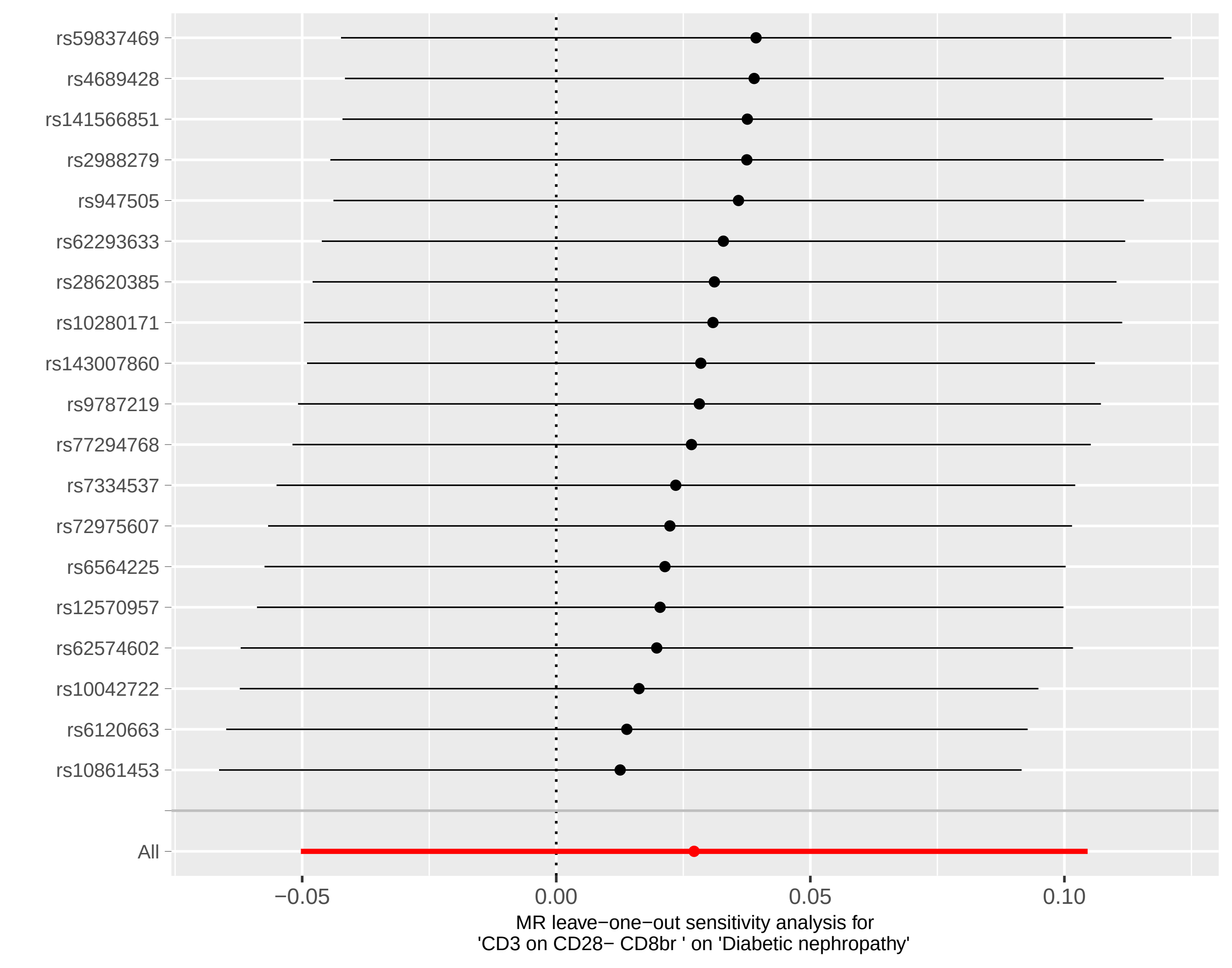
rs59837469
rs4689428
rs141566851
rs2988279
rs947505
rs62293633
rs28620385
rs10280171
rs143007860
rs9787219
rs77294768
rs7334537
rs72975607
rs6564225
rs12570957
rs62574602
rs10042722
rs6120663
rs10861453
All
−0.05
0.00
0.05
0.10
MR leave−one−out sensitivity analysis for
'CD3 on CD28− CD8br ' on 'Diabetic nephropathy'

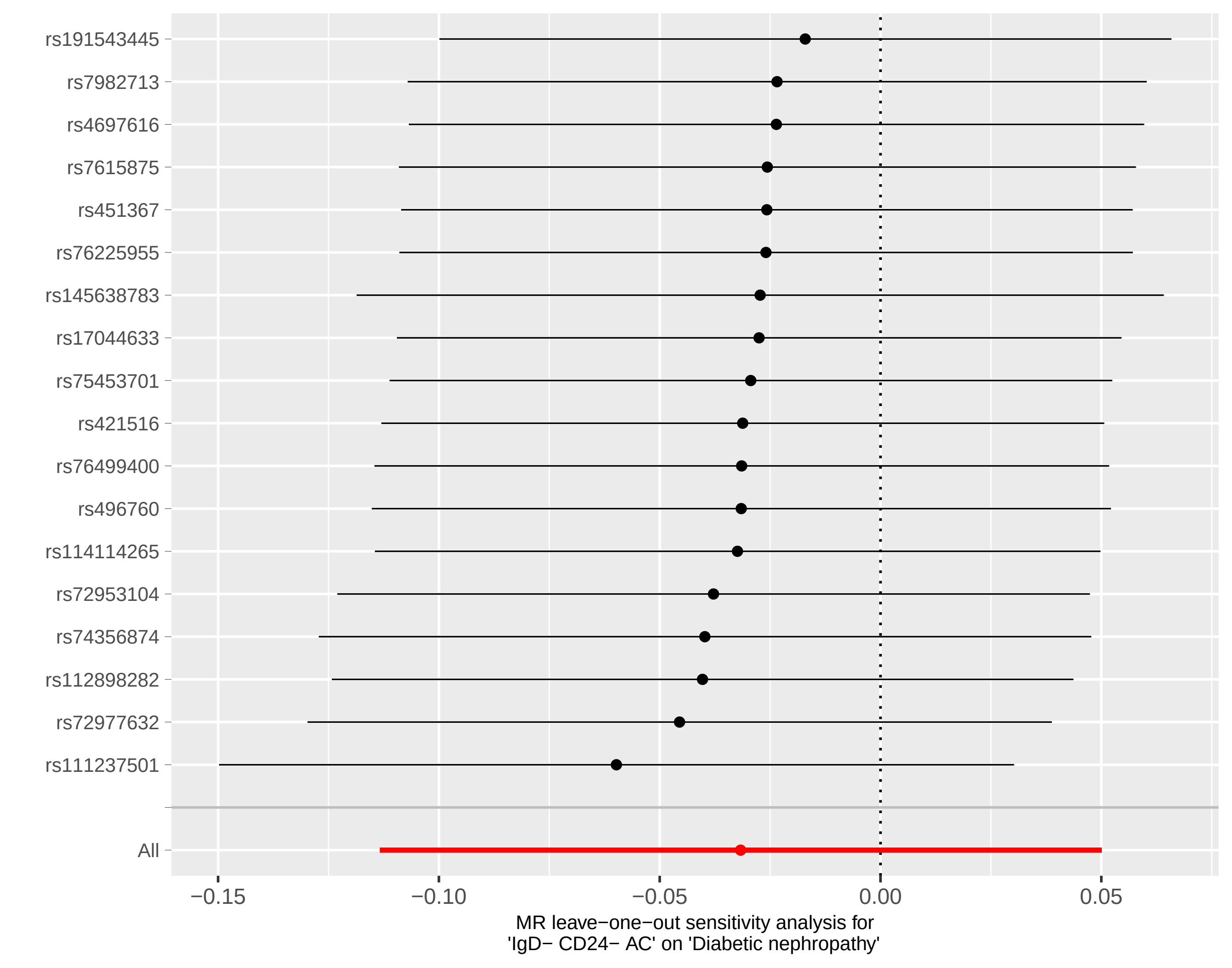
rs191543445
rs7982713
rs4697616
rs7615875
rs451367
rs76225955
rs145638783
rs17044633
rs75453701
rs421516
rs76499400
rs496760
rs114114265
rs72953104
rs74356874
rs112898282
rs72977632
rs111237501
All
−0.15
−0.10
−0.05
0.00
0.05
MR leave−one−out sensitivity analysis for
'IgD− CD24− AC' on 'Diabetic nephropathy'

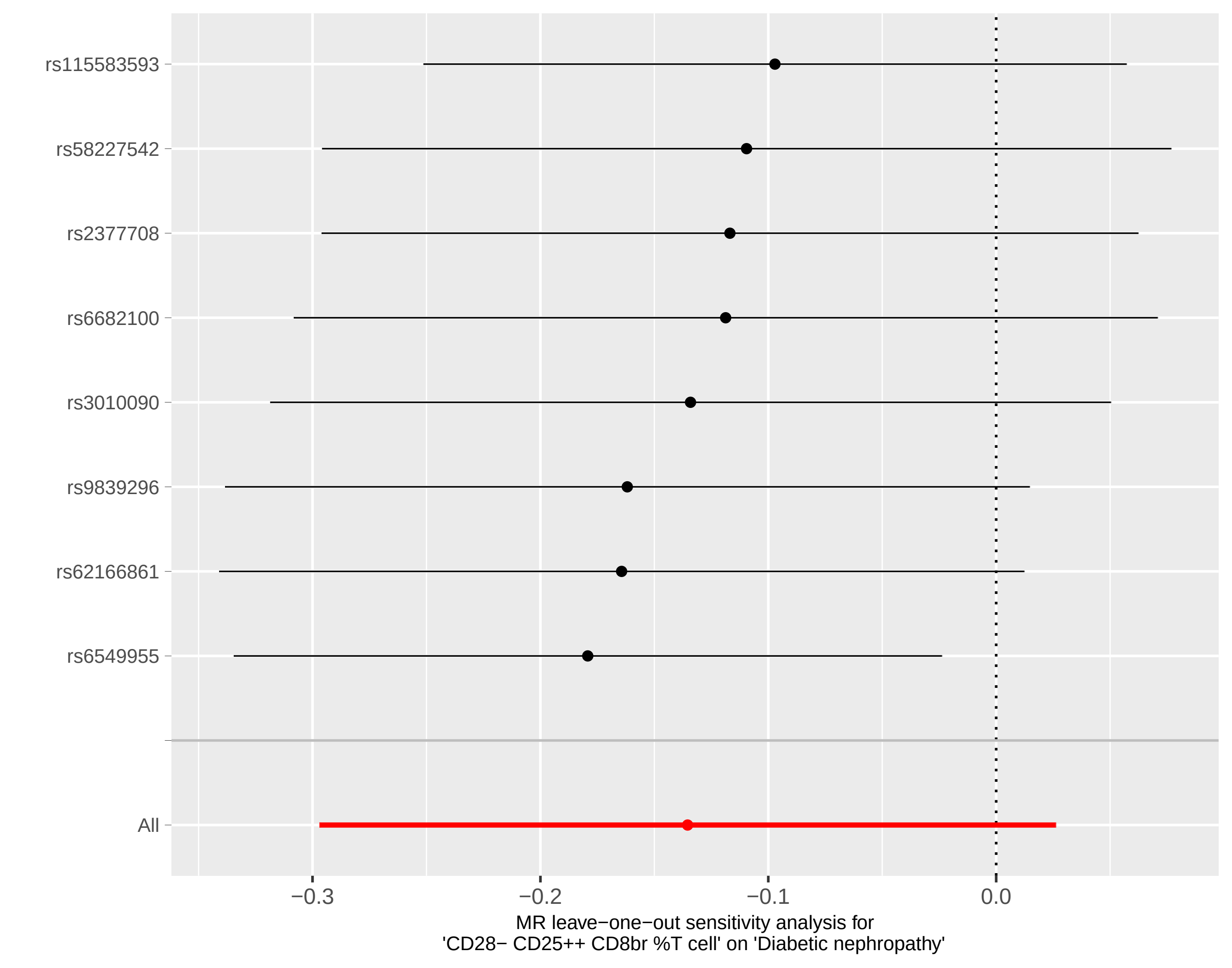
rs115583593
rs58227542
rs2377708
rs6682100
rs3010090
rs9839296
rs62166861
rs6549955
All
−0.3
−0.2
−0.1
0.0
MR leave−one−out sensitivity analysis for
'CD28− CD25++ CD8br %T cell' on 'Diabetic nephropathy'

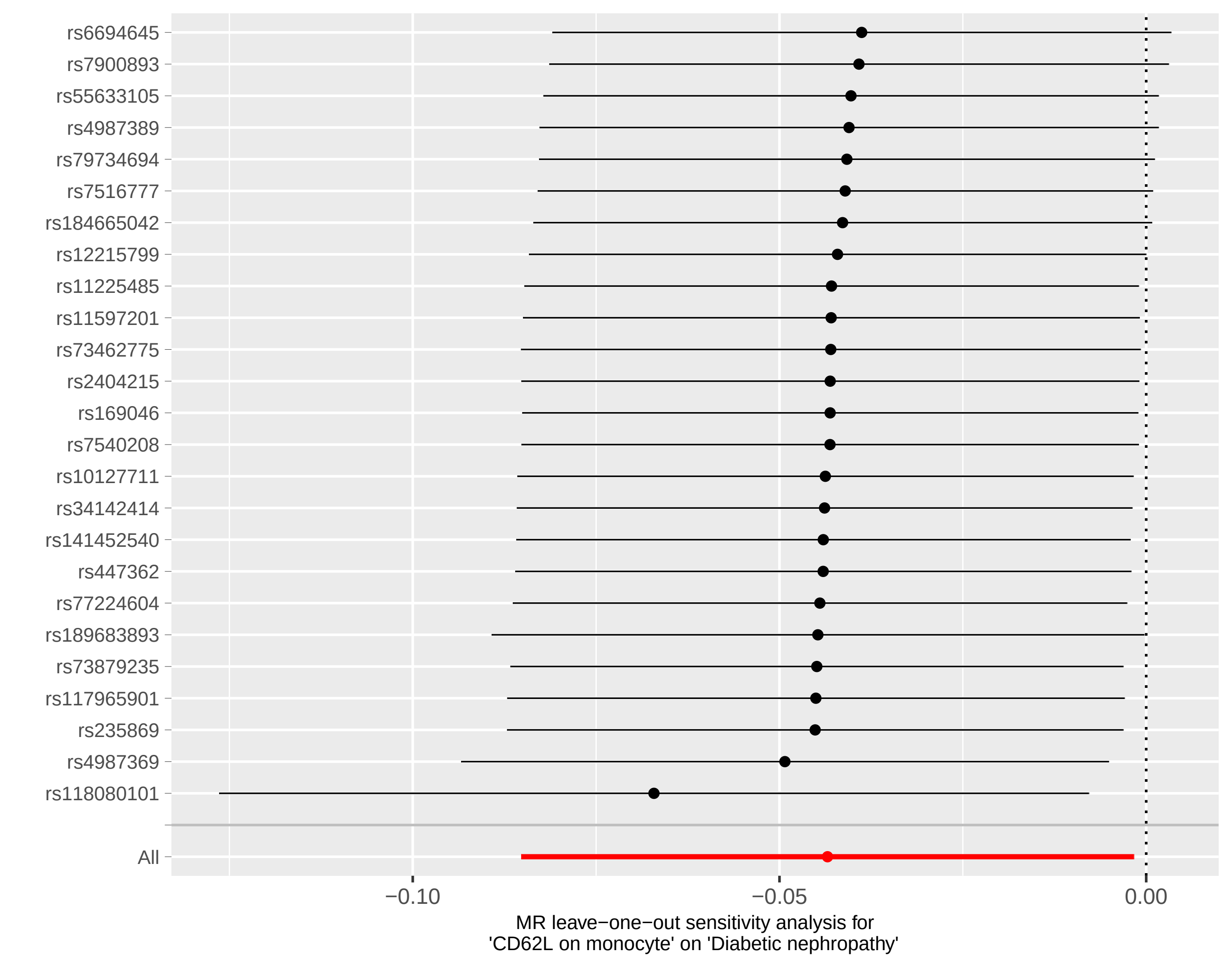

rs6694645
rs7900893
rs55633105
rs4987389
rs79734694
rs7516777
rs184665042
rs12215799
rs11225485
rs11597201
rs73462775
rs2404215
rs169046
rs7540208
rs10127711
rs34142414
rs141452540
rs447362
rs77224604
rs189683893
rs73879235
rs117965901
rs235869
rs4987369
rs118080101
All
−0.10
−0.05
0.00
MR leave−one−out sensitivity analysis for
'CD62L on monocyte' on 'Diabetic nephropathy'

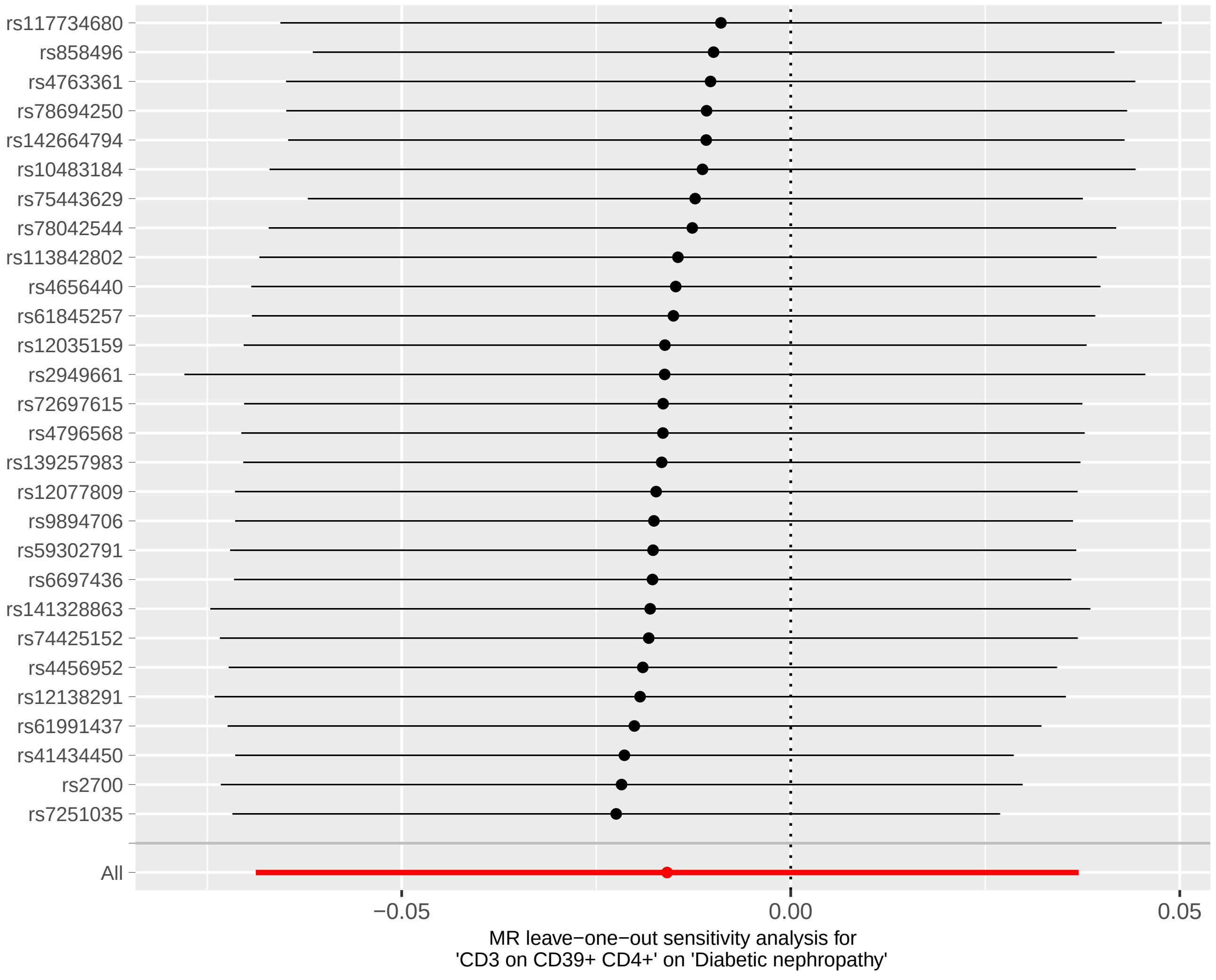

rs117734680
rs858496
rs4763361
rs78694250
rs142664794
rs10483184
rs75443629
rs78042544
rs113842802
rs4656440
rs61845257
rs12035159
rs2949661
rs72697615
rs4796568
rs139257983
rs12077809
rs9894706
rs59302791
rs6697436
rs141328863
rs74425152
rs4456952
rs12138291
rs61991437
rs41434450
rs2700
rs7251035
All
−0.05
0.00
0.05
MR leave−one−out sensitivity analysis for
'CD3 on CD39+ CD4+' on 'Diabetic nephropathy'

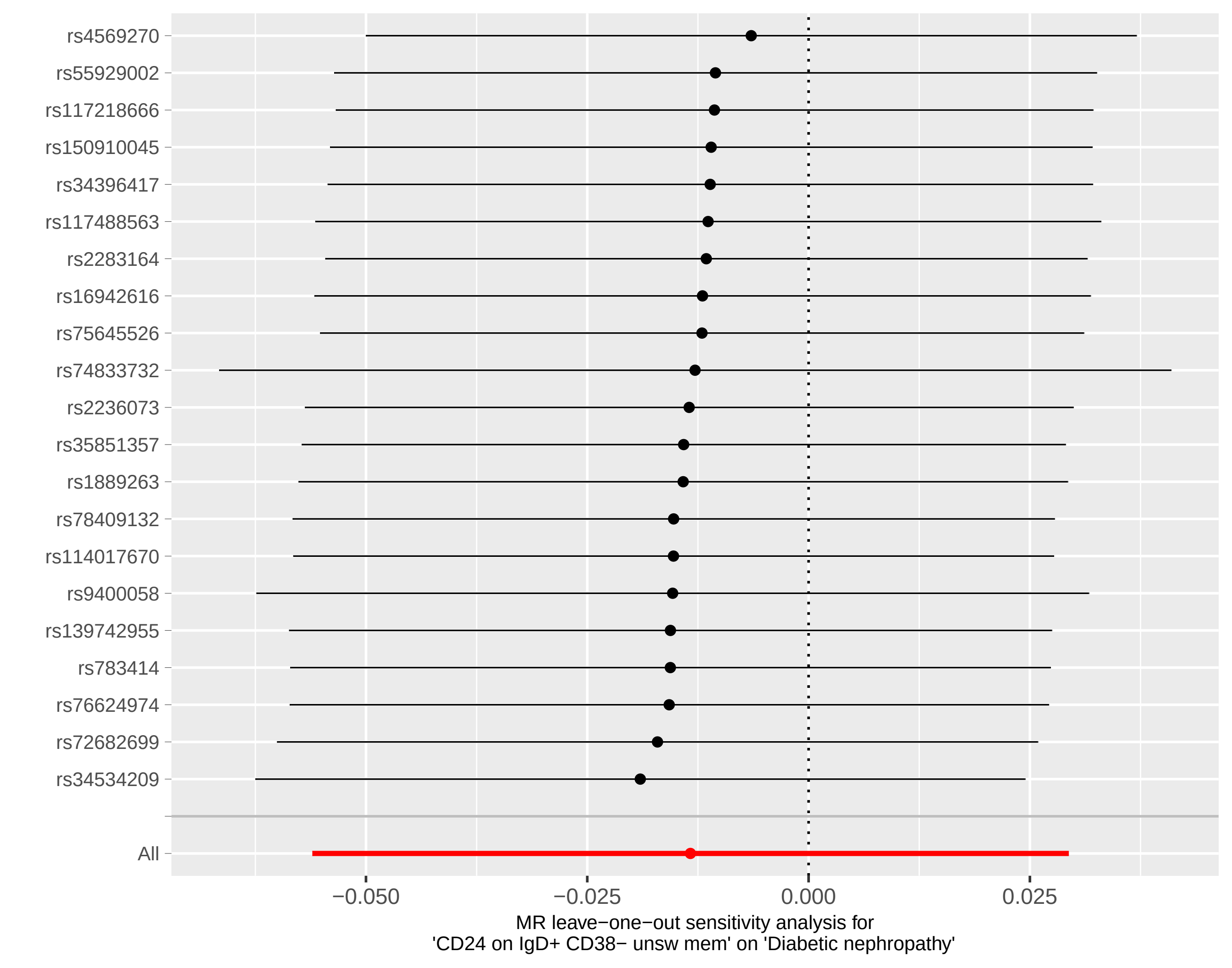
rs4569270
rs55929002
rs117218666
rs150910045
rs34396417
rs117488563
rs2283164
rs16942616
rs75645526
rs74833732
rs2236073
rs35851357
rs1889263
rs78409132
rs114017670
rs9400058
rs139742955
rs783414
rs76624974
rs72682699
rs34534209
All
−0.050
−0.025
0.000
0.025
MR leave−one−out sensitivity analysis for
'CD24 on IgD+ CD38− unsw mem' on 'Diabetic nephropathy'

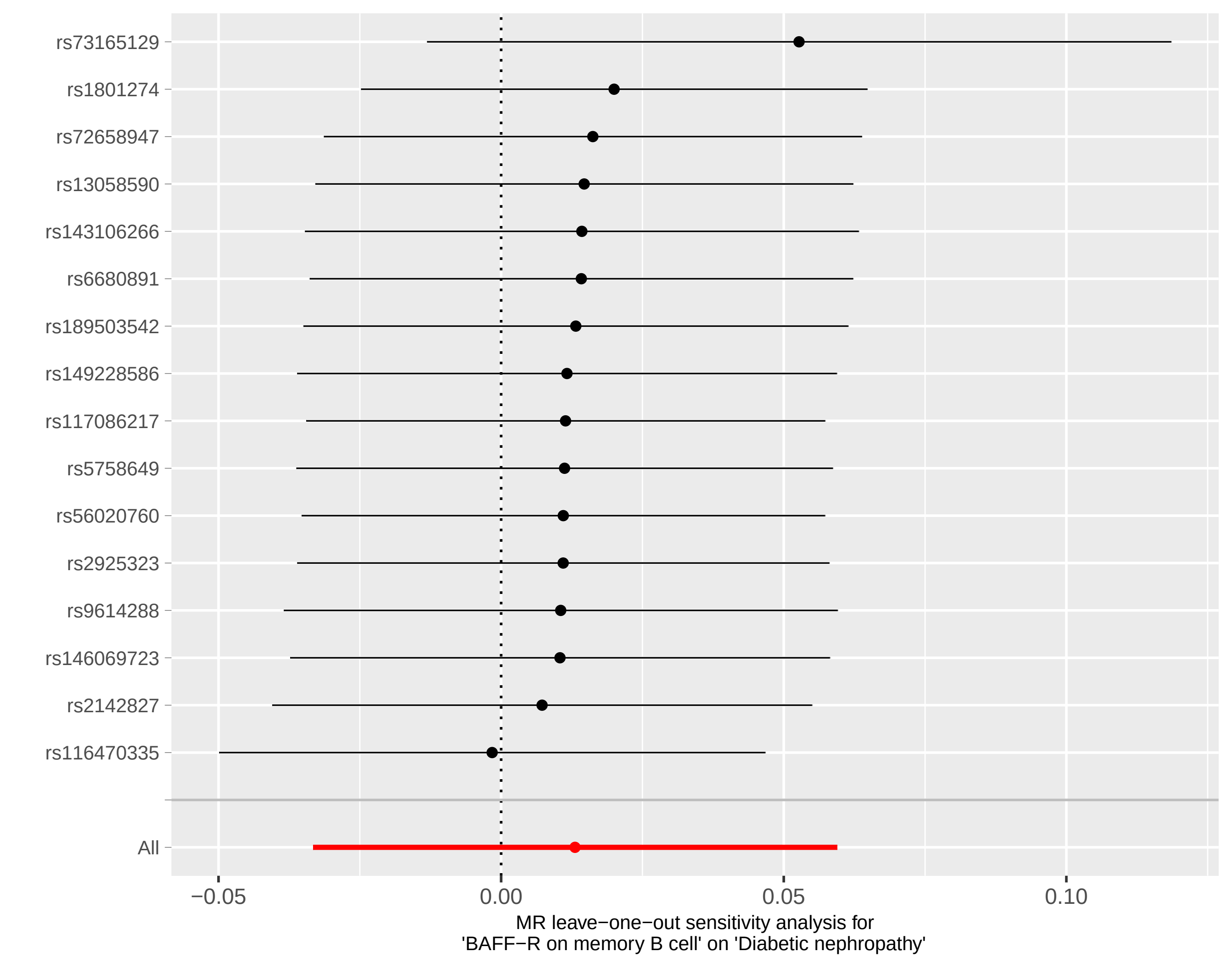
rs73165129
rs1801274
rs72658947
rs13058590
rs143106266
rs6680891
rs189503542
rs149228586
rs117086217
rs5758649
rs56020760
rs2925323
rs9614288
rs146069723
rs2142827
rs116470335
All
−0.05
0.00
0.05
0.10
MR leave−one−out sensitivity analysis for
'BAFF−R on memory B cell' on 'Diabetic nephropathy'

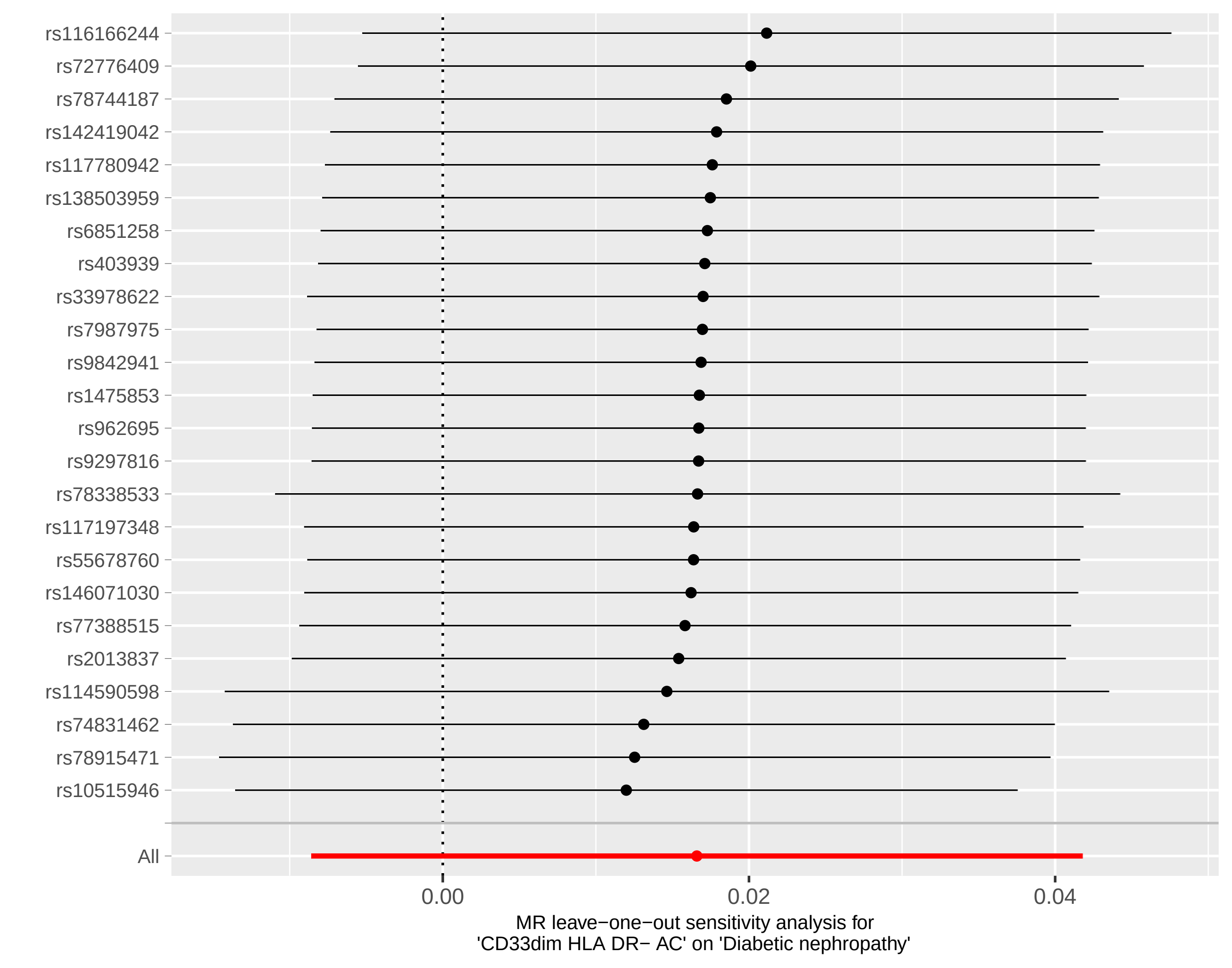
rs116166244
rs72776409
rs78744187
rs142419042
rs117780942
rs138503959
rs6851258
rs403939
rs33978622
rs7987975
rs9842941
rs1475853
rs962695
rs9297816
rs78338533
rs117197348
rs55678760
rs146071030
rs77388515
rs2013837
rs114590598
rs74831462
rs78915471
rs10515946
All
0.00
0.02
0.04
MR leave−one−out sensitivity analysis for
'CD33dim HLA DR− AC' on 'Diabetic nephropathy'

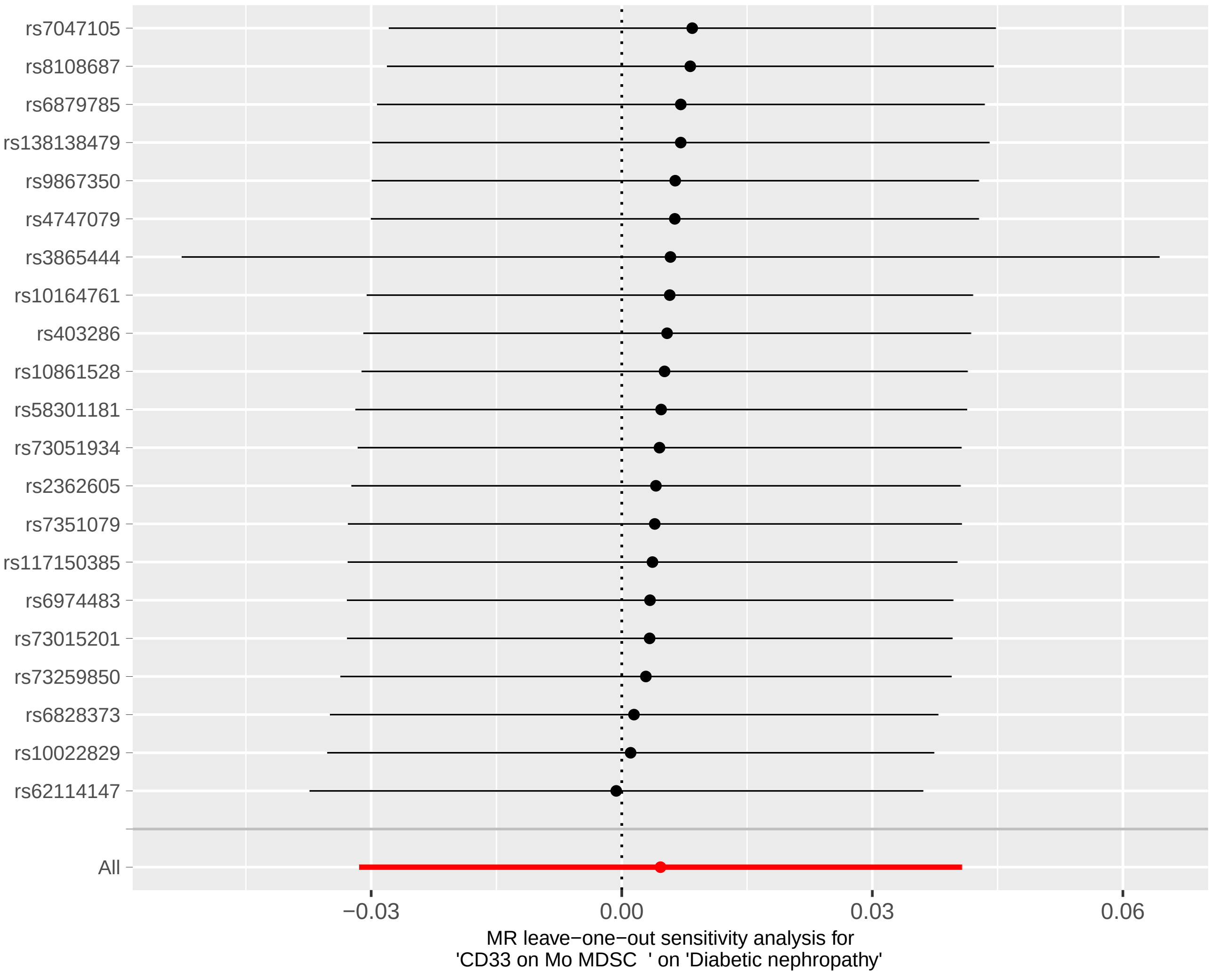
rs7047105
rs8108687
rs6879785
rs138138479
rs9867350
rs4747079
rs3865444
rs10164761
rs403286
rs10861528
rs58301181
rs73051934
rs2362605
rs7351079
rs117150385
rs6974483
rs73015201
rs73259850
rs6828373
rs10022829
rs62114147
All
−0.03
0.00
0.03
0.06
MR leave−one−out sensitivity analysis for
'CD33 on Mo MDSC ' on 'Diabetic nephropathy'

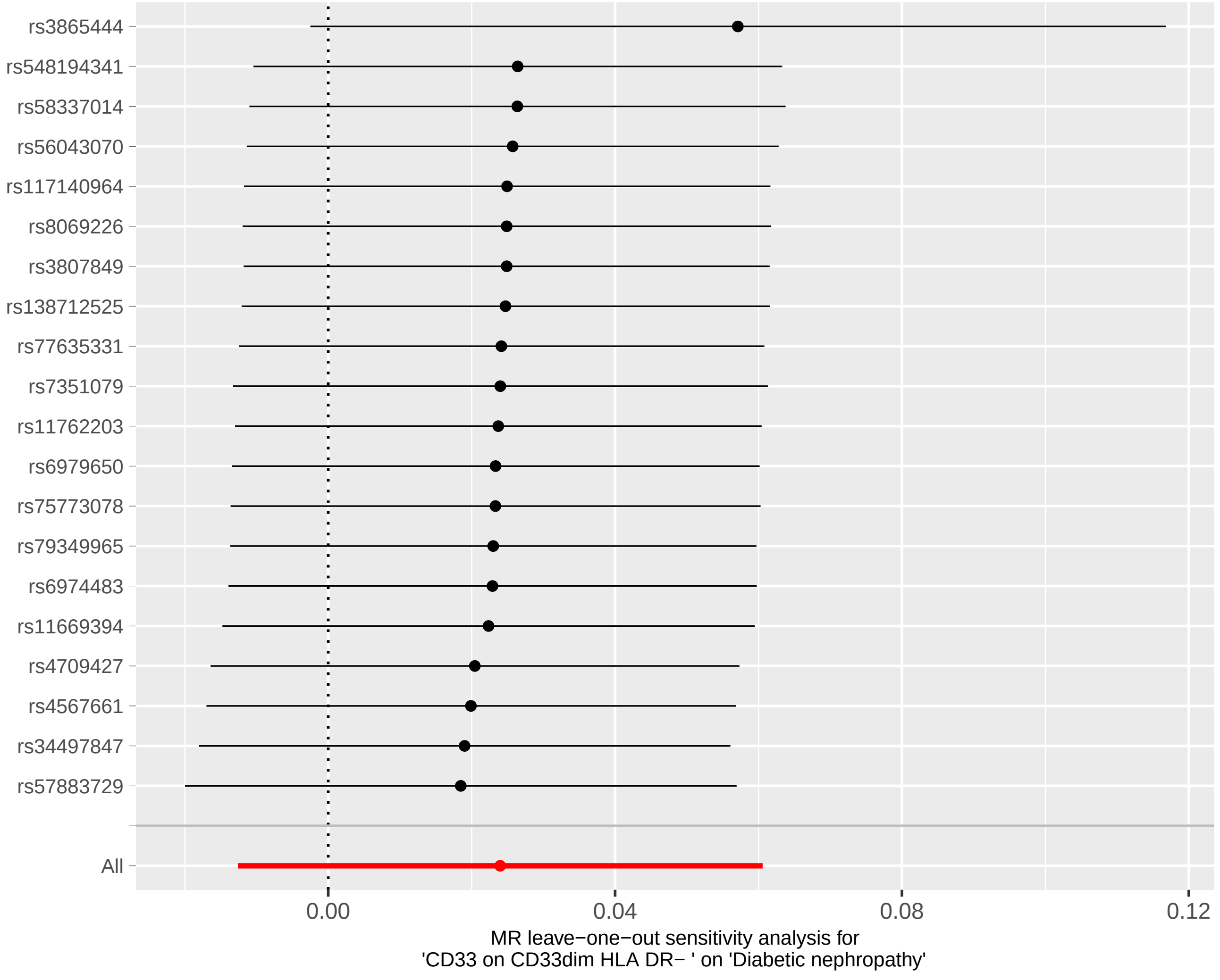
rs3865444
rs548194341
rs58337014
rs56043070
rs117140964
rs8069226
rs3807849
rs138712525
rs77635331
rs7351079
rs11762203
rs6979650
rs75773078
rs79349965
rs6974483
rs11669394
rs4709427
rs4567661
rs34497847
rs57883729
All
0.00
0.04
0.08
0.12
MR leave−one−out sensitivity analysis for
'CD33 on CD33dim HLA DR− ' on 'Diabetic nephropathy'

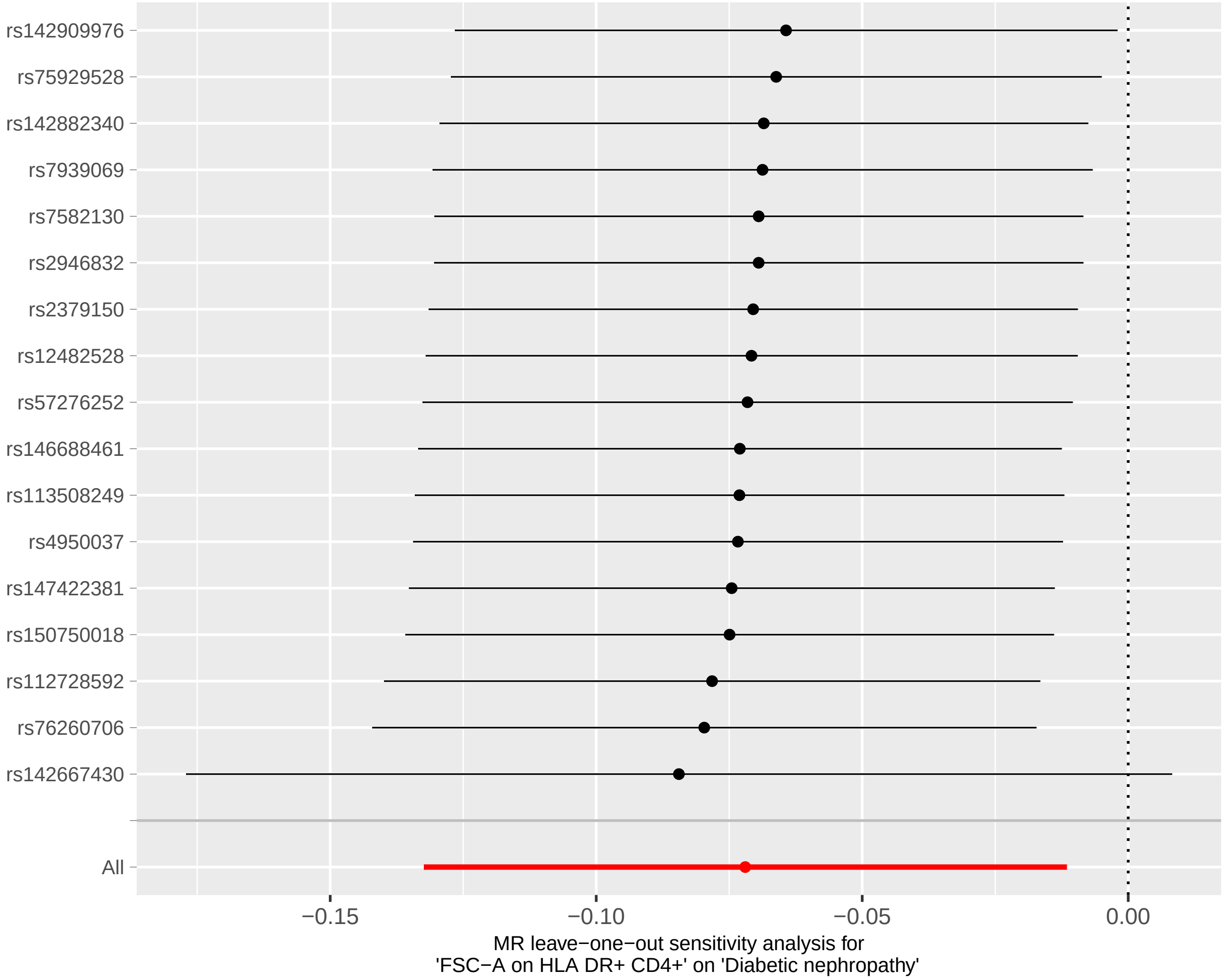
rs142909976
rs75929528
rs142882340
rs7939069
rs7582130
rs2946832
rs2379150
rs12482528
rs57276252
rs146688461
rs113508249
rs4950037
rs147422381
rs150750018
rs112728592
rs76260706
rs142667430
All
−0.15
−0.10
−0.05
0.00
MR leave−one−out sensitivity analysis for
'FSC−A on HLA DR+ CD4+' on 'Diabetic nephropathy'

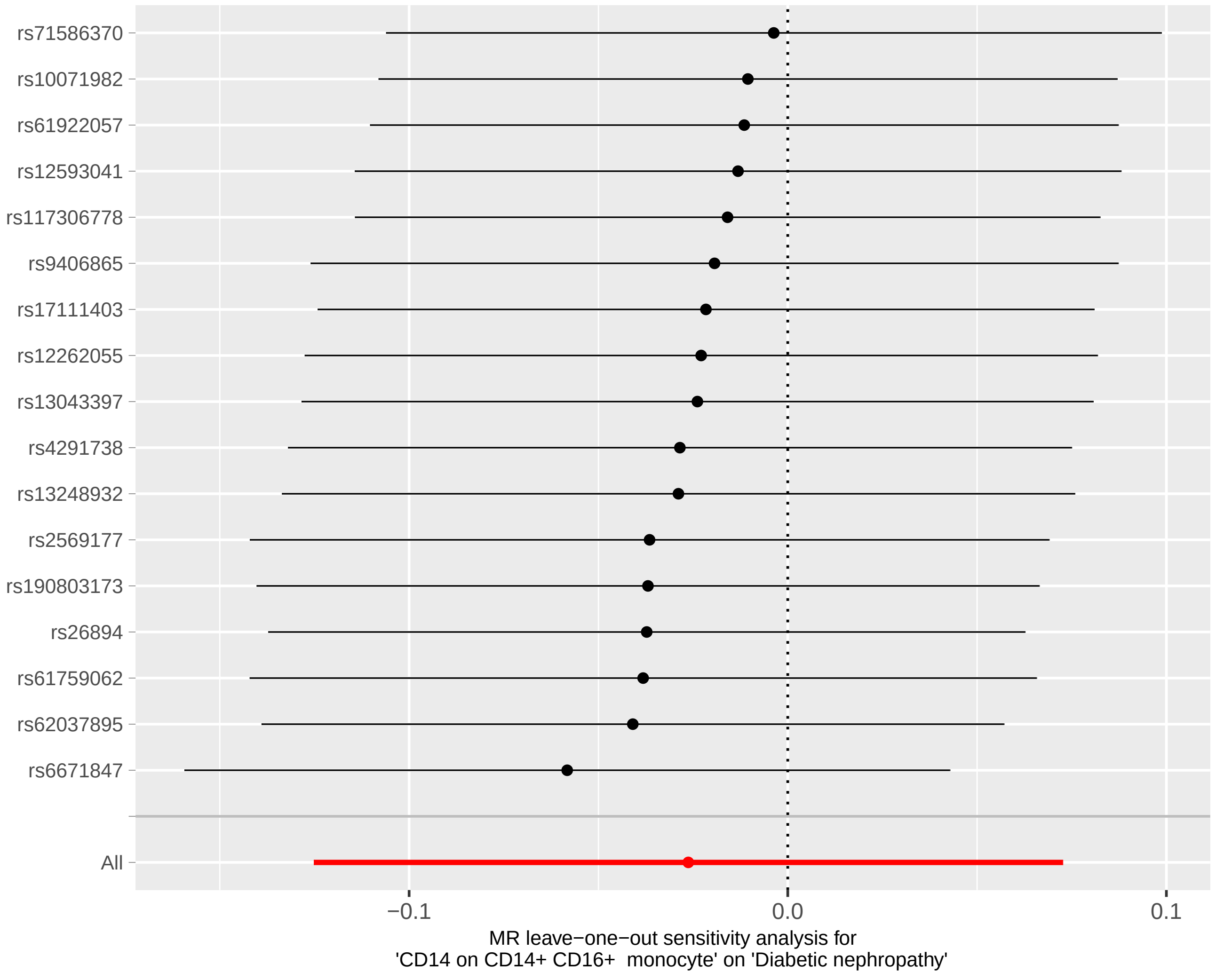
rs71586370
rs10071982
rs61922057
rs12593041
rs117306778
rs9406865
rs17111403
rs12262055
rs13043397
rs4291738
rs13248932
rs2569177
rs190803173
rs26894
rs61759062
rs62037895
rs6671847
All
−0.1
0.0
0.1
MR leave−one−out sensitivity analysis for
'CD14 on CD14+ CD16+ monocyte' on 'Diabetic nephropathy'

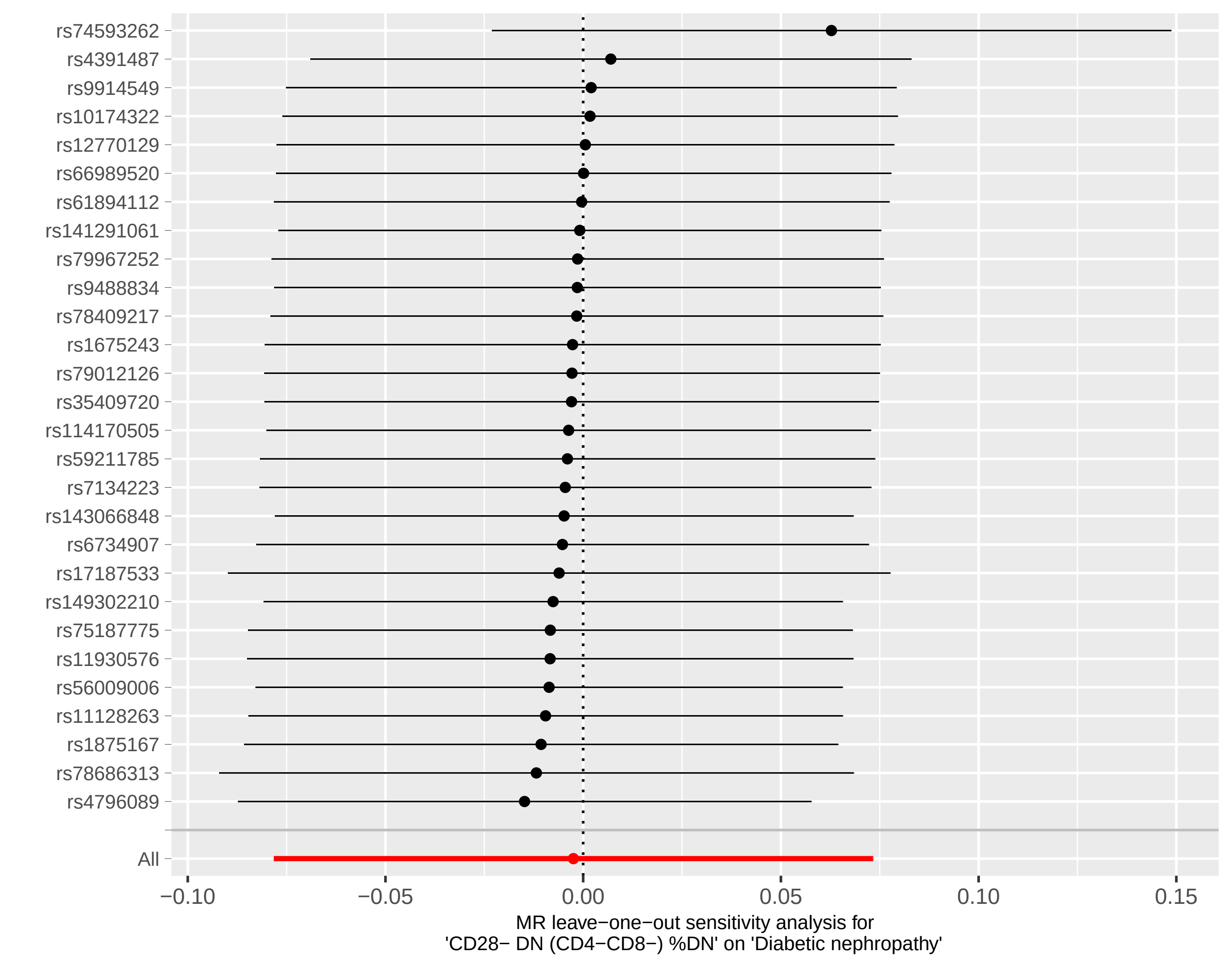

rs74593262
rs4391487
rs9914549
rs10174322
rs12770129
rs66989520
rs61894112
rs141291061
rs79967252
rs9488834
rs78409217
rs1675243
rs79012126
rs35409720
rs114170505
rs59211785
rs7134223
rs143066848
rs6734907
rs17187533
rs149302210
rs75187775
rs11930576
rs56009006
rs11128263
rs1875167
rs78686313
rs4796089
All
−0.10
−0.05
0.00
0.05
0.10
0.15
MR leave−one−out sensitivity analysis for
'CD28− DN (CD4−CD8−) %DN' on 'Diabetic nephropathy'

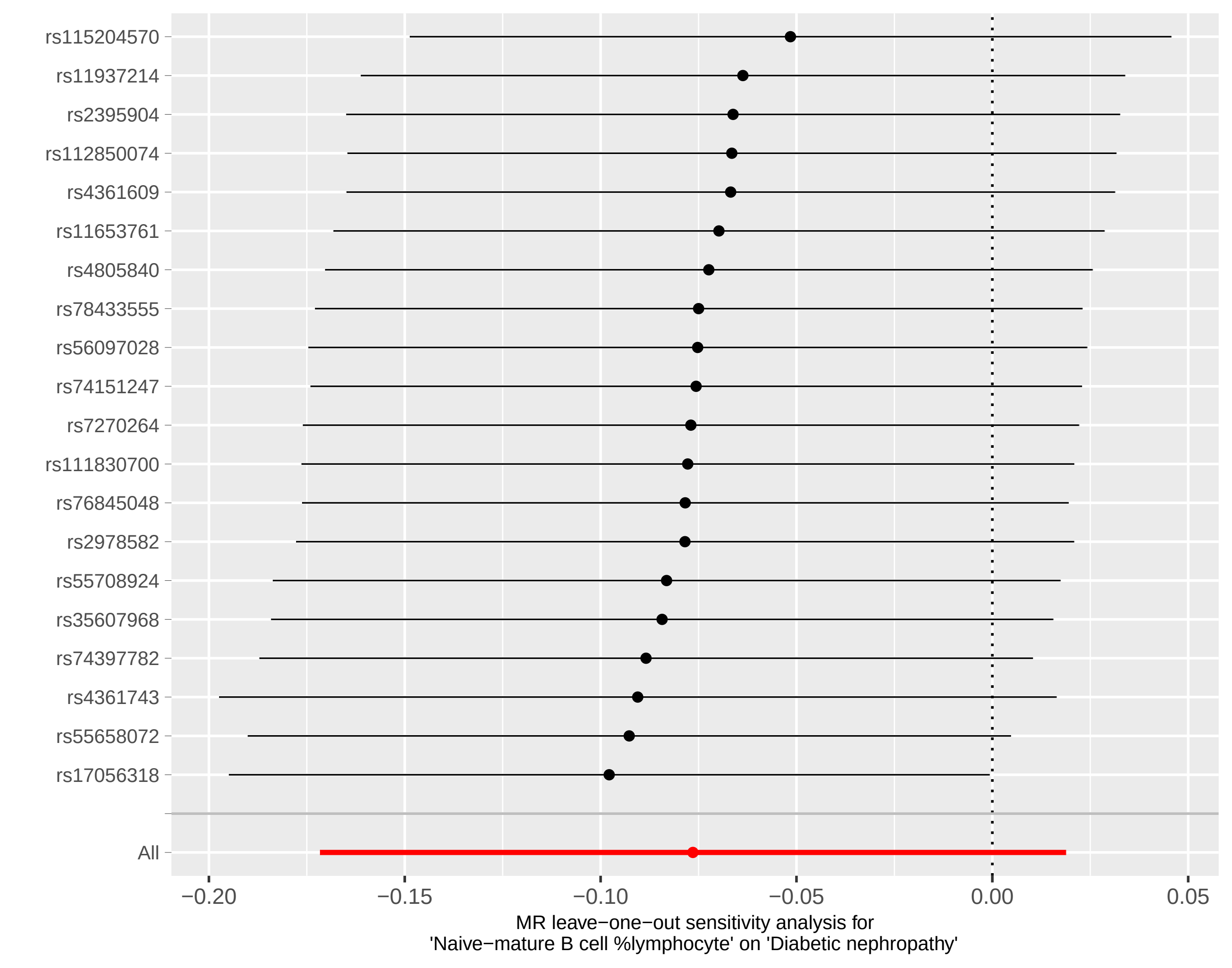
rs115204570
rs11937214
rs2395904
rs112850074
rs4361609
rs11653761
rs4805840
rs78433555
rs56097028
rs74151247
rs7270264
rs111830700
rs76845048
rs2978582
rs55708924
rs35607968
rs74397782
rs4361743
rs55658072
rs17056318
All
−0.20
−0.15
−0.10
−0.05
0.00
0.05
MR leave−one−out sensitivity analysis for
'Naive−mature B cell %lymphocyte' on 'Diabetic nephropathy'

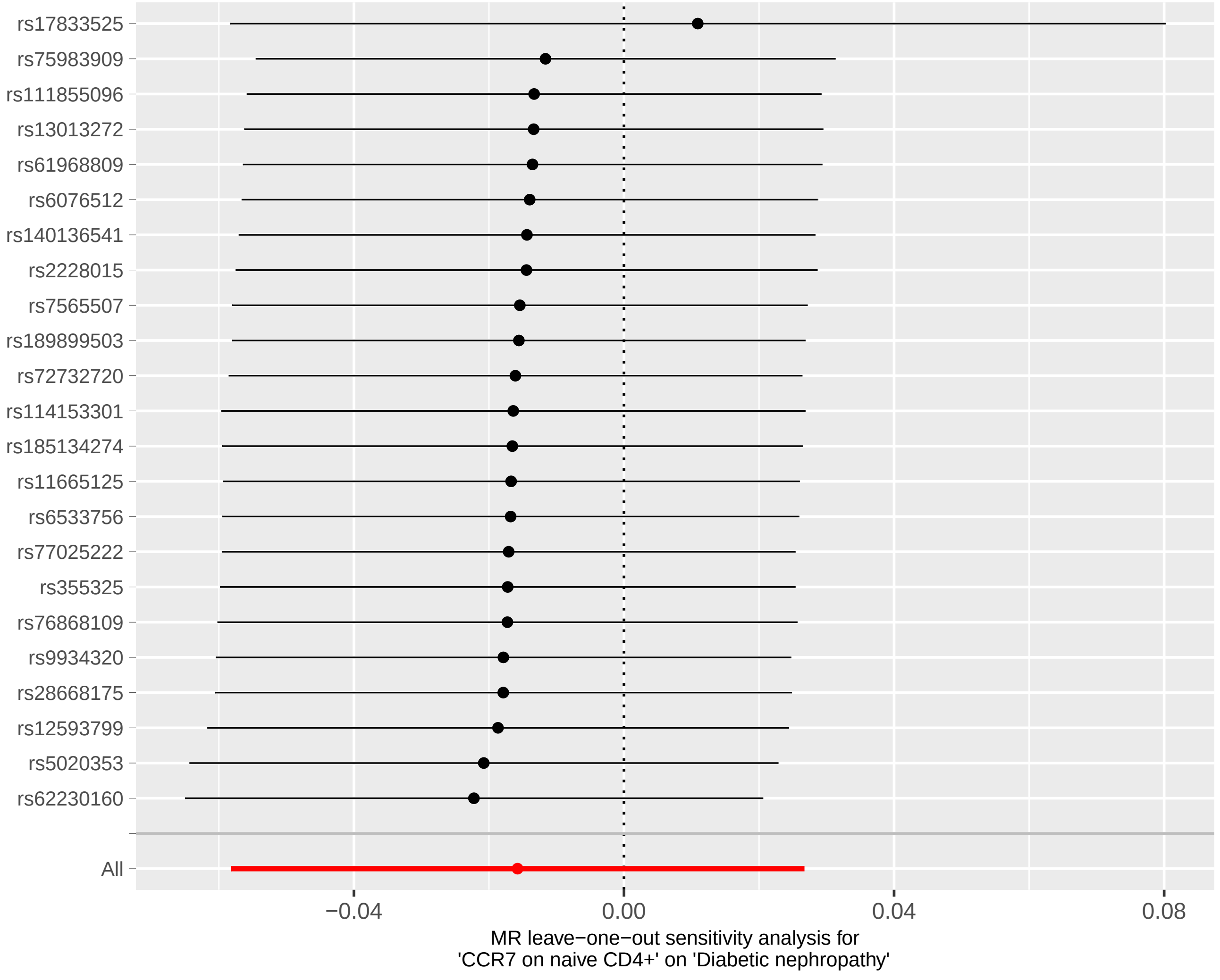
rs17833525
rs75983909
rs111855096
rs13013272
rs61968809
rs6076512
rs140136541
rs2228015
rs7565507
rs189899503
rs72732720
rs114153301
rs185134274
rs11665125
rs6533756
rs77025222
rs355325
rs76868109
rs9934320
rs28668175
rs12593799
rs5020353
rs62230160
All
−0.04
0.00
0.04
0.08
MR leave−one−out sensitivity analysis for
'CCR7 on naive CD4+' on 'Diabetic nephropathy'

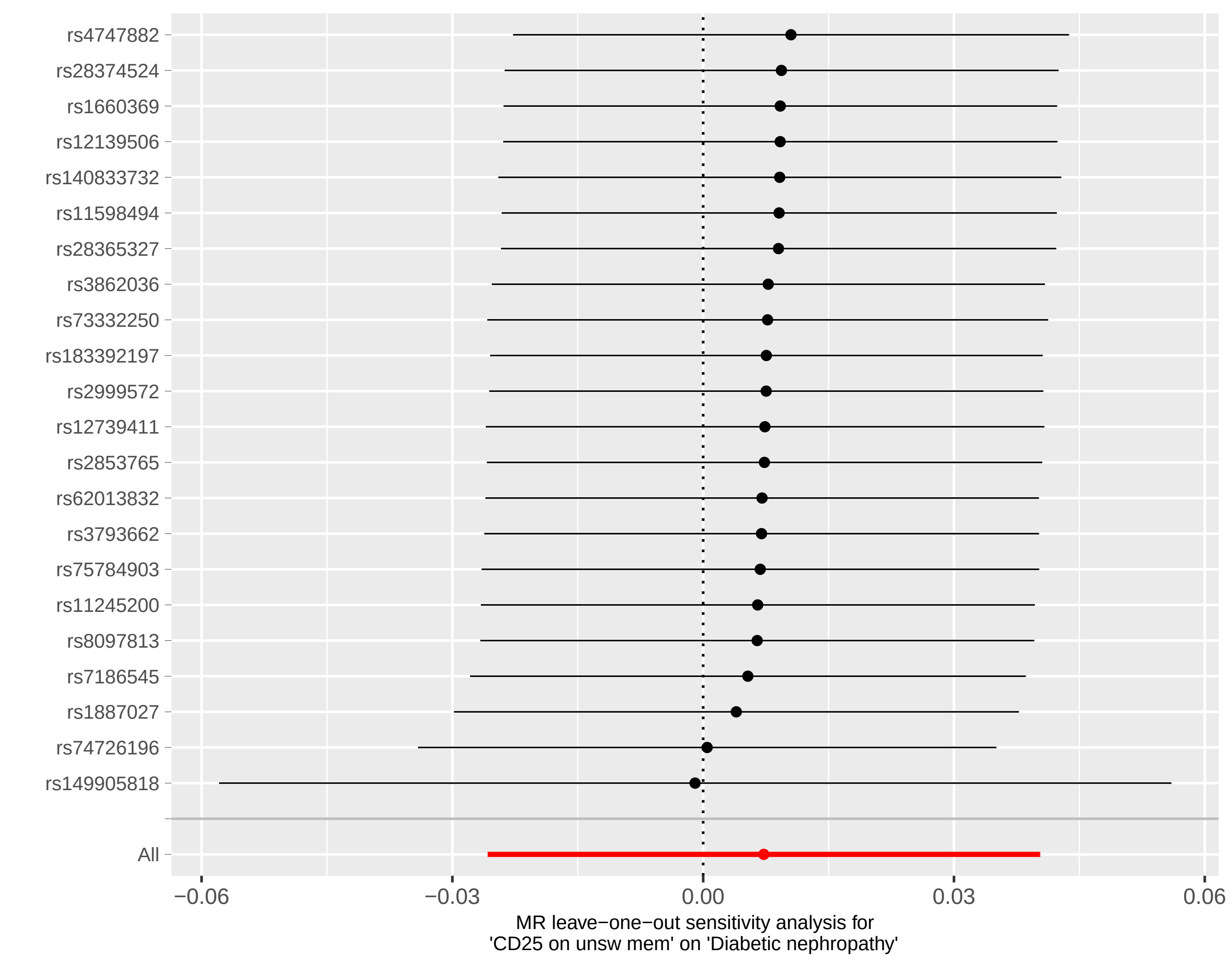

rs4747882
rs28374524
rs1660369
rs12139506
rs140833732
rs11598494
rs28365327
rs3862036
rs73332250
rs183392197
rs2999572
rs12739411
rs2853765
rs62013832
rs3793662
rs75784903
rs11245200
rs8097813
rs7186545
rs1887027
rs74726196
rs149905818
All
−0.06
−0.03
0.00
0.03
0.06
MR leave−one−out sensitivity analysis for
'CD25 on unsw mem' on 'Diabetic nephropathy'

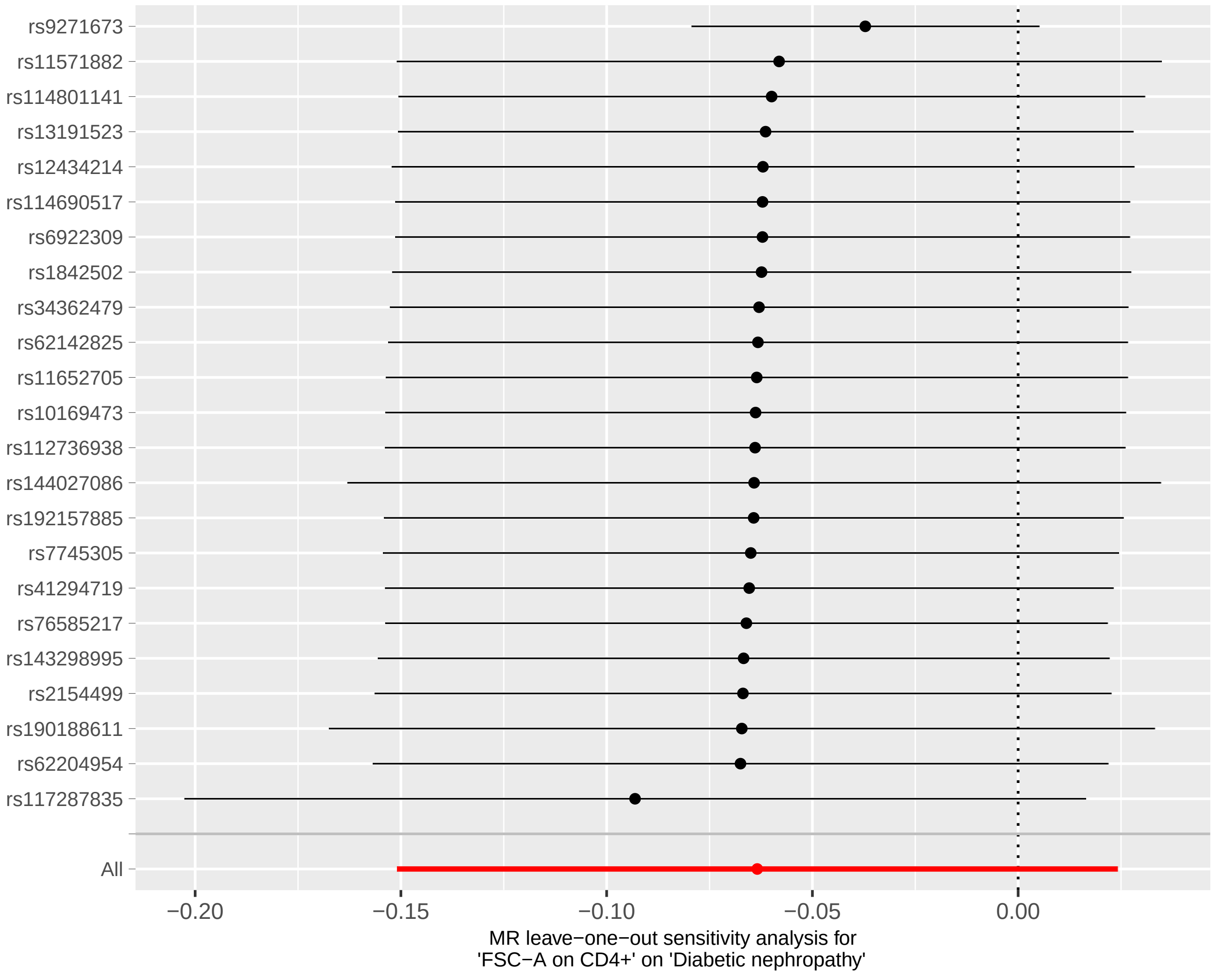
rs9271673
rs11571882
rs114801141
rs13191523
rs12434214
rs114690517
rs6922309
rs1842502
rs34362479
rs62142825
rs11652705
rs10169473
rs112736938
rs144027086
rs192157885
rs7745305
rs41294719
rs76585217
rs143298995
rs2154499
rs190188611
rs62204954
rs117287835
All
−0.20
−0.15
−0.10
−0.05
0.00
MR leave−one−out sensitivity analysis for
'FSC−A on CD4+' on 'Diabetic nephropathy'

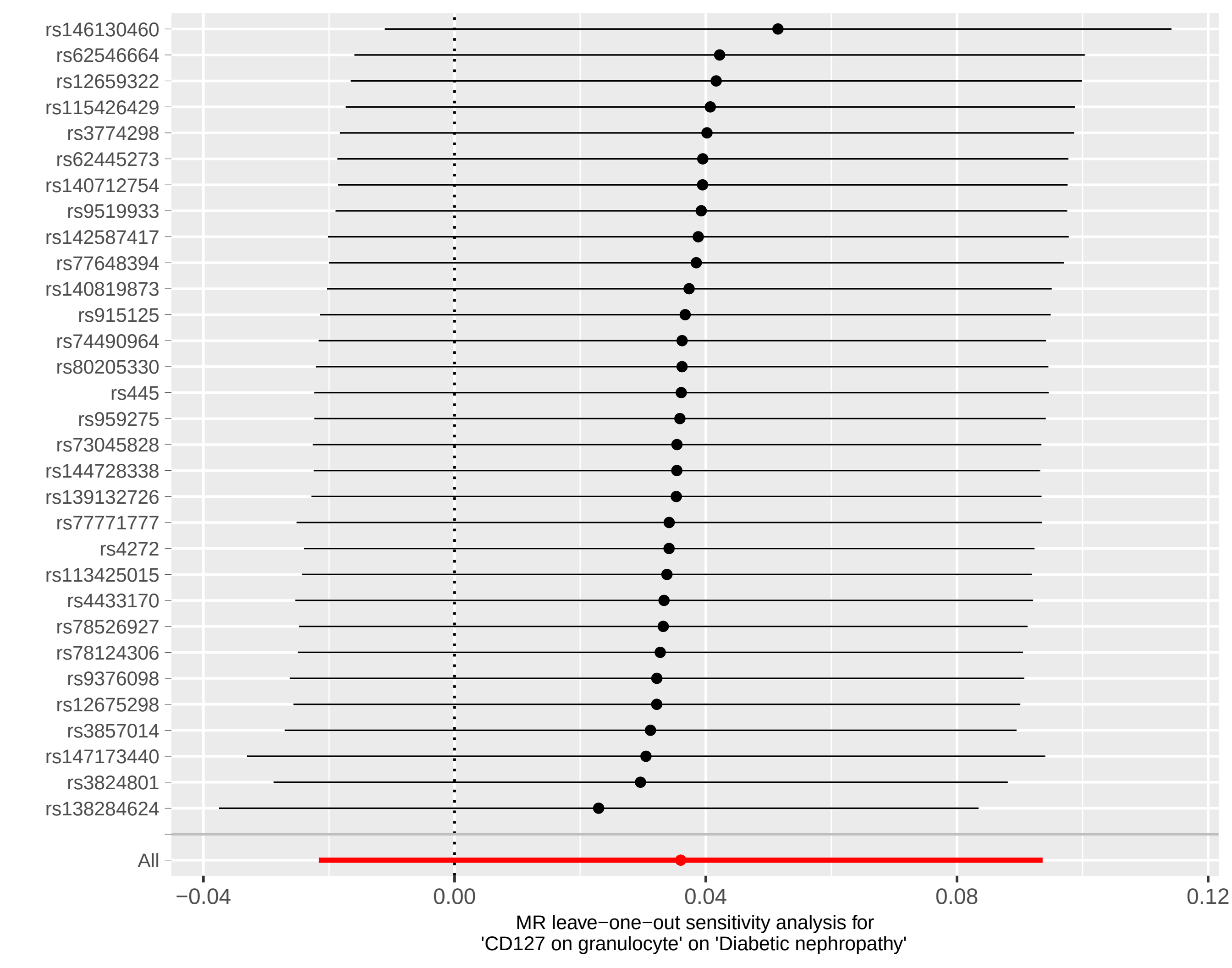
rs146130460
rs62546664
rs12659322
rs115426429
rs3774298
rs62445273
rs140712754
rs9519933
rs142587417
rs77648394
rs140819873
rs915125
rs74490964
rs80205330
rs445
rs959275
rs73045828
rs144728338
rs139132726
rs77771777
rs4272
rs113425015
rs4433170
rs78526927
rs78124306
rs9376098
rs12675298
rs3857014
rs147173440
rs3824801
rs138284624
All
−0.04
0.00
0.04
0.08
0.12
MR leave−one−out sensitivity analysis for
'CD127 on granulocyte' on 'Diabetic nephropathy'

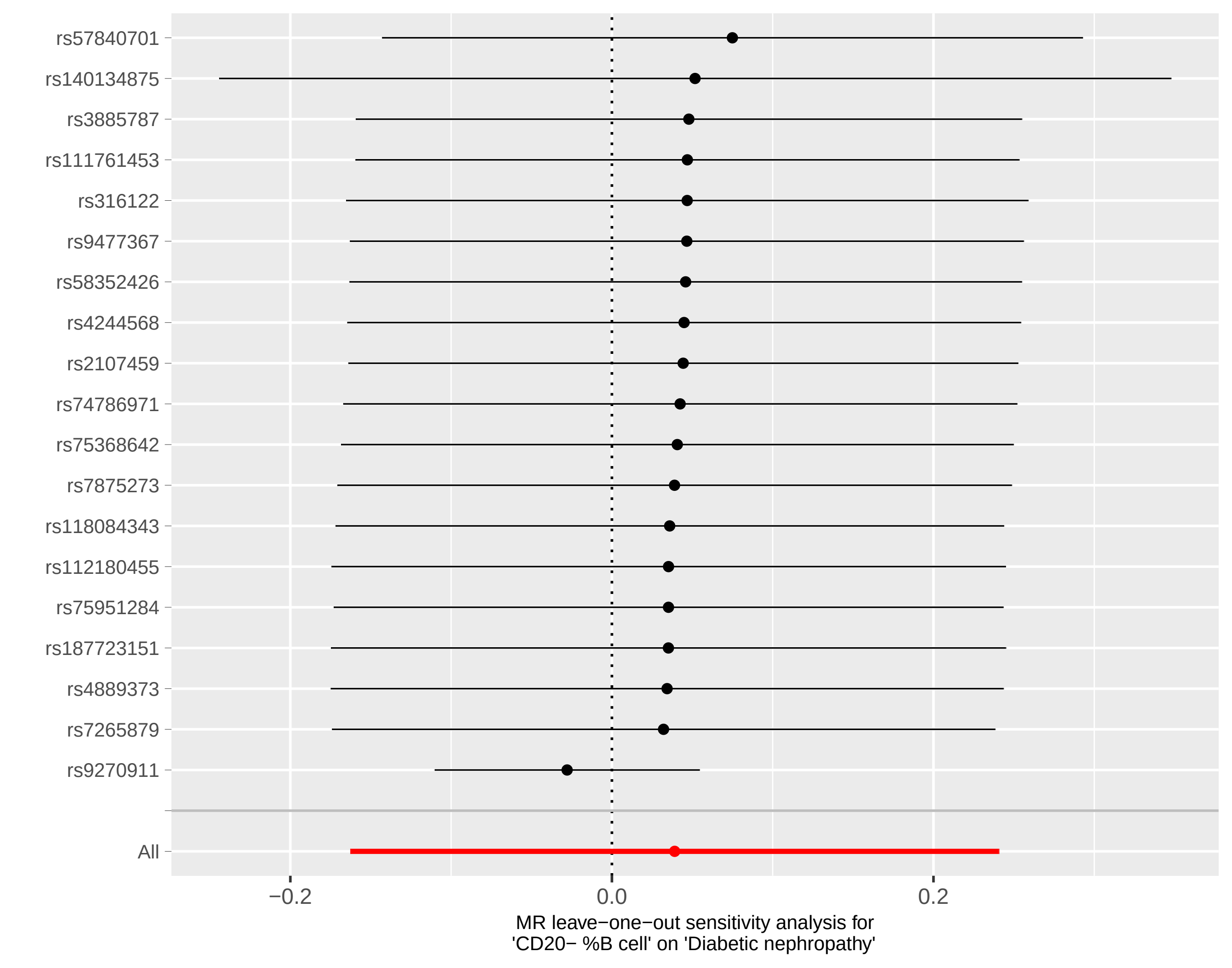

rs57840701
rs140134875
rs3885787
rs111761453
rs316122
rs9477367
rs58352426
rs4244568
rs2107459
rs74786971
rs75368642
rs7875273
rs118084343
rs112180455
rs75951284
rs187723151
rs4889373
rs7265879
rs9270911
All
−0.2
0.0
0.2
MR leave−one−out sensitivity analysis for
'CD20− %B cell' on 'Diabetic nephropathy'

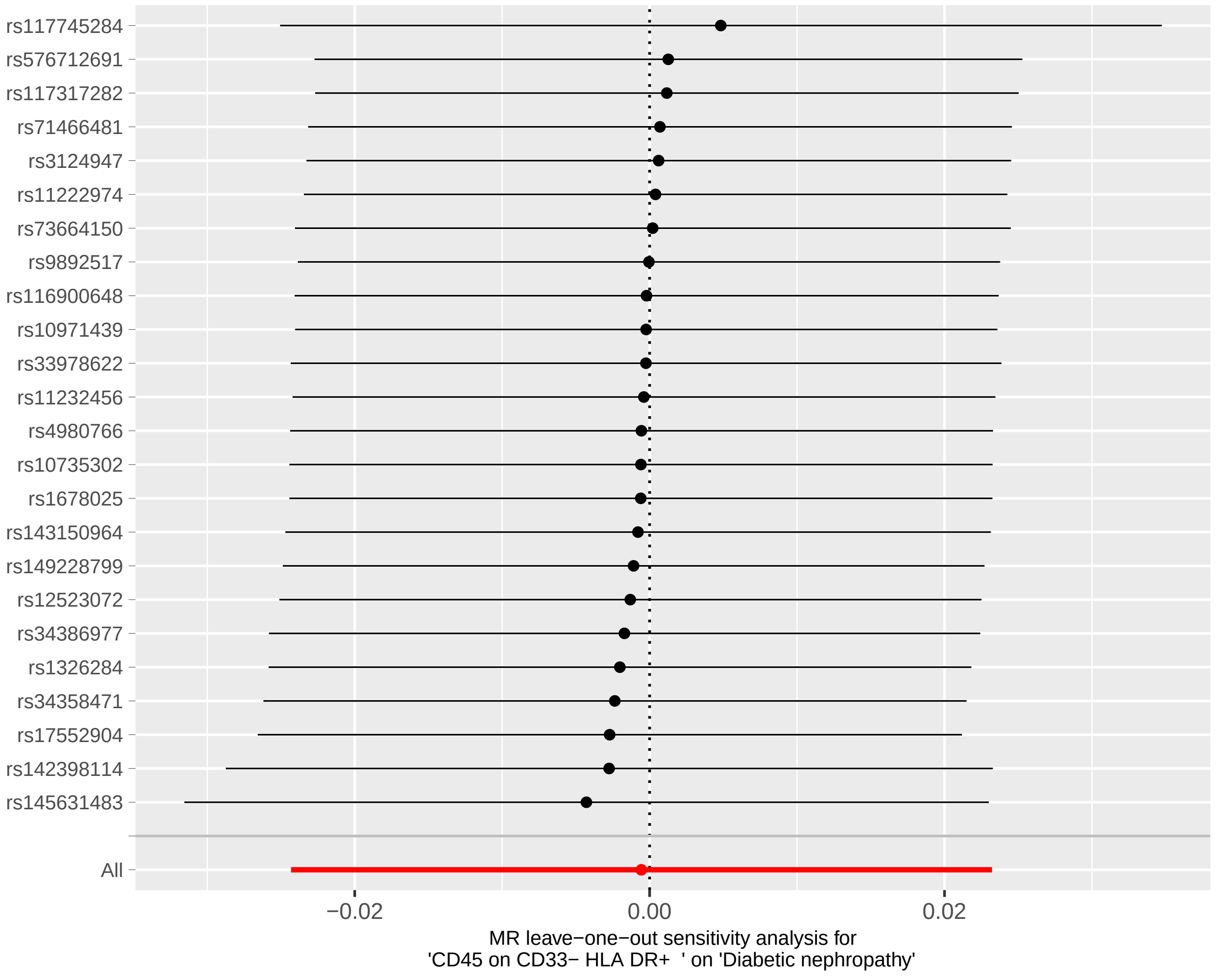

rs117745284
rs576712691
rs117317282
rs71466481
rs3124947
rs11222974
rs73664150
rs9892517
rs116900648
rs10971439
rs33978622
rs11232456
rs4980766
rs10735302
rs1678025
rs143150964
rs149228799
rs12523072
rs34386977
rs1326284
rs34358471
rs17552904
rs142398114
rs145631483
All
−0.02
0.00
0.02
MR leave−one−out sensitivity analysis for
'CD45 on CD33− HLA DR+ ' on 'Diabetic nephropathy'

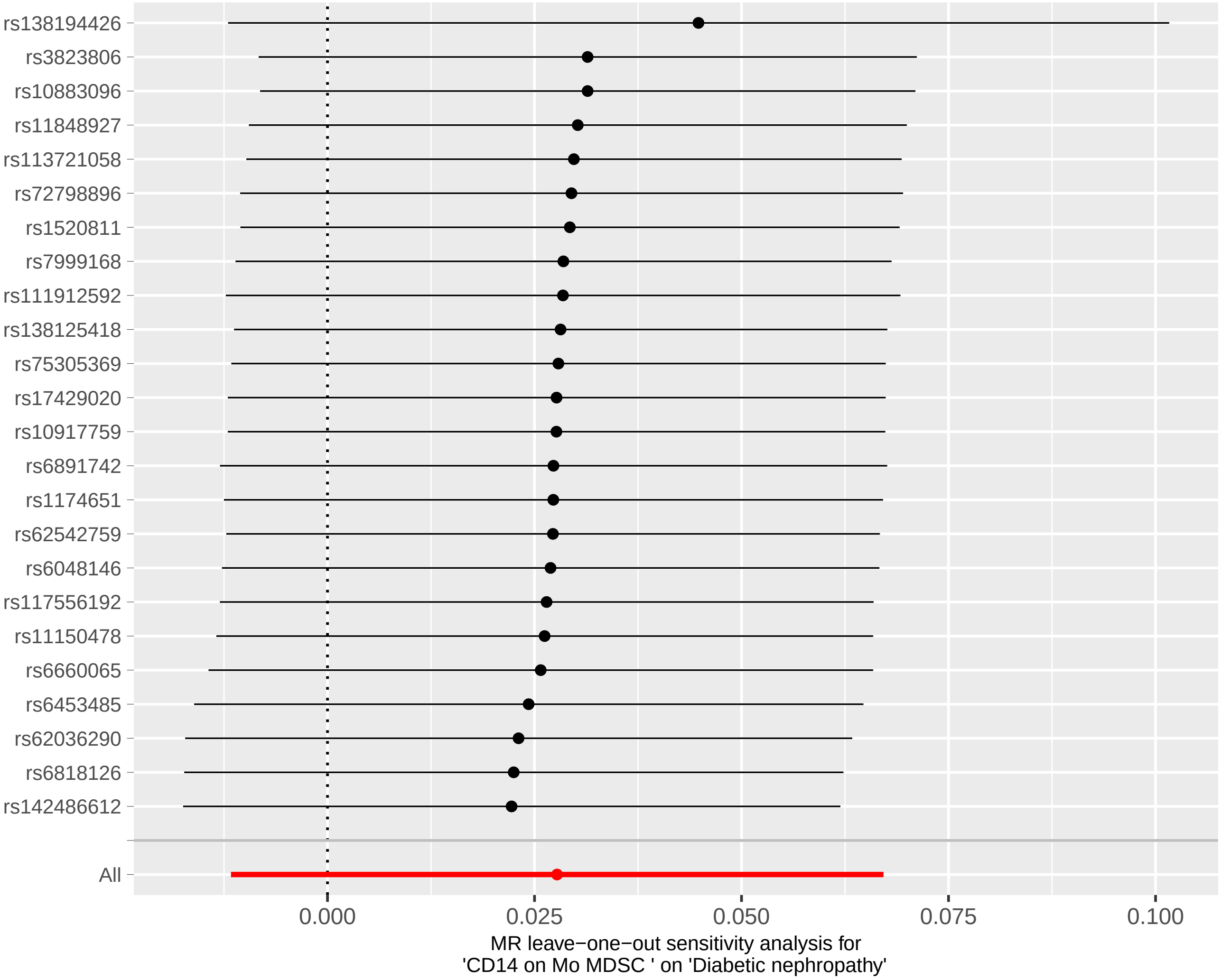
rs138194426
rs3823806
rs10883096
rs11848927
rs113721058
rs72798896
rs1520811
rs7999168
rs111912592
rs138125418
rs75305369
rs17429020
rs10917759
rs6891742
rs1174651
rs62542759
rs6048146
rs117556192
rs11150478
rs6660065
rs6453485
rs62036290
rs6818126
rs142486612
All
0.000
0.025
0.050
0.075
0.100
MR leave−one−out sensitivity analysis for
'CD14 on Mo MDSC ' on 'Diabetic nephropathy'

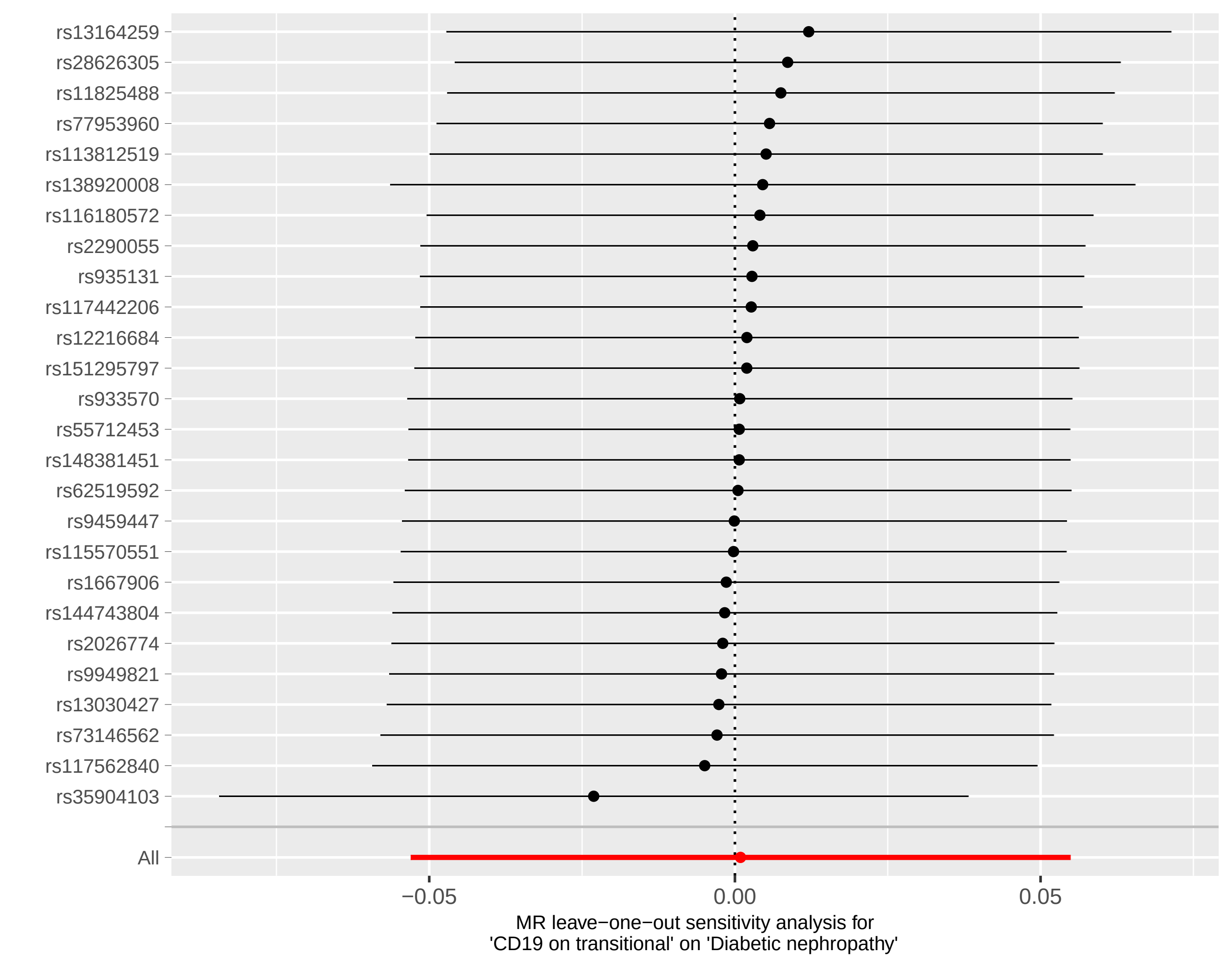
rs13164259
rs28626305
rs11825488
rs77953960
rs113812519
rs138920008
rs116180572
rs2290055
rs935131
rs117442206
rs12216684
rs151295797
rs933570
rs55712453
rs148381451
rs62519592
rs9459447
rs115570551
rs1667906
rs144743804
rs2026774
rs9949821
rs13030427
rs73146562
rs117562840
rs35904103
All
−0.05
0.00
0.05
MR leave−one−out sensitivity analysis for
'CD19 on transitional' on 'Diabetic nephropathy'

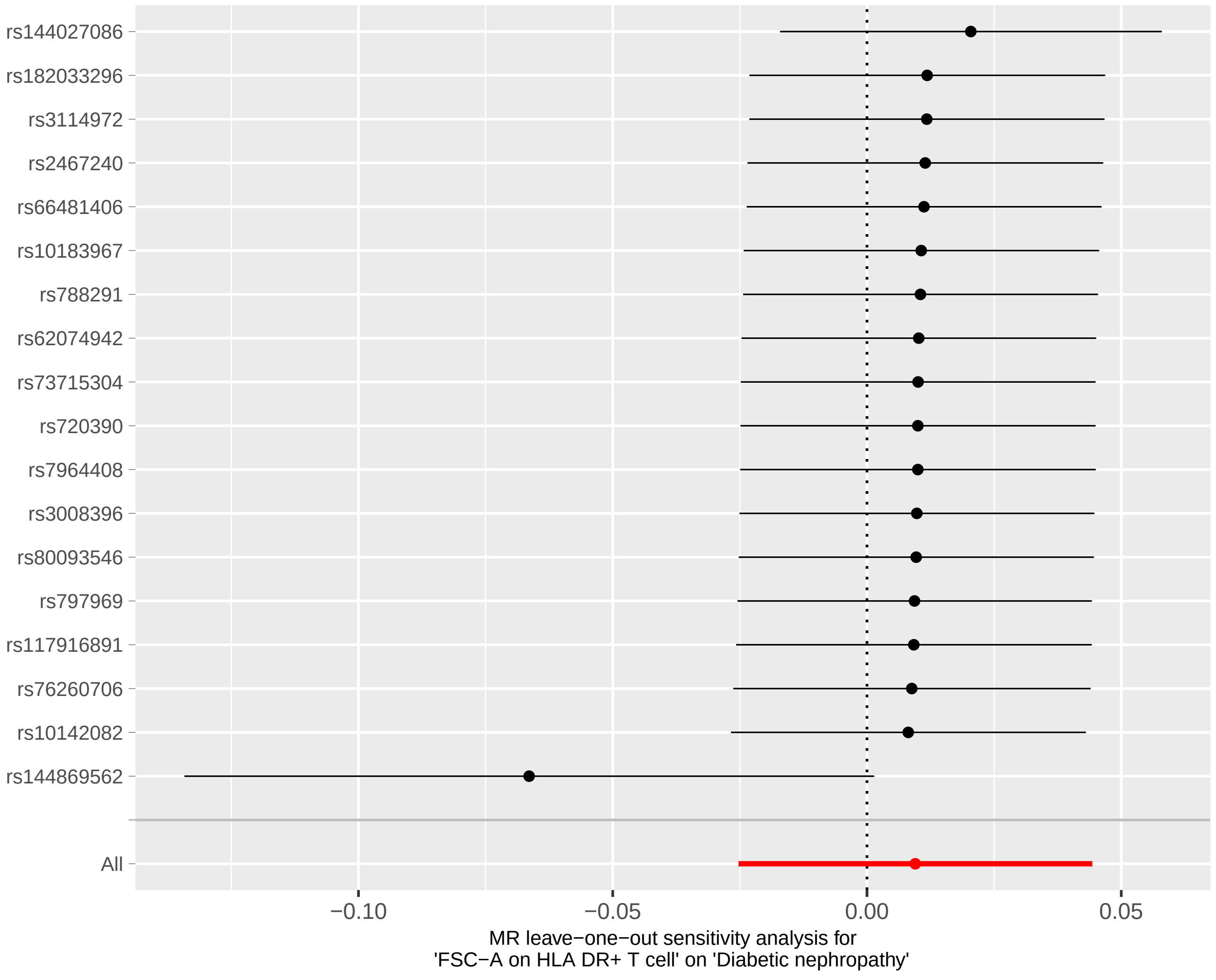

rs144027086
rs182033296
rs3114972
rs2467240
rs66481406
rs10183967
rs788291
rs62074942
rs73715304
rs720390
rs7964408
rs3008396
rs80093546
rs797969
rs117916891
rs76260706
rs10142082
rs144869562
All
−0.10
−0.05
0.00
0.05
MR leave−one−out sensitivity analysis for
'FSC−A on HLA DR+ T cell' on 'Diabetic nephropathy'

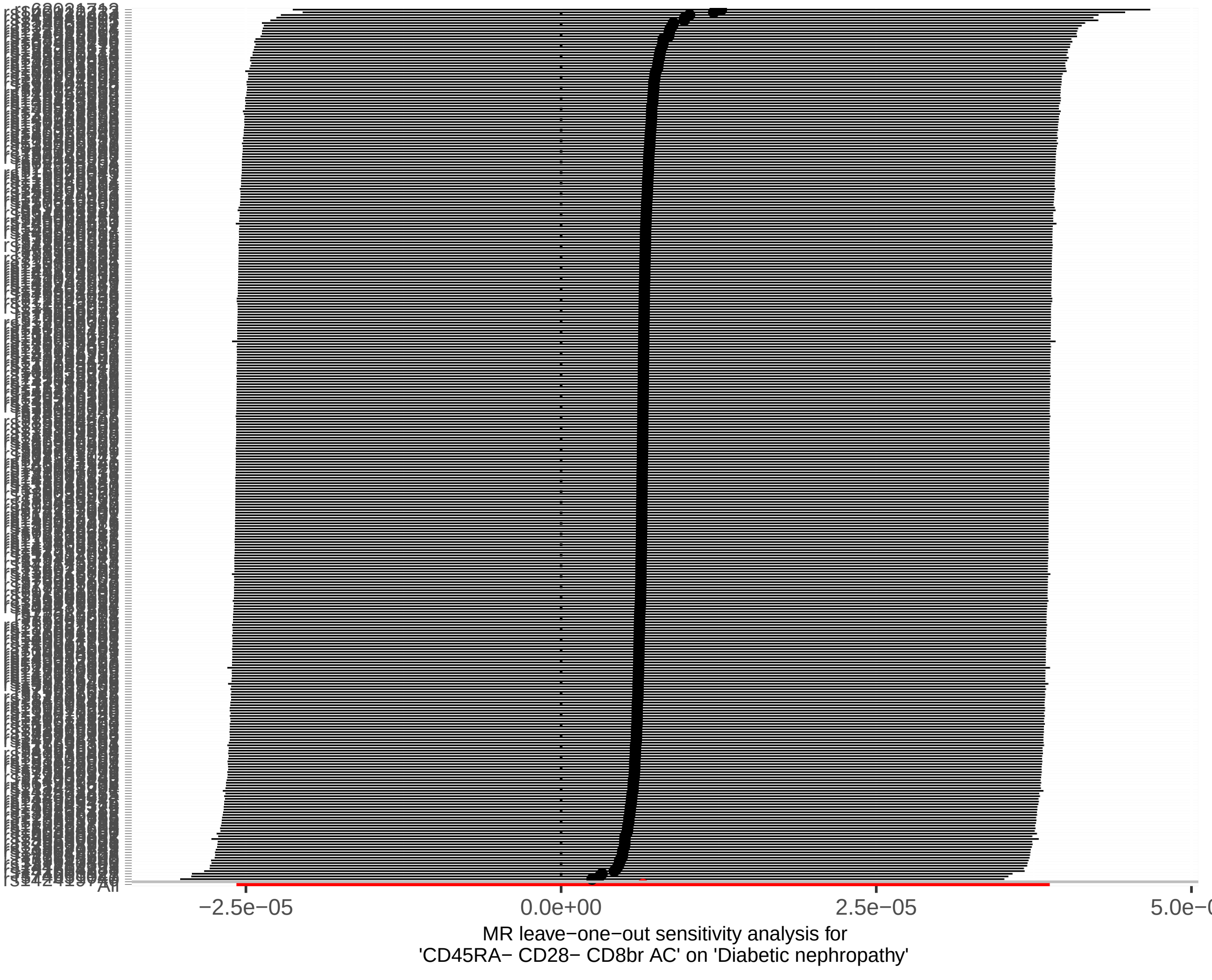
All
−2.5e−05
0.0e+00
2.5e−05
MR leave−one−out sensitivity analysis for
'CD45RA− CD28− CD8br AC' on 'Diabetic nephropathy'

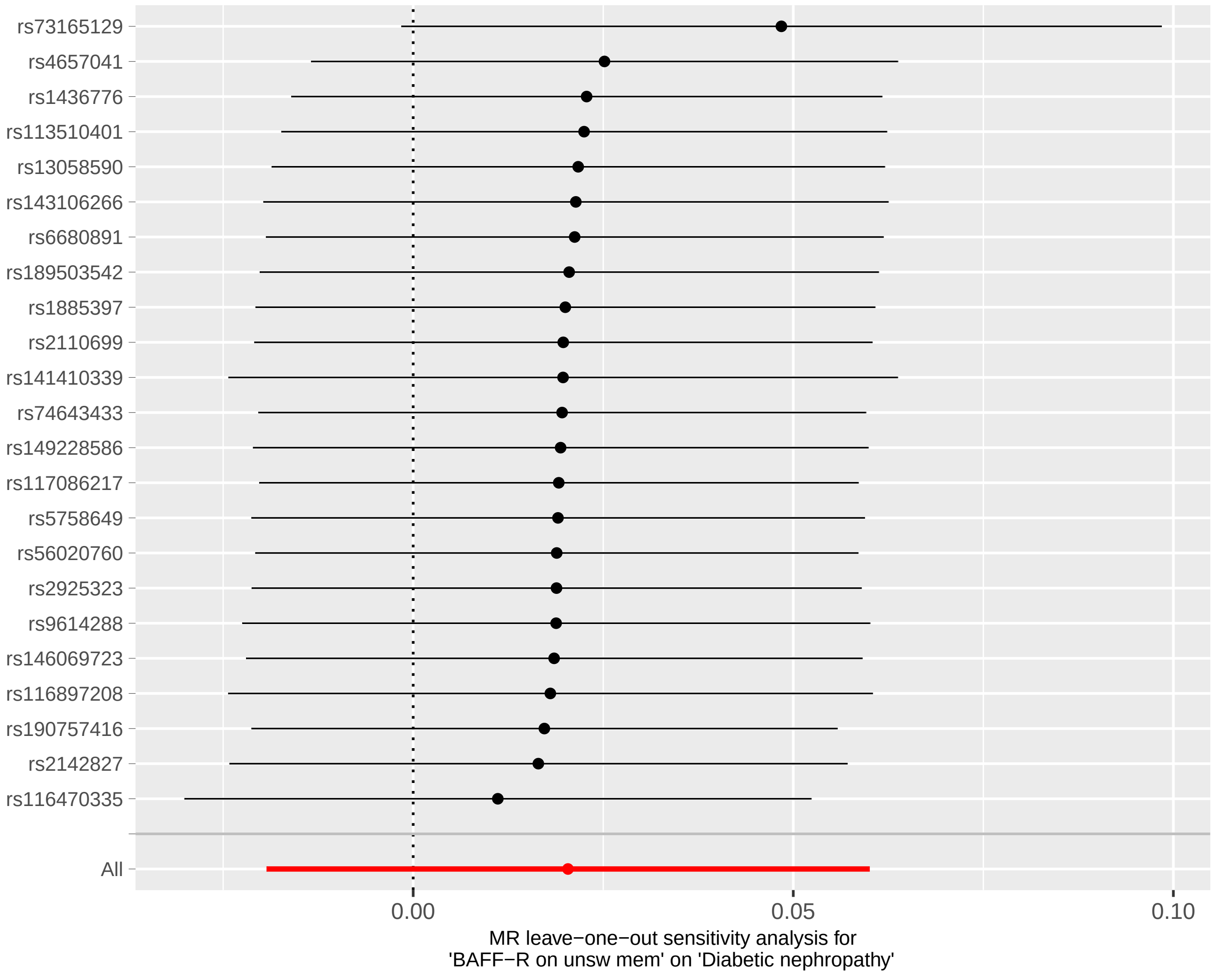

rs73165129
rs4657041
rs1436776
rs113510401
rs13058590
rs143106266
rs6680891
rs189503542
rs1885397
rs2110699
rs141410339
rs74643433
rs149228586
rs117086217
rs5758649
rs56020760
rs2925323
rs9614288
rs146069723
rs116897208
rs190757416
rs2142827
rs116470335
All
0.00
0.05
0.10
MR leave−one−out sensitivity analysis for
'BAFF−R on unsw mem' on 'Diabetic nephropathy'

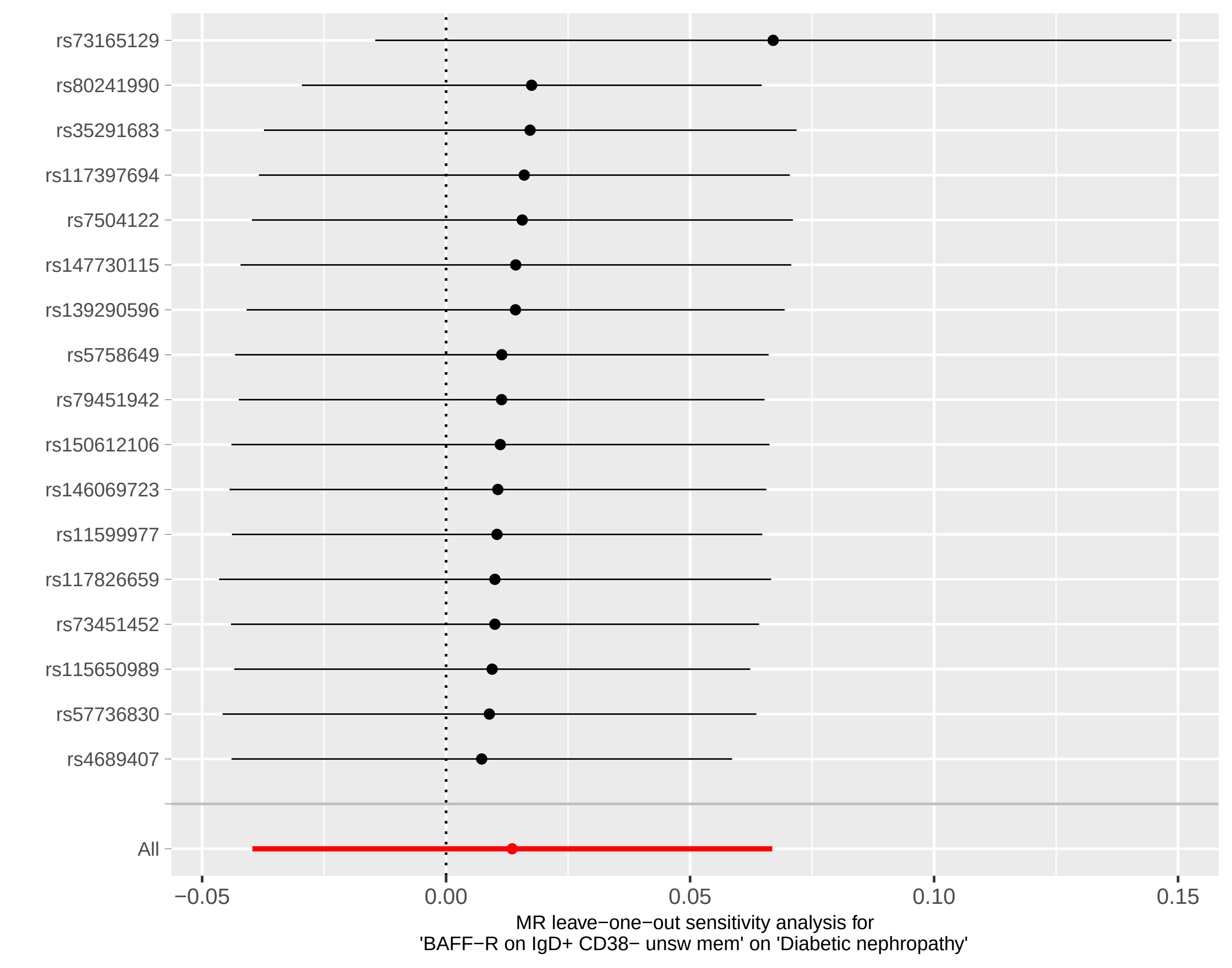
rs73165129
rs80241990
rs35291683
rs117397694
rs7504122
rs147730115
rs139290596
rs5758649
rs79451942
rs150612106
rs146069723
rs11599977
rs117826659
rs73451452
rs115650989
rs57736830
rs4689407
All
−0.05
0.00
0.05
0.10
0.15
MR leave−one−out sensitivity analysis for
'BAFF−R on IgD+ CD38− unsw mem' on 'Diabetic nephropathy'

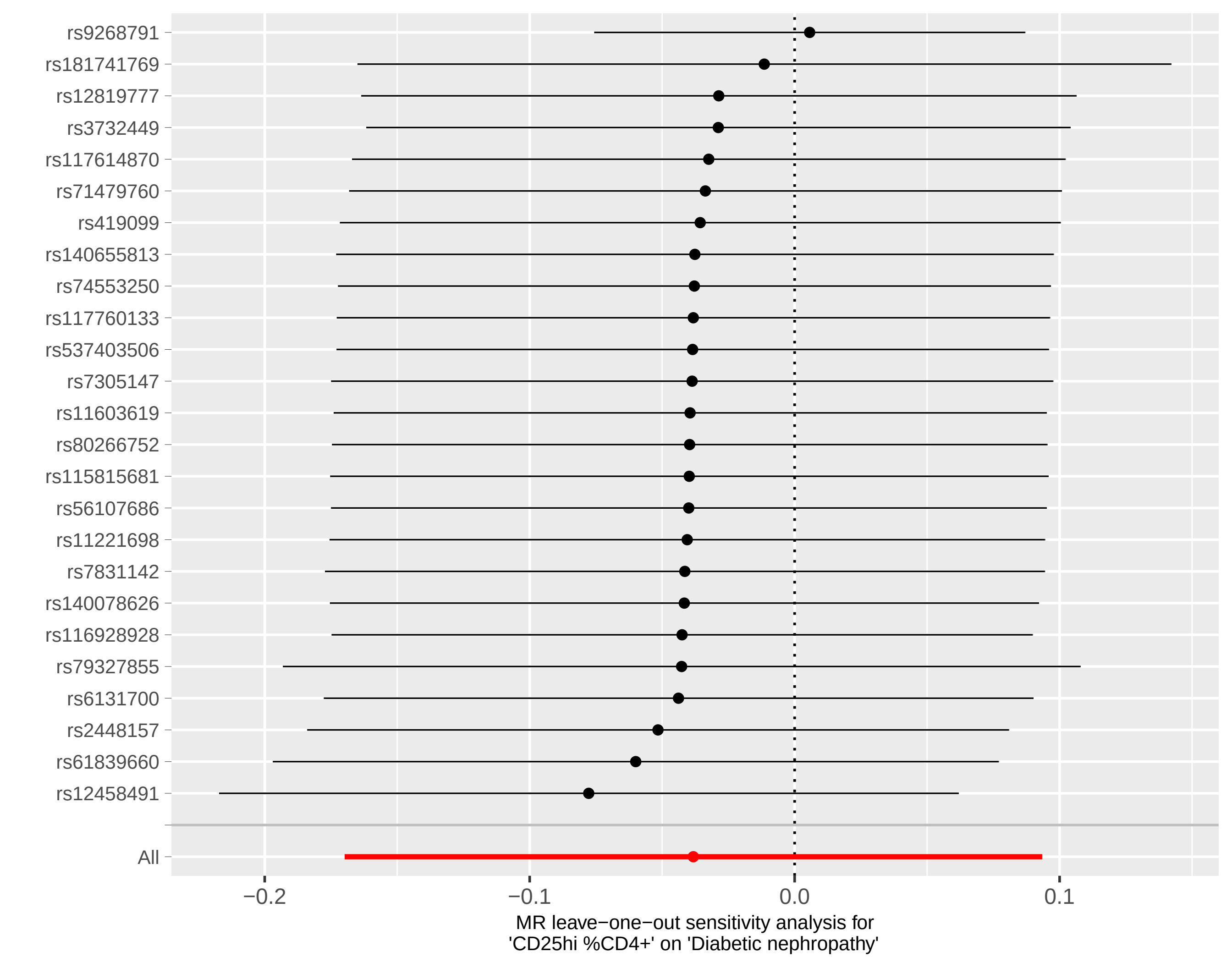
rs9268791
rs181741769
rs12819777
rs3732449
rs117614870
rs71479760
rs419099
rs140655813
rs74553250
rs117760133
rs537403506
rs7305147
rs11603619
rs80266752
rs115815681
rs56107686
rs11221698
rs7831142
rs140078626
rs116928928
rs79327855
rs6131700
rs2448157
rs61839660
rs12458491
All
−0.2
−0.1
0.0
0.1
MR leave−one−out sensitivity analysis for
'CD25hi %CD4+' on 'Diabetic nephropathy'

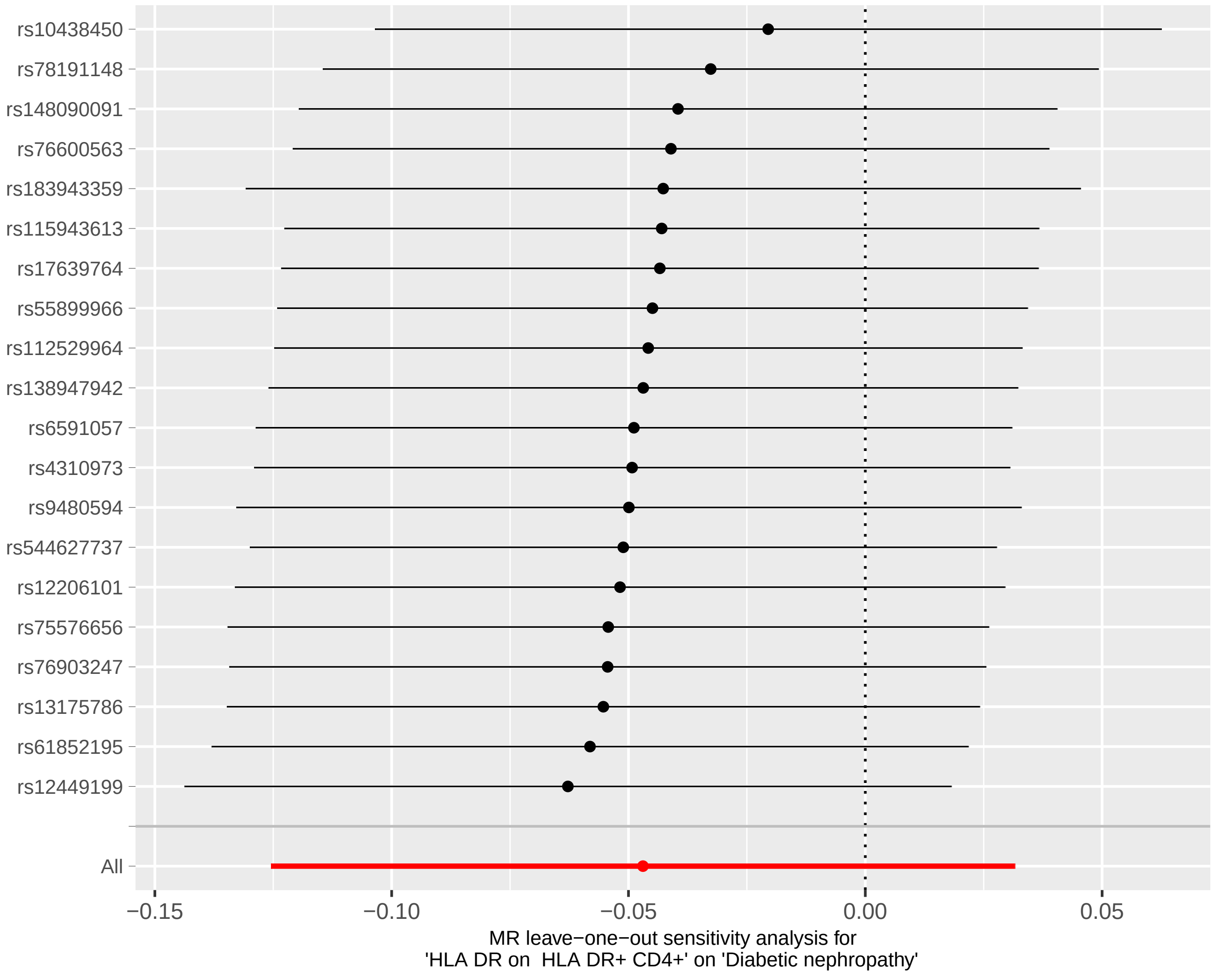
rs10438450
rs78191148
rs148090091
rs76600563
rs183943359
rs115943613
rs17639764
rs55899966
rs112529964
rs138947942
rs6591057
rs4310973
rs9480594
rs544627737
rs12206101
rs75576656
rs76903247
rs13175786
rs61852195
rs12449199
All
−0.15
−0.10
−0.05
0.00
0.05
MR leave−one−out sensitivity analysis for
'HLA DR on HLA DR+ CD4+' on 'Diabetic nephropathy'

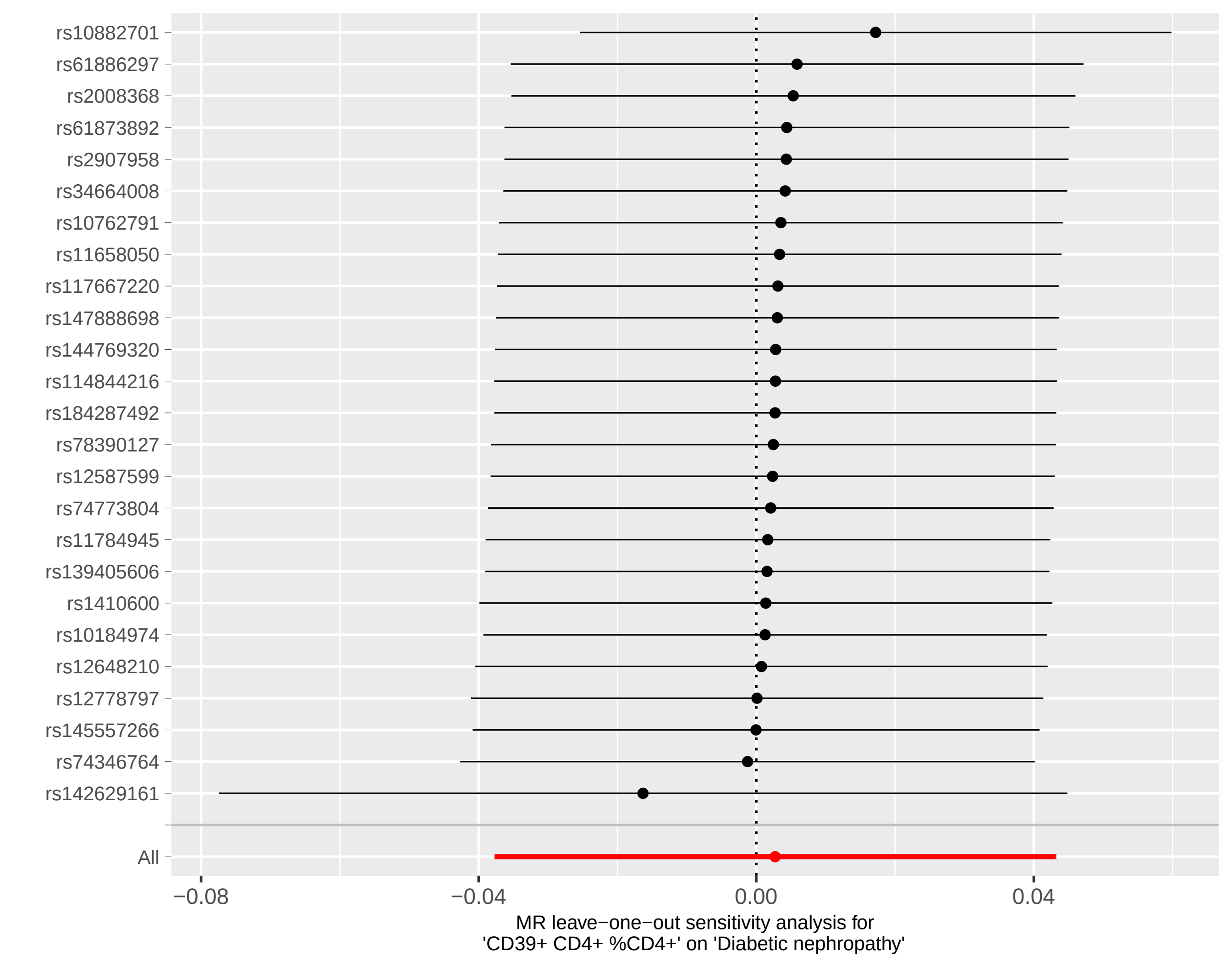

rs10882701
rs61886297
rs2008368
rs61873892
rs2907958
rs34664008
rs10762791
rs11658050
rs117667220
rs147888698
rs144769320
rs114844216
rs184287492
rs78390127
rs12587599
rs74773804
rs11784945
rs139405606
rs1410600
rs10184974
rs12648210
rs12778797
rs145557266
rs74346764
rs142629161
All
−0.08
−0.04
0.00
0.04
MR leave−one−out sensitivity analysis for
'CD39+ CD4+ %CD4+' on 'Diabetic nephropathy'

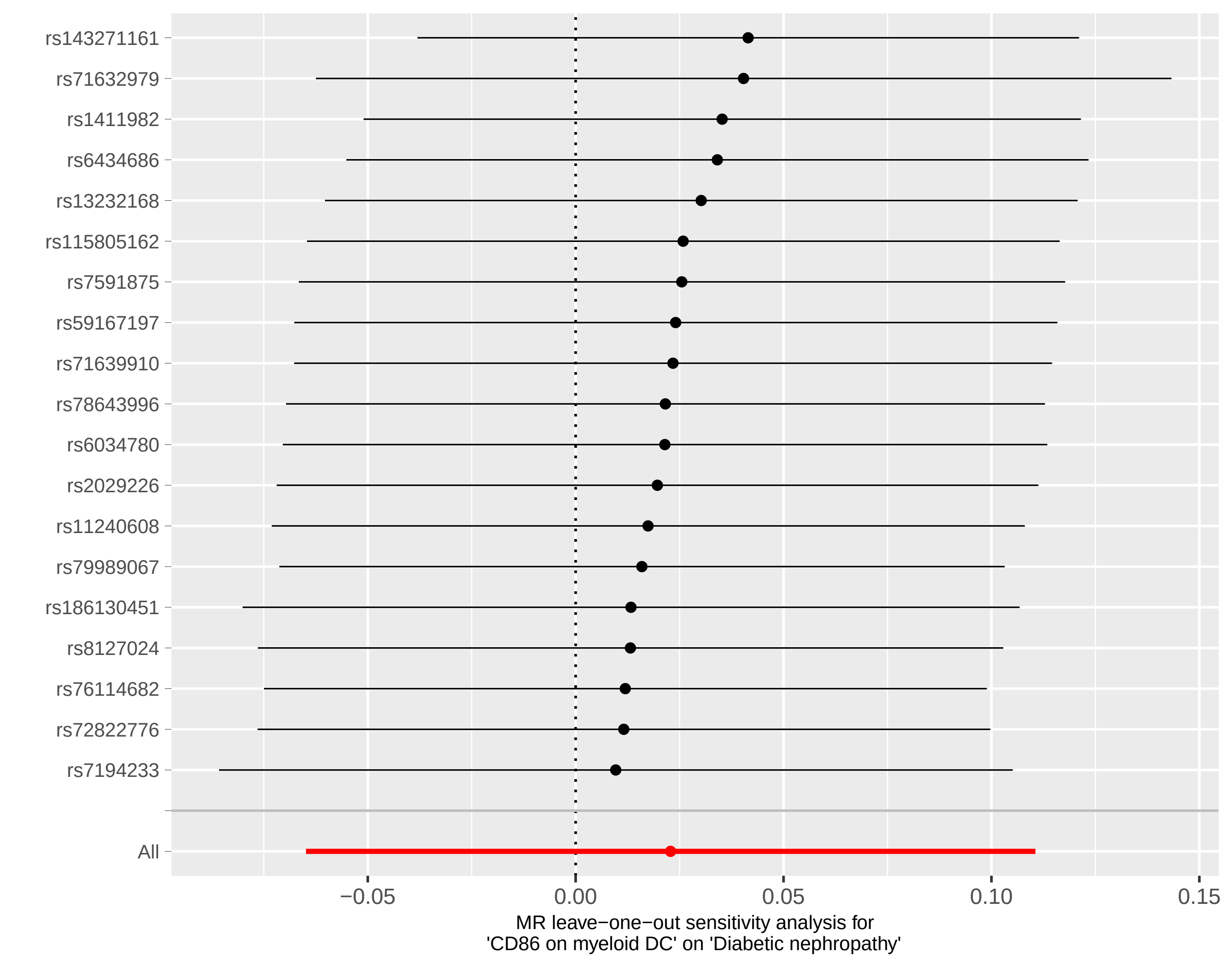
rs143271161
rs71632979
rs1411982
rs6434686
rs13232168
rs115805162
rs7591875
rs59167197
rs71639910
rs78643996
rs6034780
rs2029226
rs11240608
rs79989067
rs186130451
rs8127024
rs76114682
rs72822776
rs7194233
All
−0.05
0.00
0.05
0.10
0.15
MR leave−one−out sensitivity analysis for
'CD86 on myeloid DC' on 'Diabetic nephropathy'

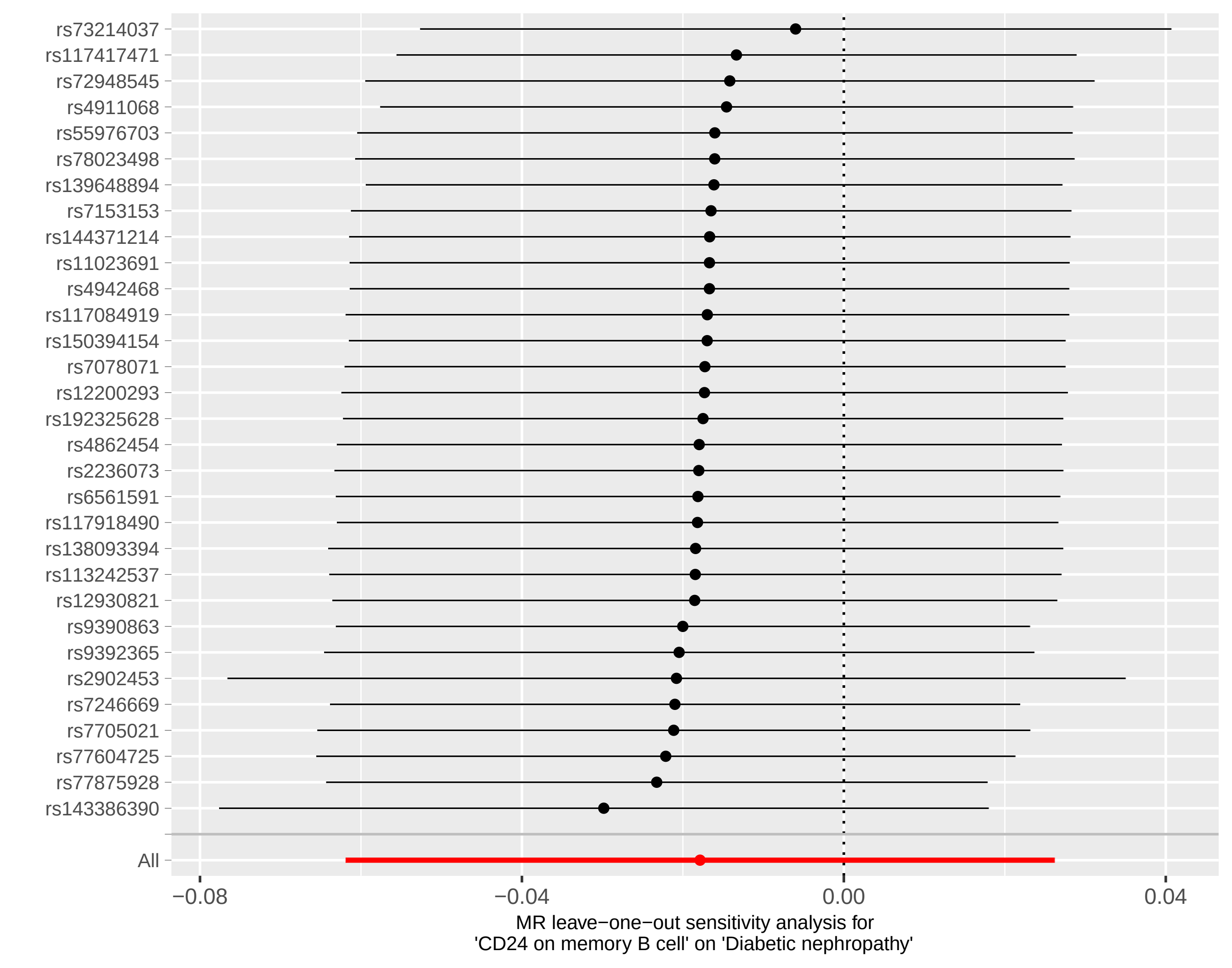
rs73214037
rs117417471
rs72948545
rs4911068
rs55976703
rs78023498
rs139648894
rs7153153
rs144371214
rs11023691
rs4942468
rs117084919
rs150394154
rs7078071
rs12200293
rs192325628
rs4862454
rs2236073
rs6561591
rs117918490
rs138093394
rs113242537
rs12930821
rs9390863
rs9392365
rs2902453
rs7246669
rs7705021
rs77604725
rs77875928
rs143386390
All
−0.08
−0.04
0.00
0.04
MR leave−one−out sensitivity analysis for
'CD24 on memory B cell' on 'Diabetic nephropathy'

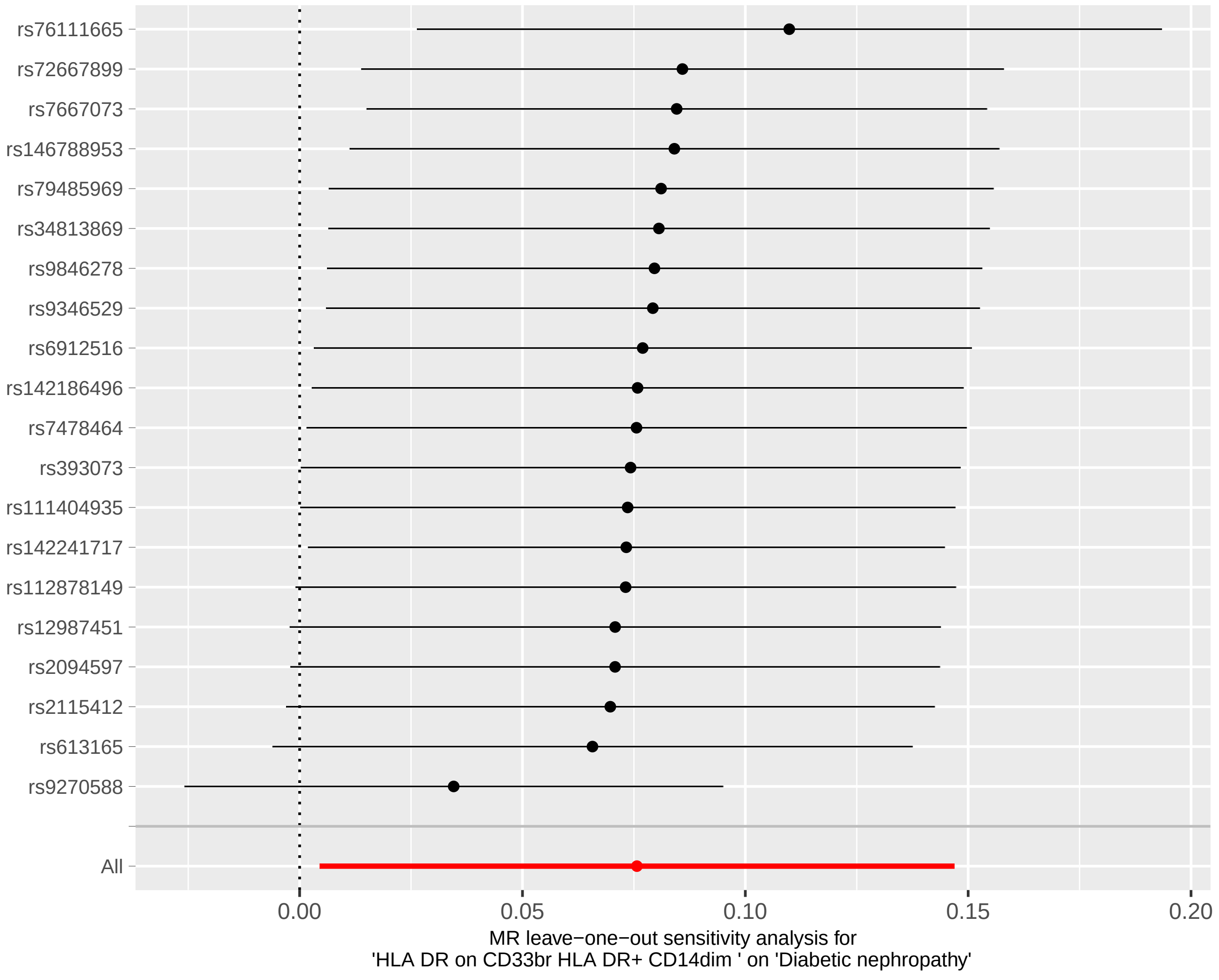

rs76111665
rs72667899
rs7667073
rs146788953
rs79485969
rs34813869
rs9846278
rs9346529
rs6912516
rs142186496
rs7478464
rs393073
rs111404935
rs142241717
rs112878149
rs12987451
rs2094597
rs2115412
rs613165
rs9270588
All
0.00
0.05
0.10
0.15
0.20
MR leave−one−out sensitivity analysis for
'HLA DR on CD33br HLA DR+ CD14dim ' on 'Diabetic nephropathy'

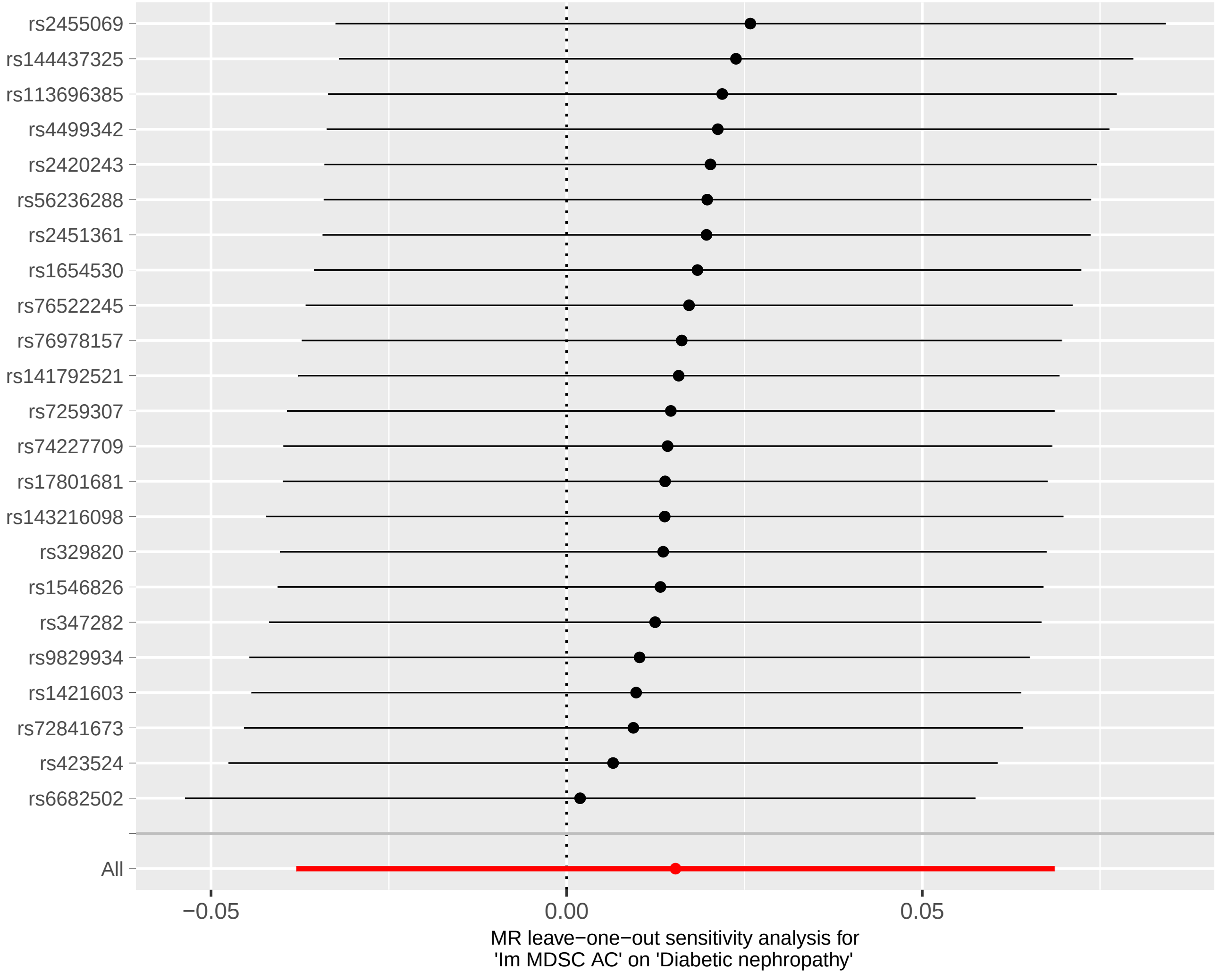
rs2455069
rs144437325
rs113696385
rs4499342
rs2420243
rs56236288
rs2451361
rs1654530
rs76522245
rs76978157
rs141792521
rs7259307
rs74227709
rs17801681
rs143216098
rs329820
rs1546826
rs347282
rs9829934
rs1421603
rs72841673
rs423524
rs6682502
All
−0.05
0.00
0.05
MR leave−one−out sensitivity analysis for
'Im MDSC AC' on 'Diabetic nephropathy'

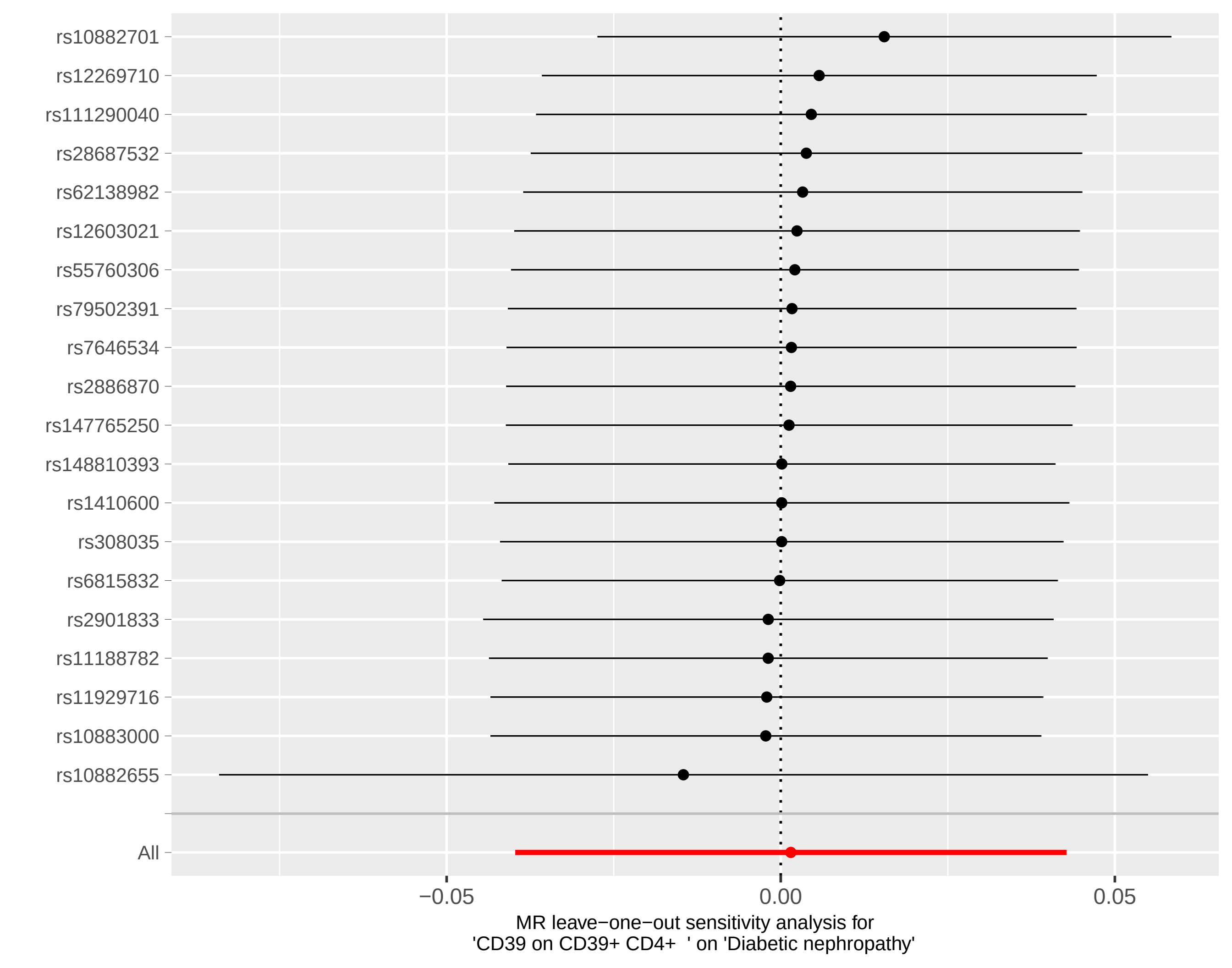
rs10882701
rs12269710
rs111290040
rs28687532
rs62138982
rs12603021
rs55760306
rs79502391
rs7646534
rs2886870
rs147765250
rs148810393
rs1410600
rs308035
rs6815832
rs2901833
rs11188782
rs11929716
rs10883000
rs10882655
All
−0.05
0.00
0.05
MR leave−one−out sensitivity analysis for
'CD39 on CD39+ CD4+ ' on 'Diabetic nephropathy'

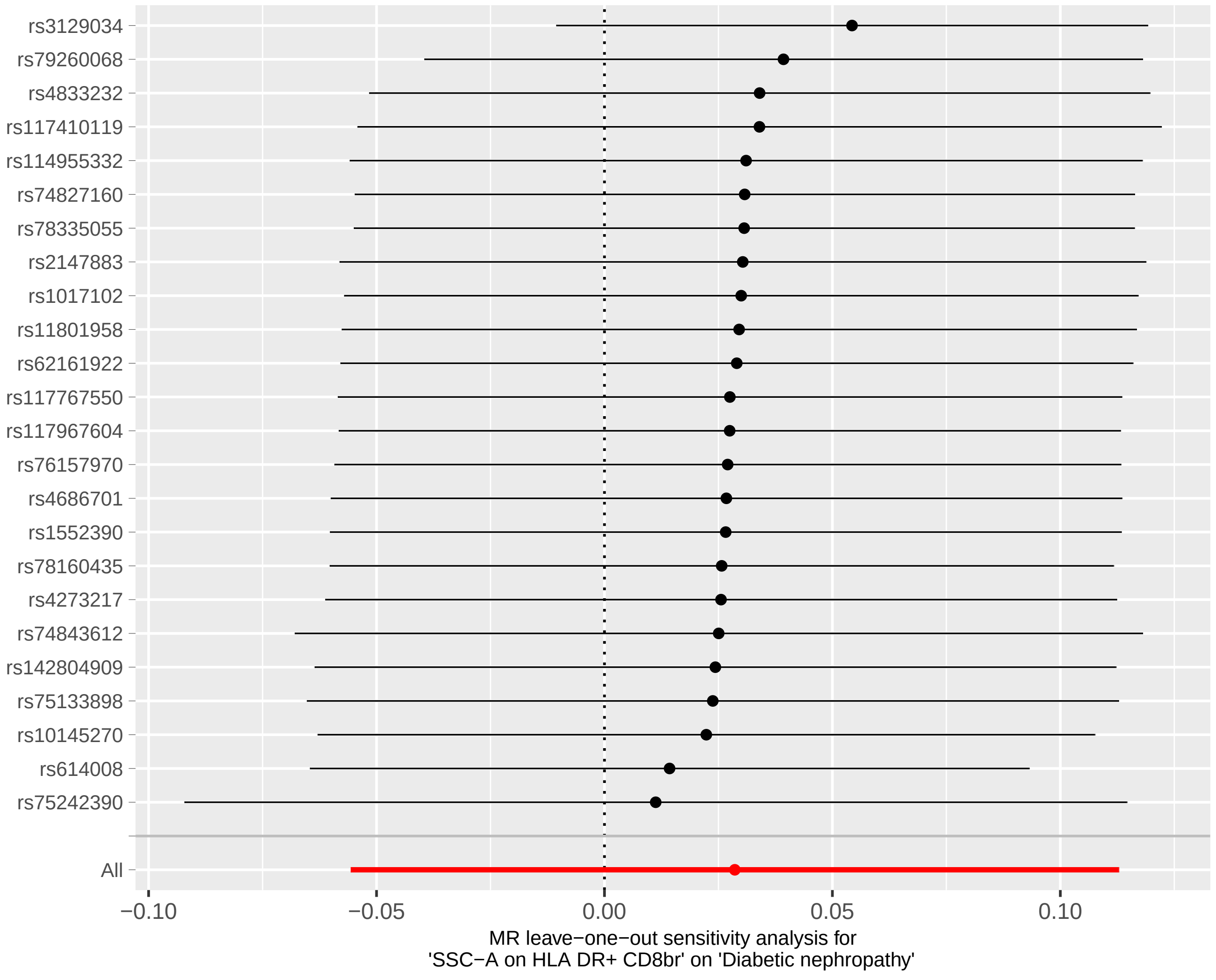
rs3129034
rs79260068
rs4833232
rs117410119
rs114955332
rs74827160
rs78335055
rs2147883
rs1017102
rs11801958
rs62161922
rs117767550
rs117967604
rs76157970
rs4686701
rs1552390
rs78160435
rs4273217
rs74843612
rs142804909
rs75133898
rs10145270
rs614008
rs75242390
All
−0.10
−0.05
0.00
0.05
0.10
MR leave−one−out sensitivity analysis for
'SSC−A on HLA DR+ CD8br' on 'Diabetic nephropathy'

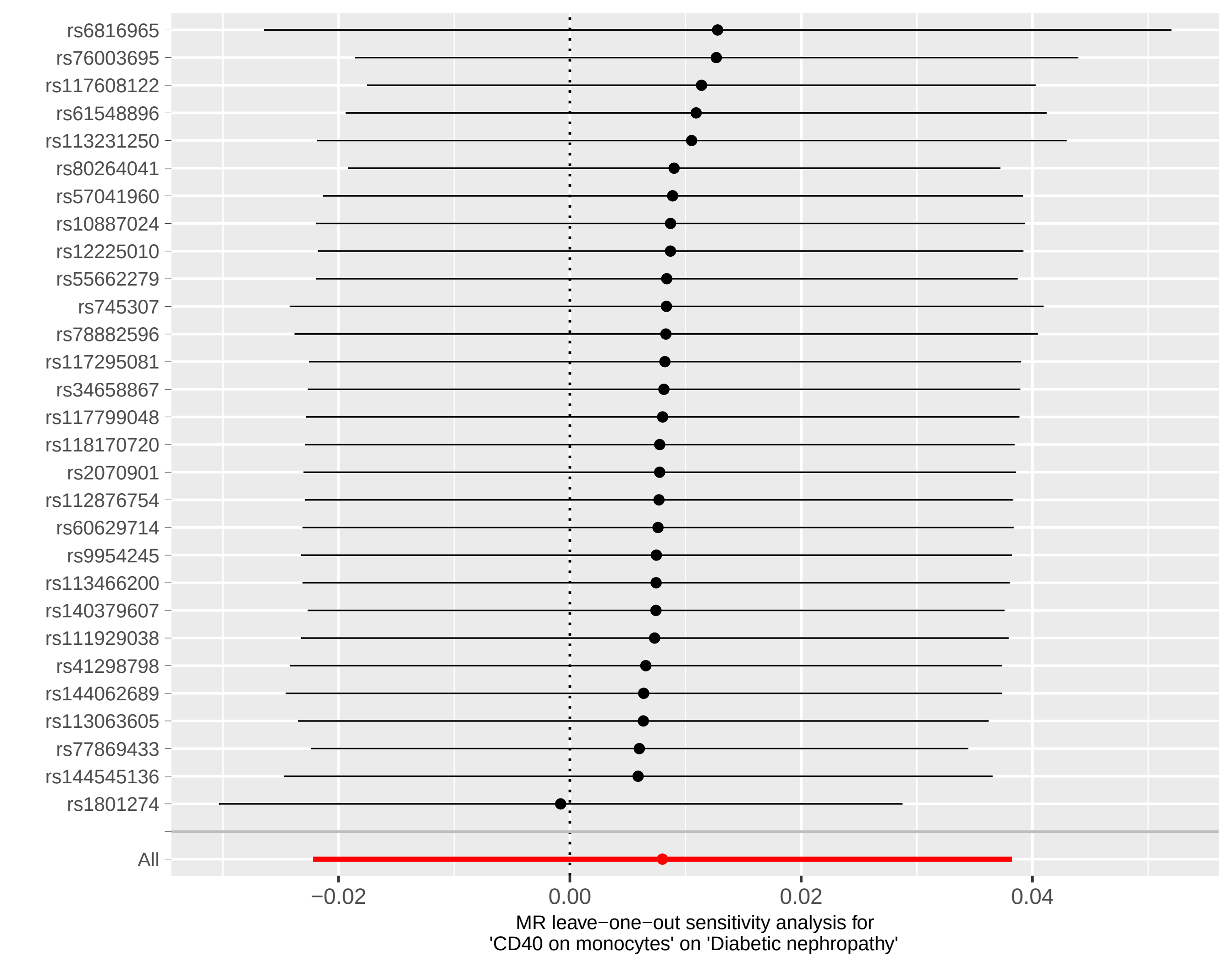
rs6816965
rs76003695
rs117608122
rs61548896
rs113231250
rs80264041
rs57041960
rs10887024
rs12225010
rs55662279
rs745307
rs78882596
rs117295081
rs34658867
rs117799048
rs118170720
rs2070901
rs112876754
rs60629714
rs9954245
rs113466200
rs140379607
rs111929038
rs41298798
rs144062689
rs113063605
rs77869433
rs144545136
rs1801274
All
−0.02
0.00
0.02
0.04
MR leave−one−out sensitivity analysis for
'CD40 on monocytes' on 'Diabetic nephropathy'

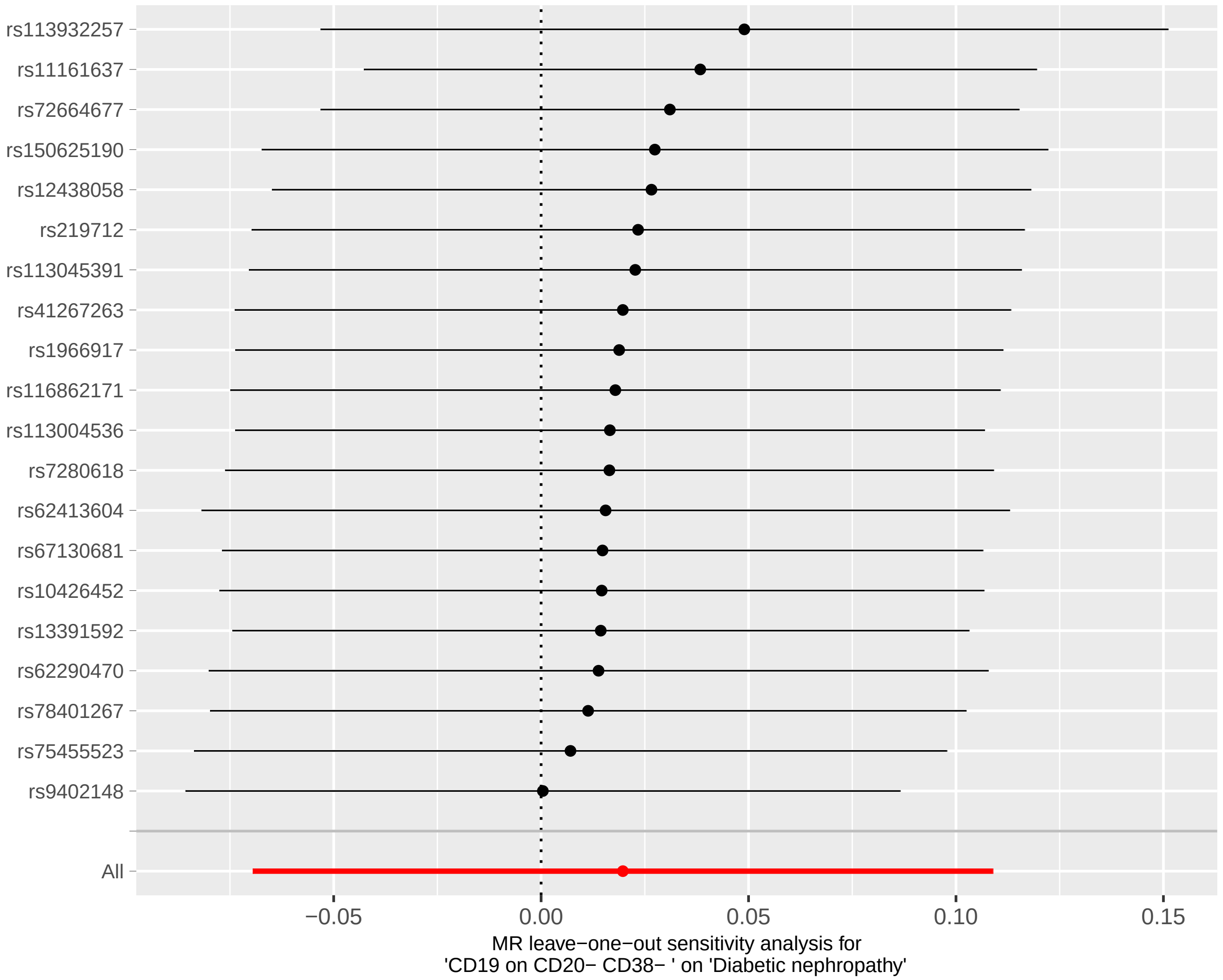
rs113932257
rs11161637
rs72664677
rs150625190
rs12438058
rs219712
rs113045391
rs41267263
rs1966917
rs116862171
rs113004536
rs7280618
rs62413604
rs67130681
rs10426452
rs13391592
rs62290470
rs78401267
rs75455523
rs9402148
All
−0.05
0.00
0.05
0.10
0.15
MR leave−one−out sensitivity analysis for
'CD19 on CD20− CD38− ' on 'Diabetic nephropathy'

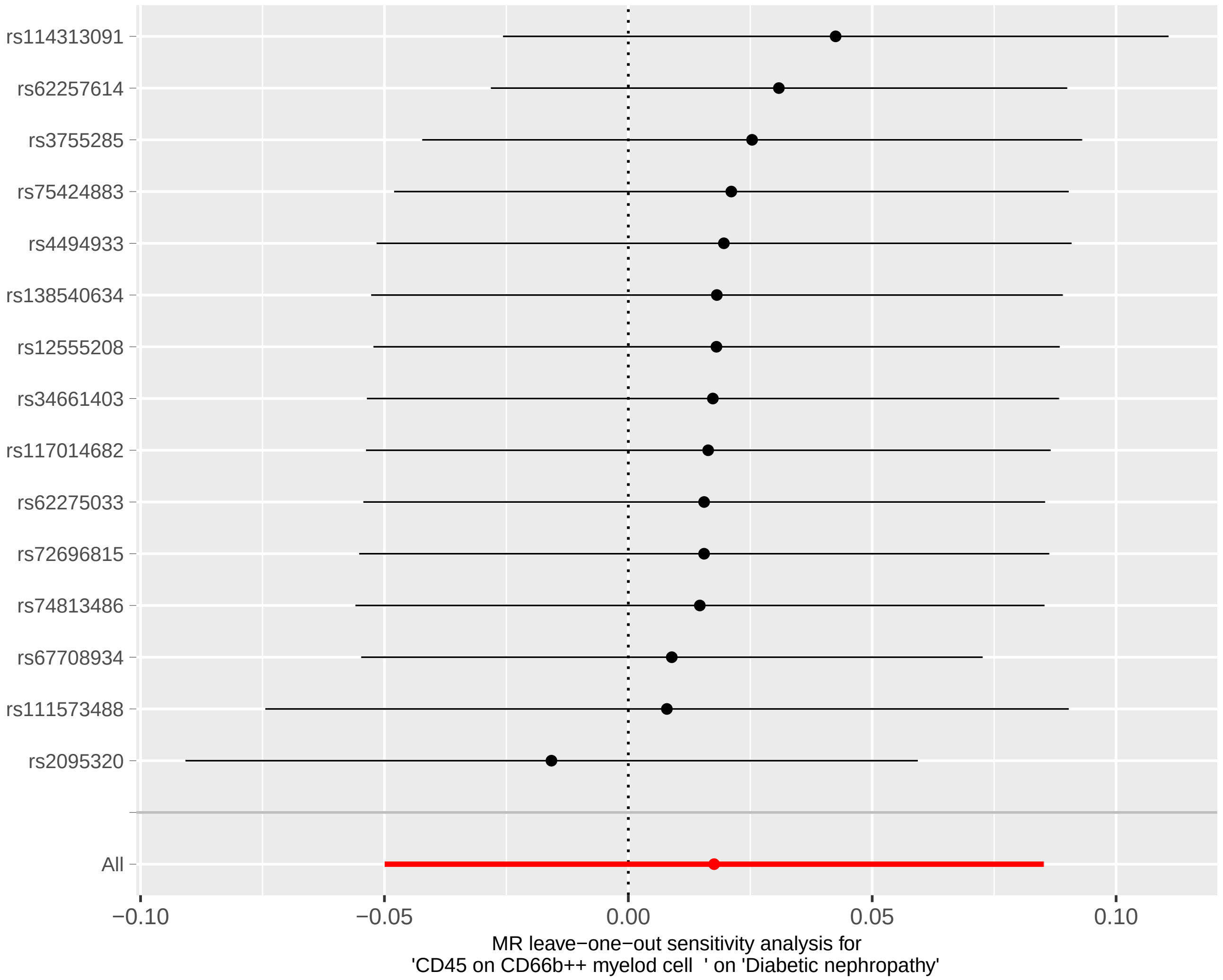
rs114313091
rs62257614
rs3755285
rs75424883
rs4494933
rs138540634
rs12555208
rs34661403
rs117014682
rs62275033
rs72696815
rs74813486
rs67708934
rs111573488
rs2095320
All
−0.10
−0.05
0.00
0.05
0.10
MR leave−one−out sensitivity analysis for
'CD45 on CD66b++ myelod cell ' on 'Diabetic nephropathy'

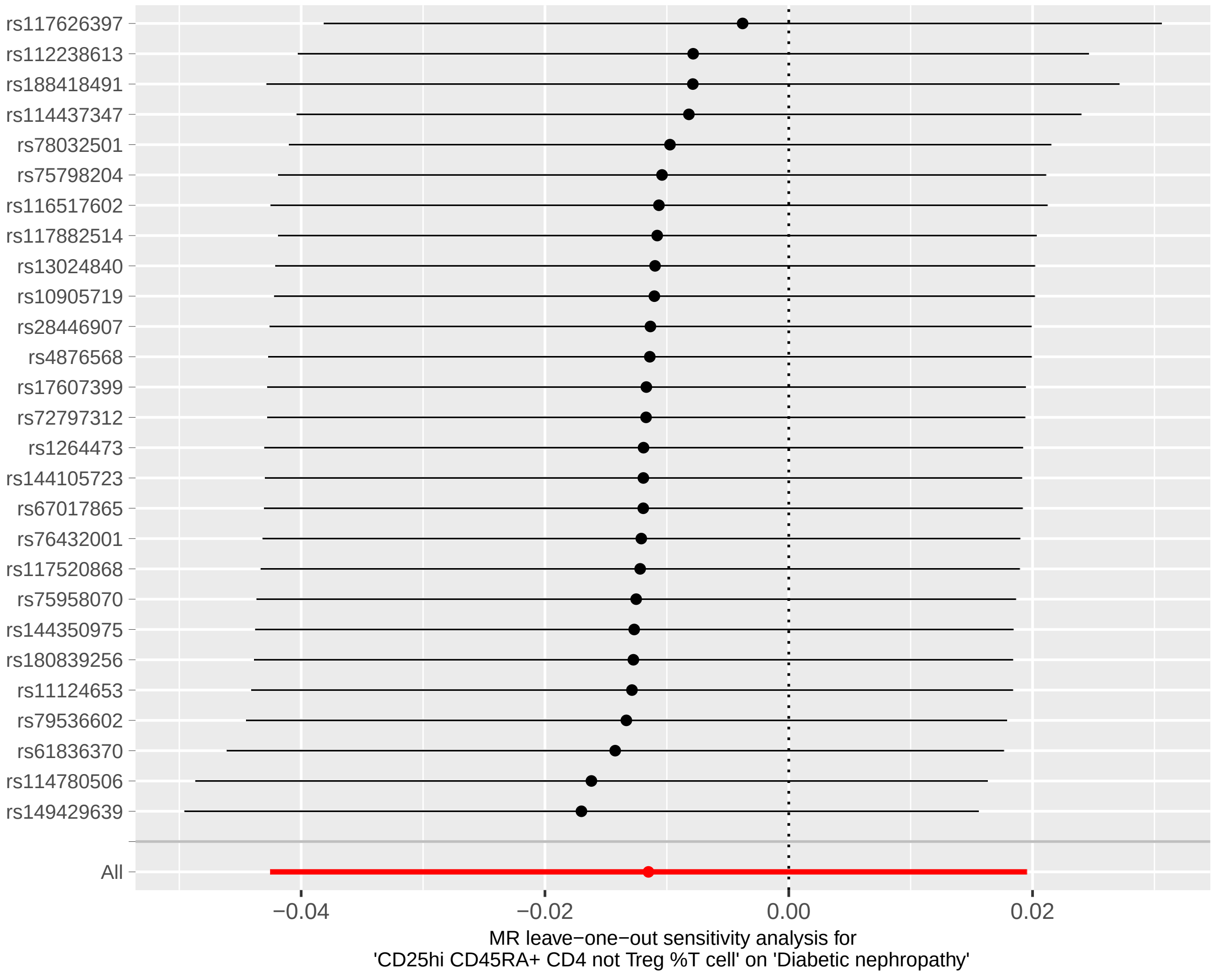
rs117626397
rs112238613
rs188418491
rs114437347
rs78032501
rs75798204
rs116517602
rs117882514
rs13024840
rs10905719
rs28446907
rs4876568
rs17607399
rs72797312
rs1264473
rs144105723
rs67017865
rs76432001
rs117520868
rs75958070
rs144350975
rs180839256
rs11124653
rs79536602
rs61836370
rs114780506
rs149429639
All
−0.04
−0.02
0.00
0.02
MR leave−one−out sensitivity analysis for
'CD25hi CD45RA+ CD4 not Treg %T cell' on 'Diabetic nephropathy'

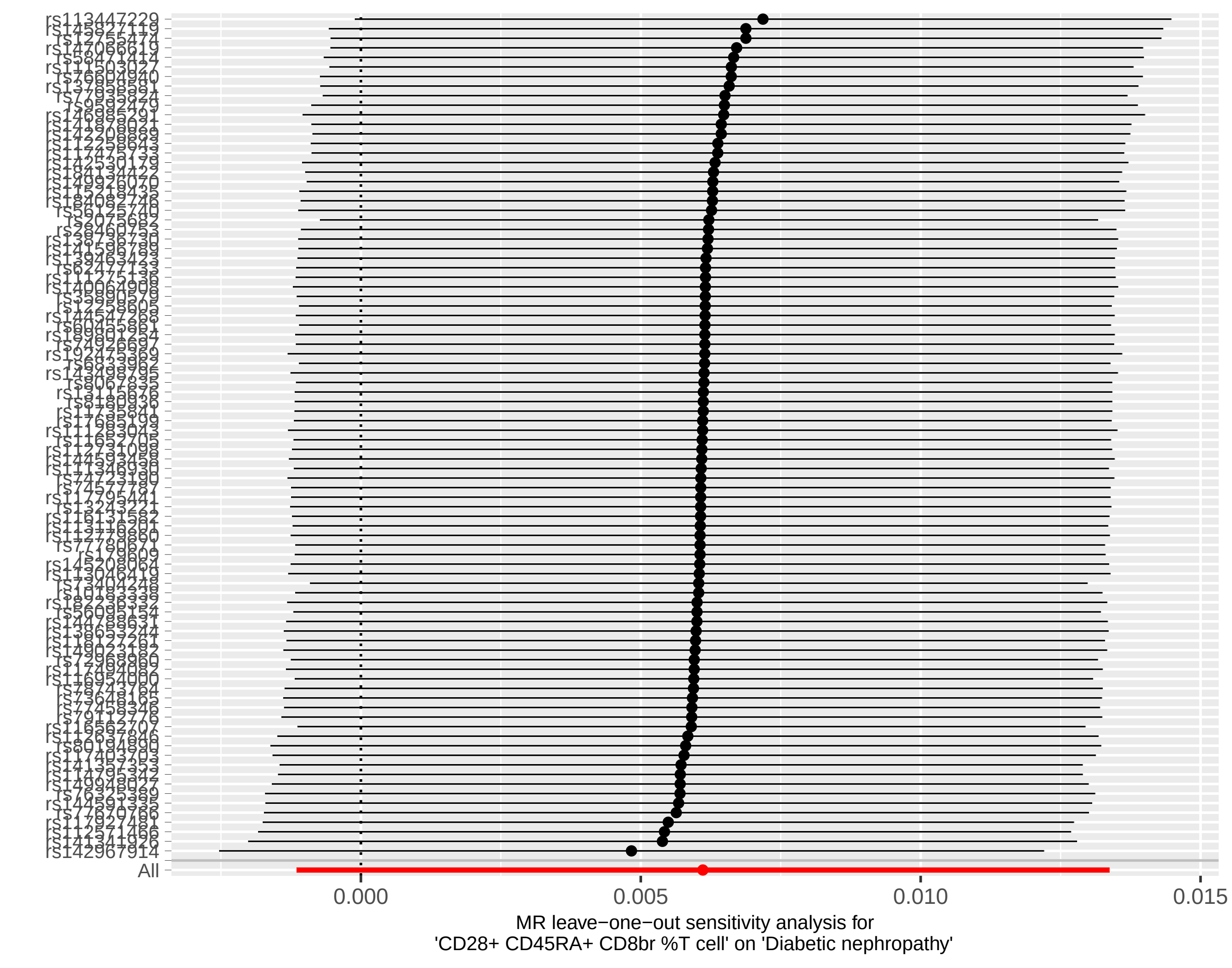
rs113447229
rs142967914
All
0.000
0.005
0.010
0.015
MR leave−one−out sensitivity analysis for
'CD28+ CD45RA+ CD8br %T cell' on 'Diabetic nephropathy'

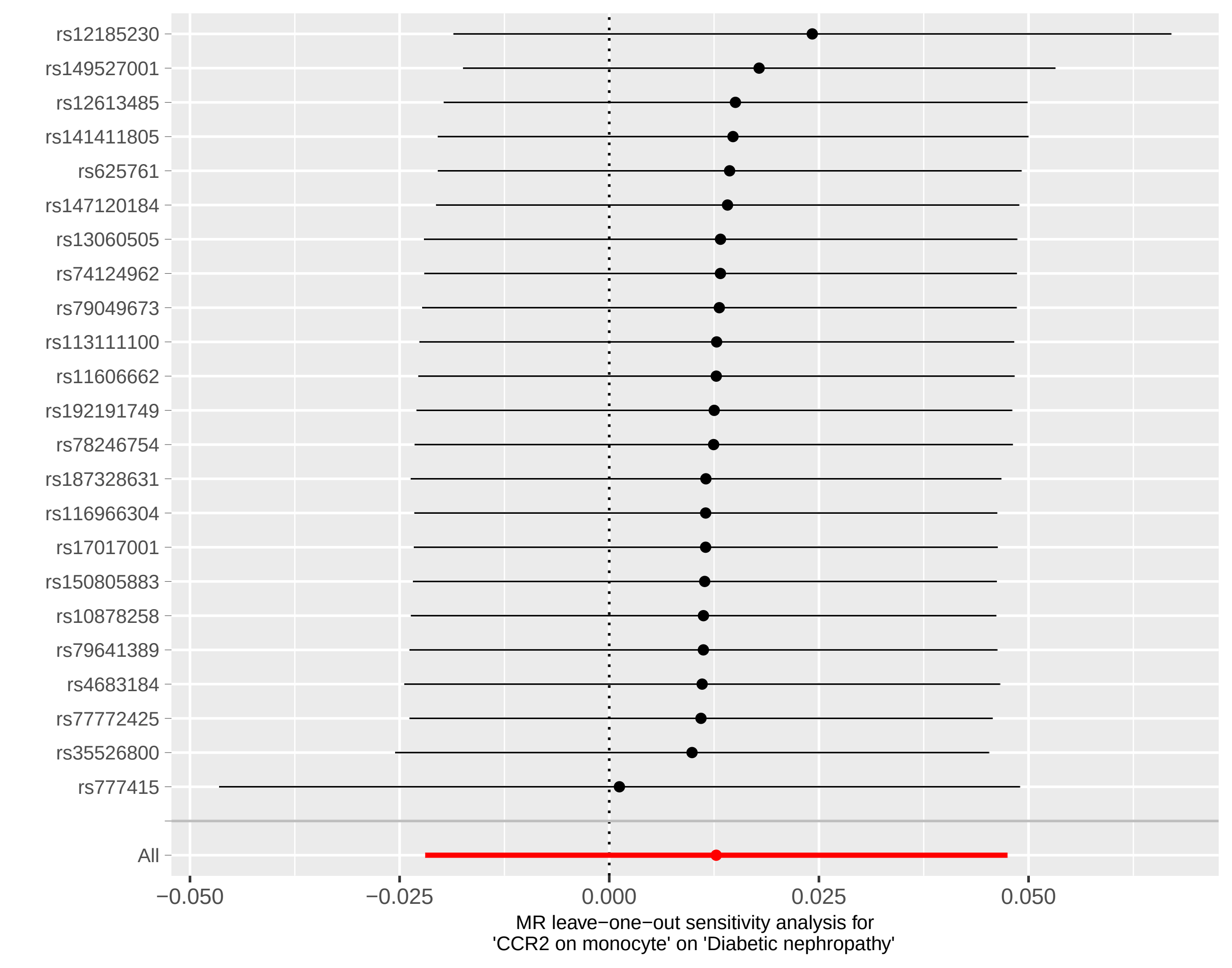
rs12185230
rs149527001
rs12613485
rs141411805
rs625761
rs147120184
rs13060505
rs74124962
rs79049673
rs113111100
rs11606662
rs192191749
rs78246754
rs187328631
rs116966304
rs17017001
rs150805883
rs10878258
rs79641389
rs4683184
rs77772425
rs35526800
rs777415
All
−0.050
−0.025
0.000
0.025
0.050
MR leave−one−out sensitivity analysis for
'CCR2 on monocyte' on 'Diabetic nephropathy'

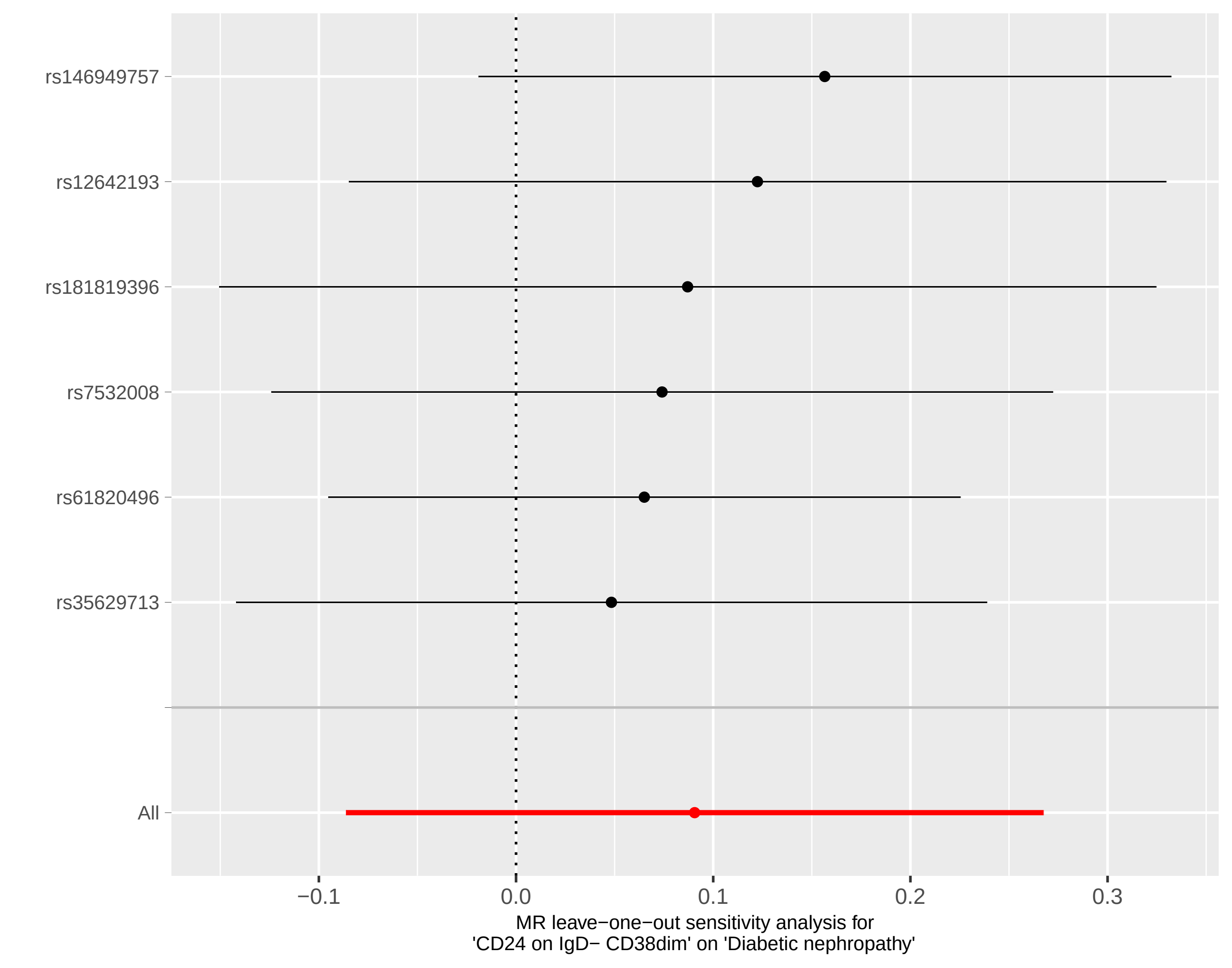
rs146949757
rs12642193
rs181819396
rs7532008
rs61820496
rs35629713
All
−0.1
0.0
0.1
0.2
0.3
MR leave−one−out sensitivity analysis for
'CD24 on IgD− CD38dim' on 'Diabetic nephropathy'

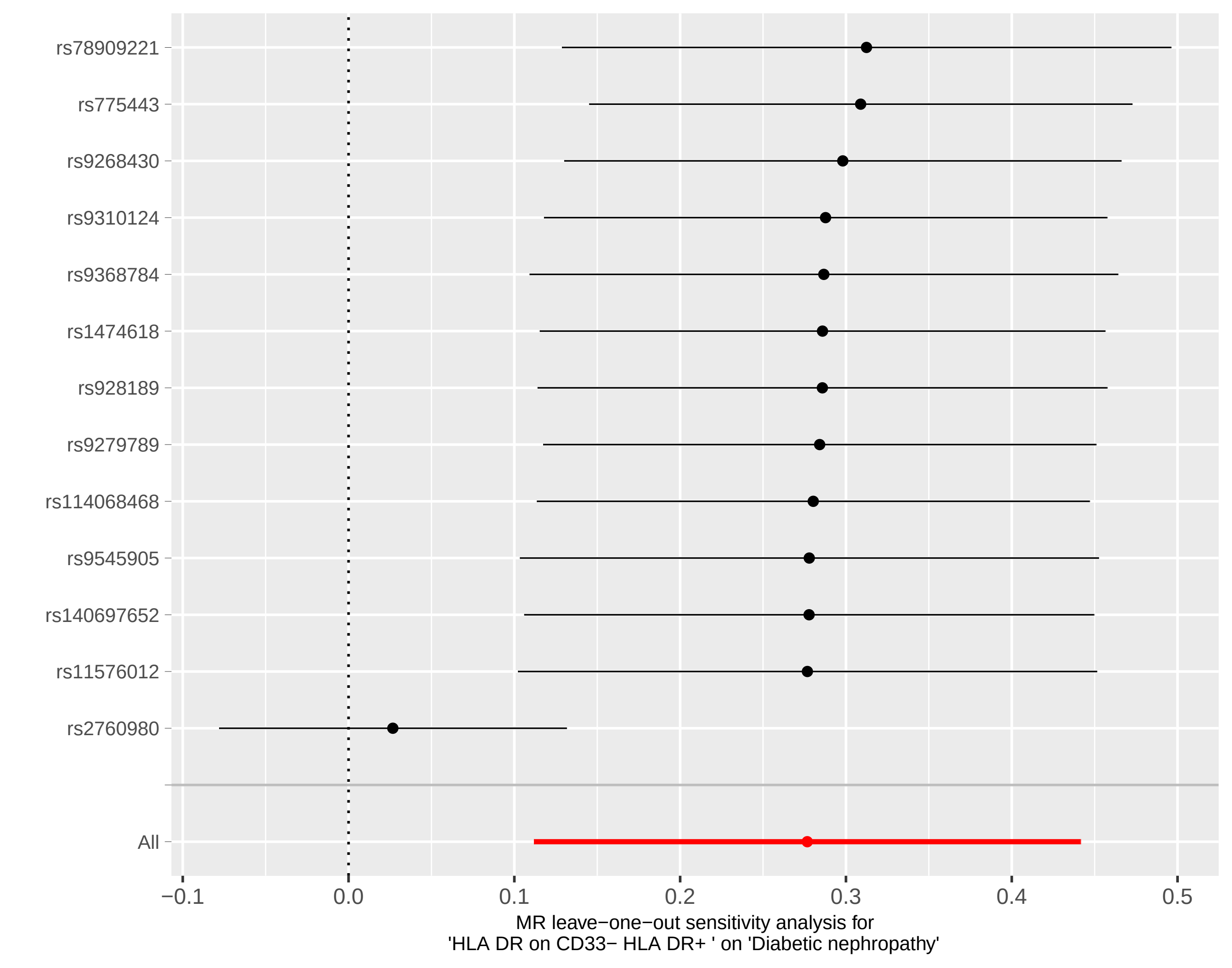
rs78909221
rs775443
rs9268430
rs9310124
rs9368784
rs1474618
rs928189
rs9279789
rs114068468
rs9545905
rs140697652
rs11576012
rs2760980
All
−0.1
0.0
0.1
0.2
0.3
0.4
0.5
MR leave−one−out sensitivity analysis for
'HLA DR on CD33− HLA DR+ ' on 'Diabetic nephropathy'

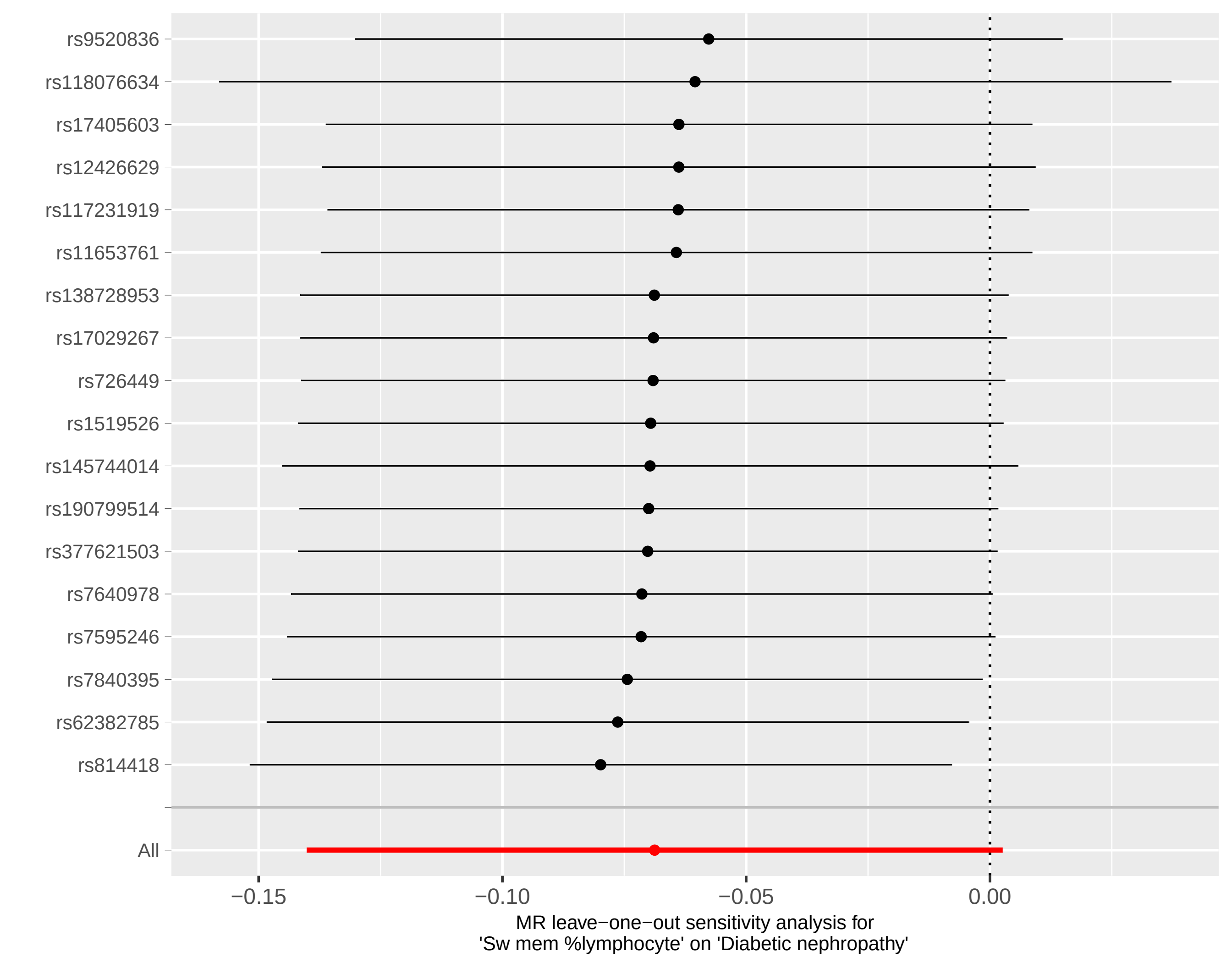
rs9520836
rs118076634
rs17405603
rs12426629
rs117231919
rs11653761
rs138728953
rs17029267
rs726449
rs1519526
rs145744014
rs190799514
rs377621503
rs7640978
rs7595246
rs7840395
rs62382785
rs814418
All
−0.15
−0.10
−0.05
0.00
MR leave−one−out sensitivity analysis for
'Sw mem %lymphocyte' on 'Diabetic nephropathy'

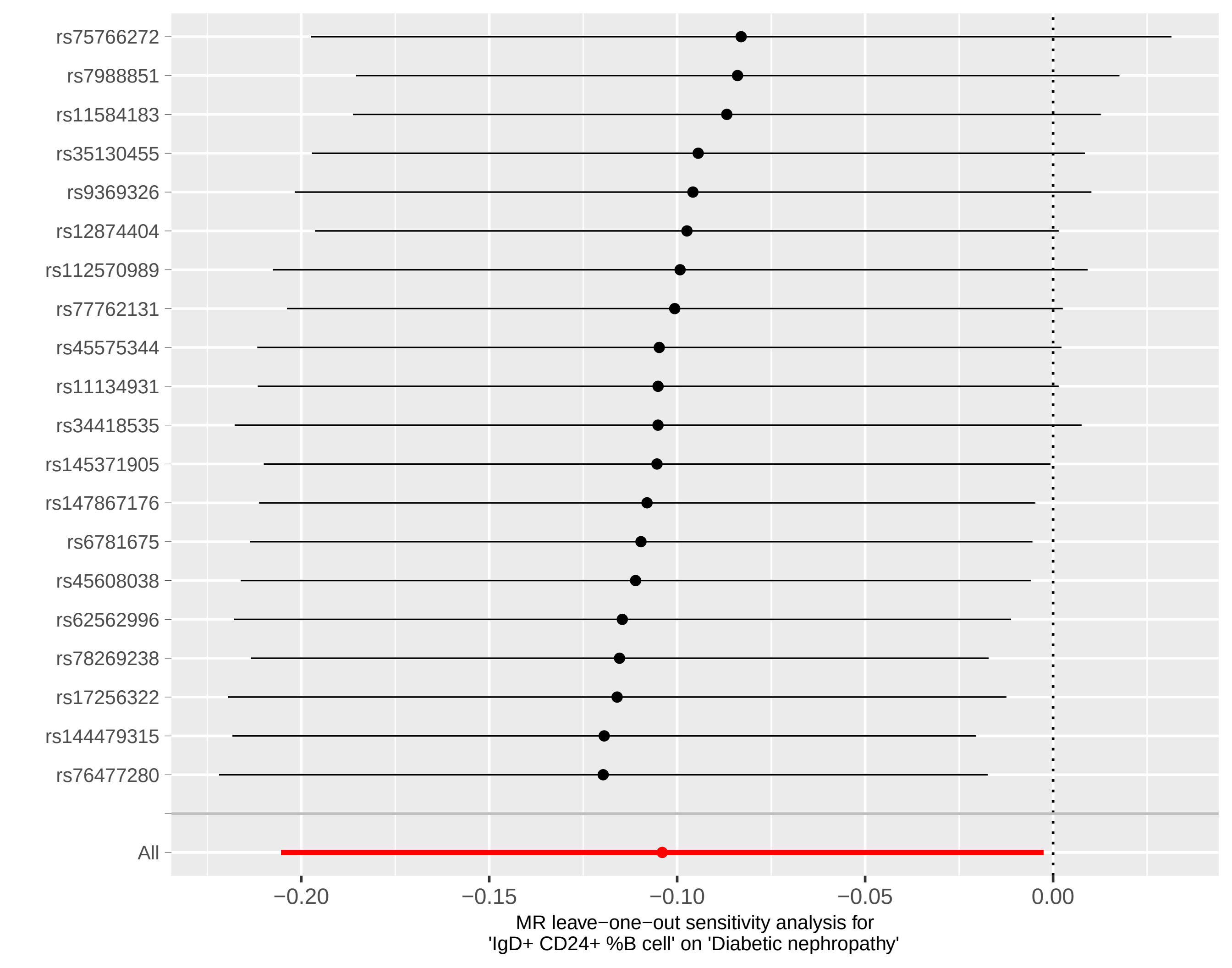
rs75766272
rs7988851
rs11584183
rs35130455
rs9369326
rs12874404
rs112570989
rs77762131
rs45575344
rs11134931
rs34418535
rs145371905
rs147867176
rs6781675
rs45608038
rs62562996
rs78269238
rs17256322
rs144479315
rs76477280
All
−0.20
−0.15
−0.10
−0.05
0.00
MR leave−one−out sensitivity analysis for
'IgD+ CD24+ %B cell' on 'Diabetic nephropathy'

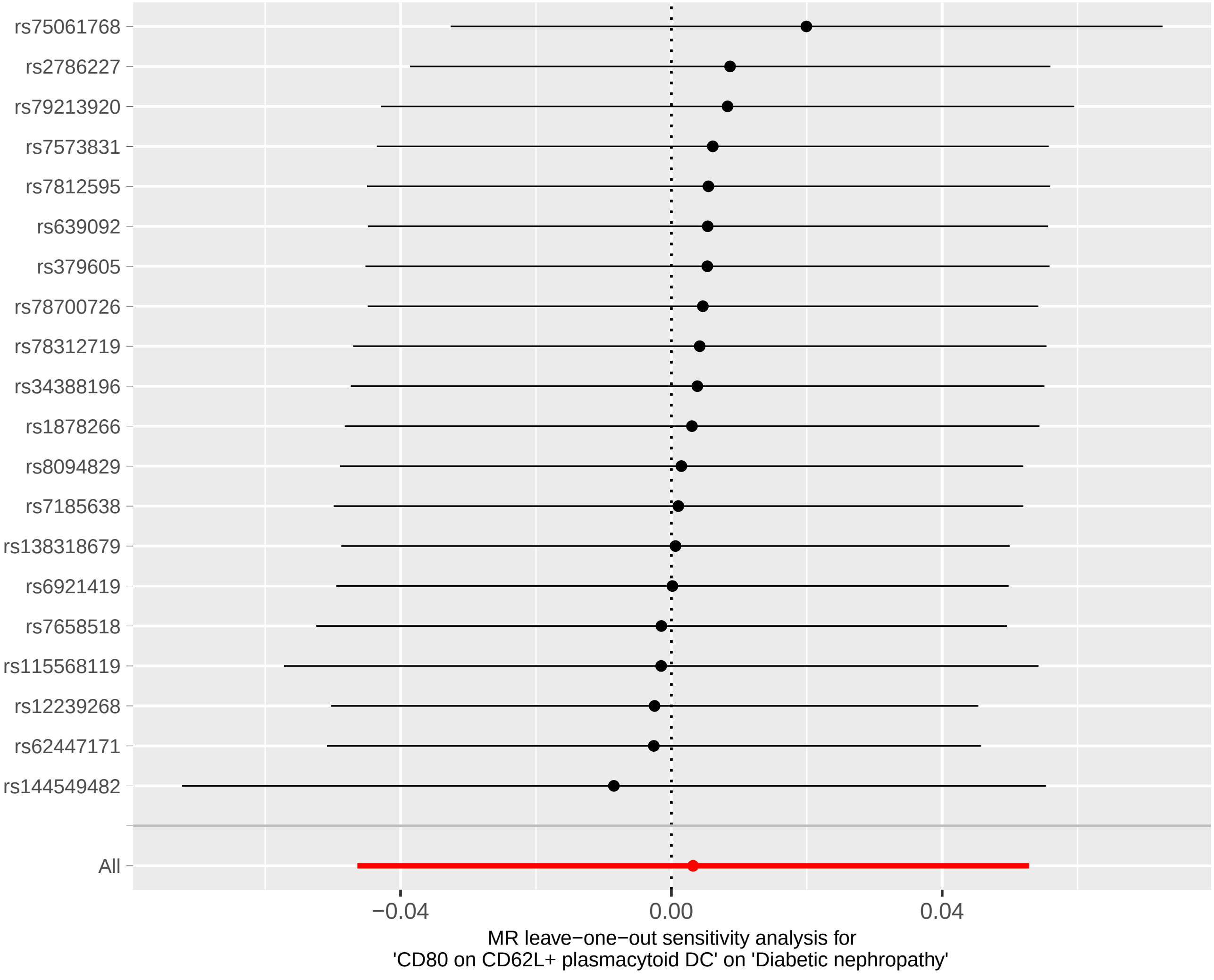
rs75061768
rs2786227
rs79213920
rs7573831
rs7812595
rs639092
rs379605
rs78700726
rs78312719
rs34388196
rs1878266
rs8094829
rs7185638
rs138318679
rs6921419
rs7658518
rs115568119
rs12239268
rs62447171
rs144549482
All
−0.04
0.00
0.04
MR leave−one−out sensitivity analysis for
'CD80 on CD62L+ plasmacytoid DC' on 'Diabetic nephropathy'

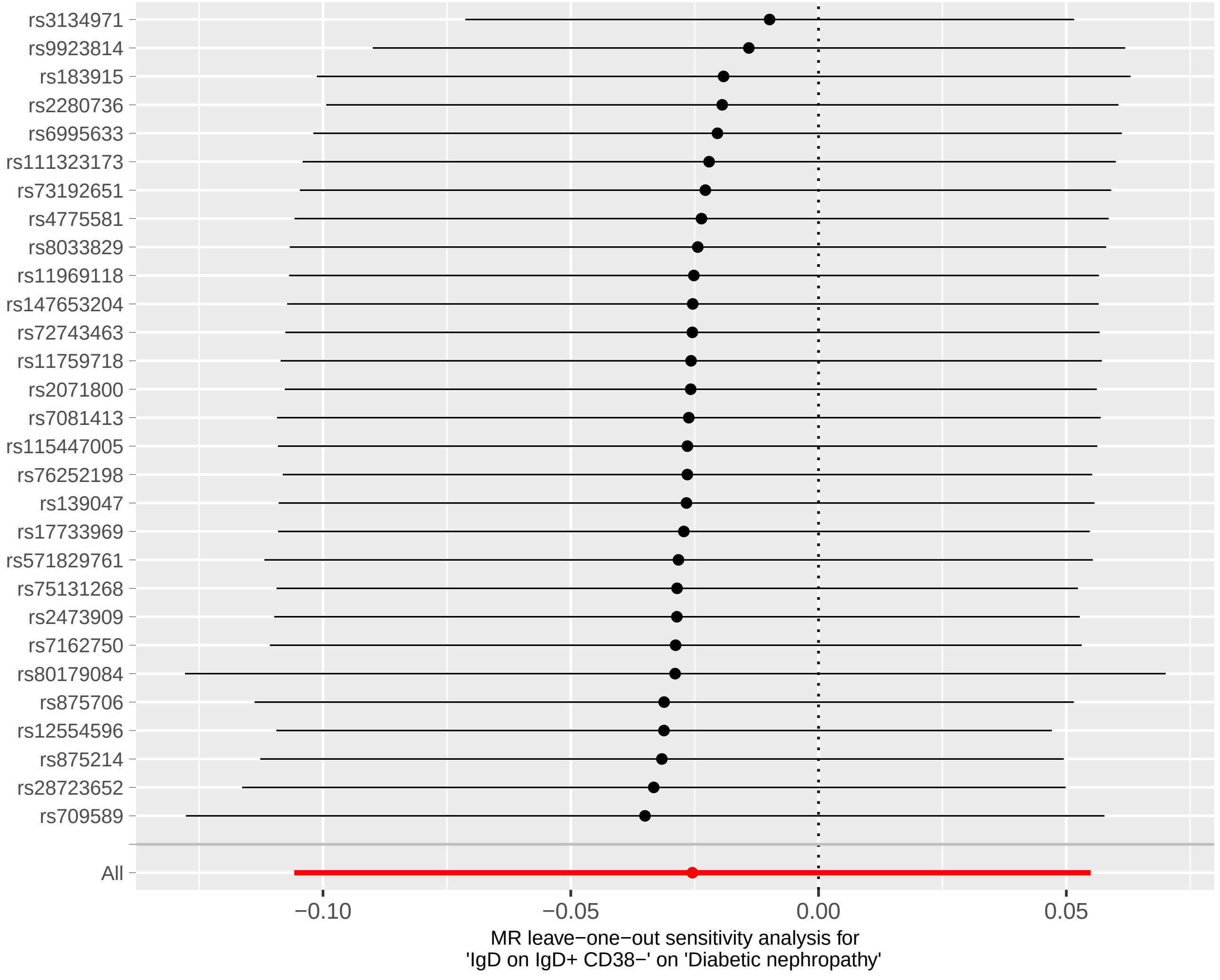

rs3134971
rs9923814
rs183915
rs2280736
rs6995633
rs111323173
rs73192651
rs4775581
rs8033829
rs11969118
rs147653204
rs72743463
rs11759718
rs2071800
rs7081413
rs115447005
rs76252198
rs139047
rs17733969
rs571829761
rs75131268
rs2473909
rs7162750
rs80179084
rs875706
rs12554596
rs875214
rs28723652
rs709589
All
−0.10
−0.05
0.00
0.05
MR leave−one−out sensitivity analysis for
'IgD on IgD+ CD38−' on 'Diabetic nephropathy'

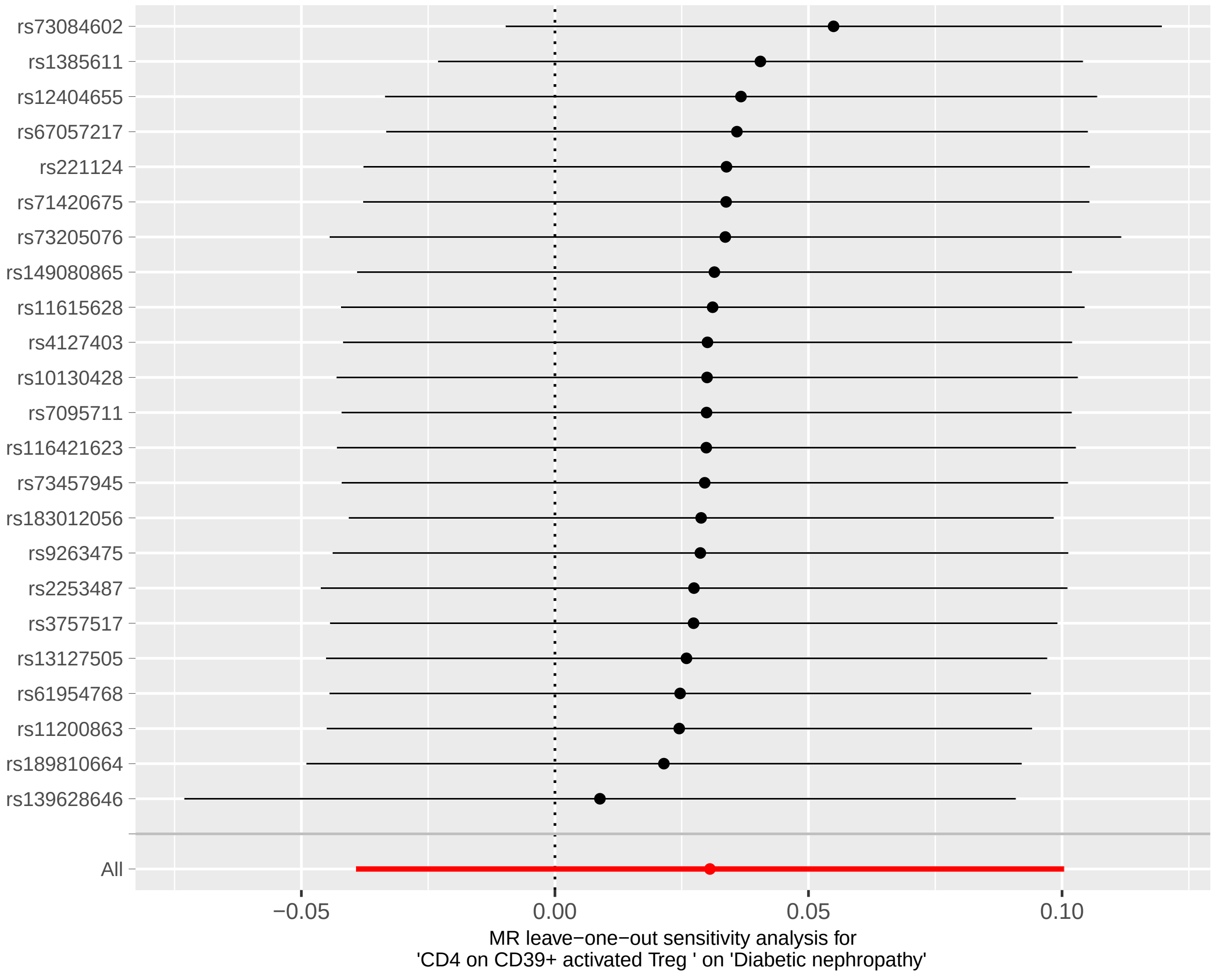

rs73084602
rs1385611
rs12404655
rs67057217
rs221124
rs71420675
rs73205076
rs149080865
rs11615628
rs4127403
rs10130428
rs7095711
rs116421623
rs73457945
rs183012056
rs9263475
rs2253487
rs3757517
rs13127505
rs61954768
rs11200863
rs189810664
rs139628646
All
−0.05
0.00
0.05
0.10
MR leave−one−out sensitivity analysis for
'CD4 on CD39+ activated Treg ' on 'Diabetic nephropathy'

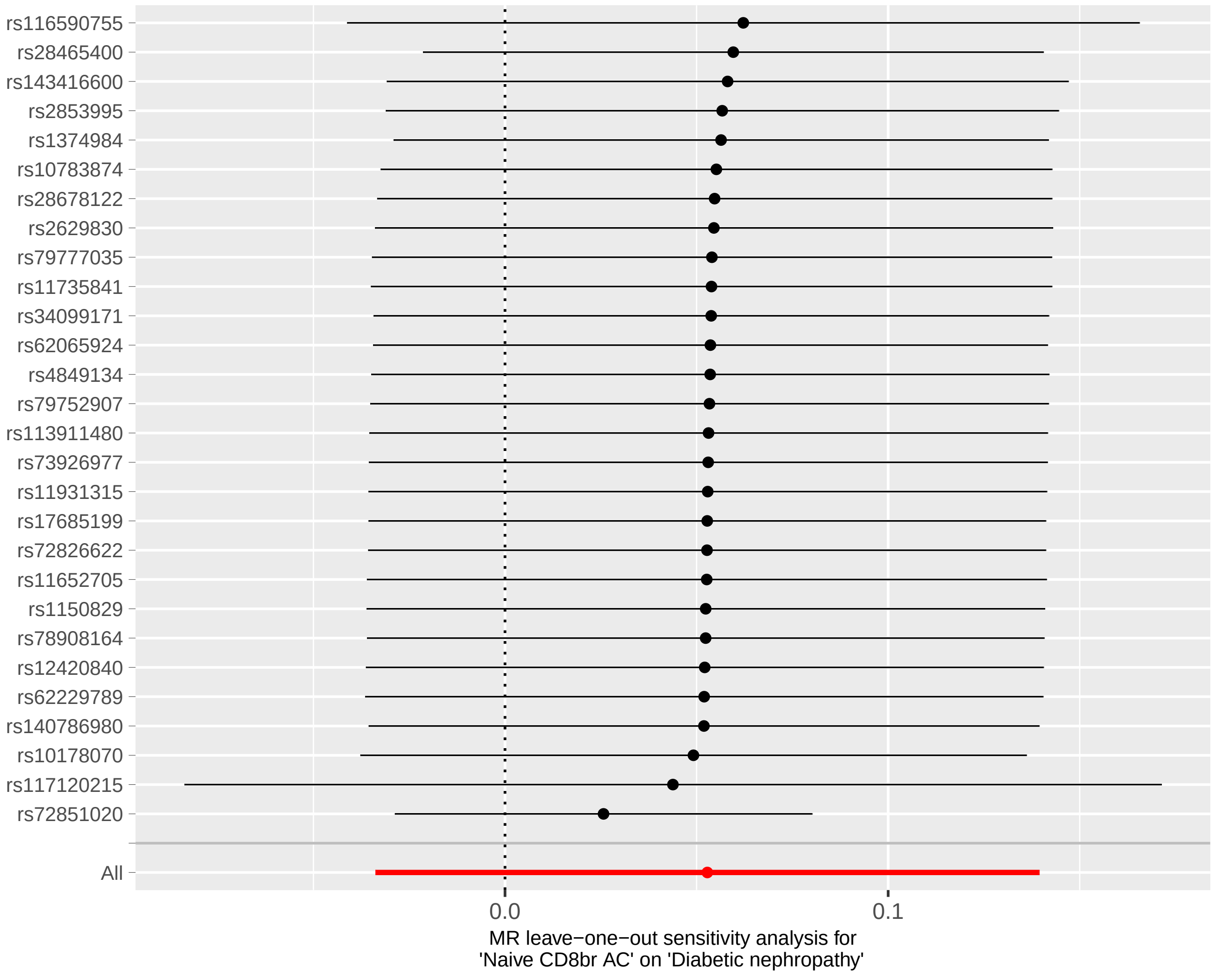

rs116590755
rs28465400
rs143416600
rs2853995
rs1374984
rs10783874
rs28678122
rs2629830
rs79777035
rs11735841
rs34099171
rs62065924
rs4849134
rs79752907
rs113911480
rs73926977
rs11931315
rs17685199
rs72826622
rs11652705
rs1150829
rs78908164
rs12420840
rs62229789
rs140786980
rs10178070
rs117120215
rs72851020
All
0.0
0.1
MR leave−one−out sensitivity analysis for
'Naive CD8br AC' on 'Diabetic nephropathy'

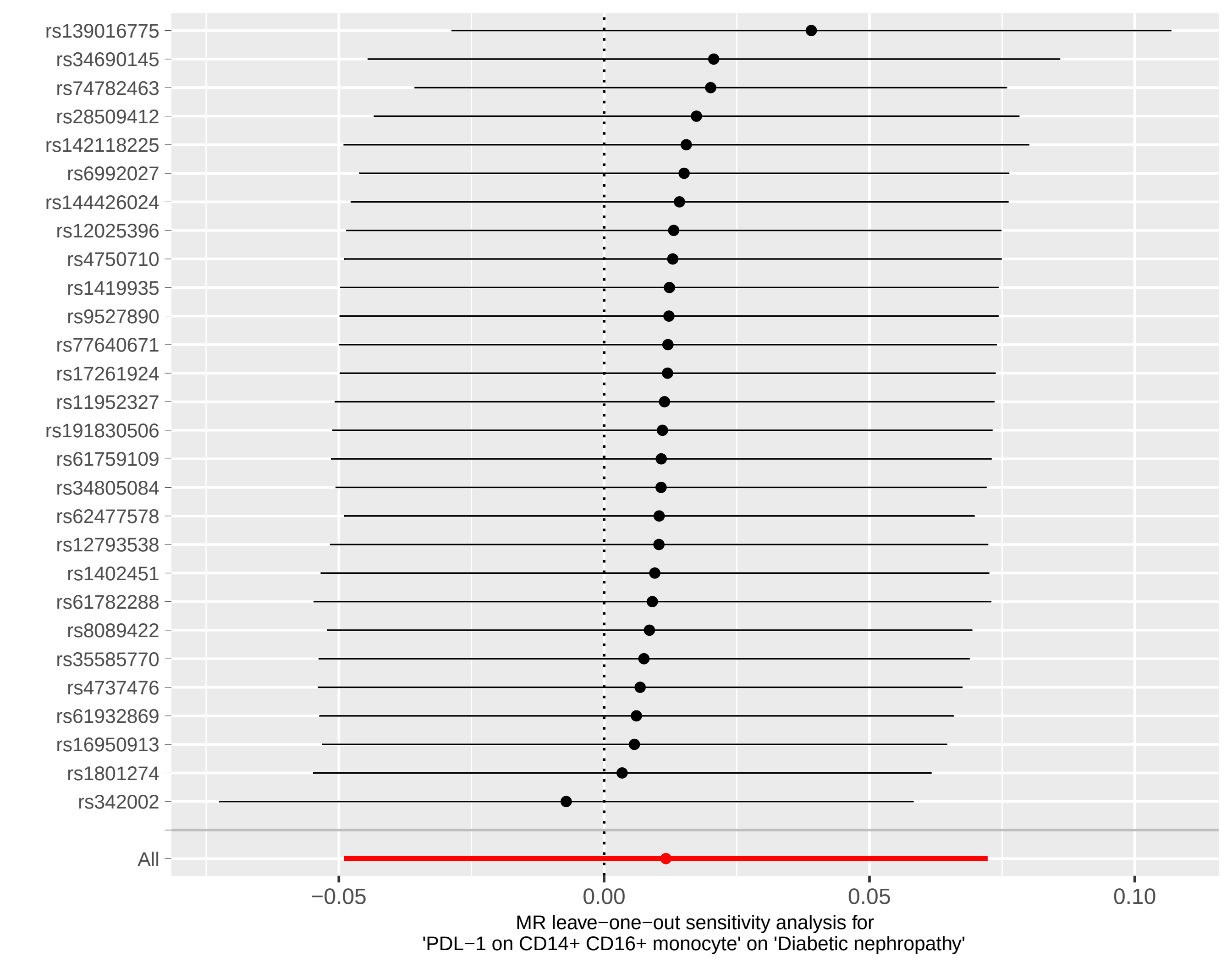
rs139016775
rs34690145
rs74782463
rs28509412
rs142118225
rs6992027
rs144426024
rs12025396
rs4750710
rs1419935
rs9527890
rs77640671
rs17261924
rs11952327
rs191830506
rs61759109
rs34805084
rs62477578
rs12793538
rs1402451
rs61782288
rs8089422
rs35585770
rs4737476
rs61932869
rs16950913
rs1801274
rs342002
All
−0.05
0.00
0.05
0.10
MR leave−one−out sensitivity analysis for
'PDL−1 on CD14+ CD16+ monocyte' on 'Diabetic nephropathy'

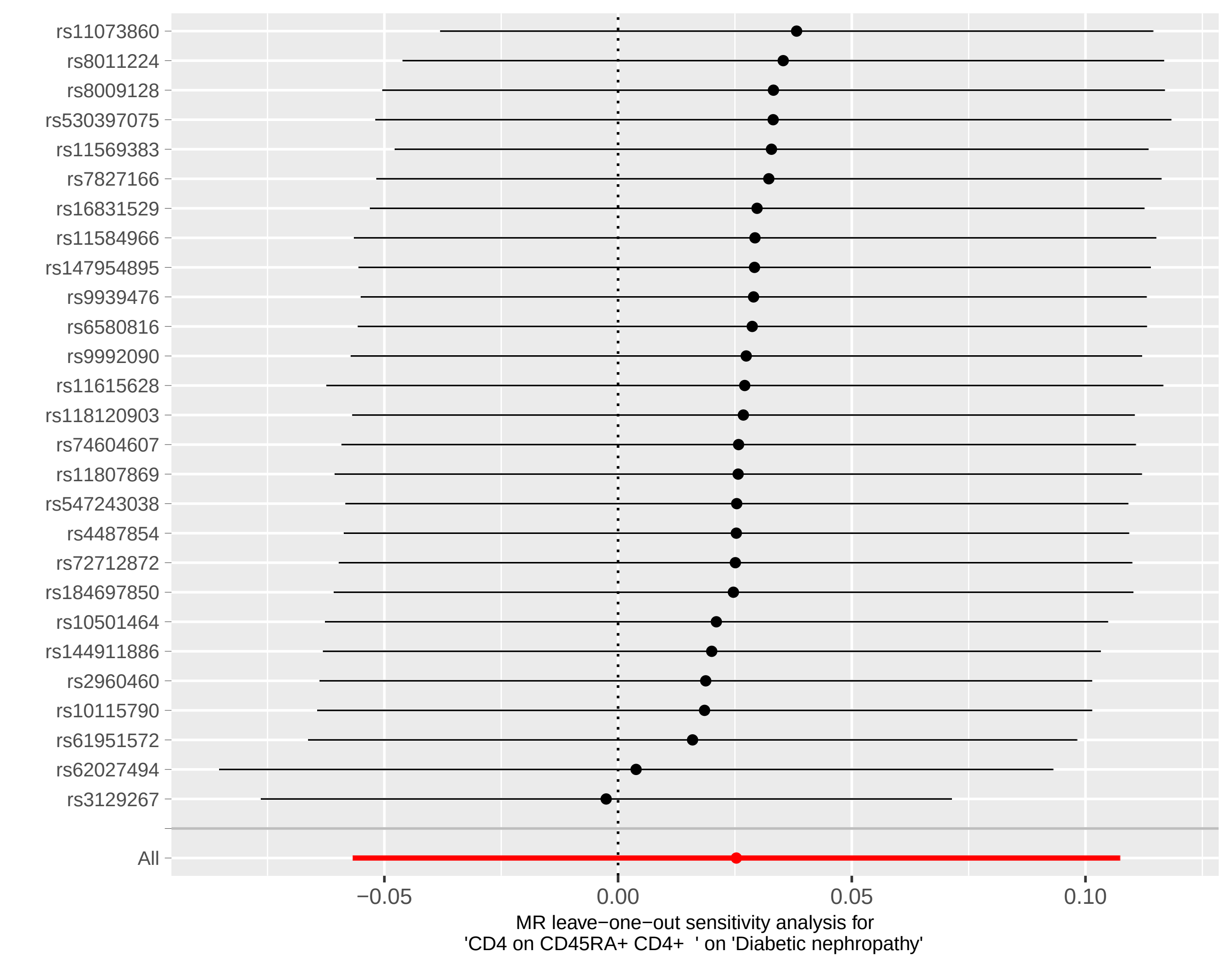
rs11073860
rs8011224
rs8009128
rs530397075
rs11569383
rs7827166
rs16831529
rs11584966
rs147954895
rs9939476
rs6580816
rs9992090
rs11615628
rs118120903
rs74604607
rs11807869
rs547243038
rs4487854
rs72712872
rs184697850
rs10501464
rs144911886
rs2960460
rs10115790
rs61951572
rs62027494
rs3129267
All
−0.05
0.00
0.05
0.10
MR leave−one−out sensitivity analysis for
'CD4 on CD45RA+ CD4+ ' on 'Diabetic nephropathy'

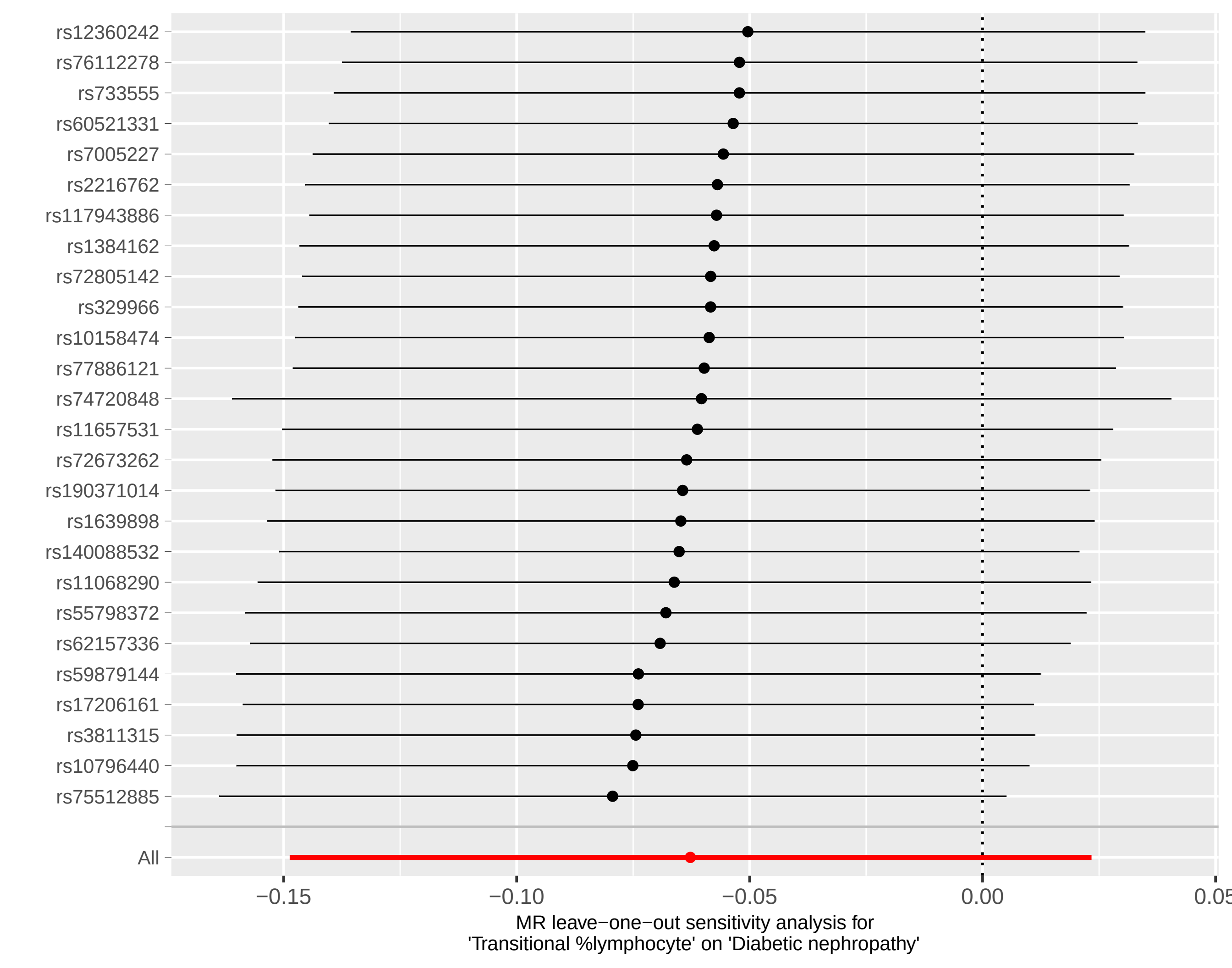

rs12360242
rs76112278
rs733555
rs60521331
rs7005227
rs2216762
rs117943886
rs1384162
rs72805142
rs329966
rs10158474
rs77886121
rs74720848
rs11657531
rs72673262
rs190371014
rs1639898
rs140088532
rs11068290
rs55798372
rs62157336
rs59879144
rs17206161
rs3811315
rs10796440
rs75512885
All
−0.15
−0.10
−0.05
0.00
MR leave−one−out sensitivity analysis for
'Transitional %lymphocyte' on 'Diabetic nephropathy'

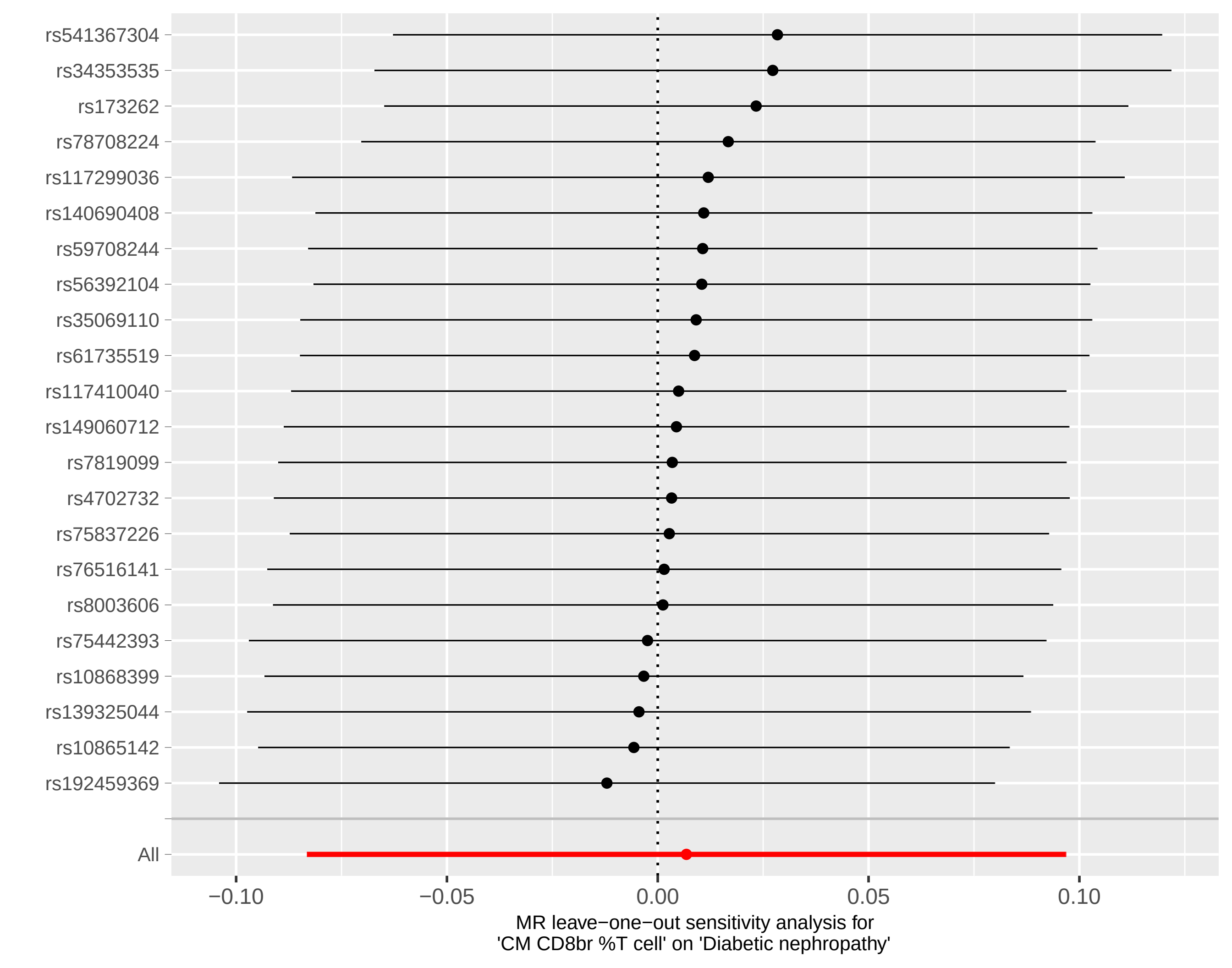
rs541367304
rs34353535
rs173262
rs78708224
rs117299036
rs140690408
rs59708244
rs56392104
rs35069110
rs61735519
rs117410040
rs149060712
rs7819099
rs4702732
rs75837226
rs76516141
rs8003606
rs75442393
rs10868399
rs139325044
rs10865142
rs192459369
All
−0.10
−0.05
0.00
0.05
0.10
MR leave−one−out sensitivity analysis for
'CM CD8br %T cell' on 'Diabetic nephropathy'

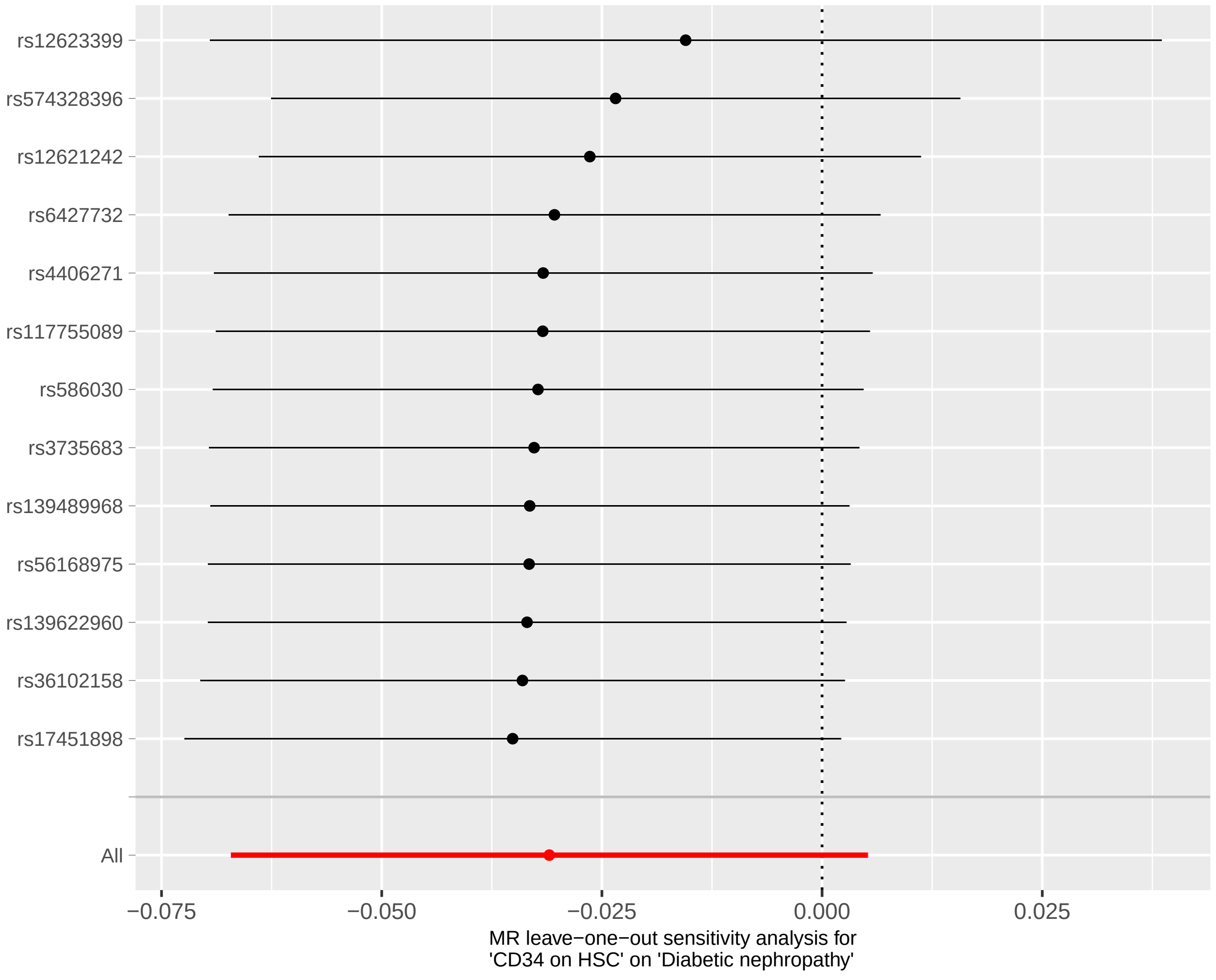

rs12623399
rs574328396
rs12621242
rs6427732
rs4406271
rs117755089
rs586030
rs3735683
rs139489968
rs56168975
rs139622960
rs36102158
rs17451898
All
−0.075
−0.050
−0.025
0.000
0.025
MR leave−one−out sensitivity analysis for
'CD34 on HSC' on 'Diabetic nephropathy'

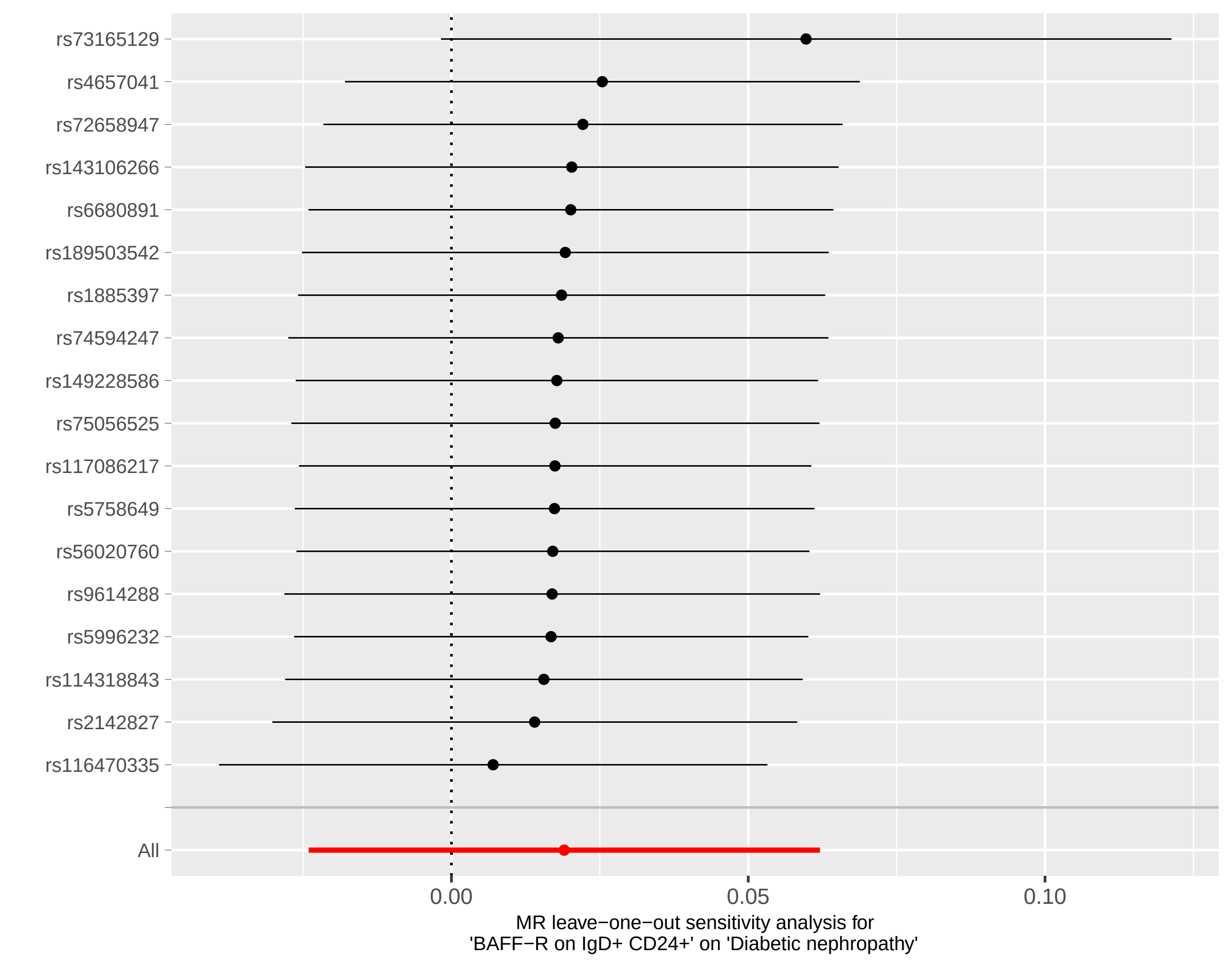

rs73165129
rs4657041
rs72658947
rs143106266
rs6680891
rs189503542
rs1885397
rs74594247
rs149228586
rs75056525
rs117086217
rs5758649
rs56020760
rs9614288
rs5996232
rs114318843
rs2142827
rs116470335
All
0.00
0.05
0.10
MR leave−one−out sensitivity analysis for
'BAFF−R on IgD+ CD24+' on 'Diabetic nephropathy'

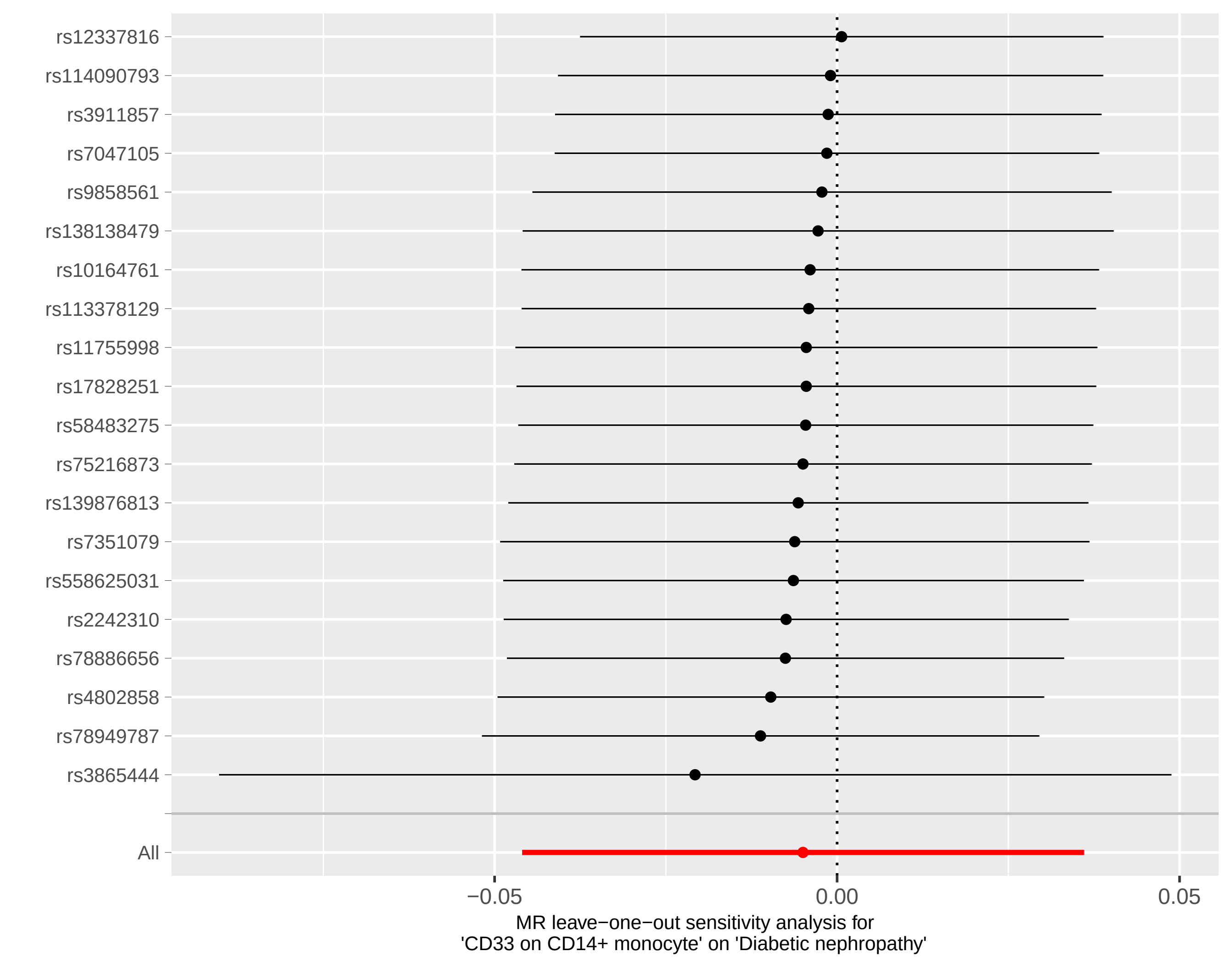

rs12337816
rs114090793
rs3911857
rs7047105
rs9858561
rs138138479
rs10164761
rs113378129
rs11755998
rs17828251
rs58483275
rs75216873
rs139876813
rs7351079
rs558625031
rs2242310
rs78886656
rs4802858
rs78949787
rs3865444
All
−0.05
0.00
0.05
MR leave−one−out sensitivity analysis for
'CD33 on CD14+ monocyte' on 'Diabetic nephropathy'

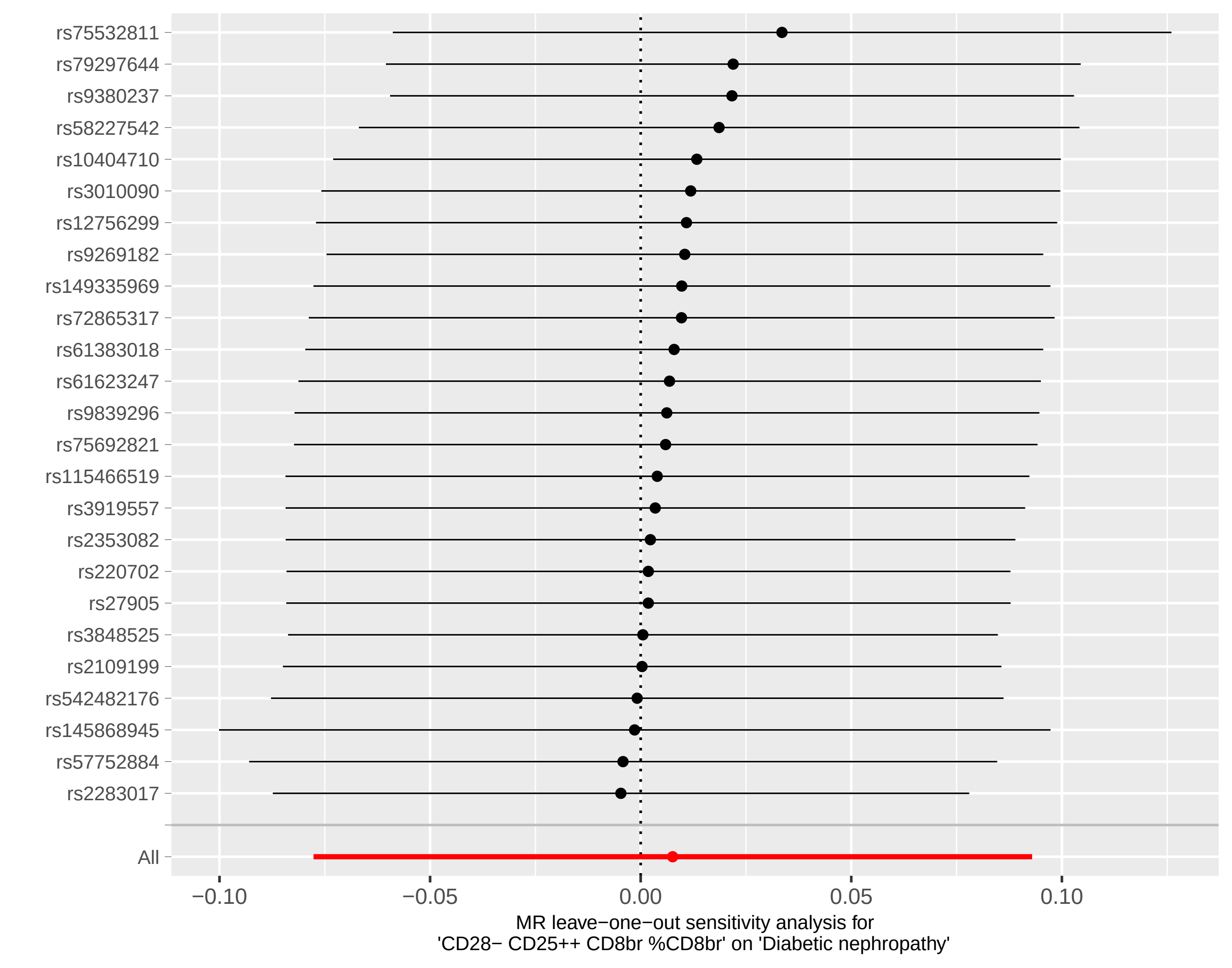

rs75532811
rs79297644
rs9380237
rs58227542
rs10404710
rs3010090
rs12756299
rs9269182
rs149335969
rs72865317
rs61383018
rs61623247
rs9839296
rs75692821
rs115466519
rs3919557
rs2353082
rs220702
rs27905
rs3848525
rs2109199
rs542482176
rs145868945
rs57752884
rs2283017
All
−0.10
−0.05
0.00
0.05
0.10
MR leave−one−out sensitivity analysis for
'CD28− CD25++ CD8br %CD8br' on 'Diabetic nephropathy'

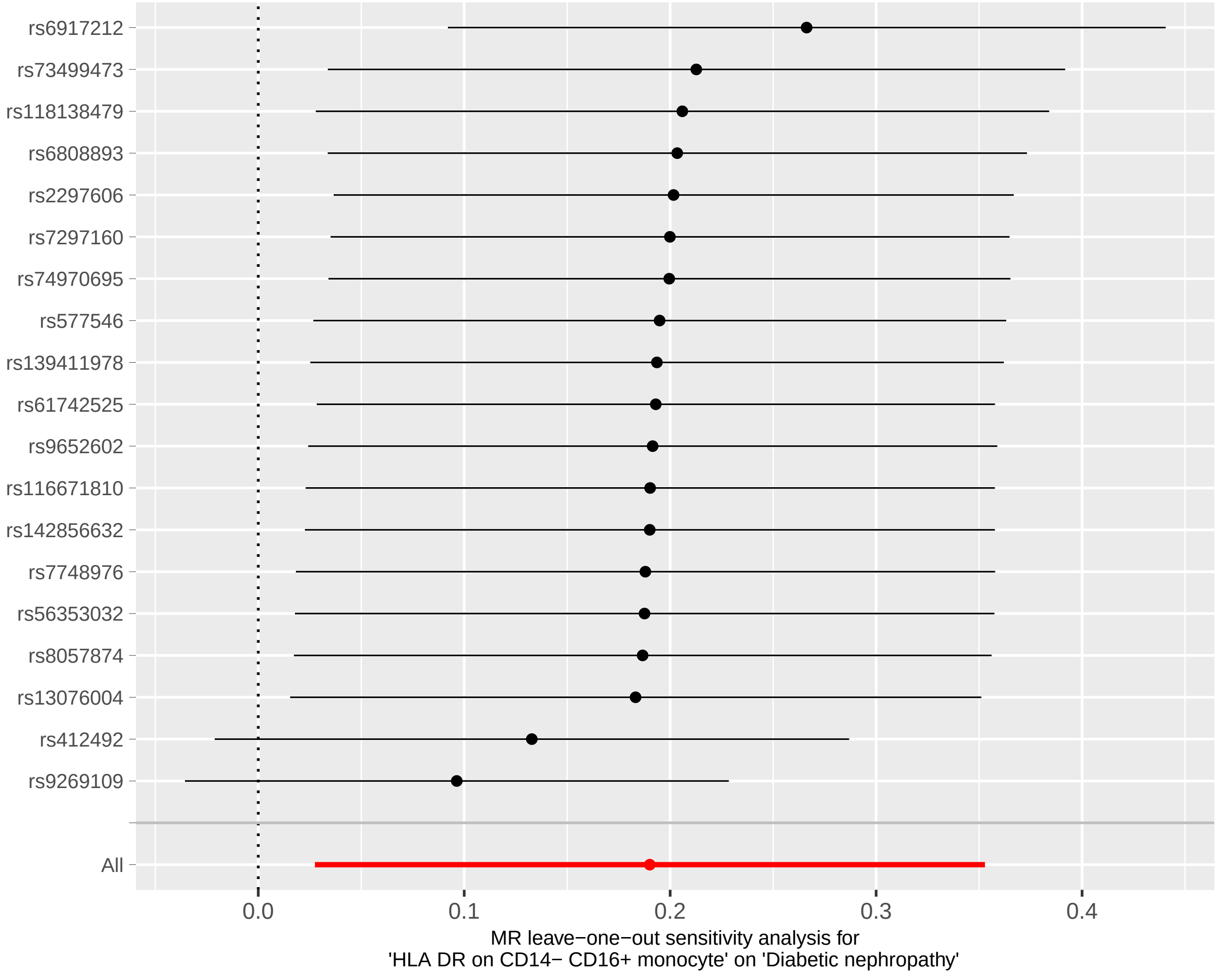
rs6917212
rs73499473
rs118138479
rs6808893
rs2297606
rs7297160
rs74970695
rs577546
rs139411978
rs61742525
rs9652602
rs116671810
rs142856632
rs7748976
rs56353032
rs8057874
rs13076004
rs412492
rs9269109
All
0.0
0.1
0.2
0.3
0.4
MR leave−one−out sensitivity analysis for
'HLA DR on CD14− CD16+ monocyte' on 'Diabetic nephropathy'

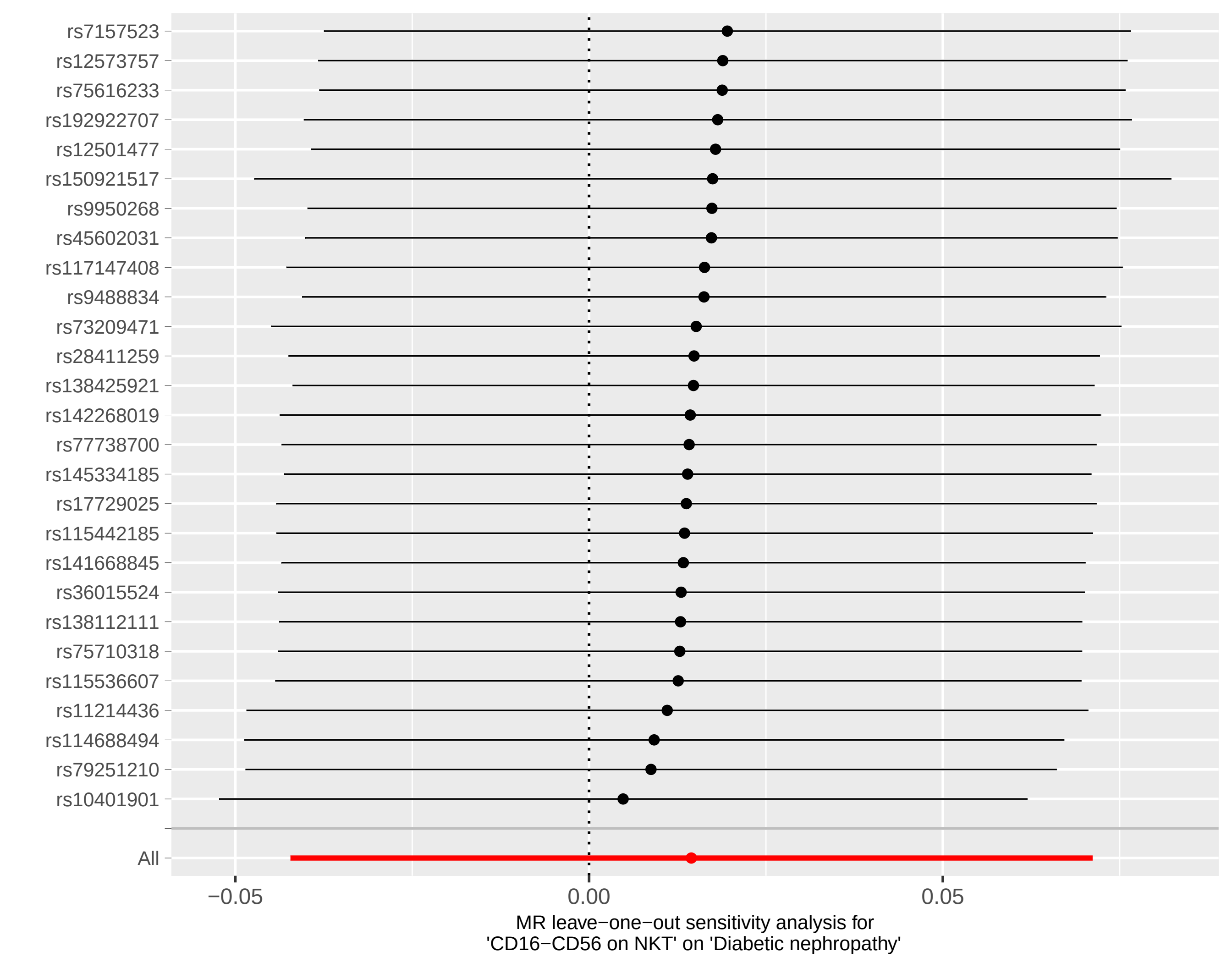
rs7157523
rs12573757
rs75616233
rs192922707
rs12501477
rs150921517
rs9950268
rs45602031
rs117147408
rs9488834
rs73209471
rs28411259
rs138425921
rs142268019
rs77738700
rs145334185
rs17729025
rs115442185
rs141668845
rs36015524
rs138112111
rs75710318
rs115536607
rs11214436
rs114688494
rs79251210
rs10401901
All
−0.05
0.00
0.05
MR leave−one−out sensitivity analysis for
'CD16−CD56 on NKT' on 'Diabetic nephropathy'

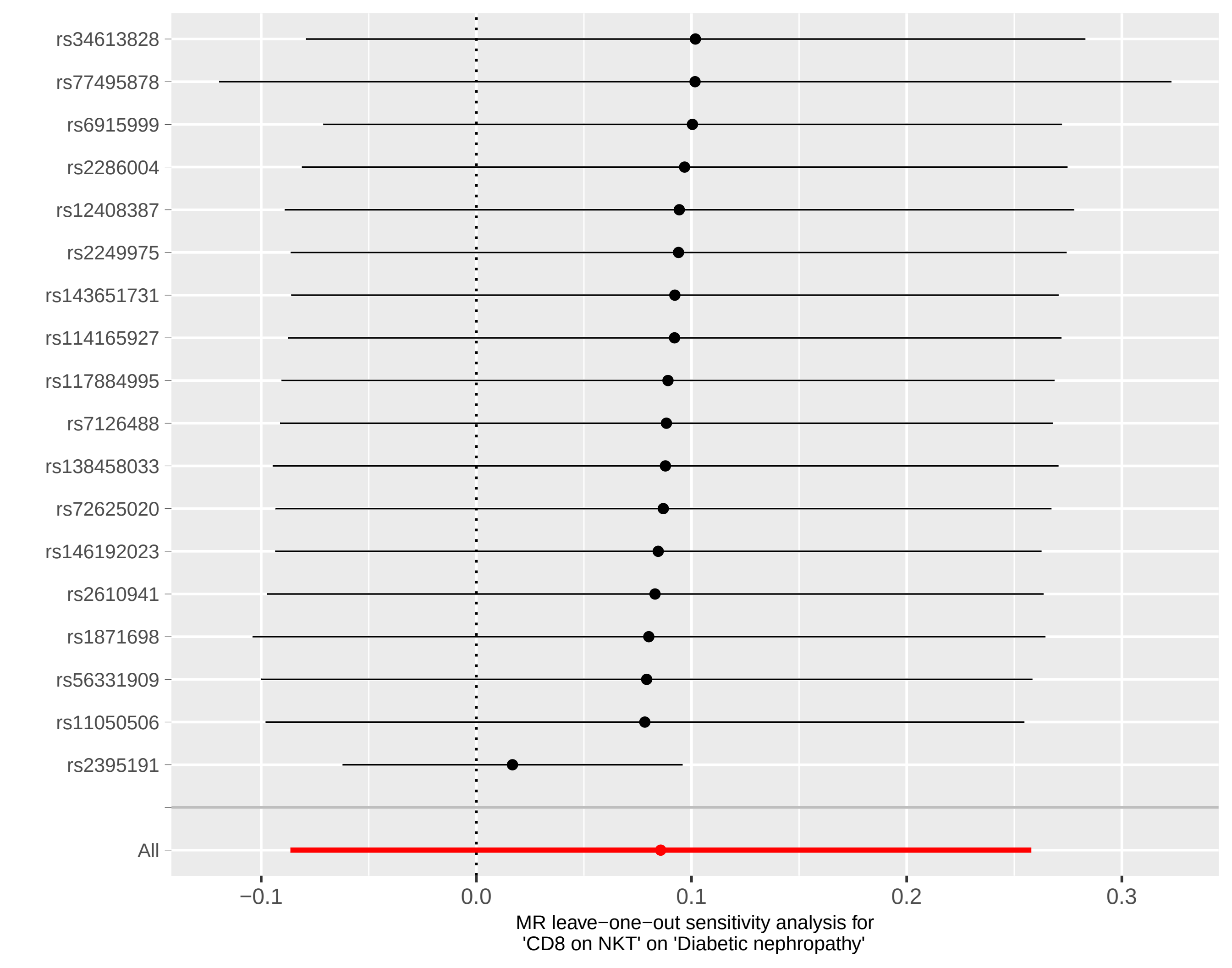

rs34613828
rs77495878
rs6915999
rs2286004
rs12408387
rs2249975
rs143651731
rs114165927
rs117884995
rs7126488
rs138458033
rs72625020
rs146192023
rs2610941
rs1871698
rs56331909
rs11050506
rs2395191
All
−0.1
0.0
0.1
0.2
0.3
MR leave−one−out sensitivity analysis for
'CD8 on NKT' on 'Diabetic nephropathy'

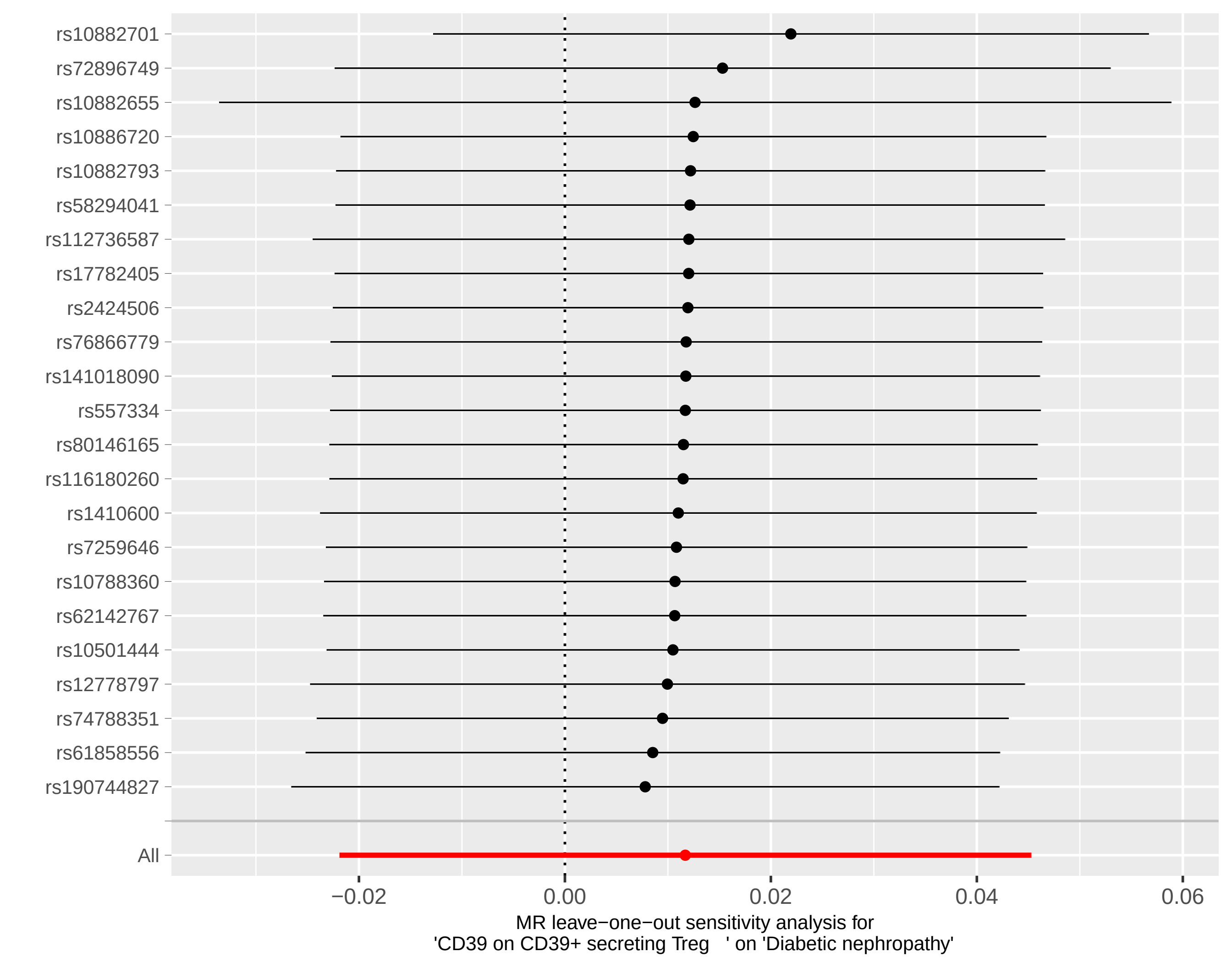

rs10882701
rs72896749
rs10882655
rs10886720
rs10882793
rs58294041
rs112736587
rs17782405
rs2424506
rs76866779
rs141018090
rs557334
rs80146165
rs116180260
rs1410600
rs7259646
rs10788360
rs62142767
rs10501444
rs12778797
rs74788351
rs61858556
rs190744827
All
−0.02
0.00
0.02
0.04
0.06
MR leave−one−out sensitivity analysis for
'CD39 on CD39+ secreting Treg ' on 'Diabetic nephropathy'

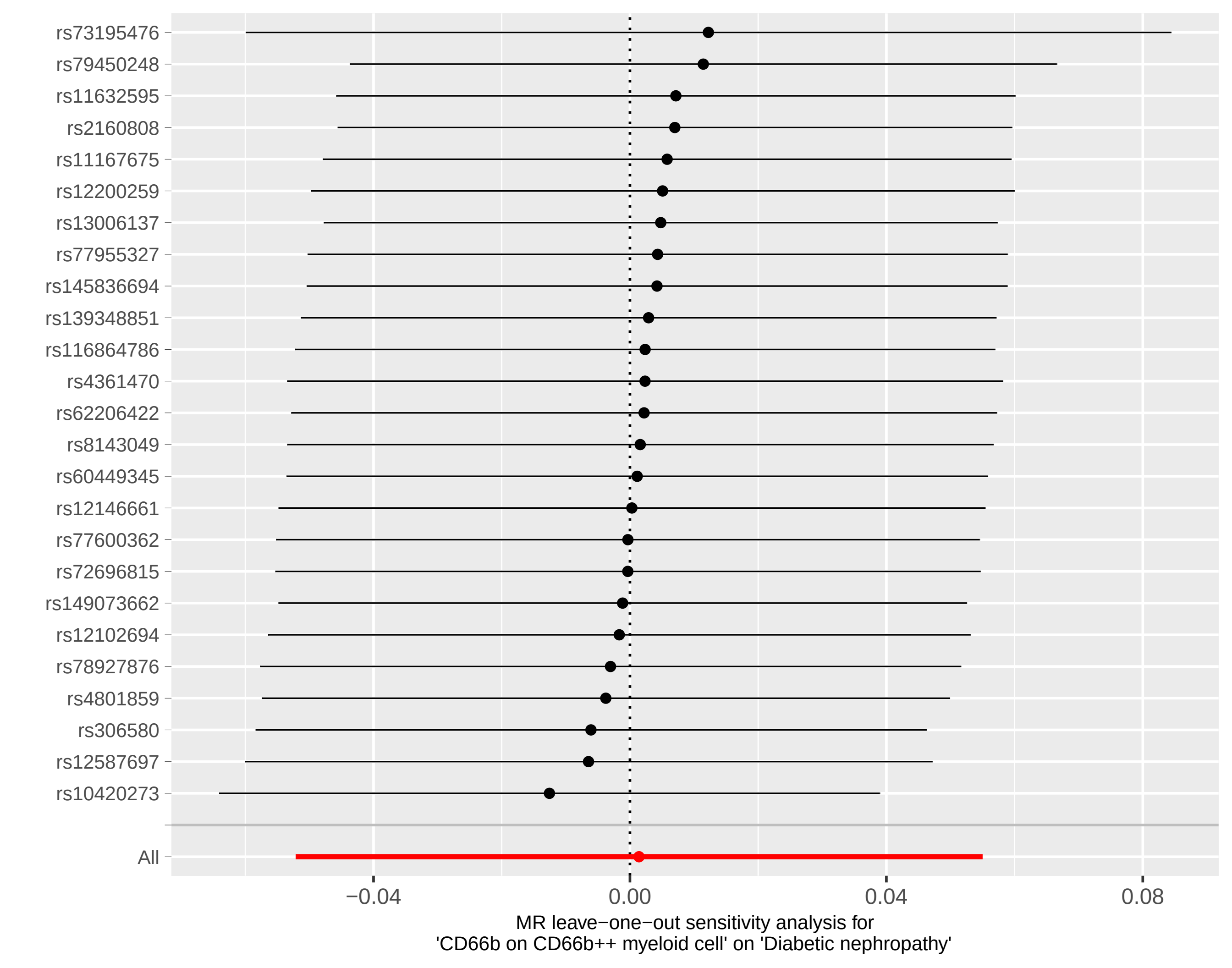
rs73195476
rs79450248
rs11632595
rs2160808
rs11167675
rs12200259
rs13006137
rs77955327
rs145836694
rs139348851
rs116864786
rs4361470
rs62206422
rs8143049
rs60449345
rs12146661
rs77600362
rs72696815
rs149073662
rs12102694
rs78927876
rs4801859
rs306580
rs12587697
rs10420273
All
−0.04
0.00
0.04
0.08
MR leave−one−out sensitivity analysis for
'CD66b on CD66b++ myeloid cell' on 'Diabetic nephropathy'

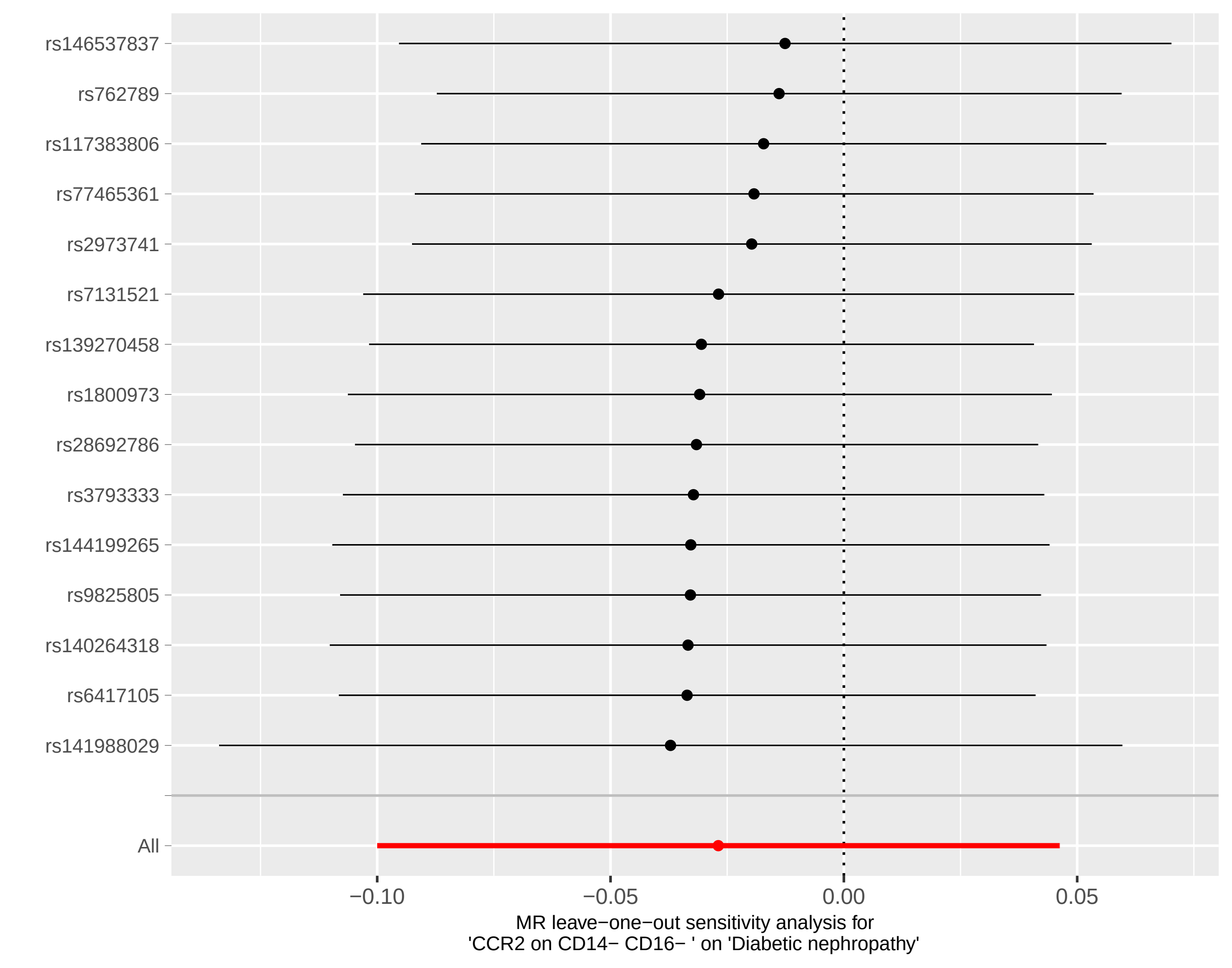
rs146537837
rs762789
rs117383806
rs77465361
rs2973741
rs7131521
rs139270458
rs1800973
rs28692786
rs3793333
rs144199265
rs9825805
rs140264318
rs6417105
rs141988029
All
−0.10
−0.05
0.00
0.05
MR leave−one−out sensitivity analysis for
'CCR2 on CD14− CD16− ' on 'Diabetic nephropathy'

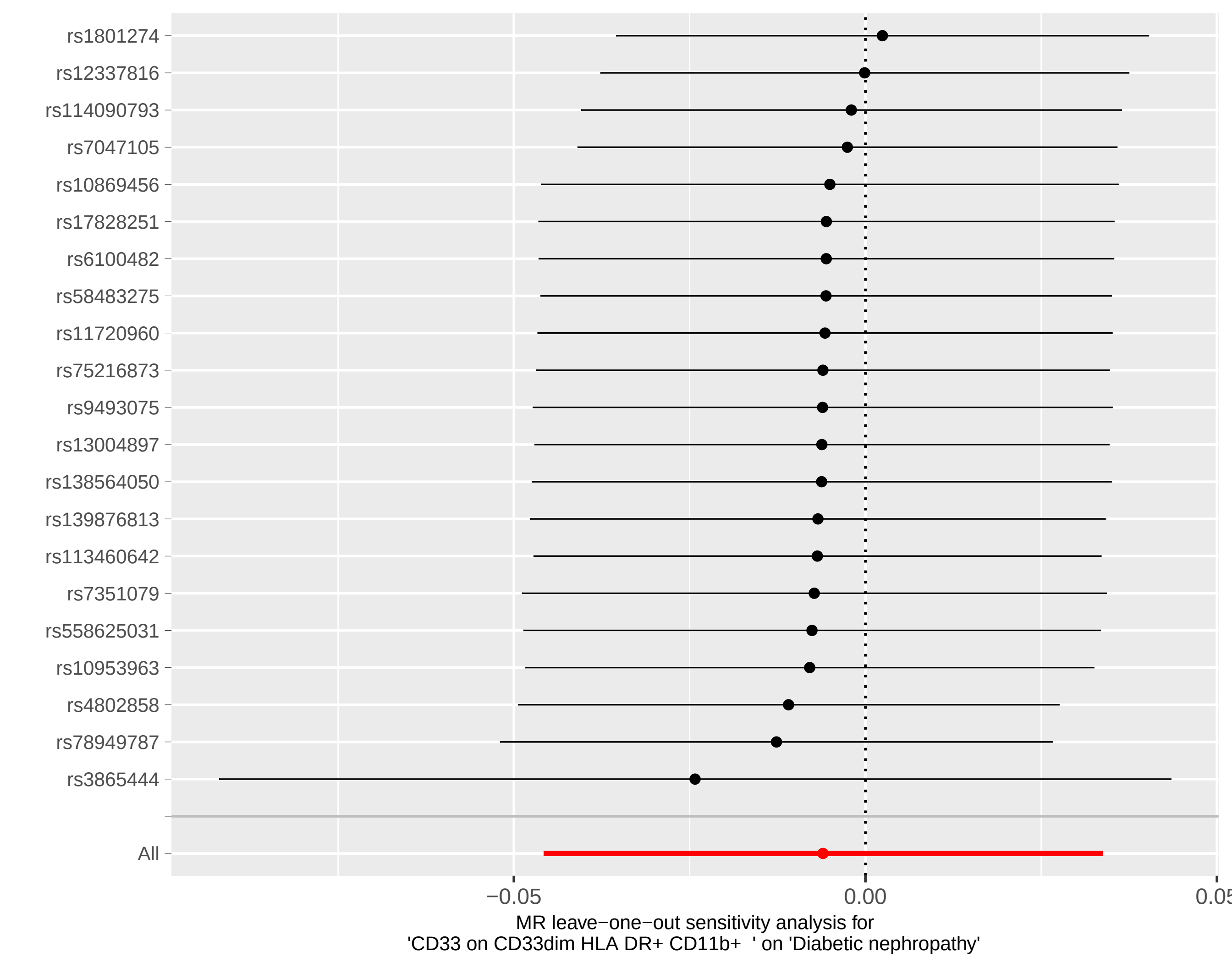

rs1801274
rs12337816
rs114090793
rs7047105
rs10869456
rs17828251
rs6100482
rs58483275
rs11720960
rs75216873
rs9493075
rs13004897
rs138564050
rs139876813
rs113460642
rs7351079
rs558625031
rs10953963
rs4802858
rs78949787
rs3865444
All
−0.05
0.00
0.05
MR leave−one−out sensitivity analysis for
'CD33 on CD33dim HLA DR+ CD11b+ ' on 'Diabetic nephropathy'

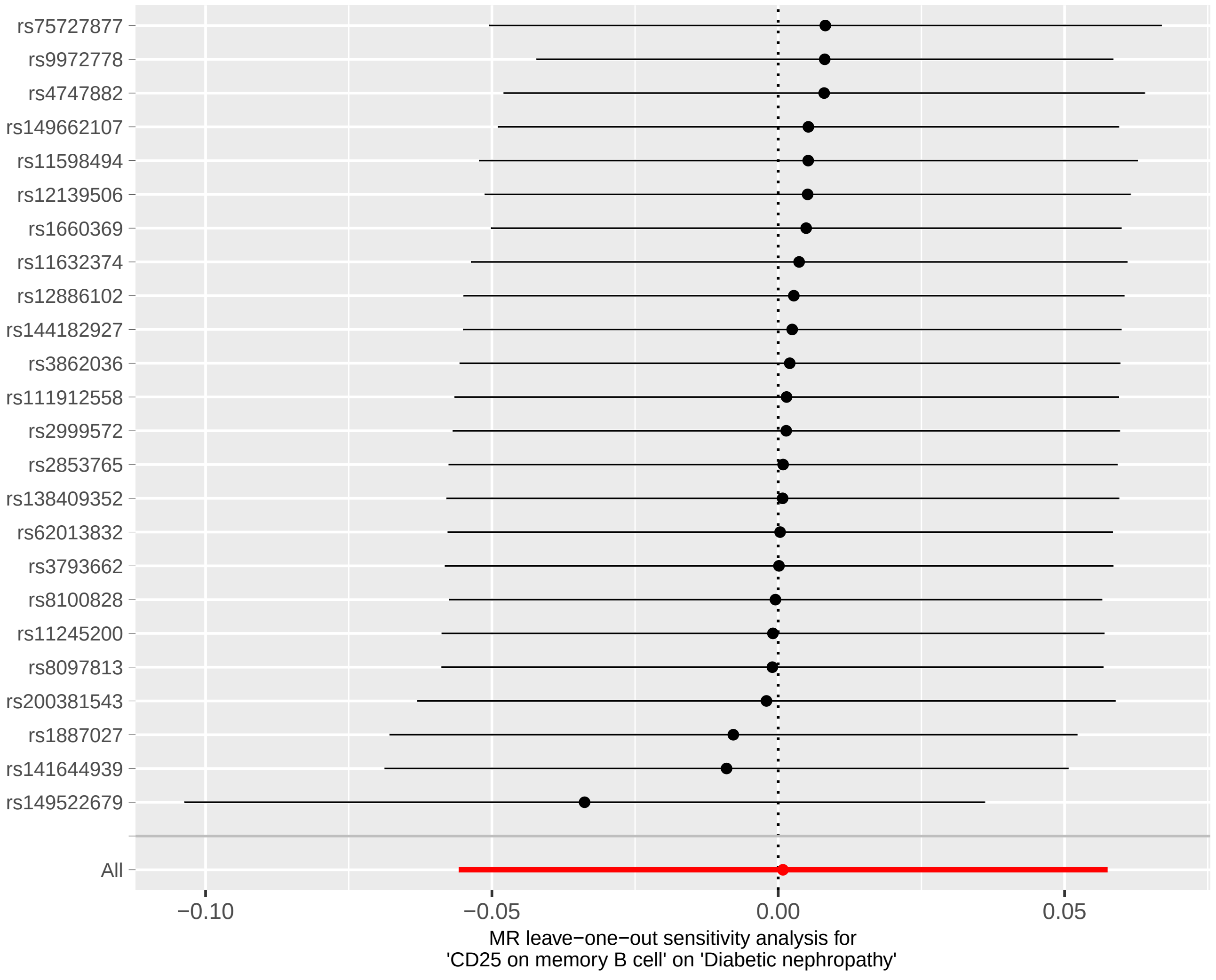

rs75727877
rs9972778
rs4747882
rs149662107
rs11598494
rs12139506
rs1660369
rs11632374
rs12886102
rs144182927
rs3862036
rs111912558
rs2999572
rs2853765
rs138409352
rs62013832
rs3793662
rs8100828
rs11245200
rs8097813
rs200381543
rs1887027
rs141644939
rs149522679
All
−0.10
−0.05
0.00
0.05
MR leave−one−out sensitivity analysis for
'CD25 on memory B cell' on 'Diabetic nephropathy'

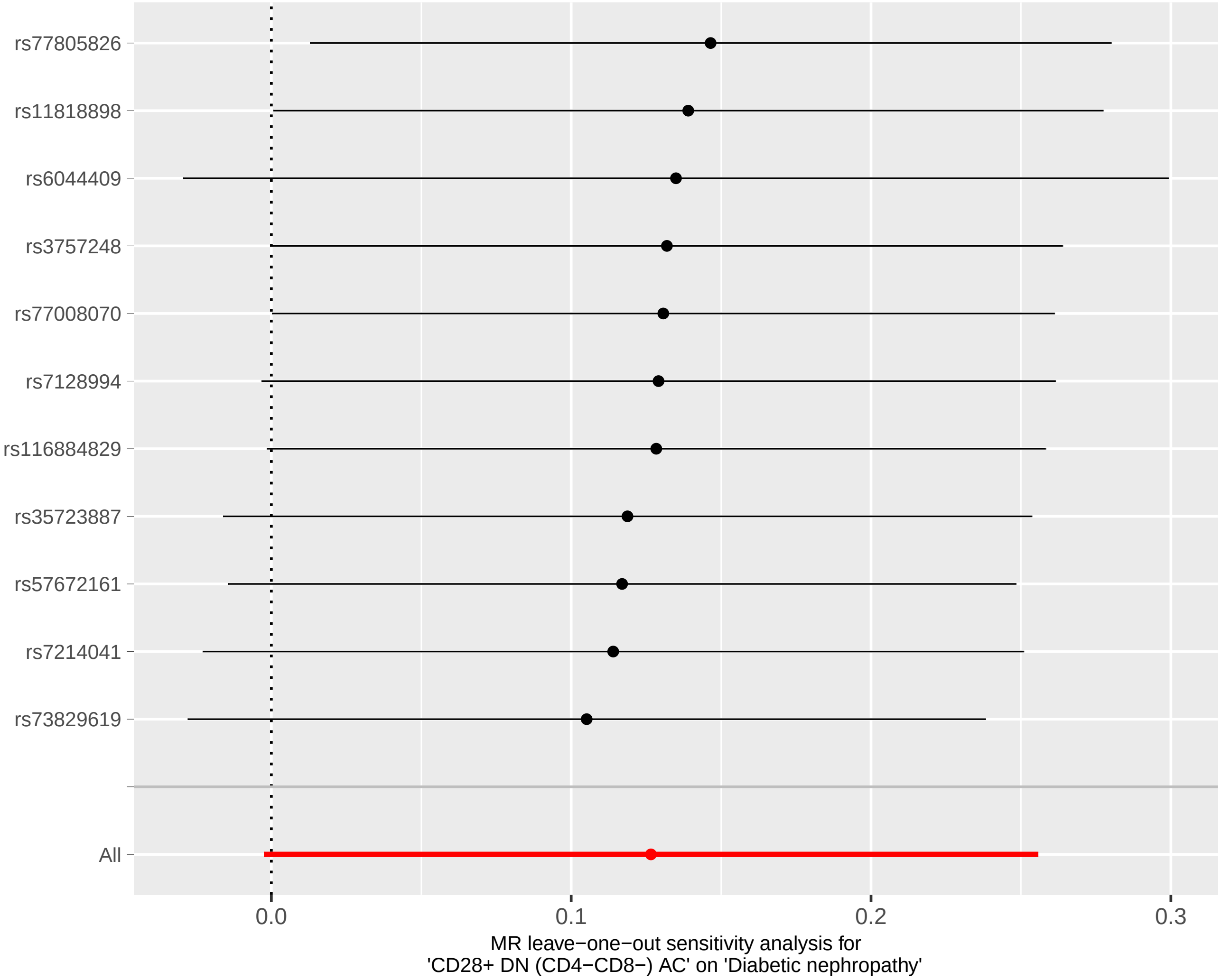
rs77805826
rs11818898
rs6044409
rs3757248
rs77008070
rs7128994
rs116884829
rs35723887
rs57672161
rs7214041
rs73829619
All
0.0
0.1
0.2
0.3
MR leave−one−out sensitivity analysis for
'CD28+ DN (CD4−CD8−) AC' on 'Diabetic nephropathy'

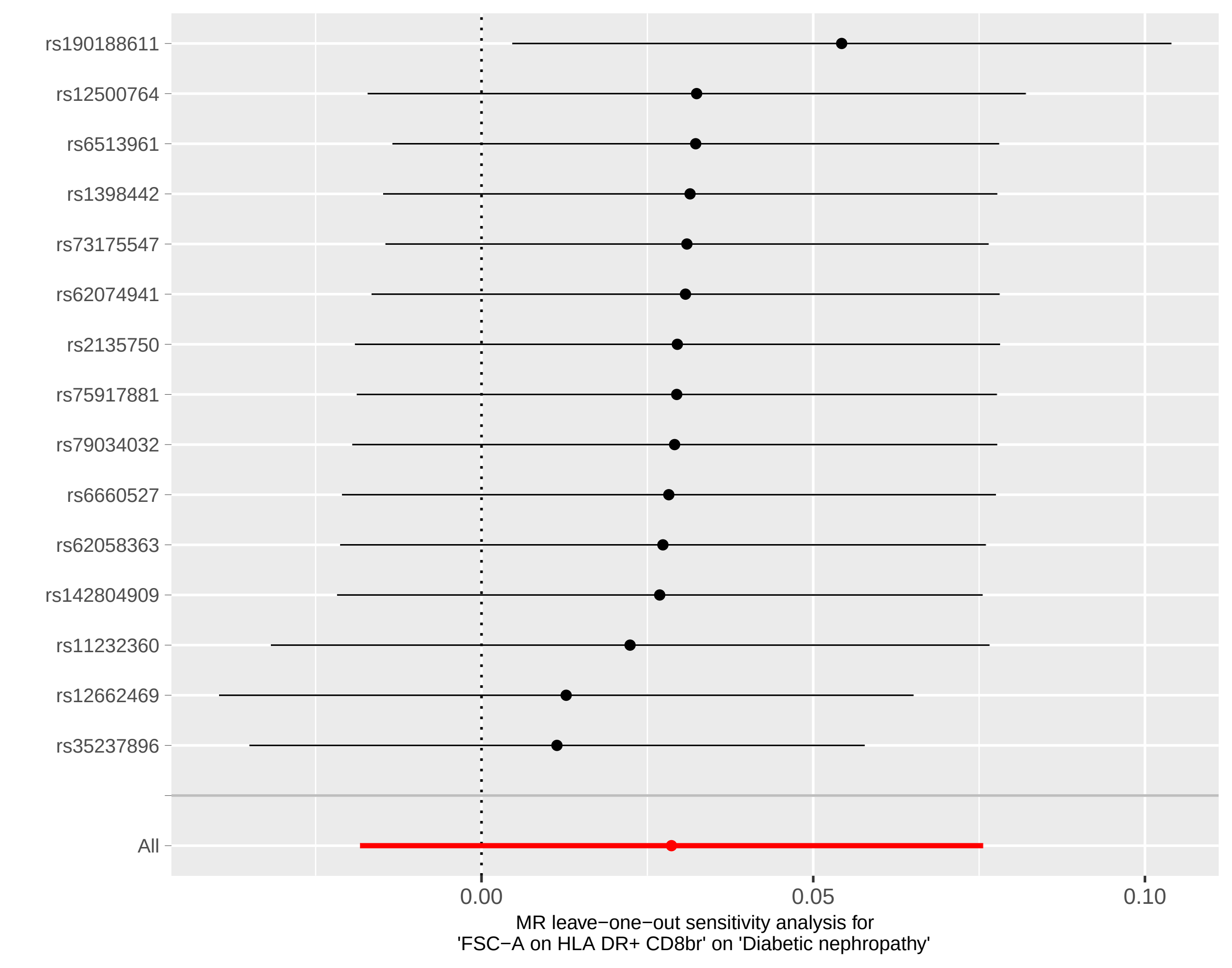
rs190188611
rs12500764
rs6513961
rs1398442
rs73175547
rs62074941
rs2135750
rs75917881
rs79034032
rs6660527
rs62058363
rs142804909
rs11232360
rs12662469
rs35237896
All
0.00
0.05
0.10
MR leave−one−out sensitivity analysis for
'FSC−A on HLA DR+ CD8br' on 'Diabetic nephropathy'

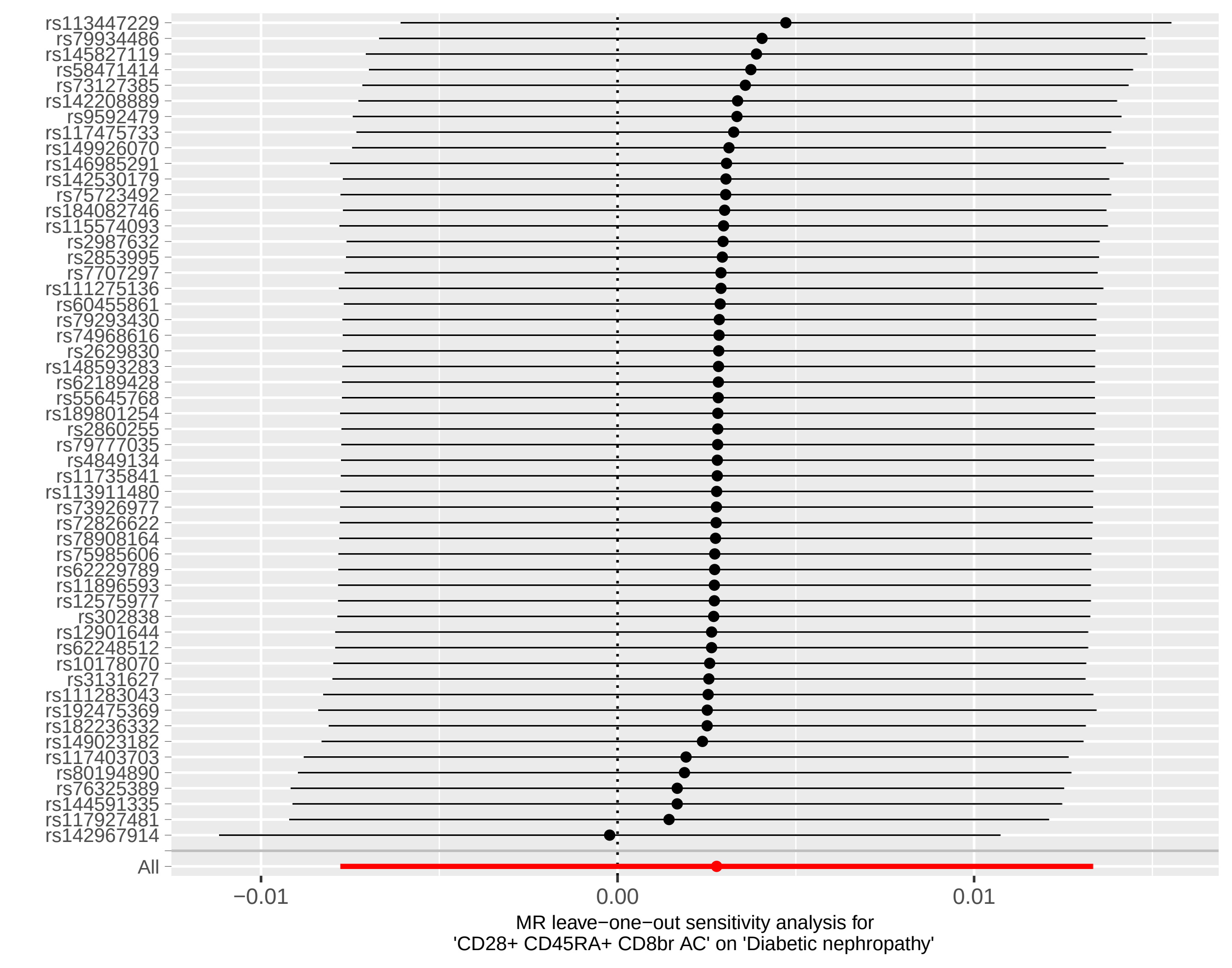
rs113447229
rs79934486
rs145827119
rs58471414
rs73127385
rs142208889
rs9592479
rs117475733
rs149926070
rs146985291
rs142530179
rs75723492
rs184082746
rs115574093
rs2987632
rs2853995
rs7707297
rs111275136
rs60455861
rs79293430
rs74968616
rs2629830
rs148593283
rs62189428
rs55645768
rs189801254
rs2860255
rs79777035
rs4849134
rs11735841
rs113911480
rs73926977
rs72826622
rs78908164
rs75985606
rs62229789
rs11896593
rs12575977
rs302838
rs12901644
rs62248512
rs10178070
rs3131627
rs111283043
rs192475369
rs182236332
rs149023182
rs117403703
rs80194890
rs76325389
rs144591335
rs117927481
rs142967914
All
−0.01
0.00
0.01
MR leave−one−out sensitivity analysis for
'CD28+ CD45RA+ CD8br AC' on 'Diabetic nephropathy'

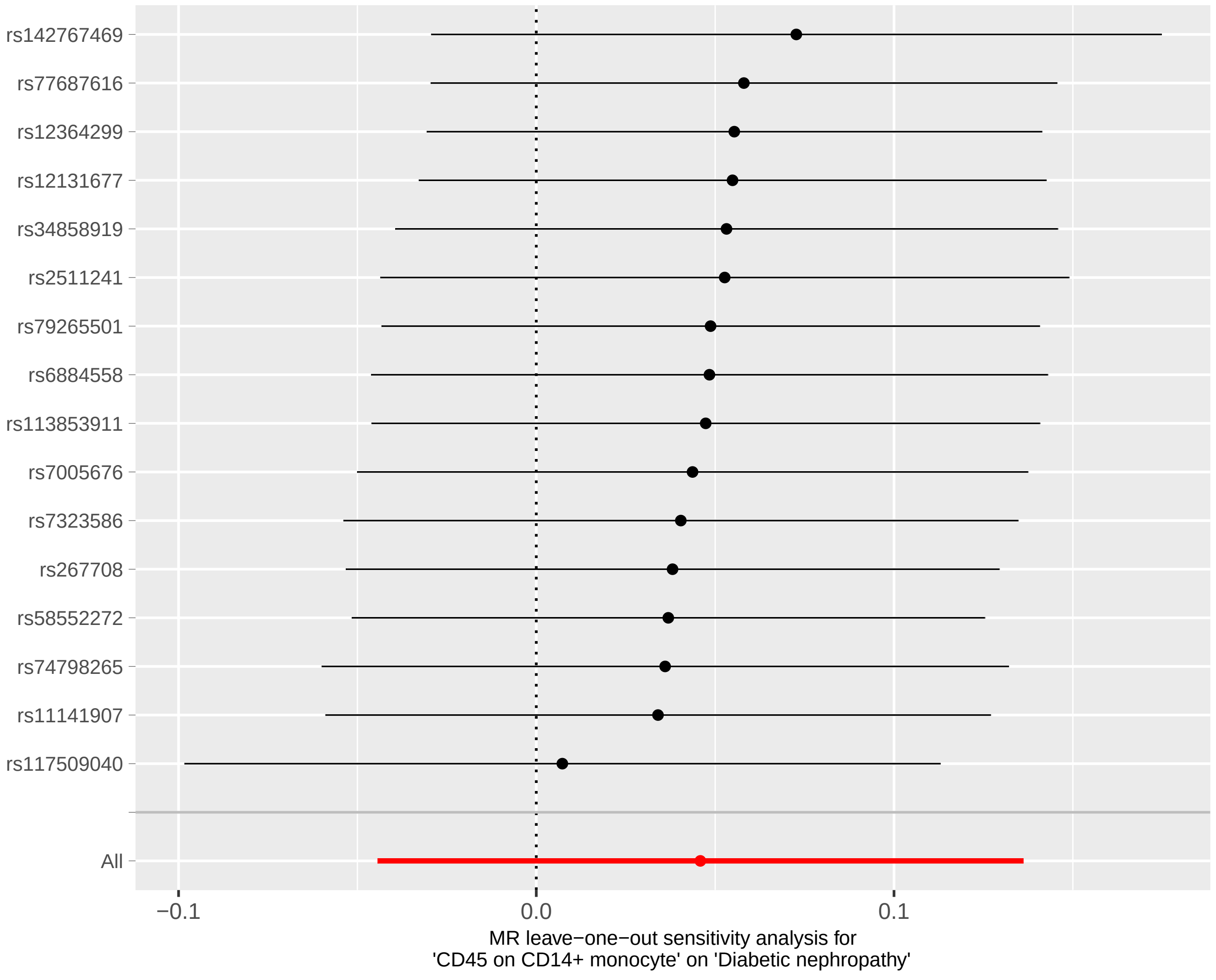
rs142767469
rs77687616
rs12364299
rs12131677
rs34858919
rs2511241
rs79265501
rs6884558
rs113853911
rs7005676
rs7323586
rs267708
rs58552272
rs74798265
rs11141907
rs117509040
All
−0.1
0.0
0.1
MR leave−one−out sensitivity analysis for
'CD45 on CD14+ monocyte' on 'Diabetic nephropathy'

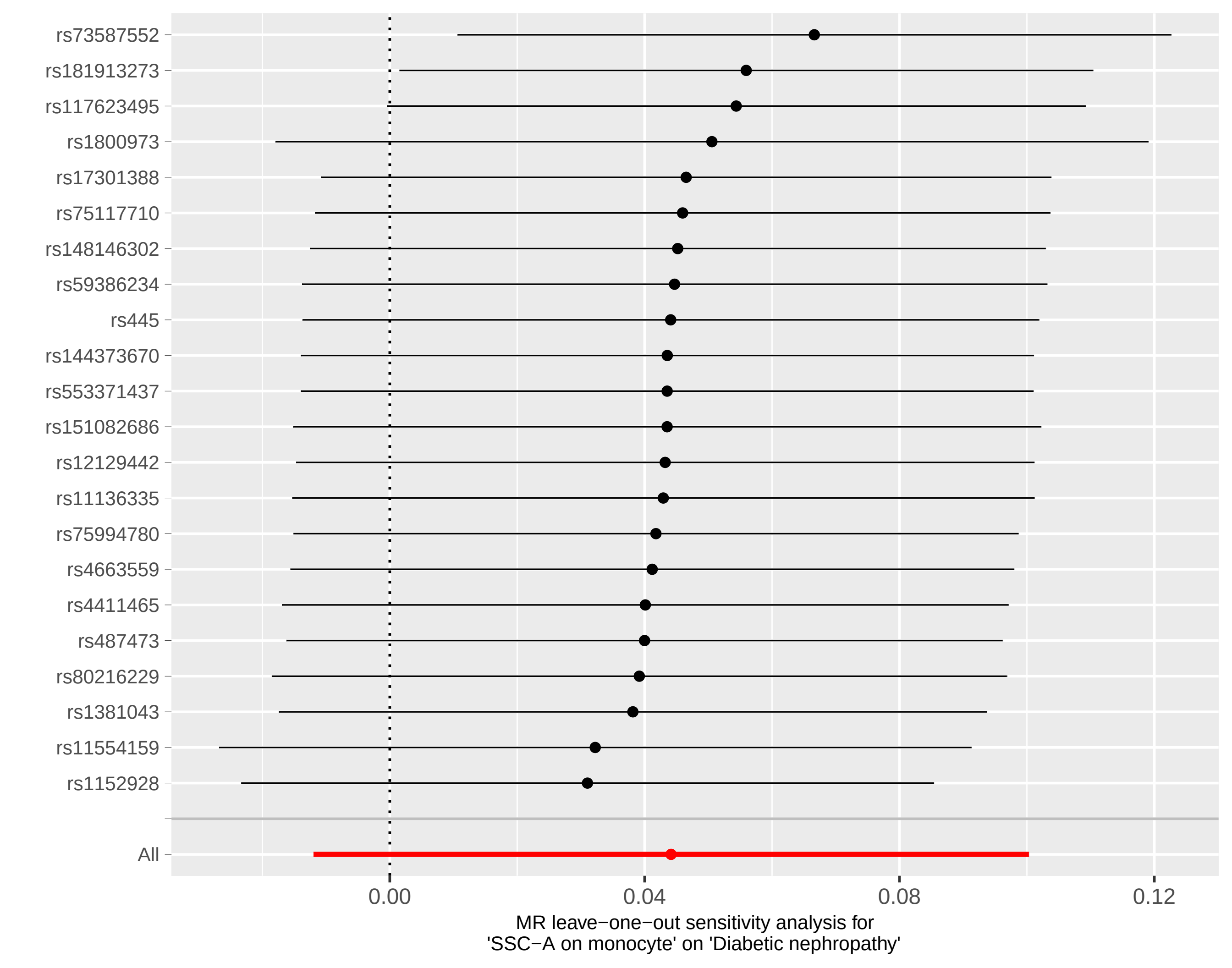

rs73587552
rs181913273
rs117623495
rs1800973
rs17301388
rs75117710
rs148146302
rs59386234
rs445
rs144373670
rs553371437
rs151082686
rs12129442
rs11136335
rs75994780
rs4663559
rs4411465
rs487473
rs80216229
rs1381043
rs11554159
rs1152928
All
0.00
0.04
0.08
0.12
MR leave−one−out sensitivity analysis for
'SSC−A on monocyte' on 'Diabetic nephropathy'

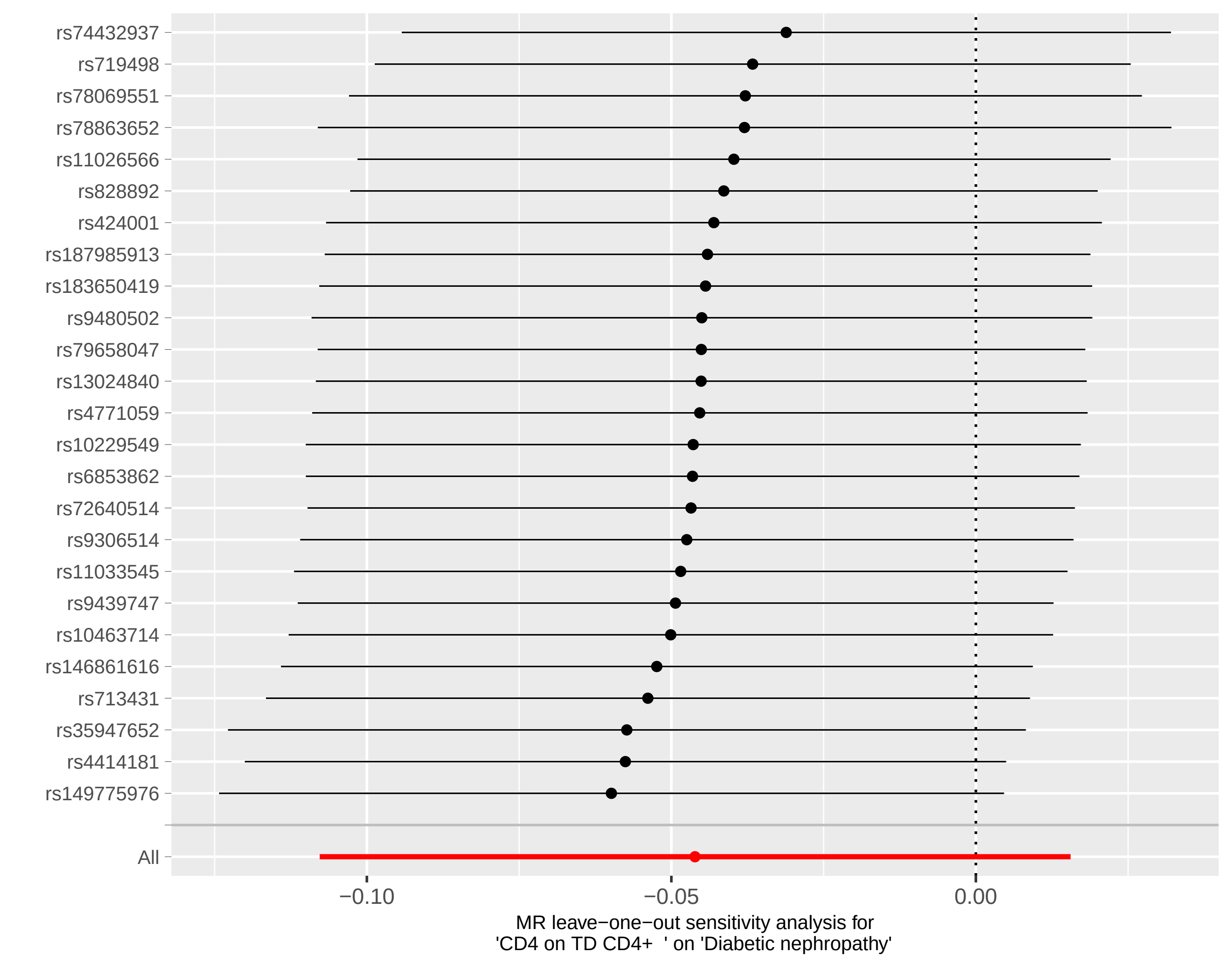

rs74432937
rs719498
rs78069551
rs78863652
rs11026566
rs828892
rs424001
rs187985913
rs183650419
rs9480502
rs79658047
rs13024840
rs4771059
rs10229549
rs6853862
rs72640514
rs9306514
rs11033545
rs9439747
rs10463714
rs146861616
rs713431
rs35947652
rs4414181
rs149775976
All
−0.10
−0.05
0.00
MR leave−one−out sensitivity analysis for
'CD4 on TD CD4+ ' on 'Diabetic nephropathy'

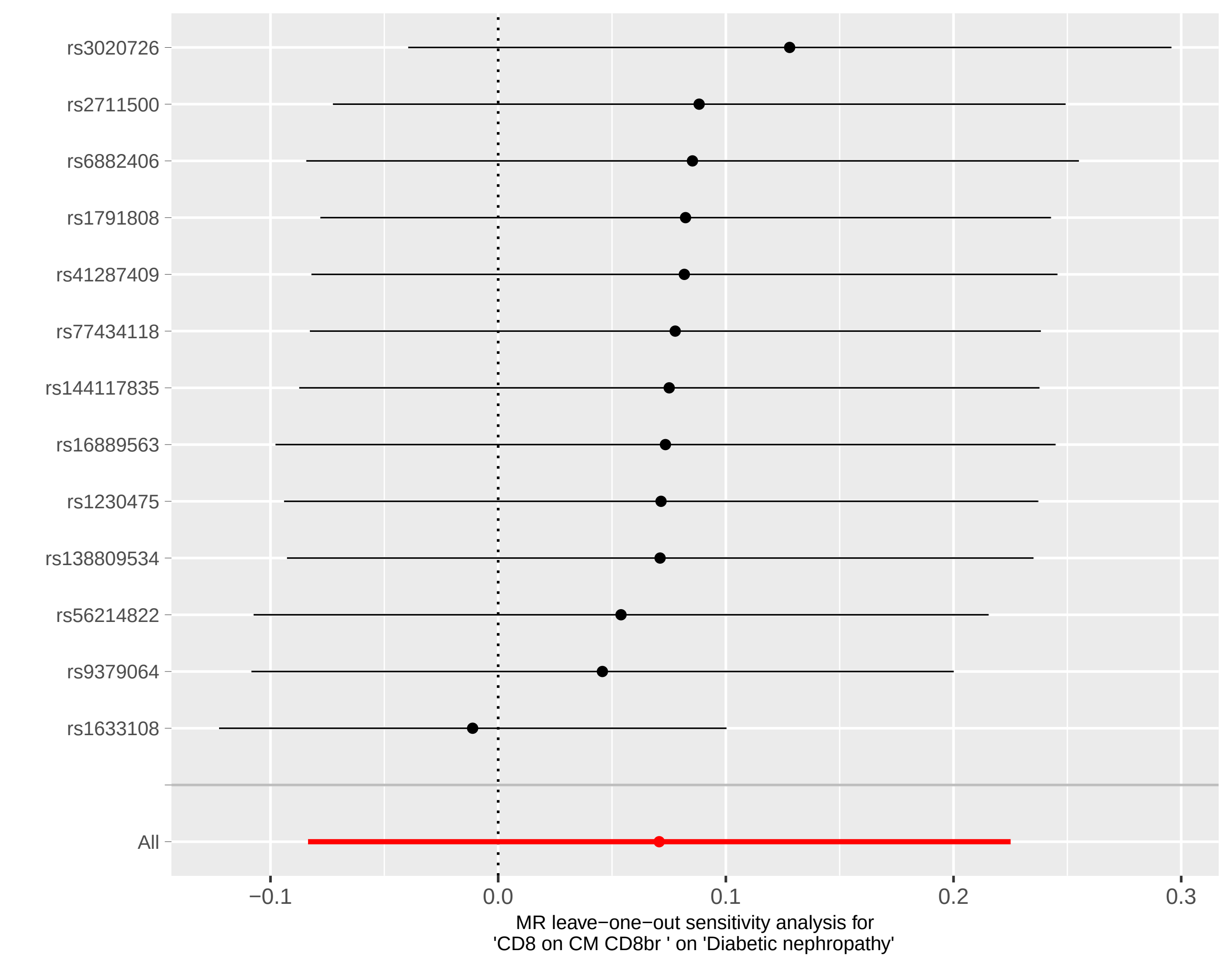
rs3020726
rs2711500
rs6882406
rs1791808
rs41287409
rs77434118
rs144117835
rs16889563
rs1230475
rs138809534
rs56214822
rs9379064
rs1633108
All
−0.1
0.0
0.1
0.2
0.3
MR leave−one−out sensitivity analysis for
'CD8 on CM CD8br ' on 'Diabetic nephropathy'

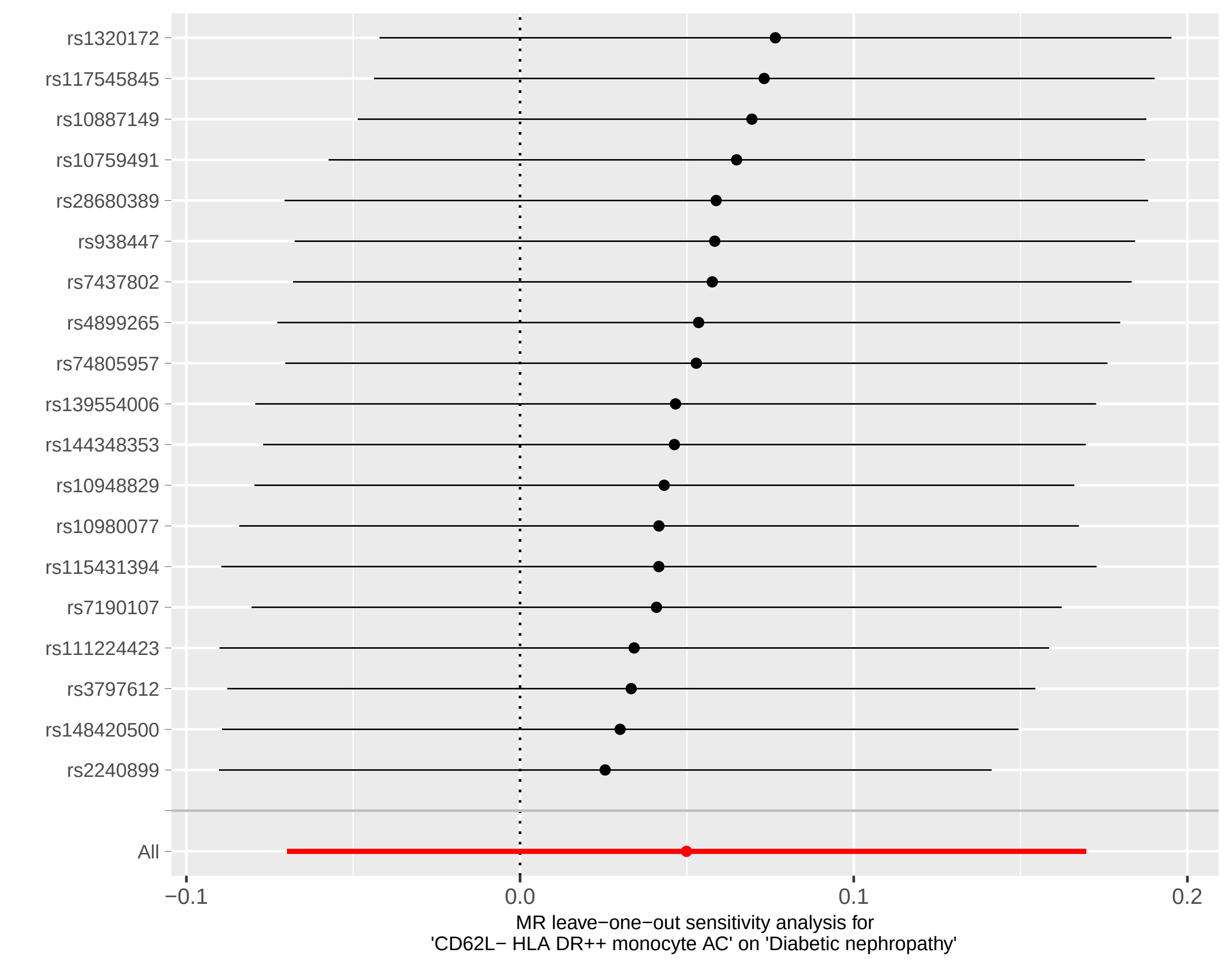
rs1320172
rs117545845
rs10887149
rs10759491
rs28680389
rs938447
rs7437802
rs4899265
rs74805957
rs139554006
rs144348353
rs10948829
rs10980077
rs115431394
rs7190107
rs111224423
rs3797612
rs148420500
rs2240899
All
−0.1
0.0
0.1
0.2
MR leave−one−out sensitivity analysis for
'CD62L− HLA DR++ monocyte AC' on 'Diabetic nephropathy'

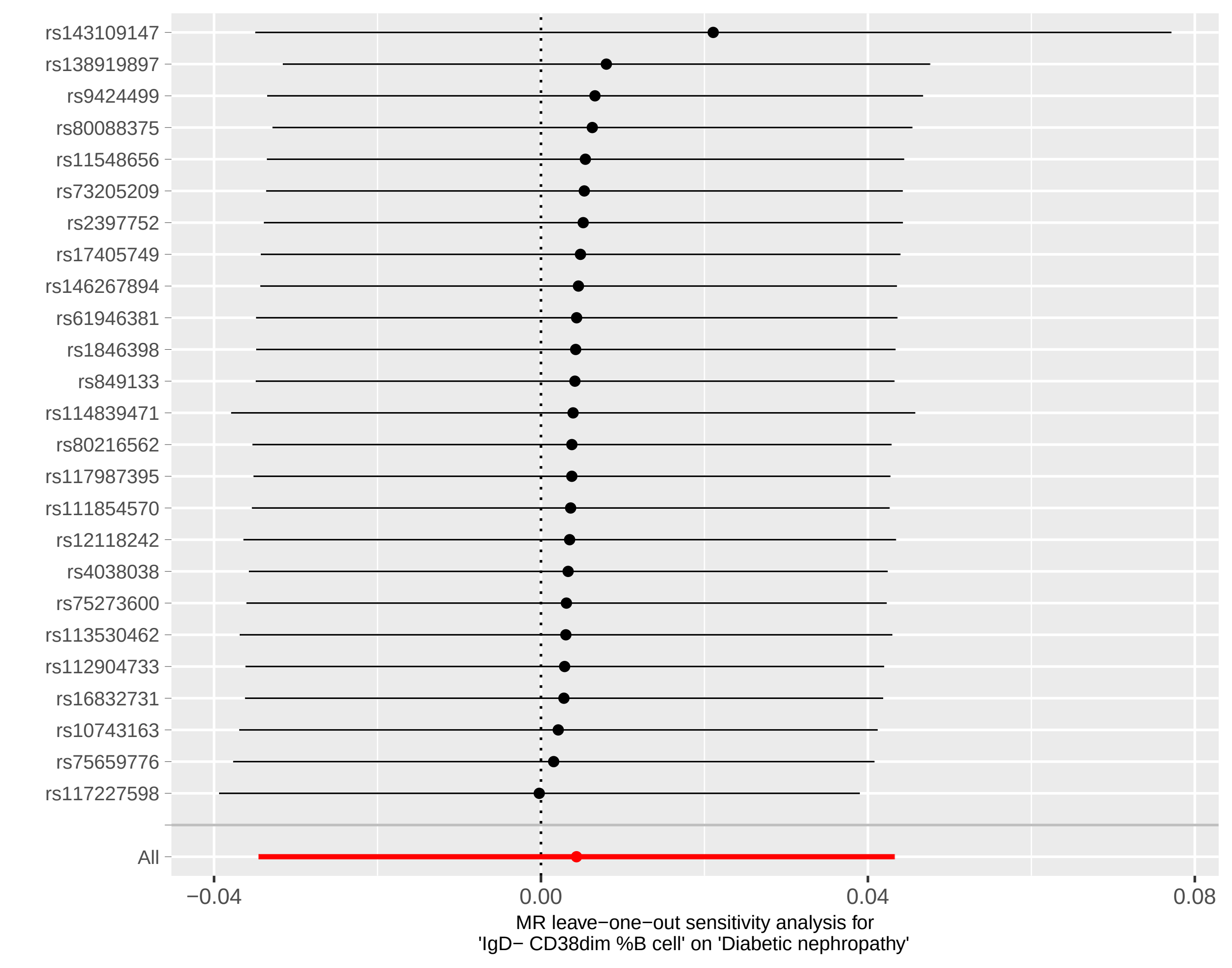
rs143109147
rs138919897
rs9424499
rs80088375
rs11548656
rs73205209
rs2397752
rs17405749
rs146267894
rs61946381
rs1846398
rs849133
rs114839471
rs80216562
rs117987395
rs111854570
rs12118242
rs4038038
rs75273600
rs113530462
rs112904733
rs16832731
rs10743163
rs75659776
rs117227598
All
−0.04
0.00
0.04
0.08
MR leave−one−out sensitivity analysis for
'IgD− CD38dim %B cell' on 'Diabetic nephropathy'

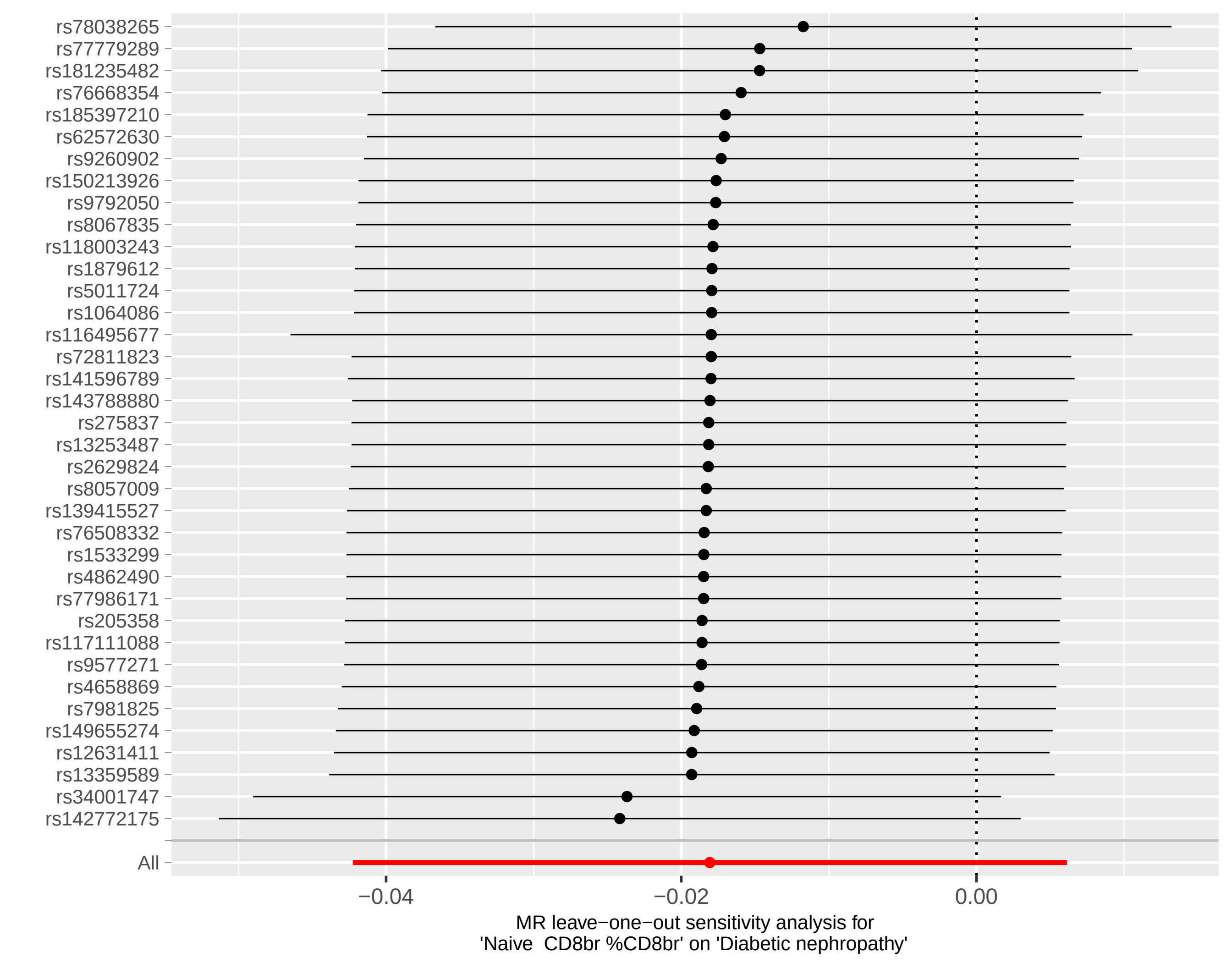
rs78038265
rs77779289
rs181235482
rs76668354
rs185397210
rs62572630
rs9260902
rs150213926
rs9792050
rs8067835
rs118003243
rs1879612
rs5011724
rs1064086
rs116495677
rs72811823
rs141596789
rs143788880
rs275837
rs13253487
rs2629824
rs8057009
rs139415527
rs76508332
rs1533299
rs4862490
rs77986171
rs205358
rs117111088
rs9577271
rs4658869
rs7981825
rs149655274
rs12631411
rs13359589
rs34001747
rs142772175
All
−0.04
−0.02
0.00
MR leave−one−out sensitivity analysis for
'Naive CD8br %CD8br' on 'Diabetic nephropathy'

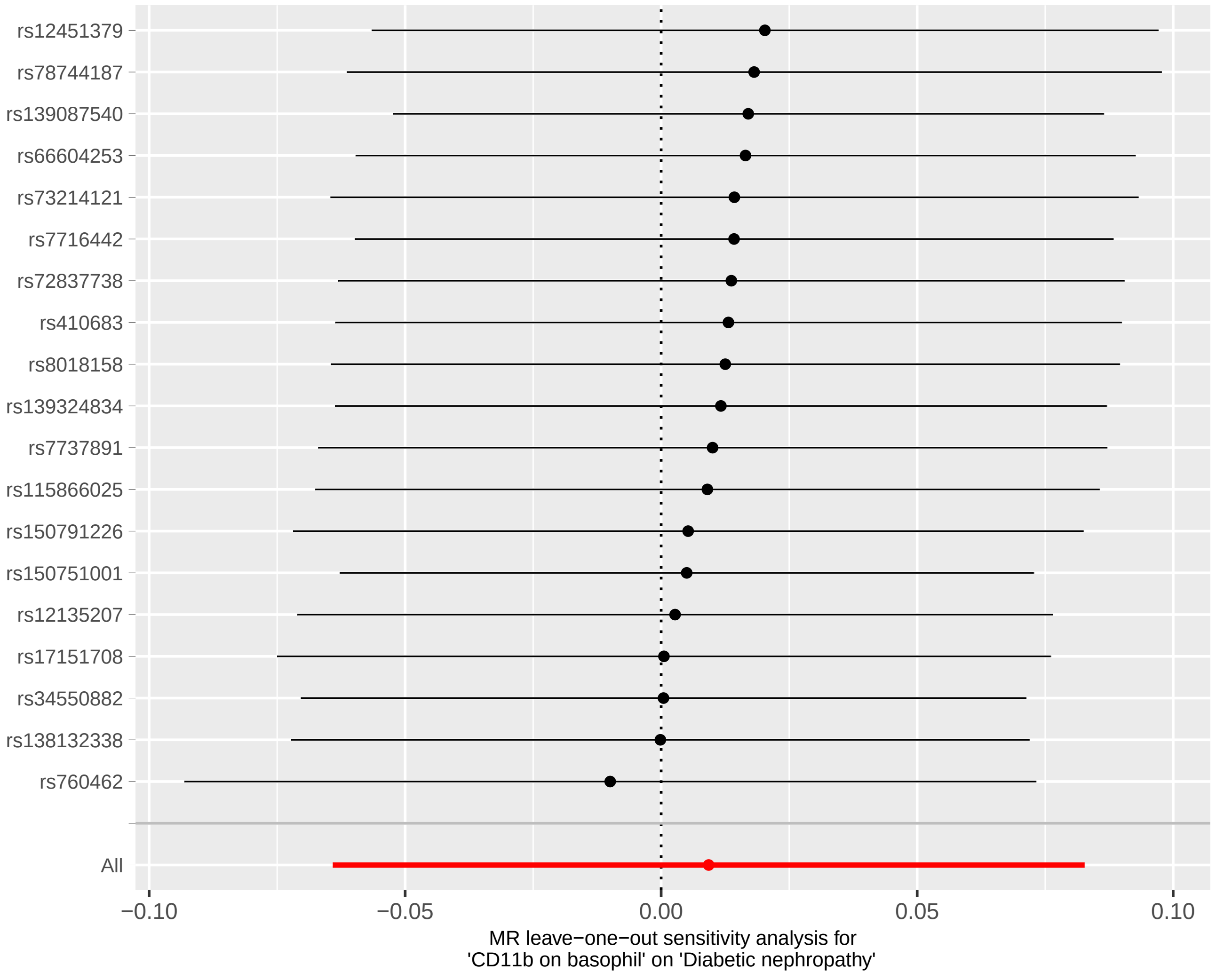
rs12451379
rs78744187
rs139087540
rs66604253
rs73214121
rs7716442
rs72837738
rs410683
rs8018158
rs139324834
rs7737891
rs115866025
rs150791226
rs150751001
rs12135207
rs17151708
rs34550882
rs138132338
rs760462
All
−0.10
−0.05
0.00
0.05
0.10
MR leave−one−out sensitivity analysis for
'CD11b on basophil' on 'Diabetic nephropathy'

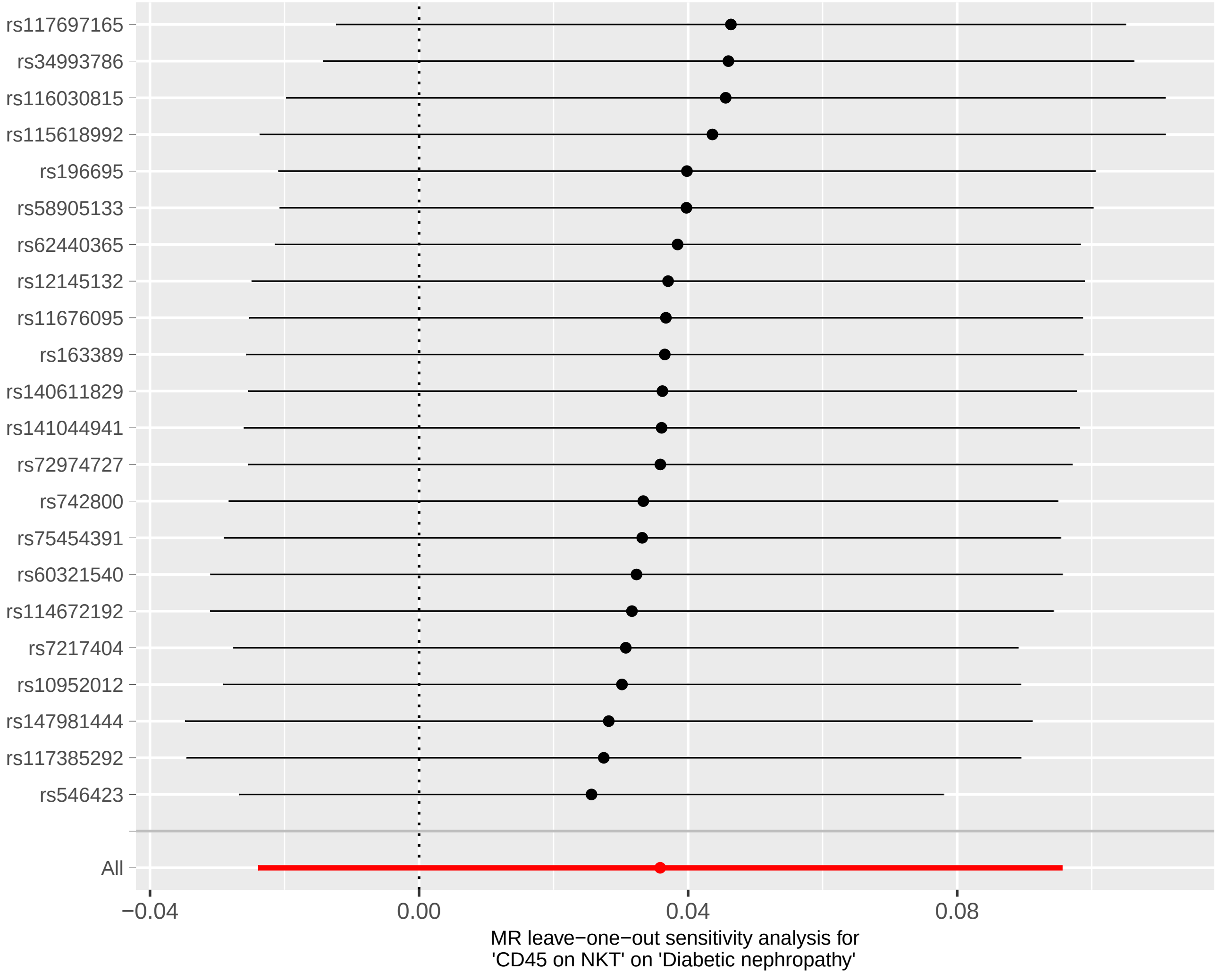
rs117697165
rs34993786
rs116030815
rs115618992
rs196695
rs58905133
rs62440365
rs12145132
rs11676095
rs163389
rs140611829
rs141044941
rs72974727
rs742800
rs75454391
rs60321540
rs114672192
rs7217404
rs10952012
rs147981444
rs117385292
rs546423
All
−0.04
0.00
0.04
0.08
MR leave−one−out sensitivity analysis for
'CD45 on NKT' on 'Diabetic nephropathy'

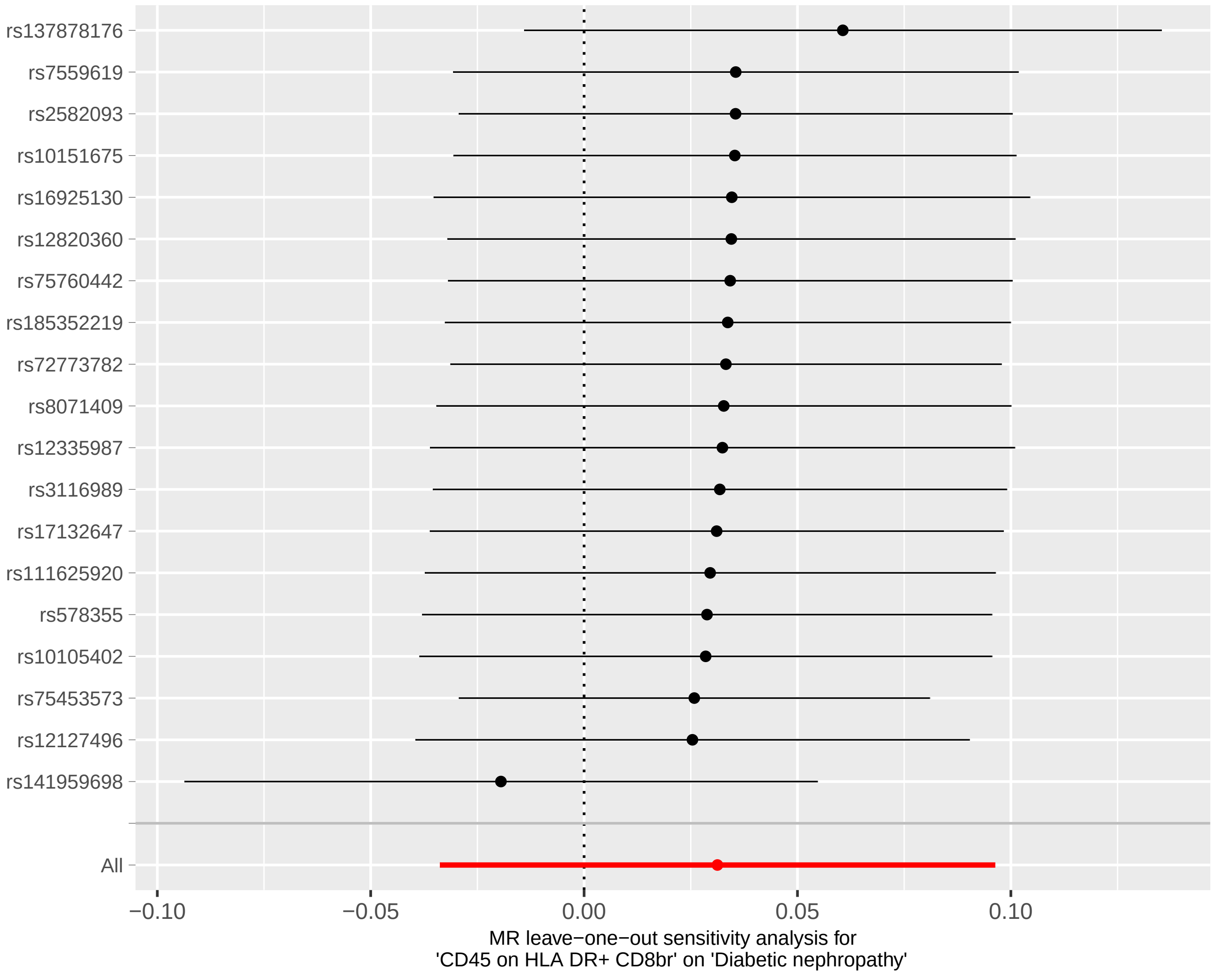
rs137878176
rs7559619
rs2582093
rs10151675
rs16925130
rs12820360
rs75760442
rs185352219
rs72773782
rs8071409
rs12335987
rs3116989
rs17132647
rs111625920
rs578355
rs10105402
rs75453573
rs12127496
rs141959698
All
−0.10
−0.05
0.00
0.05
0.10
MR leave−one−out sensitivity analysis for
'CD45 on HLA DR+ CD8br' on 'Diabetic nephropathy'

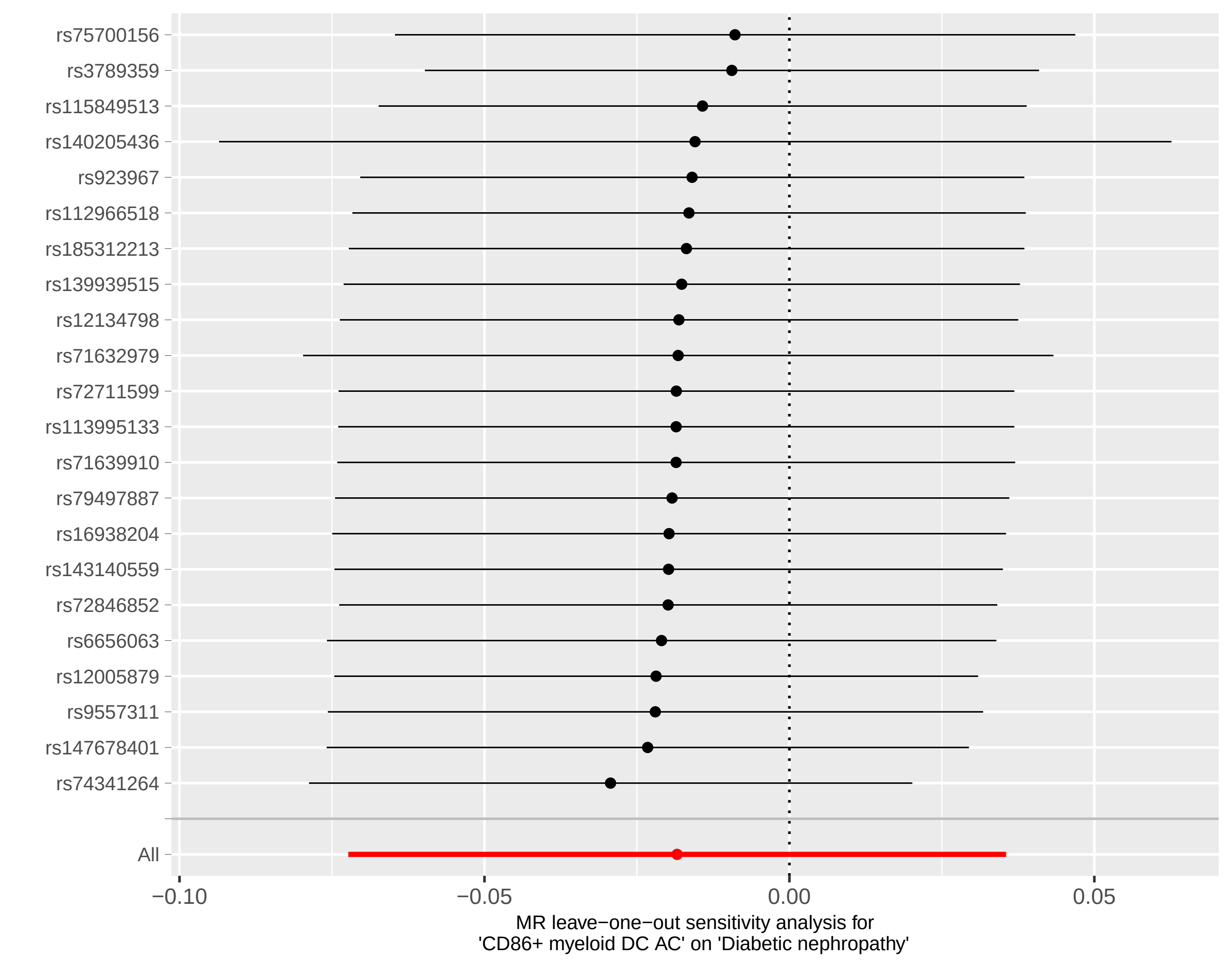
rs75700156
rs3789359
rs115849513
rs140205436
rs923967
rs112966518
rs185312213
rs139939515
rs12134798
rs71632979
rs72711599
rs113995133
rs71639910
rs79497887
rs16938204
rs143140559
rs72846852
rs6656063
rs12005879
rs9557311
rs147678401
rs74341264
All
−0.10
−0.05
0.00
0.05
MR leave−one−out sensitivity analysis for
'CD86+ myeloid DC AC' on 'Diabetic nephropathy'

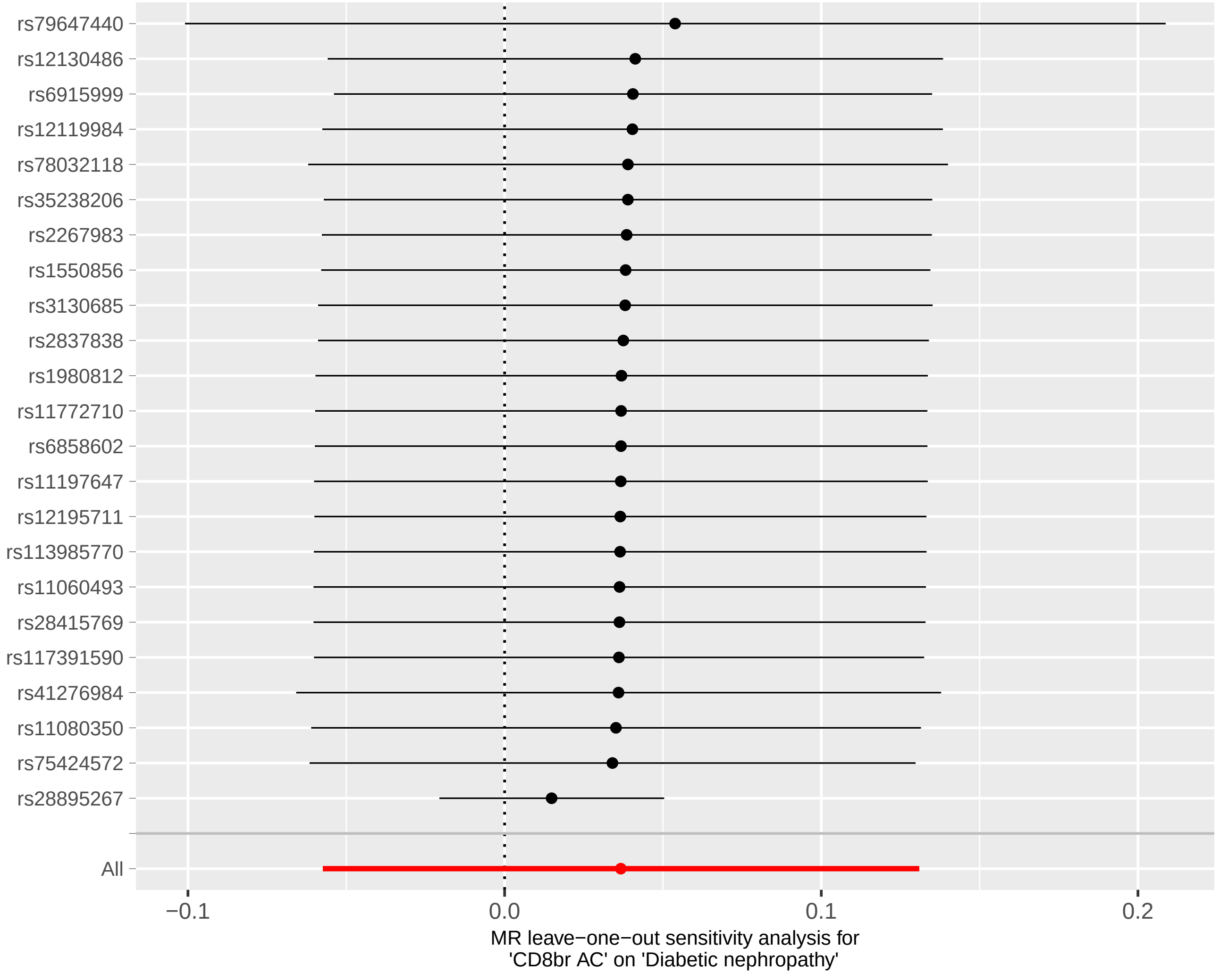
rs79647440
rs12130486
rs6915999
rs12119984
rs78032118
rs35238206
rs2267983
rs1550856
rs3130685
rs2837838
rs1980812
rs11772710
rs6858602
rs11197647
rs12195711
rs113985770
rs11060493
rs28415769
rs117391590
rs41276984
rs11080350
rs75424572
rs28895267
All
−0.1
0.0
0.1
0.2
MR leave−one−out sensitivity analysis for
'CD8br AC' on 'Diabetic nephropathy'

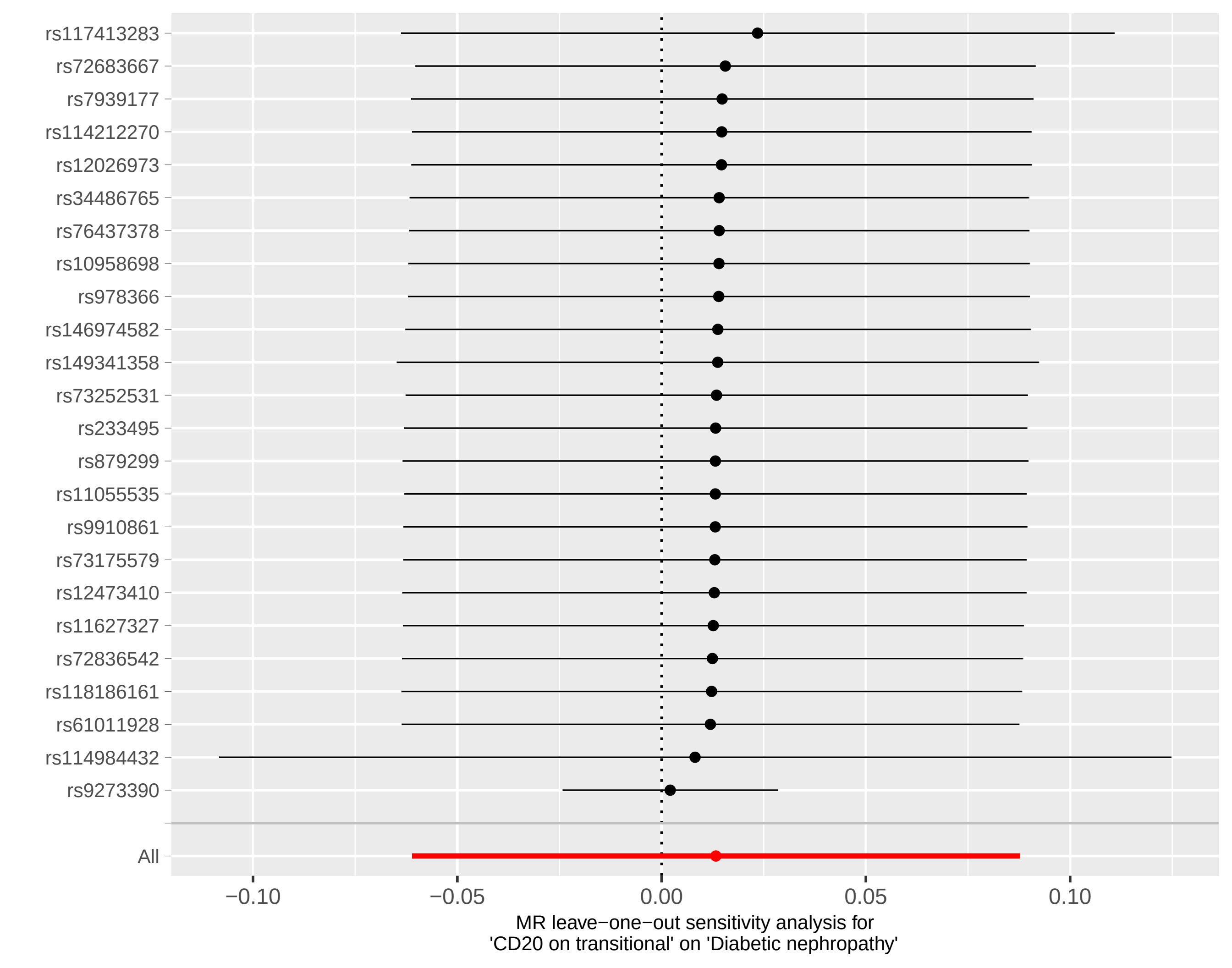
rs117413283
rs72683667
rs7939177
rs114212270
rs12026973
rs34486765
rs76437378
rs10958698
rs978366
rs146974582
rs149341358
rs73252531
rs233495
rs879299
rs11055535
rs9910861
rs73175579
rs12473410
rs11627327
rs72836542
rs118186161
rs61011928
rs114984432
rs9273390
All
−0.10
−0.05
0.00
0.05
0.10
MR leave−one−out sensitivity analysis for
'CD20 on transitional' on 'Diabetic nephropathy'

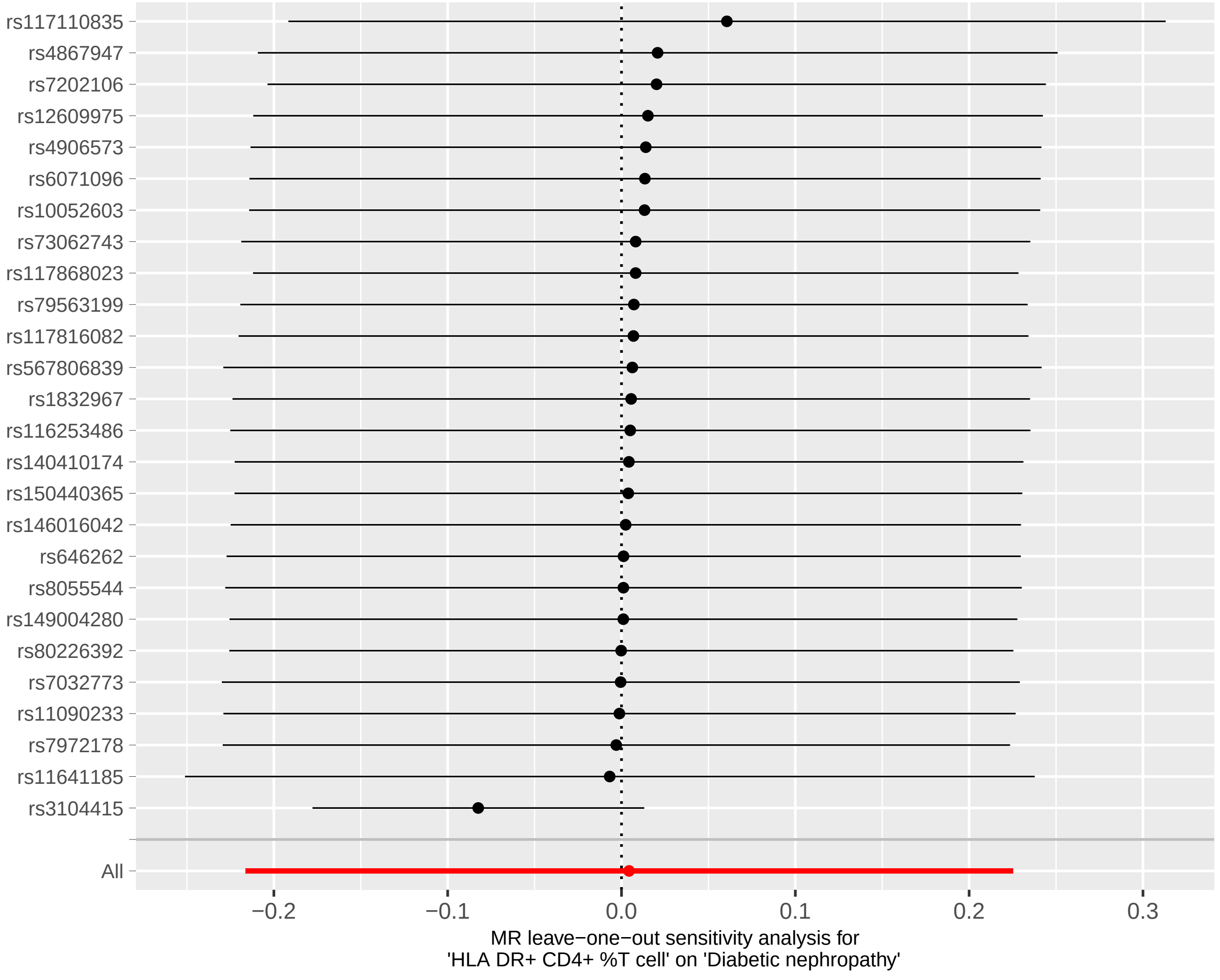
rs117110835
rs4867947
rs7202106
rs12609975
rs4906573
rs6071096
rs10052603
rs73062743
rs117868023
rs79563199
rs117816082
rs567806839
rs1832967
rs116253486
rs140410174
rs150440365
rs146016042
rs646262
rs8055544
rs149004280
rs80226392
rs7032773
rs11090233
rs7972178
rs11641185
rs3104415
All
−0.2
−0.1
0.0
0.1
0.2
0.3
MR leave−one−out sensitivity analysis for
'HLA DR+ CD4+ %T cell' on 'Diabetic nephropathy'

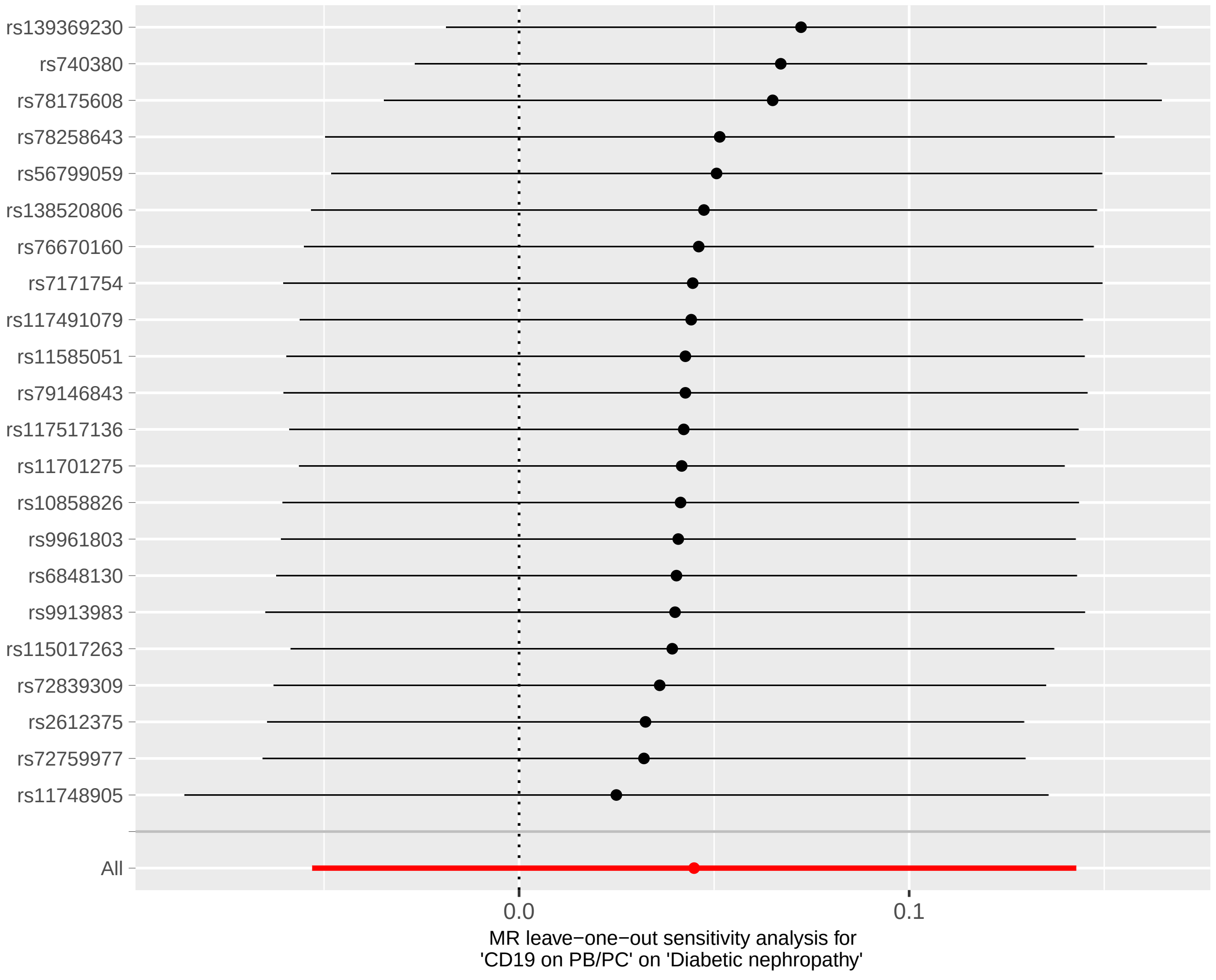

rs139369230
rs740380
rs78175608
rs78258643
rs56799059
rs138520806
rs76670160
rs7171754
rs117491079
rs11585051
rs79146843
rs117517136
rs11701275
rs10858826
rs9961803
rs6848130
rs9913983
rs115017263
rs72839309
rs2612375
rs72759977
rs11748905
All
0.0
0.1
MR leave−one−out sensitivity analysis for
'CD19 on PB/PC' on 'Diabetic nephropathy'

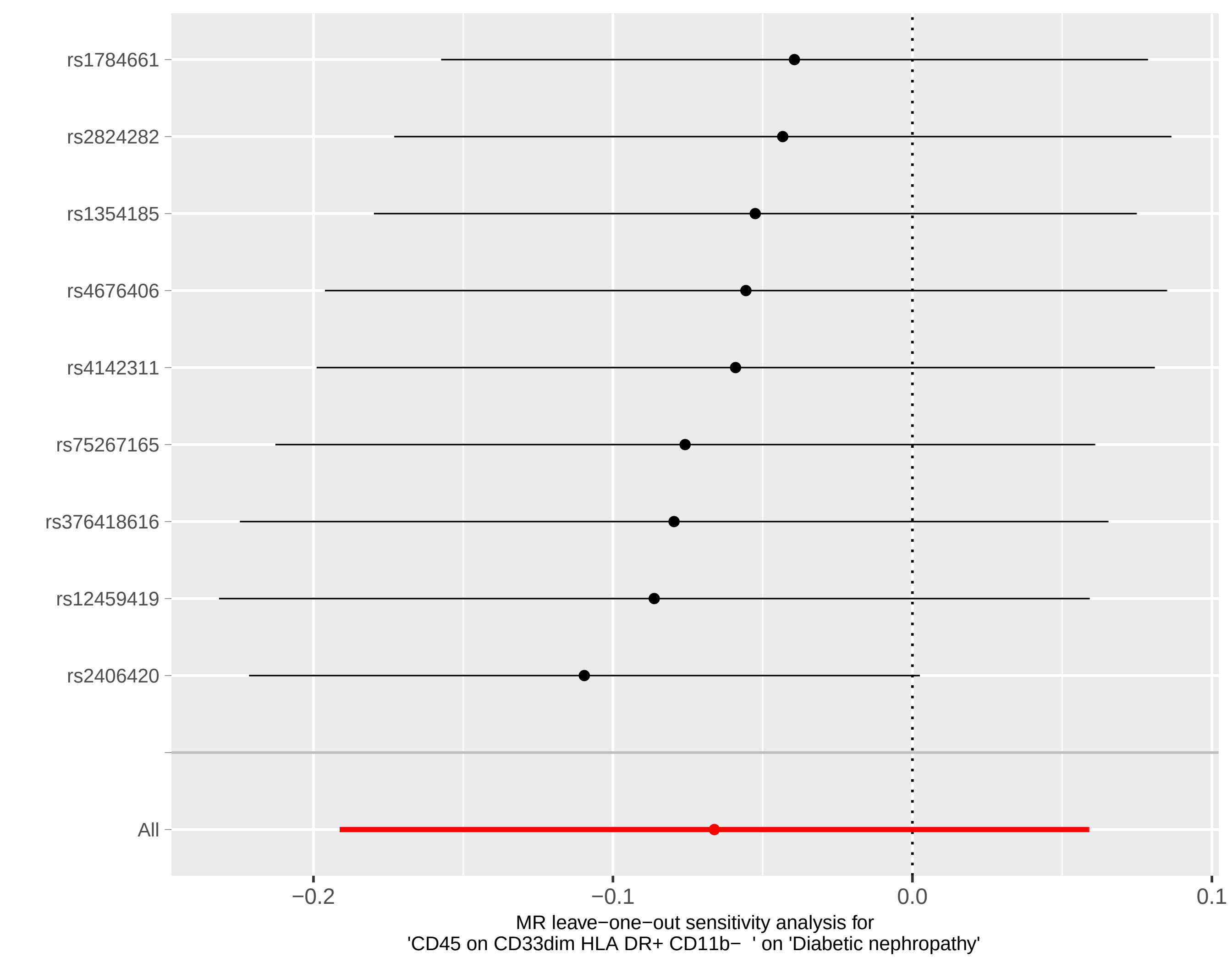
rs1784661
rs2824282
rs1354185
rs4676406
rs4142311
rs75267165
rs376418616
rs12459419
rs2406420
All
−0.2
−0.1
0.0
0.1
MR leave−one−out sensitivity analysis for
'CD45 on CD33dim HLA DR+ CD11b− ' on 'Diabetic nephropathy'

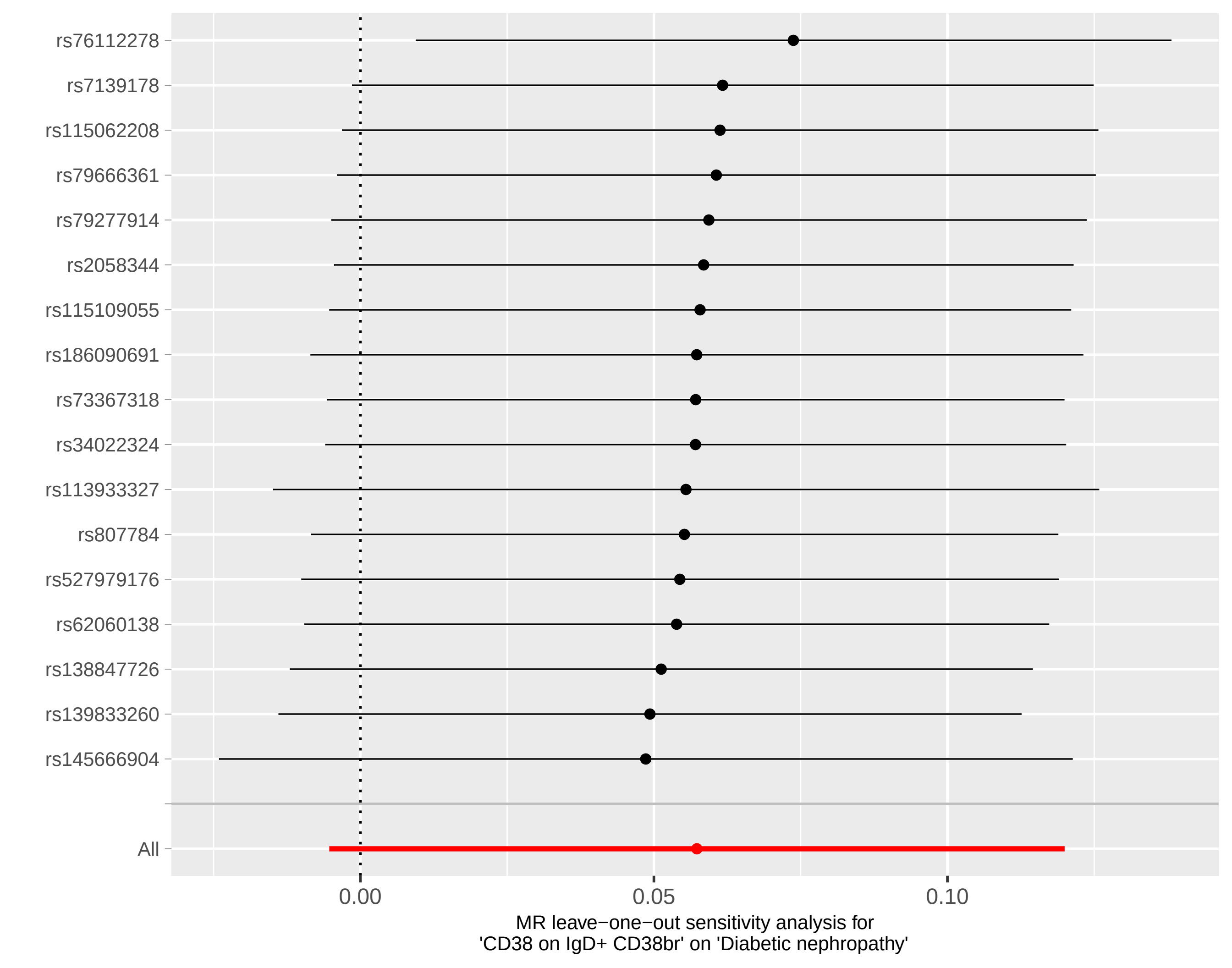

rs76112278
rs7139178
rs115062208
rs79666361
rs79277914
rs2058344
rs115109055
rs186090691
rs73367318
rs34022324
rs113933327
rs807784
rs527979176
rs62060138
rs138847726
rs139833260
rs145666904
All
0.00
0.05
0.10
MR leave−one−out sensitivity analysis for
'CD38 on IgD+ CD38br' on 'Diabetic nephropathy'

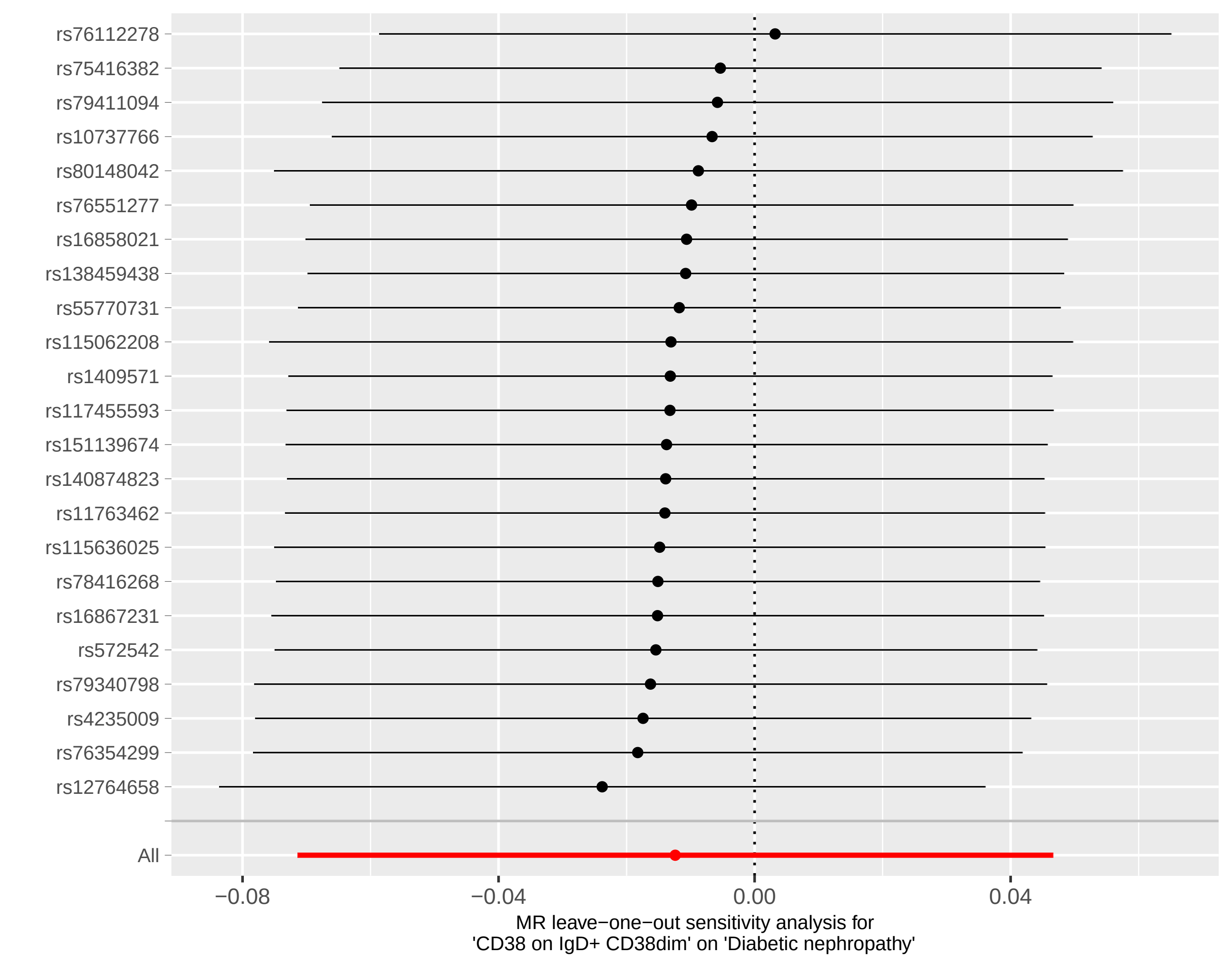
rs76112278
rs75416382
rs79411094
rs10737766
rs80148042
rs76551277
rs16858021
rs138459438
rs55770731
rs115062208
rs1409571
rs117455593
rs151139674
rs140874823
rs11763462
rs115636025
rs78416268
rs16867231
rs572542
rs79340798
rs4235009
rs76354299
rs12764658
All
−0.08
−0.04
0.00
0.04
MR leave−one−out sensitivity analysis for
'CD38 on IgD+ CD38dim' on 'Diabetic nephropathy'

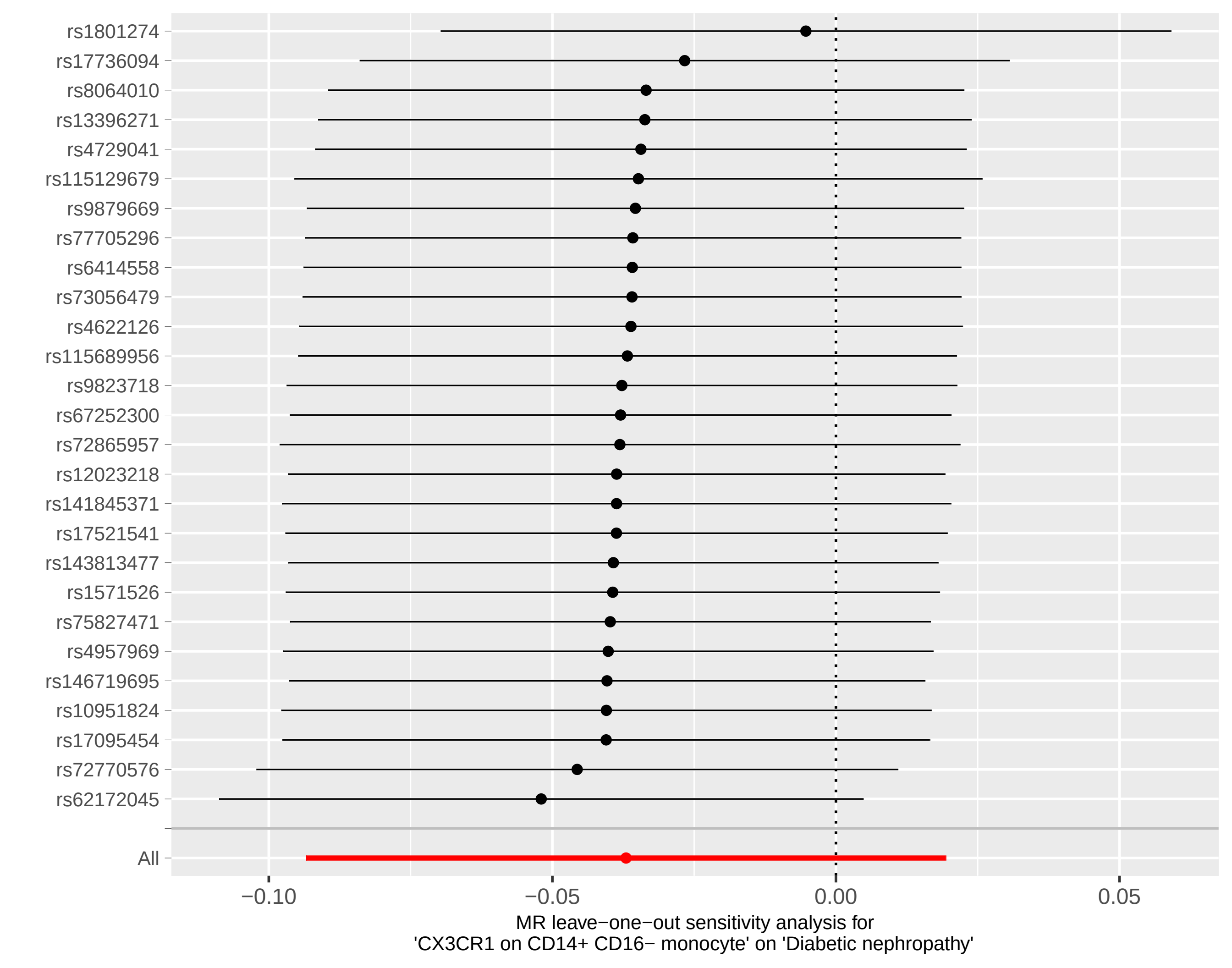

rs1801274
rs17736094
rs8064010
rs13396271
rs4729041
rs115129679
rs9879669
rs77705296
rs6414558
rs73056479
rs4622126
rs115689956
rs9823718
rs67252300
rs72865957
rs12023218
rs141845371
rs17521541
rs143813477
rs1571526
rs75827471
rs4957969
rs146719695
rs10951824
rs17095454
rs72770576
rs62172045
All
−0.10
−0.05
0.00
0.05
MR leave−one−out sensitivity analysis for
'CX3CR1 on CD14+ CD16− monocyte' on 'Diabetic nephropathy'

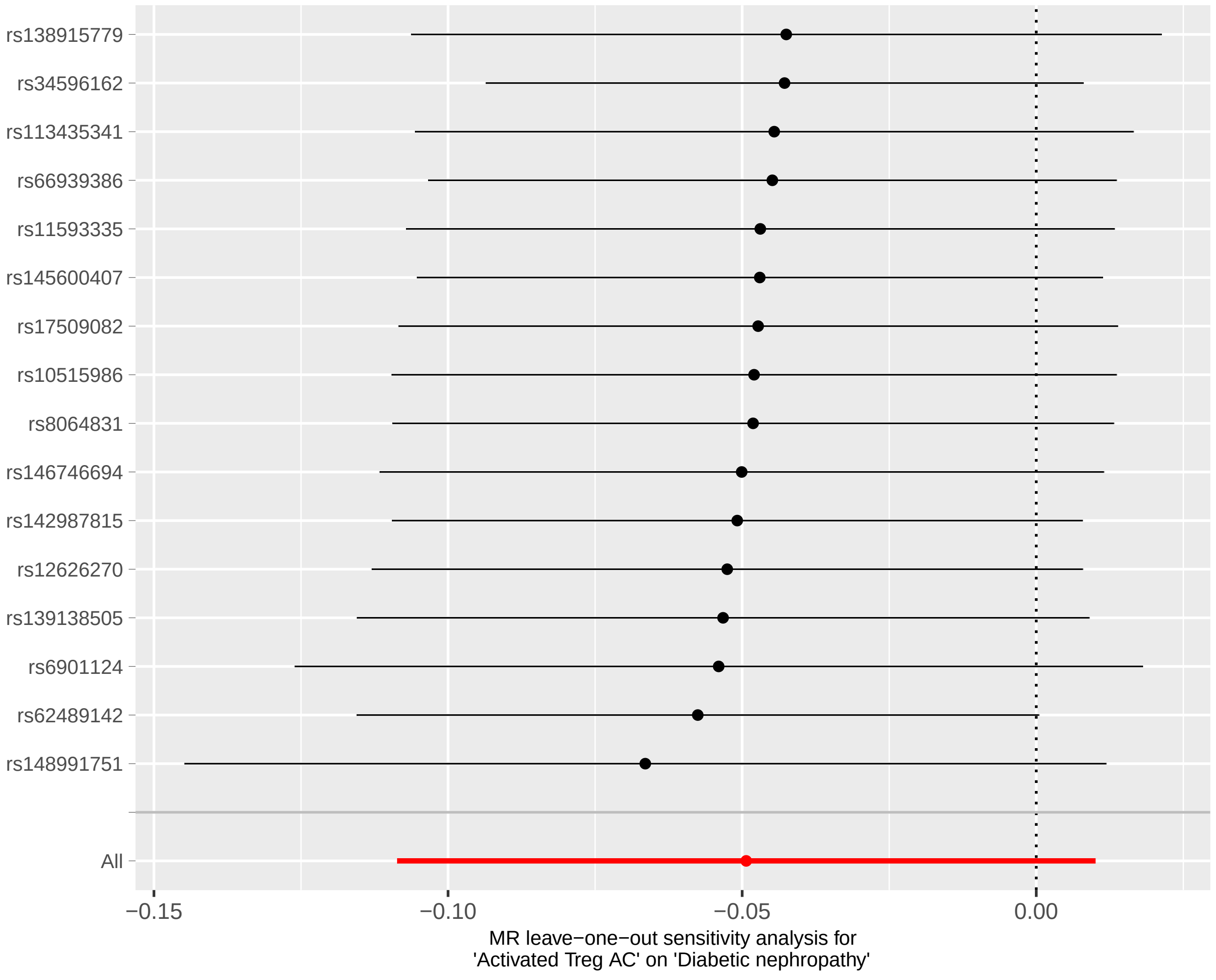

rs138915779
rs34596162
rs113435341
rs66939386
rs11593335
rs145600407
rs17509082
rs10515986
rs8064831
rs146746694
rs142987815
rs12626270
rs139138505
rs6901124
rs62489142
rs148991751
All
−0.15
−0.10
−0.05
0.00
MR leave−one−out sensitivity analysis for
'Activated Treg AC' on 'Diabetic nephropathy'

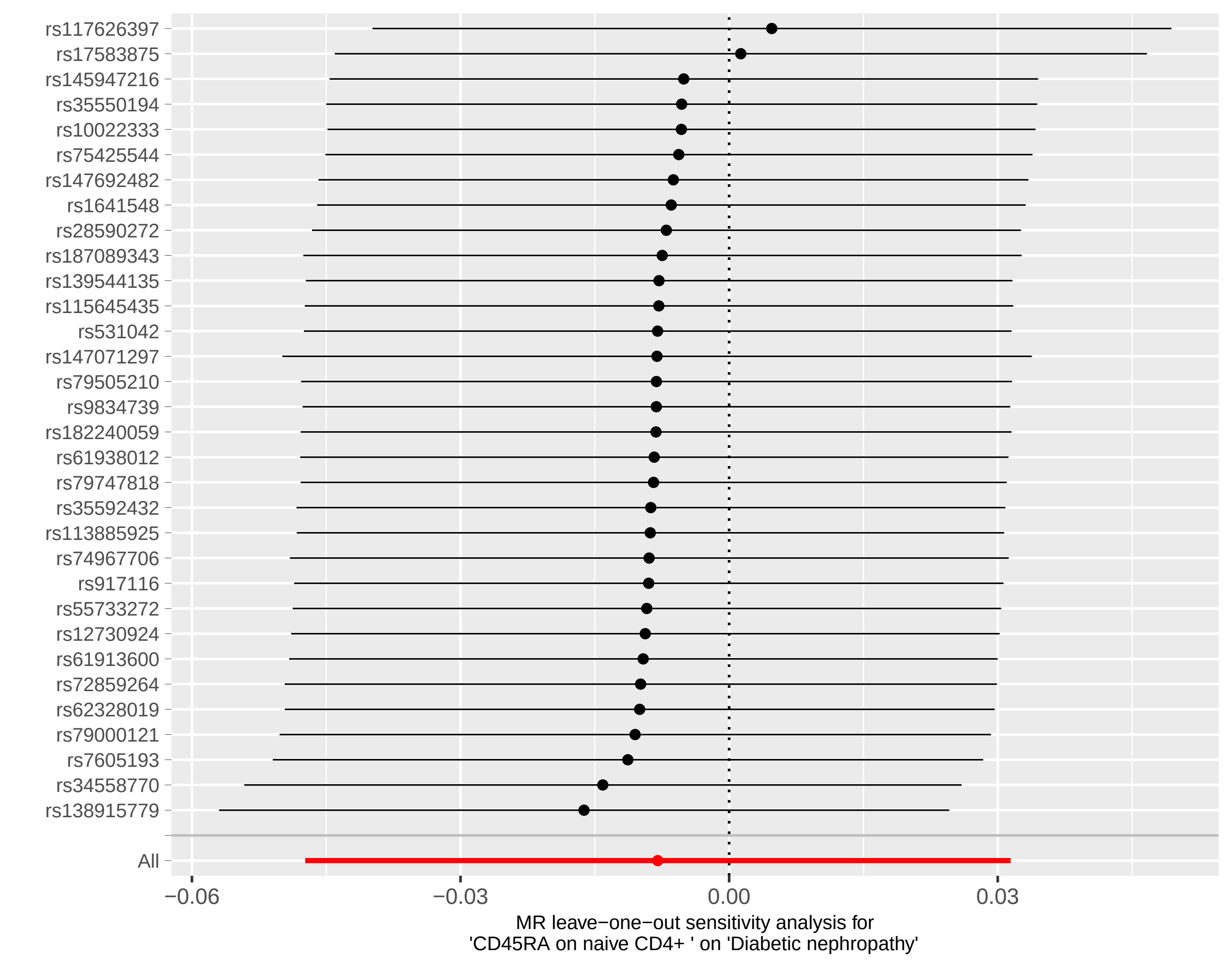
rs117626397
rs17583875
rs145947216
rs35550194
rs10022333
rs75425544
rs147692482
rs1641548
rs28590272
rs187089343
rs139544135
rs115645435
rs531042
rs147071297
rs79505210
rs9834739
rs182240059
rs61938012
rs79747818
rs35592432
rs113885925
rs74967706
rs917116
rs55733272
rs12730924
rs61913600
rs72859264
rs62328019
rs79000121
rs7605193
rs34558770
rs138915779
All
−0.06
−0.03
0.00
0.03
MR leave−one−out sensitivity analysis for
'CD45RA on naive CD4+ ' on 'Diabetic nephropathy'

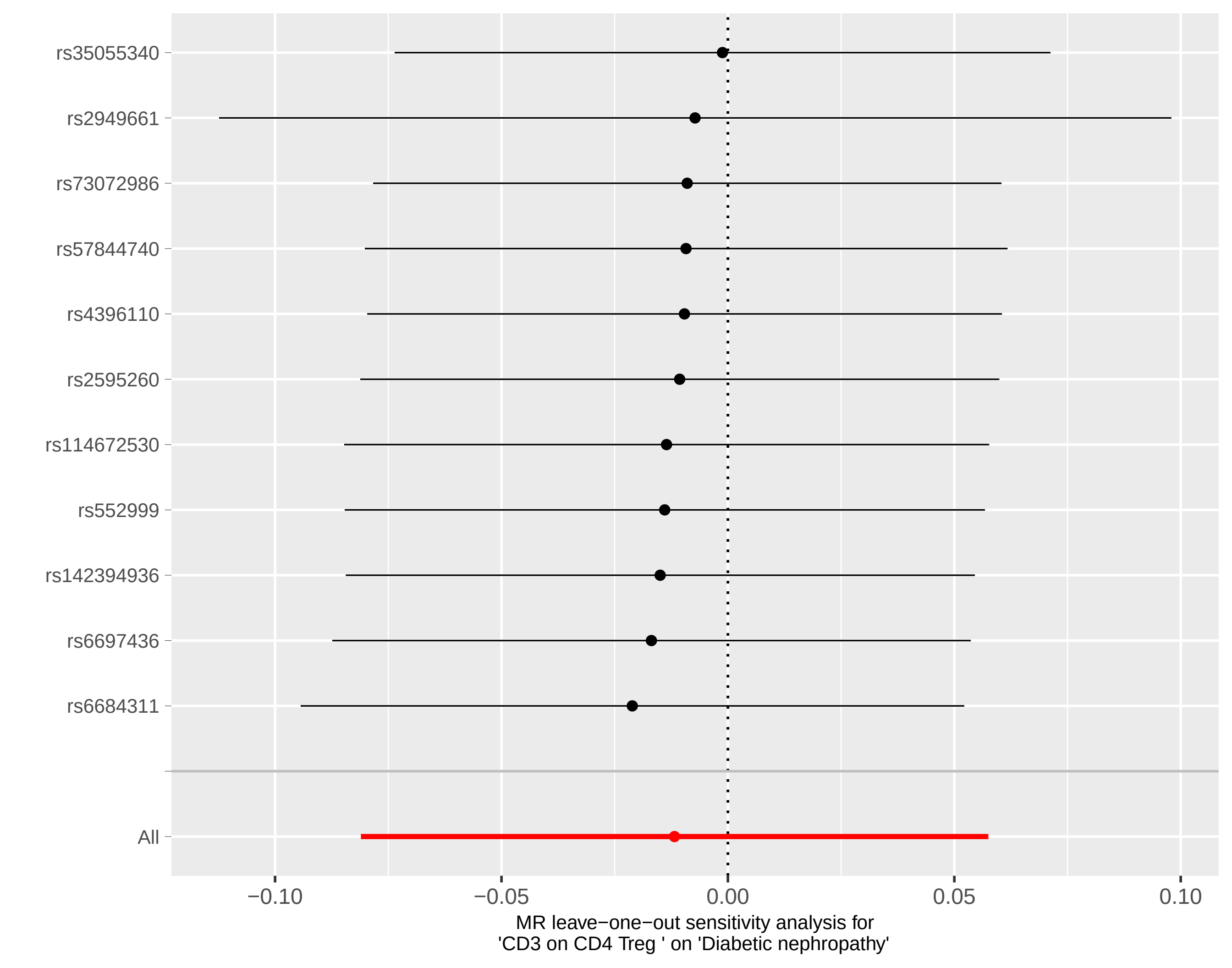
rs35055340
rs2949661
rs73072986
rs57844740
rs4396110
rs2595260
rs114672530
rs552999
rs142394936
rs6697436
rs6684311
All
−0.10
−0.05
0.00
0.05
0.10
MR leave−one−out sensitivity analysis for
'CD3 on CD4 Treg ' on 'Diabetic nephropathy'

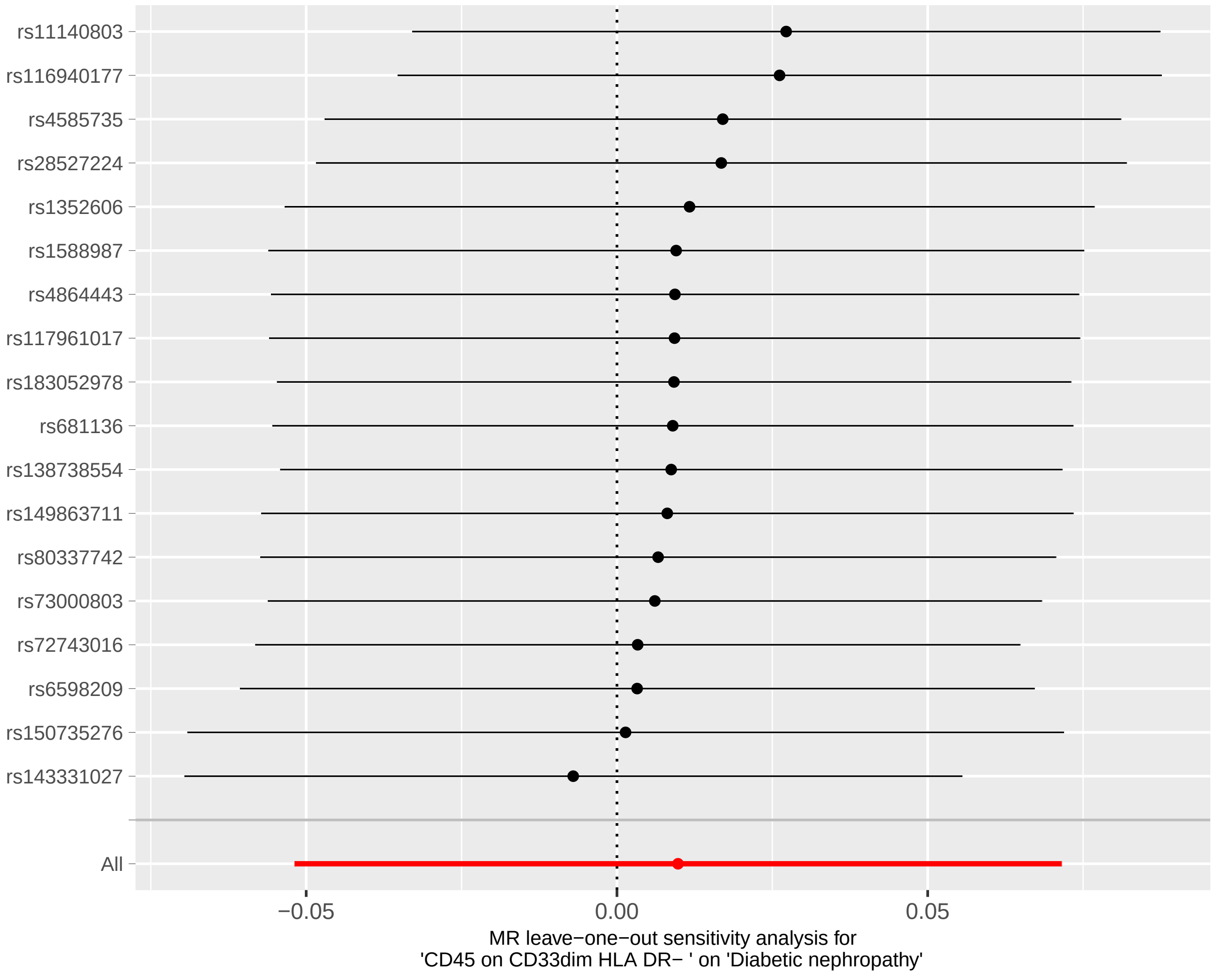
rs11140803
rs116940177
rs4585735
rs28527224
rs1352606
rs1588987
rs4864443
rs117961017
rs183052978
rs681136
rs138738554
rs149863711
rs80337742
rs73000803
rs72743016
rs6598209
rs150735276
rs143331027
All
−0.05
0.00
0.05
MR leave−one−out sensitivity analysis for
'CD45 on CD33dim HLA DR− ' on 'Diabetic nephropathy'

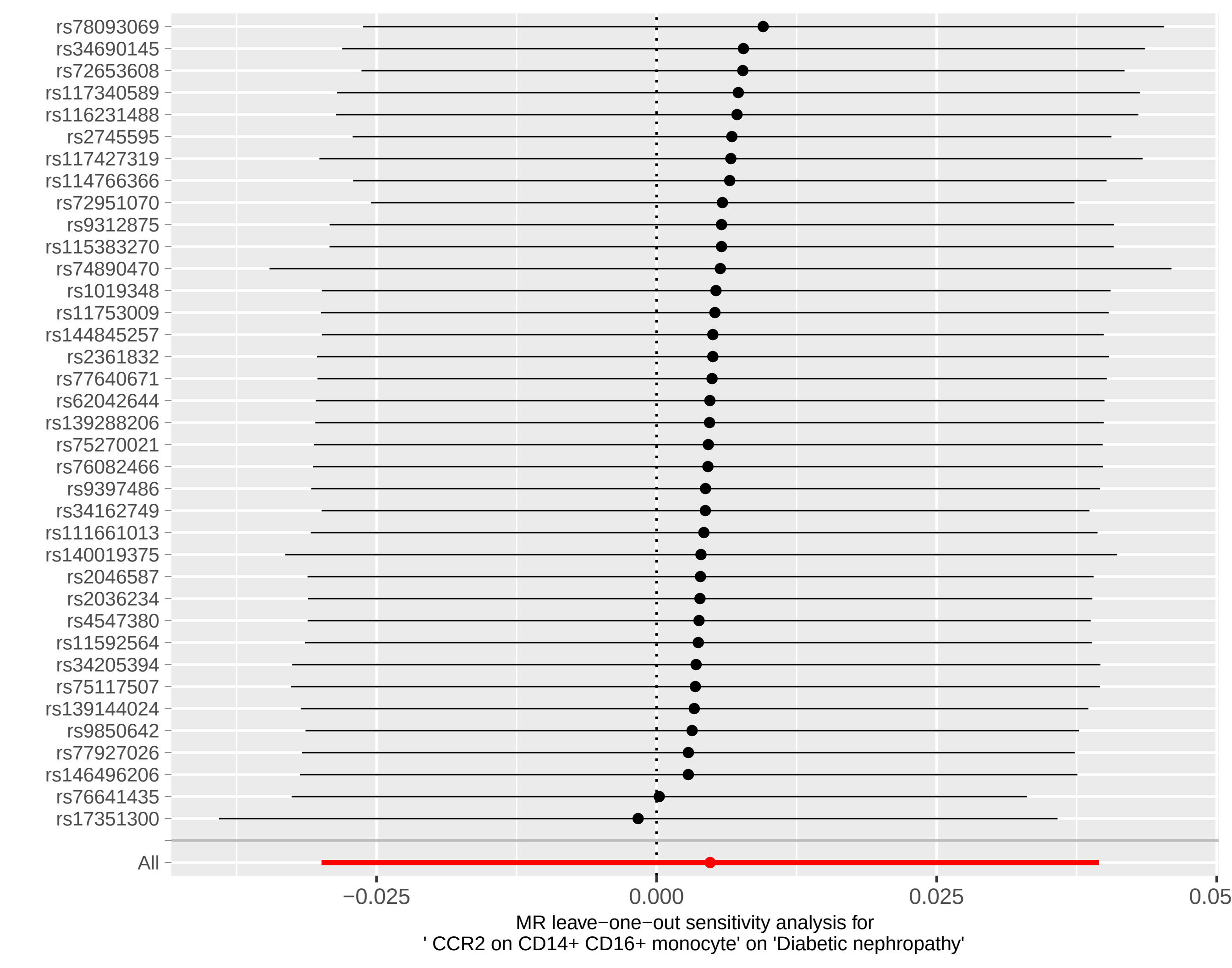
rs78093069
rs34690145
rs72653608
rs117340589
rs116231488
rs2745595
rs117427319
rs114766366
rs72951070
rs9312875
rs115383270
rs74890470
rs1019348
rs11753009
rs144845257
rs2361832
rs77640671
rs62042644
rs139288206
rs75270021
rs76082466
rs9397486
rs34162749
rs111661013
rs140019375
rs2046587
rs2036234
rs4547380
rs11592564
rs34205394
rs75117507
rs139144024
rs9850642
rs77927026
rs146496206
rs76641435
rs17351300
All
−0.025
0.000
0.025
0.05
MR leave−one−out sensitivity analysis for
' CCR2 on CD14+ CD16+ monocyte' on 'Diabetic nephropathy'

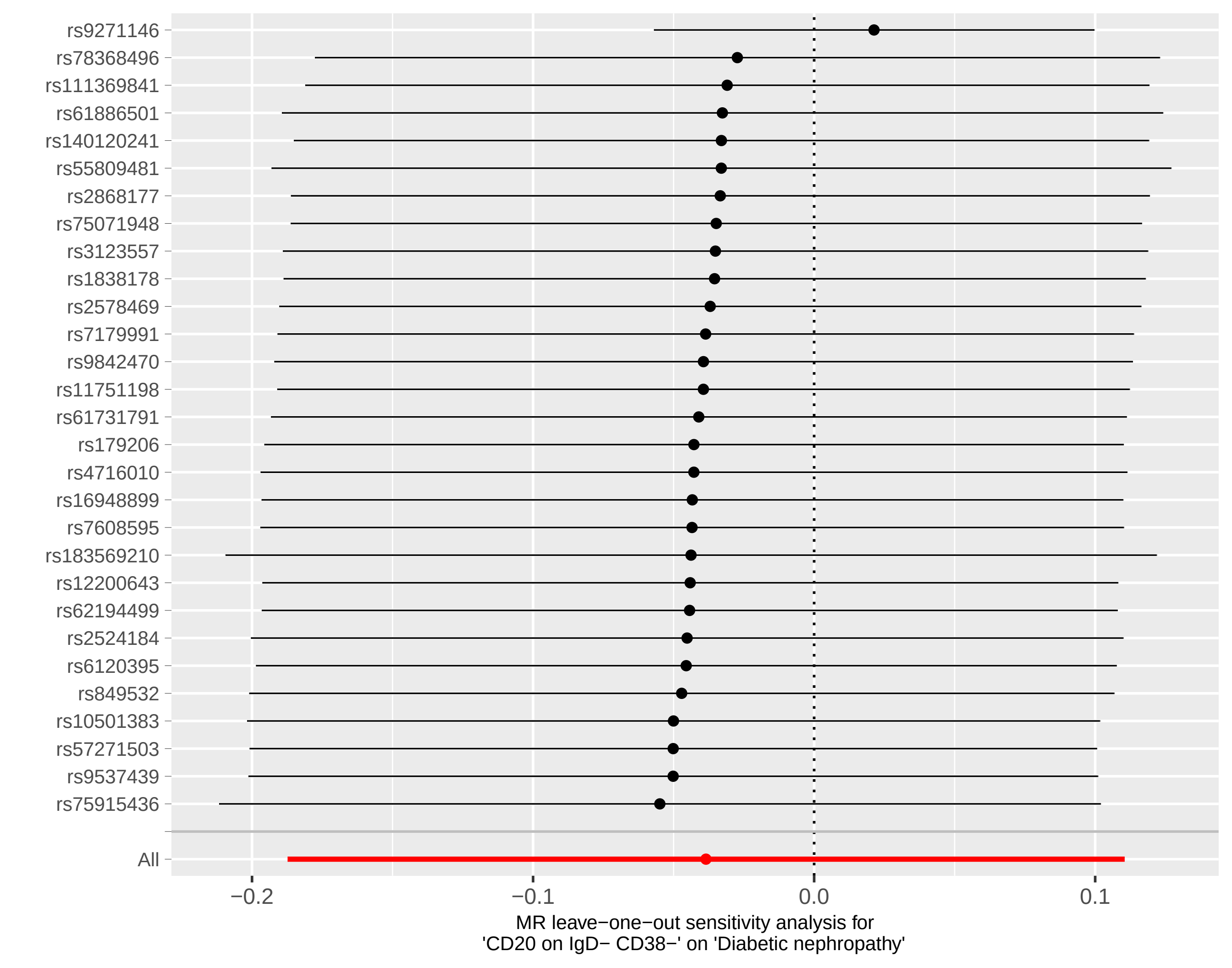
rs9271146
rs78368496
rs111369841
rs61886501
rs140120241
rs55809481
rs2868177
rs75071948
rs3123557
rs1838178
rs2578469
rs7179991
rs9842470
rs11751198
rs61731791
rs179206
rs4716010
rs16948899
rs7608595
rs183569210
rs12200643
rs62194499
rs2524184
rs6120395
rs849532
rs10501383
rs57271503
rs9537439
rs75915436
All
−0.2
−0.1
0.0
0.1
MR leave−one−out sensitivity analysis for
'CD20 on IgD− CD38−' on 'Diabetic nephropathy'

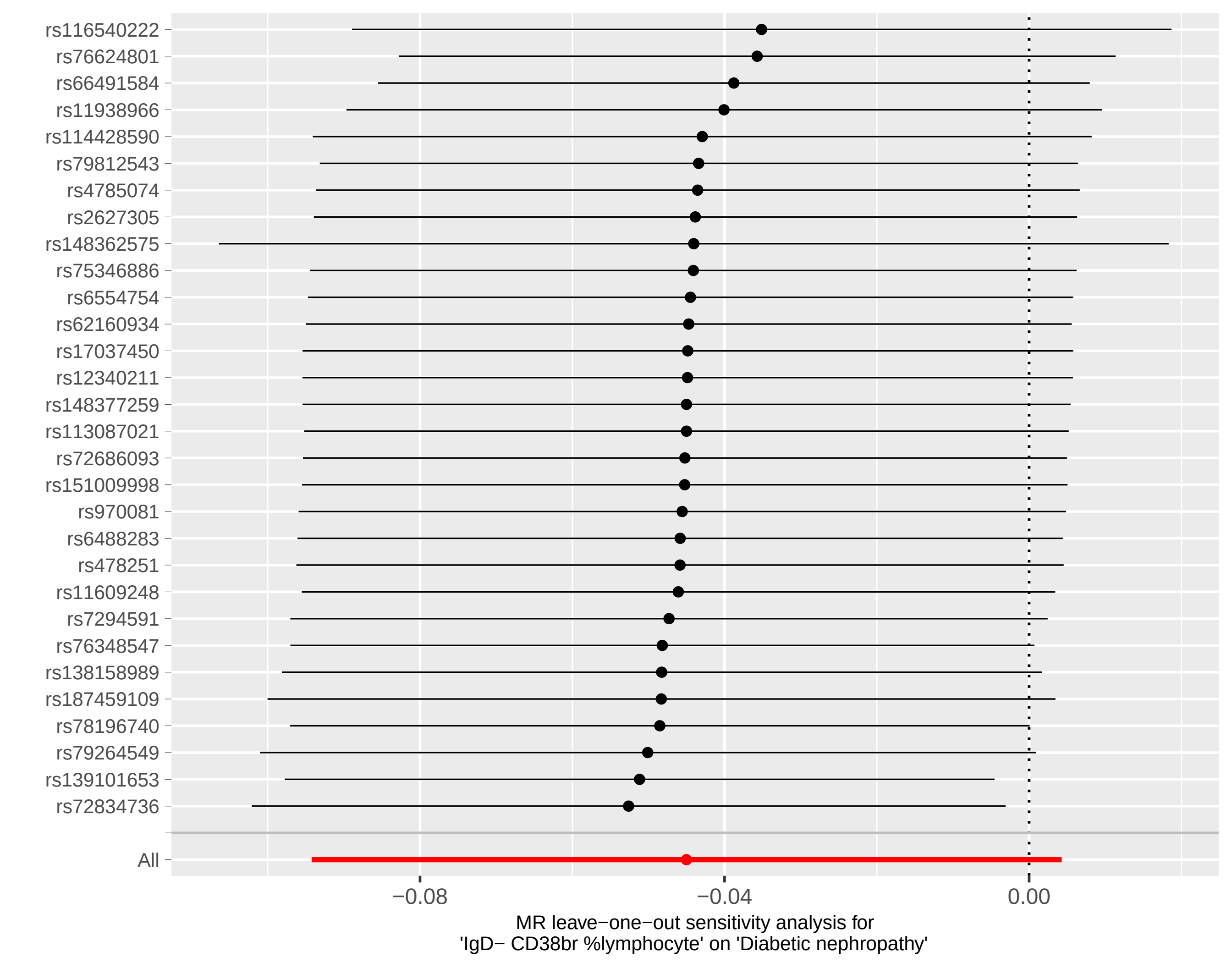
rs116540222
rs76624801
rs66491584
rs11938966
rs114428590
rs79812543
rs4785074
rs2627305
rs148362575
rs75346886
rs6554754
rs62160934
rs17037450
rs12340211
rs148377259
rs113087021
rs72686093
rs151009998
rs970081
rs6488283
rs478251
rs11609248
rs7294591
rs76348547
rs138158989
rs187459109
rs78196740
rs79264549
rs139101653
rs72834736
All
−0.08
−0.04
0.00
MR leave−one−out sensitivity analysis for
'IgD− CD38br %lymphocyte' on 'Diabetic nephropathy'

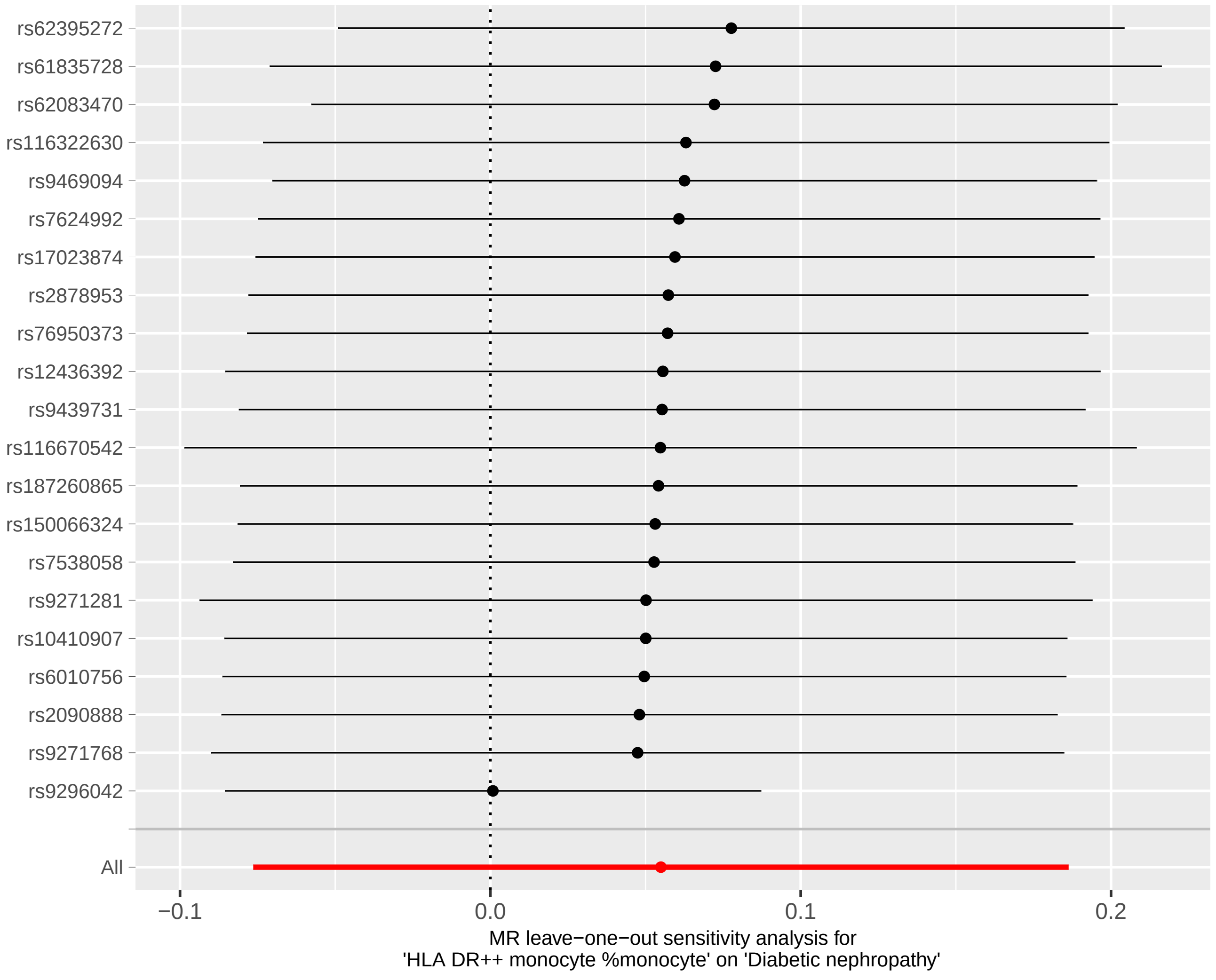

rs62395272
rs61835728
rs62083470
rs116322630
rs9469094
rs7624992
rs17023874
rs2878953
rs76950373
rs12436392
rs9439731
rs116670542
rs187260865
rs150066324
rs7538058
rs9271281
rs10410907
rs6010756
rs2090888
rs9271768
rs9296042
All
−0.1
0.0
0.1
0.2
MR leave−one−out sensitivity analysis for
'HLA DR++ monocyte %monocyte' on 'Diabetic nephropathy'

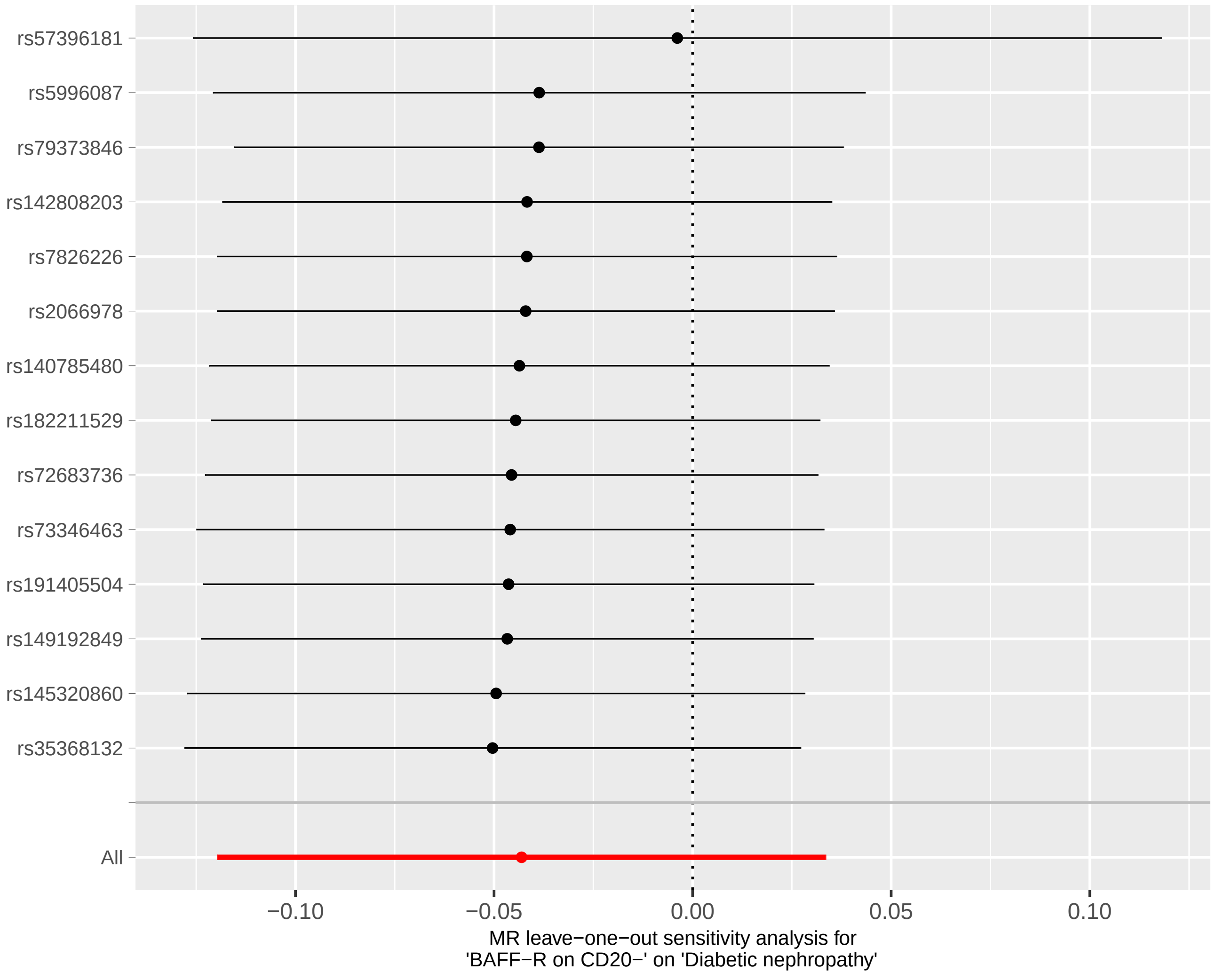
rs57396181
rs5996087
rs79373846
rs142808203
rs7826226
rs2066978
rs140785480
rs182211529
rs72683736
rs73346463
rs191405504
rs149192849
rs145320860
rs35368132
All
−0.10
−0.05
0.00
0.05
0.10
MR leave−one−out sensitivity analysis for
'BAFF−R on CD20−' on 'Diabetic nephropathy'

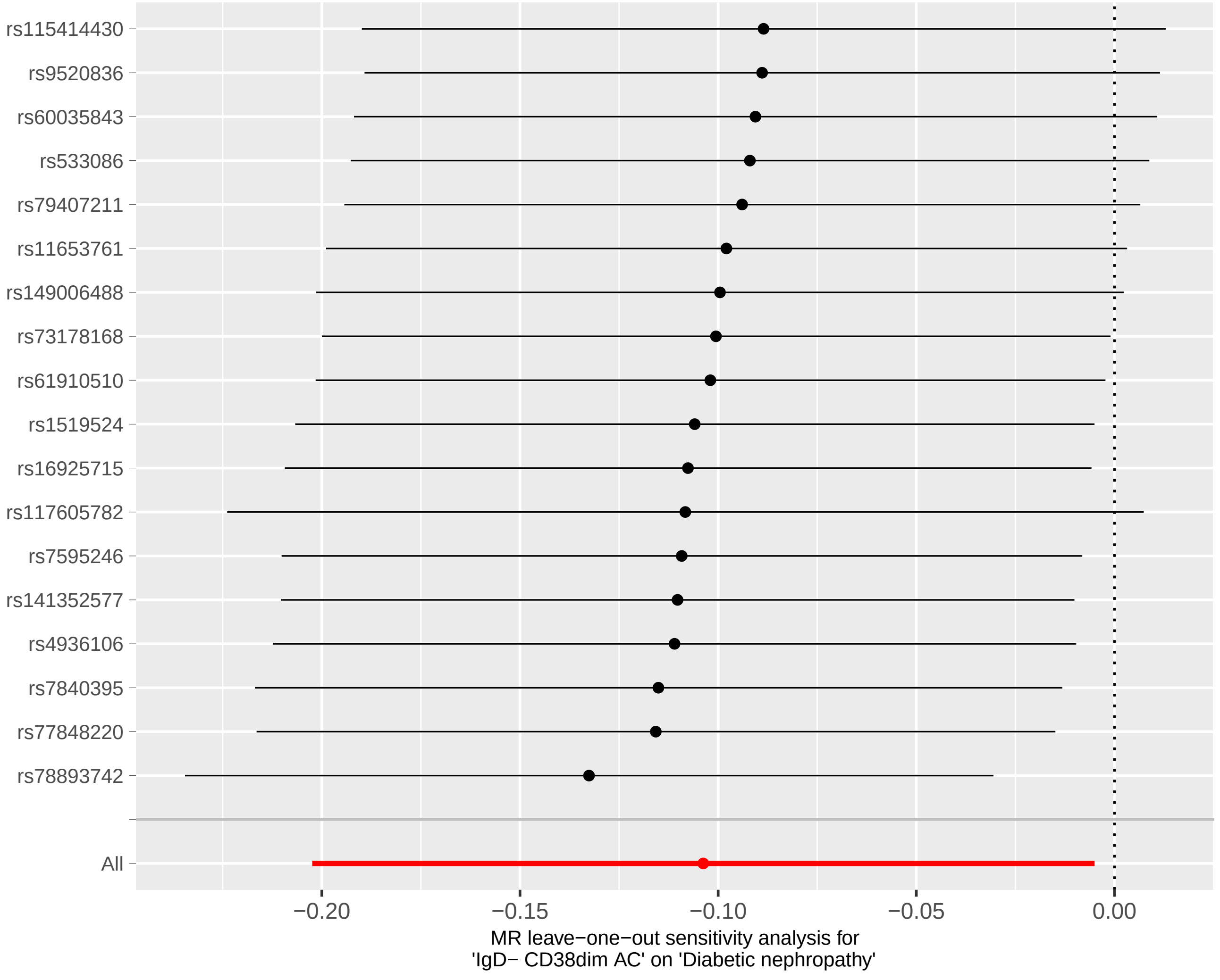

rs115414430
rs9520836
rs60035843
rs533086
rs79407211
rs11653761
rs149006488
rs73178168
rs61910510
rs1519524
rs16925715
rs117605782
rs7595246
rs141352577
rs4936106
rs7840395
rs77848220
rs78893742
All
−0.20
−0.15
−0.10
−0.05
0.00
MR leave−one−out sensitivity analysis for
'IgD− CD38dim AC' on 'Diabetic nephropathy'

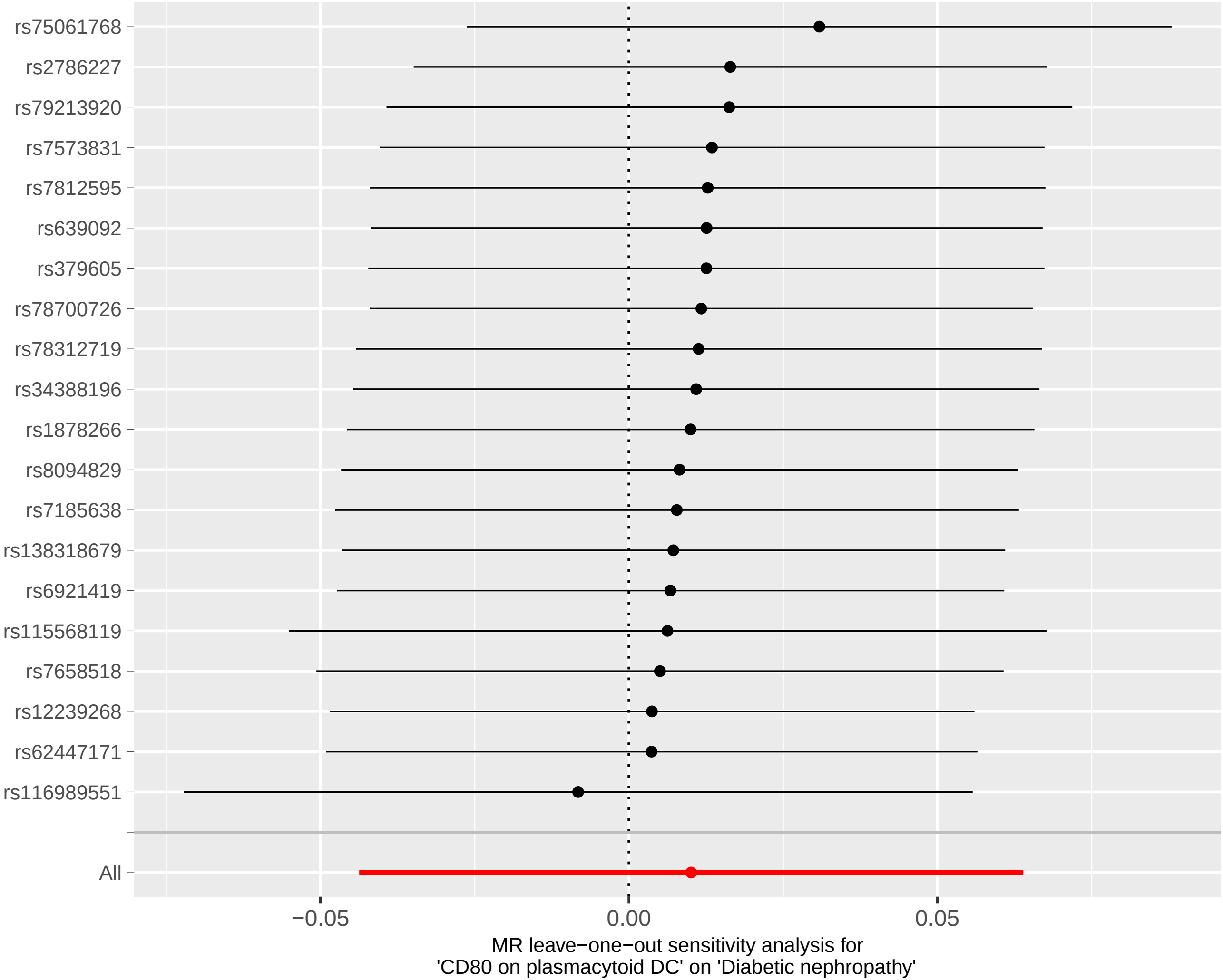

rs75061768
rs2786227
rs79213920
rs7573831
rs7812595
rs639092
rs379605
rs78700726
rs78312719
rs34388196
rs1878266
rs8094829
rs7185638
rs138318679
rs6921419
rs115568119
rs7658518
rs12239268
rs62447171
rs116989551
All
−0.05
0.00
0.05
MR leave−one−out sensitivity analysis for
'CD80 on plasmacytoid DC' on 'Diabetic nephropathy'

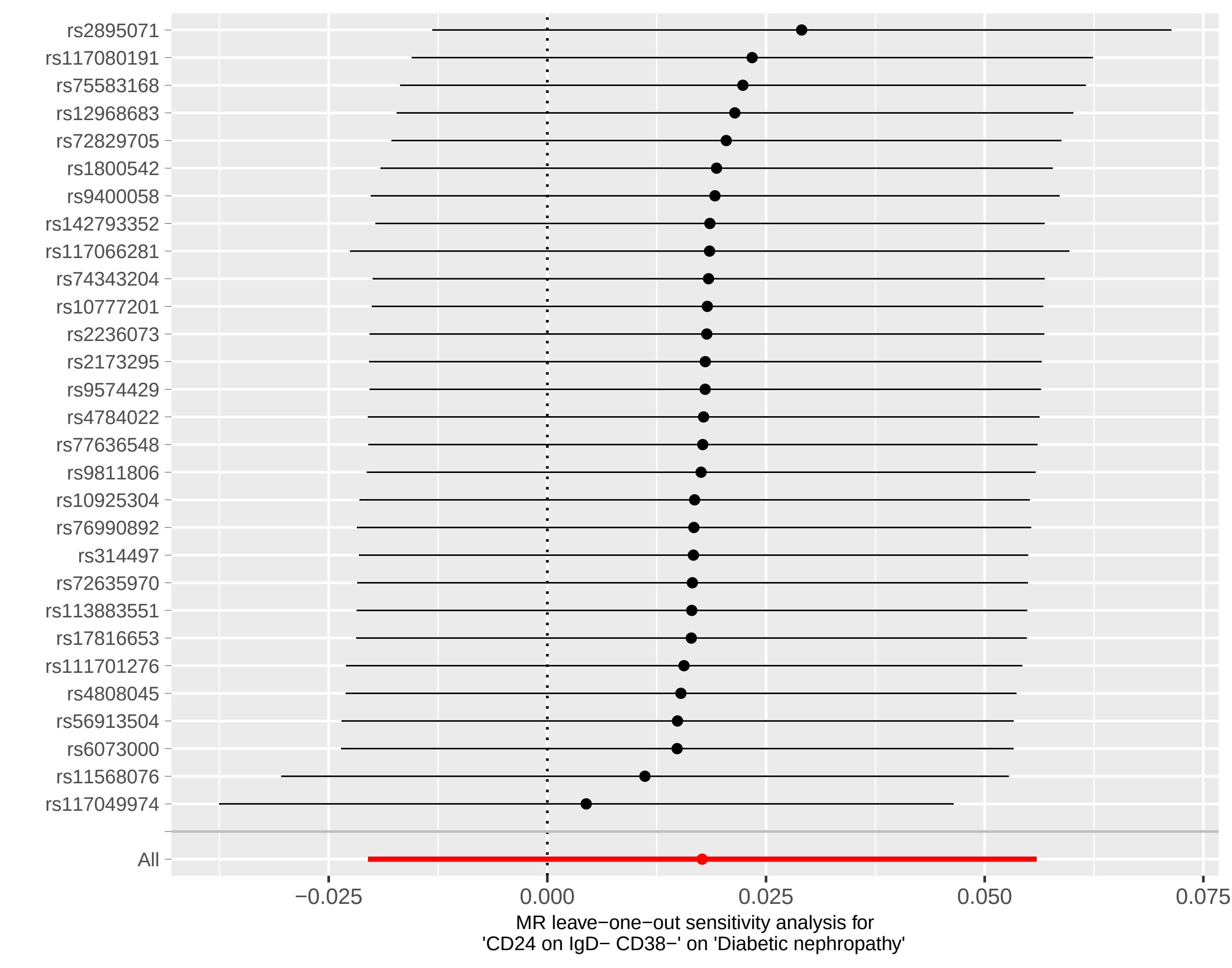
rs2895071
rs117080191
rs75583168
rs12968683
rs72829705
rs1800542
rs9400058
rs142793352
rs117066281
rs74343204
rs10777201
rs2236073
rs2173295
rs9574429
rs4784022
rs77636548
rs9811806
rs10925304
rs76990892
rs314497
rs72635970
rs113883551
rs17816653
rs111701276
rs4808045
rs56913504
rs6073000
rs11568076
rs117049974
All
−0.025
0.000
0.025
0.050
0.075
MR leave−one−out sensitivity analysis for
'CD24 on IgD− CD38−' on 'Diabetic nephropathy'

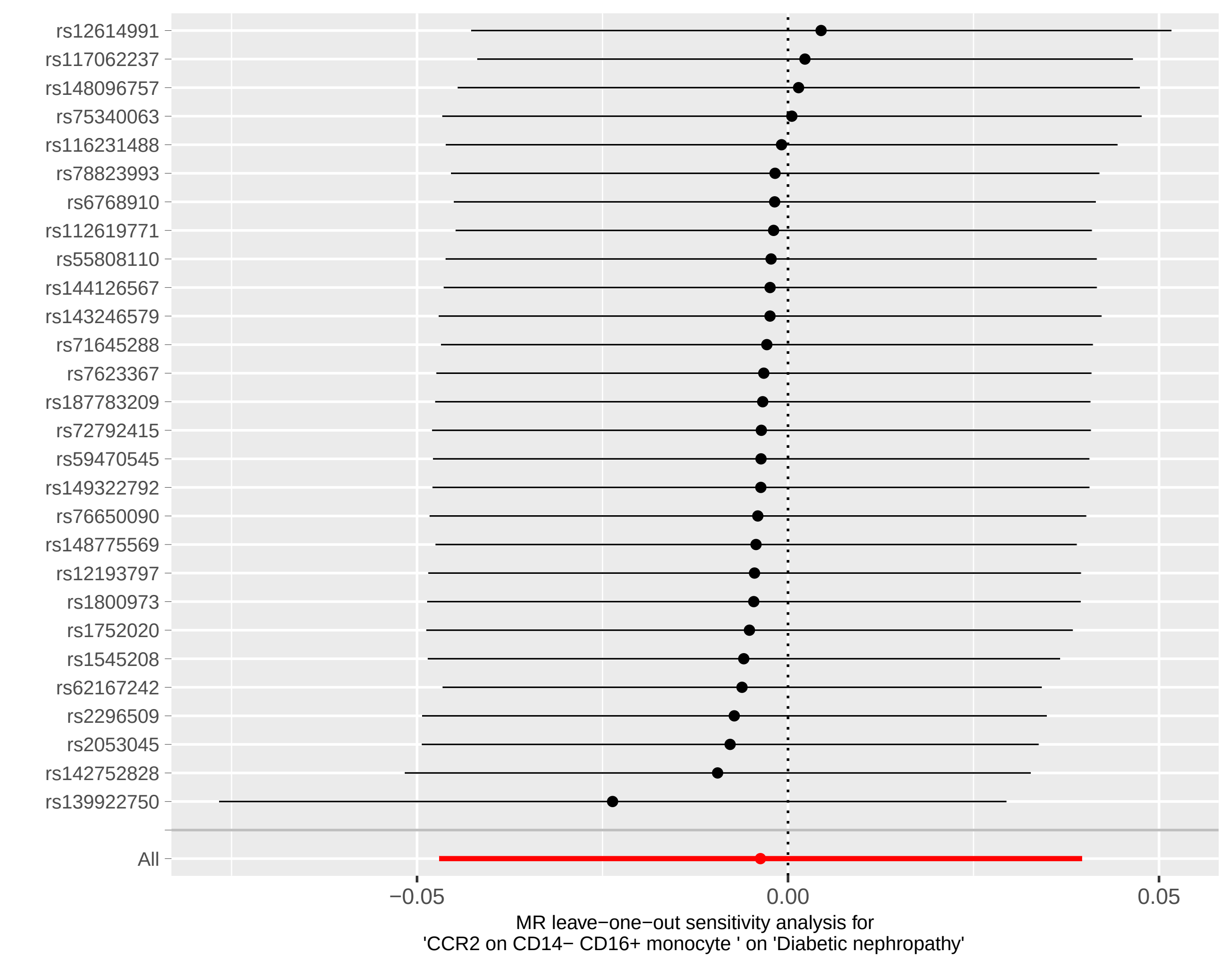

rs12614991
rs117062237
rs148096757
rs75340063
rs116231488
rs78823993
rs6768910
rs112619771
rs55808110
rs144126567
rs143246579
rs71645288
rs7623367
rs187783209
rs72792415
rs59470545
rs149322792
rs76650090
rs148775569
rs12193797
rs1800973
rs1752020
rs1545208
rs62167242
rs2296509
rs2053045
rs142752828
rs139922750
All
−0.05
0.00
0.05
MR leave−one−out sensitivity analysis for
'CCR2 on CD14− CD16+ monocyte ' on 'Diabetic nephropathy'

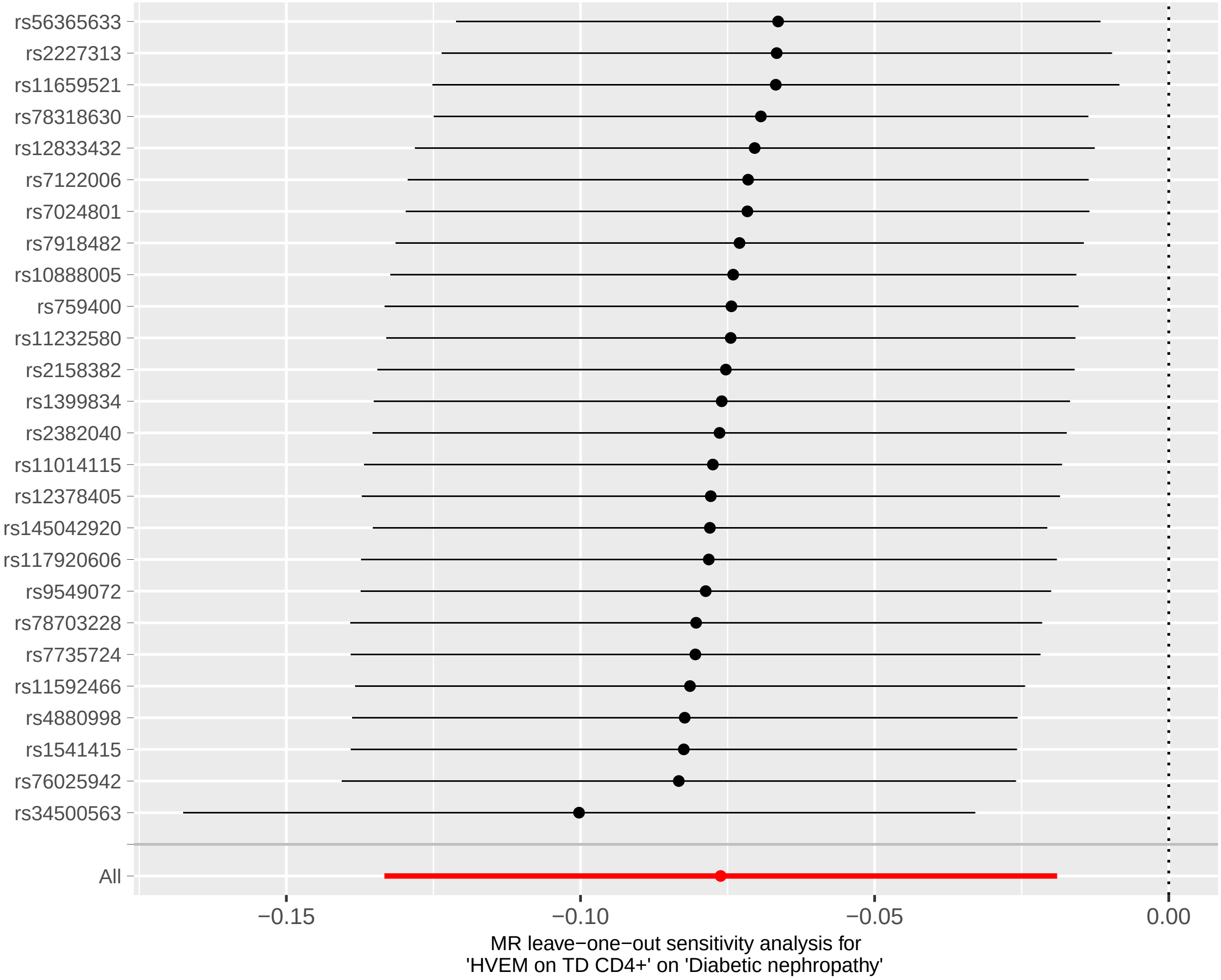

rs56365633
rs2227313
rs11659521
rs78318630
rs12833432
rs7122006
rs7024801
rs7918482
rs10888005
rs759400
rs11232580
rs2158382
rs1399834
rs2382040
rs11014115
rs12378405
rs145042920
rs117920606
rs9549072
rs78703228
rs7735724
rs11592466
rs4880998
rs1541415
rs76025942
rs34500563
All
−0.15
−0.10
−0.05
0.00
MR leave−one−out sensitivity analysis for
'HVEM on TD CD4+' on 'Diabetic nephropathy'

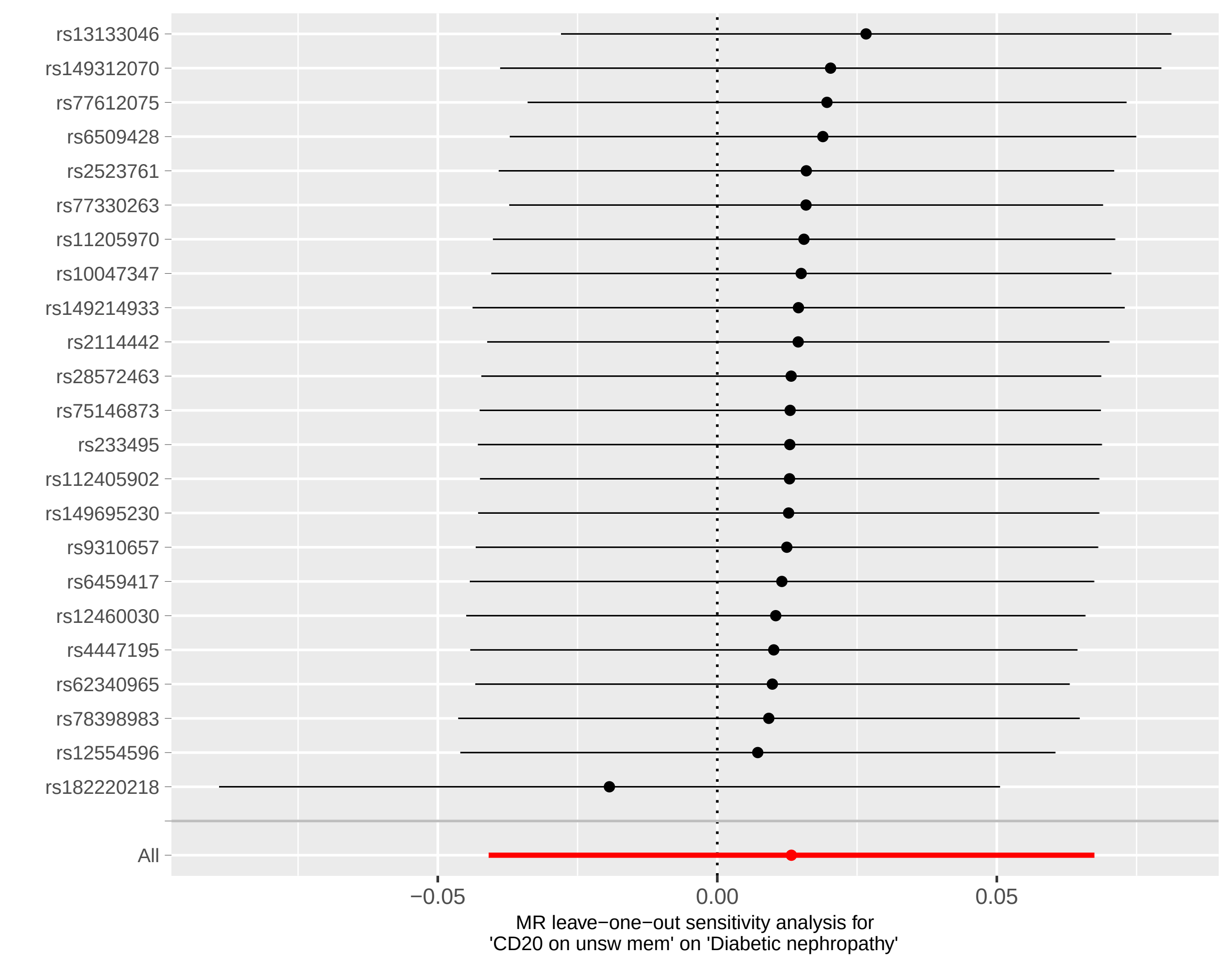
rs13133046
rs149312070
rs77612075
rs6509428
rs2523761
rs77330263
rs11205970
rs10047347
rs149214933
rs2114442
rs28572463
rs75146873
rs233495
rs112405902
rs149695230
rs9310657
rs6459417
rs12460030
rs4447195
rs62340965
rs78398983
rs12554596
rs182220218
All
−0.05
0.00
0.05
MR leave−one−out sensitivity analysis for
'CD20 on unsw mem' on 'Diabetic nephropathy'

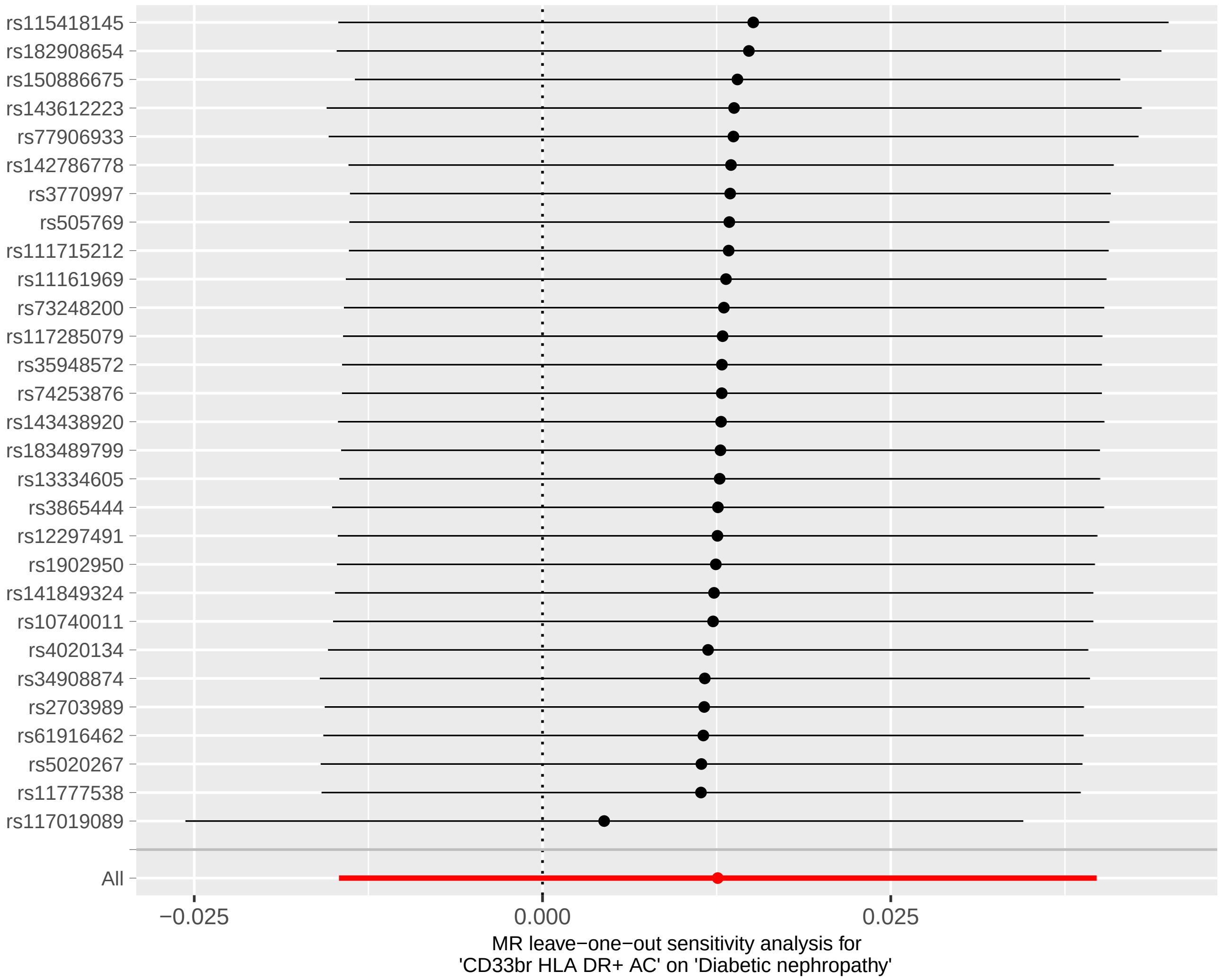
rs115418145
rs182908654
rs150886675
rs143612223
rs77906933
rs142786778
rs3770997
rs505769
rs111715212
rs11161969
rs73248200
rs117285079
rs35948572
rs74253876
rs143438920
rs183489799
rs13334605
rs3865444
rs12297491
rs1902950
rs141849324
rs10740011
rs4020134
rs34908874
rs2703989
rs61916462
rs5020267
rs11777538
rs117019089
All
−0.025
0.000
0.025
MR leave−one−out sensitivity analysis for
'CD33br HLA DR+ AC' on 'Diabetic nephropathy'

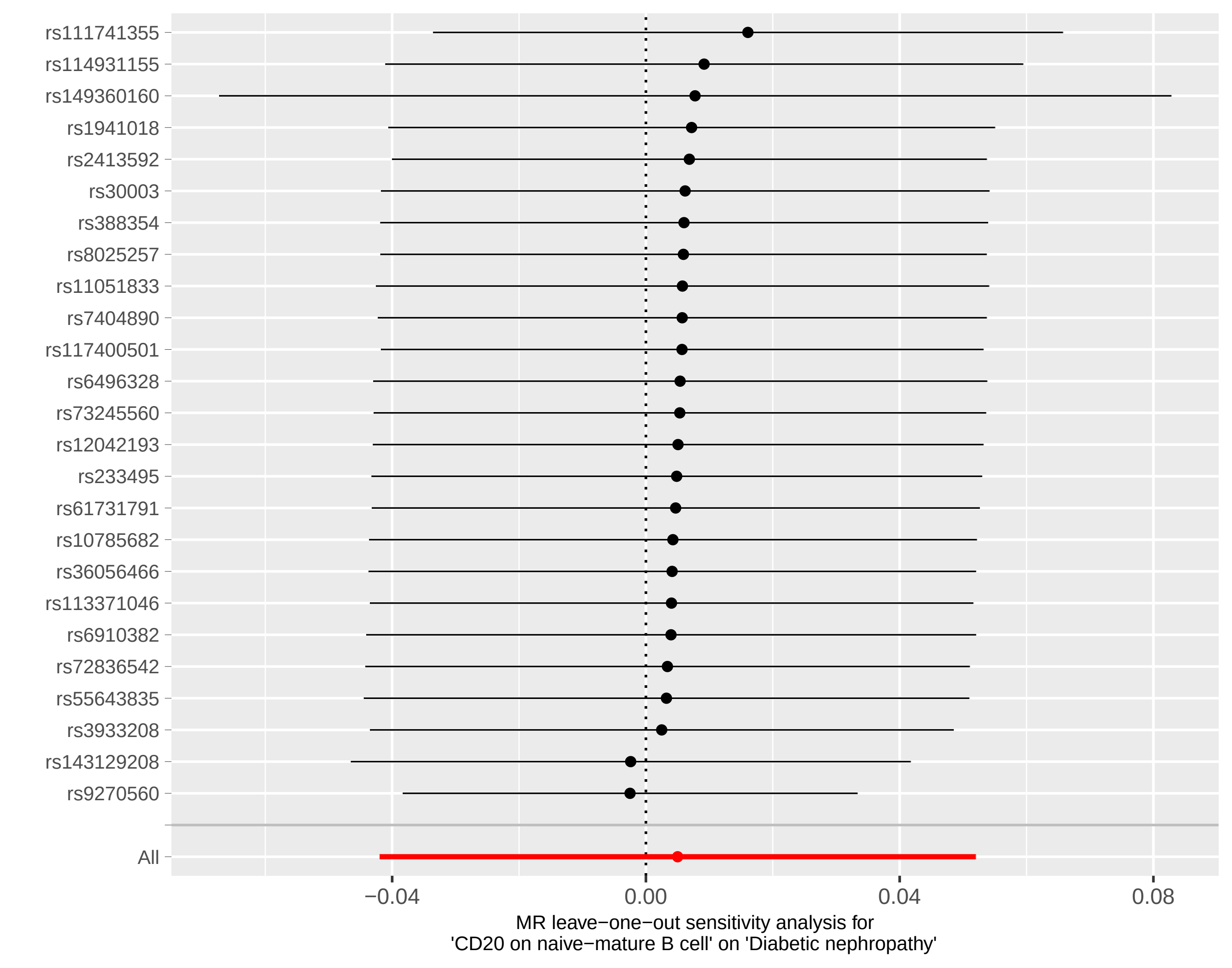
rs111741355
rs114931155
rs149360160
rs1941018
rs2413592
rs30003
rs388354
rs8025257
rs11051833
rs7404890
rs117400501
rs6496328
rs73245560
rs12042193
rs233495
rs61731791
rs10785682
rs36056466
rs113371046
rs6910382
rs72836542
rs55643835
rs3933208
rs143129208
rs9270560
All
−0.04
0.00
0.04
0.08
MR leave−one−out sensitivity analysis for
'CD20 on naive−mature B cell' on 'Diabetic nephropathy'

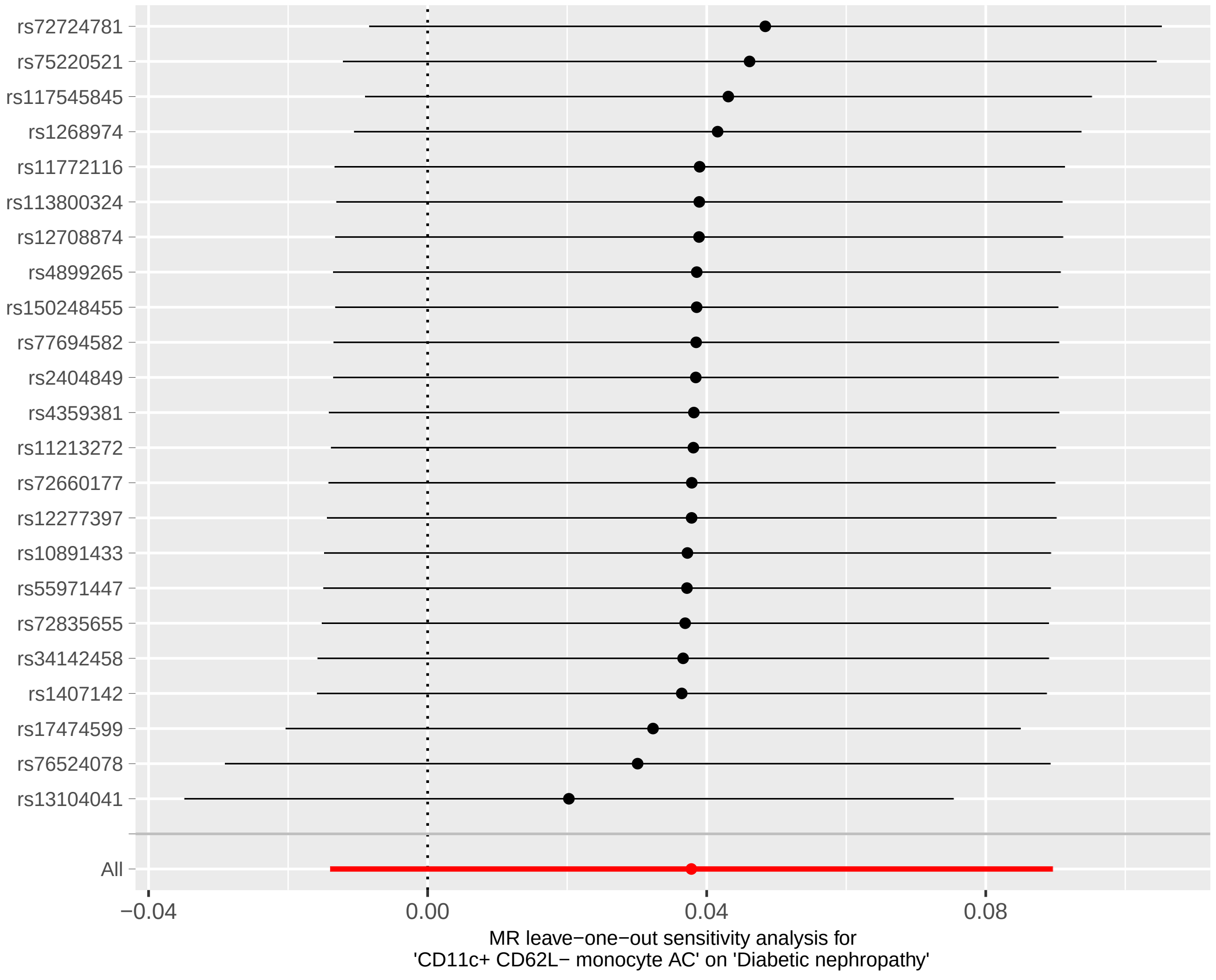
rs72724781
rs75220521
rs117545845
rs1268974
rs11772116
rs113800324
rs12708874
rs4899265
rs150248455
rs77694582
rs2404849
rs4359381
rs11213272
rs72660177
rs12277397
rs10891433
rs55971447
rs72835655
rs34142458
rs1407142
rs17474599
rs76524078
rs13104041
All
−0.04
0.00
0.04
0.08
MR leave−one−out sensitivity analysis for
'CD11c+ CD62L− monocyte AC' on 'Diabetic nephropathy'

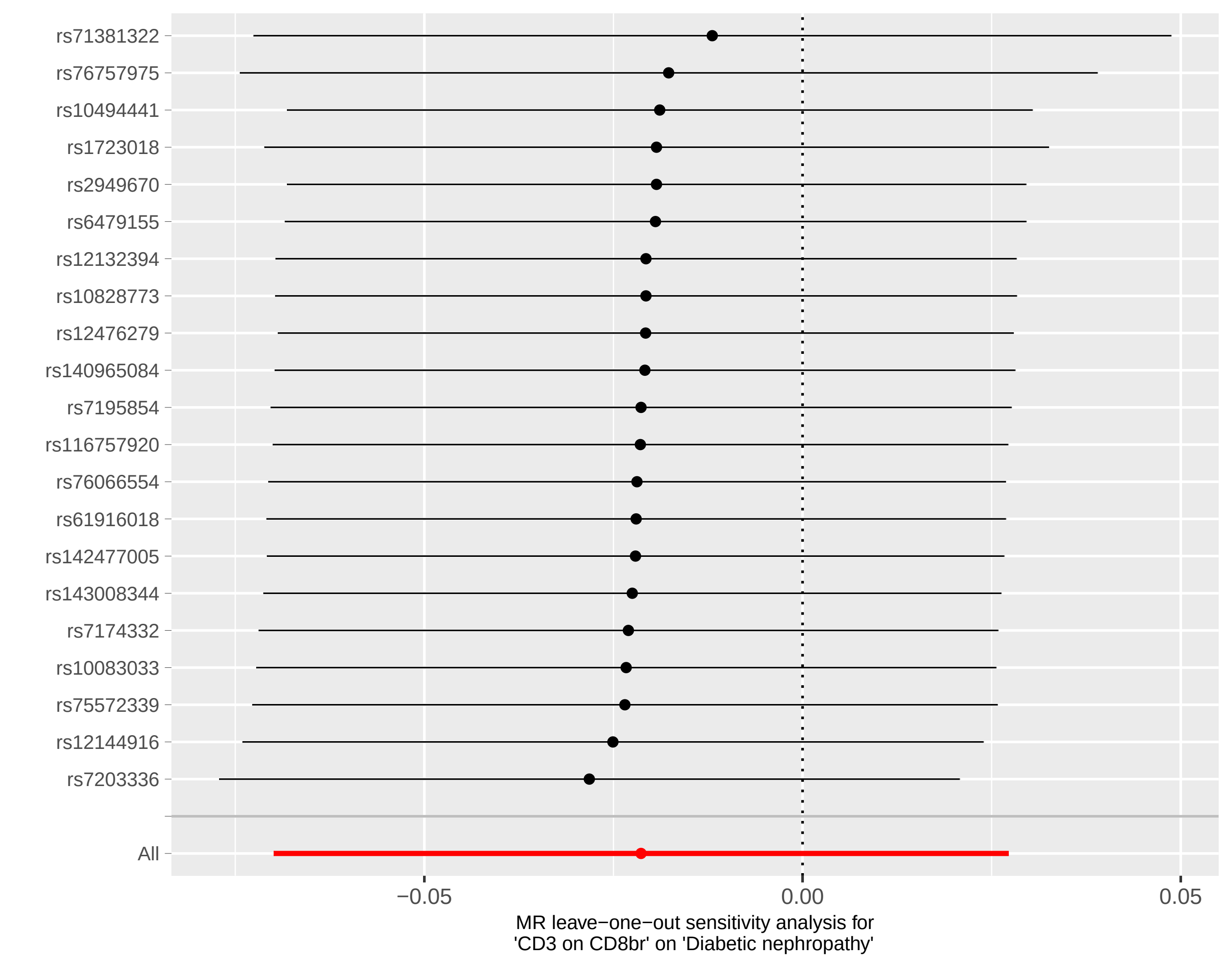

rs71381322
rs76757975
rs10494441
rs1723018
rs2949670
rs6479155
rs12132394
rs10828773
rs12476279
rs140965084
rs7195854
rs116757920
rs76066554
rs61916018
rs142477005
rs143008344
rs7174332
rs10083033
rs75572339
rs12144916
rs7203336
All
−0.05
0.00
0.05
MR leave−one−out sensitivity analysis for
'CD3 on CD8br' on 'Diabetic nephropathy'

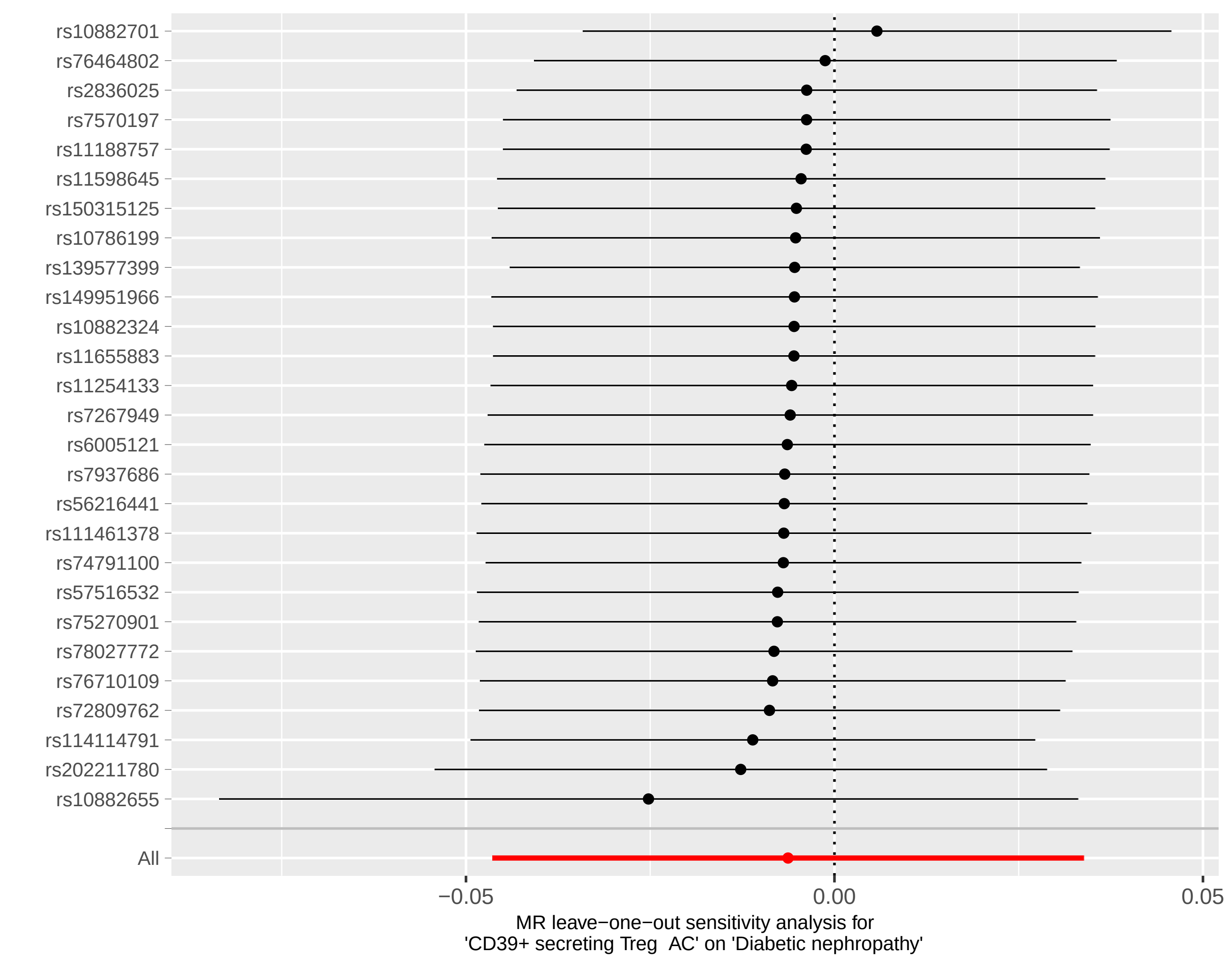
rs10882701
rs76464802
rs2836025
rs7570197
rs11188757
rs11598645
rs150315125
rs10786199
rs139577399
rs149951966
rs10882324
rs11655883
rs11254133
rs7267949
rs6005121
rs7937686
rs56216441
rs111461378
rs74791100
rs57516532
rs75270901
rs78027772
rs76710109
rs72809762
rs114114791
rs202211780
rs10882655
All
−0.05
0.00
0.05
MR leave−one−out sensitivity analysis for
'CD39+ secreting Treg AC' on 'Diabetic nephropathy'

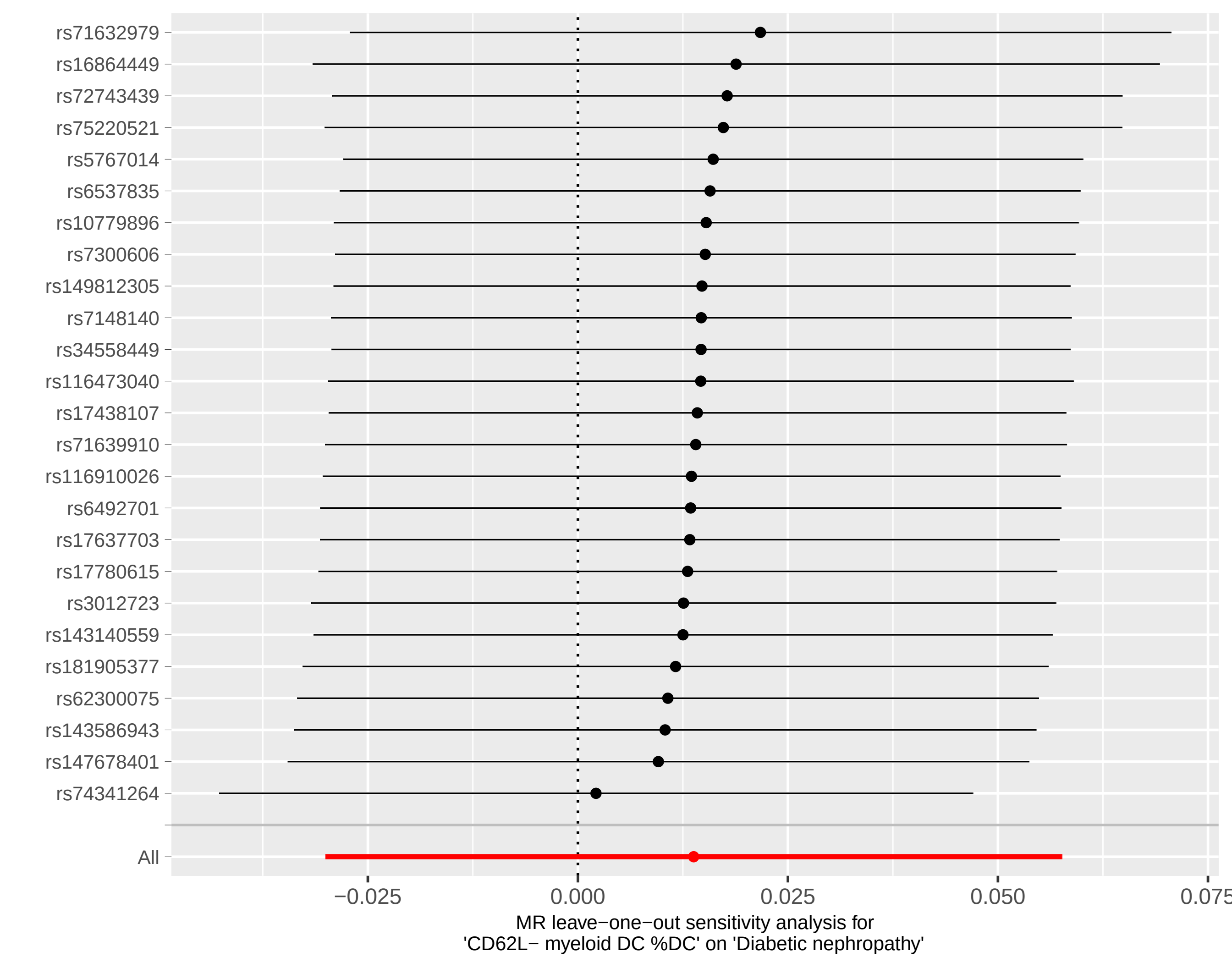

rs71632979
rs16864449
rs72743439
rs75220521
rs5767014
rs6537835
rs10779896
rs7300606
rs149812305
rs7148140
rs34558449
rs116473040
rs17438107
rs71639910
rs116910026
rs6492701
rs17637703
rs17780615
rs3012723
rs143140559
rs181905377
rs62300075
rs143586943
rs147678401
rs74341264
All
−0.025
0.000
0.025
0.050
0.075
MR leave−one−out sensitivity analysis for
'CD62L− myeloid DC %DC' on 'Diabetic nephropathy'

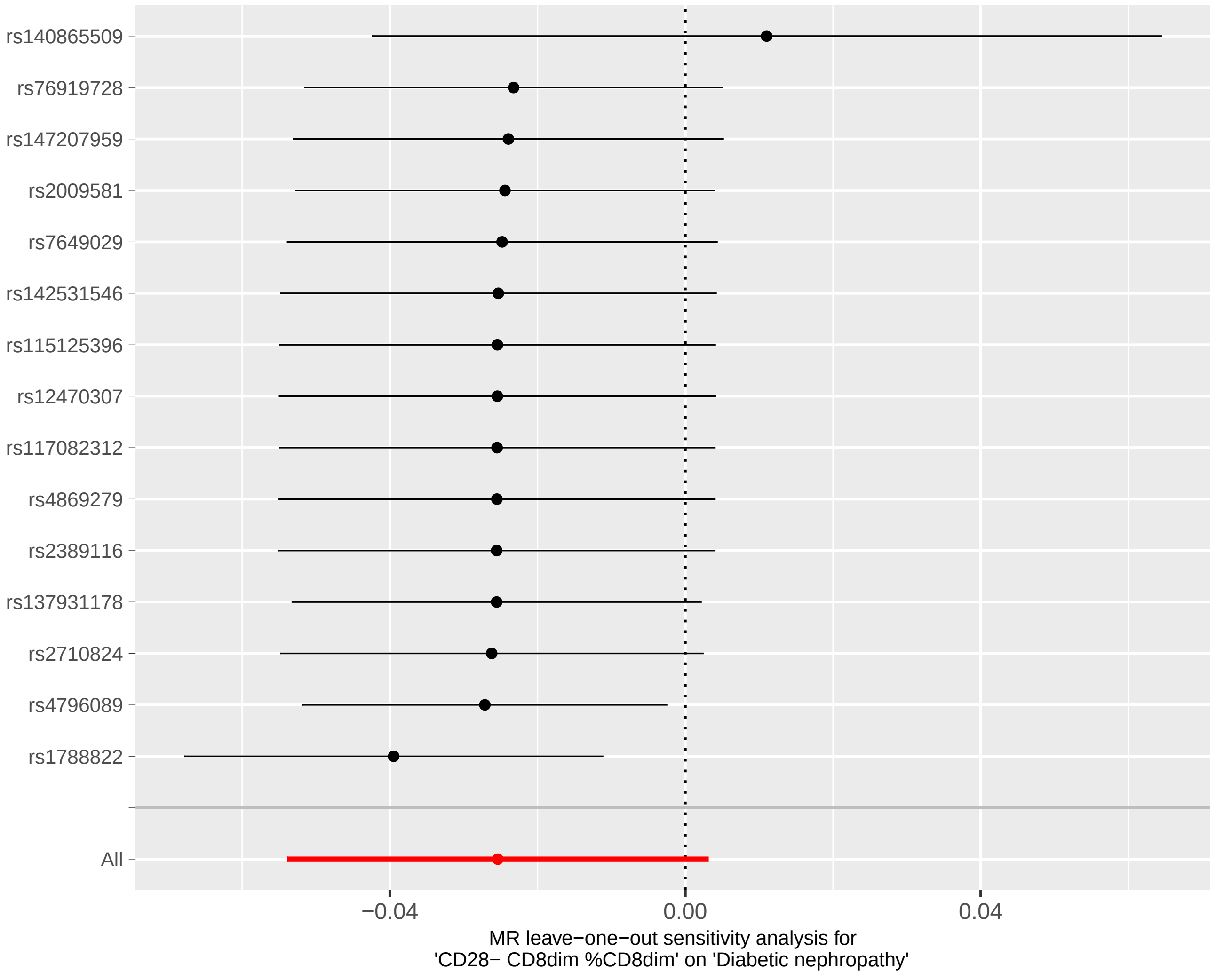
rs140865509
rs76919728
rs147207959
rs2009581
rs7649029
rs142531546
rs115125396
rs12470307
rs117082312
rs4869279
rs2389116
rs137931178
rs2710824
rs4796089
rs1788822
All
−0.04
0.00
0.04
MR leave−one−out sensitivity analysis for
'CD28− CD8dim %CD8dim' on 'Diabetic nephropathy'

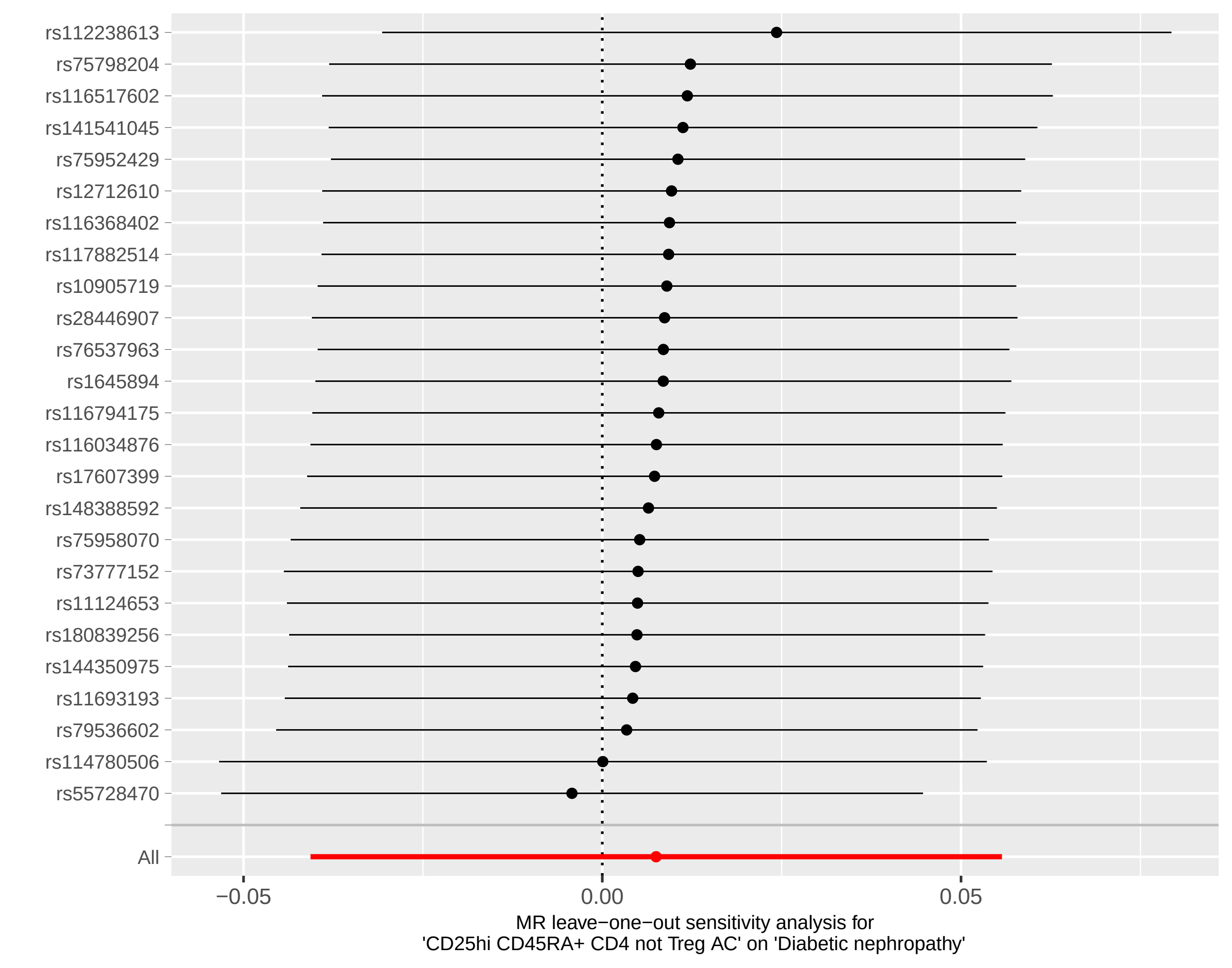
rs112238613
rs75798204
rs116517602
rs141541045
rs75952429
rs12712610
rs116368402
rs117882514
rs10905719
rs28446907
rs76537963
rs1645894
rs116794175
rs116034876
rs17607399
rs148388592
rs75958070
rs73777152
rs11124653
rs180839256
rs144350975
rs11693193
rs79536602
rs114780506
rs55728470
All
−0.05
0.00
0.05
MR leave−one−out sensitivity analysis for
'CD25hi CD45RA+ CD4 not Treg AC' on 'Diabetic nephropathy'

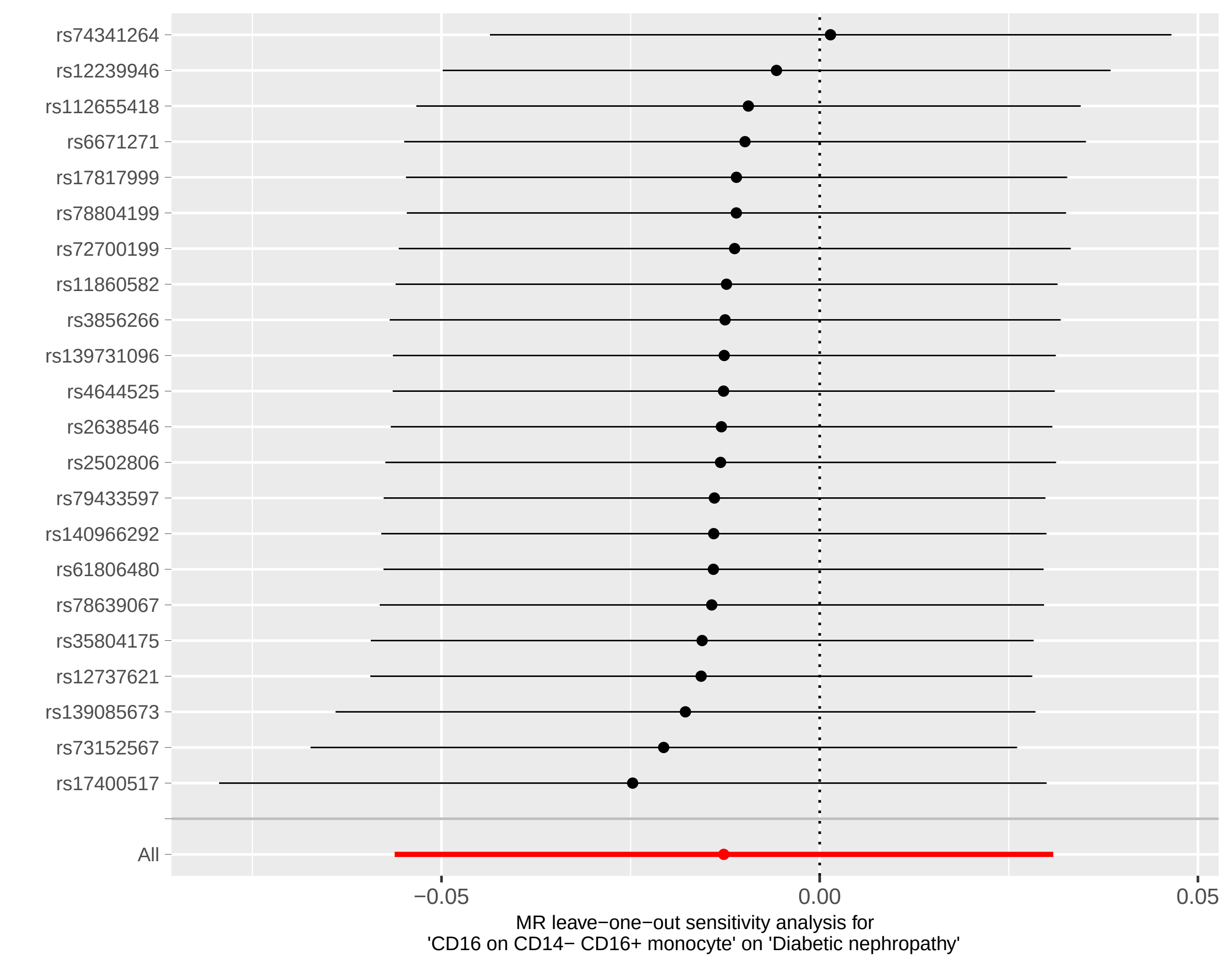
rs74341264
rs12239946
rs112655418
rs6671271
rs17817999
rs78804199
rs72700199
rs11860582
rs3856266
rs139731096
rs4644525
rs2638546
rs2502806
rs79433597
rs140966292
rs61806480
rs78639067
rs35804175
rs12737621
rs139085673
rs73152567
rs17400517
All
−0.05
0.00
0.05
MR leave−one−out sensitivity analysis for
'CD16 on CD14− CD16+ monocyte' on 'Diabetic nephropathy'

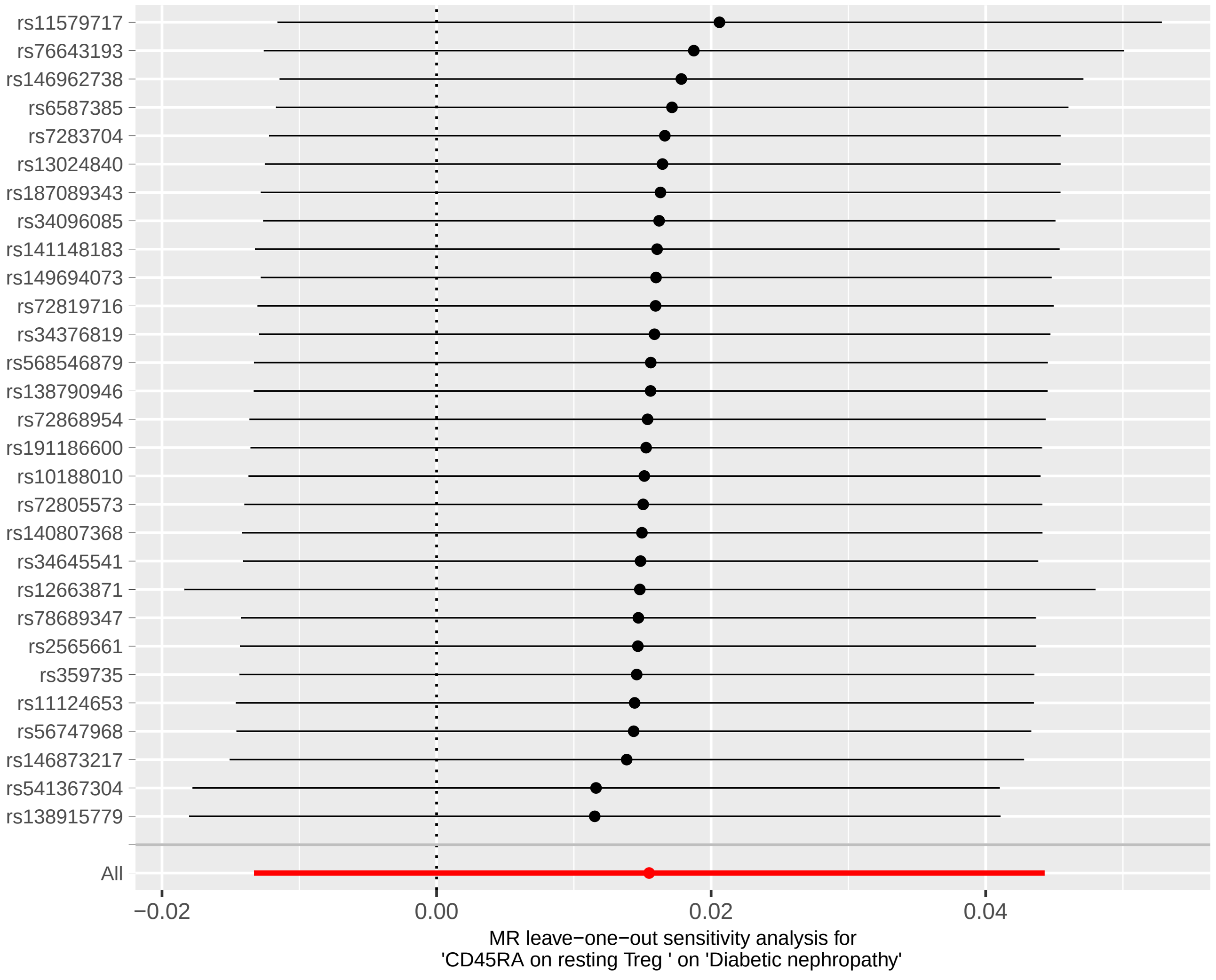
rs11579717
rs76643193
rs146962738
rs6587385
rs7283704
rs13024840
rs187089343
rs34096085
rs141148183
rs149694073
rs72819716
rs34376819
rs568546879
rs138790946
rs72868954
rs191186600
rs10188010
rs72805573
rs140807368
rs34645541
rs12663871
rs78689347
rs2565661
rs359735
rs11124653
rs56747968
rs146873217
rs541367304
rs138915779
All
−0.02
0.00
0.02
0.04
MR leave−one−out sensitivity analysis for
'CD45RA on resting Treg ' on 'Diabetic nephropathy'

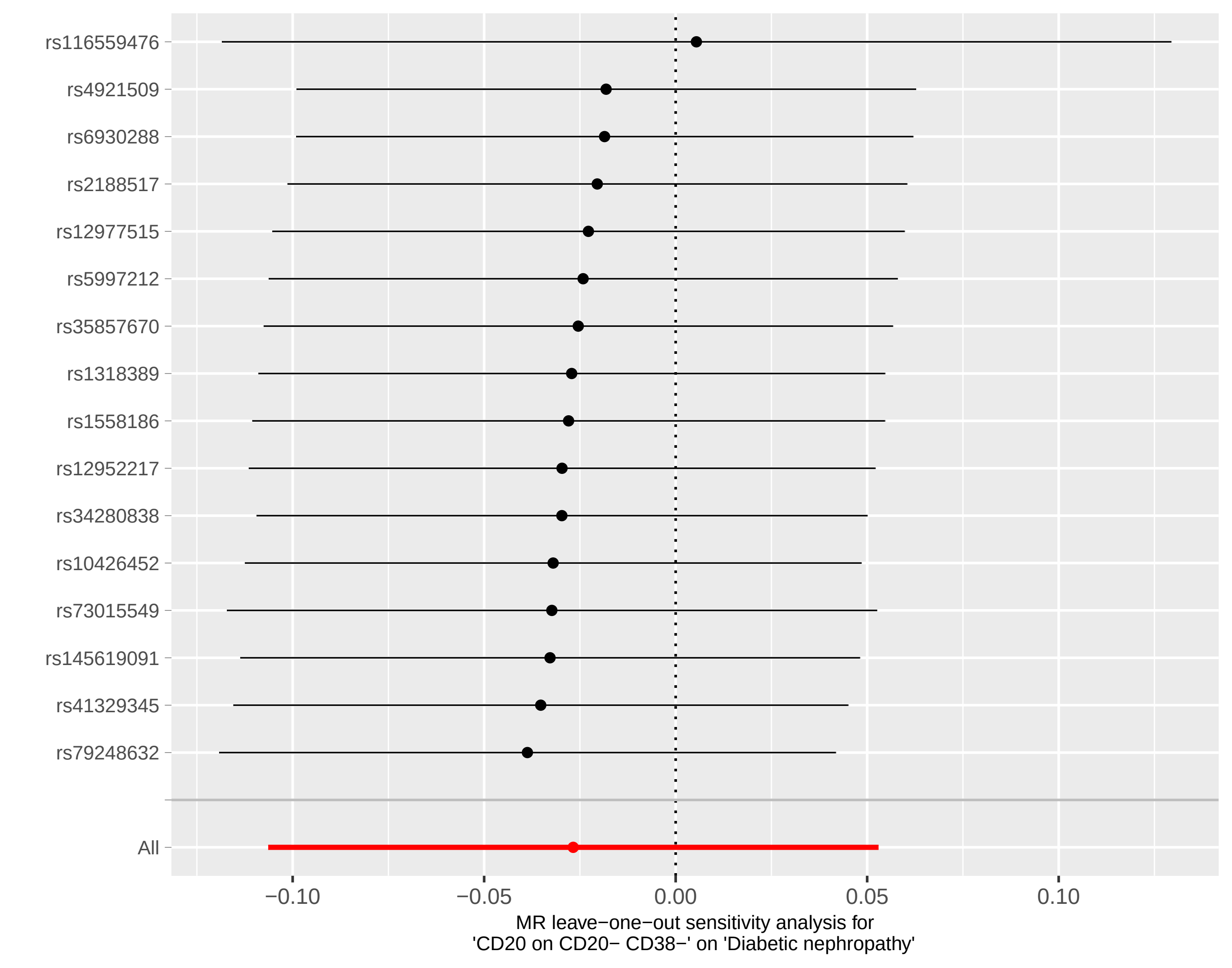
rs116559476
rs4921509
rs6930288
rs2188517
rs12977515
rs5997212
rs35857670
rs1318389
rs1558186
rs12952217
rs34280838
rs10426452
rs73015549
rs145619091
rs41329345
rs79248632
All
−0.10
−0.05
0.00
0.05
0.10
MR leave−one−out sensitivity analysis for
'CD20 on CD20− CD38−' on 'Diabetic nephropathy'

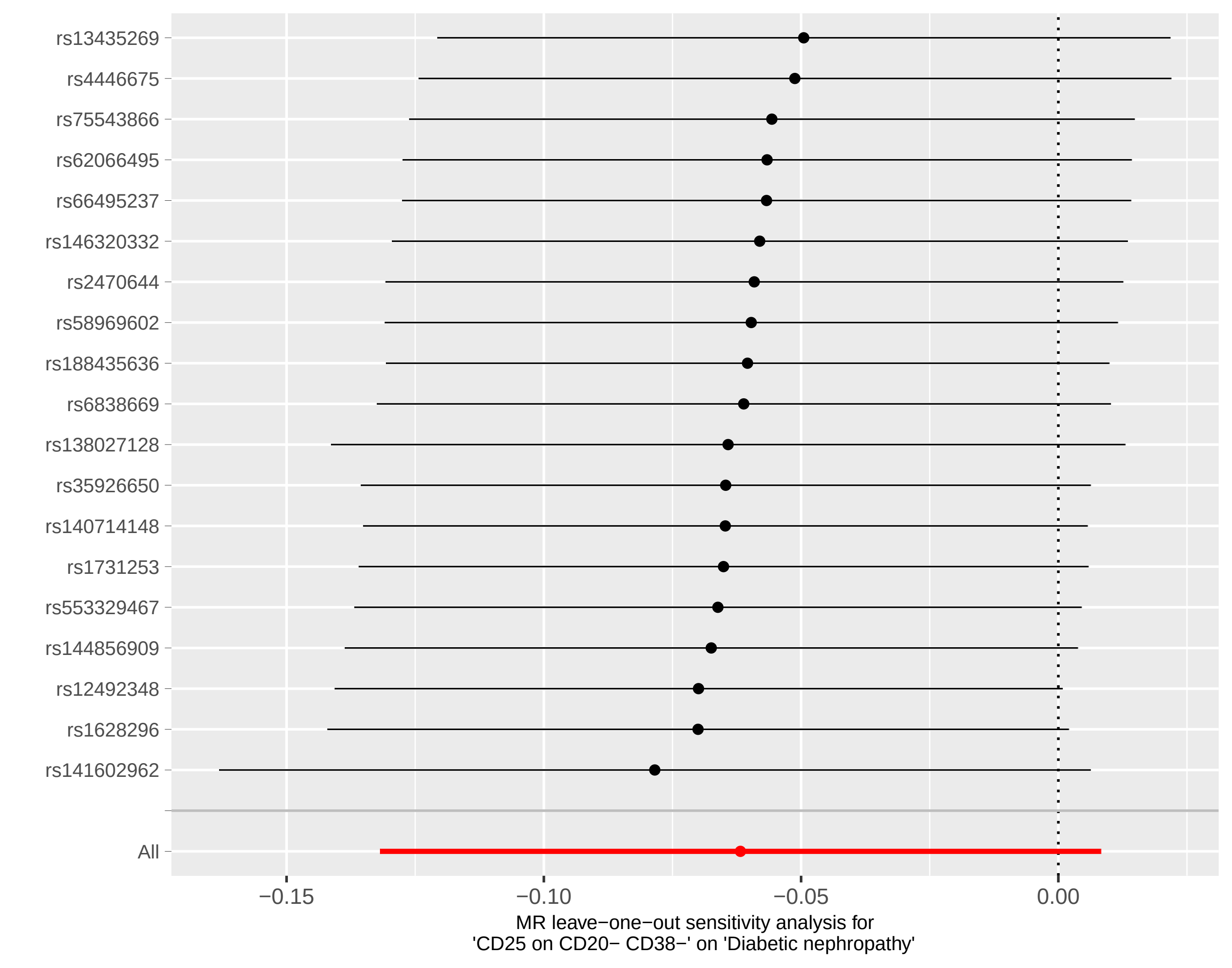
rs13435269
rs4446675
rs75543866
rs62066495
rs66495237
rs146320332
rs2470644
rs58969602
rs188435636
rs6838669
rs138027128
rs35926650
rs140714148
rs1731253
rs553329467
rs144856909
rs12492348
rs1628296
rs141602962
All
−0.15
−0.10
−0.05
0.00
MR leave−one−out sensitivity analysis for
'CD25 on CD20− CD38−' on 'Diabetic nephropathy'

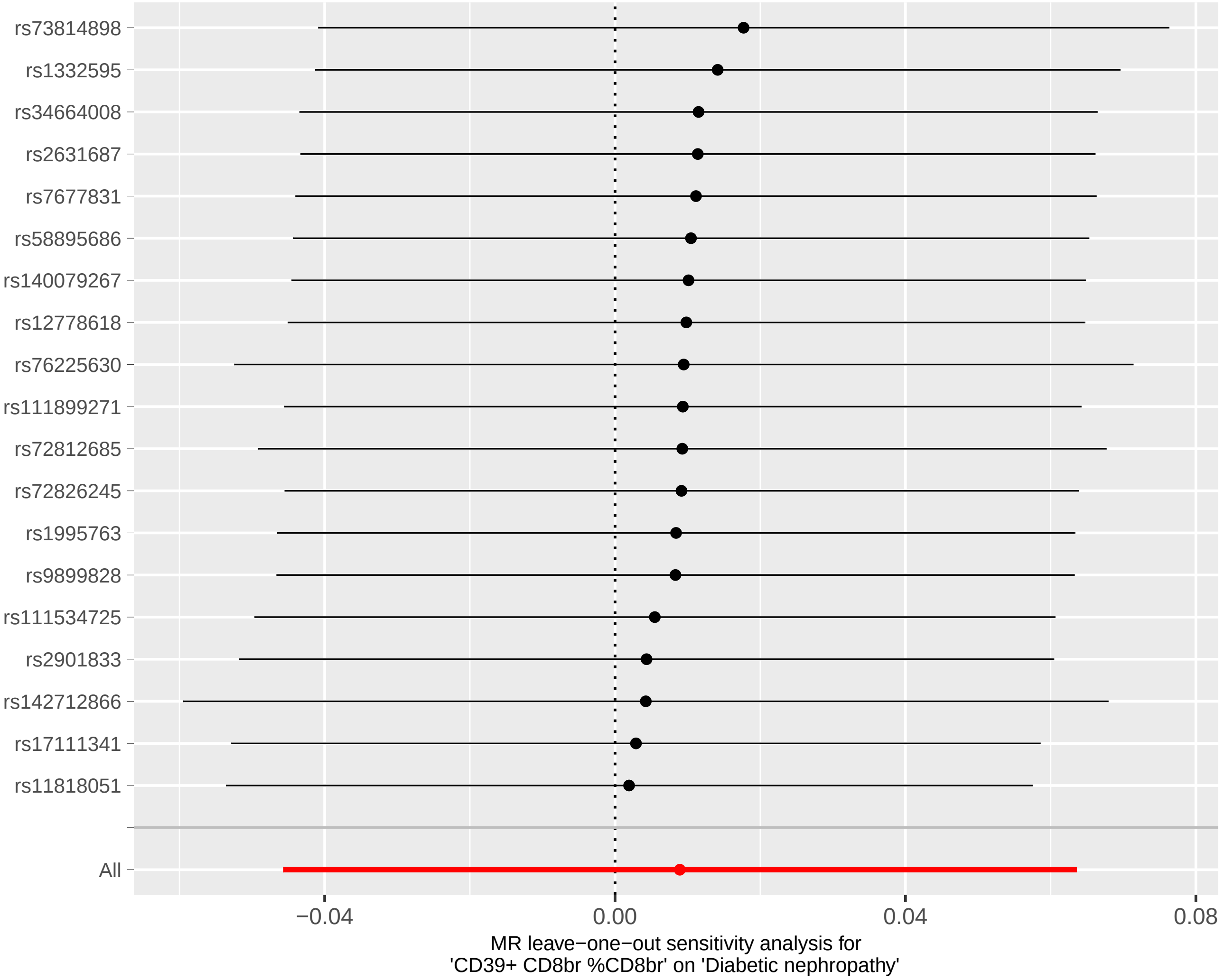
rs73814898
rs1332595
rs34664008
rs2631687
rs7677831
rs58895686
rs140079267
rs12778618
rs76225630
rs111899271
rs72812685
rs72826245
rs1995763
rs9899828
rs111534725
rs2901833
rs142712866
rs17111341
rs11818051
All
−0.04
0.00
0.04
0.08
MR leave−one−out sensitivity analysis for
'CD39+ CD8br %CD8br' on 'Diabetic nephropathy'

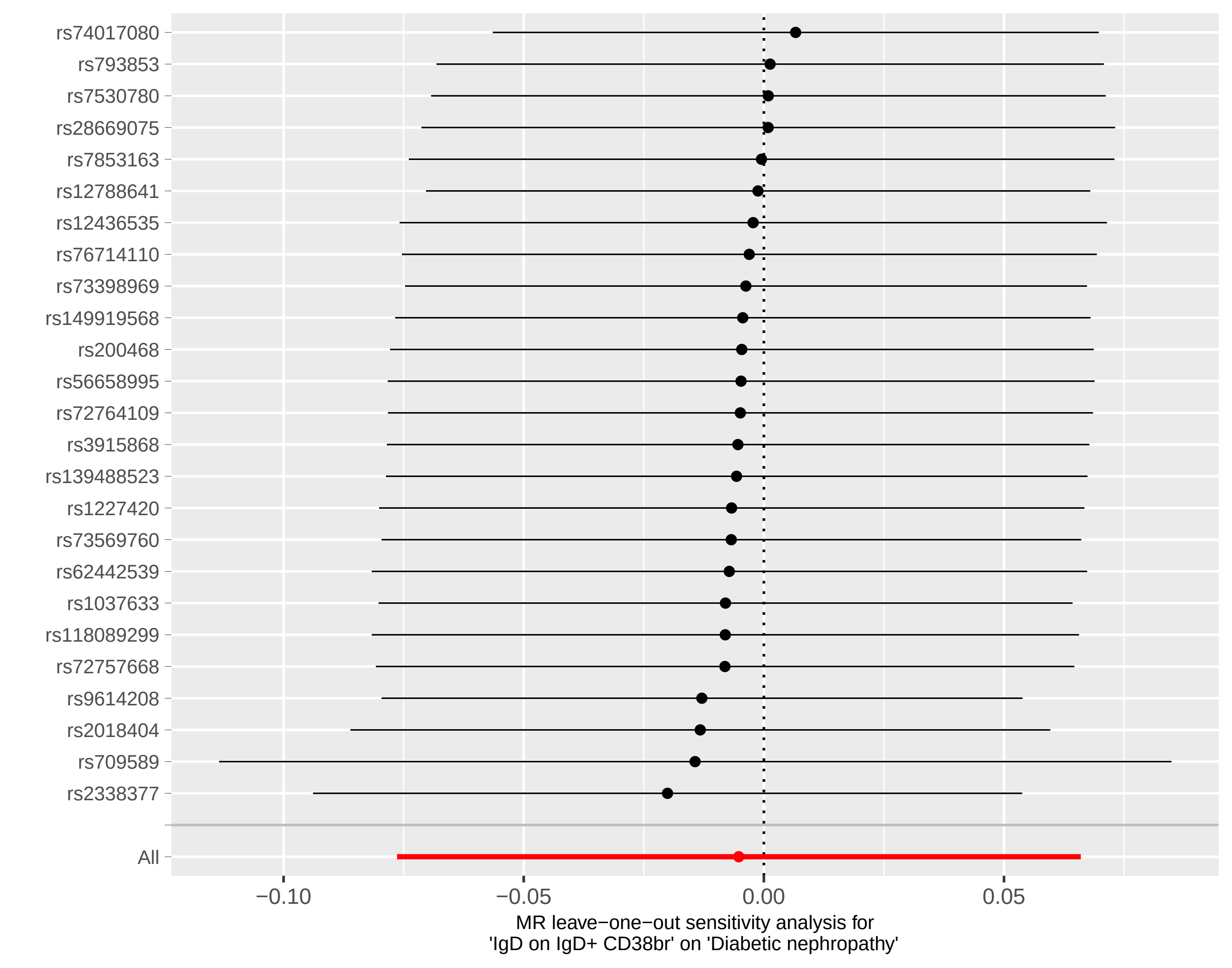

rs74017080
rs793853
rs7530780
rs28669075
rs7853163
rs12788641
rs12436535
rs76714110
rs73398969
rs149919568
rs200468
rs56658995
rs72764109
rs3915868
rs139488523
rs1227420
rs73569760
rs62442539
rs1037633
rs118089299
rs72757668
rs9614208
rs2018404
rs709589
rs2338377
All
−0.10
−0.05
0.00
0.05
MR leave−one−out sensitivity analysis for
'IgD on IgD+ CD38br' on 'Diabetic nephropathy'

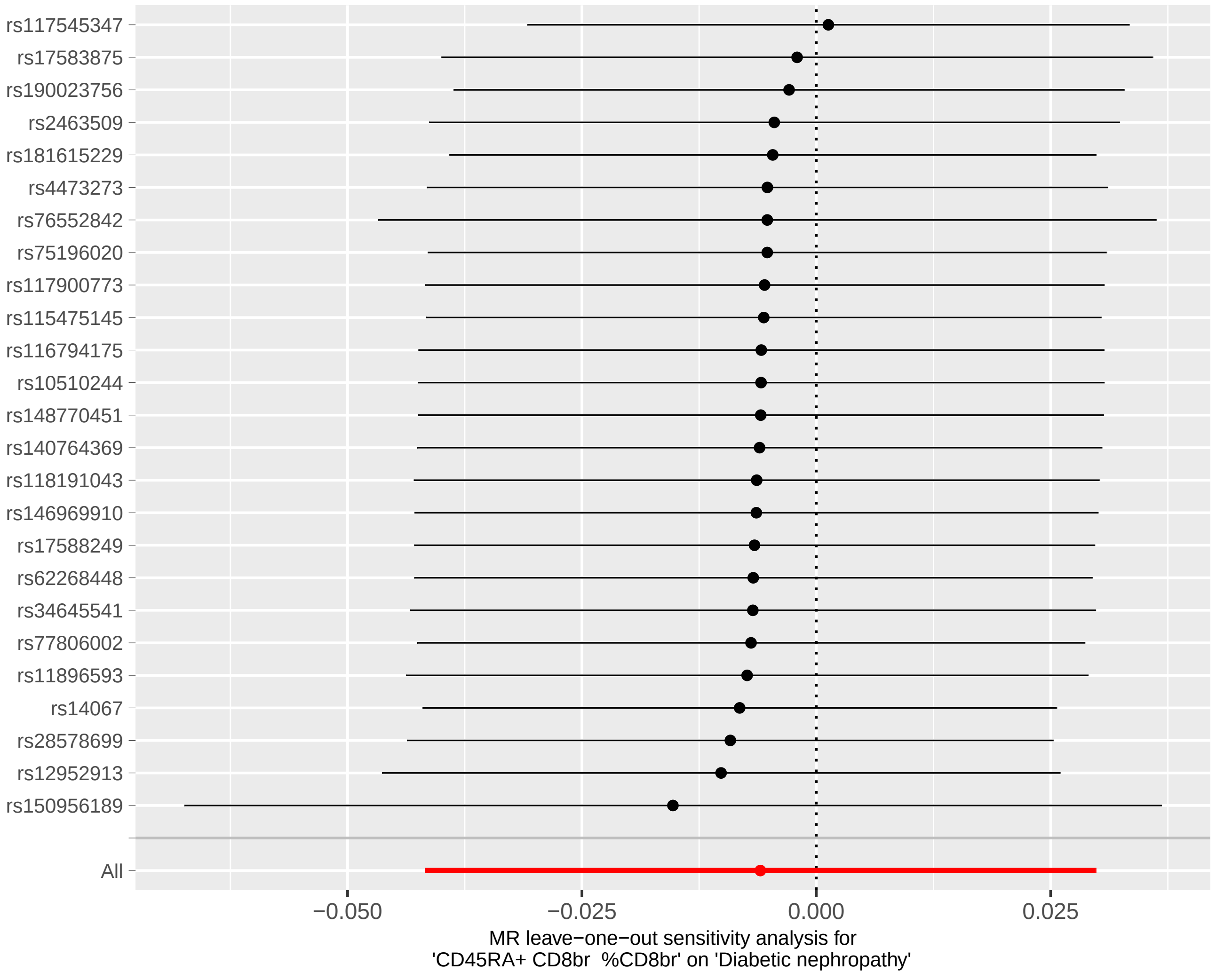
rs117545347
rs17583875
rs190023756
rs2463509
rs181615229
rs4473273
rs76552842
rs75196020
rs117900773
rs115475145
rs116794175
rs10510244
rs148770451
rs140764369
rs118191043
rs146969910
rs17588249
rs62268448
rs34645541
rs77806002
rs11896593
rs14067
rs28578699
rs12952913
rs150956189
All
−0.050
−0.025
0.000
0.025
MR leave−one−out sensitivity analysis for
'CD45RA+ CD8br %CD8br' on 'Diabetic nephropathy'

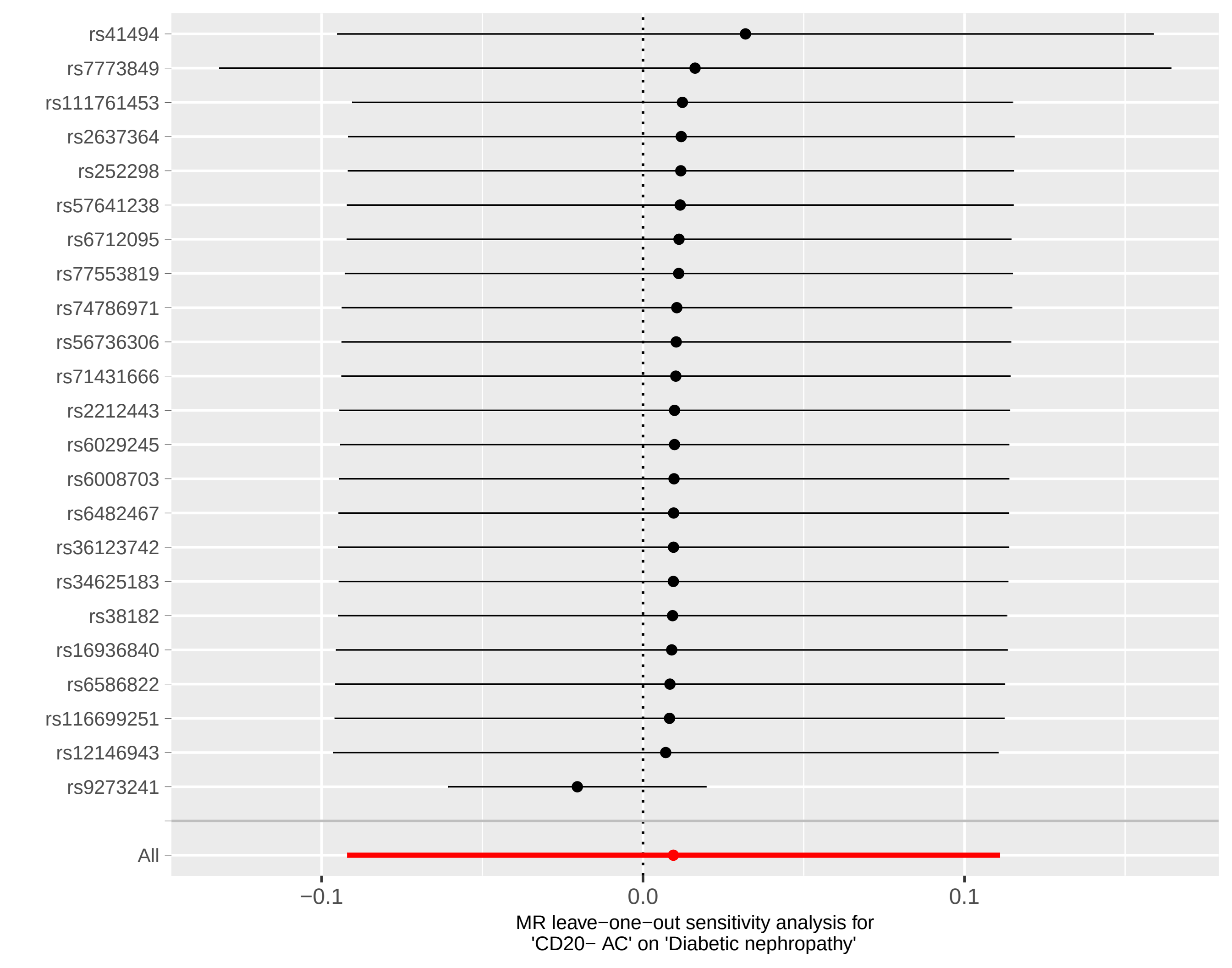

rs41494
rs7773849
rs111761453
rs2637364
rs252298
rs57641238
rs6712095
rs77553819
rs74786971
rs56736306
rs71431666
rs2212443
rs6029245
rs6008703
rs6482467
rs36123742
rs34625183
rs38182
rs16936840
rs6586822
rs116699251
rs12146943
rs9273241
All
−0.1
0.0
0.1
MR leave−one−out sensitivity analysis for
'CD20− AC' on 'Diabetic nephropathy'

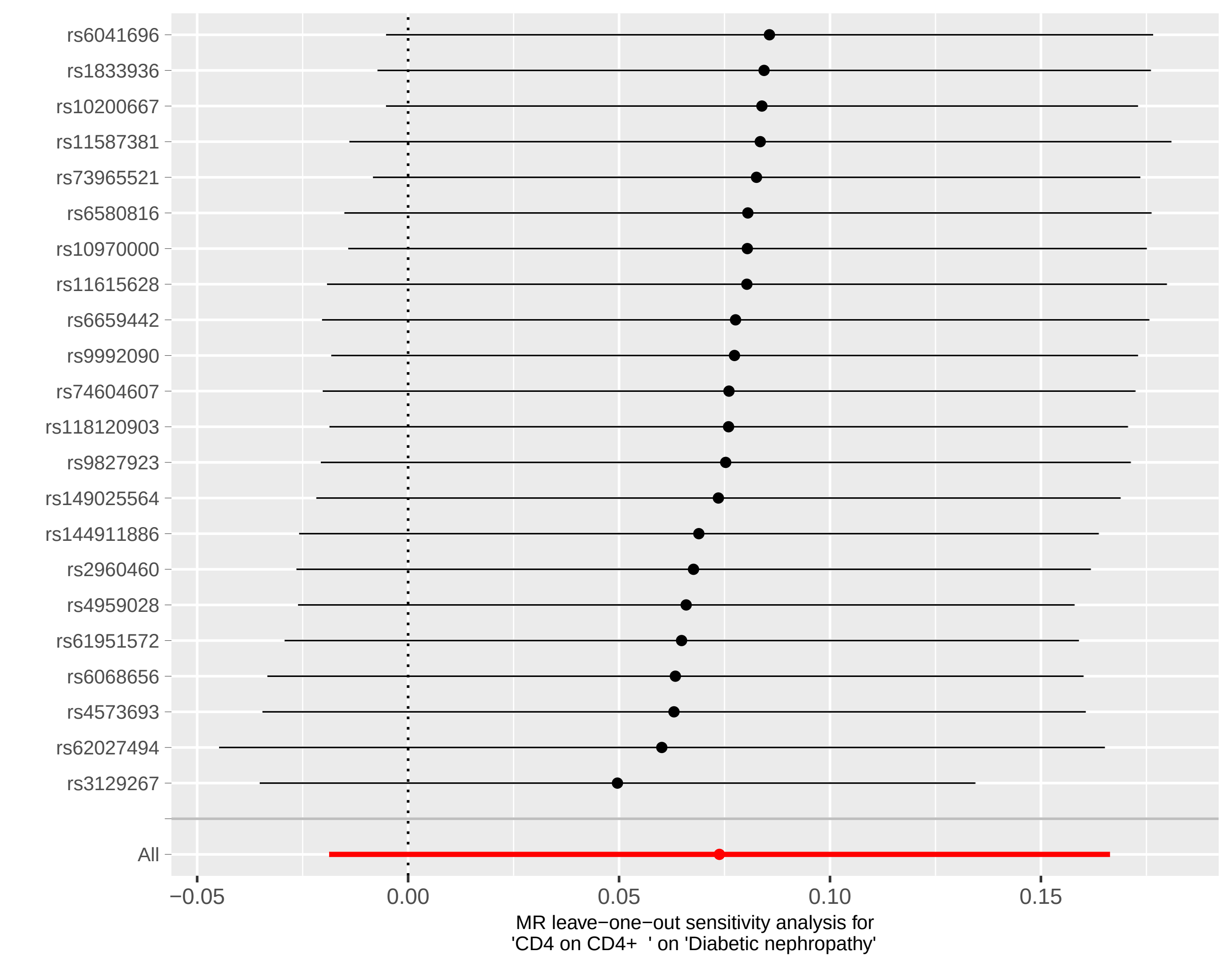
rs6041696
rs1833936
rs10200667
rs11587381
rs73965521
rs6580816
rs10970000
rs11615628
rs6659442
rs9992090
rs74604607
rs118120903
rs9827923
rs149025564
rs144911886
rs2960460
rs4959028
rs61951572
rs6068656
rs4573693
rs62027494
rs3129267
All
−0.05
0.00
0.05
0.10
0.15
MR leave−one−out sensitivity analysis for
'CD4 on CD4+ ' on 'Diabetic nephropathy'

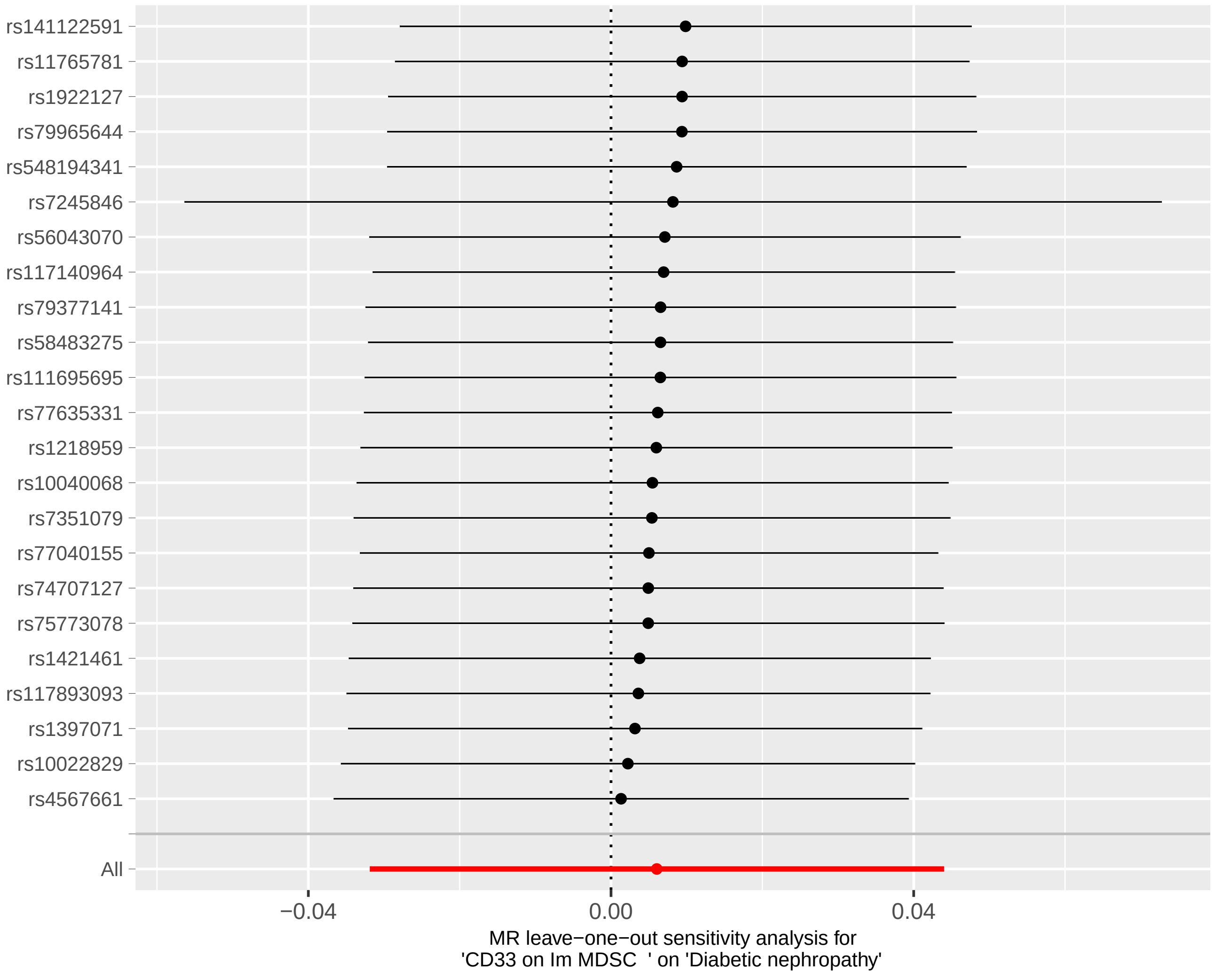

rs141122591
rs11765781
rs1922127
rs79965644
rs548194341
rs7245846
rs56043070
rs117140964
rs79377141
rs58483275
rs111695695
rs77635331
rs1218959
rs10040068
rs7351079
rs77040155
rs74707127
rs75773078
rs1421461
rs117893093
rs1397071
rs10022829
rs4567661
All
−0.04
0.00
0.04
MR leave−one−out sensitivity analysis for
'CD33 on Im MDSC ' on 'Diabetic nephropathy'

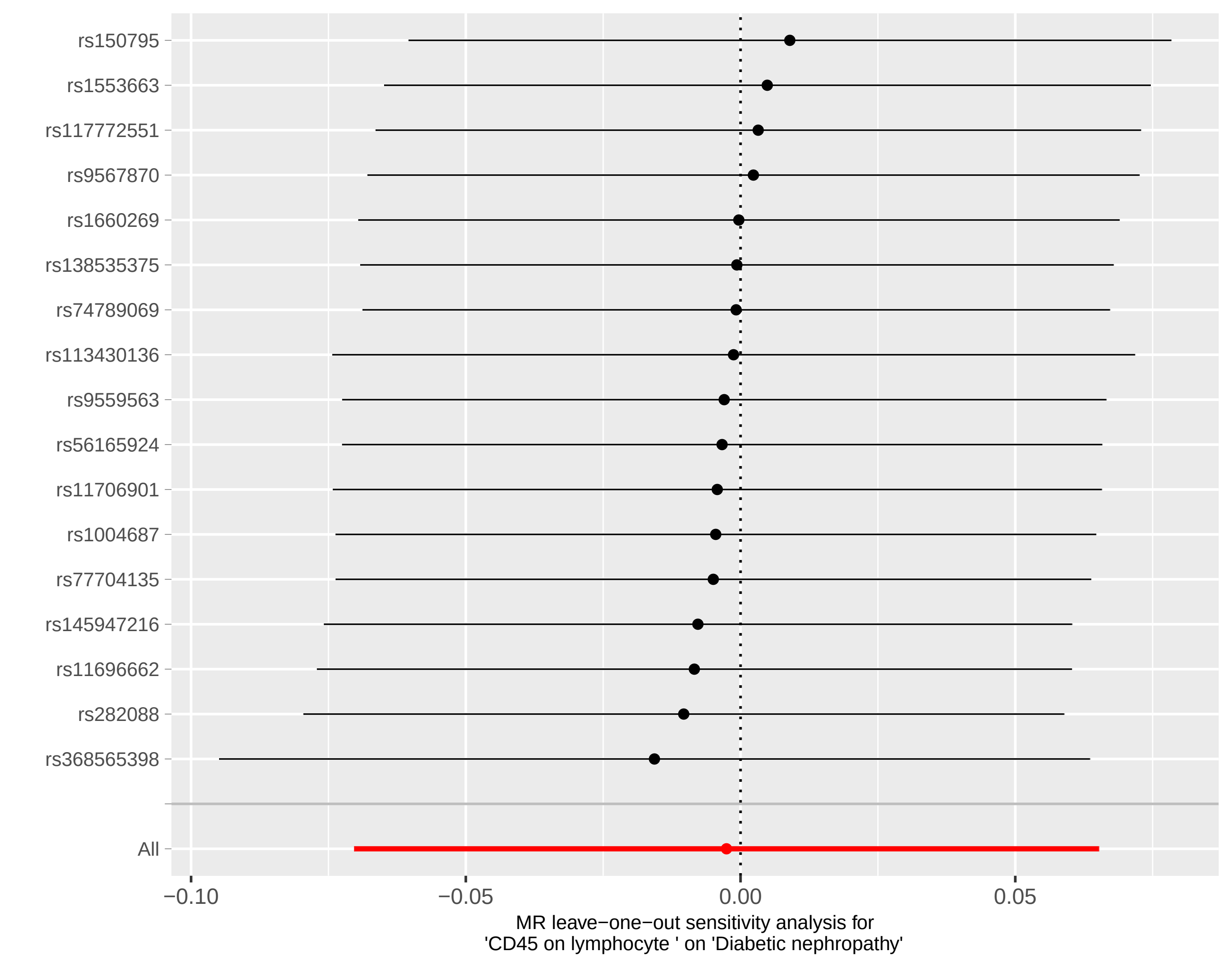
rs150795
rs1553663
rs117772551
rs9567870
rs1660269
rs138535375
rs74789069
rs113430136
rs9559563
rs56165924
rs11706901
rs1004687
rs77704135
rs145947216
rs11696662
rs282088
rs368565398
All
−0.10
−0.05
0.00
0.05
MR leave−one−out sensitivity analysis for
'CD45 on lymphocyte ' on 'Diabetic nephropathy'

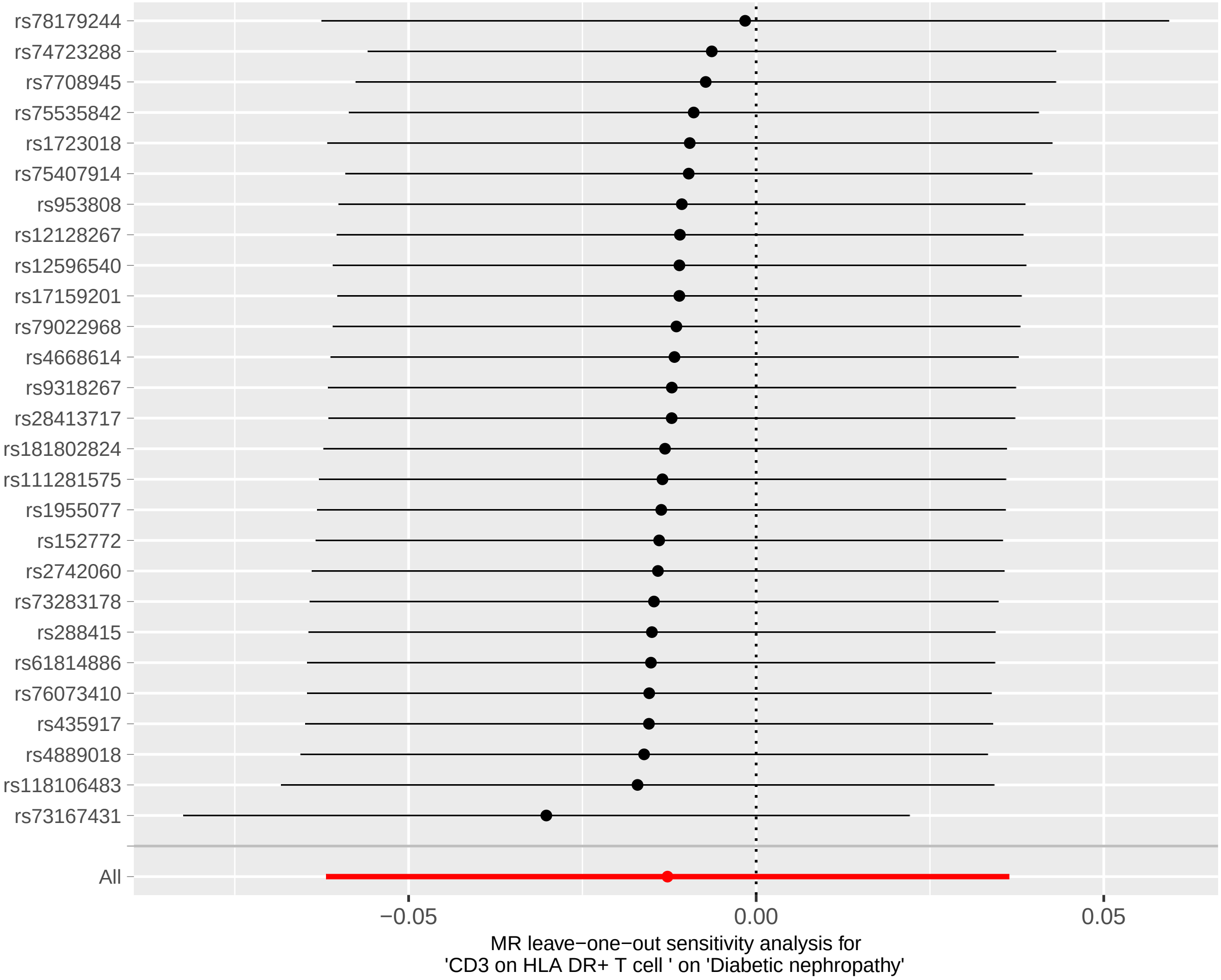
rs78179244
rs74723288
rs7708945
rs75535842
rs1723018
rs75407914
rs953808
rs12128267
rs12596540
rs17159201
rs79022968
rs4668614
rs9318267
rs28413717
rs181802824
rs111281575
rs1955077
rs152772
rs2742060
rs73283178
rs288415
rs61814886
rs76073410
rs435917
rs4889018
rs118106483
rs73167431
All
−0.05
0.00
0.05
MR leave−one−out sensitivity analysis for
'CD3 on HLA DR+ T cell ' on 'Diabetic nephropathy'

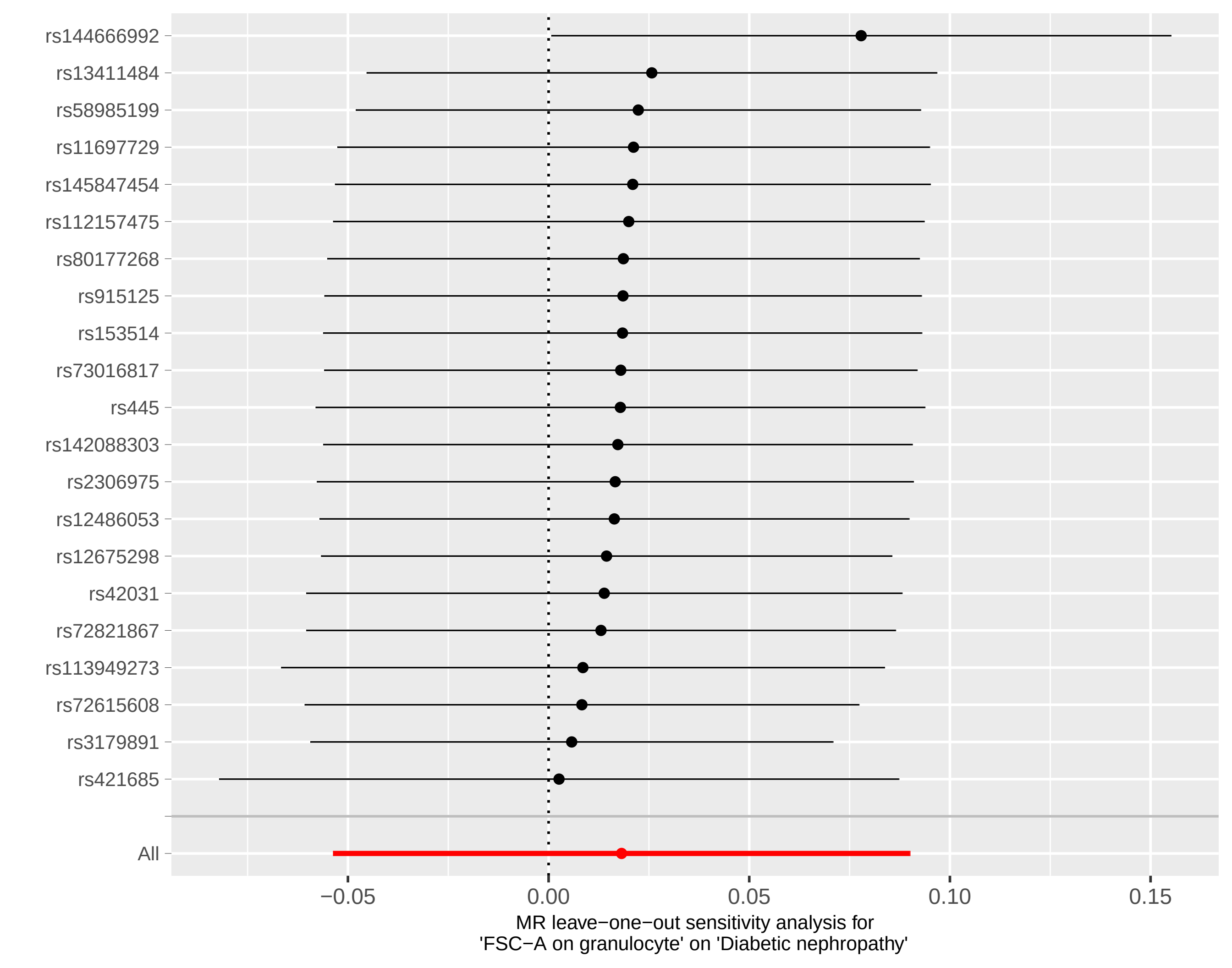
rs144666992
rs13411484
rs58985199
rs11697729
rs145847454
rs112157475
rs80177268
rs915125
rs153514
rs73016817
rs445
rs142088303
rs2306975
rs12486053
rs12675298
rs42031
rs72821867
rs113949273
rs72615608
rs3179891
rs421685
All
−0.05
0.00
0.05
0.10
0.15
MR leave−one−out sensitivity analysis for
'FSC−A on granulocyte' on 'Diabetic nephropathy'

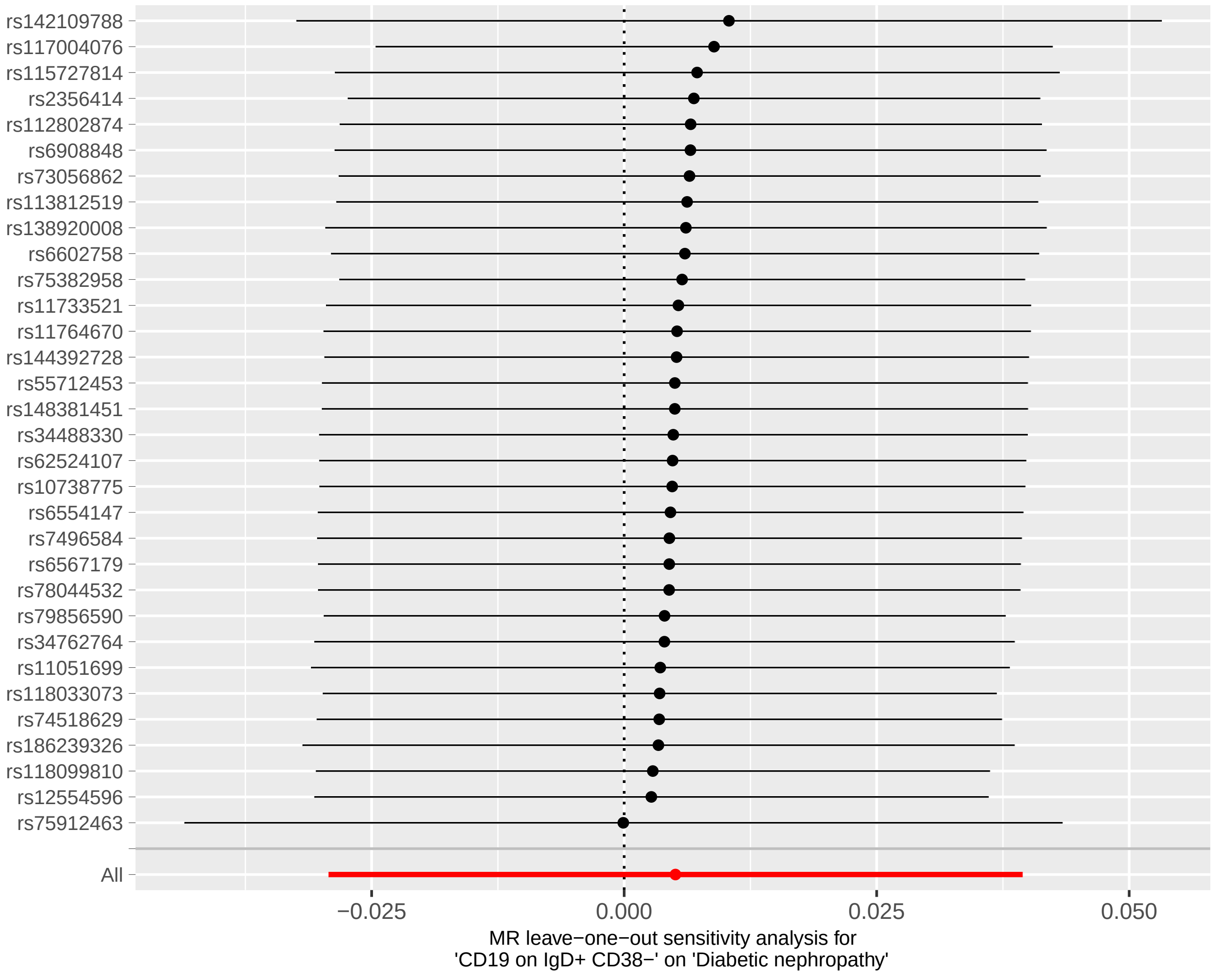
rs142109788
rs117004076
rs115727814
rs2356414
rs112802874
rs6908848
rs73056862
rs113812519
rs138920008
rs6602758
rs75382958
rs11733521
rs11764670
rs144392728
rs55712453
rs148381451
rs34488330
rs62524107
rs10738775
rs6554147
rs7496584
rs6567179
rs78044532
rs79856590
rs34762764
rs11051699
rs118033073
rs74518629
rs186239326
rs118099810
rs12554596
rs75912463
All
−0.025
0.000
0.025
0.050
MR leave−one−out sensitivity analysis for
'CD19 on IgD+ CD38−' on 'Diabetic nephropathy'

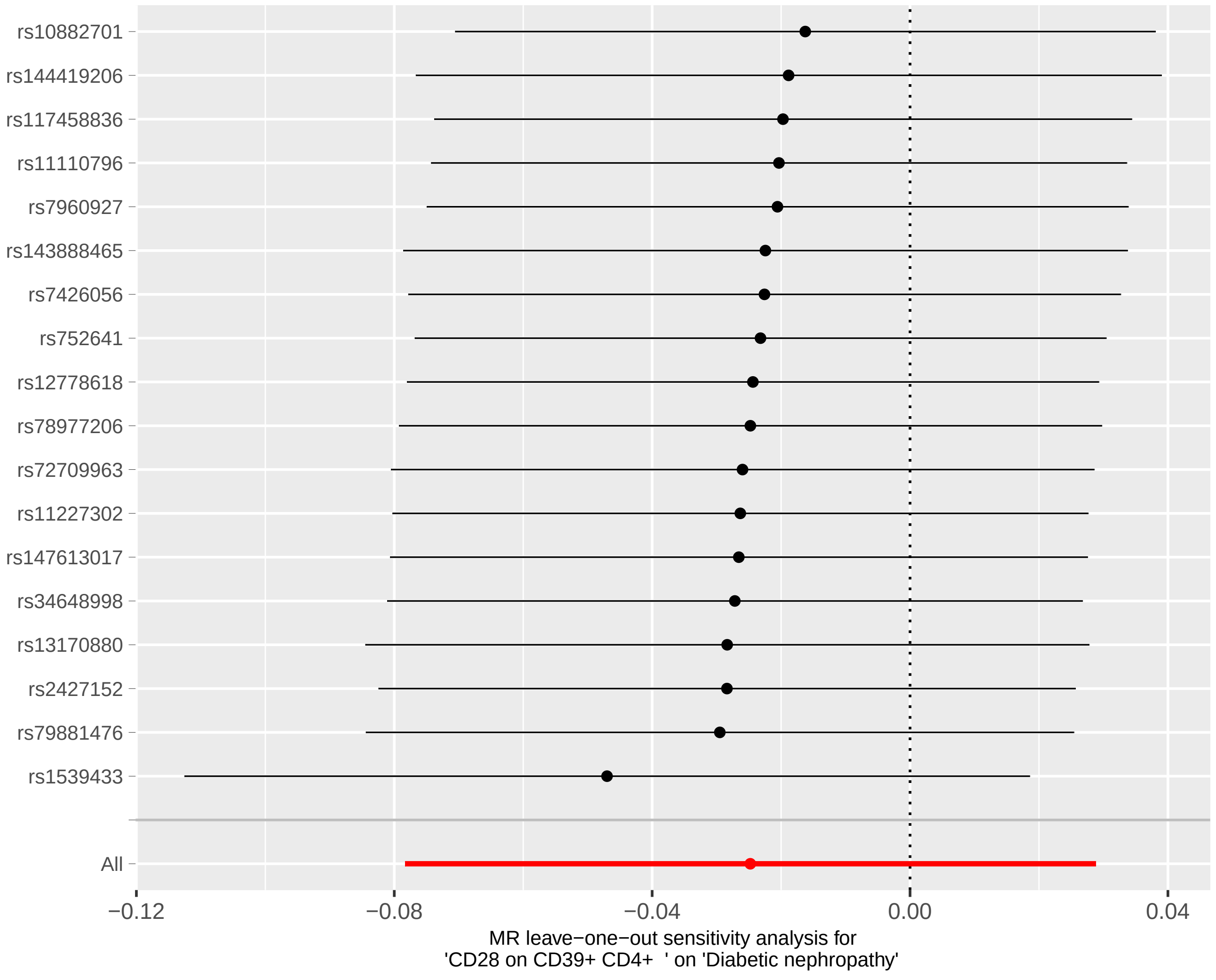
rs10882701
rs144419206
rs117458836
rs11110796
rs7960927
rs143888465
rs7426056
rs752641
rs12778618
rs78977206
rs72709963
rs11227302
rs147613017
rs34648998
rs13170880
rs2427152
rs79881476
rs1539433
All
−0.12
−0.08
−0.04
0.00
0.04
MR leave−one−out sensitivity analysis for
'CD28 on CD39+ CD4+ ' on 'Diabetic nephropathy'

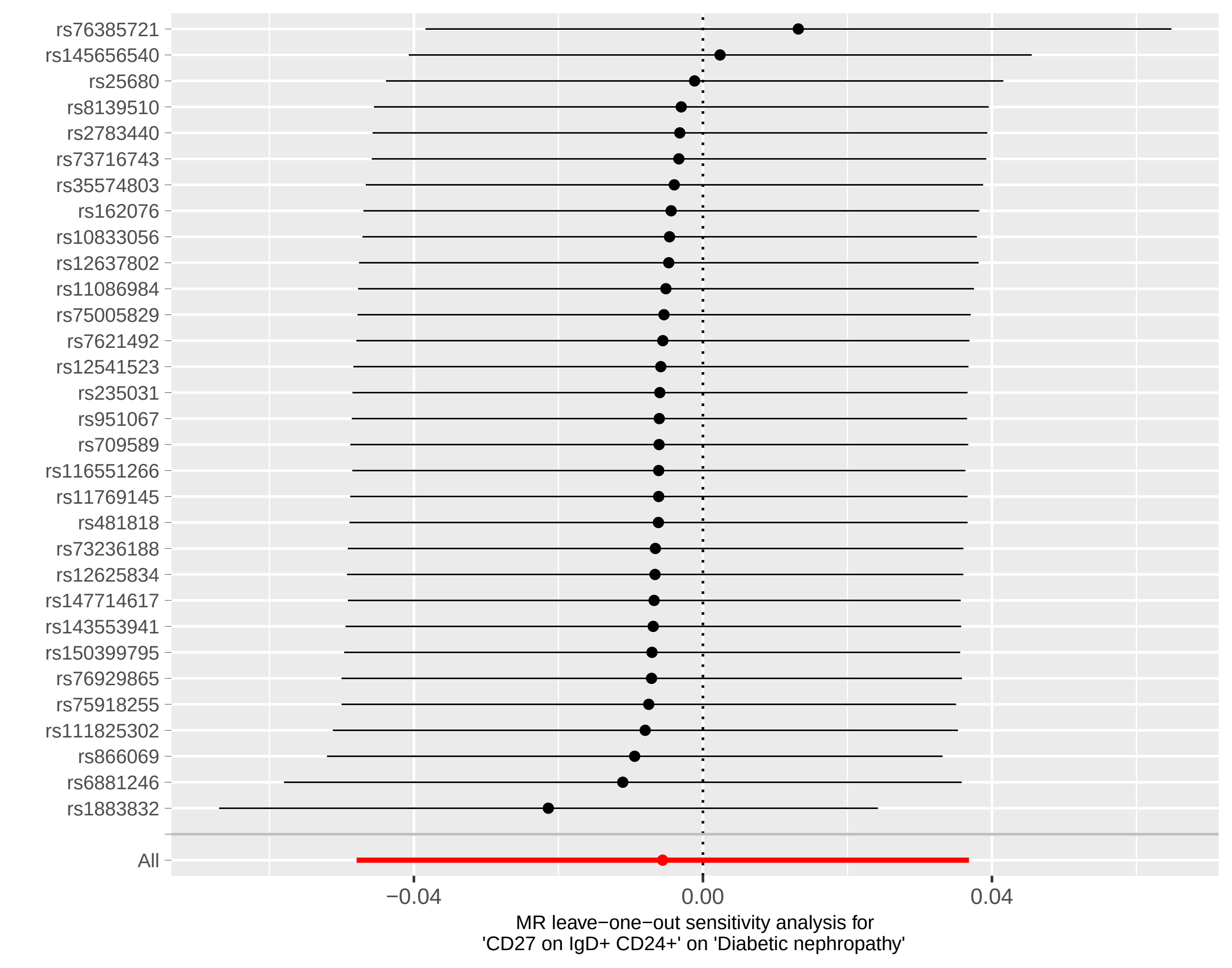

rs76385721
rs145656540
rs25680
rs8139510
rs2783440
rs73716743
rs35574803
rs162076
rs10833056
rs12637802
rs11086984
rs75005829
rs7621492
rs12541523
rs235031
rs951067
rs709589
rs116551266
rs11769145
rs481818
rs73236188
rs12625834
rs147714617
rs143553941
rs150399795
rs76929865
rs75918255
rs111825302
rs866069
rs6881246
rs1883832
All
−0.04
0.00
0.04
MR leave−one−out sensitivity analysis for
'CD27 on IgD+ CD24+' on 'Diabetic nephropathy'

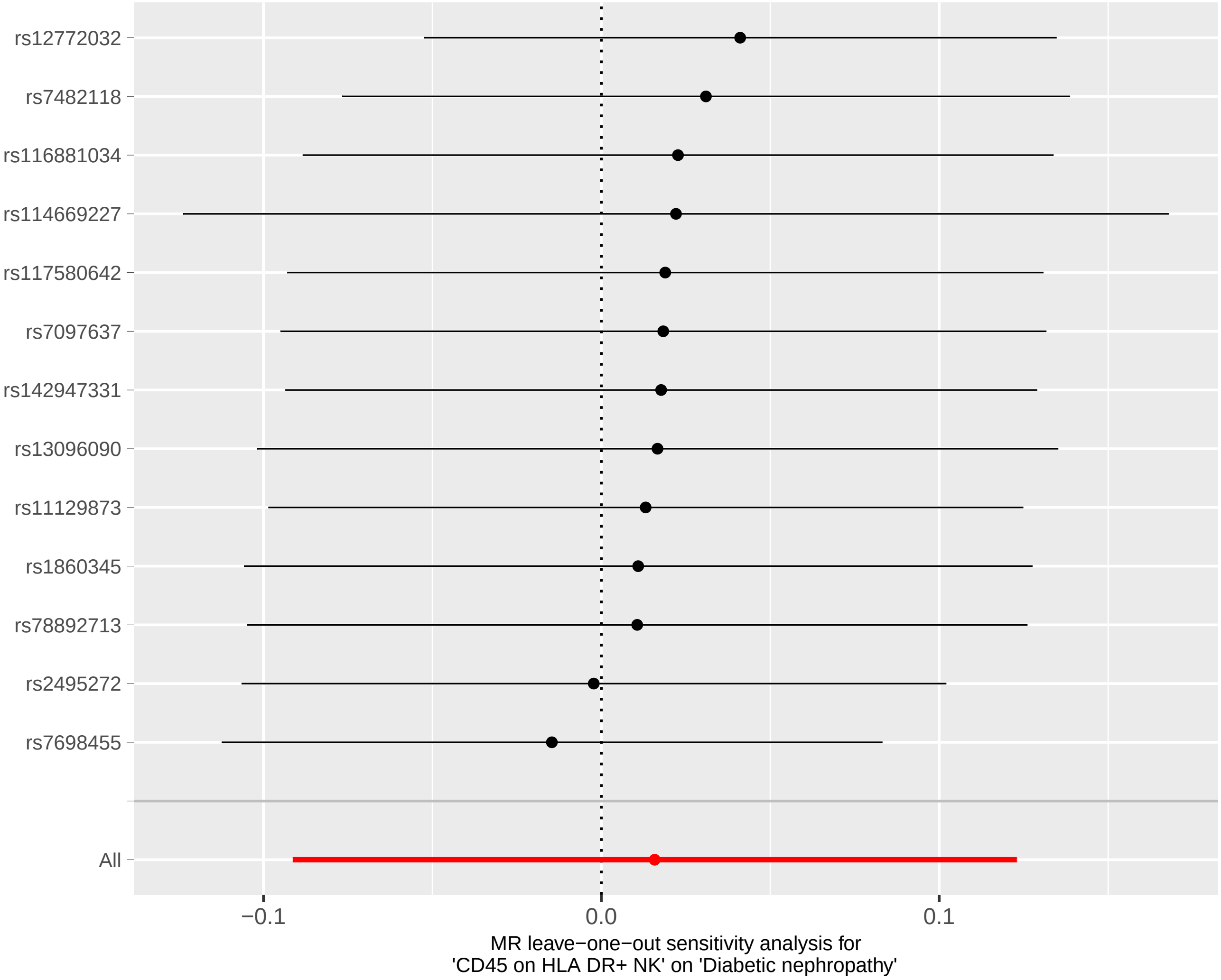
rs12772032
rs7482118
rs116881034
rs114669227
rs117580642
rs7097637
rs142947331
rs13096090
rs11129873
rs1860345
rs78892713
rs2495272
rs7698455
All
−0.1
0.0
0.1
MR leave−one−out sensitivity analysis for
'CD45 on HLA DR+ NK' on 'Diabetic nephropathy'

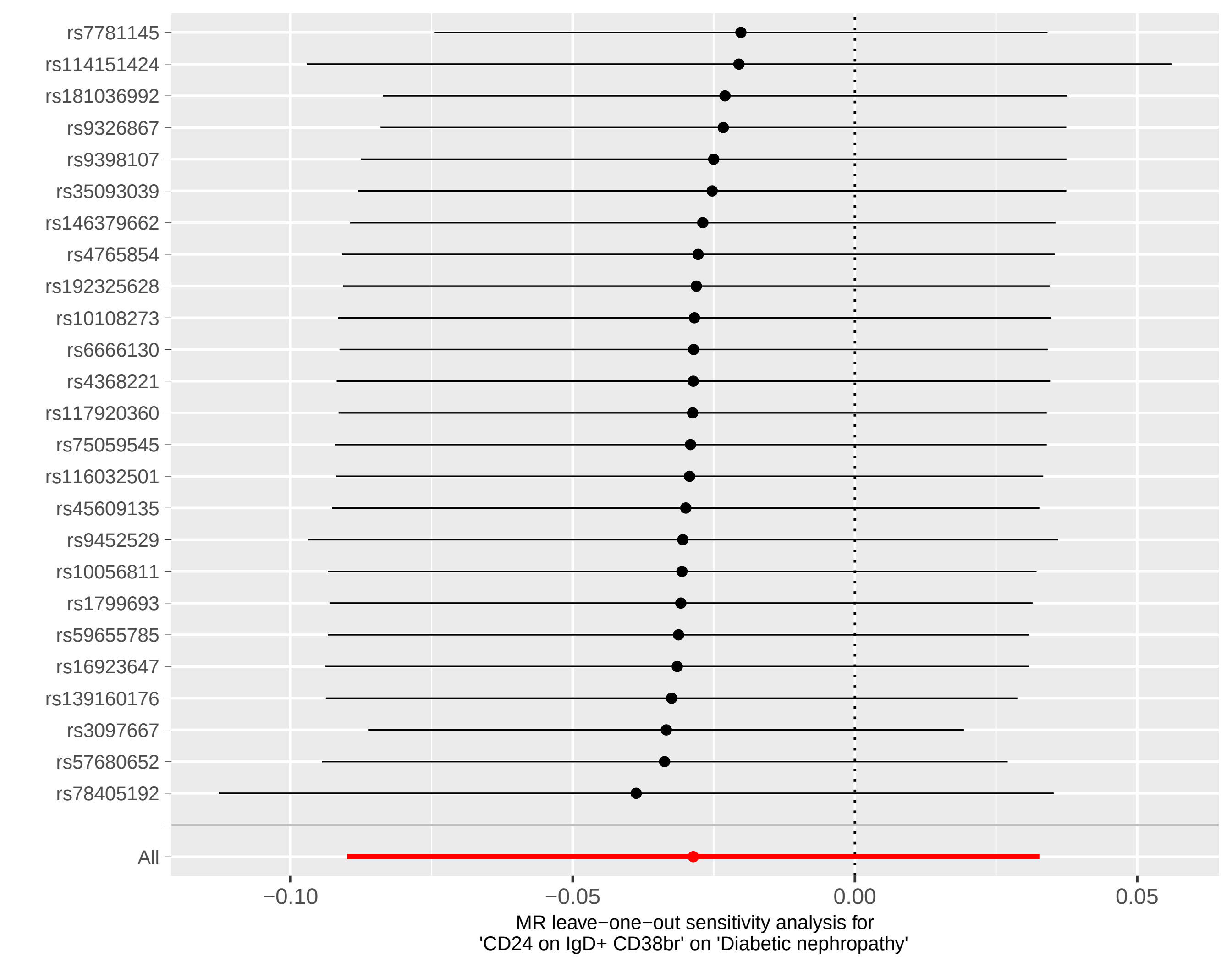

rs7781145
rs114151424
rs181036992
rs9326867
rs9398107
rs35093039
rs146379662
rs4765854
rs192325628
rs10108273
rs6666130
rs4368221
rs117920360
rs75059545
rs116032501
rs45609135
rs9452529
rs10056811
rs1799693
rs59655785
rs16923647
rs139160176
rs3097667
rs57680652
rs78405192
All
−0.10
−0.05
0.00
0.05
MR leave−one−out sensitivity analysis for
'CD24 on IgD+ CD38br' on 'Diabetic nephropathy'

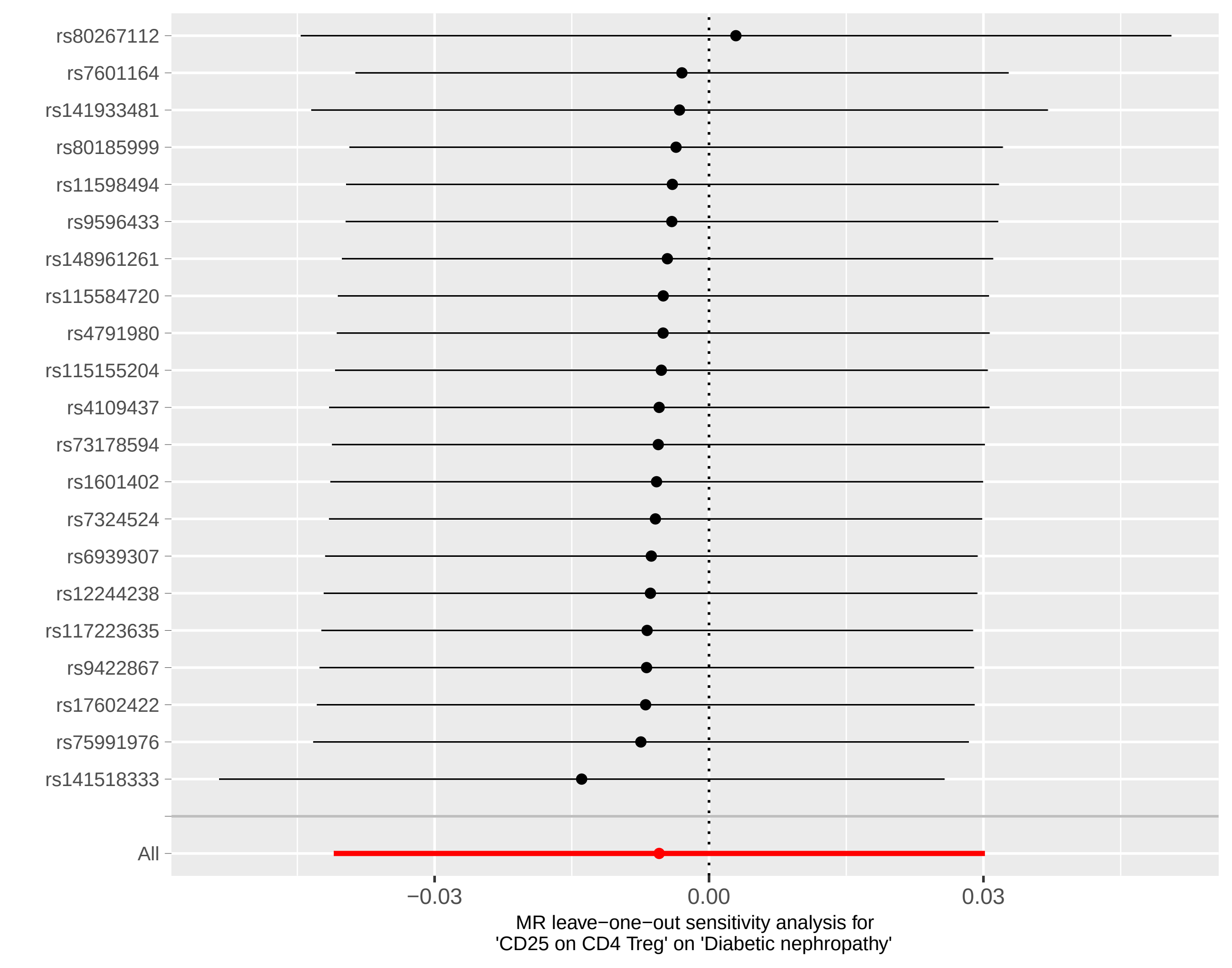
rs80267112
rs7601164
rs141933481
rs80185999
rs11598494
rs9596433
rs148961261
rs115584720
rs4791980
rs115155204
rs4109437
rs73178594
rs1601402
rs7324524
rs6939307
rs12244238
rs117223635
rs9422867
rs17602422
rs75991976
rs141518333
All
−0.03
0.00
0.03
MR leave−one−out sensitivity analysis for
'CD25 on CD4 Treg' on 'Diabetic nephropathy'

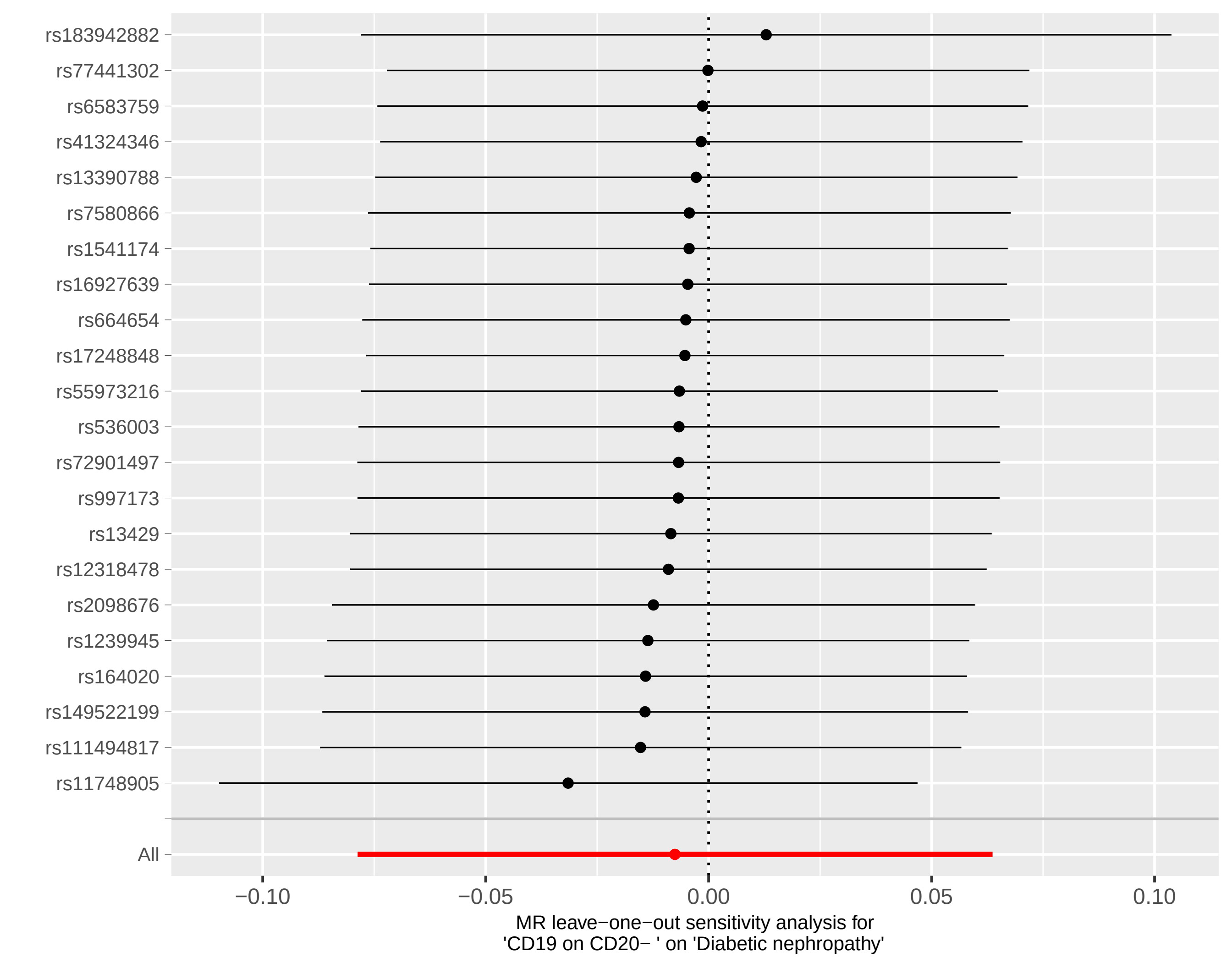

rs183942882
rs77441302
rs6583759
rs41324346
rs13390788
rs7580866
rs1541174
rs16927639
rs664654
rs17248848
rs55973216
rs536003
rs72901497
rs997173
rs13429
rs12318478
rs2098676
rs1239945
rs164020
rs149522199
rs111494817
rs11748905
All
−0.10
−0.05
0.00
0.05
0.10
MR leave−one−out sensitivity analysis for
'CD19 on CD20− ' on 'Diabetic nephropathy'

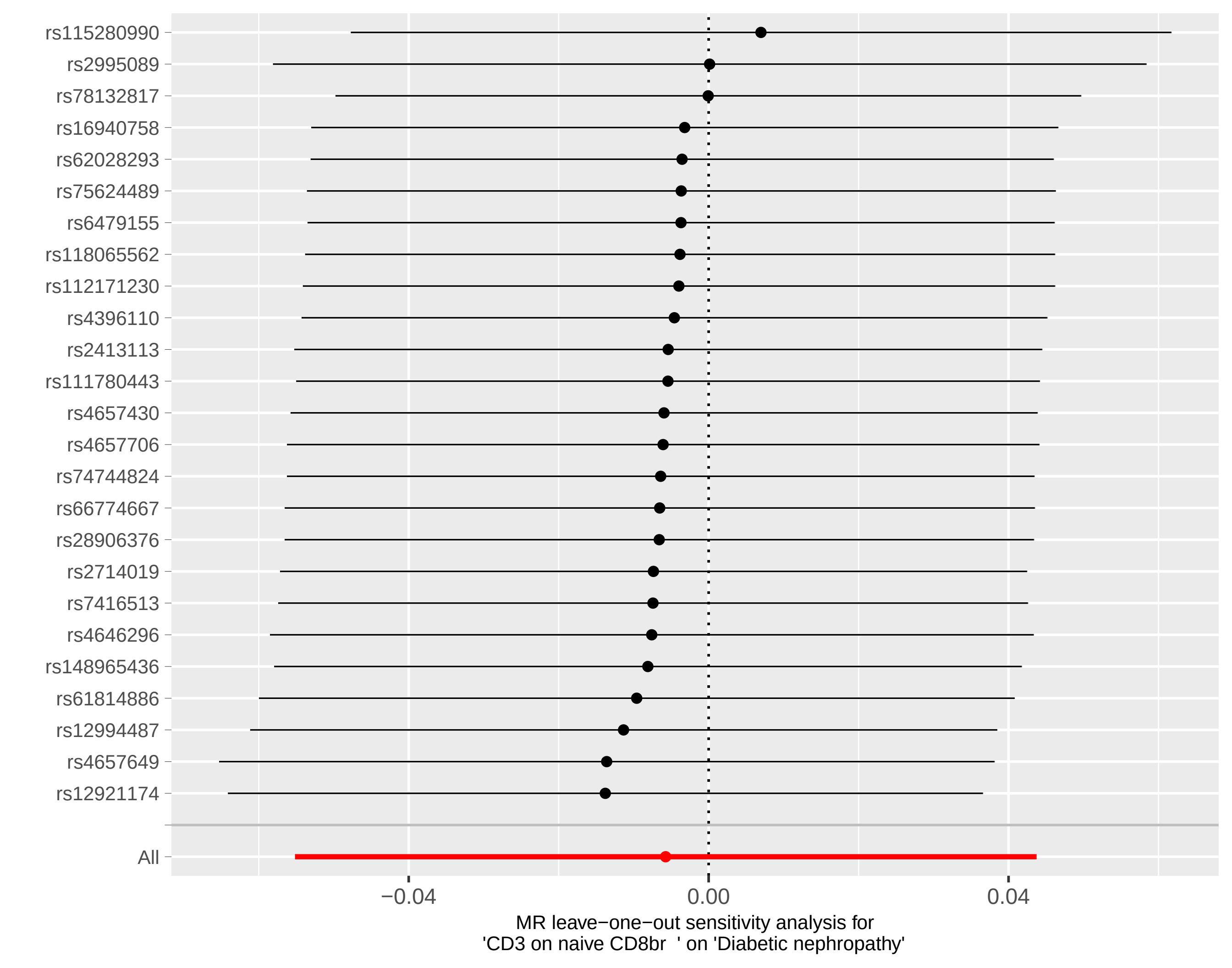
rs115280990
rs2995089
rs78132817
rs16940758
rs62028293
rs75624489
rs6479155
rs118065562
rs112171230
rs4396110
rs2413113
rs111780443
rs4657430
rs4657706
rs74744824
rs66774667
rs28906376
rs2714019
rs7416513
rs4646296
rs148965436
rs61814886
rs12994487
rs4657649
rs12921174
All
−0.04
0.00
0.04
MR leave−one−out sensitivity analysis for
'CD3 on naive CD8br ' on 'Diabetic nephropathy'

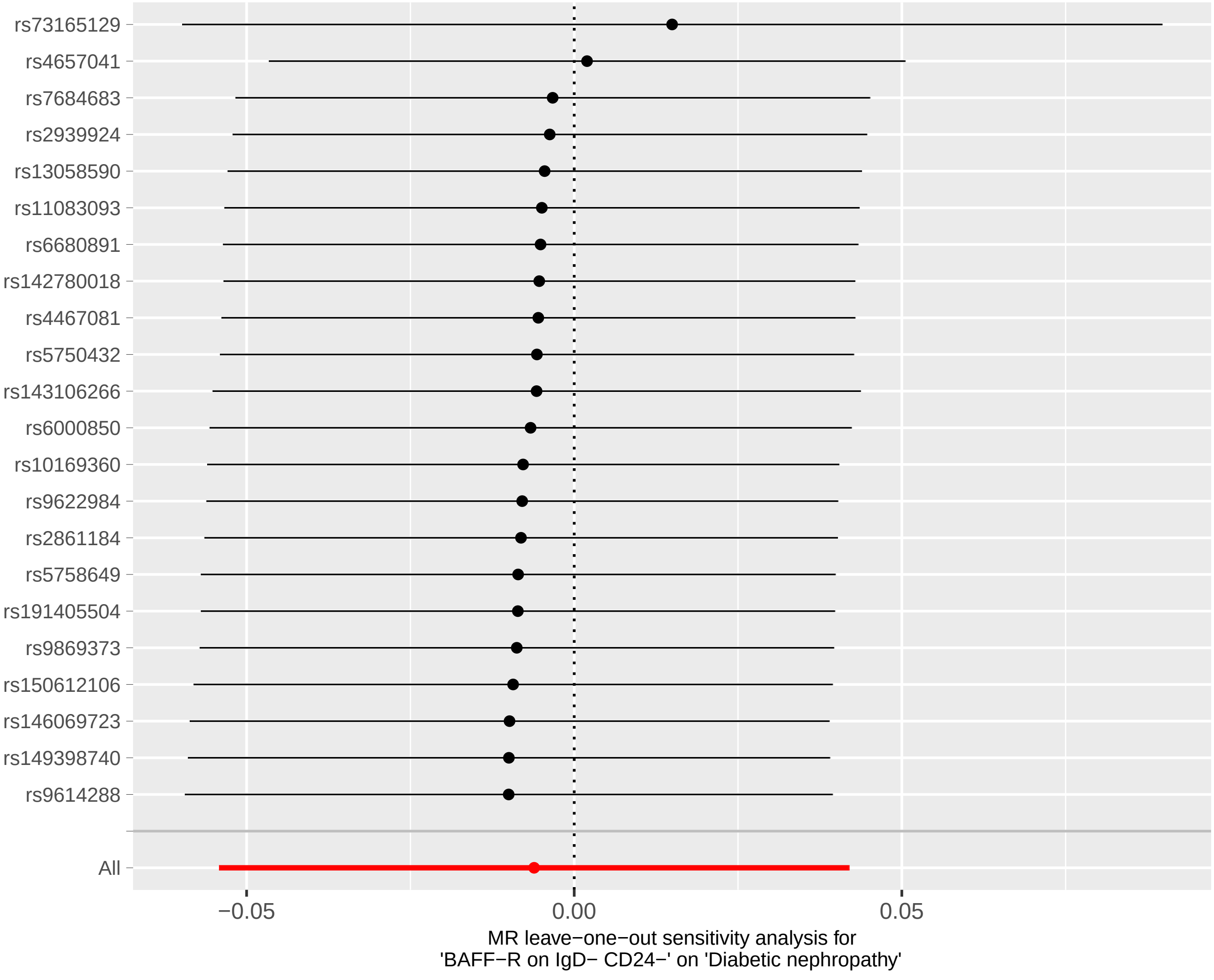
rs73165129
rs4657041
rs7684683
rs2939924
rs13058590
rs11083093
rs6680891
rs142780018
rs4467081
rs5750432
rs143106266
rs6000850
rs10169360
rs9622984
rs2861184
rs5758649
rs191405504
rs9869373
rs150612106
rs146069723
rs149398740
rs9614288
All
−0.05
0.00
0.05
MR leave−one−out sensitivity analysis for
'BAFF−R on IgD− CD24−' on 'Diabetic nephropathy'

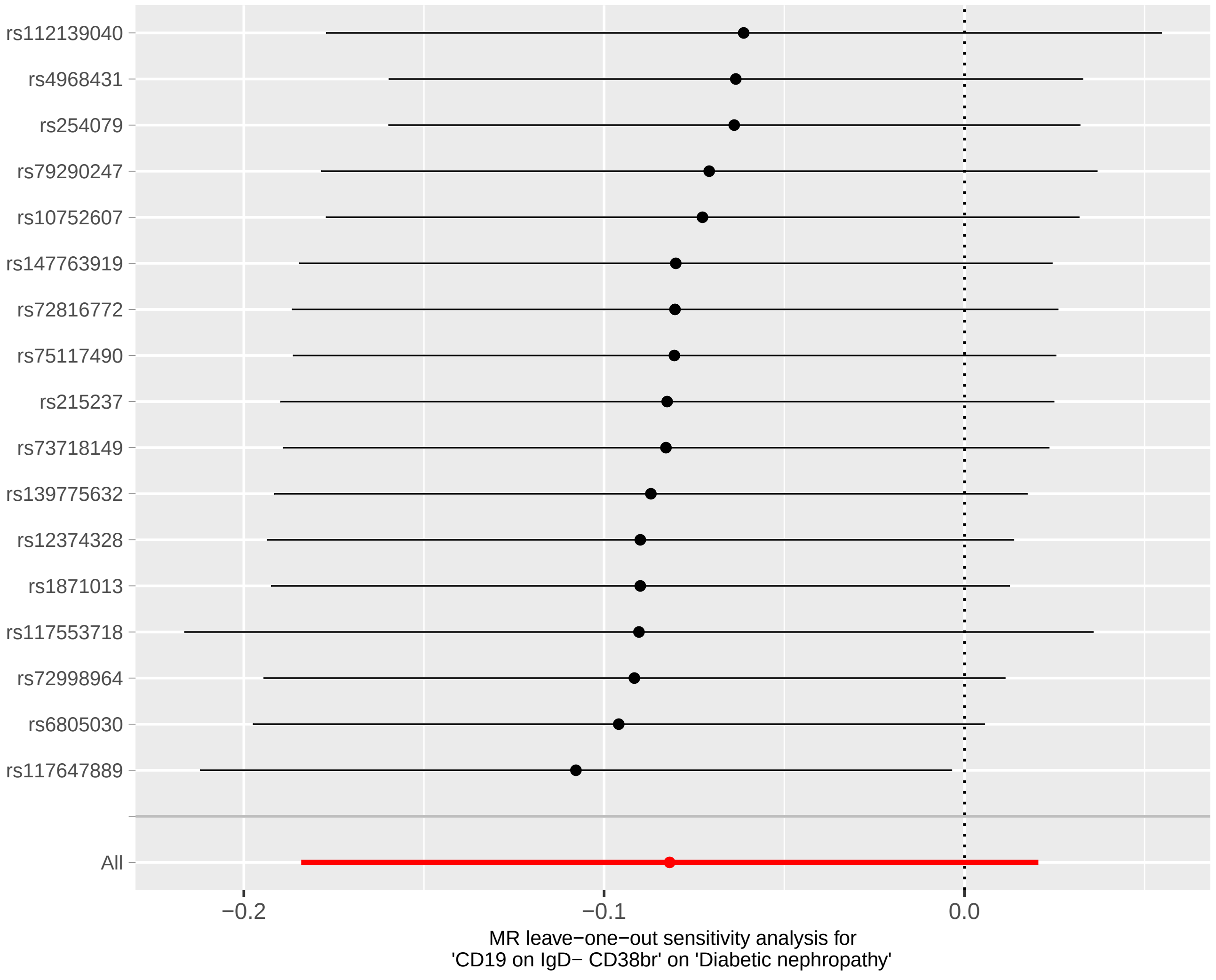
rs112139040
rs4968431
rs254079
rs79290247
rs10752607
rs147763919
rs72816772
rs75117490
rs215237
rs73718149
rs139775632
rs12374328
rs1871013
rs117553718
rs72998964
rs6805030
rs117647889
All
−0.2
−0.1
0.0
MR leave−one−out sensitivity analysis for
'CD19 on IgD− CD38br' on 'Diabetic nephropathy'

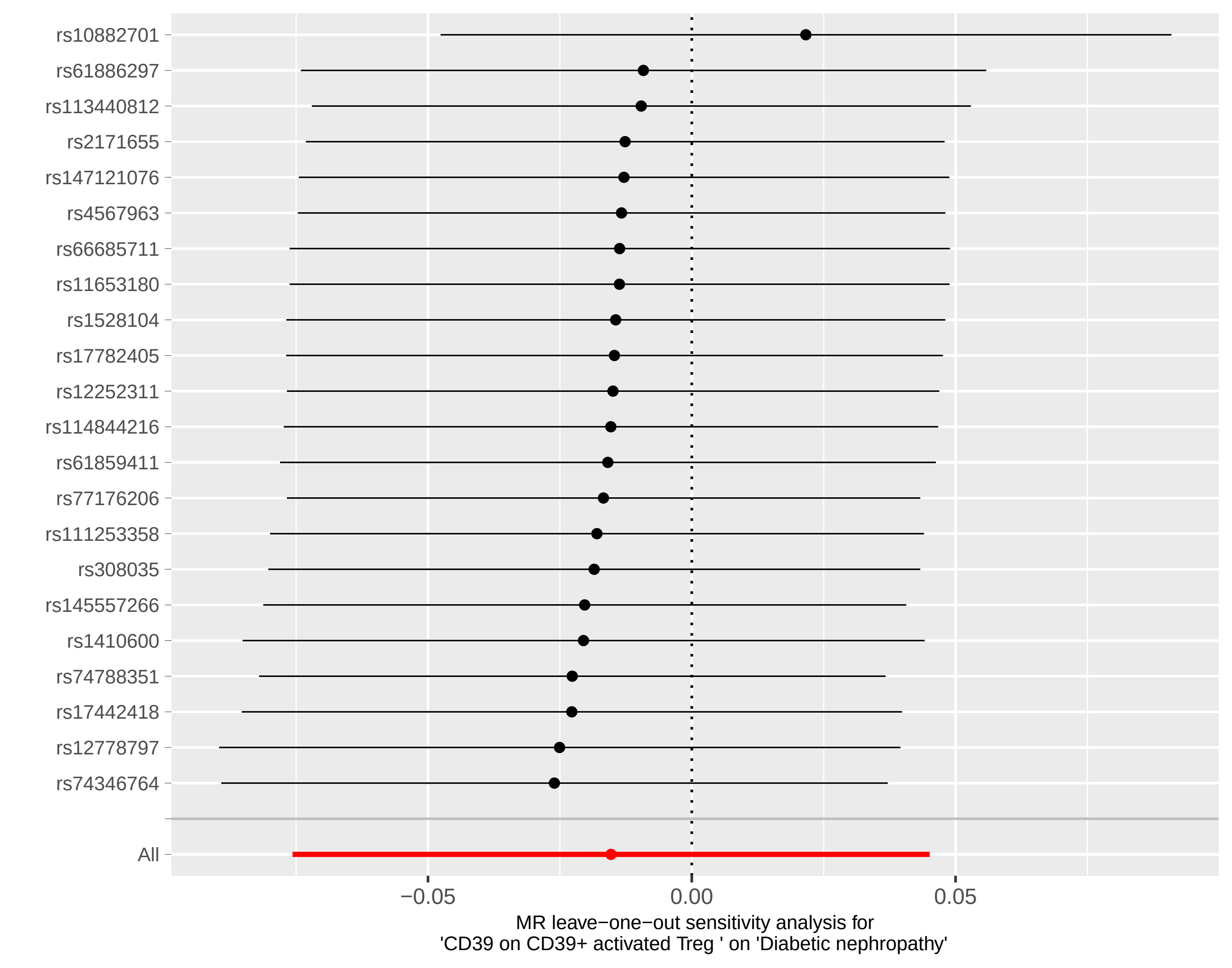
rs10882701
rs61886297
rs113440812
rs2171655
rs147121076
rs4567963
rs66685711
rs11653180
rs1528104
rs17782405
rs12252311
rs114844216
rs61859411
rs77176206
rs111253358
rs308035
rs145557266
rs1410600
rs74788351
rs17442418
rs12778797
rs74346764
All
−0.05
0.00
0.05
MR leave−one−out sensitivity analysis for
'CD39 on CD39+ activated Treg ' on 'Diabetic nephropathy'

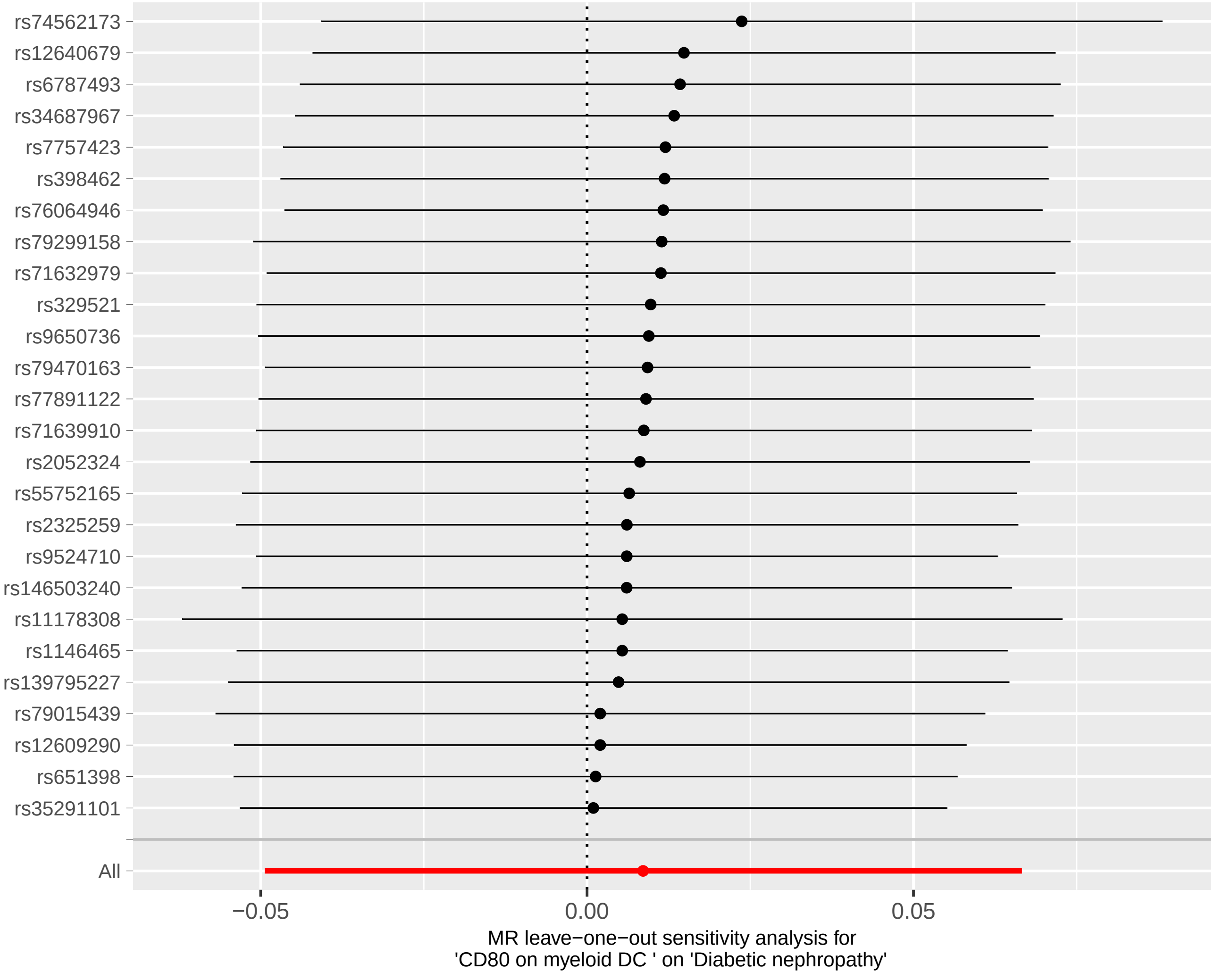

rs74562173
rs12640679
rs6787493
rs34687967
rs7757423
rs398462
rs76064946
rs79299158
rs71632979
rs329521
rs9650736
rs79470163
rs77891122
rs71639910
rs2052324
rs55752165
rs2325259
rs9524710
rs146503240
rs11178308
rs1146465
rs139795227
rs79015439
rs12609290
rs651398
rs35291101
All
−0.05
0.00
0.05
MR leave−one−out sensitivity analysis for
'CD80 on myeloid DC ' on 'Diabetic nephropathy'

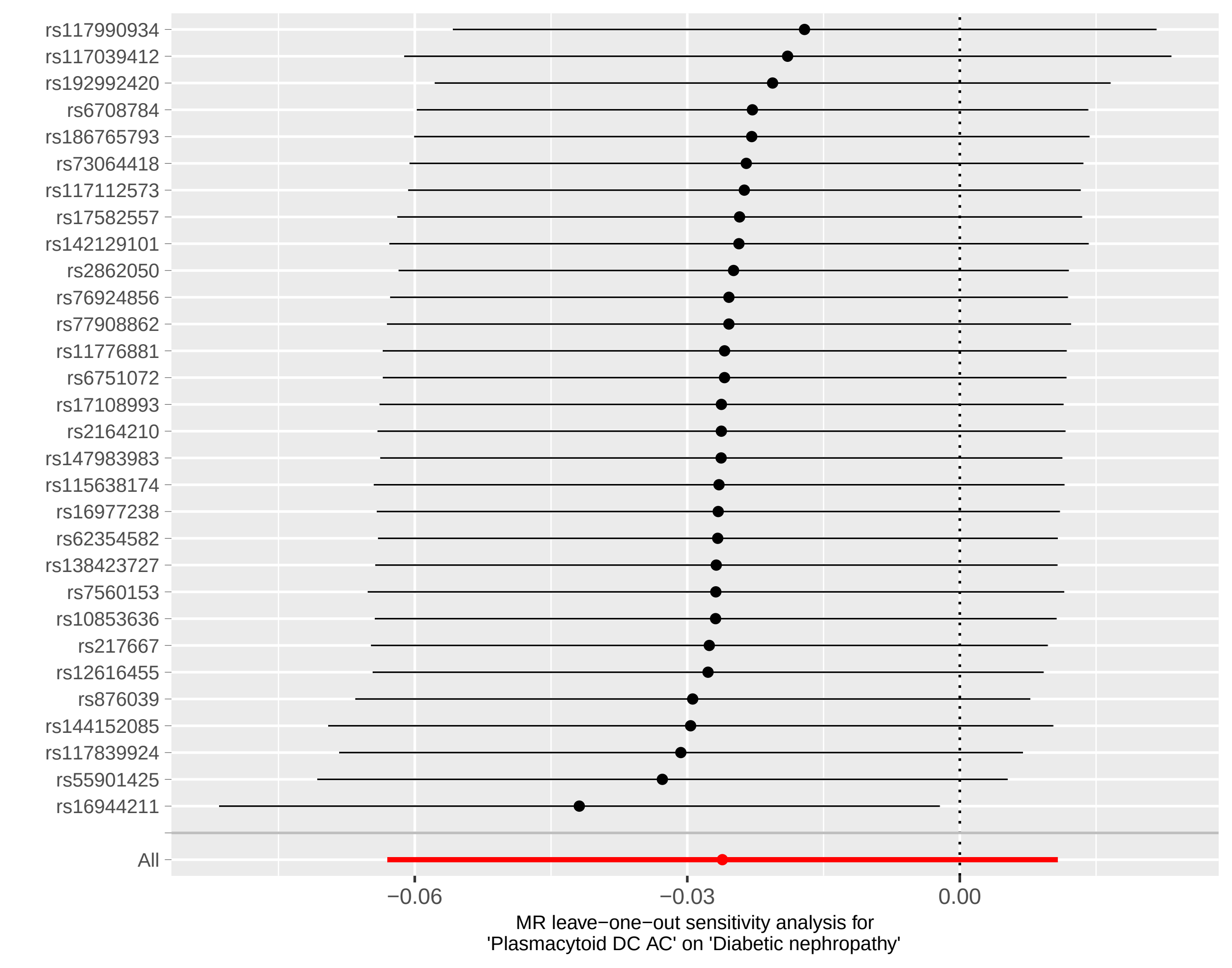
rs117990934
rs117039412
rs192992420
rs6708784
rs186765793
rs73064418
rs117112573
rs17582557
rs142129101
rs2862050
rs76924856
rs77908862
rs11776881
rs6751072
rs17108993
rs2164210
rs147983983
rs115638174
rs16977238
rs62354582
rs138423727
rs7560153
rs10853636
rs217667
rs12616455
rs876039
rs144152085
rs117839924
rs55901425
rs16944211
All
−0.06
−0.03
0.00
MR leave−one−out sensitivity analysis for
'Plasmacytoid DC AC' on 'Diabetic nephropathy'

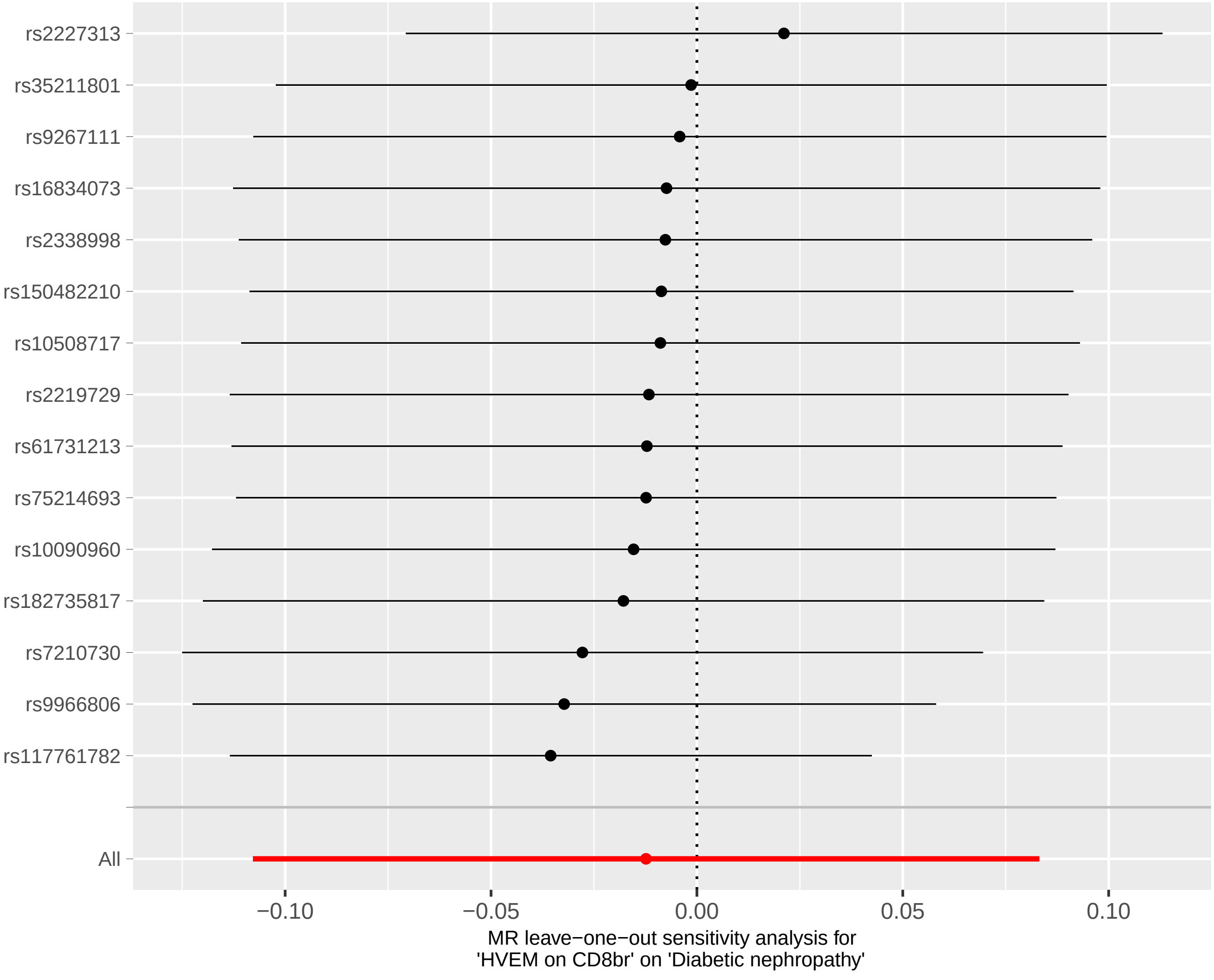
rs2227313
rs35211801
rs9267111
rs16834073
rs2338998
rs150482210
rs10508717
rs2219729
rs61731213
rs75214693
rs10090960
rs182735817
rs7210730
rs9966806
rs117761782
All
−0.10
−0.05
0.00
0.05
0.10
MR leave−one−out sensitivity analysis for
'HVEM on CD8br' on 'Diabetic nephropathy'

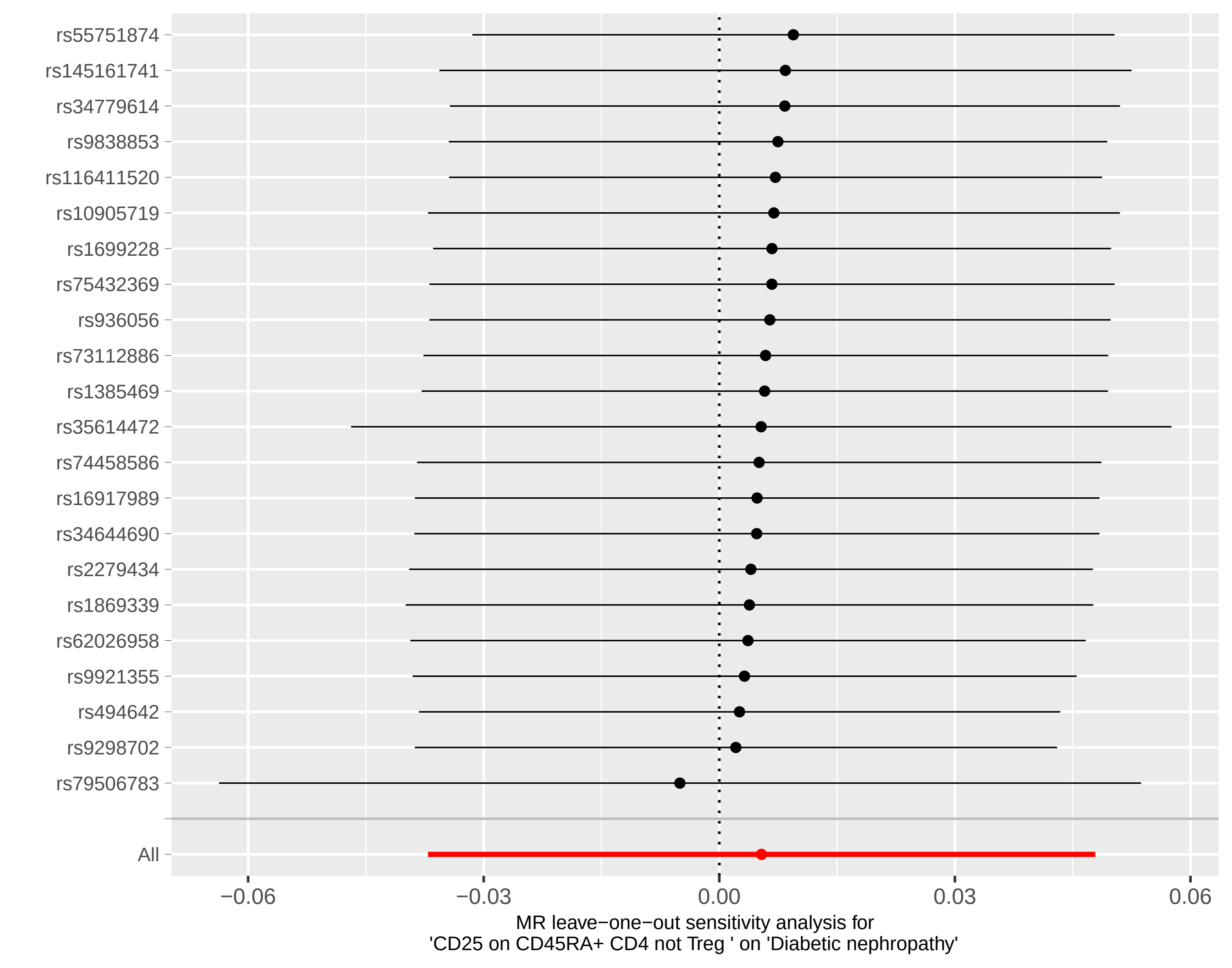
rs55751874
rs145161741
rs34779614
rs9838853
rs116411520
rs10905719
rs1699228
rs75432369
rs936056
rs73112886
rs1385469
rs35614472
rs74458586
rs16917989
rs34644690
rs2279434
rs1869339
rs62026958
rs9921355
rs494642
rs9298702
rs79506783
All
−0.06
−0.03
0.00
0.03
0.06
MR leave−one−out sensitivity analysis for
'CD25 on CD45RA+ CD4 not Treg ' on 'Diabetic nephropathy'

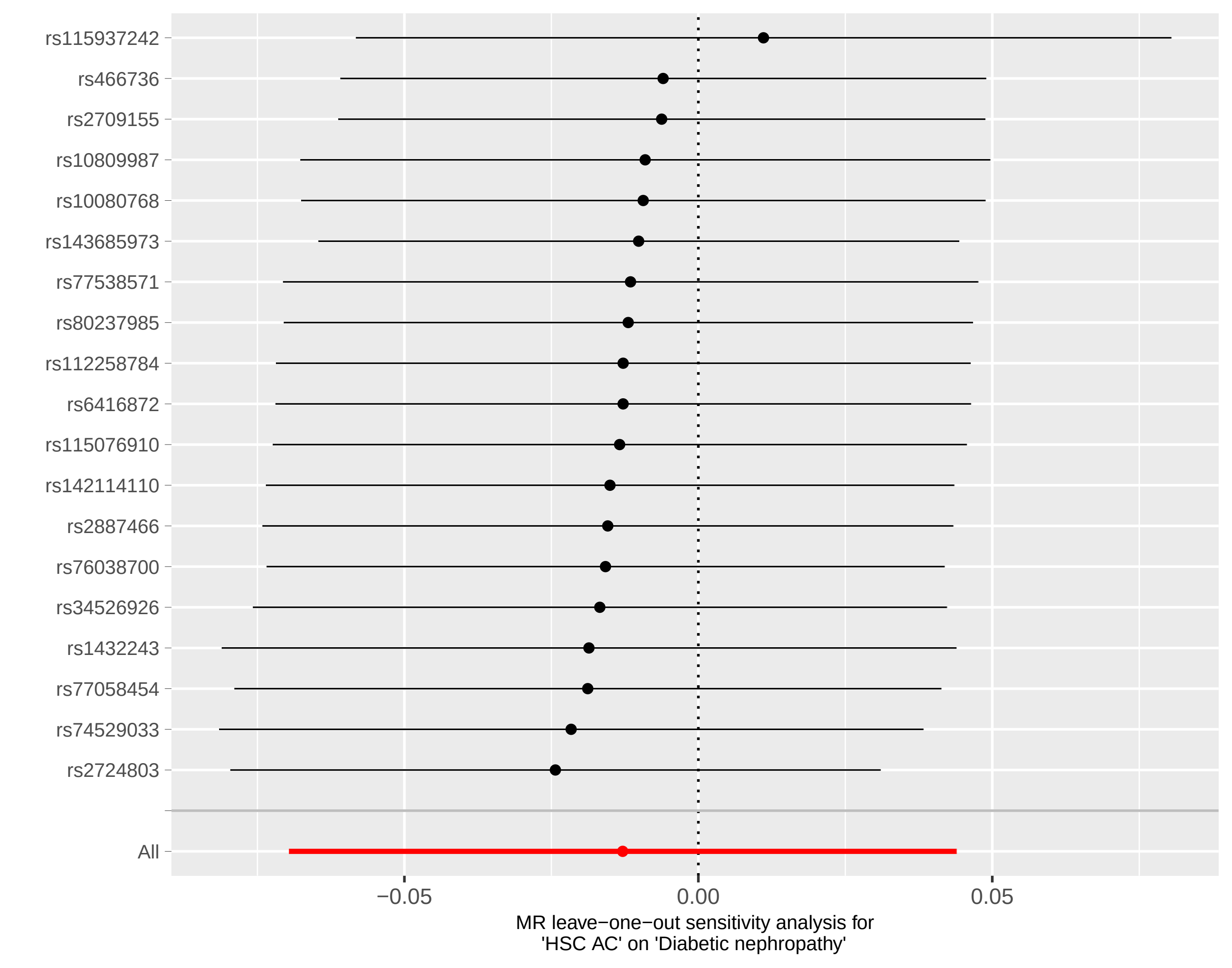

rs115937242
rs466736
rs2709155
rs10809987
rs10080768
rs143685973
rs77538571
rs80237985
rs112258784
rs6416872
rs115076910
rs142114110
rs2887466
rs76038700
rs34526926
rs1432243
rs77058454
rs74529033
rs2724803
All
−0.05
0.00
0.05
MR leave−one−out sensitivity analysis for
'HSC AC' on 'Diabetic nephropathy'

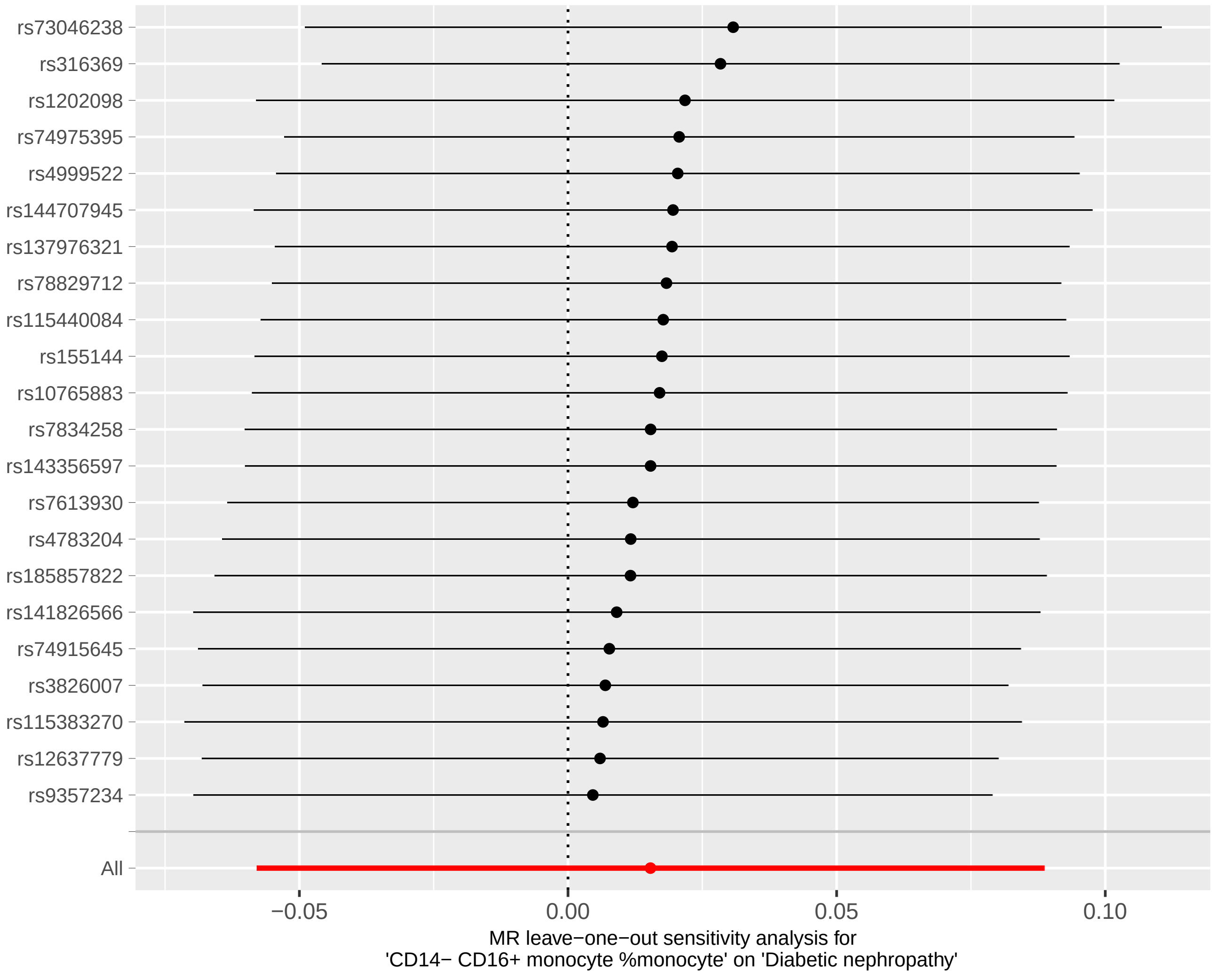
rs73046238
rs316369
rs1202098
rs74975395
rs4999522
rs144707945
rs137976321
rs78829712
rs115440084
rs155144
rs10765883
rs7834258
rs143356597
rs7613930
rs4783204
rs185857822
rs141826566
rs74915645
rs3826007
rs115383270
rs12637779
rs9357234
All
−0.05
0.00
0.05
0.10
MR leave−one−out sensitivity analysis for
'CD14− CD16+ monocyte %monocyte' on 'Diabetic nephropathy'

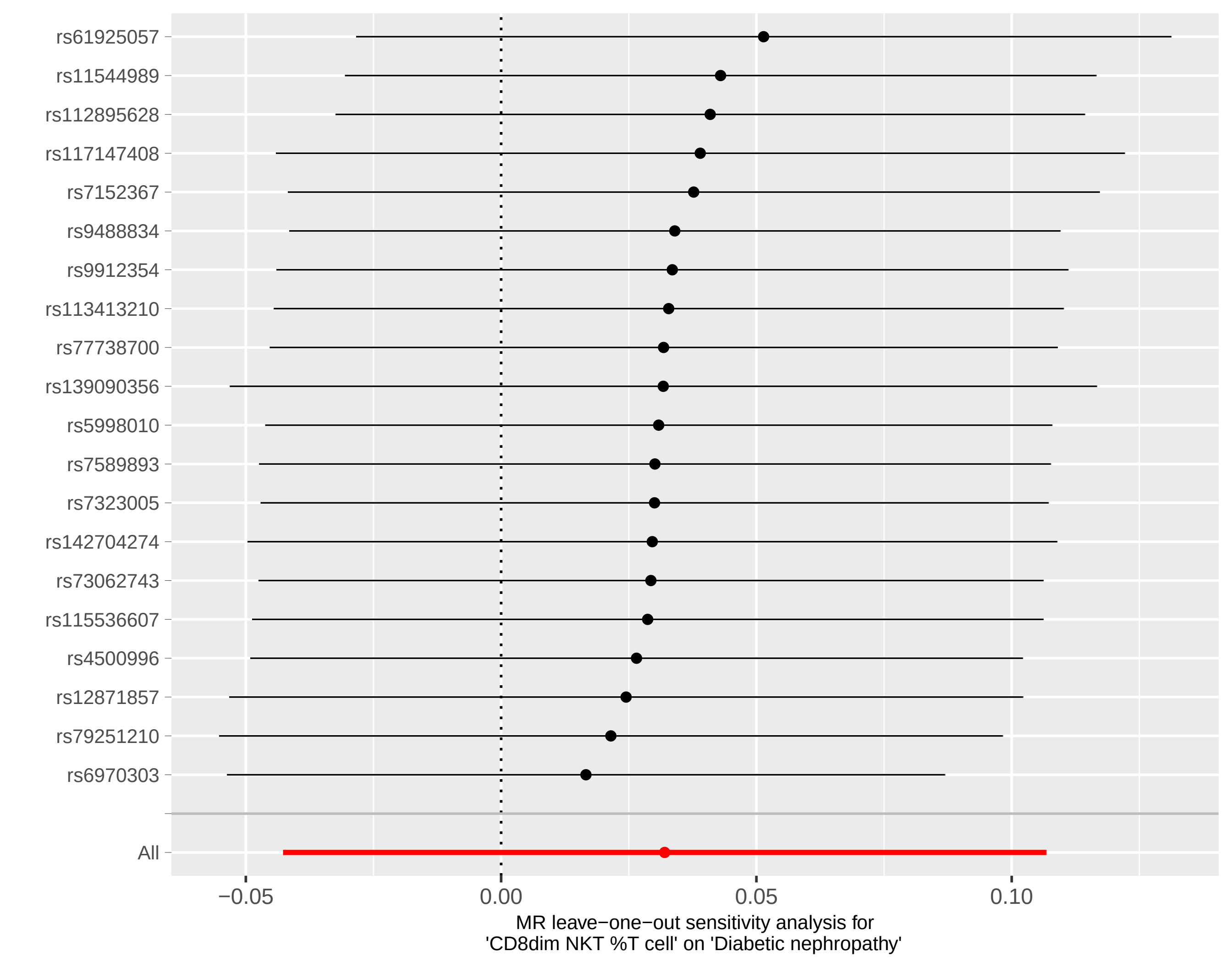

rs61925057
rs11544989
rs112895628
rs117147408
rs7152367
rs9488834
rs9912354
rs113413210
rs77738700
rs139090356
rs5998010
rs7589893
rs7323005
rs142704274
rs73062743
rs115536607
rs4500996
rs12871857
rs79251210
rs6970303
All
−0.05
0.00
0.05
0.10
MR leave−one−out sensitivity analysis for
'CD8dim NKT %T cell' on 'Diabetic nephropathy'

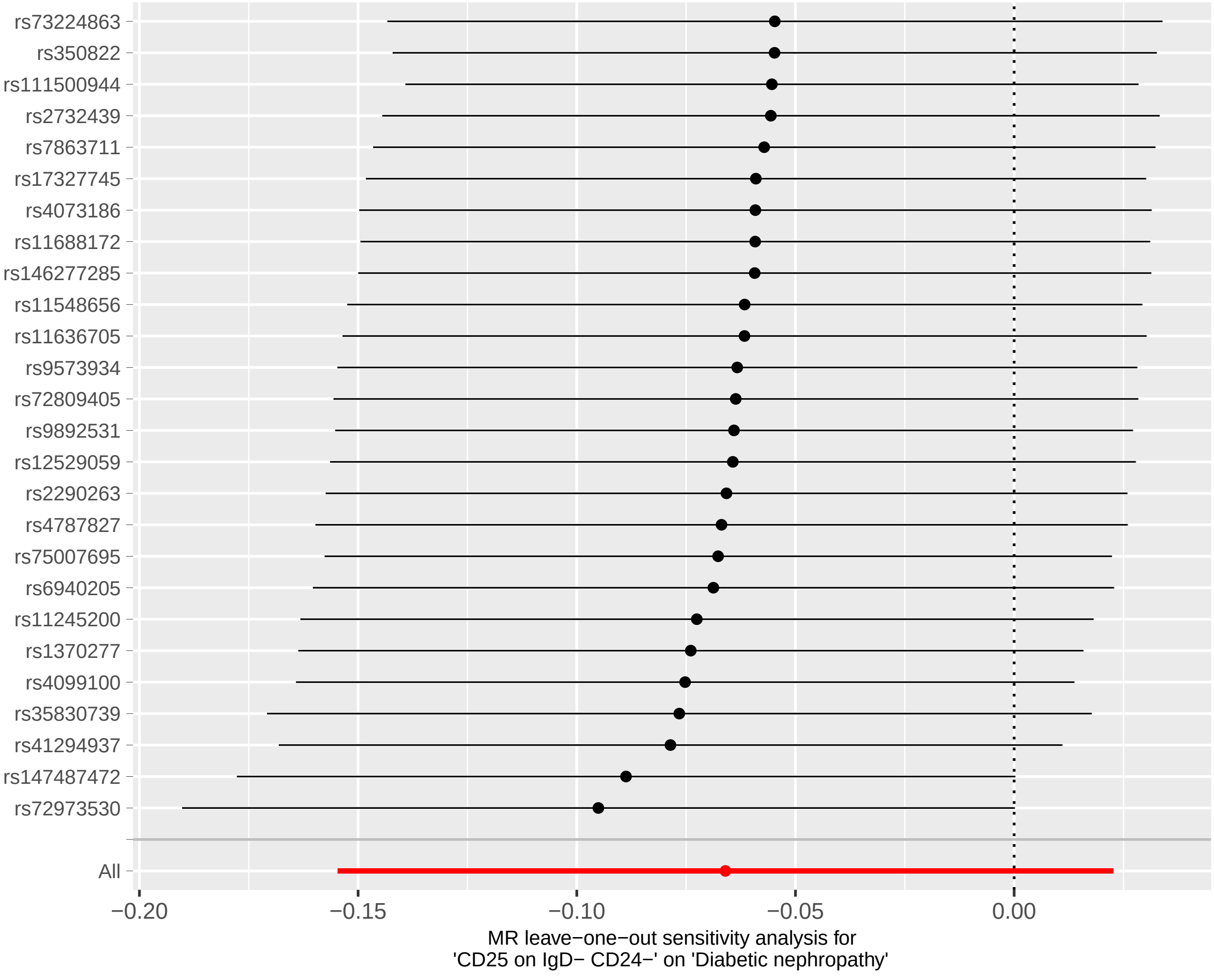
rs73224863
rs350822
rs111500944
rs2732439
rs7863711
rs17327745
rs4073186
rs11688172
rs146277285
rs11548656
rs11636705
rs9573934
rs72809405
rs9892531
rs12529059
rs2290263
rs4787827
rs75007695
rs6940205
rs11245200
rs1370277
rs4099100
rs35830739
rs41294937
rs147487472
rs72973530
All
−0.20
−0.15
−0.10
−0.05
0.00
MR leave−one−out sensitivity analysis for
'CD25 on IgD− CD24−' on 'Diabetic nephropathy'

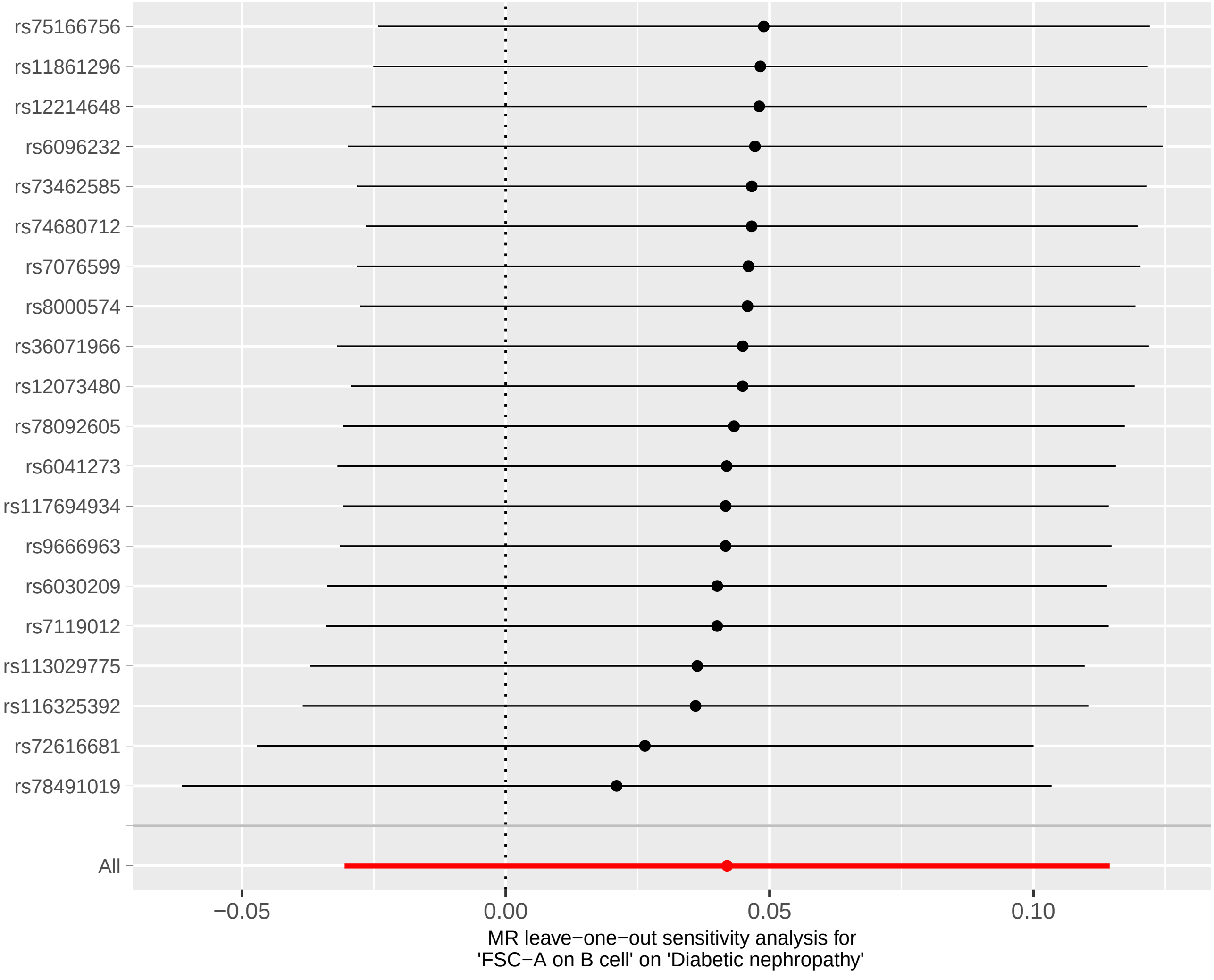
rs75166756
rs11861296
rs12214648
rs6096232
rs73462585
rs74680712
rs7076599
rs8000574
rs36071966
rs12073480
rs78092605
rs6041273
rs117694934
rs9666963
rs6030209
rs7119012
rs113029775
rs116325392
rs72616681
rs78491019
All
−0.05
0.00
0.05
0.10
MR leave−one−out sensitivity analysis for
'FSC−A on B cell' on 'Diabetic nephropathy'

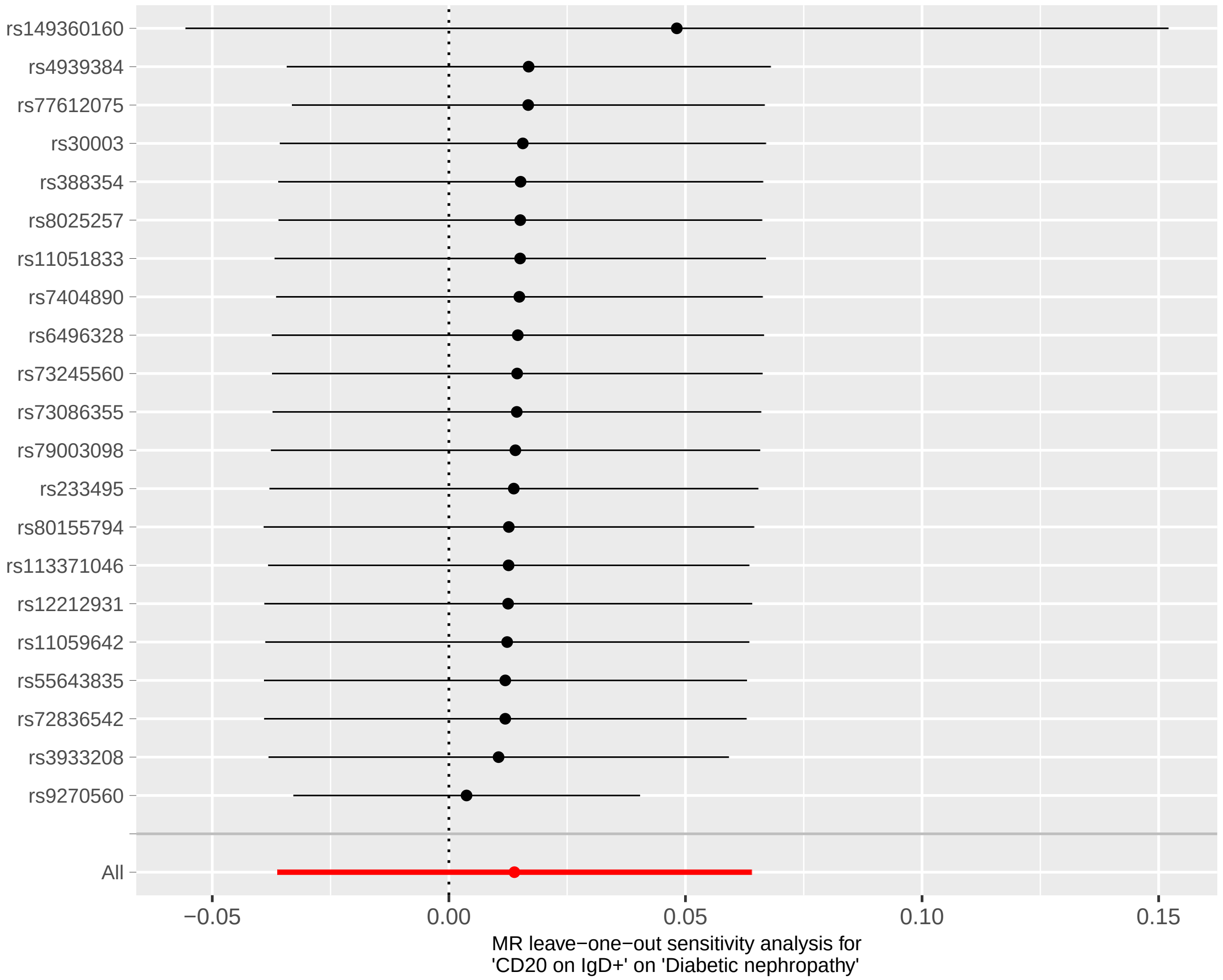
rs149360160
rs4939384
rs77612075
rs30003
rs388354
rs8025257
rs11051833
rs7404890
rs6496328
rs73245560
rs73086355
rs79003098
rs233495
rs80155794
rs113371046
rs12212931
rs11059642
rs55643835
rs72836542
rs3933208
rs9270560
All
−0.05
0.00
0.05
0.10
0.15
MR leave−one−out sensitivity analysis for
'CD20 on IgD+' on 'Diabetic nephropathy'

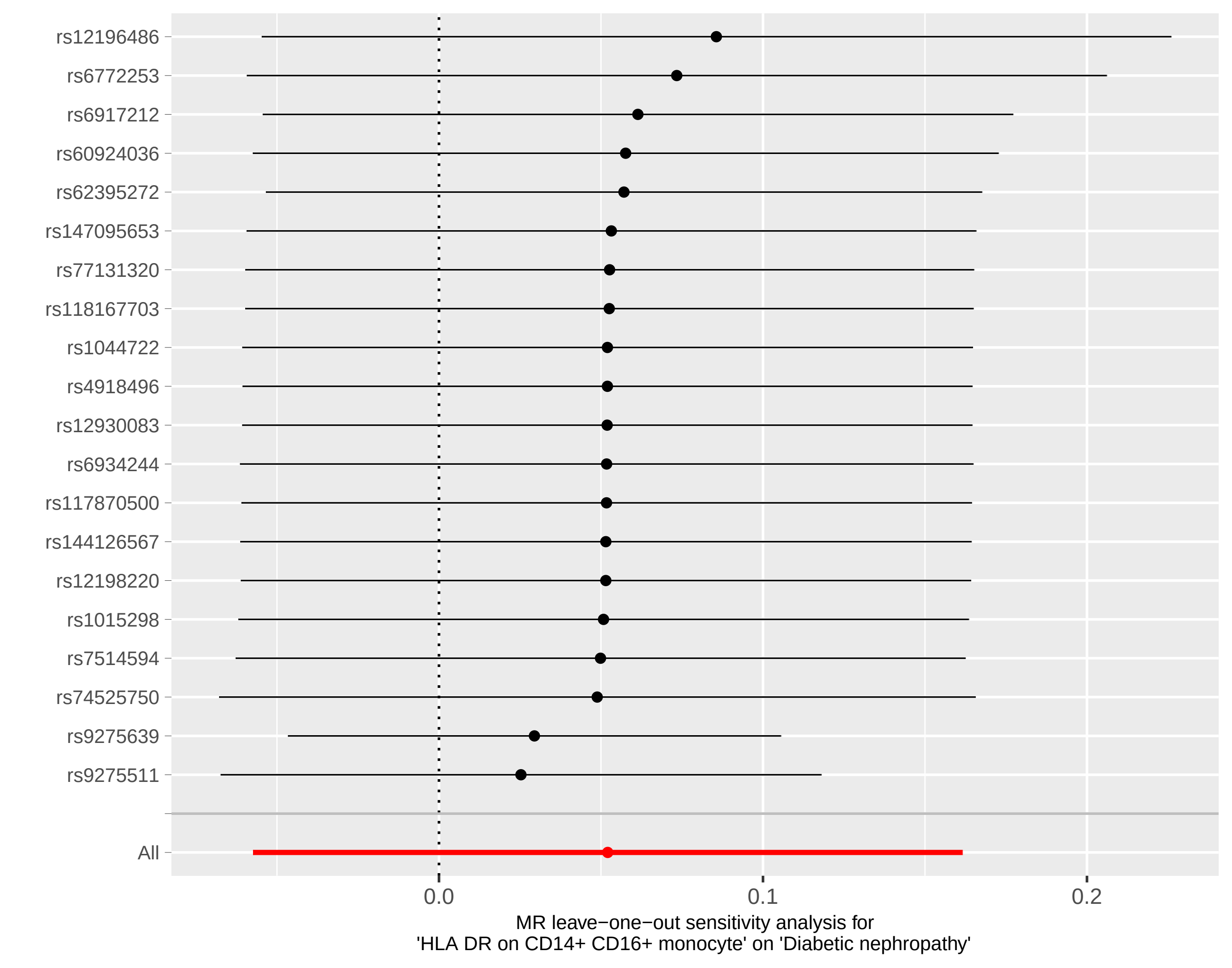

rs12196486
rs6772253
rs6917212
rs60924036
rs62395272
rs147095653
rs77131320
rs118167703
rs1044722
rs4918496
rs12930083
rs6934244
rs117870500
rs144126567
rs12198220
rs1015298
rs7514594
rs74525750
rs9275639
rs9275511
All
0.0
0.1
0.2
MR leave−one−out sensitivity analysis for
'HLA DR on CD14+ CD16+ monocyte' on 'Diabetic nephropathy'

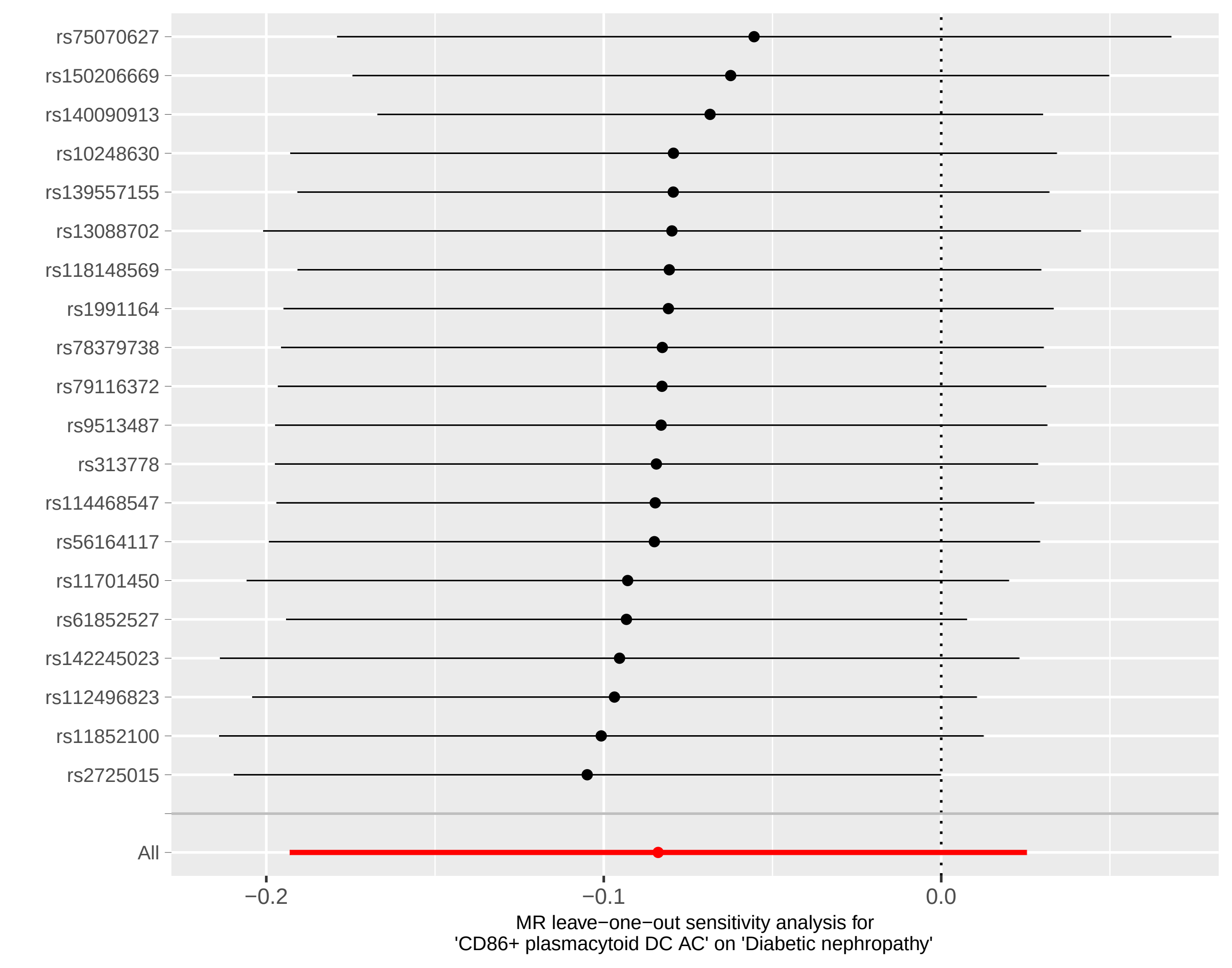
rs75070627
rs150206669
rs140090913
rs10248630
rs139557155
rs13088702
rs118148569
rs1991164
rs78379738
rs79116372
rs9513487
rs313778
rs114468547
rs56164117
rs11701450
rs61852527
rs142245023
rs112496823
rs11852100
rs2725015
All
−0.2
−0.1
0.0
MR leave−one−out sensitivity analysis for
'CD86+ plasmacytoid DC AC' on 'Diabetic nephropathy'

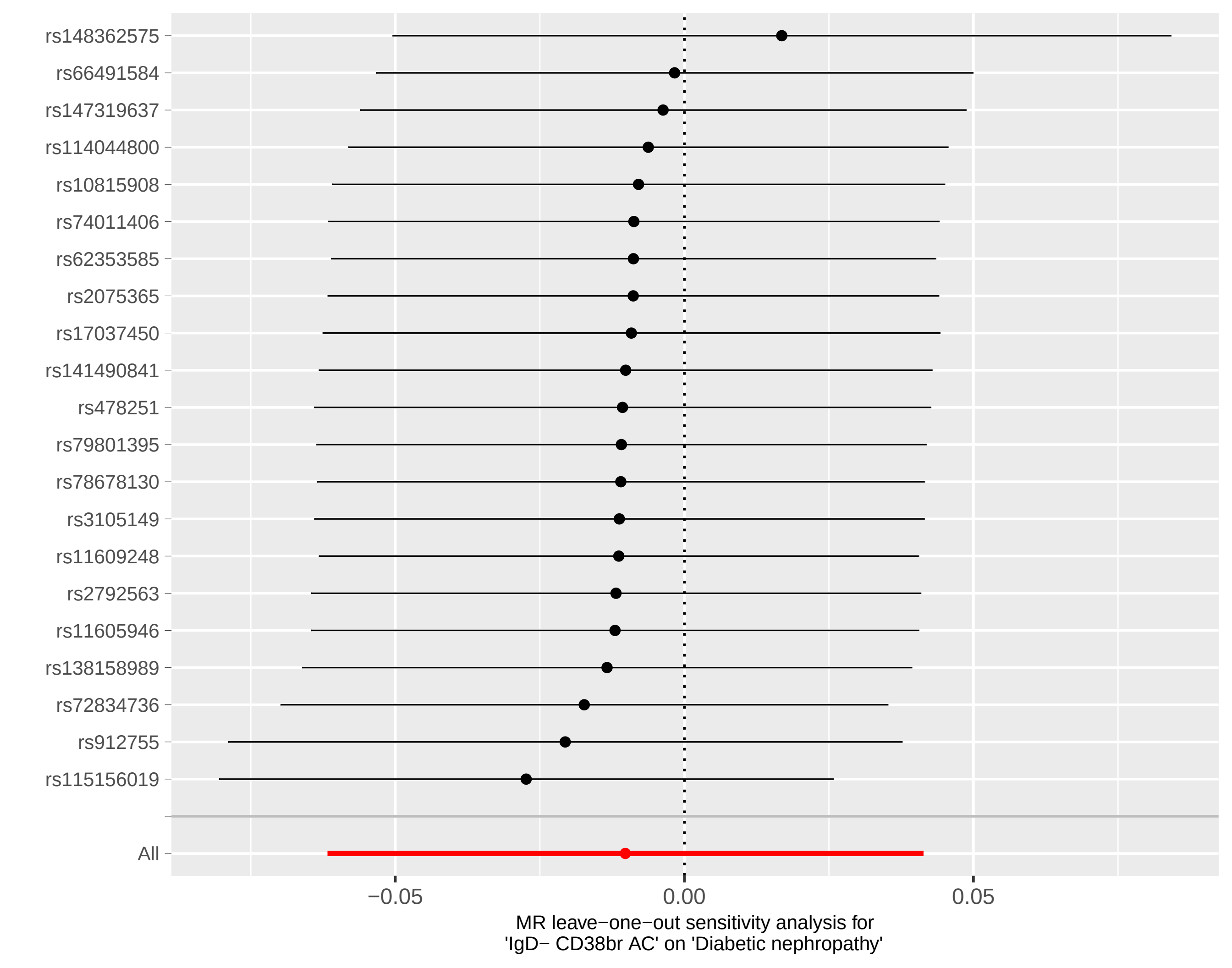

rs148362575
rs66491584
rs147319637
rs114044800
rs10815908
rs74011406
rs62353585
rs2075365
rs17037450
rs141490841
rs478251
rs79801395
rs78678130
rs3105149
rs11609248
rs2792563
rs11605946
rs138158989
rs72834736
rs912755
rs115156019
All
−0.05
0.00
0.05
MR leave−one−out sensitivity analysis for
'IgD− CD38br AC' on 'Diabetic nephropathy'

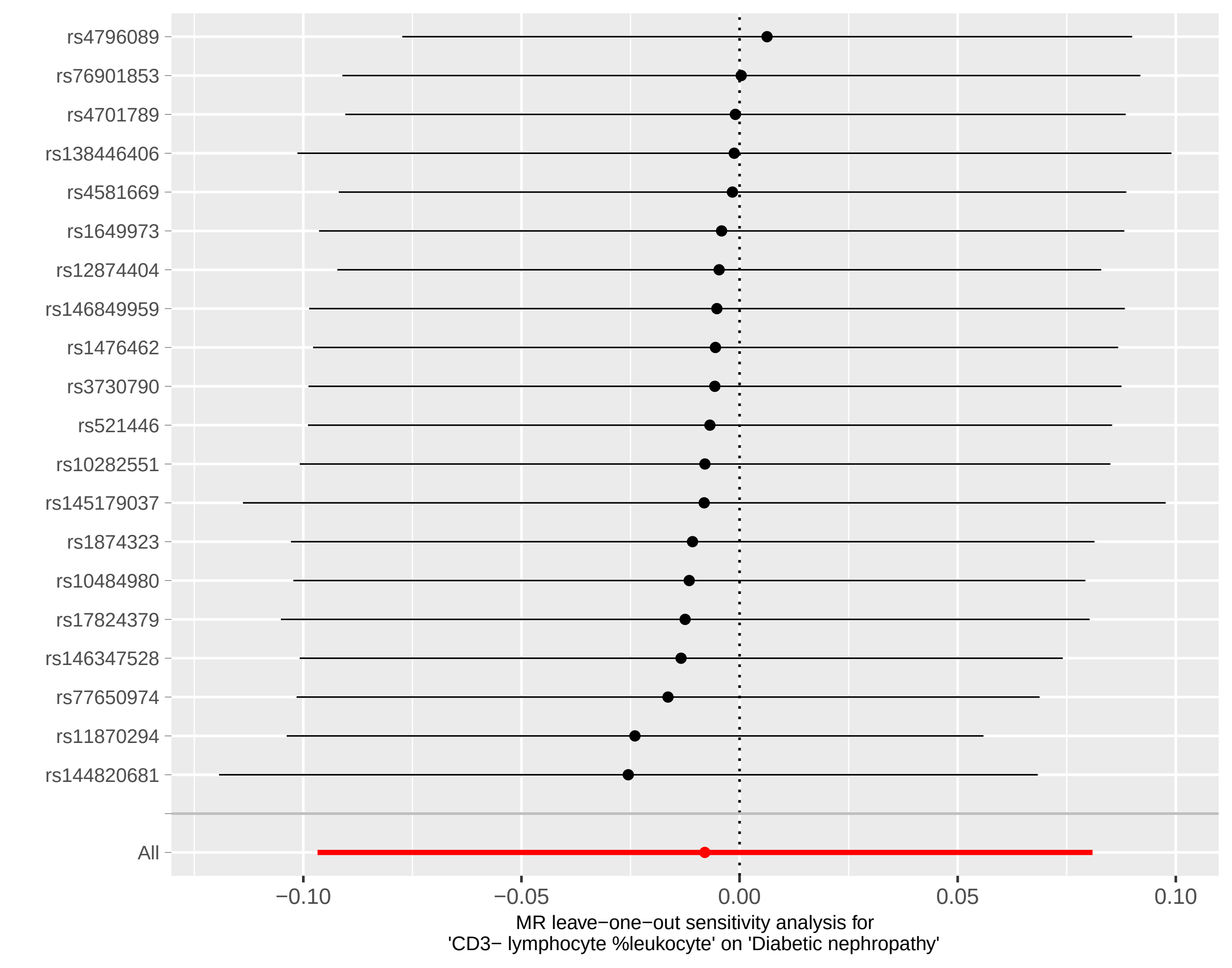

rs4796089
rs76901853
rs4701789
rs138446406
rs4581669
rs1649973
rs12874404
rs146849959
rs1476462
rs3730790
rs521446
rs10282551
rs145179037
rs1874323
rs10484980
rs17824379
rs146347528
rs77650974
rs11870294
rs144820681
All
−0.10
−0.05
0.00
0.05
0.10
MR leave−one−out sensitivity analysis for
'CD3− lymphocyte %leukocyte' on 'Diabetic nephropathy'

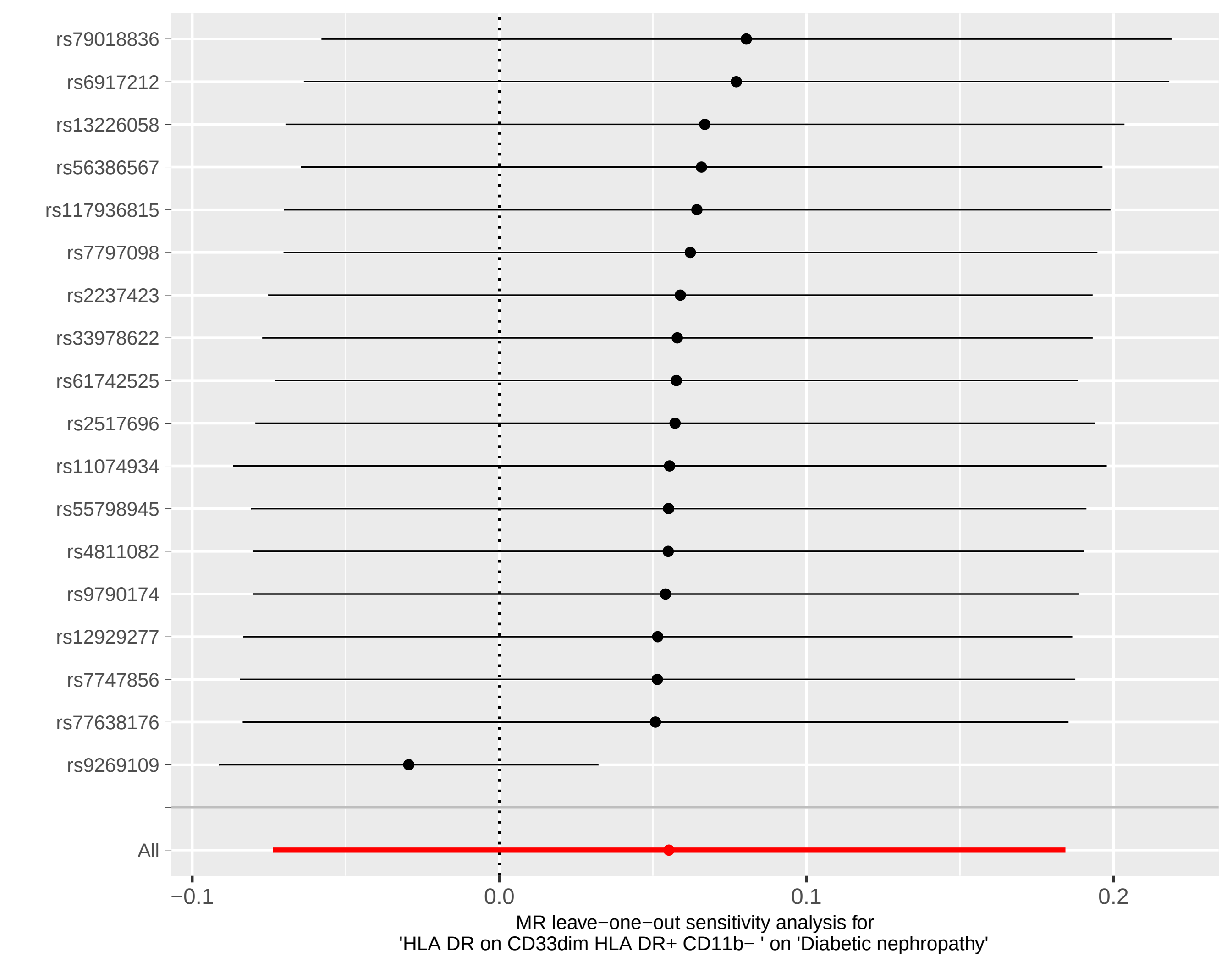
rs79018836
rs6917212
rs13226058
rs56386567
rs117936815
rs7797098
rs2237423
rs33978622
rs61742525
rs2517696
rs11074934
rs55798945
rs4811082
rs9790174
rs12929277
rs7747856
rs77638176
rs9269109
All
−0.1
0.0
0.1
0.2
MR leave−one−out sensitivity analysis for
'HLA DR on CD33dim HLA DR+ CD11b− ' on 'Diabetic nephropathy'

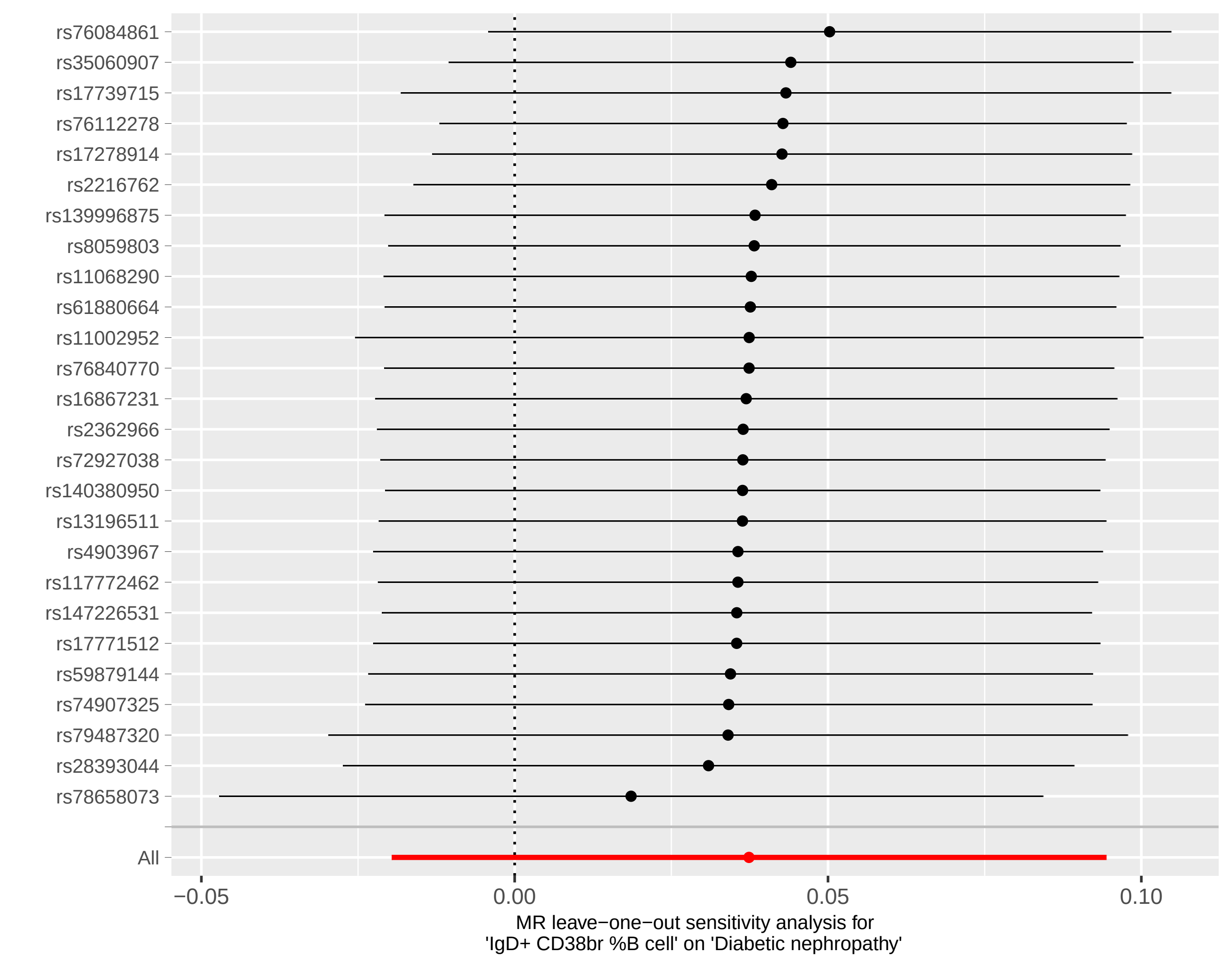

rs76084861
rs35060907
rs17739715
rs76112278
rs17278914
rs2216762
rs139996875
rs8059803
rs11068290
rs61880664
rs11002952
rs76840770
rs16867231
rs2362966
rs72927038
rs140380950
rs13196511
rs4903967
rs117772462
rs147226531
rs17771512
rs59879144
rs74907325
rs79487320
rs28393044
rs78658073
All
−0.05
0.00
0.05
0.10
MR leave−one−out sensitivity analysis for
'IgD+ CD38br %B cell' on 'Diabetic nephropathy'

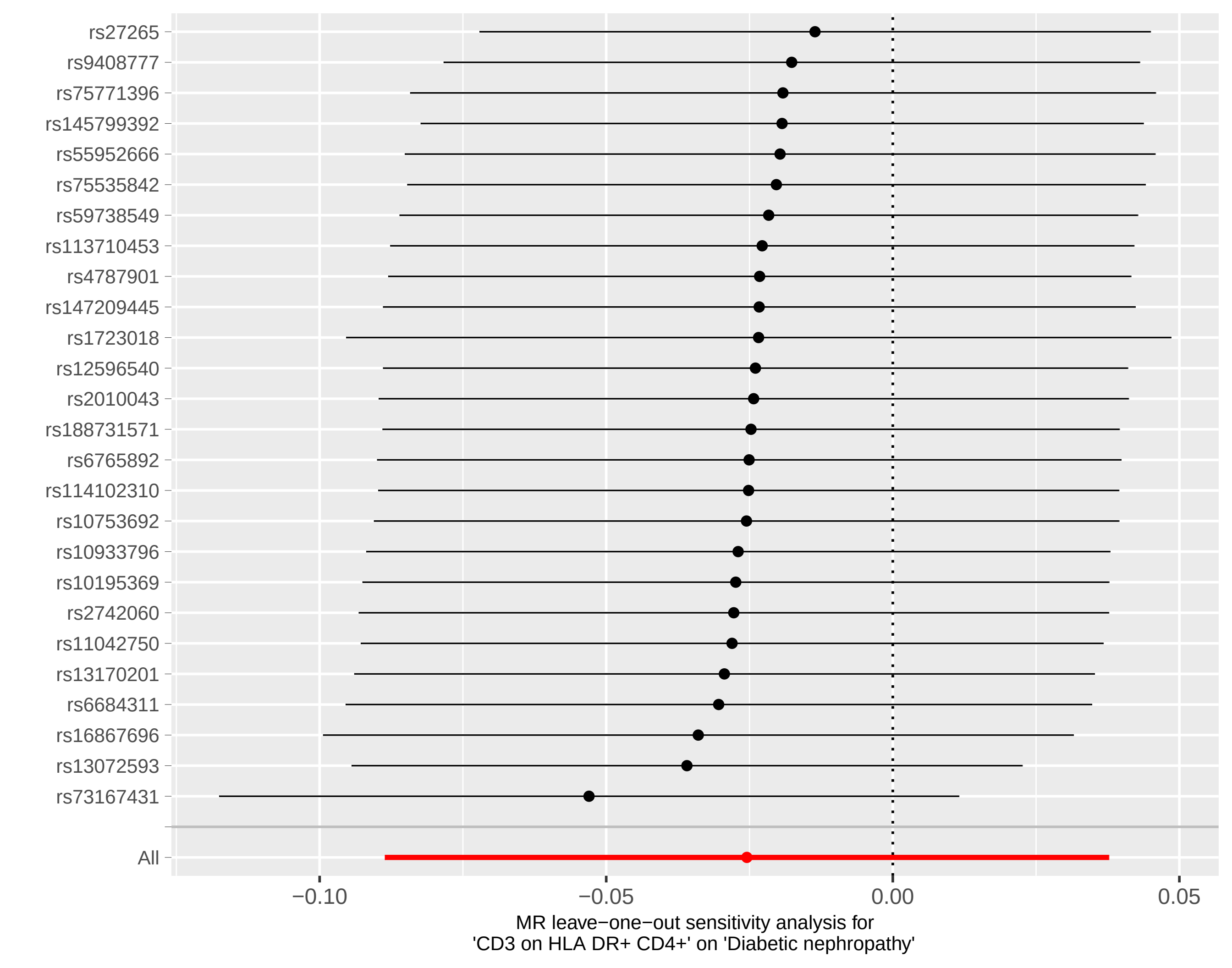
rs27265
rs9408777
rs75771396
rs145799392
rs55952666
rs75535842
rs59738549
rs113710453
rs4787901
rs147209445
rs1723018
rs12596540
rs2010043
rs188731571
rs6765892
rs114102310
rs10753692
rs10933796
rs10195369
rs2742060
rs11042750
rs13170201
rs6684311
rs16867696
rs13072593
rs73167431
All
−0.10
−0.05
0.00
0.05
MR leave−one−out sensitivity analysis for
'CD3 on HLA DR+ CD4+' on 'Diabetic nephropathy'

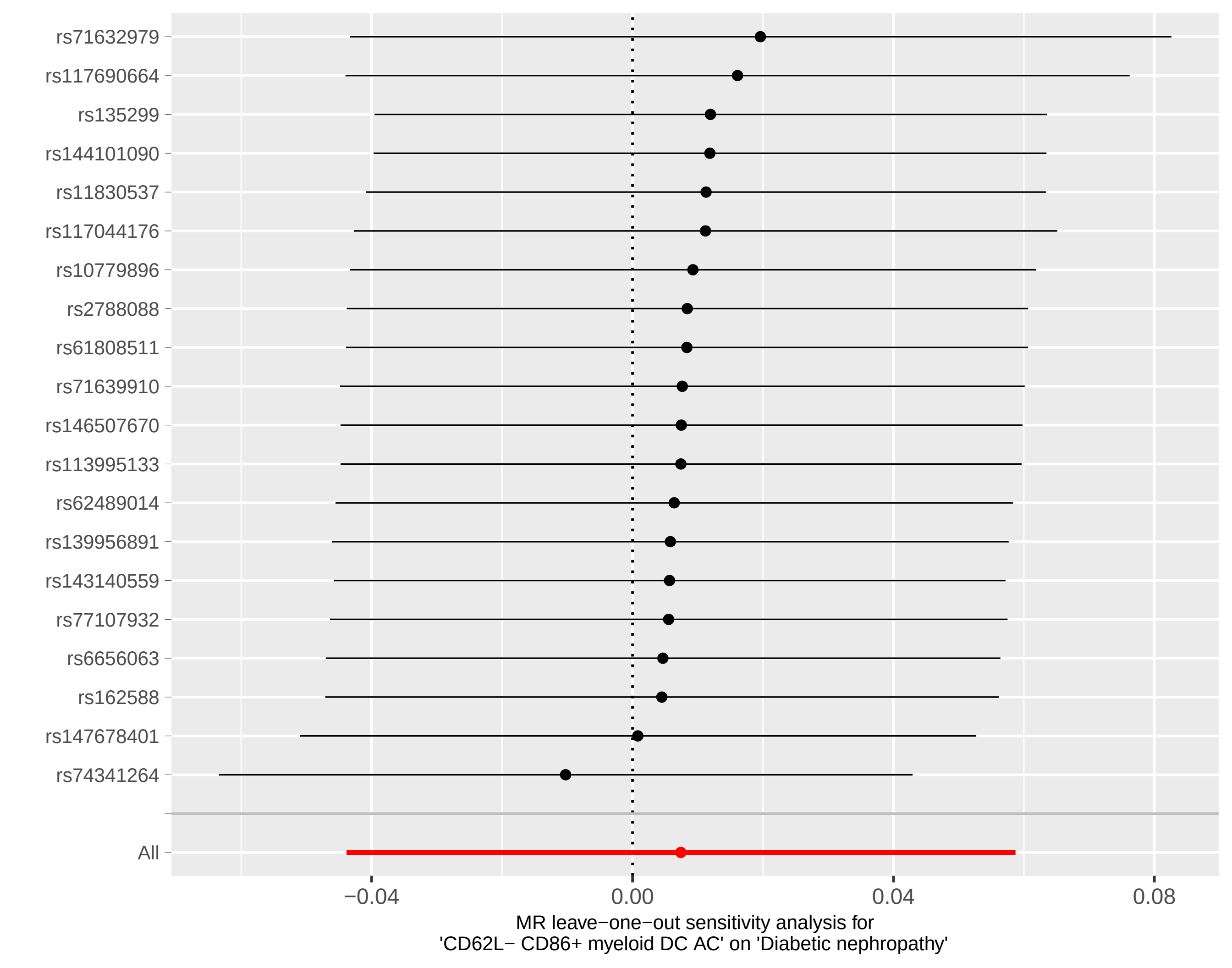

rs71632979
rs117690664
rs135299
rs144101090
rs11830537
rs117044176
rs10779896
rs2788088
rs61808511
rs71639910
rs146507670
rs113995133
rs62489014
rs139956891
rs143140559
rs77107932
rs6656063
rs162588
rs147678401
rs74341264
All
−0.04
0.00
0.04
0.08
MR leave−one−out sensitivity analysis for
'CD62L− CD86+ myeloid DC AC' on 'Diabetic nephropathy'

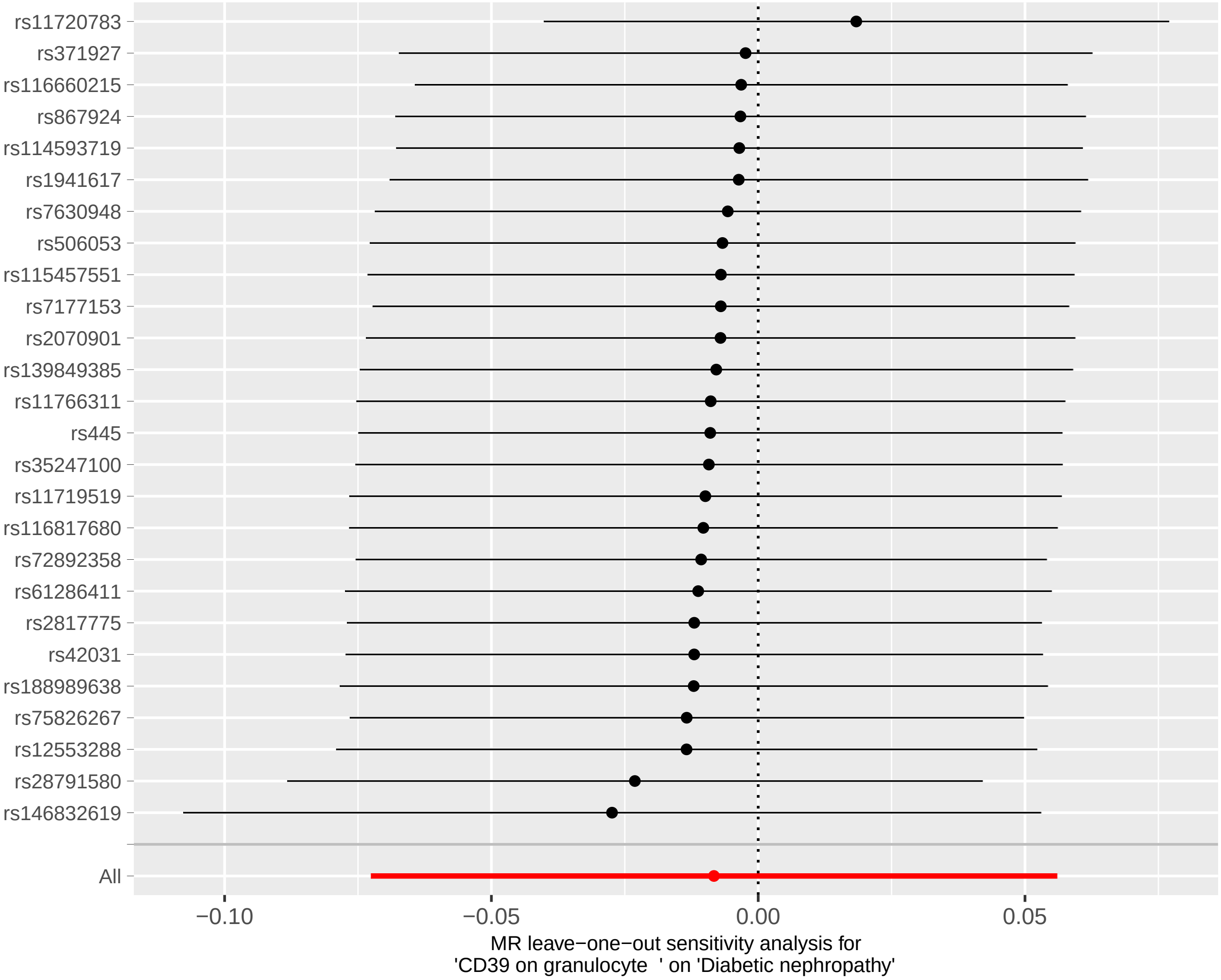

rs11720783
rs371927
rs116660215
rs867924
rs114593719
rs1941617
rs7630948
rs506053
rs115457551
rs7177153
rs2070901
rs139849385
rs11766311
rs445
rs35247100
rs11719519
rs116817680
rs72892358
rs61286411
rs2817775
rs42031
rs188989638
rs75826267
rs12553288
rs28791580
rs146832619
All
−0.10
−0.05
0.00
0.05
MR leave−one−out sensitivity analysis for
'CD39 on granulocyte ' on 'Diabetic nephropathy'

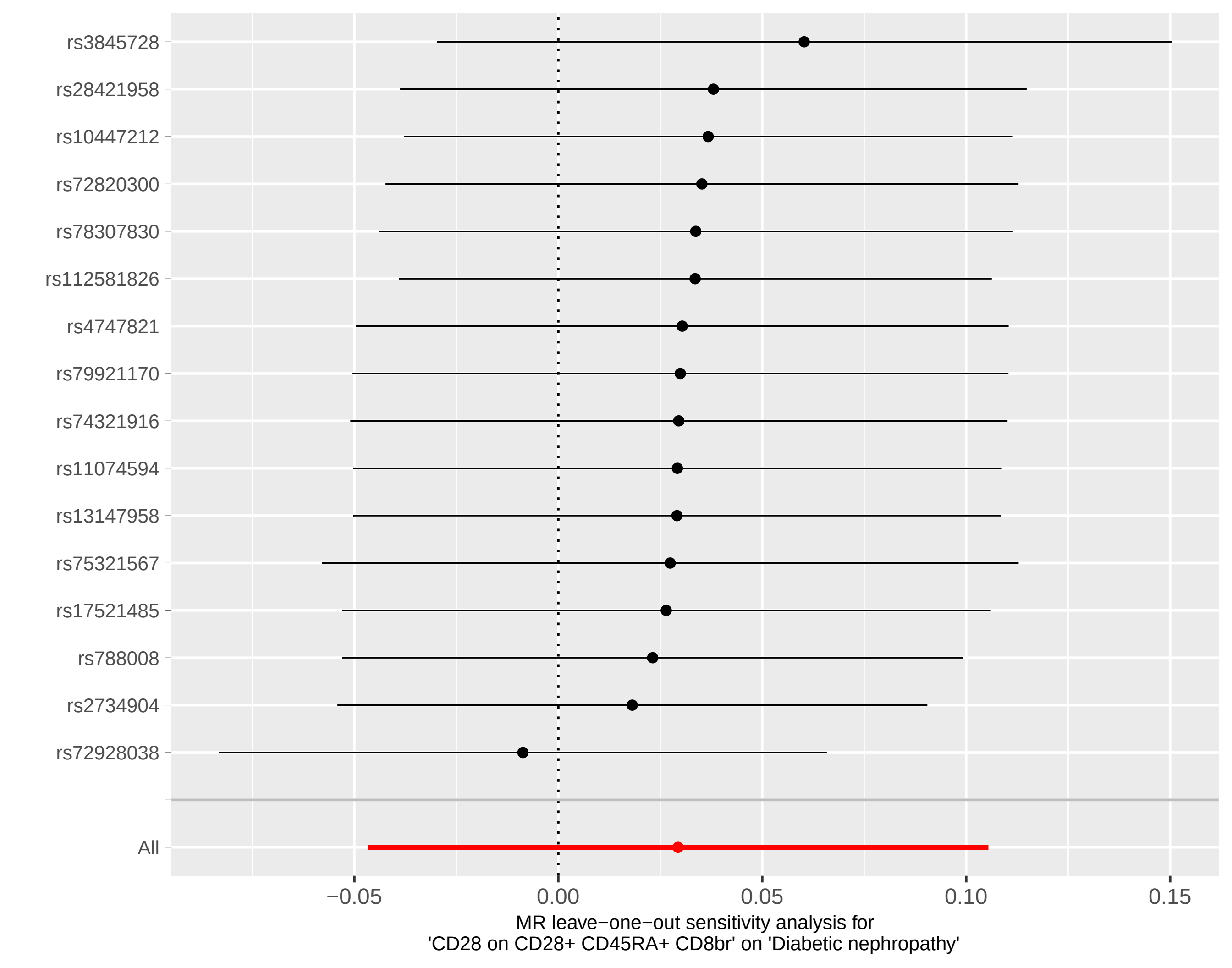
rs3845728
rs28421958
rs10447212
rs72820300
rs78307830
rs112581826
rs4747821
rs79921170
rs74321916
rs11074594
rs13147958
rs75321567
rs17521485
rs788008
rs2734904
rs72928038
All
−0.05
0.00
0.05
0.10
0.15
MR leave−one−out sensitivity analysis for
'CD28 on CD28+ CD45RA+ CD8br' on 'Diabetic nephropathy'

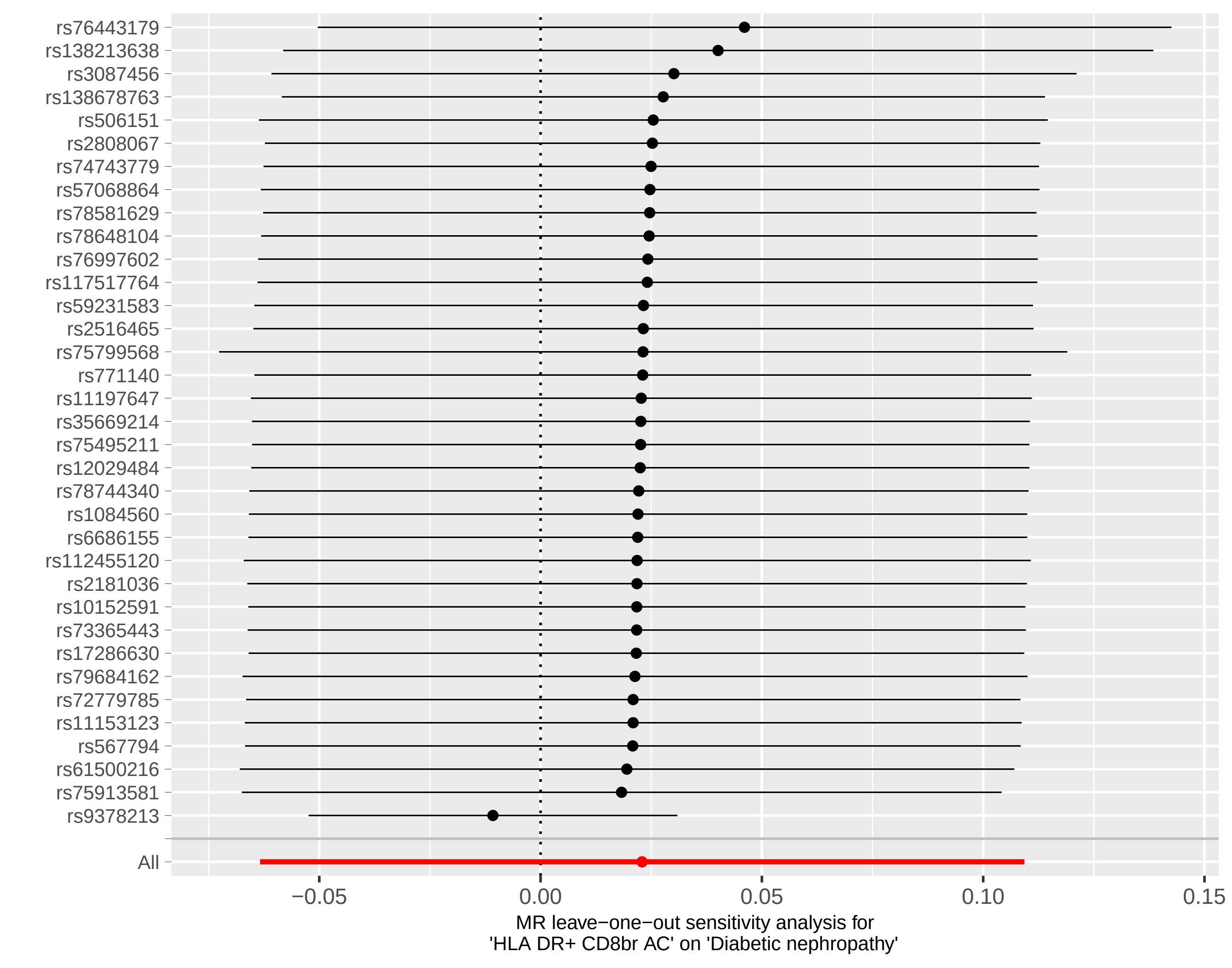
rs76443179
rs138213638
rs3087456
rs138678763
rs506151
rs2808067
rs74743779
rs57068864
rs78581629
rs78648104
rs76997602
rs117517764
rs59231583
rs2516465
rs75799568
rs771140
rs11197647
rs35669214
rs75495211
rs12029484
rs78744340
rs1084560
rs6686155
rs112455120
rs2181036
rs10152591
rs73365443
rs17286630
rs79684162
rs72779785
rs11153123
rs567794
rs61500216
rs75913581
rs9378213
All
−0.05
0.00
0.05
0.10
0.15
MR leave−one−out sensitivity analysis for
'HLA DR+ CD8br AC' on 'Diabetic nephropathy'

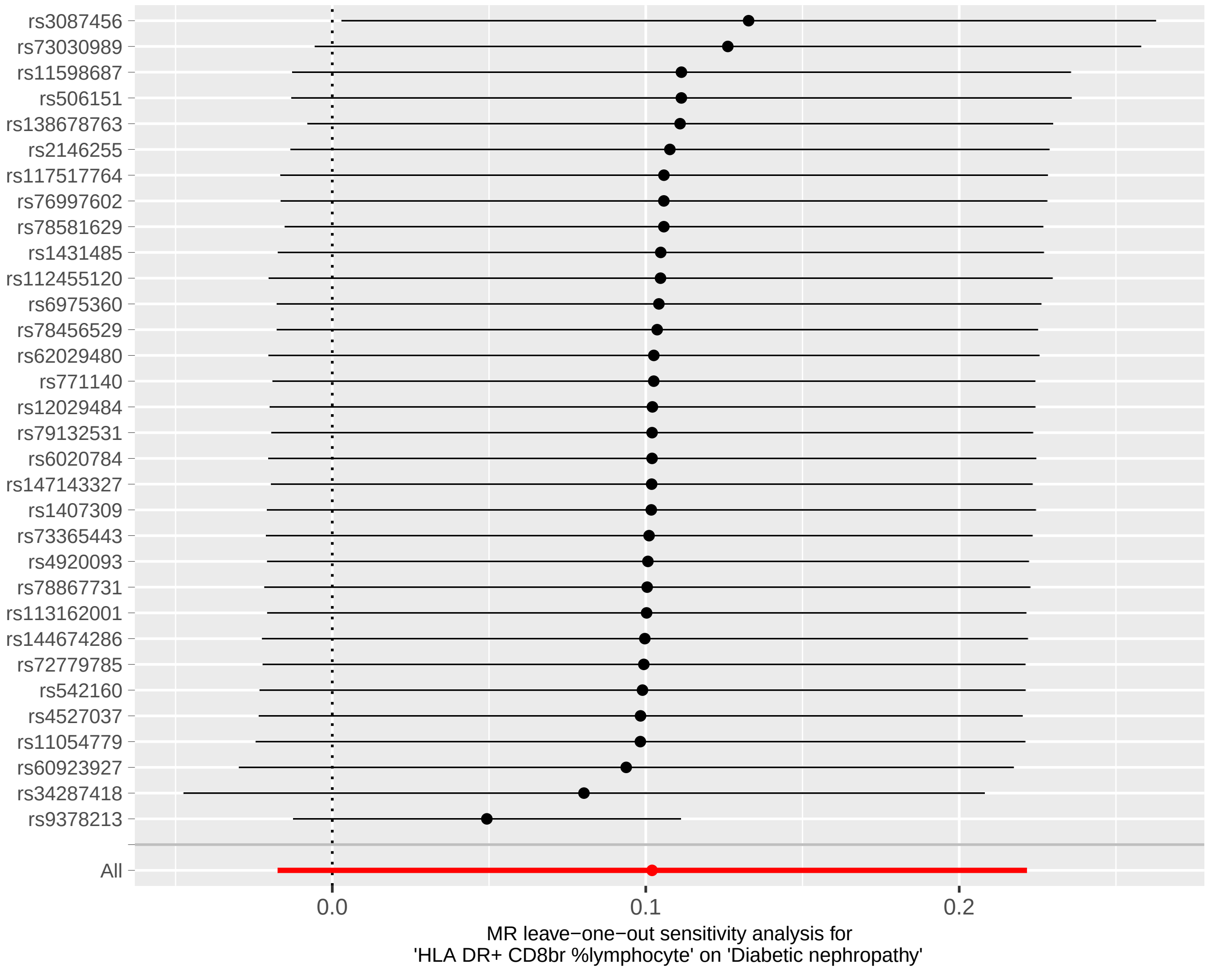

rs3087456
rs73030989
rs11598687
rs506151
rs138678763
rs2146255
rs117517764
rs76997602
rs78581629
rs1431485
rs112455120
rs6975360
rs78456529
rs62029480
rs771140
rs12029484
rs79132531
rs6020784
rs147143327
rs1407309
rs73365443
rs4920093
rs78867731
rs113162001
rs144674286
rs72779785
rs542160
rs4527037
rs11054779
rs60923927
rs34287418
rs9378213
All
0.0
0.1
0.2
MR leave−one−out sensitivity analysis for
'HLA DR+ CD8br %lymphocyte' on 'Diabetic nephropathy'

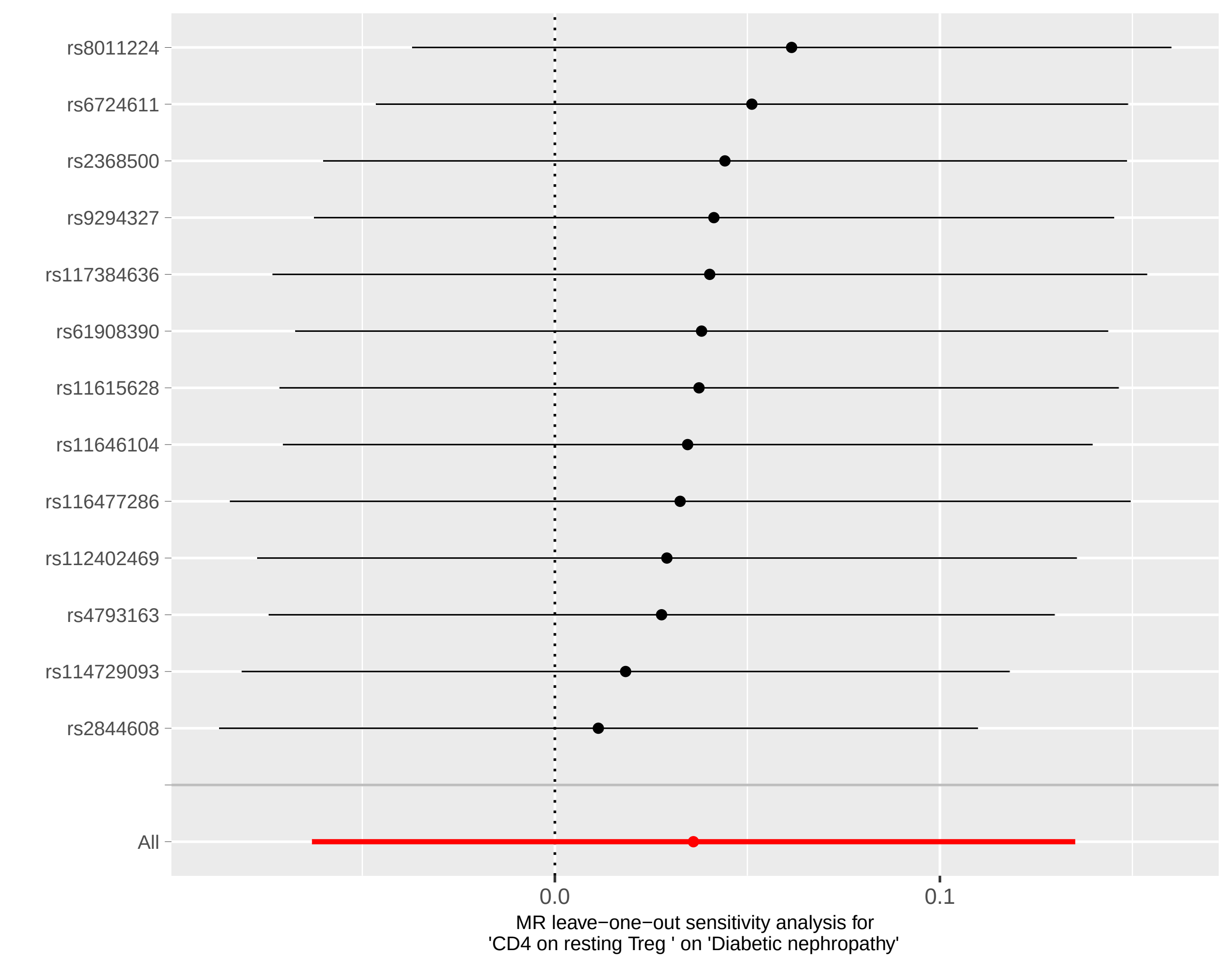
rs8011224
rs6724611
rs2368500
rs9294327
rs117384636
rs61908390
rs11615628
rs11646104
rs116477286
rs112402469
rs4793163
rs114729093
rs2844608
All
0.0
0.1
MR leave−one−out sensitivity analysis for
'CD4 on resting Treg ' on 'Diabetic nephropathy'

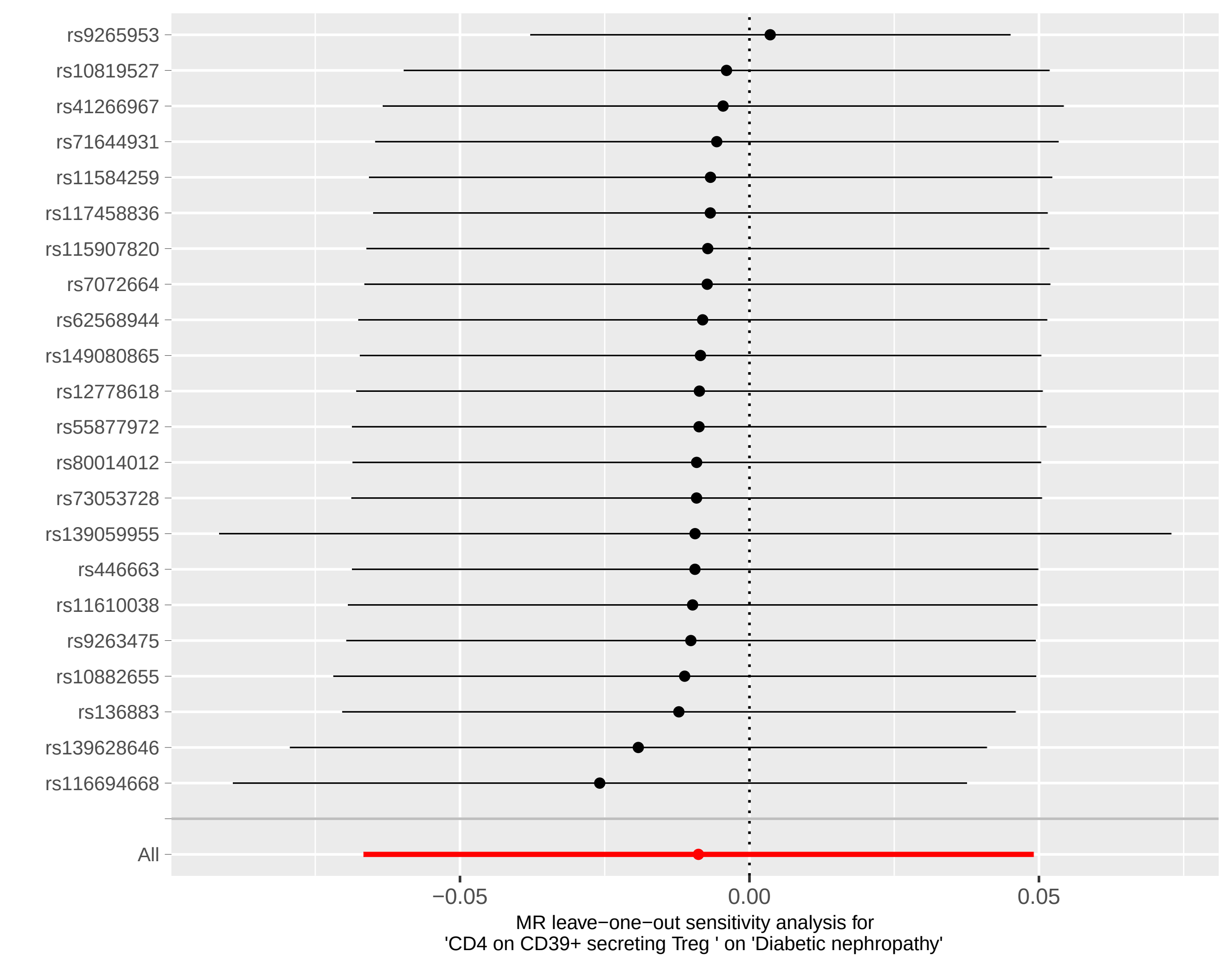

rs9265953
rs10819527
rs41266967
rs71644931
rs11584259
rs117458836
rs115907820
rs7072664
rs62568944
rs149080865
rs12778618
rs55877972
rs80014012
rs73053728
rs139059955
rs446663
rs11610038
rs9263475
rs10882655
rs136883
rs139628646
rs116694668
All
−0.05
0.00
0.05
MR leave−one−out sensitivity analysis for
'CD4 on CD39+ secreting Treg ' on 'Diabetic nephropathy'

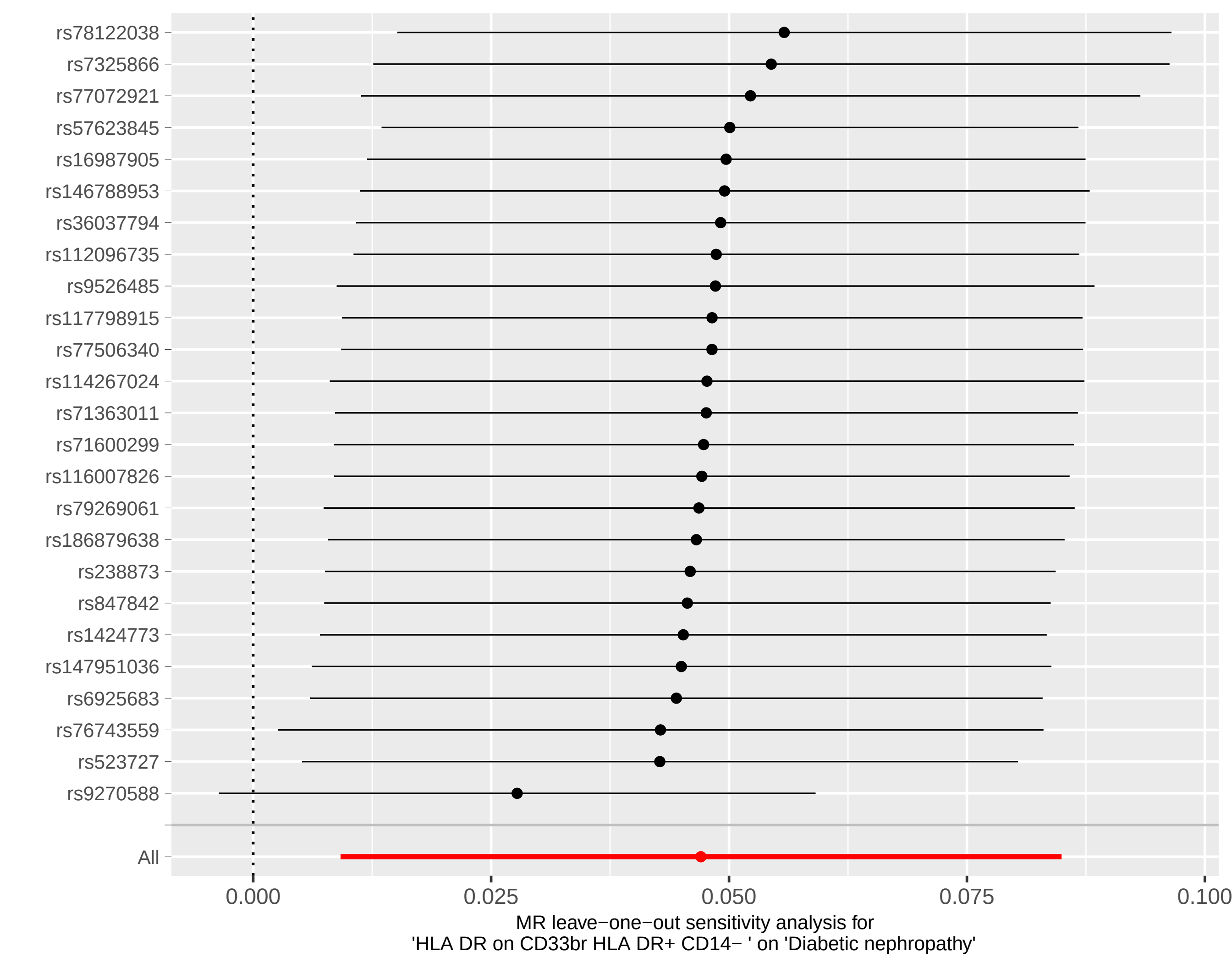
rs78122038
rs7325866
rs77072921
rs57623845
rs16987905
rs146788953
rs36037794
rs112096735
rs9526485
rs117798915
rs77506340
rs114267024
rs71363011
rs71600299
rs116007826
rs79269061
rs186879638
rs238873
rs847842
rs1424773
rs147951036
rs6925683
rs76743559
rs523727
rs9270588
All
0.000
0.025
0.050
0.075
0.100
MR leave−one−out sensitivity analysis for
'HLA DR on CD33br HLA DR+ CD14− ' on 'Diabetic nephropathy'

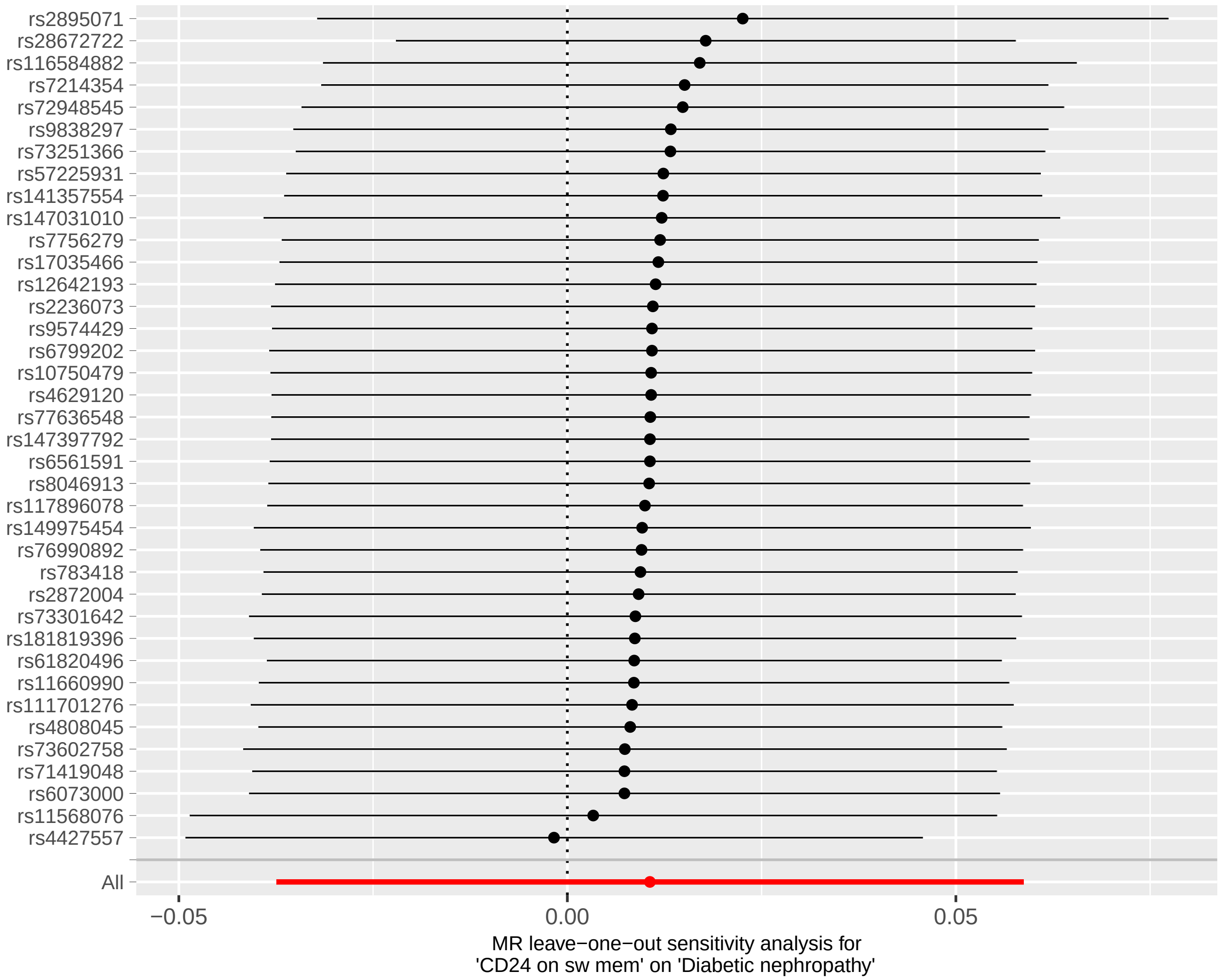
rs2895071
rs28672722
rs116584882
rs7214354
rs72948545
rs9838297
rs73251366
rs57225931
rs141357554
rs147031010
rs7756279
rs17035466
rs12642193
rs2236073
rs9574429
rs6799202
rs10750479
rs4629120
rs77636548
rs147397792
rs6561591
rs8046913
rs117896078
rs149975454
rs76990892
rs783418
rs2872004
rs73301642
rs181819396
rs61820496
rs11660990
rs111701276
rs4808045
rs73602758
rs71419048
rs6073000
rs11568076
rs4427557
All
−0.05
0.00
0.05
MR leave−one−out sensitivity analysis for
'CD24 on sw mem' on 'Diabetic nephropathy'

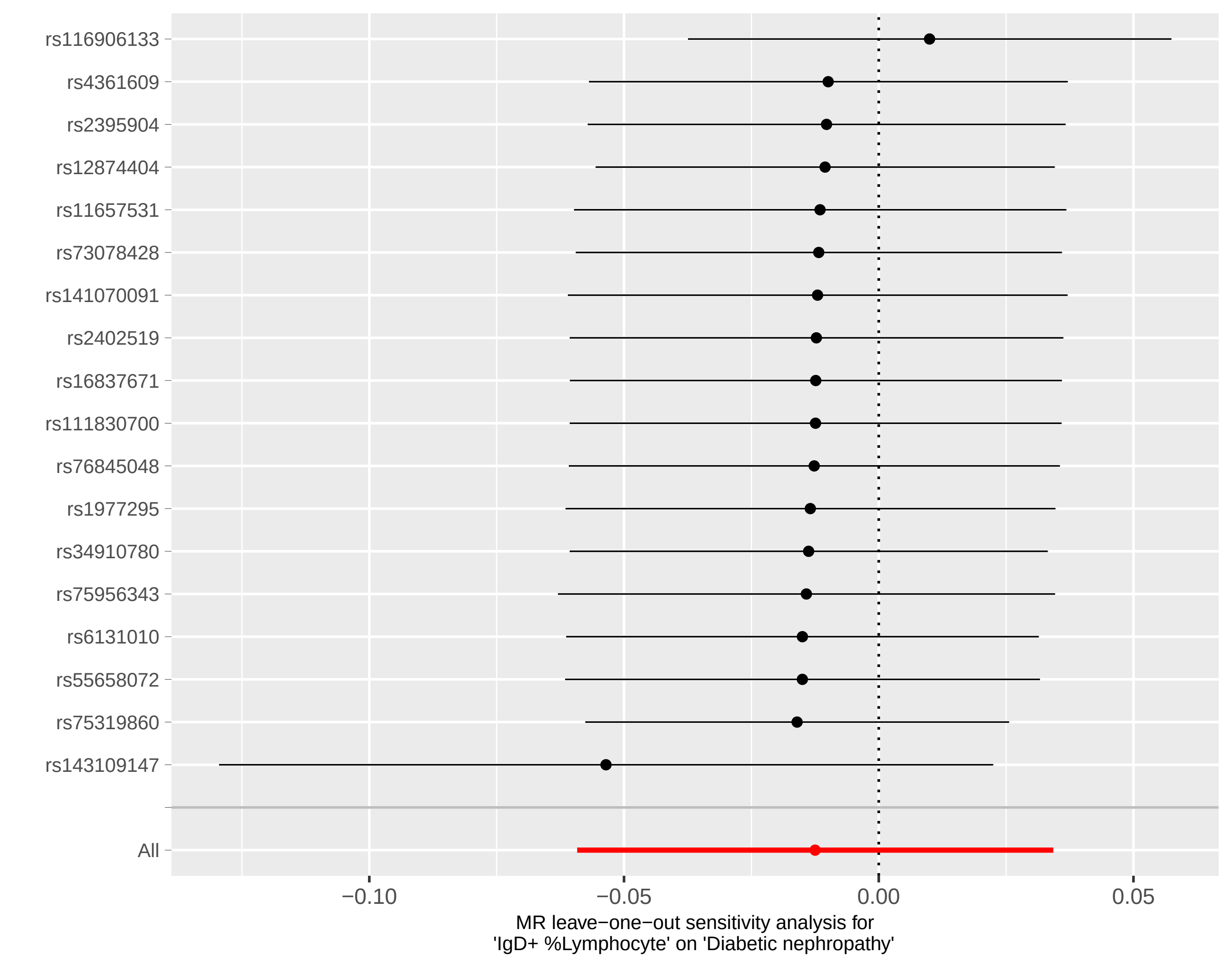
rs116906133
rs4361609
rs2395904
rs12874404
rs11657531
rs73078428
rs141070091
rs2402519
rs16837671
rs111830700
rs76845048
rs1977295
rs34910780
rs75956343
rs6131010
rs55658072
rs75319860
rs143109147
All
−0.10
−0.05
0.00
0.05
MR leave−one−out sensitivity analysis for
'IgD+ %Lymphocyte' on 'Diabetic nephropathy'

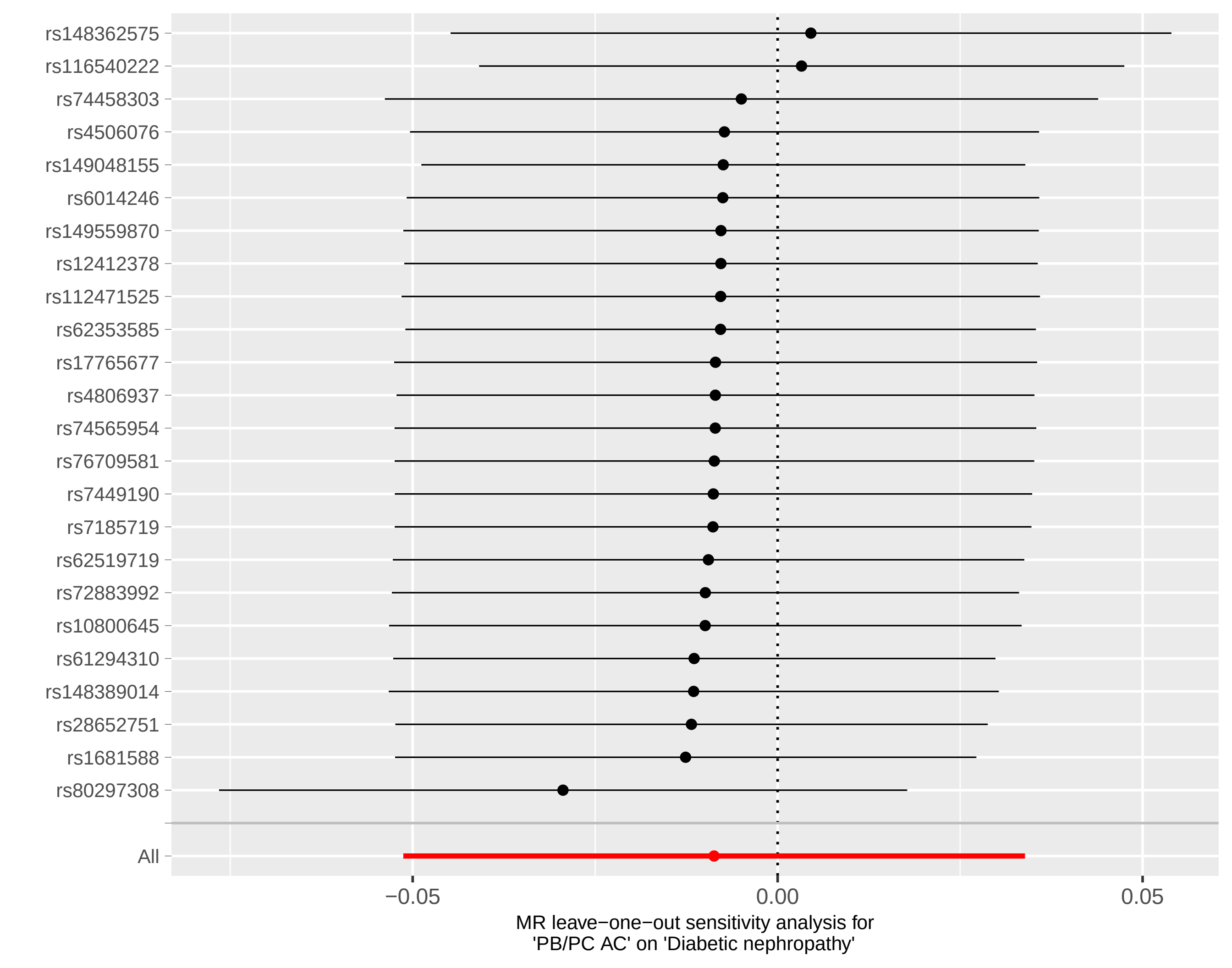

rs148362575
rs116540222
rs74458303
rs4506076
rs149048155
rs6014246
rs149559870
rs12412378
rs112471525
rs62353585
rs17765677
rs4806937
rs74565954
rs76709581
rs7449190
rs7185719
rs62519719
rs72883992
rs10800645
rs61294310
rs148389014
rs28652751
rs1681588
rs80297308
All
−0.05
0.00
0.05
MR leave−one−out sensitivity analysis for
'PB/PC AC' on 'Diabetic nephropathy'

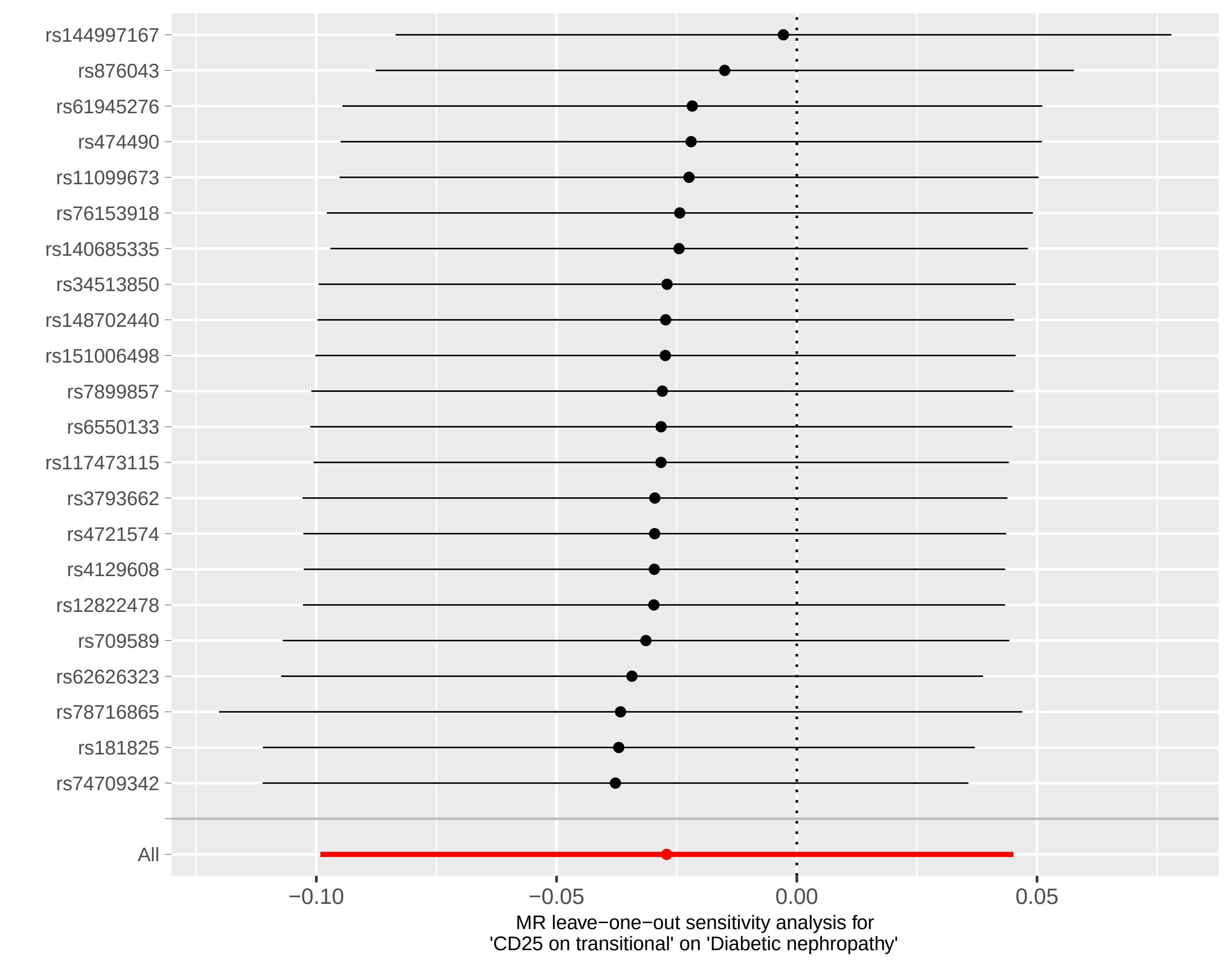

rs144997167
rs876043
rs61945276
rs474490
rs11099673
rs76153918
rs140685335
rs34513850
rs148702440
rs151006498
rs7899857
rs6550133
rs117473115
rs3793662
rs4721574
rs4129608
rs12822478
rs709589
rs62626323
rs78716865
rs181825
rs74709342
All
−0.10
−0.05
0.00
0.05
MR leave−one−out sensitivity analysis for
'CD25 on transitional' on 'Diabetic nephropathy'

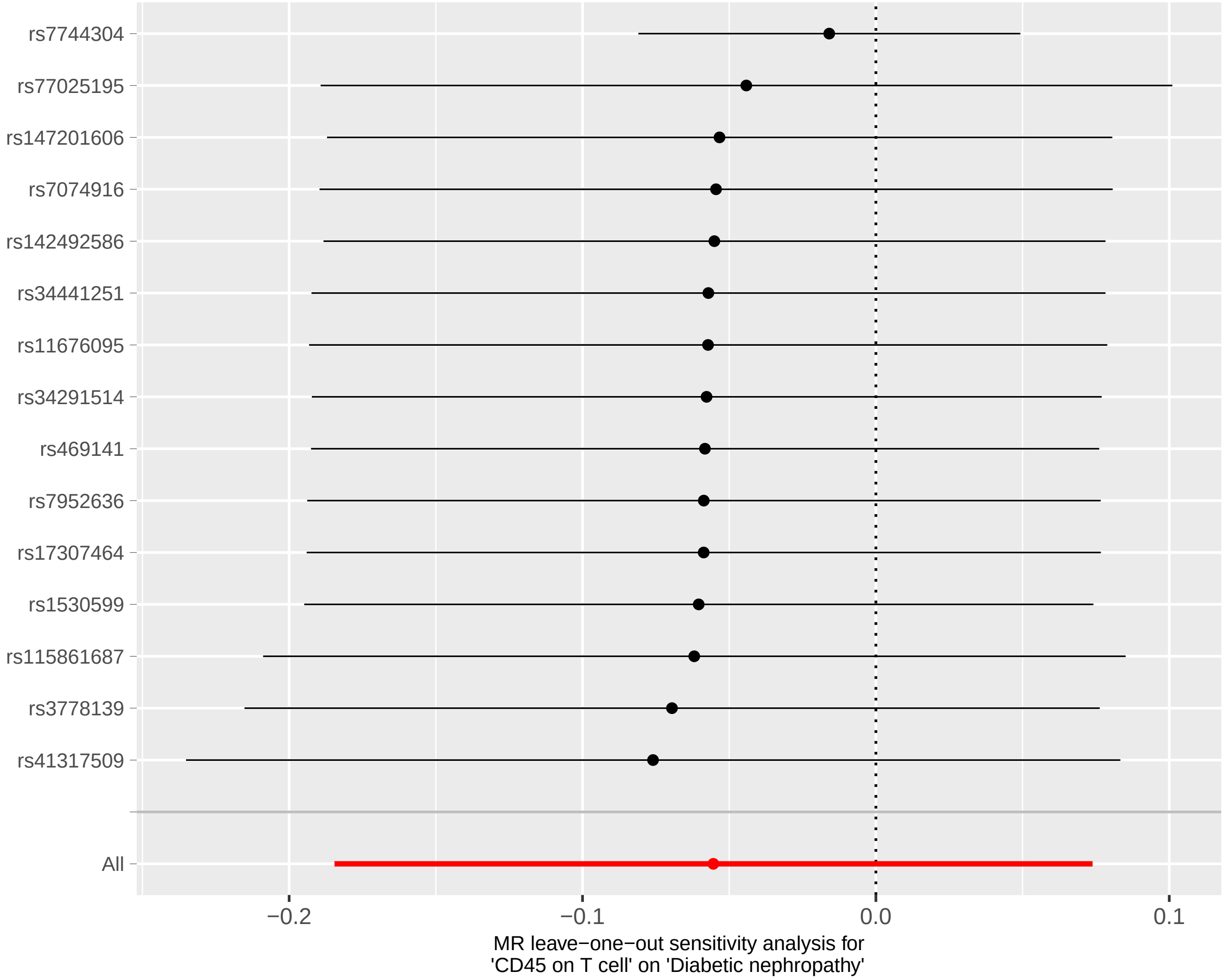

rs7744304
rs77025195
rs147201606
rs7074916
rs142492586
rs34441251
rs11676095
rs34291514
rs469141
rs7952636
rs17307464
rs1530599
rs115861687
rs3778139
rs41317509
All
−0.2
−0.1
0.0
0.1
MR leave−one−out sensitivity analysis for
'CD45 on T cell' on 'Diabetic nephropathy'

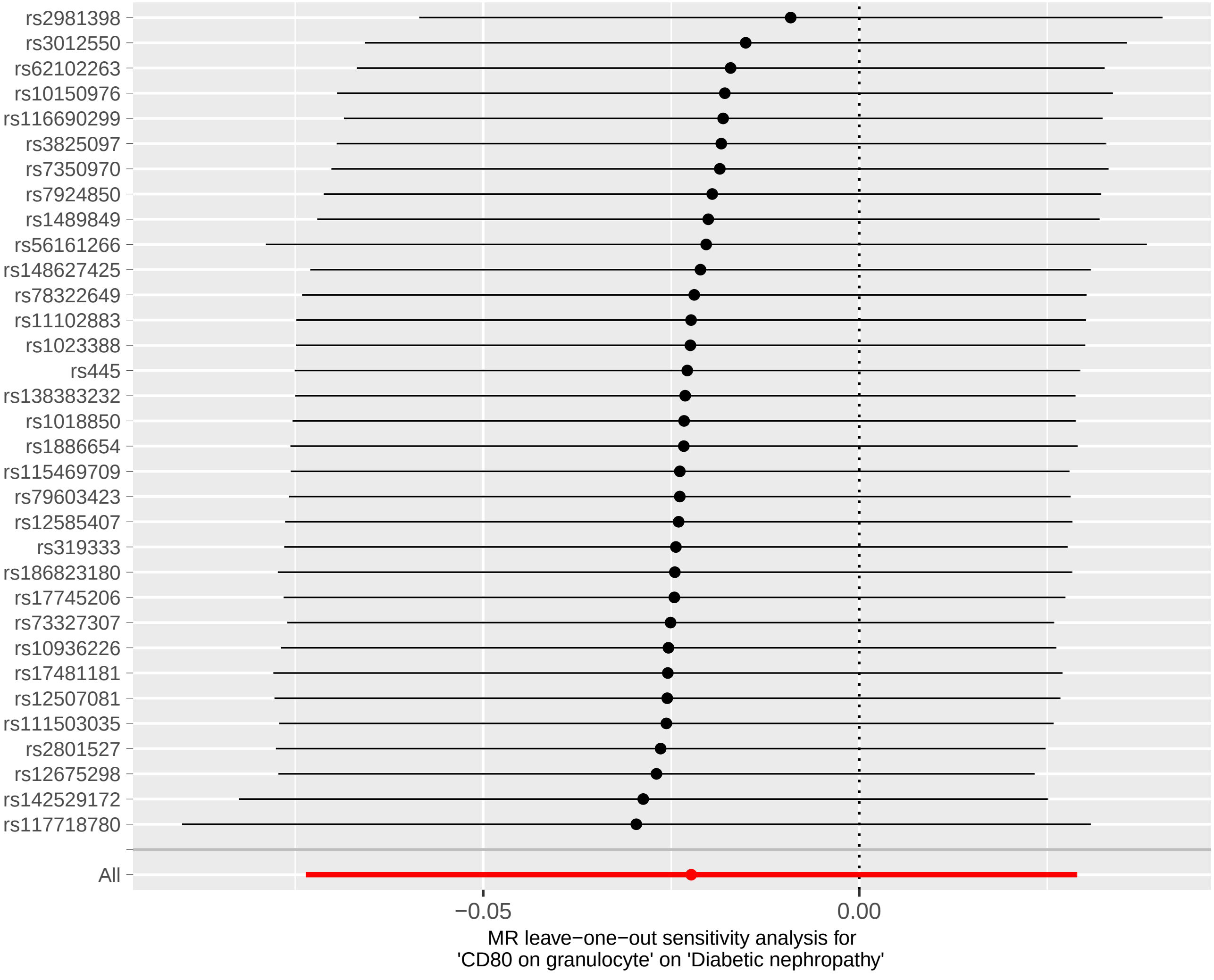
rs2981398
rs3012550
rs62102263
rs10150976
rs116690299
rs3825097
rs7350970
rs7924850
rs1489849
rs56161266
rs148627425
rs78322649
rs11102883
rs1023388
rs445
rs138383232
rs1018850
rs1886654
rs115469709
rs79603423
rs12585407
rs319333
rs186823180
rs17745206
rs73327307
rs10936226
rs17481181
rs12507081
rs111503035
rs2801527
rs12675298
rs142529172
rs117718780
All
−0.05
0.00
MR leave−one−out sensitivity analysis for
'CD80 on granulocyte' on 'Diabetic nephropathy'

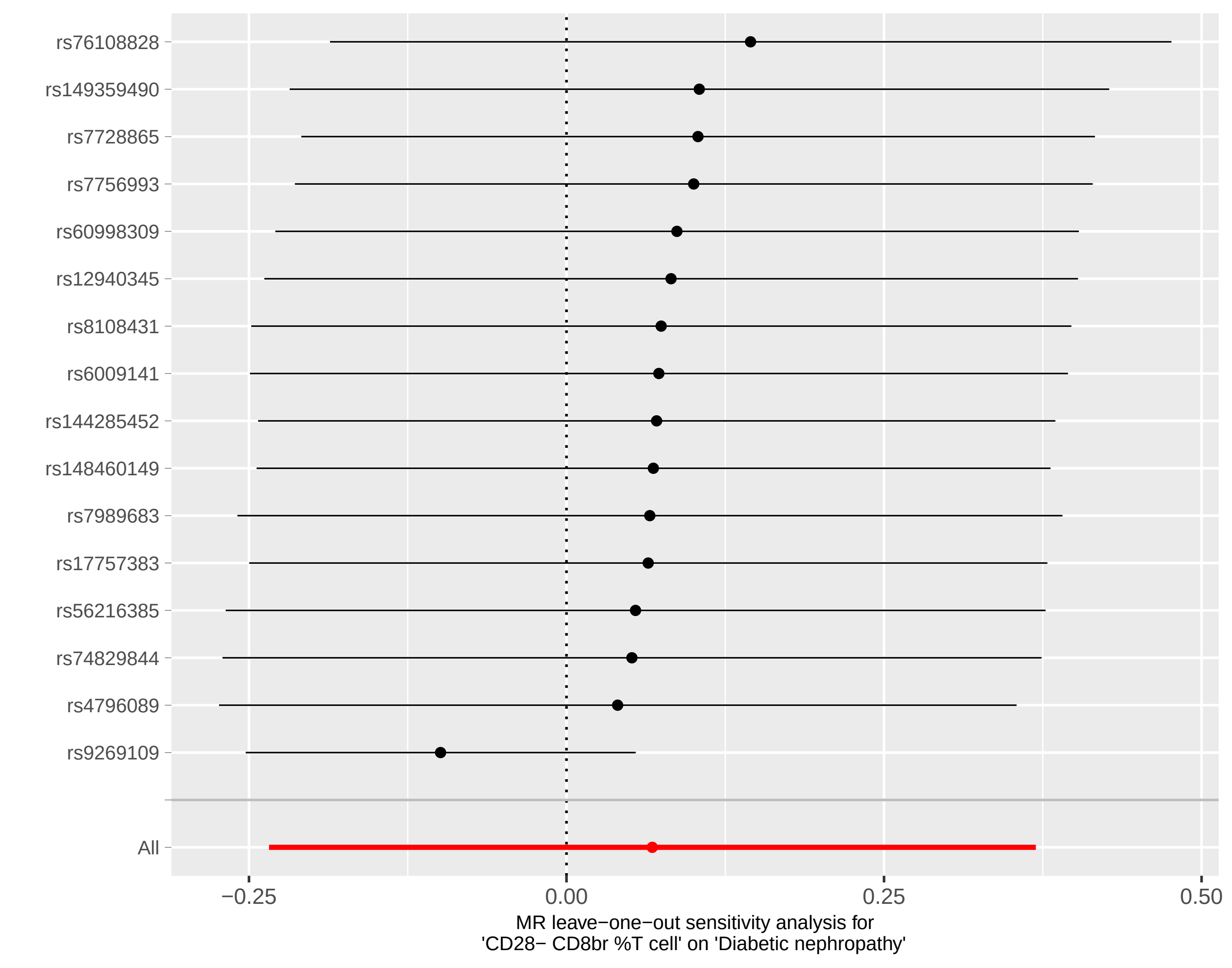
rs76108828
rs149359490
rs7728865
rs7756993
rs60998309
rs12940345
rs8108431
rs6009141
rs144285452
rs148460149
rs7989683
rs17757383
rs56216385
rs74829844
rs4796089
rs9269109
All
−0.25
0.00
0.25
0.50
MR leave−one−out sensitivity analysis for
'CD28− CD8br %T cell' on 'Diabetic nephropathy'

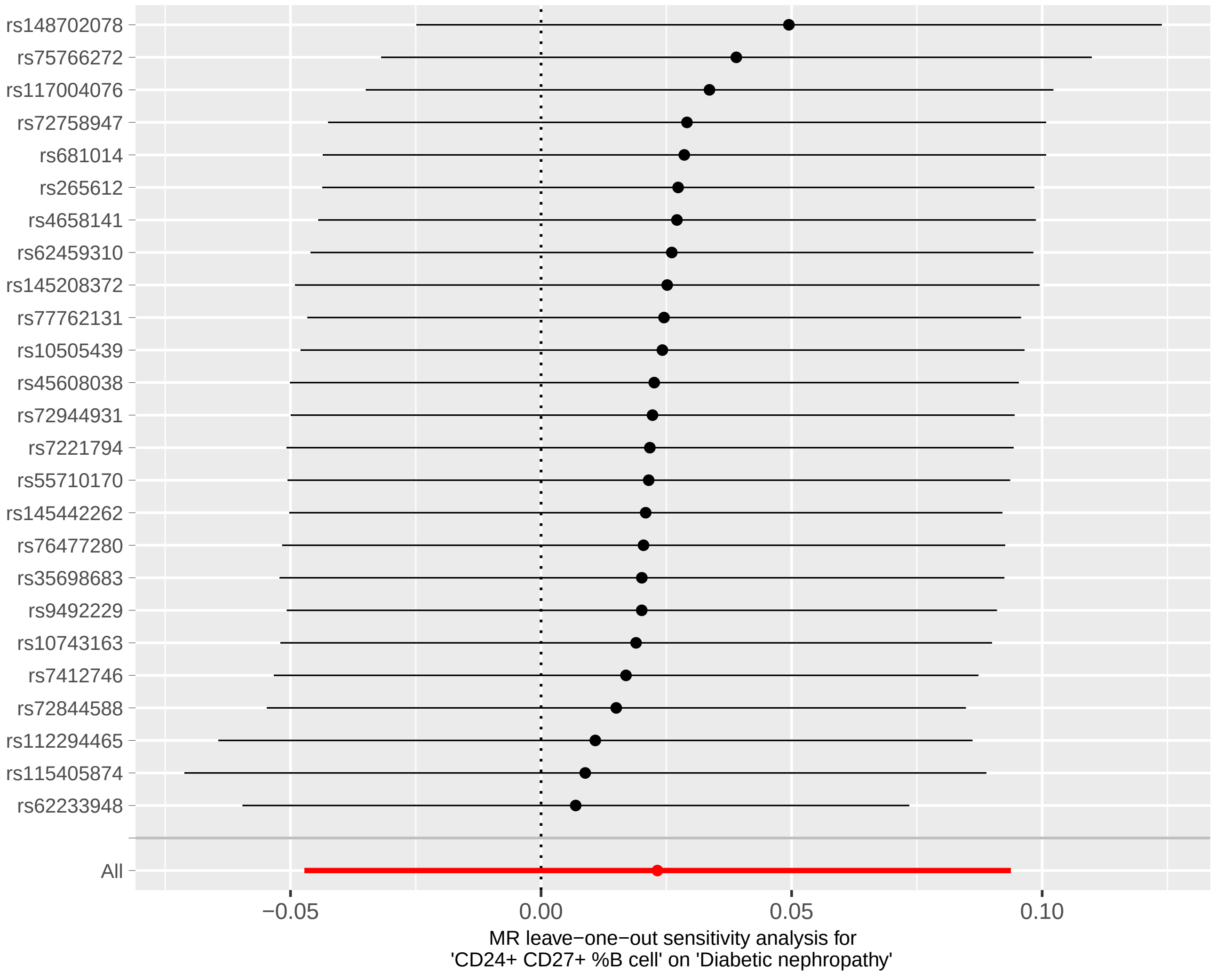
rs148702078
rs75766272
rs117004076
rs72758947
rs681014
rs265612
rs4658141
rs62459310
rs145208372
rs77762131
rs10505439
rs45608038
rs72944931
rs7221794
rs55710170
rs145442262
rs76477280
rs35698683
rs9492229
rs10743163
rs7412746
rs72844588
rs112294465
rs115405874
rs62233948
All
−0.05
0.00
0.05
0.10
MR leave−one−out sensitivity analysis for
'CD24+ CD27+ %B cell' on 'Diabetic nephropathy'

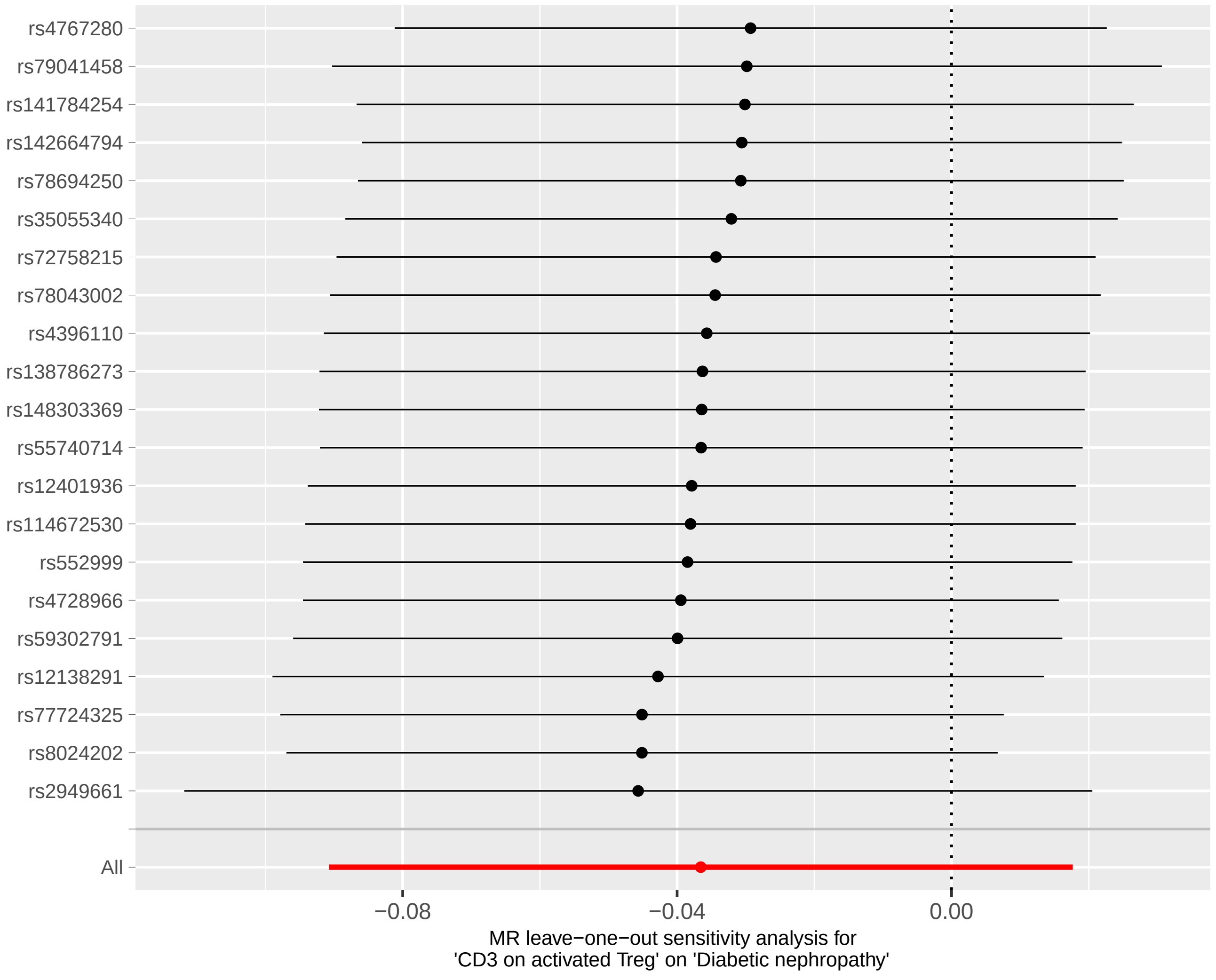
rs4767280
rs79041458
rs141784254
rs142664794
rs78694250
rs35055340
rs72758215
rs78043002
rs4396110
rs138786273
rs148303369
rs55740714
rs12401936
rs114672530
rs552999
rs4728966
rs59302791
rs12138291
rs77724325
rs8024202
rs2949661
All
−0.08
−0.04
0.00
MR leave−one−out sensitivity analysis for
'CD3 on activated Treg' on 'Diabetic nephropathy'

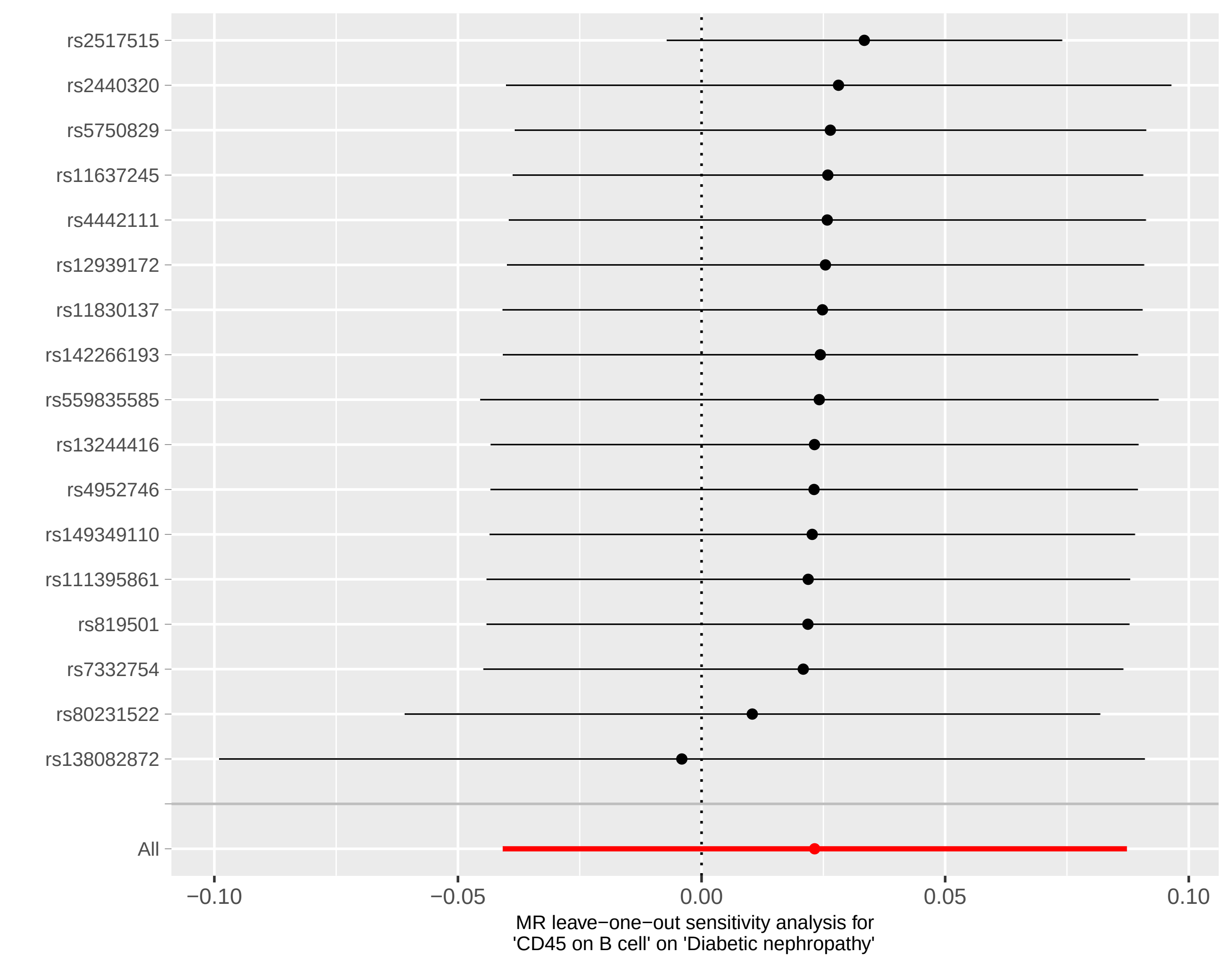

rs2517515
rs2440320
rs5750829
rs11637245
rs4442111
rs12939172
rs11830137
rs142266193
rs559835585
rs13244416
rs4952746
rs149349110
rs111395861
rs819501
rs7332754
rs80231522
rs138082872
All
−0.10
−0.05
0.00
0.05
0.10
MR leave−one−out sensitivity analysis for
'CD45 on B cell' on 'Diabetic nephropathy'

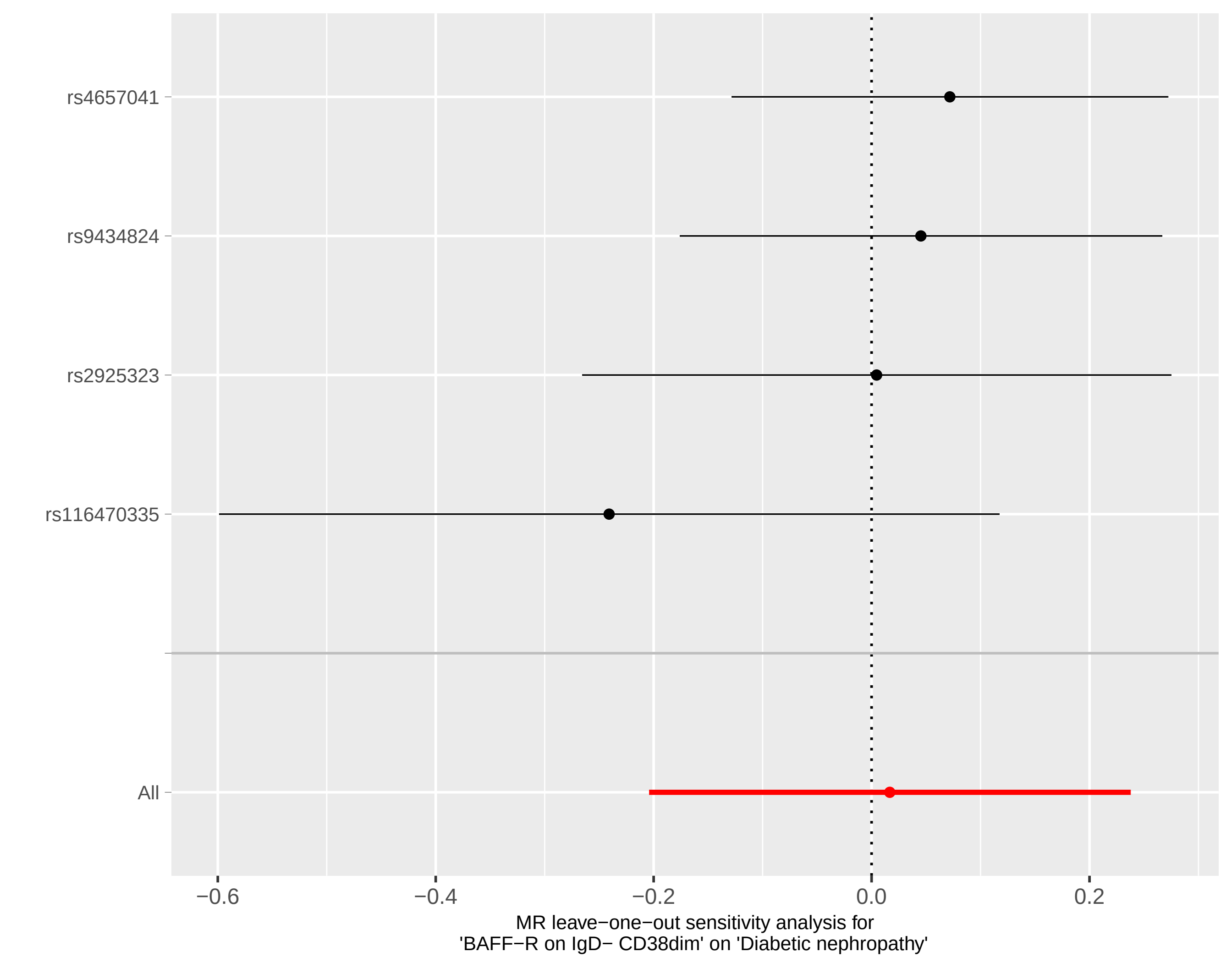
rs4657041
rs9434824
rs2925323
rs116470335
All
−0.6
−0.4
−0.2
0.0
0.2
MR leave−one−out sensitivity analysis for
'BAFF−R on IgD− CD38dim' on 'Diabetic nephropathy'

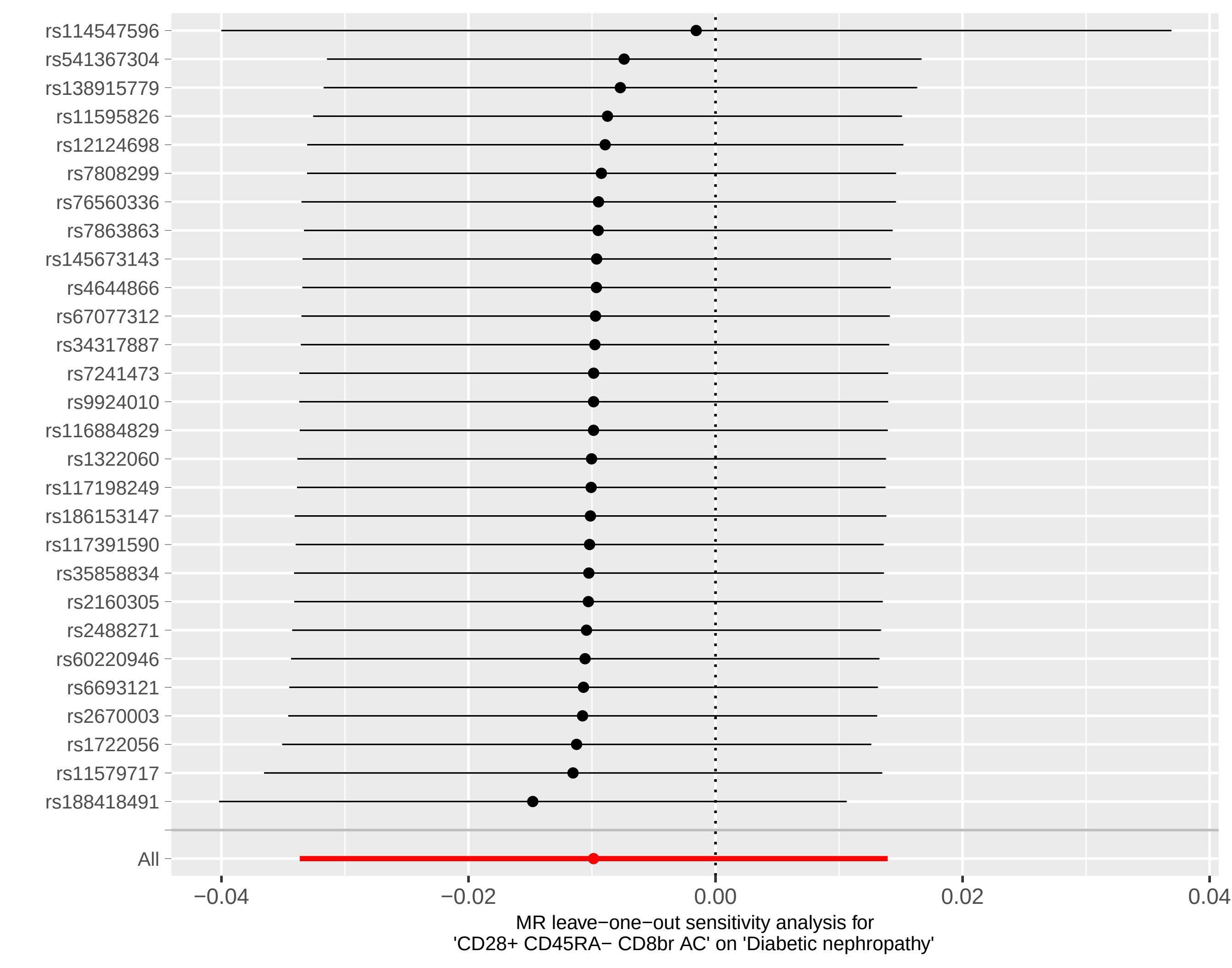

rs114547596
rs541367304
rs138915779
rs11595826
rs12124698
rs7808299
rs76560336
rs7863863
rs145673143
rs4644866
rs67077312
rs34317887
rs7241473
rs9924010
rs116884829
rs1322060
rs117198249
rs186153147
rs117391590
rs35858834
rs2160305
rs2488271
rs60220946
rs6693121
rs2670003
rs1722056
rs11579717
rs188418491
All
−0.04
−0.02
0.00
0.02
0.04
MR leave−one−out sensitivity analysis for
'CD28+ CD45RA− CD8br AC' on 'Diabetic nephropathy'

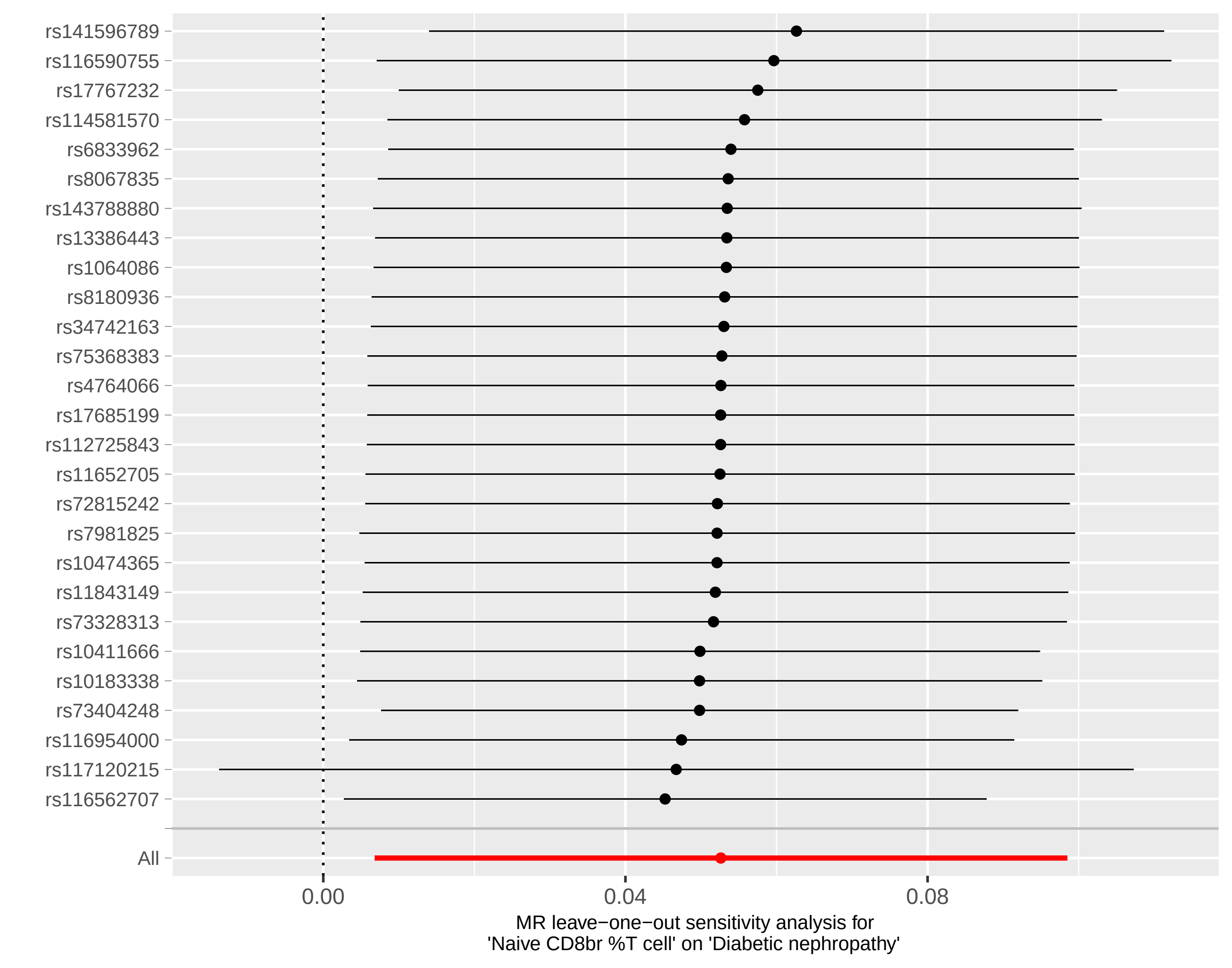
rs141596789
rs116590755
rs17767232
rs114581570
rs6833962
rs8067835
rs143788880
rs13386443
rs1064086
rs8180936
rs34742163
rs75368383
rs4764066
rs17685199
rs112725843
rs11652705
rs72815242
rs7981825
rs10474365
rs11843149
rs73328313
rs10411666
rs10183338
rs73404248
rs116954000
rs117120215
rs116562707
All
0.00
0.04
0.08
MR leave−one−out sensitivity analysis for
'Naive CD8br %T cell' on 'Diabetic nephropathy'

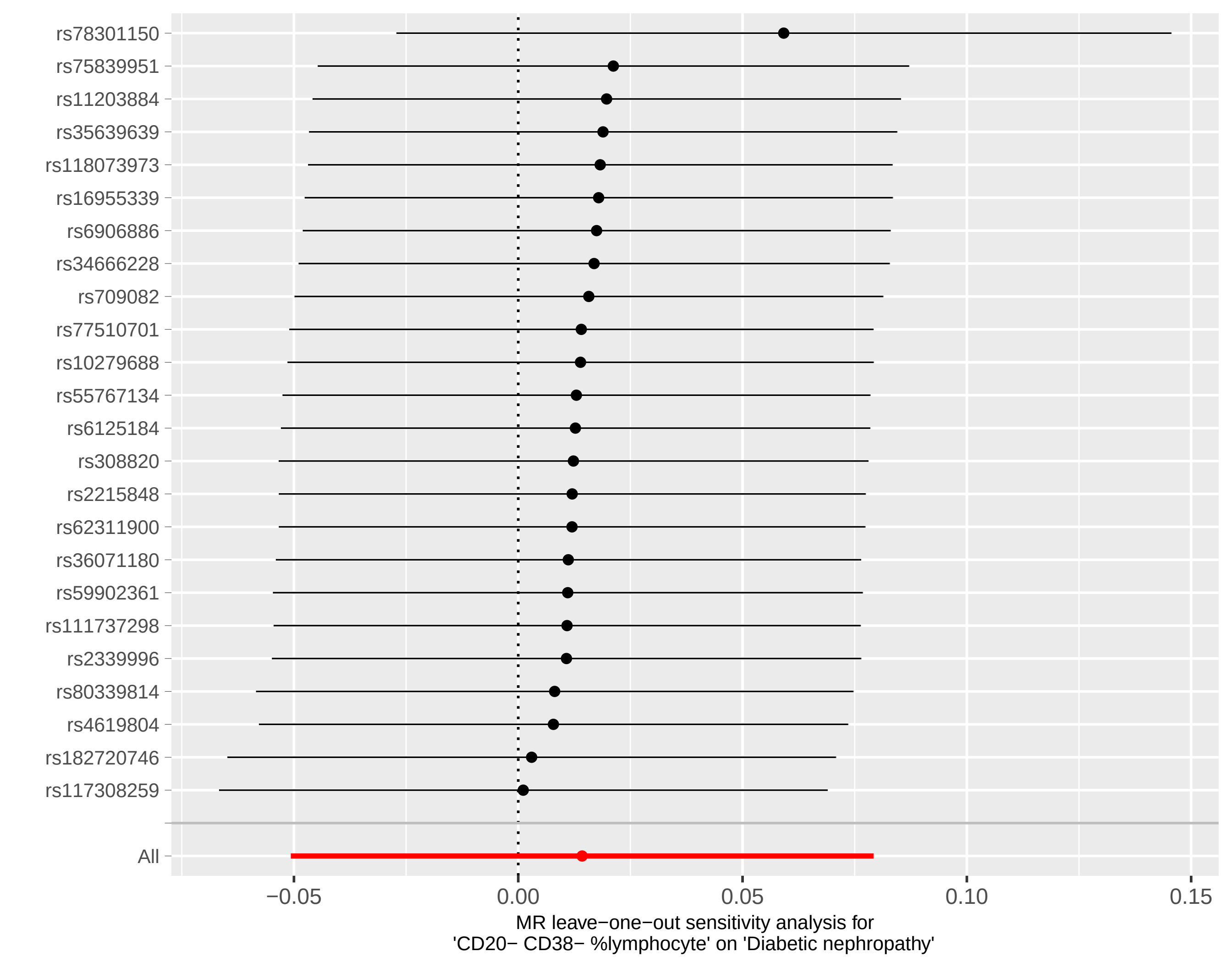

rs78301150
rs75839951
rs11203884
rs35639639
rs118073973
rs16955339
rs6906886
rs34666228
rs709082
rs77510701
rs10279688
rs55767134
rs6125184
rs308820
rs2215848
rs62311900
rs36071180
rs59902361
rs111737298
rs2339996
rs80339814
rs4619804
rs182720746
rs117308259
All
−0.05
0.00
0.05
0.10
0.15
MR leave−one−out sensitivity analysis for
'CD20− CD38− %lymphocyte' on 'Diabetic nephropathy'

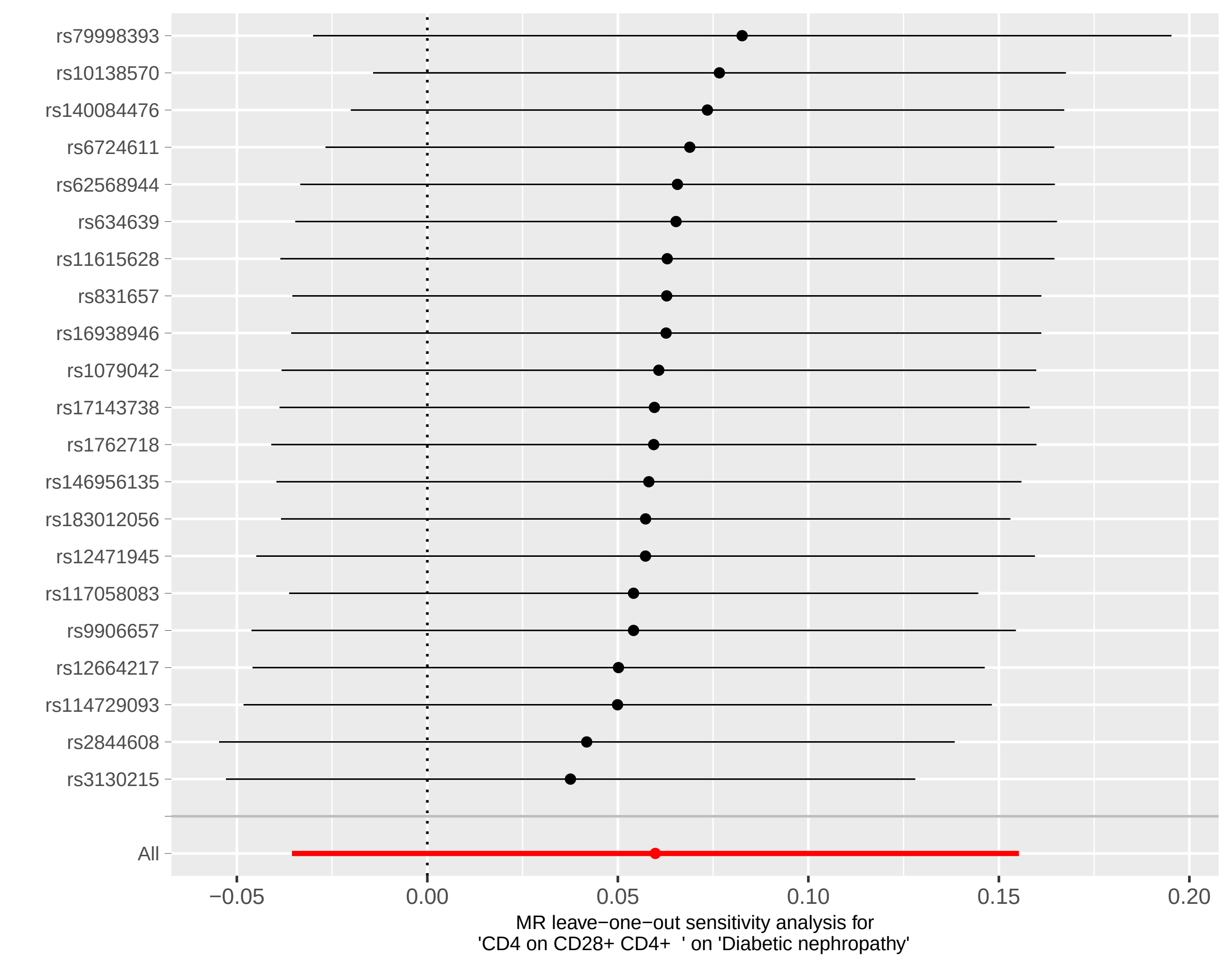
rs79998393
rs10138570
rs140084476
rs6724611
rs62568944
rs634639
rs11615628
rs831657
rs16938946
rs1079042
rs17143738
rs1762718
rs146956135
rs183012056
rs12471945
rs117058083
rs9906657
rs12664217
rs114729093
rs2844608
rs3130215
All
−0.05
0.00
0.05
0.10
0.15
0.20
MR leave−one−out sensitivity analysis for
'CD4 on CD28+ CD4+ ' on 'Diabetic nephropathy'

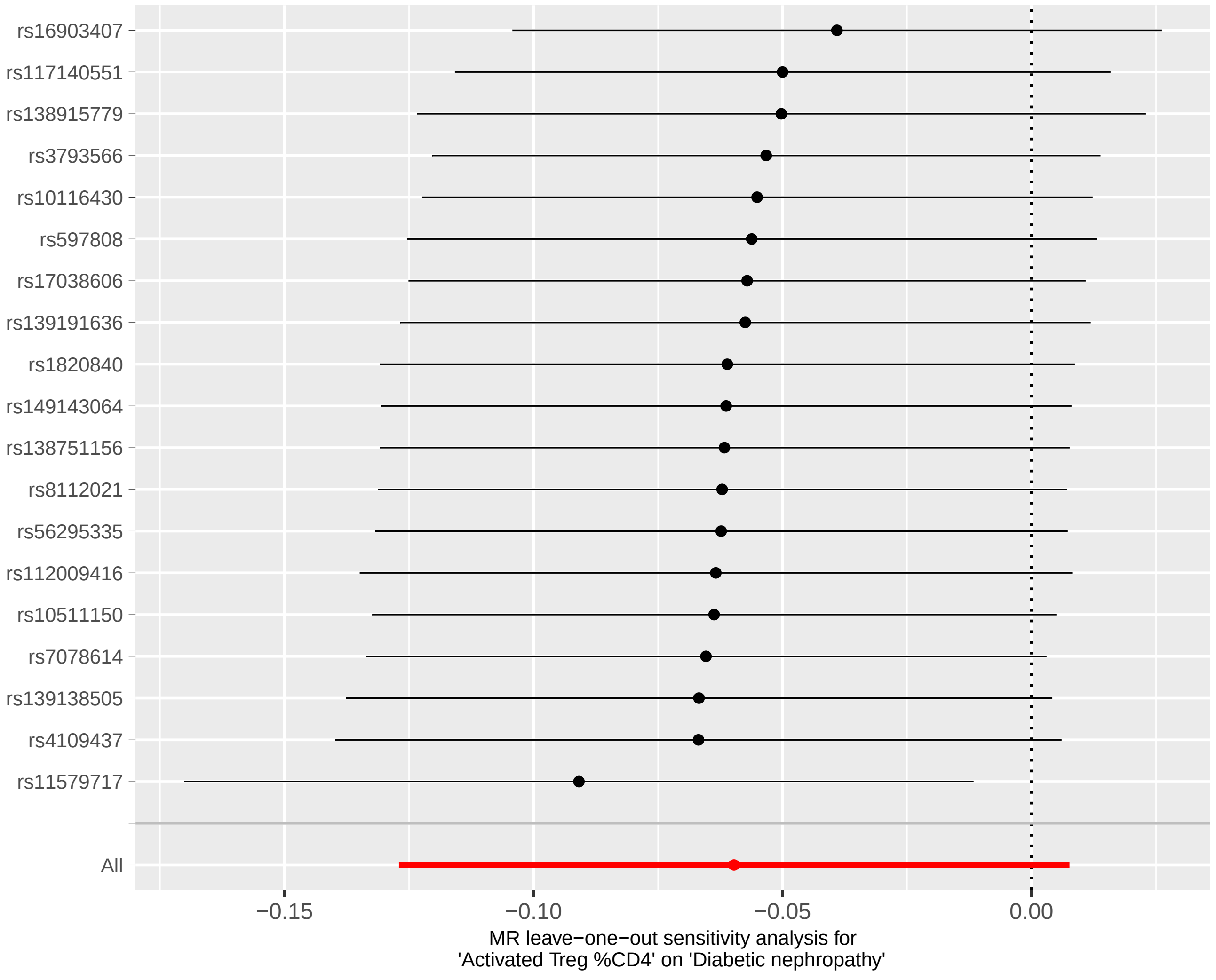
rs16903407
rs117140551
rs138915779
rs3793566
rs10116430
rs597808
rs17038606
rs139191636
rs1820840
rs149143064
rs138751156
rs8112021
rs56295335
rs112009416
rs10511150
rs7078614
rs139138505
rs4109437
rs11579717
All
−0.15
−0.10
−0.05
0.00
MR leave−one−out sensitivity analysis for
'Activated Treg %CD4' on 'Diabetic nephropathy'

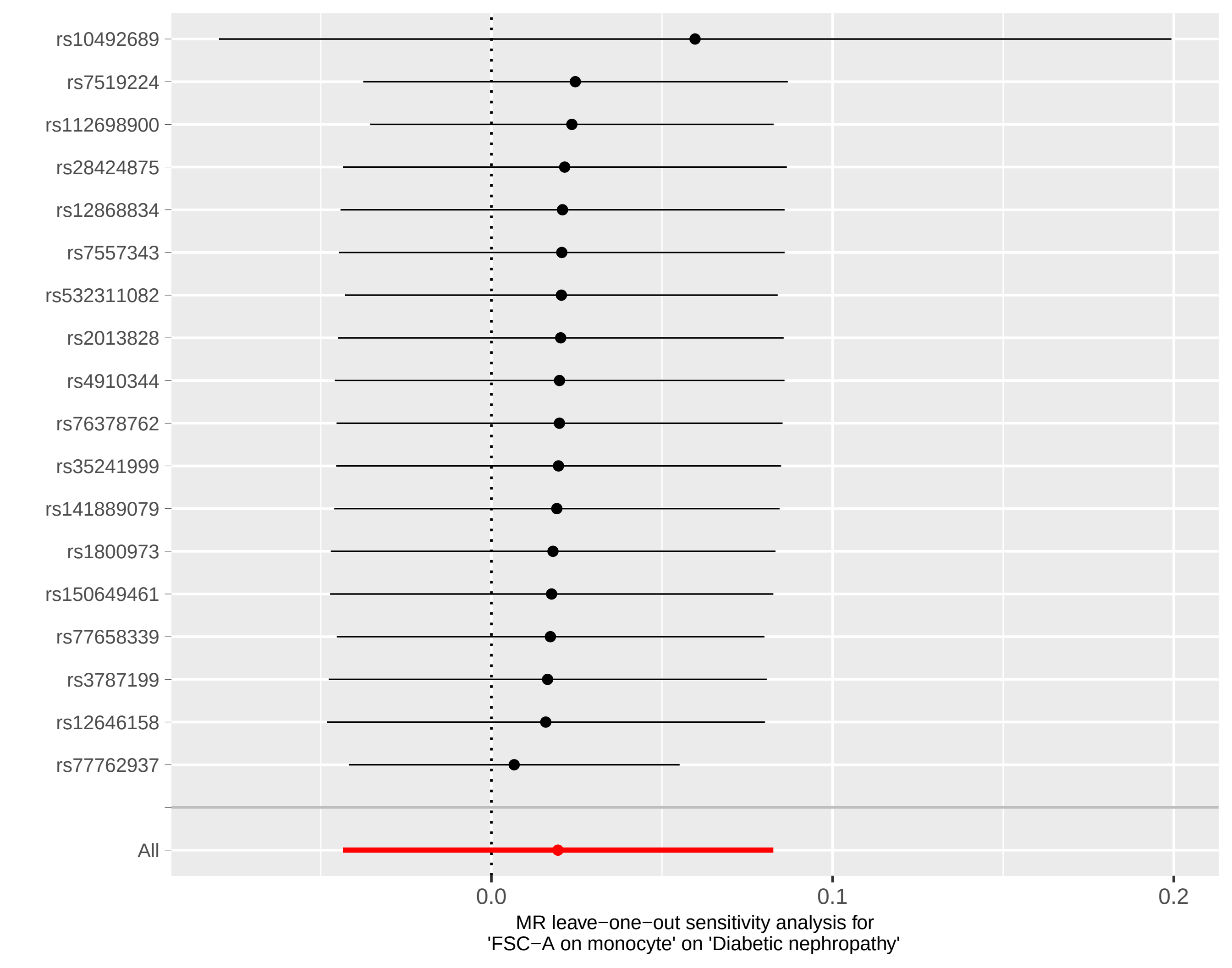

rs10492689
rs7519224
rs112698900
rs28424875
rs12868834
rs7557343
rs532311082
rs2013828
rs4910344
rs76378762
rs35241999
rs141889079
rs1800973
rs150649461
rs77658339
rs3787199
rs12646158
rs77762937
All
0.0
0.1
0.2
MR leave−one−out sensitivity analysis for
'FSC−A on monocyte' on 'Diabetic nephropathy'

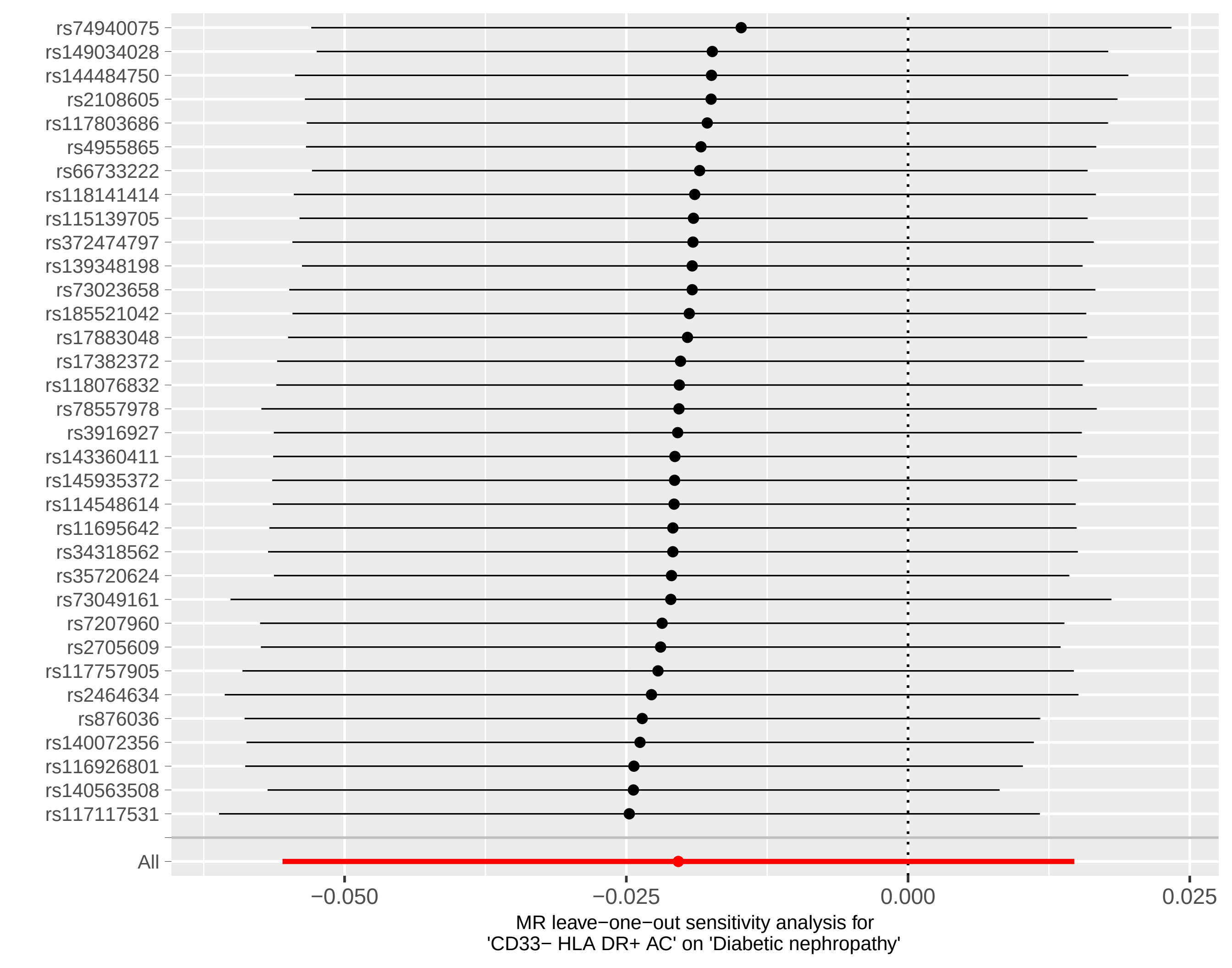
rs74940075
rs149034028
rs144484750
rs2108605
rs117803686
rs4955865
rs66733222
rs118141414
rs115139705
rs372474797
rs139348198
rs73023658
rs185521042
rs17883048
rs17382372
rs118076832
rs78557978
rs3916927
rs143360411
rs145935372
rs114548614
rs11695642
rs34318562
rs35720624
rs73049161
rs7207960
rs2705609
rs117757905
rs2464634
rs876036
rs140072356
rs116926801
rs140563508
rs117117531
All
−0.050
−0.025
0.000
0.025
MR leave−one−out sensitivity analysis for
'CD33− HLA DR+ AC' on 'Diabetic nephropathy'

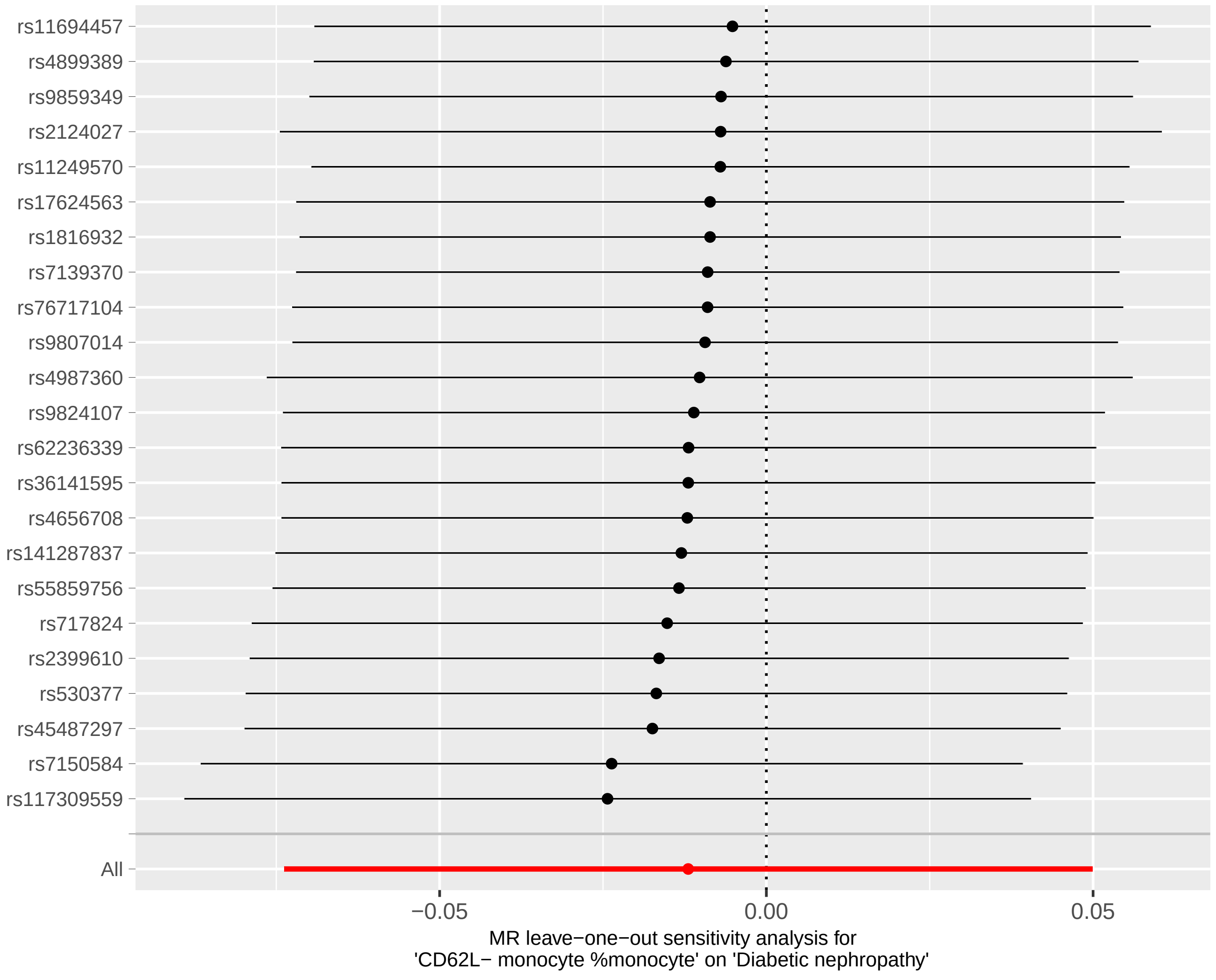
rs11694457
rs4899389
rs9859349
rs2124027
rs11249570
rs17624563
rs1816932
rs7139370
rs76717104
rs9807014
rs4987360
rs9824107
rs62236339
rs36141595
rs4656708
rs141287837
rs55859756
rs717824
rs2399610
rs530377
rs45487297
rs7150584
rs117309559
All
−0.05
0.00
0.05
MR leave−one−out sensitivity analysis for
'CD62L− monocyte %monocyte' on 'Diabetic nephropathy'

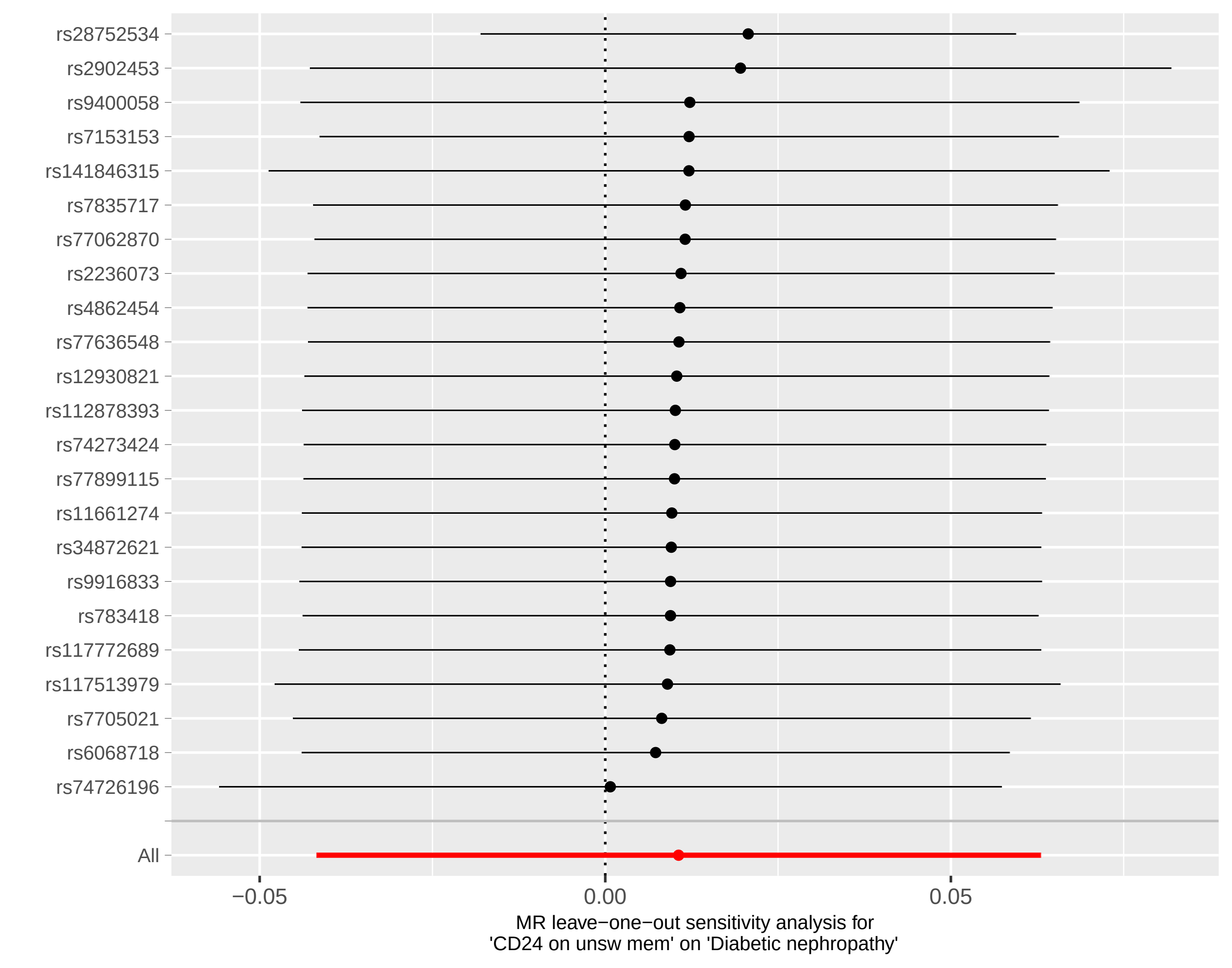

rs28752534
rs2902453
rs9400058
rs7153153
rs141846315
rs7835717
rs77062870
rs2236073
rs4862454
rs77636548
rs12930821
rs112878393
rs74273424
rs77899115
rs11661274
rs34872621
rs9916833
rs783418
rs117772689
rs117513979
rs7705021
rs6068718
rs74726196
All
−0.05
0.00
0.05
MR leave−one−out sensitivity analysis for
'CD24 on unsw mem' on 'Diabetic nephropathy'

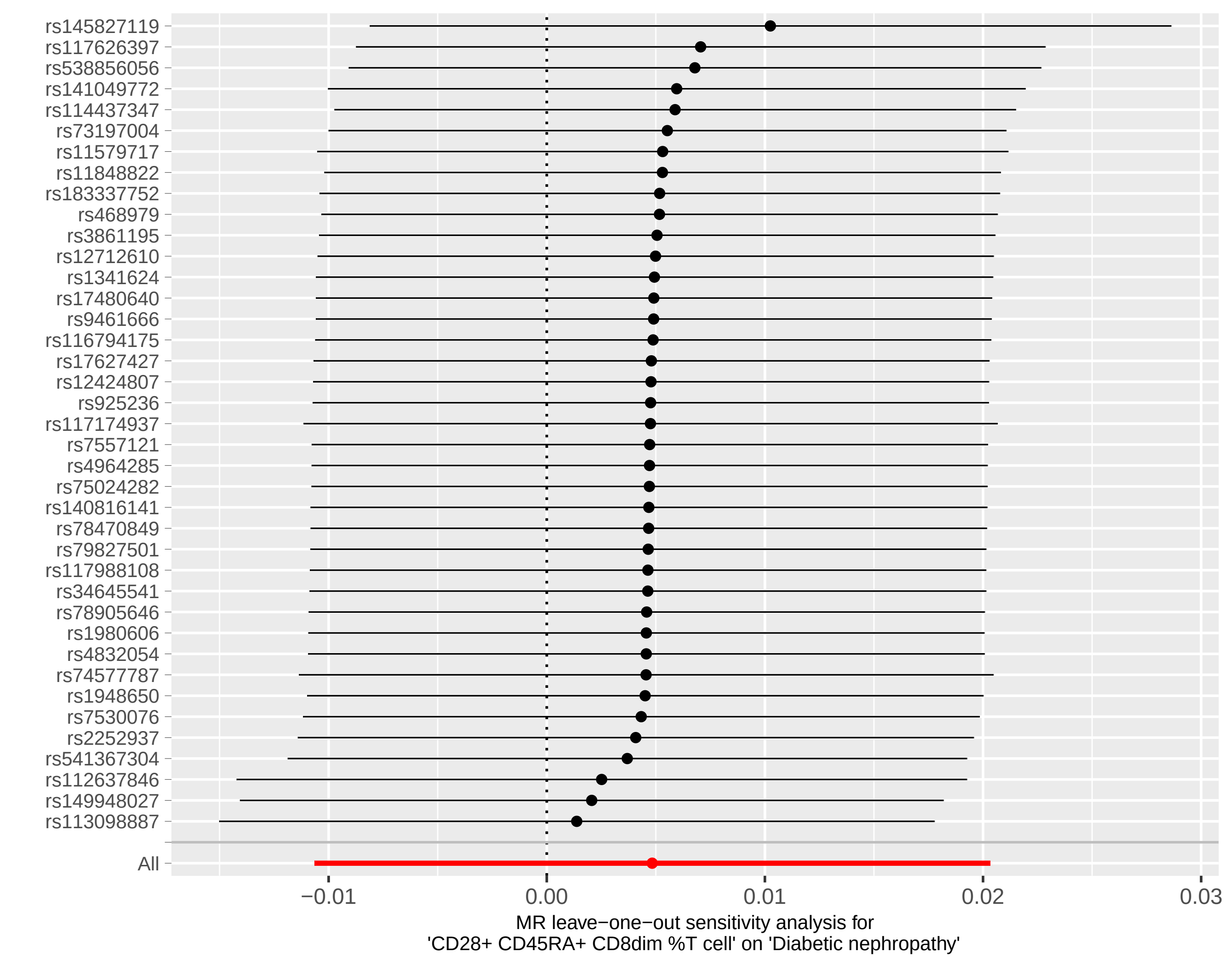

rs145827119
rs117626397
rs538856056
rs141049772
rs114437347
rs73197004
rs11579717
rs11848822
rs183337752
rs468979
rs3861195
rs12712610
rs1341624
rs17480640
rs9461666
rs116794175
rs17627427
rs12424807
rs925236
rs117174937
rs7557121
rs4964285
rs75024282
rs140816141
rs78470849
rs79827501
rs117988108
rs34645541
rs78905646
rs1980606
rs4832054
rs74577787
rs1948650
rs7530076
rs2252937
rs541367304
rs112637846
rs149948027
rs113098887
All
−0.01
0.00
0.01
0.02
0.03
MR leave−one−out sensitivity analysis for
'CD28+ CD45RA+ CD8dim %T cell' on 'Diabetic nephropathy'

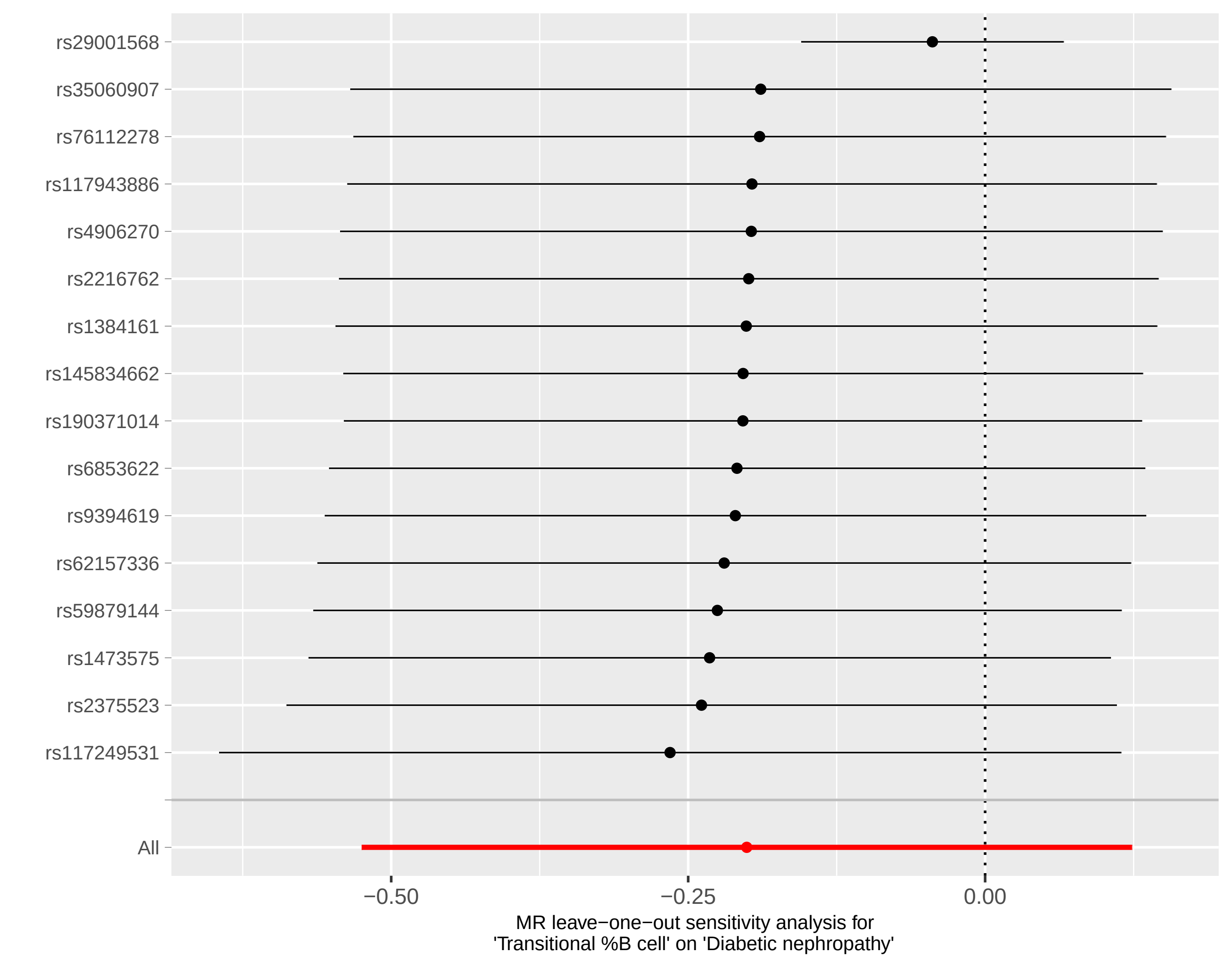
rs29001568
rs35060907
rs76112278
rs117943886
rs4906270
rs2216762
rs1384161
rs145834662
rs190371014
rs6853622
rs9394619
rs62157336
rs59879144
rs1473575
rs2375523
rs117249531
All
−0.50
−0.25
0.00
MR leave−one−out sensitivity analysis for
'Transitional %B cell' on 'Diabetic nephropathy'

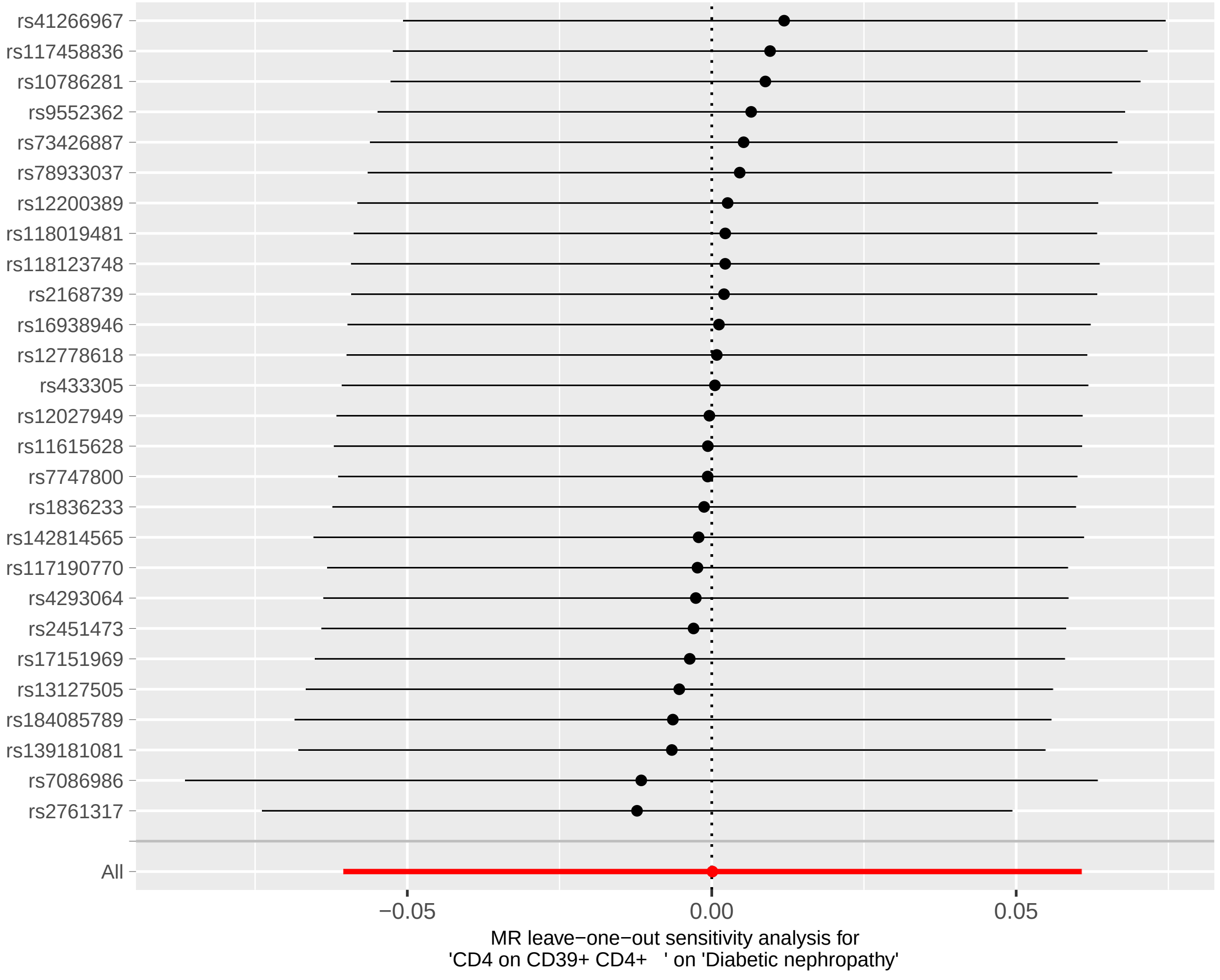
rs41266967
rs117458836
rs10786281
rs9552362
rs73426887
rs78933037
rs12200389
rs118019481
rs118123748
rs2168739
rs16938946
rs12778618
rs433305
rs12027949
rs11615628
rs7747800
rs1836233
rs142814565
rs117190770
rs4293064
rs2451473
rs17151969
rs13127505
rs184085789
rs139181081
rs7086986
rs2761317
All
−0.05
0.00
0.05
MR leave−one−out sensitivity analysis for
'CD4 on CD39+ CD4+ ' on 'Diabetic nephropathy'

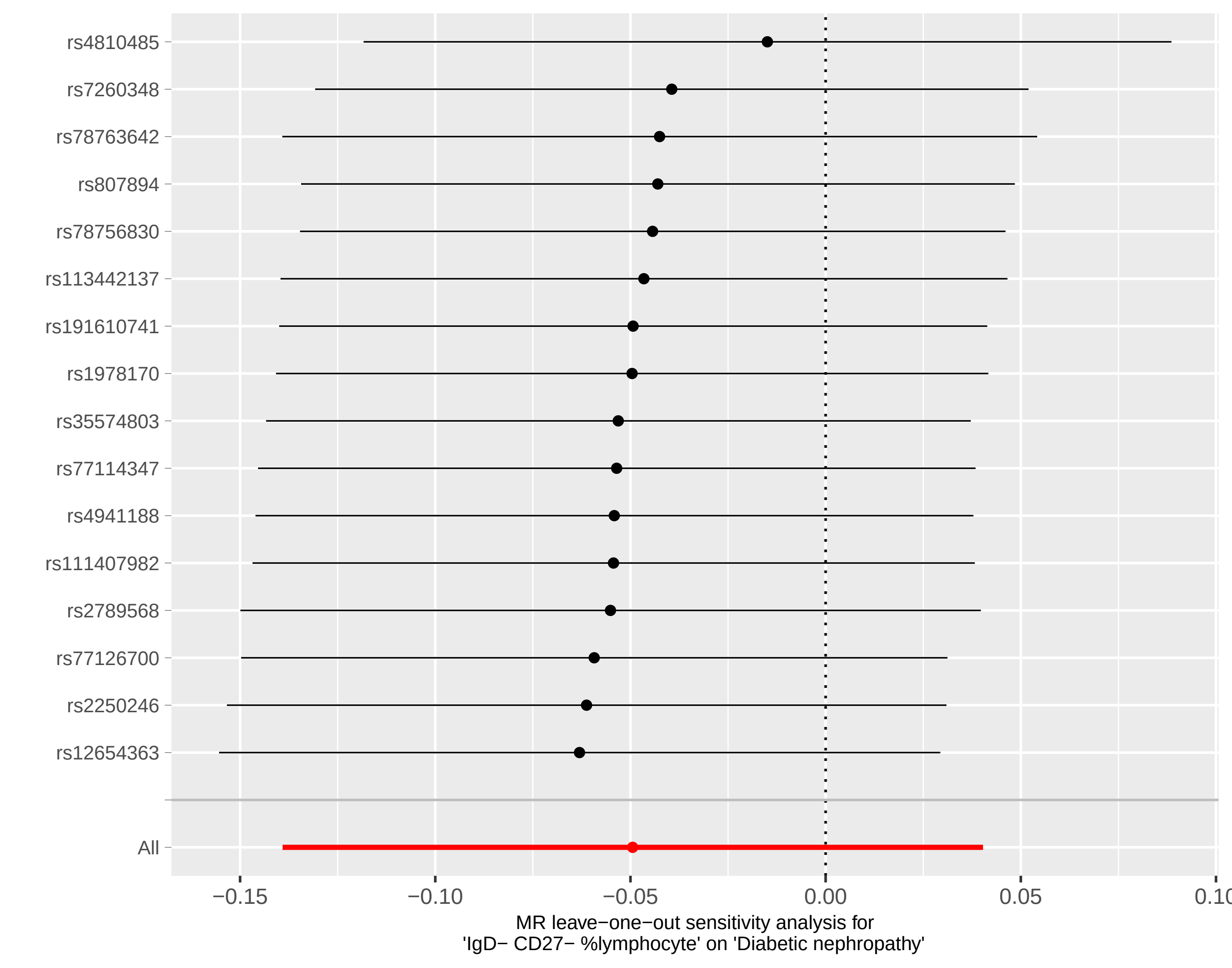

rs4810485
rs7260348
rs78763642
rs807894
rs78756830
rs113442137
rs191610741
rs1978170
rs35574803
rs77114347
rs4941188
rs111407982
rs2789568
rs77126700
rs2250246
rs12654363
All
−0.15
−0.10
−0.05
0.00
0.05
0.10
MR leave−one−out sensitivity analysis for
'IgD− CD27− %lymphocyte' on 'Diabetic nephropathy'

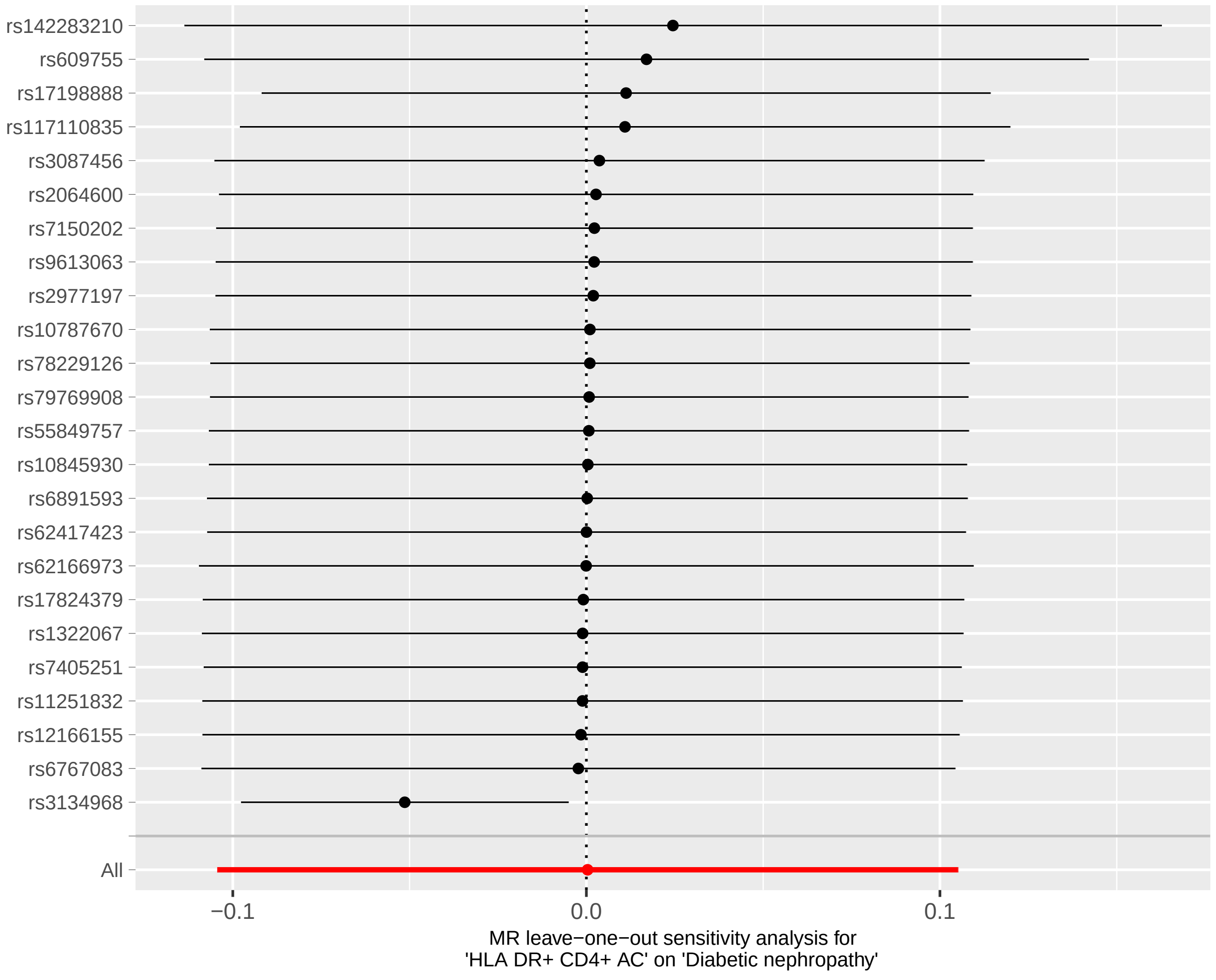

rs142283210
rs609755
rs17198888
rs117110835
rs3087456
rs2064600
rs7150202
rs9613063
rs2977197
rs10787670
rs78229126
rs79769908
rs55849757
rs10845930
rs6891593
rs62417423
rs62166973
rs17824379
rs1322067
rs7405251
rs11251832
rs12166155
rs6767083
rs3134968
All
−0.1
0.0
0.1
MR leave−one−out sensitivity analysis for
'HLA DR+ CD4+ AC' on 'Diabetic nephropathy'

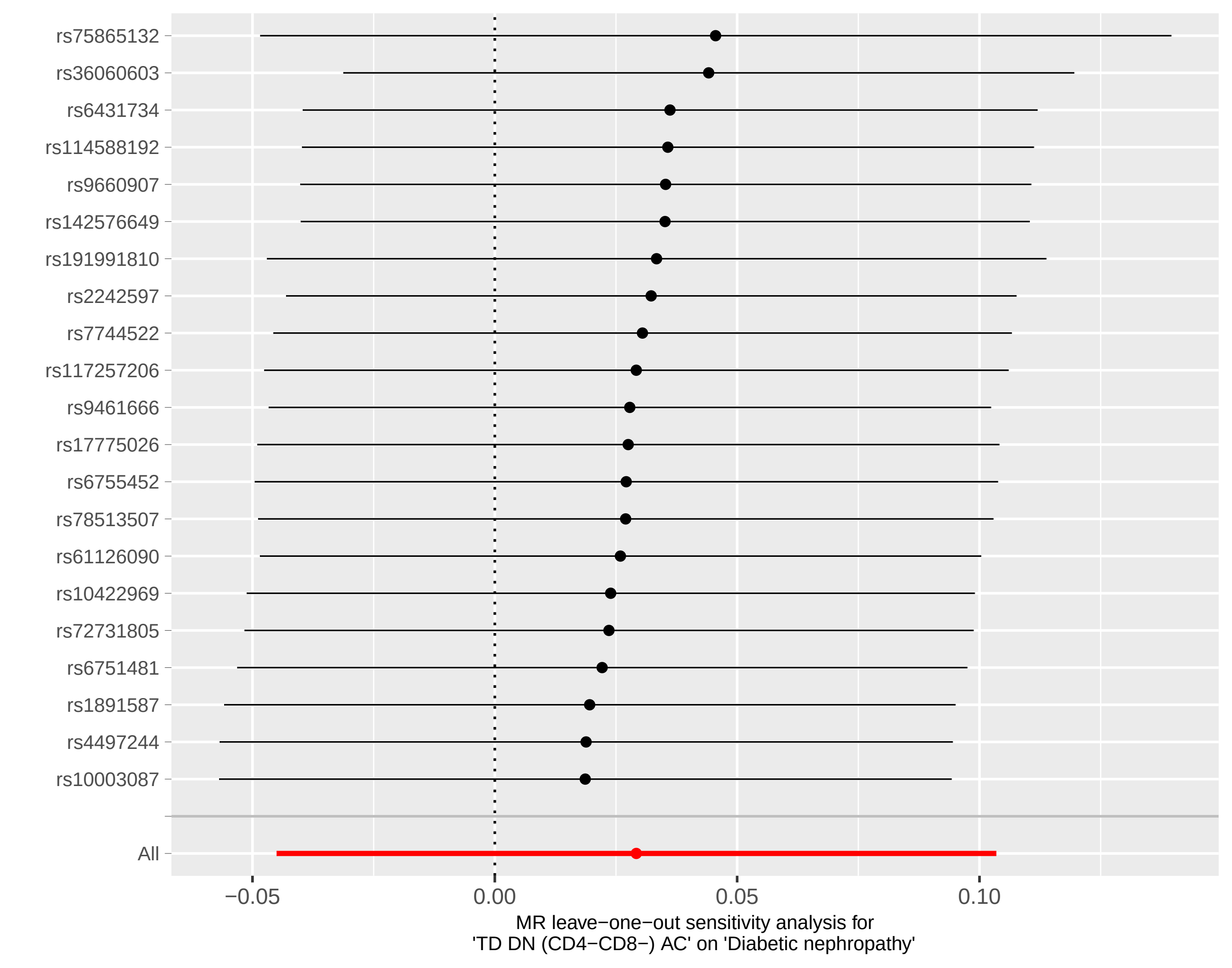

rs75865132
rs36060603
rs6431734
rs114588192
rs9660907
rs142576649
rs191991810
rs2242597
rs7744522
rs117257206
rs9461666
rs17775026
rs6755452
rs78513507
rs61126090
rs10422969
rs72731805
rs6751481
rs1891587
rs4497244
rs10003087
All
−0.05
0.00
0.05
0.10
MR leave−one−out sensitivity analysis for
'TD DN (CD4−CD8−) AC' on 'Diabetic nephropathy'

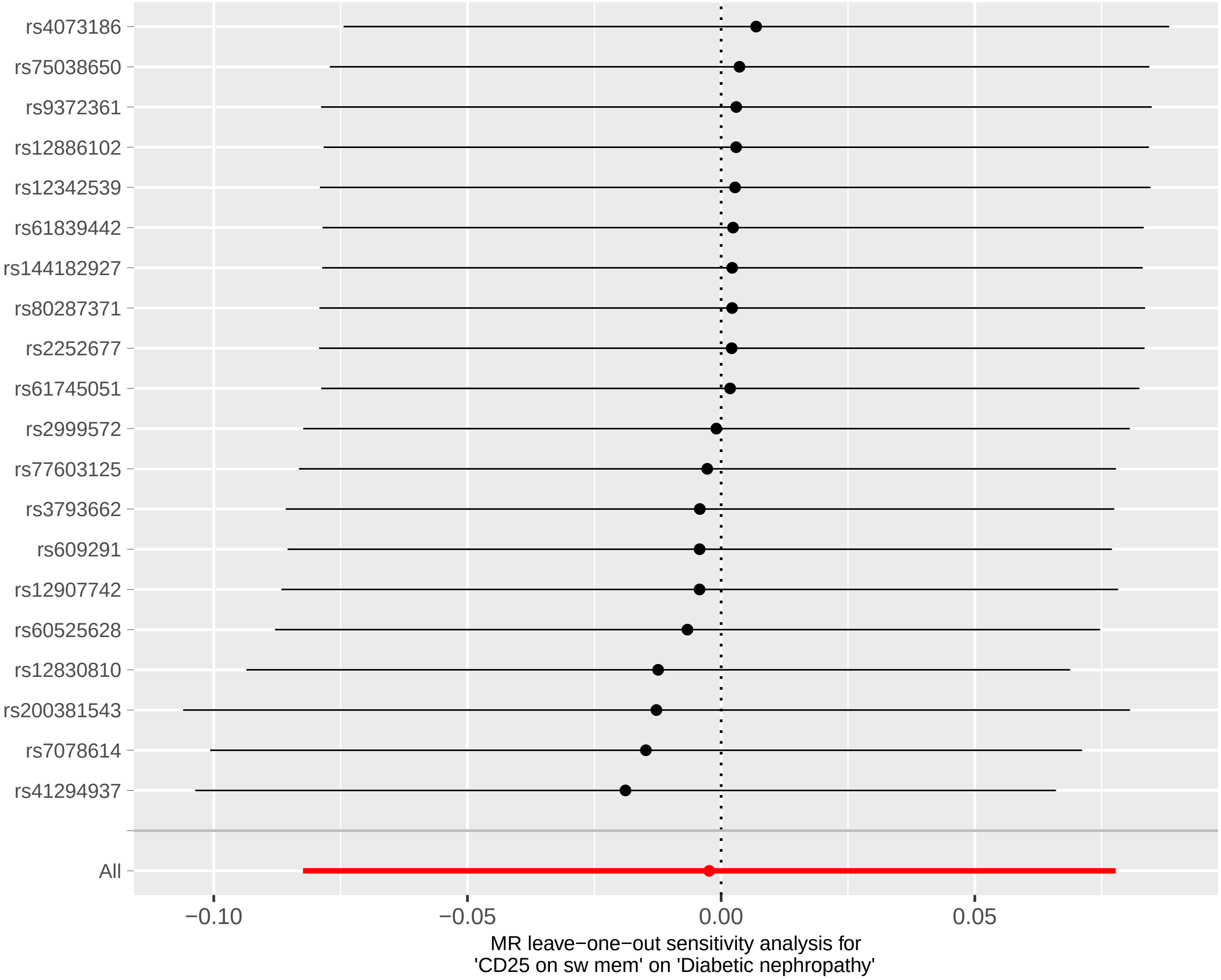

rs4073186
rs75038650
rs9372361
rs12886102
rs12342539
rs61839442
rs144182927
rs80287371
rs2252677
rs61745051
rs2999572
rs77603125
rs3793662
rs609291
rs12907742
rs60525628
rs12830810
rs200381543
rs7078614
rs41294937
All
−0.10
−0.05
0.00
0.05
MR leave−one−out sensitivity analysis for
'CD25 on sw mem' on 'Diabetic nephropathy'

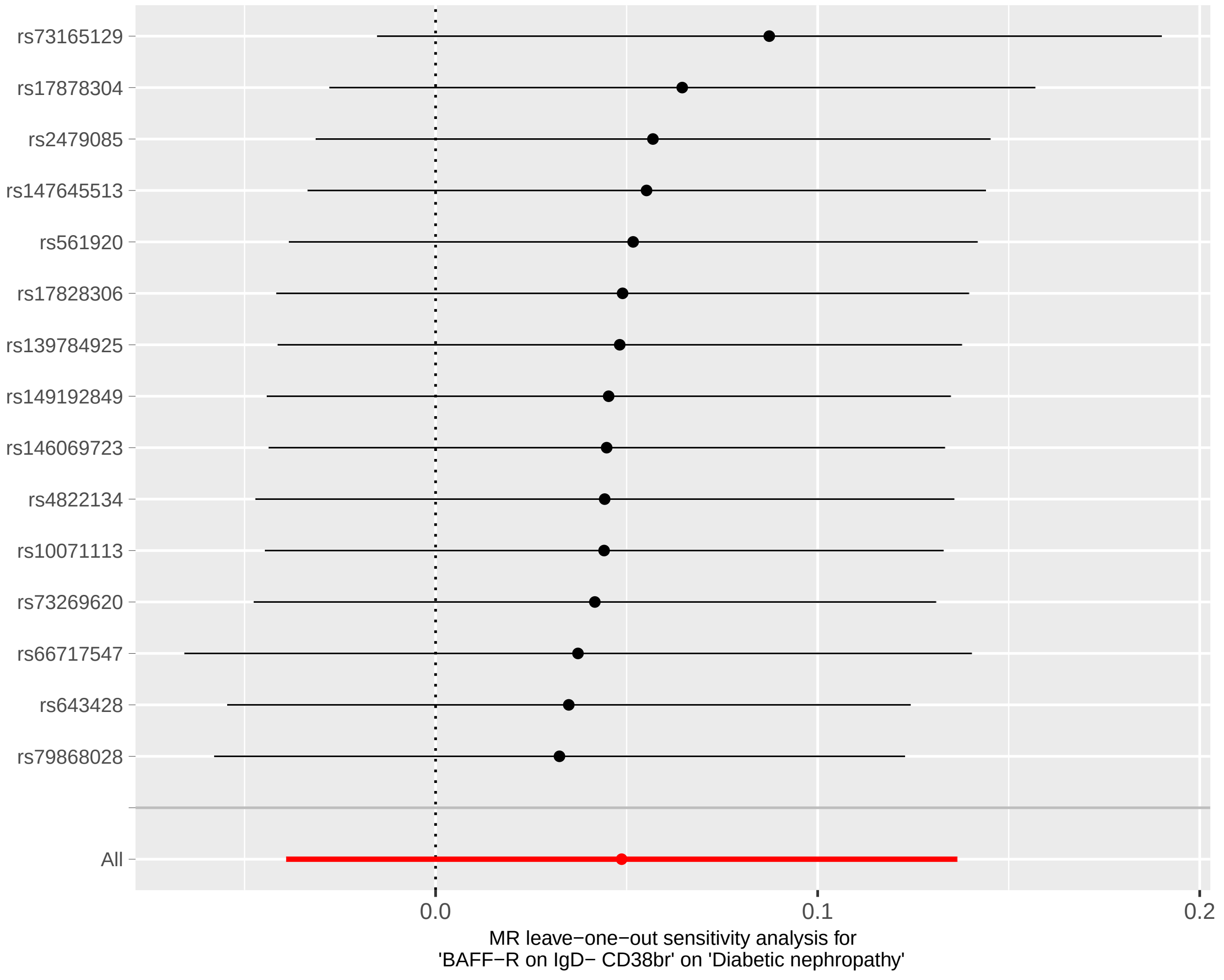
rs73165129
rs17878304
rs2479085
rs147645513
rs561920
rs17828306
rs139784925
rs149192849
rs146069723
rs4822134
rs10071113
rs73269620
rs66717547
rs643428
rs79868028
All
0.0
0.1
0.2
MR leave−one−out sensitivity analysis for
'BAFF−R on IgD− CD38br' on 'Diabetic nephropathy'

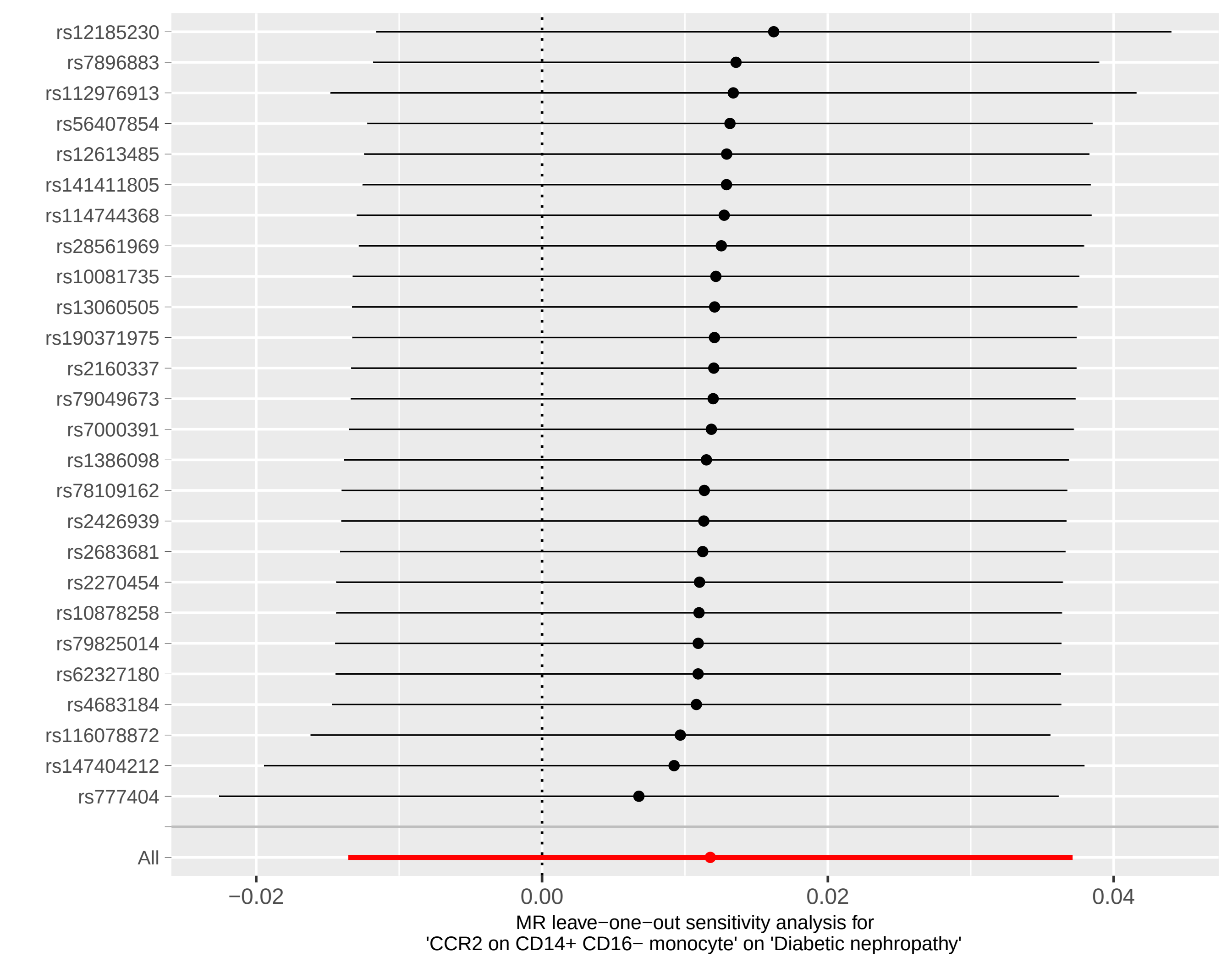
rs12185230
rs7896883
rs112976913
rs56407854
rs12613485
rs141411805
rs114744368
rs28561969
rs10081735
rs13060505
rs190371975
rs2160337
rs79049673
rs7000391
rs1386098
rs78109162
rs2426939
rs2683681
rs2270454
rs10878258
rs79825014
rs62327180
rs4683184
rs116078872
rs147404212
rs777404
All
−0.02
0.00
0.02
0.04
MR leave−one−out sensitivity analysis for
'CCR2 on CD14+ CD16− monocyte' on 'Diabetic nephropathy'

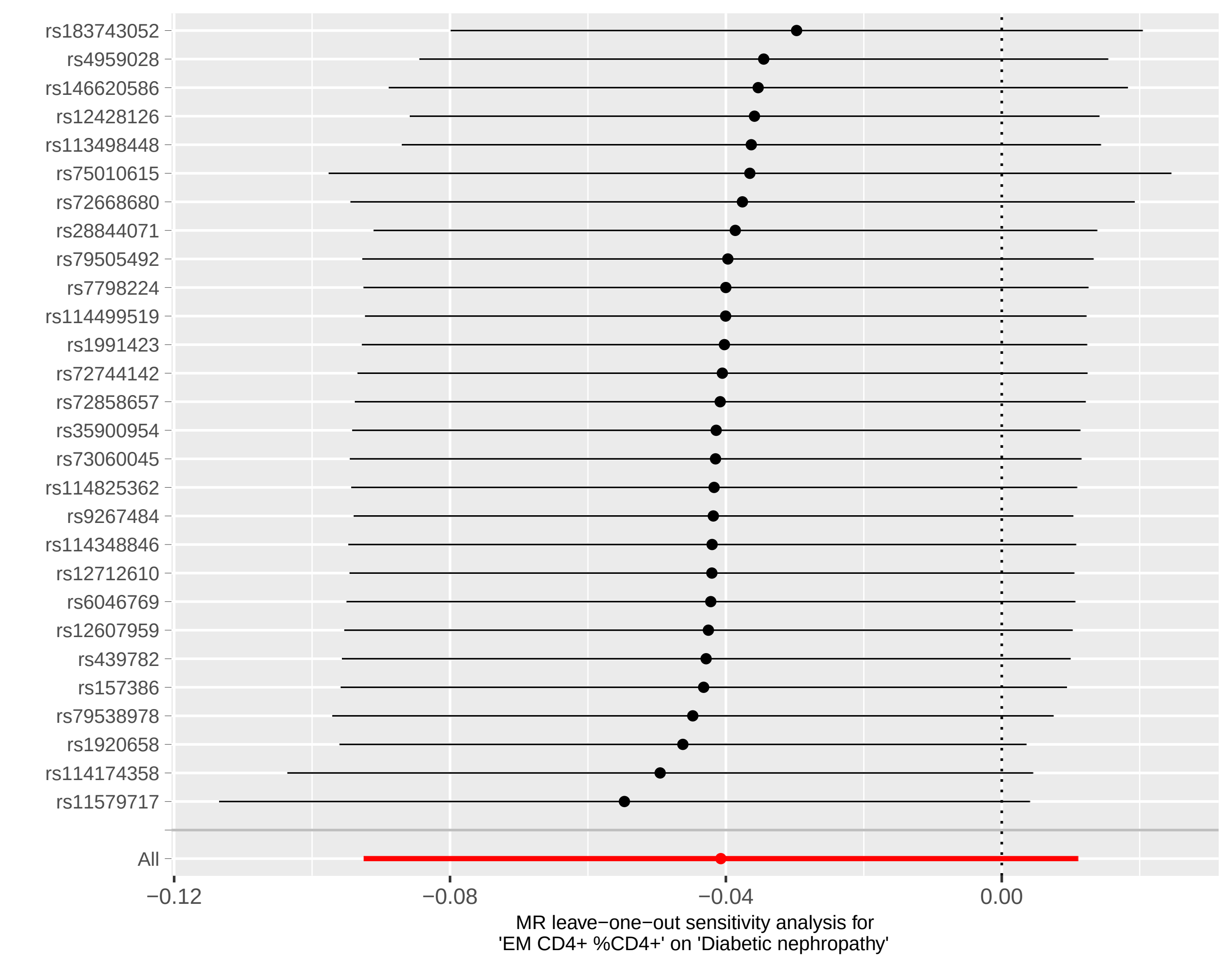
rs183743052
rs4959028
rs146620586
rs12428126
rs113498448
rs75010615
rs72668680
rs28844071
rs79505492
rs7798224
rs114499519
rs1991423
rs72744142
rs72858657
rs35900954
rs73060045
rs114825362
rs9267484
rs114348846
rs12712610
rs6046769
rs12607959
rs439782
rs157386
rs79538978
rs1920658
rs114174358
rs11579717
All
−0.12
−0.08
−0.04
0.00
MR leave−one−out sensitivity analysis for
'EM CD4+ %CD4+' on 'Diabetic nephropathy'

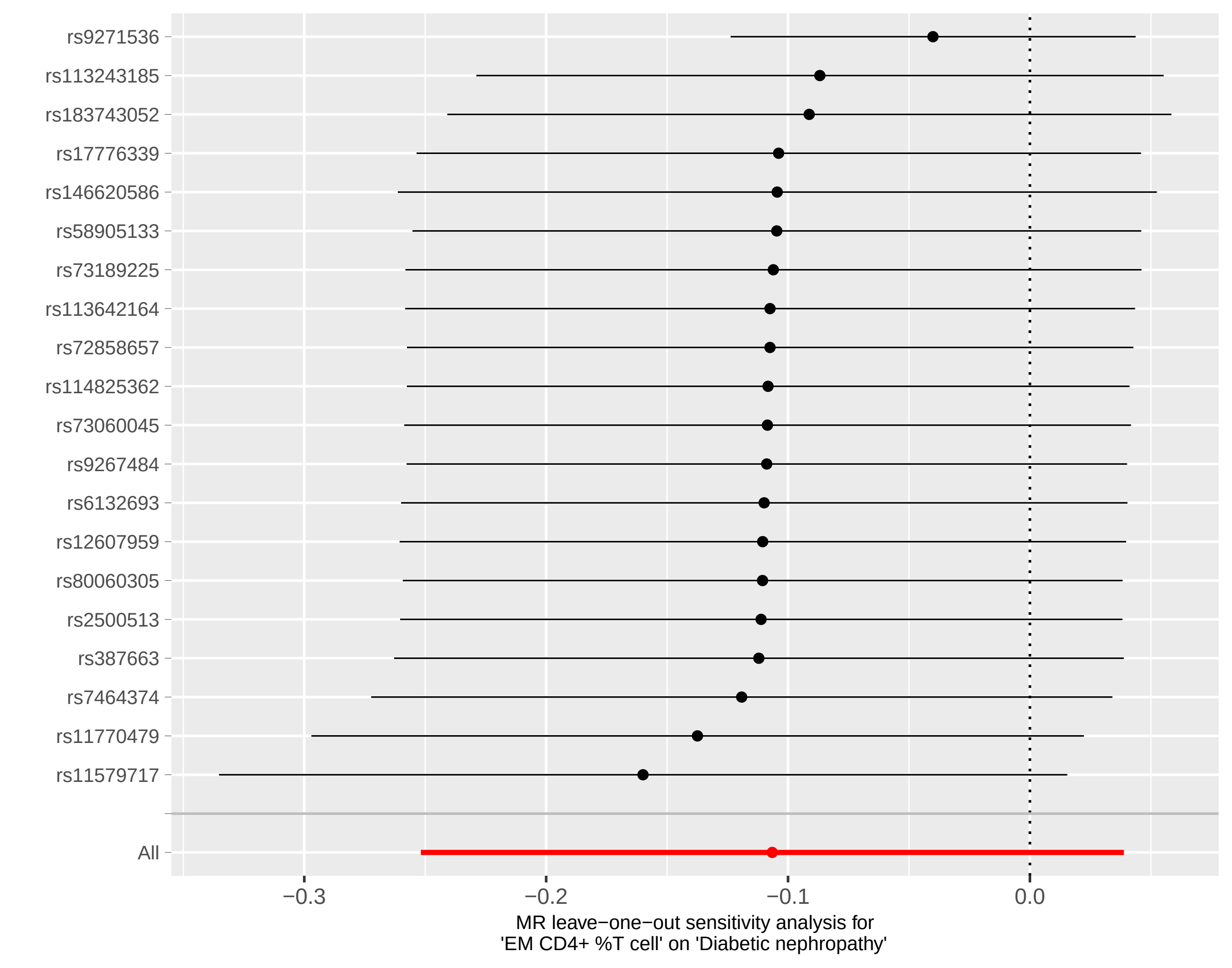

rs9271536
rs113243185
rs183743052
rs17776339
rs146620586
rs58905133
rs73189225
rs113642164
rs72858657
rs114825362
rs73060045
rs9267484
rs6132693
rs12607959
rs80060305
rs2500513
rs387663
rs7464374
rs11770479
rs11579717
All
−0.3
−0.2
−0.1
0.0
MR leave−one−out sensitivity analysis for
'EM CD4+ %T cell' on 'Diabetic nephropathy'

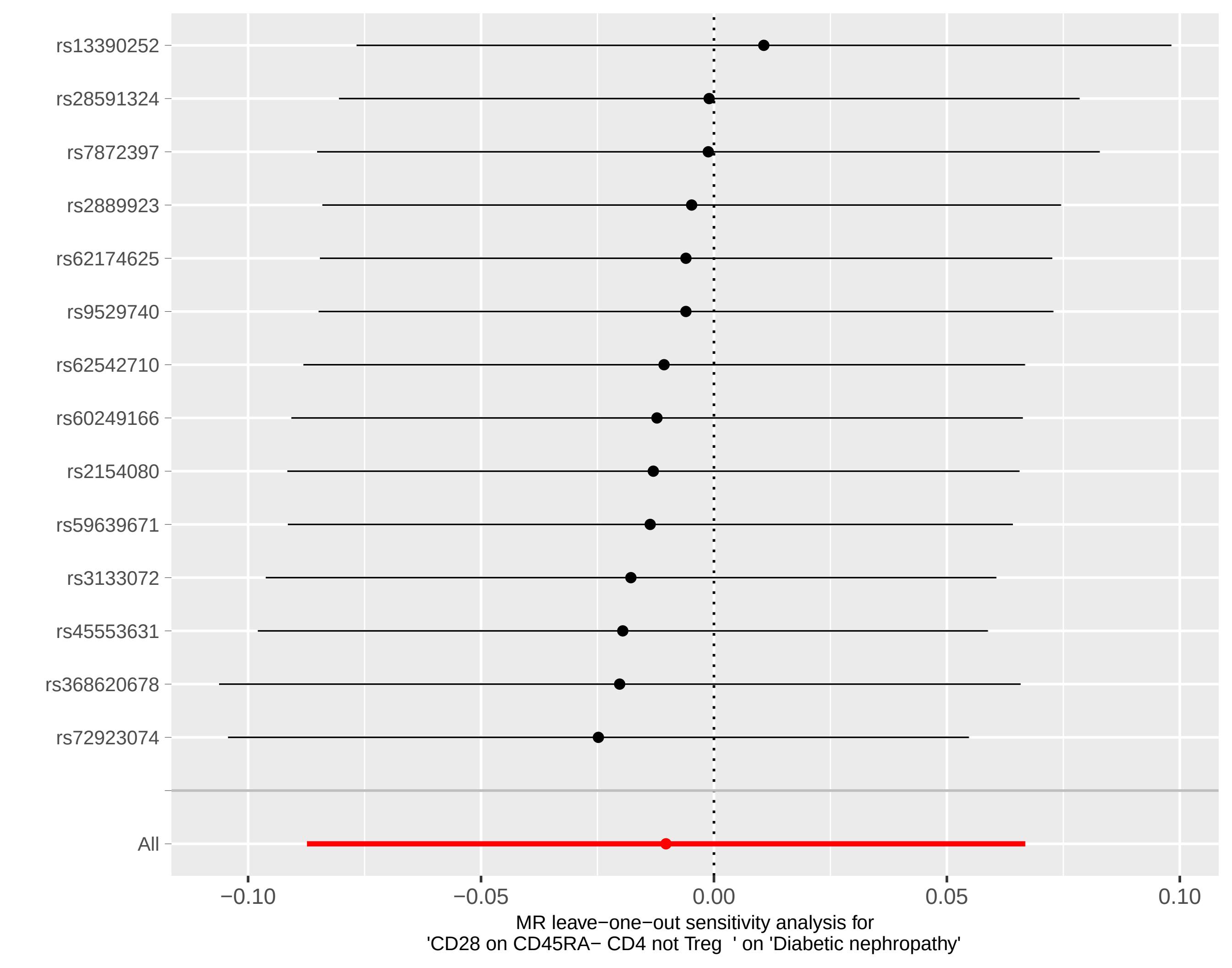
rs13390252
rs28591324
rs7872397
rs2889923
rs62174625
rs9529740
rs62542710
rs60249166
rs2154080
rs59639671
rs3133072
rs45553631
rs368620678
rs72923074
All
−0.10
−0.05
0.00
0.05
0.10
MR leave−one−out sensitivity analysis for
'CD28 on CD45RA− CD4 not Treg ' on 'Diabetic nephropathy'

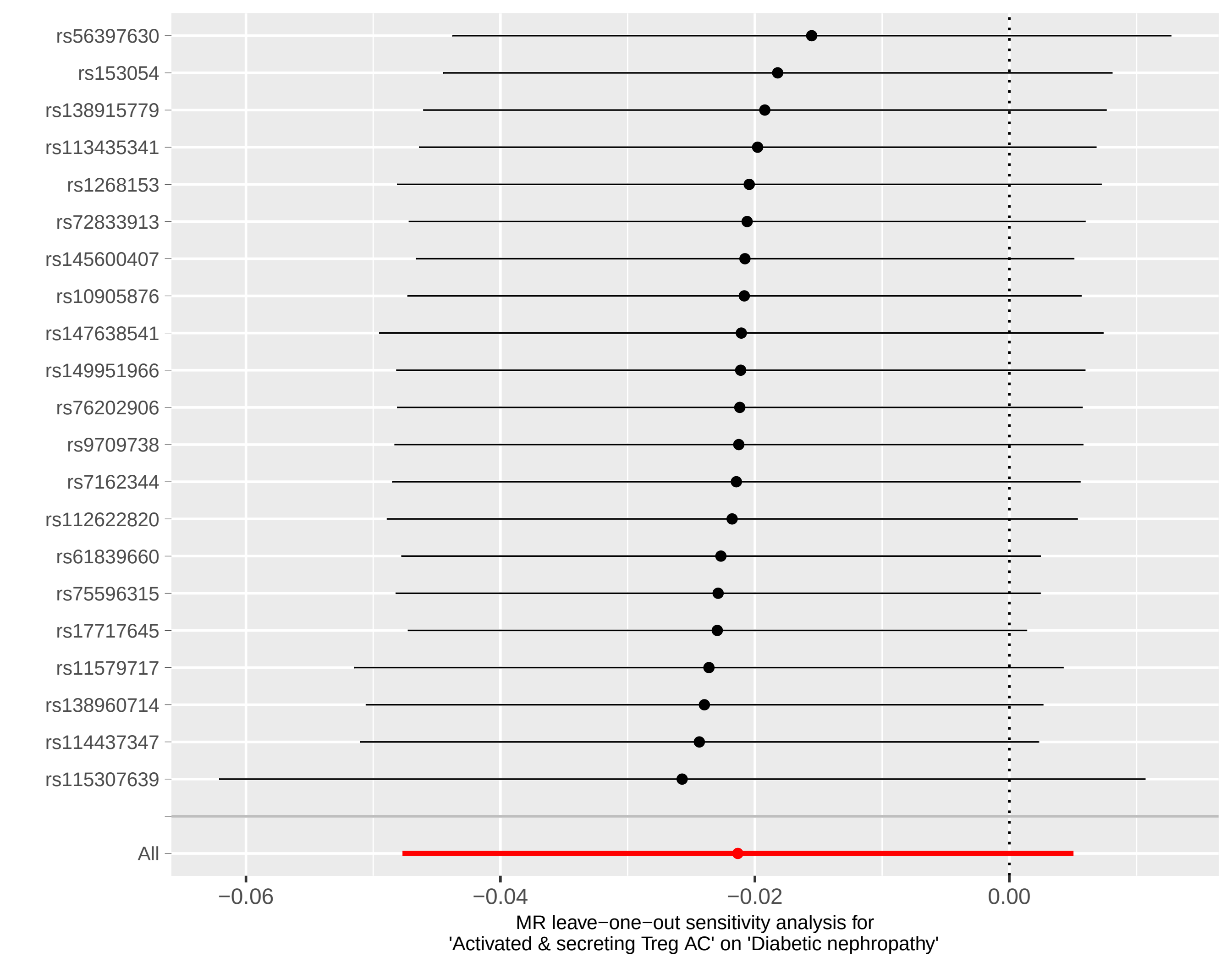
rs56397630
rs153054
rs138915779
rs113435341
rs1268153
rs72833913
rs145600407
rs10905876
rs147638541
rs149951966
rs76202906
rs9709738
rs7162344
rs112622820
rs61839660
rs75596315
rs17717645
rs11579717
rs138960714
rs114437347
rs115307639
All
−0.06
−0.04
−0.02
0.00
MR leave−one−out sensitivity analysis for
'Activated & secreting Treg AC' on 'Diabetic nephropathy'

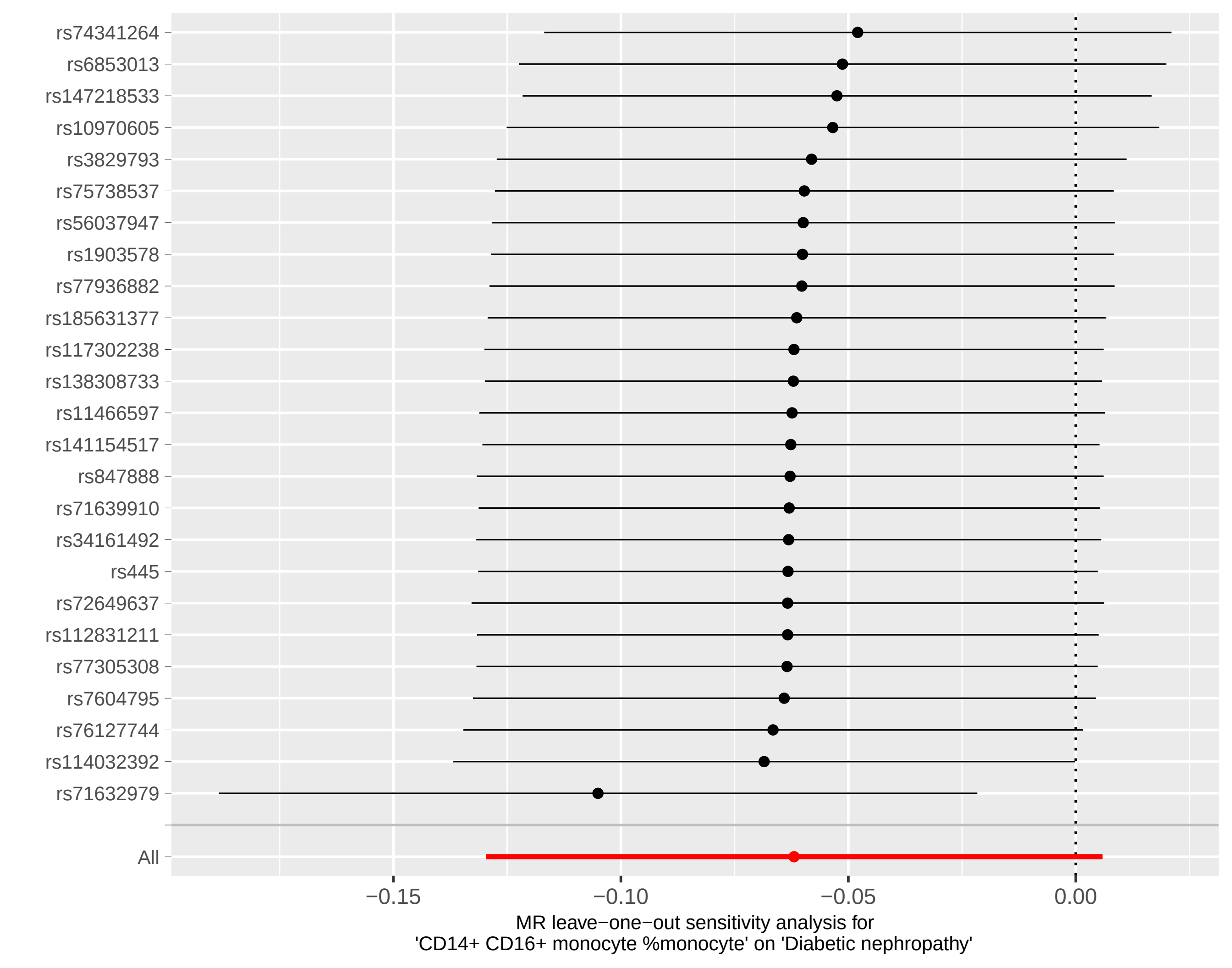
rs74341264
rs6853013
rs147218533
rs10970605
rs3829793
rs75738537
rs56037947
rs1903578
rs77936882
rs185631377
rs117302238
rs138308733
rs11466597
rs141154517
rs847888
rs71639910
rs34161492
rs445
rs72649637
rs112831211
rs77305308
rs7604795
rs76127744
rs114032392
rs71632979
All
−0.15
−0.10
−0.05
0.00
MR leave−one−out sensitivity analysis for
'CD14+ CD16+ monocyte %monocyte' on 'Diabetic nephropathy'

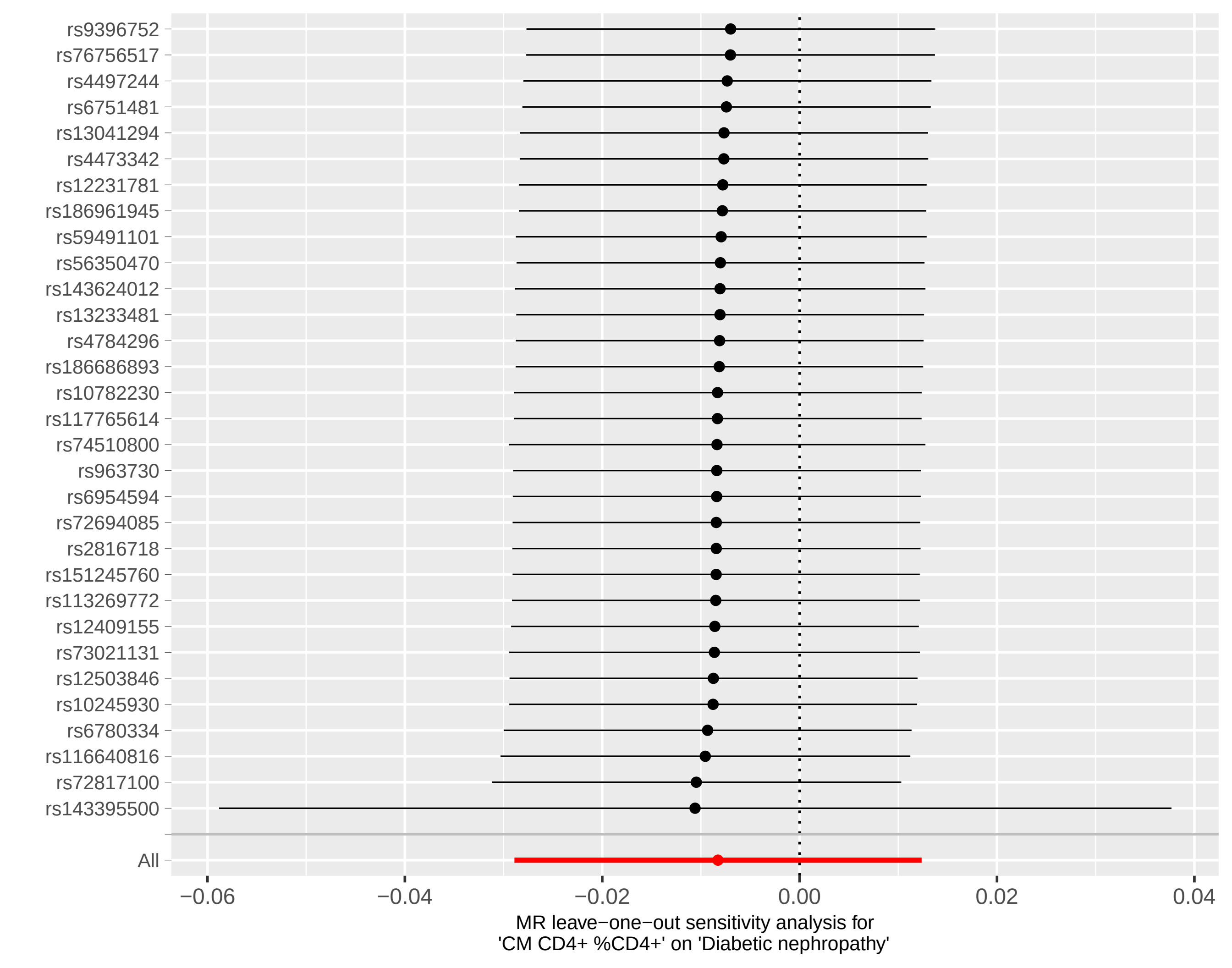
rs9396752
rs76756517
rs4497244
rs6751481
rs13041294
rs4473342
rs12231781
rs186961945
rs59491101
rs56350470
rs143624012
rs13233481
rs4784296
rs186686893
rs10782230
rs117765614
rs74510800
rs963730
rs6954594
rs72694085
rs2816718
rs151245760
rs113269772
rs12409155
rs73021131
rs12503846
rs10245930
rs6780334
rs116640816
rs72817100
rs143395500
All
−0.06
−0.04
−0.02
0.00
0.02
0.04
MR leave−one−out sensitivity analysis for
'CM CD4+ %CD4+' on 'Diabetic nephropathy'

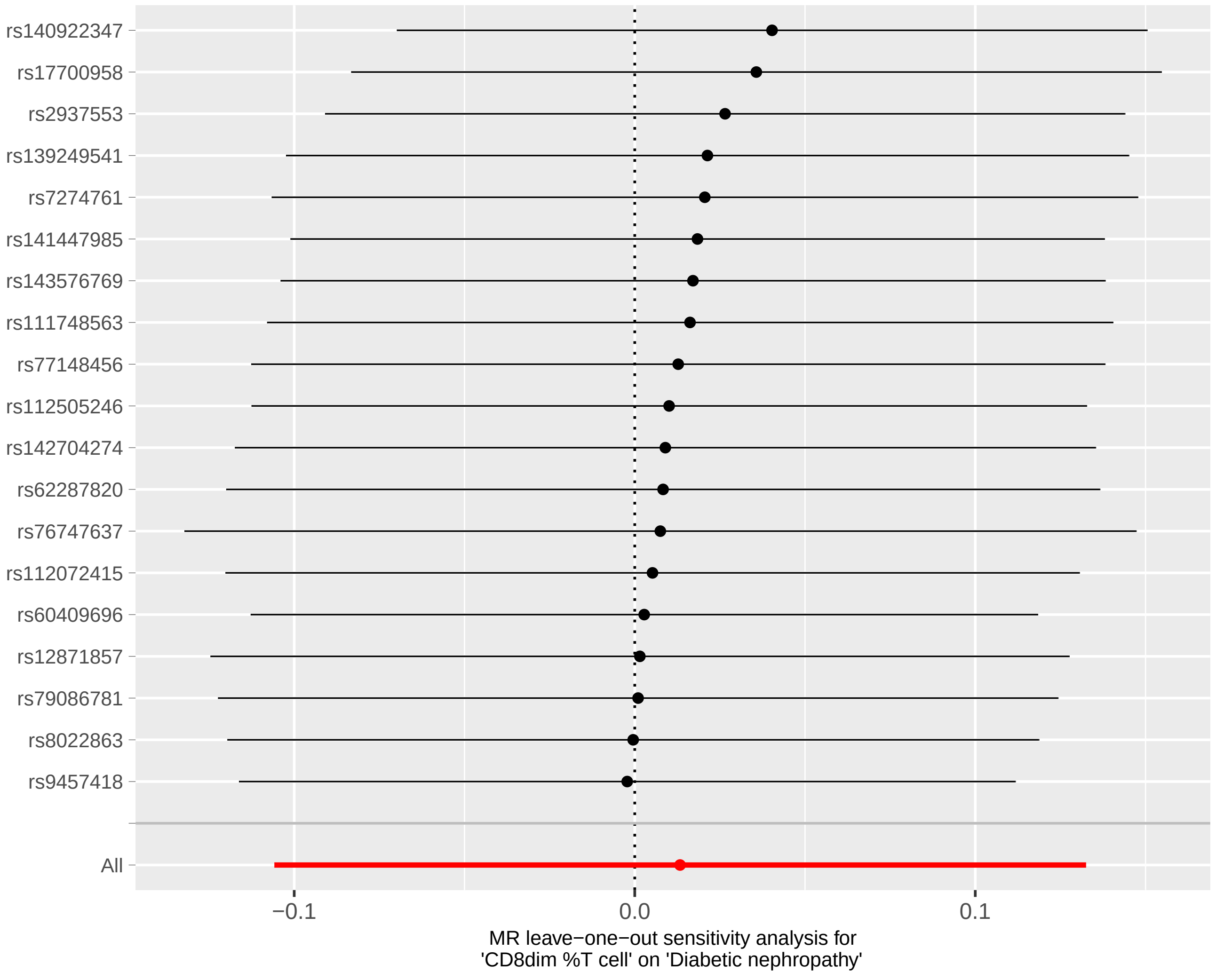
rs140922347
rs17700958
rs2937553
rs139249541
rs7274761
rs141447985
rs143576769
rs111748563
rs77148456
rs112505246
rs142704274
rs62287820
rs76747637
rs112072415
rs60409696
rs12871857
rs79086781
rs8022863
rs9457418
All
−0.1
0.0
0.1
MR leave−one−out sensitivity analysis for
'CD8dim %T cell' on 'Diabetic nephropathy'

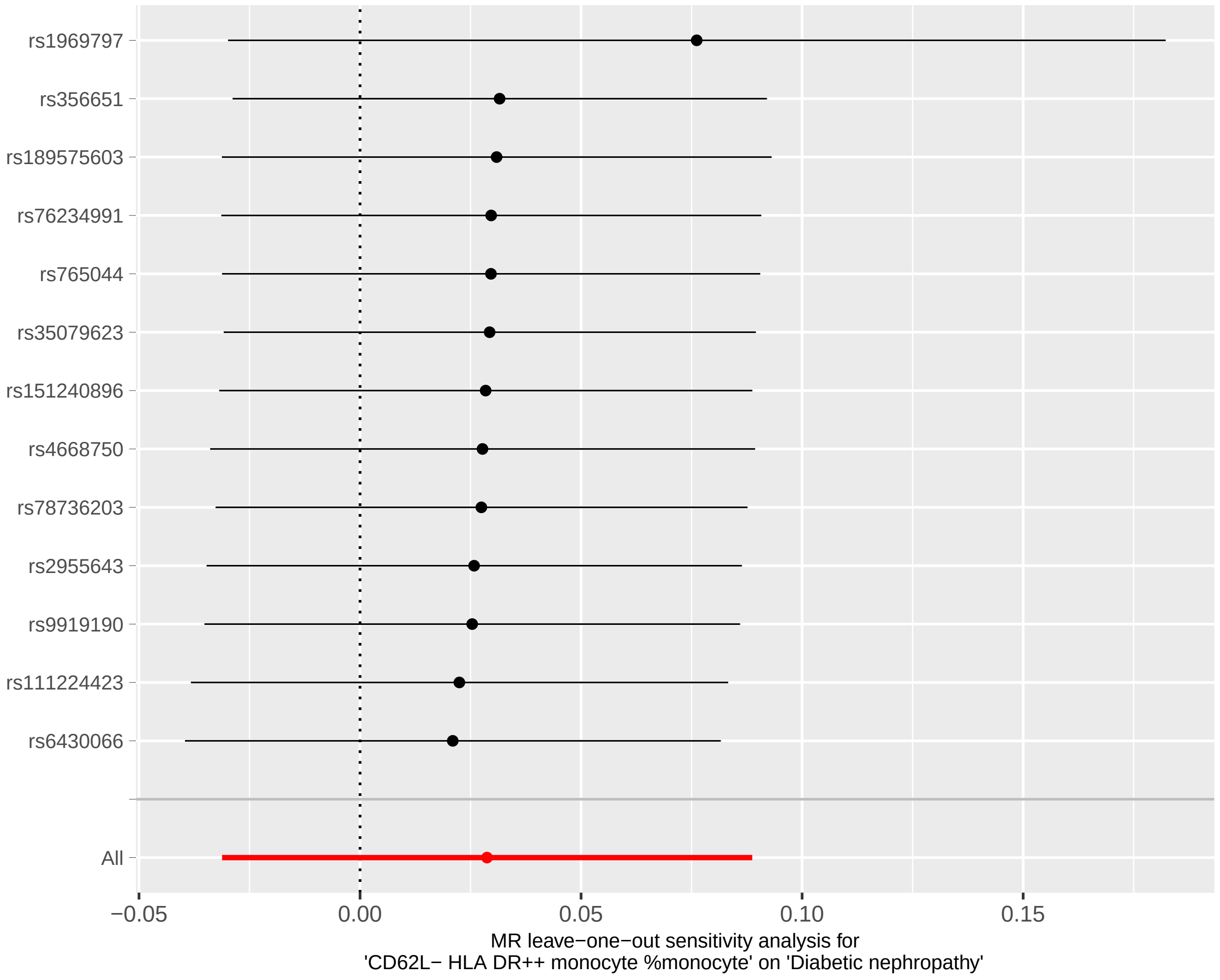
rs1969797
rs356651
rs189575603
rs76234991
rs765044
rs35079623
rs151240896
rs4668750
rs78736203
rs2955643
rs9919190
rs111224423
rs6430066
All
−0.05
0.00
0.05
0.10
0.15
MR leave−one−out sensitivity analysis for
'CD62L− HLA DR++ monocyte %monocyte' on 'Diabetic nephropathy'

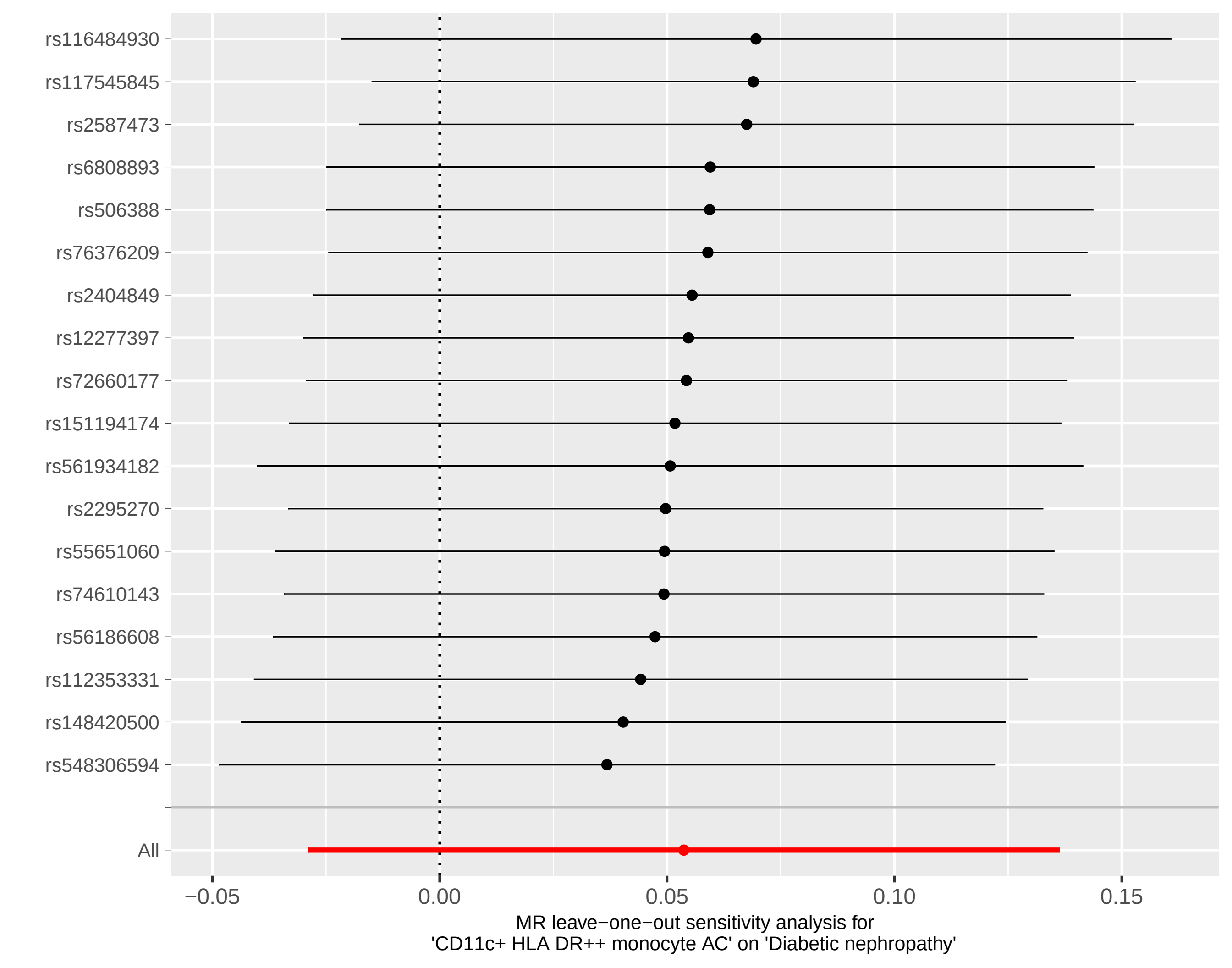
rs116484930
rs117545845
rs2587473
rs6808893
rs506388
rs76376209
rs2404849
rs12277397
rs72660177
rs151194174
rs561934182
rs2295270
rs55651060
rs74610143
rs56186608
rs112353331
rs148420500
rs548306594
All
−0.05
0.00
0.05
0.10
0.15
MR leave−one−out sensitivity analysis for
'CD11c+ HLA DR++ monocyte AC' on 'Diabetic nephropathy'

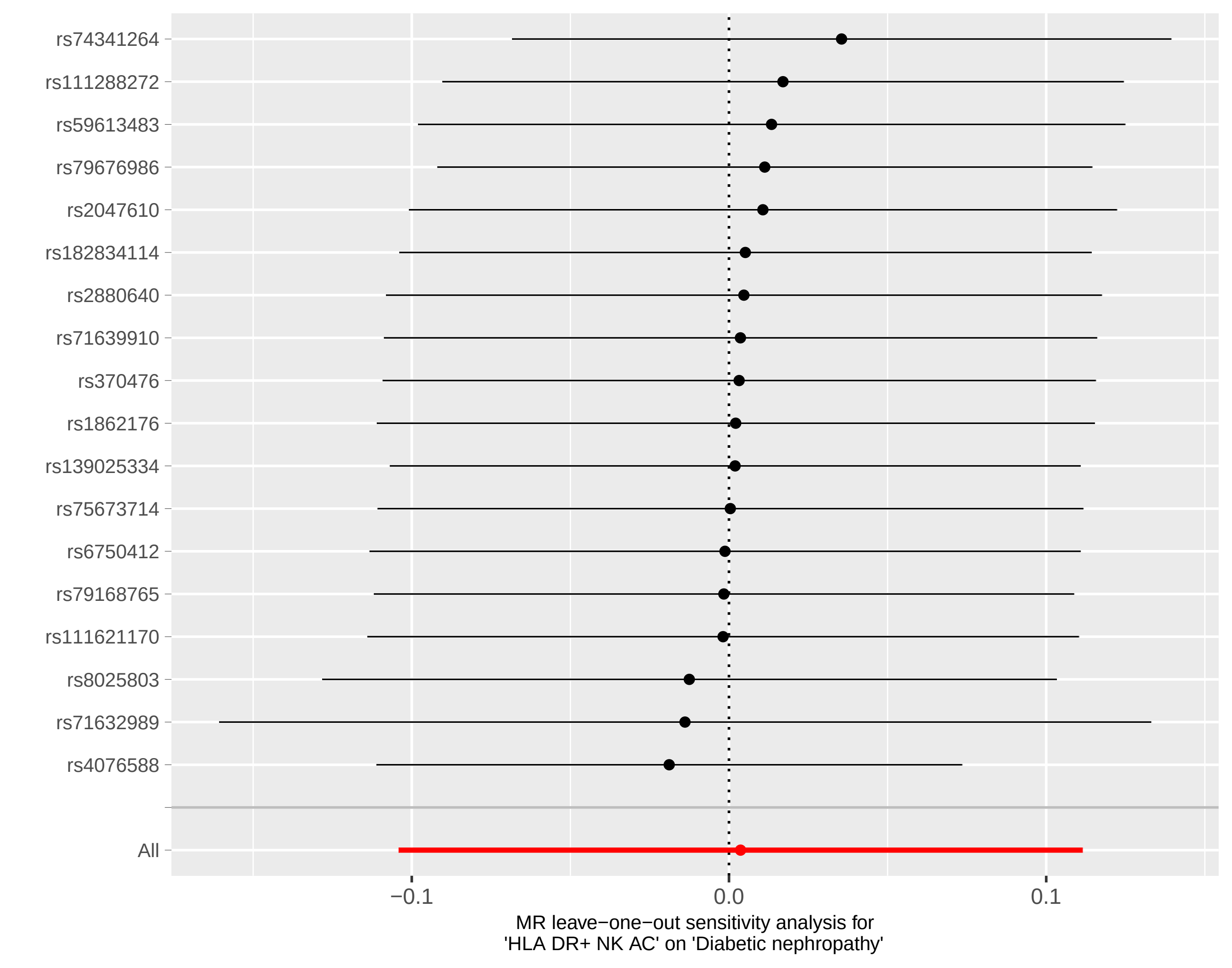
rs74341264
rs111288272
rs59613483
rs79676986
rs2047610
rs182834114
rs2880640
rs71639910
rs370476
rs1862176
rs139025334
rs75673714
rs6750412
rs79168765
rs111621170
rs8025803
rs71632989
rs4076588
All
−0.1
0.0
0.1
MR leave−one−out sensitivity analysis for
'HLA DR+ NK AC' on 'Diabetic nephropathy'

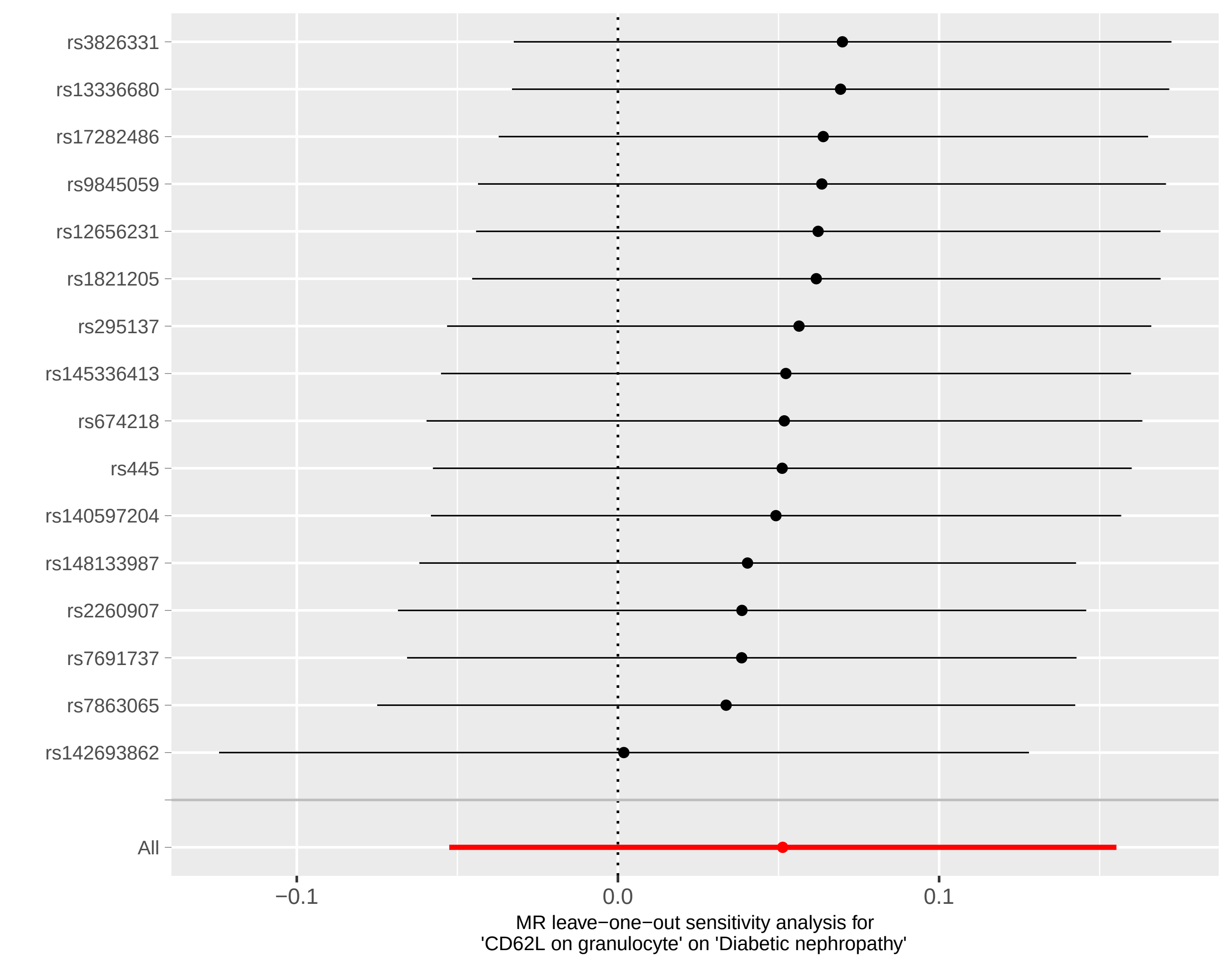
rs3826331
rs13336680
rs17282486
rs9845059
rs12656231
rs1821205
rs295137
rs145336413
rs674218
rs445
rs140597204
rs148133987
rs2260907
rs7691737
rs7863065
rs142693862
All
−0.1
0.0
0.1
MR leave−one−out sensitivity analysis for
'CD62L on granulocyte' on 'Diabetic nephropathy'

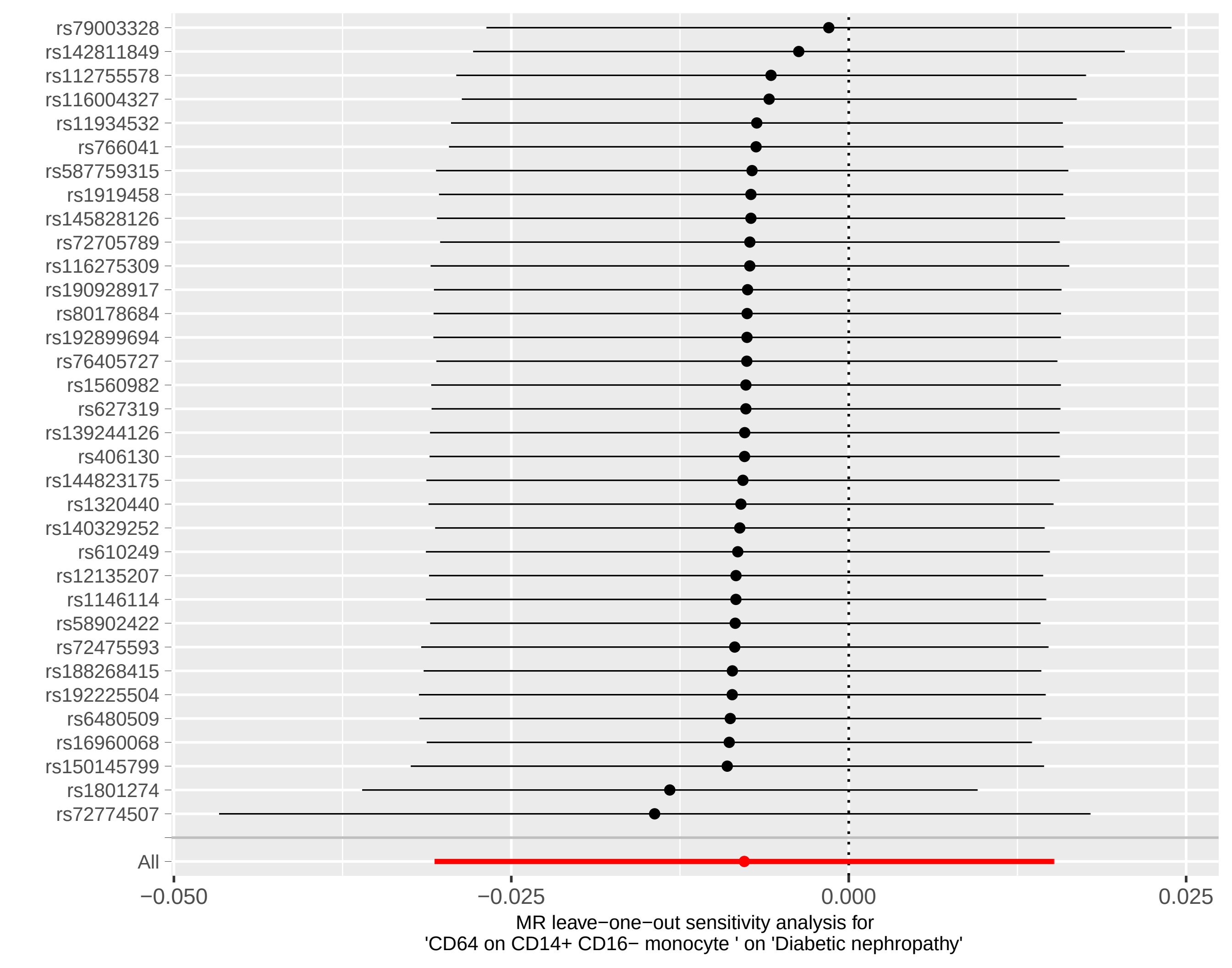
rs79003328
rs142811849
rs112755578
rs116004327
rs11934532
rs766041
rs587759315
rs1919458
rs145828126
rs72705789
rs116275309
rs190928917
rs80178684
rs192899694
rs76405727
rs1560982
rs627319
rs139244126
rs406130
rs144823175
rs1320440
rs140329252
rs610249
rs12135207
rs1146114
rs58902422
rs72475593
rs188268415
rs192225504
rs6480509
rs16960068
rs150145799
rs1801274
rs72774507
All
−0.050
−0.025
0.000
0.025
MR leave−one−out sensitivity analysis for
'CD64 on CD14+ CD16− monocyte ' on 'Diabetic nephropathy'

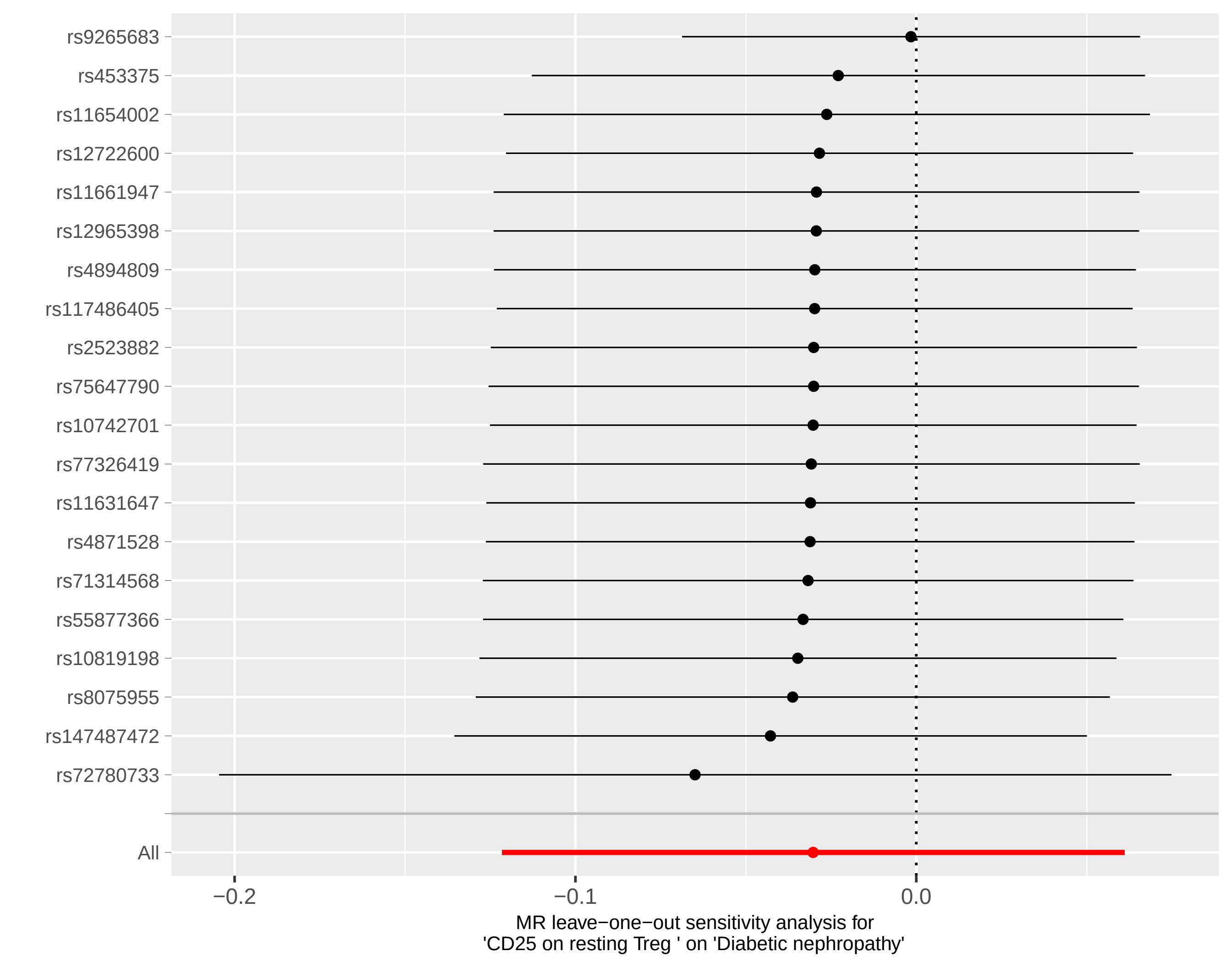

rs9265683
rs453375
rs11654002
rs12722600
rs11661947
rs12965398
rs4894809
rs117486405
rs2523882
rs75647790
rs10742701
rs77326419
rs11631647
rs4871528
rs71314568
rs55877366
rs10819198
rs8075955
rs147487472
rs72780733
All
−0.2
−0.1
0.0
MR leave−one−out sensitivity analysis for
'CD25 on resting Treg ' on 'Diabetic nephropathy'

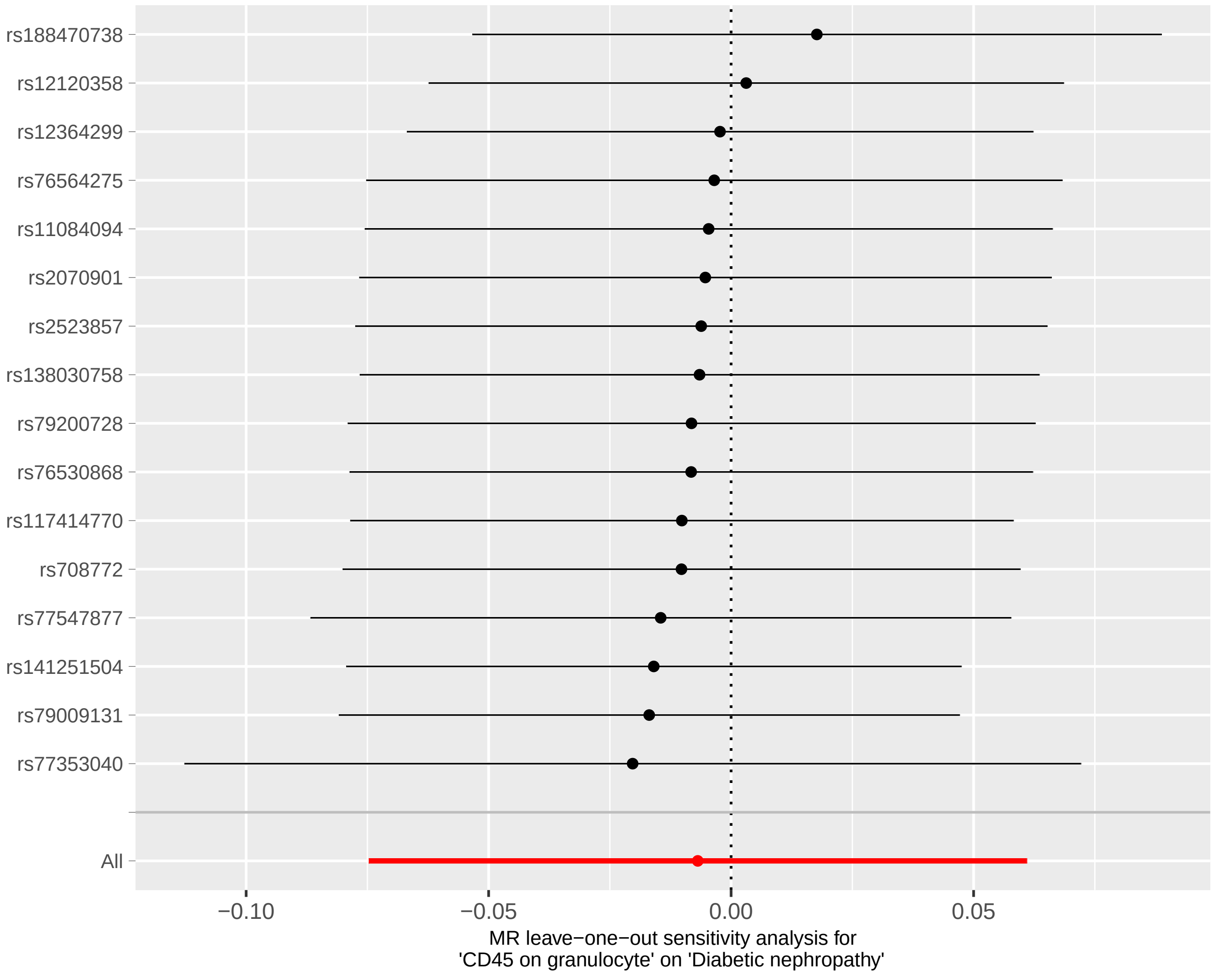
rs188470738
rs12120358
rs12364299
rs76564275
rs11084094
rs2070901
rs2523857
rs138030758
rs79200728
rs76530868
rs117414770
rs708772
rs77547877
rs141251504
rs79009131
rs77353040
All
−0.10
−0.05
0.00
0.05
MR leave−one−out sensitivity analysis for
'CD45 on granulocyte' on 'Diabetic nephropathy'

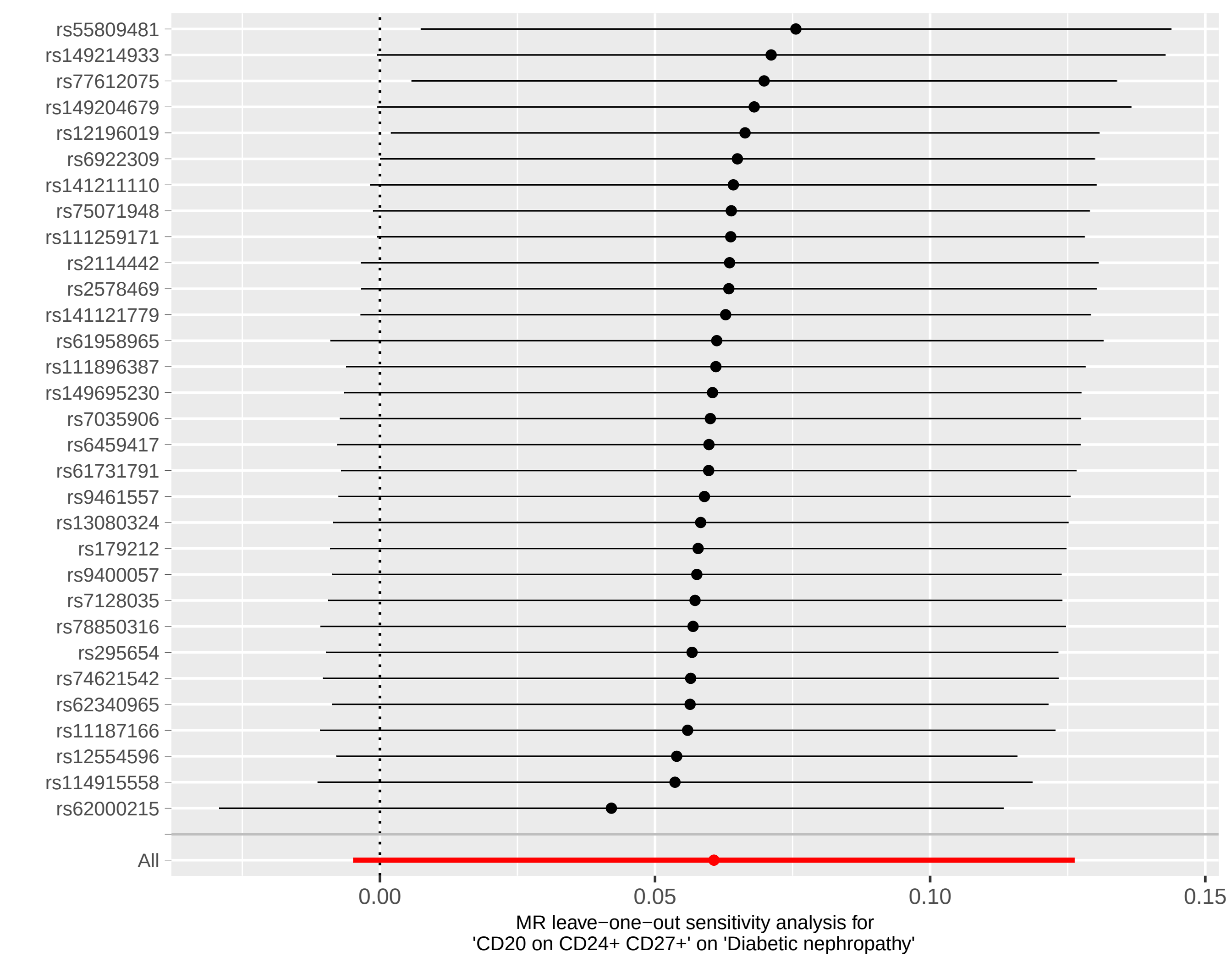
rs55809481
rs149214933
rs77612075
rs149204679
rs12196019
rs6922309
rs141211110
rs75071948
rs111259171
rs2114442
rs2578469
rs141121779
rs61958965
rs111896387
rs149695230
rs7035906
rs6459417
rs61731791
rs9461557
rs13080324
rs179212
rs9400057
rs7128035
rs78850316
rs295654
rs74621542
rs62340965
rs11187166
rs12554596
rs114915558
rs62000215
All
0.00
0.05
0.10
0.15
MR leave−one−out sensitivity analysis for
'CD20 on CD24+ CD27+' on 'Diabetic nephropathy'

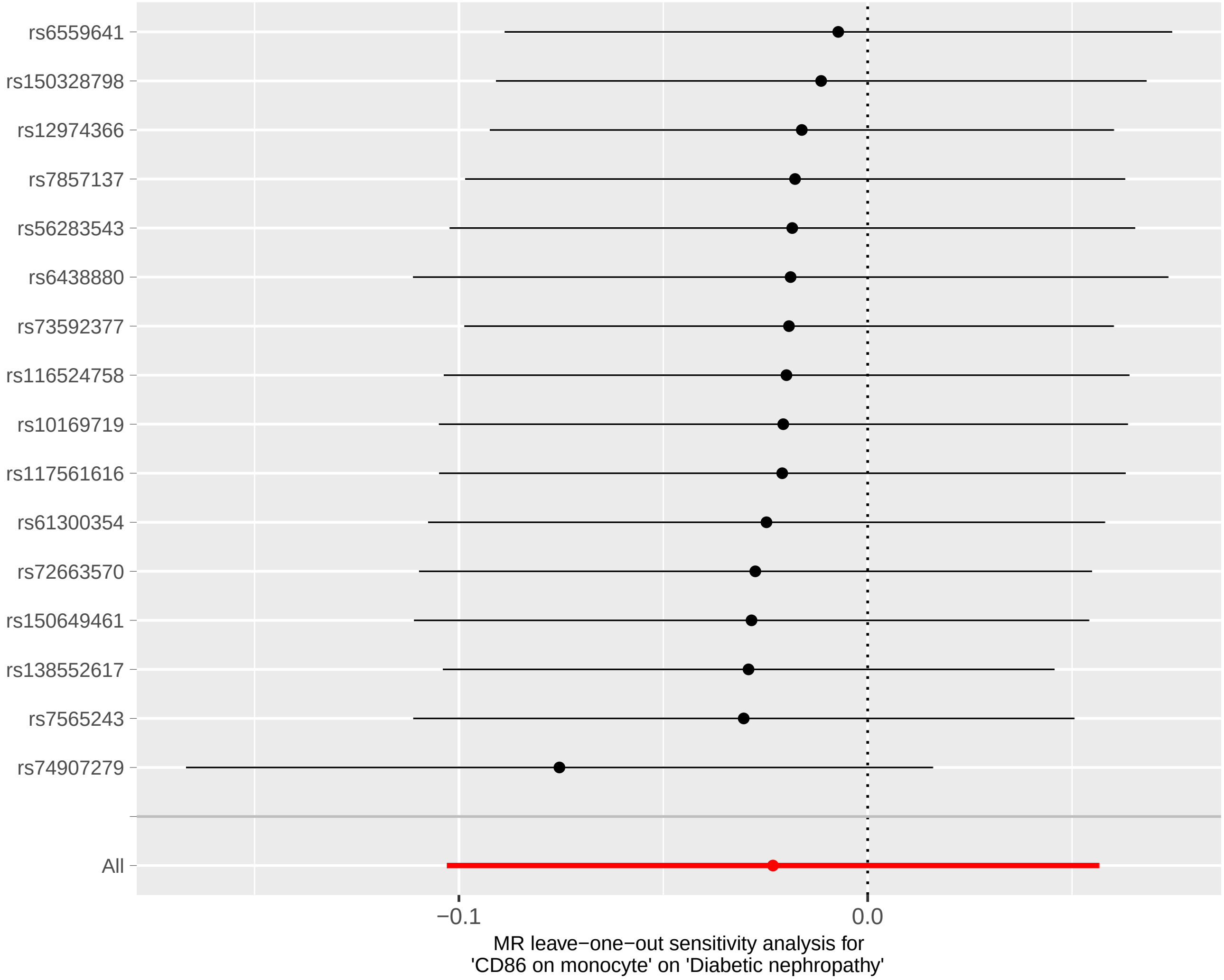
rs6559641
rs150328798
rs12974366
rs7857137
rs56283543
rs6438880
rs73592377
rs116524758
rs10169719
rs117561616
rs61300354
rs72663570
rs150649461
rs138552617
rs7565243
rs74907279
All
−0.1
0.0
MR leave−one−out sensitivity analysis for
'CD86 on monocyte' on 'Diabetic nephropathy'

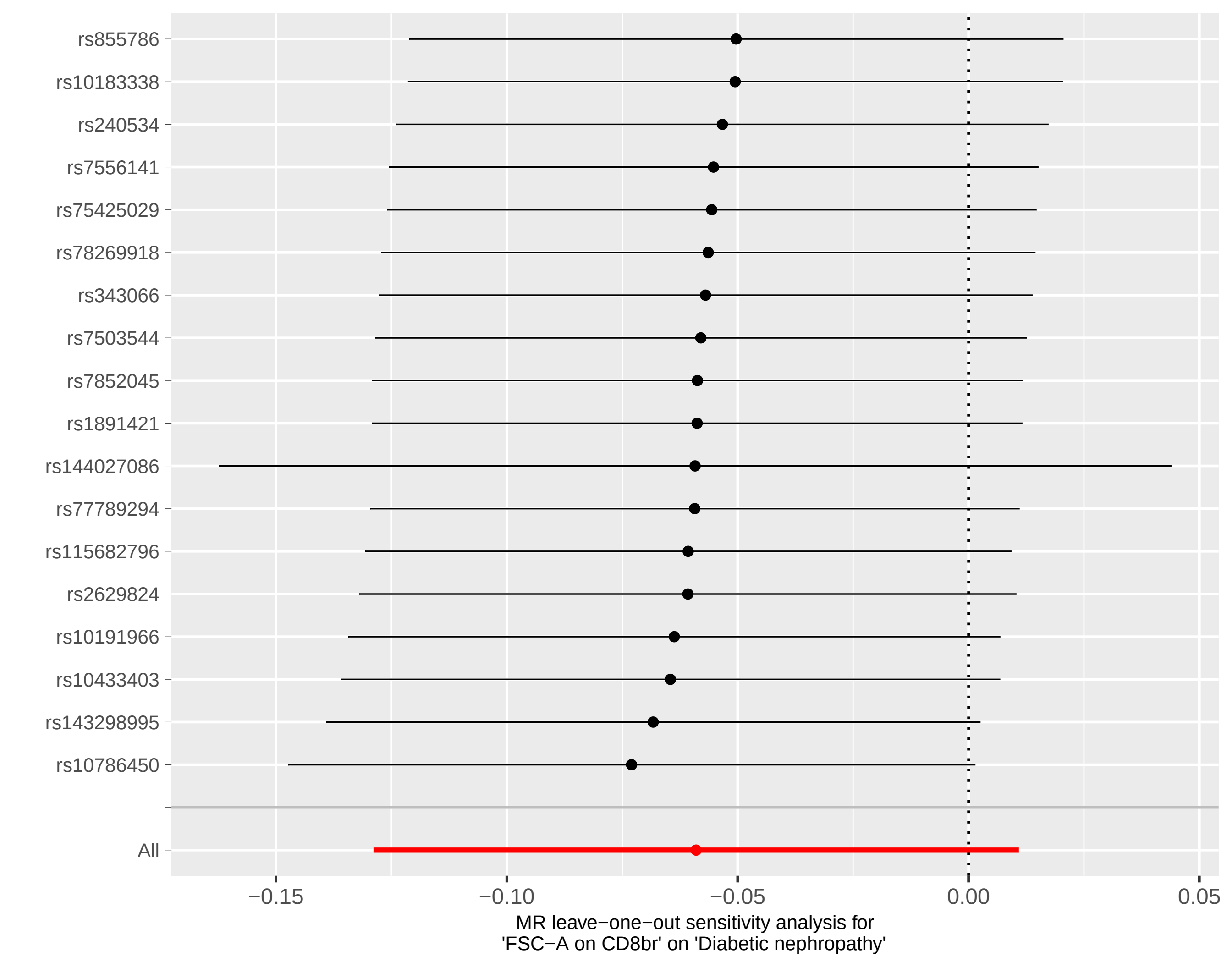
rs855786
rs10183338
rs240534
rs7556141
rs75425029
rs78269918
rs343066
rs7503544
rs7852045
rs1891421
rs144027086
rs77789294
rs115682796
rs2629824
rs10191966
rs10433403
rs143298995
rs10786450
All
−0.15
−0.10
−0.05
0.00
0.05
MR leave−one−out sensitivity analysis for
'FSC−A on CD8br' on 'Diabetic nephropathy'

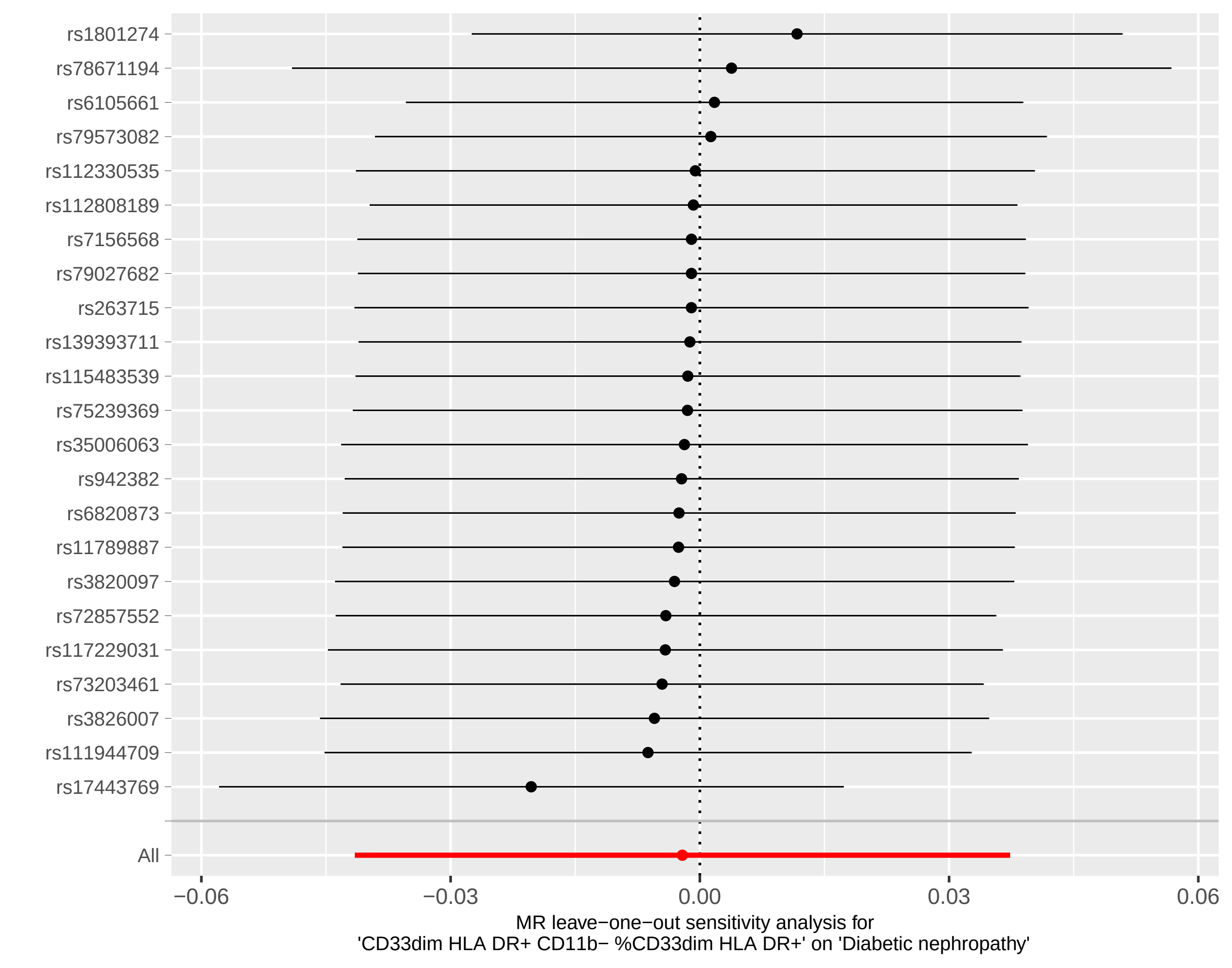

rs1801274
rs78671194
rs6105661
rs79573082
rs112330535
rs112808189
rs7156568
rs79027682
rs263715
rs139393711
rs115483539
rs75239369
rs35006063
rs942382
rs6820873
rs11789887
rs3820097
rs72857552
rs117229031
rs73203461
rs3826007
rs111944709
rs17443769
All
−0.06
−0.03
0.00
0.03
0.06
MR leave−one−out sensitivity analysis for
'CD33dim HLA DR+ CD11b− %CD33dim HLA DR+' on 'Diabetic nephropathy'

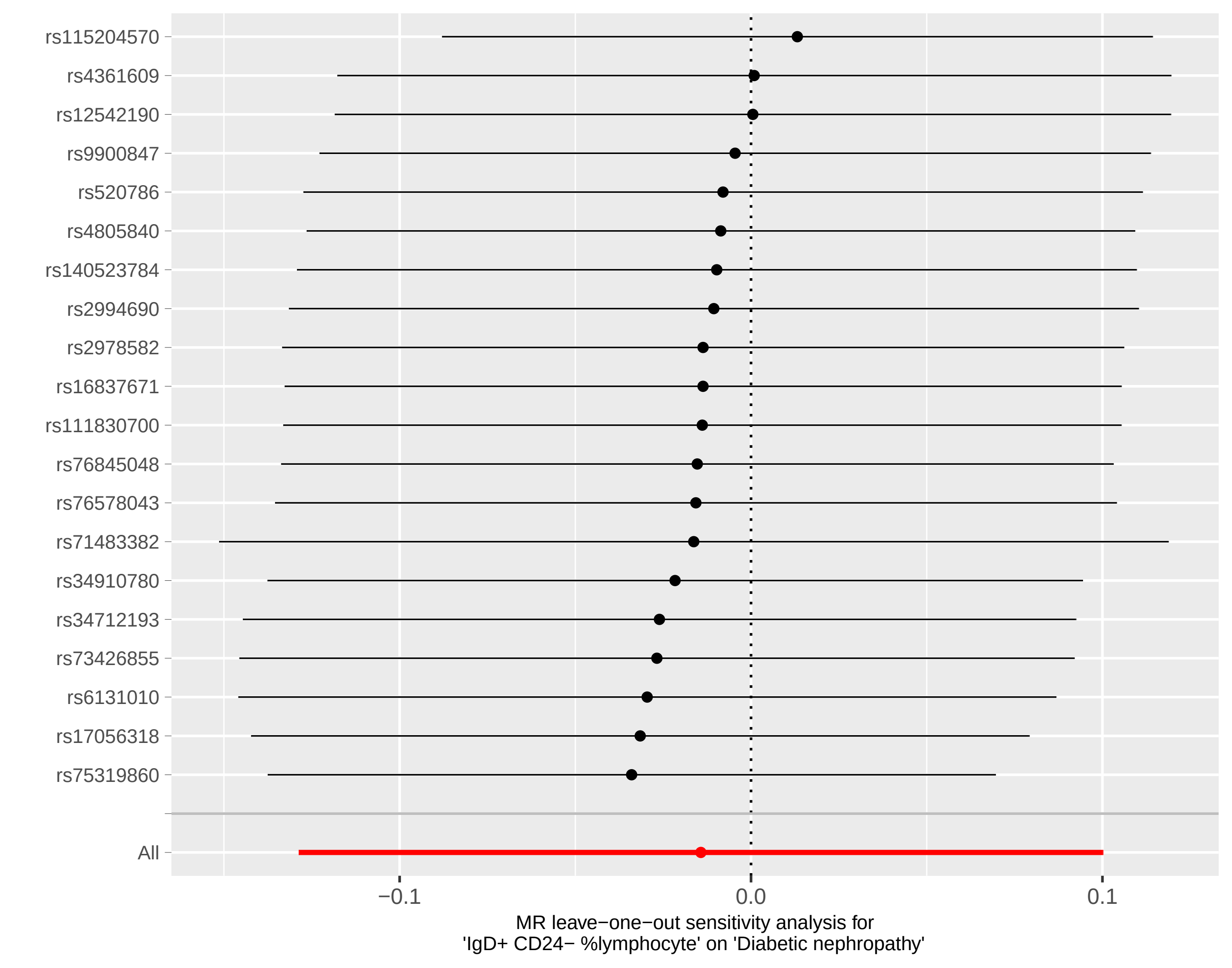

rs115204570
rs4361609
rs12542190
rs9900847
rs520786
rs4805840
rs140523784
rs2994690
rs2978582
rs16837671
rs111830700
rs76845048
rs76578043
rs71483382
rs34910780
rs34712193
rs73426855
rs6131010
rs17056318
rs75319860
All
−0.1
0.0
0.1
MR leave−one−out sensitivity analysis for
'IgD+ CD24− %lymphocyte' on 'Diabetic nephropathy'

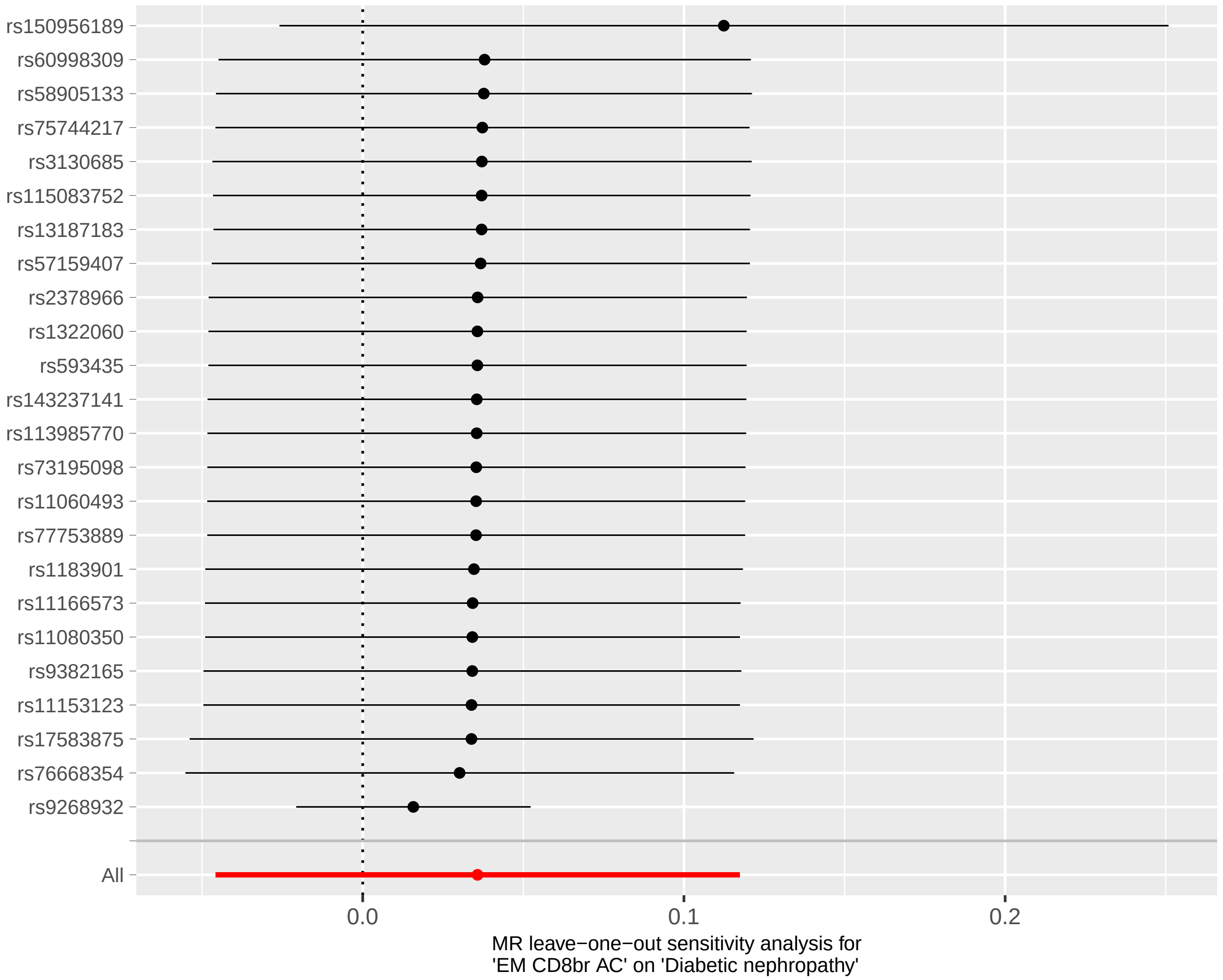
rs150956189
rs60998309
rs58905133
rs75744217
rs3130685
rs115083752
rs13187183
rs57159407
rs2378966
rs1322060
rs593435
rs143237141
rs113985770
rs73195098
rs11060493
rs77753889
rs1183901
rs11166573
rs11080350
rs9382165
rs11153123
rs17583875
rs76668354
rs9268932
All
0.0
0.1
0.2
MR leave−one−out sensitivity analysis for
'EM CD8br AC' on 'Diabetic nephropathy'

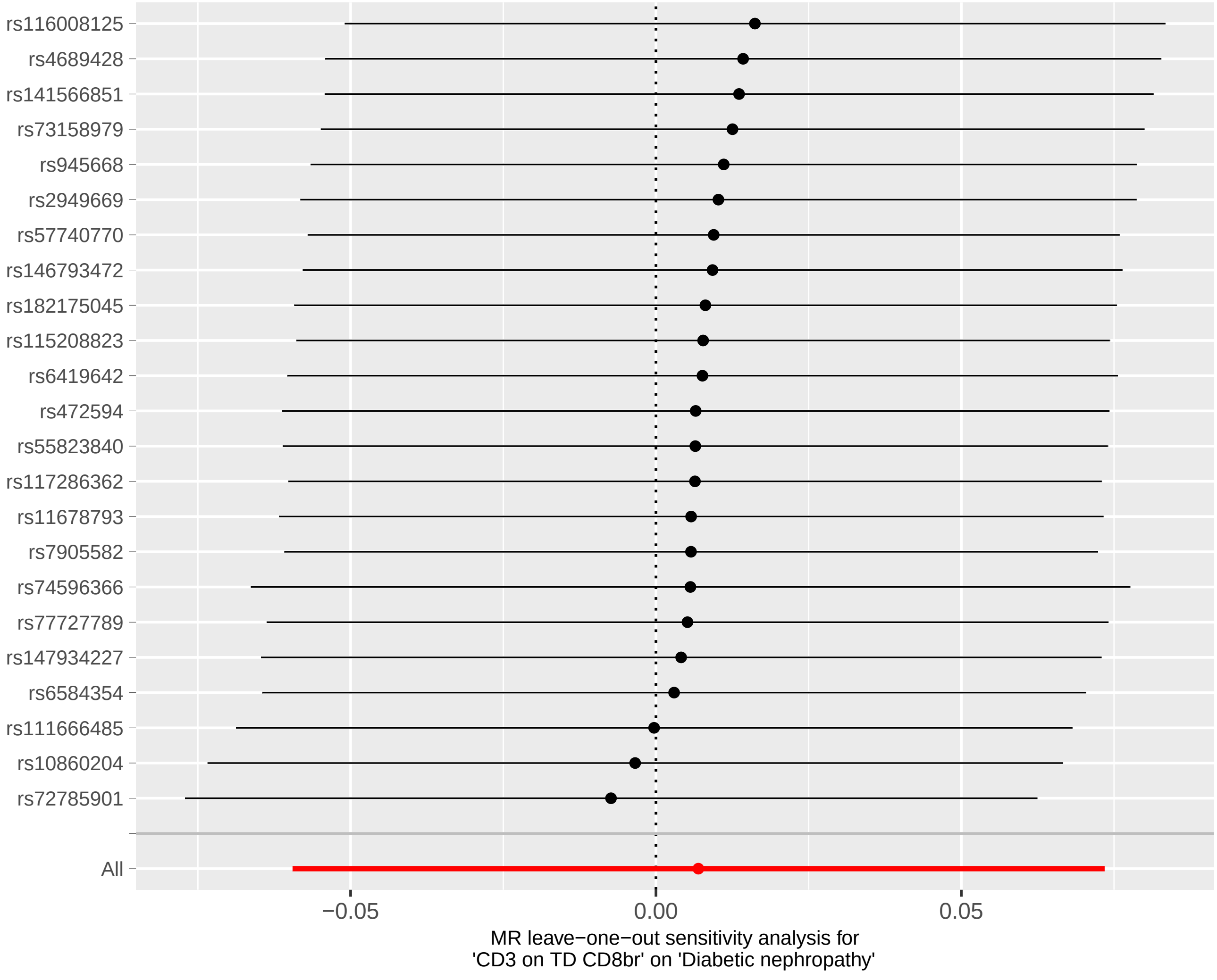
rs116008125
rs4689428
rs141566851
rs73158979
rs945668
rs2949669
rs57740770
rs146793472
rs182175045
rs115208823
rs6419642
rs472594
rs55823840
rs117286362
rs11678793
rs7905582
rs74596366
rs77727789
rs147934227
rs6584354
rs111666485
rs10860204
rs72785901
All
−0.05
0.00
0.05
MR leave−one−out sensitivity analysis for
'CD3 on TD CD8br' on 'Diabetic nephropathy'

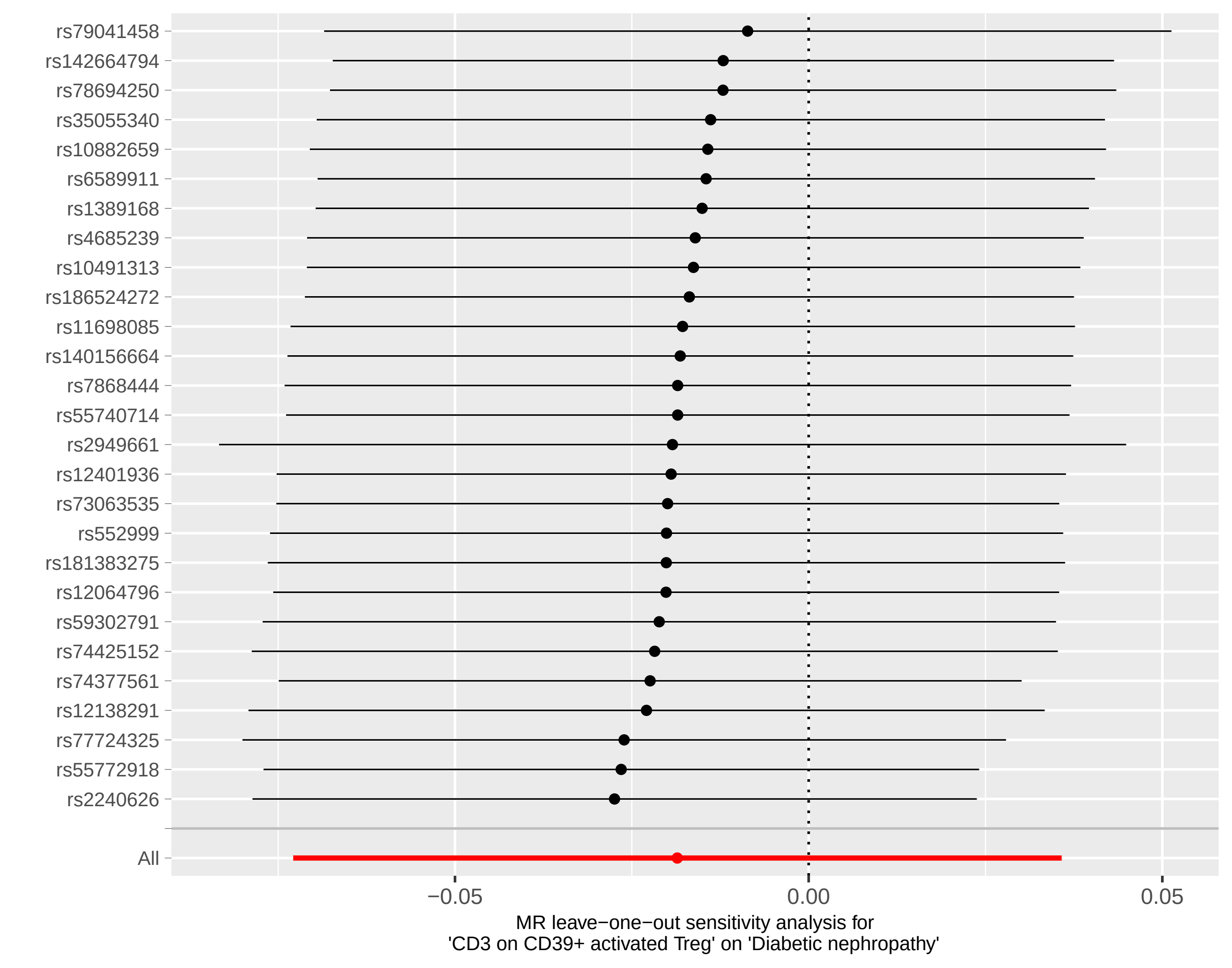
rs79041458
rs142664794
rs78694250
rs35055340
rs10882659
rs6589911
rs1389168
rs4685239
rs10491313
rs186524272
rs11698085
rs140156664
rs7868444
rs55740714
rs2949661
rs12401936
rs73063535
rs552999
rs181383275
rs12064796
rs59302791
rs74425152
rs74377561
rs12138291
rs77724325
rs55772918
rs2240626
All
−0.05
0.00
0.05
MR leave−one−out sensitivity analysis for
'CD3 on CD39+ activated Treg' on 'Diabetic nephropathy'

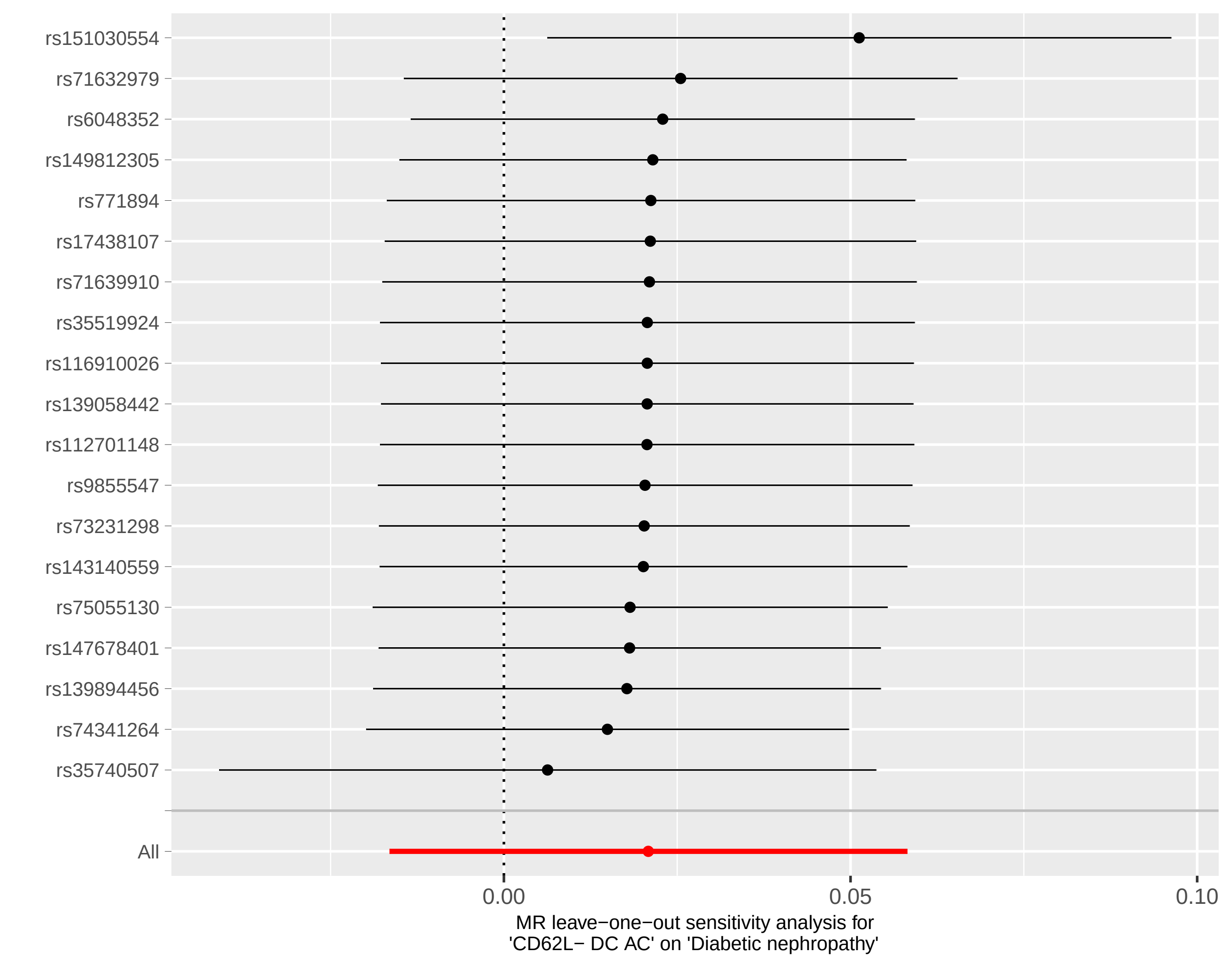
rs151030554
rs71632979
rs6048352
rs149812305
rs771894
rs17438107
rs71639910
rs35519924
rs116910026
rs139058442
rs112701148
rs9855547
rs73231298
rs143140559
rs75055130
rs147678401
rs139894456
rs74341264
rs35740507
All
0.00
0.05
0.10
MR leave−one−out sensitivity analysis for
'CD62L− DC AC' on 'Diabetic nephropathy'

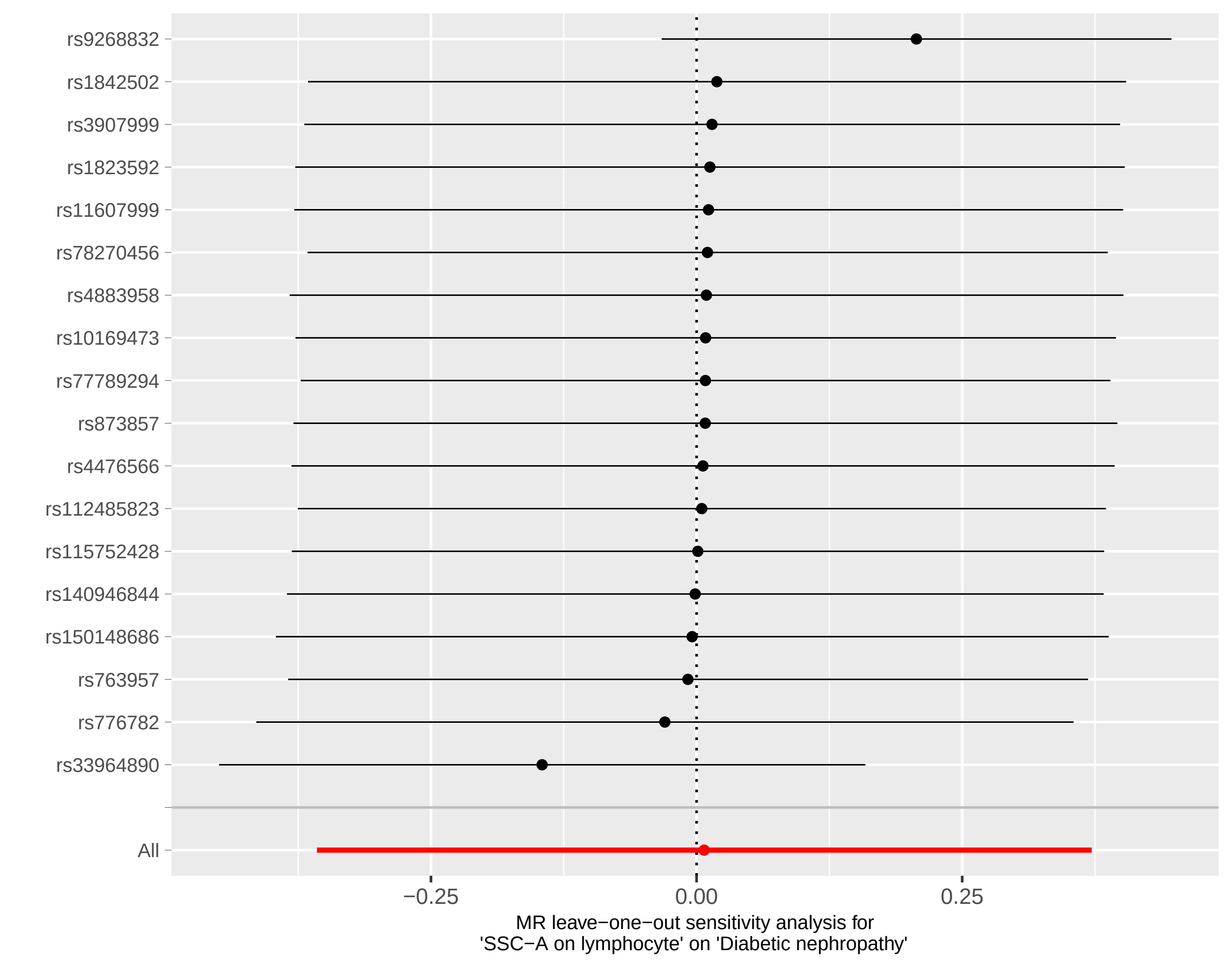
rs9268832
rs1842502
rs3907999
rs1823592
rs11607999
rs78270456
rs4883958
rs10169473
rs77789294
rs873857
rs4476566
rs112485823
rs115752428
rs140946844
rs150148686
rs763957
rs776782
rs33964890
All
−0.25
0.00
0.25
MR leave−one−out sensitivity analysis for
'SSC−A on lymphocyte' on 'Diabetic nephropathy'

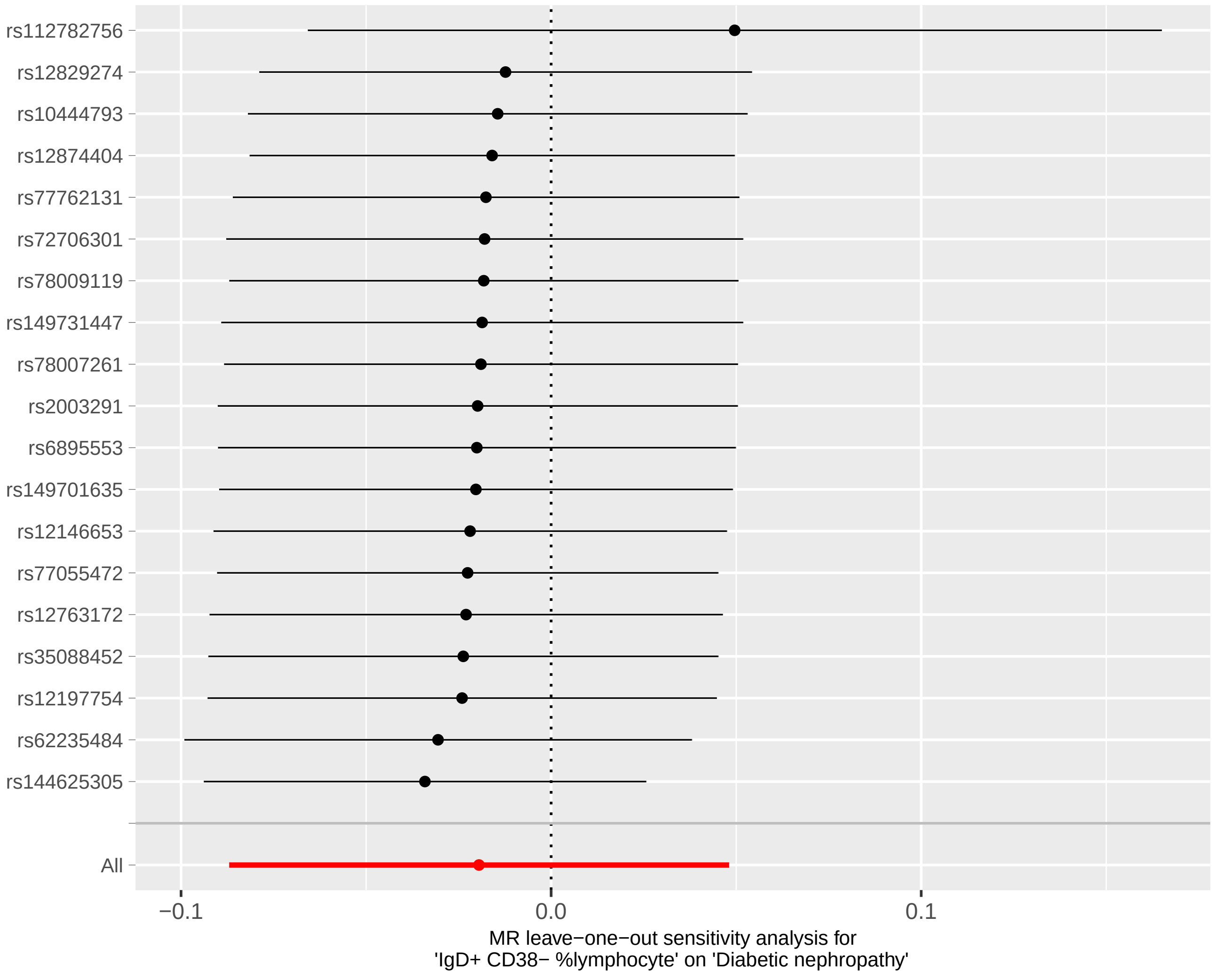

rs112782756
rs12829274
rs10444793
rs12874404
rs77762131
rs72706301
rs78009119
rs149731447
rs78007261
rs2003291
rs6895553
rs149701635
rs12146653
rs77055472
rs12763172
rs35088452
rs12197754
rs62235484
rs144625305
All
−0.1
0.0
0.1
MR leave−one−out sensitivity analysis for
'IgD+ CD38− %lymphocyte' on 'Diabetic nephropathy'

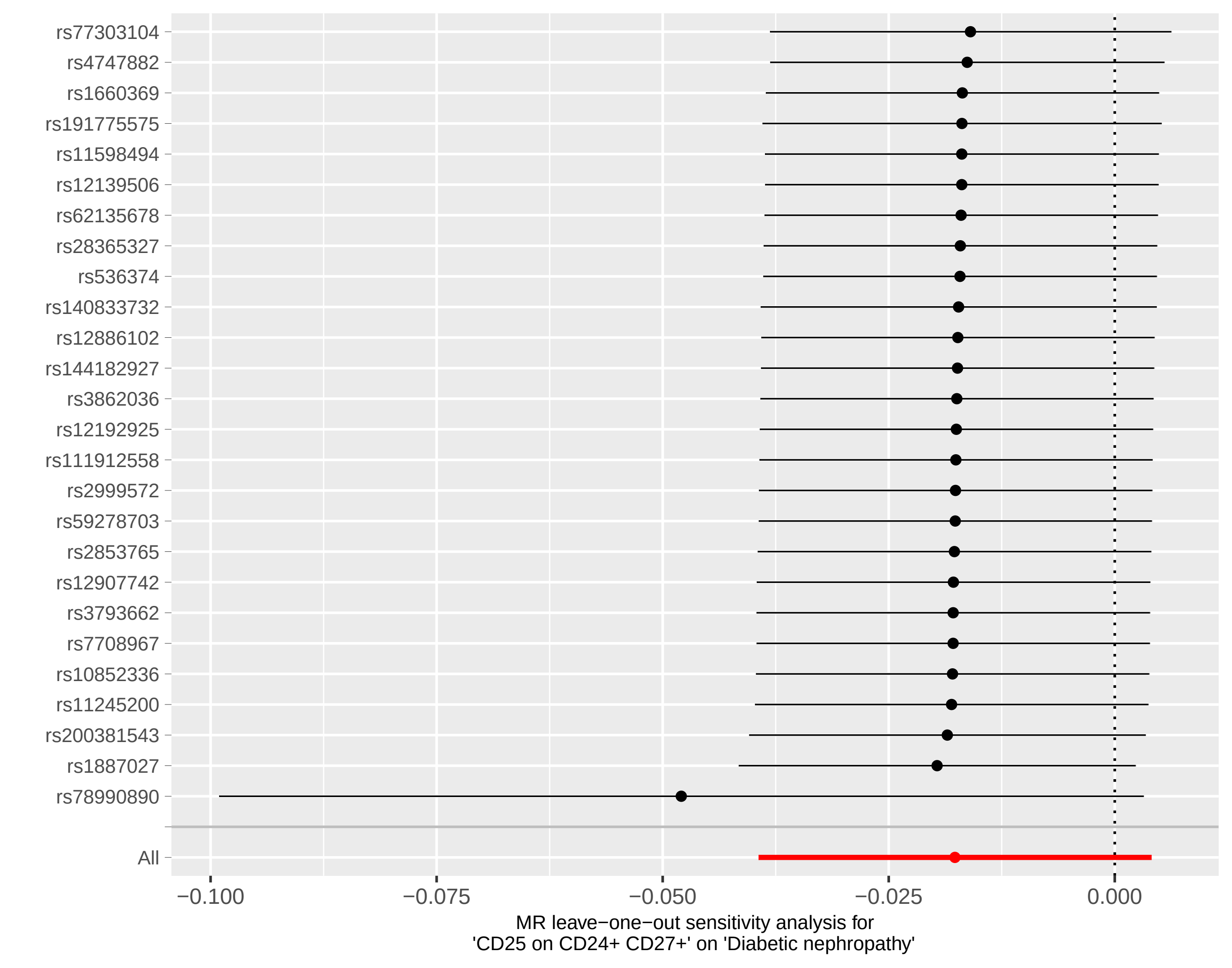

rs77303104
rs4747882
rs1660369
rs191775575
rs11598494
rs12139506
rs62135678
rs28365327
rs536374
rs140833732
rs12886102
rs144182927
rs3862036
rs12192925
rs111912558
rs2999572
rs59278703
rs2853765
rs12907742
rs3793662
rs7708967
rs10852336
rs11245200
rs200381543
rs1887027
rs78990890
All
−0.100
−0.075
−0.050
−0.025
0.000
MR leave−one−out sensitivity analysis for
'CD25 on CD24+ CD27+' on 'Diabetic nephropathy'

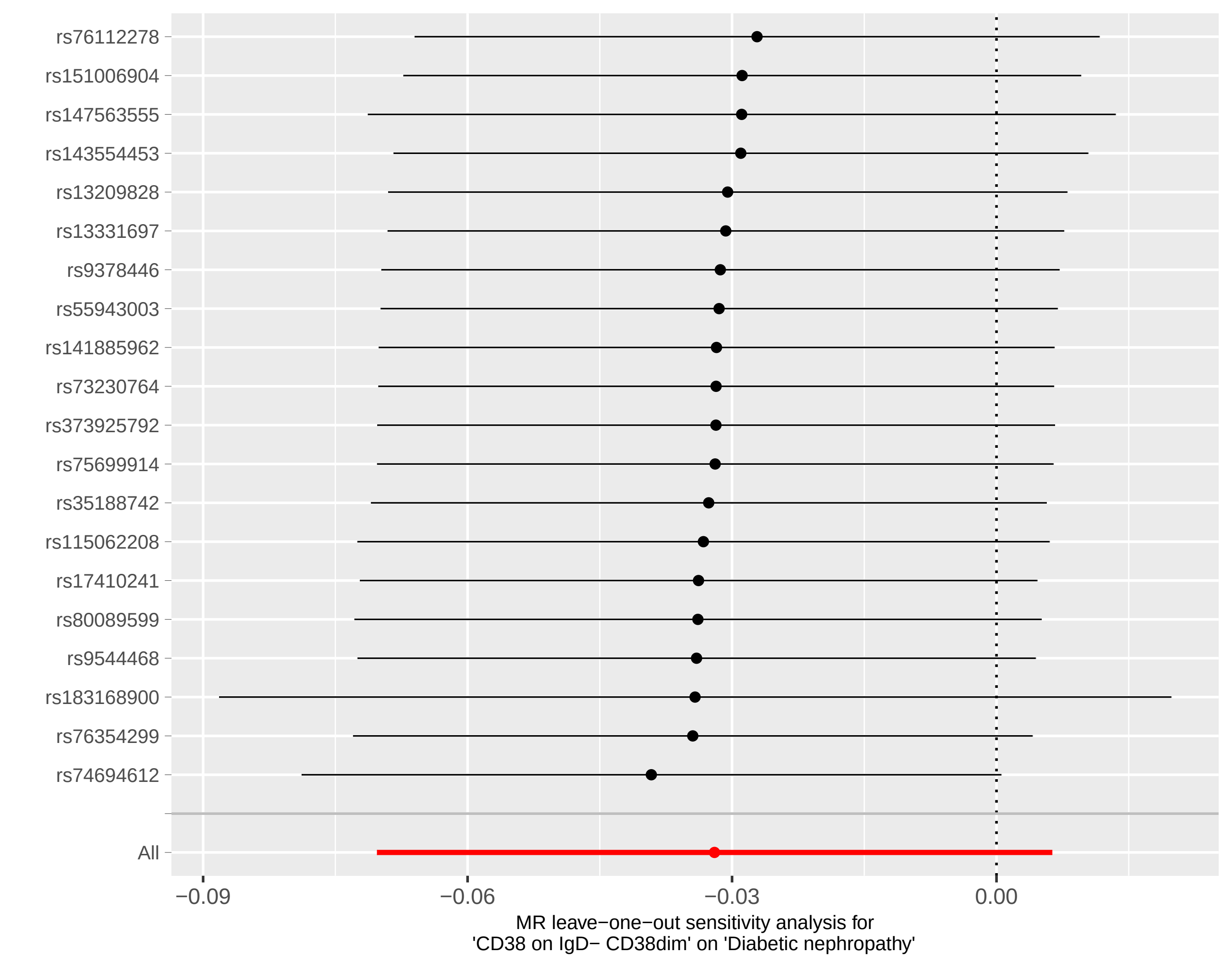
rs76112278
rs151006904
rs147563555
rs143554453
rs13209828
rs13331697
rs9378446
rs55943003
rs141885962
rs73230764
rs373925792
rs75699914
rs35188742
rs115062208
rs17410241
rs80089599
rs9544468
rs183168900
rs76354299
rs74694612
All
−0.09
−0.06
−0.03
0.00
MR leave−one−out sensitivity analysis for
'CD38 on IgD− CD38dim' on 'Diabetic nephropathy'

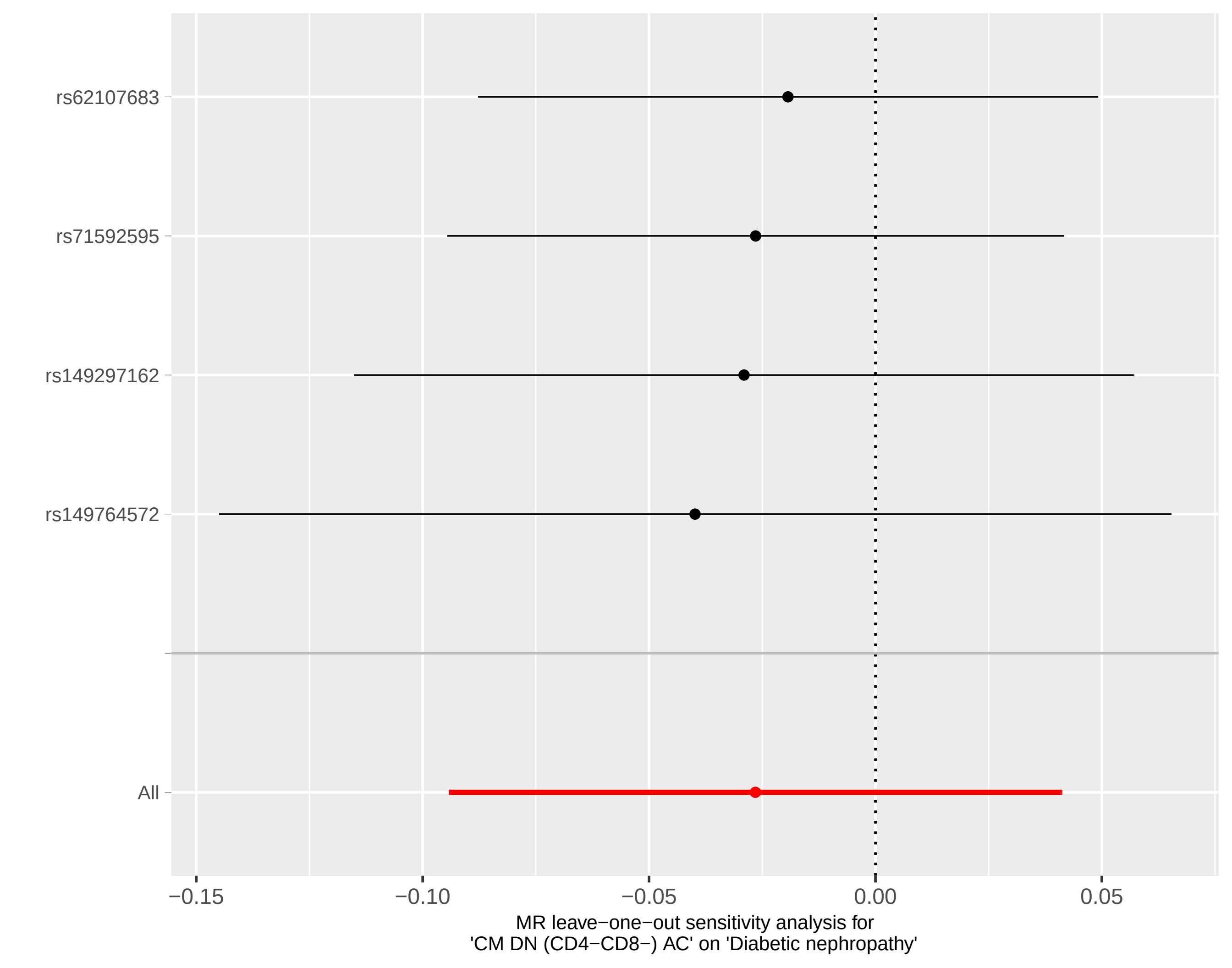
rs62107683
rs71592595
rs149297162
rs149764572
All
−0.15
−0.10
−0.05
0.00
0.05
MR leave−one−out sensitivity analysis for
'CM DN (CD4−CD8−) AC' on 'Diabetic nephropathy'

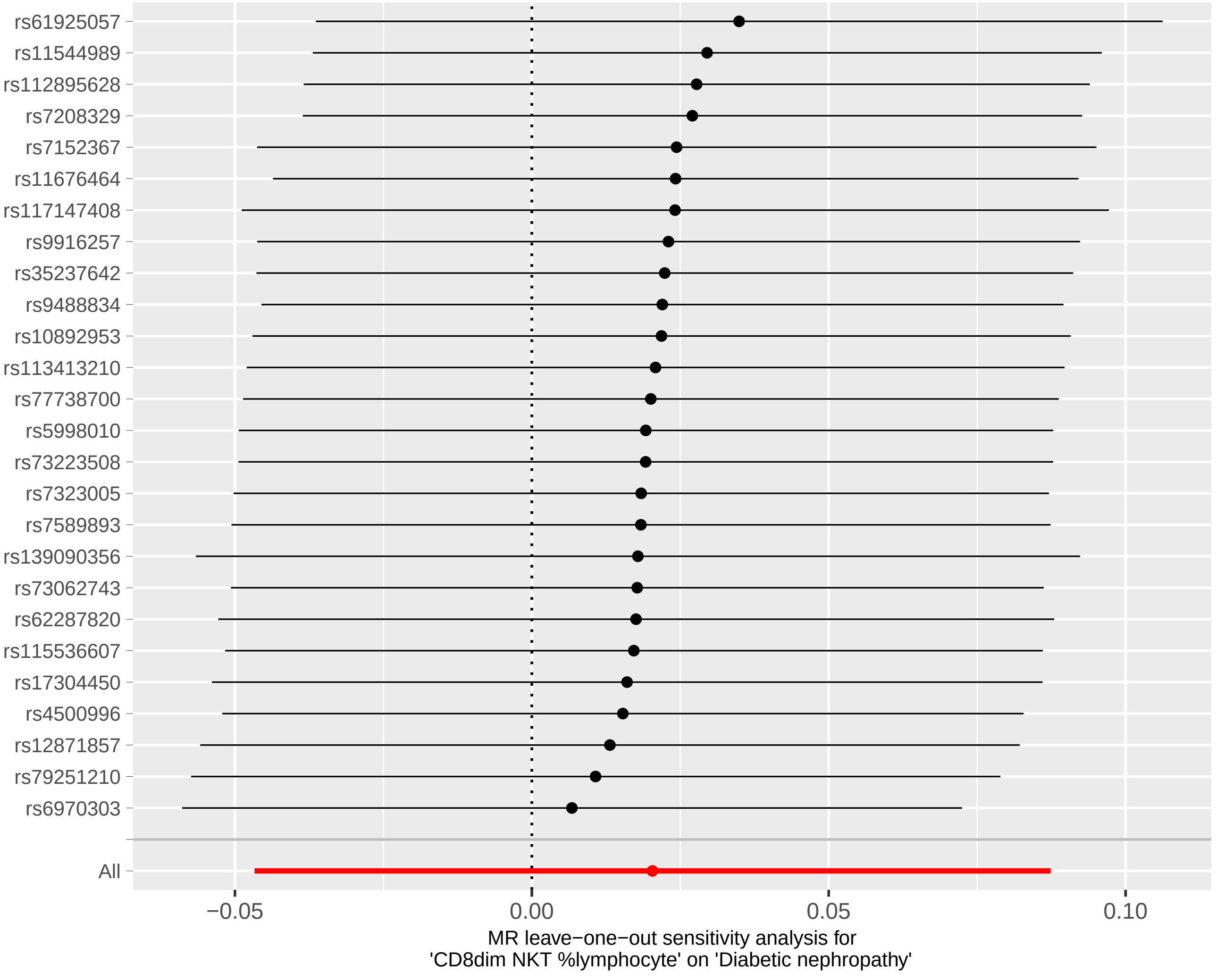
rs61925057
rs11544989
rs112895628
rs7208329
rs7152367
rs11676464
rs117147408
rs9916257
rs35237642
rs9488834
rs10892953
rs113413210
rs77738700
rs5998010
rs73223508
rs7323005
rs7589893
rs139090356
rs73062743
rs62287820
rs115536607
rs17304450
rs4500996
rs12871857
rs79251210
rs6970303
All
−0.05
0.00
0.05
0.10
MR leave−one−out sensitivity analysis for
'CD8dim NKT %lymphocyte' on 'Diabetic nephropathy'

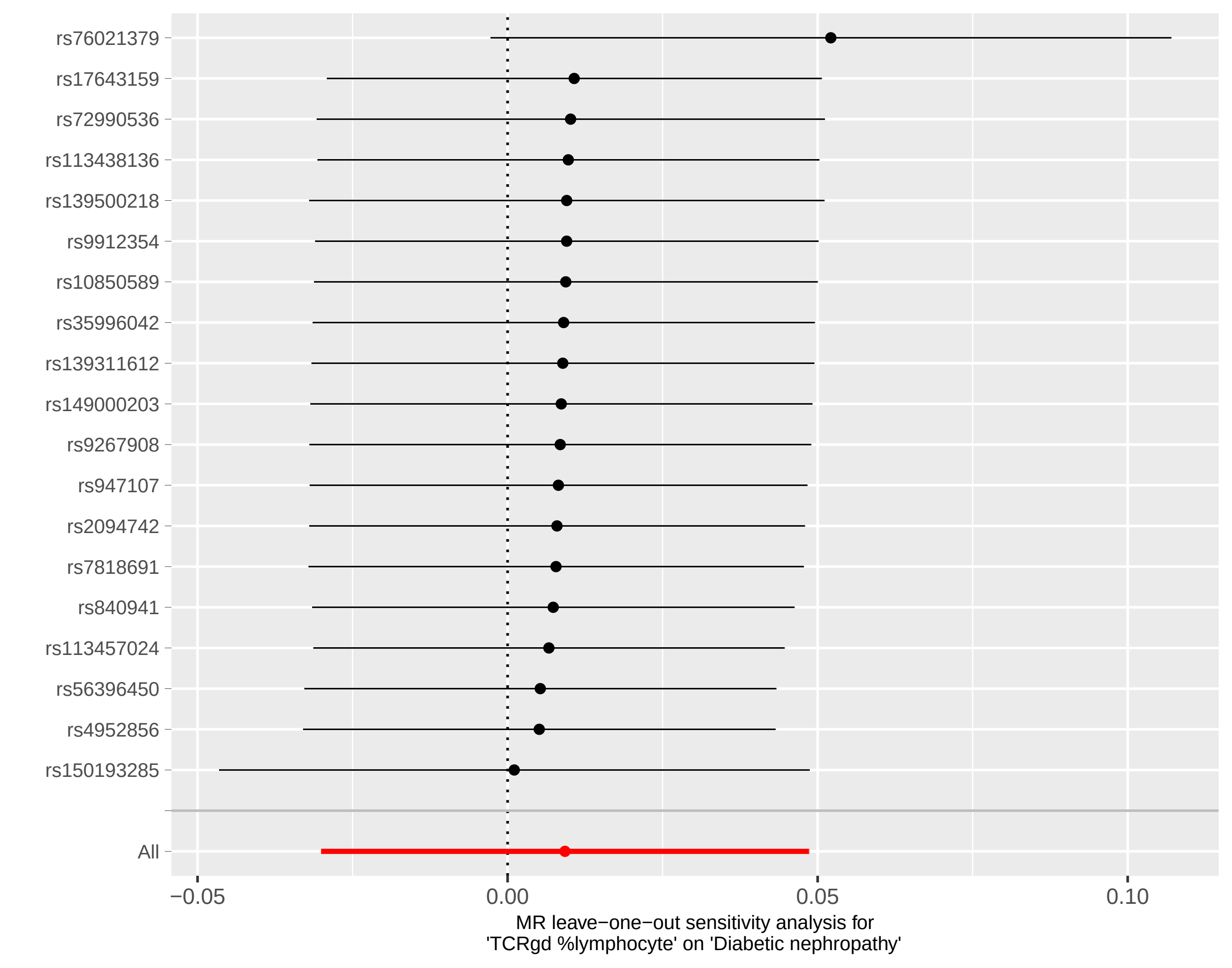

rs76021379
rs17643159
rs72990536
rs113438136
rs139500218
rs9912354
rs10850589
rs35996042
rs139311612
rs149000203
rs9267908
rs947107
rs2094742
rs7818691
rs840941
rs113457024
rs56396450
rs4952856
rs150193285
All
−0.05
0.00
0.05
0.10
MR leave−one−out sensitivity analysis for
'TCRgd %lymphocyte' on 'Diabetic nephropathy'

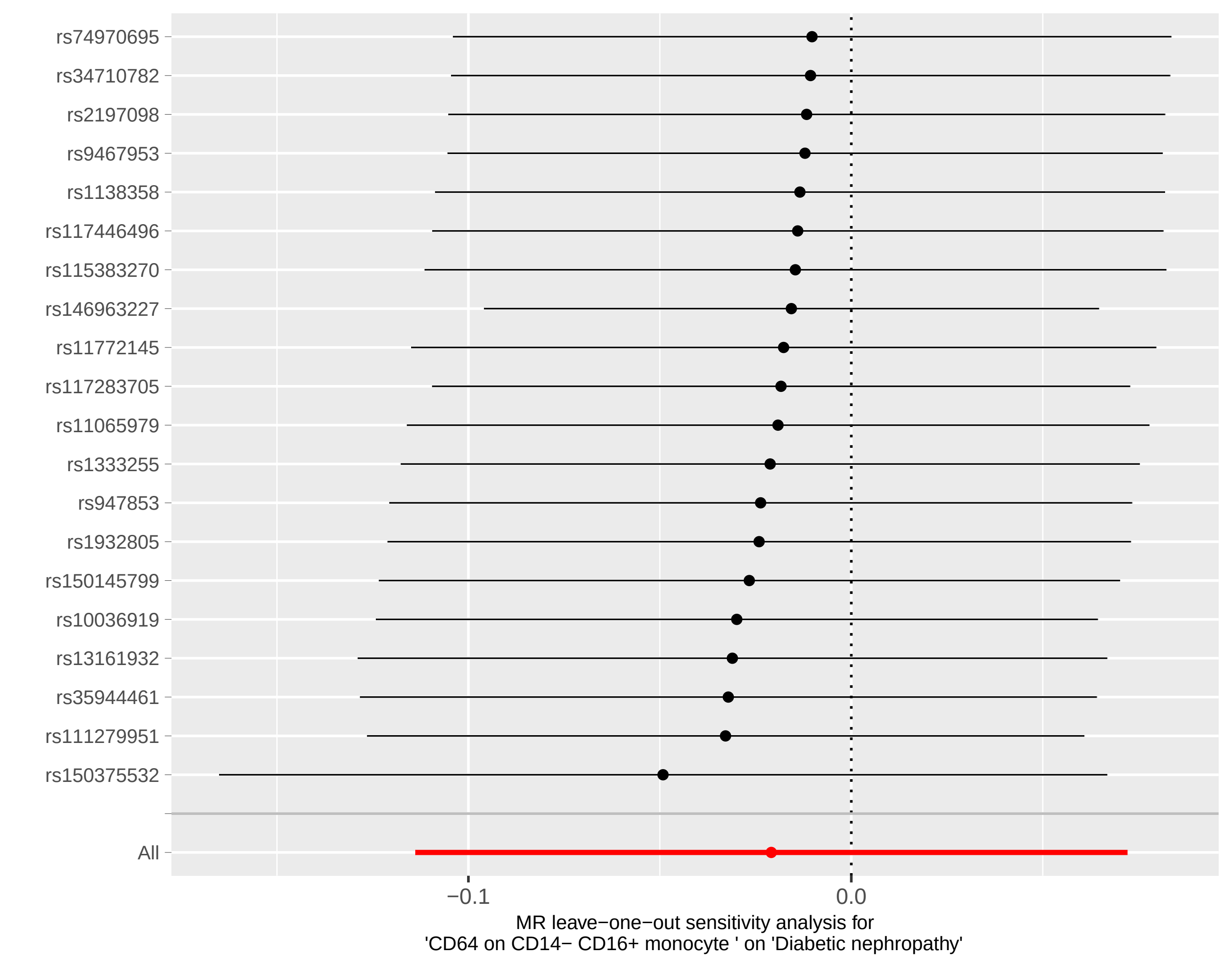
rs74970695
rs34710782
rs2197098
rs9467953
rs1138358
rs117446496
rs115383270
rs146963227
rs11772145
rs117283705
rs11065979
rs1333255
rs947853
rs1932805
rs150145799
rs10036919
rs13161932
rs35944461
rs111279951
rs150375532
All
−0.1
0.0
MR leave−one−out sensitivity analysis for
'CD64 on CD14− CD16+ monocyte ' on 'Diabetic nephropathy'

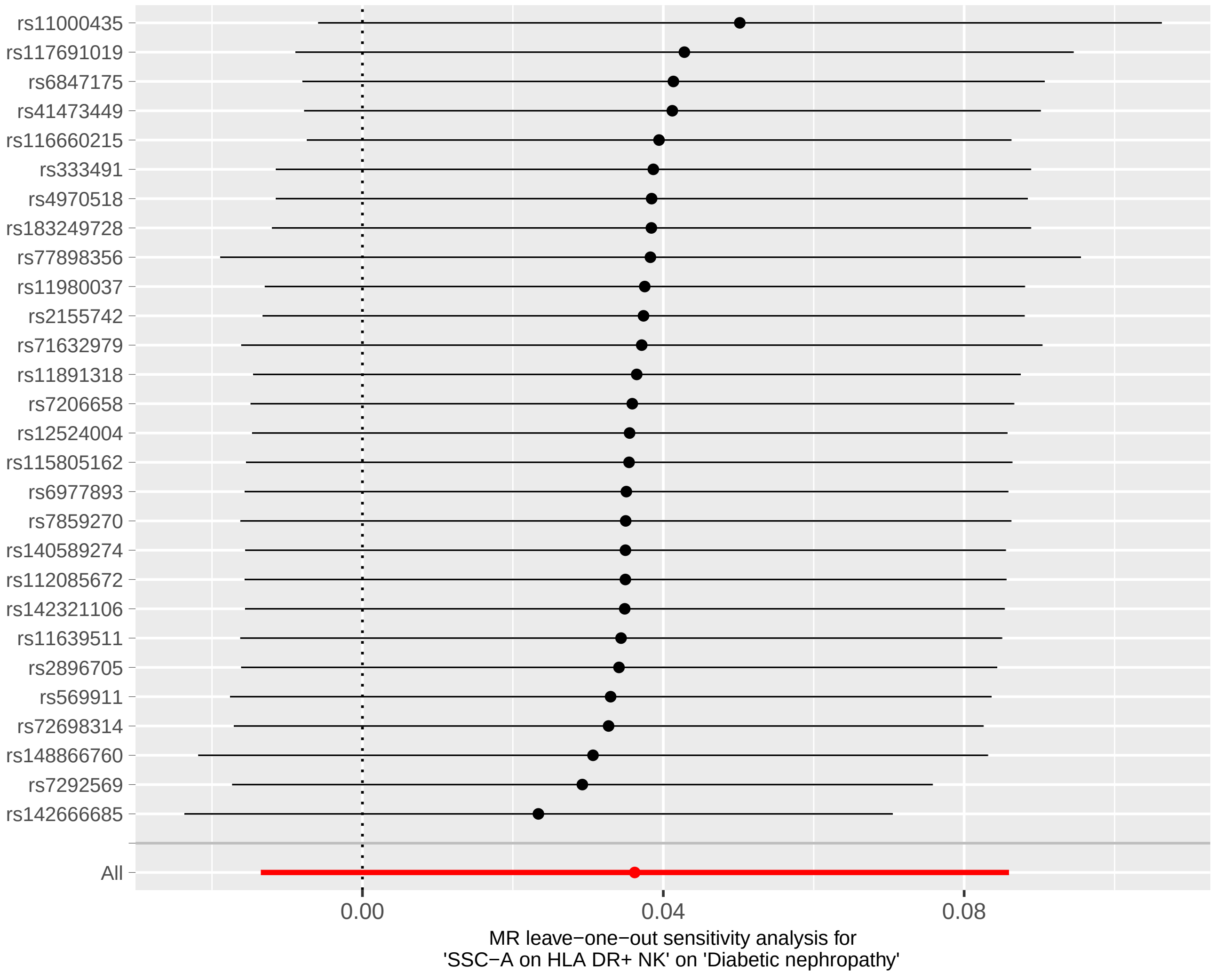
rs11000435
rs117691019
rs6847175
rs41473449
rs116660215
rs333491
rs4970518
rs183249728
rs77898356
rs11980037
rs2155742
rs71632979
rs11891318
rs7206658
rs12524004
rs115805162
rs6977893
rs7859270
rs140589274
rs112085672
rs142321106
rs11639511
rs2896705
rs569911
rs72698314
rs148866760
rs7292569
rs142666685
All
0.00
0.04
0.08
MR leave−one−out sensitivity analysis for
'SSC−A on HLA DR+ NK' on 'Diabetic nephropathy'

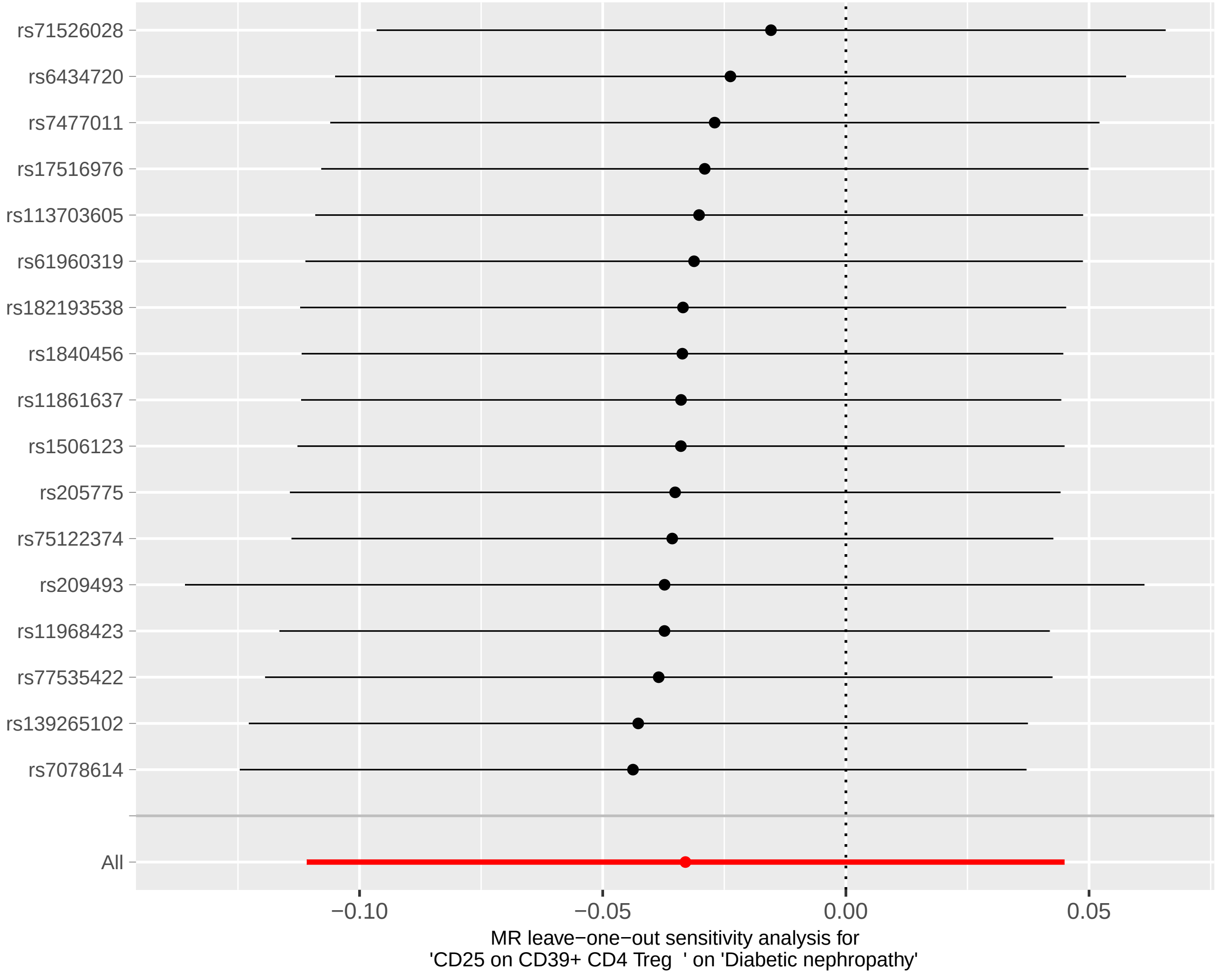

rs71526028
rs6434720
rs7477011
rs17516976
rs113703605
rs61960319
rs182193538
rs1840456
rs11861637
rs1506123
rs205775
rs75122374
rs209493
rs11968423
rs77535422
rs139265102
rs7078614
All
−0.10
−0.05
0.00
0.05
MR leave−one−out sensitivity analysis for
'CD25 on CD39+ CD4 Treg ' on 'Diabetic nephropathy'

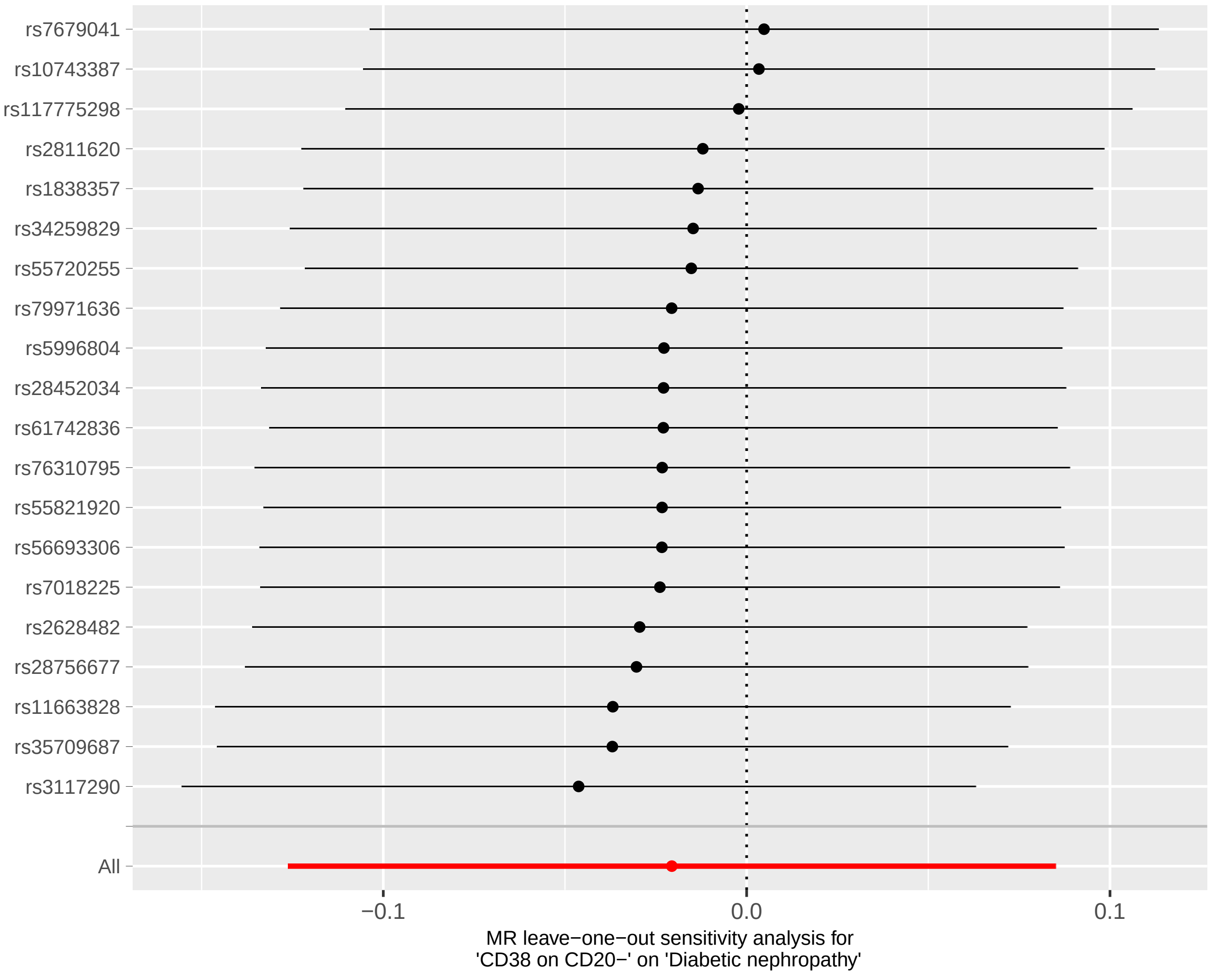

rs7679041
rs10743387
rs117775298
rs2811620
rs1838357
rs34259829
rs55720255
rs79971636
rs5996804
rs28452034
rs61742836
rs76310795
rs55821920
rs56693306
rs7018225
rs2628482
rs28756677
rs11663828
rs35709687
rs3117290
All
−0.1
0.0
0.1
MR leave−one−out sensitivity analysis for
'CD38 on CD20−' on 'Diabetic nephropathy'

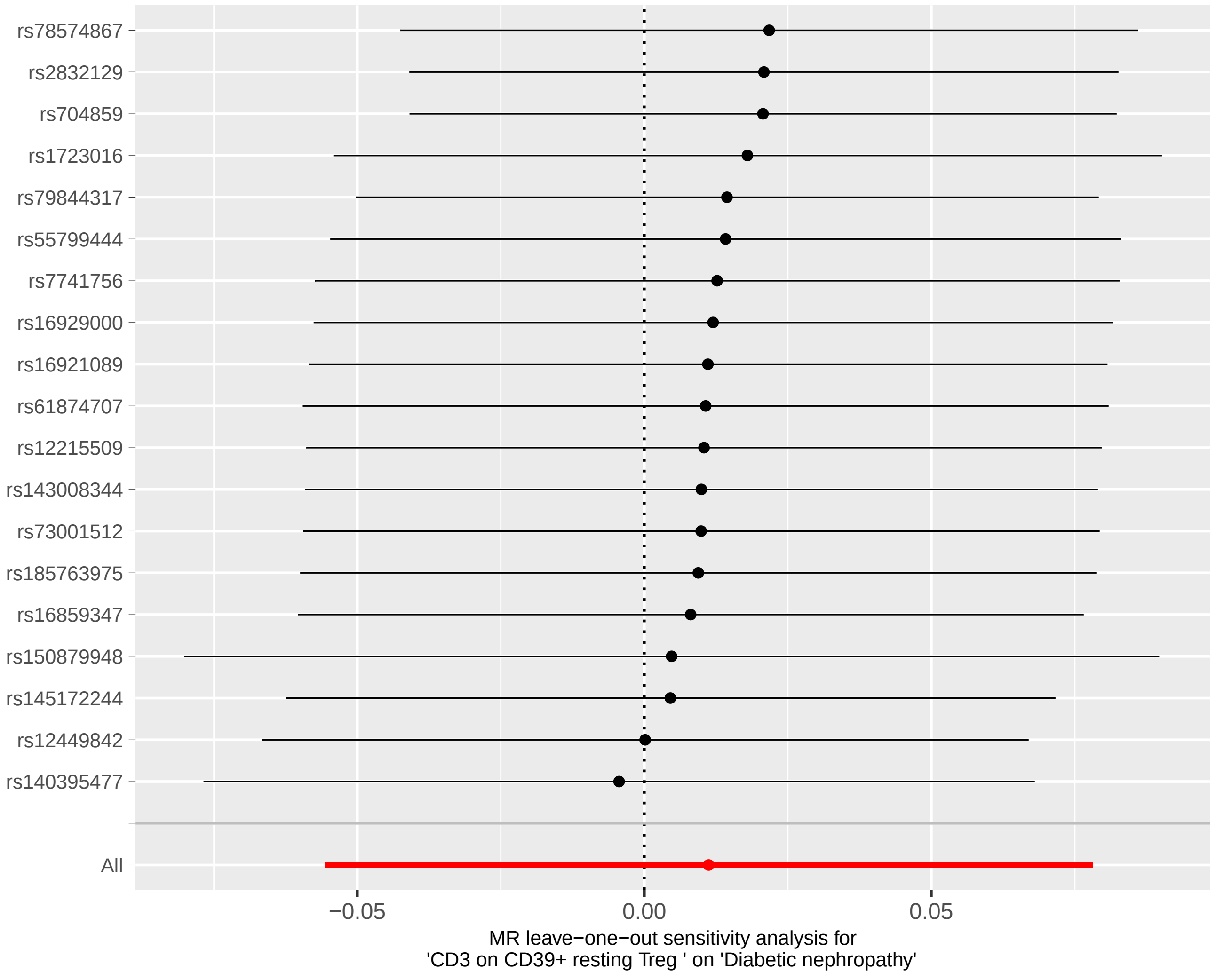

rs78574867
rs2832129
rs704859
rs1723016
rs79844317
rs55799444
rs7741756
rs16929000
rs16921089
rs61874707
rs12215509
rs143008344
rs73001512
rs185763975
rs16859347
rs150879948
rs145172244
rs12449842
rs140395477
All
−0.05
0.00
0.05
MR leave−one−out sensitivity analysis for
'CD3 on CD39+ resting Treg ' on 'Diabetic nephropathy'

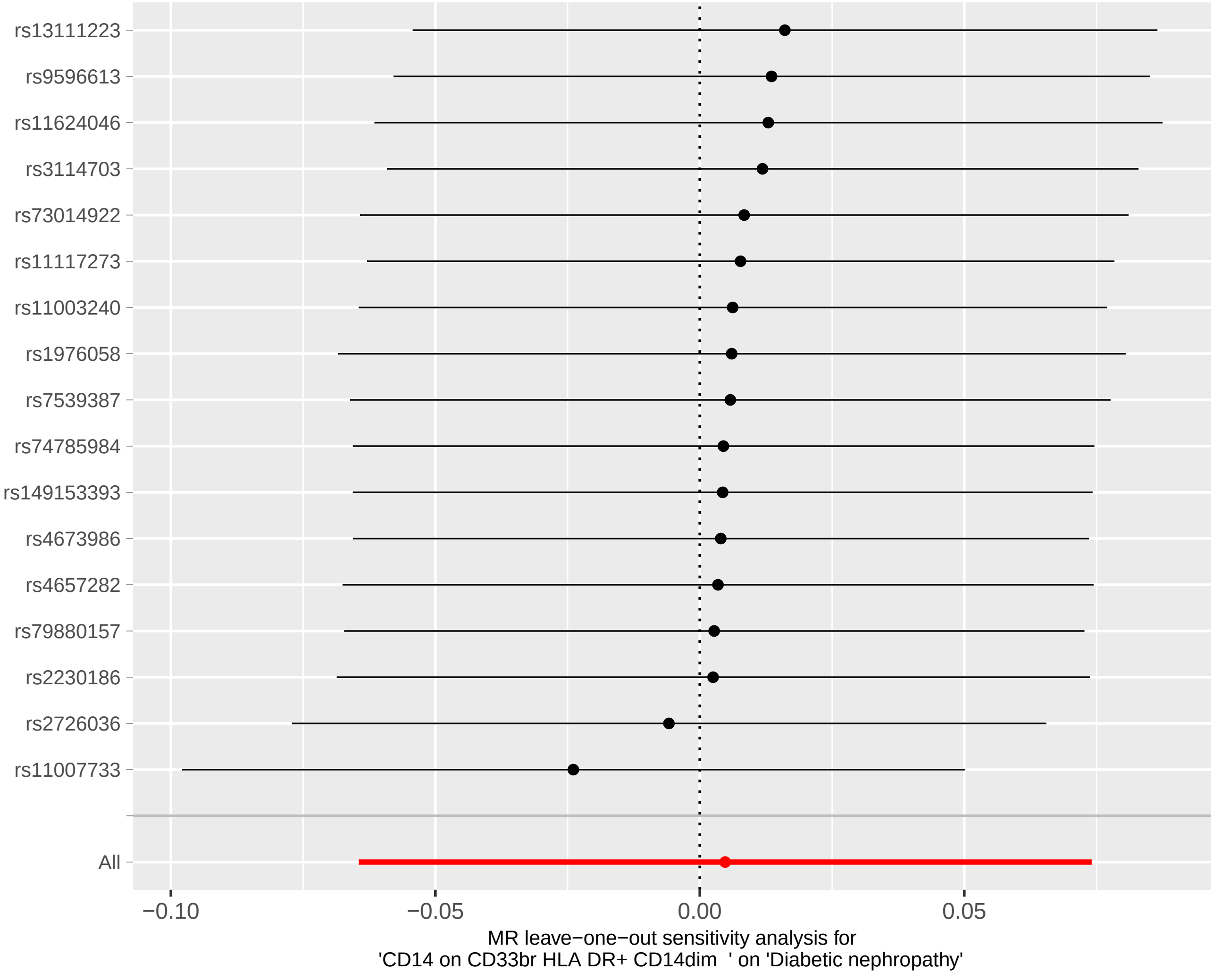
rs13111223
rs9596613
rs11624046
rs3114703
rs73014922
rs11117273
rs11003240
rs1976058
rs7539387
rs74785984
rs149153393
rs4673986
rs4657282
rs79880157
rs2230186
rs2726036
rs11007733
All
−0.10
−0.05
0.00
0.05
MR leave−one−out sensitivity analysis for
'CD14 on CD33br HLA DR+ CD14dim ' on 'Diabetic nephropathy'

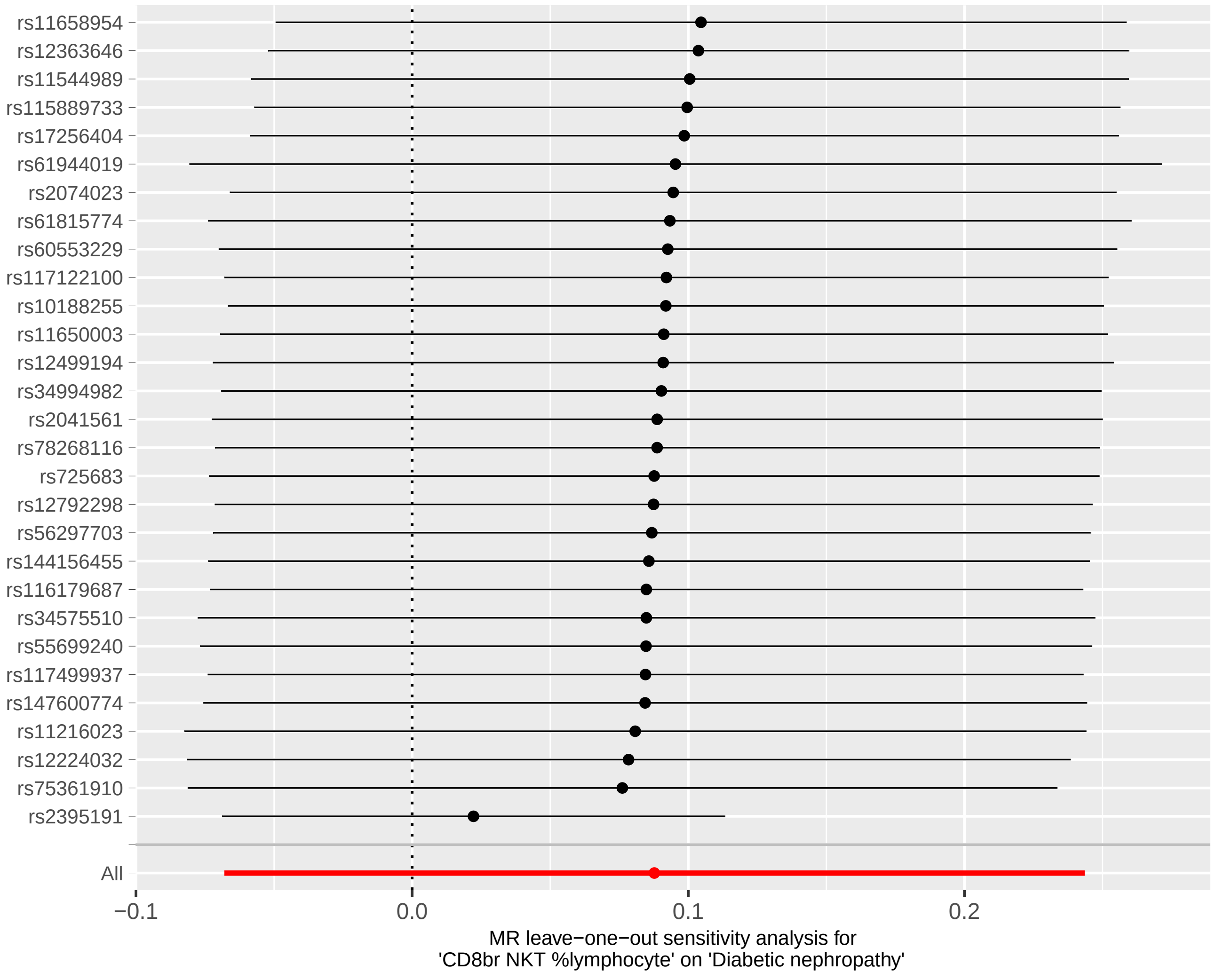

rs11658954
rs12363646
rs11544989
rs115889733
rs17256404
rs61944019
rs2074023
rs61815774
rs60553229
rs117122100
rs10188255
rs11650003
rs12499194
rs34994982
rs2041561
rs78268116
rs725683
rs12792298
rs56297703
rs144156455
rs116179687
rs34575510
rs55699240
rs117499937
rs147600774
rs11216023
rs12224032
rs75361910
rs2395191
All
−0.1
0.0
0.1
0.2
MR leave−one−out sensitivity analysis for
'CD8br NKT %lymphocyte' on 'Diabetic nephropathy'

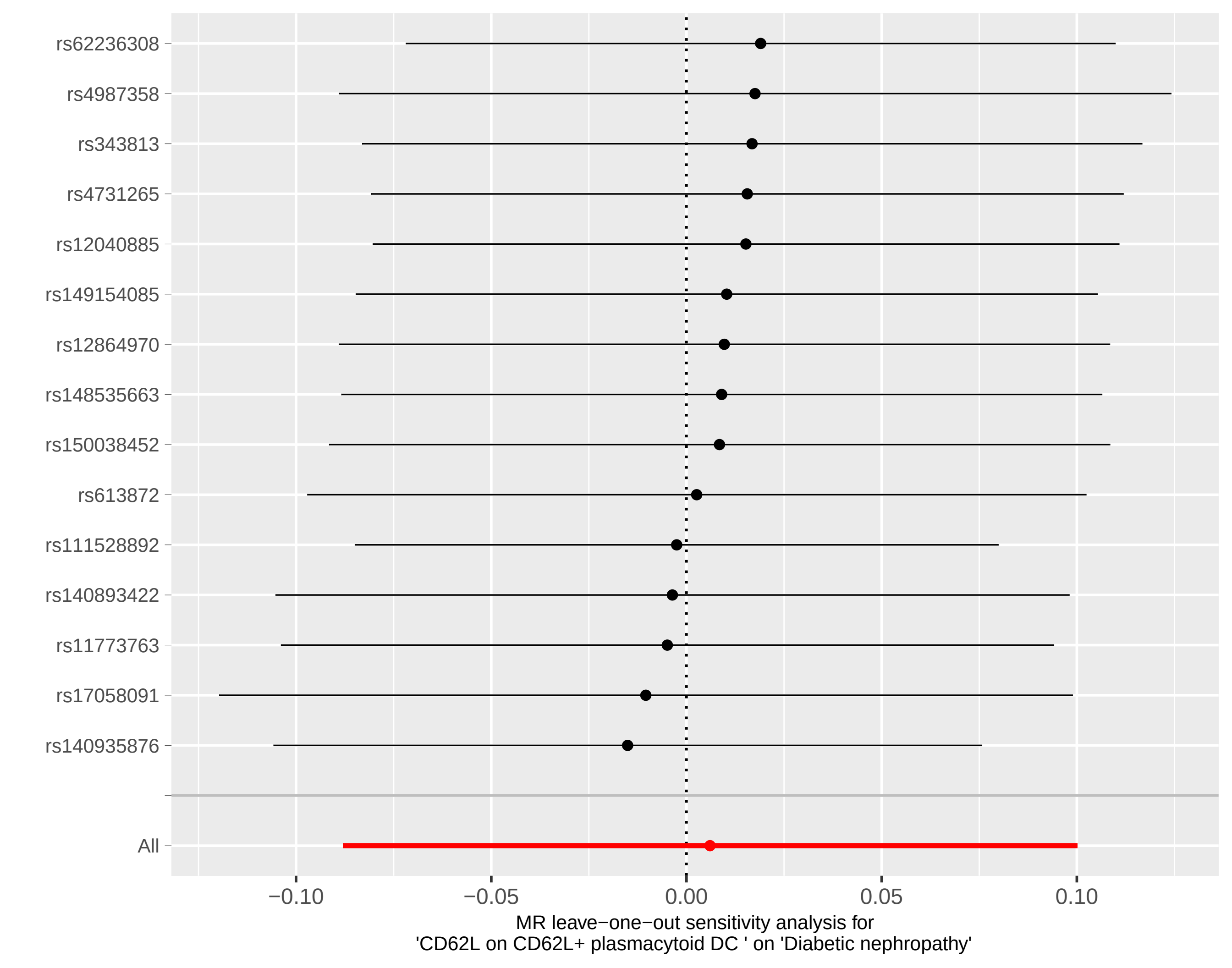
rs62236308
rs4987358
rs343813
rs4731265
rs12040885
rs149154085
rs12864970
rs148535663
rs150038452
rs613872
rs111528892
rs140893422
rs11773763
rs17058091
rs140935876
All
−0.10
−0.05
0.00
0.05
0.10
MR leave−one−out sensitivity analysis for
'CD62L on CD62L+ plasmacytoid DC ' on 'Diabetic nephropathy'

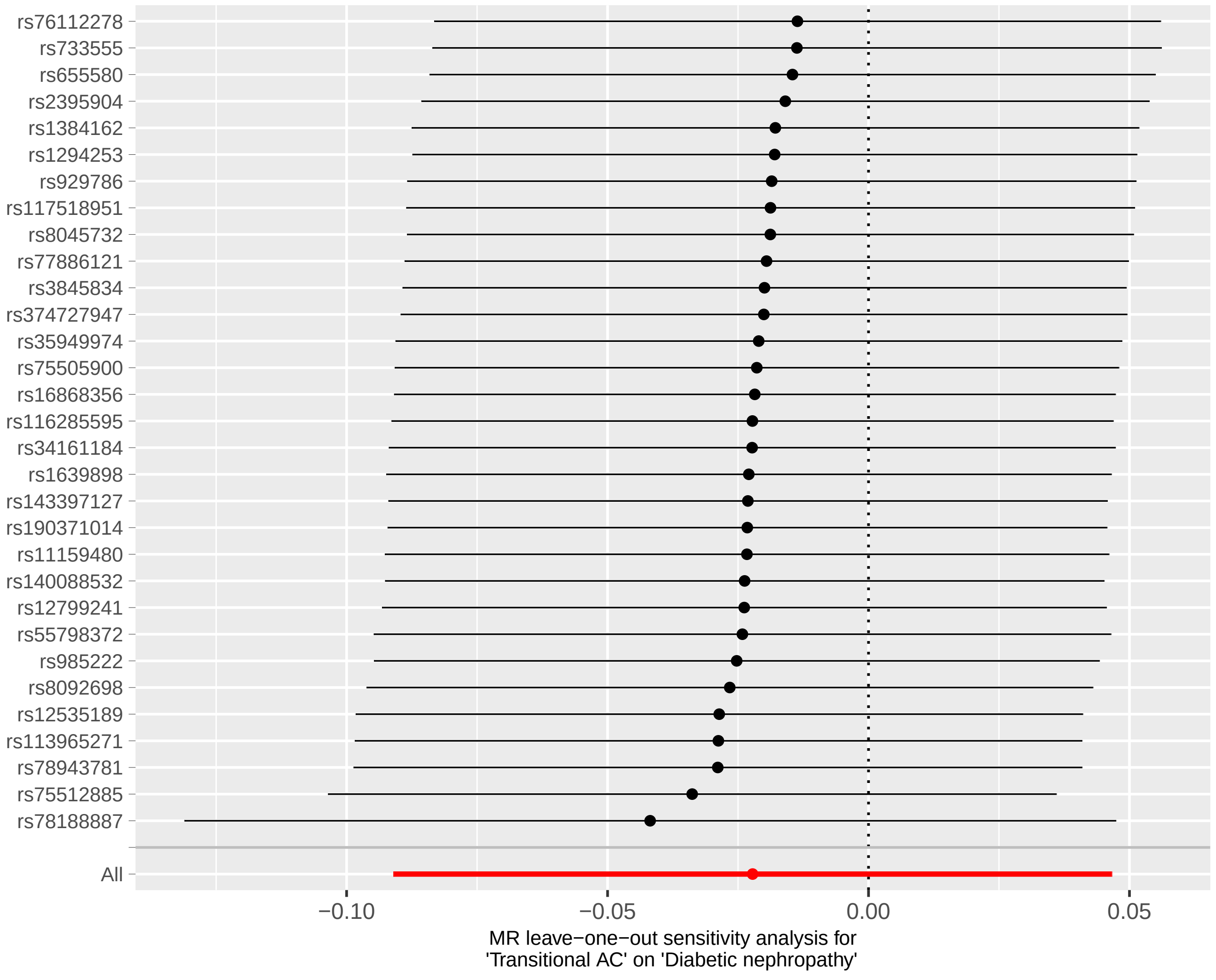
rs76112278
rs733555
rs655580
rs2395904
rs1384162
rs1294253
rs929786
rs117518951
rs8045732
rs77886121
rs3845834
rs374727947
rs35949974
rs75505900
rs16868356
rs116285595
rs34161184
rs1639898
rs143397127
rs190371014
rs11159480
rs140088532
rs12799241
rs55798372
rs985222
rs8092698
rs12535189
rs113965271
rs78943781
rs75512885
rs78188887
All
−0.10
−0.05
0.00
0.05
MR leave−one−out sensitivity analysis for
'Transitional AC' on 'Diabetic nephropathy'

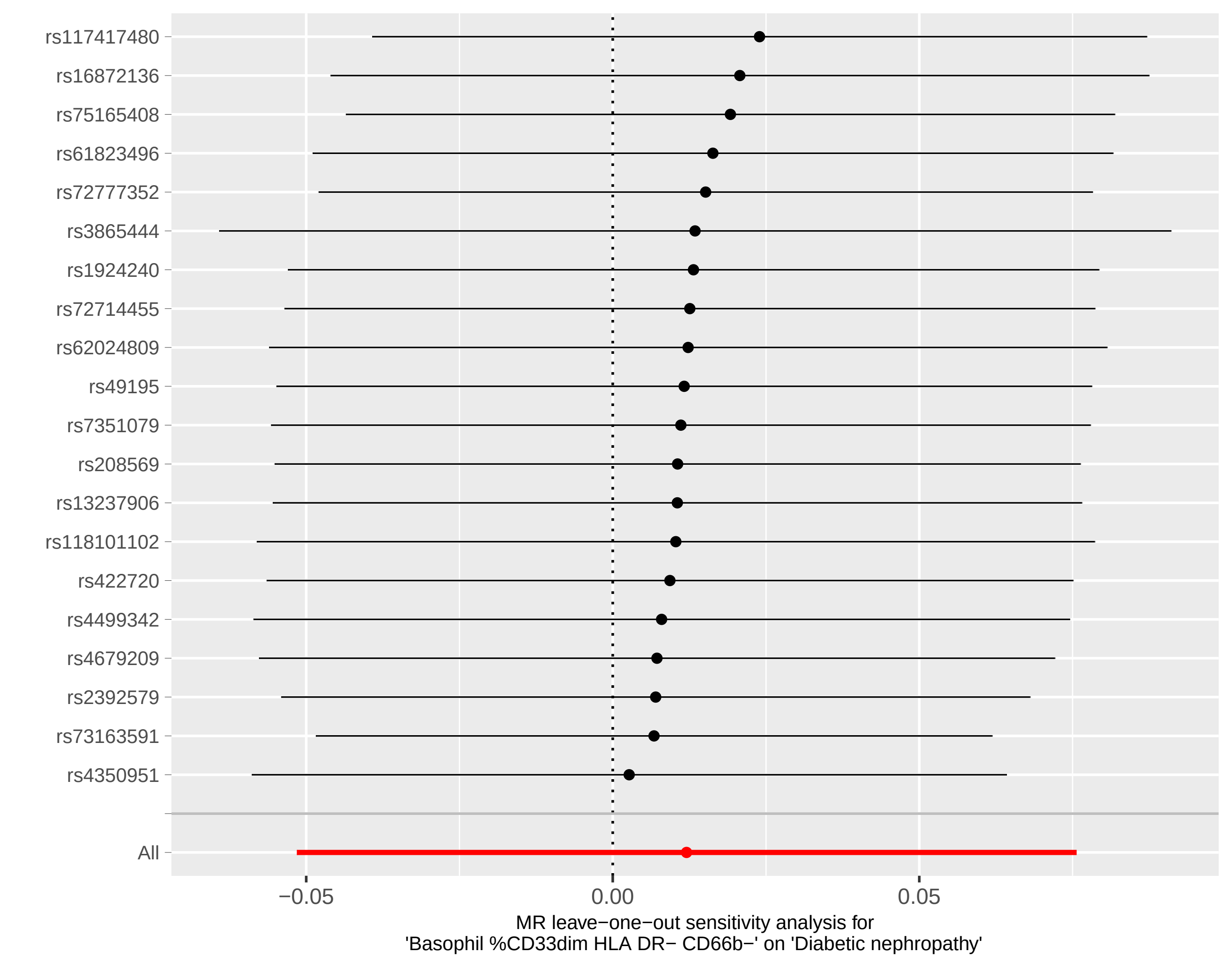
rs117417480
rs16872136
rs75165408
rs61823496
rs72777352
rs3865444
rs1924240
rs72714455
rs62024809
rs49195
rs7351079
rs208569
rs13237906
rs118101102
rs422720
rs4499342
rs4679209
rs2392579
rs73163591
rs4350951
All
−0.05
0.00
0.05
MR leave−one−out sensitivity analysis for
'Basophil %CD33dim HLA DR− CD66b−' on 'Diabetic nephropathy'

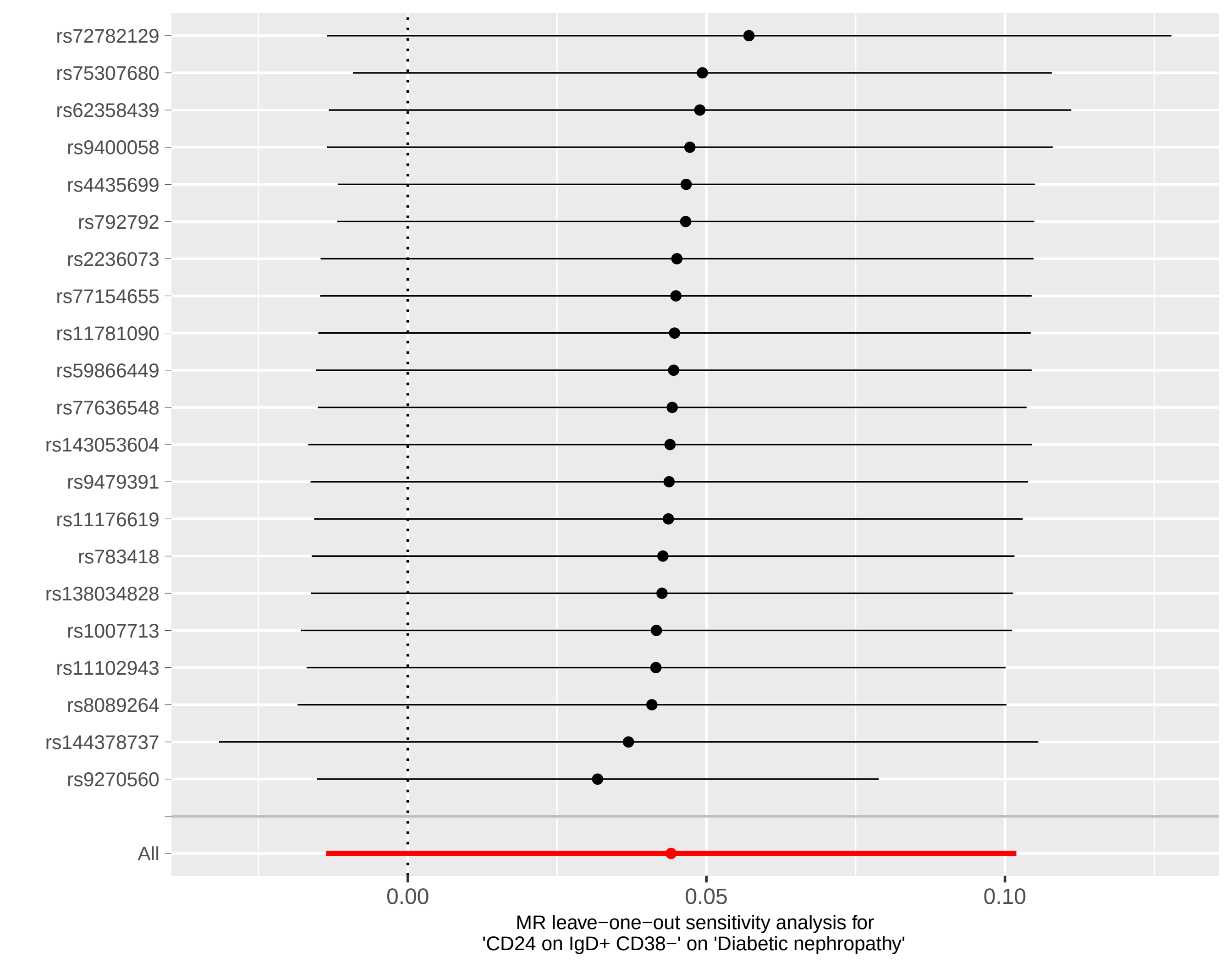
rs72782129
rs75307680
rs62358439
rs9400058
rs4435699
rs792792
rs2236073
rs77154655
rs11781090
rs59866449
rs77636548
rs143053604
rs9479391
rs11176619
rs783418
rs138034828
rs1007713
rs11102943
rs8089264
rs144378737
rs9270560
All
0.00
0.05
0.10
MR leave−one−out sensitivity analysis for
'CD24 on IgD+ CD38−' on 'Diabetic nephropathy'

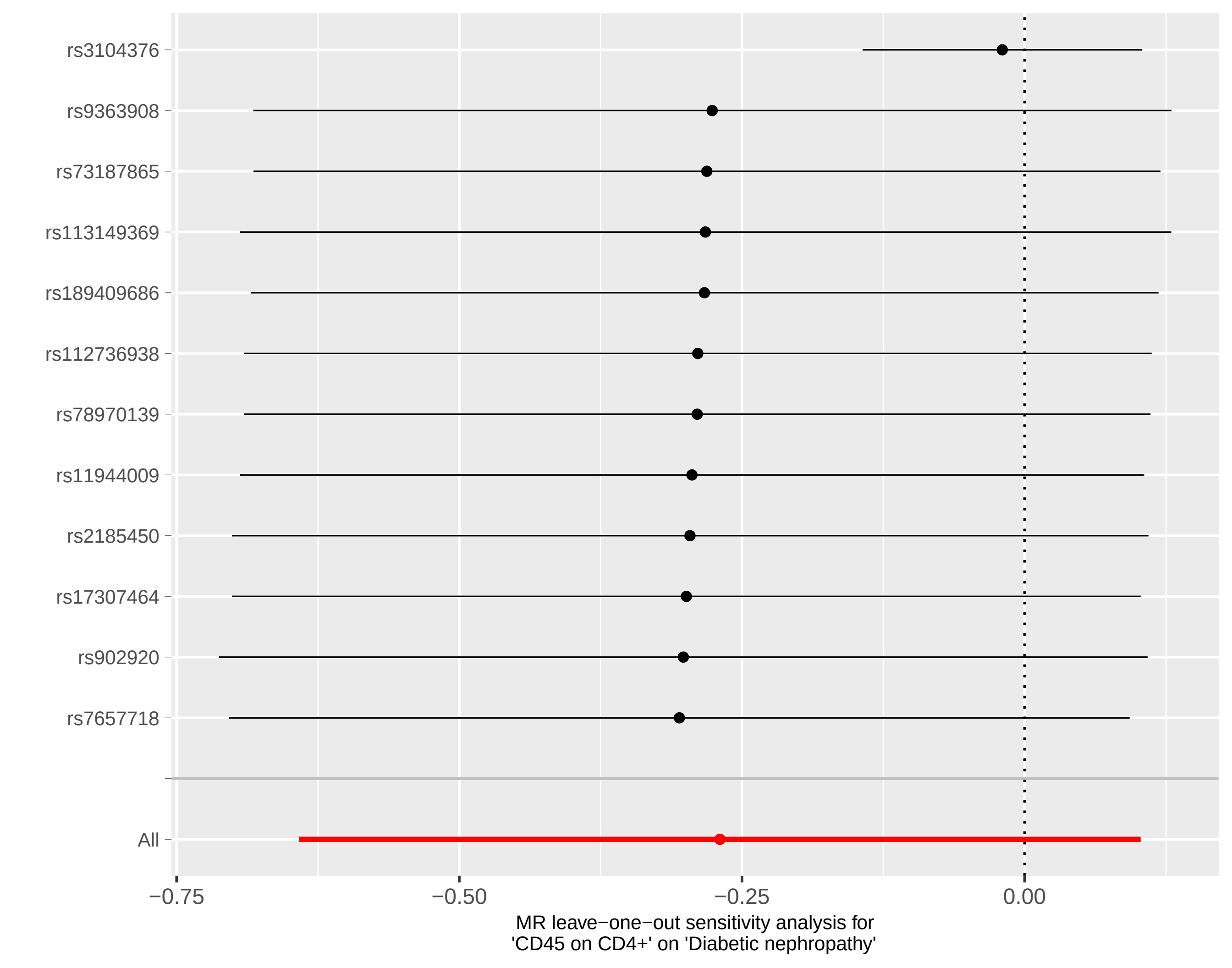
rs3104376
rs9363908
rs73187865
rs113149369
rs189409686
rs112736938
rs78970139
rs11944009
rs2185450
rs17307464
rs902920
rs7657718
All
−0.75
−0.50
−0.25
0.00
MR leave−one−out sensitivity analysis for
'CD45 on CD4+' on 'Diabetic nephropathy'

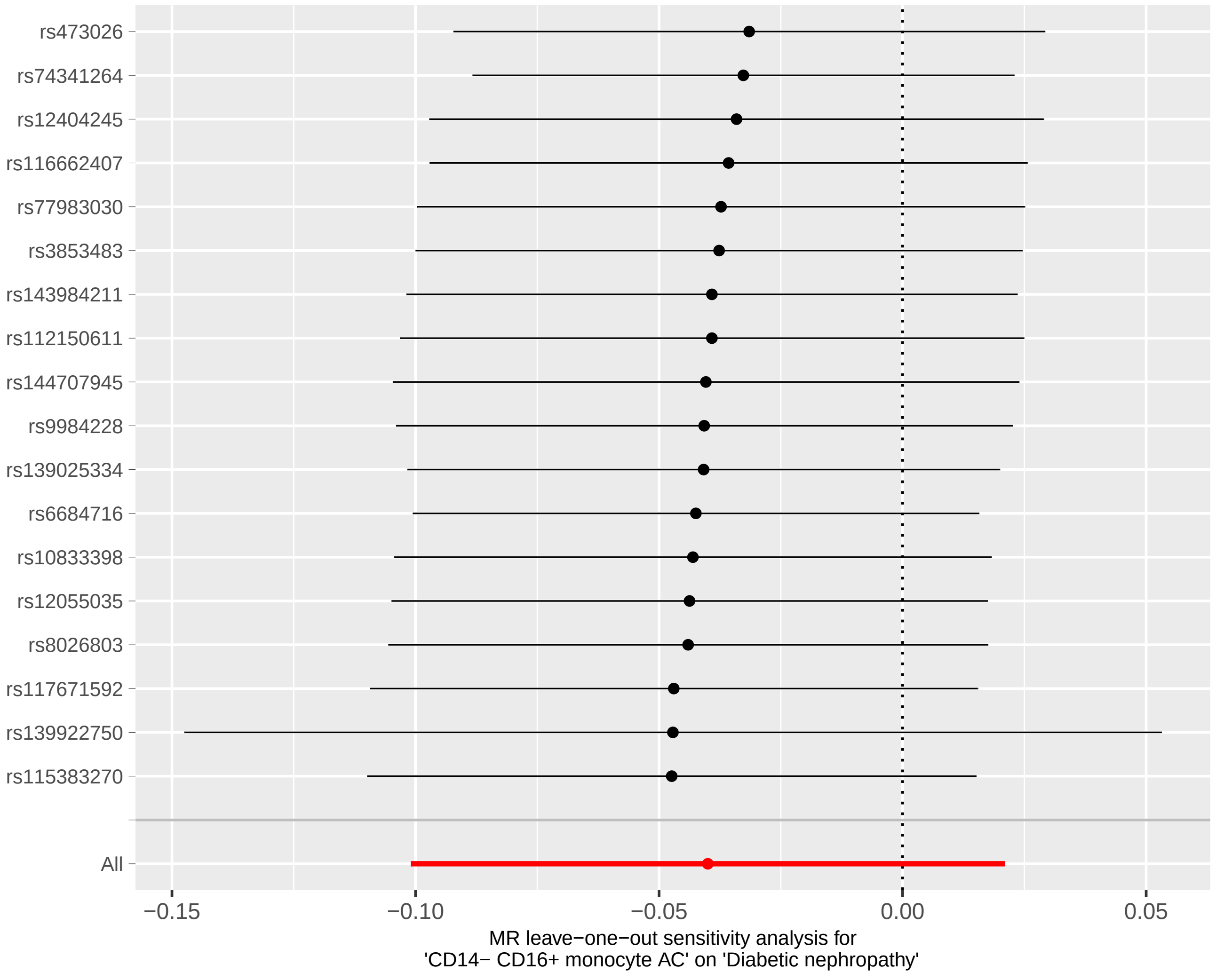
rs473026
rs74341264
rs12404245
rs116662407
rs77983030
rs3853483
rs143984211
rs112150611
rs144707945
rs9984228
rs139025334
rs6684716
rs10833398
rs12055035
rs8026803
rs117671592
rs139922750
rs115383270
All
−0.15
−0.10
−0.05
0.00
0.05
MR leave−one−out sensitivity analysis for
'CD14− CD16+ monocyte AC' on 'Diabetic nephropathy'

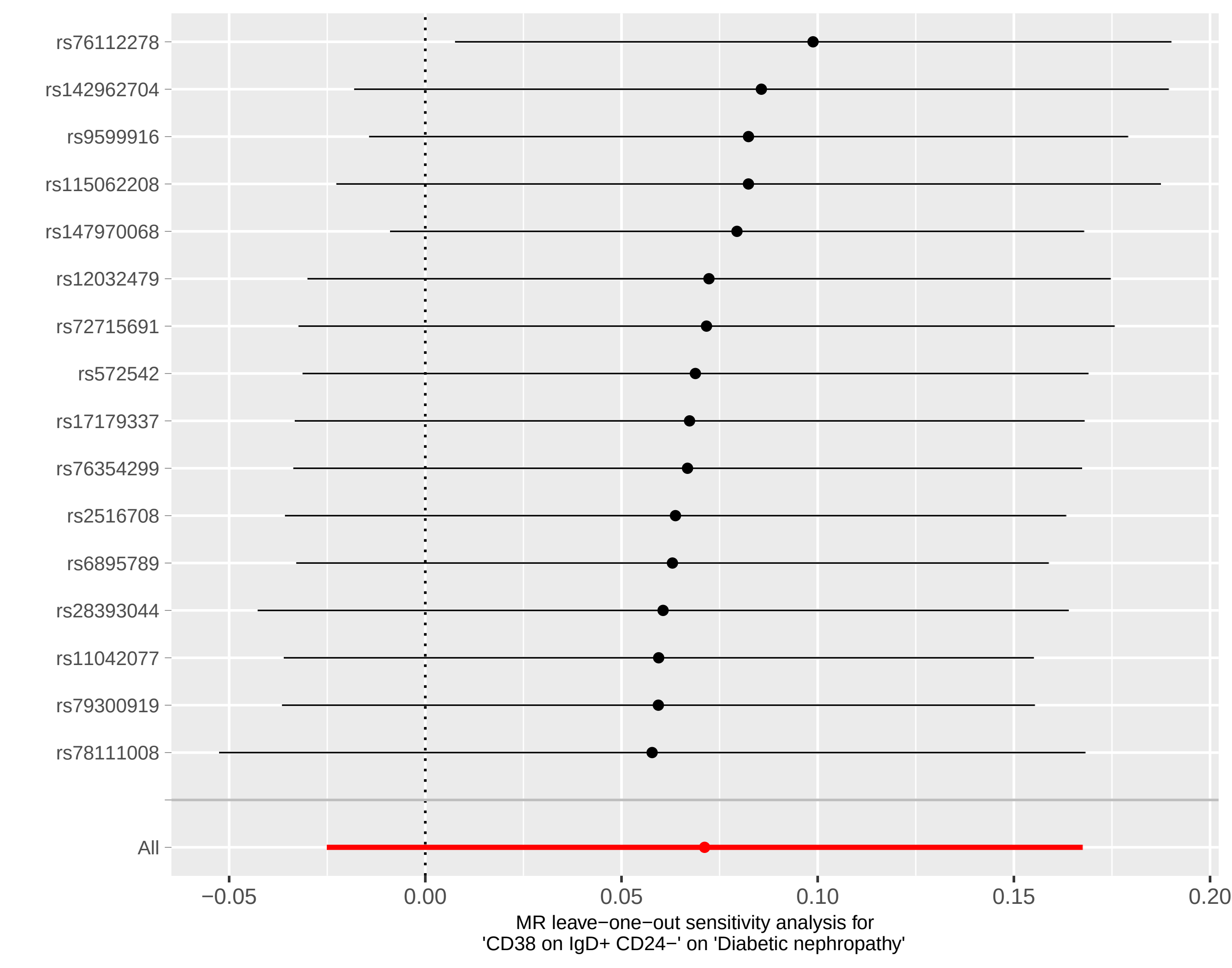

rs76112278
rs142962704
rs9599916
rs115062208
rs147970068
rs12032479
rs72715691
rs572542
rs17179337
rs76354299
rs2516708
rs6895789
rs28393044
rs11042077
rs79300919
rs78111008
All
−0.05
0.00
0.05
0.10
0.15
0.20
MR leave−one−out sensitivity analysis for
'CD38 on IgD+ CD24−' on 'Diabetic nephropathy'

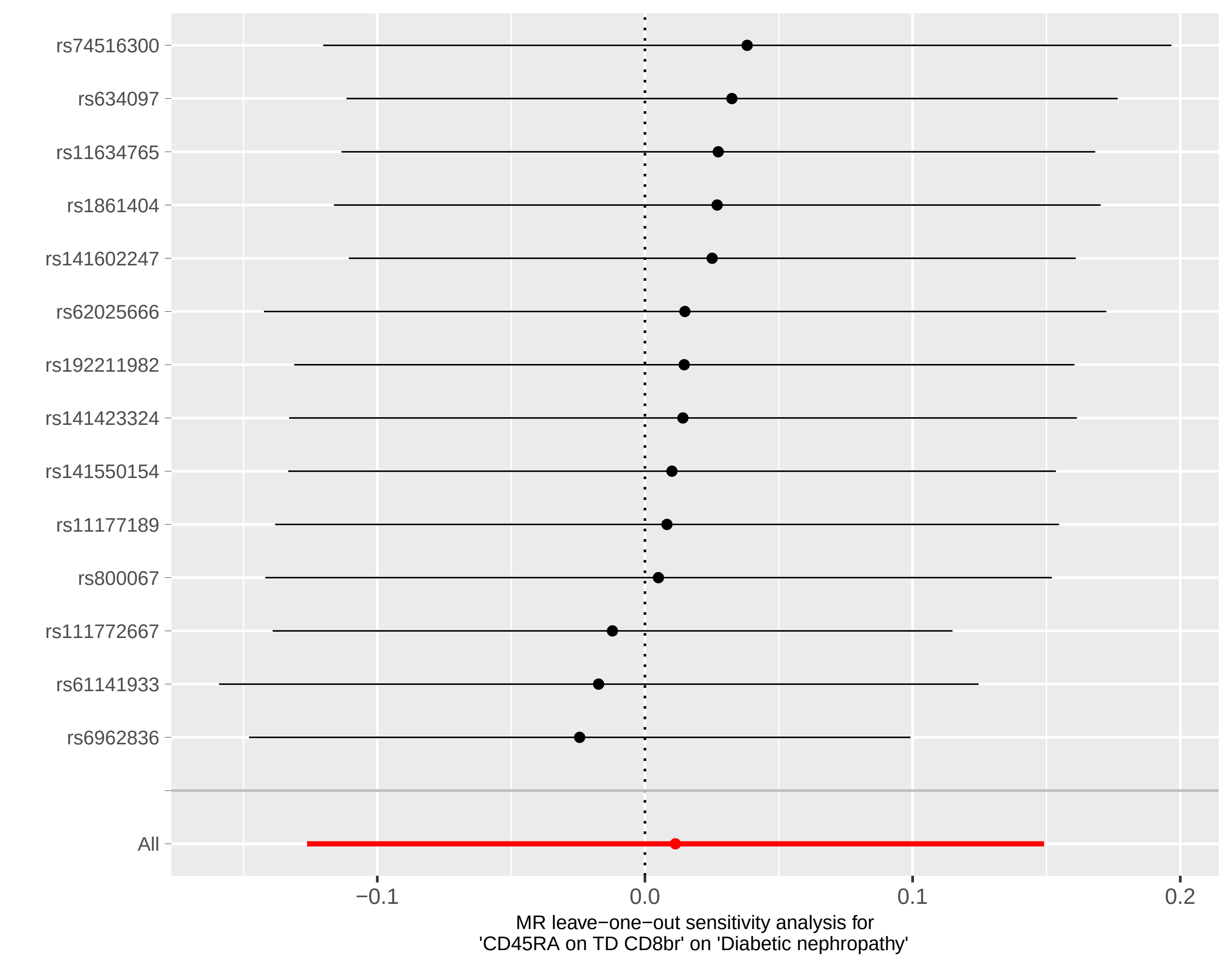
rs74516300
rs634097
rs11634765
rs1861404
rs141602247
rs62025666
rs192211982
rs141423324
rs141550154
rs11177189
rs800067
rs111772667
rs61141933
rs6962836
All
−0.1
0.0
0.1
0.2
MR leave−one−out sensitivity analysis for
'CD45RA on TD CD8br' on 'Diabetic nephropathy'

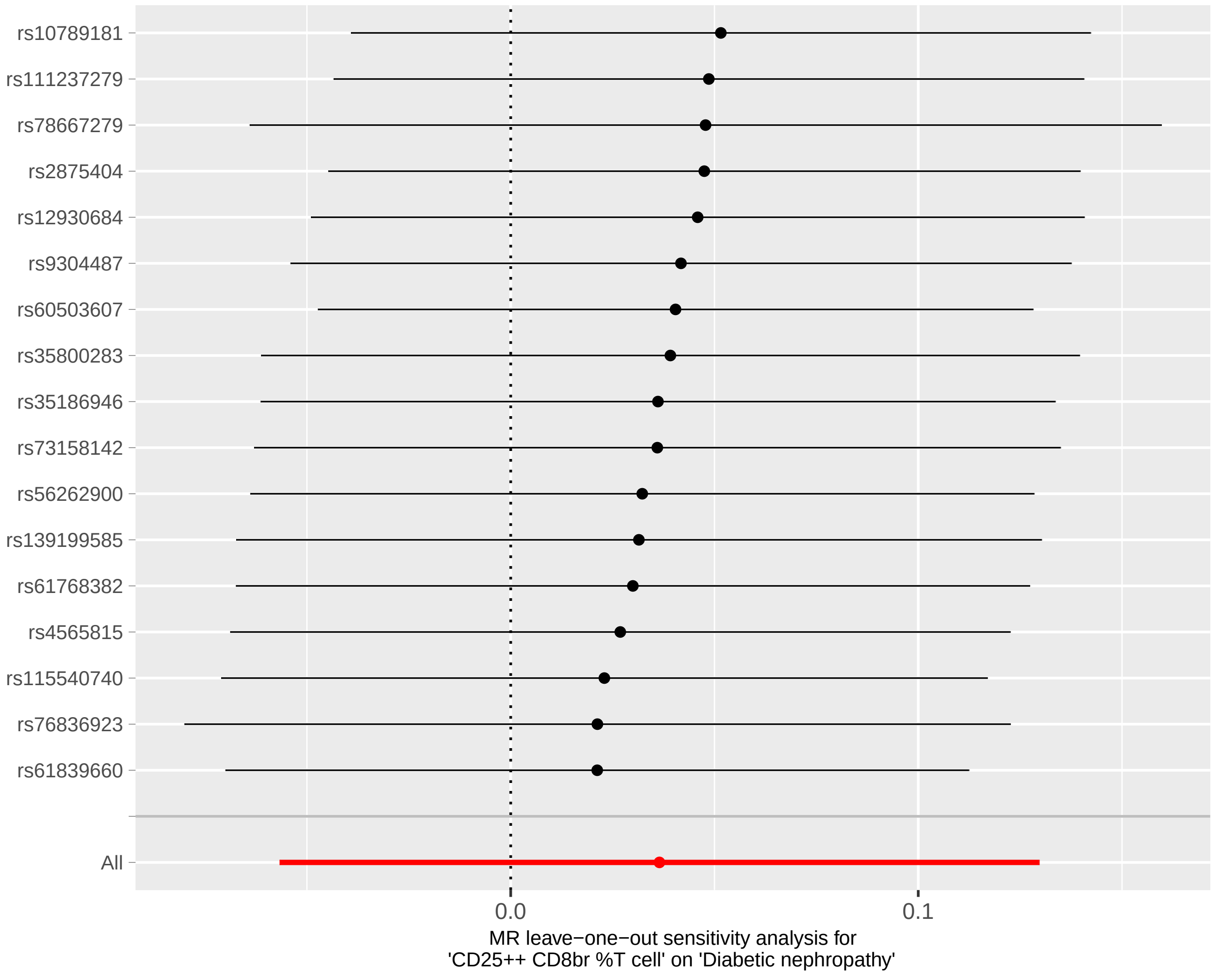
rs10789181
rs111237279
rs78667279
rs2875404
rs12930684
rs9304487
rs60503607
rs35800283
rs35186946
rs73158142
rs56262900
rs139199585
rs61768382
rs4565815
rs115540740
rs76836923
rs61839660
All
0.0
0.1
MR leave−one−out sensitivity analysis for
'CD25++ CD8br %T cell' on 'Diabetic nephropathy'

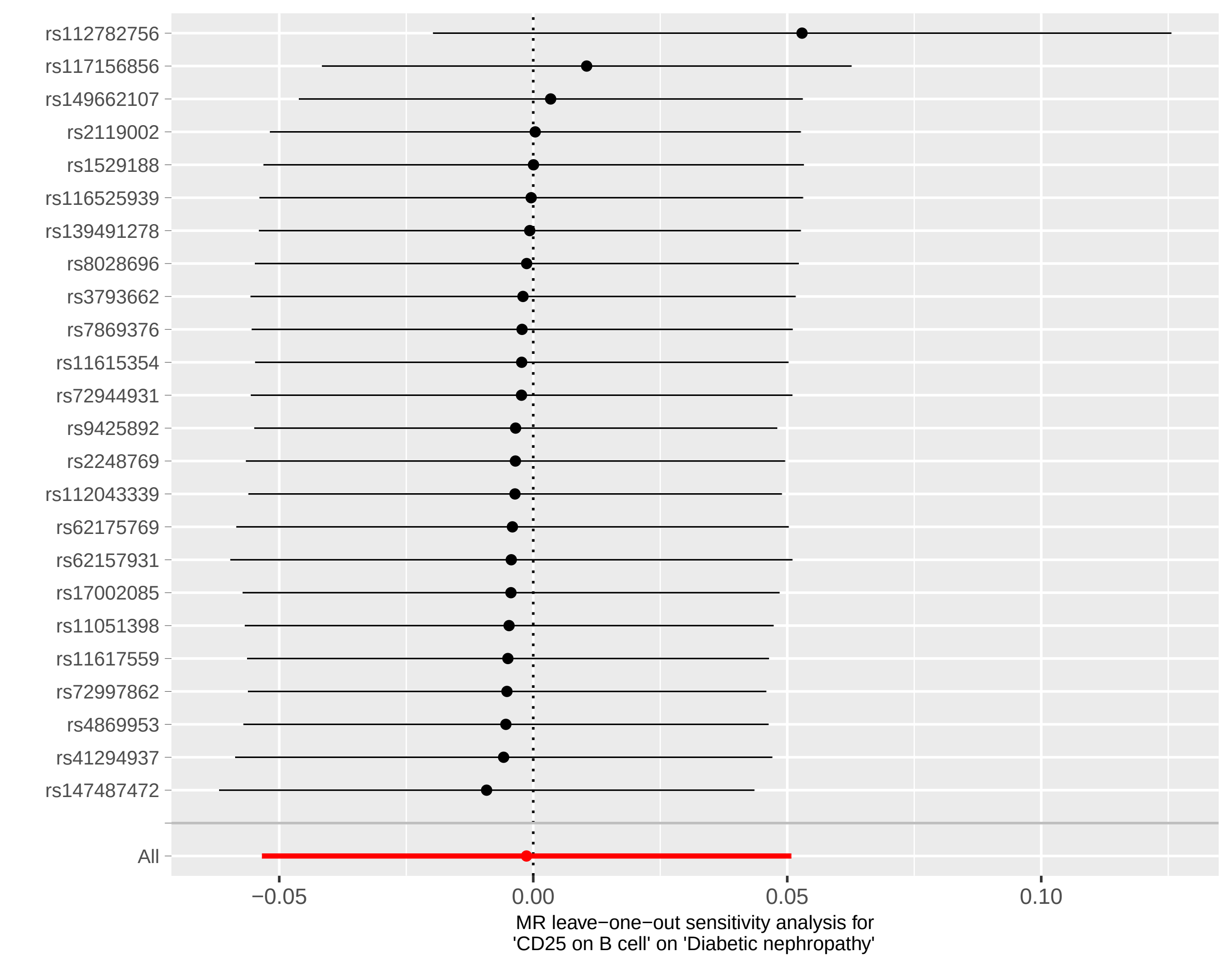
rs112782756
rs117156856
rs149662107
rs2119002
rs1529188
rs116525939
rs139491278
rs8028696
rs3793662
rs7869376
rs11615354
rs72944931
rs9425892
rs2248769
rs112043339
rs62175769
rs62157931
rs17002085
rs11051398
rs11617559
rs72997862
rs4869953
rs41294937
rs147487472
All
−0.05
0.00
0.05
0.10
MR leave−one−out sensitivity analysis for
'CD25 on B cell' on 'Diabetic nephropathy'

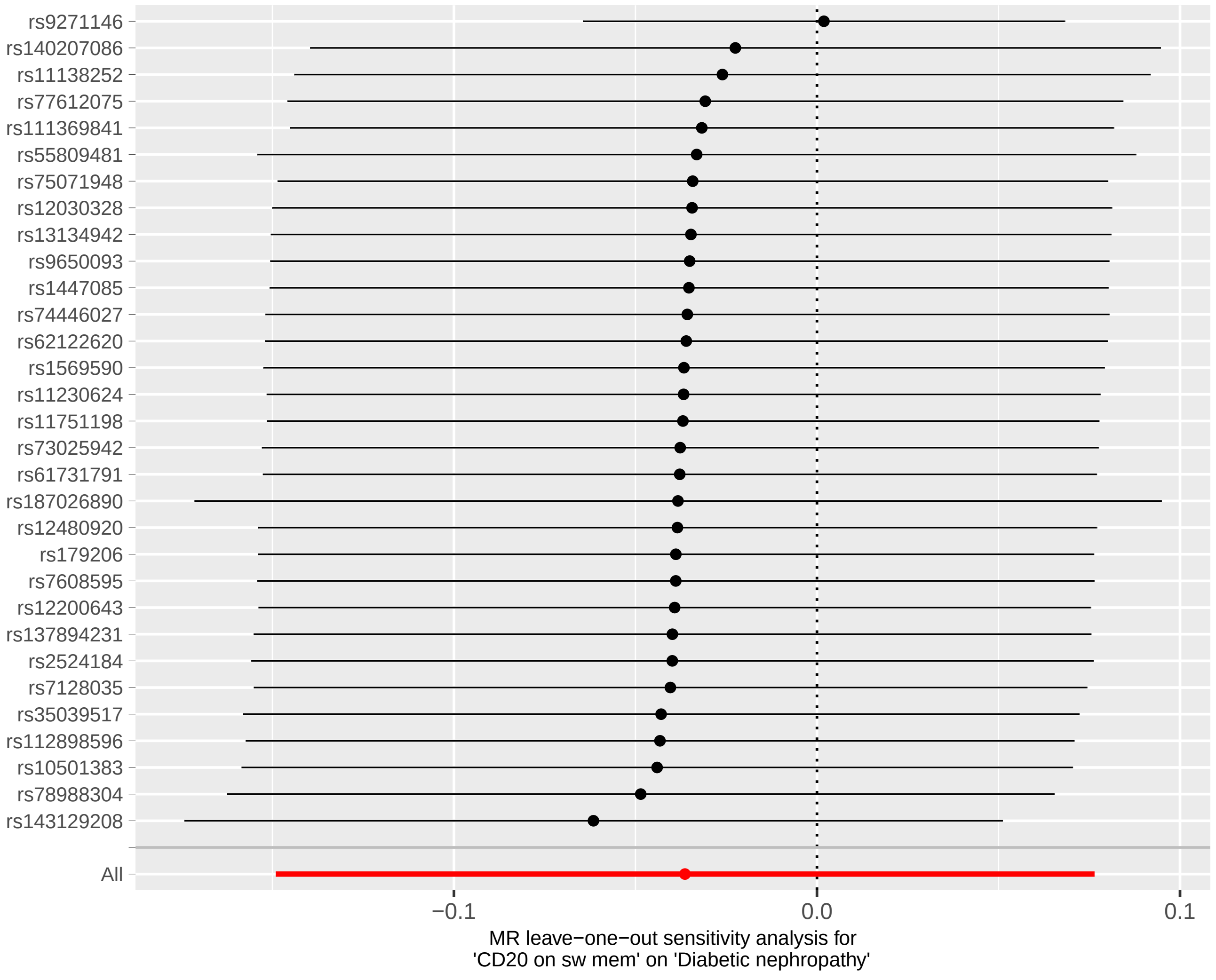
rs9271146
rs140207086
rs11138252
rs77612075
rs111369841
rs55809481
rs75071948
rs12030328
rs13134942
rs9650093
rs1447085
rs74446027
rs62122620
rs1569590
rs11230624
rs11751198
rs73025942
rs61731791
rs187026890
rs12480920
rs179206
rs7608595
rs12200643
rs137894231
rs2524184
rs7128035
rs35039517
rs112898596
rs10501383
rs78988304
rs143129208
All
−0.1
0.0
0.1
MR leave−one−out sensitivity analysis for
'CD20 on sw mem' on 'Diabetic nephropathy'

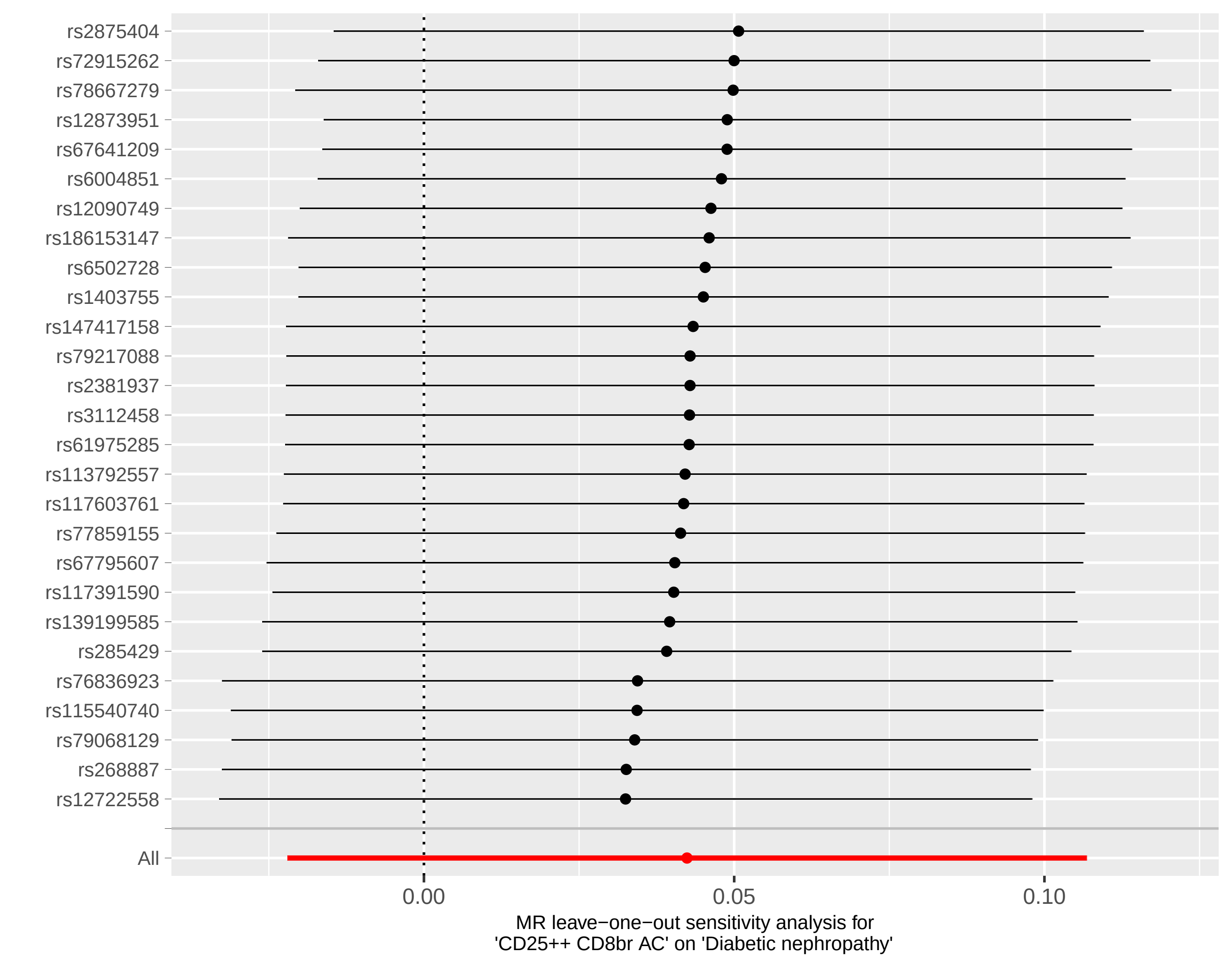
rs2875404
rs72915262
rs78667279
rs12873951
rs67641209
rs6004851
rs12090749
rs186153147
rs6502728
rs1403755
rs147417158
rs79217088
rs2381937
rs3112458
rs61975285
rs113792557
rs117603761
rs77859155
rs67795607
rs117391590
rs139199585
rs285429
rs76836923
rs115540740
rs79068129
rs268887
rs12722558
All
0.00
0.05
0.10
MR leave−one−out sensitivity analysis for
'CD25++ CD8br AC' on 'Diabetic nephropathy'

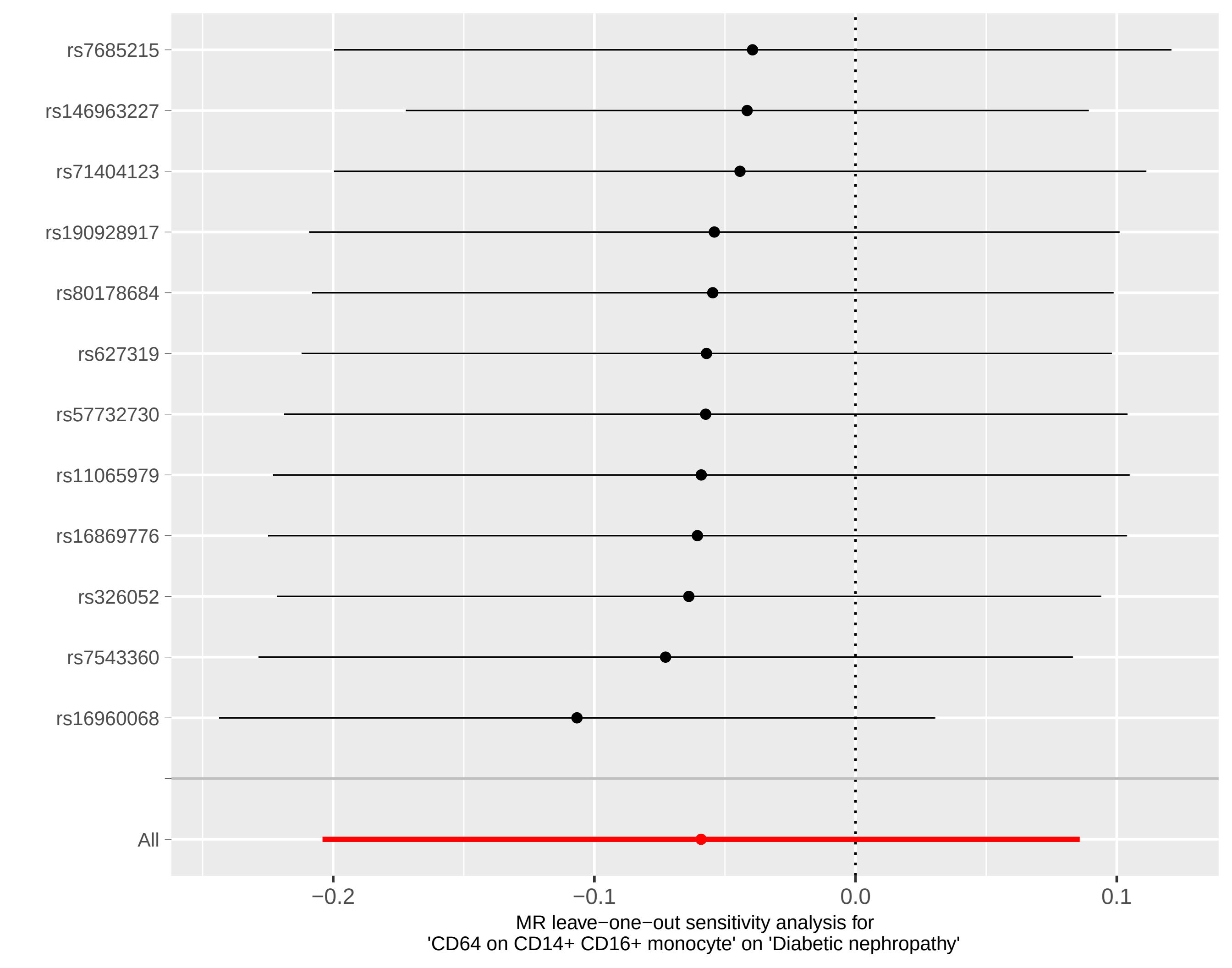

rs7685215
rs146963227
rs71404123
rs190928917
rs80178684
rs627319
rs57732730
rs11065979
rs16869776
rs326052
rs7543360
rs16960068
All
−0.2
−0.1
0.0
0.1
MR leave−one−out sensitivity analysis for
'CD64 on CD14+ CD16+ monocyte' on 'Diabetic nephropathy'

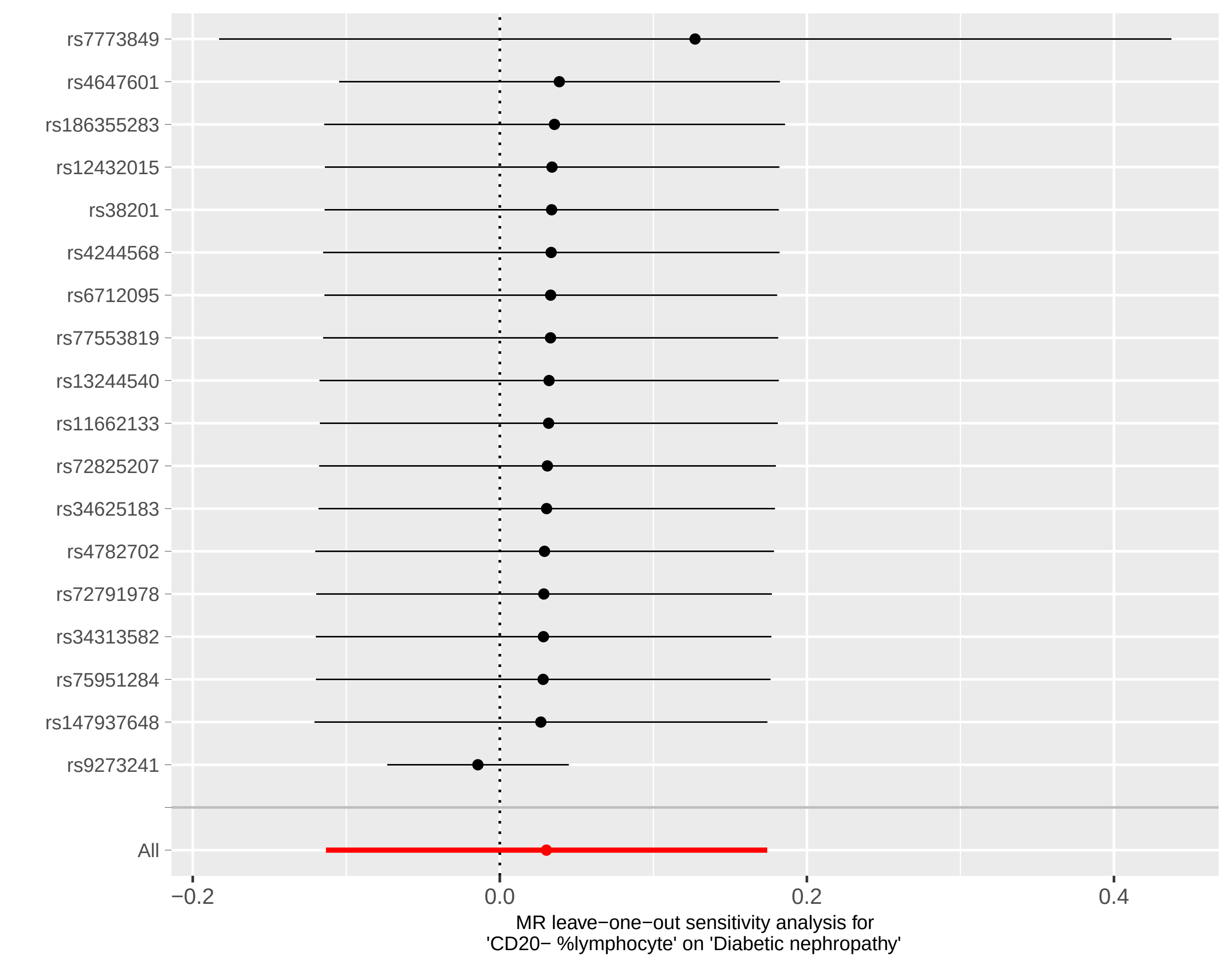
rs7773849
rs4647601
rs186355283
rs12432015
rs38201
rs4244568
rs6712095
rs77553819
rs13244540
rs11662133
rs72825207
rs34625183
rs4782702
rs72791978
rs34313582
rs75951284
rs147937648
rs9273241
All
−0.2
0.0
0.2
0.4
MR leave−one−out sensitivity analysis for
'CD20− %lymphocyte' on 'Diabetic nephropathy'

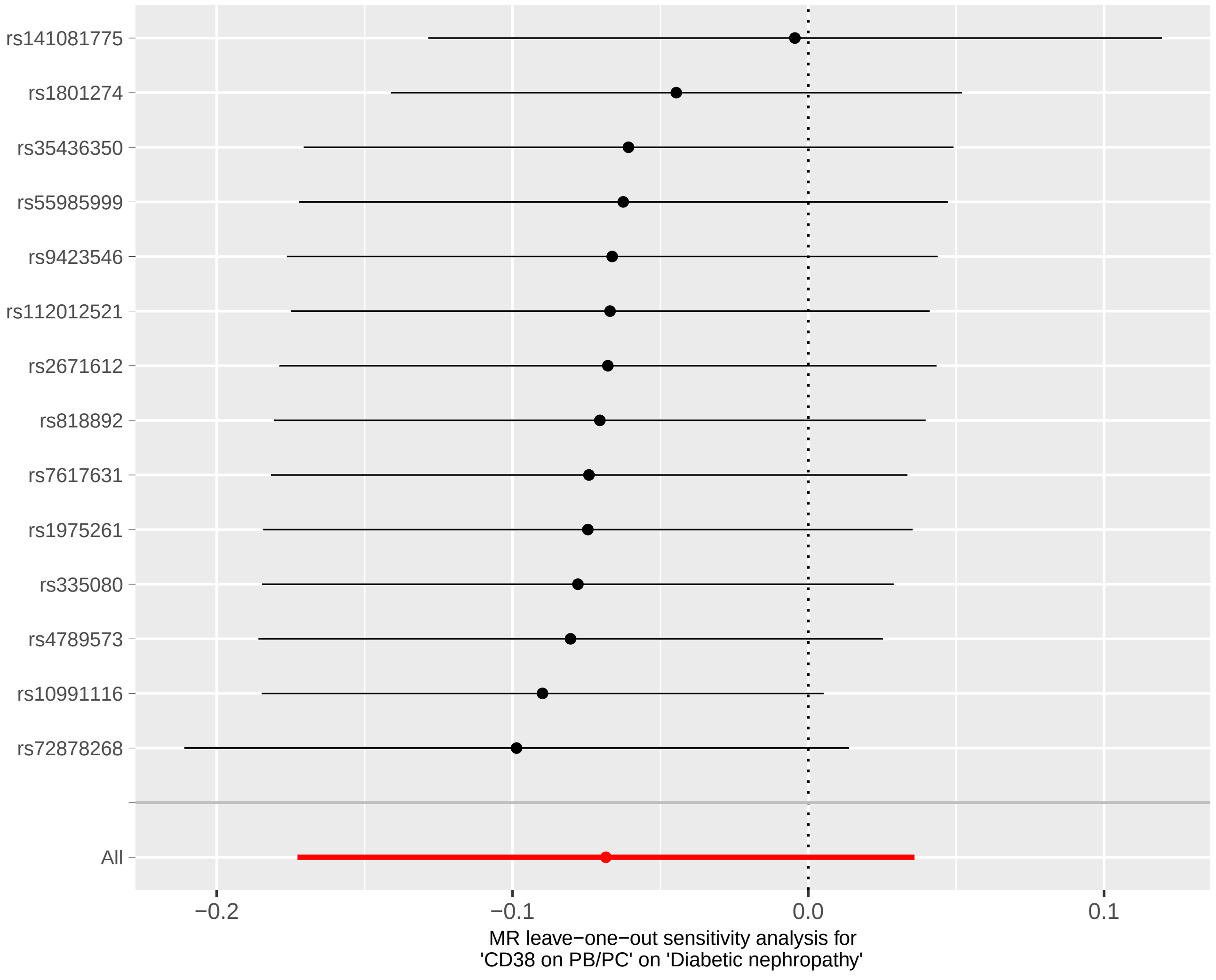

rs141081775
rs1801274
rs35436350
rs55985999
rs9423546
rs112012521
rs2671612
rs818892
rs7617631
rs1975261
rs335080
rs4789573
rs10991116
rs72878268
All
−0.2
−0.1
0.0
0.1
MR leave−one−out sensitivity analysis for
'CD38 on PB/PC' on 'Diabetic nephropathy'

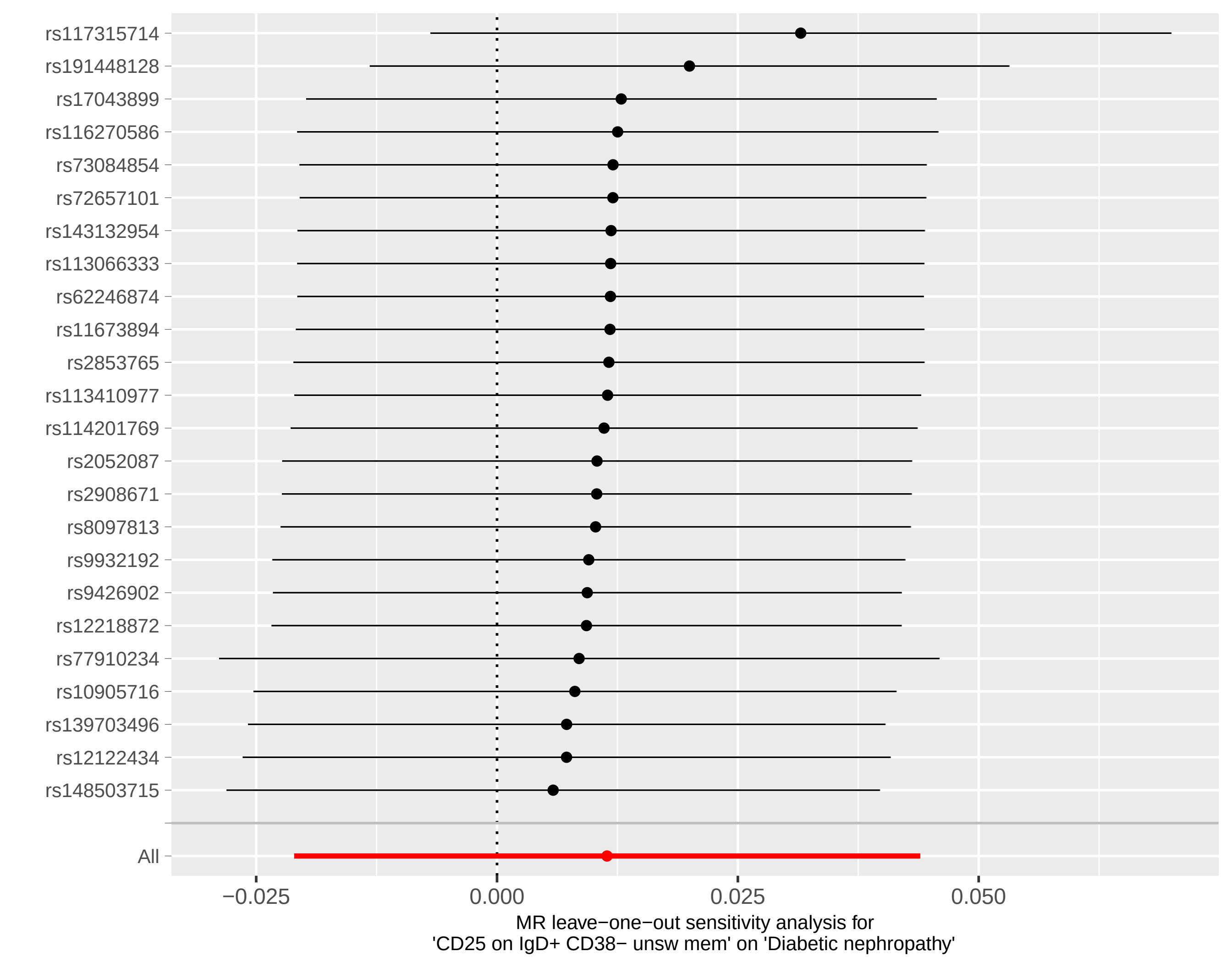
rs117315714
rs191448128
rs17043899
rs116270586
rs73084854
rs72657101
rs143132954
rs113066333
rs62246874
rs11673894
rs2853765
rs113410977
rs114201769
rs2052087
rs2908671
rs8097813
rs9932192
rs9426902
rs12218872
rs77910234
rs10905716
rs139703496
rs12122434
rs148503715
All
−0.025
0.000
0.025
0.050
MR leave−one−out sensitivity analysis for
'CD25 on IgD+ CD38− unsw mem' on 'Diabetic nephropathy'

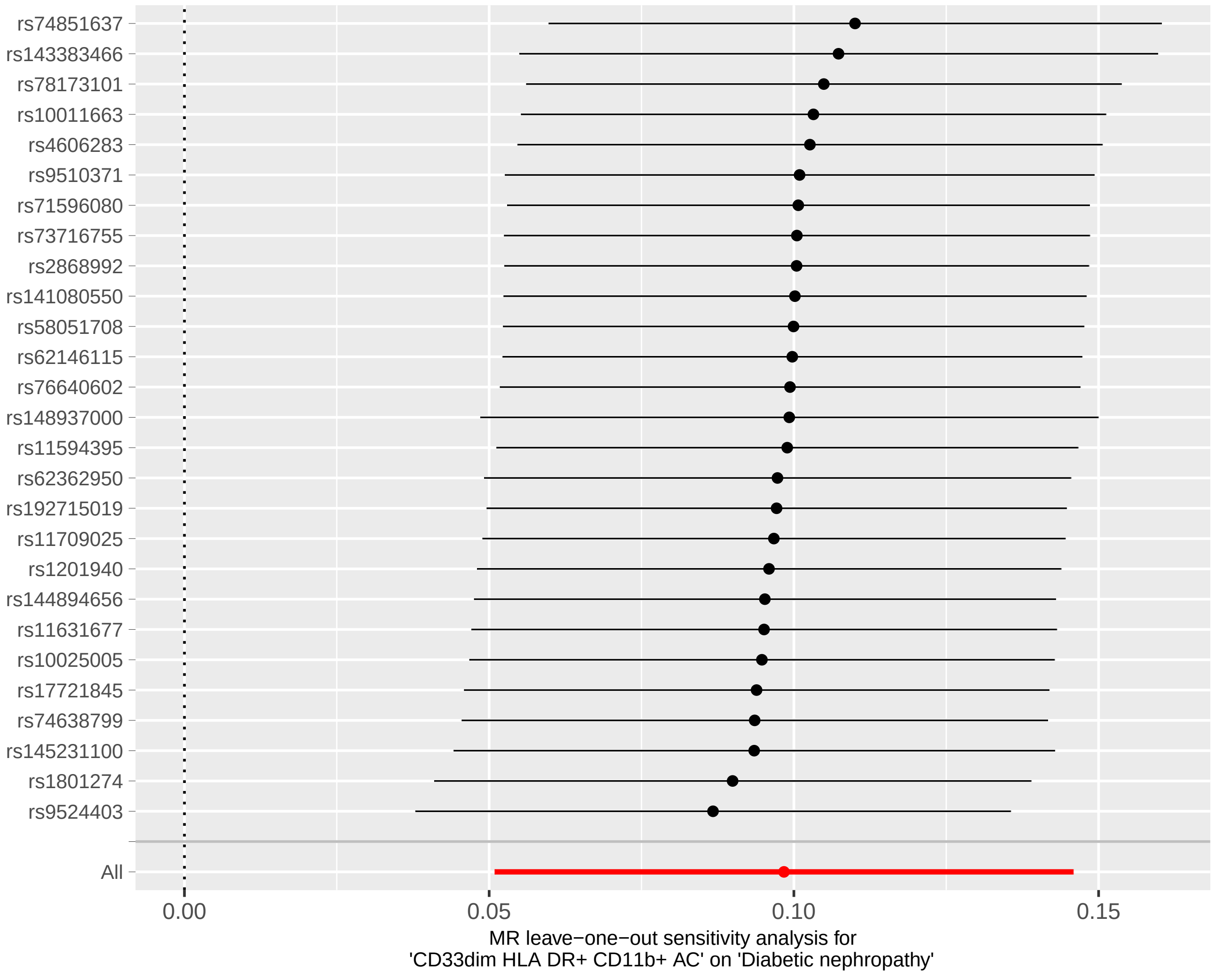

rs74851637
rs143383466
rs78173101
rs10011663
rs4606283
rs9510371
rs71596080
rs73716755
rs2868992
rs141080550
rs58051708
rs62146115
rs76640602
rs148937000
rs11594395
rs62362950
rs192715019
rs11709025
rs1201940
rs144894656
rs11631677
rs10025005
rs17721845
rs74638799
rs145231100
rs1801274
rs9524403
All
0.00
0.05
0.10
0.15
MR leave−one−out sensitivity analysis for
'CD33dim HLA DR+ CD11b+ AC' on 'Diabetic nephropathy'

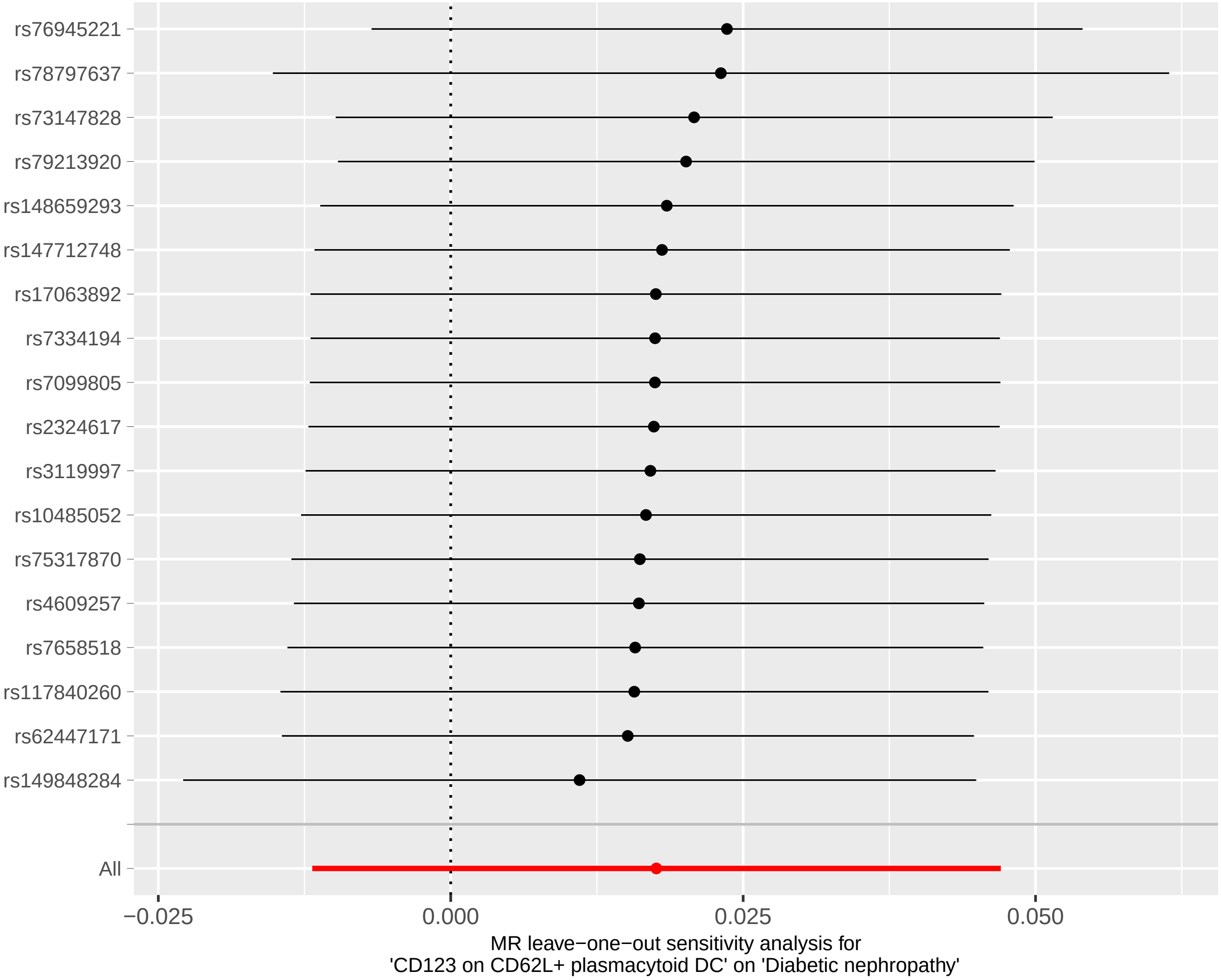
rs76945221
rs78797637
rs73147828
rs79213920
rs148659293
rs147712748
rs17063892
rs7334194
rs7099805
rs2324617
rs3119997
rs10485052
rs75317870
rs4609257
rs7658518
rs117840260
rs62447171
rs149848284
All
−0.025
0.000
0.025
0.050
MR leave−one−out sensitivity analysis for
'CD123 on CD62L+ plasmacytoid DC' on 'Diabetic nephropathy'

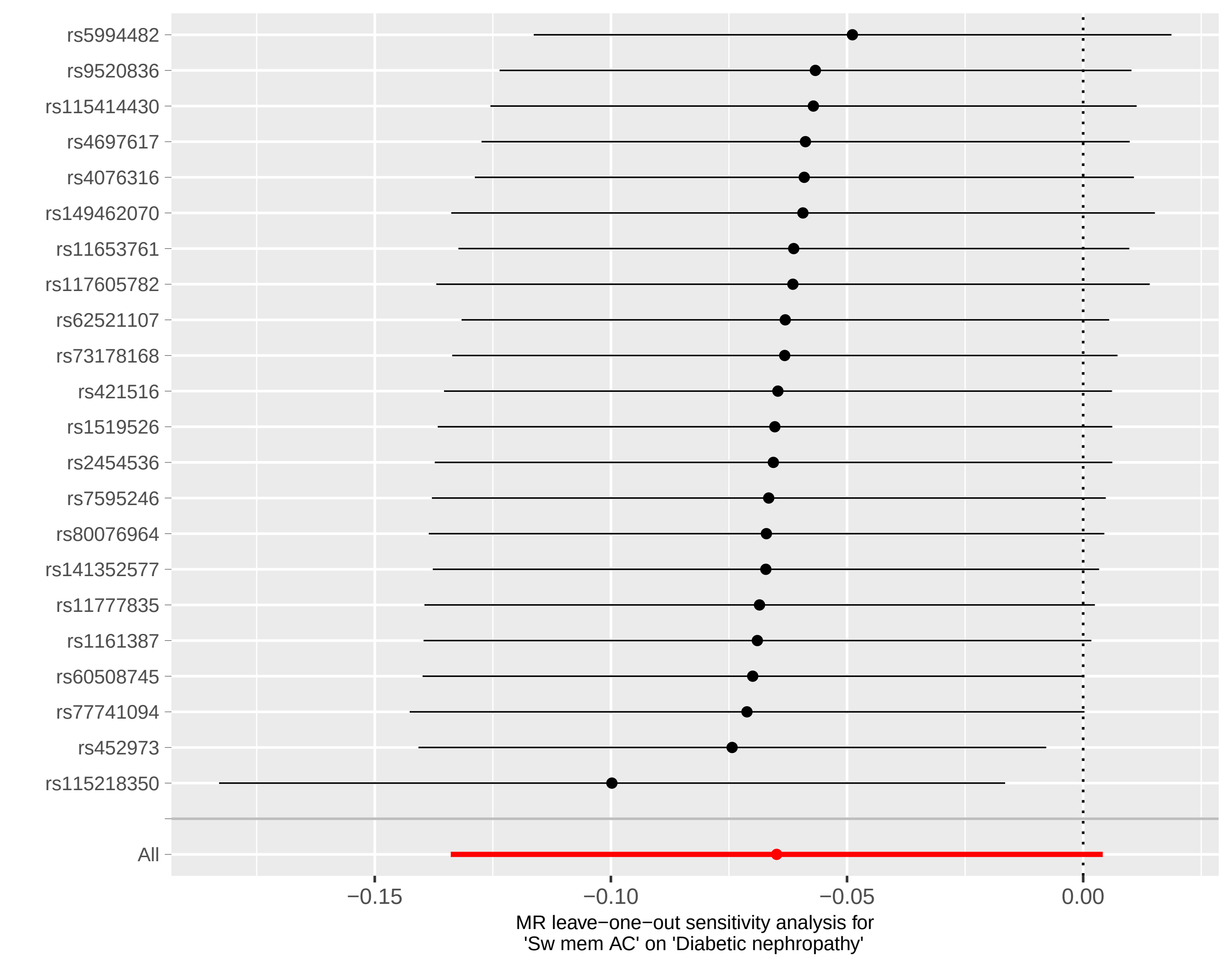

rs5994482
rs9520836
rs115414430
rs4697617
rs4076316
rs149462070
rs11653761
rs117605782
rs62521107
rs73178168
rs421516
rs1519526
rs2454536
rs7595246
rs80076964
rs141352577
rs11777835
rs1161387
rs60508745
rs77741094
rs452973
rs115218350
All
−0.15
−0.10
−0.05
0.00
MR leave−one−out sensitivity analysis for
'Sw mem AC' on 'Diabetic nephropathy'

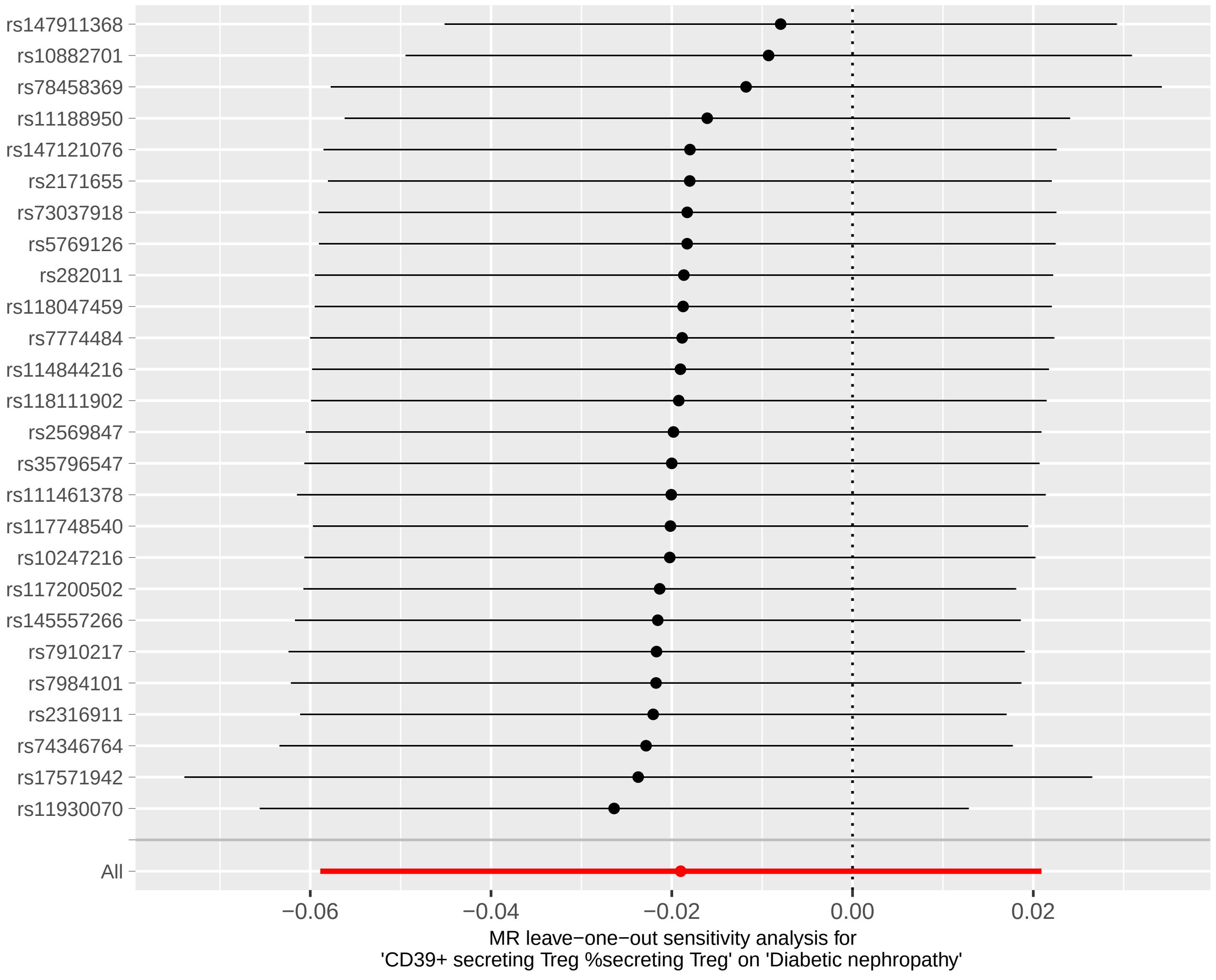
rs147911368
rs10882701
rs78458369
rs11188950
rs147121076
rs2171655
rs73037918
rs5769126
rs282011
rs118047459
rs7774484
rs114844216
rs118111902
rs2569847
rs35796547
rs111461378
rs117748540
rs10247216
rs117200502
rs145557266
rs7910217
rs7984101
rs2316911
rs74346764
rs17571942
rs11930070
All
−0.06
−0.04
−0.02
0.00
0.02
MR leave−one−out sensitivity analysis for
'CD39+ secreting Treg %secreting Treg' on 'Diabetic nephropathy'

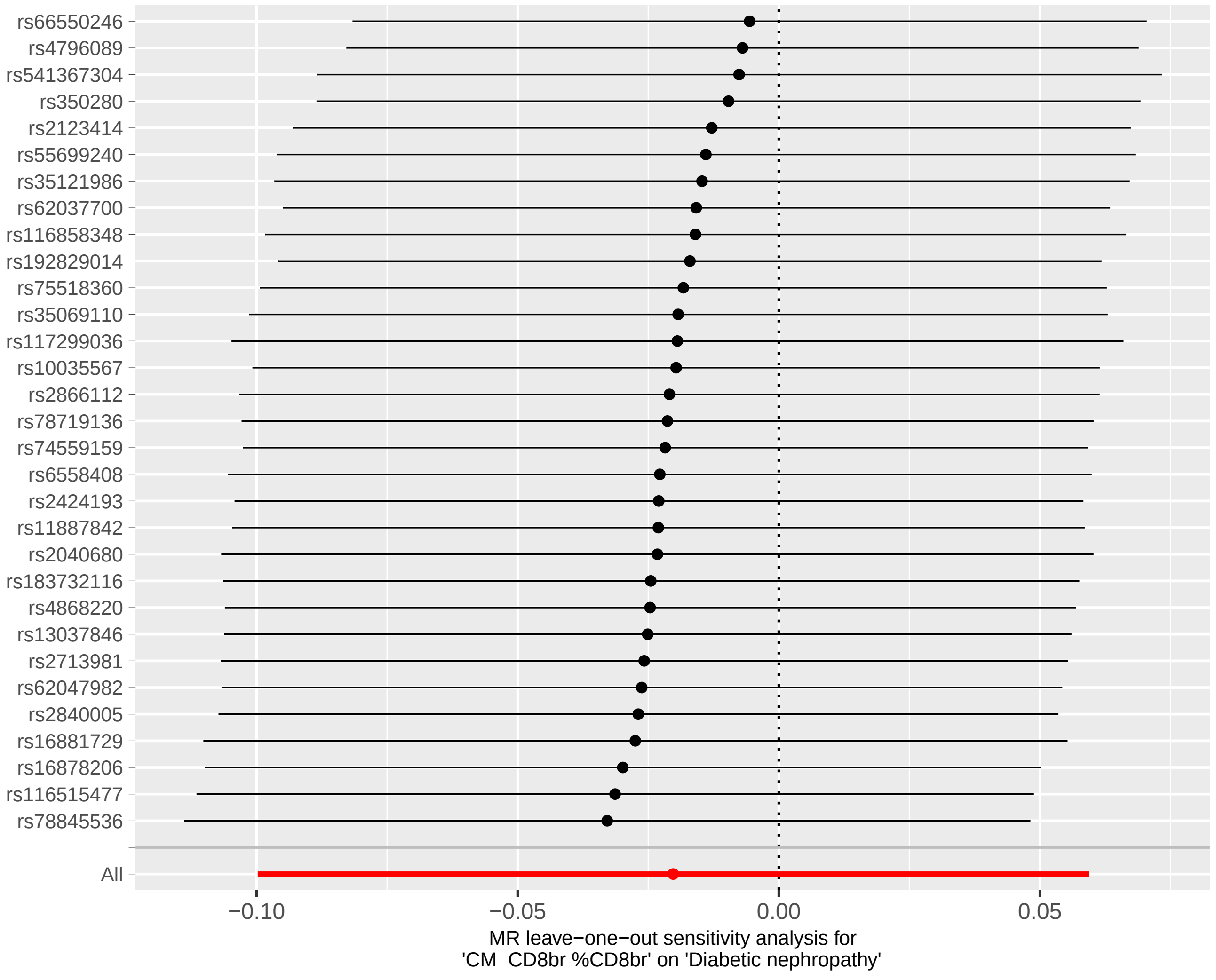
rs66550246
rs4796089
rs541367304
rs350280
rs2123414
rs55699240
rs35121986
rs62037700
rs116858348
rs192829014
rs75518360
rs35069110
rs117299036
rs10035567
rs2866112
rs78719136
rs74559159
rs6558408
rs2424193
rs11887842
rs2040680
rs183732116
rs4868220
rs13037846
rs2713981
rs62047982
rs2840005
rs16881729
rs16878206
rs116515477
rs78845536
All
−0.10
−0.05
0.00
0.05
MR leave−one−out sensitivity analysis for
'CM CD8br %CD8br' on 'Diabetic nephropathy'

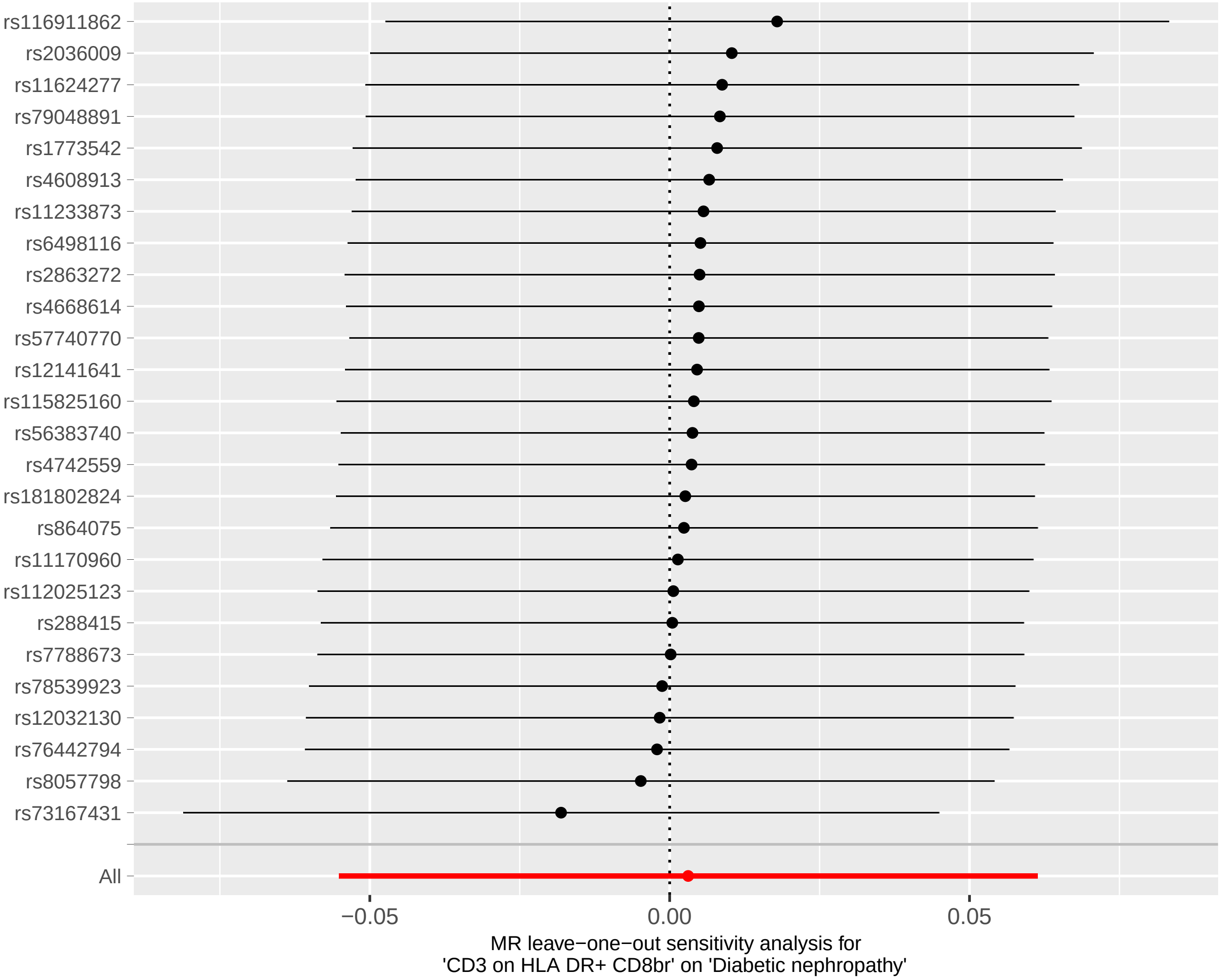
rs116911862
rs2036009
rs11624277
rs79048891
rs1773542
rs4608913
rs11233873
rs6498116
rs2863272
rs4668614
rs57740770
rs12141641
rs115825160
rs56383740
rs4742559
rs181802824
rs864075
rs11170960
rs112025123
rs288415
rs7788673
rs78539923
rs12032130
rs76442794
rs8057798
rs73167431
All
−0.05
0.00
0.05
MR leave−one−out sensitivity analysis for
'CD3 on HLA DR+ CD8br' on 'Diabetic nephropathy'

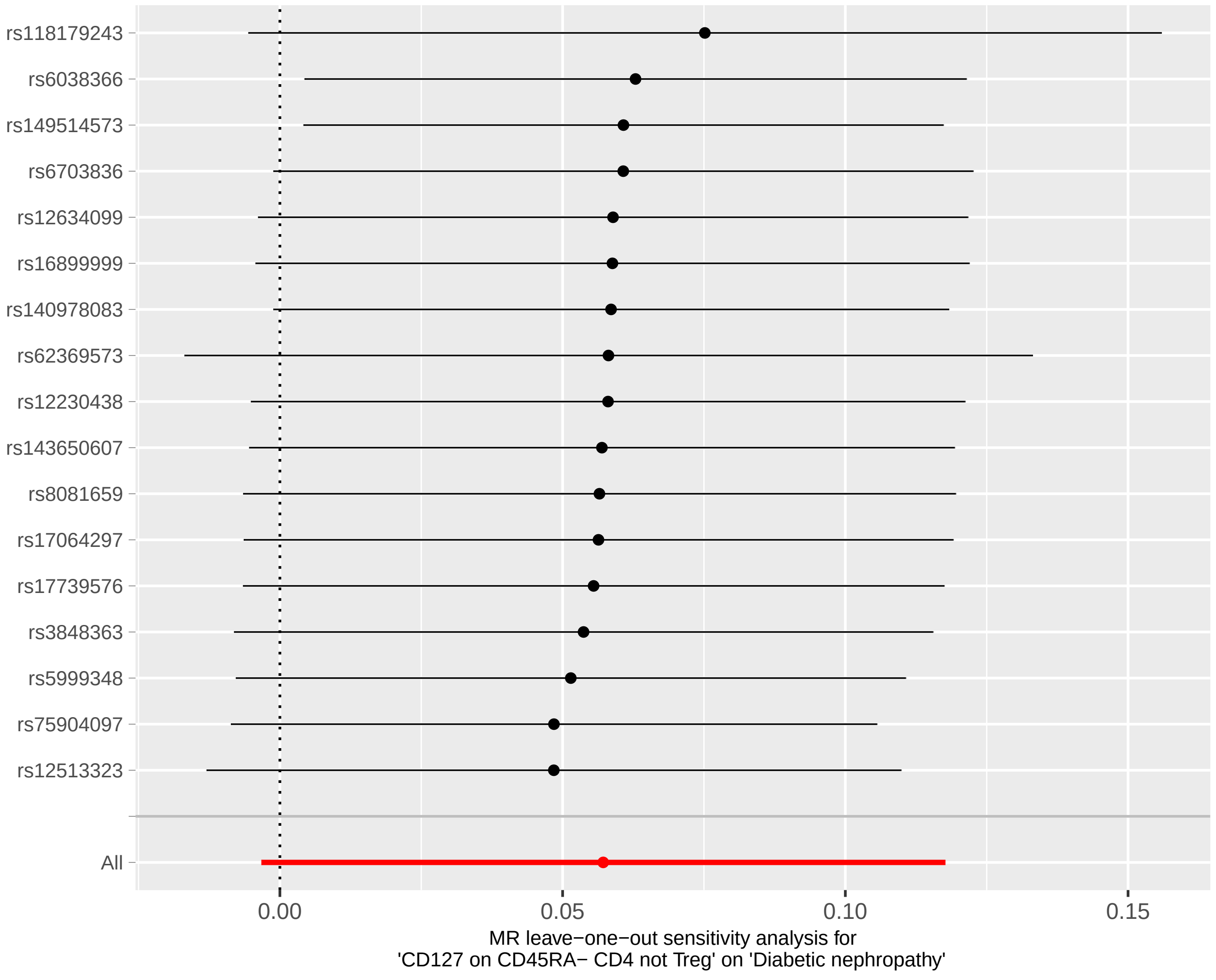
rs118179243
rs6038366
rs149514573
rs6703836
rs12634099
rs16899999
rs140978083
rs62369573
rs12230438
rs143650607
rs8081659
rs17064297
rs17739576
rs3848363
rs5999348
rs75904097
rs12513323
All
0.00
0.05
0.10
0.15
MR leave−one−out sensitivity analysis for
'CD127 on CD45RA− CD4 not Treg' on 'Diabetic nephropathy'

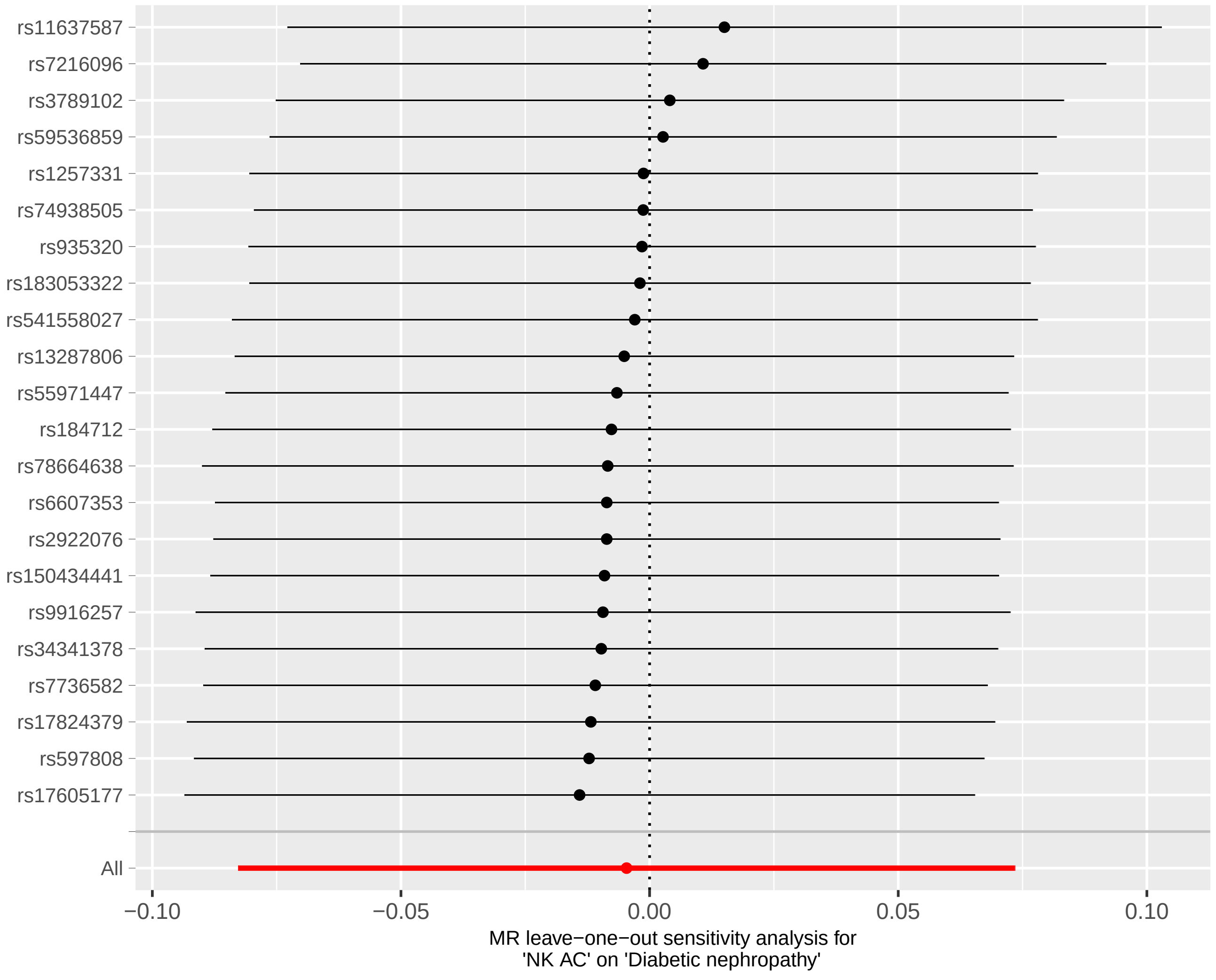
rs11637587
rs7216096
rs3789102
rs59536859
rs1257331
rs74938505
rs935320
rs183053322
rs541558027
rs13287806
rs55971447
rs184712
rs78664638
rs6607353
rs2922076
rs150434441
rs9916257
rs34341378
rs7736582
rs17824379
rs597808
rs17605177
All
−0.10
−0.05
0.00
0.05
0.10
MR leave−one−out sensitivity analysis for
'NK AC' on 'Diabetic nephropathy'

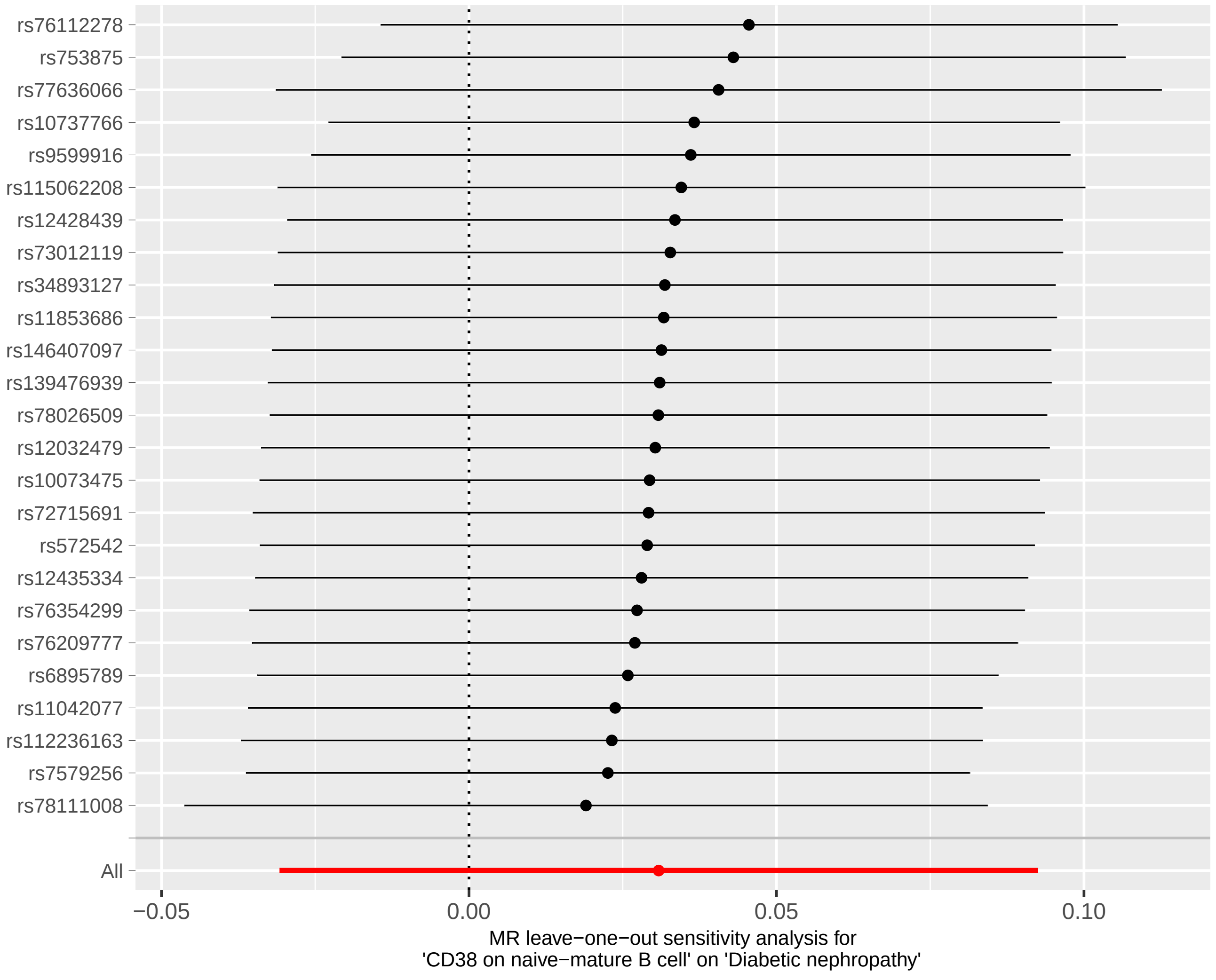
rs76112278
rs753875
rs77636066
rs10737766
rs9599916
rs115062208
rs12428439
rs73012119
rs34893127
rs11853686
rs146407097
rs139476939
rs78026509
rs12032479
rs10073475
rs72715691
rs572542
rs12435334
rs76354299
rs76209777
rs6895789
rs11042077
rs112236163
rs7579256
rs78111008
All
−0.05
0.00
0.05
0.10
MR leave−one−out sensitivity analysis for
'CD38 on naive−mature B cell' on 'Diabetic nephropathy'

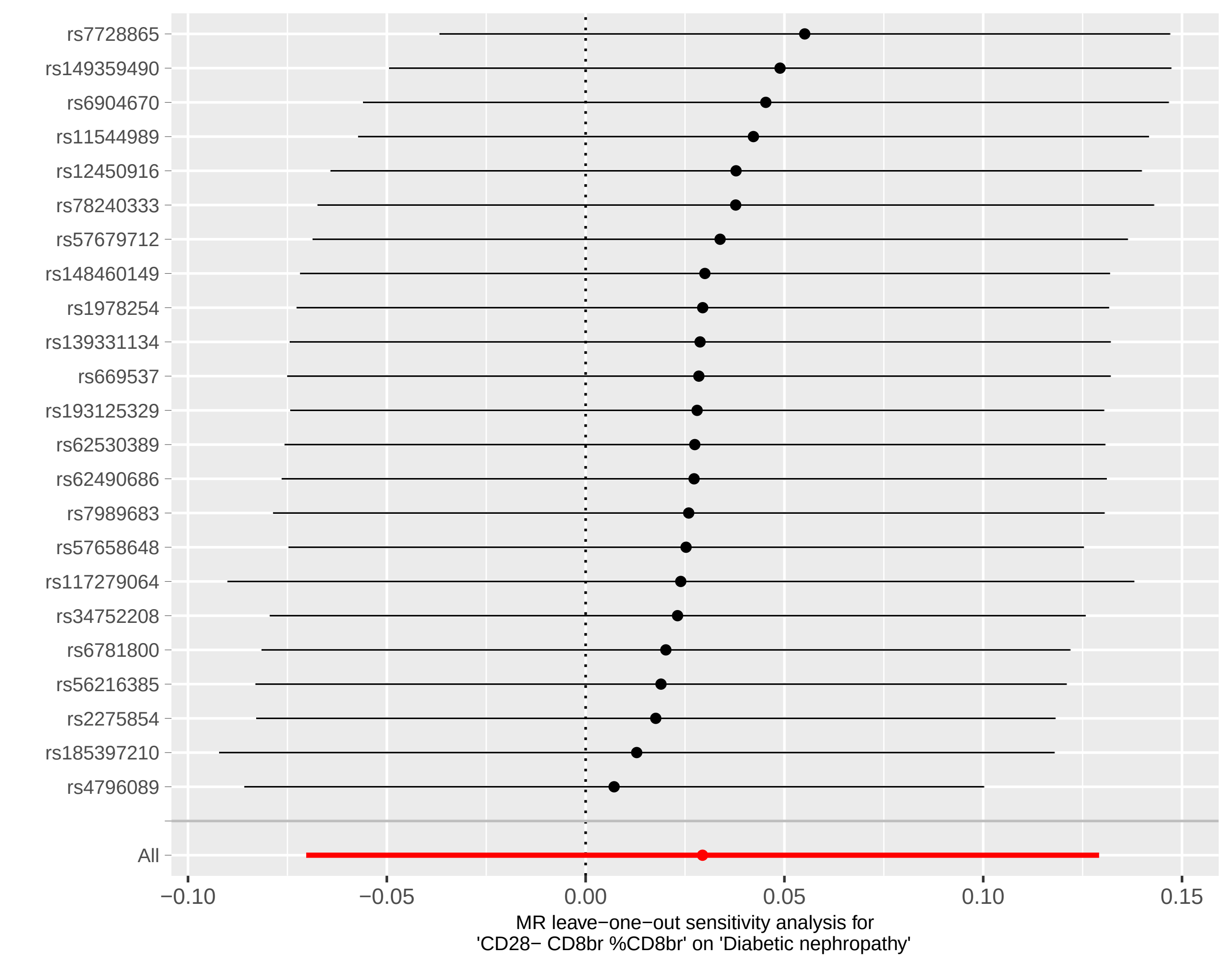

rs7728865
rs149359490
rs6904670
rs11544989
rs12450916
rs78240333
rs57679712
rs148460149
rs1978254
rs139331134
rs669537
rs193125329
rs62530389
rs62490686
rs7989683
rs57658648
rs117279064
rs34752208
rs6781800
rs56216385
rs2275854
rs185397210
rs4796089
All
−0.10
−0.05
0.00
0.05
0.10
0.15
MR leave−one−out sensitivity analysis for
'CD28− CD8br %CD8br' on 'Diabetic nephropathy'

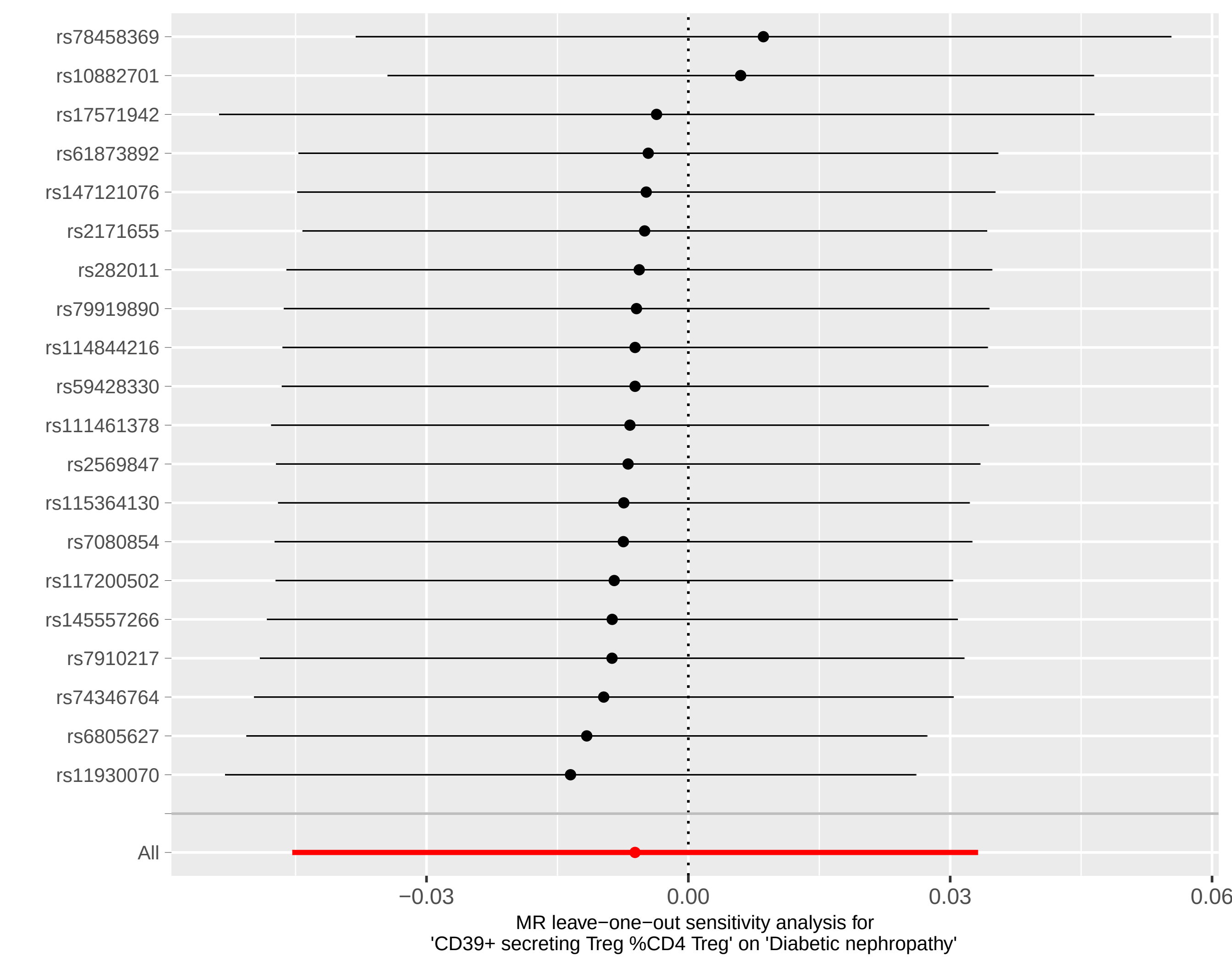
rs78458369
rs10882701
rs17571942
rs61873892
rs147121076
rs2171655
rs282011
rs79919890
rs114844216
rs59428330
rs111461378
rs2569847
rs115364130
rs7080854
rs117200502
rs145557266
rs7910217
rs74346764
rs6805627
rs11930070
All
−0.03
0.00
0.03
0.06
MR leave−one−out sensitivity analysis for
'CD39+ secreting Treg %CD4 Treg' on 'Diabetic nephropathy'

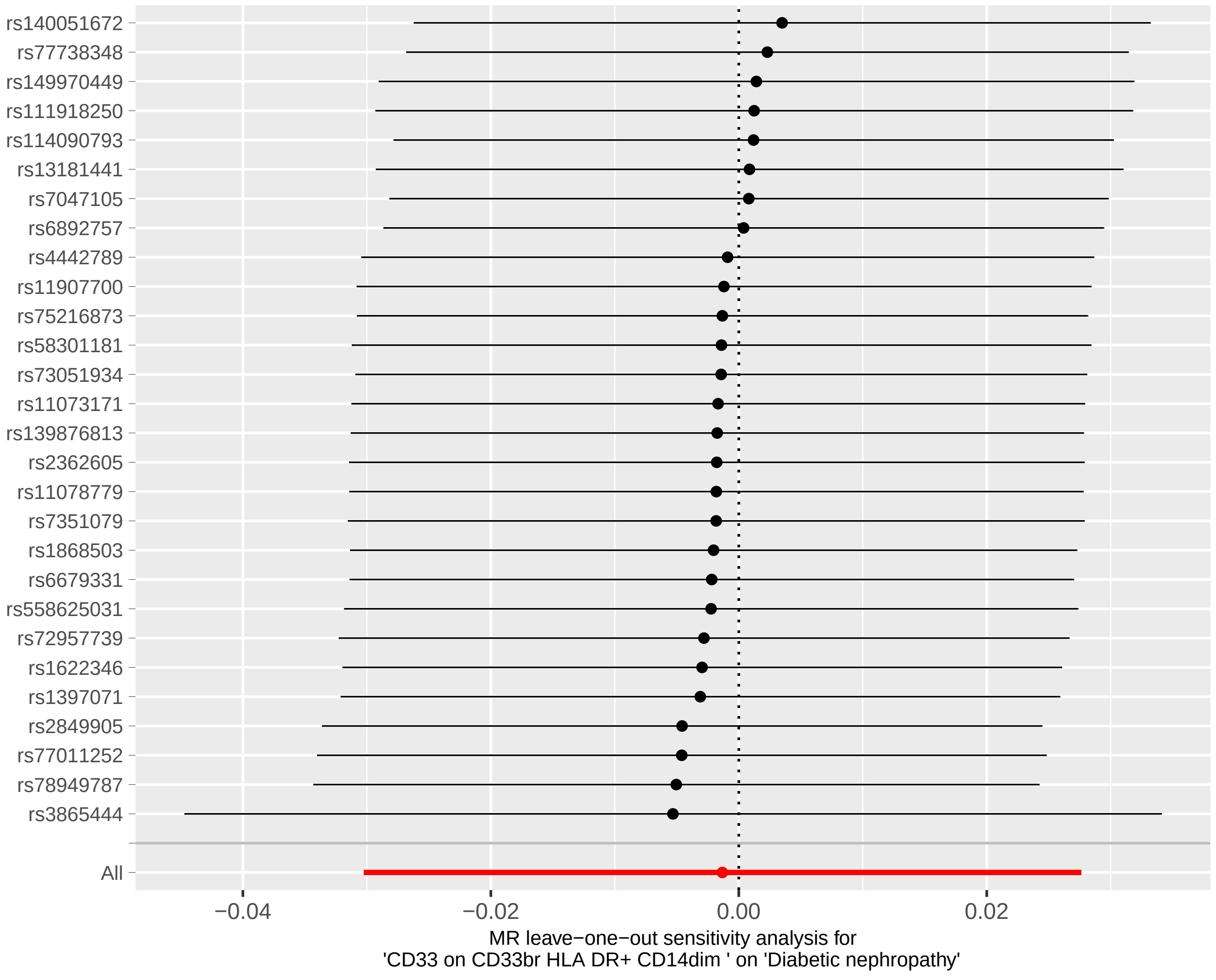
rs140051672
rs77738348
rs149970449
rs111918250
rs114090793
rs13181441
rs7047105
rs6892757
rs4442789
rs11907700
rs75216873
rs58301181
rs73051934
rs11073171
rs139876813
rs2362605
rs11078779
rs7351079
rs1868503
rs6679331
rs558625031
rs72957739
rs1622346
rs1397071
rs2849905
rs77011252
rs78949787
rs3865444
All
−0.04
−0.02
0.00
0.02
MR leave−one−out sensitivity analysis for
'CD33 on CD33br HLA DR+ CD14dim ' on 'Diabetic nephropathy'

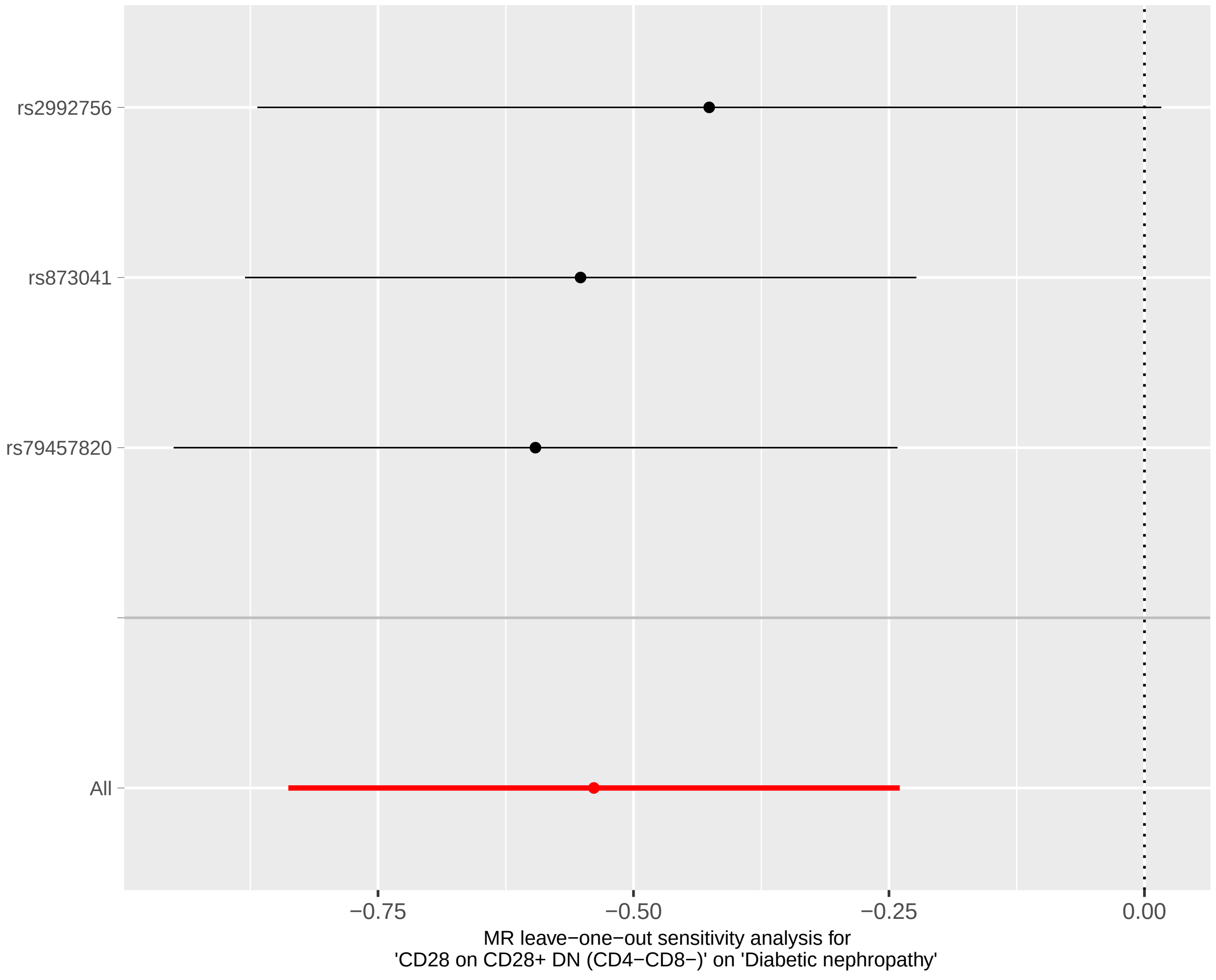
rs2992756
rs873041
rs79457820
All
−0.75
−0.50
−0.25
0.00
MR leave−one−out sensitivity analysis for
'CD28 on CD28+ DN (CD4−CD8−)' on 'Diabetic nephropathy'

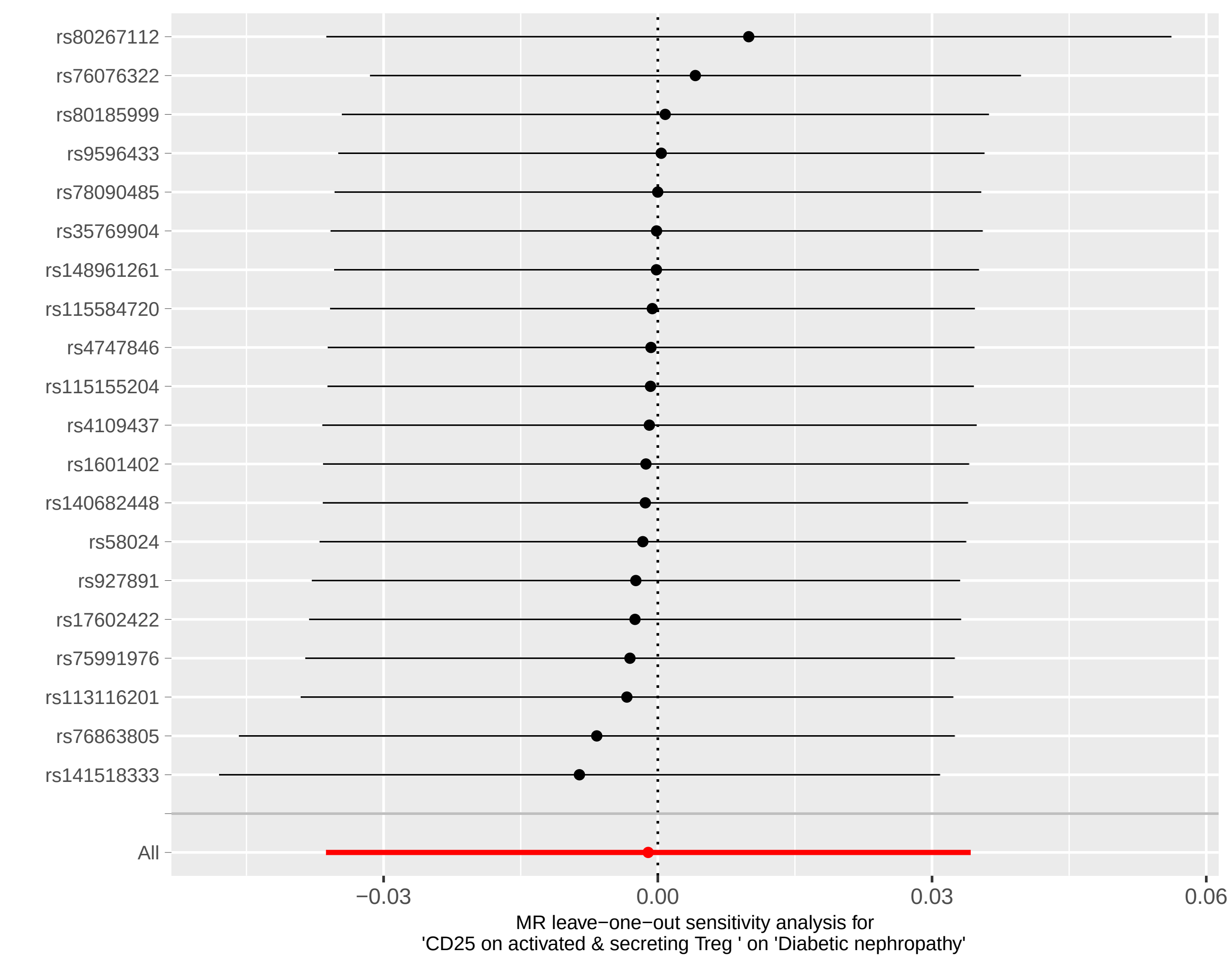

rs80267112
rs76076322
rs80185999
rs9596433
rs78090485
rs35769904
rs148961261
rs115584720
rs4747846
rs115155204
rs4109437
rs1601402
rs140682448
rs58024
rs927891
rs17602422
rs75991976
rs113116201
rs76863805
rs141518333
All
−0.03
0.00
0.03
0.06
MR leave−one−out sensitivity analysis for
'CD25 on activated & secreting Treg ' on 'Diabetic nephropathy'

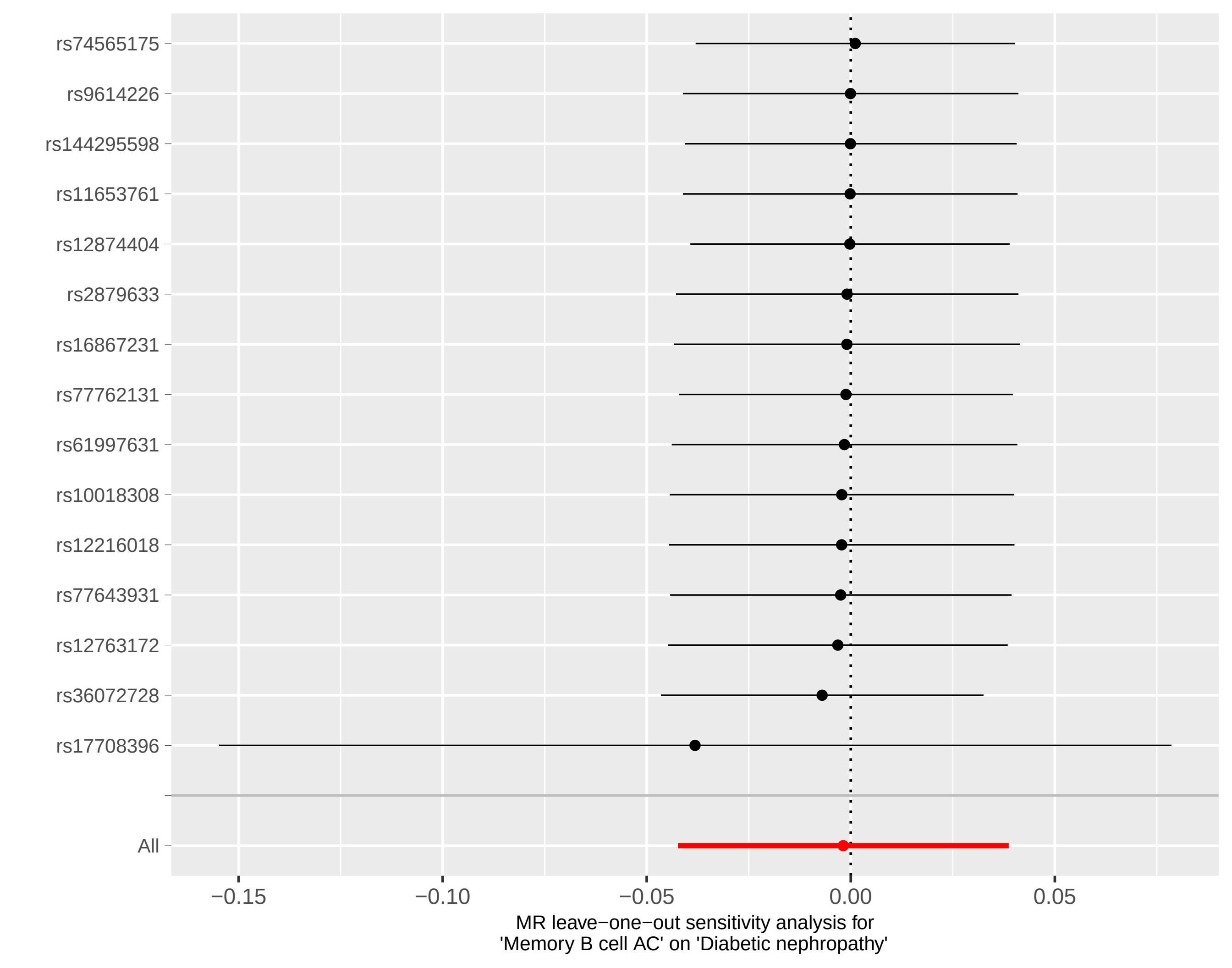
rs74565175
rs9614226
rs144295598
rs11653761
rs12874404
rs2879633
rs16867231
rs77762131
rs61997631
rs10018308
rs12216018
rs77643931
rs12763172
rs36072728
rs17708396
All
−0.15
−0.10
−0.05
0.00
0.05
MR leave−one−out sensitivity analysis for
'Memory B cell AC' on 'Diabetic nephropathy'

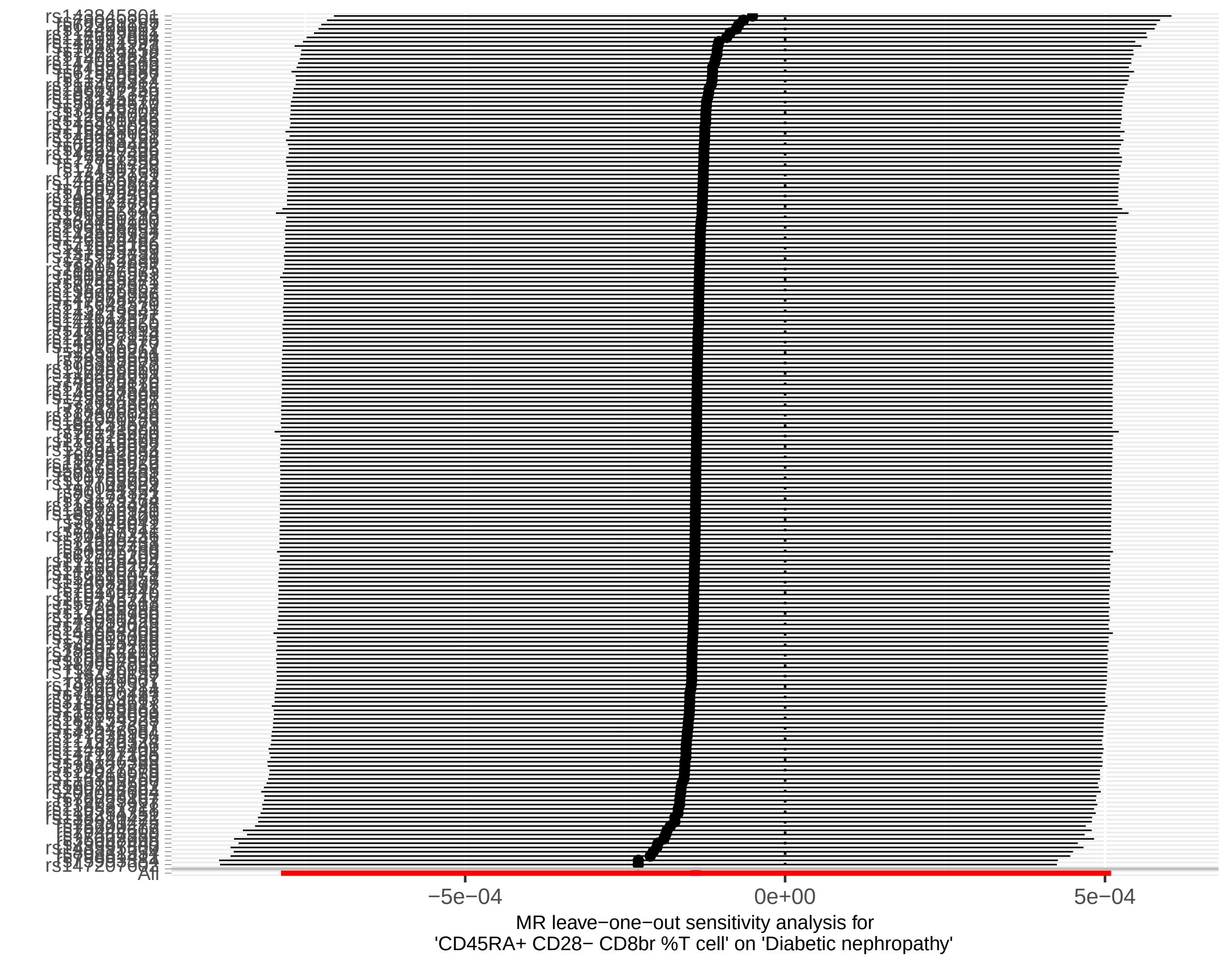
All
−5e−04
0e+00
5e−04
MR leave−one−out sensitivity analysis for
'CD45RA+ CD28− CD8br %T cell' on 'Diabetic nephropathy'

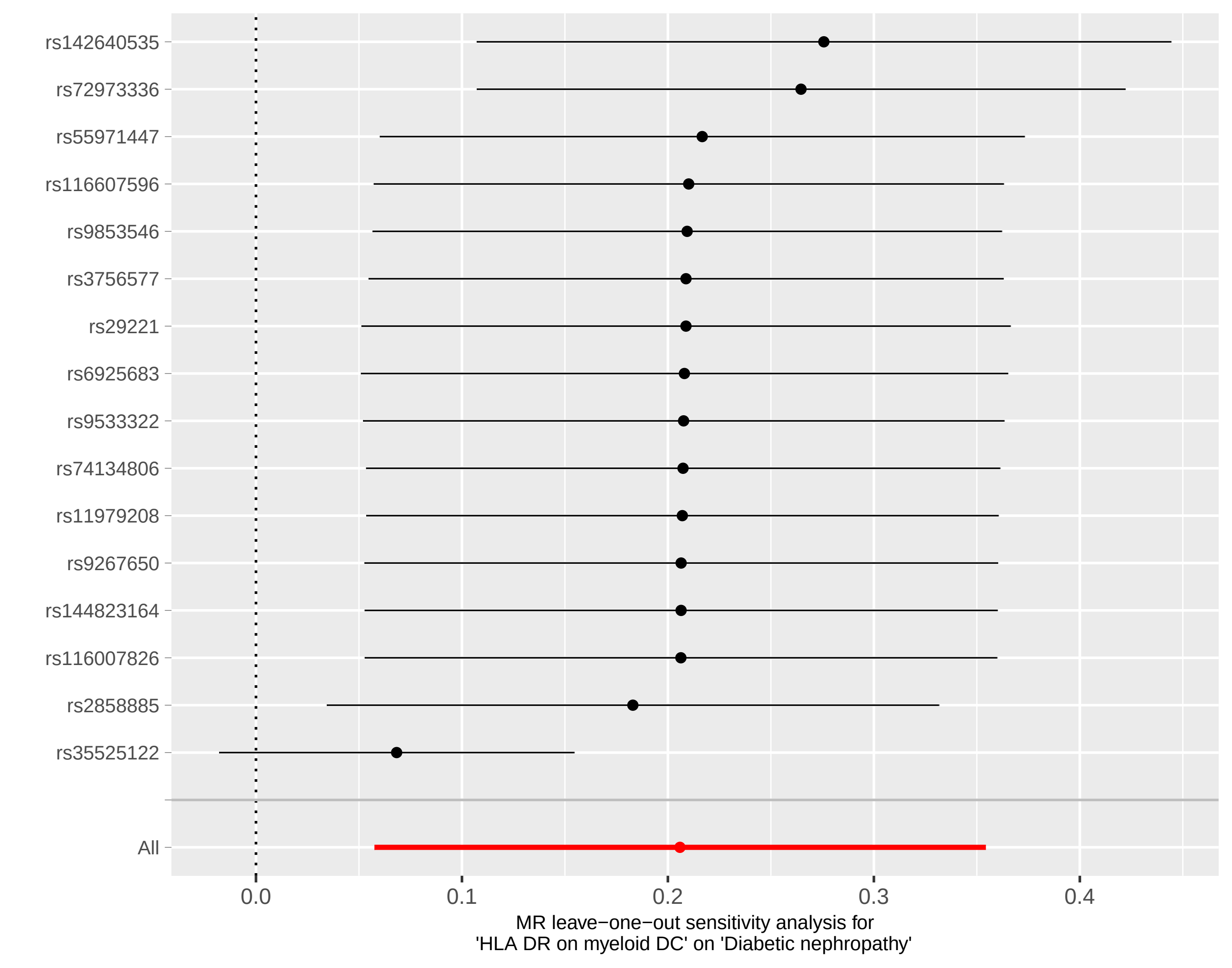
rs142640535
rs72973336
rs55971447
rs116607596
rs9853546
rs3756577
rs29221
rs6925683
rs9533322
rs74134806
rs11979208
rs9267650
rs144823164
rs116007826
rs2858885
rs35525122
All
0.0
0.1
0.2
0.3
0.4
MR leave−one−out sensitivity analysis for
'HLA DR on myeloid DC' on 'Diabetic nephropathy'

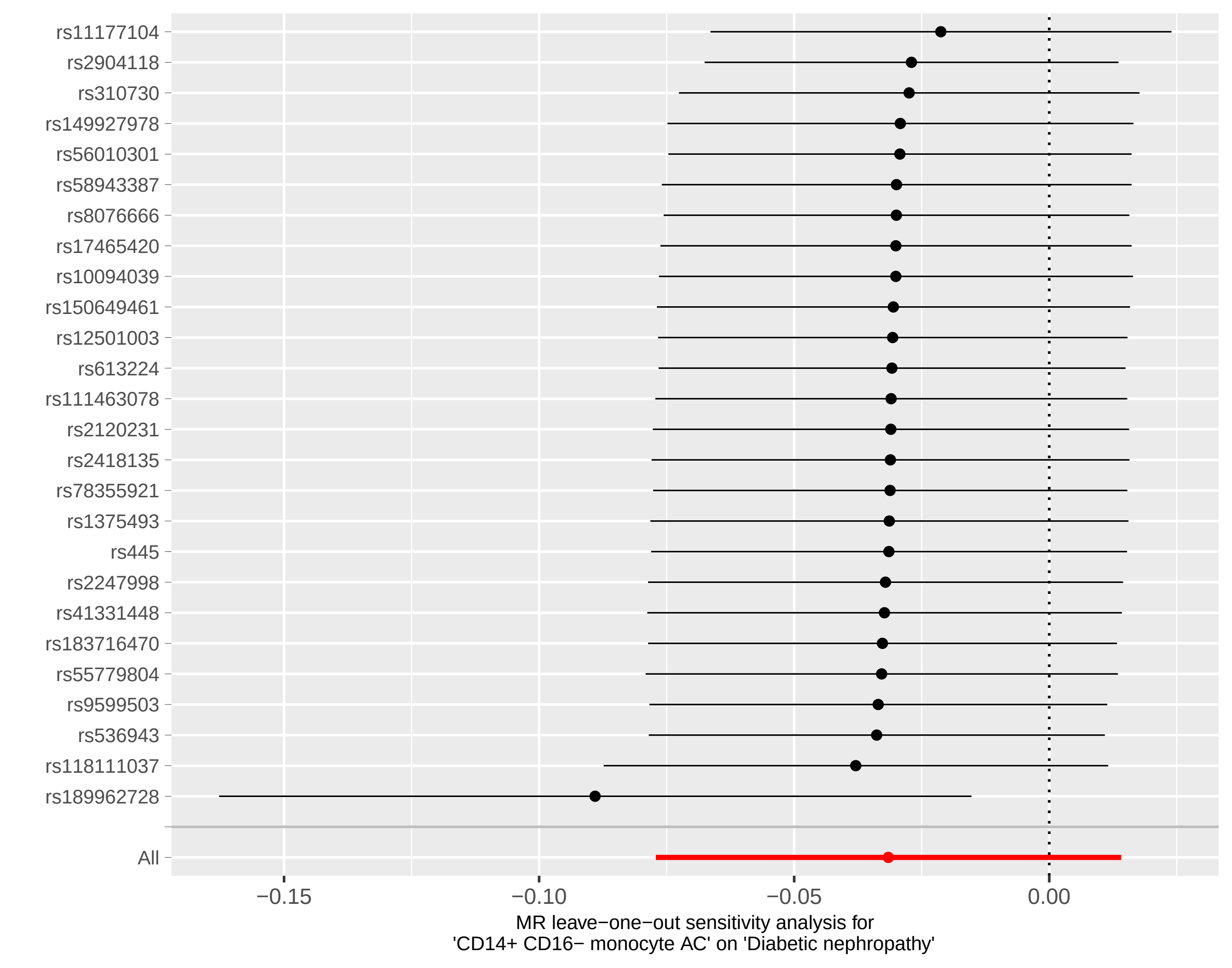

rs11177104
rs2904118
rs310730
rs149927978
rs56010301
rs58943387
rs8076666
rs17465420
rs10094039
rs150649461
rs12501003
rs613224
rs111463078
rs2120231
rs2418135
rs78355921
rs1375493
rs445
rs2247998
rs41331448
rs183716470
rs55779804
rs9599503
rs536943
rs118111037
rs189962728
All
−0.15
−0.10
−0.05
0.00
MR leave−one−out sensitivity analysis for
'CD14+ CD16− monocyte AC' on 'Diabetic nephropathy'

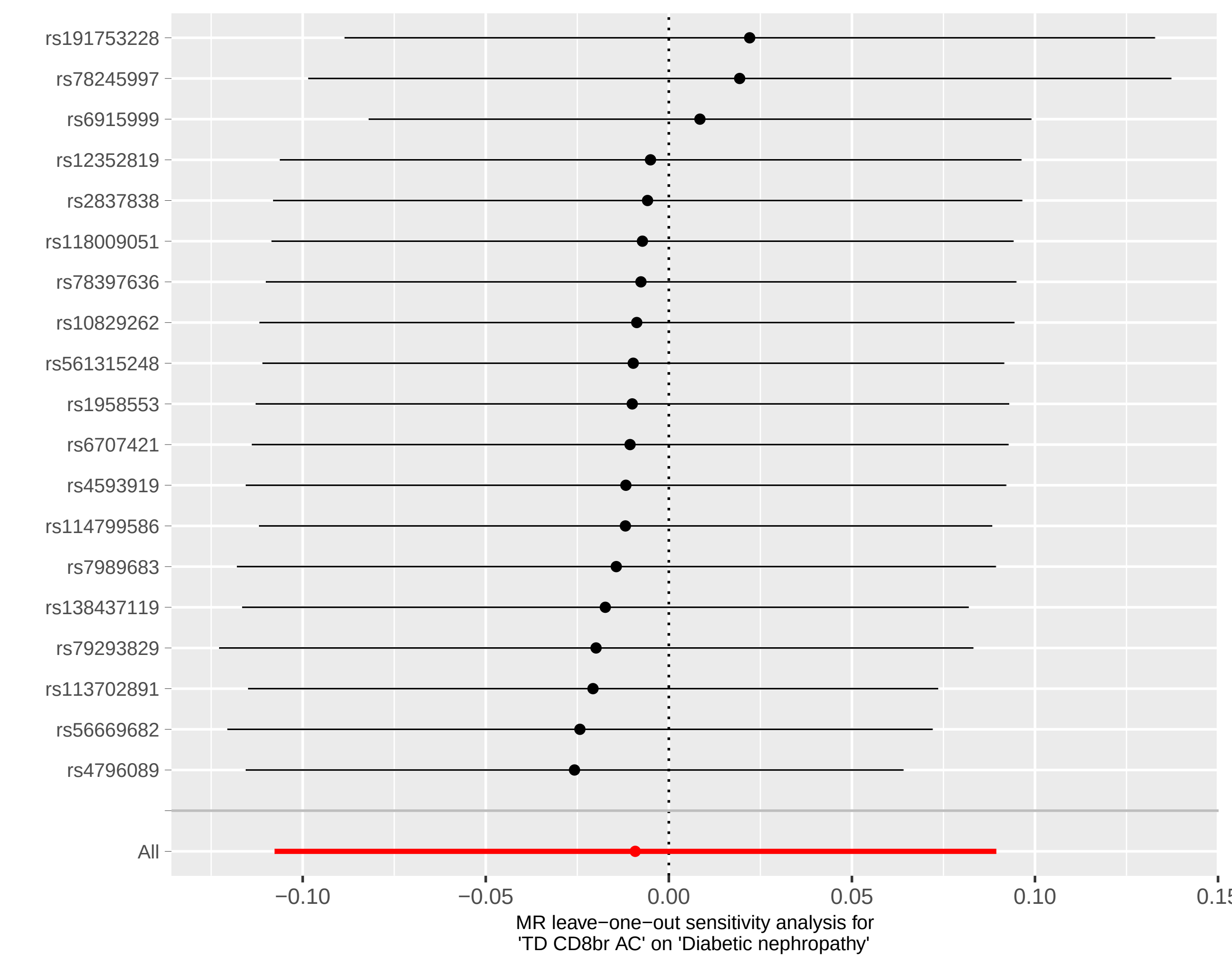
rs191753228
rs78245997
rs6915999
rs12352819
rs2837838
rs118009051
rs78397636
rs10829262
rs561315248
rs1958553
rs6707421
rs4593919
rs114799586
rs7989683
rs138437119
rs79293829
rs113702891
rs56669682
rs4796089
All
−0.10
−0.05
0.00
0.05
0.10
MR leave−one−out sensitivity analysis for
'TD CD8br AC' on 'Diabetic nephropathy'

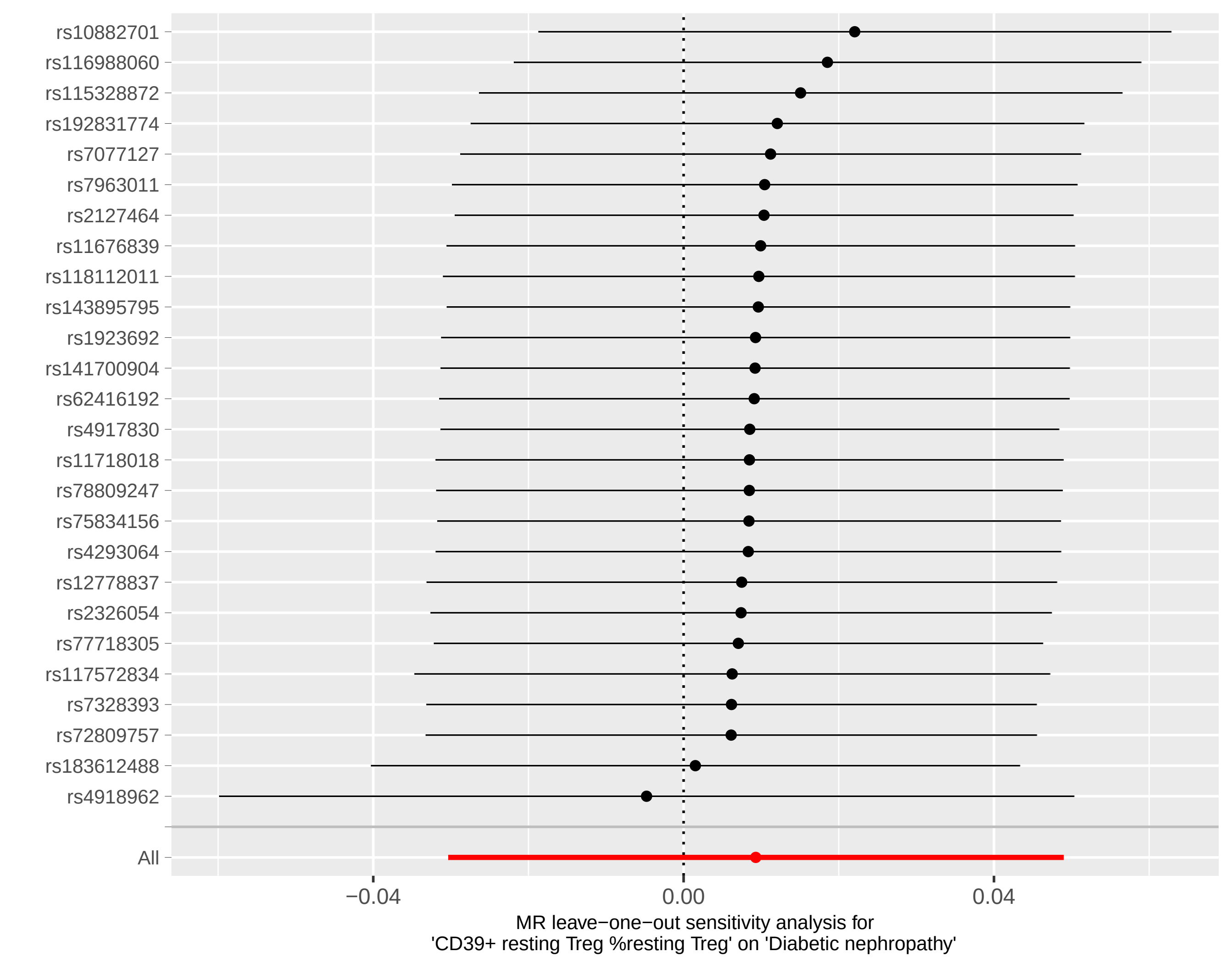
rs10882701
rs116988060
rs115328872
rs192831774
rs7077127
rs7963011
rs2127464
rs11676839
rs118112011
rs143895795
rs1923692
rs141700904
rs62416192
rs4917830
rs11718018
rs78809247
rs75834156
rs4293064
rs12778837
rs2326054
rs77718305
rs117572834
rs7328393
rs72809757
rs183612488
rs4918962
All
−0.04
0.00
0.04
MR leave−one−out sensitivity analysis for
'CD39+ resting Treg %resting Treg' on 'Diabetic nephropathy'

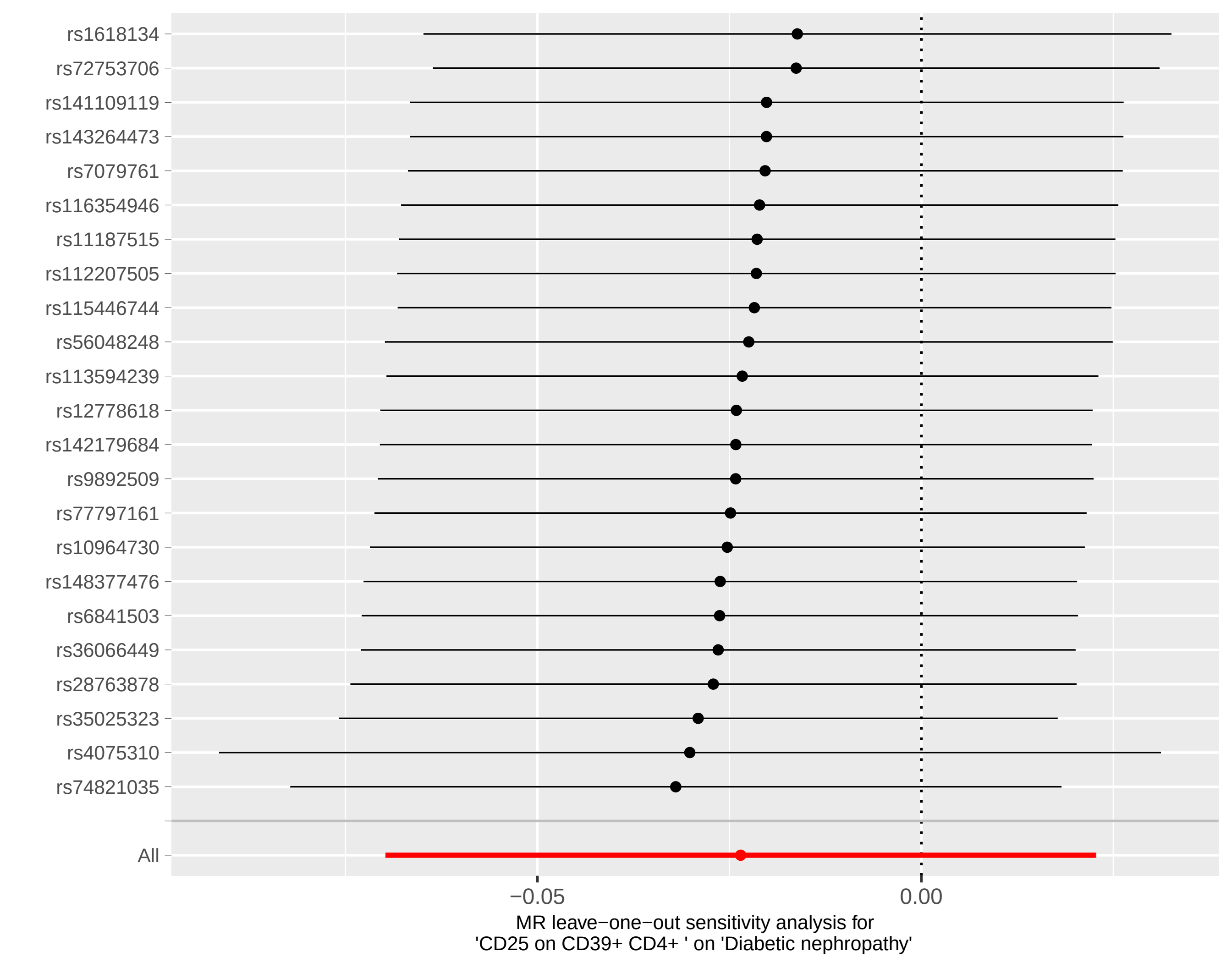
rs1618134
rs72753706
rs141109119
rs143264473
rs7079761
rs116354946
rs11187515
rs112207505
rs115446744
rs56048248
rs113594239
rs12778618
rs142179684
rs9892509
rs77797161
rs10964730
rs148377476
rs6841503
rs36066449
rs28763878
rs35025323
rs4075310
rs74821035
All
−0.05
0.00
MR leave−one−out sensitivity analysis for
'CD25 on CD39+ CD4+ ' on 'Diabetic nephropathy'

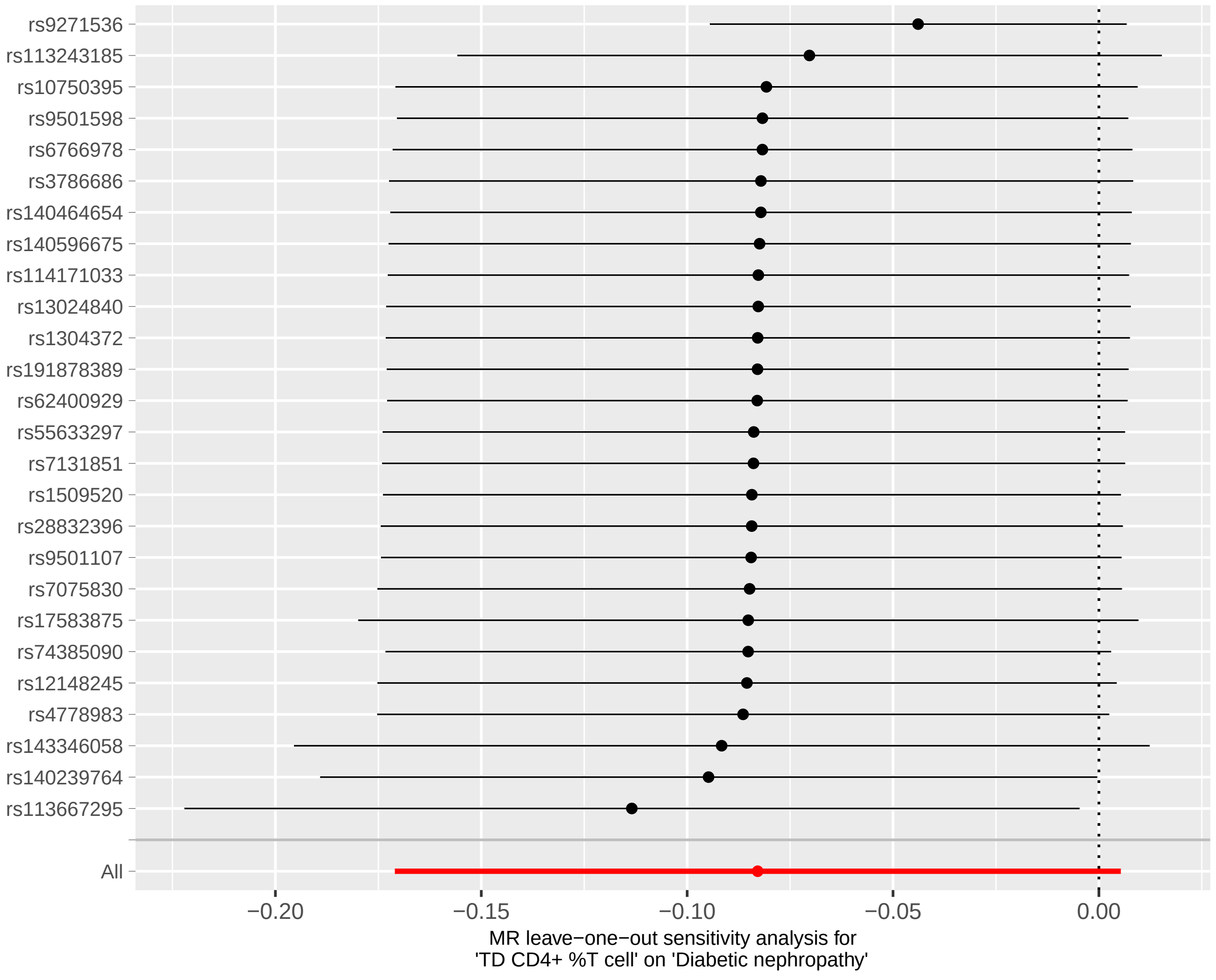

rs9271536
rs113243185
rs10750395
rs9501598
rs6766978
rs3786686
rs140464654
rs140596675
rs114171033
rs13024840
rs1304372
rs191878389
rs62400929
rs55633297
rs7131851
rs1509520
rs28832396
rs9501107
rs7075830
rs17583875
rs74385090
rs12148245
rs4778983
rs143346058
rs140239764
rs113667295
All
−0.20
−0.15
−0.10
−0.05
0.00
MR leave−one−out sensitivity analysis for
'TD CD4+ %T cell' on 'Diabetic nephropathy'

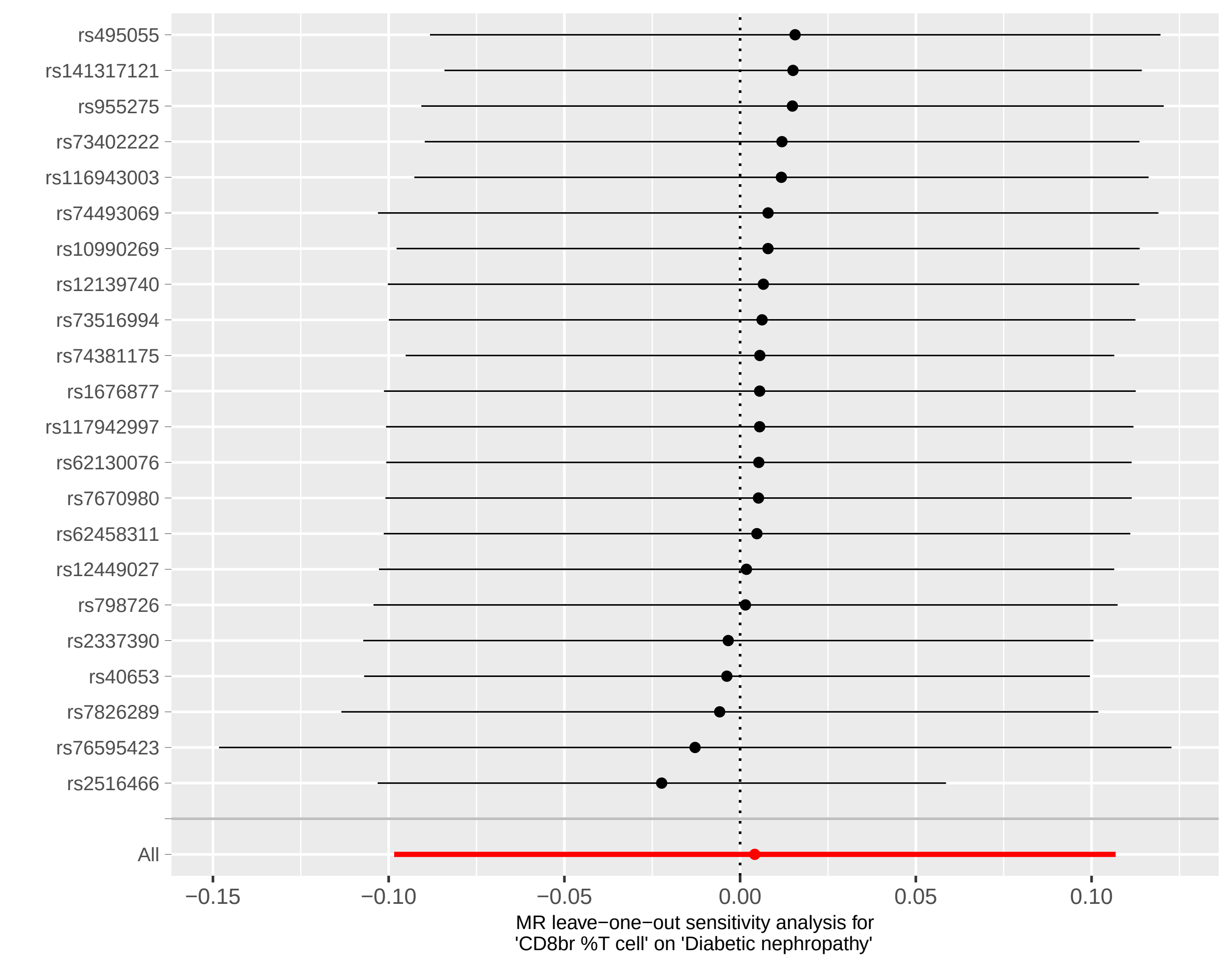
rs495055
rs141317121
rs955275
rs73402222
rs116943003
rs74493069
rs10990269
rs12139740
rs73516994
rs74381175
rs1676877
rs117942997
rs62130076
rs7670980
rs62458311
rs12449027
rs798726
rs2337390
rs40653
rs7826289
rs76595423
rs2516466
All
−0.15
−0.10
−0.05
0.00
0.05
0.10
MR leave−one−out sensitivity analysis for
'CD8br %T cell' on 'Diabetic nephropathy'

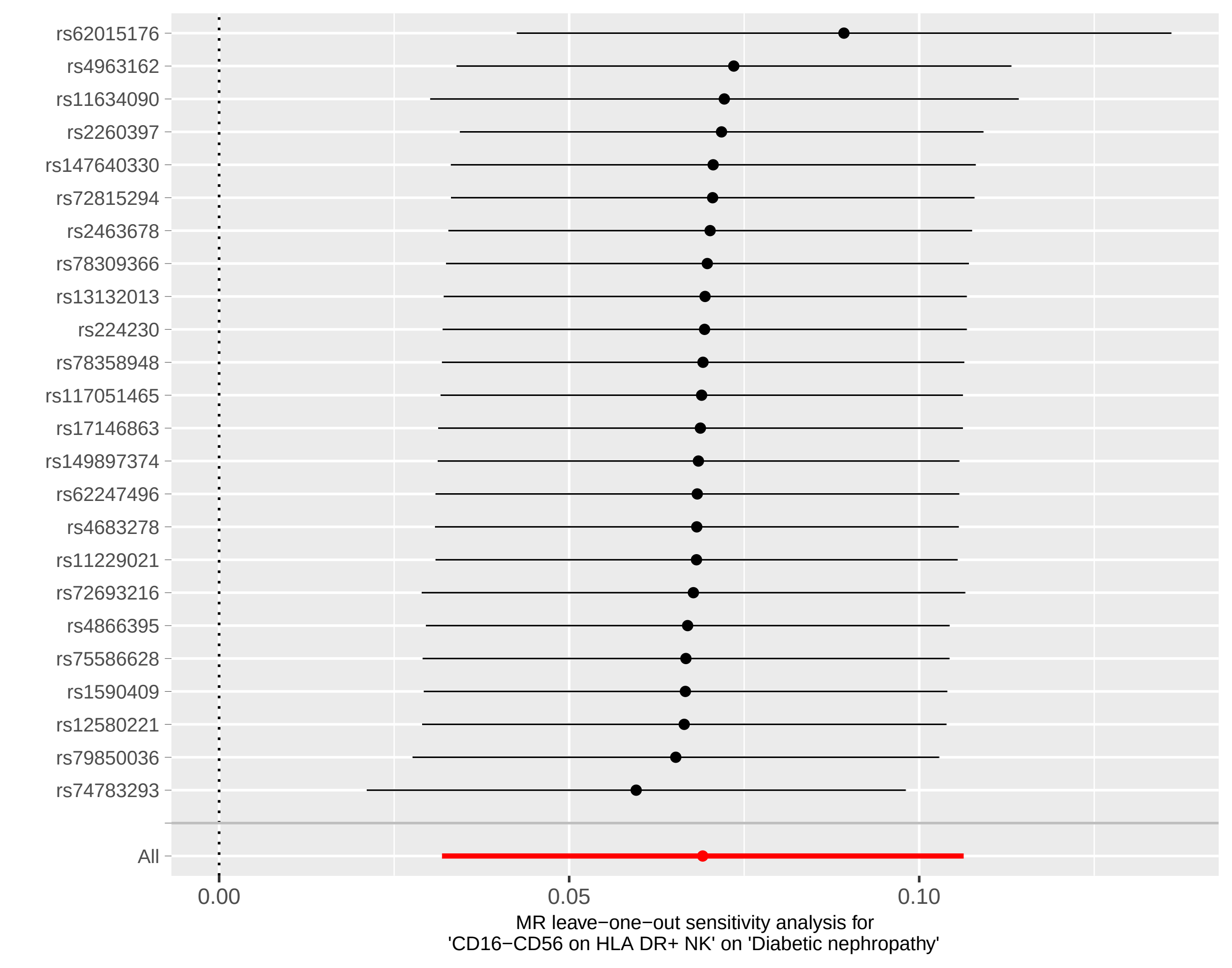
rs62015176
rs4963162
rs11634090
rs2260397
rs147640330
rs72815294
rs2463678
rs78309366
rs13132013
rs224230
rs78358948
rs117051465
rs17146863
rs149897374
rs62247496
rs4683278
rs11229021
rs72693216
rs4866395
rs75586628
rs1590409
rs12580221
rs79850036
rs74783293
All
0.00
0.05
0.10
MR leave−one−out sensitivity analysis for
'CD16−CD56 on HLA DR+ NK' on 'Diabetic nephropathy'

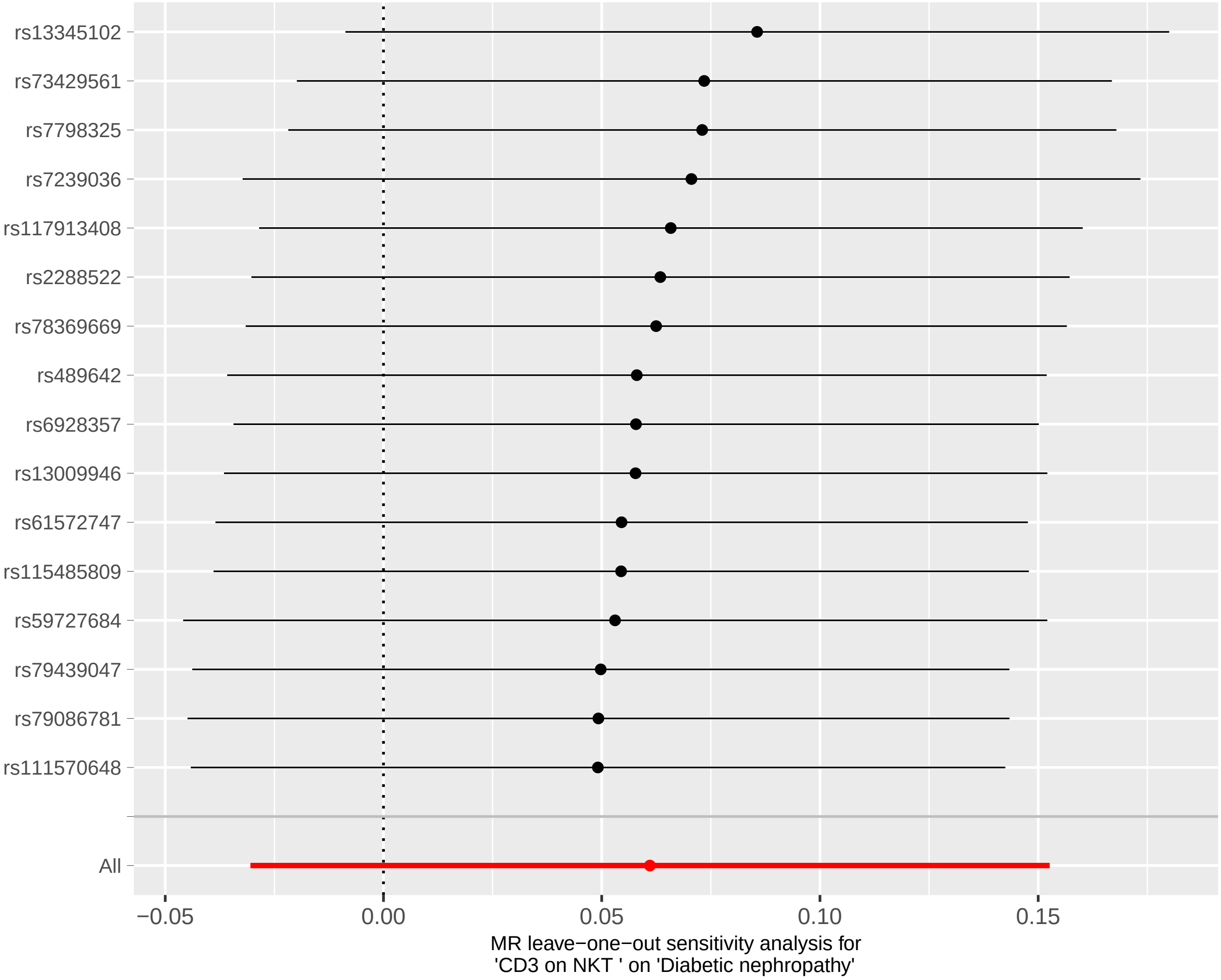
rs13345102
rs73429561
rs7798325
rs7239036
rs117913408
rs2288522
rs78369669
rs489642
rs6928357
rs13009946
rs61572747
rs115485809
rs59727684
rs79439047
rs79086781
rs111570648
All
−0.05
0.00
0.05
0.10
0.15
MR leave−one−out sensitivity analysis for
'CD3 on NKT ' on 'Diabetic nephropathy'

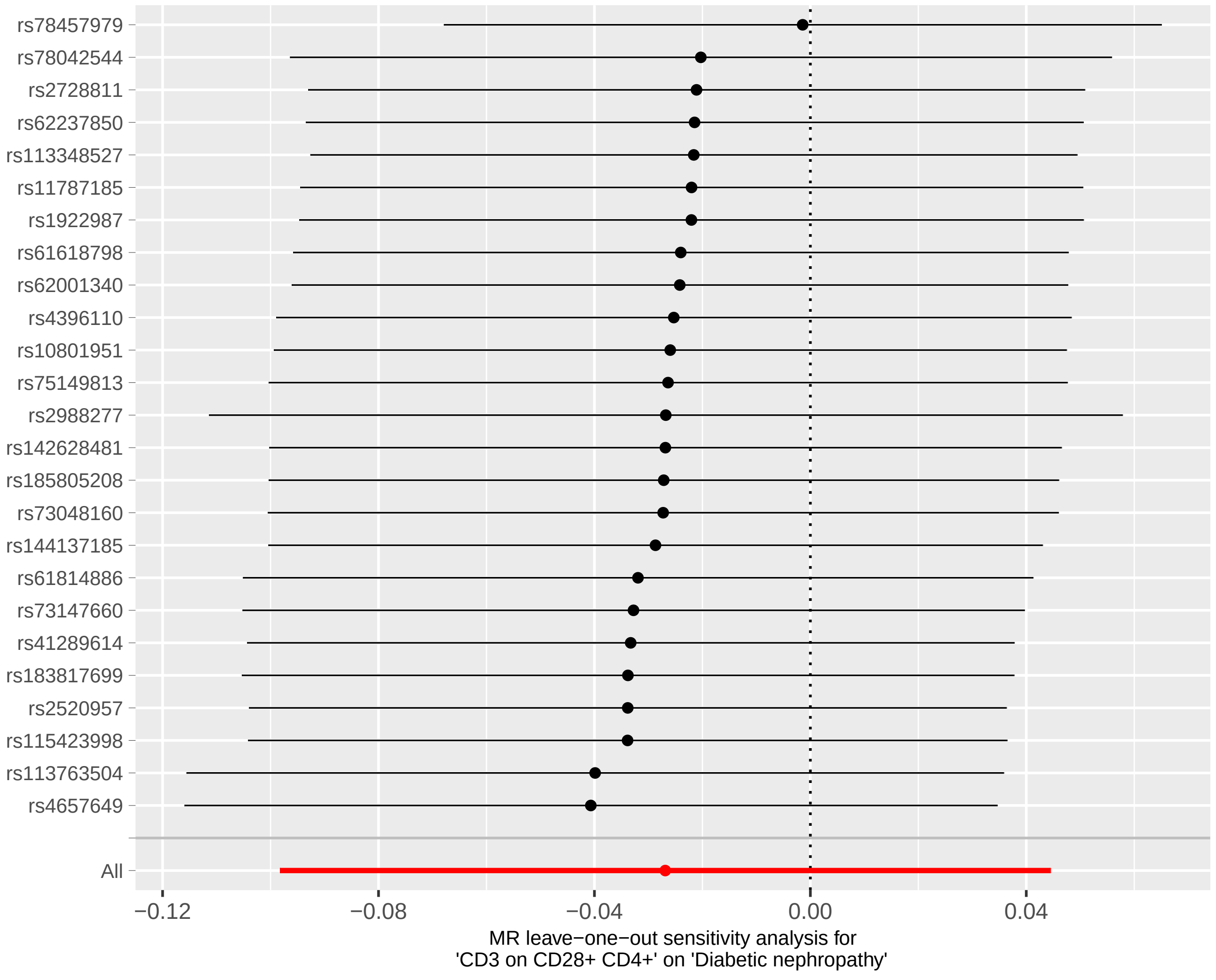

rs78457979
rs78042544
rs2728811
rs62237850
rs113348527
rs11787185
rs1922987
rs61618798
rs62001340
rs4396110
rs10801951
rs75149813
rs2988277
rs142628481
rs185805208
rs73048160
rs144137185
rs61814886
rs73147660
rs41289614
rs183817699
rs2520957
rs115423998
rs113763504
rs4657649
All
−0.12
−0.08
−0.04
0.00
0.04
MR leave−one−out sensitivity analysis for
'CD3 on CD28+ CD4+' on 'Diabetic nephropathy'

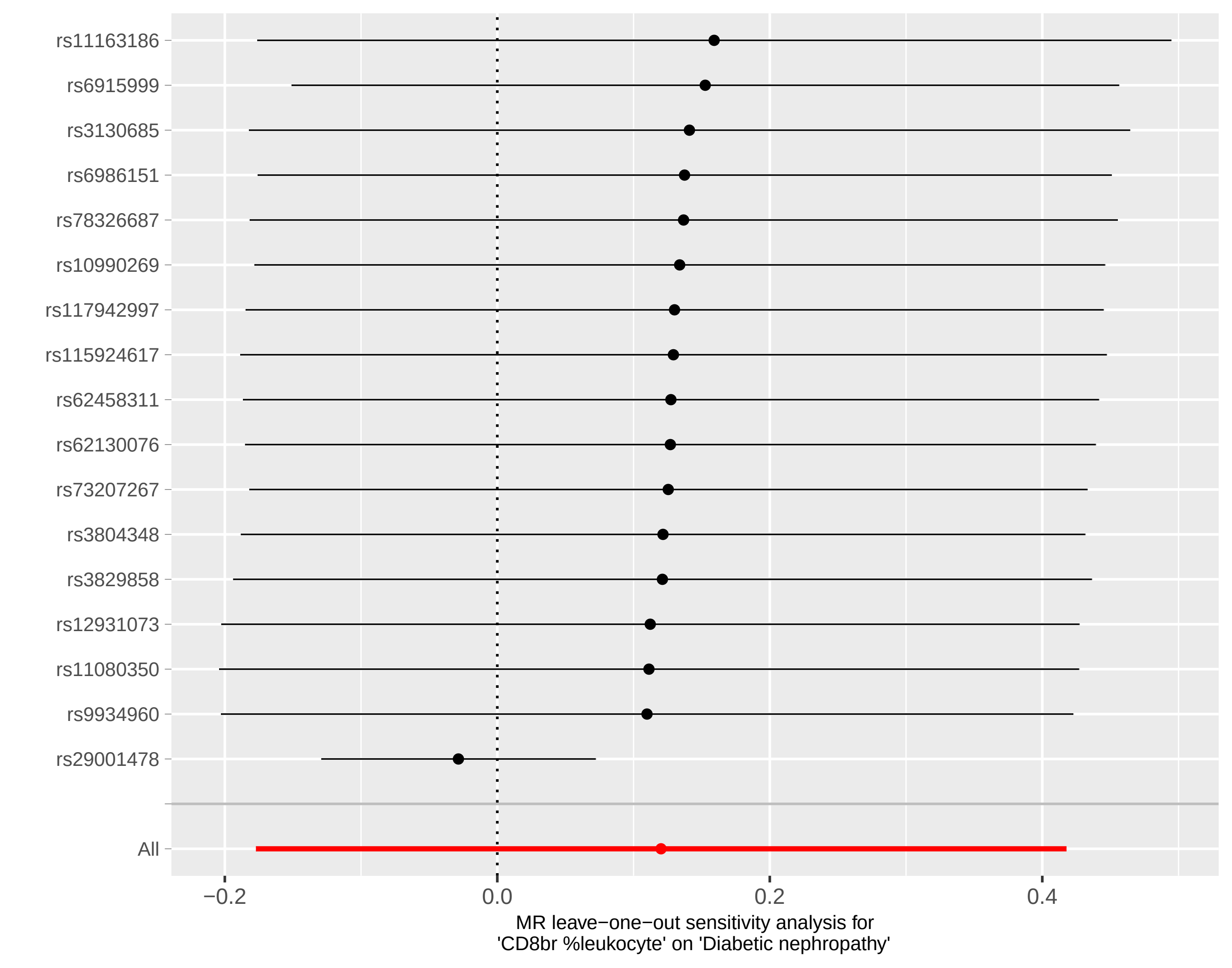
rs11163186
rs6915999
rs3130685
rs6986151
rs78326687
rs10990269
rs117942997
rs115924617
rs62458311
rs62130076
rs73207267
rs3804348
rs3829858
rs12931073
rs11080350
rs9934960
rs29001478
All
−0.2
0.0
0.2
0.4
MR leave−one−out sensitivity analysis for
'CD8br %leukocyte' on 'Diabetic nephropathy'

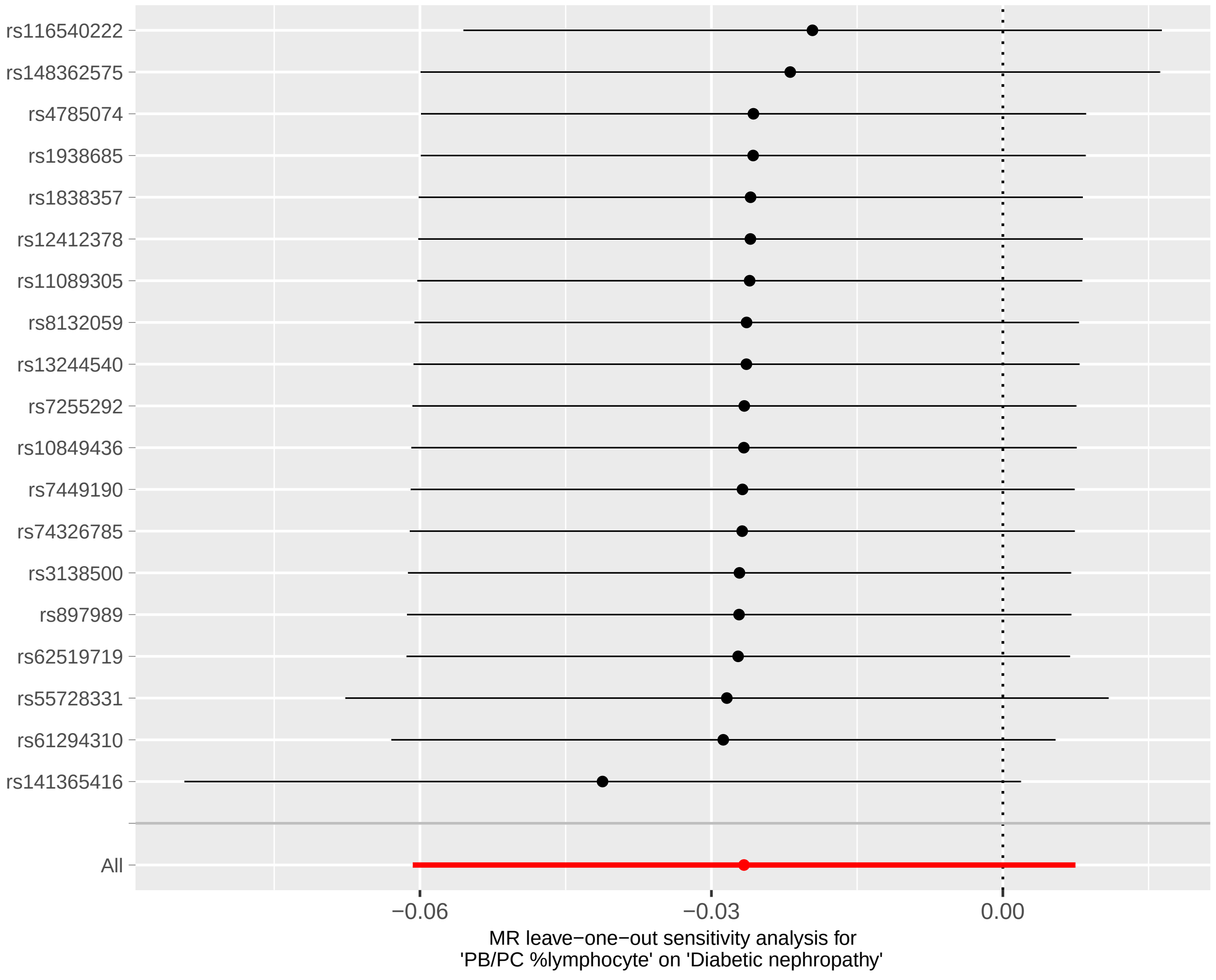
rs116540222
rs148362575
rs4785074
rs1938685
rs1838357
rs12412378
rs11089305
rs8132059
rs13244540
rs7255292
rs10849436
rs7449190
rs74326785
rs3138500
rs897989
rs62519719
rs55728331
rs61294310
rs141365416
All
−0.06
−0.03
0.00
MR leave−one−out sensitivity analysis for
'PB/PC %lymphocyte' on 'Diabetic nephropathy'

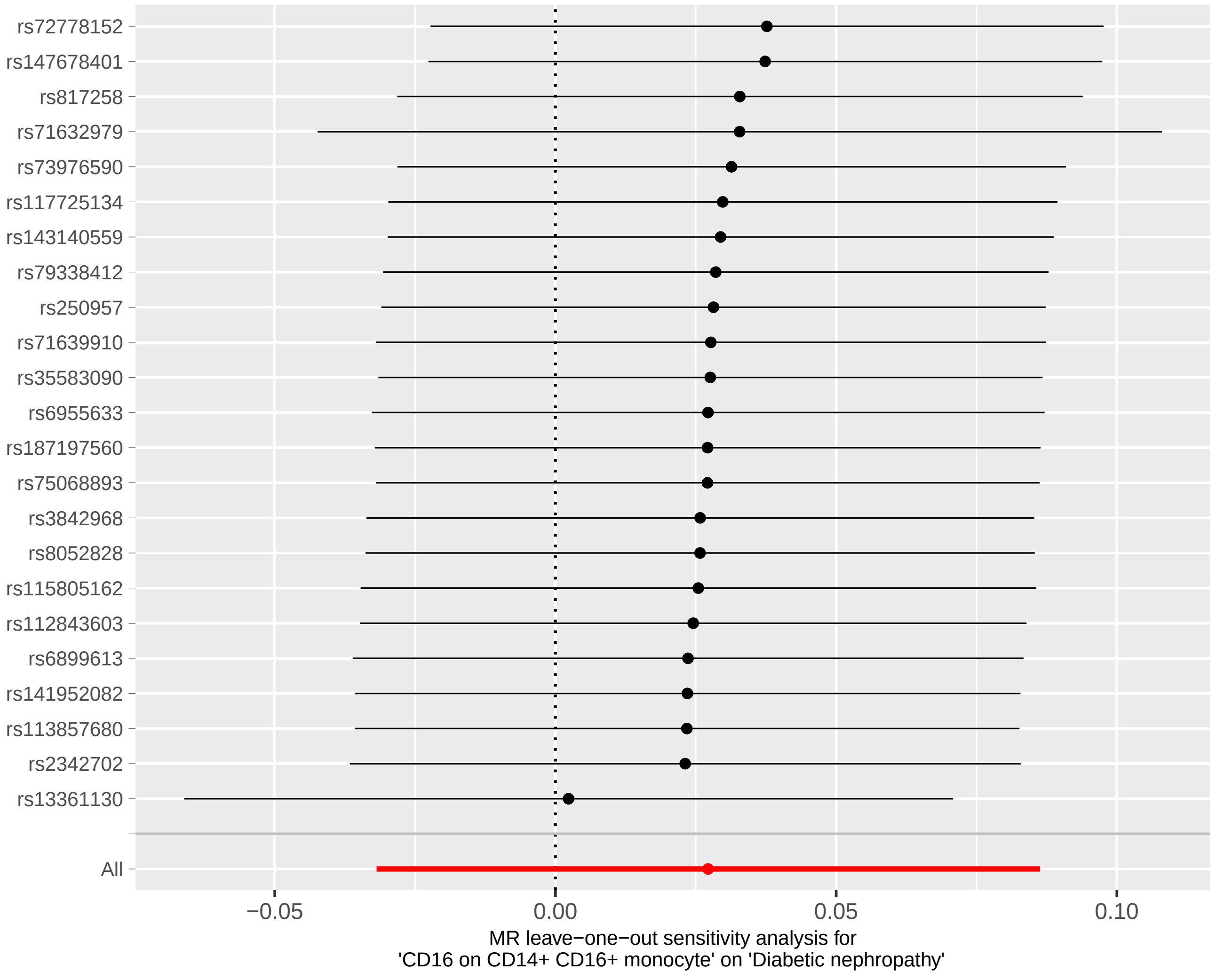
rs72778152
rs147678401
rs817258
rs71632979
rs73976590
rs117725134
rs143140559
rs79338412
rs250957
rs71639910
rs35583090
rs6955633
rs187197560
rs75068893
rs3842968
rs8052828
rs115805162
rs112843603
rs6899613
rs141952082
rs113857680
rs2342702
rs13361130
All
−0.05
0.00
0.05
0.10
MR leave−one−out sensitivity analysis for
'CD16 on CD14+ CD16+ monocyte' on 'Diabetic nephropathy'

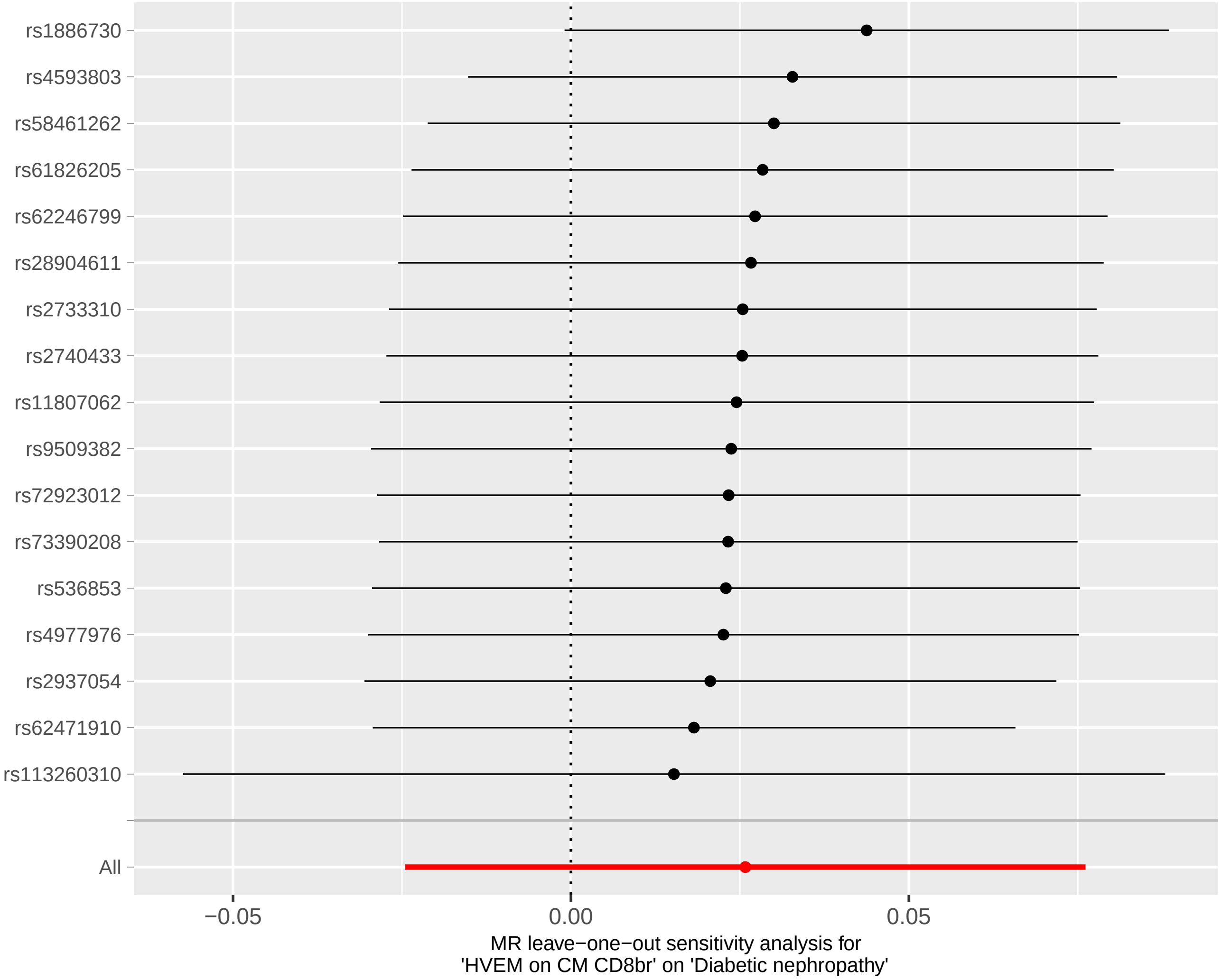

rs1886730
rs4593803
rs58461262
rs61826205
rs62246799
rs28904611
rs2733310
rs2740433
rs11807062
rs9509382
rs72923012
rs73390208
rs536853
rs4977976
rs2937054
rs62471910
rs113260310
All
−0.05
0.00
0.05
MR leave−one−out sensitivity analysis for
'HVEM on CM CD8br' on 'Diabetic nephropathy'

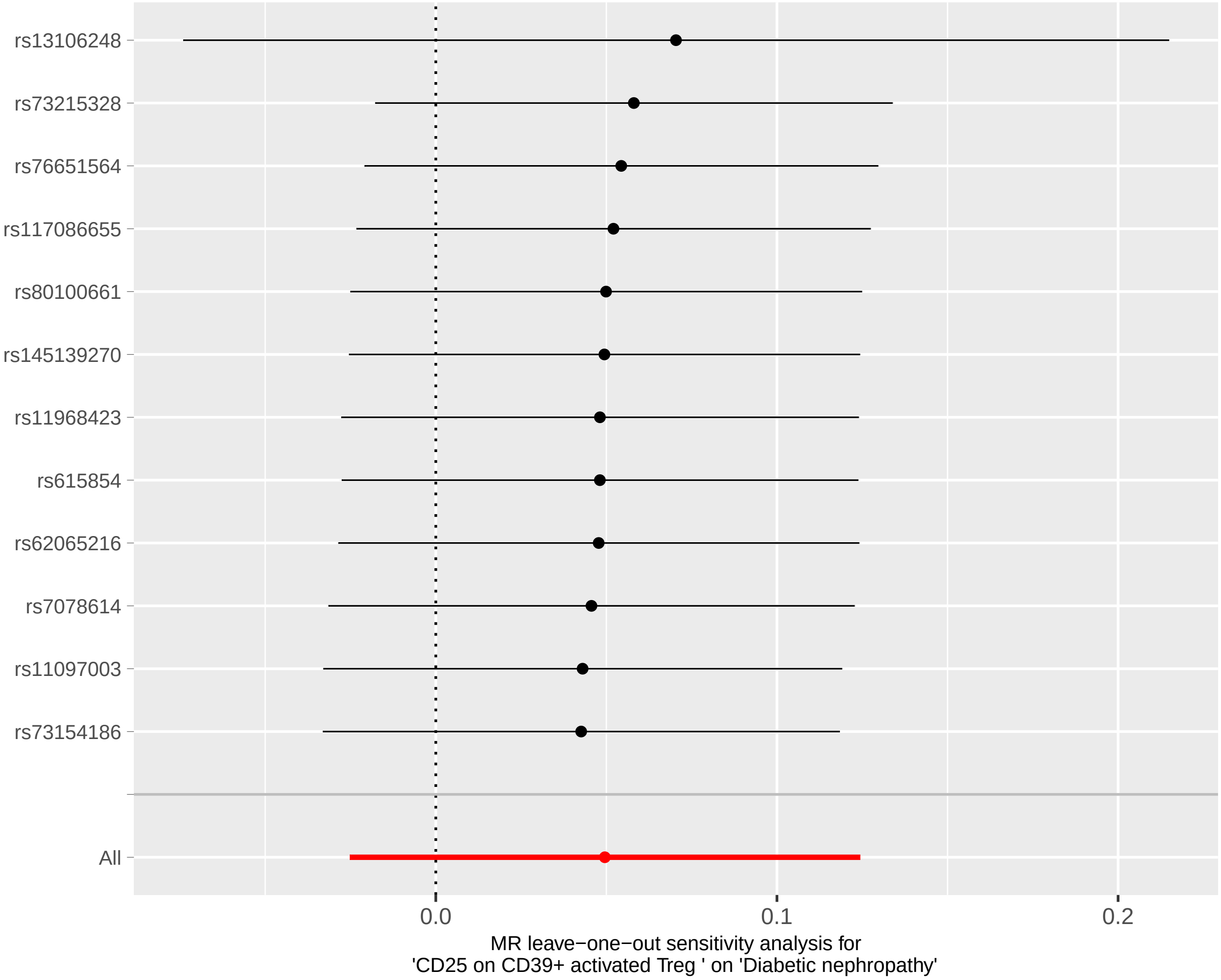
rs13106248
rs73215328
rs76651564
rs117086655
rs80100661
rs145139270
rs11968423
rs615854
rs62065216
rs7078614
rs11097003
rs73154186
All
0.0
0.1
0.2
MR leave−one−out sensitivity analysis for
'CD25 on CD39+ activated Treg ' on 'Diabetic nephropathy'

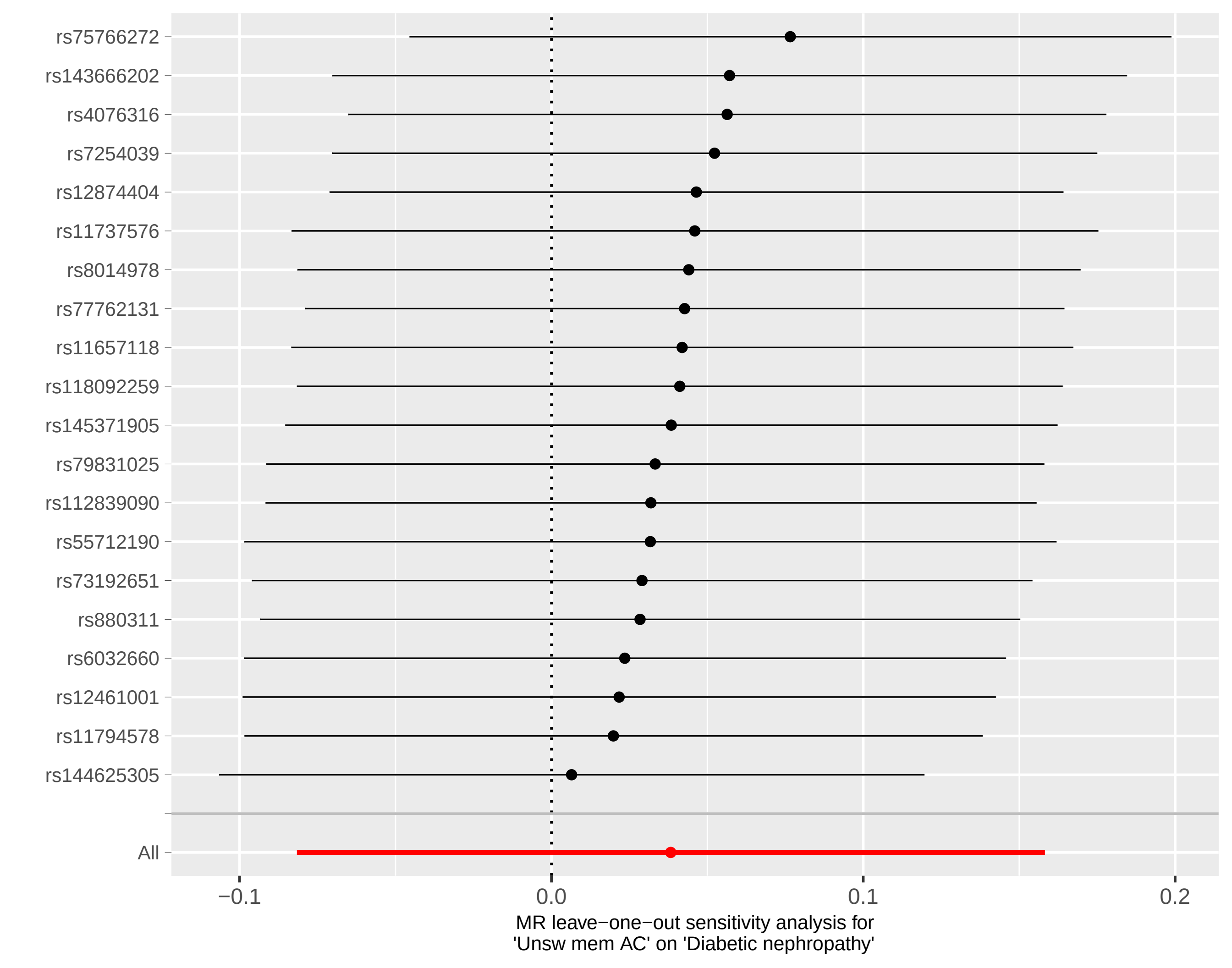
rs75766272
rs143666202
rs4076316
rs7254039
rs12874404
rs11737576
rs8014978
rs77762131
rs11657118
rs118092259
rs145371905
rs79831025
rs112839090
rs55712190
rs73192651
rs880311
rs6032660
rs12461001
rs11794578
rs144625305
All
−0.1
0.0
0.1
0.2
MR leave−one−out sensitivity analysis for
'Unsw mem AC' on 'Diabetic nephropathy'

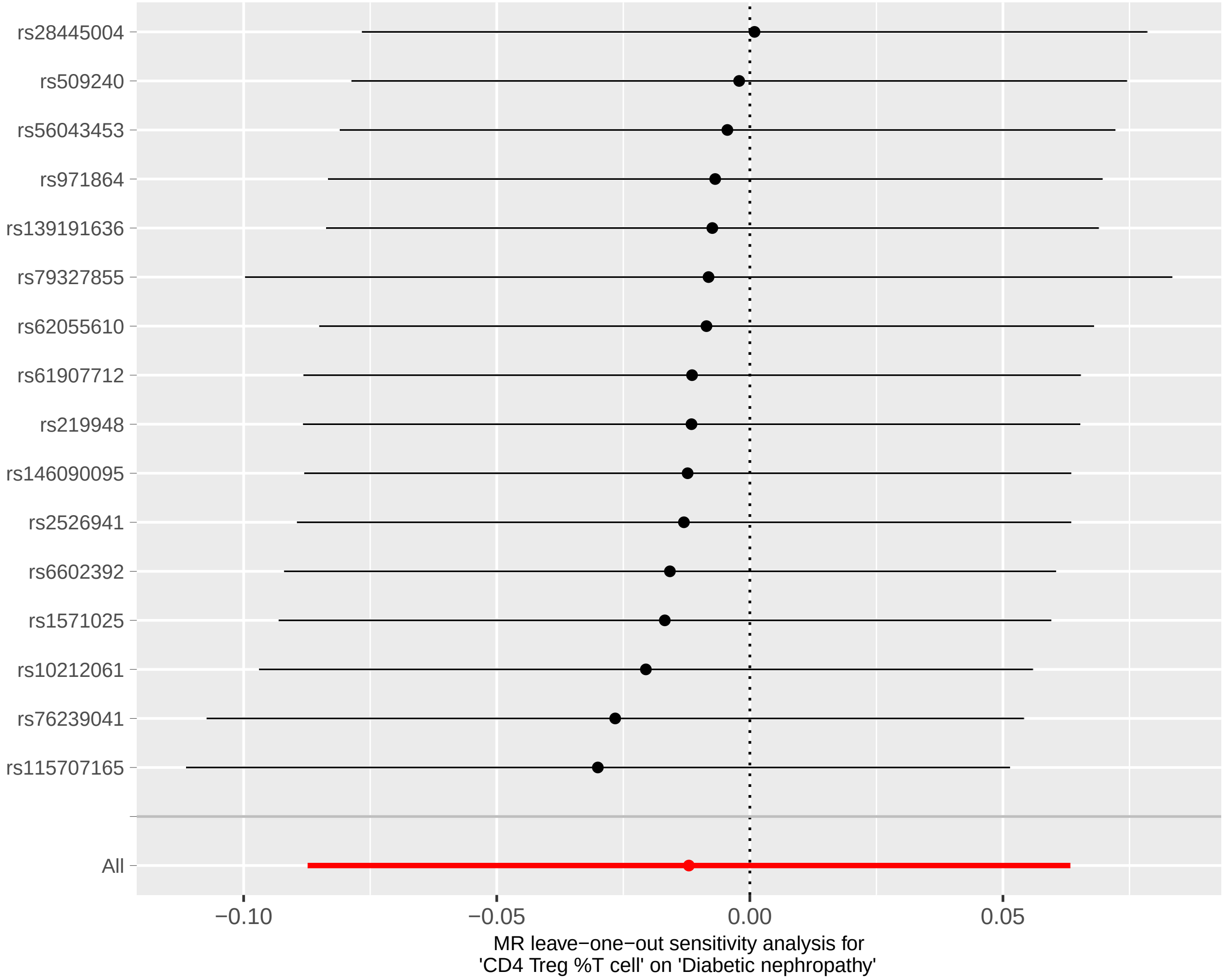
rs28445004
rs509240
rs56043453
rs971864
rs139191636
rs79327855
rs62055610
rs61907712
rs219948
rs146090095
rs2526941
rs6602392
rs1571025
rs10212061
rs76239041
rs115707165
All
−0.10
−0.05
0.00
0.05
MR leave−one−out sensitivity analysis for
'CD4 Treg %T cell' on 'Diabetic nephropathy'

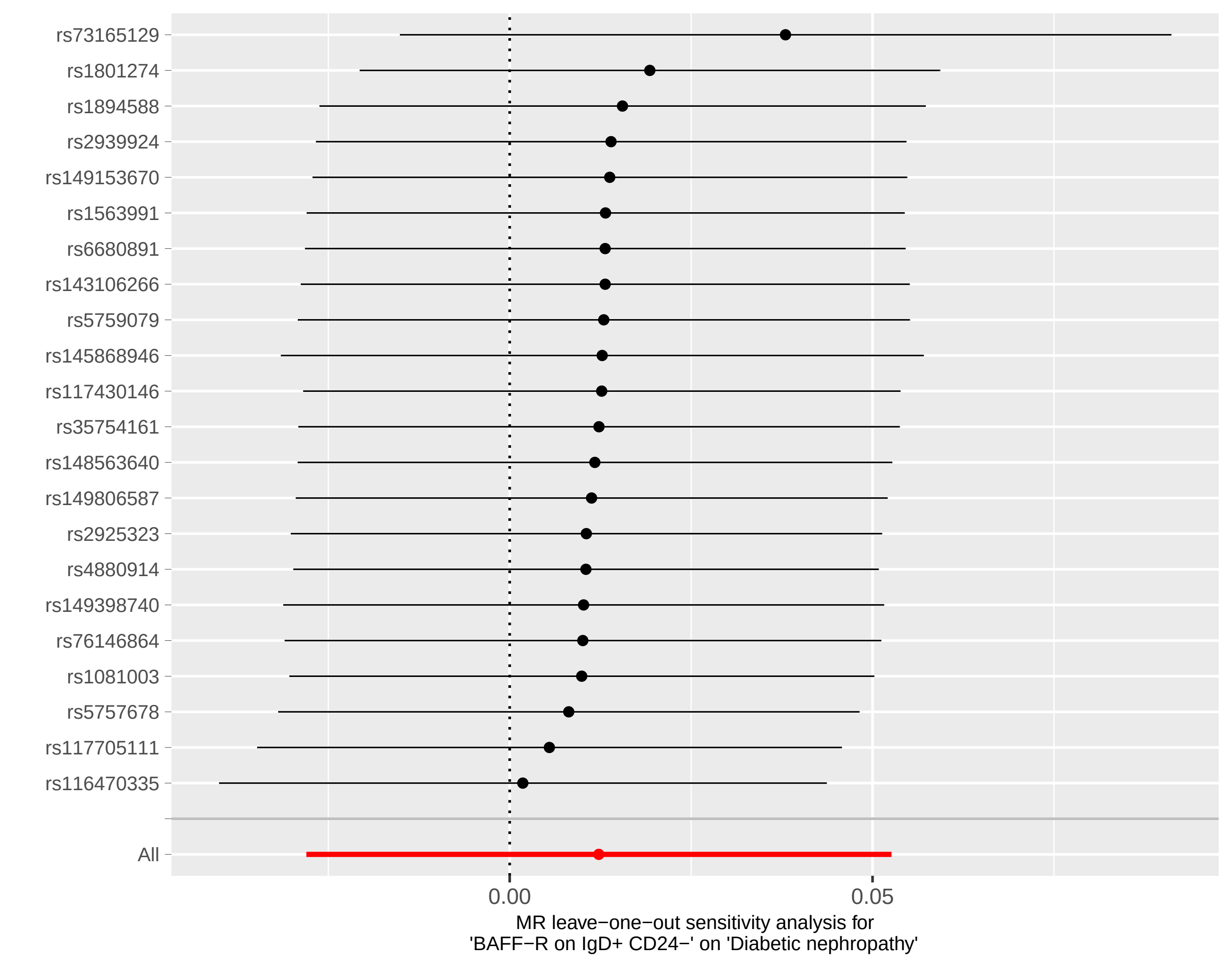

rs73165129
rs1801274
rs1894588
rs2939924
rs149153670
rs1563991
rs6680891
rs143106266
rs5759079
rs145868946
rs117430146
rs35754161
rs148563640
rs149806587
rs2925323
rs4880914
rs149398740
rs76146864
rs1081003
rs5757678
rs117705111
rs116470335
All
0.00
0.05
MR leave−one−out sensitivity analysis for
'BAFF−R on IgD+ CD24−' on 'Diabetic nephropathy'

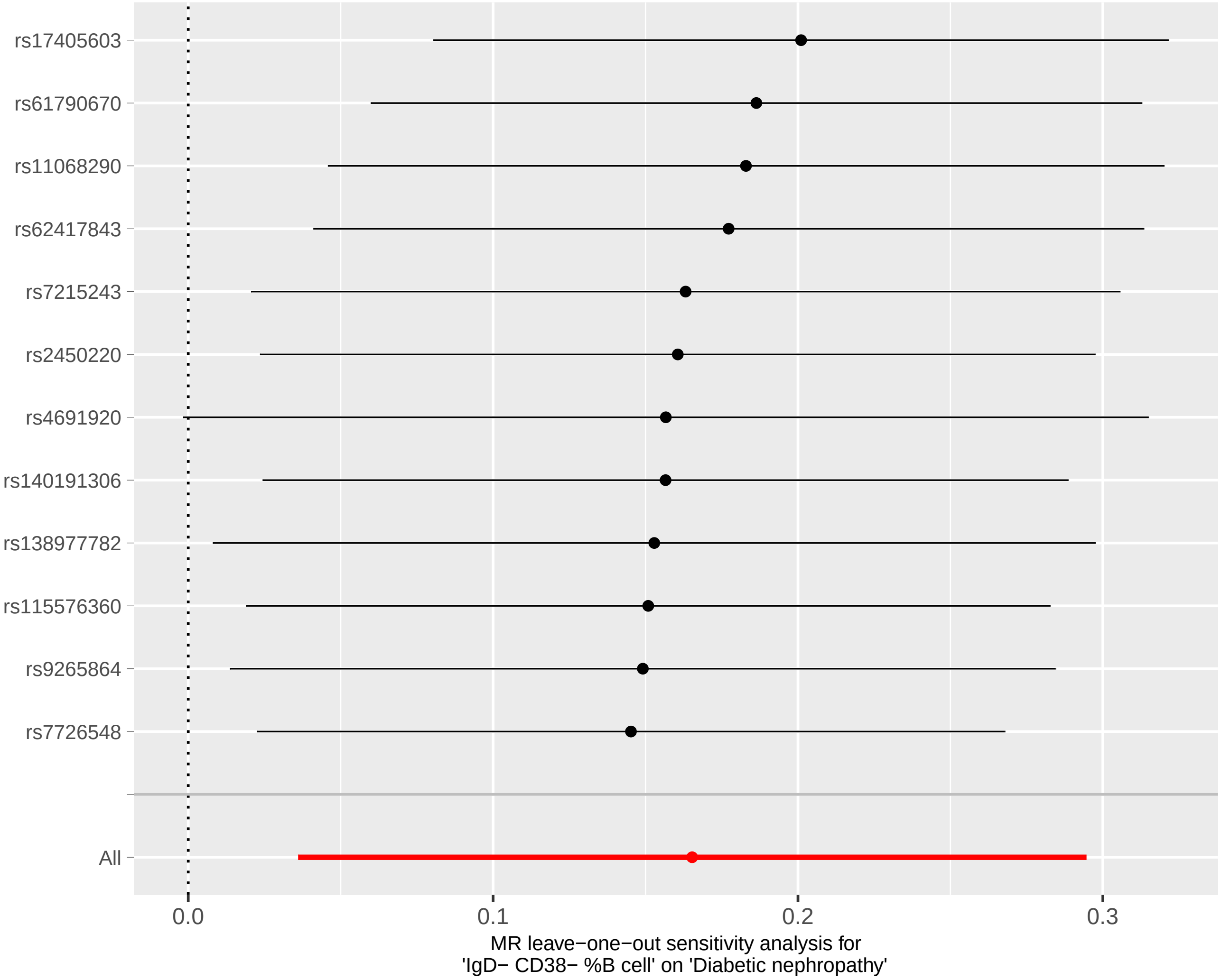
rs17405603
rs61790670
rs11068290
rs62417843
rs7215243
rs2450220
rs4691920
rs140191306
rs138977782
rs115576360
rs9265864
rs7726548
All
0.0
0.1
0.2
0.3
MR leave−one−out sensitivity analysis for
'IgD− CD38− %B cell' on 'Diabetic nephropathy'

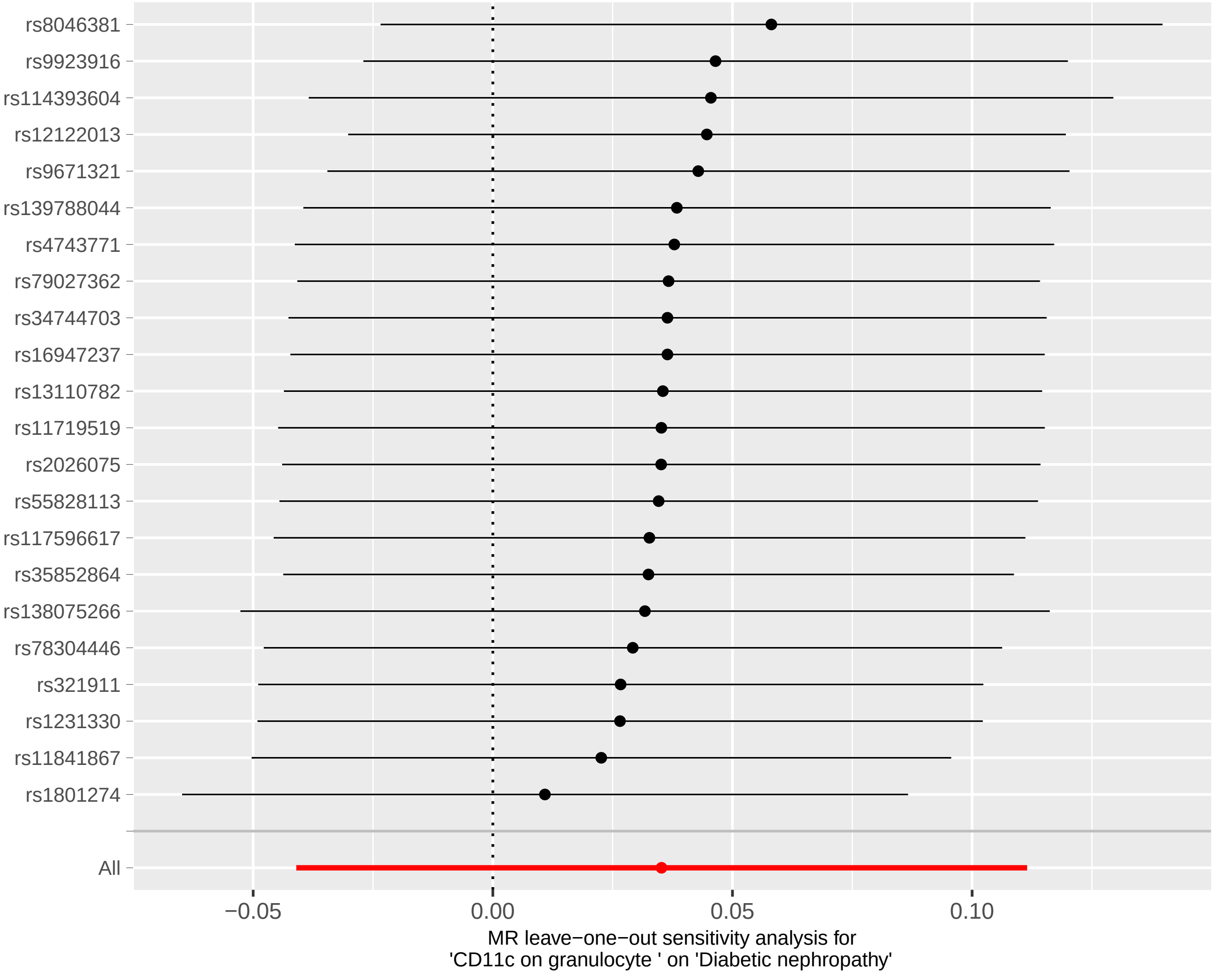
rs8046381
rs9923916
rs114393604
rs12122013
rs9671321
rs139788044
rs4743771
rs79027362
rs34744703
rs16947237
rs13110782
rs11719519
rs2026075
rs55828113
rs117596617
rs35852864
rs138075266
rs78304446
rs321911
rs1231330
rs11841867
rs1801274
All
−0.05
0.00
0.05
0.10
MR leave−one−out sensitivity analysis for
'CD11c on granulocyte ' on 'Diabetic nephropathy'

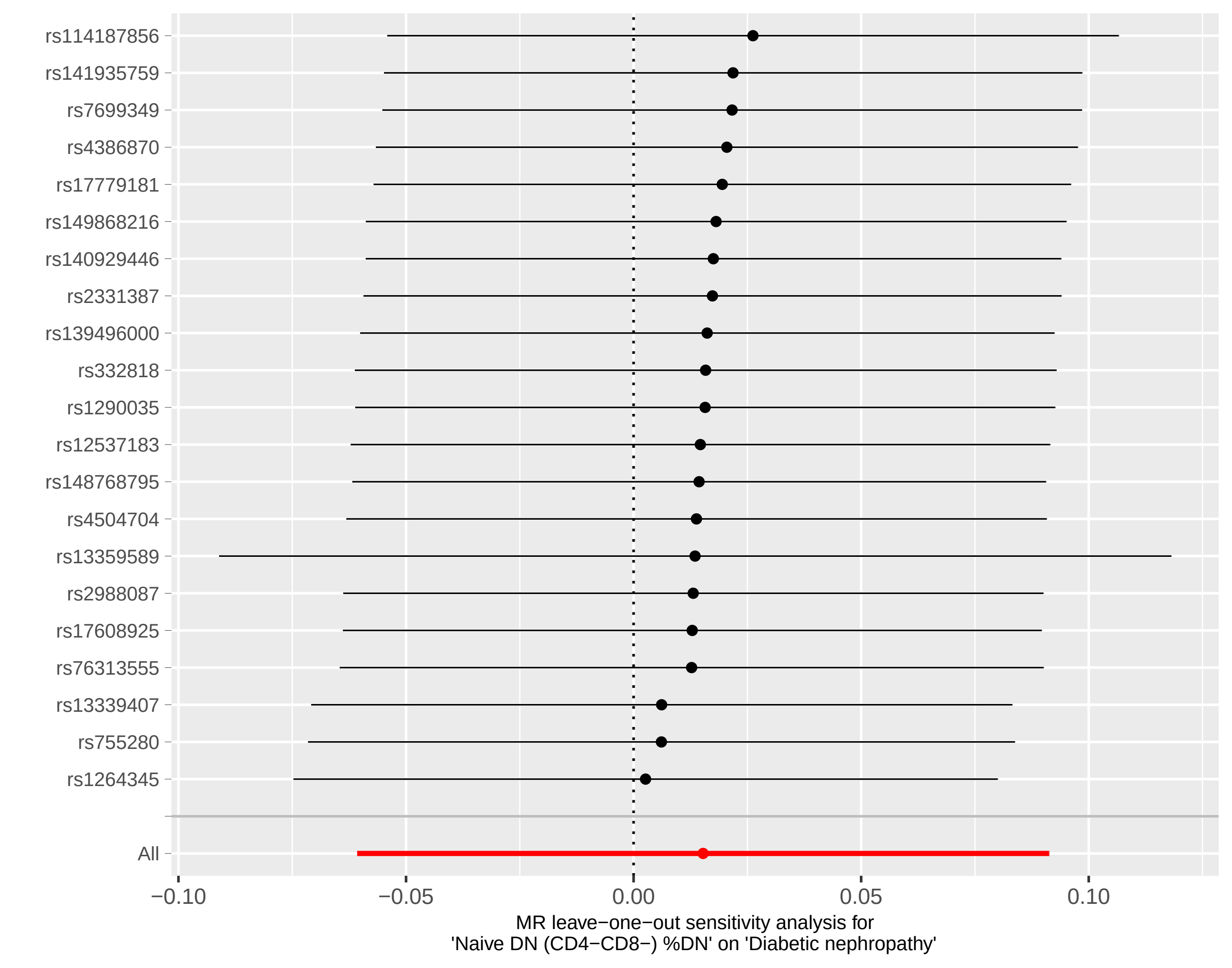
rs114187856
rs141935759
rs7699349
rs4386870
rs17779181
rs149868216
rs140929446
rs2331387
rs139496000
rs332818
rs1290035
rs12537183
rs148768795
rs4504704
rs13359589
rs2988087
rs17608925
rs76313555
rs13339407
rs755280
rs1264345
All
−0.10
−0.05
0.00
0.05
0.10
MR leave−one−out sensitivity analysis for
'Naive DN (CD4−CD8−) %DN' on 'Diabetic nephropathy'

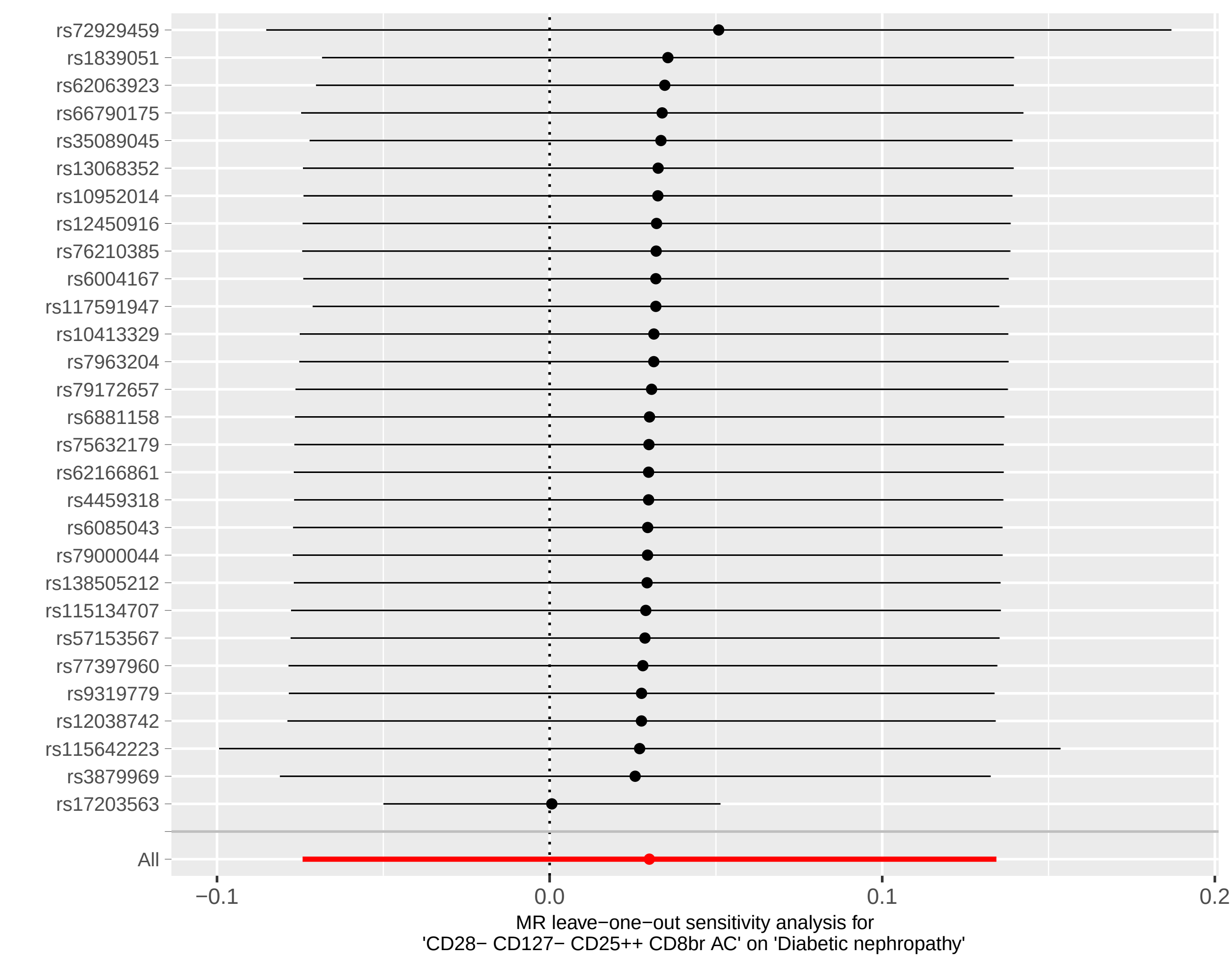

rs72929459
rs1839051
rs62063923
rs66790175
rs35089045
rs13068352
rs10952014
rs12450916
rs76210385
rs6004167
rs117591947
rs10413329
rs7963204
rs79172657
rs6881158
rs75632179
rs62166861
rs4459318
rs6085043
rs79000044
rs138505212
rs115134707
rs57153567
rs77397960
rs9319779
rs12038742
rs115642223
rs3879969
rs17203563
All
−0.1
0.0
0.1
0.2
MR leave−one−out sensitivity analysis for
'CD28− CD127− CD25++ CD8br AC' on 'Diabetic nephropathy'

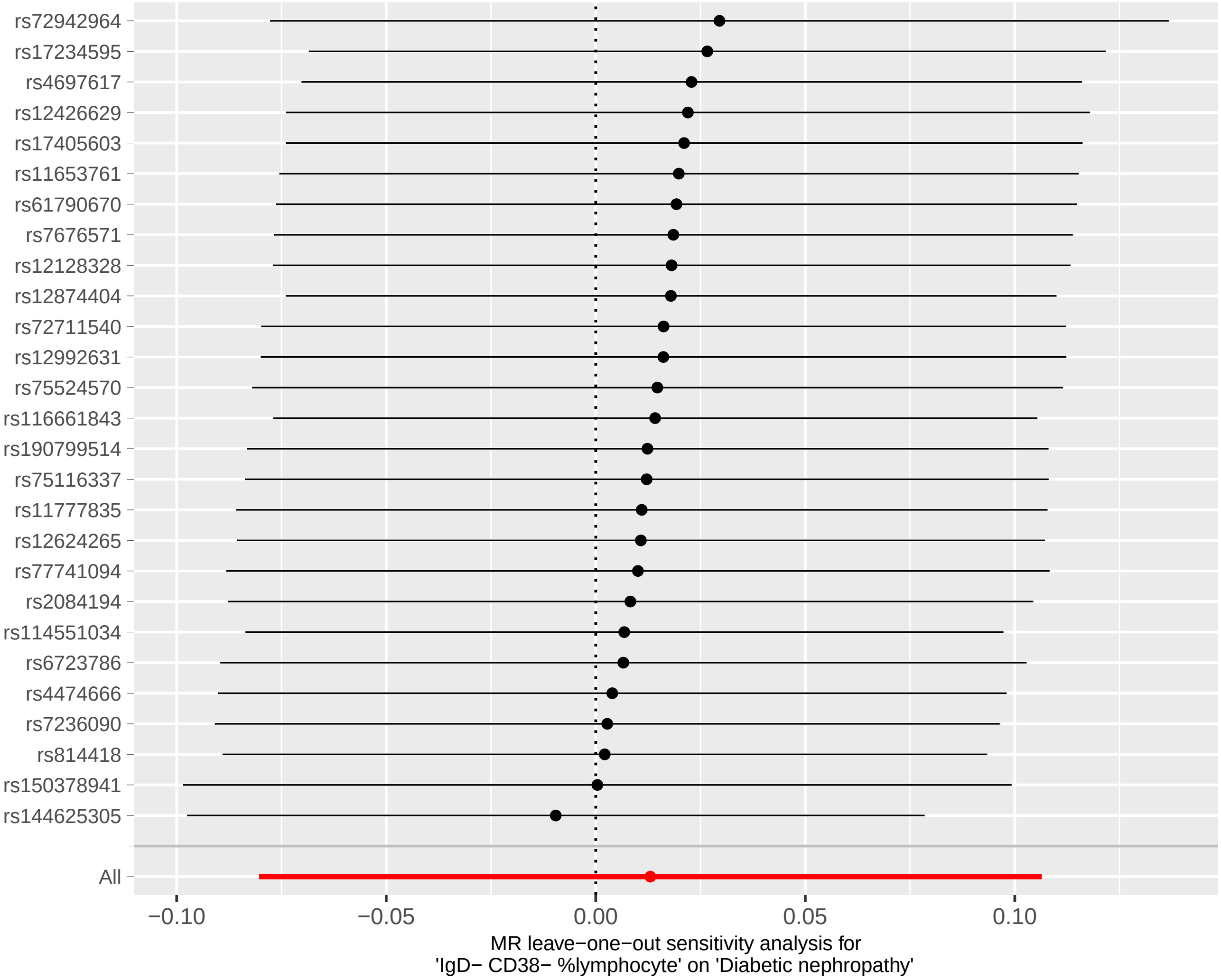
rs72942964
rs17234595
rs4697617
rs12426629
rs17405603
rs11653761
rs61790670
rs7676571
rs12128328
rs12874404
rs72711540
rs12992631
rs75524570
rs116661843
rs190799514
rs75116337
rs11777835
rs12624265
rs77741094
rs2084194
rs114551034
rs6723786
rs4474666
rs7236090
rs814418
rs150378941
rs144625305
All
−0.10
−0.05
0.00
0.05
0.10
MR leave−one−out sensitivity analysis for
'IgD− CD38− %lymphocyte' on 'Diabetic nephropathy'

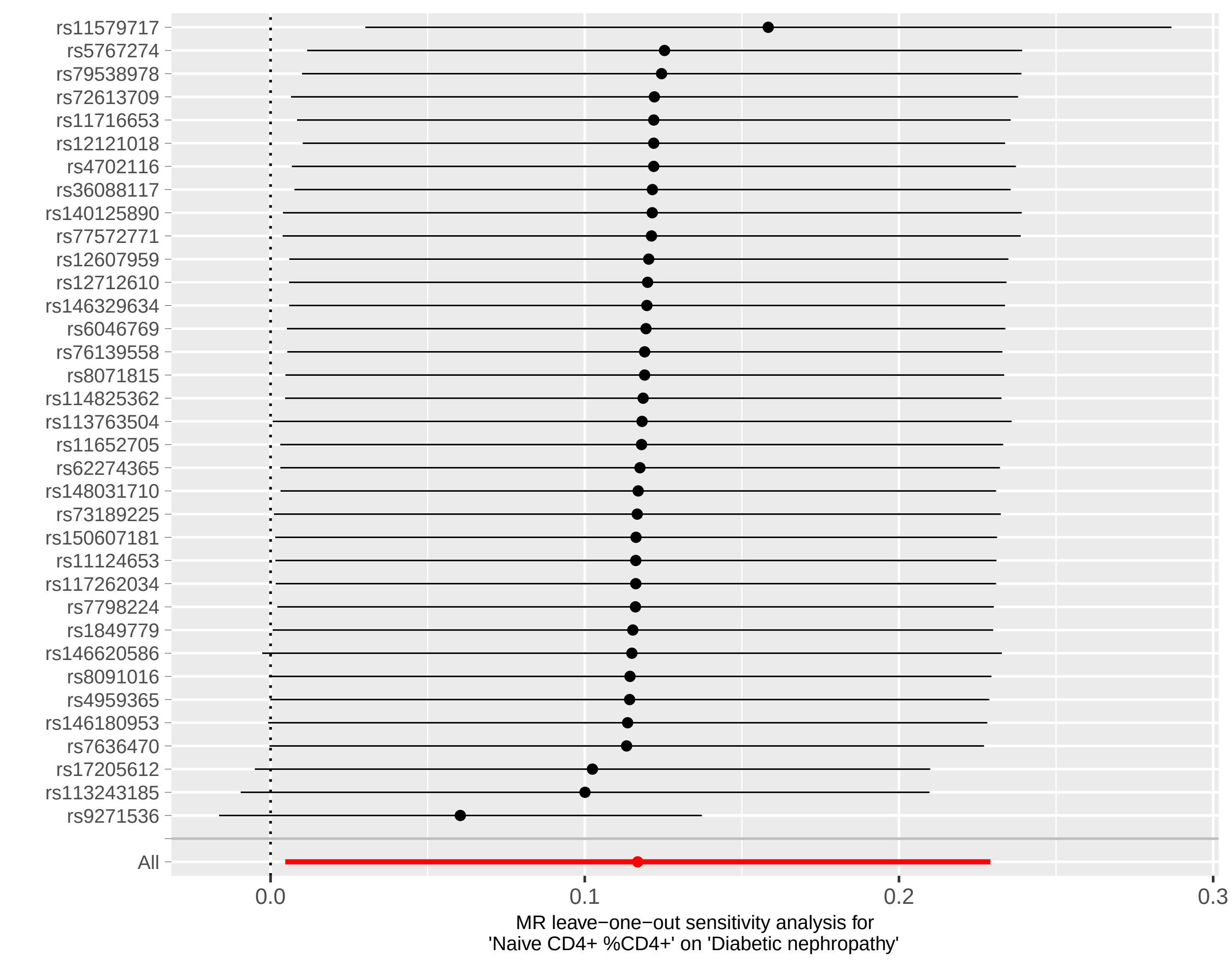

rs11579717
rs5767274
rs79538978
rs72613709
rs11716653
rs12121018
rs4702116
rs36088117
rs140125890
rs77572771
rs12607959
rs12712610
rs146329634
rs6046769
rs76139558
rs8071815
rs114825362
rs113763504
rs11652705
rs62274365
rs148031710
rs73189225
rs150607181
rs11124653
rs117262034
rs7798224
rs1849779
rs146620586
rs8091016
rs4959365
rs146180953
rs7636470
rs17205612
rs113243185
rs9271536
All
0.0
0.1
0.2
0.3
MR leave−one−out sensitivity analysis for
'Naive CD4+ %CD4+' on 'Diabetic nephropathy'

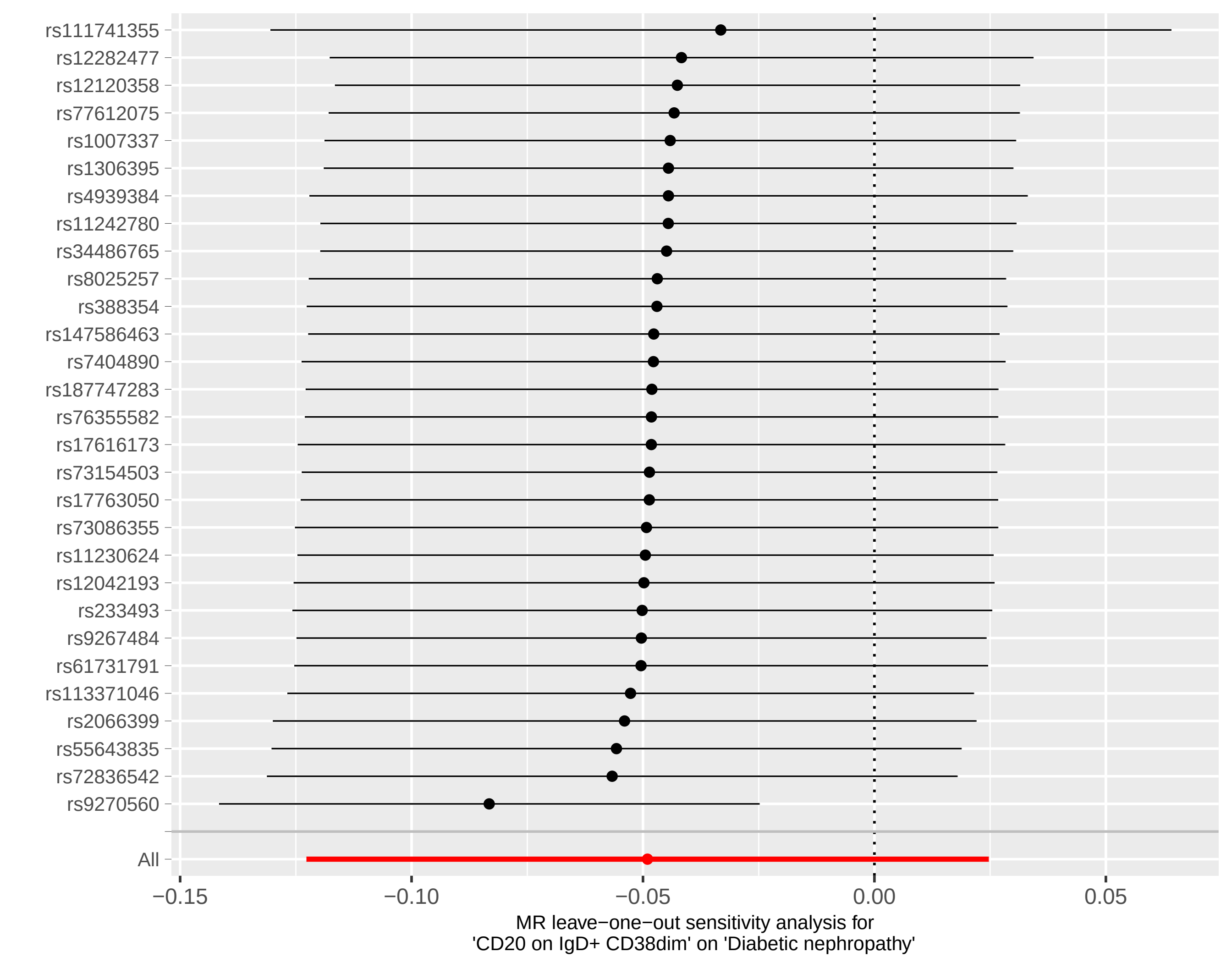

rs111741355
rs12282477
rs12120358
rs77612075
rs1007337
rs1306395
rs4939384
rs11242780
rs34486765
rs8025257
rs388354
rs147586463
rs7404890
rs187747283
rs76355582
rs17616173
rs73154503
rs17763050
rs73086355
rs11230624
rs12042193
rs233493
rs9267484
rs61731791
rs113371046
rs2066399
rs55643835
rs72836542
rs9270560
All
−0.15
−0.10
−0.05
0.00
0.05
MR leave−one−out sensitivity analysis for
'CD20 on IgD+ CD38dim' on 'Diabetic nephropathy'

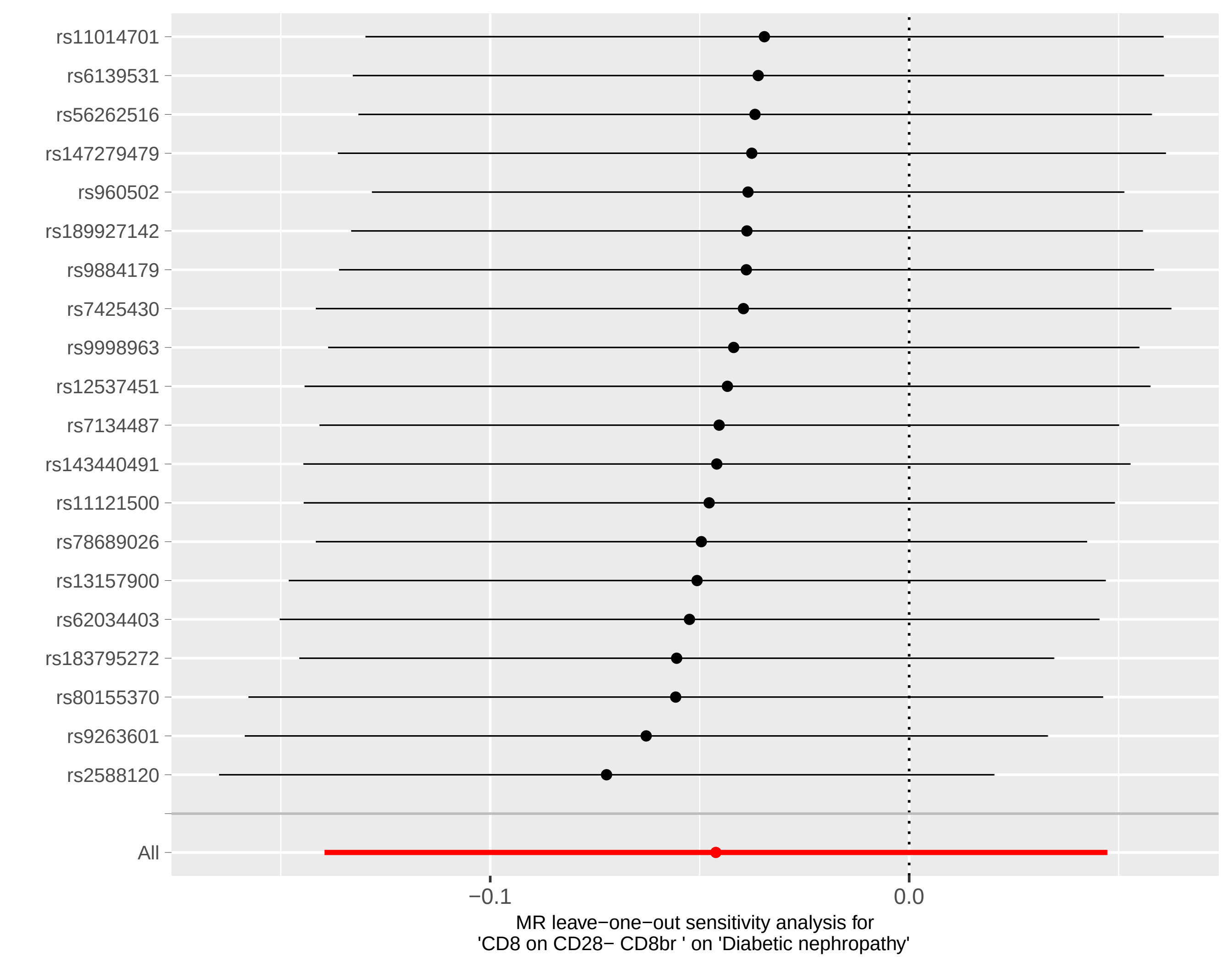
rs11014701
rs6139531
rs56262516
rs147279479
rs960502
rs189927142
rs9884179
rs7425430
rs9998963
rs12537451
rs7134487
rs143440491
rs11121500
rs78689026
rs13157900
rs62034403
rs183795272
rs80155370
rs9263601
rs2588120
All
−0.1
0.0
MR leave−one−out sensitivity analysis for
'CD8 on CD28− CD8br ' on 'Diabetic nephropathy'

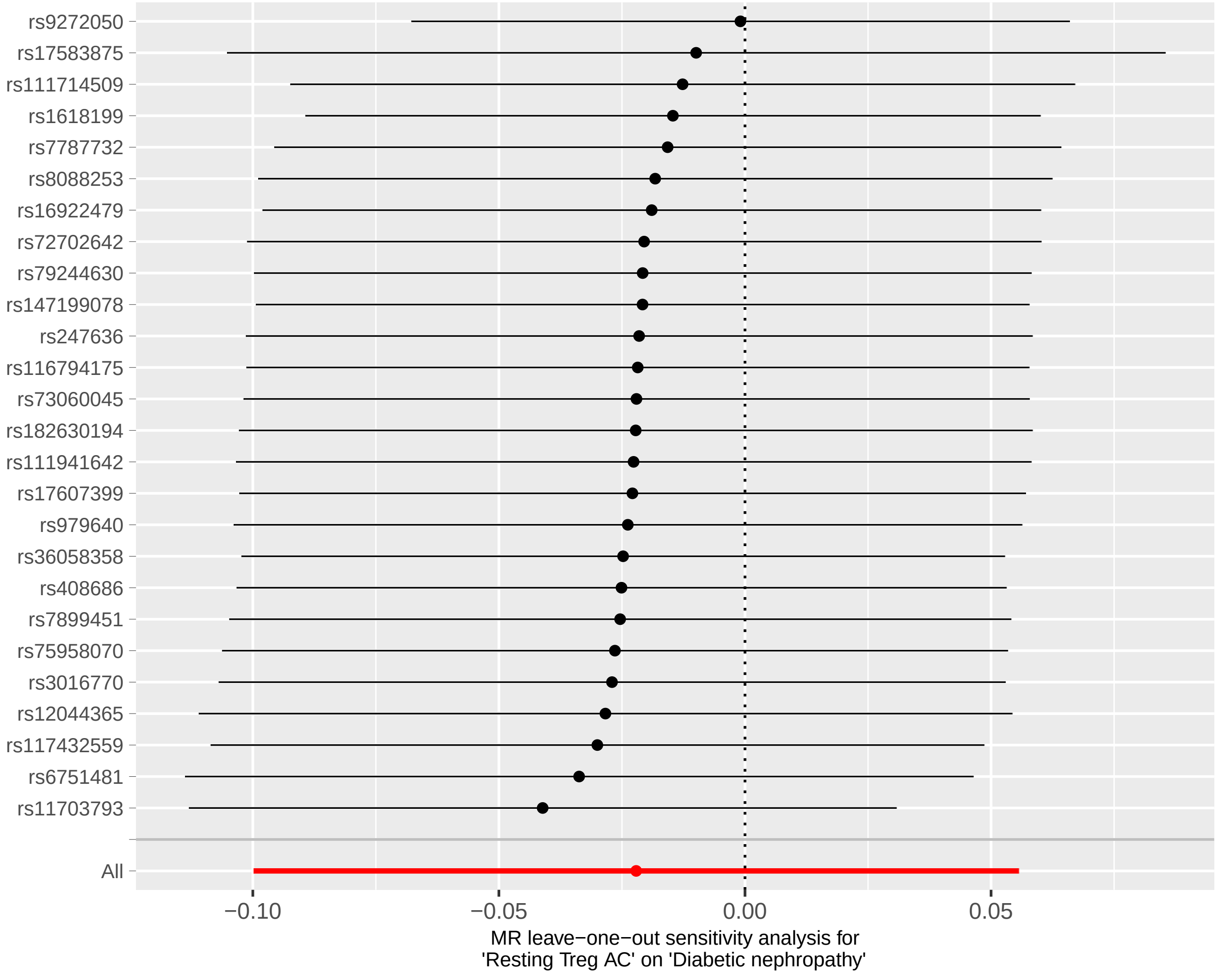
rs9272050
rs17583875
rs111714509
rs1618199
rs7787732
rs8088253
rs16922479
rs72702642
rs79244630
rs147199078
rs247636
rs116794175
rs73060045
rs182630194
rs111941642
rs17607399
rs979640
rs36058358
rs408686
rs7899451
rs75958070
rs3016770
rs12044365
rs117432559
rs6751481
rs11703793
All
−0.10
−0.05
0.00
0.05
MR leave−one−out sensitivity analysis for
'Resting Treg AC' on 'Diabetic nephropathy'

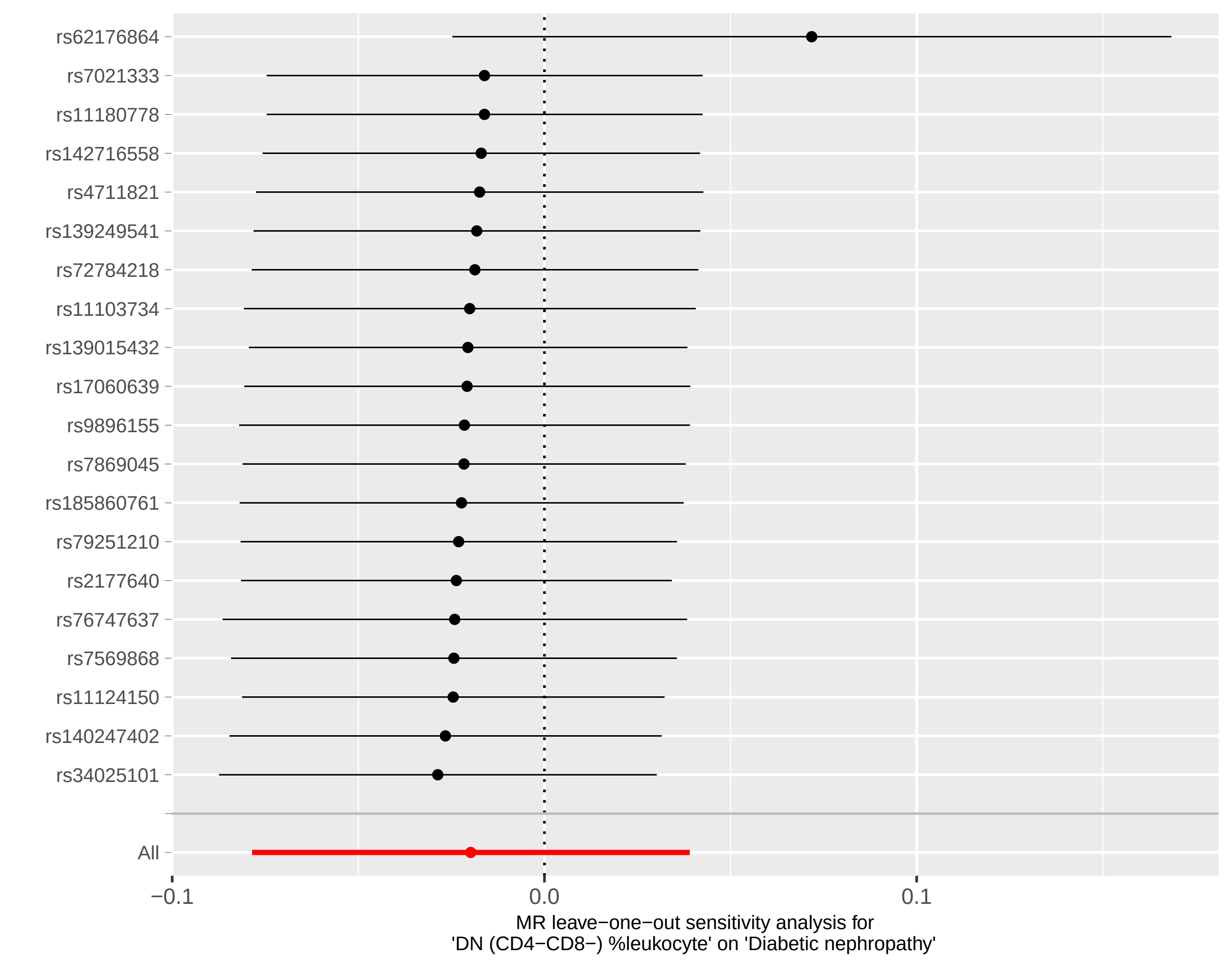
rs62176864
rs7021333
rs11180778
rs142716558
rs4711821
rs139249541
rs72784218
rs11103734
rs139015432
rs17060639
rs9896155
rs7869045
rs185860761
rs79251210
rs2177640
rs76747637
rs7569868
rs11124150
rs140247402
rs34025101
All
−0.1
0.0
0.1
MR leave−one−out sensitivity analysis for
'DN (CD4−CD8−) %leukocyte' on 'Diabetic nephropathy'

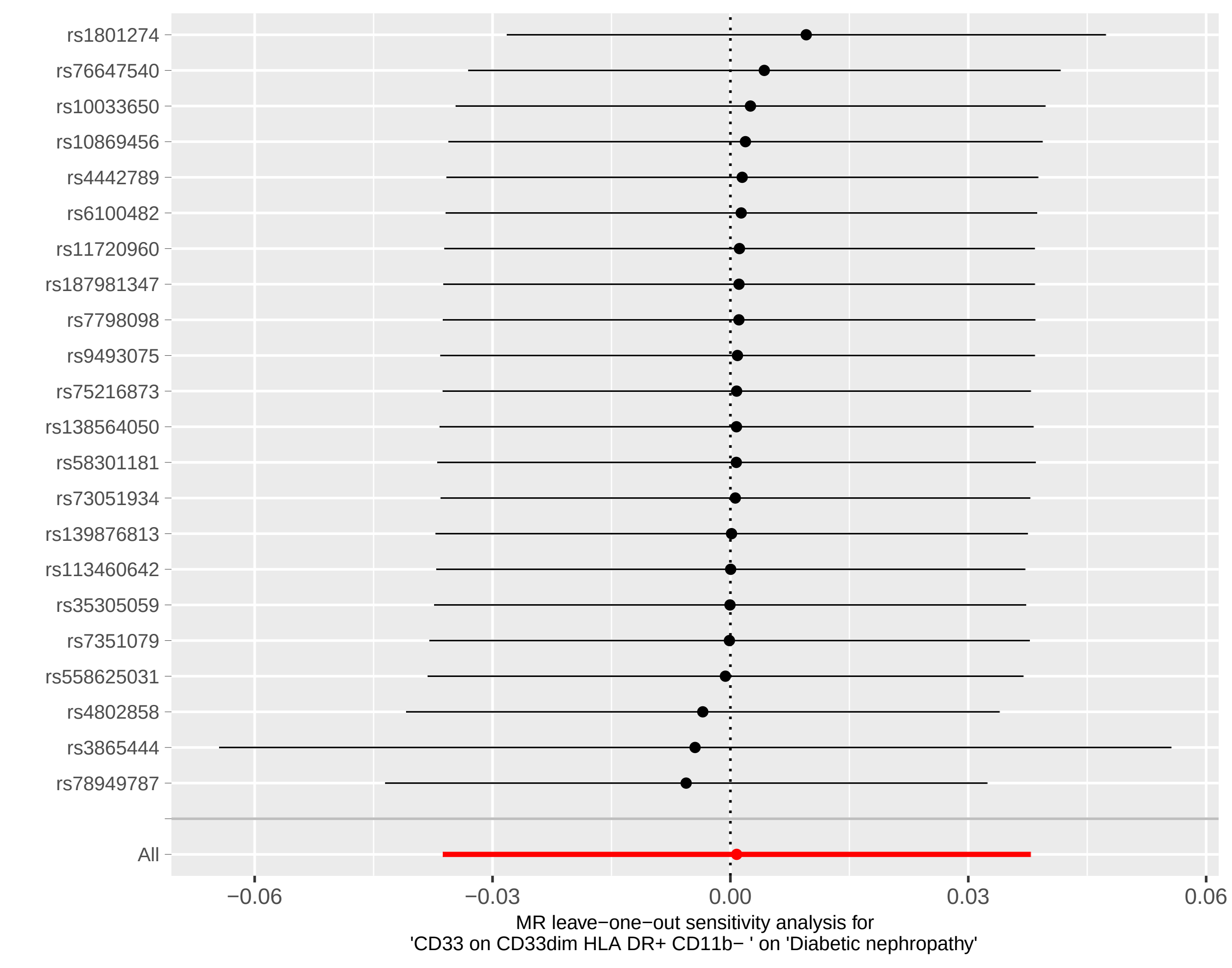
rs1801274
rs76647540
rs10033650
rs10869456
rs4442789
rs6100482
rs11720960
rs187981347
rs7798098
rs9493075
rs75216873
rs138564050
rs58301181
rs73051934
rs139876813
rs113460642
rs35305059
rs7351079
rs558625031
rs4802858
rs3865444
rs78949787
All
−0.06
−0.03
0.00
0.03
0.06
MR leave−one−out sensitivity analysis for
'CD33 on CD33dim HLA DR+ CD11b− ' on 'Diabetic nephropathy'

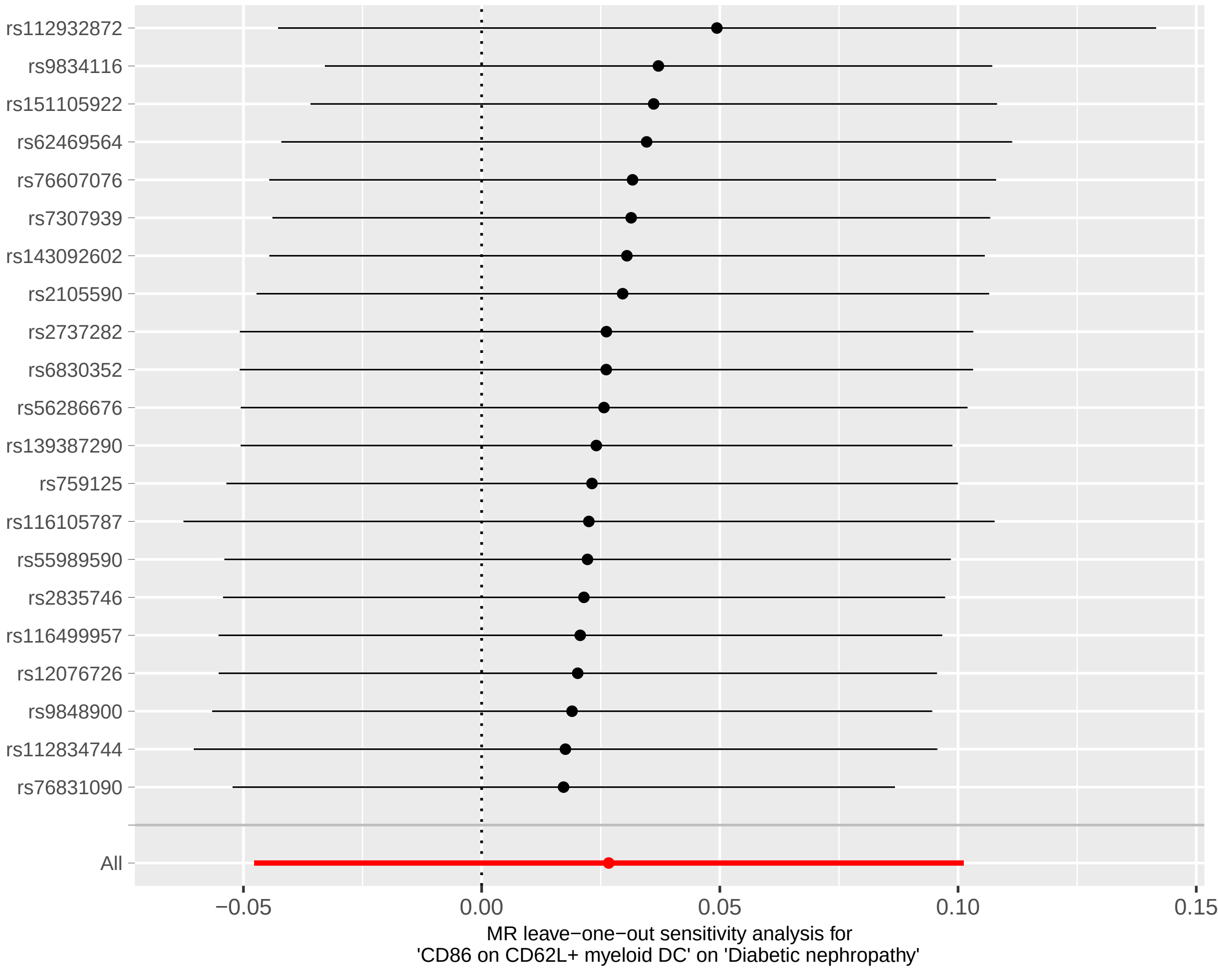
rs112932872
rs9834116
rs151105922
rs62469564
rs76607076
rs7307939
rs143092602
rs2105590
rs2737282
rs6830352
rs56286676
rs139387290
rs759125
rs116105787
rs55989590
rs2835746
rs116499957
rs12076726
rs9848900
rs112834744
rs76831090
All
−0.05
0.00
0.05
0.10
0.15
MR leave−one−out sensitivity analysis for
'CD86 on CD62L+ myeloid DC' on 'Diabetic nephropathy'

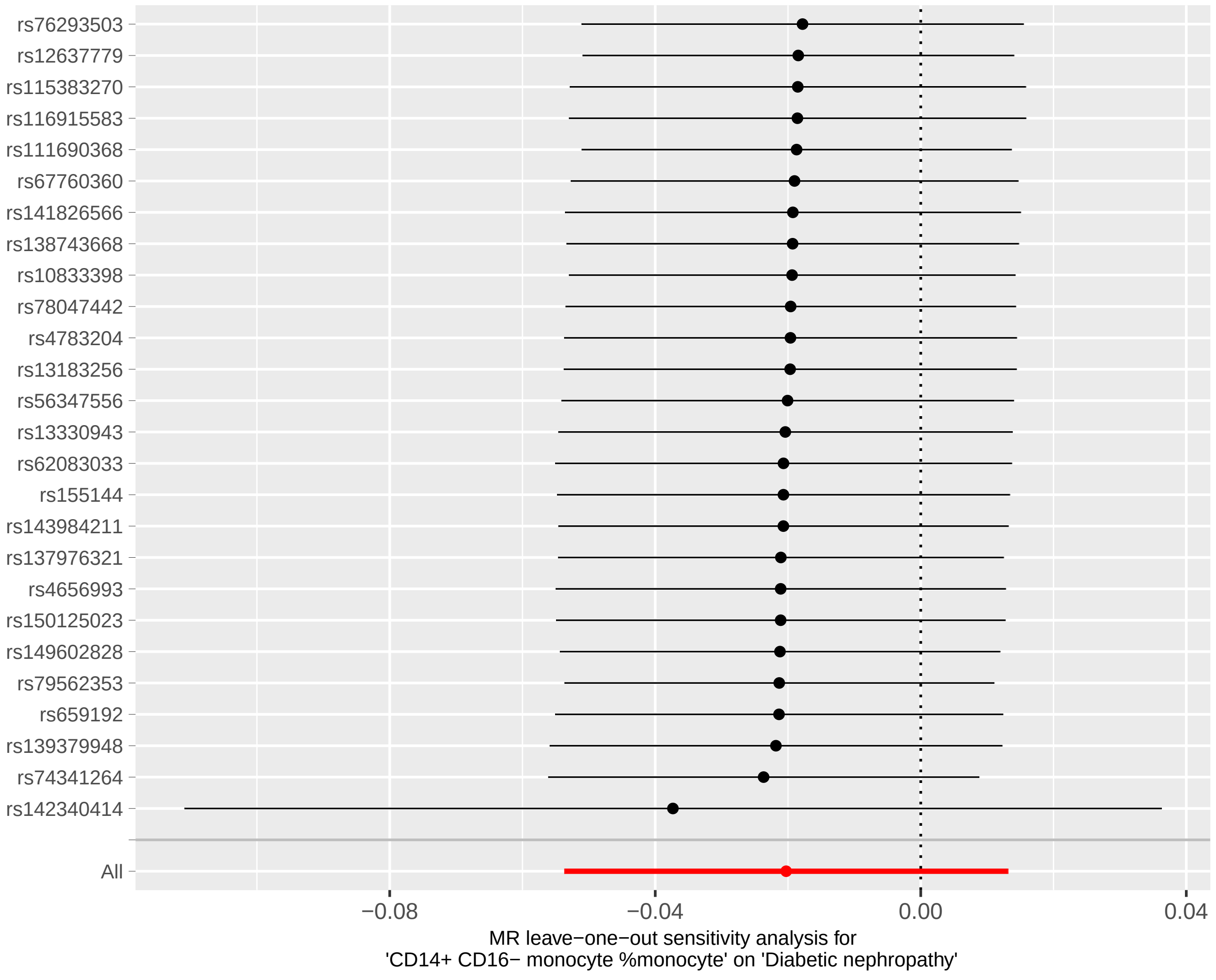
rs76293503
rs12637779
rs115383270
rs116915583
rs111690368
rs67760360
rs141826566
rs138743668
rs10833398
rs78047442
rs4783204
rs13183256
rs56347556
rs13330943
rs62083033
rs155144
rs143984211
rs137976321
rs4656993
rs150125023
rs149602828
rs79562353
rs659192
rs139379948
rs74341264
rs142340414
All
−0.08
−0.04
0.00
0.04
MR leave−one−out sensitivity analysis for
'CD14+ CD16− monocyte %monocyte' on 'Diabetic nephropathy'

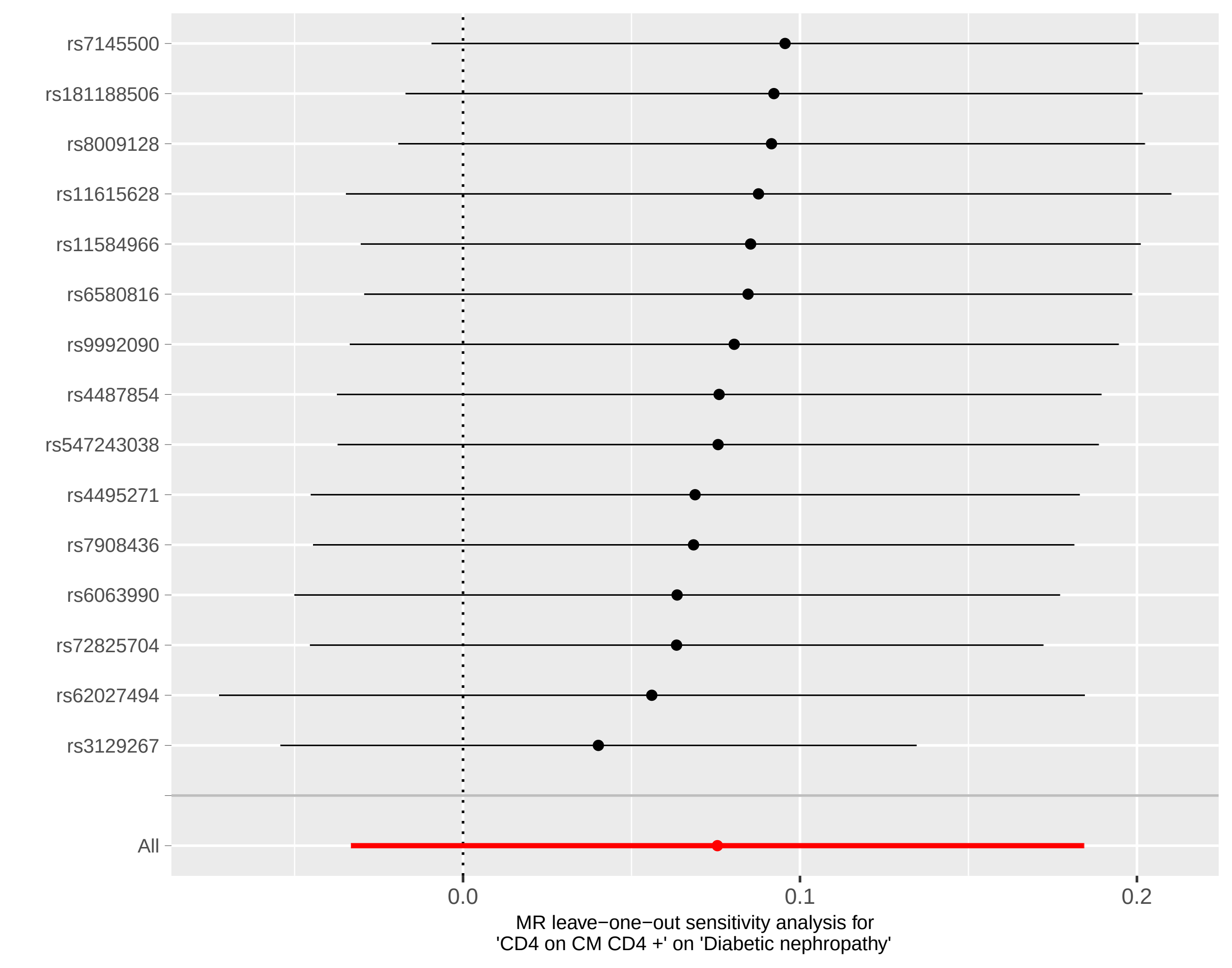
rs7145500
rs181188506
rs8009128
rs11615628
rs11584966
rs6580816
rs9992090
rs4487854
rs547243038
rs4495271
rs7908436
rs6063990
rs72825704
rs62027494
rs3129267
All
0.0
0.1
0.2
MR leave−one−out sensitivity analysis for
'CD4 on CM CD4 +' on 'Diabetic nephropathy'

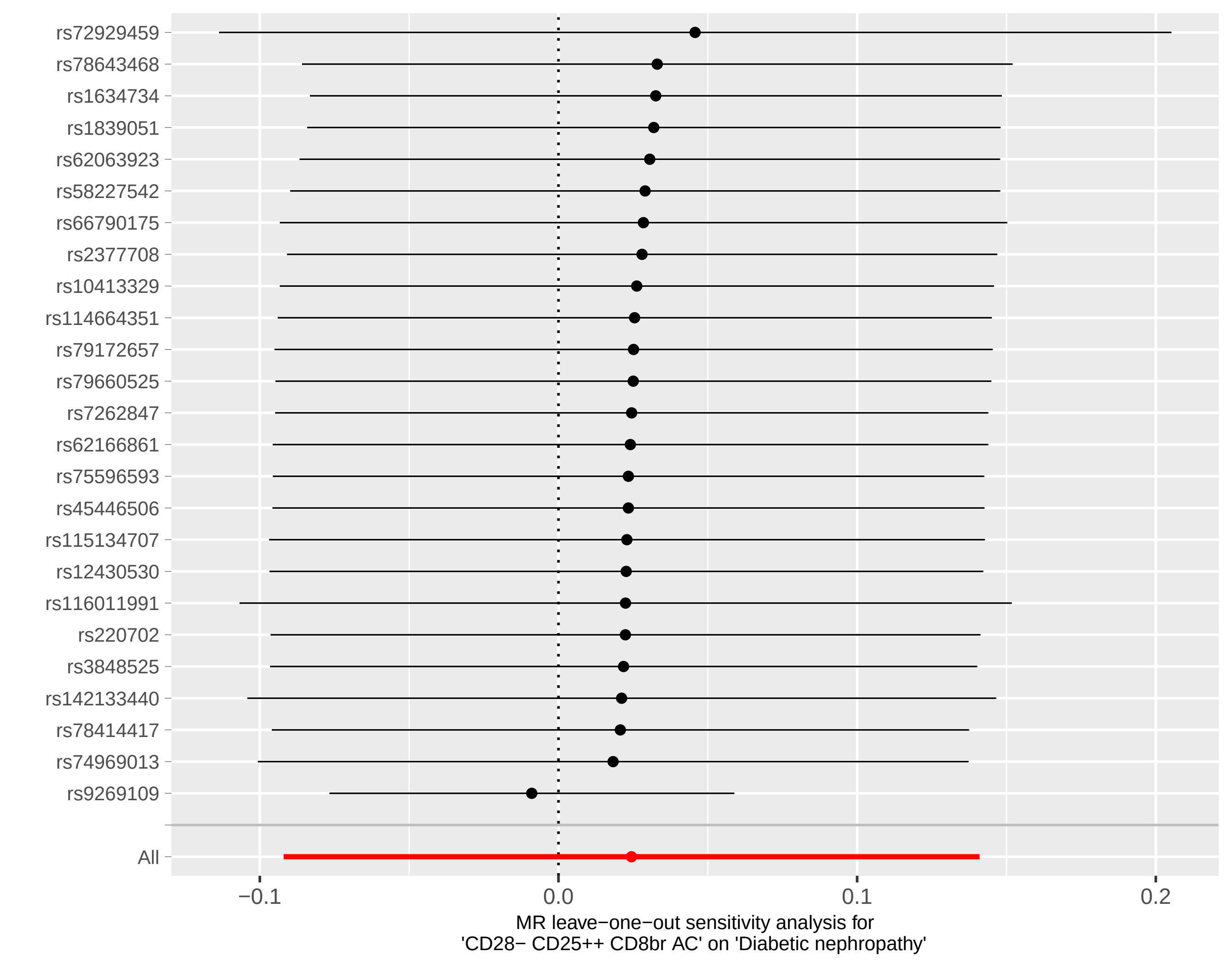
rs72929459
rs78643468
rs1634734
rs1839051
rs62063923
rs58227542
rs66790175
rs2377708
rs10413329
rs114664351
rs79172657
rs79660525
rs7262847
rs62166861
rs75596593
rs45446506
rs115134707
rs12430530
rs116011991
rs220702
rs3848525
rs142133440
rs78414417
rs74969013
rs9269109
All
−0.1
0.0
0.1
0.2
MR leave−one−out sensitivity analysis for
'CD28− CD25++ CD8br AC' on 'Diabetic nephropathy'

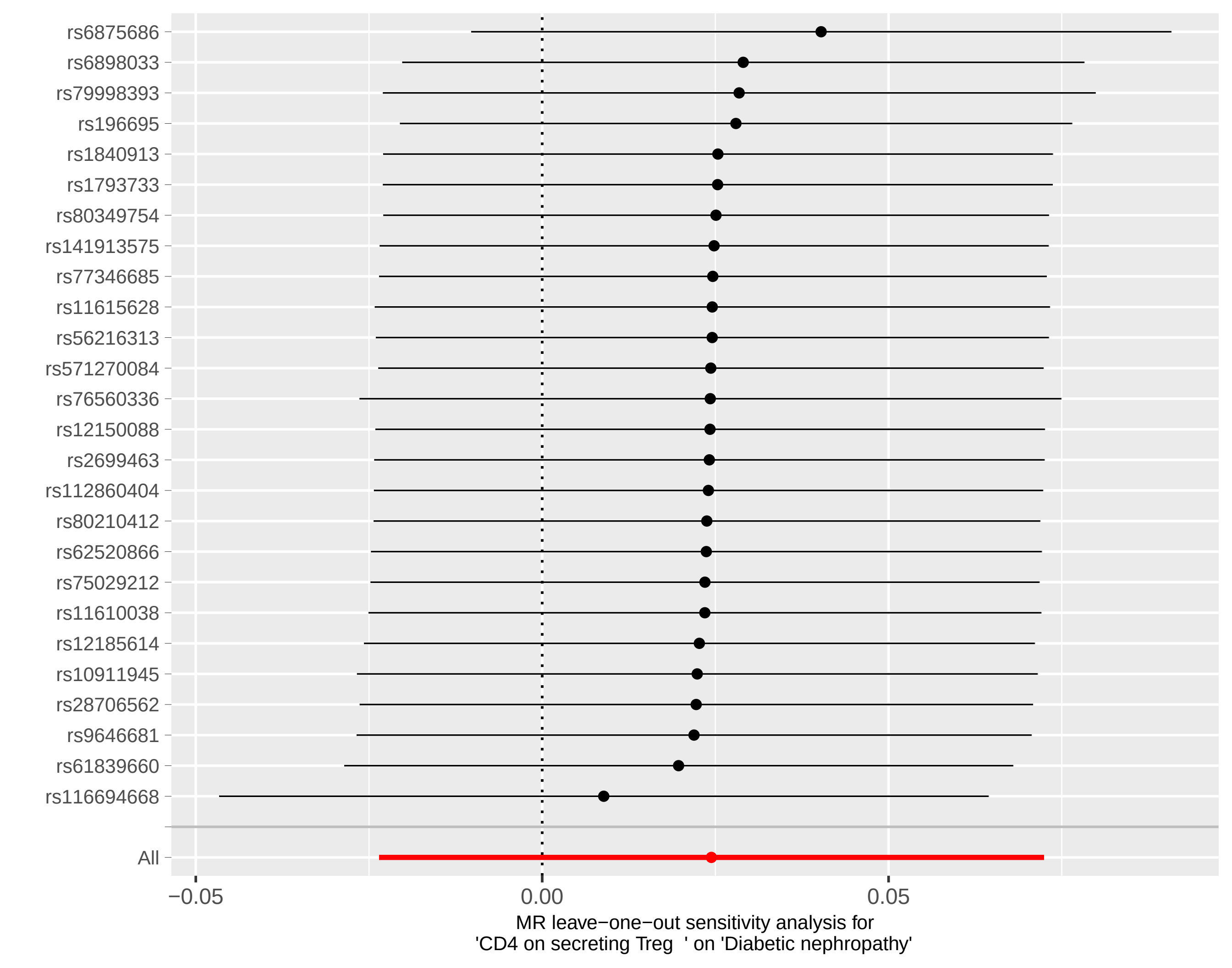

rs6875686
rs6898033
rs79998393
rs196695
rs1840913
rs1793733
rs80349754
rs141913575
rs77346685
rs11615628
rs56216313
rs571270084
rs76560336
rs12150088
rs2699463
rs112860404
rs80210412
rs62520866
rs75029212
rs11610038
rs12185614
rs10911945
rs28706562
rs9646681
rs61839660
rs116694668
All
−0.05
0.00
0.05
MR leave−one−out sensitivity analysis for
'CD4 on secreting Treg ' on 'Diabetic nephropathy'

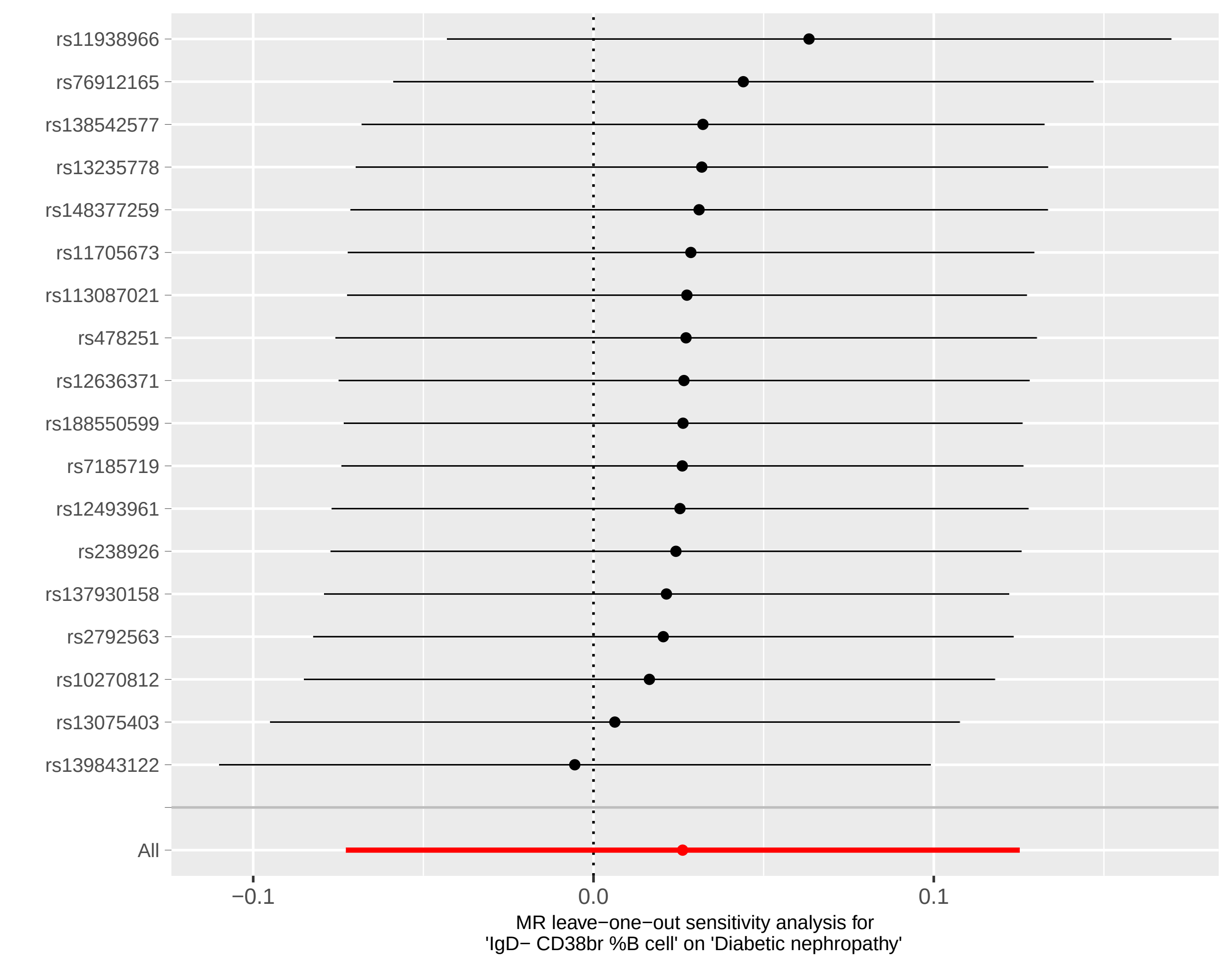

rs11938966
rs76912165
rs138542577
rs13235778
rs148377259
rs11705673
rs113087021
rs478251
rs12636371
rs188550599
rs7185719
rs12493961
rs238926
rs137930158
rs2792563
rs10270812
rs13075403
rs139843122
All
−0.1
0.0
0.1
MR leave−one−out sensitivity analysis for
'IgD− CD38br %B cell' on 'Diabetic nephropathy'

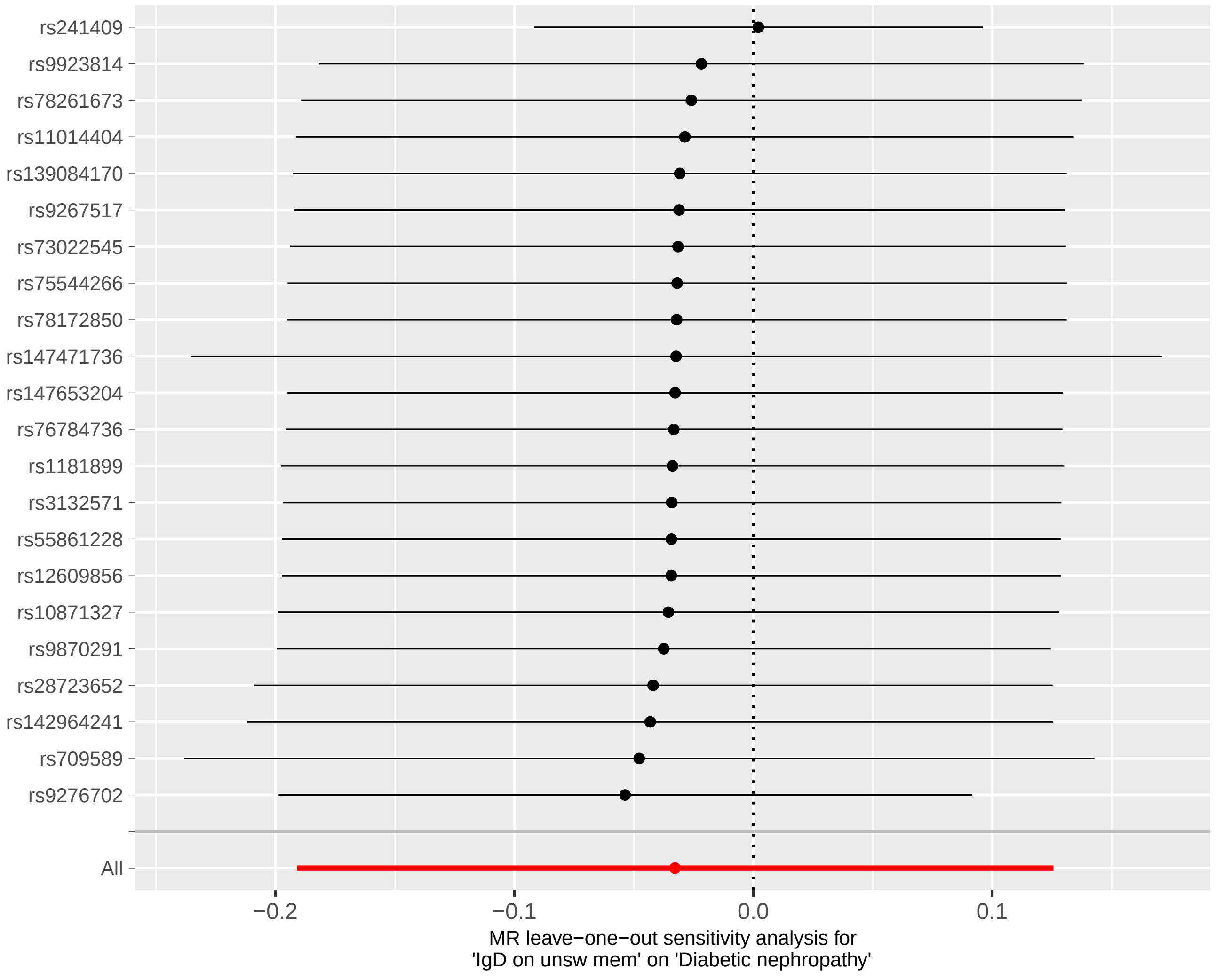
rs241409
rs9923814
rs78261673
rs11014404
rs139084170
rs9267517
rs73022545
rs75544266
rs78172850
rs147471736
rs147653204
rs76784736
rs1181899
rs3132571
rs55861228
rs12609856
rs10871327
rs9870291
rs28723652
rs142964241
rs709589
rs9276702
All
−0.2
−0.1
0.0
0.1
MR leave−one−out sensitivity analysis for
'IgD on unsw mem' on 'Diabetic nephropathy'

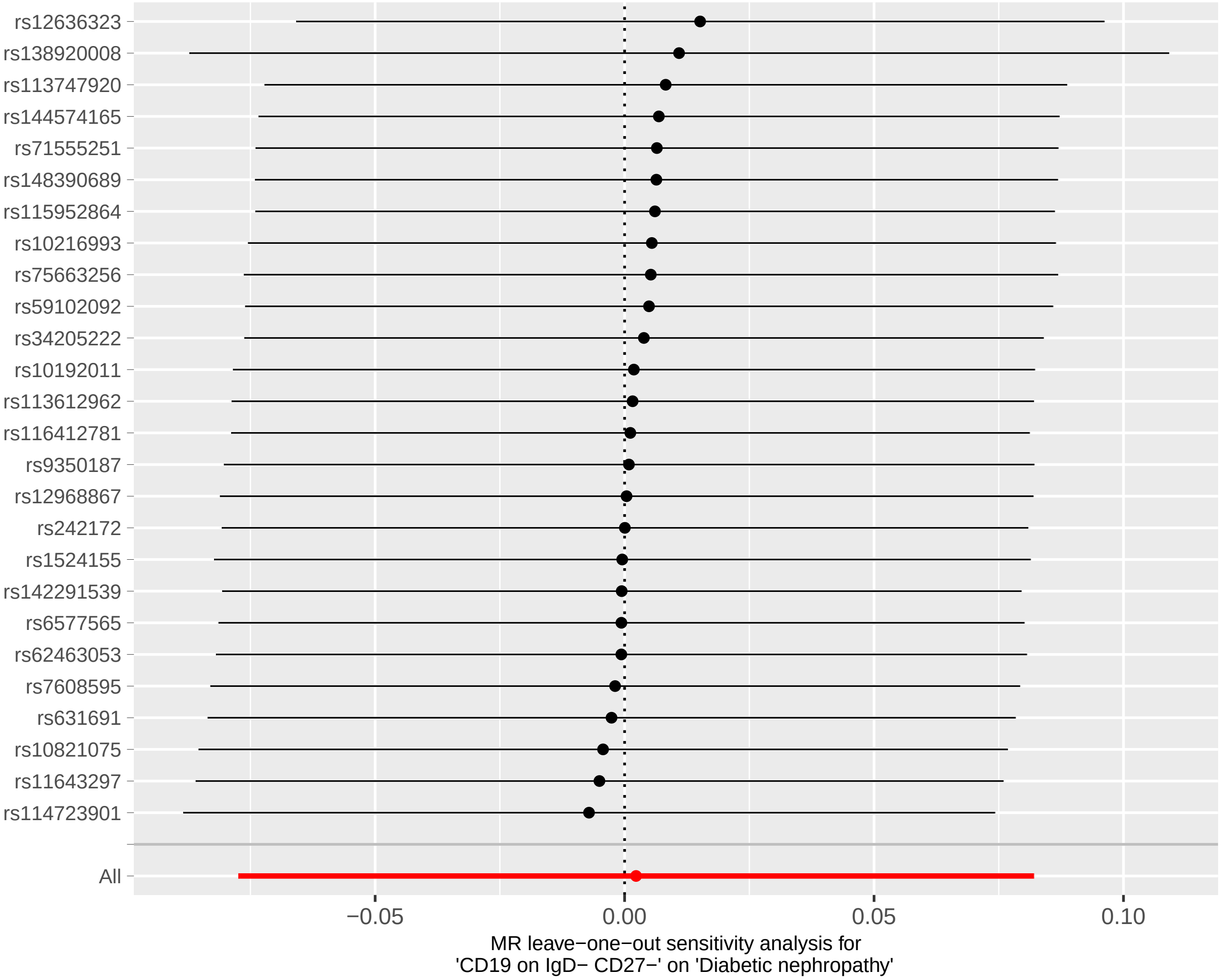
rs12636323
rs138920008
rs113747920
rs144574165
rs71555251
rs148390689
rs115952864
rs10216993
rs75663256
rs59102092
rs34205222
rs10192011
rs113612962
rs116412781
rs9350187
rs12968867
rs242172
rs1524155
rs142291539
rs6577565
rs62463053
rs7608595
rs631691
rs10821075
rs11643297
rs114723901
All
−0.05
0.00
0.05
0.10
MR leave−one−out sensitivity analysis for
'CD19 on IgD− CD27−' on 'Diabetic nephropathy'

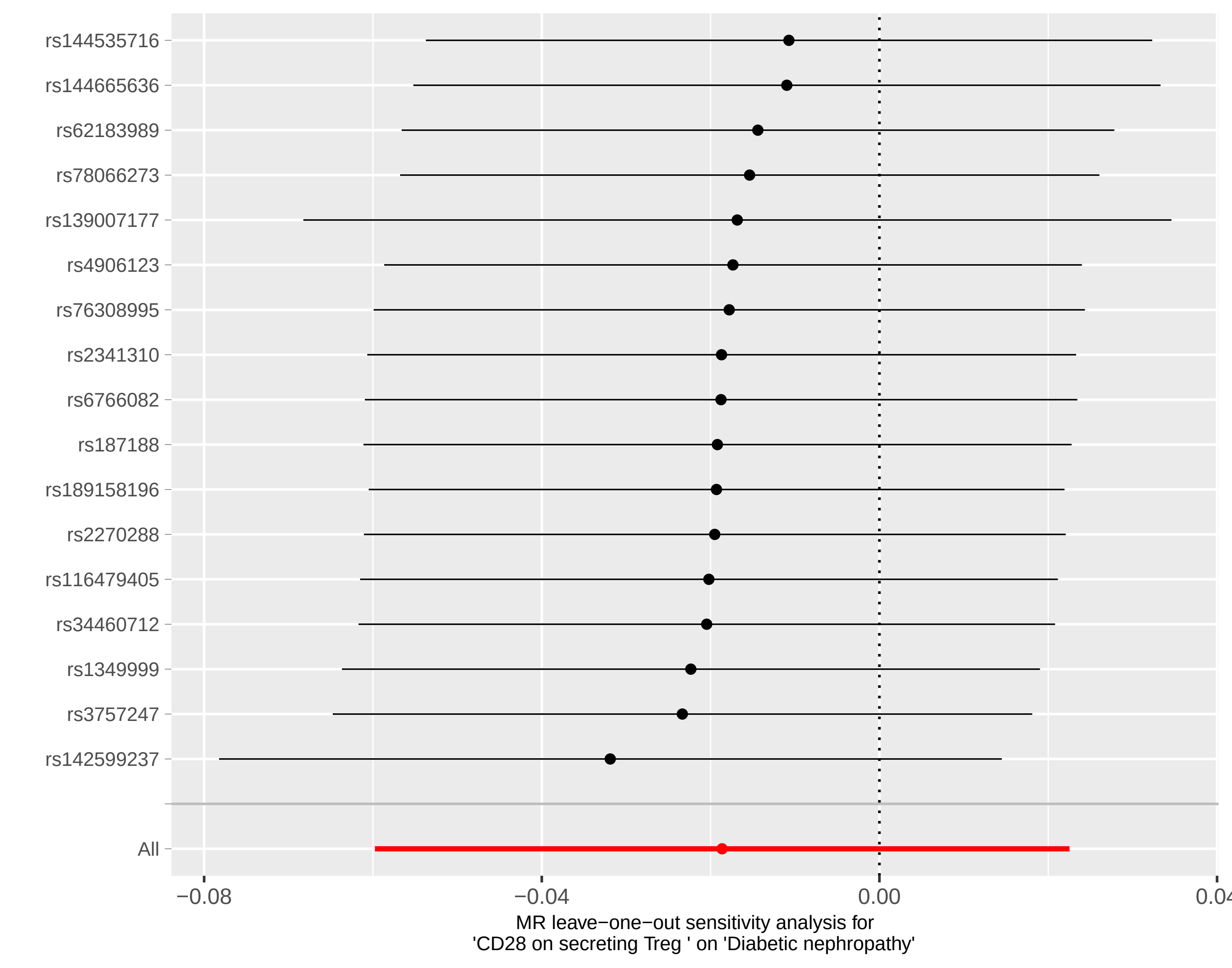
rs144535716
rs144665636
rs62183989
rs78066273
rs139007177
rs4906123
rs76308995
rs2341310
rs6766082
rs187188
rs189158196
rs2270288
rs116479405
rs34460712
rs1349999
rs3757247
rs142599237
All
−0.08
−0.04
0.00
MR leave−one−out sensitivity analysis for
'CD28 on secreting Treg ' on 'Diabetic nephropathy'

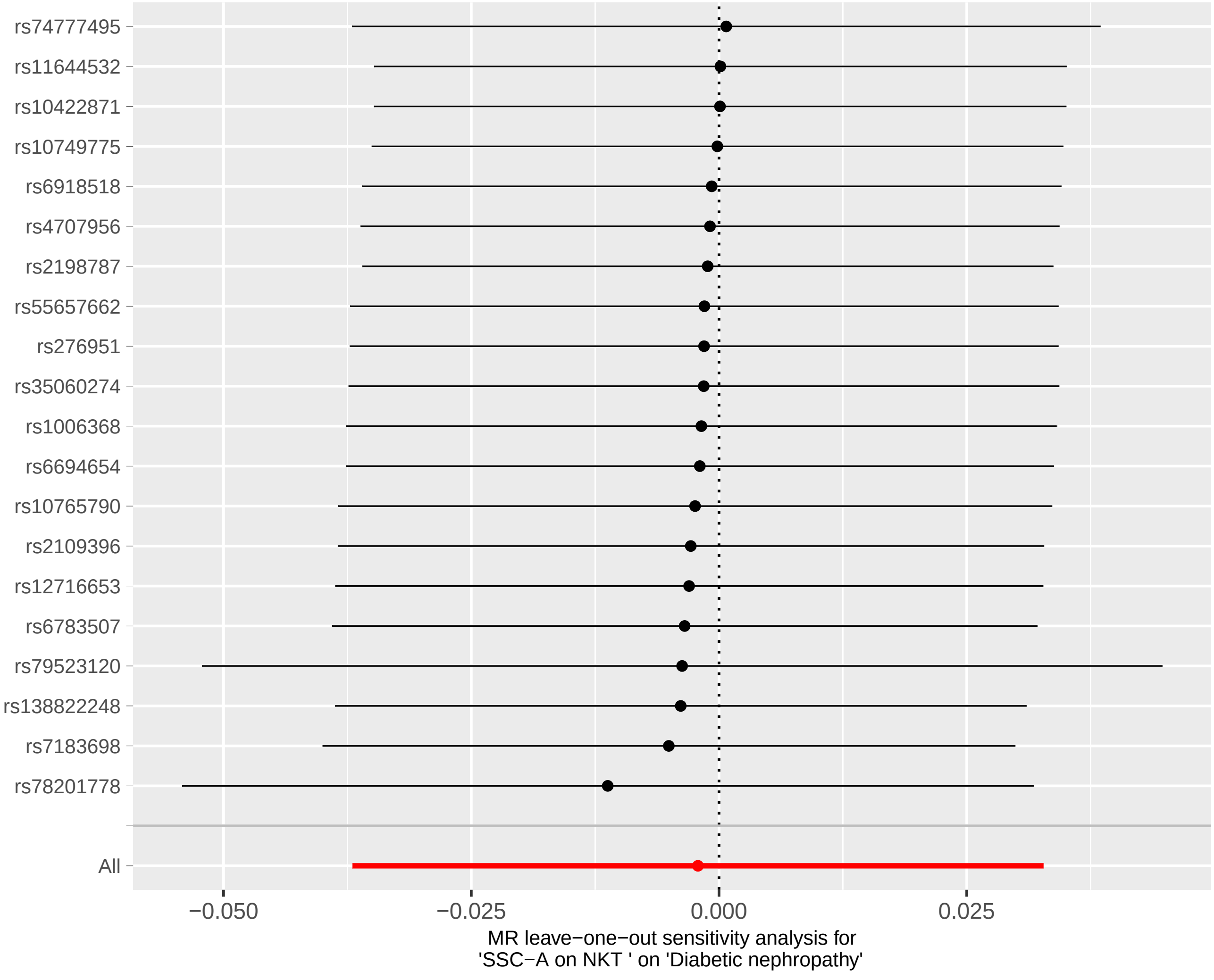

rs74777495
rs11644532
rs10422871
rs10749775
rs6918518
rs4707956
rs2198787
rs55657662
rs276951
rs35060274
rs1006368
rs6694654
rs10765790
rs2109396
rs12716653
rs6783507
rs79523120
rs138822248
rs7183698
rs78201778
All
−0.050
−0.025
0.000
0.025
MR leave−one−out sensitivity analysis for
'SSC−A on NKT ' on 'Diabetic nephropathy'

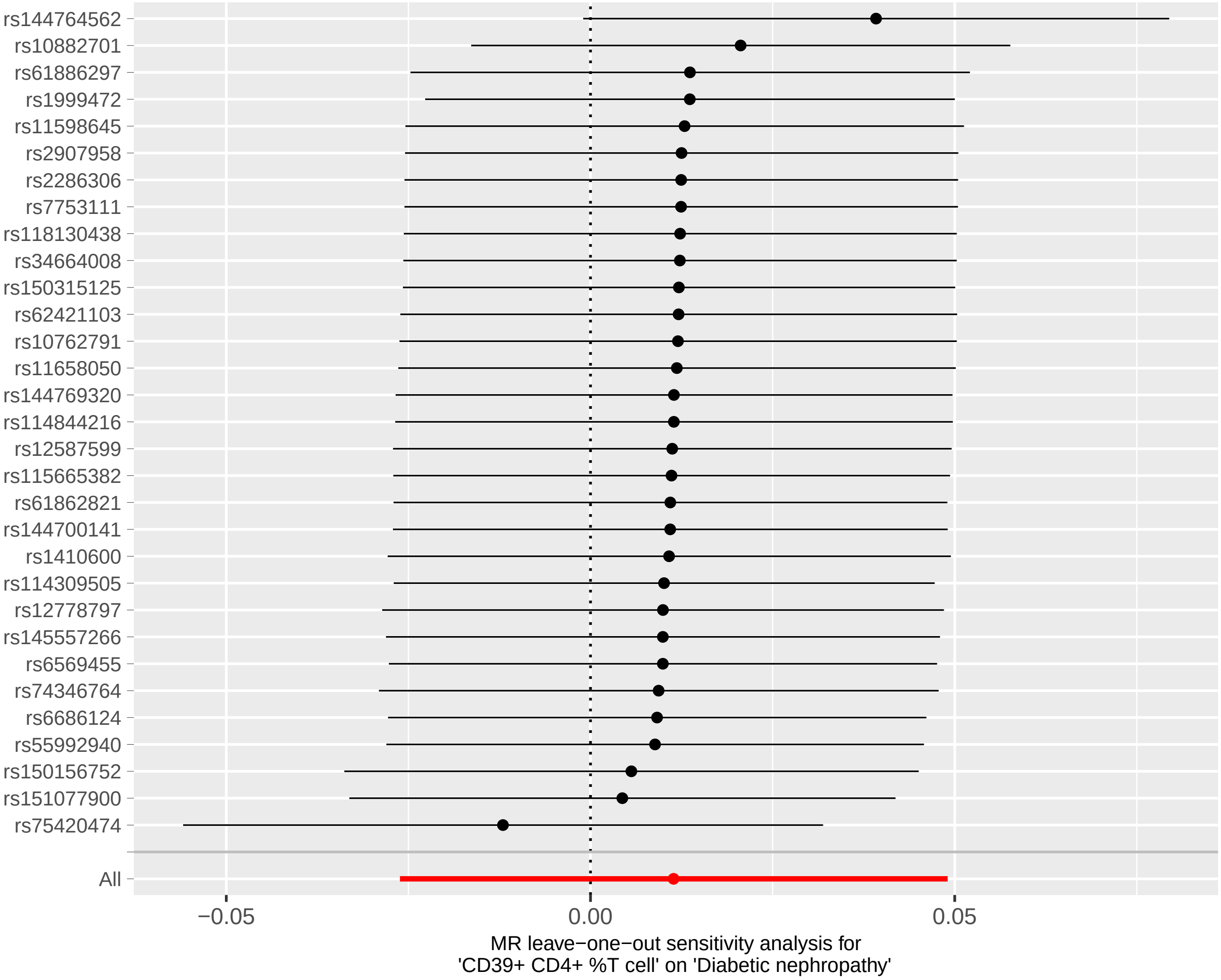
rs144764562
rs10882701
rs61886297
rs1999472
rs11598645
rs2907958
rs2286306
rs7753111
rs118130438
rs34664008
rs150315125
rs62421103
rs10762791
rs11658050
rs144769320
rs114844216
rs12587599
rs115665382
rs61862821
rs144700141
rs1410600
rs114309505
rs12778797
rs145557266
rs6569455
rs74346764
rs6686124
rs55992940
rs150156752
rs151077900
rs75420474
All
−0.05
0.00
0.05
MR leave−one−out sensitivity analysis for
'CD39+ CD4+ %T cell' on 'Diabetic nephropathy'

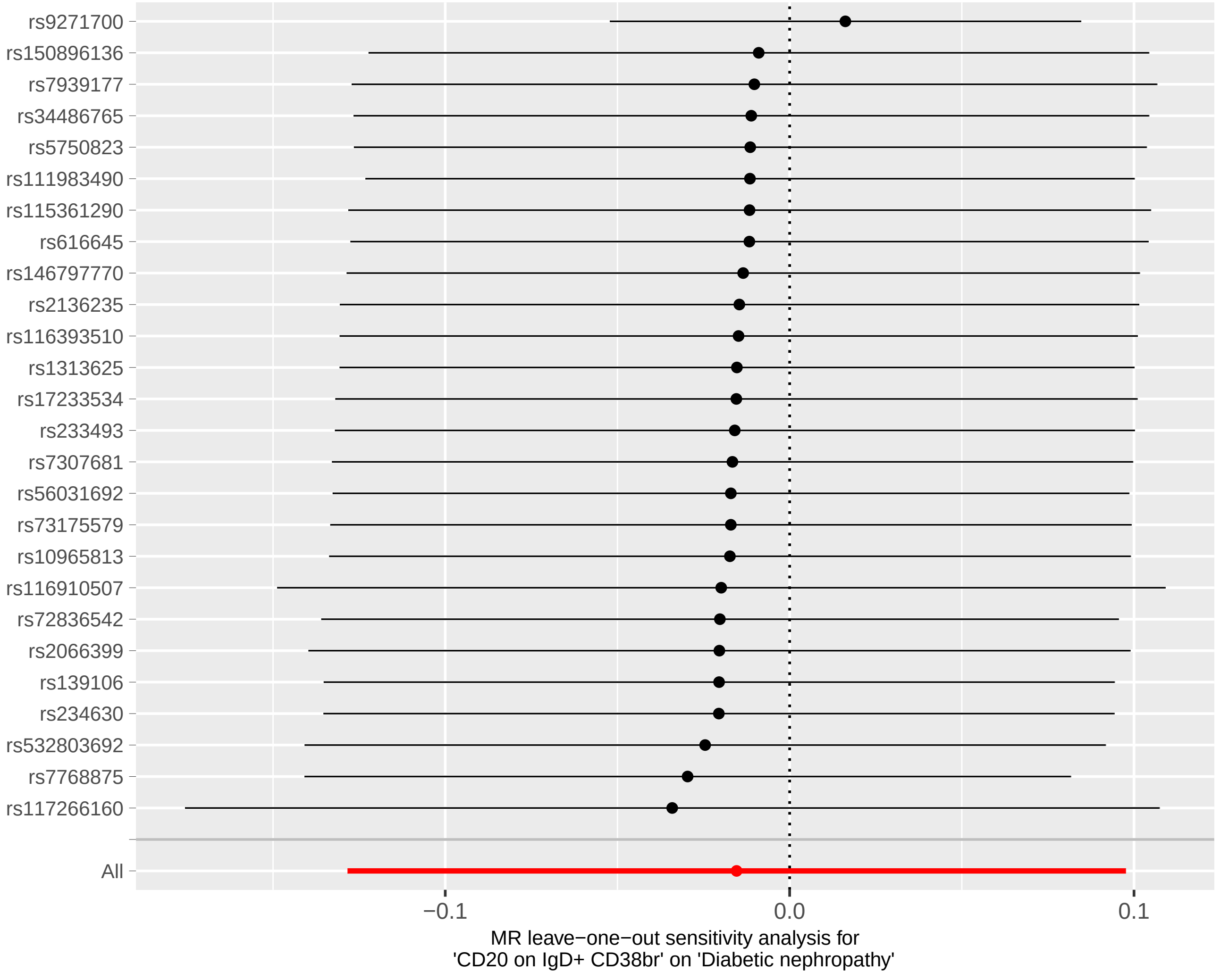
rs9271700
rs150896136
rs7939177
rs34486765
rs5750823
rs111983490
rs115361290
rs616645
rs146797770
rs2136235
rs116393510
rs1313625
rs17233534
rs233493
rs7307681
rs56031692
rs73175579
rs10965813
rs116910507
rs72836542
rs2066399
rs139106
rs234630
rs532803692
rs7768875
rs117266160
All
−0.1
0.0
0.1
MR leave−one−out sensitivity analysis for
'CD20 on IgD+ CD38br' on 'Diabetic nephropathy'

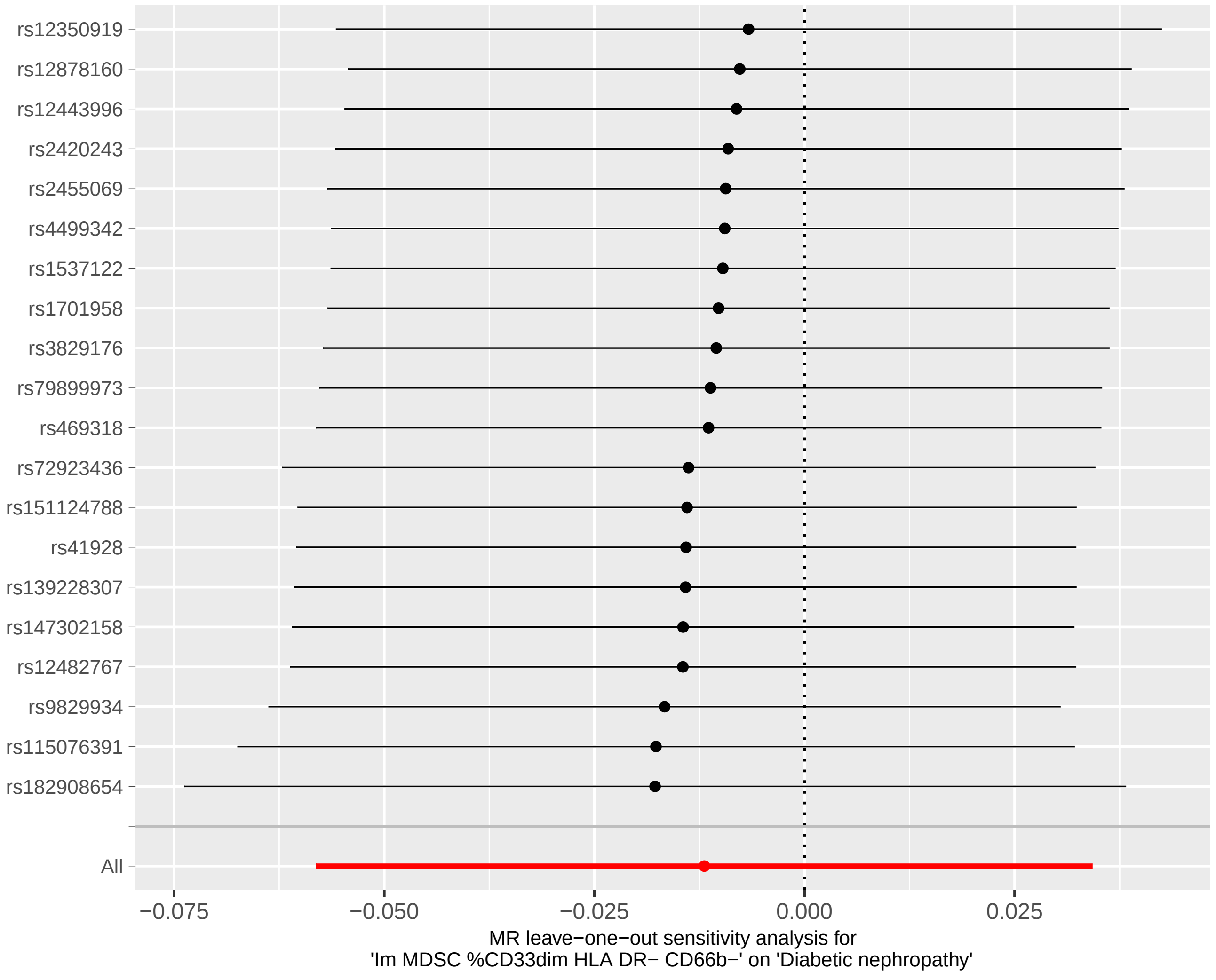
rs12350919
rs12878160
rs12443996
rs2420243
rs2455069
rs4499342
rs1537122
rs1701958
rs3829176
rs79899973
rs469318
rs72923436
rs151124788
rs41928
rs139228307
rs147302158
rs12482767
rs9829934
rs115076391
rs182908654
All
−0.075
−0.050
−0.025
0.000
0.025
MR leave−one−out sensitivity analysis for
'Im MDSC %CD33dim HLA DR− CD66b−' on 'Diabetic nephropathy'

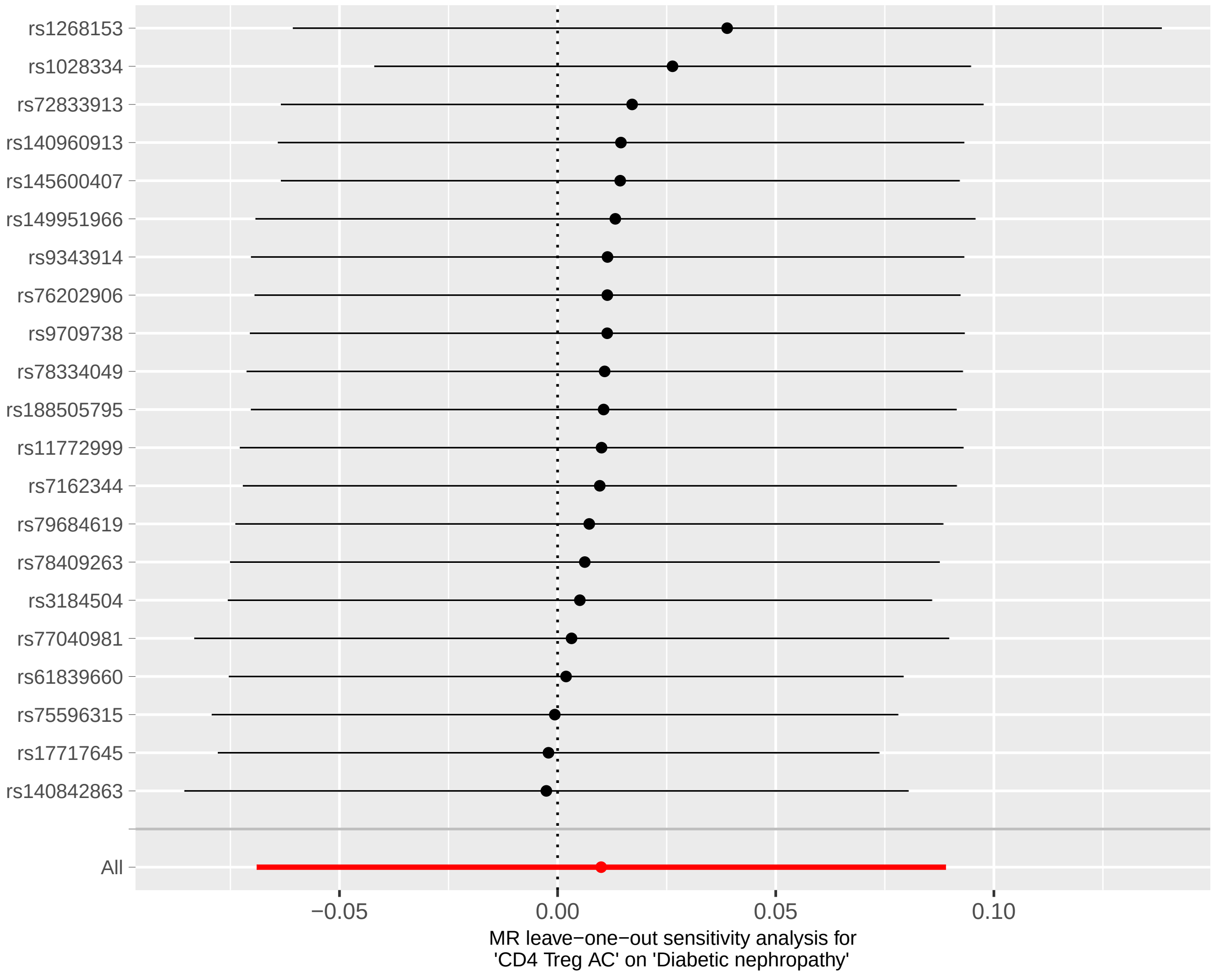
rs1268153
rs1028334
rs72833913
rs140960913
rs145600407
rs149951966
rs9343914
rs76202906
rs9709738
rs78334049
rs188505795
rs11772999
rs7162344
rs79684619
rs78409263
rs3184504
rs77040981
rs61839660
rs75596315
rs17717645
rs140842863
All
−0.05
0.00
0.05
0.10
MR leave−one−out sensitivity analysis for
'CD4 Treg AC' on 'Diabetic nephropathy'

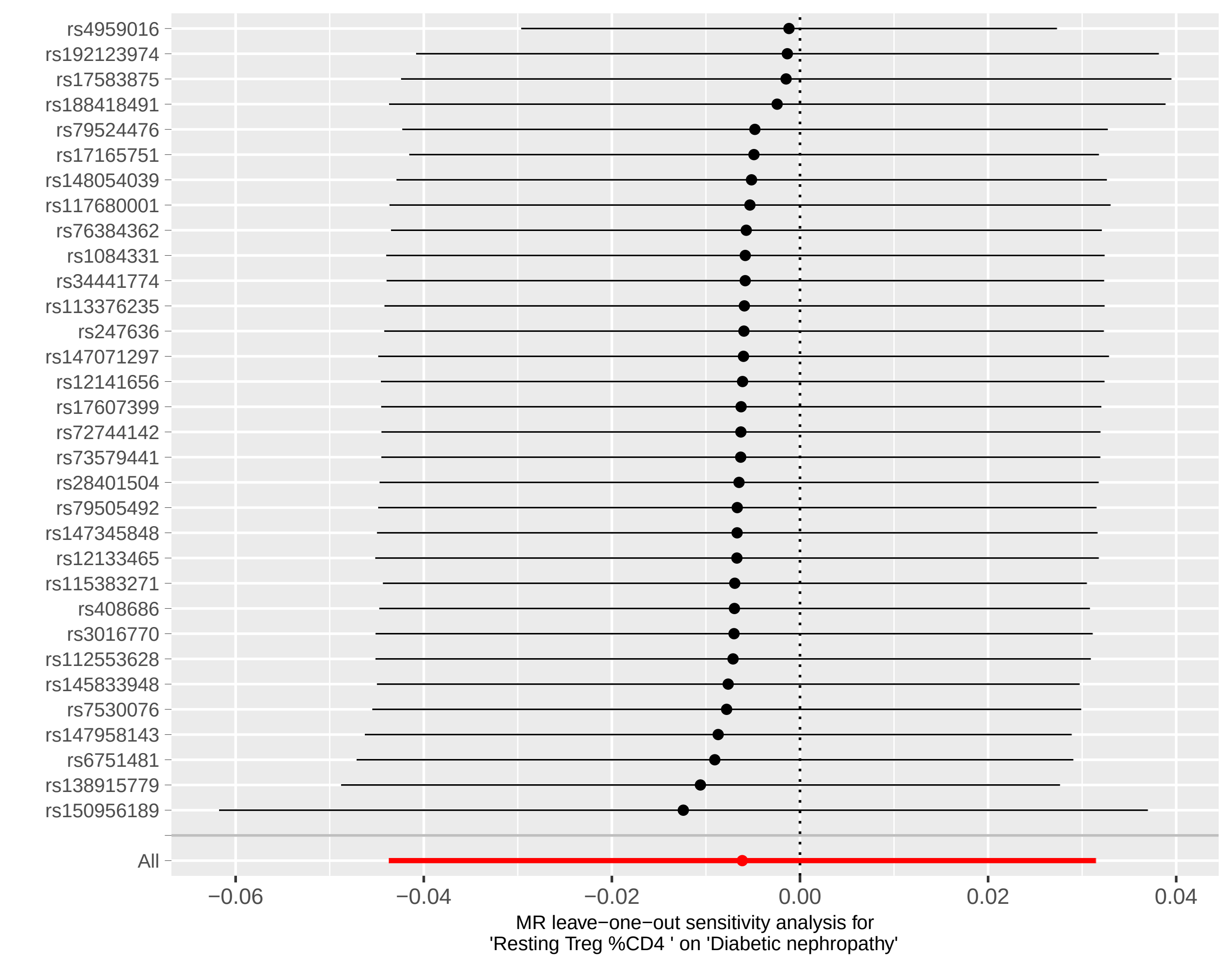

rs4959016
rs192123974
rs17583875
rs188418491
rs79524476
rs17165751
rs148054039
rs117680001
rs76384362
rs1084331
rs34441774
rs113376235
rs247636
rs147071297
rs12141656
rs17607399
rs72744142
rs73579441
rs28401504
rs79505492
rs147345848
rs12133465
rs115383271
rs408686
rs3016770
rs112553628
rs145833948
rs7530076
rs147958143
rs6751481
rs138915779
rs150956189
All
−0.06
−0.04
−0.02
0.00
0.02
0.04
MR leave−one−out sensitivity analysis for
'Resting Treg %CD4 ' on 'Diabetic nephropathy'

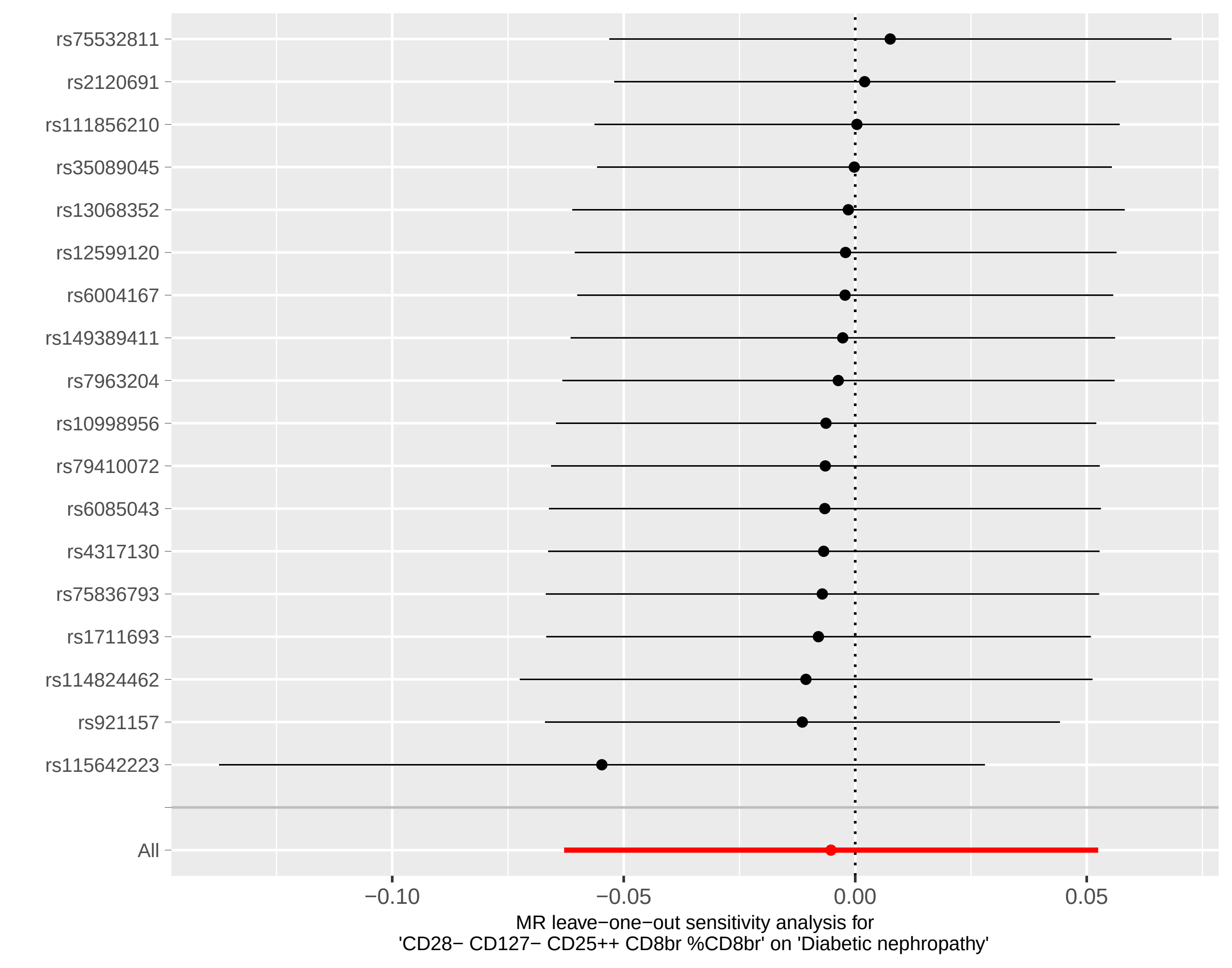
rs75532811
rs2120691
rs111856210
rs35089045
rs13068352
rs12599120
rs6004167
rs149389411
rs7963204
rs10998956
rs79410072
rs6085043
rs4317130
rs75836793
rs1711693
rs114824462
rs921157
rs115642223
All
−0.10
−0.05
0.00
0.05
MR leave−one−out sensitivity analysis for
'CD28− CD127− CD25++ CD8br %CD8br' on 'Diabetic nephropathy'

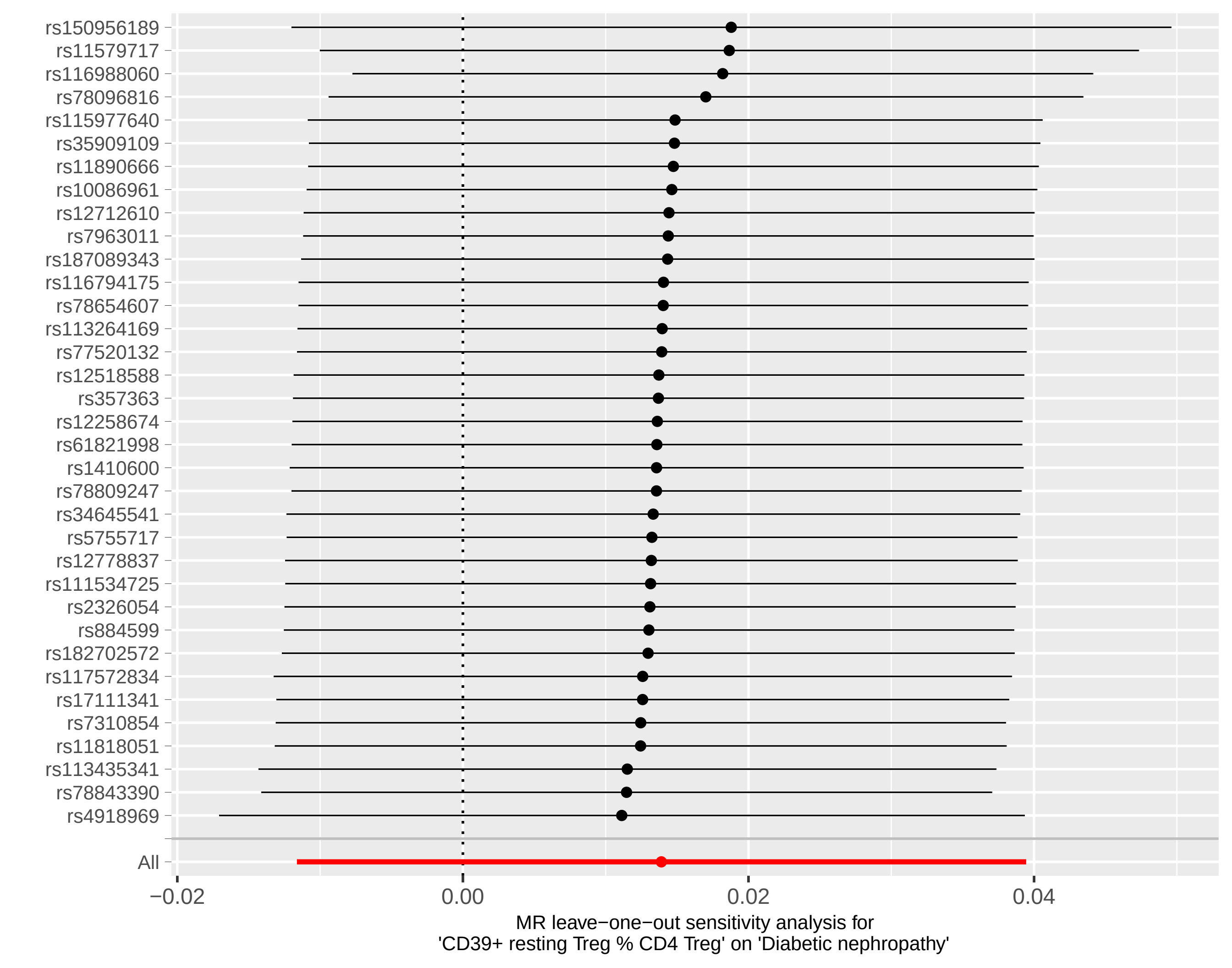

rs150956189
rs11579717
rs116988060
rs78096816
rs115977640
rs35909109
rs11890666
rs10086961
rs12712610
rs7963011
rs187089343
rs116794175
rs78654607
rs113264169
rs77520132
rs12518588
rs357363
rs12258674
rs61821998
rs1410600
rs78809247
rs34645541
rs5755717
rs12778837
rs111534725
rs2326054
rs884599
rs182702572
rs117572834
rs17111341
rs7310854
rs11818051
rs113435341
rs78843390
rs4918969
All
−0.02
0.00
0.02
0.04
MR leave−one−out sensitivity analysis for
'CD39+ resting Treg % CD4 Treg' on 'Diabetic nephropathy'

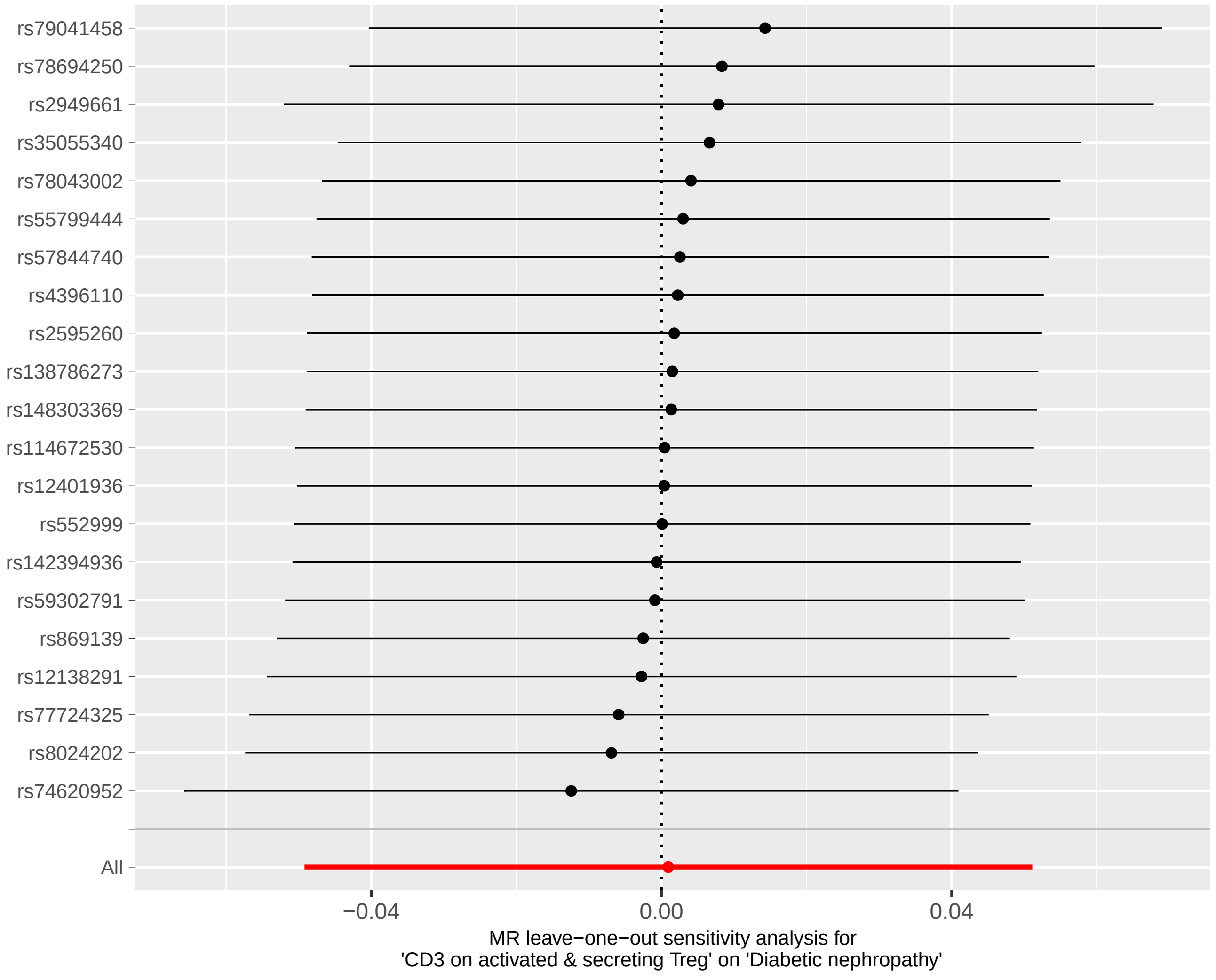
rs79041458
rs78694250
rs2949661
rs35055340
rs78043002
rs55799444
rs57844740
rs4396110
rs2595260
rs138786273
rs148303369
rs114672530
rs12401936
rs552999
rs142394936
rs59302791
rs869139
rs12138291
rs77724325
rs8024202
rs74620952
All
−0.04
0.00
0.04
MR leave−one−out sensitivity analysis for
'CD3 on activated & secreting Treg' on 'Diabetic nephropathy'

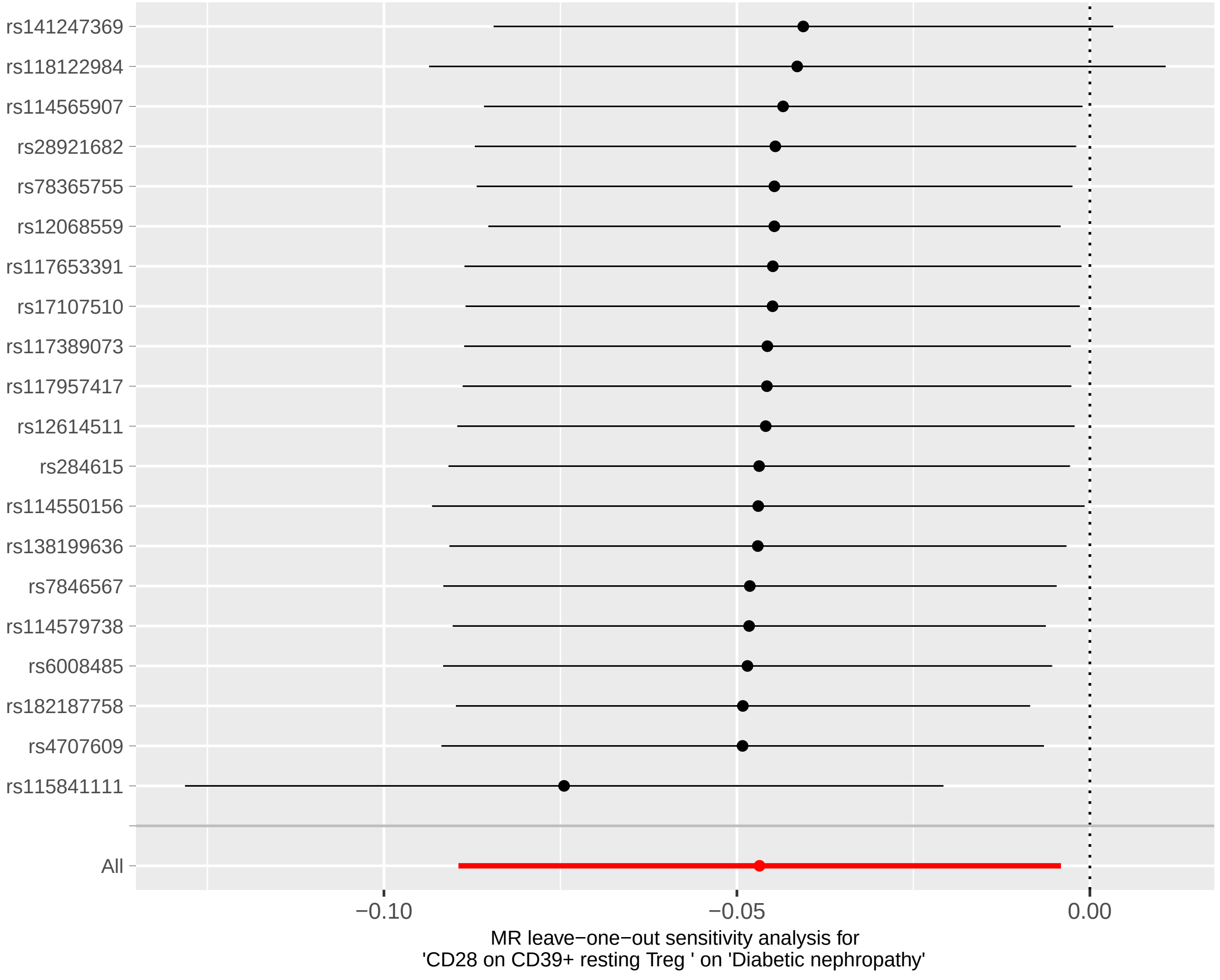
rs141247369
rs118122984
rs114565907
rs28921682
rs78365755
rs12068559
rs117653391
rs17107510
rs117389073
rs117957417
rs12614511
rs284615
rs114550156
rs138199636
rs7846567
rs114579738
rs6008485
rs182187758
rs4707609
rs115841111
All
−0.10
−0.05
0.00
MR leave−one−out sensitivity analysis for
'CD28 on CD39+ resting Treg ' on 'Diabetic nephropathy'

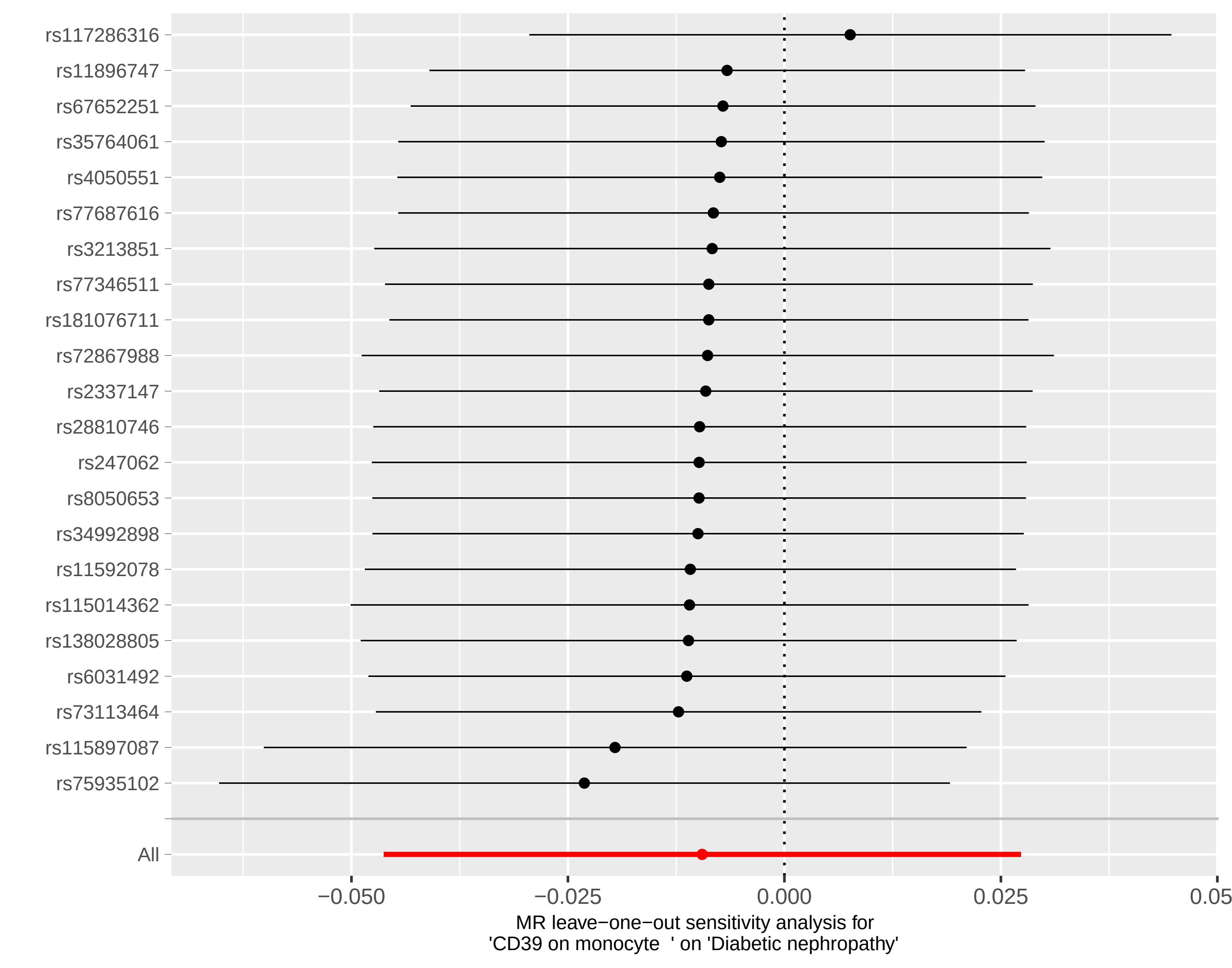

rs117286316
rs11896747
rs67652251
rs35764061
rs4050551
rs77687616
rs3213851
rs77346511
rs181076711
rs72867988
rs2337147
rs28810746
rs247062
rs8050653
rs34992898
rs11592078
rs115014362
rs138028805
rs6031492
rs73113464
rs115897087
rs75935102
All
−0.050
−0.025
0.000
0.025
0.05
MR leave−one−out sensitivity analysis for
'CD39 on monocyte ' on 'Diabetic nephropathy'

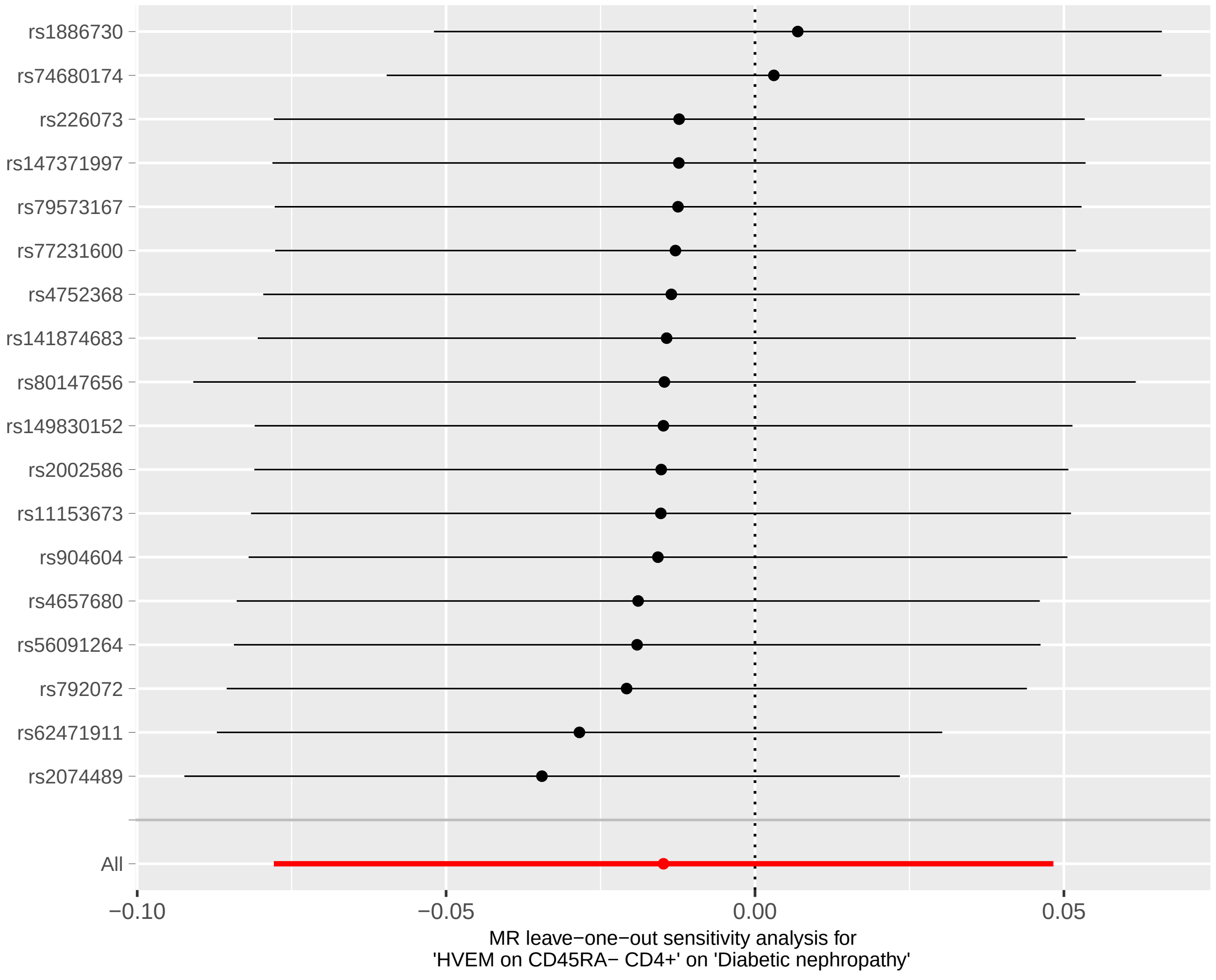
rs1886730
rs74680174
rs226073
rs147371997
rs79573167
rs77231600
rs4752368
rs141874683
rs80147656
rs149830152
rs2002586
rs11153673
rs904604
rs4657680
rs56091264
rs792072
rs62471911
rs2074489
All
−0.10
−0.05
0.00
0.05
MR leave−one−out sensitivity analysis for
'HVEM on CD45RA− CD4+' on 'Diabetic nephropathy'

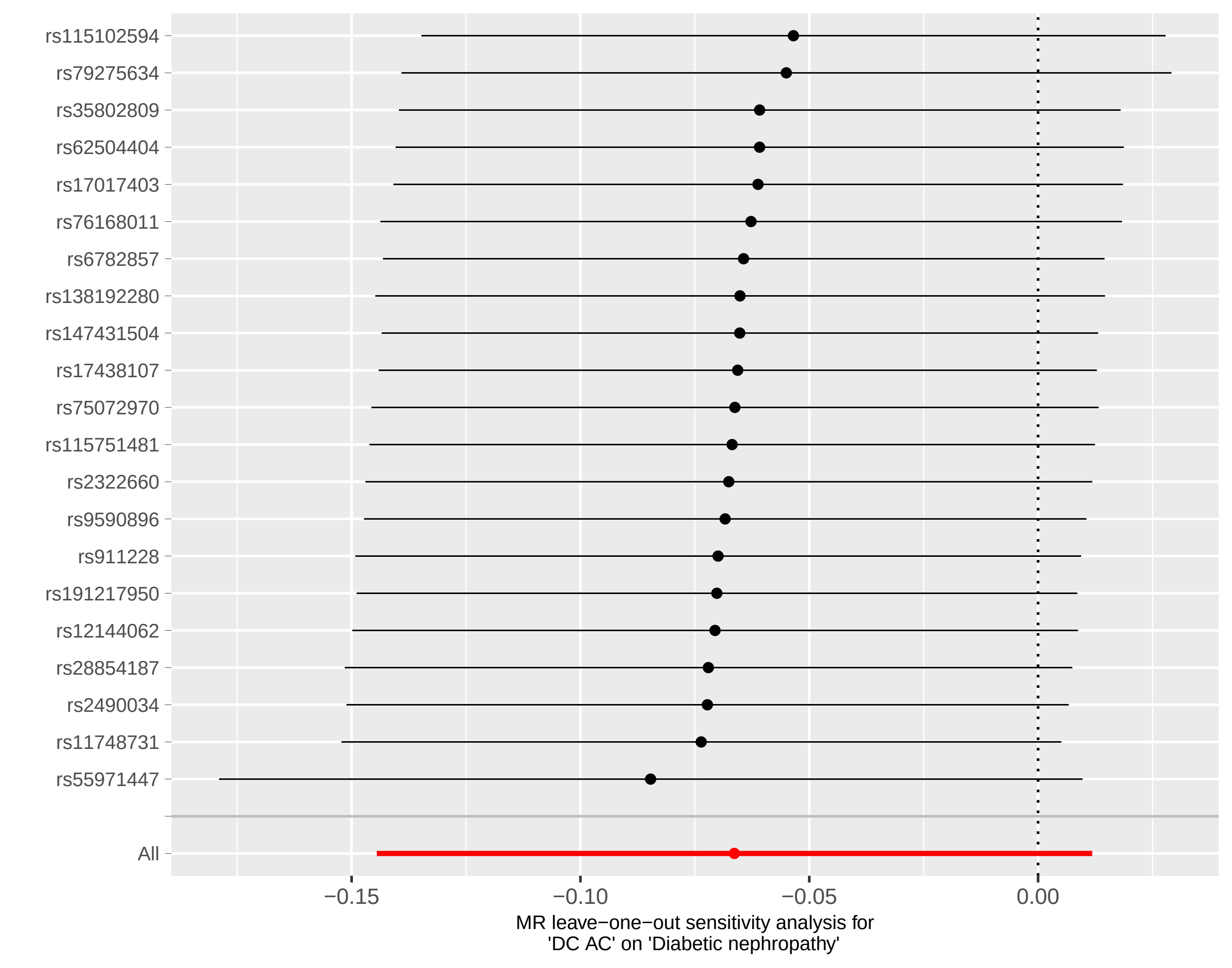
rs115102594
rs79275634
rs35802809
rs62504404
rs17017403
rs76168011
rs6782857
rs138192280
rs147431504
rs17438107
rs75072970
rs115751481
rs2322660
rs9590896
rs911228
rs191217950
rs12144062
rs28854187
rs2490034
rs11748731
rs55971447
All
−0.15
−0.10
−0.05
0.00
MR leave−one−out sensitivity analysis for
'DC AC' on 'Diabetic nephropathy'

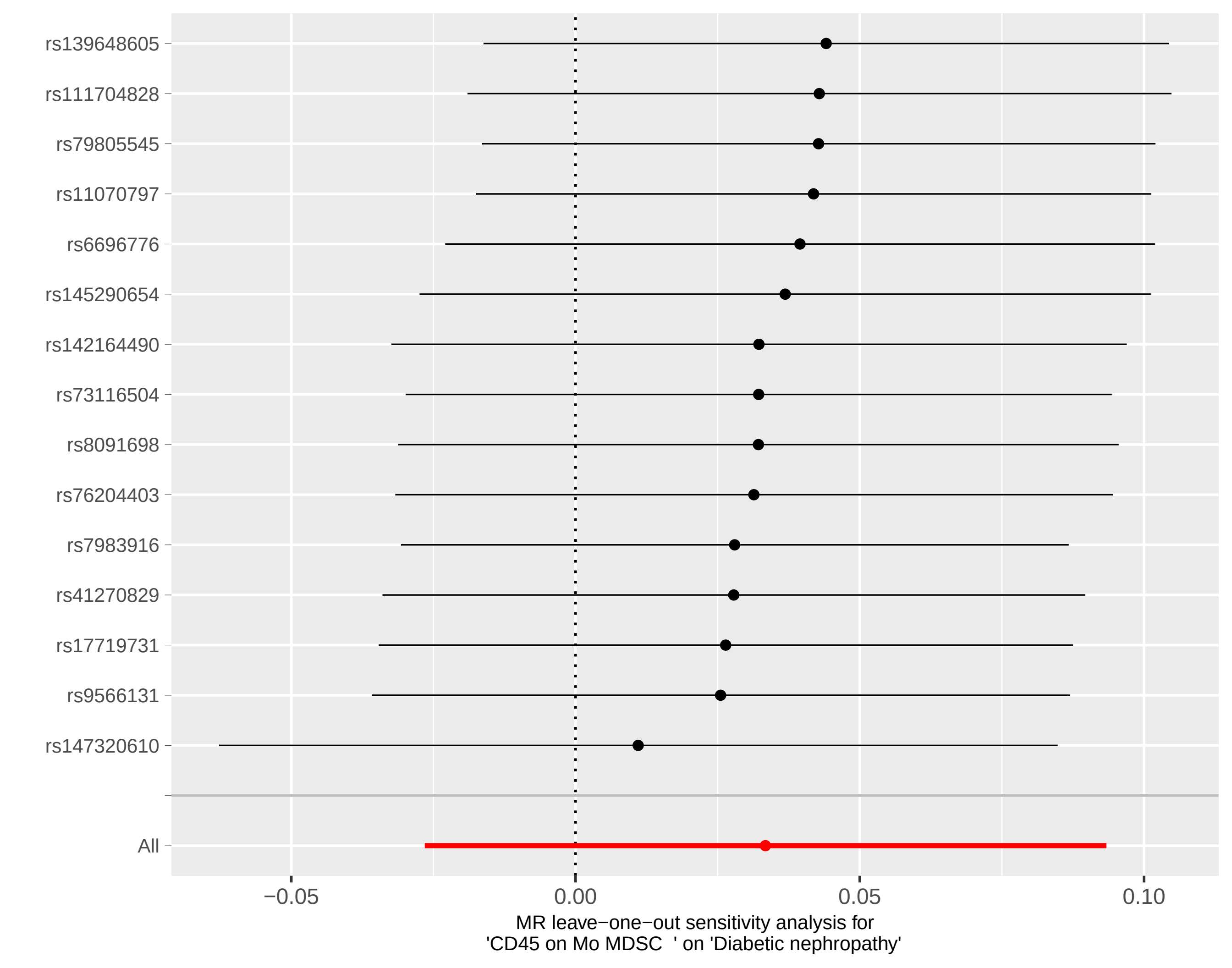
rs139648605
rs111704828
rs79805545
rs11070797
rs6696776
rs145290654
rs142164490
rs73116504
rs8091698
rs76204403
rs7983916
rs41270829
rs17719731
rs9566131
rs147320610
All
−0.05
0.00
0.05
0.10
MR leave−one−out sensitivity analysis for
'CD45 on Mo MDSC ' on 'Diabetic nephropathy'

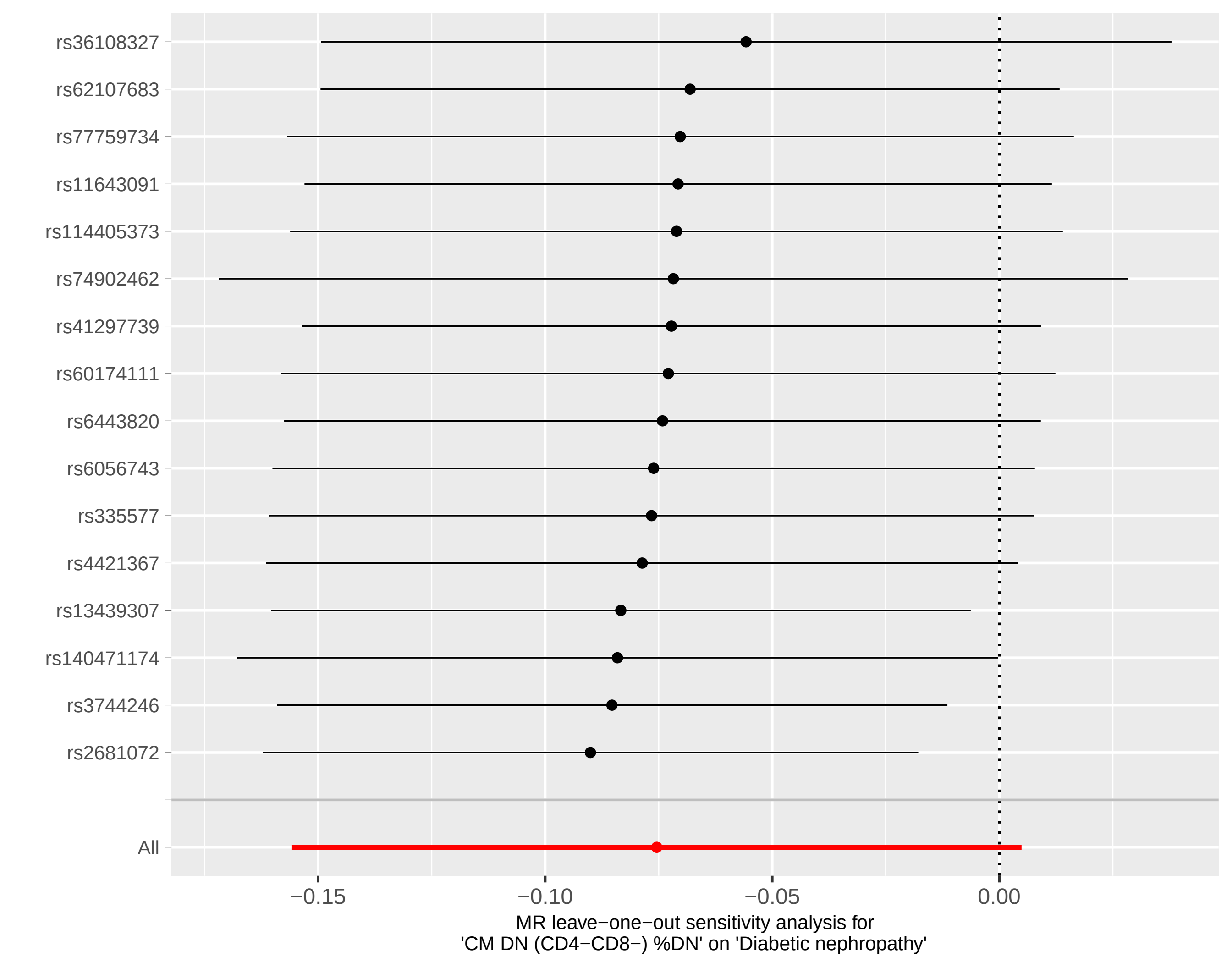
rs36108327
rs62107683
rs77759734
rs11643091
rs114405373
rs74902462
rs41297739
rs60174111
rs6443820
rs6056743
rs335577
rs4421367
rs13439307
rs140471174
rs3744246
rs2681072
All
−0.15
−0.10
−0.05
0.00
MR leave−one−out sensitivity analysis for
'CM DN (CD4−CD8−) %DN' on 'Diabetic nephropathy'

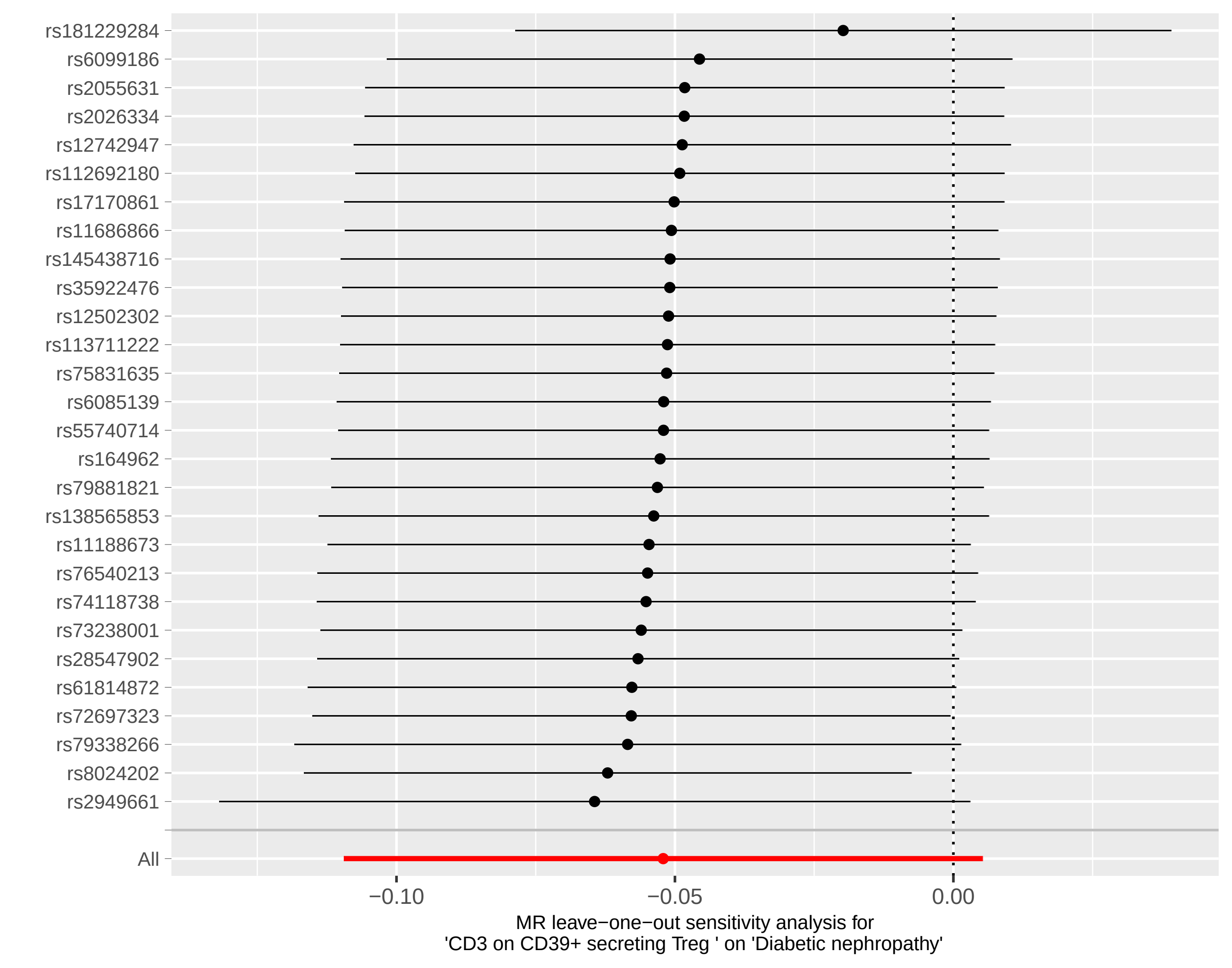

rs181229284
rs6099186
rs2055631
rs2026334
rs12742947
rs112692180
rs17170861
rs11686866
rs145438716
rs35922476
rs12502302
rs113711222
rs75831635
rs6085139
rs55740714
rs164962
rs79881821
rs138565853
rs11188673
rs76540213
rs74118738
rs73238001
rs28547902
rs61814872
rs72697323
rs79338266
rs8024202
rs2949661
All
−0.10
−0.05
0.00
MR leave−one−out sensitivity analysis for
'CD3 on CD39+ secreting Treg ' on 'Diabetic nephropathy'

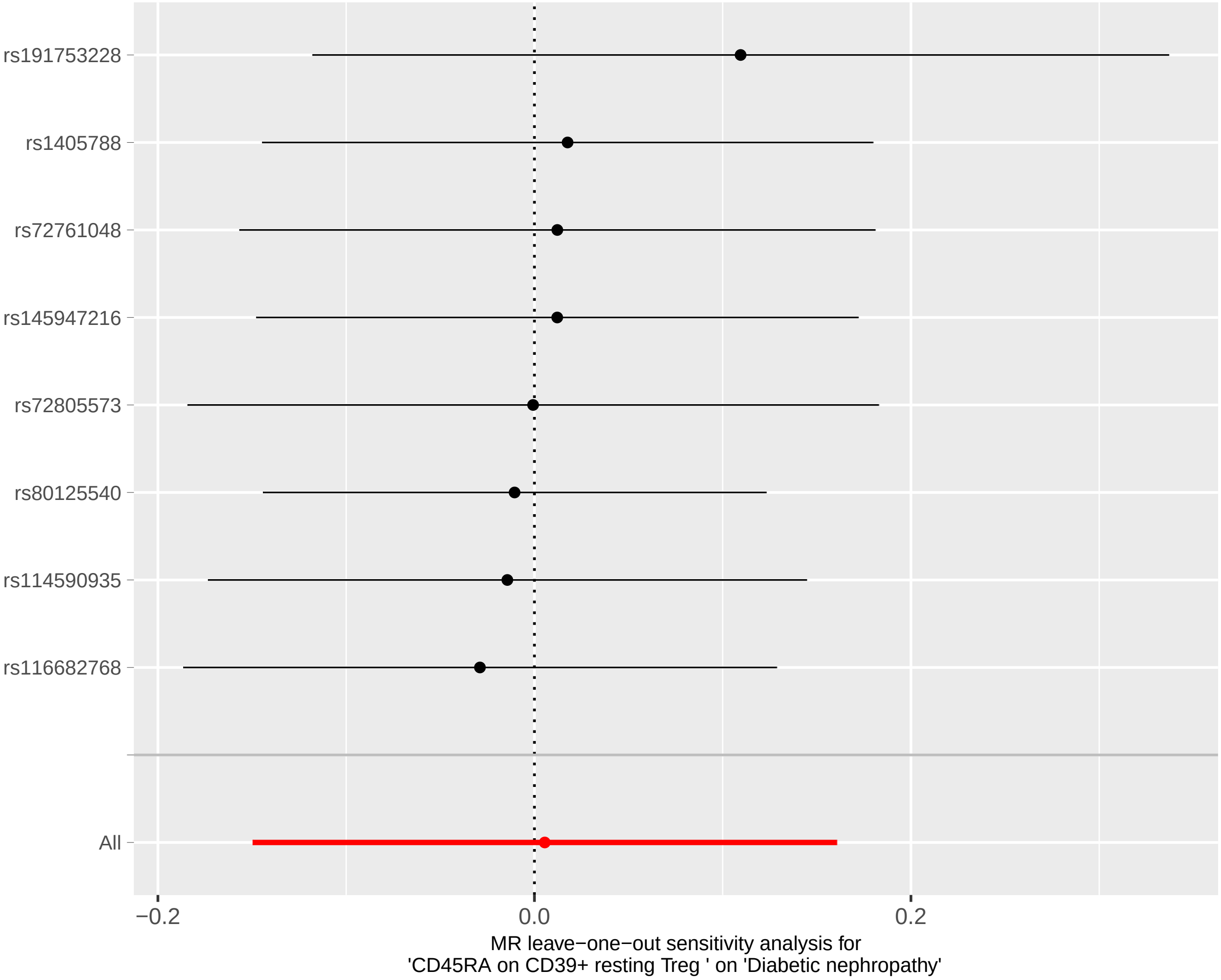

rs191753228
rs1405788
rs72761048
rs145947216
rs72805573
rs80125540
rs114590935
rs116682768
All
−0.2
0.0
0.2
MR leave−one−out sensitivity analysis for
'CD45RA on CD39+ resting Treg ' on 'Diabetic nephropathy'

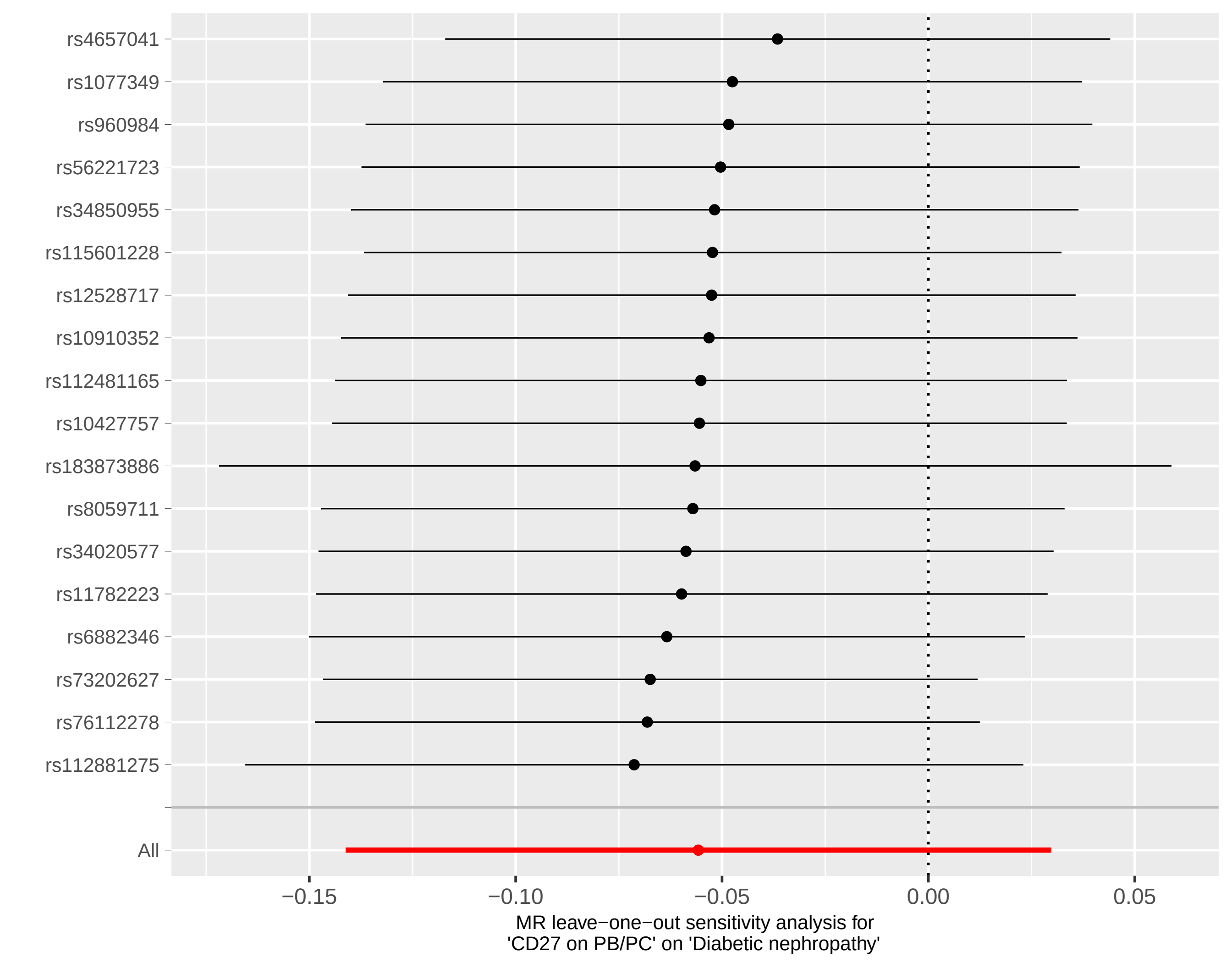
rs4657041
rs1077349
rs960984
rs56221723
rs34850955
rs115601228
rs12528717
rs10910352
rs112481165
rs10427757
rs183873886
rs8059711
rs34020577
rs11782223
rs6882346
rs73202627
rs76112278
rs112881275
All
−0.15
−0.10
−0.05
0.00
0.05
MR leave−one−out sensitivity analysis for
'CD27 on PB/PC' on 'Diabetic nephropathy'

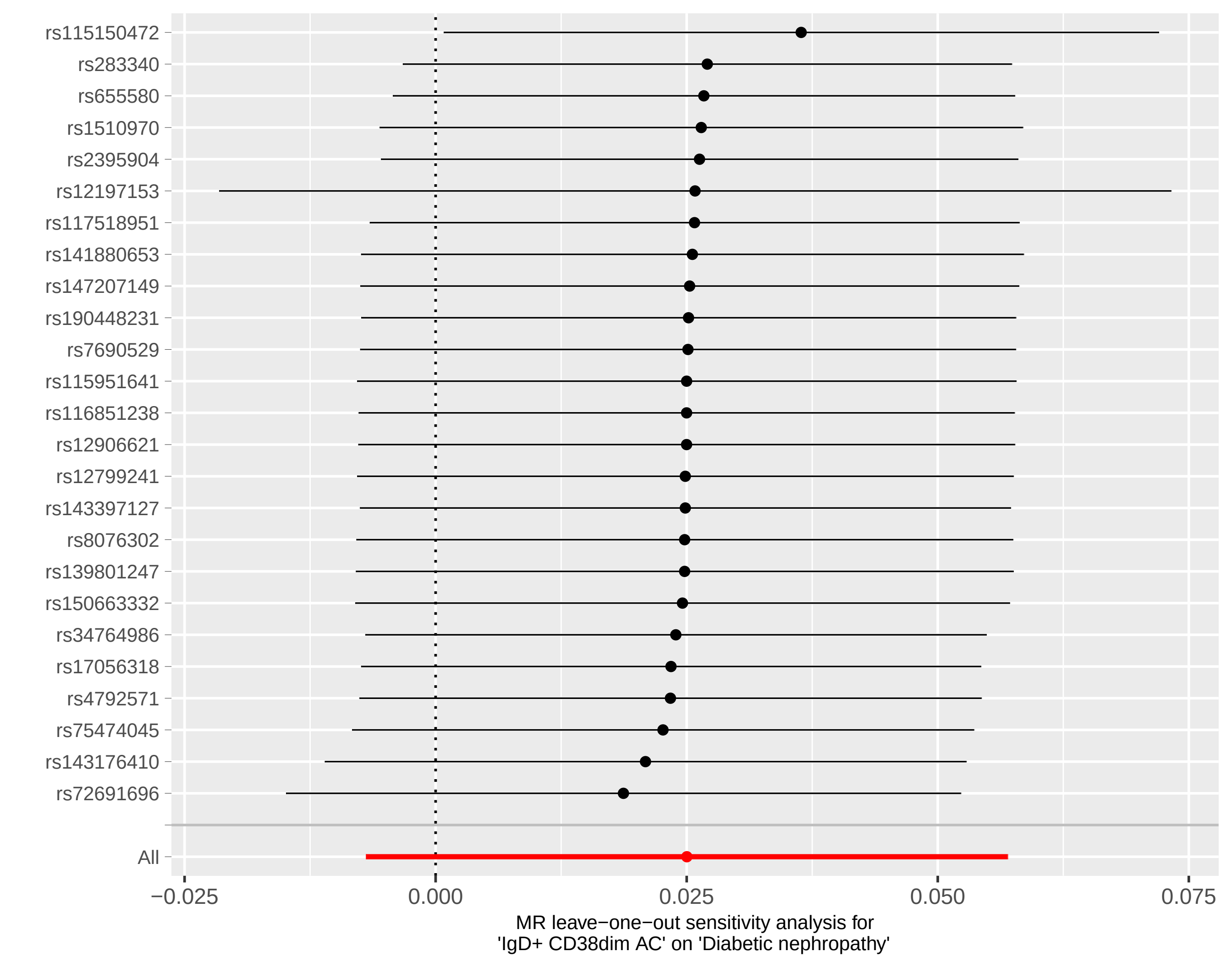

rs115150472
rs283340
rs655580
rs1510970
rs2395904
rs12197153
rs117518951
rs141880653
rs147207149
rs190448231
rs7690529
rs115951641
rs116851238
rs12906621
rs12799241
rs143397127
rs8076302
rs139801247
rs150663332
rs34764986
rs17056318
rs4792571
rs75474045
rs143176410
rs72691696
All
−0.025
0.000
0.025
0.050
0.075
MR leave−one−out sensitivity analysis for
'IgD+ CD38dim AC' on 'Diabetic nephropathy'

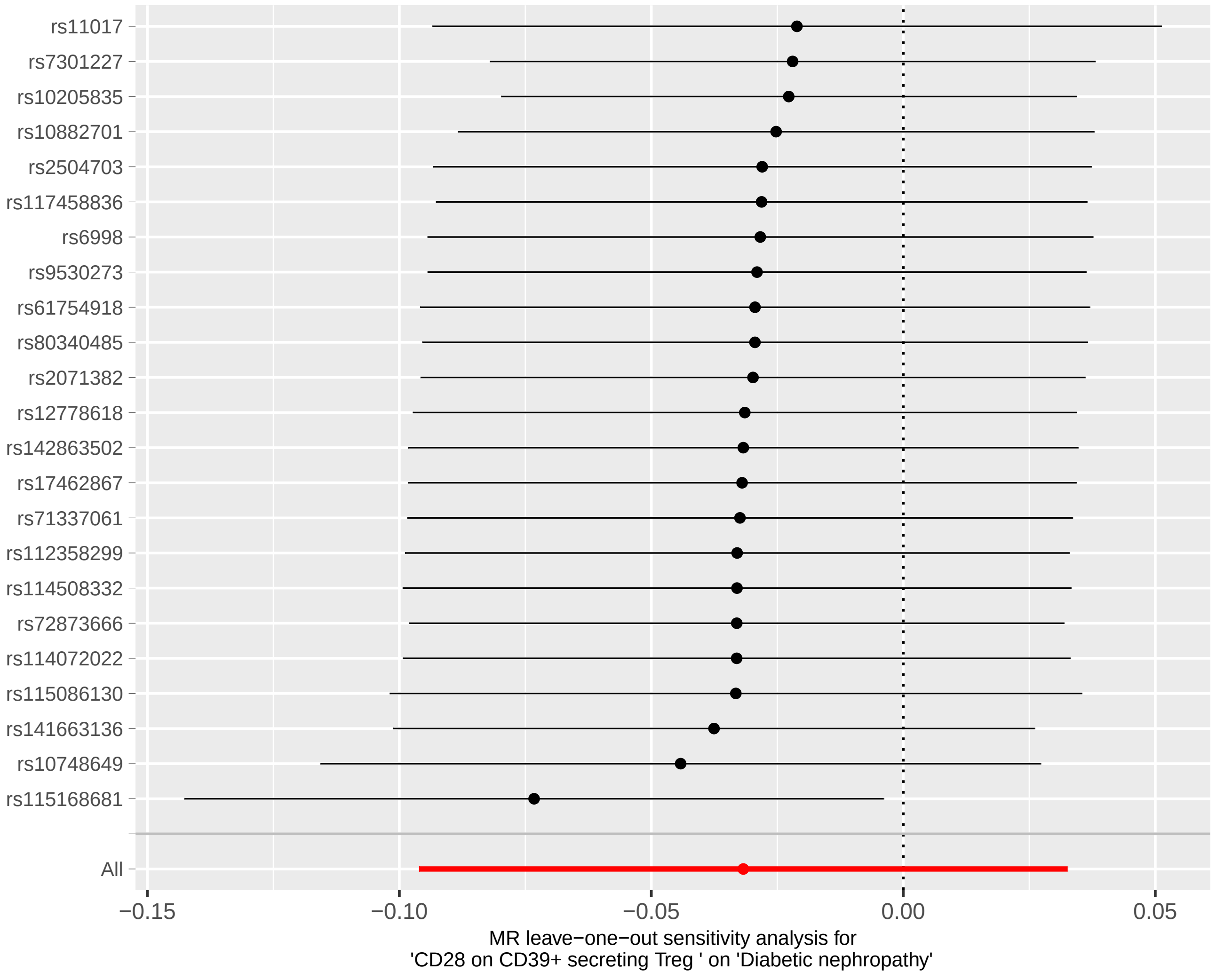
rs11017
rs7301227
rs10205835
rs10882701
rs2504703
rs117458836
rs6998
rs9530273
rs61754918
rs80340485
rs2071382
rs12778618
rs142863502
rs17462867
rs71337061
rs112358299
rs114508332
rs72873666
rs114072022
rs115086130
rs141663136
rs10748649
rs115168681
All
−0.15
−0.10
−0.05
0.00
0.05
MR leave−one−out sensitivity analysis for
'CD28 on CD39+ secreting Treg ' on 'Diabetic nephropathy'

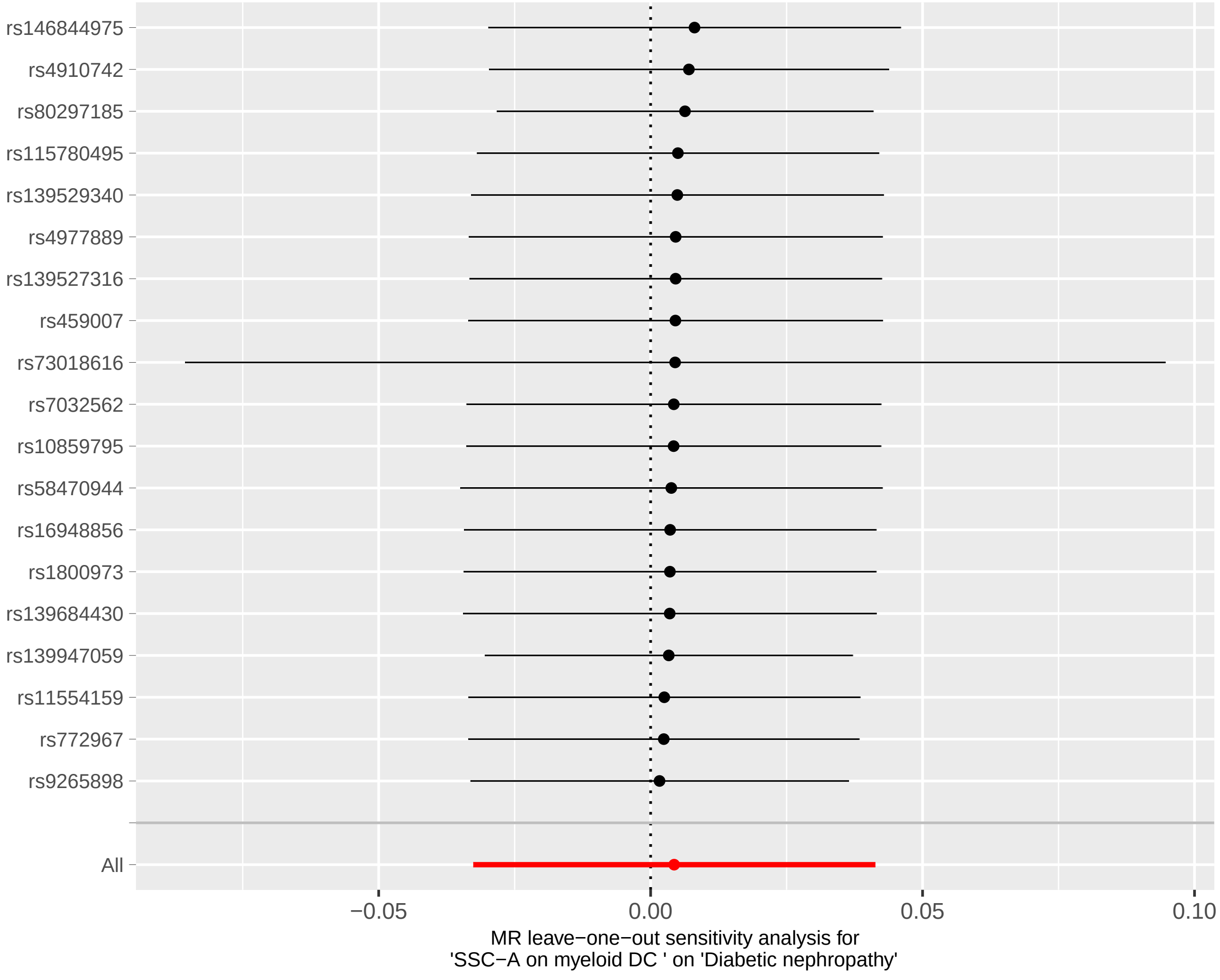
rs146844975
rs4910742
rs80297185
rs115780495
rs139529340
rs4977889
rs139527316
rs459007
rs73018616
rs7032562
rs10859795
rs58470944
rs16948856
rs1800973
rs139684430
rs139947059
rs11554159
rs772967
rs9265898
All
−0.05
0.00
0.05
0.10
MR leave−one−out sensitivity analysis for
'SSC−A on myeloid DC ' on 'Diabetic nephropathy'

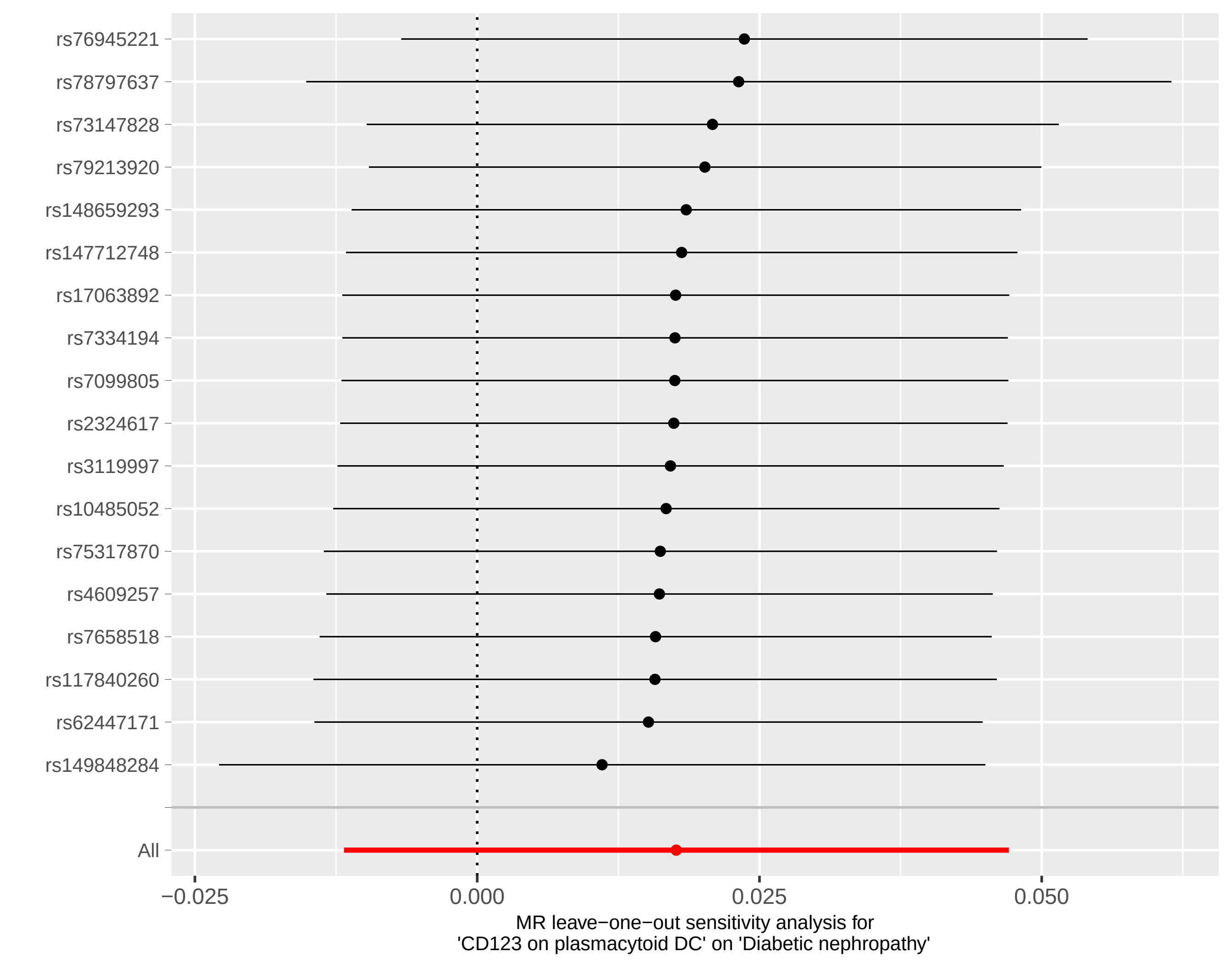
rs76945221
rs78797637
rs73147828
rs79213920
rs148659293
rs147712748
rs17063892
rs7334194
rs7099805
rs2324617
rs3119997
rs10485052
rs75317870
rs4609257
rs7658518
rs117840260
rs62447171
rs149848284
All
−0.025
0.000
0.025
0.050
MR leave−one−out sensitivity analysis for
'CD123 on plasmacytoid DC' on 'Diabetic nephropathy'

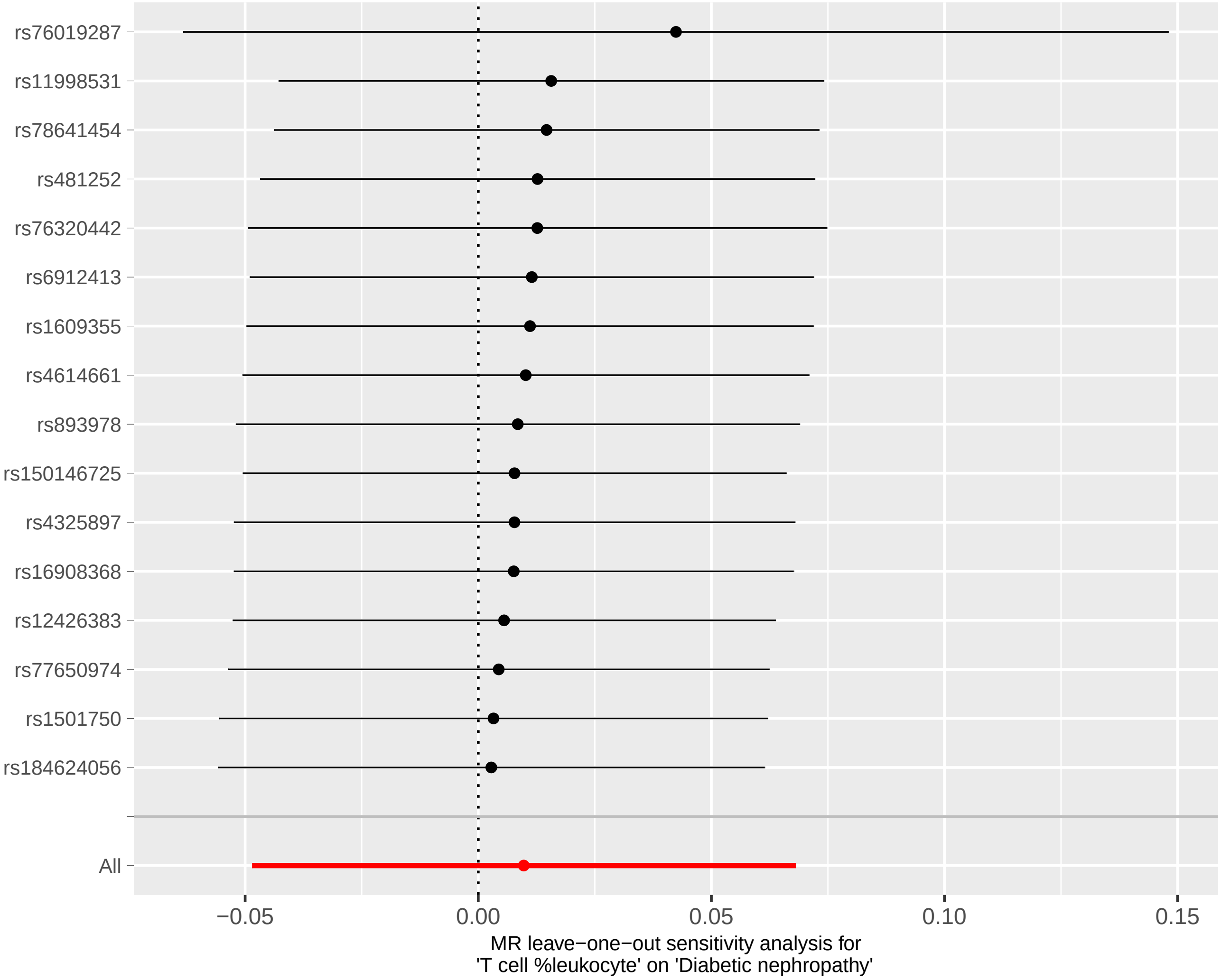
rs76019287
rs11998531
rs78641454
rs481252
rs76320442
rs6912413
rs1609355
rs4614661
rs893978
rs150146725
rs4325897
rs16908368
rs12426383
rs77650974
rs1501750
rs184624056
All
−0.05
0.00
0.05
0.10
0.15
MR leave−one−out sensitivity analysis for
'T cell %leukocyte' on 'Diabetic nephropathy'

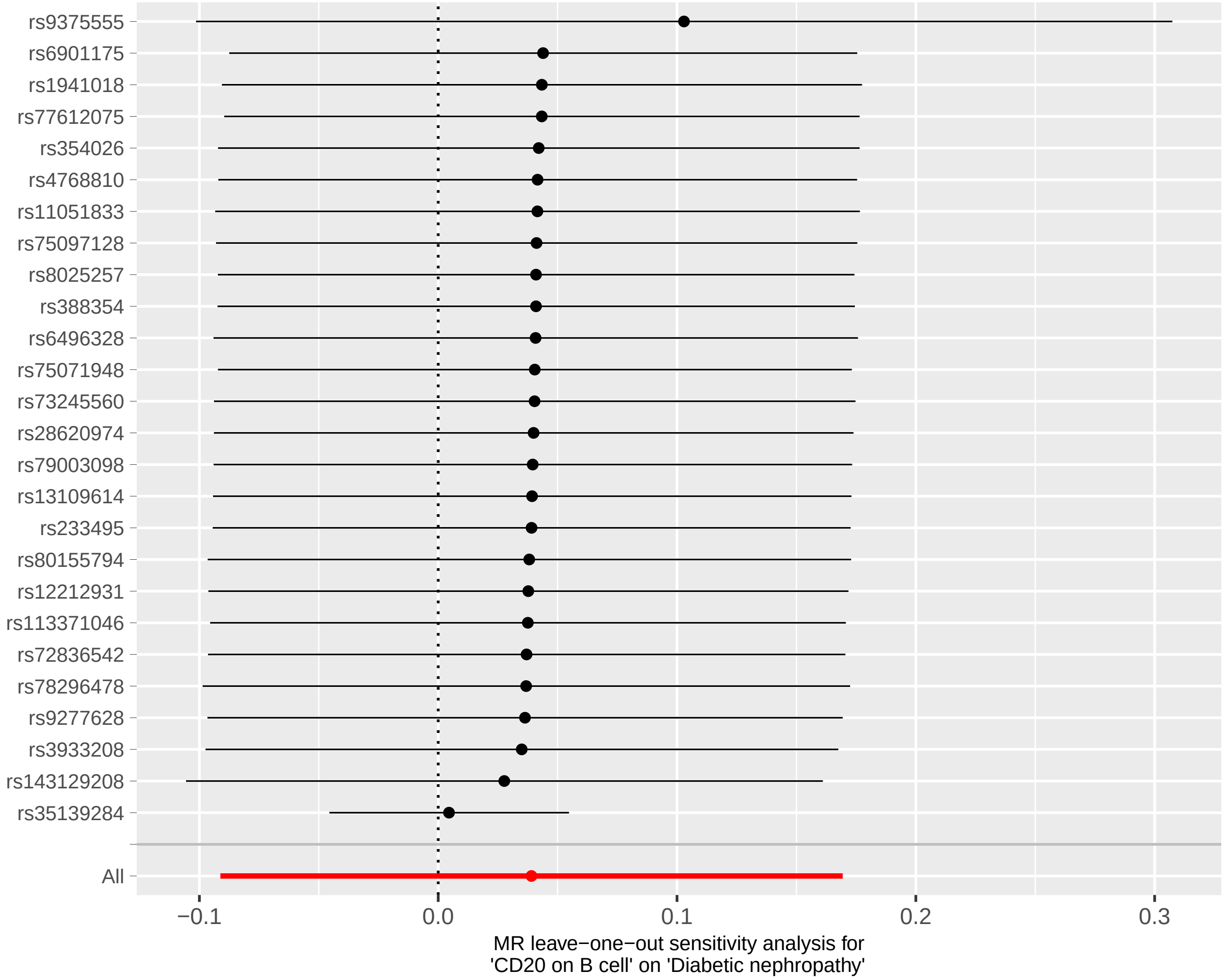
rs9375555
rs6901175
rs1941018
rs77612075
rs354026
rs4768810
rs11051833
rs75097128
rs8025257
rs388354
rs6496328
rs75071948
rs73245560
rs28620974
rs79003098
rs13109614
rs233495
rs80155794
rs12212931
rs113371046
rs72836542
rs78296478
rs9277628
rs3933208
rs143129208
rs35139284
All
−0.1
0.0
0.1
0.2
0.3
MR leave−one−out sensitivity analysis for
'CD20 on B cell' on 'Diabetic nephropathy'

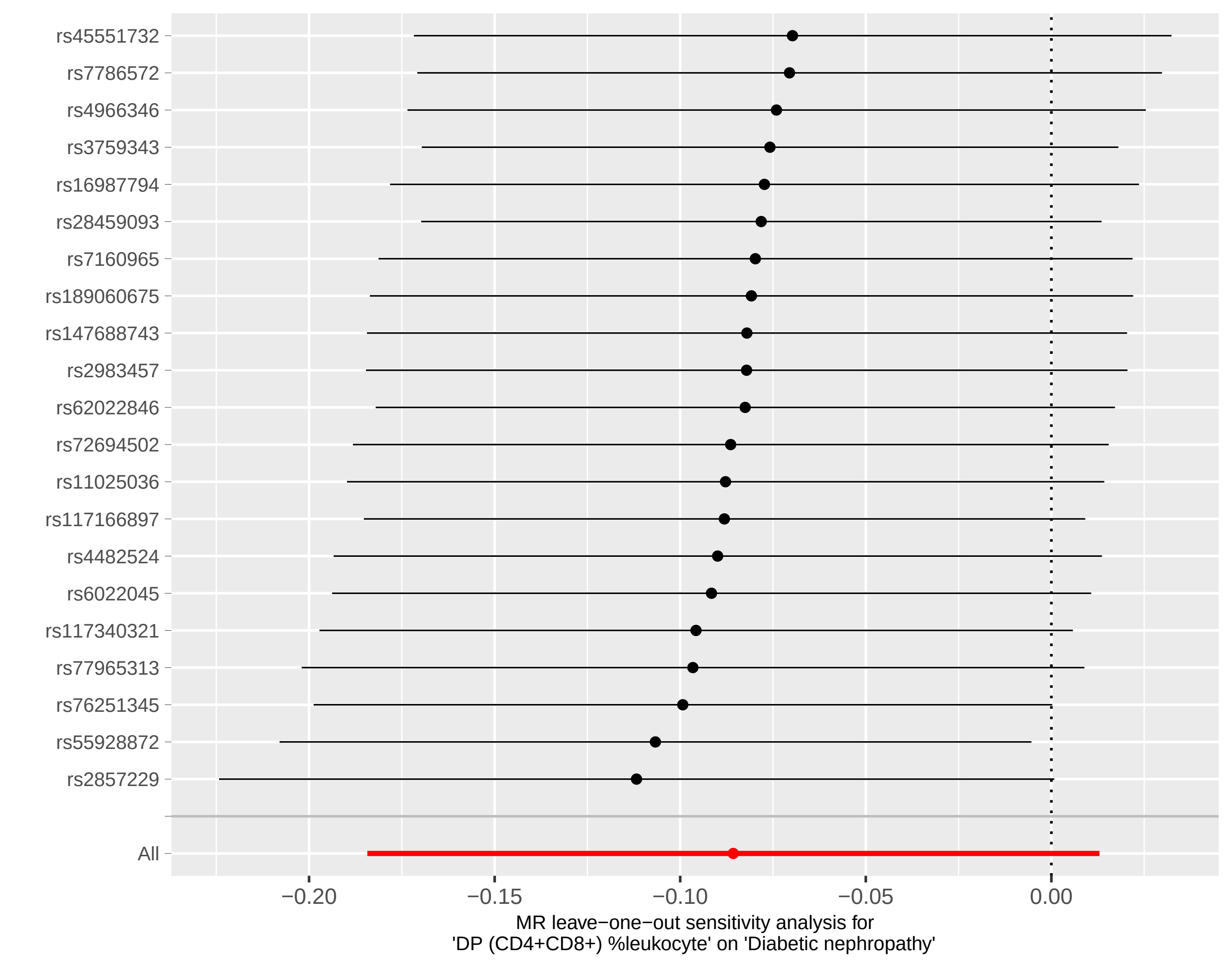
rs45551732
rs7786572
rs4966346
rs3759343
rs16987794
rs28459093
rs7160965
rs189060675
rs147688743
rs2983457
rs62022846
rs72694502
rs11025036
rs117166897
rs4482524
rs6022045
rs117340321
rs77965313
rs76251345
rs55928872
rs2857229
All
−0.20
−0.15
−0.10
−0.05
0.00
MR leave−one−out sensitivity analysis for
'DP (CD4+CD8+) %leukocyte' on 'Diabetic nephropathy'

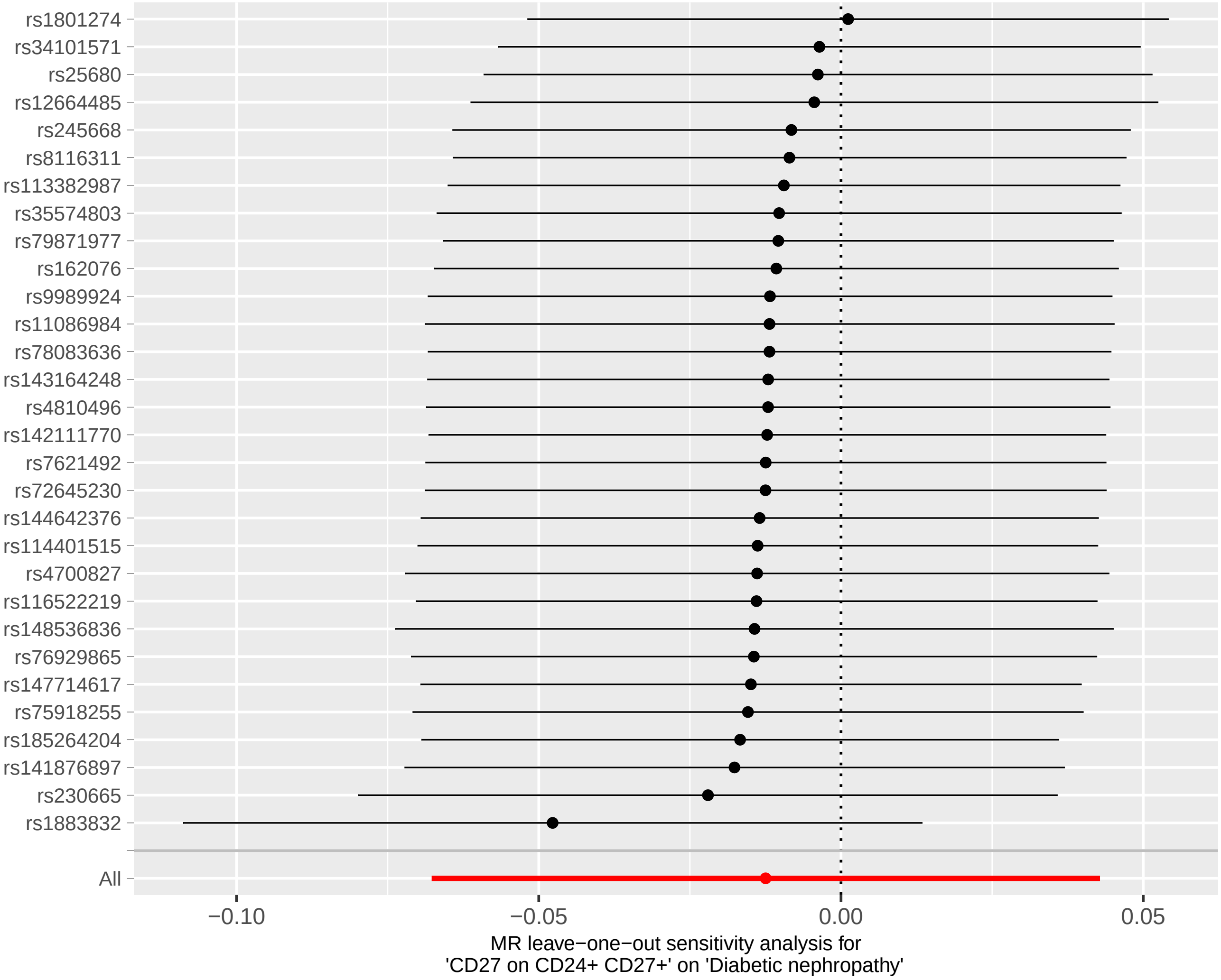
rs1801274
rs34101571
rs25680
rs12664485
rs245668
rs8116311
rs113382987
rs35574803
rs79871977
rs162076
rs9989924
rs11086984
rs78083636
rs143164248
rs4810496
rs142111770
rs7621492
rs72645230
rs144642376
rs114401515
rs4700827
rs116522219
rs148536836
rs76929865
rs147714617
rs75918255
rs185264204
rs141876897
rs230665
rs1883832
All
−0.10
−0.05
0.00
0.05
MR leave−one−out sensitivity analysis for
'CD27 on CD24+ CD27+' on 'Diabetic nephropathy'

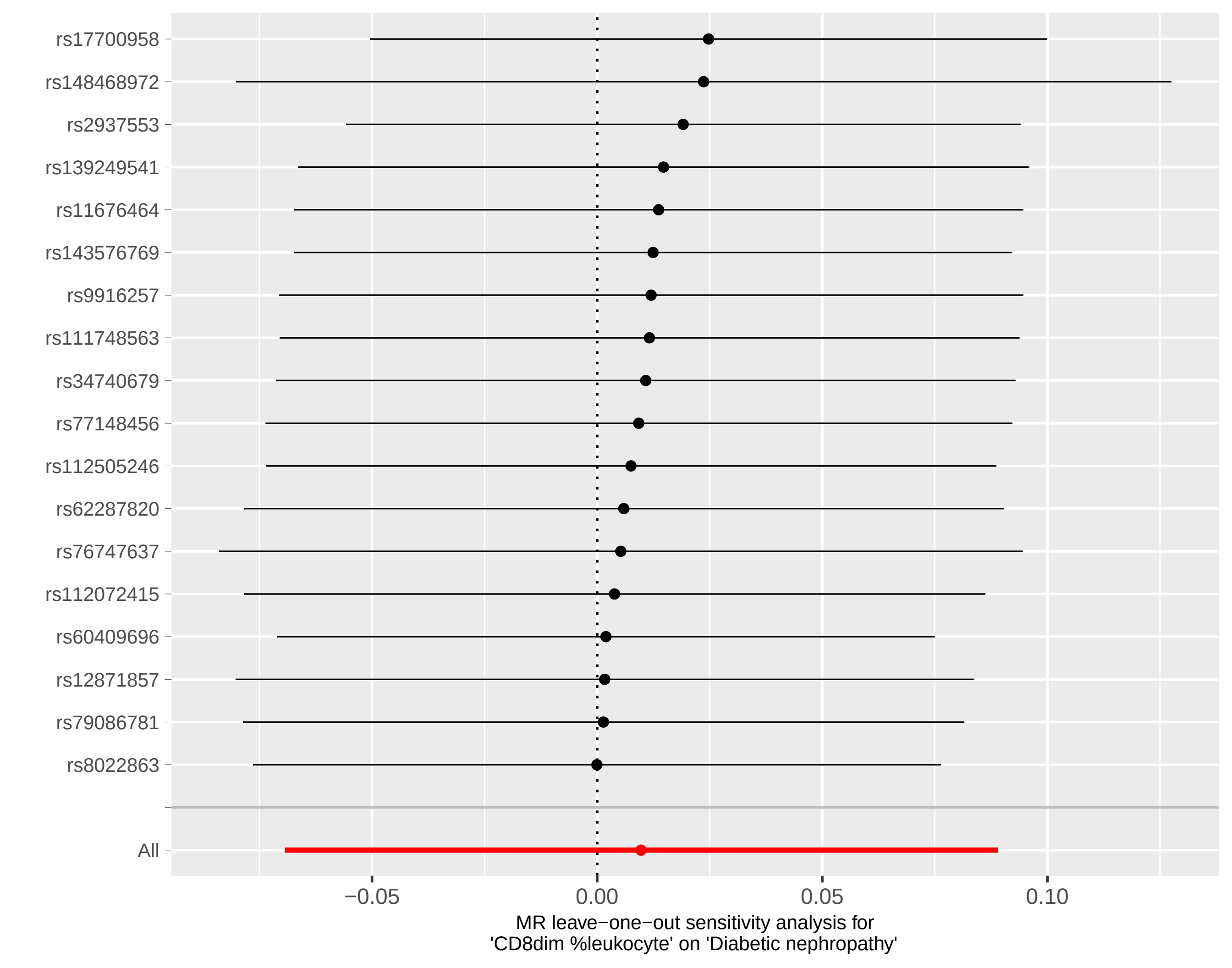

rs17700958
rs148468972
rs2937553
rs139249541
rs11676464
rs143576769
rs9916257
rs111748563
rs34740679
rs77148456
rs112505246
rs62287820
rs76747637
rs112072415
rs60409696
rs12871857
rs79086781
rs8022863
All
−0.05
0.00
0.05
0.10
MR leave−one−out sensitivity analysis for
'CD8dim %leukocyte' on 'Diabetic nephropathy'

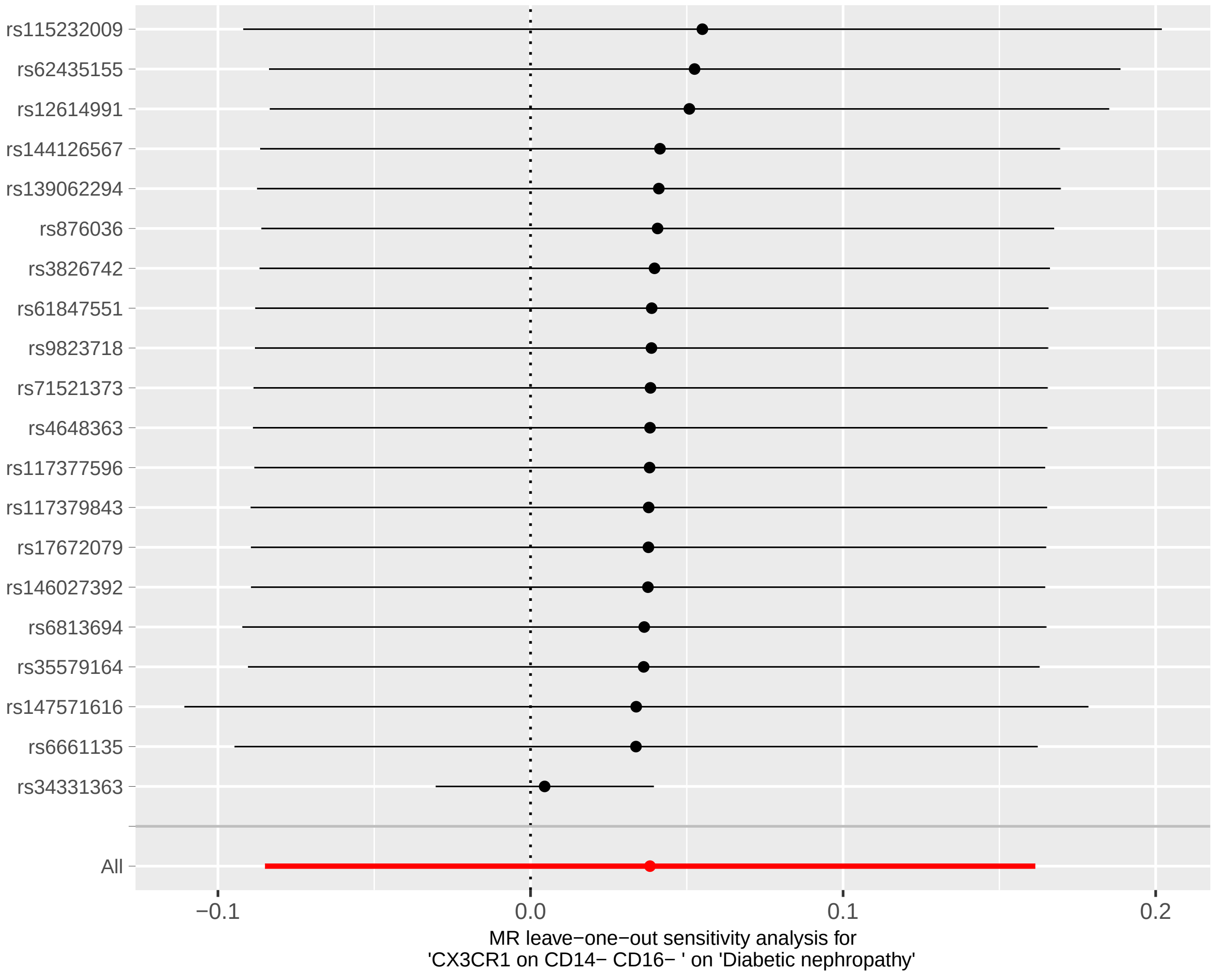

rs115232009
rs62435155
rs12614991
rs144126567
rs139062294
rs876036
rs3826742
rs61847551
rs9823718
rs71521373
rs4648363
rs117377596
rs117379843
rs17672079
rs146027392
rs6813694
rs35579164
rs147571616
rs6661135
rs34331363
All
−0.1
0.0
0.1
0.2
MR leave−one−out sensitivity analysis for
'CX3CR1 on CD14− CD16− ' on 'Diabetic nephropathy'

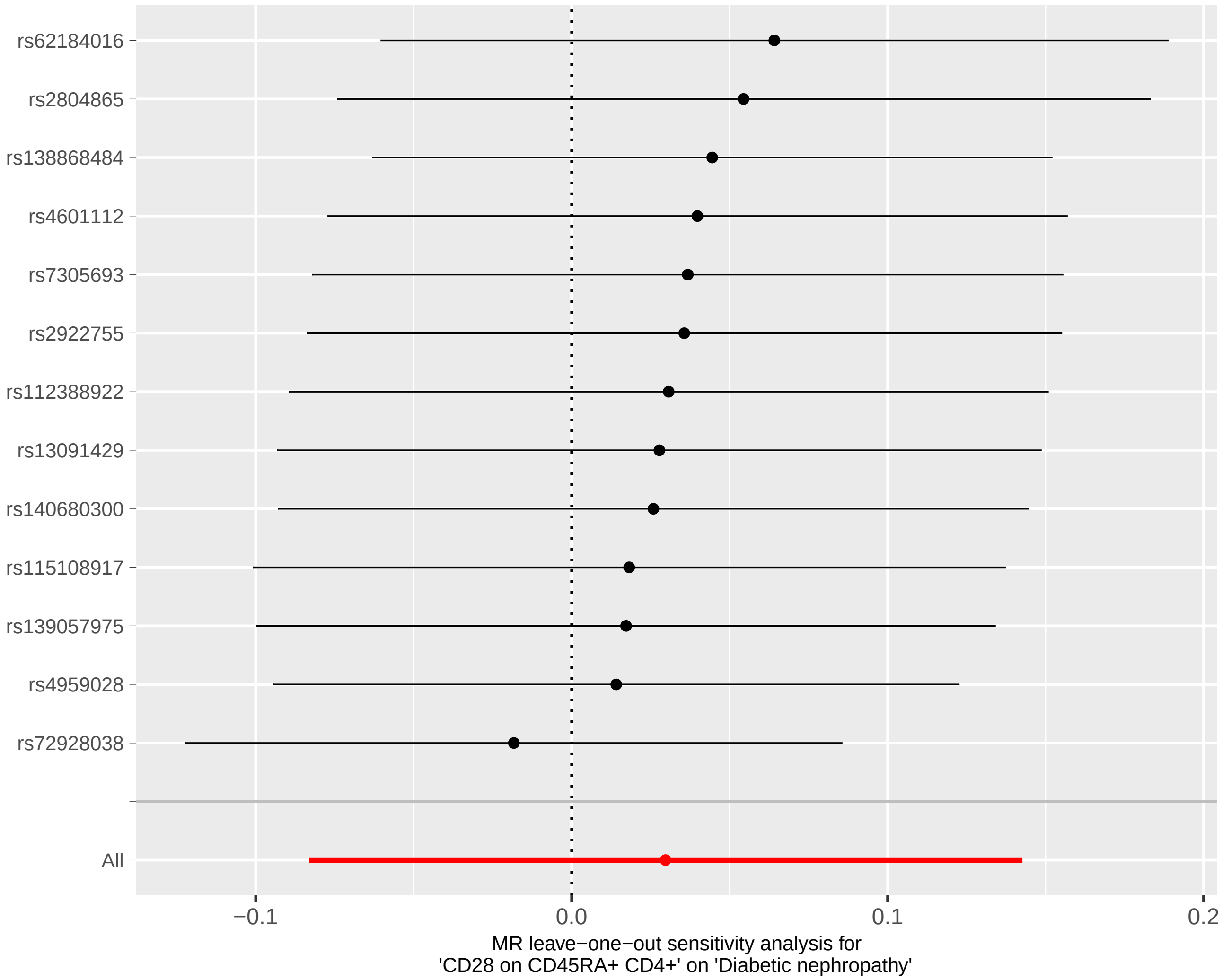

rs62184016
rs2804865
rs138868484
rs4601112
rs7305693
rs2922755
rs112388922
rs13091429
rs140680300
rs115108917
rs139057975
rs4959028
rs72928038
All
−0.1
0.0
0.1
0.2
MR leave−one−out sensitivity analysis for
'CD28 on CD45RA+ CD4+' on 'Diabetic nephropathy'

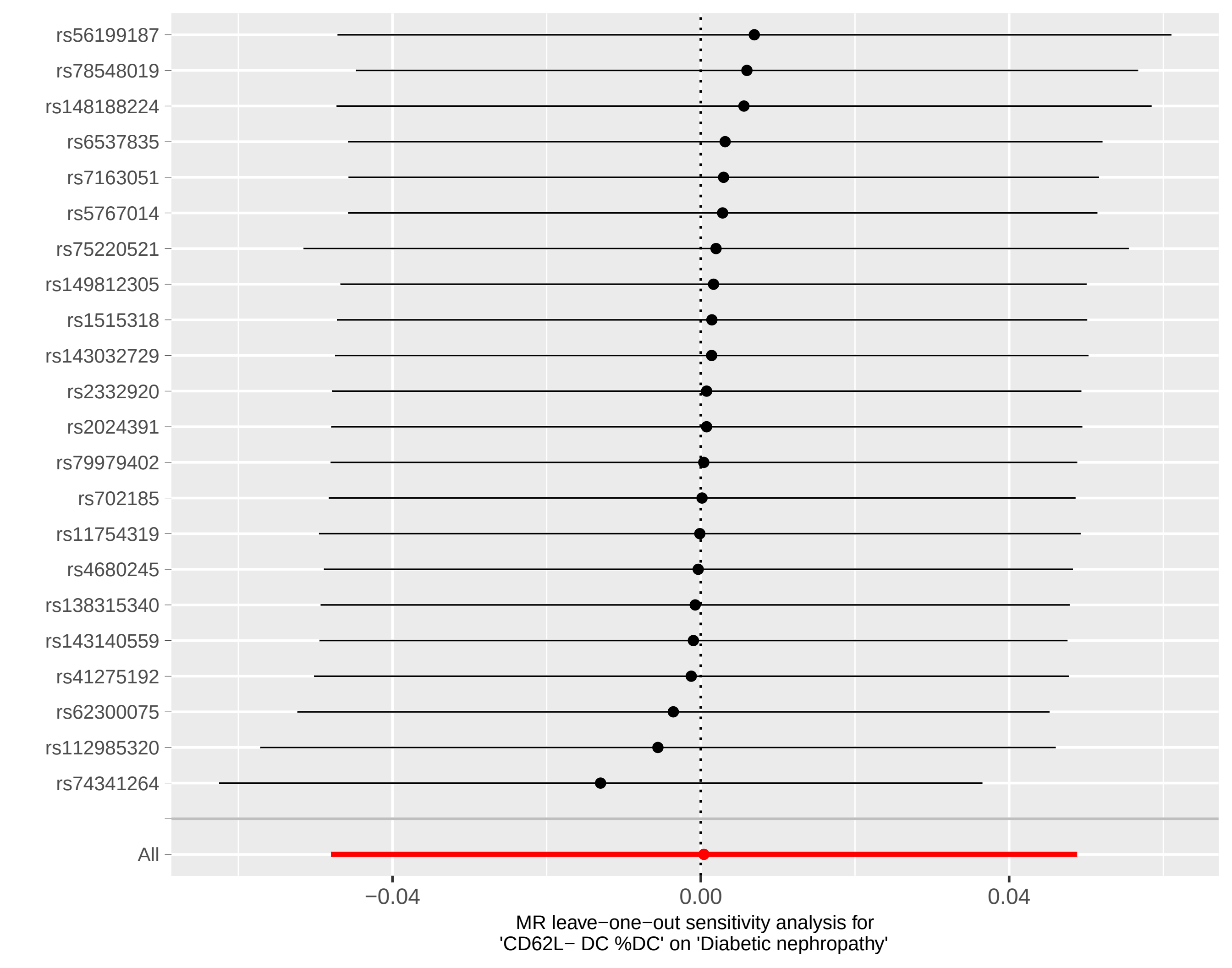
rs56199187
rs78548019
rs148188224
rs6537835
rs7163051
rs5767014
rs75220521
rs149812305
rs1515318
rs143032729
rs2332920
rs2024391
rs79979402
rs702185
rs11754319
rs4680245
rs138315340
rs143140559
rs41275192
rs62300075
rs112985320
rs74341264
All
−0.04
0.00
0.04
MR leave−one−out sensitivity analysis for
'CD62L− DC %DC' on 'Diabetic nephropathy'

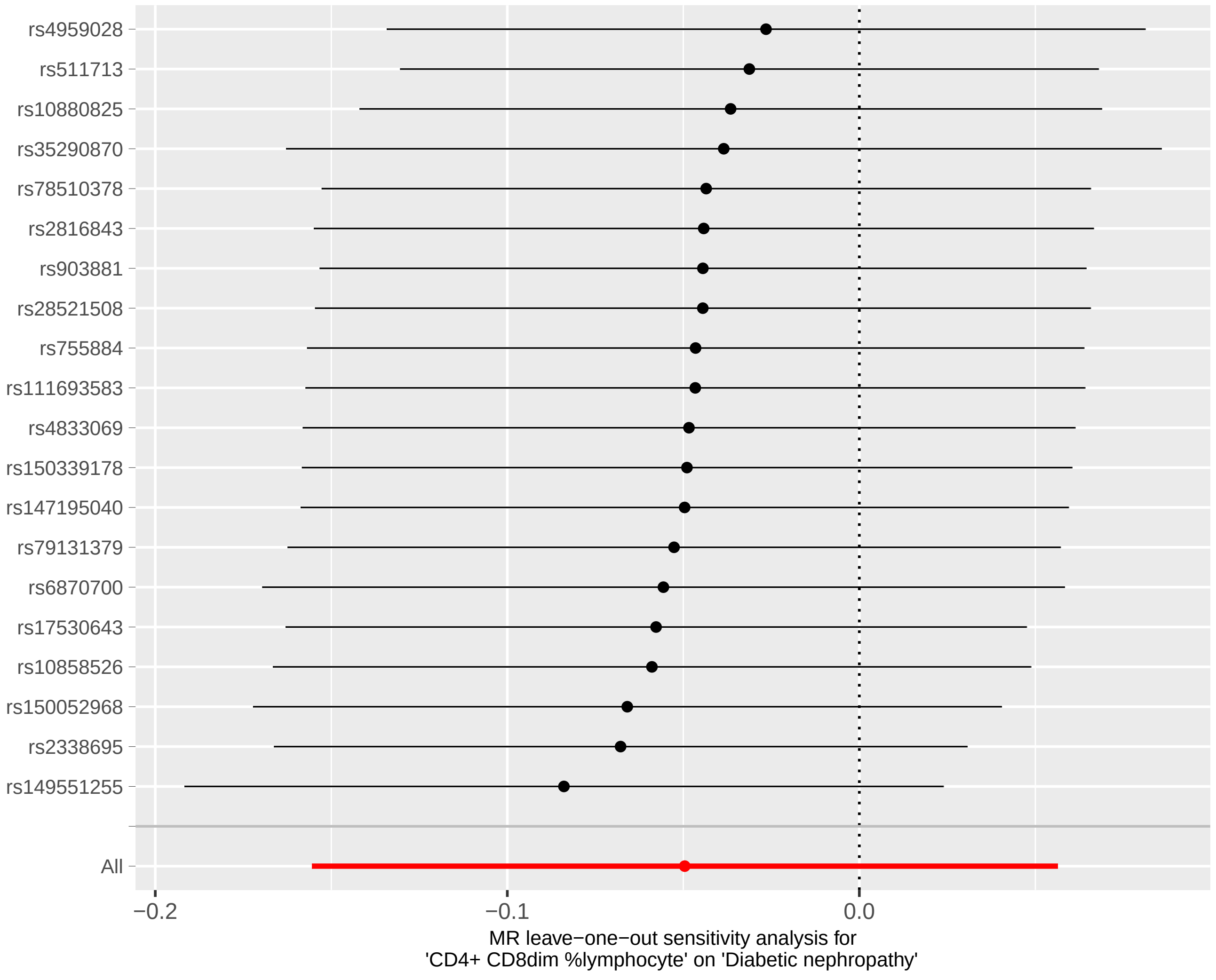
rs4959028
rs511713
rs10880825
rs35290870
rs78510378
rs2816843
rs903881
rs28521508
rs755884
rs111693583
rs4833069
rs150339178
rs147195040
rs79131379
rs6870700
rs17530643
rs10858526
rs150052968
rs2338695
rs149551255
All
−0.2
−0.1
0.0
MR leave−one−out sensitivity analysis for
'CD4+ CD8dim %lymphocyte' on 'Diabetic nephropathy'

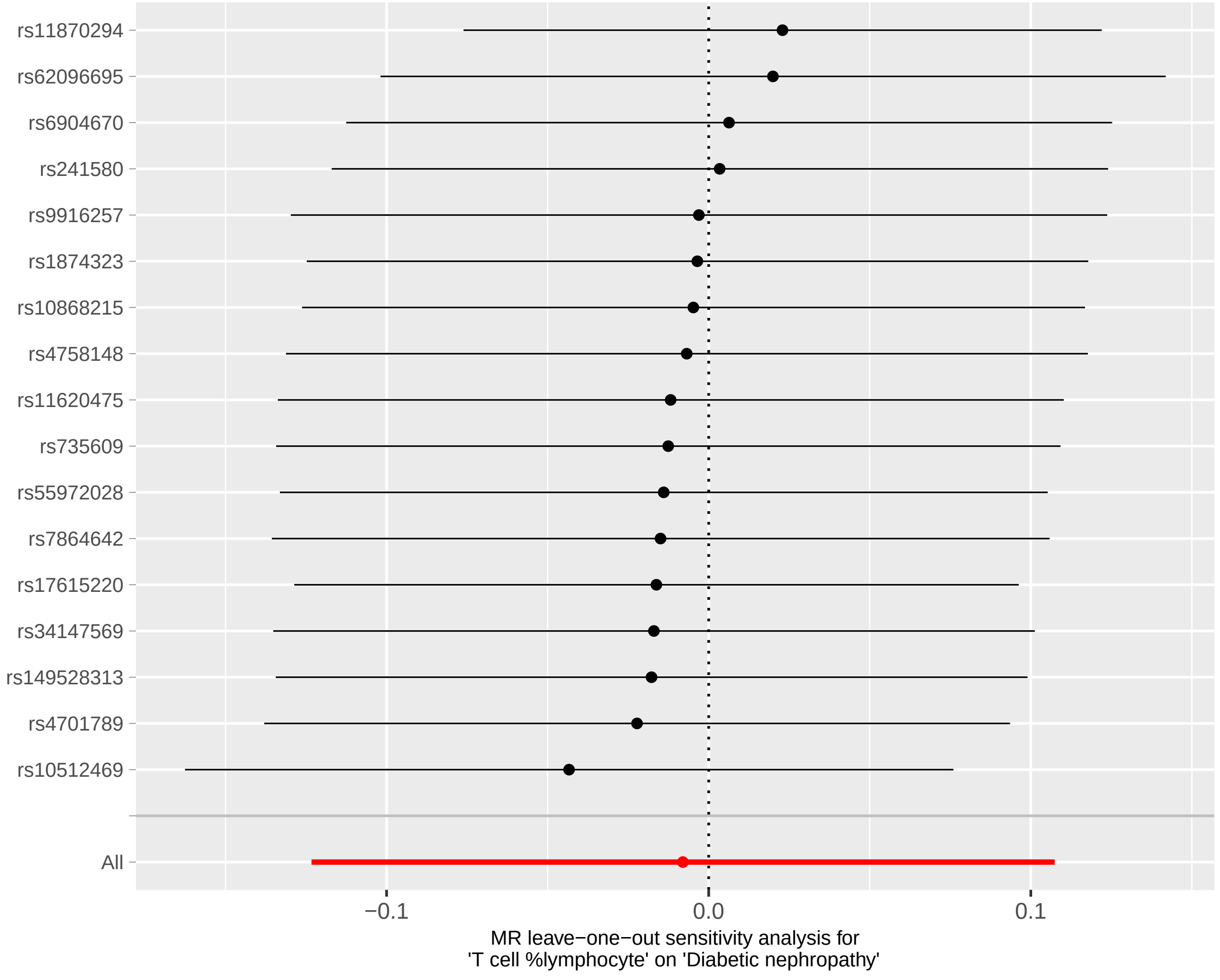

rs11870294
rs62096695
rs6904670
rs241580
rs9916257
rs1874323
rs10868215
rs4758148
rs11620475
rs735609
rs55972028
rs7864642
rs17615220
rs34147569
rs149528313
rs4701789
rs10512469
All
−0.1
0.0
0.1
MR leave−one−out sensitivity analysis for
'T cell %lymphocyte' on 'Diabetic nephropathy'

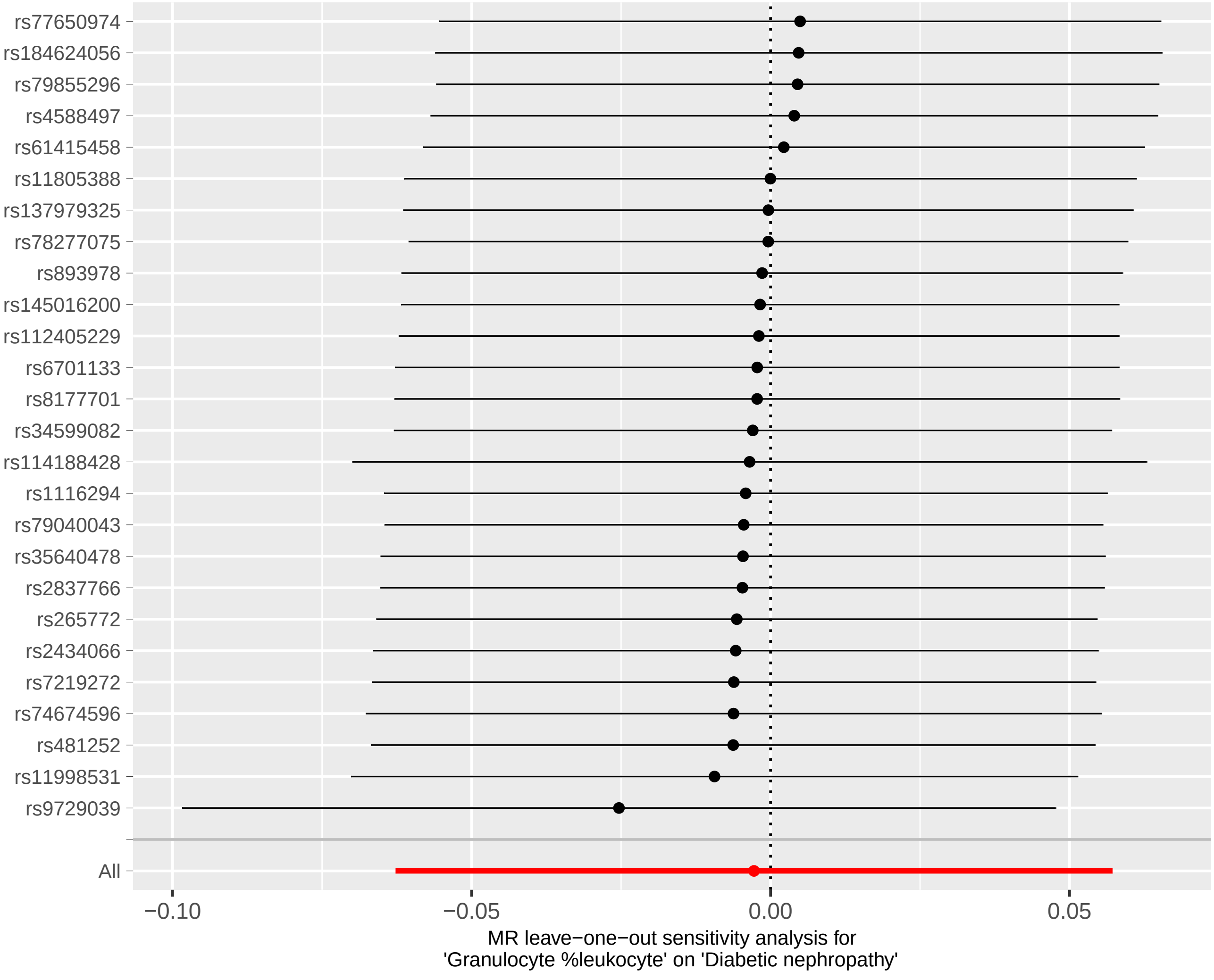
rs77650974
rs184624056
rs79855296
rs4588497
rs61415458
rs11805388
rs137979325
rs78277075
rs893978
rs145016200
rs112405229
rs6701133
rs8177701
rs34599082
rs114188428
rs1116294
rs79040043
rs35640478
rs2837766
rs265772
rs2434066
rs7219272
rs74674596
rs481252
rs11998531
rs9729039
All
−0.10
−0.05
0.00
0.05
MR leave−one−out sensitivity analysis for
'Granulocyte %leukocyte' on 'Diabetic nephropathy'

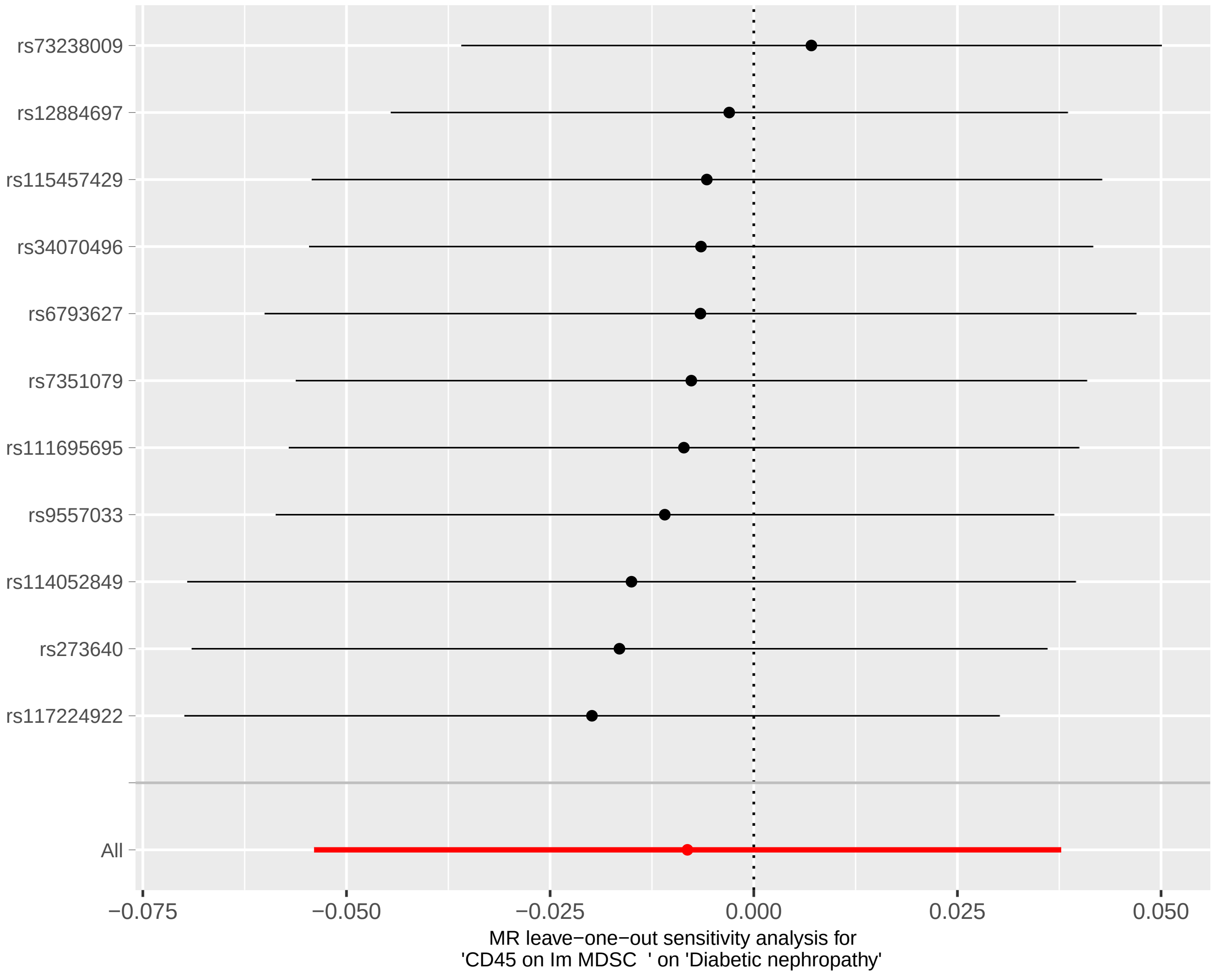
rs73238009
rs12884697
rs115457429
rs34070496
rs6793627
rs7351079
rs111695695
rs9557033
rs114052849
rs273640
rs117224922
All
−0.075
−0.050
−0.025
0.000
0.025
0.050
MR leave−one−out sensitivity analysis for
'CD45 on Im MDSC ' on 'Diabetic nephropathy'

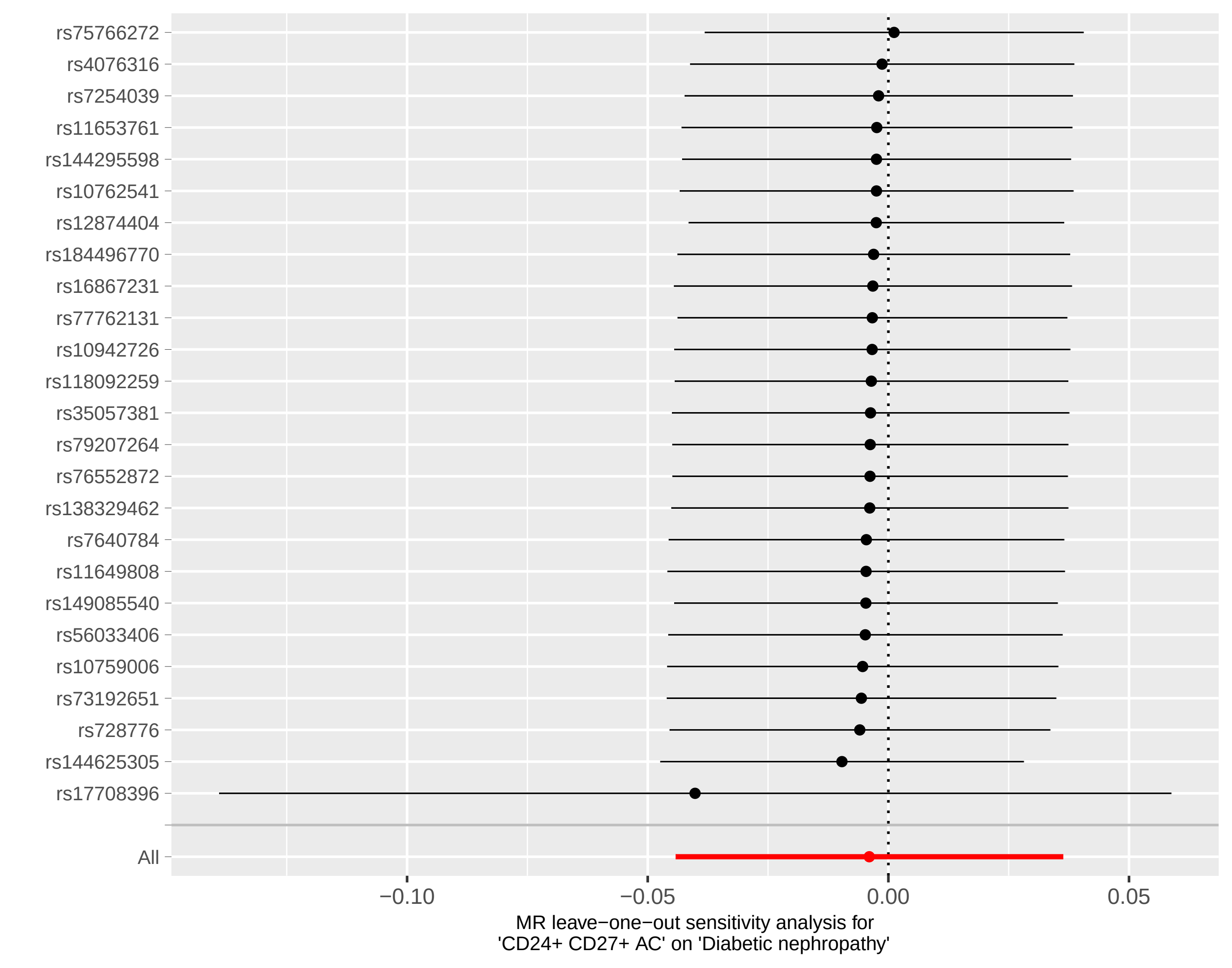
rs75766272
rs4076316
rs7254039
rs11653761
rs144295598
rs10762541
rs12874404
rs184496770
rs16867231
rs77762131
rs10942726
rs118092259
rs35057381
rs79207264
rs76552872
rs138329462
rs7640784
rs11649808
rs149085540
rs56033406
rs10759006
rs73192651
rs728776
rs144625305
rs17708396
All
−0.10
−0.05
0.00
0.05
MR leave−one−out sensitivity analysis for
'CD24+ CD27+ AC' on 'Diabetic nephropathy'

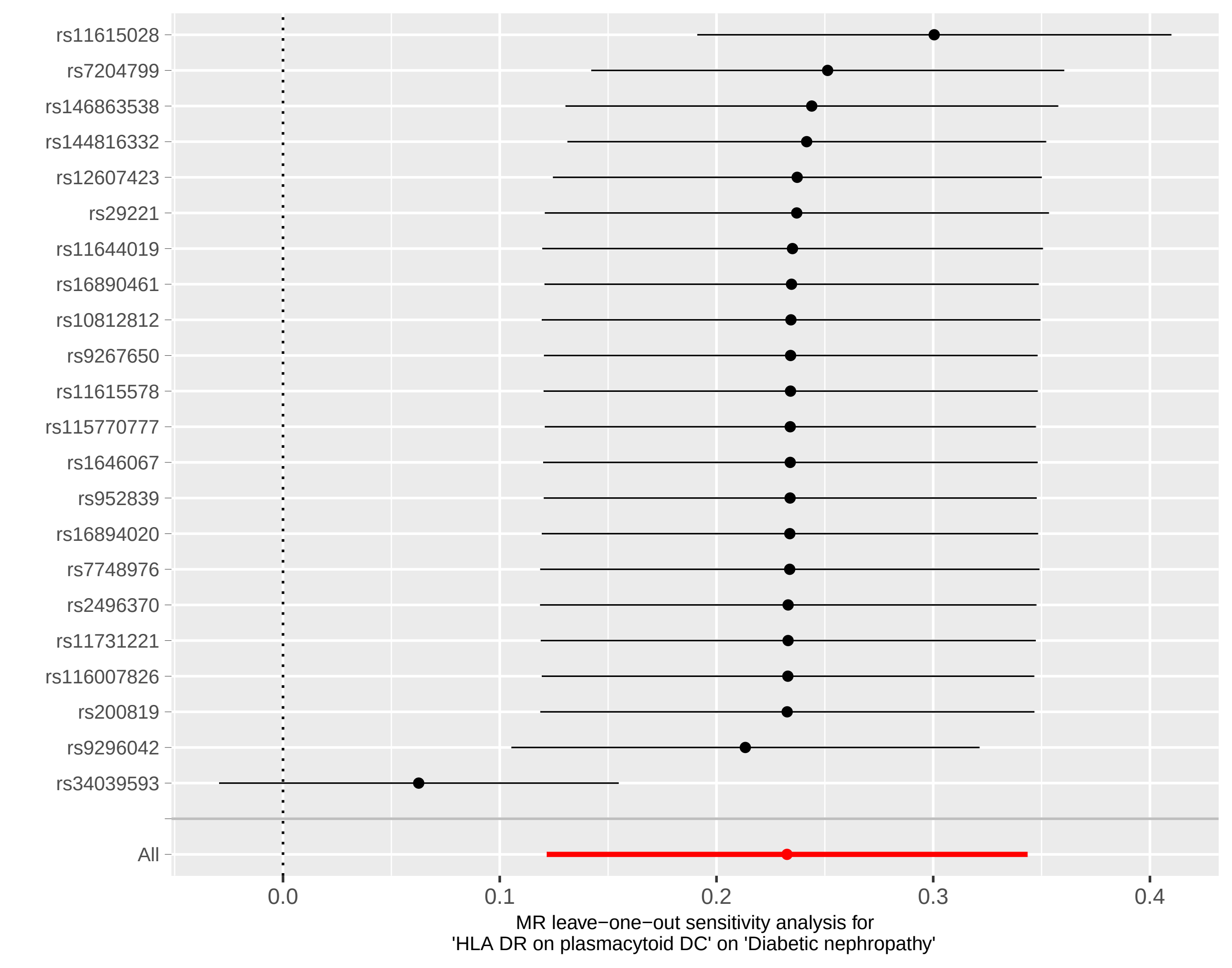
rs11615028
rs7204799
rs146863538
rs144816332
rs12607423
rs29221
rs11644019
rs16890461
rs10812812
rs9267650
rs11615578
rs115770777
rs1646067
rs952839
rs16894020
rs7748976
rs2496370
rs11731221
rs116007826
rs200819
rs9296042
rs34039593
All
0.0
0.1
0.2
0.3
0.4
MR leave−one−out sensitivity analysis for
'HLA DR on plasmacytoid DC' on 'Diabetic nephropathy'

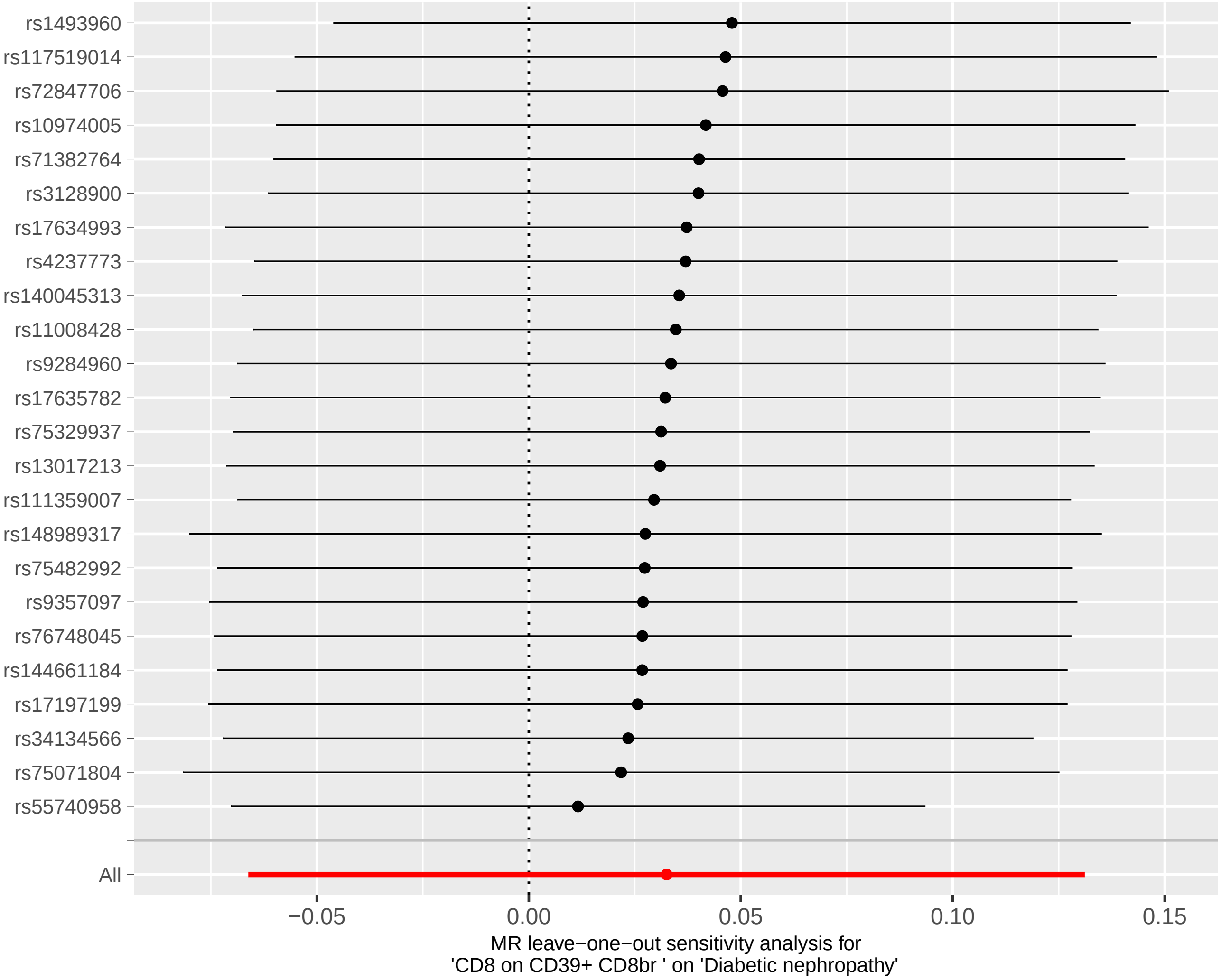
rs1493960
rs117519014
rs72847706
rs10974005
rs71382764
rs3128900
rs17634993
rs4237773
rs140045313
rs11008428
rs9284960
rs17635782
rs75329937
rs13017213
rs111359007
rs148989317
rs75482992
rs9357097
rs76748045
rs144661184
rs17197199
rs34134566
rs75071804
rs55740958
All
−0.05
0.00
0.05
0.10
0.15
MR leave−one−out sensitivity analysis for
'CD8 on CD39+ CD8br ' on 'Diabetic nephropathy'

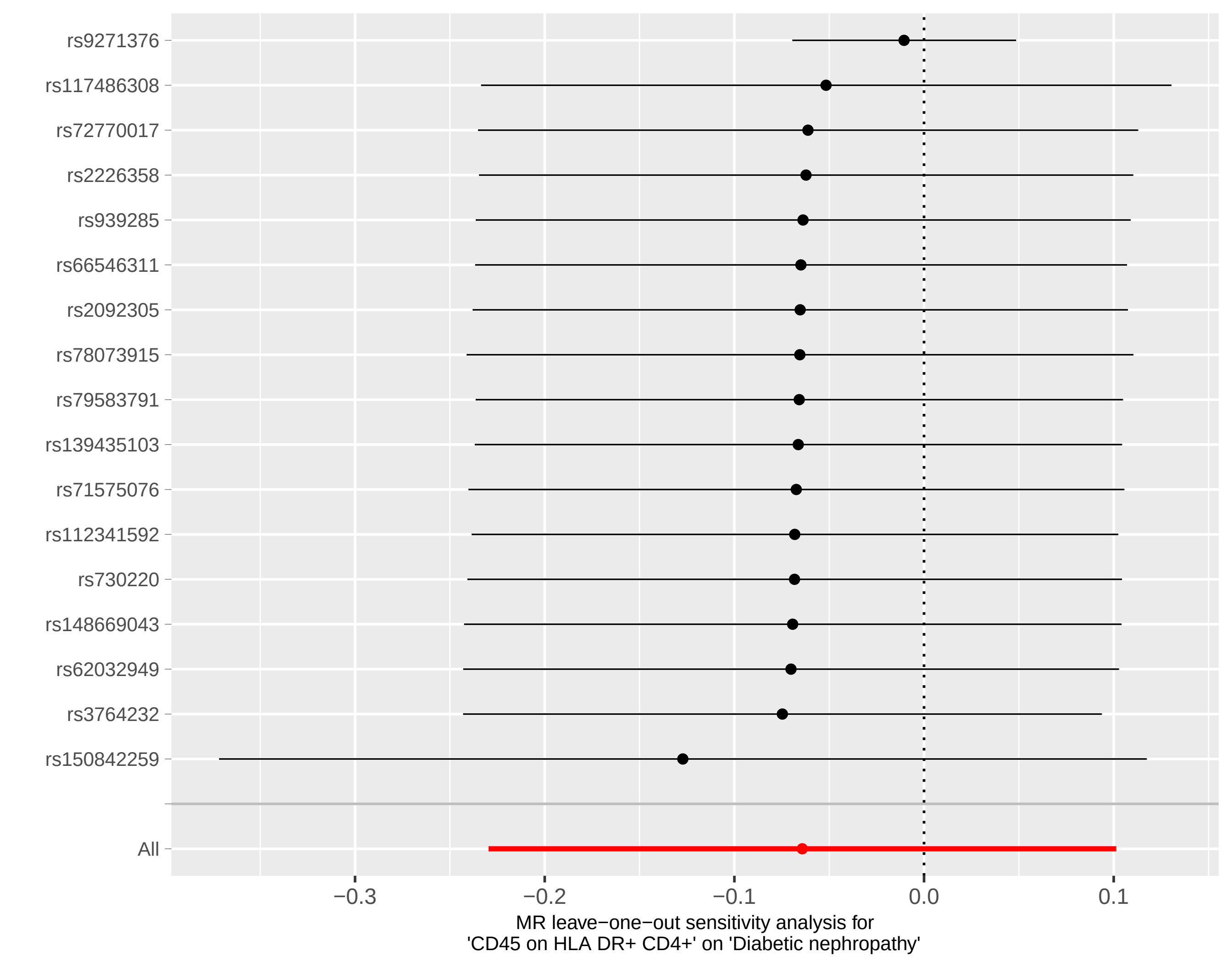
rs9271376
rs117486308
rs72770017
rs2226358
rs939285
rs66546311
rs2092305
rs78073915
rs79583791
rs139435103
rs71575076
rs112341592
rs730220
rs148669043
rs62032949
rs3764232
rs150842259
All
−0.3
−0.2
−0.1
0.0
0.1
MR leave−one−out sensitivity analysis for
'CD45 on HLA DR+ CD4+' on 'Diabetic nephropathy'

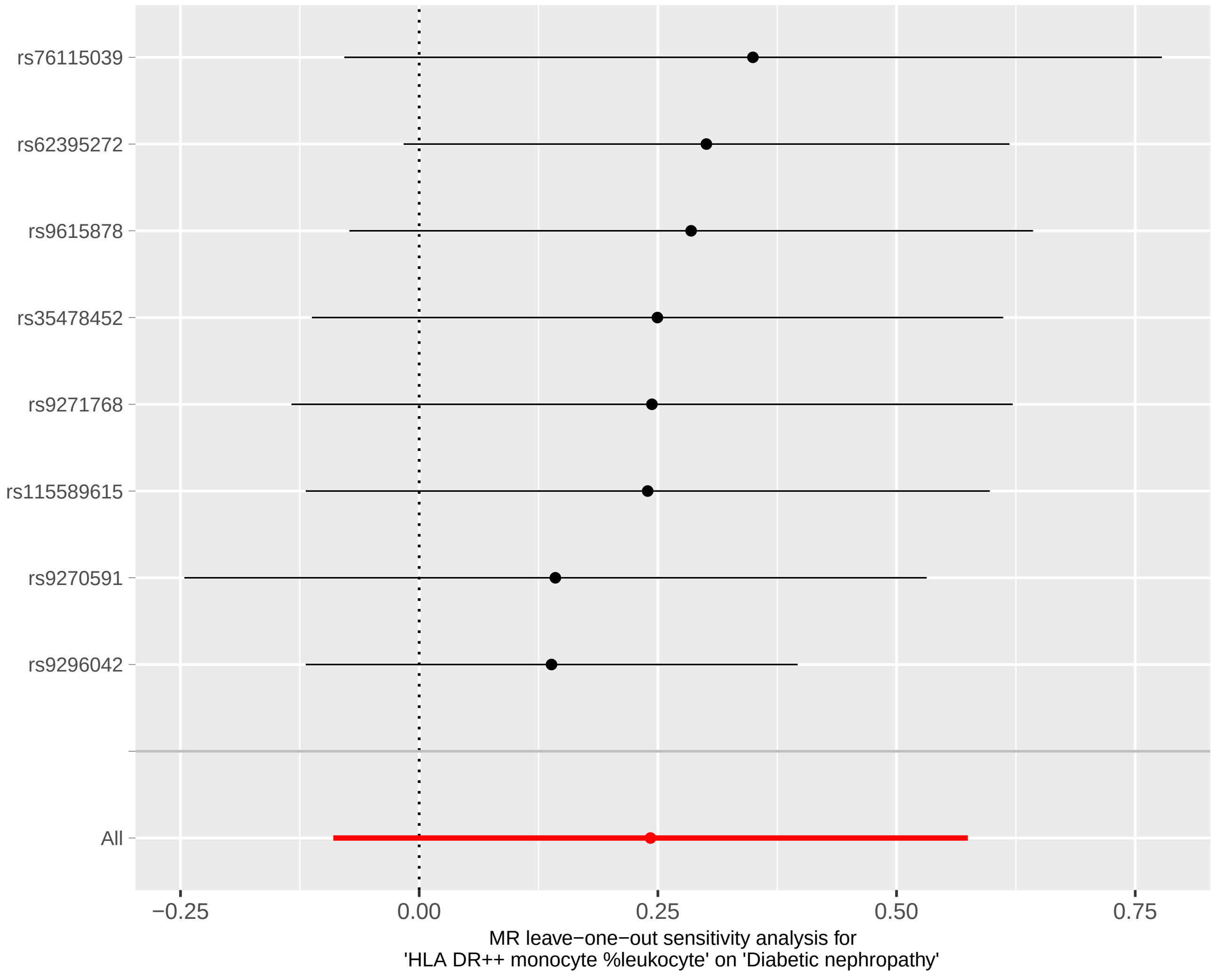

rs76115039
rs62395272
rs9615878
rs35478452
rs9271768
rs115589615
rs9270591
rs9296042
All
−0.25
0.00
0.25
0.50
0.75
MR leave−one−out sensitivity analysis for
'HLA DR++ monocyte %leukocyte' on 'Diabetic nephropathy'

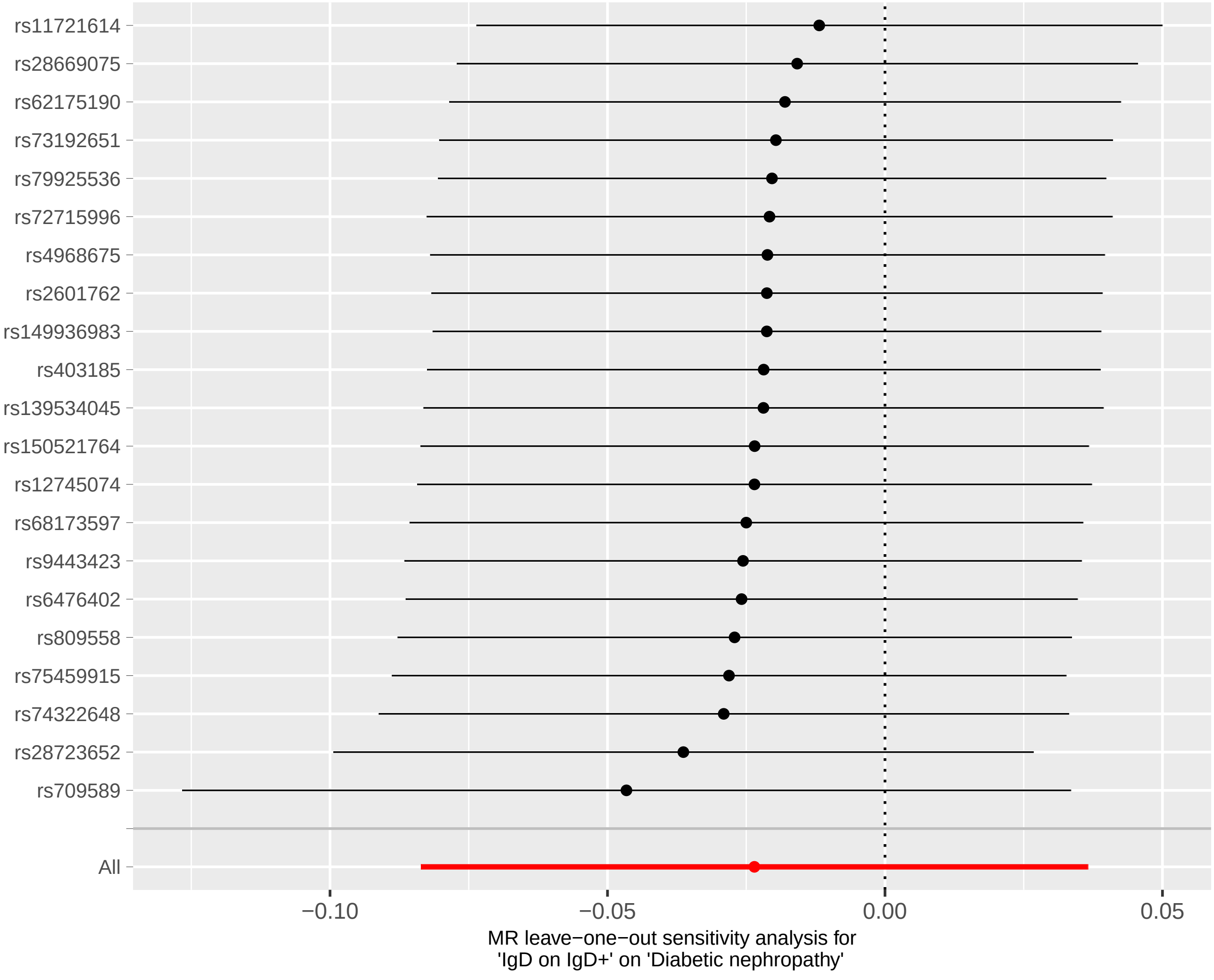
rs11721614
rs28669075
rs62175190
rs73192651
rs79925536
rs72715996
rs4968675
rs2601762
rs149936983
rs403185
rs139534045
rs150521764
rs12745074
rs68173597
rs9443423
rs6476402
rs809558
rs75459915
rs74322648
rs28723652
rs709589
All
−0.10
−0.05
0.00
0.05
MR leave−one−out sensitivity analysis for
'IgD on IgD+' on 'Diabetic nephropathy'

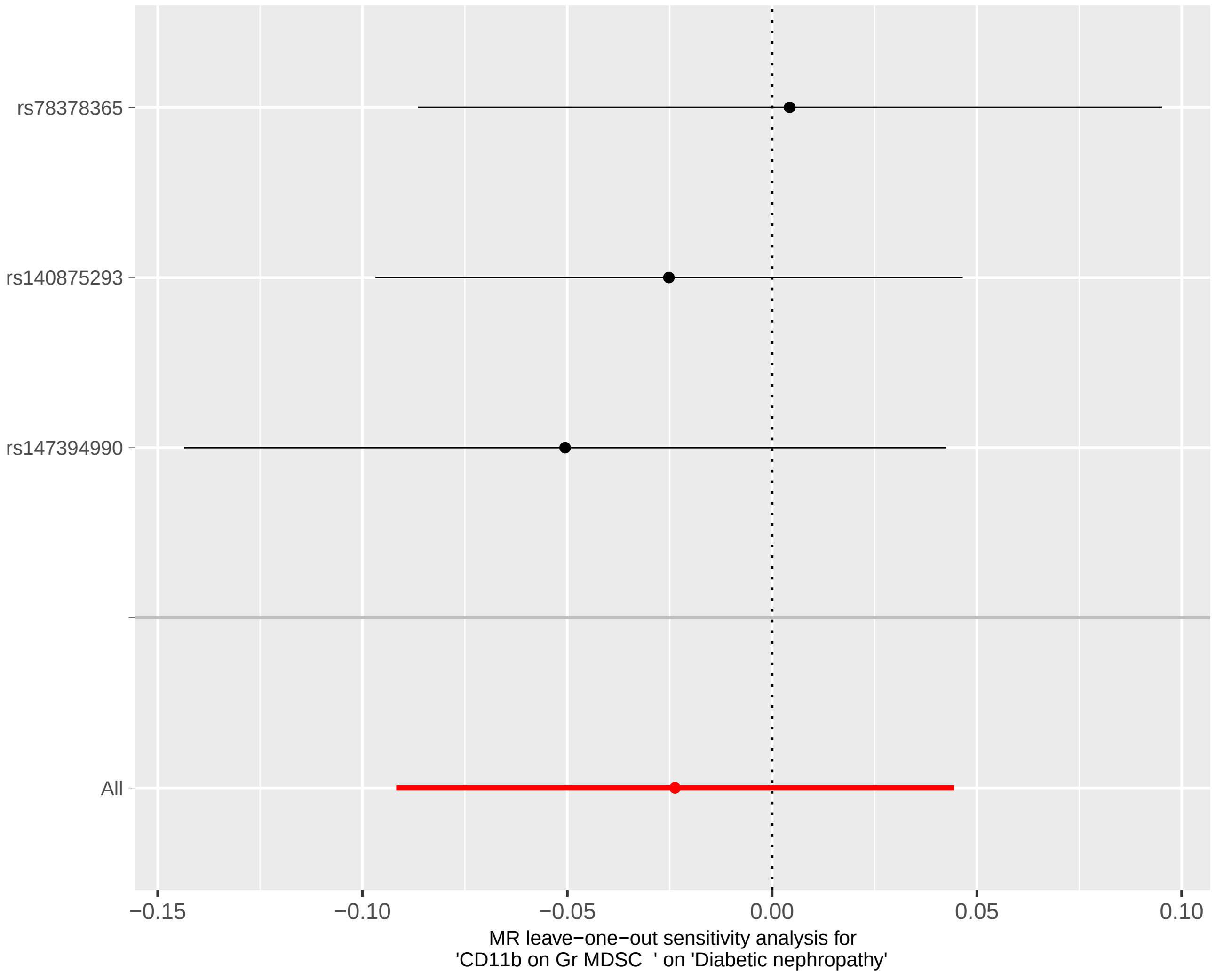
rs78378365
rs140875293
rs147394990
All
−0.15
−0.10
−0.05
0.00
0.05
0.10
MR leave−one−out sensitivity analysis for
'CD11b on Gr MDSC ' on 'Diabetic nephropathy'

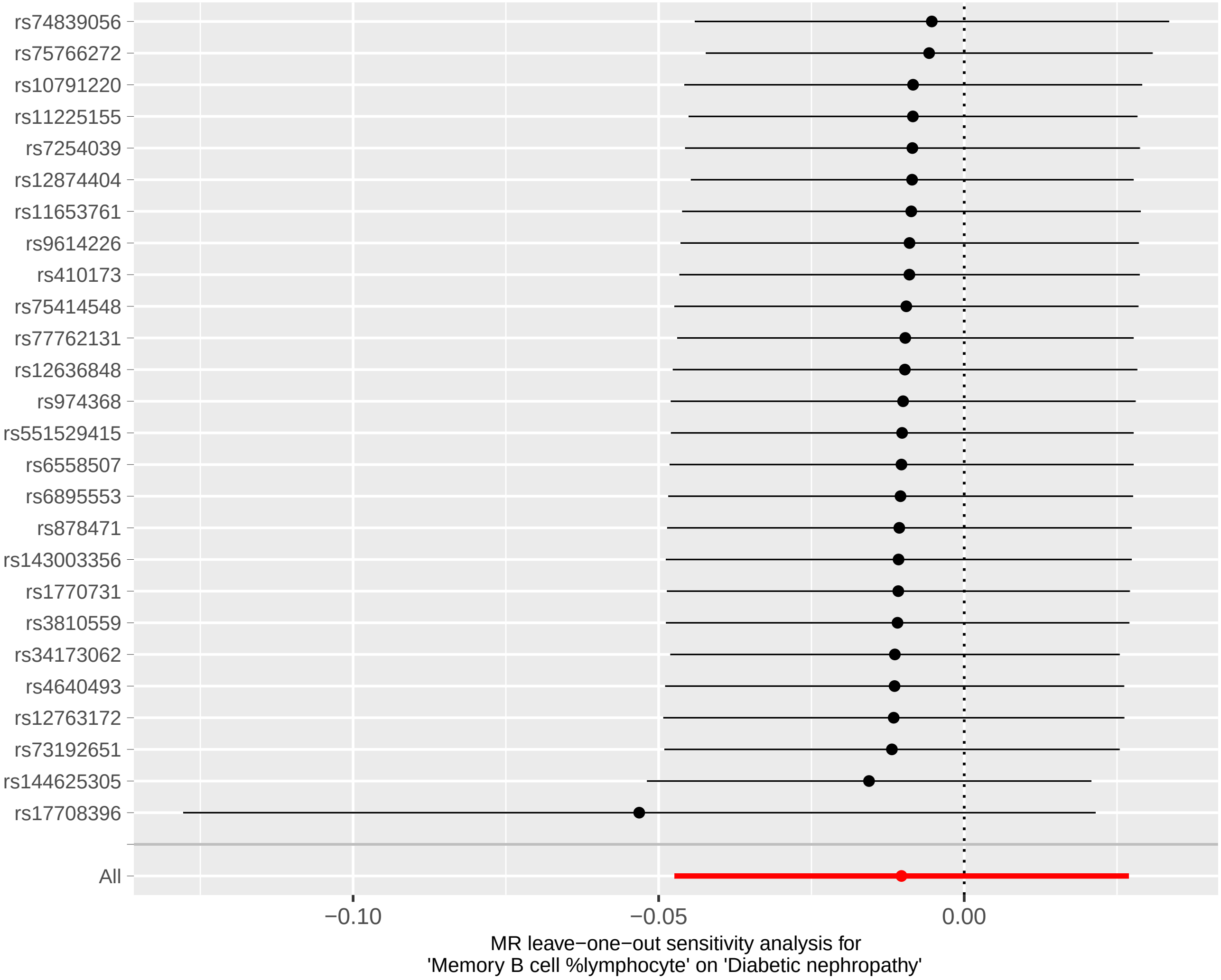

rs74839056
rs75766272
rs10791220
rs11225155
rs7254039
rs12874404
rs11653761
rs9614226
rs410173
rs75414548
rs77762131
rs12636848
rs974368
rs551529415
rs6558507
rs6895553
rs878471
rs143003356
rs1770731
rs3810559
rs34173062
rs4640493
rs12763172
rs73192651
rs144625305
rs17708396
All
−0.10
−0.05
0.00
MR leave−one−out sensitivity analysis for
'Memory B cell %lymphocyte' on 'Diabetic nephropathy'

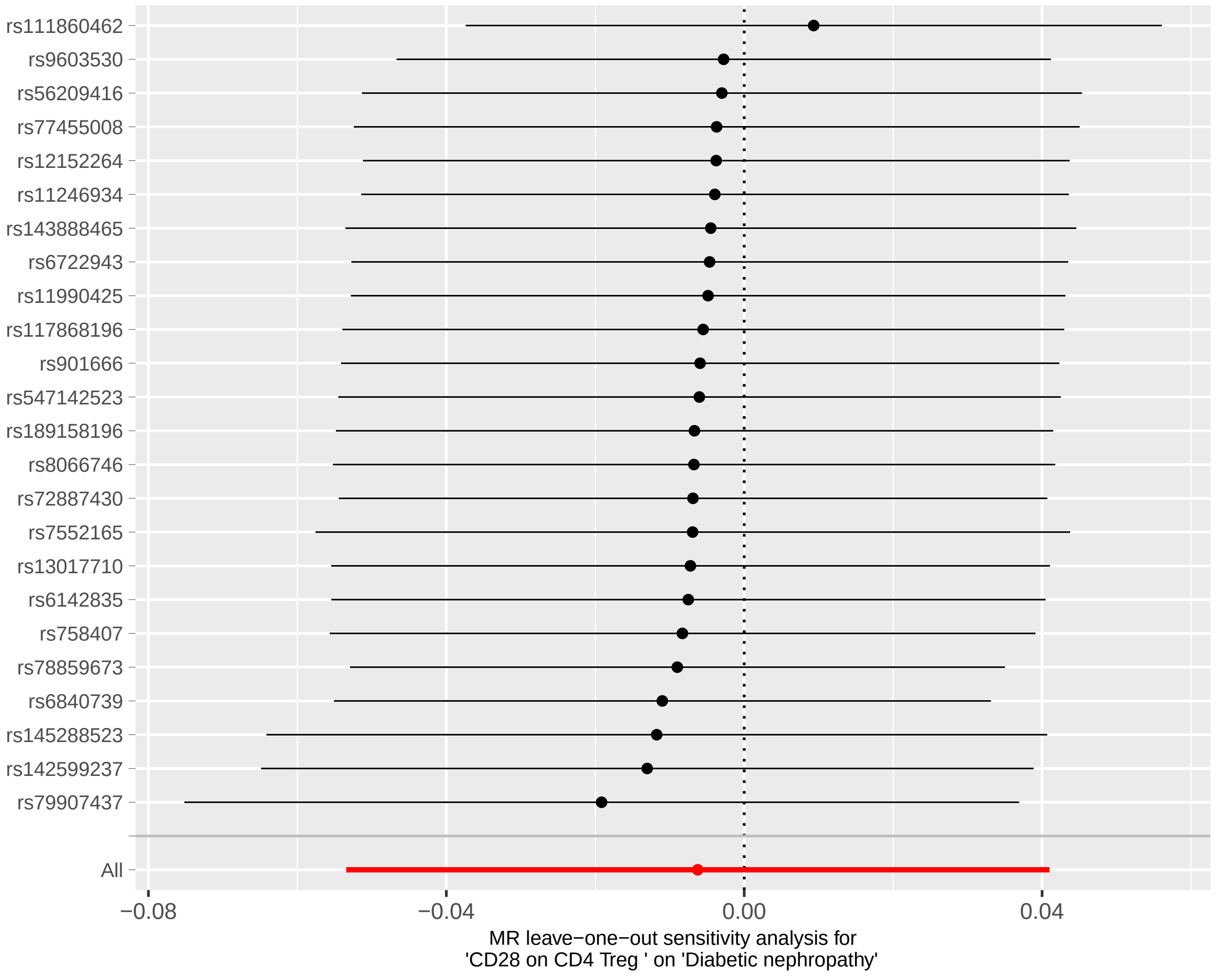
rs111860462
rs9603530
rs56209416
rs77455008
rs12152264
rs11246934
rs143888465
rs6722943
rs11990425
rs117868196
rs901666
rs547142523
rs189158196
rs8066746
rs72887430
rs7552165
rs13017710
rs6142835
rs758407
rs78859673
rs6840739
rs145288523
rs142599237
rs79907437
All
−0.08
−0.04
0.00
0.04
MR leave−one−out sensitivity analysis for
'CD28 on CD4 Treg ' on 'Diabetic nephropathy'

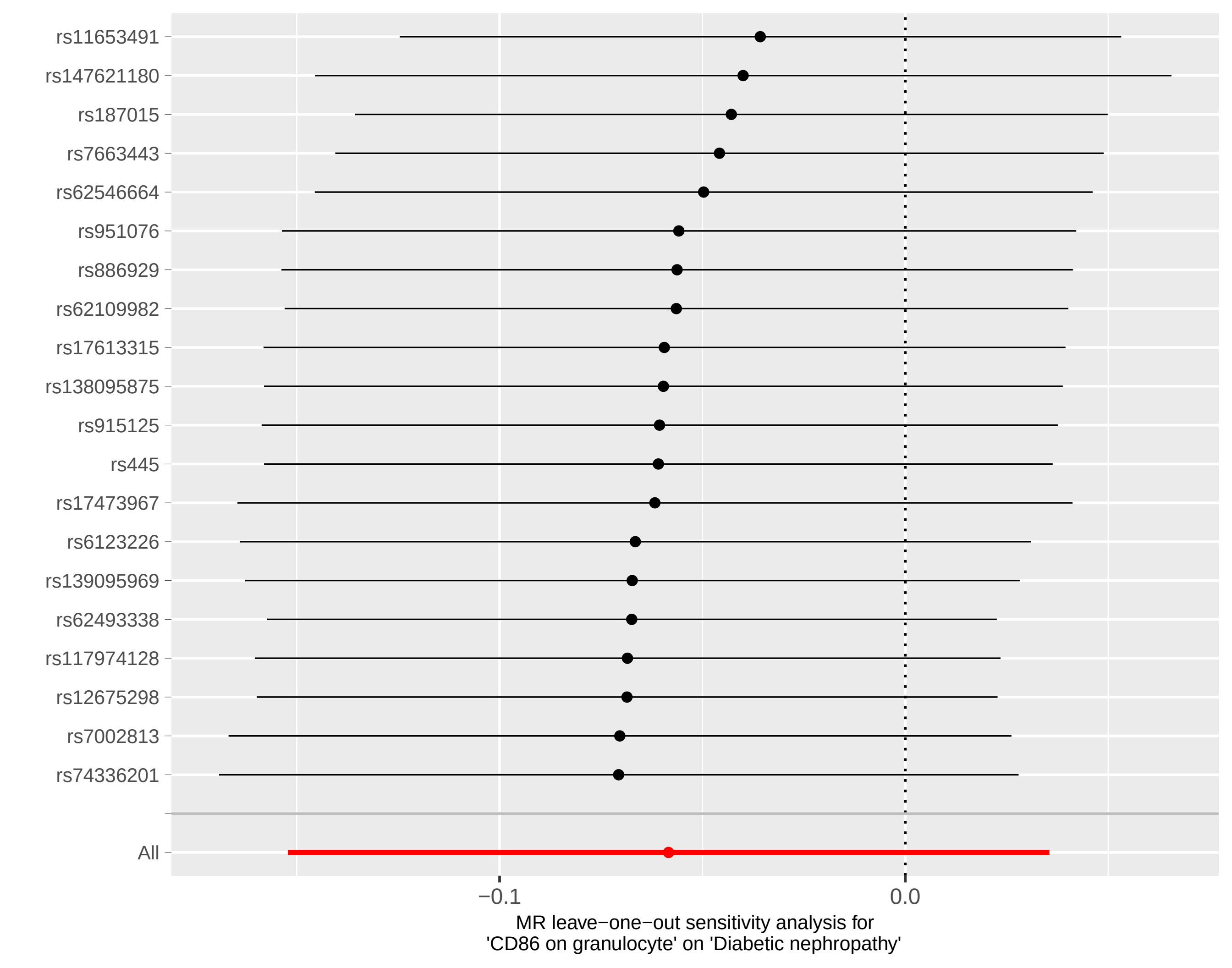
rs11653491
rs147621180
rs187015
rs7663443
rs62546664
rs951076
rs886929
rs62109982
rs17613315
rs138095875
rs915125
rs445
rs17473967
rs6123226
rs139095969
rs62493338
rs117974128
rs12675298
rs7002813
rs74336201
All
−0.1
0.0
MR leave−one−out sensitivity analysis for
'CD86 on granulocyte' on 'Diabetic nephropathy'

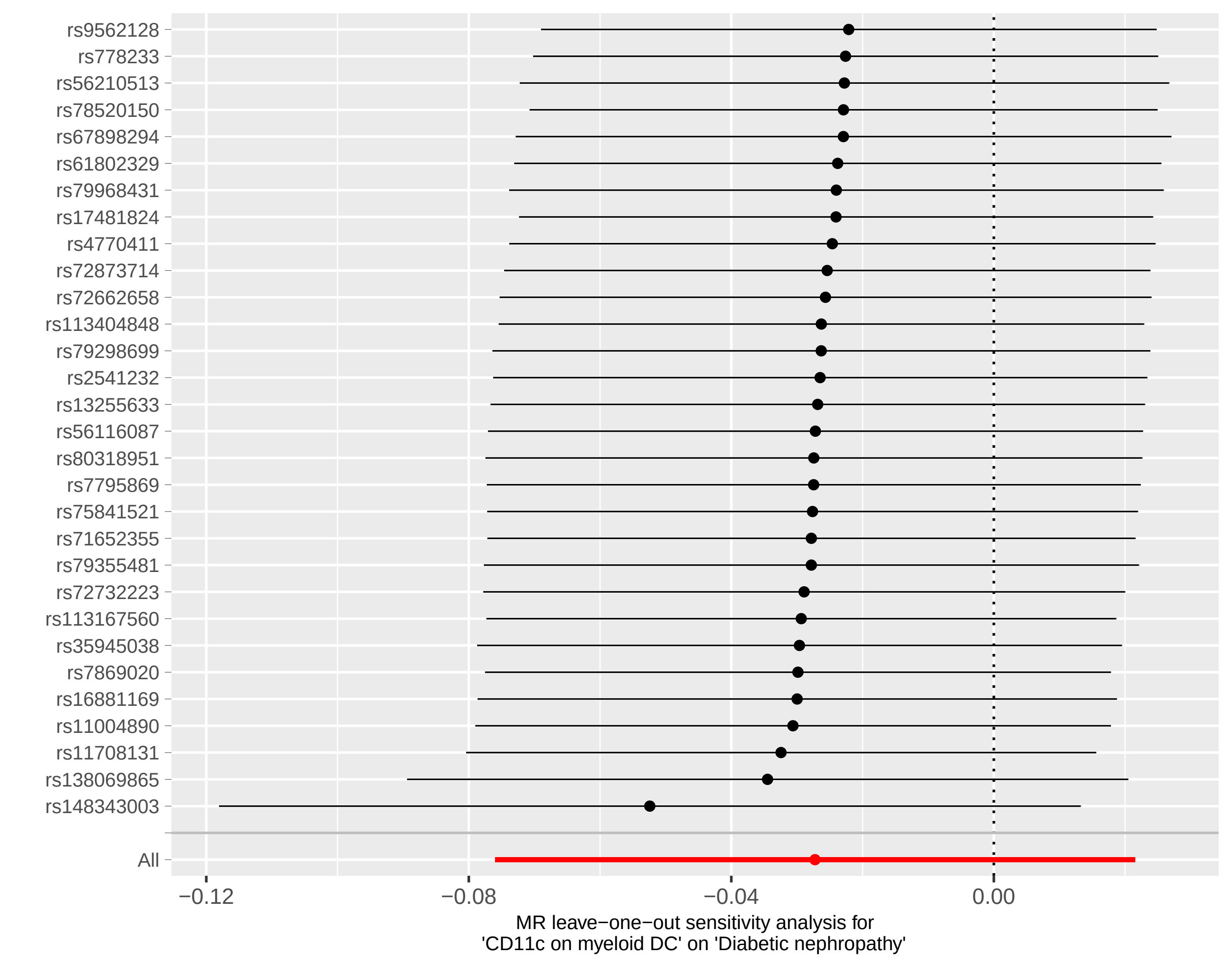
rs9562128
rs778233
rs56210513
rs78520150
rs67898294
rs61802329
rs79968431
rs17481824
rs4770411
rs72873714
rs72662658
rs113404848
rs79298699
rs2541232
rs13255633
rs56116087
rs80318951
rs7795869
rs75841521
rs71652355
rs79355481
rs72732223
rs113167560
rs35945038
rs7869020
rs16881169
rs11004890
rs11708131
rs138069865
rs148343003
All
−0.12
−0.08
−0.04
0.00
MR leave−one−out sensitivity analysis for
'CD11c on myeloid DC' on 'Diabetic nephropathy'

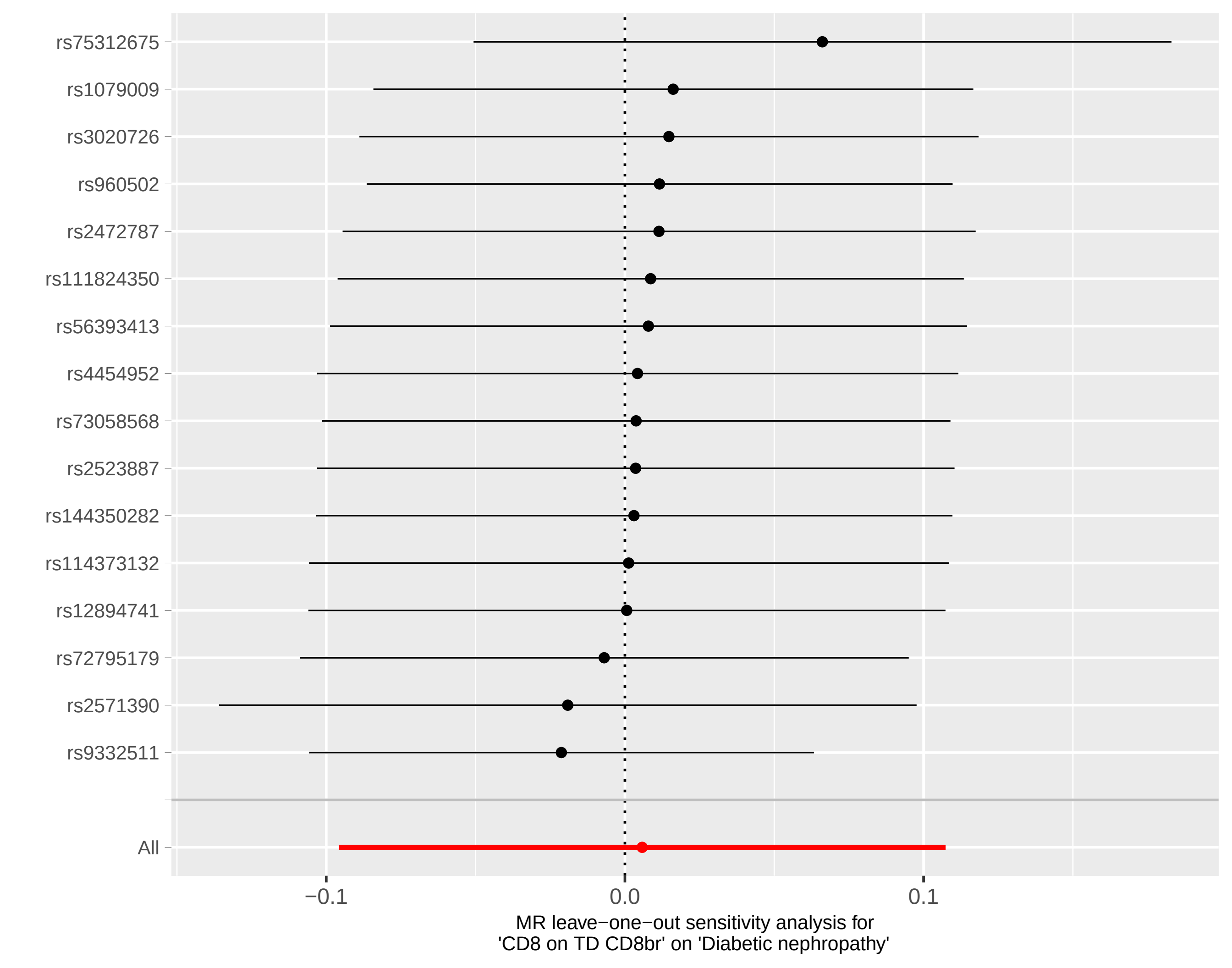
rs75312675
rs1079009
rs3020726
rs960502
rs2472787
rs111824350
rs56393413
rs4454952
rs73058568
rs2523887
rs144350282
rs114373132
rs12894741
rs72795179
rs2571390
rs9332511
All
−0.1
0.0
0.1
MR leave−one−out sensitivity analysis for
'CD8 on TD CD8br' on 'Diabetic nephropathy'

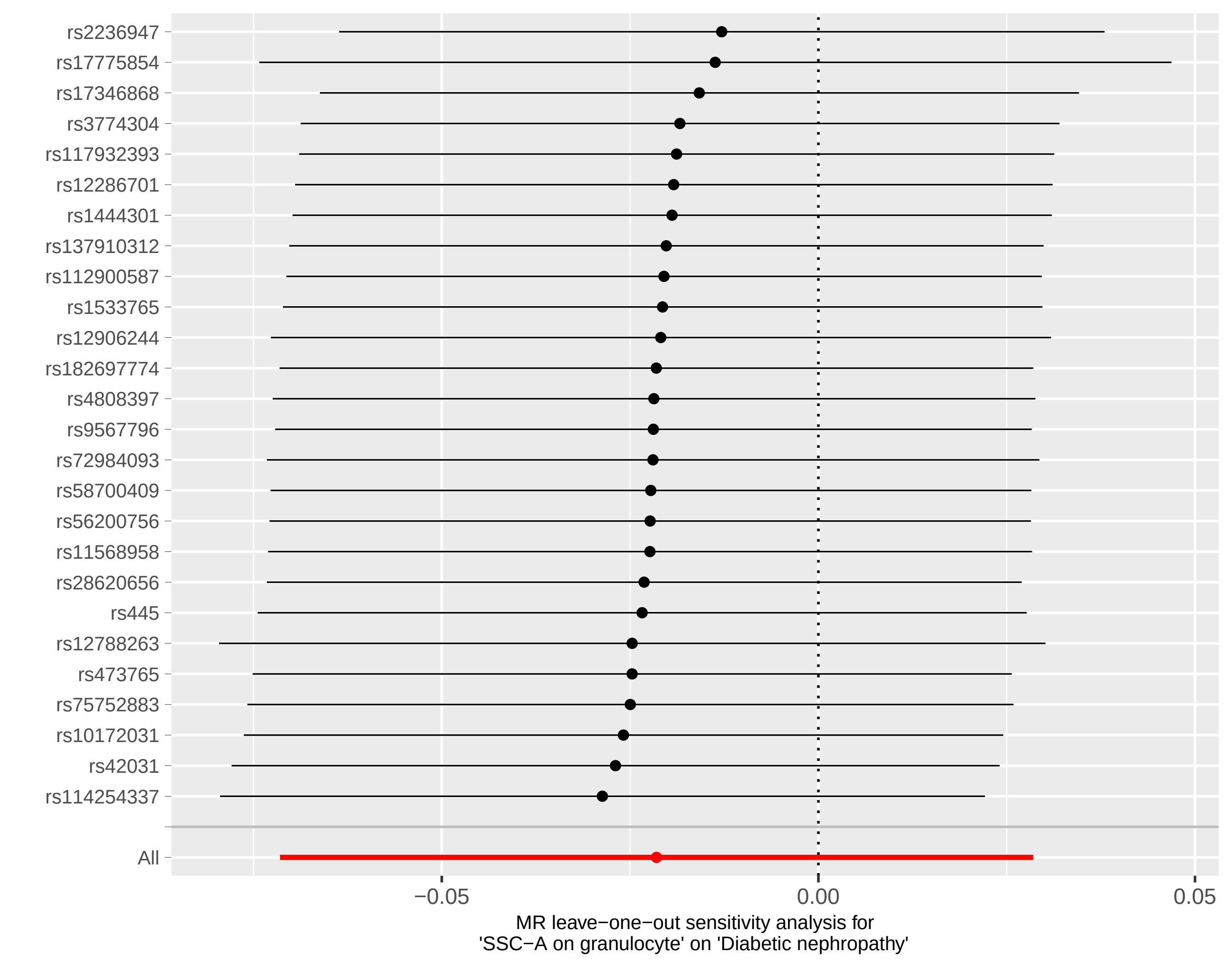
rs2236947
rs17775854
rs17346868
rs3774304
rs117932393
rs12286701
rs1444301
rs137910312
rs112900587
rs1533765
rs12906244
rs182697774
rs4808397
rs9567796
rs72984093
rs58700409
rs56200756
rs11568958
rs28620656
rs445
rs12788263
rs473765
rs75752883
rs10172031
rs42031
rs114254337
All
−0.05
0.00
0.05
MR leave−one−out sensitivity analysis for
'SSC−A on granulocyte' on 'Diabetic nephropathy'

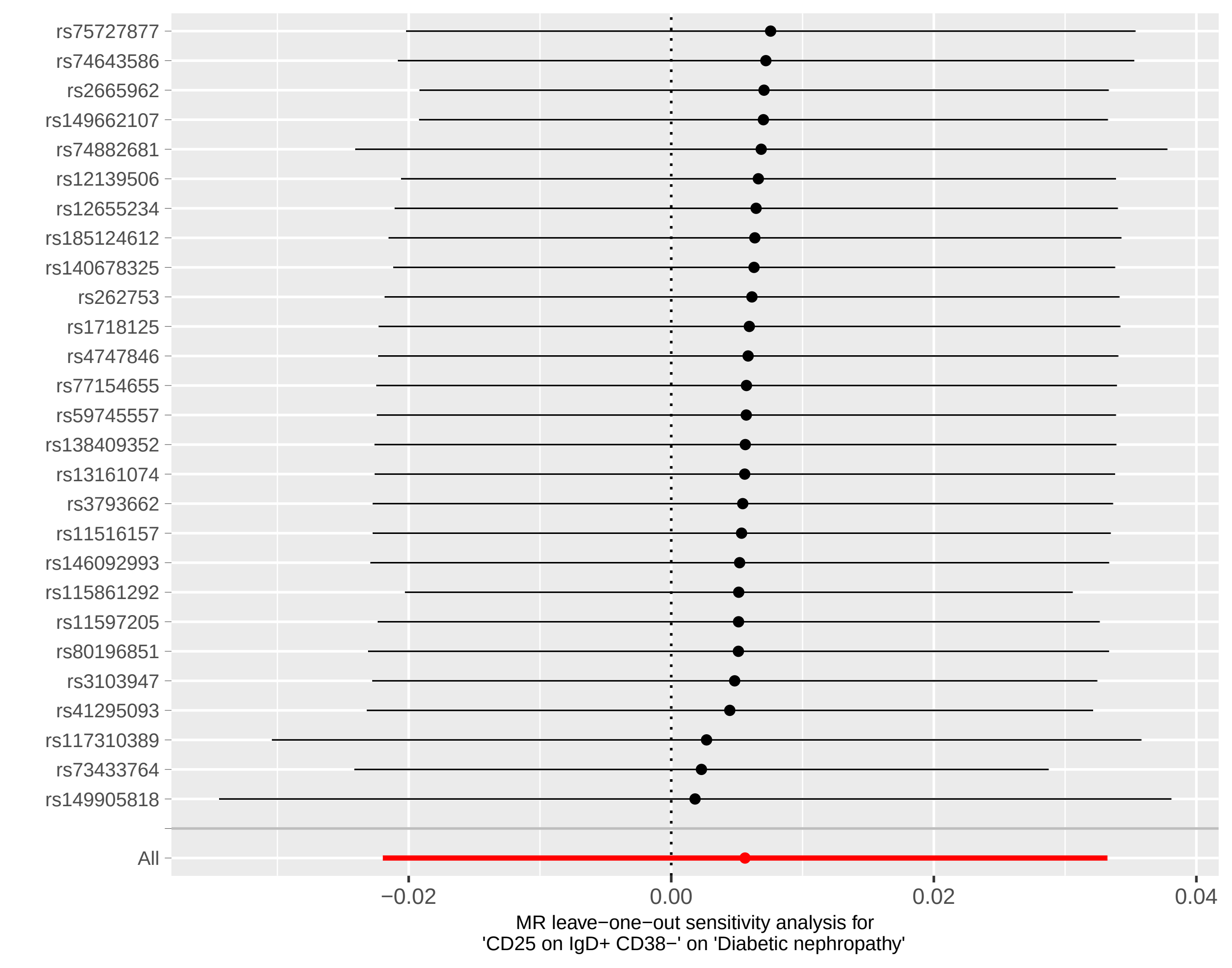

rs75727877
rs74643586
rs2665962
rs149662107
rs74882681
rs12139506
rs12655234
rs185124612
rs140678325
rs262753
rs1718125
rs4747846
rs77154655
rs59745557
rs138409352
rs13161074
rs3793662
rs11516157
rs146092993
rs115861292
rs11597205
rs80196851
rs3103947
rs41295093
rs117310389
rs73433764
rs149905818
All
−0.02
0.00
0.02
0.04
MR leave−one−out sensitivity analysis for
'CD25 on IgD+ CD38−' on 'Diabetic nephropathy'

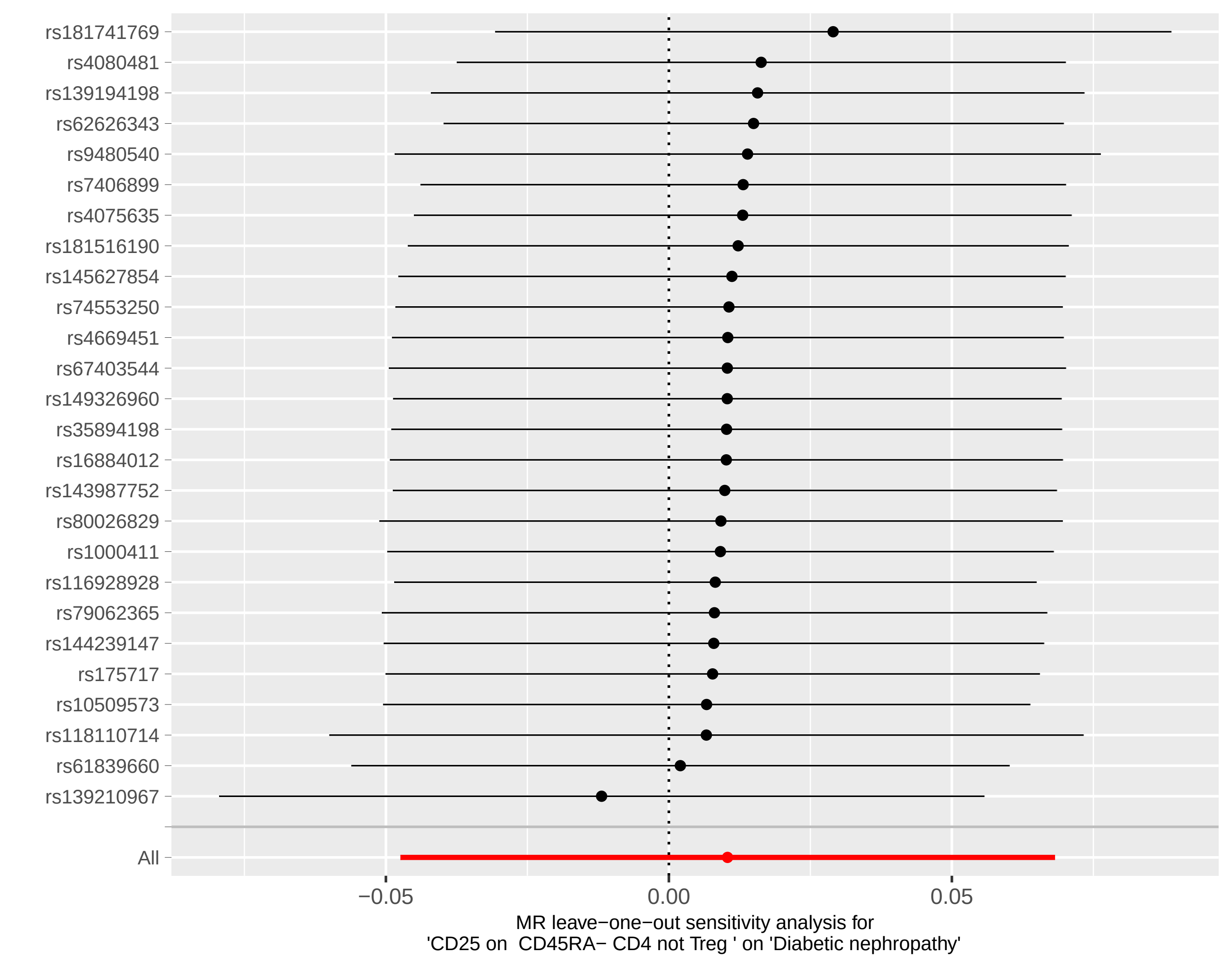
rs181741769
rs4080481
rs139194198
rs62626343
rs9480540
rs7406899
rs4075635
rs181516190
rs145627854
rs74553250
rs4669451
rs67403544
rs149326960
rs35894198
rs16884012
rs143987752
rs80026829
rs1000411
rs116928928
rs79062365
rs144239147
rs175717
rs10509573
rs118110714
rs61839660
rs139210967
All
−0.05
0.00
0.05
MR leave−one−out sensitivity analysis for
'CD25 on CD45RA− CD4 not Treg ' on 'Diabetic nephropathy'
