# Supplementary material for: Monocyte human leukocyte antigen-DR-mediated diabetic nephropathy progression is a promising therapeutic target
Source: Front Endocrinol (Lausanne). 2025 Dec 9;16:1733139. doi: 10.3389/fendo.2025.1733139 (PMC12722863; doi:10.3389/fendo.2025.1733139)
Supplement: Supplementary Figure 3 — Forest plots of all MR analyses of 731 immunophenotypes on DN. MR, mendelian randomization; DN, diabetic nephropathy. [file DataSheet3.pdf]

Supplementary Figure 3： Forest plots of all MR analyses of 731 immunophenotypes on diabetic nephropathy

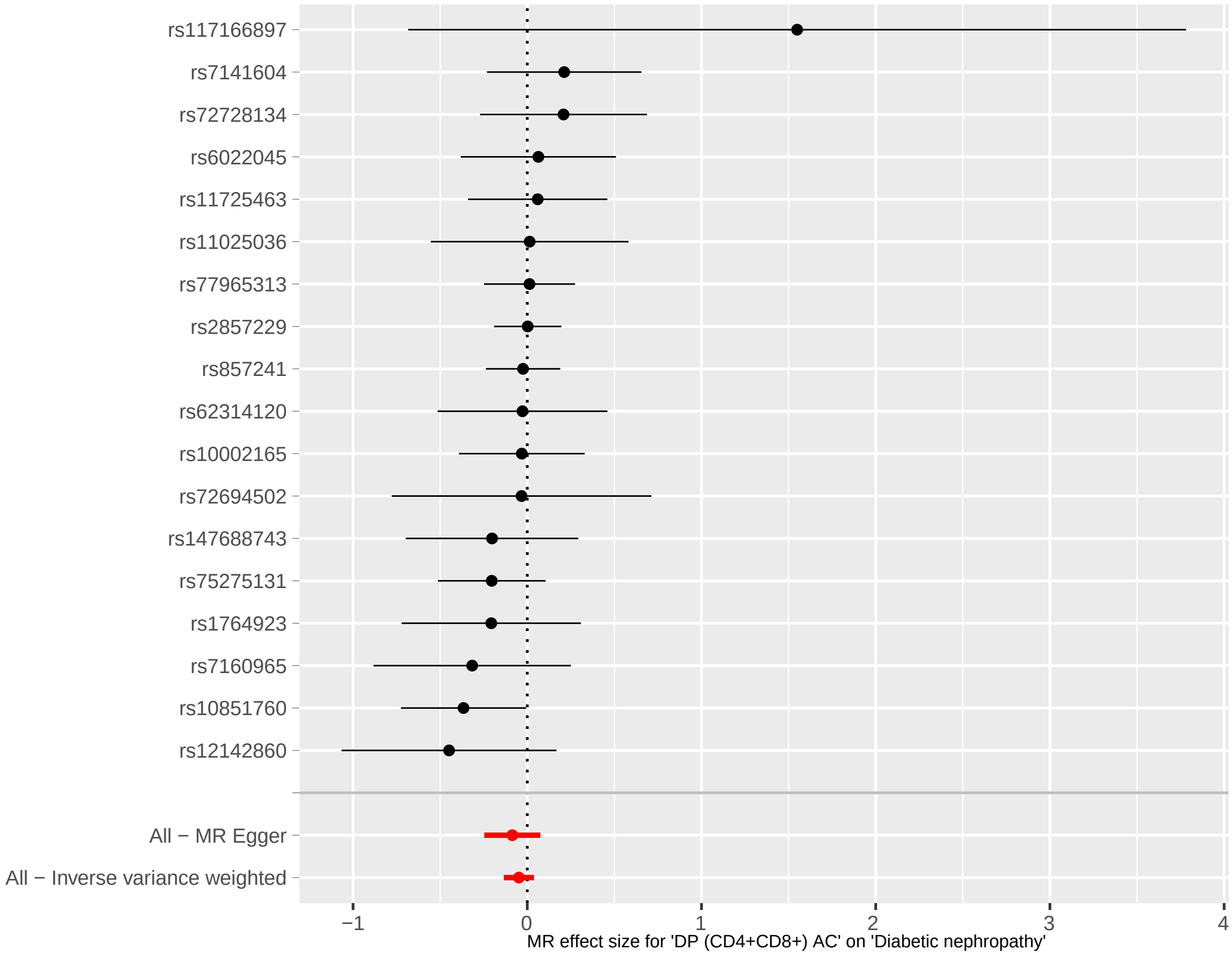

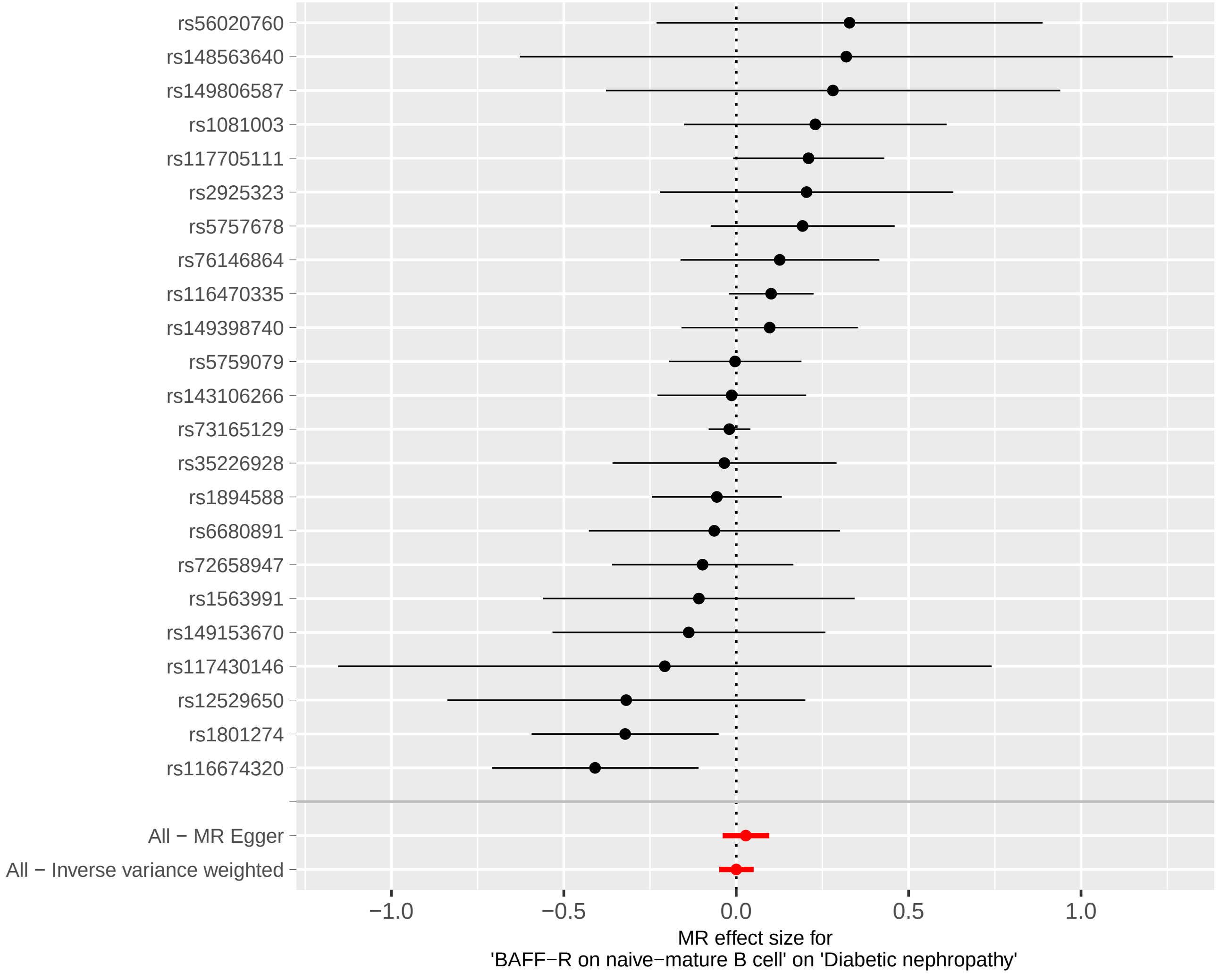
rs56020760
rs148563640
rs149806587
rs1081003
rs117705111
rs2925323
rs5757678
rs76146864
rs116470335
rs149398740
rs5759079
rs143106266
rs73165129
rs35226928
rs1894588
rs6680891
rs72658947
rs1563991
rs149153670
rs117430146
rs12529650
rs1801274
rs116674320
All − MR Egger
All − Inverse variance weighted
−1.0
−0.5
0.0
0.5
1.0
MR effect size for
'BAFF−R on naive−mature B cell' on 'Diabetic nephropathy'

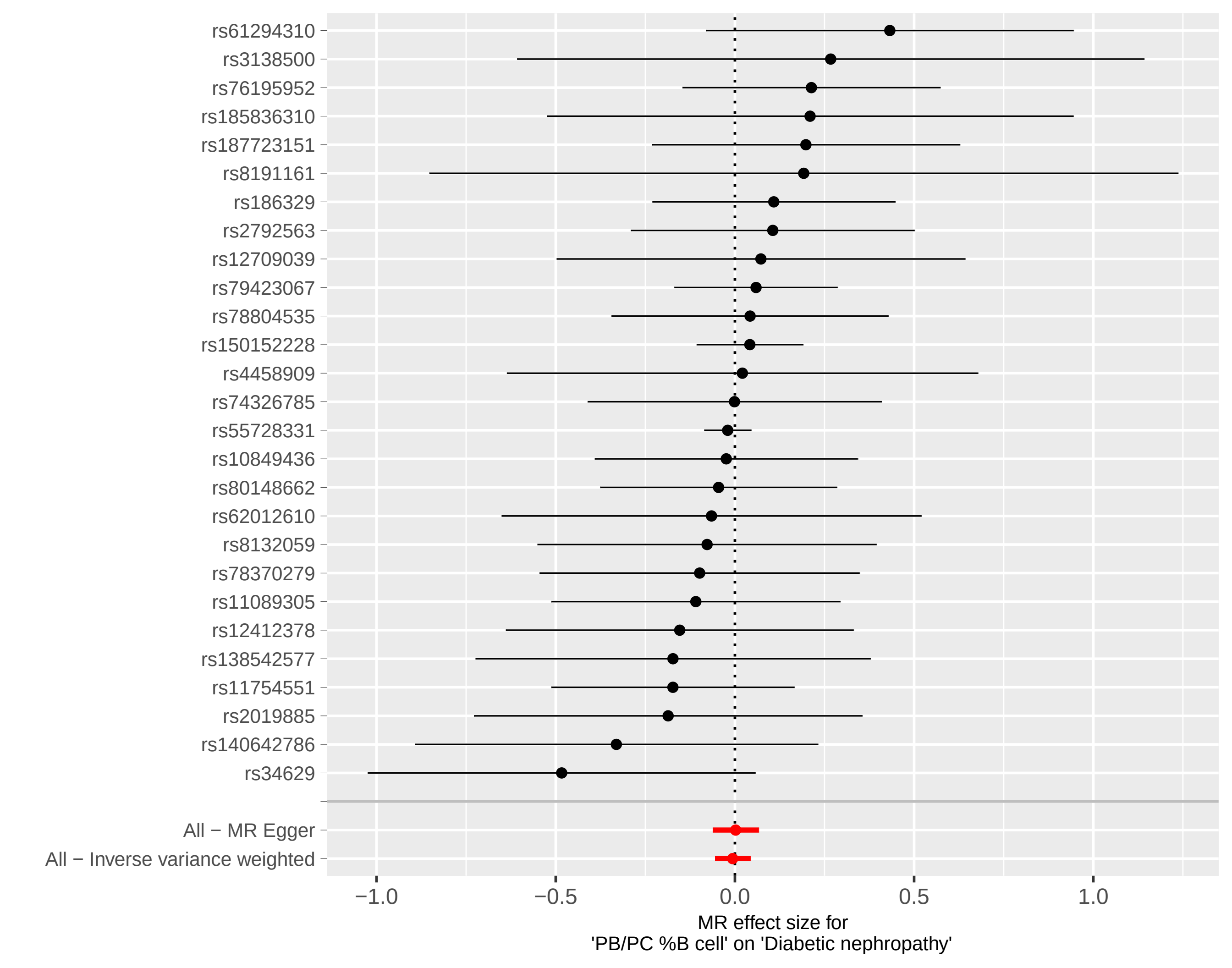
rs61294310
rs3138500
rs76195952
rs185836310
rs187723151
rs8191161
rs186329
rs2792563
rs12709039
rs79423067
rs78804535
rs150152228
rs4458909
rs74326785
rs55728331
rs10849436
rs80148662
rs62012610
rs8132059
rs78370279
rs11089305
rs12412378
rs138542577
rs11754551
rs2019885
rs140642786
rs34629
All − MR Egger
All − Inverse variance weighted
−1.0
−0.5
0.0
0.5
1.0
MR effect size for
'PB/PC %B cell' on 'Diabetic nephropathy'

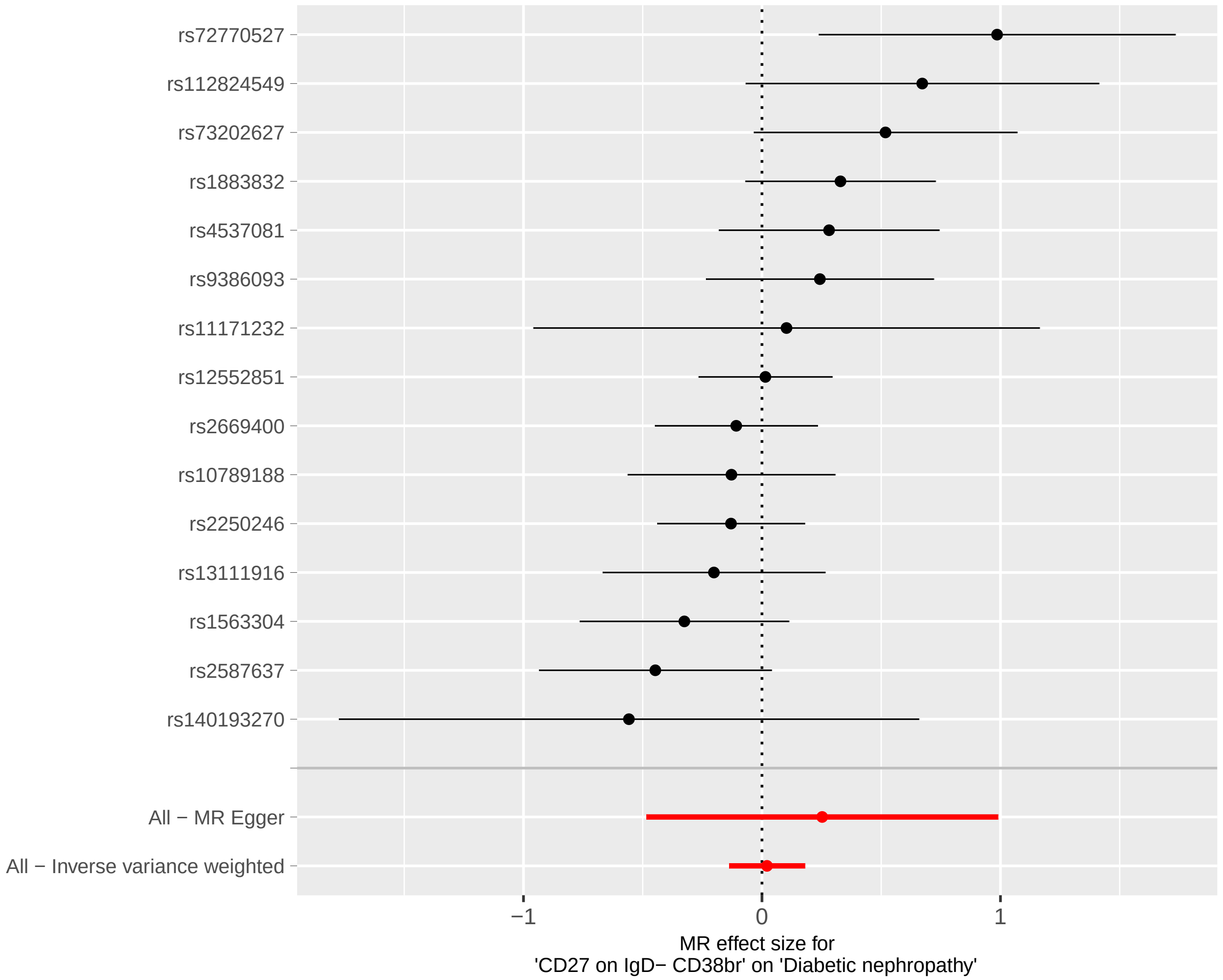

rs72770527
rs112824549
rs73202627
rs1883832
rs4537081
rs9386093
rs11171232
rs12552851
rs2669400
rs10789188
rs2250246
rs13111916
rs1563304
rs2587637
rs140193270
All − MR Egger
All − Inverse variance weighted
−1
0
1
MR effect size for
'CD27 on IgD− CD38br' on 'Diabetic nephropathy'

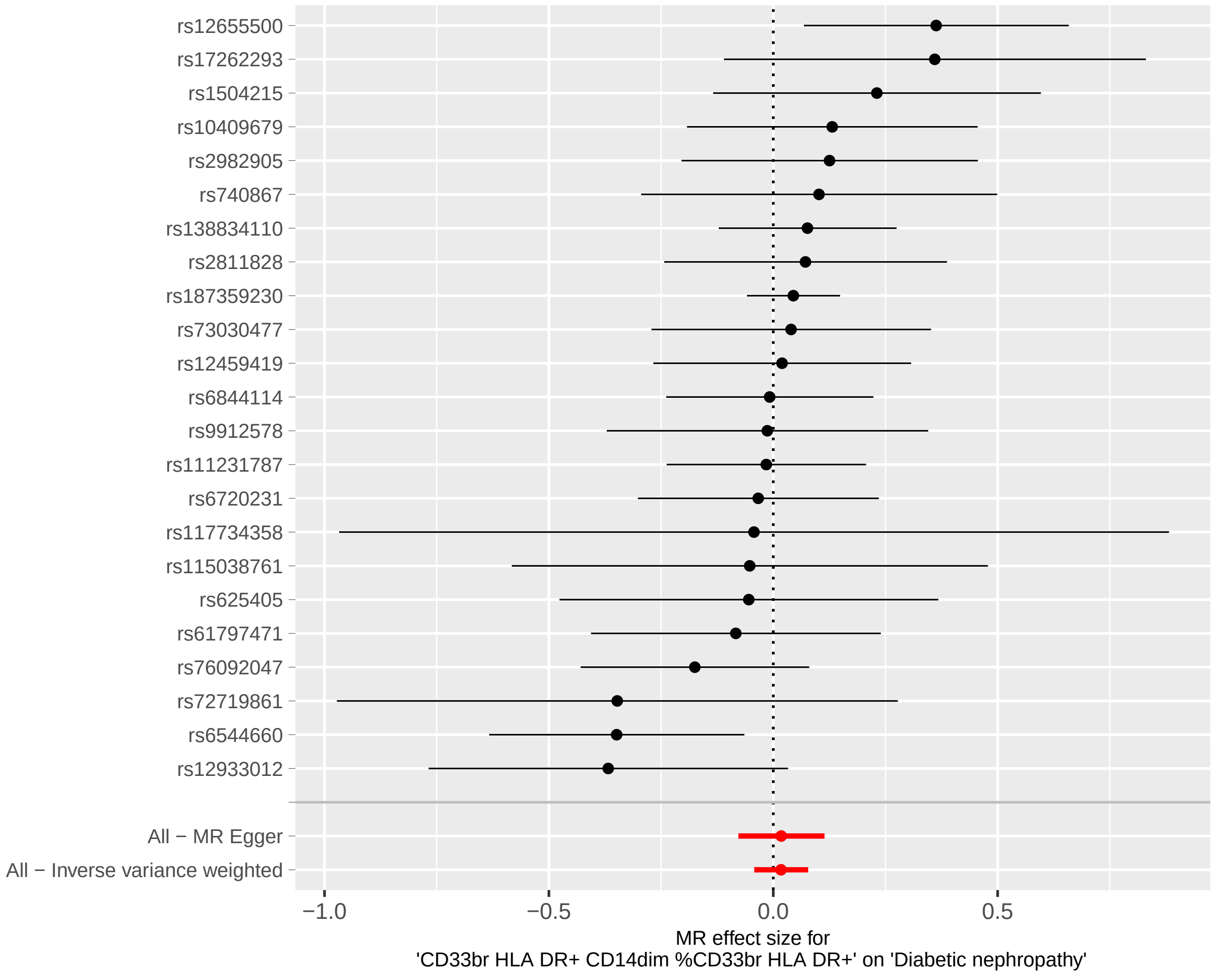
rs12655500
rs17262293
rs1504215
rs10409679
rs2982905
rs740867
rs138834110
rs2811828
rs187359230
rs73030477
rs12459419
rs6844114
rs9912578
rs111231787
rs6720231
rs117734358
rs115038761
rs625405
rs61797471
rs76092047
rs72719861
rs6544660
rs12933012
All − MR Egger
All − Inverse variance weighted
−1.0
−0.5
0.0
0.5
MR effect size for
'CD33br HLA DR+ CD14dim %CD33br HLA DR+' on 'Diabetic nephropathy'

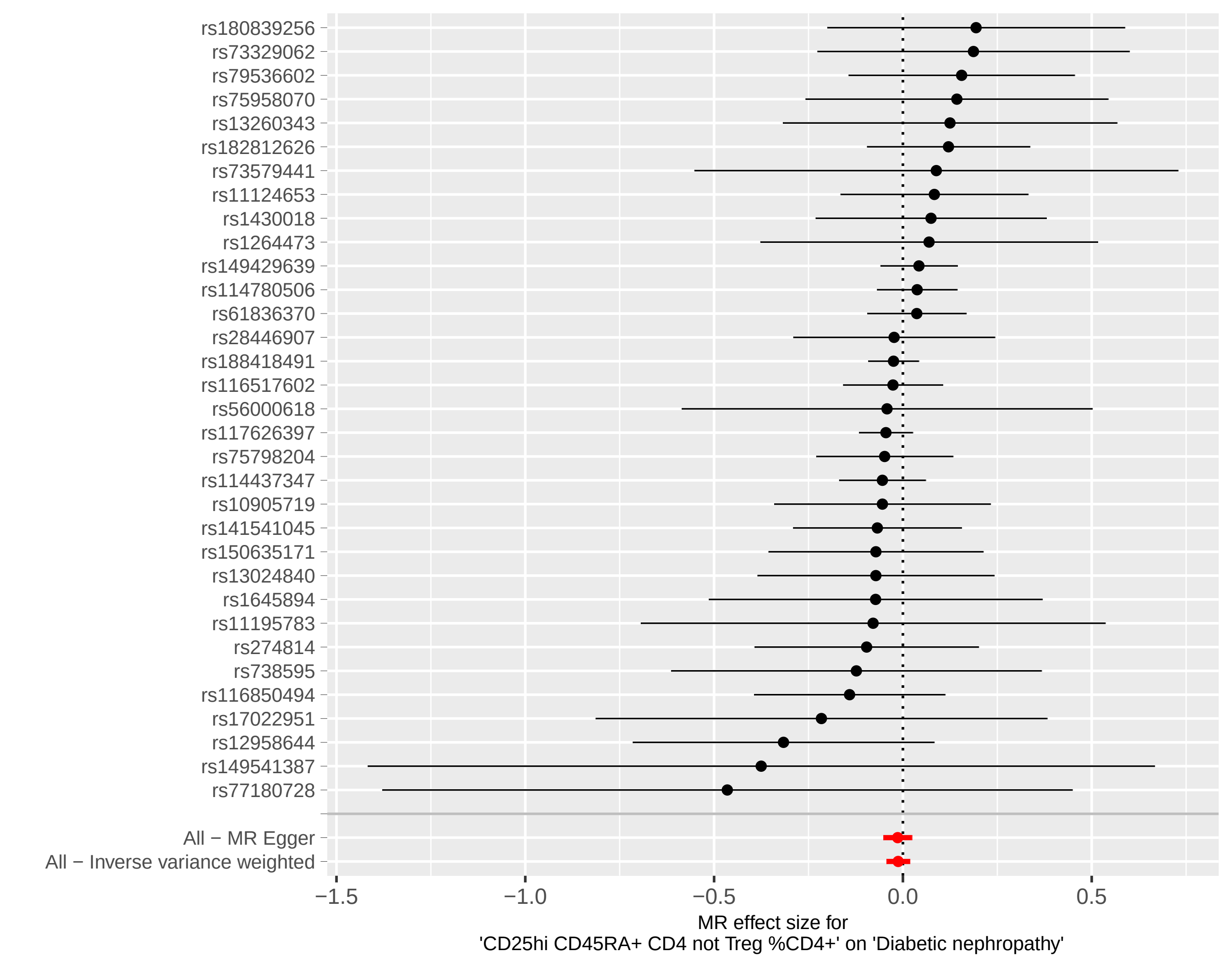

rs180839256
rs73329062
rs79536602
rs75958070
rs13260343
rs182812626
rs73579441
rs11124653
rs1430018
rs1264473
rs149429639
rs114780506
rs61836370
rs28446907
rs188418491
rs116517602
rs56000618
rs117626397
rs75798204
rs114437347
rs10905719
rs141541045
rs150635171
rs13024840
rs1645894
rs11195783
rs274814
rs738595
rs116850494
rs17022951
rs12958644
rs149541387
rs77180728
All − MR Egger
All − Inverse variance weighted
−1.5
−1.0
−0.5
0.0
0.5
MR effect size for
'CD25hi CD45RA+ CD4 not Treg %CD4+' on 'Diabetic nephropathy'

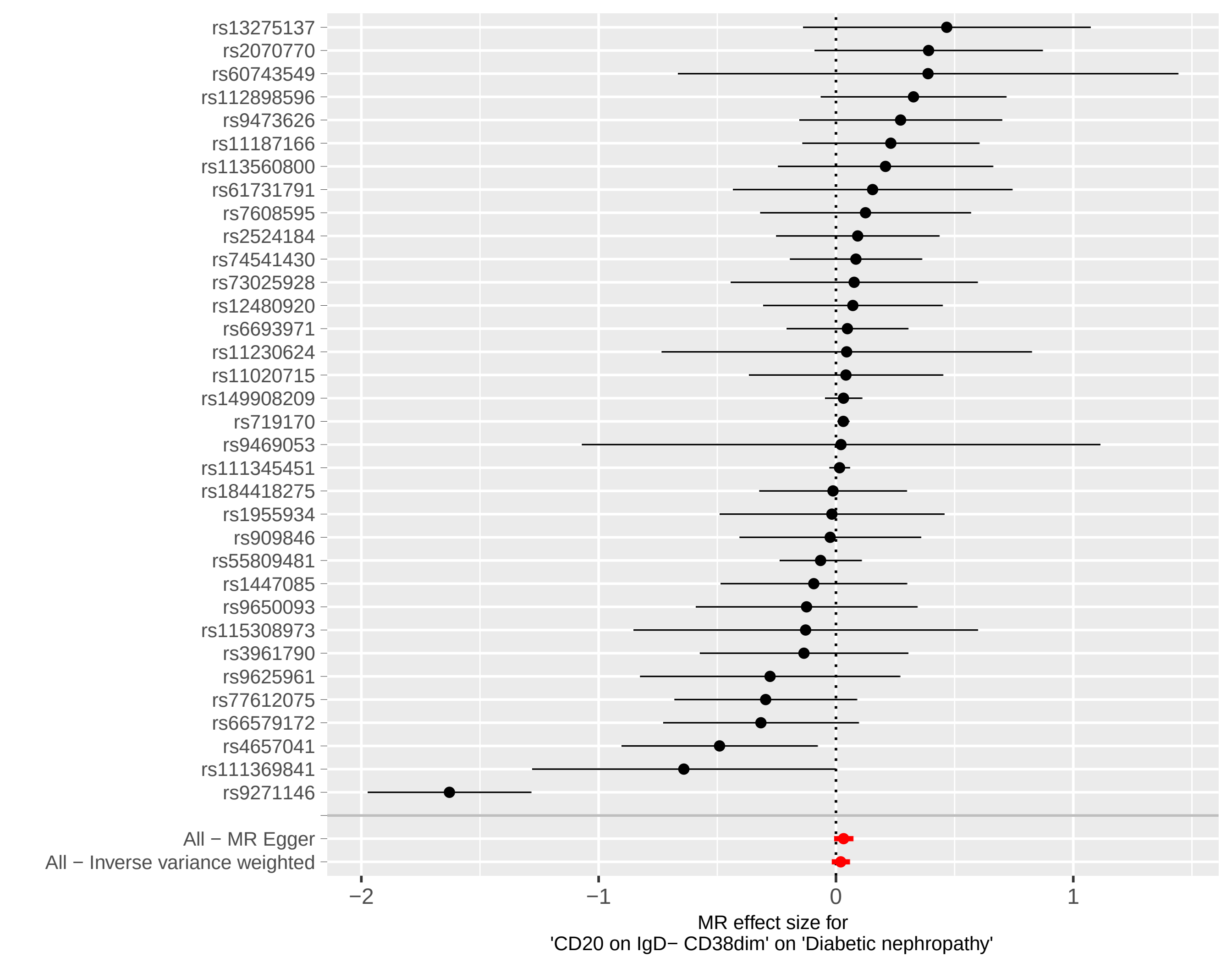
rs13275137
rs2070770
rs60743549
rs112898596
rs9473626
rs11187166
rs113560800
rs61731791
rs7608595
rs2524184
rs74541430
rs73025928
rs12480920
rs6693971
rs11230624
rs11020715
rs149908209
rs719170
rs9469053
rs111345451
rs184418275
rs1955934
rs909846
rs55809481
rs1447085
rs9650093
rs115308973
rs3961790
rs9625961
rs77612075
rs66579172
rs4657041
rs111369841
rs9271146
All − MR Egger
All − Inverse variance weighted
−2
−1
0
1
MR effect size for
'CD20 on IgD− CD38dim' on 'Diabetic nephropathy'

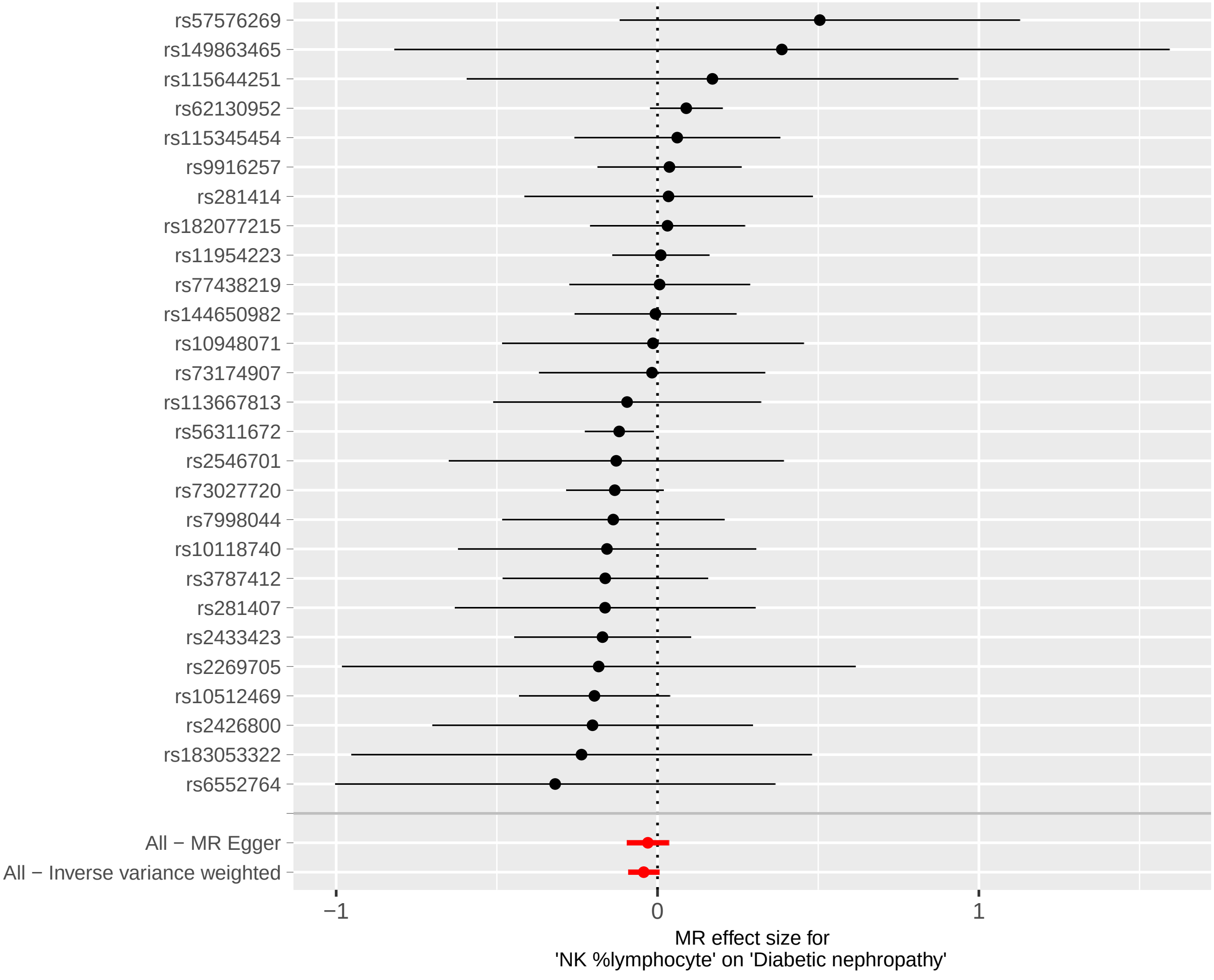

rs57576269
rs149863465
rs115644251
rs62130952
rs115345454
rs9916257
rs281414
rs182077215
rs11954223
rs77438219
rs144650982
rs10948071
rs73174907
rs113667813
rs56311672
rs2546701
rs73027720
rs7998044
rs10118740
rs3787412
rs281407
rs2433423
rs2269705
rs10512469
rs2426800
rs183053322
rs6552764
All − MR Egger
All − Inverse variance weighted
−1
0
1
MR effect size for
'NK %lymphocyte' on 'Diabetic nephropathy'

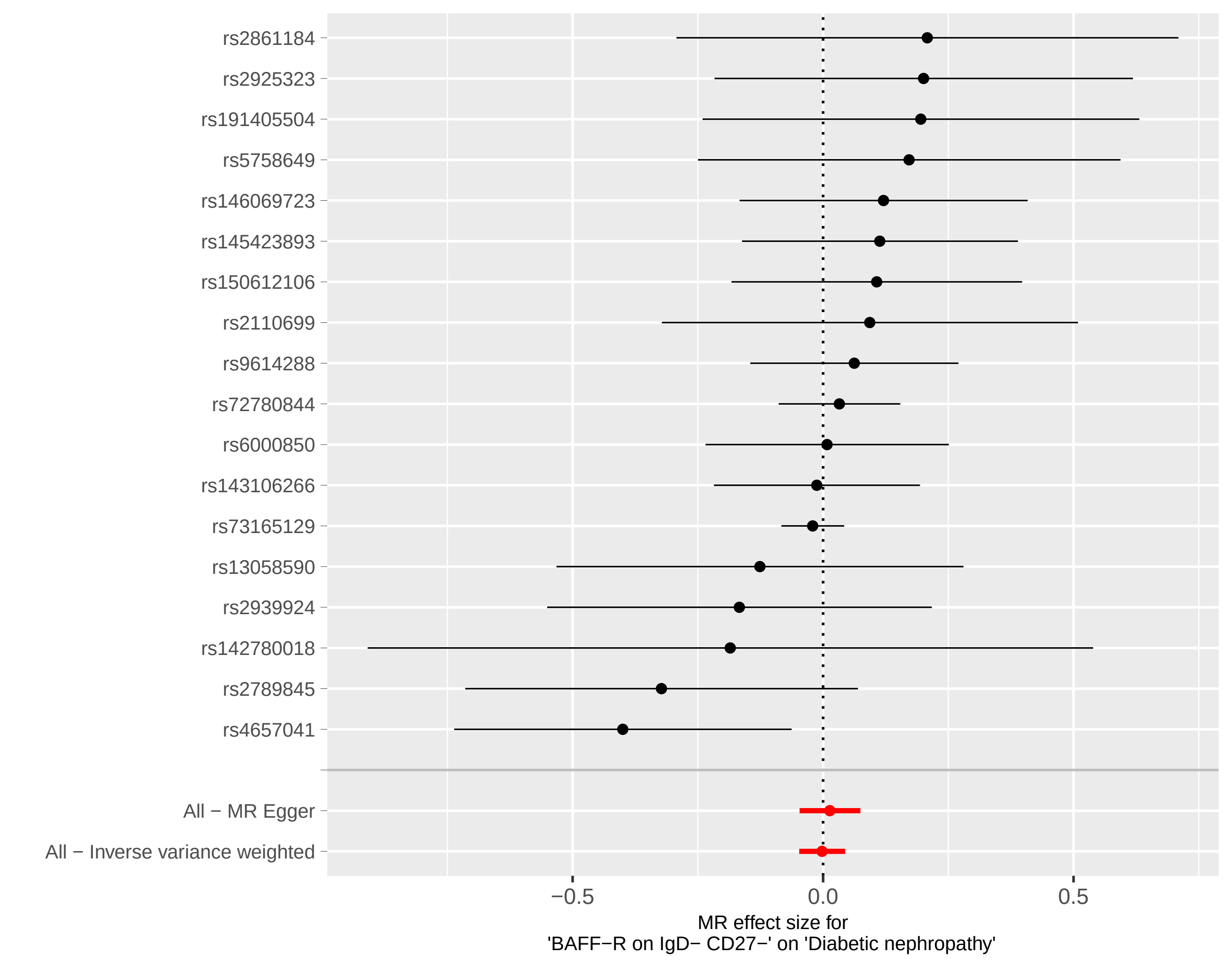
rs2861184
rs2925323
rs191405504
rs5758649
rs146069723
rs145423893
rs150612106
rs2110699
rs9614288
rs72780844
rs6000850
rs143106266
rs73165129
rs13058590
rs2939924
rs142780018
rs2789845
rs4657041
All − MR Egger
All − Inverse variance weighted
−0.5
0.0
0.5
MR effect size for
'BAFF−R on IgD− CD27−' on 'Diabetic nephropathy'

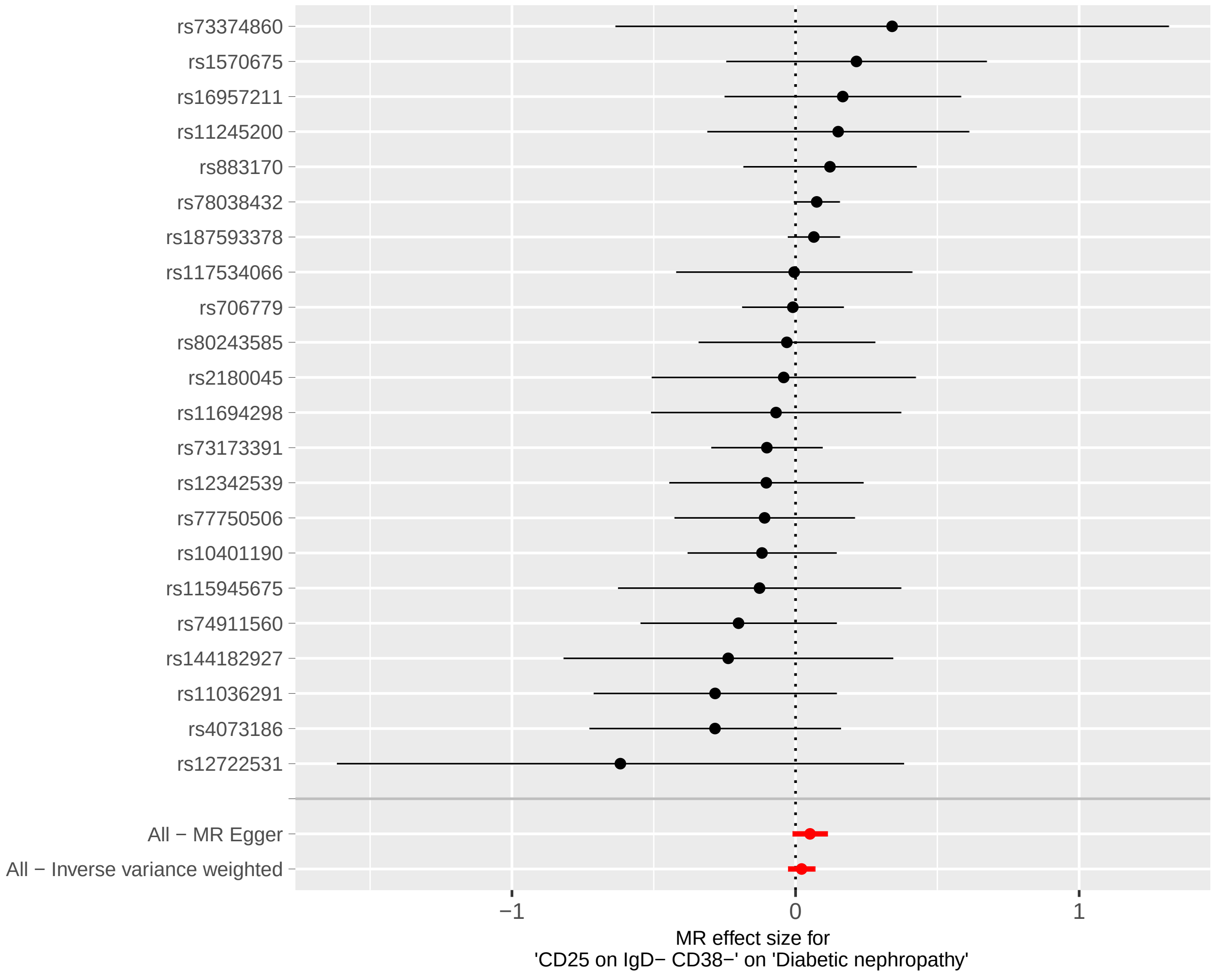
rs73374860
rs1570675
rs16957211
rs11245200
rs883170
rs78038432
rs187593378
rs117534066
rs706779
rs80243585
rs2180045
rs11694298
rs73173391
rs12342539
rs77750506
rs10401190
rs115945675
rs74911560
rs144182927
rs11036291
rs4073186
rs12722531
All − MR Egger
All − Inverse variance weighted
−1
0
1
MR effect size for
'CD25 on IgD− CD38−' on 'Diabetic nephropathy'

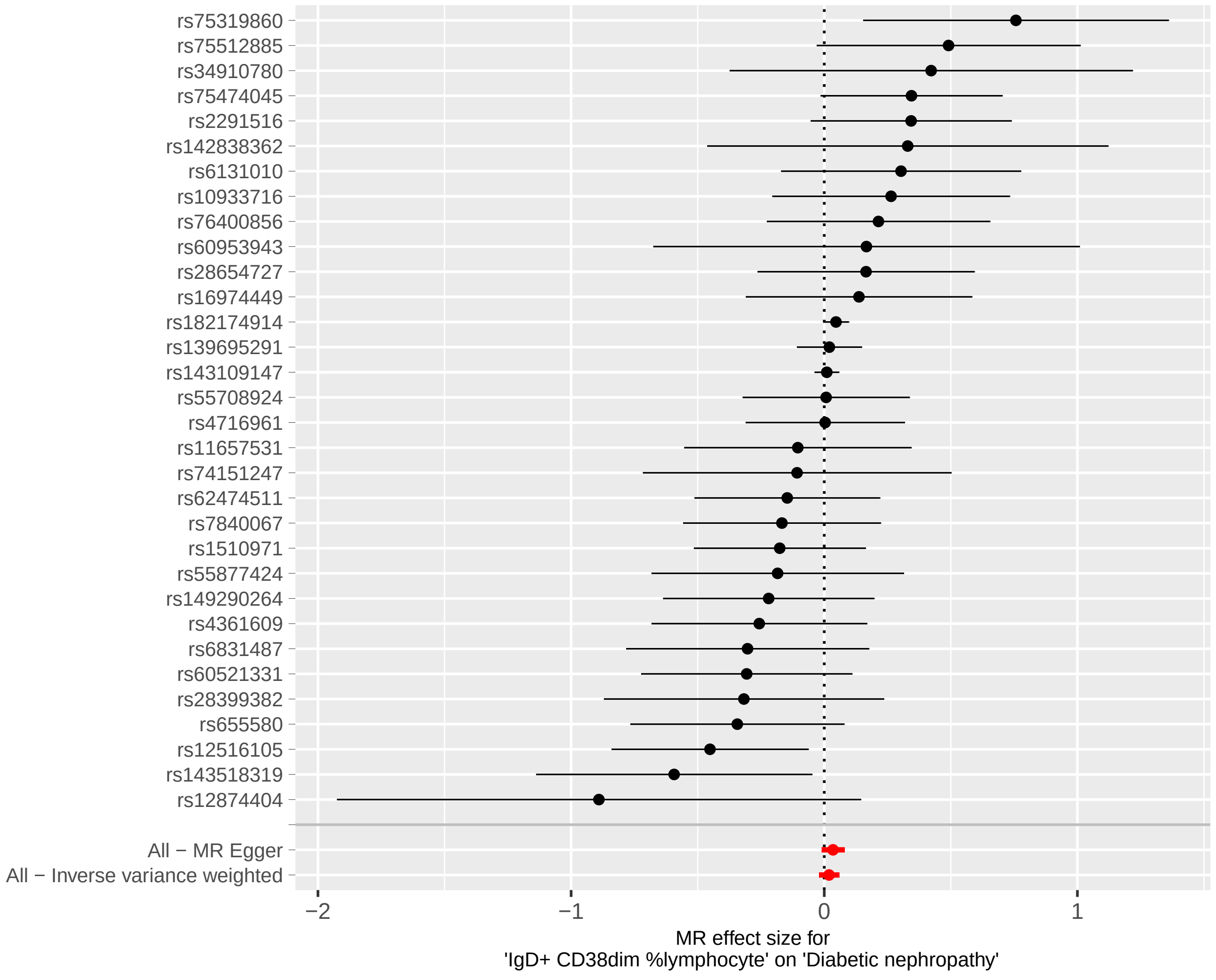
rs75319860
rs75512885
rs34910780
rs75474045
rs2291516
rs142838362
rs6131010
rs10933716
rs76400856
rs60953943
rs28654727
rs16974449
rs182174914
rs139695291
rs143109147
rs55708924
rs4716961
rs11657531
rs74151247
rs62474511
rs7840067
rs1510971
rs55877424
rs149290264
rs4361609
rs6831487
rs60521331
rs28399382
rs655580
rs12516105
rs143518319
rs12874404
All − MR Egger
All − Inverse variance weighted
−2
−1
0
1
MR effect size for
'IgD+ CD38dim %lymphocyte' on 'Diabetic nephropathy'

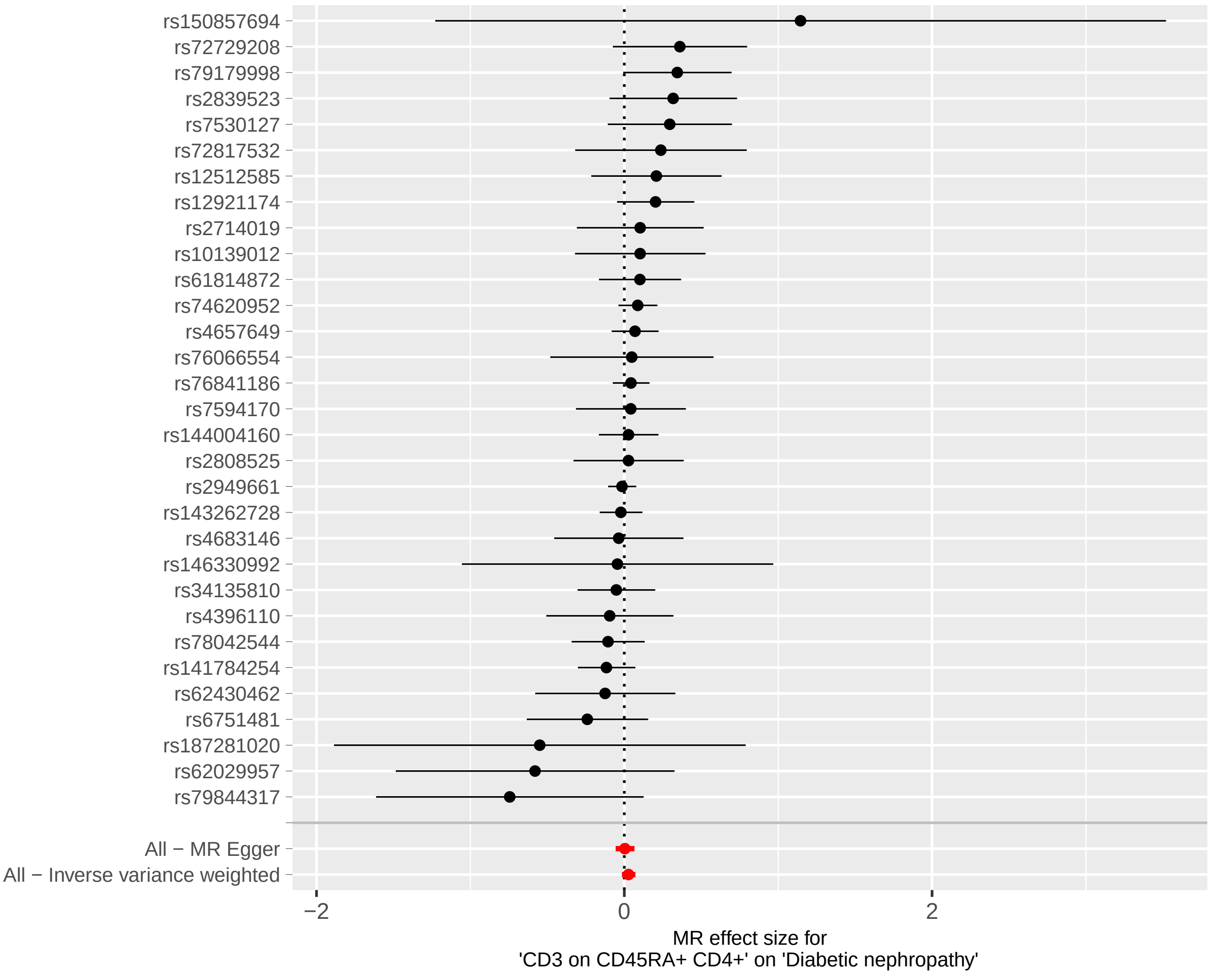

rs150857694
rs72729208
rs79179998
rs2839523
rs7530127
rs72817532
rs12512585
rs12921174
rs2714019
rs10139012
rs61814872
rs74620952
rs4657649
rs76066554
rs76841186
rs7594170
rs144004160
rs2808525
rs2949661
rs143262728
rs4683146
rs146330992
rs34135810
rs4396110
rs78042544
rs141784254
rs62430462
rs6751481
rs187281020
rs62029957
rs79844317
All − MR Egger
All − Inverse variance weighted
−2
0
2
MR effect size for
'CD3 on CD45RA+ CD4+' on 'Diabetic nephropathy'

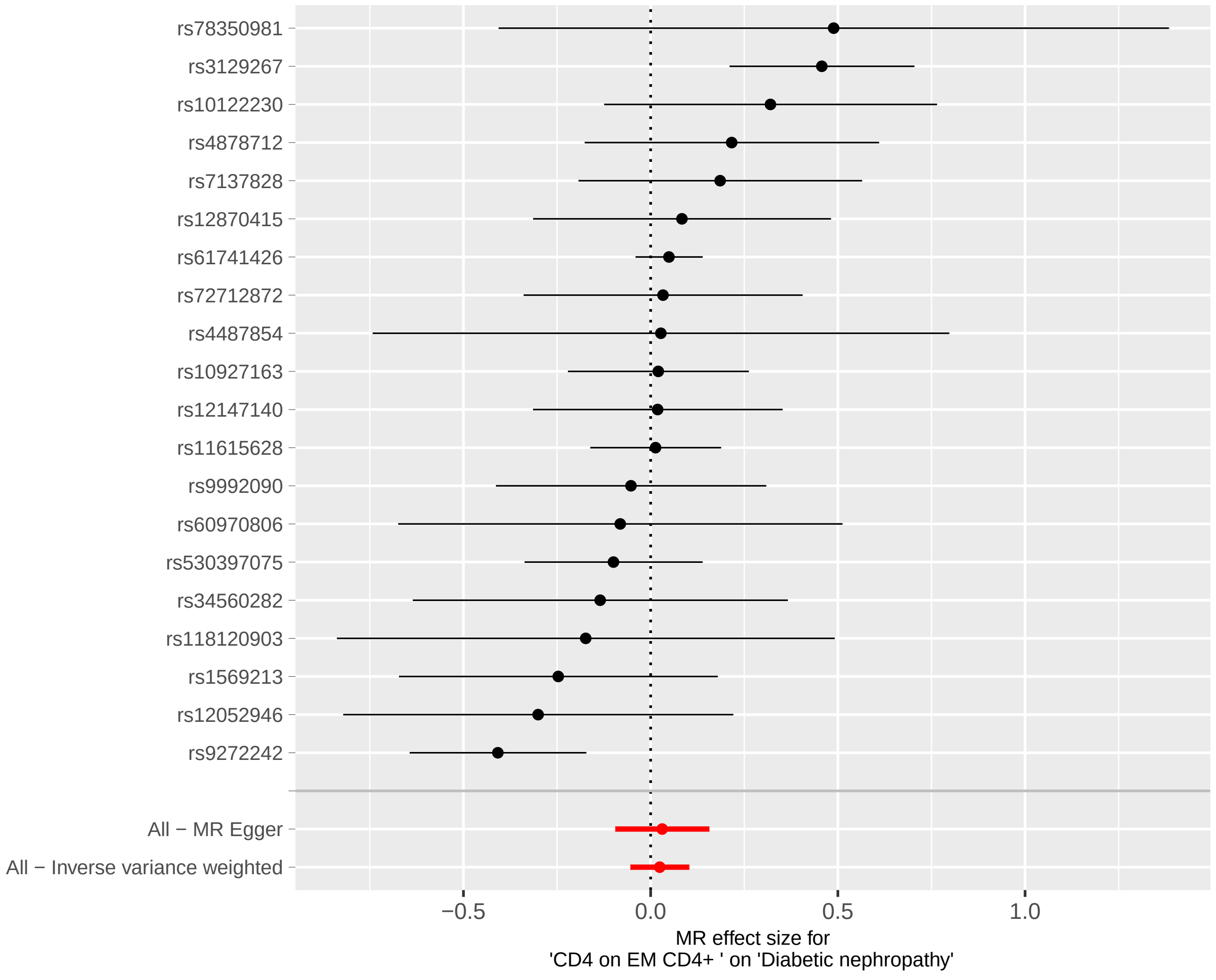

rs78350981
rs3129267
rs10122230
rs4878712
rs7137828
rs12870415
rs61741426
rs72712872
rs4487854
rs10927163
rs12147140
rs11615628
rs9992090
rs60970806
rs530397075
rs34560282
rs118120903
rs1569213
rs12052946
rs9272242
All − MR Egger
All − Inverse variance weighted
−0.5
0.0
0.5
1.0
MR effect size for
'CD4 on EM CD4+ ' on 'Diabetic nephropathy'

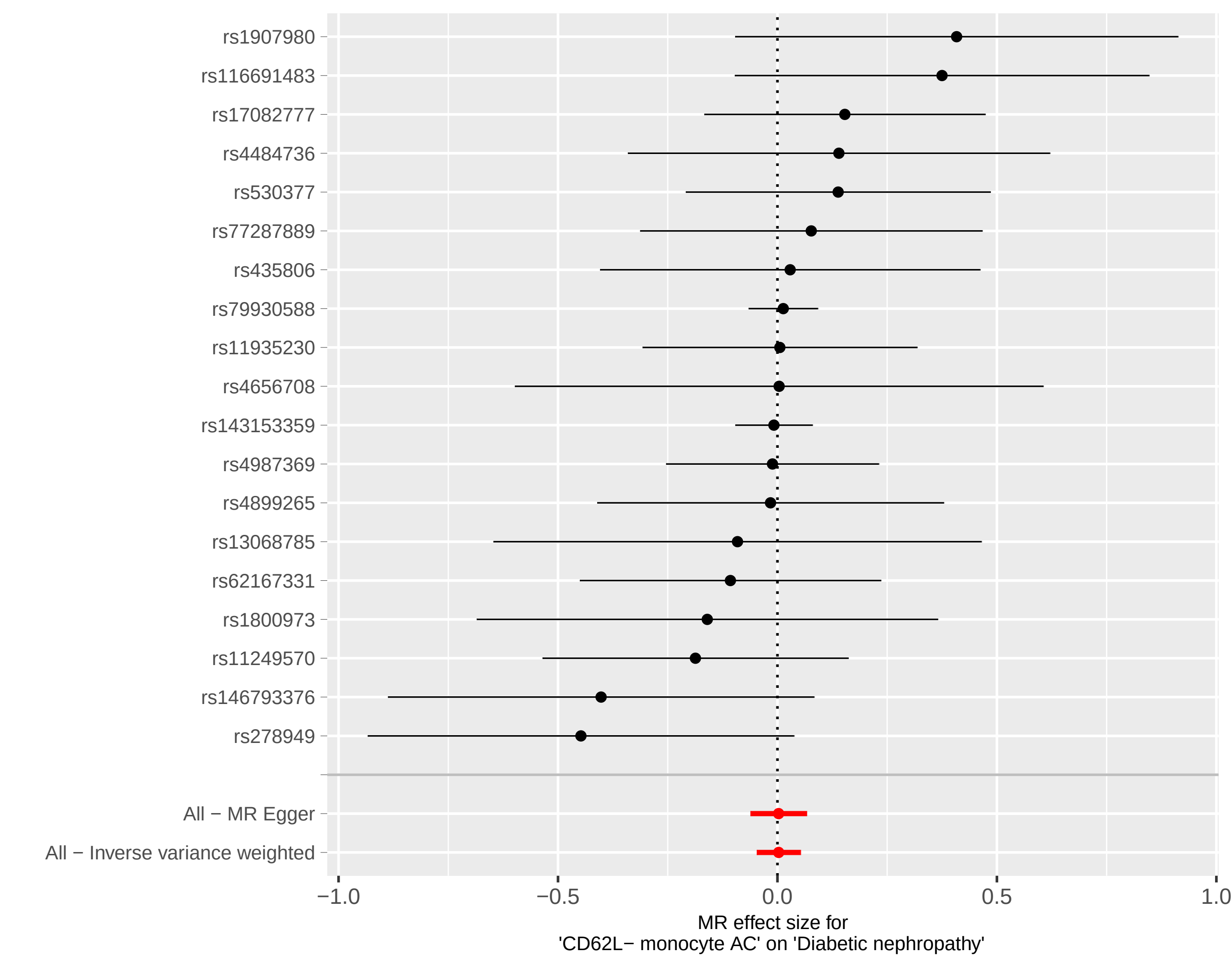
rs1907980
rs116691483
rs17082777
rs4484736
rs530377
rs77287889
rs435806
rs79930588
rs11935230
rs4656708
rs143153359
rs4987369
rs4899265
rs13068785
rs62167331
rs1800973
rs11249570
rs146793376
rs278949
All − MR Egger
All − Inverse variance weighted
−1.0
−0.5
0.0
0.5
1.0
MR effect size for
'CD62L− monocyte AC' on 'Diabetic nephropathy'

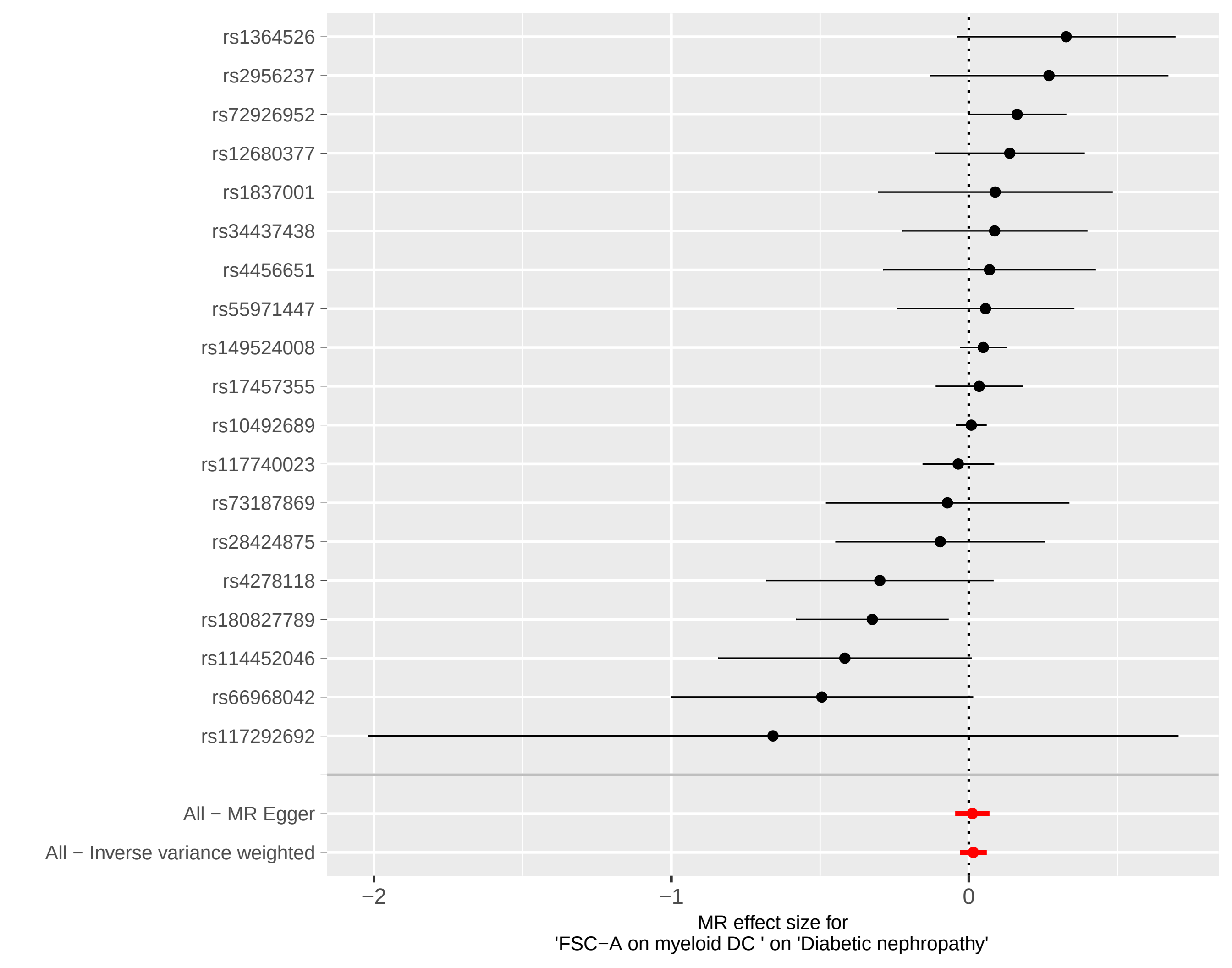
rs1364526
rs2956237
rs72926952
rs12680377
rs1837001
rs34437438
rs4456651
rs55971447
rs149524008
rs17457355
rs10492689
rs117740023
rs73187869
rs28424875
rs4278118
rs180827789
rs114452046
rs66968042
rs117292692
All − MR Egger
All − Inverse variance weighted
−2
−1
0
MR effect size for
'FSC−A on myeloid DC ' on 'Diabetic nephropathy'

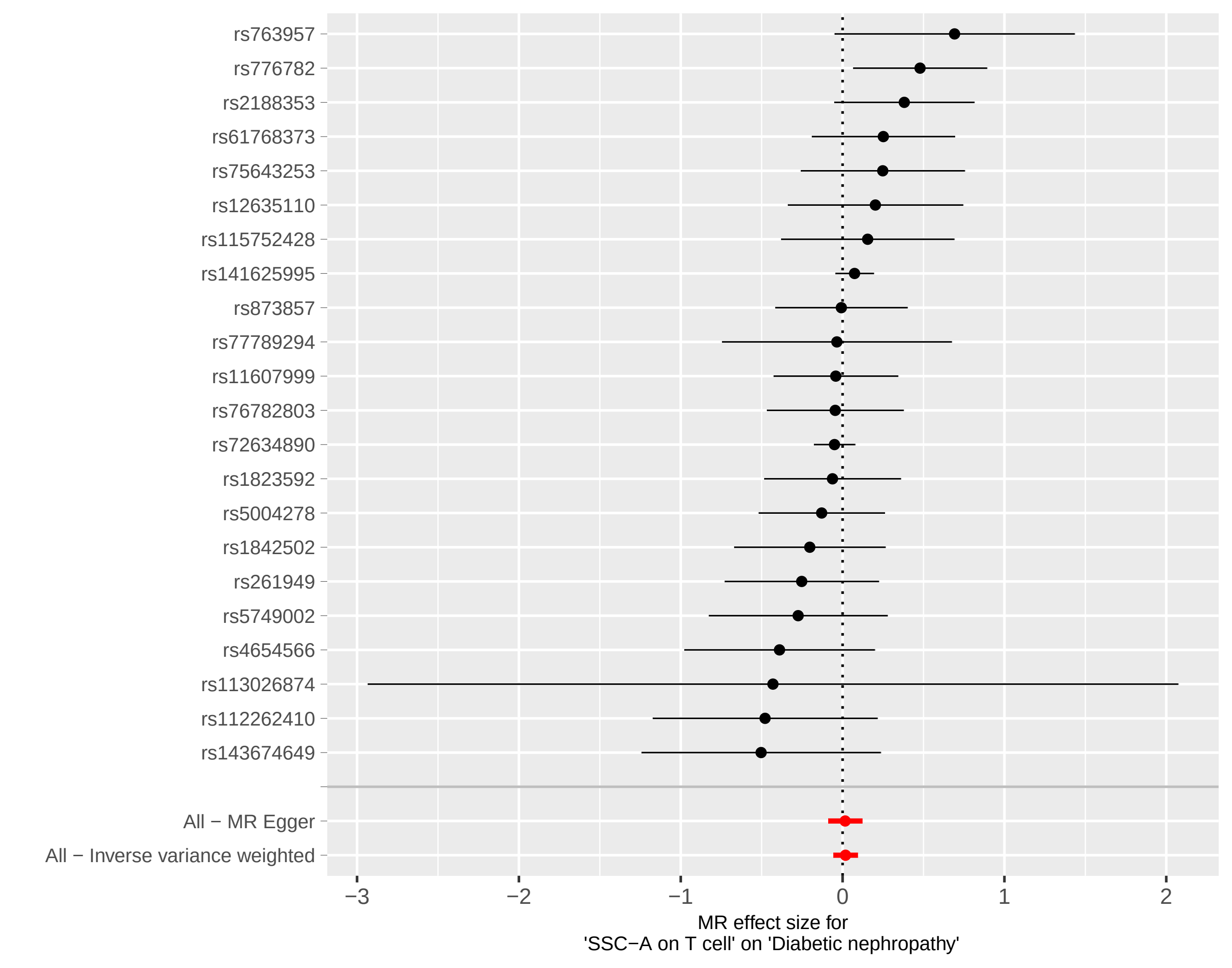
rs763957
rs776782
rs2188353
rs61768373
rs75643253
rs12635110
rs115752428
rs141625995
rs873857
rs77789294
rs11607999
rs76782803
rs72634890
rs1823592
rs5004278
rs1842502
rs261949
rs5749002
rs4654566
rs113026874
rs112262410
rs143674649
All − MR Egger
All − Inverse variance weighted
−3
−2
−1
0
1
2
MR effect size for
'SSC−A on T cell' on 'Diabetic nephropathy'

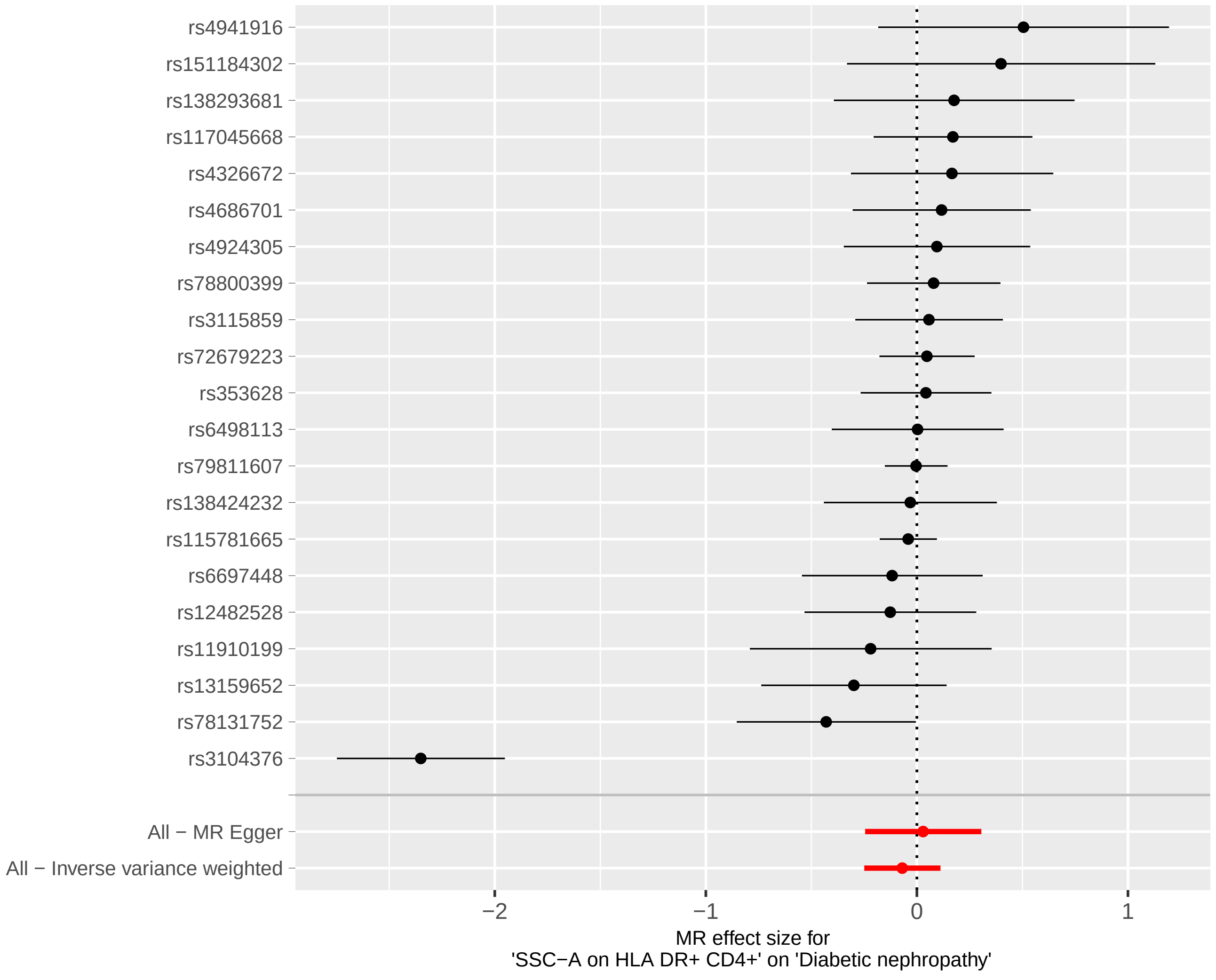
rs4941916
rs151184302
rs138293681
rs117045668
rs4326672
rs4686701
rs4924305
rs78800399
rs3115859
rs72679223
rs353628
rs6498113
rs79811607
rs138424232
rs115781665
rs6697448
rs12482528
rs11910199
rs13159652
rs78131752
rs3104376
All − MR Egger
All − Inverse variance weighted
−2
−1
0
1
MR effect size for
'SSC−A on HLA DR+ CD4+' on 'Diabetic nephropathy'

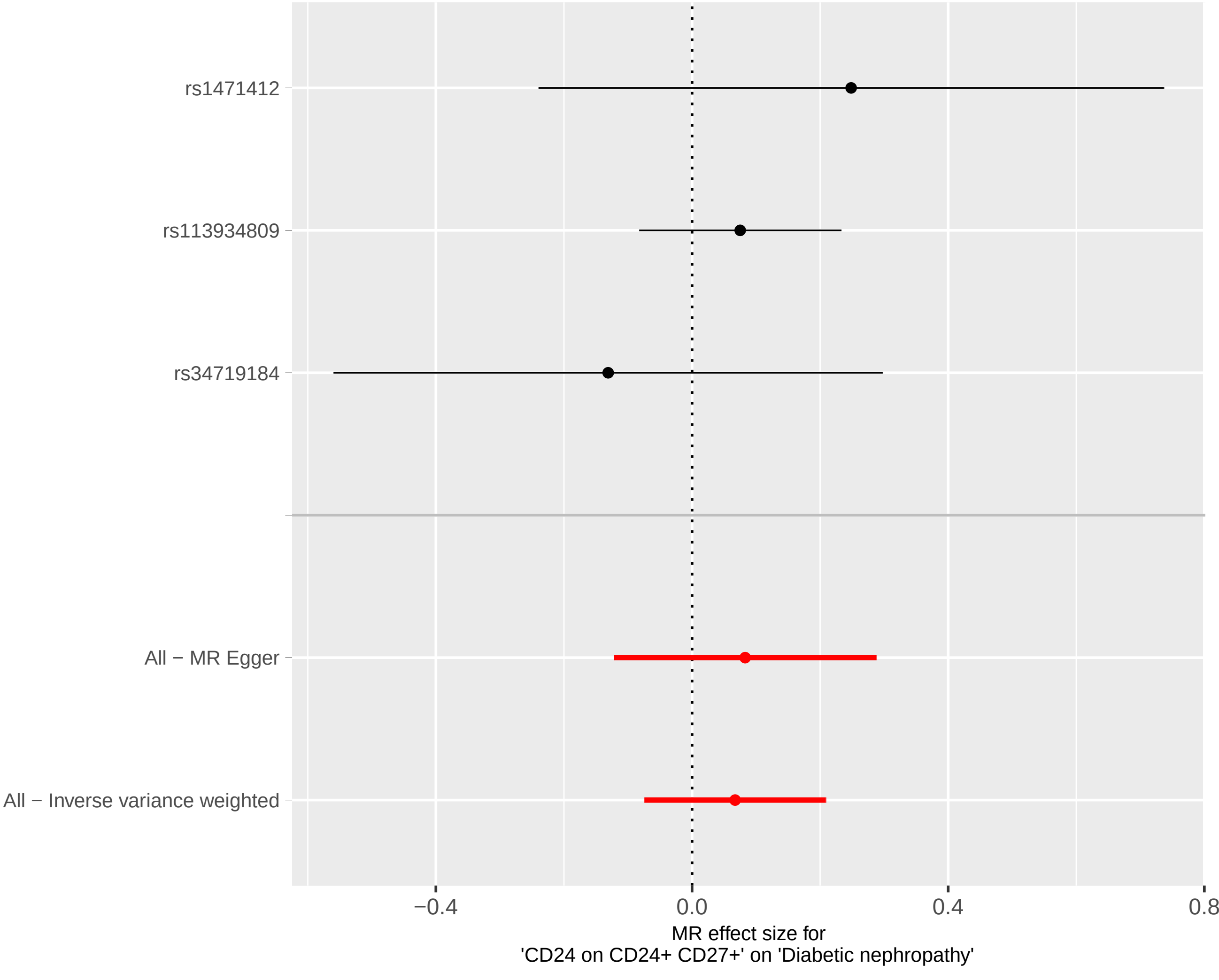
rs1471412
rs113934809
rs34719184
All − MR Egger
All − Inverse variance weighted
−0.4
0.0
0.4
0.8
MR effect size for
'CD24 on CD24+ CD27+' on 'Diabetic nephropathy'

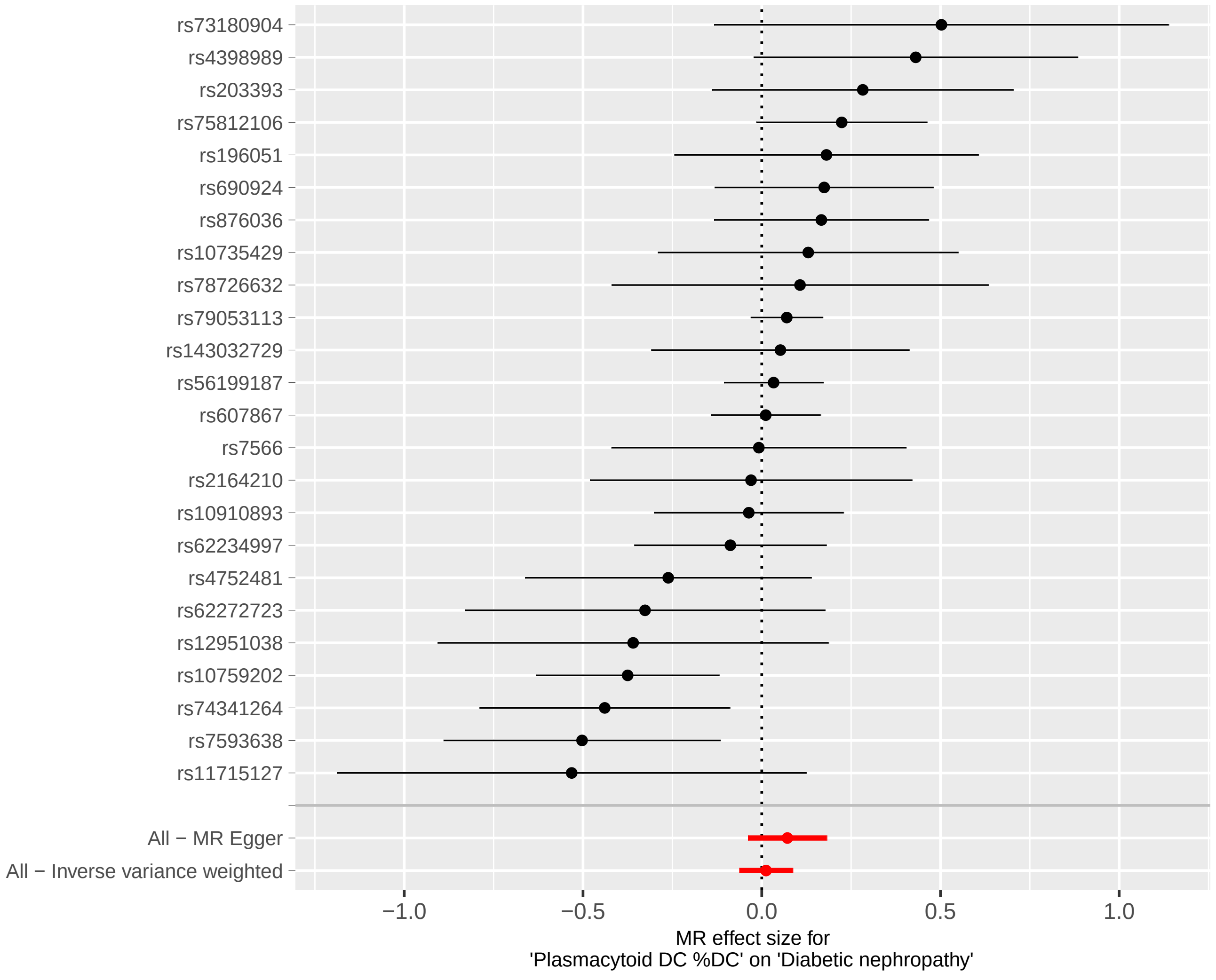
rs73180904
rs4398989
rs203393
rs75812106
rs196051
rs690924
rs876036
rs10735429
rs78726632
rs79053113
rs143032729
rs56199187
rs607867
rs7566
rs2164210
rs10910893
rs62234997
rs4752481
rs62272723
rs12951038
rs10759202
rs74341264
rs7593638
rs11715127
All − MR Egger
All − Inverse variance weighted
−1.0
−0.5
0.0
0.5
1.0
MR effect size for
'Plasmacytoid DC %DC' on 'Diabetic nephropathy'

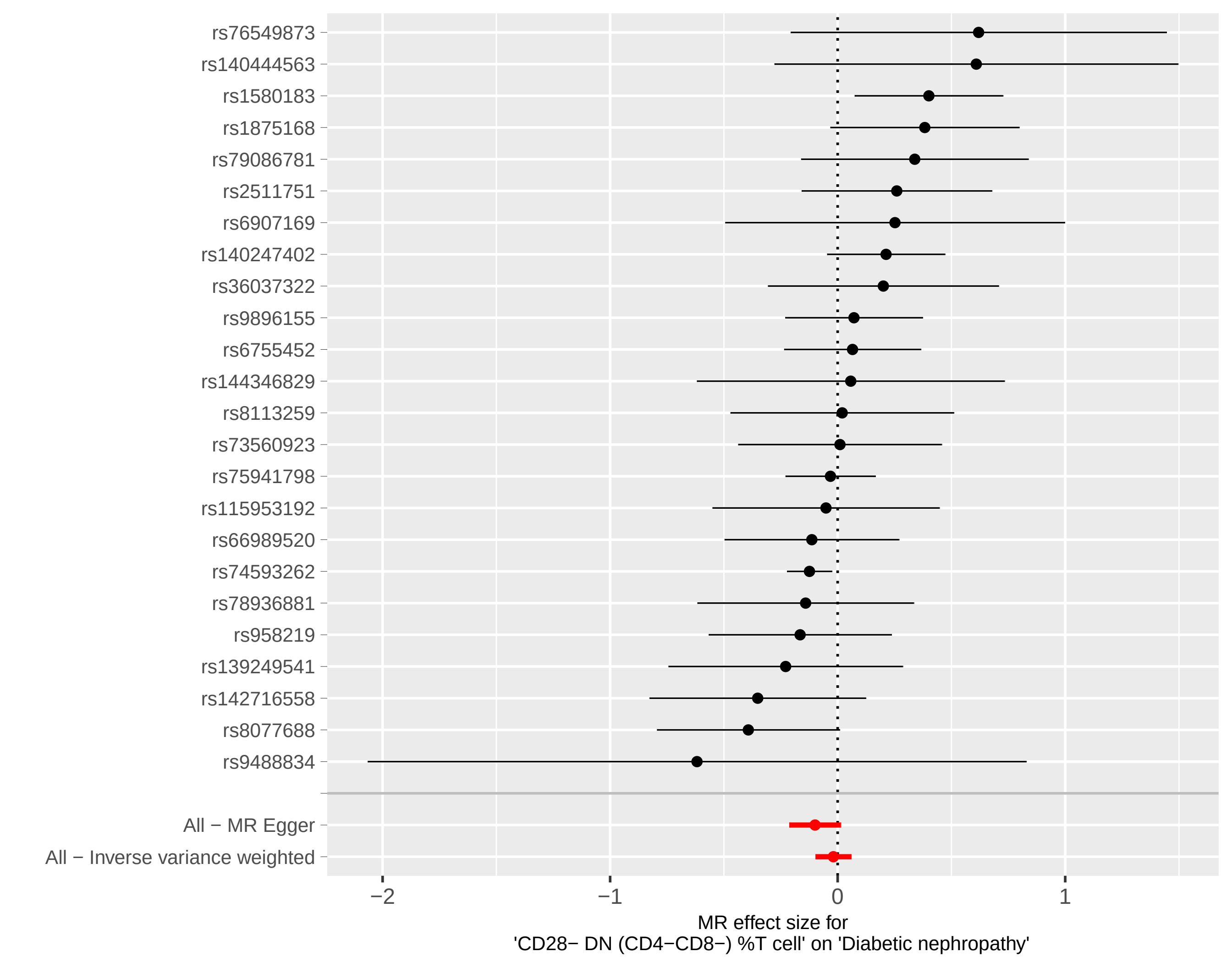
rs76549873
rs140444563
rs1580183
rs1875168
rs79086781
rs2511751
rs6907169
rs140247402
rs36037322
rs9896155
rs6755452
rs144346829
rs8113259
rs73560923
rs75941798
rs115953192
rs66989520
rs74593262
rs78936881
rs958219
rs139249541
rs142716558
rs8077688
rs9488834
All − MR Egger
All − Inverse variance weighted
−2
−1
0
1
MR effect size for
'CD28− DN (CD4−CD8−) %T cell' on 'Diabetic nephropathy'

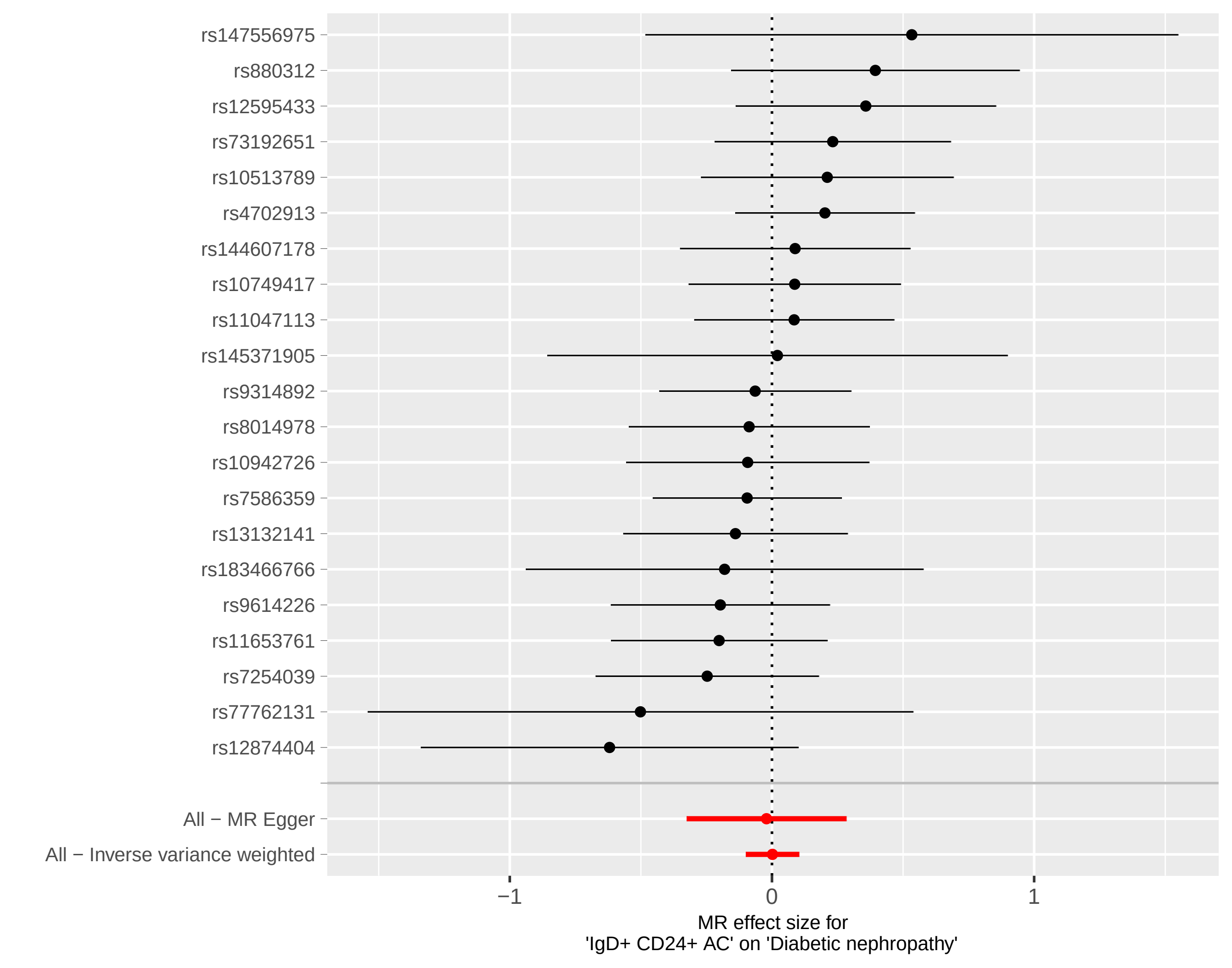
rs147556975
rs880312
rs12595433
rs73192651
rs10513789
rs4702913
rs144607178
rs10749417
rs11047113
rs145371905
rs9314892
rs8014978
rs10942726
rs7586359
rs13132141
rs183466766
rs9614226
rs11653761
rs7254039
rs77762131
rs12874404
All − MR Egger
All − Inverse variance weighted
−1
0
1
MR effect size for
'IgD+ CD24+ AC' on 'Diabetic nephropathy'

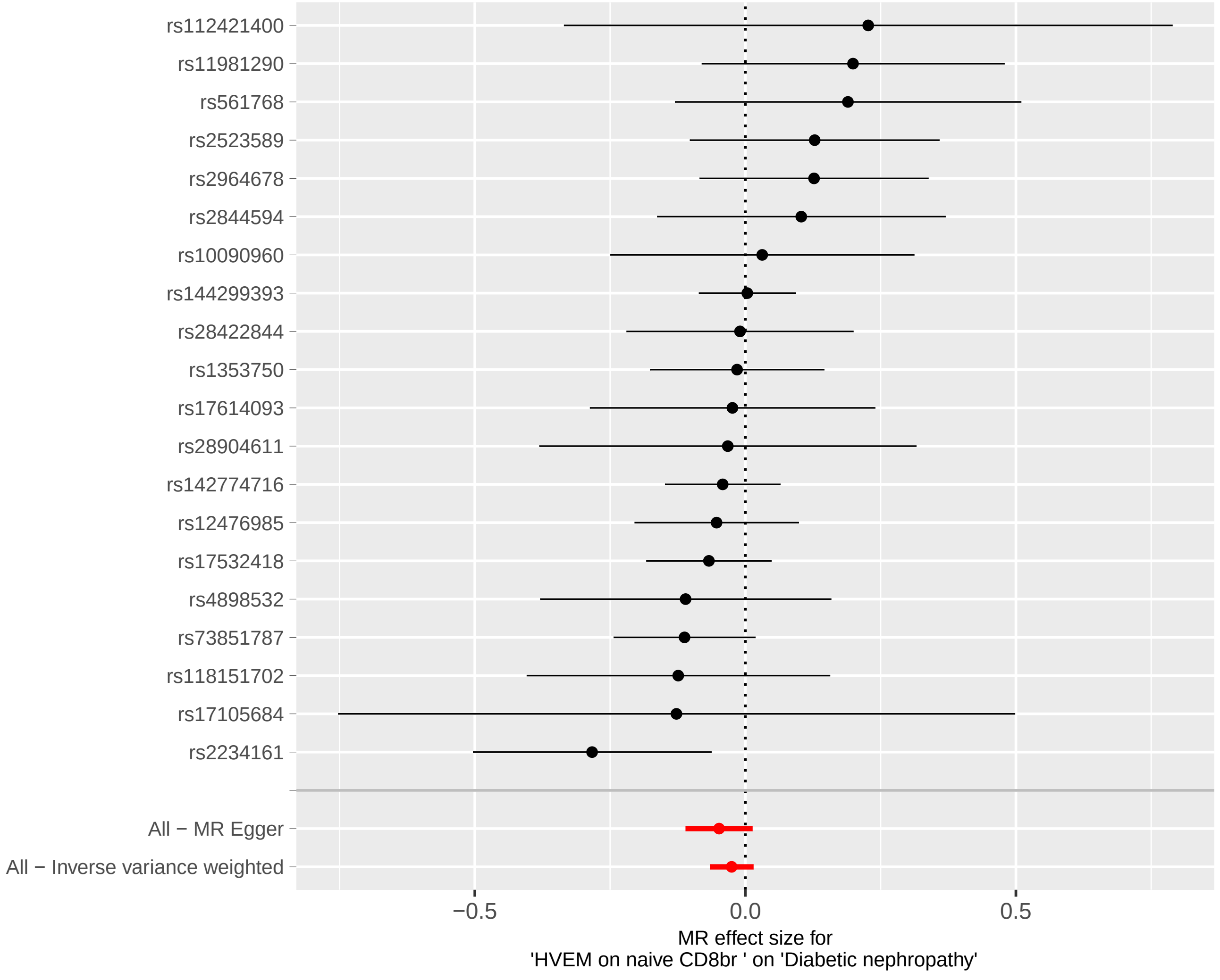
rs112421400
rs11981290
rs561768
rs2523589
rs2964678
rs2844594
rs10090960
rs144299393
rs28422844
rs1353750
rs17614093
rs28904611
rs142774716
rs12476985
rs17532418
rs4898532
rs73851787
rs118151702
rs17105684
rs2234161
All − MR Egger
All − Inverse variance weighted
−0.5
0.0
0.5
MR effect size for
'HVEM on naive CD8br ' on 'Diabetic nephropathy'

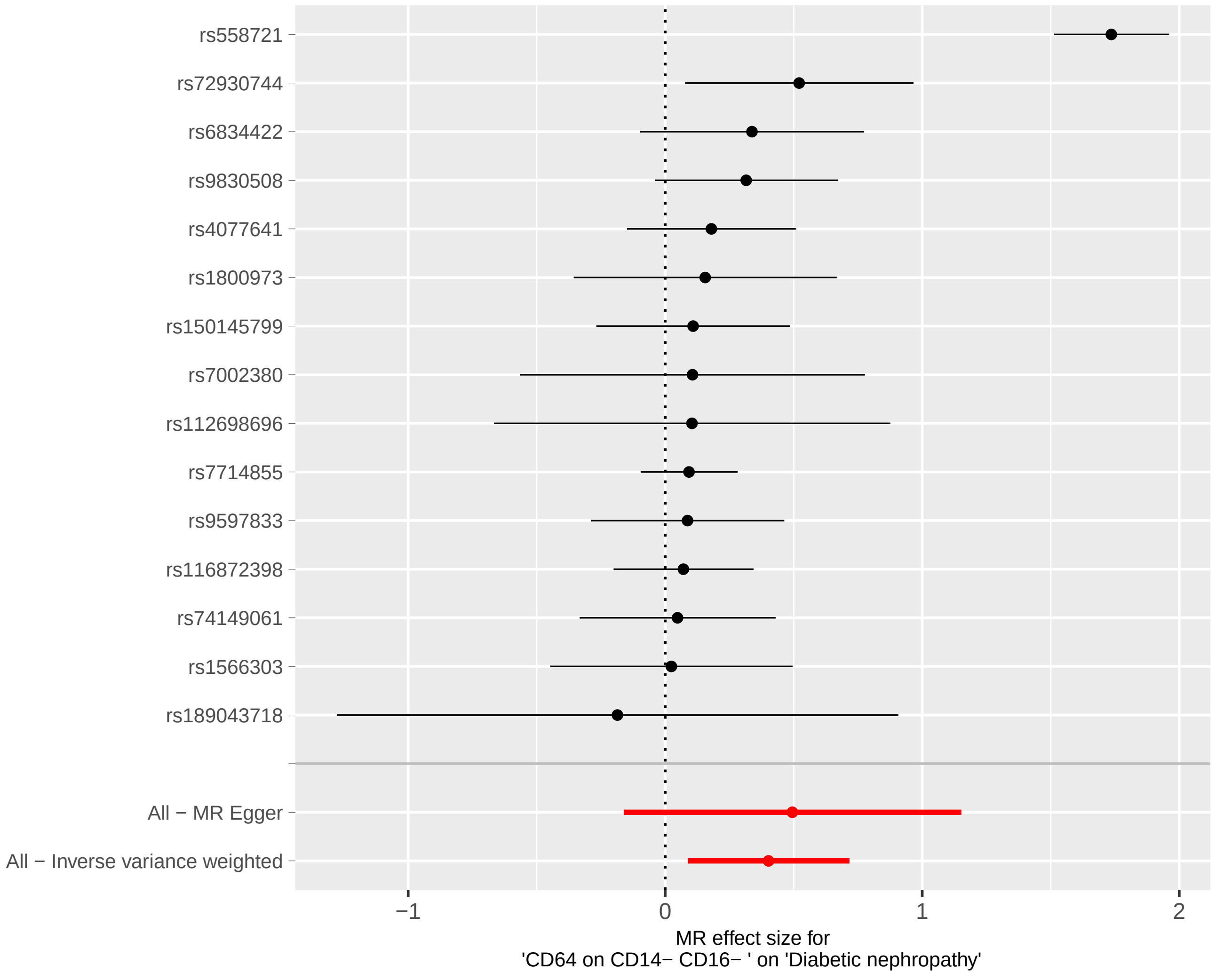
rs558721
rs72930744
rs6834422
rs9830508
rs4077641
rs1800973
rs150145799
rs7002380
rs112698696
rs7714855
rs9597833
rs116872398
rs74149061
rs1566303
rs189043718
All − MR Egger
All − Inverse variance weighted
−1
0
1
2
MR effect size for
'CD64 on CD14− CD16− ' on 'Diabetic nephropathy'

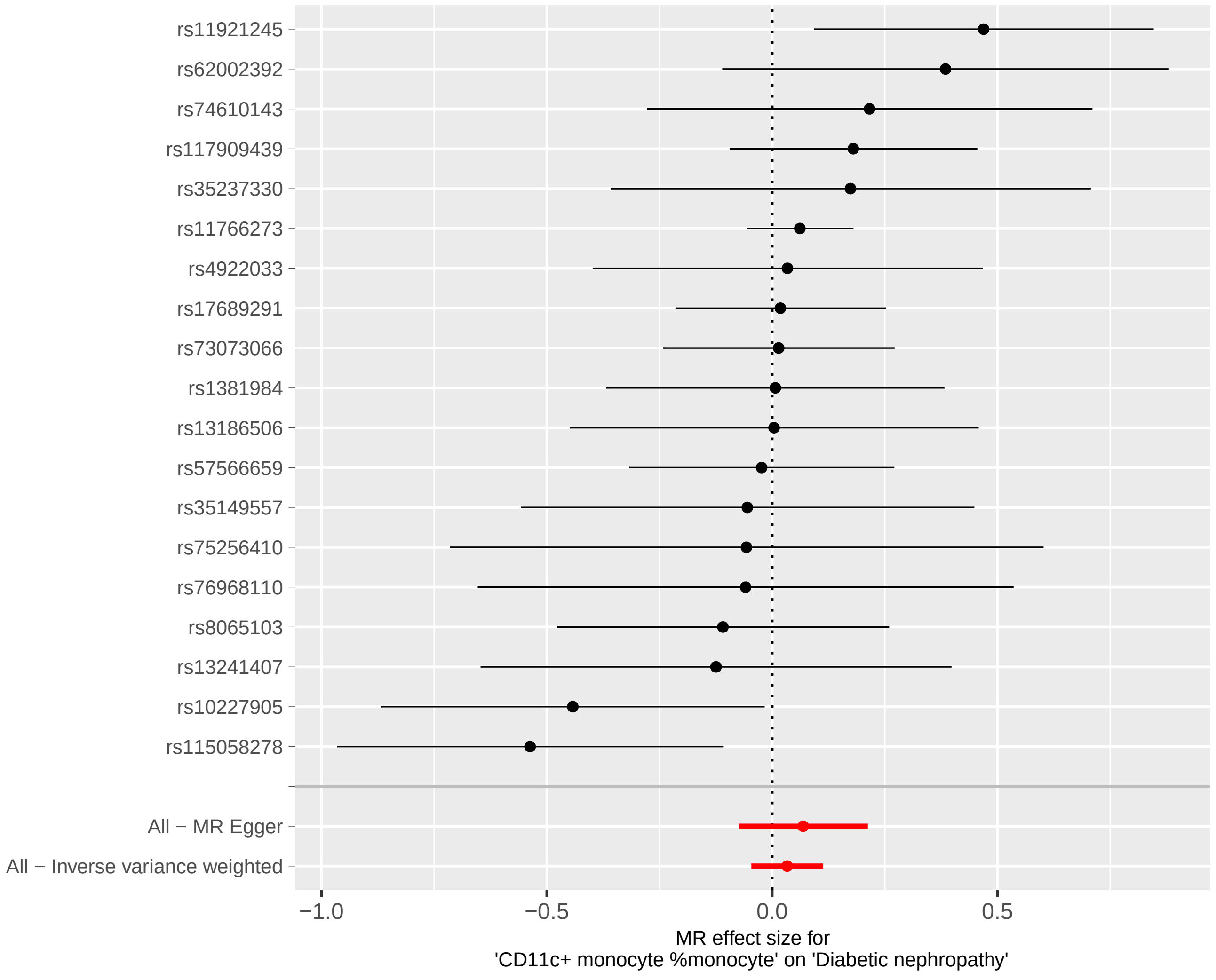
rs11921245
rs62002392
rs74610143
rs117909439
rs35237330
rs11766273
rs4922033
rs17689291
rs73073066
rs1381984
rs13186506
rs57566659
rs35149557
rs75256410
rs76968110
rs8065103
rs13241407
rs10227905
rs115058278
All − MR Egger
All − Inverse variance weighted
−1.0
−0.5
0.0
0.5
MR effect size for
'CD11c+ monocyte %monocyte' on 'Diabetic nephropathy'

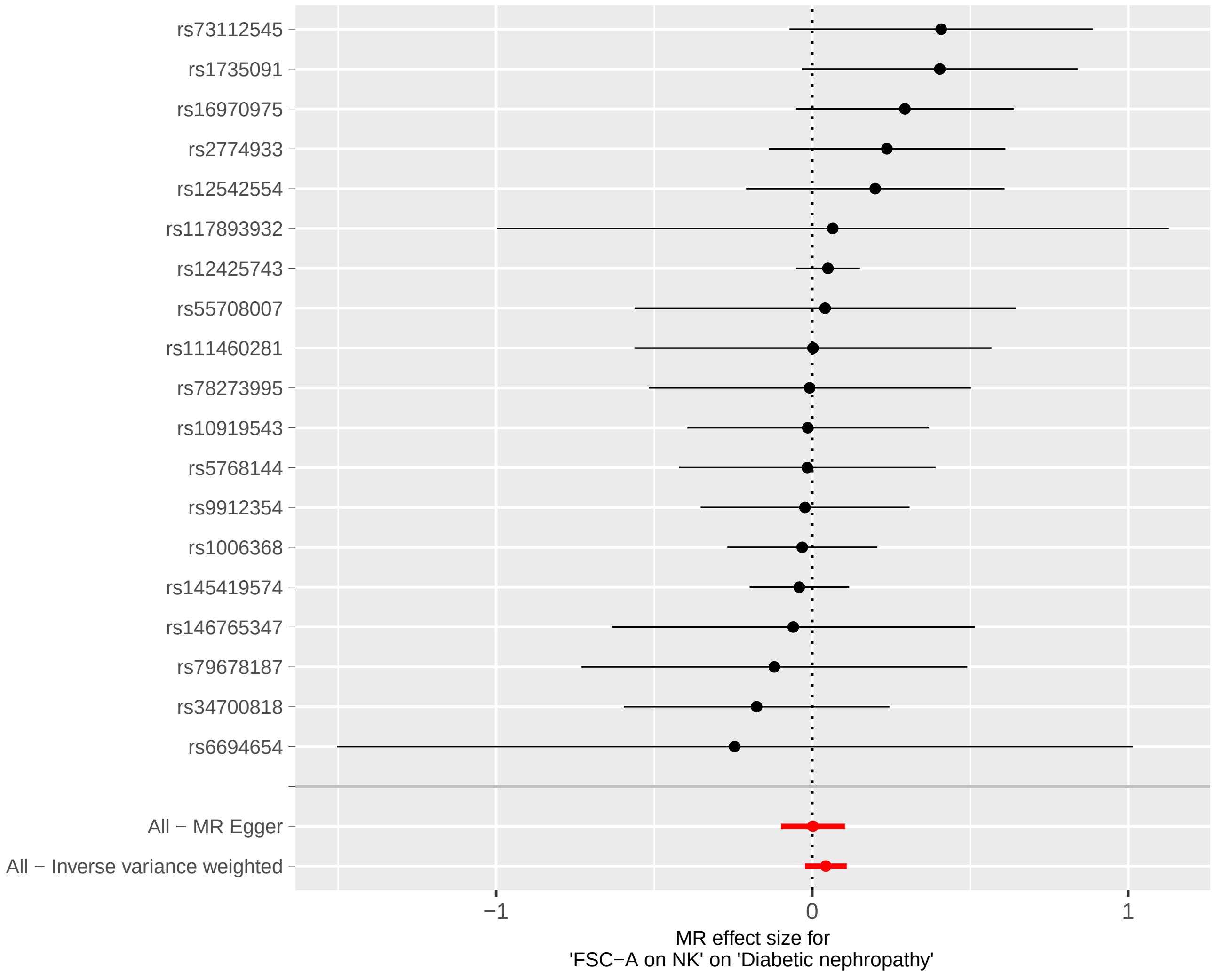
rs73112545
rs1735091
rs16970975
rs2774933
rs12542554
rs117893932
rs12425743
rs55708007
rs111460281
rs78273995
rs10919543
rs5768144
rs9912354
rs1006368
rs145419574
rs146765347
rs79678187
rs34700818
rs6694654
All − MR Egger
All − Inverse variance weighted
−1
0
1
MR effect size for
'FSC−A on NK' on 'Diabetic nephropathy'

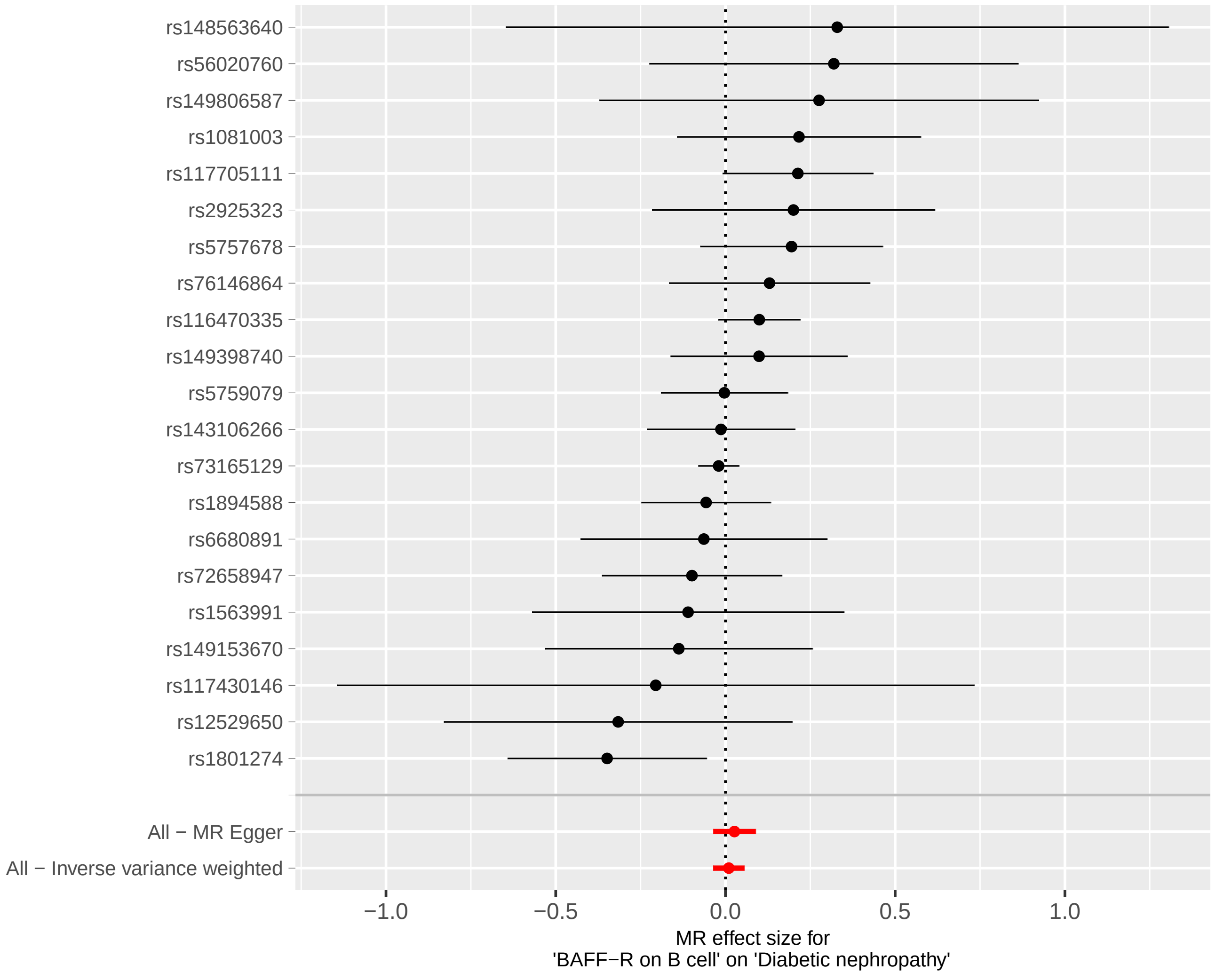
rs148563640
rs56020760
rs149806587
rs1081003
rs117705111
rs2925323
rs5757678
rs76146864
rs116470335
rs149398740
rs5759079
rs143106266
rs73165129
rs1894588
rs6680891
rs72658947
rs1563991
rs149153670
rs117430146
rs12529650
rs1801274
All − MR Egger
All − Inverse variance weighted
−1.0
−0.5
0.0
0.5
1.0
MR effect size for
'BAFF−R on B cell' on 'Diabetic nephropathy'

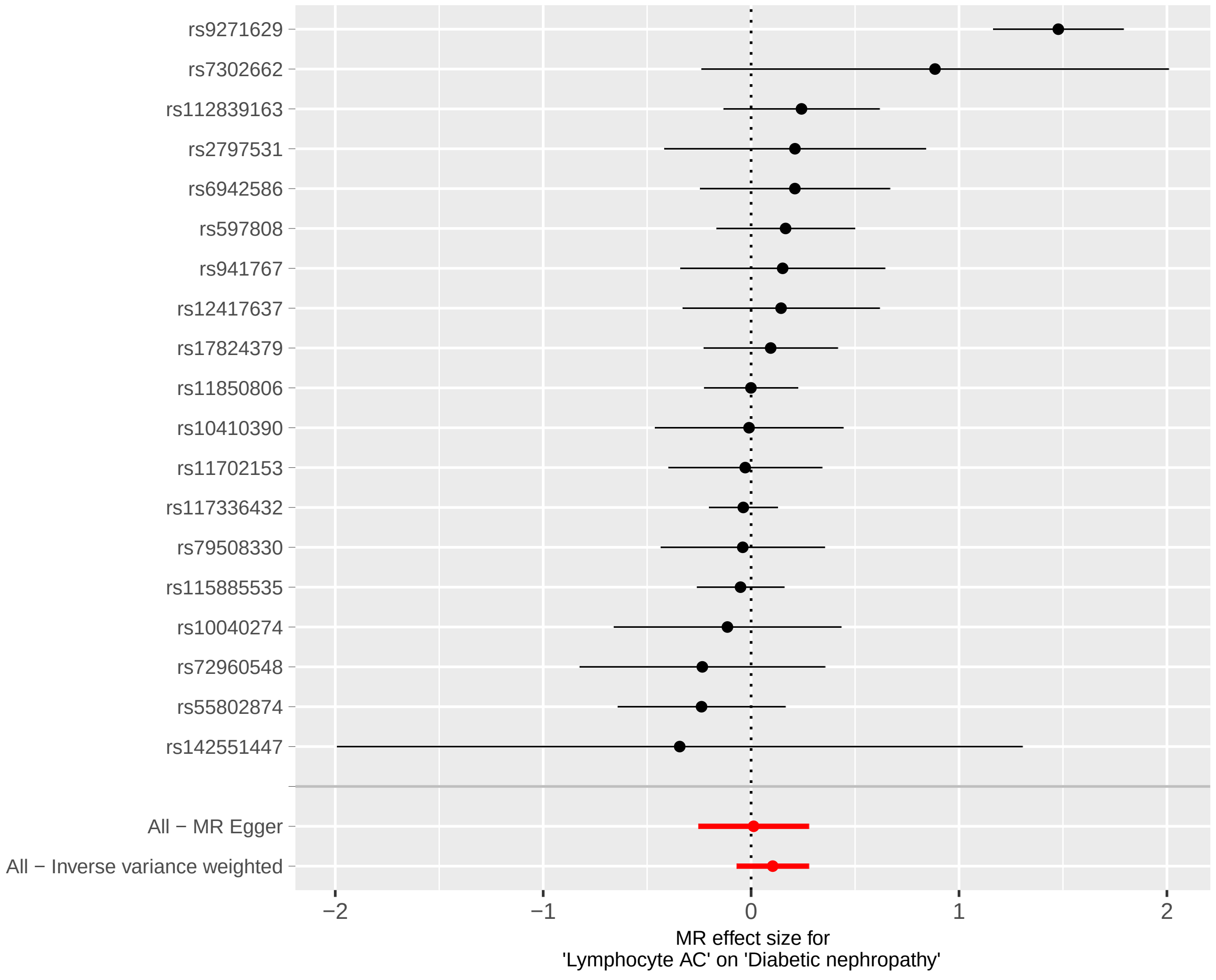
rs9271629
rs7302662
rs112839163
rs2797531
rs6942586
rs597808
rs941767
rs12417637
rs17824379
rs11850806
rs10410390
rs11702153
rs117336432
rs79508330
rs115885535
rs10040274
rs72960548
rs55802874
rs142551447
All − MR Egger
All − Inverse variance weighted
−2
−1
0
1
2
MR effect size for
'Lymphocyte AC' on 'Diabetic nephropathy'

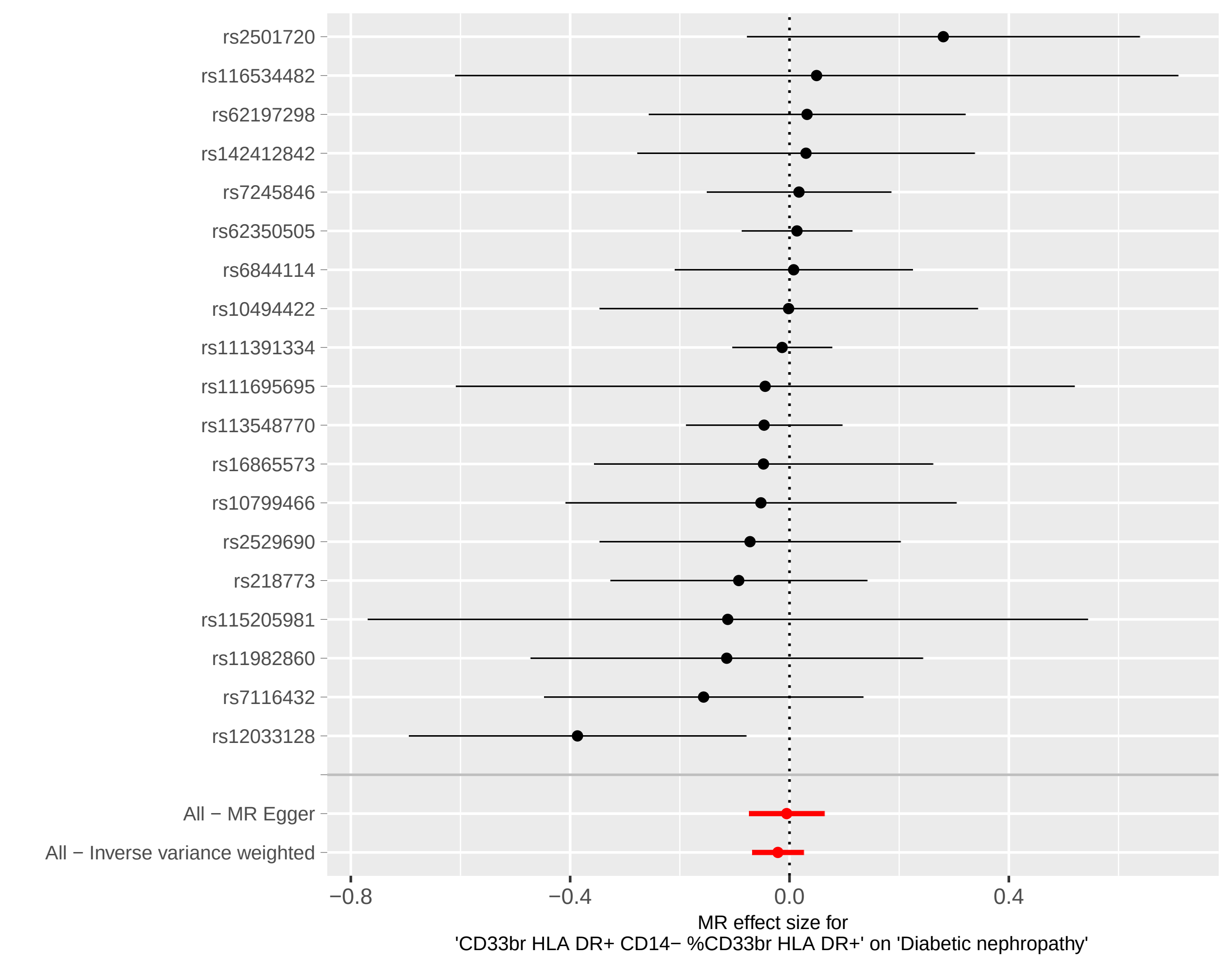
rs2501720
rs116534482
rs62197298
rs142412842
rs7245846
rs62350505
rs6844114
rs10494422
rs111391334
rs111695695
rs113548770
rs16865573
rs10799466
rs2529690
rs218773
rs115205981
rs11982860
rs7116432
rs12033128
All − MR Egger
All − Inverse variance weighted
−0.8
−0.4
0.0
0.4
MR effect size for
'CD33br HLA DR+ CD14− %CD33br HLA DR+' on 'Diabetic nephropathy'

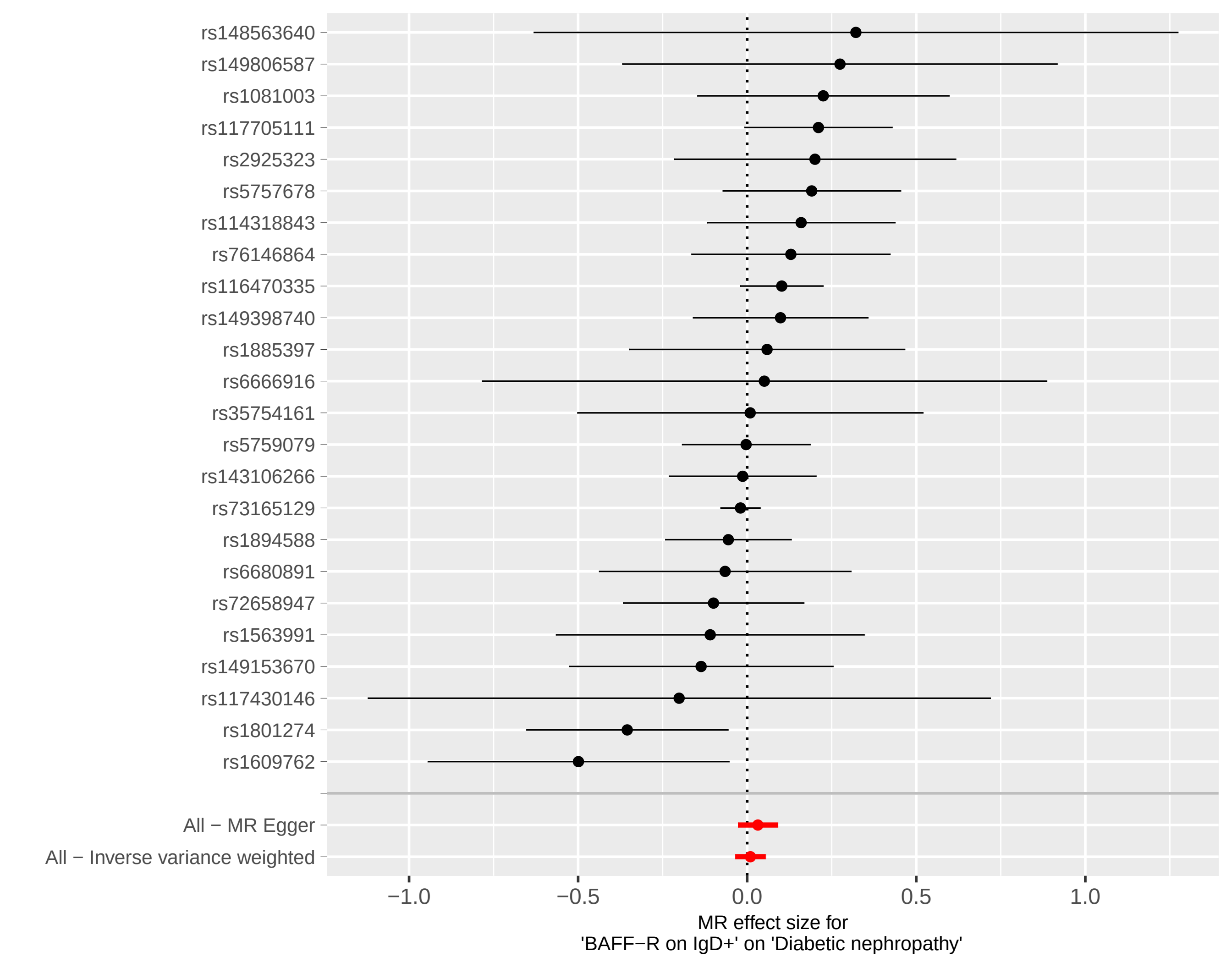
rs148563640
rs149806587
rs1081003
rs117705111
rs2925323
rs5757678
rs114318843
rs76146864
rs116470335
rs149398740
rs1885397
rs6666916
rs35754161
rs5759079
rs143106266
rs73165129
rs1894588
rs6680891
rs72658947
rs1563991
rs149153670
rs117430146
rs1801274
rs1609762
All − MR Egger
All − Inverse variance weighted
−1.0
−0.5
0.0
0.5
1.0
MR effect size for
'BAFF−R on IgD+' on 'Diabetic nephropathy'

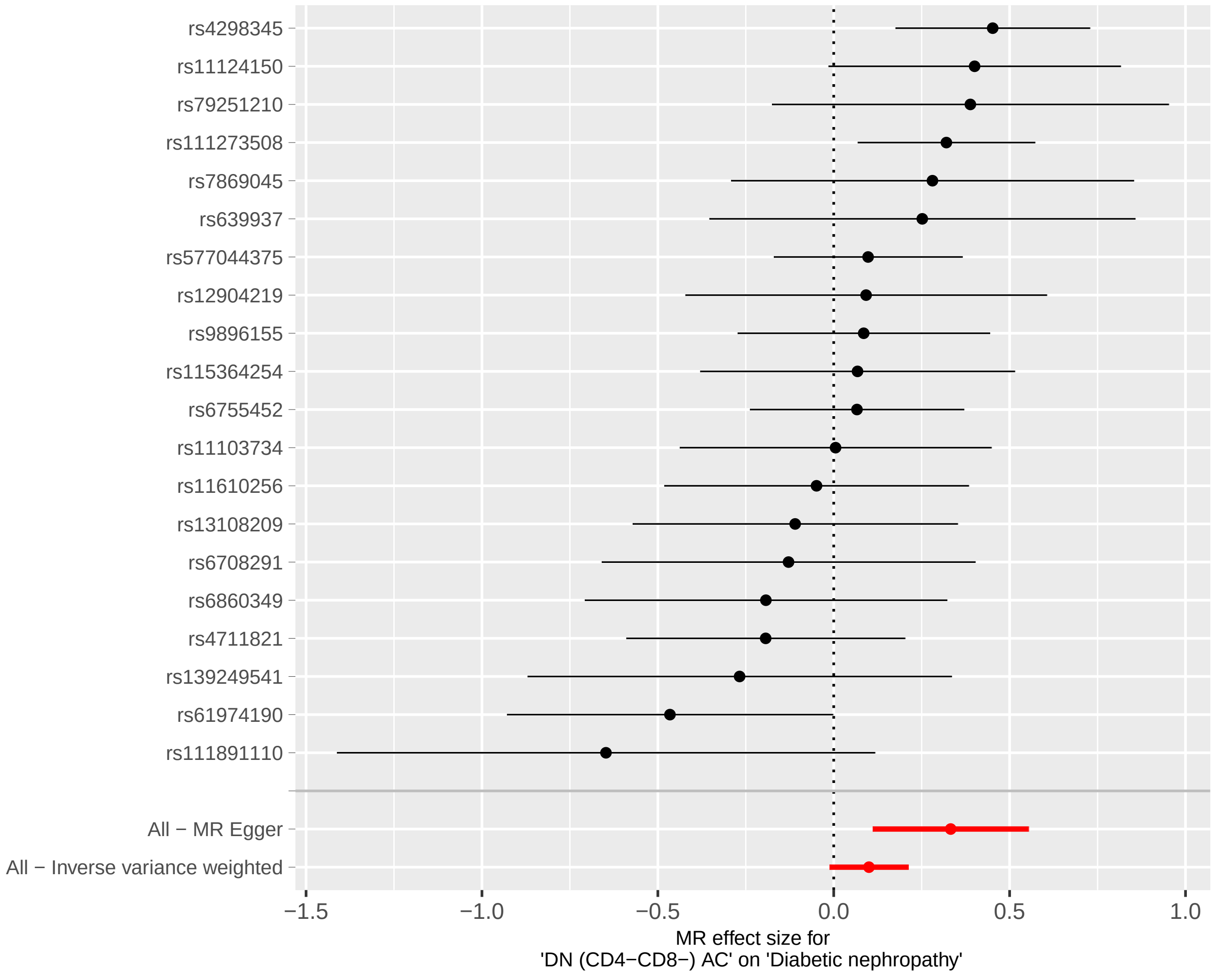

rs4298345
rs11124150
rs79251210
rs111273508
rs7869045
rs639937
rs577044375
rs12904219
rs9896155
rs115364254
rs6755452
rs11103734
rs11610256
rs13108209
rs6708291
rs6860349
rs4711821
rs139249541
rs61974190
rs111891110
All − MR Egger
All − Inverse variance weighted
−1.5
−1.0
−0.5
0.0
0.5
1.0
MR effect size for
'DN (CD4−CD8−) AC' on 'Diabetic nephropathy'

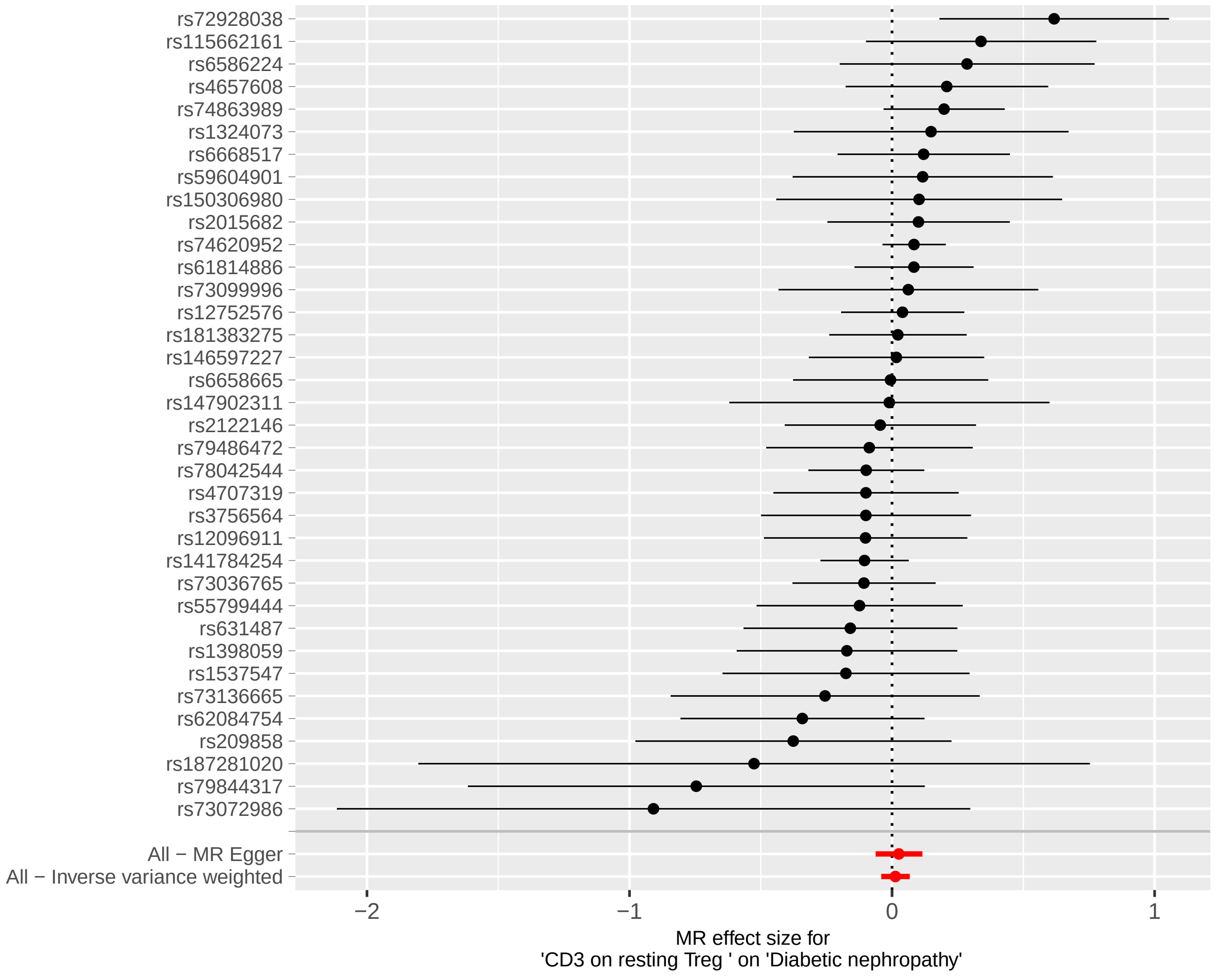
rs72928038
rs115662161
rs6586224
rs4657608
rs74863989
rs1324073
rs6668517
rs59604901
rs150306980
rs2015682
rs74620952
rs61814886
rs73099996
rs12752576
rs181383275
rs146597227
rs6658665
rs147902311
rs2122146
rs79486472
rs78042544
rs4707319
rs3756564
rs12096911
rs141784254
rs73036765
rs55799444
rs631487
rs1398059
rs1537547
rs73136665
rs62084754
rs209858
rs187281020
rs79844317
rs73072986
All − MR Egger
All − Inverse variance weighted
−2
−1
0
1
MR effect size for
'CD3 on resting Treg ' on 'Diabetic nephropathy'

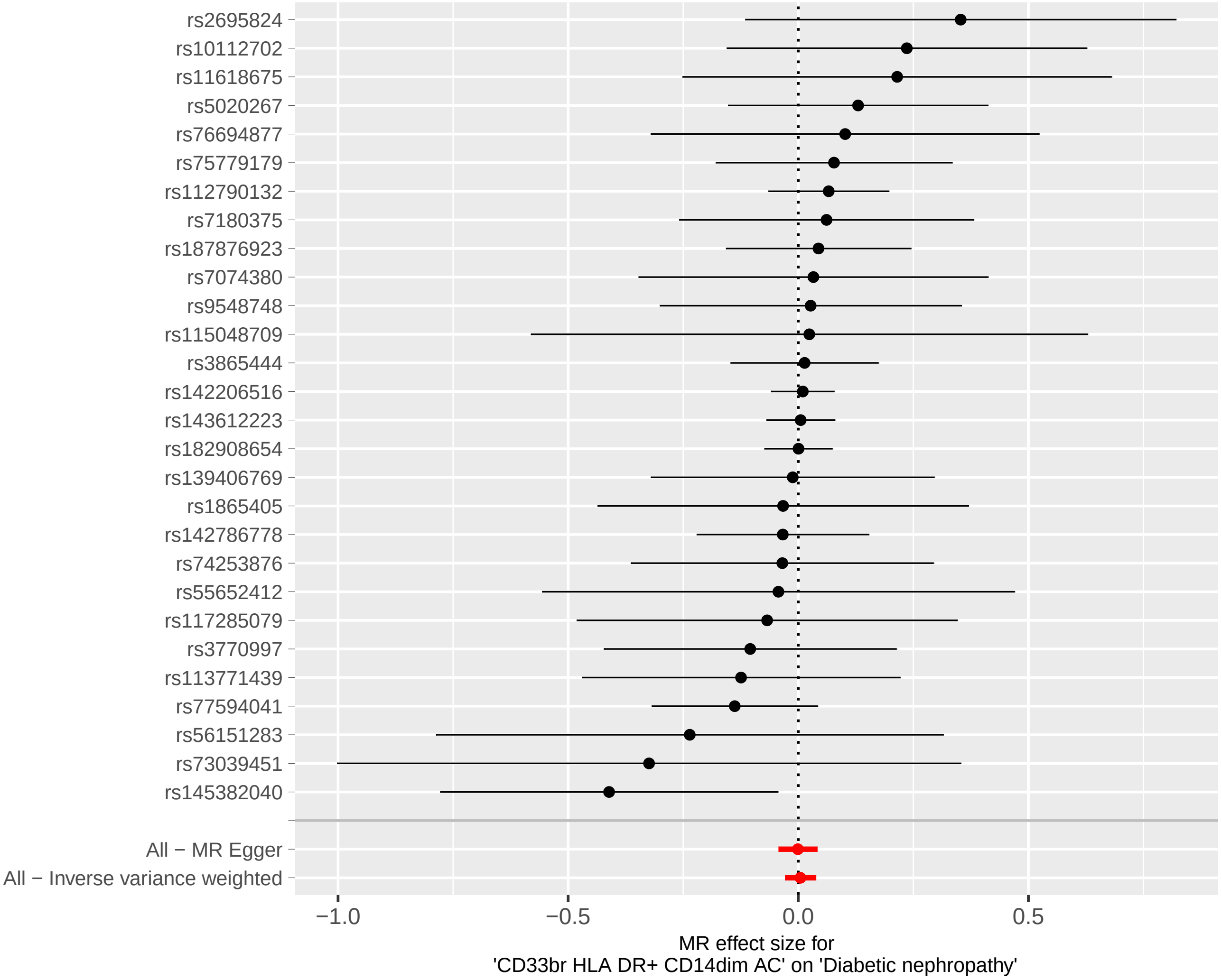
rs2695824
rs10112702
rs11618675
rs5020267
rs76694877
rs75779179
rs112790132
rs7180375
rs187876923
rs7074380
rs9548748
rs115048709
rs3865444
rs142206516
rs143612223
rs182908654
rs139406769
rs1865405
rs142786778
rs74253876
rs55652412
rs117285079
rs3770997
rs113771439
rs77594041
rs56151283
rs73039451
rs145382040
All − MR Egger
All − Inverse variance weighted
−1.0
−0.5
0.0
0.5
MR effect size for
'CD33br HLA DR+ CD14dim AC' on 'Diabetic nephropathy'

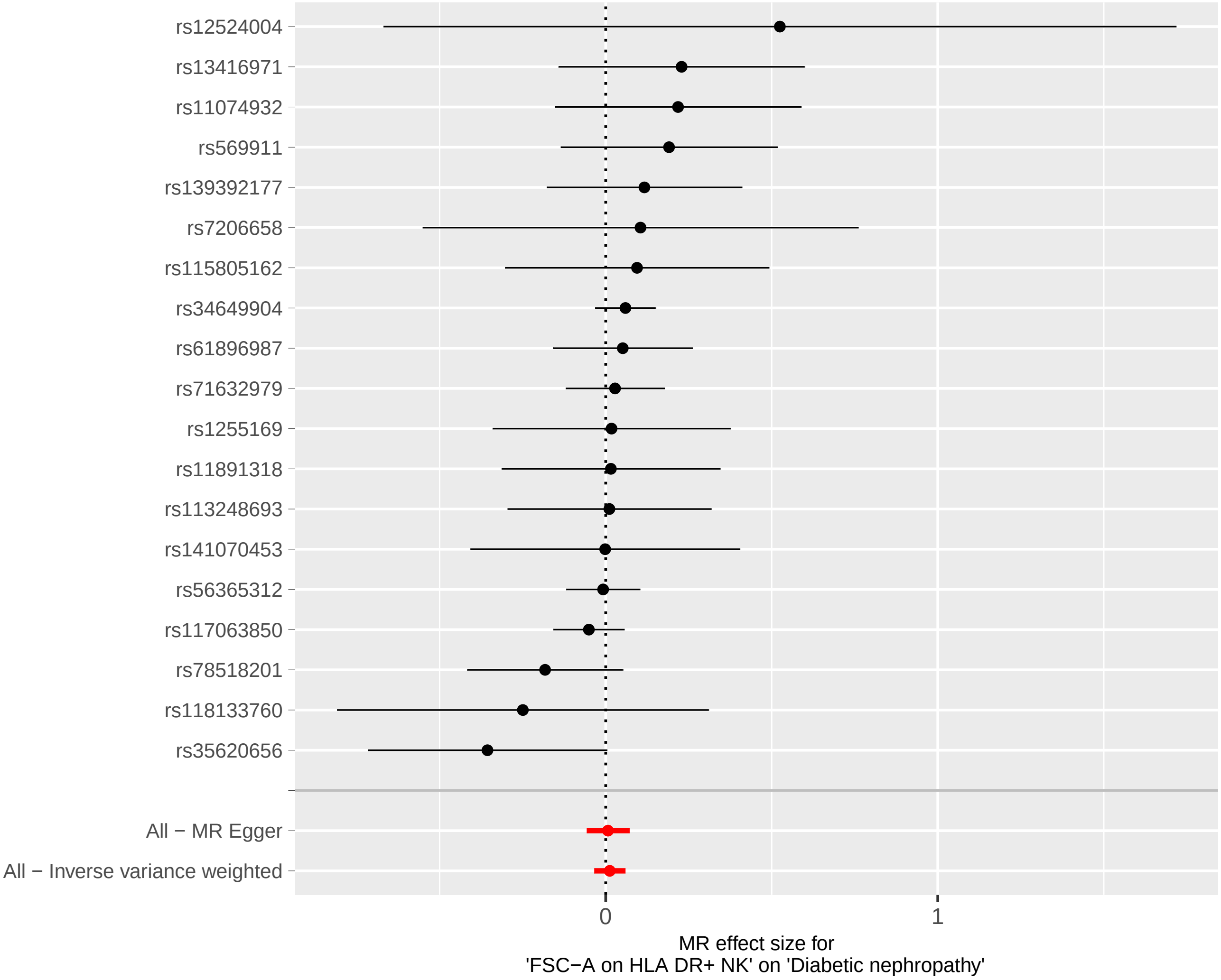
rs12524004
rs13416971
rs11074932
rs569911
rs139392177
rs7206658
rs115805162
rs34649904
rs61896987
rs71632979
rs1255169
rs11891318
rs113248693
rs141070453
rs56365312
rs117063850
rs78518201
rs118133760
rs35620656
All − MR Egger
All − Inverse variance weighted
0
1
MR effect size for
'FSC−A on HLA DR+ NK' on 'Diabetic nephropathy'

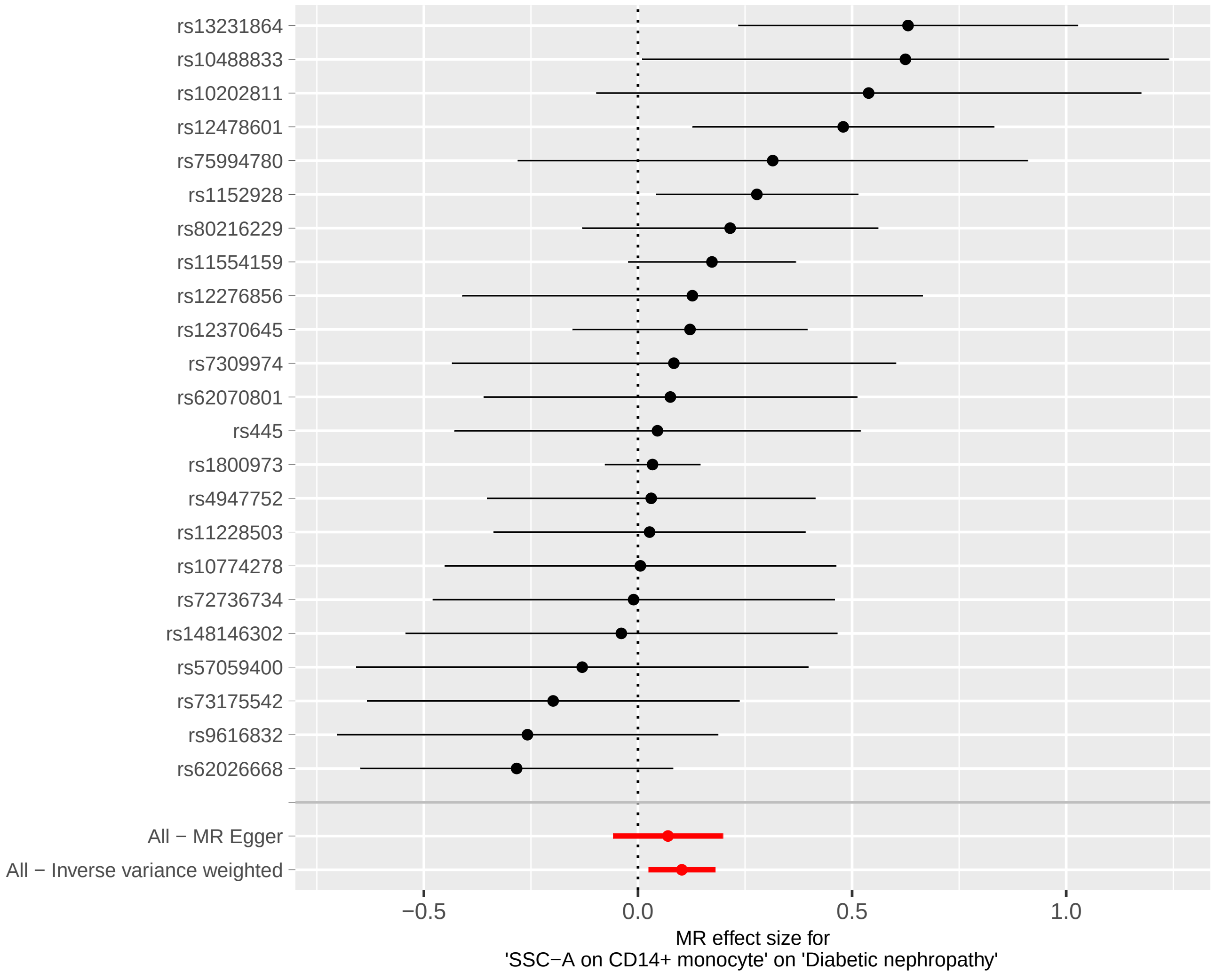
rs13231864
rs10488833
rs10202811
rs12478601
rs75994780
rs1152928
rs80216229
rs11554159
rs12276856
rs12370645
rs7309974
rs62070801
rs445
rs1800973
rs4947752
rs11228503
rs10774278
rs72736734
rs148146302
rs57059400
rs73175542
rs9616832
rs62026668
All − MR Egger
All − Inverse variance weighted
−0.5
0.0
0.5
1.0
MR effect size for
'SSC−A on CD14+ monocyte' on 'Diabetic nephropathy'

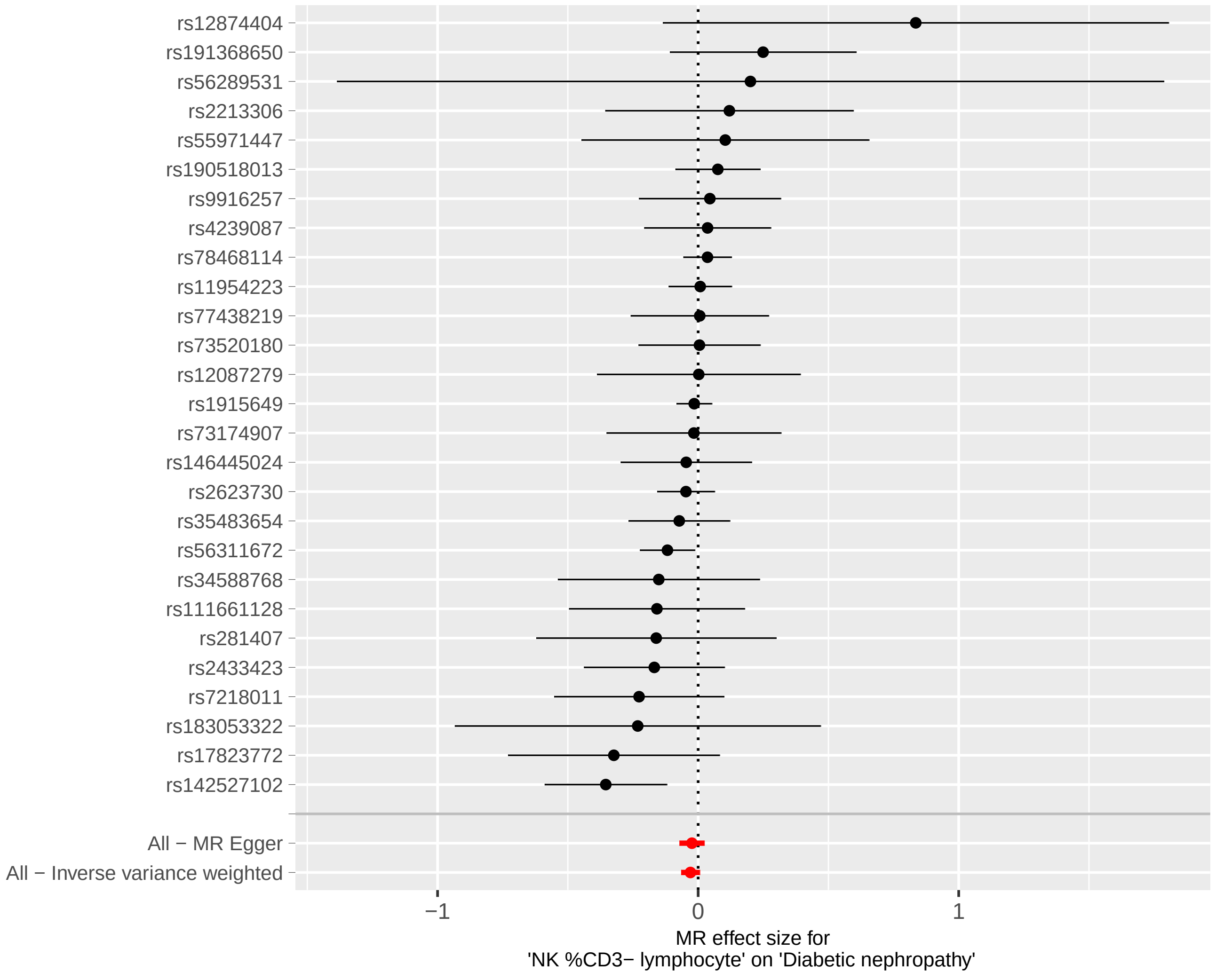
rs12874404
rs191368650
rs56289531
rs2213306
rs55971447
rs190518013
rs9916257
rs4239087
rs78468114
rs11954223
rs77438219
rs73520180
rs12087279
rs1915649
rs73174907
rs146445024
rs2623730
rs35483654
rs56311672
rs34588768
rs111661128
rs281407
rs2433423
rs7218011
rs183053322
rs17823772
rs142527102
All − MR Egger
All − Inverse variance weighted
−1
0
1
MR effect size for
'NK %CD3− lymphocyte' on 'Diabetic nephropathy'

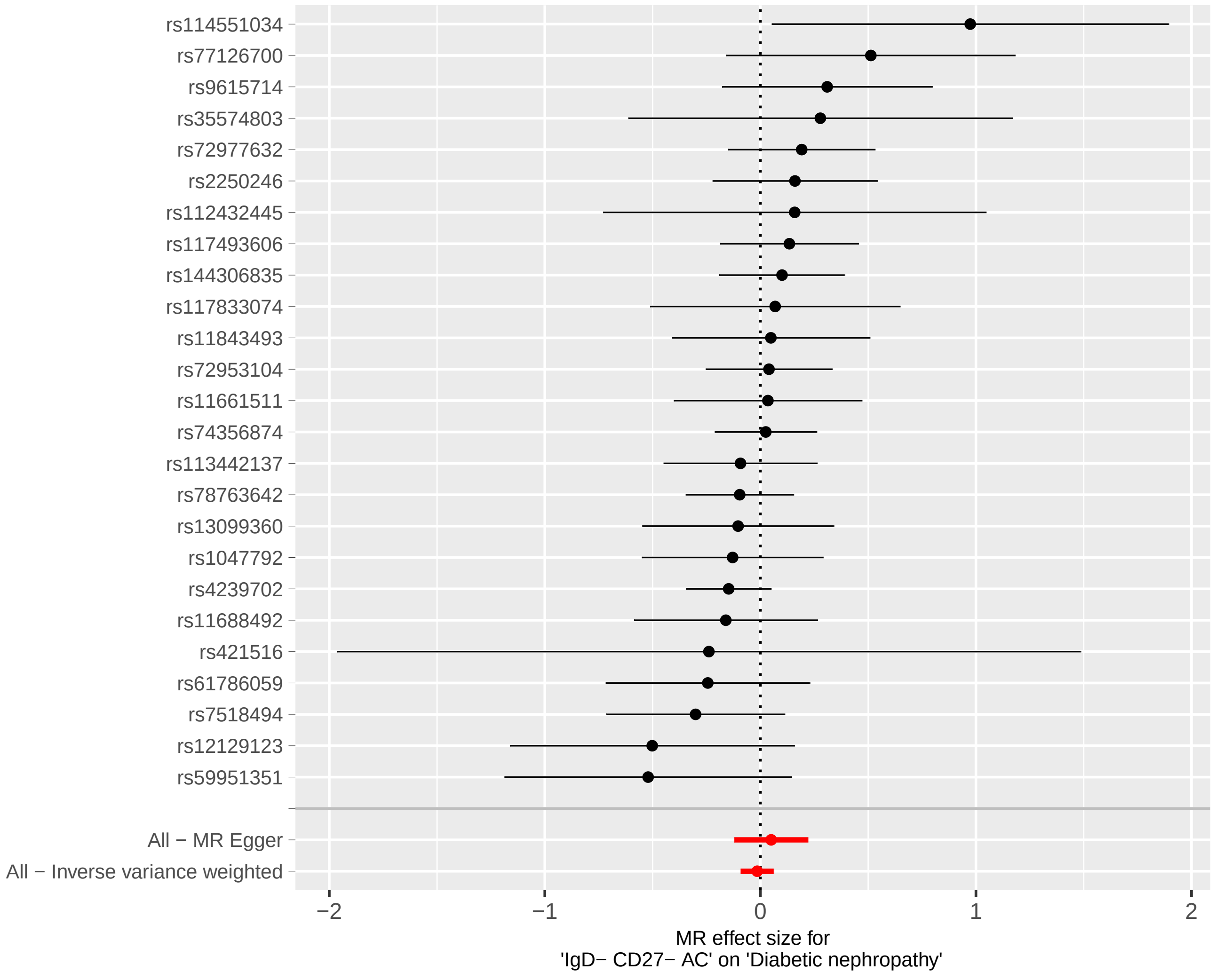
rs114551034
rs77126700
rs9615714
rs35574803
rs72977632
rs2250246
rs112432445
rs117493606
rs144306835
rs117833074
rs11843493
rs72953104
rs11661511
rs74356874
rs113442137
rs78763642
rs13099360
rs1047792
rs4239702
rs11688492
rs421516
rs61786059
rs7518494
rs12129123
rs59951351
All − MR Egger
All − Inverse variance weighted
−2
−1
0
1
2
MR effect size for
'IgD− CD27− AC' on 'Diabetic nephropathy'

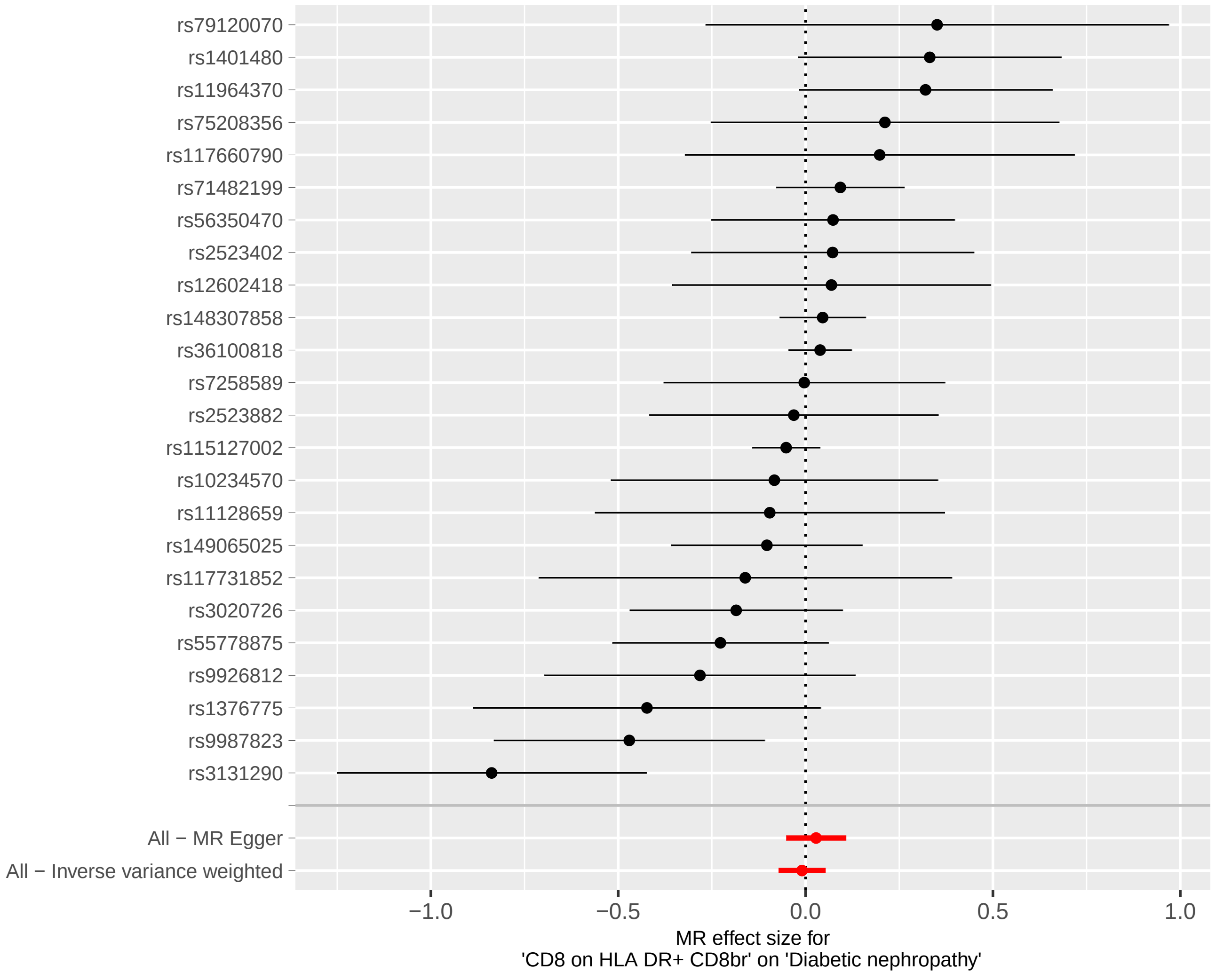
rs79120070
rs1401480
rs11964370
rs75208356
rs117660790
rs71482199
rs56350470
rs2523402
rs12602418
rs148307858
rs36100818
rs7258589
rs2523882
rs115127002
rs10234570
rs11128659
rs149065025
rs117731852
rs3020726
rs55778875
rs9926812
rs1376775
rs9987823
rs3131290
All − MR Egger
All − Inverse variance weighted
−1.0
−0.5
0.0
0.5
1.0
MR effect size for
'CD8 on HLA DR+ CD8br' on 'Diabetic nephropathy'

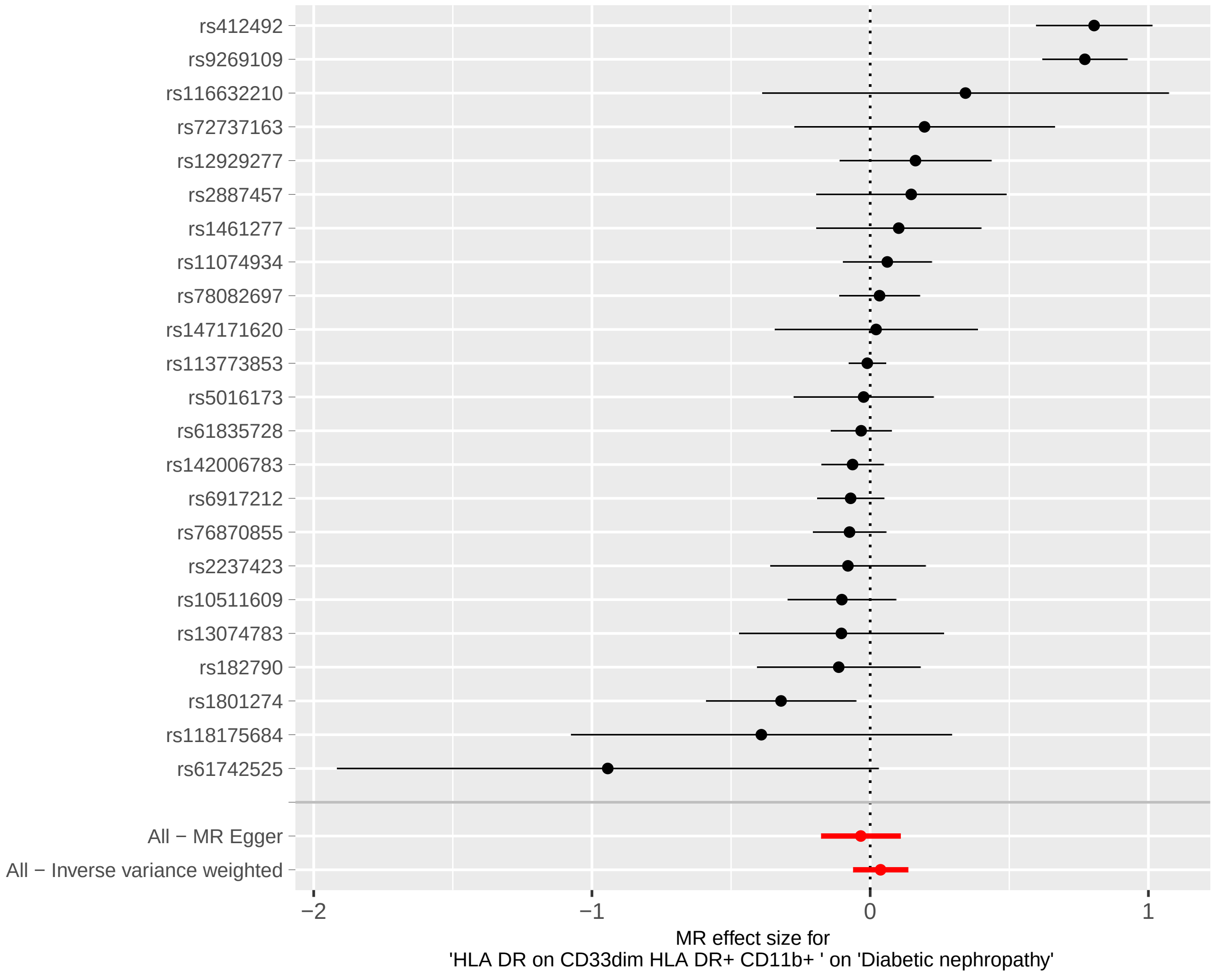

rs412492
rs9269109
rs116632210
rs72737163
rs12929277
rs2887457
rs1461277
rs11074934
rs78082697
rs147171620
rs113773853
rs5016173
rs61835728
rs142006783
rs6917212
rs76870855
rs2237423
rs10511609
rs13074783
rs182790
rs1801274
rs118175684
rs61742525
All − MR Egger
All − Inverse variance weighted
−2
−1
0
1
MR effect size for
'HLA DR on CD33dim HLA DR+ CD11b+ ' on 'Diabetic nephropathy'

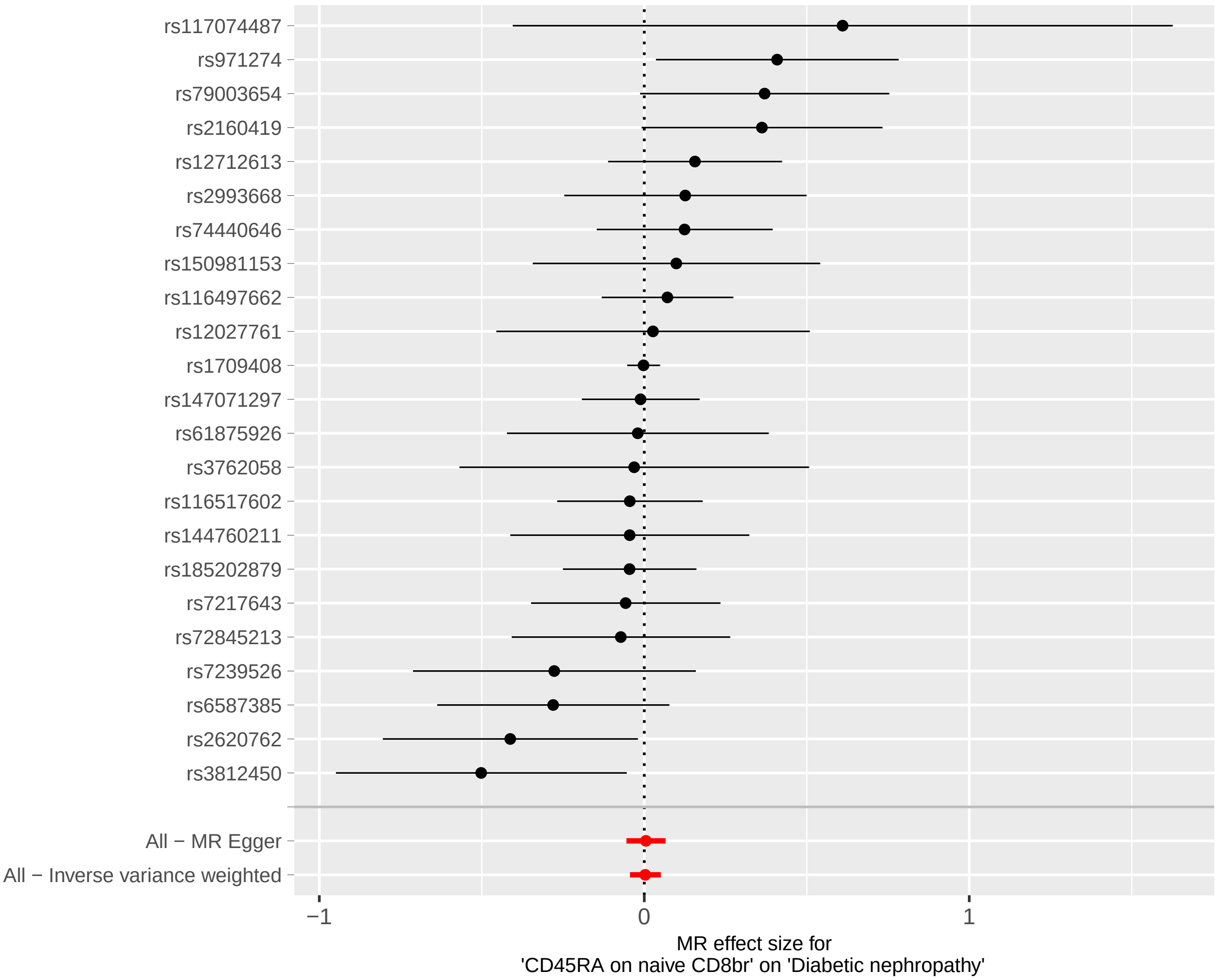
rs117074487
rs971274
rs79003654
rs2160419
rs12712613
rs2993668
rs74440646
rs150981153
rs116497662
rs12027761
rs1709408
rs147071297
rs61875926
rs3762058
rs116517602
rs144760211
rs185202879
rs7217643
rs72845213
rs7239526
rs6587385
rs2620762
rs3812450
All − MR Egger
All − Inverse variance weighted
−1
0
1
MR effect size for
'CD45RA on naive CD8br' on 'Diabetic nephropathy'

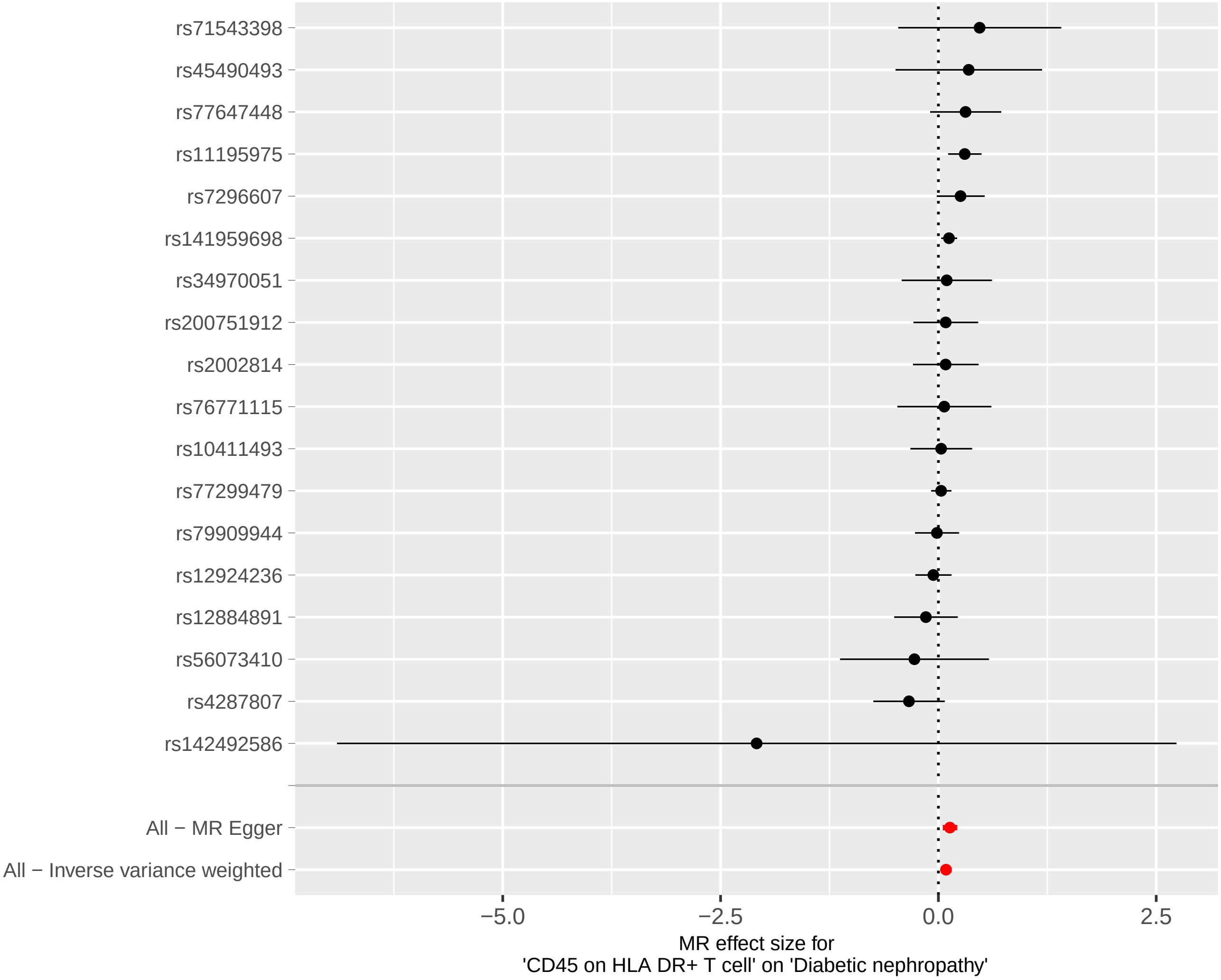
rs71543398
rs45490493
rs77647448
rs11195975
rs7296607
rs141959698
rs34970051
rs200751912
rs2002814
rs76771115
rs10411493
rs77299479
rs79909944
rs12924236
rs12884891
rs56073410
rs4287807
rs142492586
All − MR Egger
All − Inverse variance weighted
−5.0
−2.5
0.0
2.5
MR effect size for
'CD45 on HLA DR+ T cell' on 'Diabetic nephropathy'

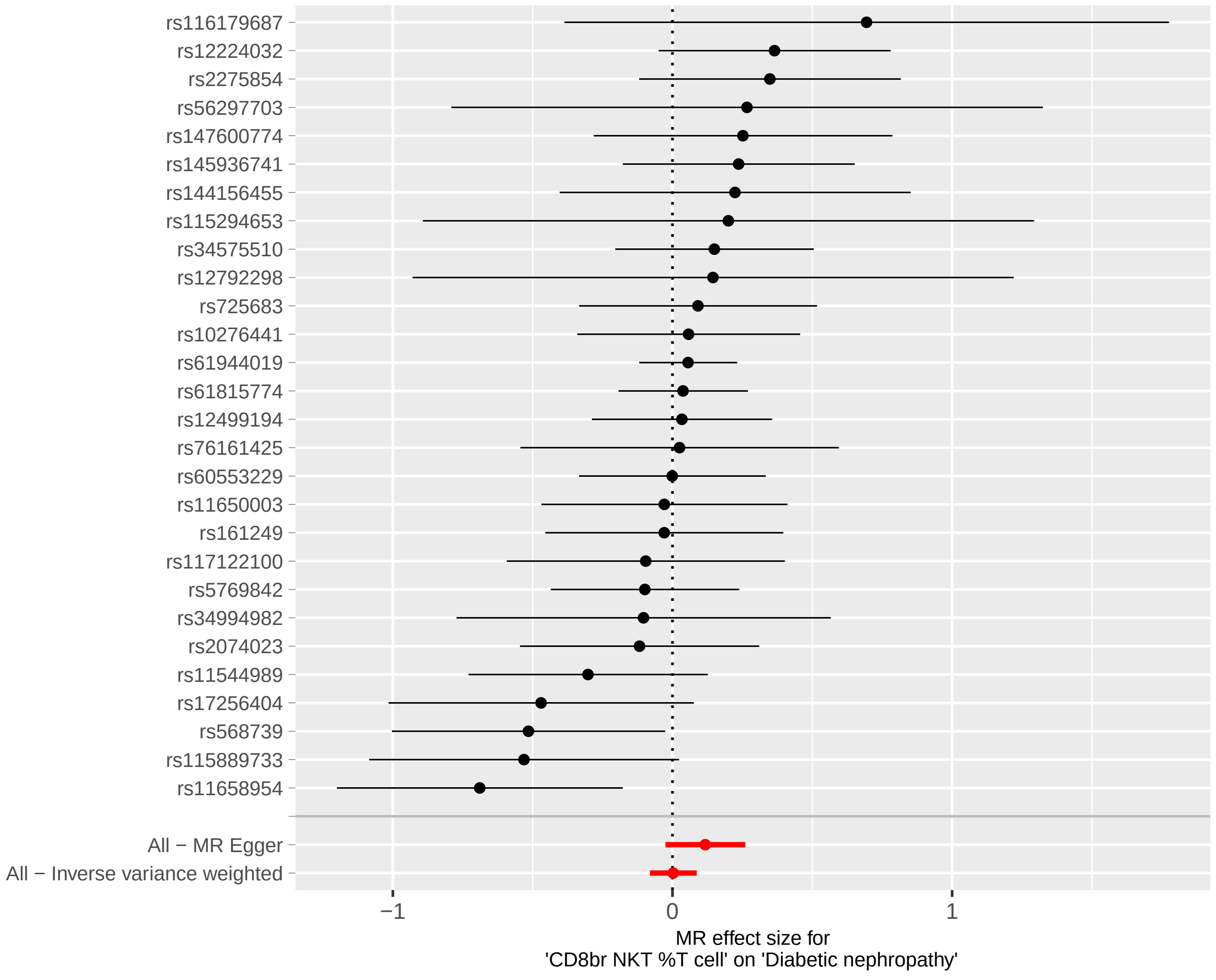
rs116179687
rs12224032
rs2275854
rs56297703
rs147600774
rs145936741
rs144156455
rs115294653
rs34575510
rs12792298
rs725683
rs10276441
rs61944019
rs61815774
rs12499194
rs76161425
rs60553229
rs11650003
rs161249
rs117122100
rs5769842
rs34994982
rs2074023
rs11544989
rs17256404
rs568739
rs115889733
rs11658954
All − MR Egger
All − Inverse variance weighted
−1
0
1
MR effect size for
'CD8br NKT %T cell' on 'Diabetic nephropathy'

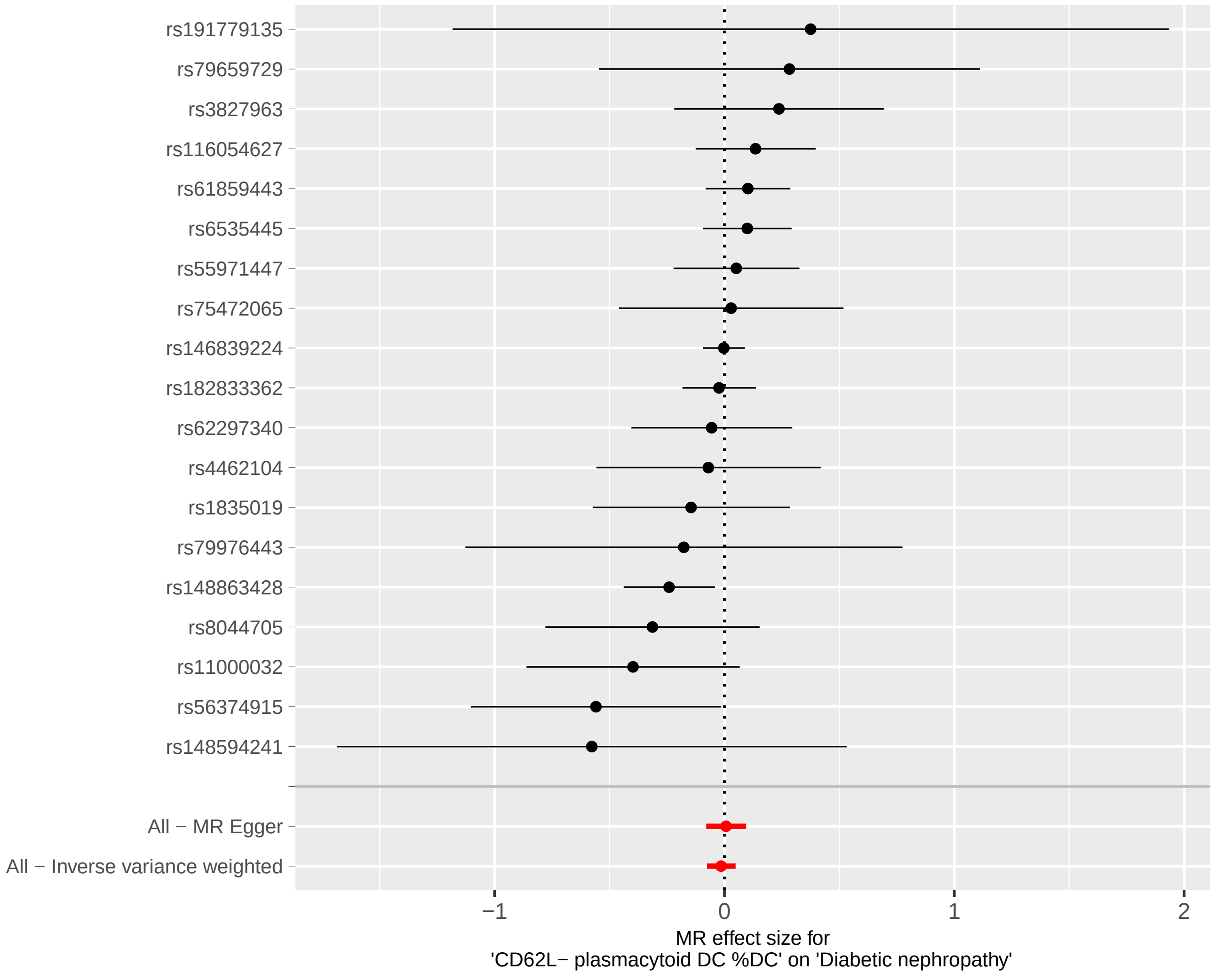

rs191779135
rs79659729
rs3827963
rs116054627
rs61859443
rs6535445
rs55971447
rs75472065
rs146839224
rs182833362
rs62297340
rs4462104
rs1835019
rs79976443
rs148863428
rs8044705
rs11000032
rs56374915
rs148594241
All − MR Egger
All − Inverse variance weighted
−1
0
1
2
MR effect size for
'CD62L− plasmacytoid DC %DC' on 'Diabetic nephropathy'

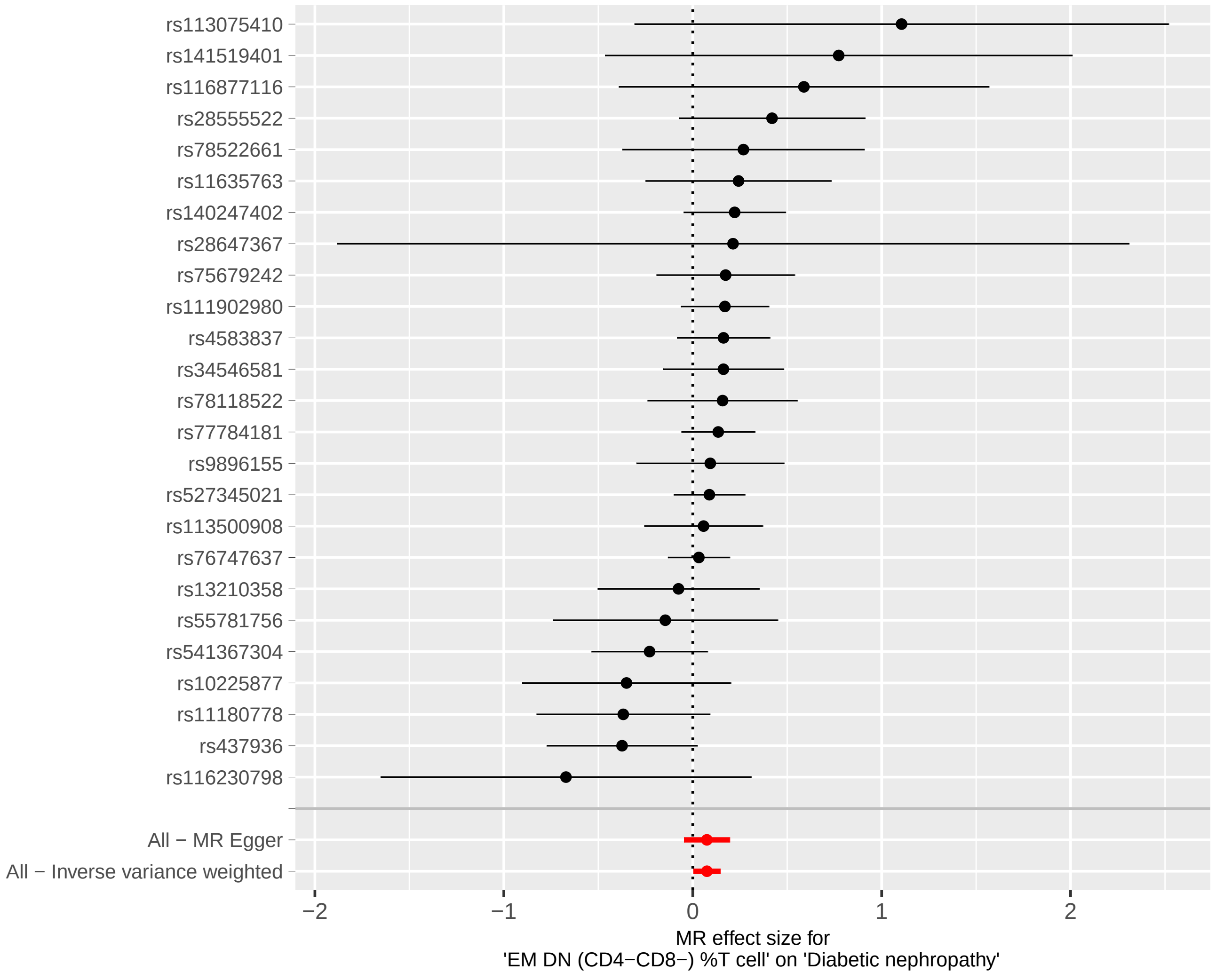
rs113075410
rs141519401
rs116877116
rs28555522
rs78522661
rs11635763
rs140247402
rs28647367
rs75679242
rs111902980
rs4583837
rs34546581
rs78118522
rs77784181
rs9896155
rs527345021
rs113500908
rs76747637
rs13210358
rs55781756
rs541367304
rs10225877
rs11180778
rs437936
rs116230798
All − MR Egger
All − Inverse variance weighted
−2
−1
0
1
2
MR effect size for
'EM DN (CD4−CD8−) %T cell' on 'Diabetic nephropathy'

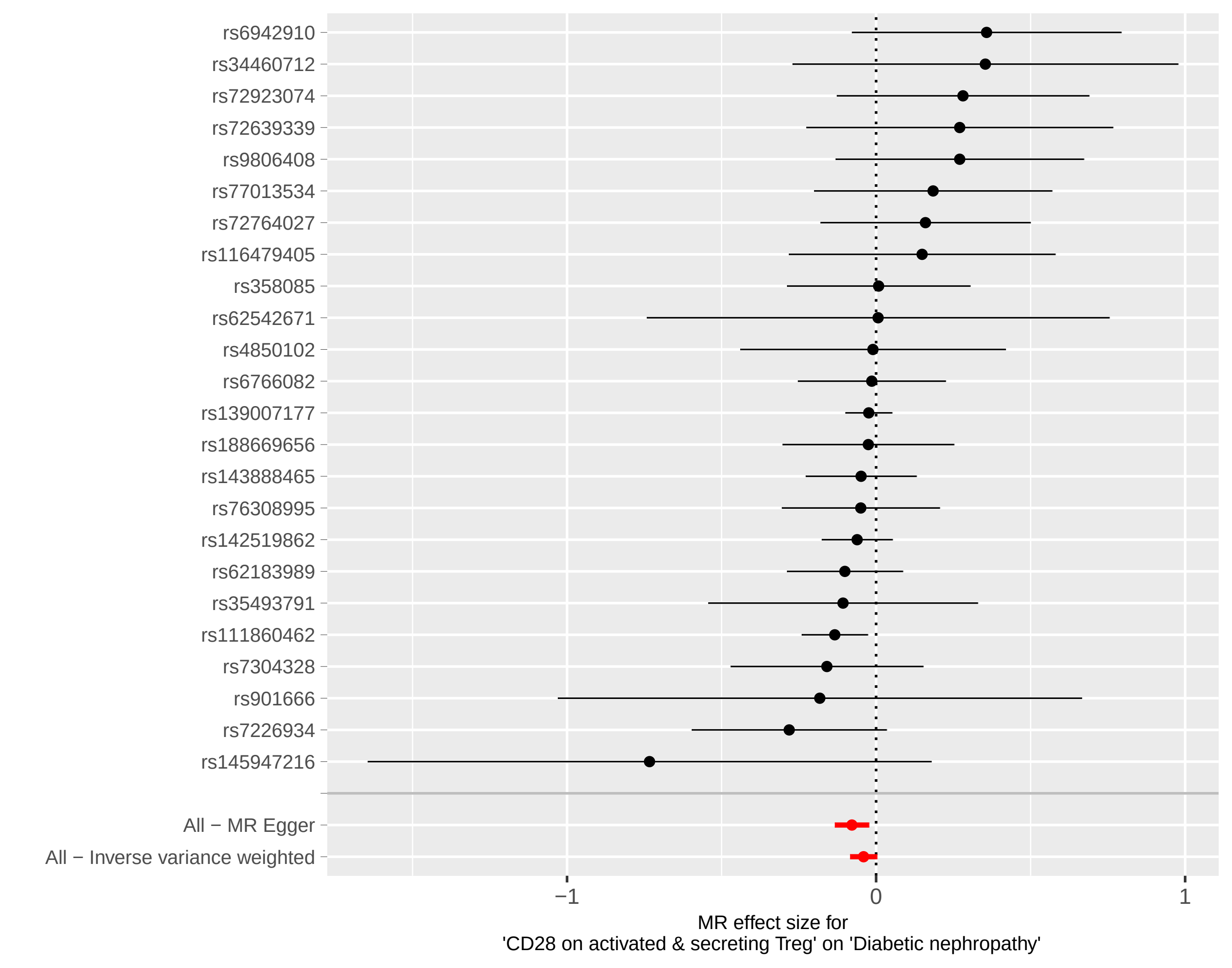
rs6942910
rs34460712
rs72923074
rs72639339
rs9806408
rs77013534
rs72764027
rs116479405
rs358085
rs62542671
rs4850102
rs6766082
rs139007177
rs188669656
rs143888465
rs76308995
rs142519862
rs62183989
rs35493791
rs111860462
rs7304328
rs901666
rs7226934
rs145947216
All − MR Egger
All − Inverse variance weighted
−1
0
1
MR effect size for
'CD28 on activated & secreting Treg' on 'Diabetic nephropathy'

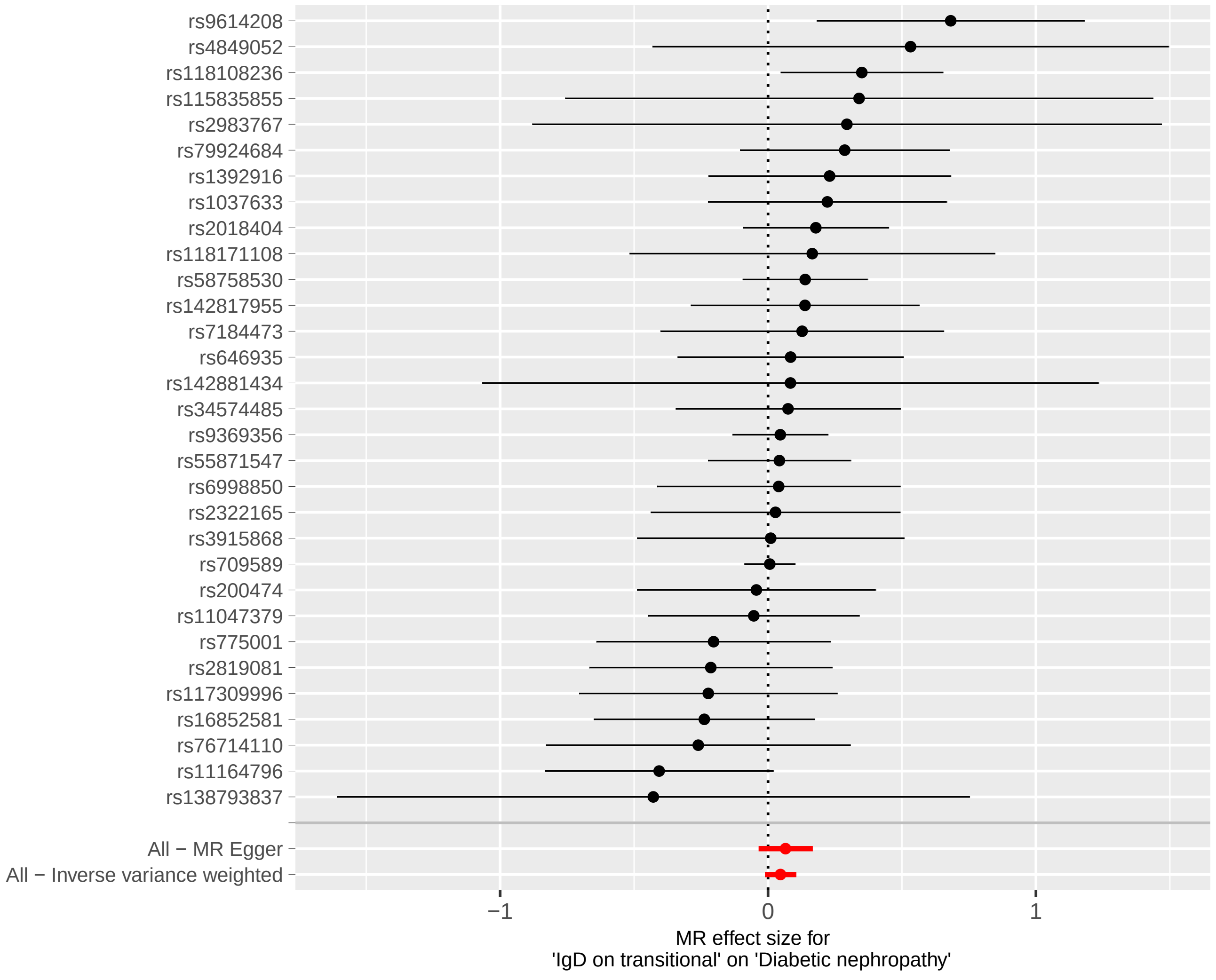

rs9614208
rs4849052
rs118108236
rs115835855
rs2983767
rs79924684
rs1392916
rs1037633
rs2018404
rs118171108
rs58758530
rs142817955
rs7184473
rs646935
rs142881434
rs34574485
rs9369356
rs55871547
rs6998850
rs2322165
rs3915868
rs709589
rs200474
rs11047379
rs775001
rs2819081
rs117309996
rs16852581
rs76714110
rs11164796
rs138793837
All − MR Egger
All − Inverse variance weighted
−1
0
1
MR effect size for
'IgD on transitional' on 'Diabetic nephropathy'

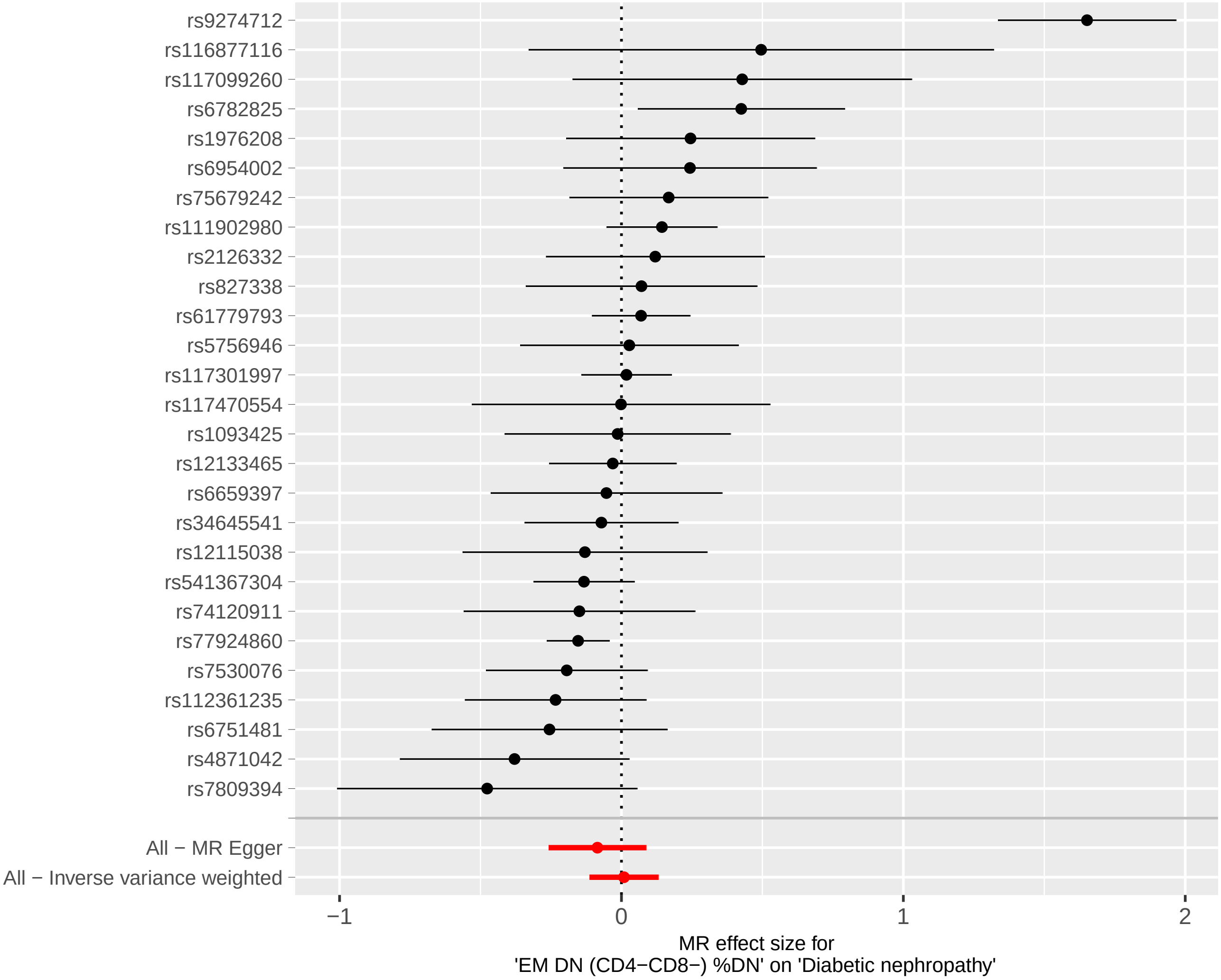
rs9274712
rs116877116
rs117099260
rs6782825
rs1976208
rs6954002
rs75679242
rs111902980
rs2126332
rs827338
rs61779793
rs5756946
rs117301997
rs117470554
rs1093425
rs12133465
rs6659397
rs34645541
rs12115038
rs541367304
rs74120911
rs77924860
rs7530076
rs112361235
rs6751481
rs4871042
rs7809394
All − MR Egger
All − Inverse variance weighted
−1
0
1
2
MR effect size for
'EM DN (CD4−CD8−) %DN' on 'Diabetic nephropathy'

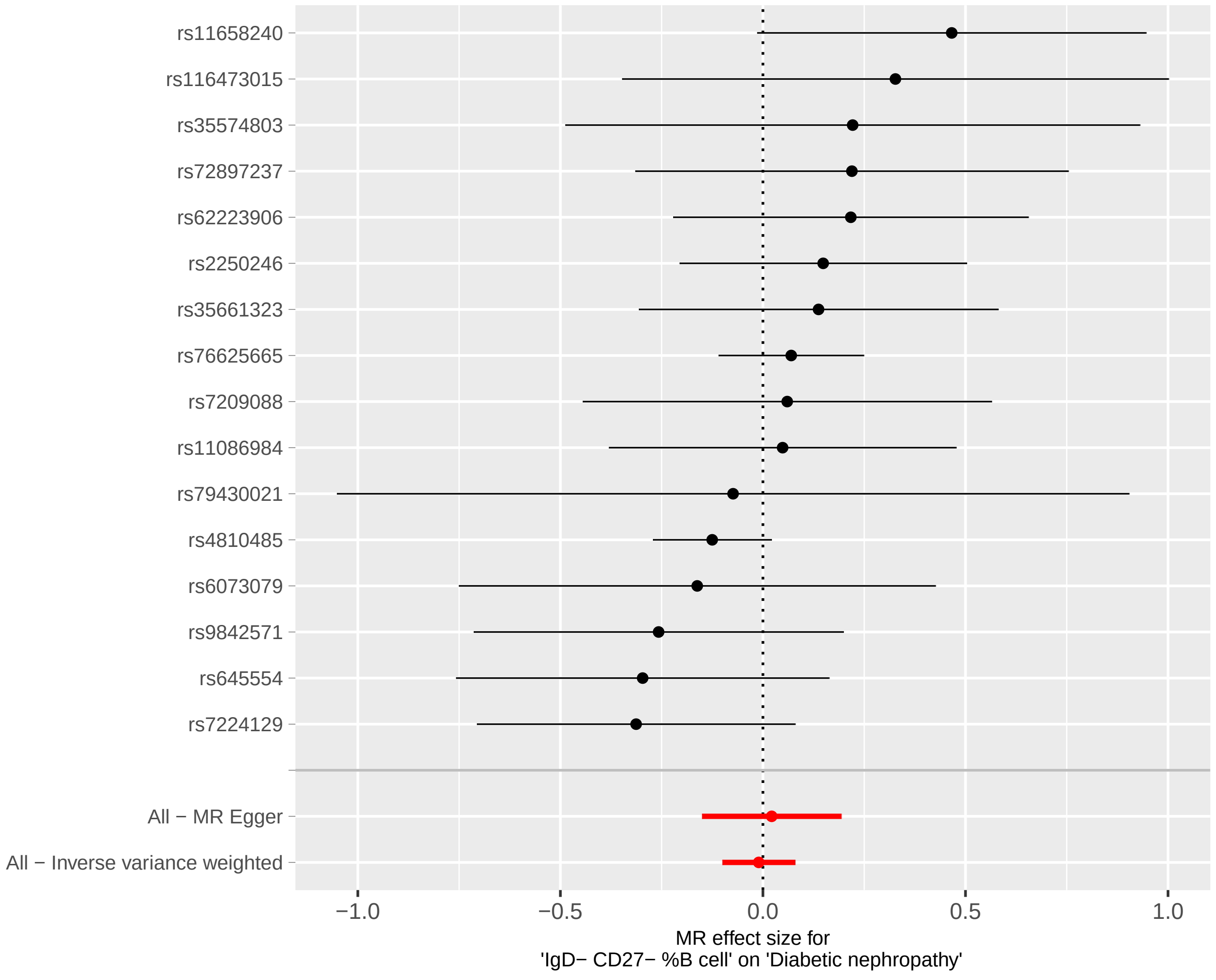

rs11658240
rs116473015
rs35574803
rs72897237
rs62223906
rs2250246
rs35661323
rs76625665
rs7209088
rs11086984
rs79430021
rs4810485
rs6073079
rs9842571
rs645554
rs7224129
All − MR Egger
All − Inverse variance weighted
−1.0
−0.5
0.0
0.5
1.0
MR effect size for
'IgD− CD27− %B cell' on 'Diabetic nephropathy'

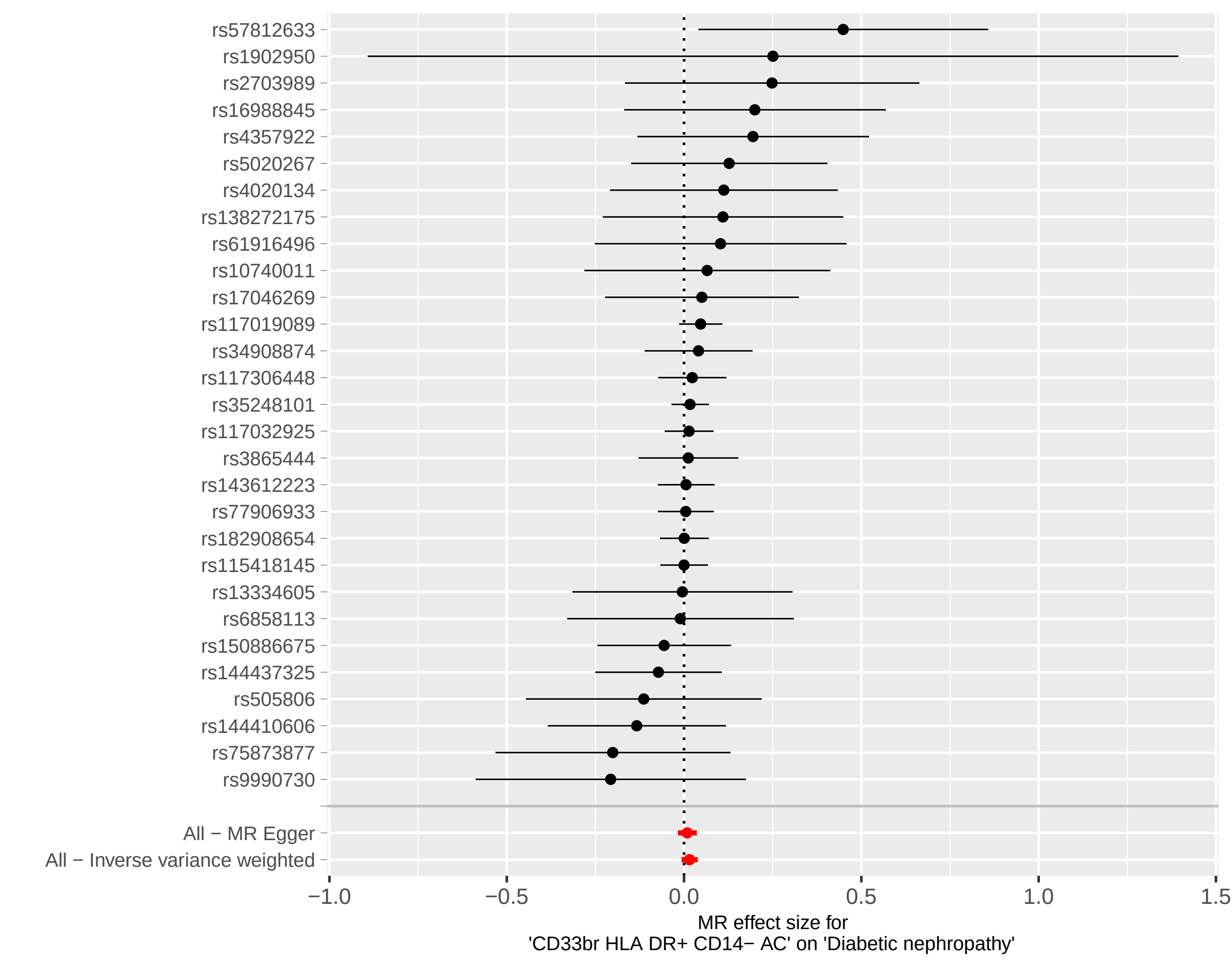
rs57812633
rs1902950
rs2703989
rs16988845
rs4357922
rs5020267
rs4020134
rs138272175
rs61916496
rs10740011
rs17046269
rs117019089
rs34908874
rs117306448
rs35248101
rs117032925
rs3865444
rs143612223
rs77906933
rs182908654
rs115418145
rs13334605
rs6858113
rs150886675
rs144437325
rs505806
rs144410606
rs75873877
rs9990730
All − MR Egger
All − Inverse variance weighted
−1.0
−0.5
0.0
0.5
1.0
1.5
MR effect size for
'CD33br HLA DR+ CD14− AC' on 'Diabetic nephropathy'

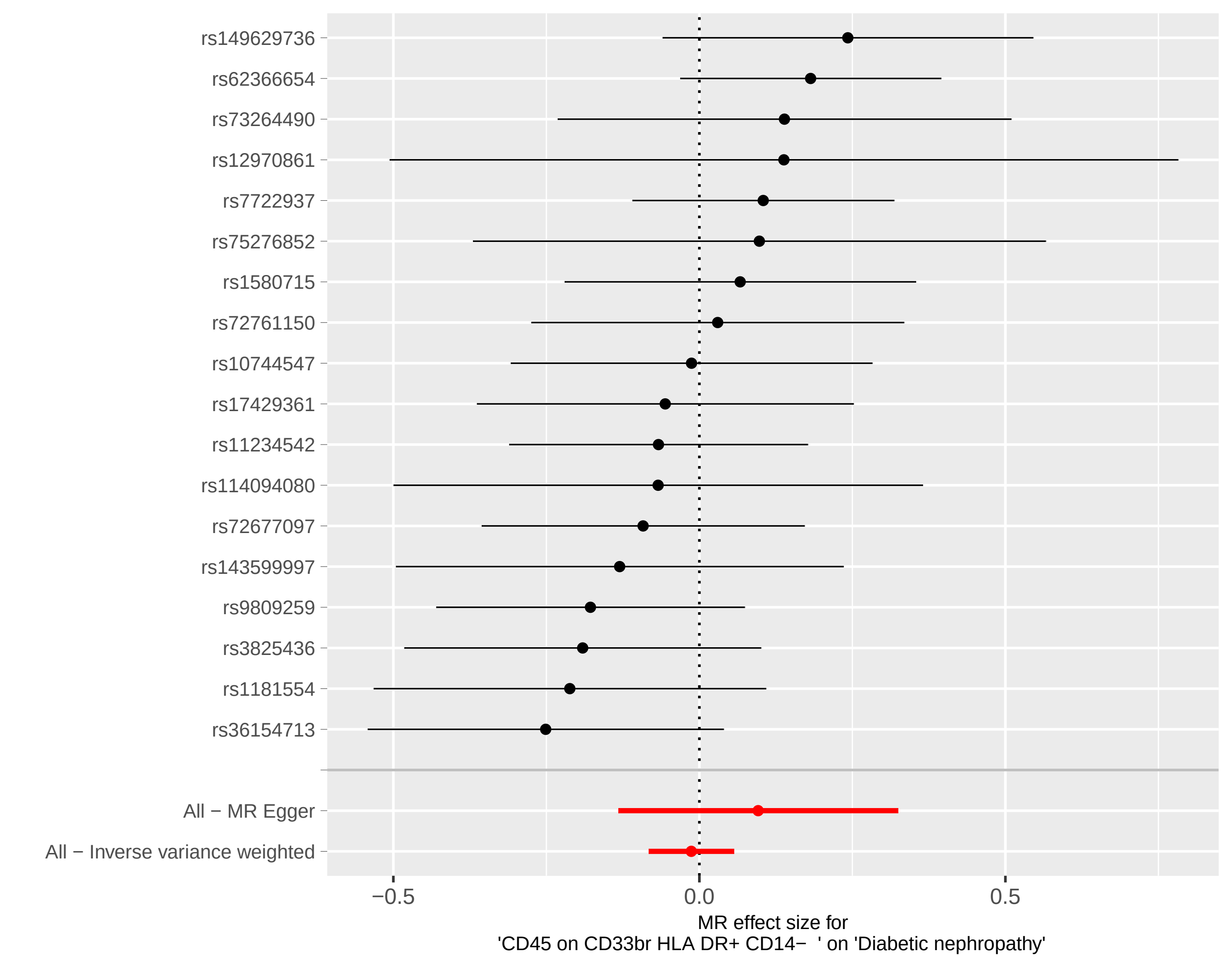
rs149629736
rs62366654
rs73264490
rs12970861
rs7722937
rs75276852
rs1580715
rs72761150
rs10744547
rs17429361
rs11234542
rs114094080
rs72677097
rs143599997
rs9809259
rs3825436
rs1181554
rs36154713
All − MR Egger
All − Inverse variance weighted
−0.5
0.0
0.5
MR effect size for
'CD45 on CD33br HLA DR+ CD14− ' on 'Diabetic nephropathy'

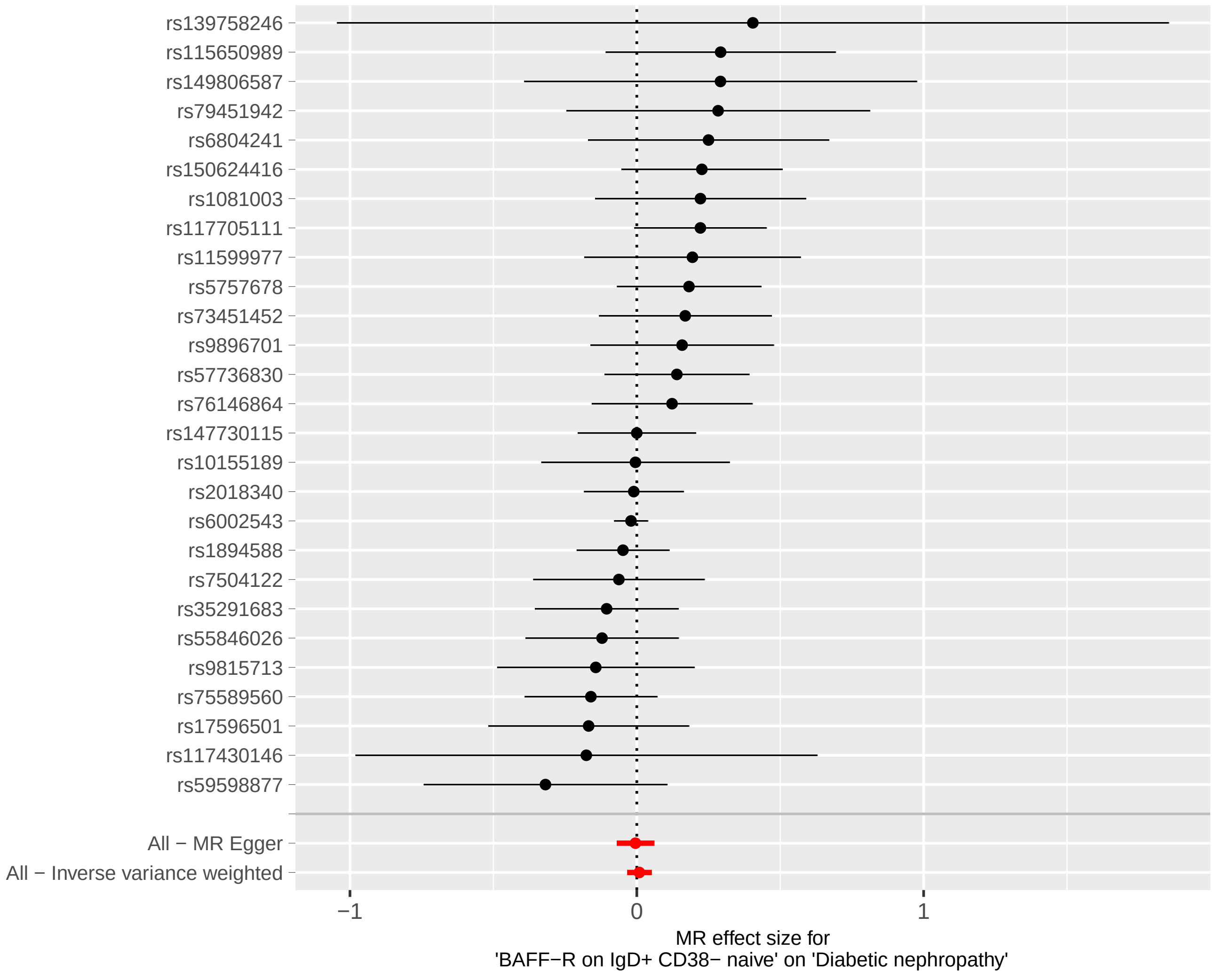

rs139758246
rs115650989
rs149806587
rs79451942
rs6804241
rs150624416
rs1081003
rs117705111
rs11599977
rs5757678
rs73451452
rs9896701
rs57736830
rs76146864
rs147730115
rs10155189
rs2018340
rs6002543
rs1894588
rs7504122
rs35291683
rs55846026
rs9815713
rs75589560
rs17596501
rs117430146
rs59598877
All − MR Egger
All − Inverse variance weighted
−1
0
1
MR effect size for
'BAFF−R on IgD+ CD38− naive' on 'Diabetic nephropathy'

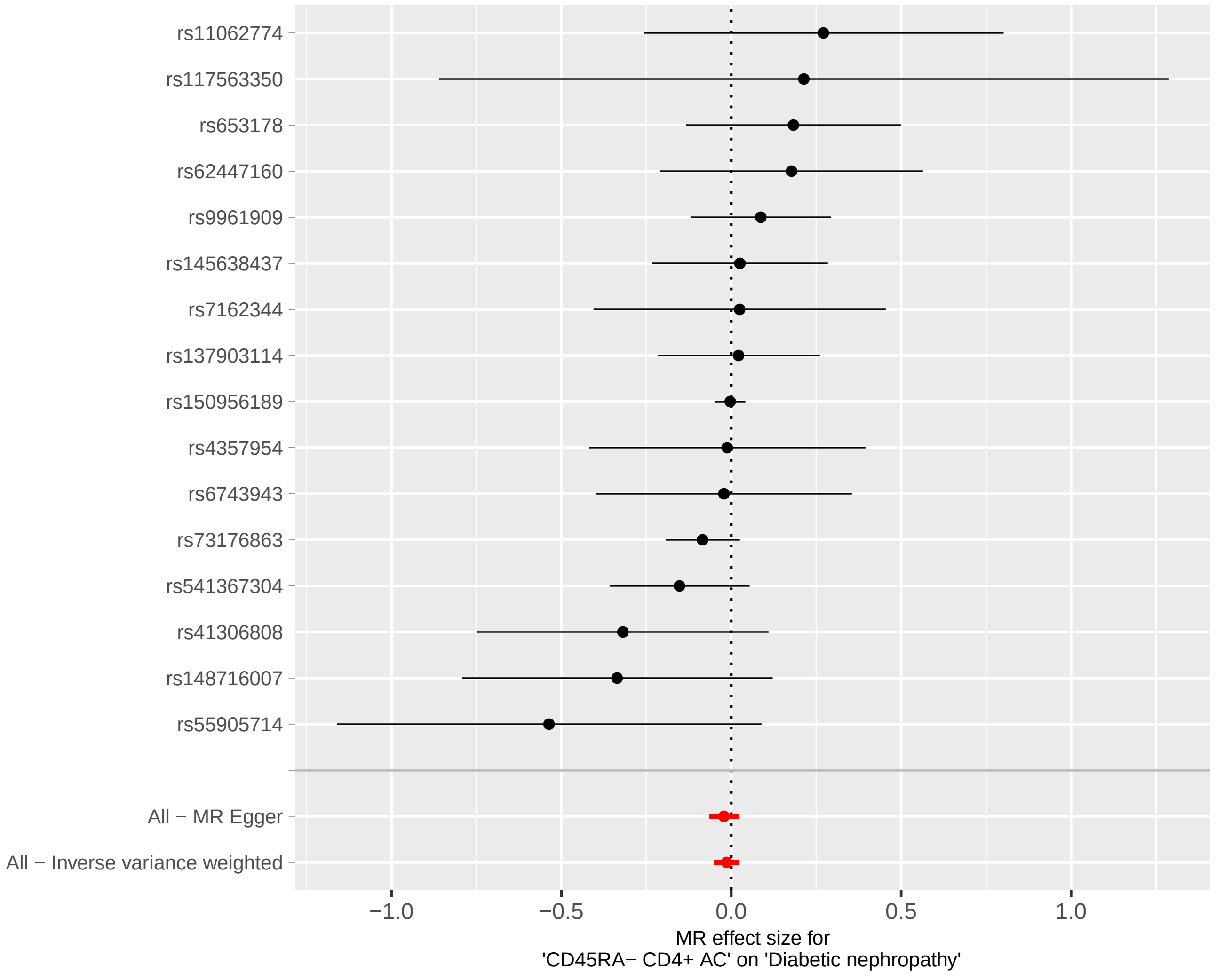
rs11062774
rs117563350
rs653178
rs62447160
rs9961909
rs145638437
rs7162344
rs137903114
rs150956189
rs4357954
rs6743943
rs73176863
rs541367304
rs41306808
rs148716007
rs55905714
All − MR Egger
All − Inverse variance weighted
−1.0
−0.5
0.0
0.5
1.0
MR effect size for
'CD45RA− CD4+ AC' on 'Diabetic nephropathy'

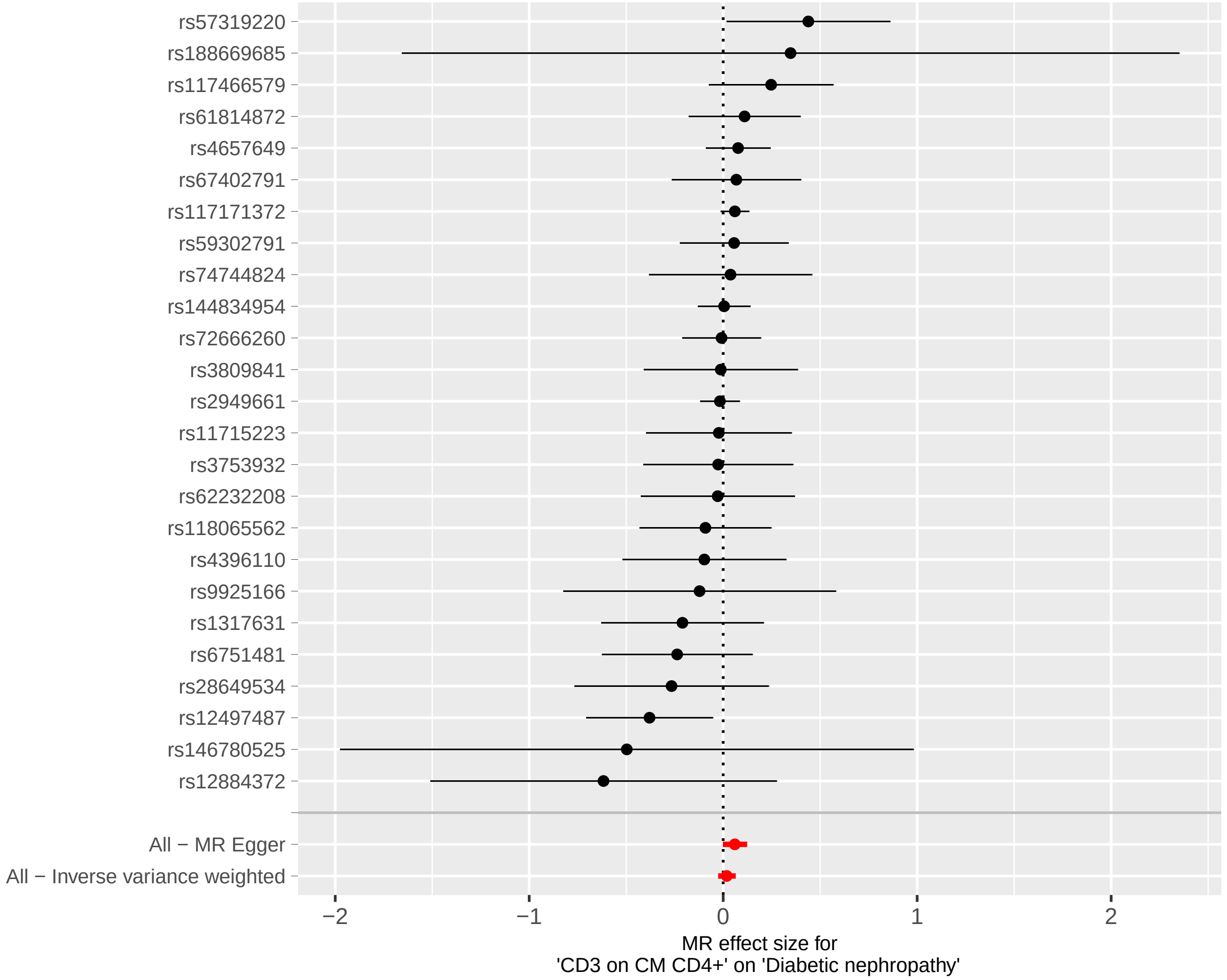
rs57319220
rs188669685
rs117466579
rs61814872
rs4657649
rs67402791
rs117171372
rs59302791
rs74744824
rs144834954
rs72666260
rs3809841
rs2949661
rs11715223
rs3753932
rs62232208
rs118065562
rs4396110
rs9925166
rs1317631
rs6751481
rs28649534
rs12497487
rs146780525
rs12884372
All − MR Egger
All − Inverse variance weighted
−2
−1
0
1
2
MR effect size for
'CD3 on CM CD4+' on 'Diabetic nephropathy'

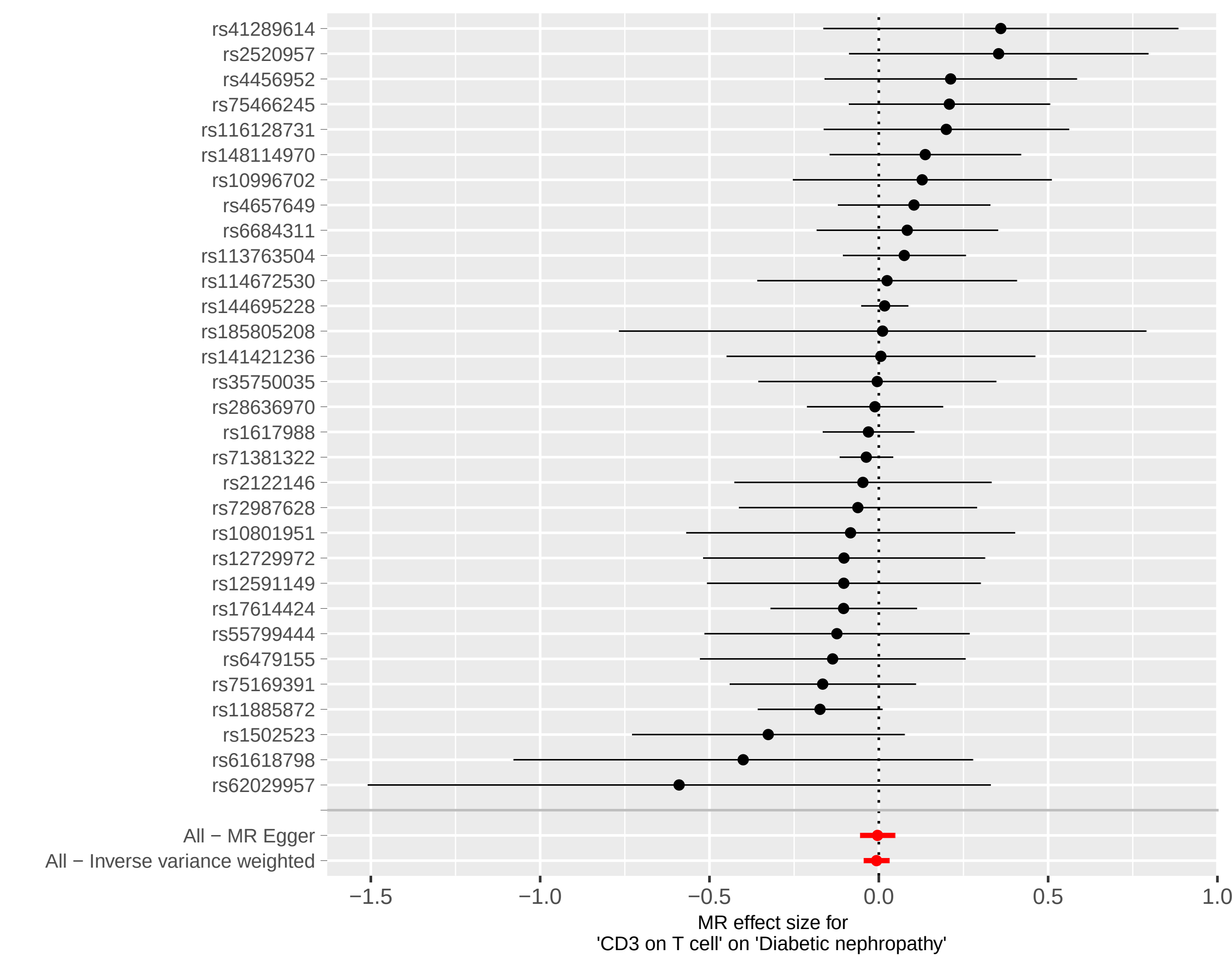
rs41289614
rs2520957
rs4456952
rs75466245
rs116128731
rs148114970
rs10996702
rs4657649
rs6684311
rs113763504
rs114672530
rs144695228
rs185805208
rs141421236
rs35750035
rs28636970
rs1617988
rs71381322
rs2122146
rs72987628
rs10801951
rs12729972
rs12591149
rs17614424
rs55799444
rs6479155
rs75169391
rs11885872
rs1502523
rs61618798
rs62029957
All − MR Egger
All − Inverse variance weighted
−1.5
−1.0
−0.5
0.0
0.5
1.0
MR effect size for
'CD3 on T cell' on 'Diabetic nephropathy'

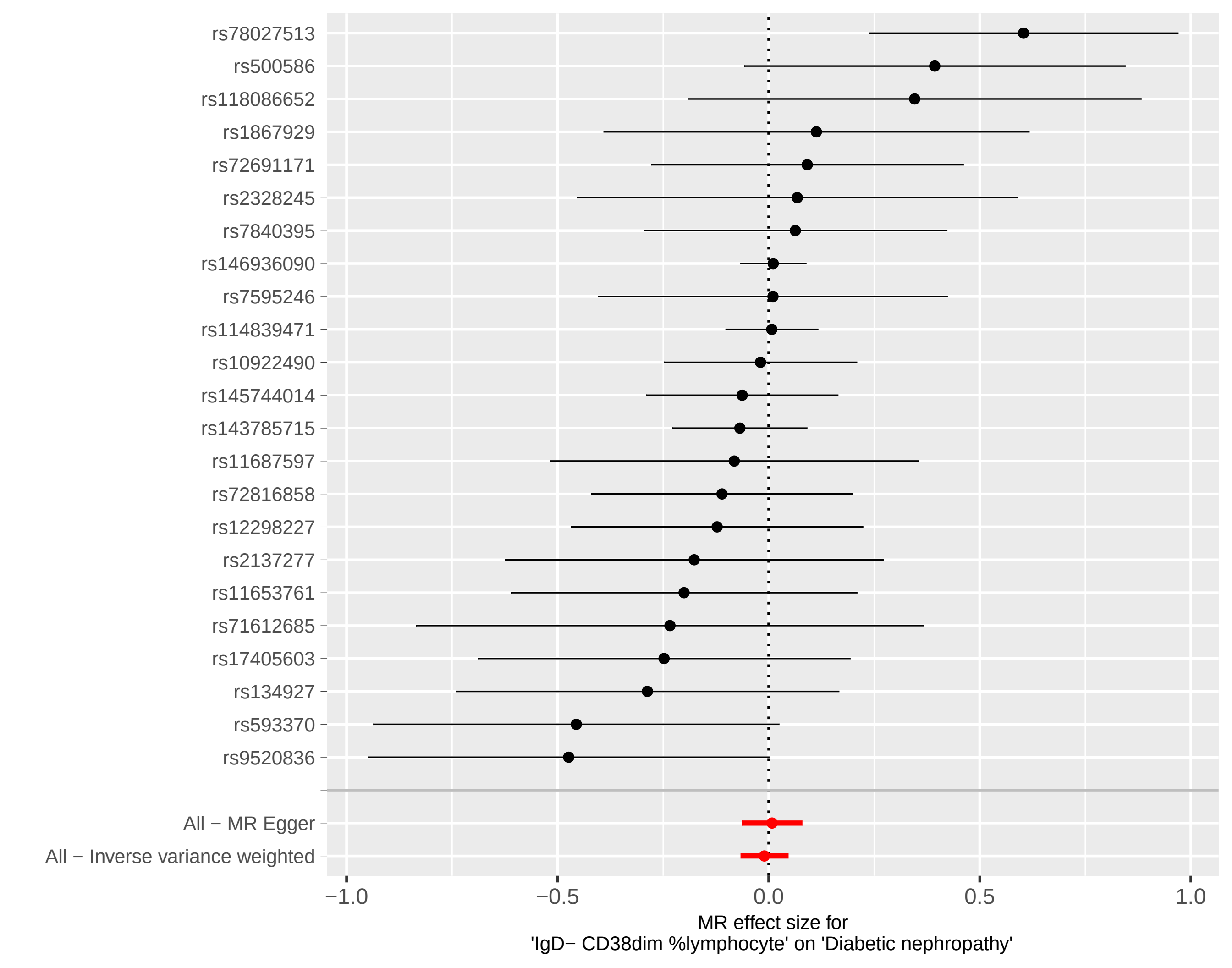

rs78027513
rs500586
rs118086652
rs1867929
rs72691171
rs2328245
rs7840395
rs146936090
rs7595246
rs114839471
rs10922490
rs145744014
rs143785715
rs11687597
rs72816858
rs12298227
rs2137277
rs11653761
rs71612685
rs17405603
rs134927
rs593370
rs9520836
All − MR Egger
All − Inverse variance weighted
−1.0
−0.5
0.0
0.5
1.0
MR effect size for
'IgD− CD38dim %lymphocyte' on 'Diabetic nephropathy'

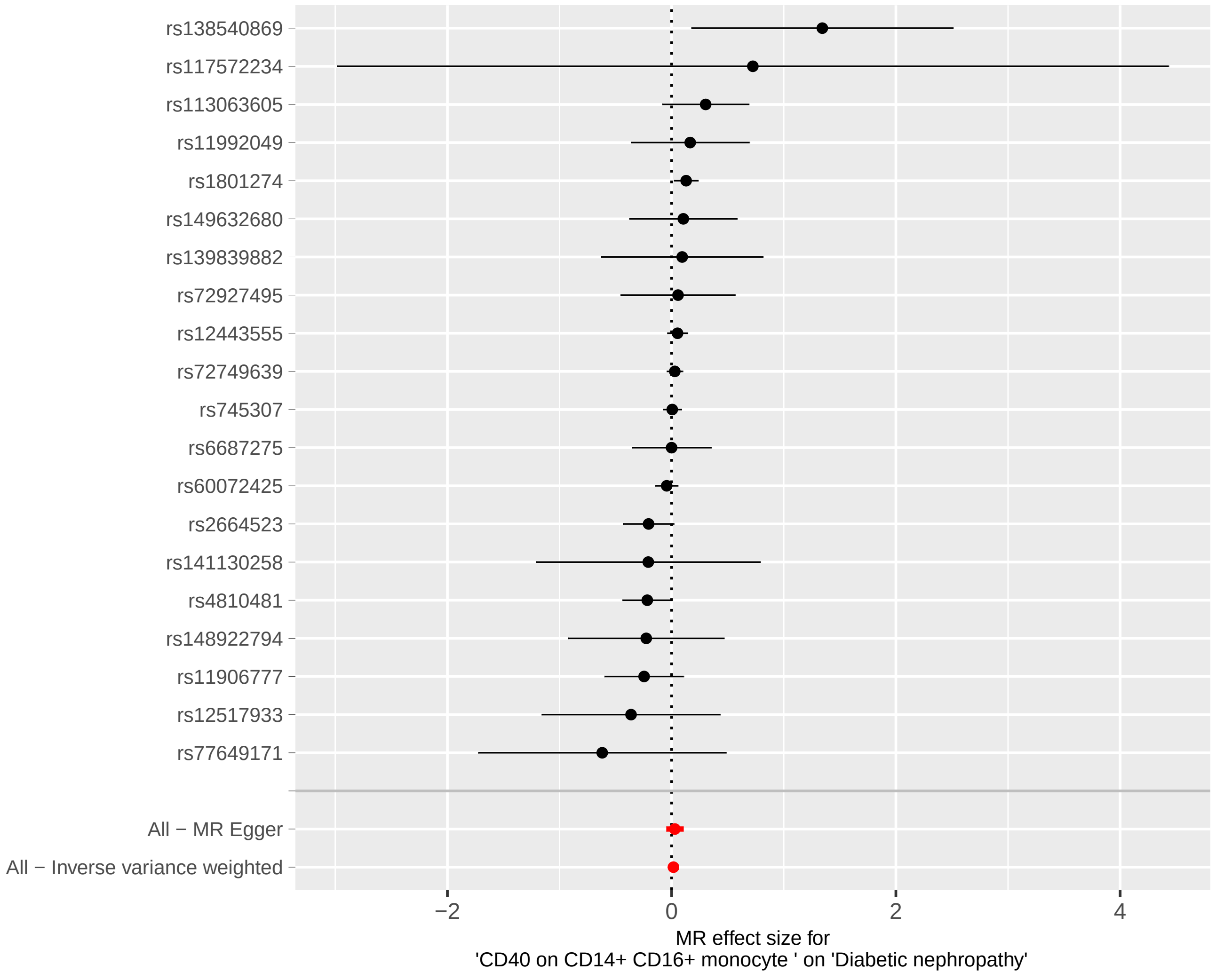

rs138540869
rs117572234
rs113063605
rs11992049
rs1801274
rs149632680
rs139839882
rs72927495
rs12443555
rs72749639
rs745307
rs6687275
rs60072425
rs2664523
rs141130258
rs4810481
rs148922794
rs11906777
rs12517933
rs77649171
All − MR Egger
All − Inverse variance weighted
−2
0
2
4
MR effect size for
'CD40 on CD14+ CD16+ monocyte ' on 'Diabetic nephropathy'

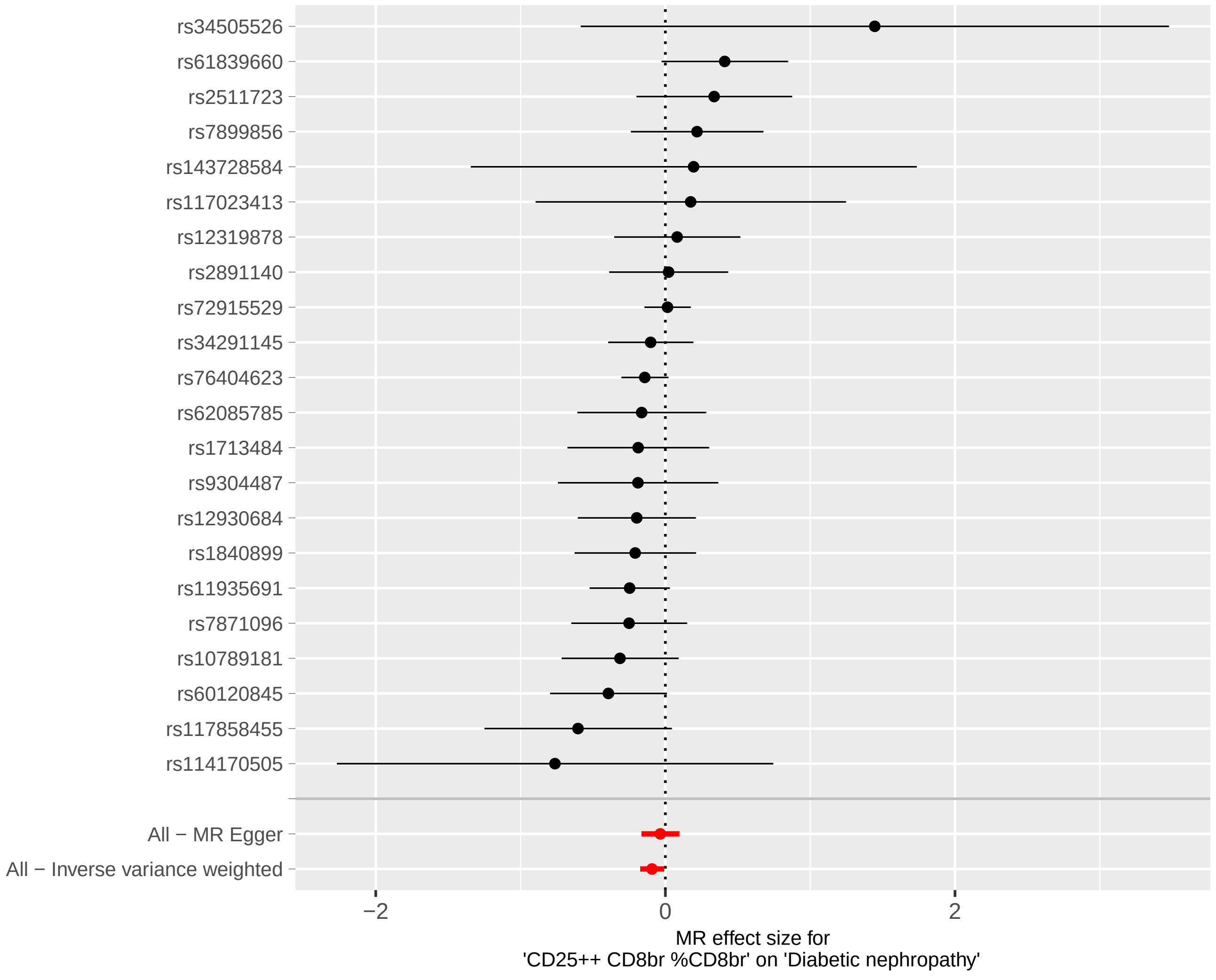

rs34505526
rs61839660
rs2511723
rs7899856
rs143728584
rs117023413
rs12319878
rs2891140
rs72915529
rs34291145
rs76404623
rs62085785
rs1713484
rs9304487
rs12930684
rs1840899
rs11935691
rs7871096
rs10789181
rs60120845
rs117858455
rs114170505
All − MR Egger
All − Inverse variance weighted
−2
0
2
MR effect size for
'CD25++ CD8br %CD8br' on 'Diabetic nephropathy'

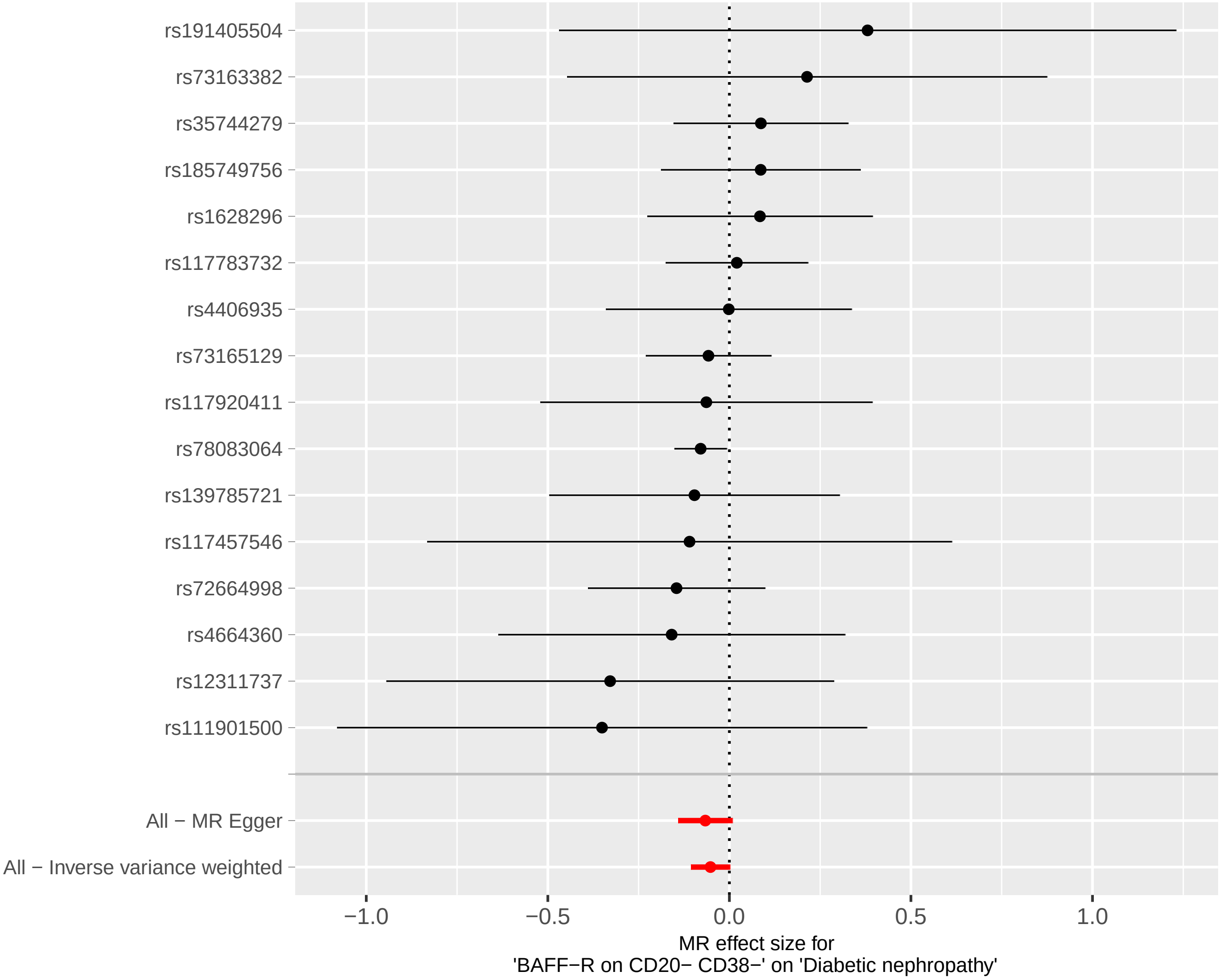

rs191405504
rs73163382
rs35744279
rs185749756
rs1628296
rs117783732
rs4406935
rs73165129
rs117920411
rs78083064
rs139785721
rs117457546
rs72664998
rs4664360
rs12311737
rs111901500
All − MR Egger
All − Inverse variance weighted
−1.0
−0.5
0.0
0.5
1.0
MR effect size for
'BAFF−R on CD20− CD38−' on 'Diabetic nephropathy'

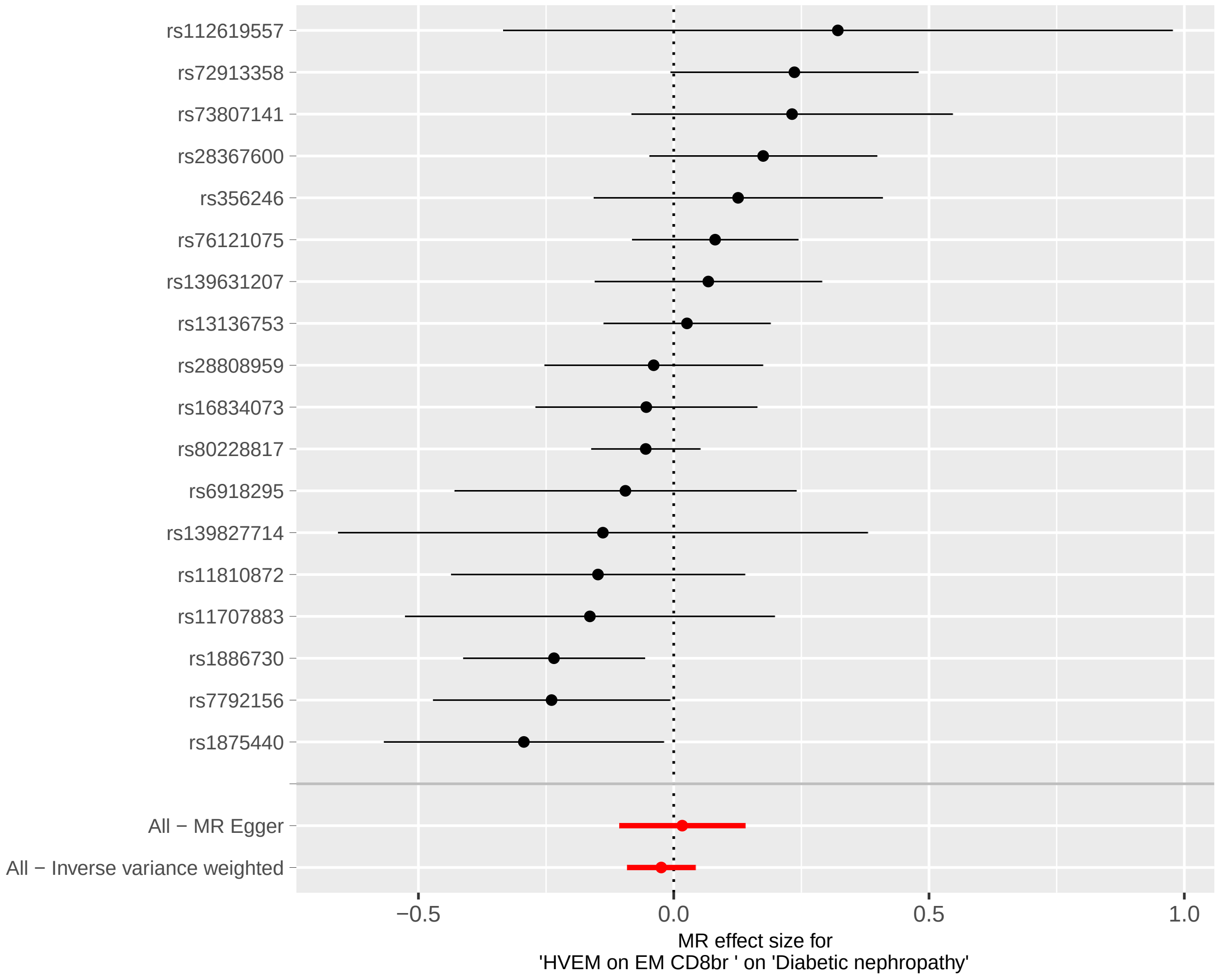
rs112619557
rs72913358
rs73807141
rs28367600
rs356246
rs76121075
rs139631207
rs13136753
rs28808959
rs16834073
rs80228817
rs6918295
rs139827714
rs11810872
rs11707883
rs1886730
rs7792156
rs1875440
All − MR Egger
All − Inverse variance weighted
−0.5
0.0
0.5
1.0
MR effect size for
'HVEM on EM CD8br ' on 'Diabetic nephropathy'

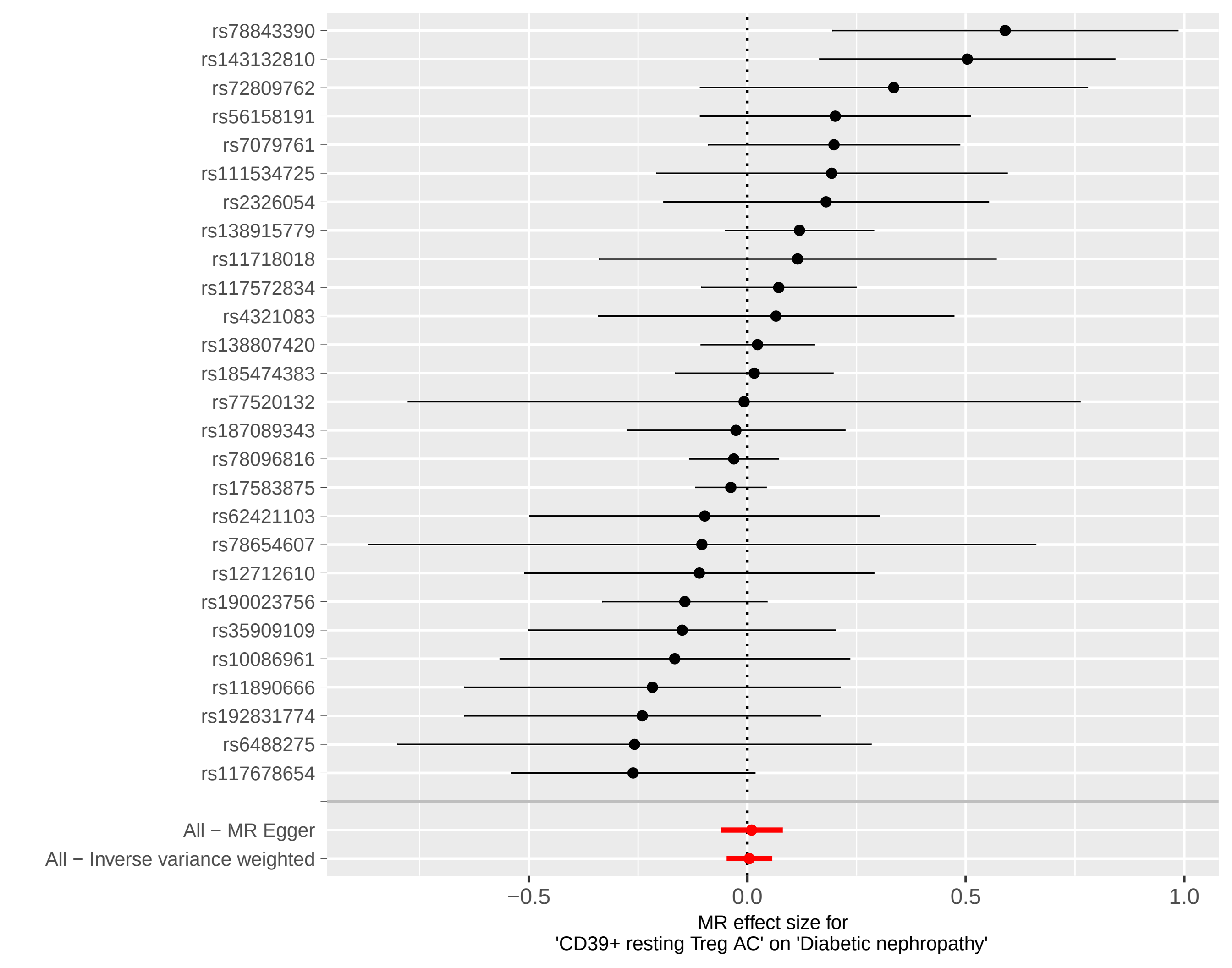

rs78843390
rs143132810
rs72809762
rs56158191
rs7079761
rs111534725
rs2326054
rs138915779
rs11718018
rs117572834
rs4321083
rs138807420
rs185474383
rs77520132
rs187089343
rs78096816
rs17583875
rs62421103
rs78654607
rs12712610
rs190023756
rs35909109
rs10086961
rs11890666
rs192831774
rs6488275
rs117678654
All − MR Egger
All − Inverse variance weighted
−0.5
0.0
0.5
1.0
MR effect size for
'CD39+ resting Treg AC' on 'Diabetic nephropathy'

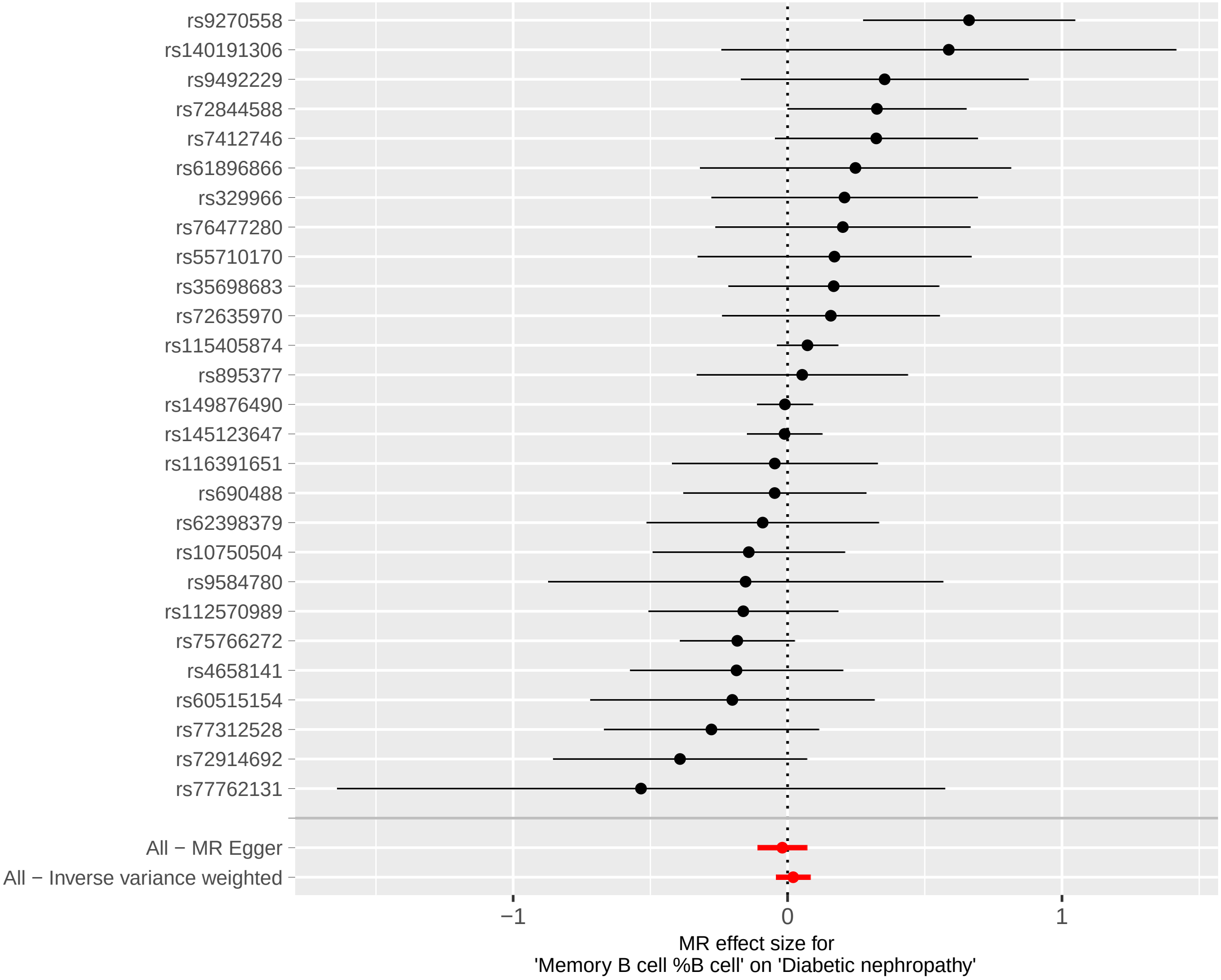

rs9270558
rs140191306
rs9492229
rs72844588
rs7412746
rs61896866
rs329966
rs76477280
rs55710170
rs35698683
rs72635970
rs115405874
rs895377
rs149876490
rs145123647
rs116391651
rs690488
rs62398379
rs10750504
rs9584780
rs112570989
rs75766272
rs4658141
rs60515154
rs77312528
rs72914692
rs77762131
All − MR Egger
All − Inverse variance weighted
−1
0
1
MR effect size for
'Memory B cell %B cell' on 'Diabetic nephropathy'

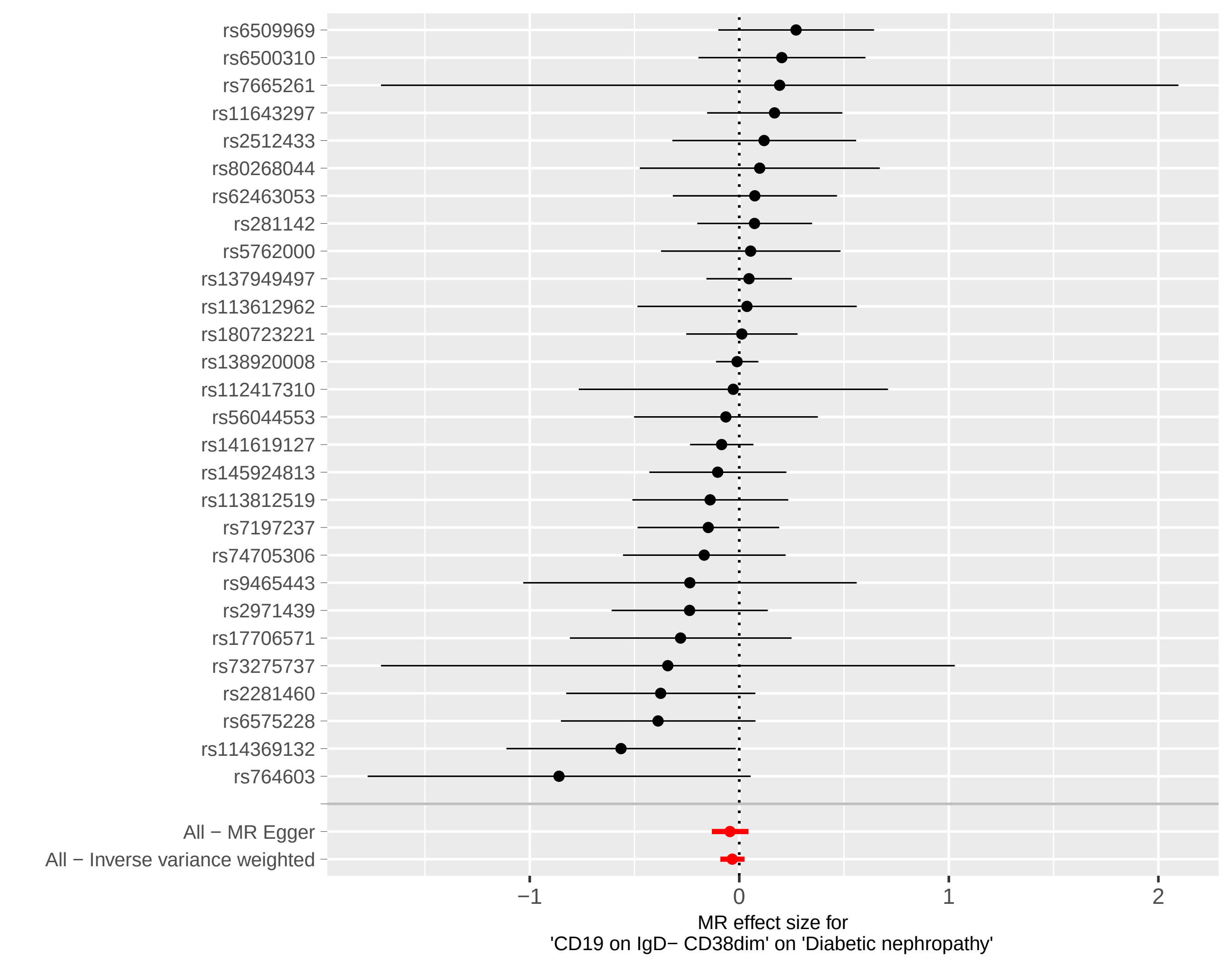

rs6509969
rs6500310
rs7665261
rs11643297
rs2512433
rs80268044
rs62463053
rs281142
rs5762000
rs137949497
rs113612962
rs180723221
rs138920008
rs112417310
rs56044553
rs141619127
rs145924813
rs113812519
rs7197237
rs74705306
rs9465443
rs2971439
rs17706571
rs73275737
rs2281460
rs6575228
rs114369132
rs764603
All − MR Egger
All − Inverse variance weighted
−1
0
1
2
MR effect size for
'CD19 on IgD− CD38dim' on 'Diabetic nephropathy'

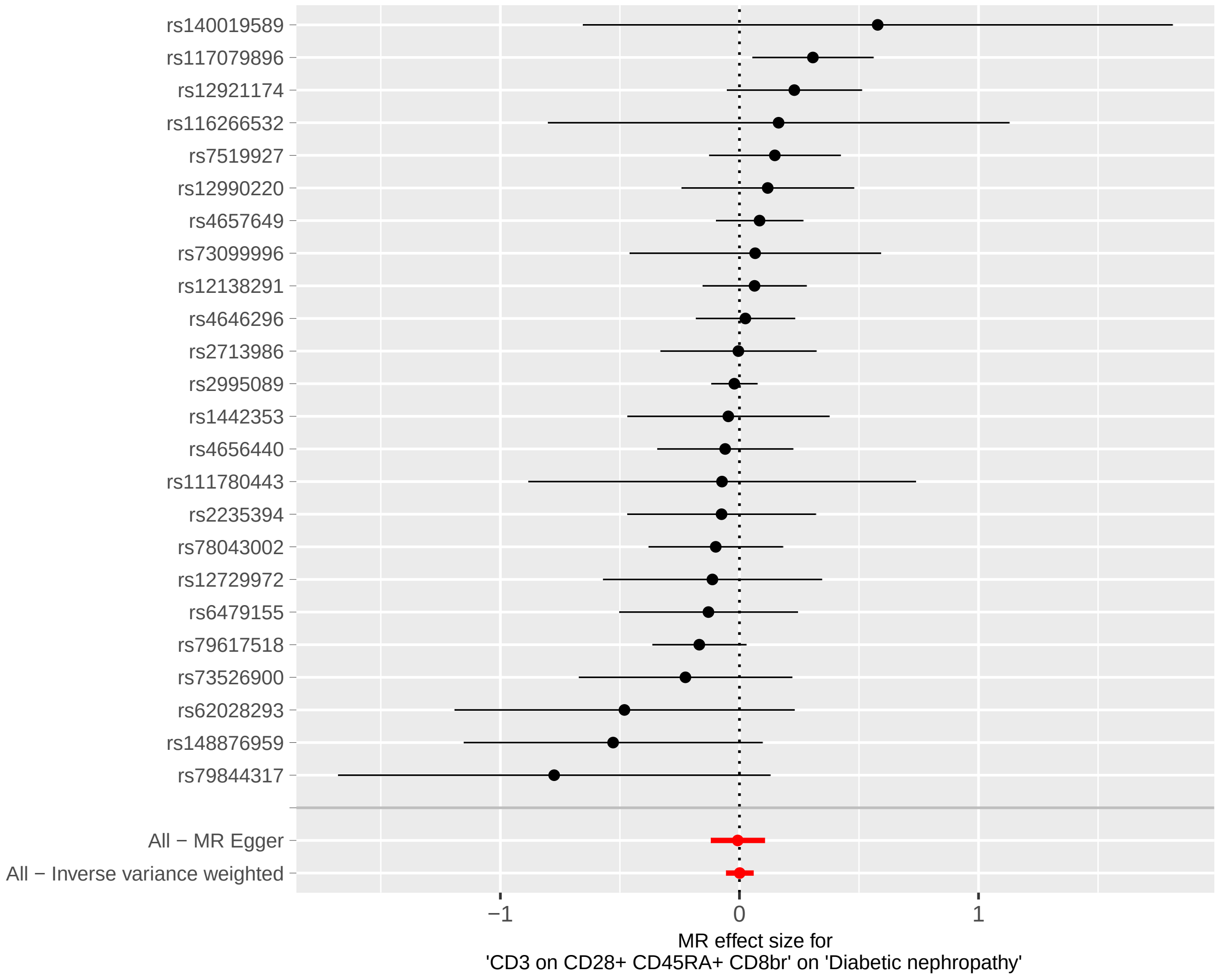
rs140019589
rs117079896
rs12921174
rs116266532
rs7519927
rs12990220
rs4657649
rs73099996
rs12138291
rs4646296
rs2713986
rs2995089
rs1442353
rs4656440
rs111780443
rs2235394
rs78043002
rs12729972
rs6479155
rs79617518
rs73526900
rs62028293
rs148876959
rs79844317
All − MR Egger
All − Inverse variance weighted
−1
0
1
MR effect size for
'CD3 on CD28+ CD45RA+ CD8br' on 'Diabetic nephropathy'

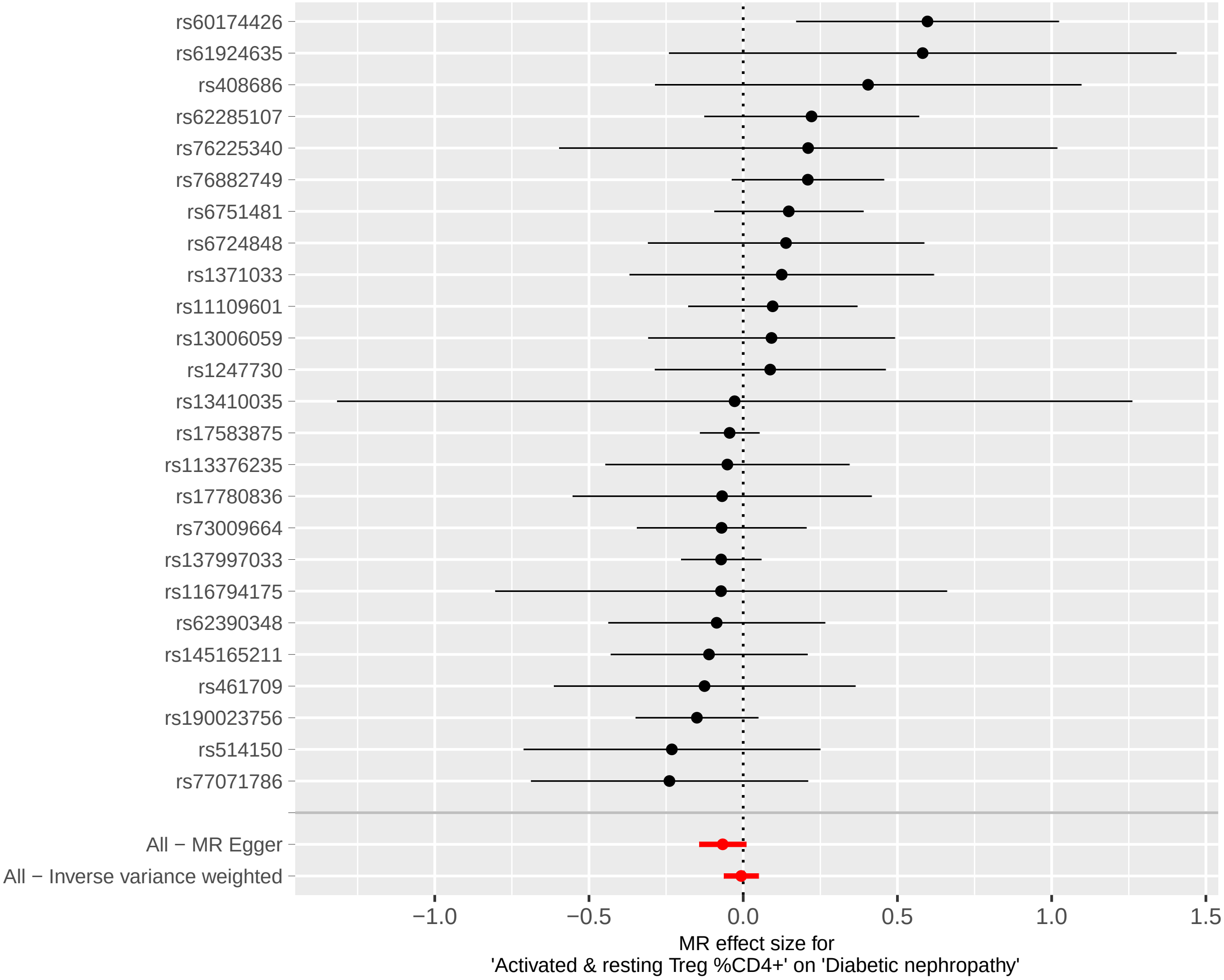

rs60174426
rs61924635
rs408686
rs62285107
rs76225340
rs76882749
rs6751481
rs6724848
rs1371033
rs11109601
rs13006059
rs1247730
rs13410035
rs17583875
rs113376235
rs17780836
rs73009664
rs137997033
rs116794175
rs62390348
rs145165211
rs461709
rs190023756
rs514150
rs77071786
All − MR Egger
All − Inverse variance weighted
−1.0
−0.5
0.0
0.5
1.0
1.5
MR effect size for
'Activated & resting Treg %CD4+' on 'Diabetic nephropathy'

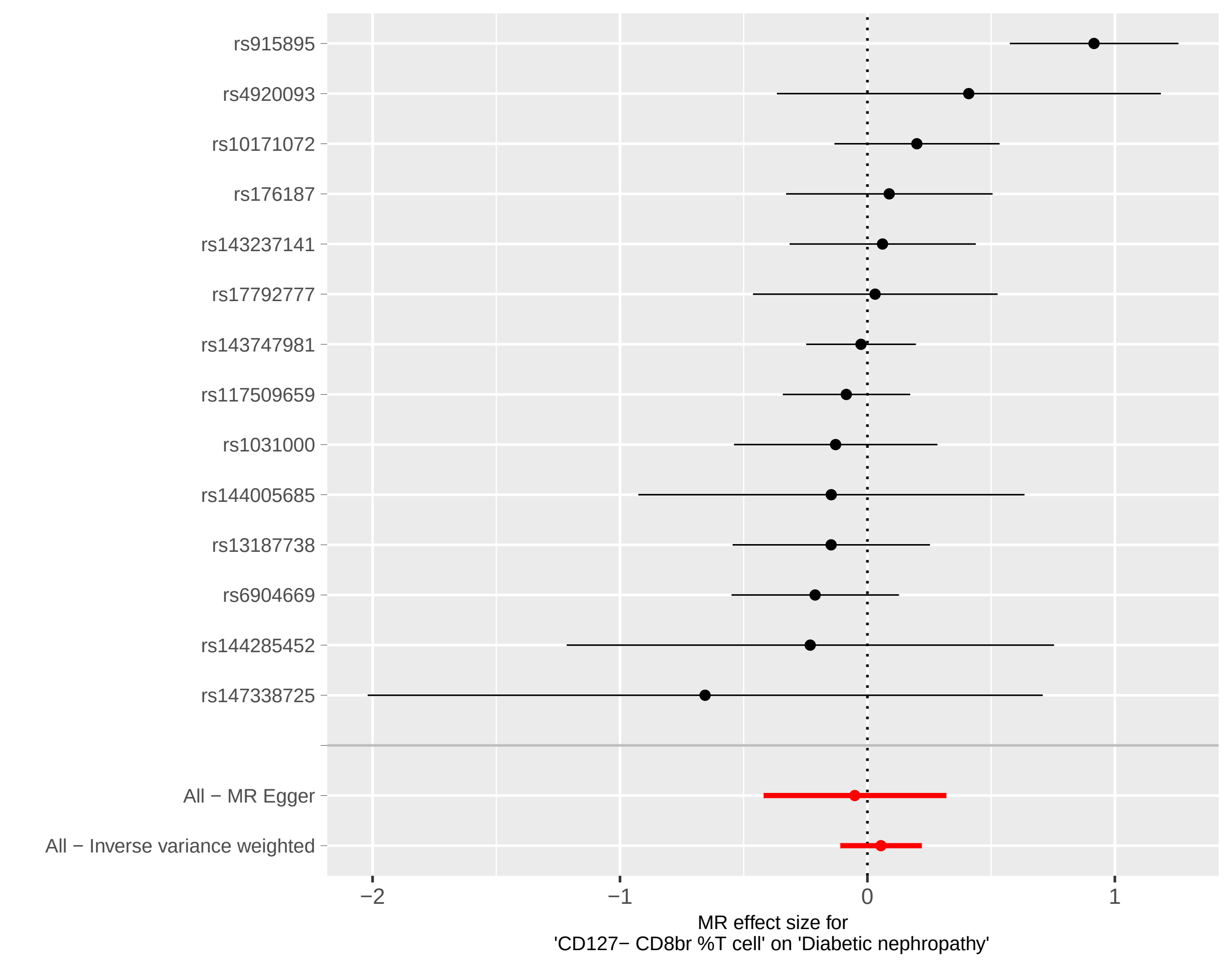
rs915895
rs4920093
rs10171072
rs176187
rs143237141
rs17792777
rs143747981
rs117509659
rs1031000
rs144005685
rs13187738
rs6904669
rs144285452
rs147338725
All − MR Egger
All − Inverse variance weighted
−2
−1
0
1
MR effect size for
'CD127− CD8br %T cell' on 'Diabetic nephropathy'

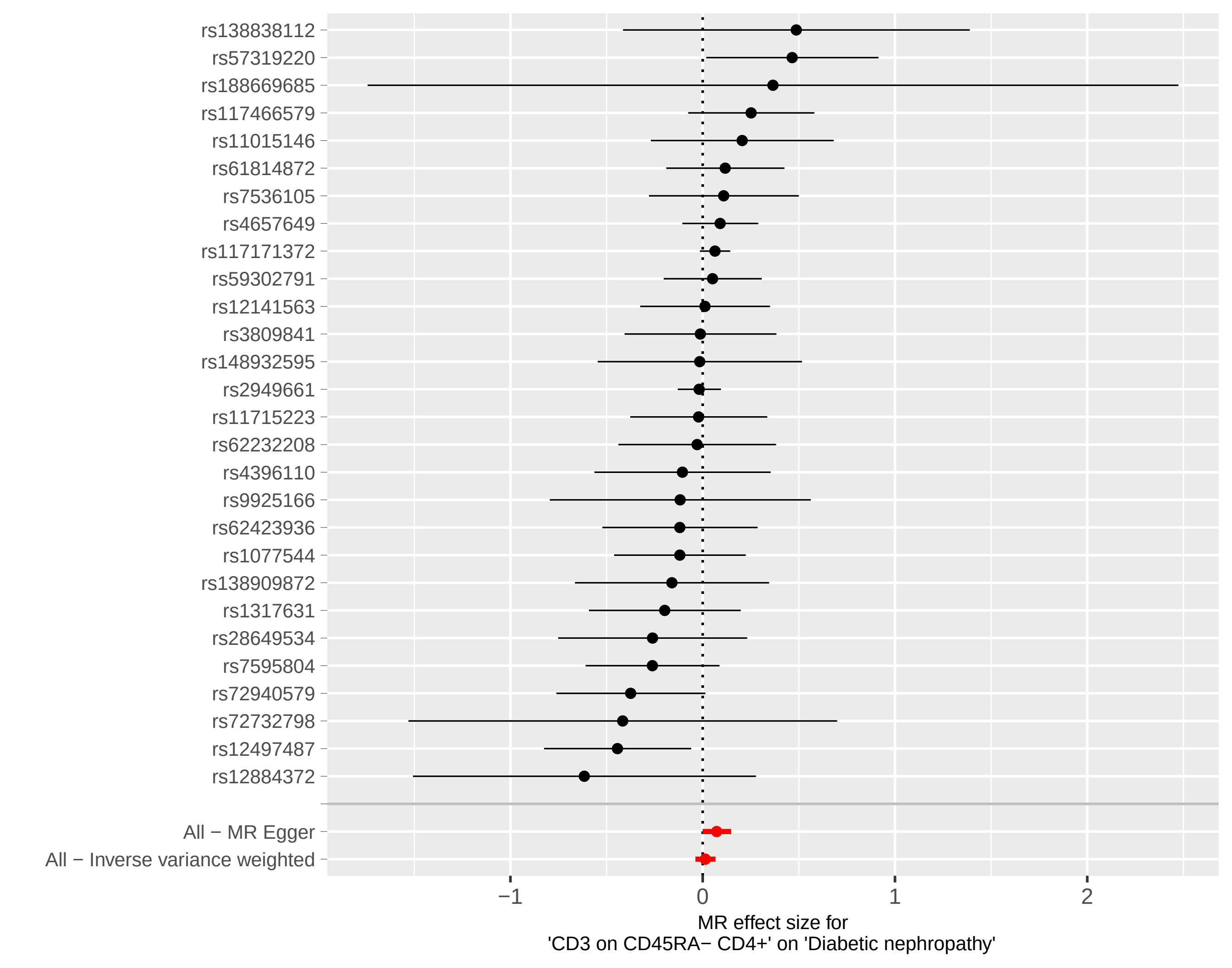

rs138838112
rs57319220
rs188669685
rs117466579
rs11015146
rs61814872
rs7536105
rs4657649
rs117171372
rs59302791
rs12141563
rs3809841
rs148932595
rs2949661
rs11715223
rs62232208
rs4396110
rs9925166
rs62423936
rs1077544
rs138909872
rs1317631
rs28649534
rs7595804
rs72940579
rs72732798
rs12497487
rs12884372
All − MR Egger
All − Inverse variance weighted
−1
0
1
2
MR effect size for
'CD3 on CD45RA− CD4+' on 'Diabetic nephropathy'

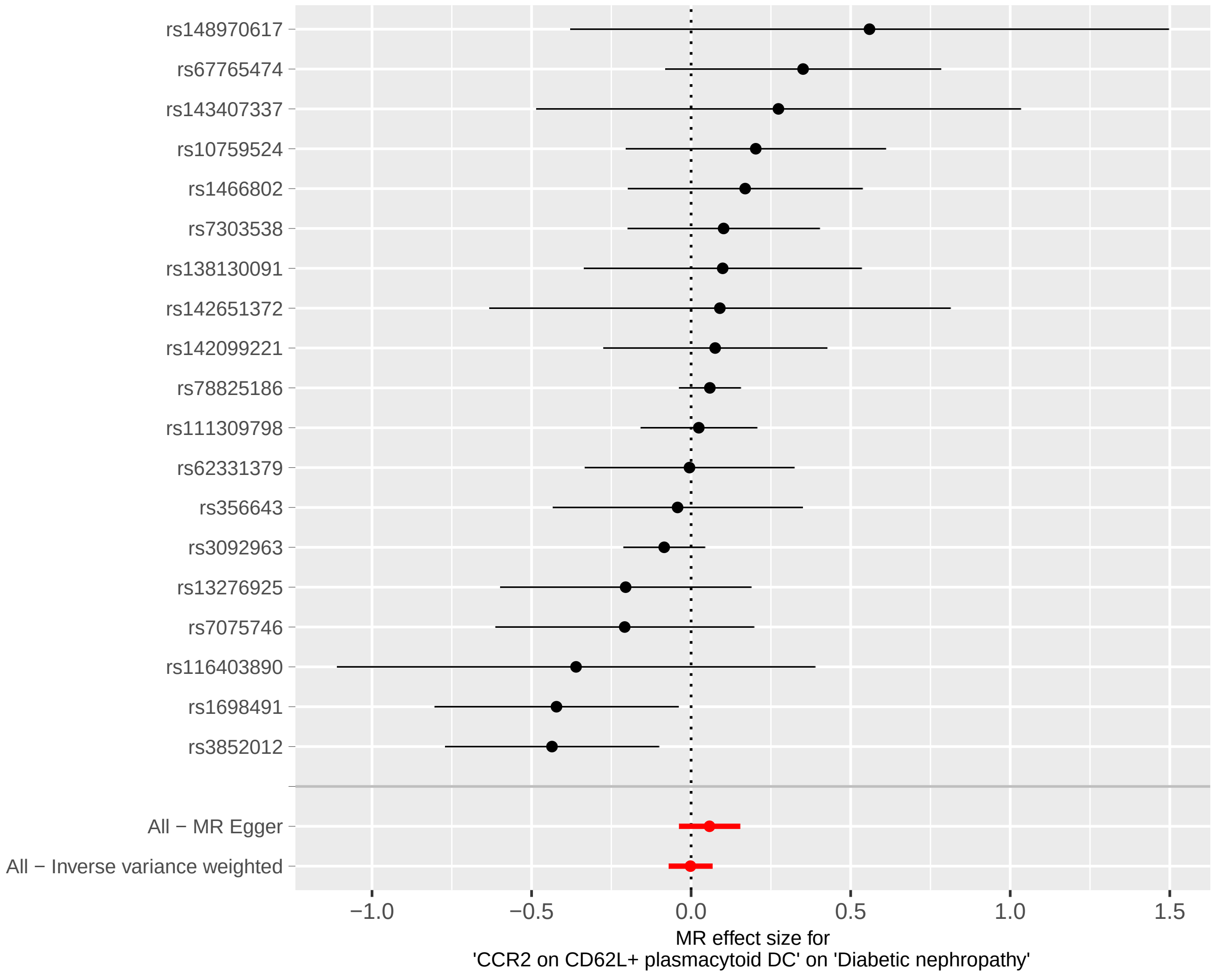
rs148970617
rs67765474
rs143407337
rs10759524
rs1466802
rs7303538
rs138130091
rs142651372
rs142099221
rs78825186
rs111309798
rs62331379
rs356643
rs3092963
rs13276925
rs7075746
rs116403890
rs1698491
rs3852012
All − MR Egger
All − Inverse variance weighted
−1.0
−0.5
0.0
0.5
1.0
1.5
MR effect size for
'CCR2 on CD62L+ plasmacytoid DC' on 'Diabetic nephropathy'

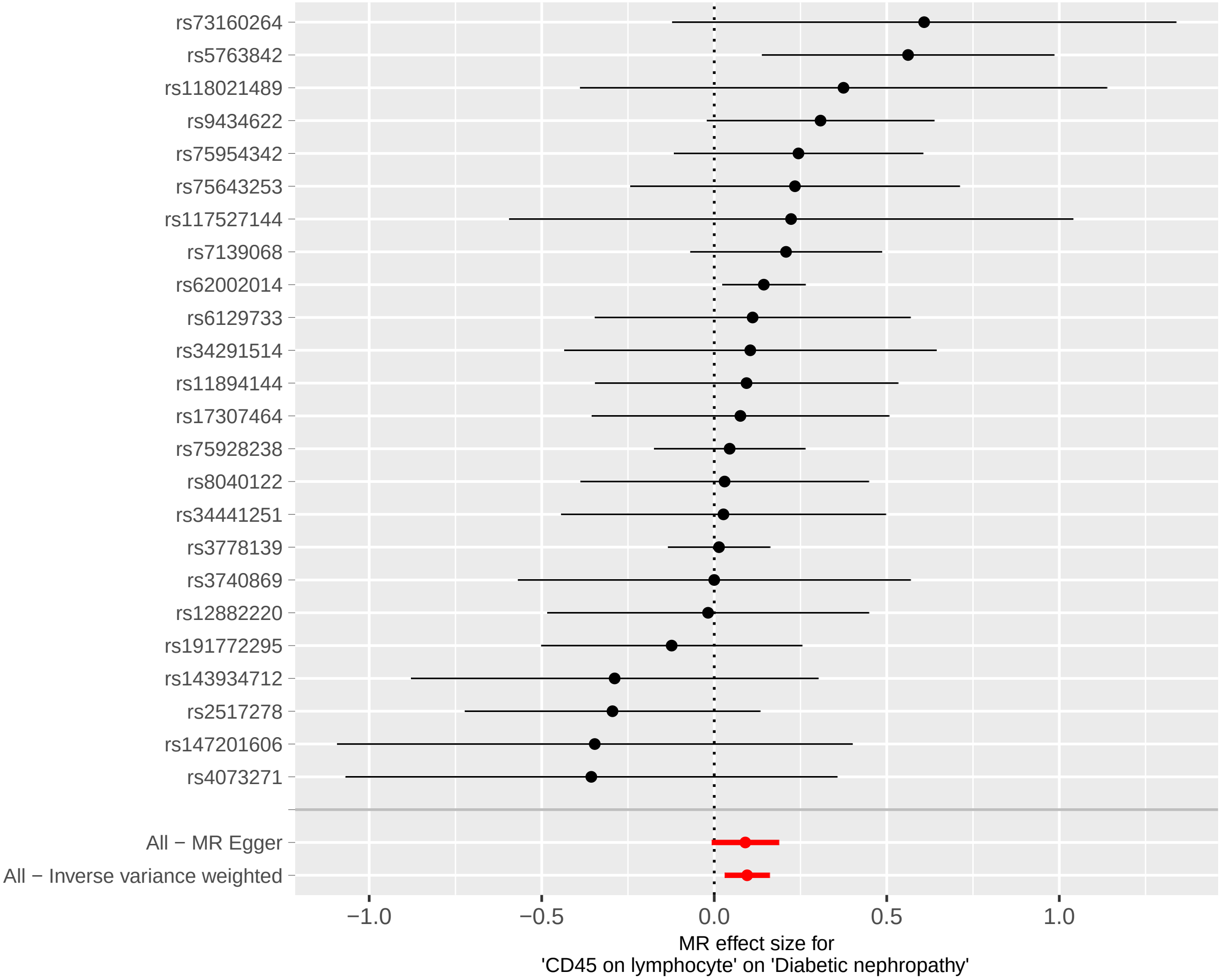
rs73160264
rs5763842
rs118021489
rs9434622
rs75954342
rs75643253
rs117527144
rs7139068
rs62002014
rs6129733
rs34291514
rs11894144
rs17307464
rs75928238
rs8040122
rs34441251
rs3778139
rs3740869
rs12882220
rs191772295
rs143934712
rs2517278
rs147201606
rs4073271
All − MR Egger
All − Inverse variance weighted
−1.0
−0.5
0.0
0.5
1.0
MR effect size for
'CD45 on lymphocyte' on 'Diabetic nephropathy'

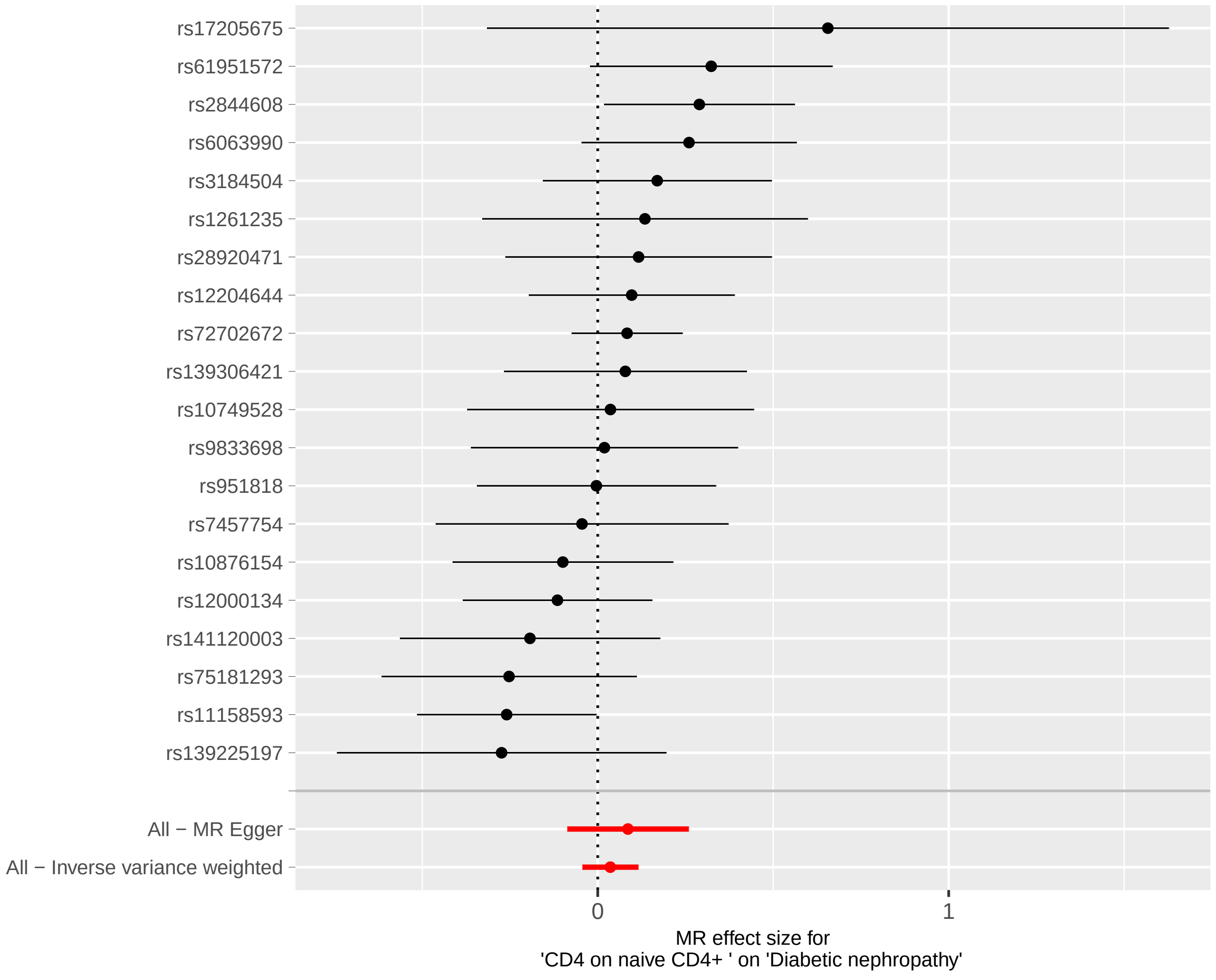
rs17205675
rs61951572
rs2844608
rs6063990
rs3184504
rs1261235
rs28920471
rs12204644
rs72702672
rs139306421
rs10749528
rs9833698
rs951818
rs7457754
rs10876154
rs12000134
rs141120003
rs75181293
rs11158593
rs139225197
All − MR Egger
All − Inverse variance weighted
0
1
MR effect size for
'CD4 on naive CD4+ ' on 'Diabetic nephropathy'

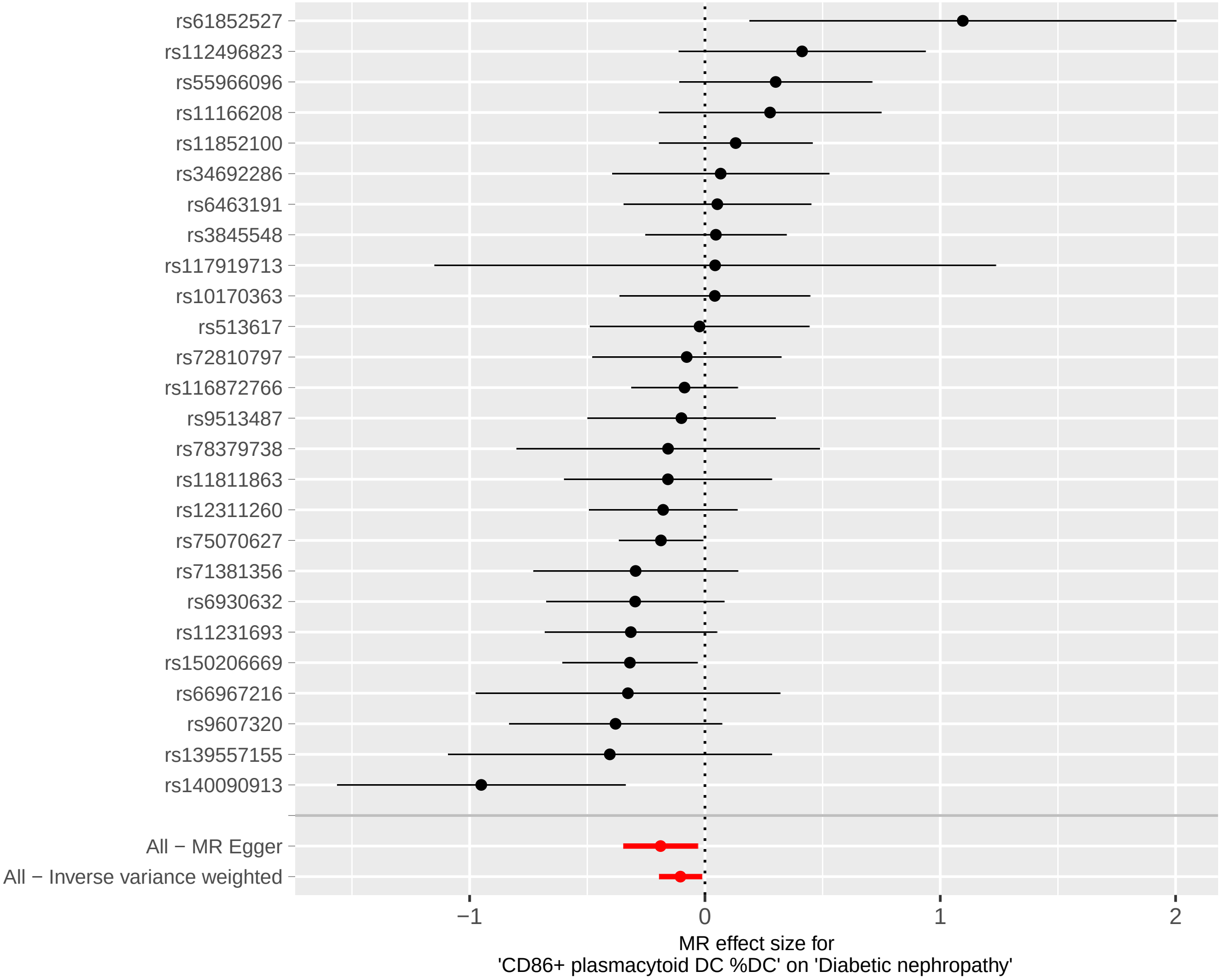
rs61852527
rs112496823
rs55966096
rs11166208
rs11852100
rs34692286
rs6463191
rs3845548
rs117919713
rs10170363
rs513617
rs72810797
rs116872766
rs9513487
rs78379738
rs11811863
rs12311260
rs75070627
rs71381356
rs6930632
rs11231693
rs150206669
rs66967216
rs9607320
rs139557155
rs140090913
All − MR Egger
All − Inverse variance weighted
−1
0
1
2
MR effect size for
'CD86+ plasmacytoid DC %DC' on 'Diabetic nephropathy'

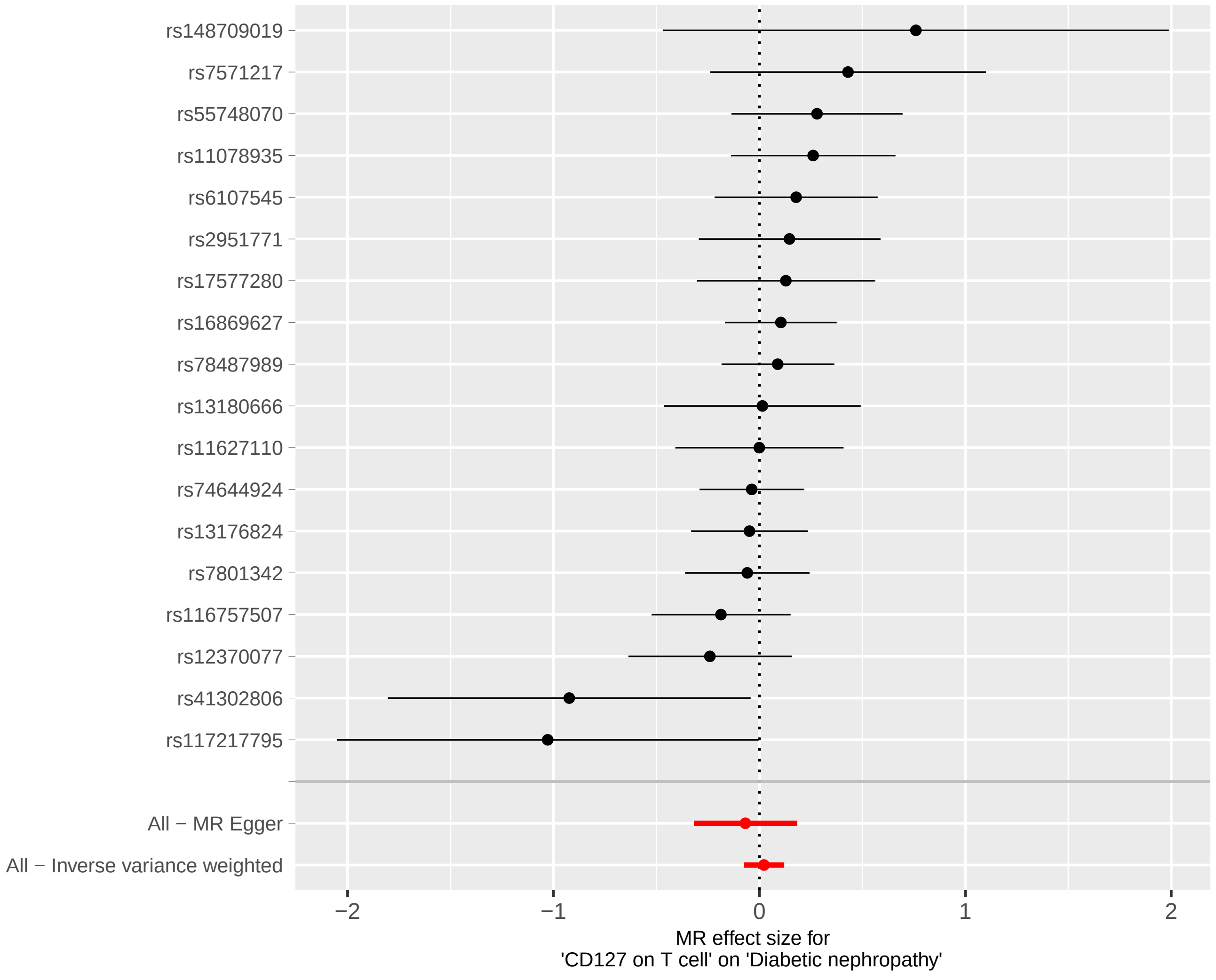
rs148709019
rs7571217
rs55748070
rs11078935
rs6107545
rs2951771
rs17577280
rs16869627
rs78487989
rs13180666
rs11627110
rs74644924
rs13176824
rs7801342
rs116757507
rs12370077
rs41302806
rs117217795
All − MR Egger
All − Inverse variance weighted
−2
−1
0
1
2
MR effect size for
'CD127 on T cell' on 'Diabetic nephropathy'

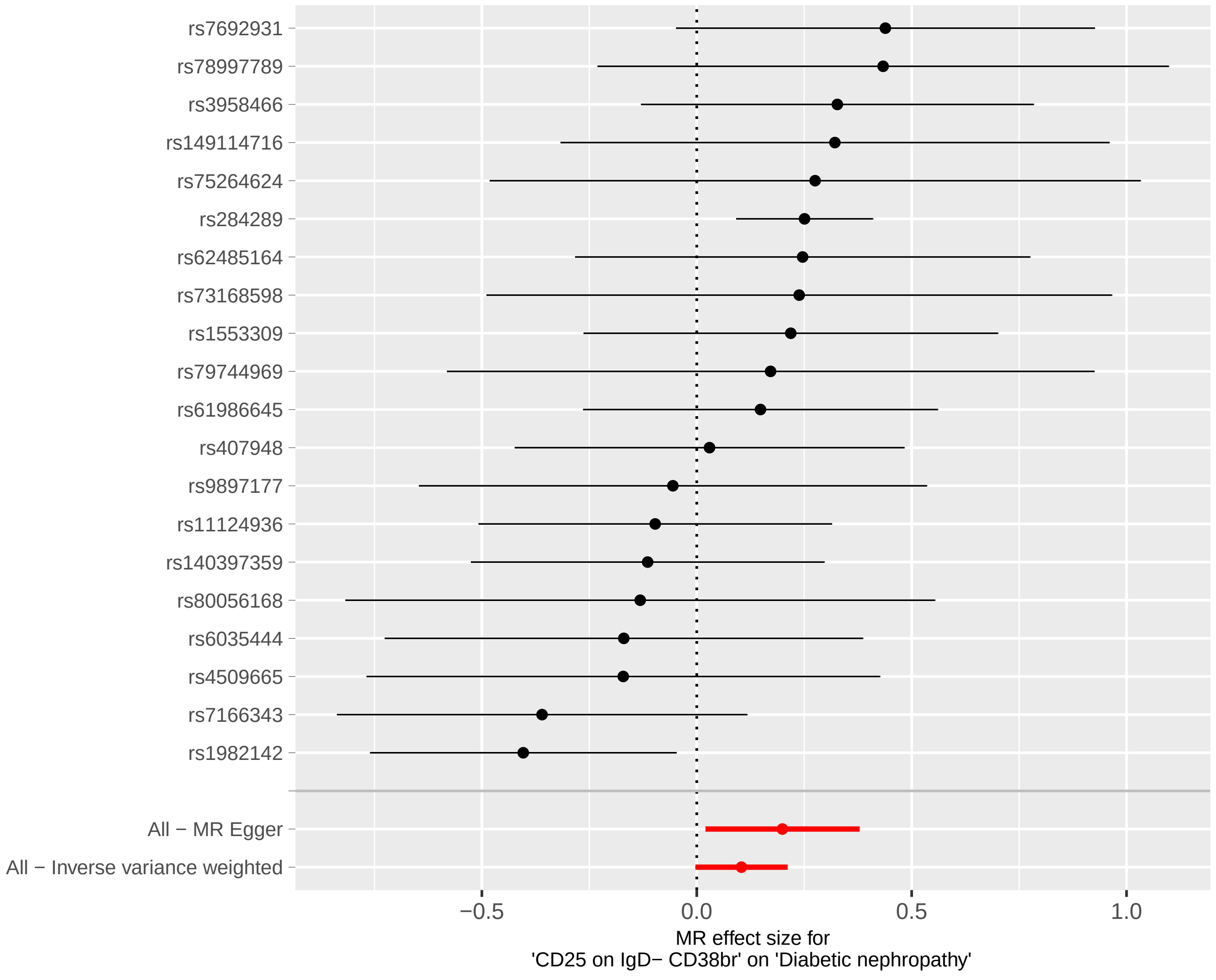
rs7692931
rs78997789
rs3958466
rs149114716
rs75264624
rs284289
rs62485164
rs73168598
rs1553309
rs79744969
rs61986645
rs407948
rs9897177
rs11124936
rs140397359
rs80056168
rs6035444
rs4509665
rs7166343
rs1982142
All − MR Egger
All − Inverse variance weighted
−0.5
0.0
0.5
1.0
MR effect size for
'CD25 on IgD− CD38br' on 'Diabetic nephropathy'

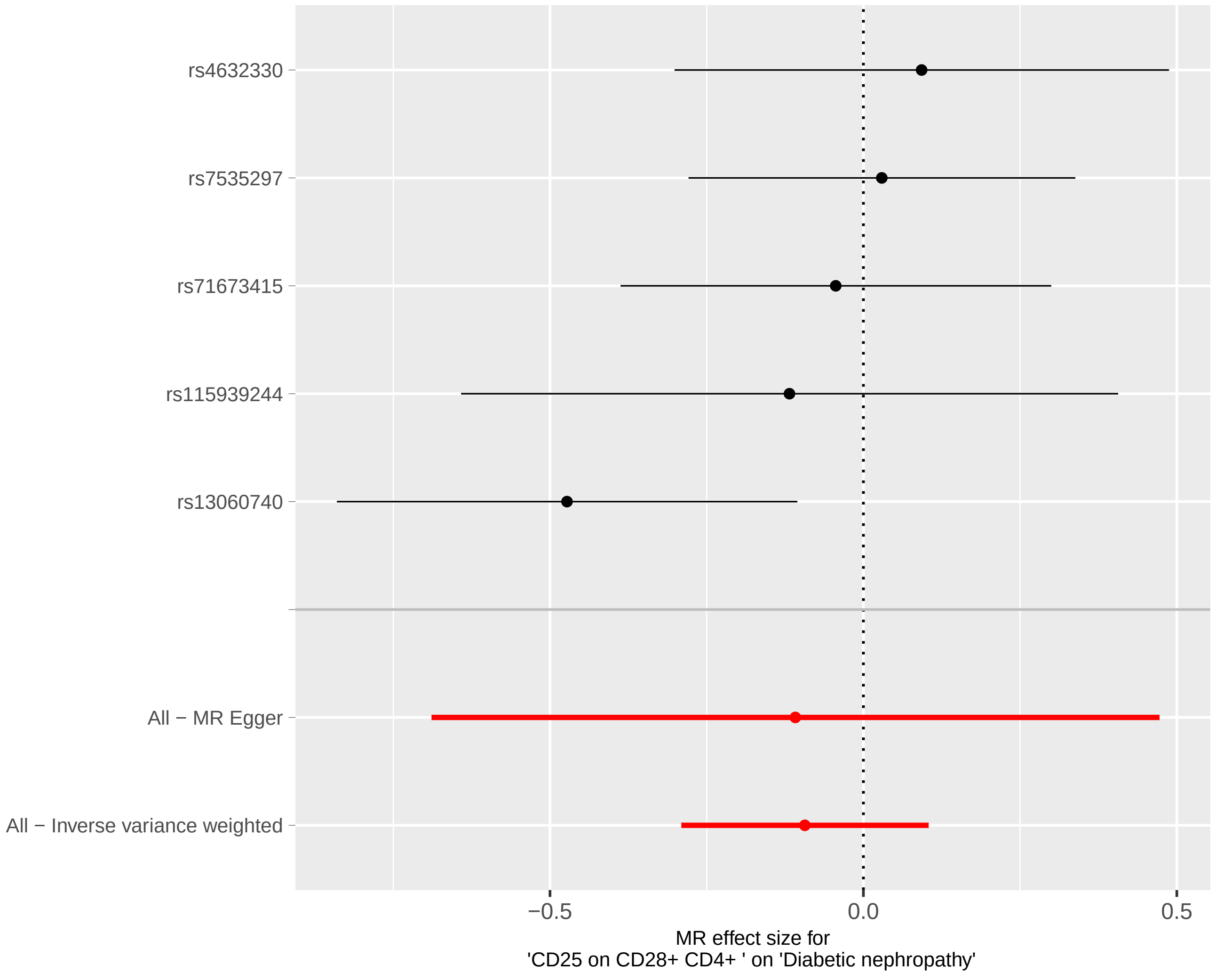
rs4632330
rs7535297
rs71673415
rs115939244
rs13060740
All − MR Egger
All − Inverse variance weighted
−0.5
0.0
0.5
MR effect size for
'CD25 on CD28+ CD4+ ' on 'Diabetic nephropathy'

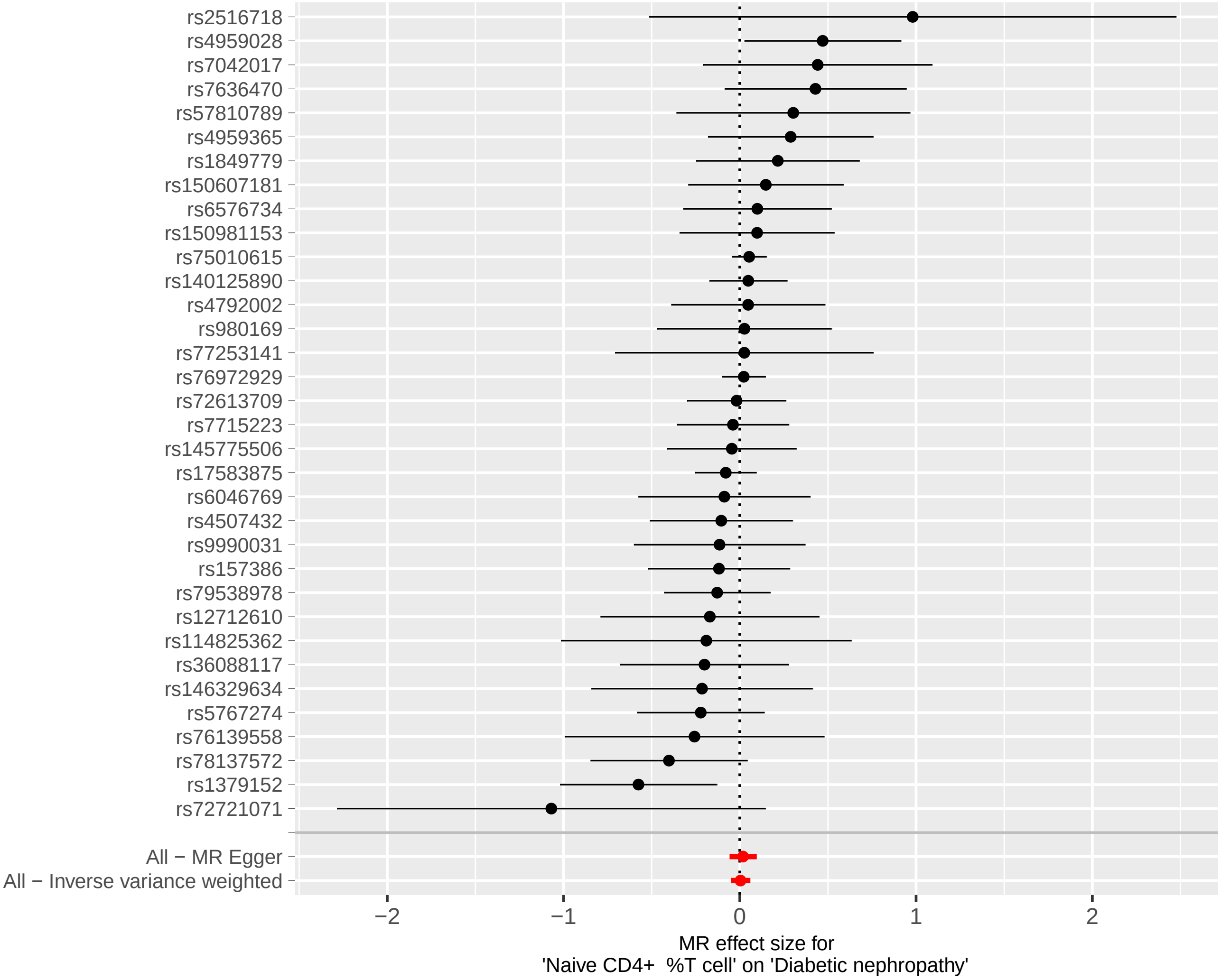
rs2516718
rs4959028
rs7042017
rs7636470
rs57810789
rs4959365
rs1849779
rs150607181
rs6576734
rs150981153
rs75010615
rs140125890
rs4792002
rs980169
rs77253141
rs76972929
rs72613709
rs7715223
rs145775506
rs17583875
rs6046769
rs4507432
rs9990031
rs157386
rs79538978
rs12712610
rs114825362
rs36088117
rs146329634
rs5767274
rs76139558
rs78137572
rs1379152
rs72721071
All − MR Egger
All − Inverse variance weighted
−2
−1
0
1
2
MR effect size for
'Naive CD4+ %T cell' on 'Diabetic nephropathy'

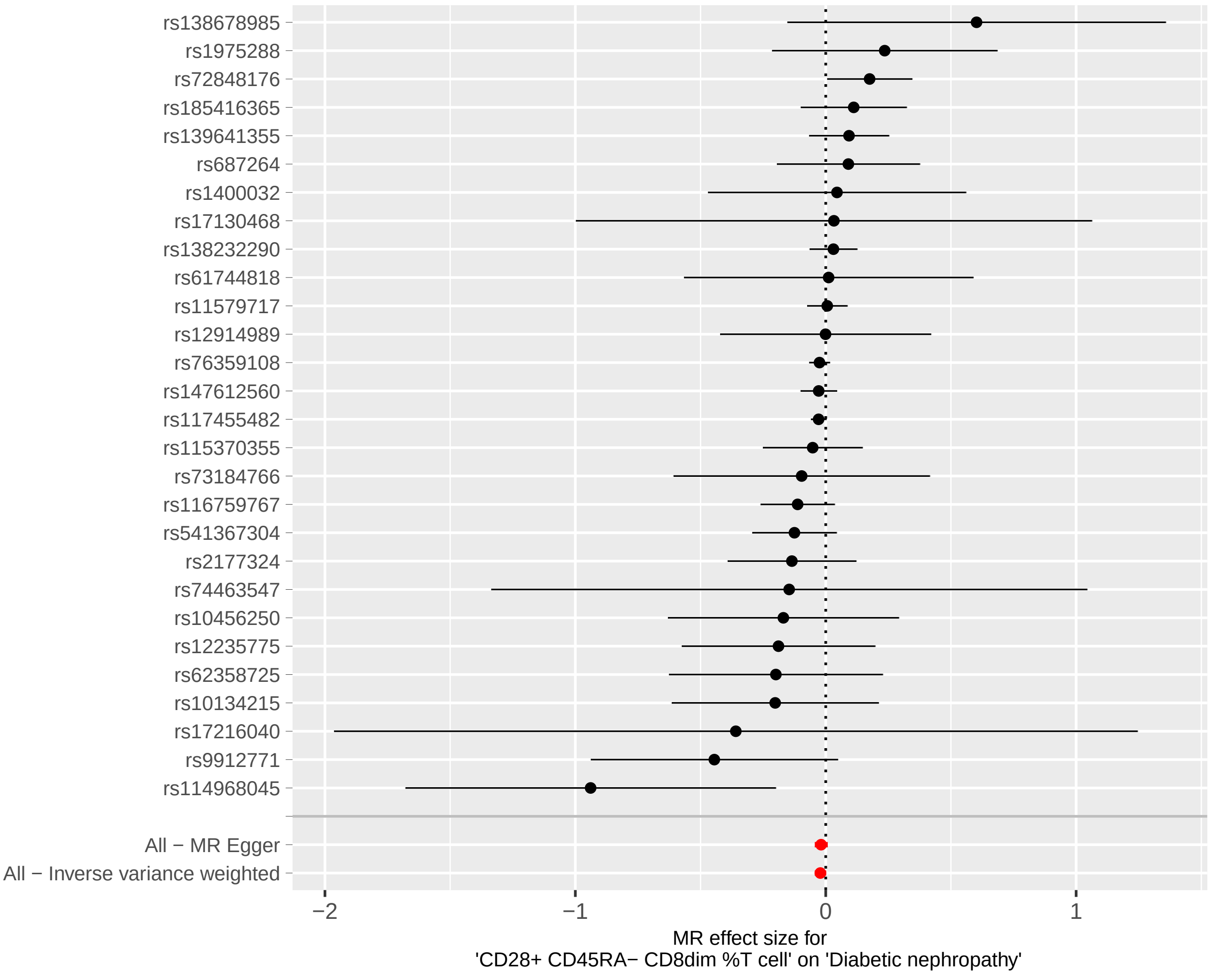
rs138678985
rs1975288
rs72848176
rs185416365
rs139641355
rs687264
rs1400032
rs17130468
rs138232290
rs61744818
rs11579717
rs12914989
rs76359108
rs147612560
rs117455482
rs115370355
rs73184766
rs116759767
rs541367304
rs2177324
rs74463547
rs10456250
rs12235775
rs62358725
rs10134215
rs17216040
rs9912771
rs114968045
All − MR Egger
All − Inverse variance weighted
−2
−1
0
1
MR effect size for
'CD28+ CD45RA− CD8dim %T cell' on 'Diabetic nephropathy'

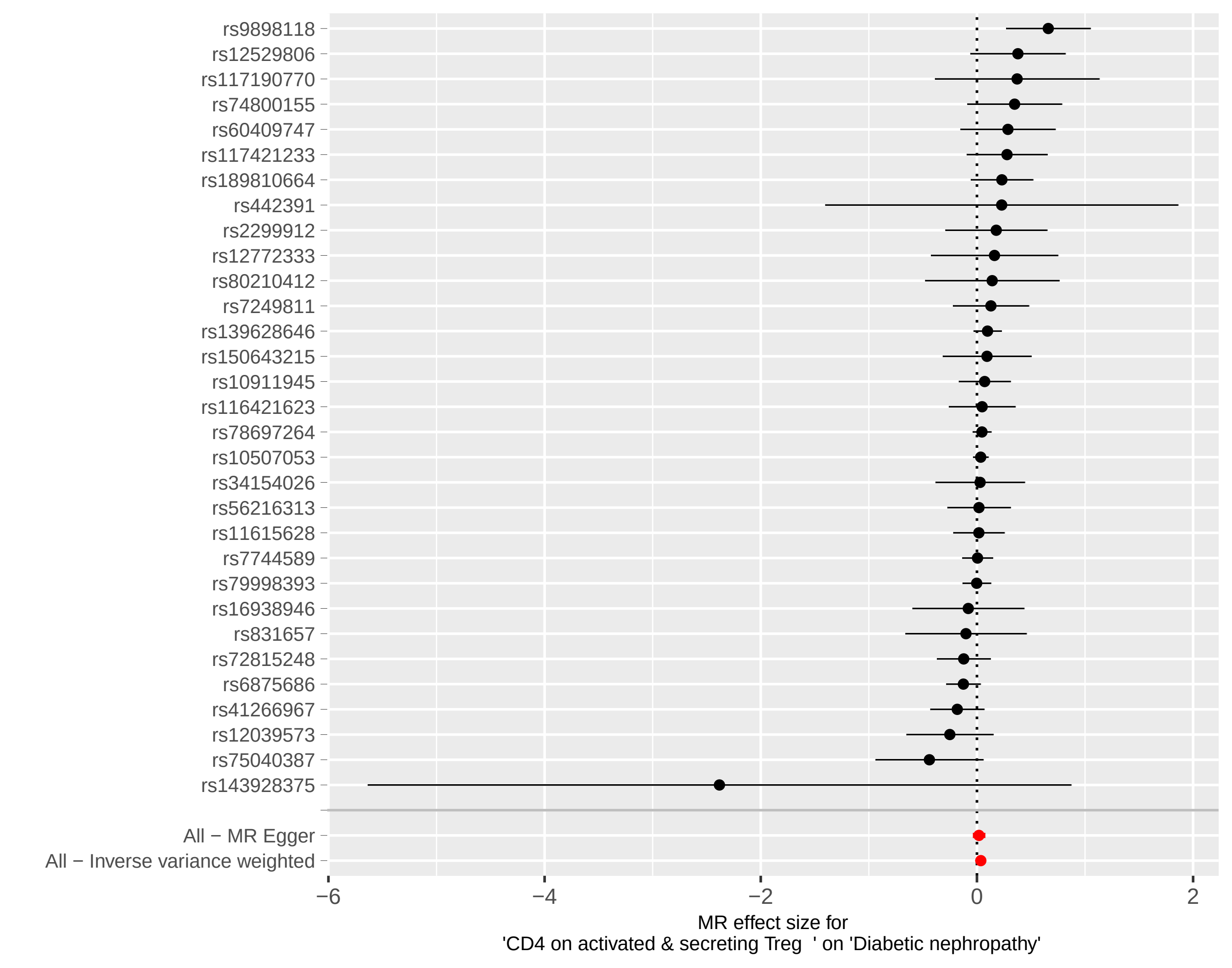
rs9898118
rs12529806
rs117190770
rs74800155
rs60409747
rs117421233
rs189810664
rs442391
rs2299912
rs12772333
rs80210412
rs7249811
rs139628646
rs150643215
rs10911945
rs116421623
rs78697264
rs10507053
rs34154026
rs56216313
rs11615628
rs7744589
rs79998393
rs16938946
rs831657
rs72815248
rs6875686
rs41266967
rs12039573
rs75040387
rs143928375
All − MR Egger
All − Inverse variance weighted
−6
−4
−2
0
2
MR effect size for
'CD4 on activated & secreting Treg ' on 'Diabetic nephropathy'

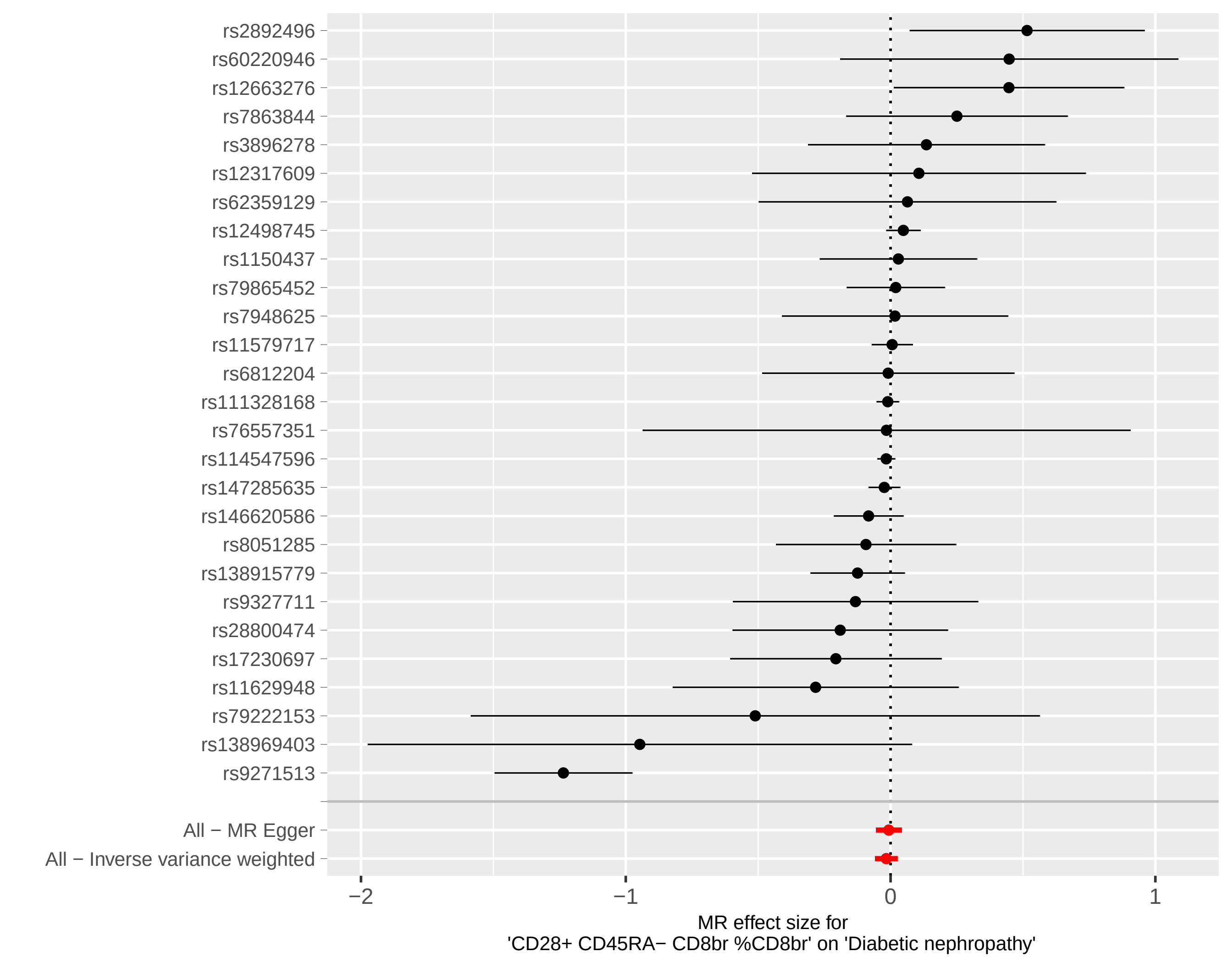
rs2892496
rs60220946
rs12663276
rs7863844
rs3896278
rs12317609
rs62359129
rs12498745
rs1150437
rs79865452
rs7948625
rs11579717
rs6812204
rs111328168
rs76557351
rs114547596
rs147285635
rs146620586
rs8051285
rs138915779
rs9327711
rs28800474
rs17230697
rs11629948
rs79222153
rs138969403
rs9271513
All − MR Egger
All − Inverse variance weighted
−2
−1
0
1
MR effect size for
'CD28+ CD45RA− CD8br %CD8br' on 'Diabetic nephropathy'

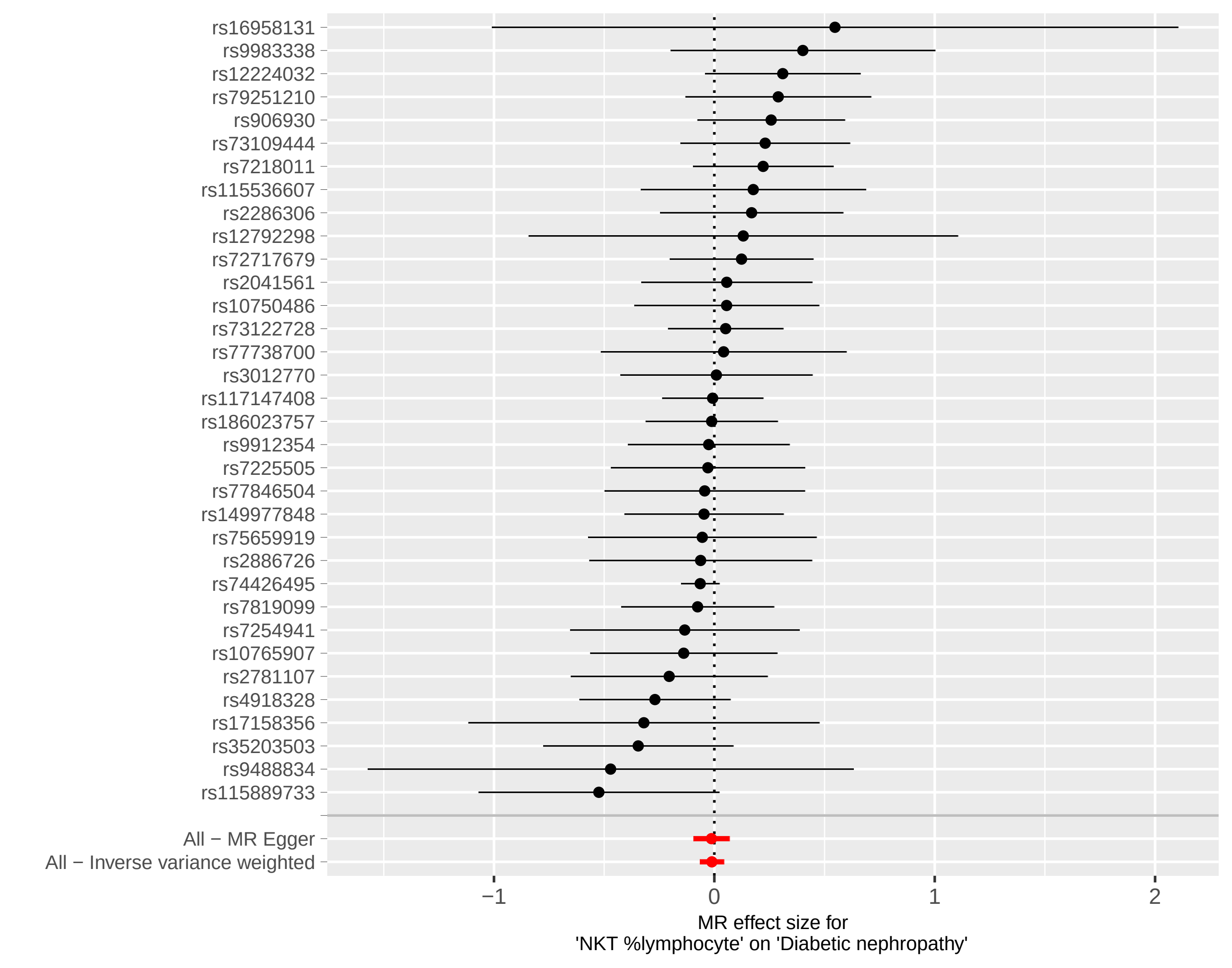
rs16958131
rs9983338
rs12224032
rs79251210
rs906930
rs73109444
rs7218011
rs115536607
rs2286306
rs12792298
rs72717679
rs2041561
rs10750486
rs73122728
rs77738700
rs3012770
rs117147408
rs186023757
rs9912354
rs7225505
rs77846504
rs149977848
rs75659919
rs2886726
rs74426495
rs7819099
rs7254941
rs10765907
rs2781107
rs4918328
rs17158356
rs35203503
rs9488834
rs115889733
All − MR Egger
All − Inverse variance weighted
−1
0
1
2
MR effect size for
'NKT %lymphocyte' on 'Diabetic nephropathy'

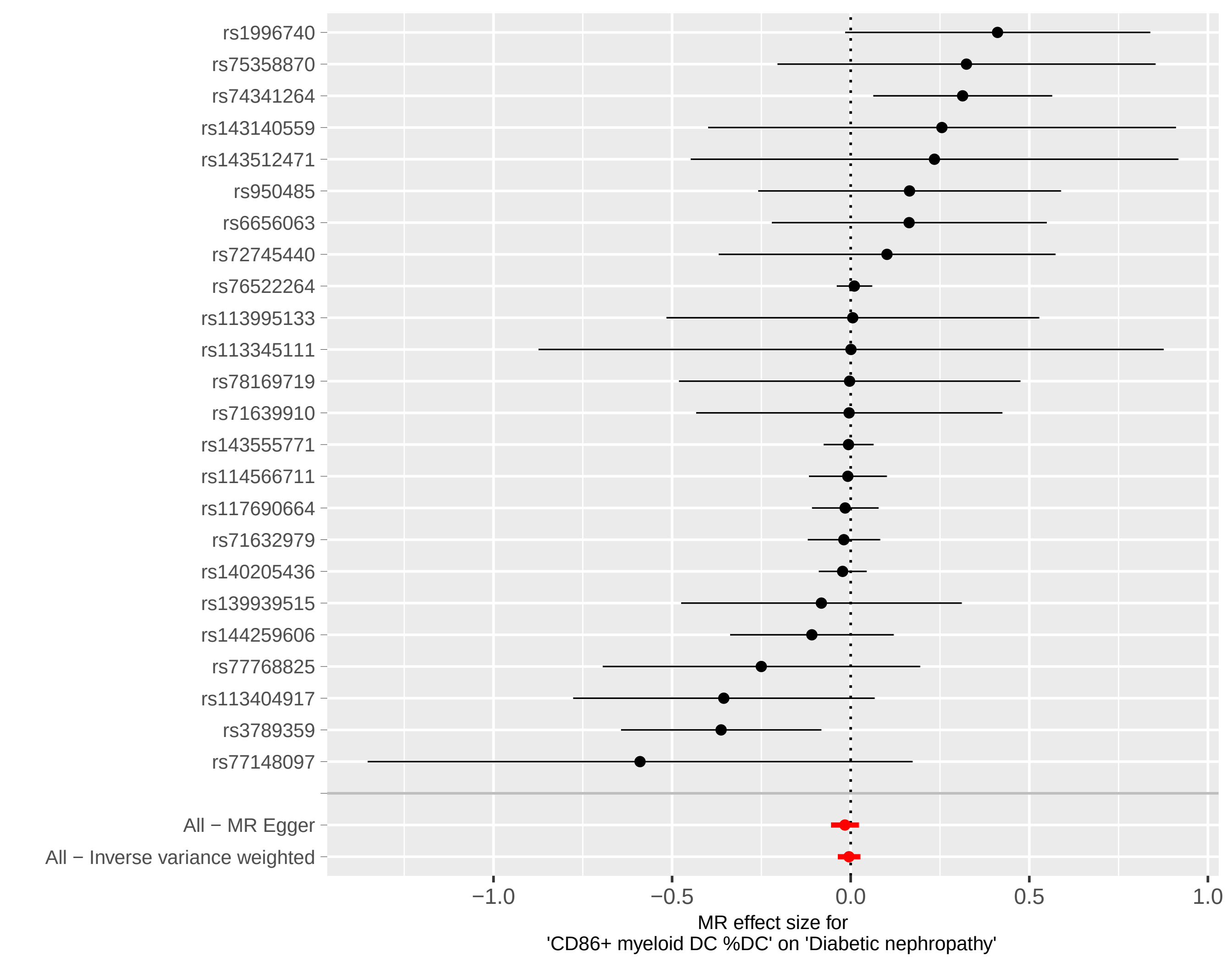
rs1996740
rs75358870
rs74341264
rs143140559
rs143512471
rs950485
rs6656063
rs72745440
rs76522264
rs113995133
rs113345111
rs78169719
rs71639910
rs143555771
rs114566711
rs117690664
rs71632979
rs140205436
rs139939515
rs144259606
rs77768825
rs113404917
rs3789359
rs77148097
All − MR Egger
All − Inverse variance weighted
−1.0
−0.5
0.0
0.5
1.0
MR effect size for
'CD86+ myeloid DC %DC' on 'Diabetic nephropathy'

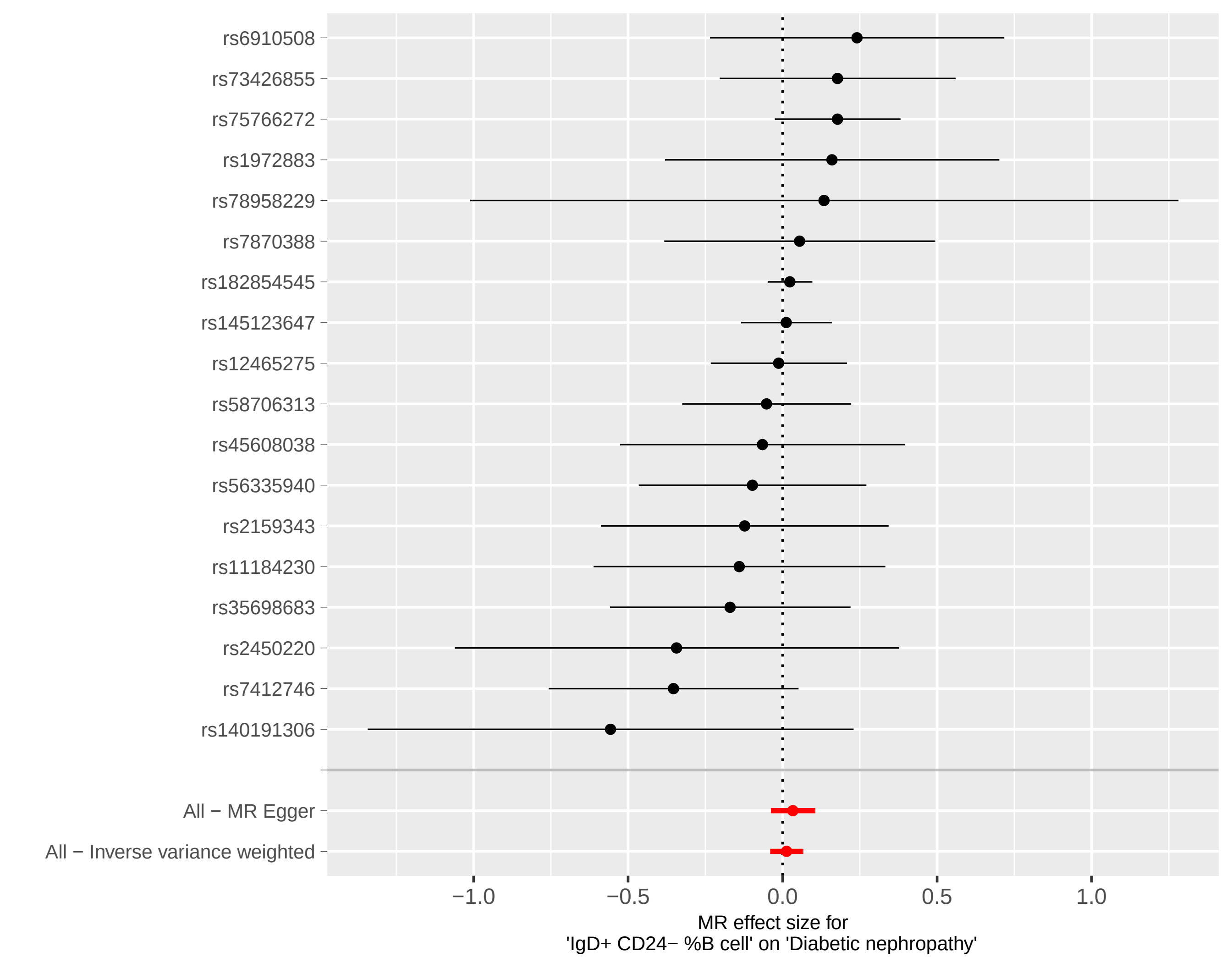
rs6910508
rs73426855
rs75766272
rs1972883
rs78958229
rs7870388
rs182854545
rs145123647
rs12465275
rs58706313
rs45608038
rs56335940
rs2159343
rs11184230
rs35698683
rs2450220
rs7412746
rs140191306
All − MR Egger
All − Inverse variance weighted
−1.0
−0.5
0.0
0.5
1.0
MR effect size for
'IgD+ CD24− %B cell' on 'Diabetic nephropathy'

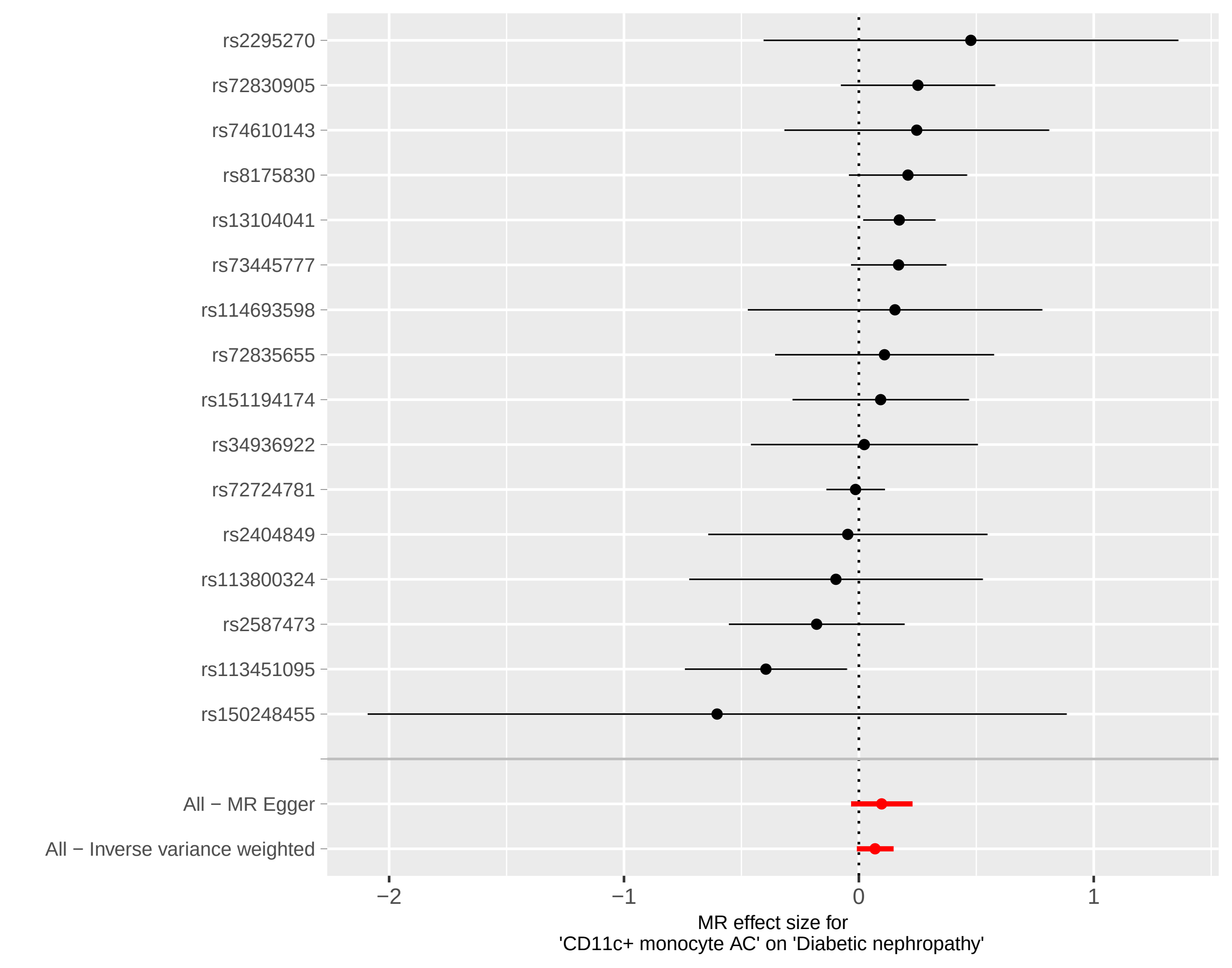

rs2295270
rs72830905
rs74610143
rs8175830
rs13104041
rs73445777
rs114693598
rs72835655
rs151194174
rs34936922
rs72724781
rs2404849
rs113800324
rs2587473
rs113451095
rs150248455
All − MR Egger
All − Inverse variance weighted
−2
−1
0
1
MR effect size for
'CD11c+ monocyte AC' on 'Diabetic nephropathy'

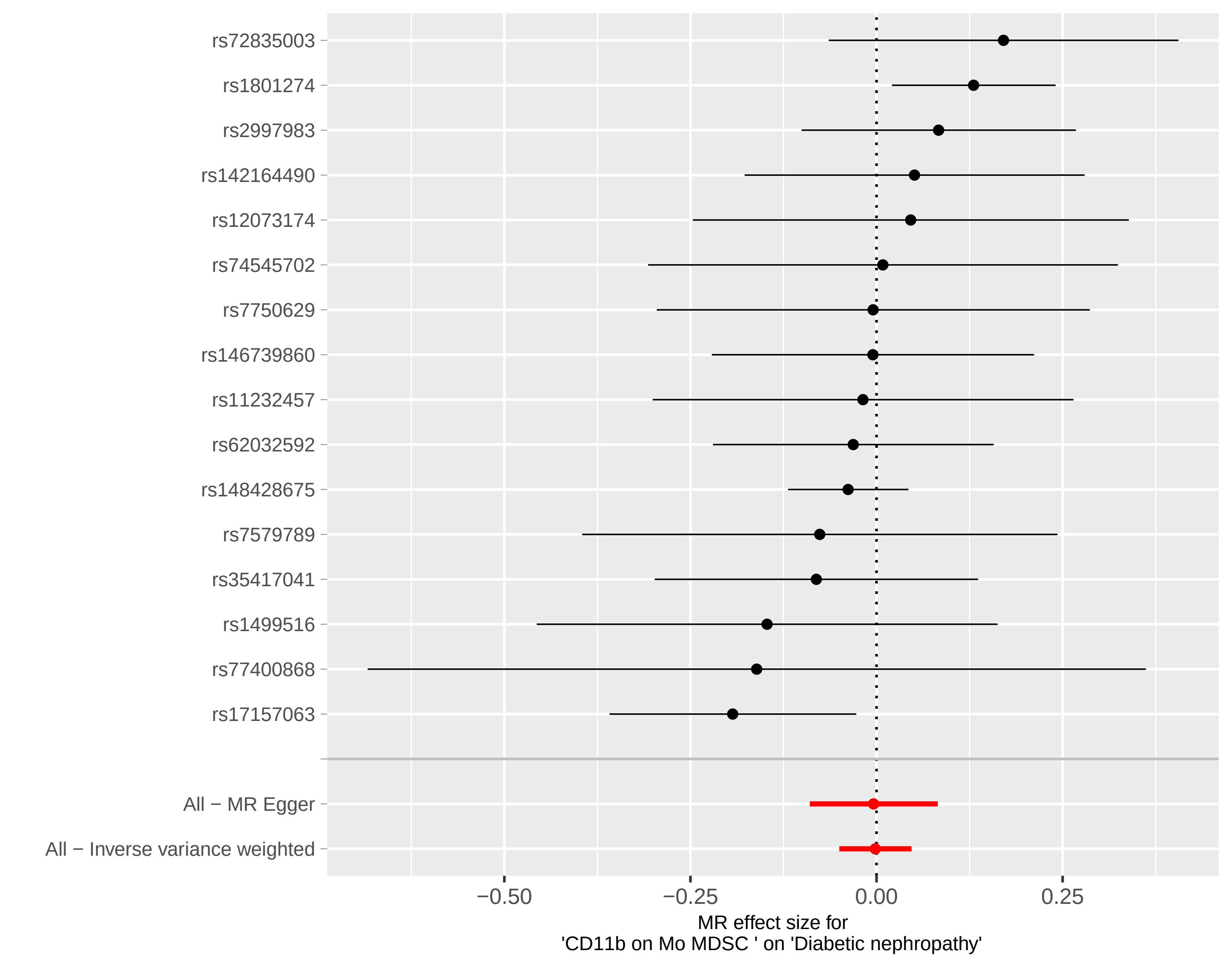
rs72835003
rs1801274
rs2997983
rs142164490
rs12073174
rs74545702
rs7750629
rs146739860
rs11232457
rs62032592
rs148428675
rs7579789
rs35417041
rs1499516
rs77400868
rs17157063
All − MR Egger
All − Inverse variance weighted
−0.50
−0.25
0.00
0.25
MR effect size for
'CD11b on Mo MDSC ' on 'Diabetic nephropathy'

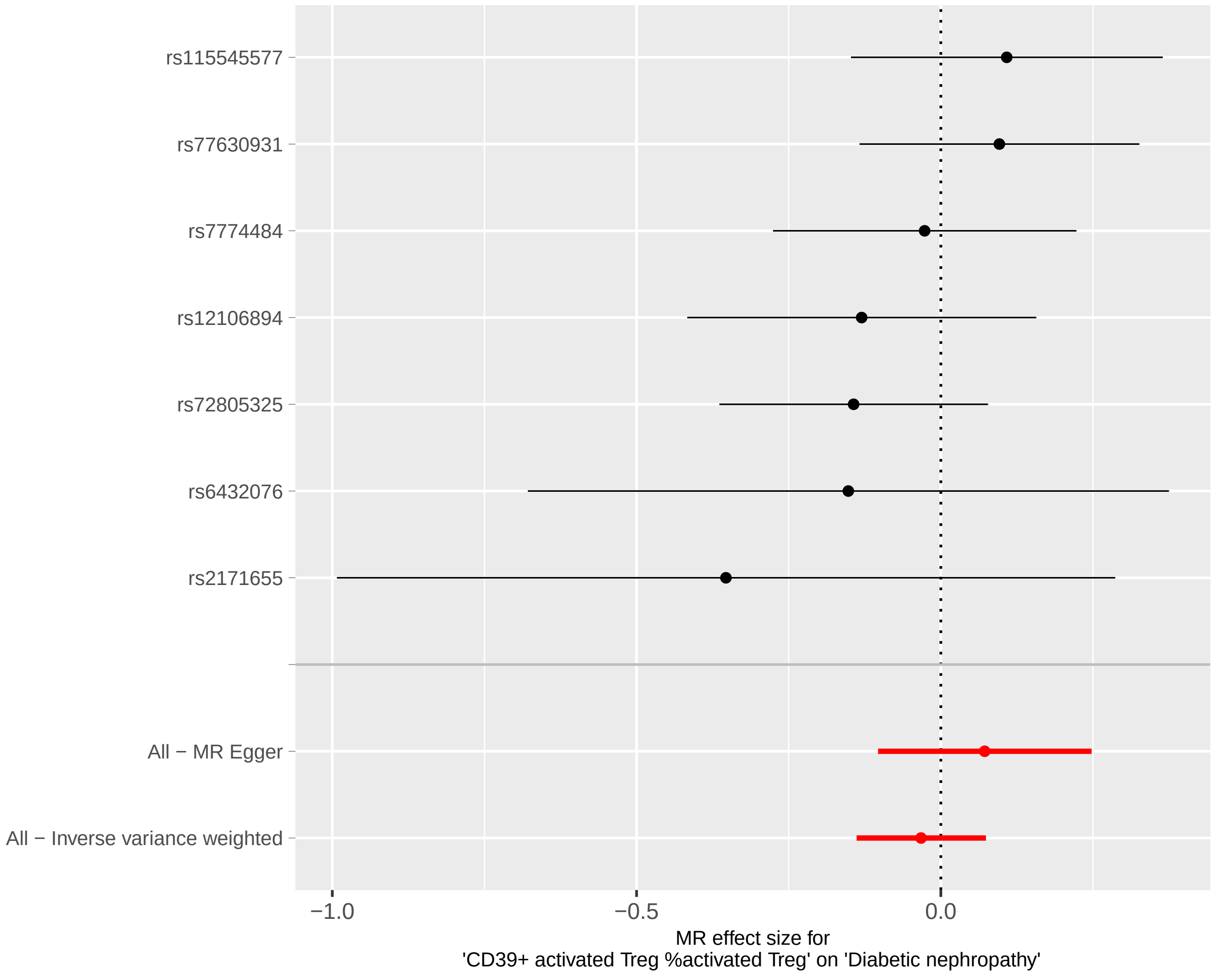
rs115545577
rs77630931
rs7774484
rs12106894
rs72805325
rs6432076
rs2171655
All − MR Egger
All − Inverse variance weighted
−1.0
−0.5
0.0
MR effect size for
'CD39+ activated Treg %activated Treg' on 'Diabetic nephropathy'

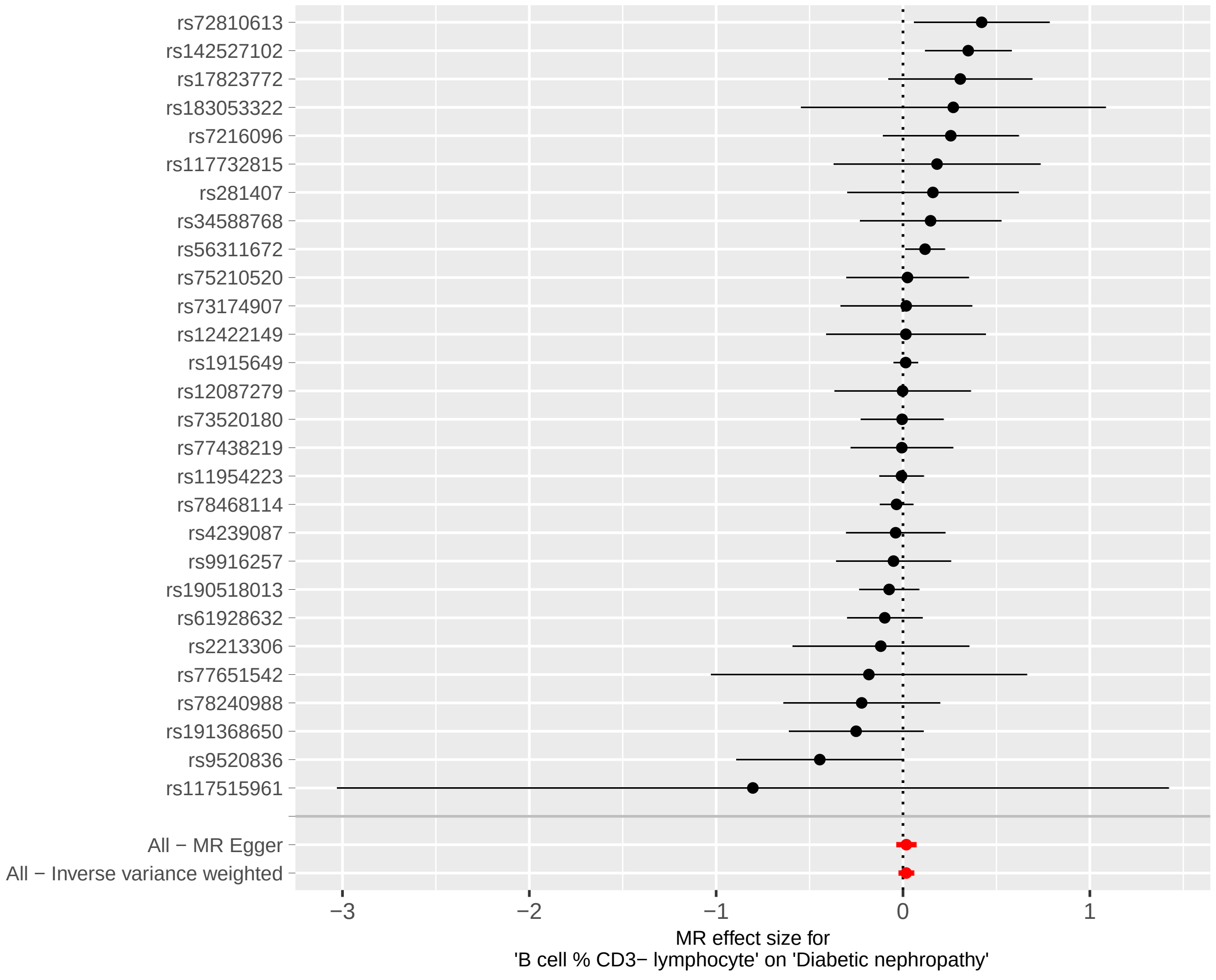
rs72810613
rs142527102
rs17823772
rs183053322
rs7216096
rs117732815
rs281407
rs34588768
rs56311672
rs75210520
rs73174907
rs12422149
rs1915649
rs12087279
rs73520180
rs77438219
rs11954223
rs78468114
rs4239087
rs9916257
rs190518013
rs61928632
rs2213306
rs77651542
rs78240988
rs191368650
rs9520836
rs117515961
All − MR Egger
All − Inverse variance weighted
−3
−2
−1
0
1
MR effect size for
'B cell % CD3− lymphocyte' on 'Diabetic nephropathy'

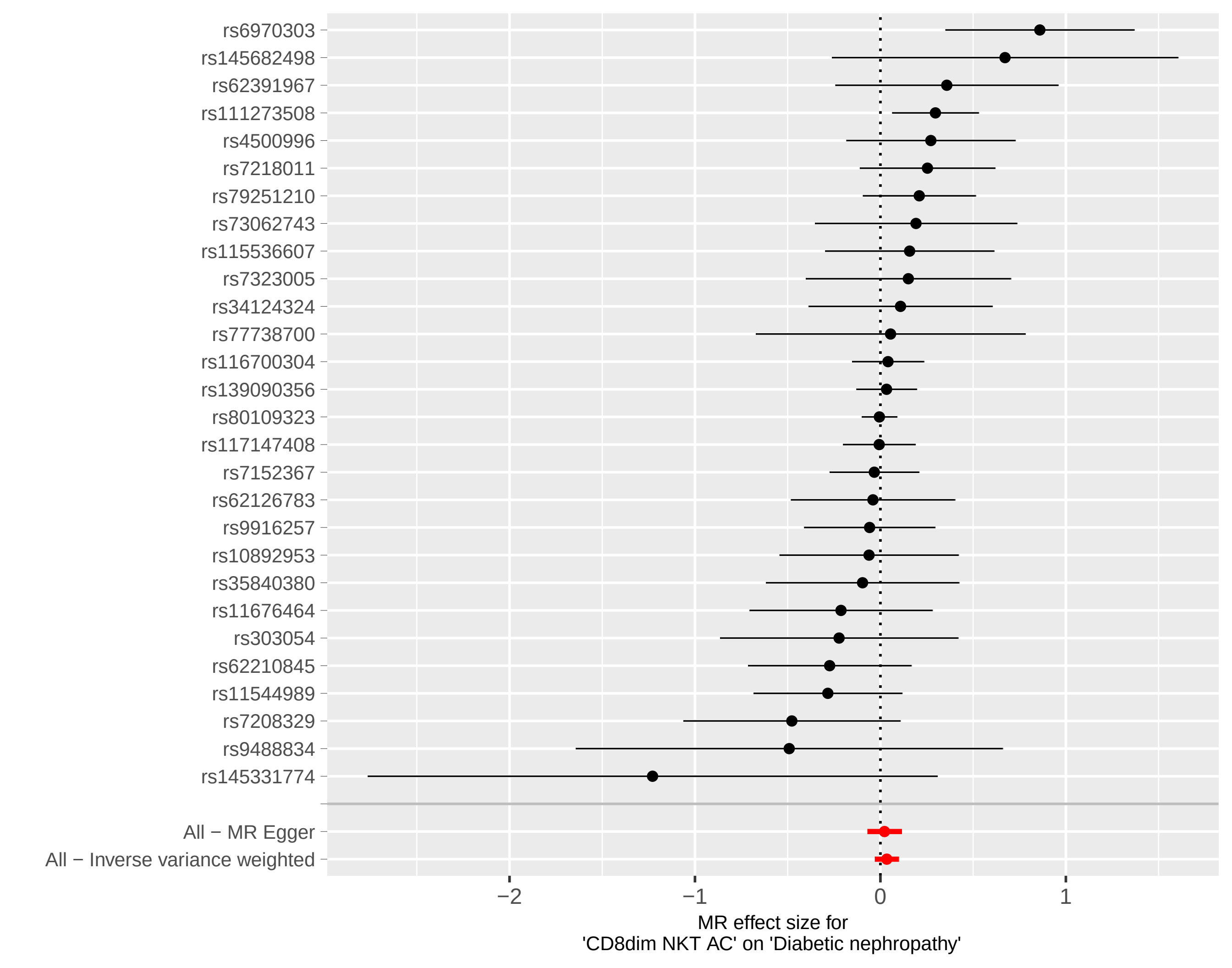
rs6970303
rs145682498
rs62391967
rs111273508
rs4500996
rs7218011
rs79251210
rs73062743
rs115536607
rs7323005
rs34124324
rs77738700
rs116700304
rs139090356
rs80109323
rs117147408
rs7152367
rs62126783
rs9916257
rs10892953
rs35840380
rs11676464
rs303054
rs62210845
rs11544989
rs7208329
rs9488834
rs145331774
All − MR Egger
All − Inverse variance weighted
−2
−1
0
1
MR effect size for
'CD8dim NKT AC' on 'Diabetic nephropathy'

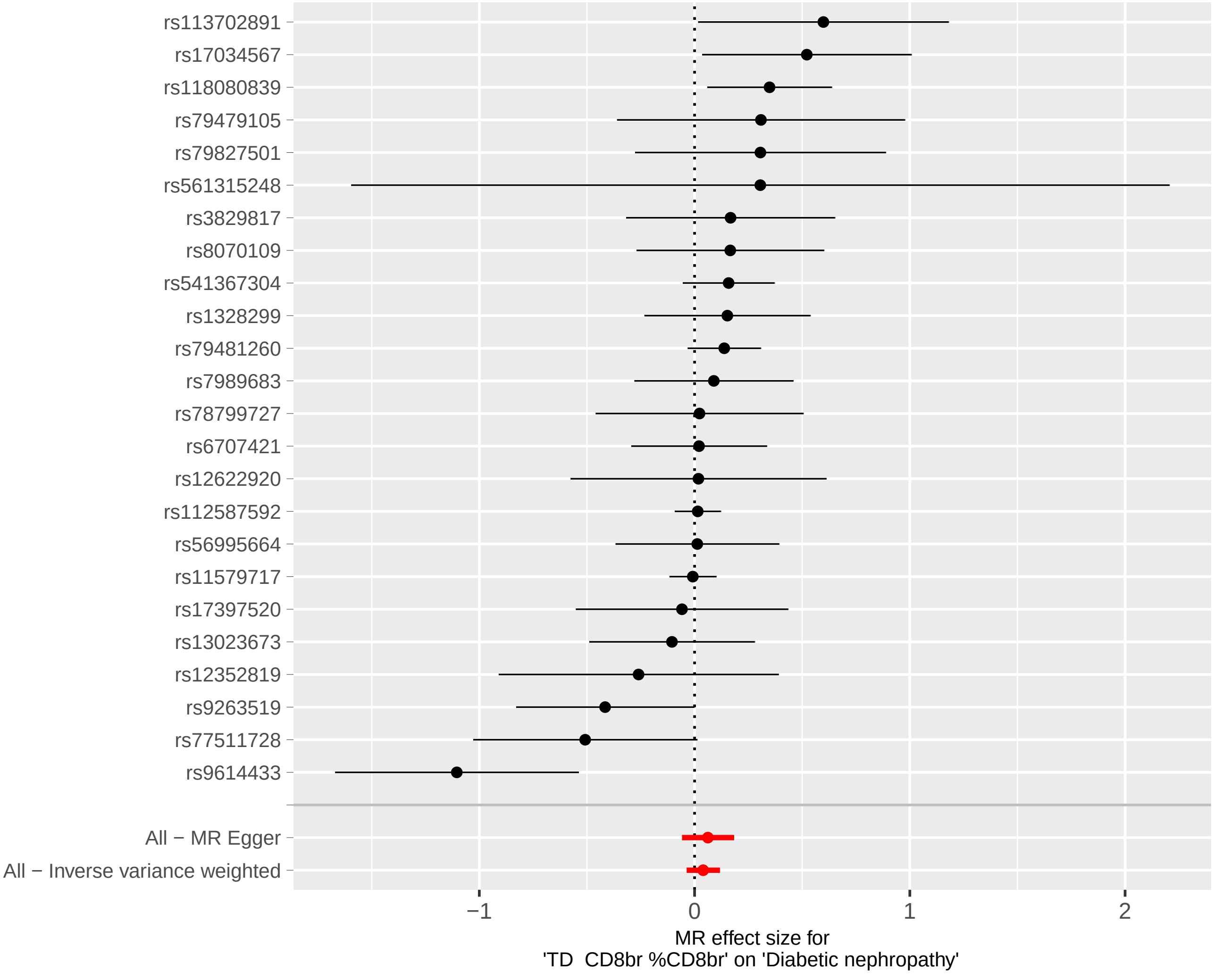

rs113702891
rs17034567
rs118080839
rs79479105
rs79827501
rs561315248
rs3829817
rs8070109
rs541367304
rs1328299
rs79481260
rs7989683
rs78799727
rs6707421
rs12622920
rs112587592
rs56995664
rs11579717
rs17397520
rs13023673
rs12352819
rs9263519
rs77511728
rs9614433
All − MR Egger
All − Inverse variance weighted
−1
0
1
2
MR effect size for
'TD CD8br %CD8br' on 'Diabetic nephropathy'

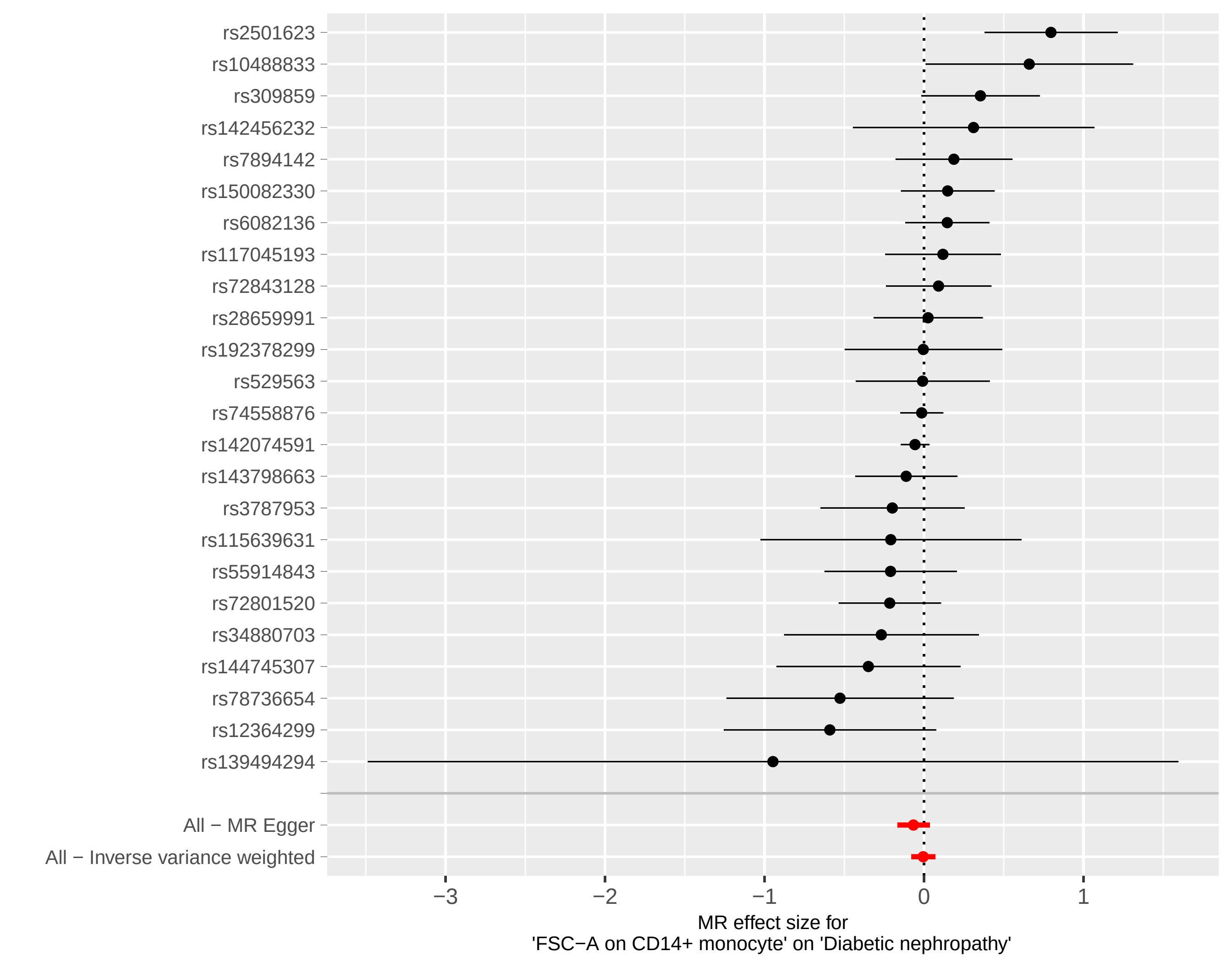

rs2501623
rs10488833
rs309859
rs142456232
rs7894142
rs150082330
rs6082136
rs117045193
rs72843128
rs28659991
rs192378299
rs529563
rs74558876
rs142074591
rs143798663
rs3787953
rs115639631
rs55914843
rs72801520
rs34880703
rs144745307
rs78736654
rs12364299
rs139494294
All − MR Egger
All − Inverse variance weighted
−3
−2
−1
0
1
MR effect size for
'FSC−A on CD14+ monocyte' on 'Diabetic nephropathy'

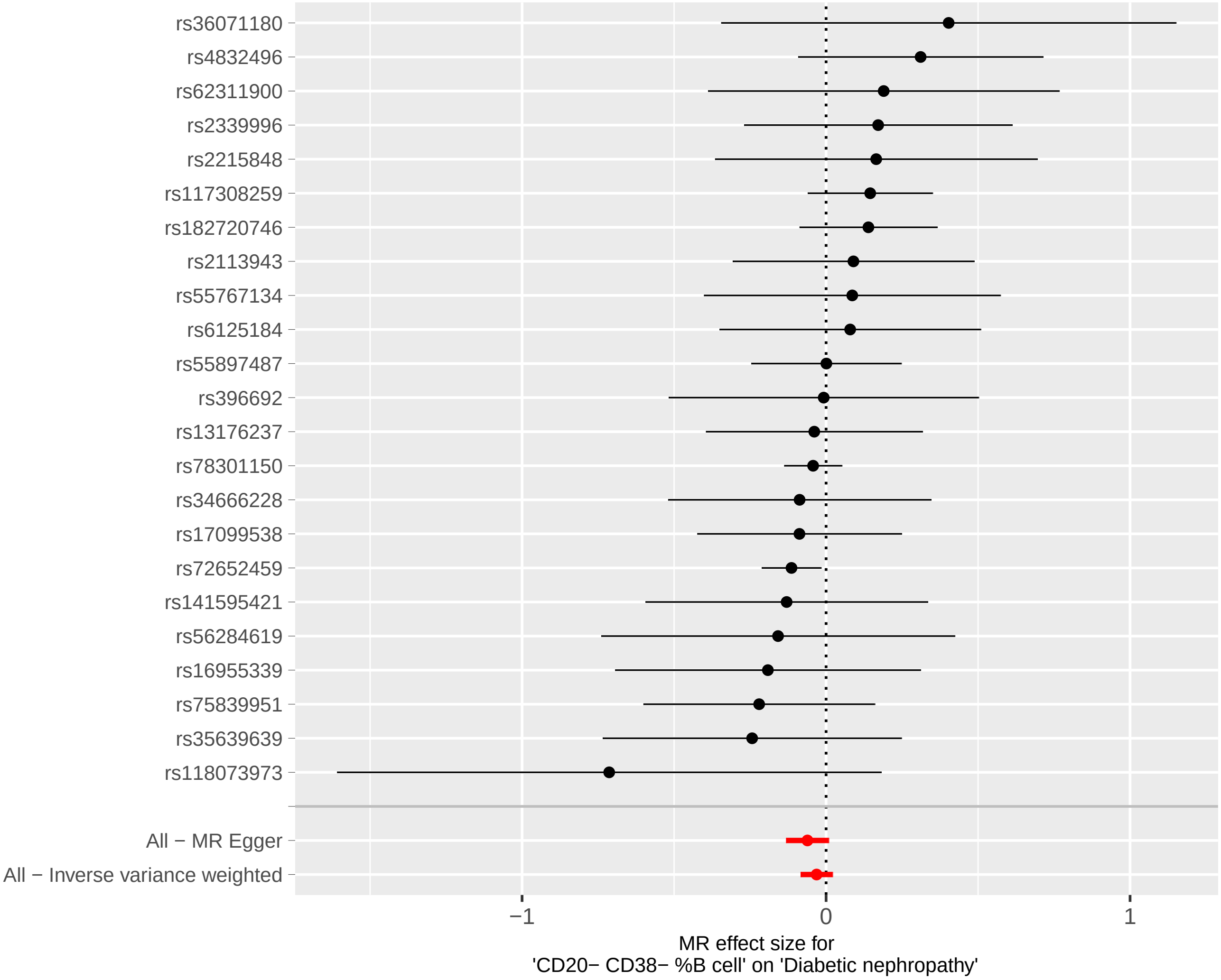
rs36071180
rs4832496
rs62311900
rs2339996
rs2215848
rs117308259
rs182720746
rs2113943
rs55767134
rs6125184
rs55897487
rs396692
rs13176237
rs78301150
rs34666228
rs17099538
rs72652459
rs141595421
rs56284619
rs16955339
rs75839951
rs35639639
rs118073973
All − MR Egger
All − Inverse variance weighted
−1
0
1
MR effect size for
'CD20− CD38− %B cell' on 'Diabetic nephropathy'

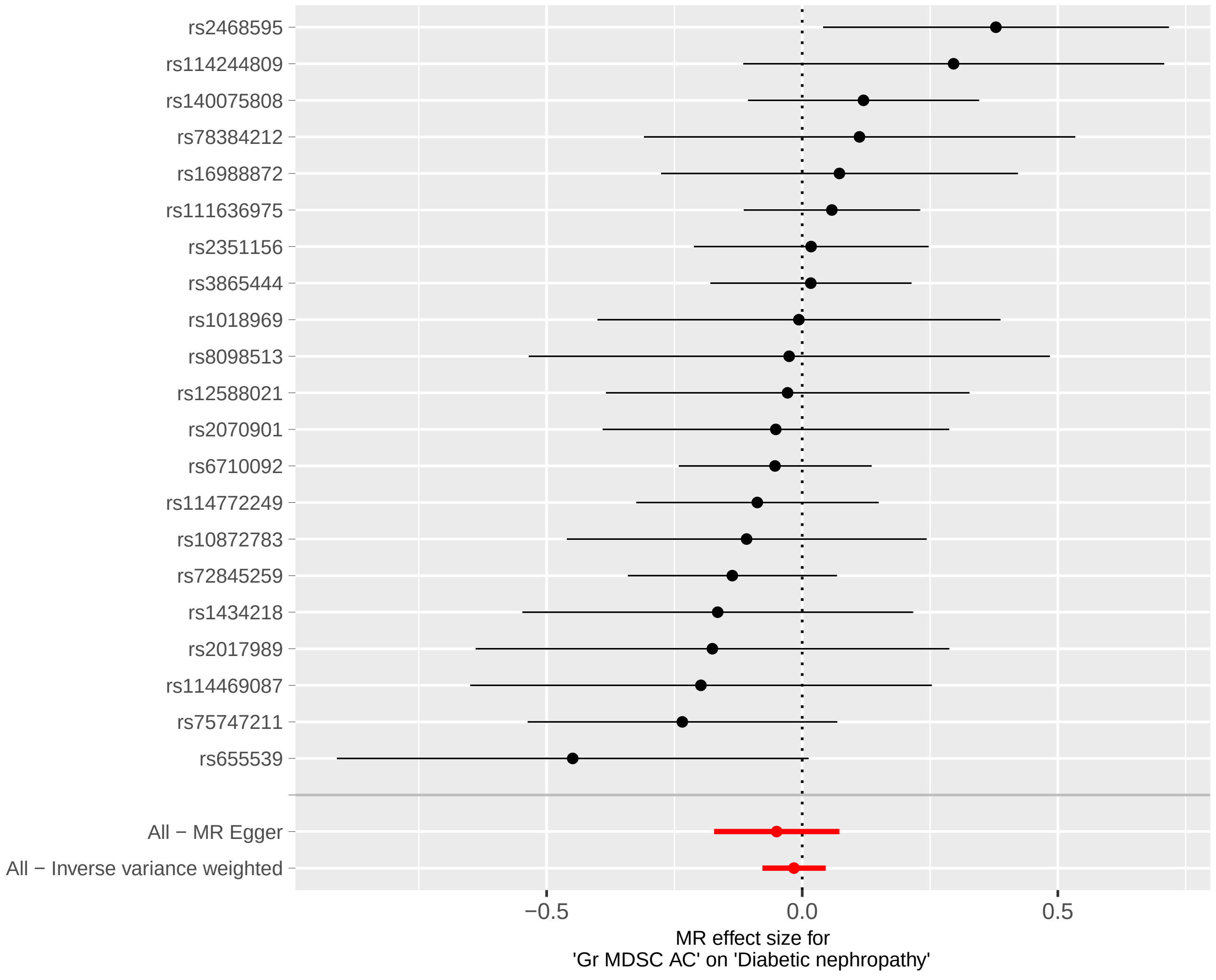
rs2468595
rs114244809
rs140075808
rs78384212
rs16988872
rs111636975
rs2351156
rs3865444
rs1018969
rs8098513
rs12588021
rs2070901
rs6710092
rs114772249
rs10872783
rs72845259
rs1434218
rs2017989
rs114469087
rs75747211
rs655539
All − MR Egger
All − Inverse variance weighted
−0.5
0.0
0.5
MR effect size for
'Gr MDSC AC' on 'Diabetic nephropathy'

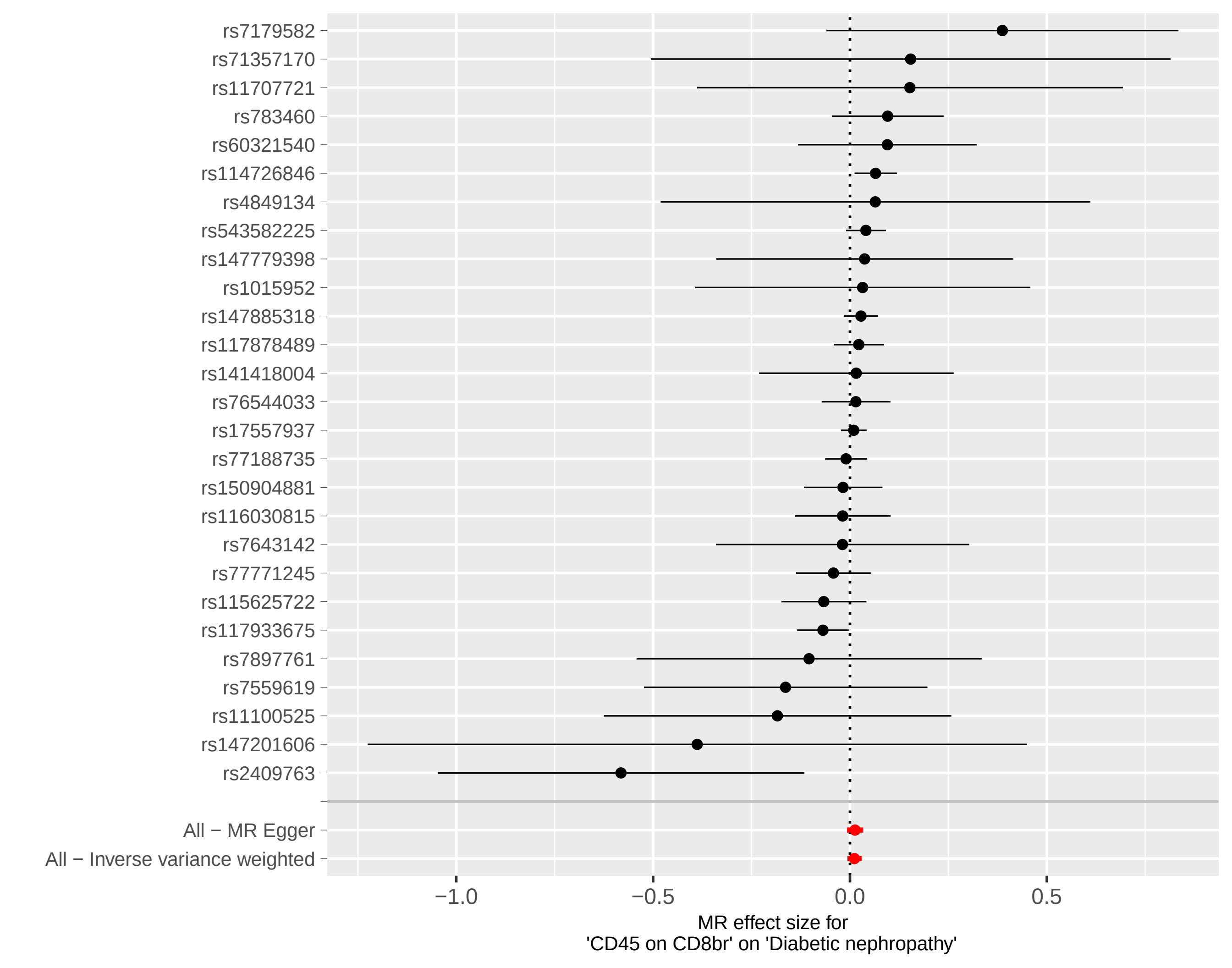
rs7179582
rs71357170
rs11707721
rs783460
rs60321540
rs114726846
rs4849134
rs543582225
rs147779398
rs1015952
rs147885318
rs117878489
rs141418004
rs76544033
rs17557937
rs77188735
rs150904881
rs116030815
rs7643142
rs77771245
rs115625722
rs117933675
rs7897761
rs7559619
rs11100525
rs147201606
rs2409763
All − MR Egger
All − Inverse variance weighted
−1.0
−0.5
0.0
0.5
MR effect size for
'CD45 on CD8br' on 'Diabetic nephropathy'

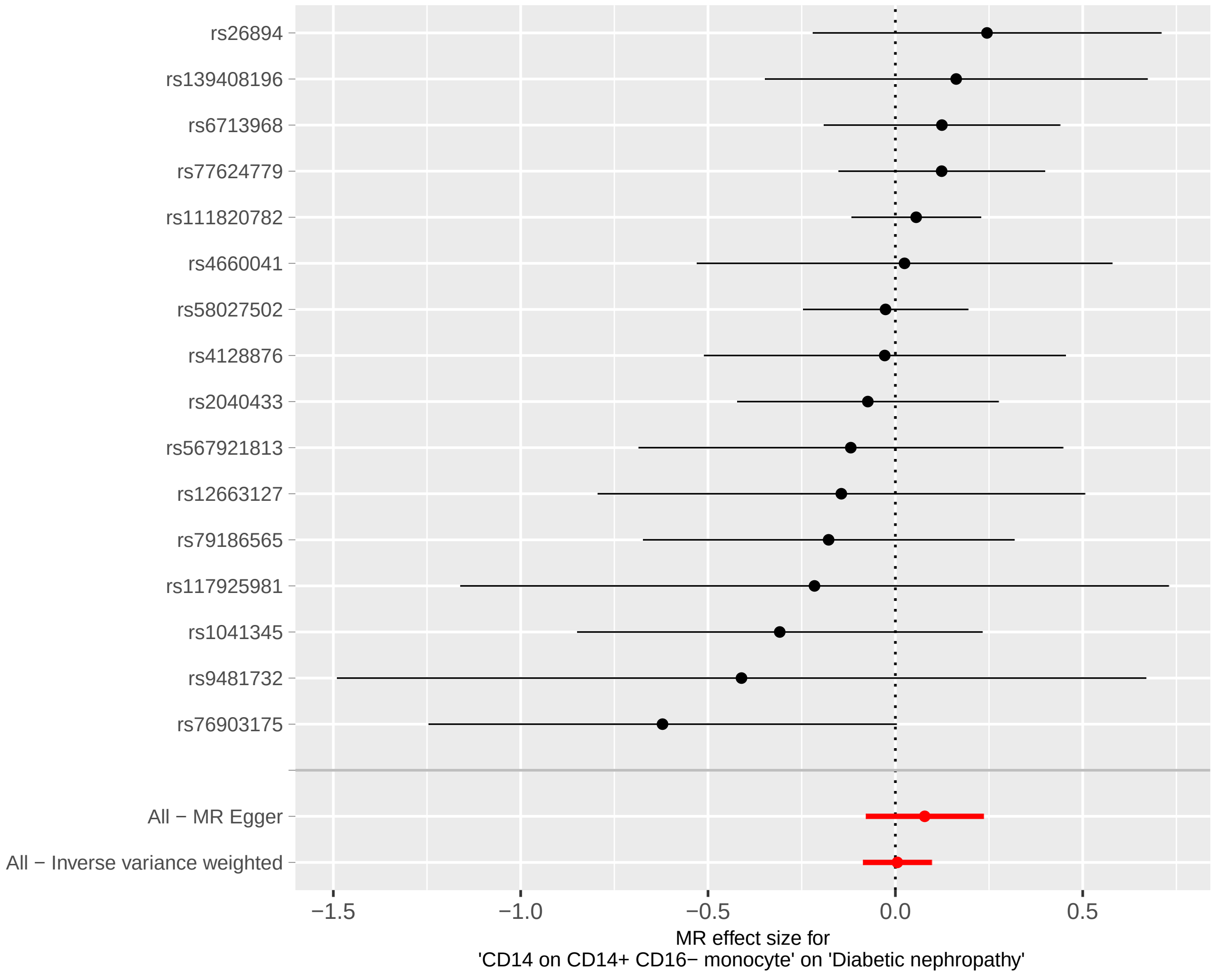
rs26894
rs139408196
rs6713968
rs77624779
rs111820782
rs4660041
rs58027502
rs4128876
rs2040433
rs567921813
rs12663127
rs79186565
rs117925981
rs1041345
rs9481732
rs76903175
All − MR Egger
All − Inverse variance weighted
−1.5
−1.0
−0.5
0.0
0.5
MR effect size for
'CD14 on CD14+ CD16− monocyte' on 'Diabetic nephropathy'

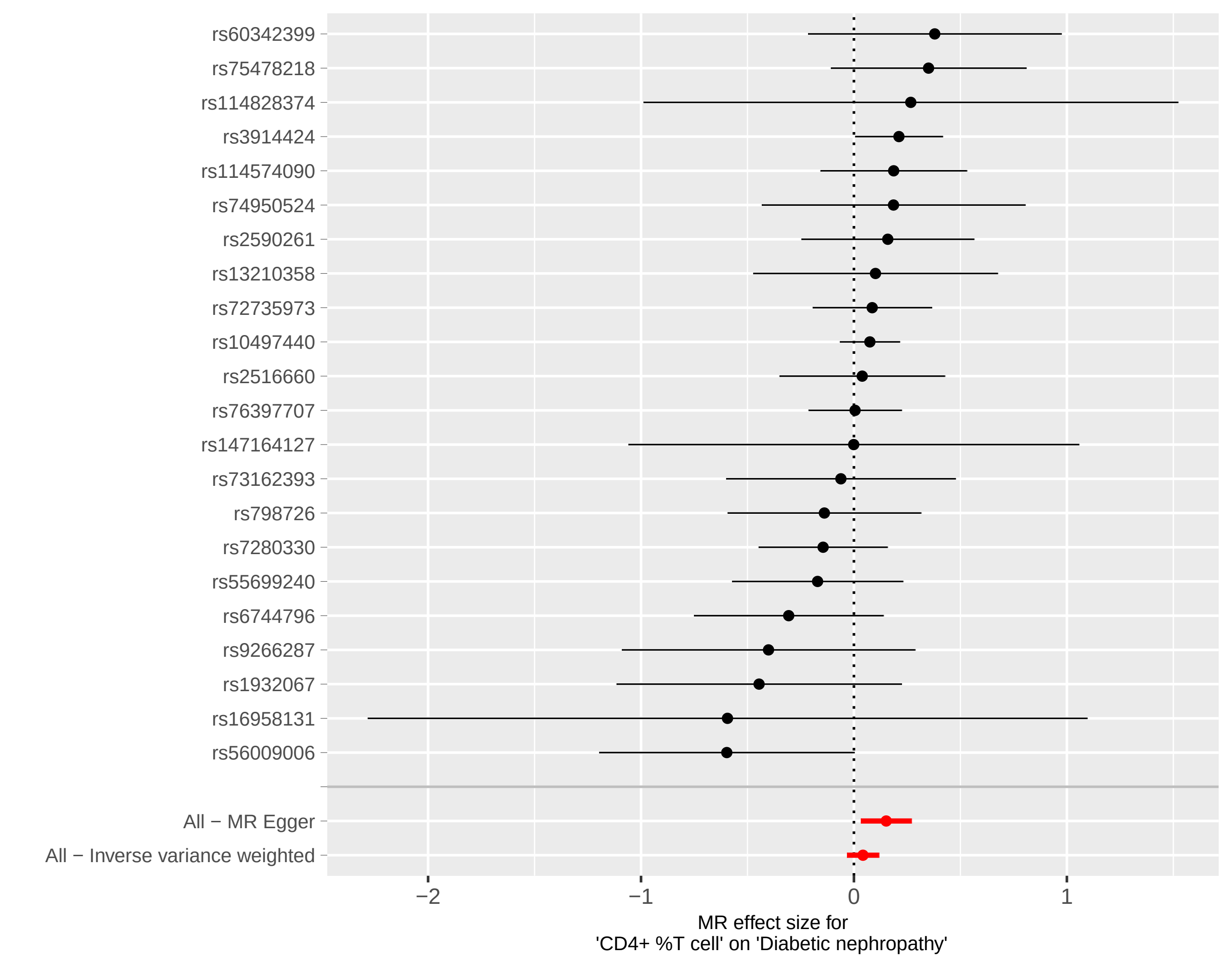

rs60342399
rs75478218
rs114828374
rs3914424
rs114574090
rs74950524
rs2590261
rs13210358
rs72735973
rs10497440
rs2516660
rs76397707
rs147164127
rs73162393
rs798726
rs7280330
rs55699240
rs6744796
rs9266287
rs1932067
rs16958131
rs56009006
All − MR Egger
All − Inverse variance weighted
−2
−1
0
1
MR effect size for
'CD4+ %T cell' on 'Diabetic nephropathy'

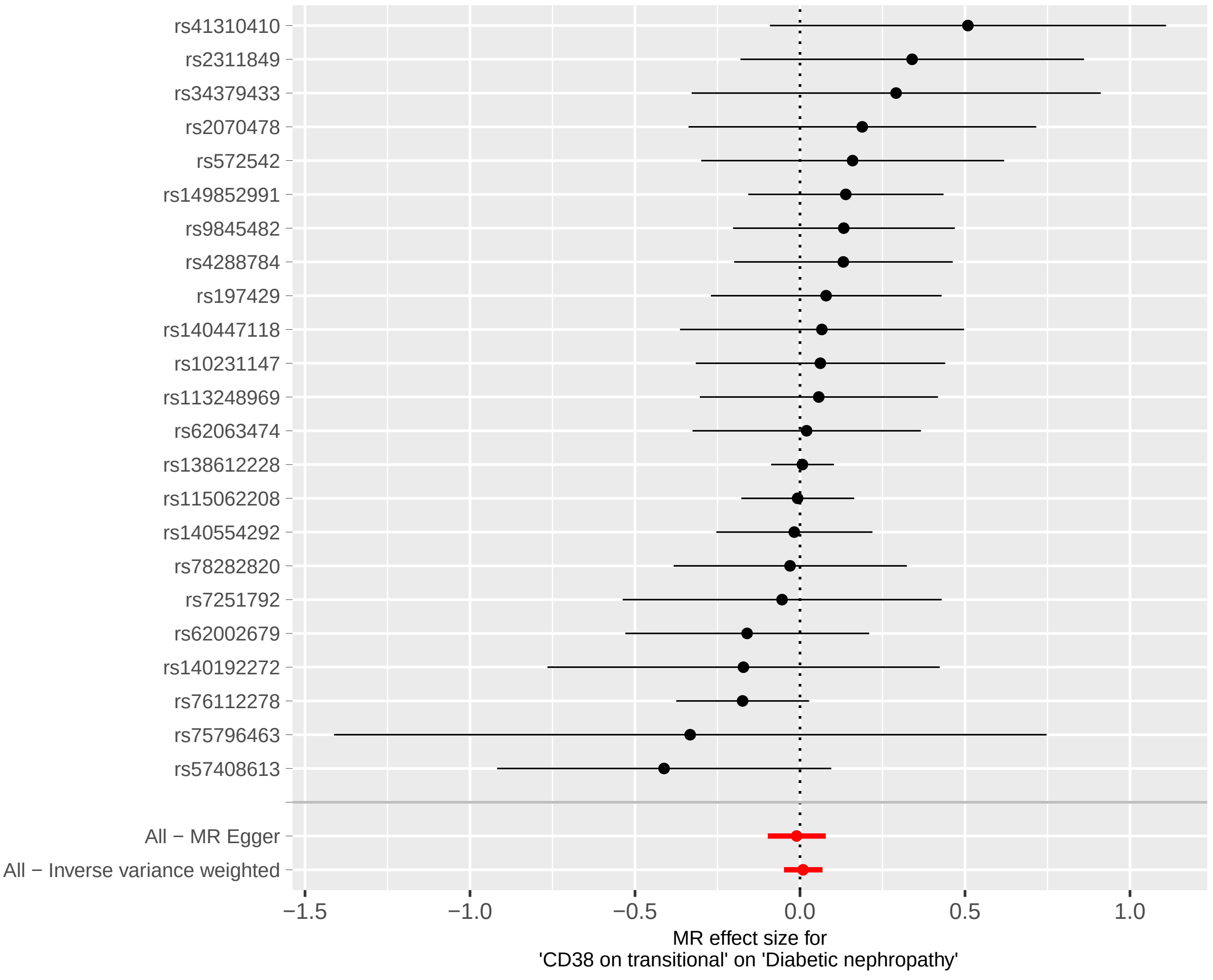
rs41310410
rs2311849
rs34379433
rs2070478
rs572542
rs149852991
rs9845482
rs4288784
rs197429
rs140447118
rs10231147
rs113248969
rs62063474
rs138612228
rs115062208
rs140554292
rs78282820
rs7251792
rs62002679
rs140192272
rs76112278
rs75796463
rs57408613
All − MR Egger
All − Inverse variance weighted
−1.5
−1.0
−0.5
0.0
0.5
1.0
MR effect size for
'CD38 on transitional' on 'Diabetic nephropathy'

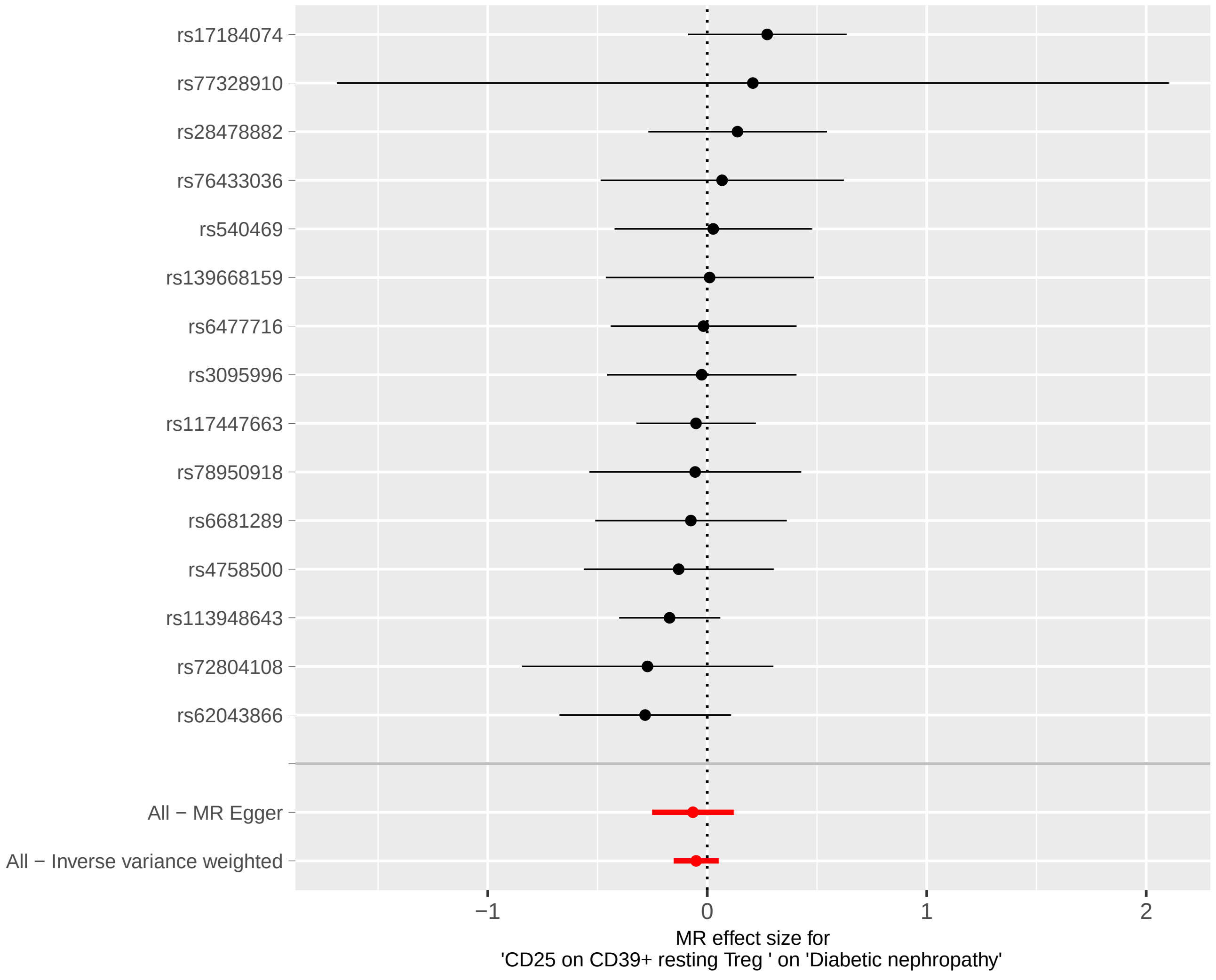
rs17184074
rs77328910
rs28478882
rs76433036
rs540469
rs139668159
rs6477716
rs3095996
rs117447663
rs78950918
rs6681289
rs4758500
rs113948643
rs72804108
rs62043866
All − MR Egger
All − Inverse variance weighted
−1
0
1
2
MR effect size for
'CD25 on CD39+ resting Treg ' on 'Diabetic nephropathy'

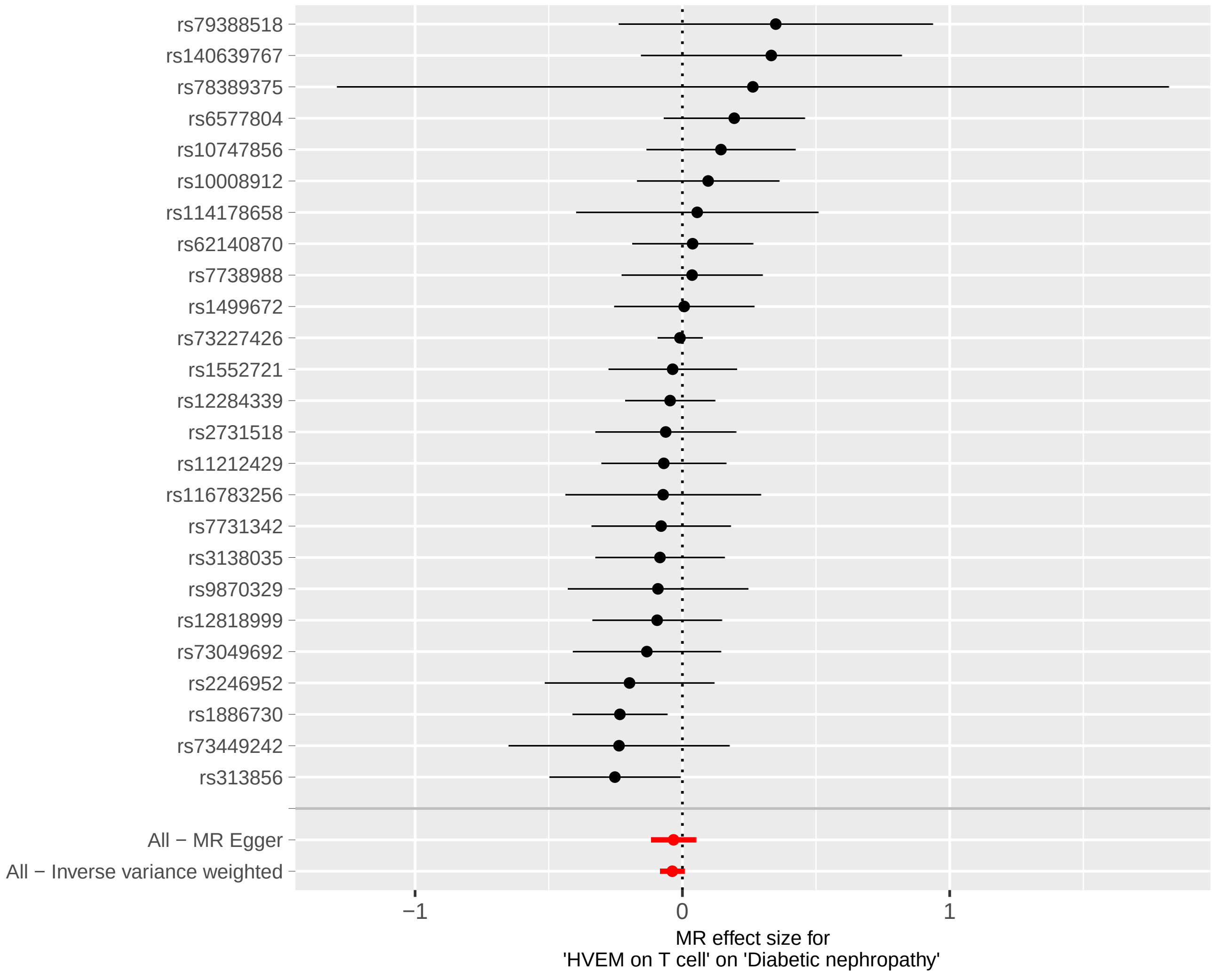
rs79388518
rs140639767
rs78389375
rs6577804
rs10747856
rs10008912
rs114178658
rs62140870
rs7738988
rs1499672
rs73227426
rs1552721
rs12284339
rs2731518
rs11212429
rs116783256
rs7731342
rs3138035
rs9870329
rs12818999
rs73049692
rs2246952
rs1886730
rs73449242
rs313856
All − MR Egger
All − Inverse variance weighted
−1
0
1
MR effect size for
'HVEM on T cell' on 'Diabetic nephropathy'

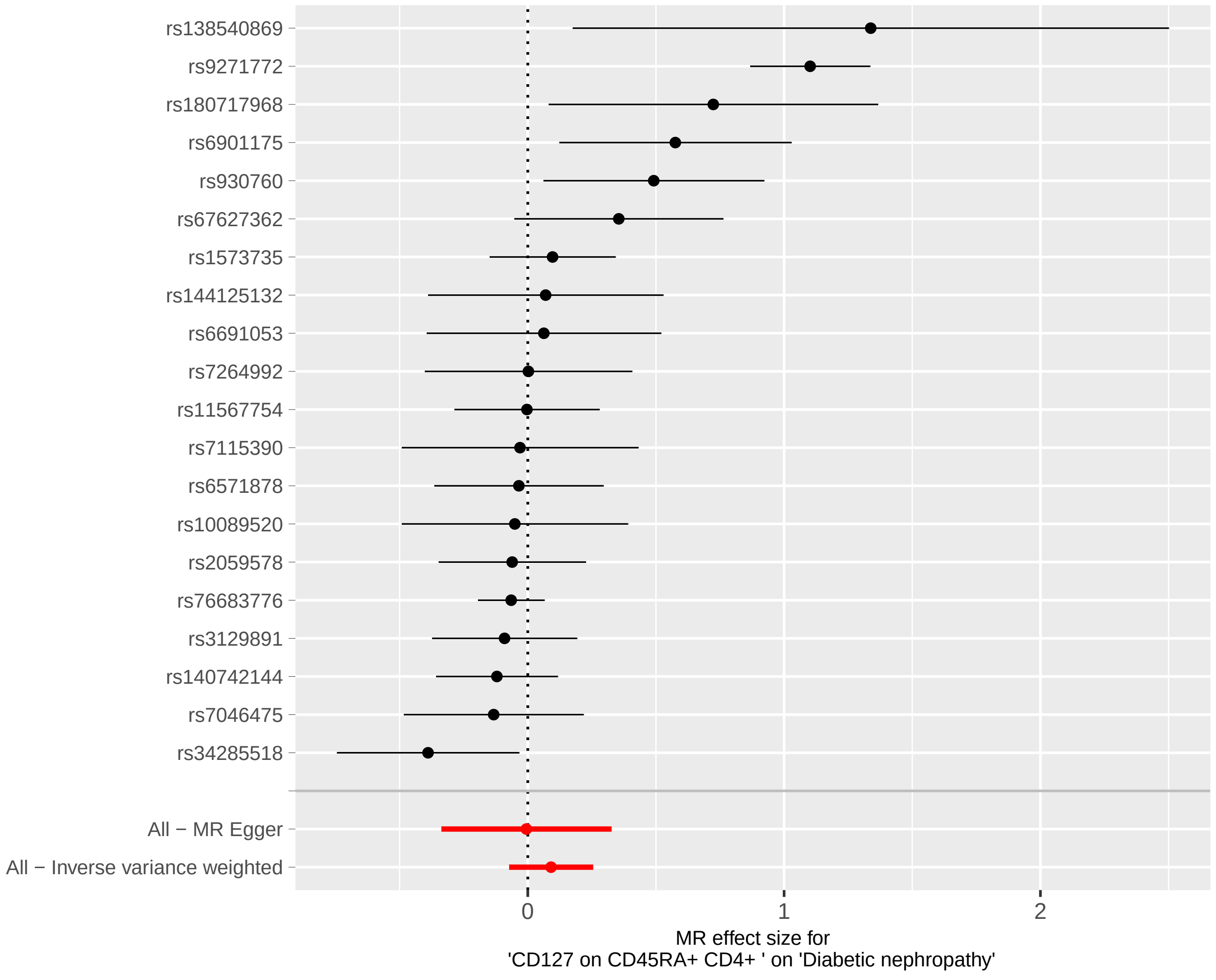
rs138540869
rs9271772
rs180717968
rs6901175
rs930760
rs67627362
rs1573735
rs144125132
rs6691053
rs7264992
rs11567754
rs7115390
rs6571878
rs10089520
rs2059578
rs76683776
rs3129891
rs140742144
rs7046475
rs34285518
All − MR Egger
All − Inverse variance weighted
0
1
2
MR effect size for
'CD127 on CD45RA+ CD4+ ' on 'Diabetic nephropathy'

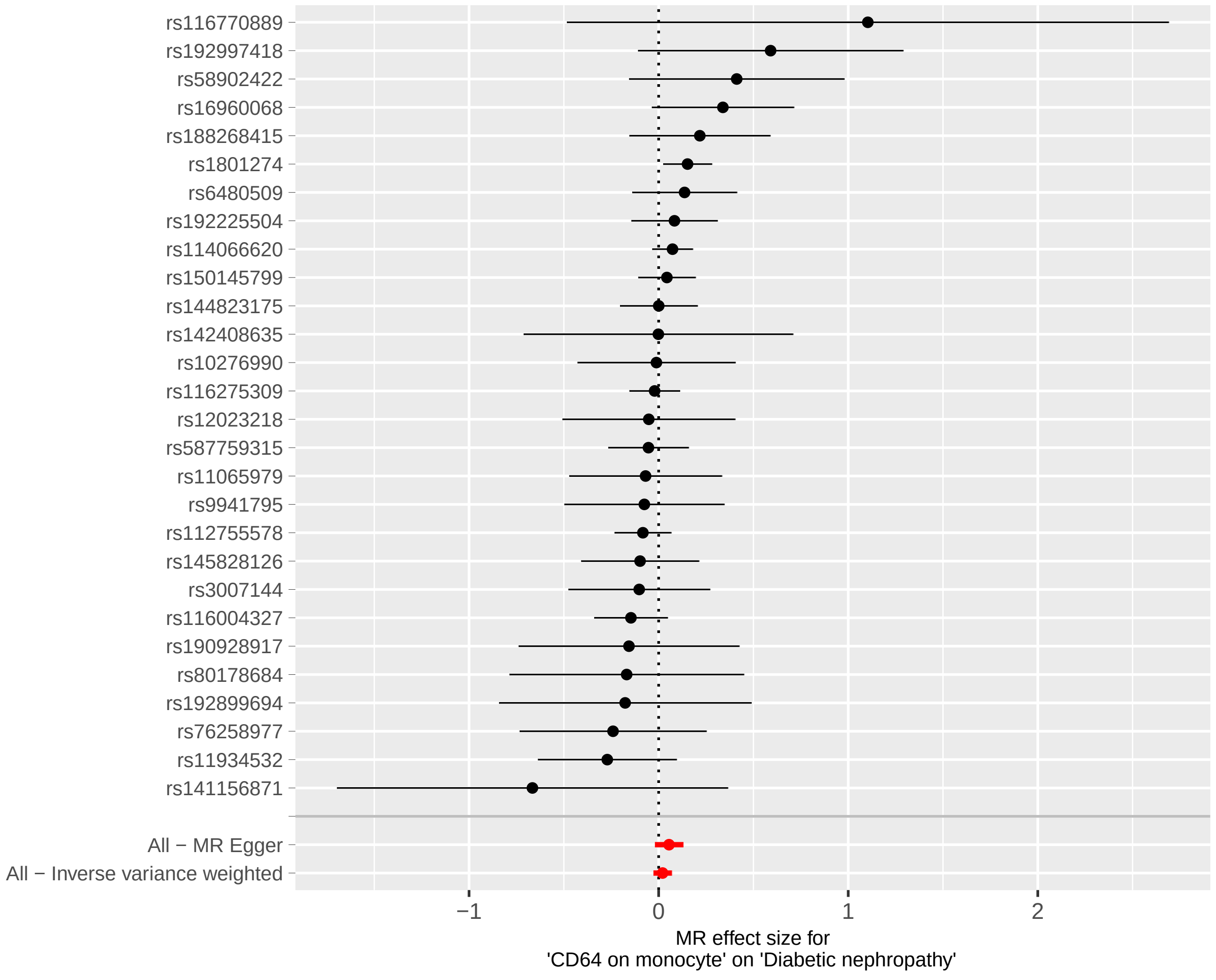
rs116770889
rs192997418
rs58902422
rs16960068
rs188268415
rs1801274
rs6480509
rs192225504
rs114066620
rs150145799
rs144823175
rs142408635
rs10276990
rs116275309
rs12023218
rs587759315
rs11065979
rs9941795
rs112755578
rs145828126
rs3007144
rs116004327
rs190928917
rs80178684
rs192899694
rs76258977
rs11934532
rs141156871
All − MR Egger
All − Inverse variance weighted
−1
0
1
2
MR effect size for
'CD64 on monocyte' on 'Diabetic nephropathy'

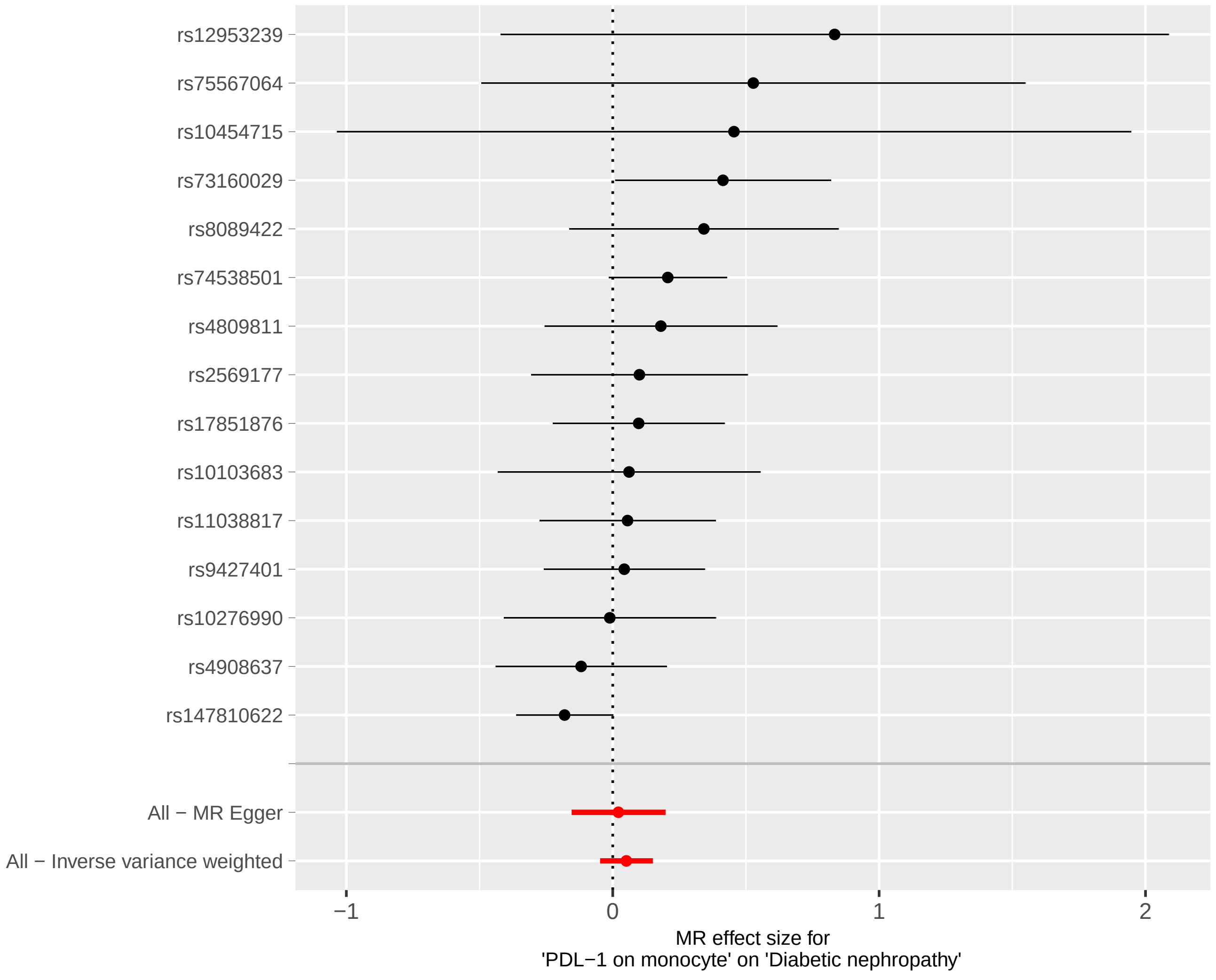

rs12953239
rs75567064
rs10454715
rs73160029
rs8089422
rs74538501
rs4809811
rs2569177
rs17851876
rs10103683
rs11038817
rs9427401
rs10276990
rs4908637
rs147810622
All − MR Egger
All − Inverse variance weighted
−1
0
1
2
MR effect size for
'PDL−1 on monocyte' on 'Diabetic nephropathy'

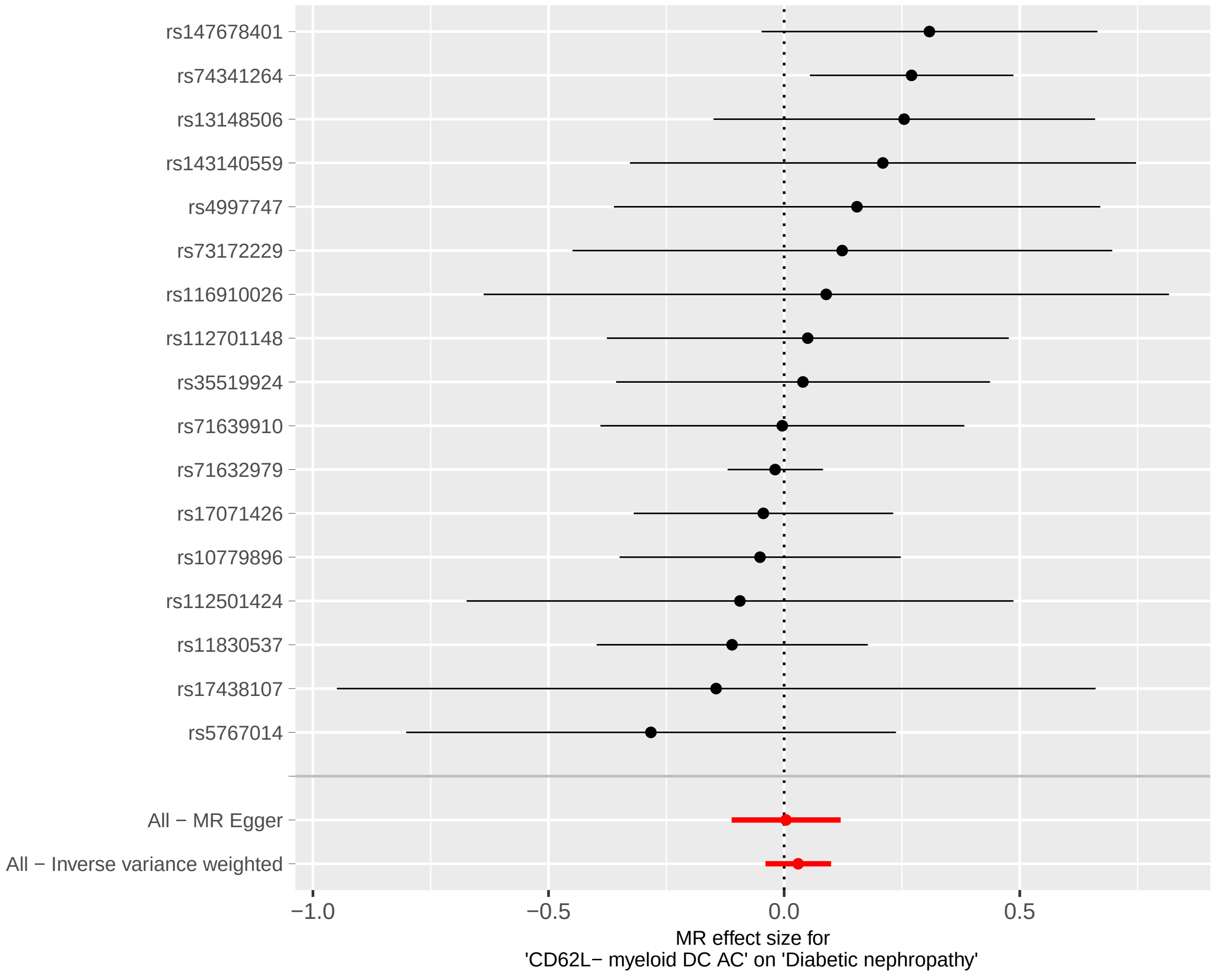

rs147678401
rs74341264
rs13148506
rs143140559
rs4997747
rs73172229
rs116910026
rs112701148
rs35519924
rs71639910
rs71632979
rs17071426
rs10779896
rs112501424
rs11830537
rs17438107
rs5767014
All − MR Egger
All − Inverse variance weighted
−1.0
−0.5
0.0
0.5
MR effect size for
'CD62L− myeloid DC AC' on 'Diabetic nephropathy'

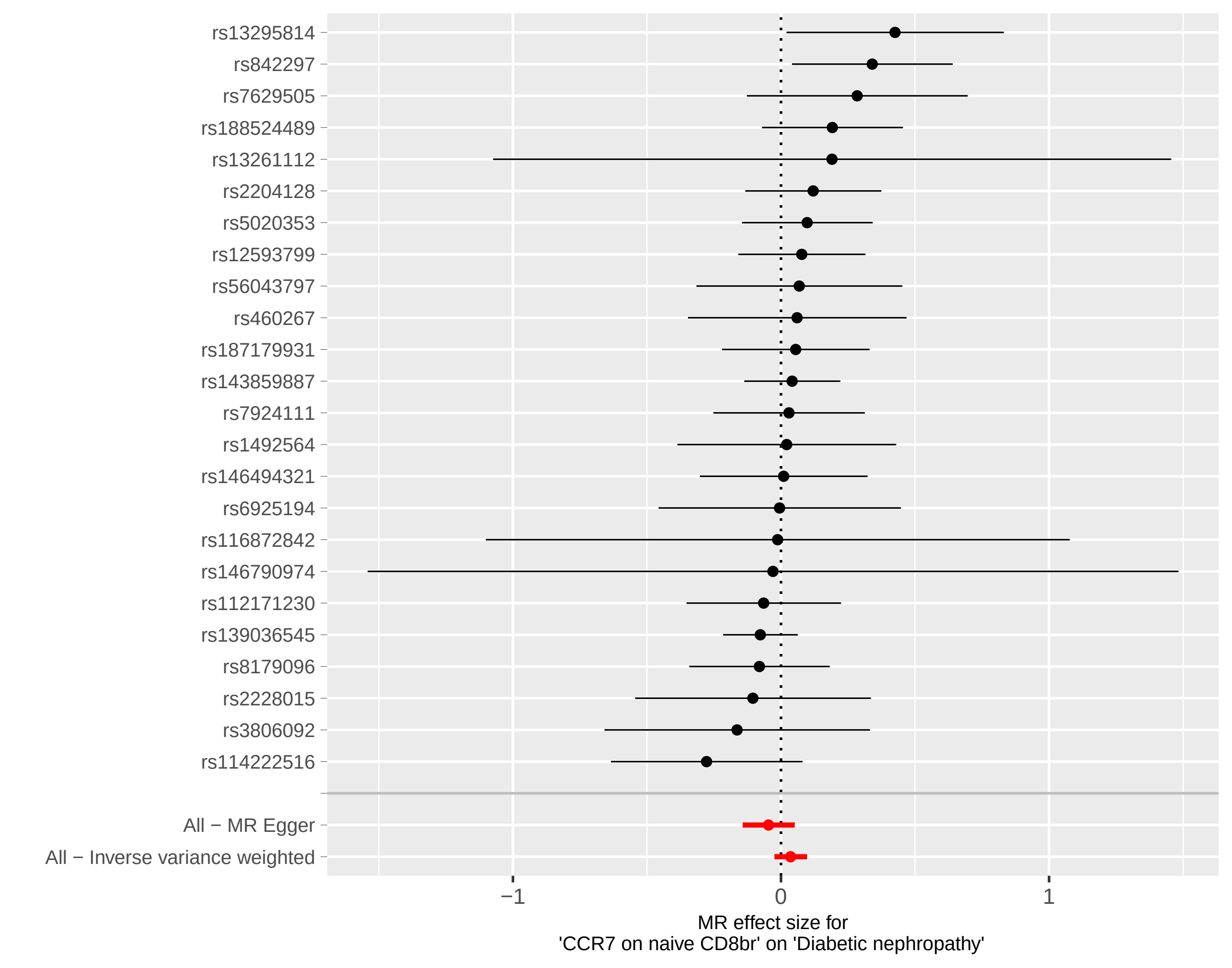

rs13295814
rs842297
rs7629505
rs188524489
rs13261112
rs2204128
rs5020353
rs12593799
rs56043797
rs460267
rs187179931
rs143859887
rs7924111
rs1492564
rs146494321
rs6925194
rs116872842
rs146790974
rs112171230
rs139036545
rs8179096
rs2228015
rs3806092
rs114222516
All − MR Egger
All − Inverse variance weighted
−1
0
1
MR effect size for
'CCR7 on naive CD8br' on 'Diabetic nephropathy'

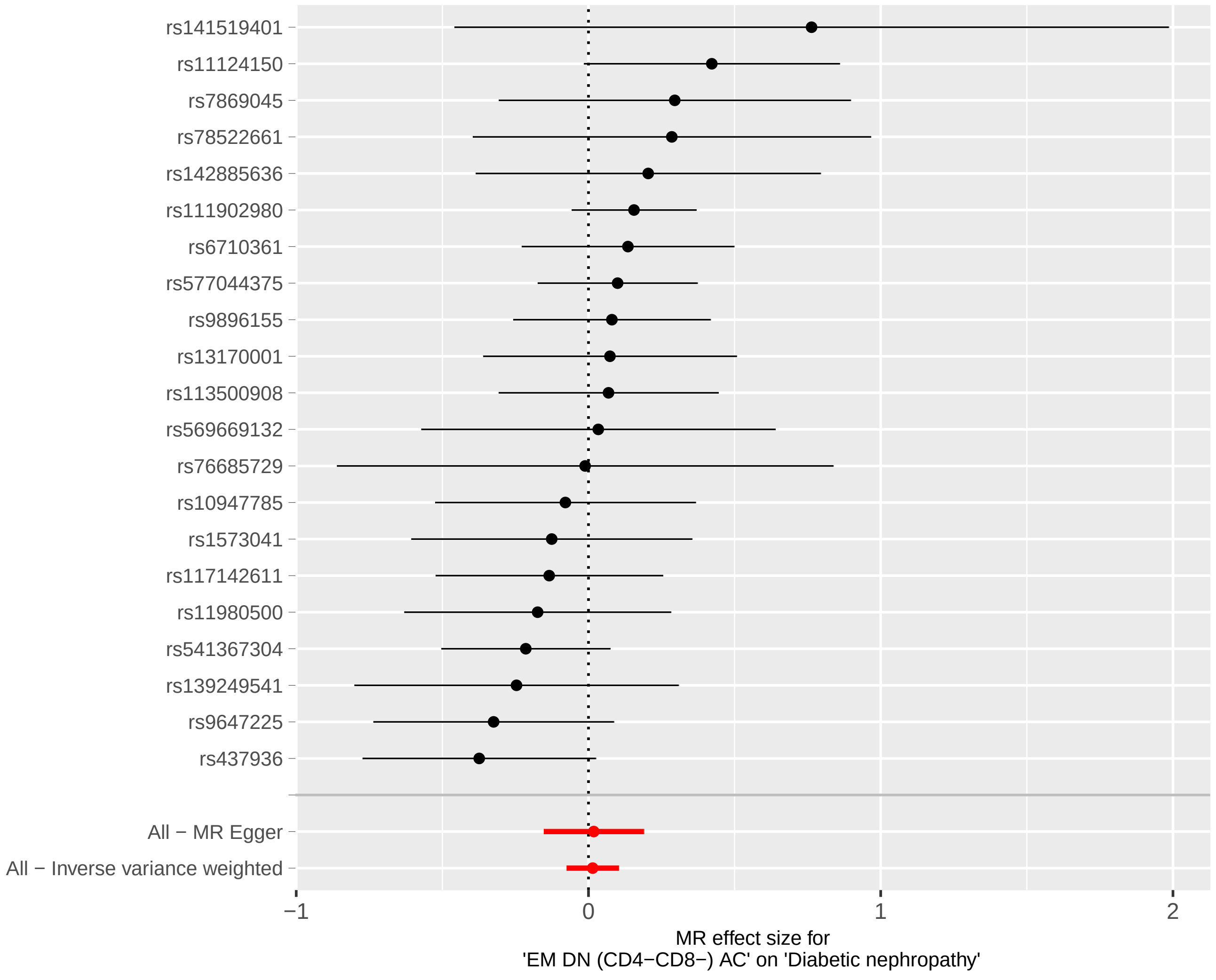

rs141519401
rs11124150
rs7869045
rs78522661
rs142885636
rs111902980
rs6710361
rs577044375
rs9896155
rs13170001
rs113500908
rs569669132
rs76685729
rs10947785
rs1573041
rs117142611
rs11980500
rs541367304
rs139249541
rs9647225
rs437936
All − MR Egger
All − Inverse variance weighted
−1
0
1
2
MR effect size for
'EM DN (CD4−CD8−) AC' on 'Diabetic nephropathy'

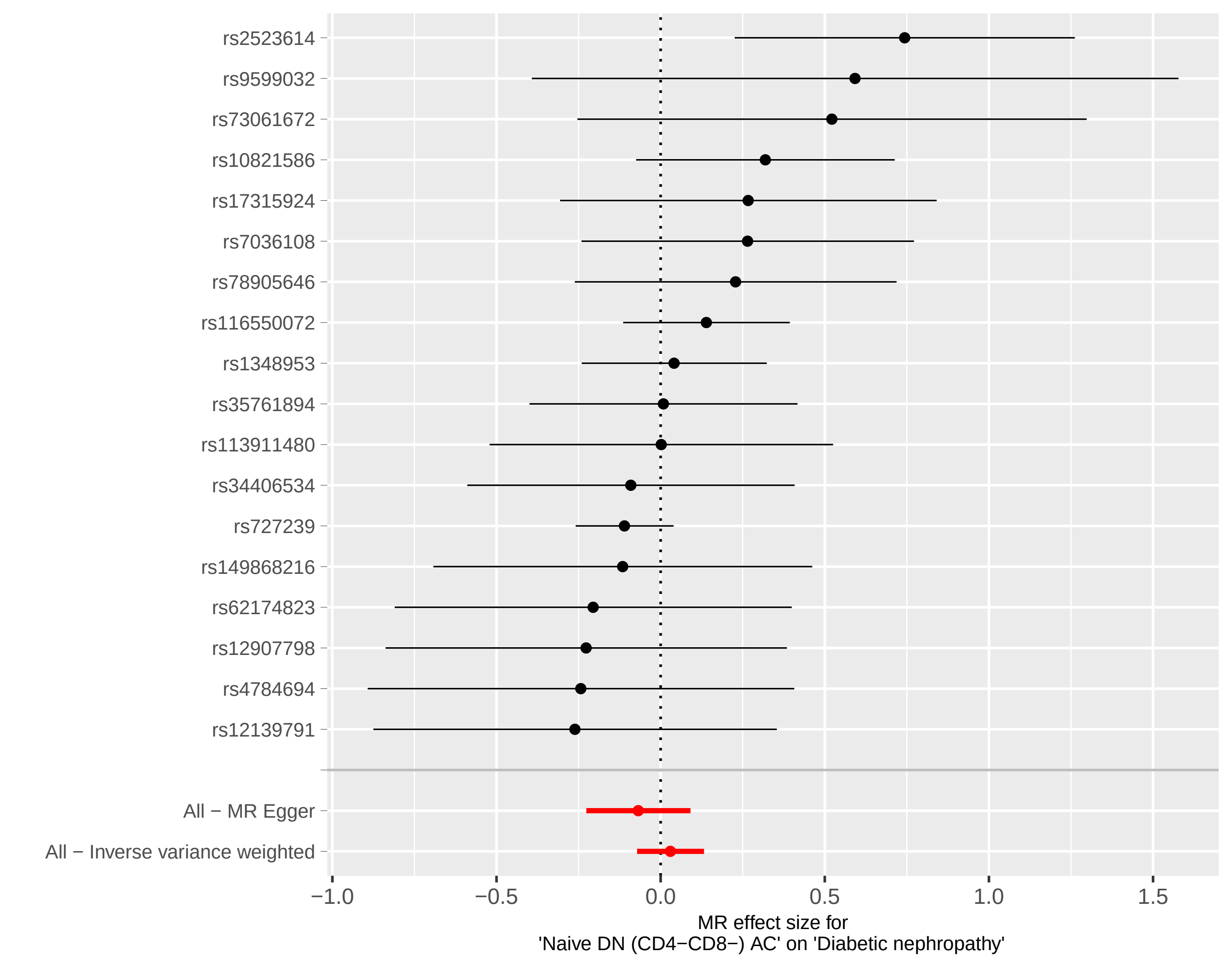
rs2523614
rs9599032
rs73061672
rs10821586
rs17315924
rs7036108
rs78905646
rs116550072
rs1348953
rs35761894
rs113911480
rs34406534
rs727239
rs149868216
rs62174823
rs12907798
rs4784694
rs12139791
All − MR Egger
All − Inverse variance weighted
−1.0
−0.5
0.0
0.5
1.0
1.5
MR effect size for
'Naive DN (CD4−CD8−) AC' on 'Diabetic nephropathy'

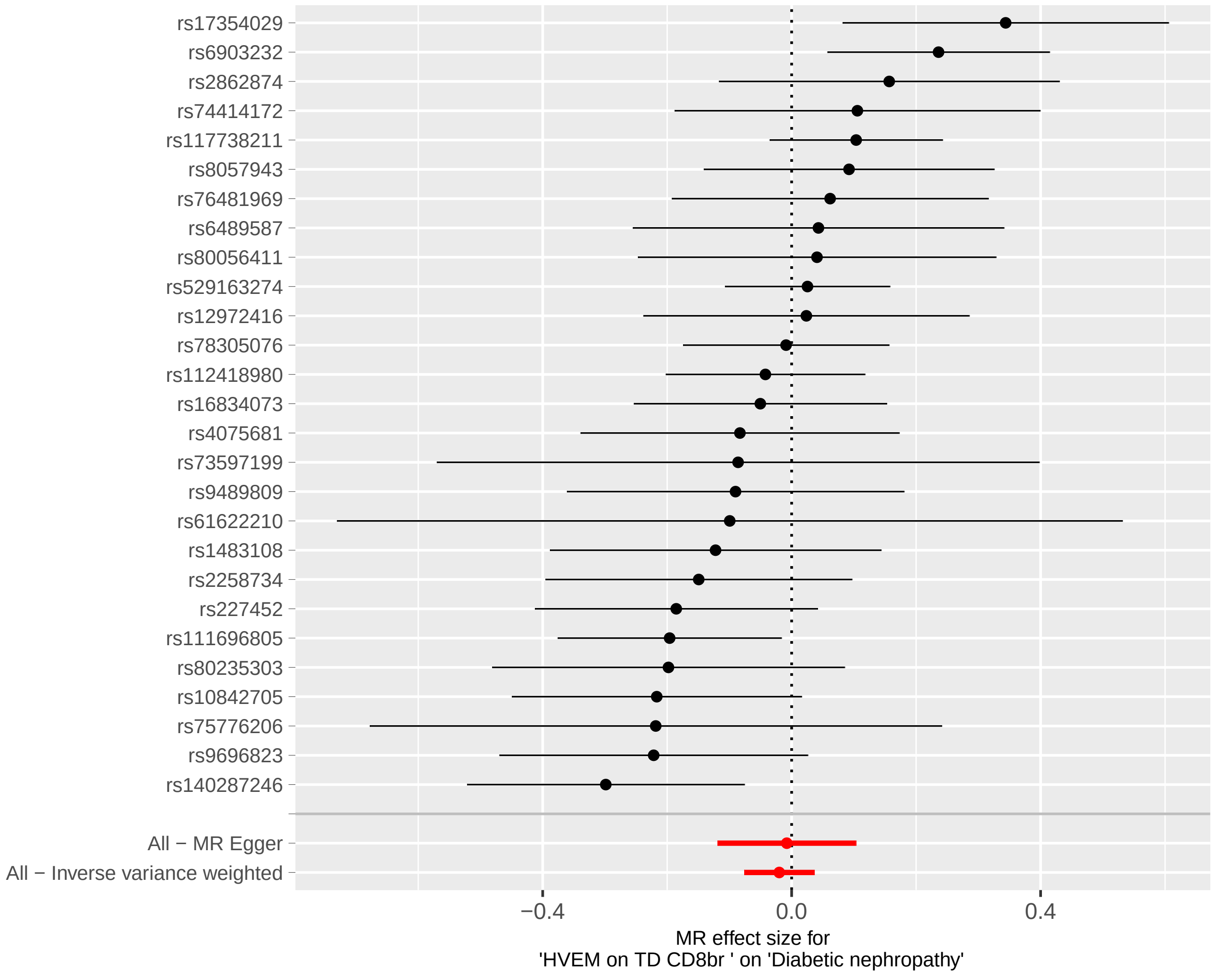
rs17354029
rs6903232
rs2862874
rs74414172
rs117738211
rs8057943
rs76481969
rs6489587
rs80056411
rs529163274
rs12972416
rs78305076
rs112418980
rs16834073
rs4075681
rs73597199
rs9489809
rs61622210
rs1483108
rs2258734
rs227452
rs111696805
rs80235303
rs10842705
rs75776206
rs9696823
rs140287246
All − MR Egger
All − Inverse variance weighted
−0.4
0.0
0.4
MR effect size for
'HVEM on TD CD8br ' on 'Diabetic nephropathy'

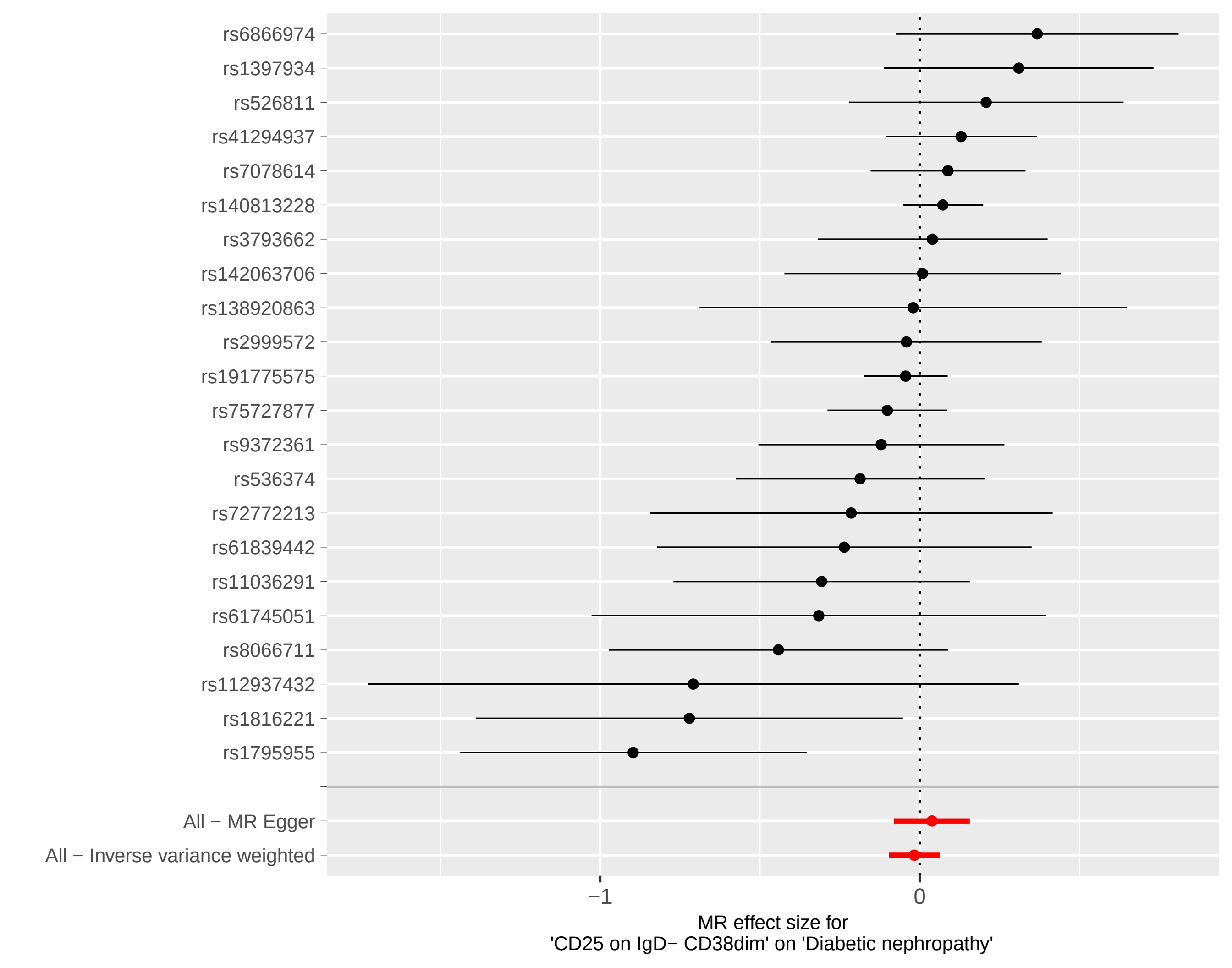

rs6866974
rs1397934
rs526811
rs41294937
rs7078614
rs140813228
rs3793662
rs142063706
rs138920863
rs2999572
rs191775575
rs75727877
rs9372361
rs536374
rs72772213
rs61839442
rs11036291
rs61745051
rs8066711
rs112937432
rs1816221
rs1795955
All − MR Egger
All − Inverse variance weighted
−1
0
MR effect size for
'CD25 on IgD− CD38dim' on 'Diabetic nephropathy'

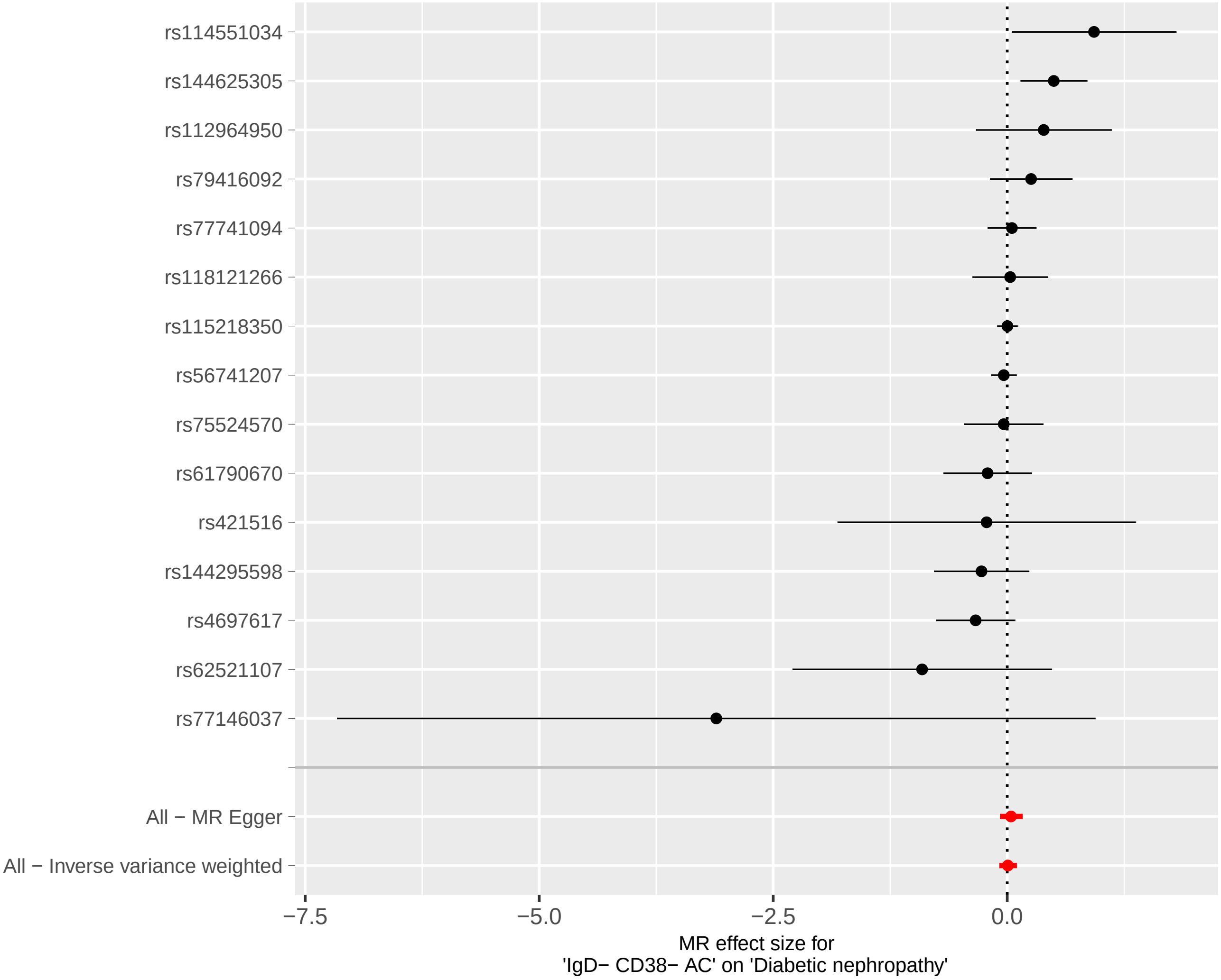
rs114551034
rs144625305
rs112964950
rs79416092
rs77741094
rs118121266
rs115218350
rs56741207
rs75524570
rs61790670
rs421516
rs144295598
rs4697617
rs62521107
rs77146037
All − MR Egger
All − Inverse variance weighted
−7.5
−5.0
−2.5
0.0
MR effect size for
'IgD− CD38− AC' on 'Diabetic nephropathy'

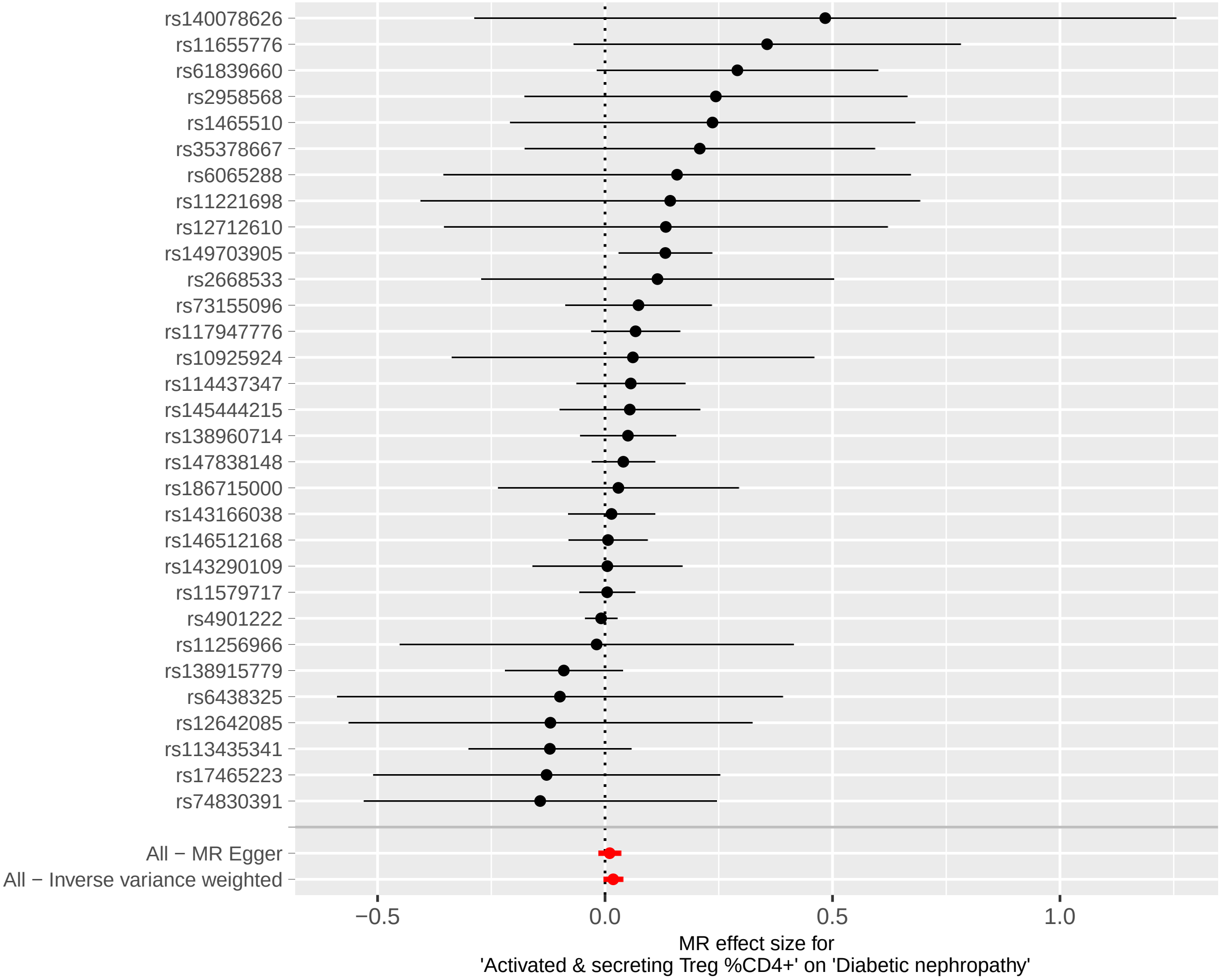
rs140078626
rs11655776
rs61839660
rs2958568
rs1465510
rs35378667
rs6065288
rs11221698
rs12712610
rs149703905
rs2668533
rs73155096
rs117947776
rs10925924
rs114437347
rs145444215
rs138960714
rs147838148
rs186715000
rs143166038
rs146512168
rs143290109
rs11579717
rs4901222
rs11256966
rs138915779
rs6438325
rs12642085
rs113435341
rs17465223
rs74830391
All − MR Egger
All − Inverse variance weighted
−0.5
0.0
0.5
1.0
MR effect size for
'Activated & secreting Treg %CD4+' on 'Diabetic nephropathy'

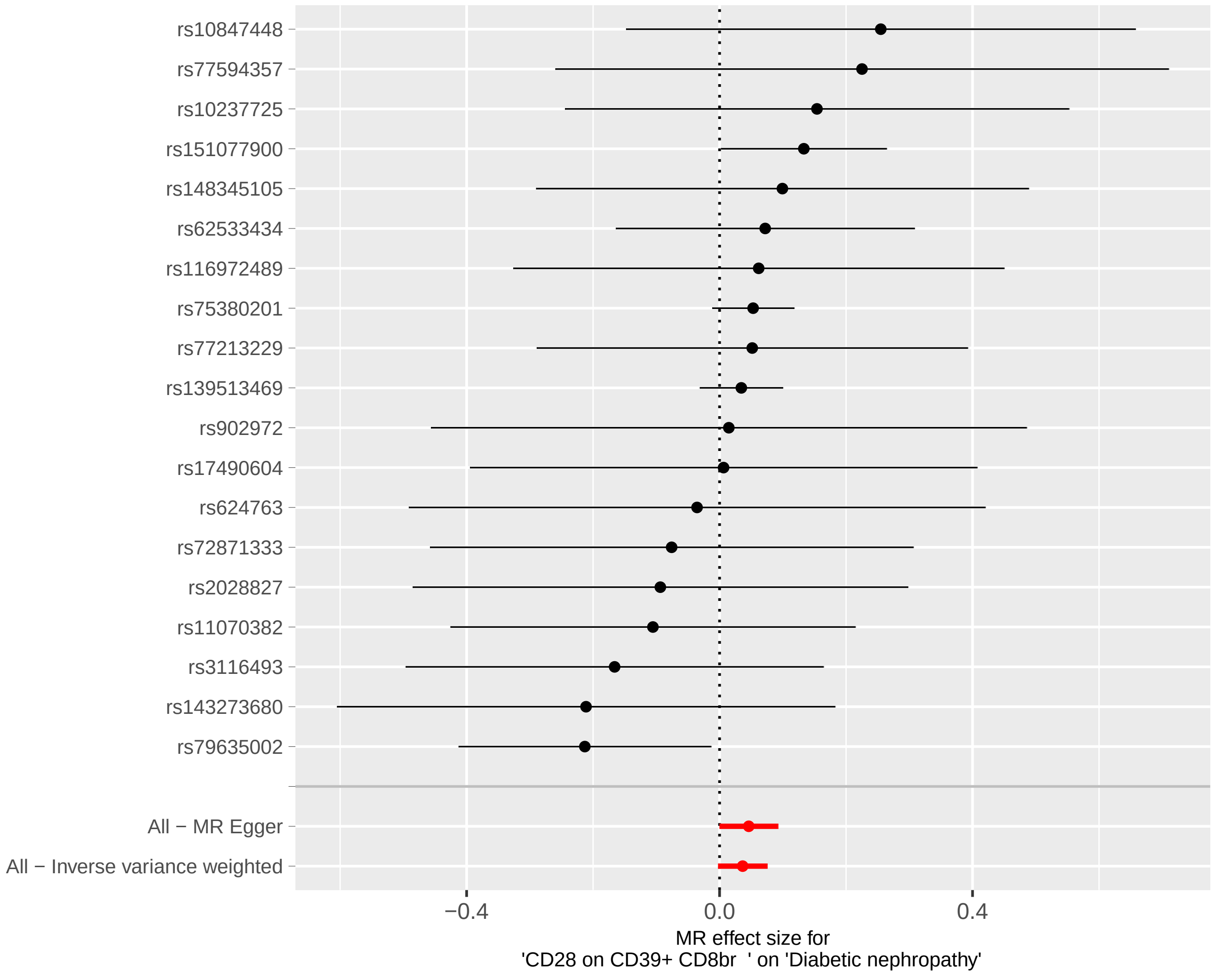

rs10847448
rs77594357
rs10237725
rs151077900
rs148345105
rs62533434
rs116972489
rs75380201
rs77213229
rs139513469
rs902972
rs17490604
rs624763
rs72871333
rs2028827
rs11070382
rs3116493
rs143273680
rs79635002
All − MR Egger
All − Inverse variance weighted
−0.4
0.0
0.4
MR effect size for
'CD28 on CD39+ CD8br ' on 'Diabetic nephropathy'

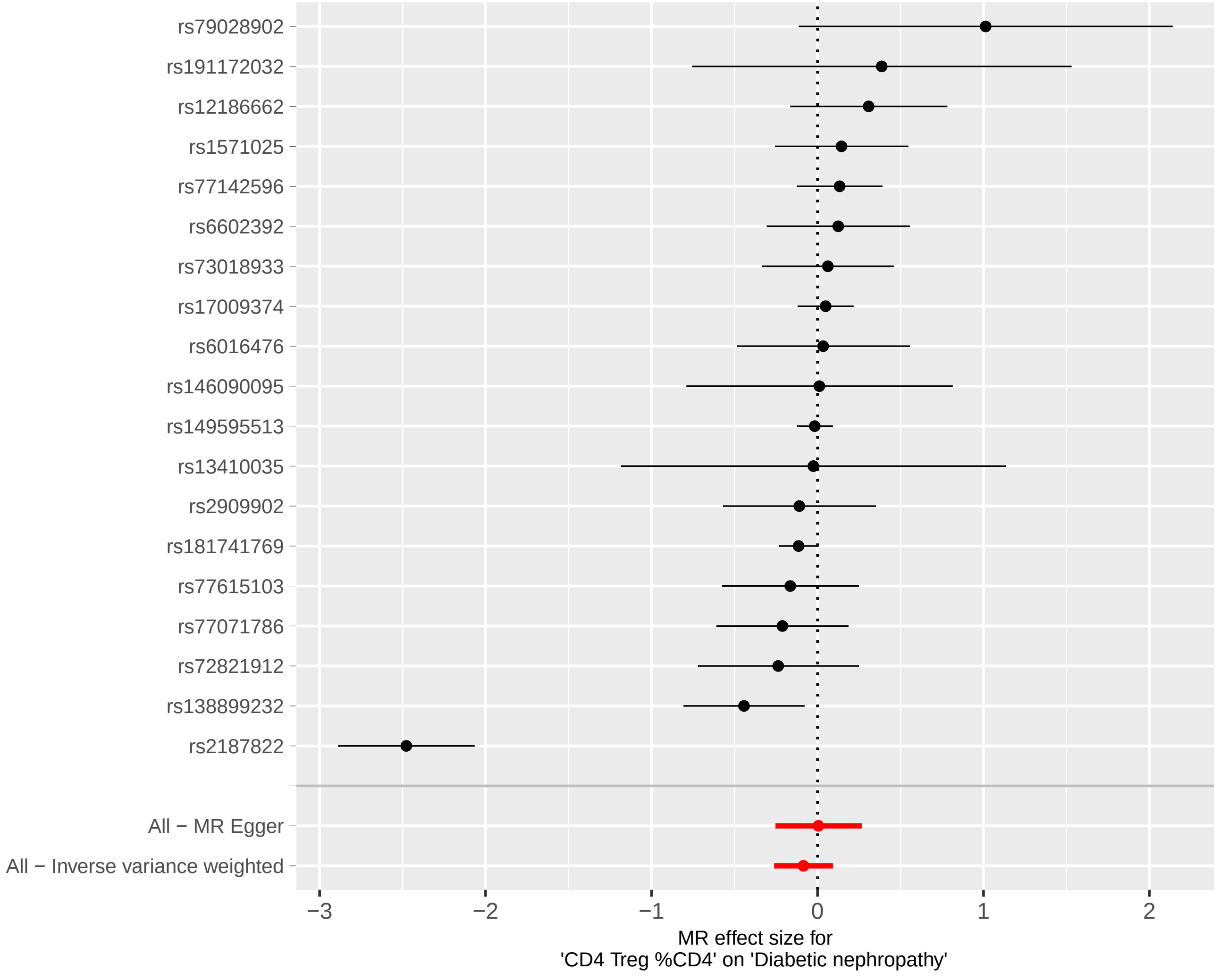
rs79028902
rs191172032
rs12186662
rs1571025
rs77142596
rs6602392
rs73018933
rs17009374
rs6016476
rs146090095
rs149595513
rs13410035
rs2909902
rs181741769
rs77615103
rs77071786
rs72821912
rs138899232
rs2187822
All − MR Egger
All − Inverse variance weighted
−3
−2
−1
0
1
2
MR effect size for
'CD4 Treg %CD4' on 'Diabetic nephropathy'

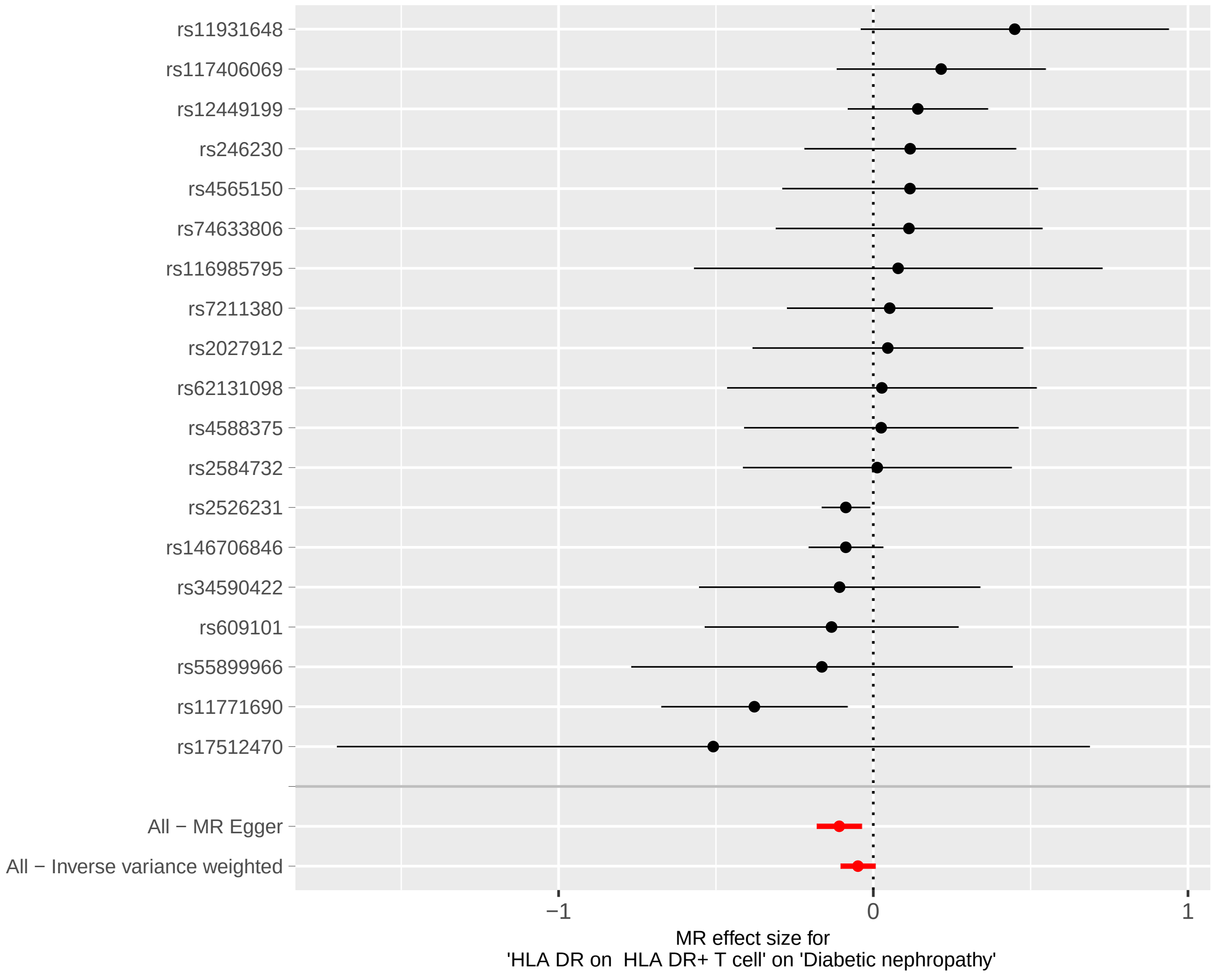
rs11931648
rs117406069
rs12449199
rs246230
rs4565150
rs74633806
rs116985795
rs7211380
rs2027912
rs62131098
rs4588375
rs2584732
rs2526231
rs146706846
rs34590422
rs609101
rs55899966
rs11771690
rs17512470
All − MR Egger
All − Inverse variance weighted
−1
0
1
MR effect size for
'HLA DR on HLA DR+ T cell' on 'Diabetic nephropathy'

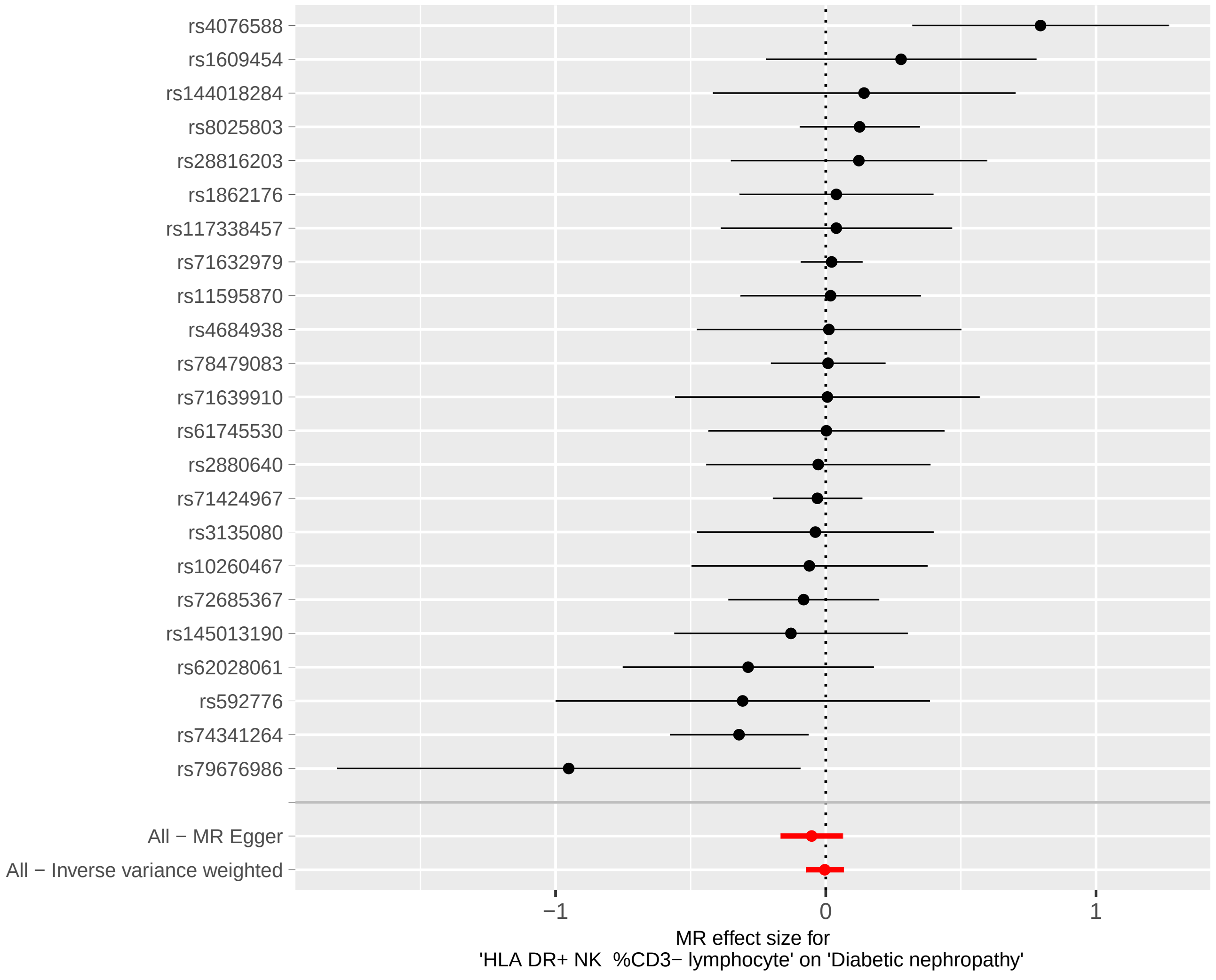
rs4076588
rs1609454
rs144018284
rs8025803
rs28816203
rs1862176
rs117338457
rs71632979
rs11595870
rs4684938
rs78479083
rs71639910
rs61745530
rs2880640
rs71424967
rs3135080
rs10260467
rs72685367
rs145013190
rs62028061
rs592776
rs74341264
rs79676986
All − MR Egger
All − Inverse variance weighted
−1
0
1
MR effect size for
'HLA DR+ NK %CD3− lymphocyte' on 'Diabetic nephropathy'

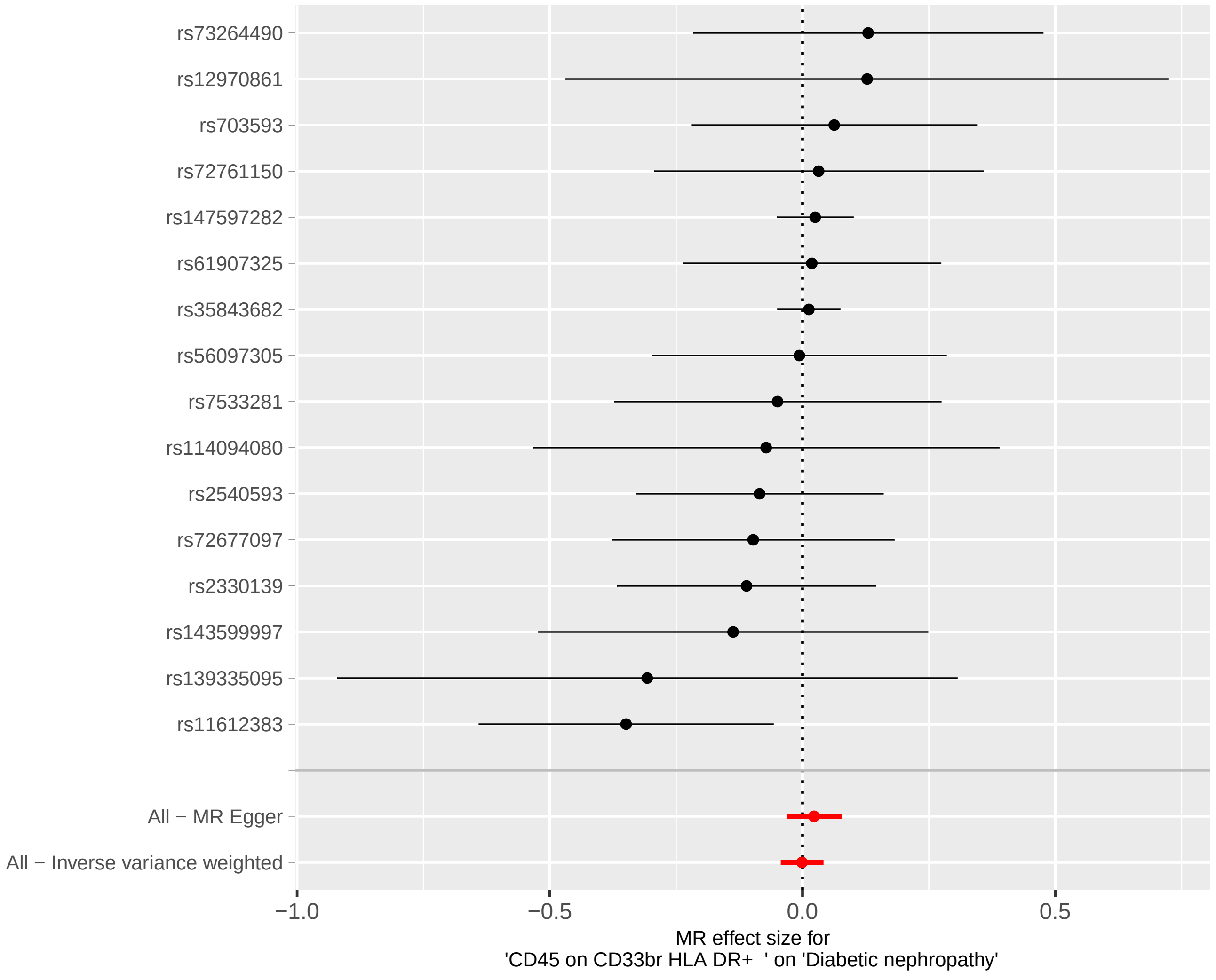
rs73264490
rs12970861
rs703593
rs72761150
rs147597282
rs61907325
rs35843682
rs56097305
rs7533281
rs114094080
rs2540593
rs72677097
rs2330139
rs143599997
rs139335095
rs11612383
All − MR Egger
All − Inverse variance weighted
−1.0
−0.5
0.0
0.5
MR effect size for
'CD45 on CD33br HLA DR+ ' on 'Diabetic nephropathy'

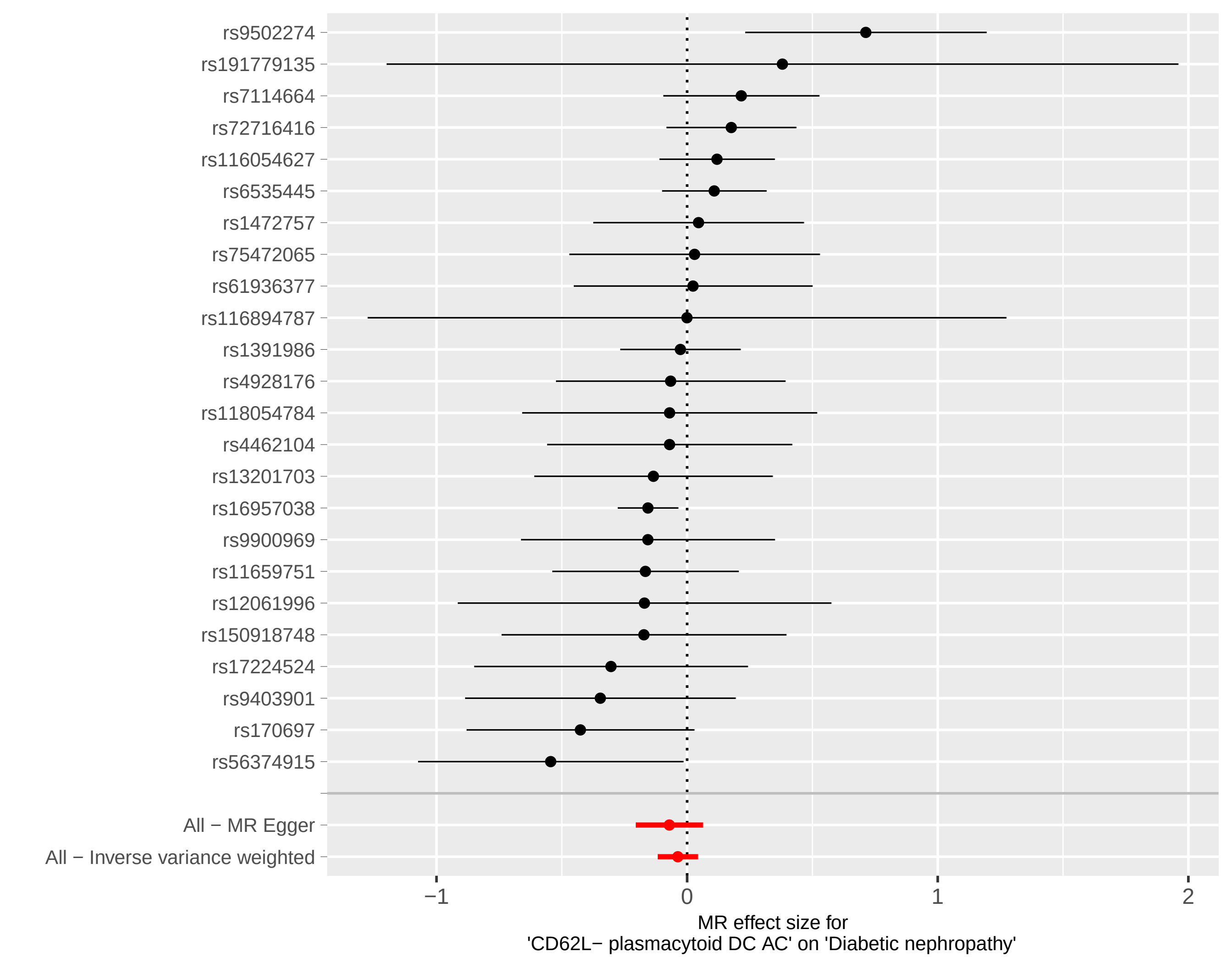
rs9502274
rs191779135
rs7114664
rs72716416
rs116054627
rs6535445
rs1472757
rs75472065
rs61936377
rs116894787
rs1391986
rs4928176
rs118054784
rs4462104
rs13201703
rs16957038
rs9900969
rs11659751
rs12061996
rs150918748
rs17224524
rs9403901
rs170697
rs56374915
All − MR Egger
All − Inverse variance weighted
−1
0
1
2
MR effect size for
'CD62L− plasmacytoid DC AC' on 'Diabetic nephropathy'

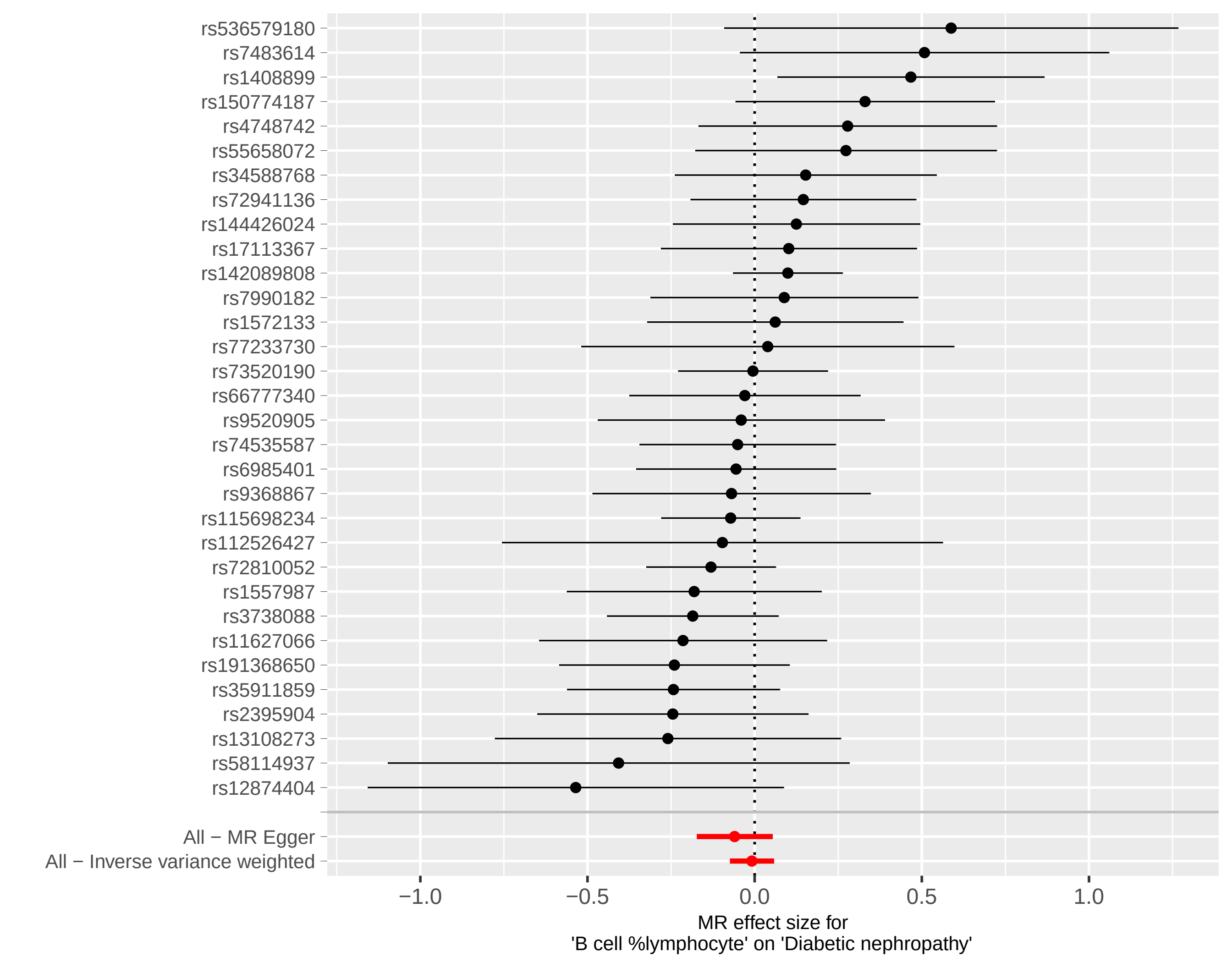
rs536579180
rs7483614
rs1408899
rs150774187
rs4748742
rs55658072
rs34588768
rs72941136
rs144426024
rs17113367
rs142089808
rs7990182
rs1572133
rs77233730
rs73520190
rs66777340
rs9520905
rs74535587
rs6985401
rs9368867
rs115698234
rs112526427
rs72810052
rs1557987
rs3738088
rs11627066
rs191368650
rs35911859
rs2395904
rs13108273
rs58114937
rs12874404
All − MR Egger
All − Inverse variance weighted
−1.0
−0.5
0.0
0.5
1.0
MR effect size for
'B cell %lymphocyte' on 'Diabetic nephropathy'

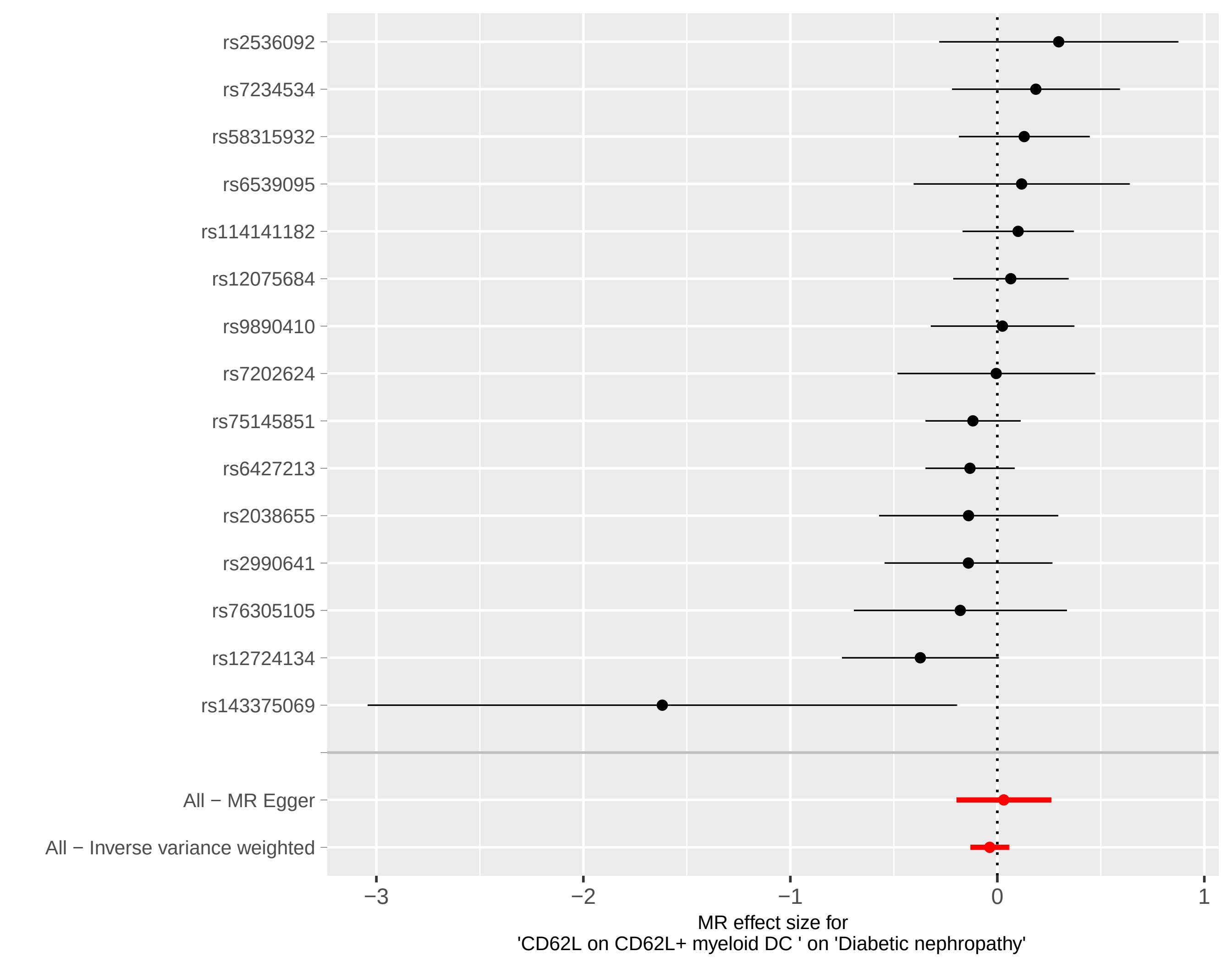
rs2536092
rs7234534
rs58315932
rs6539095
rs114141182
rs12075684
rs9890410
rs7202624
rs75145851
rs6427213
rs2038655
rs2990641
rs76305105
rs12724134
rs143375069
All − MR Egger
All − Inverse variance weighted
−3
−2
−1
0
1
MR effect size for
'CD62L on CD62L+ myeloid DC ' on 'Diabetic nephropathy'

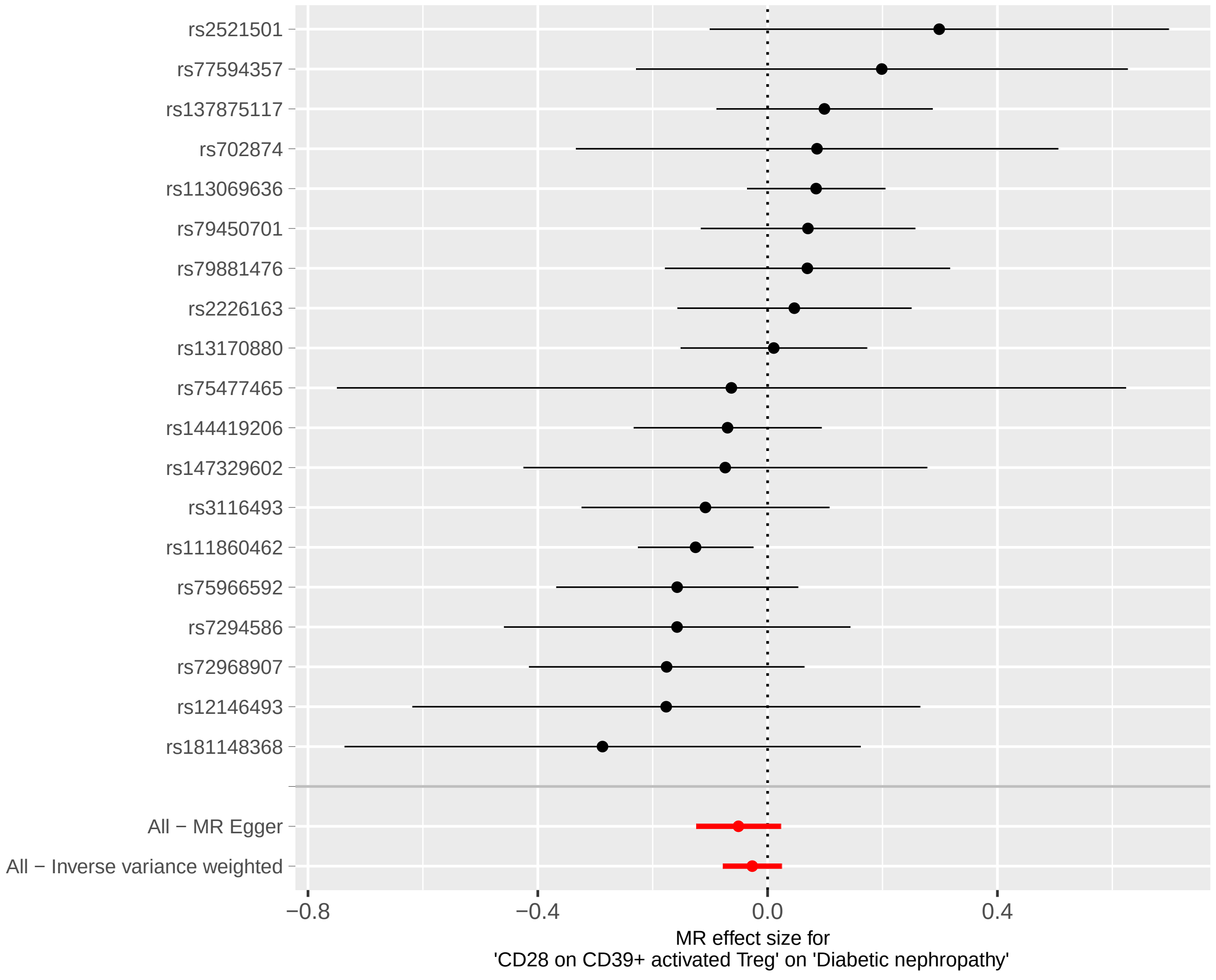

rs2521501
rs77594357
rs137875117
rs702874
rs113069636
rs79450701
rs79881476
rs2226163
rs13170880
rs75477465
rs144419206
rs147329602
rs3116493
rs111860462
rs75966592
rs7294586
rs72968907
rs12146493
rs181148368
All − MR Egger
All − Inverse variance weighted
−0.8
−0.4
0.0
0.4
MR effect size for
'CD28 on CD39+ activated Treg' on 'Diabetic nephropathy'

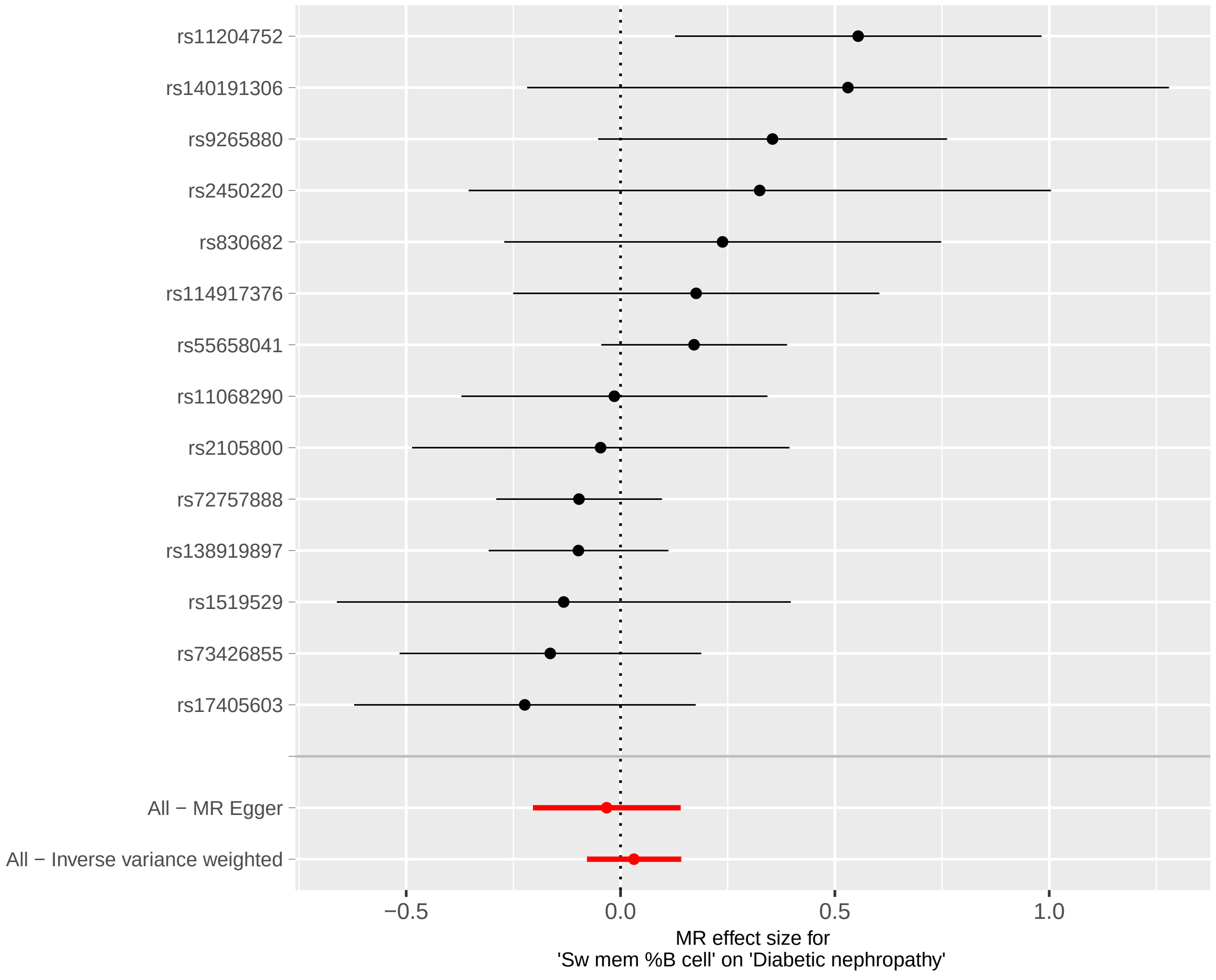
rs11204752
rs140191306
rs9265880
rs2450220
rs830682
rs114917376
rs55658041
rs11068290
rs2105800
rs72757888
rs138919897
rs1519529
rs73426855
rs17405603
All − MR Egger
All − Inverse variance weighted
−0.5
0.0
0.5
1.0
MR effect size for
'Sw mem %B cell' on 'Diabetic nephropathy'

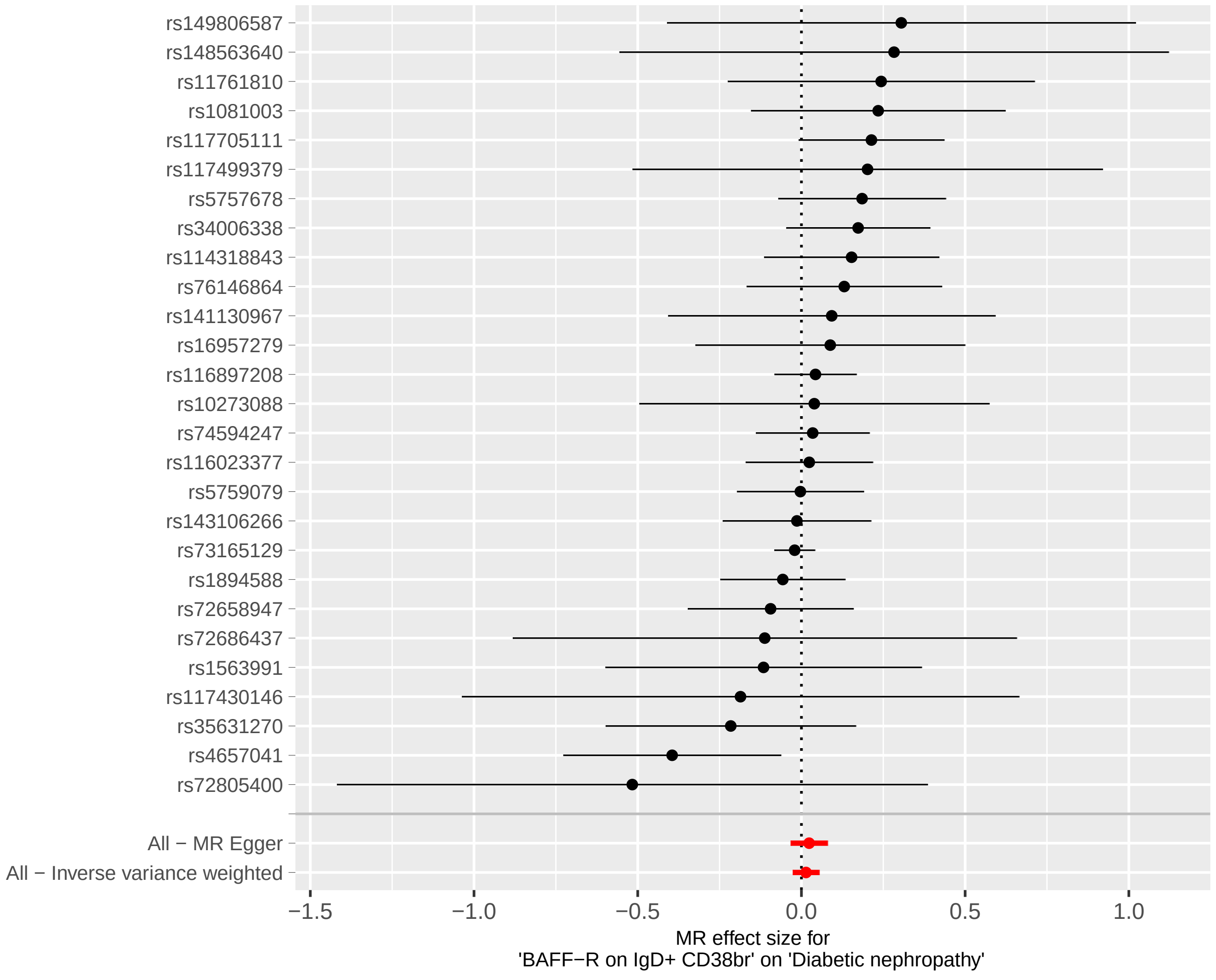
rs149806587
rs148563640
rs11761810
rs1081003
rs117705111
rs117499379
rs5757678
rs34006338
rs114318843
rs76146864
rs141130967
rs16957279
rs116897208
rs10273088
rs74594247
rs116023377
rs5759079
rs143106266
rs73165129
rs1894588
rs72658947
rs72686437
rs1563991
rs117430146
rs35631270
rs4657041
rs72805400
All − MR Egger
All − Inverse variance weighted
−1.5
−1.0
−0.5
0.0
0.5
1.0
MR effect size for
'BAFF−R on IgD+ CD38br' on 'Diabetic nephropathy'

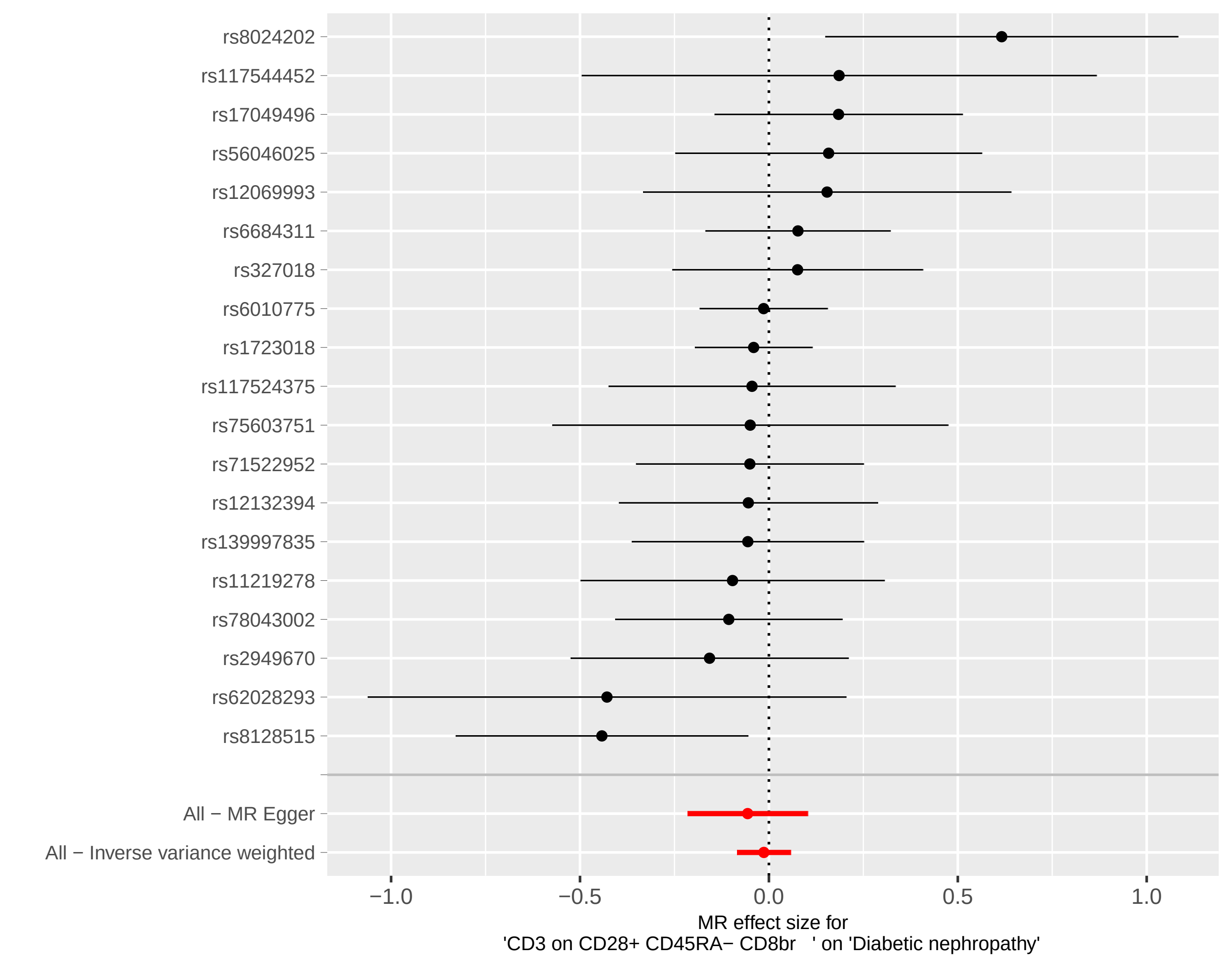
rs8024202
rs117544452
rs17049496
rs56046025
rs12069993
rs6684311
rs327018
rs6010775
rs1723018
rs117524375
rs75603751
rs71522952
rs12132394
rs139997835
rs11219278
rs78043002
rs2949670
rs62028293
rs8128515
All − MR Egger
All − Inverse variance weighted
−1.0
−0.5
0.0
0.5
1.0
MR effect size for
'CD3 on CD28+ CD45RA− CD8br ' on 'Diabetic nephropathy'

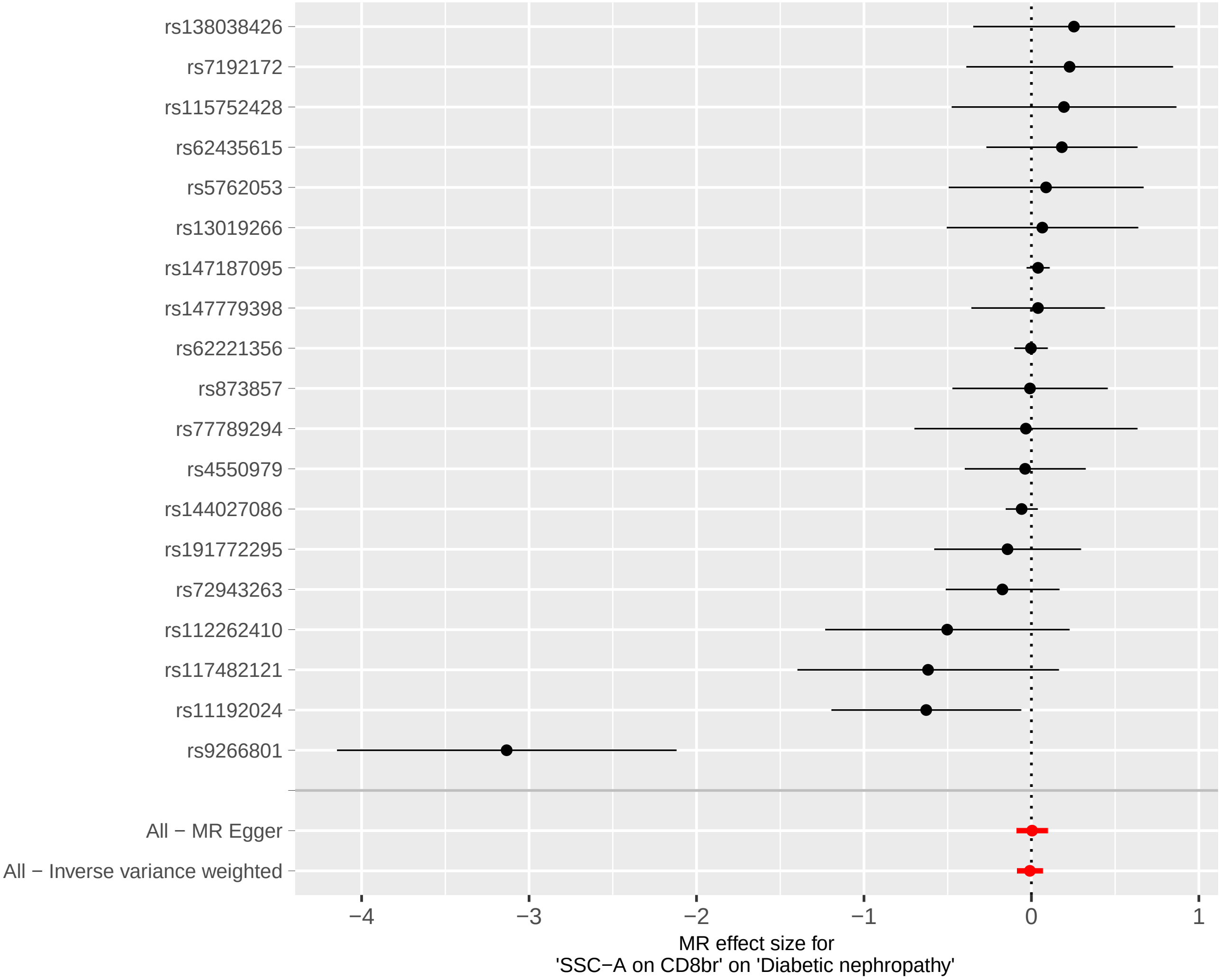
rs138038426
rs7192172
rs115752428
rs62435615
rs5762053
rs13019266
rs147187095
rs147779398
rs62221356
rs873857
rs77789294
rs4550979
rs144027086
rs191772295
rs72943263
rs112262410
rs117482121
rs11192024
rs9266801
All − MR Egger
All − Inverse variance weighted
−4
−3
−2
−1
0
1
MR effect size for
'SSC−A on CD8br' on 'Diabetic nephropathy'

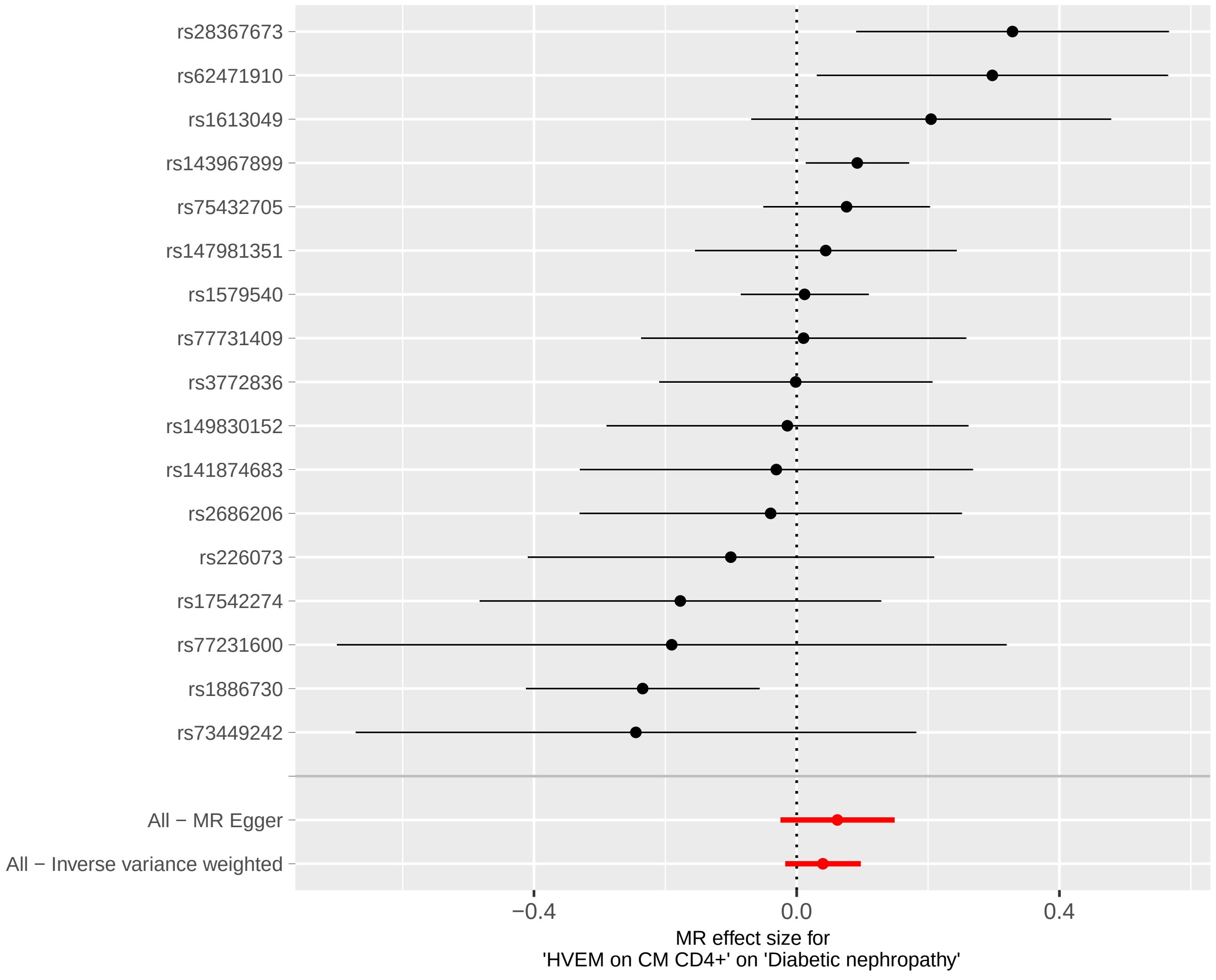
rs28367673
rs62471910
rs1613049
rs143967899
rs75432705
rs147981351
rs1579540
rs77731409
rs3772836
rs149830152
rs141874683
rs2686206
rs226073
rs17542274
rs77231600
rs1886730
rs73449242
All − MR Egger
All − Inverse variance weighted
−0.4
0.0
0.4
MR effect size for
'HVEM on CM CD4+' on 'Diabetic nephropathy'

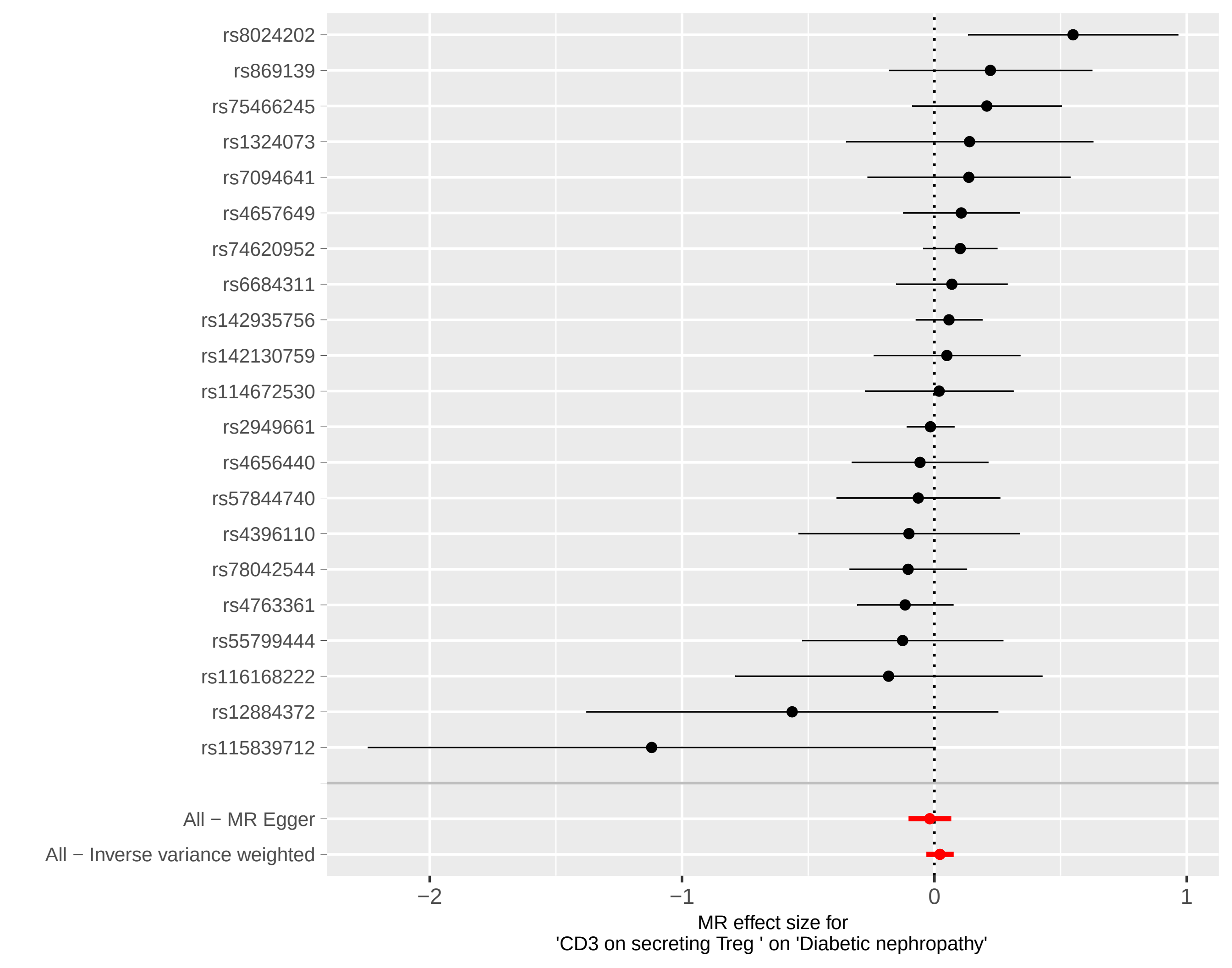

rs8024202
rs869139
rs75466245
rs1324073
rs7094641
rs4657649
rs74620952
rs6684311
rs142935756
rs142130759
rs114672530
rs2949661
rs4656440
rs57844740
rs4396110
rs78042544
rs4763361
rs55799444
rs116168222
rs12884372
rs115839712
All − MR Egger
All − Inverse variance weighted
−2
−1
0
1
MR effect size for
'CD3 on secreting Treg ' on 'Diabetic nephropathy'

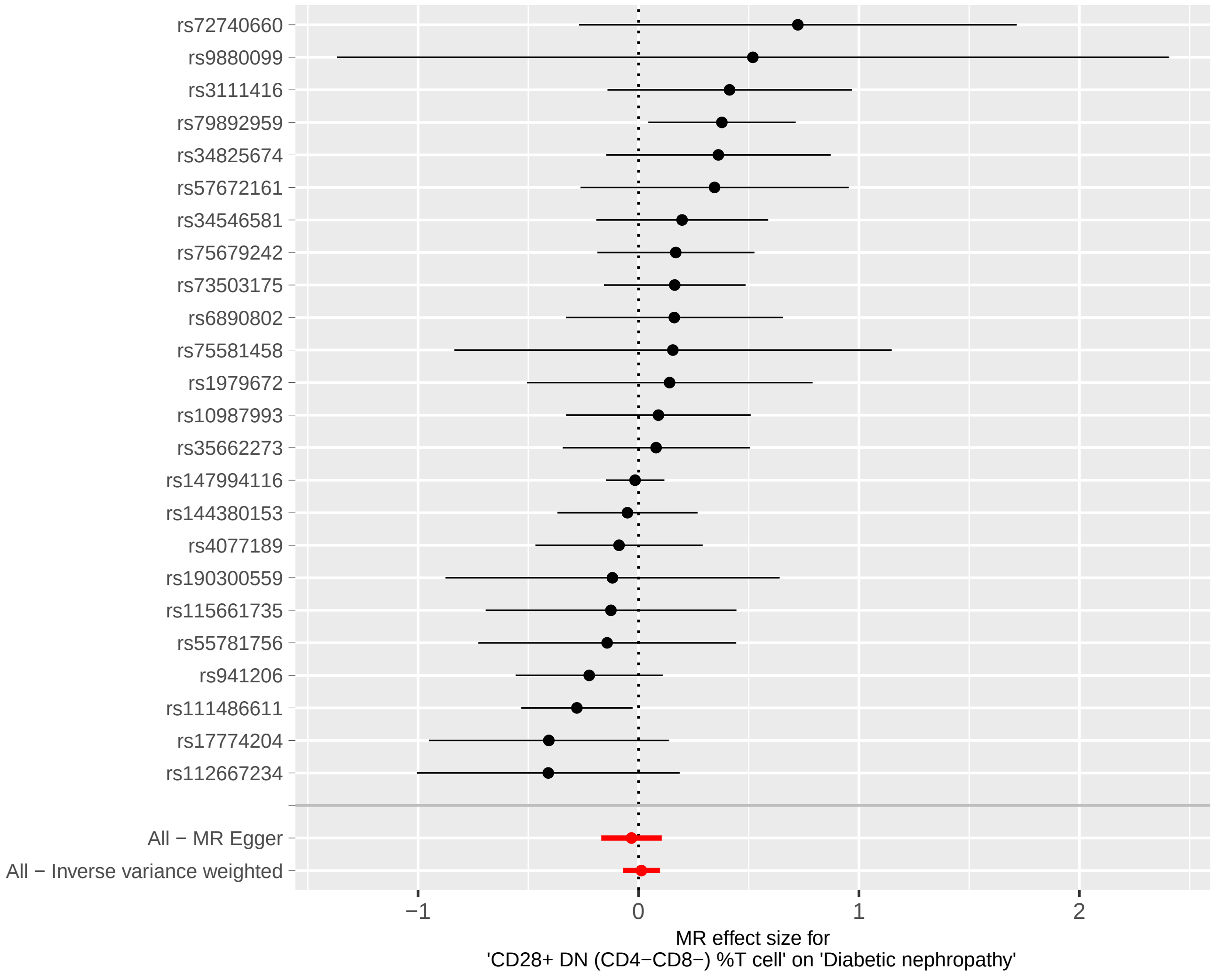
rs72740660
rs9880099
rs3111416
rs79892959
rs34825674
rs57672161
rs34546581
rs75679242
rs73503175
rs6890802
rs75581458
rs1979672
rs10987993
rs35662273
rs147994116
rs144380153
rs4077189
rs190300559
rs115661735
rs55781756
rs941206
rs111486611
rs17774204
rs112667234
All − MR Egger
All − Inverse variance weighted
−1
0
1
2
MR effect size for
'CD28+ DN (CD4−CD8−) %T cell' on 'Diabetic nephropathy'

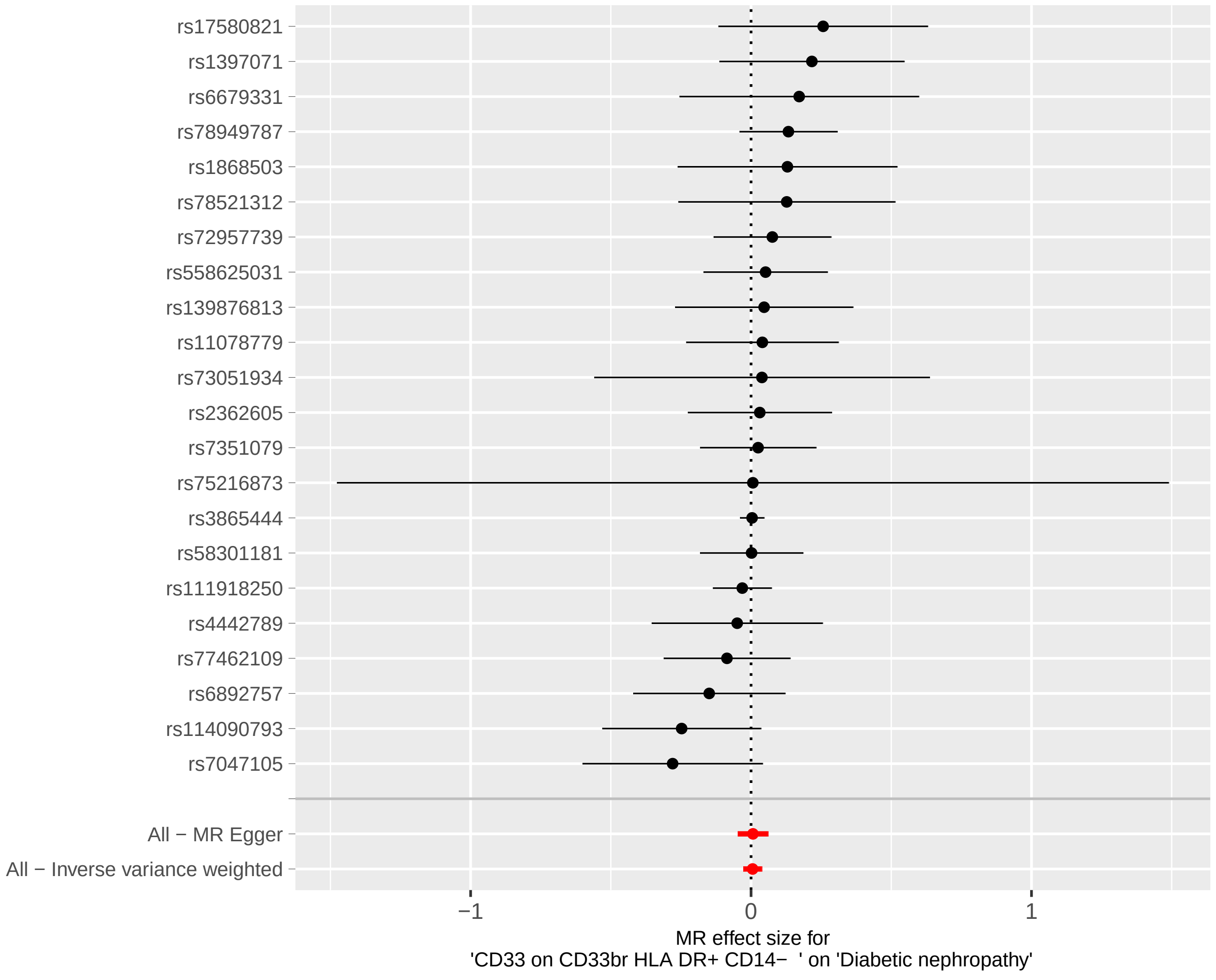
rs17580821
rs1397071
rs6679331
rs78949787
rs1868503
rs78521312
rs72957739
rs558625031
rs139876813
rs11078779
rs73051934
rs2362605
rs7351079
rs75216873
rs3865444
rs58301181
rs111918250
rs4442789
rs77462109
rs6892757
rs114090793
rs7047105
All − MR Egger
All − Inverse variance weighted
−1
0
1
MR effect size for
'CD33 on CD33br HLA DR+ CD14− ' on 'Diabetic nephropathy'

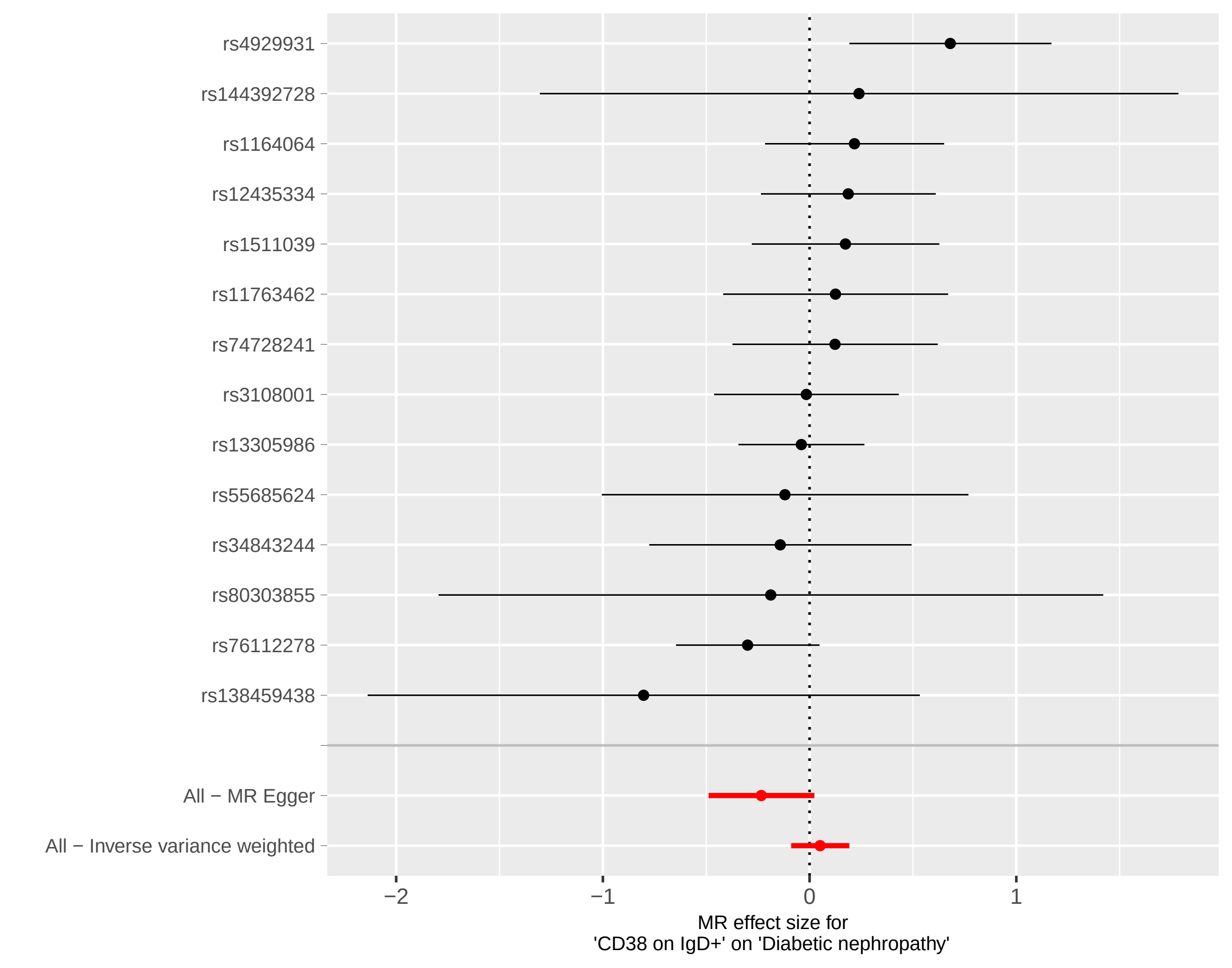
rs4929931
rs144392728
rs1164064
rs12435334
rs1511039
rs11763462
rs74728241
rs3108001
rs13305986
rs55685624
rs34843244
rs80303855
rs76112278
rs138459438
All − MR Egger
All − Inverse variance weighted
−2
−1
0
1
MR effect size for
'CD38 on IgD+' on 'Diabetic nephropathy'

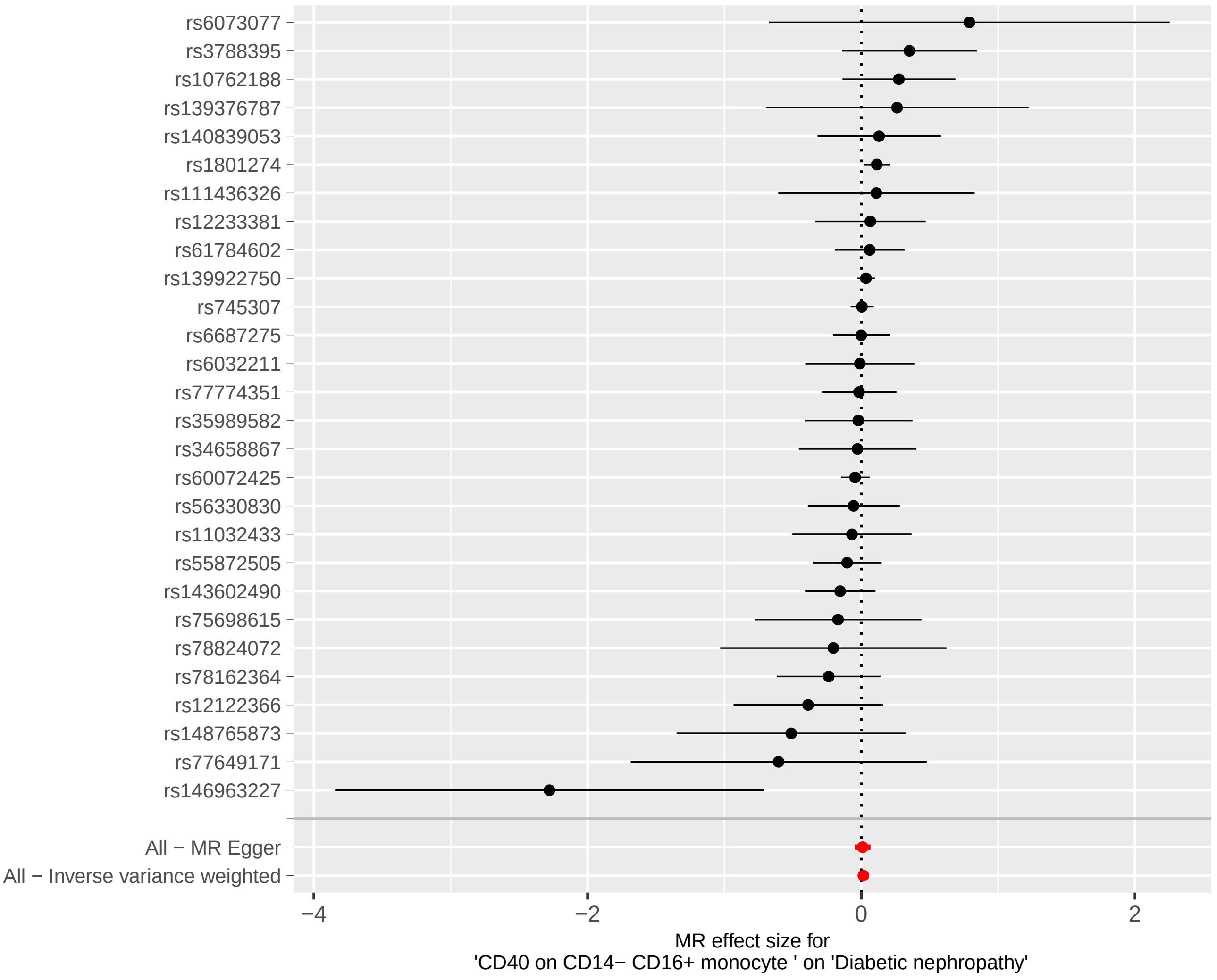

rs6073077
rs3788395
rs10762188
rs139376787
rs140839053
rs1801274
rs111436326
rs12233381
rs61784602
rs139922750
rs745307
rs6687275
rs6032211
rs77774351
rs35989582
rs34658867
rs60072425
rs56330830
rs11032433
rs55872505
rs143602490
rs75698615
rs78824072
rs78162364
rs12122366
rs148765873
rs77649171
rs146963227
All − MR Egger
All − Inverse variance weighted
−4
−2
0
2
MR effect size for
'CD40 on CD14− CD16+ monocyte ' on 'Diabetic nephropathy'

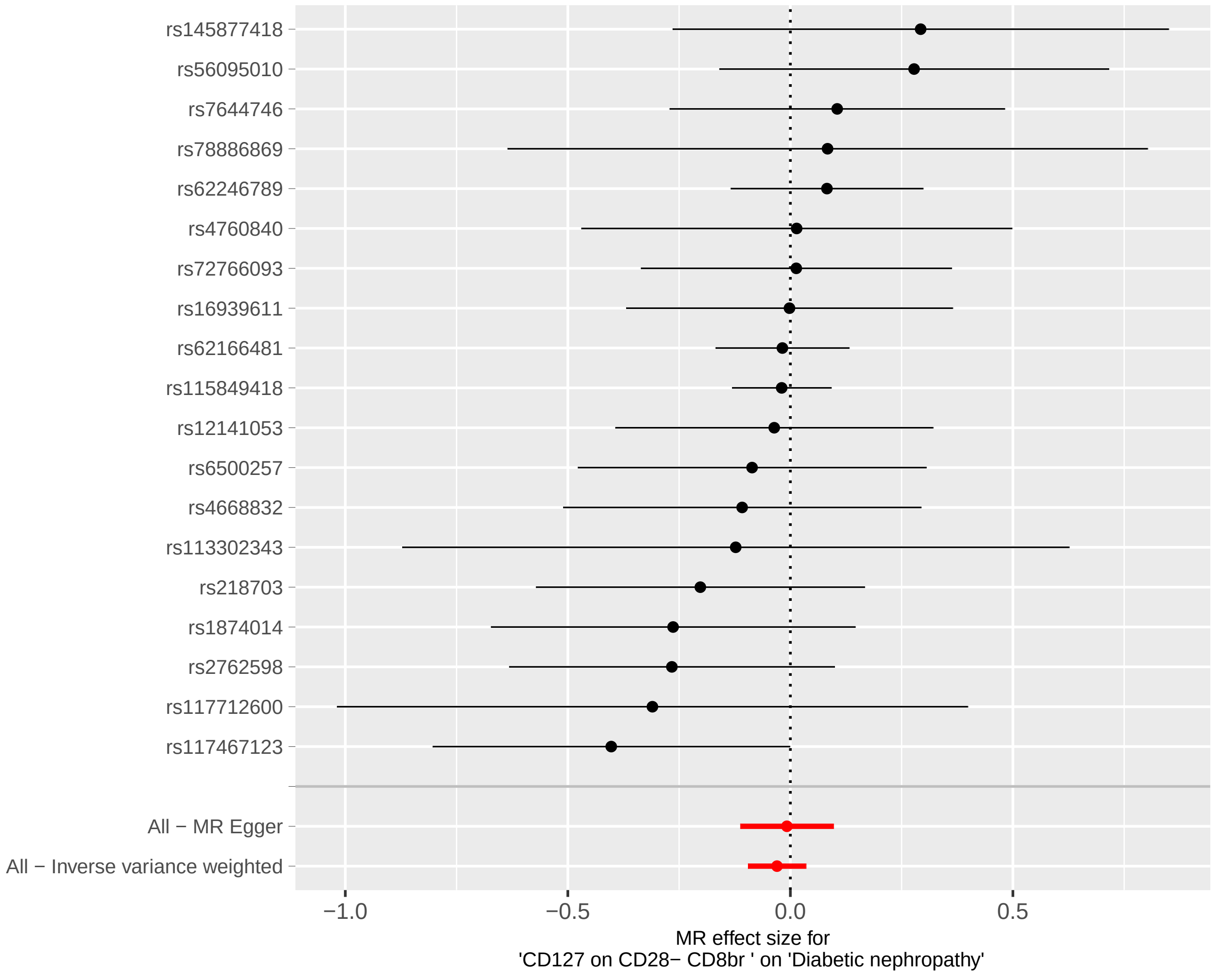
rs145877418
rs56095010
rs7644746
rs78886869
rs62246789
rs4760840
rs72766093
rs16939611
rs62166481
rs115849418
rs12141053
rs6500257
rs4668832
rs113302343
rs218703
rs1874014
rs2762598
rs117712600
rs117467123
All − MR Egger
All − Inverse variance weighted
−1.0
−0.5
0.0
0.5
MR effect size for
'CD127 on CD28− CD8br ' on 'Diabetic nephropathy'

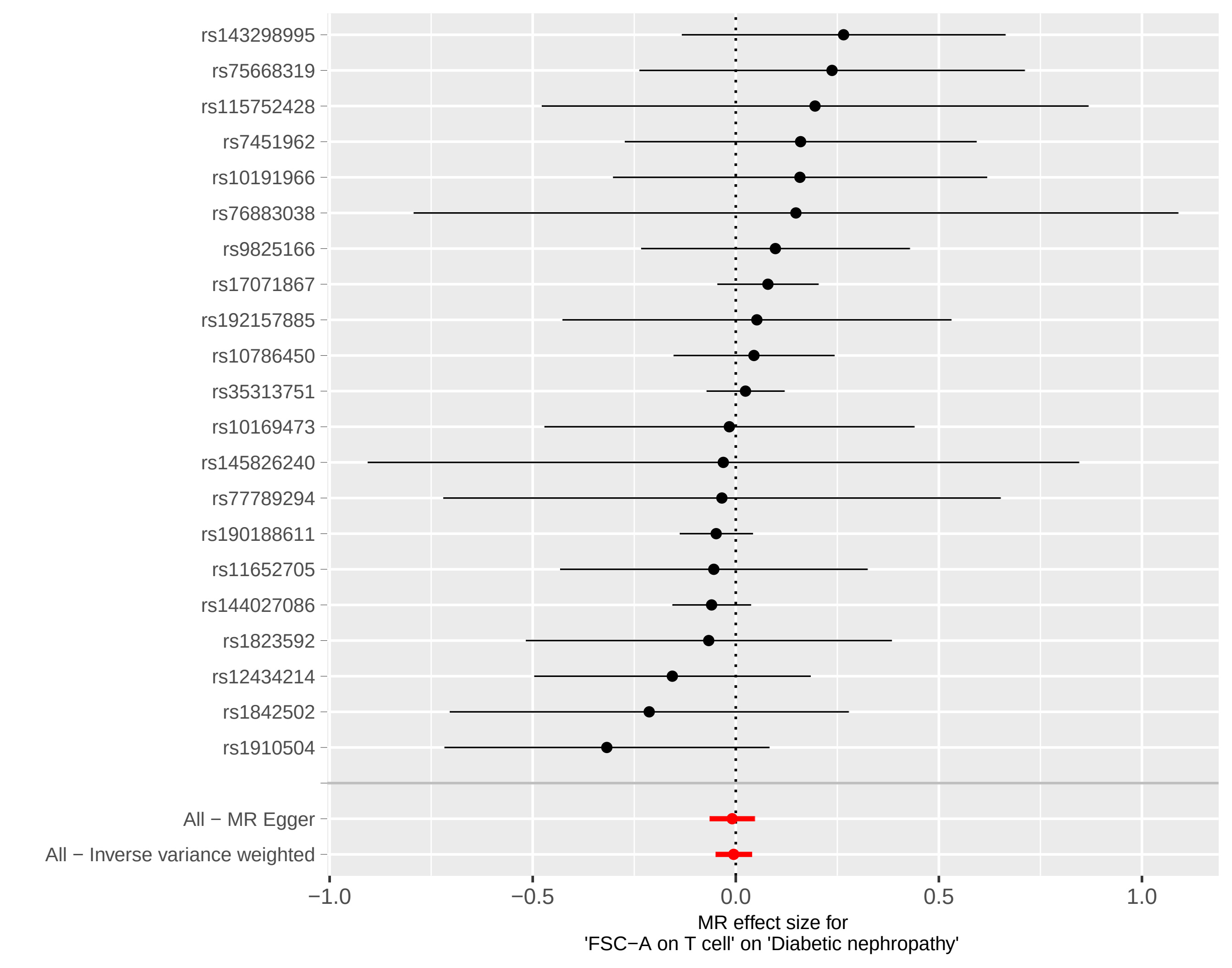
rs143298995
rs75668319
rs115752428
rs7451962
rs10191966
rs76883038
rs9825166
rs17071867
rs192157885
rs10786450
rs35313751
rs10169473
rs145826240
rs77789294
rs190188611
rs11652705
rs144027086
rs1823592
rs12434214
rs1842502
rs1910504
All − MR Egger
All − Inverse variance weighted
−1.0
−0.5
0.0
0.5
1.0
MR effect size for
'FSC−A on T cell' on 'Diabetic nephropathy'

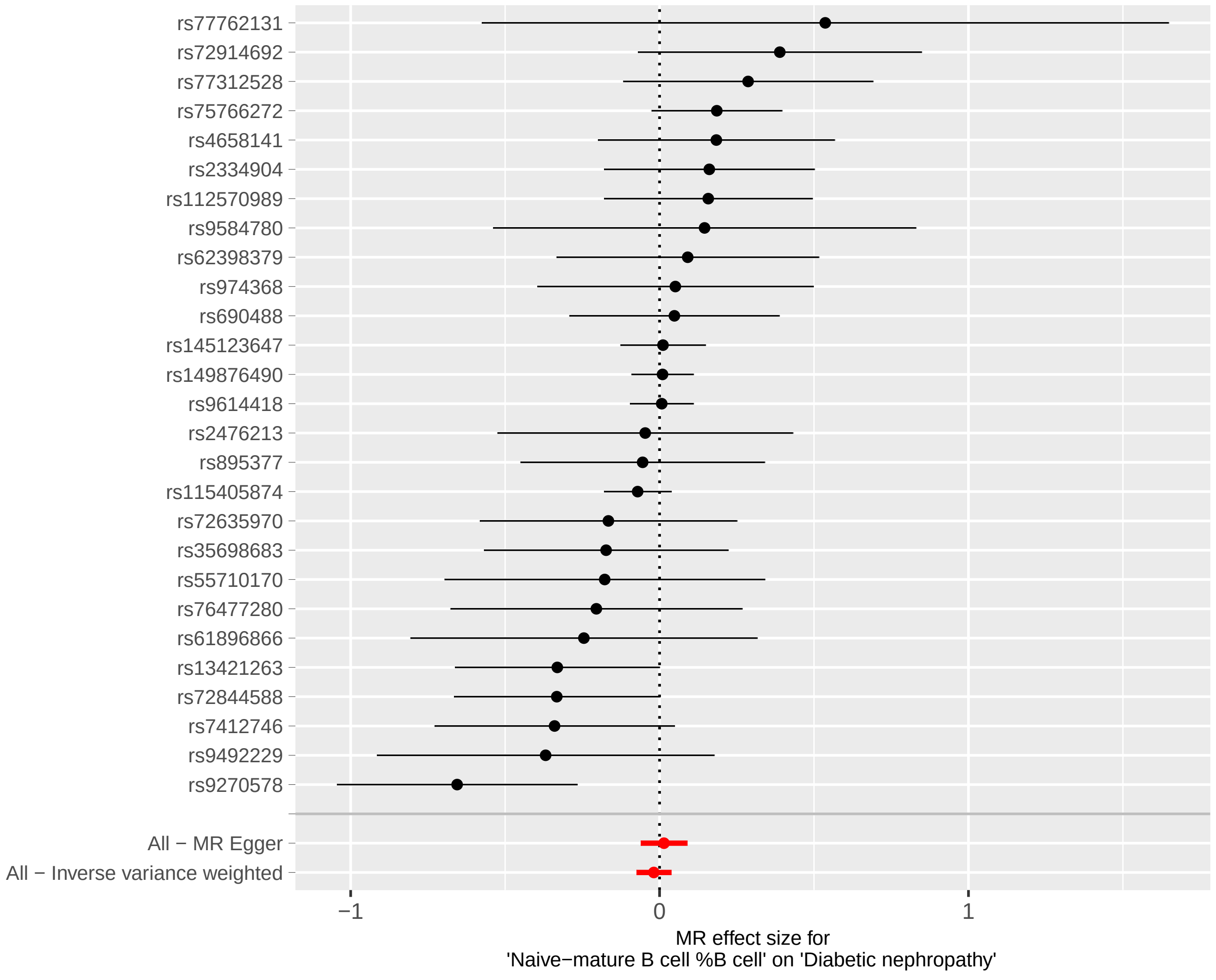
rs77762131
rs72914692
rs77312528
rs75766272
rs4658141
rs2334904
rs112570989
rs9584780
rs62398379
rs974368
rs690488
rs145123647
rs149876490
rs9614418
rs2476213
rs895377
rs115405874
rs72635970
rs35698683
rs55710170
rs76477280
rs61896866
rs13421263
rs72844588
rs7412746
rs9492229
rs9270578
All − MR Egger
All − Inverse variance weighted
−1
0
1
MR effect size for
'Naive−mature B cell %B cell' on 'Diabetic nephropathy'

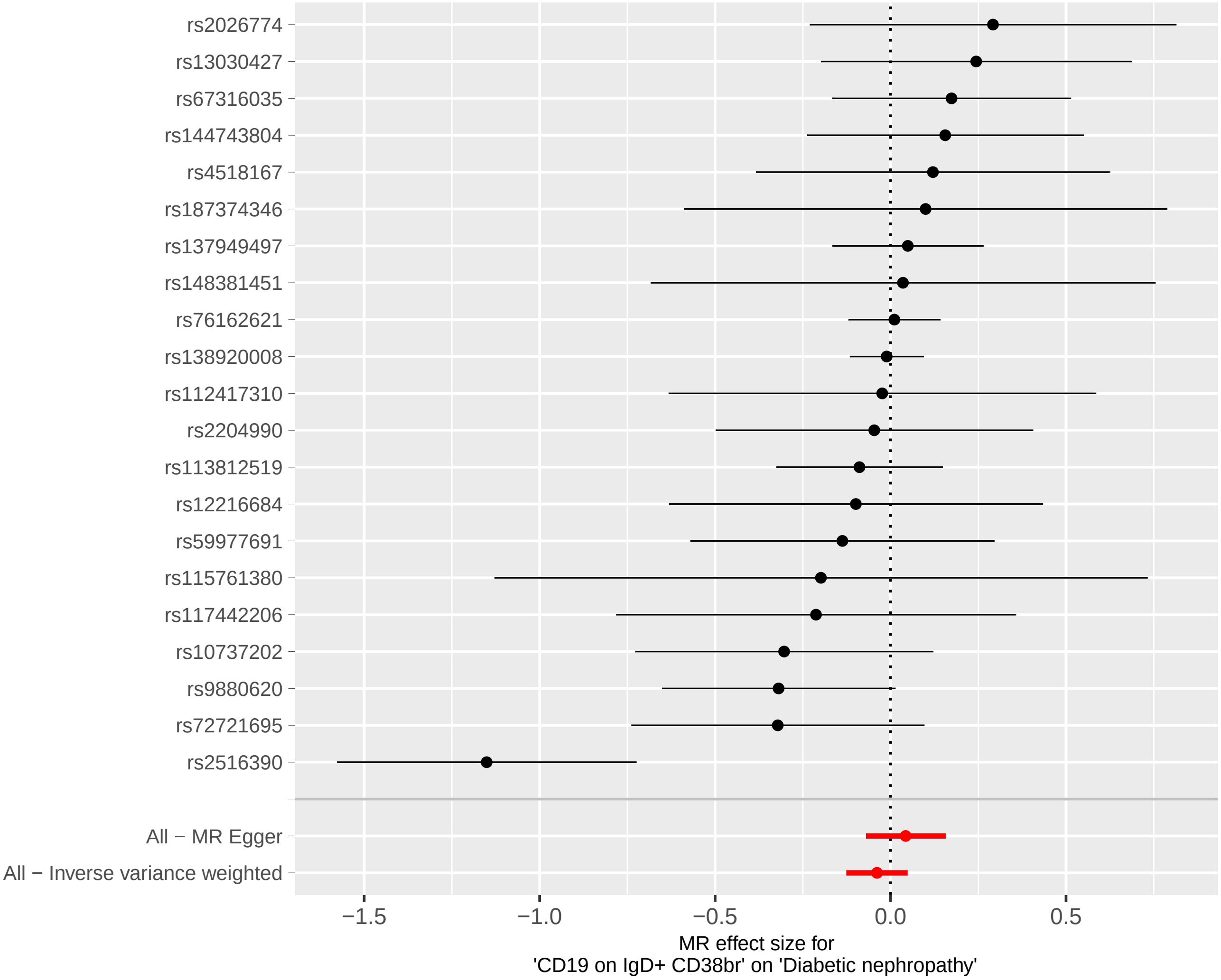

rs2026774
rs13030427
rs67316035
rs144743804
rs4518167
rs187374346
rs137949497
rs148381451
rs76162621
rs138920008
rs112417310
rs2204990
rs113812519
rs12216684
rs59977691
rs115761380
rs117442206
rs10737202
rs9880620
rs72721695
rs2516390
All − MR Egger
All − Inverse variance weighted
−1.5
−1.0
−0.5
0.0
0.5
MR effect size for
'CD19 on IgD+ CD38br' on 'Diabetic nephropathy'

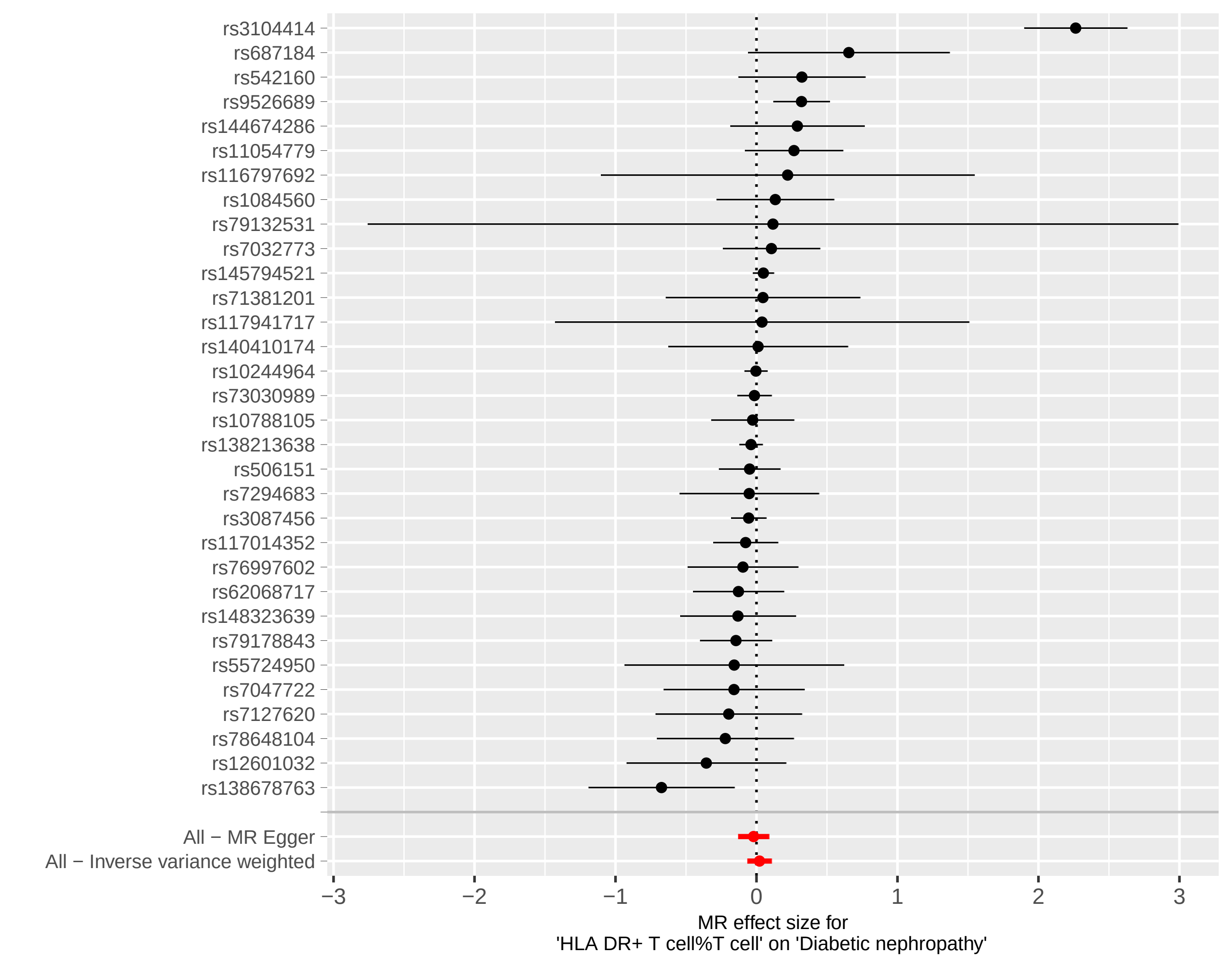
rs3104414
rs687184
rs542160
rs9526689
rs144674286
rs11054779
rs116797692
rs1084560
rs79132531
rs7032773
rs145794521
rs71381201
rs117941717
rs140410174
rs10244964
rs73030989
rs10788105
rs138213638
rs506151
rs7294683
rs3087456
rs117014352
rs76997602
rs62068717
rs148323639
rs79178843
rs55724950
rs7047722
rs7127620
rs78648104
rs12601032
rs138678763
All − MR Egger
All − Inverse variance weighted
−3
−2
−1
0
1
2
3
MR effect size for
'HLA DR+ T cell%T cell' on 'Diabetic nephropathy'

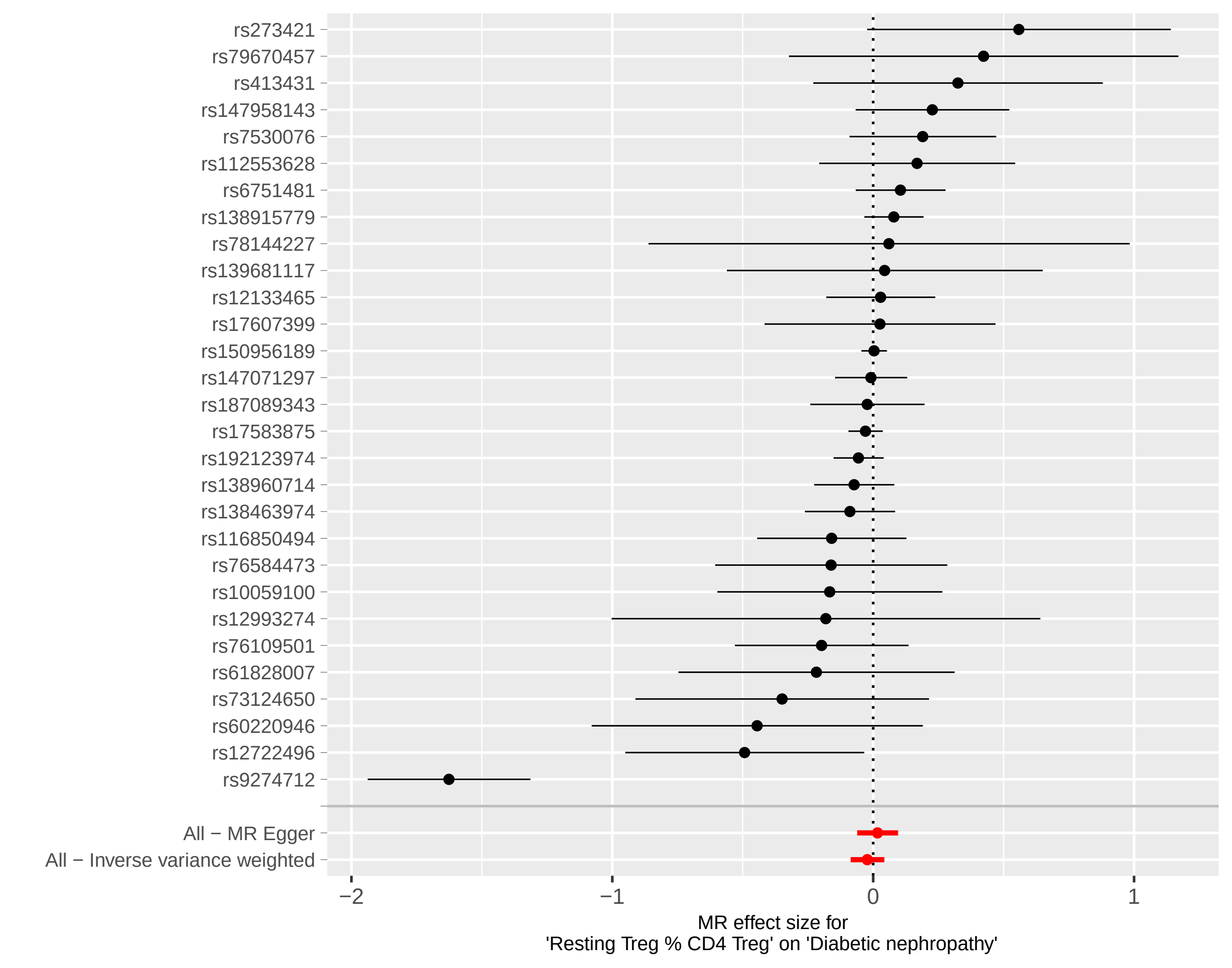

rs273421
rs79670457
rs413431
rs147958143
rs7530076
rs112553628
rs6751481
rs138915779
rs78144227
rs139681117
rs12133465
rs17607399
rs150956189
rs147071297
rs187089343
rs17583875
rs192123974
rs138960714
rs138463974
rs116850494
rs76584473
rs10059100
rs12993274
rs76109501
rs61828007
rs73124650
rs60220946
rs12722496
rs9274712
All − MR Egger
All − Inverse variance weighted
−2
−1
0
1
MR effect size for
'Resting Treg % CD4 Treg' on 'Diabetic nephropathy'

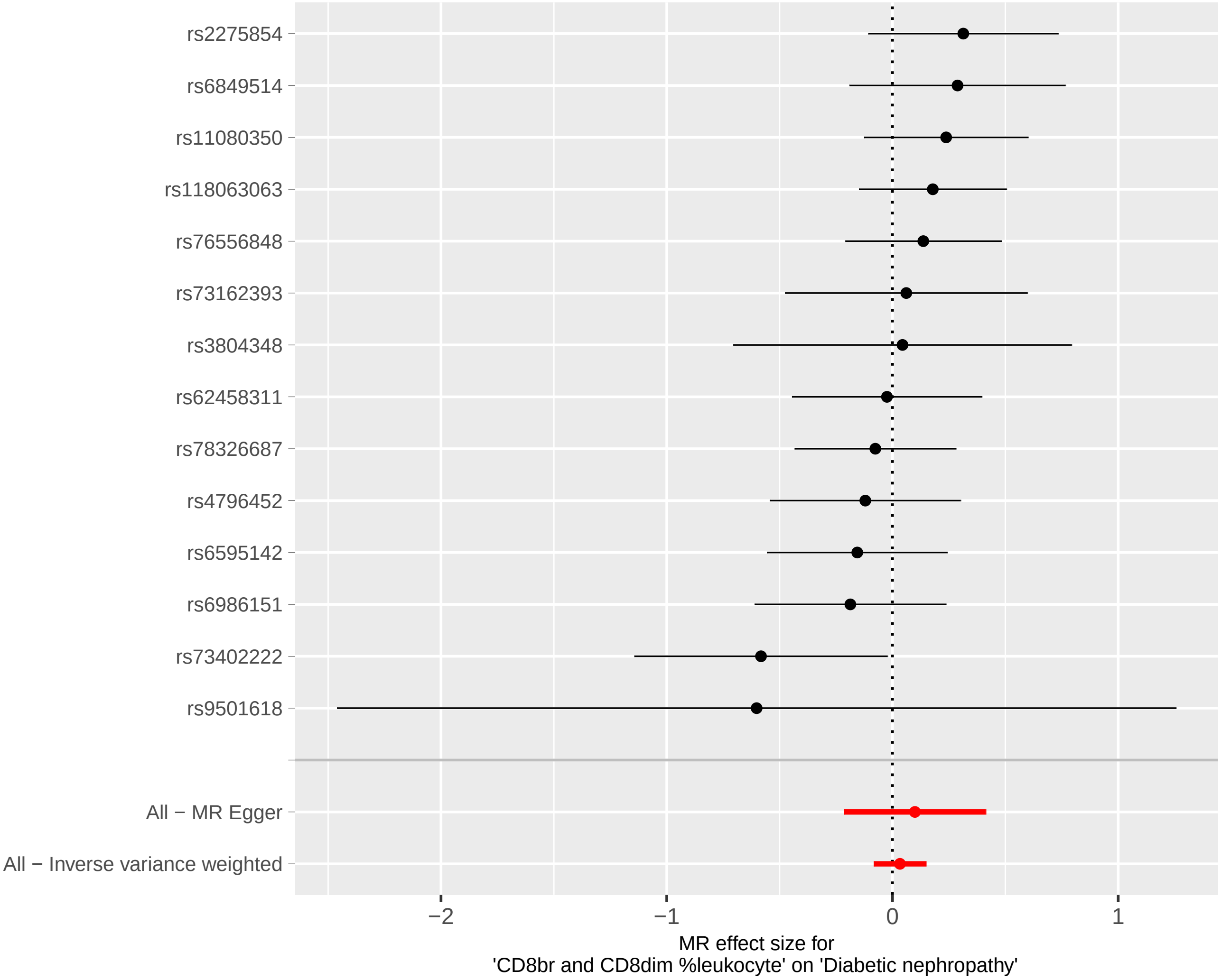

rs2275854
rs6849514
rs11080350
rs118063063
rs76556848
rs73162393
rs3804348
rs62458311
rs78326687
rs4796452
rs6595142
rs6986151
rs73402222
rs9501618
All − MR Egger
All − Inverse variance weighted
−2
−1
0
1
MR effect size for
'CD8br and CD8dim %leukocyte' on 'Diabetic nephropathy'

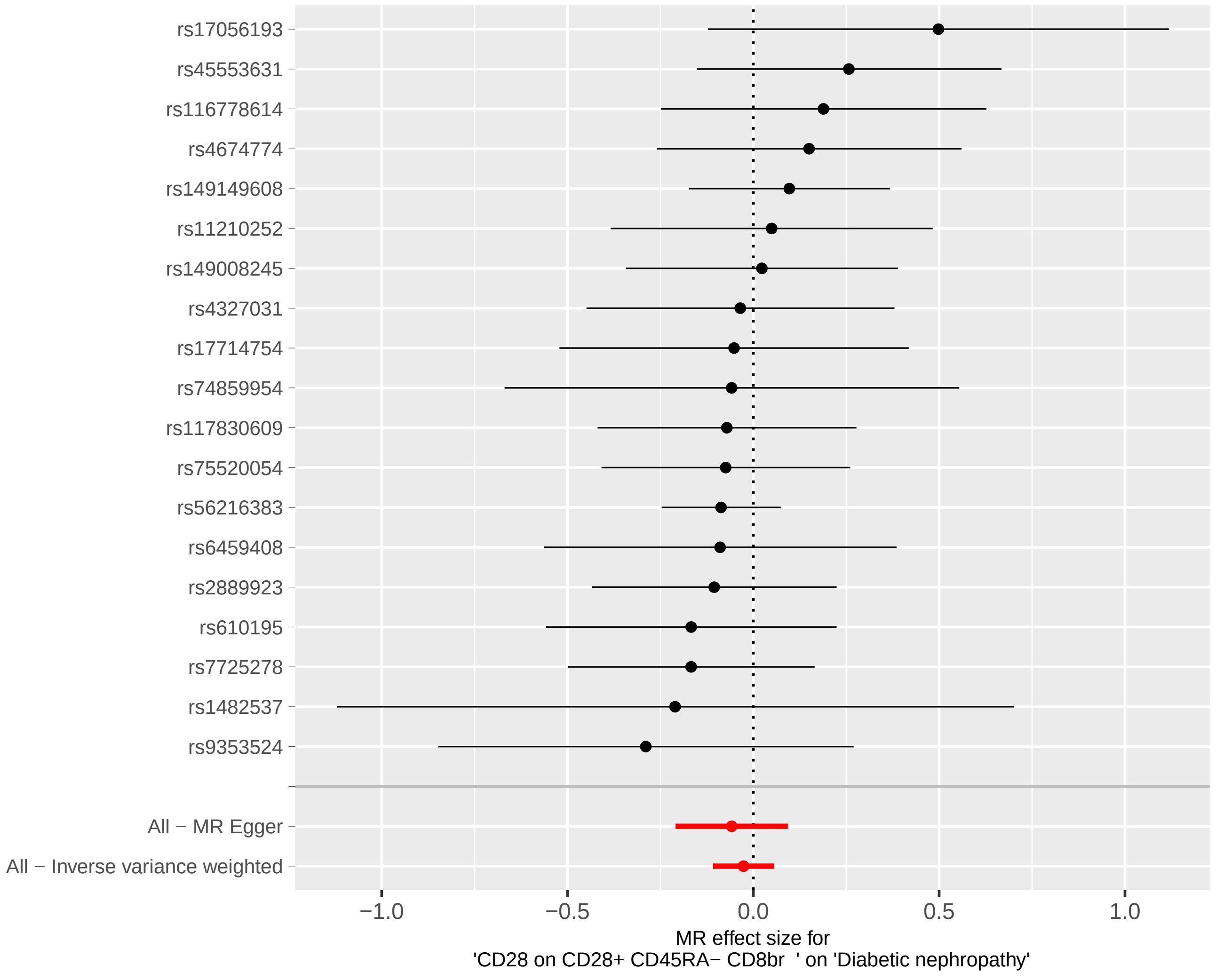
rs17056193
rs45553631
rs116778614
rs4674774
rs149149608
rs11210252
rs149008245
rs4327031
rs17714754
rs74859954
rs117830609
rs75520054
rs56216383
rs6459408
rs2889923
rs610195
rs7725278
rs1482537
rs9353524
All − MR Egger
All − Inverse variance weighted
−1.0
−0.5
0.0
0.5
1.0
MR effect size for
'CD28 on CD28+ CD45RA− CD8br ' on 'Diabetic nephropathy'

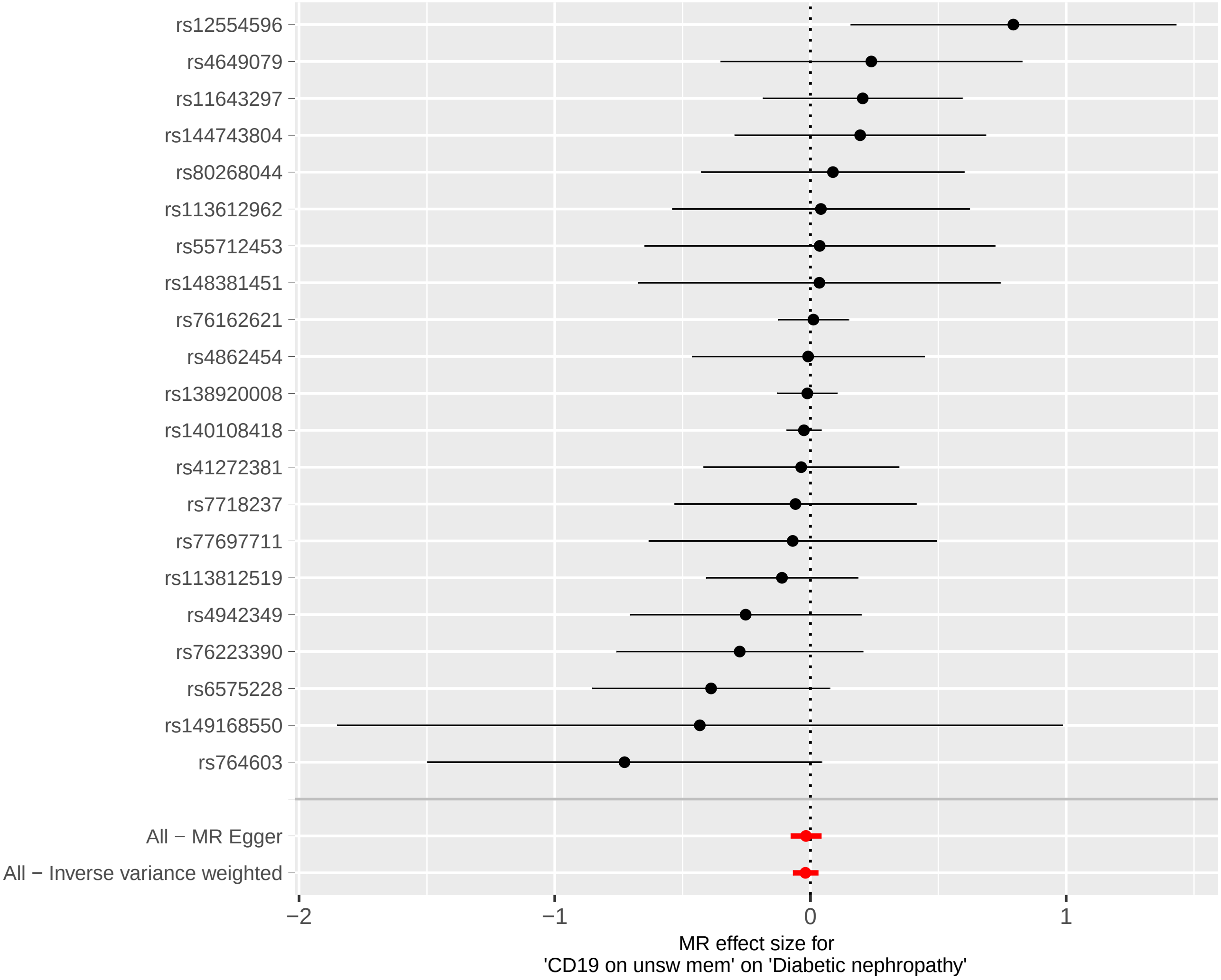
rs12554596
rs4649079
rs11643297
rs144743804
rs80268044
rs113612962
rs55712453
rs148381451
rs76162621
rs4862454
rs138920008
rs140108418
rs41272381
rs7718237
rs77697711
rs113812519
rs4942349
rs76223390
rs6575228
rs149168550
rs764603
All − MR Egger
All − Inverse variance weighted
−2
−1
0
1
MR effect size for
'CD19 on unsw mem' on 'Diabetic nephropathy'

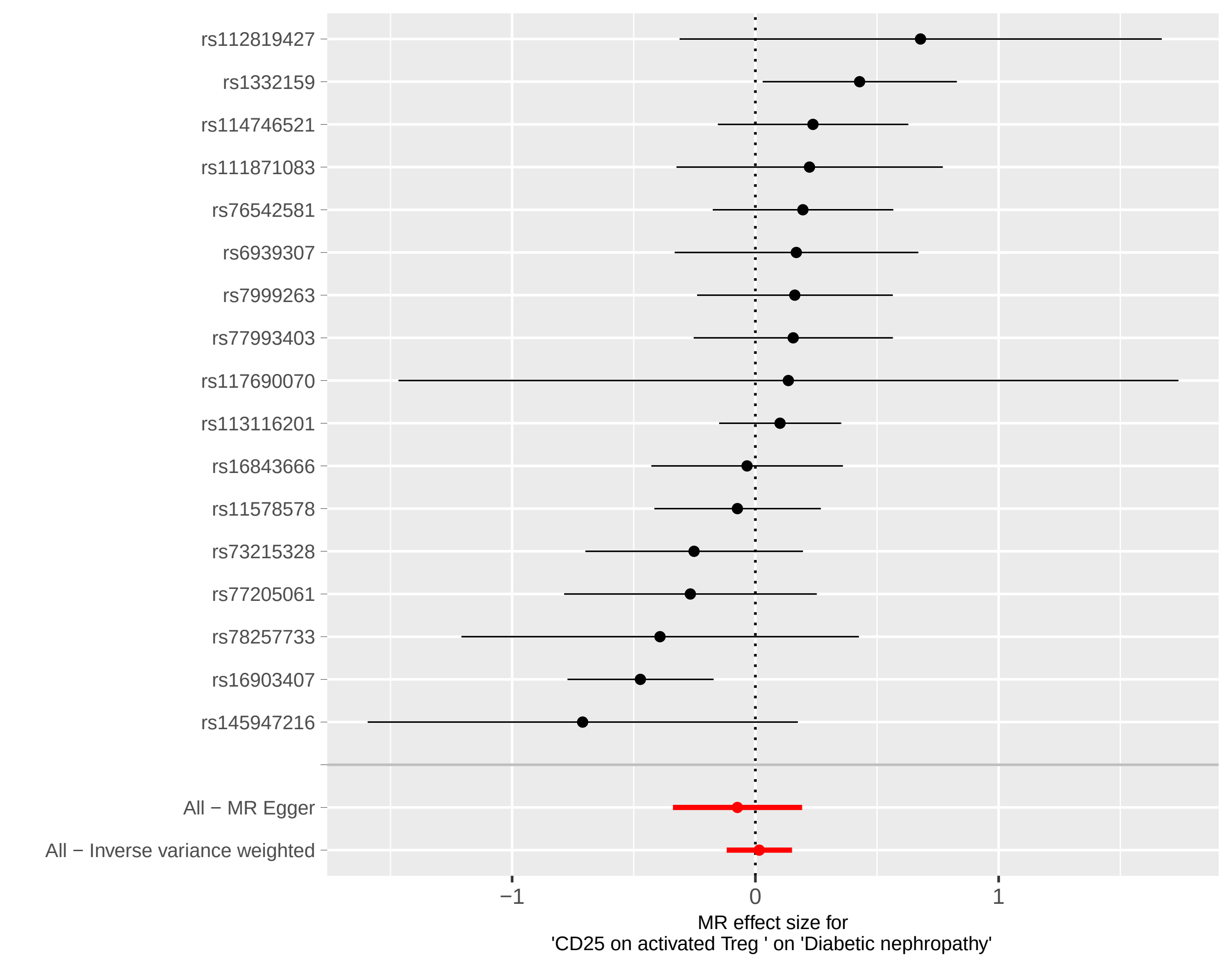
rs112819427
rs1332159
rs114746521
rs111871083
rs76542581
rs6939307
rs7999263
rs77993403
rs117690070
rs113116201
rs16843666
rs11578578
rs73215328
rs77205061
rs78257733
rs16903407
rs145947216
All − MR Egger
All − Inverse variance weighted
−1
0
1
MR effect size for
'CD25 on activated Treg ' on 'Diabetic nephropathy'

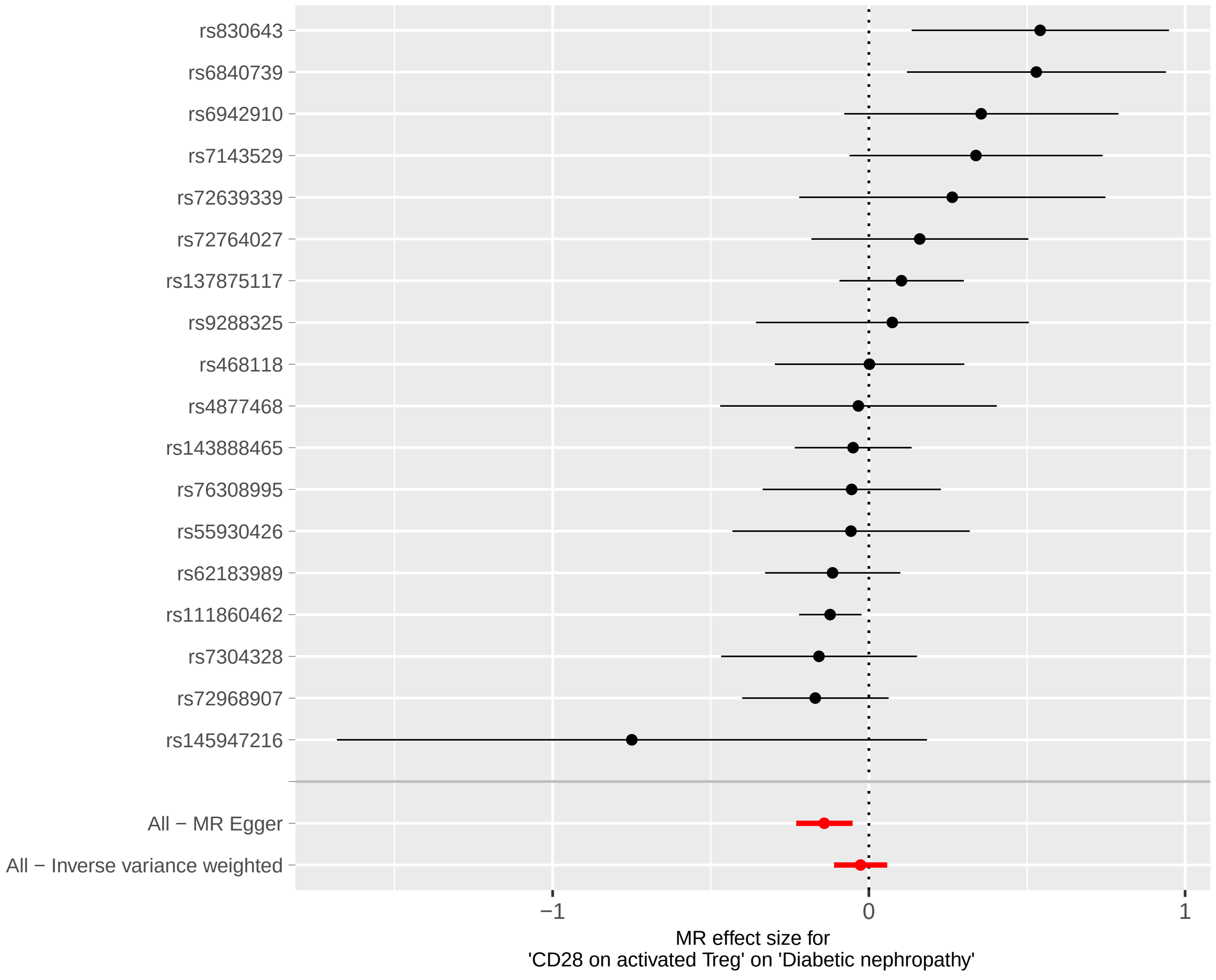
rs830643
rs6840739
rs6942910
rs7143529
rs72639339
rs72764027
rs137875117
rs9288325
rs468118
rs4877468
rs143888465
rs76308995
rs55930426
rs62183989
rs111860462
rs7304328
rs72968907
rs145947216
All − MR Egger
All − Inverse variance weighted
−1
0
1
MR effect size for
'CD28 on activated Treg' on 'Diabetic nephropathy'

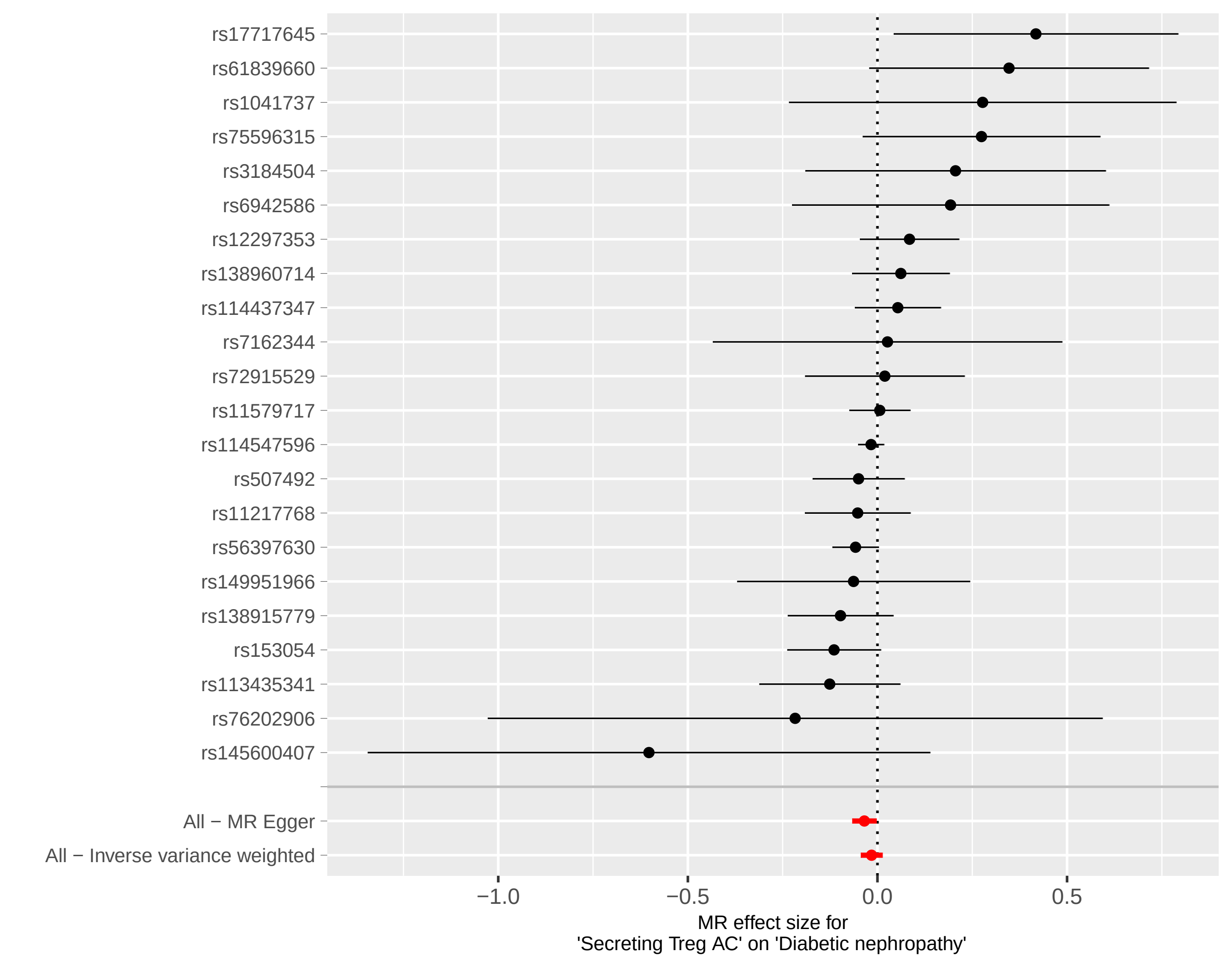
rs17717645
rs61839660
rs1041737
rs75596315
rs3184504
rs6942586
rs12297353
rs138960714
rs114437347
rs7162344
rs72915529
rs11579717
rs114547596
rs507492
rs11217768
rs56397630
rs149951966
rs138915779
rs153054
rs113435341
rs76202906
rs145600407
All − MR Egger
All − Inverse variance weighted
−1.0
−0.5
0.0
0.5
MR effect size for
'Secreting Treg AC' on 'Diabetic nephropathy'

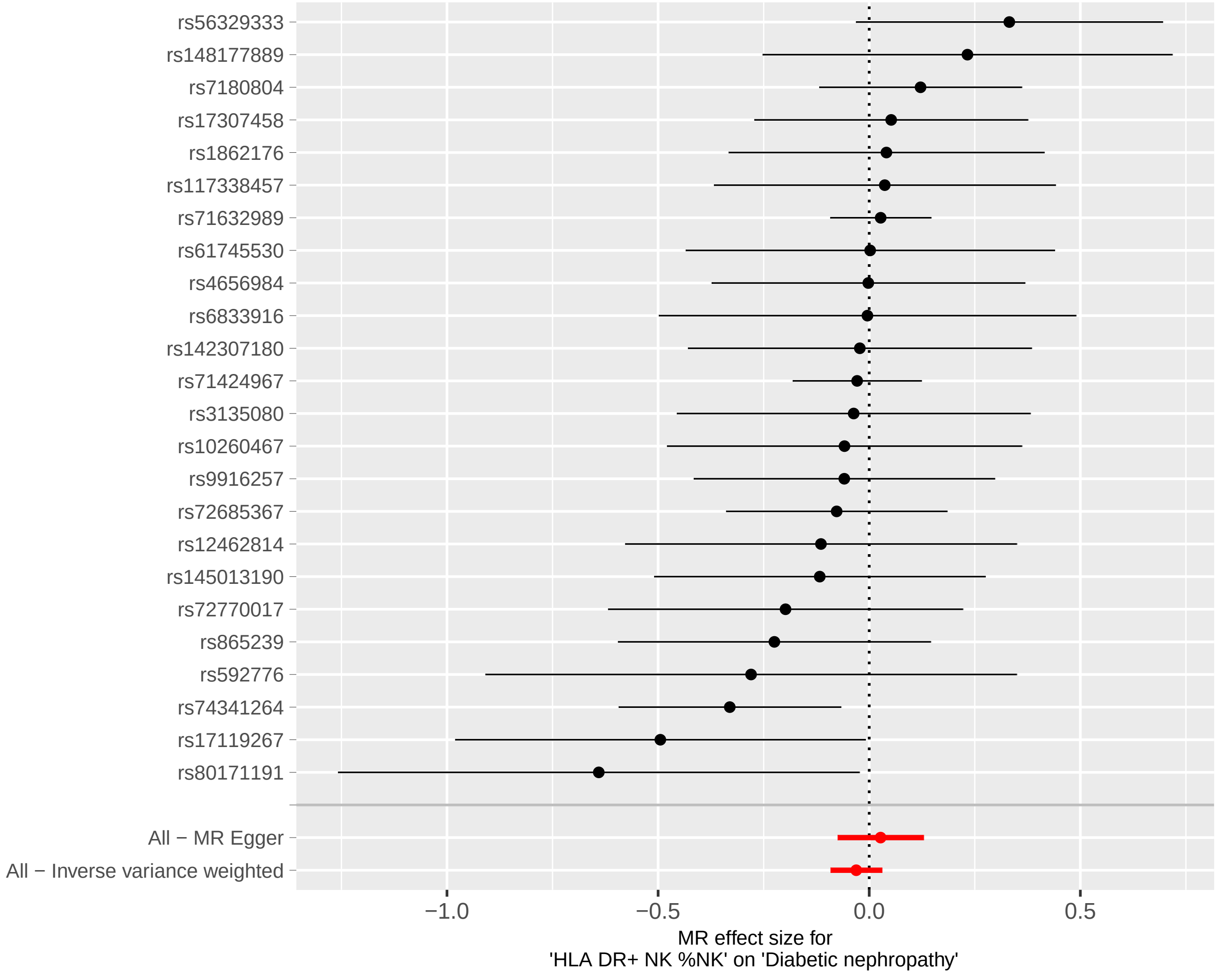
rs56329333
rs148177889
rs7180804
rs17307458
rs1862176
rs117338457
rs71632989
rs61745530
rs4656984
rs6833916
rs142307180
rs71424967
rs3135080
rs10260467
rs9916257
rs72685367
rs12462814
rs145013190
rs72770017
rs865239
rs592776
rs74341264
rs17119267
rs80171191
All − MR Egger
All − Inverse variance weighted
−1.0
−0.5
0.0
0.5
MR effect size for
'HLA DR+ NK %NK' on 'Diabetic nephropathy'

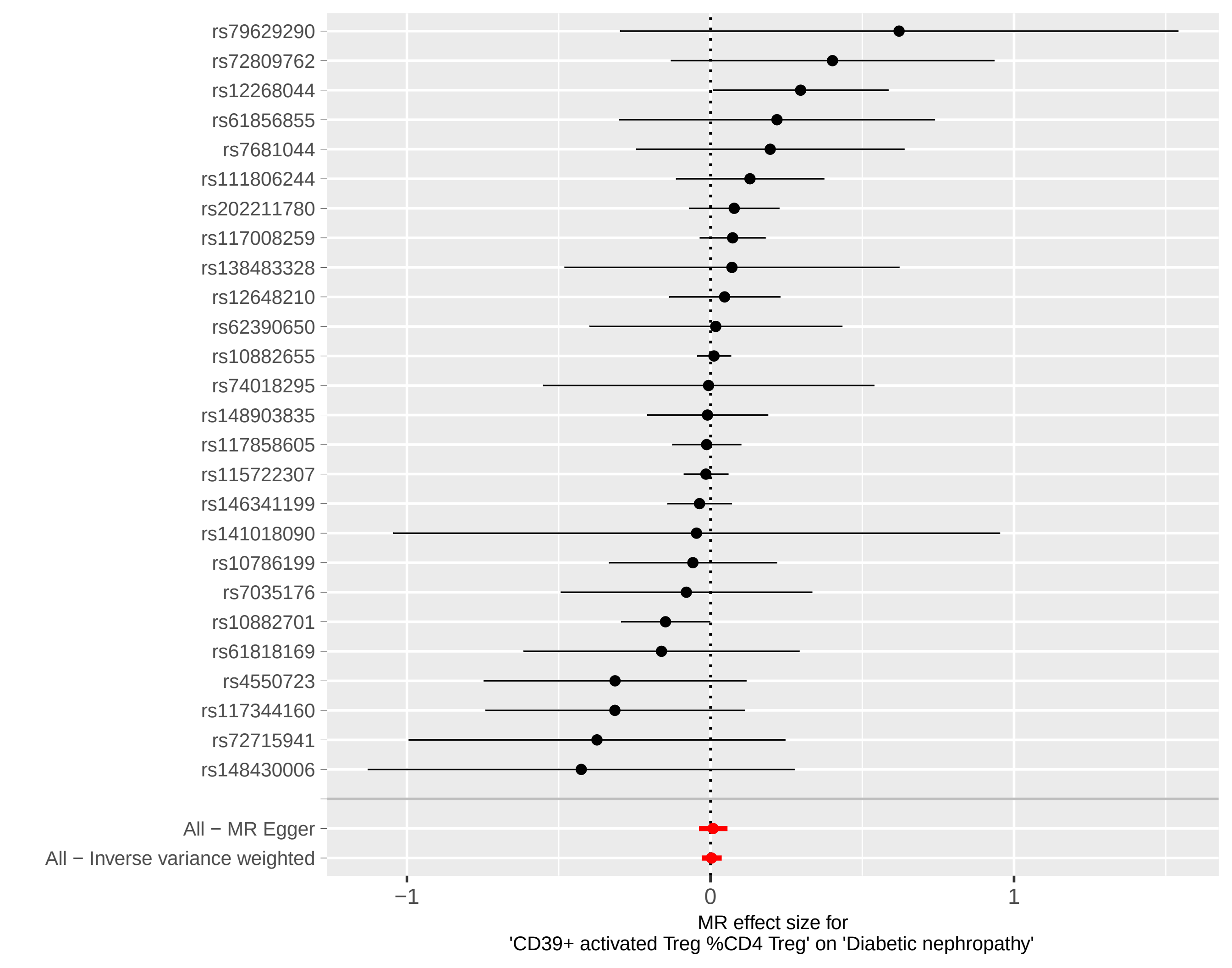
rs79629290
rs72809762
rs12268044
rs61856855
rs7681044
rs111806244
rs202211780
rs117008259
rs138483328
rs12648210
rs62390650
rs10882655
rs74018295
rs148903835
rs117858605
rs115722307
rs146341199
rs141018090
rs10786199
rs7035176
rs10882701
rs61818169
rs4550723
rs117344160
rs72715941
rs148430006
All − MR Egger
All − Inverse variance weighted
−1
0
1
MR effect size for
'CD39+ activated Treg %CD4 Treg' on 'Diabetic nephropathy'

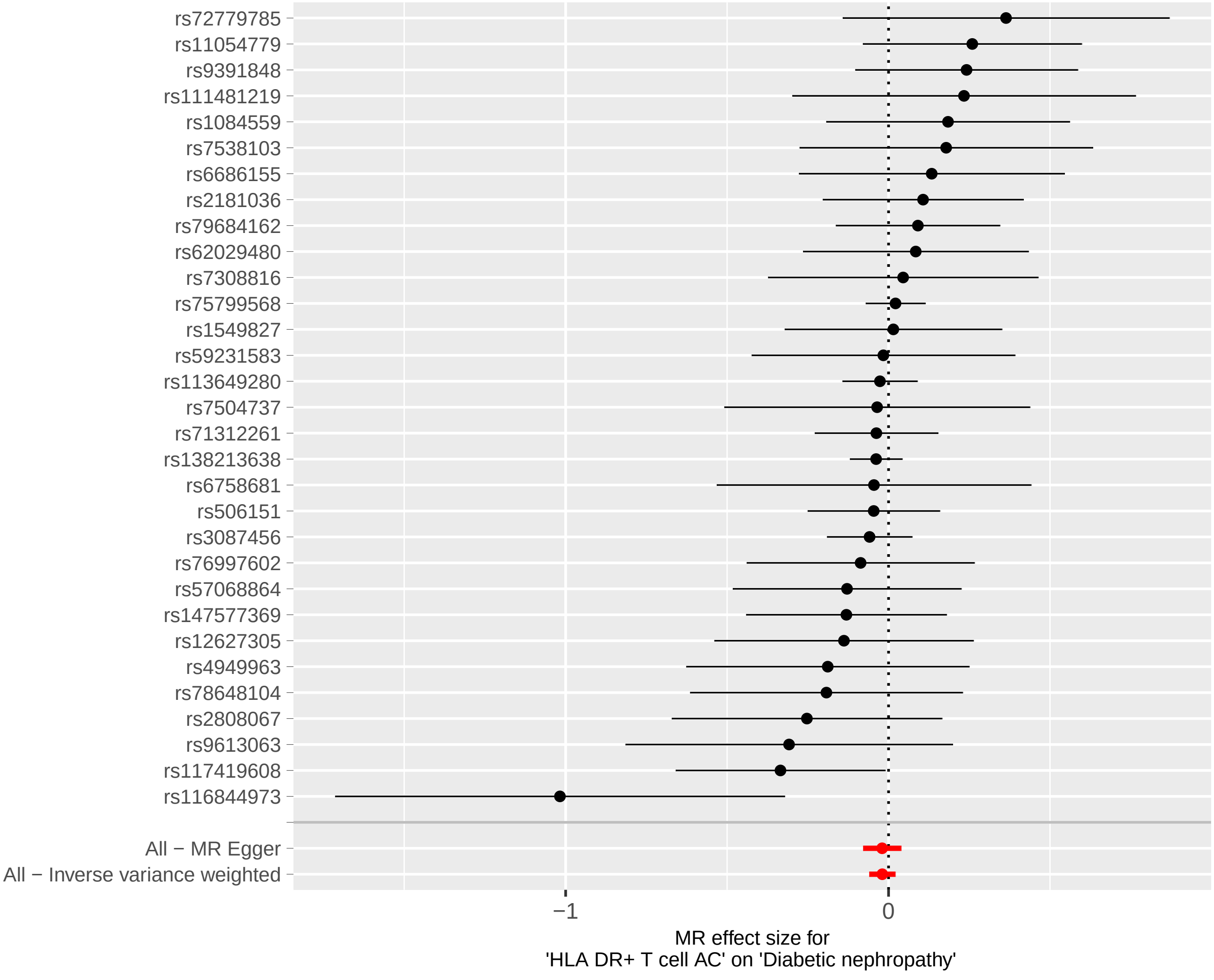
rs72779785
rs11054779
rs9391848
rs111481219
rs1084559
rs7538103
rs6686155
rs2181036
rs79684162
rs62029480
rs7308816
rs75799568
rs1549827
rs59231583
rs113649280
rs7504737
rs71312261
rs138213638
rs6758681
rs506151
rs3087456
rs76997602
rs57068864
rs147577369
rs12627305
rs4949963
rs78648104
rs2808067
rs9613063
rs117419608
rs116844973
All − MR Egger
All − Inverse variance weighted
−1
0
MR effect size for
'HLA DR+ T cell AC' on 'Diabetic nephropathy'

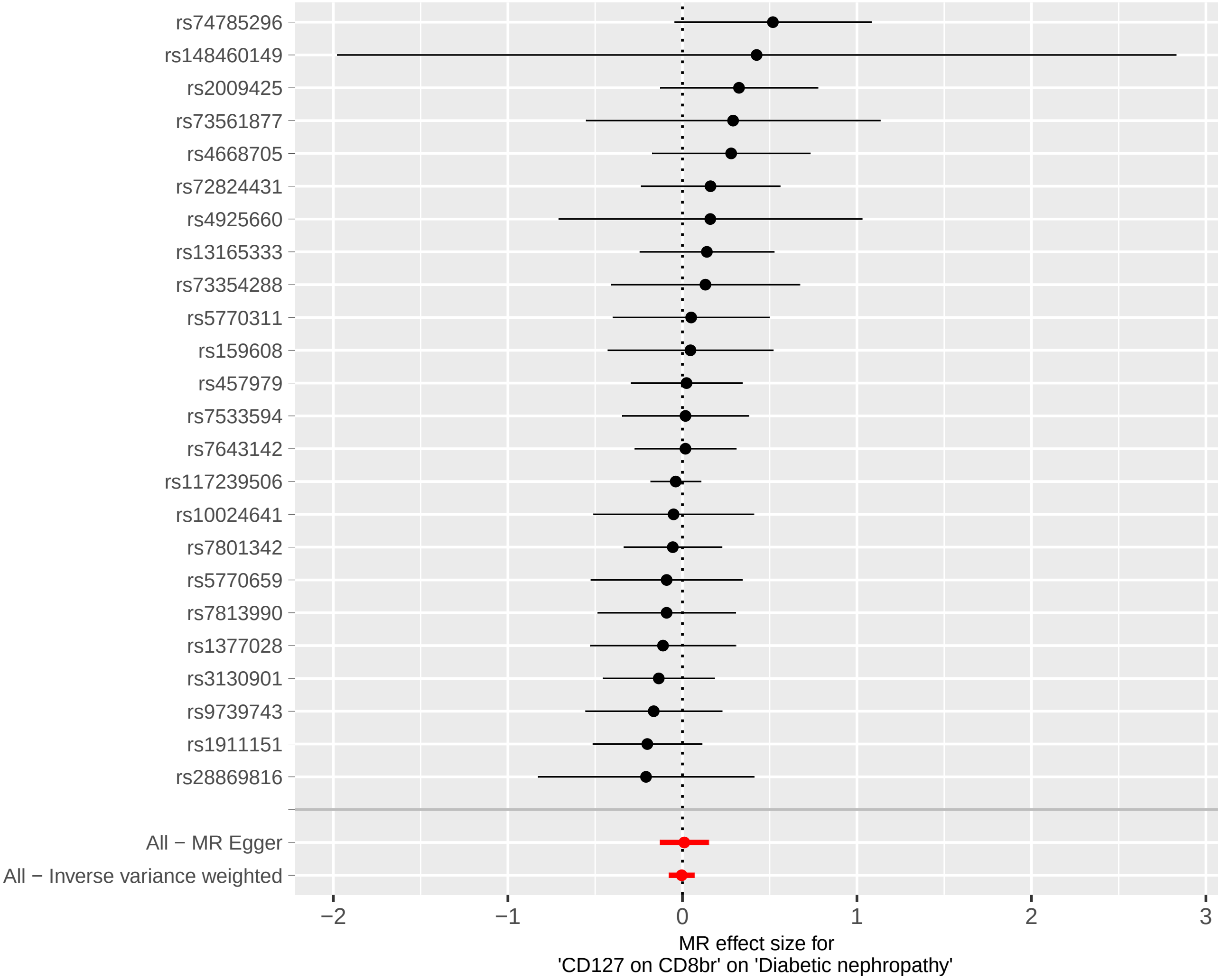
rs74785296
rs148460149
rs2009425
rs73561877
rs4668705
rs72824431
rs4925660
rs13165333
rs73354288
rs5770311
rs159608
rs457979
rs7533594
rs7643142
rs117239506
rs10024641
rs7801342
rs5770659
rs7813990
rs1377028
rs3130901
rs9739743
rs1911151
rs28869816
All − MR Egger
All − Inverse variance weighted
−2
−1
0
1
2
3
MR effect size for
'CD127 on CD8br' on 'Diabetic nephropathy'

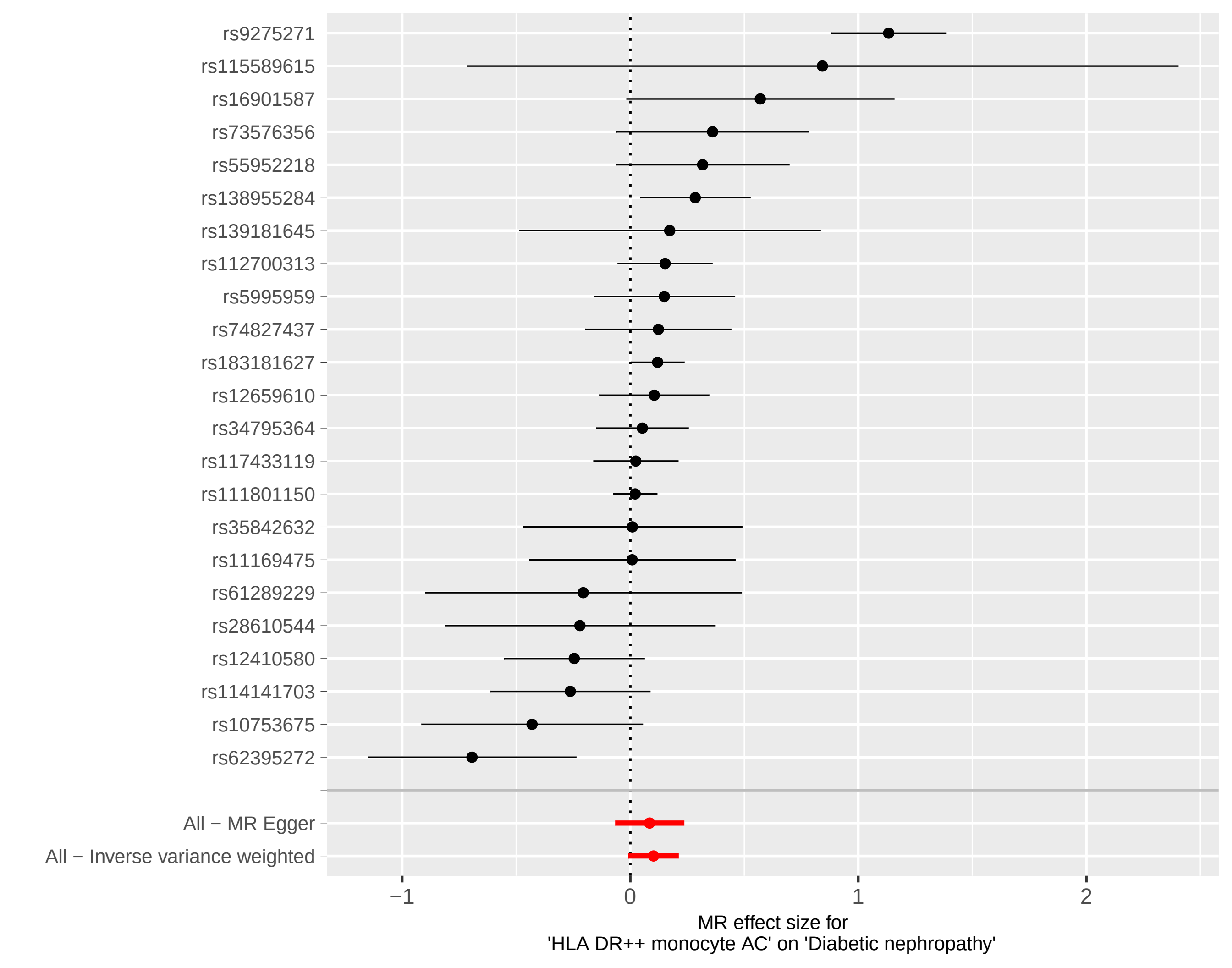
rs9275271
rs115589615
rs16901587
rs73576356
rs55952218
rs138955284
rs139181645
rs112700313
rs5995959
rs74827437
rs183181627
rs12659610
rs34795364
rs117433119
rs111801150
rs35842632
rs11169475
rs61289229
rs28610544
rs12410580
rs114141703
rs10753675
rs62395272
All − MR Egger
All − Inverse variance weighted
−1
0
1
2
MR effect size for
'HLA DR++ monocyte AC' on 'Diabetic nephropathy'

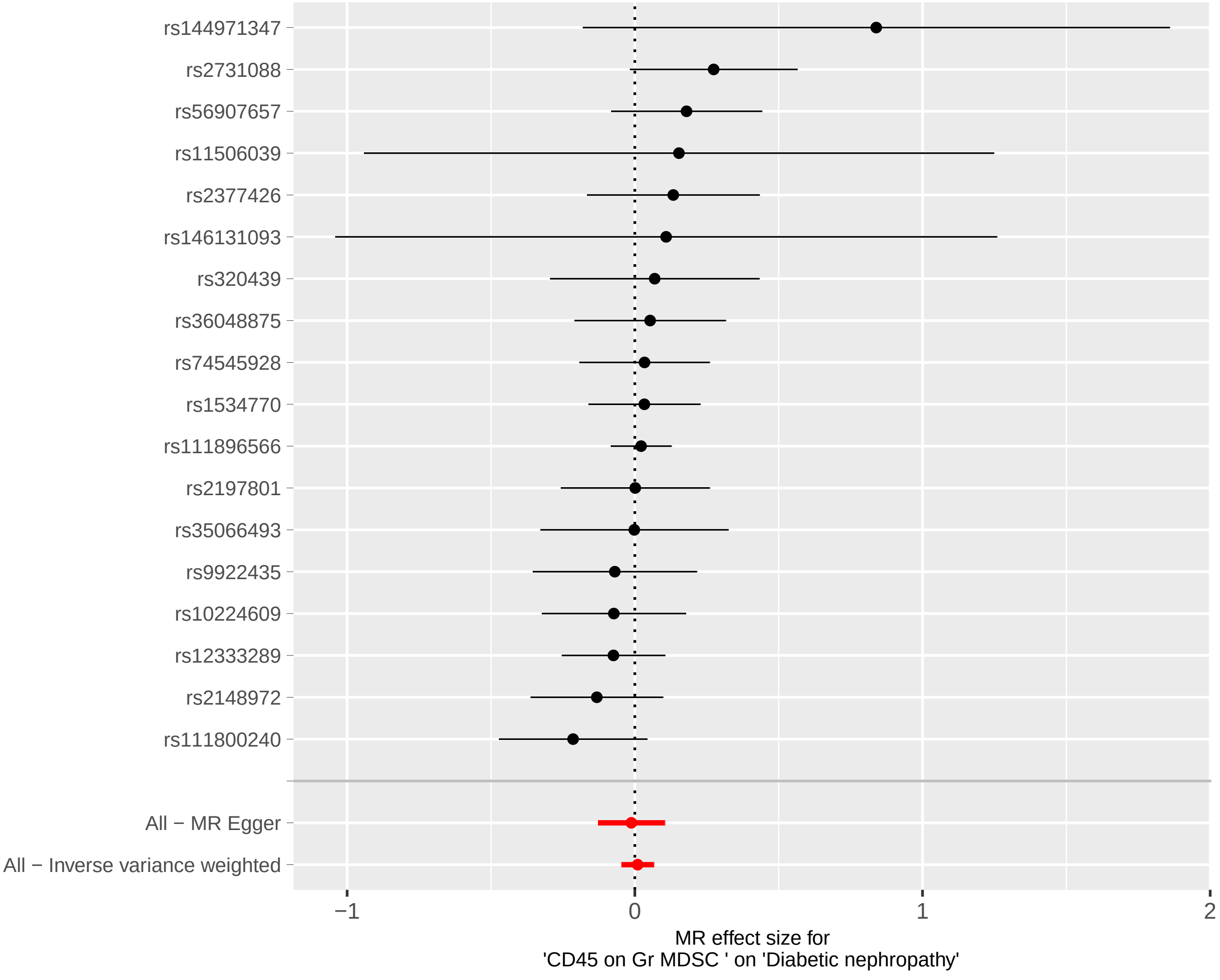
rs144971347
rs2731088
rs56907657
rs11506039
rs2377426
rs146131093
rs320439
rs36048875
rs74545928
rs1534770
rs111896566
rs2197801
rs35066493
rs9922435
rs10224609
rs12333289
rs2148972
rs111800240
All − MR Egger
All − Inverse variance weighted
−1
0
1
2
MR effect size for
'CD45 on Gr MDSC ' on 'Diabetic nephropathy'

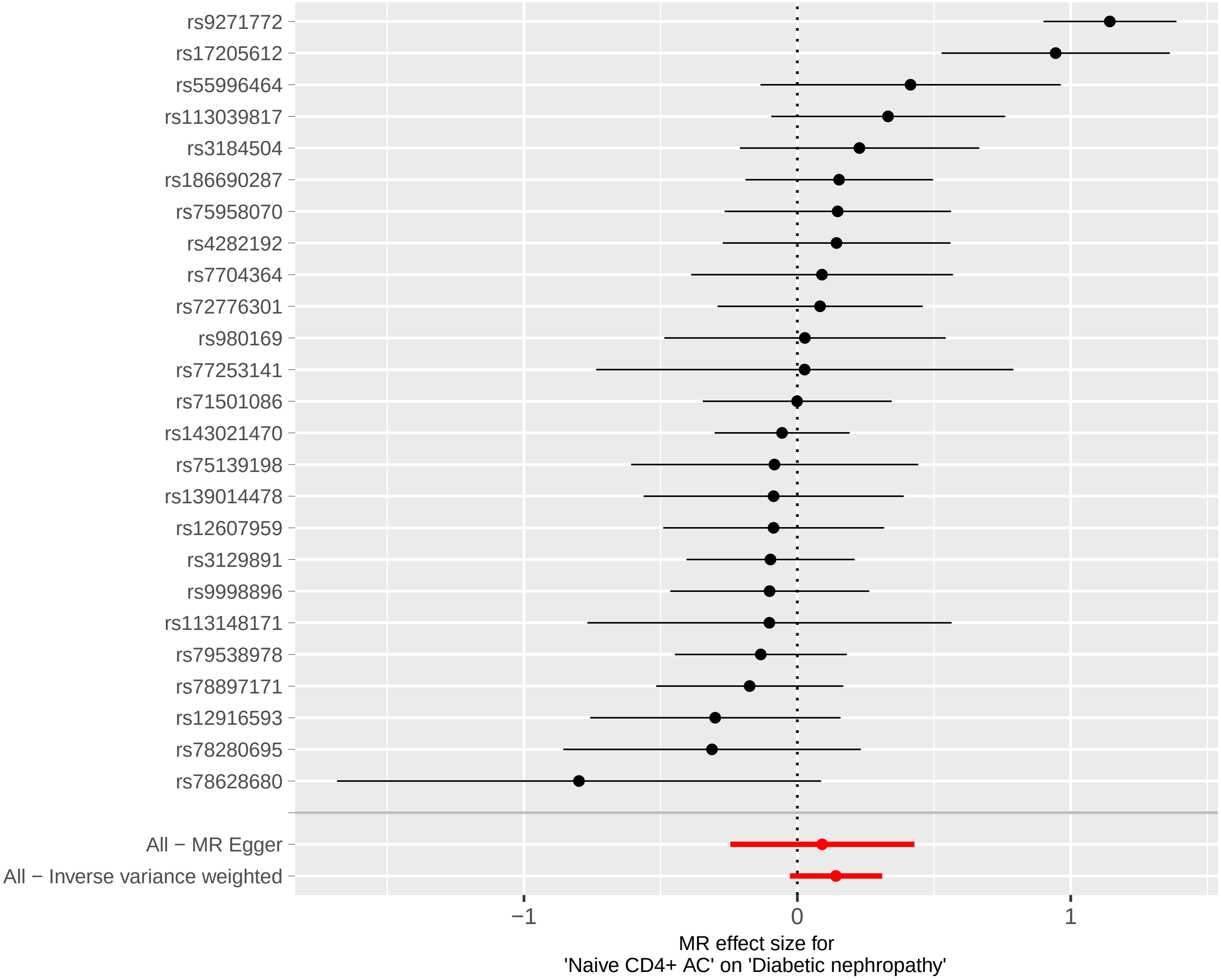

rs9271772
rs17205612
rs55996464
rs113039817
rs3184504
rs186690287
rs75958070
rs4282192
rs7704364
rs72776301
rs980169
rs77253141
rs71501086
rs143021470
rs75139198
rs139014478
rs12607959
rs3129891
rs9998896
rs113148171
rs79538978
rs78897171
rs12916593
rs78280695
rs78628680
All − MR Egger
All − Inverse variance weighted
−1
0
1
MR effect size for
'Naive CD4+ AC' on 'Diabetic nephropathy'

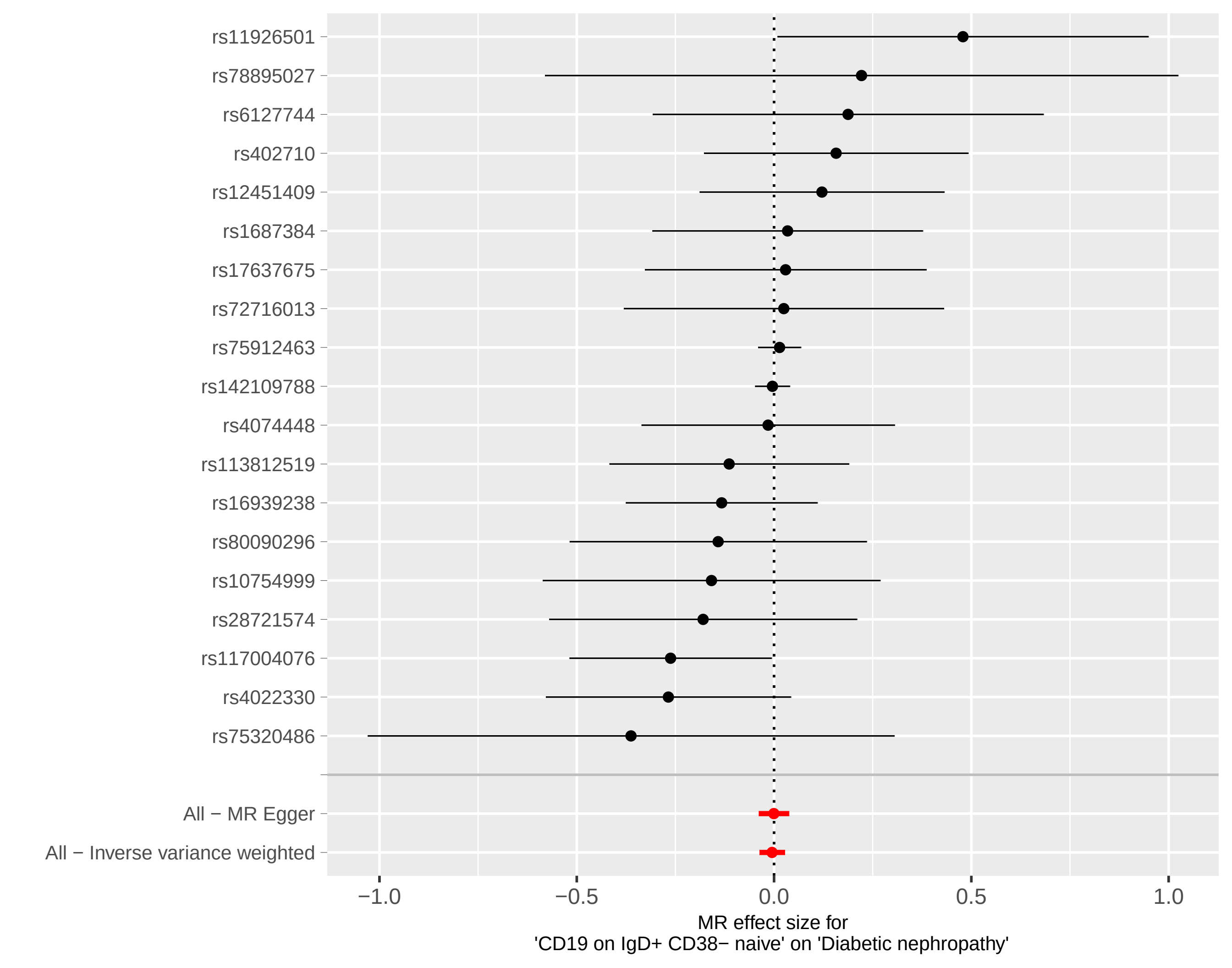
rs11926501
rs78895027
rs6127744
rs402710
rs12451409
rs1687384
rs17637675
rs72716013
rs75912463
rs142109788
rs4074448
rs113812519
rs16939238
rs80090296
rs10754999
rs28721574
rs117004076
rs4022330
rs75320486
All − MR Egger
All − Inverse variance weighted
−1.0
−0.5
0.0
0.5
1.0
MR effect size for
'CD19 on IgD+ CD38− naive' on 'Diabetic nephropathy'

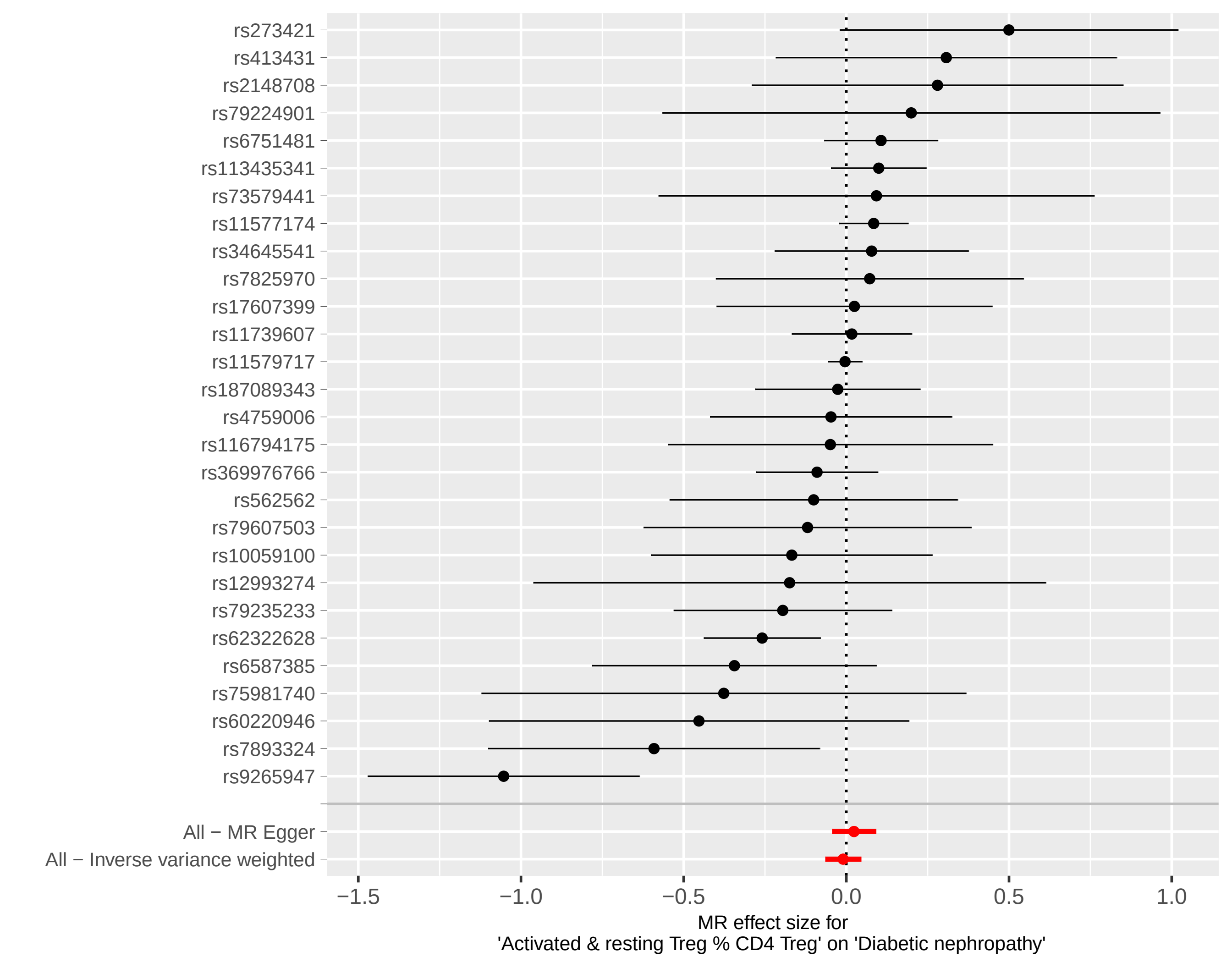
rs273421
rs413431
rs2148708
rs79224901
rs6751481
rs113435341
rs73579441
rs11577174
rs34645541
rs7825970
rs17607399
rs11739607
rs11579717
rs187089343
rs4759006
rs116794175
rs369976766
rs562562
rs79607503
rs10059100
rs12993274
rs79235233
rs62322628
rs6587385
rs75981740
rs60220946
rs7893324
rs9265947
All − MR Egger
All − Inverse variance weighted
−1.5
−1.0
−0.5
0.0
0.5
1.0
MR effect size for
'Activated & resting Treg % CD4 Treg' on 'Diabetic nephropathy'

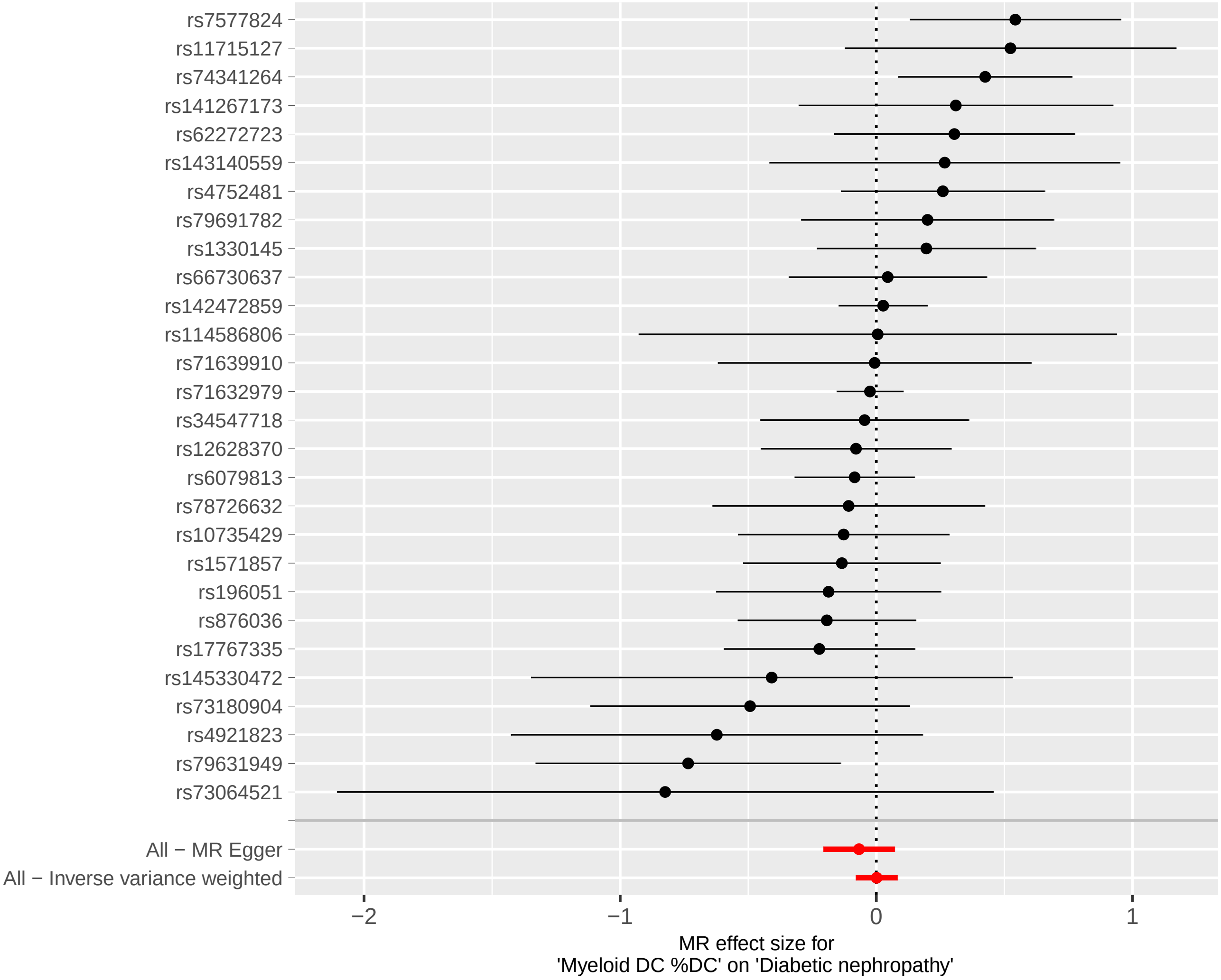

rs7577824
rs11715127
rs74341264
rs141267173
rs62272723
rs143140559
rs4752481
rs79691782
rs1330145
rs66730637
rs142472859
rs114586806
rs71639910
rs71632979
rs34547718
rs12628370
rs6079813
rs78726632
rs10735429
rs1571857
rs196051
rs876036
rs17767335
rs145330472
rs73180904
rs4921823
rs79631949
rs73064521
All − MR Egger
All − Inverse variance weighted
−2
−1
0
1
MR effect size for
'Myeloid DC %DC' on 'Diabetic nephropathy'

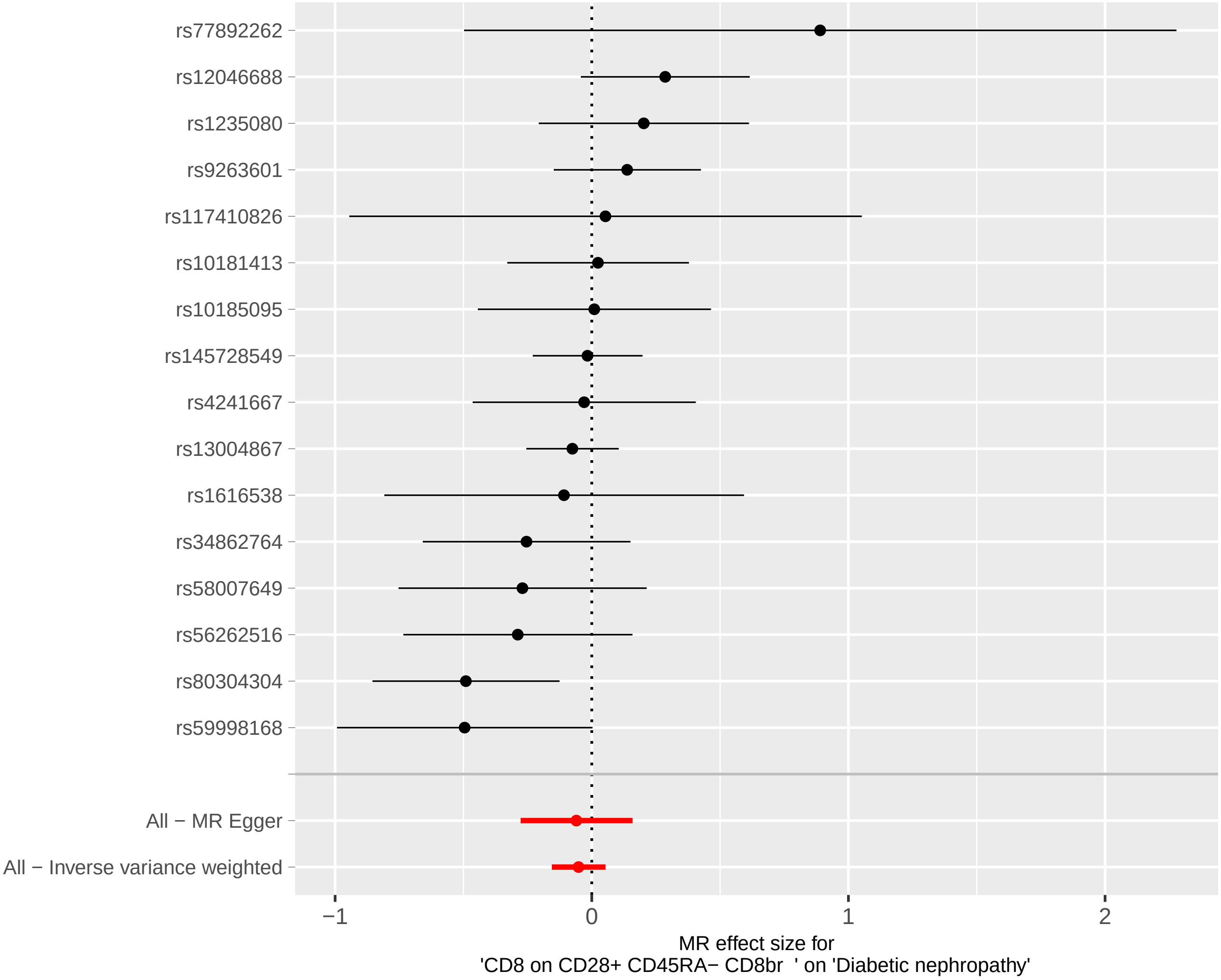

rs77892262
rs12046688
rs1235080
rs9263601
rs117410826
rs10181413
rs10185095
rs145728549
rs4241667
rs13004867
rs1616538
rs34862764
rs58007649
rs56262516
rs80304304
rs59998168
All − MR Egger
All − Inverse variance weighted
−1
0
1
2
MR effect size for
'CD8 on CD28+ CD45RA− CD8br ' on 'Diabetic nephropathy'

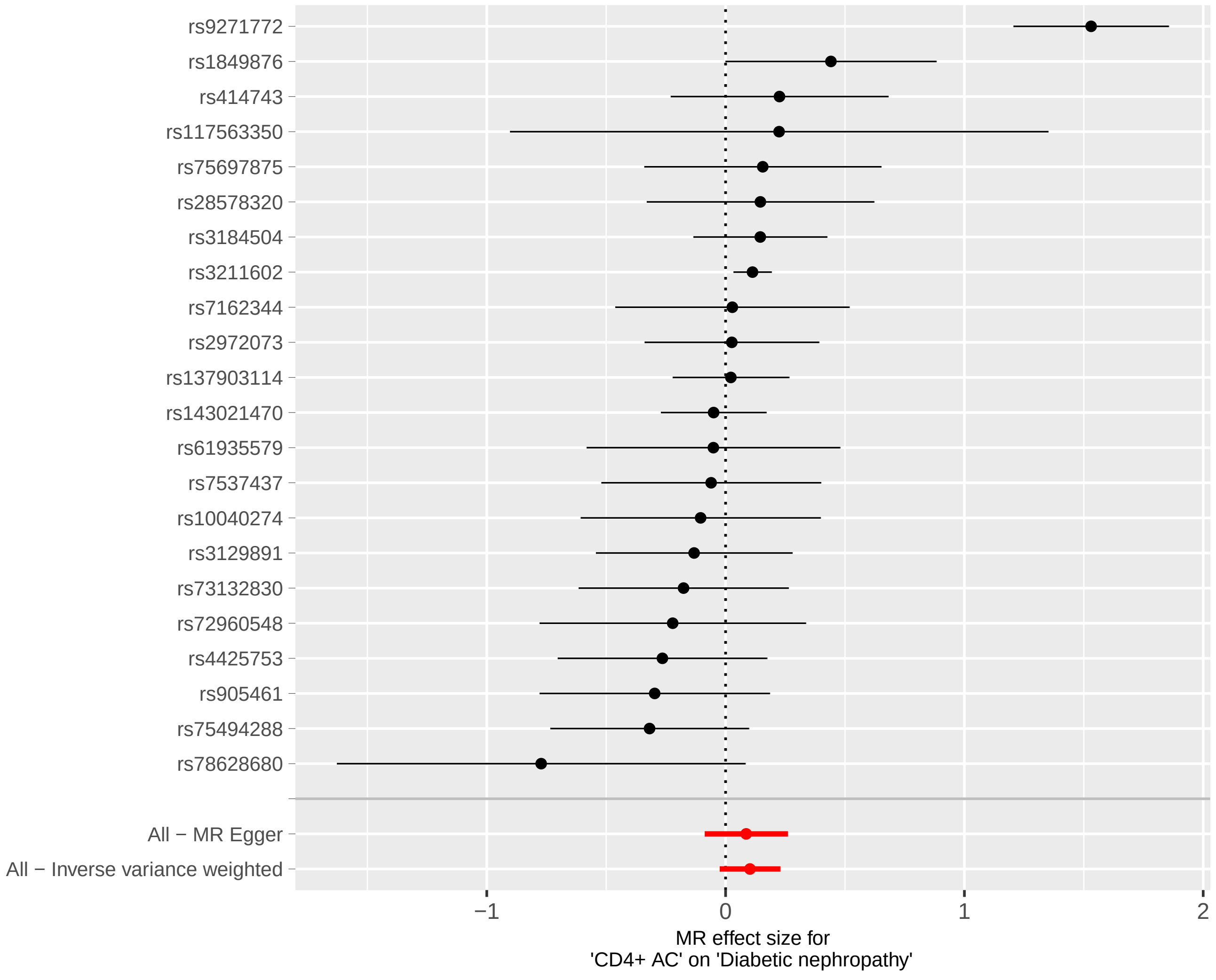
rs9271772
rs1849876
rs414743
rs117563350
rs75697875
rs28578320
rs3184504
rs3211602
rs7162344
rs2972073
rs137903114
rs143021470
rs61935579
rs7537437
rs10040274
rs3129891
rs73132830
rs72960548
rs4425753
rs905461
rs75494288
rs78628680
All − MR Egger
All − Inverse variance weighted
−1
0
1
2
MR effect size for
'CD4+ AC' on 'Diabetic nephropathy'

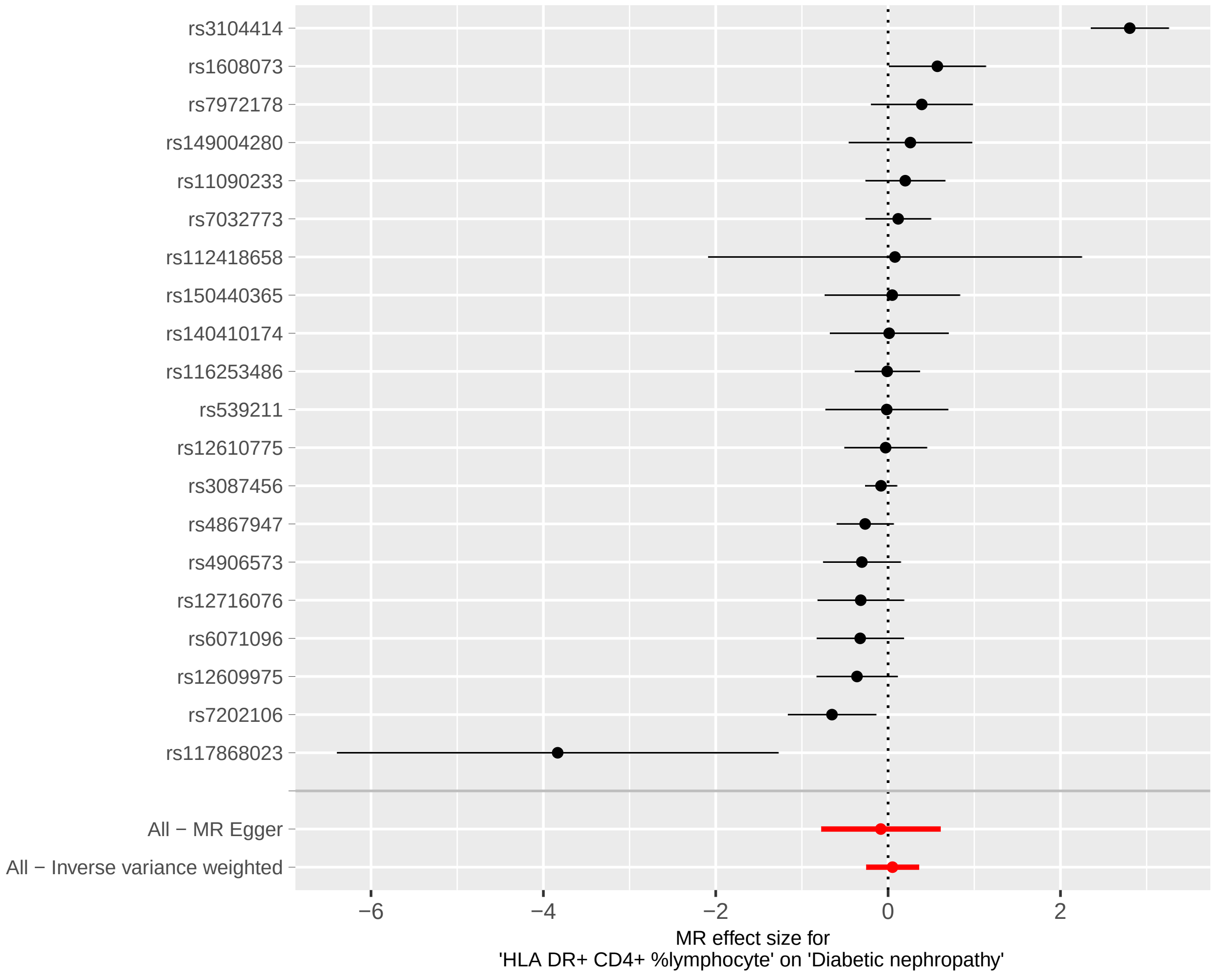
rs3104414
rs1608073
rs7972178
rs149004280
rs11090233
rs7032773
rs112418658
rs150440365
rs140410174
rs116253486
rs539211
rs12610775
rs3087456
rs4867947
rs4906573
rs12716076
rs6071096
rs12609975
rs7202106
rs117868023
All − MR Egger
All − Inverse variance weighted
−6
−4
−2
0
2
MR effect size for
'HLA DR+ CD4+ %lymphocyte' on 'Diabetic nephropathy'

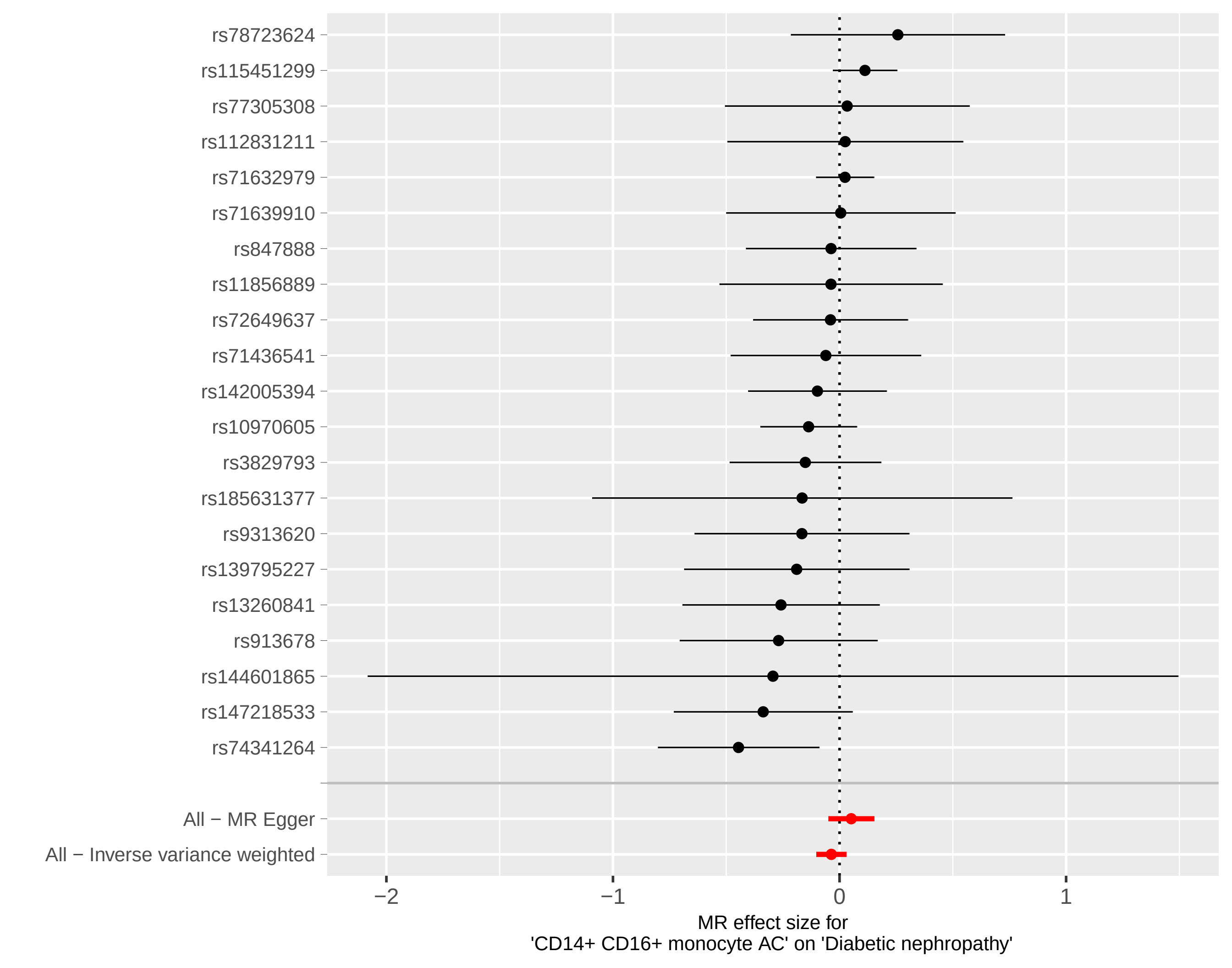
rs78723624
rs115451299
rs77305308
rs112831211
rs71632979
rs71639910
rs847888
rs11856889
rs72649637
rs71436541
rs142005394
rs10970605
rs3829793
rs185631377
rs9313620
rs139795227
rs13260841
rs913678
rs144601865
rs147218533
rs74341264
All − MR Egger
All − Inverse variance weighted
−2
−1
0
1
MR effect size for
'CD14+ CD16+ monocyte AC' on 'Diabetic nephropathy'

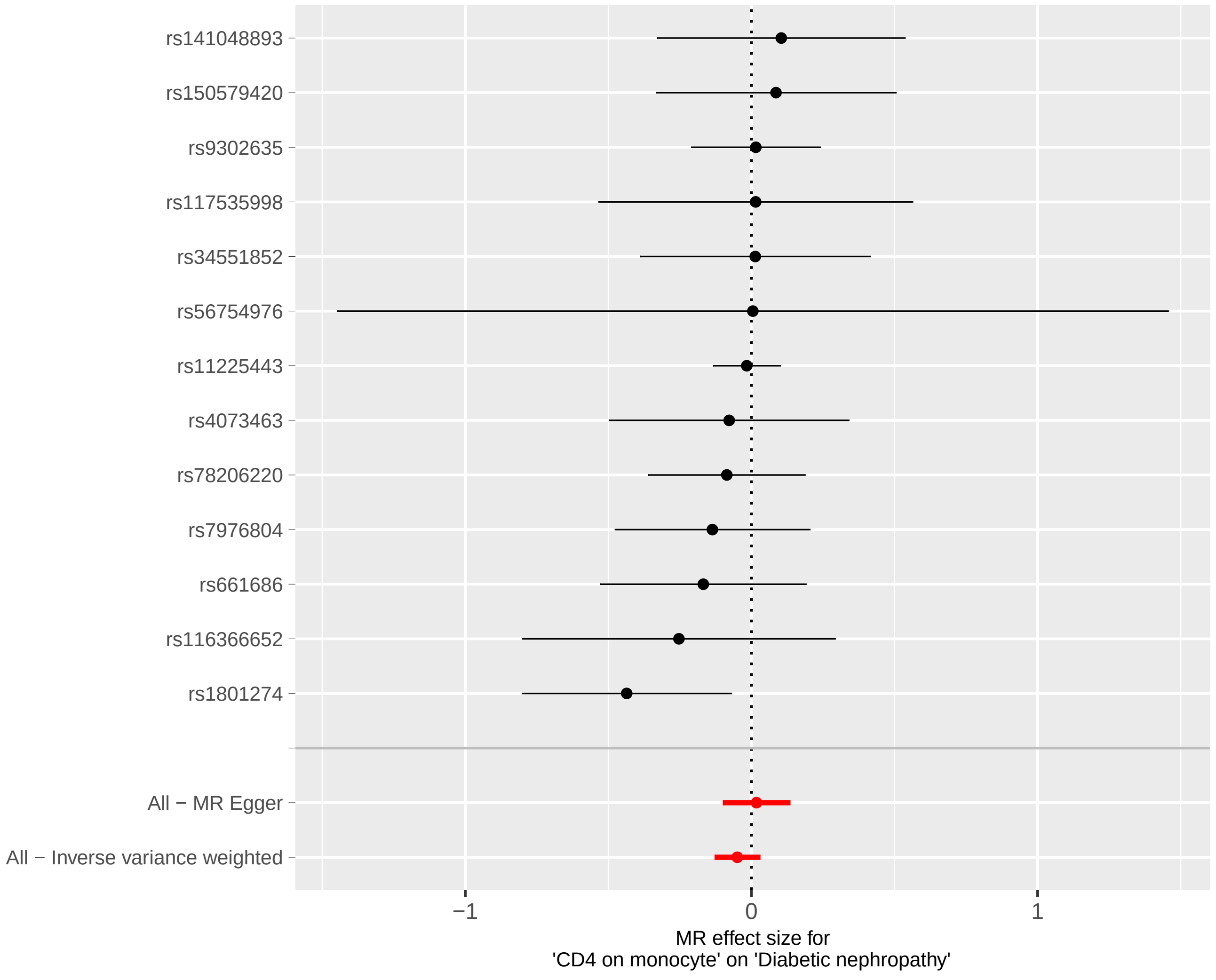
rs141048893
rs150579420
rs9302635
rs117535998
rs34551852
rs56754976
rs11225443
rs4073463
rs78206220
rs7976804
rs661686
rs116366652
rs1801274
All − MR Egger
All − Inverse variance weighted
−1
0
1
MR effect size for
'CD4 on monocyte' on 'Diabetic nephropathy'

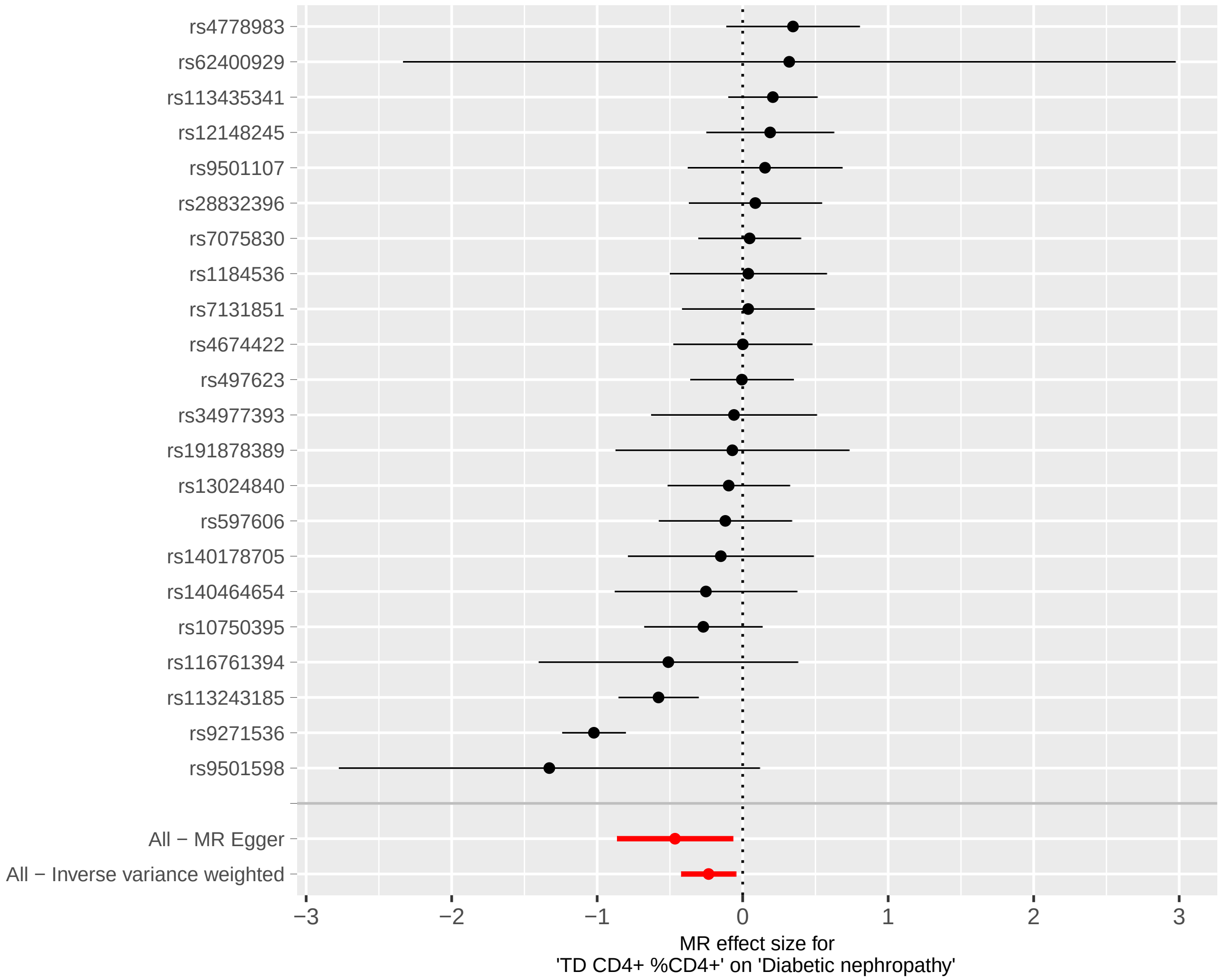
rs4778983
rs62400929
rs113435341
rs12148245
rs9501107
rs28832396
rs7075830
rs1184536
rs7131851
rs4674422
rs497623
rs34977393
rs191878389
rs13024840
rs597606
rs140178705
rs140464654
rs10750395
rs116761394
rs113243185
rs9271536
rs9501598
All − MR Egger
All − Inverse variance weighted
−3
−2
−1
0
1
2
3
MR effect size for
'TD CD4+ %CD4+' on 'Diabetic nephropathy'

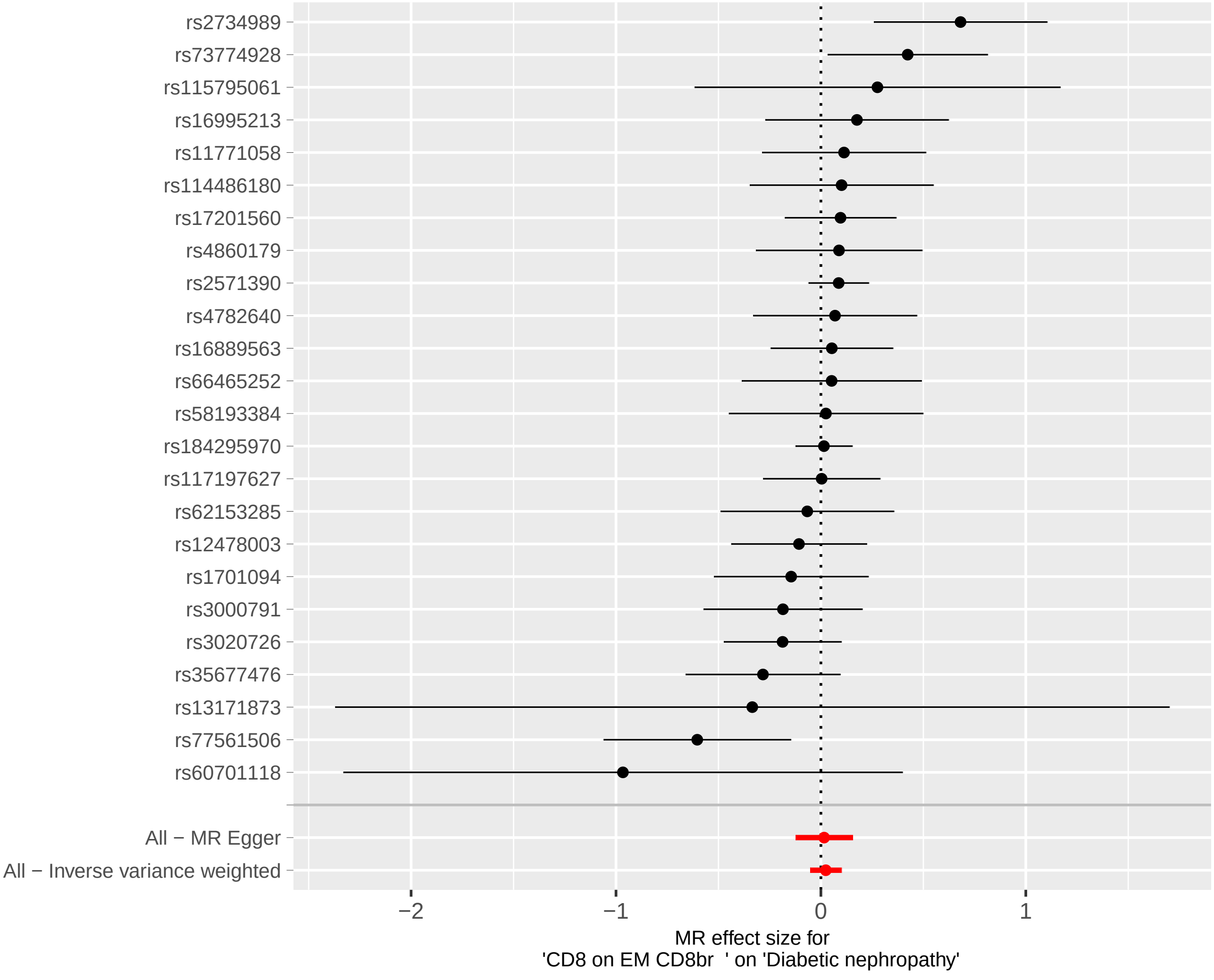
rs2734989
rs73774928
rs115795061
rs16995213
rs11771058
rs114486180
rs17201560
rs4860179
rs2571390
rs4782640
rs16889563
rs66465252
rs58193384
rs184295970
rs117197627
rs62153285
rs12478003
rs1701094
rs3000791
rs3020726
rs35677476
rs13171873
rs77561506
rs60701118
All − MR Egger
All − Inverse variance weighted
−2
−1
0
1
MR effect size for
'CD8 on EM CD8br ' on 'Diabetic nephropathy'

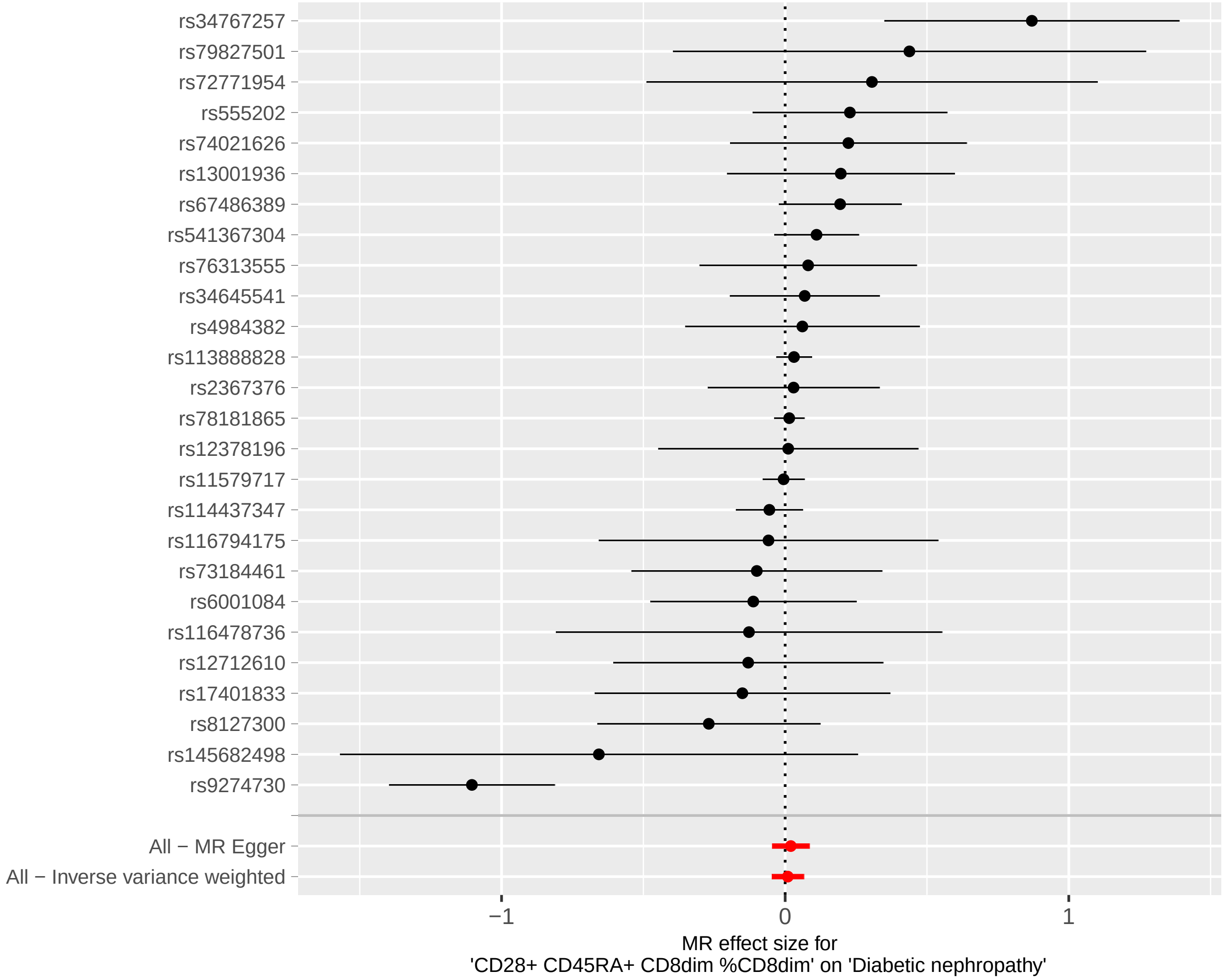
rs34767257
rs79827501
rs72771954
rs555202
rs74021626
rs13001936
rs67486389
rs541367304
rs76313555
rs34645541
rs4984382
rs113888828
rs2367376
rs78181865
rs12378196
rs11579717
rs114437347
rs116794175
rs73184461
rs6001084
rs116478736
rs12712610
rs17401833
rs8127300
rs145682498
rs9274730
All − MR Egger
All − Inverse variance weighted
−1
0
1
MR effect size for
'CD28+ CD45RA+ CD8dim %CD8dim' on 'Diabetic nephropathy'

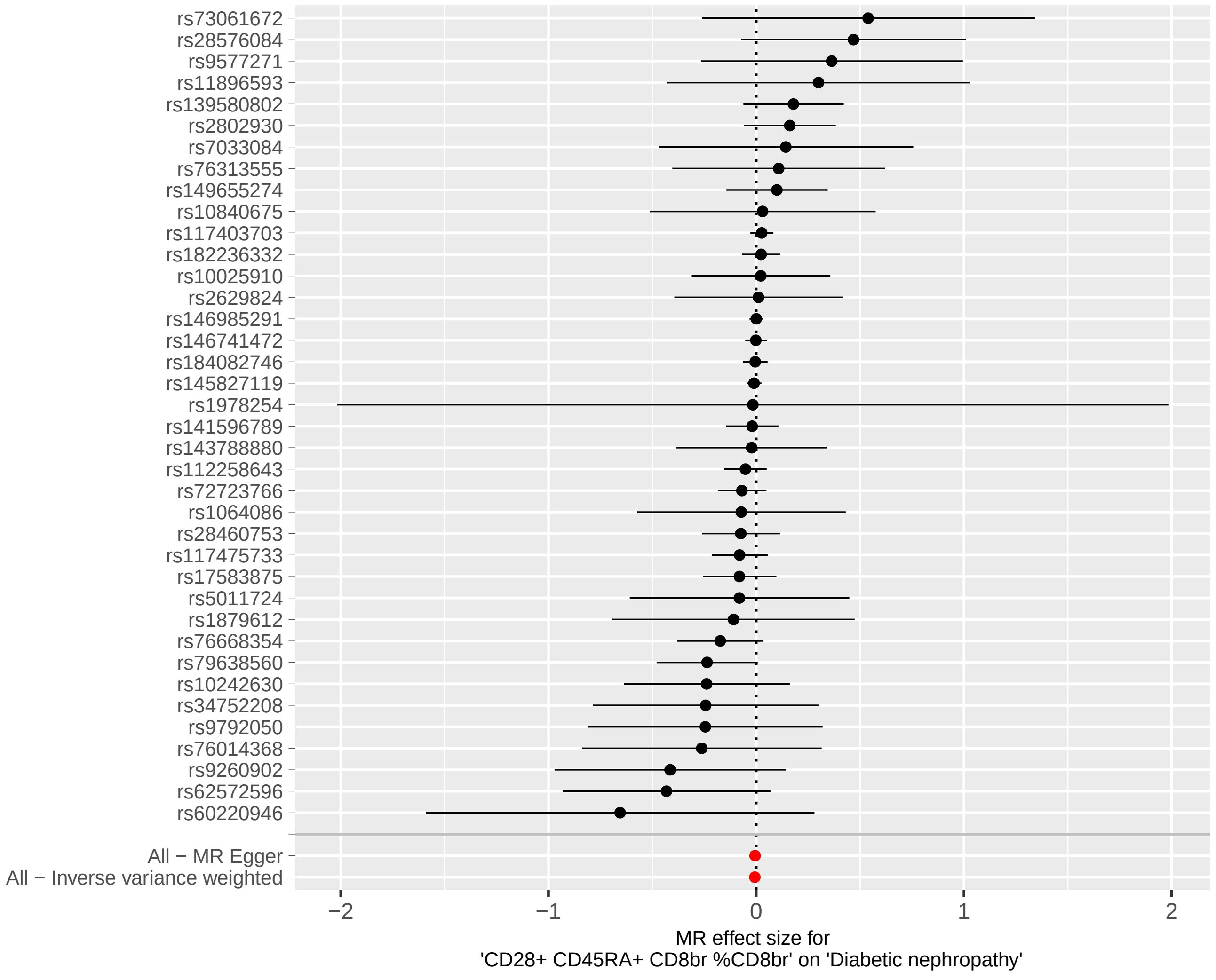
rs73061672
rs28576084
rs9577271
rs11896593
rs139580802
rs2802930
rs7033084
rs76313555
rs149655274
rs10840675
rs117403703
rs182236332
rs10025910
rs2629824
rs146985291
rs146741472
rs184082746
rs145827119
rs1978254
rs141596789
rs143788880
rs112258643
rs72723766
rs1064086
rs28460753
rs117475733
rs17583875
rs5011724
rs1879612
rs76668354
rs79638560
rs10242630
rs34752208
rs9792050
rs76014368
rs9260902
rs62572596
rs60220946
All − MR Egger
All − Inverse variance weighted
−2
−1
0
1
2
MR effect size for
'CD28+ CD45RA+ CD8br %CD8br' on 'Diabetic nephropathy'

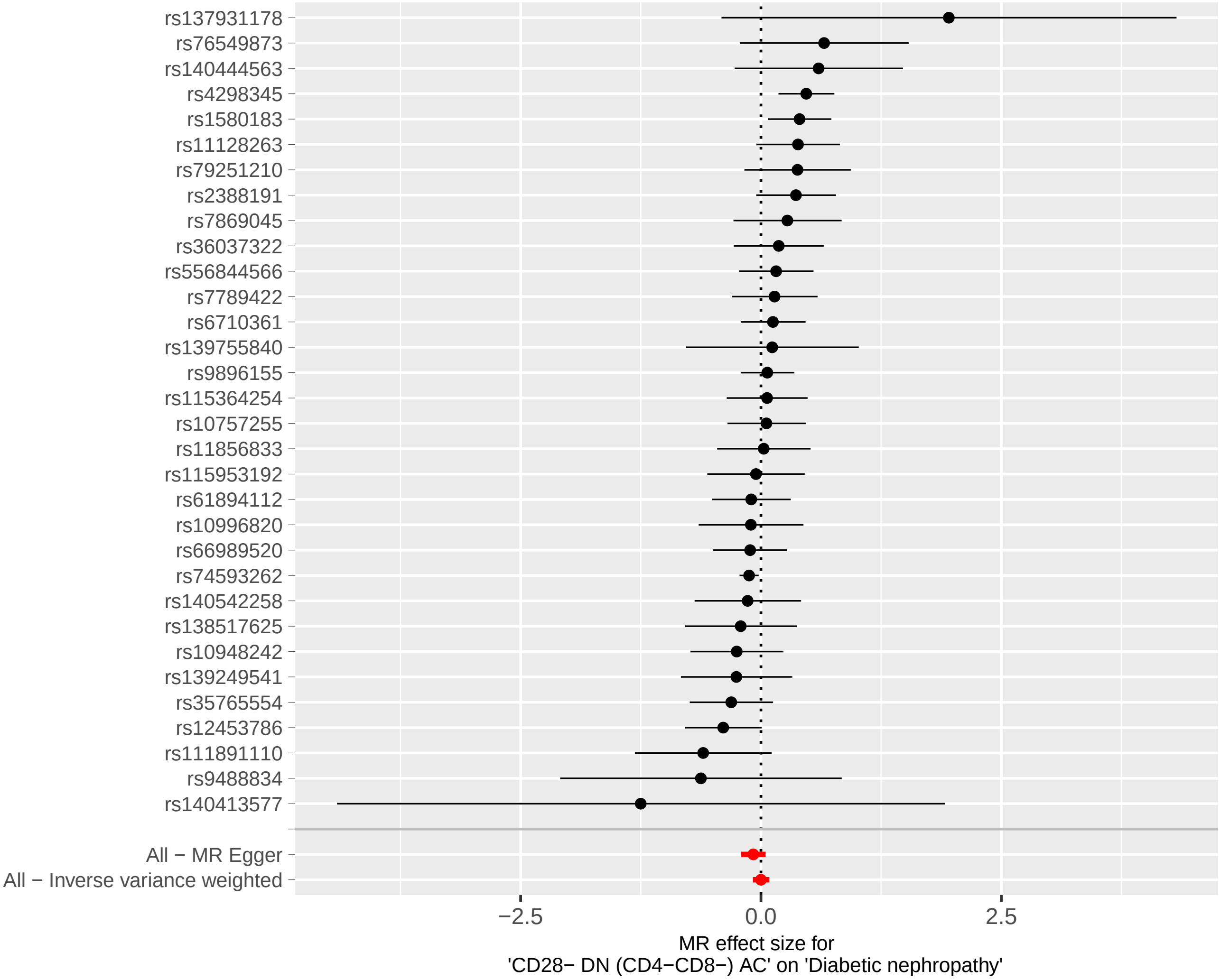
rs137931178
rs76549873
rs140444563
rs4298345
rs1580183
rs11128263
rs79251210
rs2388191
rs7869045
rs36037322
rs556844566
rs7789422
rs6710361
rs139755840
rs9896155
rs115364254
rs10757255
rs11856833
rs115953192
rs61894112
rs10996820
rs66989520
rs74593262
rs140542258
rs138517625
rs10948242
rs139249541
rs35765554
rs12453786
rs111891110
rs9488834
rs140413577
All − MR Egger
All − Inverse variance weighted
−2.5
0.0
2.5
MR effect size for
'CD28− DN (CD4−CD8−) AC' on 'Diabetic nephropathy'

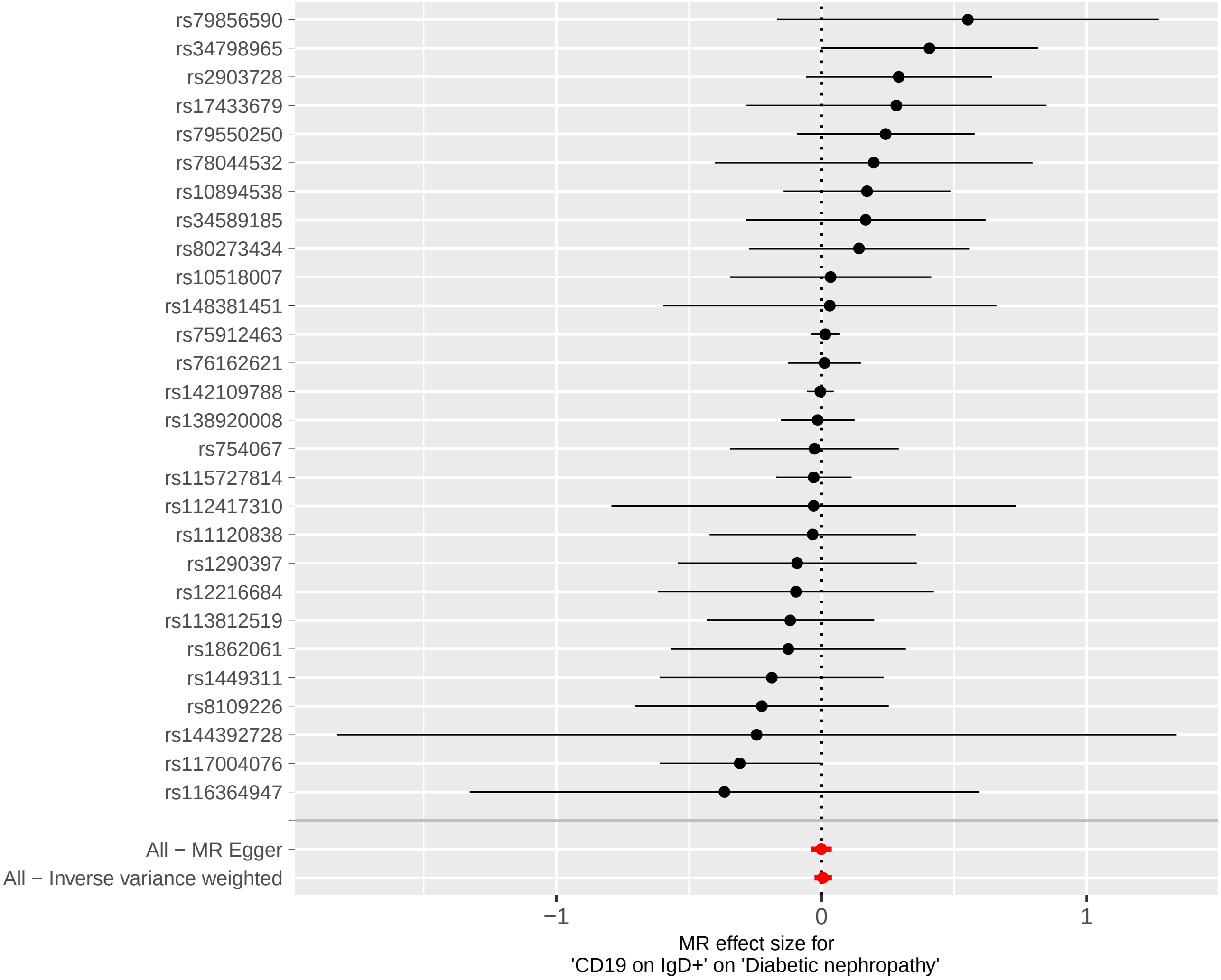
rs79856590
rs34798965
rs2903728
rs17433679
rs79550250
rs78044532
rs10894538
rs34589185
rs80273434
rs10518007
rs148381451
rs75912463
rs76162621
rs142109788
rs138920008
rs754067
rs115727814
rs112417310
rs11120838
rs1290397
rs12216684
rs113812519
rs1862061
rs1449311
rs8109226
rs144392728
rs117004076
rs116364947
All − MR Egger
All − Inverse variance weighted
−1
0
1
MR effect size for
'CD19 on IgD+' on 'Diabetic nephropathy'

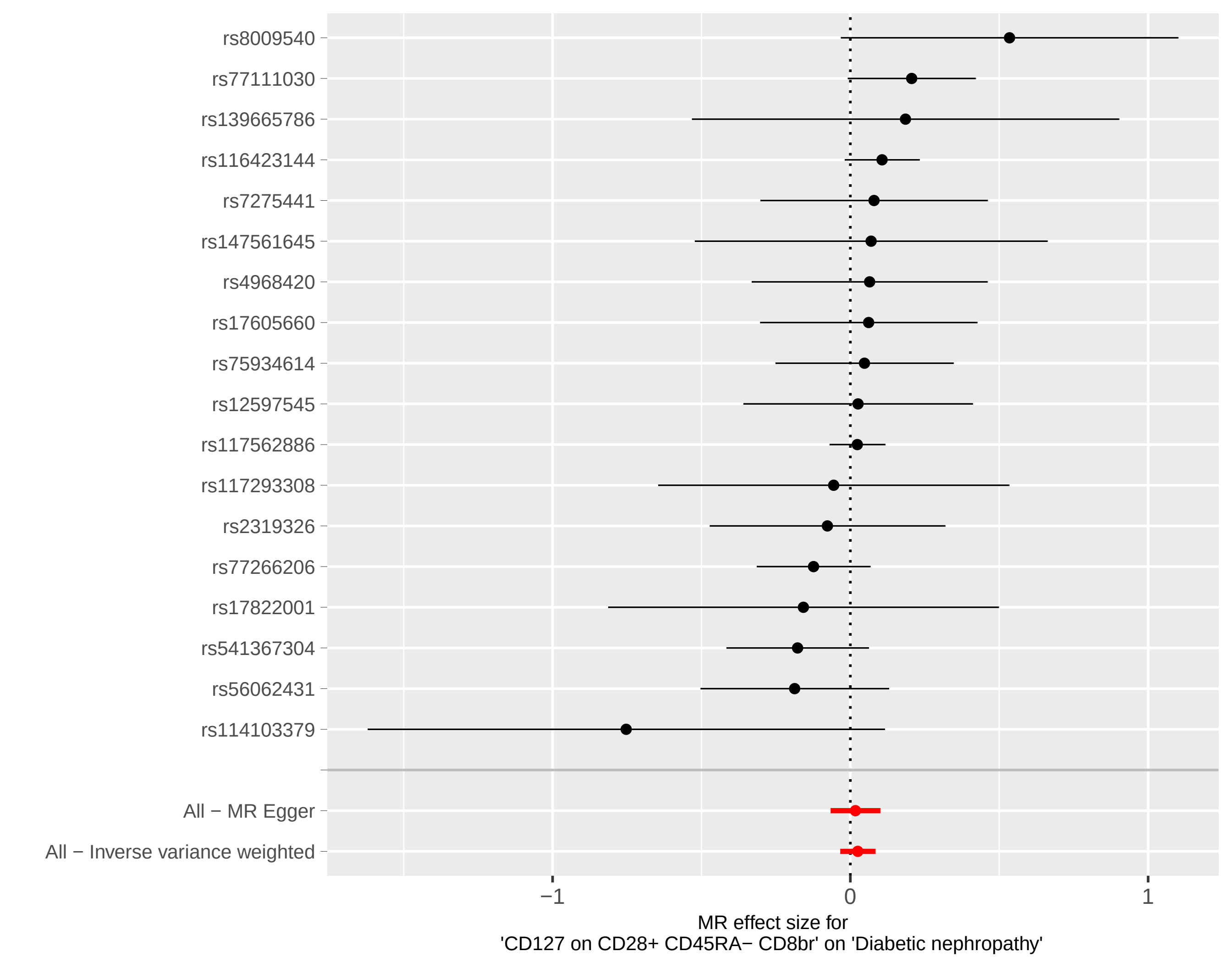
rs8009540
rs77111030
rs139665786
rs116423144
rs7275441
rs147561645
rs4968420
rs17605660
rs75934614
rs12597545
rs117562886
rs117293308
rs2319326
rs77266206
rs17822001
rs541367304
rs56062431
rs114103379
All − MR Egger
All − Inverse variance weighted
−1
0
1
MR effect size for
'CD127 on CD28+ CD45RA− CD8br' on 'Diabetic nephropathy'

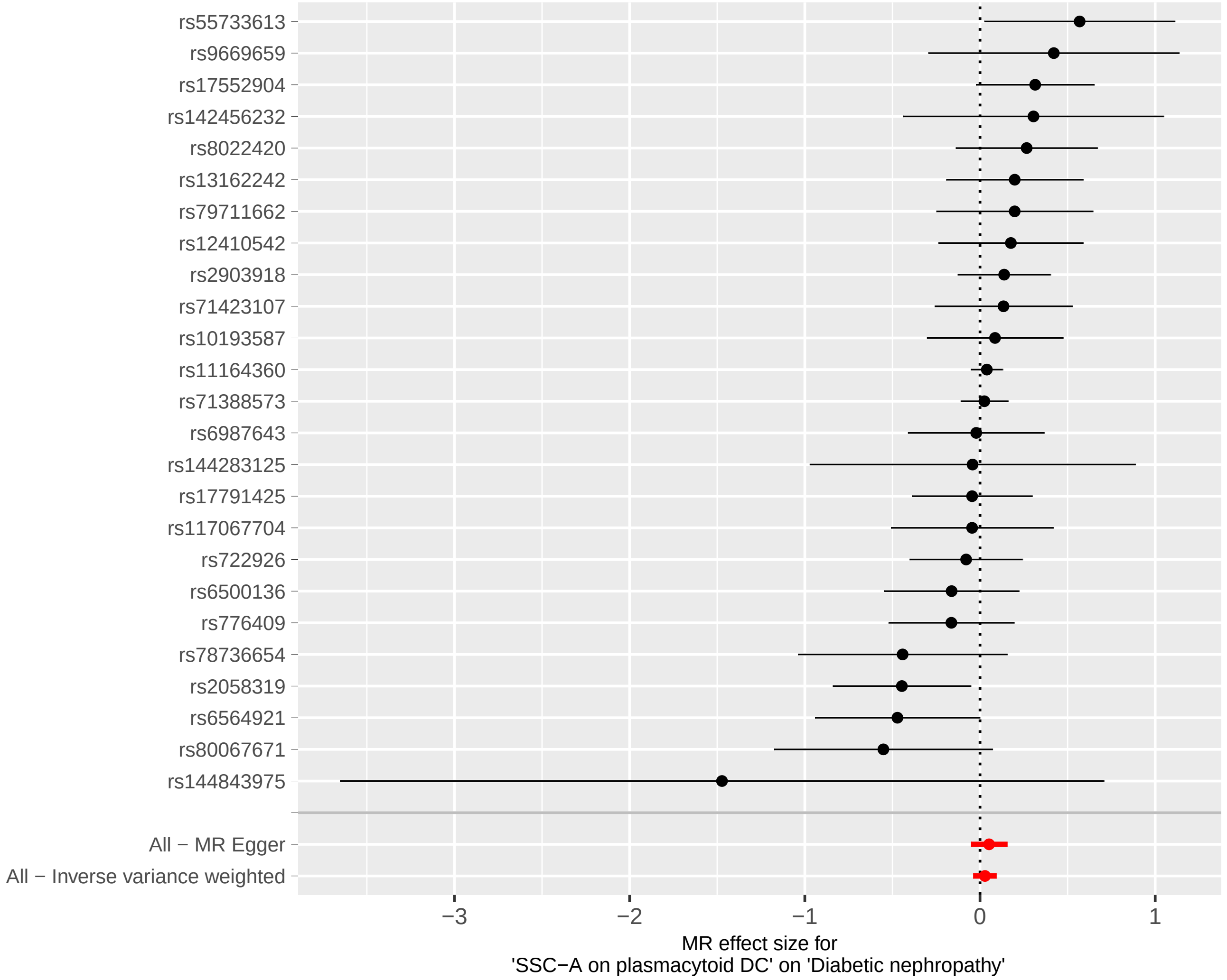

rs55733613
rs9669659
rs17552904
rs142456232
rs8022420
rs13162242
rs79711662
rs12410542
rs2903918
rs71423107
rs10193587
rs11164360
rs71388573
rs6987643
rs144283125
rs17791425
rs117067704
rs722926
rs6500136
rs776409
rs78736654
rs2058319
rs6564921
rs80067671
rs144843975
All − MR Egger
All − Inverse variance weighted
−3
−2
−1
0
1
MR effect size for
'SSC−A on plasmacytoid DC' on 'Diabetic nephropathy'

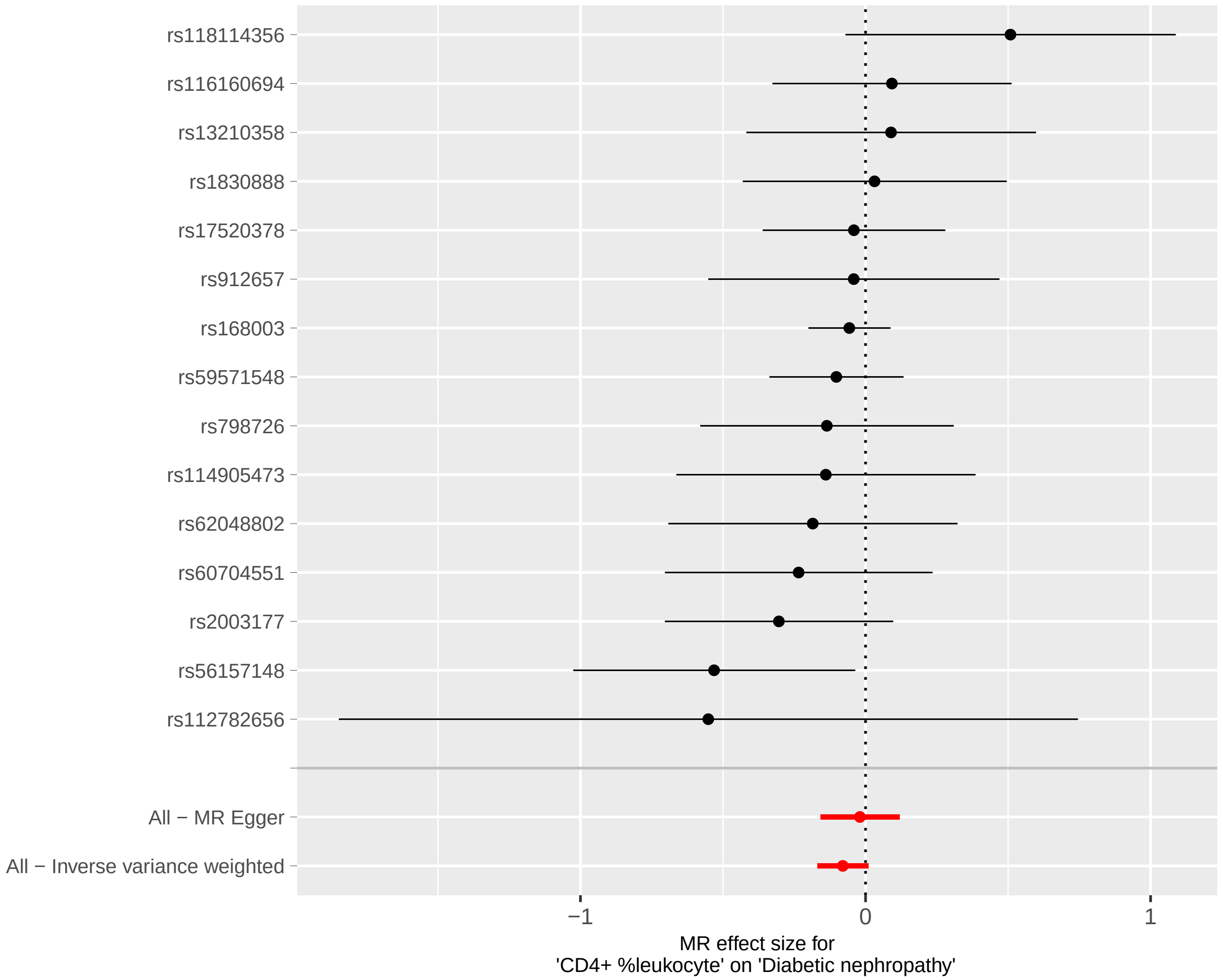

rs118114356
rs116160694
rs13210358
rs1830888
rs17520378
rs912657
rs168003
rs59571548
rs798726
rs114905473
rs62048802
rs60704551
rs2003177
rs56157148
rs112782656
All − MR Egger
All − Inverse variance weighted
−1
0
1
MR effect size for
'CD4+ %leukocyte' on 'Diabetic nephropathy'

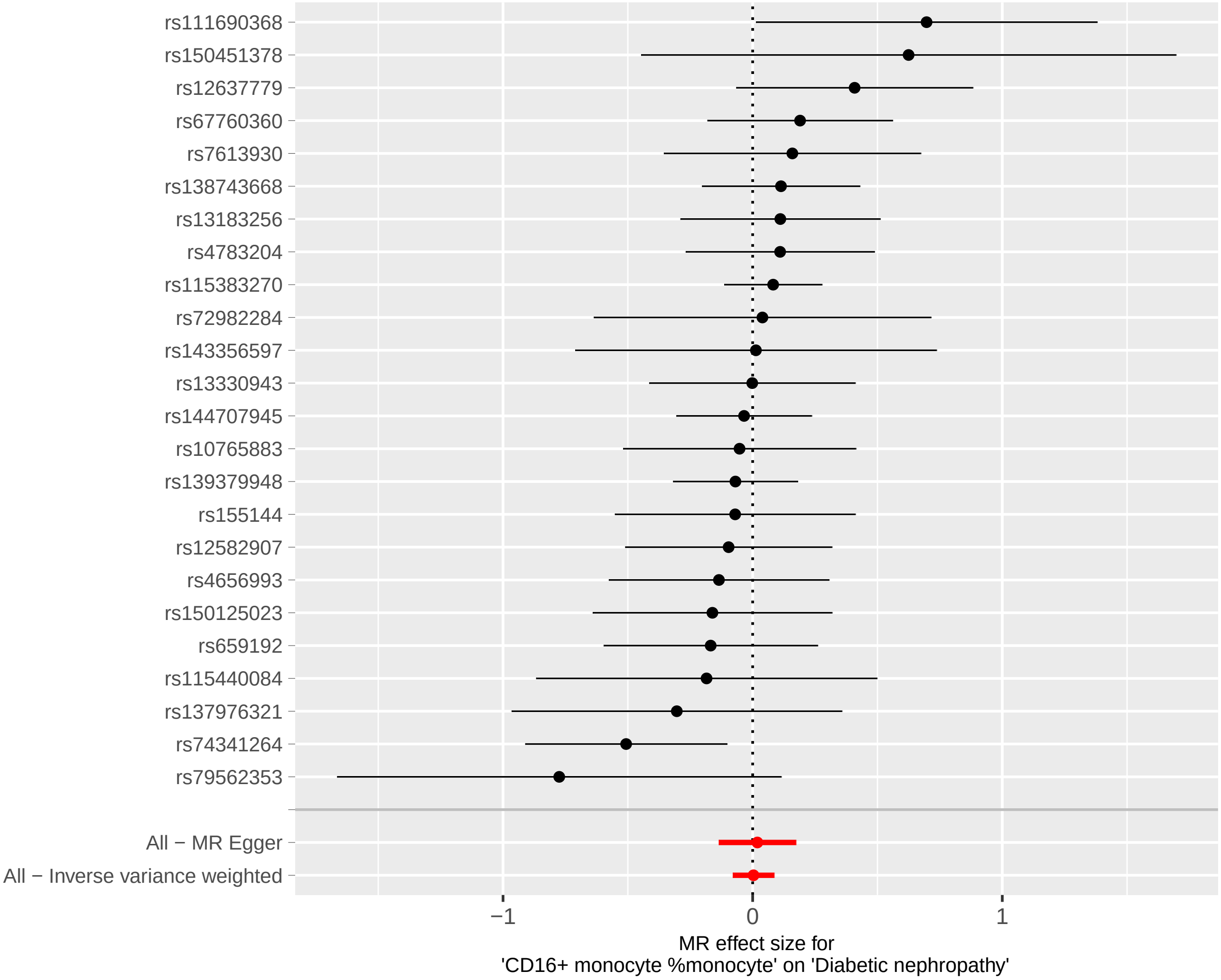

rs111690368
rs150451378
rs12637779
rs67760360
rs7613930
rs138743668
rs13183256
rs4783204
rs115383270
rs72982284
rs143356597
rs13330943
rs144707945
rs10765883
rs139379948
rs155144
rs12582907
rs4656993
rs150125023
rs659192
rs115440084
rs137976321
rs74341264
rs79562353
All − MR Egger
All − Inverse variance weighted
−1
0
1
MR effect size for
'CD16+ monocyte %monocyte' on 'Diabetic nephropathy'

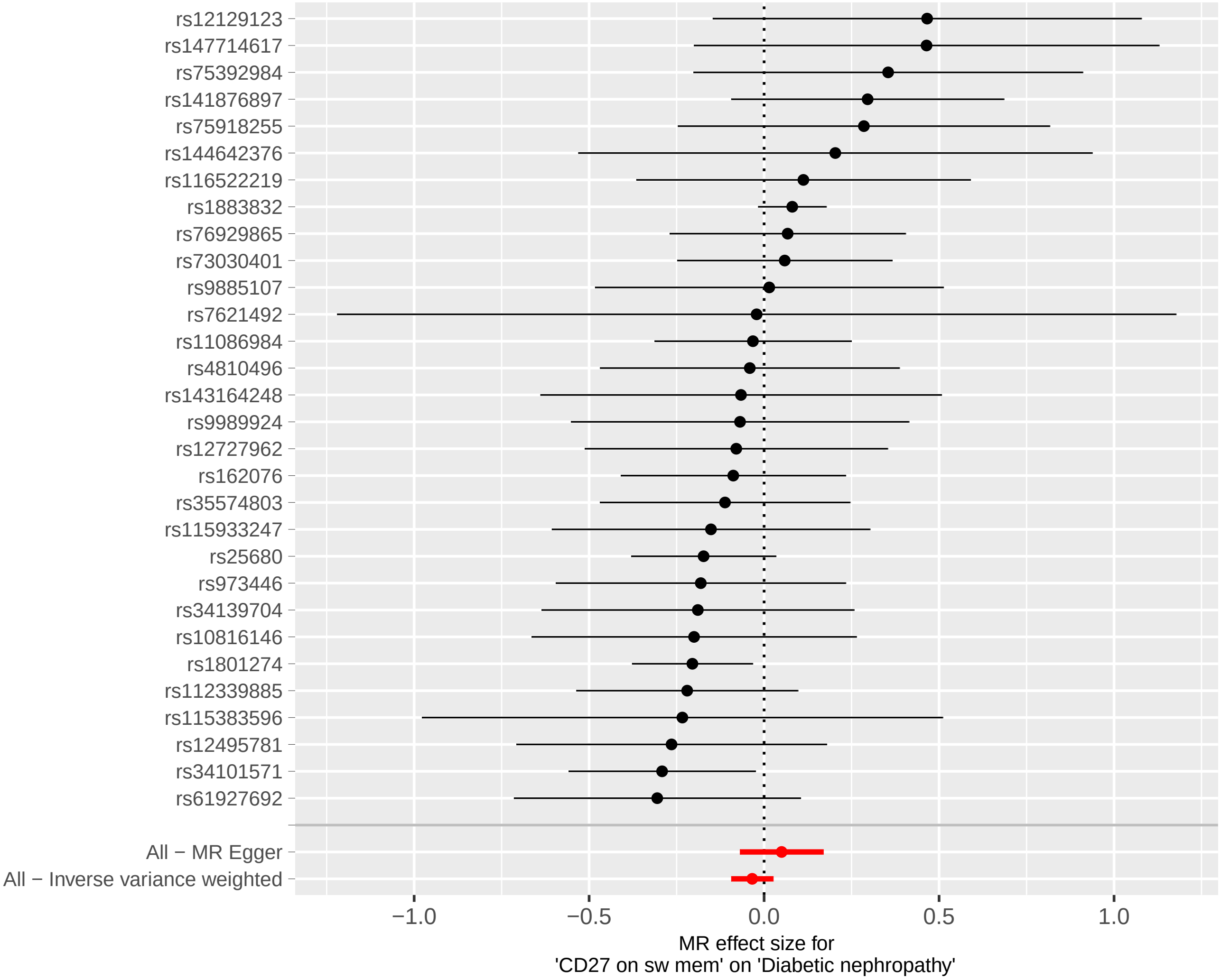
rs12129123
rs147714617
rs75392984
rs141876897
rs75918255
rs144642376
rs116522219
rs1883832
rs76929865
rs73030401
rs9885107
rs7621492
rs11086984
rs4810496
rs143164248
rs9989924
rs12727962
rs162076
rs35574803
rs115933247
rs25680
rs973446
rs34139704
rs10816146
rs1801274
rs112339885
rs115383596
rs12495781
rs34101571
rs61927692
All − MR Egger
All − Inverse variance weighted
−1.0
−0.5
0.0
0.5
1.0
MR effect size for
'CD27 on sw mem' on 'Diabetic nephropathy'

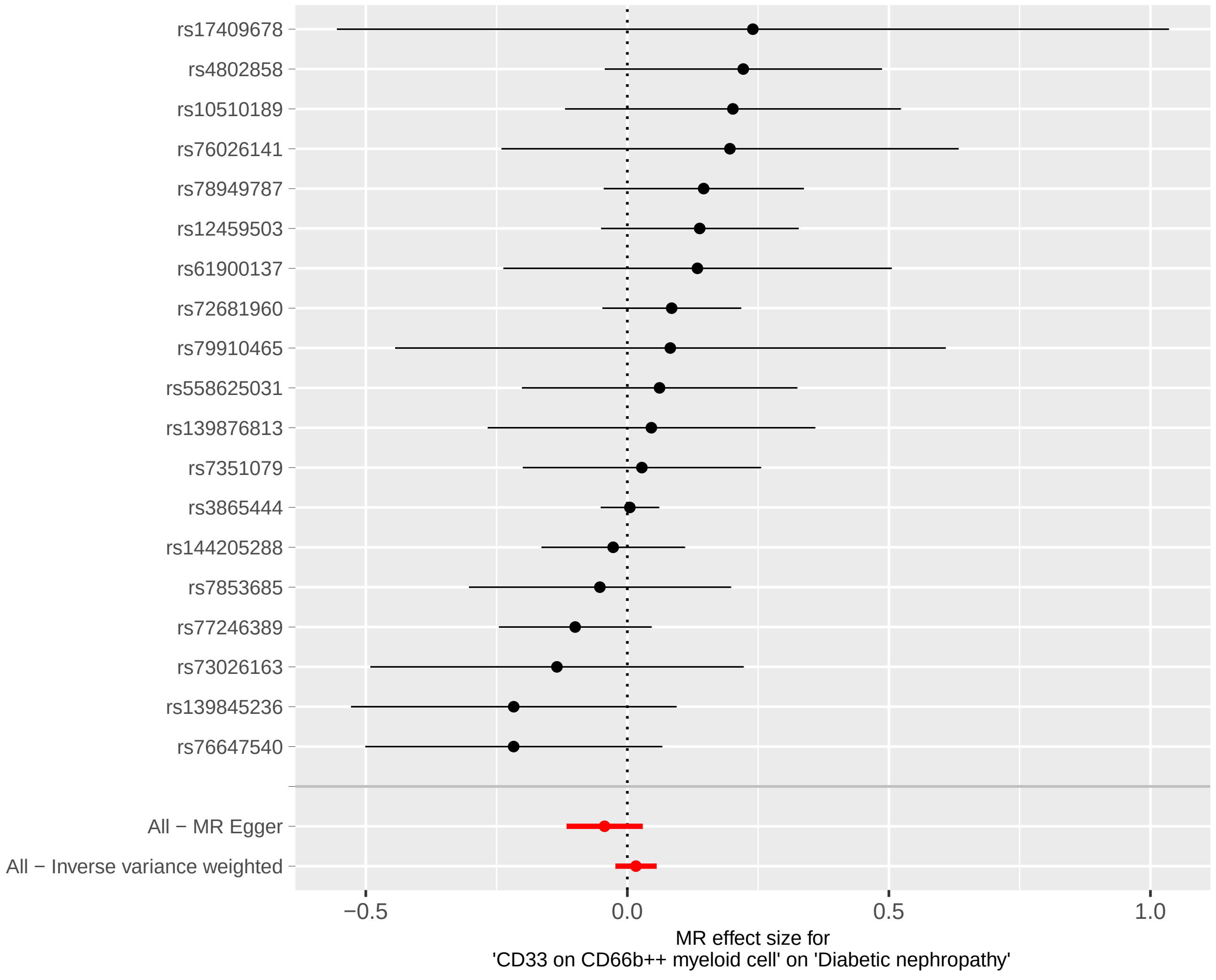

rs17409678
rs4802858
rs10510189
rs76026141
rs78949787
rs12459503
rs61900137
rs72681960
rs79910465
rs558625031
rs139876813
rs7351079
rs3865444
rs144205288
rs7853685
rs77246389
rs73026163
rs139845236
rs76647540
All − MR Egger
All − Inverse variance weighted
−0.5
0.0
0.5
1.0
MR effect size for
'CD33 on CD66b++ myeloid cell' on 'Diabetic nephropathy'

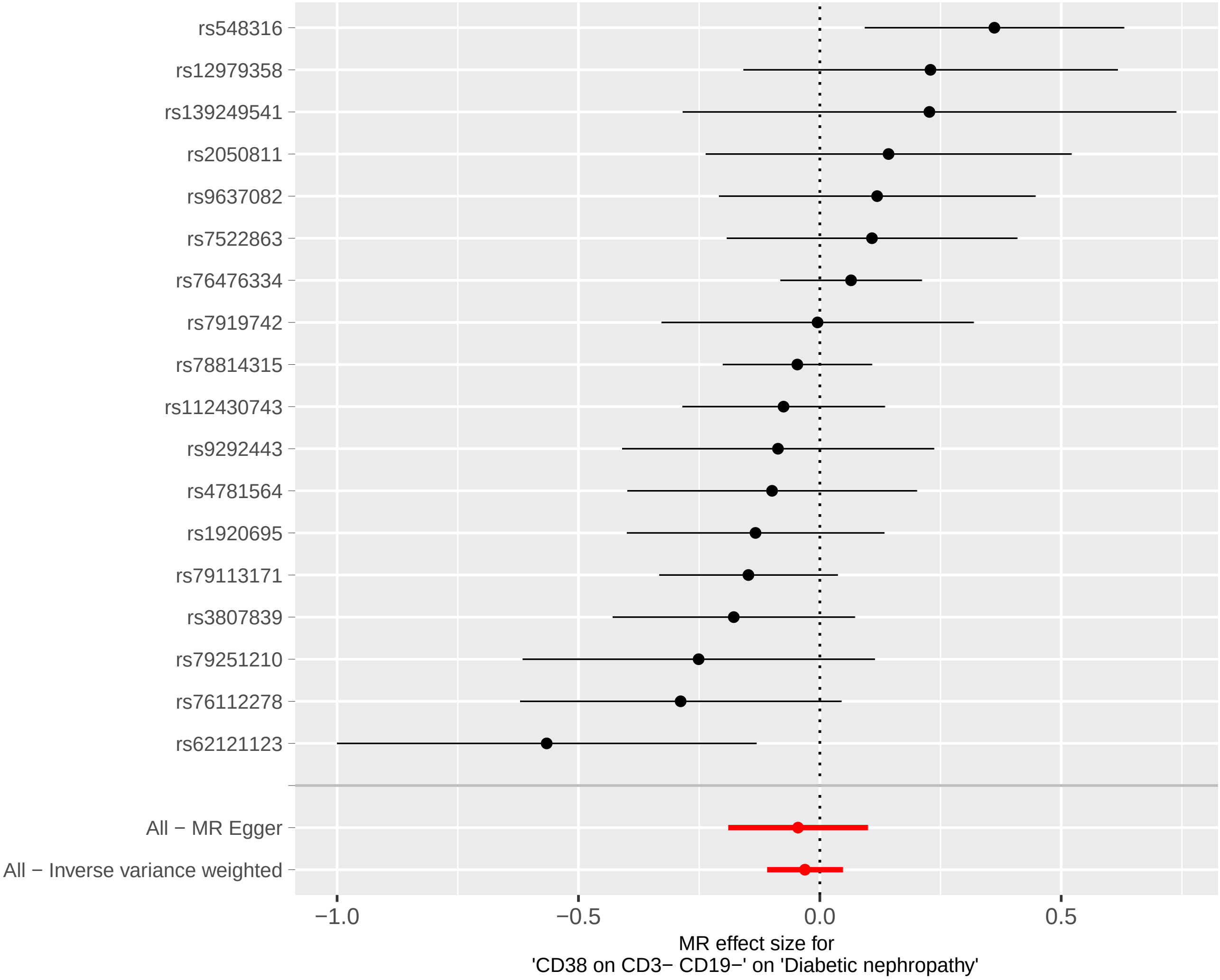
rs548316
rs12979358
rs139249541
rs2050811
rs9637082
rs7522863
rs76476334
rs7919742
rs78814315
rs112430743
rs9292443
rs4781564
rs1920695
rs79113171
rs3807839
rs79251210
rs76112278
rs62121123
All − MR Egger
All − Inverse variance weighted
−1.0
−0.5
0.0
0.5
MR effect size for
'CD38 on CD3− CD19−' on 'Diabetic nephropathy'

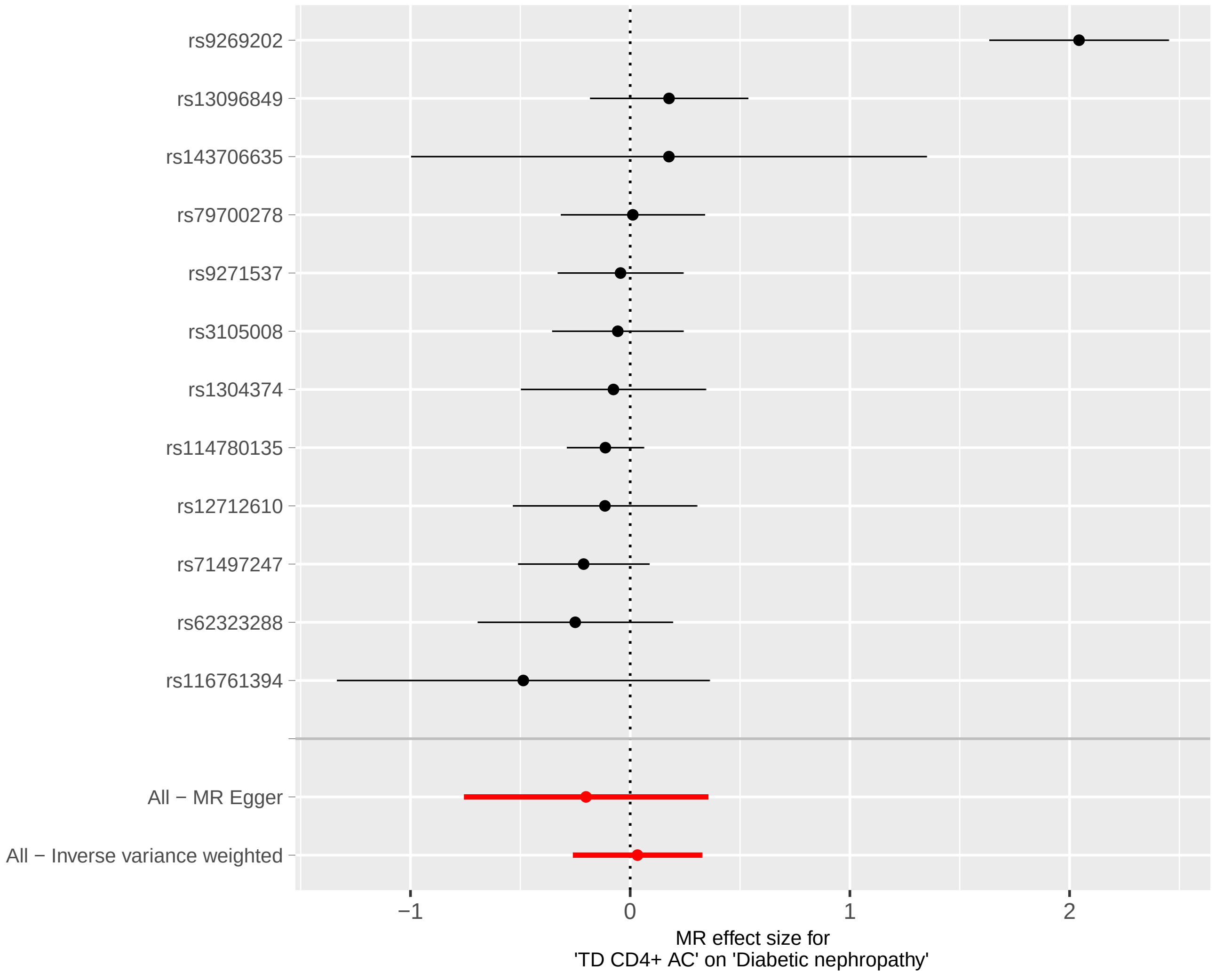
rs9269202
rs13096849
rs143706635
rs79700278
rs9271537
rs3105008
rs1304374
rs114780135
rs12712610
rs71497247
rs62323288
rs116761394
All − MR Egger
All − Inverse variance weighted
−1
0
1
2
MR effect size for
'TD CD4+ AC' on 'Diabetic nephropathy'

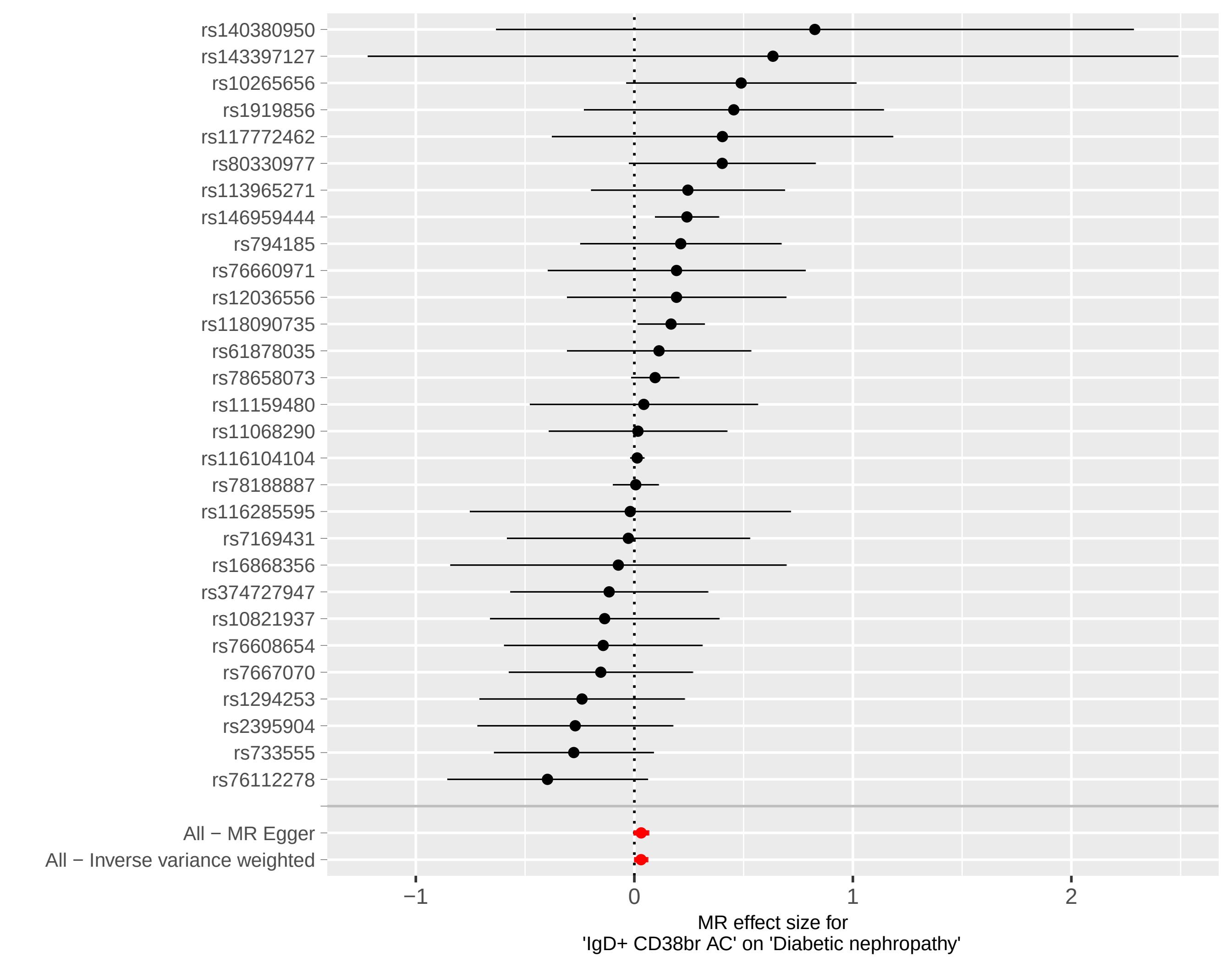
rs140380950
rs143397127
rs10265656
rs1919856
rs117772462
rs80330977
rs113965271
rs146959444
rs794185
rs76660971
rs12036556
rs118090735
rs61878035
rs78658073
rs11159480
rs11068290
rs116104104
rs78188887
rs116285595
rs7169431
rs16868356
rs374727947
rs10821937
rs76608654
rs7667070
rs1294253
rs2395904
rs733555
rs76112278
All − MR Egger
All − Inverse variance weighted
−1
0
1
2
MR effect size for
'IgD+ CD38br AC' on 'Diabetic nephropathy'

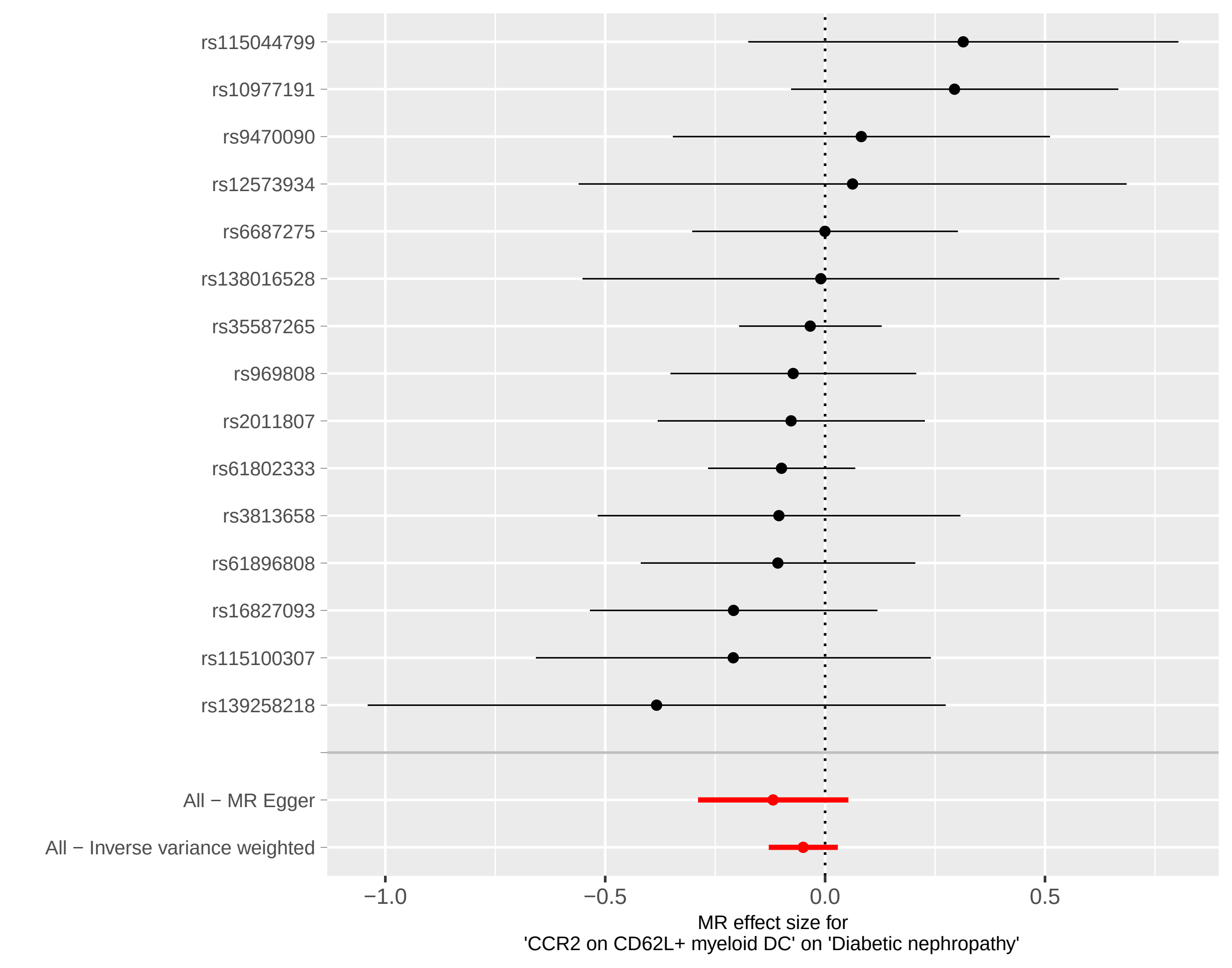
rs115044799
rs10977191
rs9470090
rs12573934
rs6687275
rs138016528
rs35587265
rs969808
rs2011807
rs61802333
rs3813658
rs61896808
rs16827093
rs115100307
rs139258218
All − MR Egger
All − Inverse variance weighted
−1.0
−0.5
0.0
0.5
MR effect size for
'CCR2 on CD62L+ myeloid DC' on 'Diabetic nephropathy'

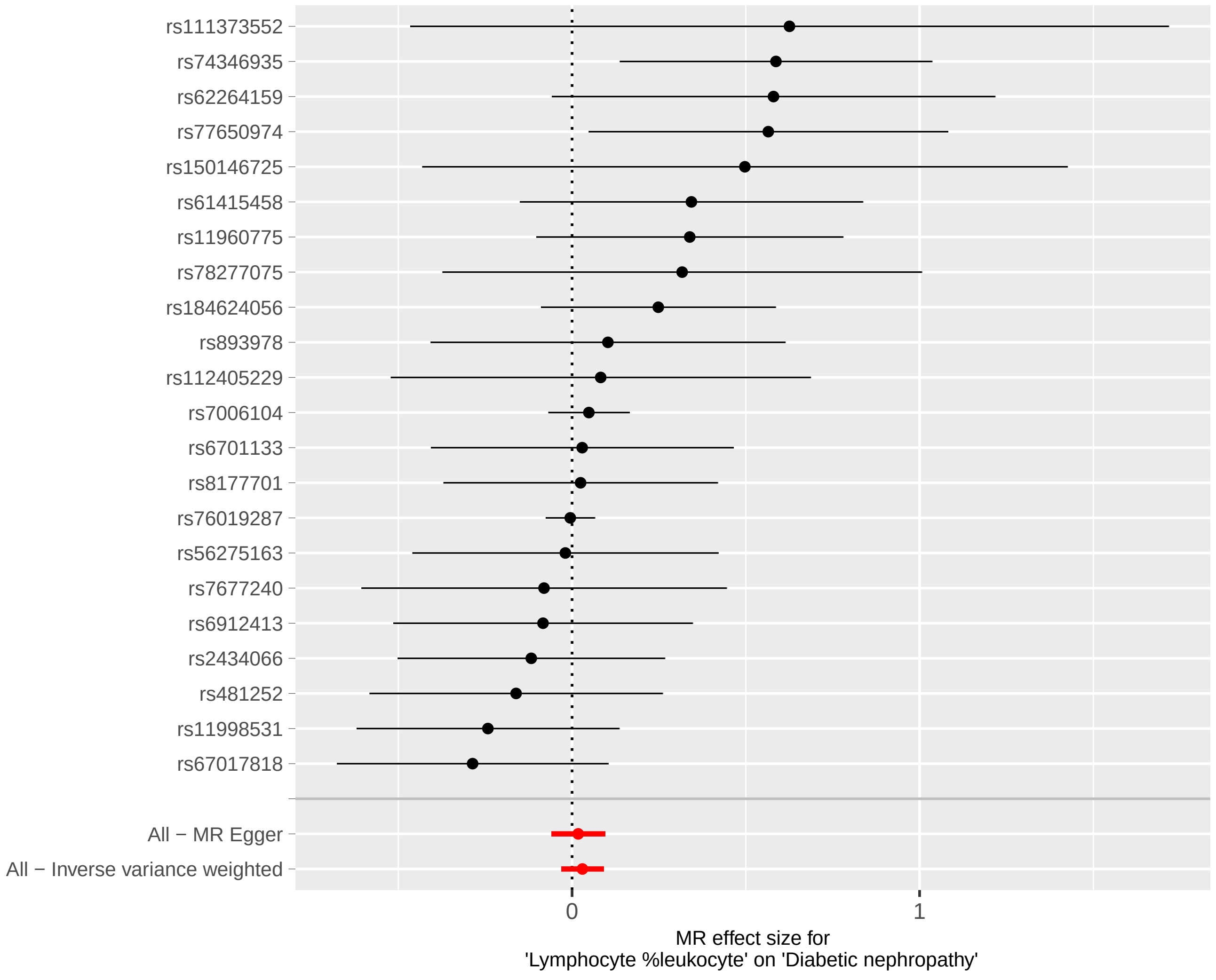

rs111373552
rs74346935
rs62264159
rs77650974
rs150146725
rs61415458
rs11960775
rs78277075
rs184624056
rs893978
rs112405229
rs7006104
rs6701133
rs8177701
rs76019287
rs56275163
rs7677240
rs6912413
rs2434066
rs481252
rs11998531
rs67017818
All − MR Egger
All − Inverse variance weighted
0
1
MR effect size for
'Lymphocyte %leukocyte' on 'Diabetic nephropathy'

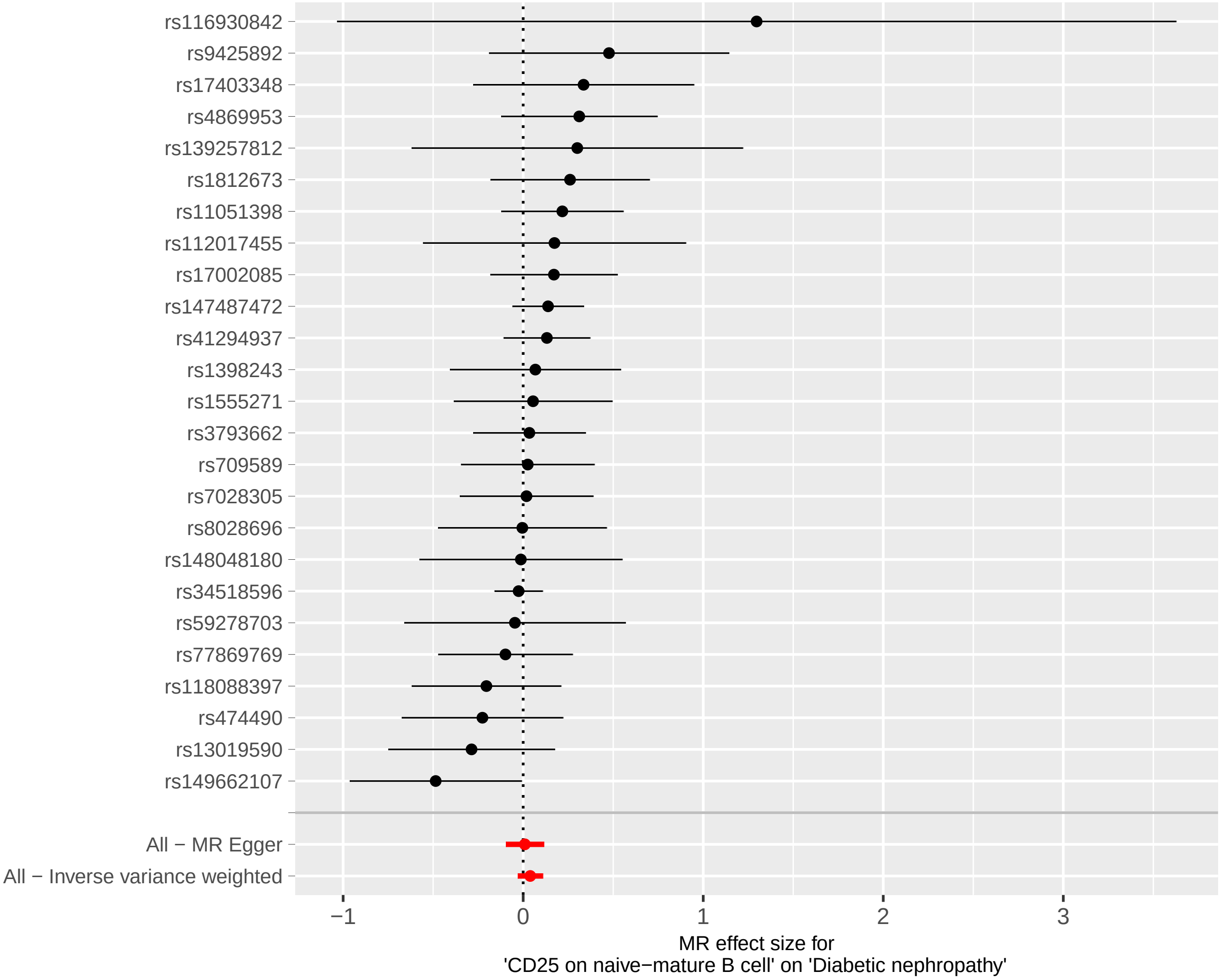

rs116930842
rs9425892
rs17403348
rs4869953
rs139257812
rs1812673
rs11051398
rs112017455
rs17002085
rs147487472
rs41294937
rs1398243
rs1555271
rs3793662
rs709589
rs7028305
rs8028696
rs148048180
rs34518596
rs59278703
rs77869769
rs118088397
rs474490
rs13019590
rs149662107
All − MR Egger
All − Inverse variance weighted
−1
0
1
2
3
MR effect size for
'CD25 on naive−mature B cell' on 'Diabetic nephropathy'

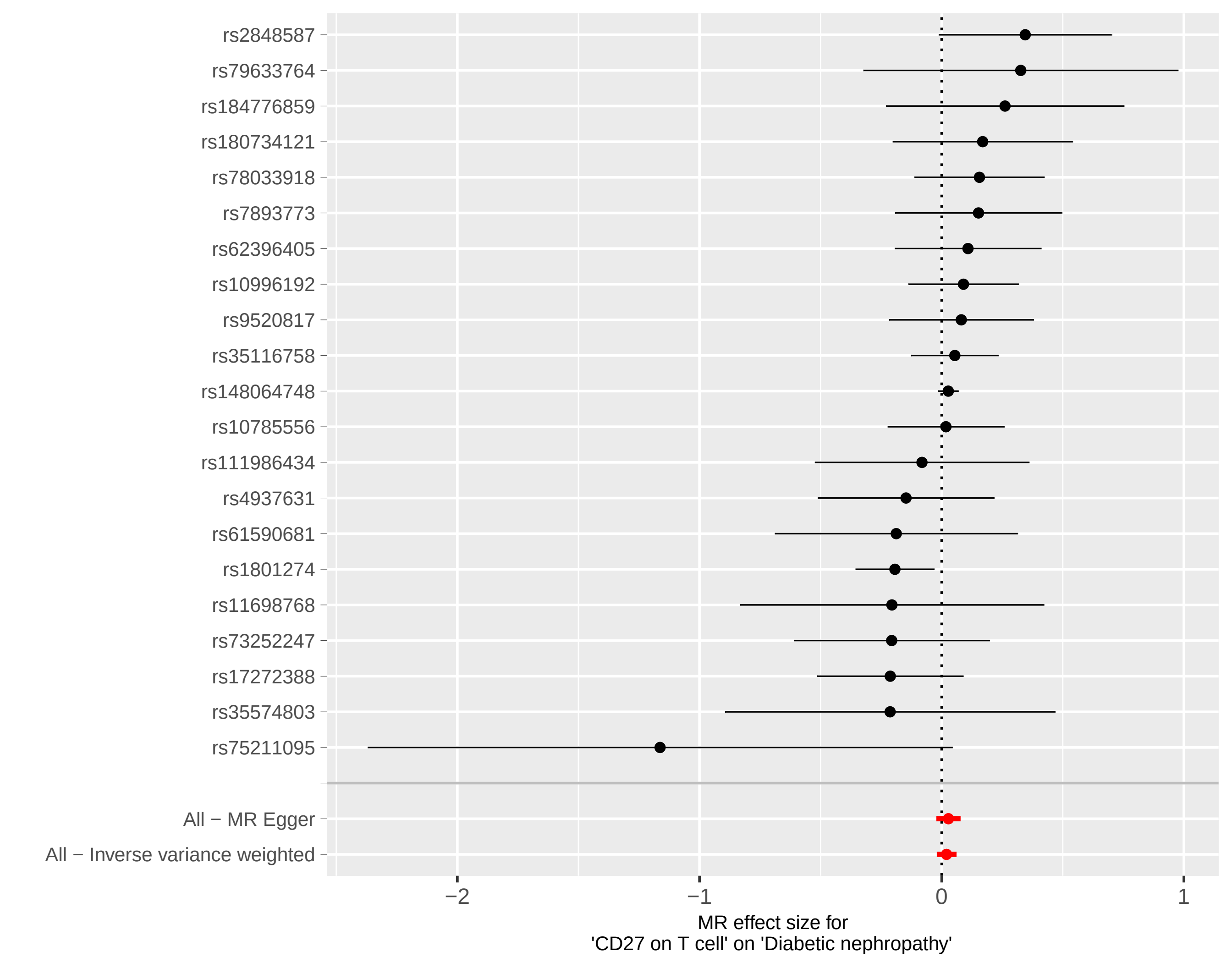
rs2848587
rs79633764
rs184776859
rs180734121
rs78033918
rs7893773
rs62396405
rs10996192
rs9520817
rs35116758
rs148064748
rs10785556
rs111986434
rs4937631
rs61590681
rs1801274
rs11698768
rs73252247
rs17272388
rs35574803
rs75211095
All − MR Egger
All − Inverse variance weighted
−2
−1
0
1
MR effect size for
'CD27 on T cell' on 'Diabetic nephropathy'

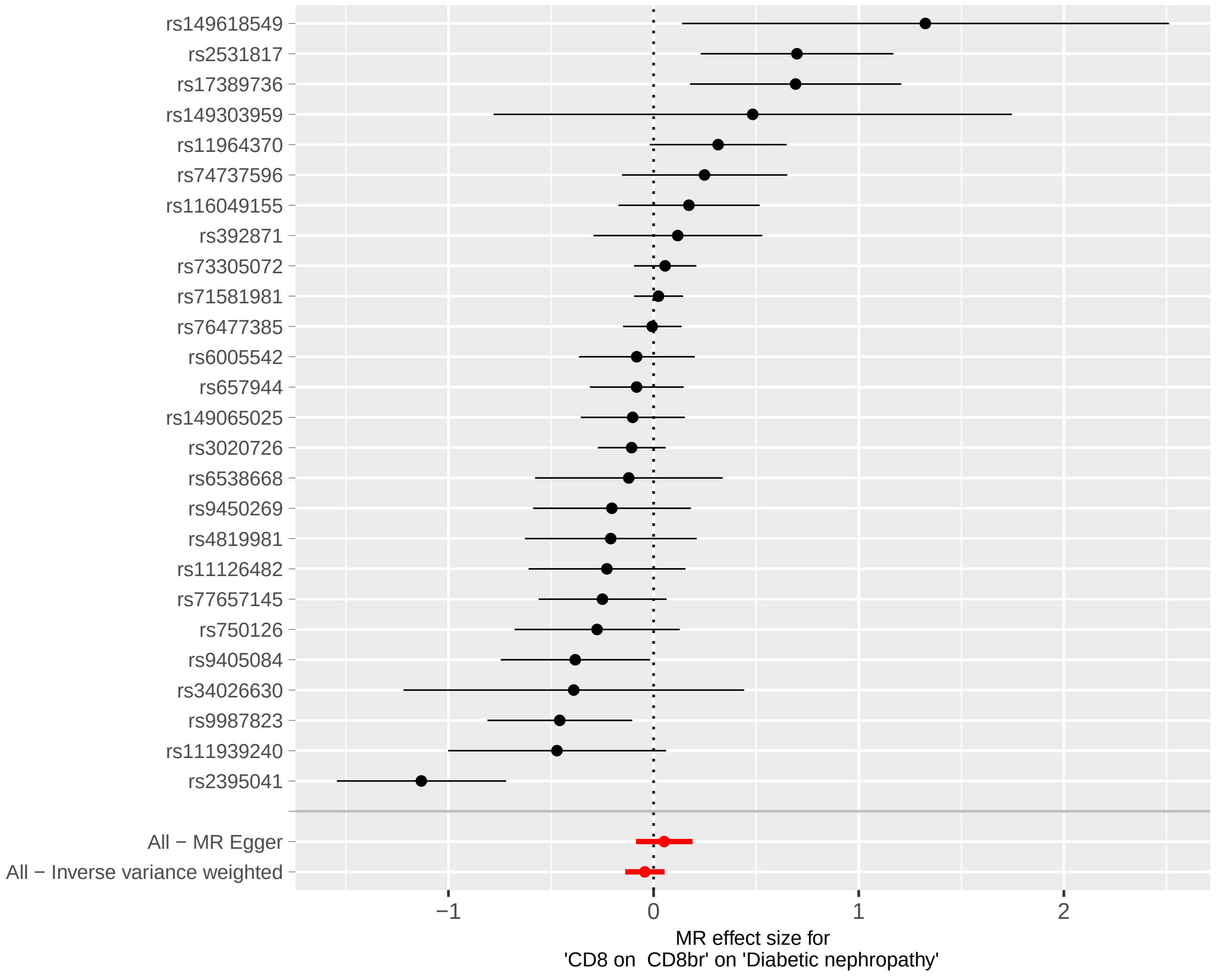
rs149618549
rs2531817
rs17389736
rs149303959
rs11964370
rs74737596
rs116049155
rs392871
rs73305072
rs71581981
rs76477385
rs6005542
rs657944
rs149065025
rs3020726
rs6538668
rs9450269
rs4819981
rs11126482
rs77657145
rs750126
rs9405084
rs34026630
rs9987823
rs111939240
rs2395041
All − MR Egger
All − Inverse variance weighted
−1
0
1
2
MR effect size for
'CD8 on CD8br' on 'Diabetic nephropathy'

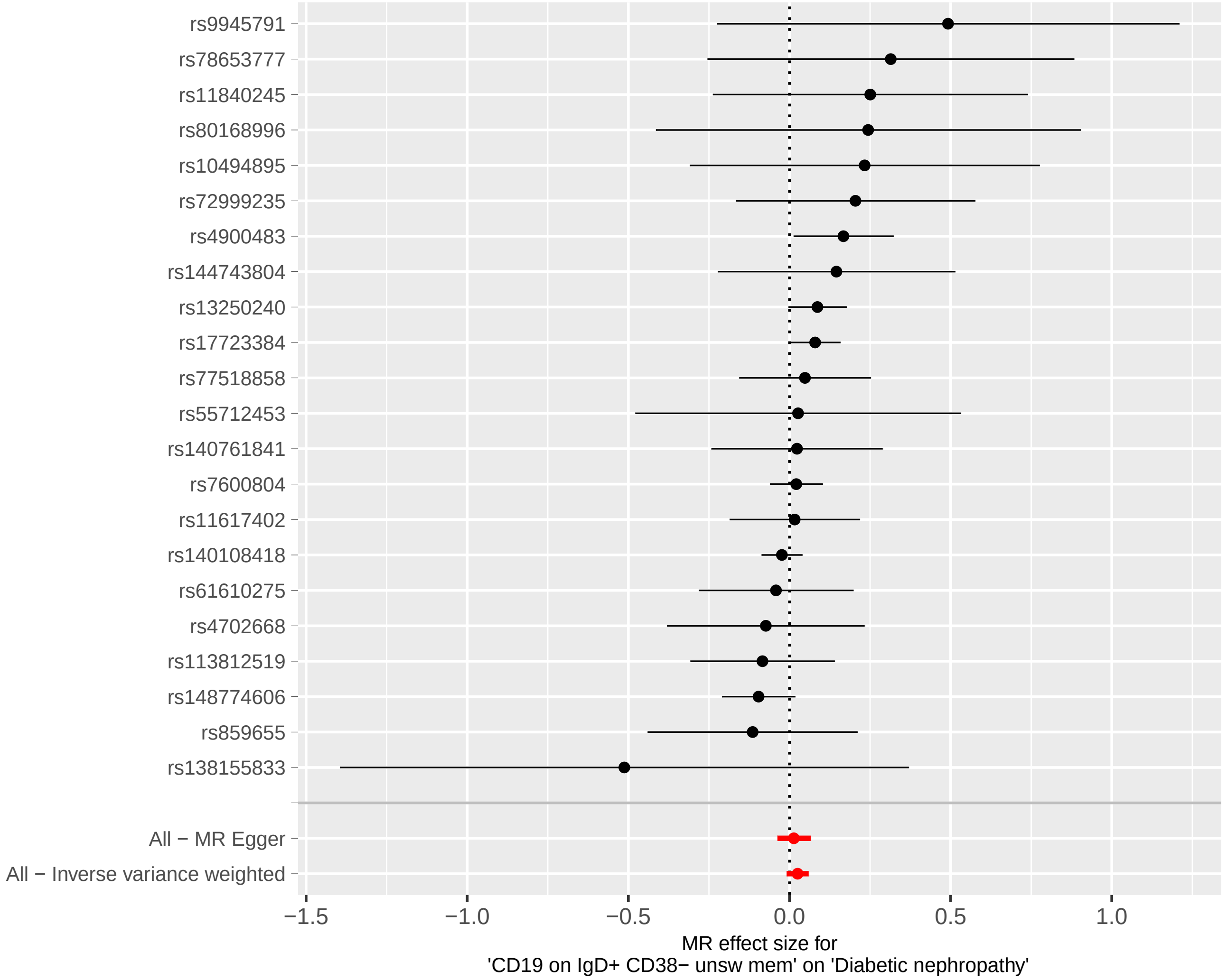

rs9945791
rs78653777
rs11840245
rs80168996
rs10494895
rs72999235
rs4900483
rs144743804
rs13250240
rs17723384
rs77518858
rs55712453
rs140761841
rs7600804
rs11617402
rs140108418
rs61610275
rs4702668
rs113812519
rs148774606
rs859655
rs138155833
All − MR Egger
All − Inverse variance weighted
−1.5
−1.0
−0.5
0.0
0.5
1.0
MR effect size for
'CD19 on IgD+ CD38− unsw mem' on 'Diabetic nephropathy'

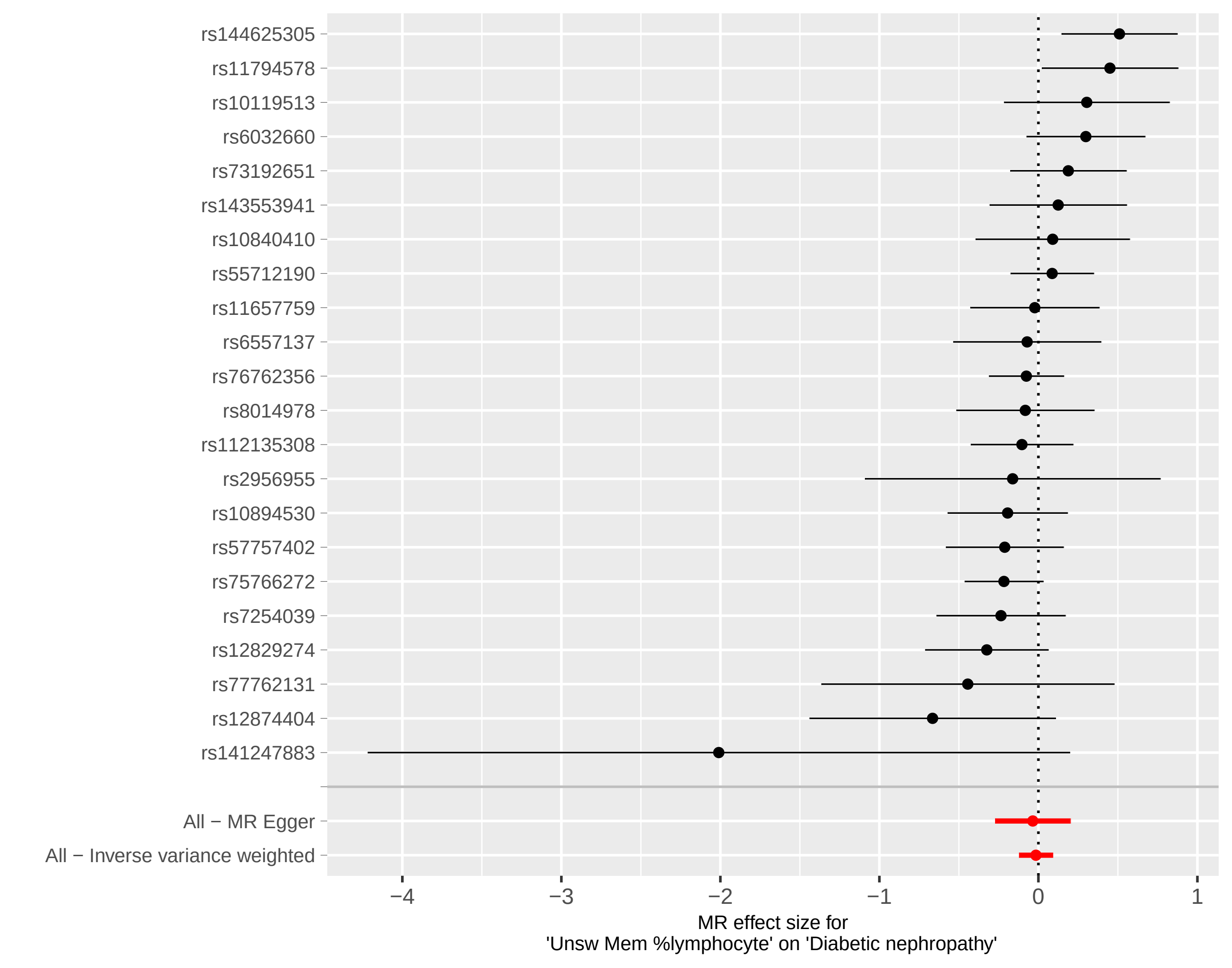
rs144625305
rs11794578
rs10119513
rs6032660
rs73192651
rs143553941
rs10840410
rs55712190
rs11657759
rs6557137
rs76762356
rs8014978
rs112135308
rs2956955
rs10894530
rs57757402
rs75766272
rs7254039
rs12829274
rs77762131
rs12874404
rs141247883
All − MR Egger
All − Inverse variance weighted
−4
−3
−2
−1
0
1
MR effect size for
'Unsw Mem %lymphocyte' on 'Diabetic nephropathy'

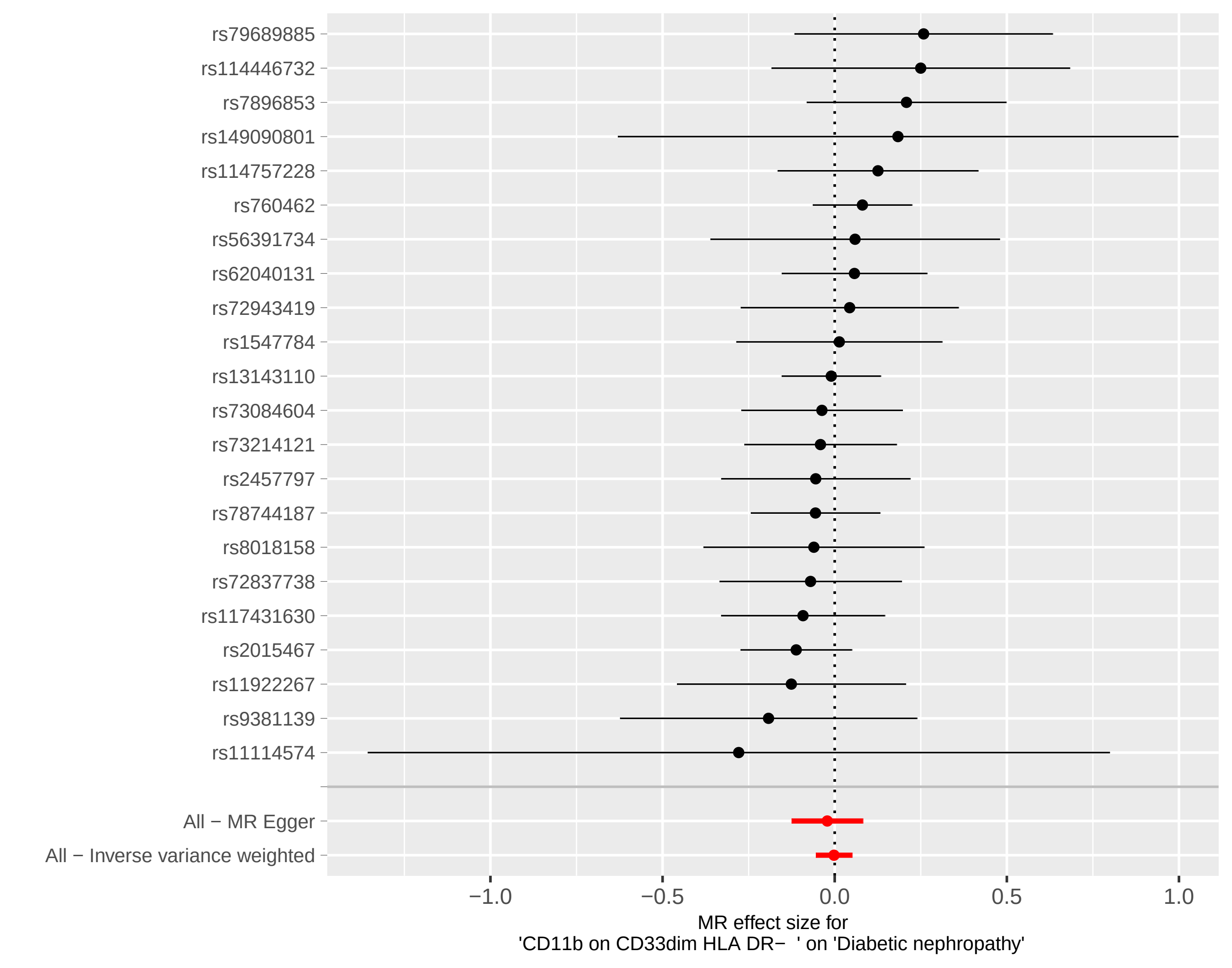
rs79689885
rs114446732
rs7896853
rs149090801
rs114757228
rs760462
rs56391734
rs62040131
rs72943419
rs1547784
rs13143110
rs73084604
rs73214121
rs2457797
rs78744187
rs8018158
rs72837738
rs117431630
rs2015467
rs11922267
rs9381139
rs11114574
All − MR Egger
All − Inverse variance weighted
−1.0
−0.5
0.0
0.5
1.0
MR effect size for
'CD11b on CD33dim HLA DR− ' on 'Diabetic nephropathy'

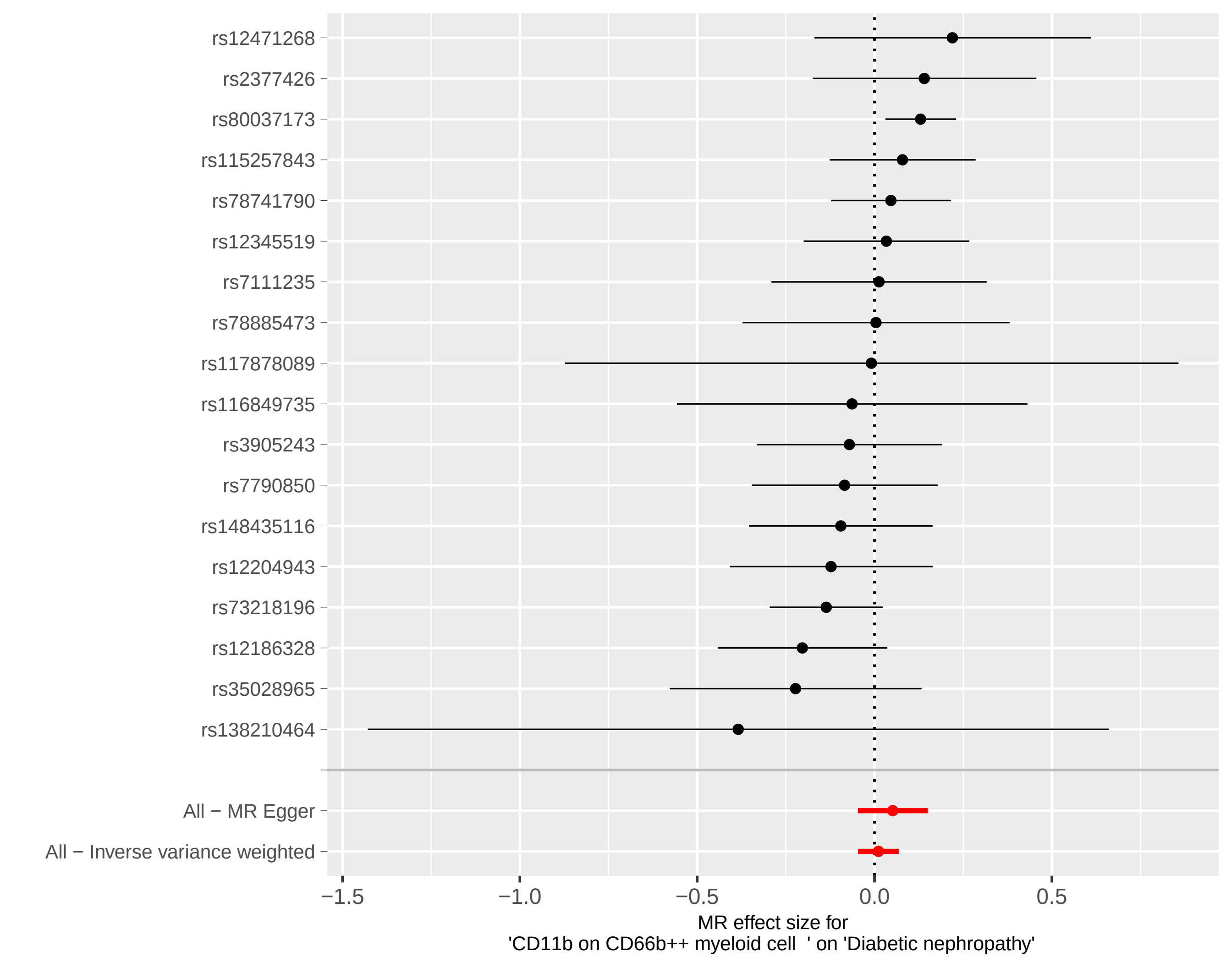

rs12471268
rs2377426
rs80037173
rs115257843
rs78741790
rs12345519
rs7111235
rs78885473
rs117878089
rs116849735
rs3905243
rs7790850
rs148435116
rs12204943
rs73218196
rs12186328
rs35028965
rs138210464
All − MR Egger
All − Inverse variance weighted
−1.5
−1.0
−0.5
0.0
0.5
MR effect size for
'CD11b on CD66b++ myeloid cell ' on 'Diabetic nephropathy'

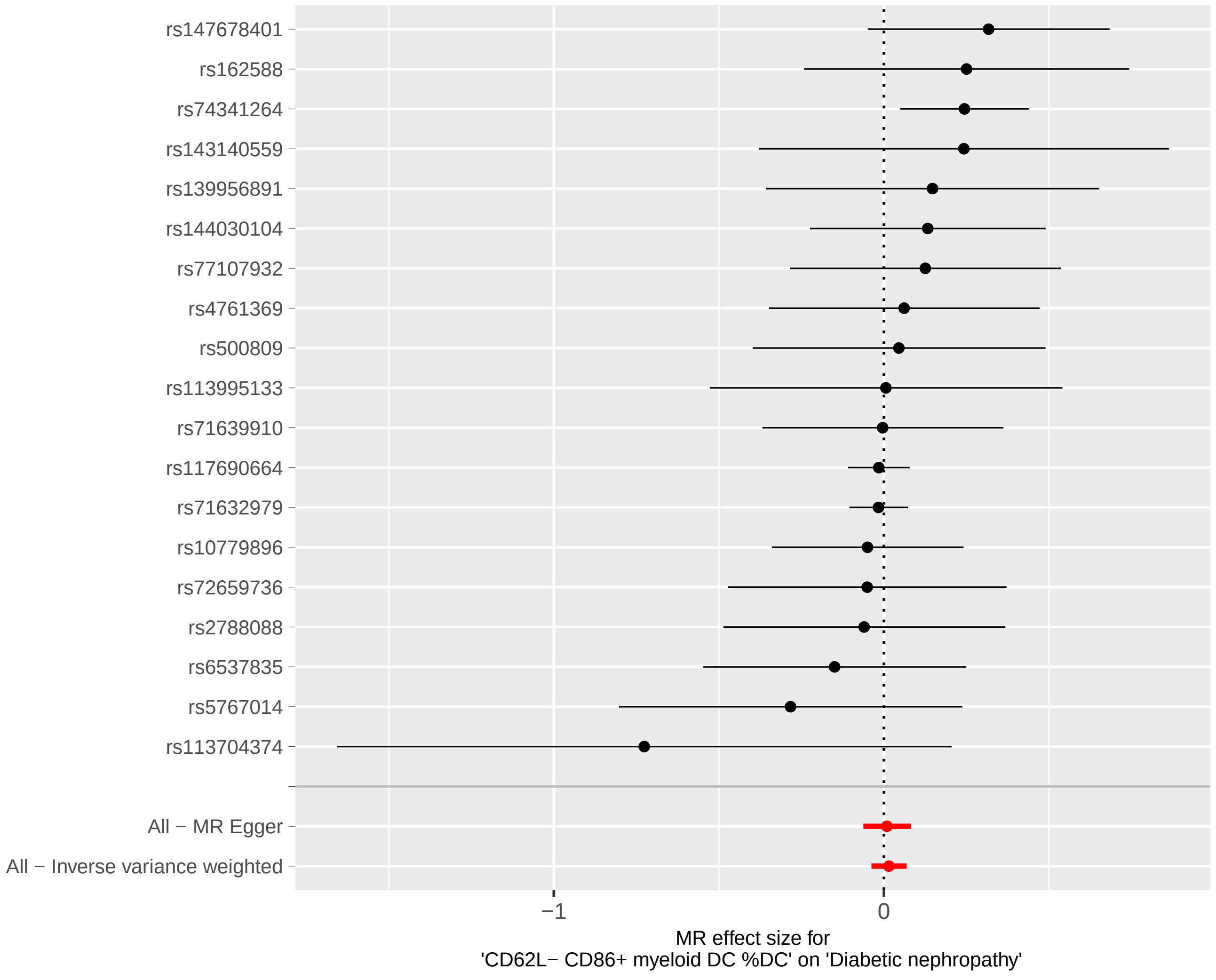
rs147678401
rs162588
rs74341264
rs143140559
rs139956891
rs144030104
rs77107932
rs4761369
rs500809
rs113995133
rs71639910
rs117690664
rs71632979
rs10779896
rs72659736
rs2788088
rs6537835
rs5767014
rs113704374
All − MR Egger
All − Inverse variance weighted
−1
0
MR effect size for
'CD62L− CD86+ myeloid DC %DC' on 'Diabetic nephropathy'

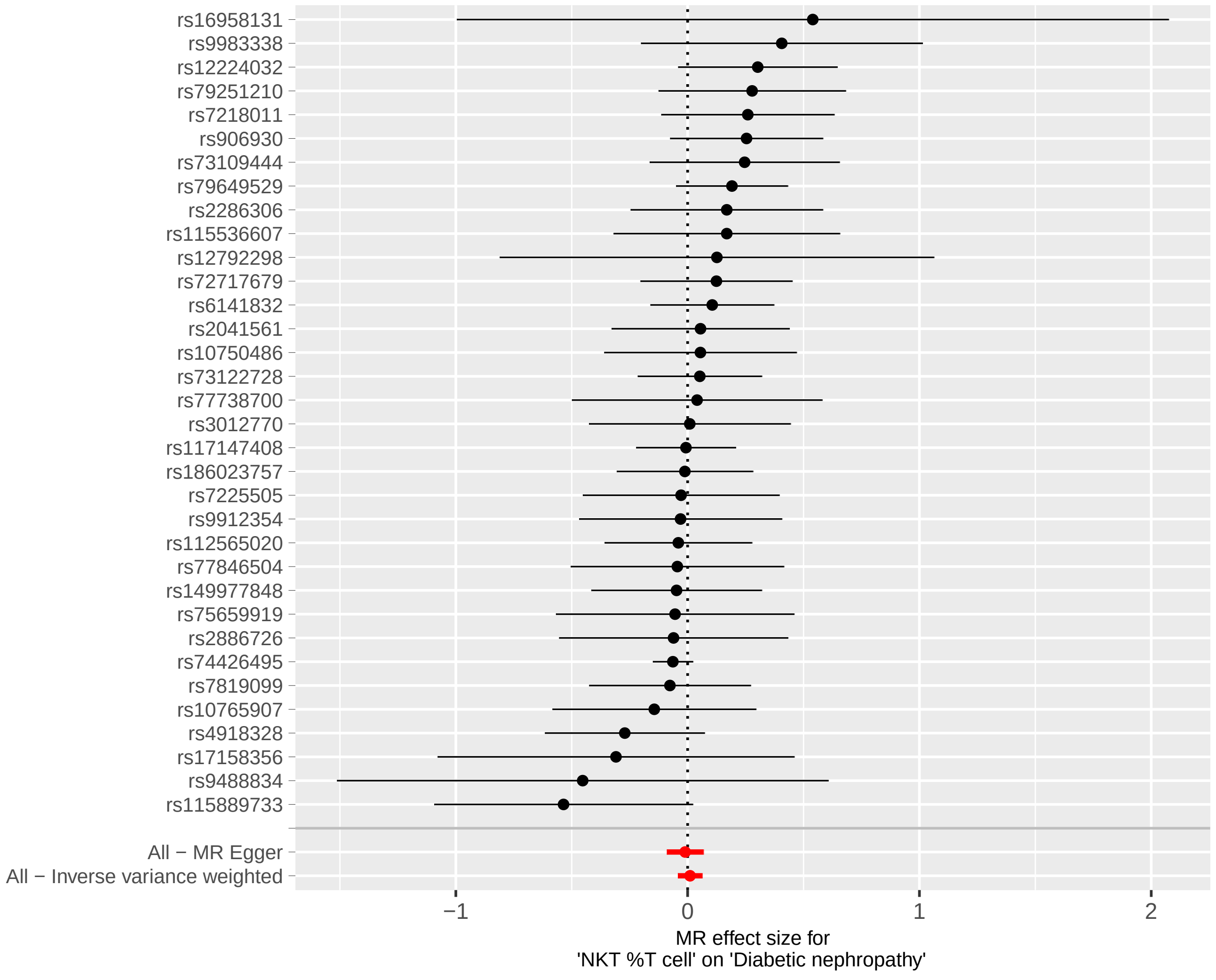
rs16958131
rs9983338
rs12224032
rs79251210
rs7218011
rs906930
rs73109444
rs79649529
rs2286306
rs115536607
rs12792298
rs72717679
rs6141832
rs2041561
rs10750486
rs73122728
rs77738700
rs3012770
rs117147408
rs186023757
rs7225505
rs9912354
rs112565020
rs77846504
rs149977848
rs75659919
rs2886726
rs74426495
rs7819099
rs10765907
rs4918328
rs17158356
rs9488834
rs115889733
All − MR Egger
All − Inverse variance weighted
−1
0
1
2
MR effect size for
'NKT %T cell' on 'Diabetic nephropathy'

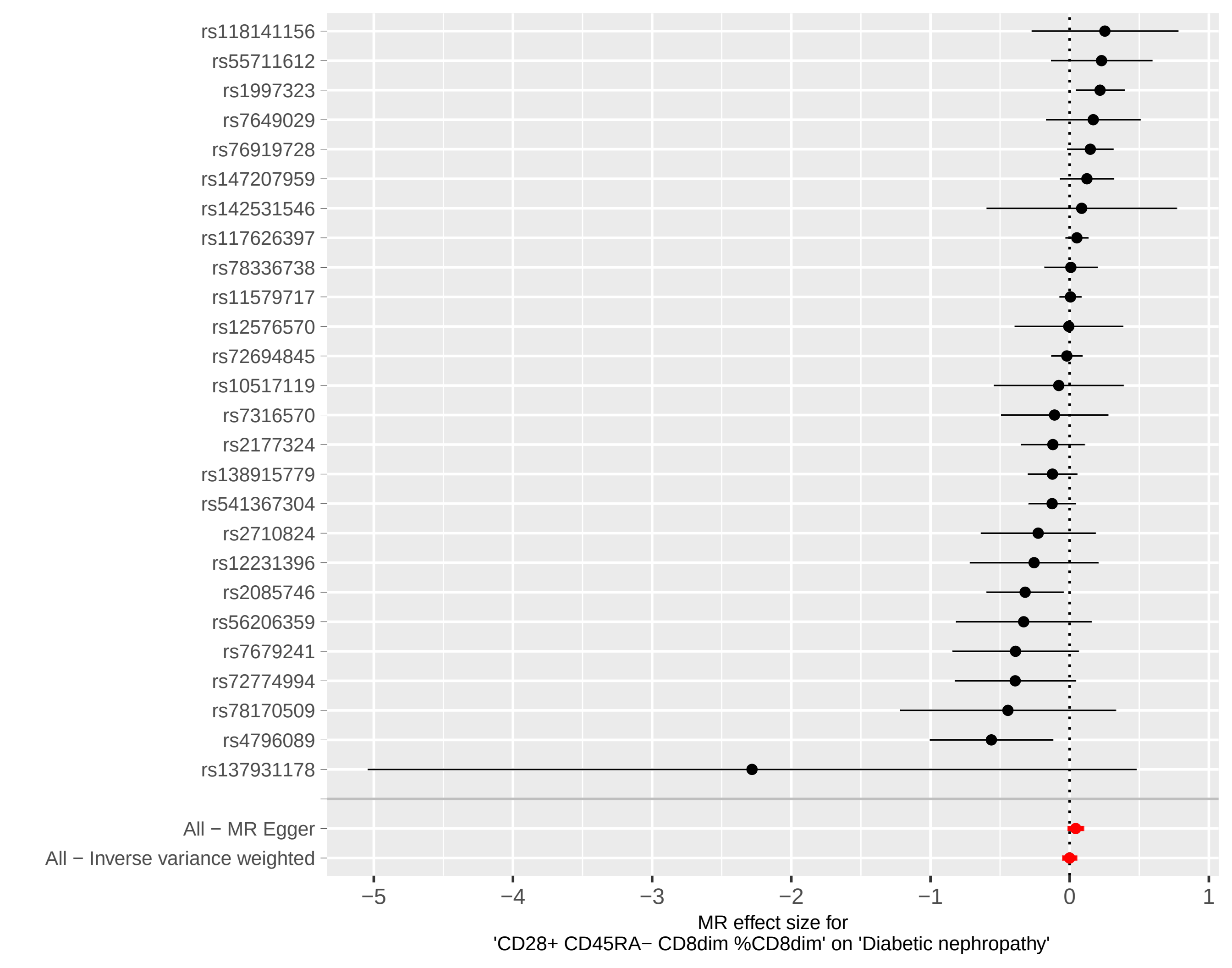
rs118141156
rs55711612
rs1997323
rs7649029
rs76919728
rs147207959
rs142531546
rs117626397
rs78336738
rs11579717
rs12576570
rs72694845
rs10517119
rs7316570
rs2177324
rs138915779
rs541367304
rs2710824
rs12231396
rs2085746
rs56206359
rs7679241
rs72774994
rs78170509
rs4796089
rs137931178
All − MR Egger
All − Inverse variance weighted
−5
−4
−3
−2
−1
0
1
MR effect size for
'CD28+ CD45RA− CD8dim %CD8dim' on 'Diabetic nephropathy'

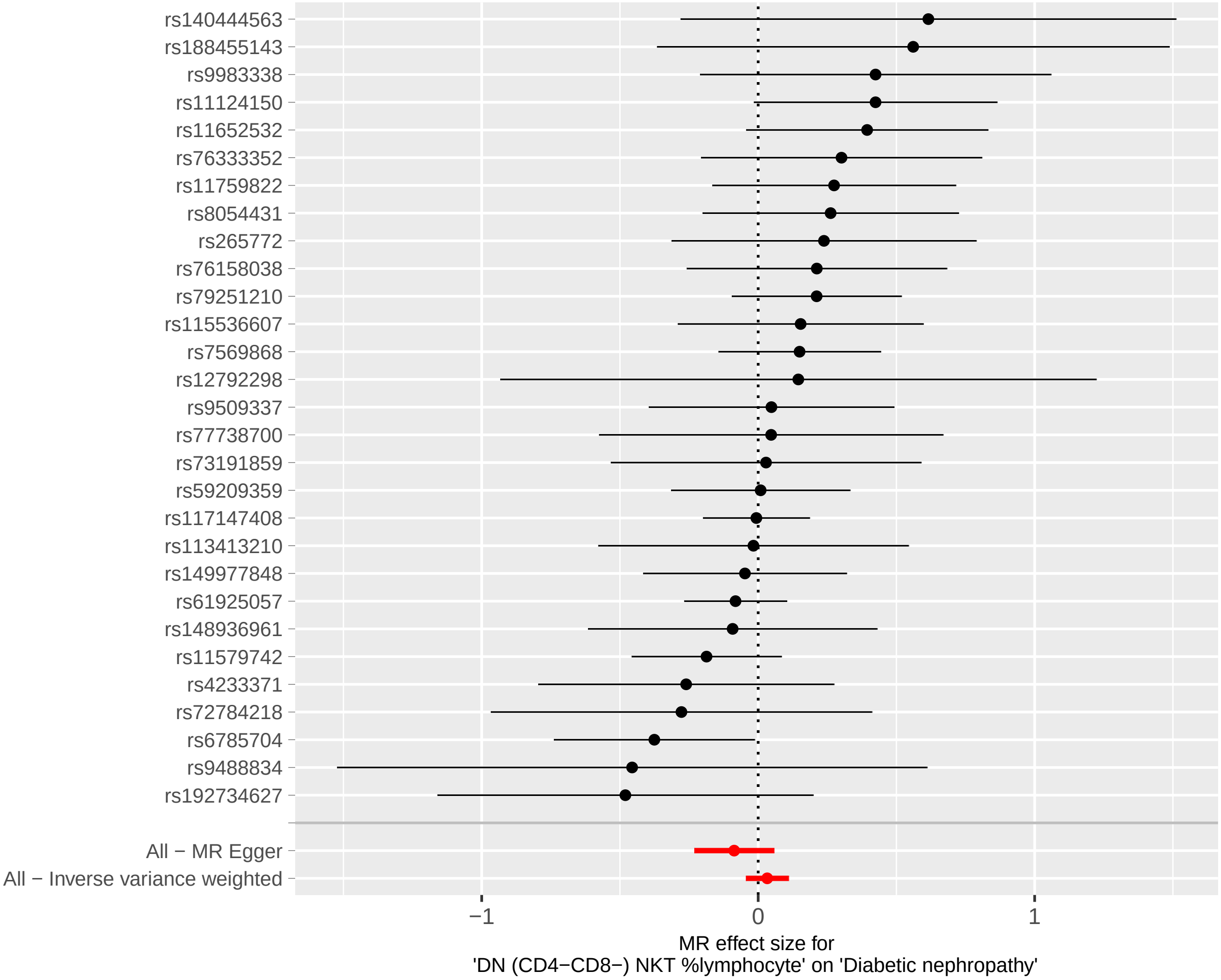
rs140444563
rs188455143
rs9983338
rs11124150
rs11652532
rs76333352
rs11759822
rs8054431
rs265772
rs76158038
rs79251210
rs115536607
rs7569868
rs12792298
rs9509337
rs77738700
rs73191859
rs59209359
rs117147408
rs113413210
rs149977848
rs61925057
rs148936961
rs11579742
rs4233371
rs72784218
rs6785704
rs9488834
rs192734627
All − MR Egger
All − Inverse variance weighted
−1
0
1
MR effect size for
'DN (CD4−CD8−) NKT %lymphocyte' on 'Diabetic nephropathy'

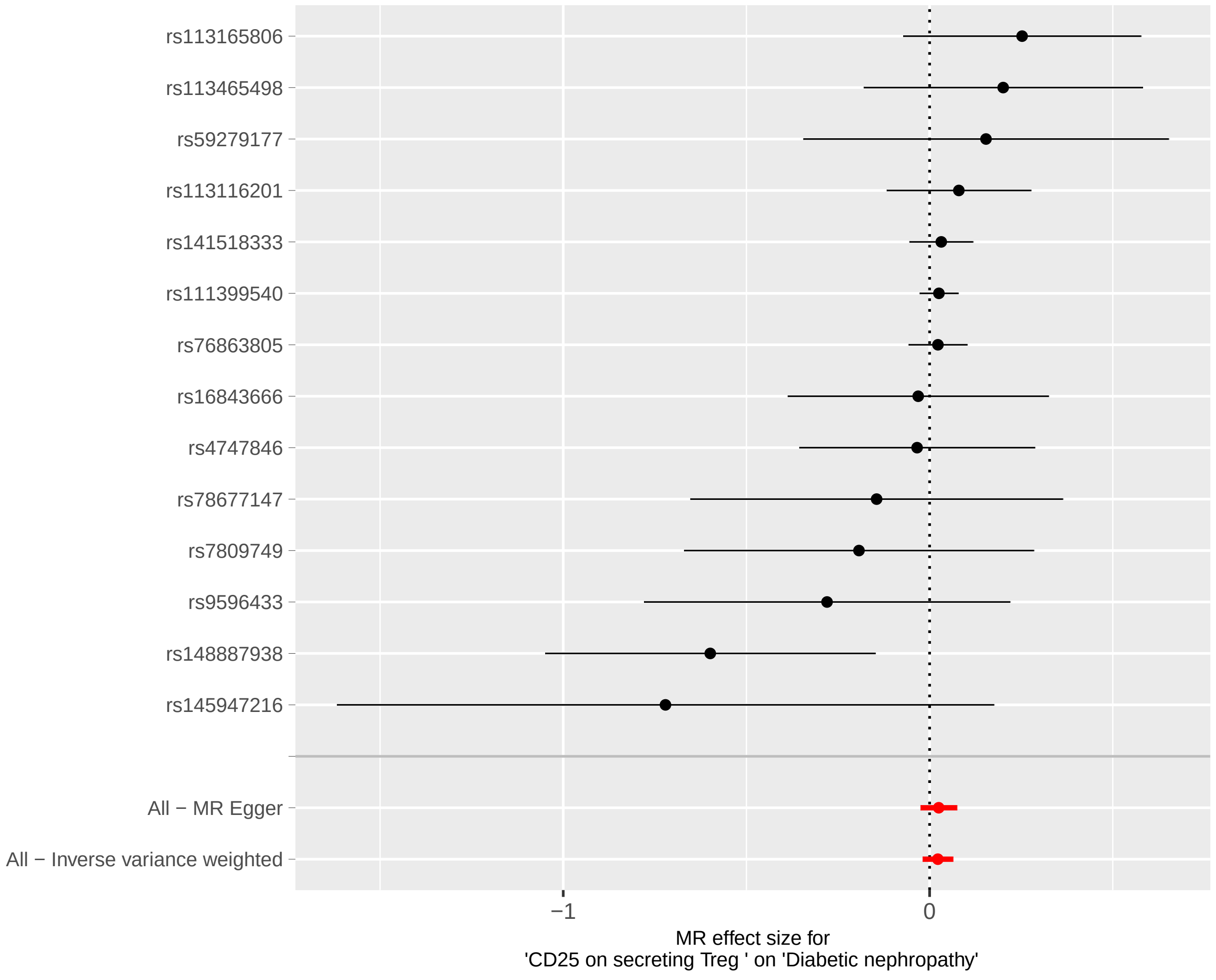
rs113165806
rs113465498
rs59279177
rs113116201
rs141518333
rs111399540
rs76863805
rs16843666
rs4747846
rs78677147
rs7809749
rs9596433
rs148887938
rs145947216
All − MR Egger
All − Inverse variance weighted
−1
0
MR effect size for
'CD25 on secreting Treg ' on 'Diabetic nephropathy'

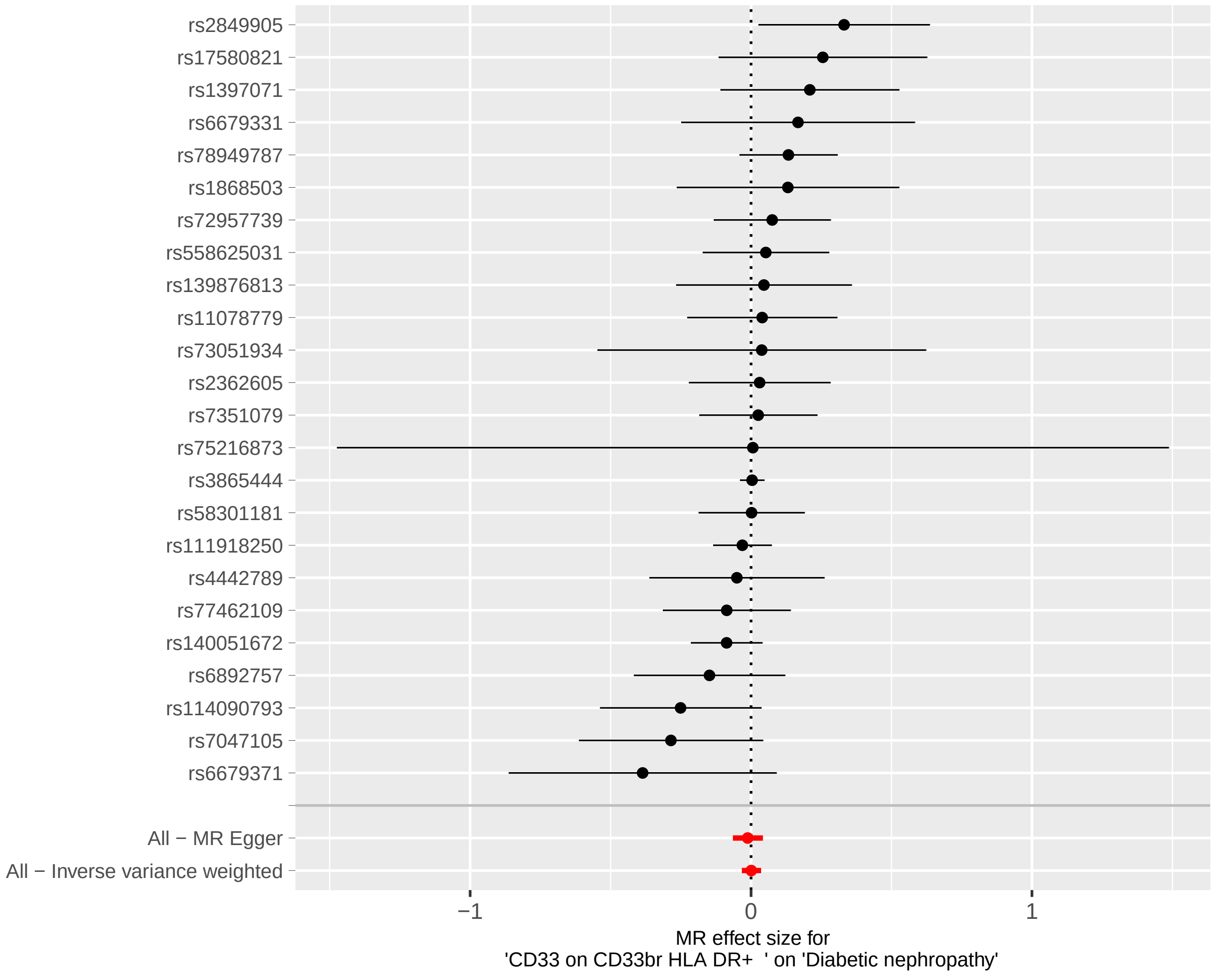
rs2849905
rs17580821
rs1397071
rs6679331
rs78949787
rs1868503
rs72957739
rs558625031
rs139876813
rs11078779
rs73051934
rs2362605
rs7351079
rs75216873
rs3865444
rs58301181
rs111918250
rs4442789
rs77462109
rs140051672
rs6892757
rs114090793
rs7047105
rs6679371
All − MR Egger
All − Inverse variance weighted
−1
0
1
MR effect size for
'CD33 on CD33br HLA DR+ ' on 'Diabetic nephropathy'

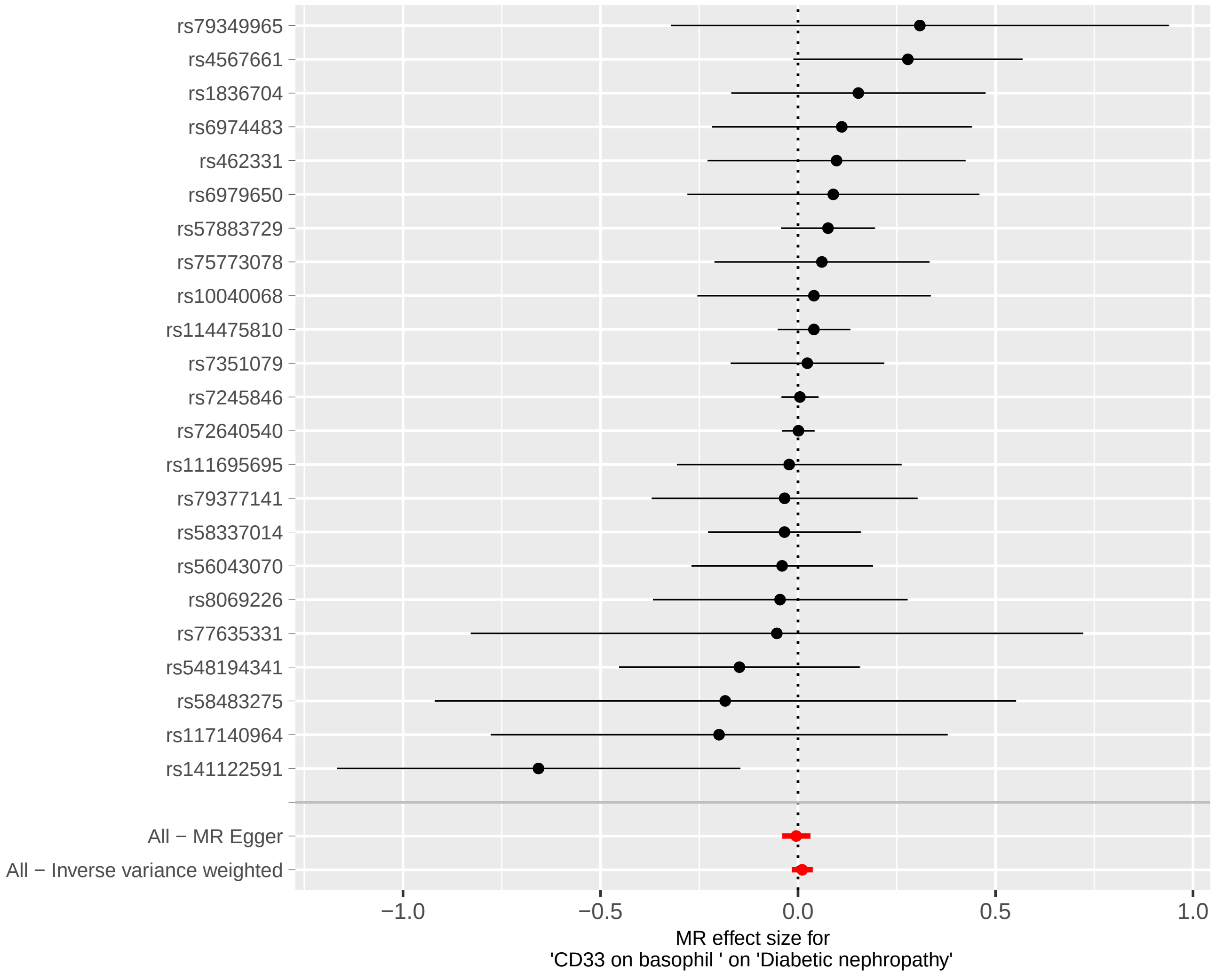

rs79349965
rs4567661
rs1836704
rs6974483
rs462331
rs6979650
rs57883729
rs75773078
rs10040068
rs114475810
rs7351079
rs7245846
rs72640540
rs111695695
rs79377141
rs58337014
rs56043070
rs8069226
rs77635331
rs548194341
rs58483275
rs117140964
rs141122591
All − MR Egger
All − Inverse variance weighted
−1.0
−0.5
0.0
0.5
1.0
MR effect size for
'CD33 on basophil ' on 'Diabetic nephropathy'

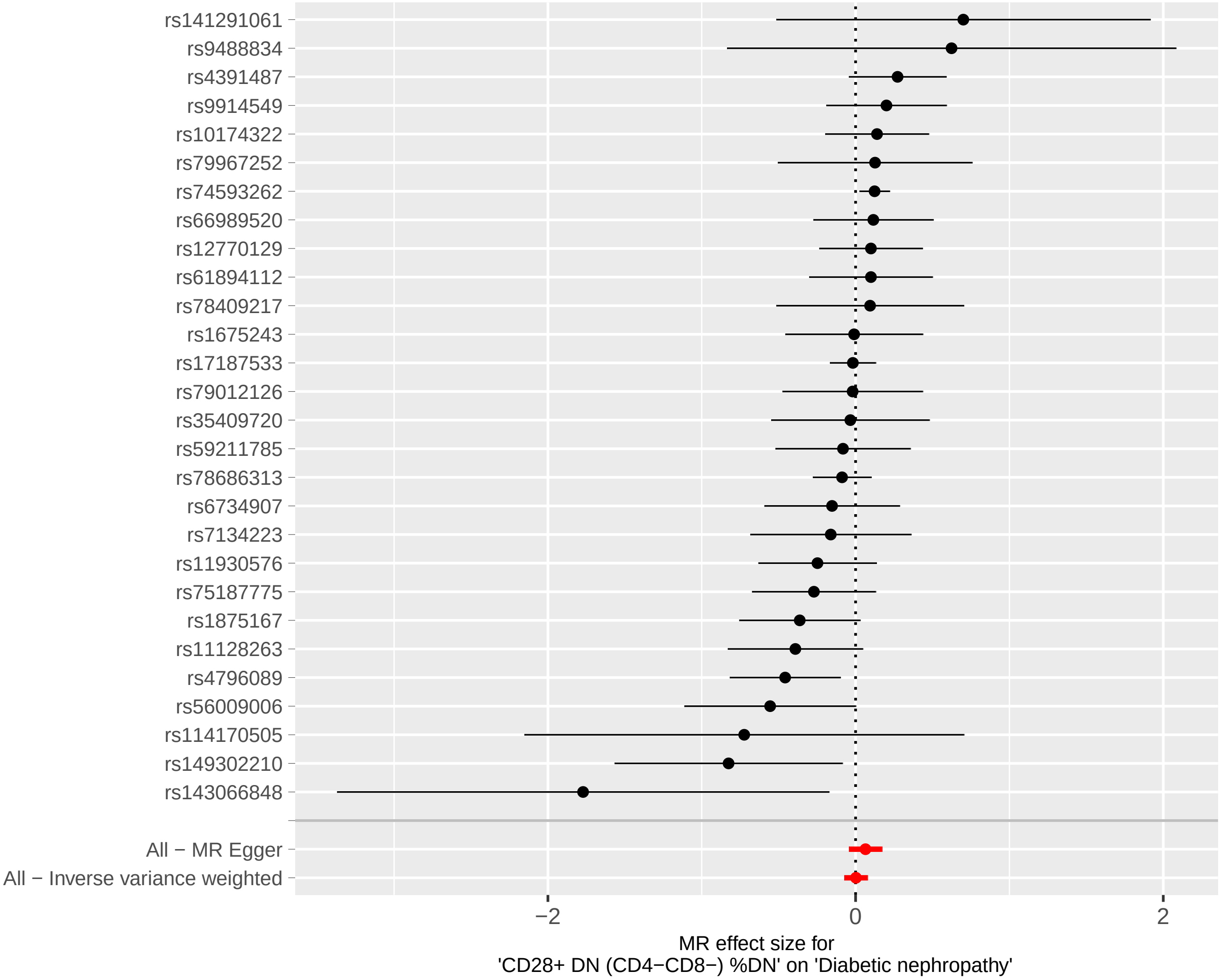
rs141291061
rs9488834
rs4391487
rs9914549
rs10174322
rs79967252
rs74593262
rs66989520
rs12770129
rs61894112
rs78409217
rs1675243
rs17187533
rs79012126
rs35409720
rs59211785
rs78686313
rs6734907
rs7134223
rs11930576
rs75187775
rs1875167
rs11128263
rs4796089
rs56009006
rs114170505
rs149302210
rs143066848
All − MR Egger
All − Inverse variance weighted
−2
0
2
MR effect size for
'CD28+ DN (CD4−CD8−) %DN' on 'Diabetic nephropathy'

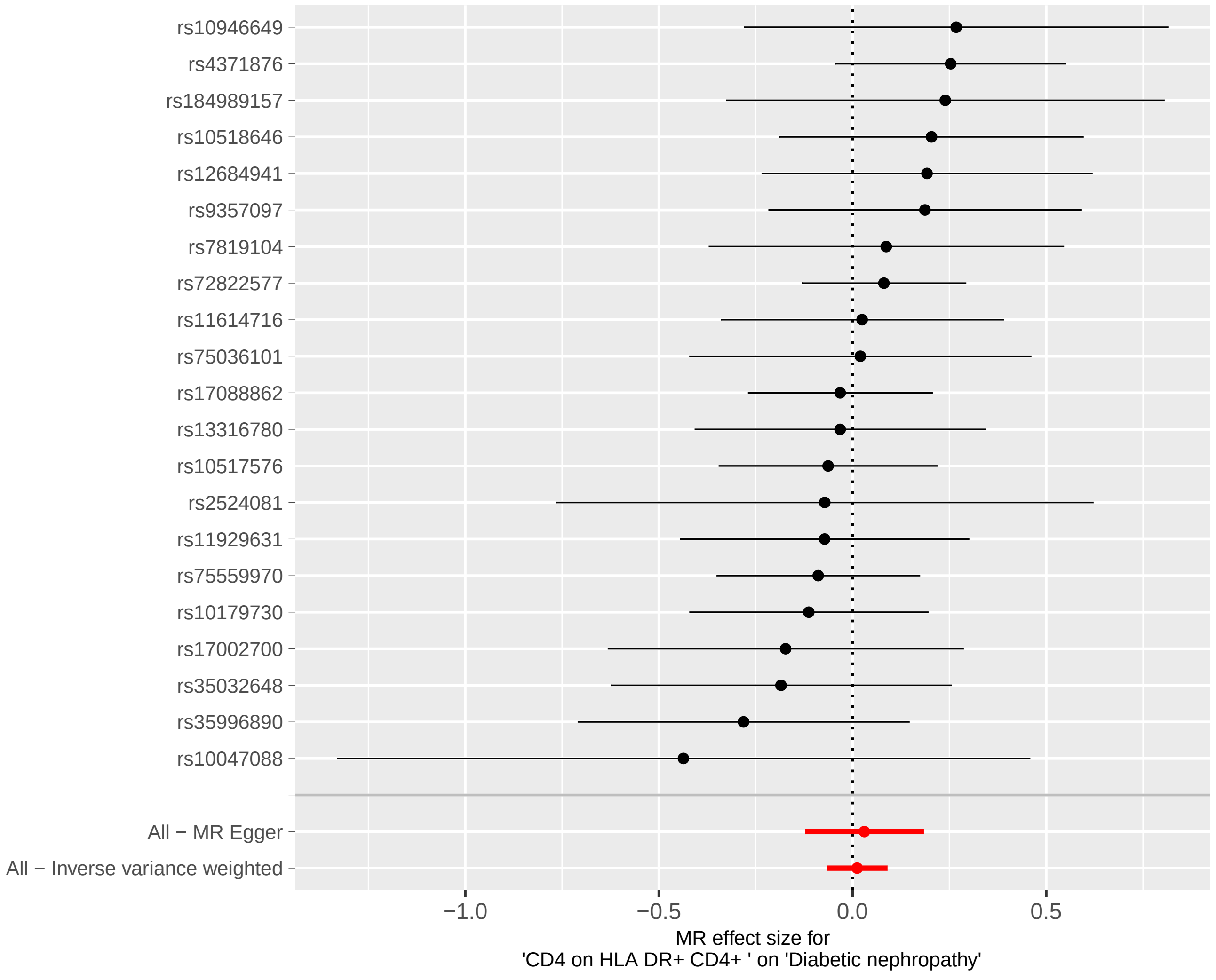
rs10946649
rs4371876
rs184989157
rs10518646
rs12684941
rs9357097
rs7819104
rs72822577
rs11614716
rs75036101
rs17088862
rs13316780
rs10517576
rs2524081
rs11929631
rs75559970
rs10179730
rs17002700
rs35032648
rs35996890
rs10047088
All − MR Egger
All − Inverse variance weighted
−1.0
−0.5
0.0
0.5
MR effect size for
'CD4 on HLA DR+ CD4+ ' on 'Diabetic nephropathy'

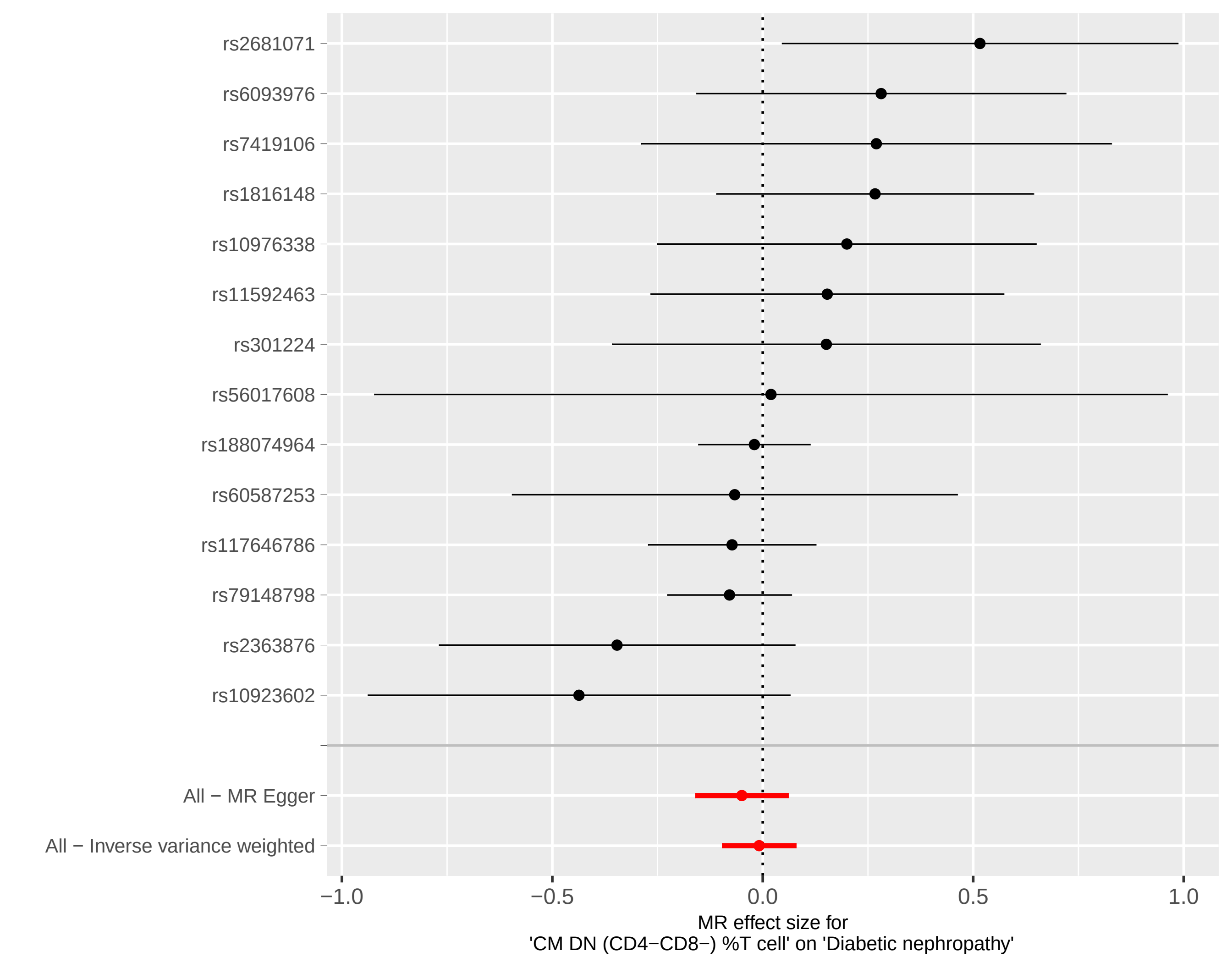

rs2681071
rs6093976
rs7419106
rs1816148
rs10976338
rs11592463
rs301224
rs56017608
rs188074964
rs60587253
rs117646786
rs79148798
rs2363876
rs10923602
All − MR Egger
All − Inverse variance weighted
−1.0
−0.5
0.0
0.5
1.0
MR effect size for
'CM DN (CD4−CD8−) %T cell' on 'Diabetic nephropathy'

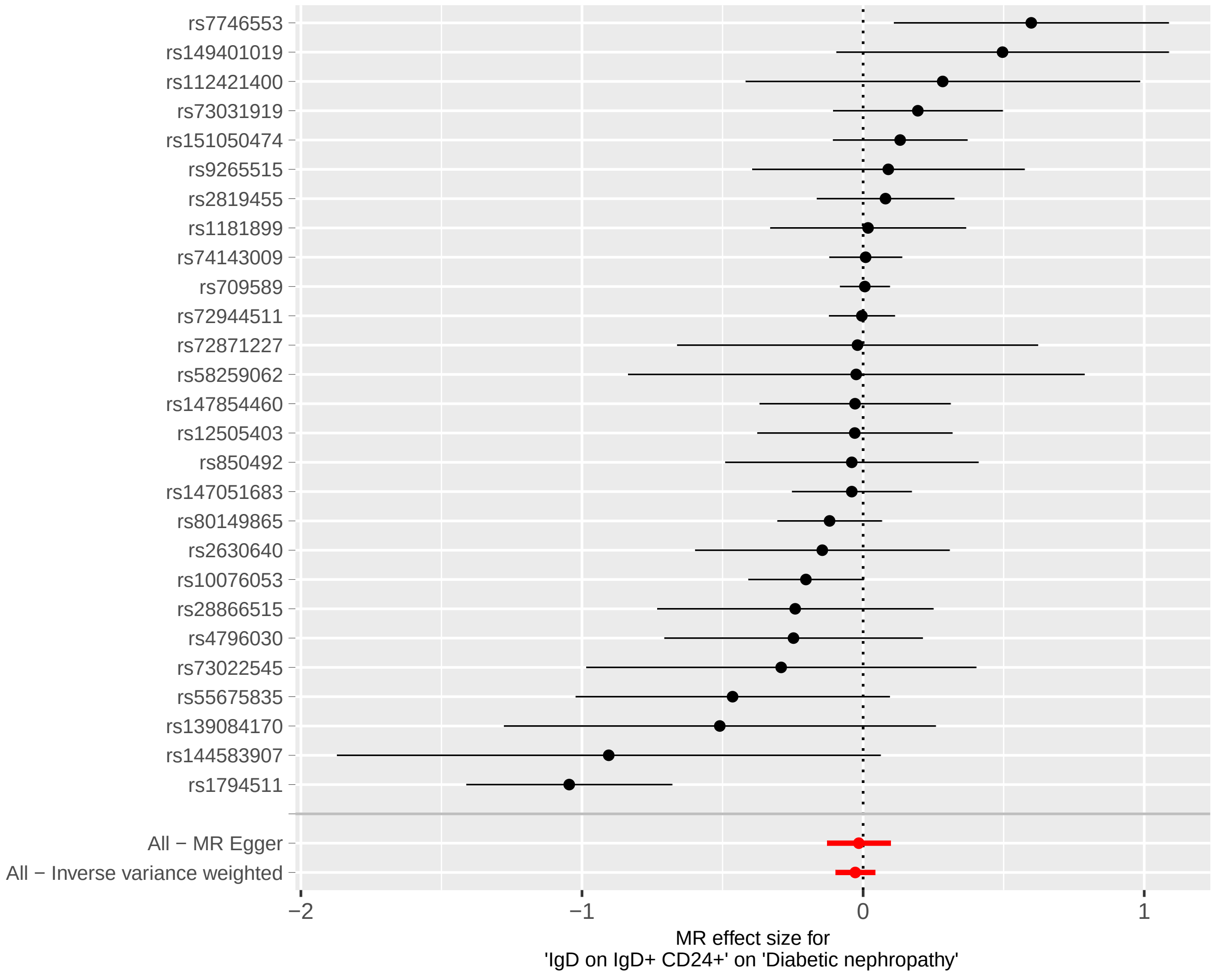
rs7746553
rs149401019
rs112421400
rs73031919
rs151050474
rs9265515
rs2819455
rs1181899
rs74143009
rs709589
rs72944511
rs72871227
rs58259062
rs147854460
rs12505403
rs850492
rs147051683
rs80149865
rs2630640
rs10076053
rs28866515
rs4796030
rs73022545
rs55675835
rs139084170
rs144583907
rs1794511
All − MR Egger
All − Inverse variance weighted
−2
−1
0
1
MR effect size for
'IgD on IgD+ CD24+' on 'Diabetic nephropathy'

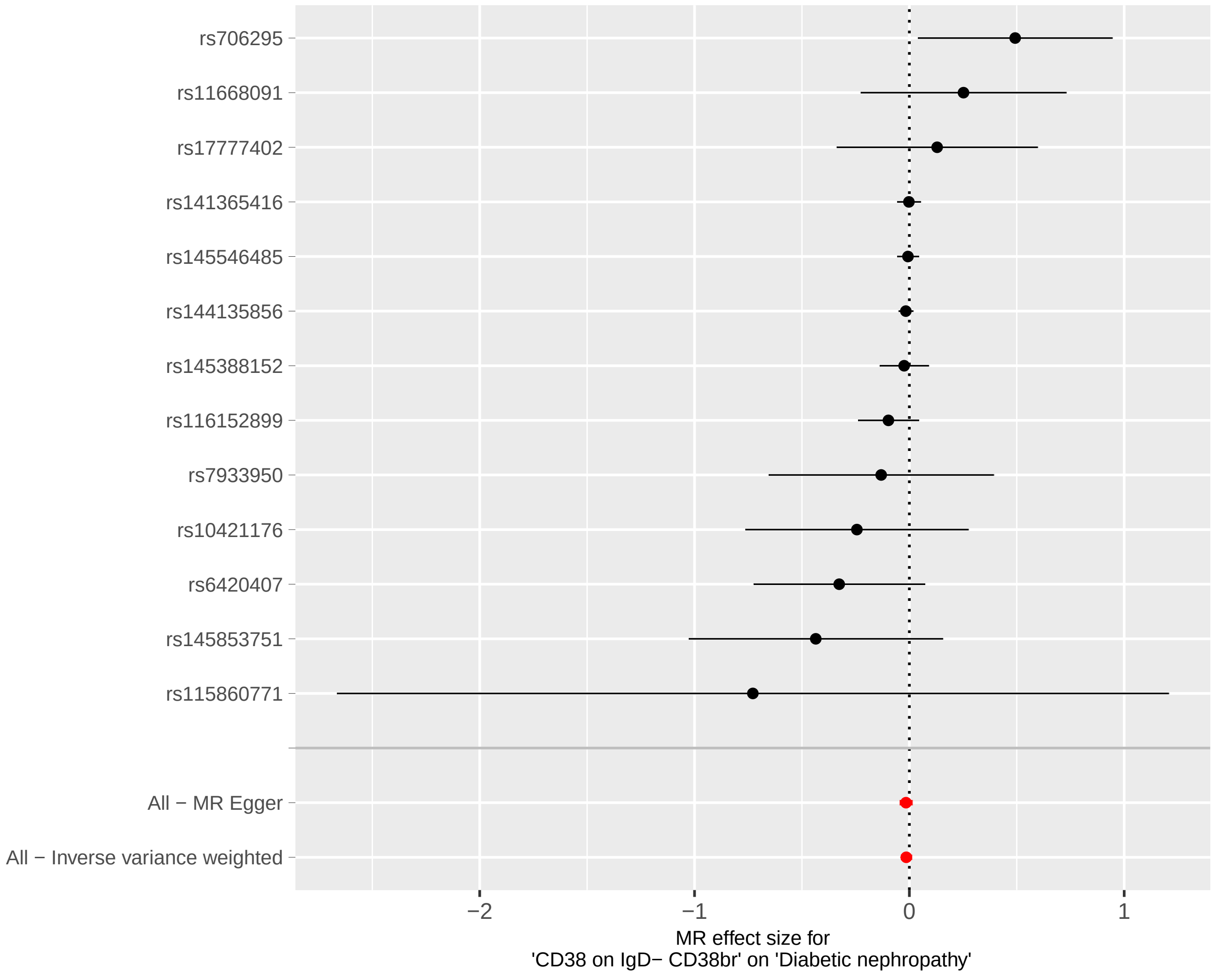

rs706295
rs11668091
rs17777402
rs141365416
rs145546485
rs144135856
rs145388152
rs116152899
rs7933950
rs10421176
rs6420407
rs145853751
rs115860771
All − MR Egger
All − Inverse variance weighted
−2
−1
0
1
MR effect size for
'CD38 on IgD− CD38br' on 'Diabetic nephropathy'

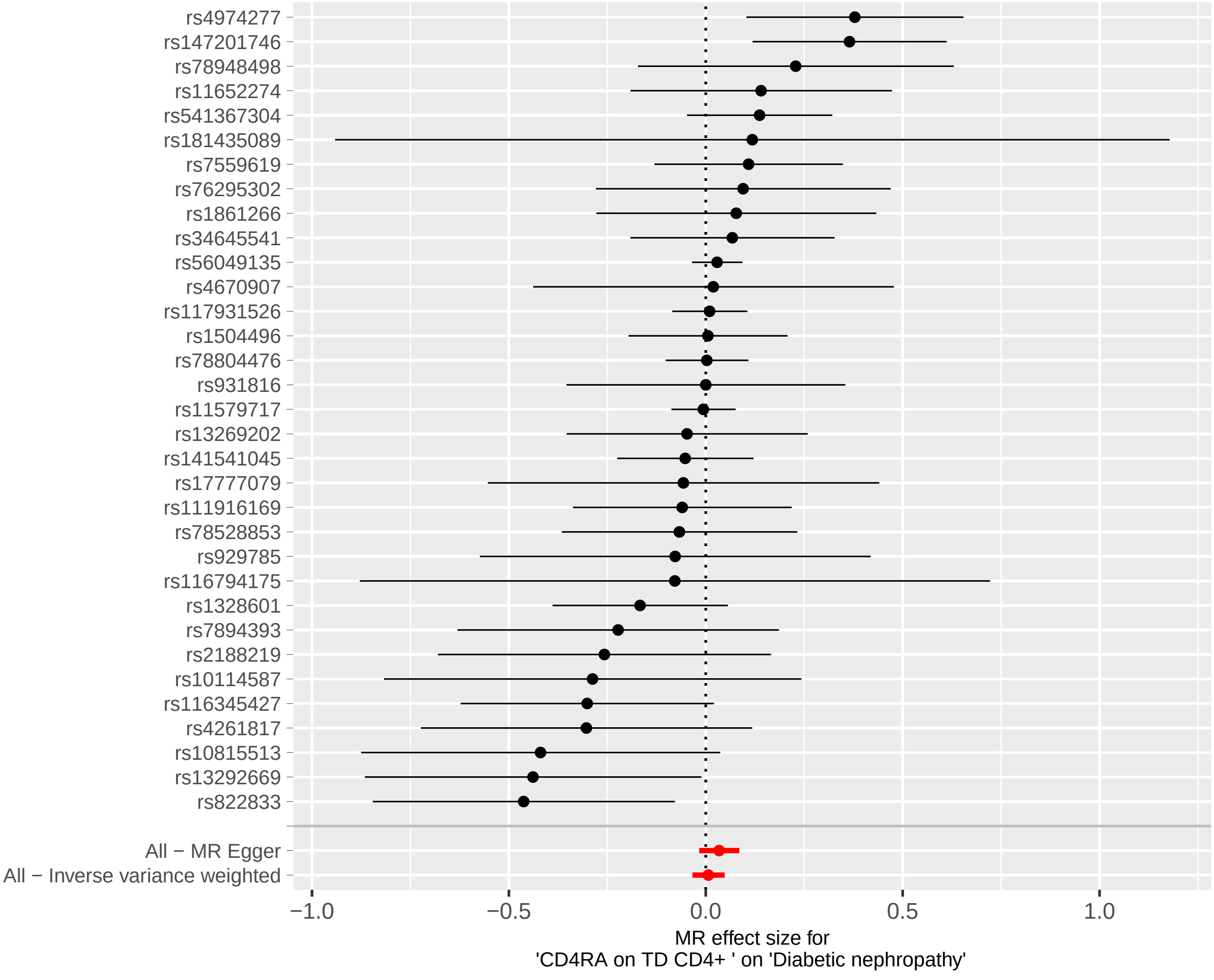
rs4974277
rs147201746
rs78948498
rs11652274
rs541367304
rs181435089
rs7559619
rs76295302
rs1861266
rs34645541
rs56049135
rs4670907
rs117931526
rs1504496
rs78804476
rs931816
rs11579717
rs13269202
rs141541045
rs17777079
rs111916169
rs78528853
rs929785
rs116794175
rs1328601
rs7894393
rs2188219
rs10114587
rs116345427
rs4261817
rs10815513
rs13292669
rs822833
All − MR Egger
All − Inverse variance weighted
−1.0
−0.5
0.0
0.5
1.0
MR effect size for
'CD4RA on TD CD4+ ' on 'Diabetic nephropathy'

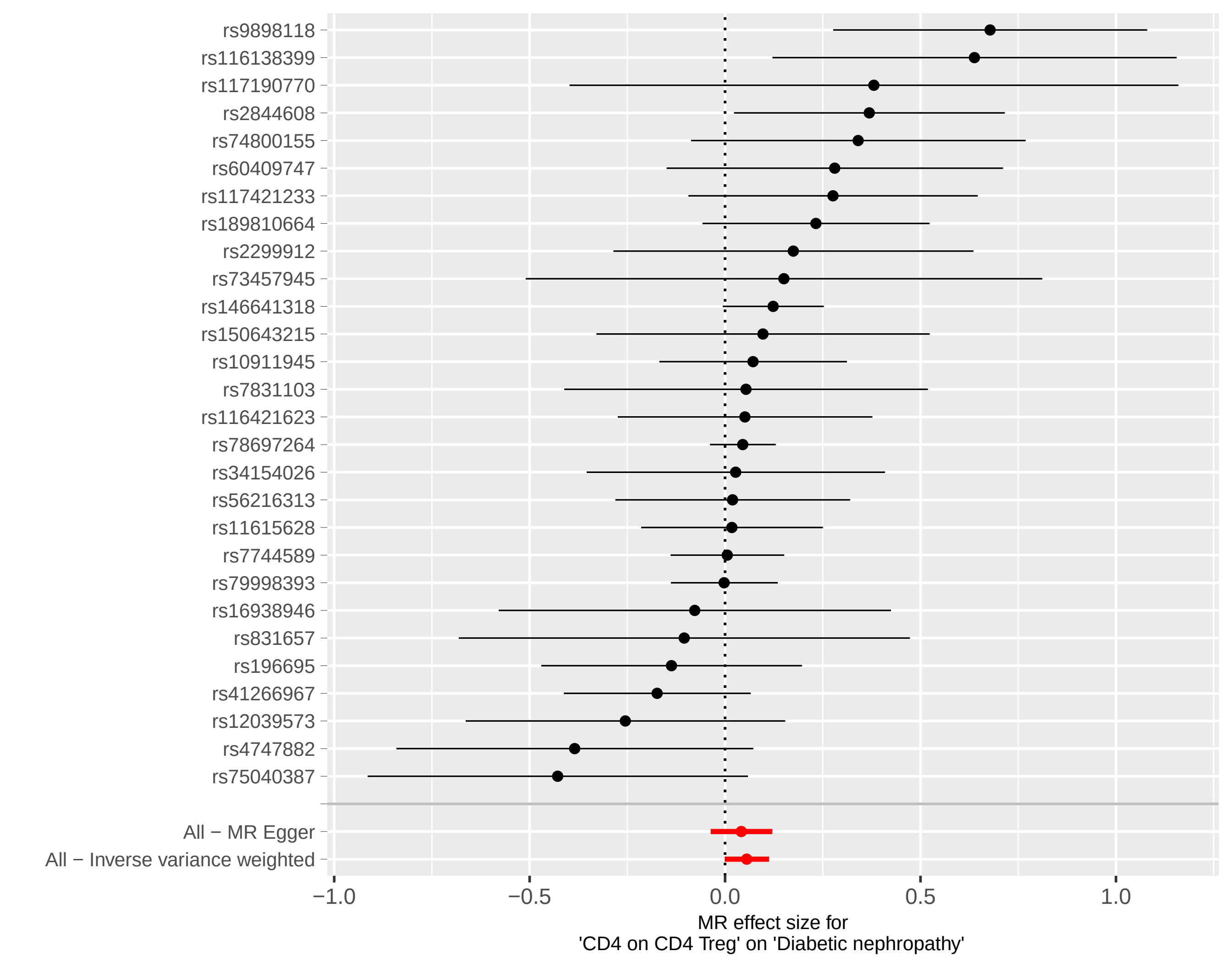
rs9898118
rs116138399
rs117190770
rs2844608
rs74800155
rs60409747
rs117421233
rs189810664
rs2299912
rs73457945
rs146641318
rs150643215
rs10911945
rs7831103
rs116421623
rs78697264
rs34154026
rs56216313
rs11615628
rs7744589
rs79998393
rs16938946
rs831657
rs196695
rs41266967
rs12039573
rs4747882
rs75040387
All − MR Egger
All − Inverse variance weighted
−1.0
−0.5
0.0
0.5
1.0
MR effect size for
'CD4 on CD4 Treg' on 'Diabetic nephropathy'

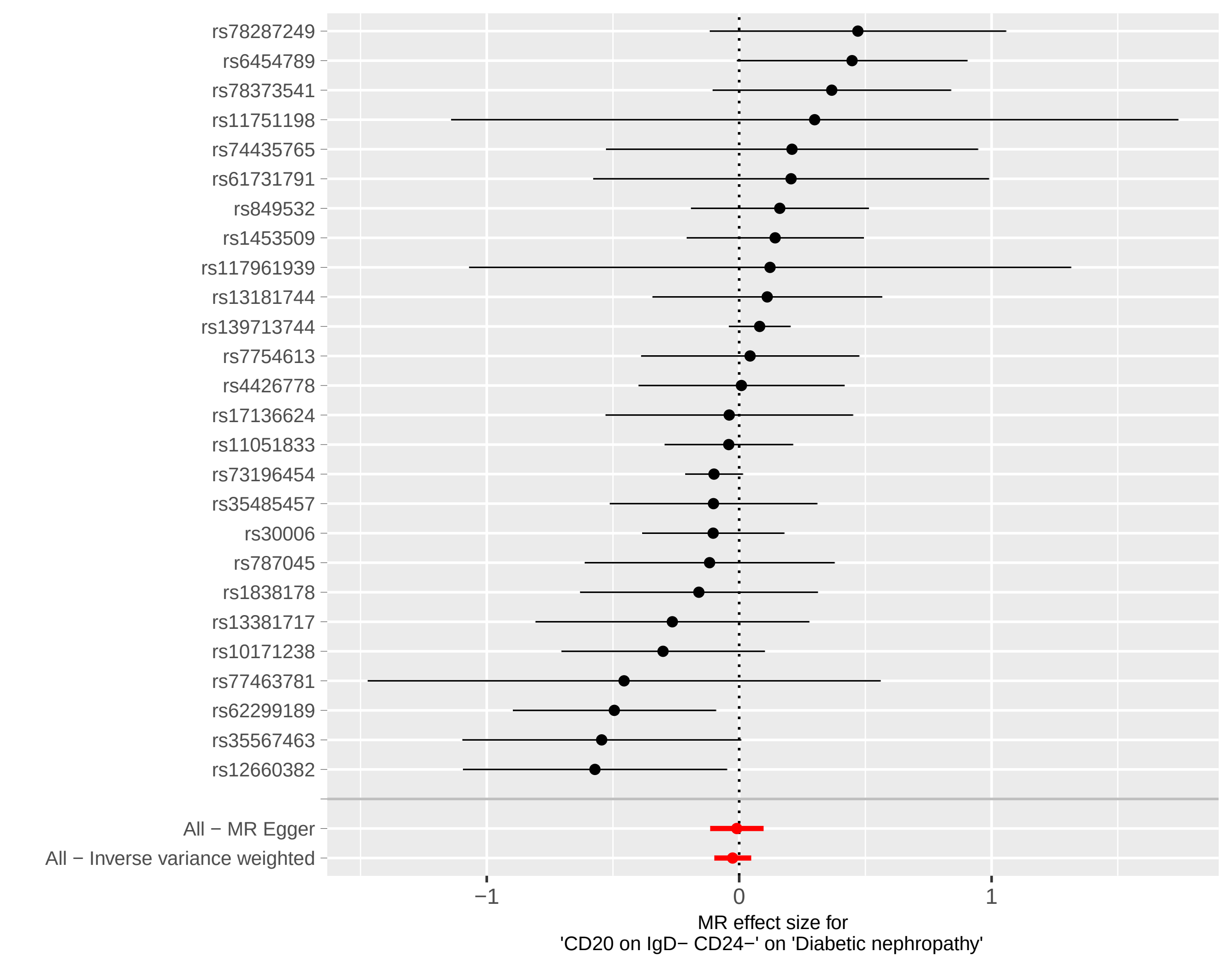

rs78287249
rs6454789
rs78373541
rs11751198
rs74435765
rs61731791
rs849532
rs1453509
rs117961939
rs13181744
rs139713744
rs7754613
rs4426778
rs17136624
rs11051833
rs73196454
rs35485457
rs30006
rs787045
rs1838178
rs13381717
rs10171238
rs77463781
rs62299189
rs35567463
rs12660382
All − MR Egger
All − Inverse variance weighted
−1
0
1
MR effect size for
'CD20 on IgD− CD24−' on 'Diabetic nephropathy'

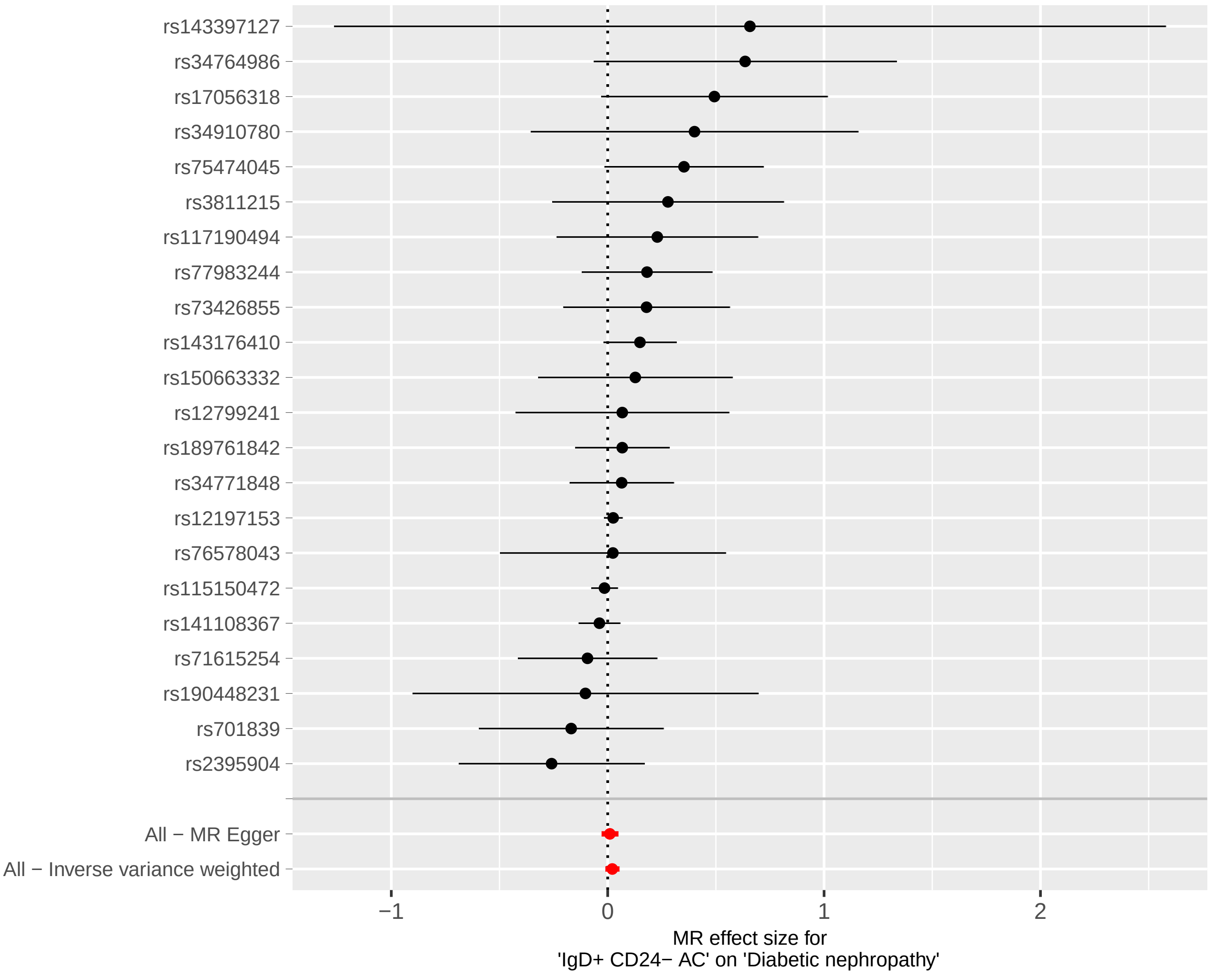
rs143397127
rs34764986
rs17056318
rs34910780
rs75474045
rs3811215
rs117190494
rs77983244
rs73426855
rs143176410
rs150663332
rs12799241
rs189761842
rs34771848
rs12197153
rs76578043
rs115150472
rs141108367
rs71615254
rs190448231
rs701839
rs2395904
All − MR Egger
All − Inverse variance weighted
−1
0
1
2
MR effect size for
'IgD+ CD24− AC' on 'Diabetic nephropathy'

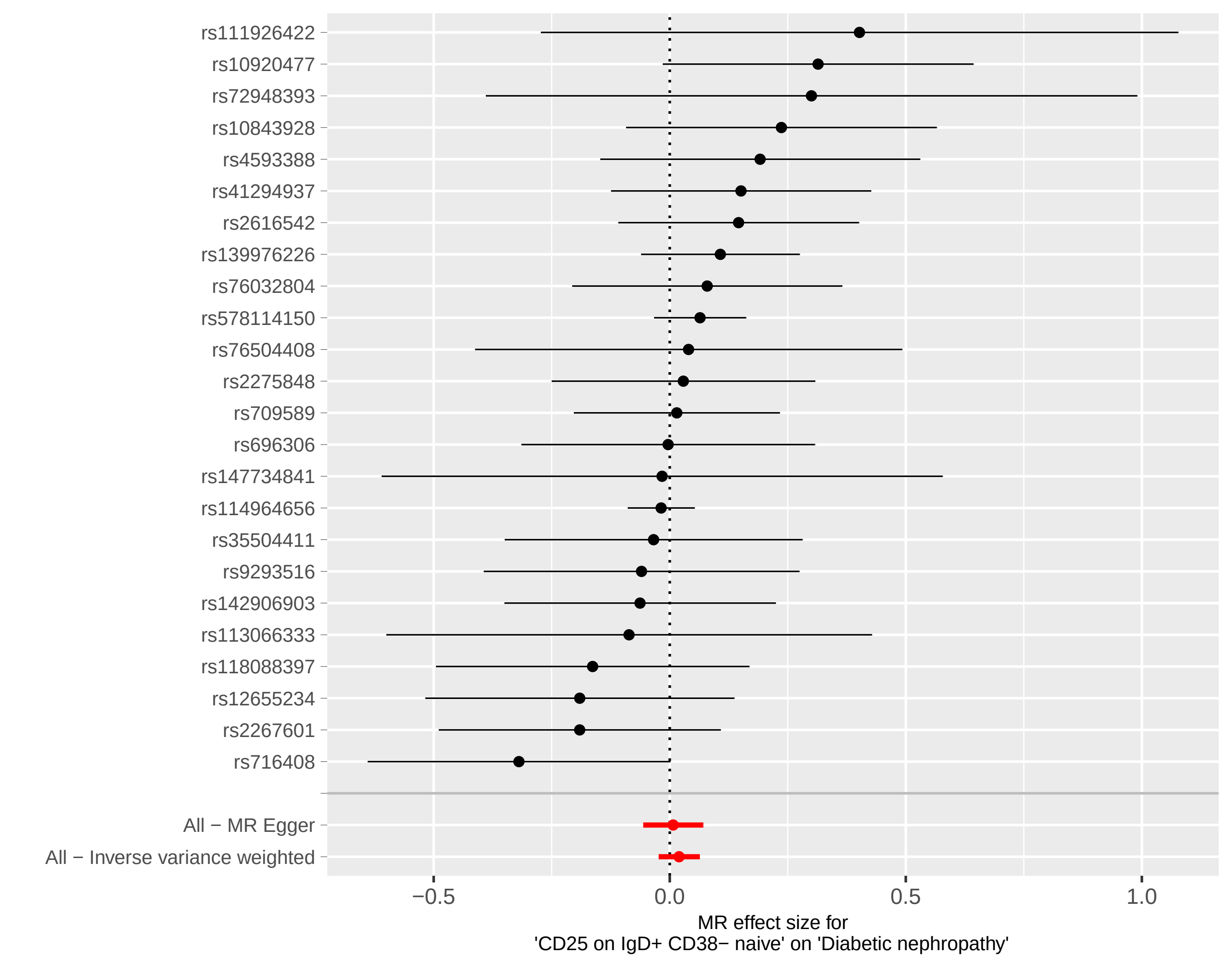

rs111926422
rs10920477
rs72948393
rs10843928
rs4593388
rs41294937
rs2616542
rs139976226
rs76032804
rs578114150
rs76504408
rs2275848
rs709589
rs696306
rs147734841
rs114964656
rs35504411
rs9293516
rs142906903
rs113066333
rs118088397
rs12655234
rs2267601
rs716408
All − MR Egger
All − Inverse variance weighted
−0.5
0.0
0.5
1.0
MR effect size for
'CD25 on IgD+ CD38− naive' on 'Diabetic nephropathy'

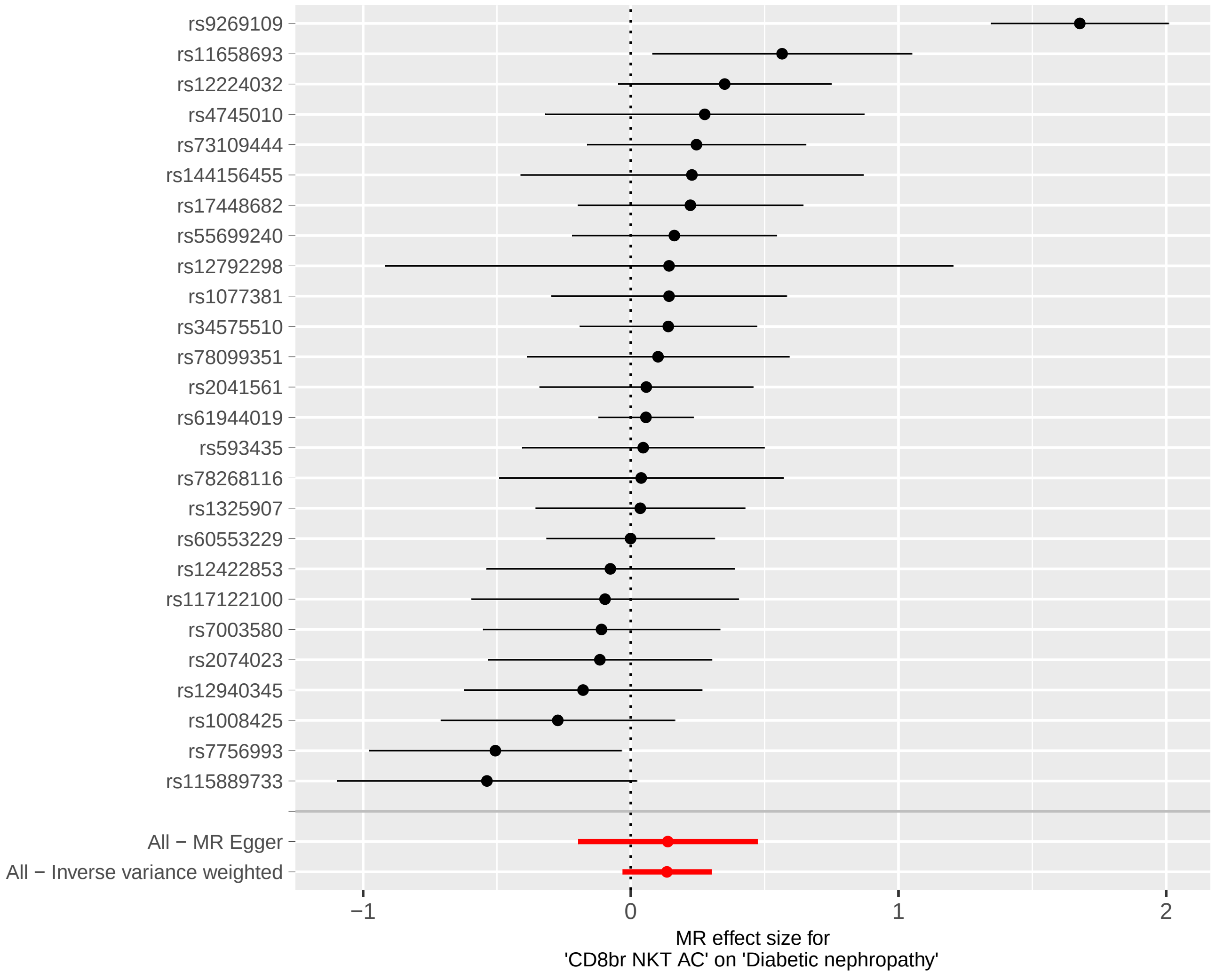
rs9269109
rs11658693
rs12224032
rs4745010
rs73109444
rs144156455
rs17448682
rs55699240
rs12792298
rs1077381
rs34575510
rs78099351
rs2041561
rs61944019
rs593435
rs78268116
rs1325907
rs60553229
rs12422853
rs117122100
rs7003580
rs2074023
rs12940345
rs1008425
rs7756993
rs115889733
All − MR Egger
All − Inverse variance weighted
−1
0
1
2
MR effect size for
'CD8br NKT AC' on 'Diabetic nephropathy'

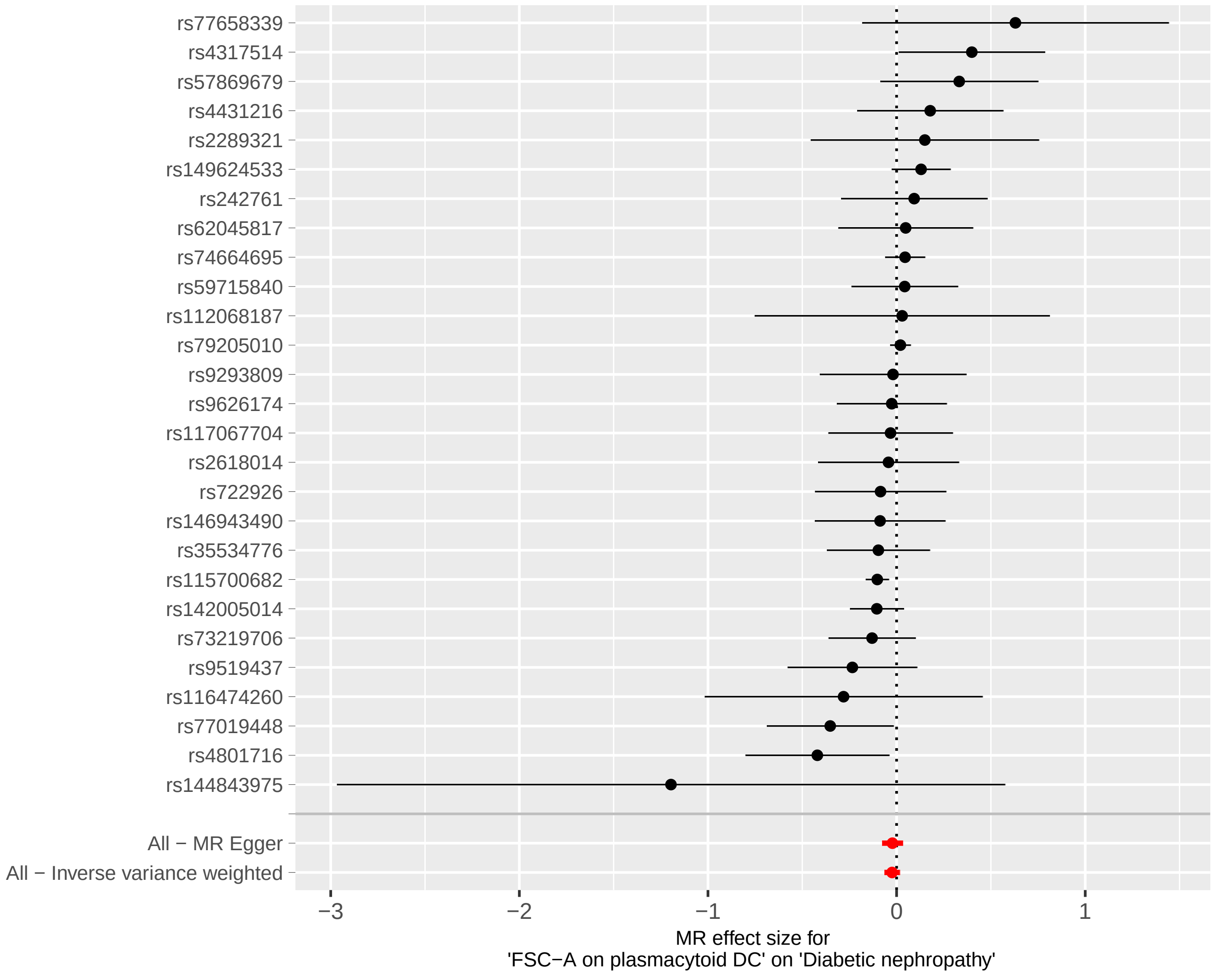
rs77658339
rs4317514
rs57869679
rs4431216
rs2289321
rs149624533
rs242761
rs62045817
rs74664695
rs59715840
rs112068187
rs79205010
rs9293809
rs9626174
rs117067704
rs2618014
rs722926
rs146943490
rs35534776
rs115700682
rs142005014
rs73219706
rs9519437
rs116474260
rs77019448
rs4801716
rs144843975
All − MR Egger
All − Inverse variance weighted
−3
−2
−1
0
1
MR effect size for
'FSC−A on plasmacytoid DC' on 'Diabetic nephropathy'

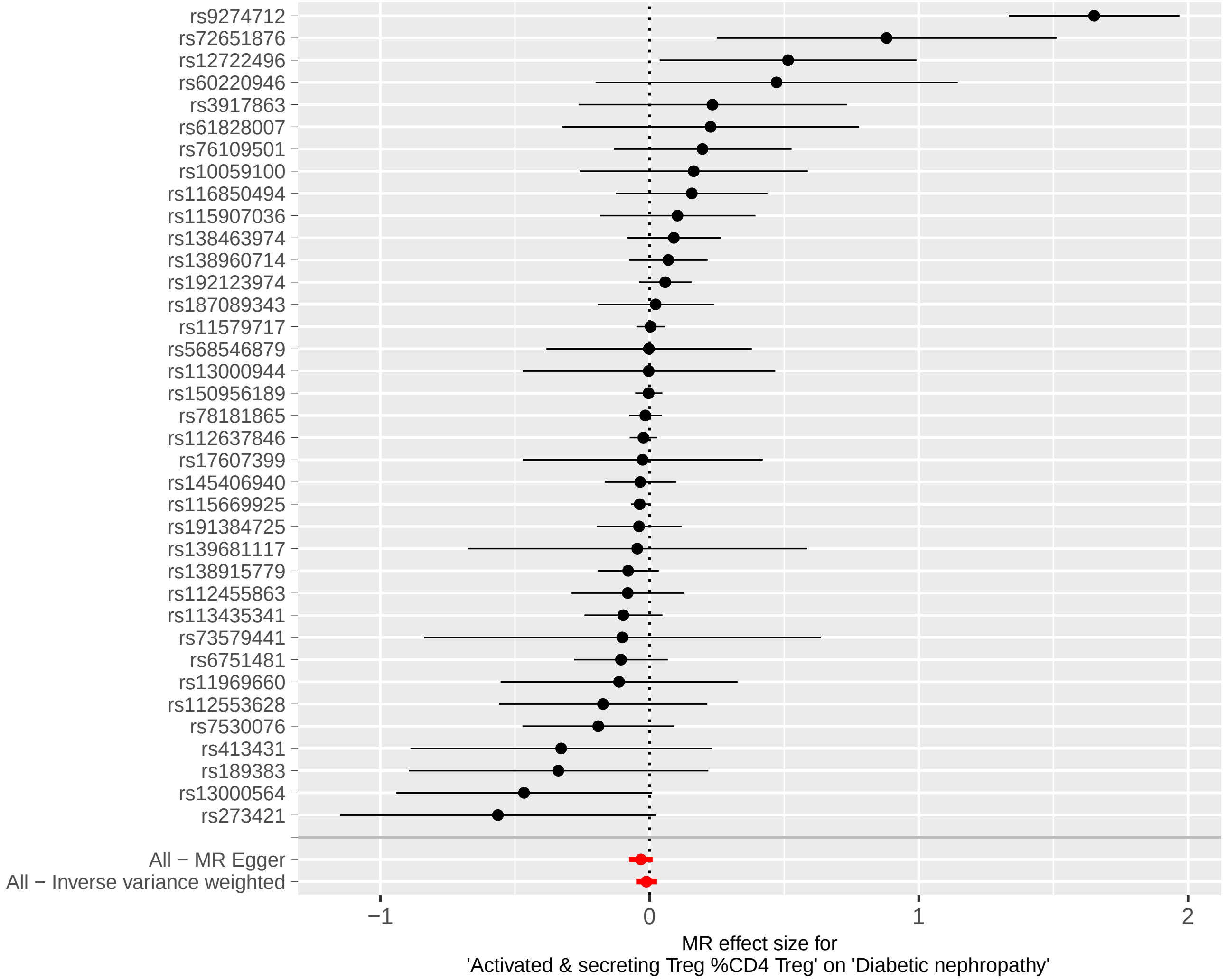
rs9274712
rs72651876
rs12722496
rs60220946
rs3917863
rs61828007
rs76109501
rs10059100
rs116850494
rs115907036
rs138463974
rs138960714
rs192123974
rs187089343
rs11579717
rs568546879
rs113000944
rs150956189
rs78181865
rs112637846
rs17607399
rs145406940
rs115669925
rs191384725
rs139681117
rs138915779
rs112455863
rs113435341
rs73579441
rs6751481
rs11969660
rs112553628
rs7530076
rs413431
rs189383
rs13000564
rs273421
All − MR Egger
All − Inverse variance weighted
−1
0
1
2
MR effect size for
'Activated & secreting Treg %CD4 Treg' on 'Diabetic nephropathy'

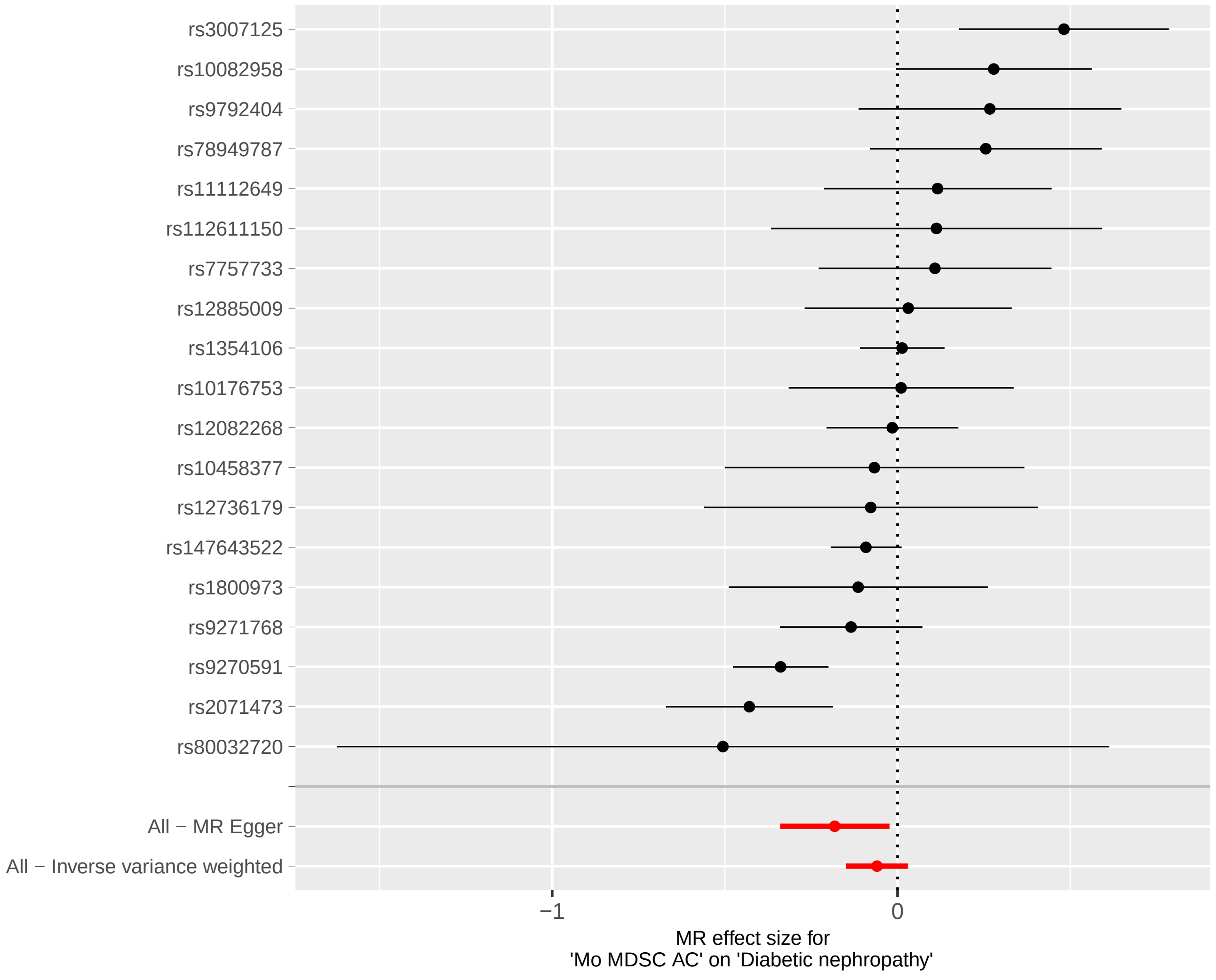

rs3007125
rs10082958
rs9792404
rs78949787
rs11112649
rs112611150
rs7757733
rs12885009
rs1354106
rs10176753
rs12082268
rs10458377
rs12736179
rs147643522
rs1800973
rs9271768
rs9270591
rs2071473
rs80032720
All − MR Egger
All − Inverse variance weighted
−1
0
MR effect size for
'Mo MDSC AC' on 'Diabetic nephropathy'

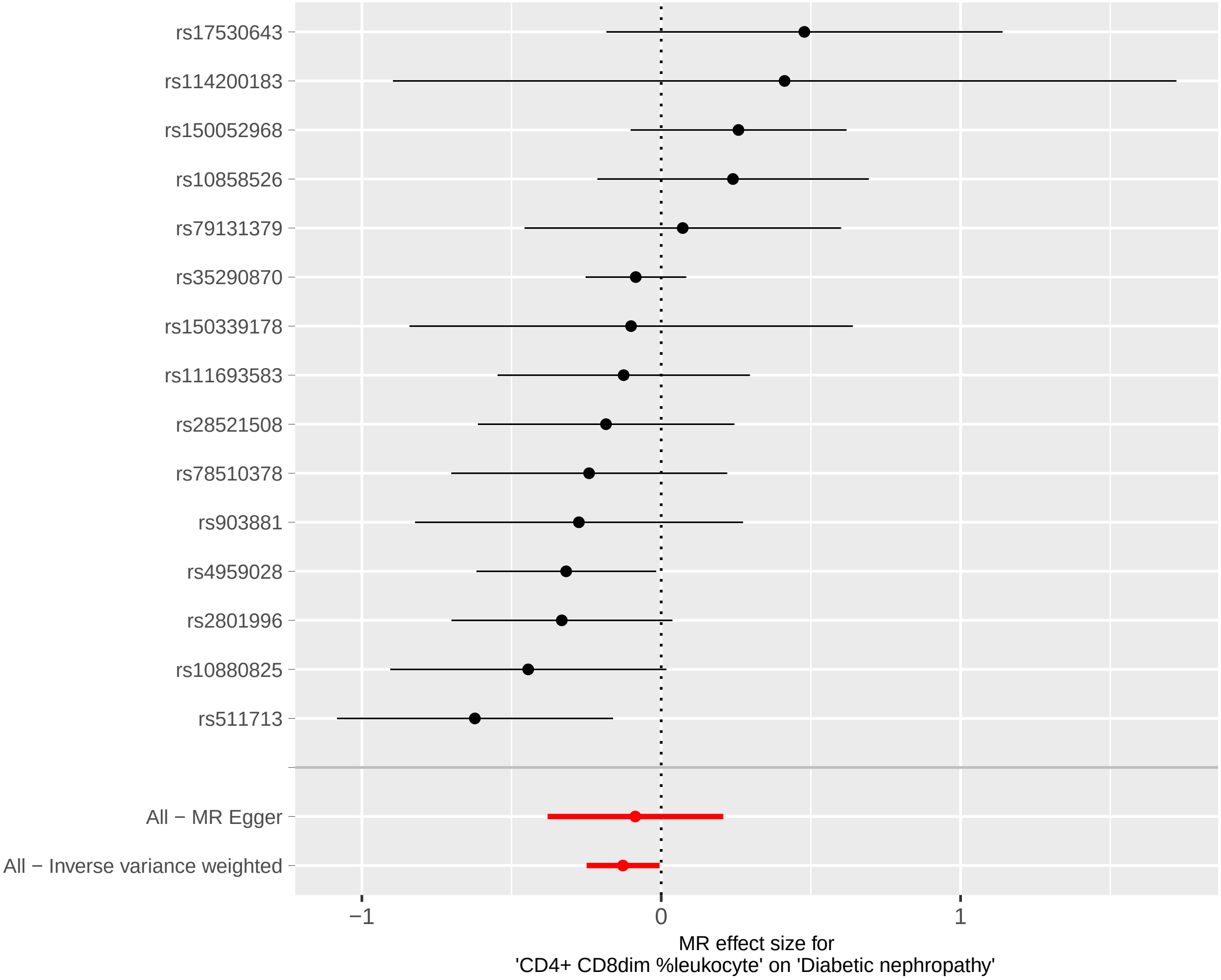
rs17530643
rs114200183
rs150052968
rs10858526
rs79131379
rs35290870
rs150339178
rs111693583
rs28521508
rs78510378
rs903881
rs4959028
rs2801996
rs10880825
rs511713
All − MR Egger
All − Inverse variance weighted
−1
0
1
MR effect size for
'CD4+ CD8dim %leukocyte' on 'Diabetic nephropathy'

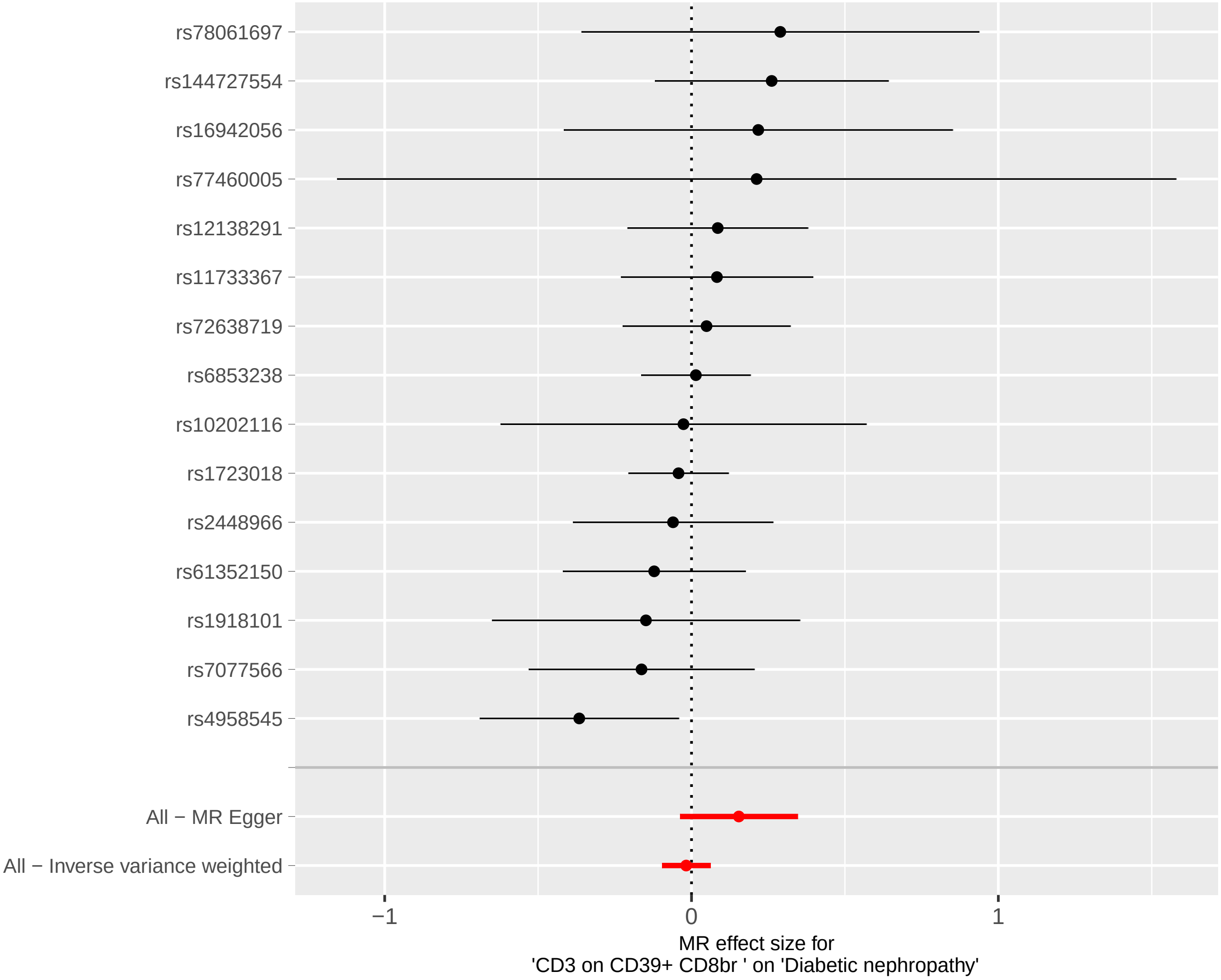
rs78061697
rs144727554
rs16942056
rs77460005
rs12138291
rs11733367
rs72638719
rs6853238
rs10202116
rs1723018
rs2448966
rs61352150
rs1918101
rs7077566
rs4958545
All − MR Egger
All − Inverse variance weighted
−1
0
1
MR effect size for
'CD3 on CD39+ CD8br ' on 'Diabetic nephropathy'

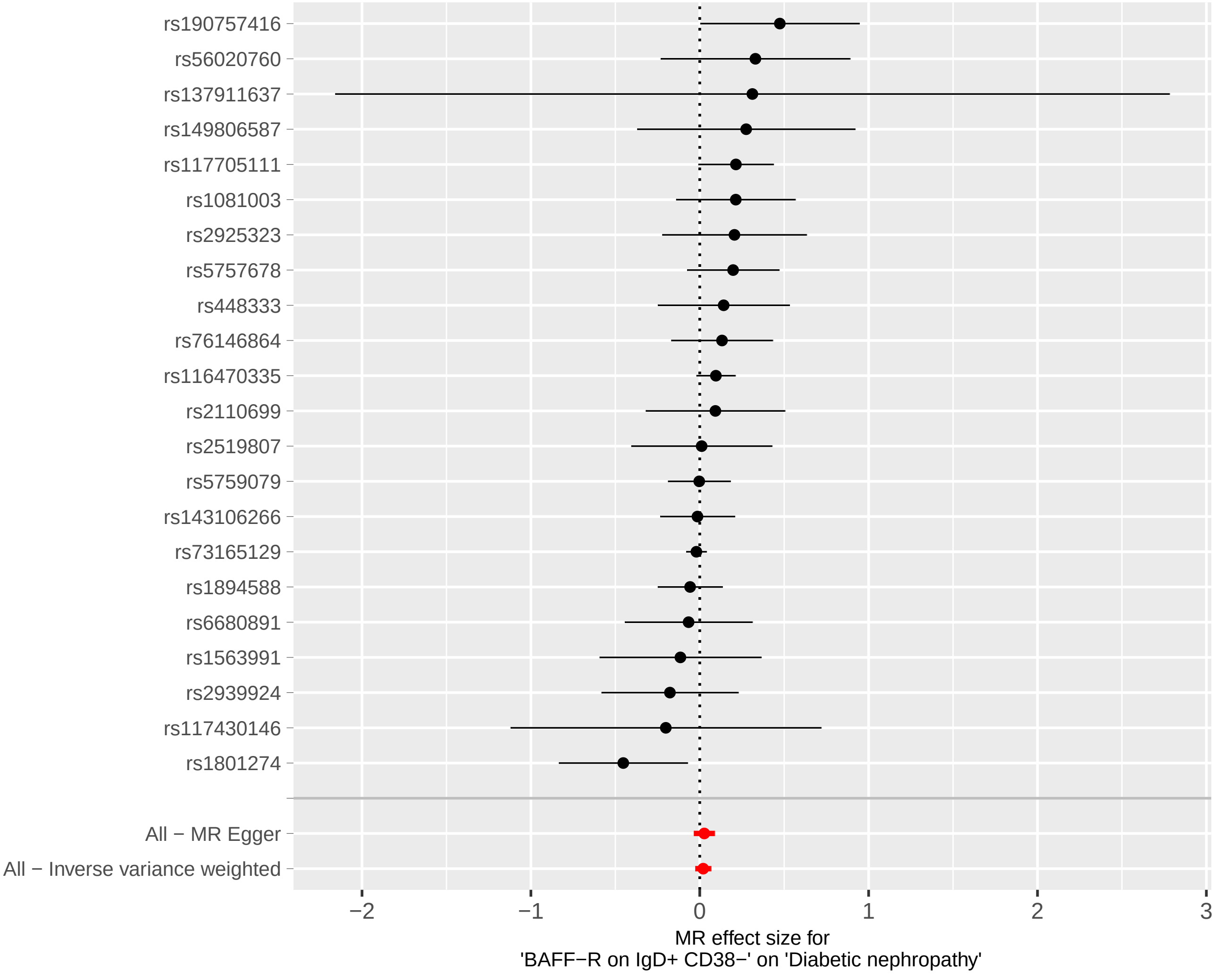

rs190757416
rs56020760
rs137911637
rs149806587
rs117705111
rs1081003
rs2925323
rs5757678
rs448333
rs76146864
rs116470335
rs2110699
rs2519807
rs5759079
rs143106266
rs73165129
rs1894588
rs6680891
rs1563991
rs2939924
rs117430146
rs1801274
All − MR Egger
All − Inverse variance weighted
−2
−1
0
1
2
3
MR effect size for
'BAFF−R on IgD+ CD38−' on 'Diabetic nephropathy'

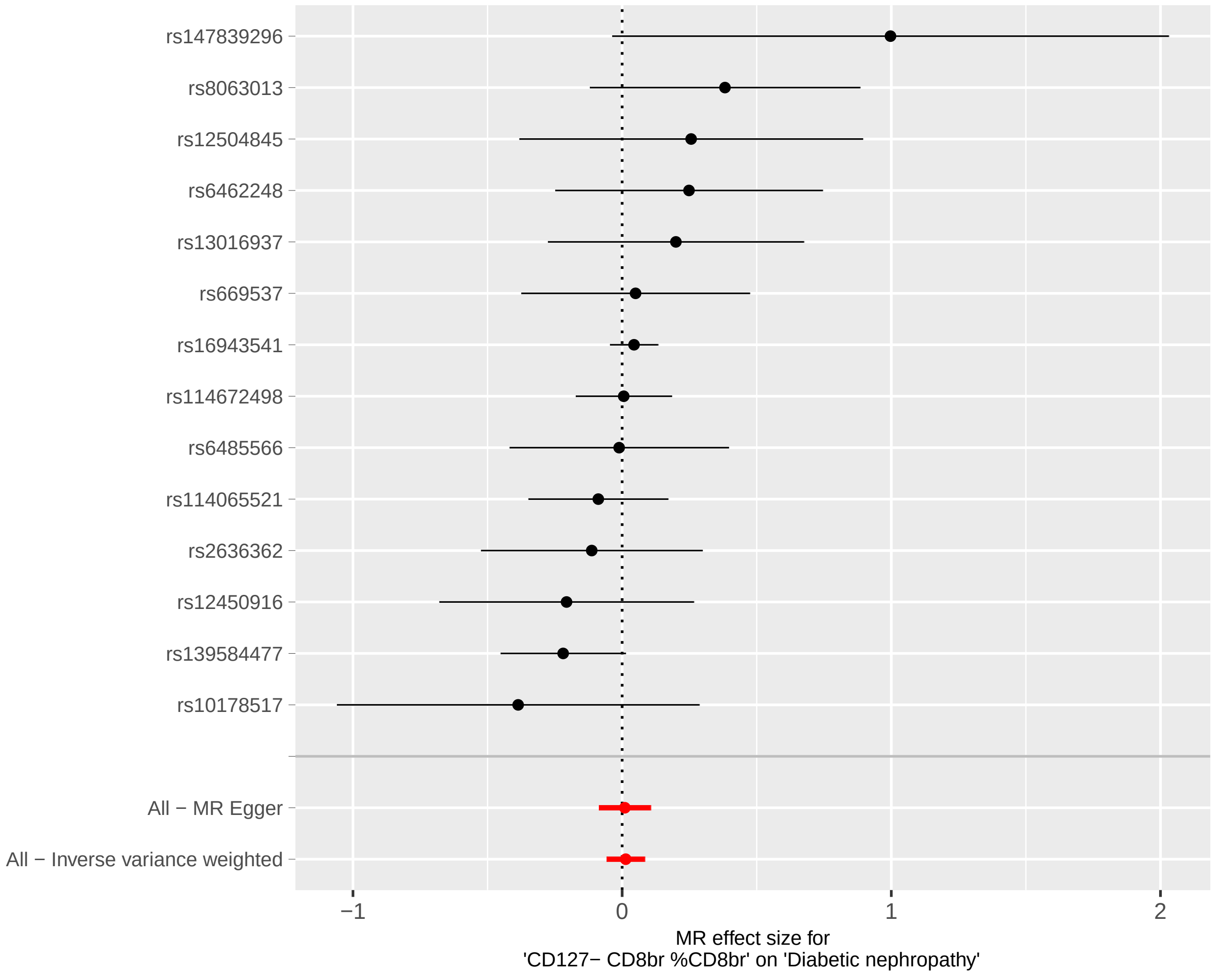
rs147839296
rs8063013
rs12504845
rs6462248
rs13016937
rs669537
rs16943541
rs114672498
rs6485566
rs114065521
rs2636362
rs12450916
rs139584477
rs10178517
All − MR Egger
All − Inverse variance weighted
−1
0
1
2
MR effect size for
'CD127− CD8br %CD8br' on 'Diabetic nephropathy'

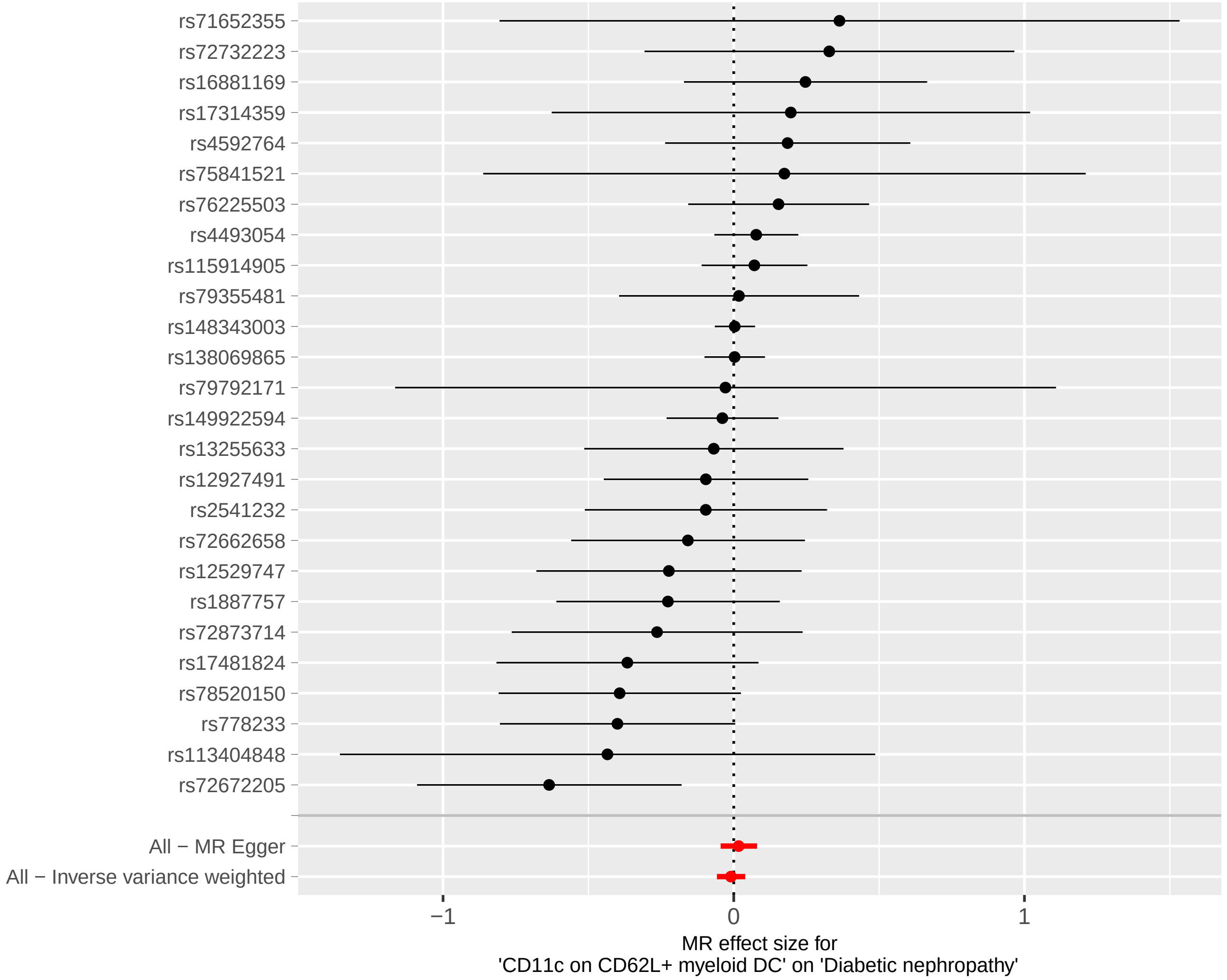

rs71652355
rs72732223
rs16881169
rs17314359
rs4592764
rs75841521
rs76225503
rs4493054
rs115914905
rs79355481
rs148343003
rs138069865
rs79792171
rs149922594
rs13255633
rs12927491
rs2541232
rs72662658
rs12529747
rs1887757
rs72873714
rs17481824
rs78520150
rs778233
rs113404848
rs72672205
All − MR Egger
All − Inverse variance weighted
−1
0
1
MR effect size for
'CD11c on CD62L+ myeloid DC' on 'Diabetic nephropathy'

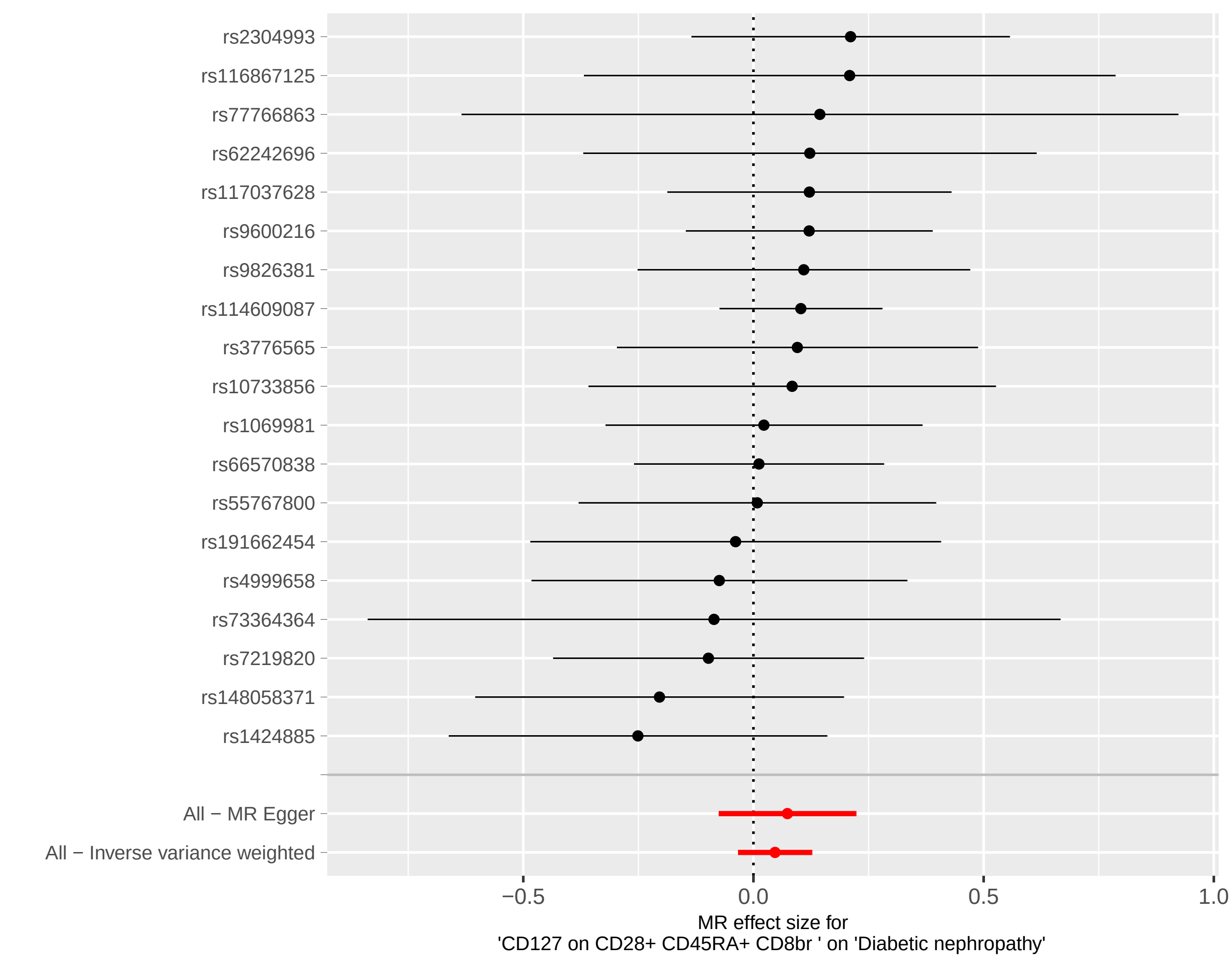
rs2304993
rs116867125
rs77766863
rs62242696
rs117037628
rs9600216
rs9826381
rs114609087
rs3776565
rs10733856
rs1069981
rs66570838
rs55767800
rs191662454
rs4999658
rs73364364
rs7219820
rs148058371
rs1424885
All − MR Egger
All − Inverse variance weighted
−0.5
0.0
0.5
1.0
MR effect size for
'CD127 on CD28+ CD45RA+ CD8br ' on 'Diabetic nephropathy'

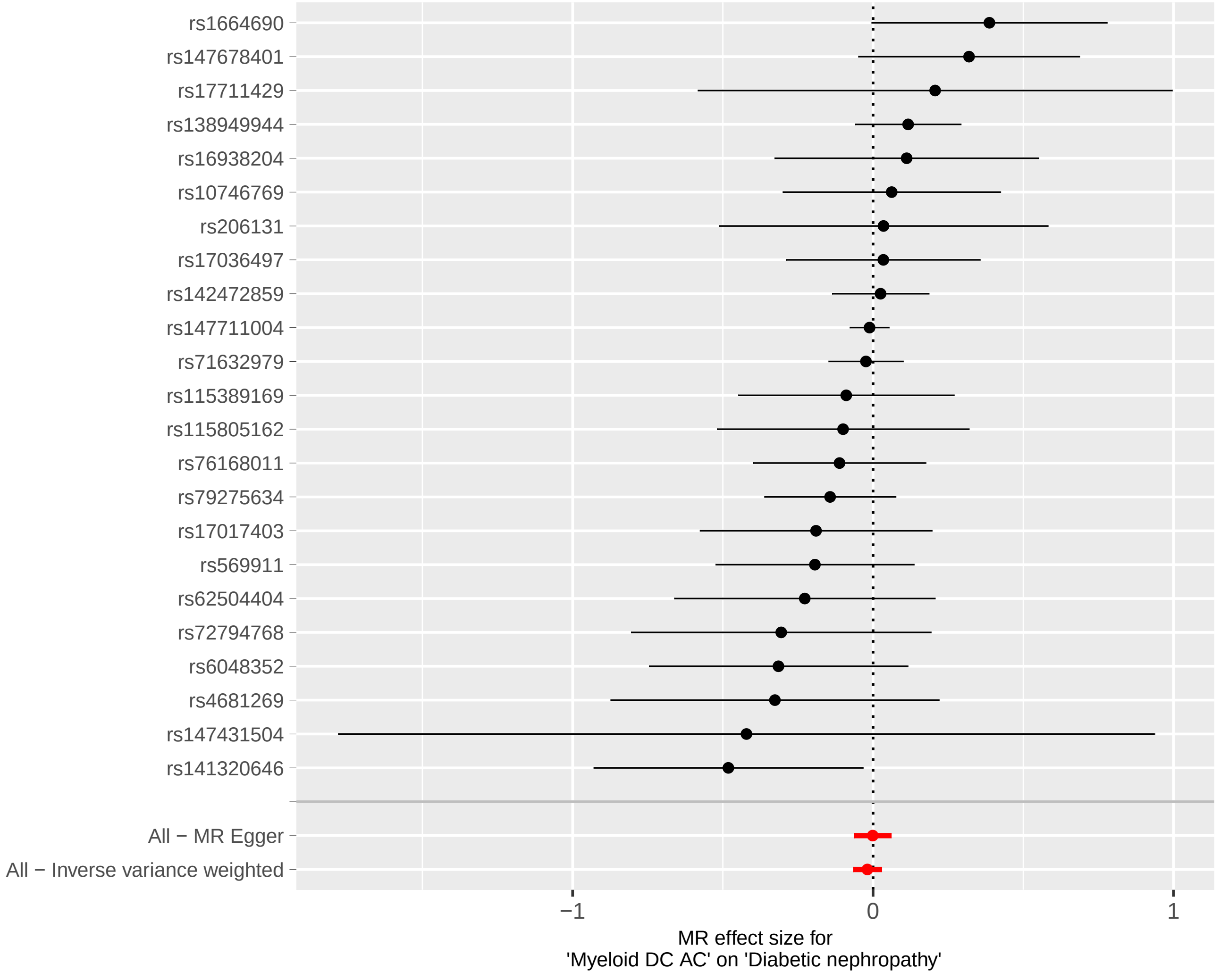
rs1664690
rs147678401
rs17711429
rs138949944
rs16938204
rs10746769
rs206131
rs17036497
rs142472859
rs147711004
rs71632979
rs115389169
rs115805162
rs76168011
rs79275634
rs17017403
rs569911
rs62504404
rs72794768
rs6048352
rs4681269
rs147431504
rs141320646
All − MR Egger
All − Inverse variance weighted
−1
0
1
MR effect size for
'Myeloid DC AC' on 'Diabetic nephropathy'

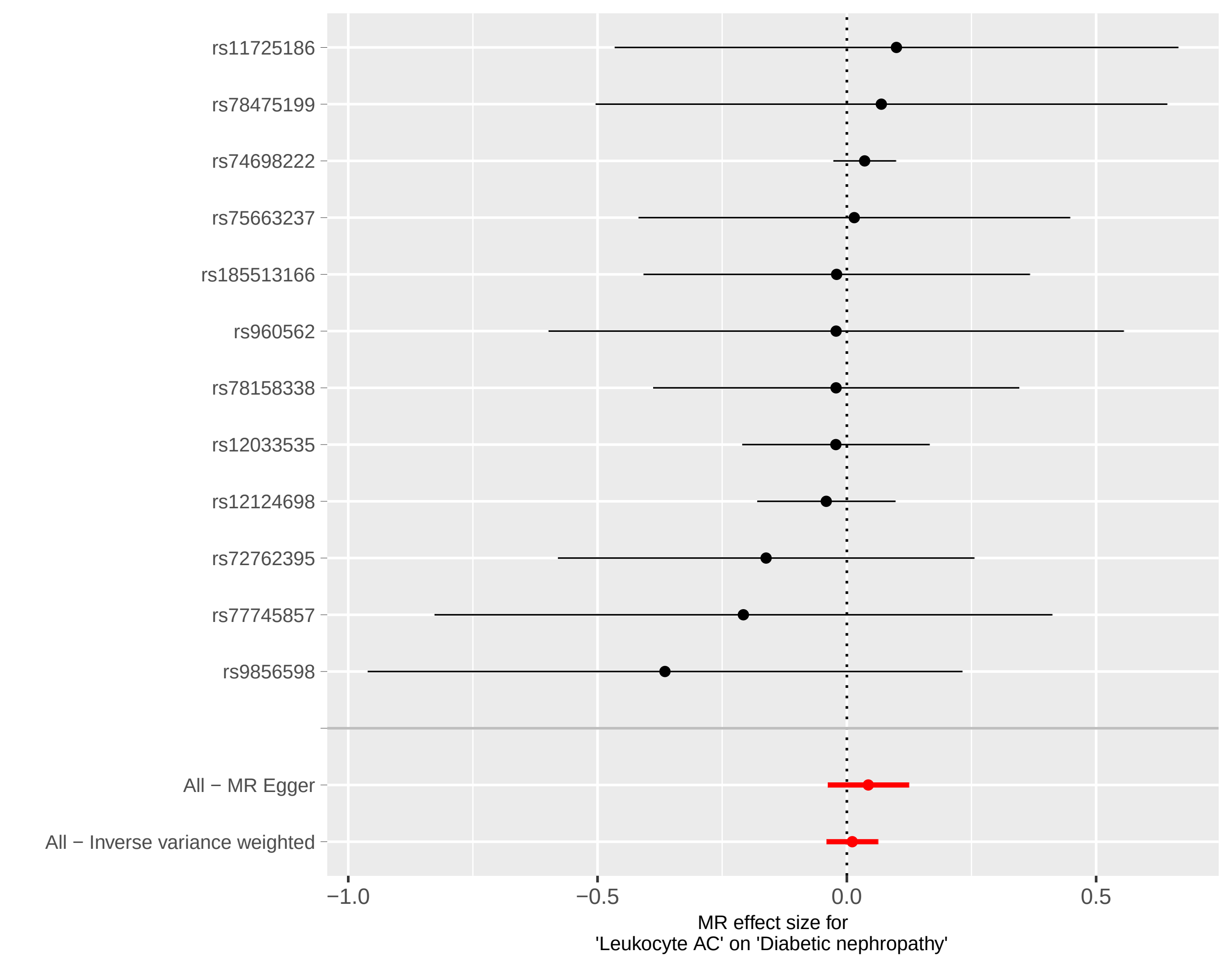

rs11725186
rs78475199
rs74698222
rs75663237
rs185513166
rs960562
rs78158338
rs12033535
rs12124698
rs72762395
rs77745857
rs9856598
All − MR Egger
All − Inverse variance weighted
−1.0
−0.5
0.0
0.5
MR effect size for
'Leukocyte AC' on 'Diabetic nephropathy'

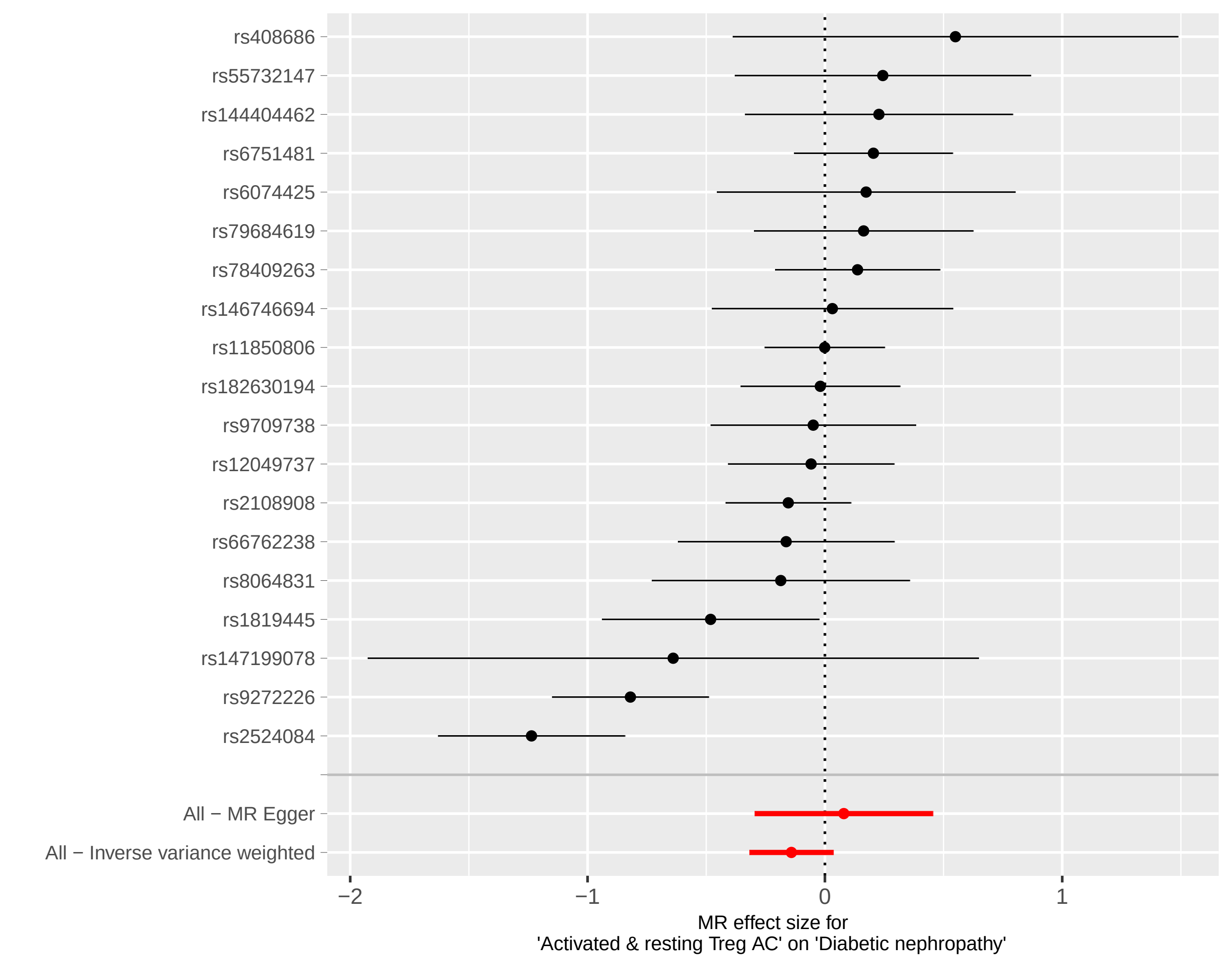
rs408686
rs55732147
rs144404462
rs6751481
rs6074425
rs79684619
rs78409263
rs146746694
rs11850806
rs182630194
rs9709738
rs12049737
rs2108908
rs66762238
rs8064831
rs1819445
rs147199078
rs9272226
rs2524084
All − MR Egger
All − Inverse variance weighted
−2
−1
0
1
MR effect size for
'Activated & resting Treg AC' on 'Diabetic nephropathy'

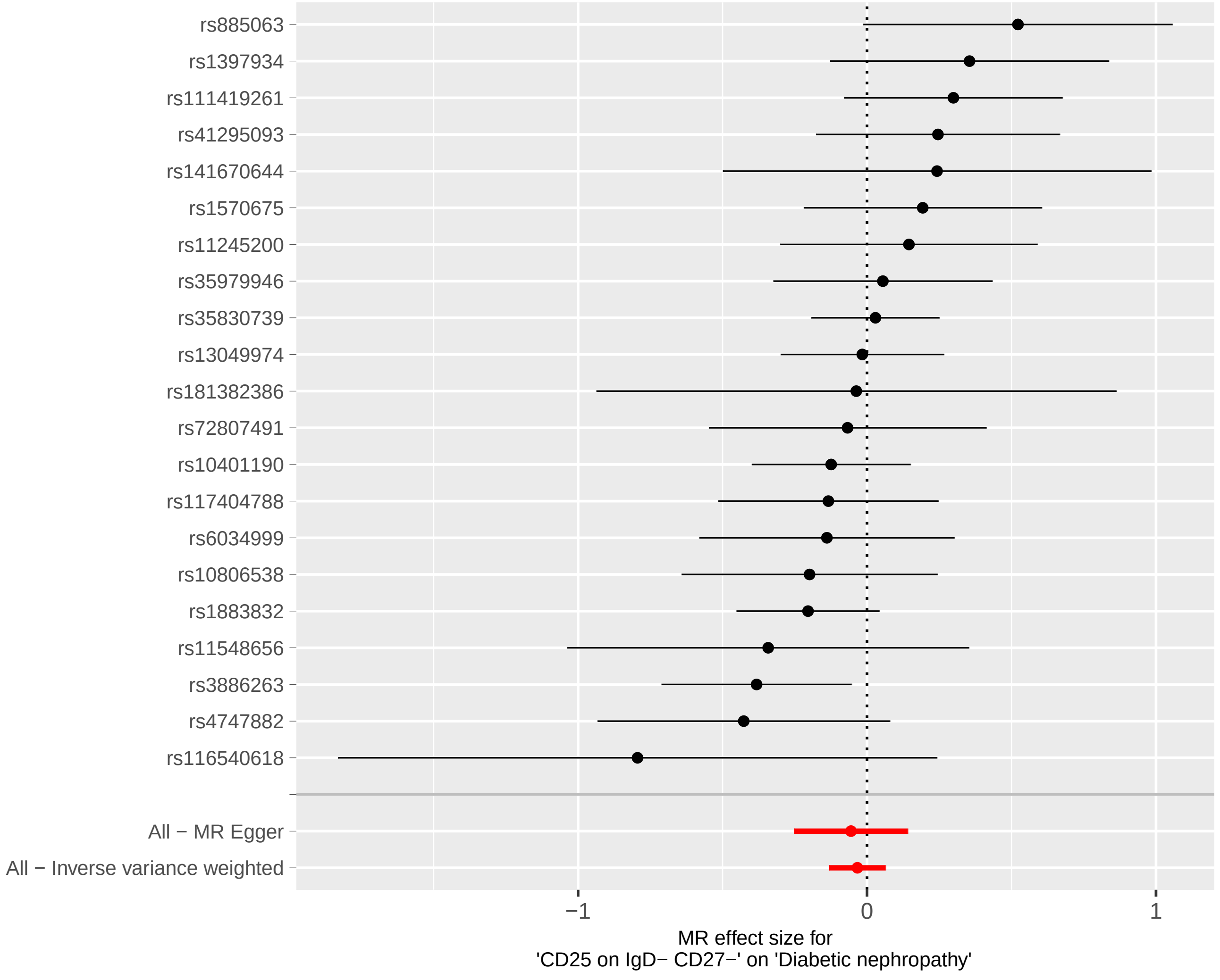
rs885063
rs1397934
rs111419261
rs41295093
rs141670644
rs1570675
rs11245200
rs35979946
rs35830739
rs13049974
rs181382386
rs72807491
rs10401190
rs117404788
rs6034999
rs10806538
rs1883832
rs11548656
rs3886263
rs4747882
rs116540618
All − MR Egger
All − Inverse variance weighted
−1
0
1
MR effect size for
'CD25 on IgD− CD27−' on 'Diabetic nephropathy'

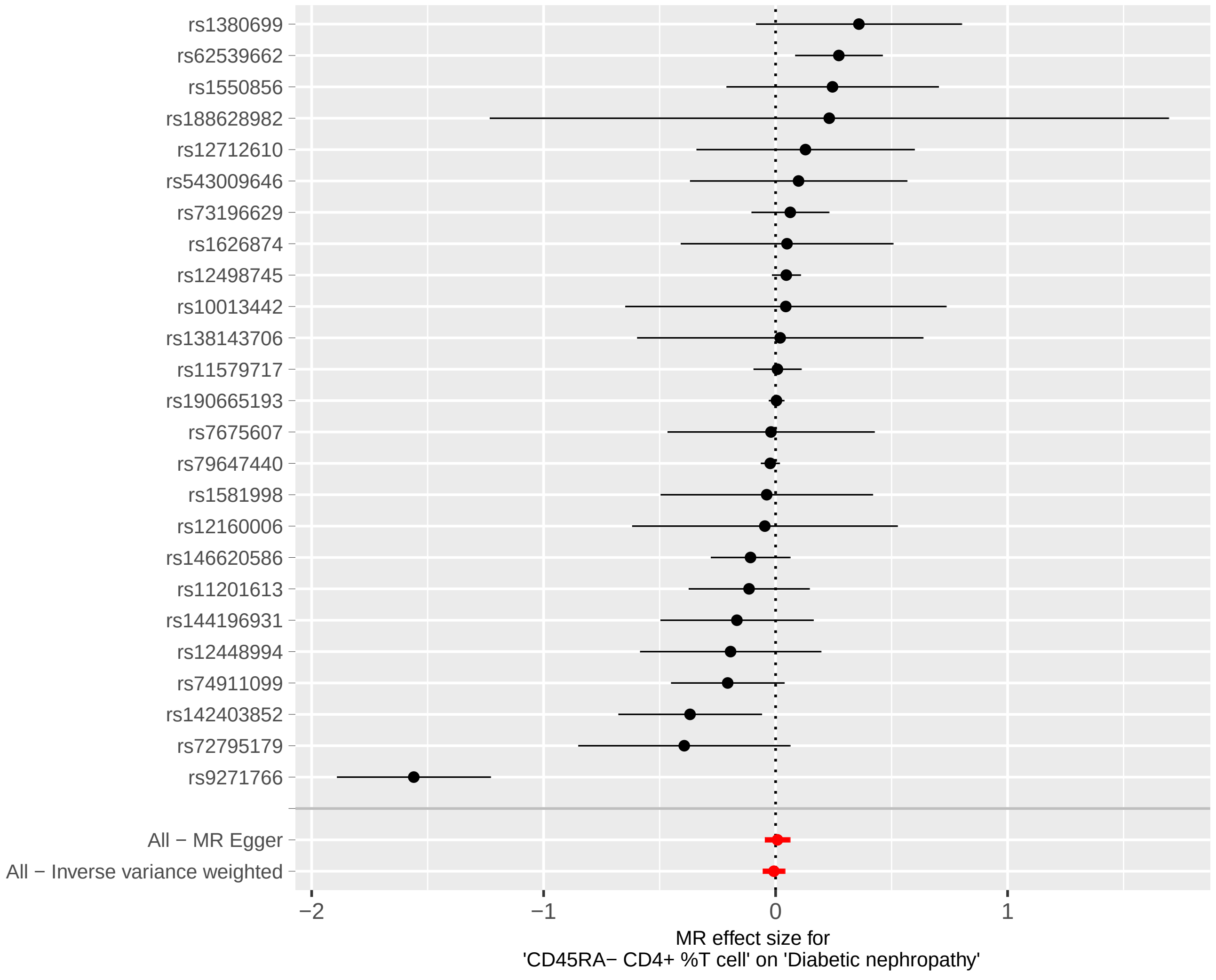

rs1380699
rs62539662
rs1550856
rs188628982
rs12712610
rs543009646
rs73196629
rs1626874
rs12498745
rs10013442
rs138143706
rs11579717
rs190665193
rs7675607
rs79647440
rs1581998
rs12160006
rs146620586
rs11201613
rs144196931
rs12448994
rs74911099
rs142403852
rs72795179
rs9271766
All − MR Egger
All − Inverse variance weighted
−2
−1
0
1
MR effect size for
'CD45RA− CD4+ %T cell' on 'Diabetic nephropathy'

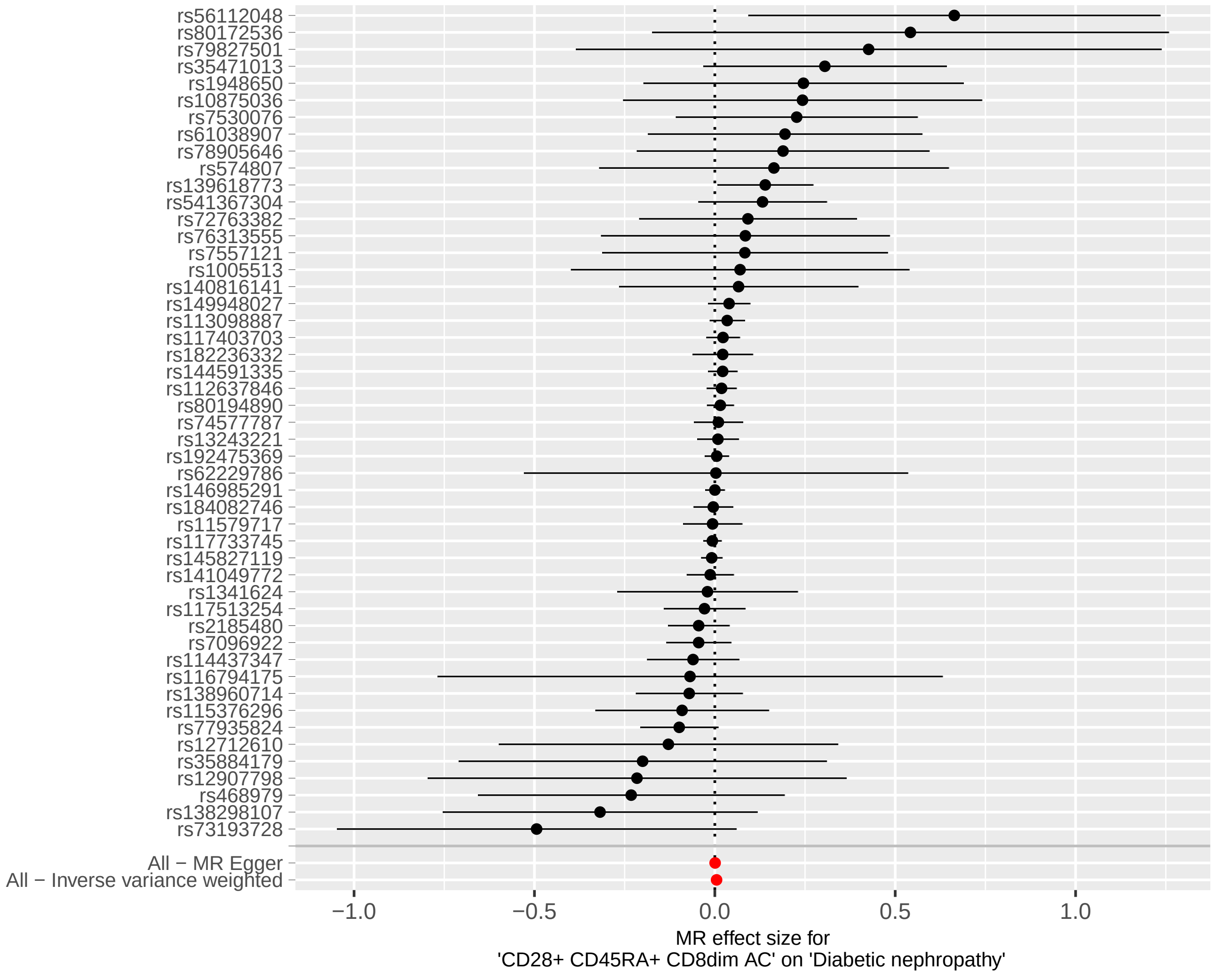
rs56112048
rs80172536
rs79827501
rs35471013
rs1948650
rs10875036
rs7530076
rs61038907
rs78905646
rs574807
rs139618773
rs541367304
rs72763382
rs76313555
rs7557121
rs1005513
rs140816141
rs149948027
rs113098887
rs117403703
rs182236332
rs144591335
rs112637846
rs80194890
rs74577787
rs13243221
rs192475369
rs62229786
rs146985291
rs184082746
rs11579717
rs117733745
rs145827119
rs141049772
rs1341624
rs117513254
rs2185480
rs7096922
rs114437347
rs116794175
rs138960714
rs115376296
rs77935824
rs12712610
rs35884179
rs12907798
rs468979
rs138298107
rs73193728
All − MR Egger
All − Inverse variance weighted
−1.0
−0.5
0.0
0.5
1.0
MR effect size for
'CD28+ CD45RA+ CD8dim AC' on 'Diabetic nephropathy'

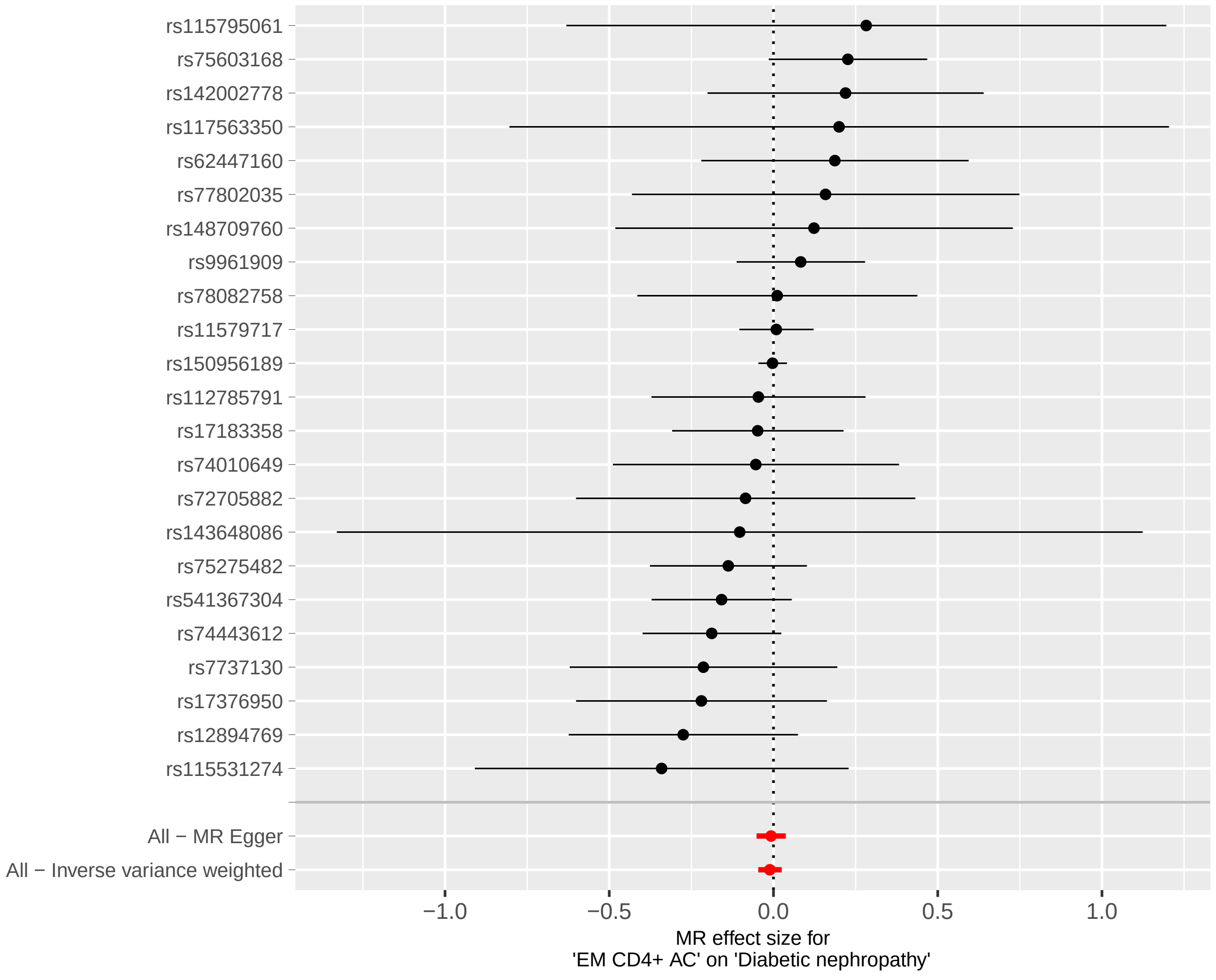
rs115795061
rs75603168
rs142002778
rs117563350
rs62447160
rs77802035
rs148709760
rs9961909
rs78082758
rs11579717
rs150956189
rs112785791
rs17183358
rs74010649
rs72705882
rs143648086
rs75275482
rs541367304
rs74443612
rs7737130
rs17376950
rs12894769
rs115531274
All − MR Egger
All − Inverse variance weighted
−1.0
−0.5
0.0
0.5
1.0
MR effect size for
'EM CD4+ AC' on 'Diabetic nephropathy'

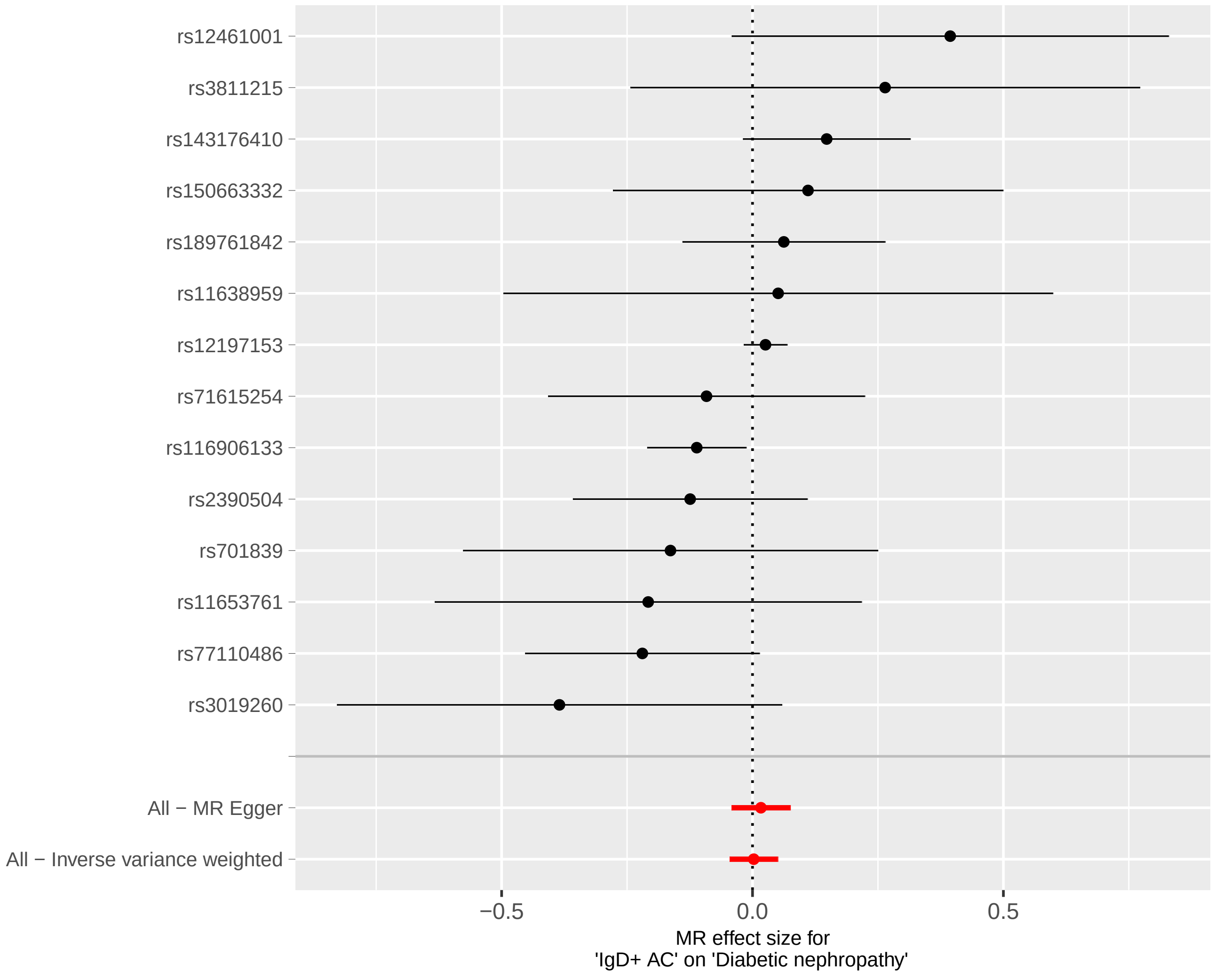

rs12461001
rs3811215
rs143176410
rs150663332
rs189761842
rs11638959
rs12197153
rs71615254
rs116906133
rs2390504
rs701839
rs11653761
rs77110486
rs3019260
All − MR Egger
All − Inverse variance weighted
−0.5
0.0
0.5
MR effect size for
'IgD+ AC' on 'Diabetic nephropathy'

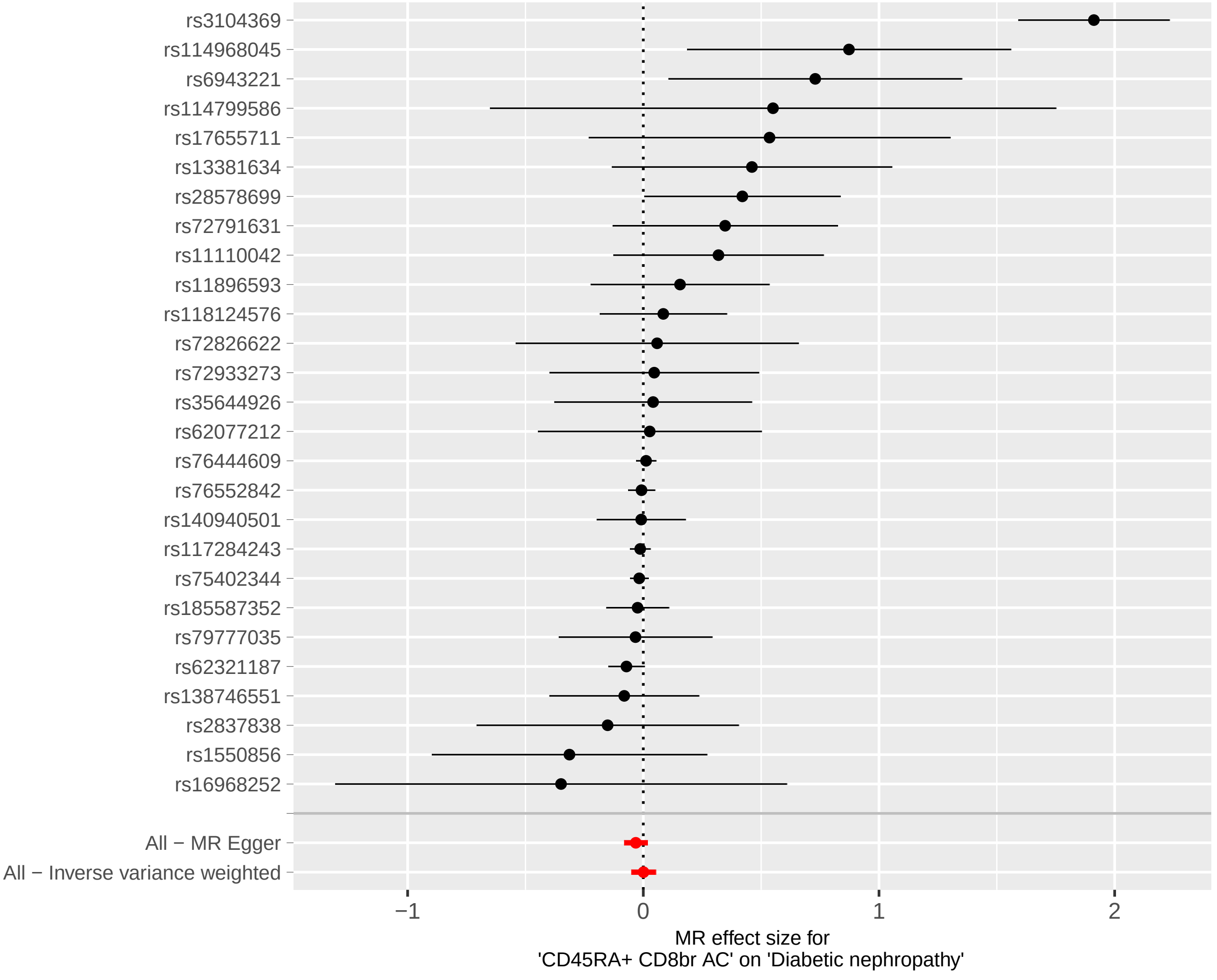
rs3104369
rs114968045
rs6943221
rs114799586
rs17655711
rs13381634
rs28578699
rs72791631
rs11110042
rs11896593
rs118124576
rs72826622
rs72933273
rs35644926
rs62077212
rs76444609
rs76552842
rs140940501
rs117284243
rs75402344
rs185587352
rs79777035
rs62321187
rs138746551
rs2837838
rs1550856
rs16968252
All − MR Egger
All − Inverse variance weighted
−1
0
1
2
MR effect size for
'CD45RA+ CD8br AC' on 'Diabetic nephropathy'

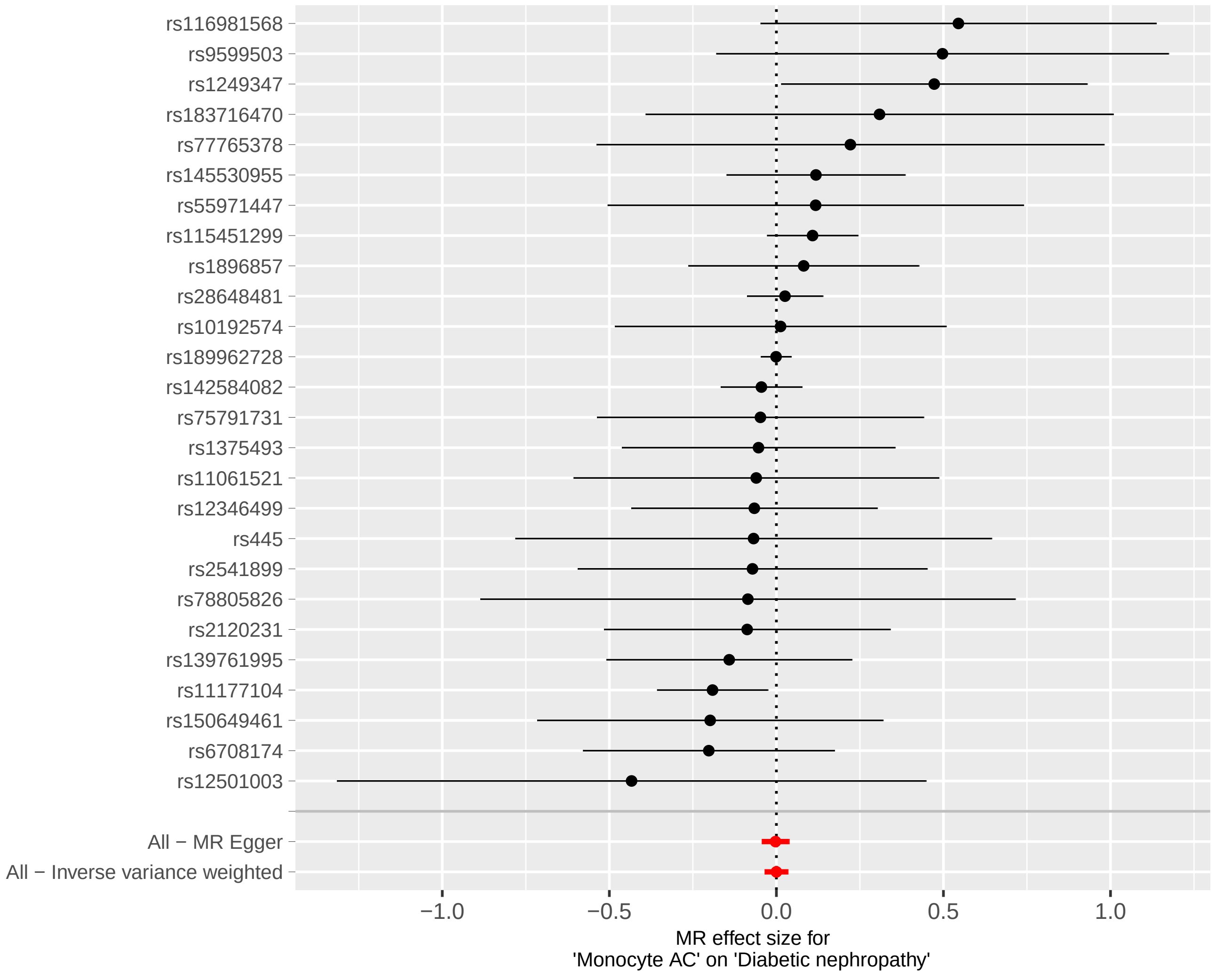
rs116981568
rs9599503
rs1249347
rs183716470
rs77765378
rs145530955
rs55971447
rs115451299
rs1896857
rs28648481
rs10192574
rs189962728
rs142584082
rs75791731
rs1375493
rs11061521
rs12346499
rs445
rs2541899
rs78805826
rs2120231
rs139761995
rs11177104
rs150649461
rs6708174
rs12501003
All − MR Egger
All − Inverse variance weighted
−1.0
−0.5
0.0
0.5
1.0
MR effect size for
'Monocyte AC' on 'Diabetic nephropathy'

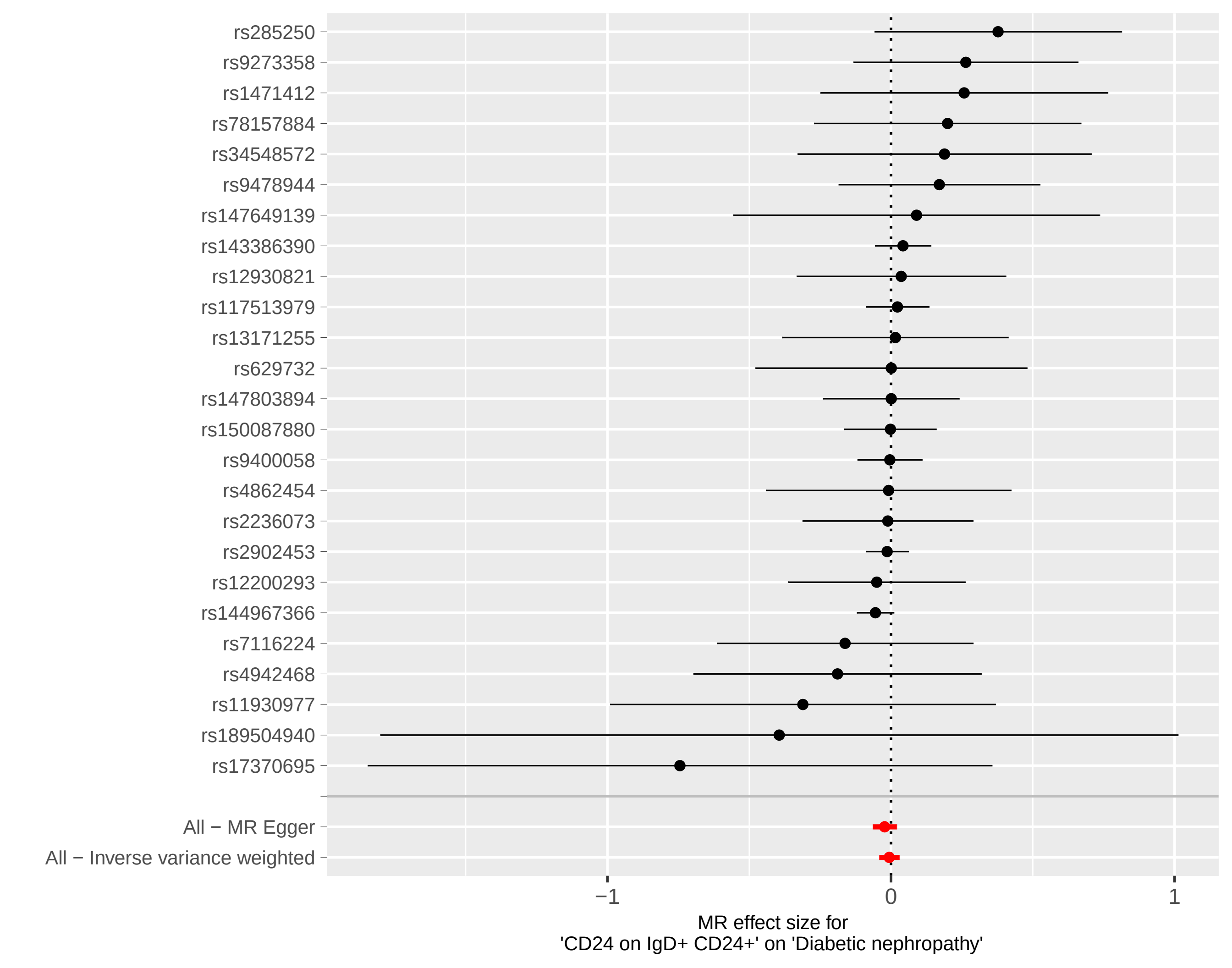
rs285250
rs9273358
rs1471412
rs78157884
rs34548572
rs9478944
rs147649139
rs143386390
rs12930821
rs117513979
rs13171255
rs629732
rs147803894
rs150087880
rs9400058
rs4862454
rs2236073
rs2902453
rs12200293
rs144967366
rs7116224
rs4942468
rs11930977
rs189504940
rs17370695
All − MR Egger
All − Inverse variance weighted
−1
0
1
MR effect size for
'CD24 on IgD+ CD24+' on 'Diabetic nephropathy'

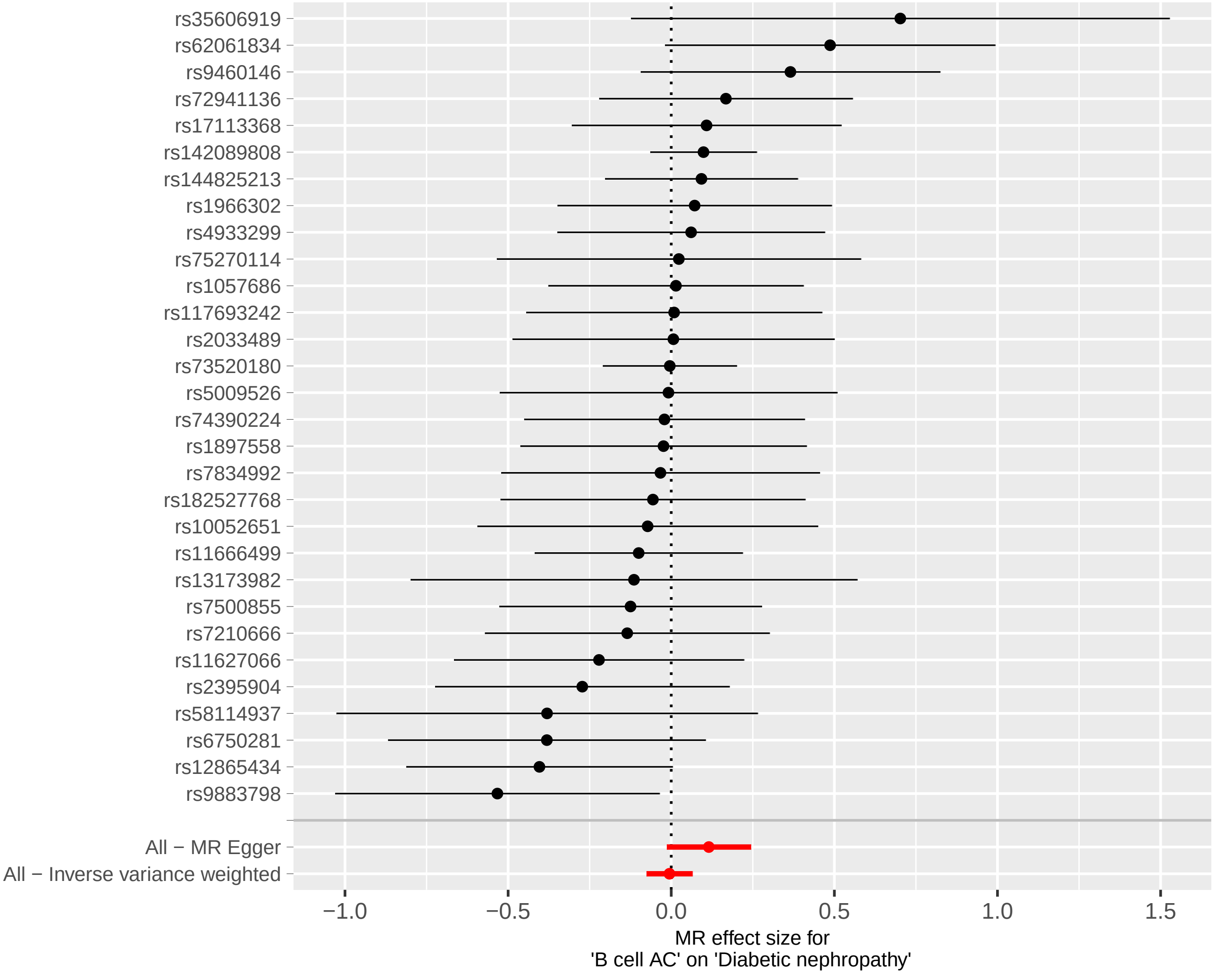
rs35606919
rs62061834
rs9460146
rs72941136
rs17113368
rs142089808
rs144825213
rs1966302
rs4933299
rs75270114
rs1057686
rs117693242
rs2033489
rs73520180
rs5009526
rs74390224
rs1897558
rs7834992
rs182527768
rs10052651
rs11666499
rs13173982
rs7500855
rs7210666
rs11627066
rs2395904
rs58114937
rs6750281
rs12865434
rs9883798
All − MR Egger
All − Inverse variance weighted
−1.0
−0.5
0.0
0.5
1.0
1.5
MR effect size for
'B cell AC' on 'Diabetic nephropathy'

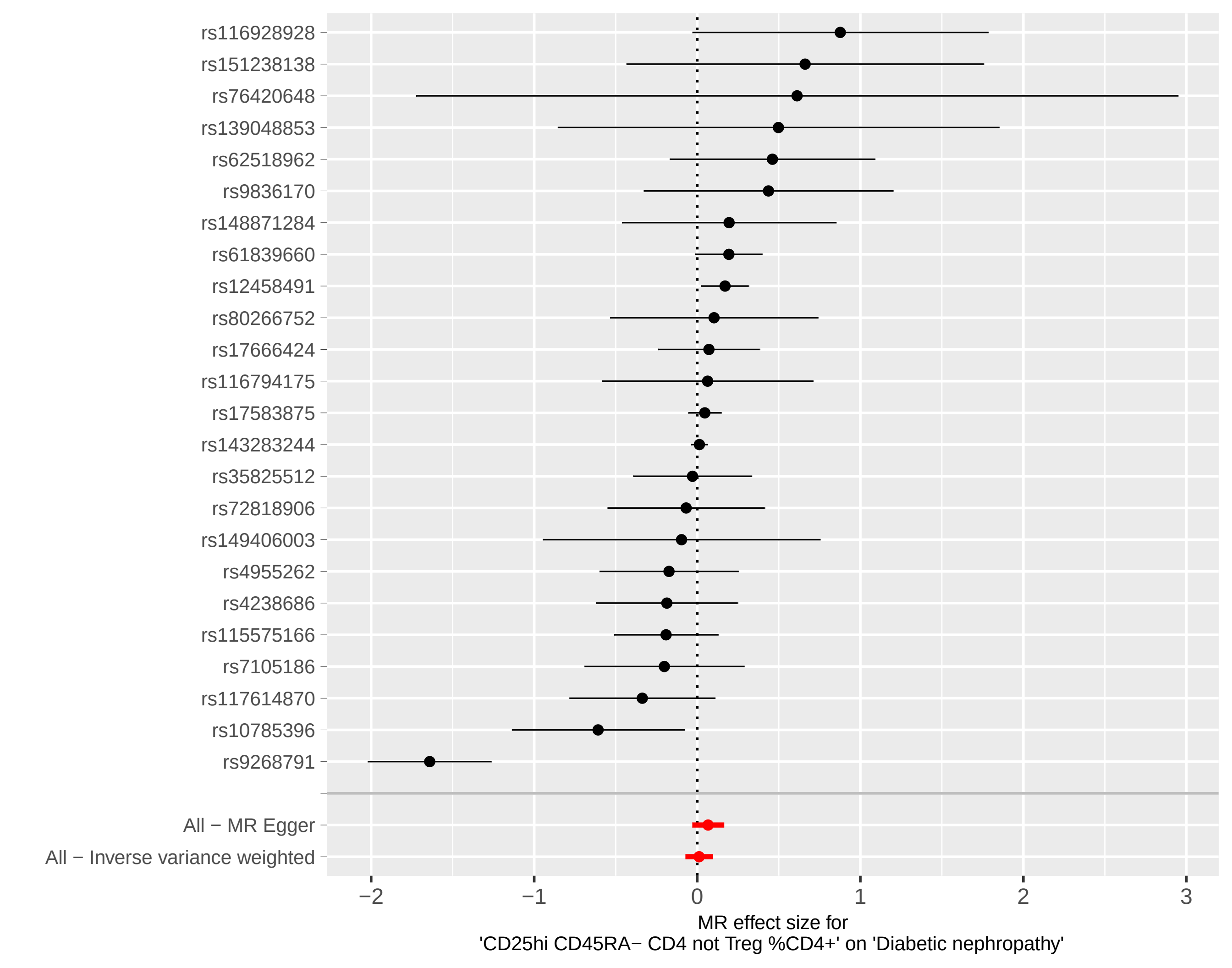
rs116928928
rs151238138
rs76420648
rs139048853
rs62518962
rs9836170
rs148871284
rs61839660
rs12458491
rs80266752
rs17666424
rs116794175
rs17583875
rs143283244
rs35825512
rs72818906
rs149406003
rs4955262
rs4238686
rs115575166
rs7105186
rs117614870
rs10785396
rs9268791
All − MR Egger
All − Inverse variance weighted
−2
−1
0
1
2
3
MR effect size for
'CD25hi CD45RA− CD4 not Treg %CD4+' on 'Diabetic nephropathy'

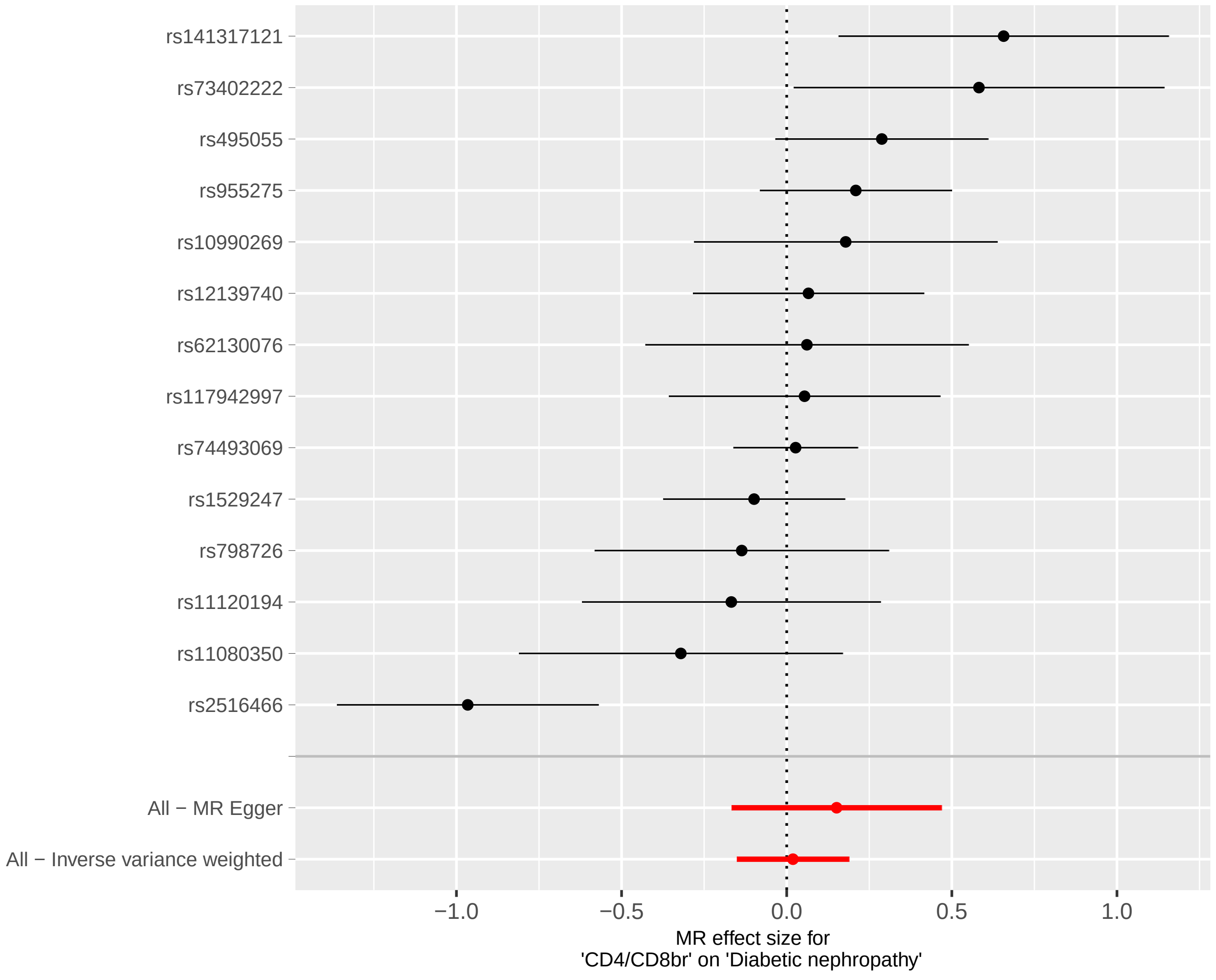

rs141317121
rs73402222
rs495055
rs955275
rs10990269
rs12139740
rs62130076
rs117942997
rs74493069
rs1529247
rs798726
rs11120194
rs11080350
rs2516466
All − MR Egger
All − Inverse variance weighted
−1.0
−0.5
0.0
0.5
1.0
MR effect size for
'CD4/CD8br' on 'Diabetic nephropathy'

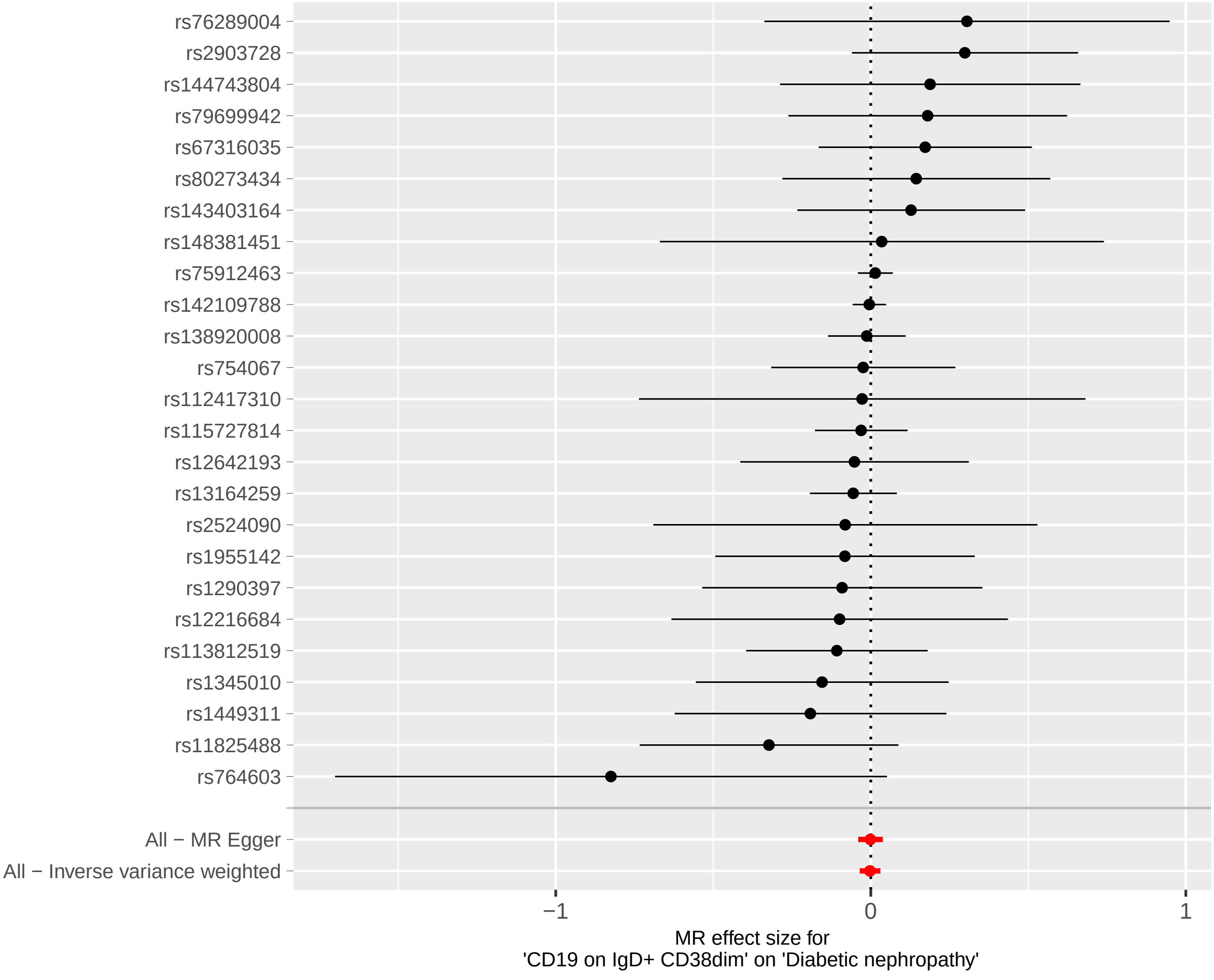
rs76289004
rs2903728
rs144743804
rs79699942
rs67316035
rs80273434
rs143403164
rs148381451
rs75912463
rs142109788
rs138920008
rs754067
rs112417310
rs115727814
rs12642193
rs13164259
rs2524090
rs1955142
rs1290397
rs12216684
rs113812519
rs1345010
rs1449311
rs11825488
rs764603
All − MR Egger
All − Inverse variance weighted
−1
0
1
MR effect size for
'CD19 on IgD+ CD38dim' on 'Diabetic nephropathy'

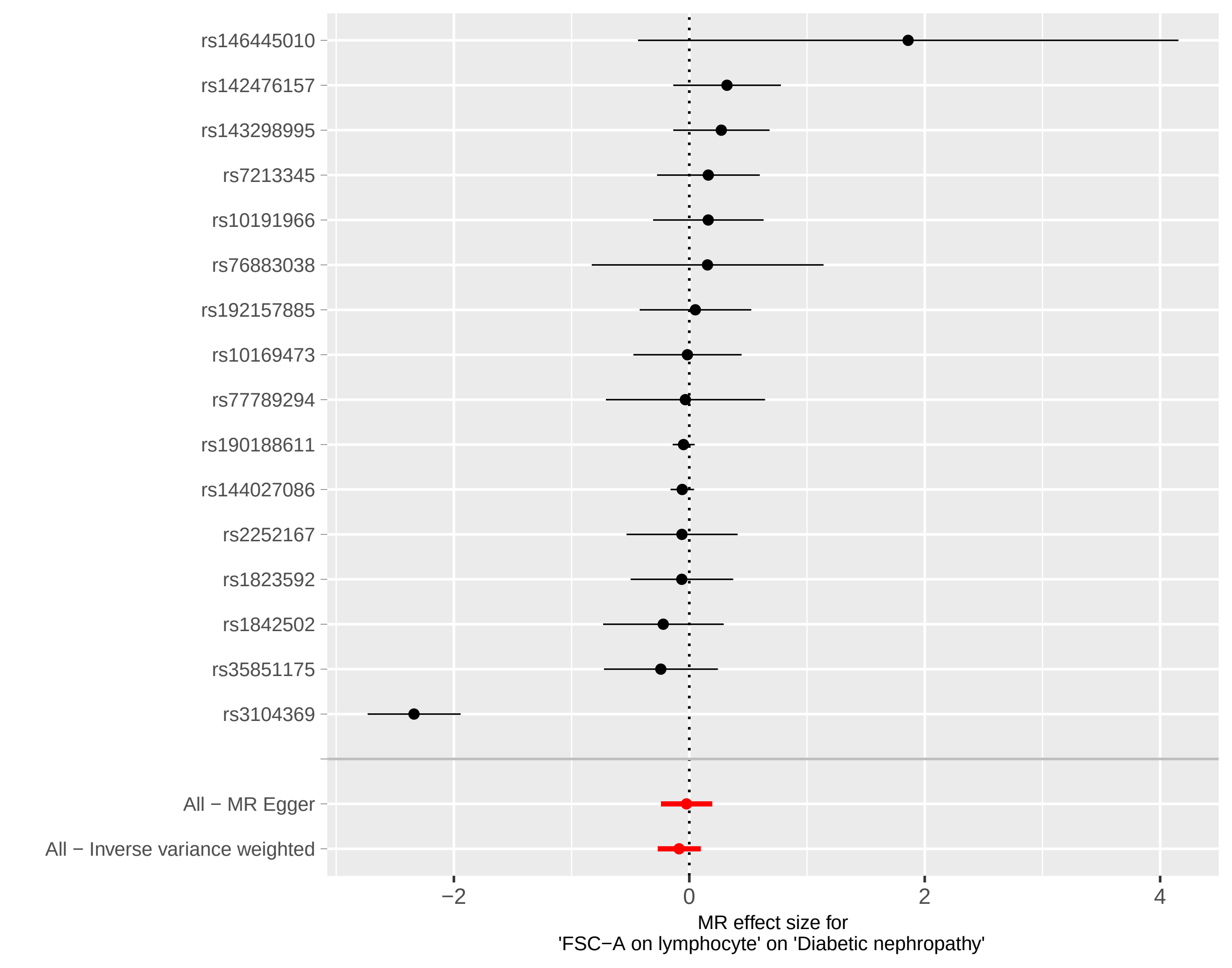
rs146445010
rs142476157
rs143298995
rs7213345
rs10191966
rs76883038
rs192157885
rs10169473
rs77789294
rs190188611
rs144027086
rs2252167
rs1823592
rs1842502
rs35851175
rs3104369
All − MR Egger
All − Inverse variance weighted
−2
0
2
4
MR effect size for
'FSC−A on lymphocyte' on 'Diabetic nephropathy'

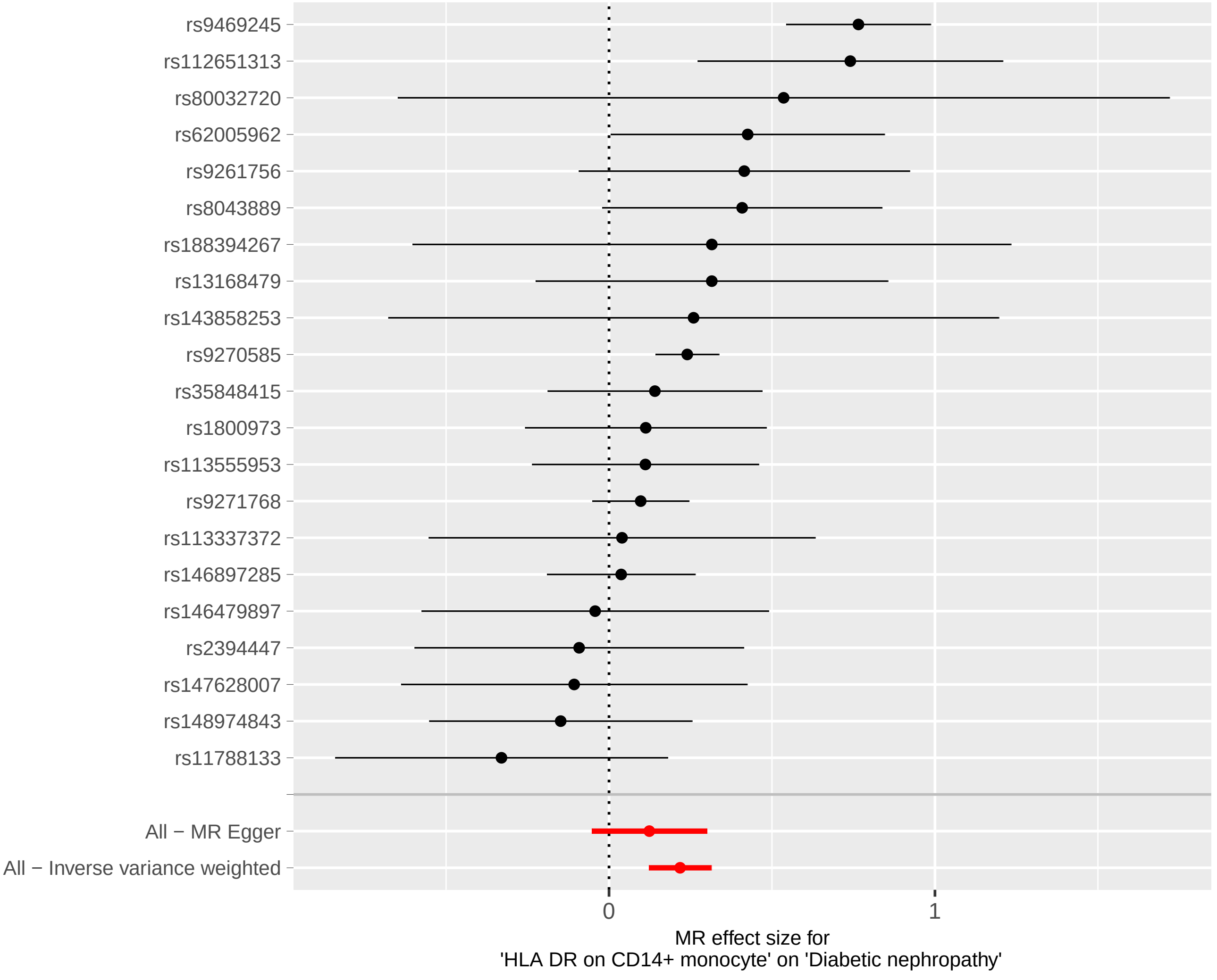
rs9469245
rs112651313
rs80032720
rs62005962
rs9261756
rs8043889
rs188394267
rs13168479
rs143858253
rs9270585
rs35848415
rs1800973
rs113555953
rs9271768
rs113337372
rs146897285
rs146479897
rs2394447
rs147628007
rs148974843
rs11788133
All − MR Egger
All − Inverse variance weighted
0
1
MR effect size for
'HLA DR on CD14+ monocyte' on 'Diabetic nephropathy'

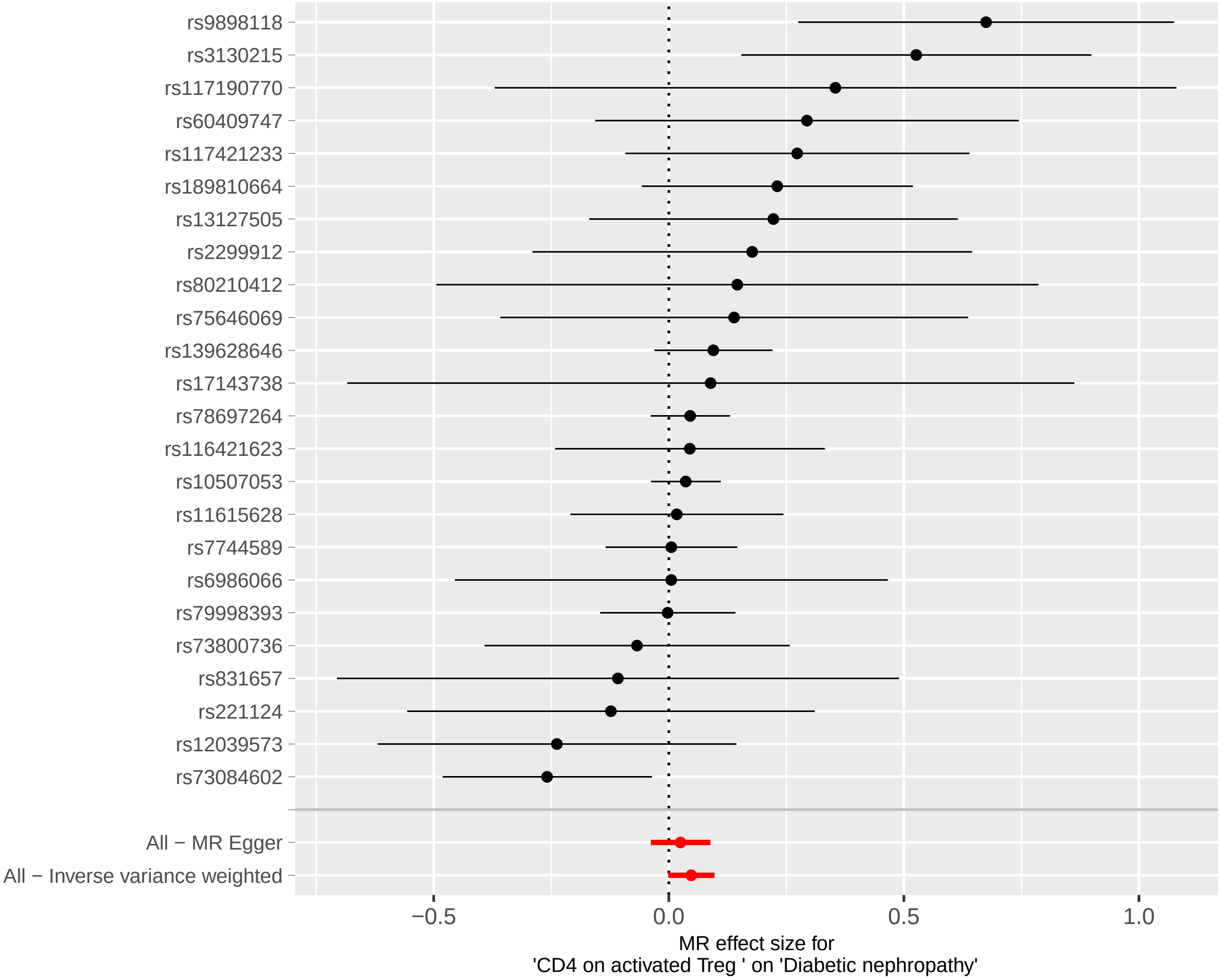

rs9898118
rs3130215
rs117190770
rs60409747
rs117421233
rs189810664
rs13127505
rs2299912
rs80210412
rs75646069
rs139628646
rs17143738
rs78697264
rs116421623
rs10507053
rs11615628
rs7744589
rs6986066
rs79998393
rs73800736
rs831657
rs221124
rs12039573
rs73084602
All − MR Egger
All − Inverse variance weighted
−0.5
0.0
0.5
1.0
MR effect size for
'CD4 on activated Treg ' on 'Diabetic nephropathy'

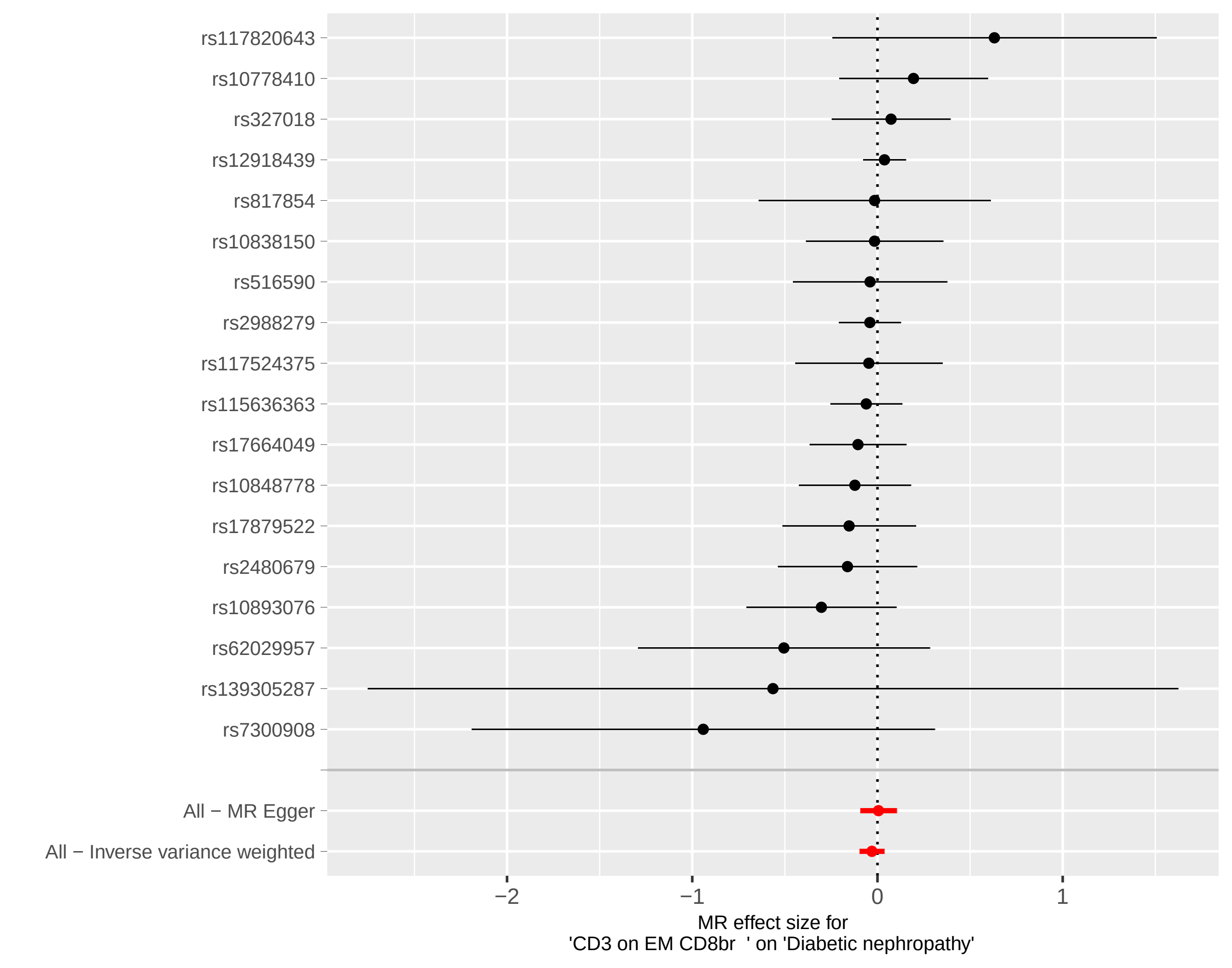
rs117820643
rs10778410
rs327018
rs12918439
rs817854
rs10838150
rs516590
rs2988279
rs117524375
rs115636363
rs17664049
rs10848778
rs17879522
rs2480679
rs10893076
rs62029957
rs139305287
rs7300908
All − MR Egger
All − Inverse variance weighted
−2
−1
0
1
MR effect size for
'CD3 on EM CD8br ' on 'Diabetic nephropathy'

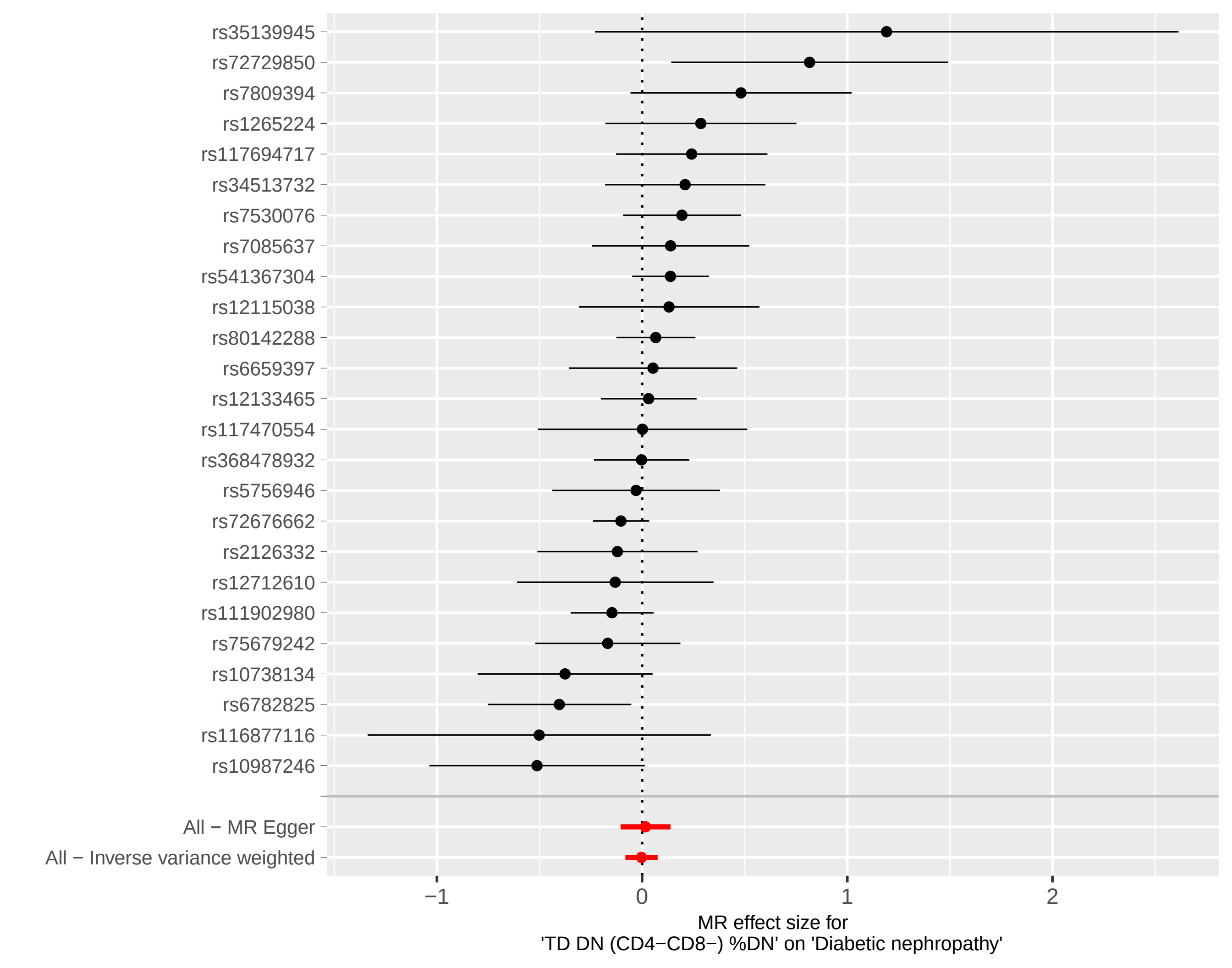
rs35139945
rs72729850
rs7809394
rs1265224
rs117694717
rs34513732
rs7530076
rs7085637
rs541367304
rs12115038
rs80142288
rs6659397
rs12133465
rs117470554
rs368478932
rs5756946
rs72676662
rs2126332
rs12712610
rs111902980
rs75679242
rs10738134
rs6782825
rs116877116
rs10987246
All − MR Egger
All − Inverse variance weighted
−1
0
1
2
MR effect size for
'TD DN (CD4−CD8−) %DN' on 'Diabetic nephropathy'

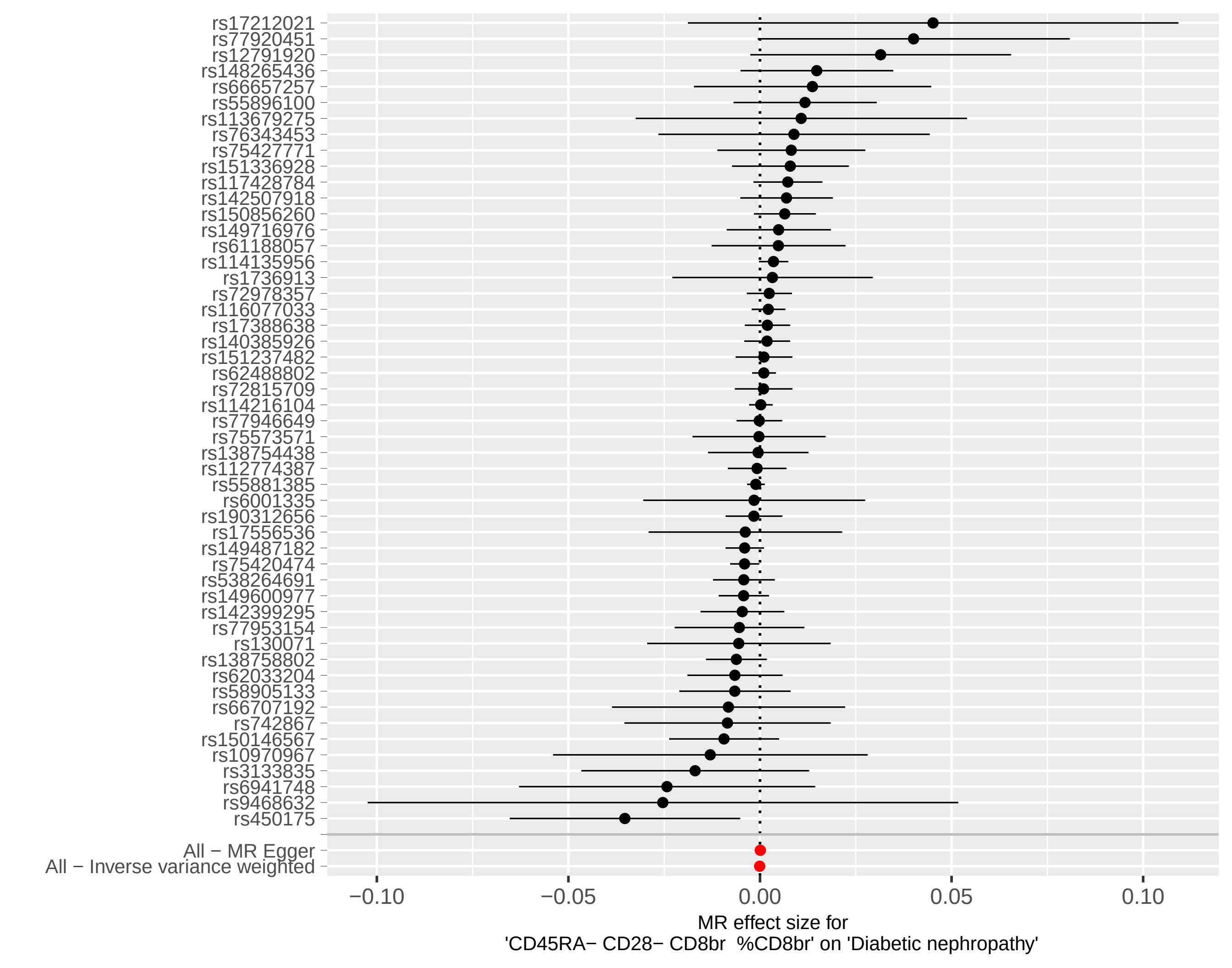
rs17212021
rs77920451
rs12791920
rs148265436
rs66657257
rs55896100
rs113679275
rs76343453
rs75427771
rs151336928
rs117428784
rs142507918
rs150856260
rs149716976
rs61188057
rs114135956
rs1736913
rs72978357
rs116077033
rs17388638
rs140385926
rs151237482
rs62488802
rs72815709
rs114216104
rs77946649
rs75573571
rs138754438
rs112774387
rs55881385
rs6001335
rs190312656
rs17556536
rs149487182
rs75420474
rs538264691
rs149600977
rs142399295
rs77953154
rs130071
rs138758802
rs62033204
rs58905133
rs66707192
rs742867
rs150146567
rs10970967
rs3133835
rs6941748
rs9468632
rs450175
All − MR Egger
All − Inverse variance weighted
−0.10
−0.05
0.00
0.05
0.10
MR effect size for
'CD45RA− CD28− CD8br %CD8br' on 'Diabetic nephropathy'

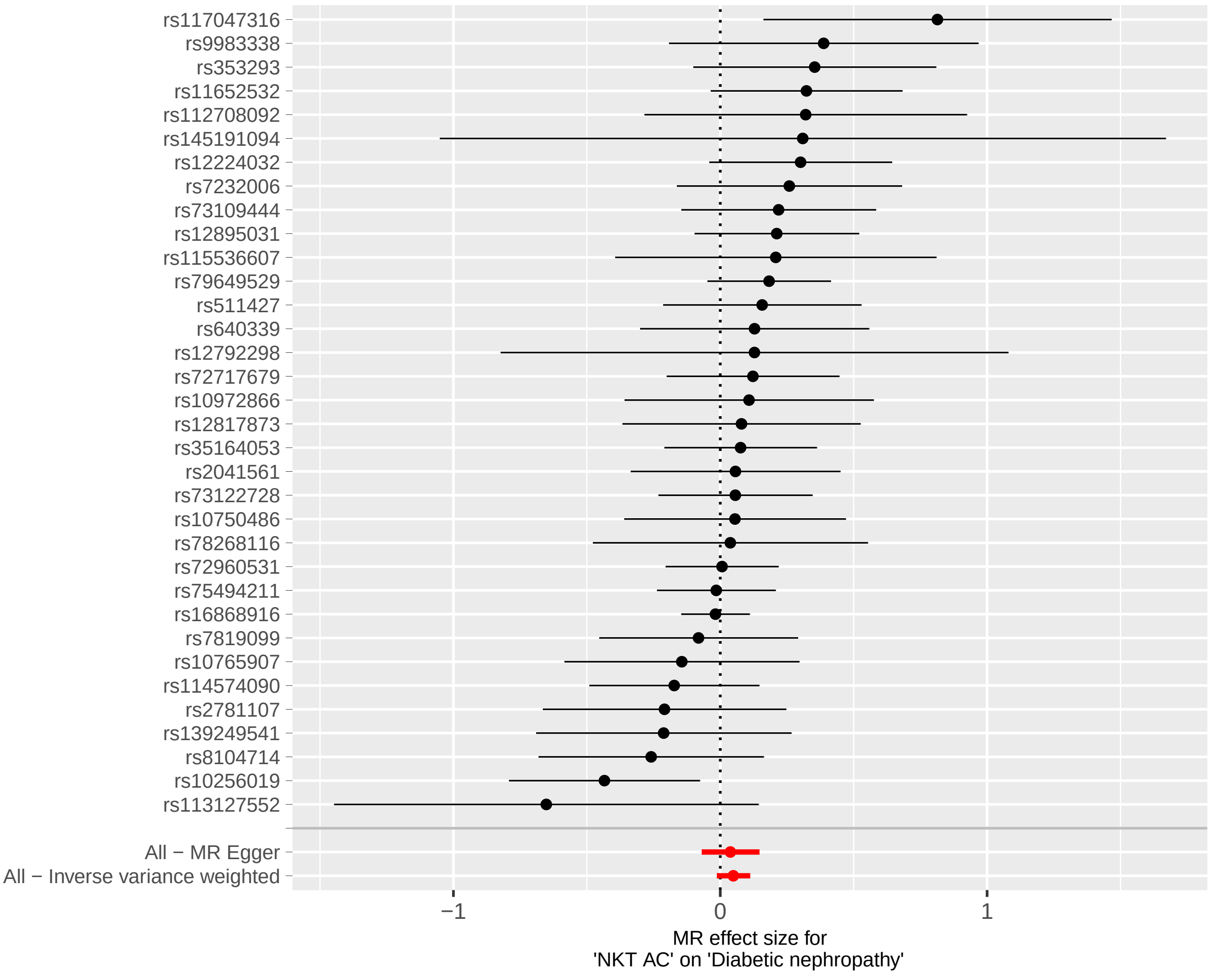
rs117047316
rs9983338
rs353293
rs11652532
rs112708092
rs145191094
rs12224032
rs7232006
rs73109444
rs12895031
rs115536607
rs79649529
rs511427
rs640339
rs12792298
rs72717679
rs10972866
rs12817873
rs35164053
rs2041561
rs73122728
rs10750486
rs78268116
rs72960531
rs75494211
rs16868916
rs7819099
rs10765907
rs114574090
rs2781107
rs139249541
rs8104714
rs10256019
rs113127552
All − MR Egger
All − Inverse variance weighted
−1
0
1
MR effect size for
'NKT AC' on 'Diabetic nephropathy'

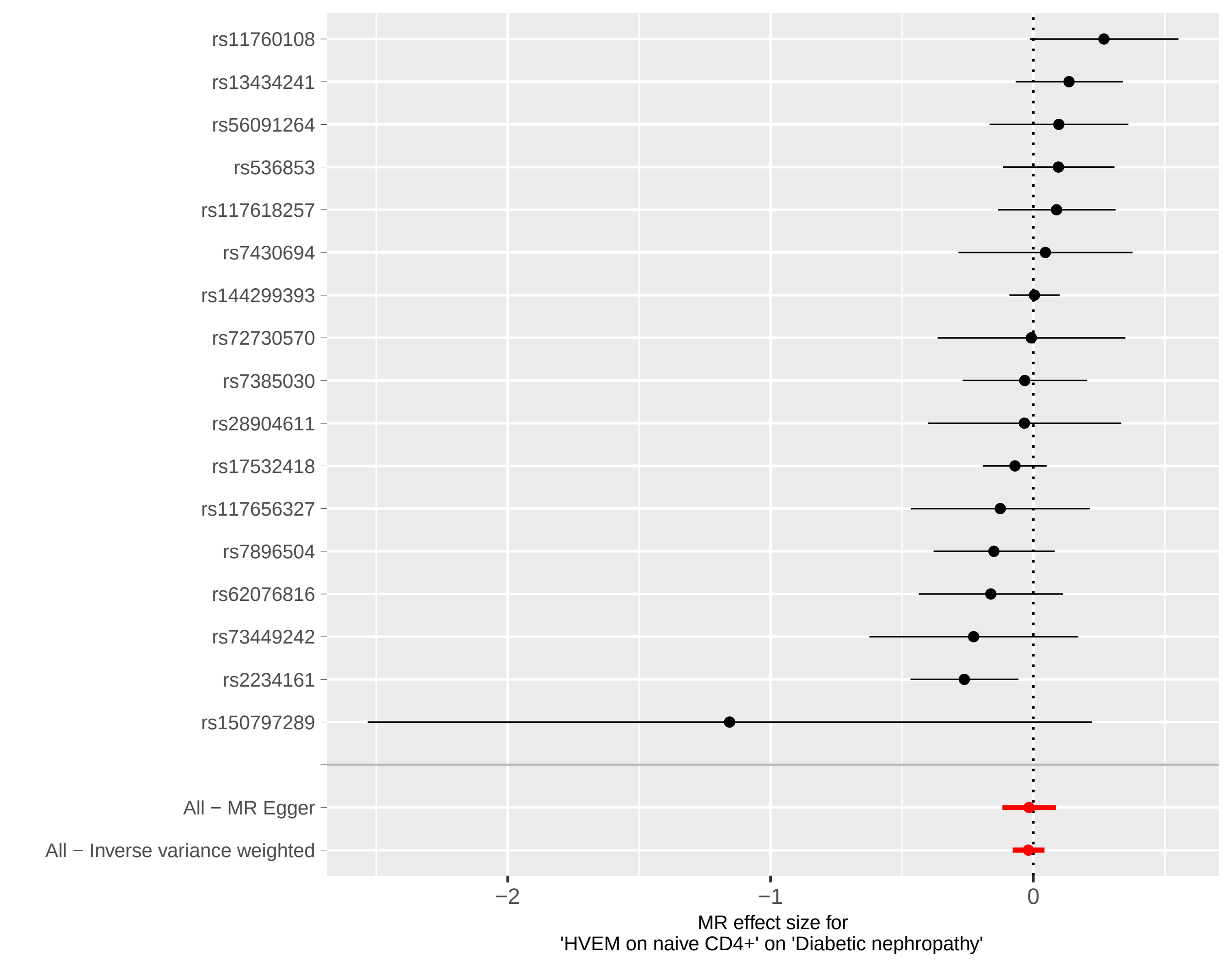
rs11760108
rs13434241
rs56091264
rs536853
rs117618257
rs7430694
rs144299393
rs72730570
rs7385030
rs28904611
rs17532418
rs117656327
rs7896504
rs62076816
rs73449242
rs2234161
rs150797289
All − MR Egger
All − Inverse variance weighted
−2
−1
0
MR effect size for
'HVEM on naive CD4+' on 'Diabetic nephropathy'

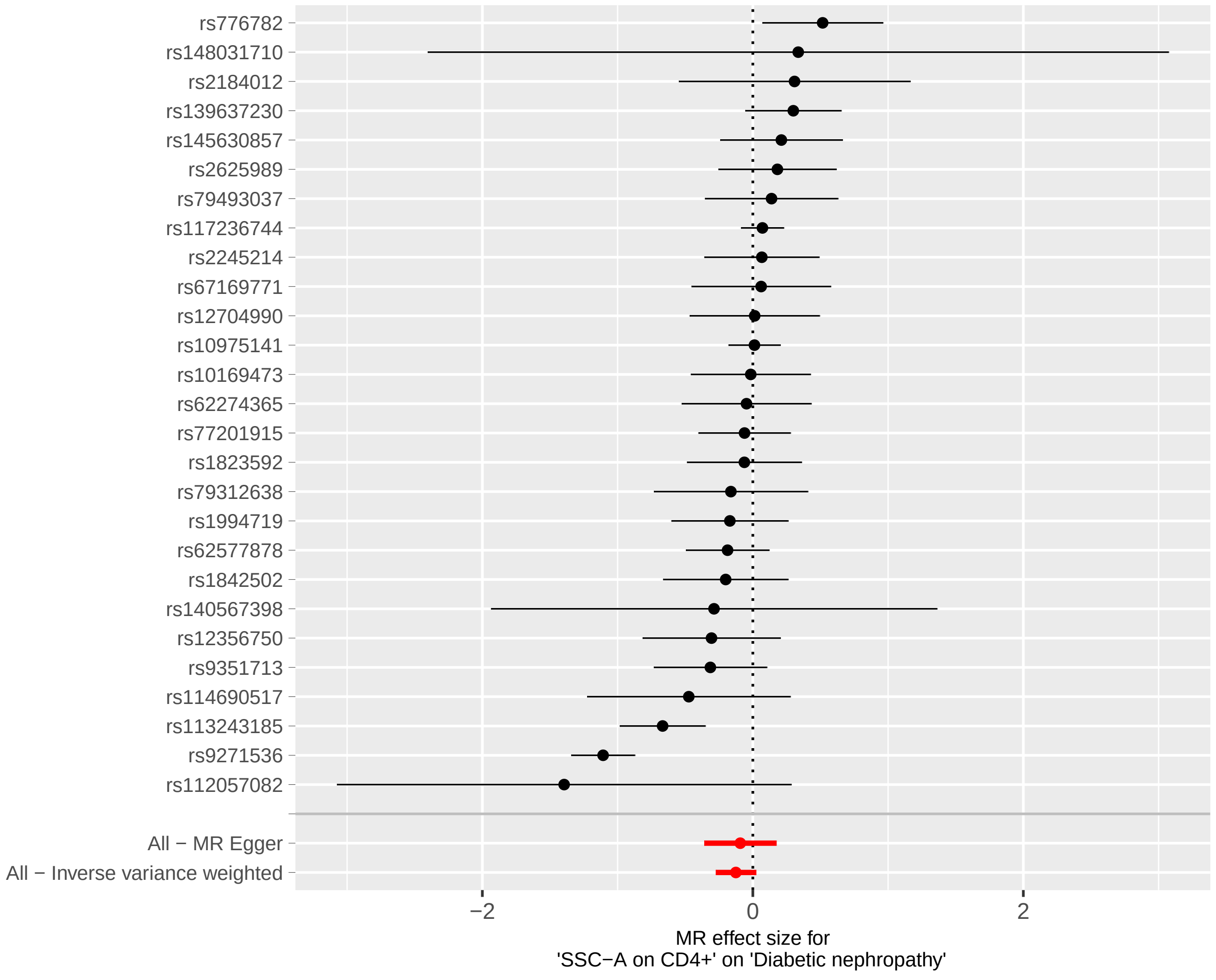
rs776782
rs148031710
rs2184012
rs139637230
rs145630857
rs2625989
rs79493037
rs117236744
rs2245214
rs67169771
rs12704990
rs10975141
rs10169473
rs62274365
rs77201915
rs1823592
rs79312638
rs1994719
rs62577878
rs1842502
rs140567398
rs12356750
rs9351713
rs114690517
rs113243185
rs9271536
rs112057082
All − MR Egger
All − Inverse variance weighted
−2
0
2
MR effect size for
'SSC−A on CD4+' on 'Diabetic nephropathy'

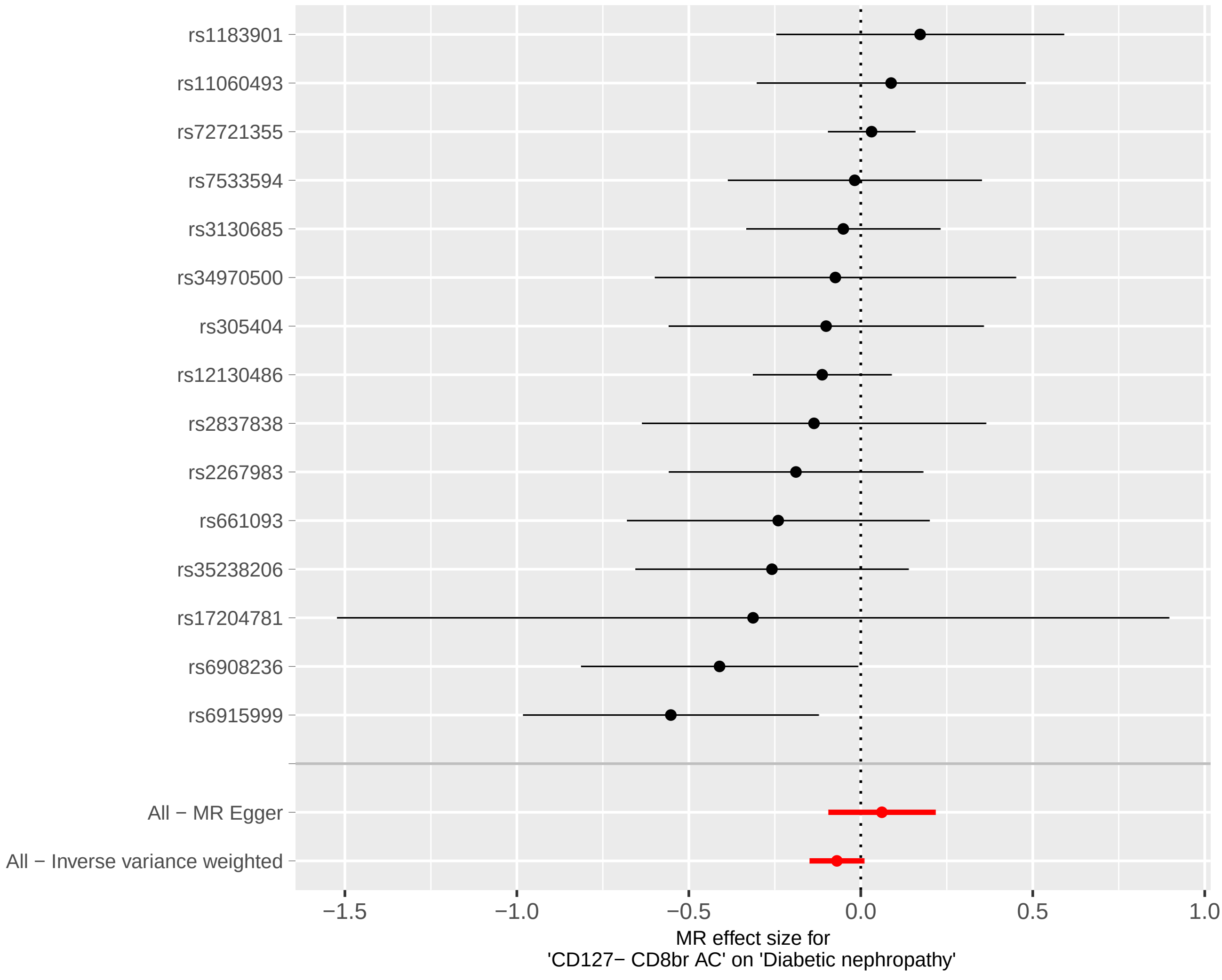
rs1183901
rs11060493
rs72721355
rs7533594
rs3130685
rs34970500
rs305404
rs12130486
rs2837838
rs2267983
rs661093
rs35238206
rs17204781
rs6908236
rs6915999
All − MR Egger
All − Inverse variance weighted
−1.5
−1.0
−0.5
0.0
0.5
1.0
MR effect size for
'CD127− CD8br AC' on 'Diabetic nephropathy'

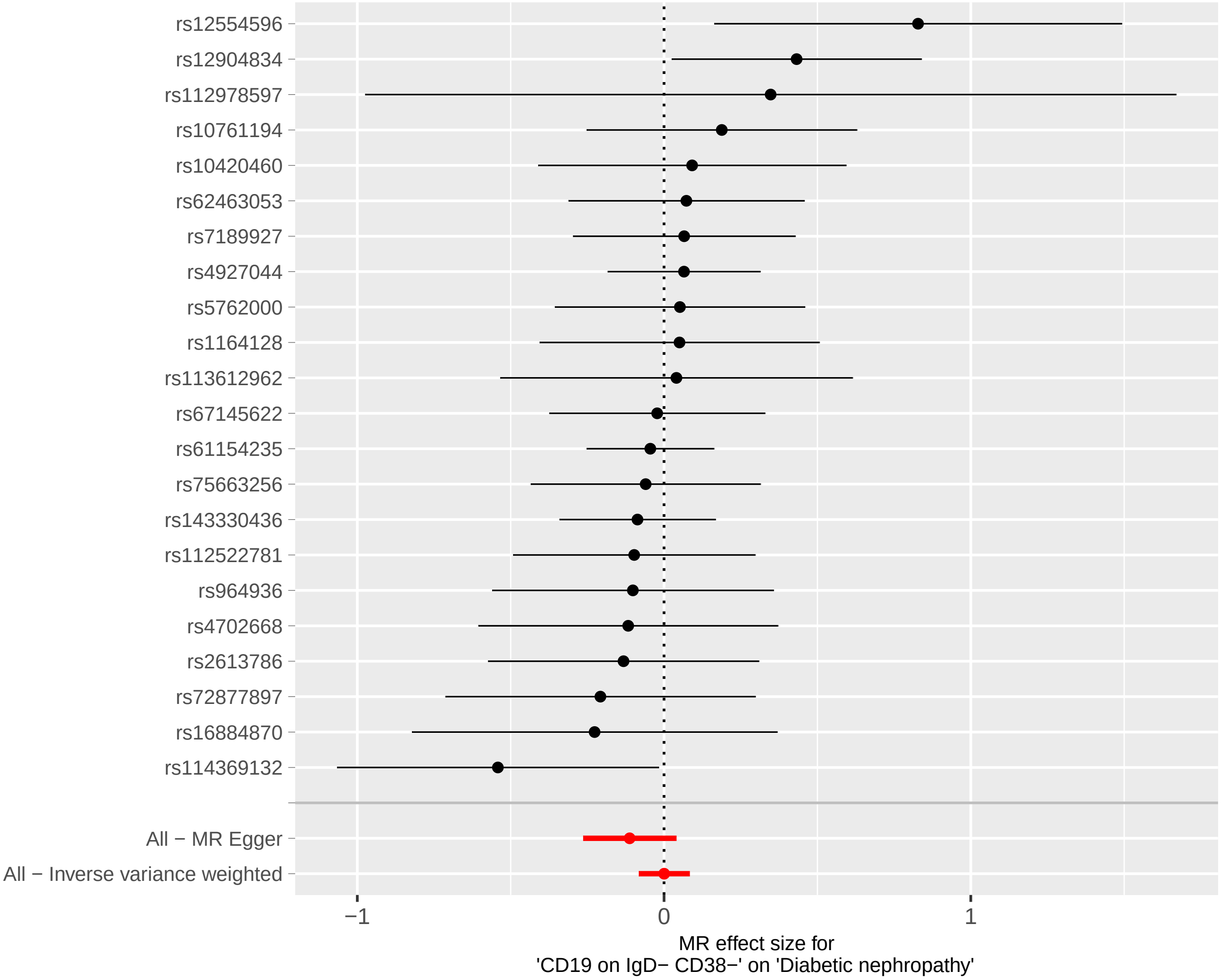
rs12554596
rs12904834
rs112978597
rs10761194
rs10420460
rs62463053
rs7189927
rs4927044
rs5762000
rs1164128
rs113612962
rs67145622
rs61154235
rs75663256
rs143330436
rs112522781
rs964936
rs4702668
rs2613786
rs72877897
rs16884870
rs114369132
All − MR Egger
All − Inverse variance weighted
−1
0
1
MR effect size for
'CD19 on IgD− CD38−' on 'Diabetic nephropathy'

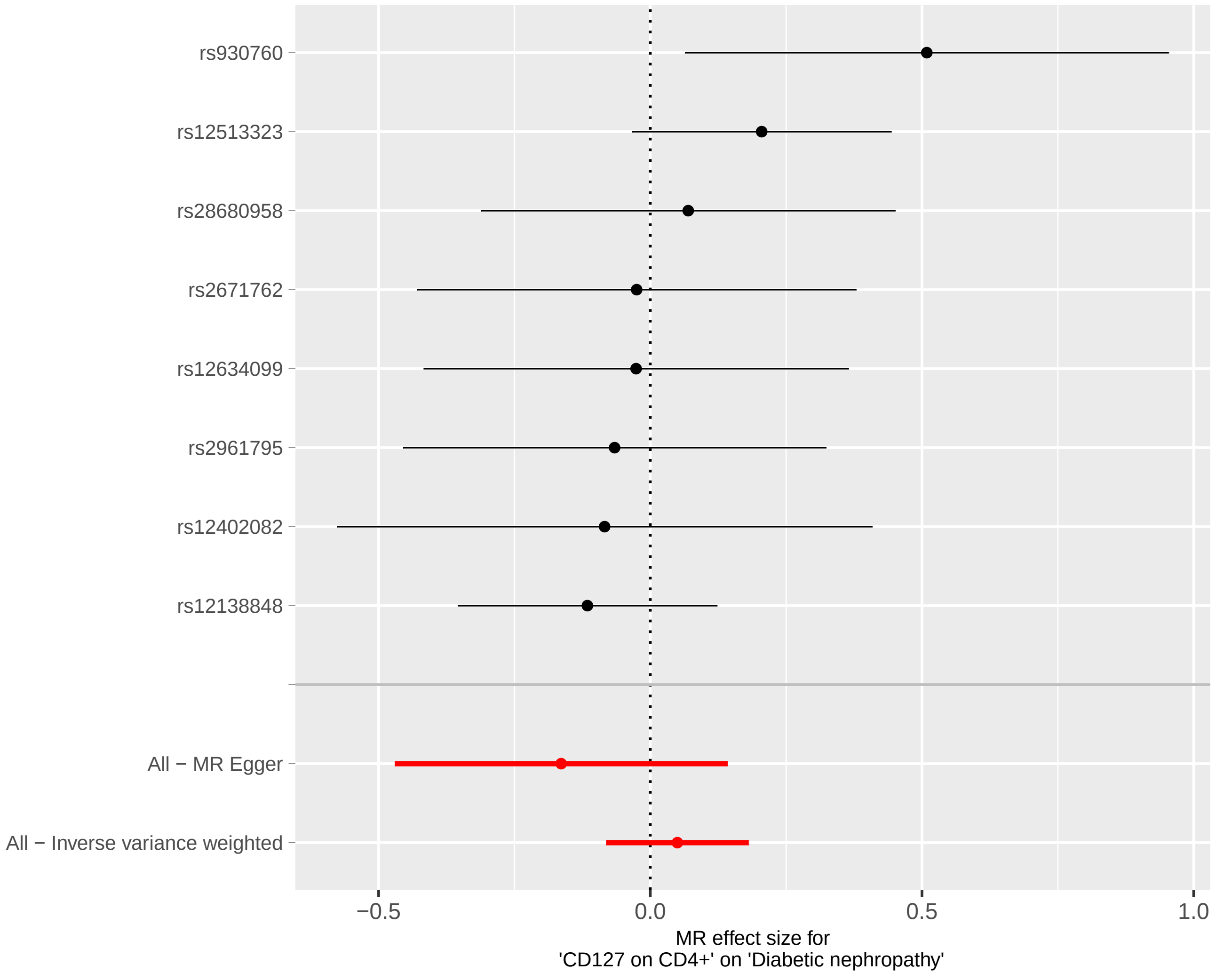

rs930760
rs12513323
rs28680958
rs2671762
rs12634099
rs2961795
rs12402082
rs12138848
All − MR Egger
All − Inverse variance weighted
−0.5
0.0
0.5
1.0
MR effect size for
'CD127 on CD4+' on 'Diabetic nephropathy'

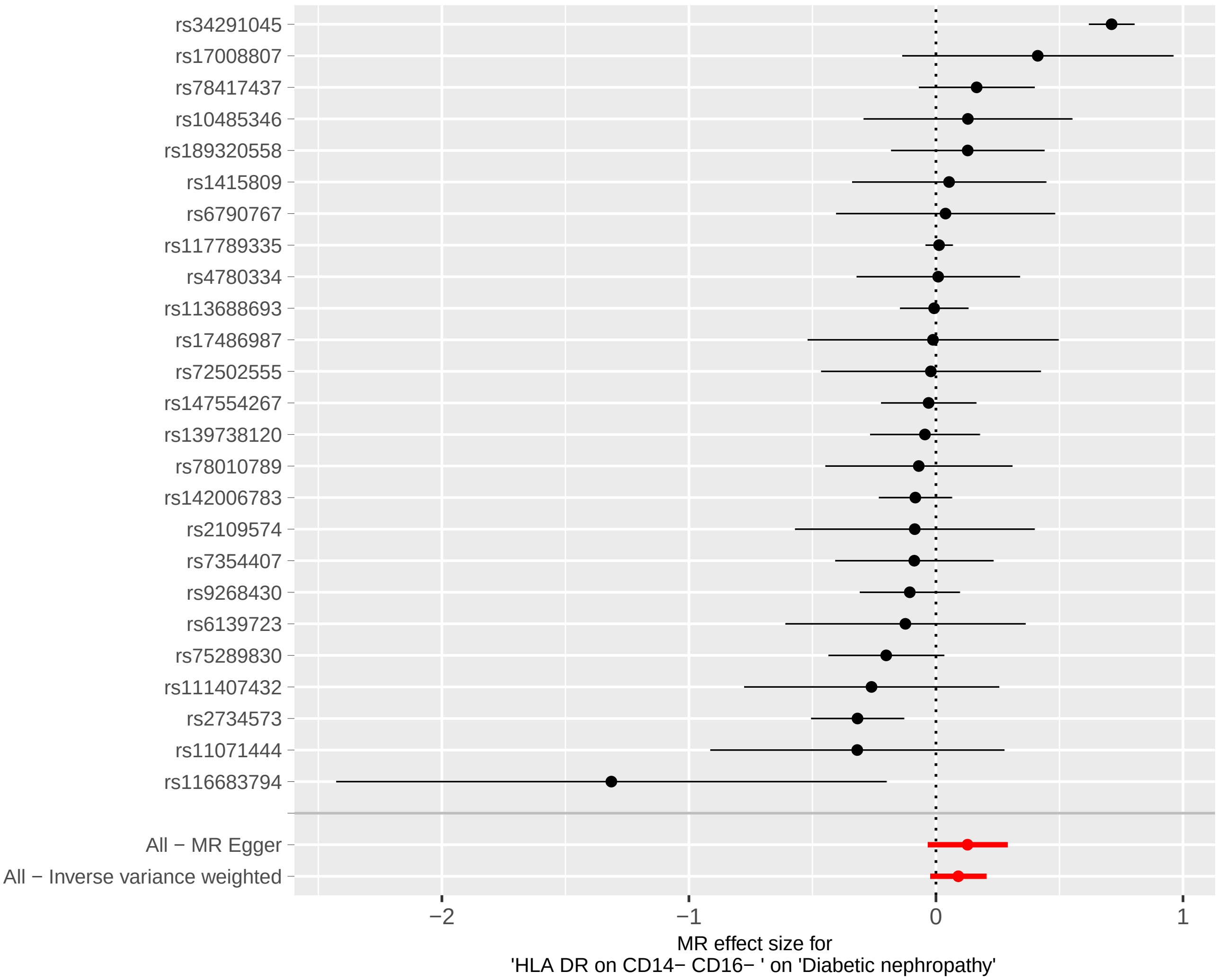
rs34291045
rs17008807
rs78417437
rs10485346
rs189320558
rs1415809
rs6790767
rs117789335
rs4780334
rs113688693
rs17486987
rs72502555
rs147554267
rs139738120
rs78010789
rs142006783
rs2109574
rs7354407
rs9268430
rs6139723
rs75289830
rs111407432
rs2734573
rs11071444
rs116683794
All − MR Egger
All − Inverse variance weighted
−2
−1
0
1
MR effect size for
'HLA DR on CD14− CD16− ' on 'Diabetic nephropathy'

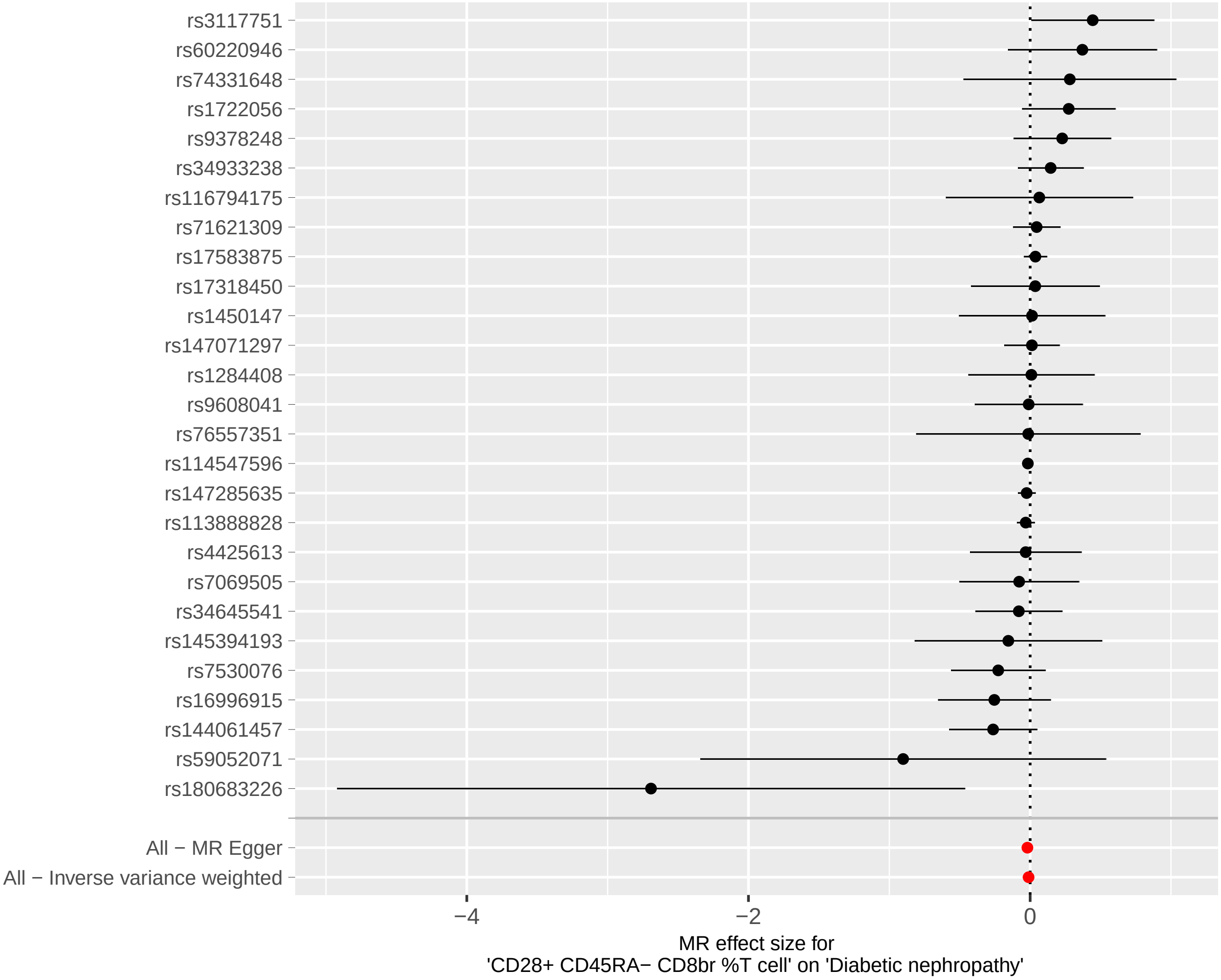

rs3117751
rs60220946
rs74331648
rs1722056
rs9378248
rs34933238
rs116794175
rs71621309
rs17583875
rs17318450
rs1450147
rs147071297
rs1284408
rs9608041
rs76557351
rs114547596
rs147285635
rs113888828
rs4425613
rs7069505
rs34645541
rs145394193
rs7530076
rs16996915
rs144061457
rs59052071
rs180683226
All − MR Egger
All − Inverse variance weighted
−4
−2
0
MR effect size for
'CD28+ CD45RA− CD8br %T cell' on 'Diabetic nephropathy'

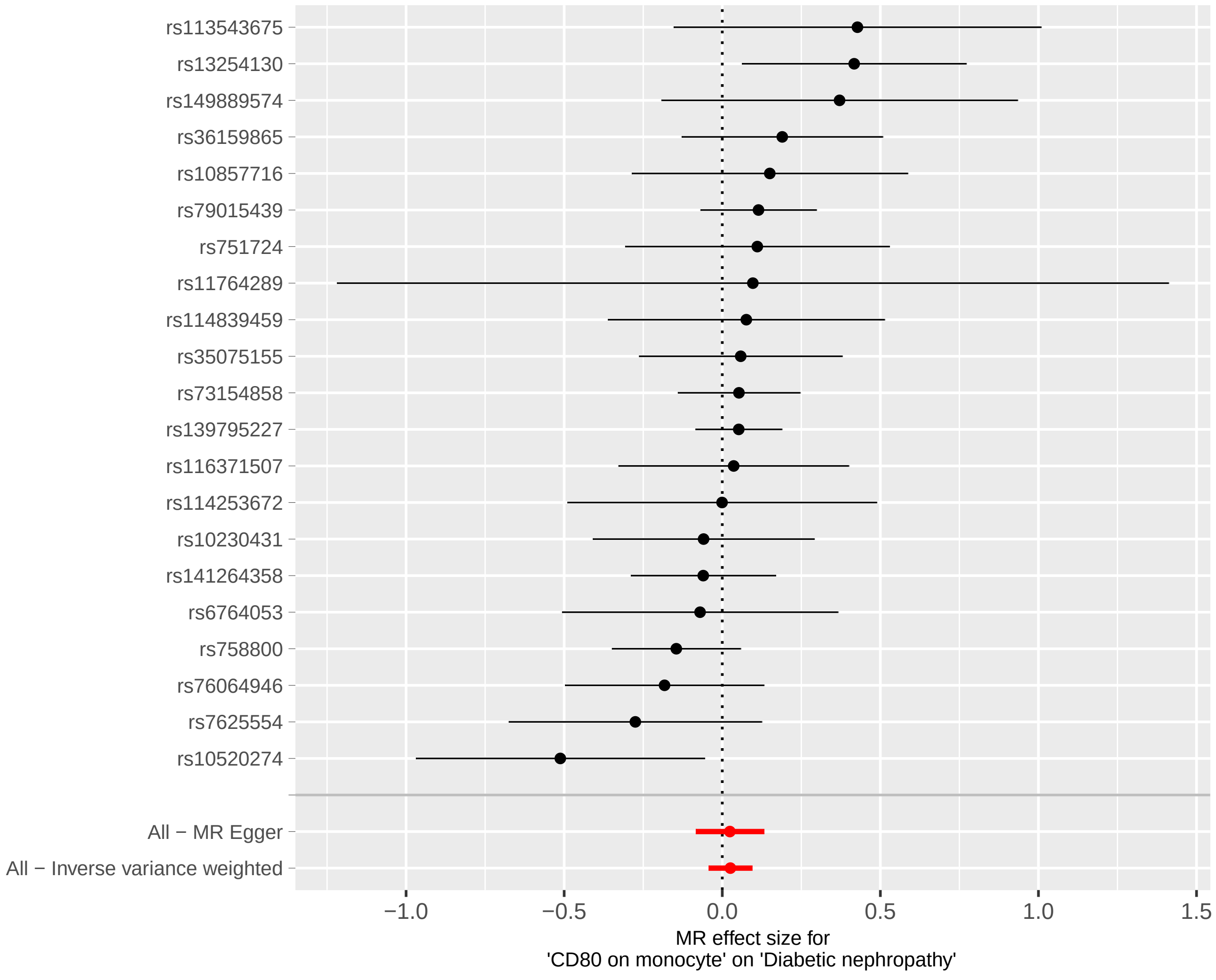
rs113543675
rs13254130
rs149889574
rs36159865
rs10857716
rs79015439
rs751724
rs11764289
rs114839459
rs35075155
rs73154858
rs139795227
rs116371507
rs114253672
rs10230431
rs141264358
rs6764053
rs758800
rs76064946
rs7625554
rs10520274
All − MR Egger
All − Inverse variance weighted
−1.0
−0.5
0.0
0.5
1.0
1.5
MR effect size for
'CD80 on monocyte' on 'Diabetic nephropathy'

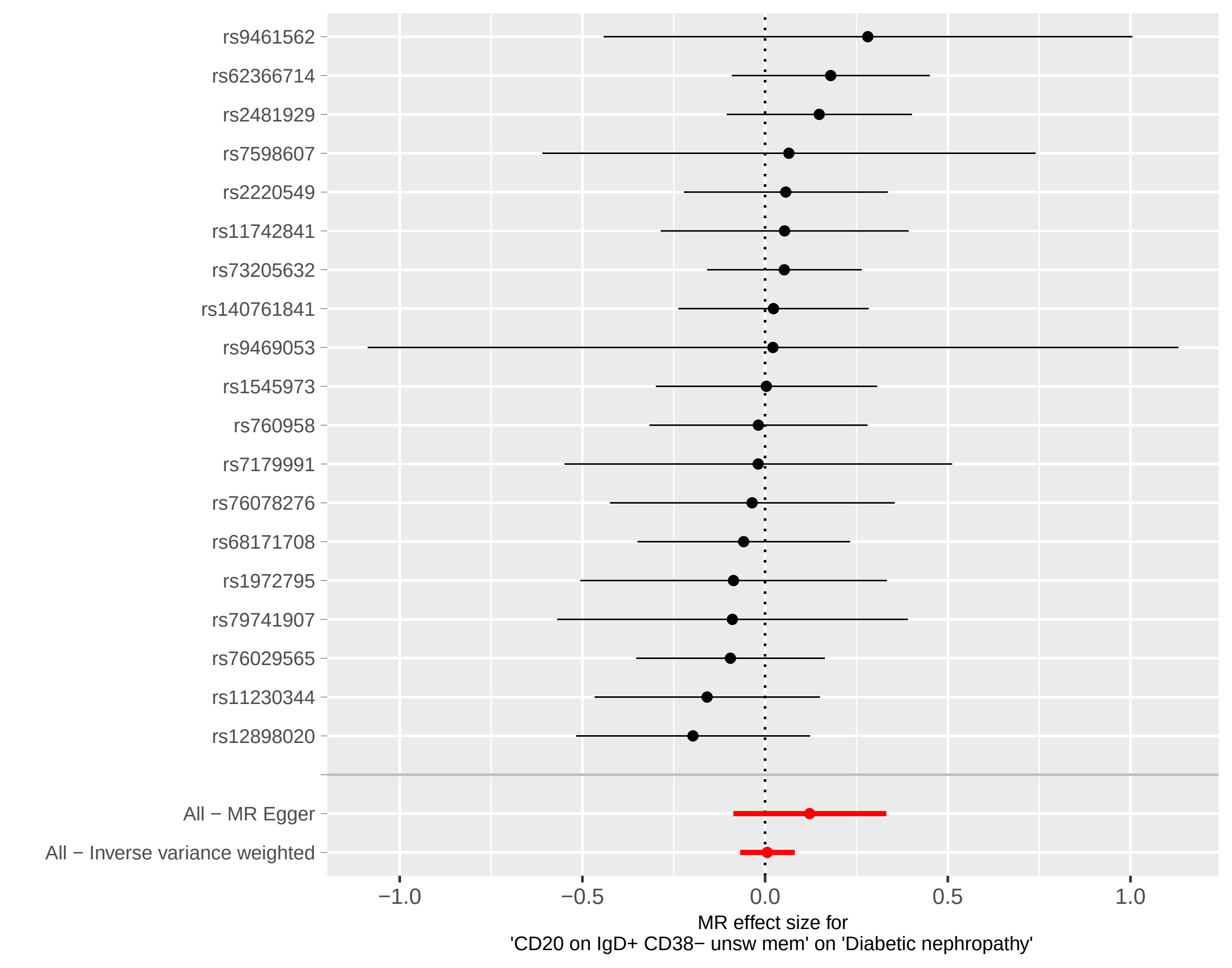

rs9461562
rs62366714
rs2481929
rs7598607
rs2220549
rs11742841
rs73205632
rs140761841
rs9469053
rs1545973
rs760958
rs7179991
rs76078276
rs68171708
rs1972795
rs79741907
rs76029565
rs11230344
rs12898020
All − MR Egger
All − Inverse variance weighted
−1.0
−0.5
0.0
0.5
1.0
MR effect size for
'CD20 on IgD+ CD38− unsw mem' on 'Diabetic nephropathy'

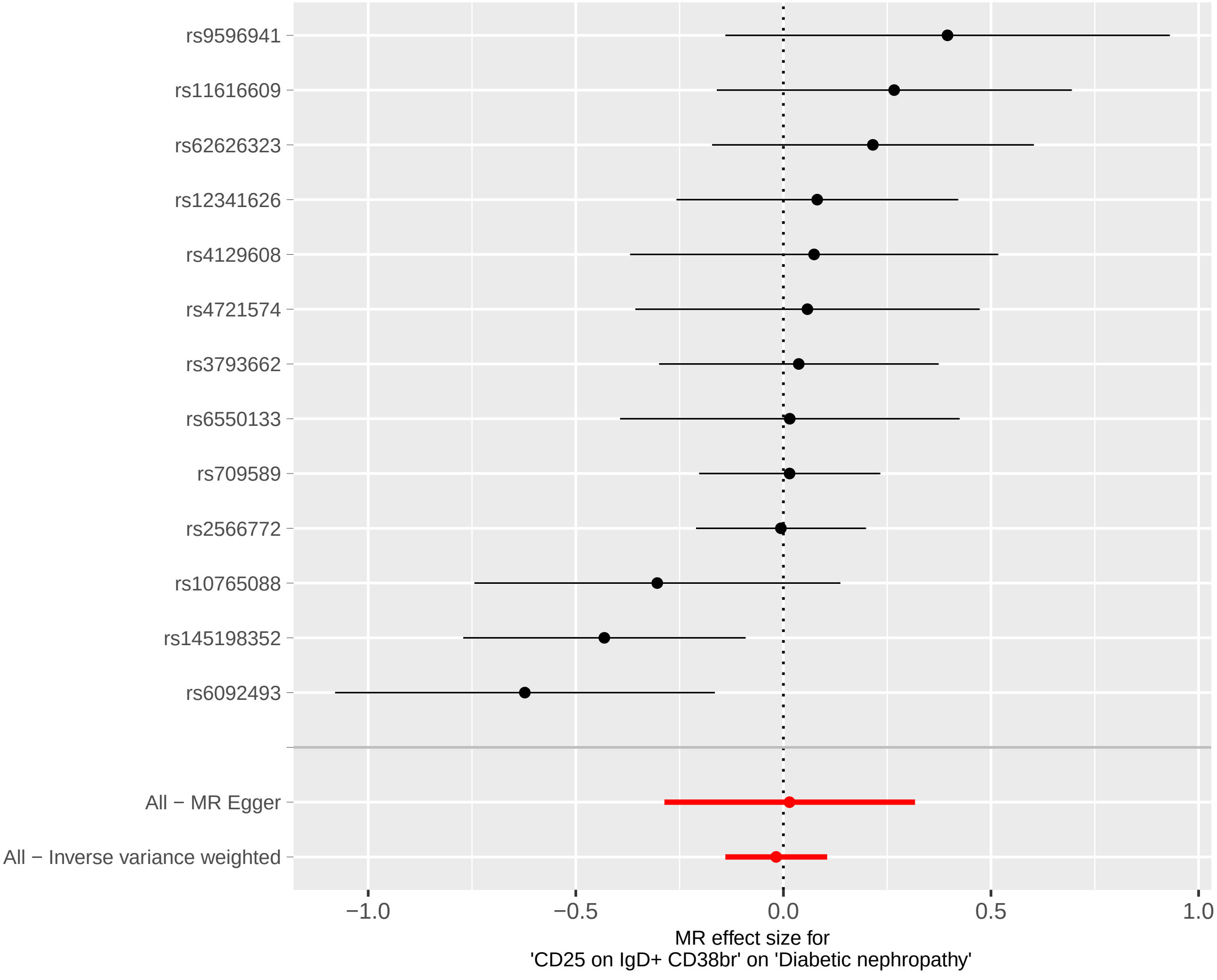

rs9596941
rs11616609
rs62626323
rs12341626
rs4129608
rs4721574
rs3793662
rs6550133
rs709589
rs2566772
rs10765088
rs145198352
rs6092493
All − MR Egger
All − Inverse variance weighted
−1.0
−0.5
0.0
0.5
1.0
MR effect size for
'CD25 on IgD+ CD38br' on 'Diabetic nephropathy'

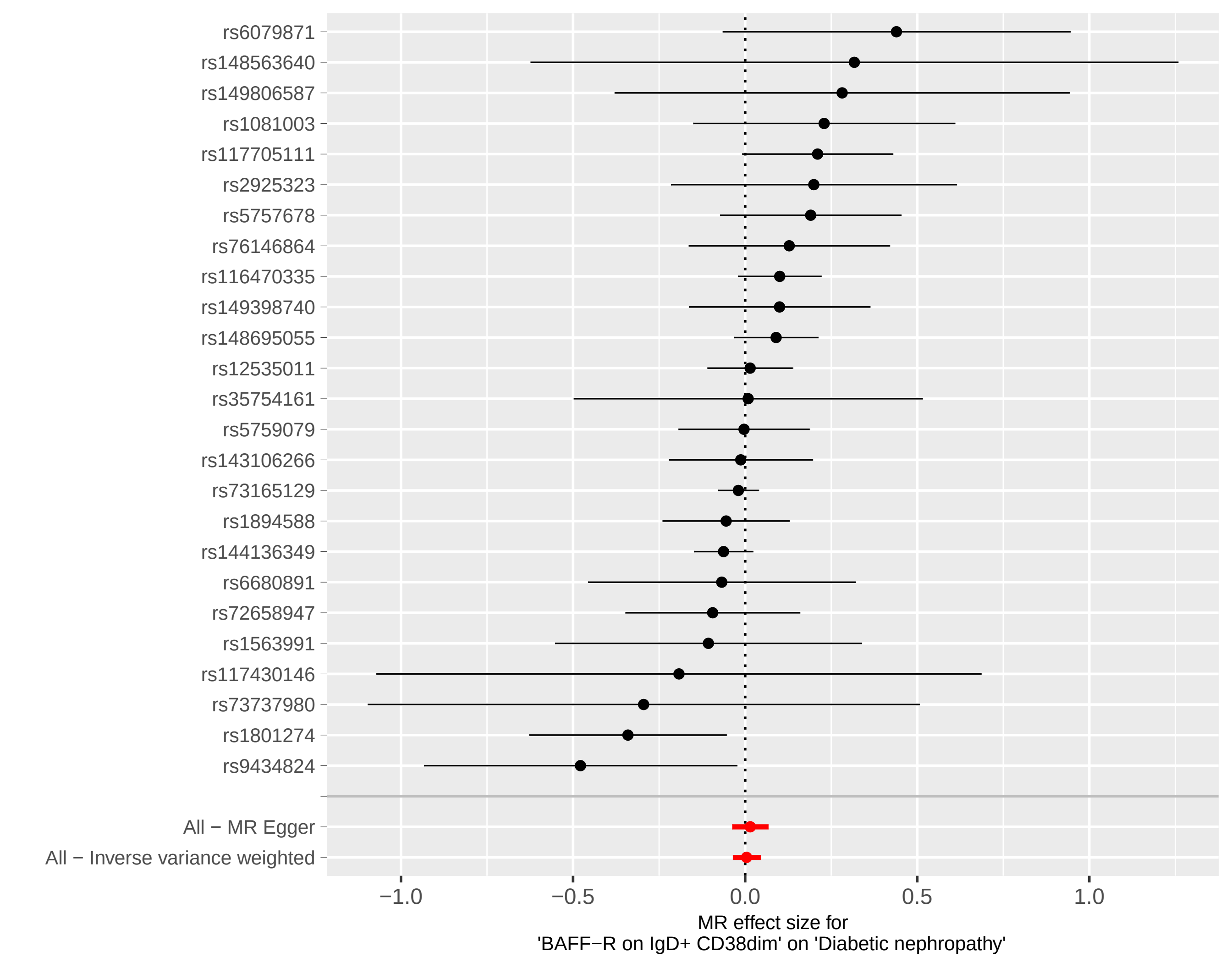

rs6079871
rs148563640
rs149806587
rs1081003
rs117705111
rs2925323
rs5757678
rs76146864
rs116470335
rs149398740
rs148695055
rs12535011
rs35754161
rs5759079
rs143106266
rs73165129
rs1894588
rs144136349
rs6680891
rs72658947
rs1563991
rs117430146
rs73737980
rs1801274
rs9434824
All − MR Egger
All − Inverse variance weighted
−1.0
−0.5
0.0
0.5
1.0
MR effect size for
'BAFF−R on IgD+ CD38dim' on 'Diabetic nephropathy'

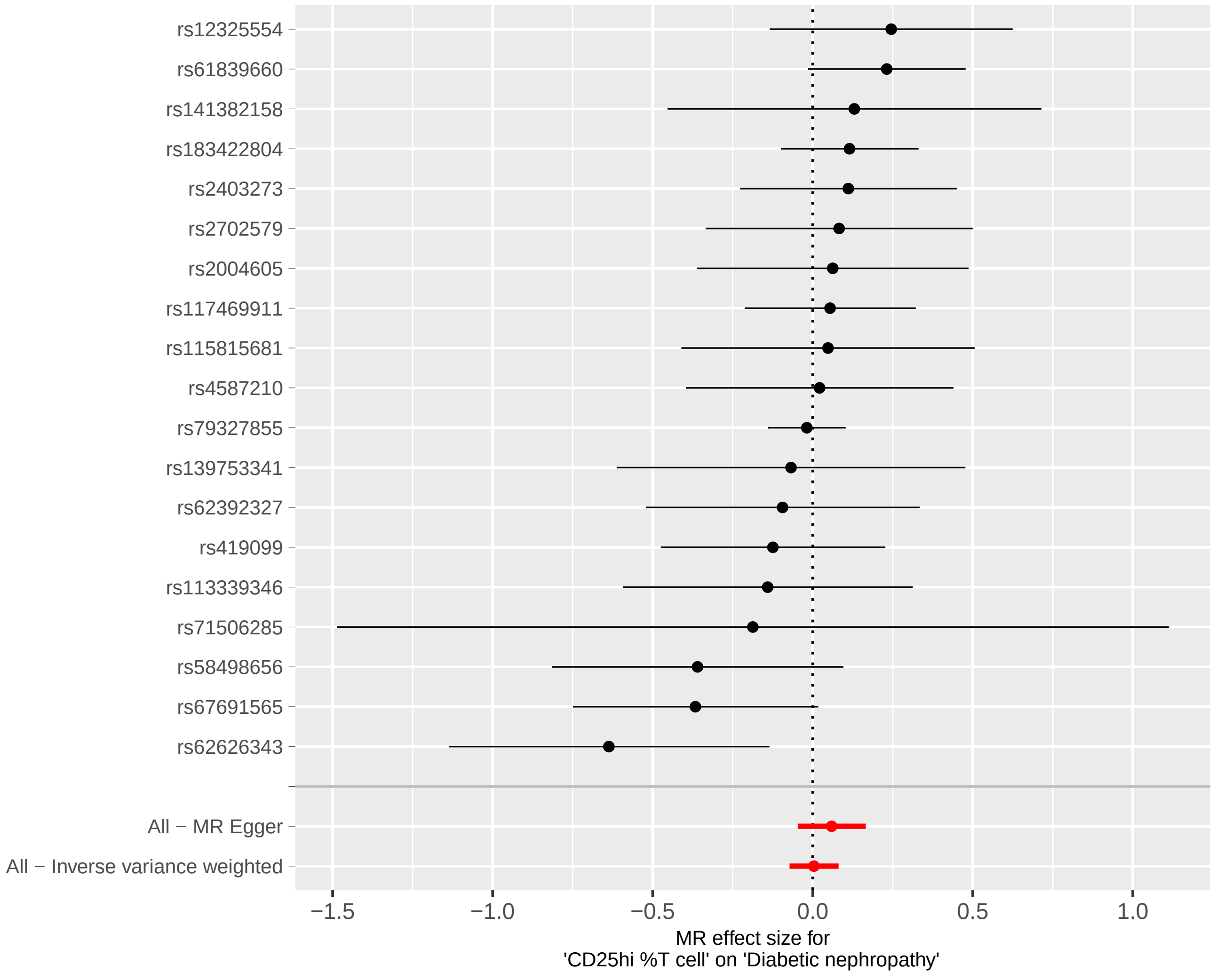
rs12325554
rs61839660
rs141382158
rs183422804
rs2403273
rs2702579
rs2004605
rs117469911
rs115815681
rs4587210
rs79327855
rs139753341
rs62392327
rs419099
rs113339346
rs71506285
rs58498656
rs67691565
rs62626343
All − MR Egger
All − Inverse variance weighted
−1.5
−1.0
−0.5
0.0
0.5
1.0
MR effect size for
'CD25hi %T cell' on 'Diabetic nephropathy'

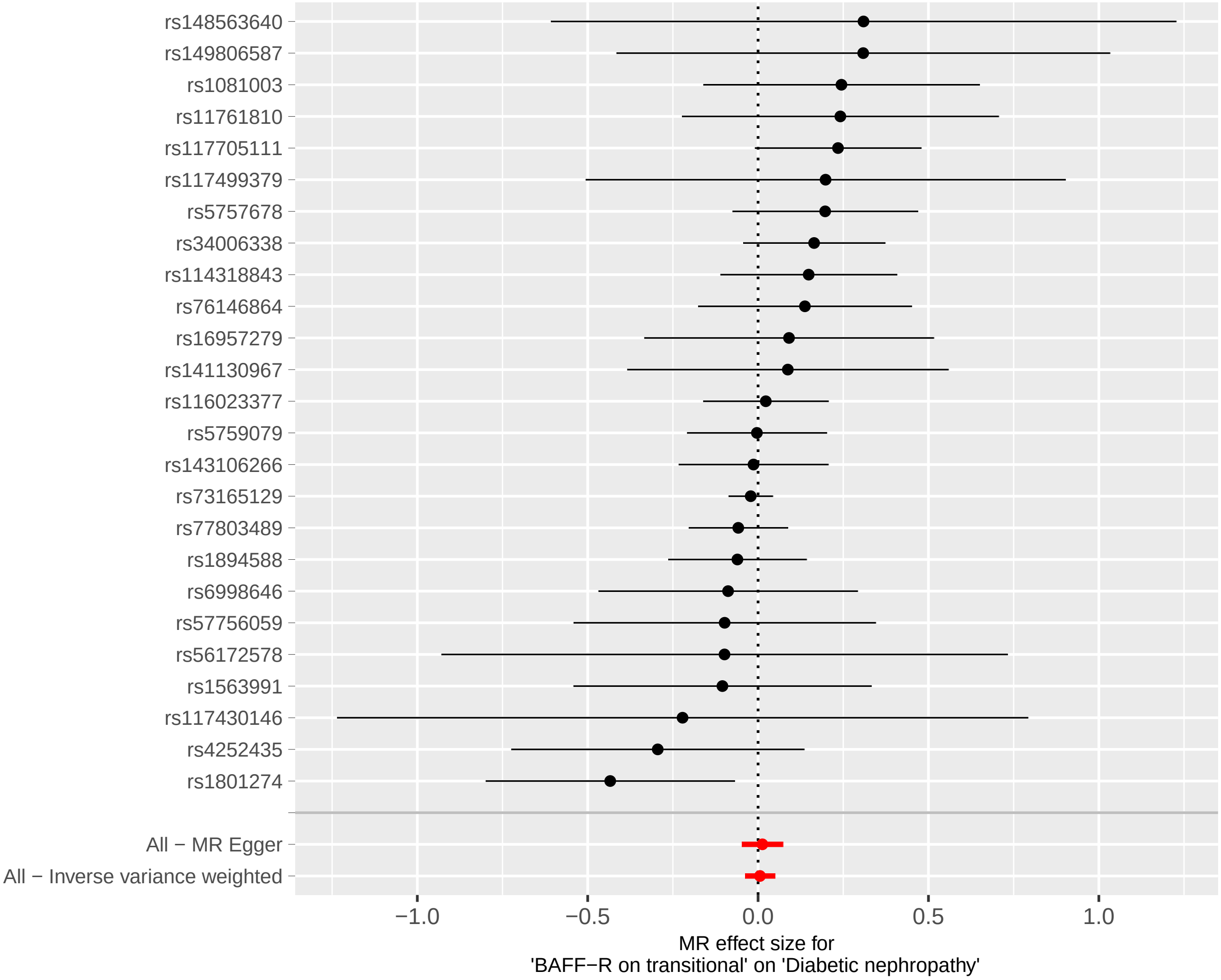
rs148563640
rs149806587
rs1081003
rs11761810
rs117705111
rs117499379
rs5757678
rs34006338
rs114318843
rs76146864
rs16957279
rs141130967
rs116023377
rs5759079
rs143106266
rs73165129
rs77803489
rs1894588
rs6998646
rs57756059
rs56172578
rs1563991
rs117430146
rs4252435
rs1801274
All − MR Egger
All − Inverse variance weighted
−1.0
−0.5
0.0
0.5
1.0
MR effect size for
'BAFF−R on transitional' on 'Diabetic nephropathy'

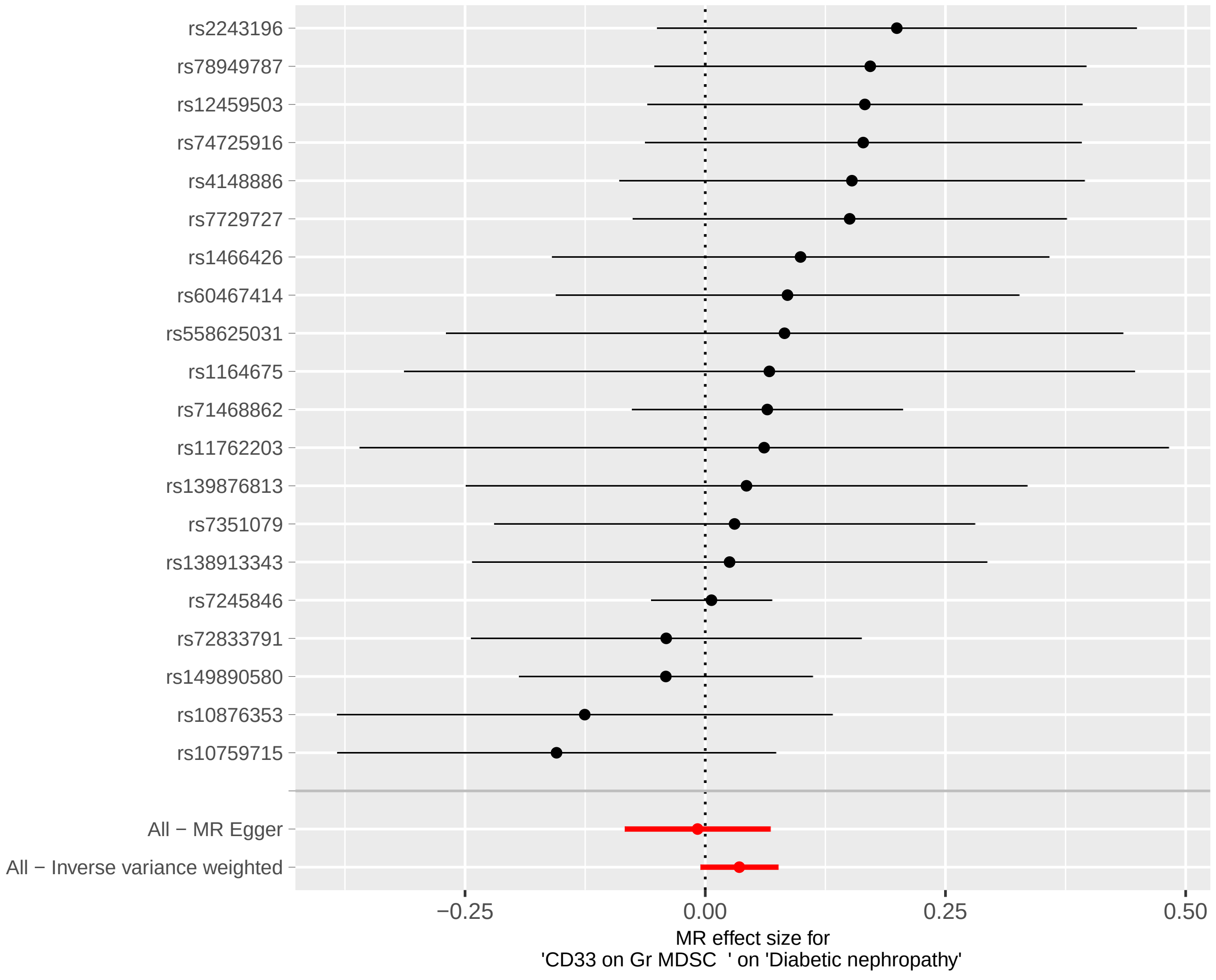
rs2243196
rs78949787
rs12459503
rs74725916
rs4148886
rs7729727
rs1466426
rs60467414
rs558625031
rs1164675
rs71468862
rs11762203
rs139876813
rs7351079
rs138913343
rs7245846
rs72833791
rs149890580
rs10876353
rs10759715
All − MR Egger
All − Inverse variance weighted
−0.25
0.00
0.25
0.50
MR effect size for
'CD33 on Gr MDSC ' on 'Diabetic nephropathy'

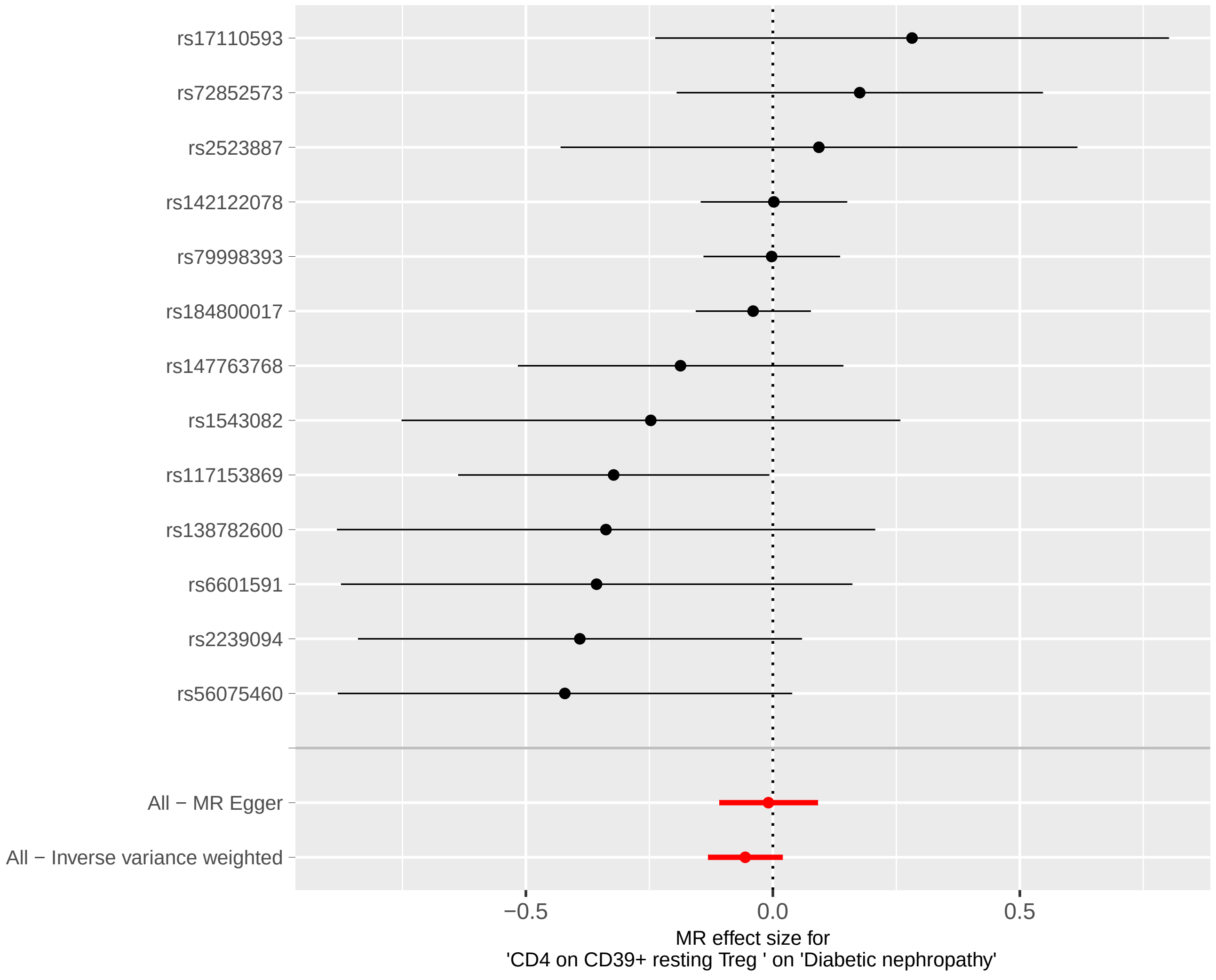
rs17110593
rs72852573
rs2523887
rs142122078
rs79998393
rs184800017
rs147763768
rs1543082
rs117153869
rs138782600
rs6601591
rs2239094
rs56075460
All − MR Egger
All − Inverse variance weighted
−0.5
0.0
0.5
MR effect size for
'CD4 on CD39+ resting Treg ' on 'Diabetic nephropathy'

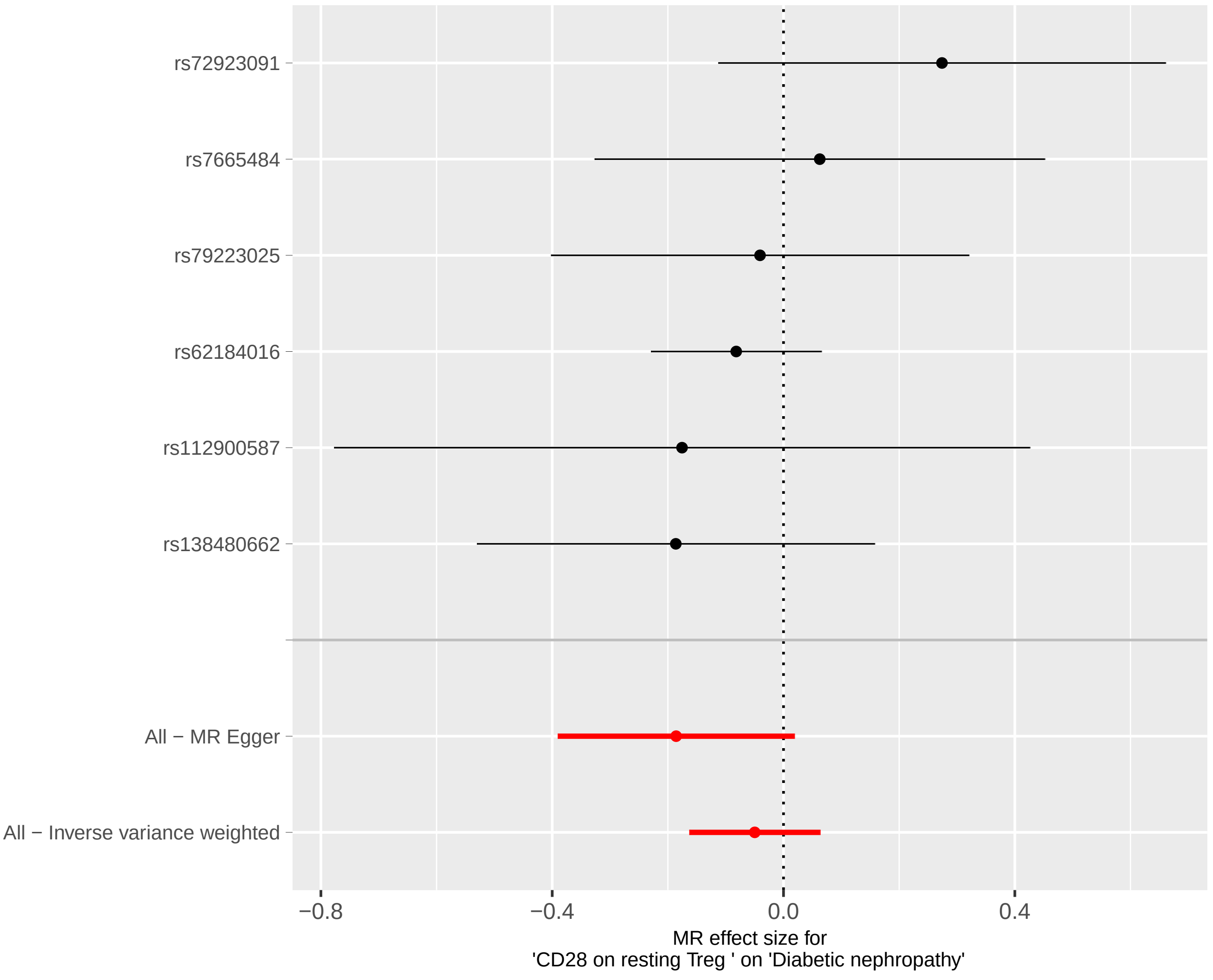
rs72923091
rs7665484
rs79223025
rs62184016
rs112900587
rs138480662
All − MR Egger
All − Inverse variance weighted
−0.8
−0.4
0.0
0.4
MR effect size for
'CD28 on resting Treg ' on 'Diabetic nephropathy'

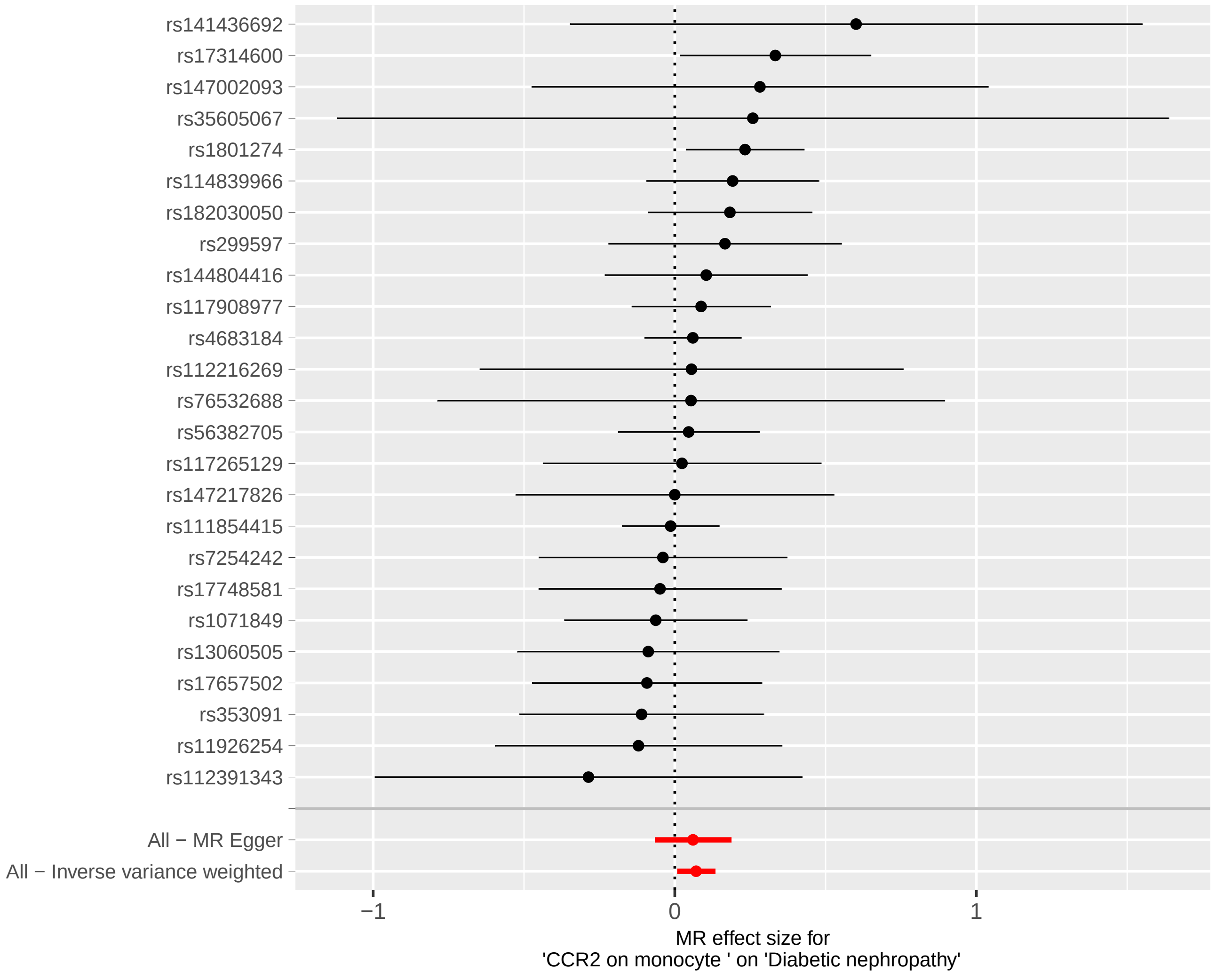
rs141436692
rs17314600
rs147002093
rs35605067
rs1801274
rs114839966
rs182030050
rs299597
rs144804416
rs117908977
rs4683184
rs112216269
rs76532688
rs56382705
rs117265129
rs147217826
rs111854415
rs7254242
rs17748581
rs1071849
rs13060505
rs17657502
rs353091
rs11926254
rs112391343
All − MR Egger
All − Inverse variance weighted
−1
0
1
MR effect size for
'CCR2 on monocyte ' on 'Diabetic nephropathy'

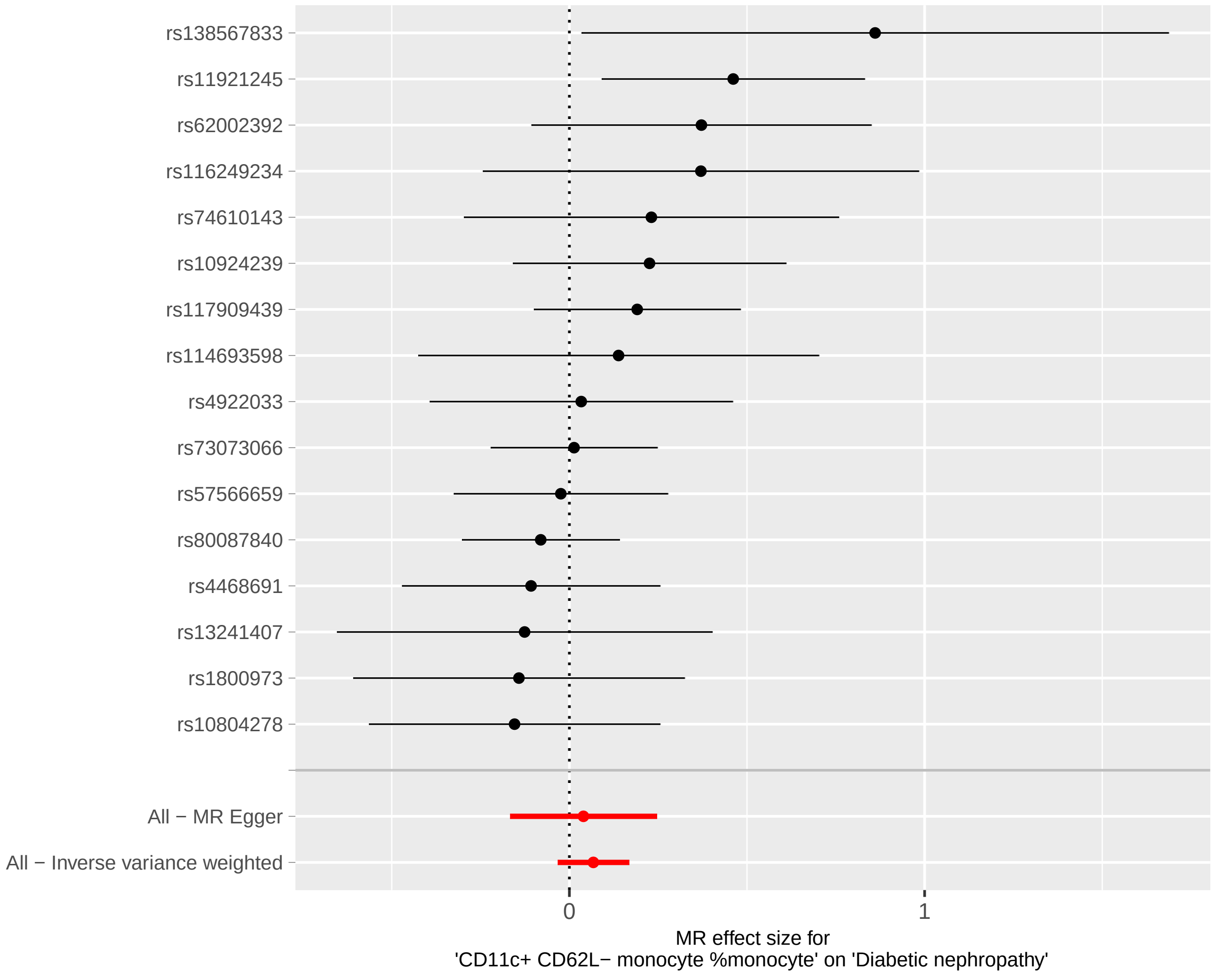
rs138567833
rs11921245
rs62002392
rs116249234
rs74610143
rs10924239
rs117909439
rs114693598
rs4922033
rs73073066
rs57566659
rs80087840
rs4468691
rs13241407
rs1800973
rs10804278
All − MR Egger
All − Inverse variance weighted
0
1
MR effect size for
'CD11c+ CD62L− monocyte %monocyte' on 'Diabetic nephropathy'

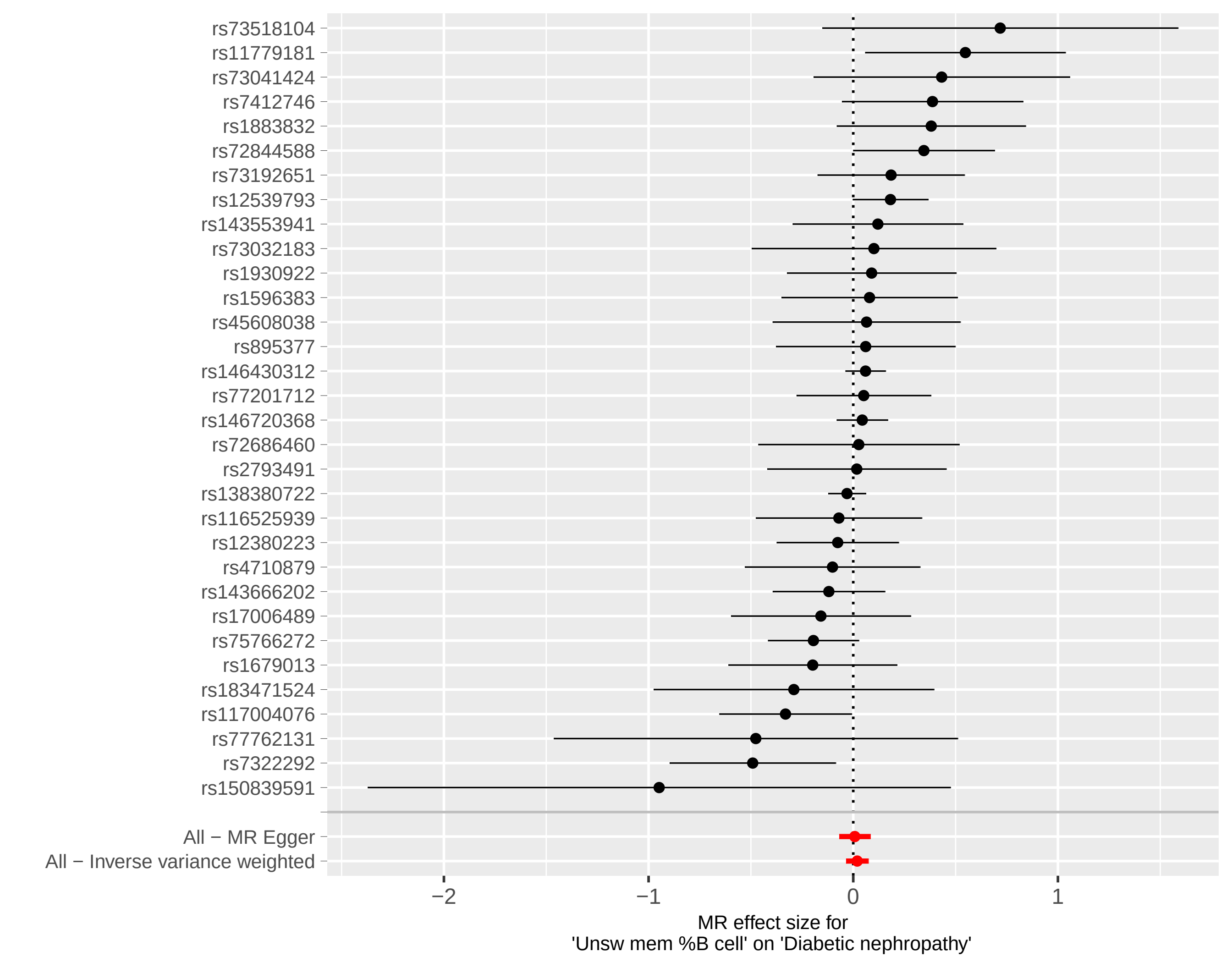
rs73518104
rs11779181
rs73041424
rs7412746
rs1883832
rs72844588
rs73192651
rs12539793
rs143553941
rs73032183
rs1930922
rs1596383
rs45608038
rs895377
rs146430312
rs77201712
rs146720368
rs72686460
rs2793491
rs138380722
rs116525939
rs12380223
rs4710879
rs143666202
rs17006489
rs75766272
rs1679013
rs183471524
rs117004076
rs77762131
rs7322292
rs150839591
All − MR Egger
All − Inverse variance weighted
−2
−1
0
1
MR effect size for
'Unsw mem %B cell' on 'Diabetic nephropathy'

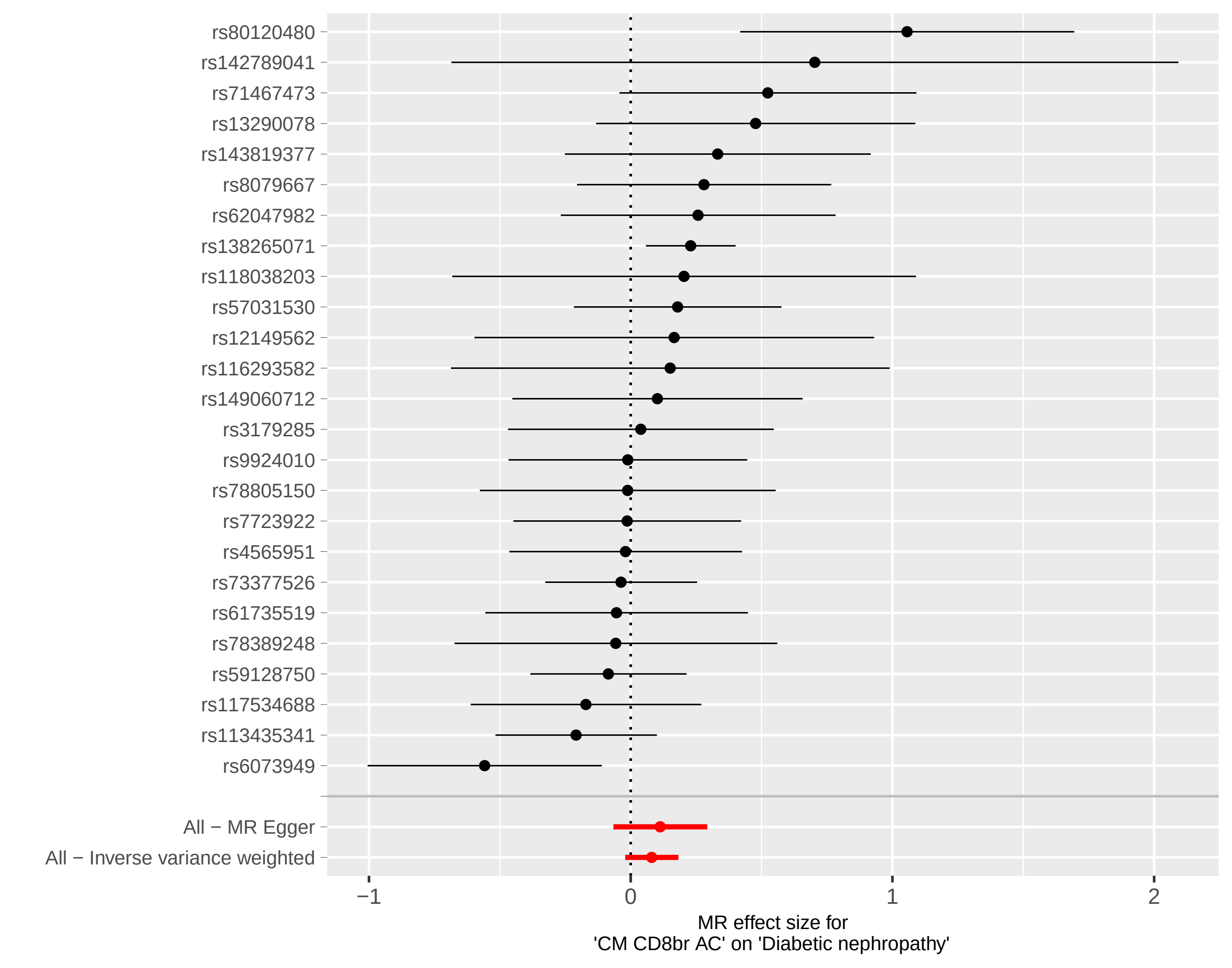

rs80120480
rs142789041
rs71467473
rs13290078
rs143819377
rs8079667
rs62047982
rs138265071
rs118038203
rs57031530
rs12149562
rs116293582
rs149060712
rs3179285
rs9924010
rs78805150
rs7723922
rs4565951
rs73377526
rs61735519
rs78389248
rs59128750
rs117534688
rs113435341
rs6073949
All − MR Egger
All − Inverse variance weighted
−1
0
1
2
MR effect size for
'CM CD8br AC' on 'Diabetic nephropathy'

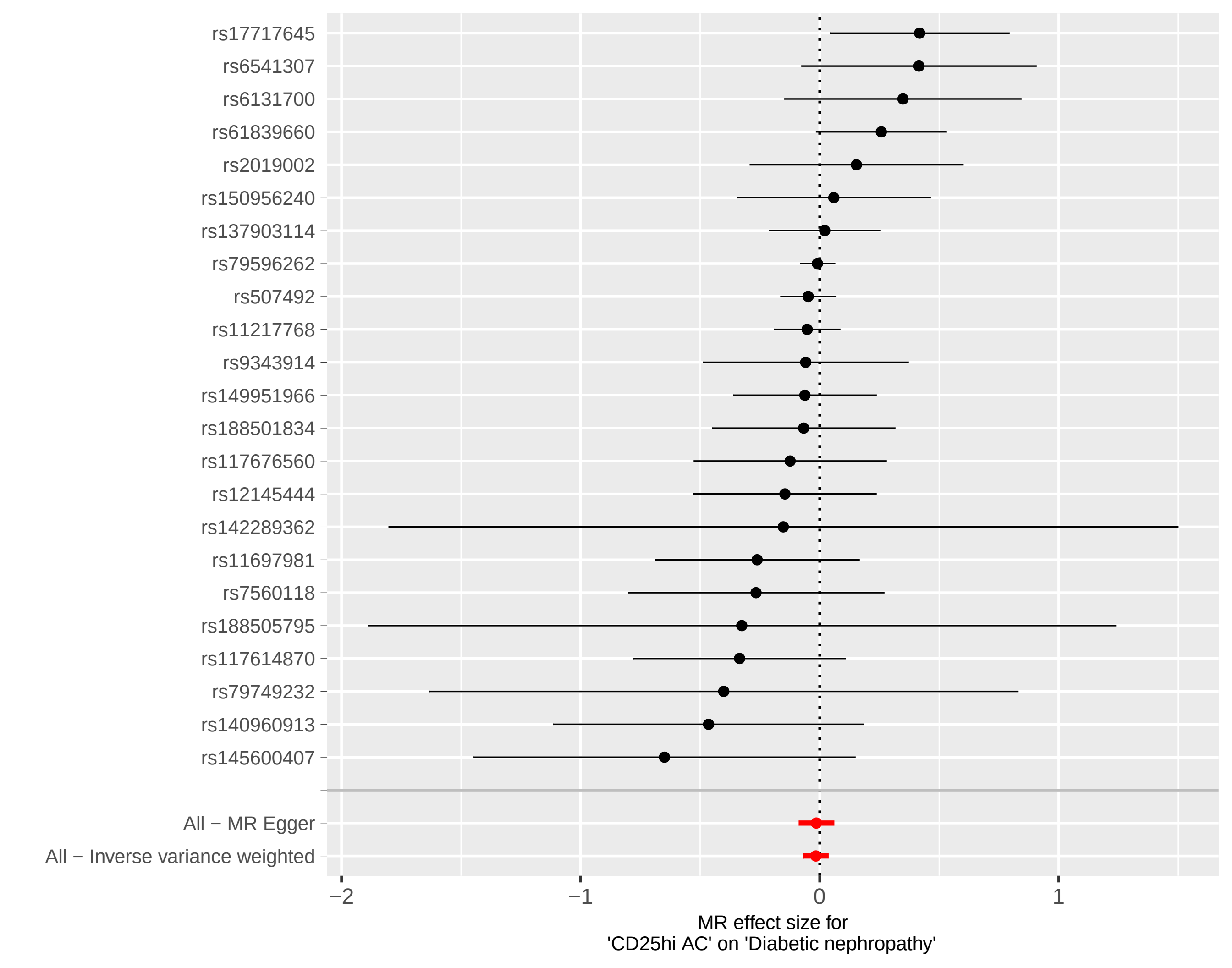
rs17717645
rs6541307
rs6131700
rs61839660
rs2019002
rs150956240
rs137903114
rs79596262
rs507492
rs11217768
rs9343914
rs149951966
rs188501834
rs117676560
rs12145444
rs142289362
rs11697981
rs7560118
rs188505795
rs117614870
rs79749232
rs140960913
rs145600407
All − MR Egger
All − Inverse variance weighted
−2
−1
0
1
MR effect size for
'CD25hi AC' on 'Diabetic nephropathy'

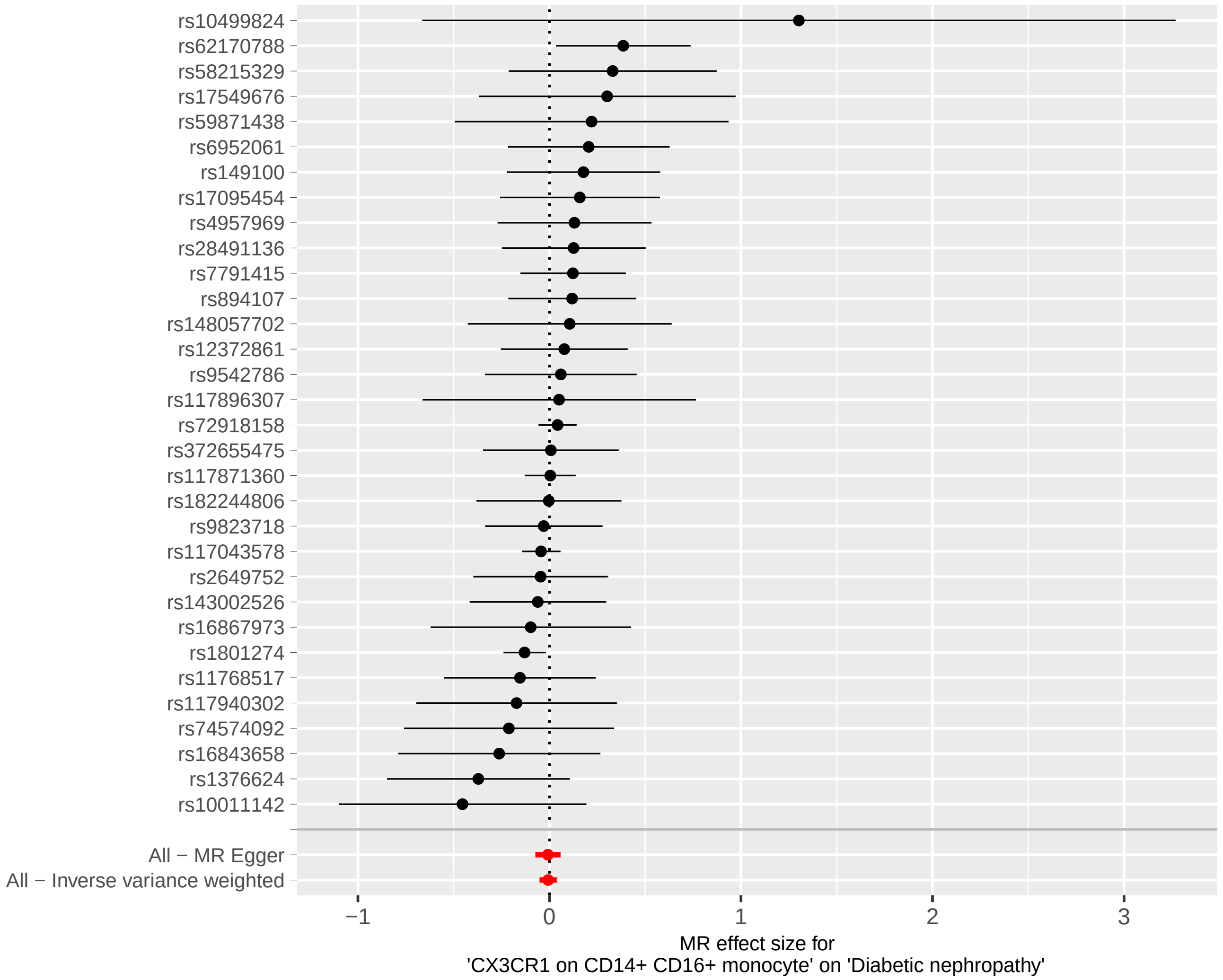
rs10499824
rs62170788
rs58215329
rs17549676
rs59871438
rs6952061
rs149100
rs17095454
rs4957969
rs28491136
rs7791415
rs894107
rs148057702
rs12372861
rs9542786
rs117896307
rs72918158
rs372655475
rs117871360
rs182244806
rs9823718
rs117043578
rs2649752
rs143002526
rs16867973
rs1801274
rs11768517
rs117940302
rs74574092
rs16843658
rs1376624
rs10011142
All − MR Egger
All − Inverse variance weighted
−1
0
1
2
3
MR effect size for
'CX3CR1 on CD14+ CD16+ monocyte' on 'Diabetic nephropathy'

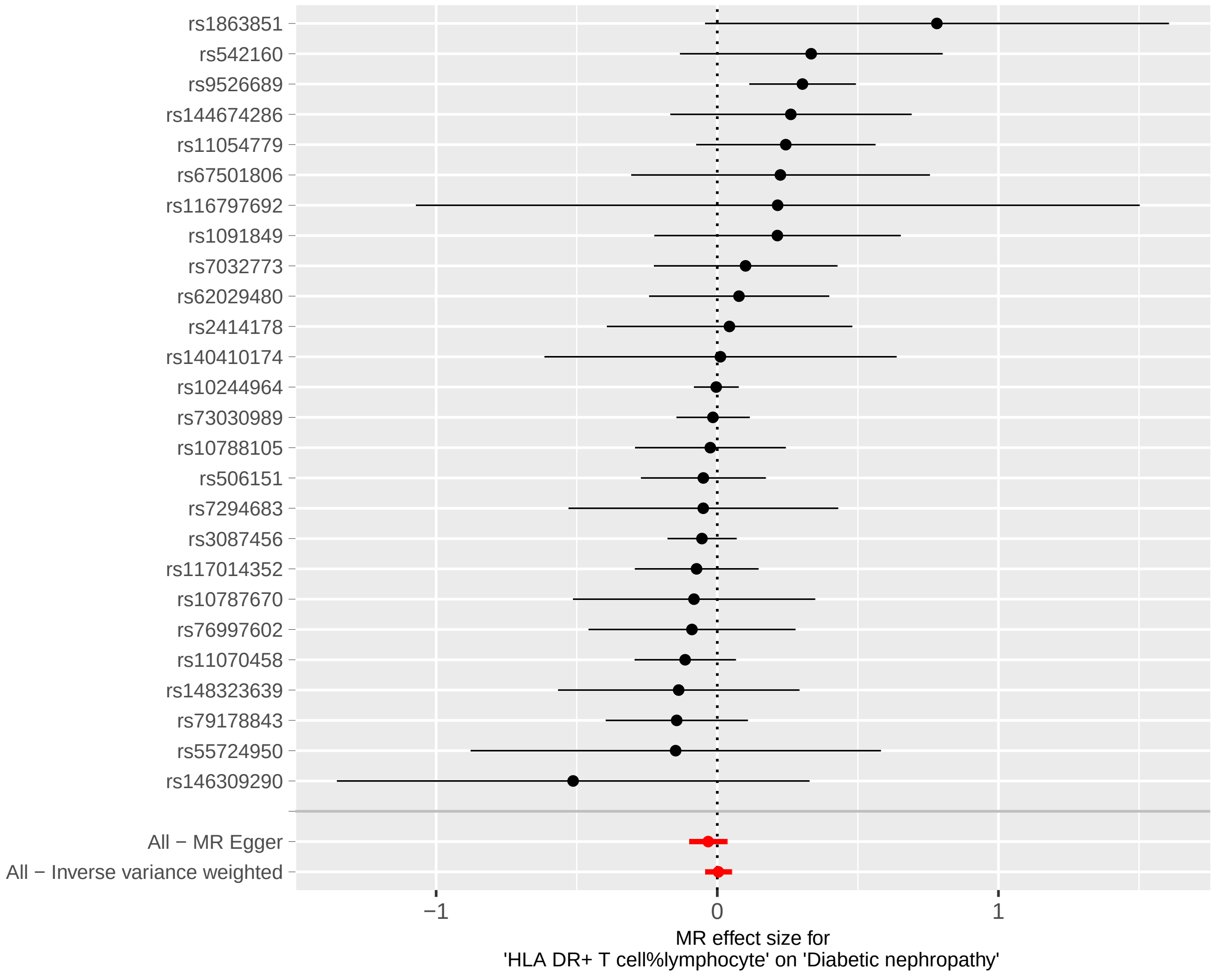
rs1863851
rs542160
rs9526689
rs144674286
rs11054779
rs67501806
rs116797692
rs1091849
rs7032773
rs62029480
rs2414178
rs140410174
rs10244964
rs73030989
rs10788105
rs506151
rs7294683
rs3087456
rs117014352
rs10787670
rs76997602
rs11070458
rs148323639
rs79178843
rs55724950
rs146309290
All − MR Egger
All − Inverse variance weighted
−1
0
1
MR effect size for
'HLA DR+ T cell%lymphocyte' on 'Diabetic nephropathy'

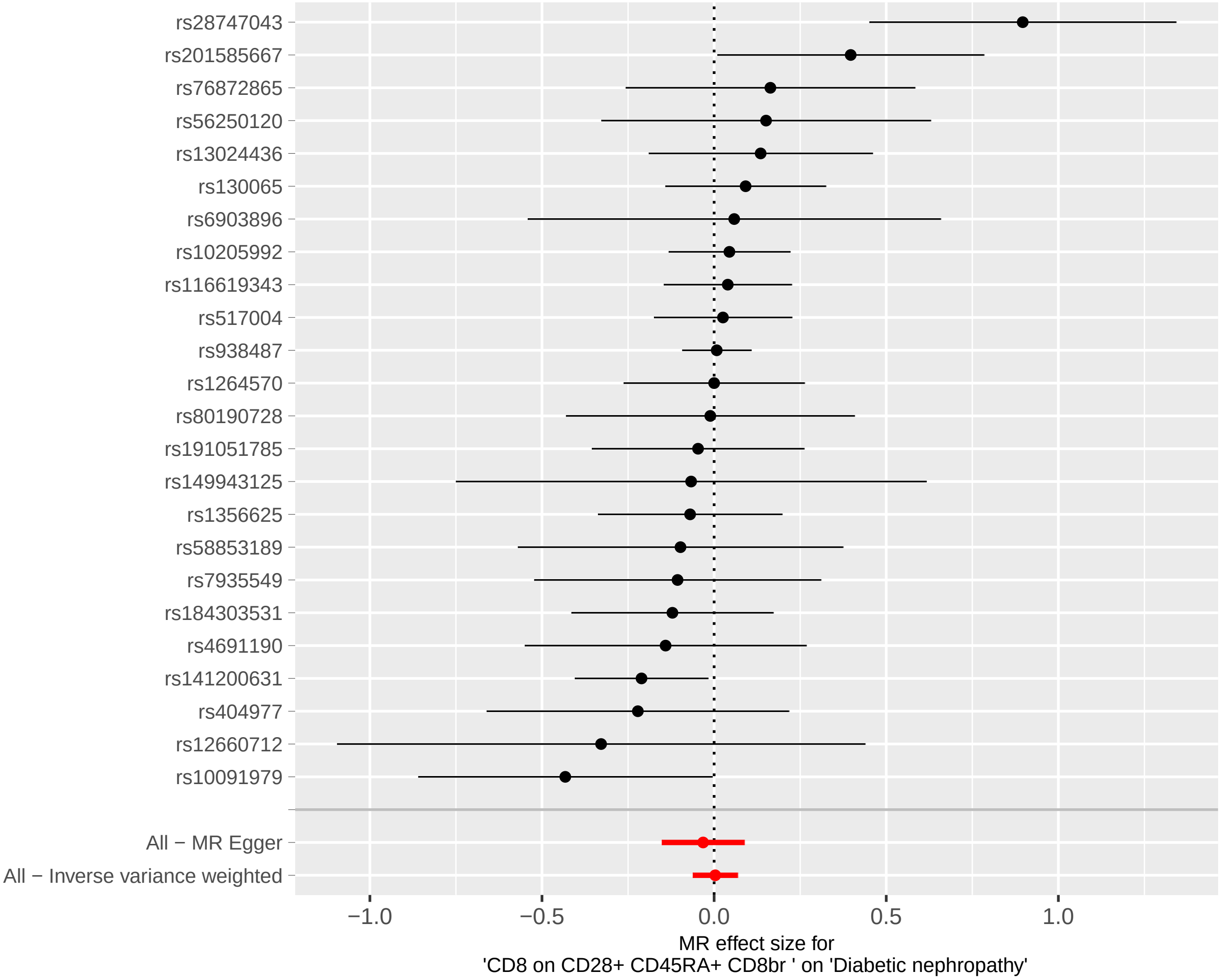
rs28747043
rs201585667
rs76872865
rs56250120
rs13024436
rs130065
rs6903896
rs10205992
rs116619343
rs517004
rs938487
rs1264570
rs80190728
rs191051785
rs149943125
rs1356625
rs58853189
rs7935549
rs184303531
rs4691190
rs141200631
rs404977
rs12660712
rs10091979
All − MR Egger
All − Inverse variance weighted
−1.0
−0.5
0.0
0.5
1.0
MR effect size for
'CD8 on CD28+ CD45RA+ CD8br ' on 'Diabetic nephropathy'

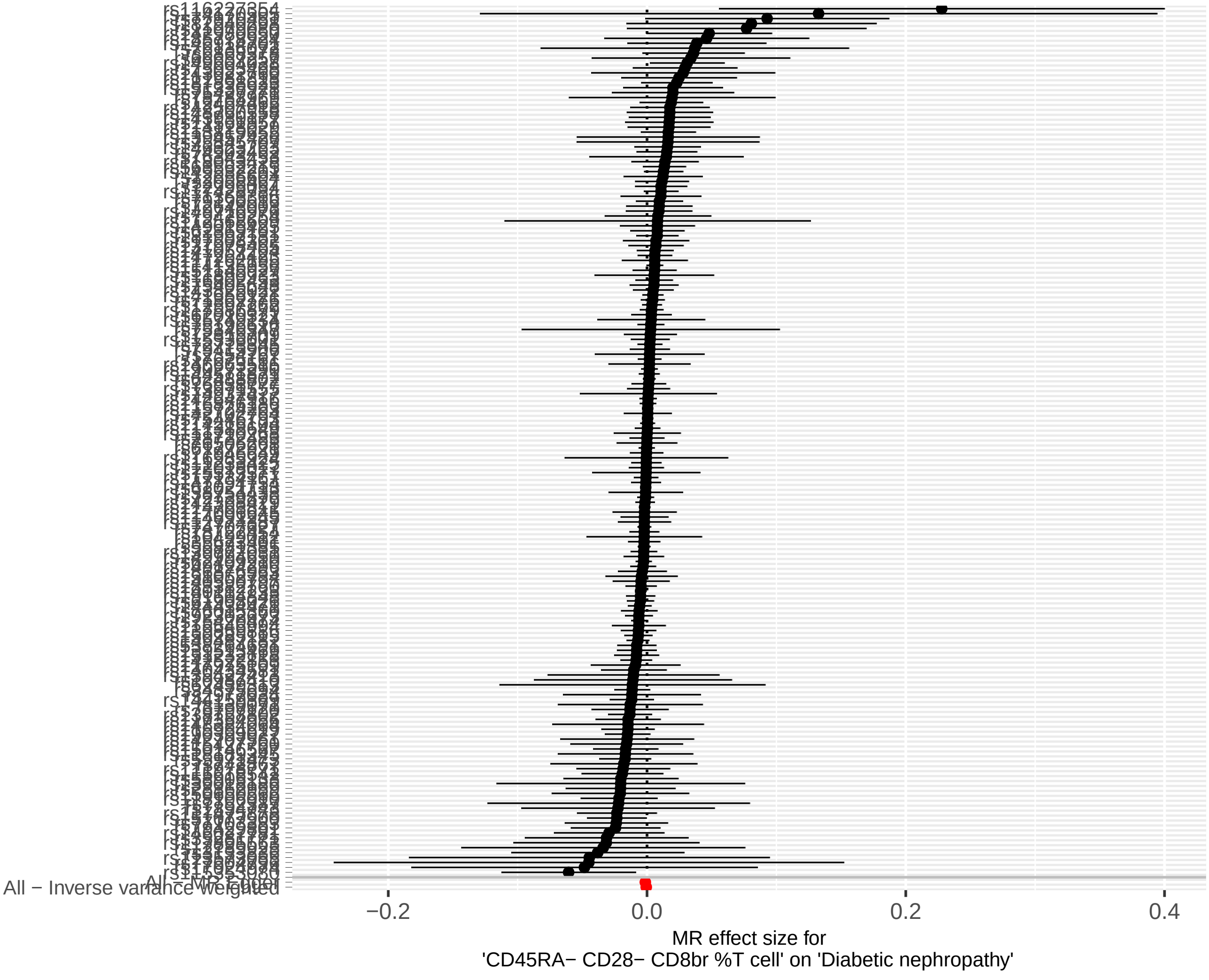
rs116227354
All − MR Egger
All − Inverse variance weighted
−0.2
0.0
0.2
0.4
MR effect size for
'CD45RA− CD28− CD8br %T cell' on 'Diabetic nephropathy'

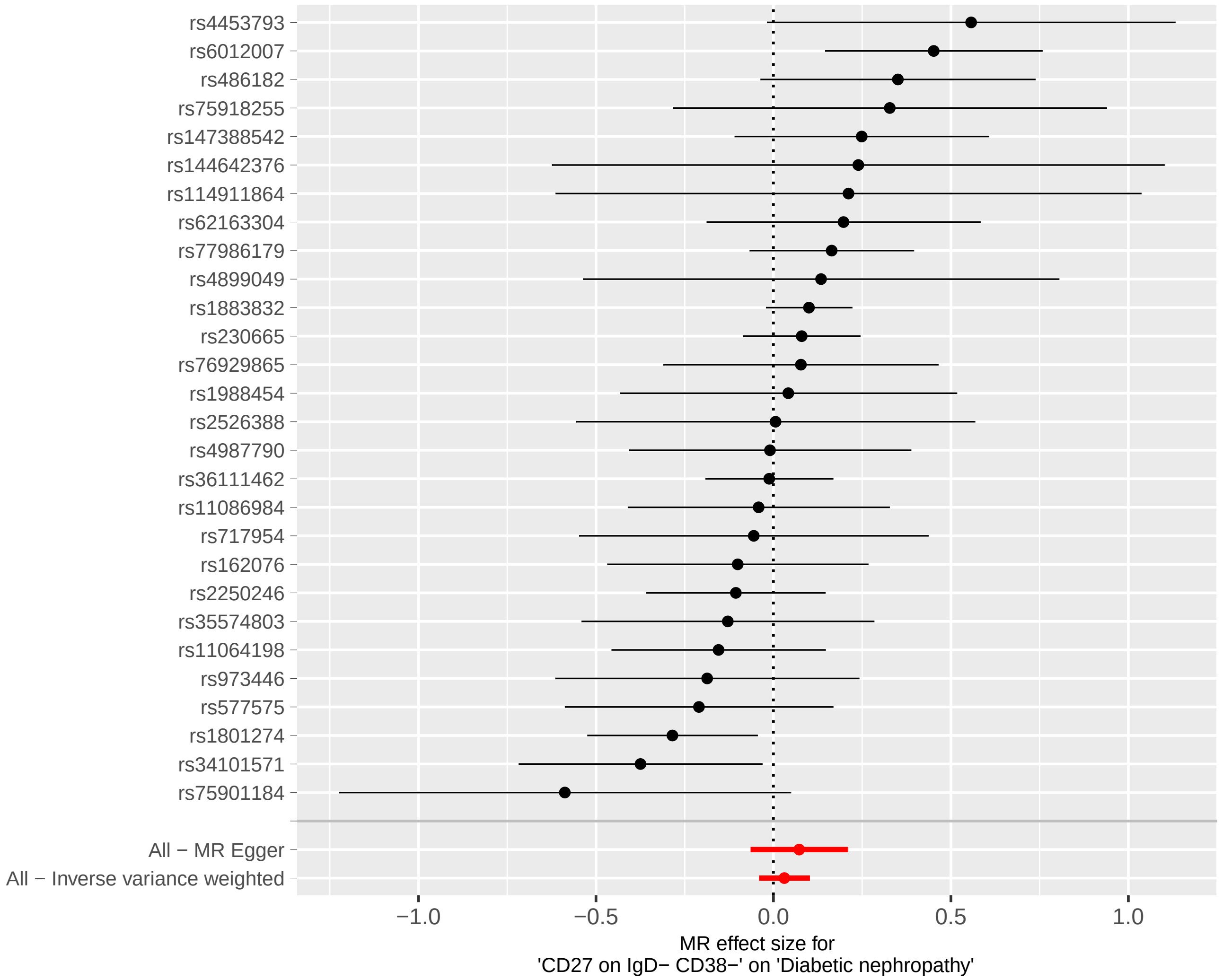

rs4453793
rs6012007
rs486182
rs75918255
rs147388542
rs144642376
rs114911864
rs62163304
rs77986179
rs4899049
rs1883832
rs230665
rs76929865
rs1988454
rs2526388
rs4987790
rs36111462
rs11086984
rs717954
rs162076
rs2250246
rs35574803
rs11064198
rs973446
rs577575
rs1801274
rs34101571
rs75901184
All − MR Egger
All − Inverse variance weighted
−1.0
−0.5
0.0
0.5
1.0
MR effect size for
'CD27 on IgD− CD38−' on 'Diabetic nephropathy'

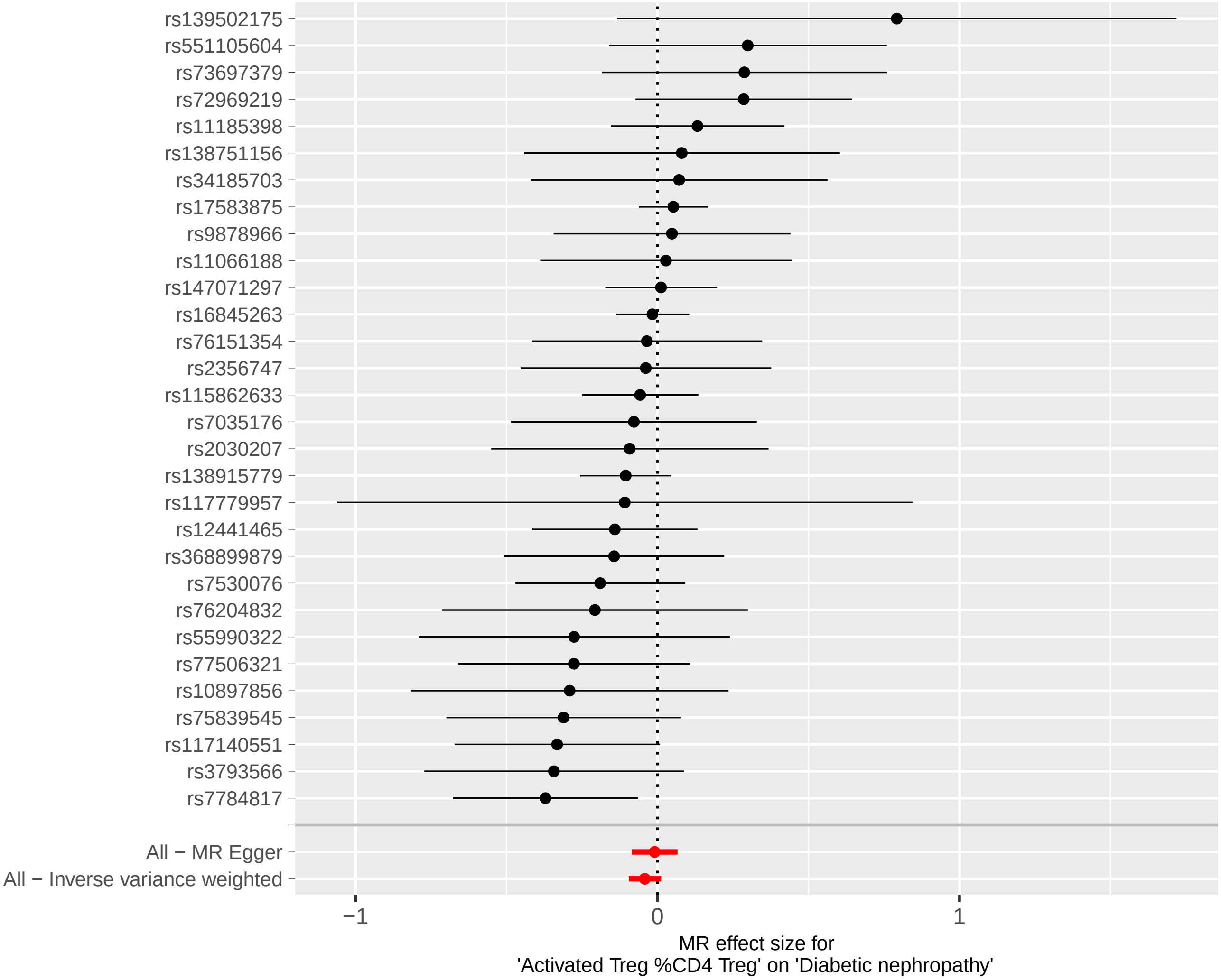
rs139502175
rs551105604
rs73697379
rs72969219
rs11185398
rs138751156
rs34185703
rs17583875
rs9878966
rs11066188
rs147071297
rs16845263
rs76151354
rs2356747
rs115862633
rs7035176
rs2030207
rs138915779
rs117779957
rs12441465
rs368899879
rs7530076
rs76204832
rs55990322
rs77506321
rs10897856
rs75839545
rs117140551
rs3793566
rs7784817
All − MR Egger
All − Inverse variance weighted
−1
0
1
MR effect size for
'Activated Treg %CD4 Treg' on 'Diabetic nephropathy'

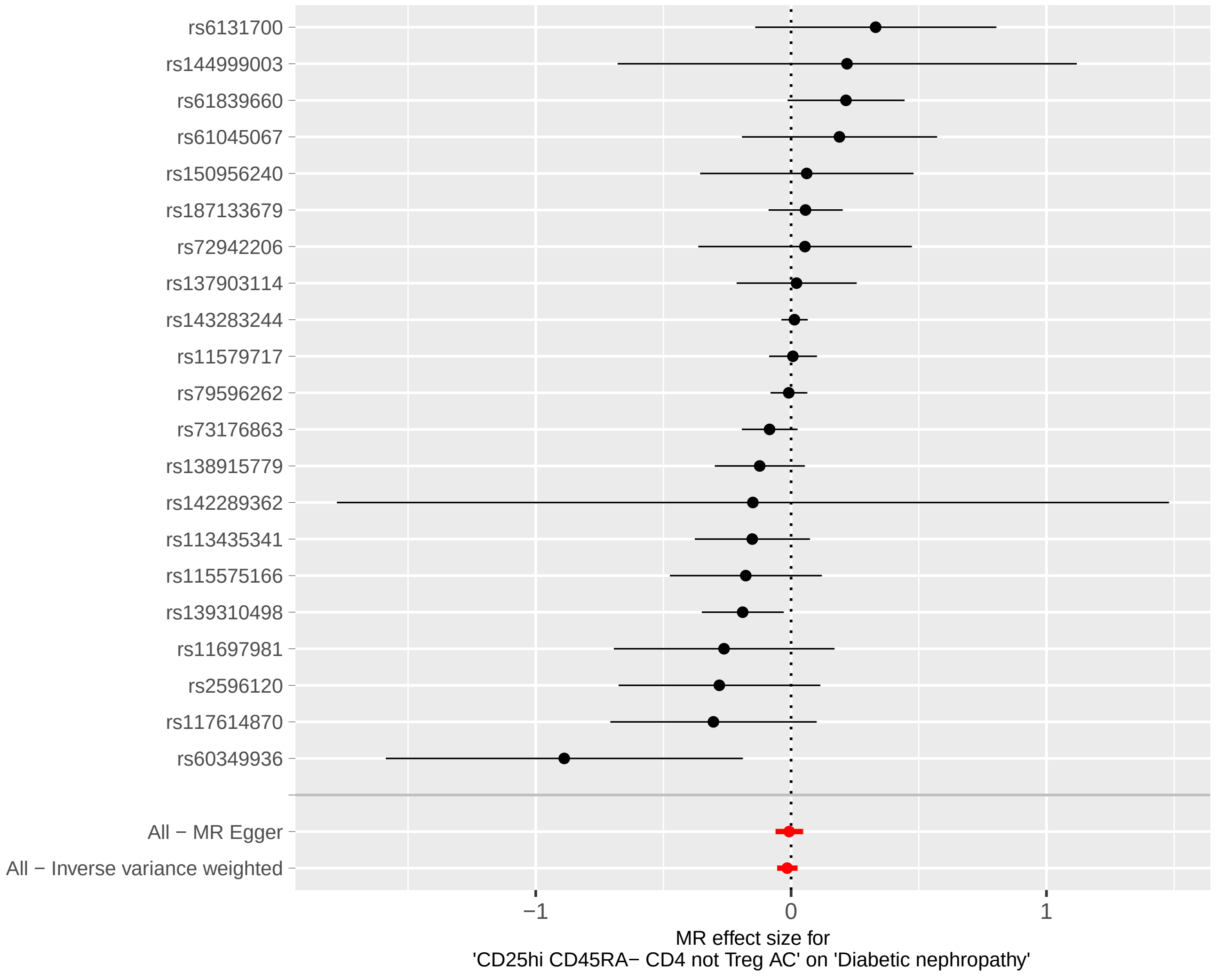
rs6131700
rs144999003
rs61839660
rs61045067
rs150956240
rs187133679
rs72942206
rs137903114
rs143283244
rs11579717
rs79596262
rs73176863
rs138915779
rs142289362
rs113435341
rs115575166
rs139310498
rs11697981
rs2596120
rs117614870
rs60349936
All − MR Egger
All − Inverse variance weighted
−1
0
1
MR effect size for
'CD25hi CD45RA− CD4 not Treg AC' on 'Diabetic nephropathy'

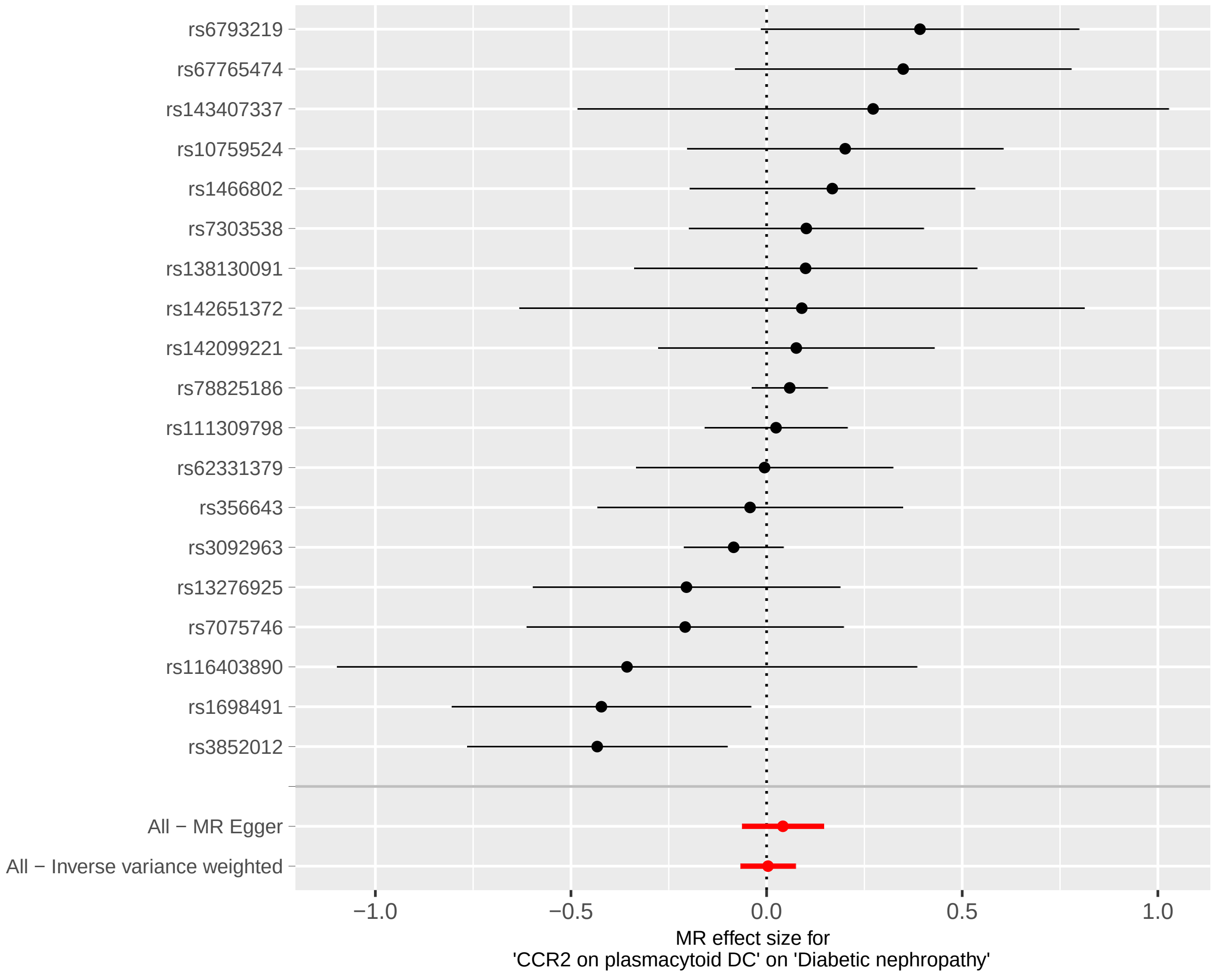
rs6793219
rs67765474
rs143407337
rs10759524
rs1466802
rs7303538
rs138130091
rs142651372
rs142099221
rs78825186
rs111309798
rs62331379
rs356643
rs3092963
rs13276925
rs7075746
rs116403890
rs1698491
rs3852012
All − MR Egger
All − Inverse variance weighted
−1.0
−0.5
0.0
0.5
1.0
MR effect size for
'CCR2 on plasmacytoid DC' on 'Diabetic nephropathy'

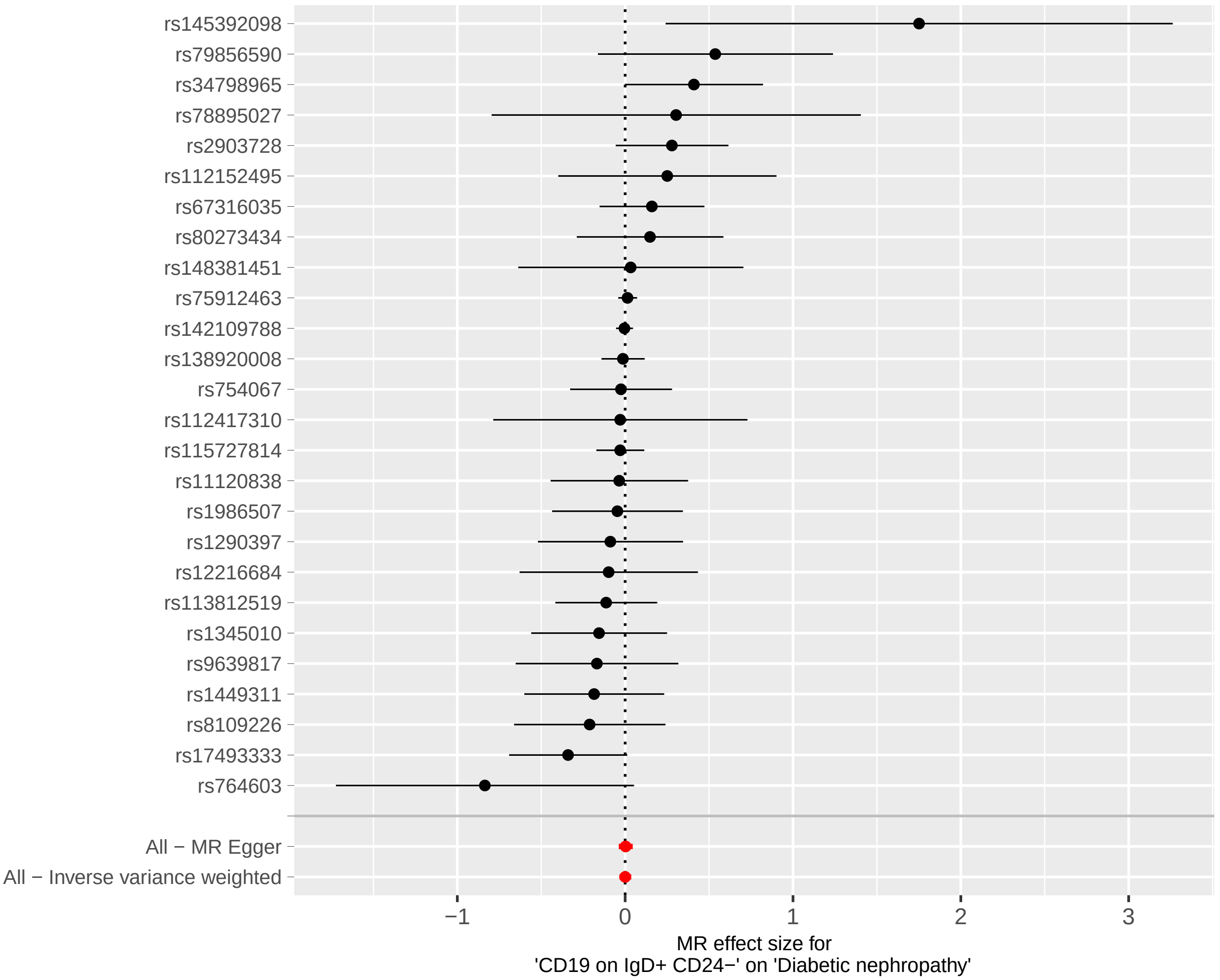
rs145392098
rs79856590
rs34798965
rs78895027
rs2903728
rs112152495
rs67316035
rs80273434
rs148381451
rs75912463
rs142109788
rs138920008
rs754067
rs112417310
rs115727814
rs11120838
rs1986507
rs1290397
rs12216684
rs113812519
rs1345010
rs9639817
rs1449311
rs8109226
rs17493333
rs764603
All − MR Egger
All − Inverse variance weighted
−1
0
1
2
3
MR effect size for
'CD19 on IgD+ CD24−' on 'Diabetic nephropathy'

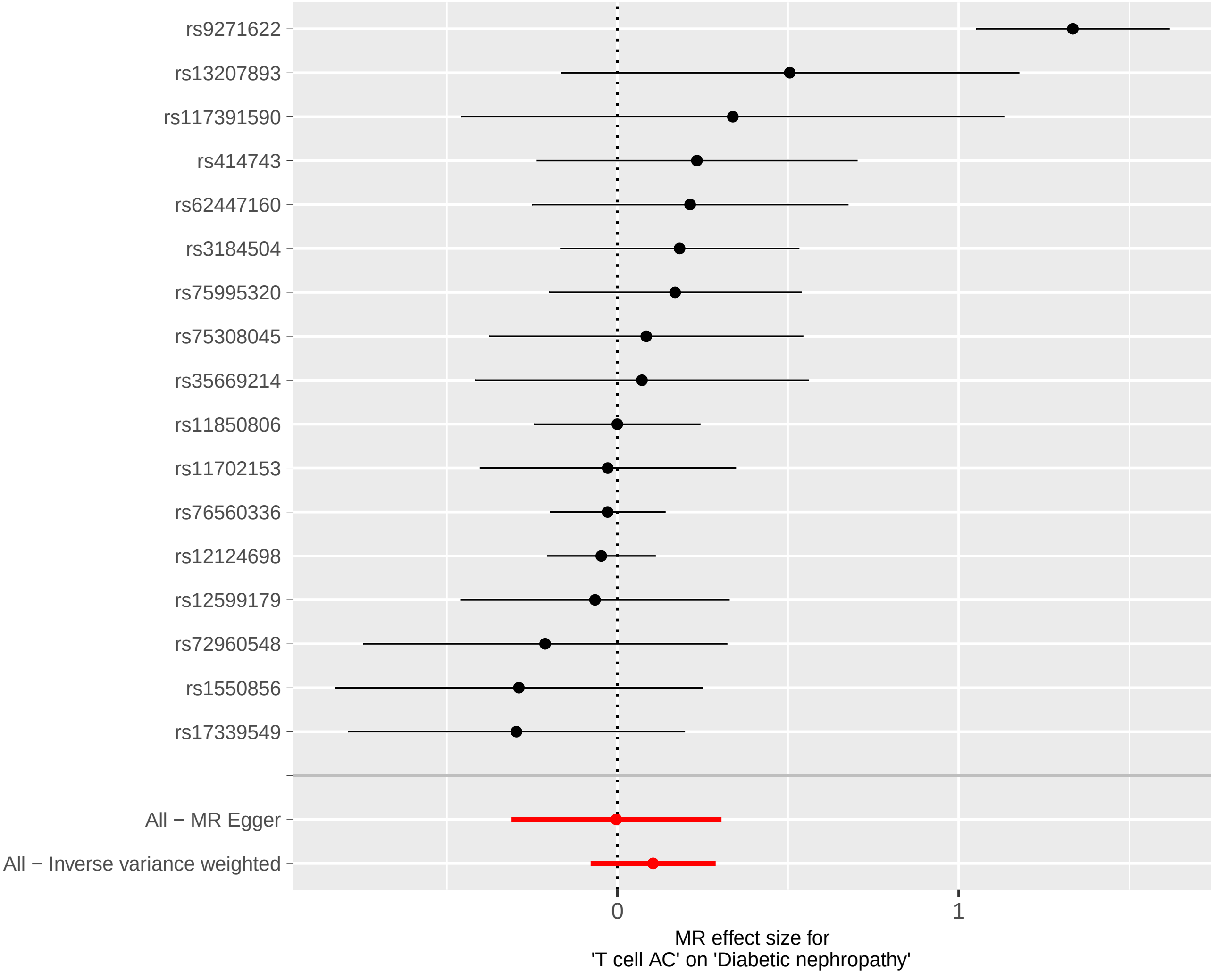

rs9271622
rs13207893
rs117391590
rs414743
rs62447160
rs3184504
rs75995320
rs75308045
rs35669214
rs11850806
rs11702153
rs76560336
rs12124698
rs12599179
rs72960548
rs1550856
rs17339549
All − MR Egger
All − Inverse variance weighted
0
1
MR effect size for
'T cell AC' on 'Diabetic nephropathy'

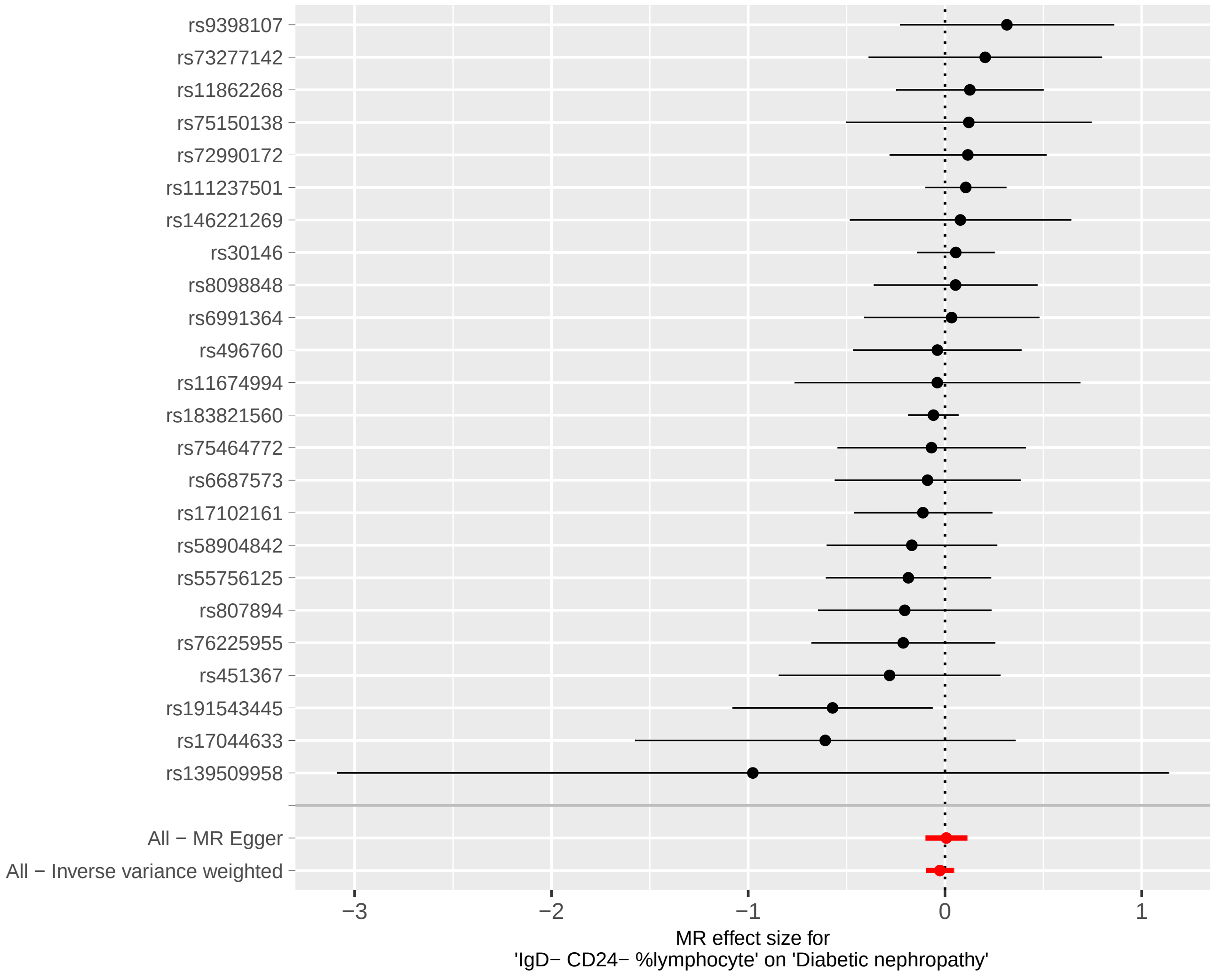

rs9398107
rs73277142
rs11862268
rs75150138
rs72990172
rs111237501
rs146221269
rs30146
rs8098848
rs6991364
rs496760
rs11674994
rs183821560
rs75464772
rs6687573
rs17102161
rs58904842
rs55756125
rs807894
rs76225955
rs451367
rs191543445
rs17044633
rs139509958
All − MR Egger
All − Inverse variance weighted
−3
−2
−1
0
1
MR effect size for
'IgD− CD24− %lymphocyte' on 'Diabetic nephropathy'

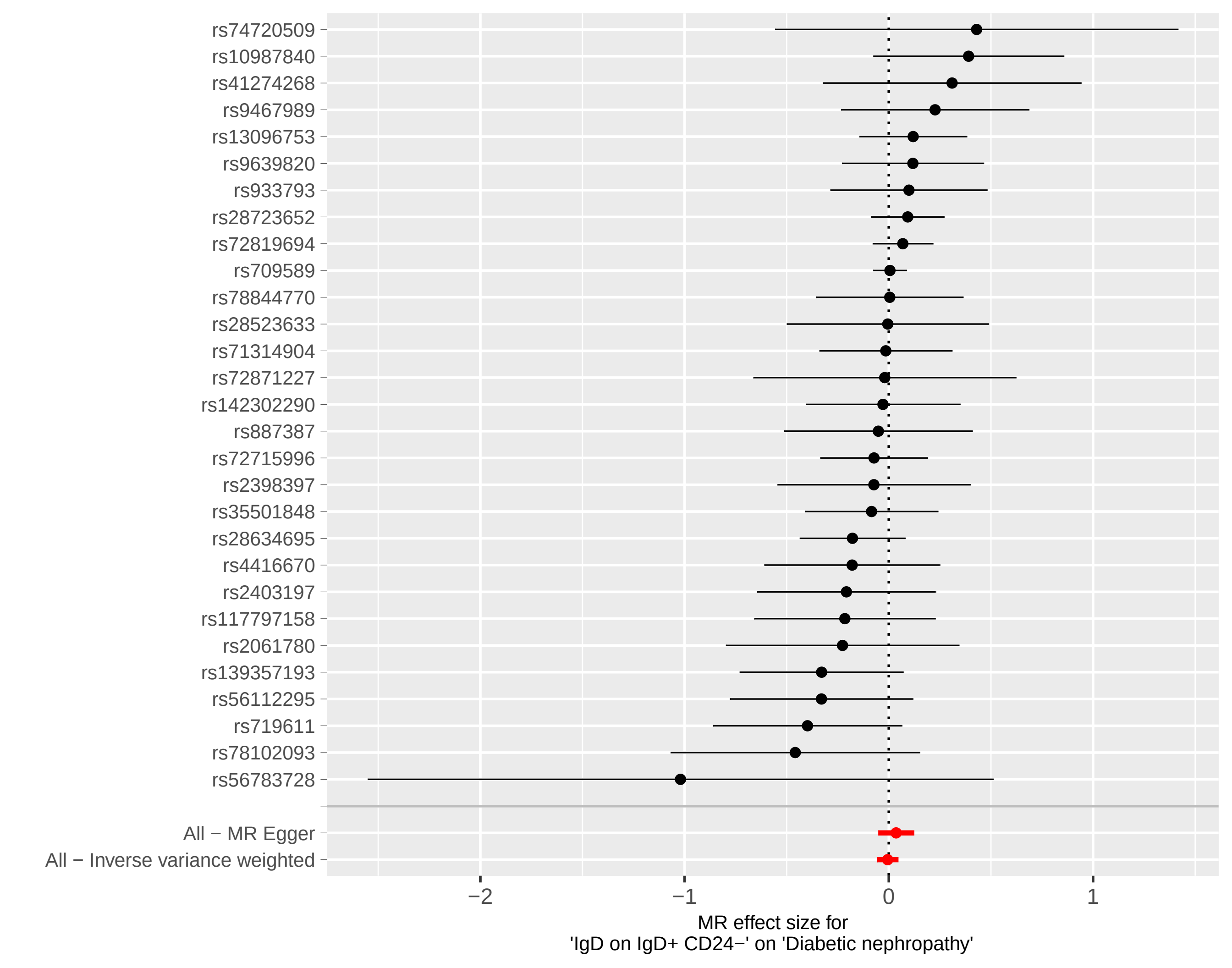
rs74720509
rs10987840
rs41274268
rs9467989
rs13096753
rs9639820
rs933793
rs28723652
rs72819694
rs709589
rs78844770
rs28523633
rs71314904
rs72871227
rs142302290
rs887387
rs72715996
rs2398397
rs35501848
rs28634695
rs4416670
rs2403197
rs117797158
rs2061780
rs139357193
rs56112295
rs719611
rs78102093
rs56783728
All − MR Egger
All − Inverse variance weighted
−2
−1
0
1
MR effect size for
'IgD on IgD+ CD24−' on 'Diabetic nephropathy'

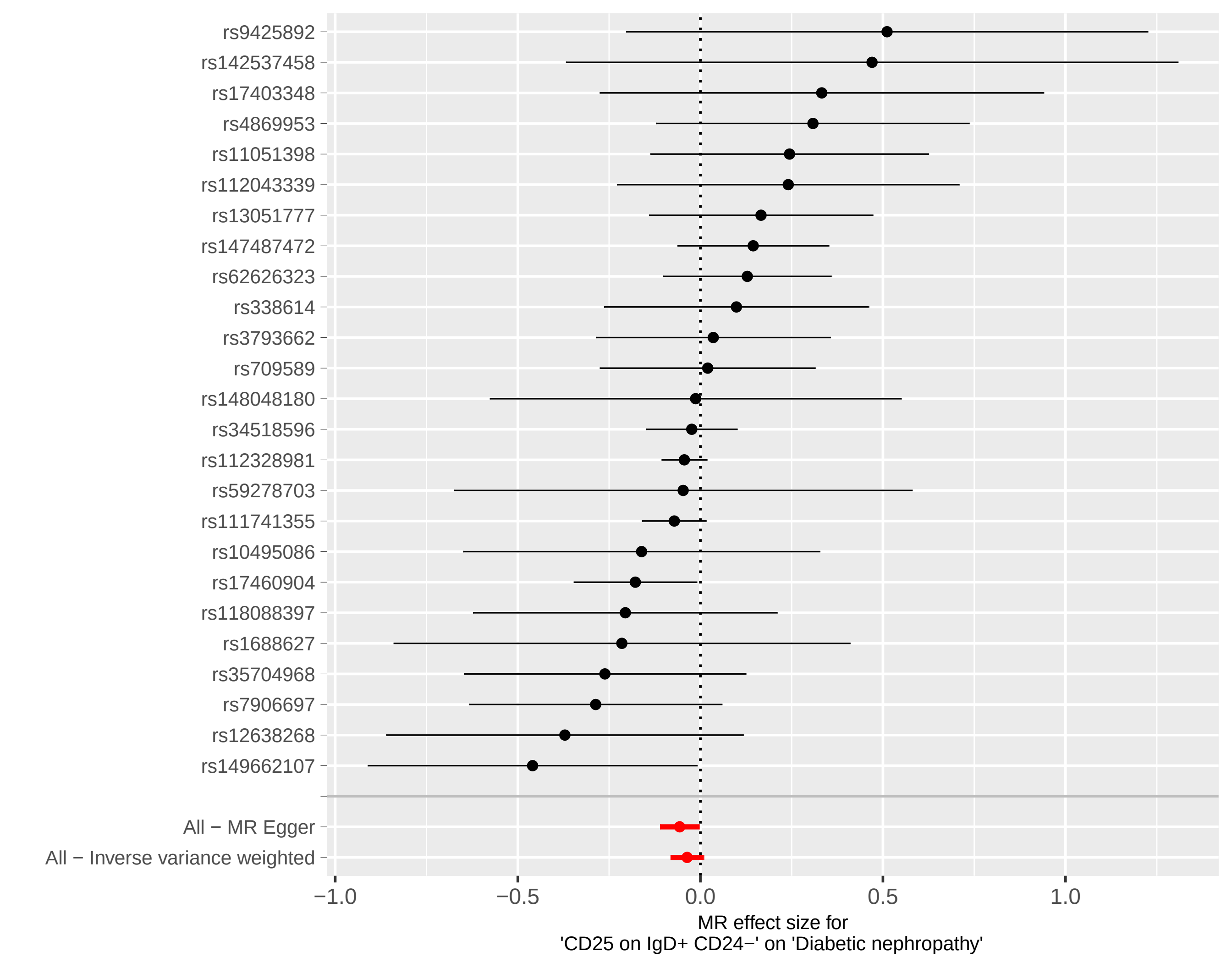
rs9425892
rs142537458
rs17403348
rs4869953
rs11051398
rs112043339
rs13051777
rs147487472
rs62626323
rs338614
rs3793662
rs709589
rs148048180
rs34518596
rs112328981
rs59278703
rs111741355
rs10495086
rs17460904
rs118088397
rs1688627
rs35704968
rs7906697
rs12638268
rs149662107
All − MR Egger
All − Inverse variance weighted
−1.0
−0.5
0.0
0.5
1.0
MR effect size for
'CD25 on IgD+ CD24−' on 'Diabetic nephropathy'

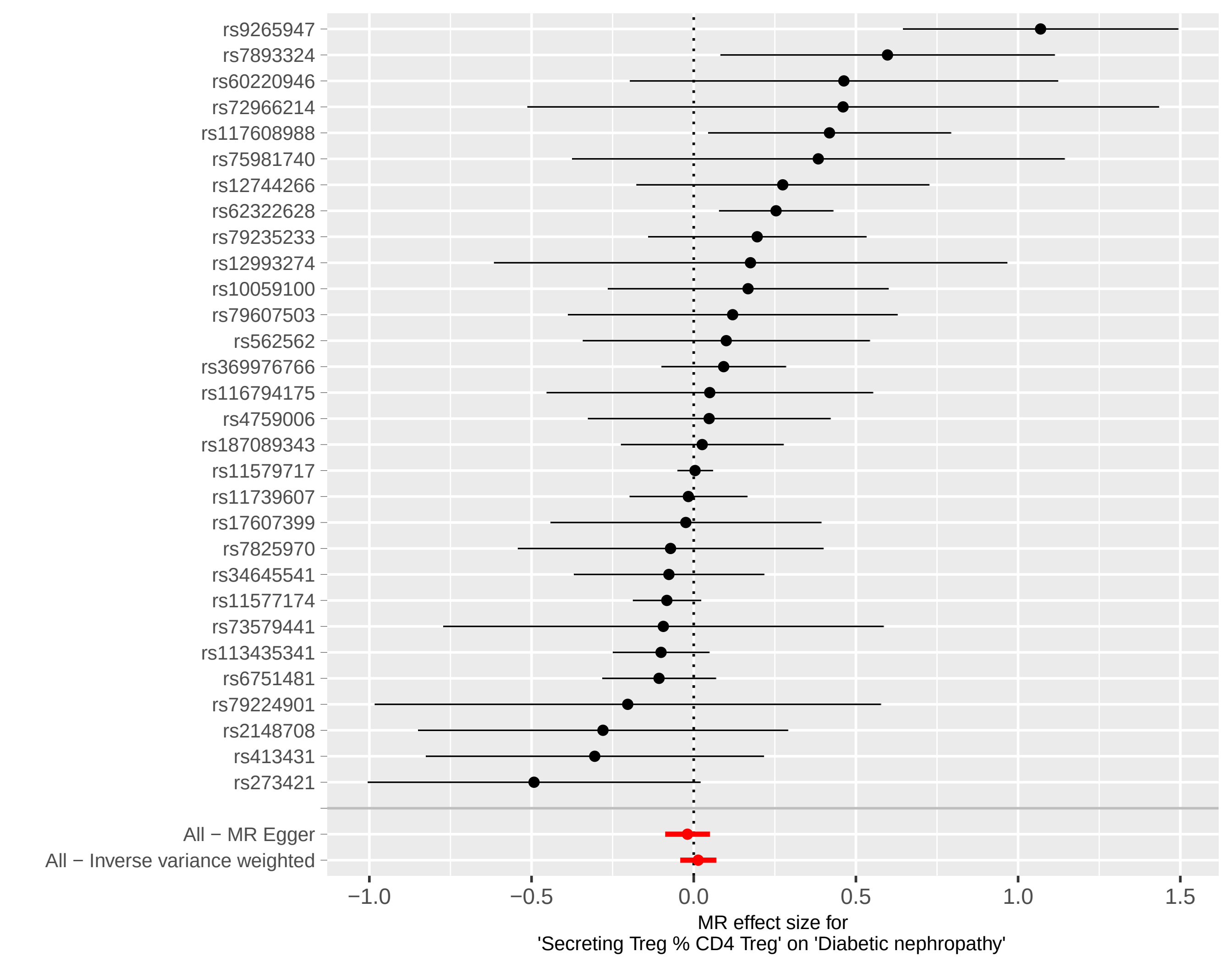
rs9265947
rs7893324
rs60220946
rs72966214
rs117608988
rs75981740
rs12744266
rs62322628
rs79235233
rs12993274
rs10059100
rs79607503
rs562562
rs369976766
rs116794175
rs4759006
rs187089343
rs11579717
rs11739607
rs17607399
rs7825970
rs34645541
rs11577174
rs73579441
rs113435341
rs6751481
rs79224901
rs2148708
rs413431
rs273421
All − MR Egger
All − Inverse variance weighted
−1.0
−0.5
0.0
0.5
1.0
1.5
MR effect size for
'Secreting Treg % CD4 Treg' on 'Diabetic nephropathy'

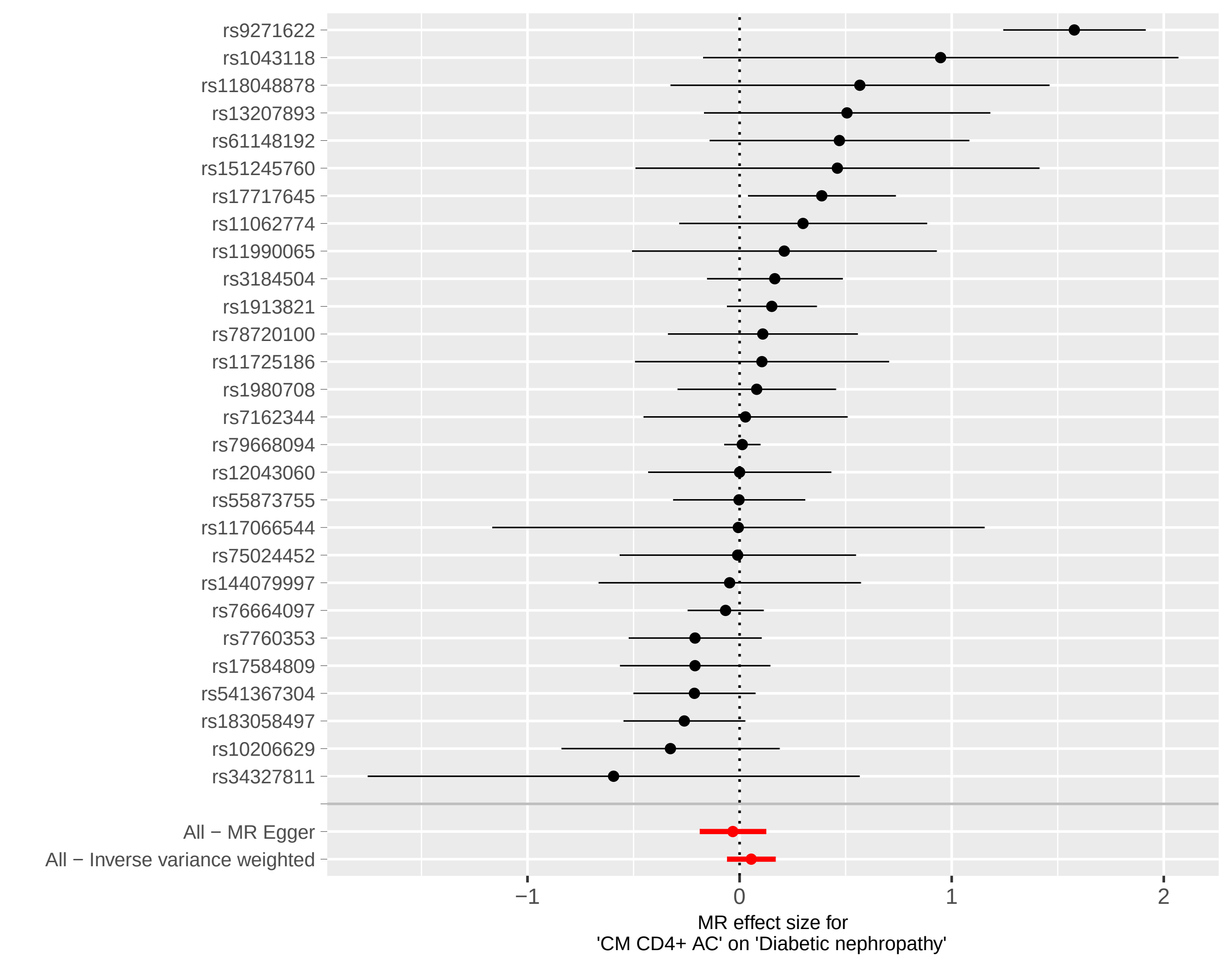

rs9271622
rs1043118
rs118048878
rs13207893
rs61148192
rs151245760
rs17717645
rs11062774
rs11990065
rs3184504
rs1913821
rs78720100
rs11725186
rs1980708
rs7162344
rs79668094
rs12043060
rs55873755
rs117066544
rs75024452
rs144079997
rs76664097
rs7760353
rs17584809
rs541367304
rs183058497
rs10206629
rs34327811
All − MR Egger
All − Inverse variance weighted
−1
0
1
2
MR effect size for
'CM CD4+ AC' on 'Diabetic nephropathy'

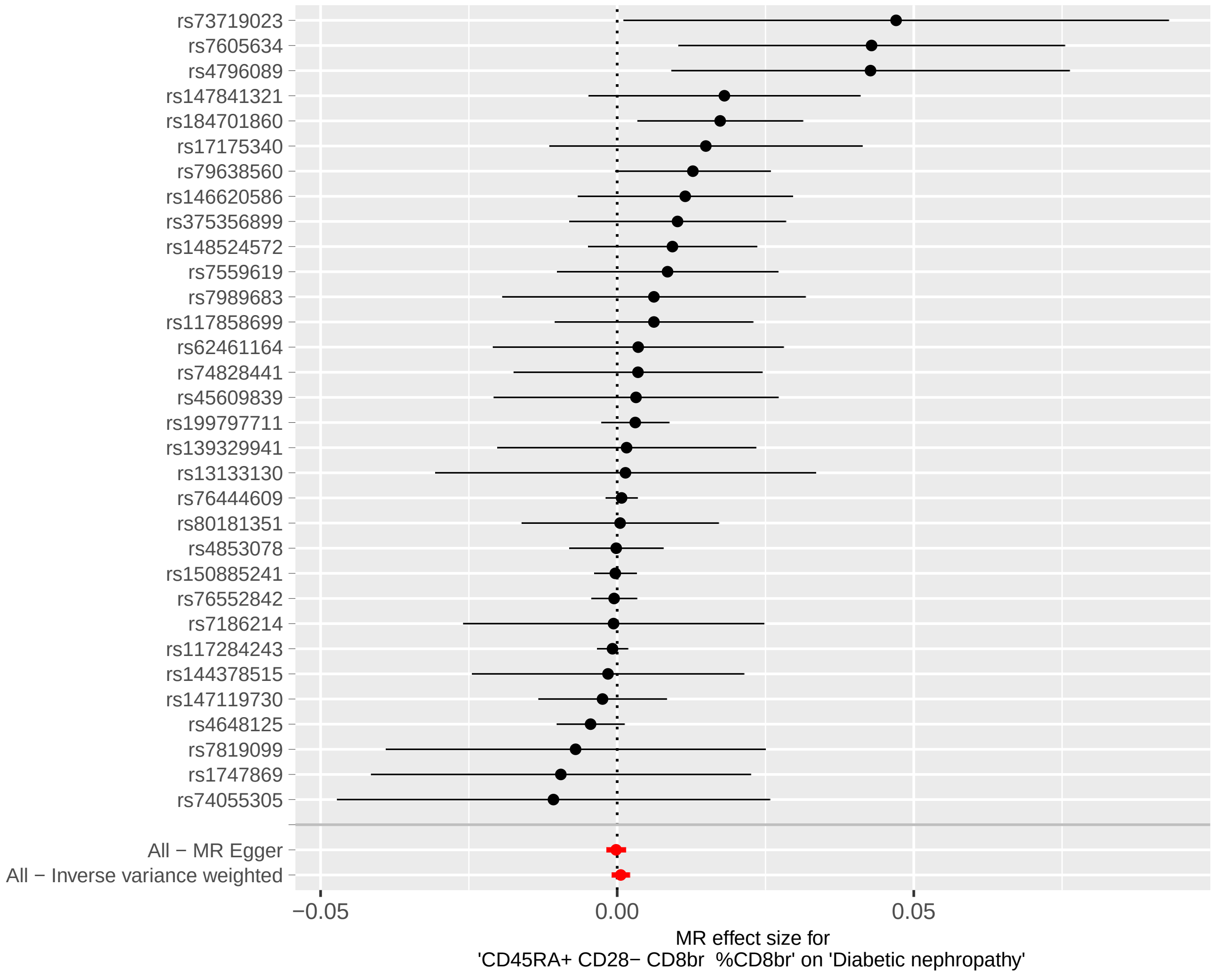
rs73719023
rs7605634
rs4796089
rs147841321
rs184701860
rs17175340
rs79638560
rs146620586
rs375356899
rs148524572
rs7559619
rs7989683
rs117858699
rs62461164
rs74828441
rs45609839
rs199797711
rs139329941
rs13133130
rs76444609
rs80181351
rs4853078
rs150885241
rs76552842
rs7186214
rs117284243
rs144378515
rs147119730
rs4648125
rs7819099
rs1747869
rs74055305
All − MR Egger
All − Inverse variance weighted
−0.05
0.00
0.05
MR effect size for
'CD45RA+ CD28− CD8br %CD8br' on 'Diabetic nephropathy'

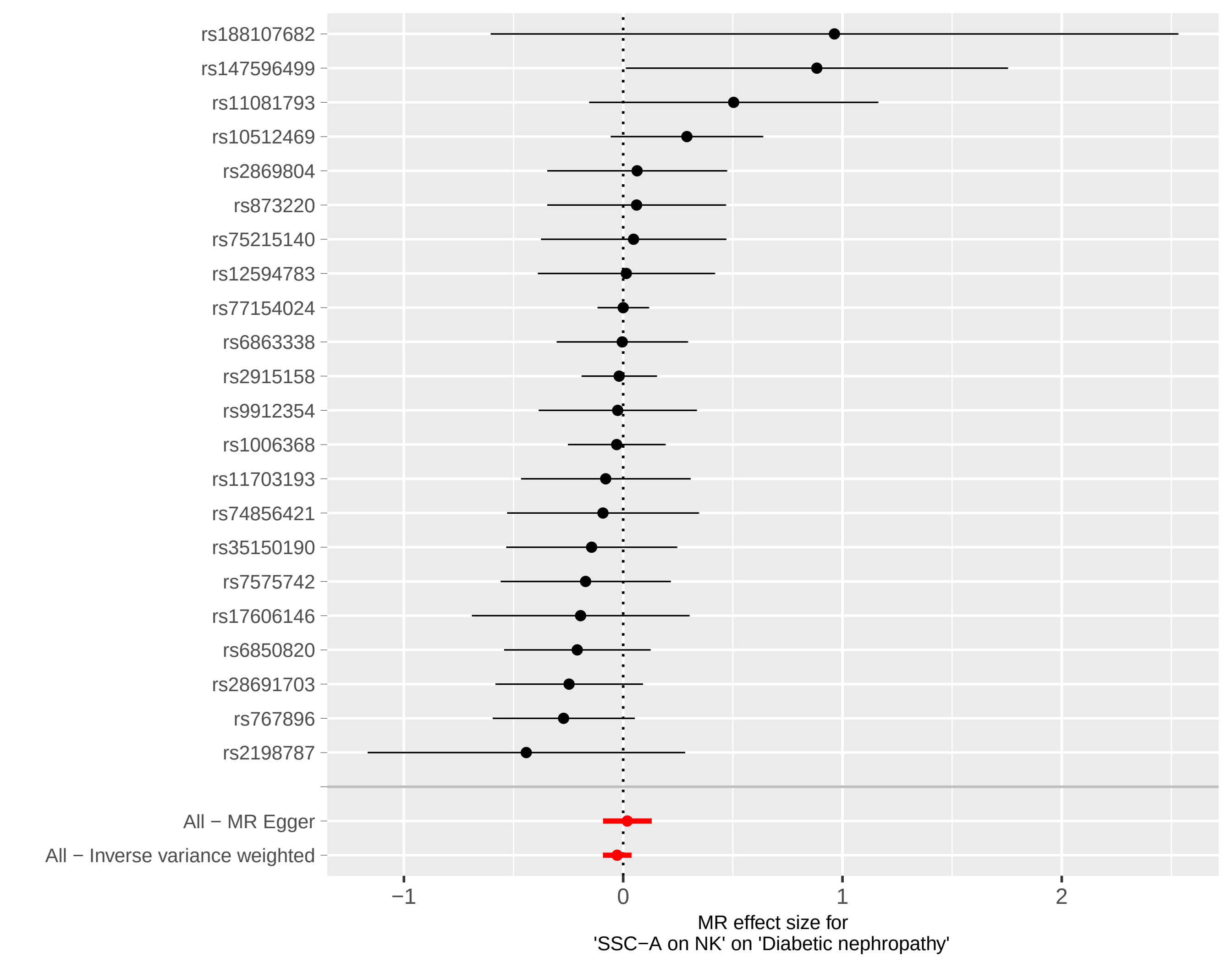
rs188107682
rs147596499
rs11081793
rs10512469
rs2869804
rs873220
rs75215140
rs12594783
rs77154024
rs6863338
rs2915158
rs9912354
rs1006368
rs11703193
rs74856421
rs35150190
rs7575742
rs17606146
rs6850820
rs28691703
rs767896
rs2198787
All − MR Egger
All − Inverse variance weighted
−1
0
1
2
MR effect size for
'SSC−A on NK' on 'Diabetic nephropathy'

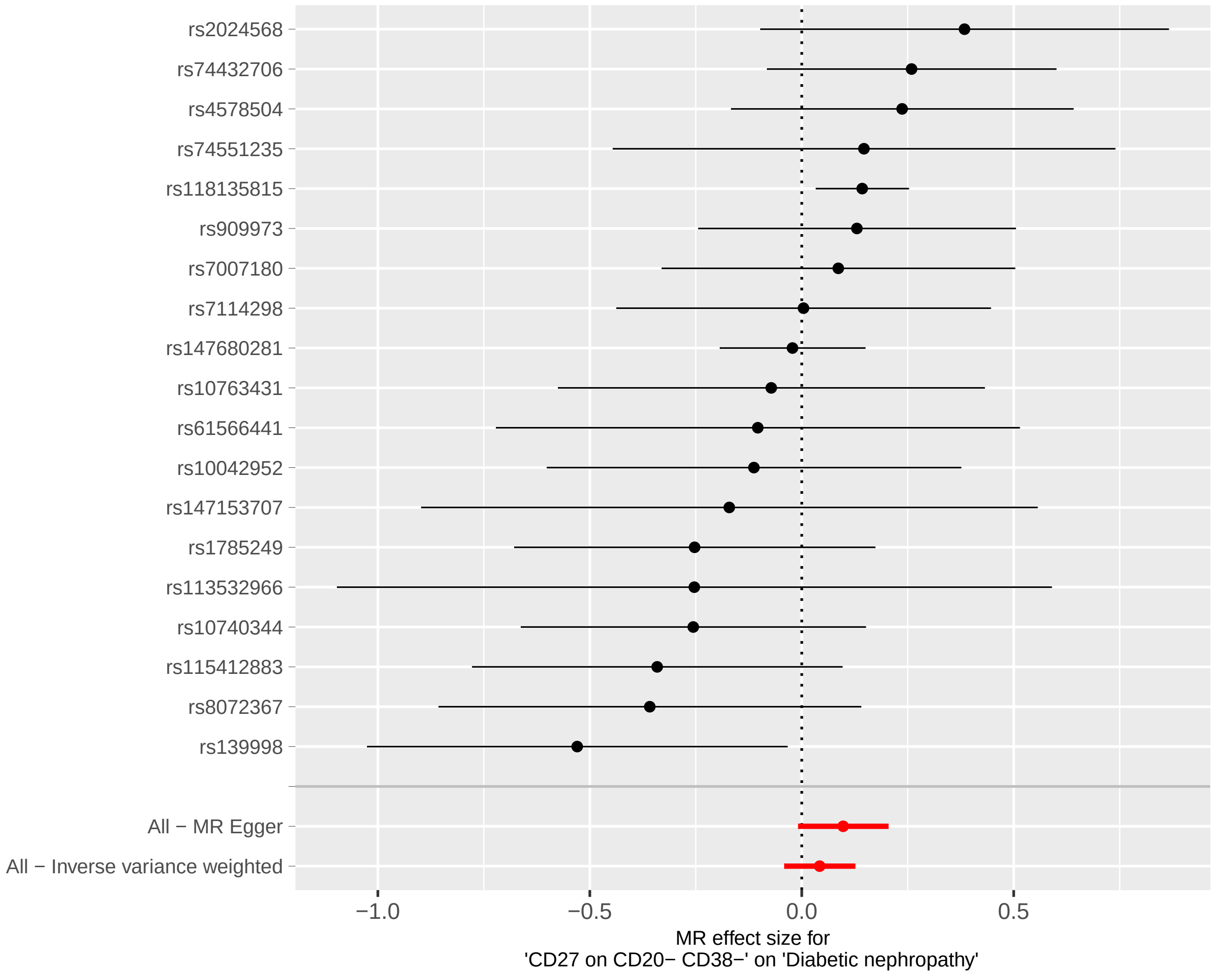
rs2024568
rs74432706
rs4578504
rs74551235
rs118135815
rs909973
rs7007180
rs7114298
rs147680281
rs10763431
rs61566441
rs10042952
rs147153707
rs1785249
rs113532966
rs10740344
rs115412883
rs8072367
rs139998
All − MR Egger
All − Inverse variance weighted
−1.0
−0.5
0.0
0.5
MR effect size for
'CD27 on CD20− CD38−' on 'Diabetic nephropathy'

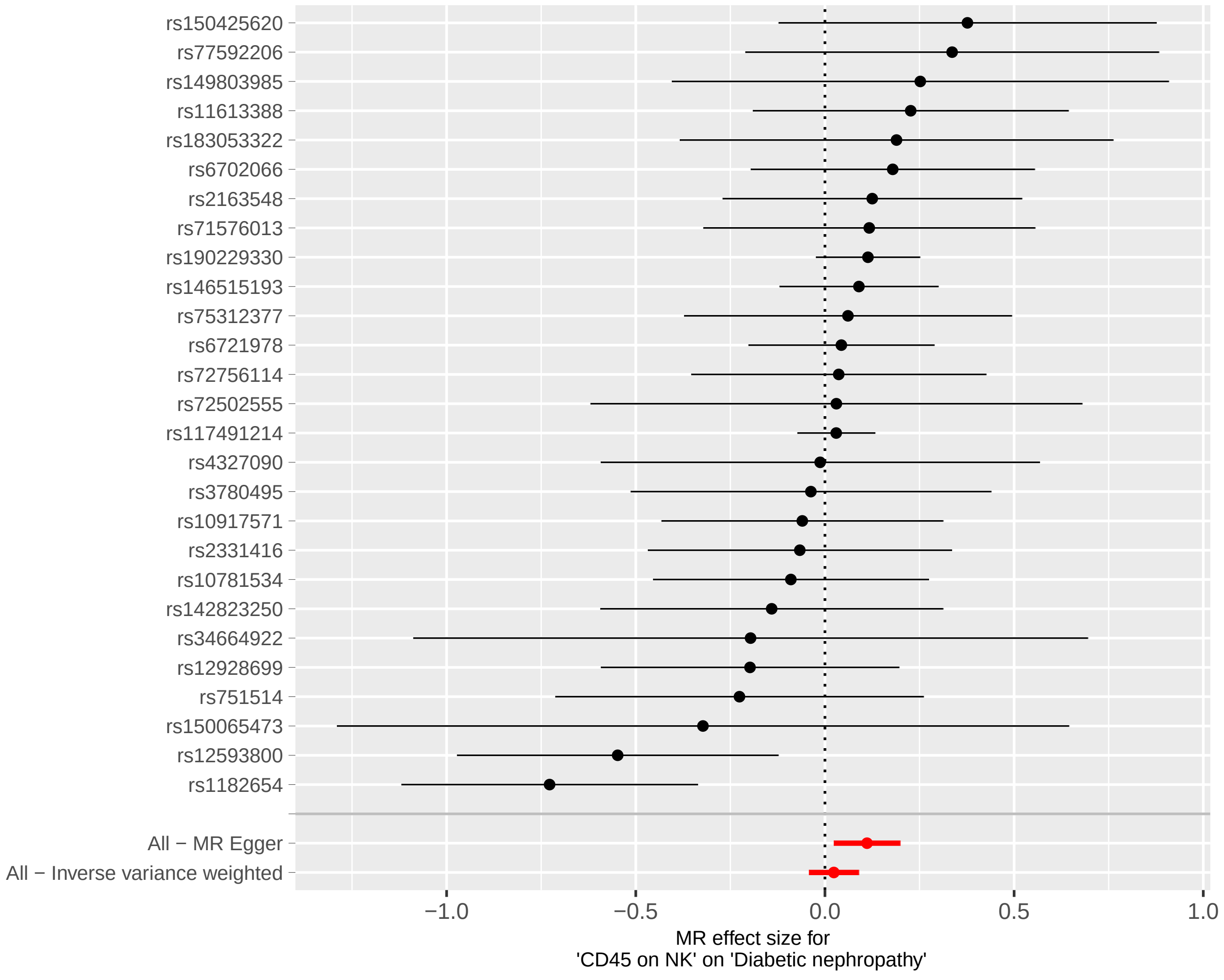

rs150425620
rs77592206
rs149803985
rs11613388
rs183053322
rs6702066
rs2163548
rs71576013
rs190229330
rs146515193
rs75312377
rs6721978
rs72756114
rs72502555
rs117491214
rs4327090
rs3780495
rs10917571
rs2331416
rs10781534
rs142823250
rs34664922
rs12928699
rs751514
rs150065473
rs12593800
rs1182654
All − MR Egger
All − Inverse variance weighted
−1.0
−0.5
0.0
0.5
1.0
MR effect size for
'CD45 on NK' on 'Diabetic nephropathy'

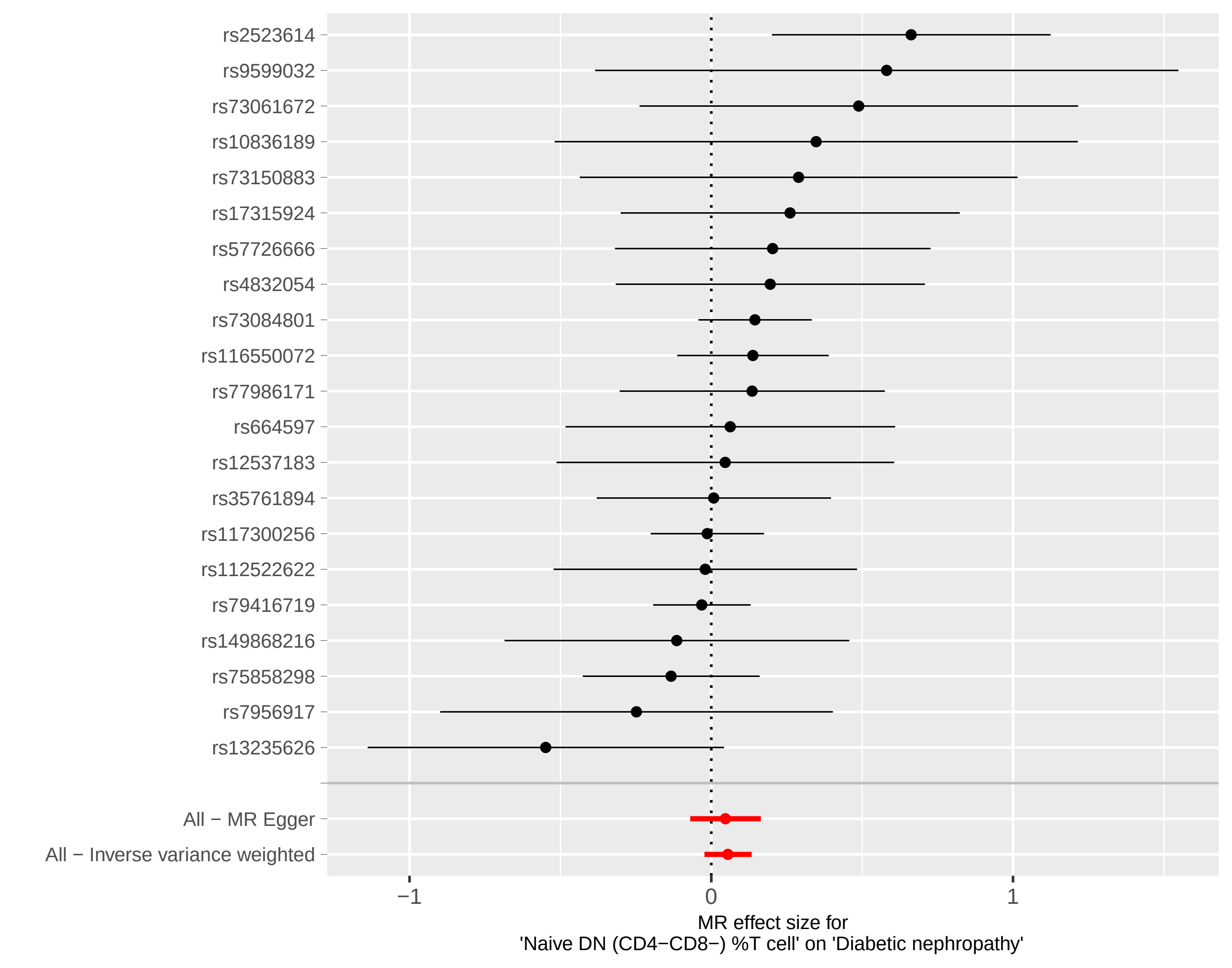
rs2523614
rs9599032
rs73061672
rs10836189
rs73150883
rs17315924
rs57726666
rs4832054
rs73084801
rs116550072
rs77986171
rs664597
rs12537183
rs35761894
rs117300256
rs112522622
rs79416719
rs149868216
rs75858298
rs7956917
rs13235626
All − MR Egger
All − Inverse variance weighted
−1
0
1
MR effect size for
'Naive DN (CD4−CD8−) %T cell' on 'Diabetic nephropathy'

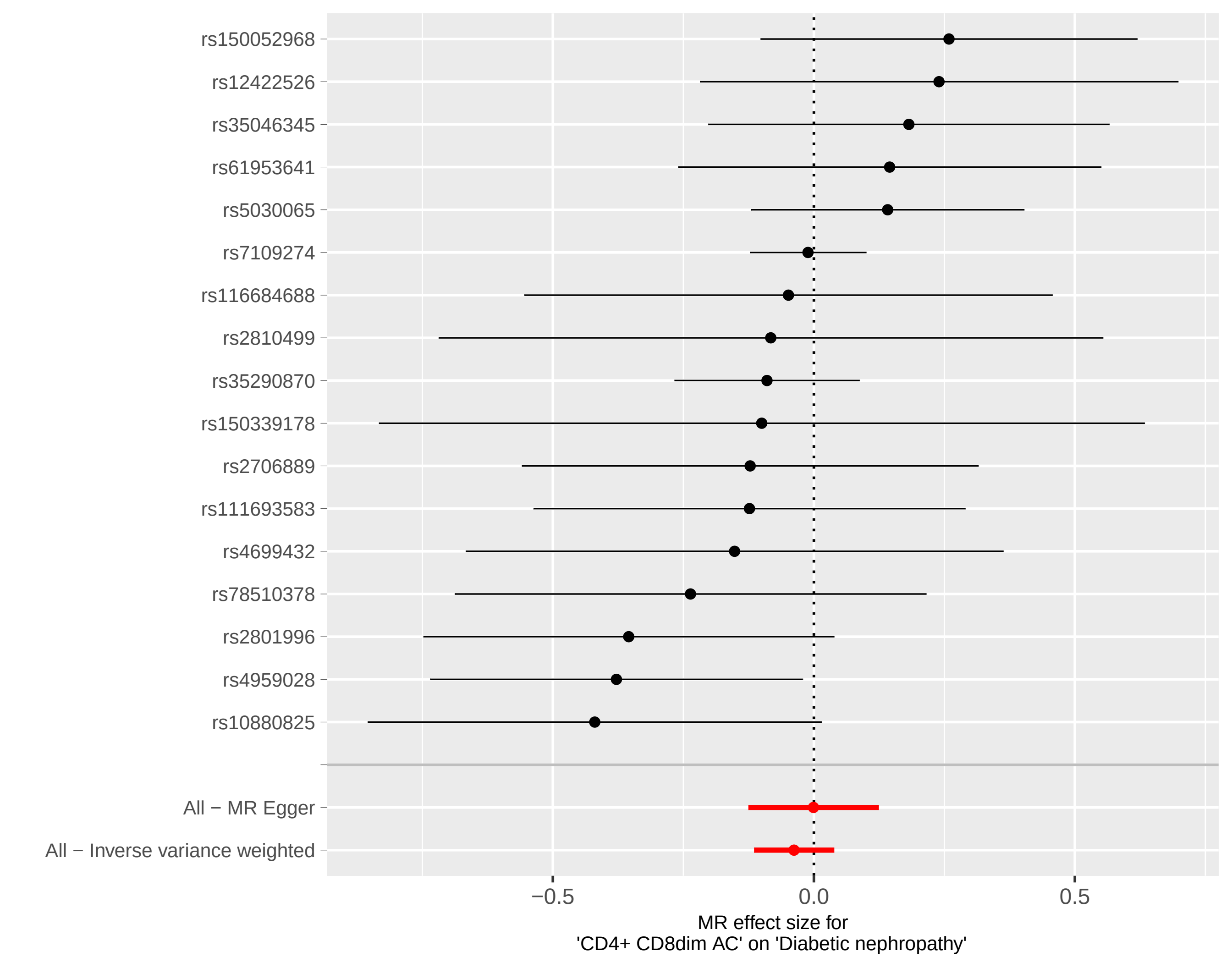
rs150052968
rs12422526
rs35046345
rs61953641
rs5030065
rs7109274
rs116684688
rs2810499
rs35290870
rs150339178
rs2706889
rs111693583
rs4699432
rs78510378
rs2801996
rs4959028
rs10880825
All − MR Egger
All − Inverse variance weighted
−0.5
0.0
0.5
MR effect size for
'CD4+ CD8dim AC' on 'Diabetic nephropathy'

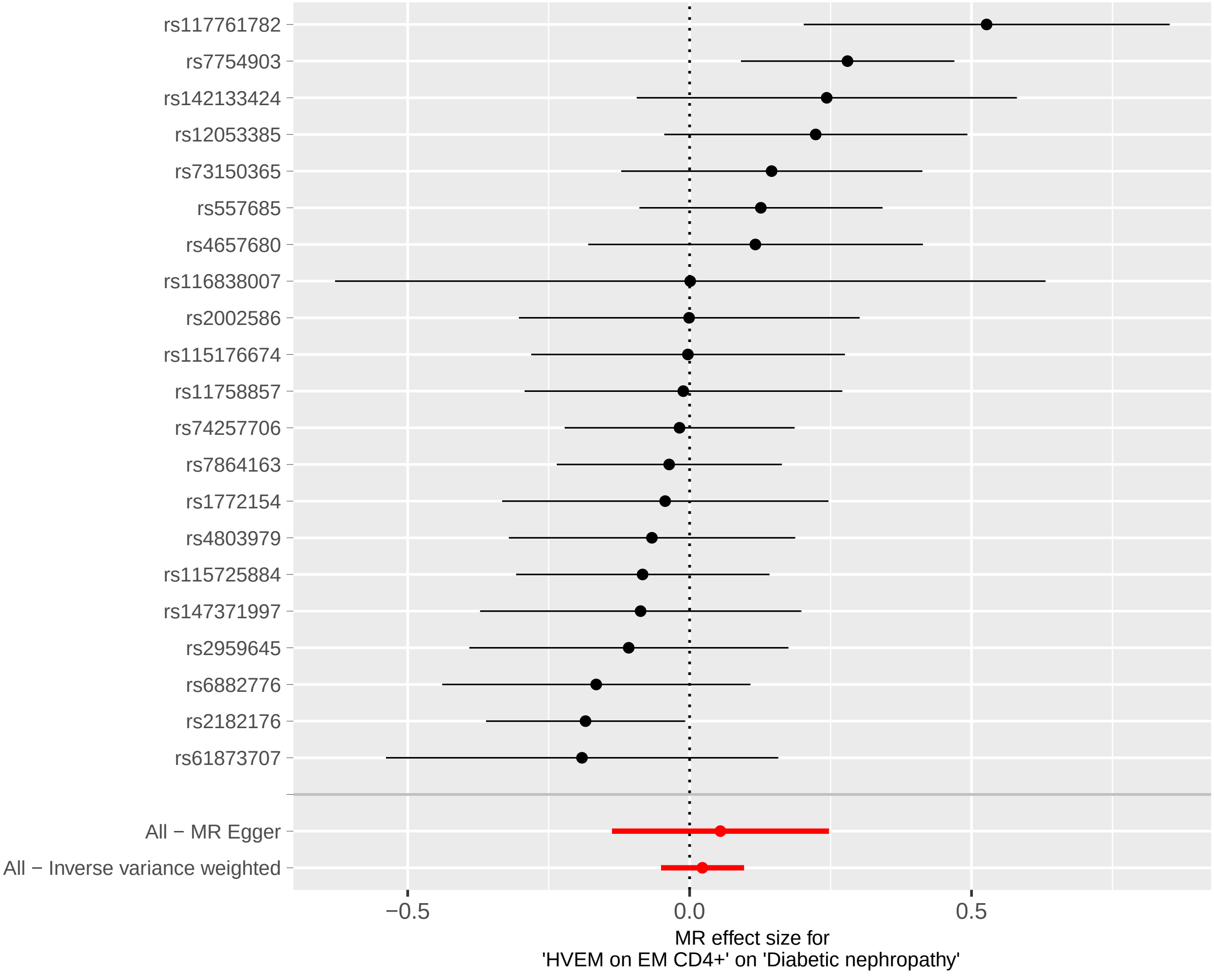

rs117761782
rs7754903
rs142133424
rs12053385
rs73150365
rs557685
rs4657680
rs116838007
rs2002586
rs115176674
rs11758857
rs74257706
rs7864163
rs1772154
rs4803979
rs115725884
rs147371997
rs2959645
rs6882776
rs2182176
rs61873707
All − MR Egger
All − Inverse variance weighted
−0.5
0.0
0.5
MR effect size for
'HVEM on EM CD4+' on 'Diabetic nephropathy'

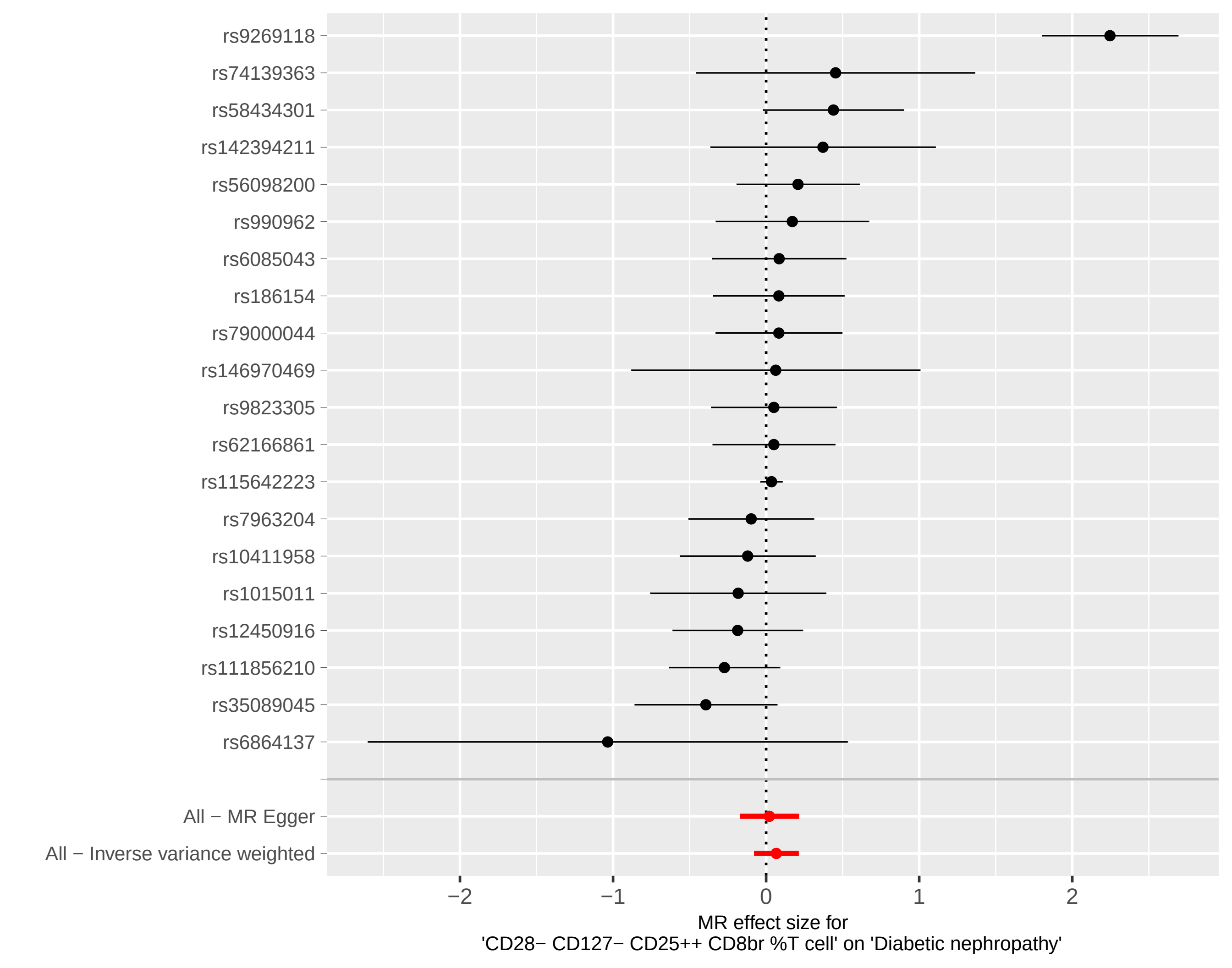
rs9269118
rs74139363
rs58434301
rs142394211
rs56098200
rs990962
rs6085043
rs186154
rs79000044
rs146970469
rs9823305
rs62166861
rs115642223
rs7963204
rs10411958
rs1015011
rs12450916
rs111856210
rs35089045
rs6864137
All − MR Egger
All − Inverse variance weighted
−2
−1
0
1
2
MR effect size for
'CD28− CD127− CD25++ CD8br %T cell' on 'Diabetic nephropathy'

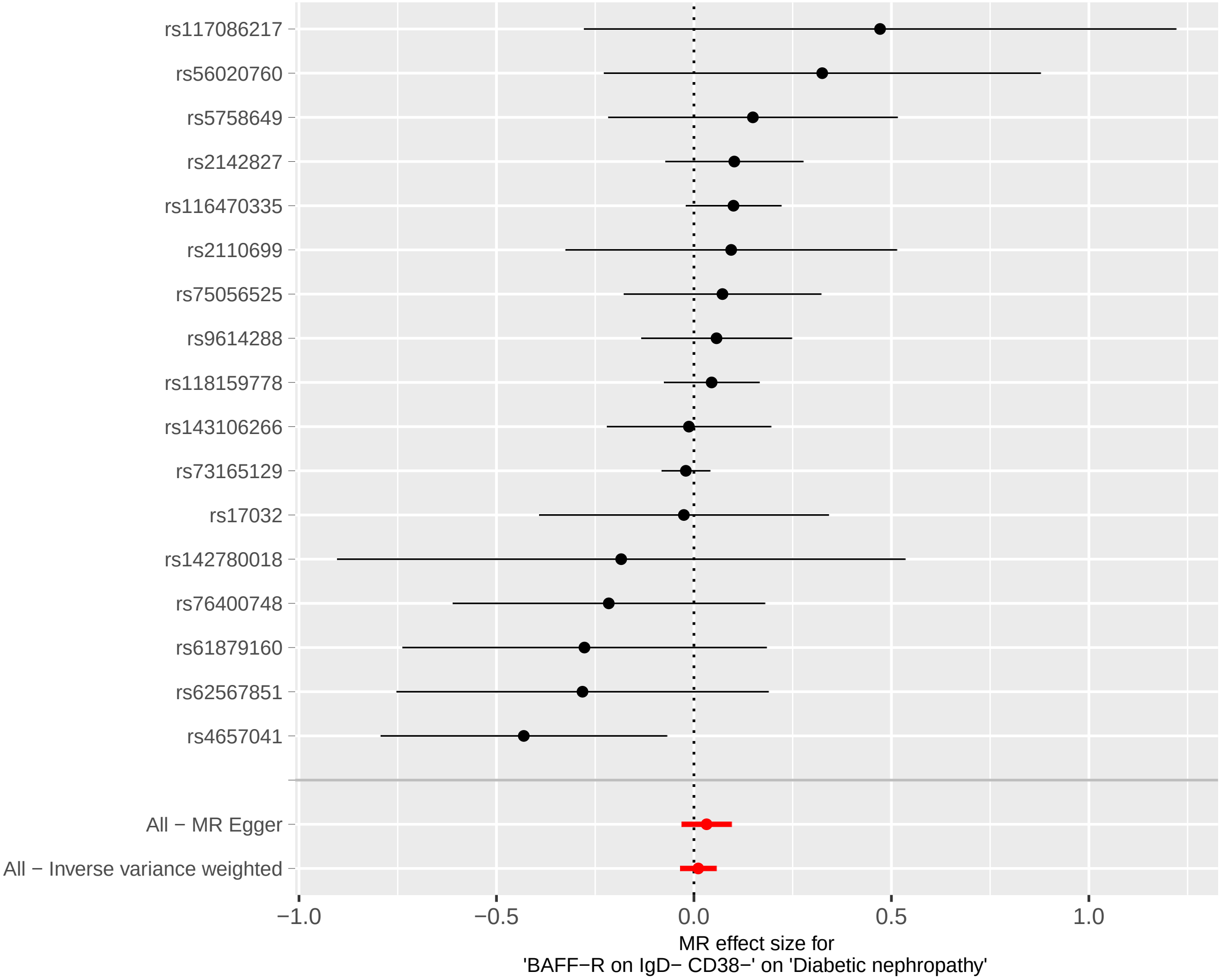

rs117086217
rs56020760
rs5758649
rs2142827
rs116470335
rs2110699
rs75056525
rs9614288
rs118159778
rs143106266
rs73165129
rs17032
rs142780018
rs76400748
rs61879160
rs62567851
rs4657041
All − MR Egger
All − Inverse variance weighted
−1.0
−0.5
0.0
0.5
1.0
MR effect size for
'BAFF−R on IgD− CD38−' on 'Diabetic nephropathy'

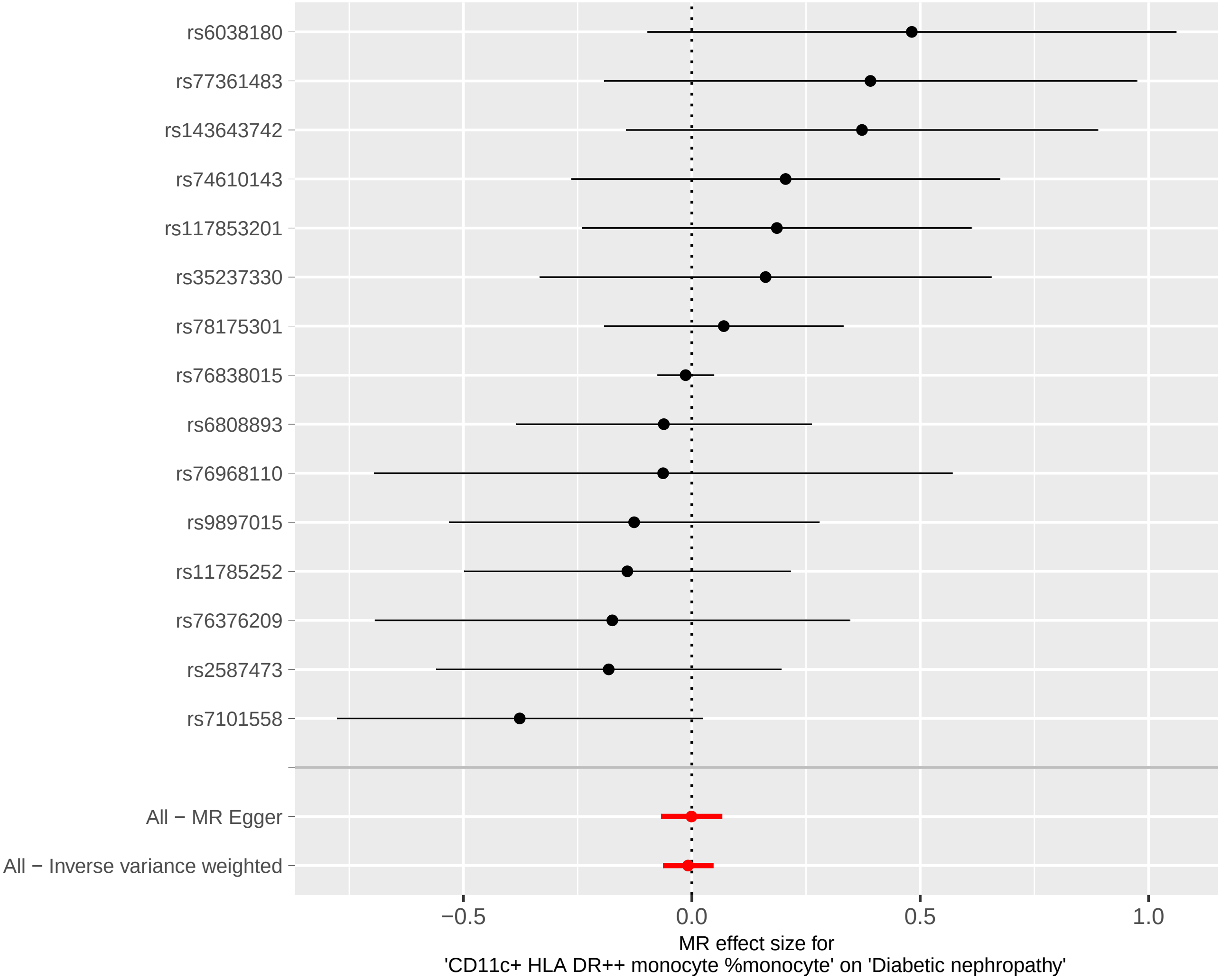
rs6038180
rs77361483
rs143643742
rs74610143
rs117853201
rs35237330
rs78175301
rs76838015
rs6808893
rs76968110
rs9897015
rs11785252
rs76376209
rs2587473
rs7101558
All − MR Egger
All − Inverse variance weighted
−0.5
0.0
0.5
1.0
MR effect size for
'CD11c+ HLA DR++ monocyte %monocyte' on 'Diabetic nephropathy'

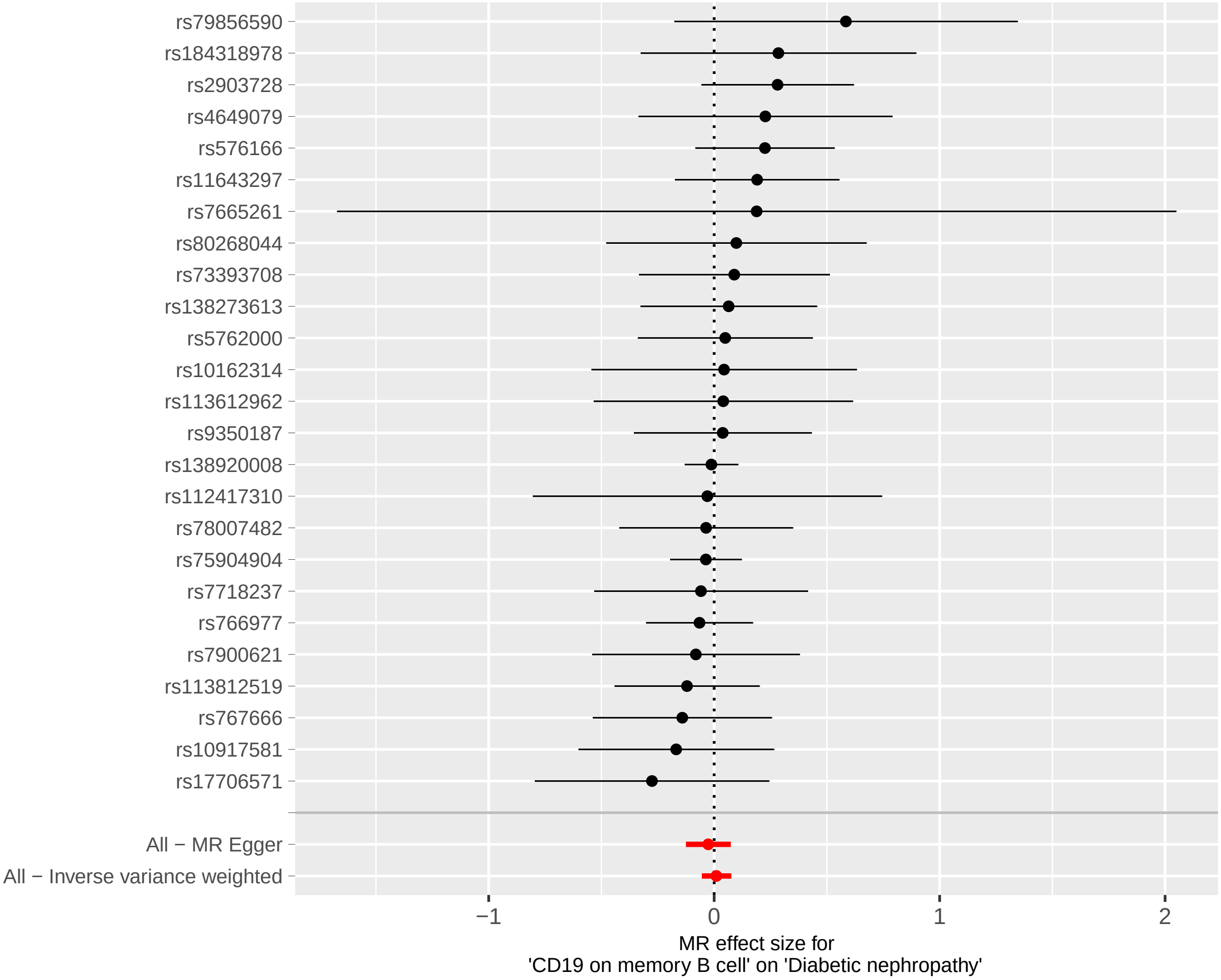

rs79856590
rs184318978
rs2903728
rs4649079
rs576166
rs11643297
rs7665261
rs80268044
rs73393708
rs138273613
rs5762000
rs10162314
rs113612962
rs9350187
rs138920008
rs112417310
rs78007482
rs75904904
rs7718237
rs766977
rs7900621
rs113812519
rs767666
rs10917581
rs17706571
All − MR Egger
All − Inverse variance weighted
−1
0
1
2
MR effect size for
'CD19 on memory B cell' on 'Diabetic nephropathy'

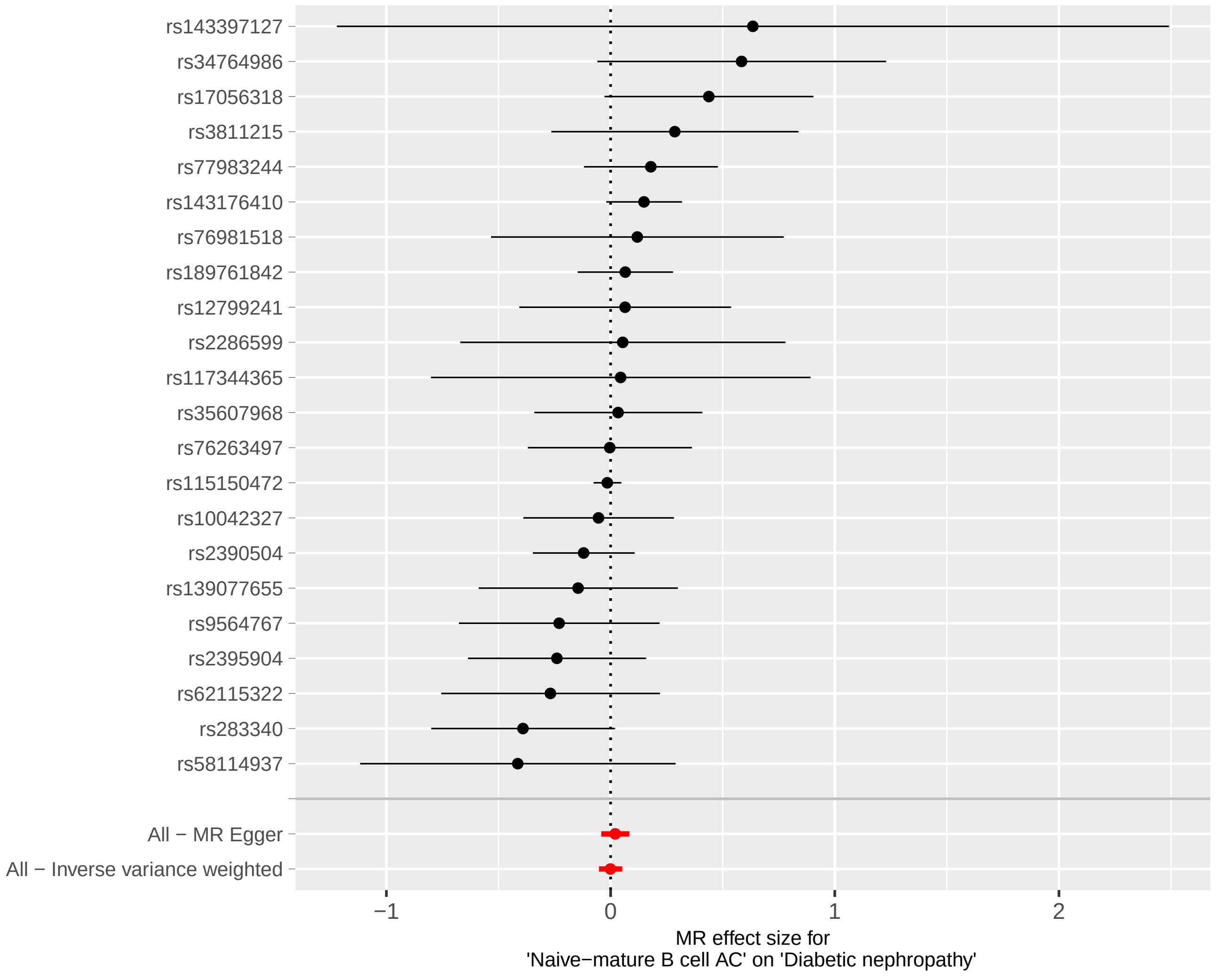

rs143397127
rs34764986
rs17056318
rs3811215
rs77983244
rs143176410
rs76981518
rs189761842
rs12799241
rs2286599
rs117344365
rs35607968
rs76263497
rs115150472
rs10042327
rs2390504
rs139077655
rs9564767
rs2395904
rs62115322
rs283340
rs58114937
All − MR Egger
All − Inverse variance weighted
−1
0
1
2
MR effect size for
'Naive−mature B cell AC' on 'Diabetic nephropathy'

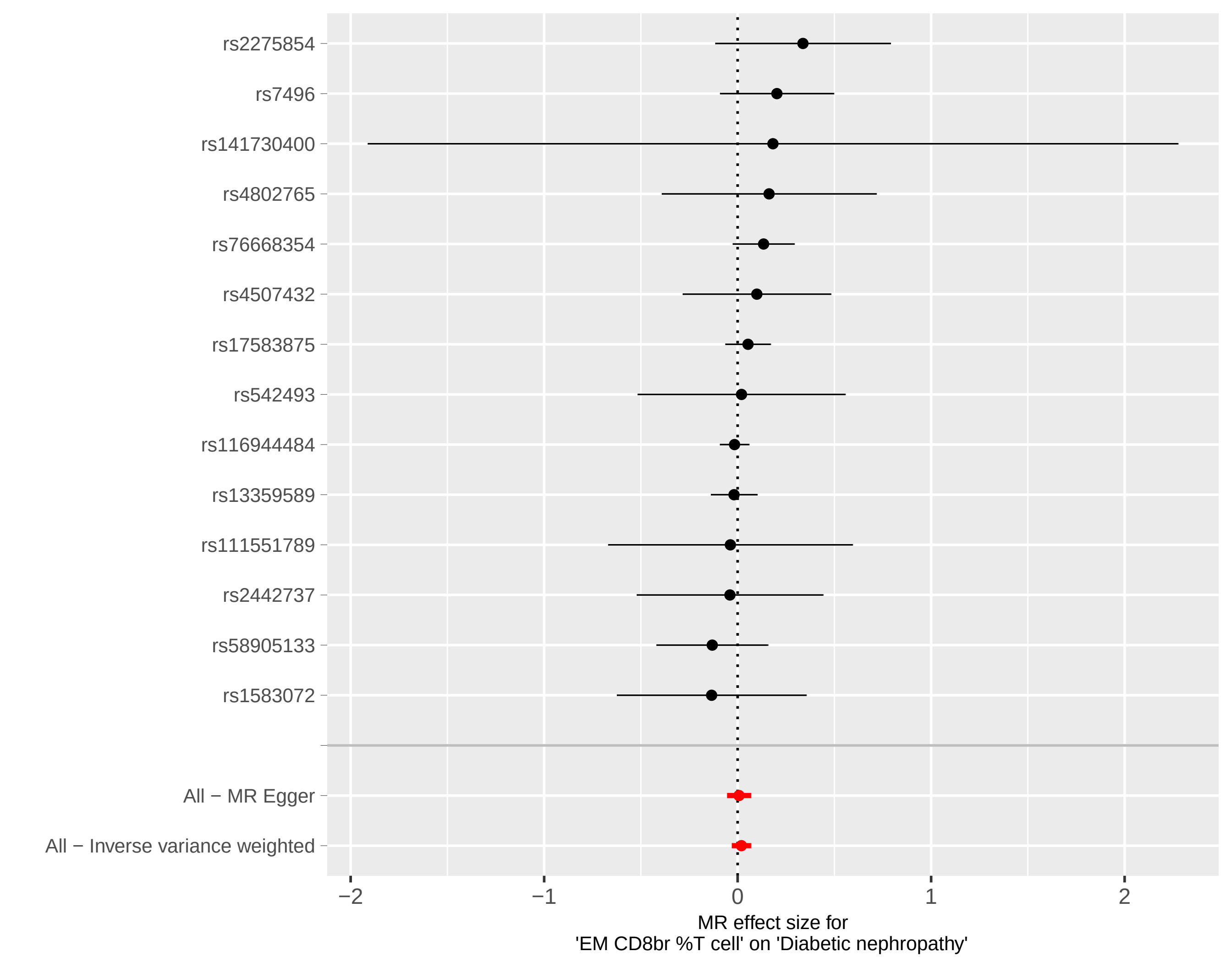
rs2275854
rs7496
rs141730400
rs4802765
rs76668354
rs4507432
rs17583875
rs542493
rs116944484
rs13359589
rs111551789
rs2442737
rs58905133
rs1583072
All − MR Egger
All − Inverse variance weighted
−2
−1
0
1
2
MR effect size for
'EM CD8br %T cell' on 'Diabetic nephropathy'

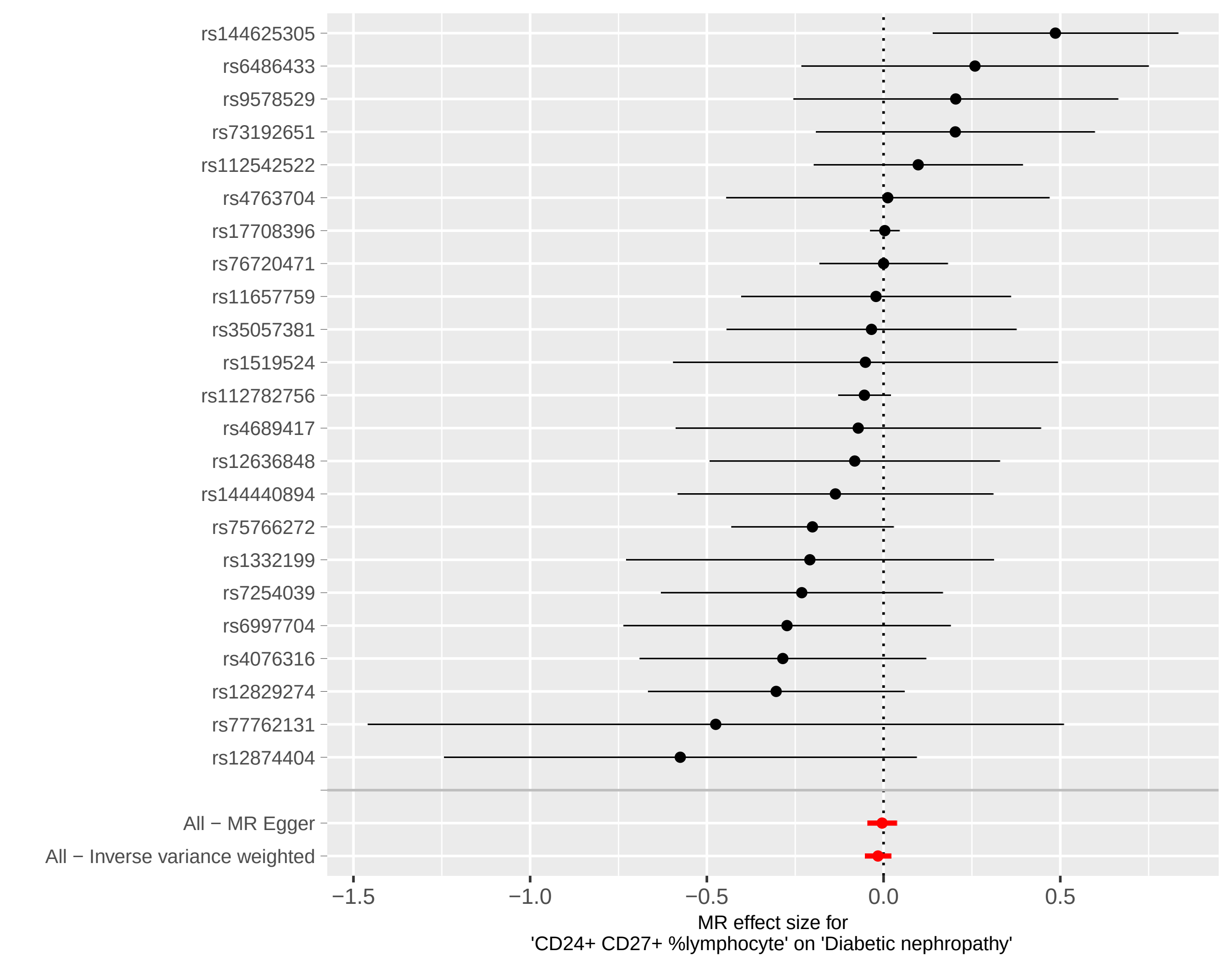
rs144625305
rs6486433
rs9578529
rs73192651
rs112542522
rs4763704
rs17708396
rs76720471
rs11657759
rs35057381
rs1519524
rs112782756
rs4689417
rs12636848
rs144440894
rs75766272
rs1332199
rs7254039
rs6997704
rs4076316
rs12829274
rs77762131
rs12874404
All − MR Egger
All − Inverse variance weighted
−1.5
−1.0
−0.5
0.0
0.5
MR effect size for
'CD24+ CD27+ %lymphocyte' on 'Diabetic nephropathy'

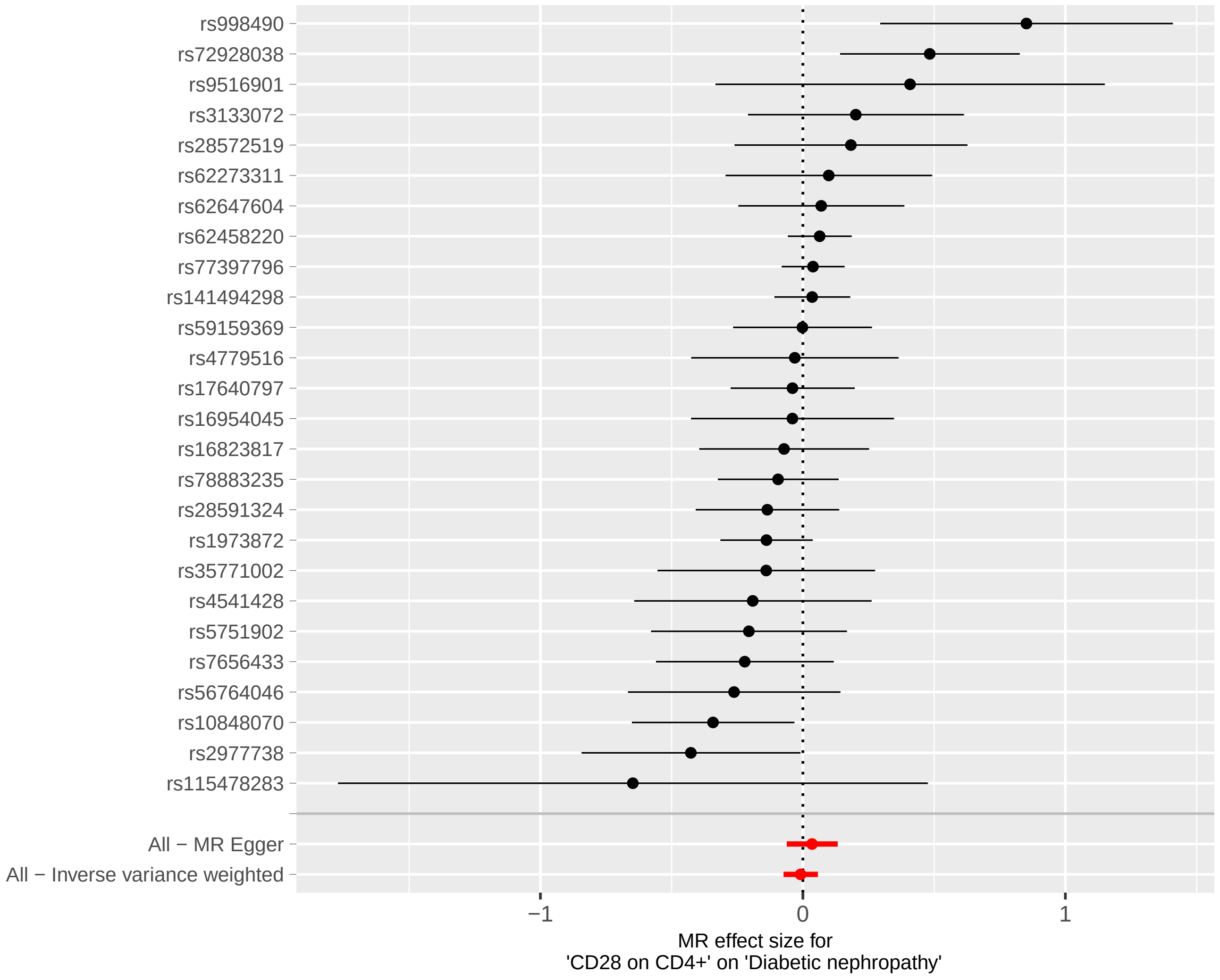

rs998490
rs72928038
rs9516901
rs3133072
rs28572519
rs62273311
rs62647604
rs62458220
rs77397796
rs141494298
rs59159369
rs4779516
rs17640797
rs16954045
rs16823817
rs78883235
rs28591324
rs1973872
rs35771002
rs4541428
rs5751902
rs7656433
rs56764046
rs10848070
rs2977738
rs115478283
All − MR Egger
All − Inverse variance weighted
−1
0
1
MR effect size for
'CD28 on CD4+' on 'Diabetic nephropathy'

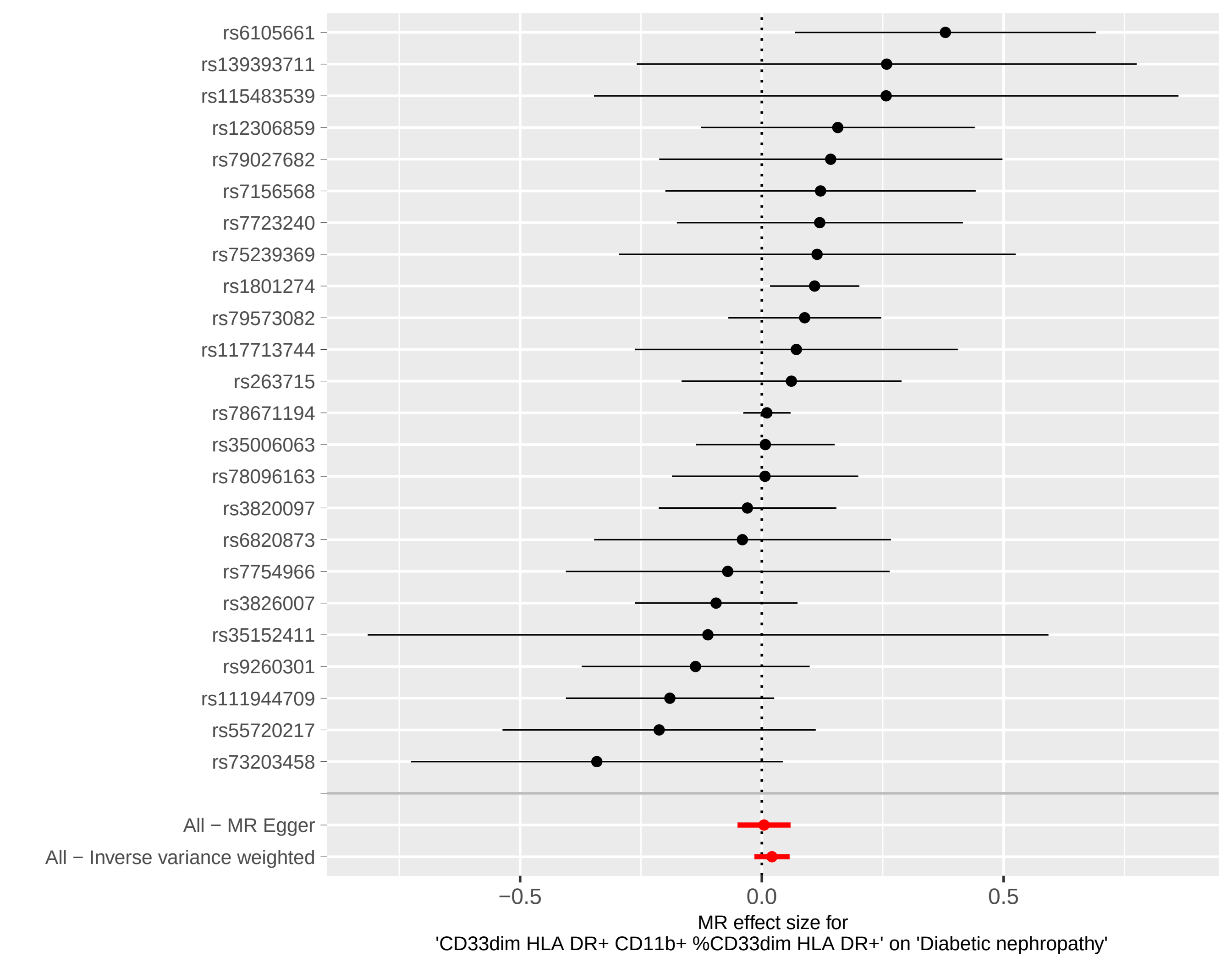
rs6105661
rs139393711
rs115483539
rs12306859
rs79027682
rs7156568
rs7723240
rs75239369
rs1801274
rs79573082
rs117713744
rs263715
rs78671194
rs35006063
rs78096163
rs3820097
rs6820873
rs7754966
rs3826007
rs35152411
rs9260301
rs111944709
rs55720217
rs73203458
All − MR Egger
All − Inverse variance weighted
−0.5
0.0
0.5
MR effect size for
'CD33dim HLA DR+ CD11b+ %CD33dim HLA DR+' on 'Diabetic nephropathy'

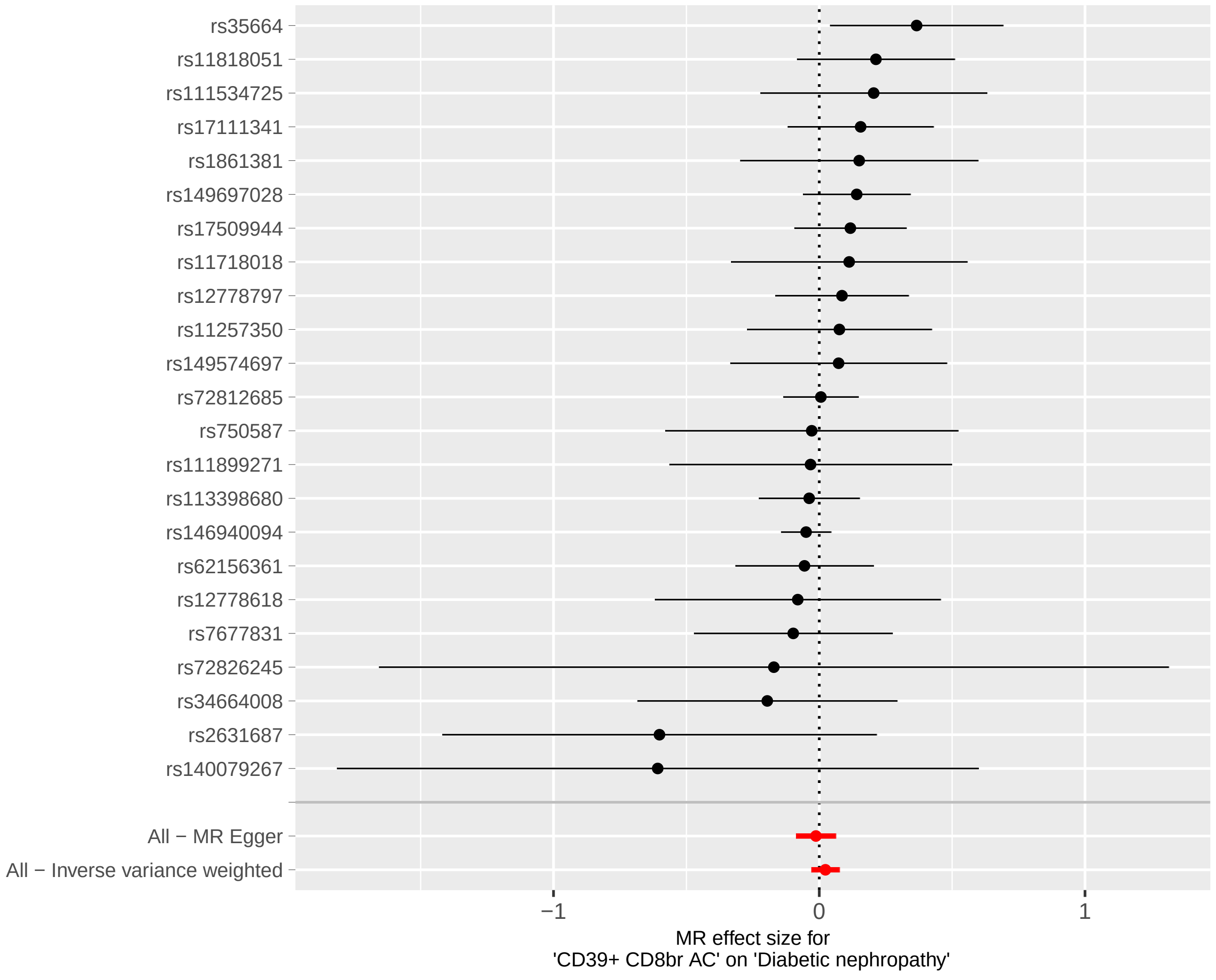

rs35664
rs11818051
rs111534725
rs17111341
rs1861381
rs149697028
rs17509944
rs11718018
rs12778797
rs11257350
rs149574697
rs72812685
rs750587
rs111899271
rs113398680
rs146940094
rs62156361
rs12778618
rs7677831
rs72826245
rs34664008
rs2631687
rs140079267
All − MR Egger
All − Inverse variance weighted
−1
0
1
MR effect size for
'CD39+ CD8br AC' on 'Diabetic nephropathy'

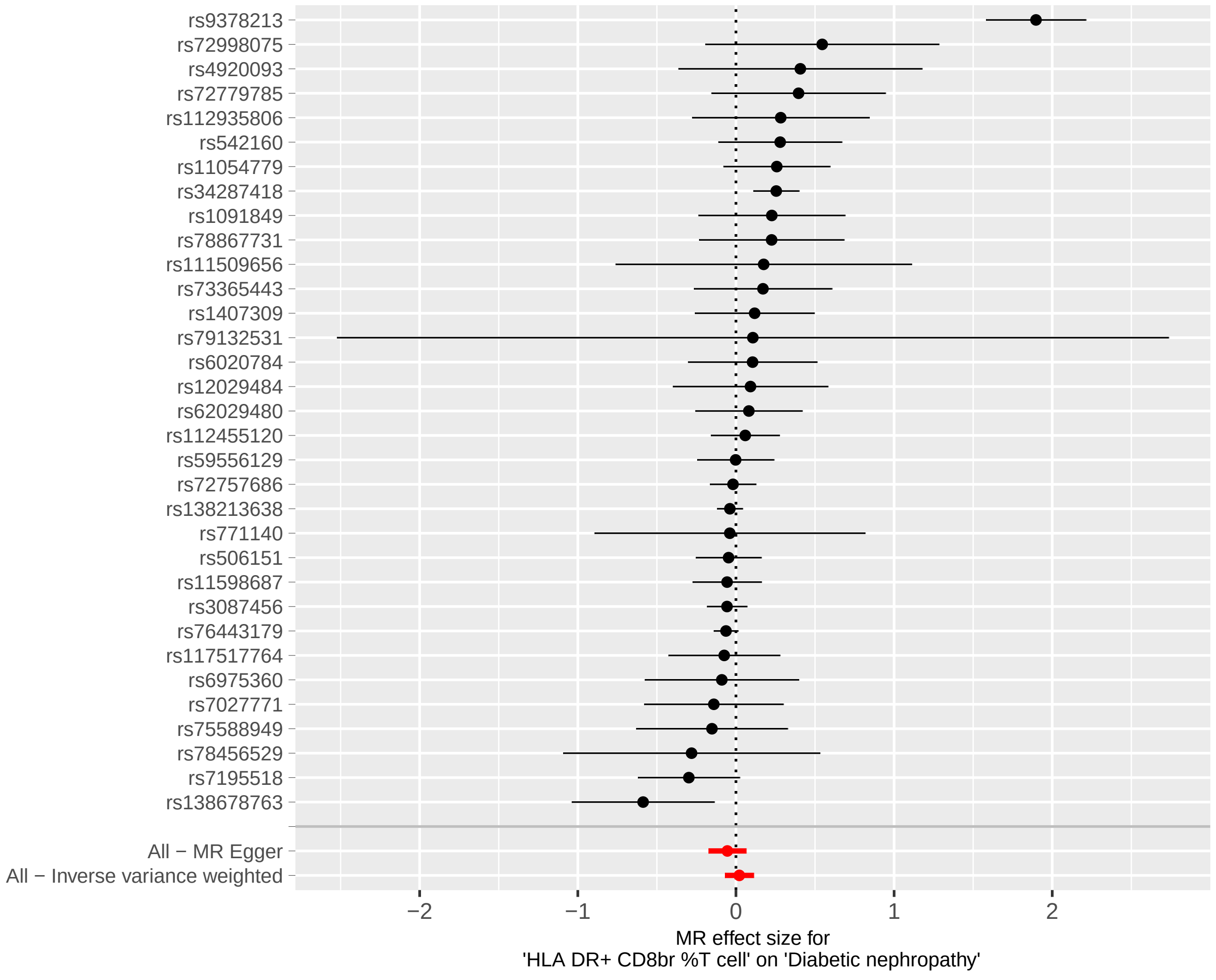
rs9378213
rs72998075
rs4920093
rs72779785
rs112935806
rs542160
rs11054779
rs34287418
rs1091849
rs78867731
rs111509656
rs73365443
rs1407309
rs79132531
rs6020784
rs12029484
rs62029480
rs112455120
rs59556129
rs72757686
rs138213638
rs771140
rs506151
rs11598687
rs3087456
rs76443179
rs117517764
rs6975360
rs7027771
rs75588949
rs78456529
rs7195518
rs138678763
All − MR Egger
All − Inverse variance weighted
−2
−1
0
1
2
MR effect size for
'HLA DR+ CD8br %T cell' on 'Diabetic nephropathy'

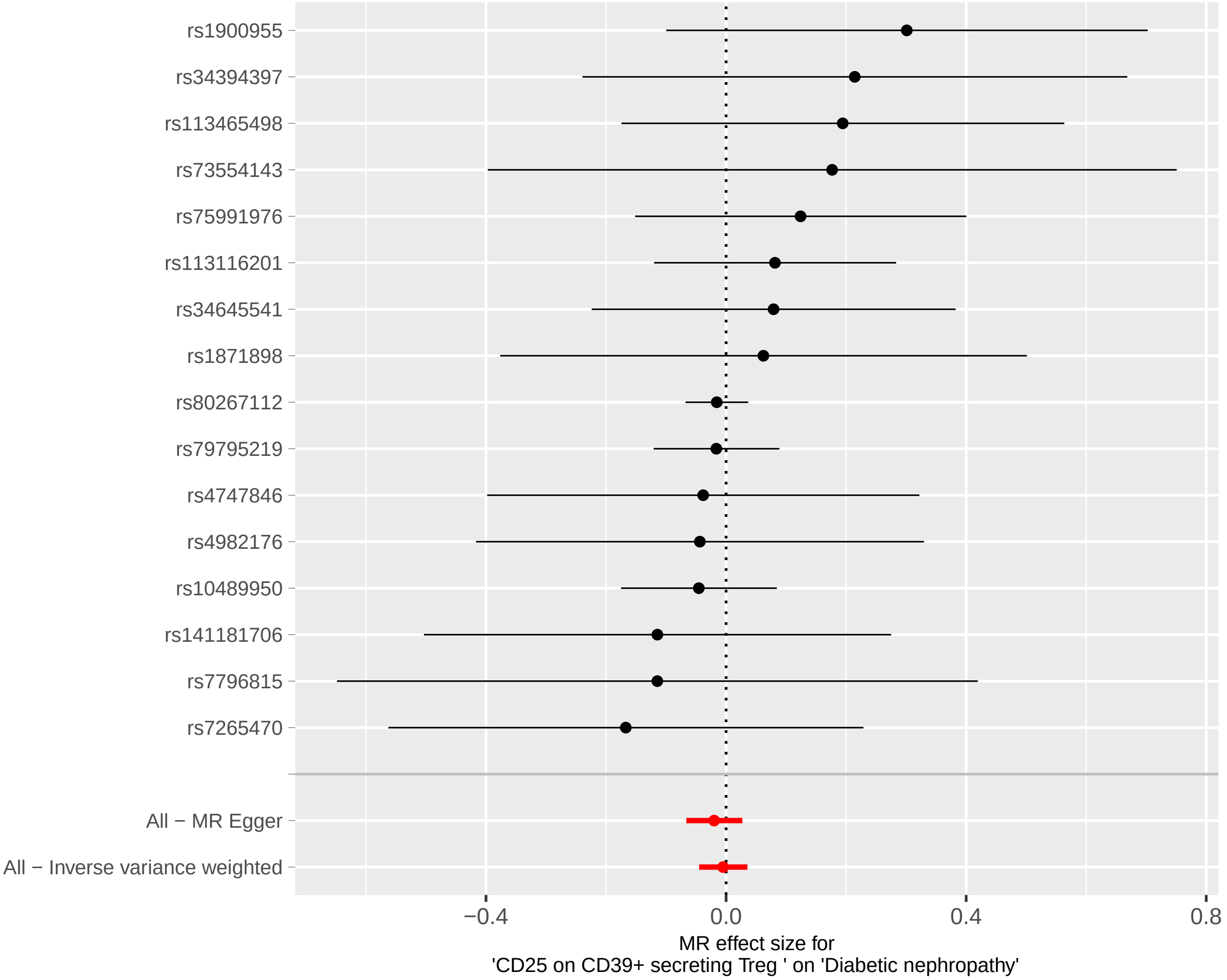
rs1900955
rs34394397
rs113465498
rs73554143
rs75991976
rs113116201
rs34645541
rs1871898
rs80267112
rs79795219
rs4747846
rs4982176
rs10489950
rs141181706
rs7796815
rs7265470
All − MR Egger
All − Inverse variance weighted
−0.4
0.0
0.4
0.8
MR effect size for
'CD25 on CD39+ secreting Treg ' on 'Diabetic nephropathy'

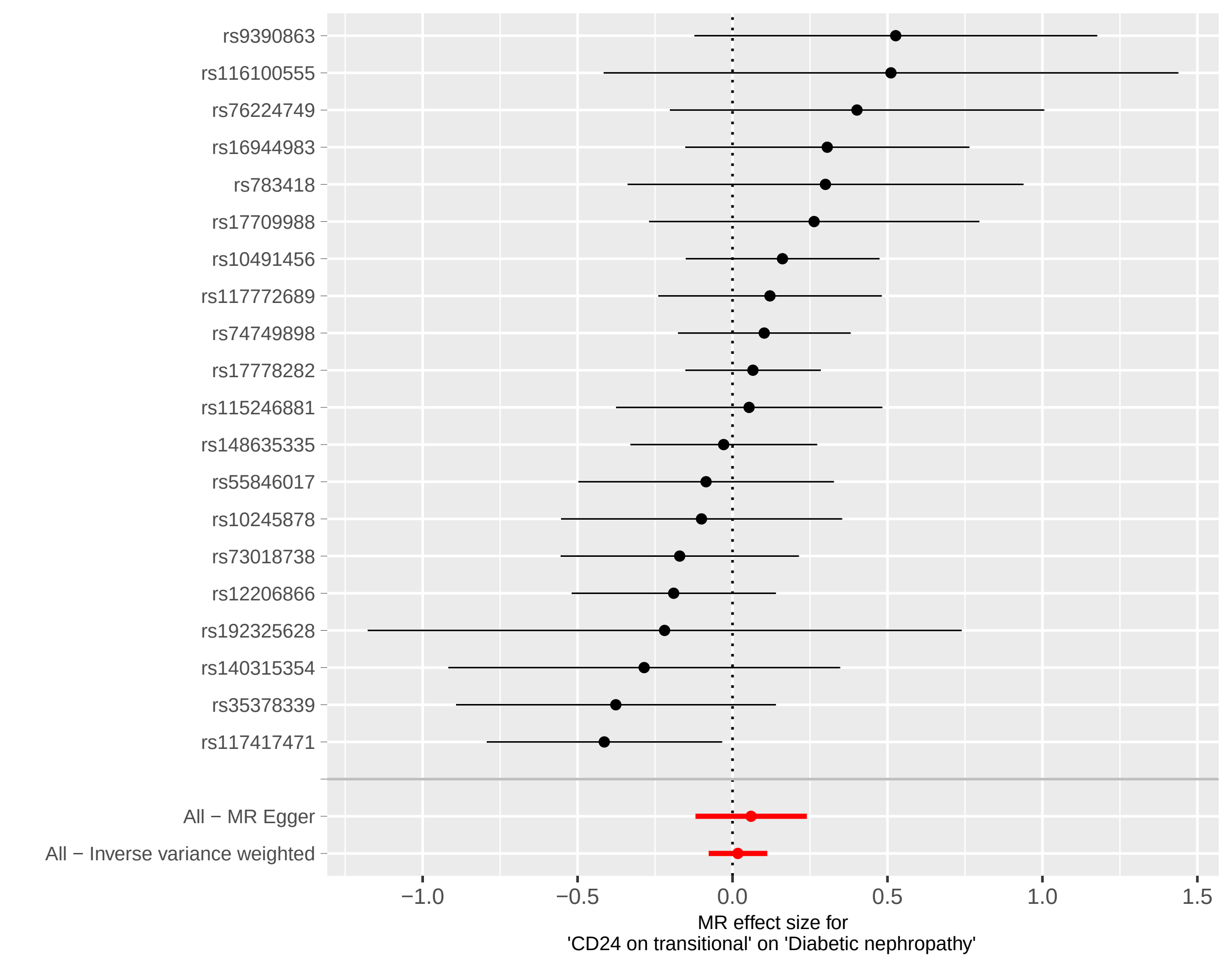

rs9390863
rs116100555
rs76224749
rs16944983
rs783418
rs17709988
rs10491456
rs117772689
rs74749898
rs17778282
rs115246881
rs148635335
rs55846017
rs10245878
rs73018738
rs12206866
rs192325628
rs140315354
rs35378339
rs117417471
All − MR Egger
All − Inverse variance weighted
−1.0
−0.5
0.0
0.5
1.0
1.5
MR effect size for
'CD24 on transitional' on 'Diabetic nephropathy'

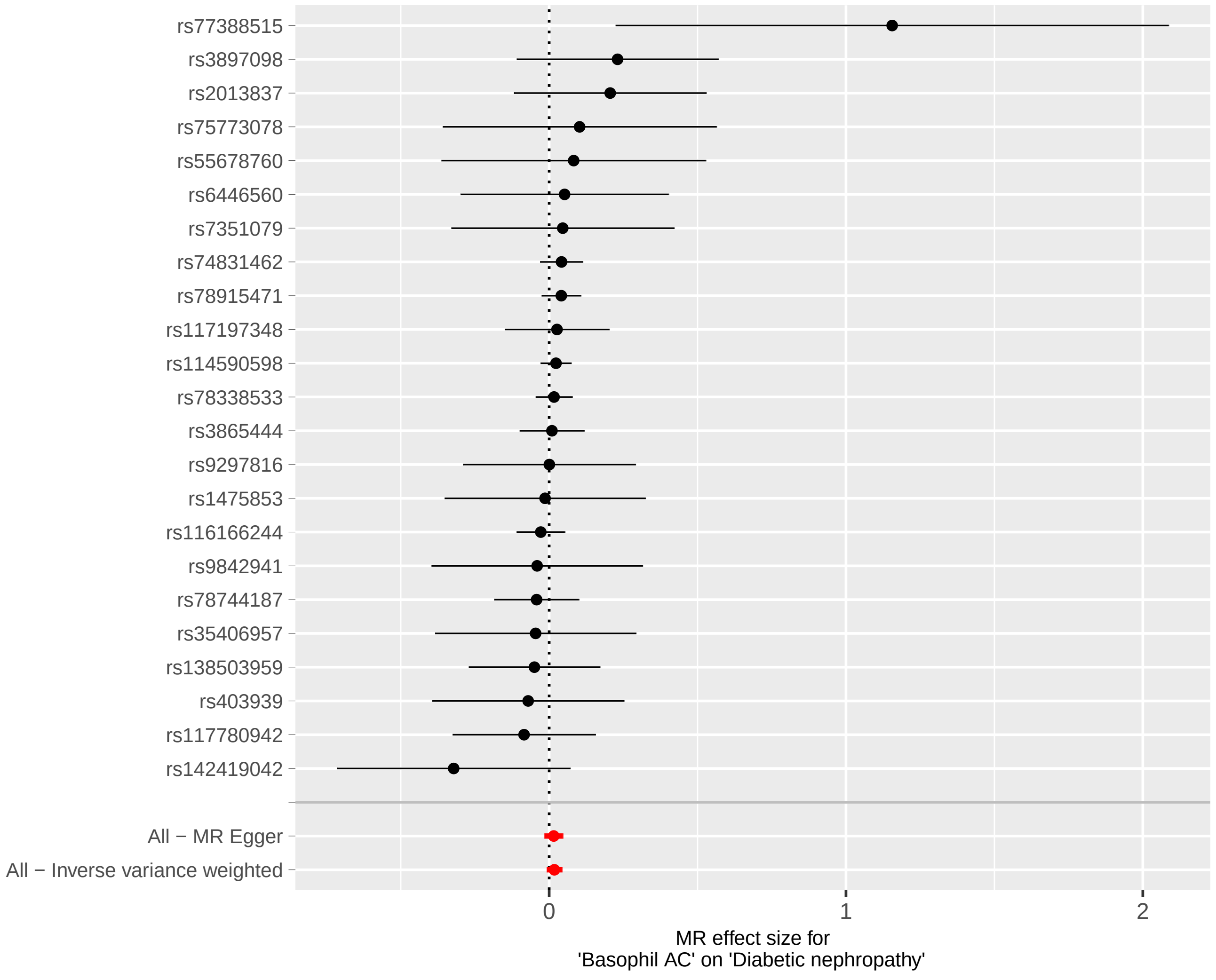
rs77388515
rs3897098
rs2013837
rs75773078
rs55678760
rs6446560
rs7351079
rs74831462
rs78915471
rs117197348
rs114590598
rs78338533
rs3865444
rs9297816
rs1475853
rs116166244
rs9842941
rs78744187
rs35406957
rs138503959
rs403939
rs117780942
rs142419042
All − MR Egger
All − Inverse variance weighted
0
1
2
MR effect size for
'Basophil AC' on 'Diabetic nephropathy'

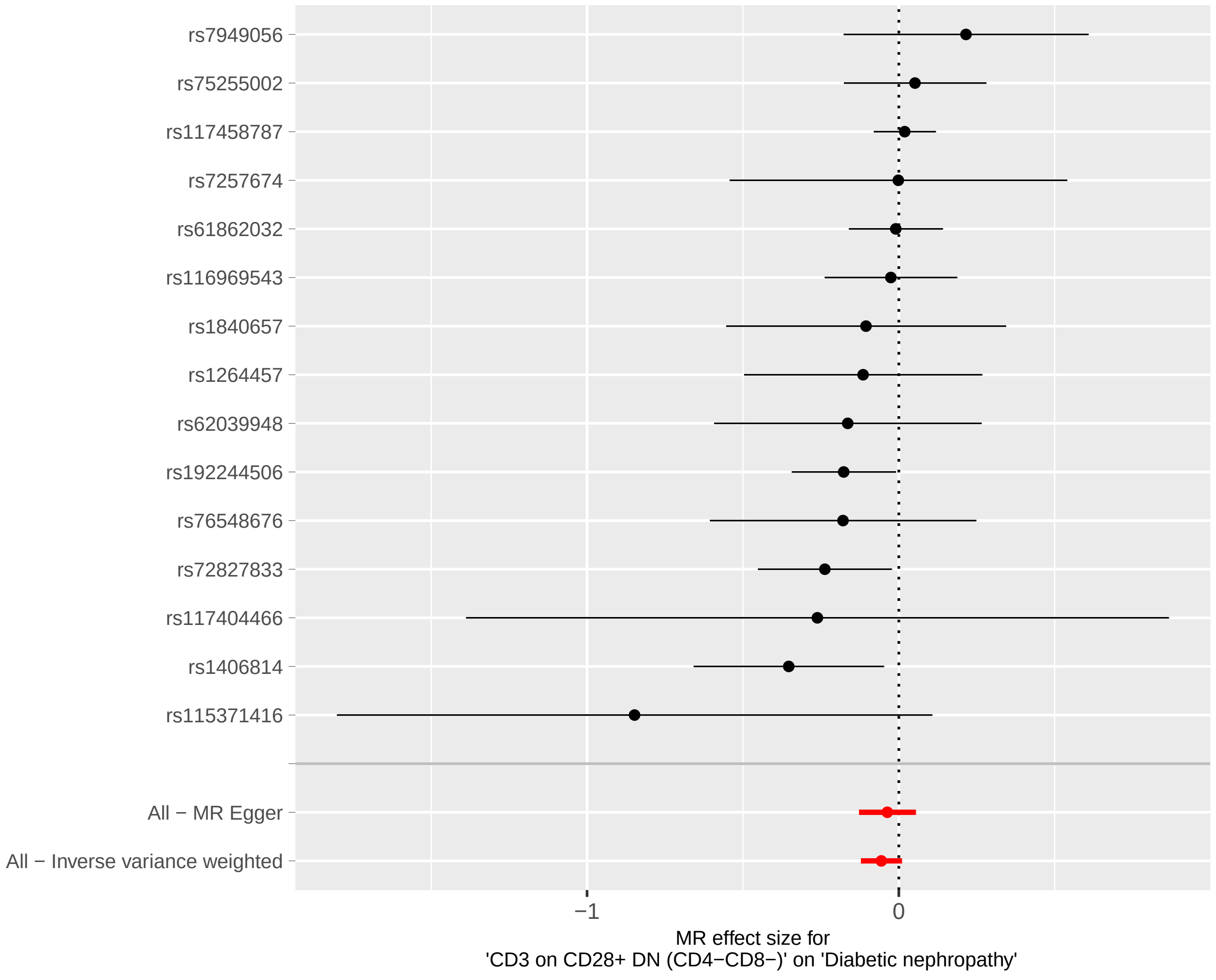
rs7949056
rs75255002
rs117458787
rs7257674
rs61862032
rs116969543
rs1840657
rs1264457
rs62039948
rs192244506
rs76548676
rs72827833
rs117404466
rs1406814
rs115371416
All − MR Egger
All − Inverse variance weighted
−1
0
MR effect size for
'CD3 on CD28+ DN (CD4−CD8−)' on 'Diabetic nephropathy'

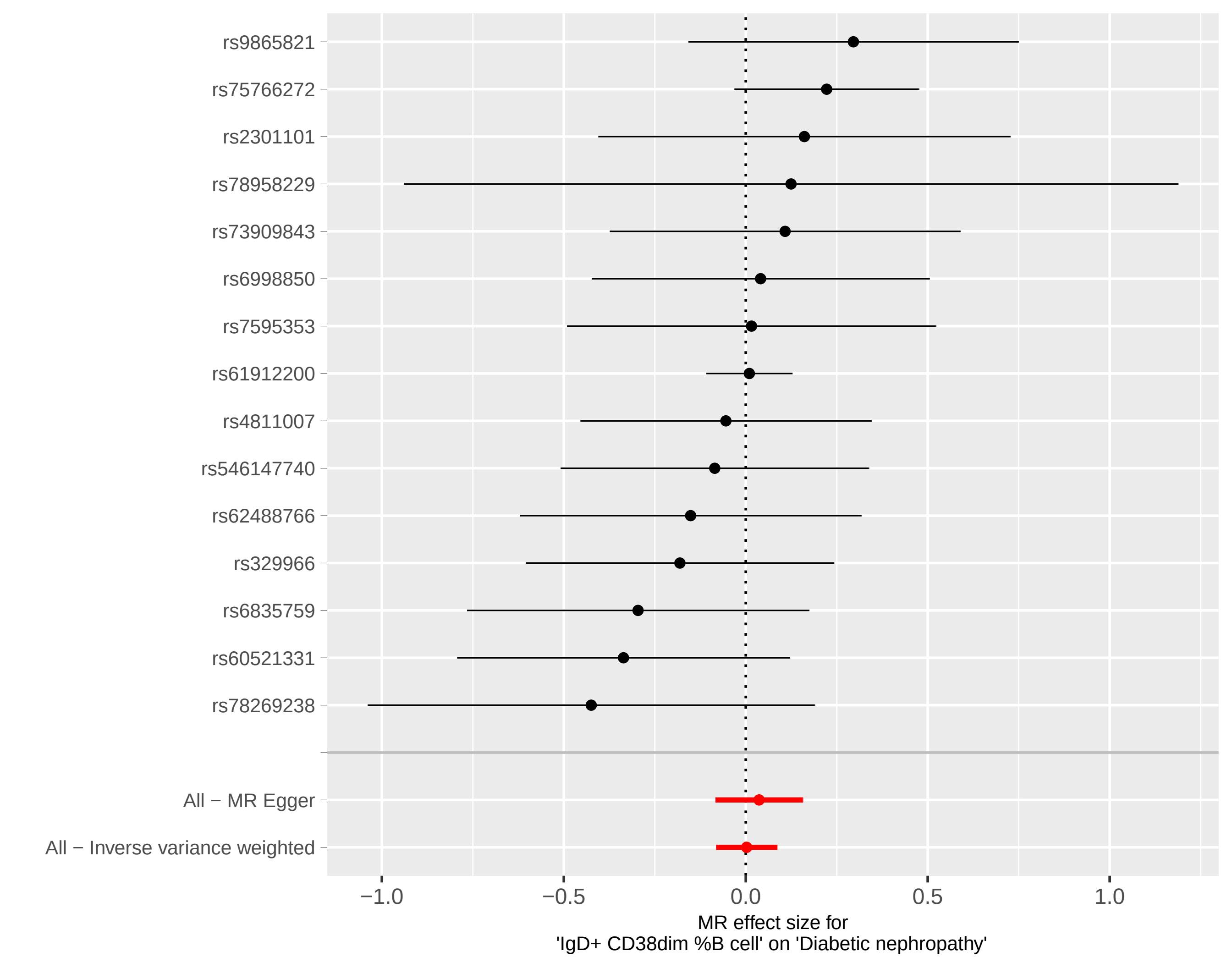

rs9865821
rs75766272
rs2301101
rs78958229
rs73909843
rs6998850
rs7595353
rs61912200
rs4811007
rs546147740
rs62488766
rs329966
rs6835759
rs60521331
rs78269238
All − MR Egger
All − Inverse variance weighted
−1.0
−0.5
0.0
0.5
1.0
MR effect size for
'IgD+ CD38dim %B cell' on 'Diabetic nephropathy'

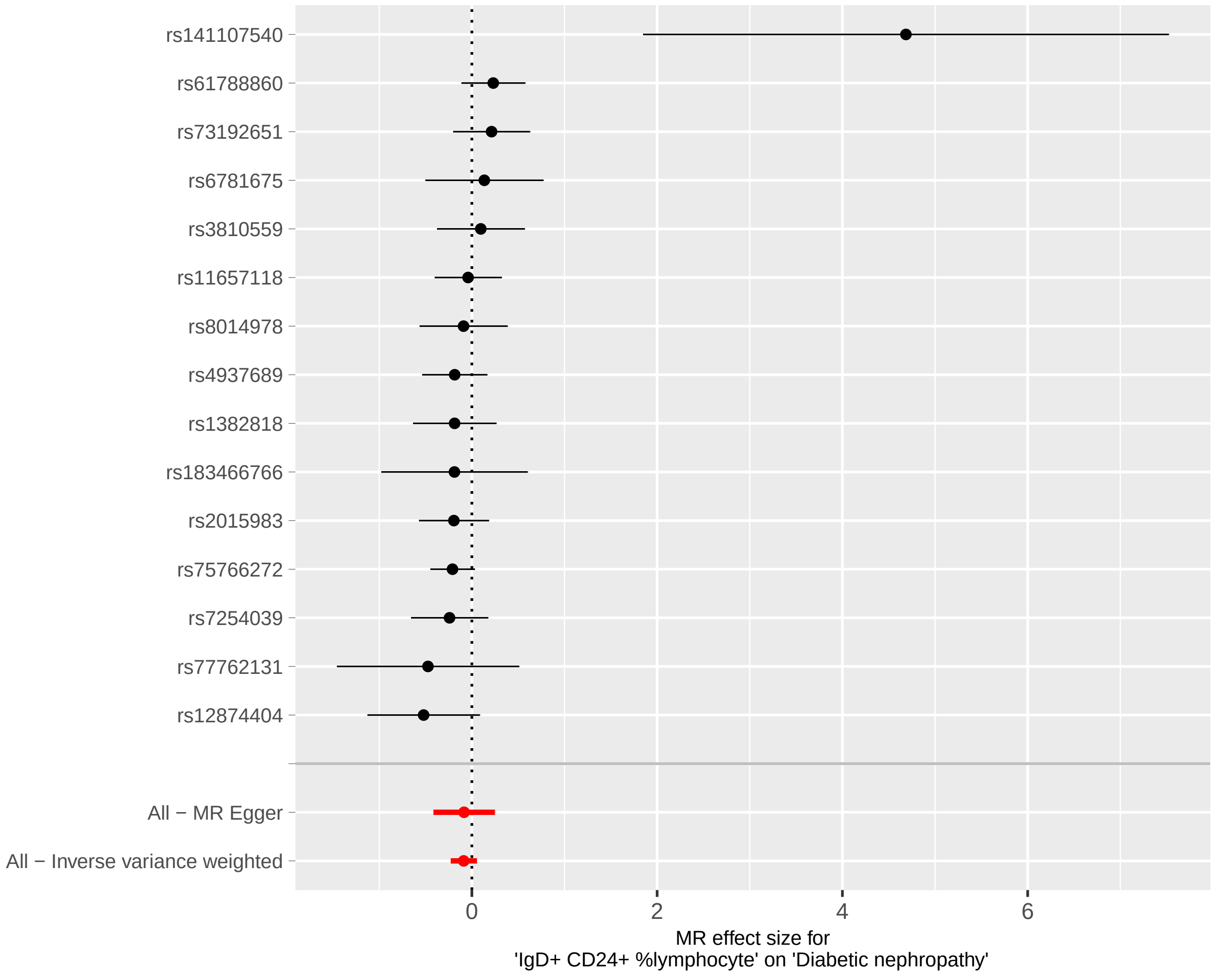
rs141107540
rs61788860
rs73192651
rs6781675
rs3810559
rs11657118
rs8014978
rs4937689
rs1382818
rs183466766
rs2015983
rs75766272
rs7254039
rs77762131
rs12874404
All − MR Egger
All − Inverse variance weighted
0
2
4
6
MR effect size for
'IgD+ CD24+ %lymphocyte' on 'Diabetic nephropathy'

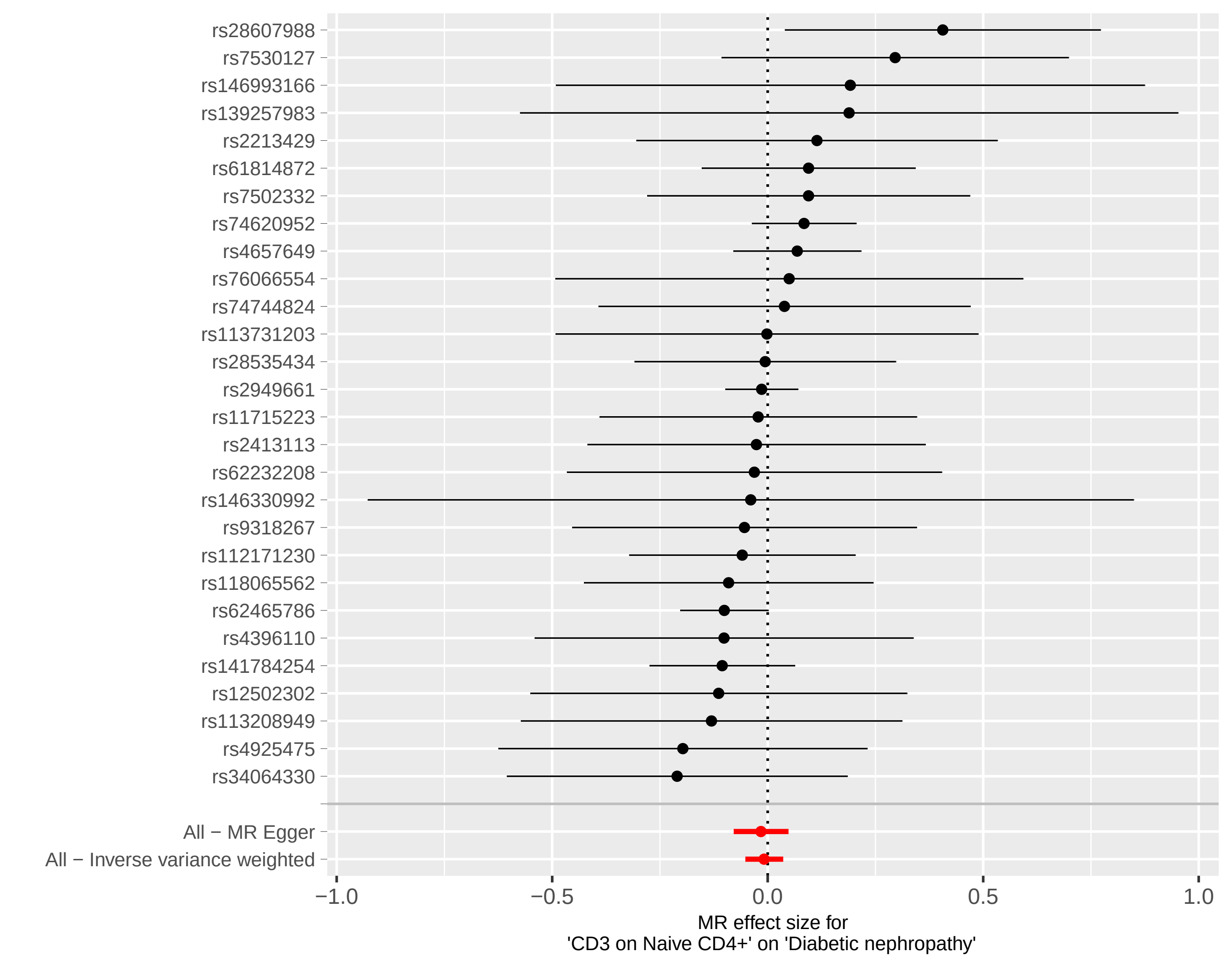

rs28607988
rs7530127
rs146993166
rs139257983
rs2213429
rs61814872
rs7502332
rs74620952
rs4657649
rs76066554
rs74744824
rs113731203
rs28535434
rs2949661
rs11715223
rs2413113
rs62232208
rs146330992
rs9318267
rs112171230
rs118065562
rs62465786
rs4396110
rs141784254
rs12502302
rs113208949
rs4925475
rs34064330
All − MR Egger
All − Inverse variance weighted
−1.0
−0.5
0.0
0.5
1.0
MR effect size for
'CD3 on Naive CD4+' on 'Diabetic nephropathy'

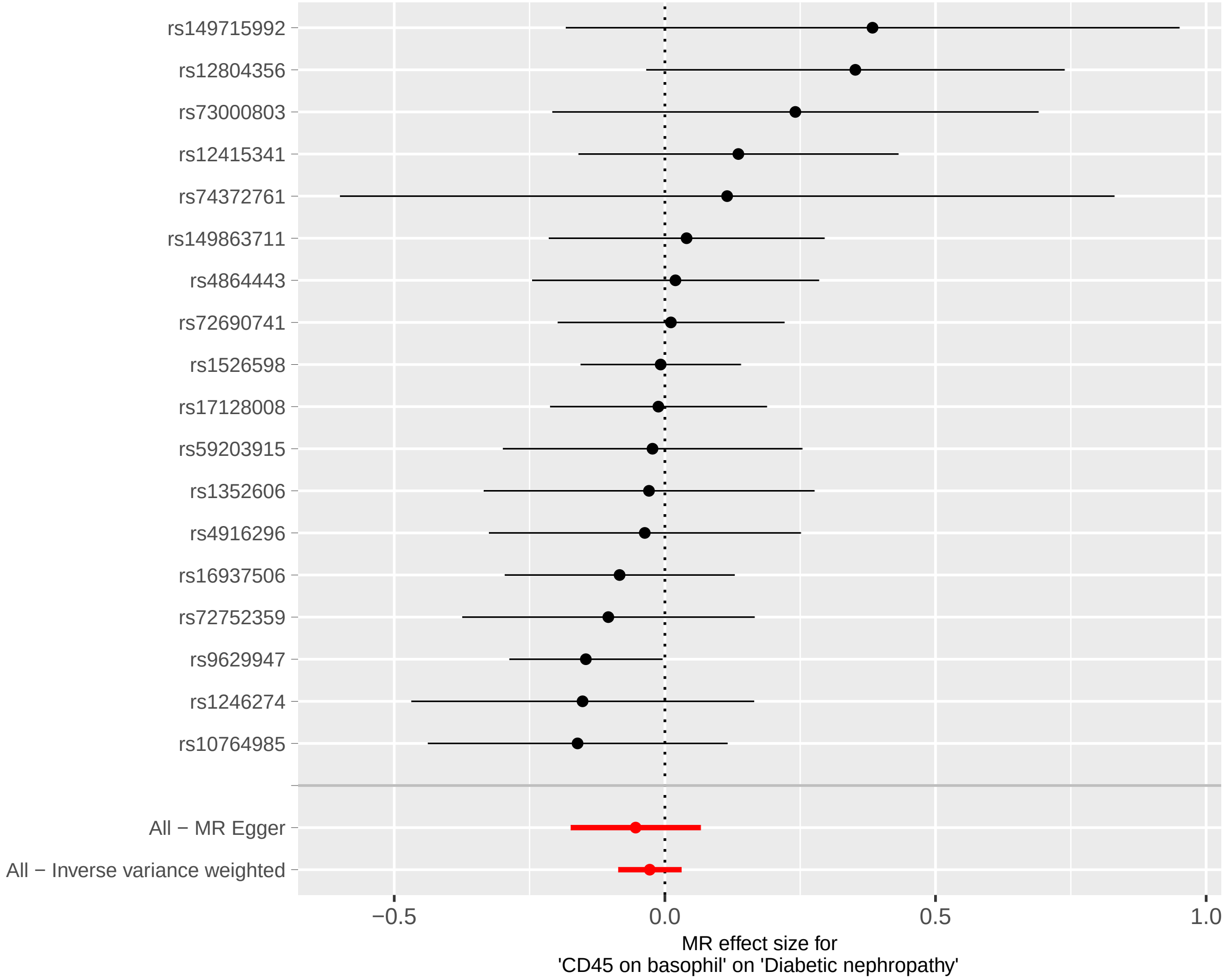

rs149715992
rs12804356
rs73000803
rs12415341
rs74372761
rs149863711
rs4864443
rs72690741
rs1526598
rs17128008
rs59203915
rs1352606
rs4916296
rs16937506
rs72752359
rs9629947
rs1246274
rs10764985
All − MR Egger
All − Inverse variance weighted
−0.5
0.0
0.5
1.0
MR effect size for
'CD45 on basophil' on 'Diabetic nephropathy'

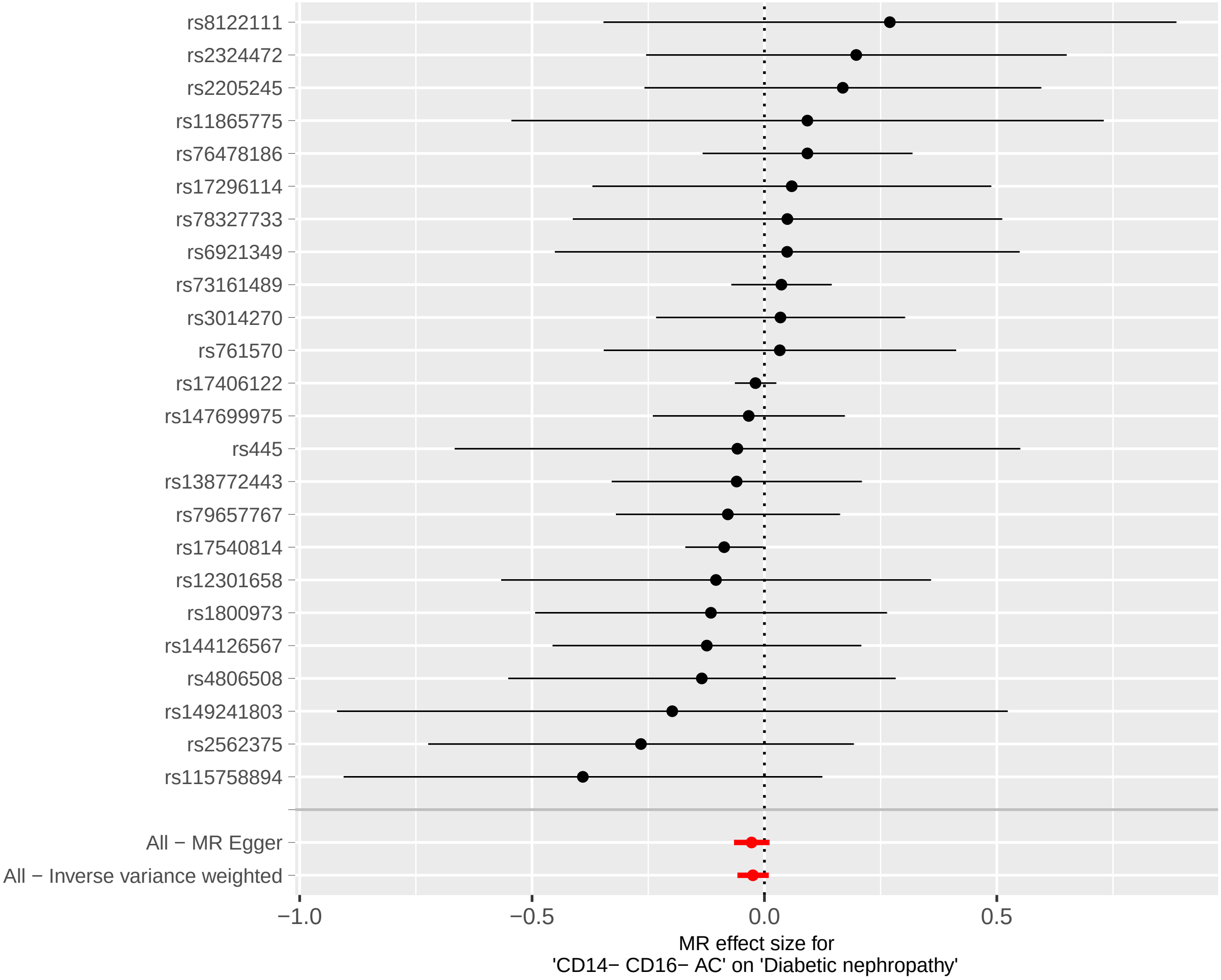

rs8122111
rs2324472
rs2205245
rs11865775
rs76478186
rs17296114
rs78327733
rs6921349
rs73161489
rs3014270
rs761570
rs17406122
rs147699975
rs445
rs138772443
rs79657767
rs17540814
rs12301658
rs1800973
rs144126567
rs4806508
rs149241803
rs2562375
rs115758894
All − MR Egger
All − Inverse variance weighted
−1.0
−0.5
0.0
0.5
MR effect size for
'CD14− CD16− AC' on 'Diabetic nephropathy'

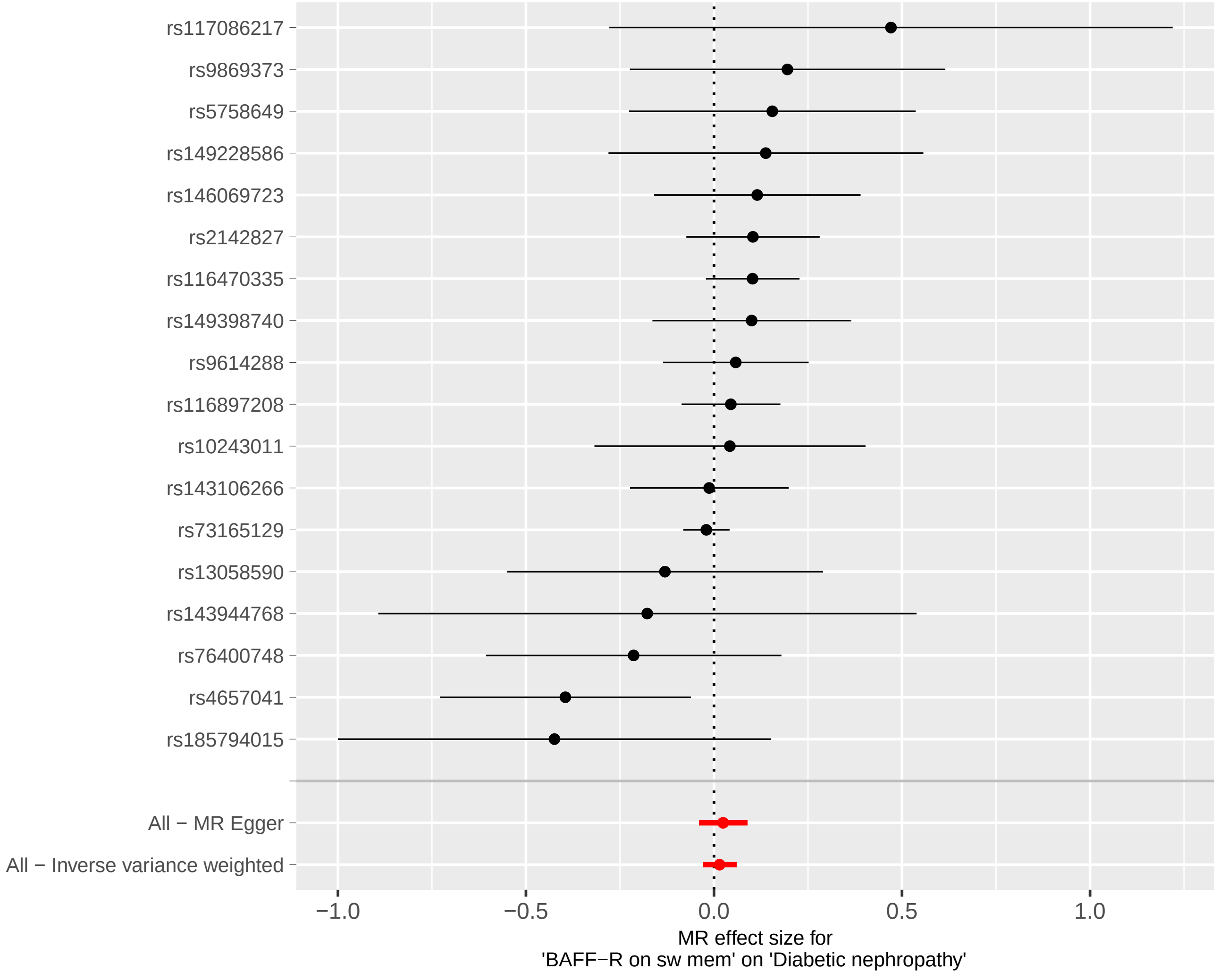
rs117086217
rs9869373
rs5758649
rs149228586
rs146069723
rs2142827
rs116470335
rs149398740
rs9614288
rs116897208
rs10243011
rs143106266
rs73165129
rs13058590
rs143944768
rs76400748
rs4657041
rs185794015
All − MR Egger
All − Inverse variance weighted
−1.0
−0.5
0.0
0.5
1.0
MR effect size for
'BAFF−R on sw mem' on 'Diabetic nephropathy'

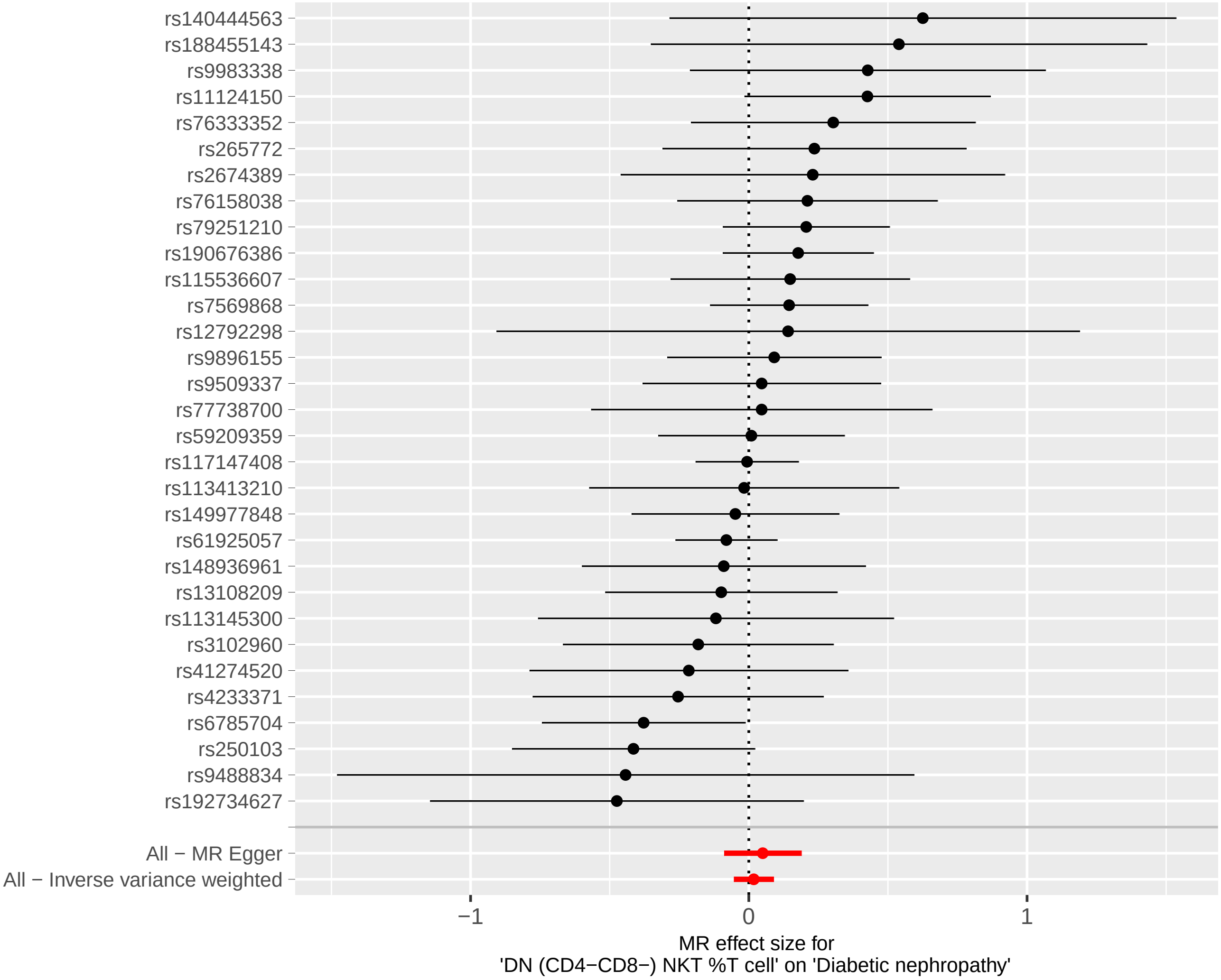
rs140444563
rs188455143
rs9983338
rs11124150
rs76333352
rs265772
rs2674389
rs76158038
rs79251210
rs190676386
rs115536607
rs7569868
rs12792298
rs9896155
rs9509337
rs77738700
rs59209359
rs117147408
rs113413210
rs149977848
rs61925057
rs148936961
rs13108209
rs113145300
rs3102960
rs41274520
rs4233371
rs6785704
rs250103
rs9488834
rs192734627
All − MR Egger
All − Inverse variance weighted
−1
0
1
MR effect size for
'DN (CD4−CD8−) NKT %T cell' on 'Diabetic nephropathy'

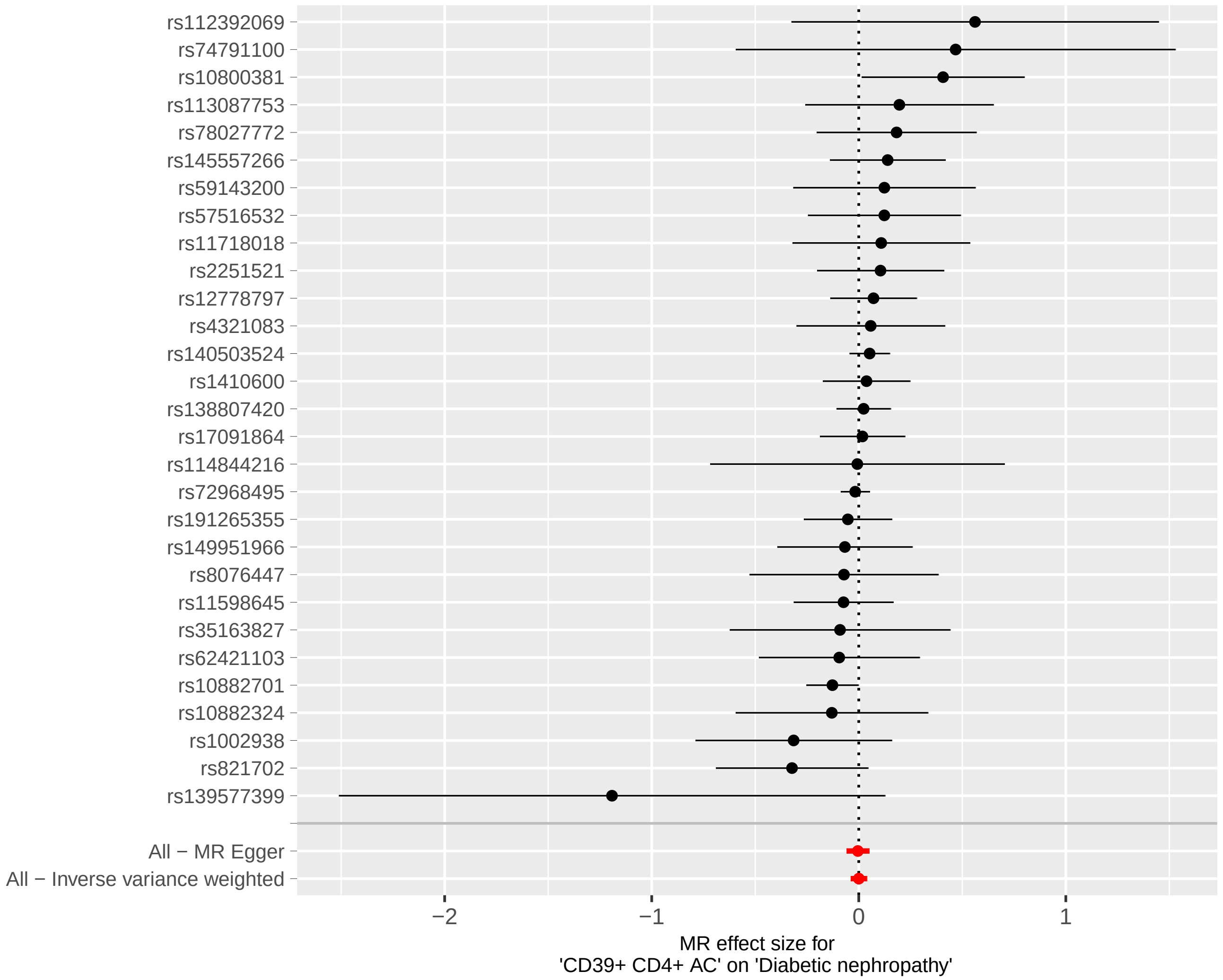
rs112392069
rs74791100
rs10800381
rs113087753
rs78027772
rs145557266
rs59143200
rs57516532
rs11718018
rs2251521
rs12778797
rs4321083
rs140503524
rs1410600
rs138807420
rs17091864
rs114844216
rs72968495
rs191265355
rs149951966
rs8076447
rs11598645
rs35163827
rs62421103
rs10882701
rs10882324
rs1002938
rs821702
rs139577399
All − MR Egger
All − Inverse variance weighted
−2
−1
0
1
MR effect size for
'CD39+ CD4+ AC' on 'Diabetic nephropathy'

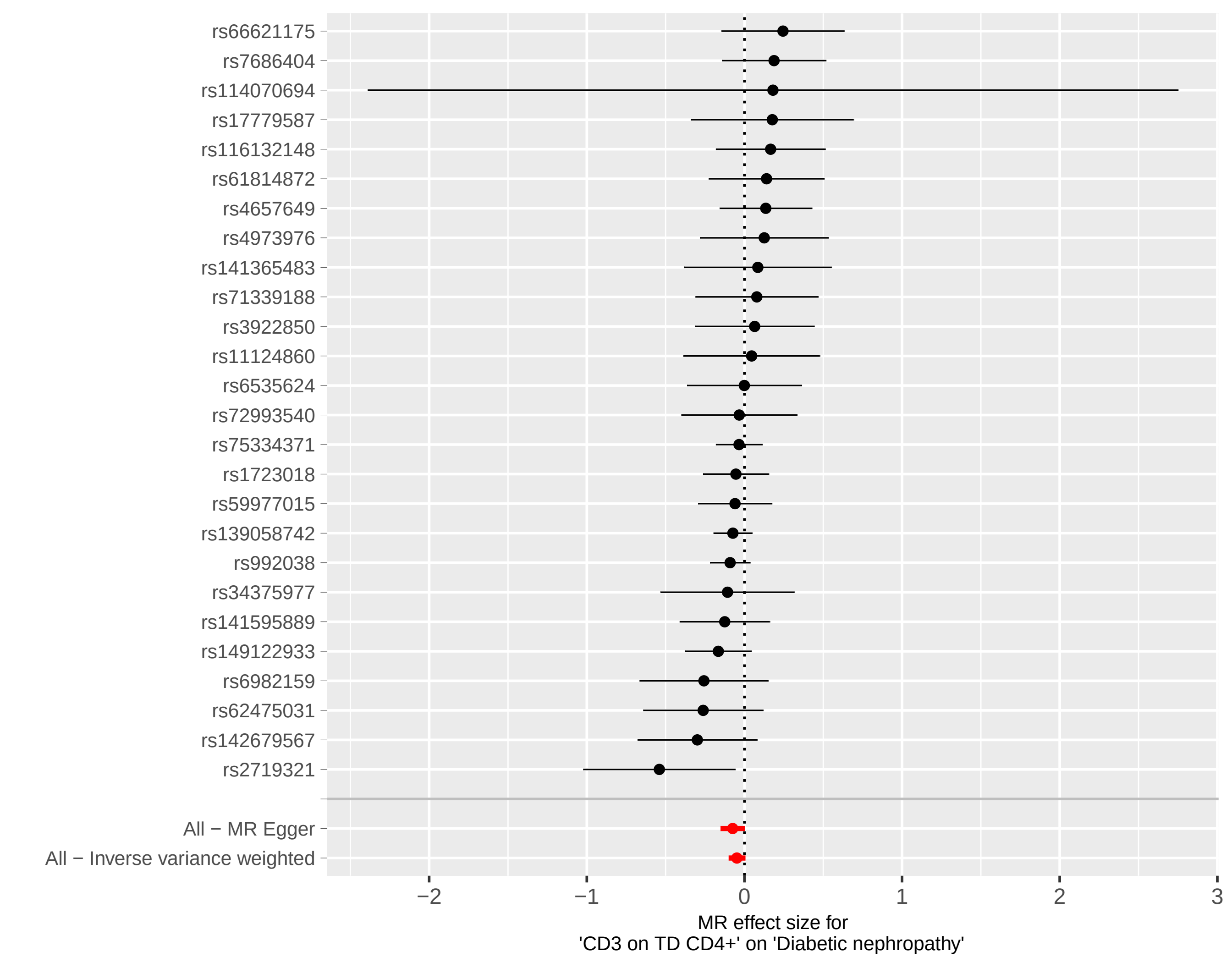
rs66621175
rs7686404
rs114070694
rs17779587
rs116132148
rs61814872
rs4657649
rs4973976
rs141365483
rs71339188
rs3922850
rs11124860
rs6535624
rs72993540
rs75334371
rs1723018
rs59977015
rs139058742
rs992038
rs34375977
rs141595889
rs149122933
rs6982159
rs62475031
rs142679567
rs2719321
All − MR Egger
All − Inverse variance weighted
−2
−1
0
1
2
3
MR effect size for
'CD3 on TD CD4+' on 'Diabetic nephropathy'

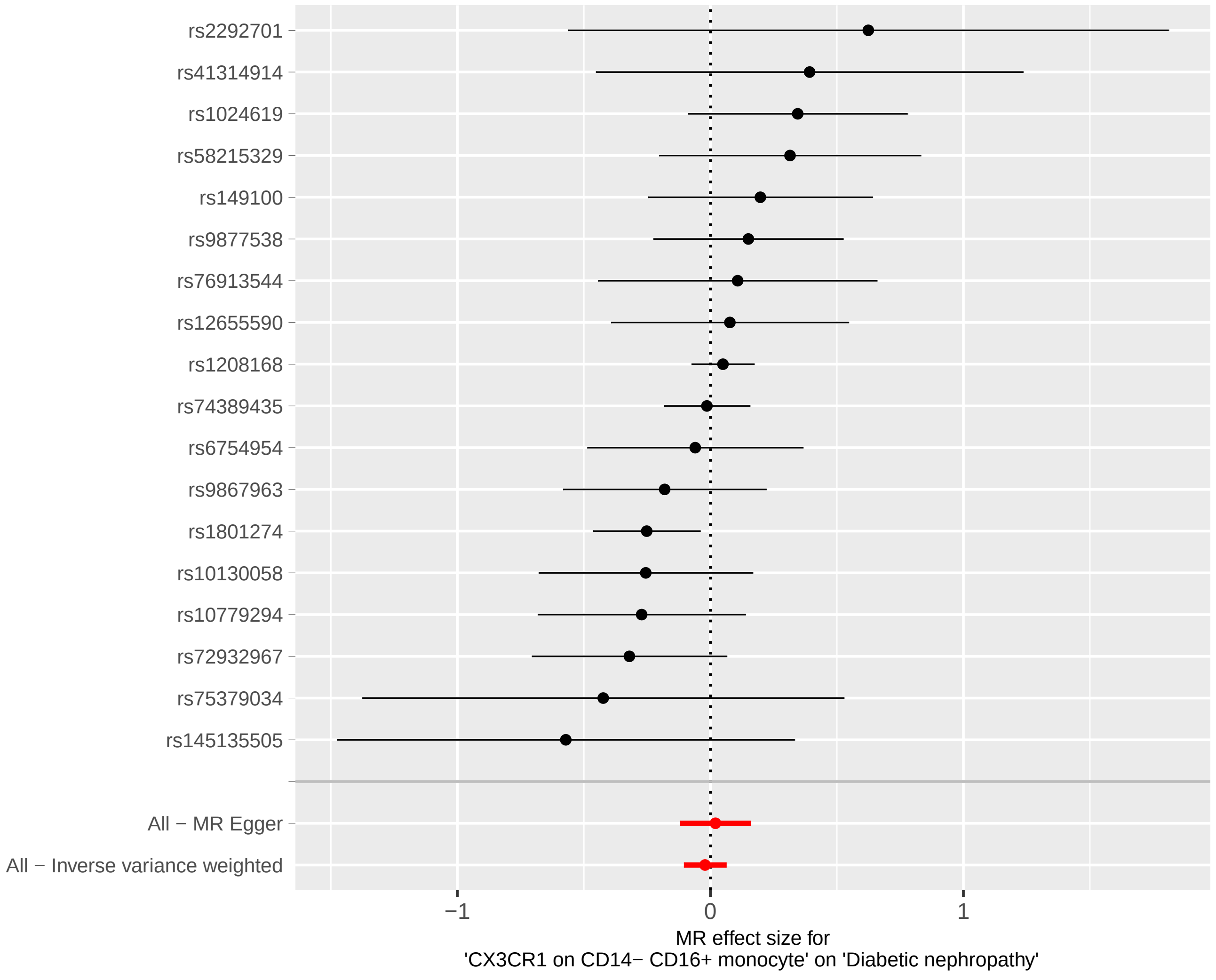
rs2292701
rs41314914
rs1024619
rs58215329
rs149100
rs9877538
rs76913544
rs12655590
rs1208168
rs74389435
rs6754954
rs9867963
rs1801274
rs10130058
rs10779294
rs72932967
rs75379034
rs145135505
All − MR Egger
All − Inverse variance weighted
−1
0
1
MR effect size for
'CX3CR1 on CD14− CD16+ monocyte' on 'Diabetic nephropathy'

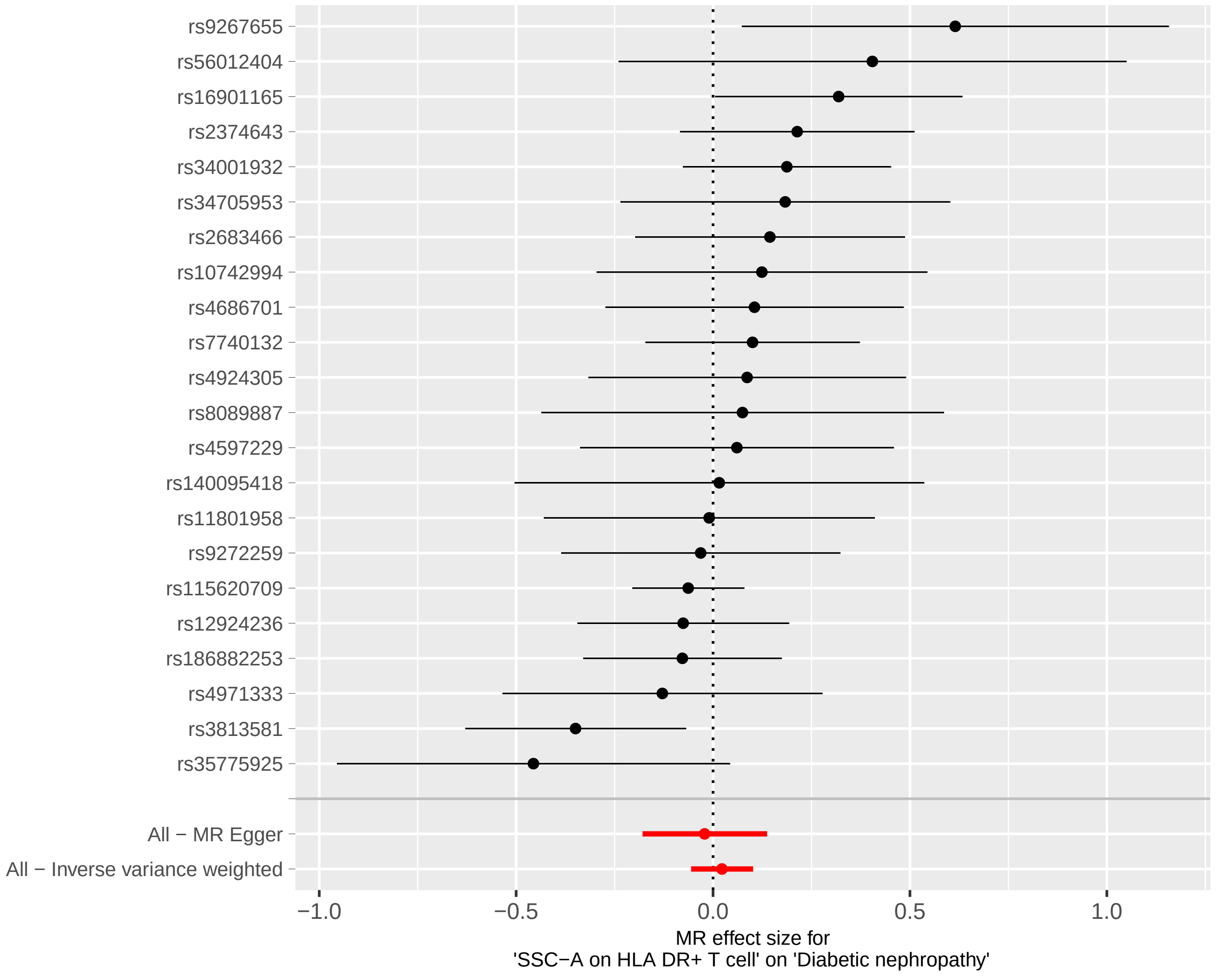
rs9267655
rs56012404
rs16901165
rs2374643
rs34001932
rs34705953
rs2683466
rs10742994
rs4686701
rs7740132
rs4924305
rs8089887
rs4597229
rs140095418
rs11801958
rs9272259
rs115620709
rs12924236
rs186882253
rs4971333
rs3813581
rs35775925
All − MR Egger
All − Inverse variance weighted
−1.0
−0.5
0.0
0.5
1.0
MR effect size for
'SSC−A on HLA DR+ T cell' on 'Diabetic nephropathy'

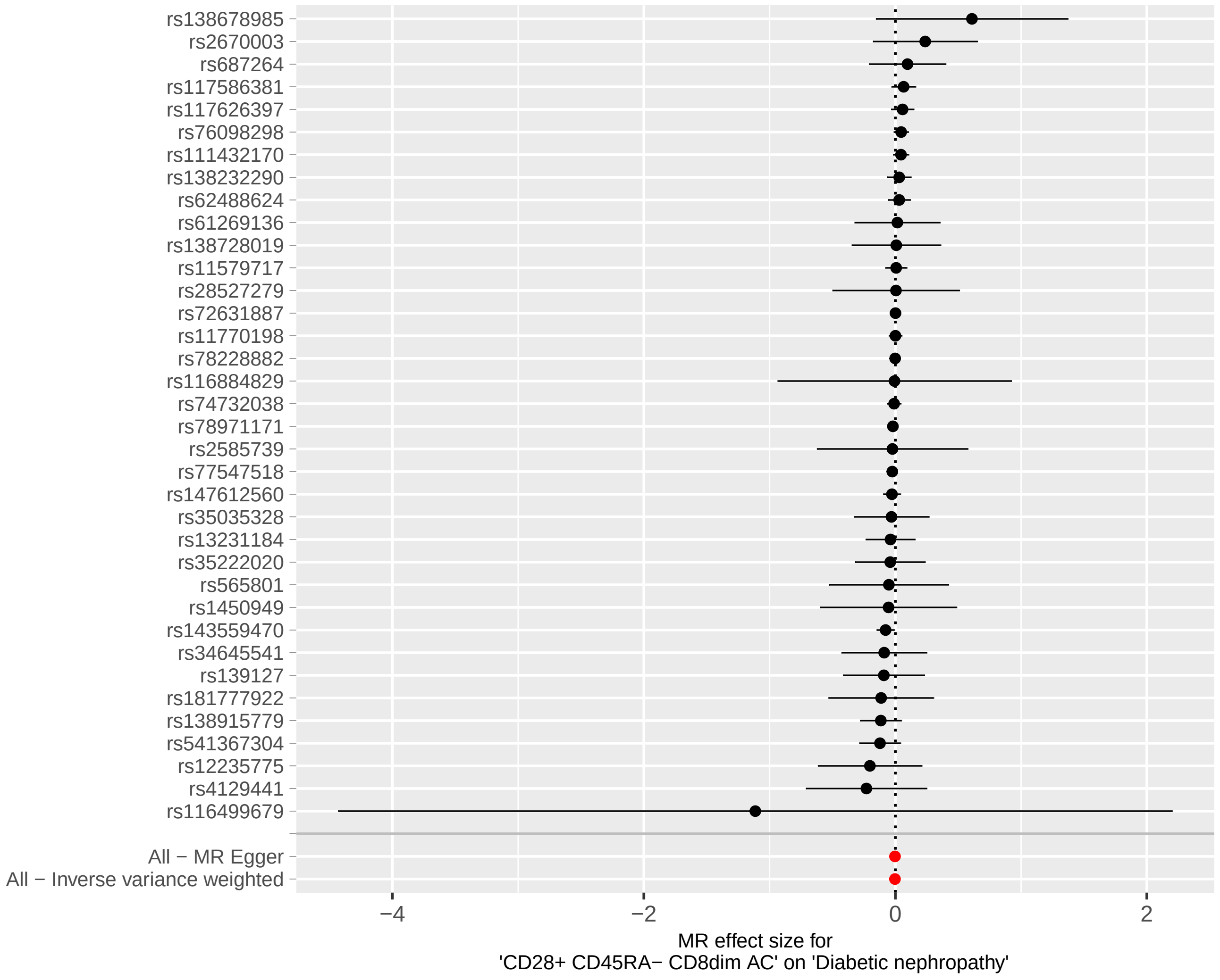
rs138678985
rs2670003
rs687264
rs117586381
rs117626397
rs76098298
rs111432170
rs138232290
rs62488624
rs61269136
rs138728019
rs11579717
rs28527279
rs72631887
rs11770198
rs78228882
rs116884829
rs74732038
rs78971171
rs2585739
rs77547518
rs147612560
rs35035328
rs13231184
rs35222020
rs565801
rs1450949
rs143559470
rs34645541
rs139127
rs181777922
rs138915779
rs541367304
rs12235775
rs4129441
rs116499679
All − MR Egger
All − Inverse variance weighted
−4
−2
0
2
MR effect size for
'CD28+ CD45RA− CD8dim AC' on 'Diabetic nephropathy'

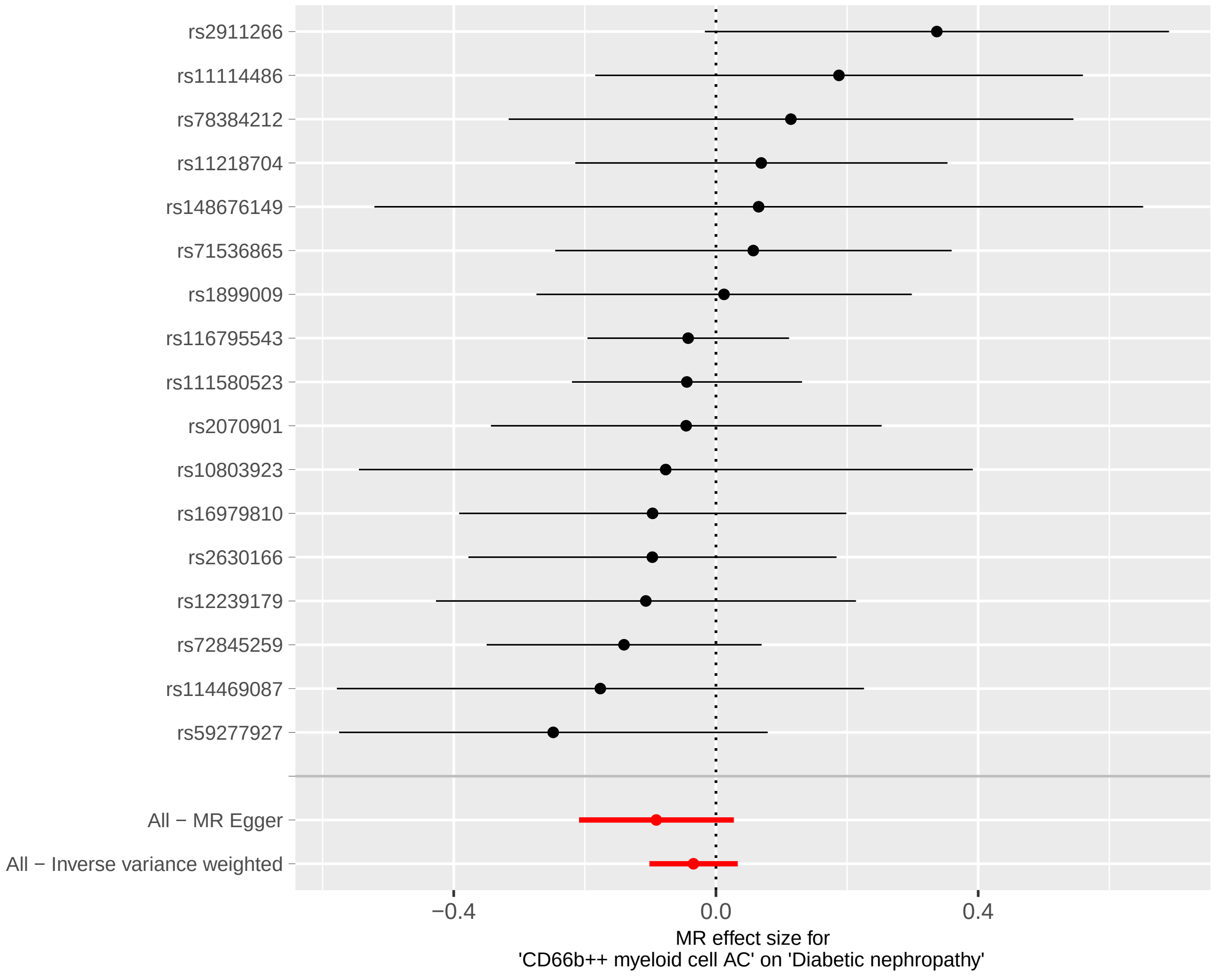

rs2911266
rs11114486
rs78384212
rs11218704
rs148676149
rs71536865
rs1899009
rs116795543
rs111580523
rs2070901
rs10803923
rs16979810
rs2630166
rs12239179
rs72845259
rs114469087
rs59277927
All − MR Egger
All − Inverse variance weighted
−0.4
0.0
0.4
MR effect size for
'CD66b++ myeloid cell AC' on 'Diabetic nephropathy'

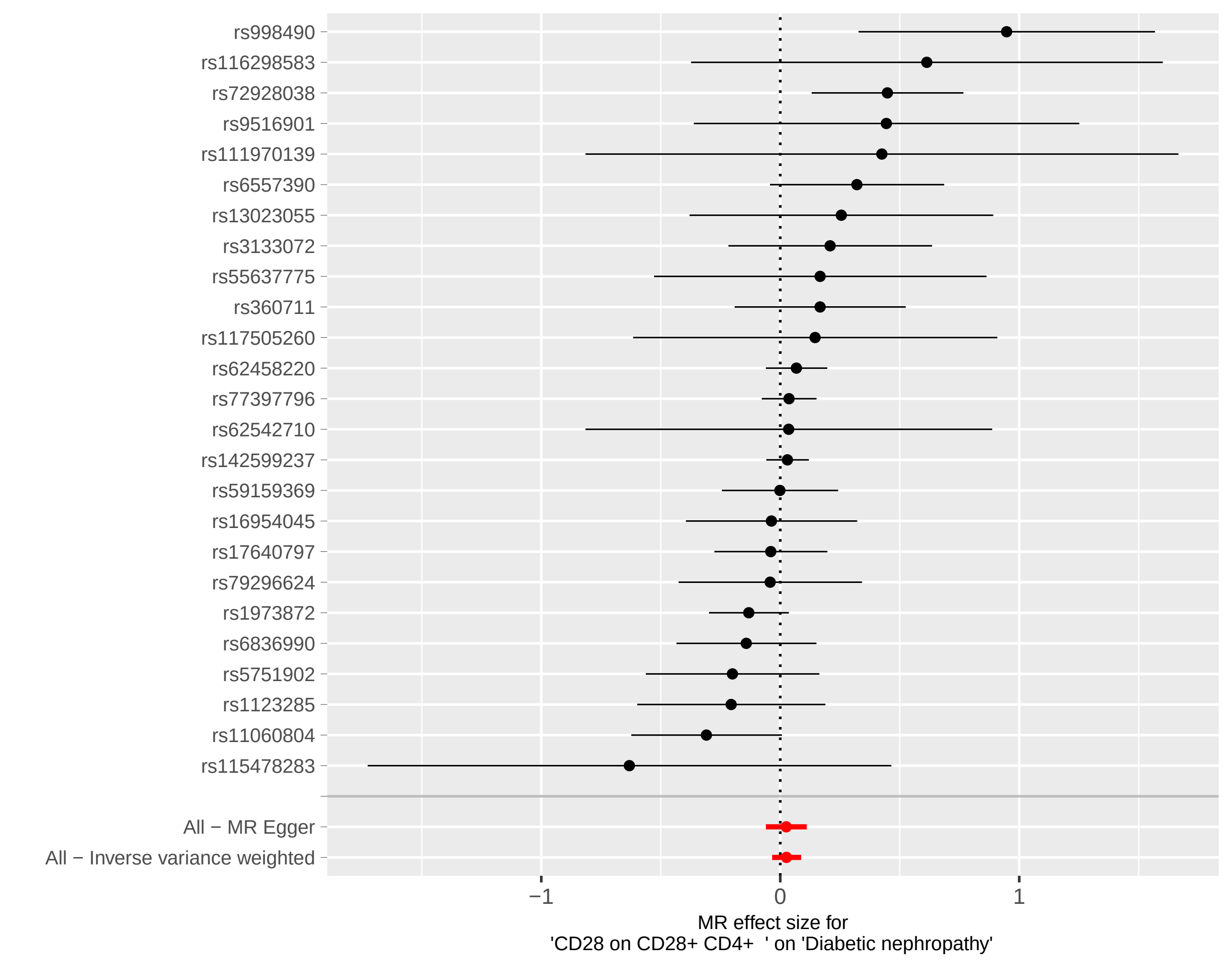

rs998490
rs116298583
rs72928038
rs9516901
rs111970139
rs6557390
rs13023055
rs3133072
rs55637775
rs360711
rs117505260
rs62458220
rs77397796
rs62542710
rs142599237
rs59159369
rs16954045
rs17640797
rs79296624
rs1973872
rs6836990
rs5751902
rs1123285
rs11060804
rs115478283
All − MR Egger
All − Inverse variance weighted
−1
0
1
MR effect size for
'CD28 on CD28+ CD4+ ' on 'Diabetic nephropathy'

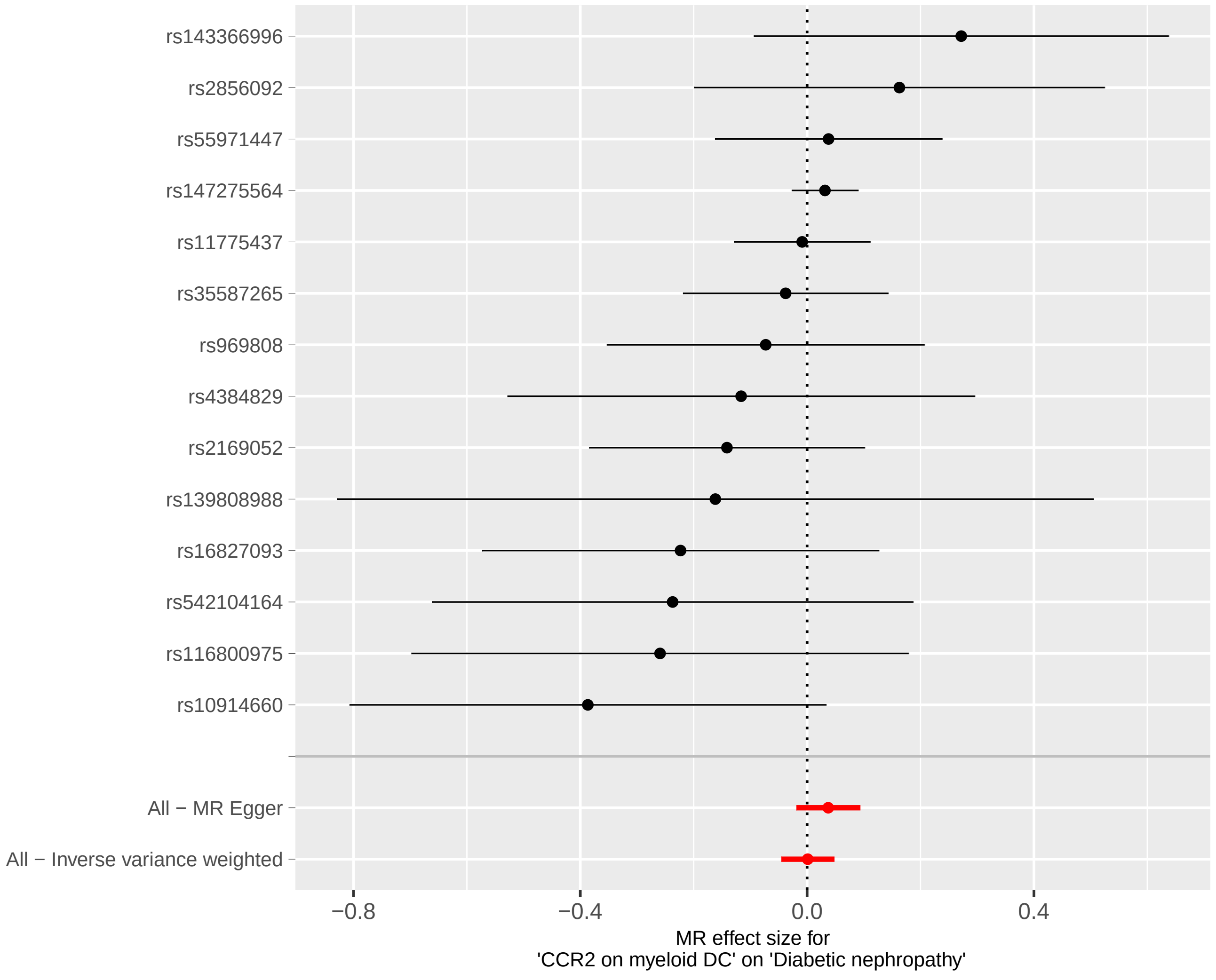
rs143366996
rs2856092
rs55971447
rs147275564
rs11775437
rs35587265
rs969808
rs4384829
rs2169052
rs139808988
rs16827093
rs542104164
rs116800975
rs10914660
All − MR Egger
All − Inverse variance weighted
−0.8
−0.4
0.0
0.4
MR effect size for
'CCR2 on myeloid DC' on 'Diabetic nephropathy'

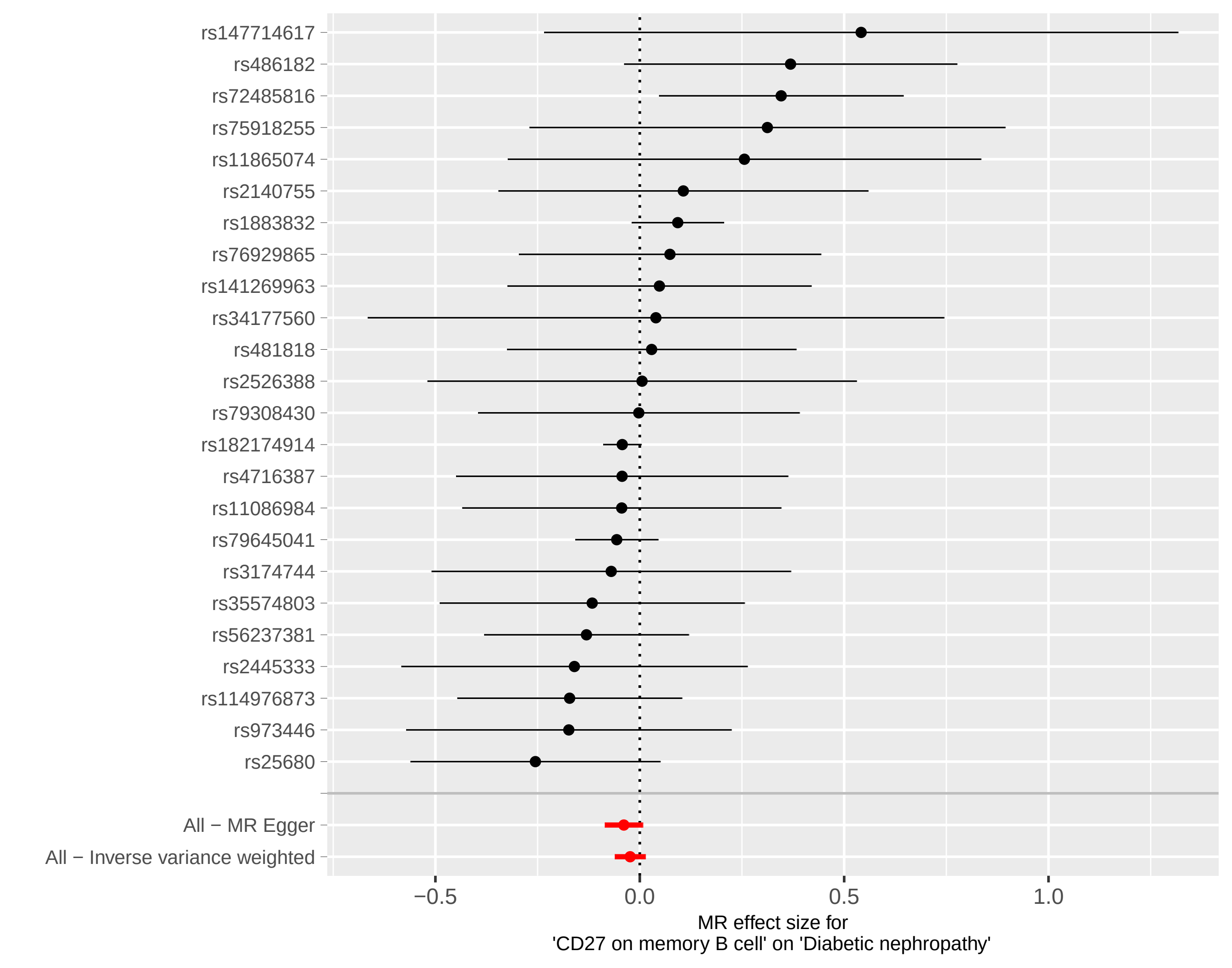

rs147714617
rs486182
rs72485816
rs75918255
rs11865074
rs2140755
rs1883832
rs76929865
rs141269963
rs34177560
rs481818
rs2526388
rs79308430
rs182174914
rs4716387
rs11086984
rs79645041
rs3174744
rs35574803
rs56237381
rs2445333
rs114976873
rs973446
rs25680
All − MR Egger
All − Inverse variance weighted
−0.5
0.0
0.5
1.0
MR effect size for
'CD27 on memory B cell' on 'Diabetic nephropathy'

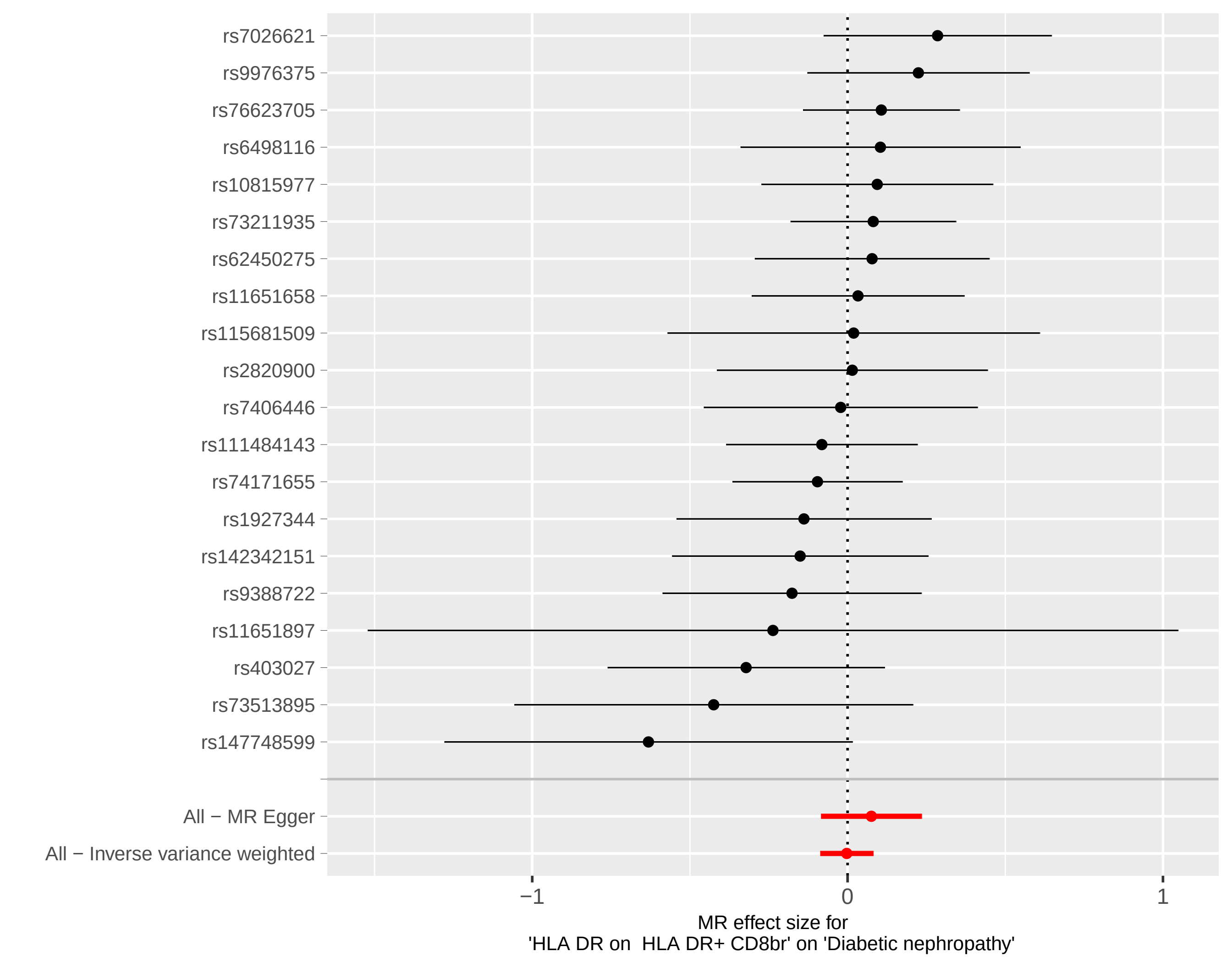
rs7026621
rs9976375
rs76623705
rs6498116
rs10815977
rs73211935
rs62450275
rs11651658
rs115681509
rs2820900
rs7406446
rs111484143
rs74171655
rs1927344
rs142342151
rs9388722
rs11651897
rs403027
rs73513895
rs147748599
All − MR Egger
All − Inverse variance weighted
−1
0
1
MR effect size for
'HLA DR on HLA DR+ CD8br' on 'Diabetic nephropathy'

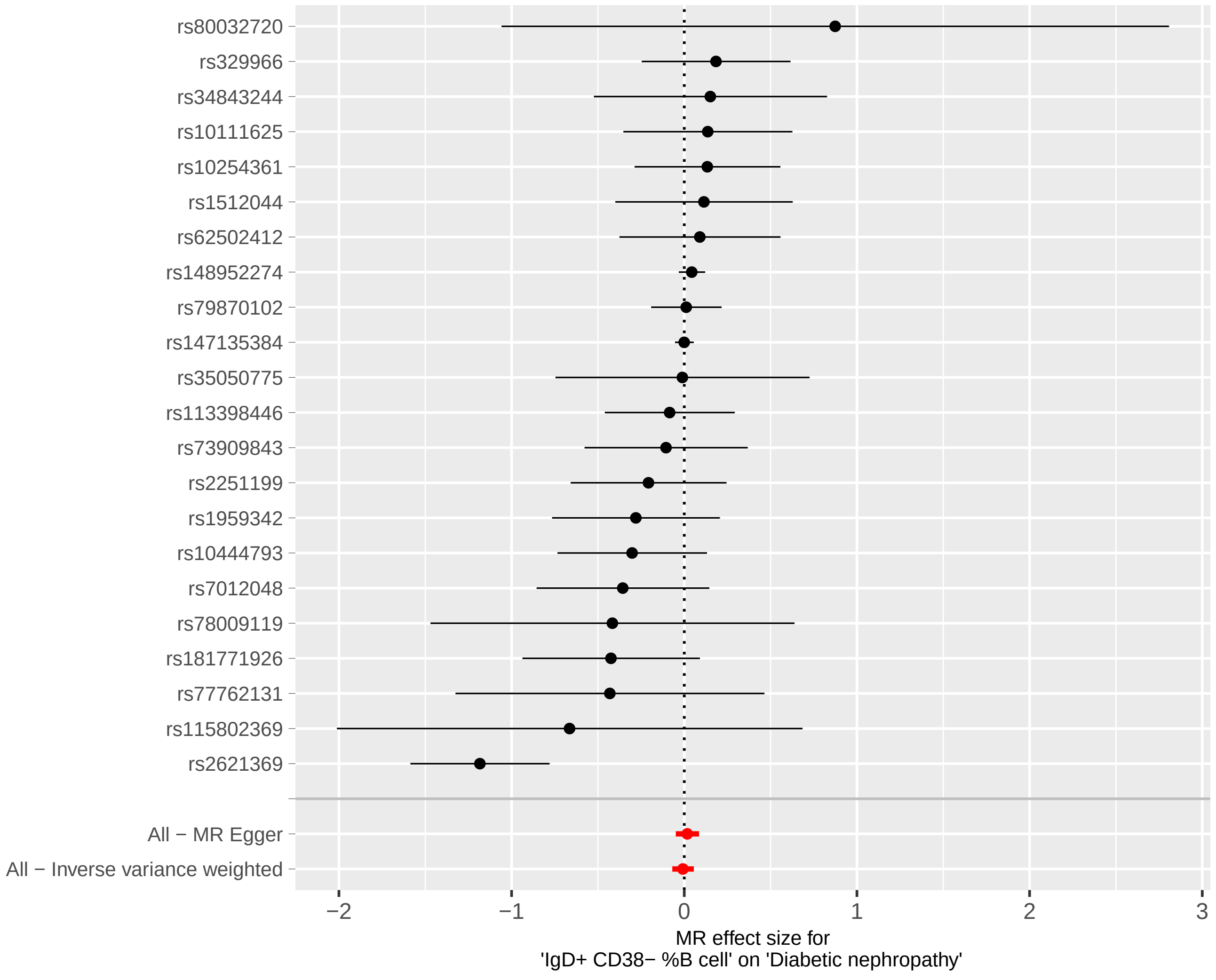

rs80032720
rs329966
rs34843244
rs10111625
rs10254361
rs1512044
rs62502412
rs148952274
rs79870102
rs147135384
rs35050775
rs113398446
rs73909843
rs2251199
rs1959342
rs10444793
rs7012048
rs78009119
rs181771926
rs77762131
rs115802369
rs2621369
All − MR Egger
All − Inverse variance weighted
−2
−1
0
1
2
3
MR effect size for
'IgD+ CD38− %B cell' on 'Diabetic nephropathy'

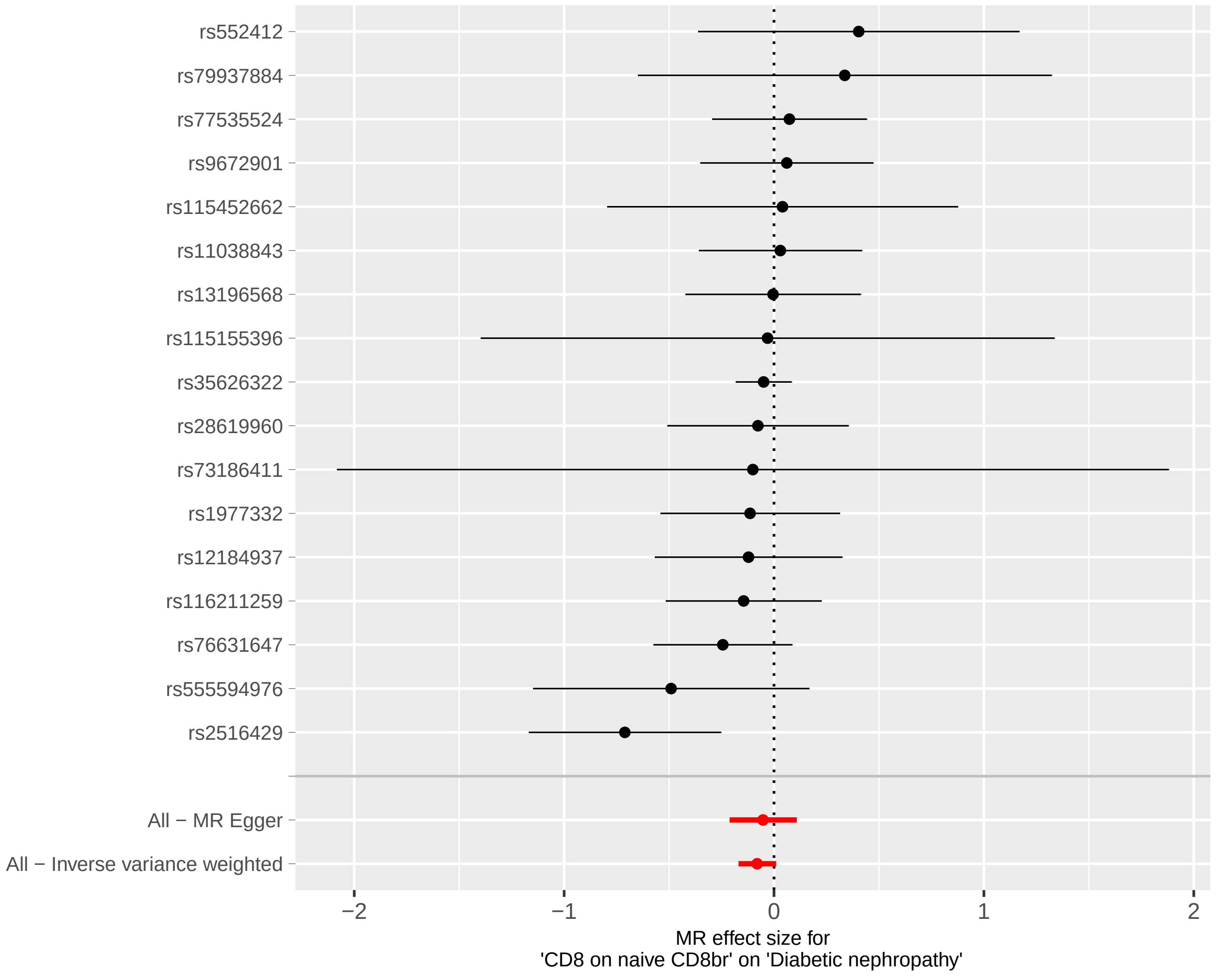

rs552412
rs79937884
rs77535524
rs9672901
rs115452662
rs11038843
rs13196568
rs115155396
rs35626322
rs28619960
rs73186411
rs1977332
rs12184937
rs116211259
rs76631647
rs555594976
rs2516429
All − MR Egger
All − Inverse variance weighted
−2
−1
0
1
2
MR effect size for
'CD8 on naive CD8br' on 'Diabetic nephropathy'

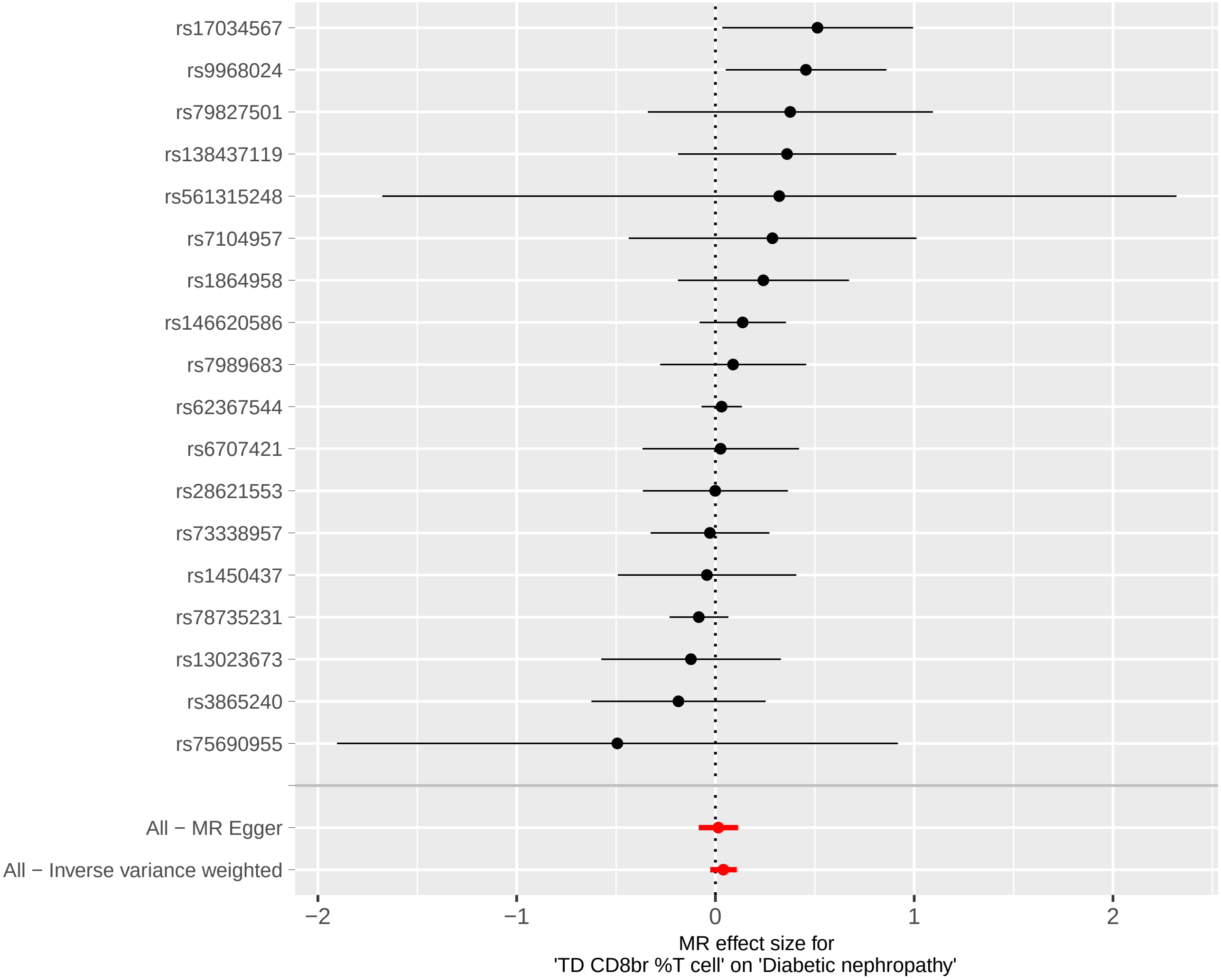
rs17034567
rs9968024
rs79827501
rs138437119
rs561315248
rs7104957
rs1864958
rs146620586
rs7989683
rs62367544
rs6707421
rs28621553
rs73338957
rs1450437
rs78735231
rs13023673
rs3865240
rs75690955
All − MR Egger
All − Inverse variance weighted
−2
−1
0
1
2
MR effect size for
'TD CD8br %T cell' on 'Diabetic nephropathy'

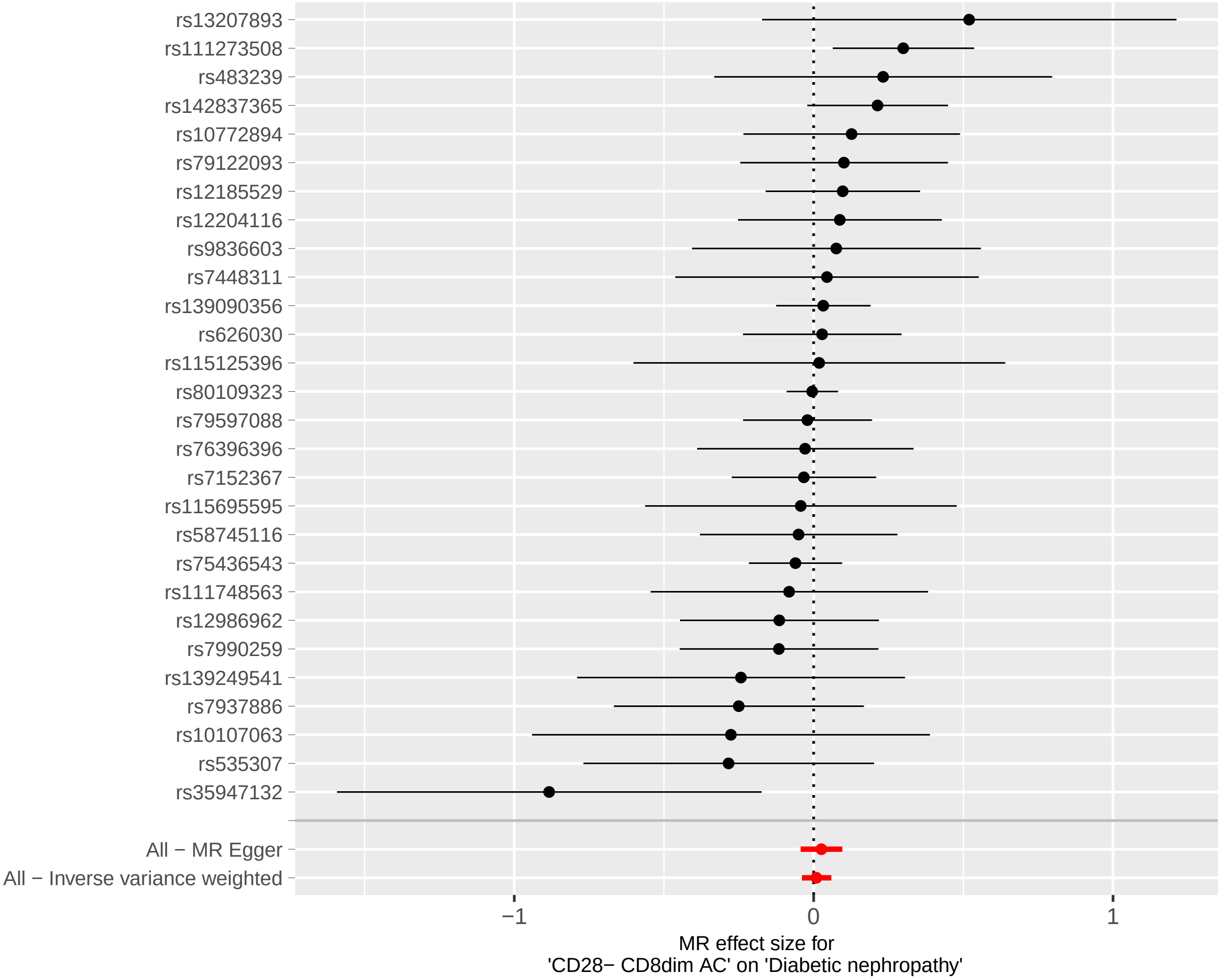
rs13207893
rs111273508
rs483239
rs142837365
rs10772894
rs79122093
rs12185529
rs12204116
rs9836603
rs7448311
rs139090356
rs626030
rs115125396
rs80109323
rs79597088
rs76396396
rs7152367
rs115695595
rs58745116
rs75436543
rs111748563
rs12986962
rs7990259
rs139249541
rs7937886
rs10107063
rs535307
rs35947132
All − MR Egger
All − Inverse variance weighted
−1
0
1
MR effect size for
'CD28− CD8dim AC' on 'Diabetic nephropathy'

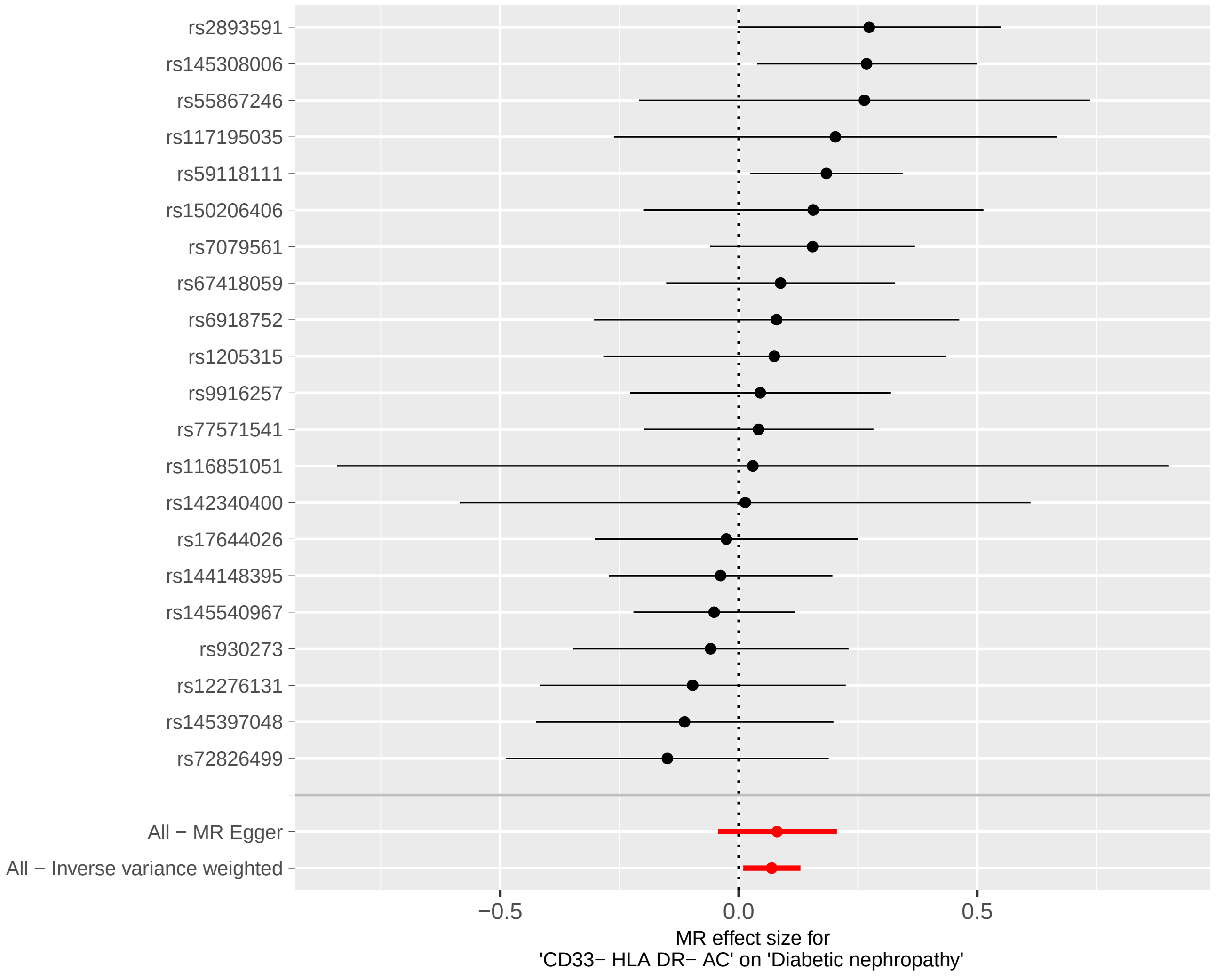
rs2893591
rs145308006
rs55867246
rs117195035
rs59118111
rs150206406
rs7079561
rs67418059
rs6918752
rs1205315
rs9916257
rs77571541
rs116851051
rs142340400
rs17644026
rs144148395
rs145540967
rs930273
rs12276131
rs145397048
rs72826499
All − MR Egger
All − Inverse variance weighted
−0.5
0.0
0.5
MR effect size for
'CD33− HLA DR− AC' on 'Diabetic nephropathy'

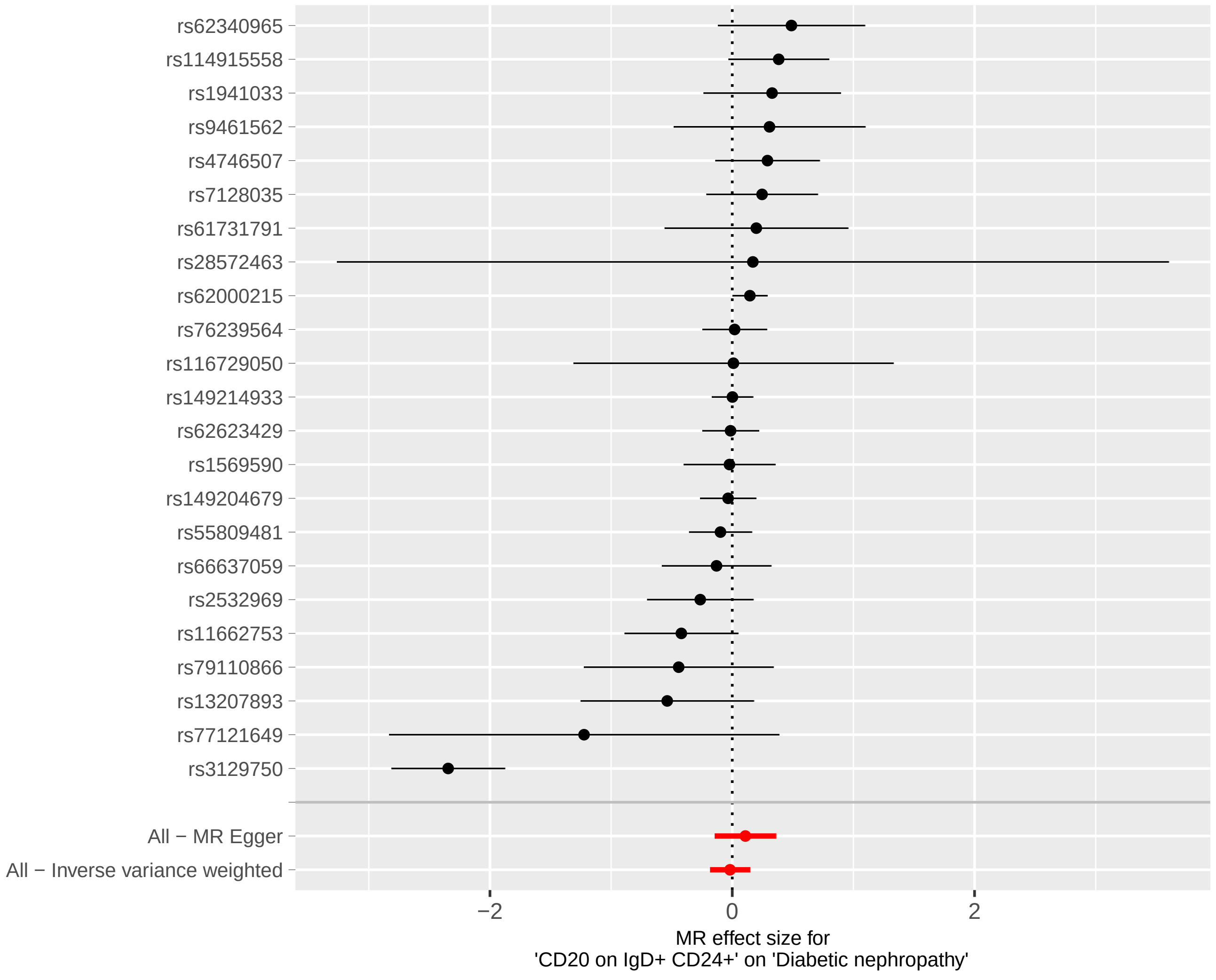
rs62340965
rs114915558
rs1941033
rs9461562
rs4746507
rs7128035
rs61731791
rs28572463
rs62000215
rs76239564
rs116729050
rs149214933
rs62623429
rs1569590
rs149204679
rs55809481
rs66637059
rs2532969
rs11662753
rs79110866
rs13207893
rs77121649
rs3129750
All − MR Egger
All − Inverse variance weighted
−2
0
2
MR effect size for
'CD20 on IgD+ CD24+' on 'Diabetic nephropathy'

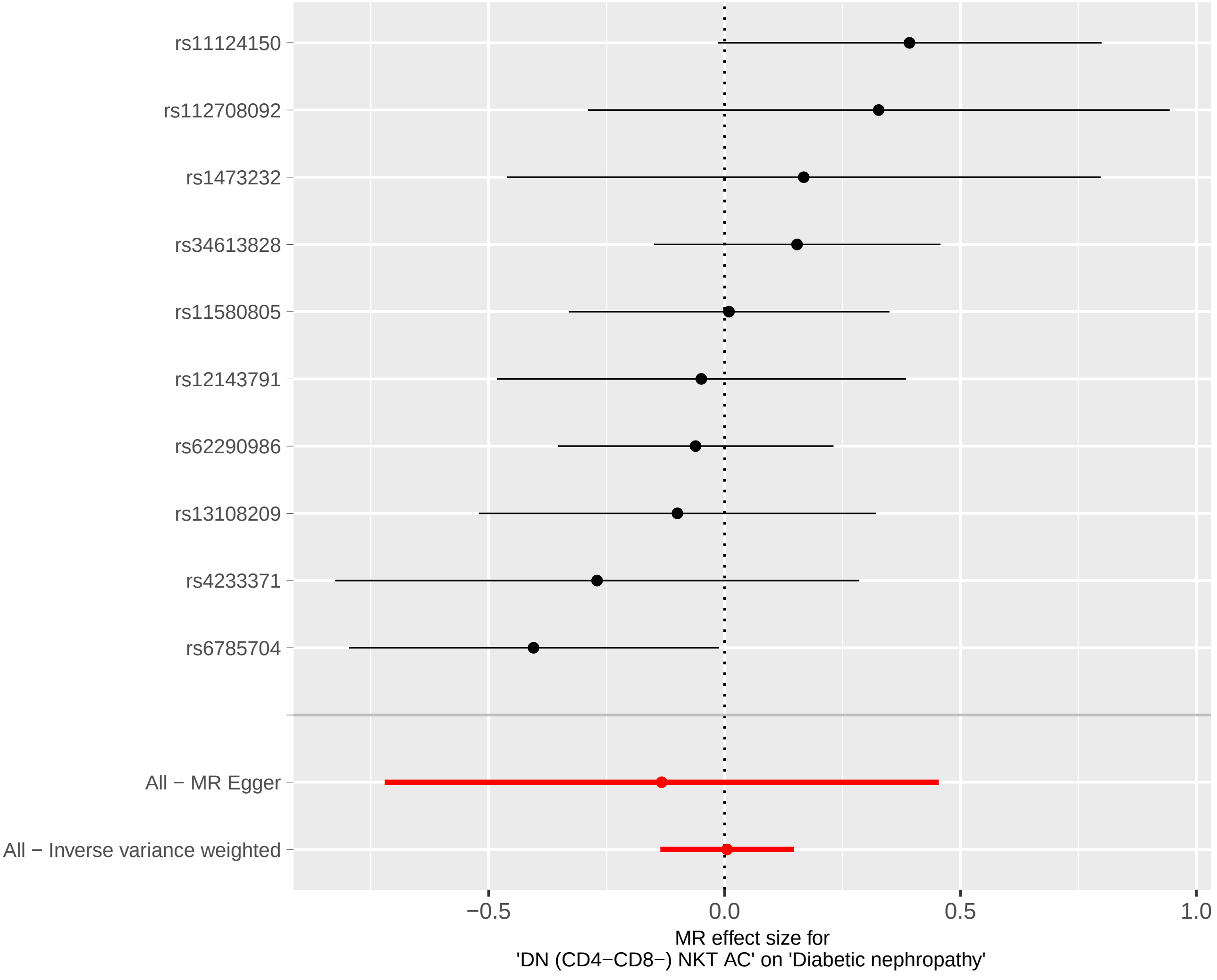
rs11124150
rs112708092
rs1473232
rs34613828
rs11580805
rs12143791
rs62290986
rs13108209
rs4233371
rs6785704
All − MR Egger
All − Inverse variance weighted
−0.5
0.0
0.5
1.0
MR effect size for
'DN (CD4−CD8−) NKT AC' on 'Diabetic nephropathy'

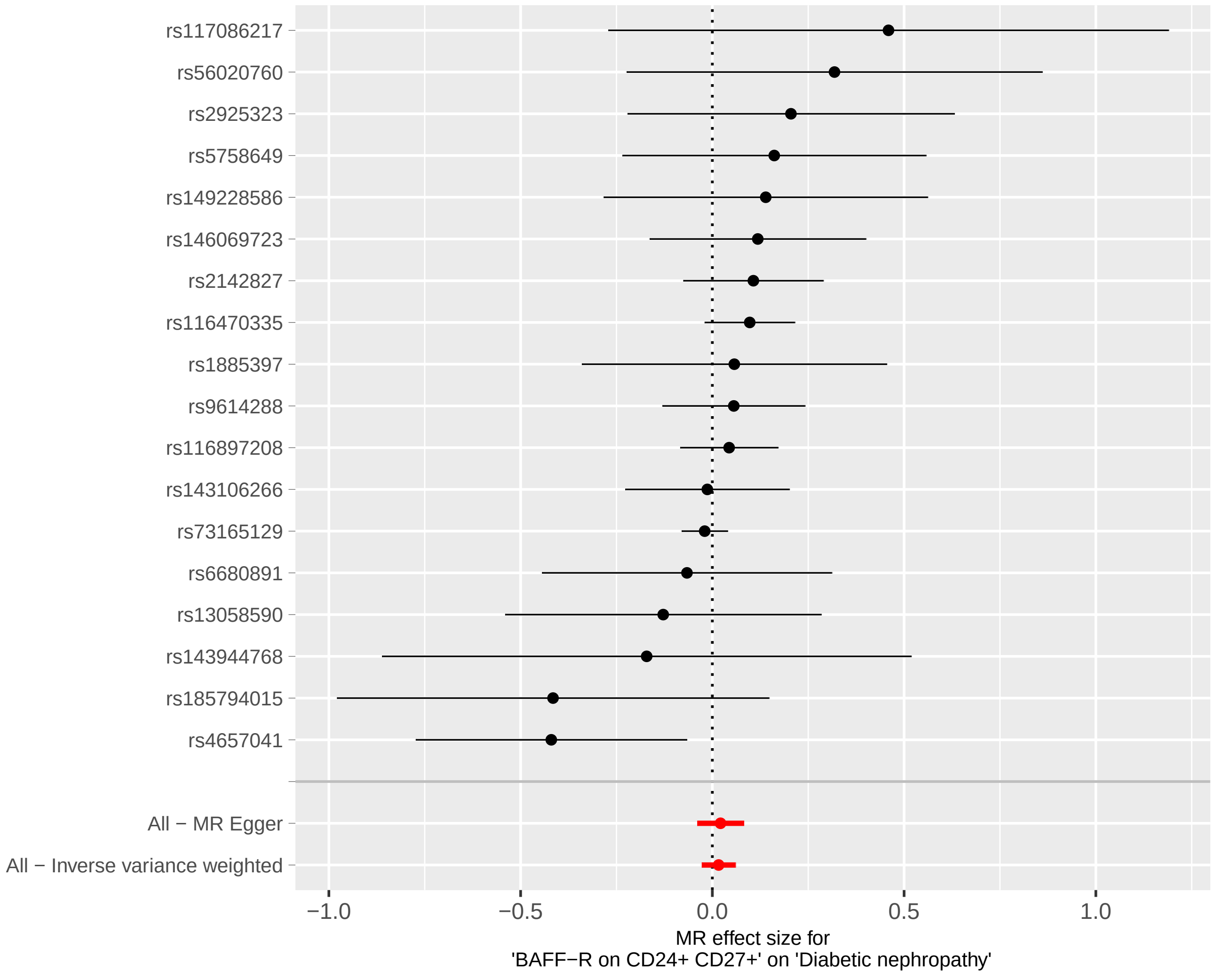
rs117086217
rs56020760
rs2925323
rs5758649
rs149228586
rs146069723
rs2142827
rs116470335
rs1885397
rs9614288
rs116897208
rs143106266
rs73165129
rs6680891
rs13058590
rs143944768
rs185794015
rs4657041
All − MR Egger
All − Inverse variance weighted
−1.0
−0.5
0.0
0.5
1.0
MR effect size for
'BAFF−R on CD24+ CD27+' on 'Diabetic nephropathy'

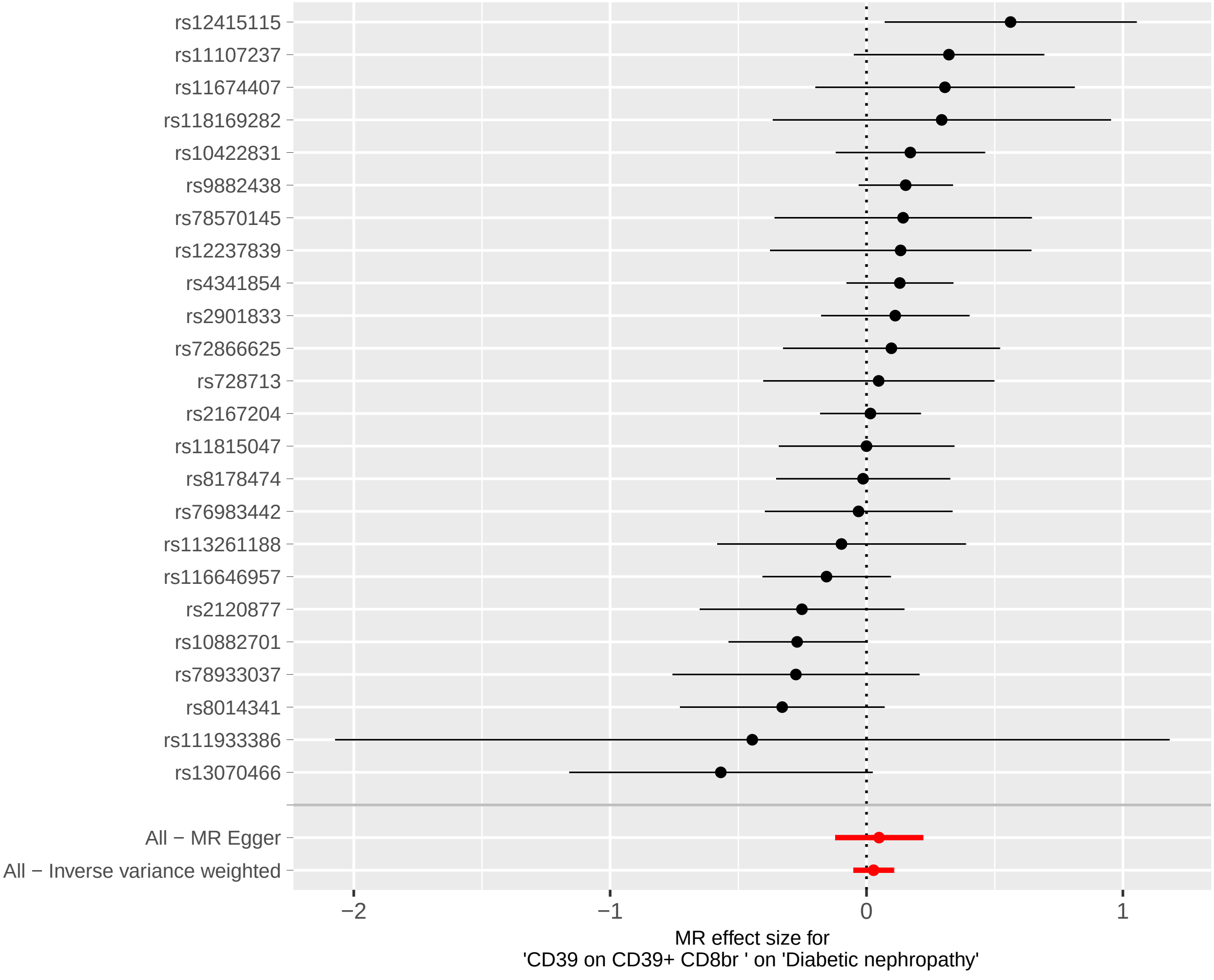
rs12415115
rs11107237
rs11674407
rs118169282
rs10422831
rs9882438
rs78570145
rs12237839
rs4341854
rs2901833
rs72866625
rs728713
rs2167204
rs11815047
rs8178474
rs76983442
rs113261188
rs116646957
rs2120877
rs10882701
rs78933037
rs8014341
rs111933386
rs13070466
All − MR Egger
All − Inverse variance weighted
−2
−1
0
1
MR effect size for
'CD39 on CD39+ CD8br ' on 'Diabetic nephropathy'

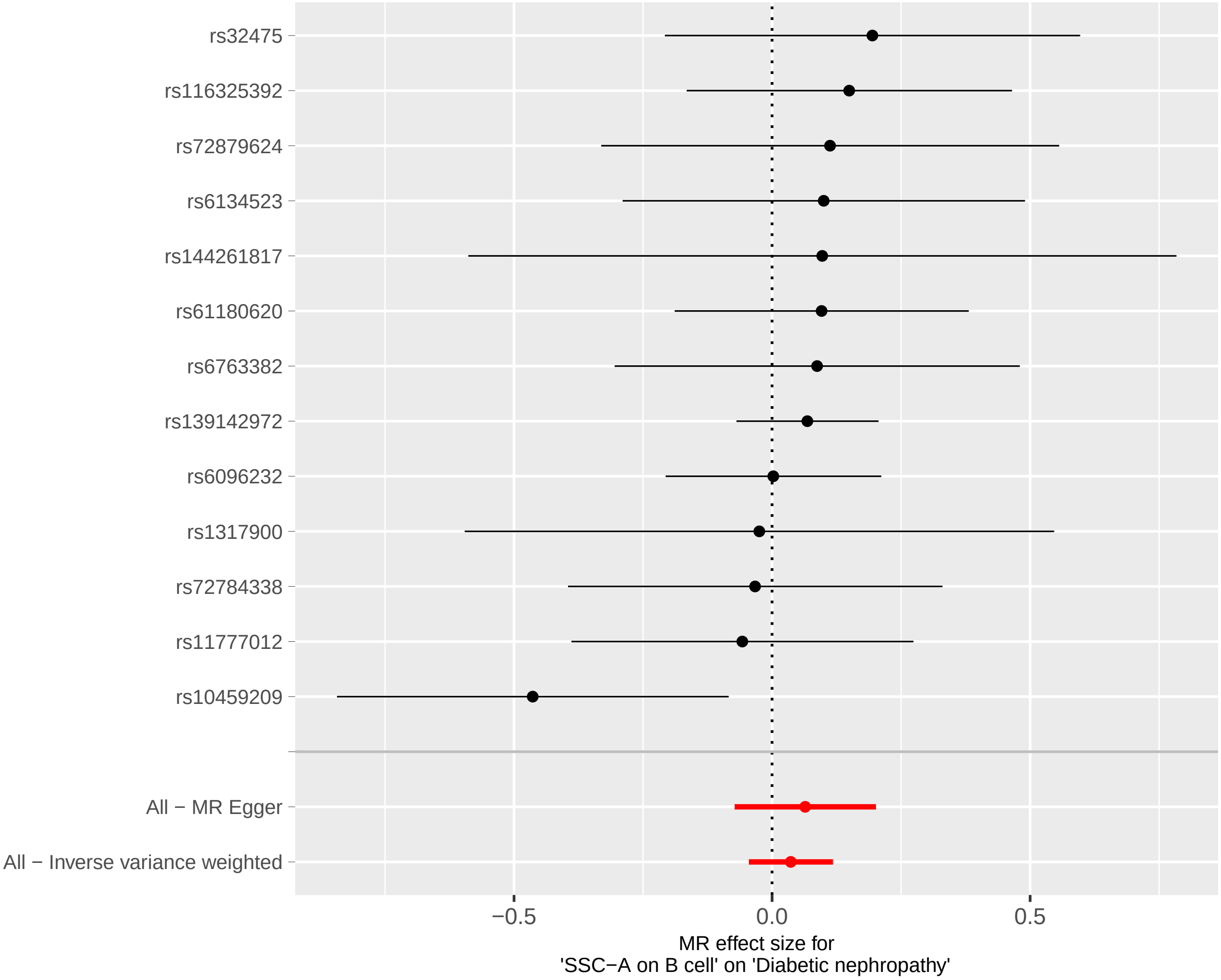

rs32475
rs116325392
rs72879624
rs6134523
rs144261817
rs61180620
rs6763382
rs139142972
rs6096232
rs1317900
rs72784338
rs11777012
rs10459209
All − MR Egger
All − Inverse variance weighted
−0.5
0.0
0.5
MR effect size for
'SSC−A on B cell' on 'Diabetic nephropathy'

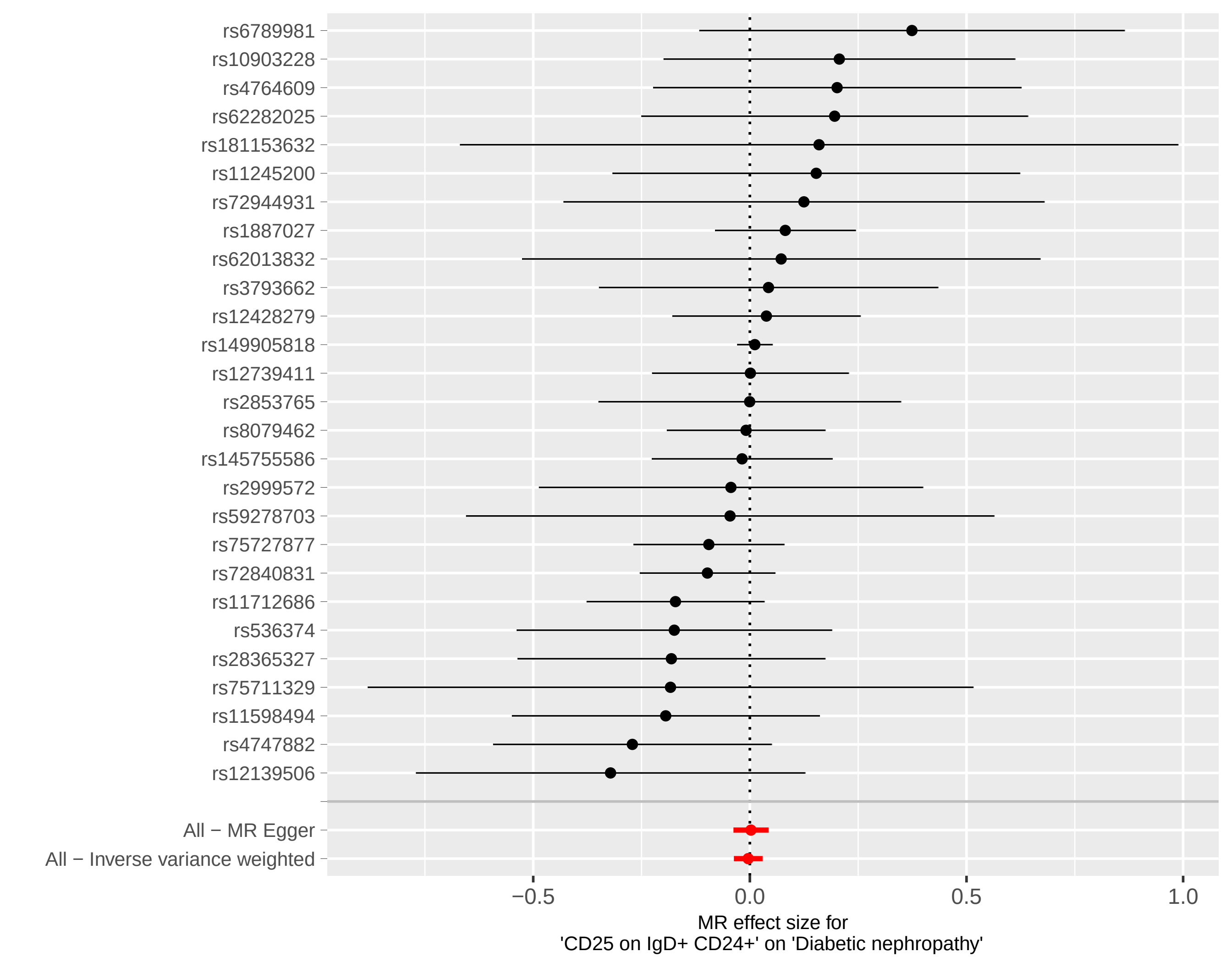

rs6789981
rs10903228
rs4764609
rs62282025
rs181153632
rs11245200
rs72944931
rs1887027
rs62013832
rs3793662
rs12428279
rs149905818
rs12739411
rs2853765
rs8079462
rs145755586
rs2999572
rs59278703
rs75727877
rs72840831
rs11712686
rs536374
rs28365327
rs75711329
rs11598494
rs4747882
rs12139506
All − MR Egger
All − Inverse variance weighted
−0.5
0.0
0.5
1.0
MR effect size for
'CD25 on IgD+ CD24+' on 'Diabetic nephropathy'

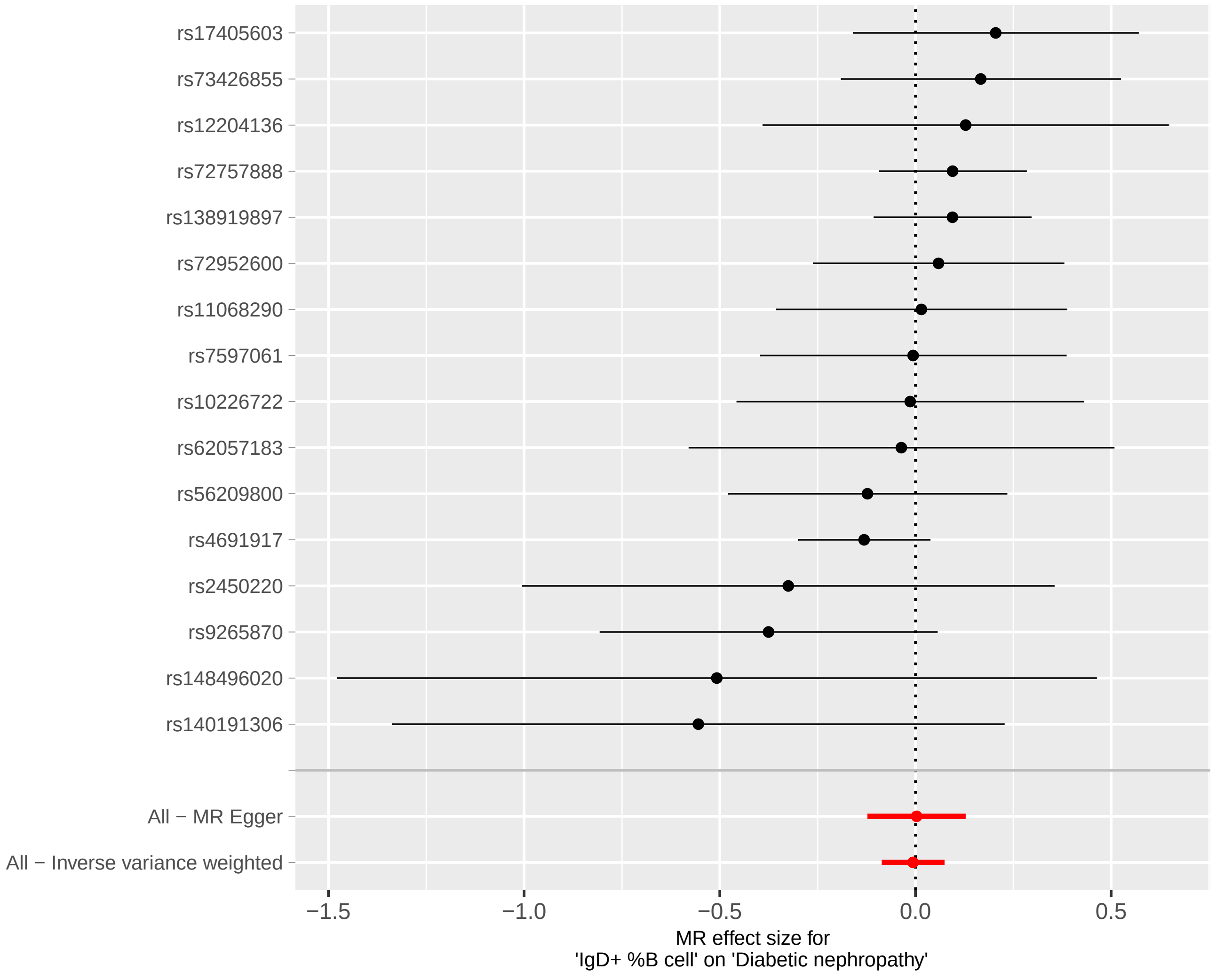
rs17405603
rs73426855
rs12204136
rs72757888
rs138919897
rs72952600
rs11068290
rs7597061
rs10226722
rs62057183
rs56209800
rs4691917
rs2450220
rs9265870
rs148496020
rs140191306
All − MR Egger
All − Inverse variance weighted
−1.5
−1.0
−0.5
0.0
0.5
MR effect size for
'IgD+ %B cell' on 'Diabetic nephropathy'

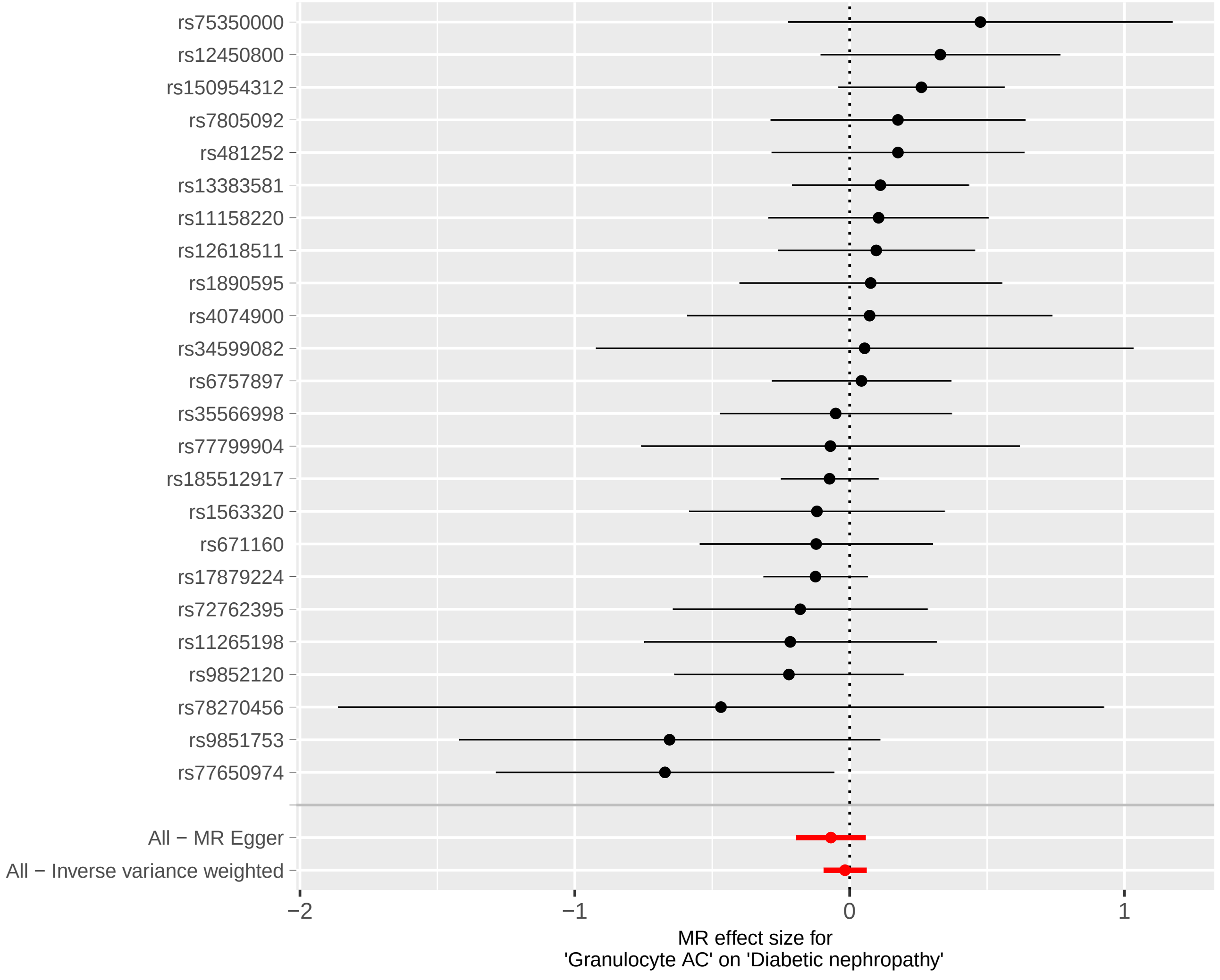
rs75350000
rs12450800
rs150954312
rs7805092
rs481252
rs13383581
rs11158220
rs12618511
rs1890595
rs4074900
rs34599082
rs6757897
rs35566998
rs77799904
rs185512917
rs1563320
rs671160
rs17879224
rs72762395
rs11265198
rs9852120
rs78270456
rs9851753
rs77650974
All − MR Egger
All − Inverse variance weighted
−2
−1
0
1
MR effect size for
'Granulocyte AC' on 'Diabetic nephropathy'

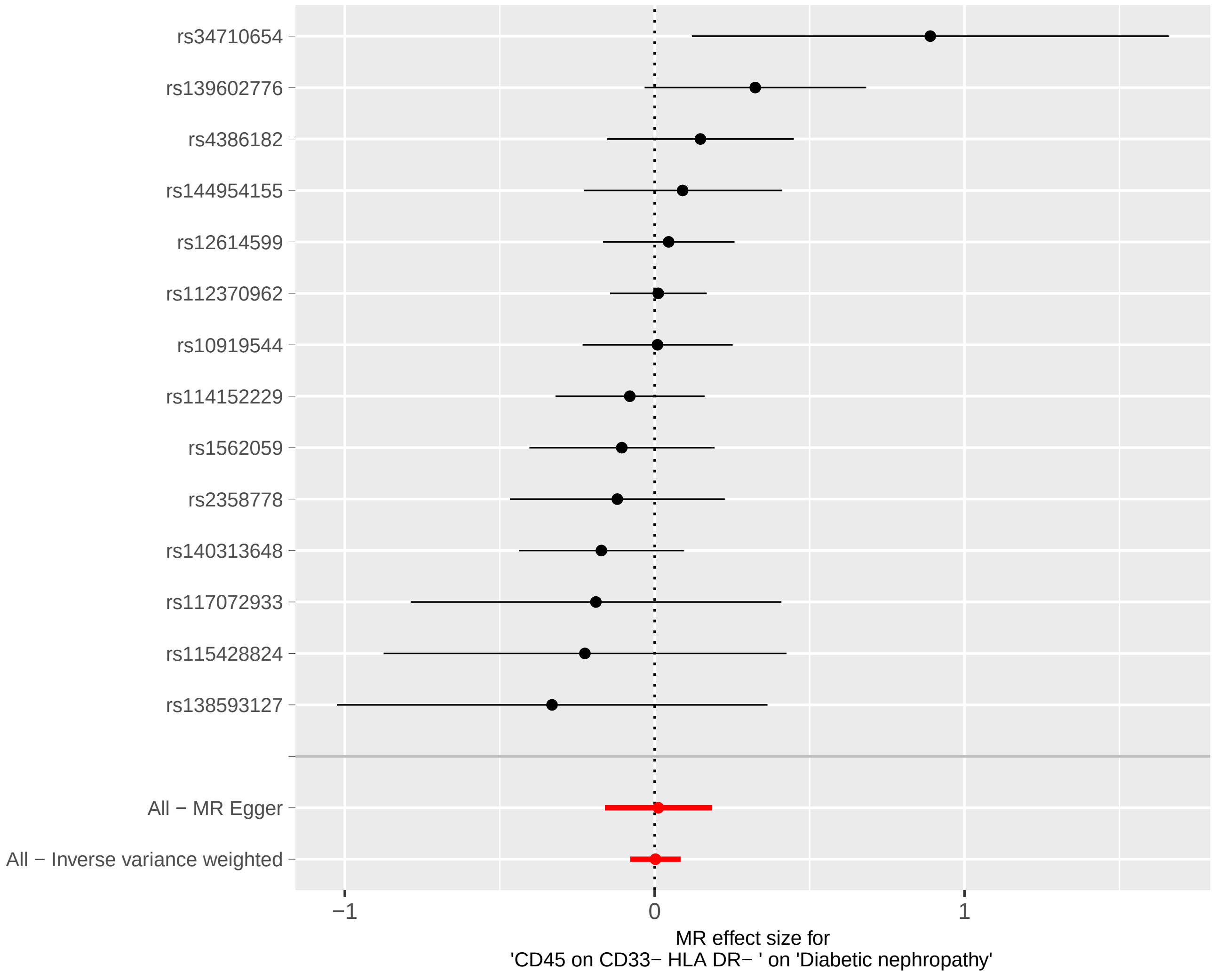

rs34710654
rs139602776
rs4386182
rs144954155
rs12614599
rs112370962
rs10919544
rs114152229
rs1562059
rs2358778
rs140313648
rs117072933
rs115428824
rs138593127
All − MR Egger
All − Inverse variance weighted
−1
0
1
MR effect size for
'CD45 on CD33− HLA DR− ' on 'Diabetic nephropathy'

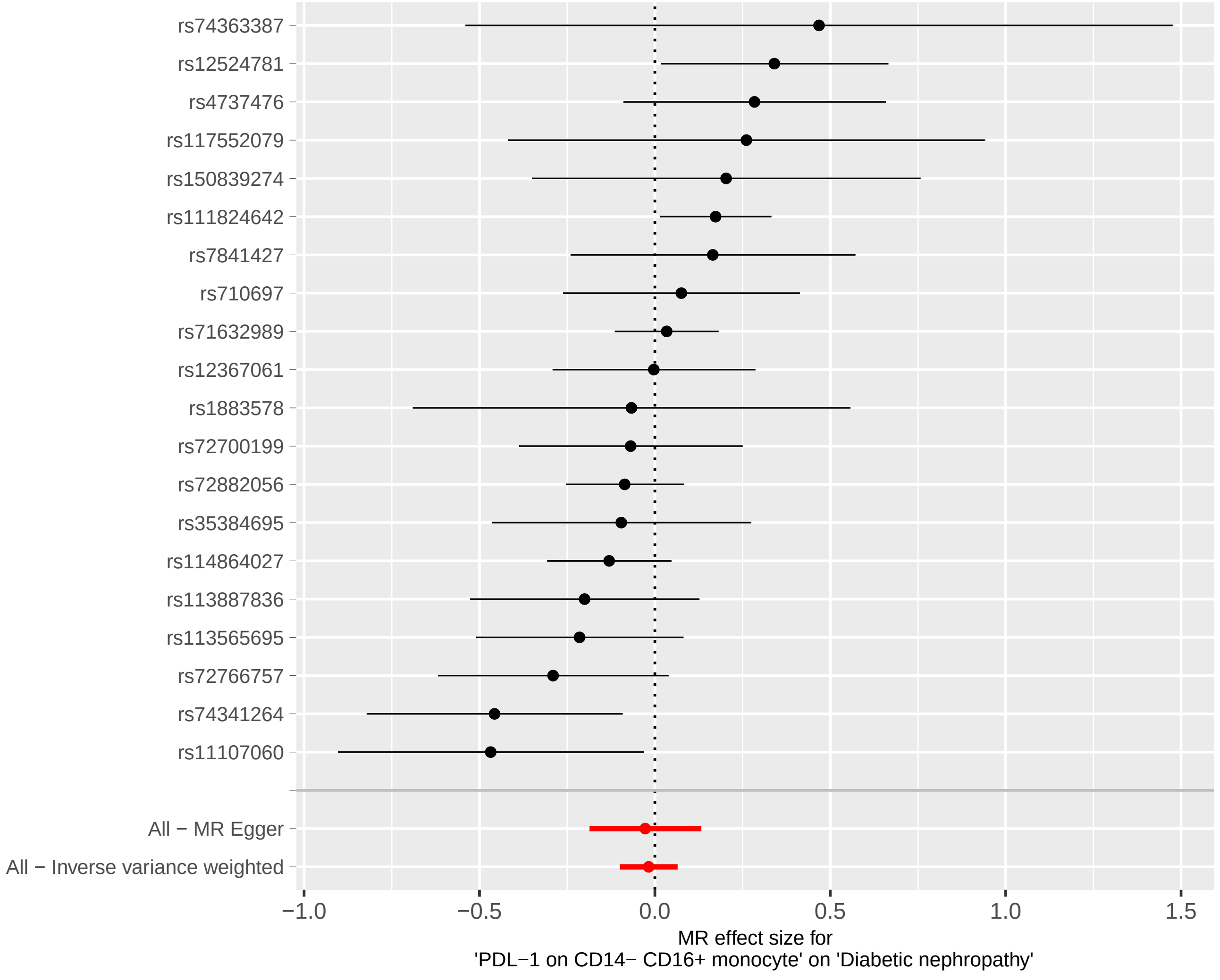

rs74363387
rs12524781
rs4737476
rs117552079
rs150839274
rs111824642
rs7841427
rs710697
rs71632989
rs12367061
rs1883578
rs72700199
rs72882056
rs35384695
rs114864027
rs113887836
rs113565695
rs72766757
rs74341264
rs11107060
All − MR Egger
All − Inverse variance weighted
−1.0
−0.5
0.0
0.5
1.0
1.5
MR effect size for
'PDL−1 on CD14− CD16+ monocyte' on 'Diabetic nephropathy'

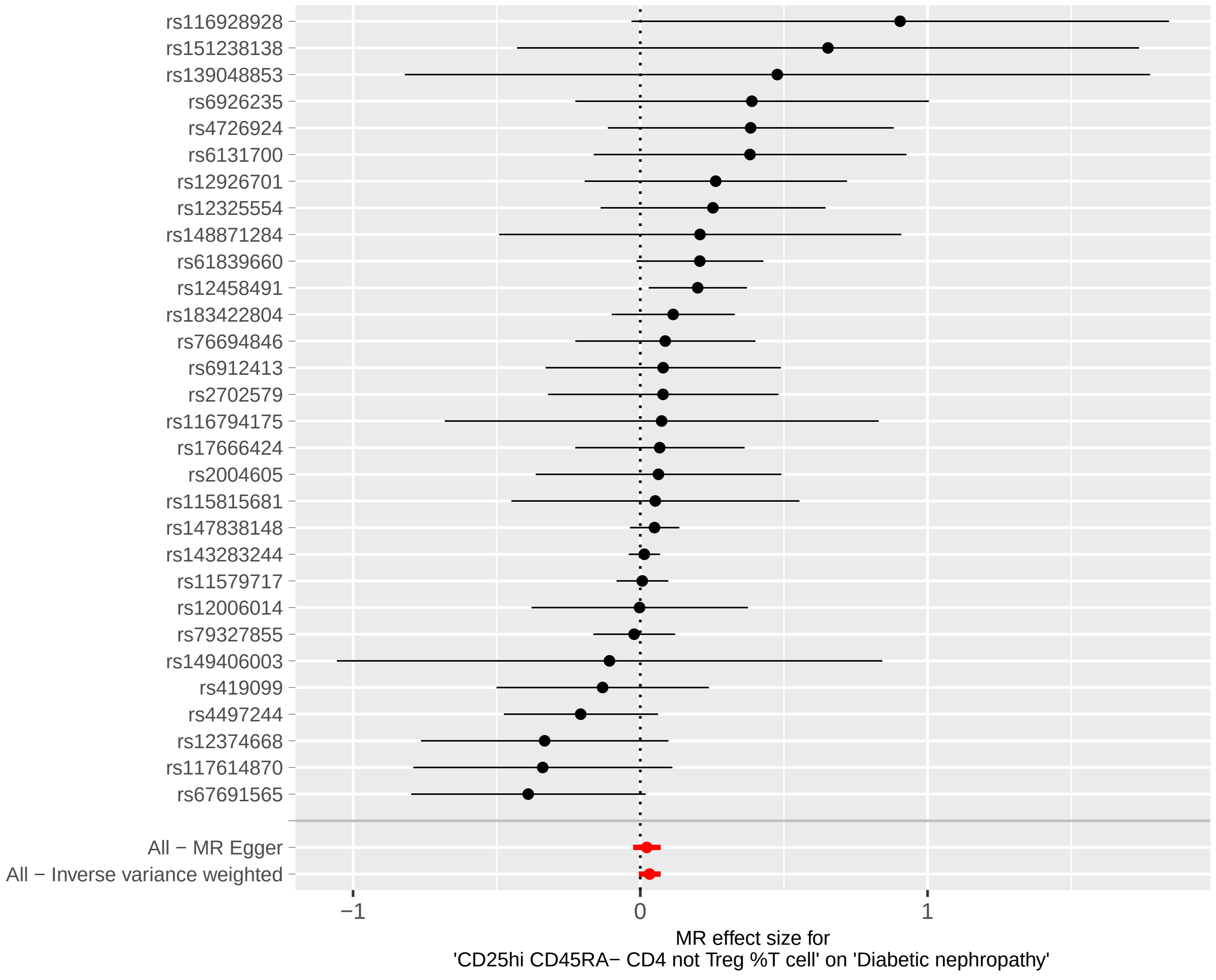
rs116928928
rs151238138
rs139048853
rs6926235
rs4726924
rs6131700
rs12926701
rs12325554
rs148871284
rs61839660
rs12458491
rs183422804
rs76694846
rs6912413
rs2702579
rs116794175
rs17666424
rs2004605
rs115815681
rs147838148
rs143283244
rs11579717
rs12006014
rs79327855
rs149406003
rs419099
rs4497244
rs12374668
rs117614870
rs67691565
All − MR Egger
All − Inverse variance weighted
−1
0
1
MR effect size for
'CD25hi CD45RA− CD4 not Treg %T cell' on 'Diabetic nephropathy'

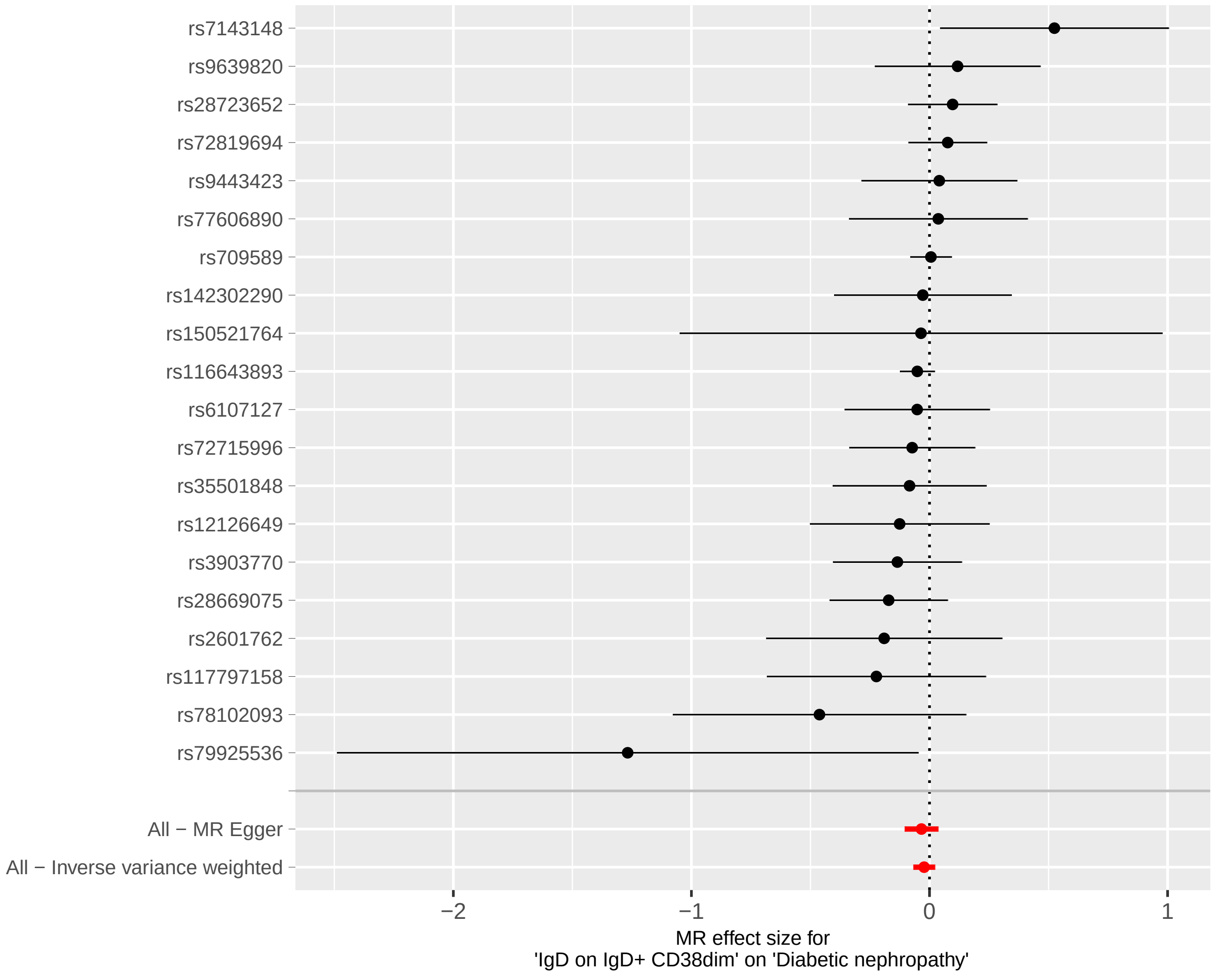
rs7143148
rs9639820
rs28723652
rs72819694
rs9443423
rs77606890
rs709589
rs142302290
rs150521764
rs116643893
rs6107127
rs72715996
rs35501848
rs12126649
rs3903770
rs28669075
rs2601762
rs117797158
rs78102093
rs79925536
All − MR Egger
All − Inverse variance weighted
−2
−1
0
1
MR effect size for
'IgD on IgD+ CD38dim' on 'Diabetic nephropathy'

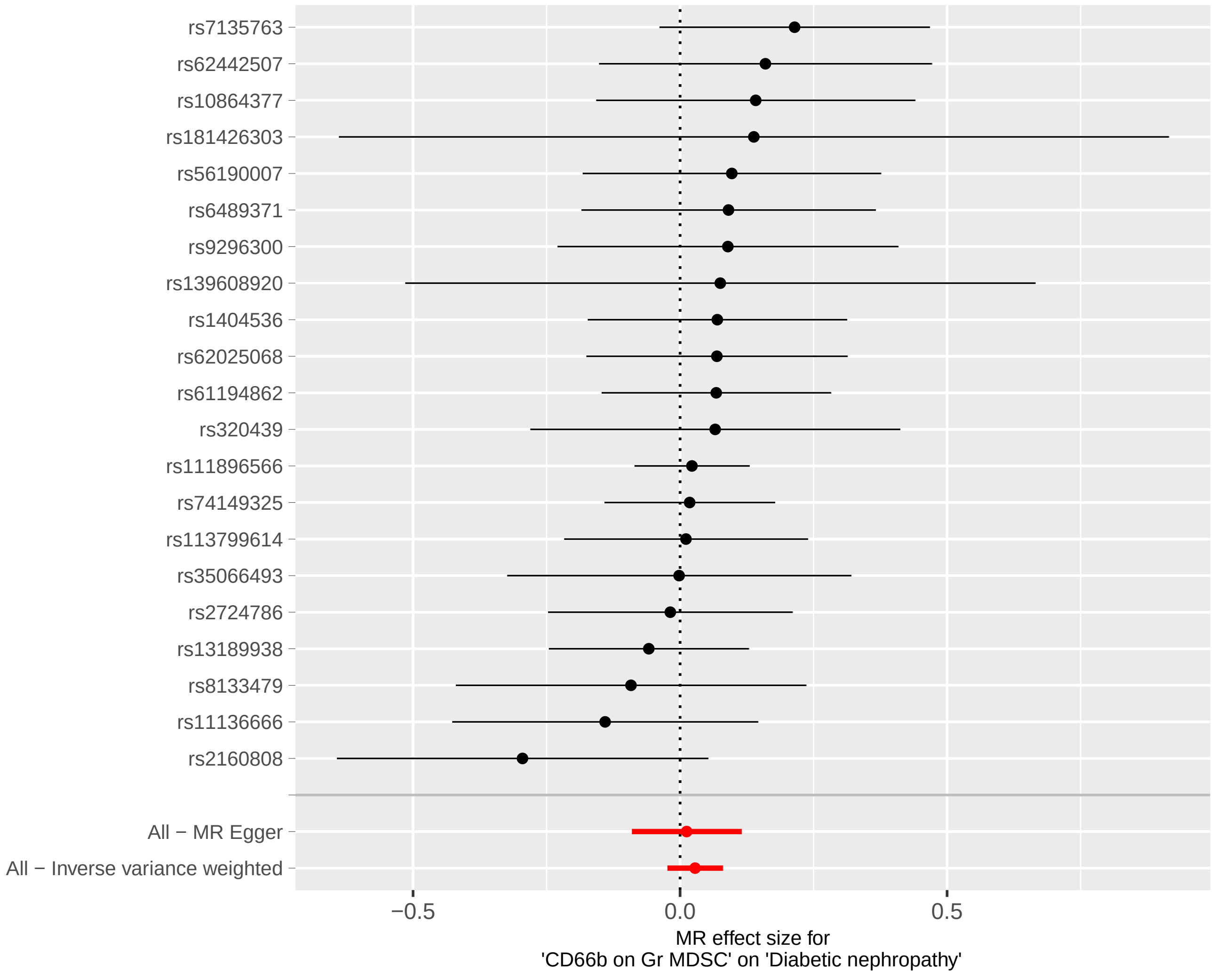
rs7135763
rs62442507
rs10864377
rs181426303
rs56190007
rs6489371
rs9296300
rs139608920
rs1404536
rs62025068
rs61194862
rs320439
rs111896566
rs74149325
rs113799614
rs35066493
rs2724786
rs13189938
rs8133479
rs11136666
rs2160808
All − MR Egger
All − Inverse variance weighted
−0.5
0.0
0.5
MR effect size for
'CD66b on Gr MDSC' on 'Diabetic nephropathy'

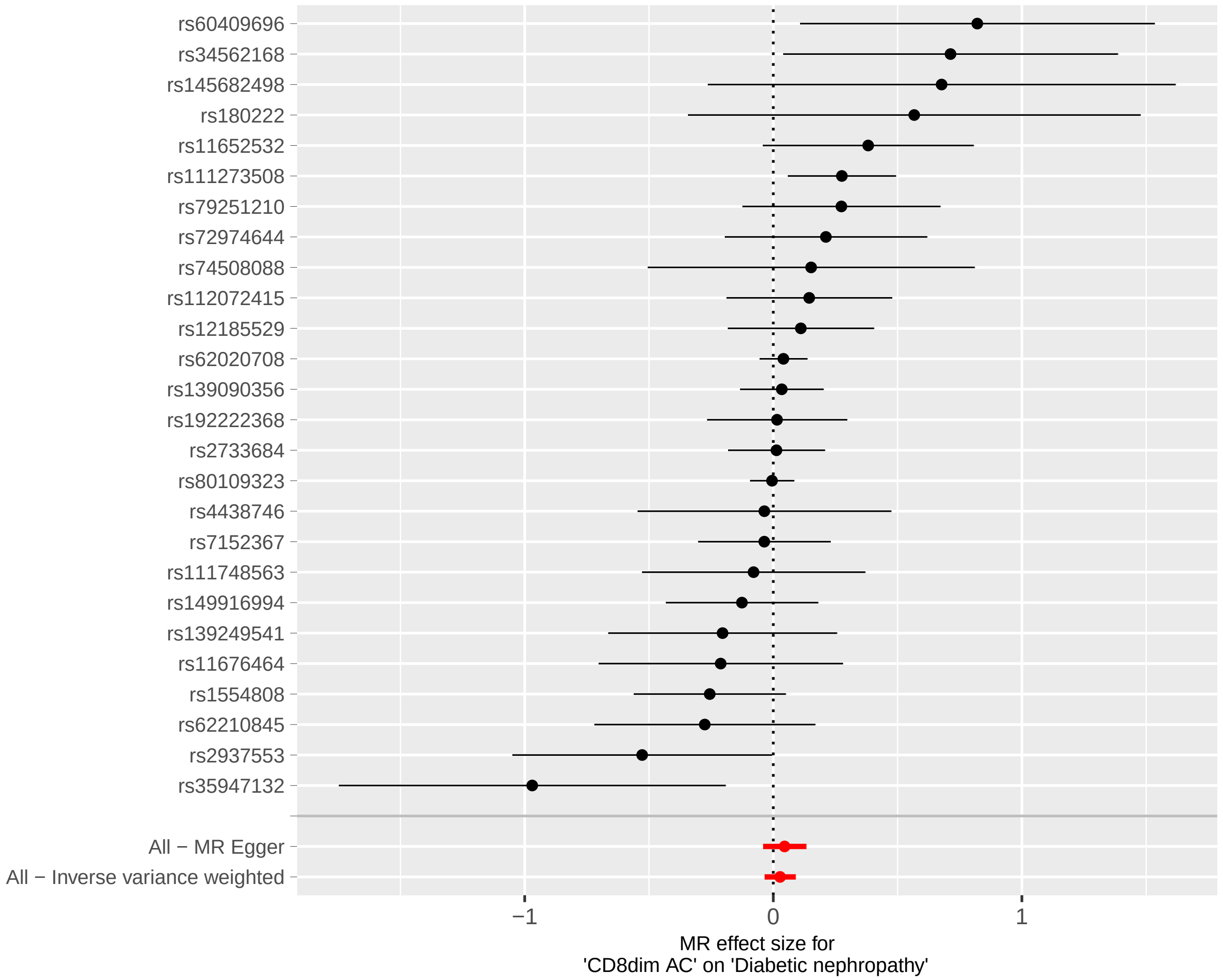
rs60409696
rs34562168
rs145682498
rs180222
rs11652532
rs111273508
rs79251210
rs72974644
rs74508088
rs112072415
rs12185529
rs62020708
rs139090356
rs192222368
rs2733684
rs80109323
rs4438746
rs7152367
rs111748563
rs149916994
rs139249541
rs11676464
rs1554808
rs62210845
rs2937553
rs35947132
All − MR Egger
All − Inverse variance weighted
−1
0
1
MR effect size for
'CD8dim AC' on 'Diabetic nephropathy'

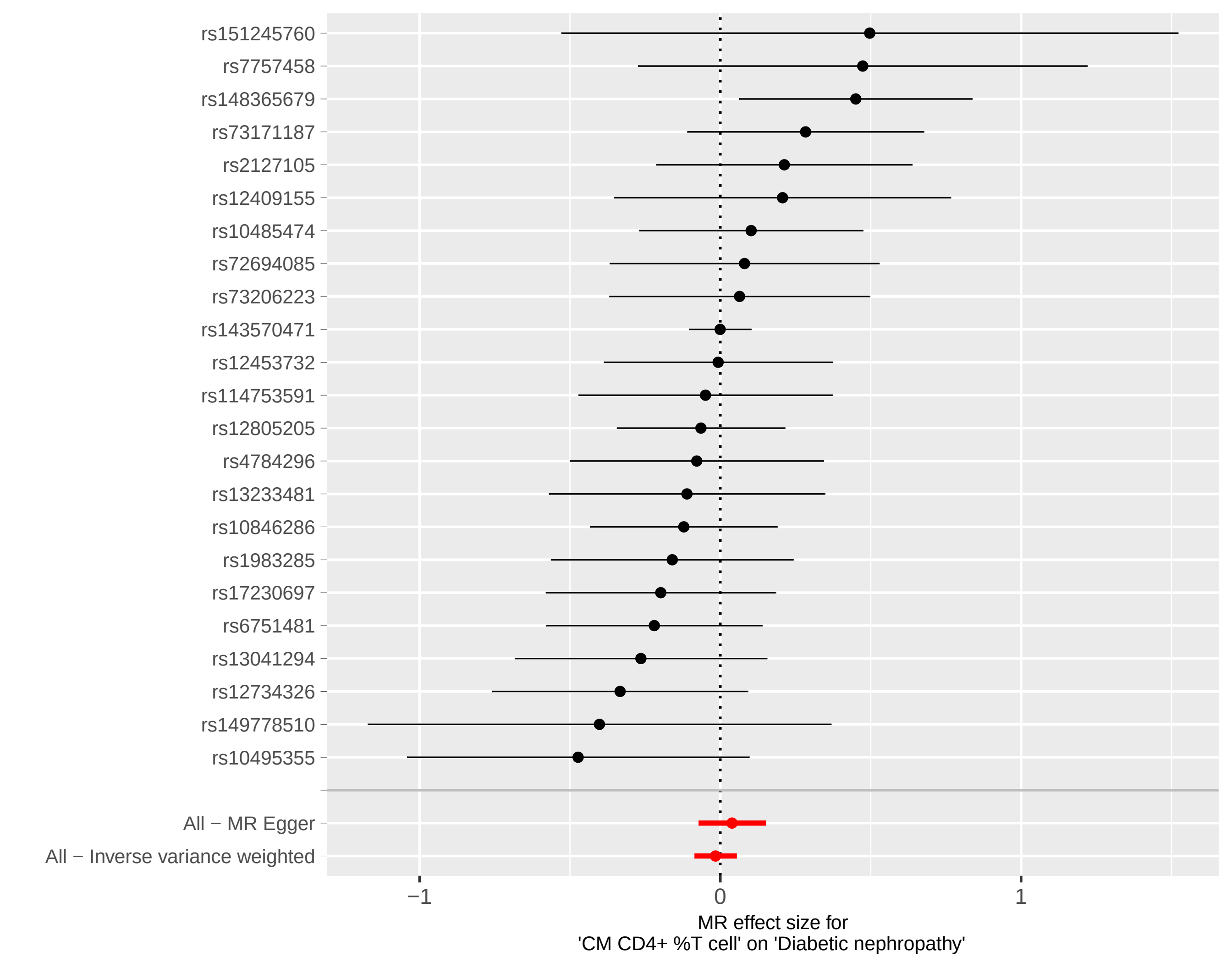
rs151245760
rs7757458
rs148365679
rs73171187
rs2127105
rs12409155
rs10485474
rs72694085
rs73206223
rs143570471
rs12453732
rs114753591
rs12805205
rs4784296
rs13233481
rs10846286
rs1983285
rs17230697
rs6751481
rs13041294
rs12734326
rs149778510
rs10495355
All − MR Egger
All − Inverse variance weighted
−1
0
1
MR effect size for
'CM CD4+ %T cell' on 'Diabetic nephropathy'

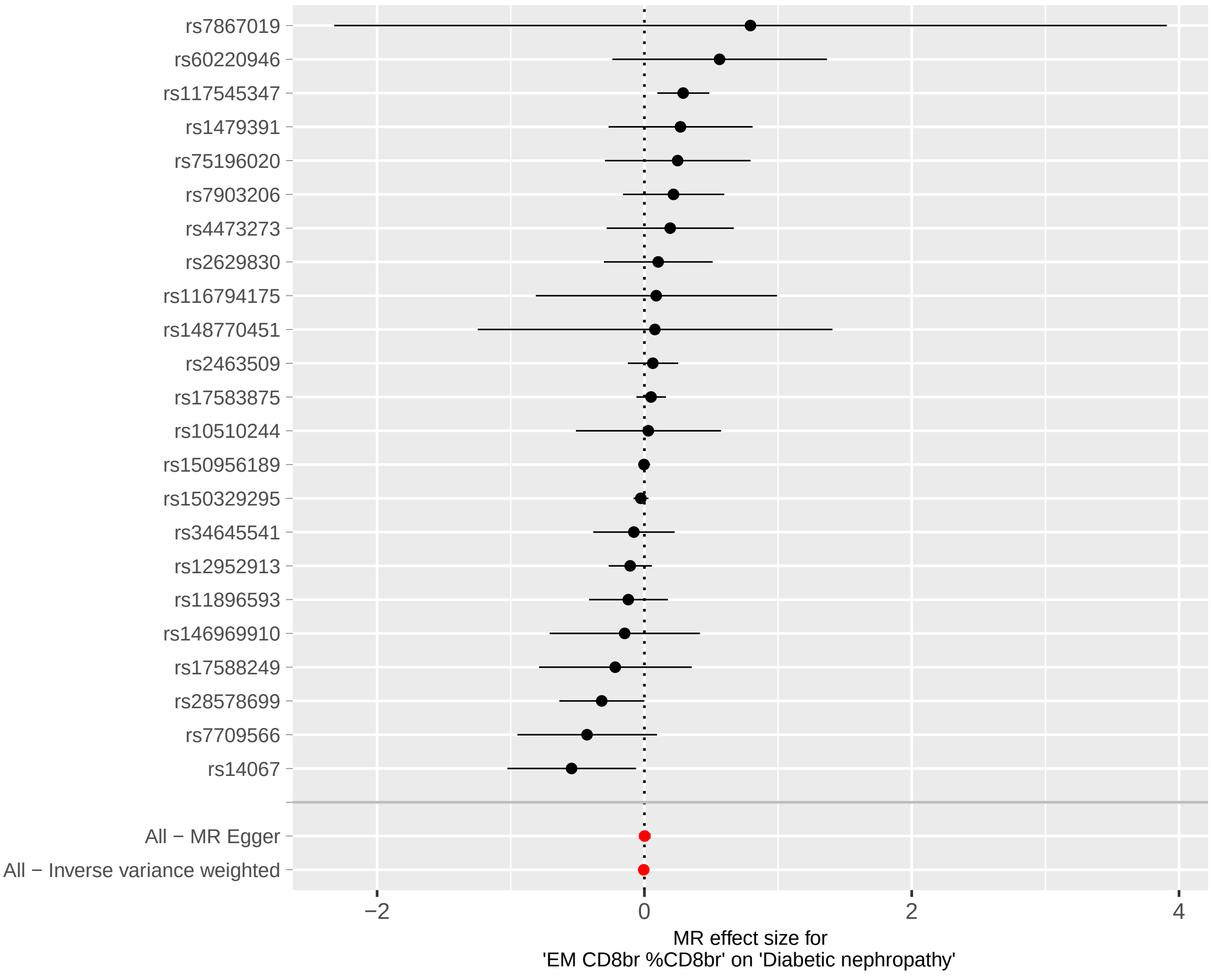
rs7867019
rs60220946
rs117545347
rs1479391
rs75196020
rs7903206
rs4473273
rs2629830
rs116794175
rs148770451
rs2463509
rs17583875
rs10510244
rs150956189
rs150329295
rs34645541
rs12952913
rs11896593
rs146969910
rs17588249
rs28578699
rs7709566
rs14067
All − MR Egger
All − Inverse variance weighted
−2
0
2
4
MR effect size for
'EM CD8br %CD8br' on 'Diabetic nephropathy'

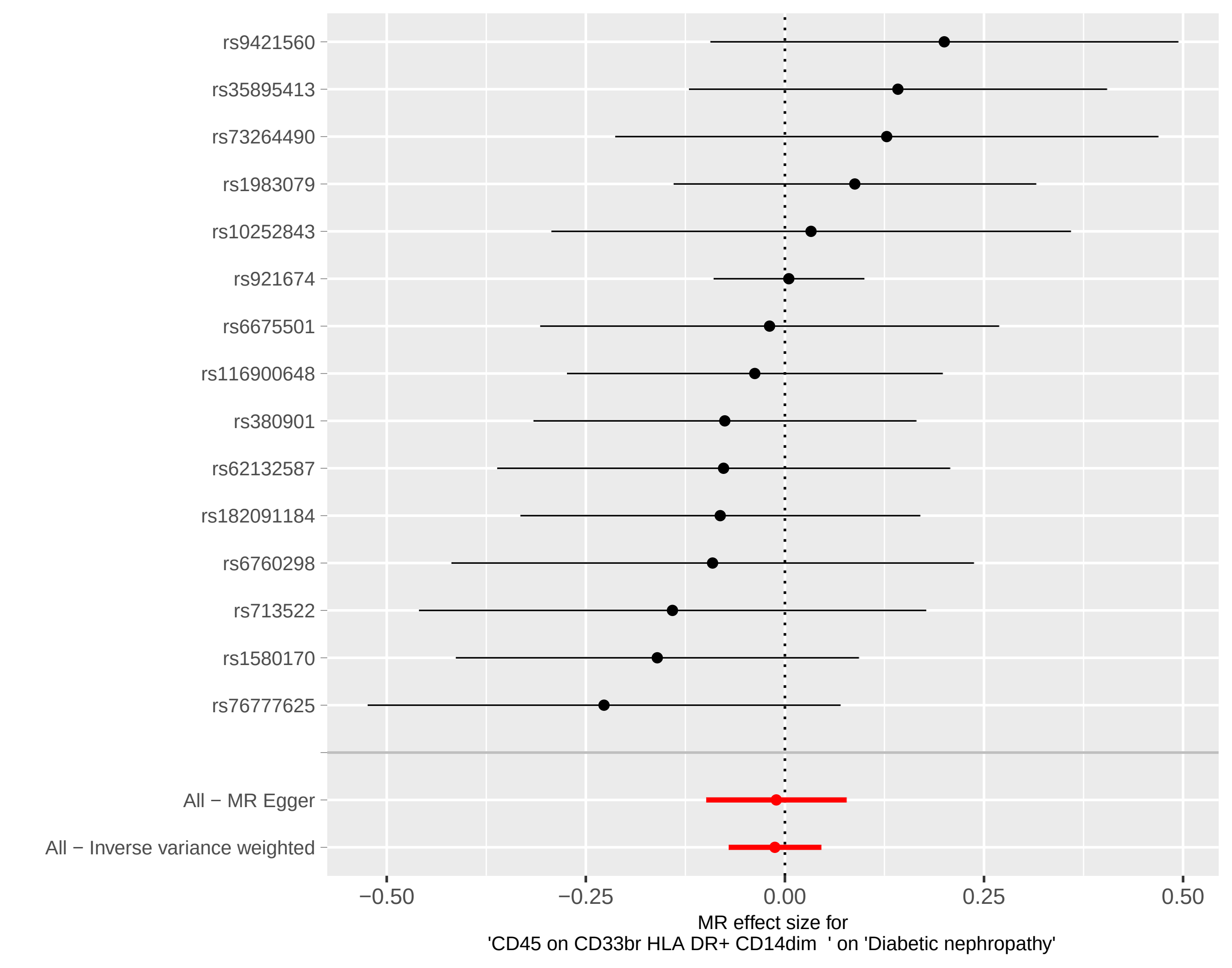

rs9421560
rs35895413
rs73264490
rs1983079
rs10252843
rs921674
rs6675501
rs116900648
rs380901
rs62132587
rs182091184
rs6760298
rs713522
rs1580170
rs76777625
All − MR Egger
All − Inverse variance weighted
−0.50
−0.25
0.00
0.25
0.50
MR effect size for
'CD45 on CD33br HLA DR+ CD14dim ' on 'Diabetic nephropathy'

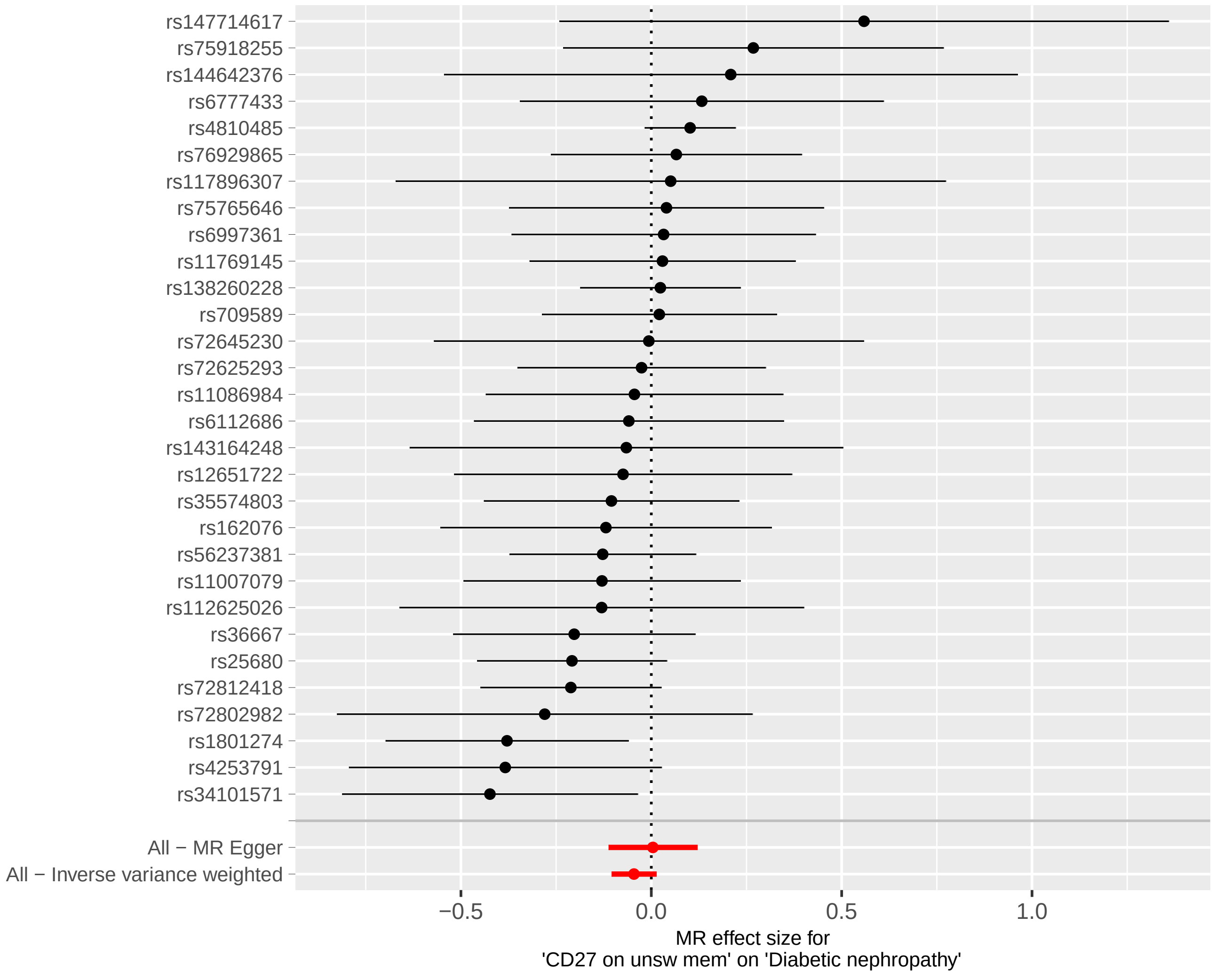
rs147714617
rs75918255
rs144642376
rs6777433
rs4810485
rs76929865
rs117896307
rs75765646
rs6997361
rs11769145
rs138260228
rs709589
rs72645230
rs72625293
rs11086984
rs6112686
rs143164248
rs12651722
rs35574803
rs162076
rs56237381
rs11007079
rs112625026
rs36667
rs25680
rs72812418
rs72802982
rs1801274
rs4253791
rs34101571
All − MR Egger
All − Inverse variance weighted
−0.5
0.0
0.5
1.0
MR effect size for
'CD27 on unsw mem' on 'Diabetic nephropathy'

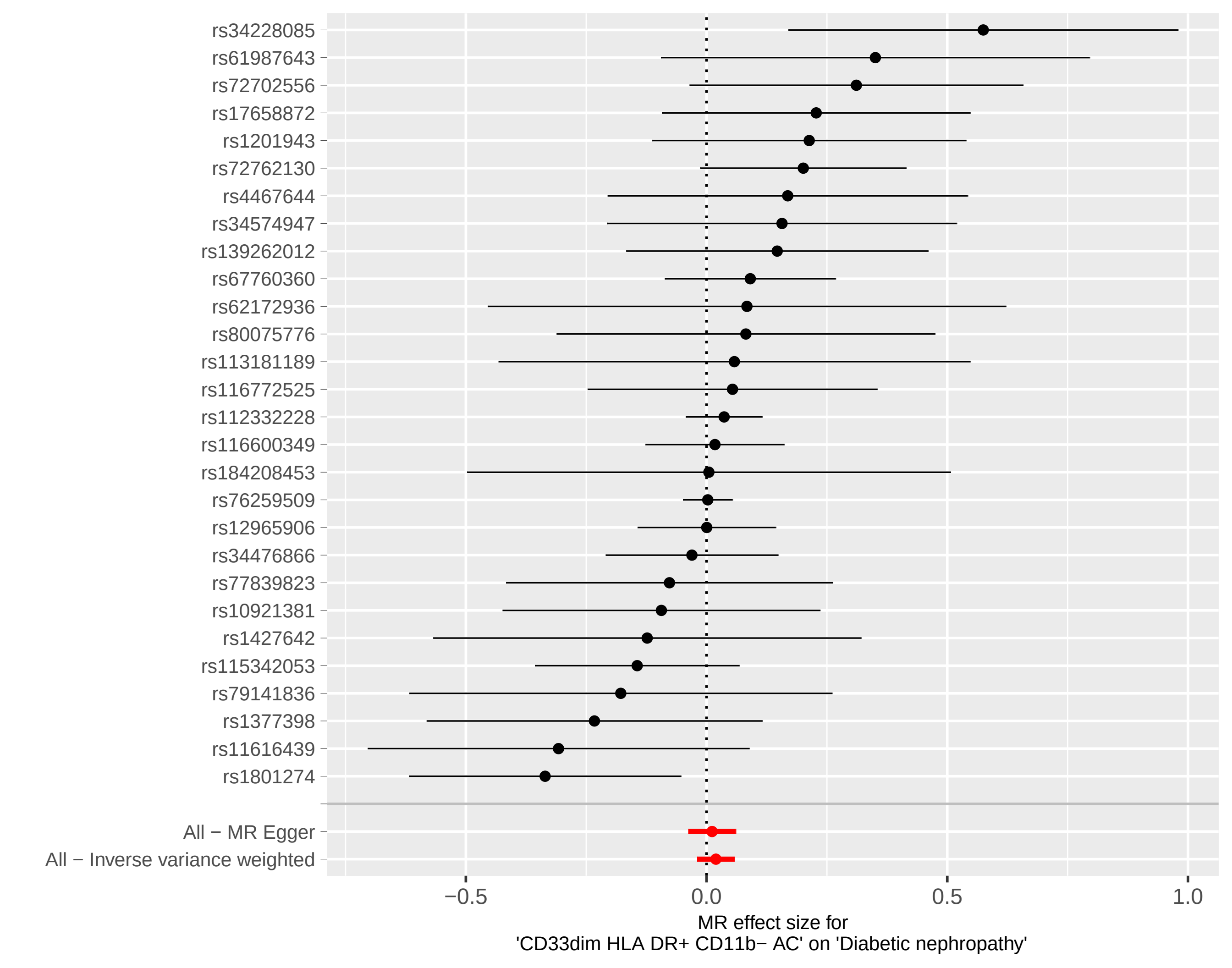

rs34228085
rs61987643
rs72702556
rs17658872
rs1201943
rs72762130
rs4467644
rs34574947
rs139262012
rs67760360
rs62172936
rs80075776
rs113181189
rs116772525
rs112332228
rs116600349
rs184208453
rs76259509
rs12965906
rs34476866
rs77839823
rs10921381
rs1427642
rs115342053
rs79141836
rs1377398
rs11616439
rs1801274
All − MR Egger
All − Inverse variance weighted
−0.5
0.0
0.5
1.0
MR effect size for
'CD33dim HLA DR+ CD11b− AC' on 'Diabetic nephropathy'

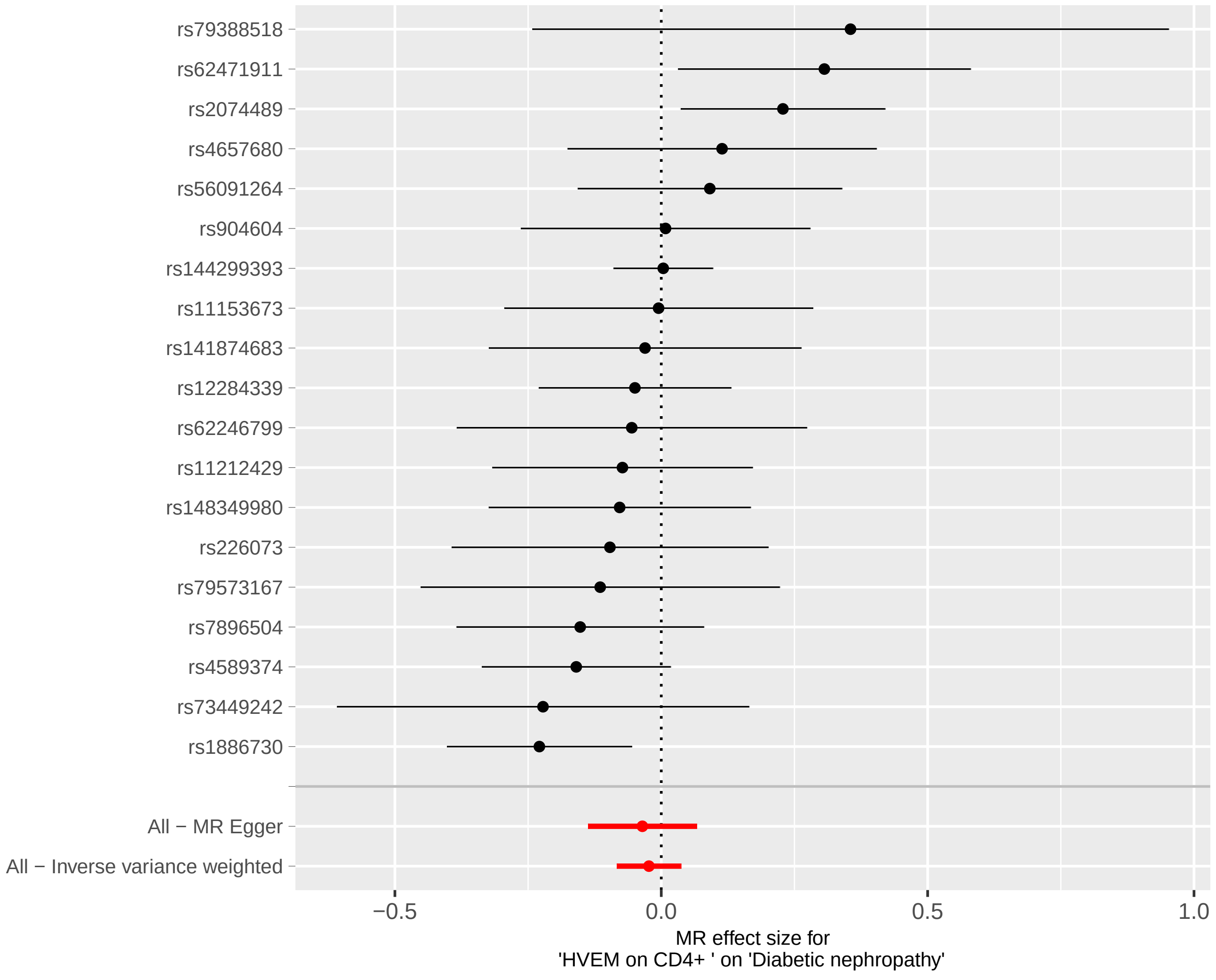
rs79388518
rs62471911
rs2074489
rs4657680
rs56091264
rs904604
rs144299393
rs11153673
rs141874683
rs12284339
rs62246799
rs11212429
rs148349980
rs226073
rs79573167
rs7896504
rs4589374
rs73449242
rs1886730
All − MR Egger
All − Inverse variance weighted
−0.5
0.0
0.5
1.0
MR effect size for
'HVEM on CD4+ ' on 'Diabetic nephropathy'

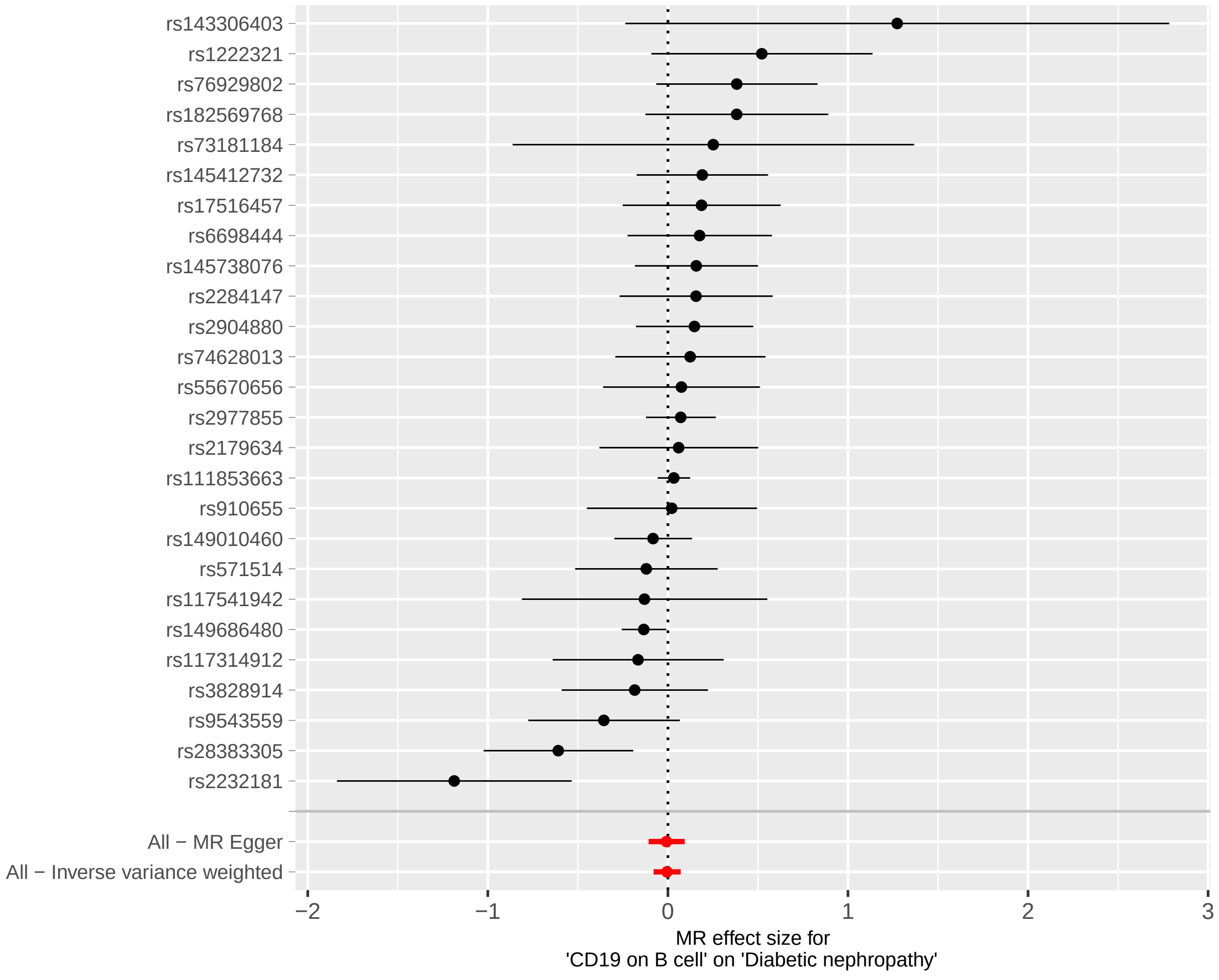
rs143306403
rs1222321
rs76929802
rs182569768
rs73181184
rs145412732
rs17516457
rs6698444
rs145738076
rs2284147
rs2904880
rs74628013
rs55670656
rs2977855
rs2179634
rs111853663
rs910655
rs149010460
rs571514
rs117541942
rs149686480
rs117314912
rs3828914
rs9543559
rs28383305
rs2232181
All − MR Egger
All − Inverse variance weighted
−2
−1
0
1
2
3
MR effect size for
'CD19 on B cell' on 'Diabetic nephropathy'

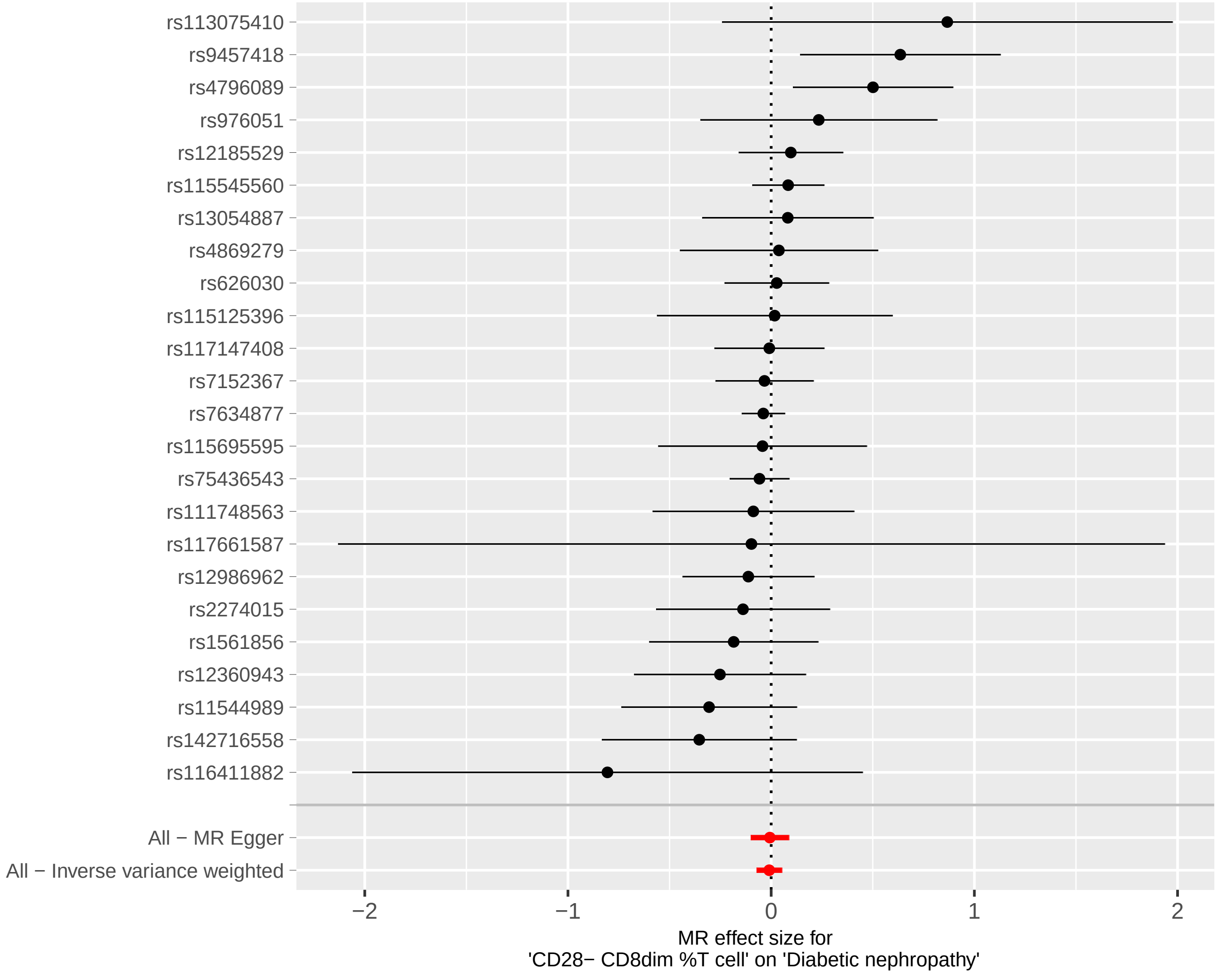
rs113075410
rs9457418
rs4796089
rs976051
rs12185529
rs115545560
rs13054887
rs4869279
rs626030
rs115125396
rs117147408
rs7152367
rs7634877
rs115695595
rs75436543
rs111748563
rs117661587
rs12986962
rs2274015
rs1561856
rs12360943
rs11544989
rs142716558
rs116411882
All − MR Egger
All − Inverse variance weighted
−2
−1
0
1
2
MR effect size for
'CD28− CD8dim %T cell' on 'Diabetic nephropathy'

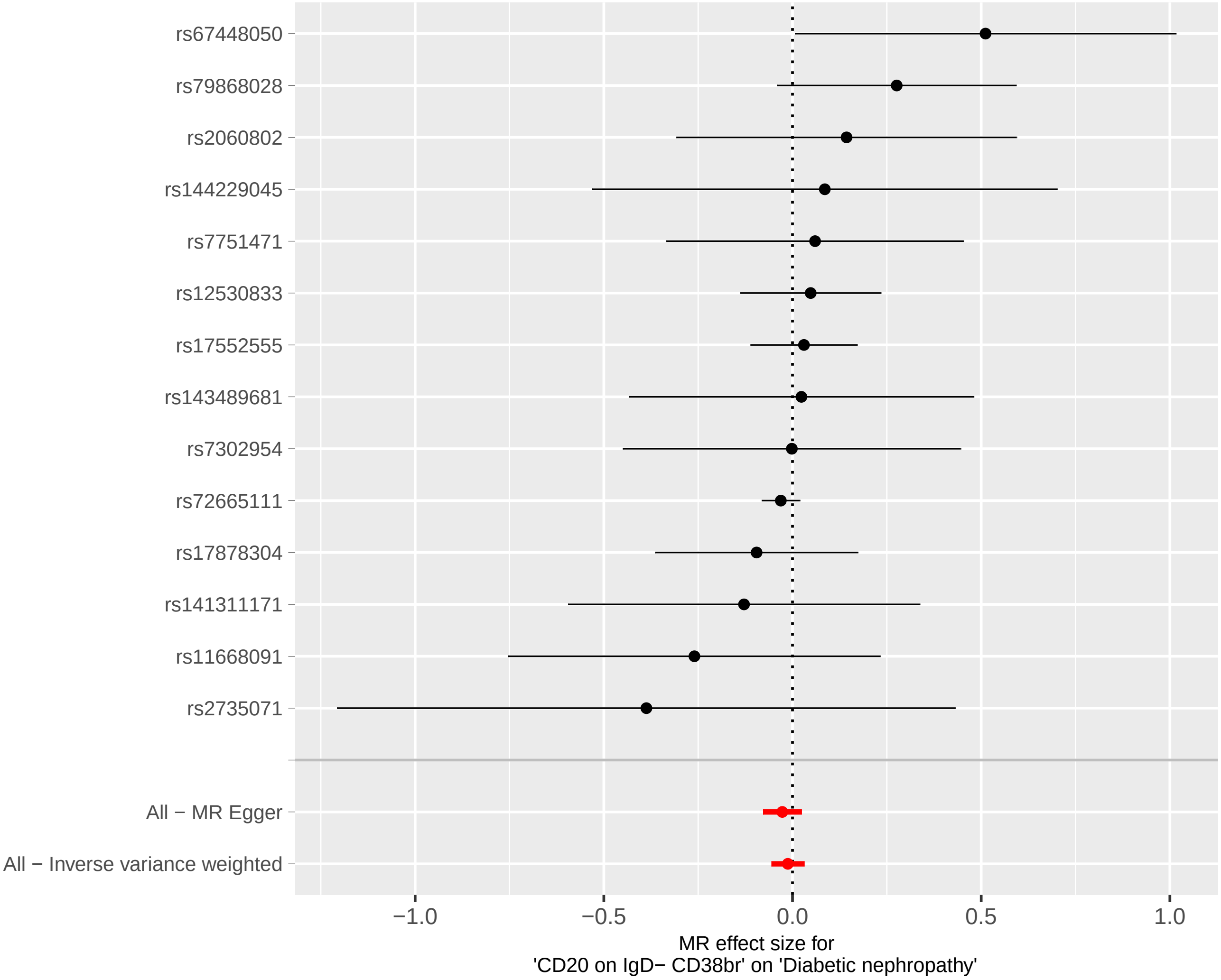
rs67448050
rs79868028
rs2060802
rs144229045
rs7751471
rs12530833
rs17552555
rs143489681
rs7302954
rs72665111
rs17878304
rs141311171
rs11668091
rs2735071
All − MR Egger
All − Inverse variance weighted
−1.0
−0.5
0.0
0.5
1.0
MR effect size for
'CD20 on IgD− CD38br' on 'Diabetic nephropathy'

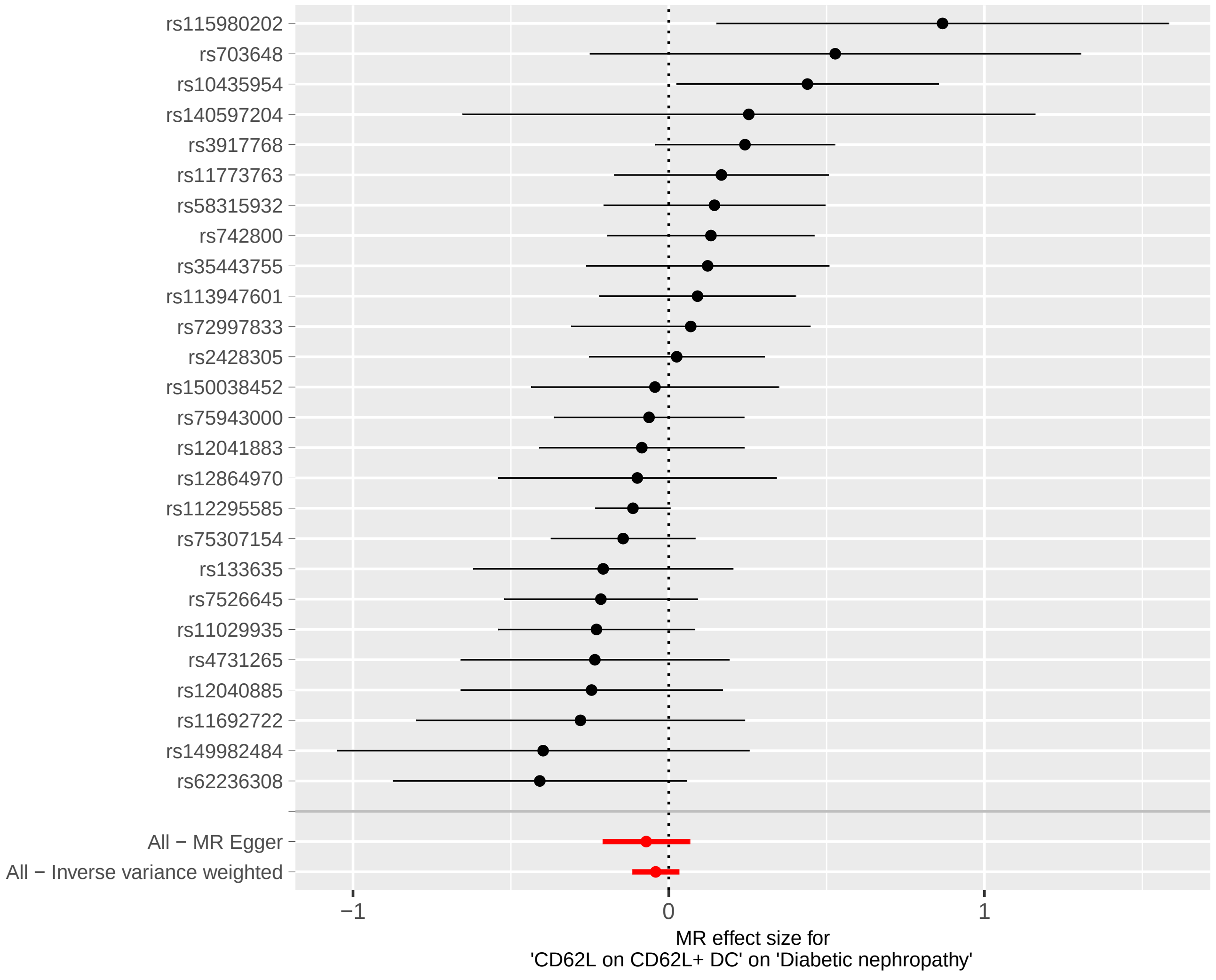
rs115980202
rs703648
rs10435954
rs140597204
rs3917768
rs11773763
rs58315932
rs742800
rs35443755
rs113947601
rs72997833
rs2428305
rs150038452
rs75943000
rs12041883
rs12864970
rs112295585
rs75307154
rs133635
rs7526645
rs11029935
rs4731265
rs12040885
rs11692722
rs149982484
rs62236308
All − MR Egger
All − Inverse variance weighted
−1
0
1
MR effect size for
'CD62L on CD62L+ DC' on 'Diabetic nephropathy'

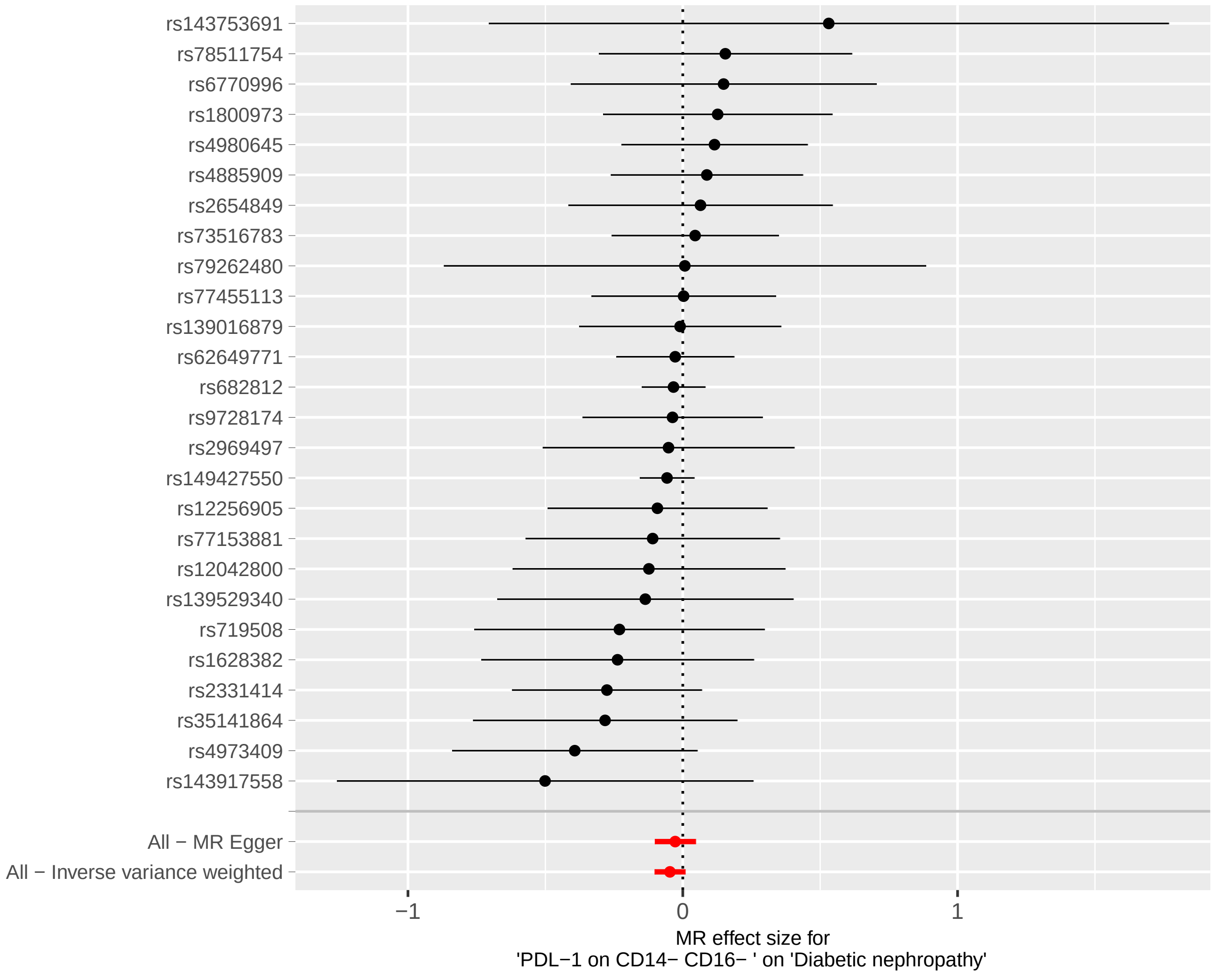

rs143753691
rs78511754
rs6770996
rs1800973
rs4980645
rs4885909
rs2654849
rs73516783
rs79262480
rs77455113
rs139016879
rs62649771
rs682812
rs9728174
rs2969497
rs149427550
rs12256905
rs77153881
rs12042800
rs139529340
rs719508
rs1628382
rs2331414
rs35141864
rs4973409
rs143917558
All − MR Egger
All − Inverse variance weighted
−1
0
1
MR effect size for
'PDL−1 on CD14− CD16− ' on 'Diabetic nephropathy'

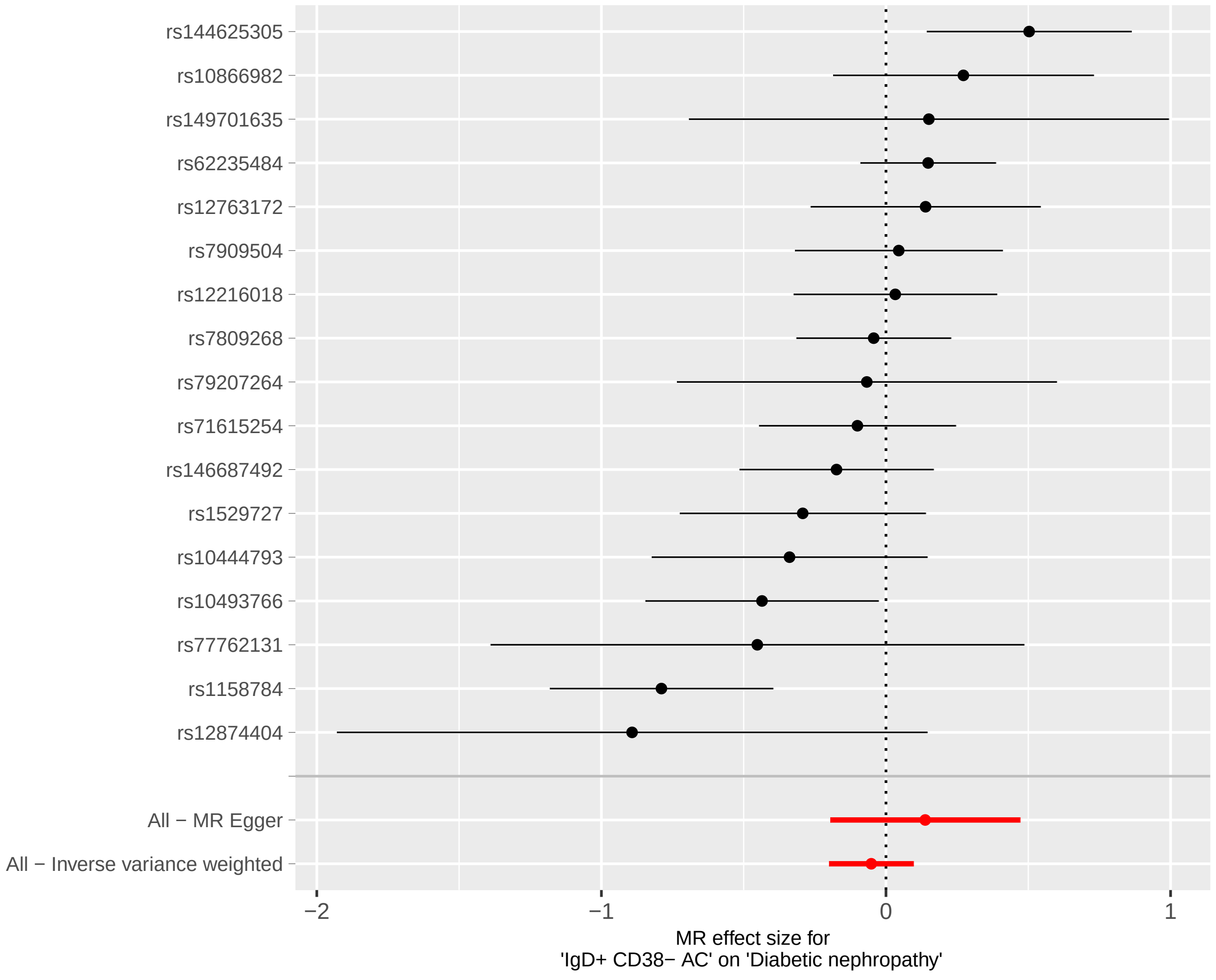
rs144625305
rs10866982
rs149701635
rs62235484
rs12763172
rs7909504
rs12216018
rs7809268
rs79207264
rs71615254
rs146687492
rs1529727
rs10444793
rs10493766
rs77762131
rs1158784
rs12874404
All − MR Egger
All − Inverse variance weighted
−2
−1
0
1
MR effect size for
'IgD+ CD38− AC' on 'Diabetic nephropathy'

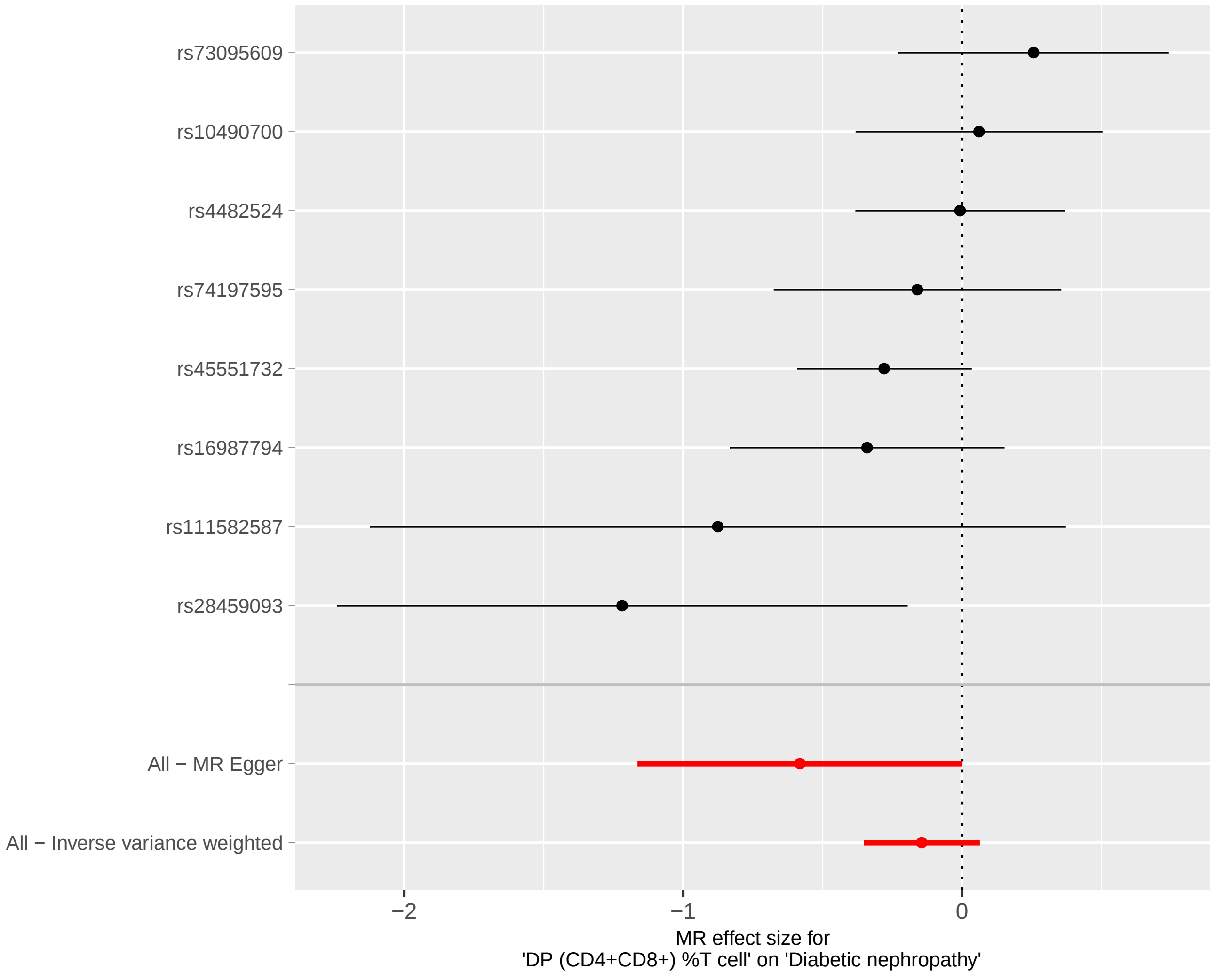
rs73095609
rs10490700
rs4482524
rs74197595
rs45551732
rs16987794
rs111582587
rs28459093
All − MR Egger
All − Inverse variance weighted
−2
−1
0
MR effect size for
'DP (CD4+CD8+) %T cell' on 'Diabetic nephropathy'

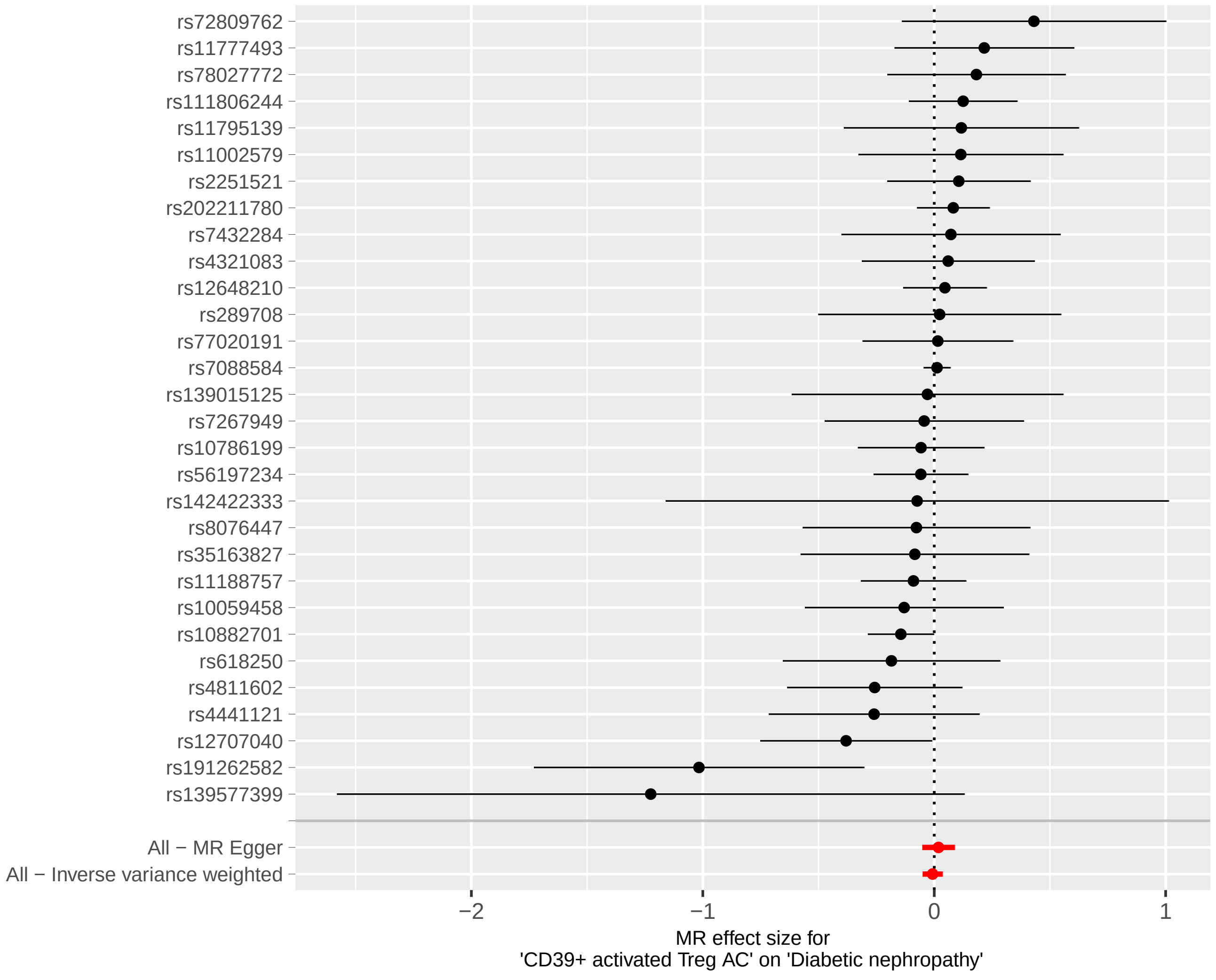
rs72809762
rs11777493
rs78027772
rs111806244
rs11795139
rs11002579
rs2251521
rs202211780
rs7432284
rs4321083
rs12648210
rs289708
rs77020191
rs7088584
rs139015125
rs7267949
rs10786199
rs56197234
rs142422333
rs8076447
rs35163827
rs11188757
rs10059458
rs10882701
rs618250
rs4811602
rs4441121
rs12707040
rs191262582
rs139577399
All − MR Egger
All − Inverse variance weighted
−2
−1
0
1
MR effect size for
'CD39+ activated Treg AC' on 'Diabetic nephropathy'

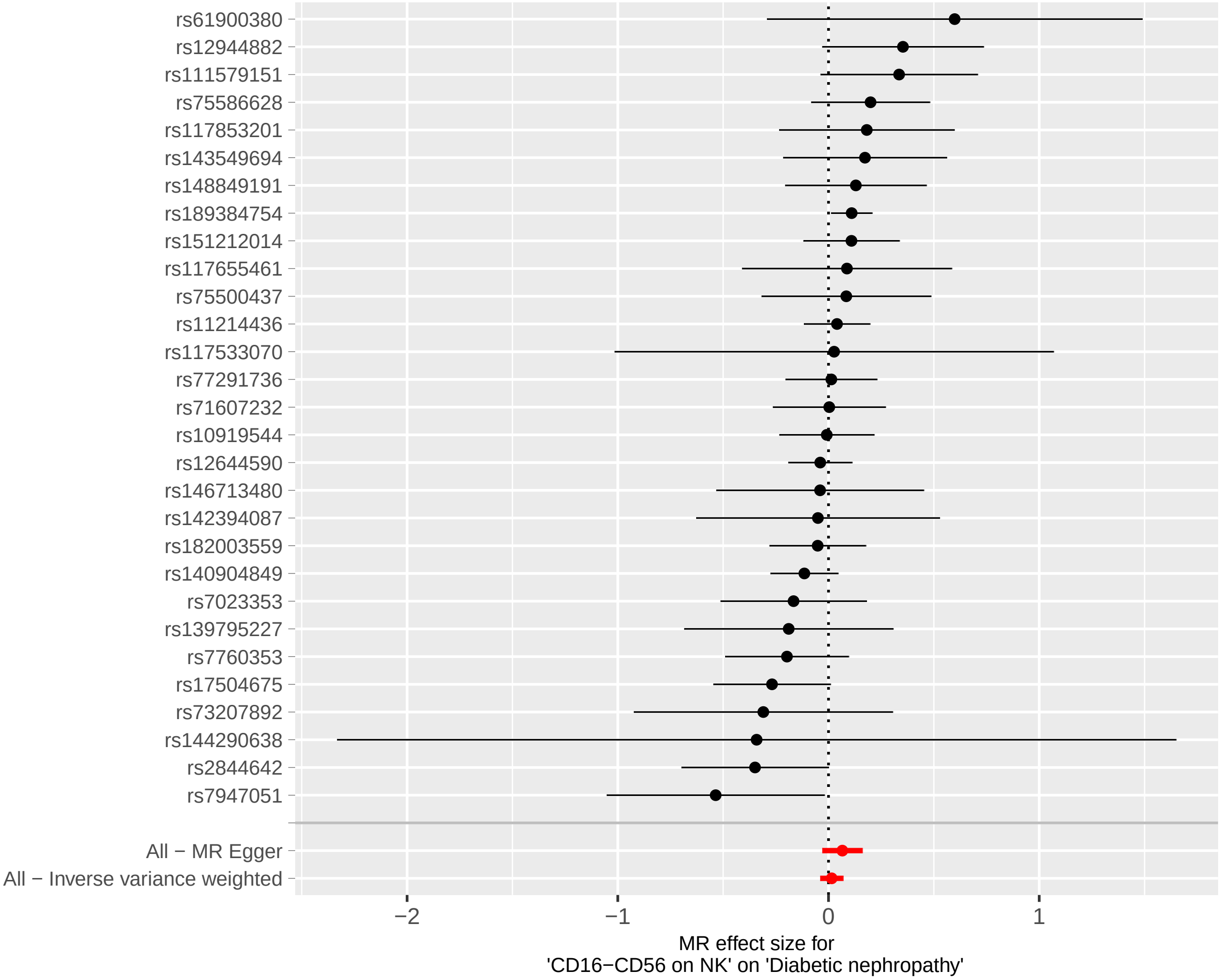
rs61900380
rs12944882
rs111579151
rs75586628
rs117853201
rs143549694
rs148849191
rs189384754
rs151212014
rs117655461
rs75500437
rs11214436
rs117533070
rs77291736
rs71607232
rs10919544
rs12644590
rs146713480
rs142394087
rs182003559
rs140904849
rs7023353
rs139795227
rs7760353
rs17504675
rs73207892
rs144290638
rs2844642
rs7947051
All − MR Egger
All − Inverse variance weighted
−2
−1
0
1
MR effect size for
'CD16−CD56 on NK' on 'Diabetic nephropathy'

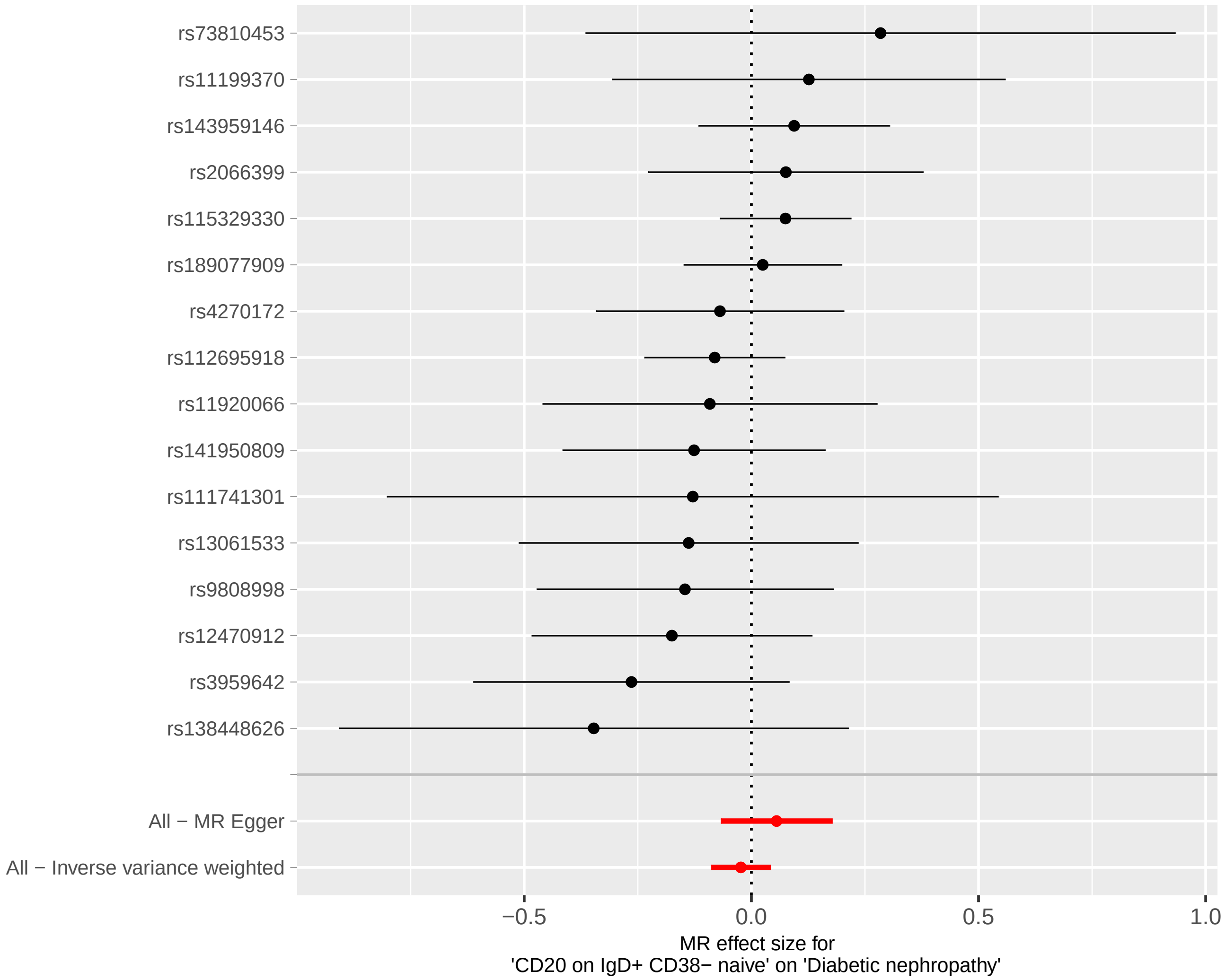
rs73810453
rs11199370
rs143959146
rs2066399
rs115329330
rs189077909
rs4270172
rs112695918
rs11920066
rs141950809
rs111741301
rs13061533
rs9808998
rs12470912
rs3959642
rs138448626
All − MR Egger
All − Inverse variance weighted
−0.5
0.0
0.5
1.0
MR effect size for
'CD20 on IgD+ CD38− naive' on 'Diabetic nephropathy'

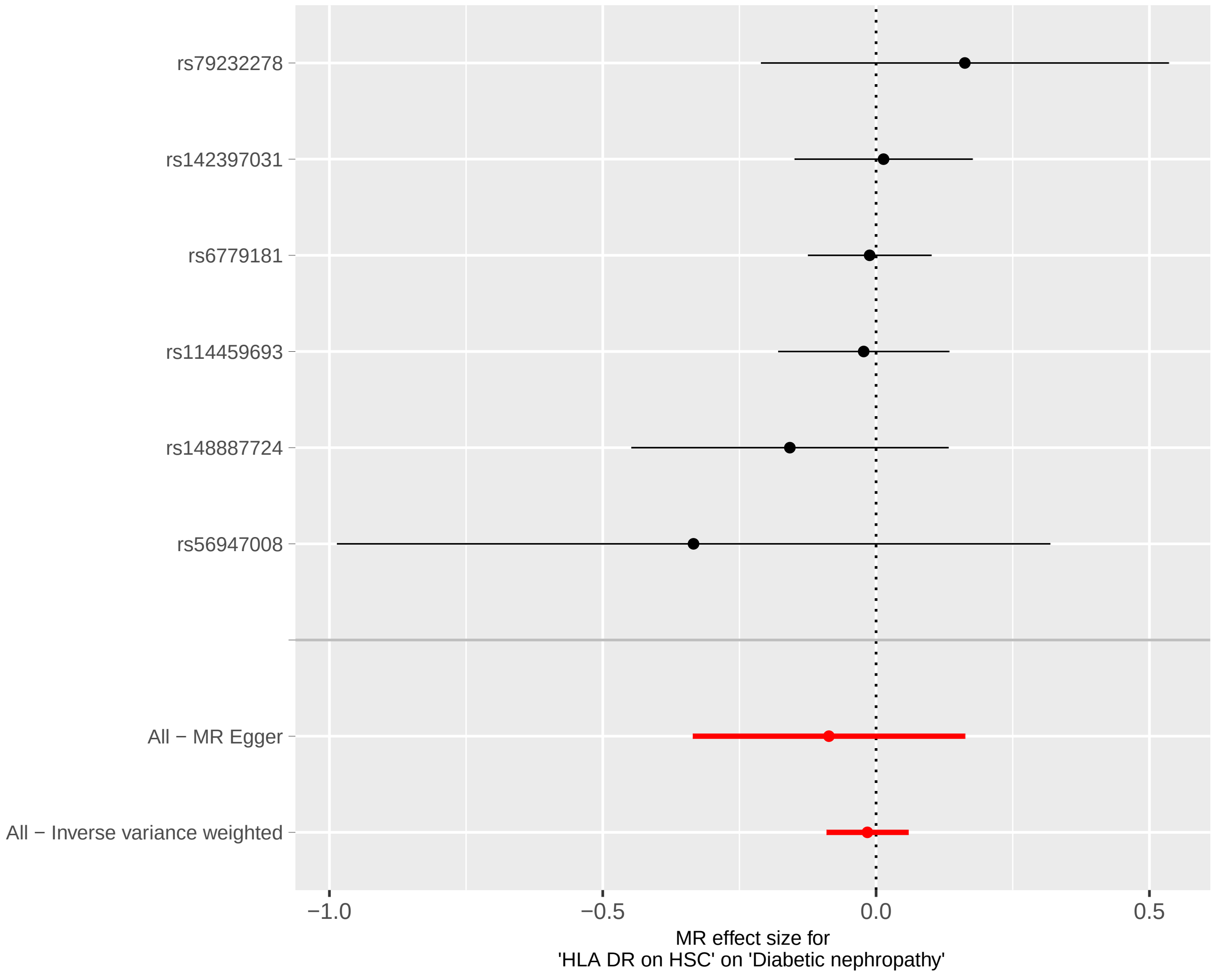

rs79232278
rs142397031
rs6779181
rs114459693
rs148887724
rs56947008
All − MR Egger
All − Inverse variance weighted
−1.0
−0.5
0.0
0.5
MR effect size for
'HLA DR on HSC' on 'Diabetic nephropathy'

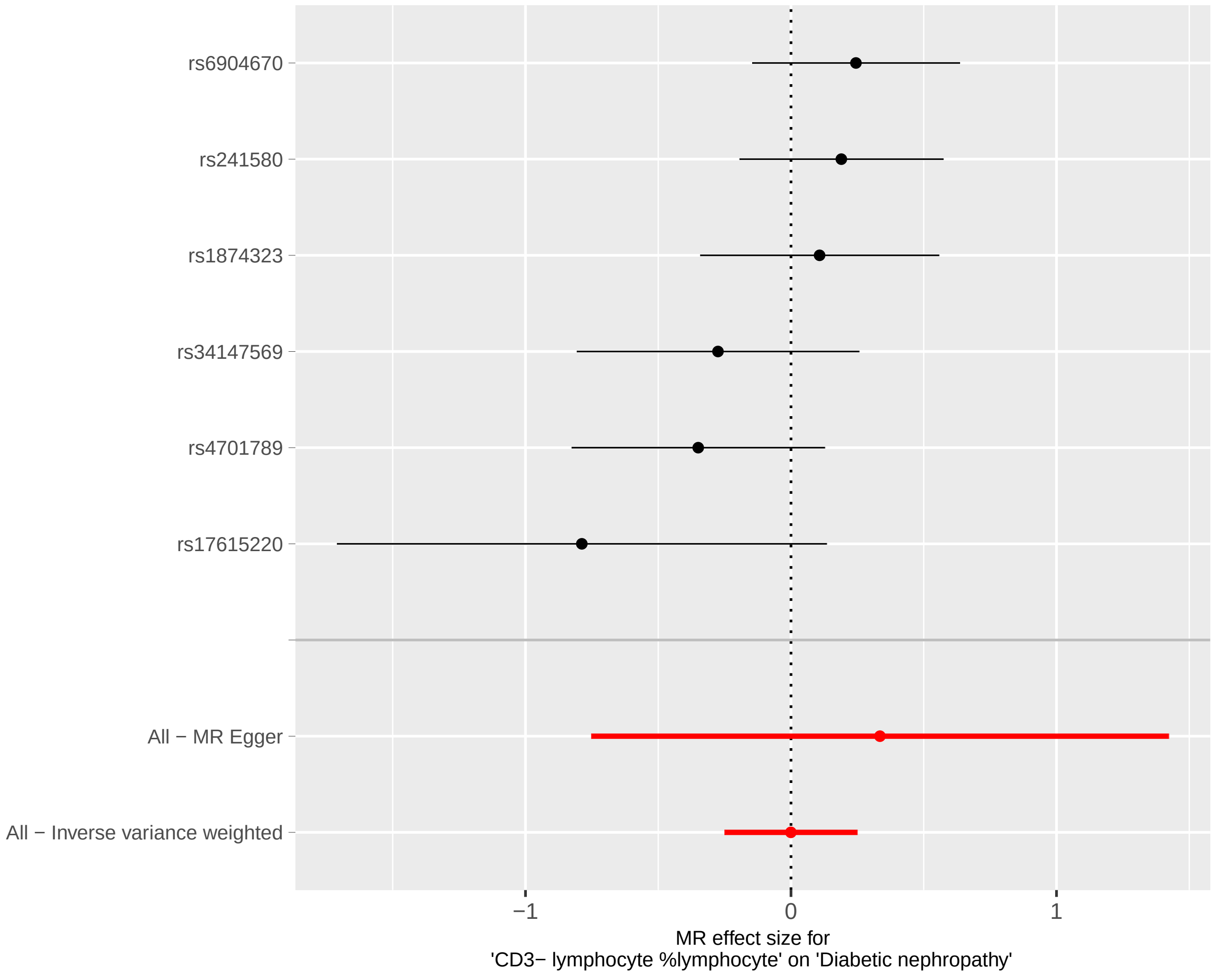
rs6904670
rs241580
rs1874323
rs34147569
rs4701789
rs17615220
All − MR Egger
All − Inverse variance weighted
−1
0
1
MR effect size for
'CD3− lymphocyte %lymphocyte' on 'Diabetic nephropathy'

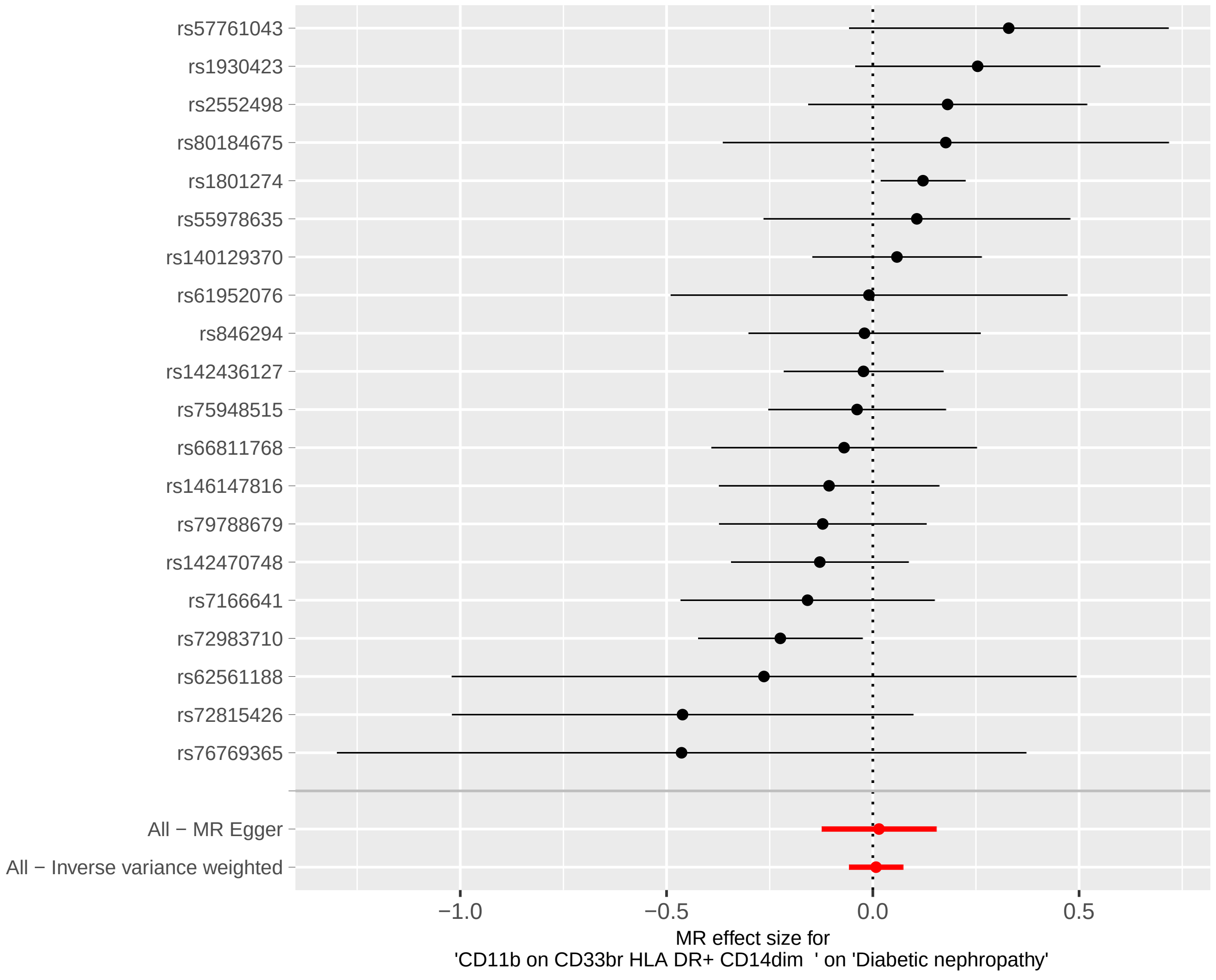

rs57761043
rs1930423
rs2552498
rs80184675
rs1801274
rs55978635
rs140129370
rs61952076
rs846294
rs142436127
rs75948515
rs66811768
rs146147816
rs79788679
rs142470748
rs7166641
rs72983710
rs62561188
rs72815426
rs76769365
All − MR Egger
All − Inverse variance weighted
−1.0
−0.5
0.0
0.5
MR effect size for
'CD11b on CD33br HLA DR+ CD14dim ' on 'Diabetic nephropathy'

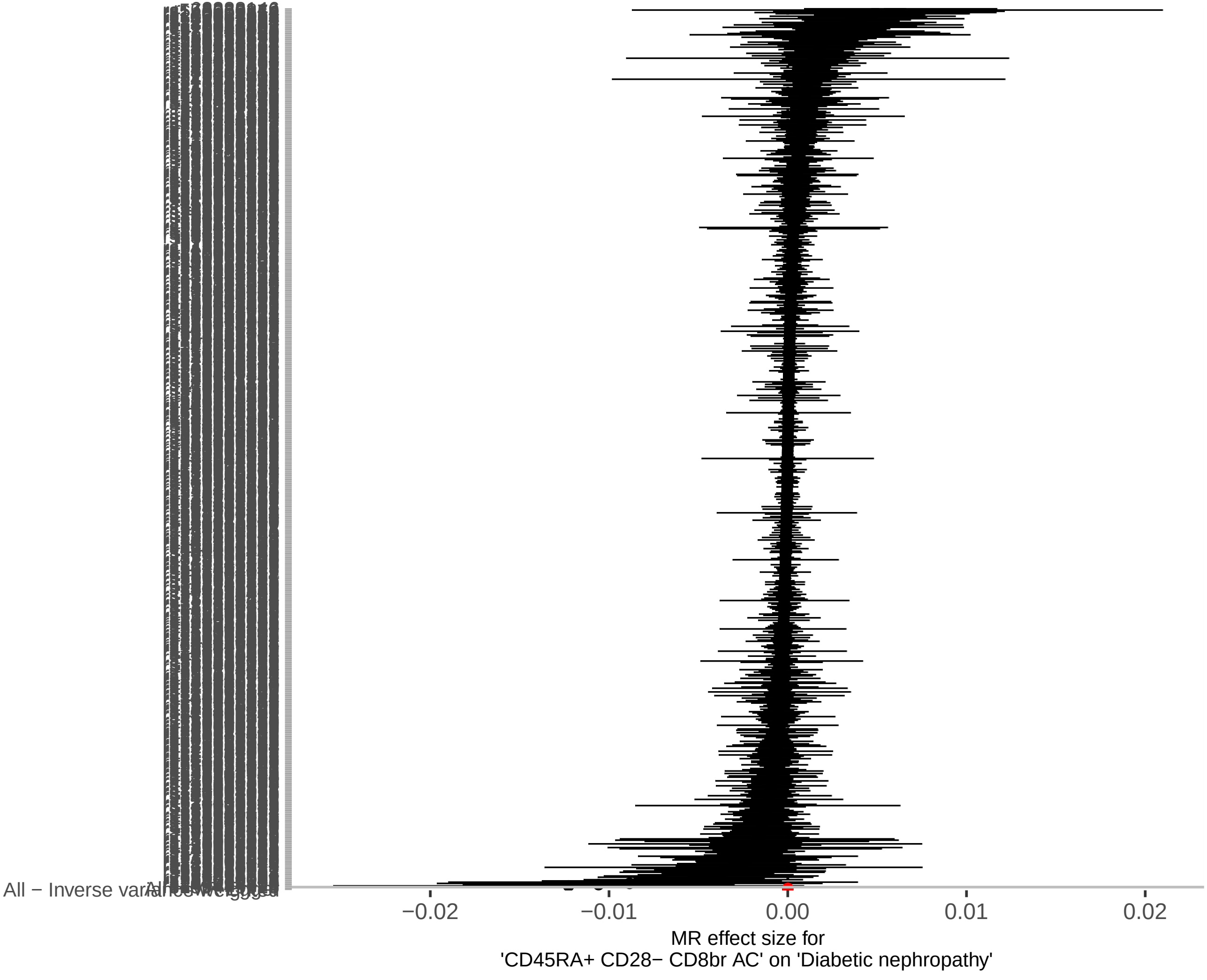

All − Inverse variance weighted
−0.02
−0.01
0.00
0.01
0.02
MR effect size for
'CD45RA+ CD28− CD8br AC' on 'Diabetic nephropathy'

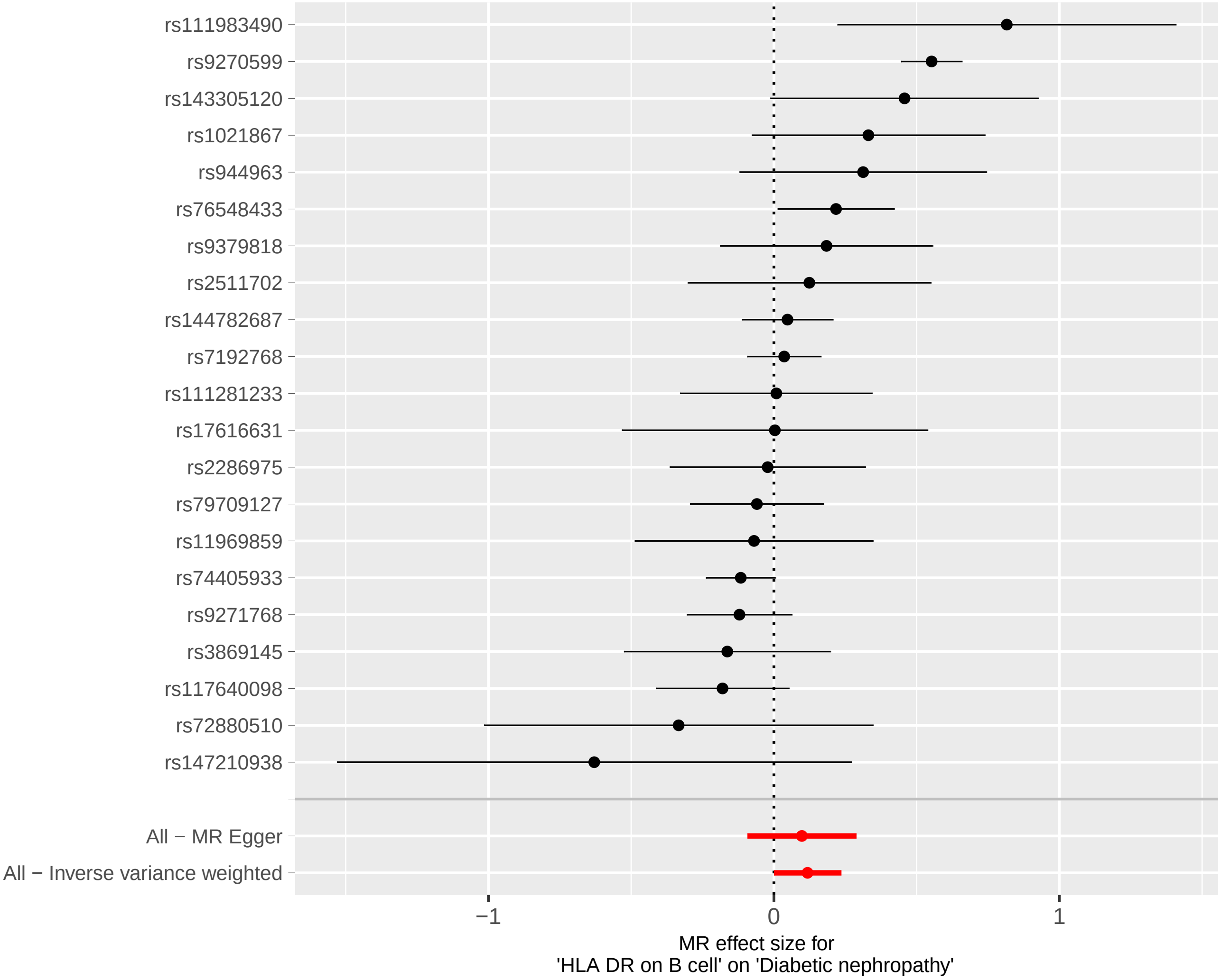

rs111983490
rs9270599
rs143305120
rs1021867
rs944963
rs76548433
rs9379818
rs2511702
rs144782687
rs7192768
rs111281233
rs17616631
rs2286975
rs79709127
rs11969859
rs74405933
rs9271768
rs3869145
rs117640098
rs72880510
rs147210938
All − MR Egger
All − Inverse variance weighted
−1
0
1
MR effect size for
'HLA DR on B cell' on 'Diabetic nephropathy'

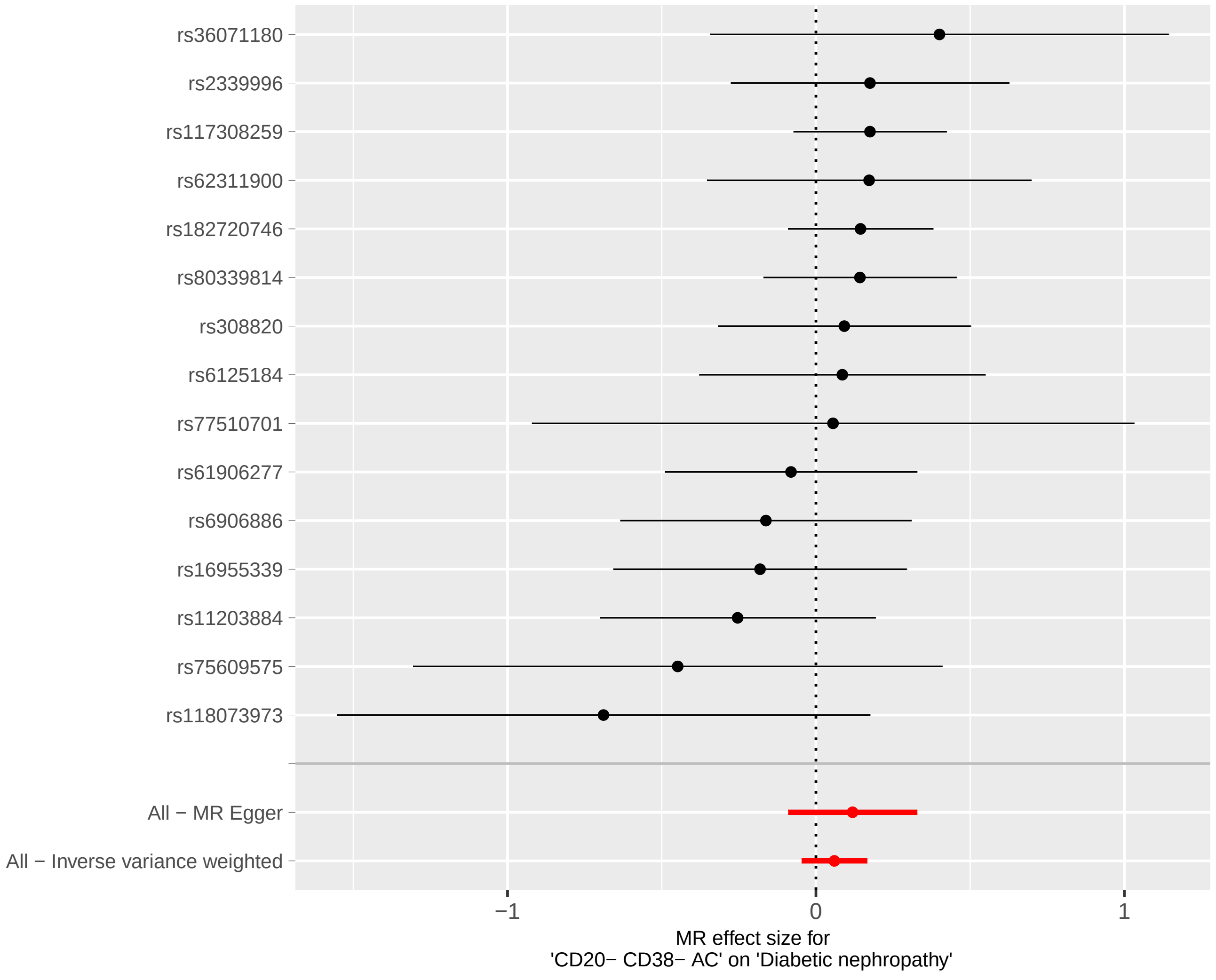
rs36071180
rs2339996
rs117308259
rs62311900
rs182720746
rs80339814
rs308820
rs6125184
rs77510701
rs61906277
rs6906886
rs16955339
rs11203884
rs75609575
rs118073973
All − MR Egger
All − Inverse variance weighted
−1
0
1
MR effect size for
'CD20− CD38− AC' on 'Diabetic nephropathy'

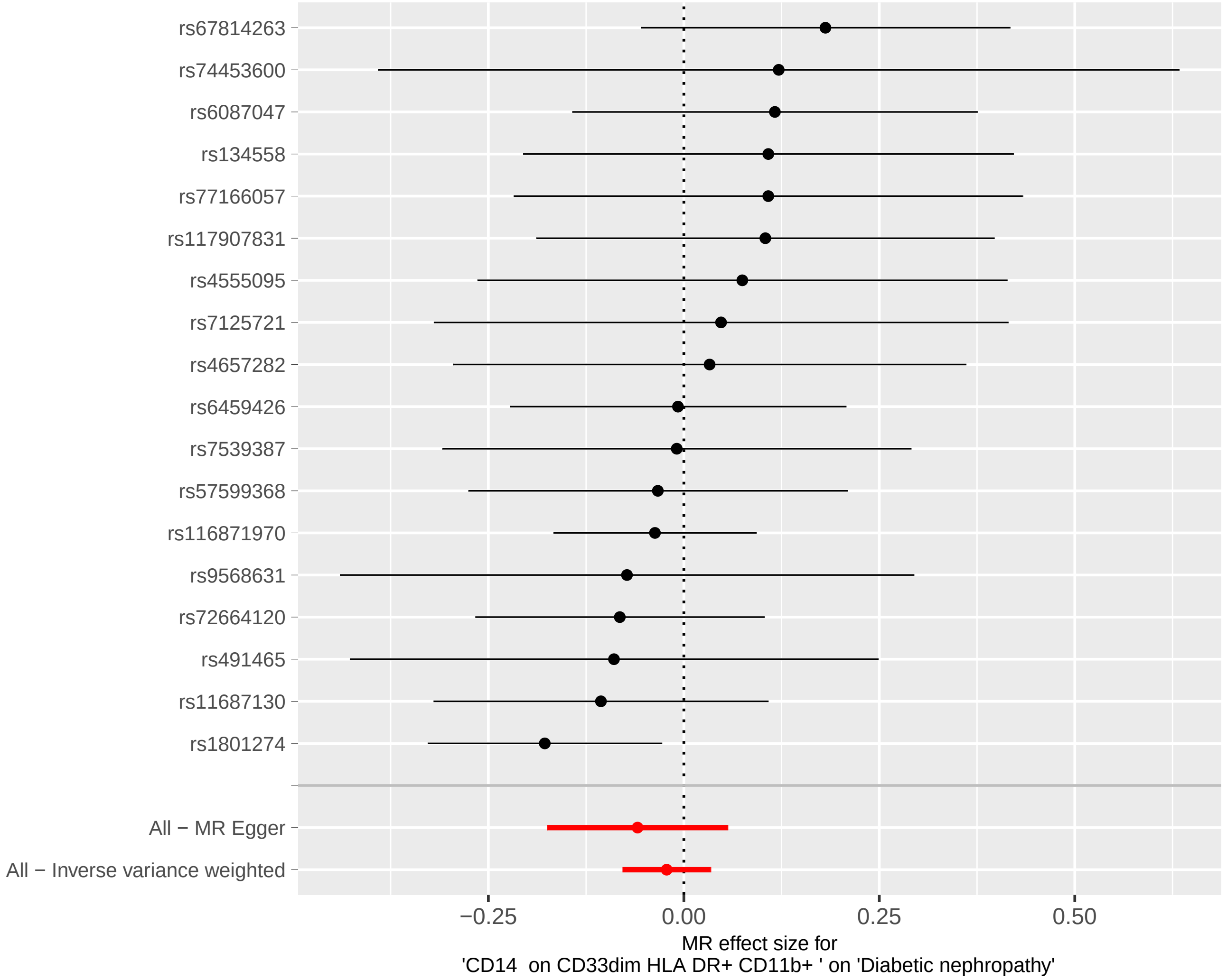
rs67814263
rs74453600
rs6087047
rs134558
rs77166057
rs117907831
rs4555095
rs7125721
rs4657282
rs6459426
rs7539387
rs57599368
rs116871970
rs9568631
rs72664120
rs491465
rs11687130
rs1801274
All − MR Egger
All − Inverse variance weighted
−0.25
0.00
0.25
0.50
MR effect size for
'CD14 on CD33dim HLA DR+ CD11b+ ' on 'Diabetic nephropathy'

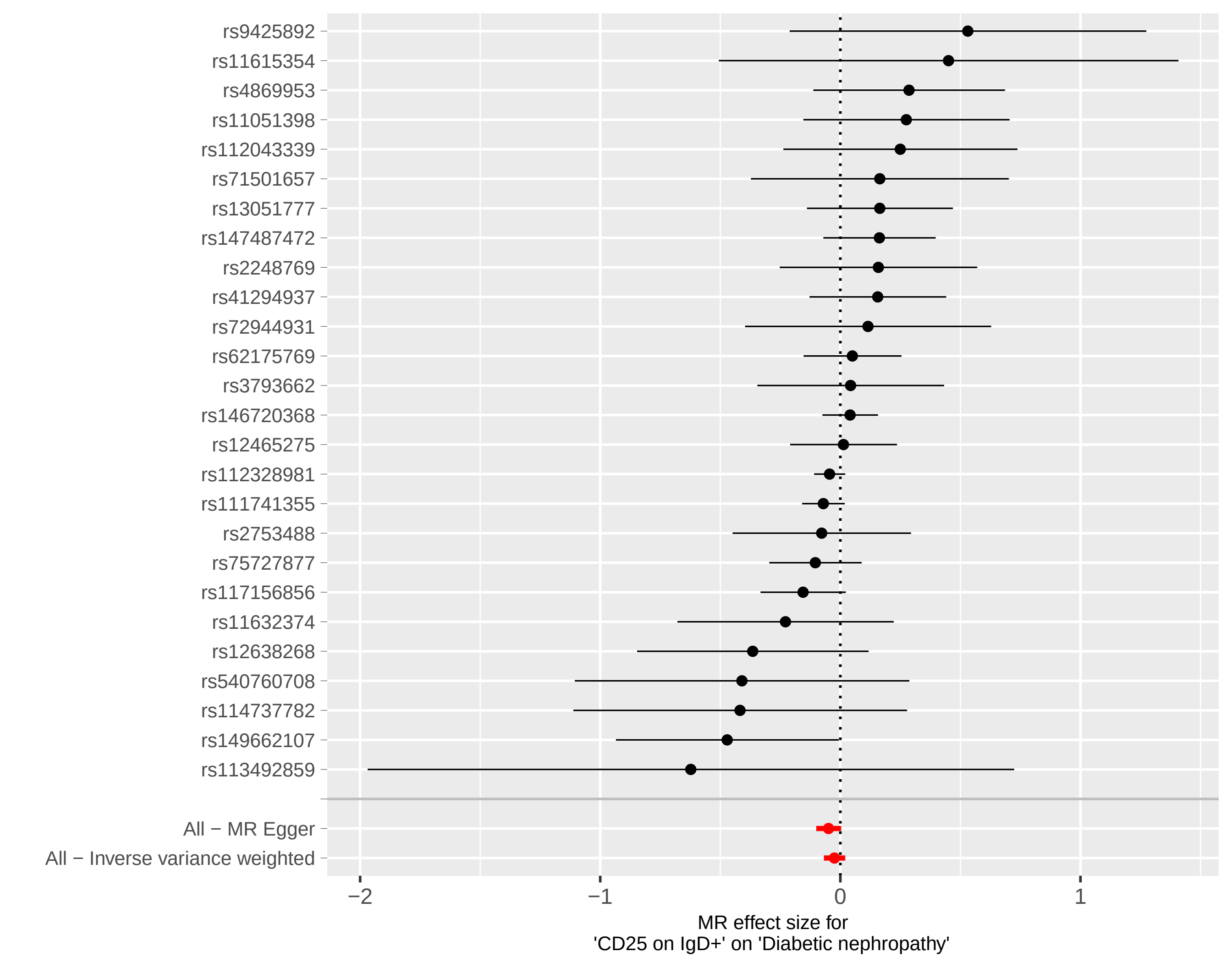

rs9425892
rs11615354
rs4869953
rs11051398
rs112043339
rs71501657
rs13051777
rs147487472
rs2248769
rs41294937
rs72944931
rs62175769
rs3793662
rs146720368
rs12465275
rs112328981
rs111741355
rs2753488
rs75727877
rs117156856
rs11632374
rs12638268
rs540760708
rs114737782
rs149662107
rs113492859
All − MR Egger
All − Inverse variance weighted
−2
−1
0
1
MR effect size for
'CD25 on IgD+' on 'Diabetic nephropathy'

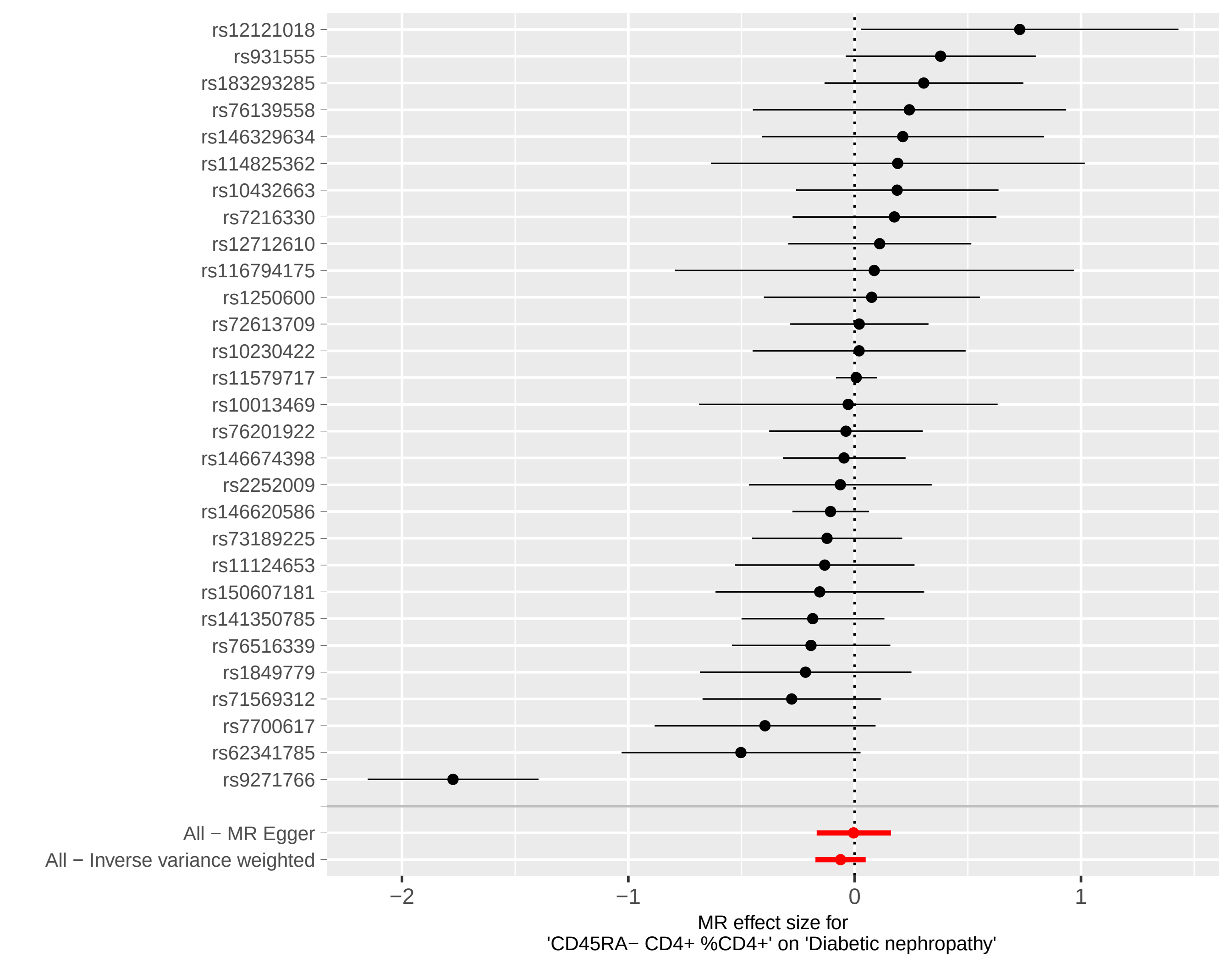
rs12121018
rs931555
rs183293285
rs76139558
rs146329634
rs114825362
rs10432663
rs7216330
rs12712610
rs116794175
rs1250600
rs72613709
rs10230422
rs11579717
rs10013469
rs76201922
rs146674398
rs2252009
rs146620586
rs73189225
rs11124653
rs150607181
rs141350785
rs76516339
rs1849779
rs71569312
rs7700617
rs62341785
rs9271766
All − MR Egger
All − Inverse variance weighted
−2
−1
0
1
MR effect size for
'CD45RA− CD4+ %CD4+' on 'Diabetic nephropathy'

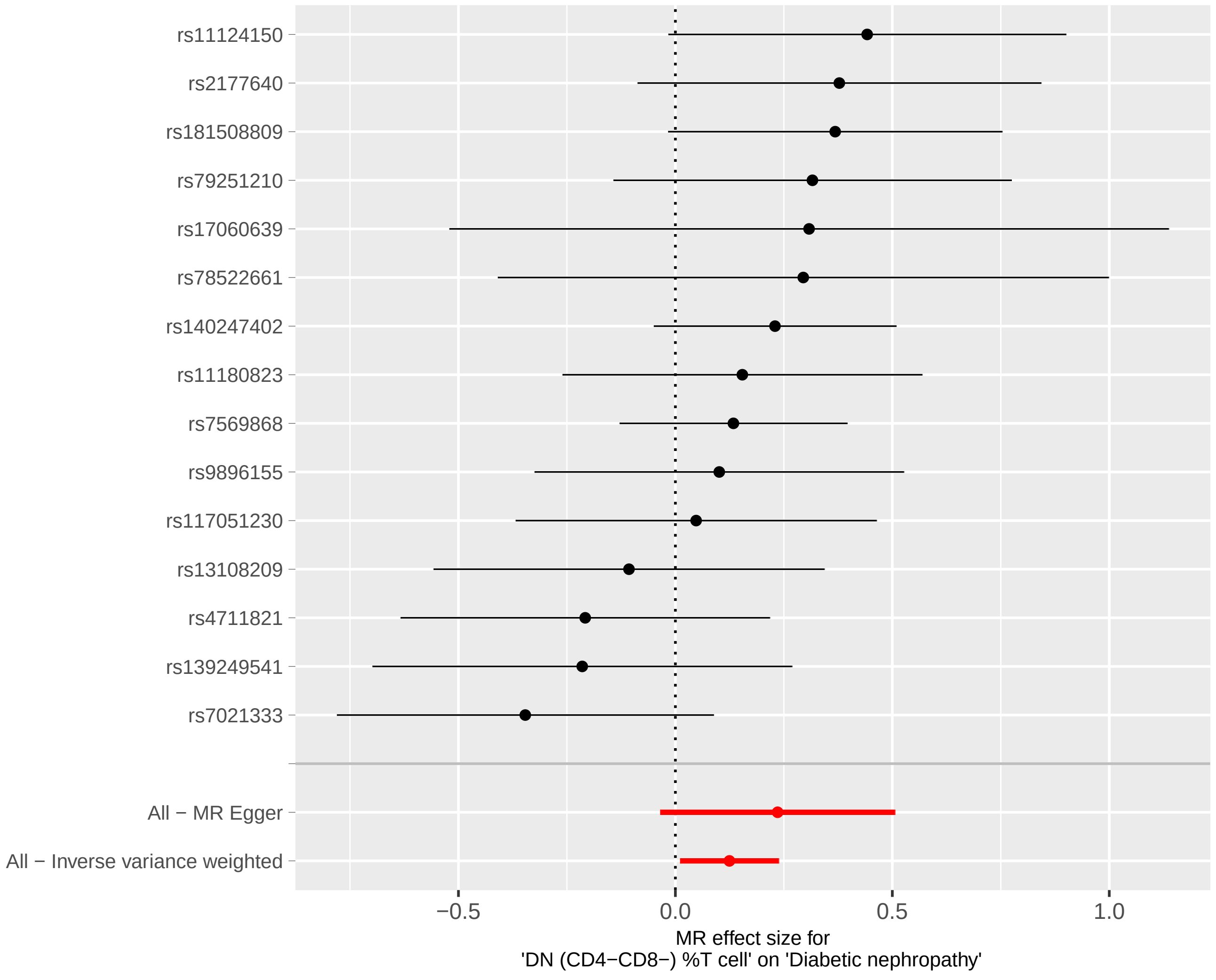
rs11124150
rs2177640
rs181508809
rs79251210
rs17060639
rs78522661
rs140247402
rs11180823
rs7569868
rs9896155
rs117051230
rs13108209
rs4711821
rs139249541
rs7021333
All − MR Egger
All − Inverse variance weighted
−0.5
0.0
0.5
1.0
MR effect size for
'DN (CD4−CD8−) %T cell' on 'Diabetic nephropathy'

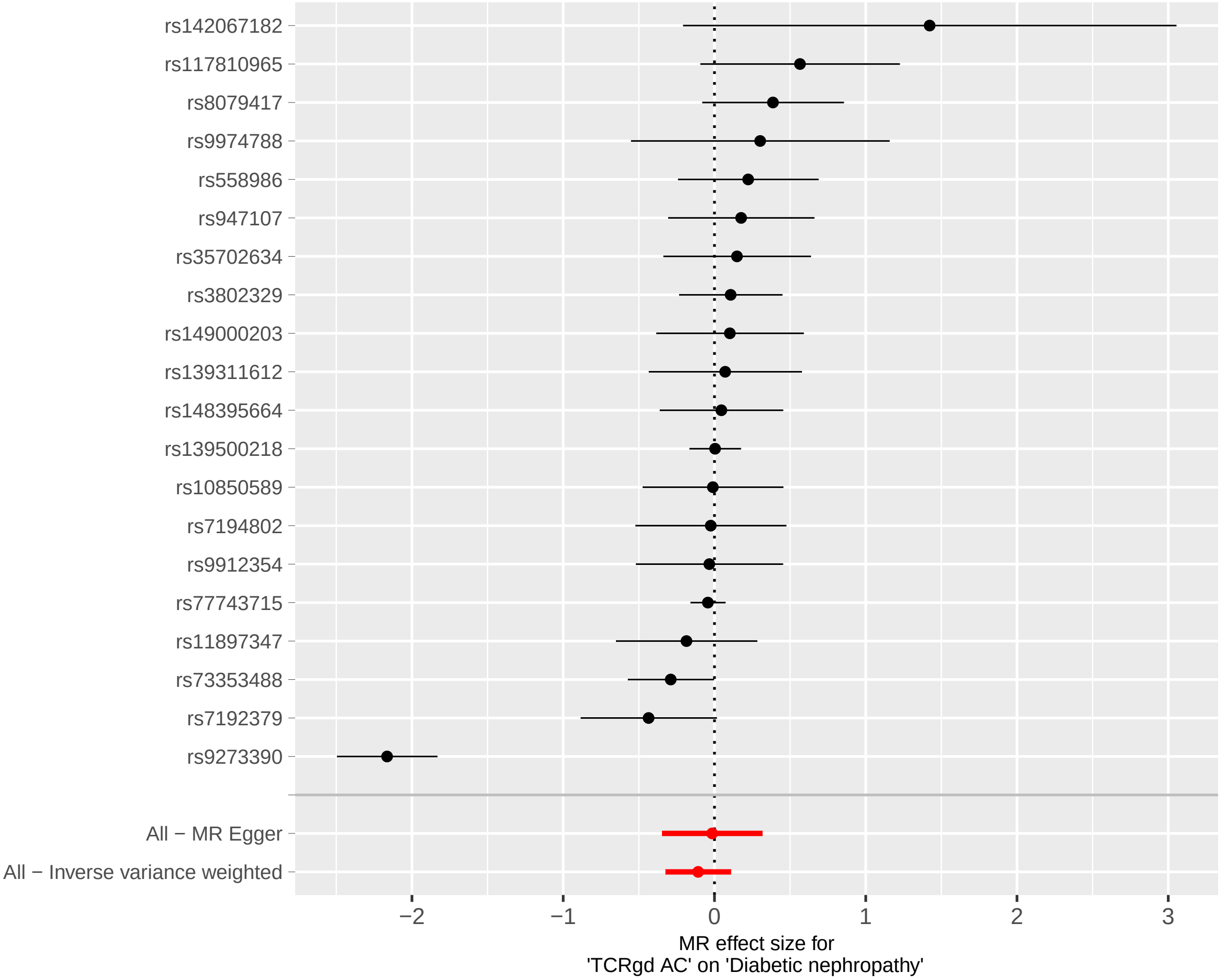

rs142067182
rs117810965
rs8079417
rs9974788
rs558986
rs947107
rs35702634
rs3802329
rs149000203
rs139311612
rs148395664
rs139500218
rs10850589
rs7194802
rs9912354
rs77743715
rs11897347
rs73353488
rs7192379
rs9273390
All − MR Egger
All − Inverse variance weighted
−2
−1
0
1
2
3
MR effect size for
'TCRgd AC' on 'Diabetic nephropathy'

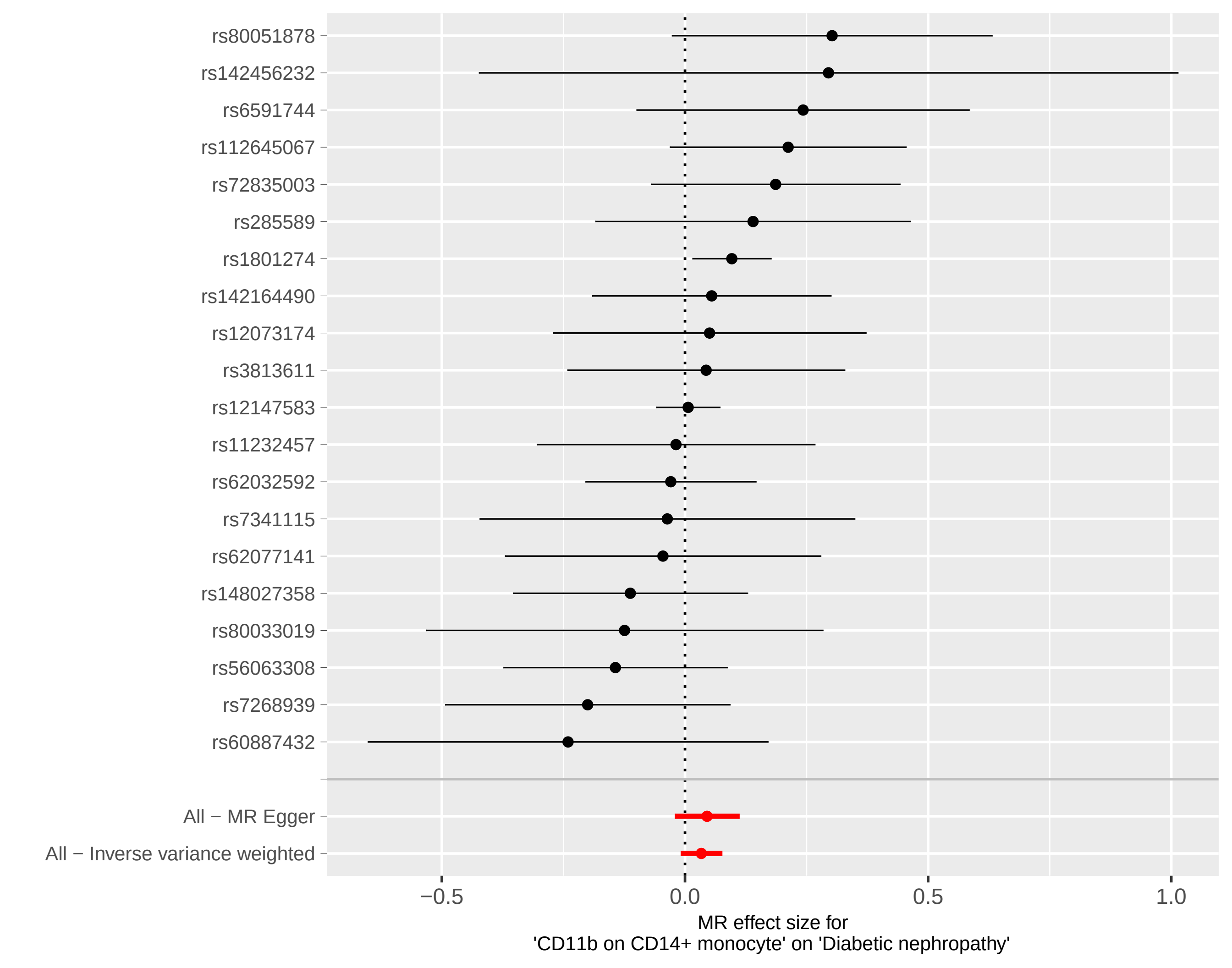
rs80051878
rs142456232
rs6591744
rs112645067
rs72835003
rs285589
rs1801274
rs142164490
rs12073174
rs3813611
rs12147583
rs11232457
rs62032592
rs7341115
rs62077141
rs148027358
rs80033019
rs56063308
rs7268939
rs60887432
All − MR Egger
All − Inverse variance weighted
−0.5
0.0
0.5
1.0
MR effect size for
'CD11b on CD14+ monocyte' on 'Diabetic nephropathy'

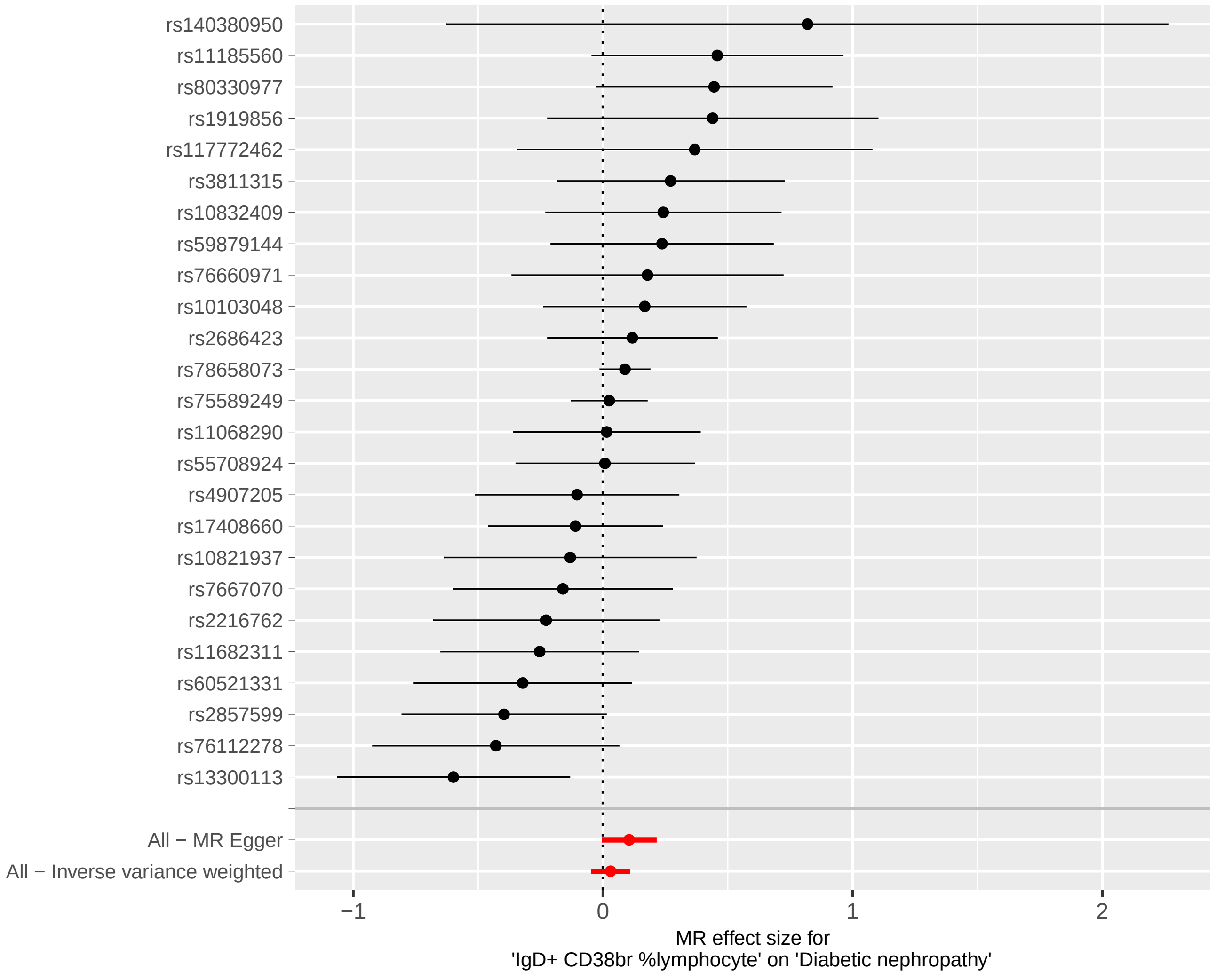
rs140380950
rs11185560
rs80330977
rs1919856
rs117772462
rs3811315
rs10832409
rs59879144
rs76660971
rs10103048
rs2686423
rs78658073
rs75589249
rs11068290
rs55708924
rs4907205
rs17408660
rs10821937
rs7667070
rs2216762
rs11682311
rs60521331
rs2857599
rs76112278
rs13300113
All − MR Egger
All − Inverse variance weighted
−1
0
1
2
MR effect size for
'IgD+ CD38br %lymphocyte' on 'Diabetic nephropathy'

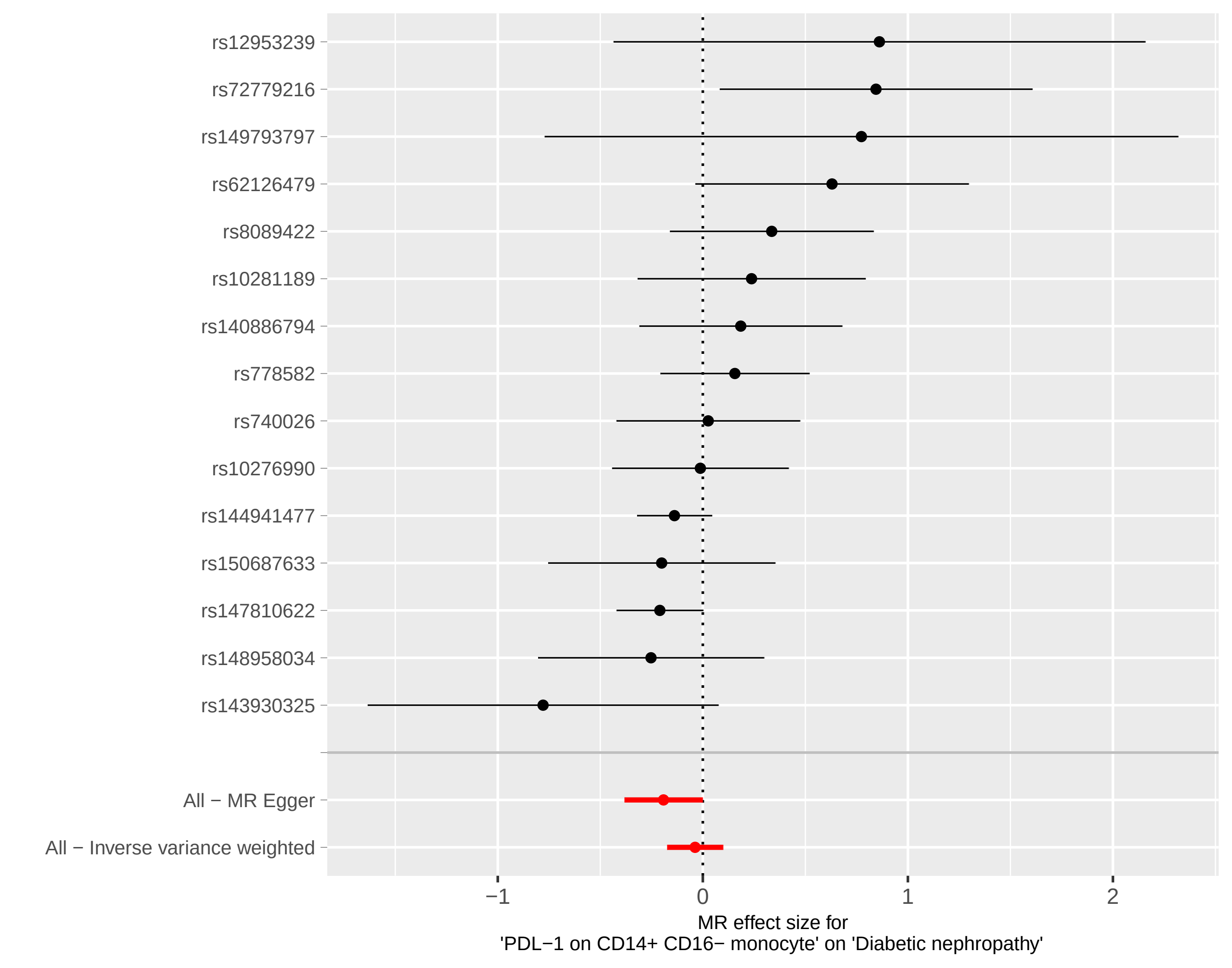
rs12953239
rs72779216
rs149793797
rs62126479
rs8089422
rs10281189
rs140886794
rs778582
rs740026
rs10276990
rs144941477
rs150687633
rs147810622
rs148958034
rs143930325
All − MR Egger
All − Inverse variance weighted
−1
0
1
2
MR effect size for
'PDL−1 on CD14+ CD16− monocyte' on 'Diabetic nephropathy'

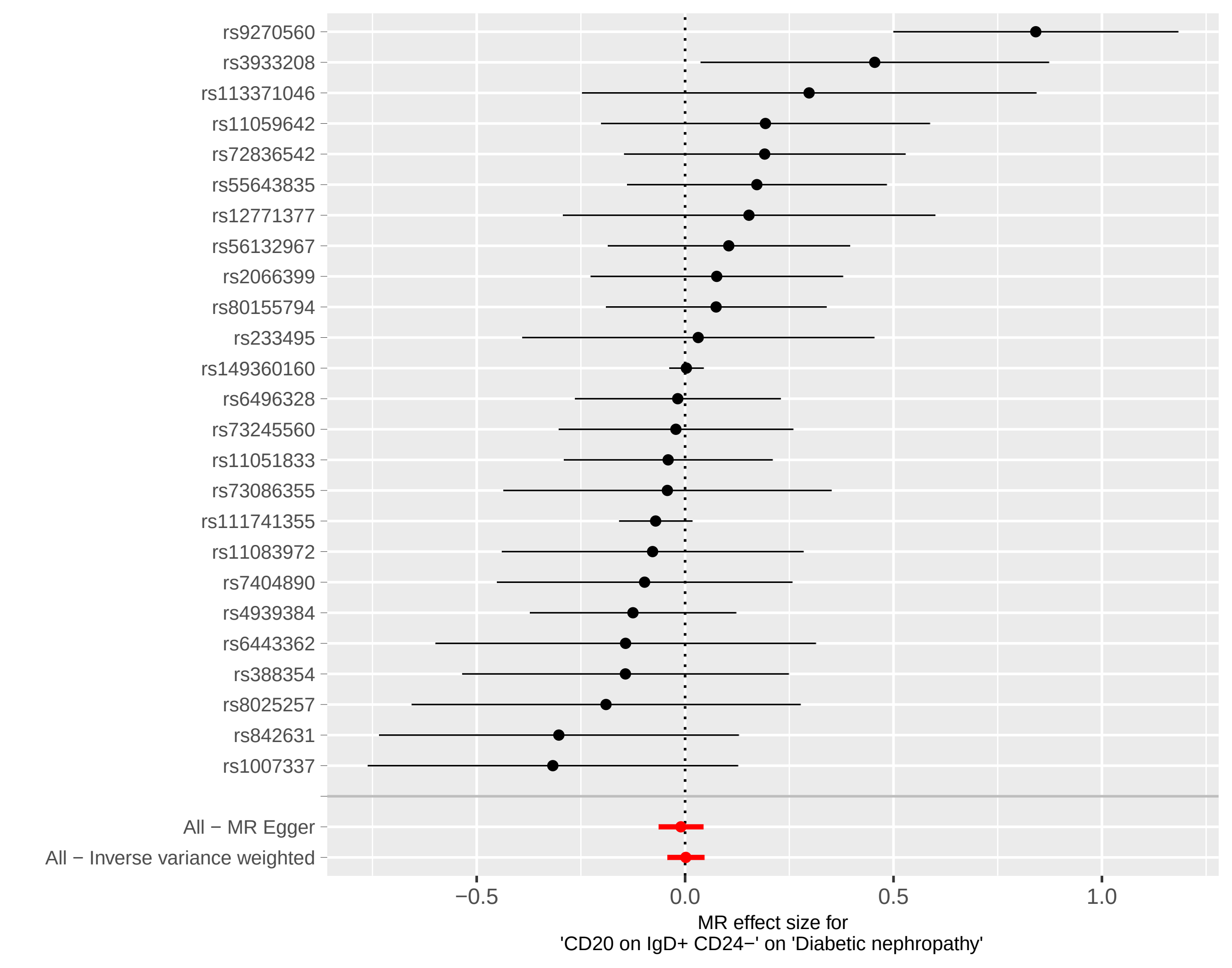

rs9270560
rs3933208
rs113371046
rs11059642
rs72836542
rs55643835
rs12771377
rs56132967
rs2066399
rs80155794
rs233495
rs149360160
rs6496328
rs73245560
rs11051833
rs73086355
rs111741355
rs11083972
rs7404890
rs4939384
rs6443362
rs388354
rs8025257
rs842631
rs1007337
All − MR Egger
All − Inverse variance weighted
−0.5
0.0
0.5
1.0
MR effect size for
'CD20 on IgD+ CD24−' on 'Diabetic nephropathy'

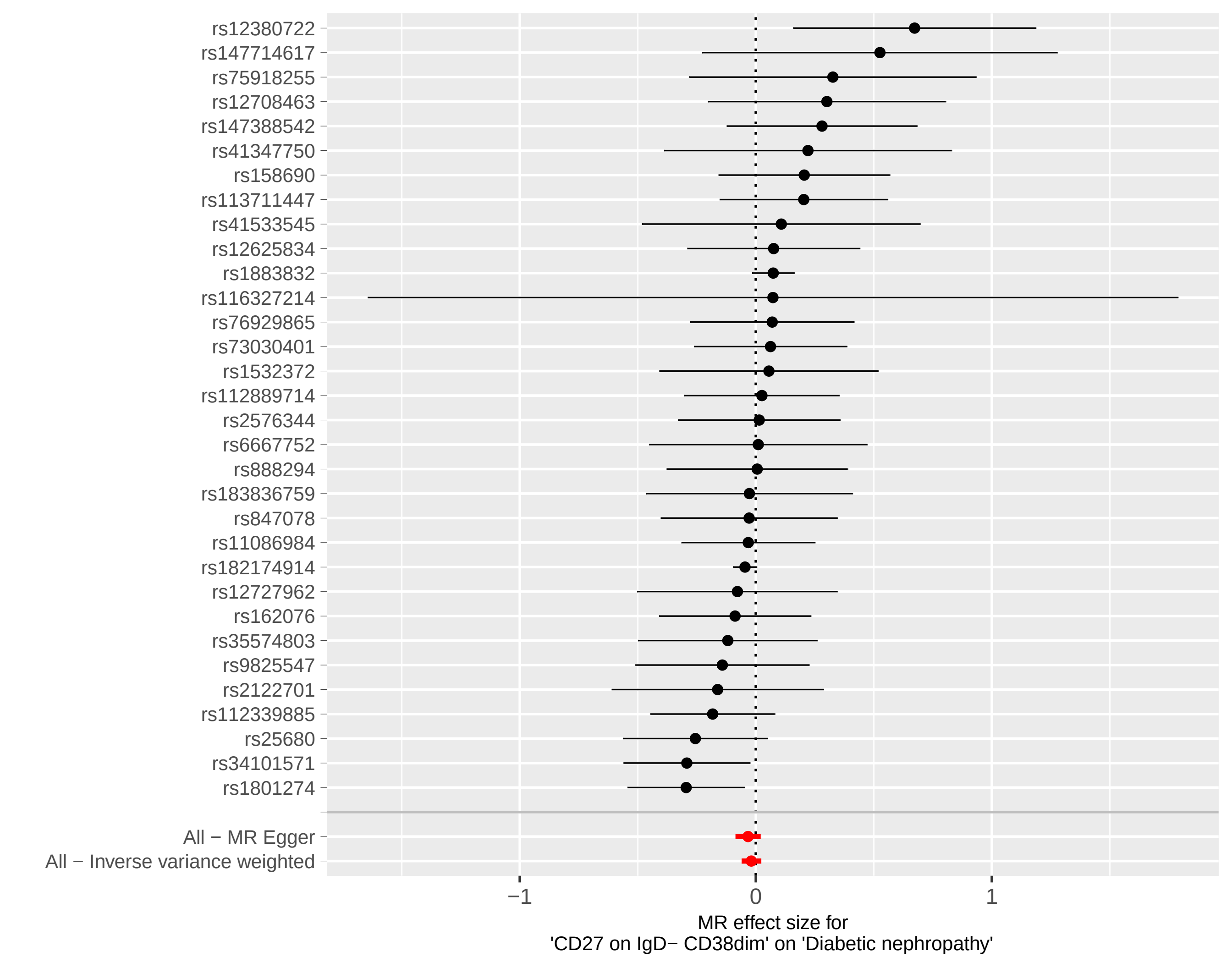
rs12380722
rs147714617
rs75918255
rs12708463
rs147388542
rs41347750
rs158690
rs113711447
rs41533545
rs12625834
rs1883832
rs116327214
rs76929865
rs73030401
rs1532372
rs112889714
rs2576344
rs6667752
rs888294
rs183836759
rs847078
rs11086984
rs182174914
rs12727962
rs162076
rs35574803
rs9825547
rs2122701
rs112339885
rs25680
rs34101571
rs1801274
All − MR Egger
All − Inverse variance weighted
−1
0
1
MR effect size for
'CD27 on IgD− CD38dim' on 'Diabetic nephropathy'

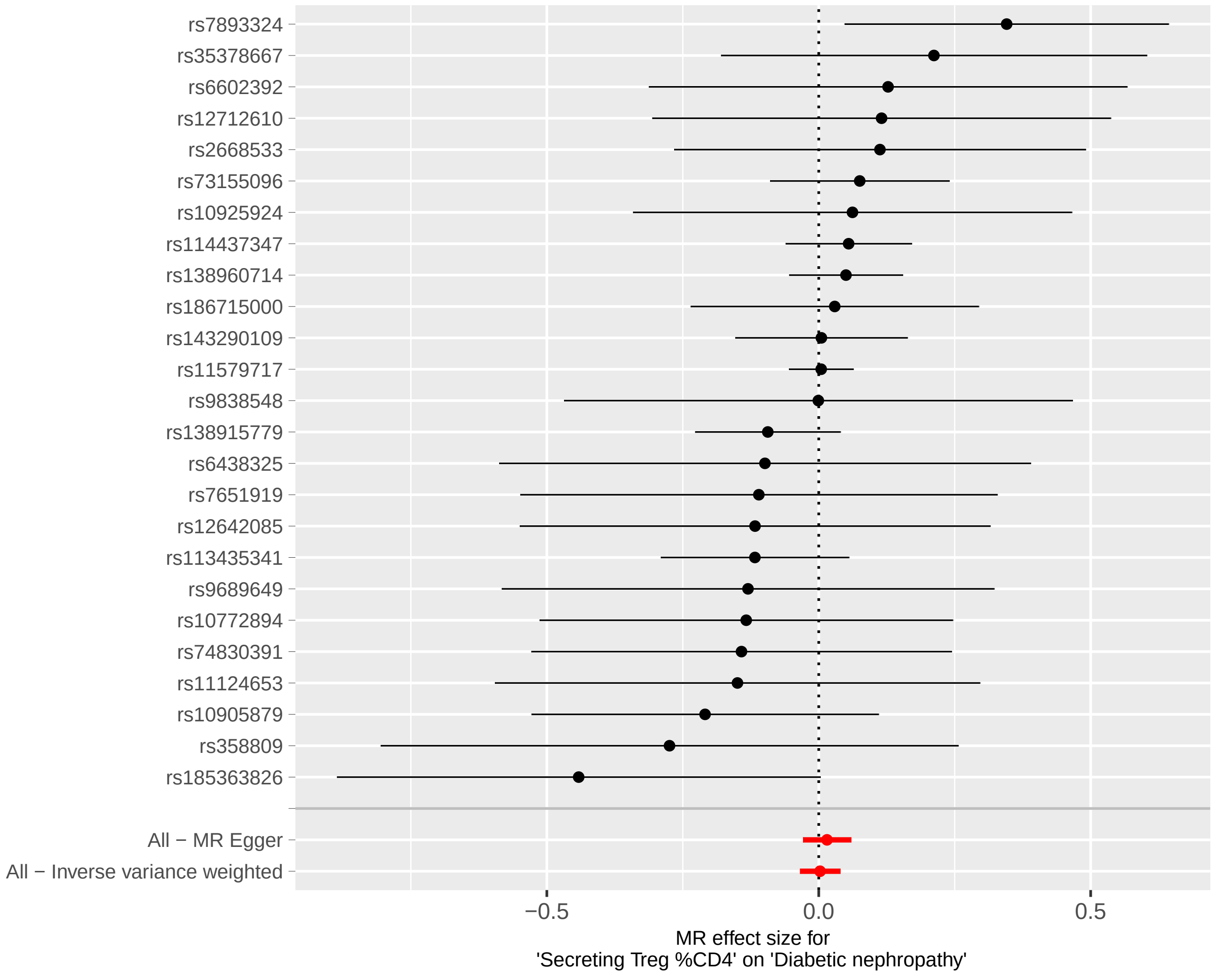

rs7893324
rs35378667
rs6602392
rs12712610
rs2668533
rs73155096
rs10925924
rs114437347
rs138960714
rs186715000
rs143290109
rs11579717
rs9838548
rs138915779
rs6438325
rs7651919
rs12642085
rs113435341
rs9689649
rs10772894
rs74830391
rs11124653
rs10905879
rs358809
rs185363826
All − MR Egger
All − Inverse variance weighted
−0.5
0.0
0.5
MR effect size for
'Secreting Treg %CD4' on 'Diabetic nephropathy'

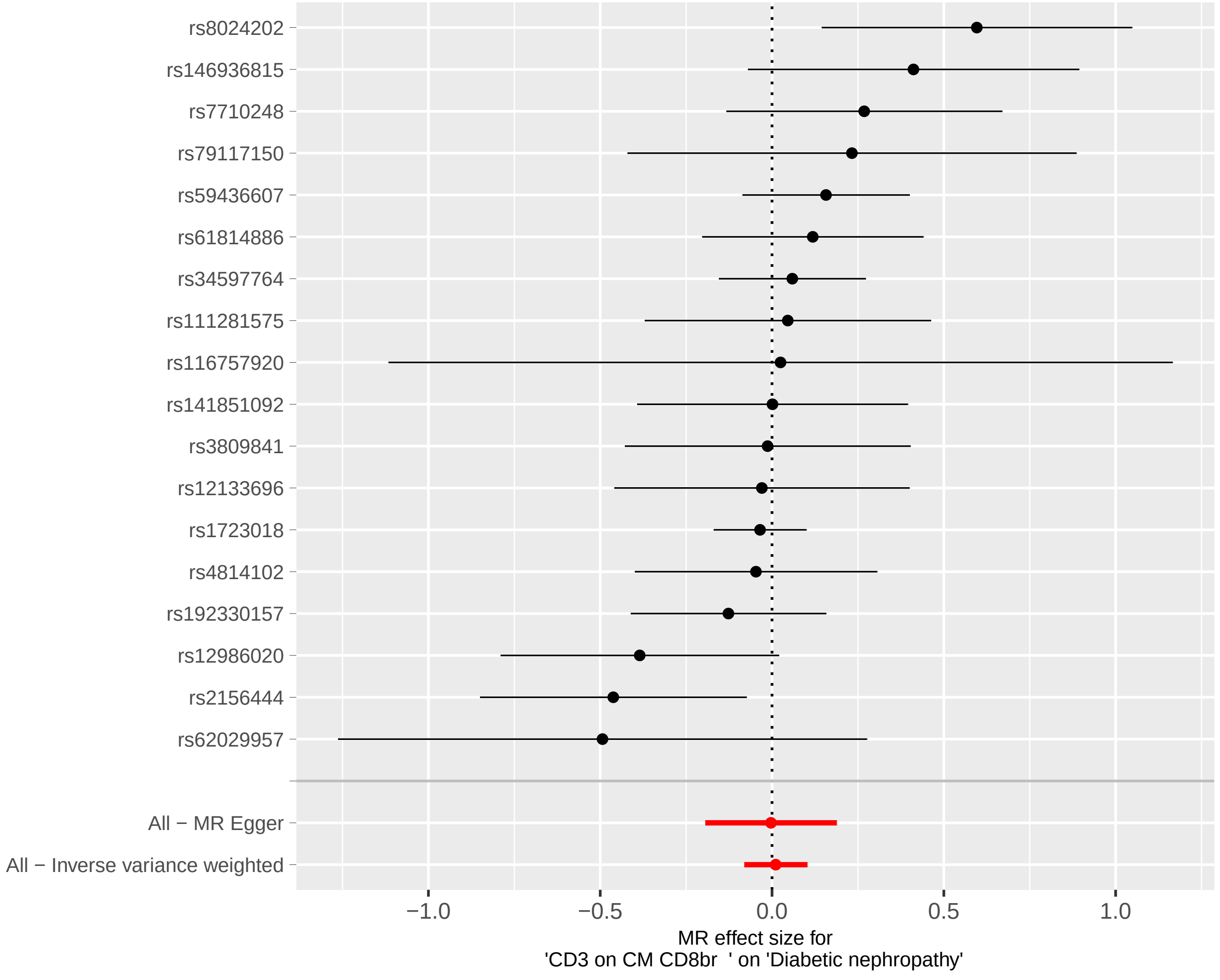
rs8024202
rs146936815
rs7710248
rs79117150
rs59436607
rs61814886
rs34597764
rs111281575
rs116757920
rs141851092
rs3809841
rs12133696
rs1723018
rs4814102
rs192330157
rs12986020
rs2156444
rs62029957
All − MR Egger
All − Inverse variance weighted
−1.0
−0.5
0.0
0.5
1.0
MR effect size for
'CD3 on CM CD8br ' on 'Diabetic nephropathy'

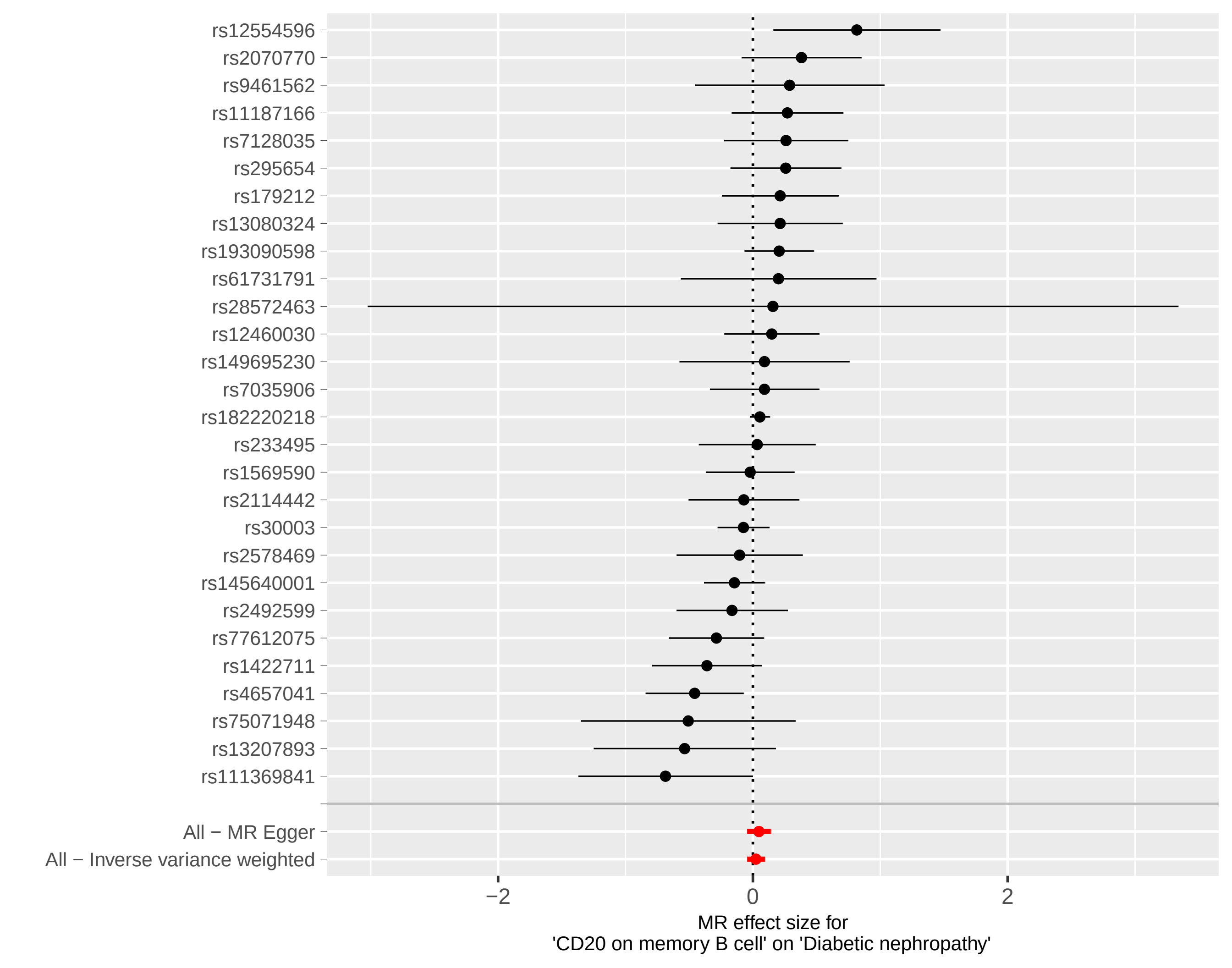

rs12554596
rs2070770
rs9461562
rs11187166
rs7128035
rs295654
rs179212
rs13080324
rs193090598
rs61731791
rs28572463
rs12460030
rs149695230
rs7035906
rs182220218
rs233495
rs1569590
rs2114442
rs30003
rs2578469
rs145640001
rs2492599
rs77612075
rs1422711
rs4657041
rs75071948
rs13207893
rs111369841
All − MR Egger
All − Inverse variance weighted
−2
0
2
MR effect size for
'CD20 on memory B cell' on 'Diabetic nephropathy'

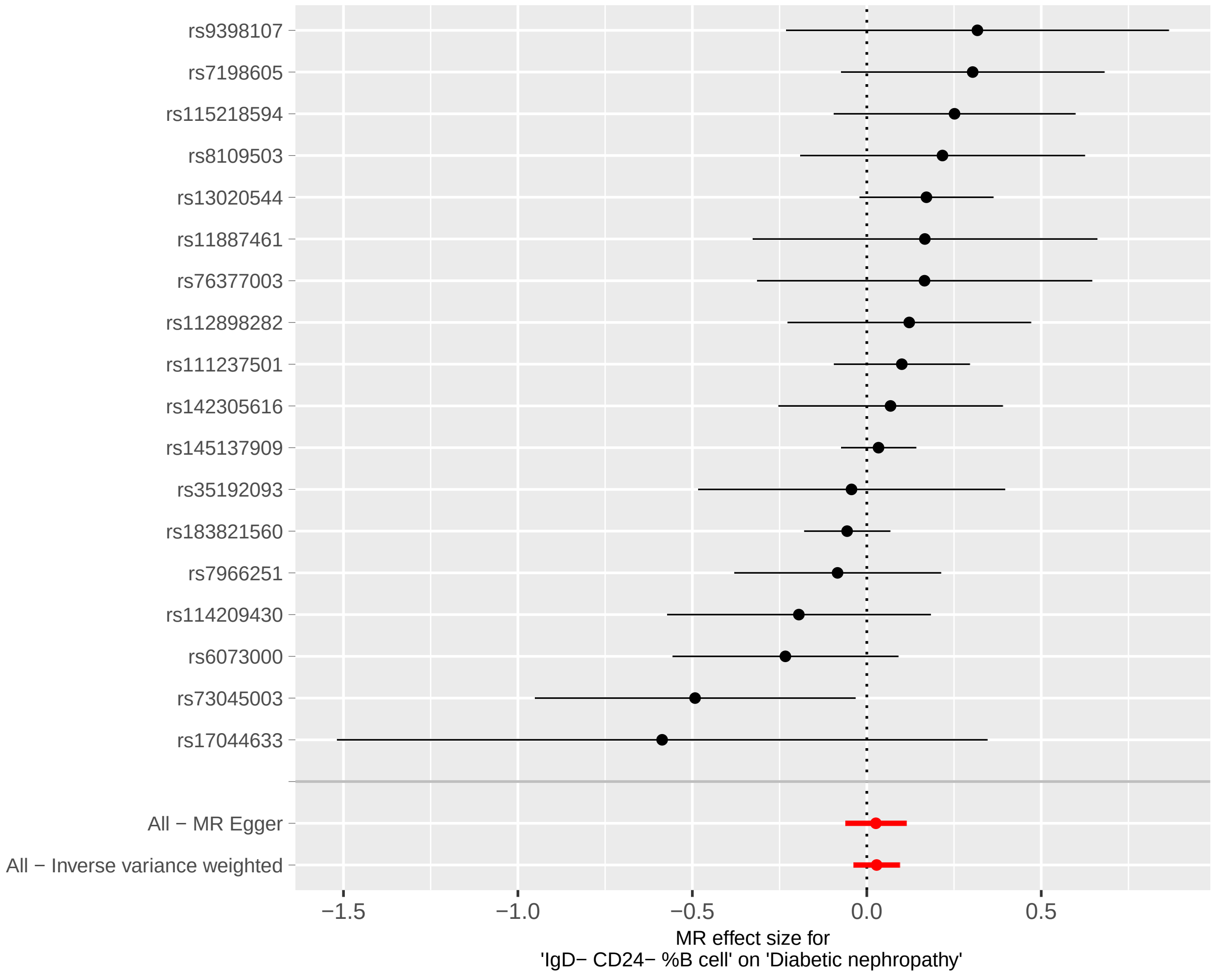

rs9398107
rs7198605
rs115218594
rs8109503
rs13020544
rs11887461
rs76377003
rs112898282
rs111237501
rs142305616
rs145137909
rs35192093
rs183821560
rs7966251
rs114209430
rs6073000
rs73045003
rs17044633
All − MR Egger
All − Inverse variance weighted
−1.5
−1.0
−0.5
0.0
0.5
MR effect size for
'IgD− CD24− %B cell' on 'Diabetic nephropathy'

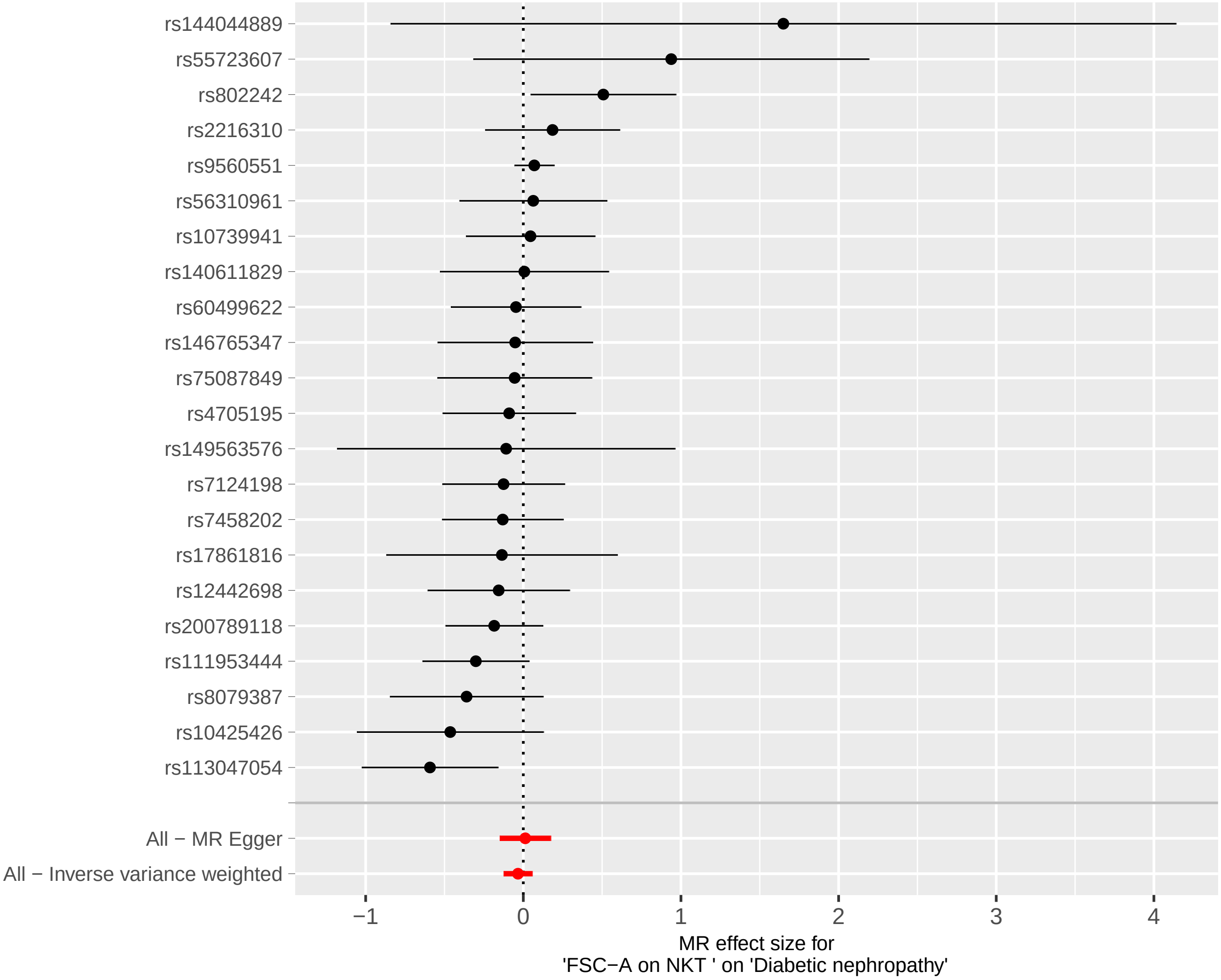
rs144044889
rs55723607
rs802242
rs2216310
rs9560551
rs56310961
rs10739941
rs140611829
rs60499622
rs146765347
rs75087849
rs4705195
rs149563576
rs7124198
rs7458202
rs17861816
rs12442698
rs200789118
rs111953444
rs8079387
rs10425426
rs113047054
All − MR Egger
All − Inverse variance weighted
−1
0
1
2
3
4
MR effect size for
'FSC−A on NKT ' on 'Diabetic nephropathy'

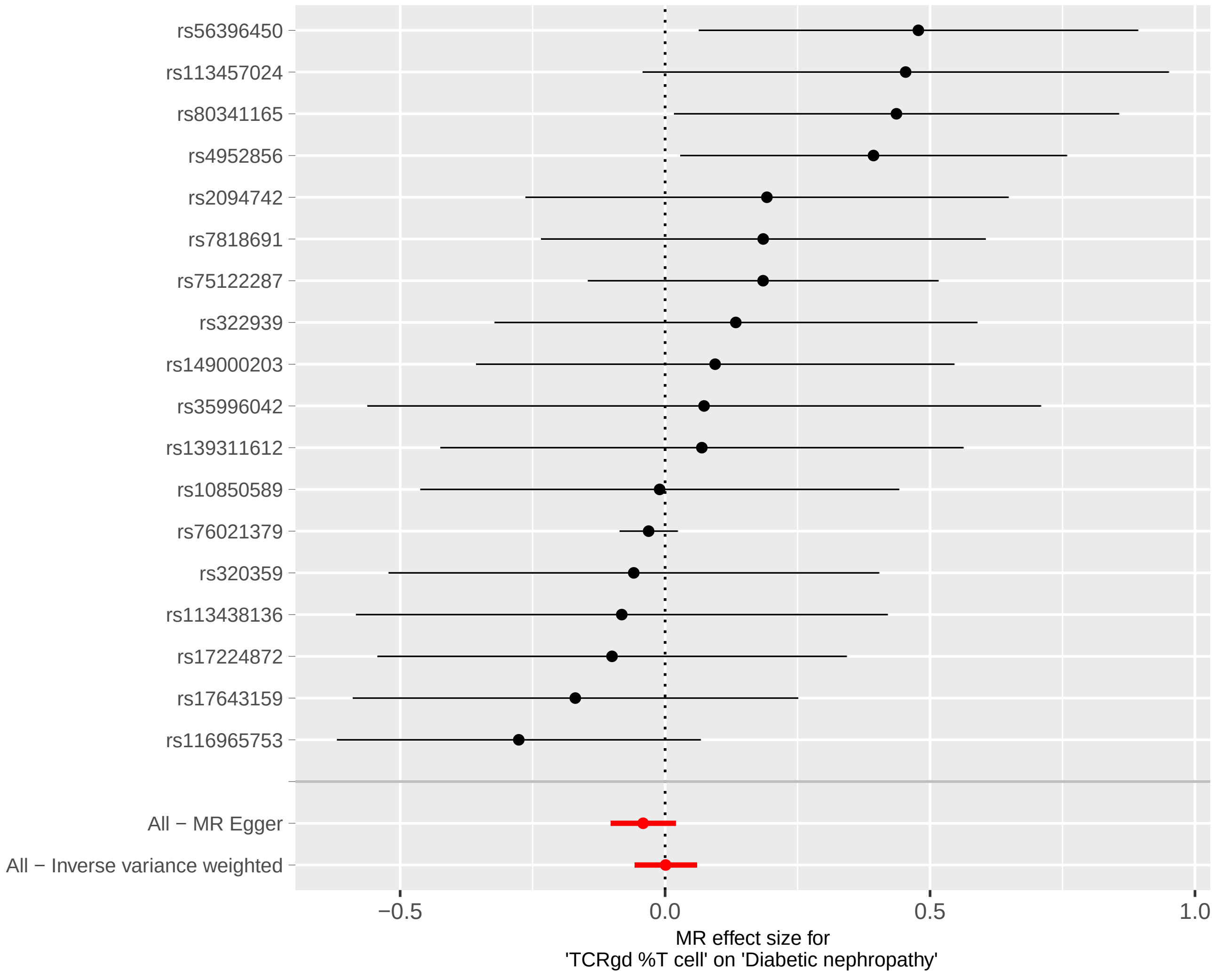
rs56396450
rs113457024
rs80341165
rs4952856
rs2094742
rs7818691
rs75122287
rs322939
rs149000203
rs35996042
rs139311612
rs10850589
rs76021379
rs320359
rs113438136
rs17224872
rs17643159
rs116965753
All − MR Egger
All − Inverse variance weighted
−0.5
0.0
0.5
1.0
MR effect size for
'TCRgd %T cell' on 'Diabetic nephropathy'

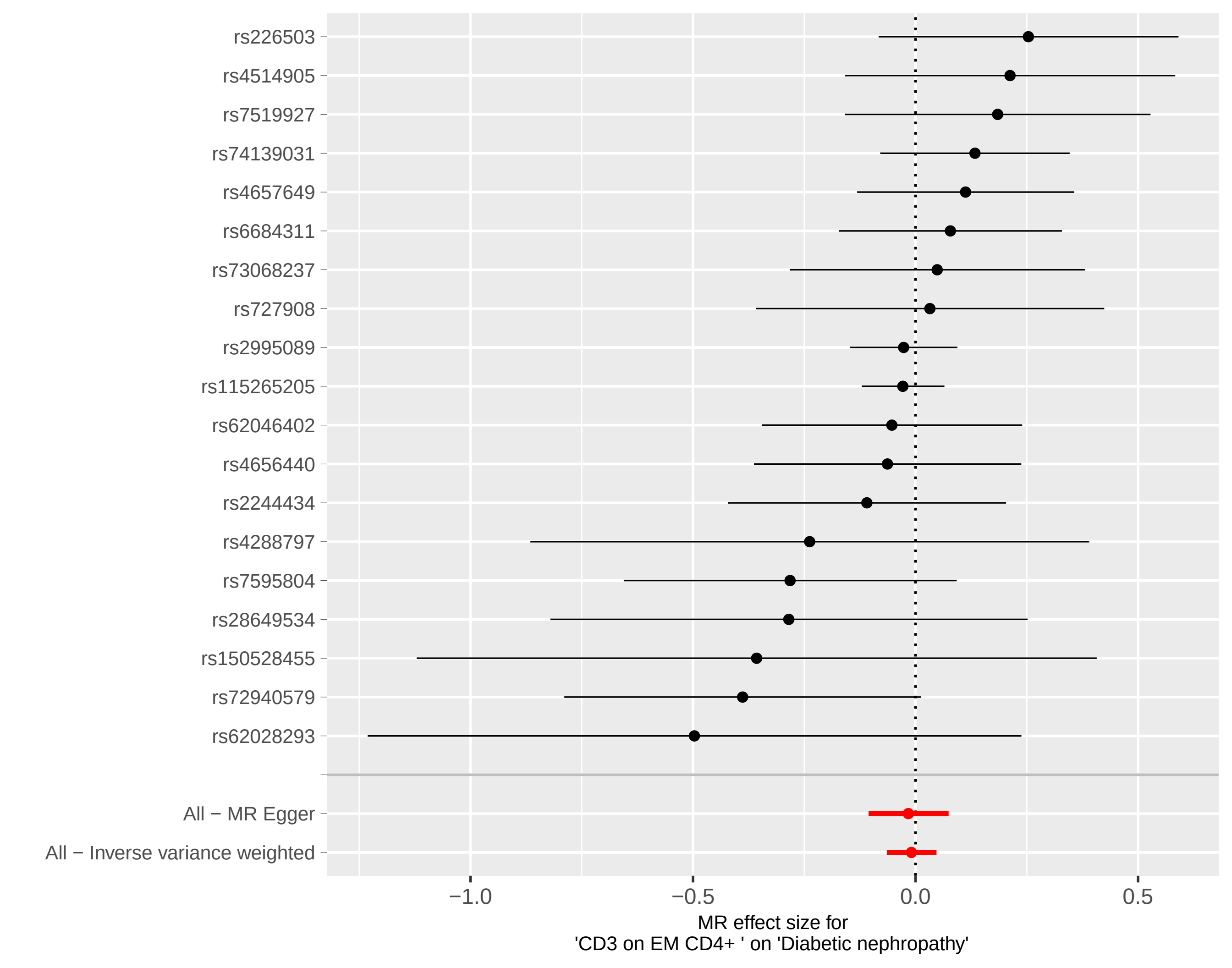
rs226503
rs4514905
rs7519927
rs74139031
rs4657649
rs6684311
rs73068237
rs727908
rs2995089
rs115265205
rs62046402
rs4656440
rs2244434
rs4288797
rs7595804
rs28649534
rs150528455
rs72940579
rs62028293
All − MR Egger
All − Inverse variance weighted
−1.0
−0.5
0.0
0.5
MR effect size for
'CD3 on EM CD4+ ' on 'Diabetic nephropathy'

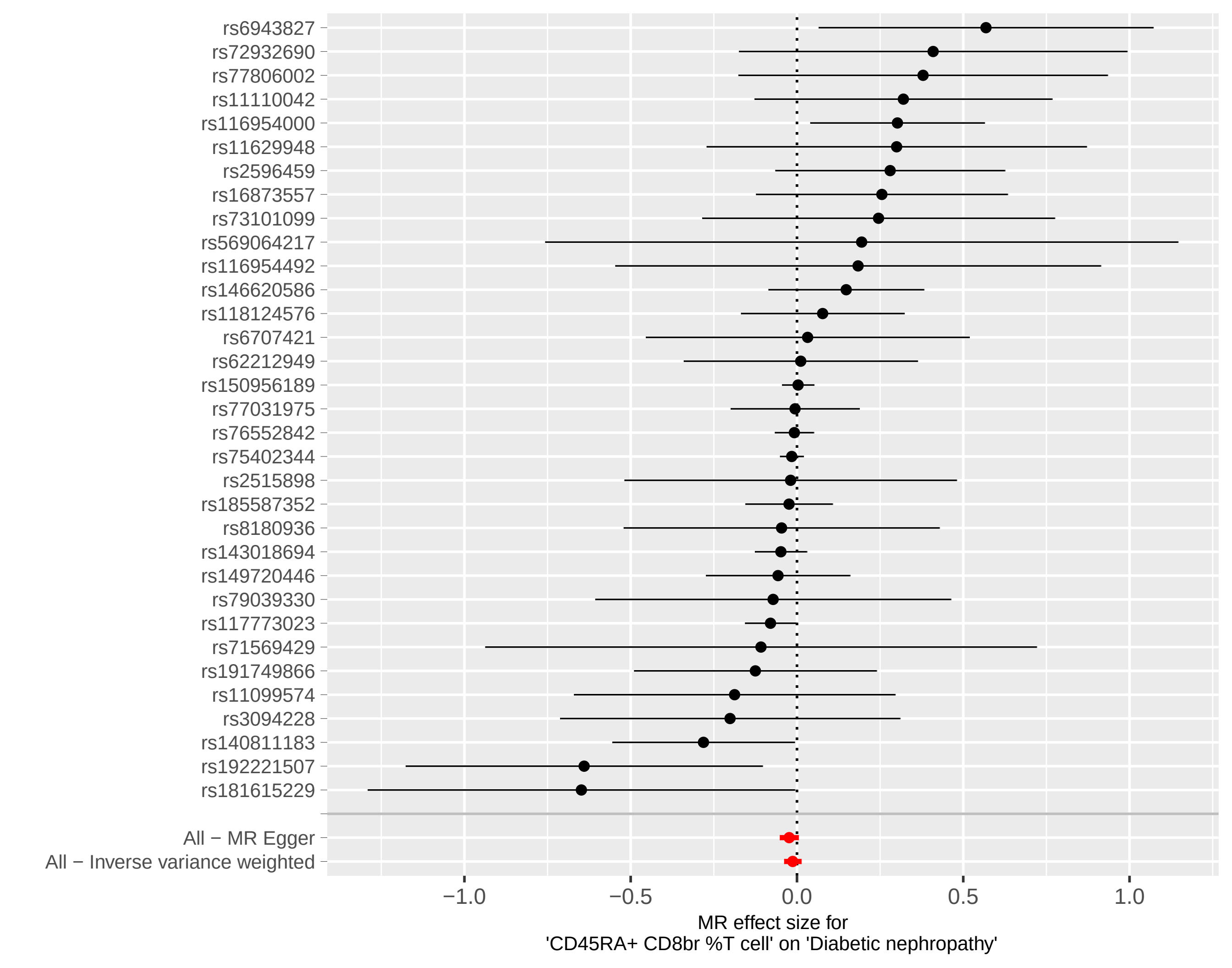
rs6943827
rs72932690
rs77806002
rs11110042
rs116954000
rs11629948
rs2596459
rs16873557
rs73101099
rs569064217
rs116954492
rs146620586
rs118124576
rs6707421
rs62212949
rs150956189
rs77031975
rs76552842
rs75402344
rs2515898
rs185587352
rs8180936
rs143018694
rs149720446
rs79039330
rs117773023
rs71569429
rs191749866
rs11099574
rs3094228
rs140811183
rs192221507
rs181615229
All − MR Egger
All − Inverse variance weighted
−1.0
−0.5
0.0
0.5
1.0
MR effect size for
'CD45RA+ CD8br %T cell' on 'Diabetic nephropathy'

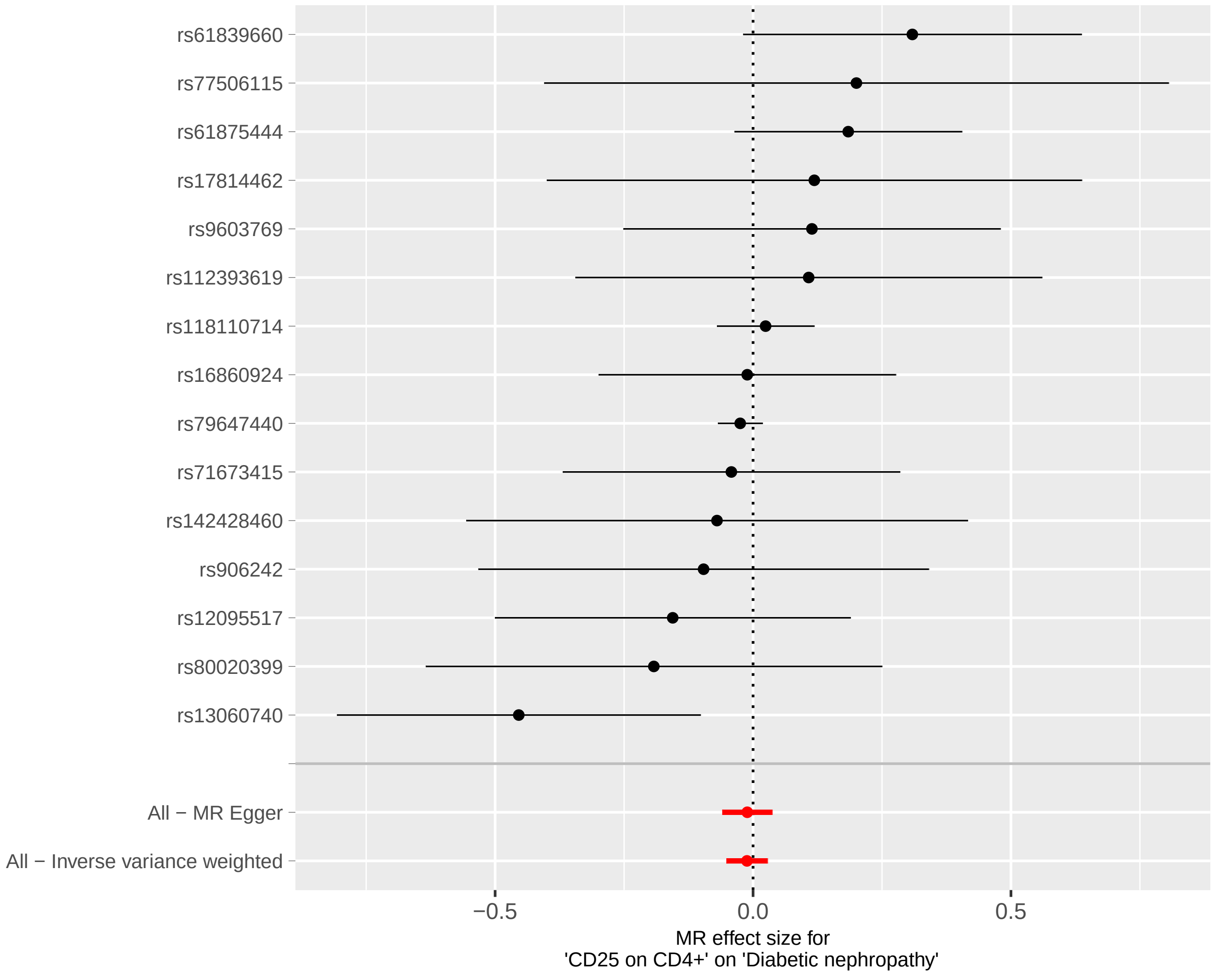

rs61839660
rs77506115
rs61875444
rs17814462
rs9603769
rs112393619
rs118110714
rs16860924
rs79647440
rs71673415
rs142428460
rs906242
rs12095517
rs80020399
rs13060740
All − MR Egger
All − Inverse variance weighted
−0.5
0.0
0.5
MR effect size for
'CD25 on CD4+' on 'Diabetic nephropathy'

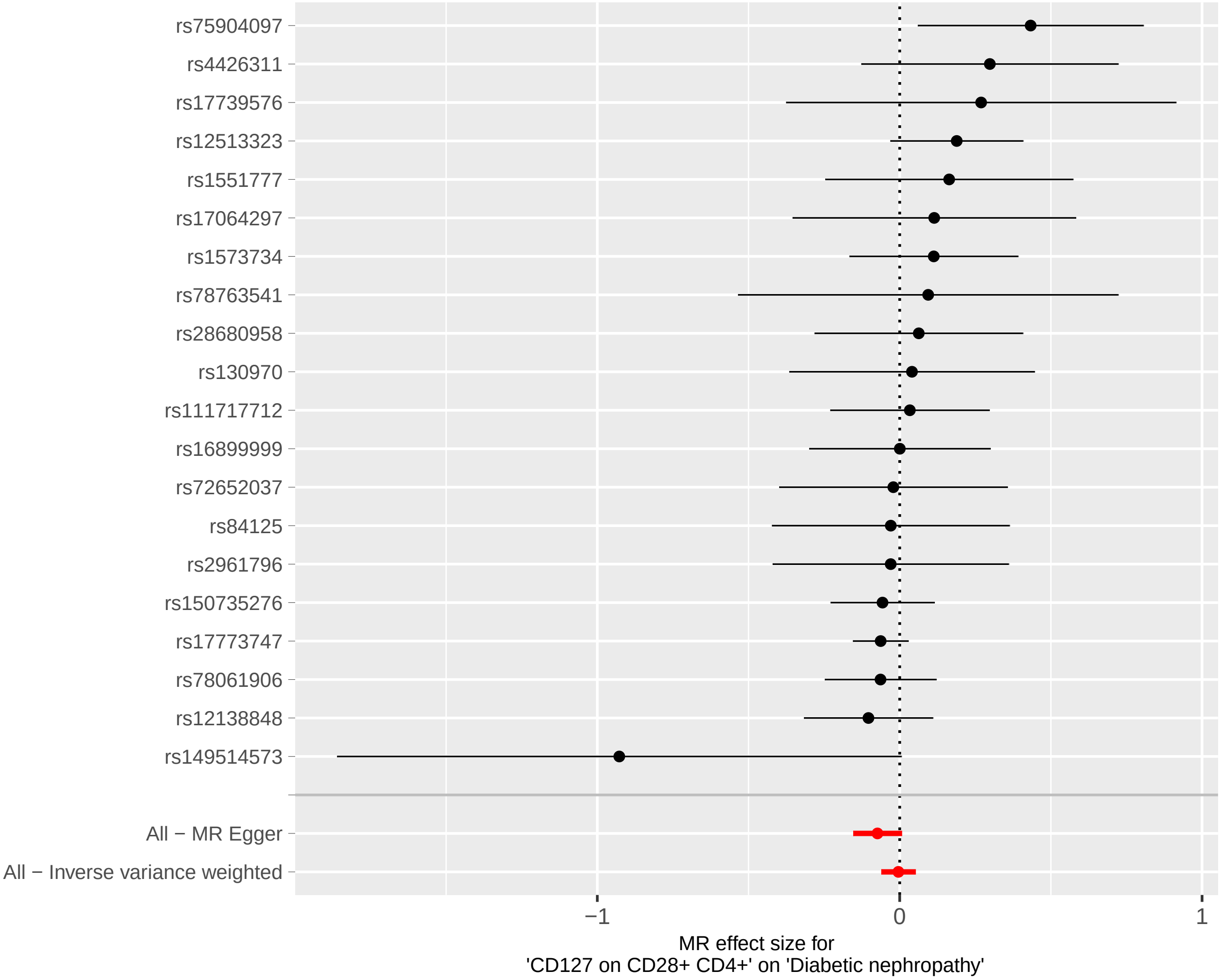
rs75904097
rs4426311
rs17739576
rs12513323
rs1551777
rs17064297
rs1573734
rs78763541
rs28680958
rs130970
rs111717712
rs16899999
rs72652037
rs84125
rs2961796
rs150735276
rs17773747
rs78061906
rs12138848
rs149514573
All − MR Egger
All − Inverse variance weighted
−1
0
1
MR effect size for
'CD127 on CD28+ CD4+' on 'Diabetic nephropathy'

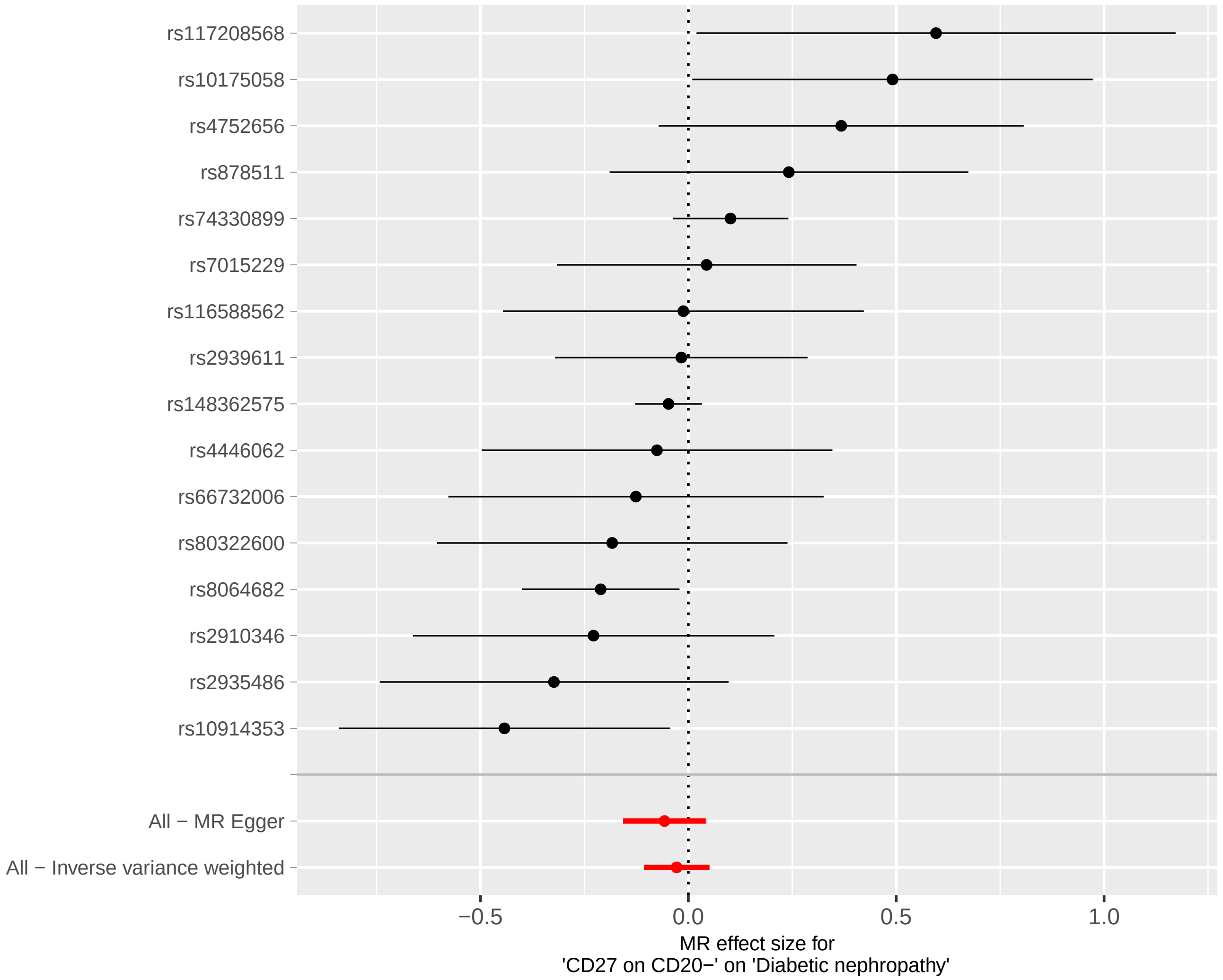
rs117208568
rs10175058
rs4752656
rs878511
rs74330899
rs7015229
rs116588562
rs2939611
rs148362575
rs4446062
rs66732006
rs80322600
rs8064682
rs2910346
rs2935486
rs10914353
All − MR Egger
All − Inverse variance weighted
−0.5
0.0
0.5
1.0
MR effect size for
'CD27 on CD20−' on 'Diabetic nephropathy'

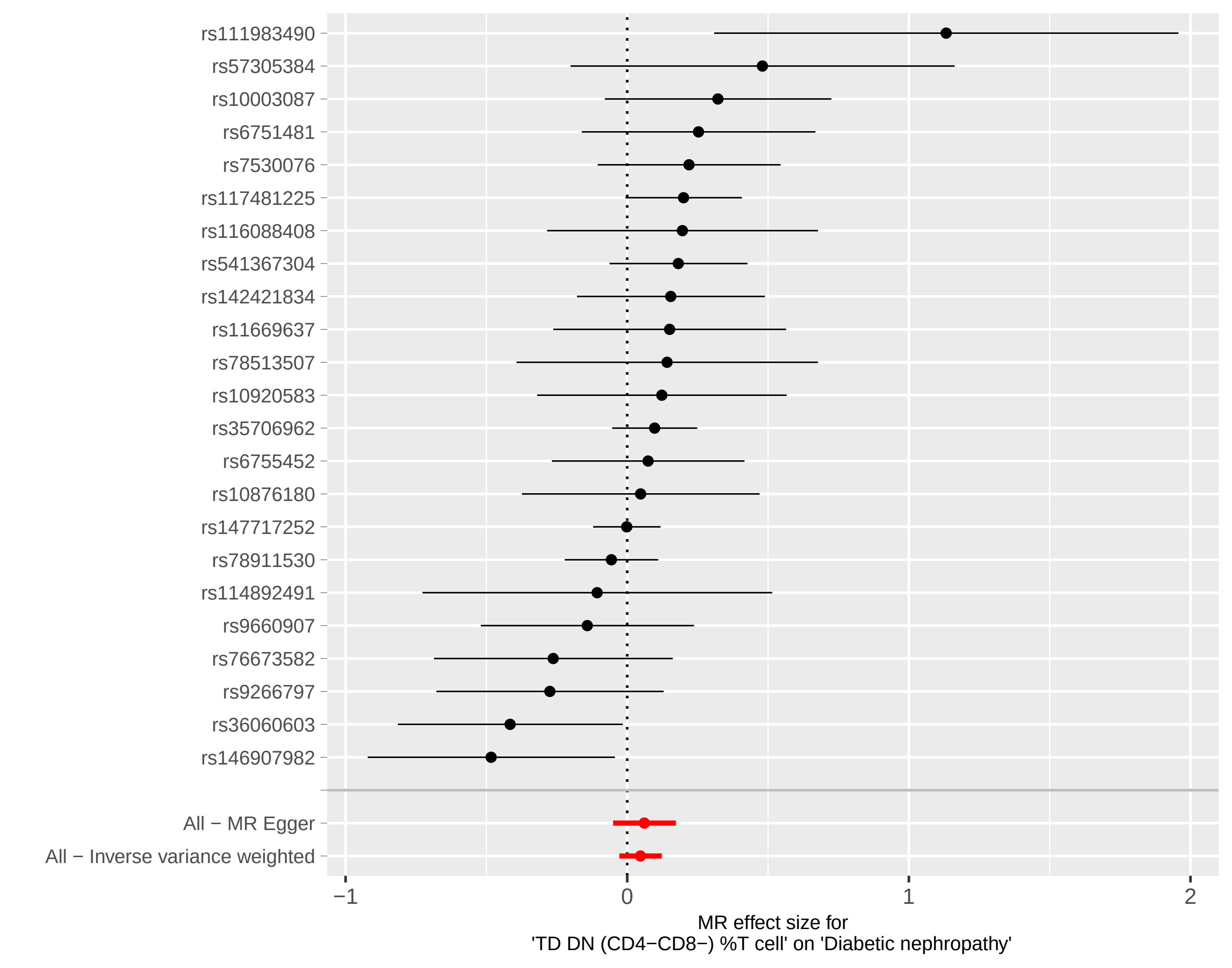
rs111983490
rs57305384
rs10003087
rs6751481
rs7530076
rs117481225
rs116088408
rs541367304
rs142421834
rs11669637
rs78513507
rs10920583
rs35706962
rs6755452
rs10876180
rs147717252
rs78911530
rs114892491
rs9660907
rs76673582
rs9266797
rs36060603
rs146907982
All − MR Egger
All − Inverse variance weighted
−1
0
1
2
MR effect size for
'TD DN (CD4−CD8−) %T cell' on 'Diabetic nephropathy'

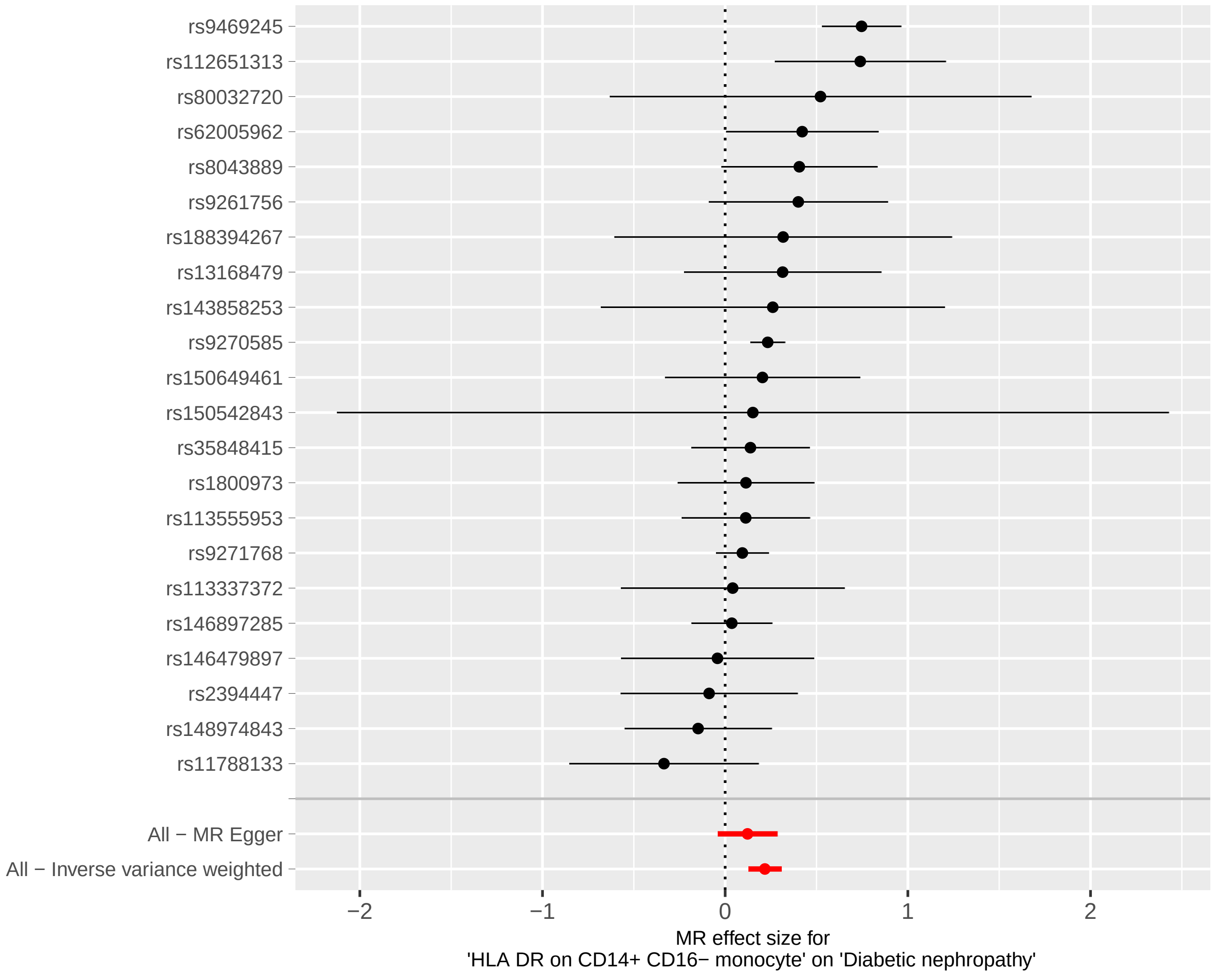

rs9469245
rs112651313
rs80032720
rs62005962
rs8043889
rs9261756
rs188394267
rs13168479
rs143858253
rs9270585
rs150649461
rs150542843
rs35848415
rs1800973
rs113555953
rs9271768
rs113337372
rs146897285
rs146479897
rs2394447
rs148974843
rs11788133
All − MR Egger
All − Inverse variance weighted
−2
−1
0
1
2
MR effect size for
'HLA DR on CD14+ CD16− monocyte' on 'Diabetic nephropathy'

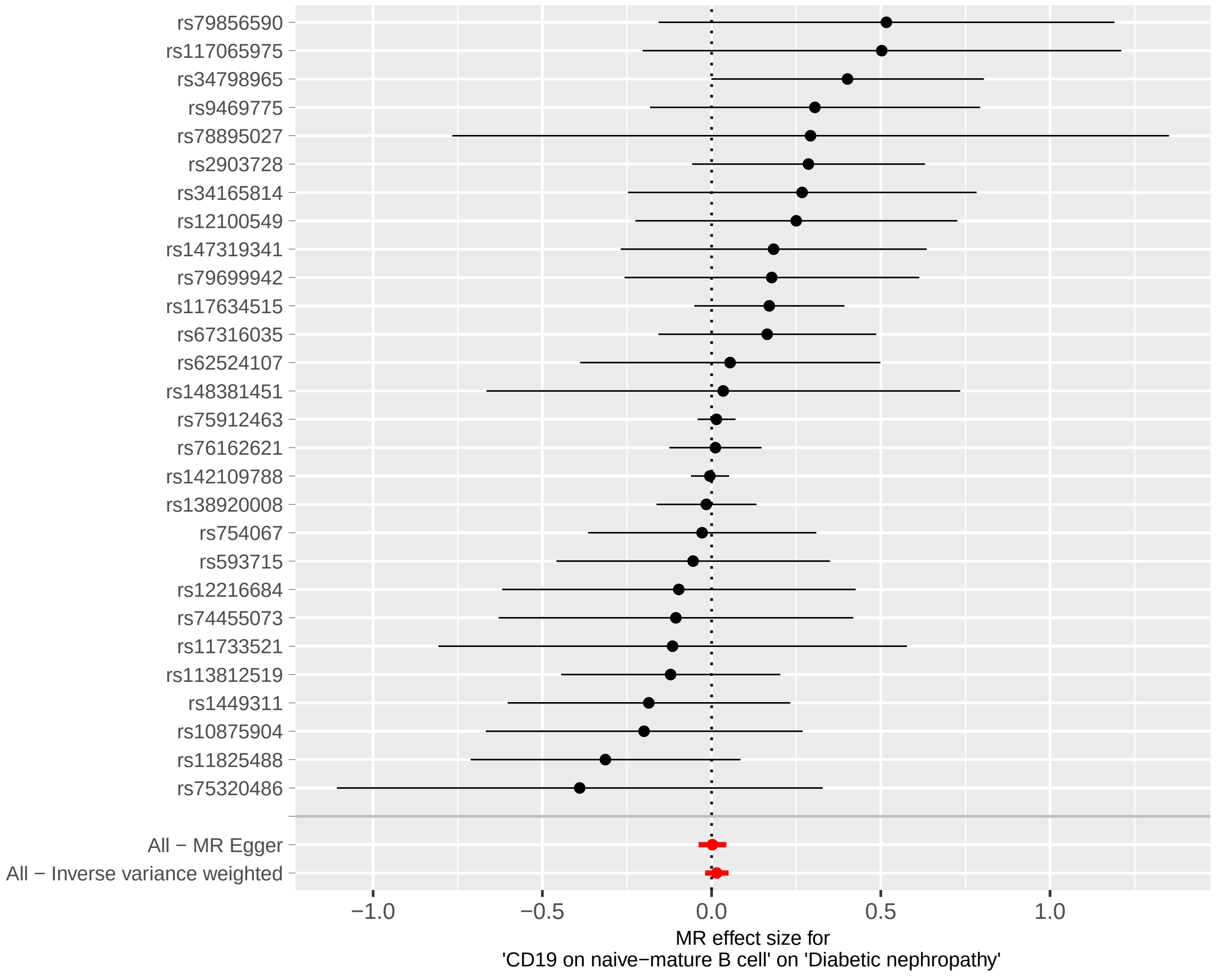

rs79856590
rs117065975
rs34798965
rs9469775
rs78895027
rs2903728
rs34165814
rs12100549
rs147319341
rs79699942
rs117634515
rs67316035
rs62524107
rs148381451
rs75912463
rs76162621
rs142109788
rs138920008
rs754067
rs593715
rs12216684
rs74455073
rs11733521
rs113812519
rs1449311
rs10875904
rs11825488
rs75320486
All − MR Egger
All − Inverse variance weighted
−1.0
−0.5
0.0
0.5
1.0
MR effect size for
'CD19 on naive−mature B cell' on 'Diabetic nephropathy'

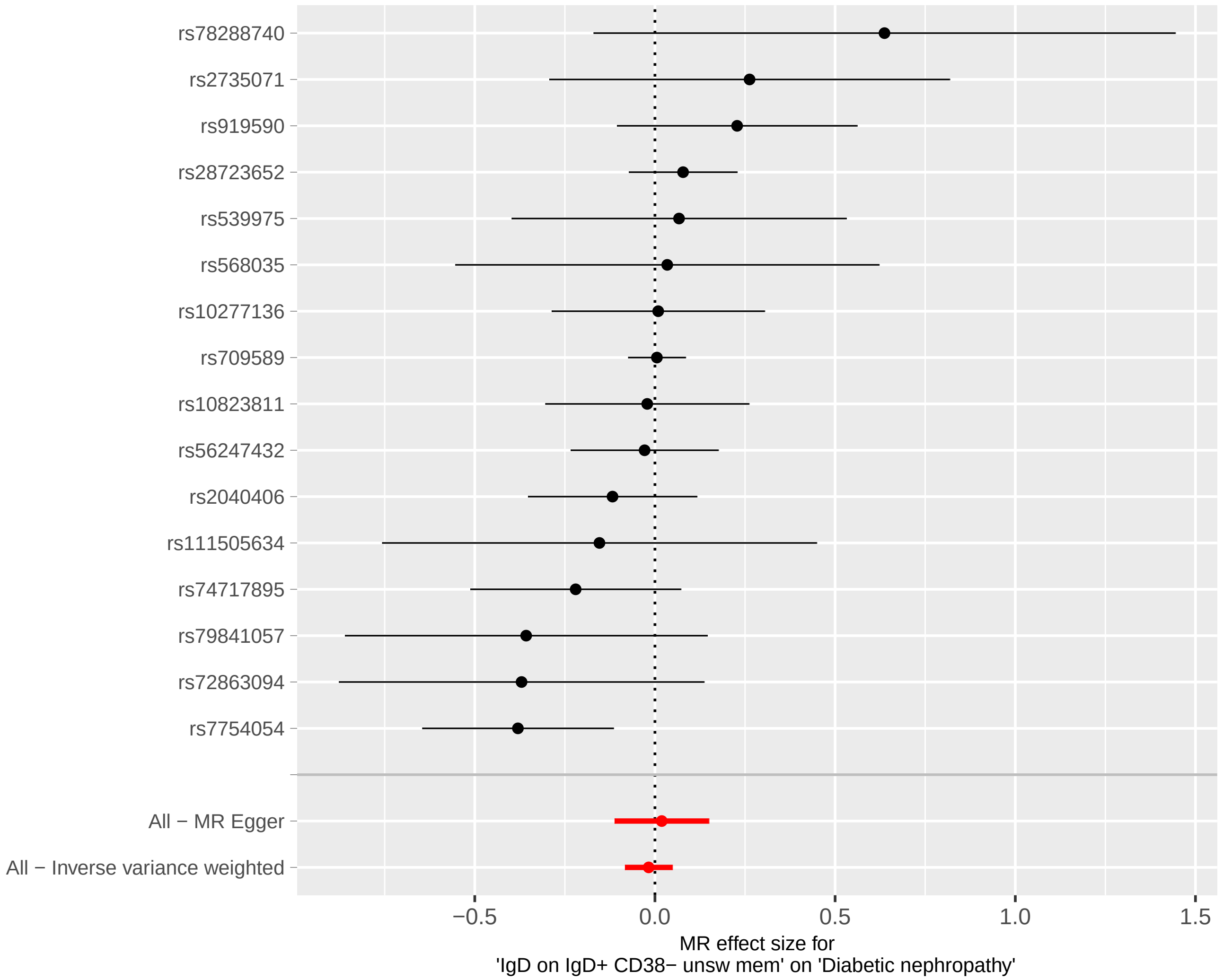
rs78288740
rs2735071
rs919590
rs28723652
rs539975
rs568035
rs10277136
rs709589
rs10823811
rs56247432
rs2040406
rs111505634
rs74717895
rs79841057
rs72863094
rs7754054
All − MR Egger
All − Inverse variance weighted
−0.5
0.0
0.5
1.0
1.5
MR effect size for
'IgD on IgD+ CD38− unsw mem' on 'Diabetic nephropathy'

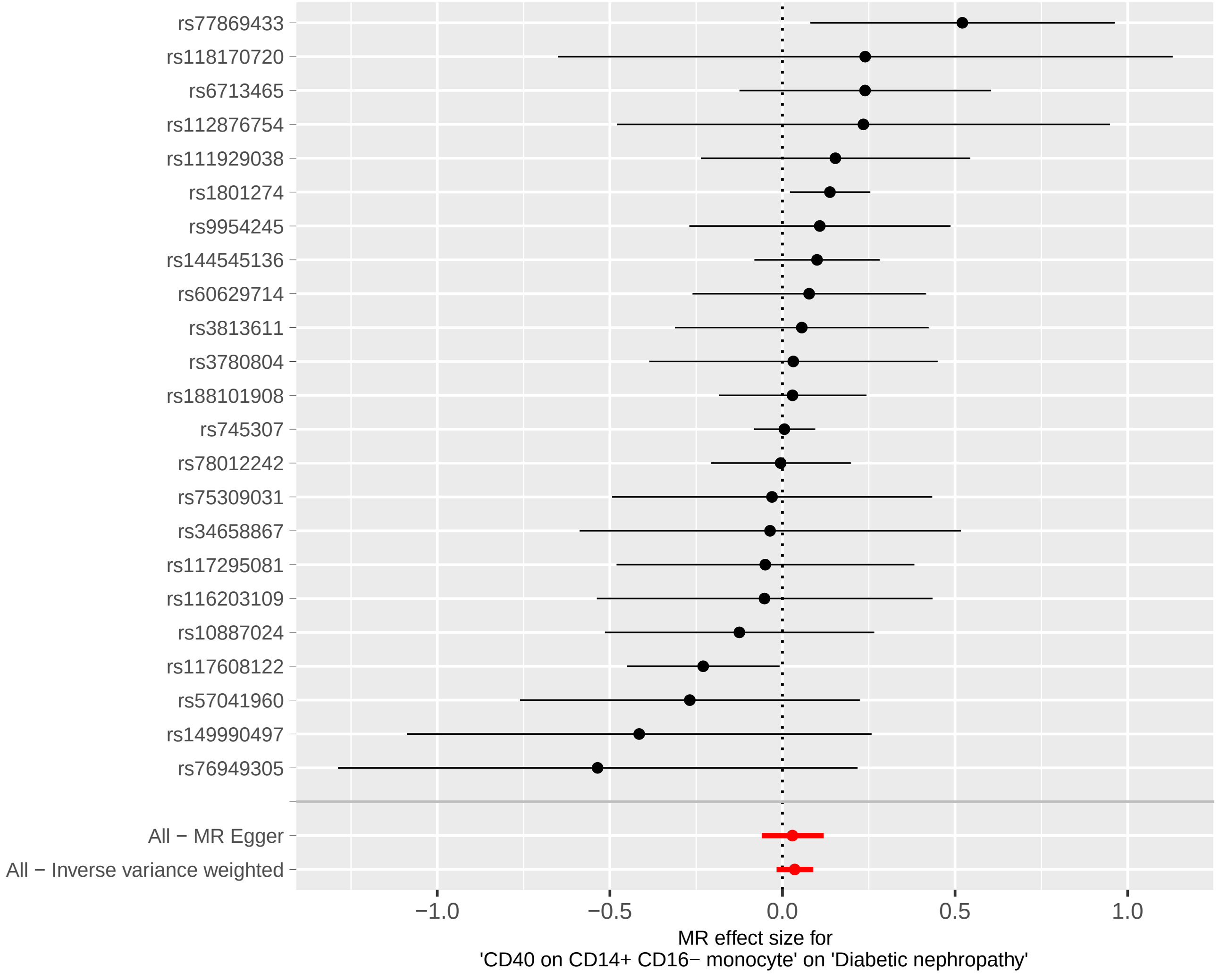

rs77869433
rs118170720
rs6713465
rs112876754
rs111929038
rs1801274
rs9954245
rs144545136
rs60629714
rs3813611
rs3780804
rs188101908
rs745307
rs78012242
rs75309031
rs34658867
rs117295081
rs116203109
rs10887024
rs117608122
rs57041960
rs149990497
rs76949305
All − MR Egger
All − Inverse variance weighted
−1.0
−0.5
0.0
0.5
1.0
MR effect size for
'CD40 on CD14+ CD16− monocyte' on 'Diabetic nephropathy'

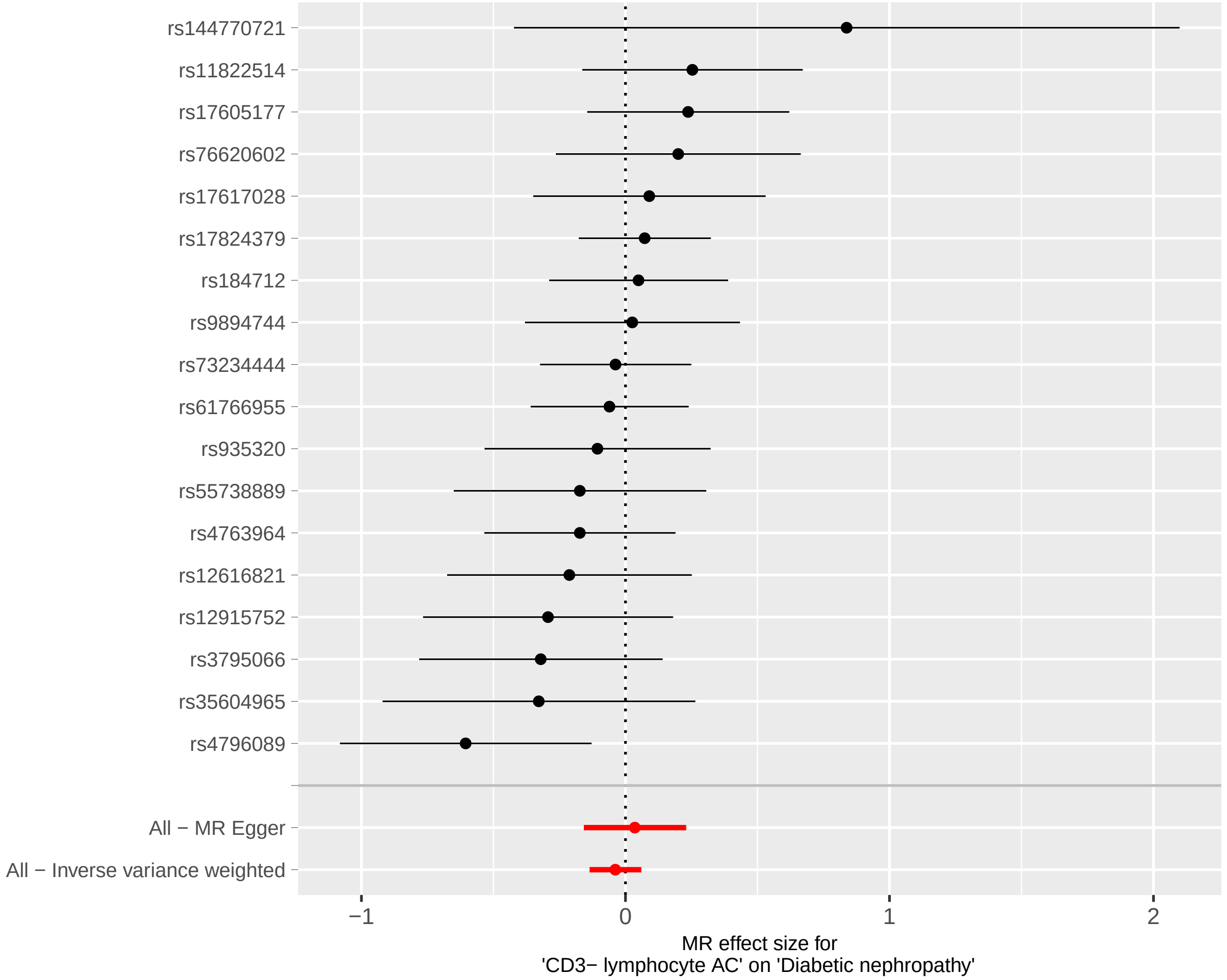
rs144770721
rs11822514
rs17605177
rs76620602
rs17617028
rs17824379
rs184712
rs9894744
rs73234444
rs61766955
rs935320
rs55738889
rs4763964
rs12616821
rs12915752
rs3795066
rs35604965
rs4796089
All − MR Egger
All − Inverse variance weighted
−1
0
1
2
MR effect size for
'CD3− lymphocyte AC' on 'Diabetic nephropathy'

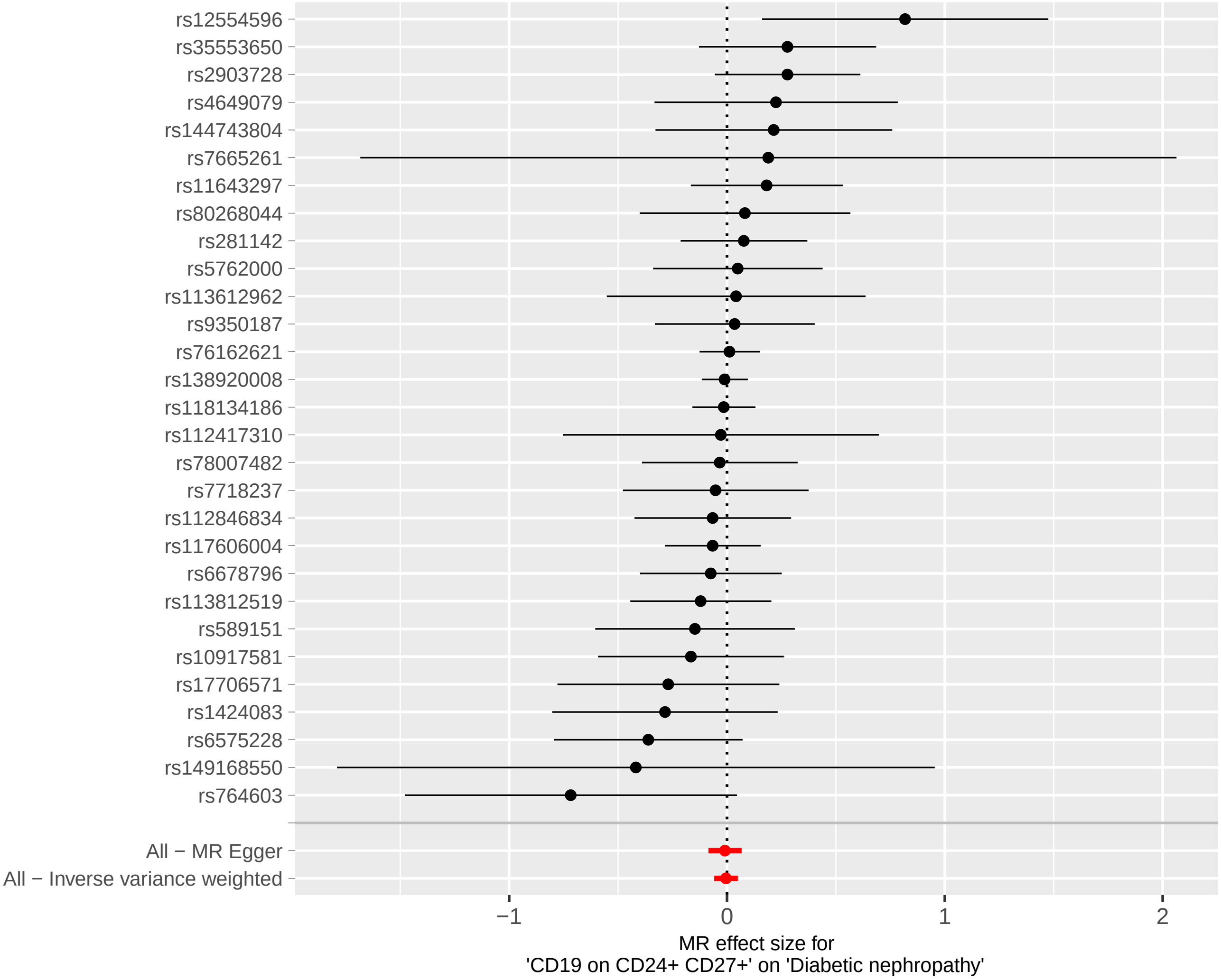

rs12554596
rs35553650
rs2903728
rs4649079
rs144743804
rs7665261
rs11643297
rs80268044
rs281142
rs5762000
rs113612962
rs9350187
rs76162621
rs138920008
rs118134186
rs112417310
rs78007482
rs7718237
rs112846834
rs117606004
rs6678796
rs113812519
rs589151
rs10917581
rs17706571
rs1424083
rs6575228
rs149168550
rs764603
All − MR Egger
All − Inverse variance weighted
−1
0
1
2
MR effect size for
'CD19 on CD24+ CD27+' on 'Diabetic nephropathy'

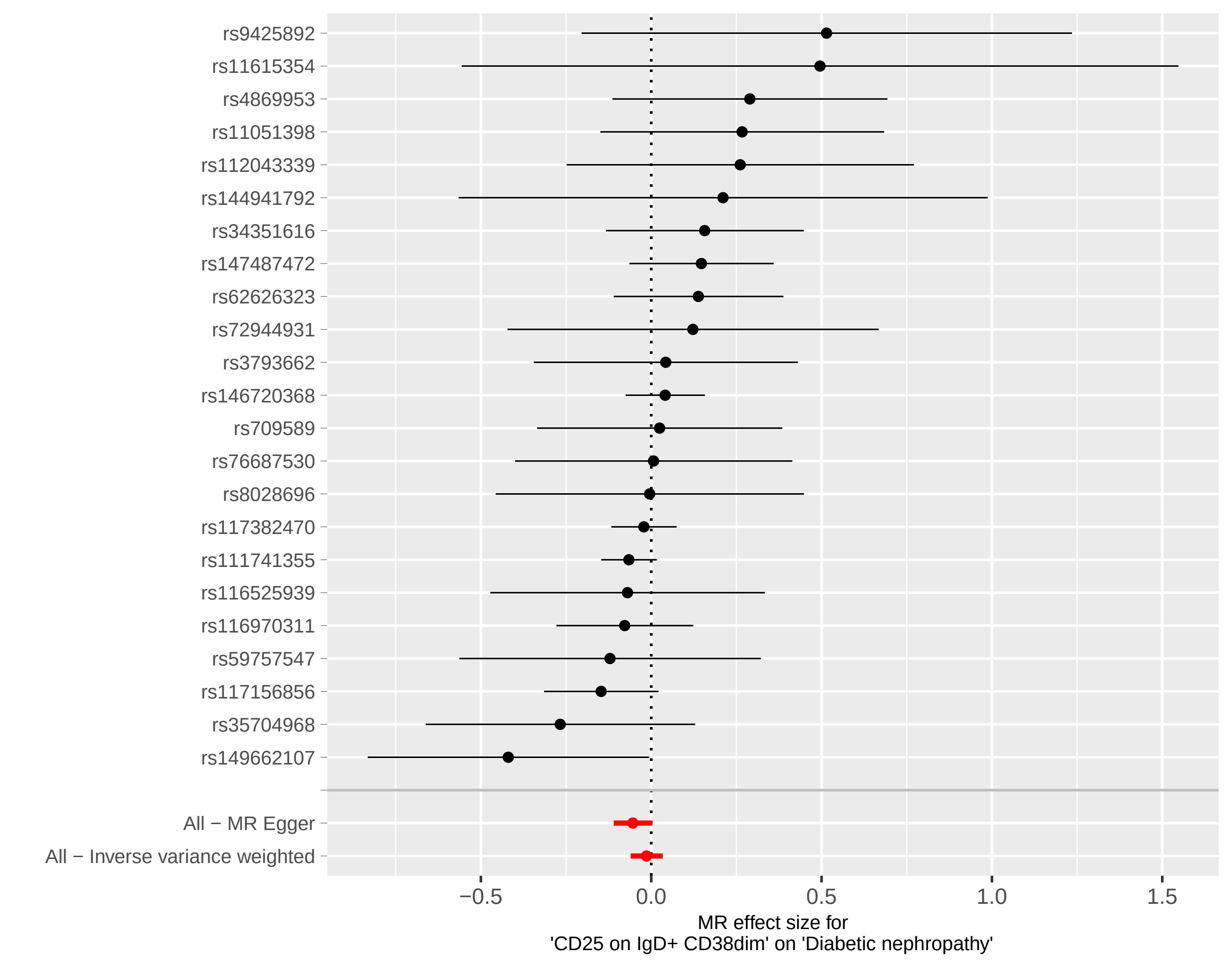
rs9425892
rs11615354
rs4869953
rs11051398
rs112043339
rs144941792
rs34351616
rs147487472
rs62626323
rs72944931
rs3793662
rs146720368
rs709589
rs76687530
rs8028696
rs117382470
rs111741355
rs116525939
rs116970311
rs59757547
rs117156856
rs35704968
rs149662107
All − MR Egger
All − Inverse variance weighted
−0.5
0.0
0.5
1.0
1.5
MR effect size for
'CD25 on IgD+ CD38dim' on 'Diabetic nephropathy'

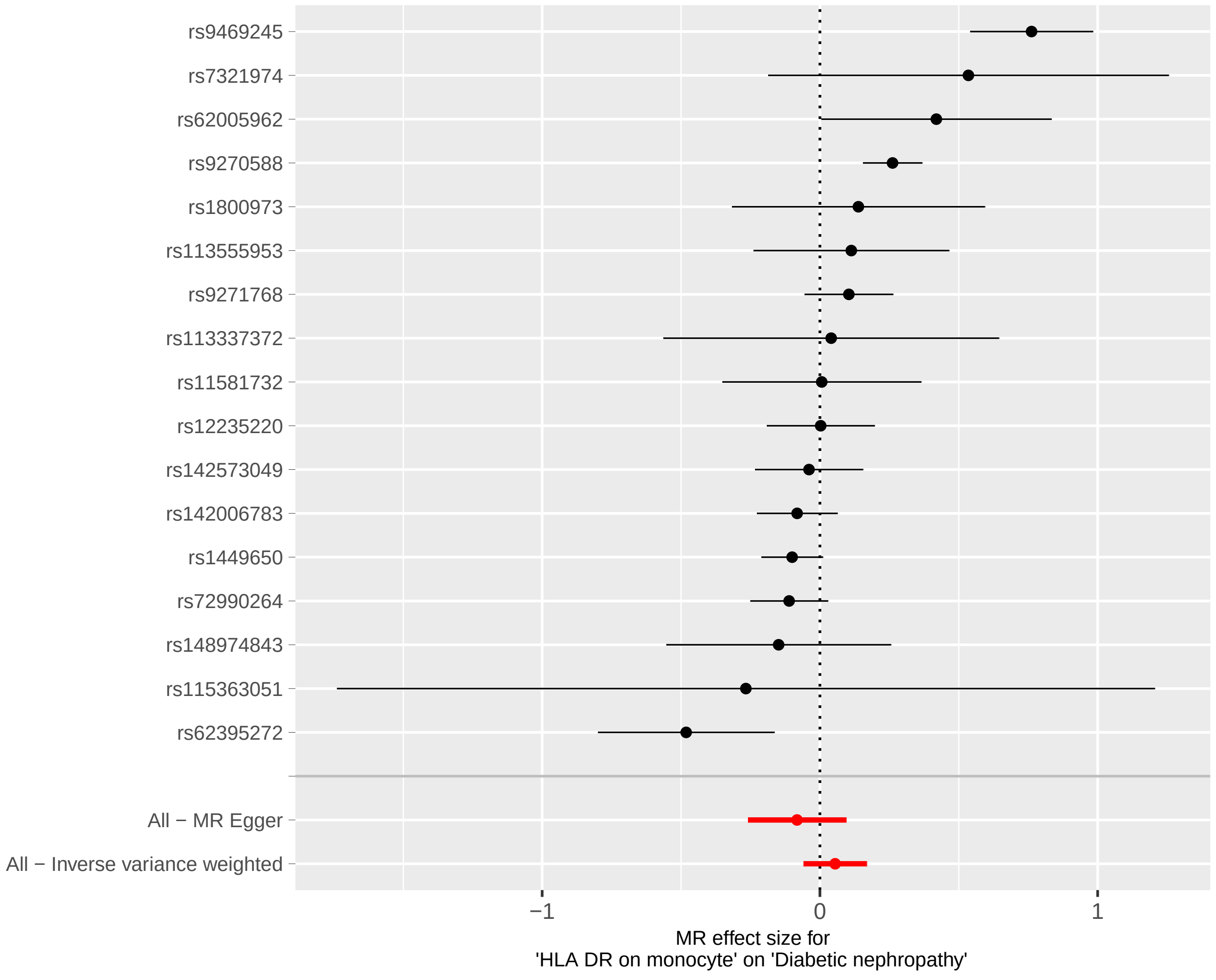
rs9469245
rs7321974
rs62005962
rs9270588
rs1800973
rs113555953
rs9271768
rs113337372
rs11581732
rs12235220
rs142573049
rs142006783
rs1449650
rs72990264
rs148974843
rs115363051
rs62395272
All − MR Egger
All − Inverse variance weighted
−1
0
1
MR effect size for
'HLA DR on monocyte' on 'Diabetic nephropathy'

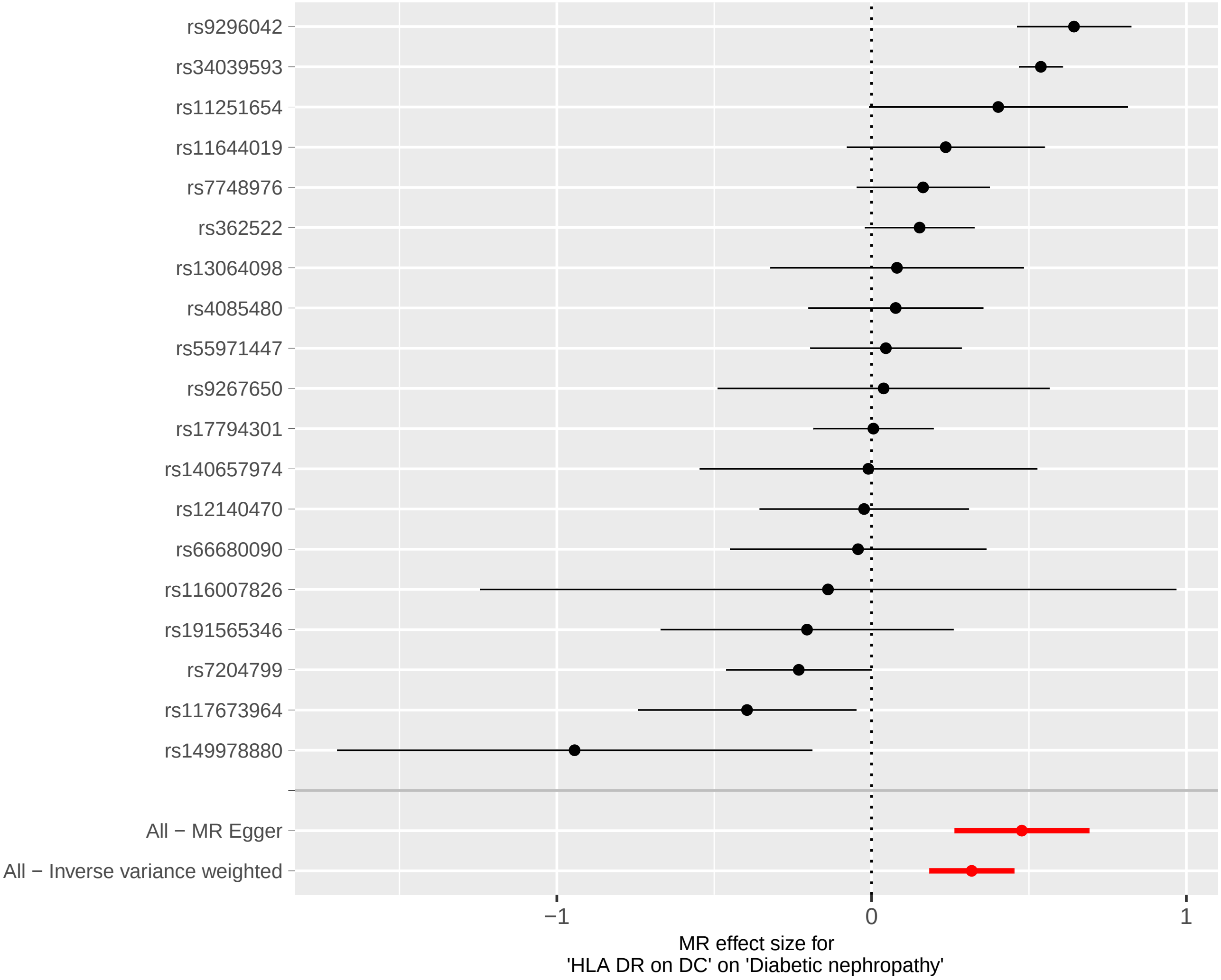

rs9296042
rs34039593
rs11251654
rs11644019
rs7748976
rs362522
rs13064098
rs4085480
rs55971447
rs9267650
rs17794301
rs140657974
rs12140470
rs66680090
rs116007826
rs191565346
rs7204799
rs117673964
rs149978880
All − MR Egger
All − Inverse variance weighted
−1
0
1
MR effect size for
'HLA DR on DC' on 'Diabetic nephropathy'

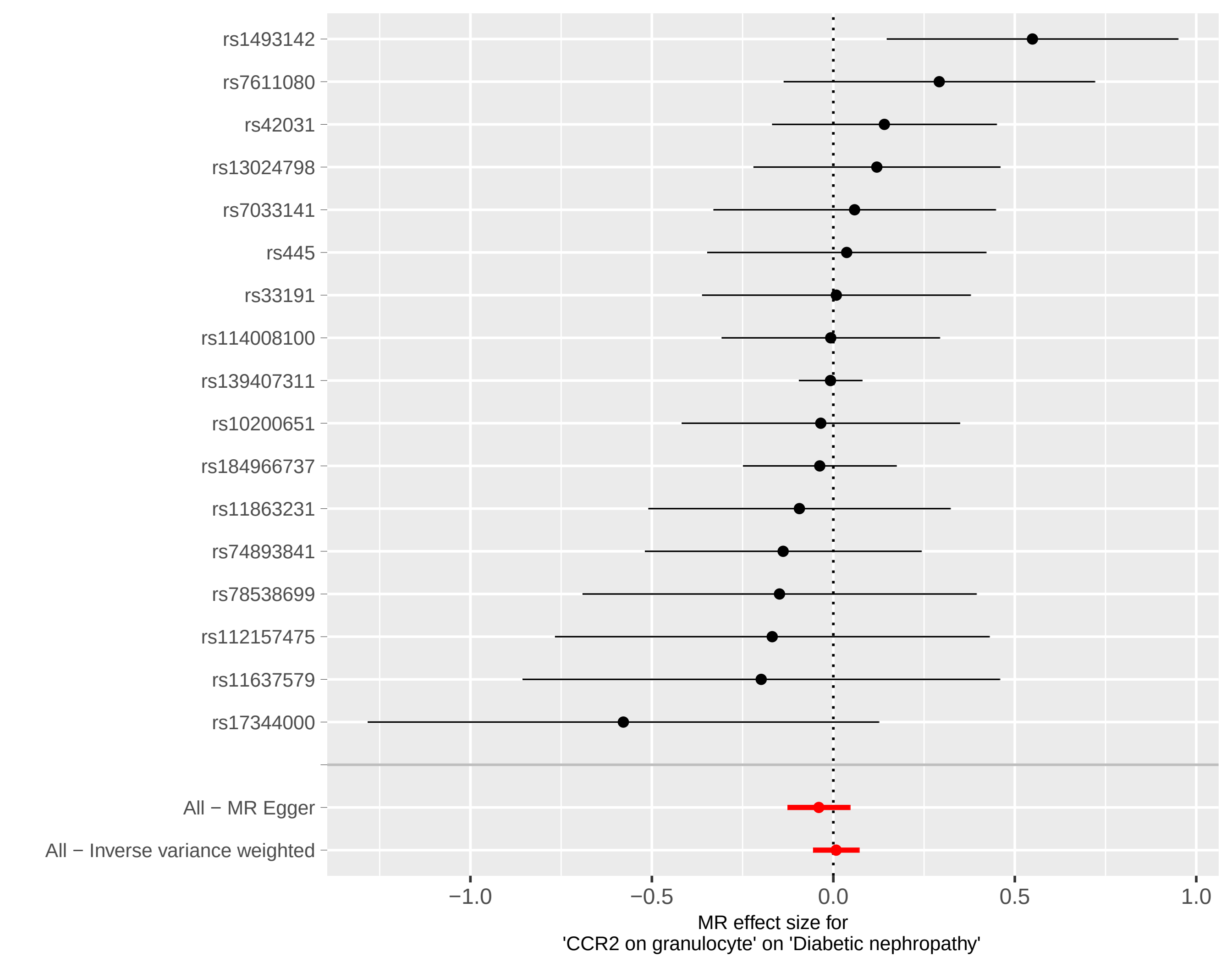
rs1493142
rs7611080
rs42031
rs13024798
rs7033141
rs445
rs33191
rs114008100
rs139407311
rs10200651
rs184966737
rs11863231
rs74893841
rs78538699
rs112157475
rs11637579
rs17344000
All − MR Egger
All − Inverse variance weighted
−1.0
−0.5
0.0
0.5
1.0
MR effect size for
'CCR2 on granulocyte' on 'Diabetic nephropathy'

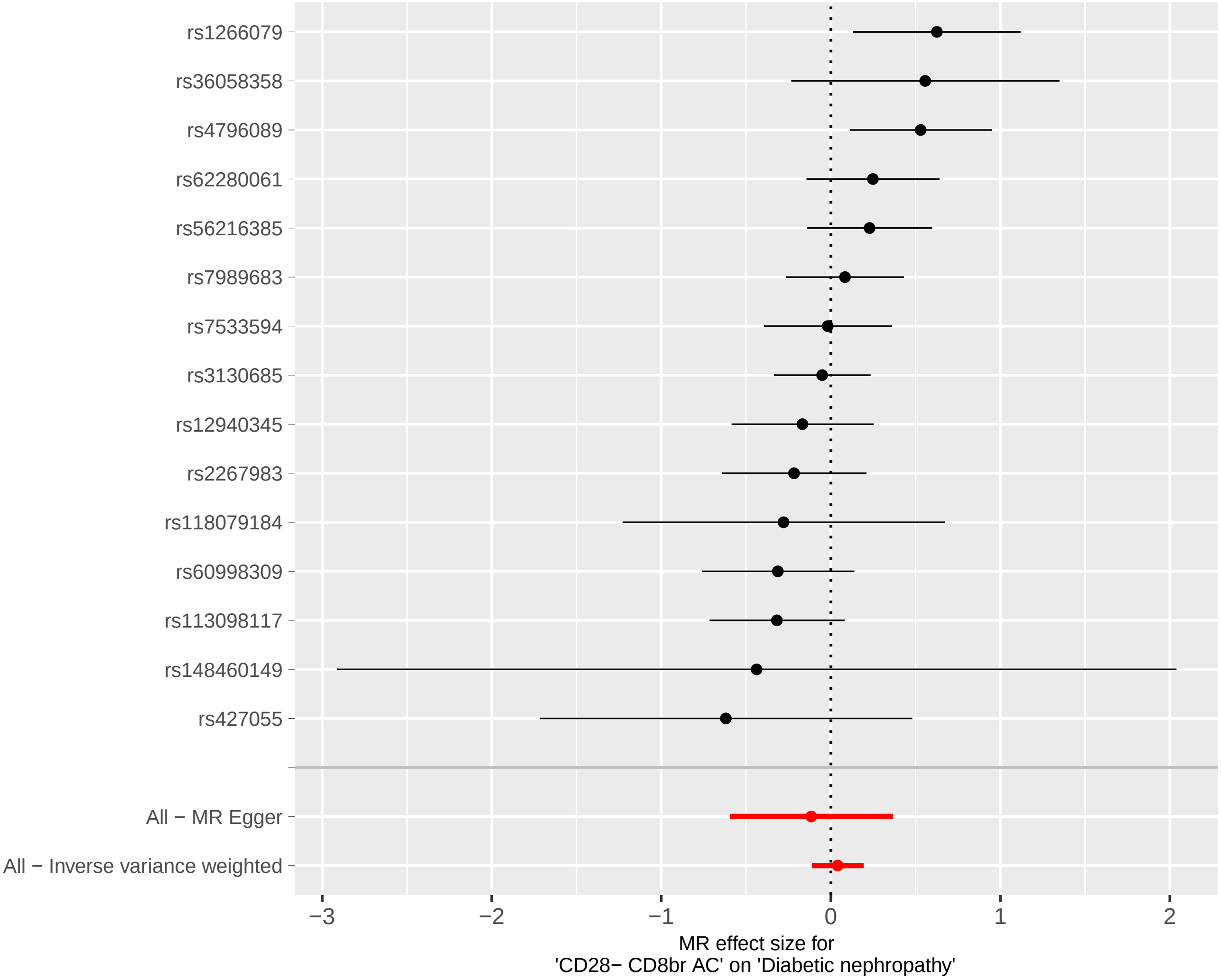
rs1266079
rs36058358
rs4796089
rs62280061
rs56216385
rs7989683
rs7533594
rs3130685
rs12940345
rs2267983
rs118079184
rs60998309
rs113098117
rs148460149
rs427055
All − MR Egger
All − Inverse variance weighted
−3
−2
−1
0
1
2
MR effect size for
'CD28− CD8br AC' on 'Diabetic nephropathy'

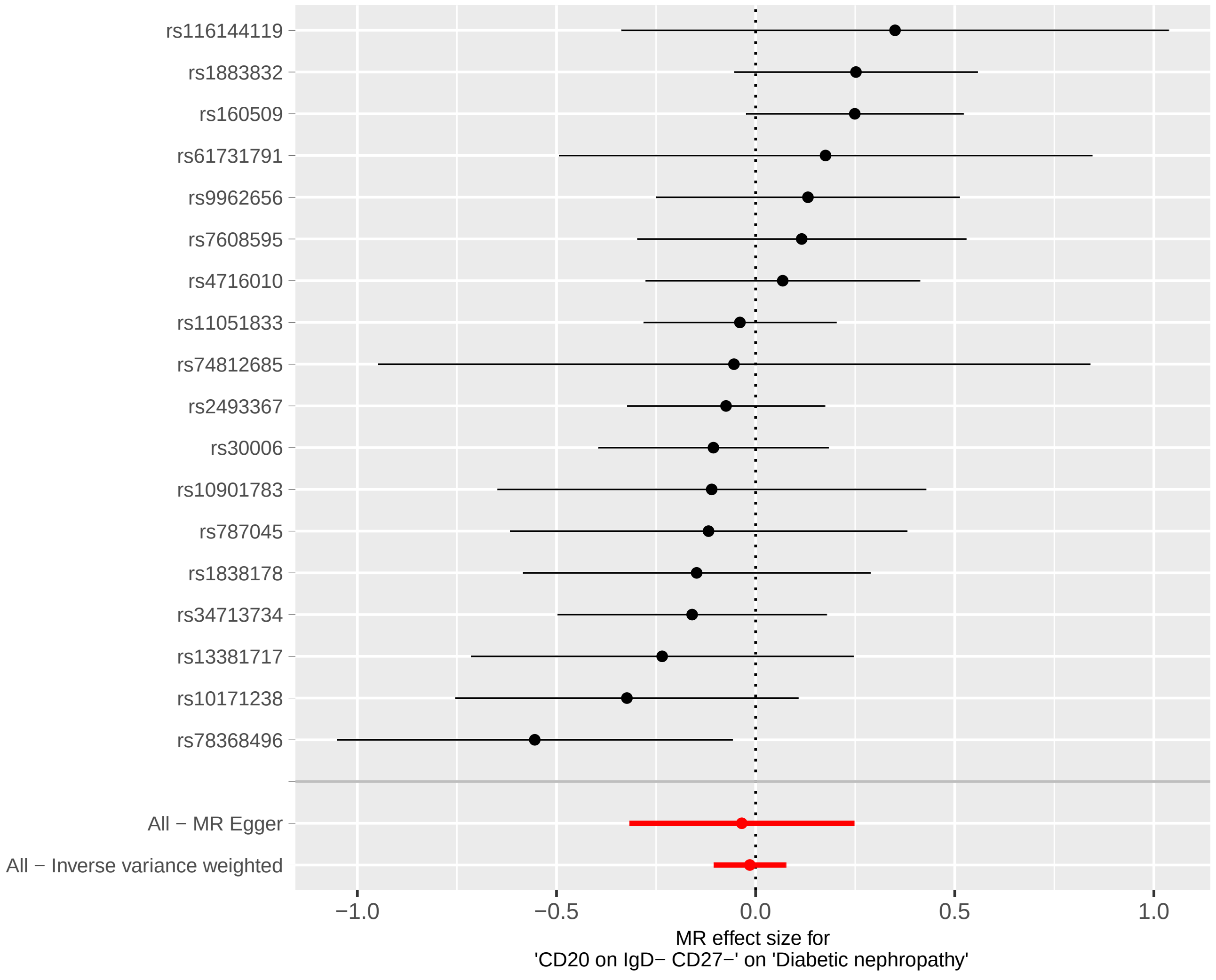

rs116144119
rs1883832
rs160509
rs61731791
rs9962656
rs7608595
rs4716010
rs11051833
rs74812685
rs2493367
rs30006
rs10901783
rs787045
rs1838178
rs34713734
rs13381717
rs10171238
rs78368496
All − MR Egger
All − Inverse variance weighted
−1.0
−0.5
0.0
0.5
1.0
MR effect size for
'CD20 on IgD− CD27−' on 'Diabetic nephropathy'

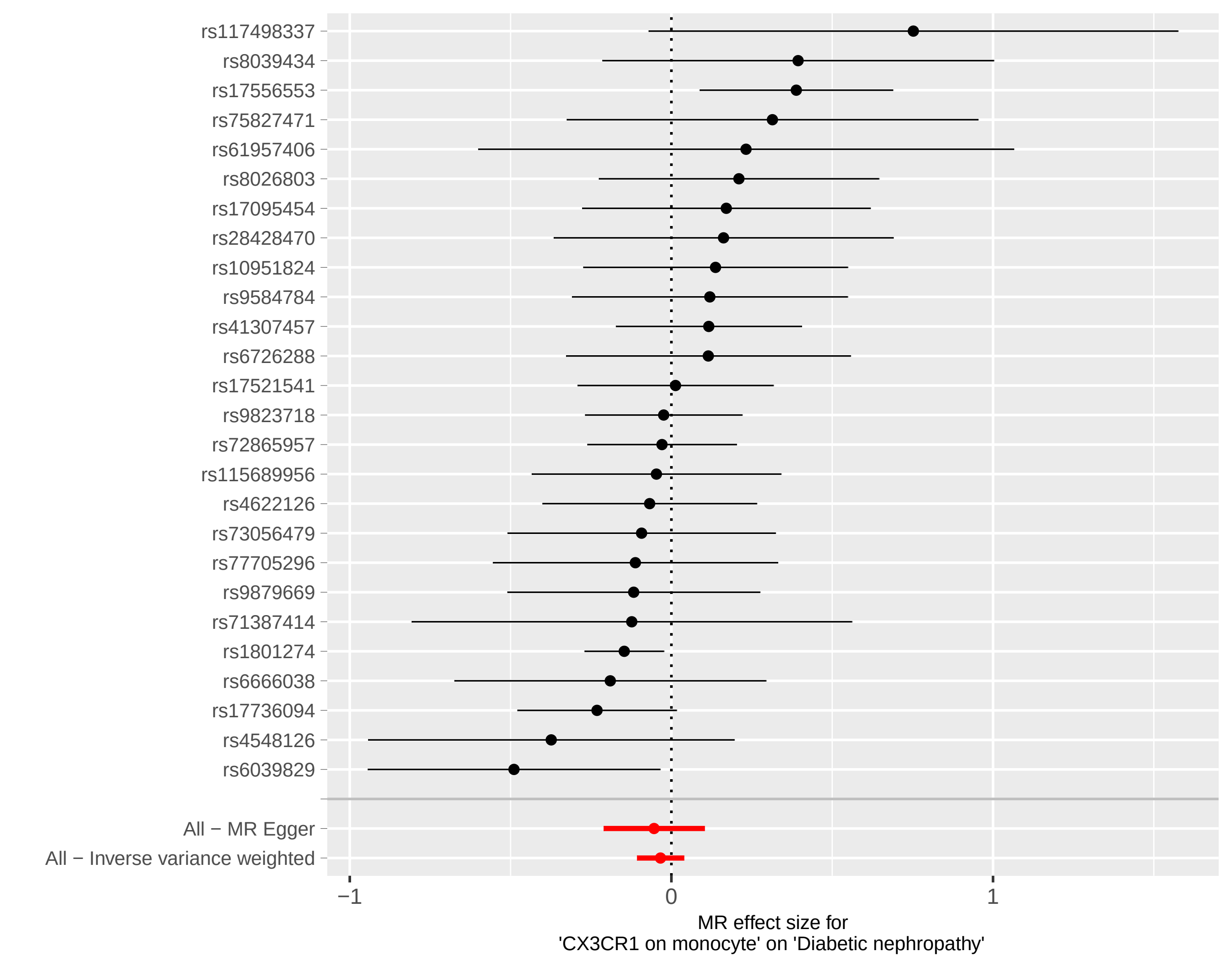

rs117498337
rs8039434
rs17556553
rs75827471
rs61957406
rs8026803
rs17095454
rs28428470
rs10951824
rs9584784
rs41307457
rs6726288
rs17521541
rs9823718
rs72865957
rs115689956
rs4622126
rs73056479
rs77705296
rs9879669
rs71387414
rs1801274
rs6666038
rs17736094
rs4548126
rs6039829
All − MR Egger
All − Inverse variance weighted
−1
0
1
MR effect size for
'CX3CR1 on monocyte' on 'Diabetic nephropathy'

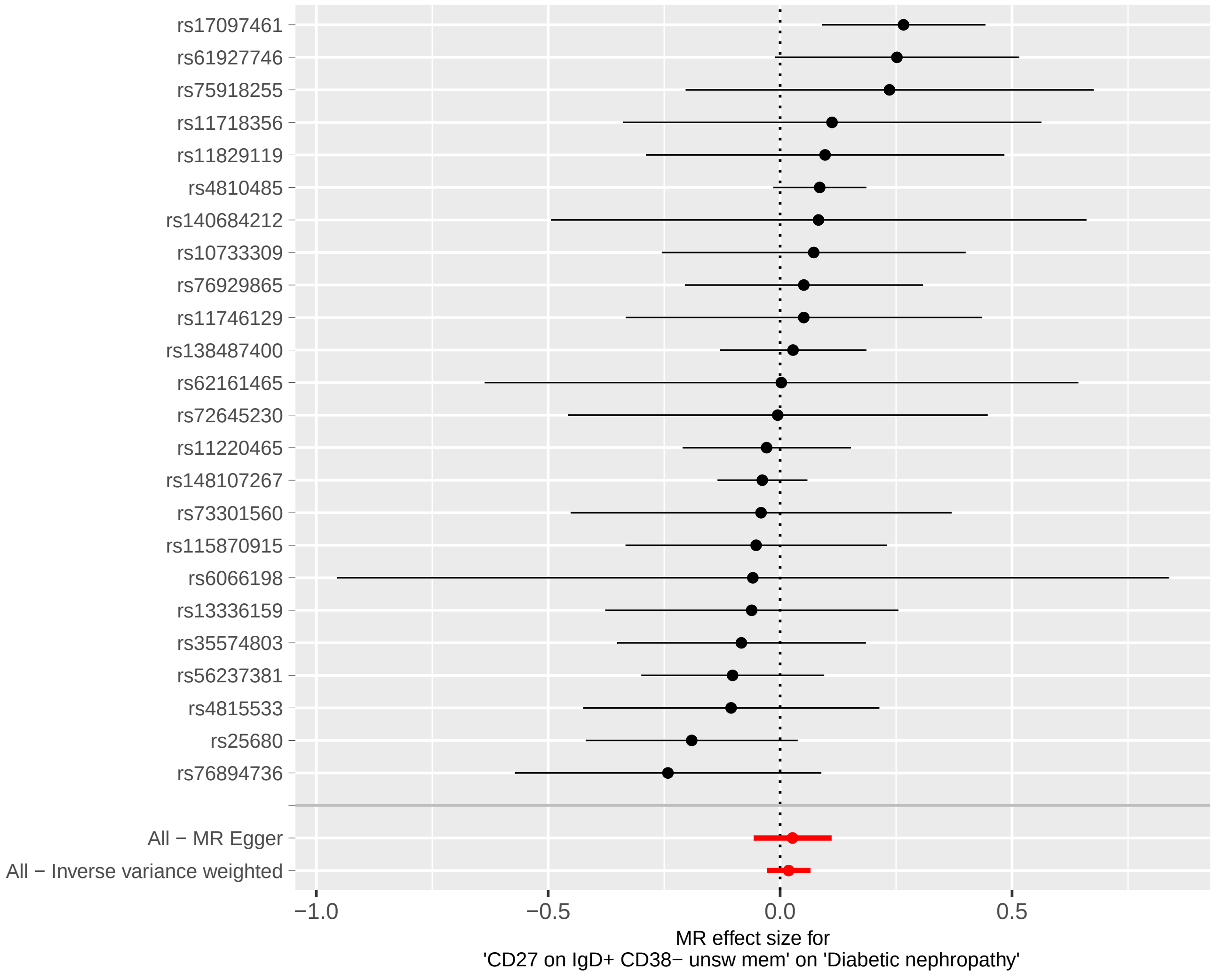
rs17097461
rs61927746
rs75918255
rs11718356
rs11829119
rs4810485
rs140684212
rs10733309
rs76929865
rs11746129
rs138487400
rs62161465
rs72645230
rs11220465
rs148107267
rs73301560
rs115870915
rs6066198
rs13336159
rs35574803
rs56237381
rs4815533
rs25680
rs76894736
All − MR Egger
All − Inverse variance weighted
−1.0
−0.5
0.0
0.5
MR effect size for
'CD27 on IgD+ CD38− unsw mem' on 'Diabetic nephropathy'

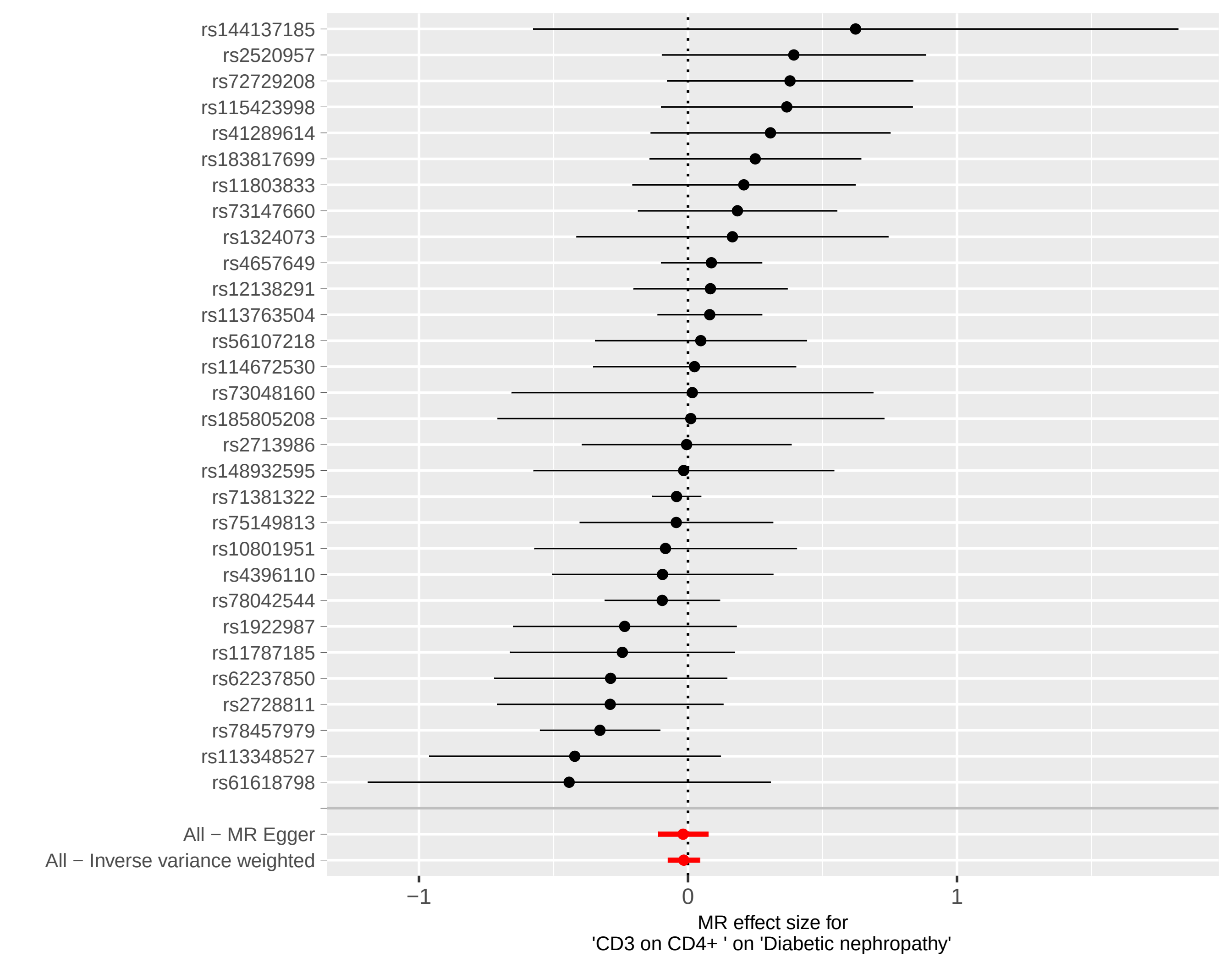

rs144137185
rs2520957
rs72729208
rs115423998
rs41289614
rs183817699
rs11803833
rs73147660
rs1324073
rs4657649
rs12138291
rs113763504
rs56107218
rs114672530
rs73048160
rs185805208
rs2713986
rs148932595
rs71381322
rs75149813
rs10801951
rs4396110
rs78042544
rs1922987
rs11787185
rs62237850
rs2728811
rs78457979
rs113348527
rs61618798
All − MR Egger
All − Inverse variance weighted
−1
0
1
MR effect size for
'CD3 on CD4+ ' on 'Diabetic nephropathy'

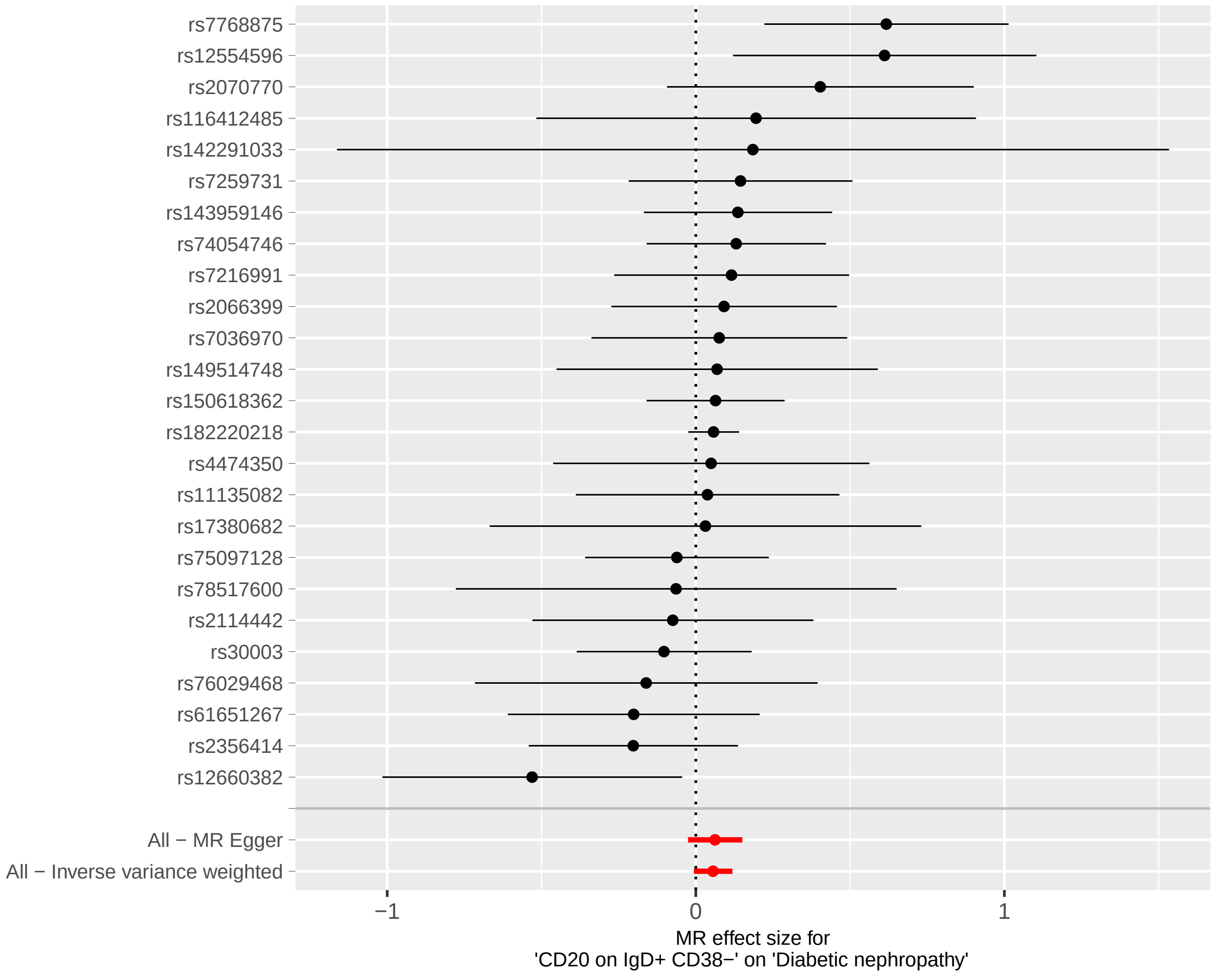

rs7768875
rs12554596
rs2070770
rs116412485
rs142291033
rs7259731
rs143959146
rs74054746
rs7216991
rs2066399
rs7036970
rs149514748
rs150618362
rs182220218
rs4474350
rs11135082
rs17380682
rs75097128
rs78517600
rs2114442
rs30003
rs76029468
rs61651267
rs2356414
rs12660382
All − MR Egger
All − Inverse variance weighted
−1
0
1
MR effect size for
'CD20 on IgD+ CD38−' on 'Diabetic nephropathy'

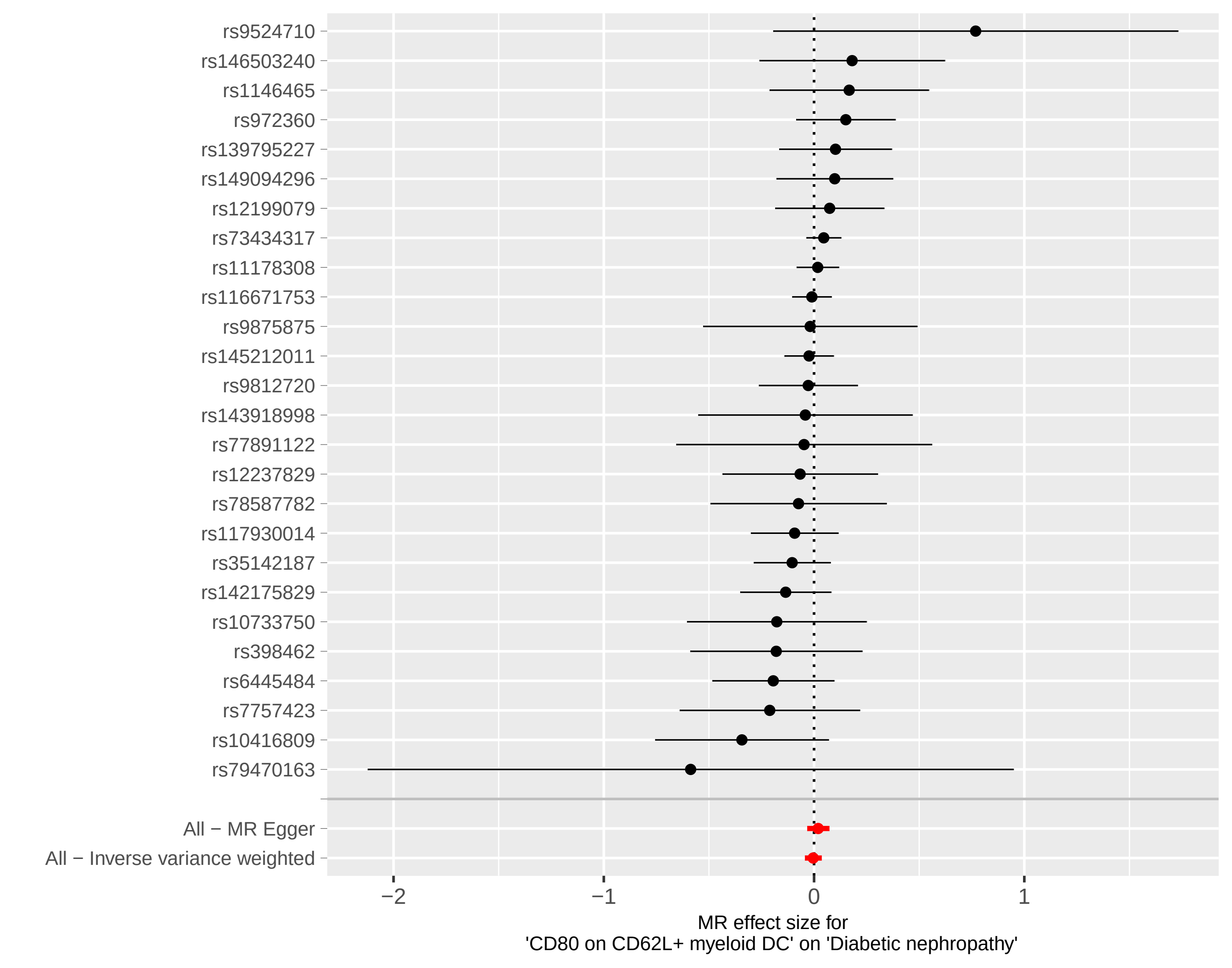
rs9524710
rs146503240
rs1146465
rs972360
rs139795227
rs149094296
rs12199079
rs73434317
rs11178308
rs116671753
rs9875875
rs145212011
rs9812720
rs143918998
rs77891122
rs12237829
rs78587782
rs117930014
rs35142187
rs142175829
rs10733750
rs398462
rs6445484
rs7757423
rs10416809
rs79470163
All − MR Egger
All − Inverse variance weighted
−2
−1
0
1
MR effect size for
'CD80 on CD62L+ myeloid DC' on 'Diabetic nephropathy'

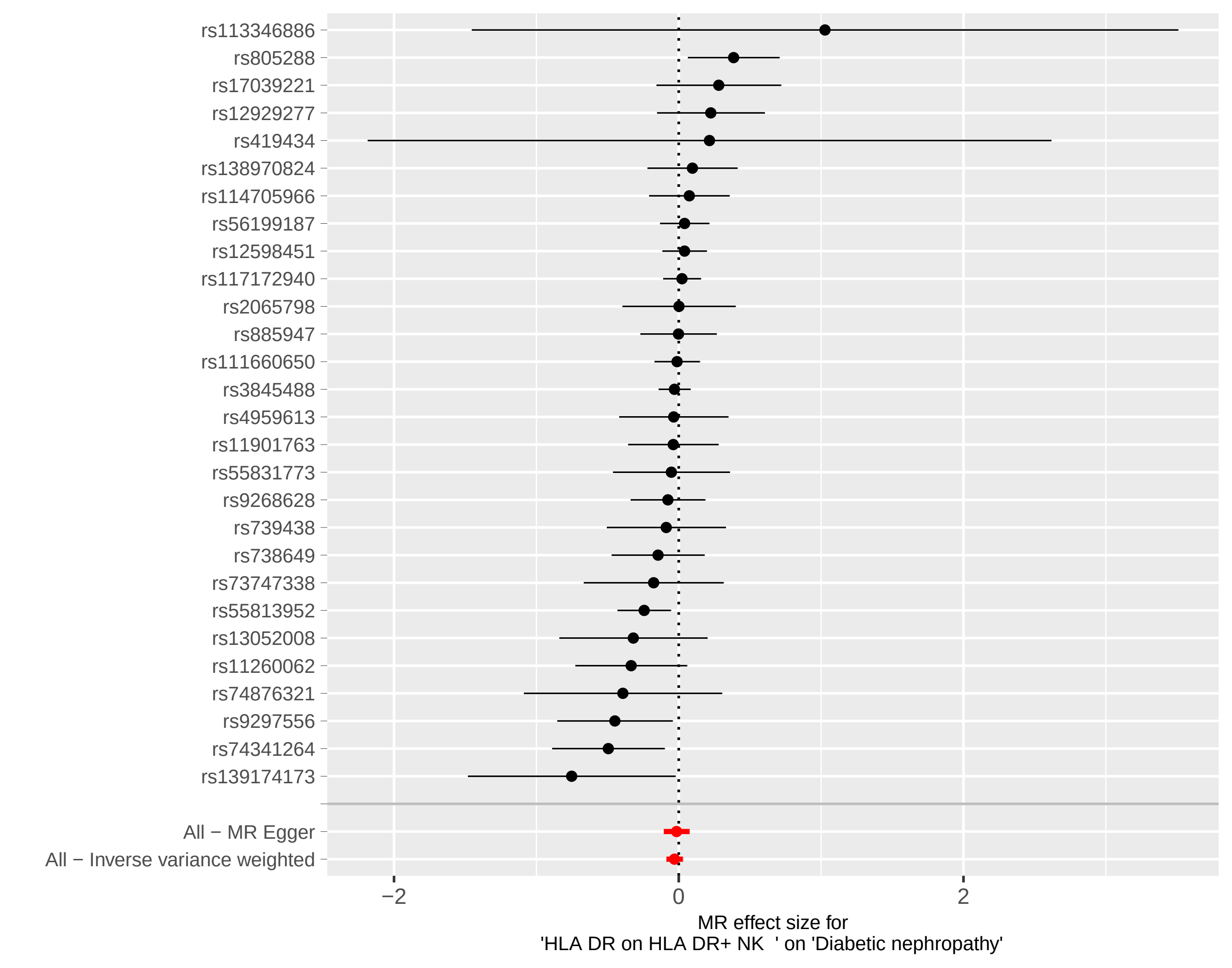
rs113346886
rs805288
rs17039221
rs12929277
rs419434
rs138970824
rs114705966
rs56199187
rs12598451
rs117172940
rs2065798
rs885947
rs111660650
rs3845488
rs4959613
rs11901763
rs55831773
rs9268628
rs739438
rs738649
rs73747338
rs55813952
rs13052008
rs11260062
rs74876321
rs9297556
rs74341264
rs139174173
All − MR Egger
All − Inverse variance weighted
−2
0
2
MR effect size for
'HLA DR on HLA DR+ NK ' on 'Diabetic nephropathy'

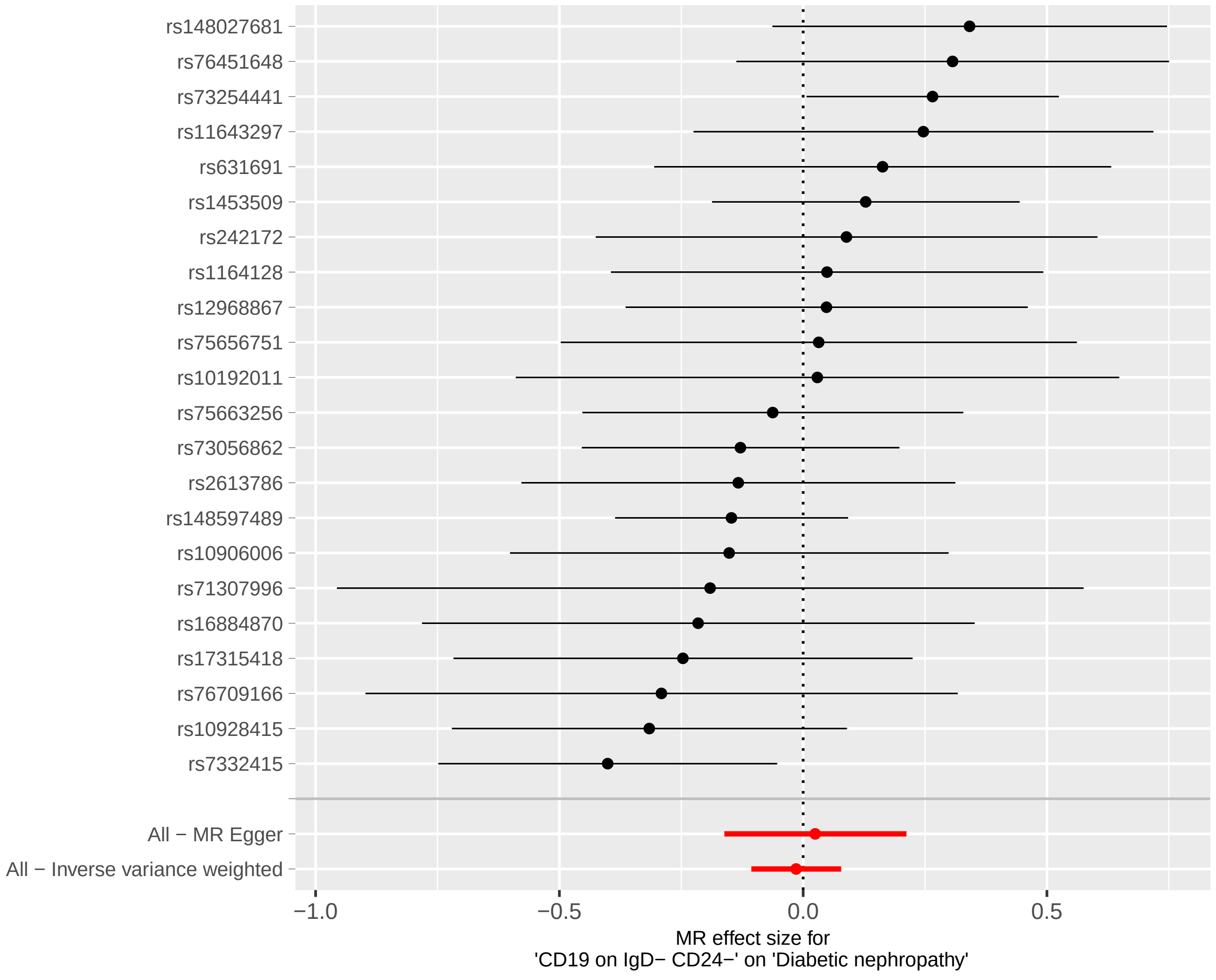
rs148027681
rs76451648
rs73254441
rs11643297
rs631691
rs1453509
rs242172
rs1164128
rs12968867
rs75656751
rs10192011
rs75663256
rs73056862
rs2613786
rs148597489
rs10906006
rs71307996
rs16884870
rs17315418
rs76709166
rs10928415
rs7332415
All − MR Egger
All − Inverse variance weighted
−1.0
−0.5
0.0
0.5
MR effect size for
'CD19 on IgD− CD24−' on 'Diabetic nephropathy'

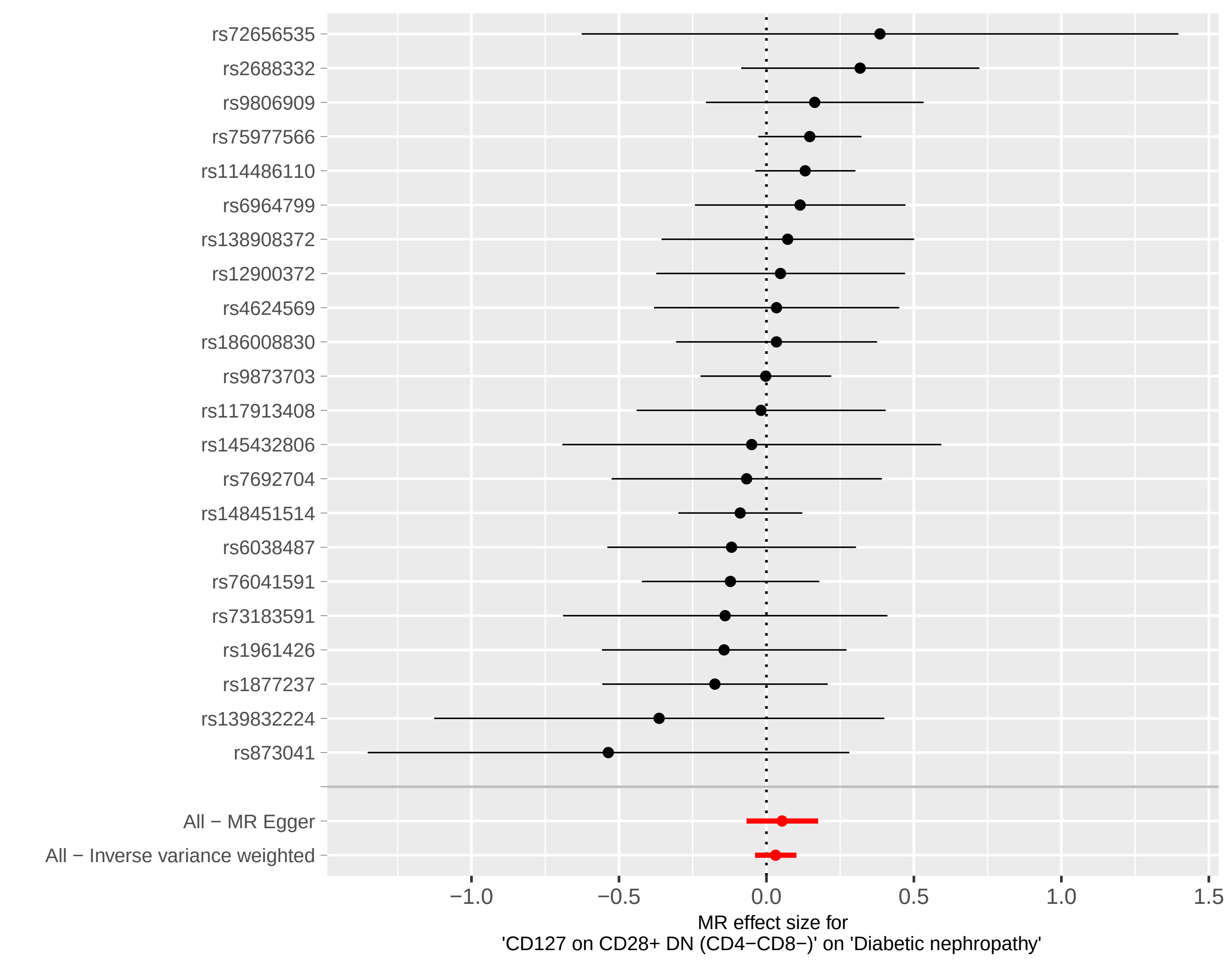
rs72656535
rs2688332
rs9806909
rs75977566
rs114486110
rs6964799
rs138908372
rs12900372
rs4624569
rs186008830
rs9873703
rs117913408
rs145432806
rs7692704
rs148451514
rs6038487
rs76041591
rs73183591
rs1961426
rs1877237
rs139832224
rs873041
All − MR Egger
All − Inverse variance weighted
−1.0
−0.5
0.0
0.5
1.0
1.5
MR effect size for
'CD127 on CD28+ DN (CD4−CD8−)' on 'Diabetic nephropathy'

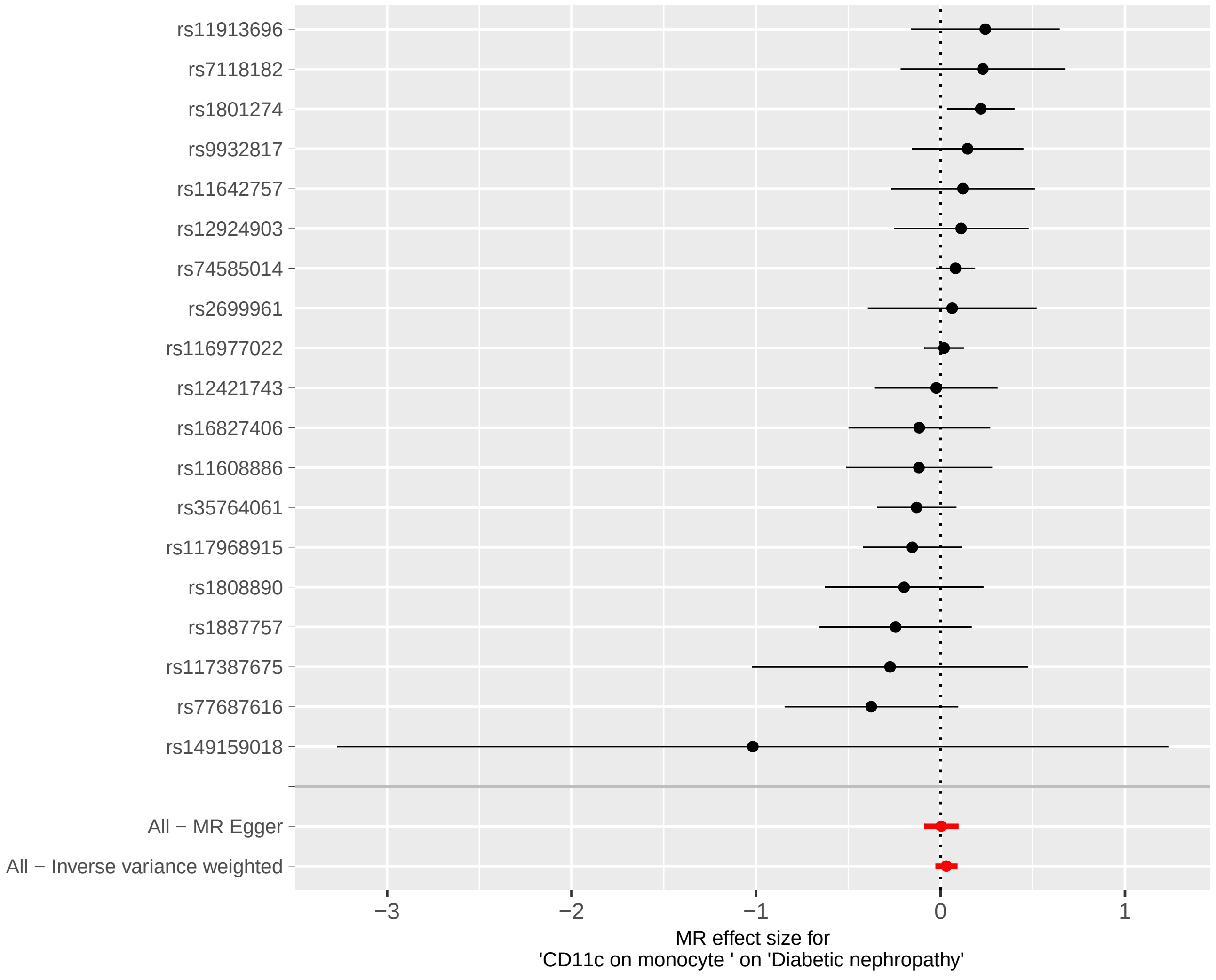
rs11913696
rs7118182
rs1801274
rs9932817
rs11642757
rs12924903
rs74585014
rs2699961
rs116977022
rs12421743
rs16827406
rs11608886
rs35764061
rs117968915
rs1808890
rs1887757
rs117387675
rs77687616
rs149159018
All − MR Egger
All − Inverse variance weighted
−3
−2
−1
0
1
MR effect size for
'CD11c on monocyte ' on 'Diabetic nephropathy'

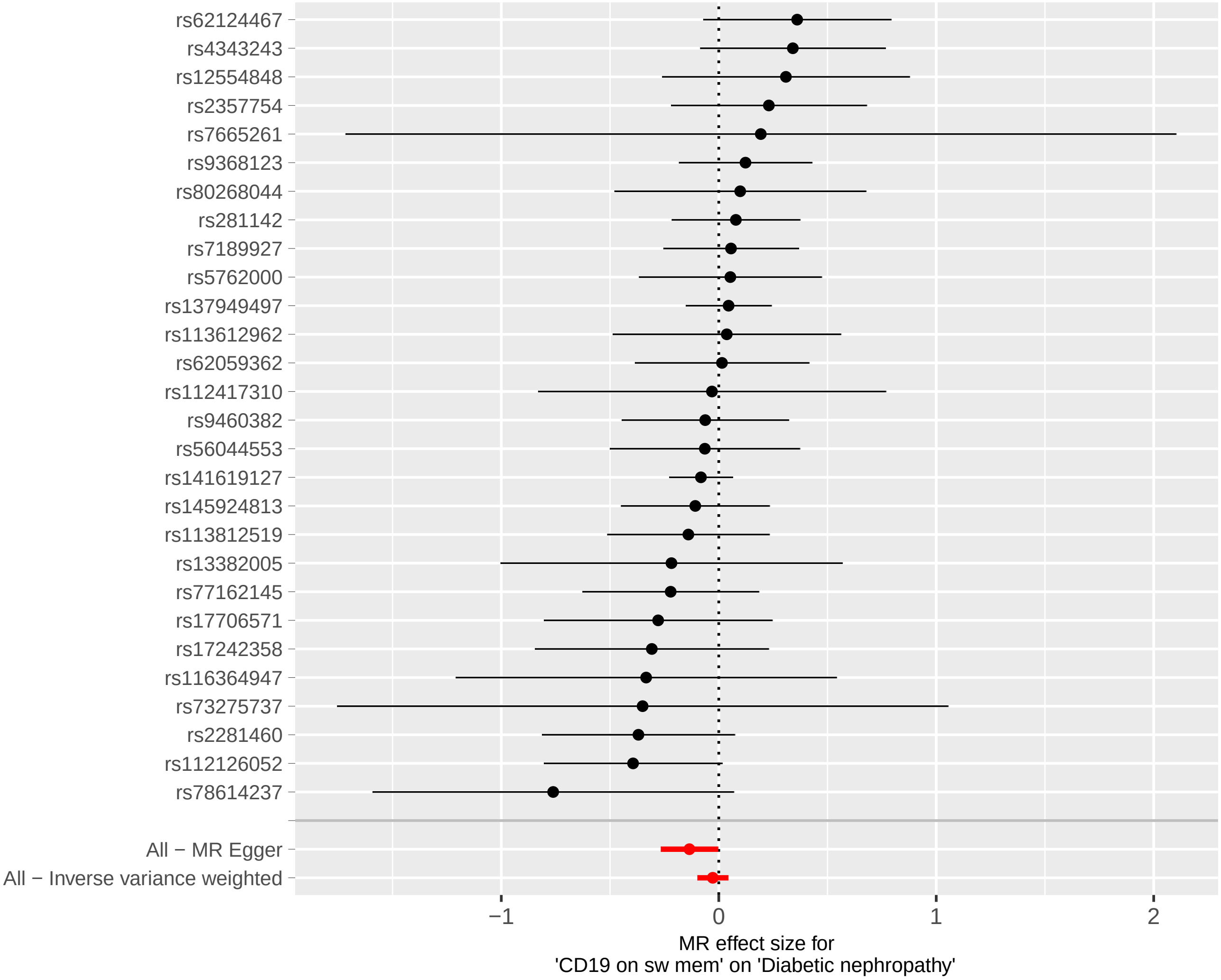
rs62124467
rs4343243
rs12554848
rs2357754
rs7665261
rs9368123
rs80268044
rs281142
rs7189927
rs5762000
rs137949497
rs113612962
rs62059362
rs112417310
rs9460382
rs56044553
rs141619127
rs145924813
rs113812519
rs13382005
rs77162145
rs17706571
rs17242358
rs116364947
rs73275737
rs2281460
rs112126052
rs78614237
All − MR Egger
All − Inverse variance weighted
−1
0
1
2
MR effect size for
'CD19 on sw mem' on 'Diabetic nephropathy'

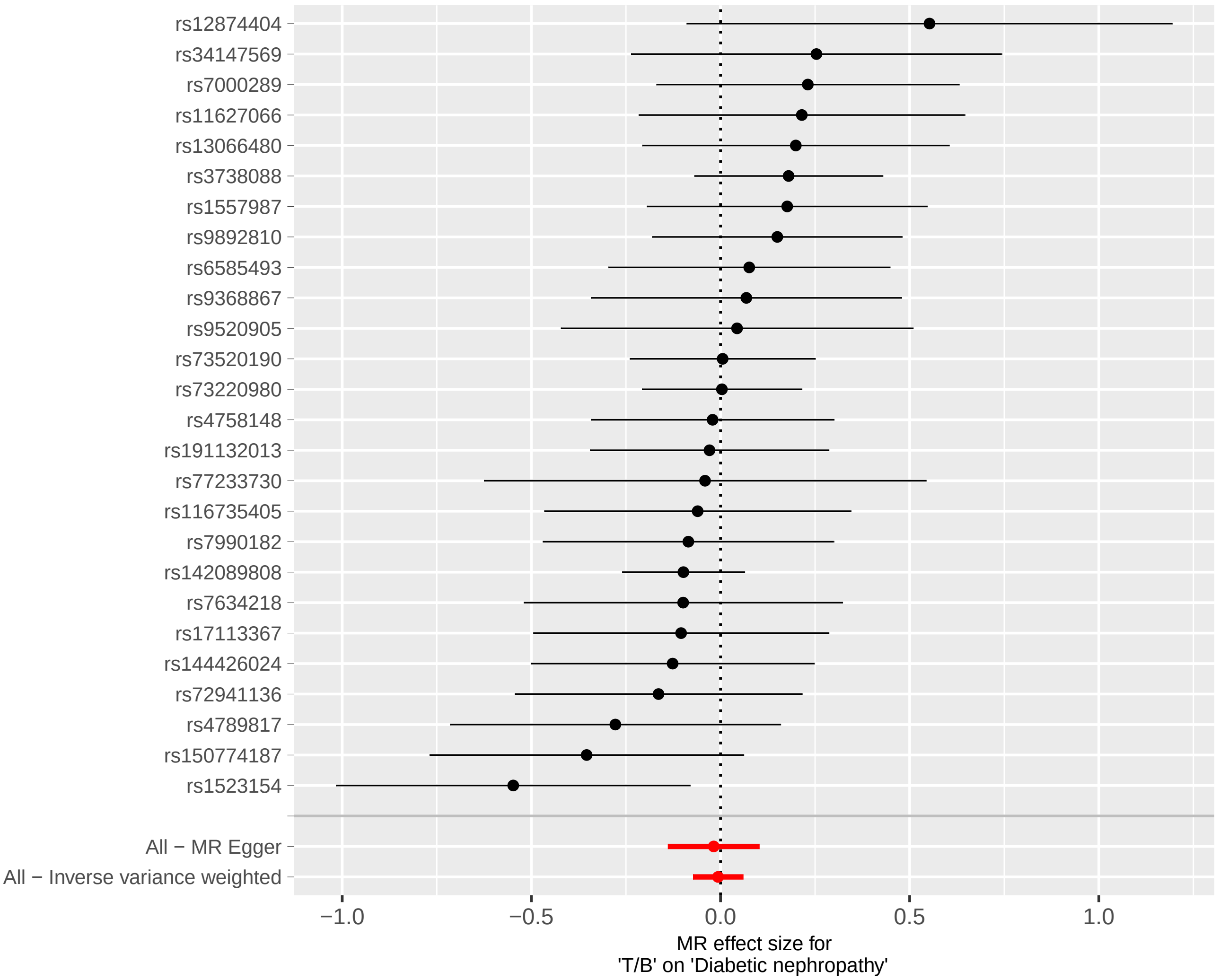
rs12874404
rs34147569
rs7000289
rs11627066
rs13066480
rs3738088
rs1557987
rs9892810
rs6585493
rs9368867
rs9520905
rs73520190
rs73220980
rs4758148
rs191132013
rs77233730
rs116735405
rs7990182
rs142089808
rs7634218
rs17113367
rs144426024
rs72941136
rs4789817
rs150774187
rs1523154
All − MR Egger
All − Inverse variance weighted
−1.0
−0.5
0.0
0.5
1.0
MR effect size for
'T/B' on 'Diabetic nephropathy'

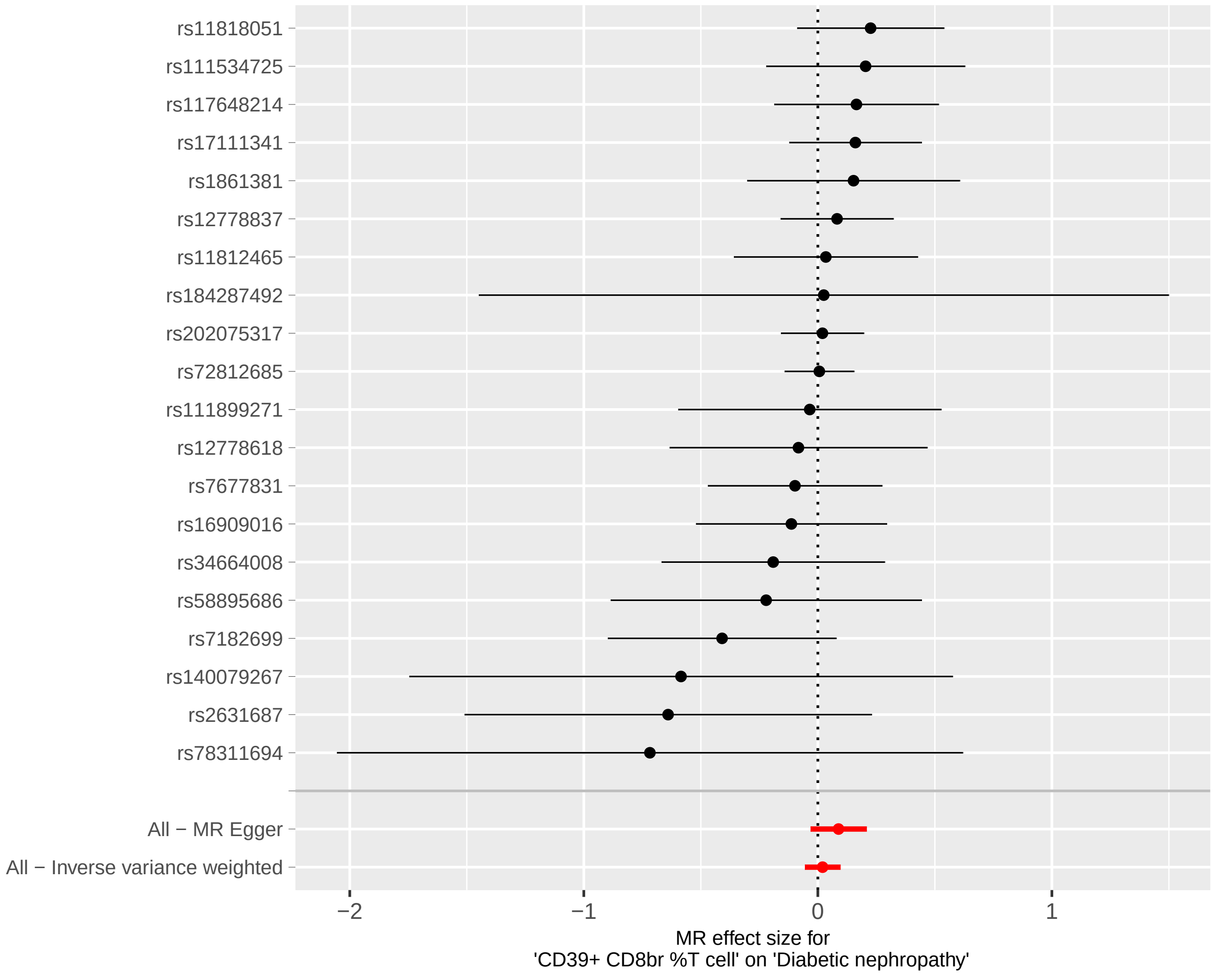

rs11818051
rs111534725
rs117648214
rs17111341
rs1861381
rs12778837
rs11812465
rs184287492
rs202075317
rs72812685
rs111899271
rs12778618
rs7677831
rs16909016
rs34664008
rs58895686
rs7182699
rs140079267
rs2631687
rs78311694
All − MR Egger
All − Inverse variance weighted
−2
−1
0
1
MR effect size for
'CD39+ CD8br %T cell' on 'Diabetic nephropathy'

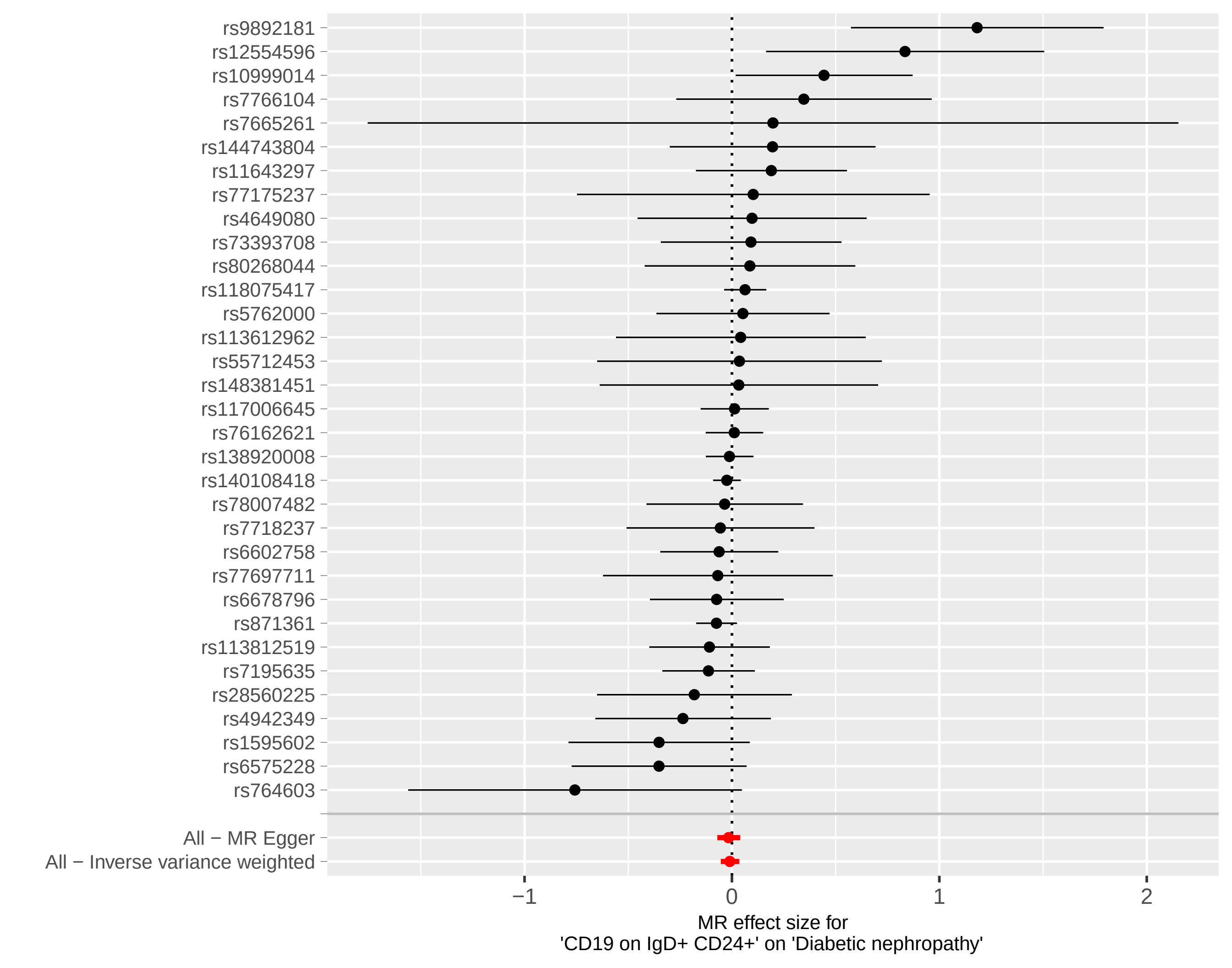
rs9892181
rs12554596
rs10999014
rs7766104
rs7665261
rs144743804
rs11643297
rs77175237
rs4649080
rs73393708
rs80268044
rs118075417
rs5762000
rs113612962
rs55712453
rs148381451
rs117006645
rs76162621
rs138920008
rs140108418
rs78007482
rs7718237
rs6602758
rs77697711
rs6678796
rs871361
rs113812519
rs7195635
rs28560225
rs4942349
rs1595602
rs6575228
rs764603
All − MR Egger
All − Inverse variance weighted
−1
0
1
2
MR effect size for
'CD19 on IgD+ CD24+' on 'Diabetic nephropathy'

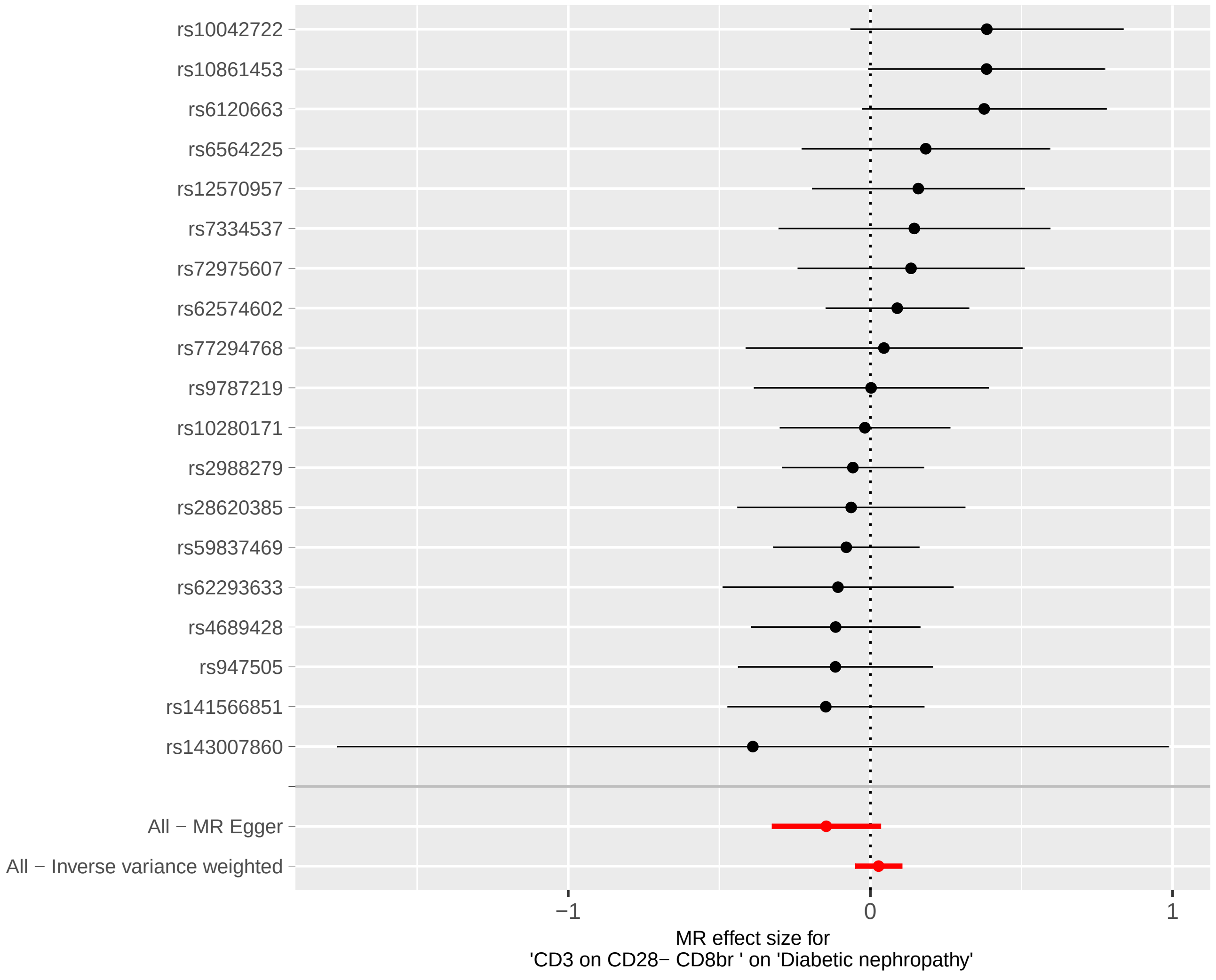
rs10042722
rs10861453
rs6120663
rs6564225
rs12570957
rs7334537
rs72975607
rs62574602
rs77294768
rs9787219
rs10280171
rs2988279
rs28620385
rs59837469
rs62293633
rs4689428
rs947505
rs141566851
rs143007860
All − MR Egger
All − Inverse variance weighted
−1
0
1
MR effect size for
'CD3 on CD28− CD8br ' on 'Diabetic nephropathy'

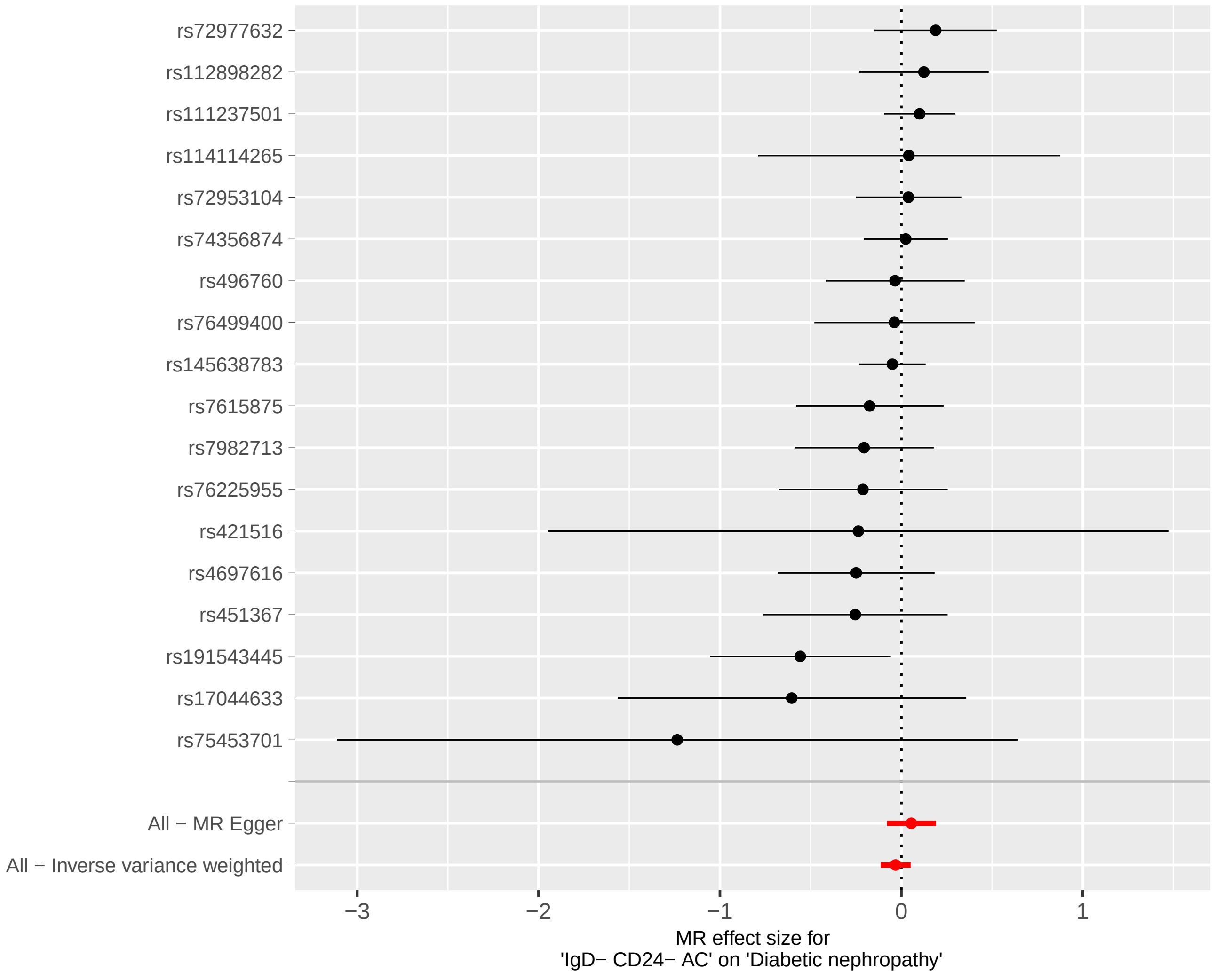

rs72977632
rs112898282
rs111237501
rs114114265
rs72953104
rs74356874
rs496760
rs76499400
rs145638783
rs7615875
rs7982713
rs76225955
rs421516
rs4697616
rs451367
rs191543445
rs17044633
rs75453701
All − MR Egger
All − Inverse variance weighted
−3
−2
−1
0
1
MR effect size for
'IgD− CD24− AC' on 'Diabetic nephropathy'

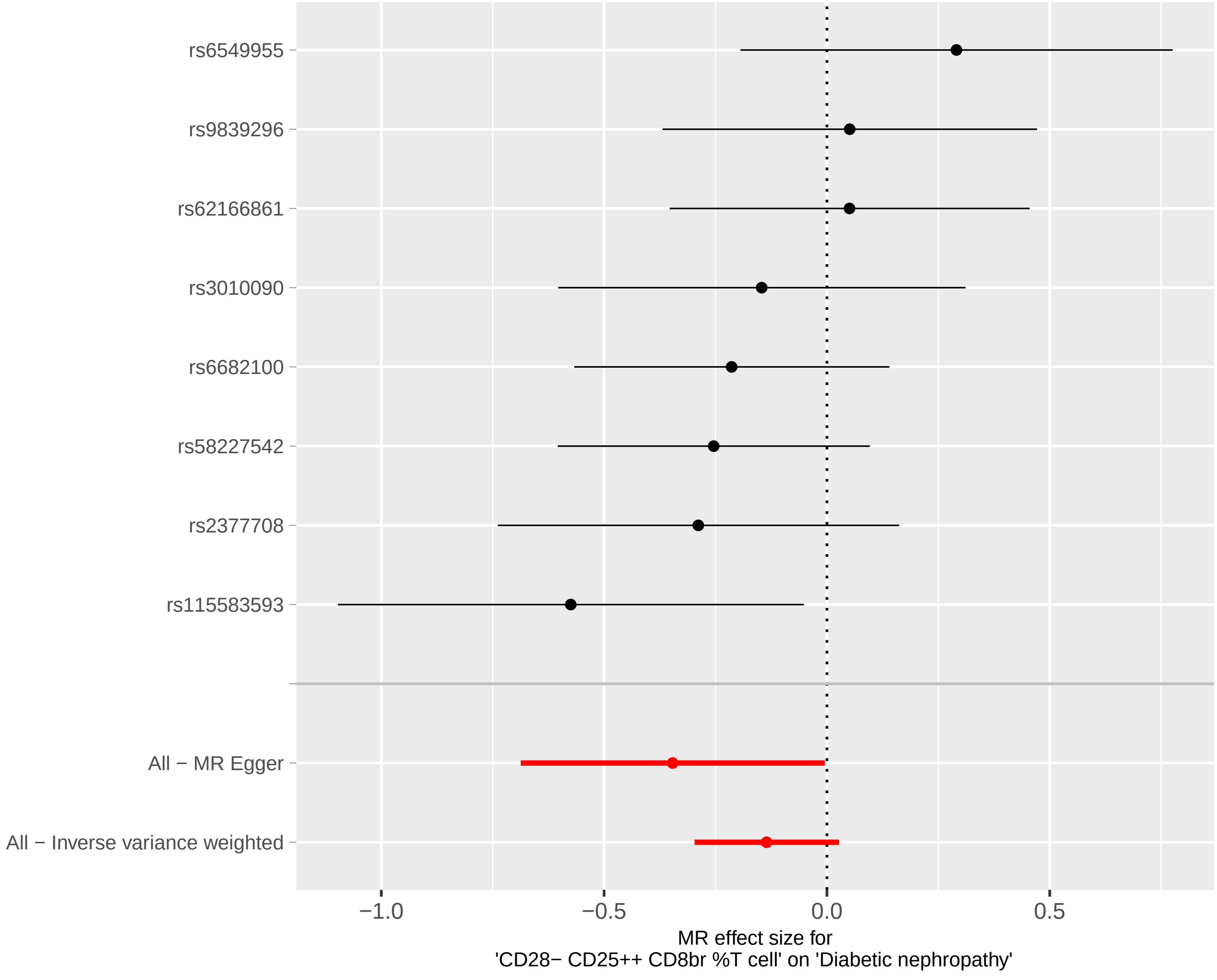

rs6549955
rs9839296
rs62166861
rs3010090
rs6682100
rs58227542
rs2377708
rs115583593
All − MR Egger
All − Inverse variance weighted
−1.0
−0.5
0.0
0.5
MR effect size for
'CD28− CD25++ CD8br %T cell' on 'Diabetic nephropathy'

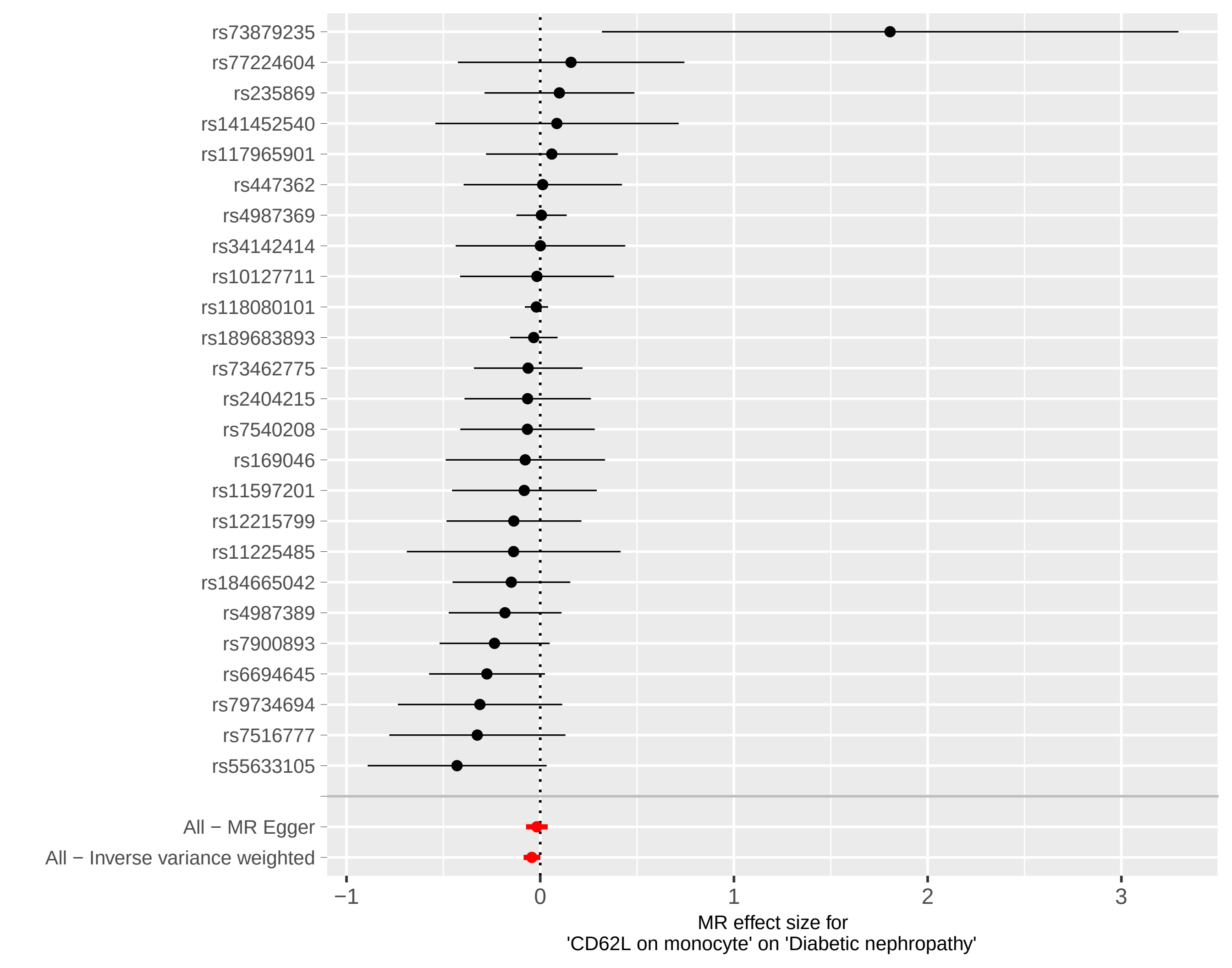
rs73879235
rs77224604
rs235869
rs141452540
rs117965901
rs447362
rs4987369
rs34142414
rs10127711
rs118080101
rs189683893
rs73462775
rs2404215
rs7540208
rs169046
rs11597201
rs12215799
rs11225485
rs184665042
rs4987389
rs7900893
rs6694645
rs79734694
rs7516777
rs55633105
All − MR Egger
All − Inverse variance weighted
−1
0
1
2
3
MR effect size for
'CD62L on monocyte' on 'Diabetic nephropathy'

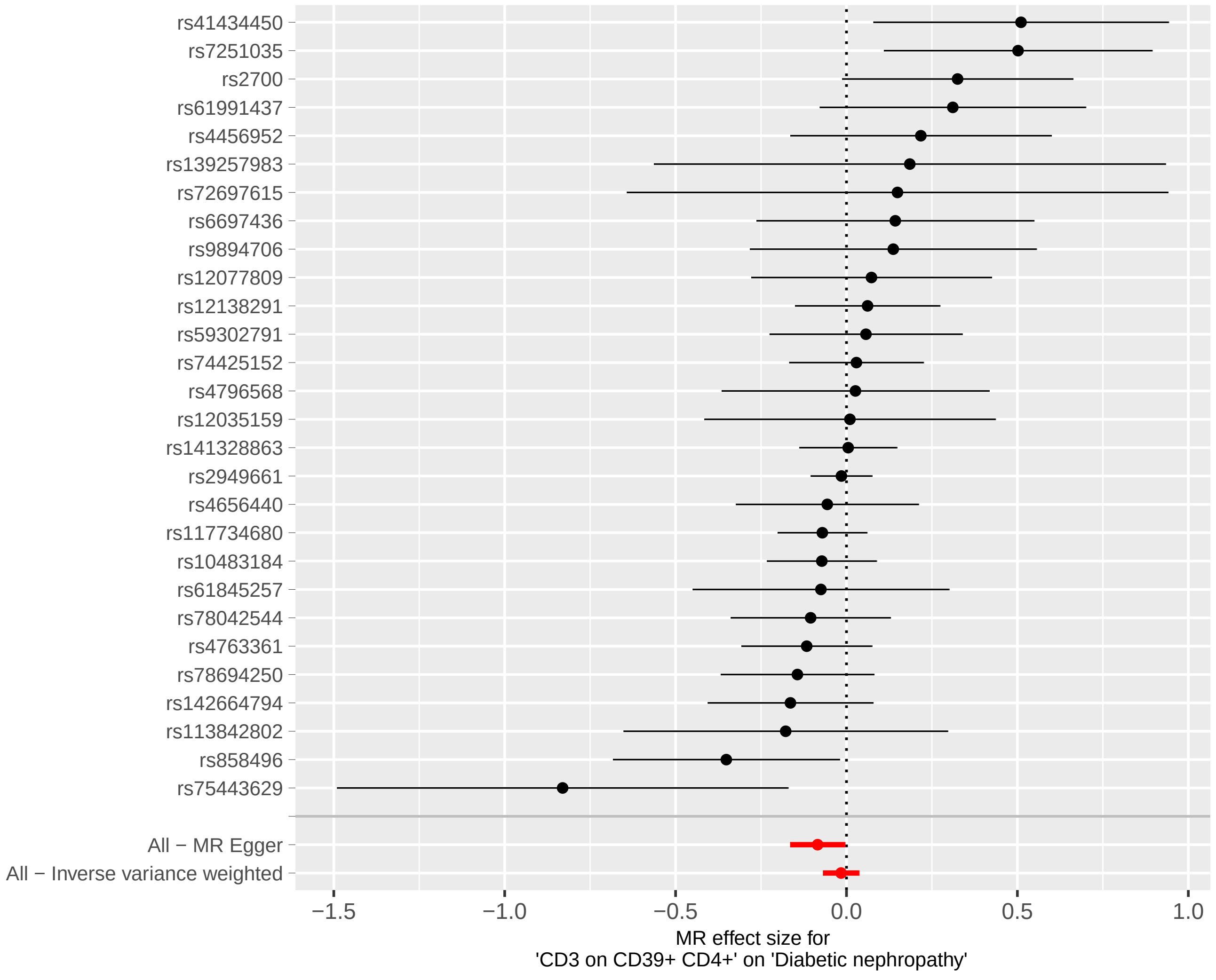

rs41434450
rs7251035
rs2700
rs61991437
rs4456952
rs139257983
rs72697615
rs6697436
rs9894706
rs12077809
rs12138291
rs59302791
rs74425152
rs4796568
rs12035159
rs141328863
rs2949661
rs4656440
rs117734680
rs10483184
rs61845257
rs78042544
rs4763361
rs78694250
rs142664794
rs113842802
rs858496
rs75443629
All − MR Egger
All − Inverse variance weighted
−1.5
−1.0
−0.5
0.0
0.5
1.0
MR effect size for
'CD3 on CD39+ CD4+' on 'Diabetic nephropathy'

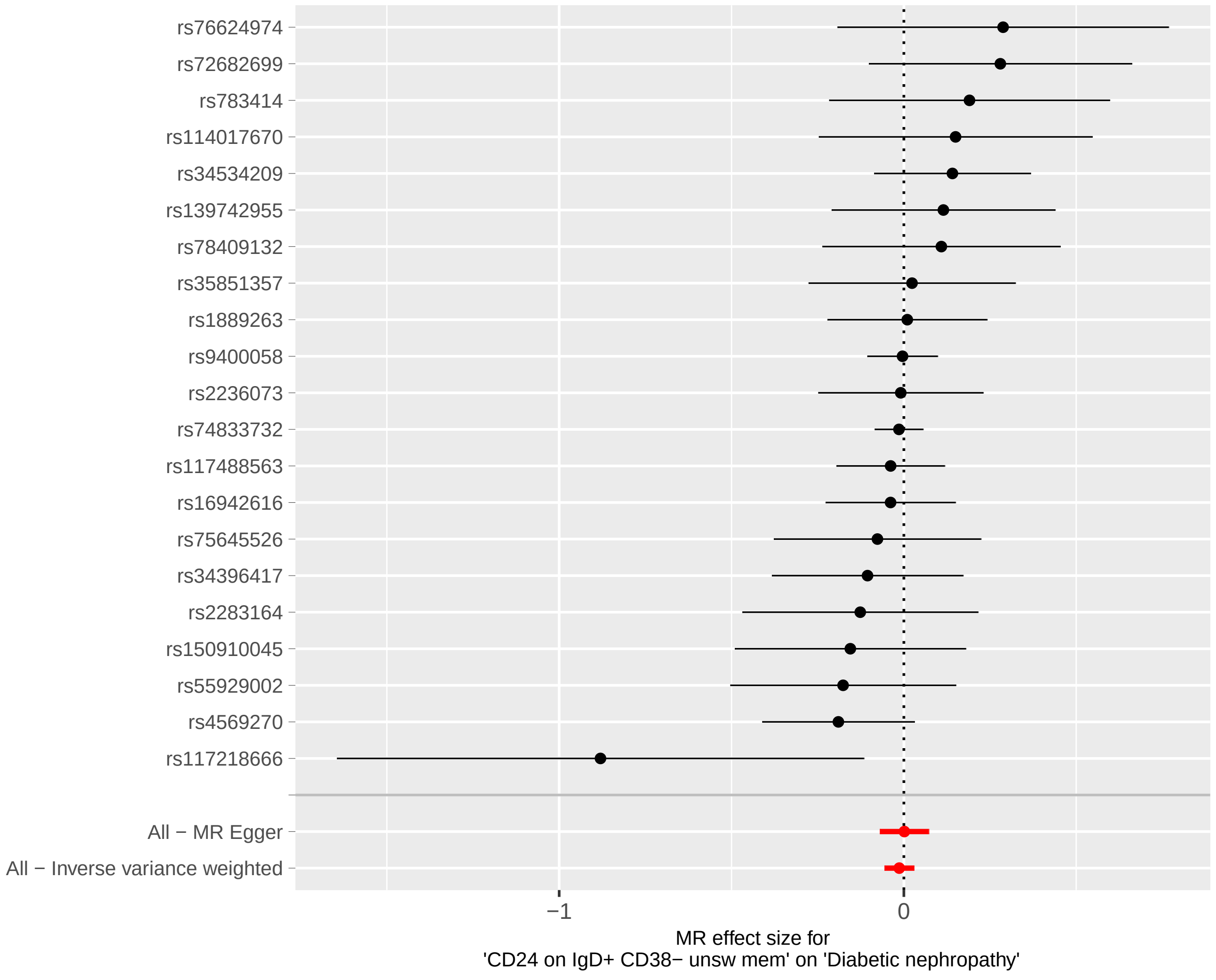
rs76624974
rs72682699
rs783414
rs114017670
rs34534209
rs139742955
rs78409132
rs35851357
rs1889263
rs9400058
rs2236073
rs74833732
rs117488563
rs16942616
rs75645526
rs34396417
rs2283164
rs150910045
rs55929002
rs4569270
rs117218666
All − MR Egger
All − Inverse variance weighted
−1
0
MR effect size for
'CD24 on IgD+ CD38− unsw mem' on 'Diabetic nephropathy'

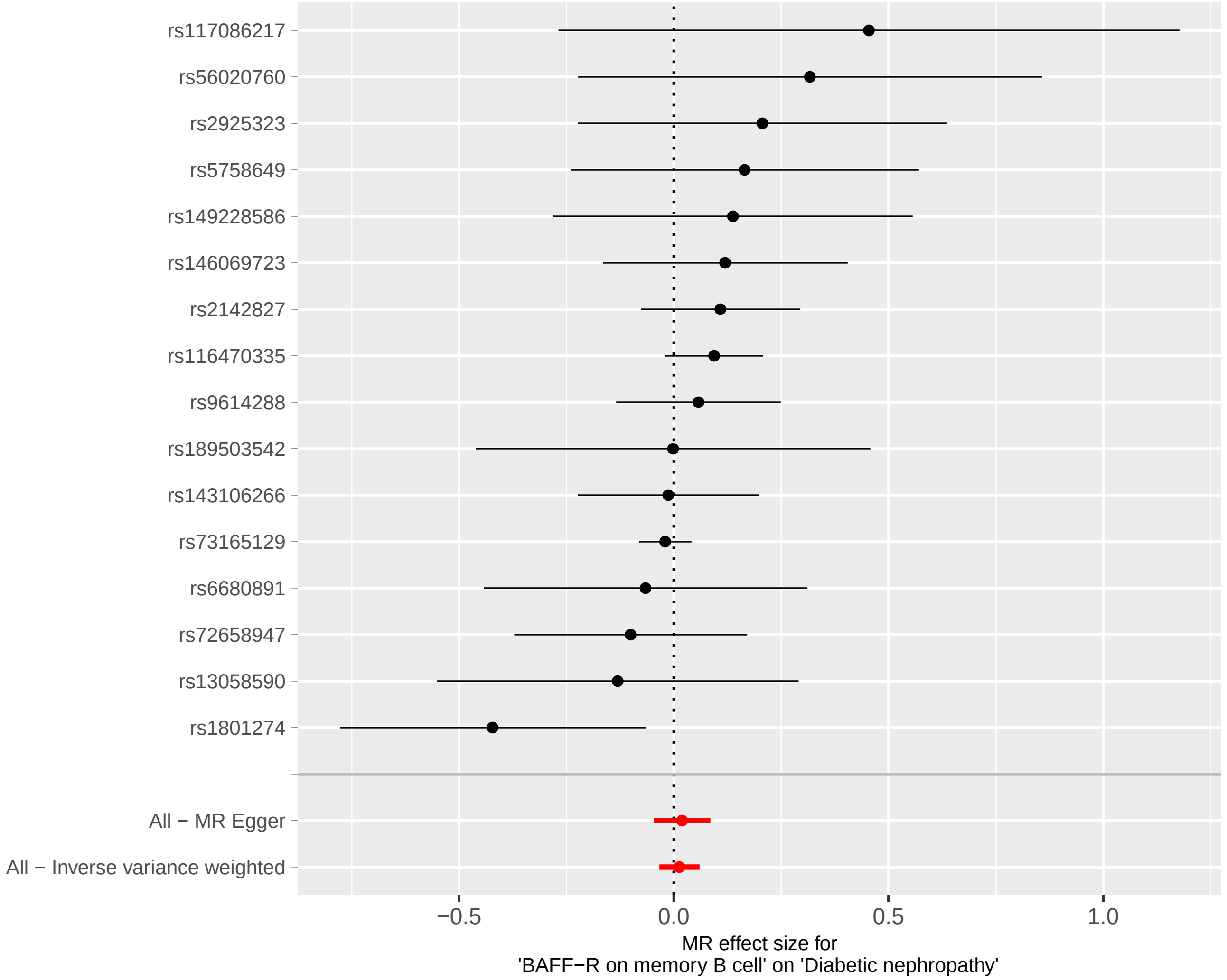

rs117086217
rs56020760
rs2925323
rs5758649
rs149228586
rs146069723
rs2142827
rs116470335
rs9614288
rs189503542
rs143106266
rs73165129
rs6680891
rs72658947
rs13058590
rs1801274
All − MR Egger
All − Inverse variance weighted
−0.5
0.0
0.5
1.0
MR effect size for
'BAFF−R on memory B cell' on 'Diabetic nephropathy'

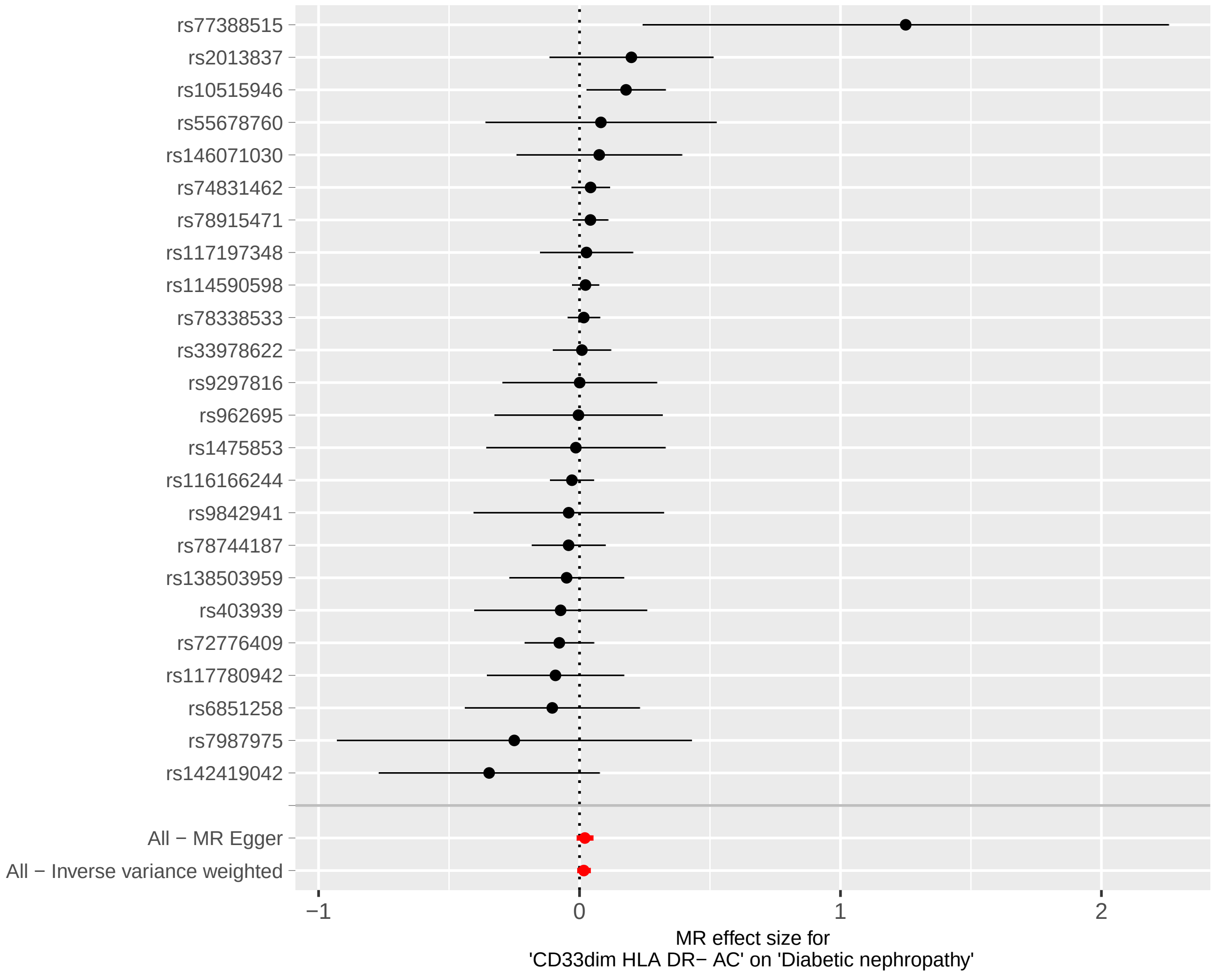

rs77388515
rs2013837
rs10515946
rs55678760
rs146071030
rs74831462
rs78915471
rs117197348
rs114590598
rs78338533
rs33978622
rs9297816
rs962695
rs1475853
rs116166244
rs9842941
rs78744187
rs138503959
rs403939
rs72776409
rs117780942
rs6851258
rs7987975
rs142419042
All − MR Egger
All − Inverse variance weighted
−1
0
1
2
MR effect size for
'CD33dim HLA DR− AC' on 'Diabetic nephropathy'

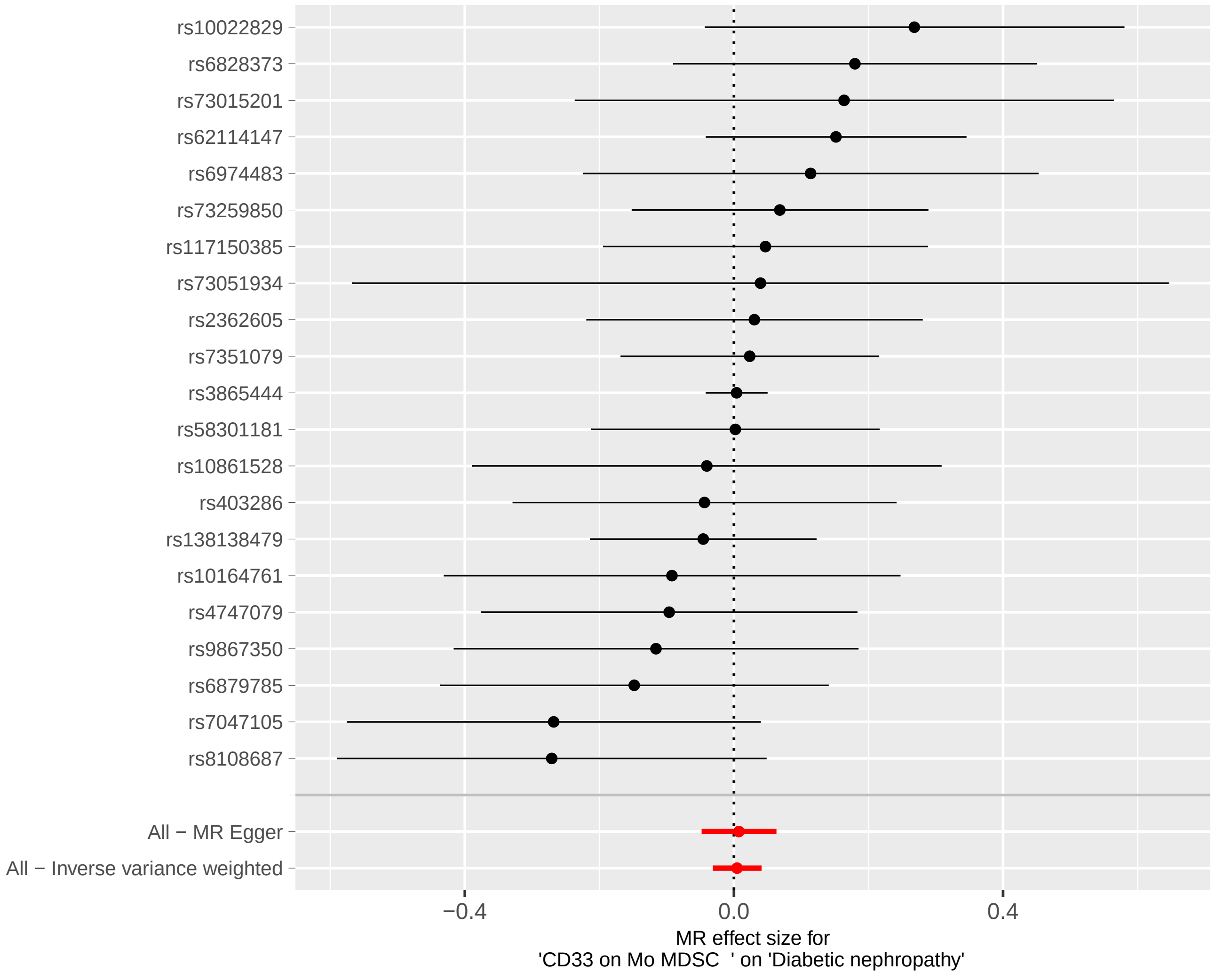

rs10022829
rs6828373
rs73015201
rs62114147
rs6974483
rs73259850
rs117150385
rs73051934
rs2362605
rs7351079
rs3865444
rs58301181
rs10861528
rs403286
rs138138479
rs10164761
rs4747079
rs9867350
rs6879785
rs7047105
rs8108687
All − MR Egger
All − Inverse variance weighted
−0.4
0.0
0.4
MR effect size for
'CD33 on Mo MDSC ' on 'Diabetic nephropathy'

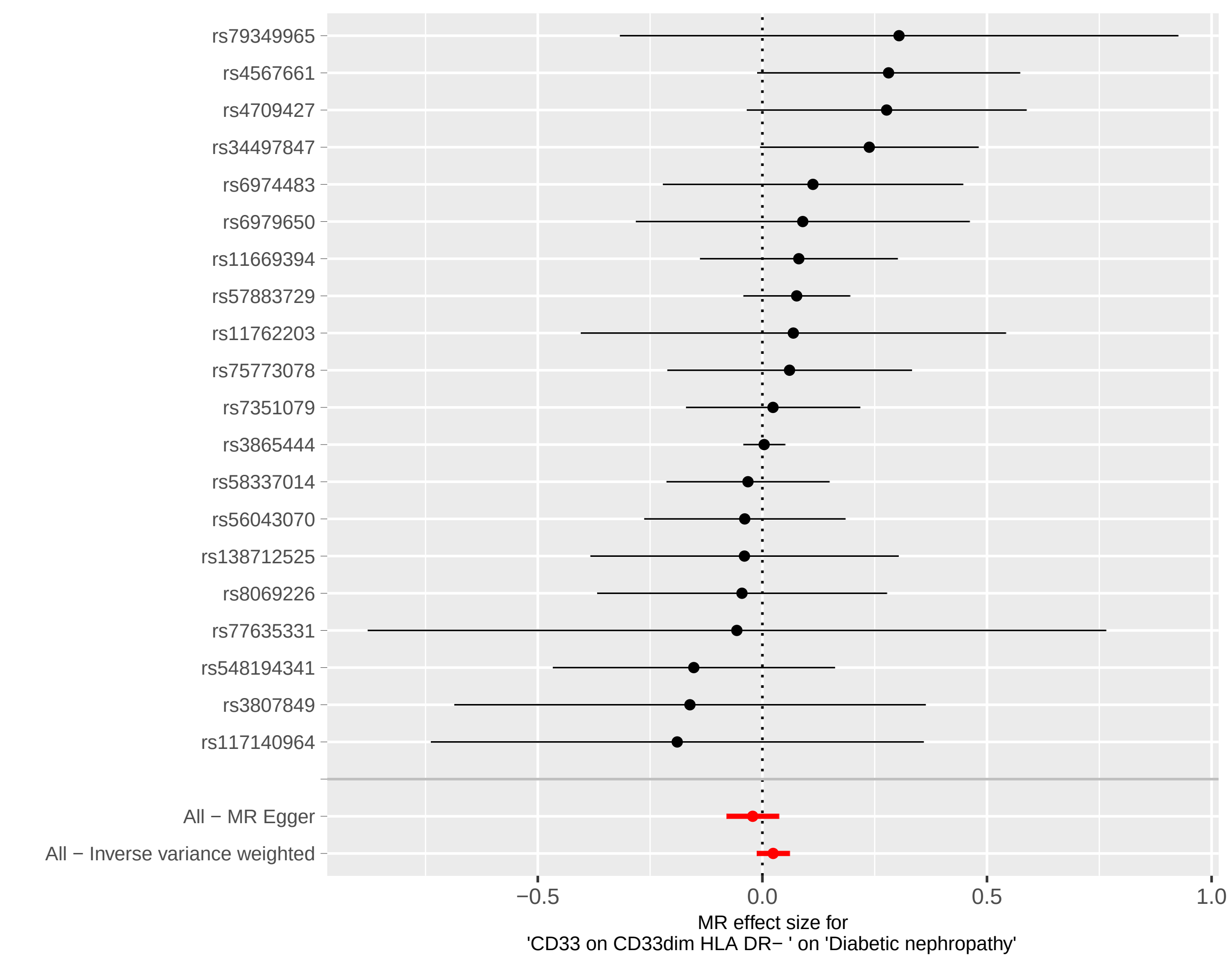
rs79349965
rs4567661
rs4709427
rs34497847
rs6974483
rs6979650
rs11669394
rs57883729
rs11762203
rs75773078
rs7351079
rs3865444
rs58337014
rs56043070
rs138712525
rs8069226
rs77635331
rs548194341
rs3807849
rs117140964
All − MR Egger
All − Inverse variance weighted
−0.5
0.0
0.5
1.0
MR effect size for
'CD33 on CD33dim HLA DR− ' on 'Diabetic nephropathy'

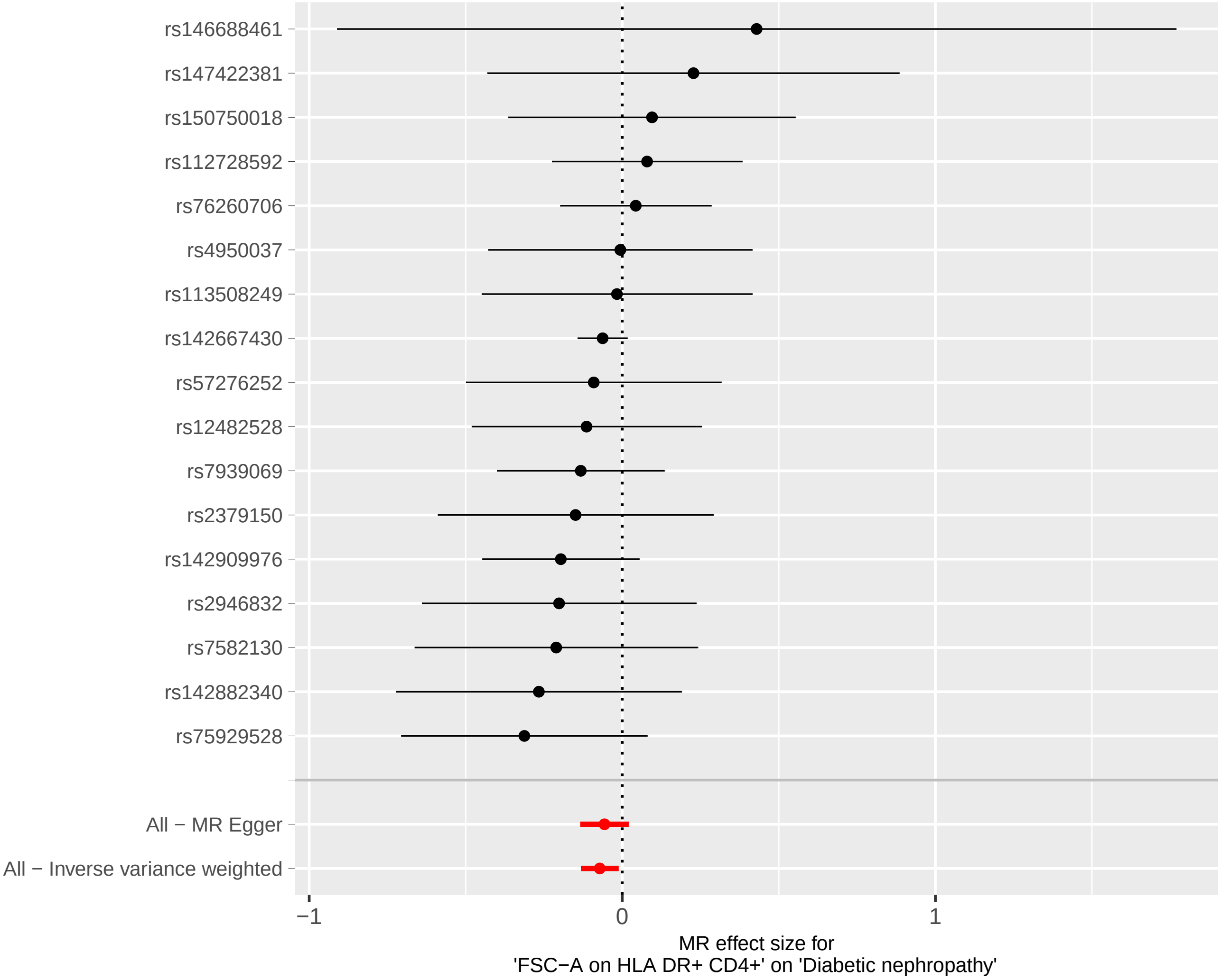

rs146688461
rs147422381
rs150750018
rs112728592
rs76260706
rs4950037
rs113508249
rs142667430
rs57276252
rs12482528
rs7939069
rs2379150
rs142909976
rs2946832
rs7582130
rs142882340
rs75929528
All − MR Egger
All − Inverse variance weighted
−1
0
1
MR effect size for
'FSC−A on HLA DR+ CD4+' on 'Diabetic nephropathy'

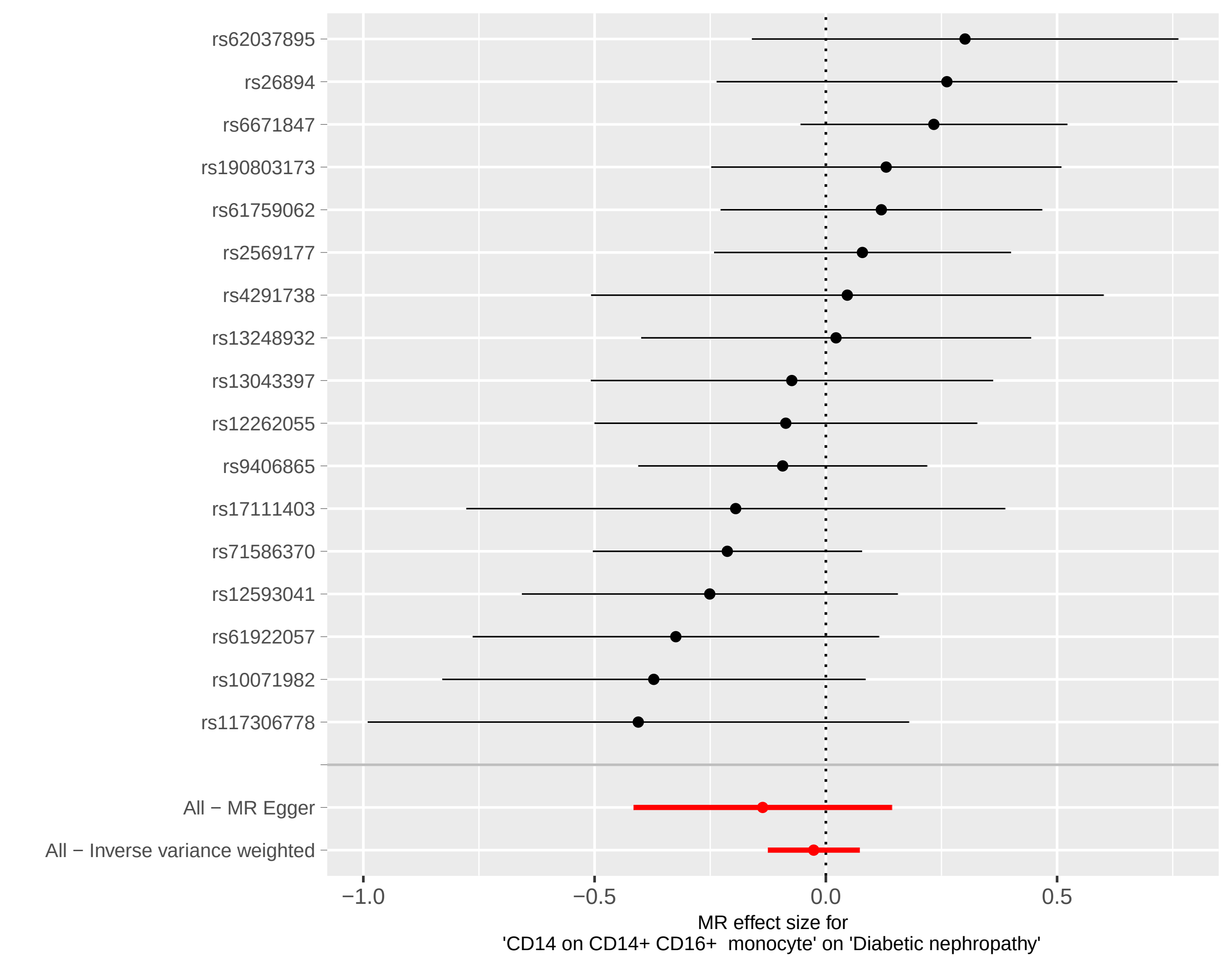

rs62037895
rs26894
rs6671847
rs190803173
rs61759062
rs2569177
rs4291738
rs13248932
rs13043397
rs12262055
rs9406865
rs17111403
rs71586370
rs12593041
rs61922057
rs10071982
rs117306778
All − MR Egger
All − Inverse variance weighted
−1.0
−0.5
0.0
0.5
MR effect size for
'CD14 on CD14+ CD16+ monocyte' on 'Diabetic nephropathy'

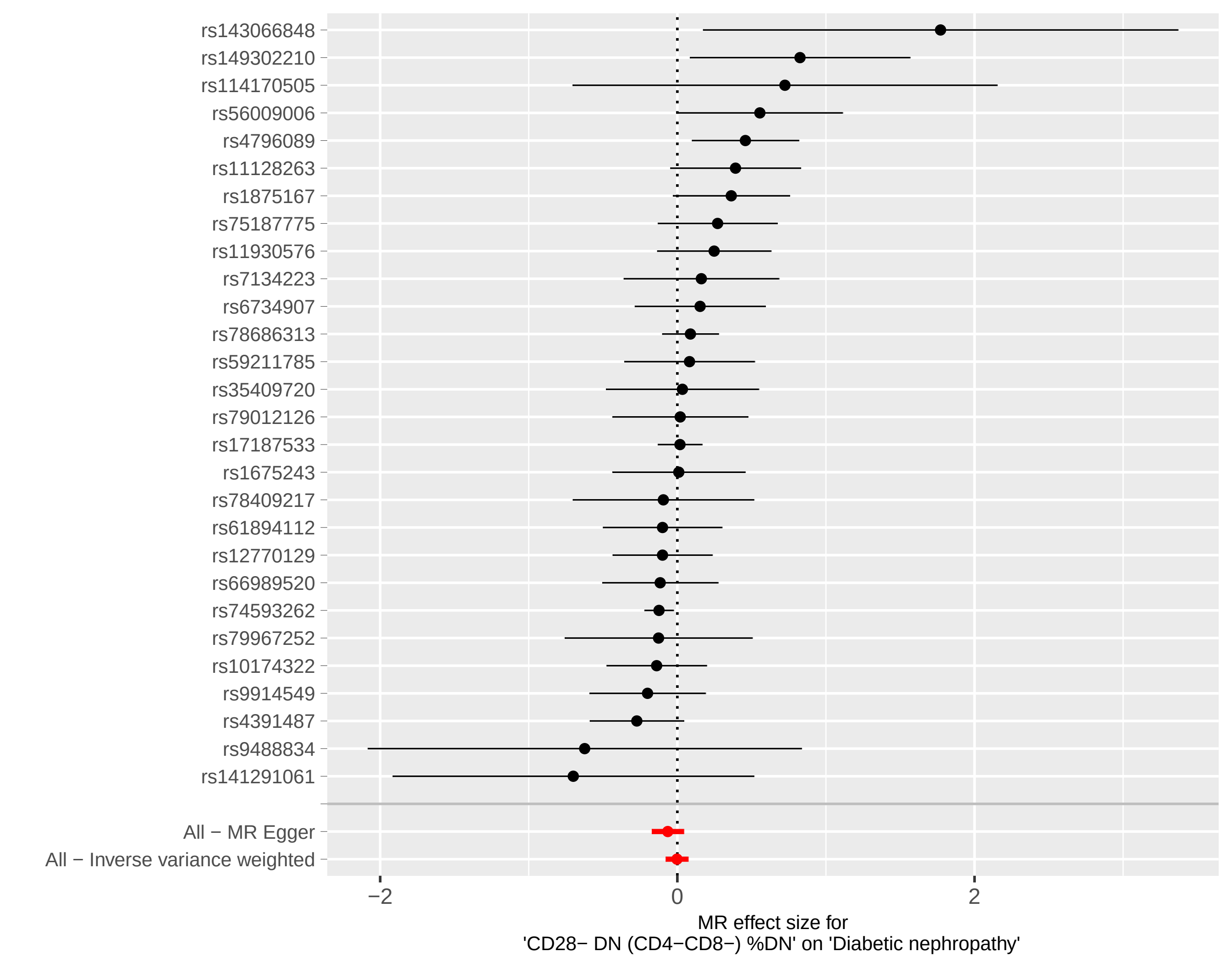

rs143066848
rs149302210
rs114170505
rs56009006
rs4796089
rs11128263
rs1875167
rs75187775
rs11930576
rs7134223
rs6734907
rs78686313
rs59211785
rs35409720
rs79012126
rs17187533
rs1675243
rs78409217
rs61894112
rs12770129
rs66989520
rs74593262
rs79967252
rs10174322
rs9914549
rs4391487
rs9488834
rs141291061
All − MR Egger
All − Inverse variance weighted
−2
0
2
MR effect size for
'CD28− DN (CD4−CD8−) %DN' on 'Diabetic nephropathy'

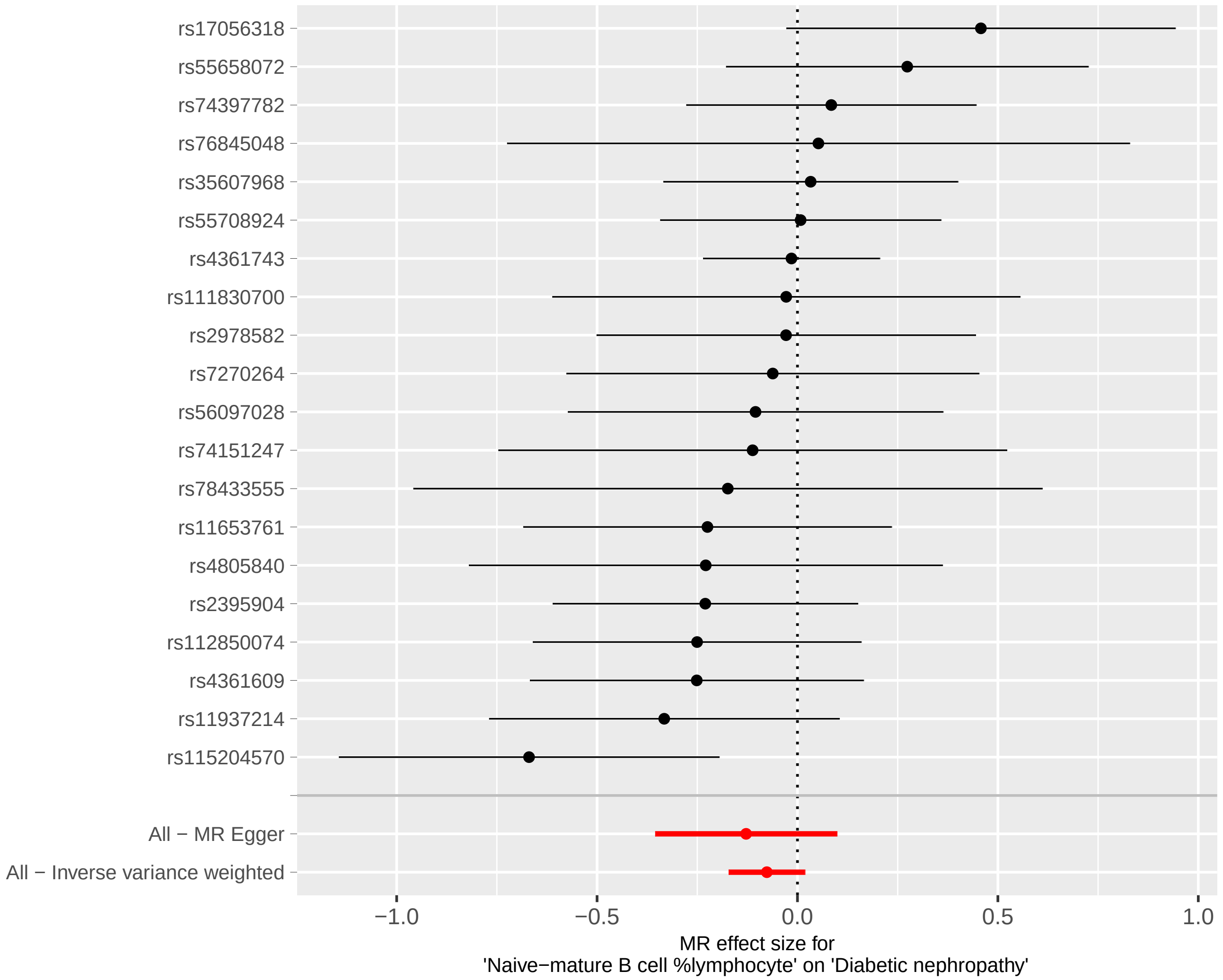

rs17056318
rs55658072
rs74397782
rs76845048
rs35607968
rs55708924
rs4361743
rs111830700
rs2978582
rs7270264
rs56097028
rs74151247
rs78433555
rs11653761
rs4805840
rs2395904
rs112850074
rs4361609
rs11937214
rs115204570
All − MR Egger
All − Inverse variance weighted
−1.0
−0.5
0.0
0.5
1.0
MR effect size for
'Naive−mature B cell %lymphocyte' on 'Diabetic nephropathy'

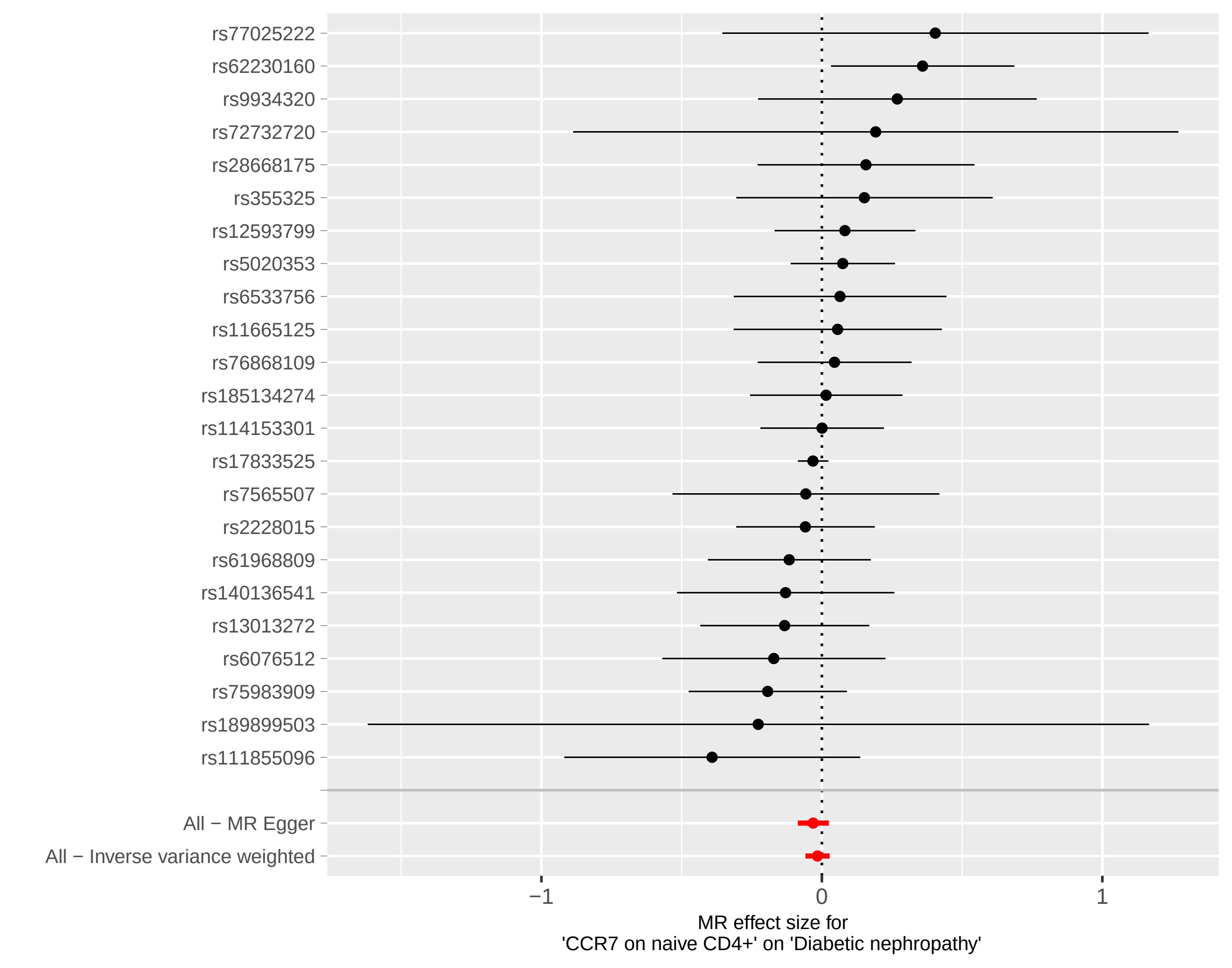

rs77025222
rs62230160
rs9934320
rs72732720
rs28668175
rs355325
rs12593799
rs5020353
rs6533756
rs11665125
rs76868109
rs185134274
rs114153301
rs17833525
rs7565507
rs2228015
rs61968809
rs140136541
rs13013272
rs6076512
rs75983909
rs189899503
rs111855096
All − MR Egger
All − Inverse variance weighted
−1
0
1
MR effect size for
'CCR7 on naive CD4+' on 'Diabetic nephropathy'

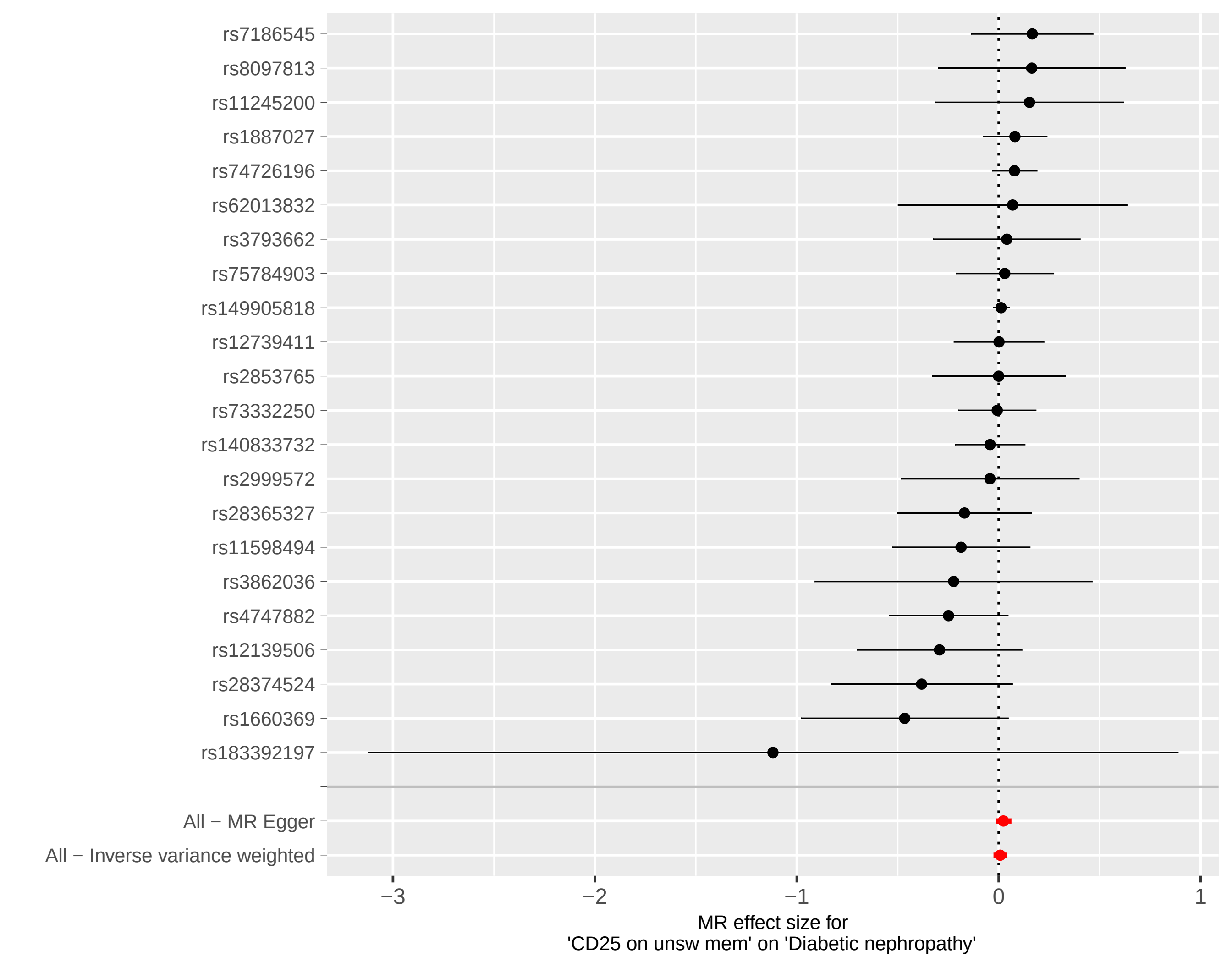

rs7186545
rs8097813
rs11245200
rs1887027
rs74726196
rs62013832
rs3793662
rs75784903
rs149905818
rs12739411
rs2853765
rs73332250
rs140833732
rs2999572
rs28365327
rs11598494
rs3862036
rs4747882
rs12139506
rs28374524
rs1660369
rs183392197
All − MR Egger
All − Inverse variance weighted
−3
−2
−1
0
1
MR effect size for
'CD25 on unsw mem' on 'Diabetic nephropathy'

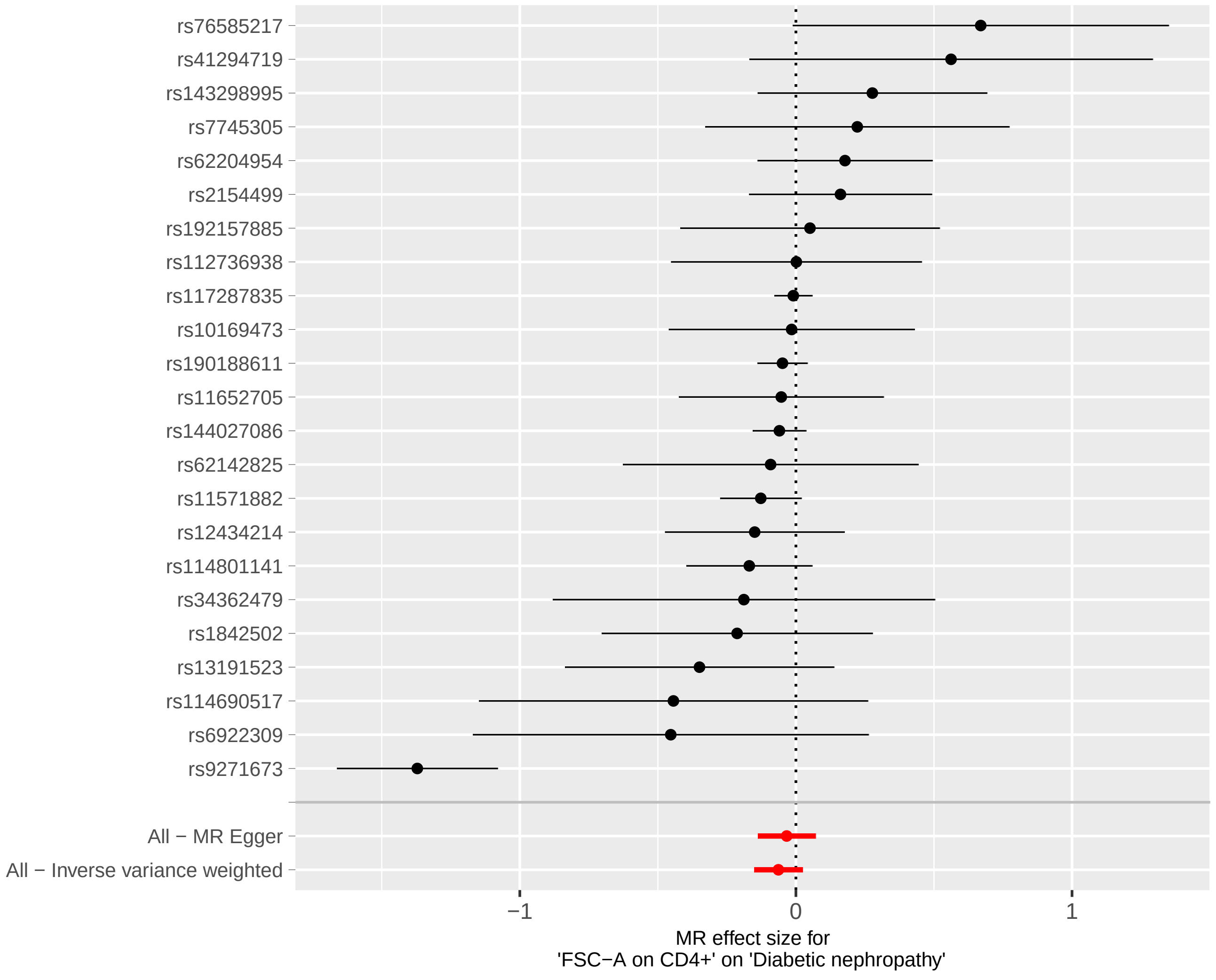
rs76585217
rs41294719
rs143298995
rs7745305
rs62204954
rs2154499
rs192157885
rs112736938
rs117287835
rs10169473
rs190188611
rs11652705
rs144027086
rs62142825
rs11571882
rs12434214
rs114801141
rs34362479
rs1842502
rs13191523
rs114690517
rs6922309
rs9271673
All − MR Egger
All − Inverse variance weighted
−1
0
1
MR effect size for
'FSC−A on CD4+' on 'Diabetic nephropathy'

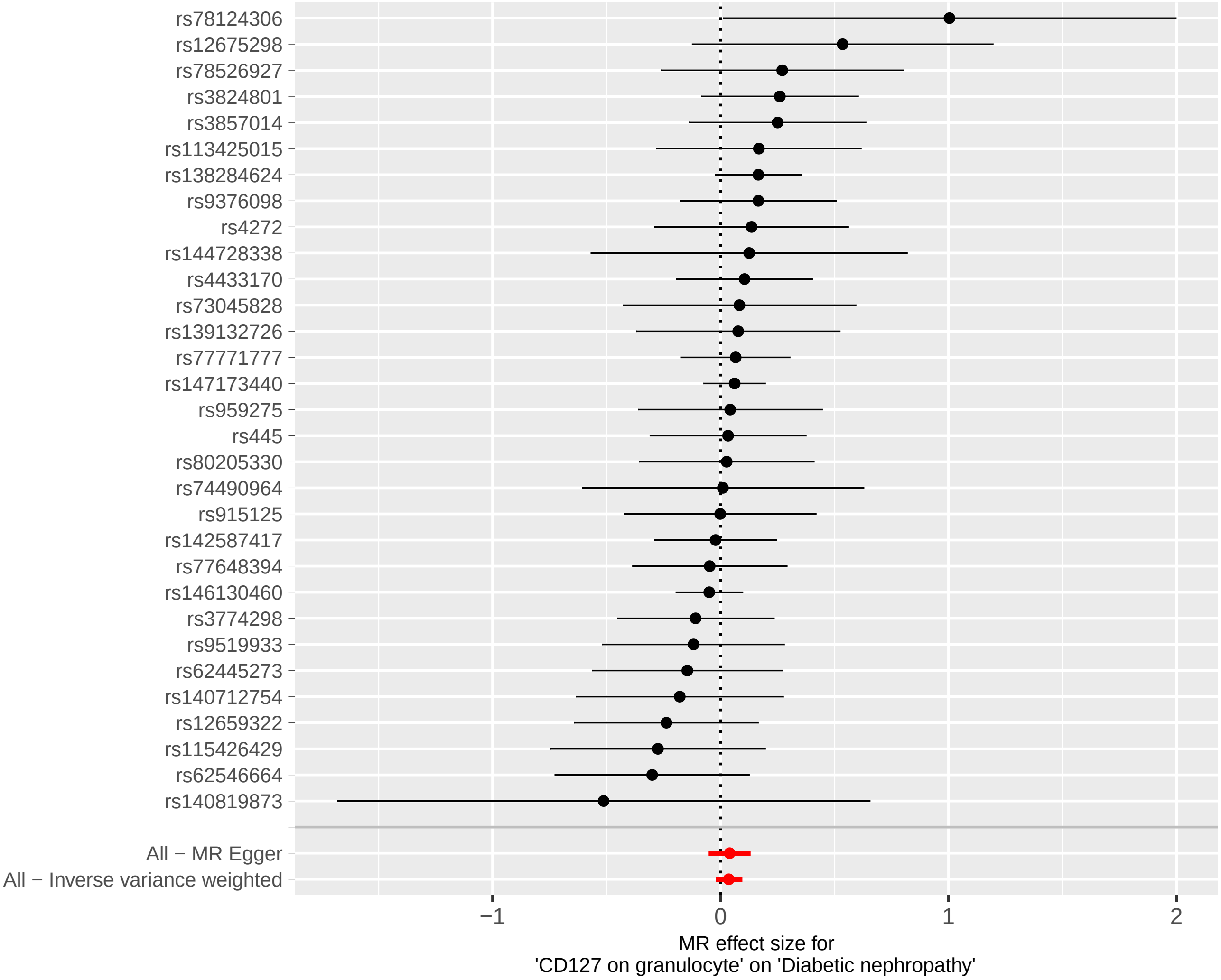
rs78124306
rs12675298
rs78526927
rs3824801
rs3857014
rs113425015
rs138284624
rs9376098
rs4272
rs144728338
rs4433170
rs73045828
rs139132726
rs77771777
rs147173440
rs959275
rs445
rs80205330
rs74490964
rs915125
rs142587417
rs77648394
rs146130460
rs3774298
rs9519933
rs62445273
rs140712754
rs12659322
rs115426429
rs62546664
rs140819873
All − MR Egger
All − Inverse variance weighted
−1
0
1
2
MR effect size for
'CD127 on granulocyte' on 'Diabetic nephropathy'

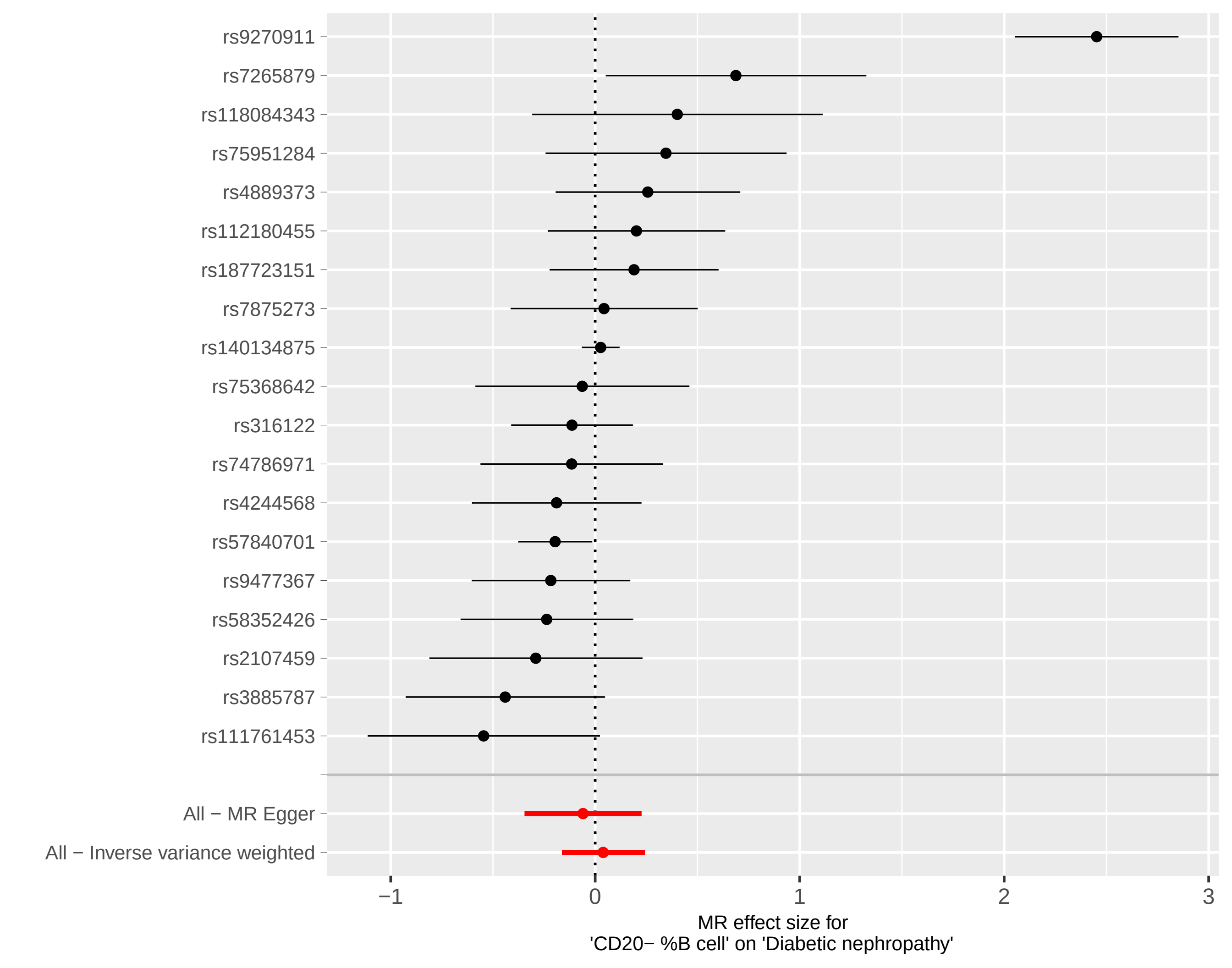
rs9270911
rs7265879
rs118084343
rs75951284
rs4889373
rs112180455
rs187723151
rs7875273
rs140134875
rs75368642
rs316122
rs74786971
rs4244568
rs57840701
rs9477367
rs58352426
rs2107459
rs3885787
rs111761453
All − MR Egger
All − Inverse variance weighted
−1
0
1
2
3
MR effect size for
'CD20− %B cell' on 'Diabetic nephropathy'

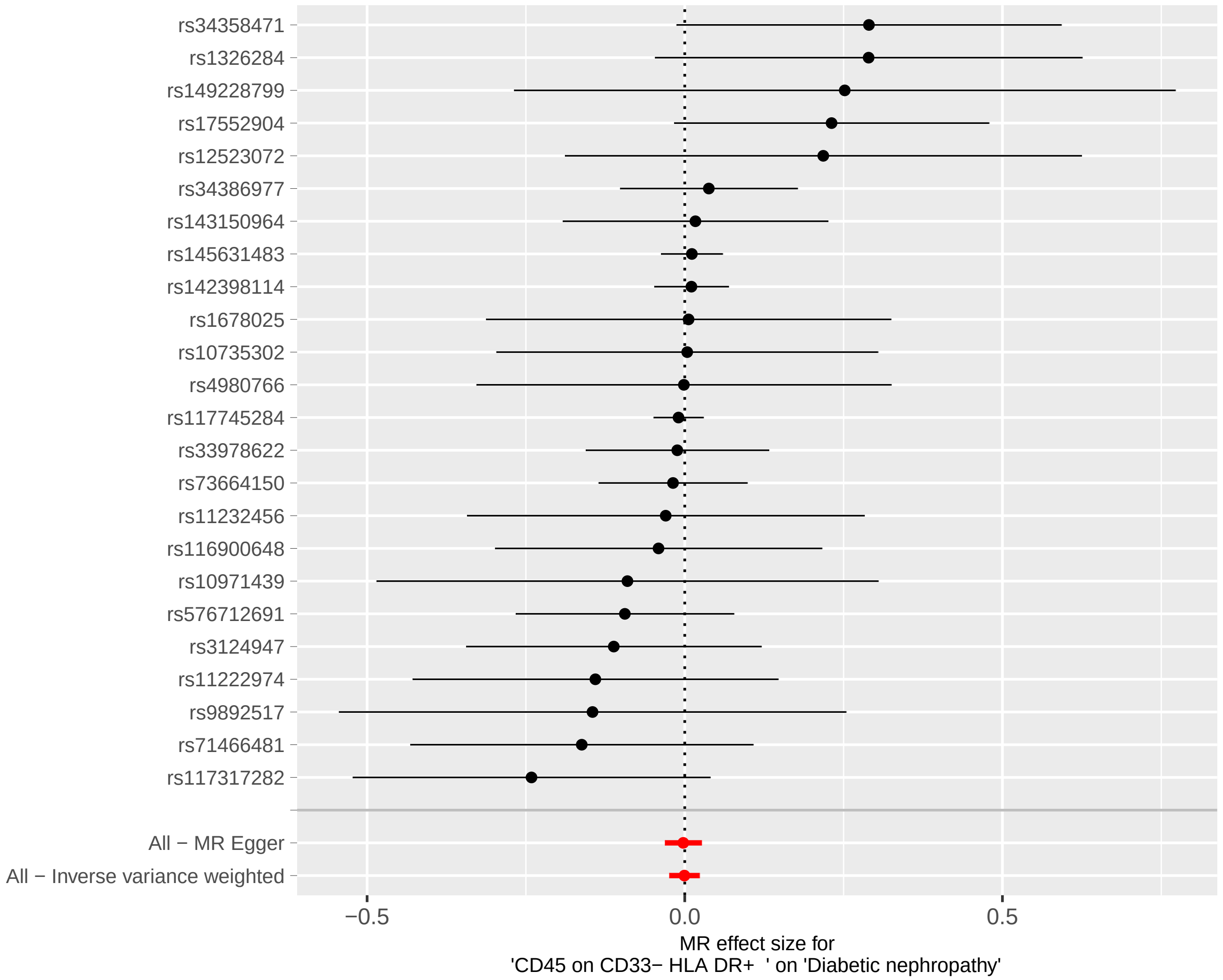

rs34358471
rs1326284
rs149228799
rs17552904
rs12523072
rs34386977
rs143150964
rs145631483
rs142398114
rs1678025
rs10735302
rs4980766
rs117745284
rs33978622
rs73664150
rs11232456
rs116900648
rs10971439
rs576712691
rs3124947
rs11222974
rs9892517
rs71466481
rs117317282
All − MR Egger
All − Inverse variance weighted
−0.5
0.0
0.5
MR effect size for
'CD45 on CD33− HLA DR+ ' on 'Diabetic nephropathy'

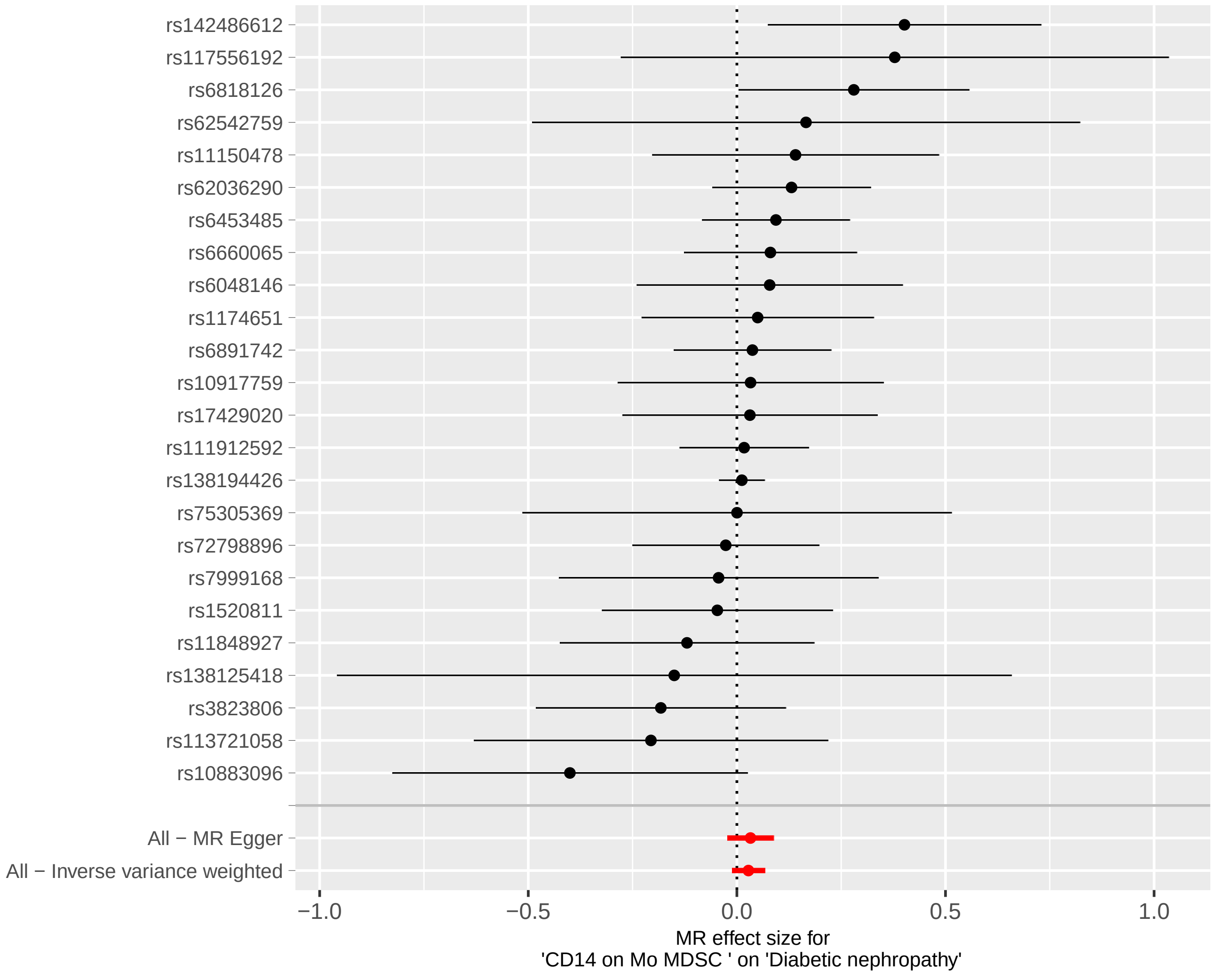
rs142486612
rs117556192
rs6818126
rs62542759
rs11150478
rs62036290
rs6453485
rs6660065
rs6048146
rs1174651
rs6891742
rs10917759
rs17429020
rs111912592
rs138194426
rs75305369
rs72798896
rs7999168
rs1520811
rs11848927
rs138125418
rs3823806
rs113721058
rs10883096
All − MR Egger
All − Inverse variance weighted
−1.0
−0.5
0.0
0.5
1.0
MR effect size for
'CD14 on Mo MDSC ' on 'Diabetic nephropathy'

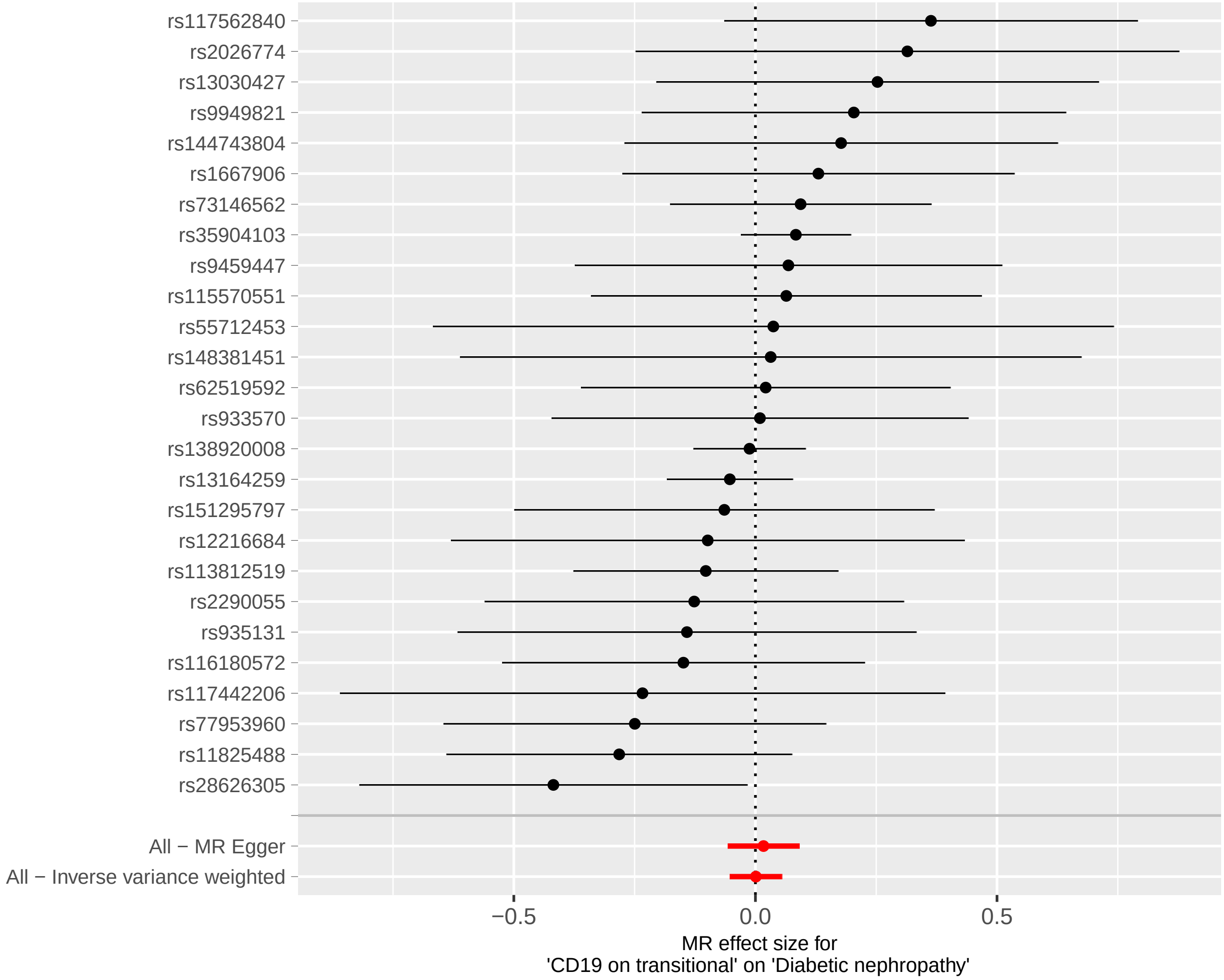
rs117562840
rs2026774
rs13030427
rs9949821
rs144743804
rs1667906
rs73146562
rs35904103
rs9459447
rs115570551
rs55712453
rs148381451
rs62519592
rs933570
rs138920008
rs13164259
rs151295797
rs12216684
rs113812519
rs2290055
rs935131
rs116180572
rs117442206
rs77953960
rs11825488
rs28626305
All − MR Egger
All − Inverse variance weighted
−0.5
0.0
0.5
MR effect size for
'CD19 on transitional' on 'Diabetic nephropathy'

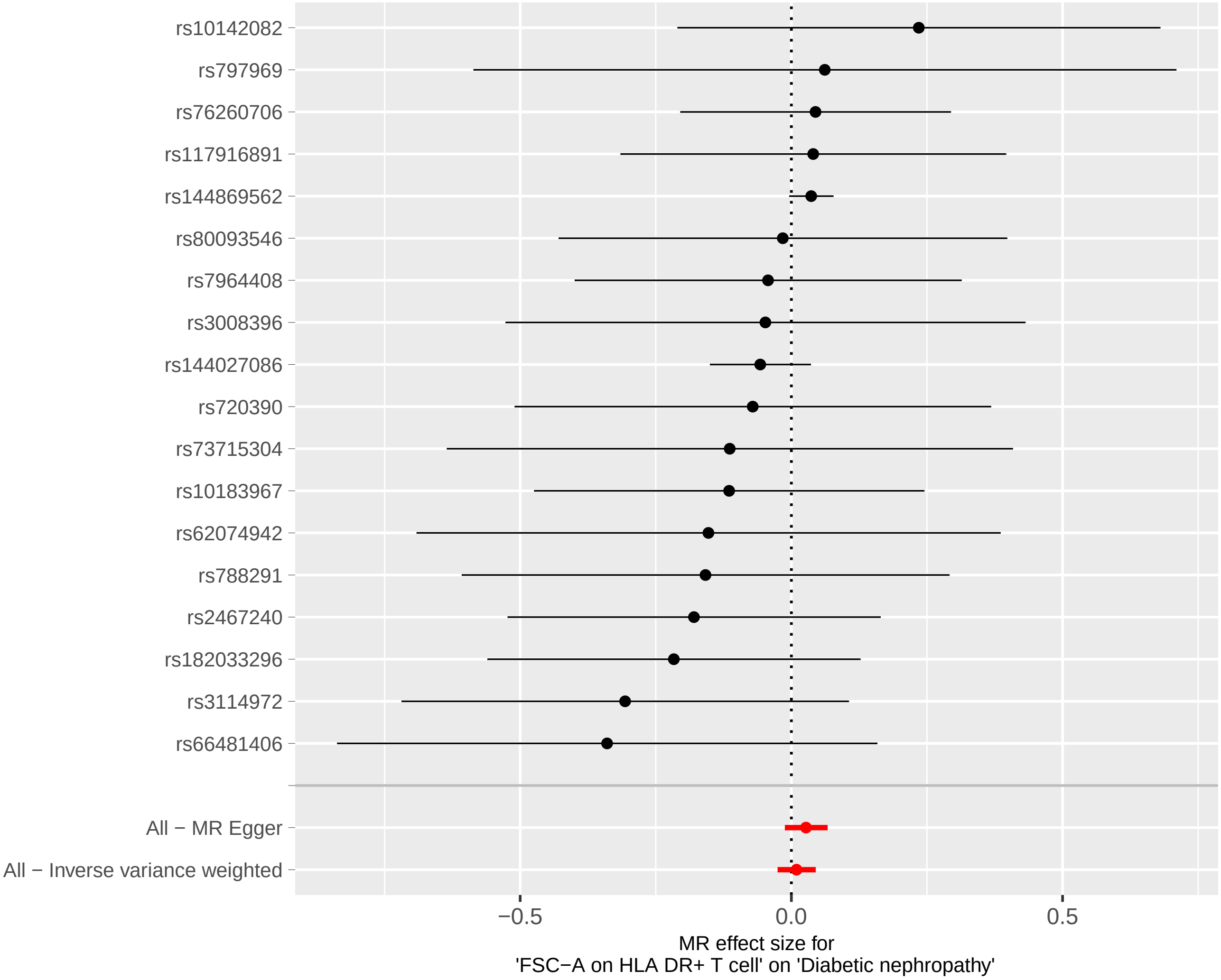
rs10142082
rs797969
rs76260706
rs117916891
rs144869562
rs80093546
rs7964408
rs3008396
rs144027086
rs720390
rs73715304
rs10183967
rs62074942
rs788291
rs2467240
rs182033296
rs3114972
rs66481406
All − MR Egger
All − Inverse variance weighted
−0.5
0.0
0.5
MR effect size for
'FSC−A on HLA DR+ T cell' on 'Diabetic nephropathy'

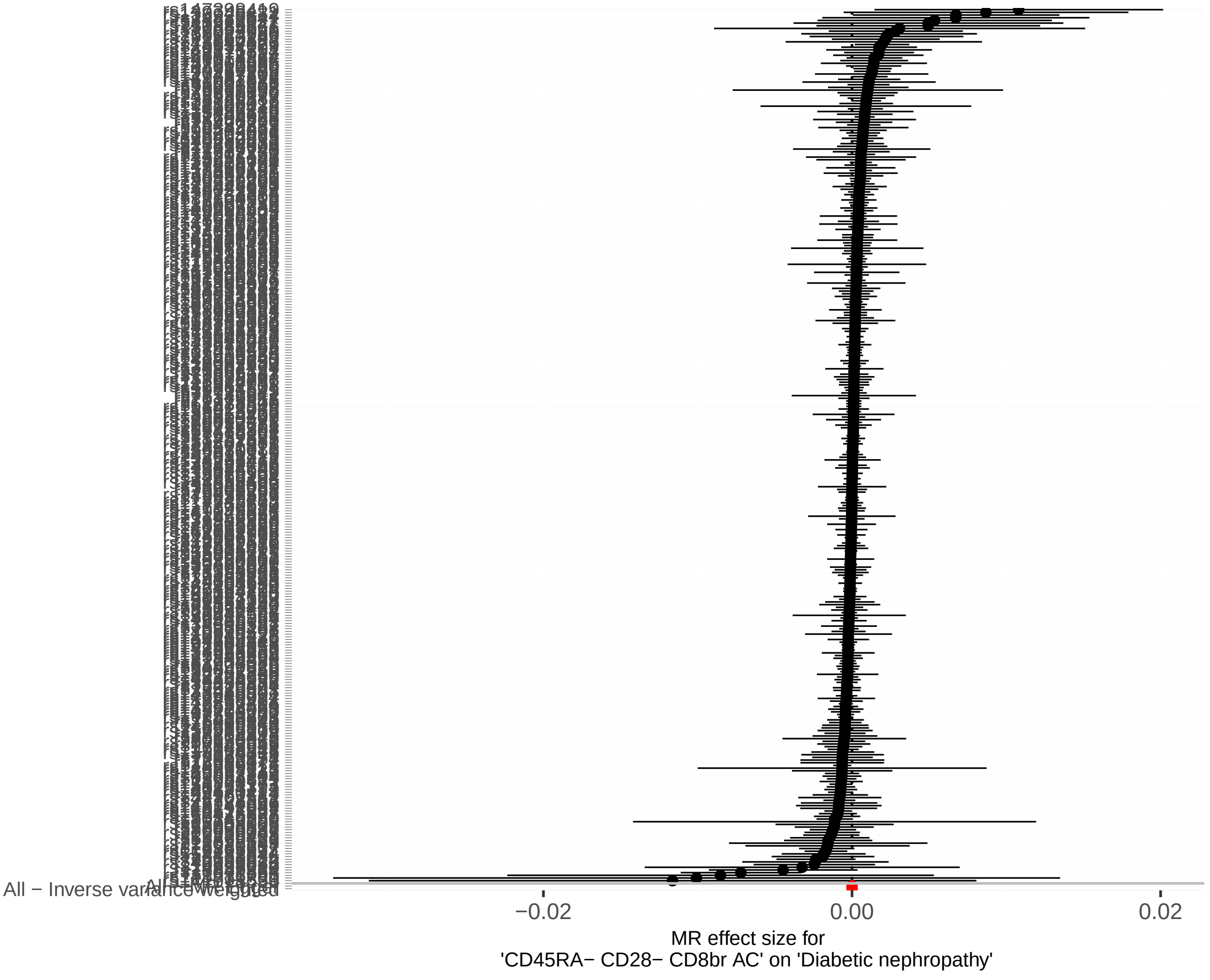
All − Inverse variance weighted
−0.02
0.00
0.02
MR effect size for
'CD45RA− CD28− CD8br AC' on 'Diabetic nephropathy'

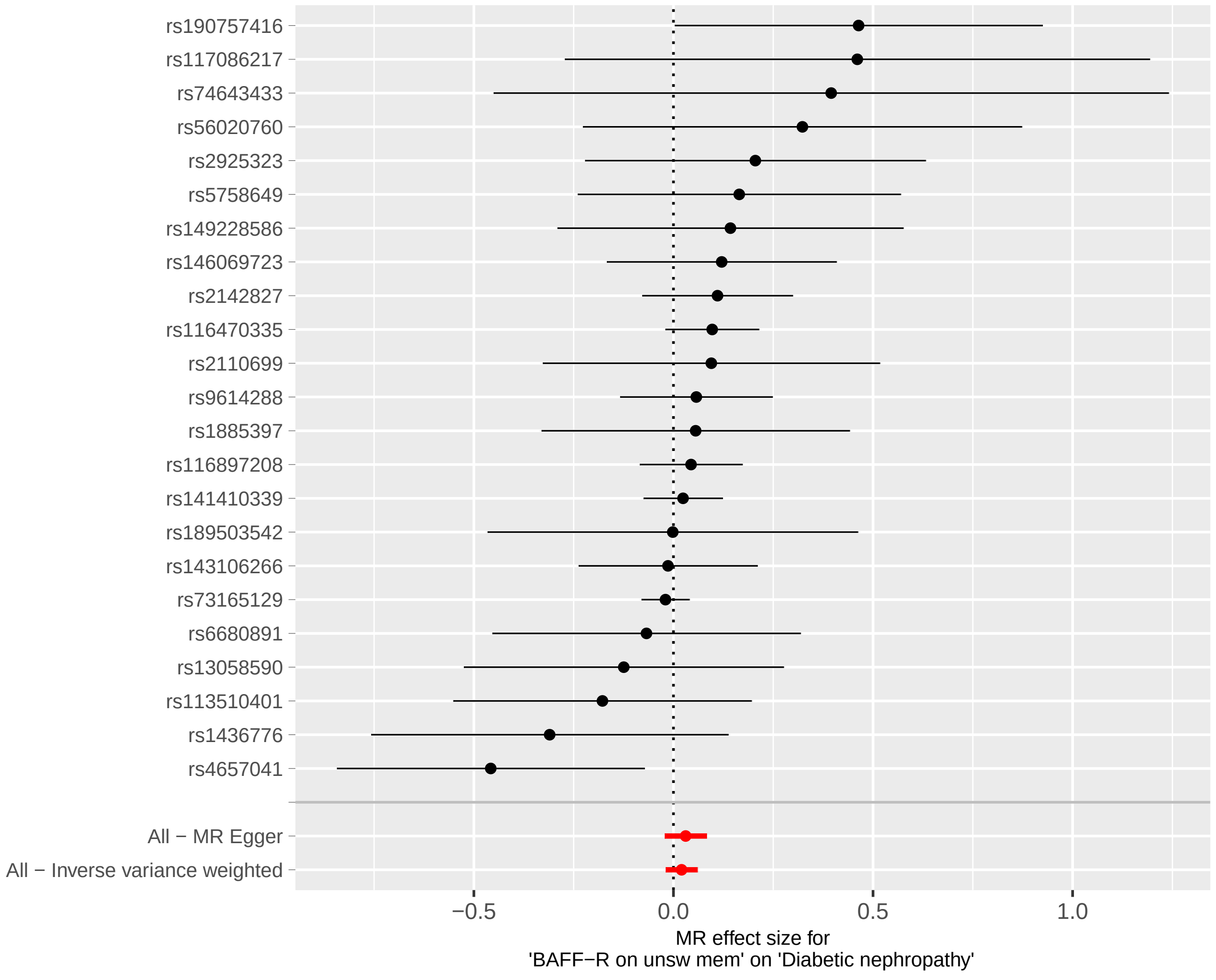
rs190757416
rs117086217
rs74643433
rs56020760
rs2925323
rs5758649
rs149228586
rs146069723
rs2142827
rs116470335
rs2110699
rs9614288
rs1885397
rs116897208
rs141410339
rs189503542
rs143106266
rs73165129
rs6680891
rs13058590
rs113510401
rs1436776
rs4657041
All − MR Egger
All − Inverse variance weighted
−0.5
0.0
0.5
1.0
MR effect size for
'BAFF−R on unsw mem' on 'Diabetic nephropathy'

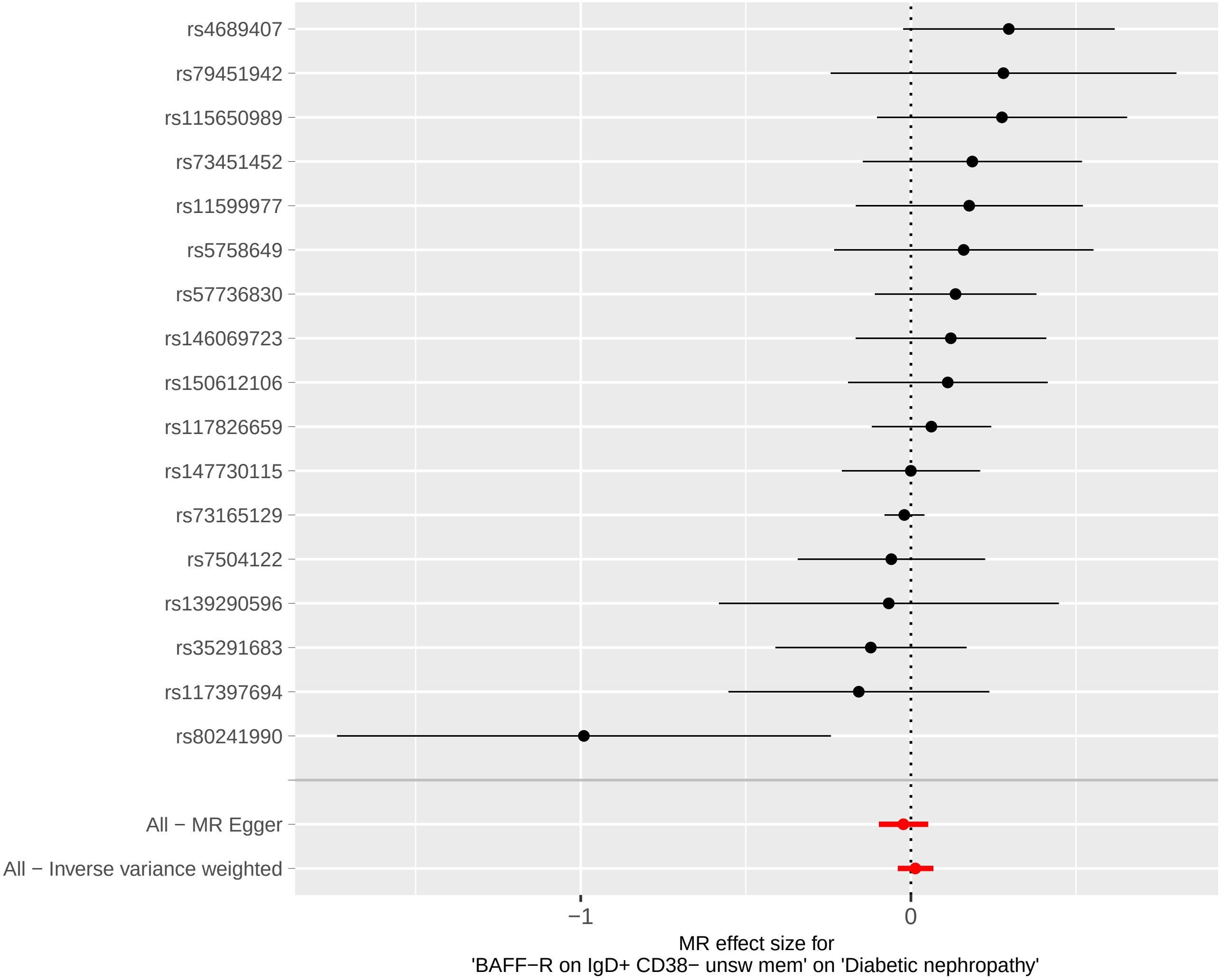
rs4689407
rs79451942
rs115650989
rs73451452
rs11599977
rs5758649
rs57736830
rs146069723
rs150612106
rs117826659
rs147730115
rs73165129
rs7504122
rs139290596
rs35291683
rs117397694
rs80241990
All − MR Egger
All − Inverse variance weighted
−1
0
MR effect size for
'BAFF−R on IgD+ CD38− unsw mem' on 'Diabetic nephropathy'

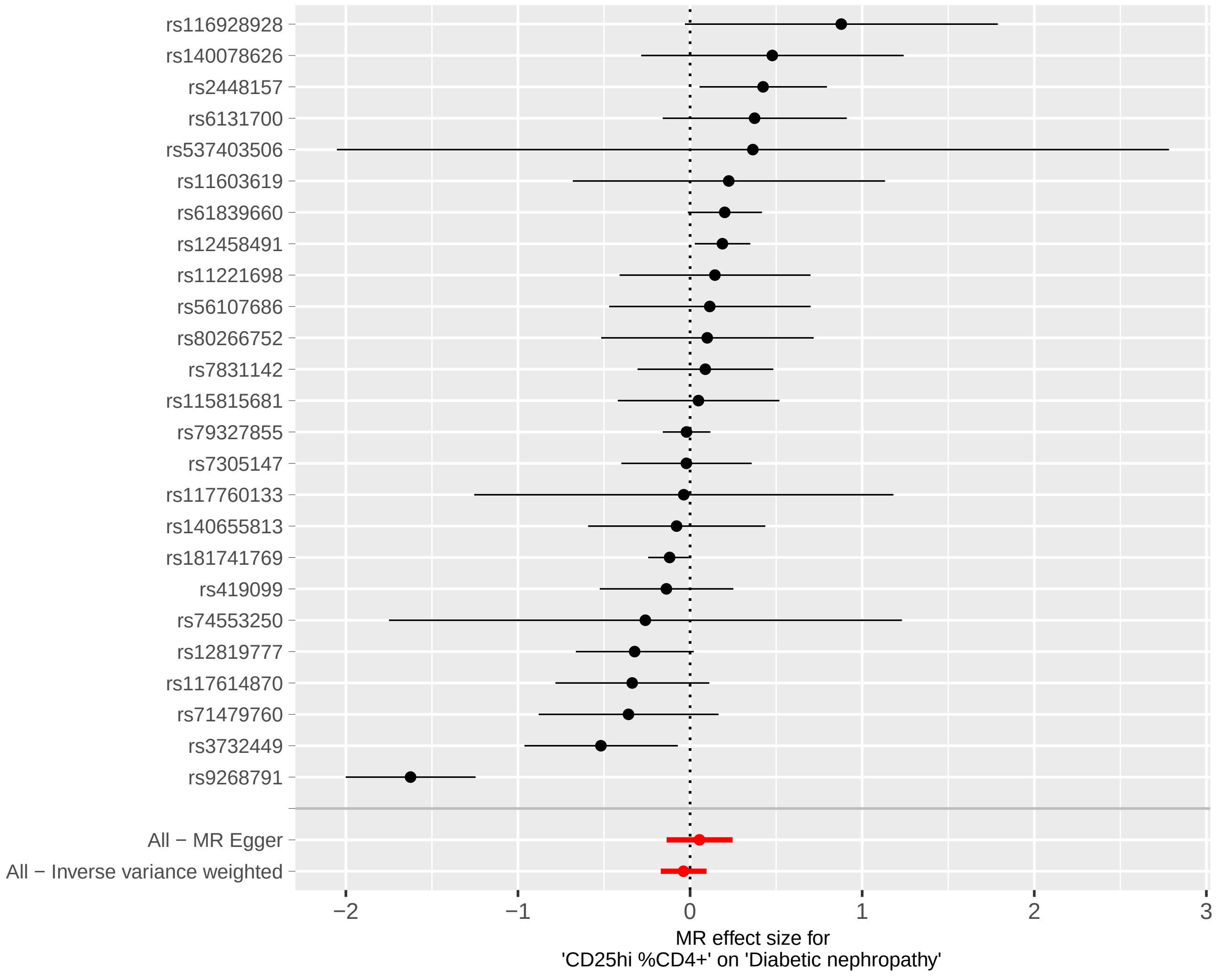

rs116928928
rs140078626
rs2448157
rs6131700
rs537403506
rs11603619
rs61839660
rs12458491
rs11221698
rs56107686
rs80266752
rs7831142
rs115815681
rs79327855
rs7305147
rs117760133
rs140655813
rs181741769
rs419099
rs74553250
rs12819777
rs117614870
rs71479760
rs3732449
rs9268791
All − MR Egger
All − Inverse variance weighted
−2
−1
0
1
2
3
MR effect size for
'CD25hi %CD4+' on 'Diabetic nephropathy'

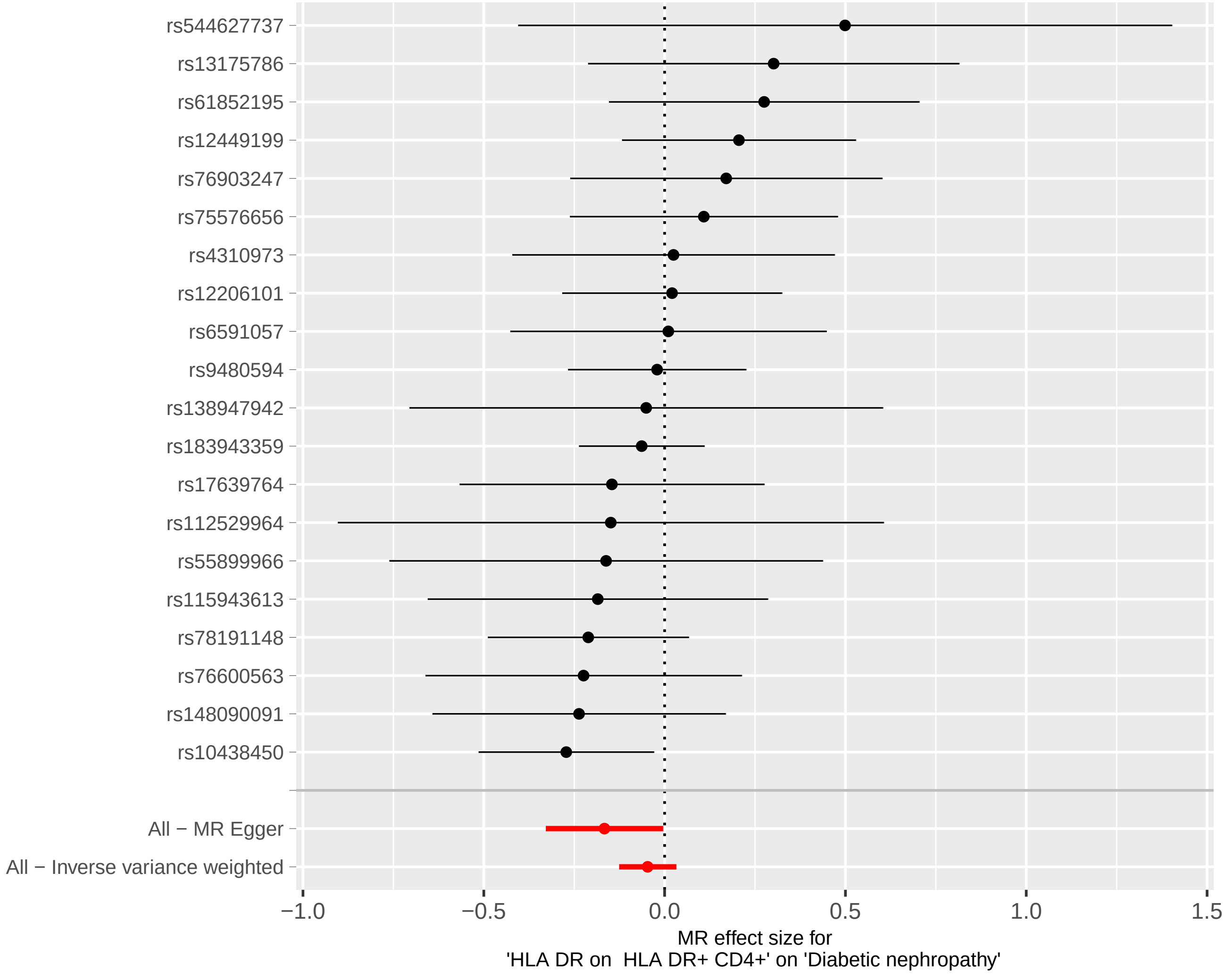

rs544627737
rs13175786
rs61852195
rs12449199
rs76903247
rs75576656
rs4310973
rs12206101
rs6591057
rs9480594
rs138947942
rs183943359
rs17639764
rs112529964
rs55899966
rs115943613
rs78191148
rs76600563
rs148090091
rs10438450
All − MR Egger
All − Inverse variance weighted
−1.0
−0.5
0.0
0.5
1.0
1.5
MR effect size for
'HLA DR on HLA DR+ CD4+' on 'Diabetic nephropathy'

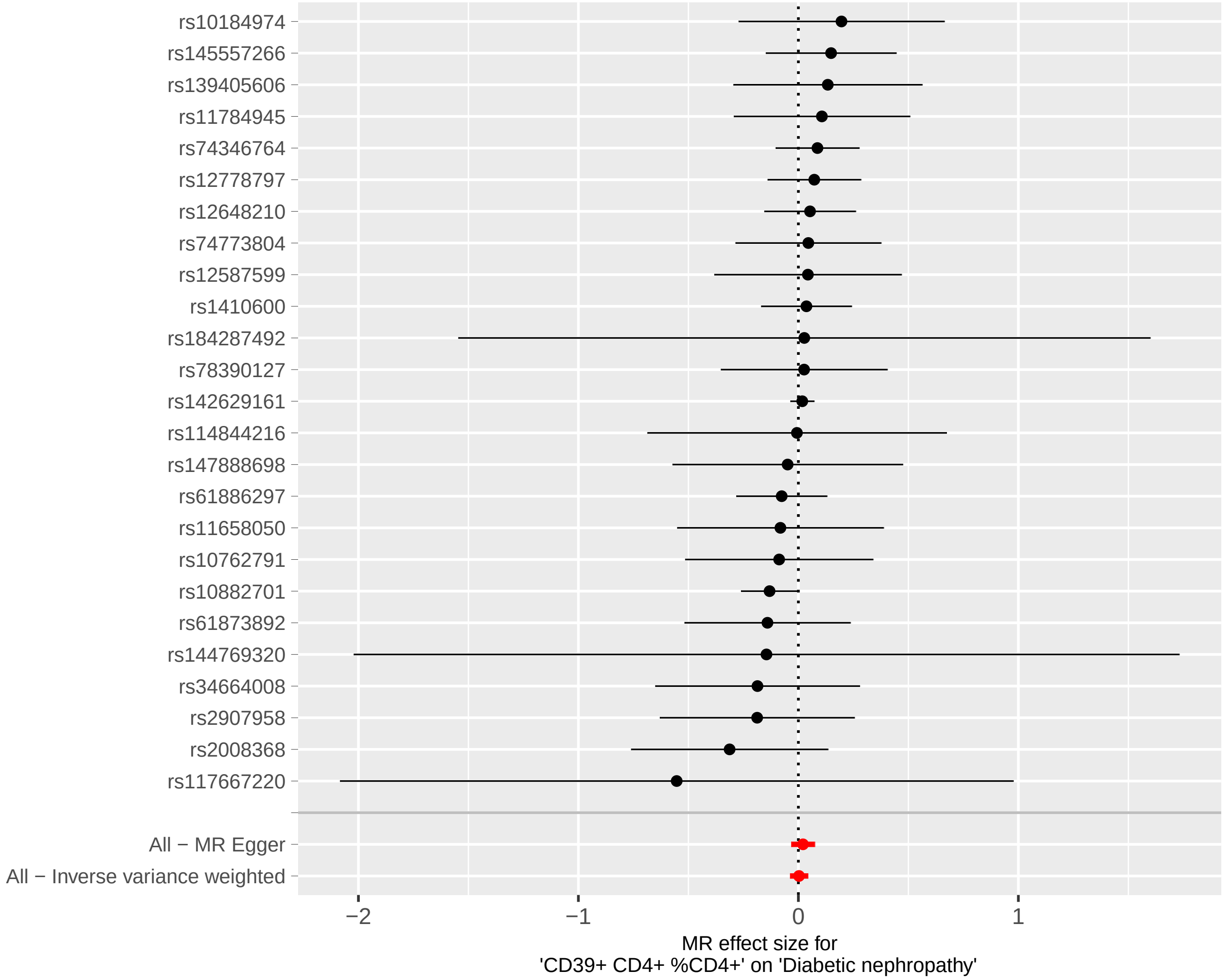
rs10184974
rs145557266
rs139405606
rs11784945
rs74346764
rs12778797
rs12648210
rs74773804
rs12587599
rs1410600
rs184287492
rs78390127
rs142629161
rs114844216
rs147888698
rs61886297
rs11658050
rs10762791
rs10882701
rs61873892
rs144769320
rs34664008
rs2907958
rs2008368
rs117667220
All − MR Egger
All − Inverse variance weighted
−2
−1
0
1
MR effect size for
'CD39+ CD4+ %CD4+' on 'Diabetic nephropathy'

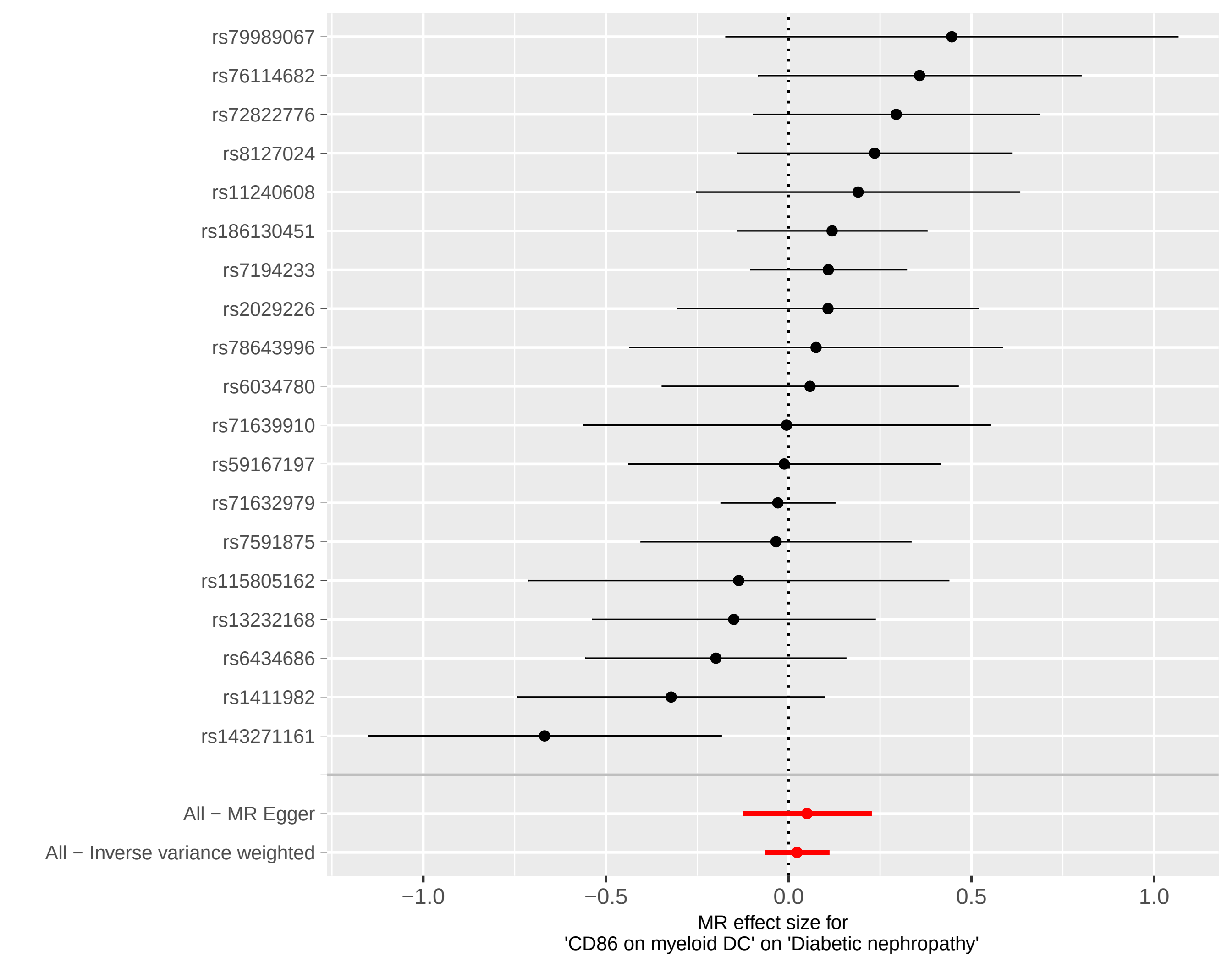
rs79989067
rs76114682
rs72822776
rs8127024
rs11240608
rs186130451
rs7194233
rs2029226
rs78643996
rs6034780
rs71639910
rs59167197
rs71632979
rs7591875
rs115805162
rs13232168
rs6434686
rs1411982
rs143271161
All − MR Egger
All − Inverse variance weighted
−1.0
−0.5
0.0
0.5
1.0
MR effect size for
'CD86 on myeloid DC' on 'Diabetic nephropathy'

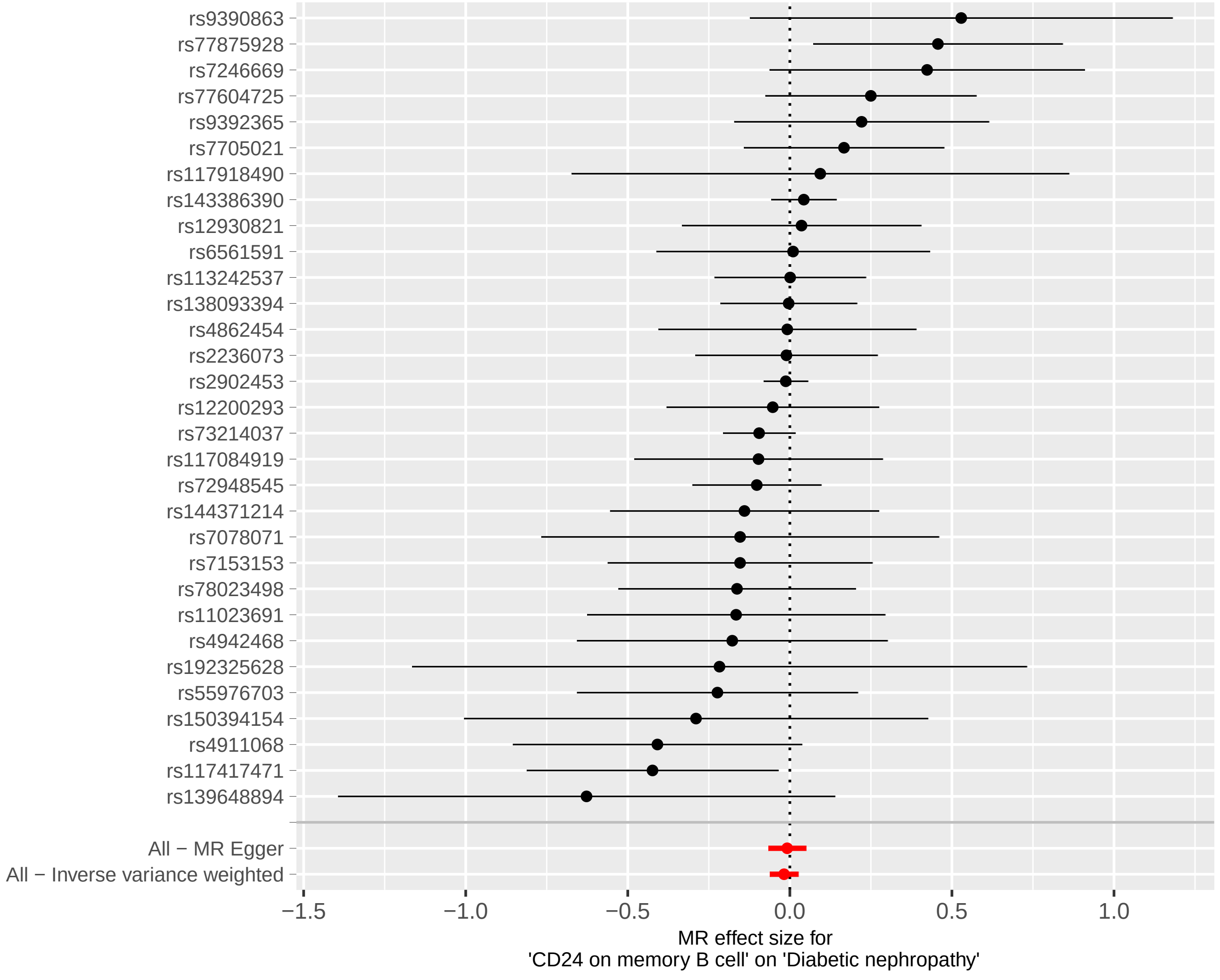

rs9390863
rs77875928
rs7246669
rs77604725
rs9392365
rs7705021
rs117918490
rs143386390
rs12930821
rs6561591
rs113242537
rs138093394
rs4862454
rs2236073
rs2902453
rs12200293
rs73214037
rs117084919
rs72948545
rs144371214
rs7078071
rs7153153
rs78023498
rs11023691
rs4942468
rs192325628
rs55976703
rs150394154
rs4911068
rs117417471
rs139648894
All − MR Egger
All − Inverse variance weighted
−1.5
−1.0
−0.5
0.0
0.5
1.0
MR effect size for
'CD24 on memory B cell' on 'Diabetic nephropathy'

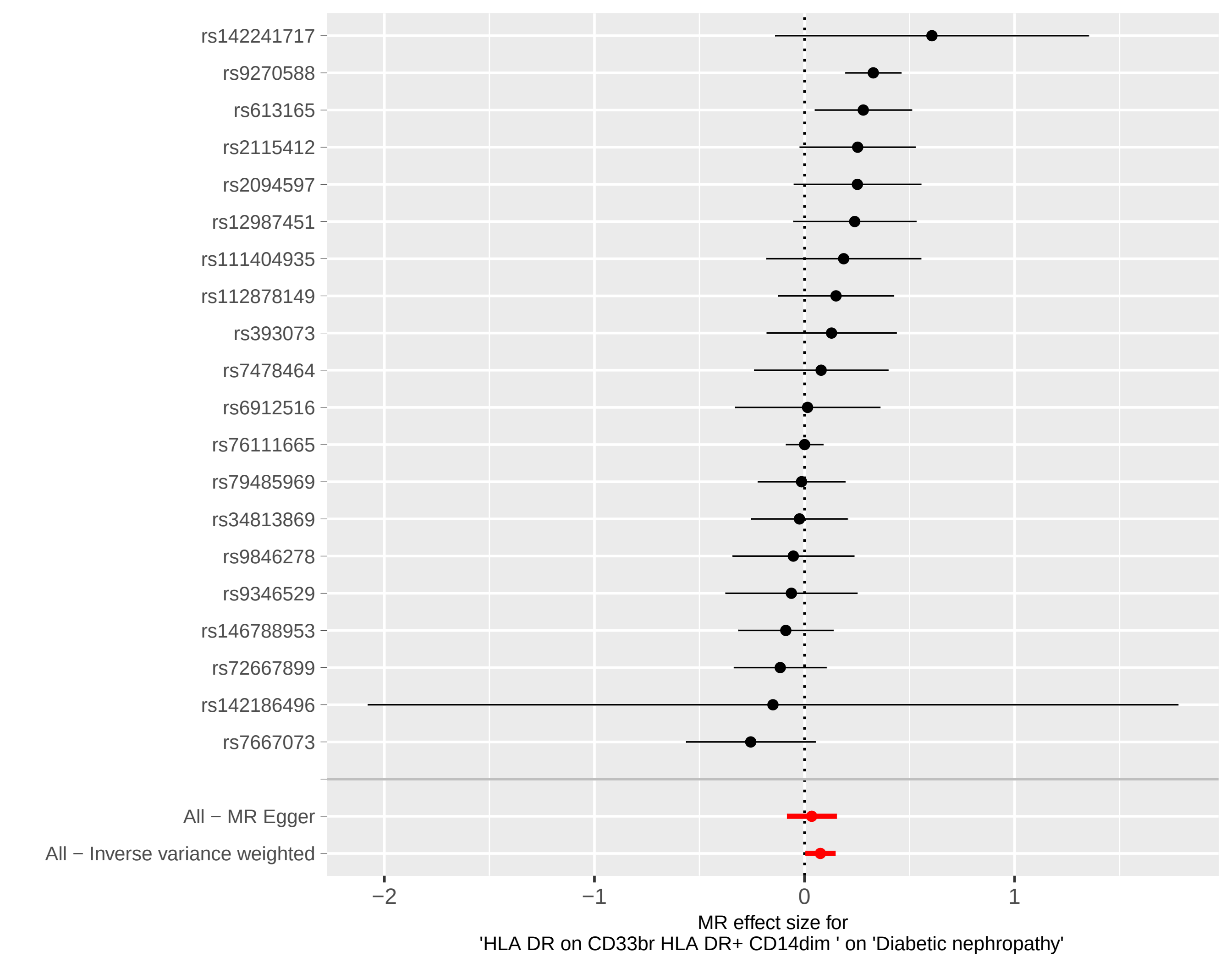

rs142241717
rs9270588
rs613165
rs2115412
rs2094597
rs12987451
rs111404935
rs112878149
rs393073
rs7478464
rs6912516
rs76111665
rs79485969
rs34813869
rs9846278
rs9346529
rs146788953
rs72667899
rs142186496
rs7667073
All − MR Egger
All − Inverse variance weighted
−2
−1
0
1
MR effect size for
'HLA DR on CD33br HLA DR+ CD14dim ' on 'Diabetic nephropathy'

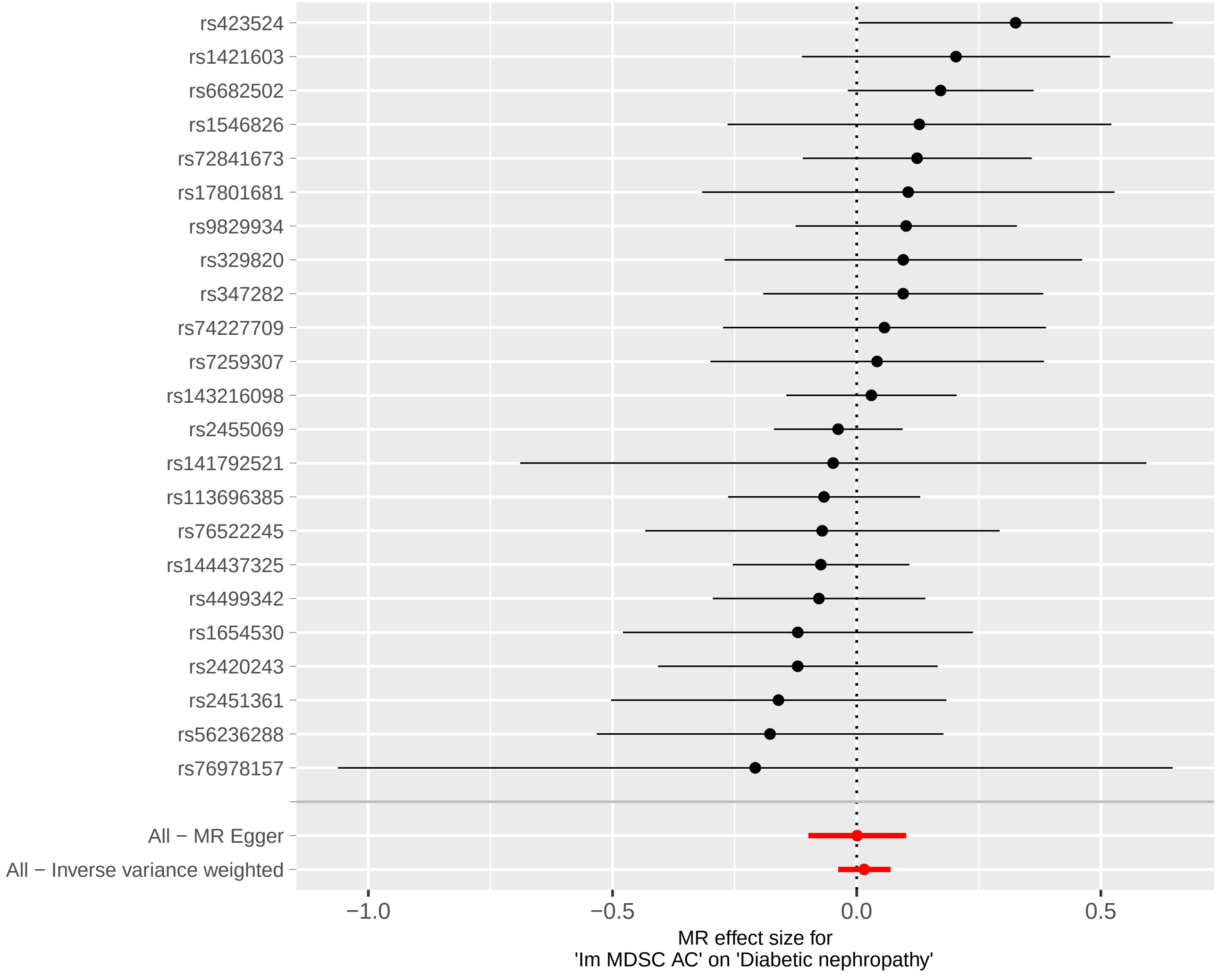
rs423524
rs1421603
rs6682502
rs1546826
rs72841673
rs17801681
rs9829934
rs329820
rs347282
rs74227709
rs7259307
rs143216098
rs2455069
rs141792521
rs113696385
rs76522245
rs144437325
rs4499342
rs1654530
rs2420243
rs2451361
rs56236288
rs76978157
All − MR Egger
All − Inverse variance weighted
−1.0
−0.5
0.0
0.5
MR effect size for
'Im MDSC AC' on 'Diabetic nephropathy'

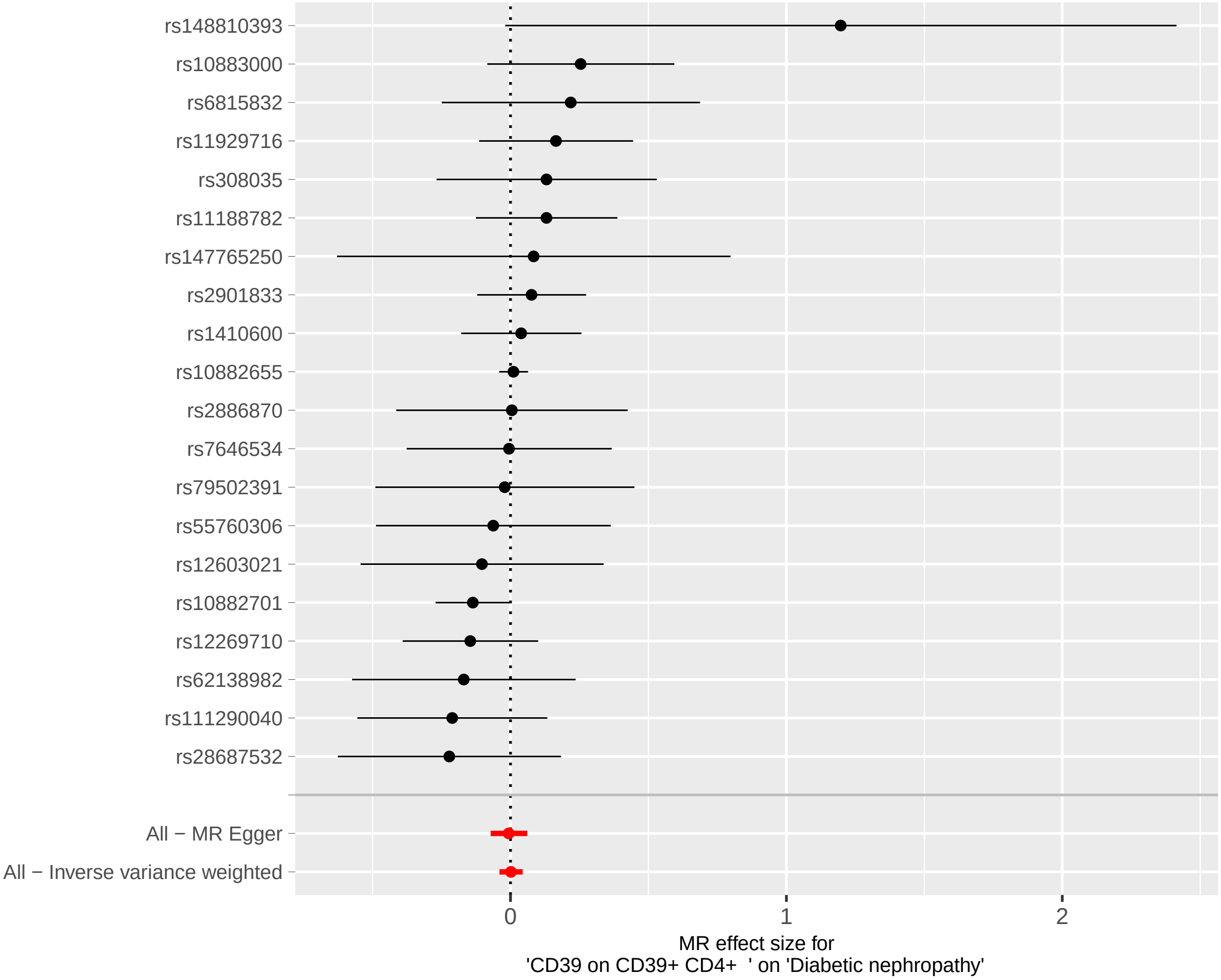
rs148810393
rs10883000
rs6815832
rs11929716
rs308035
rs11188782
rs147765250
rs2901833
rs1410600
rs10882655
rs2886870
rs7646534
rs79502391
rs55760306
rs12603021
rs10882701
rs12269710
rs62138982
rs111290040
rs28687532
All − MR Egger
All − Inverse variance weighted
0
1
2
MR effect size for
'CD39 on CD39+ CD4+ ' on 'Diabetic nephropathy'

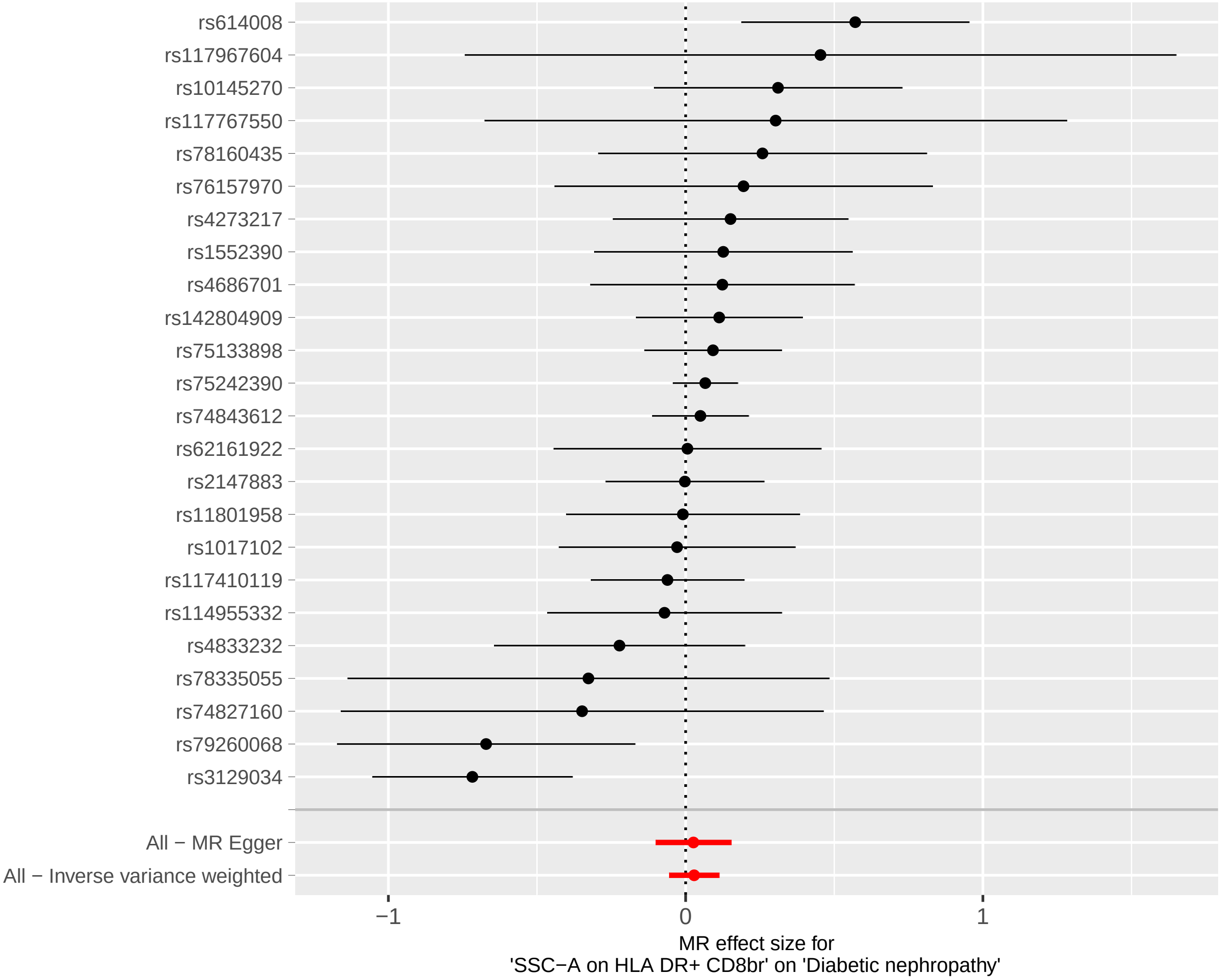
rs614008
rs117967604
rs10145270
rs117767550
rs78160435
rs76157970
rs4273217
rs1552390
rs4686701
rs142804909
rs75133898
rs75242390
rs74843612
rs62161922
rs2147883
rs11801958
rs1017102
rs117410119
rs114955332
rs4833232
rs78335055
rs74827160
rs79260068
rs3129034
All − MR Egger
All − Inverse variance weighted
−1
0
1
MR effect size for
'SSC−A on HLA DR+ CD8br' on 'Diabetic nephropathy'

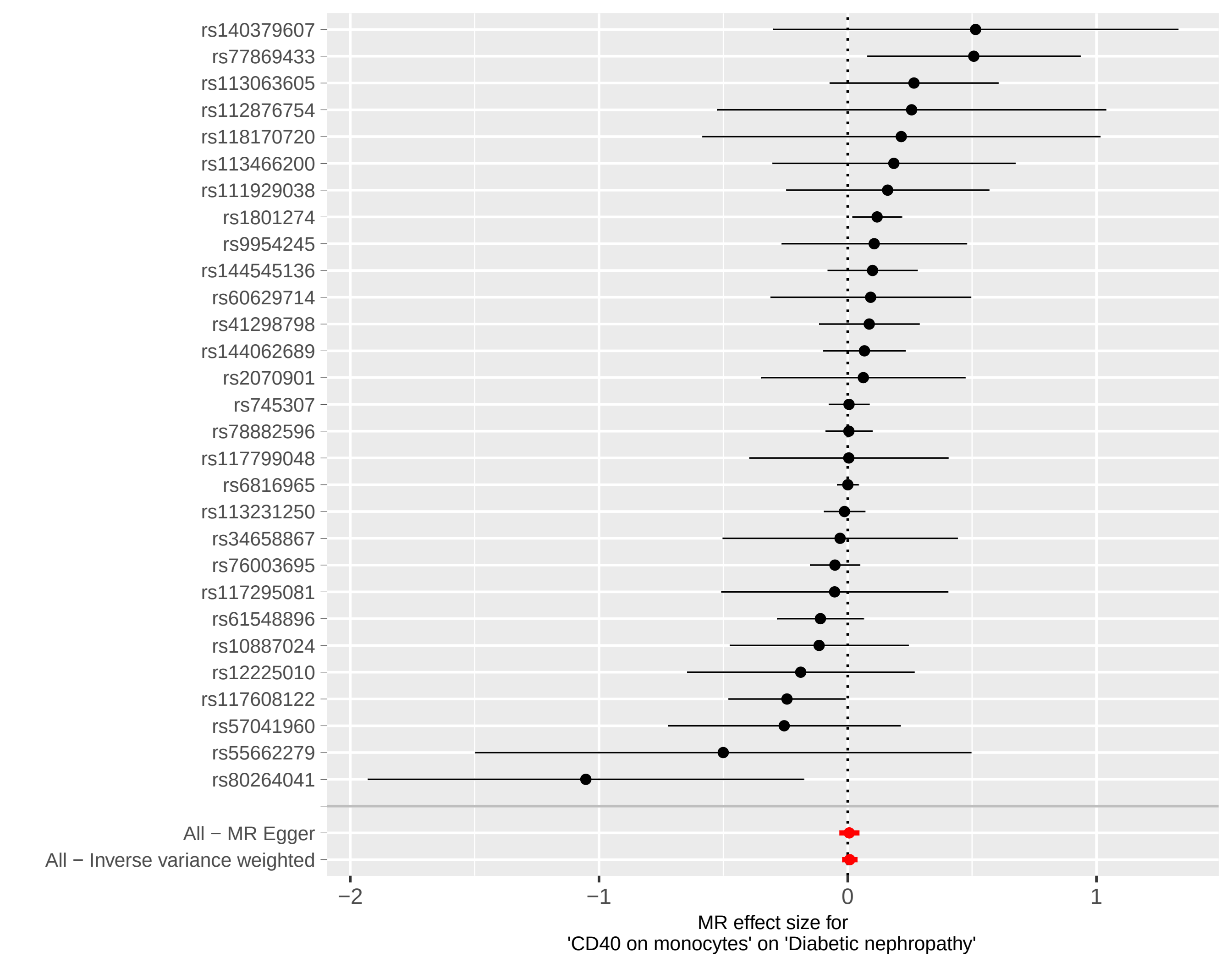

rs140379607
rs77869433
rs113063605
rs112876754
rs118170720
rs113466200
rs111929038
rs1801274
rs9954245
rs144545136
rs60629714
rs41298798
rs144062689
rs2070901
rs745307
rs78882596
rs117799048
rs6816965
rs113231250
rs34658867
rs76003695
rs117295081
rs61548896
rs10887024
rs12225010
rs117608122
rs57041960
rs55662279
rs80264041
All − MR Egger
All − Inverse variance weighted
−2
−1
0
1
MR effect size for
'CD40 on monocytes' on 'Diabetic nephropathy'

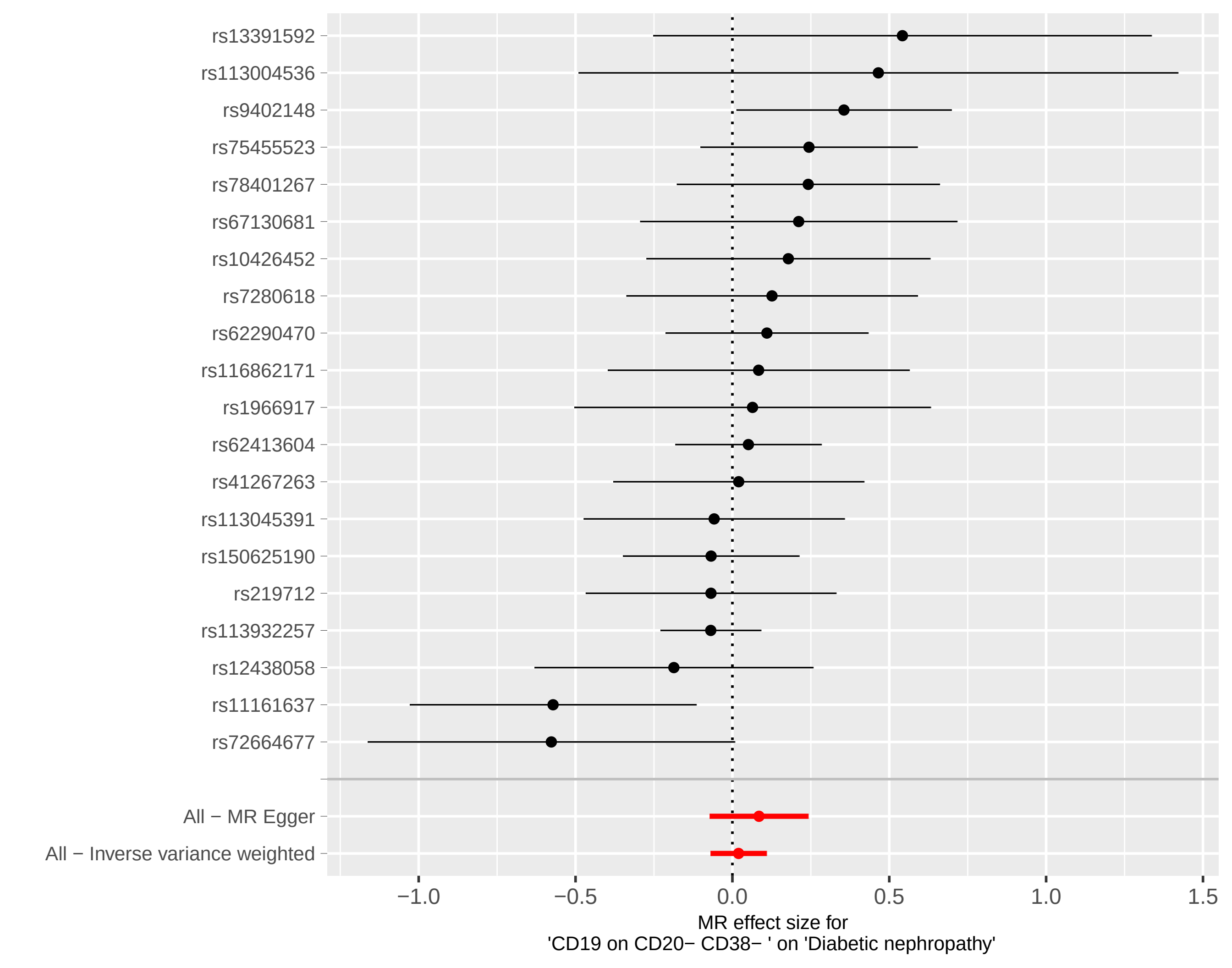

rs13391592
rs113004536
rs9402148
rs75455523
rs78401267
rs67130681
rs10426452
rs7280618
rs62290470
rs116862171
rs1966917
rs62413604
rs41267263
rs113045391
rs150625190
rs219712
rs113932257
rs12438058
rs11161637
rs72664677
All − MR Egger
All − Inverse variance weighted
−1.0
−0.5
0.0
0.5
1.0
1.5
MR effect size for
'CD19 on CD20− CD38− ' on 'Diabetic nephropathy'

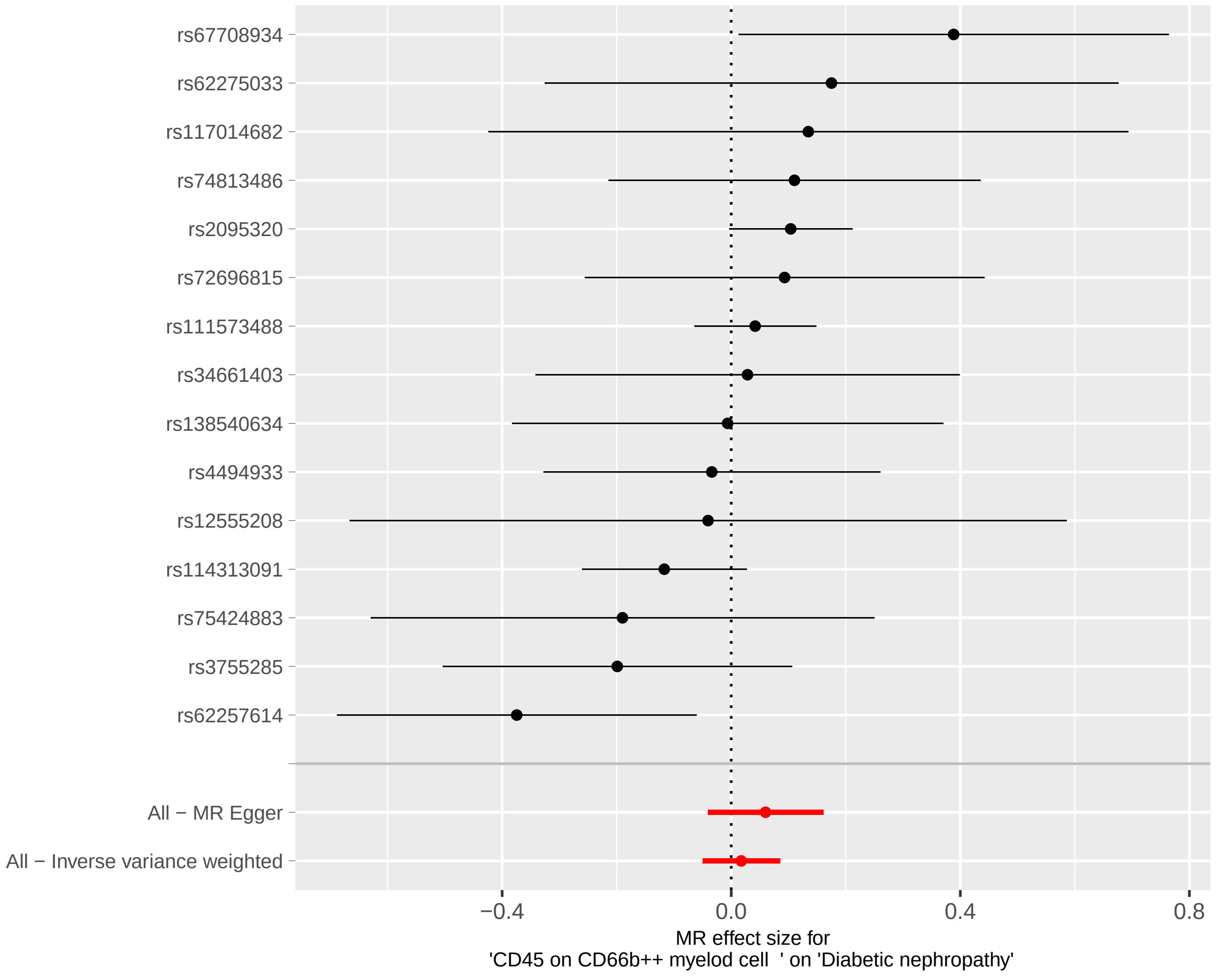
rs67708934
rs62275033
rs117014682
rs74813486
rs2095320
rs72696815
rs111573488
rs34661403
rs138540634
rs4494933
rs12555208
rs114313091
rs75424883
rs3755285
rs62257614
All − MR Egger
All − Inverse variance weighted
−0.4
0.0
0.4
0.8
MR effect size for
'CD45 on CD66b++ myelod cell ' on 'Diabetic nephropathy'

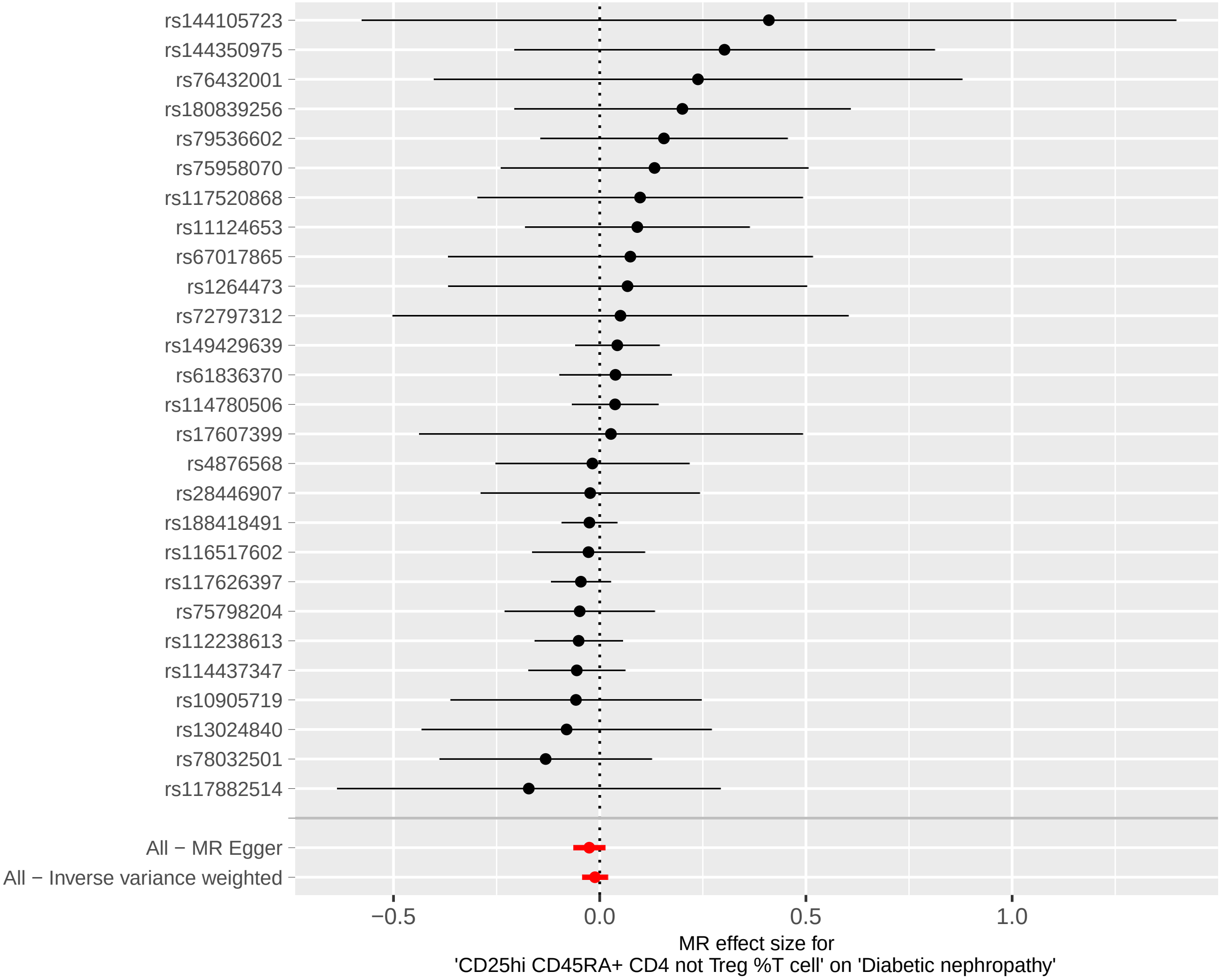
rs144105723
rs144350975
rs76432001
rs180839256
rs79536602
rs75958070
rs117520868
rs11124653
rs67017865
rs1264473
rs72797312
rs149429639
rs61836370
rs114780506
rs17607399
rs4876568
rs28446907
rs188418491
rs116517602
rs117626397
rs75798204
rs112238613
rs114437347
rs10905719
rs13024840
rs78032501
rs117882514
All − MR Egger
All − Inverse variance weighted
−0.5
0.0
0.5
1.0
MR effect size for
'CD25hi CD45RA+ CD4 not Treg %T cell' on 'Diabetic nephropathy'

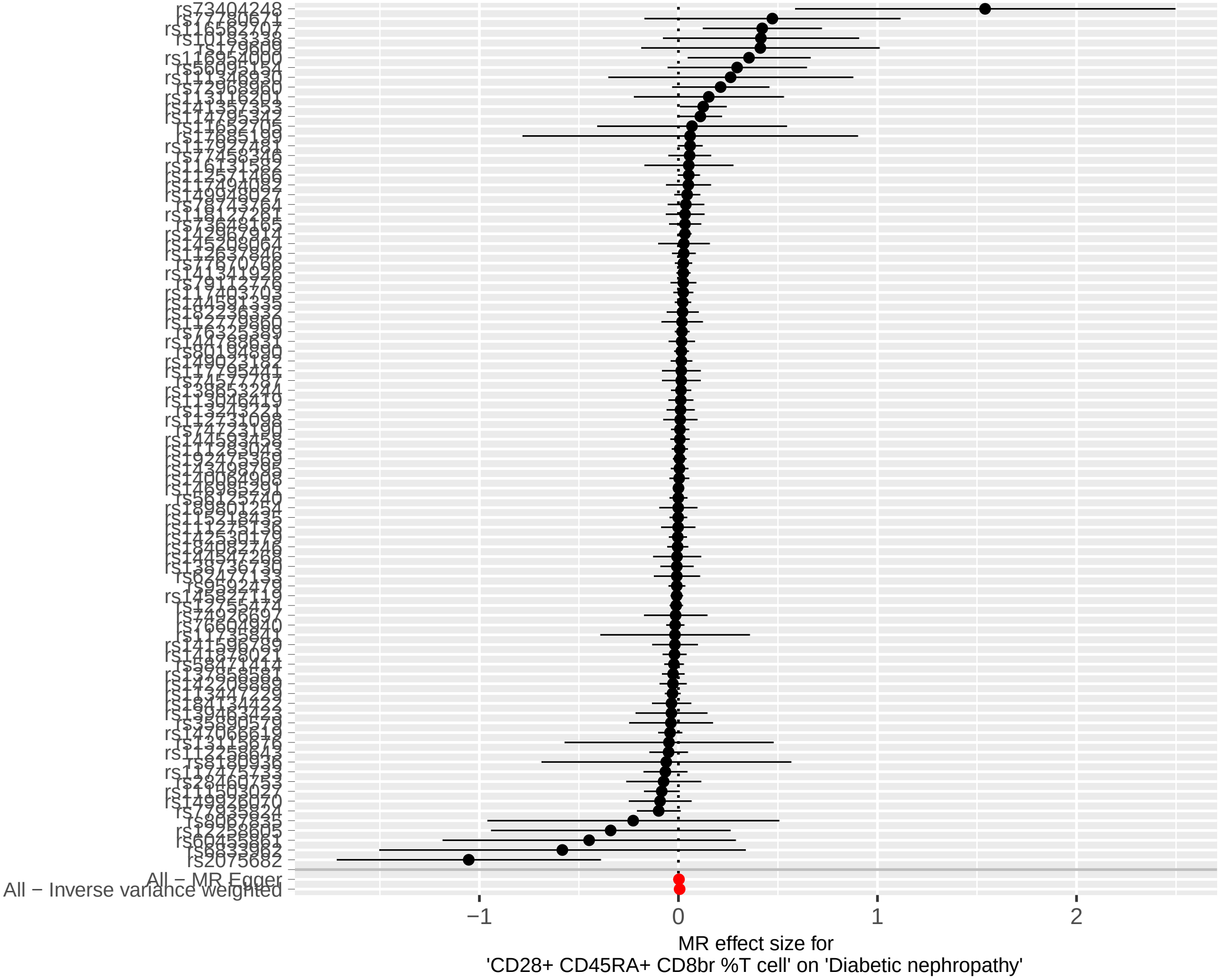
rs73404248
rs2075682
All − MR Egger
All − Inverse variance weighted
−1
0
1
2
MR effect size for
'CD28+ CD45RA+ CD8br %T cell' on 'Diabetic nephropathy'

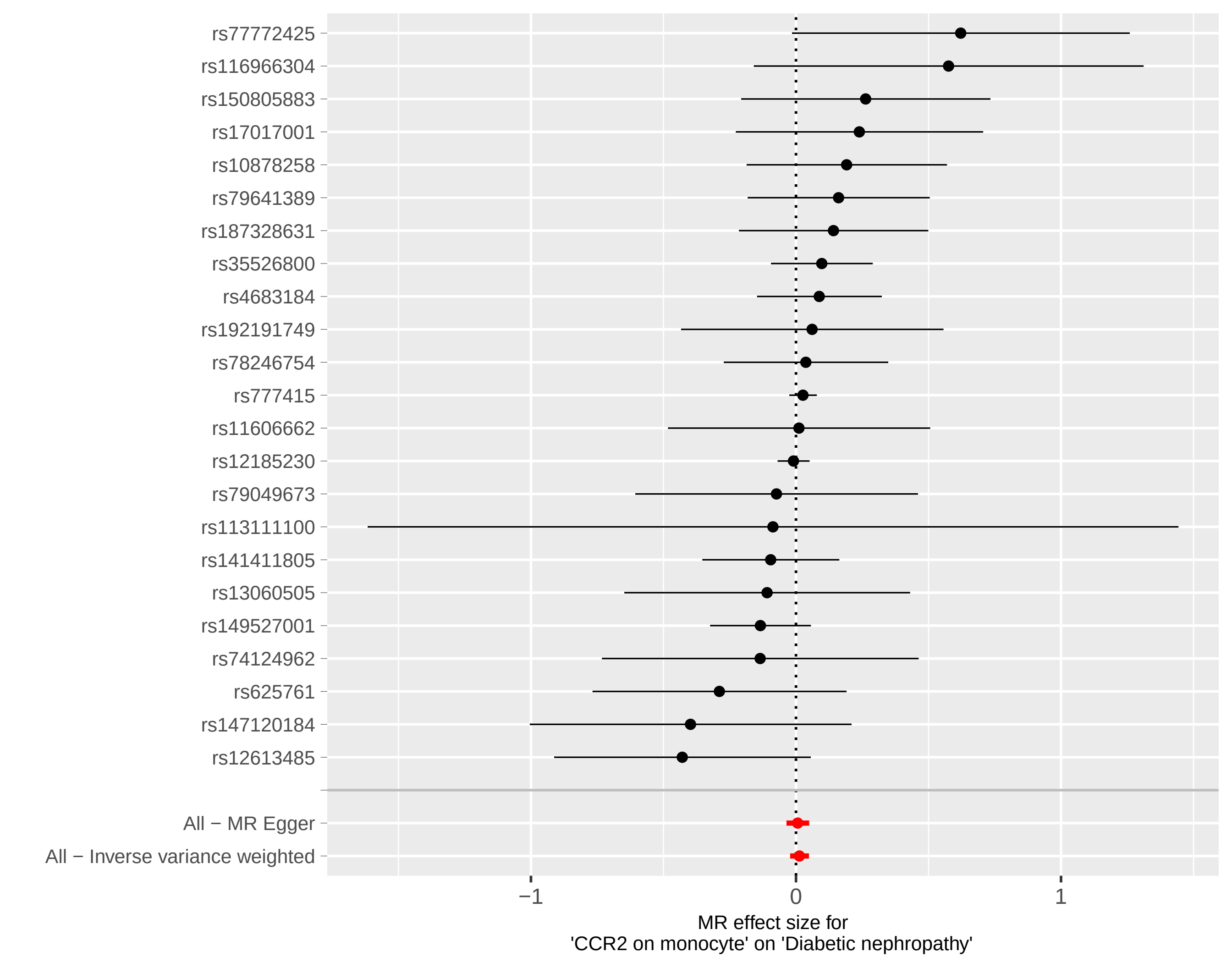
rs77772425
rs116966304
rs150805883
rs17017001
rs10878258
rs79641389
rs187328631
rs35526800
rs4683184
rs192191749
rs78246754
rs777415
rs11606662
rs12185230
rs79049673
rs113111100
rs141411805
rs13060505
rs149527001
rs74124962
rs625761
rs147120184
rs12613485
All − MR Egger
All − Inverse variance weighted
−1
0
1
MR effect size for
'CCR2 on monocyte' on 'Diabetic nephropathy'

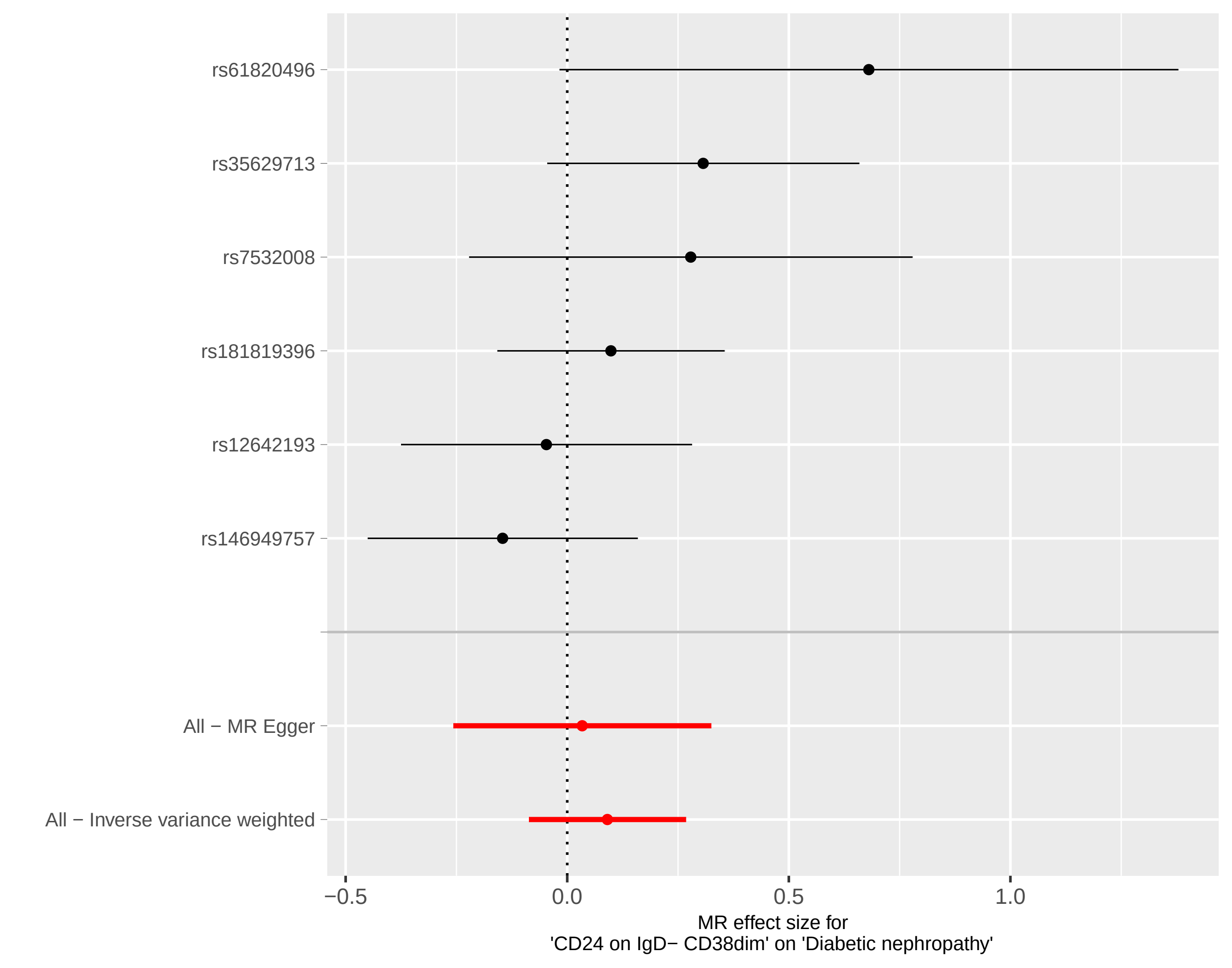
rs61820496
rs35629713
rs7532008
rs181819396
rs12642193
rs146949757
All − MR Egger
All − Inverse variance weighted
−0.5
0.0
0.5
1.0
MR effect size for
'CD24 on IgD− CD38dim' on 'Diabetic nephropathy'

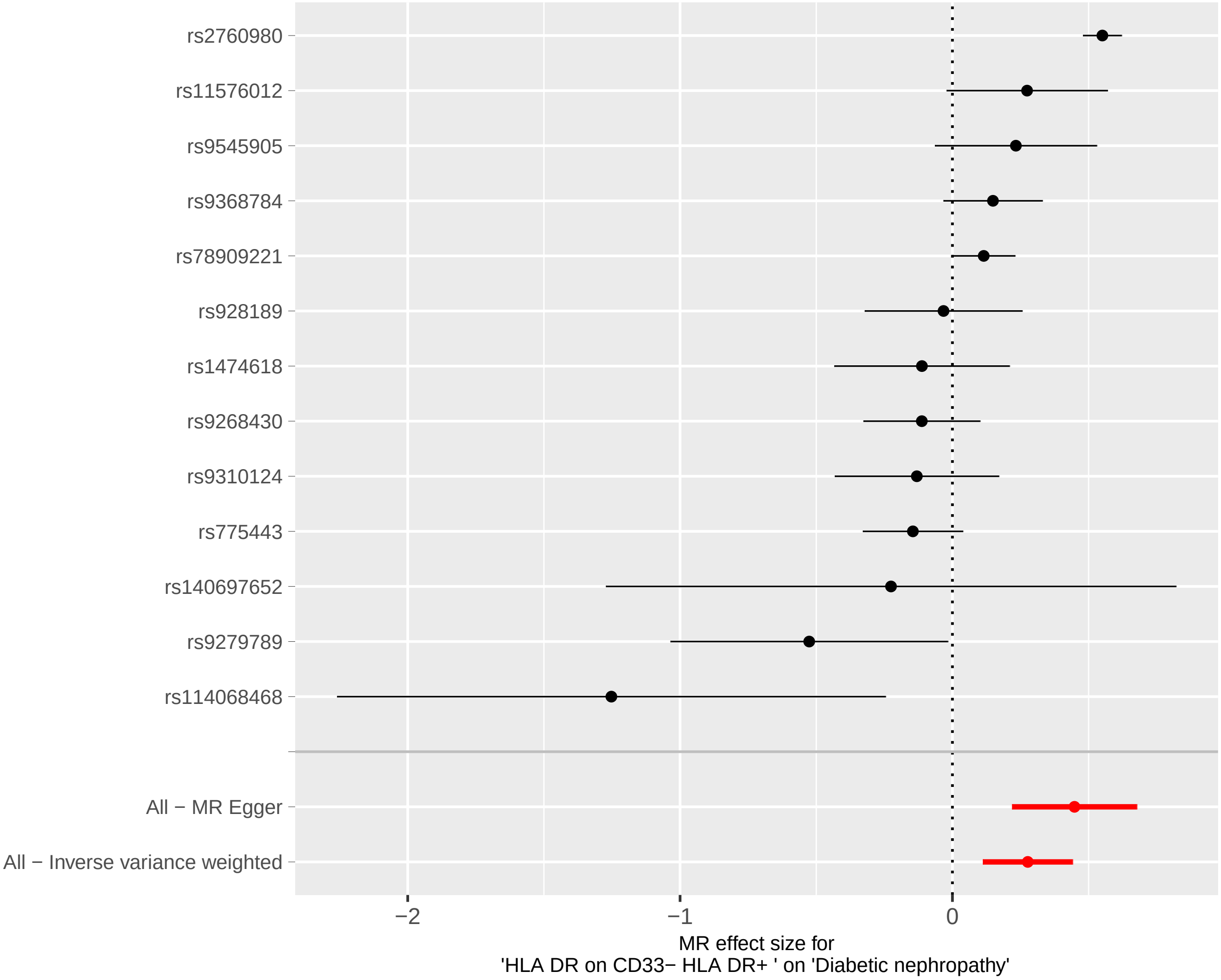
rs2760980
rs11576012
rs9545905
rs9368784
rs78909221
rs928189
rs1474618
rs9268430
rs9310124
rs775443
rs140697652
rs9279789
rs114068468
All − MR Egger
All − Inverse variance weighted
−2
−1
0
MR effect size for
'HLA DR on CD33− HLA DR+ ' on 'Diabetic nephropathy'

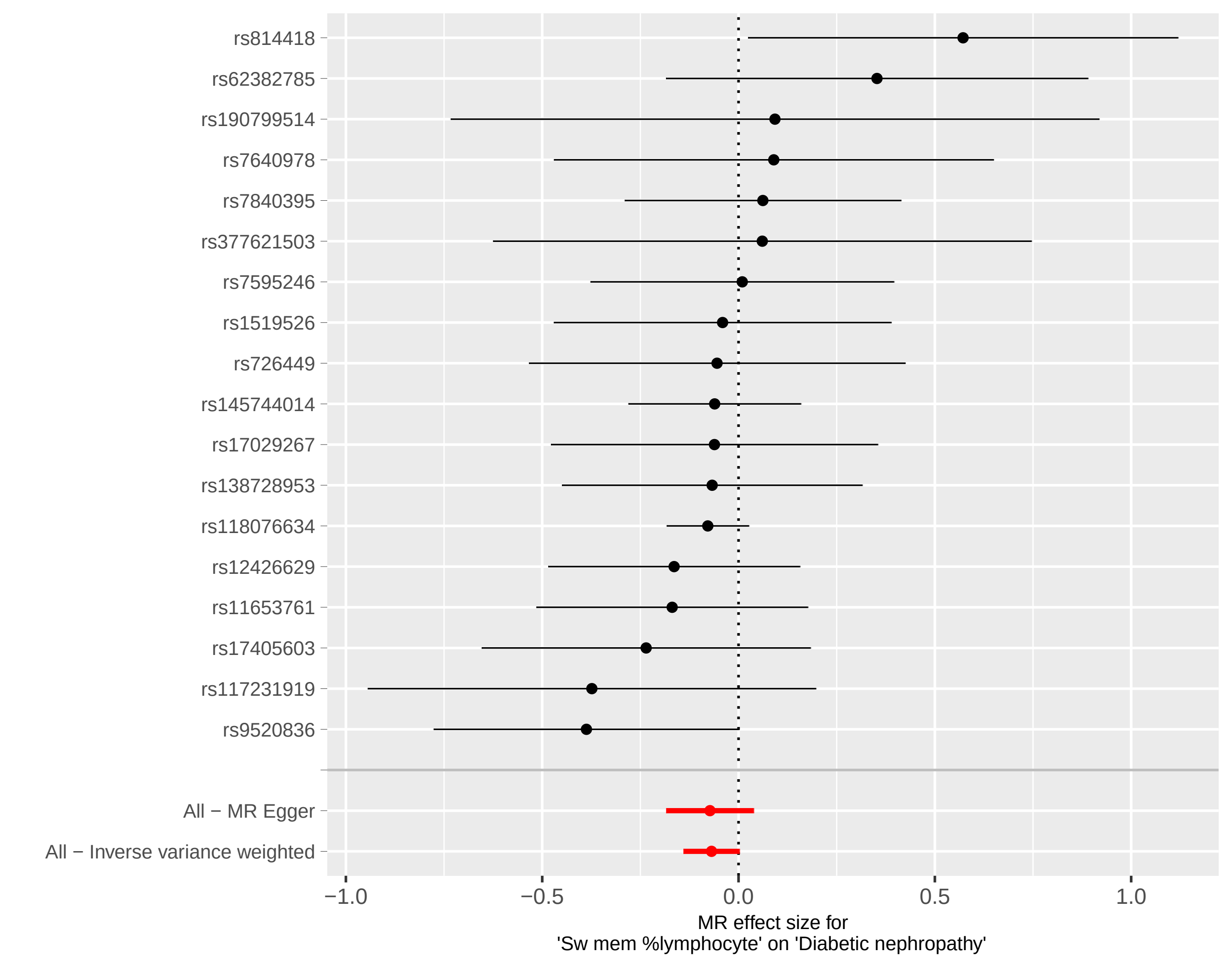
rs814418
rs62382785
rs190799514
rs7640978
rs7840395
rs377621503
rs7595246
rs1519526
rs726449
rs145744014
rs17029267
rs138728953
rs118076634
rs12426629
rs11653761
rs17405603
rs117231919
rs9520836
All − MR Egger
All − Inverse variance weighted
−1.0
−0.5
0.0
0.5
1.0
MR effect size for
'Sw mem %lymphocyte' on 'Diabetic nephropathy'

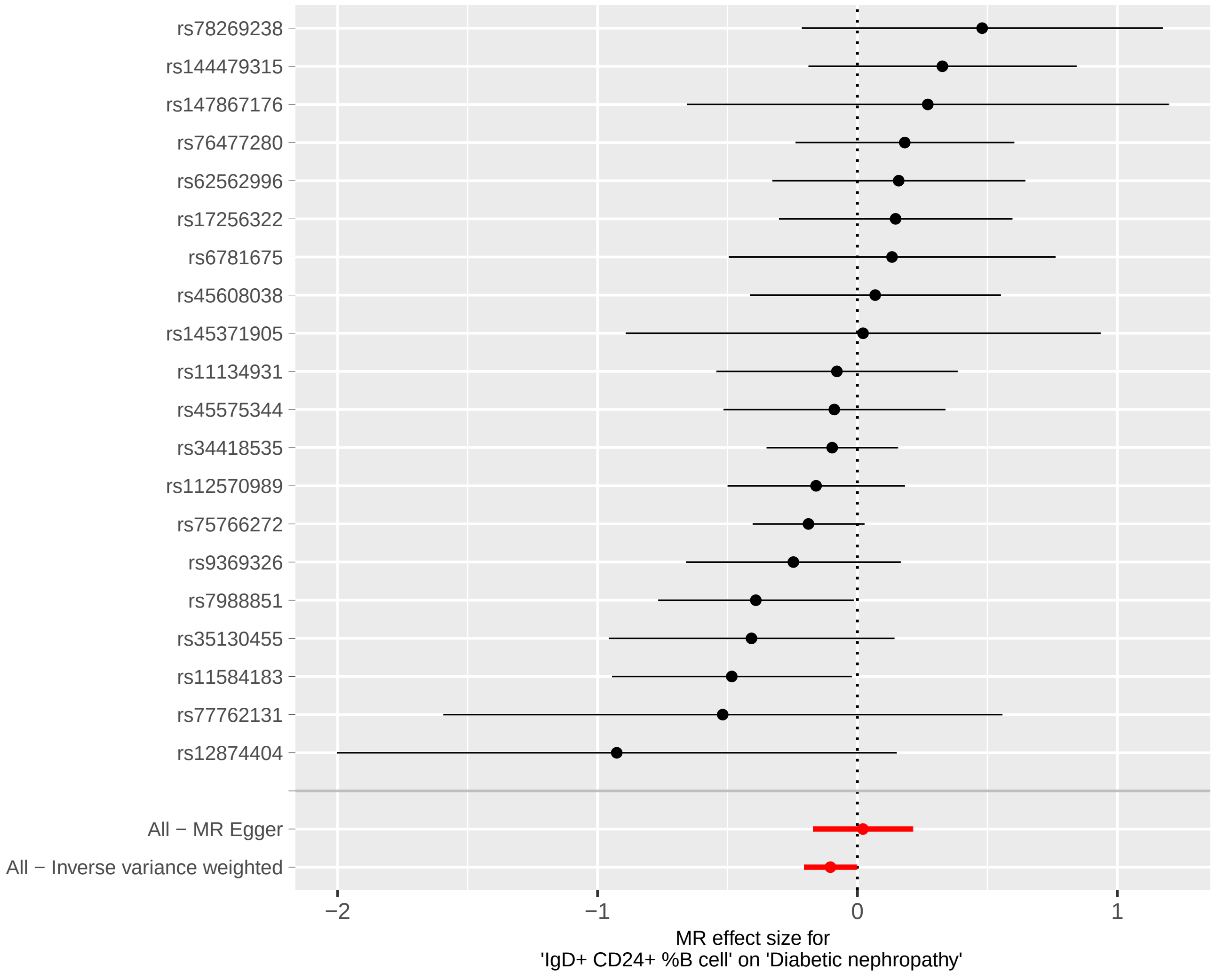
rs78269238
rs144479315
rs147867176
rs76477280
rs62562996
rs17256322
rs6781675
rs45608038
rs145371905
rs11134931
rs45575344
rs34418535
rs112570989
rs75766272
rs9369326
rs7988851
rs35130455
rs11584183
rs77762131
rs12874404
All − MR Egger
All − Inverse variance weighted
−2
−1
0
1
MR effect size for
'IgD+ CD24+ %B cell' on 'Diabetic nephropathy'

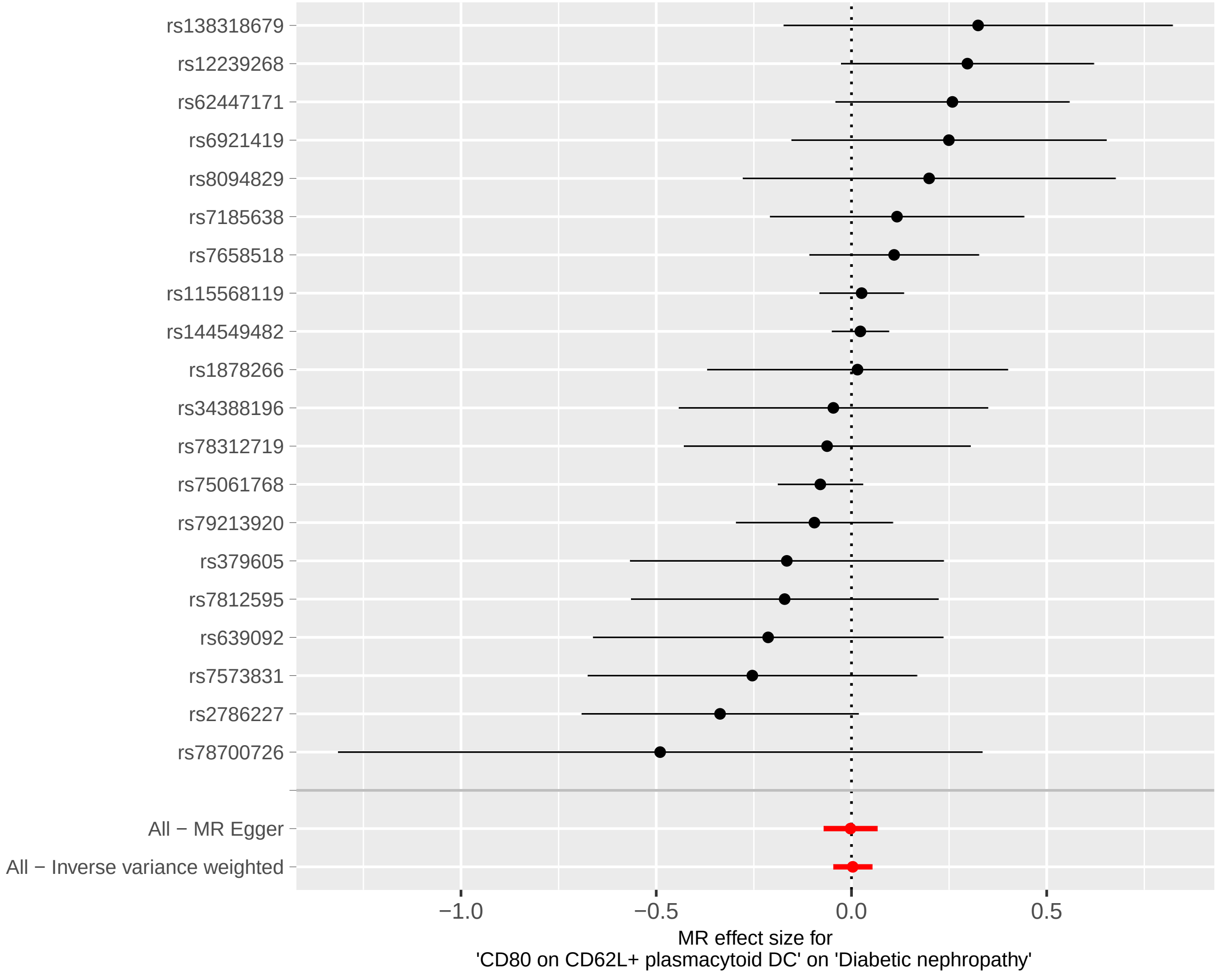
rs138318679
rs12239268
rs62447171
rs6921419
rs8094829
rs7185638
rs7658518
rs115568119
rs144549482
rs1878266
rs34388196
rs78312719
rs75061768
rs79213920
rs379605
rs7812595
rs639092
rs7573831
rs2786227
rs78700726
All − MR Egger
All − Inverse variance weighted
−1.0
−0.5
0.0
0.5
MR effect size for
'CD80 on CD62L+ plasmacytoid DC' on 'Diabetic nephropathy'

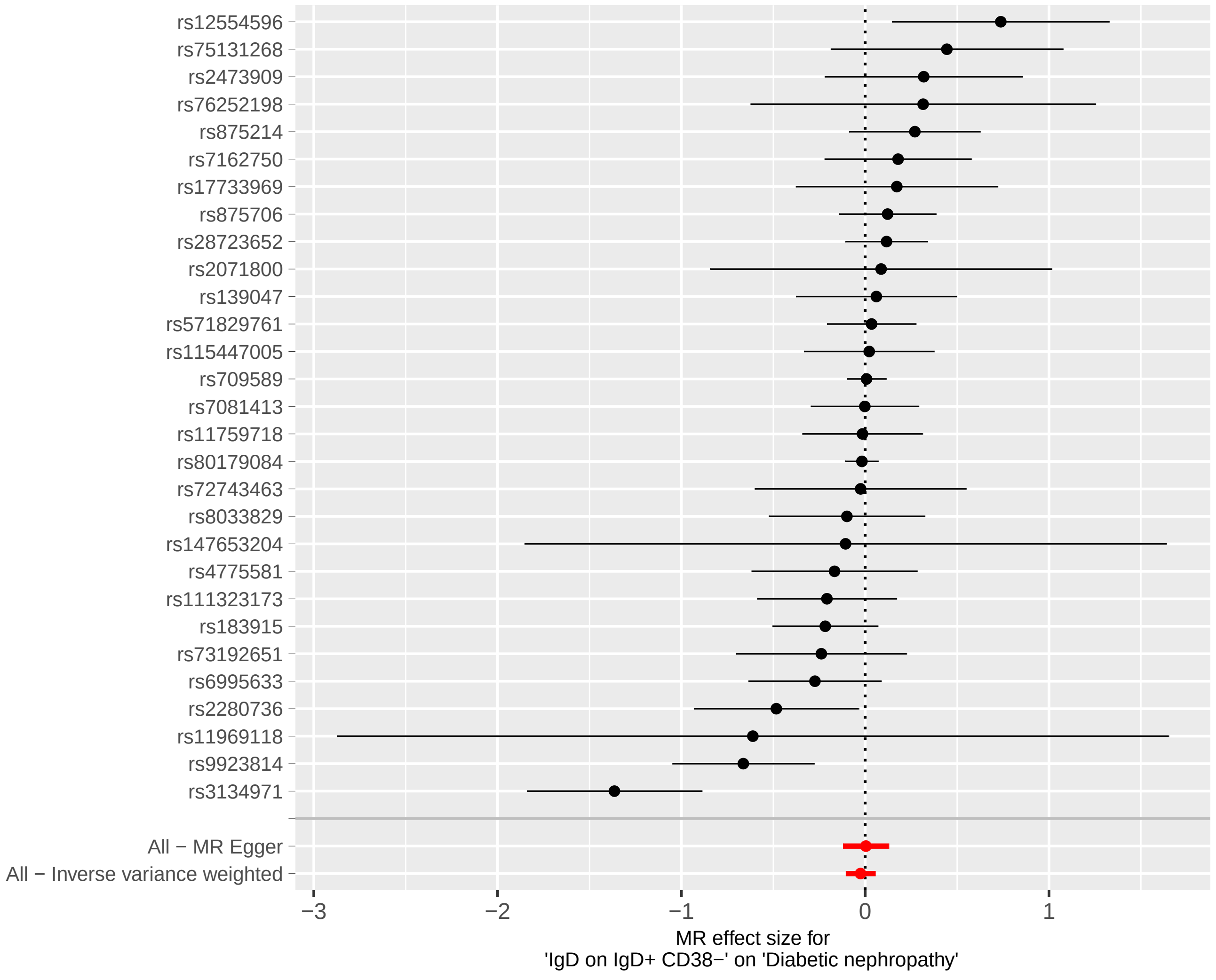

rs12554596
rs75131268
rs2473909
rs76252198
rs875214
rs7162750
rs17733969
rs875706
rs28723652
rs2071800
rs139047
rs571829761
rs115447005
rs709589
rs7081413
rs11759718
rs80179084
rs72743463
rs8033829
rs147653204
rs4775581
rs111323173
rs183915
rs73192651
rs6995633
rs2280736
rs11969118
rs9923814
rs3134971
All − MR Egger
All − Inverse variance weighted
−3
−2
−1
0
1
MR effect size for
'IgD on IgD+ CD38−' on 'Diabetic nephropathy'

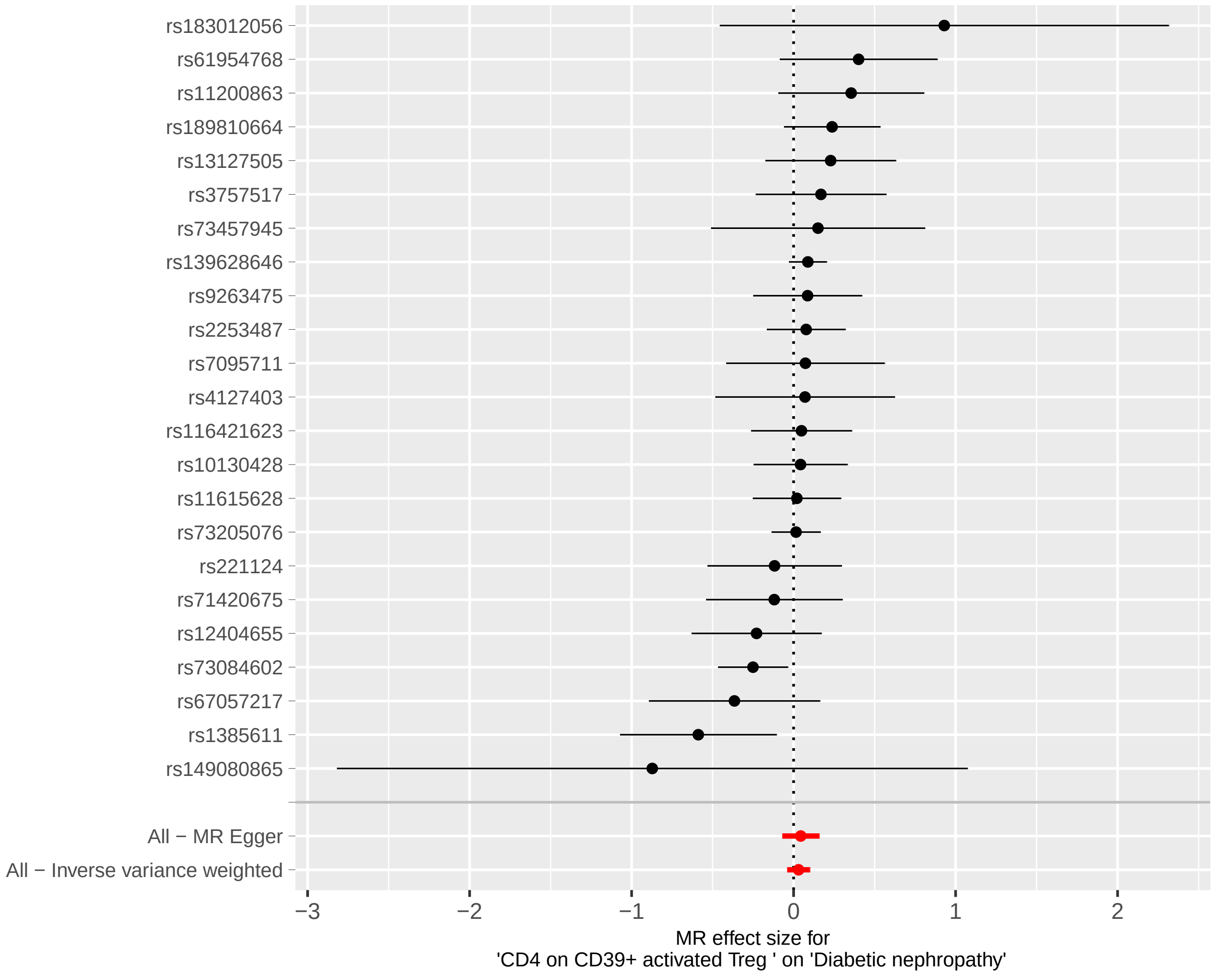

rs183012056
rs61954768
rs11200863
rs189810664
rs13127505
rs3757517
rs73457945
rs139628646
rs9263475
rs2253487
rs7095711
rs4127403
rs116421623
rs10130428
rs11615628
rs73205076
rs221124
rs71420675
rs12404655
rs73084602
rs67057217
rs1385611
rs149080865
All − MR Egger
All − Inverse variance weighted
−3
−2
−1
0
1
2
MR effect size for
'CD4 on CD39+ activated Treg ' on 'Diabetic nephropathy'

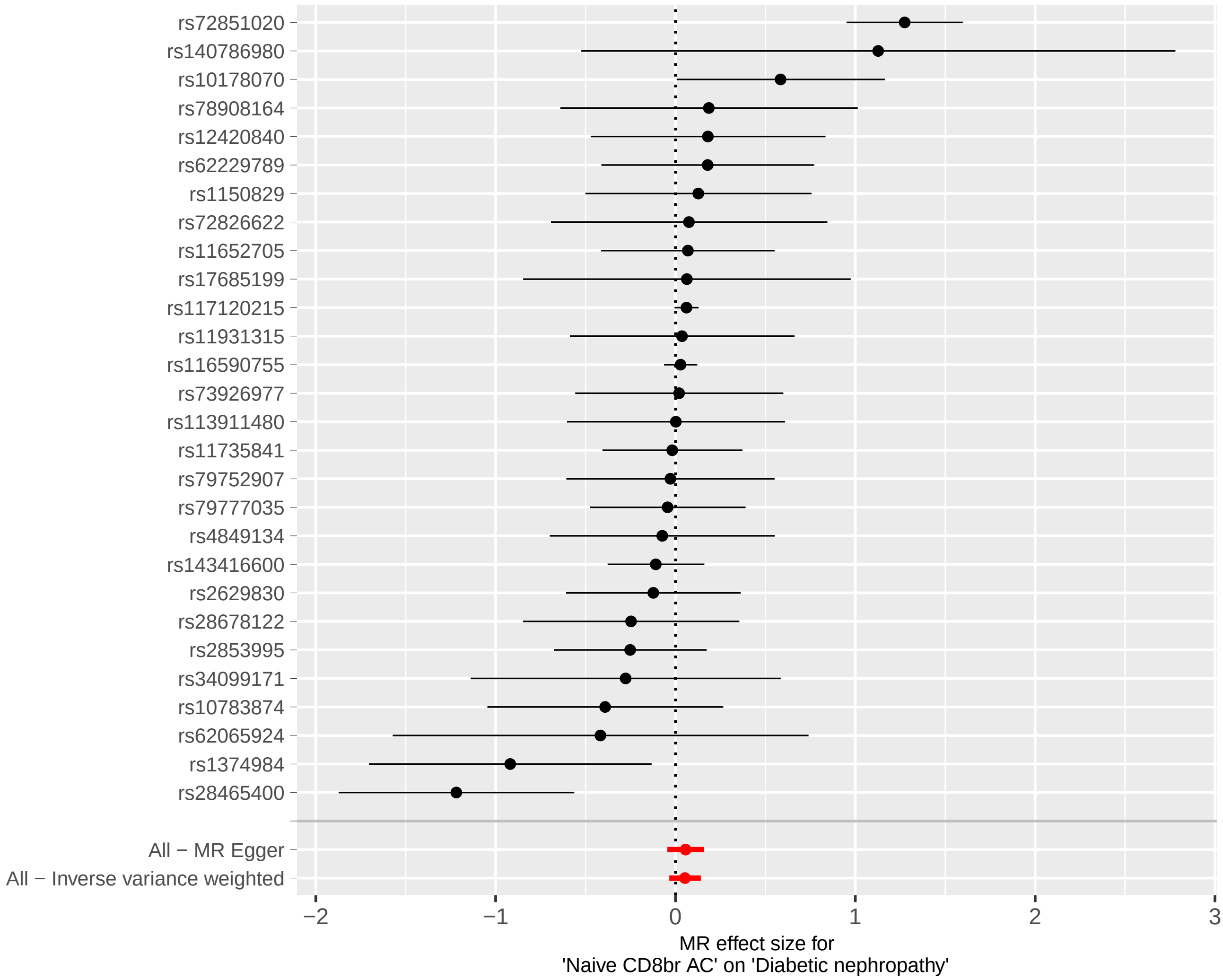
rs72851020
rs140786980
rs10178070
rs78908164
rs12420840
rs62229789
rs1150829
rs72826622
rs11652705
rs17685199
rs117120215
rs11931315
rs116590755
rs73926977
rs113911480
rs11735841
rs79752907
rs79777035
rs4849134
rs143416600
rs2629830
rs28678122
rs2853995
rs34099171
rs10783874
rs62065924
rs1374984
rs28465400
All − MR Egger
All − Inverse variance weighted
−2
−1
0
1
2
3
MR effect size for
'Naive CD8br AC' on 'Diabetic nephropathy'

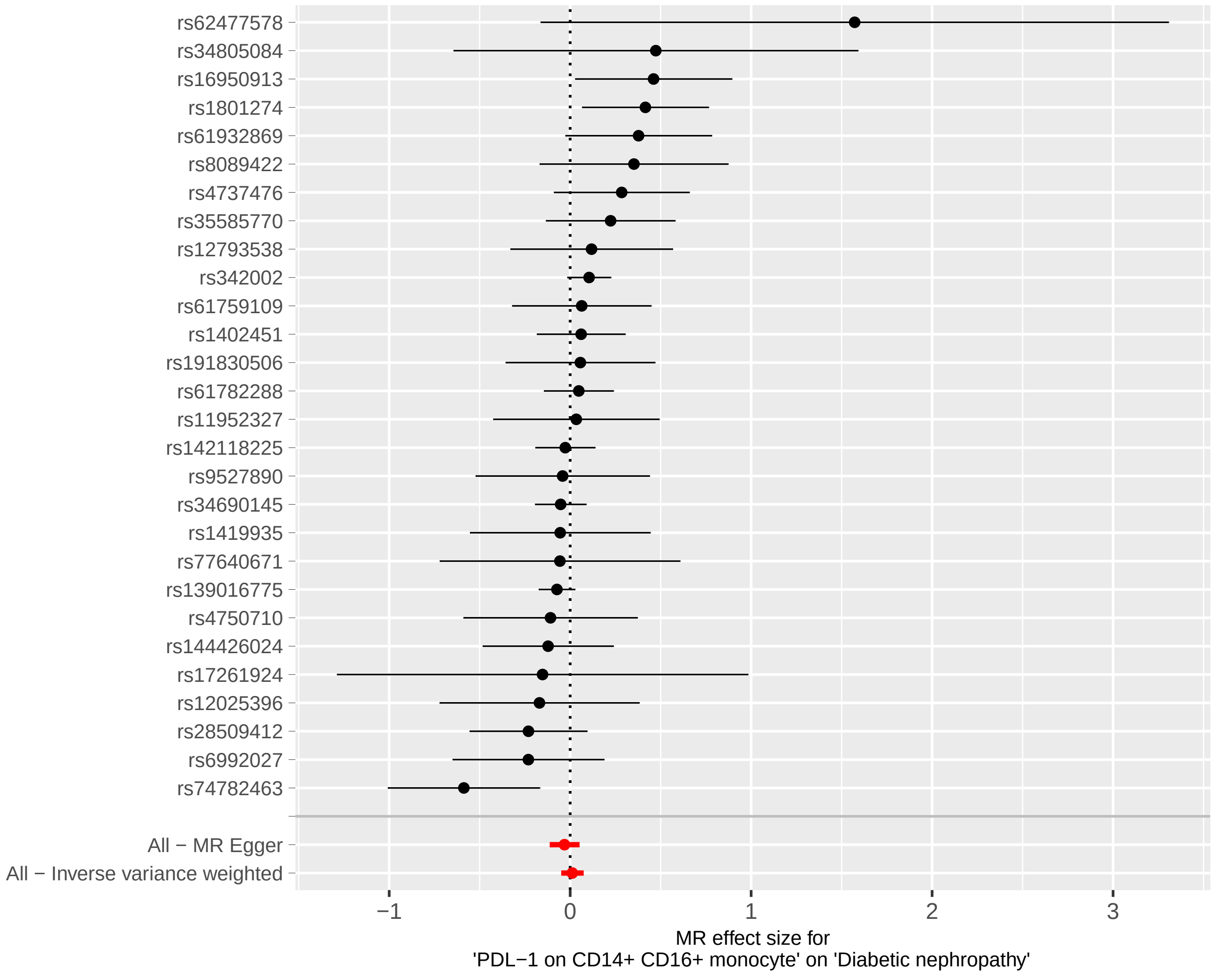

rs62477578
rs34805084
rs16950913
rs1801274
rs61932869
rs8089422
rs4737476
rs35585770
rs12793538
rs342002
rs61759109
rs1402451
rs191830506
rs61782288
rs11952327
rs142118225
rs9527890
rs34690145
rs1419935
rs77640671
rs139016775
rs4750710
rs144426024
rs17261924
rs12025396
rs28509412
rs6992027
rs74782463
All − MR Egger
All − Inverse variance weighted
−1
0
1
2
3
MR effect size for
'PDL−1 on CD14+ CD16+ monocyte' on 'Diabetic nephropathy'

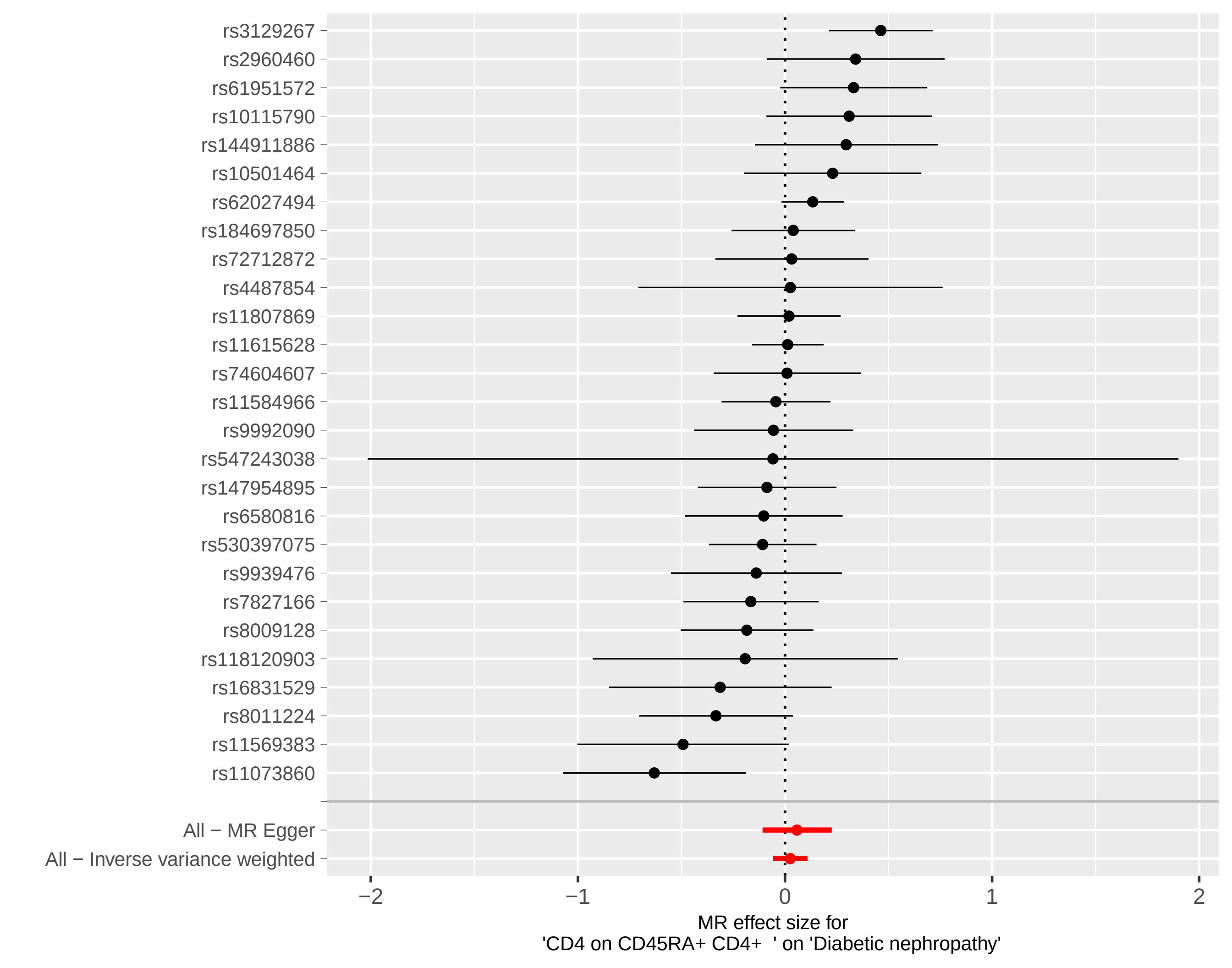
rs3129267
rs2960460
rs61951572
rs10115790
rs144911886
rs10501464
rs62027494
rs184697850
rs72712872
rs4487854
rs11807869
rs11615628
rs74604607
rs11584966
rs9992090
rs547243038
rs147954895
rs6580816
rs530397075
rs9939476
rs7827166
rs8009128
rs118120903
rs16831529
rs8011224
rs11569383
rs11073860
All − MR Egger
All − Inverse variance weighted
−2
−1
0
1
2
MR effect size for
'CD4 on CD45RA+ CD4+ ' on 'Diabetic nephropathy'

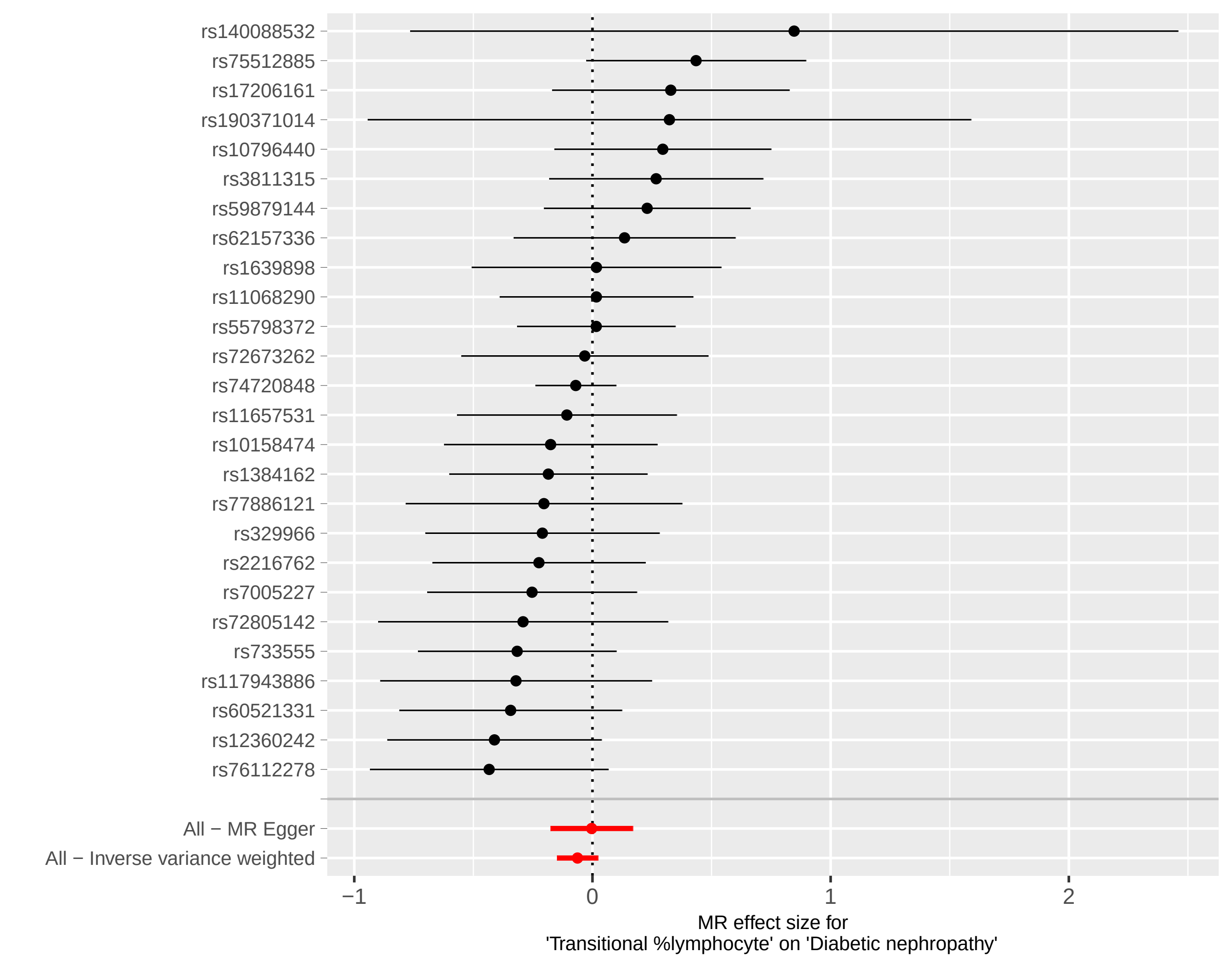

rs140088532
rs75512885
rs17206161
rs190371014
rs10796440
rs3811315
rs59879144
rs62157336
rs1639898
rs11068290
rs55798372
rs72673262
rs74720848
rs11657531
rs10158474
rs1384162
rs77886121
rs329966
rs2216762
rs7005227
rs72805142
rs733555
rs117943886
rs60521331
rs12360242
rs76112278
All − MR Egger
All − Inverse variance weighted
−1
0
1
2
MR effect size for
'Transitional %lymphocyte' on 'Diabetic nephropathy'

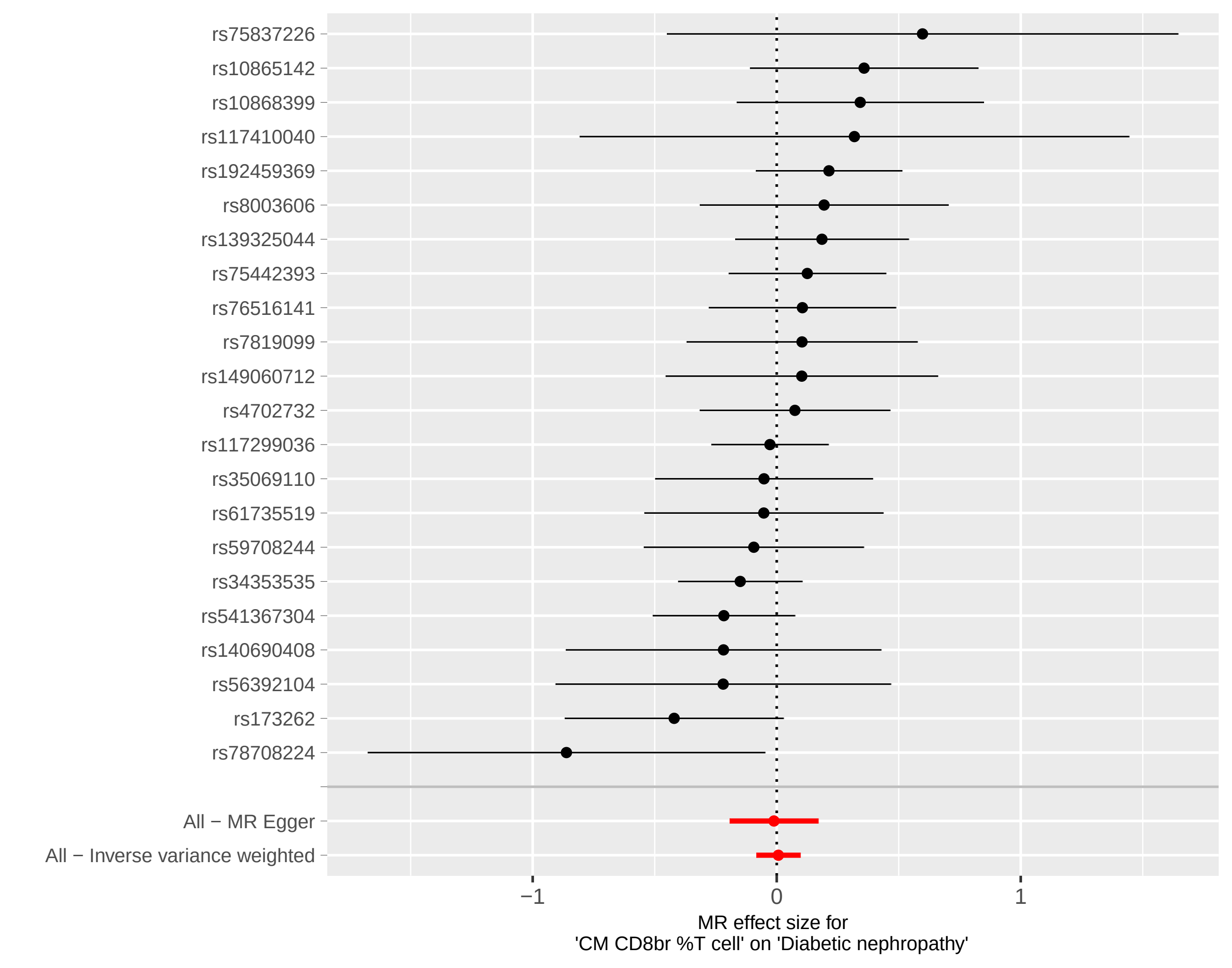

rs75837226
rs10865142
rs10868399
rs117410040
rs192459369
rs8003606
rs139325044
rs75442393
rs76516141
rs7819099
rs149060712
rs4702732
rs117299036
rs35069110
rs61735519
rs59708244
rs34353535
rs541367304
rs140690408
rs56392104
rs173262
rs78708224
All − MR Egger
All − Inverse variance weighted
−1
0
1
MR effect size for
'CM CD8br %T cell' on 'Diabetic nephropathy'

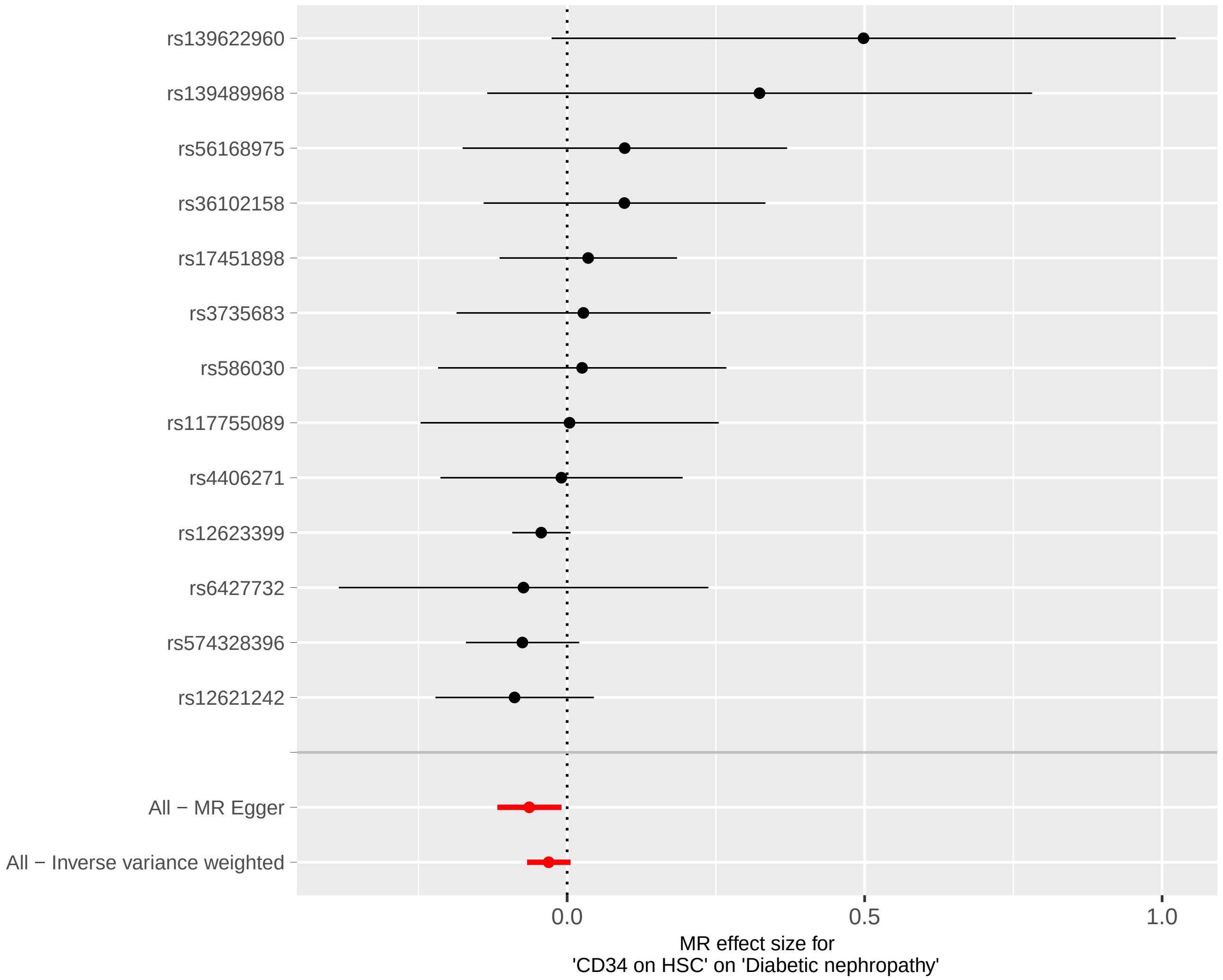

rs139622960
rs139489968
rs56168975
rs36102158
rs17451898
rs3735683
rs586030
rs117755089
rs4406271
rs12623399
rs6427732
rs574328396
rs12621242
All − MR Egger
All − Inverse variance weighted
0.0
0.5
1.0
MR effect size for
'CD34 on HSC' on 'Diabetic nephropathy'

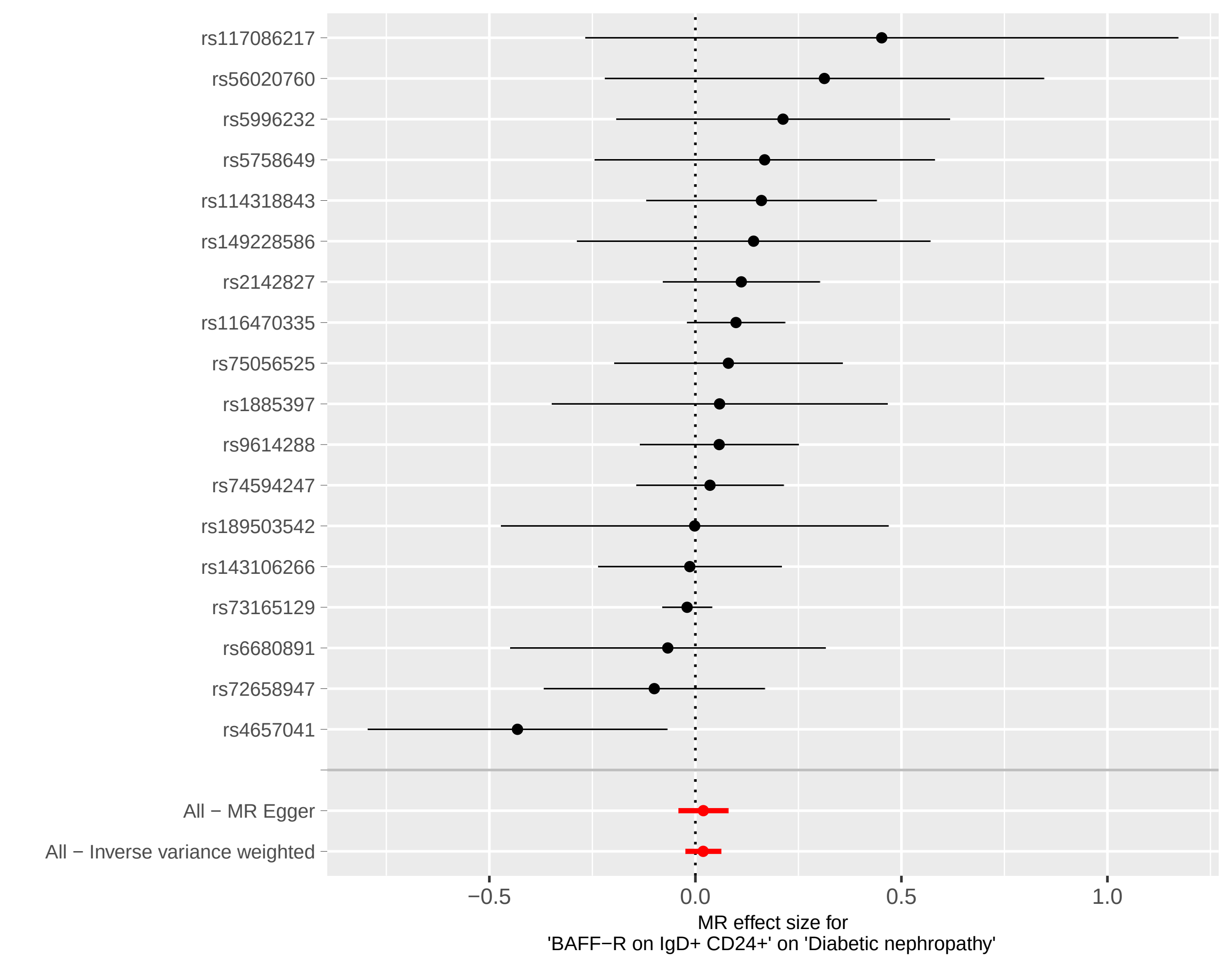
rs117086217
rs56020760
rs5996232
rs5758649
rs114318843
rs149228586
rs2142827
rs116470335
rs75056525
rs1885397
rs9614288
rs74594247
rs189503542
rs143106266
rs73165129
rs6680891
rs72658947
rs4657041
All − MR Egger
All − Inverse variance weighted
−0.5
0.0
0.5
1.0
MR effect size for
'BAFF−R on IgD+ CD24+' on 'Diabetic nephropathy'

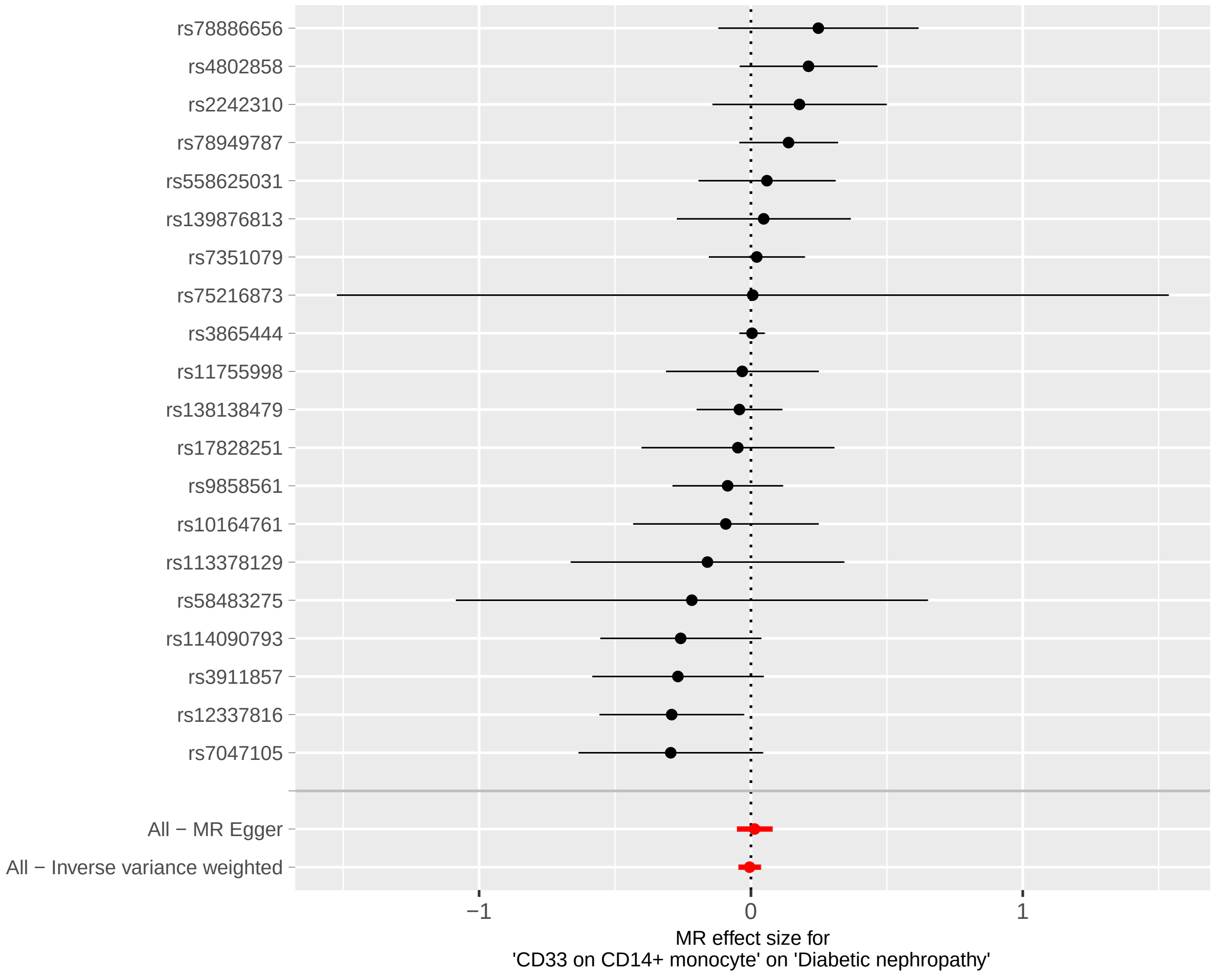

rs78886656
rs4802858
rs2242310
rs78949787
rs558625031
rs139876813
rs7351079
rs75216873
rs3865444
rs11755998
rs138138479
rs17828251
rs9858561
rs10164761
rs113378129
rs58483275
rs114090793
rs3911857
rs12337816
rs7047105
All − MR Egger
All − Inverse variance weighted
−1
0
1
MR effect size for
'CD33 on CD14+ monocyte' on 'Diabetic nephropathy'

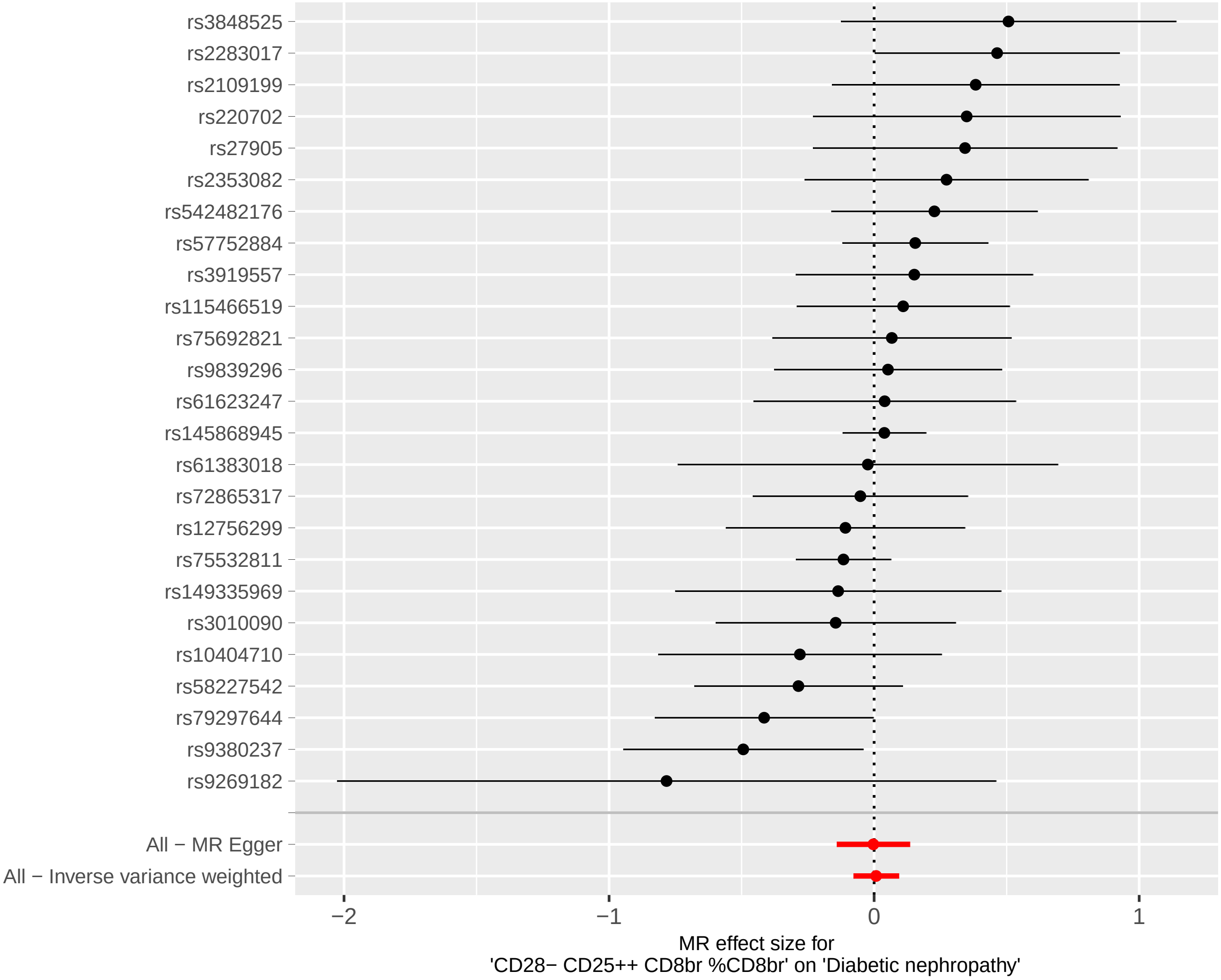
rs3848525
rs2283017
rs2109199
rs220702
rs27905
rs2353082
rs542482176
rs57752884
rs3919557
rs115466519
rs75692821
rs9839296
rs61623247
rs145868945
rs61383018
rs72865317
rs12756299
rs75532811
rs149335969
rs3010090
rs10404710
rs58227542
rs79297644
rs9380237
rs9269182
All − MR Egger
All − Inverse variance weighted
−2
−1
0
1
MR effect size for
'CD28− CD25++ CD8br %CD8br' on 'Diabetic nephropathy'

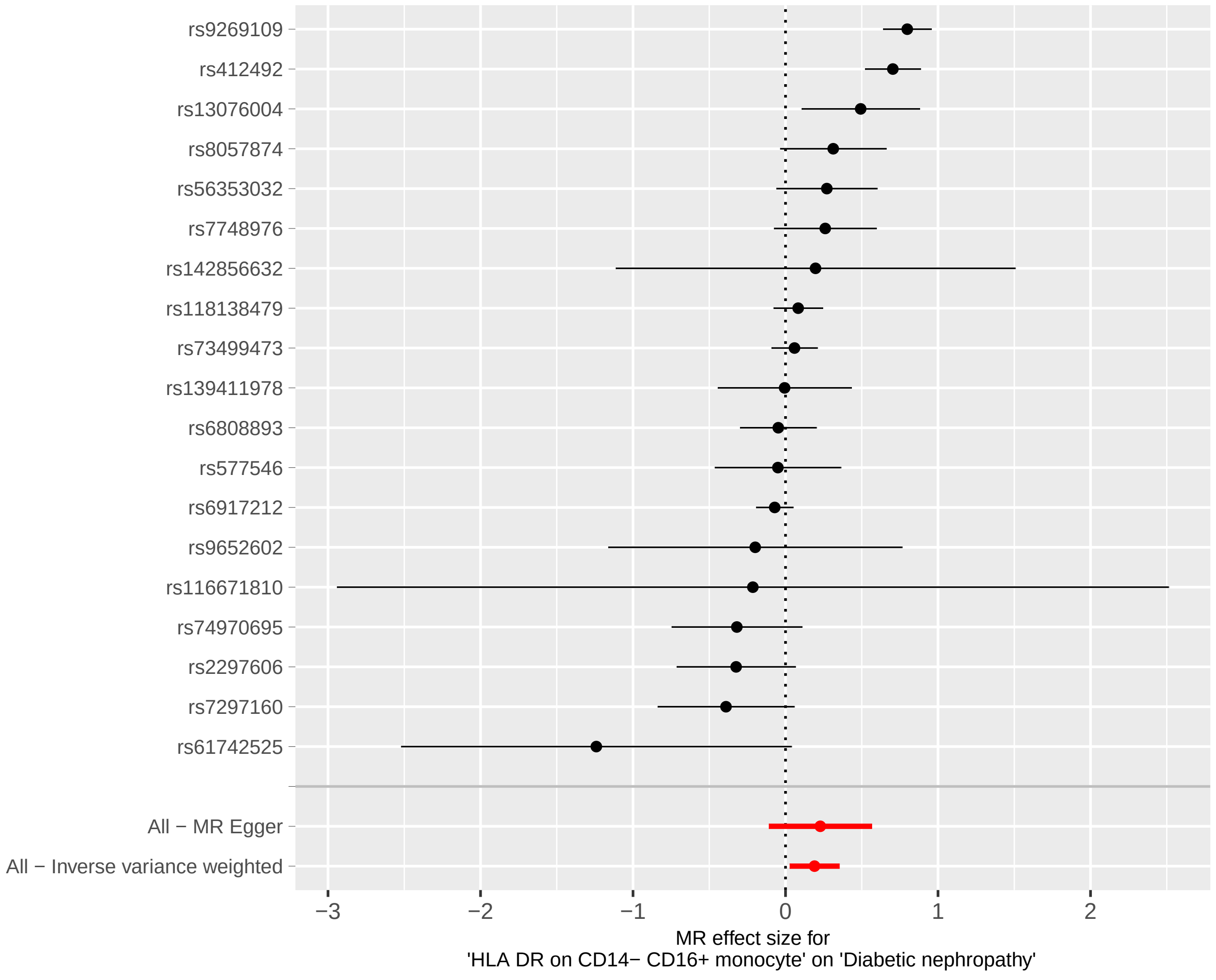

rs9269109
rs412492
rs13076004
rs8057874
rs56353032
rs7748976
rs142856632
rs118138479
rs73499473
rs139411978
rs6808893
rs577546
rs6917212
rs9652602
rs116671810
rs74970695
rs2297606
rs7297160
rs61742525
All − MR Egger
All − Inverse variance weighted
−3
−2
−1
0
1
2
MR effect size for
'HLA DR on CD14− CD16+ monocyte' on 'Diabetic nephropathy'

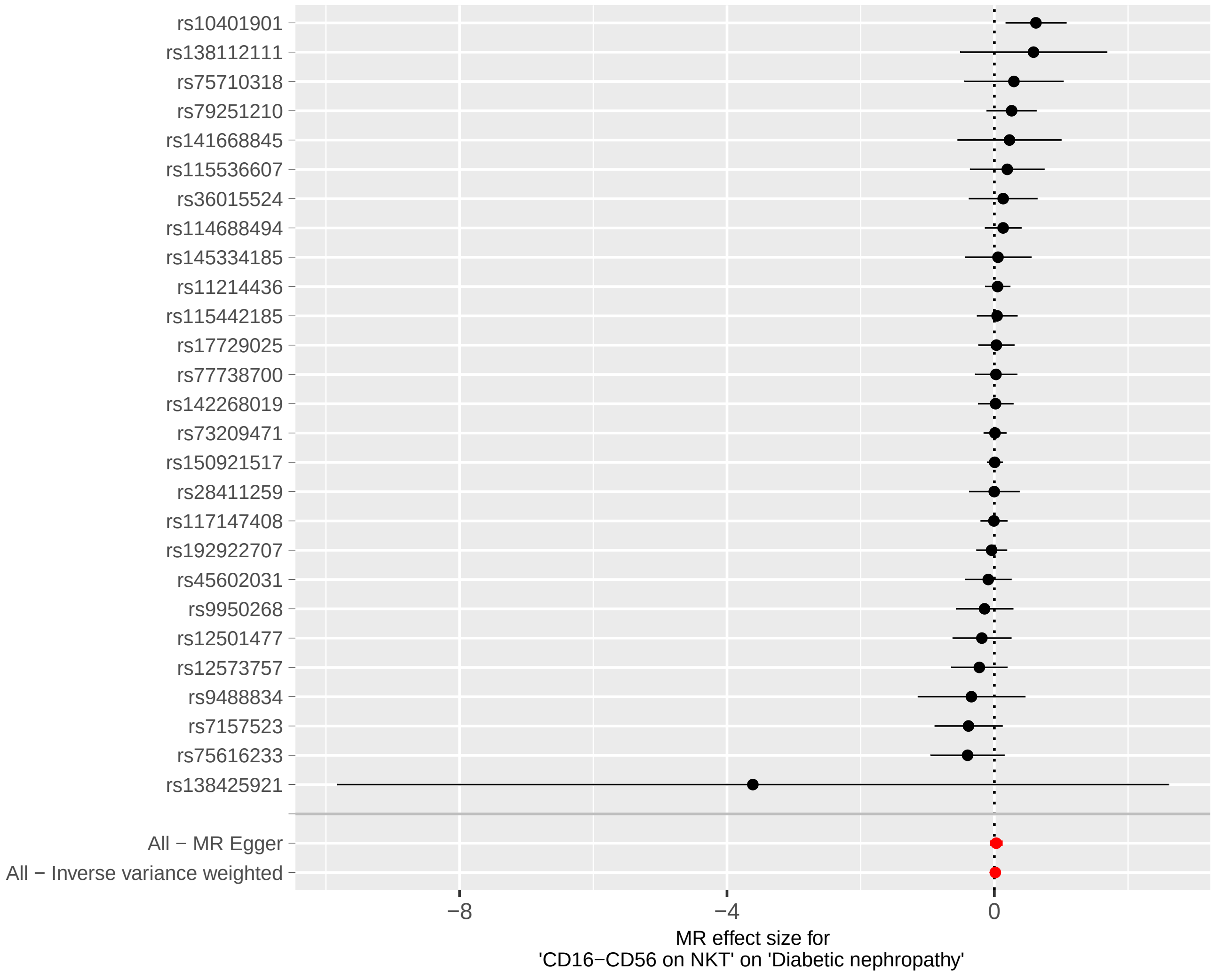

rs10401901
rs138112111
rs75710318
rs79251210
rs141668845
rs115536607
rs36015524
rs114688494
rs145334185
rs11214436
rs115442185
rs17729025
rs77738700
rs142268019
rs73209471
rs150921517
rs28411259
rs117147408
rs192922707
rs45602031
rs9950268
rs12501477
rs12573757
rs9488834
rs7157523
rs75616233
rs138425921
All − MR Egger
All − Inverse variance weighted
−8
−4
0
MR effect size for
'CD16−CD56 on NKT' on 'Diabetic nephropathy'

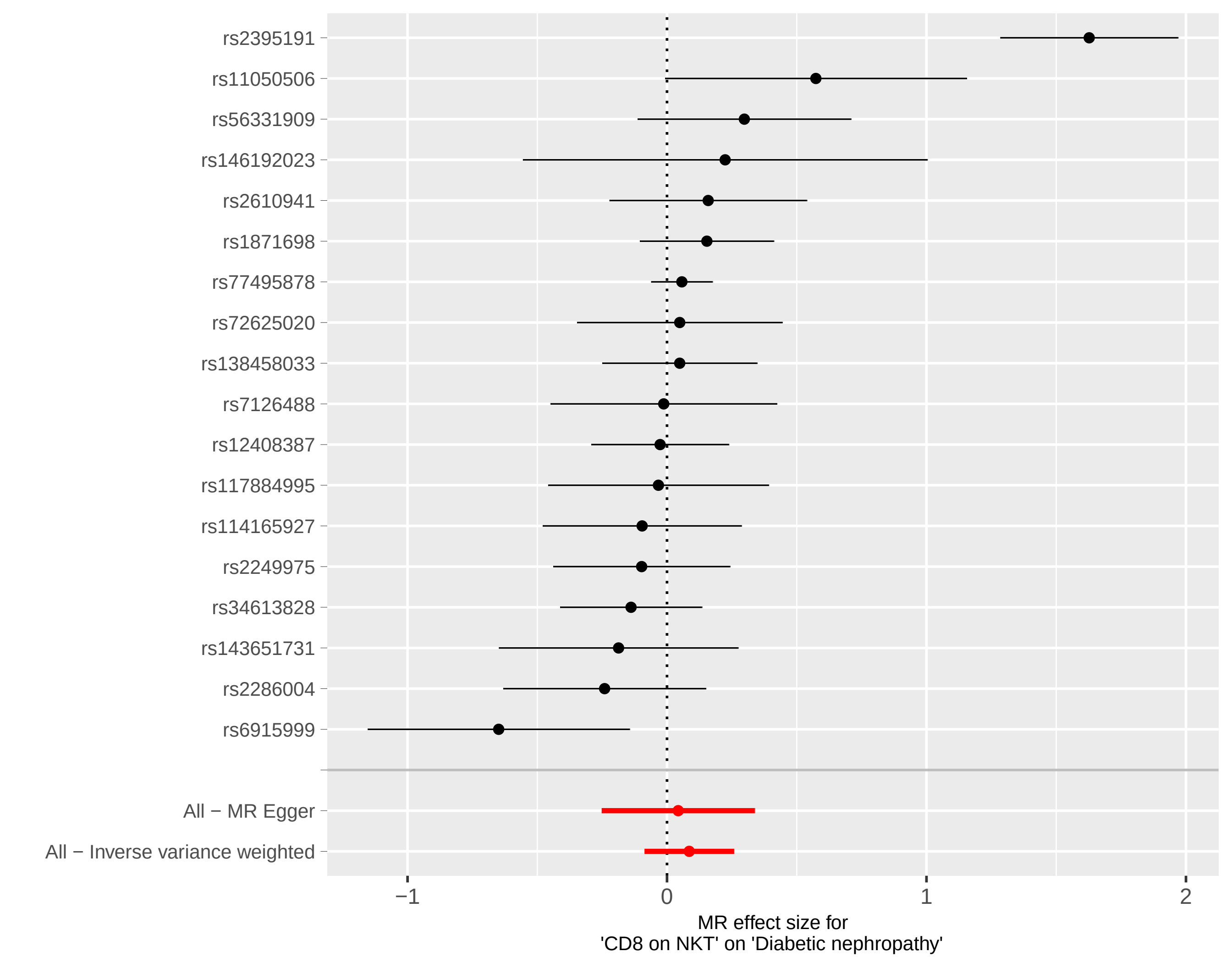
rs2395191
rs11050506
rs56331909
rs146192023
rs2610941
rs1871698
rs77495878
rs72625020
rs138458033
rs7126488
rs12408387
rs117884995
rs114165927
rs2249975
rs34613828
rs143651731
rs2286004
rs6915999
All − MR Egger
All − Inverse variance weighted
−1
0
1
2
MR effect size for
'CD8 on NKT' on 'Diabetic nephropathy'

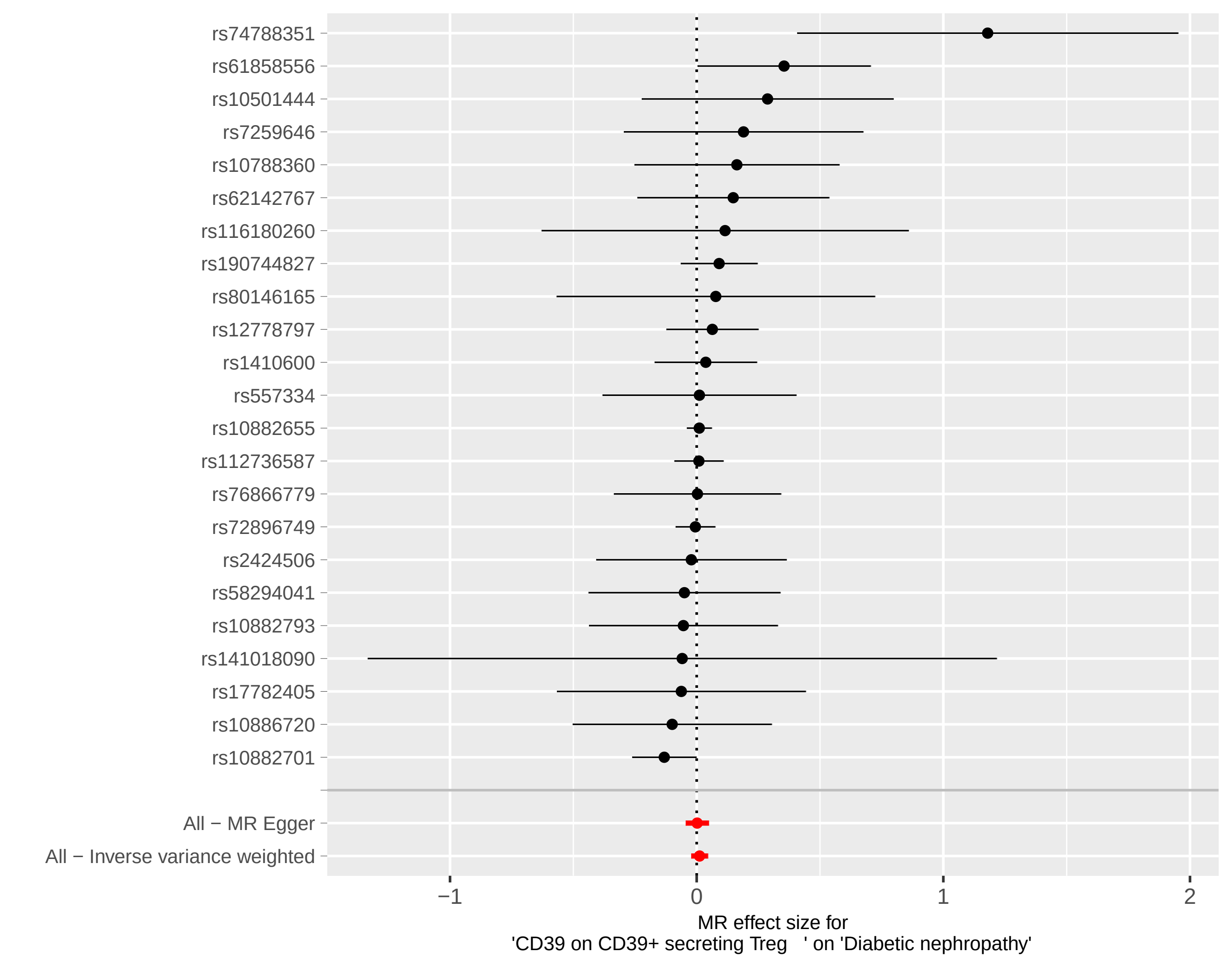

rs74788351
rs61858556
rs10501444
rs7259646
rs10788360
rs62142767
rs116180260
rs190744827
rs80146165
rs12778797
rs1410600
rs557334
rs10882655
rs112736587
rs76866779
rs72896749
rs2424506
rs58294041
rs10882793
rs141018090
rs17782405
rs10886720
rs10882701
All − MR Egger
All − Inverse variance weighted
−1
0
1
2
MR effect size for
'CD39 on CD39+ secreting Treg ' on 'Diabetic nephropathy'

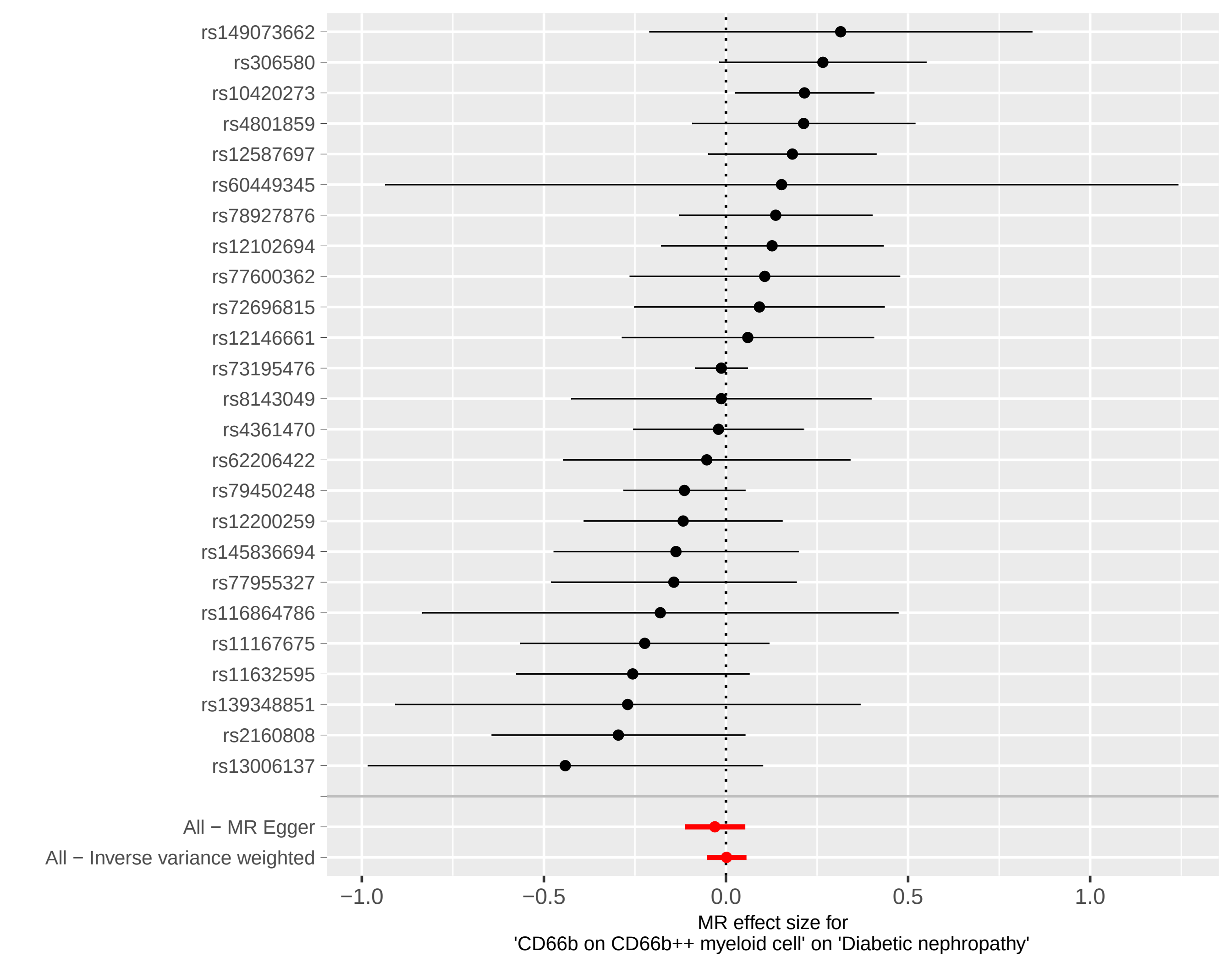
rs149073662
rs306580
rs10420273
rs4801859
rs12587697
rs60449345
rs78927876
rs12102694
rs77600362
rs72696815
rs12146661
rs73195476
rs8143049
rs4361470
rs62206422
rs79450248
rs12200259
rs145836694
rs77955327
rs116864786
rs11167675
rs11632595
rs139348851
rs2160808
rs13006137
All − MR Egger
All − Inverse variance weighted
−1.0
−0.5
0.0
0.5
1.0
MR effect size for
'CD66b on CD66b++ myeloid cell' on 'Diabetic nephropathy'

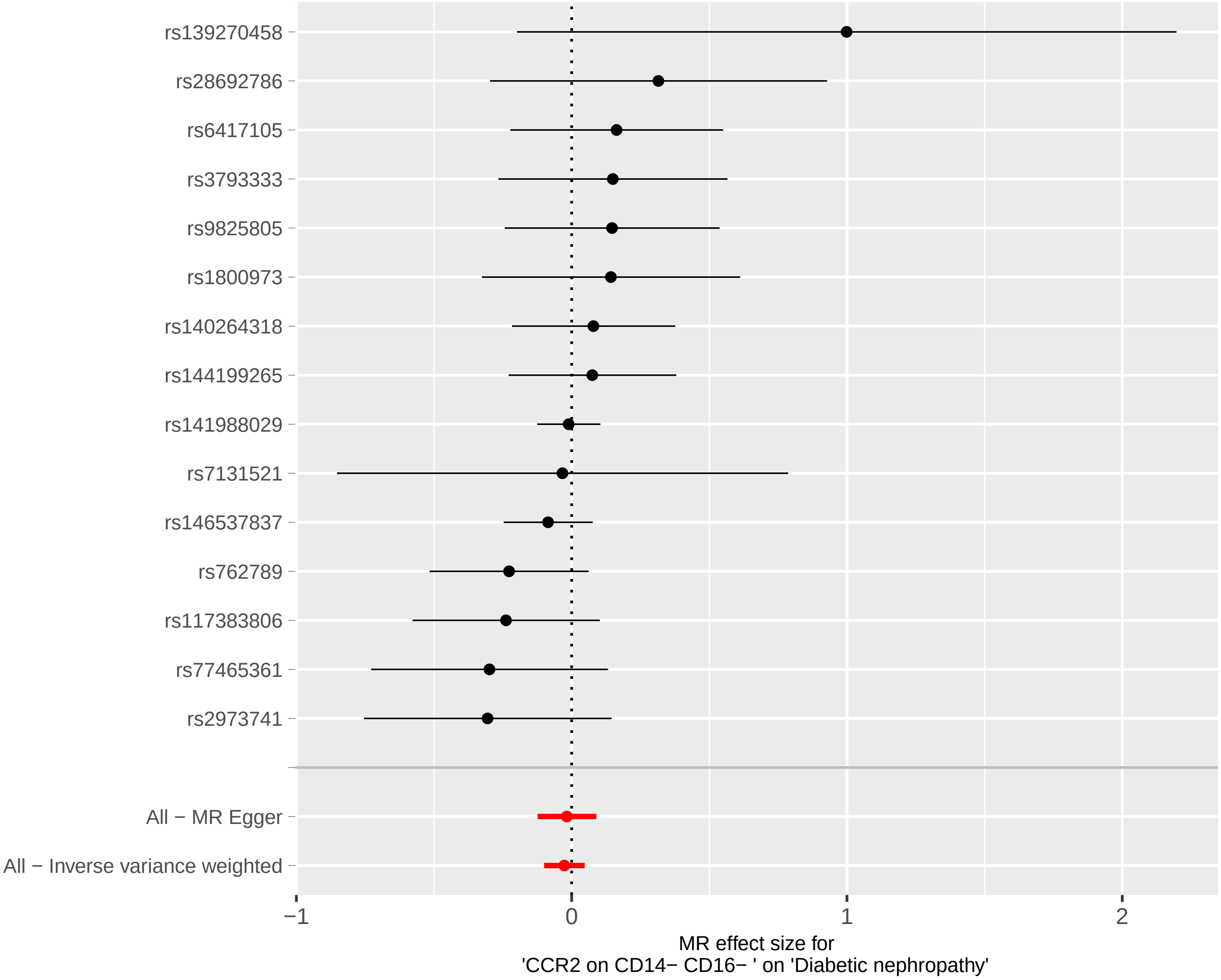
rs139270458
rs28692786
rs6417105
rs3793333
rs9825805
rs1800973
rs140264318
rs144199265
rs141988029
rs7131521
rs146537837
rs762789
rs117383806
rs77465361
rs2973741
All − MR Egger
All − Inverse variance weighted
−1
0
1
2
MR effect size for
'CCR2 on CD14− CD16− ' on 'Diabetic nephropathy'

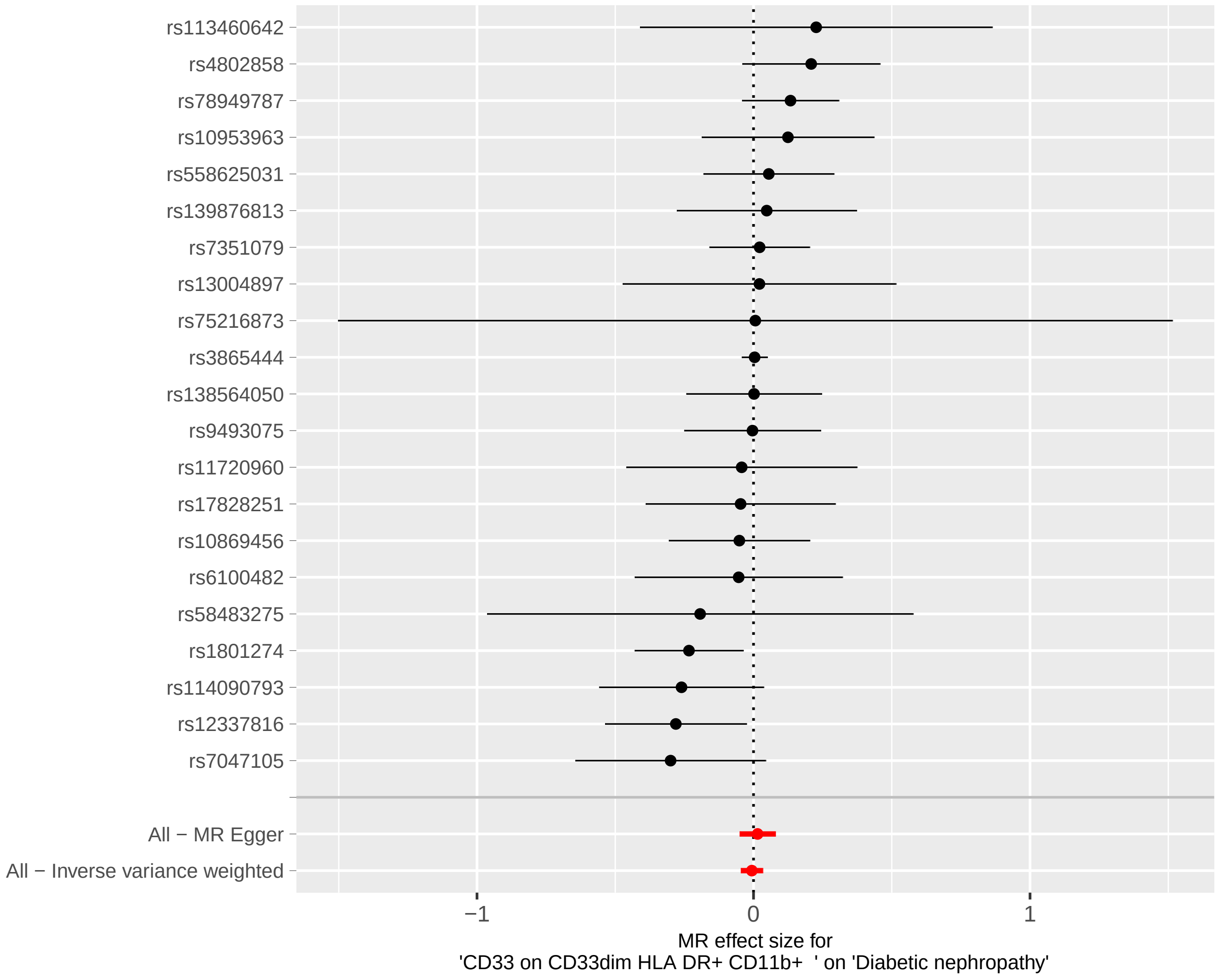
rs113460642
rs4802858
rs78949787
rs10953963
rs558625031
rs139876813
rs7351079
rs13004897
rs75216873
rs3865444
rs138564050
rs9493075
rs11720960
rs17828251
rs10869456
rs6100482
rs58483275
rs1801274
rs114090793
rs12337816
rs7047105
All − MR Egger
All − Inverse variance weighted
−1
0
1
MR effect size for
'CD33 on CD33dim HLA DR+ CD11b+ ' on 'Diabetic nephropathy'

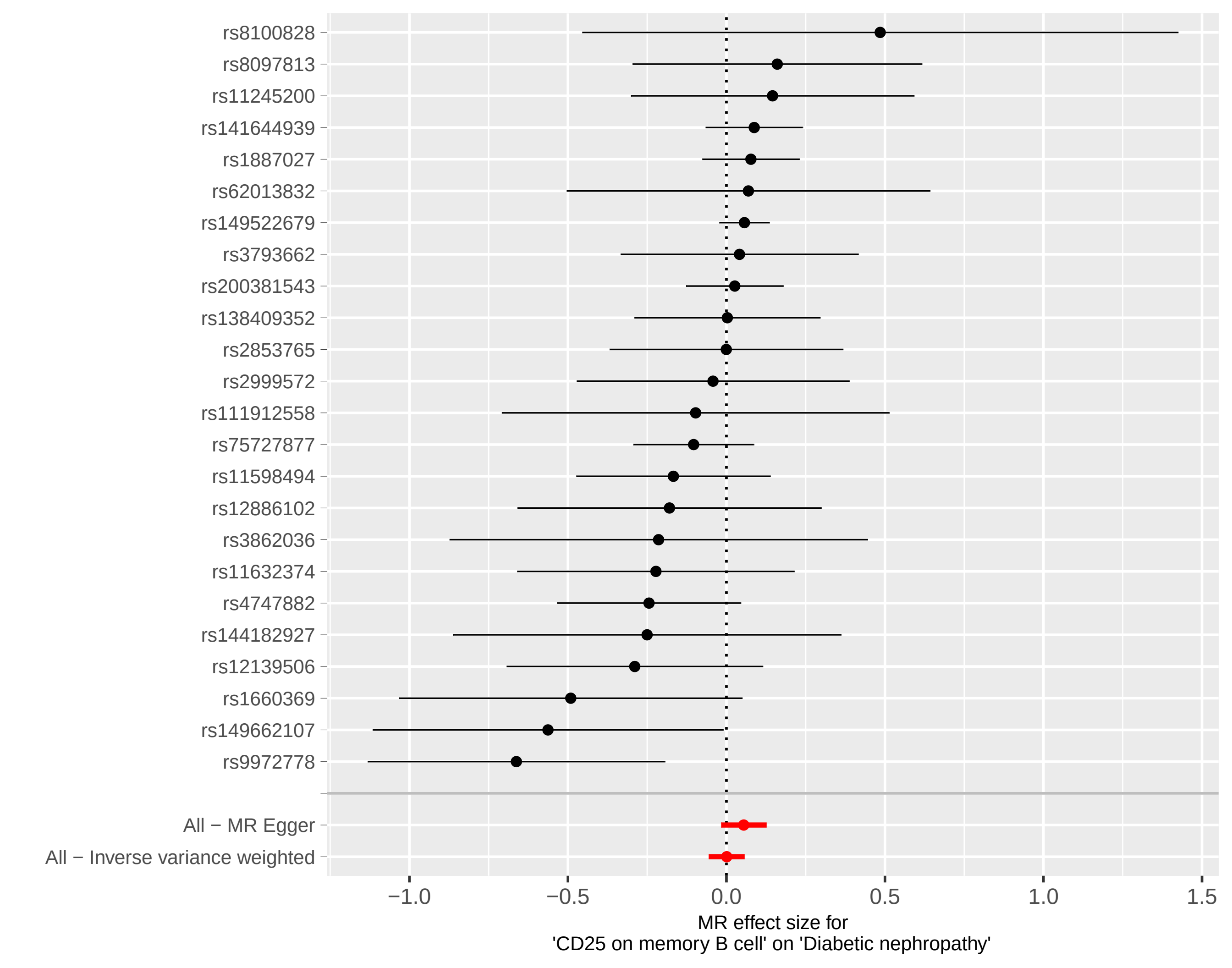
rs8100828
rs8097813
rs11245200
rs141644939
rs1887027
rs62013832
rs149522679
rs3793662
rs200381543
rs138409352
rs2853765
rs2999572
rs111912558
rs75727877
rs11598494
rs12886102
rs3862036
rs11632374
rs4747882
rs144182927
rs12139506
rs1660369
rs149662107
rs9972778
All − MR Egger
All − Inverse variance weighted
−1.0
−0.5
0.0
0.5
1.0
1.5
MR effect size for
'CD25 on memory B cell' on 'Diabetic nephropathy'

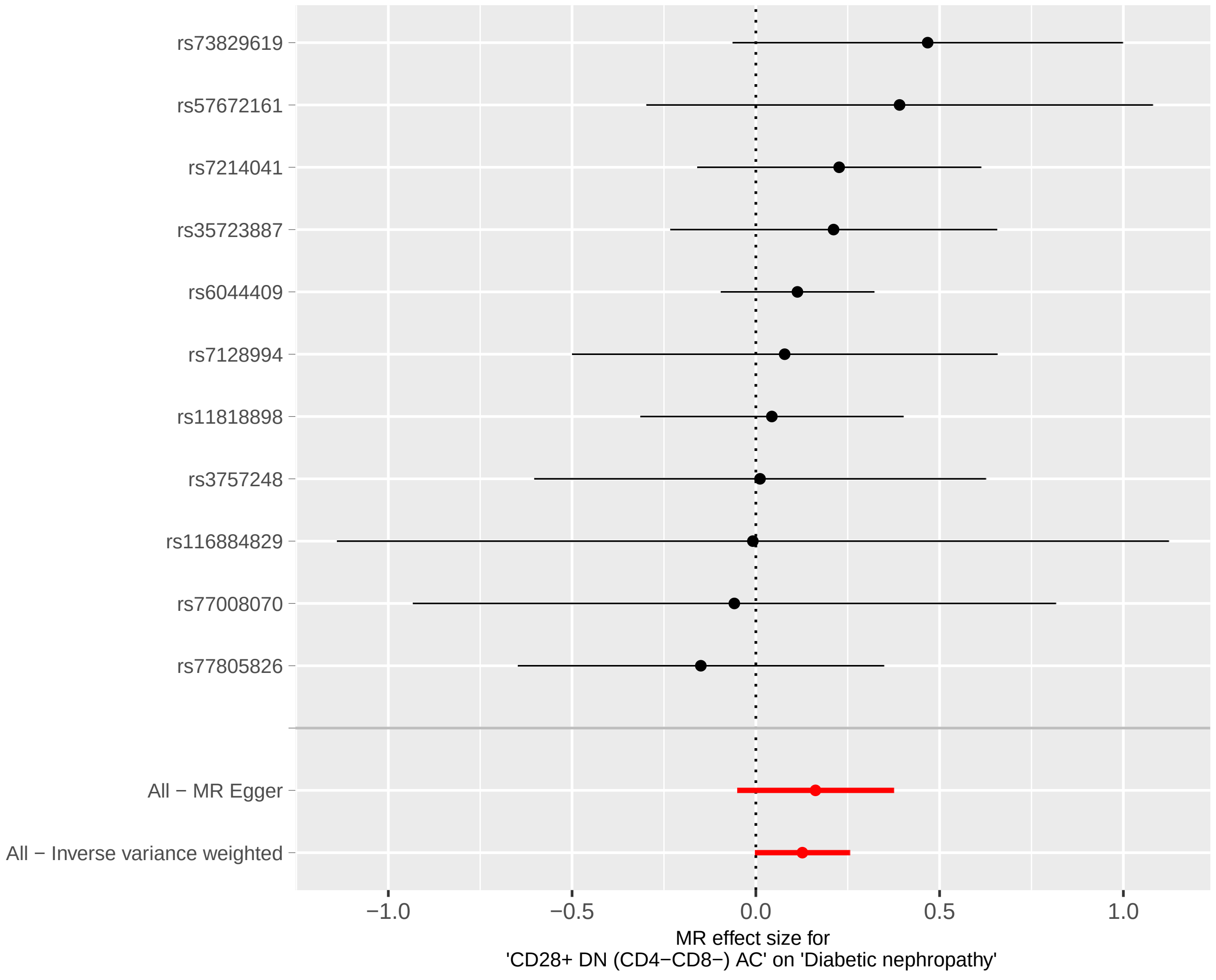

rs73829619
rs57672161
rs7214041
rs35723887
rs6044409
rs7128994
rs11818898
rs3757248
rs116884829
rs77008070
rs77805826
All − MR Egger
All − Inverse variance weighted
−1.0
−0.5
0.0
0.5
1.0
MR effect size for
'CD28+ DN (CD4−CD8−) AC' on 'Diabetic nephropathy'

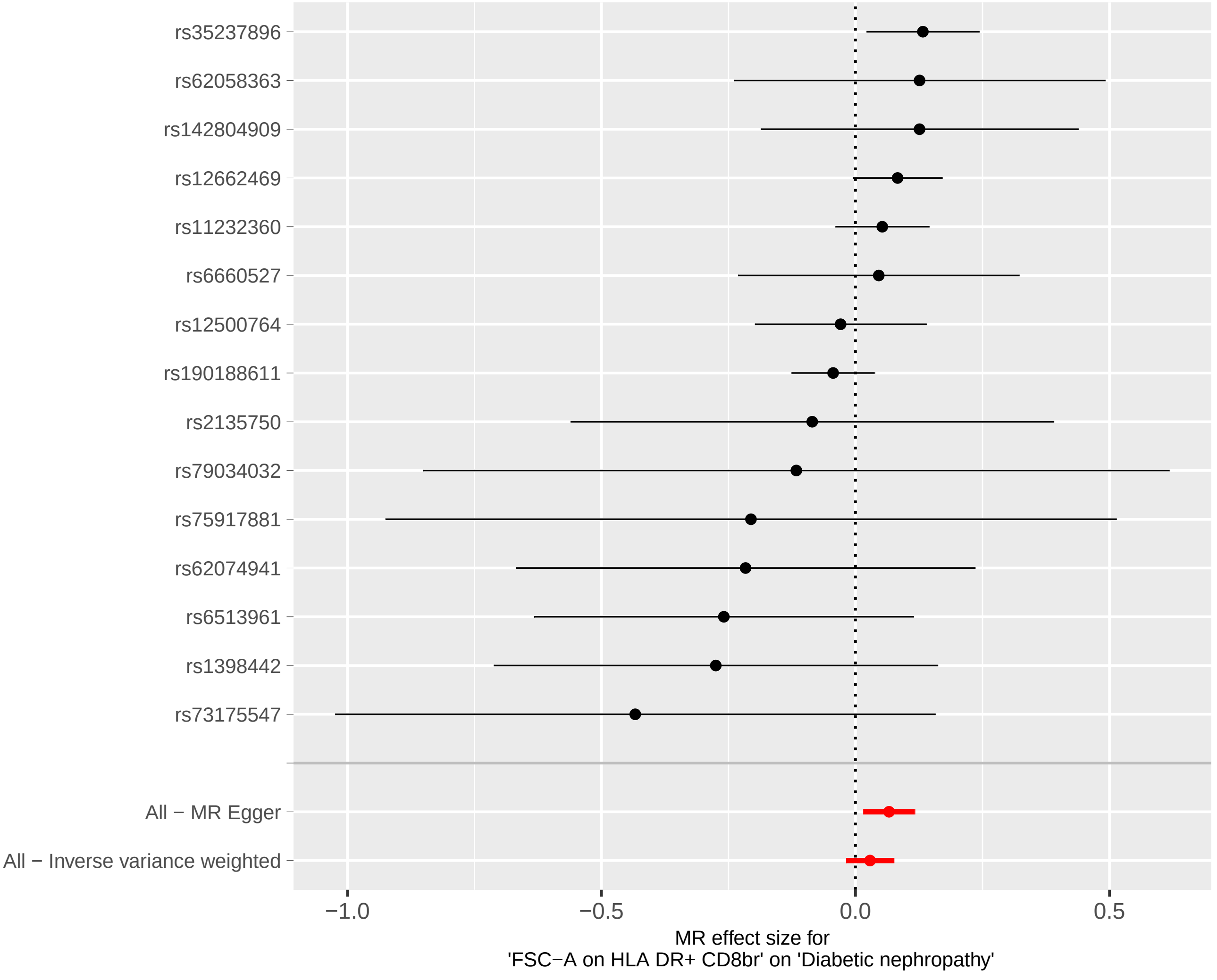

rs35237896
rs62058363
rs142804909
rs12662469
rs11232360
rs6660527
rs12500764
rs190188611
rs2135750
rs79034032
rs75917881
rs62074941
rs6513961
rs1398442
rs73175547
All − MR Egger
All − Inverse variance weighted
−1.0
−0.5
0.0
0.5
MR effect size for
'FSC−A on HLA DR+ CD8br' on 'Diabetic nephropathy'

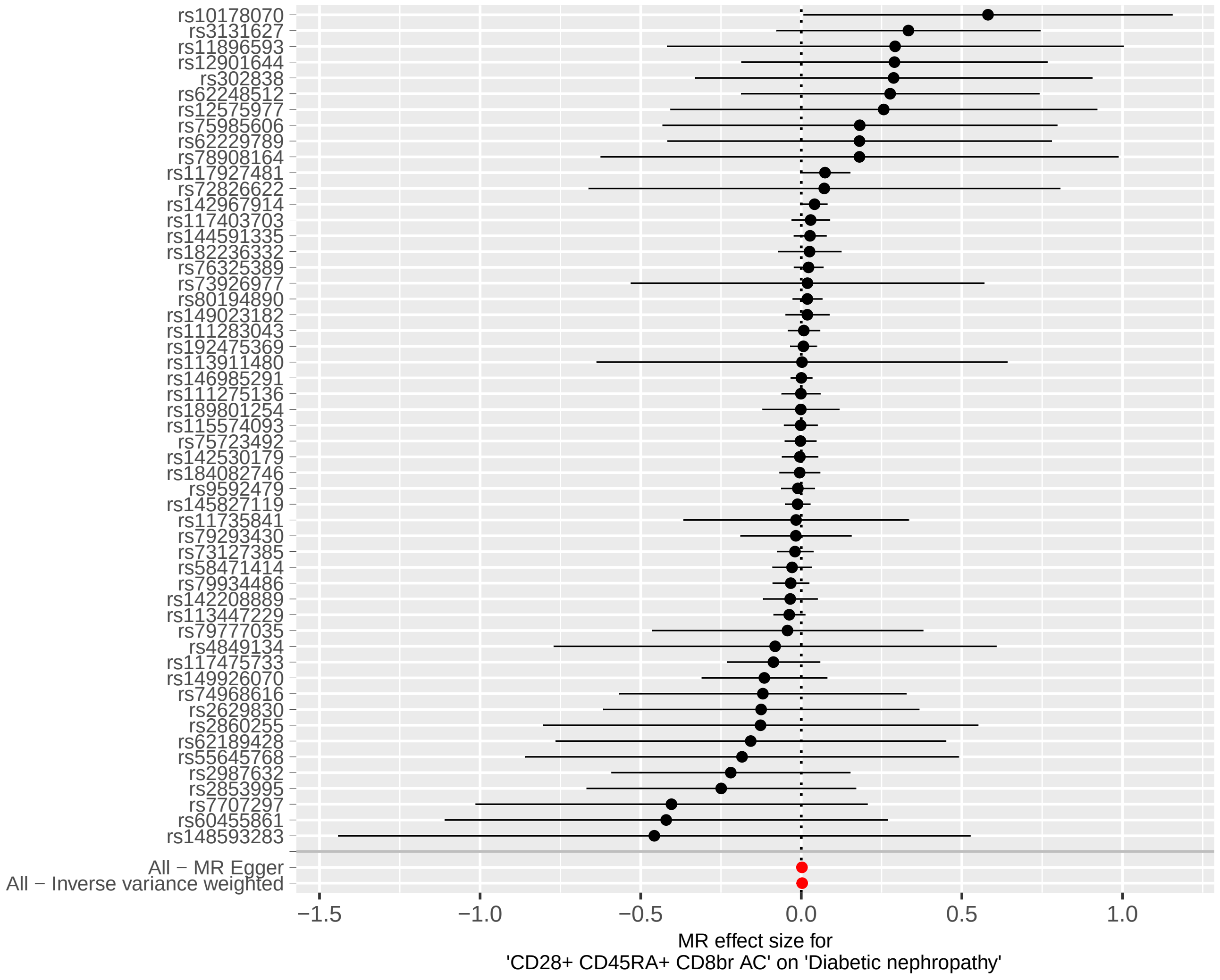
rs10178070
rs3131627
rs11896593
rs12901644
rs302838
rs62248512
rs12575977
rs75985606
rs62229789
rs78908164
rs117927481
rs72826622
rs142967914
rs117403703
rs144591335
rs182236332
rs76325389
rs73926977
rs80194890
rs149023182
rs111283043
rs192475369
rs113911480
rs146985291
rs111275136
rs189801254
rs115574093
rs75723492
rs142530179
rs184082746
rs9592479
rs145827119
rs11735841
rs79293430
rs73127385
rs58471414
rs79934486
rs142208889
rs113447229
rs79777035
rs4849134
rs117475733
rs149926070
rs74968616
rs2629830
rs2860255
rs62189428
rs55645768
rs2987632
rs2853995
rs7707297
rs60455861
rs148593283
All − MR Egger
All − Inverse variance weighted
−1.5
−1.0
−0.5
0.0
0.5
1.0
MR effect size for
'CD28+ CD45RA+ CD8br AC' on 'Diabetic nephropathy'

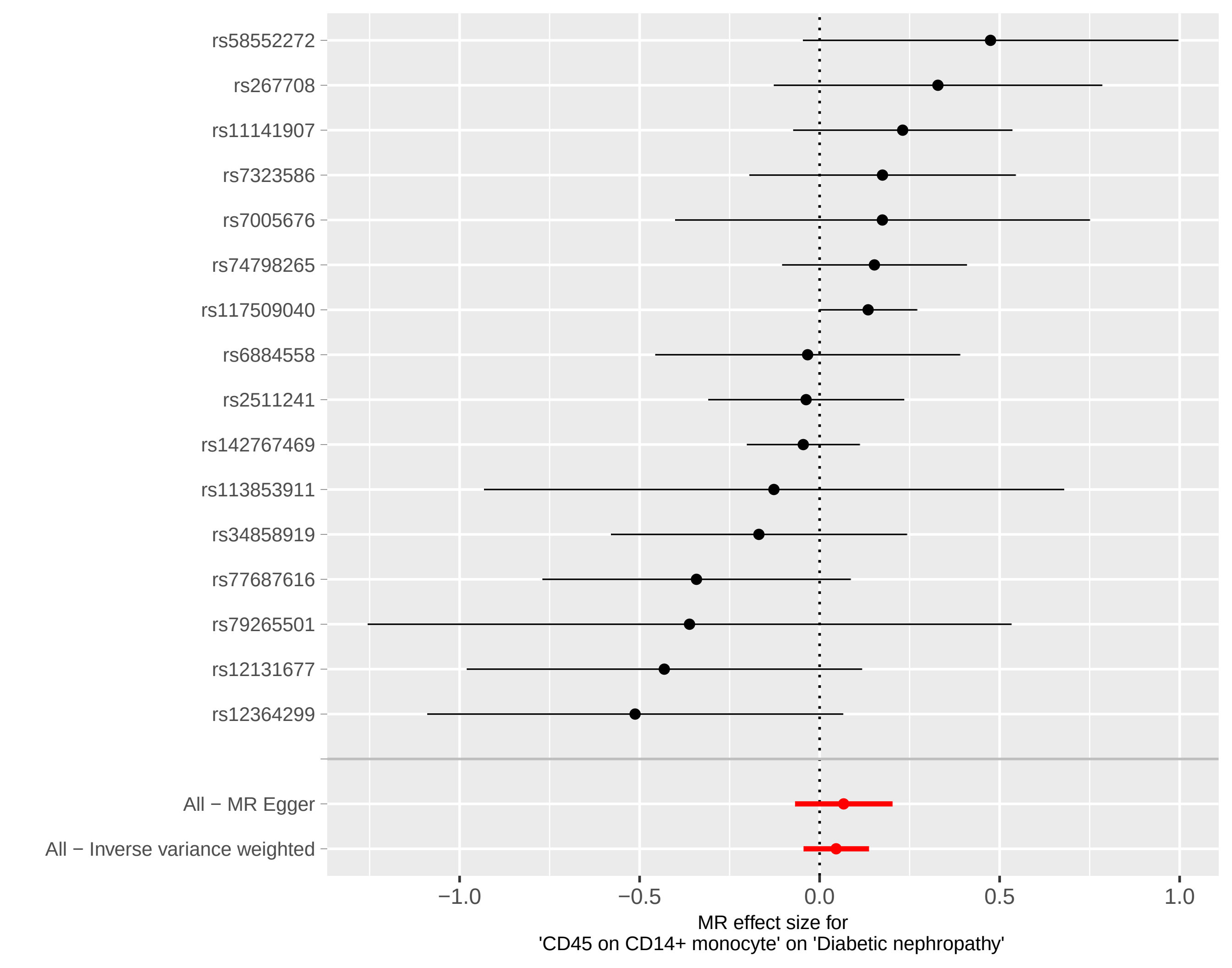

rs58552272
rs267708
rs11141907
rs7323586
rs7005676
rs74798265
rs117509040
rs6884558
rs2511241
rs142767469
rs113853911
rs34858919
rs77687616
rs79265501
rs12131677
rs12364299
All − MR Egger
All − Inverse variance weighted
−1.0
−0.5
0.0
0.5
1.0
MR effect size for
'CD45 on CD14+ monocyte' on 'Diabetic nephropathy'

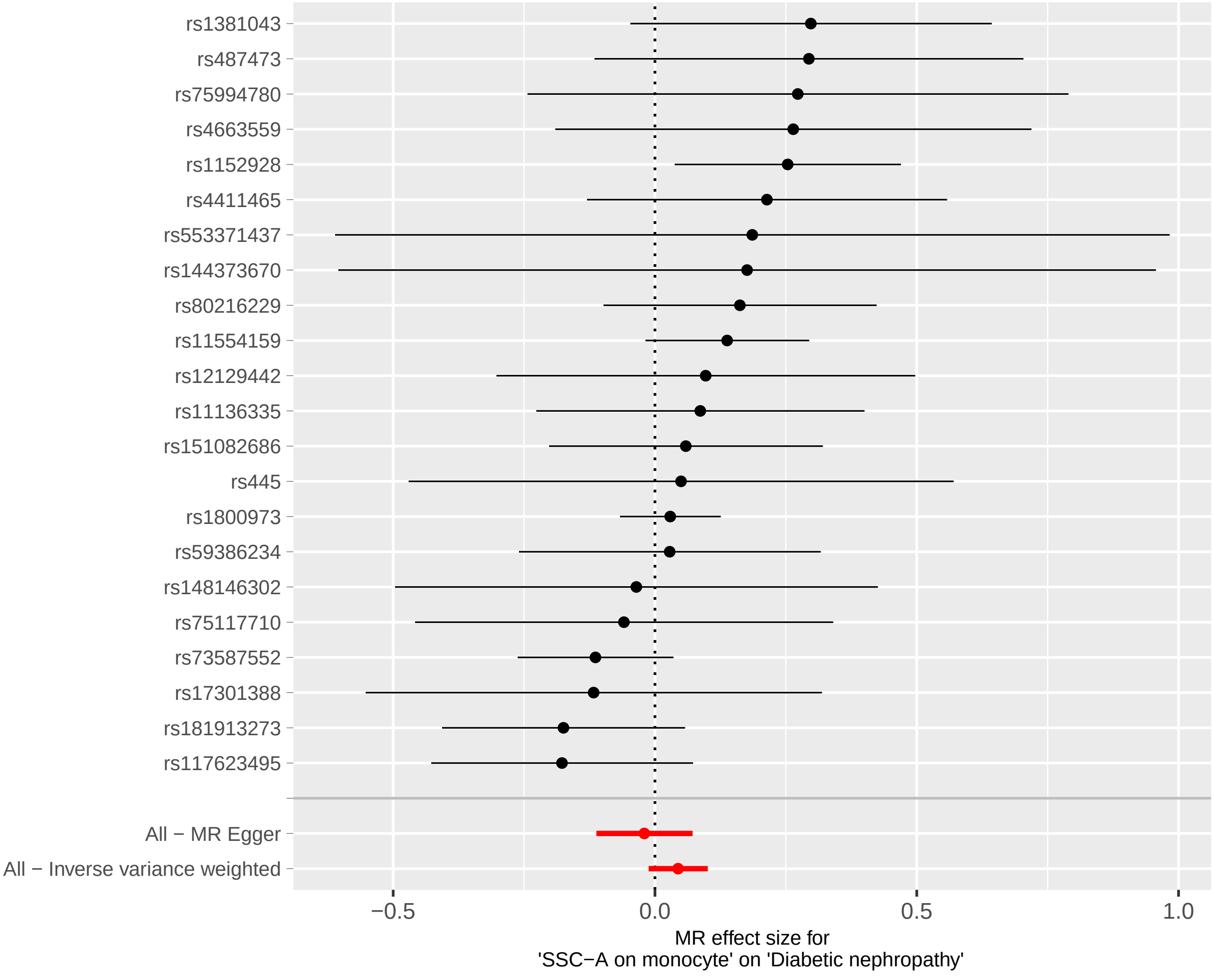

rs1381043
rs487473
rs75994780
rs4663559
rs1152928
rs4411465
rs553371437
rs144373670
rs80216229
rs11554159
rs12129442
rs11136335
rs151082686
rs445
rs1800973
rs59386234
rs148146302
rs75117710
rs73587552
rs17301388
rs181913273
rs117623495
All − MR Egger
All − Inverse variance weighted
−0.5
0.0
0.5
1.0
MR effect size for
'SSC−A on monocyte' on 'Diabetic nephropathy'

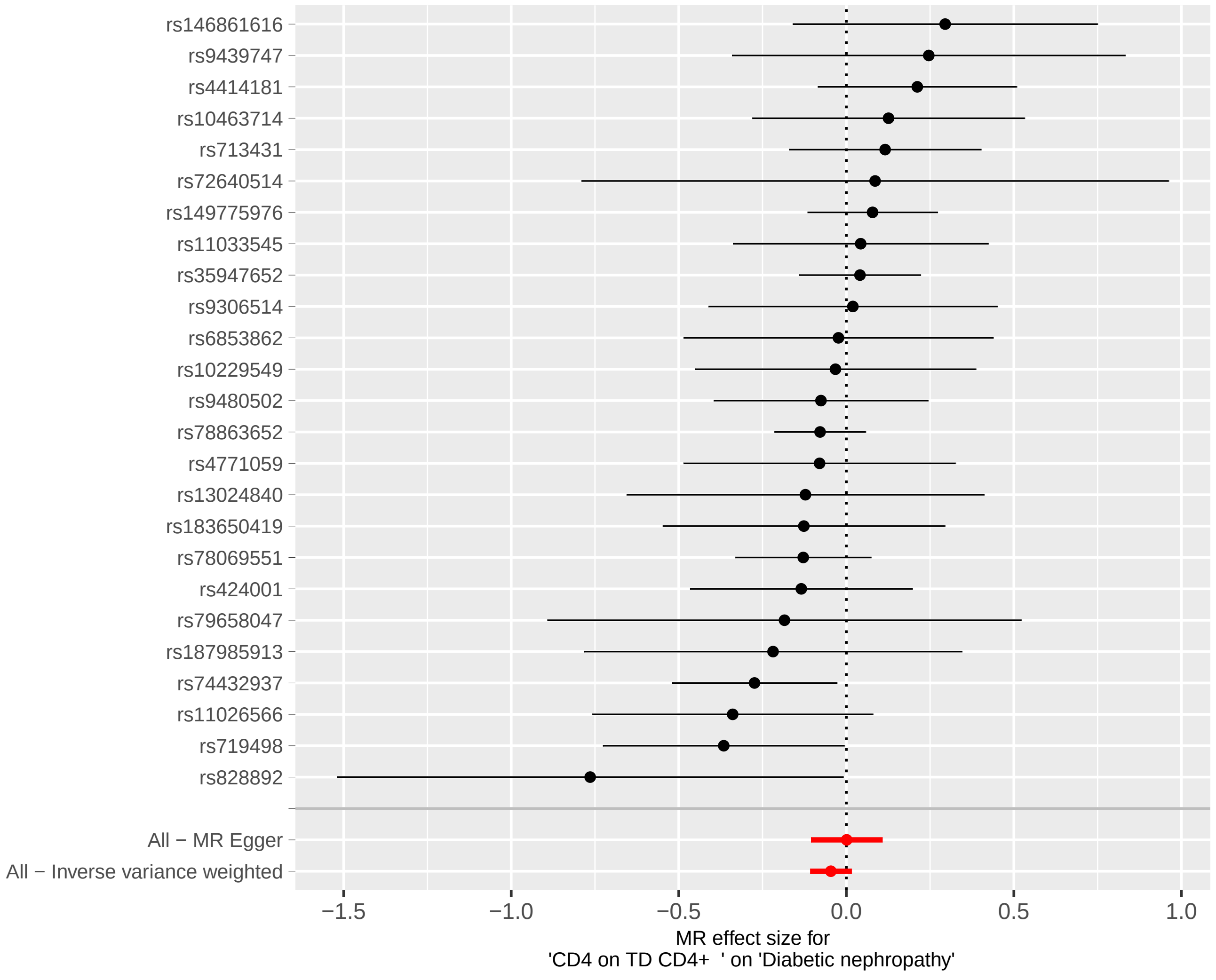
rs146861616
rs9439747
rs4414181
rs10463714
rs713431
rs72640514
rs149775976
rs11033545
rs35947652
rs9306514
rs6853862
rs10229549
rs9480502
rs78863652
rs4771059
rs13024840
rs183650419
rs78069551
rs424001
rs79658047
rs187985913
rs74432937
rs11026566
rs719498
rs828892
All − MR Egger
All − Inverse variance weighted
−1.5
−1.0
−0.5
0.0
0.5
1.0
MR effect size for
'CD4 on TD CD4+ ' on 'Diabetic nephropathy'

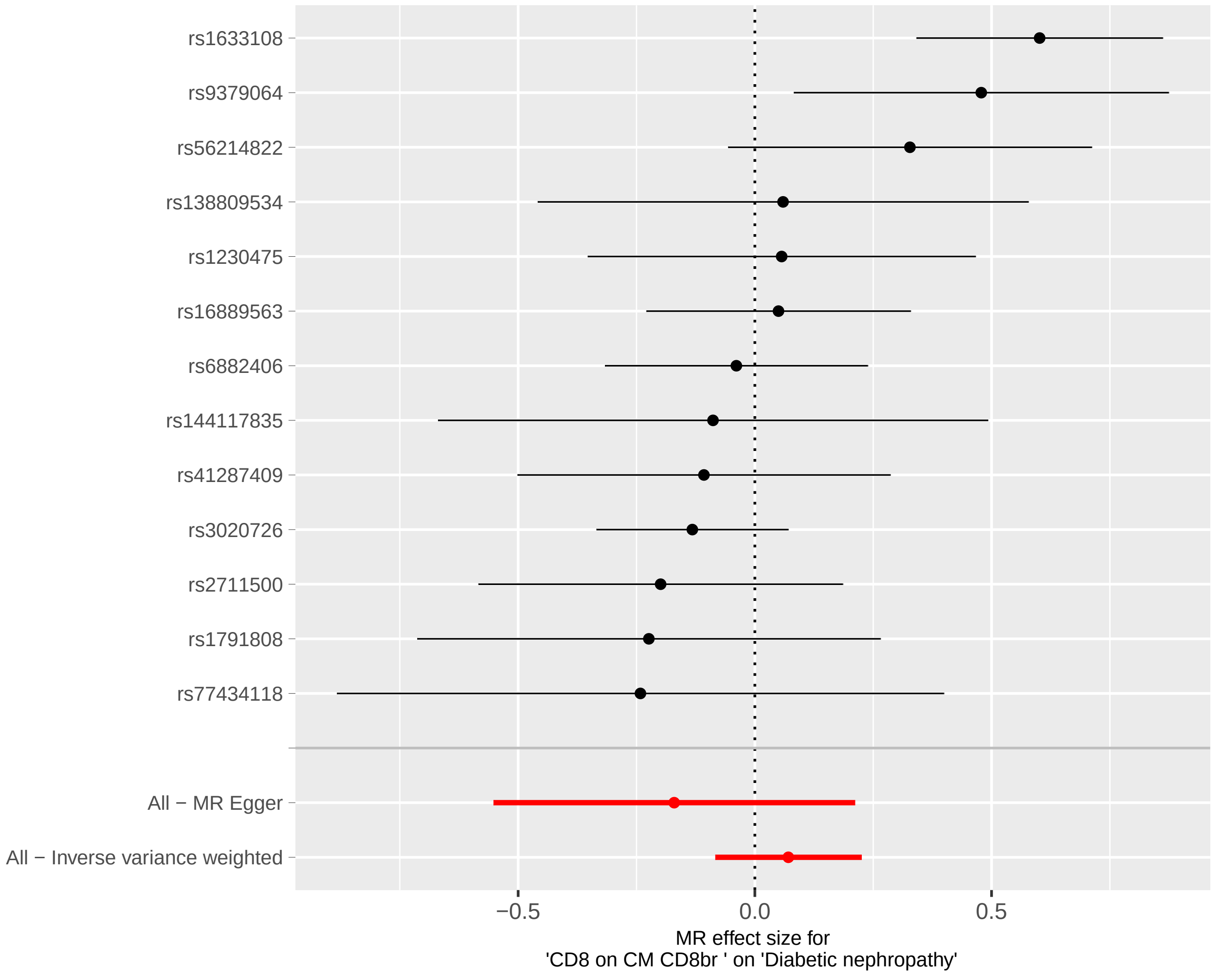
rs1633108
rs9379064
rs56214822
rs138809534
rs1230475
rs16889563
rs6882406
rs144117835
rs41287409
rs3020726
rs2711500
rs1791808
rs77434118
All − MR Egger
All − Inverse variance weighted
−0.5
0.0
0.5
MR effect size for
'CD8 on CM CD8br ' on 'Diabetic nephropathy'

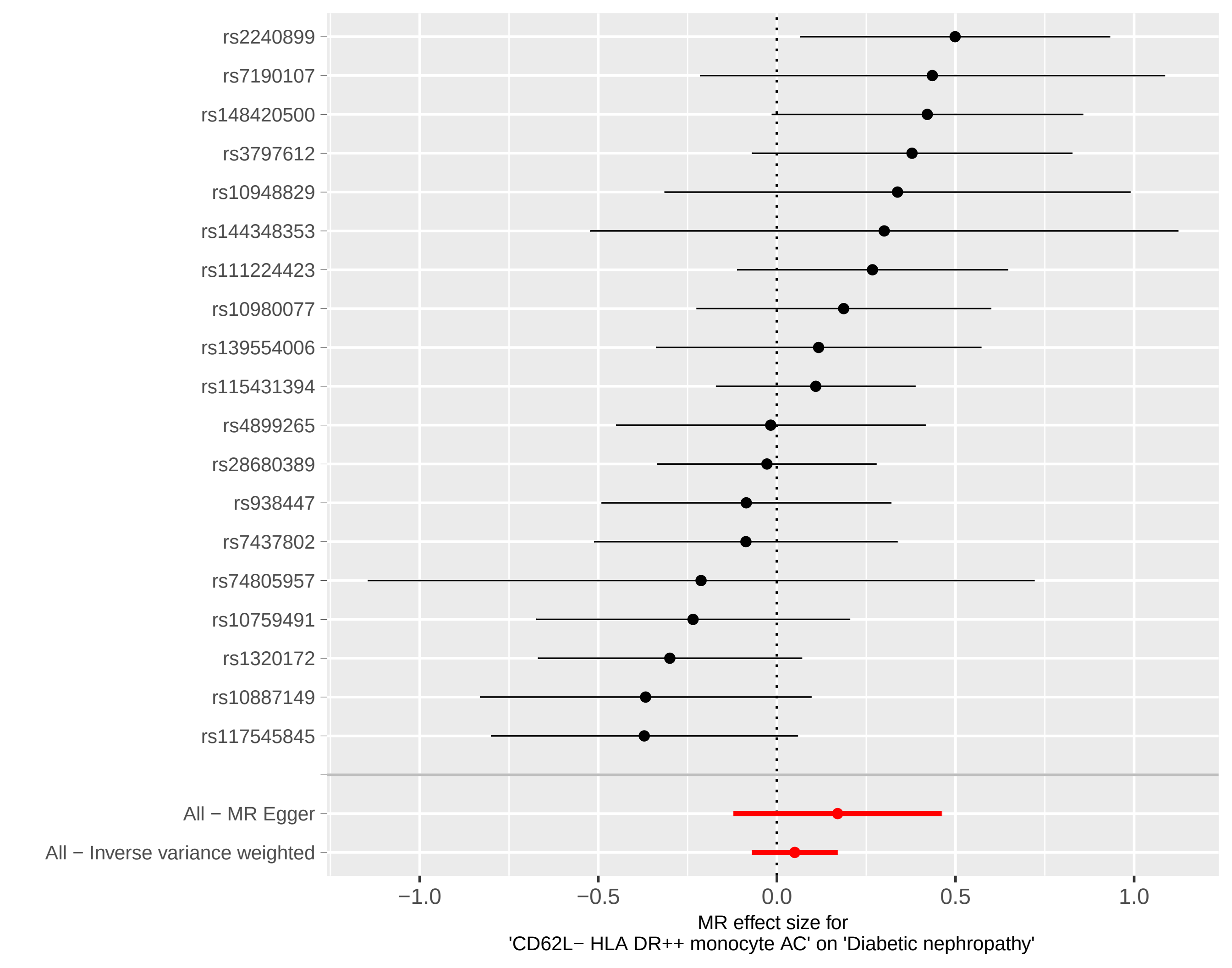

rs2240899
rs7190107
rs148420500
rs3797612
rs10948829
rs144348353
rs111224423
rs10980077
rs139554006
rs115431394
rs4899265
rs28680389
rs938447
rs7437802
rs74805957
rs10759491
rs1320172
rs10887149
rs117545845
All − MR Egger
All − Inverse variance weighted
−1.0
−0.5
0.0
0.5
1.0
MR effect size for
'CD62L− HLA DR++ monocyte AC' on 'Diabetic nephropathy'

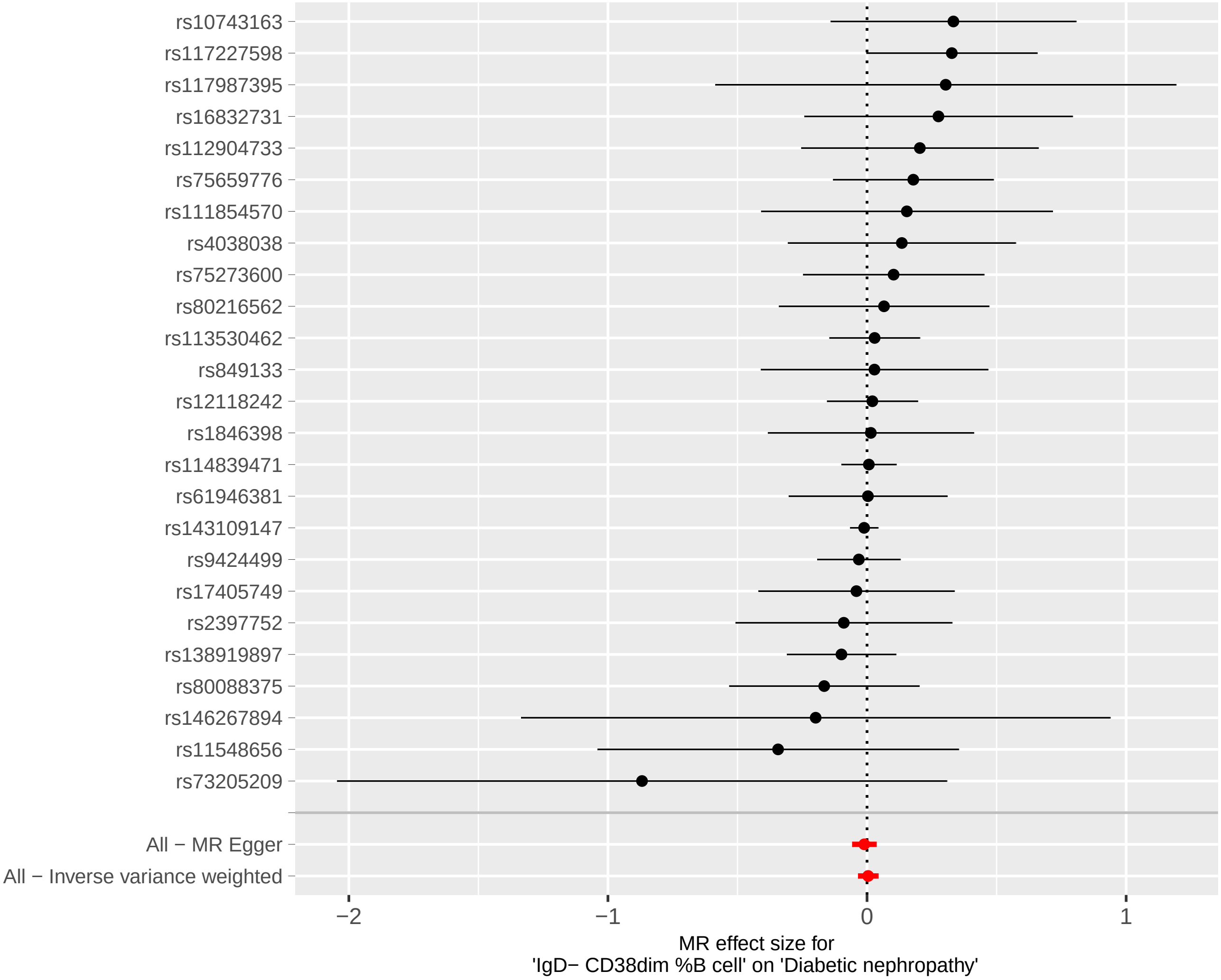
rs10743163
rs117227598
rs117987395
rs16832731
rs112904733
rs75659776
rs111854570
rs4038038
rs75273600
rs80216562
rs113530462
rs849133
rs12118242
rs1846398
rs114839471
rs61946381
rs143109147
rs9424499
rs17405749
rs2397752
rs138919897
rs80088375
rs146267894
rs11548656
rs73205209
All − MR Egger
All − Inverse variance weighted
−2
−1
0
1
MR effect size for
'IgD− CD38dim %B cell' on 'Diabetic nephropathy'

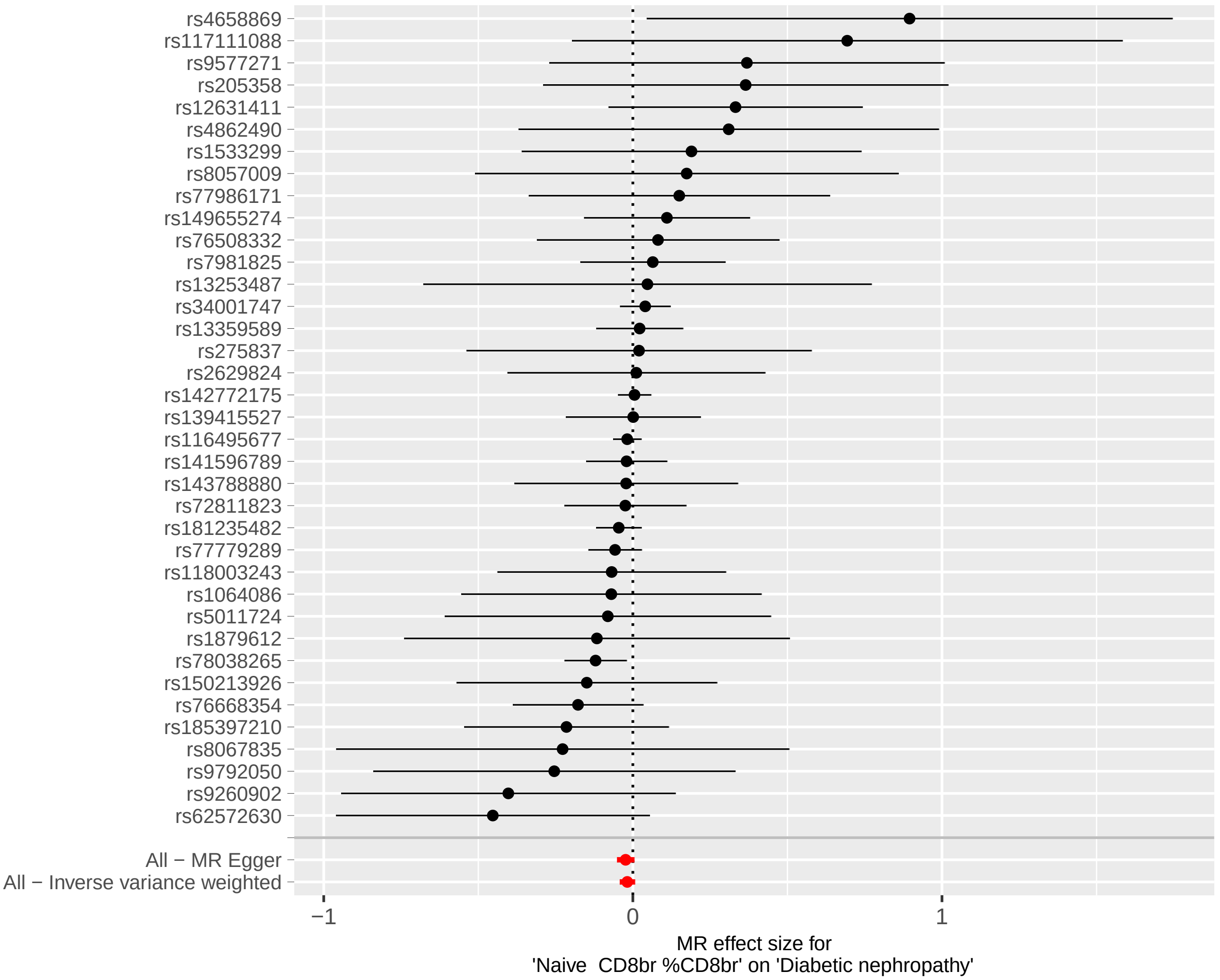

rs4658869
rs117111088
rs9577271
rs205358
rs12631411
rs4862490
rs1533299
rs8057009
rs77986171
rs149655274
rs76508332
rs7981825
rs13253487
rs34001747
rs13359589
rs275837
rs2629824
rs142772175
rs139415527
rs116495677
rs141596789
rs143788880
rs72811823
rs181235482
rs77779289
rs118003243
rs1064086
rs5011724
rs1879612
rs78038265
rs150213926
rs76668354
rs185397210
rs8067835
rs9792050
rs9260902
rs62572630
All − MR Egger
All − Inverse variance weighted
−1
0
1
MR effect size for
'Naive CD8br %CD8br' on 'Diabetic nephropathy'

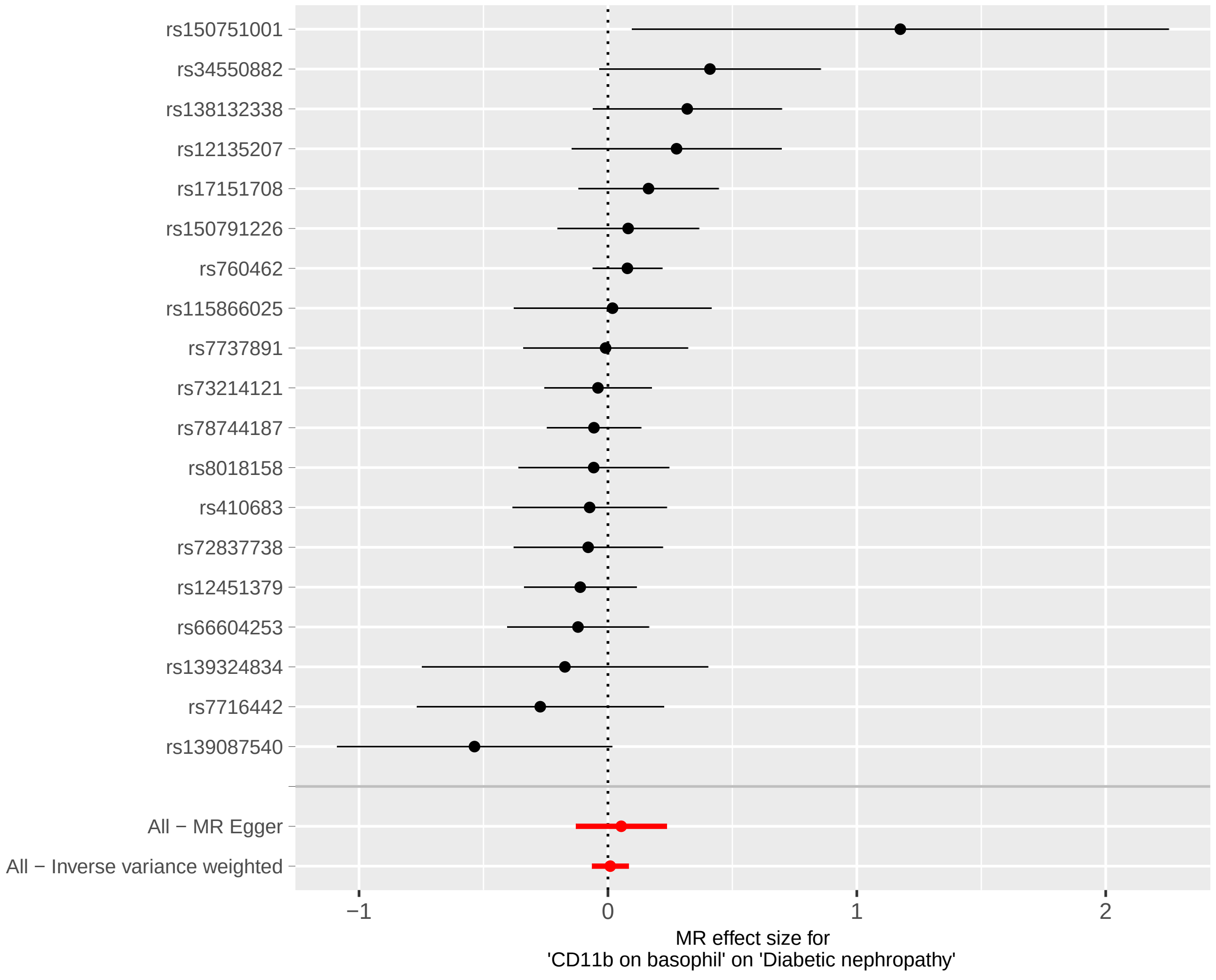
rs150751001
rs34550882
rs138132338
rs12135207
rs17151708
rs150791226
rs760462
rs115866025
rs7737891
rs73214121
rs78744187
rs8018158
rs410683
rs72837738
rs12451379
rs66604253
rs139324834
rs7716442
rs139087540
All − MR Egger
All − Inverse variance weighted
−1
0
1
2
MR effect size for
'CD11b on basophil' on 'Diabetic nephropathy'

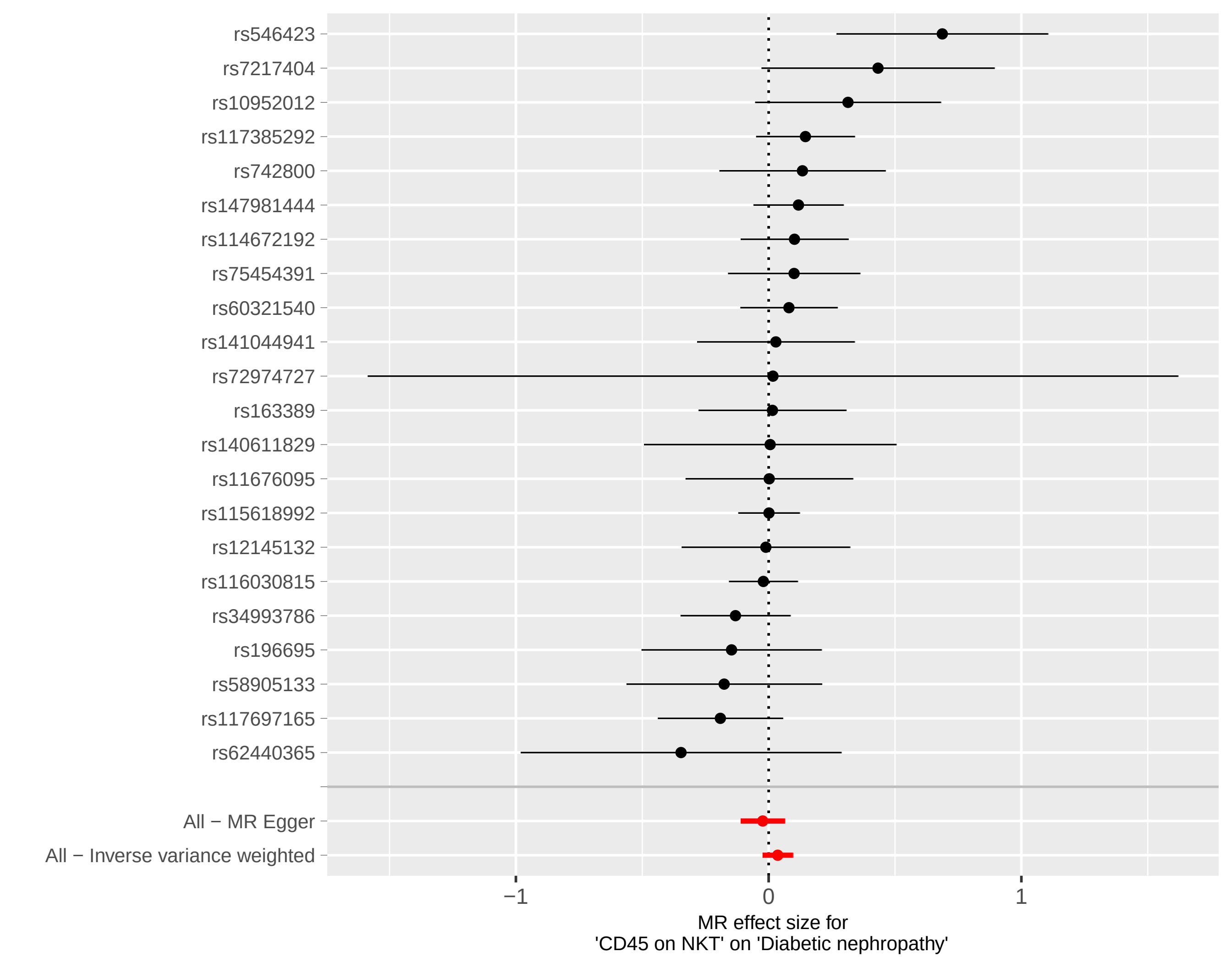
rs546423
rs7217404
rs10952012
rs117385292
rs742800
rs147981444
rs114672192
rs75454391
rs60321540
rs141044941
rs72974727
rs163389
rs140611829
rs11676095
rs115618992
rs12145132
rs116030815
rs34993786
rs196695
rs58905133
rs117697165
rs62440365
All − MR Egger
All − Inverse variance weighted
−1
0
1
MR effect size for
'CD45 on NKT' on 'Diabetic nephropathy'

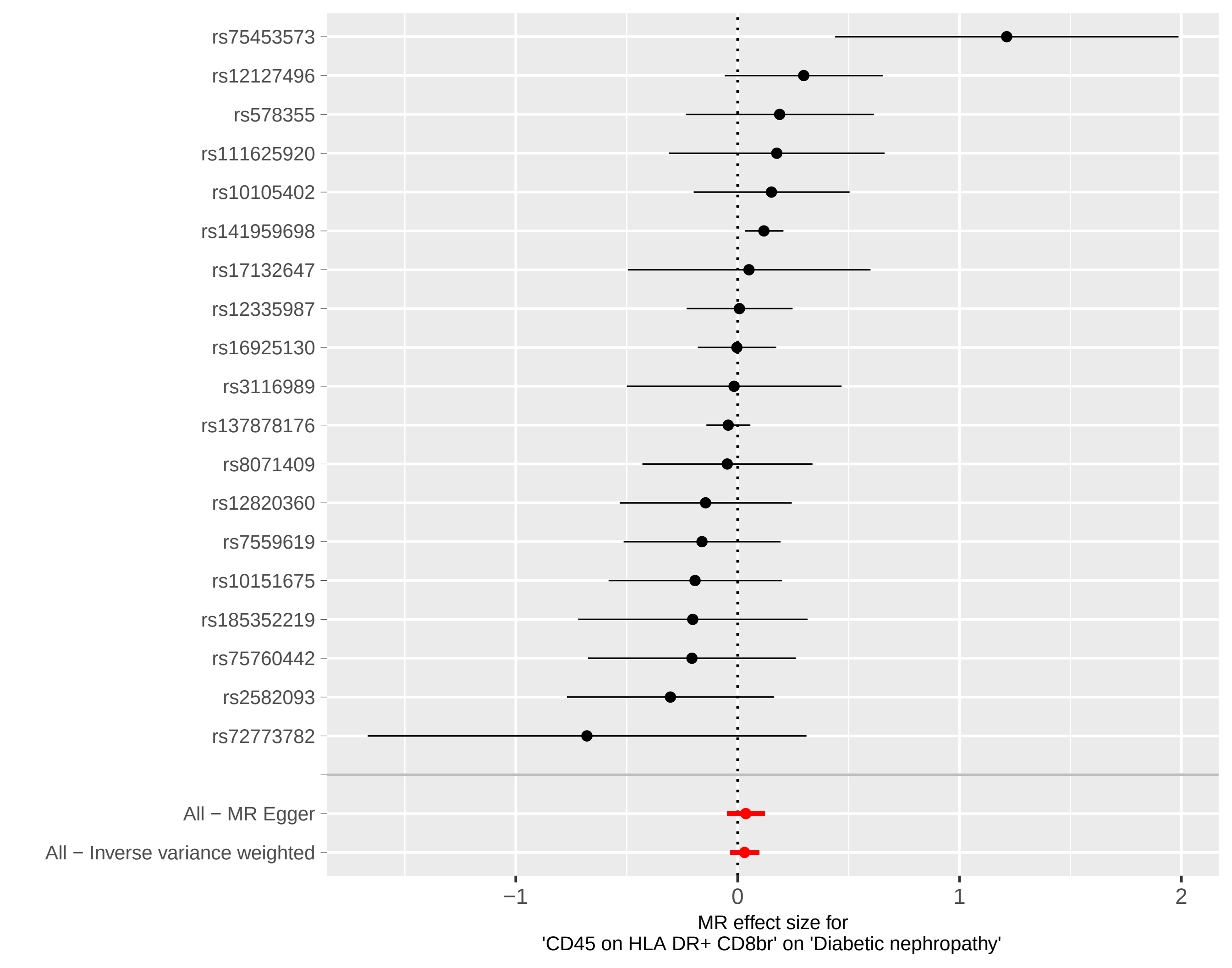
rs75453573
rs12127496
rs578355
rs111625920
rs10105402
rs141959698
rs17132647
rs12335987
rs16925130
rs3116989
rs137878176
rs8071409
rs12820360
rs7559619
rs10151675
rs185352219
rs75760442
rs2582093
rs72773782
All − MR Egger
All − Inverse variance weighted
−1
0
1
2
MR effect size for
'CD45 on HLA DR+ CD8br' on 'Diabetic nephropathy'

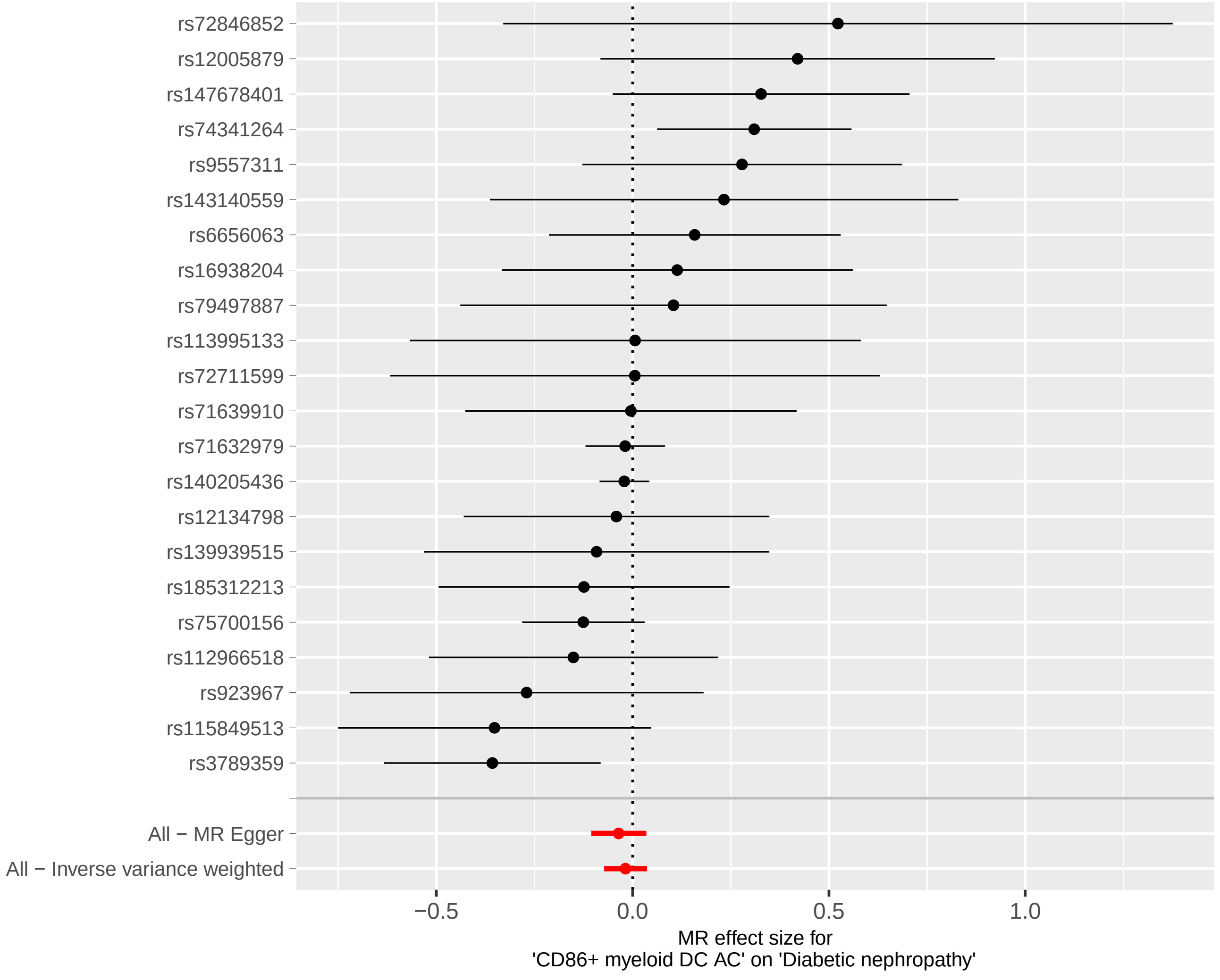
rs72846852
rs12005879
rs147678401
rs74341264
rs9557311
rs143140559
rs6656063
rs16938204
rs79497887
rs113995133
rs72711599
rs71639910
rs71632979
rs140205436
rs12134798
rs139939515
rs185312213
rs75700156
rs112966518
rs923967
rs115849513
rs3789359
All − MR Egger
All − Inverse variance weighted
−0.5
0.0
0.5
1.0
MR effect size for
'CD86+ myeloid DC AC' on 'Diabetic nephropathy'

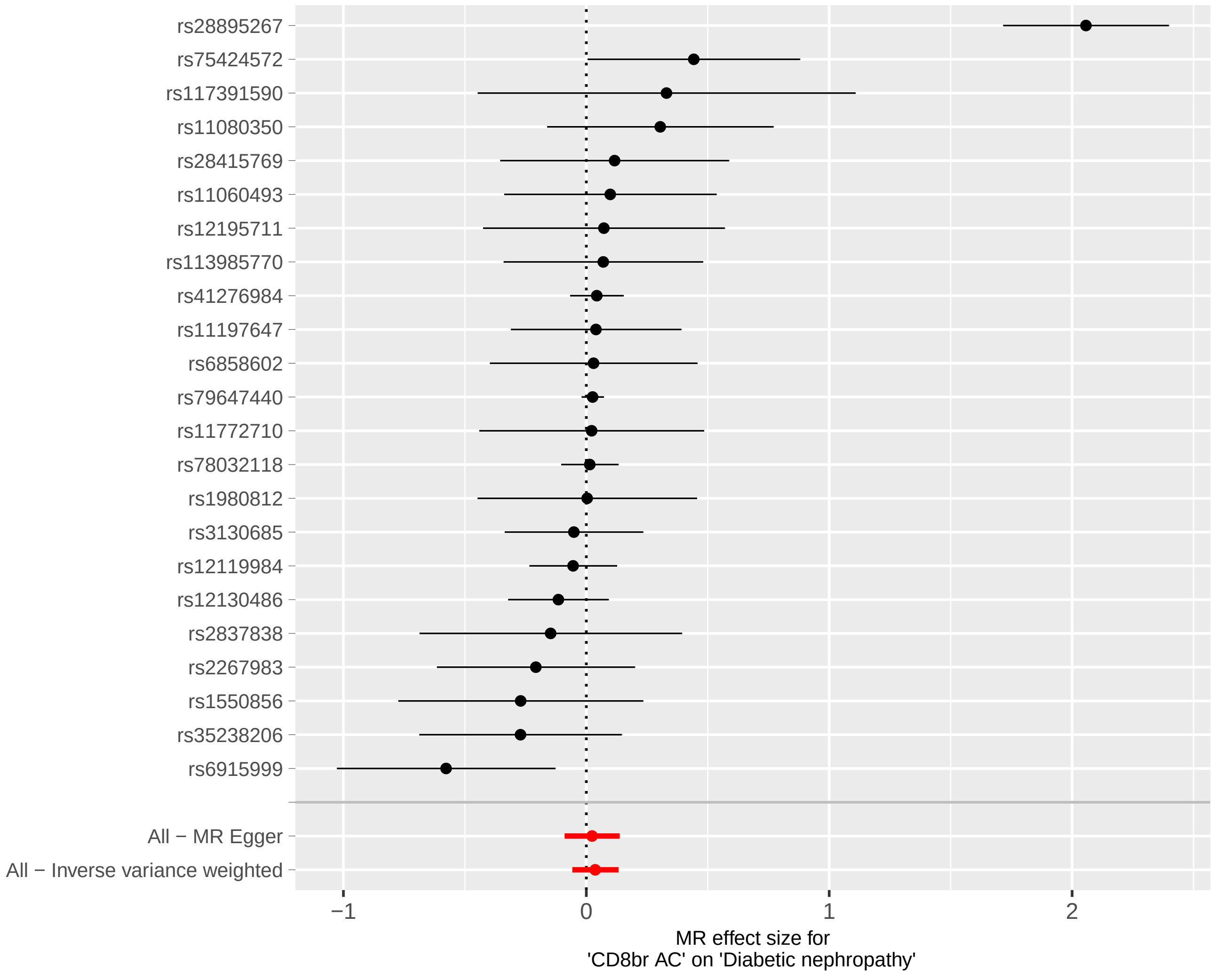
rs28895267
rs75424572
rs117391590
rs11080350
rs28415769
rs11060493
rs12195711
rs113985770
rs41276984
rs11197647
rs6858602
rs79647440
rs11772710
rs78032118
rs1980812
rs3130685
rs12119984
rs12130486
rs2837838
rs2267983
rs1550856
rs35238206
rs6915999
All − MR Egger
All − Inverse variance weighted
−1
0
1
2
MR effect size for
'CD8br AC' on 'Diabetic nephropathy'

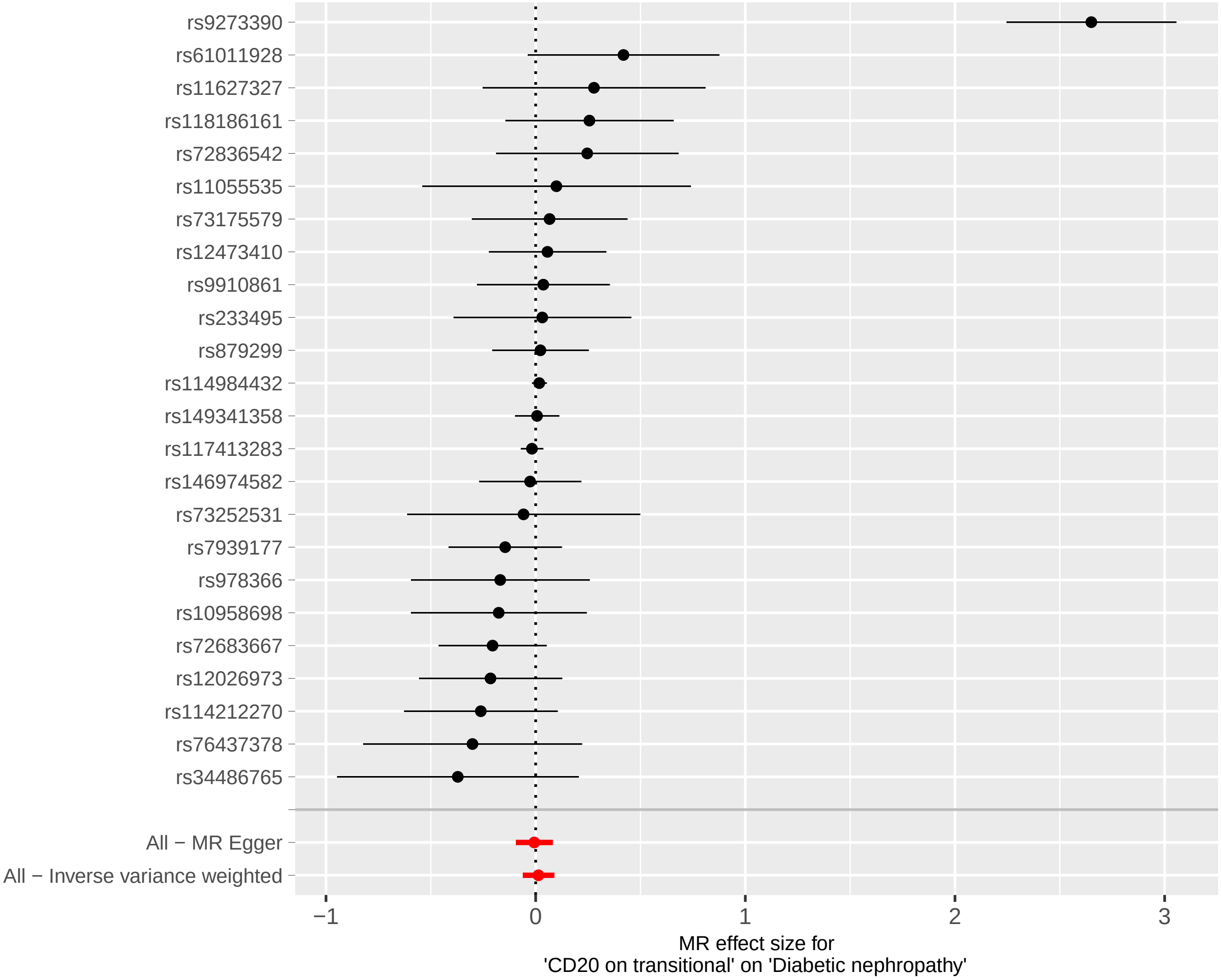
rs9273390
rs61011928
rs11627327
rs118186161
rs72836542
rs11055535
rs73175579
rs12473410
rs9910861
rs233495
rs879299
rs114984432
rs149341358
rs117413283
rs146974582
rs73252531
rs7939177
rs978366
rs10958698
rs72683667
rs12026973
rs114212270
rs76437378
rs34486765
All − MR Egger
All − Inverse variance weighted
−1
0
1
2
3
MR effect size for
'CD20 on transitional' on 'Diabetic nephropathy'

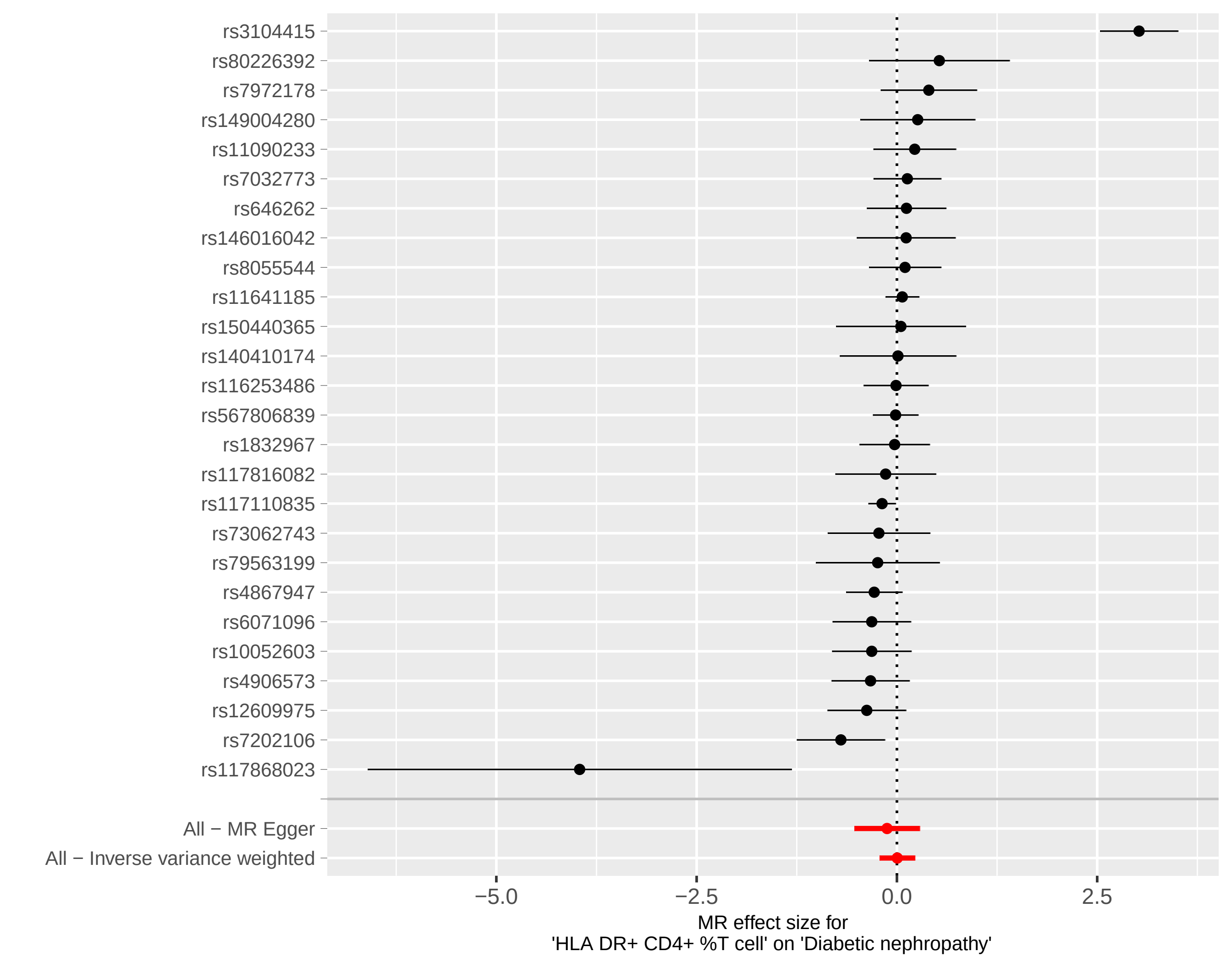
rs3104415
rs80226392
rs7972178
rs149004280
rs11090233
rs7032773
rs646262
rs146016042
rs8055544
rs11641185
rs150440365
rs140410174
rs116253486
rs567806839
rs1832967
rs117816082
rs117110835
rs73062743
rs79563199
rs4867947
rs6071096
rs10052603
rs4906573
rs12609975
rs7202106
rs117868023
All − MR Egger
All − Inverse variance weighted
−5.0
−2.5
0.0
2.5
MR effect size for
'HLA DR+ CD4+ %T cell' on 'Diabetic nephropathy'

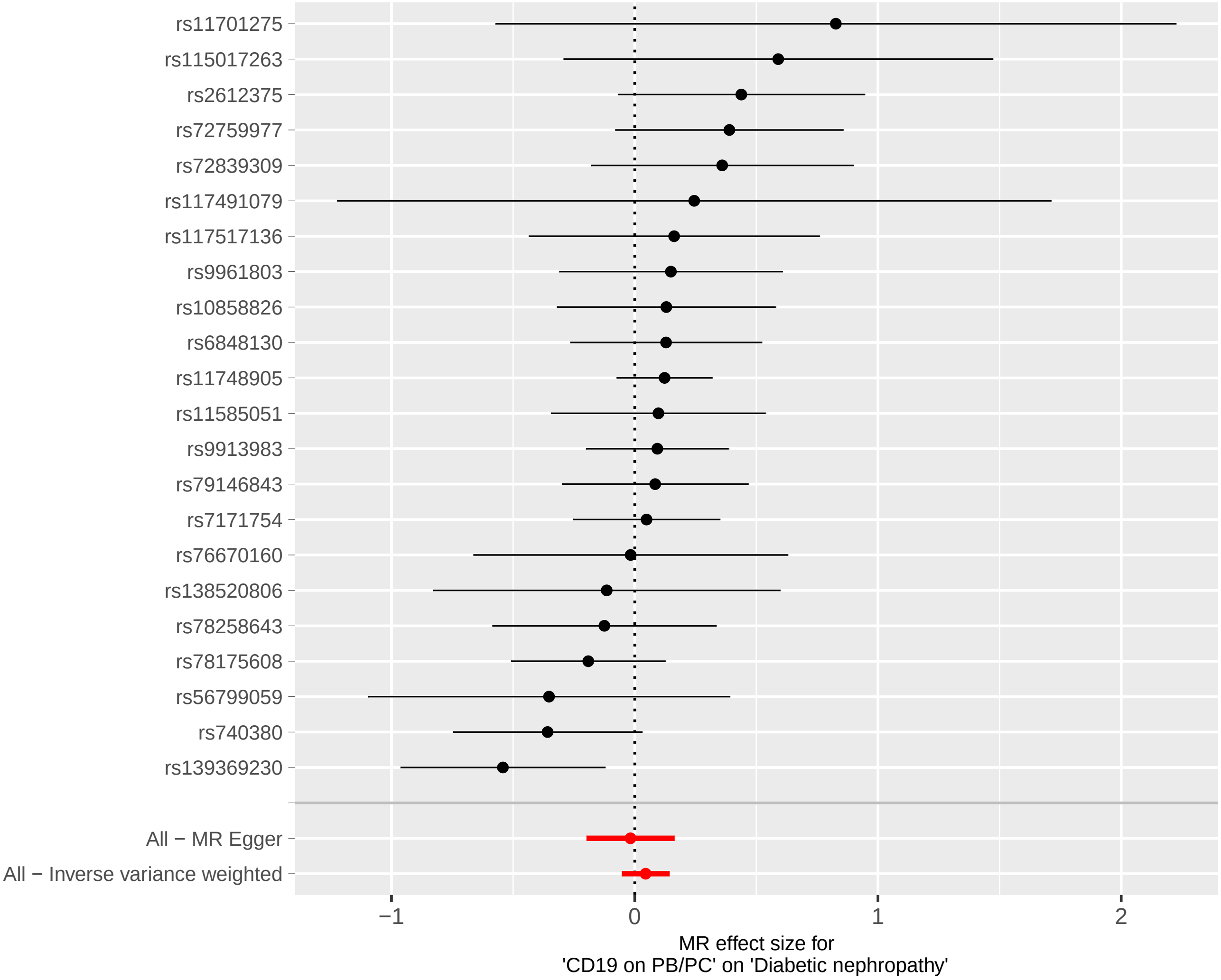
rs11701275
rs115017263
rs2612375
rs72759977
rs72839309
rs117491079
rs117517136
rs9961803
rs10858826
rs6848130
rs11748905
rs11585051
rs9913983
rs79146843
rs7171754
rs76670160
rs138520806
rs78258643
rs78175608
rs56799059
rs740380
rs139369230
All − MR Egger
All − Inverse variance weighted
−1
0
1
2
MR effect size for
'CD19 on PB/PC' on 'Diabetic nephropathy'

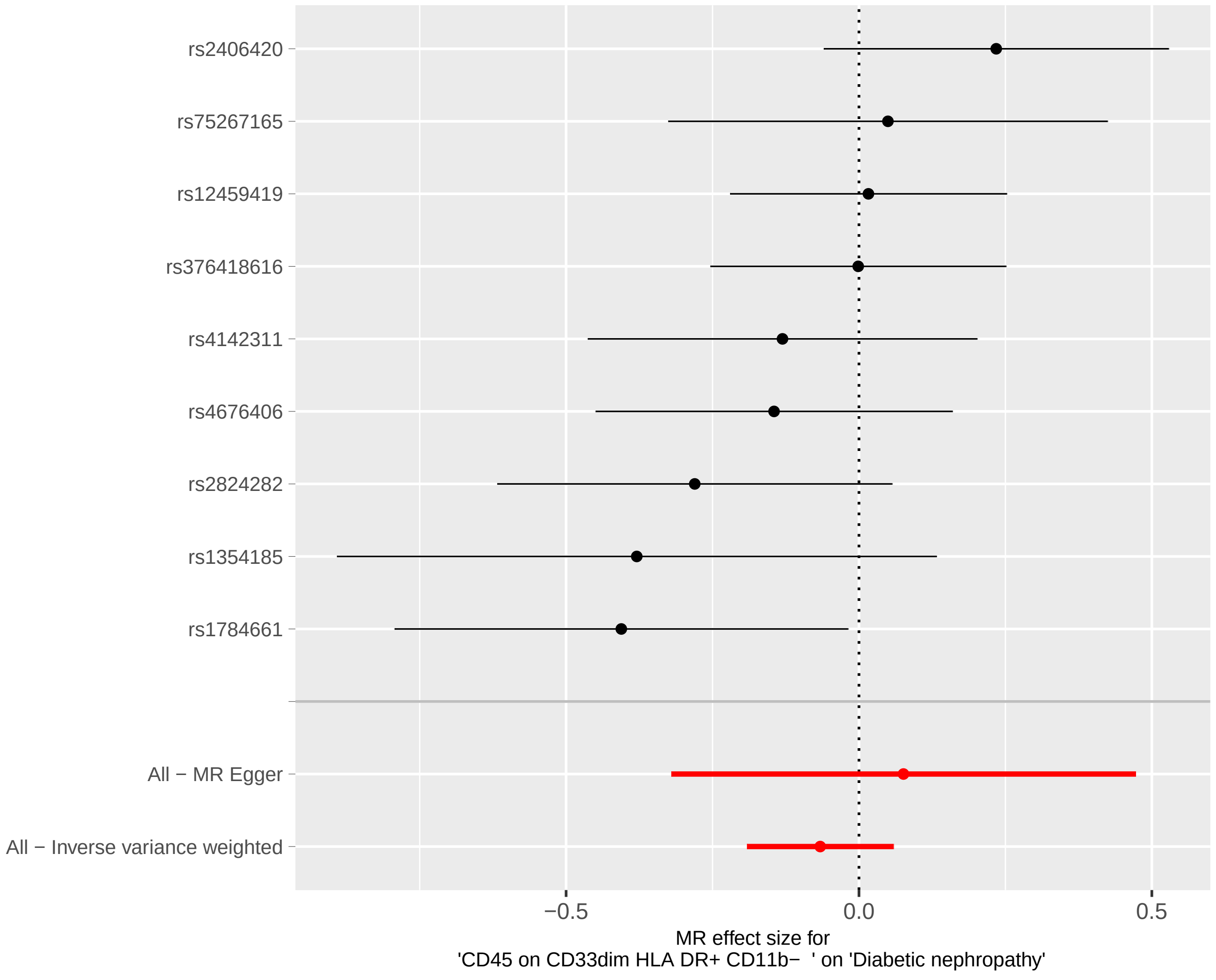
rs2406420
rs75267165
rs12459419
rs376418616
rs4142311
rs4676406
rs2824282
rs1354185
rs1784661
All − MR Egger
All − Inverse variance weighted
−0.5
0.0
0.5
MR effect size for
'CD45 on CD33dim HLA DR+ CD11b− ' on 'Diabetic nephropathy'

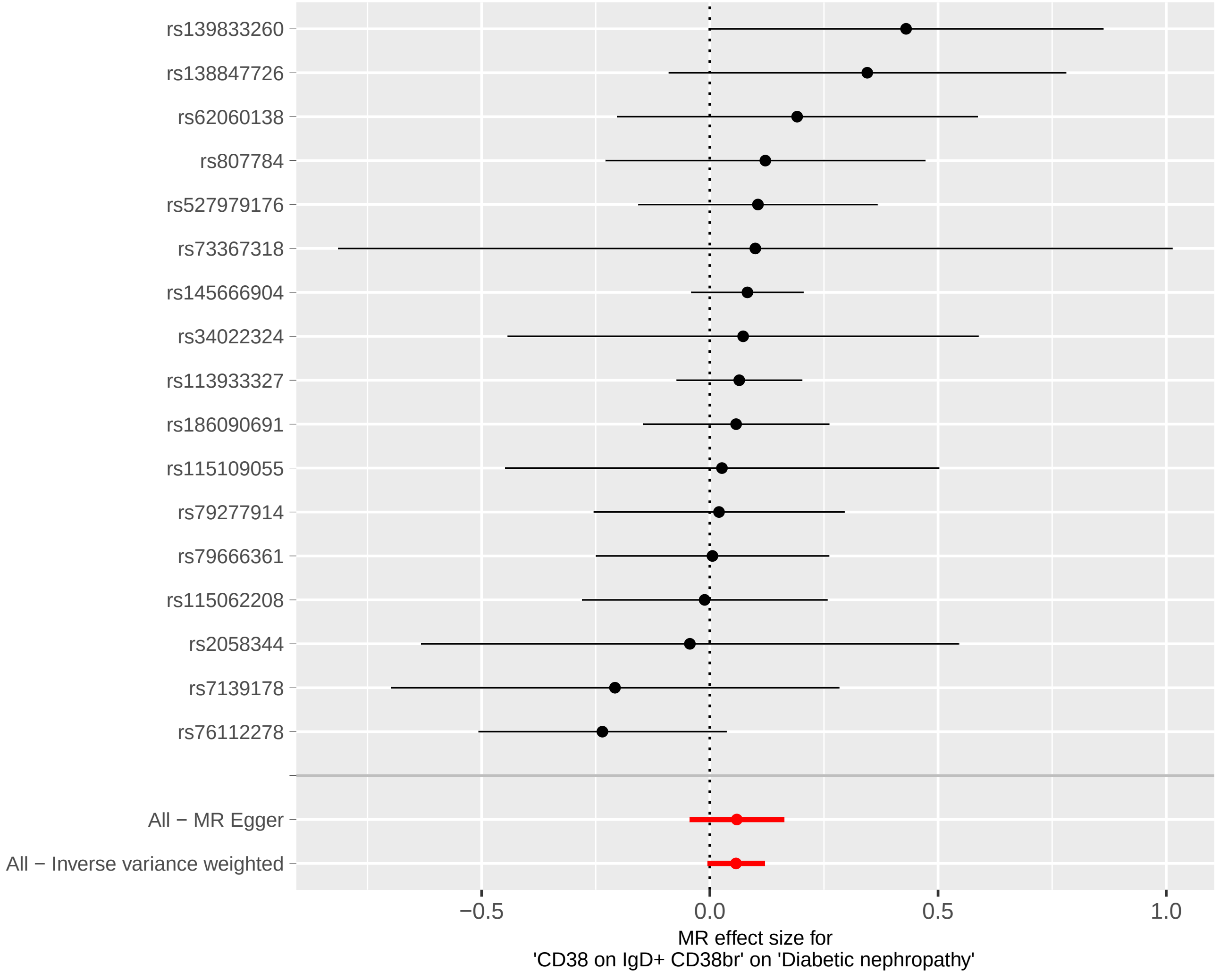

rs139833260
rs138847726
rs62060138
rs807784
rs527979176
rs73367318
rs145666904
rs34022324
rs113933327
rs186090691
rs115109055
rs79277914
rs79666361
rs115062208
rs2058344
rs7139178
rs76112278
All − MR Egger
All − Inverse variance weighted
−0.5
0.0
0.5
1.0
MR effect size for
'CD38 on IgD+ CD38br' on 'Diabetic nephropathy'

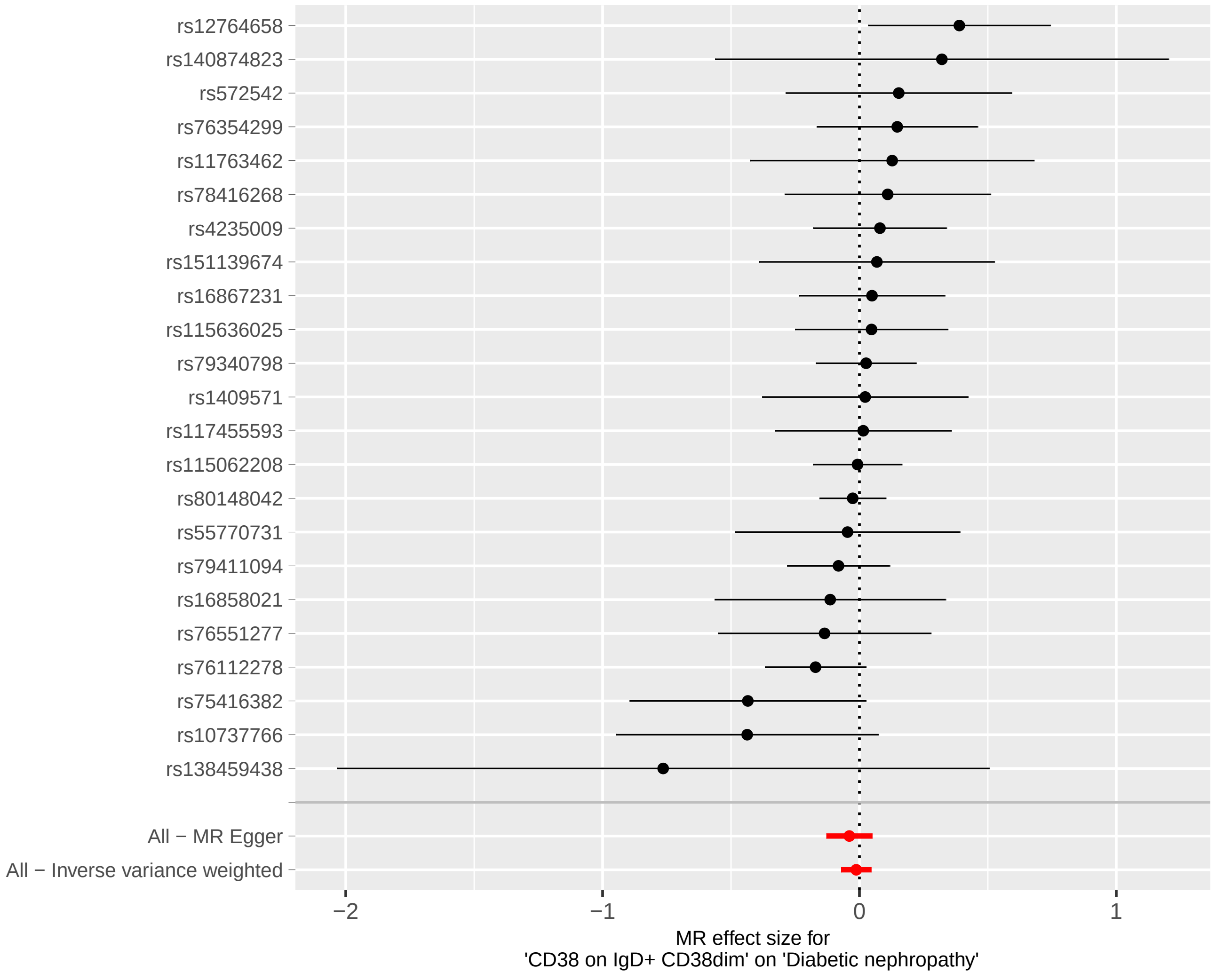
rs12764658
rs140874823
rs572542
rs76354299
rs11763462
rs78416268
rs4235009
rs151139674
rs16867231
rs115636025
rs79340798
rs1409571
rs117455593
rs115062208
rs80148042
rs55770731
rs79411094
rs16858021
rs76551277
rs76112278
rs75416382
rs10737766
rs138459438
All − MR Egger
All − Inverse variance weighted
−2
−1
0
1
MR effect size for
'CD38 on IgD+ CD38dim' on 'Diabetic nephropathy'

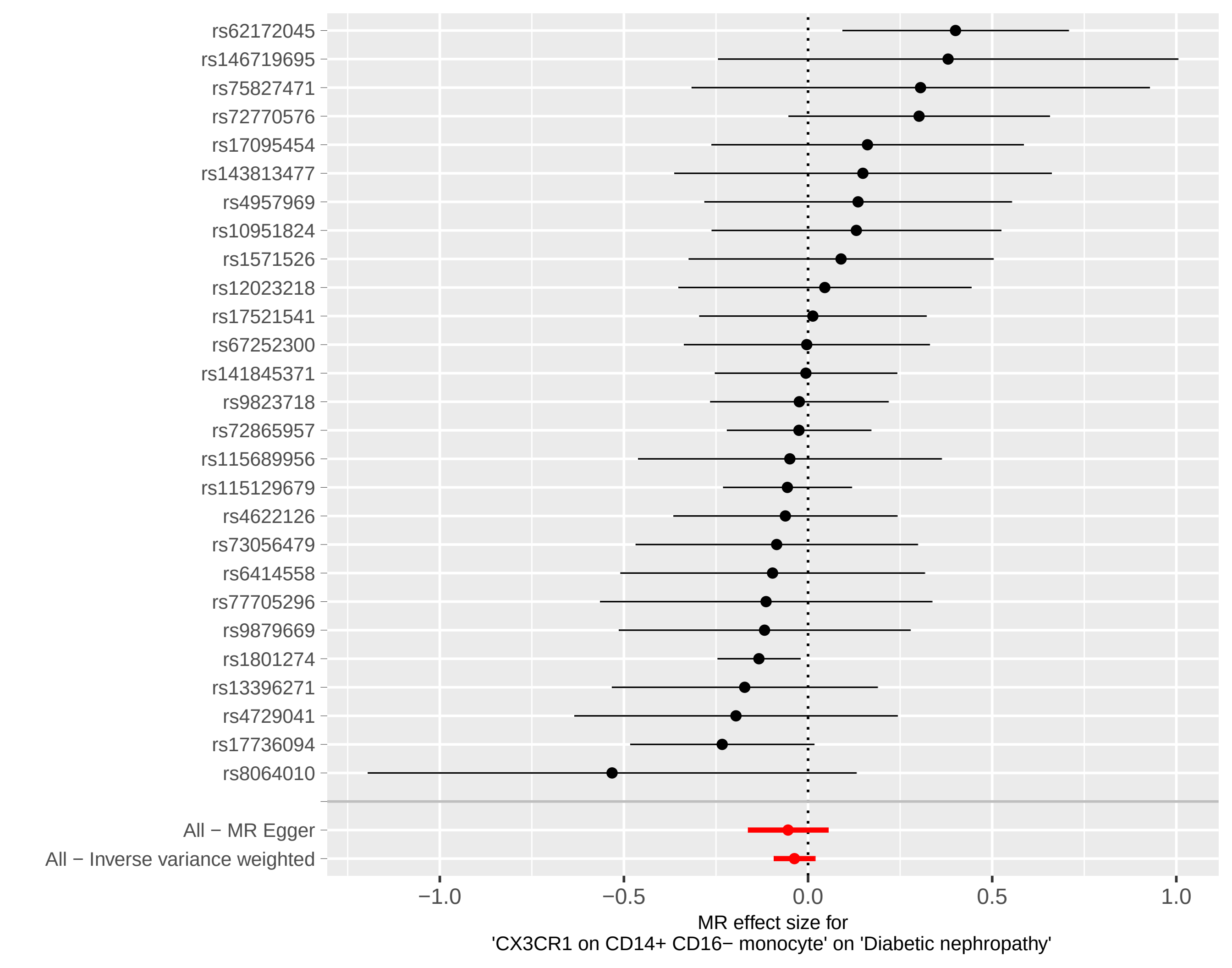
rs62172045
rs146719695
rs75827471
rs72770576
rs17095454
rs143813477
rs4957969
rs10951824
rs1571526
rs12023218
rs17521541
rs67252300
rs141845371
rs9823718
rs72865957
rs115689956
rs115129679
rs4622126
rs73056479
rs6414558
rs77705296
rs9879669
rs1801274
rs13396271
rs4729041
rs17736094
rs8064010
All − MR Egger
All − Inverse variance weighted
−1.0
−0.5
0.0
0.5
1.0
MR effect size for
'CX3CR1 on CD14+ CD16− monocyte' on 'Diabetic nephropathy'

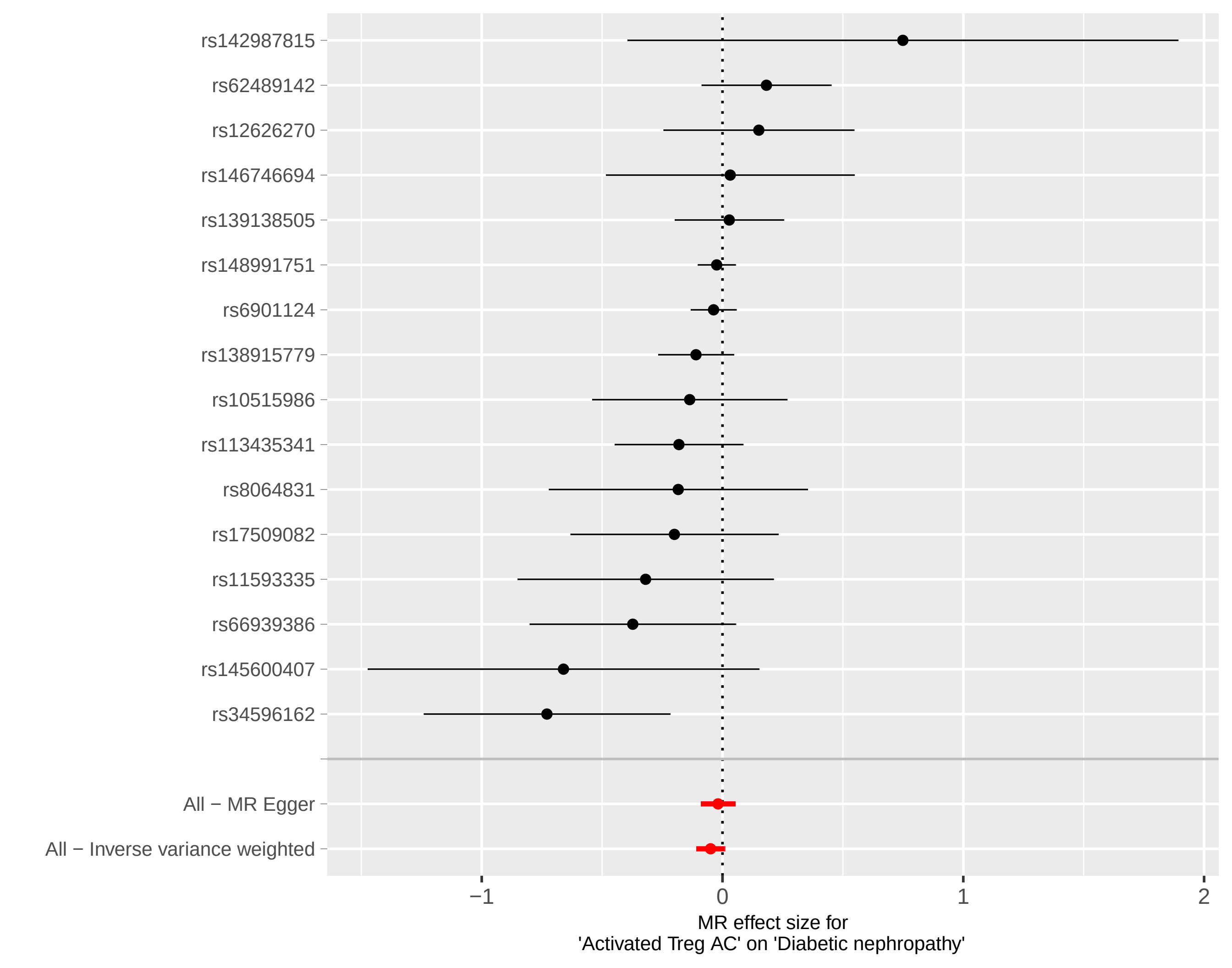
rs142987815
rs62489142
rs12626270
rs146746694
rs139138505
rs148991751
rs6901124
rs138915779
rs10515986
rs113435341
rs8064831
rs17509082
rs11593335
rs66939386
rs145600407
rs34596162
All − MR Egger
All − Inverse variance weighted
−1
0
1
2
MR effect size for
'Activated Treg AC' on 'Diabetic nephropathy'

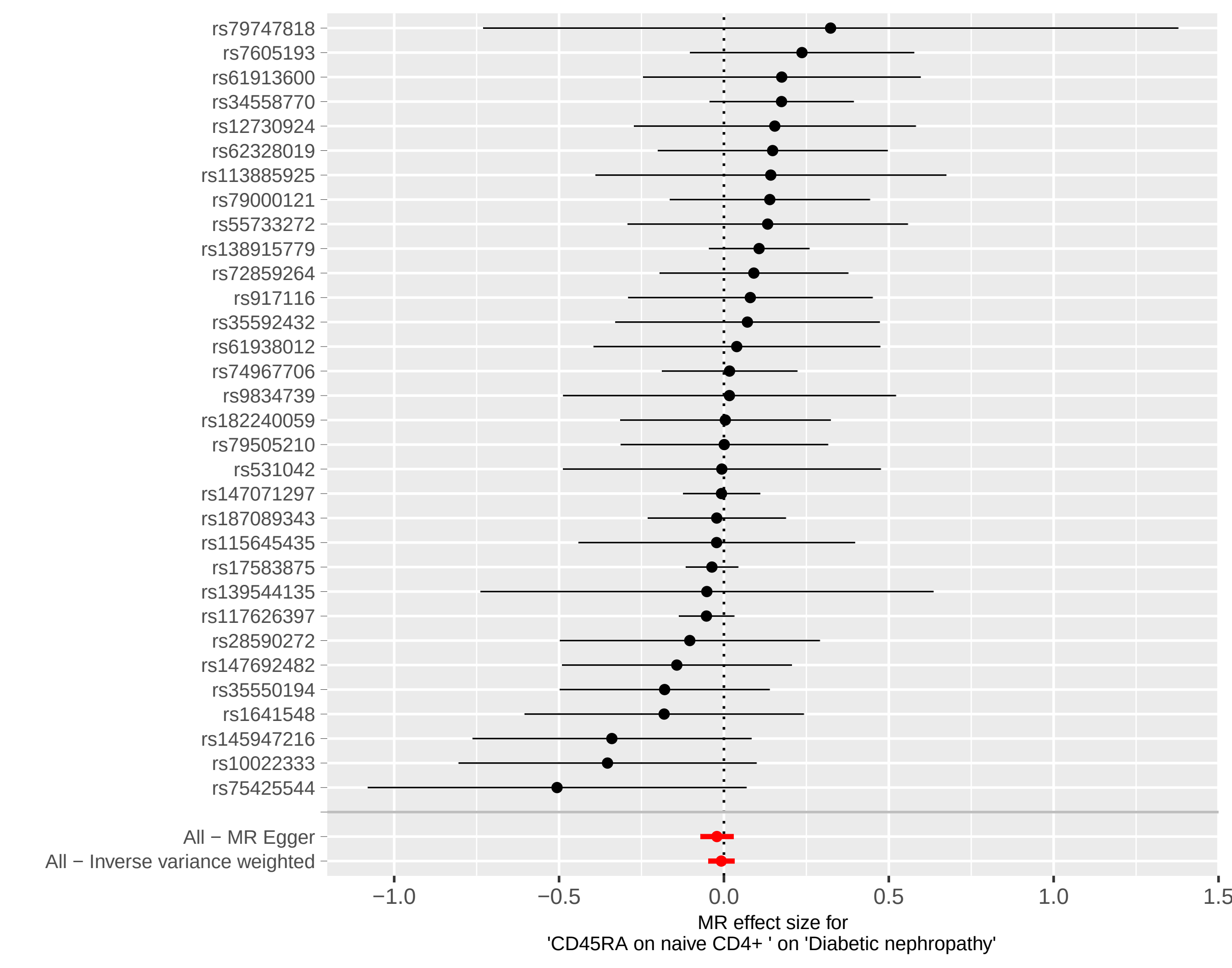
rs79747818
rs7605193
rs61913600
rs34558770
rs12730924
rs62328019
rs113885925
rs79000121
rs55733272
rs138915779
rs72859264
rs917116
rs35592432
rs61938012
rs74967706
rs9834739
rs182240059
rs79505210
rs531042
rs147071297
rs187089343
rs115645435
rs17583875
rs139544135
rs117626397
rs28590272
rs147692482
rs35550194
rs1641548
rs145947216
rs10022333
rs75425544
All − MR Egger
All − Inverse variance weighted
−1.0
−0.5
0.0
0.5
1.0
1.5
MR effect size for
'CD45RA on naive CD4+ ' on 'Diabetic nephropathy'

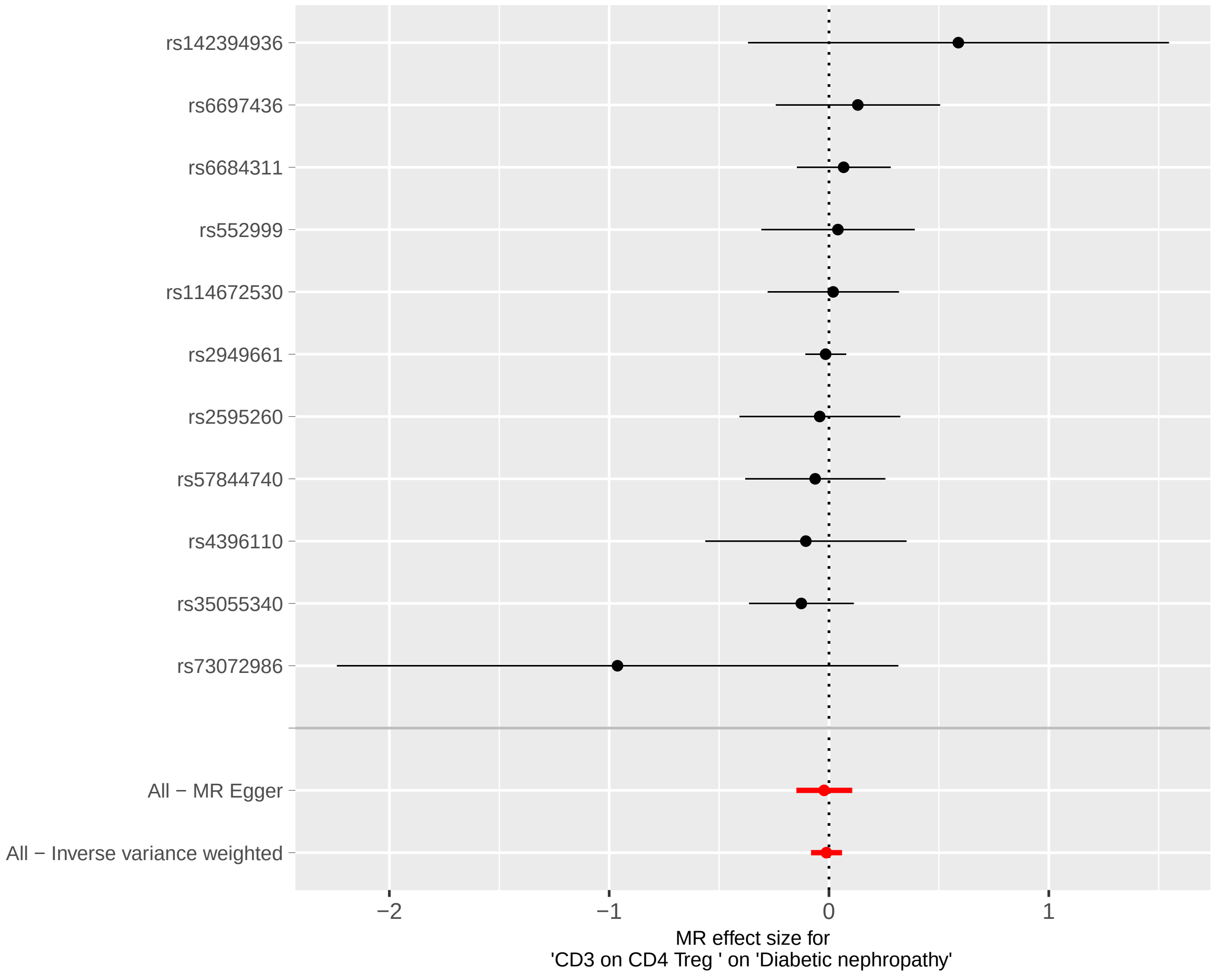
rs142394936
rs6697436
rs6684311
rs552999
rs114672530
rs2949661
rs2595260
rs57844740
rs4396110
rs35055340
rs73072986
All − MR Egger
All − Inverse variance weighted
−2
−1
0
1
MR effect size for
'CD3 on CD4 Treg ' on 'Diabetic nephropathy'

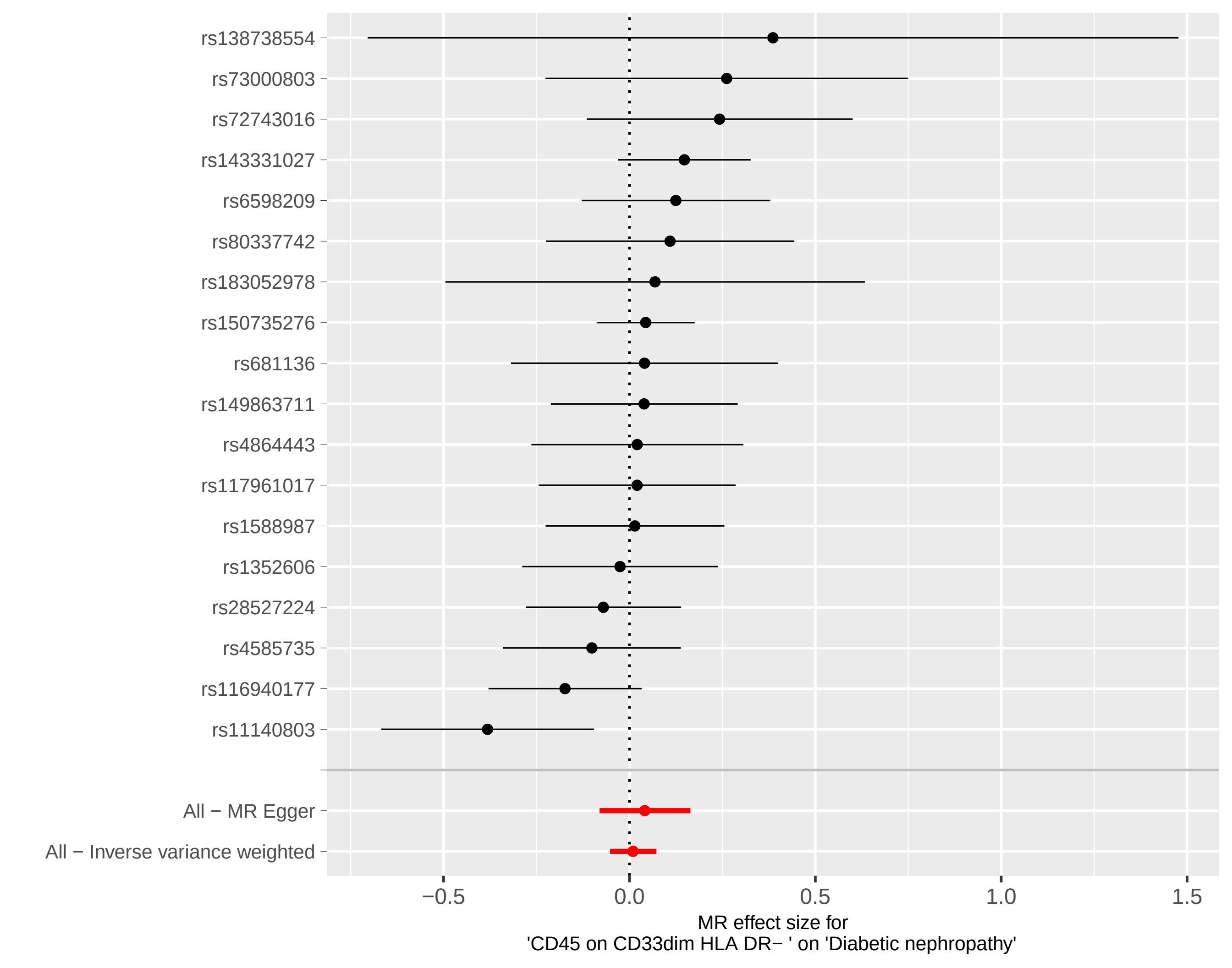
rs138738554
rs73000803
rs72743016
rs143331027
rs6598209
rs80337742
rs183052978
rs150735276
rs681136
rs149863711
rs4864443
rs117961017
rs1588987
rs1352606
rs28527224
rs4585735
rs116940177
rs11140803
All − MR Egger
All − Inverse variance weighted
−0.5
0.0
0.5
1.0
1.5
MR effect size for
'CD45 on CD33dim HLA DR− ' on 'Diabetic nephropathy'

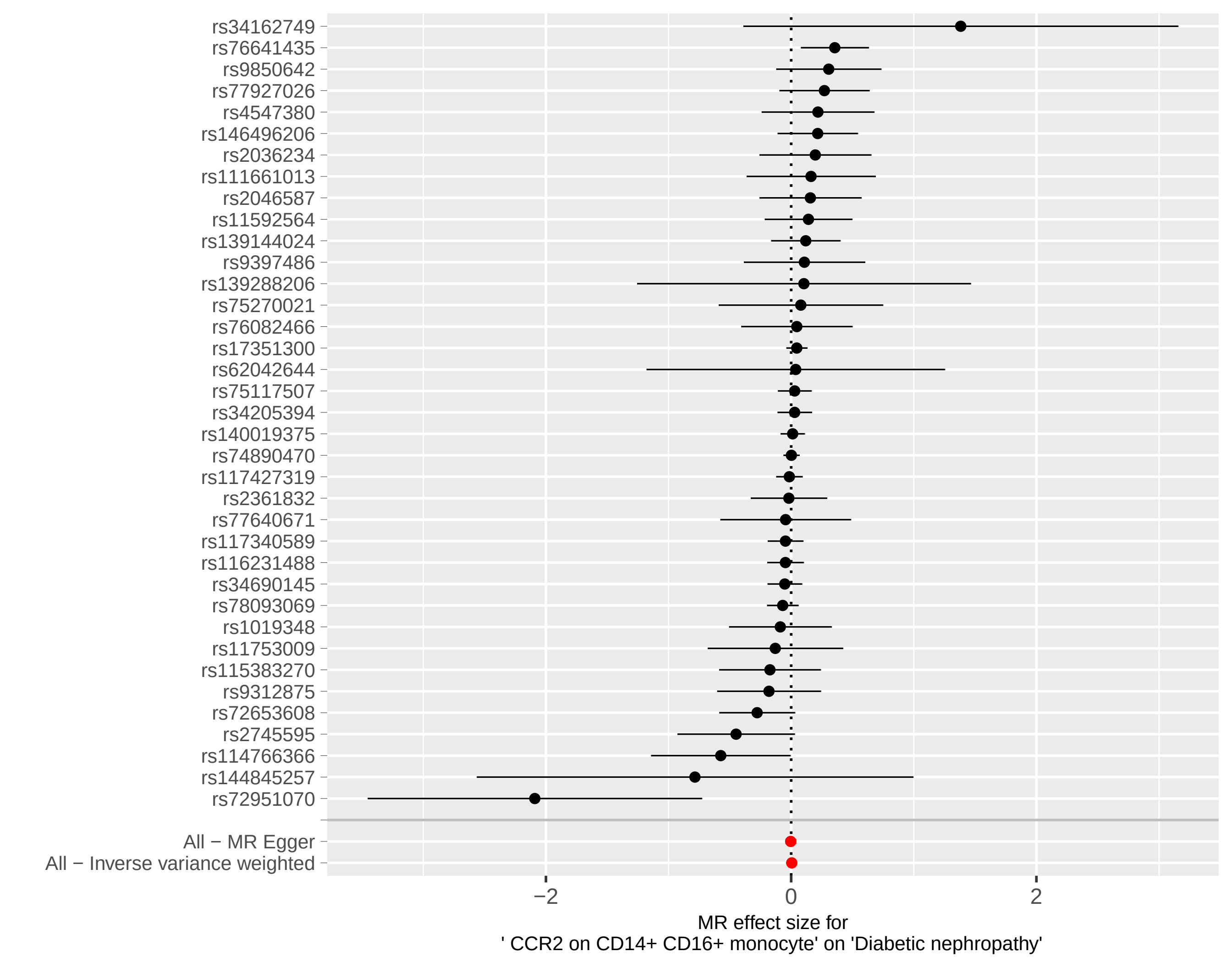
rs34162749
rs76641435
rs9850642
rs77927026
rs4547380
rs146496206
rs2036234
rs111661013
rs2046587
rs11592564
rs139144024
rs9397486
rs139288206
rs75270021
rs76082466
rs17351300
rs62042644
rs75117507
rs34205394
rs140019375
rs74890470
rs117427319
rs2361832
rs77640671
rs117340589
rs116231488
rs34690145
rs78093069
rs1019348
rs11753009
rs115383270
rs9312875
rs72653608
rs2745595
rs114766366
rs144845257
rs72951070
All − MR Egger
All − Inverse variance weighted
−2
0
2
MR effect size for
' CCR2 on CD14+ CD16+ monocyte' on 'Diabetic nephropathy'

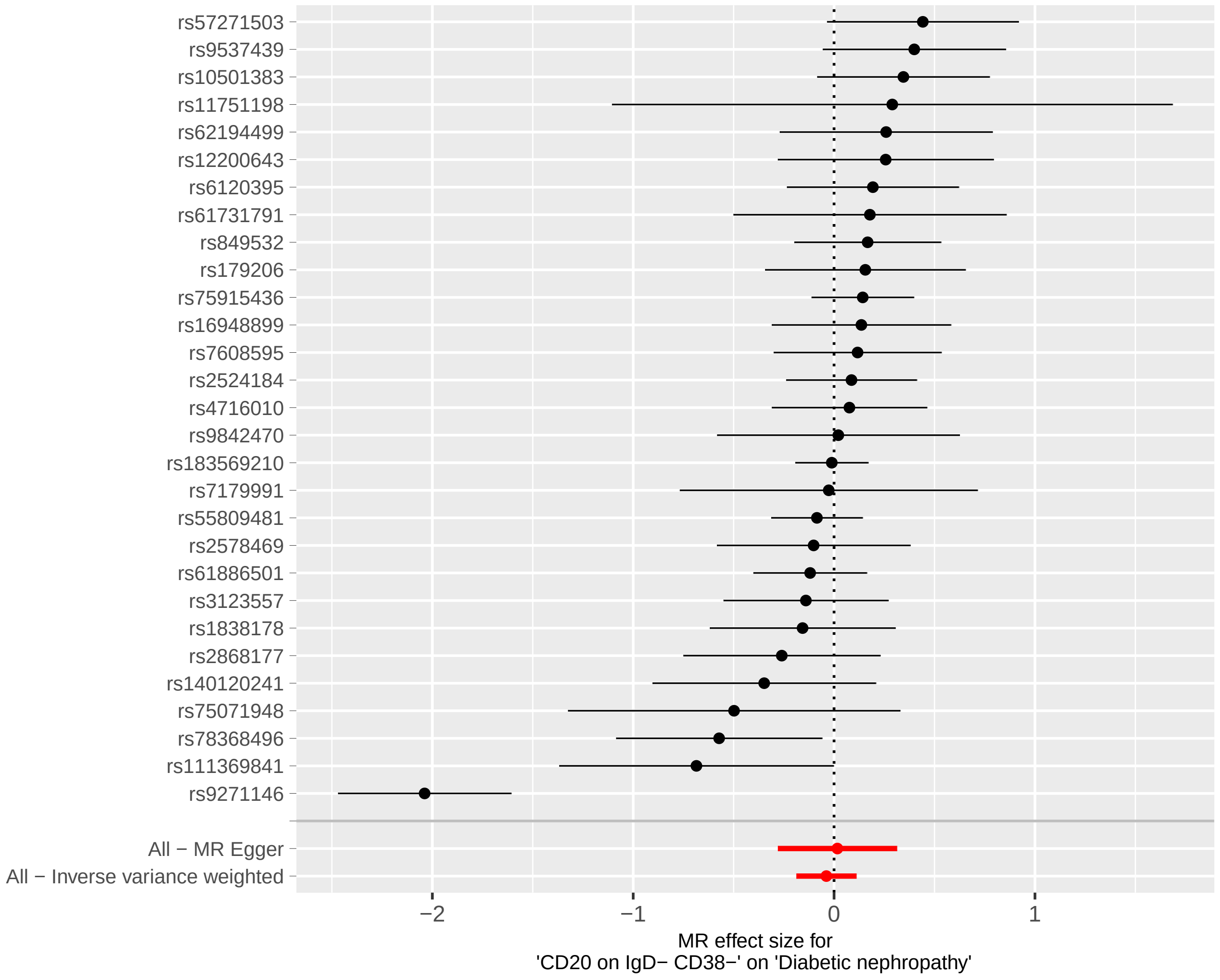
rs57271503
rs9537439
rs10501383
rs11751198
rs62194499
rs12200643
rs6120395
rs61731791
rs849532
rs179206
rs75915436
rs16948899
rs7608595
rs2524184
rs4716010
rs9842470
rs183569210
rs7179991
rs55809481
rs2578469
rs61886501
rs3123557
rs1838178
rs2868177
rs140120241
rs75071948
rs78368496
rs111369841
rs9271146
All − MR Egger
All − Inverse variance weighted
−2
−1
0
1
MR effect size for
'CD20 on IgD− CD38−' on 'Diabetic nephropathy'

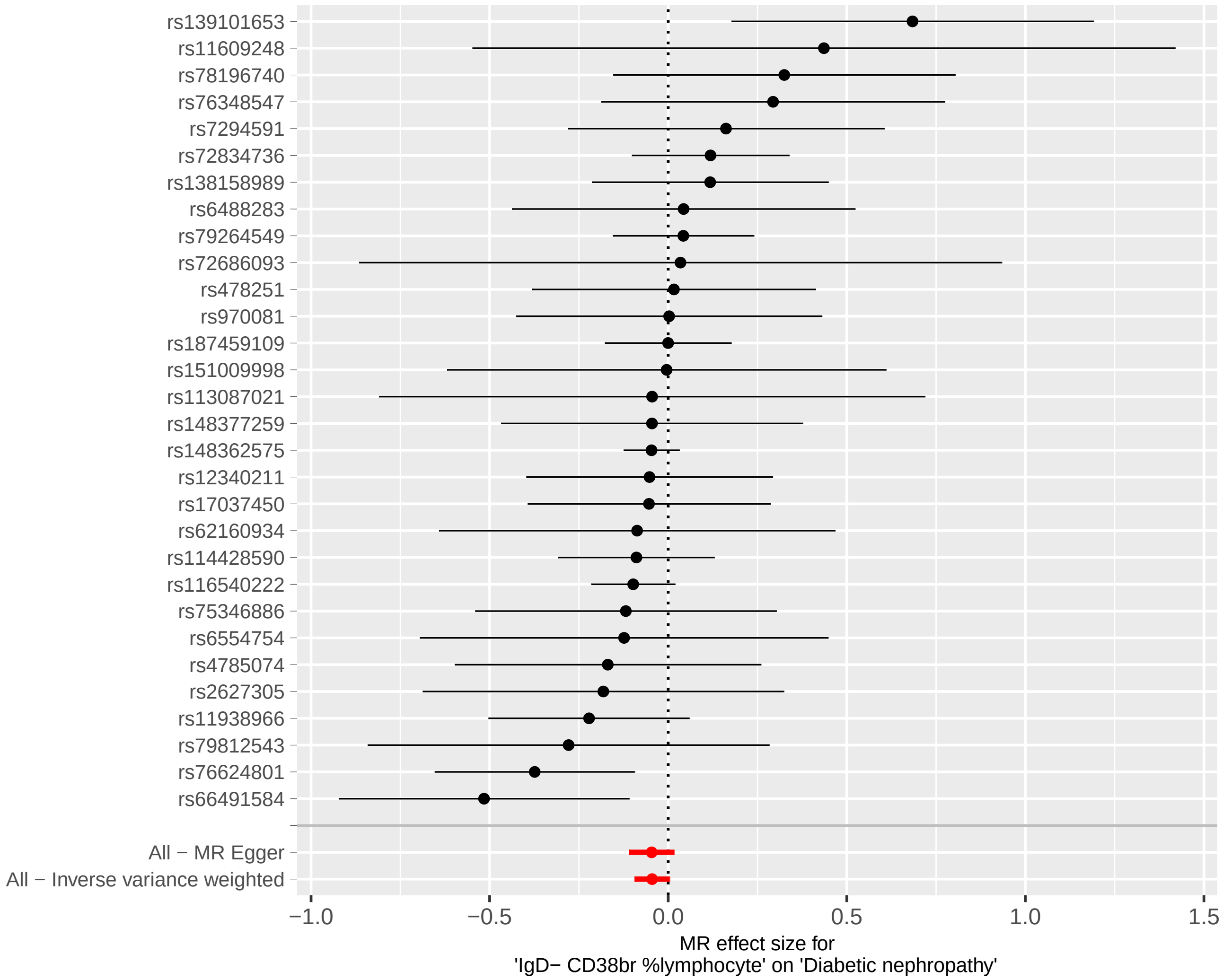

rs139101653
rs11609248
rs78196740
rs76348547
rs7294591
rs72834736
rs138158989
rs6488283
rs79264549
rs72686093
rs478251
rs970081
rs187459109
rs151009998
rs113087021
rs148377259
rs148362575
rs12340211
rs17037450
rs62160934
rs114428590
rs116540222
rs75346886
rs6554754
rs4785074
rs2627305
rs11938966
rs79812543
rs76624801
rs66491584
All − MR Egger
All − Inverse variance weighted
−1.0
−0.5
0.0
0.5
1.0
1.5
MR effect size for
'IgD− CD38br %lymphocyte' on 'Diabetic nephropathy'

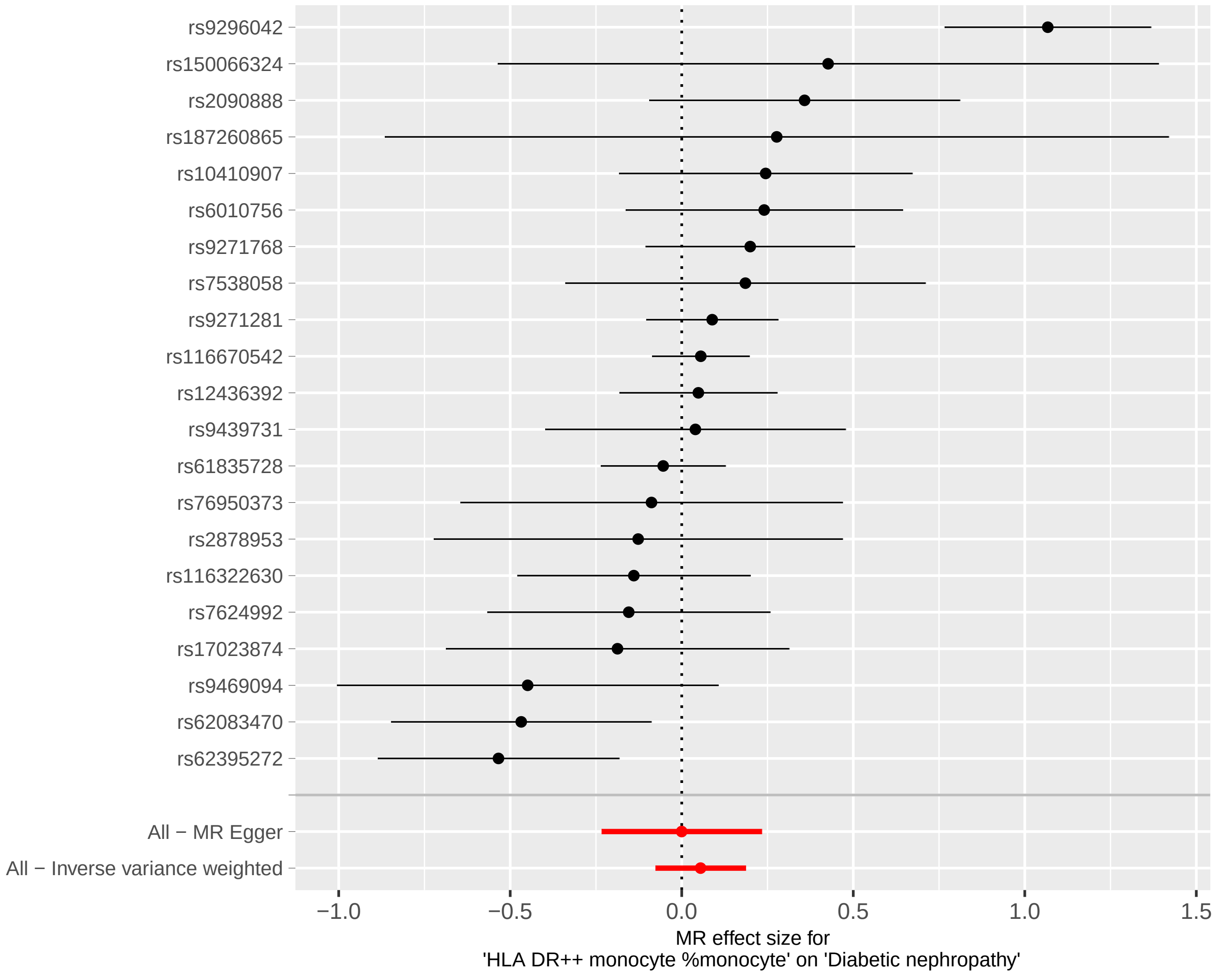
rs9296042
rs150066324
rs2090888
rs187260865
rs10410907
rs6010756
rs9271768
rs7538058
rs9271281
rs116670542
rs12436392
rs9439731
rs61835728
rs76950373
rs2878953
rs116322630
rs7624992
rs17023874
rs9469094
rs62083470
rs62395272
All − MR Egger
All − Inverse variance weighted
−1.0
−0.5
0.0
0.5
1.0
1.5
MR effect size for
'HLA DR++ monocyte %monocyte' on 'Diabetic nephropathy'

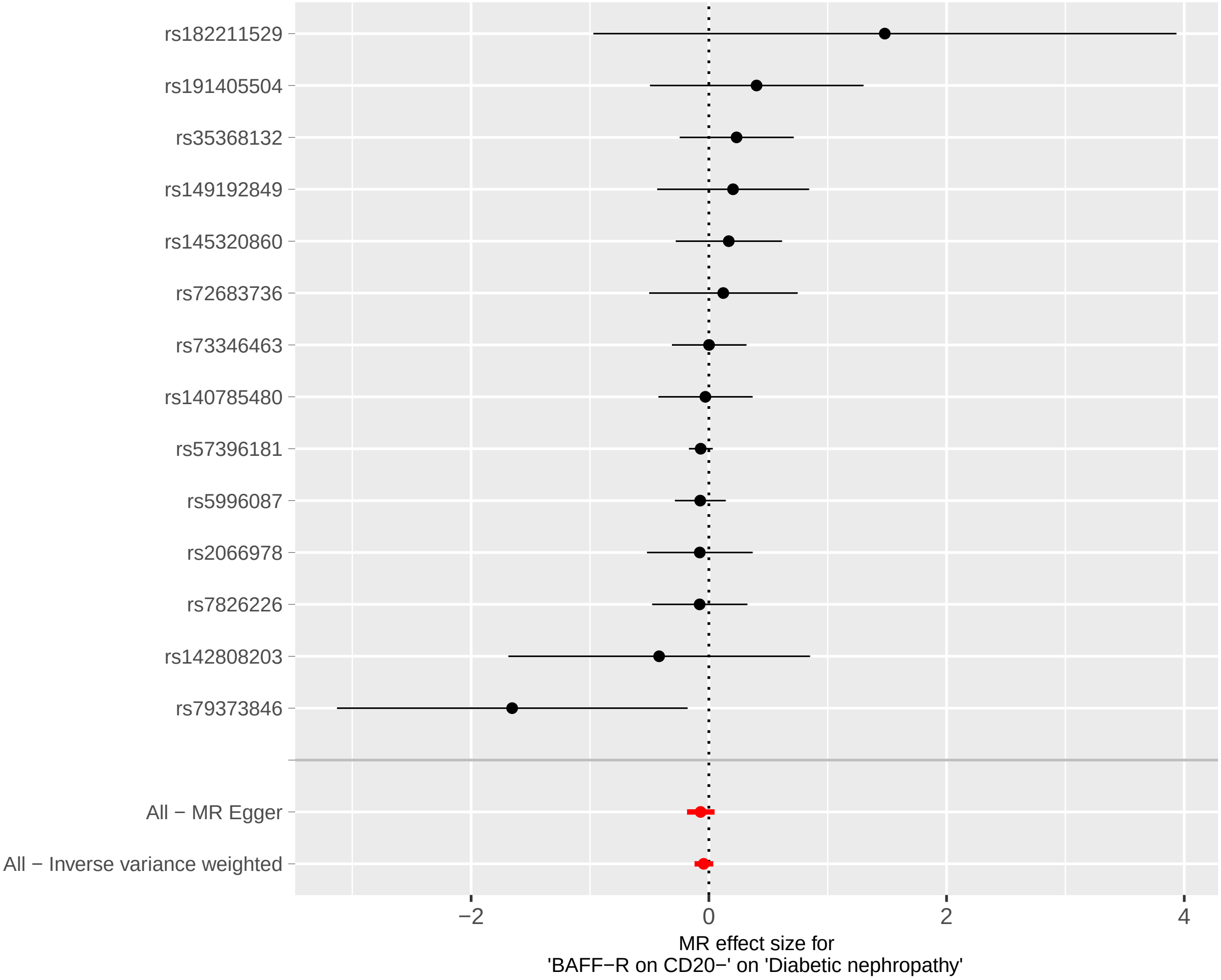

rs182211529
rs191405504
rs35368132
rs149192849
rs145320860
rs72683736
rs73346463
rs140785480
rs57396181
rs5996087
rs2066978
rs7826226
rs142808203
rs79373846
All − MR Egger
All − Inverse variance weighted
−2
0
2
4
MR effect size for
'BAFF−R on CD20−' on 'Diabetic nephropathy'

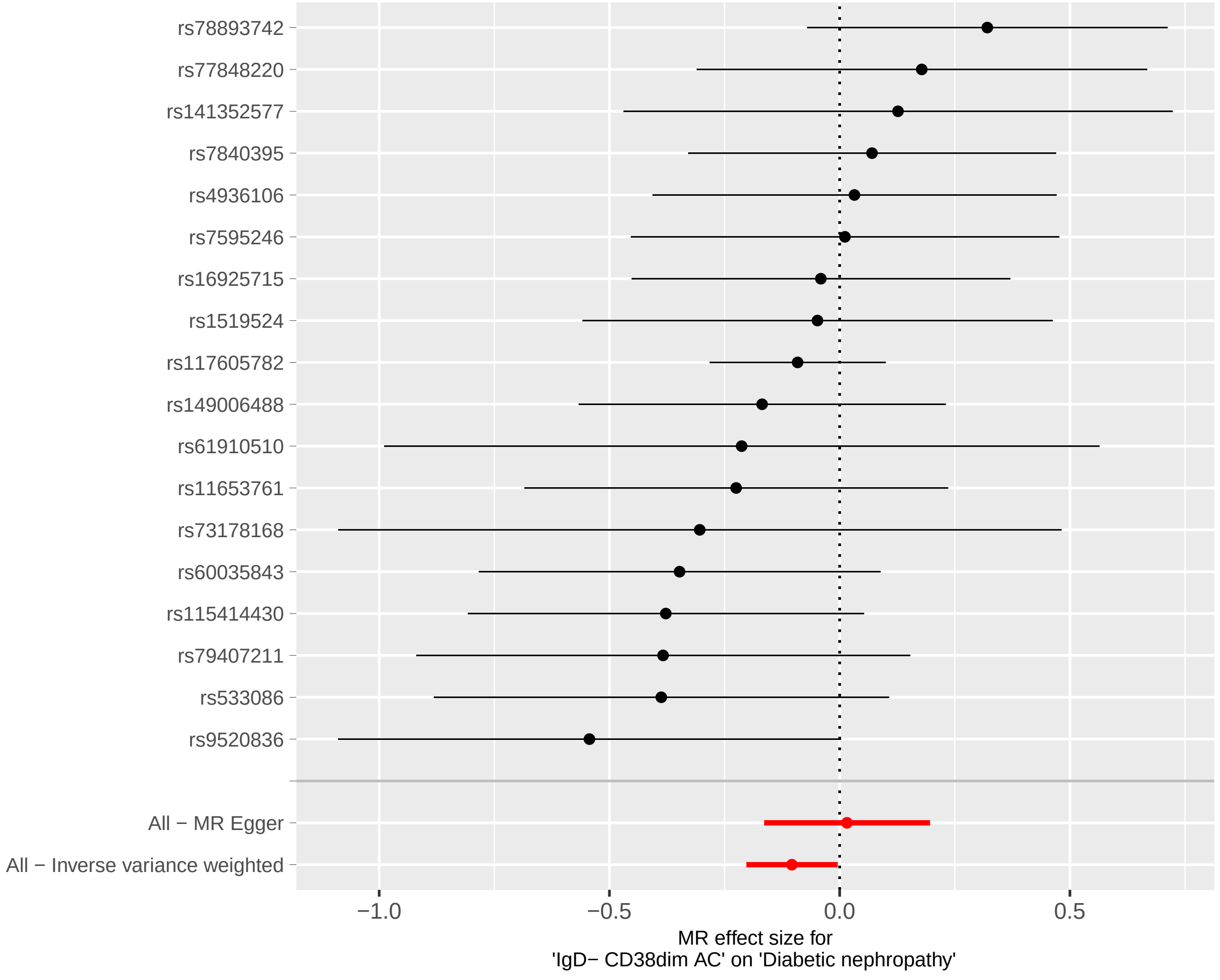

rs78893742
rs77848220
rs141352577
rs7840395
rs4936106
rs7595246
rs16925715
rs1519524
rs117605782
rs149006488
rs61910510
rs11653761
rs73178168
rs60035843
rs115414430
rs79407211
rs533086
rs9520836
All − MR Egger
All − Inverse variance weighted
−1.0
−0.5
0.0
0.5
MR effect size for
'IgD− CD38dim AC' on 'Diabetic nephropathy'

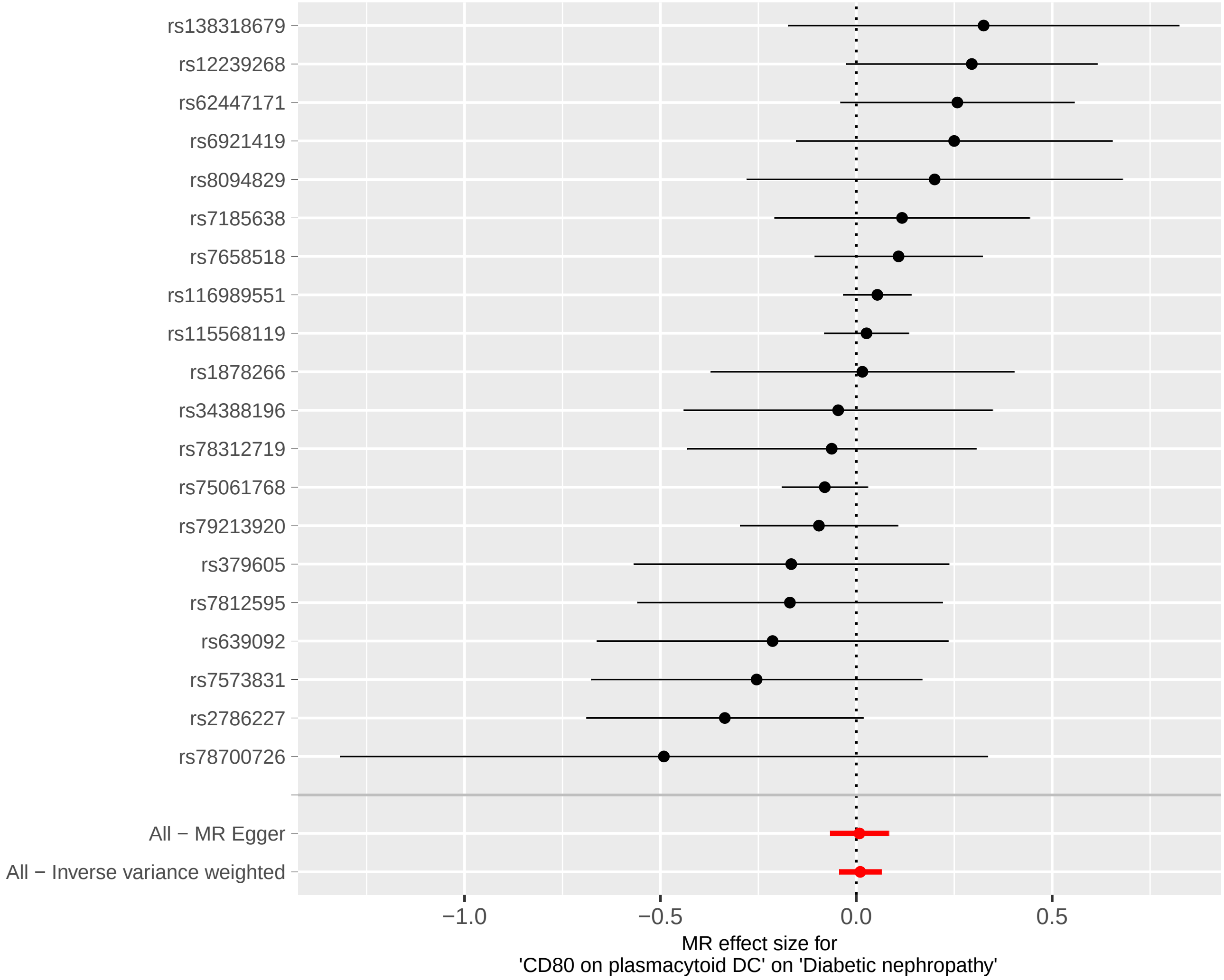
rs138318679
rs12239268
rs62447171
rs6921419
rs8094829
rs7185638
rs7658518
rs116989551
rs115568119
rs1878266
rs34388196
rs78312719
rs75061768
rs79213920
rs379605
rs7812595
rs639092
rs7573831
rs2786227
rs78700726
All − MR Egger
All − Inverse variance weighted
−1.0
−0.5
0.0
0.5
MR effect size for
'CD80 on plasmacytoid DC' on 'Diabetic nephropathy'

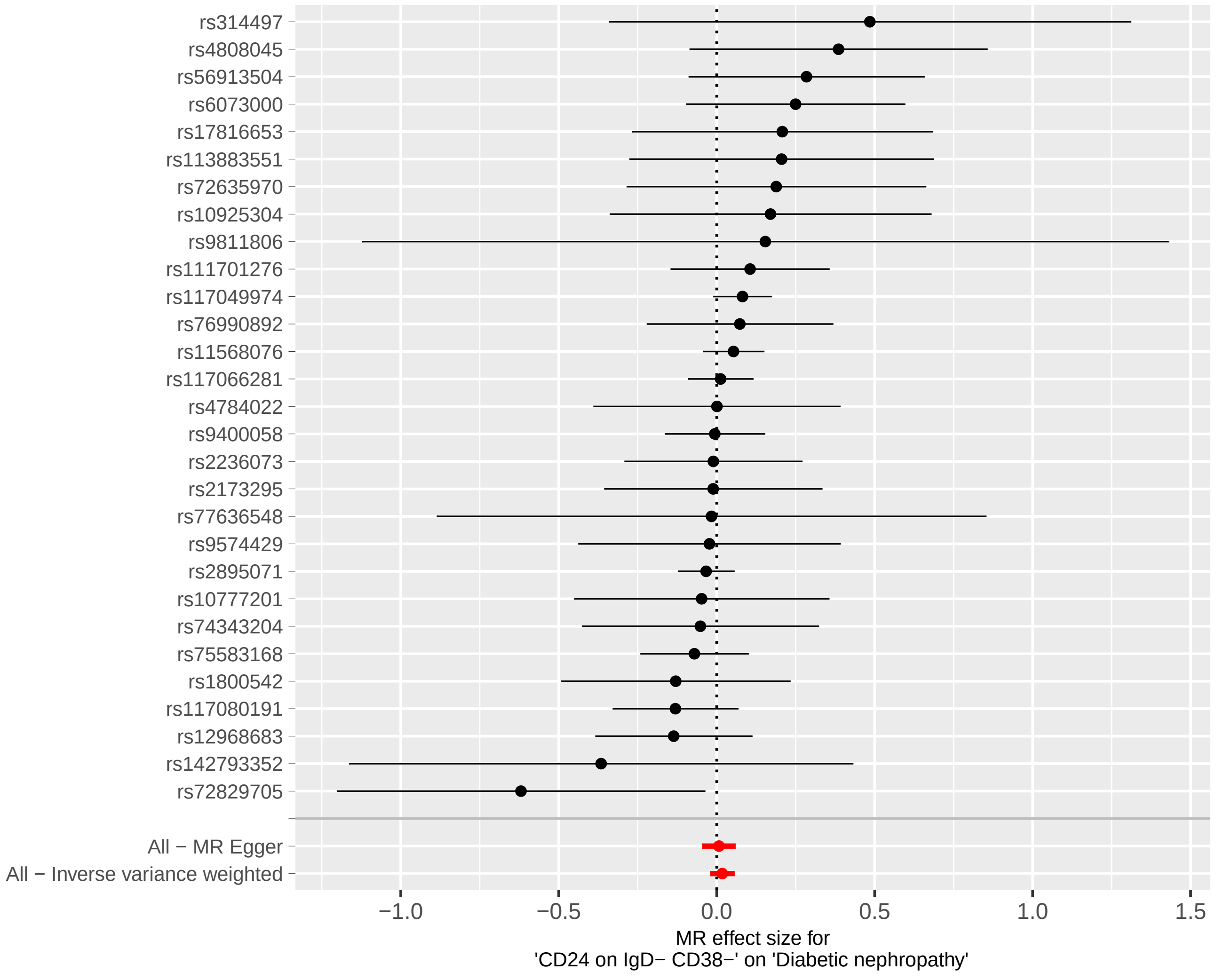
rs314497
rs4808045
rs56913504
rs6073000
rs17816653
rs113883551
rs72635970
rs10925304
rs9811806
rs111701276
rs117049974
rs76990892
rs11568076
rs117066281
rs4784022
rs9400058
rs2236073
rs2173295
rs77636548
rs9574429
rs2895071
rs10777201
rs74343204
rs75583168
rs1800542
rs117080191
rs12968683
rs142793352
rs72829705
All − MR Egger
All − Inverse variance weighted
−1.0
−0.5
0.0
0.5
1.0
1.5
MR effect size for
'CD24 on IgD− CD38−' on 'Diabetic nephropathy'

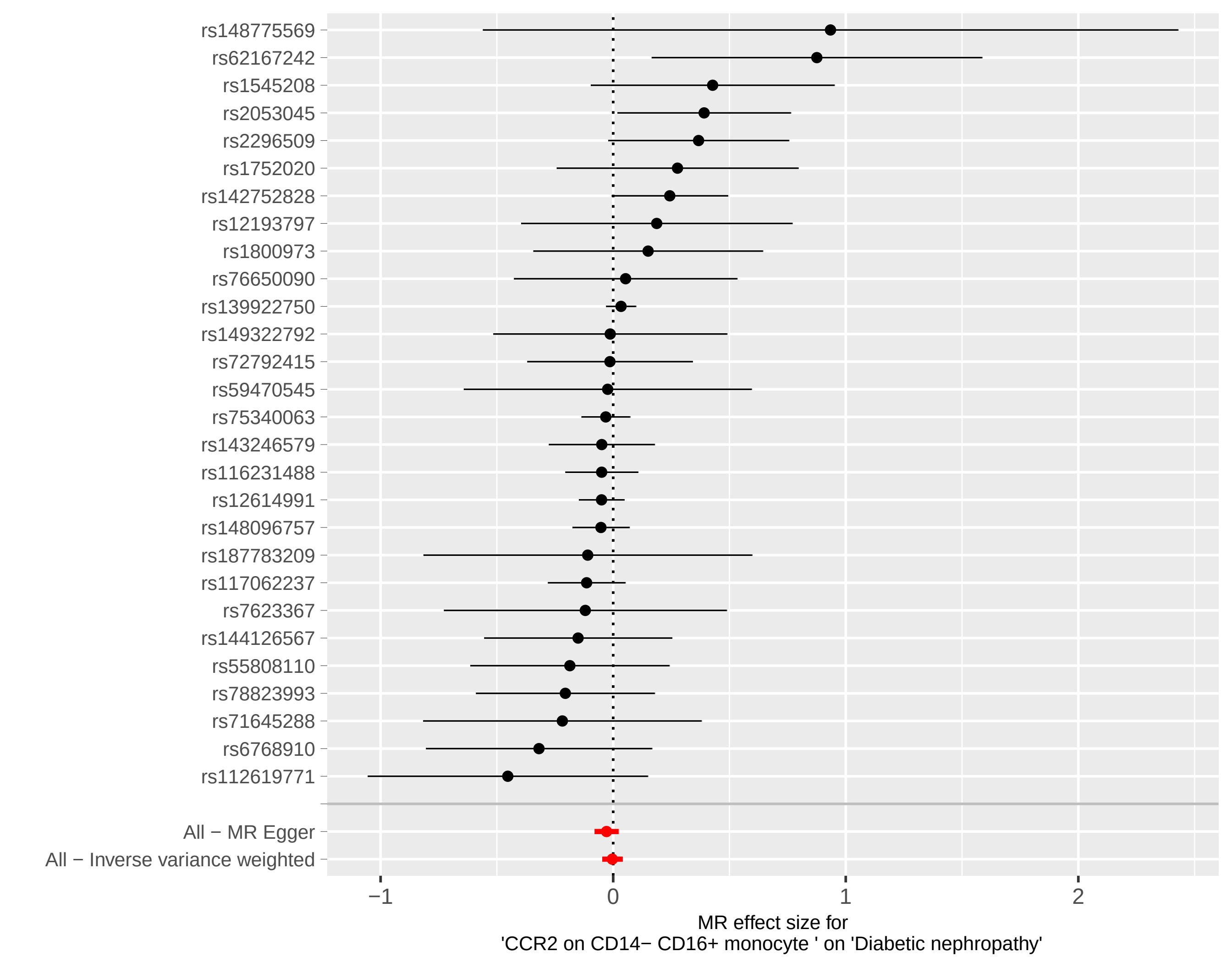
rs148775569
rs62167242
rs1545208
rs2053045
rs2296509
rs1752020
rs142752828
rs12193797
rs1800973
rs76650090
rs139922750
rs149322792
rs72792415
rs59470545
rs75340063
rs143246579
rs116231488
rs12614991
rs148096757
rs187783209
rs117062237
rs7623367
rs144126567
rs55808110
rs78823993
rs71645288
rs6768910
rs112619771
All − MR Egger
All − Inverse variance weighted
−1
0
1
2
MR effect size for
'CCR2 on CD14− CD16+ monocyte ' on 'Diabetic nephropathy'

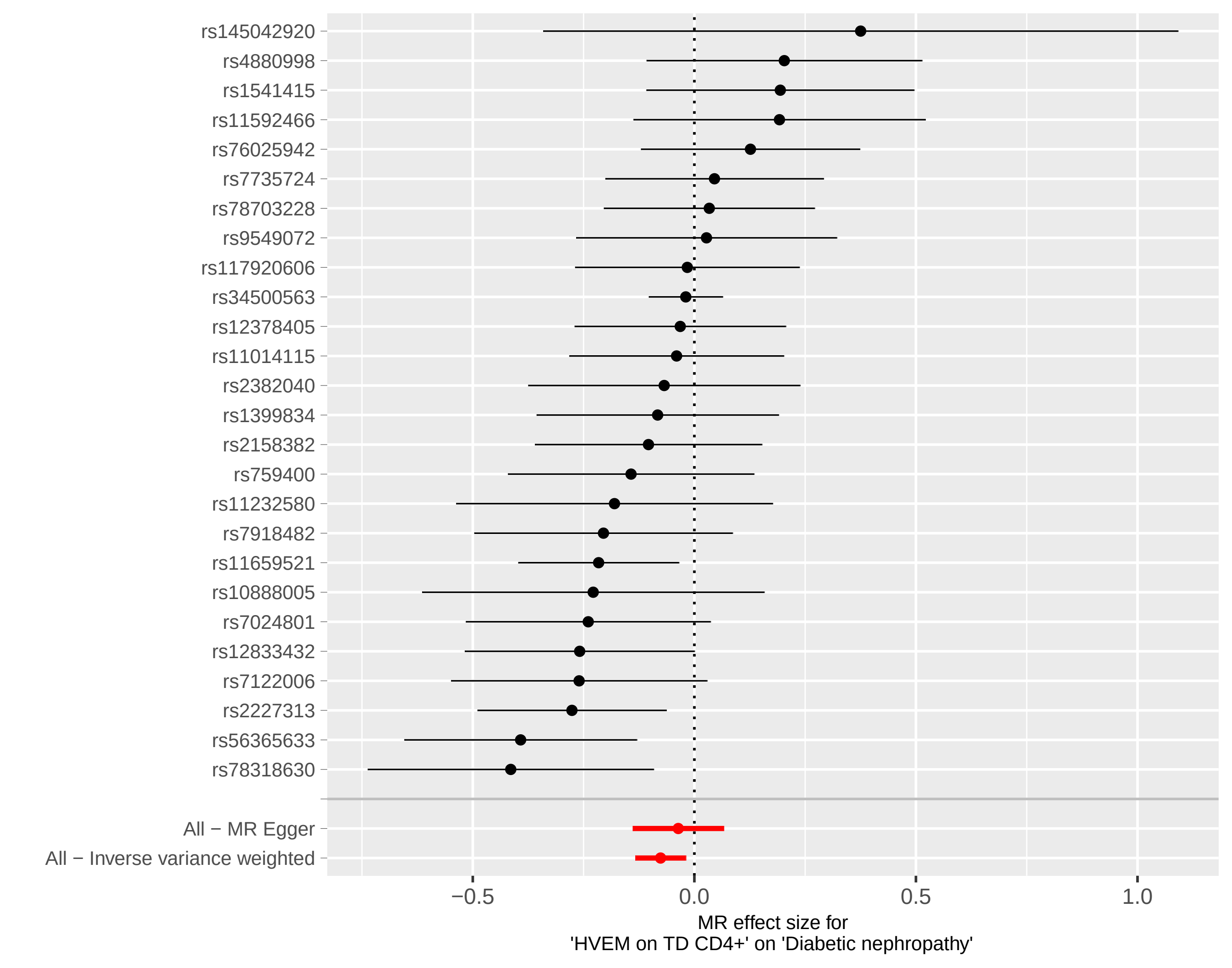
rs145042920
rs4880998
rs1541415
rs11592466
rs76025942
rs7735724
rs78703228
rs9549072
rs117920606
rs34500563
rs12378405
rs11014115
rs2382040
rs1399834
rs2158382
rs759400
rs11232580
rs7918482
rs11659521
rs10888005
rs7024801
rs12833432
rs7122006
rs2227313
rs56365633
rs78318630
All − MR Egger
All − Inverse variance weighted
−0.5
0.0
0.5
1.0
MR effect size for
'HVEM on TD CD4+' on 'Diabetic nephropathy'

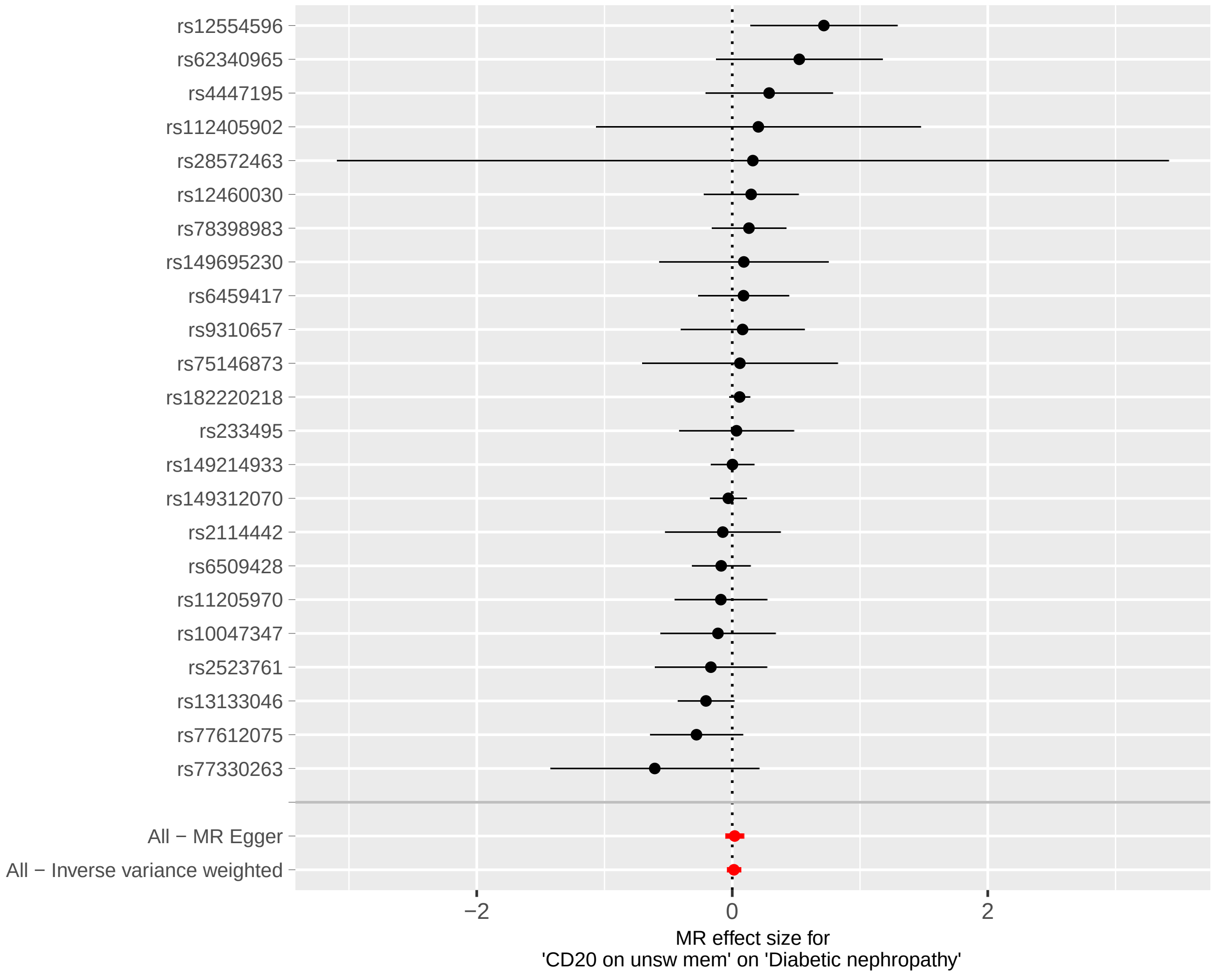

rs12554596
rs62340965
rs4447195
rs112405902
rs28572463
rs12460030
rs78398983
rs149695230
rs6459417
rs9310657
rs75146873
rs182220218
rs233495
rs149214933
rs149312070
rs2114442
rs6509428
rs11205970
rs10047347
rs2523761
rs13133046
rs77612075
rs77330263
All − MR Egger
All − Inverse variance weighted
−2
0
2
MR effect size for
'CD20 on unsw mem' on 'Diabetic nephropathy'

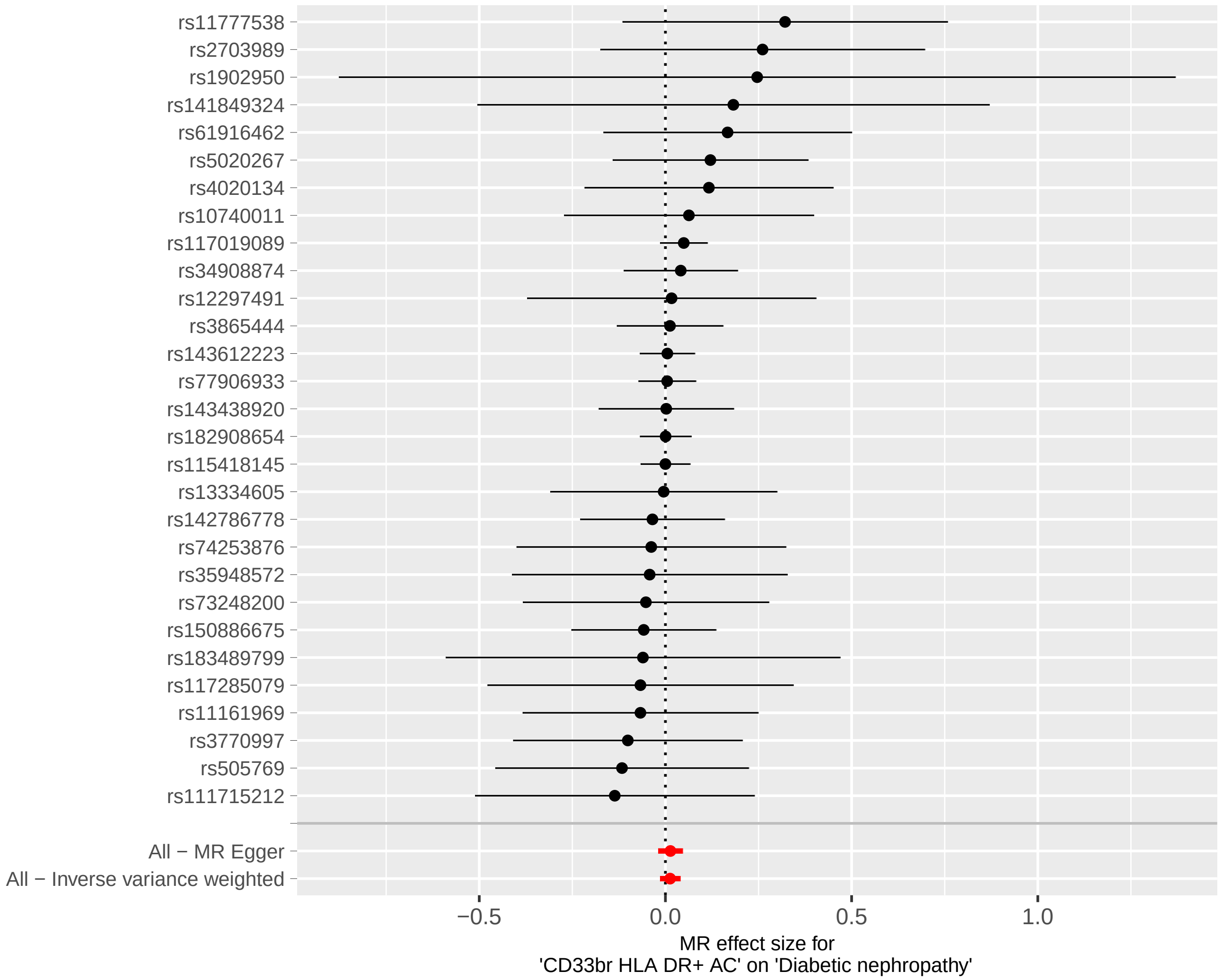

rs11777538
rs2703989
rs1902950
rs141849324
rs61916462
rs5020267
rs4020134
rs10740011
rs117019089
rs34908874
rs12297491
rs3865444
rs143612223
rs77906933
rs143438920
rs182908654
rs115418145
rs13334605
rs142786778
rs74253876
rs35948572
rs73248200
rs150886675
rs183489799
rs117285079
rs11161969
rs3770997
rs505769
rs111715212
All − MR Egger
All − Inverse variance weighted
−0.5
0.0
0.5
1.0
MR effect size for
'CD33br HLA DR+ AC' on 'Diabetic nephropathy'

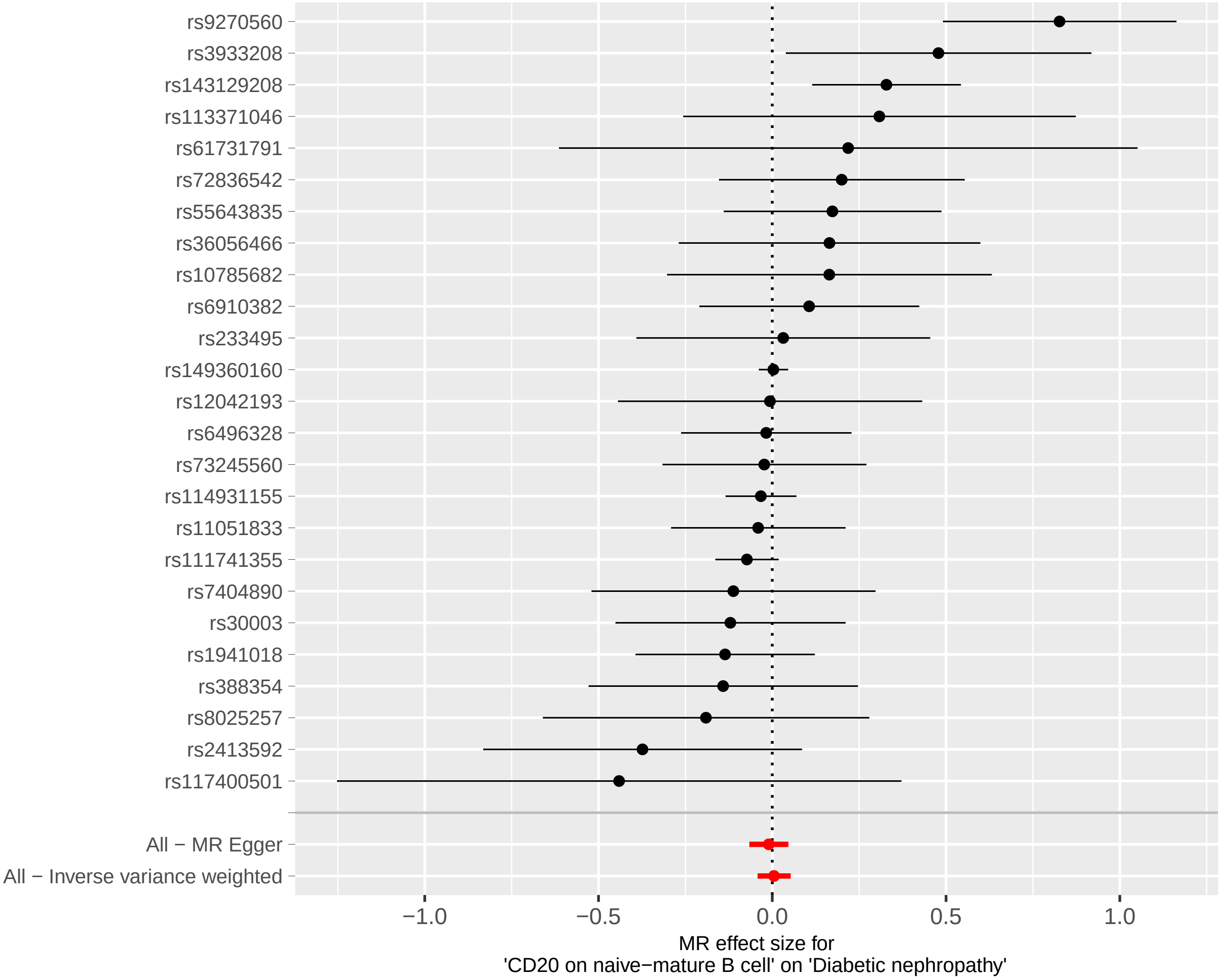
rs9270560
rs3933208
rs143129208
rs113371046
rs61731791
rs72836542
rs55643835
rs36056466
rs10785682
rs6910382
rs233495
rs149360160
rs12042193
rs6496328
rs73245560
rs114931155
rs11051833
rs111741355
rs7404890
rs30003
rs1941018
rs388354
rs8025257
rs2413592
rs117400501
All − MR Egger
All − Inverse variance weighted
−1.0
−0.5
0.0
0.5
1.0
MR effect size for
'CD20 on naive−mature B cell' on 'Diabetic nephropathy'

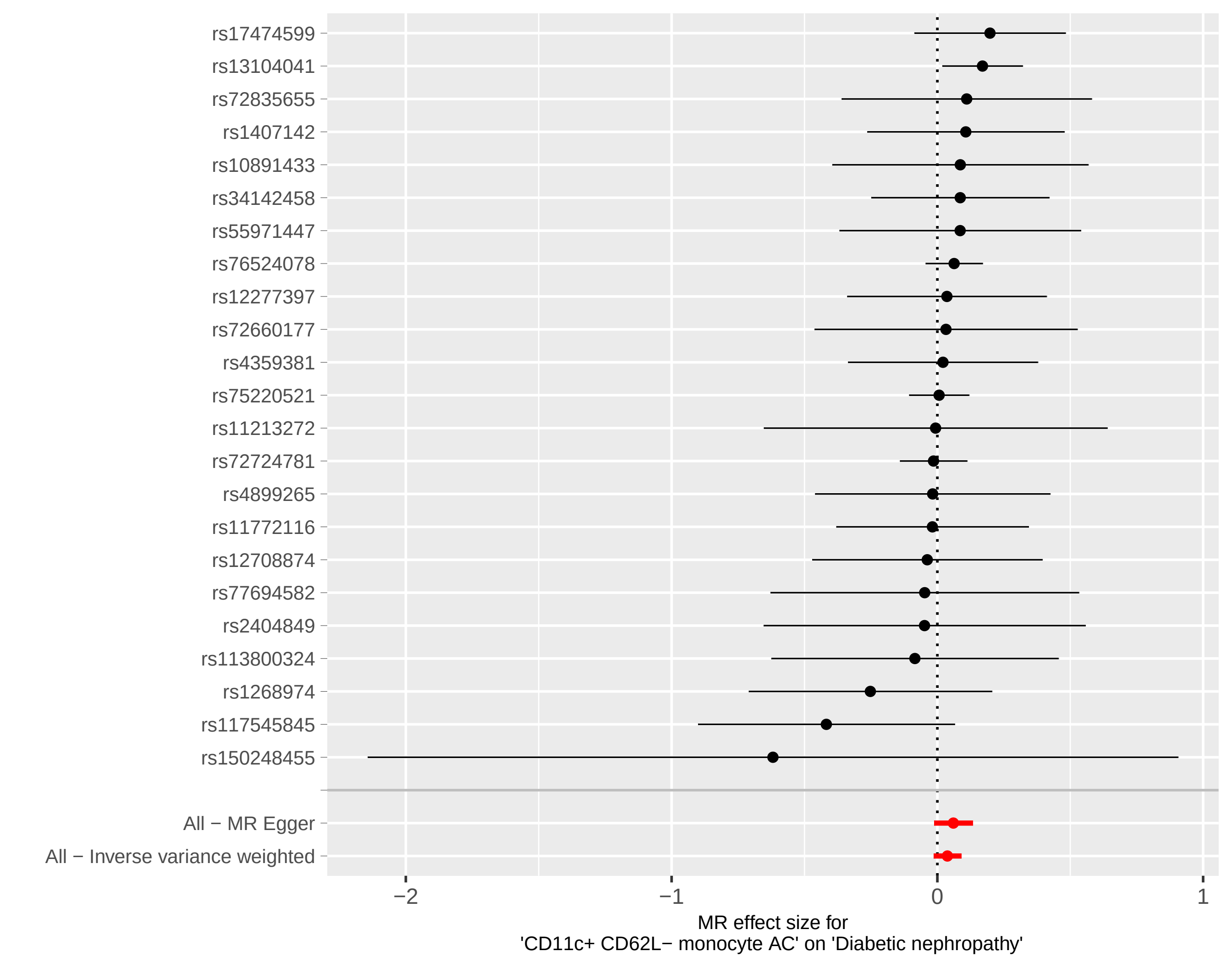
rs17474599
rs13104041
rs72835655
rs1407142
rs10891433
rs34142458
rs55971447
rs76524078
rs12277397
rs72660177
rs4359381
rs75220521
rs11213272
rs72724781
rs4899265
rs11772116
rs12708874
rs77694582
rs2404849
rs113800324
rs1268974
rs117545845
rs150248455
All − MR Egger
All − Inverse variance weighted
−2
−1
0
1
MR effect size for
'CD11c+ CD62L− monocyte AC' on 'Diabetic nephropathy'

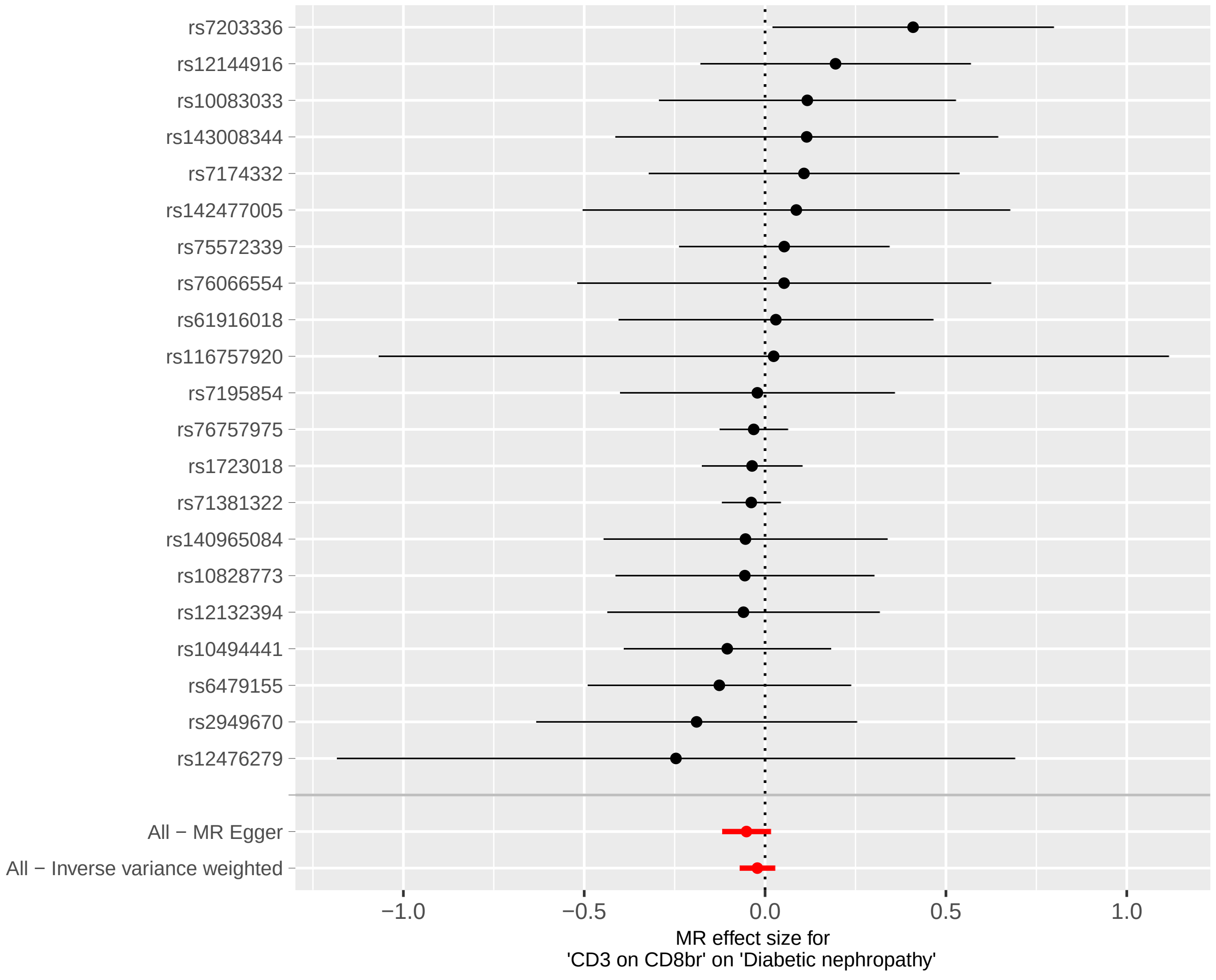

rs7203336
rs12144916
rs10083033
rs143008344
rs7174332
rs142477005
rs75572339
rs76066554
rs61916018
rs116757920
rs7195854
rs76757975
rs1723018
rs71381322
rs140965084
rs10828773
rs12132394
rs10494441
rs6479155
rs2949670
rs12476279
All − MR Egger
All − Inverse variance weighted
−1.0
−0.5
0.0
0.5
1.0
MR effect size for
'CD3 on CD8br' on 'Diabetic nephropathy'

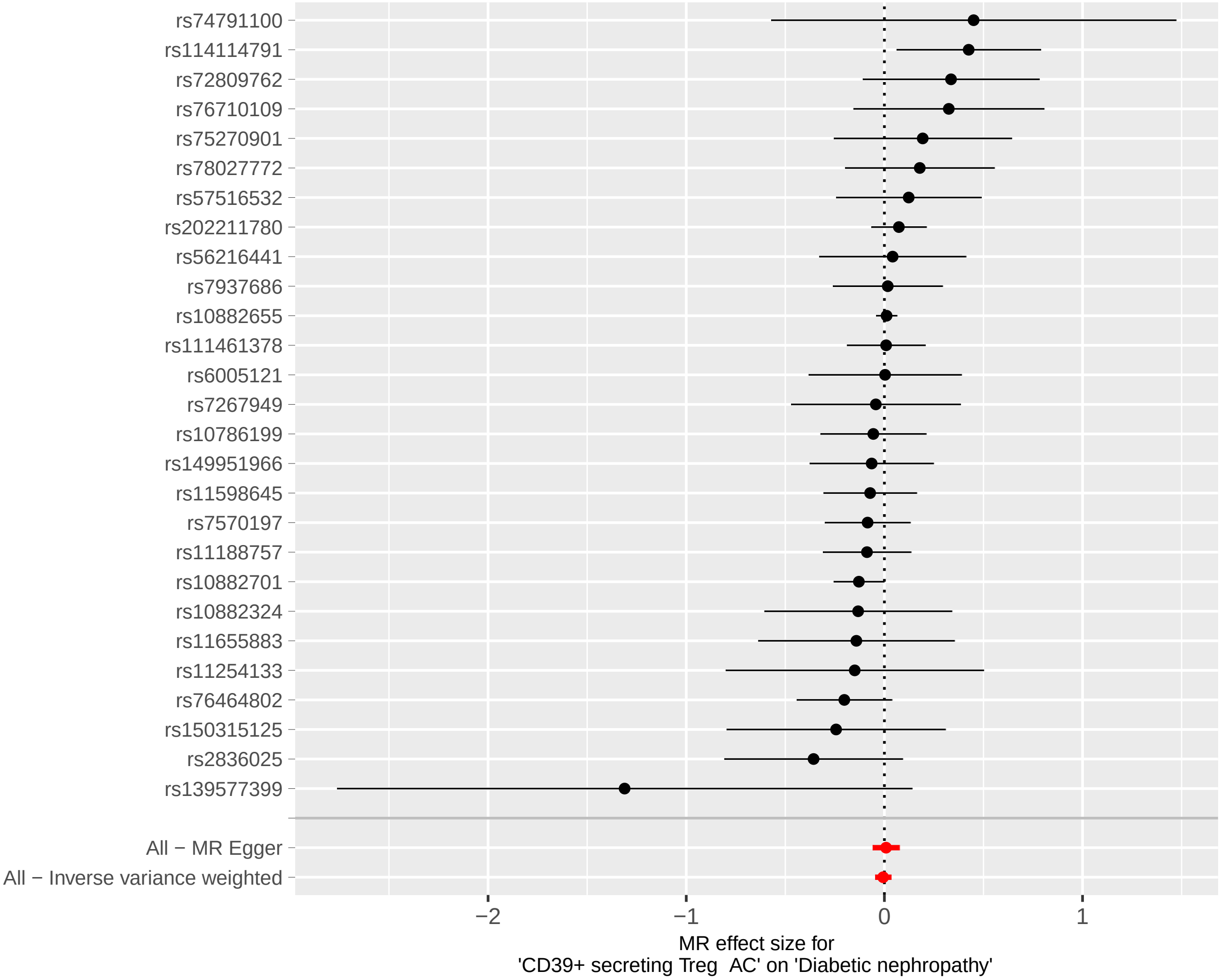

rs74791100
rs114114791
rs72809762
rs76710109
rs75270901
rs78027772
rs57516532
rs202211780
rs56216441
rs7937686
rs10882655
rs111461378
rs6005121
rs7267949
rs10786199
rs149951966
rs11598645
rs7570197
rs11188757
rs10882701
rs10882324
rs11655883
rs11254133
rs76464802
rs150315125
rs2836025
rs139577399
All − MR Egger
All − Inverse variance weighted
−2
−1
0
1
MR effect size for
'CD39+ secreting Treg AC' on 'Diabetic nephropathy'

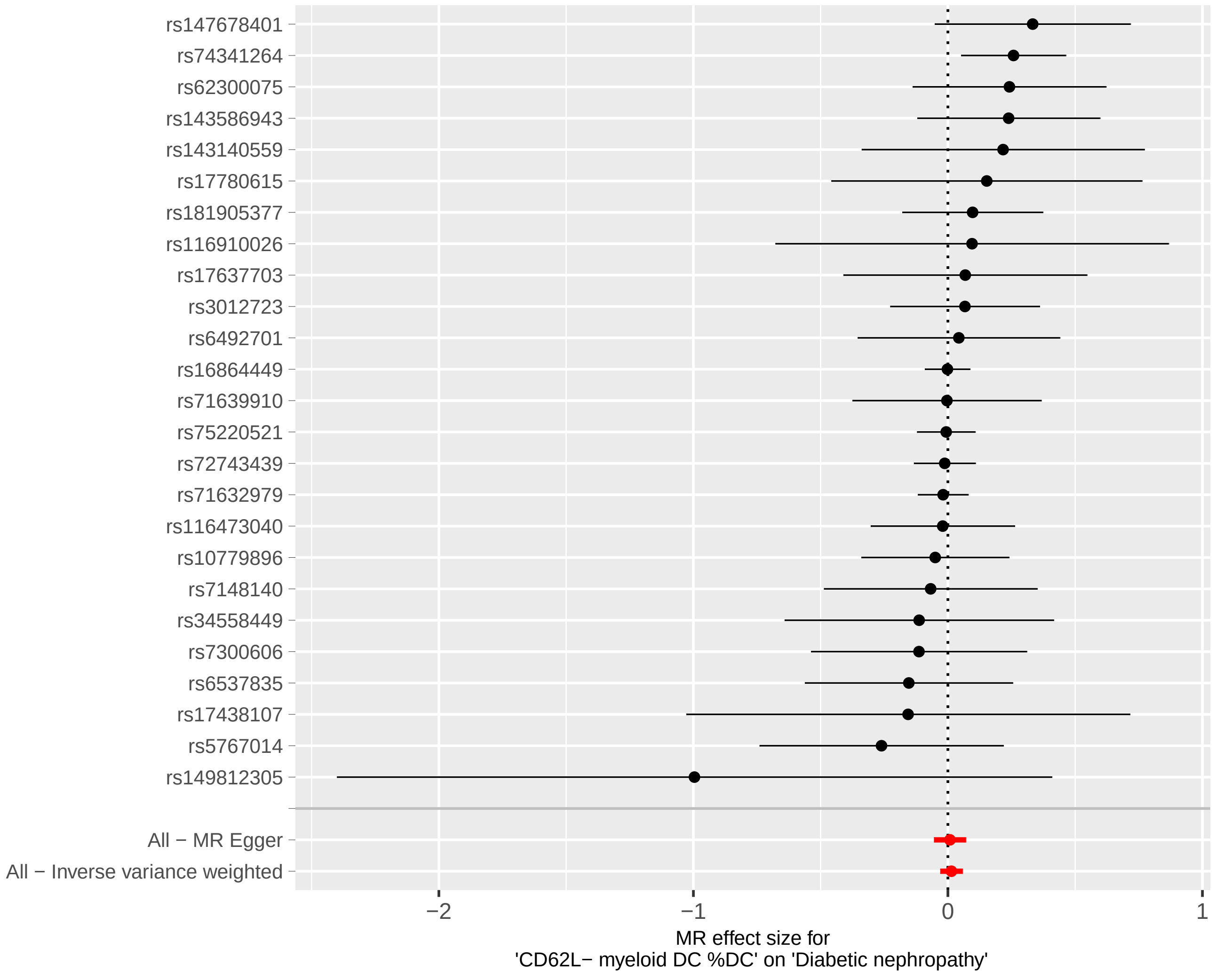
rs147678401
rs74341264
rs62300075
rs143586943
rs143140559
rs17780615
rs181905377
rs116910026
rs17637703
rs3012723
rs6492701
rs16864449
rs71639910
rs75220521
rs72743439
rs71632979
rs116473040
rs10779896
rs7148140
rs34558449
rs7300606
rs6537835
rs17438107
rs5767014
rs149812305
All − MR Egger
All − Inverse variance weighted
−2
−1
0
1
MR effect size for
'CD62L− myeloid DC %DC' on 'Diabetic nephropathy'

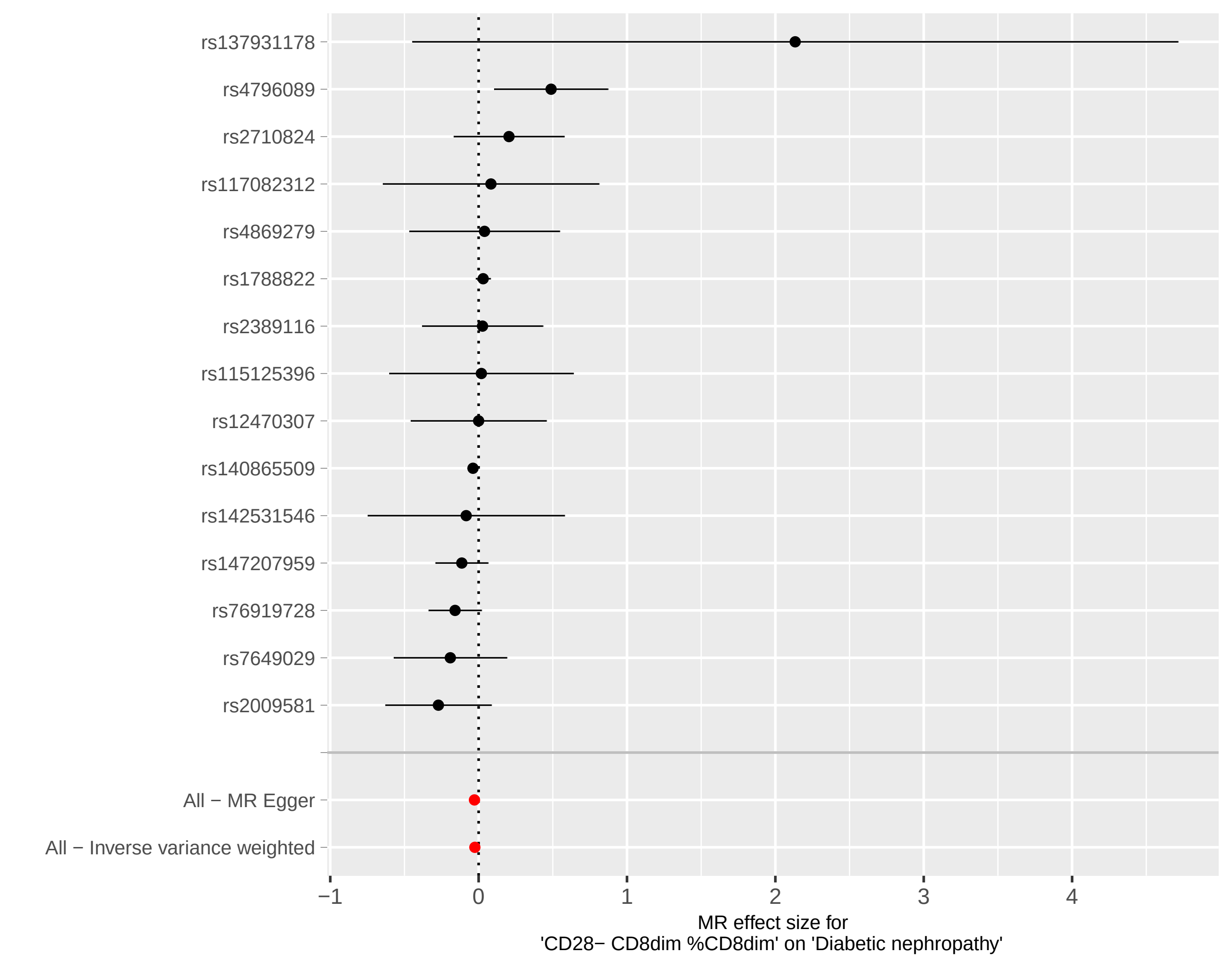
rs137931178
rs4796089
rs2710824
rs117082312
rs4869279
rs1788822
rs2389116
rs115125396
rs12470307
rs140865509
rs142531546
rs147207959
rs76919728
rs7649029
rs2009581
All − MR Egger
All − Inverse variance weighted
−1
0
1
2
3
4
MR effect size for
'CD28− CD8dim %CD8dim' on 'Diabetic nephropathy'

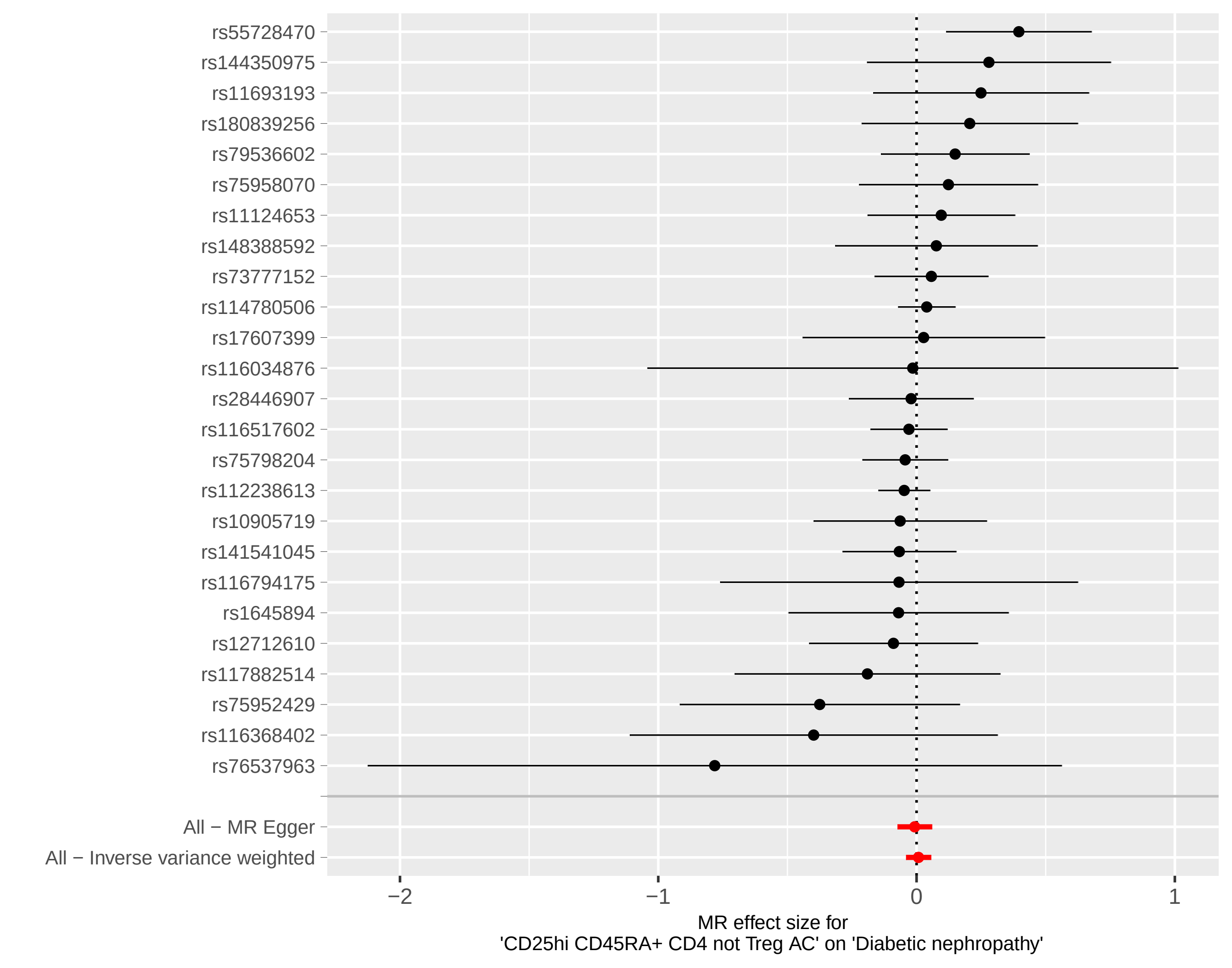

rs55728470
rs144350975
rs11693193
rs180839256
rs79536602
rs75958070
rs11124653
rs148388592
rs73777152
rs114780506
rs17607399
rs116034876
rs28446907
rs116517602
rs75798204
rs112238613
rs10905719
rs141541045
rs116794175
rs1645894
rs12712610
rs117882514
rs75952429
rs116368402
rs76537963
All − MR Egger
All − Inverse variance weighted
−2
−1
0
1
MR effect size for
'CD25hi CD45RA+ CD4 not Treg AC' on 'Diabetic nephropathy'

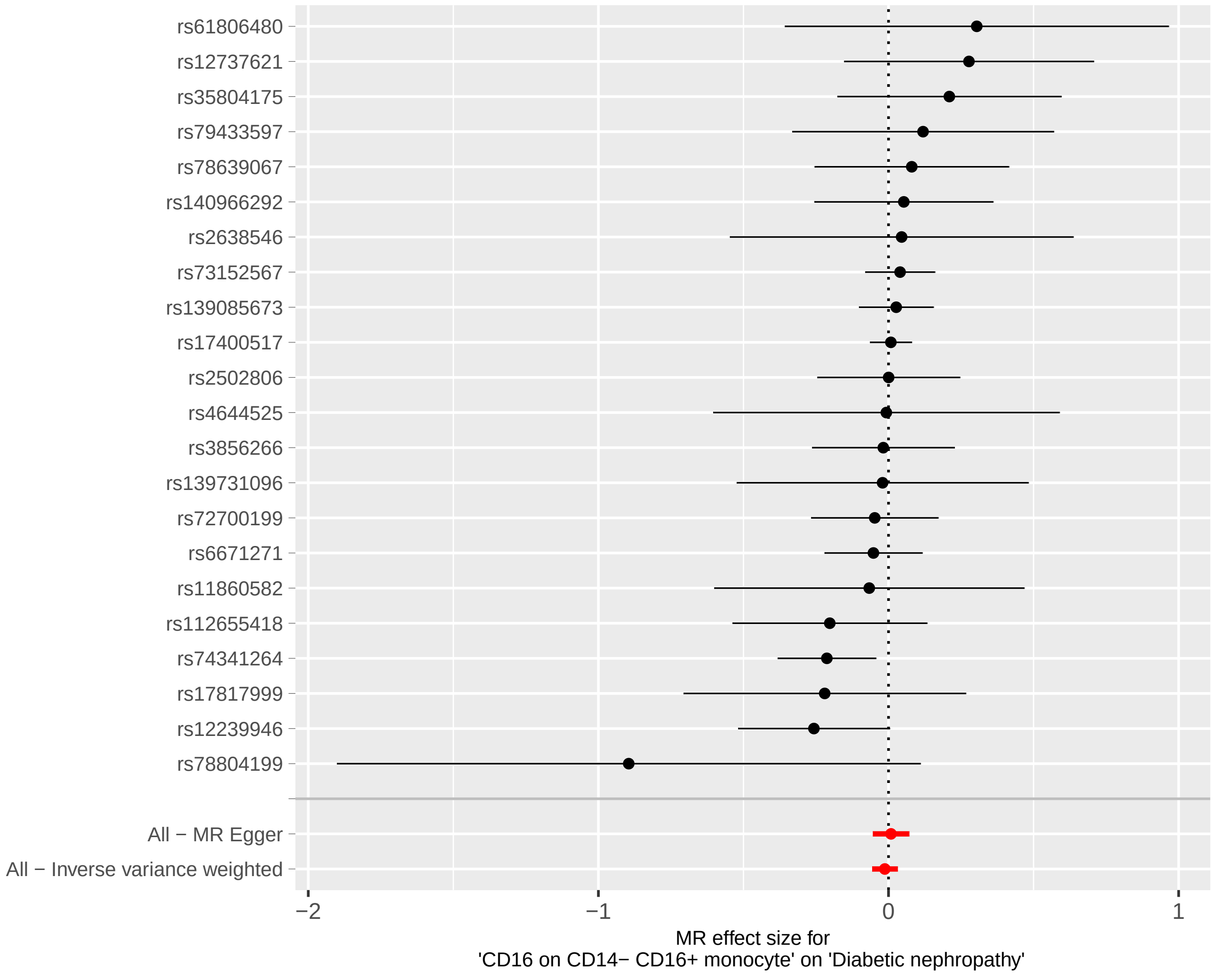
rs61806480
rs12737621
rs35804175
rs79433597
rs78639067
rs140966292
rs2638546
rs73152567
rs139085673
rs17400517
rs2502806
rs4644525
rs3856266
rs139731096
rs72700199
rs6671271
rs11860582
rs112655418
rs74341264
rs17817999
rs12239946
rs78804199
All − MR Egger
All − Inverse variance weighted
−2
−1
0
1
MR effect size for
'CD16 on CD14− CD16+ monocyte' on 'Diabetic nephropathy'

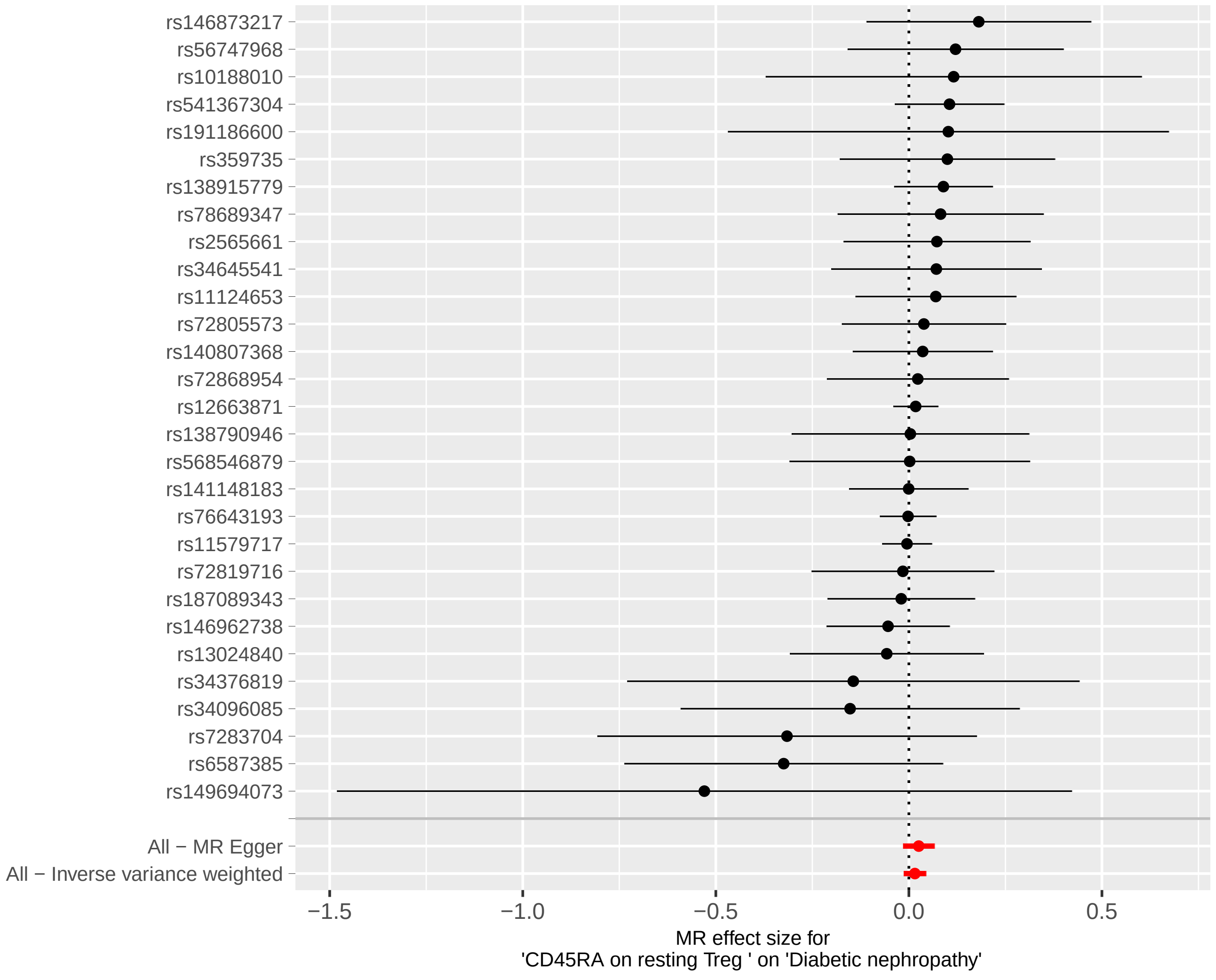
rs146873217
rs56747968
rs10188010
rs541367304
rs191186600
rs359735
rs138915779
rs78689347
rs2565661
rs34645541
rs11124653
rs72805573
rs140807368
rs72868954
rs12663871
rs138790946
rs568546879
rs141148183
rs76643193
rs11579717
rs72819716
rs187089343
rs146962738
rs13024840
rs34376819
rs34096085
rs7283704
rs6587385
rs149694073
All − MR Egger
All − Inverse variance weighted
−1.5
−1.0
−0.5
0.0
0.5
MR effect size for
'CD45RA on resting Treg ' on 'Diabetic nephropathy'

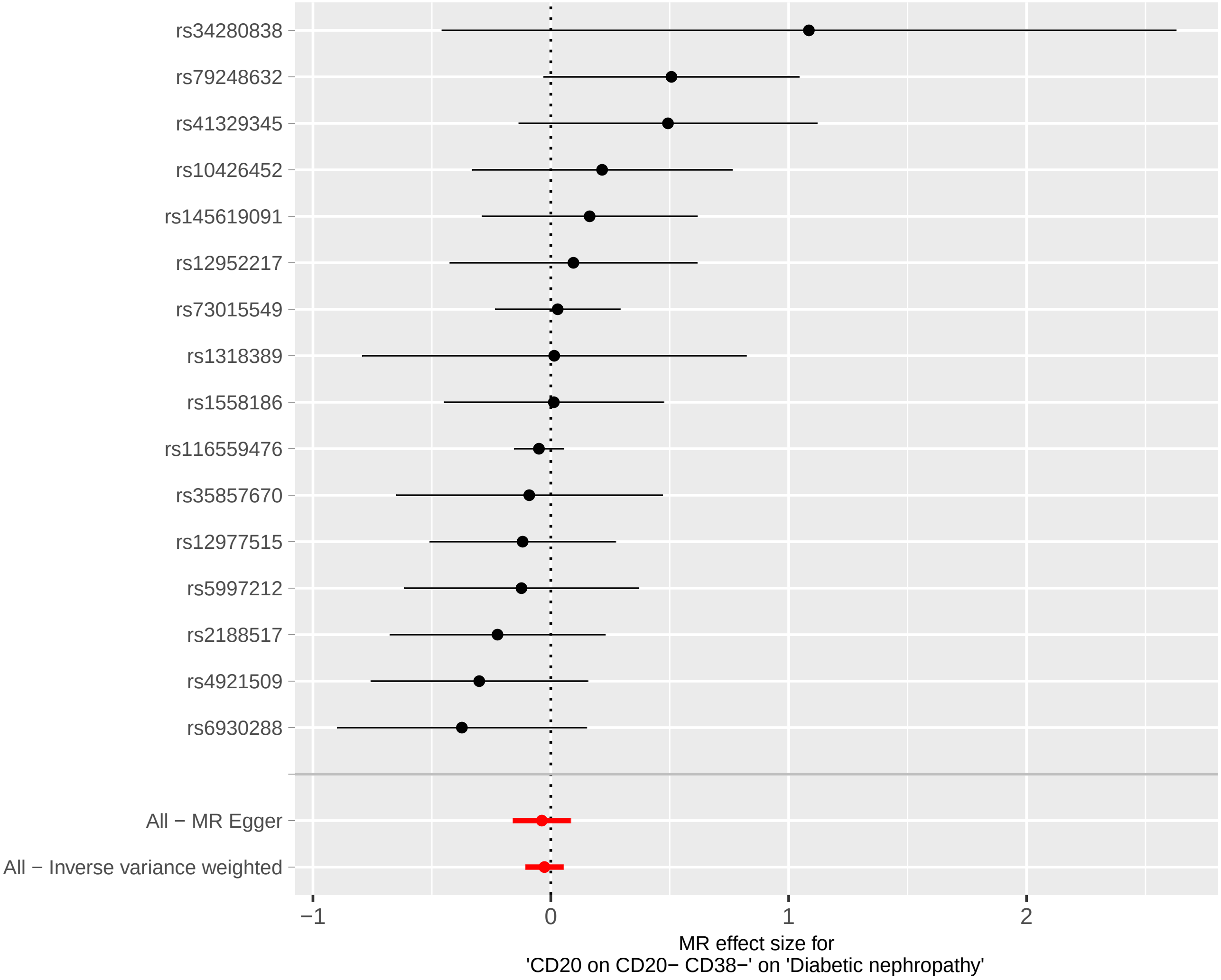
rs34280838
rs79248632
rs41329345
rs10426452
rs145619091
rs12952217
rs73015549
rs1318389
rs1558186
rs116559476
rs35857670
rs12977515
rs5997212
rs2188517
rs4921509
rs6930288
All − MR Egger
All − Inverse variance weighted
−1
0
1
2
MR effect size for
'CD20 on CD20− CD38−' on 'Diabetic nephropathy'

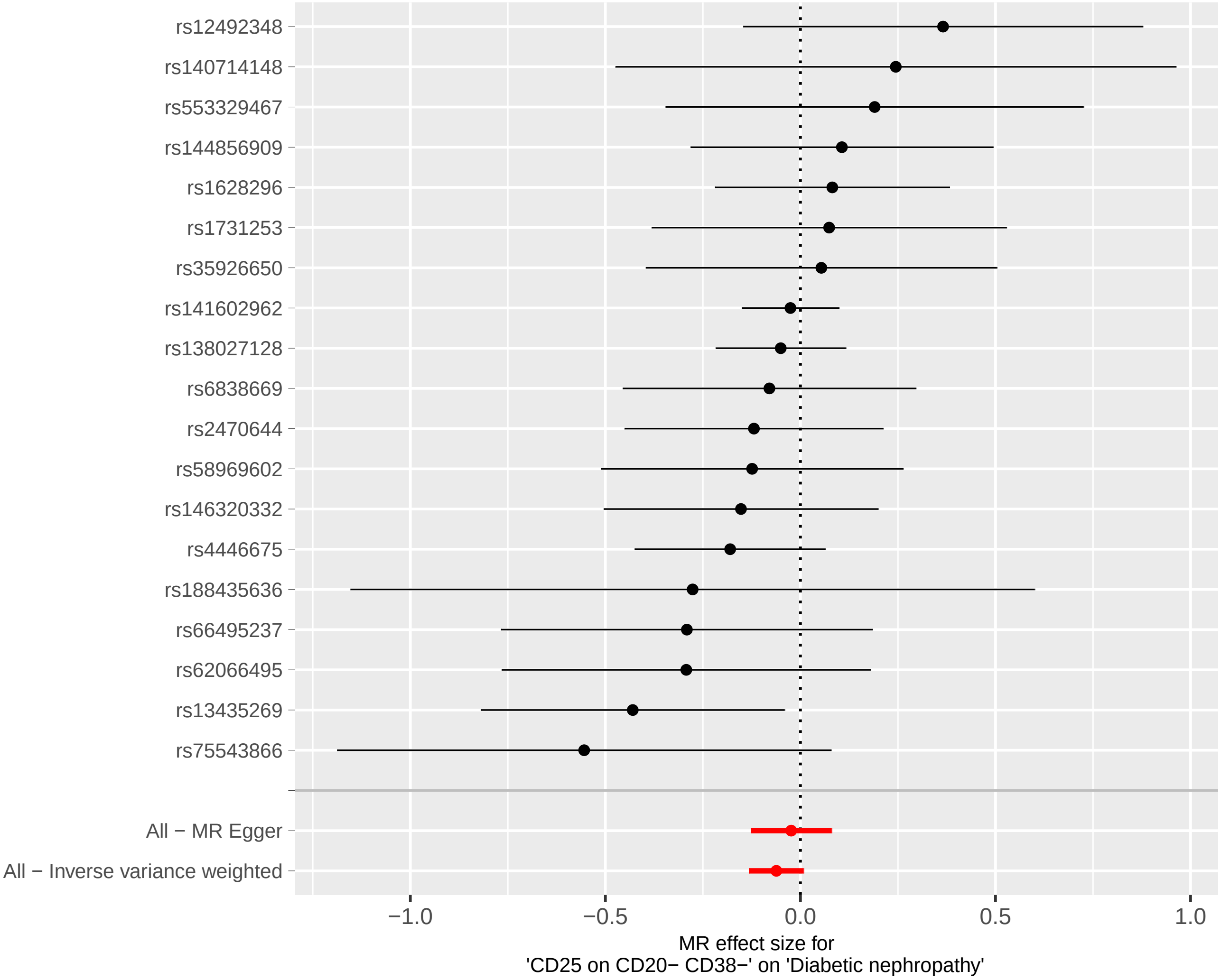
rs12492348
rs140714148
rs553329467
rs144856909
rs1628296
rs1731253
rs35926650
rs141602962
rs138027128
rs6838669
rs2470644
rs58969602
rs146320332
rs4446675
rs188435636
rs66495237
rs62066495
rs13435269
rs75543866
All − MR Egger
All − Inverse variance weighted
−1.0
−0.5
0.0
0.5
1.0
MR effect size for
'CD25 on CD20− CD38−' on 'Diabetic nephropathy'

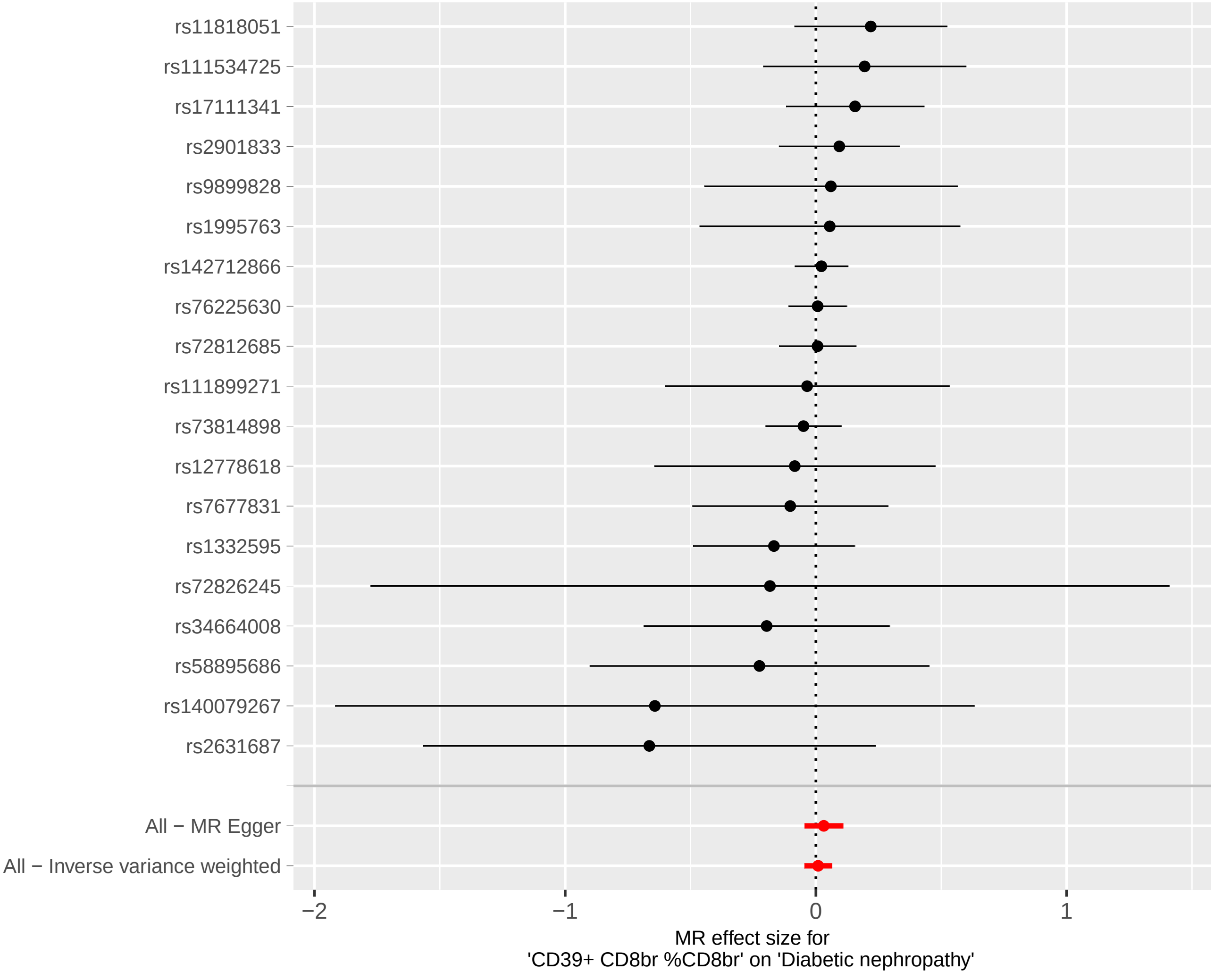
rs11818051
rs111534725
rs17111341
rs2901833
rs9899828
rs1995763
rs142712866
rs76225630
rs72812685
rs111899271
rs73814898
rs12778618
rs7677831
rs1332595
rs72826245
rs34664008
rs58895686
rs140079267
rs2631687
All − MR Egger
All − Inverse variance weighted
−2
−1
0
1
MR effect size for
'CD39+ CD8br %CD8br' on 'Diabetic nephropathy'

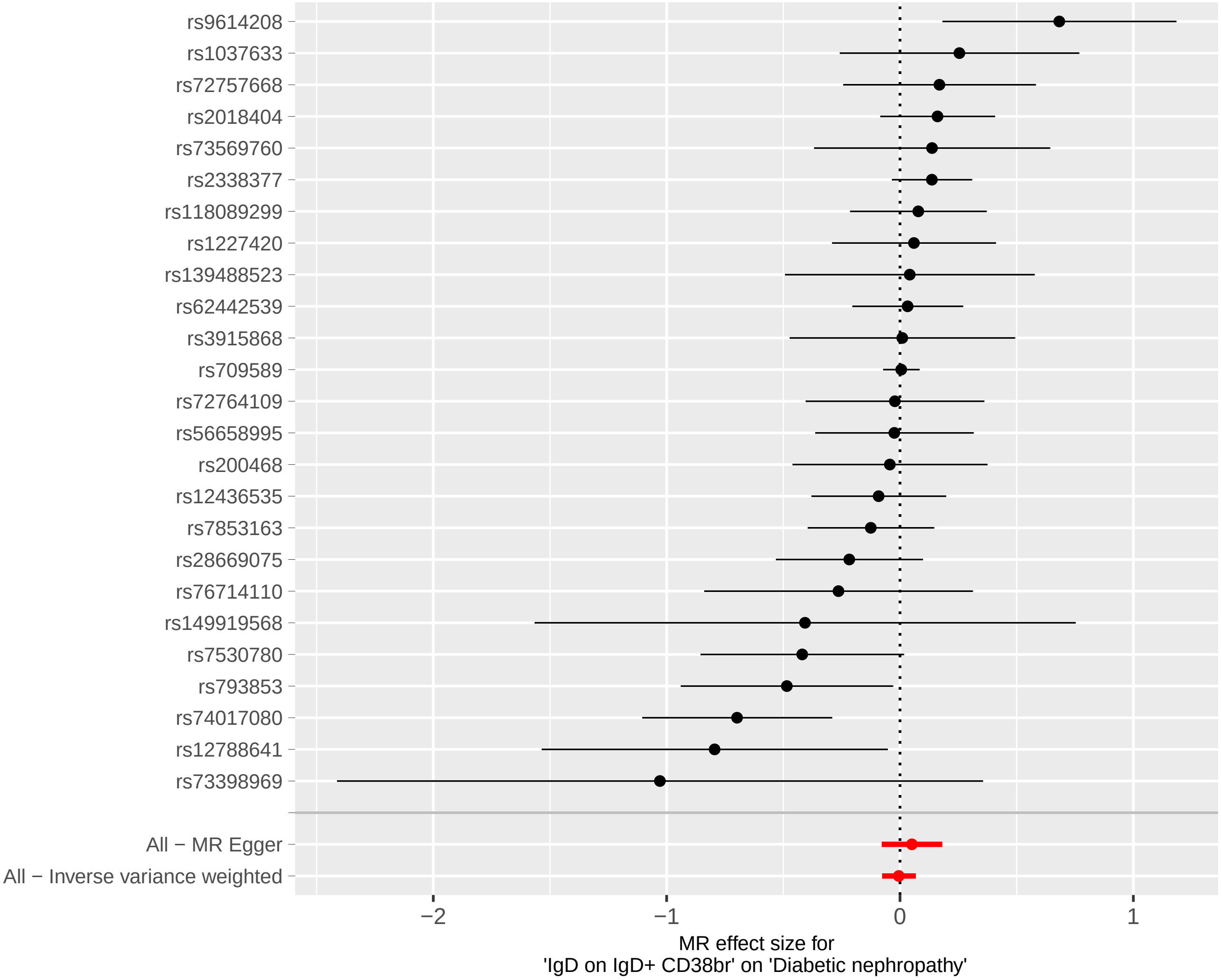

rs9614208
rs1037633
rs72757668
rs2018404
rs73569760
rs2338377
rs118089299
rs1227420
rs139488523
rs62442539
rs3915868
rs709589
rs72764109
rs56658995
rs200468
rs12436535
rs7853163
rs28669075
rs76714110
rs149919568
rs7530780
rs793853
rs74017080
rs12788641
rs73398969
All − MR Egger
All − Inverse variance weighted
−2
−1
0
1
MR effect size for
'IgD on IgD+ CD38br' on 'Diabetic nephropathy'

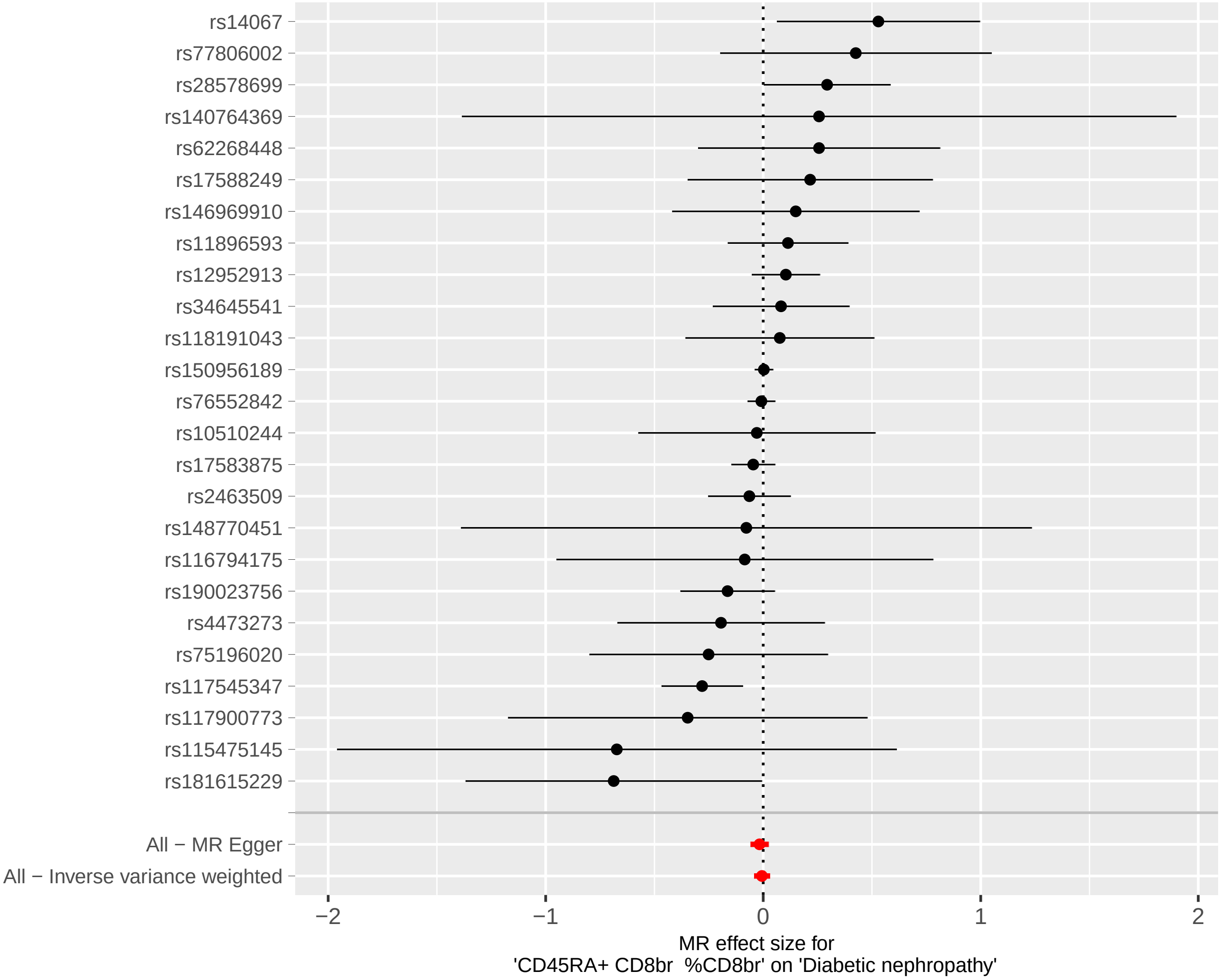

rs14067
rs77806002
rs28578699
rs140764369
rs62268448
rs17588249
rs146969910
rs11896593
rs12952913
rs34645541
rs118191043
rs150956189
rs76552842
rs10510244
rs17583875
rs2463509
rs148770451
rs116794175
rs190023756
rs4473273
rs75196020
rs117545347
rs117900773
rs115475145
rs181615229
All − MR Egger
All − Inverse variance weighted
−2
−1
0
1
2
MR effect size for
'CD45RA+ CD8br %CD8br' on 'Diabetic nephropathy'

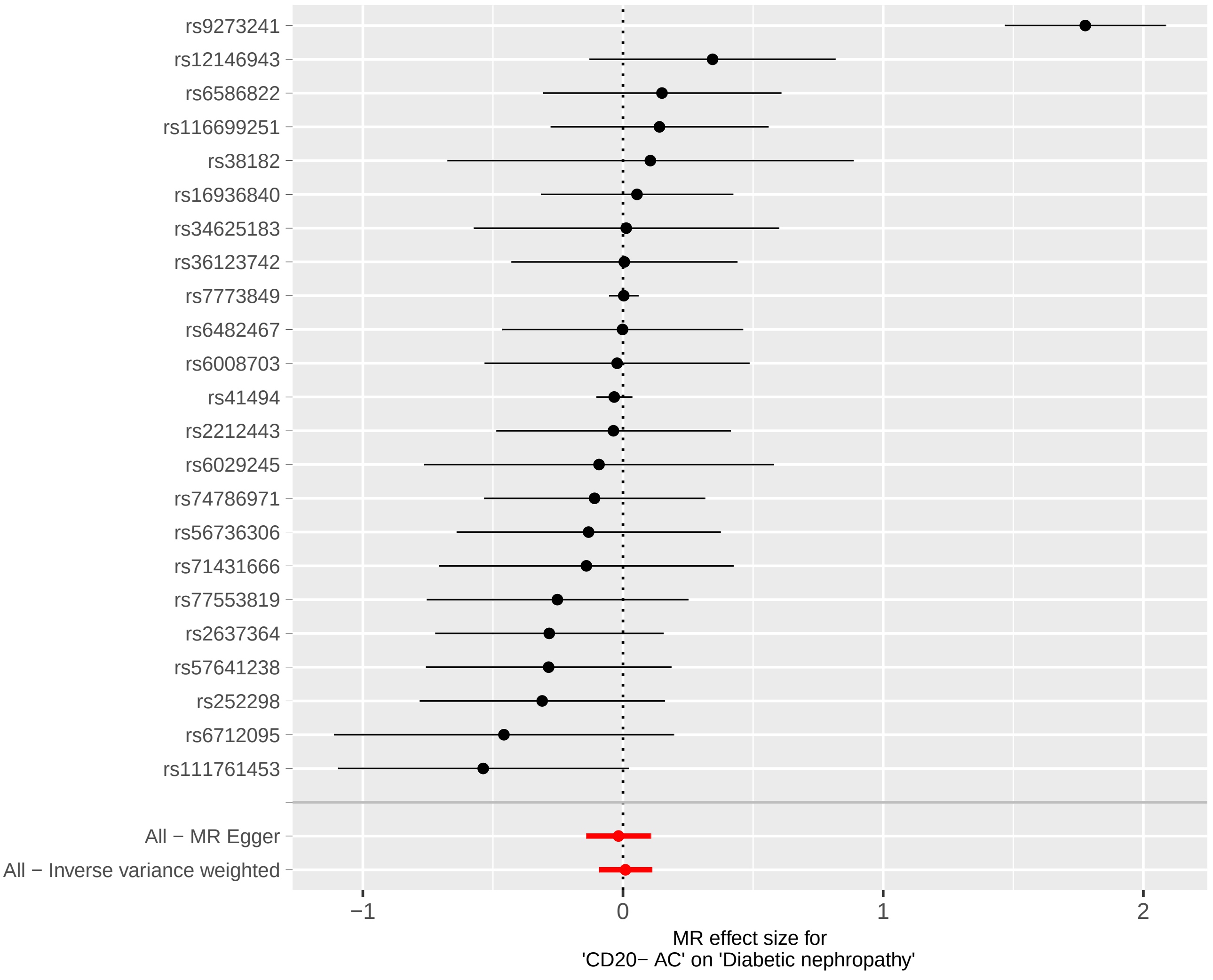
rs9273241
rs12146943
rs6586822
rs116699251
rs38182
rs16936840
rs34625183
rs36123742
rs7773849
rs6482467
rs6008703
rs41494
rs2212443
rs6029245
rs74786971
rs56736306
rs71431666
rs77553819
rs2637364
rs57641238
rs252298
rs6712095
rs111761453
All − MR Egger
All − Inverse variance weighted
−1
0
1
2
MR effect size for
'CD20− AC' on 'Diabetic nephropathy'

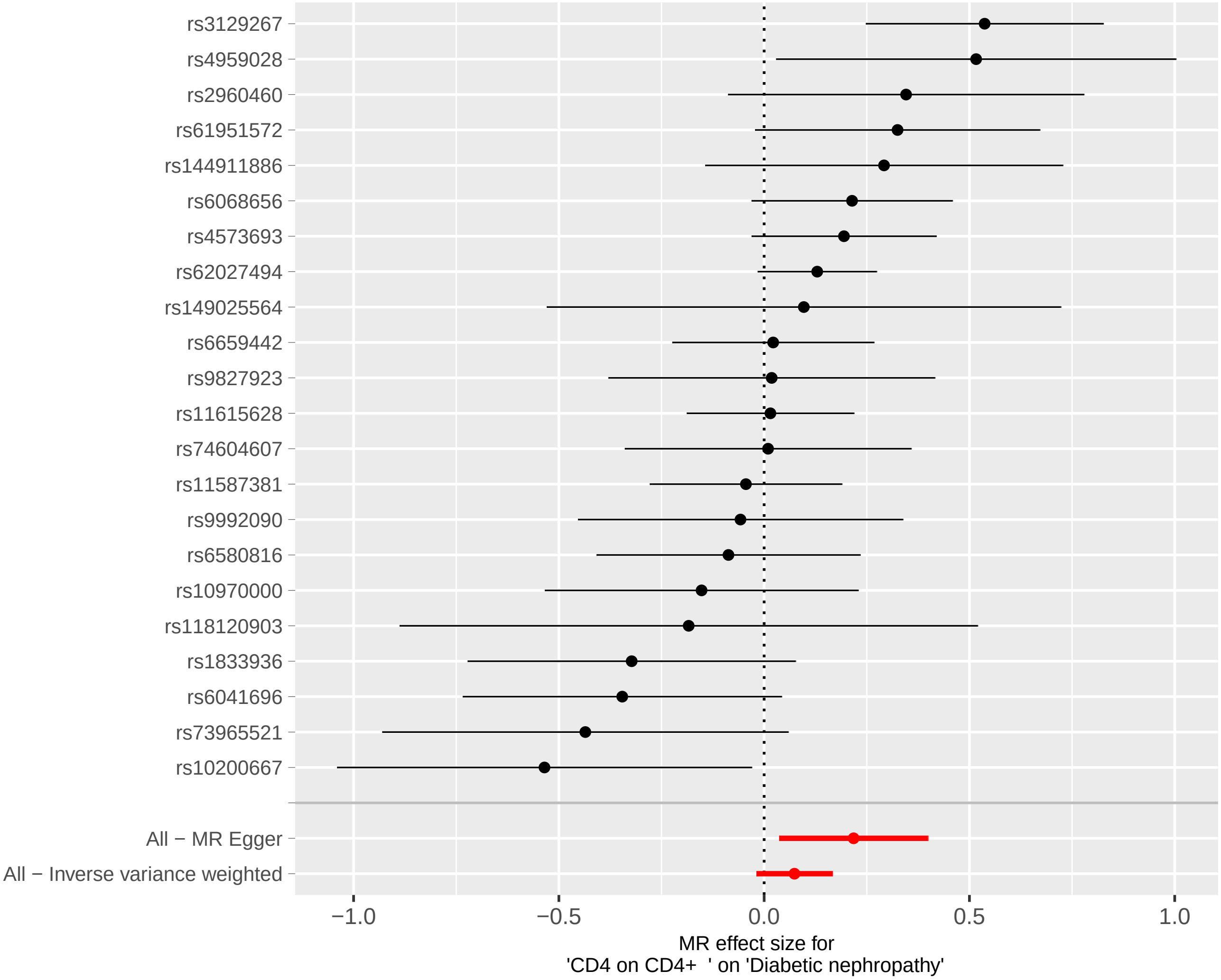

rs3129267
rs4959028
rs2960460
rs61951572
rs144911886
rs6068656
rs4573693
rs62027494
rs149025564
rs6659442
rs9827923
rs11615628
rs74604607
rs11587381
rs9992090
rs6580816
rs10970000
rs118120903
rs1833936
rs6041696
rs73965521
rs10200667
All − MR Egger
All − Inverse variance weighted
−1.0
−0.5
0.0
0.5
1.0
MR effect size for
'CD4 on CD4+ ' on 'Diabetic nephropathy'

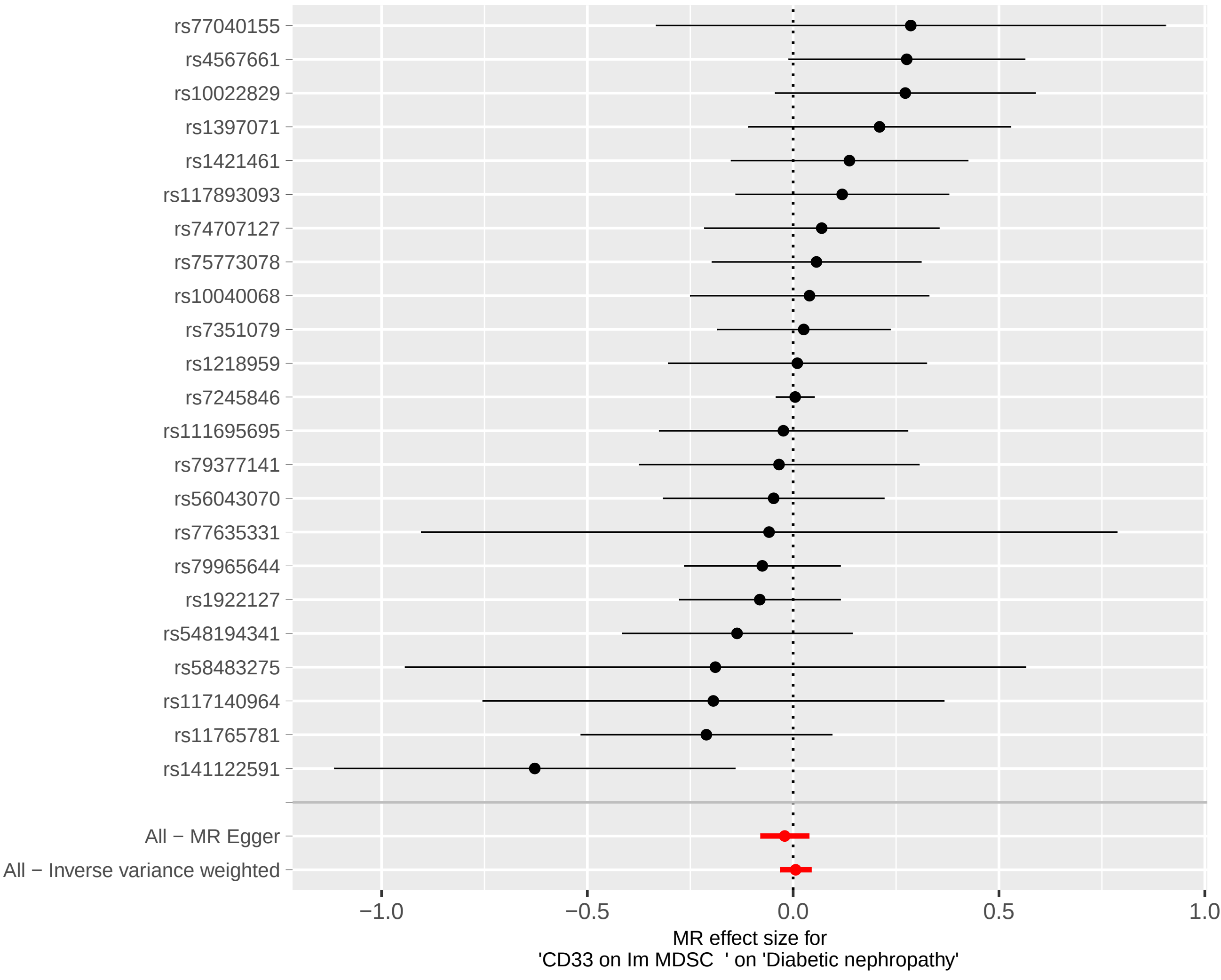
rs77040155
rs4567661
rs10022829
rs1397071
rs1421461
rs117893093
rs74707127
rs75773078
rs10040068
rs7351079
rs1218959
rs7245846
rs111695695
rs79377141
rs56043070
rs77635331
rs79965644
rs1922127
rs548194341
rs58483275
rs117140964
rs11765781
rs141122591
All − MR Egger
All − Inverse variance weighted
−1.0
−0.5
0.0
0.5
1.0
MR effect size for
'CD33 on Im MDSC ' on 'Diabetic nephropathy'

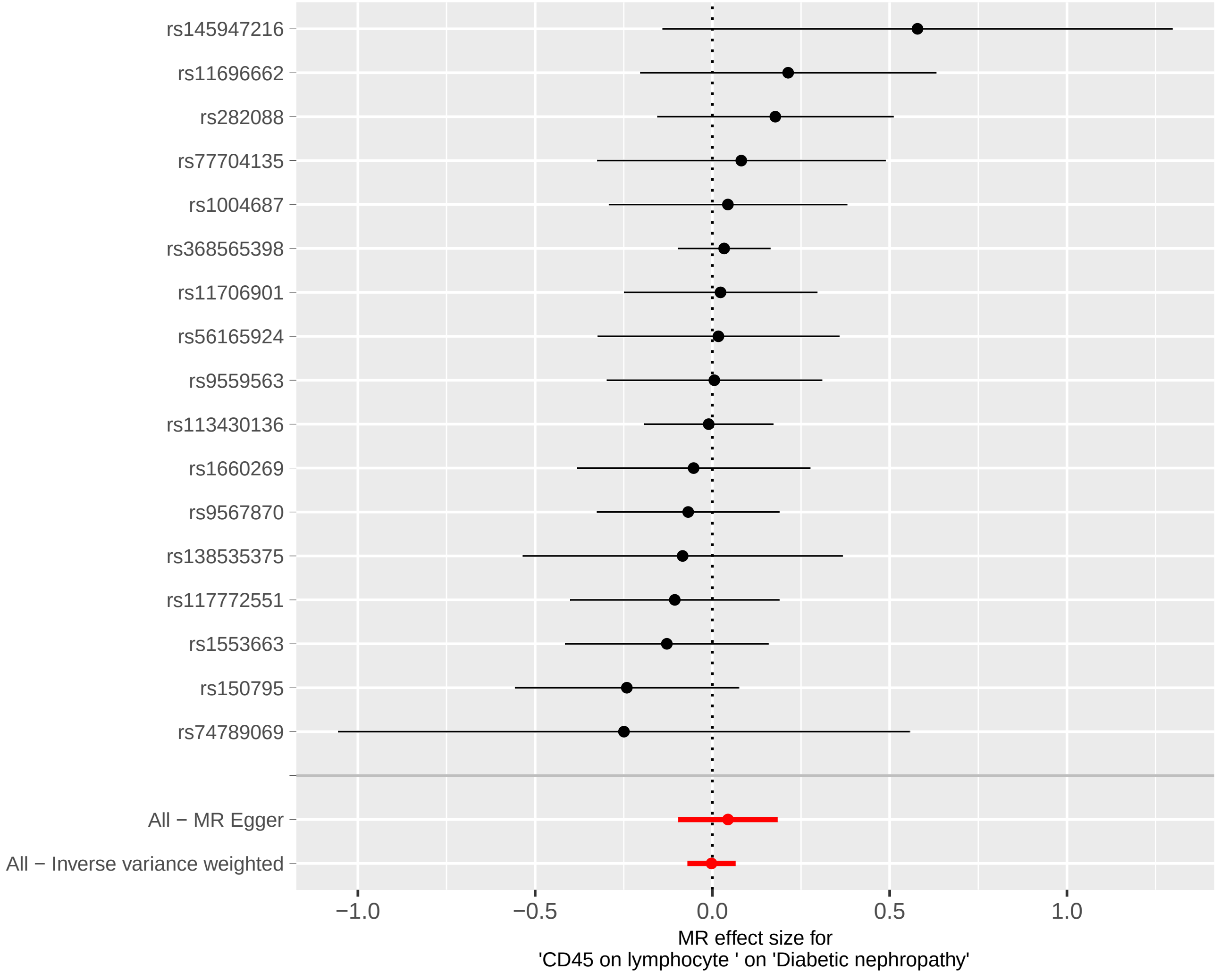
rs145947216
rs11696662
rs282088
rs77704135
rs1004687
rs368565398
rs11706901
rs56165924
rs9559563
rs113430136
rs1660269
rs9567870
rs138535375
rs117772551
rs1553663
rs150795
rs74789069
All − MR Egger
All − Inverse variance weighted
−1.0
−0.5
0.0
0.5
1.0
MR effect size for
'CD45 on lymphocyte ' on 'Diabetic nephropathy'

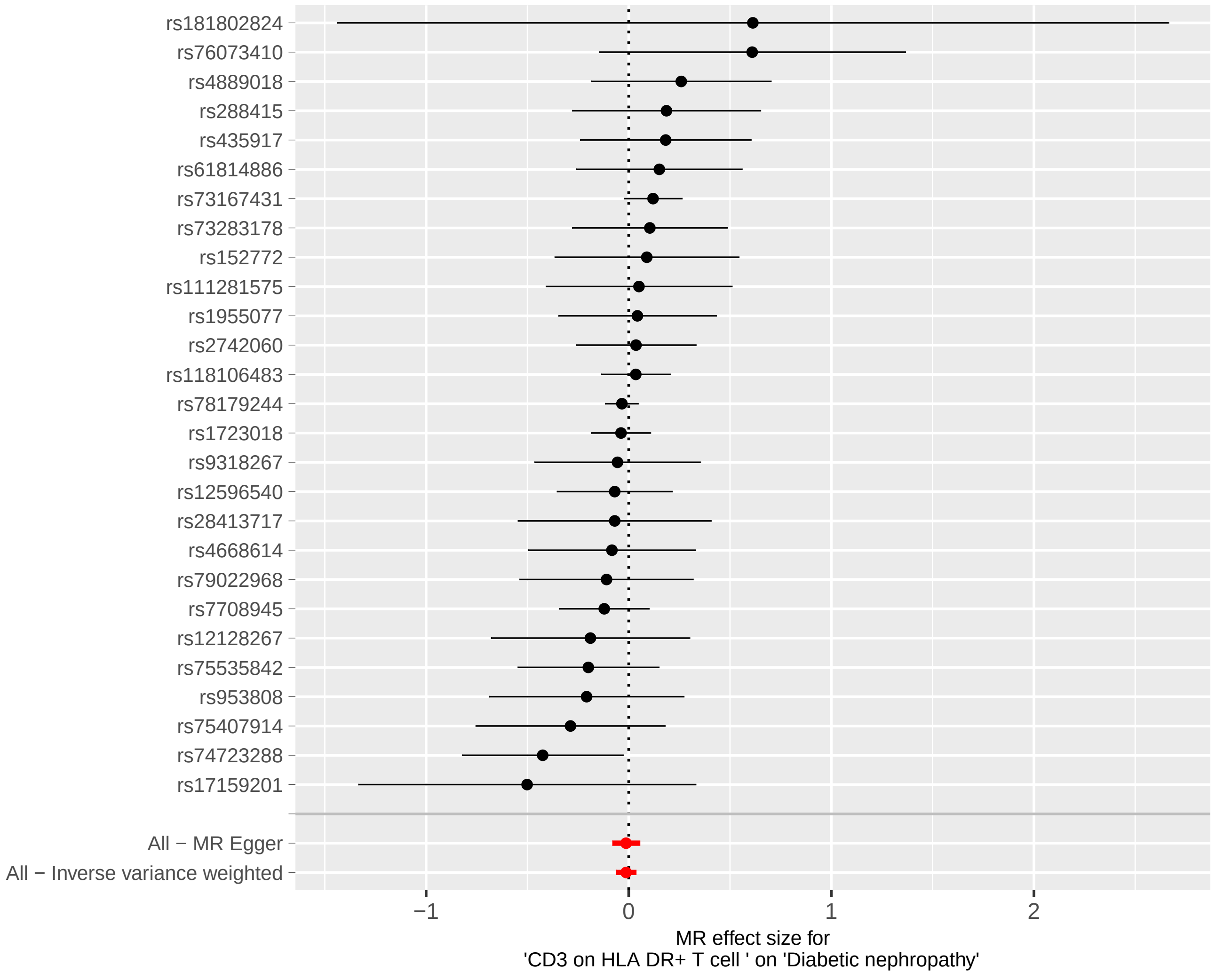

rs181802824
rs76073410
rs4889018
rs288415
rs435917
rs61814886
rs73167431
rs73283178
rs152772
rs111281575
rs1955077
rs2742060
rs118106483
rs78179244
rs1723018
rs9318267
rs12596540
rs28413717
rs4668614
rs79022968
rs7708945
rs12128267
rs75535842
rs953808
rs75407914
rs74723288
rs17159201
All − MR Egger
All − Inverse variance weighted
−1
0
1
2
MR effect size for
'CD3 on HLA DR+ T cell ' on 'Diabetic nephropathy'

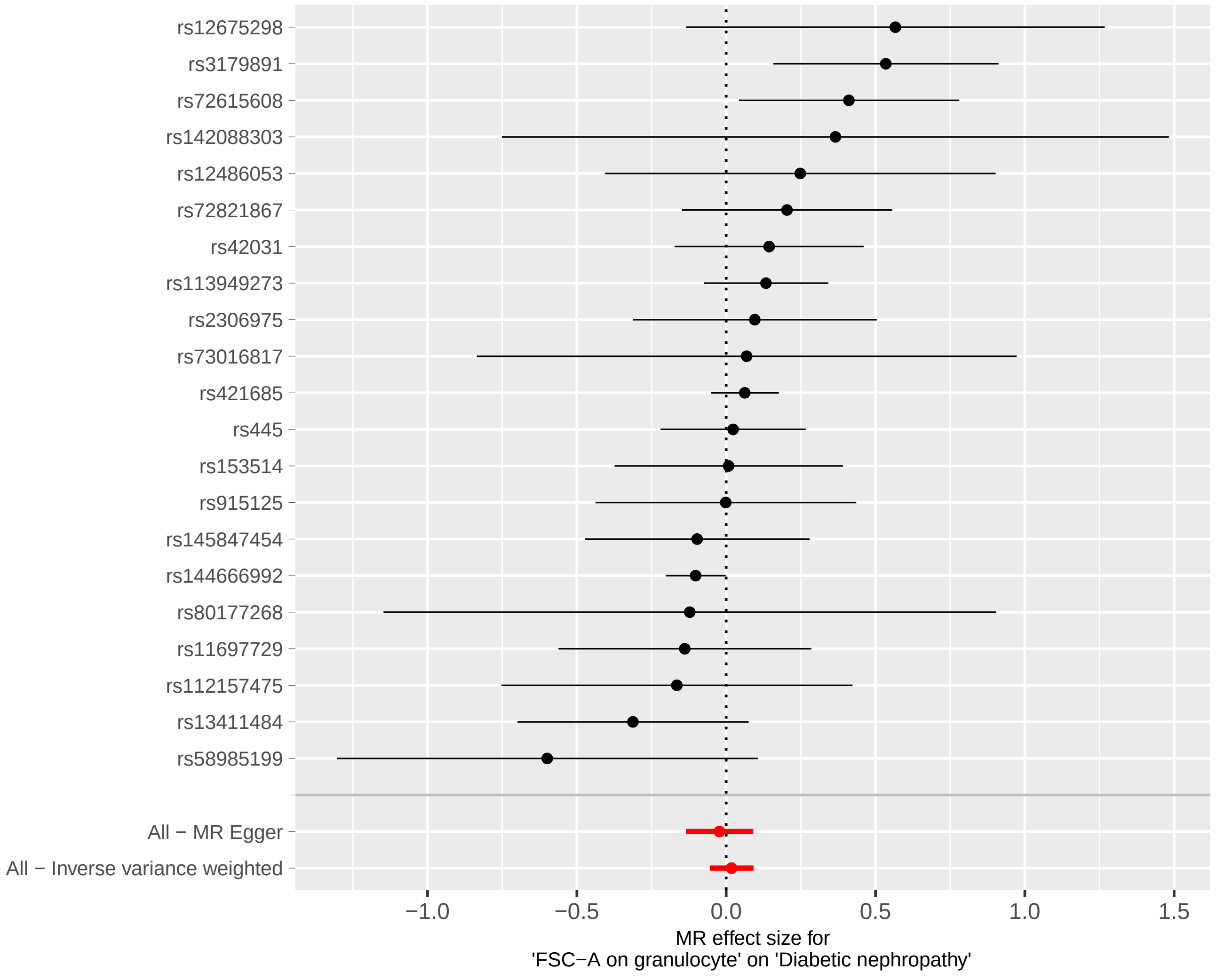
rs12675298
rs3179891
rs72615608
rs142088303
rs12486053
rs72821867
rs42031
rs113949273
rs2306975
rs73016817
rs421685
rs445
rs153514
rs915125
rs145847454
rs144666992
rs80177268
rs11697729
rs112157475
rs13411484
rs58985199
All − MR Egger
All − Inverse variance weighted
−1.0
−0.5
0.0
0.5
1.0
1.5
MR effect size for
'FSC−A on granulocyte' on 'Diabetic nephropathy'

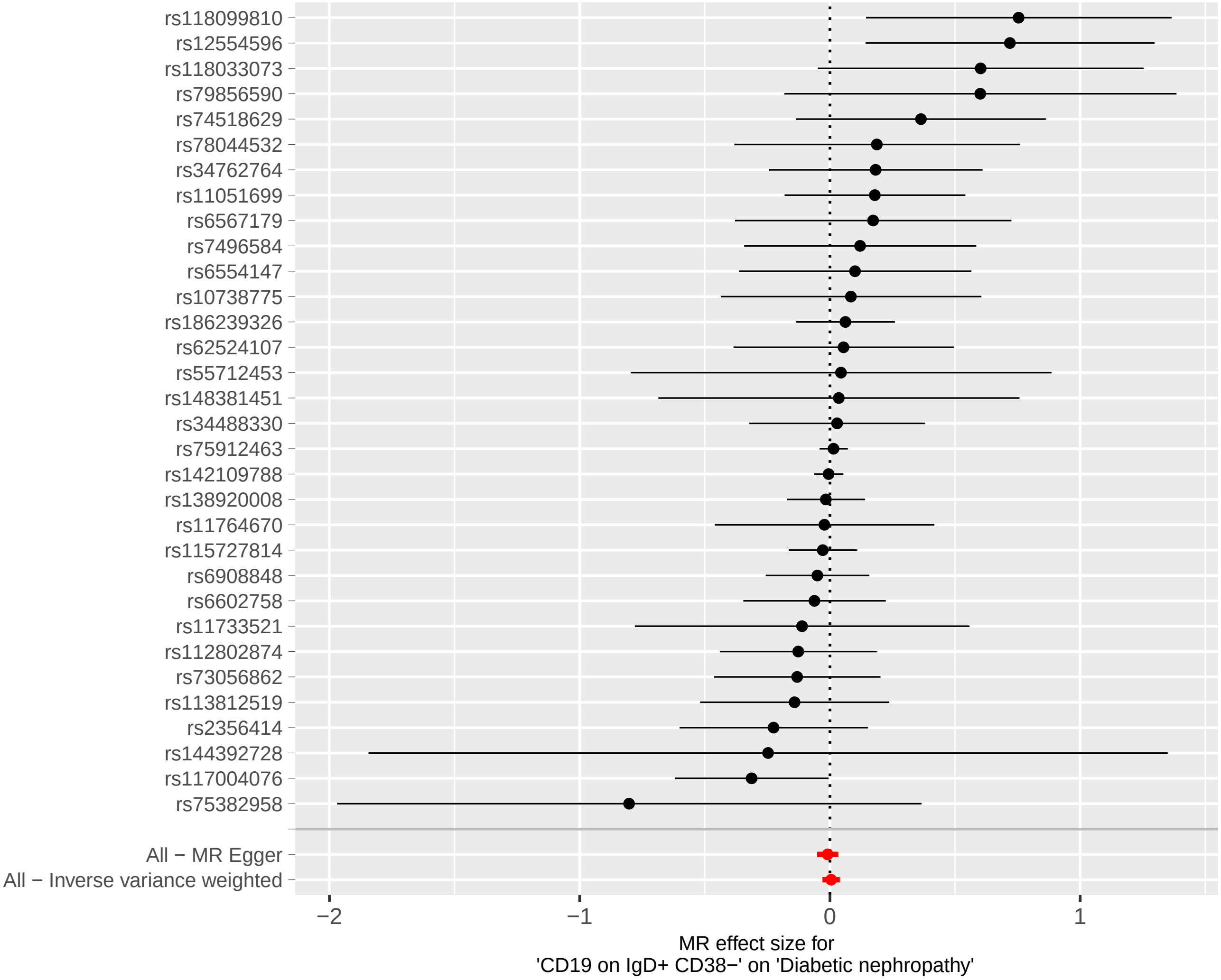
rs118099810
rs12554596
rs118033073
rs79856590
rs74518629
rs78044532
rs34762764
rs11051699
rs6567179
rs7496584
rs6554147
rs10738775
rs186239326
rs62524107
rs55712453
rs148381451
rs34488330
rs75912463
rs142109788
rs138920008
rs11764670
rs115727814
rs6908848
rs6602758
rs11733521
rs112802874
rs73056862
rs113812519
rs2356414
rs144392728
rs117004076
rs75382958
All − MR Egger
All − Inverse variance weighted
−2
−1
0
1
MR effect size for
'CD19 on IgD+ CD38−' on 'Diabetic nephropathy'

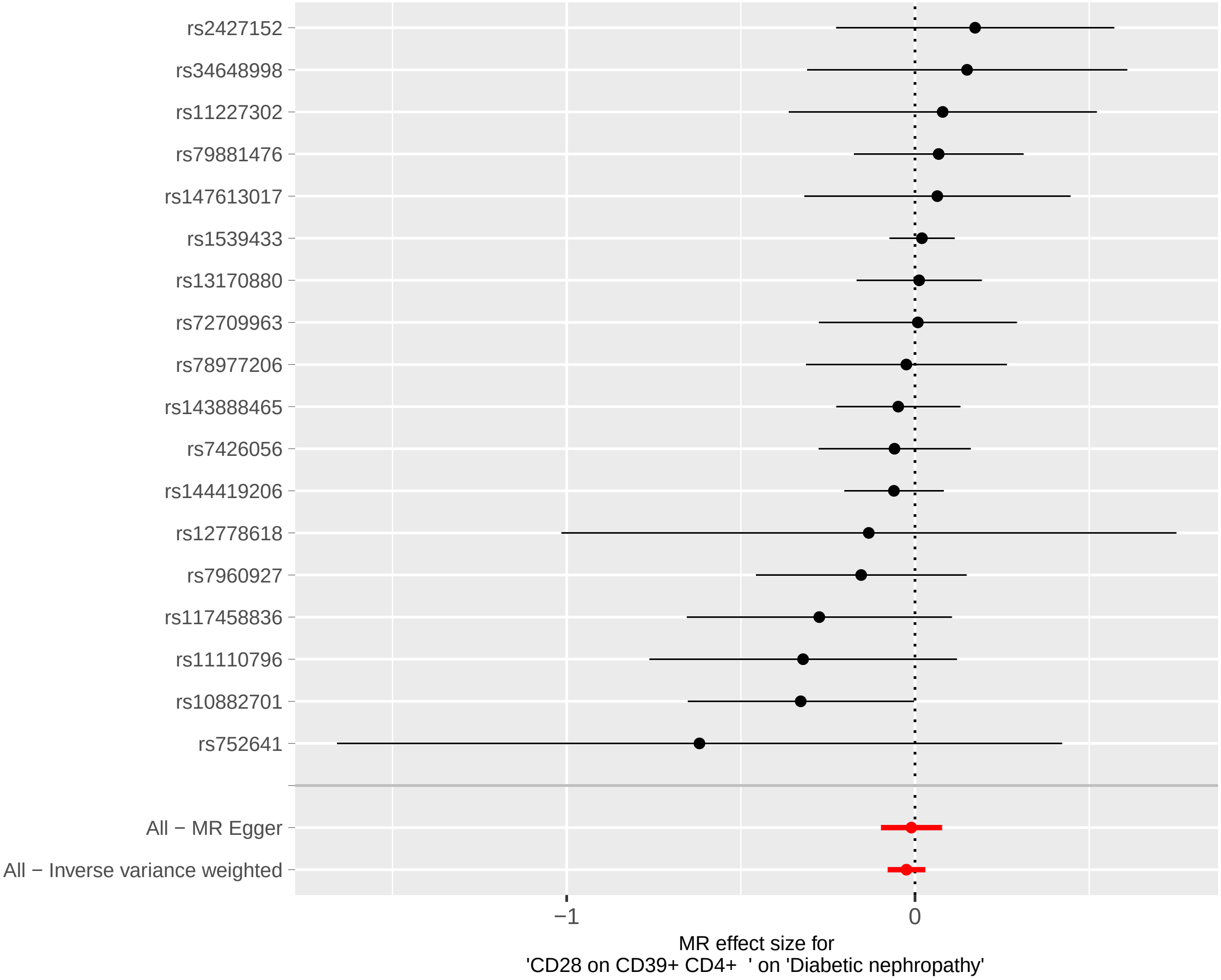
rs2427152
rs34648998
rs11227302
rs79881476
rs147613017
rs1539433
rs13170880
rs72709963
rs78977206
rs143888465
rs7426056
rs144419206
rs12778618
rs7960927
rs117458836
rs11110796
rs10882701
rs752641
All − MR Egger
All − Inverse variance weighted
−1
0
MR effect size for
'CD28 on CD39+ CD4+ ' on 'Diabetic nephropathy'

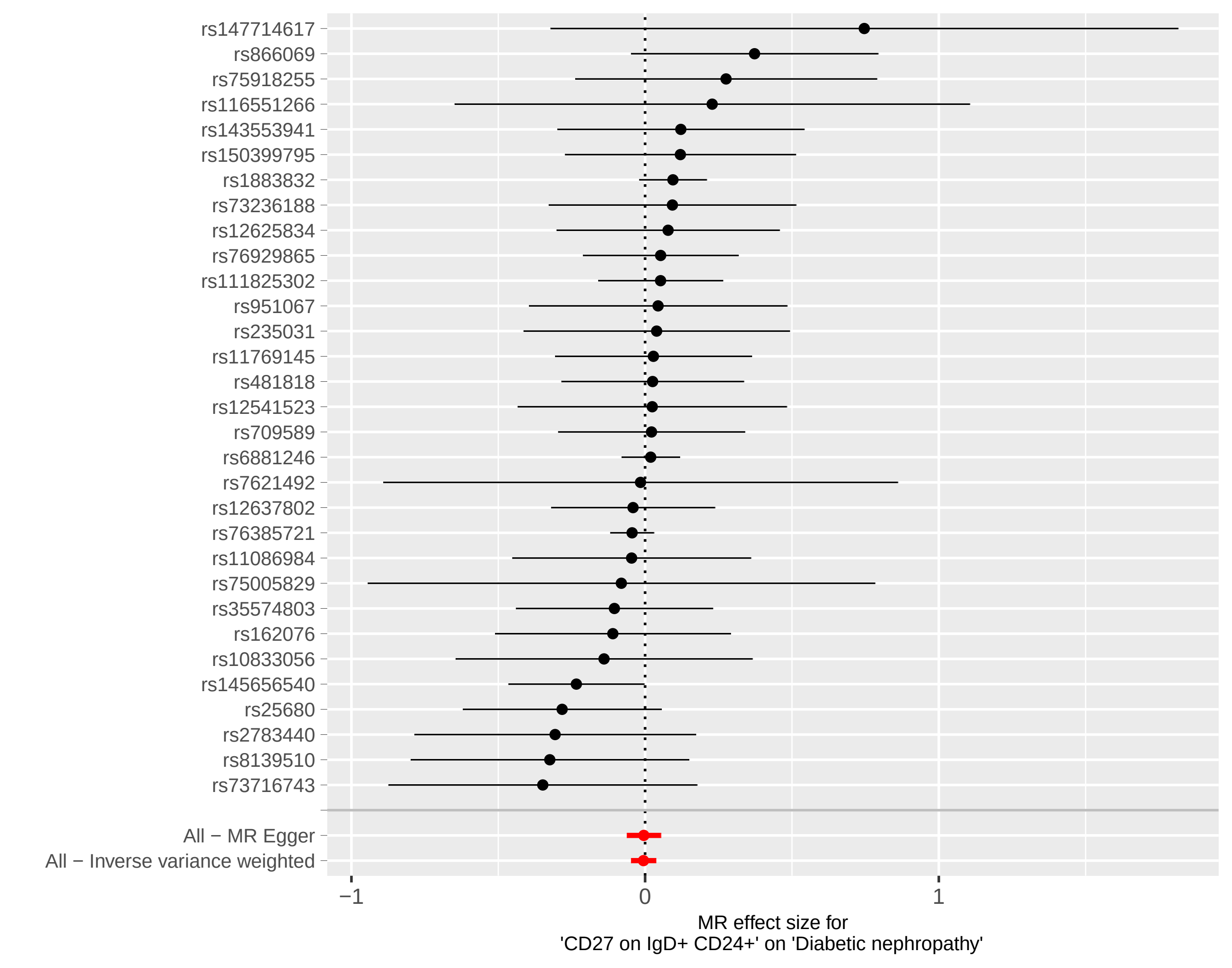
rs147714617
rs866069
rs75918255
rs116551266
rs143553941
rs150399795
rs1883832
rs73236188
rs12625834
rs76929865
rs111825302
rs951067
rs235031
rs11769145
rs481818
rs12541523
rs709589
rs6881246
rs7621492
rs12637802
rs76385721
rs11086984
rs75005829
rs35574803
rs162076
rs10833056
rs145656540
rs25680
rs2783440
rs8139510
rs73716743
All − MR Egger
All − Inverse variance weighted
−1
0
1
MR effect size for
'CD27 on IgD+ CD24+' on 'Diabetic nephropathy'

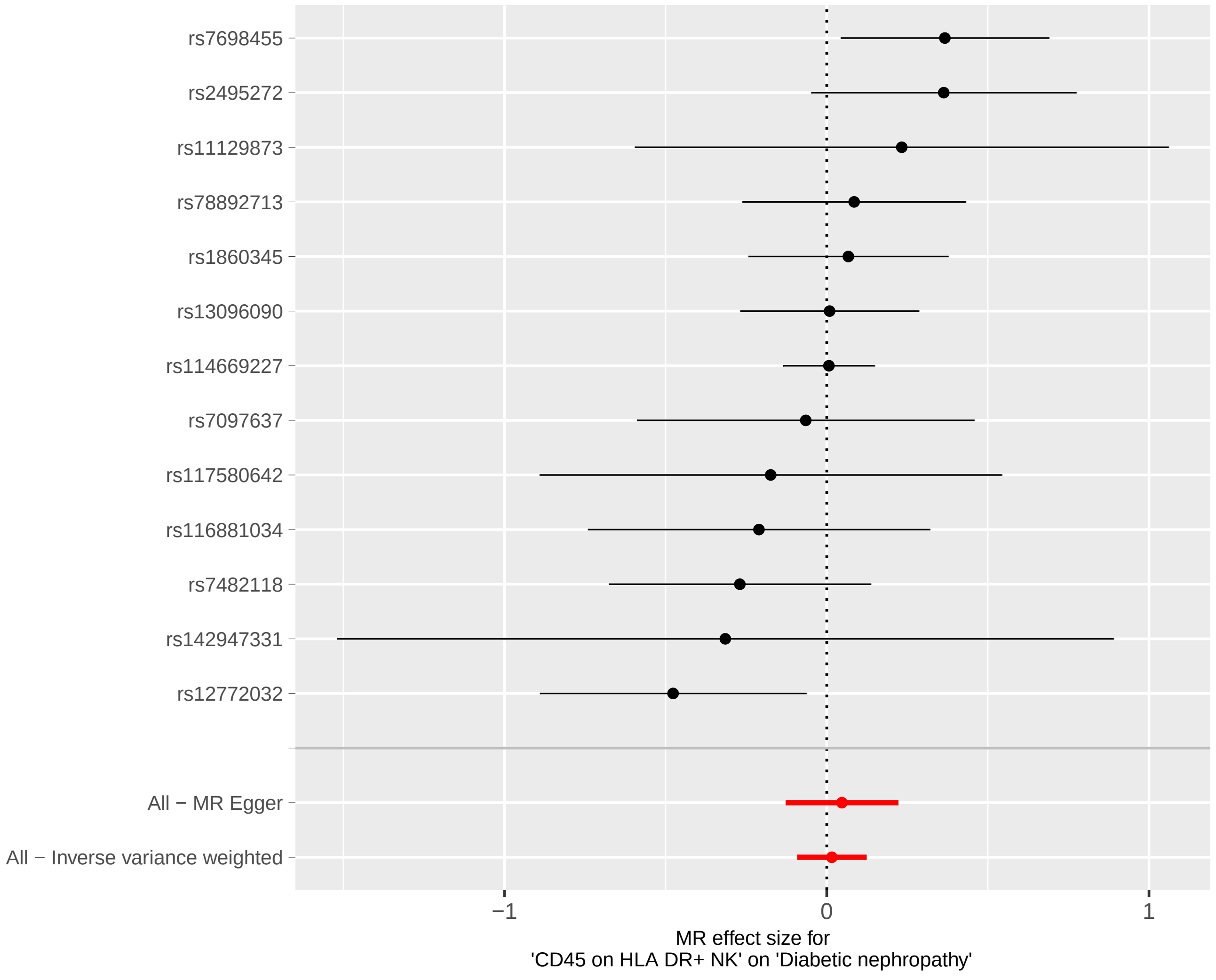
rs7698455
rs2495272
rs11129873
rs78892713
rs1860345
rs13096090
rs114669227
rs7097637
rs117580642
rs116881034
rs7482118
rs142947331
rs12772032
All − MR Egger
All − Inverse variance weighted
−1
0
1
MR effect size for
'CD45 on HLA DR+ NK' on 'Diabetic nephropathy'

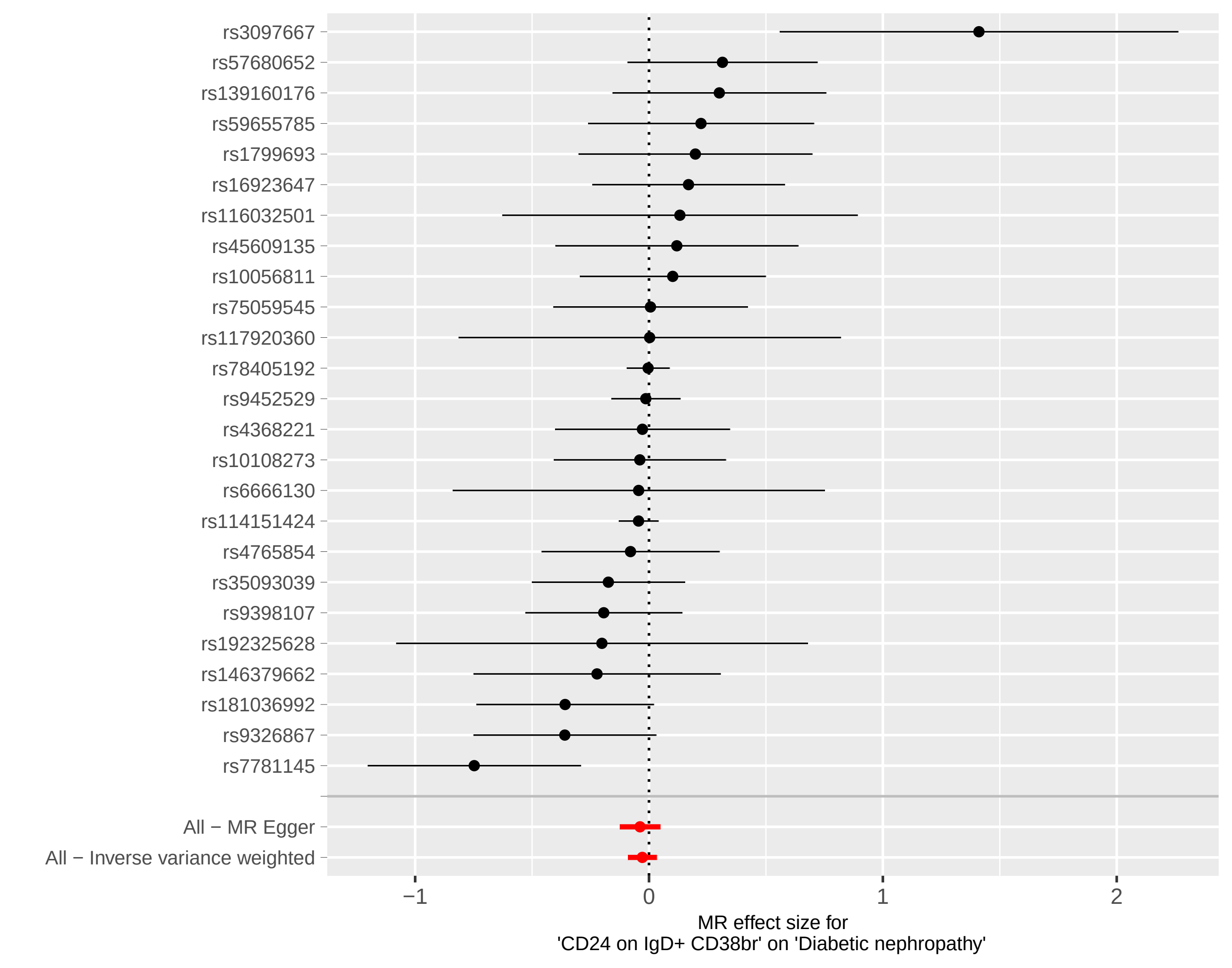
rs3097667
rs57680652
rs139160176
rs59655785
rs1799693
rs16923647
rs116032501
rs45609135
rs10056811
rs75059545
rs117920360
rs78405192
rs9452529
rs4368221
rs10108273
rs6666130
rs114151424
rs4765854
rs35093039
rs9398107
rs192325628
rs146379662
rs181036992
rs9326867
rs7781145
All − MR Egger
All − Inverse variance weighted
−1
0
1
2
MR effect size for
'CD24 on IgD+ CD38br' on 'Diabetic nephropathy'

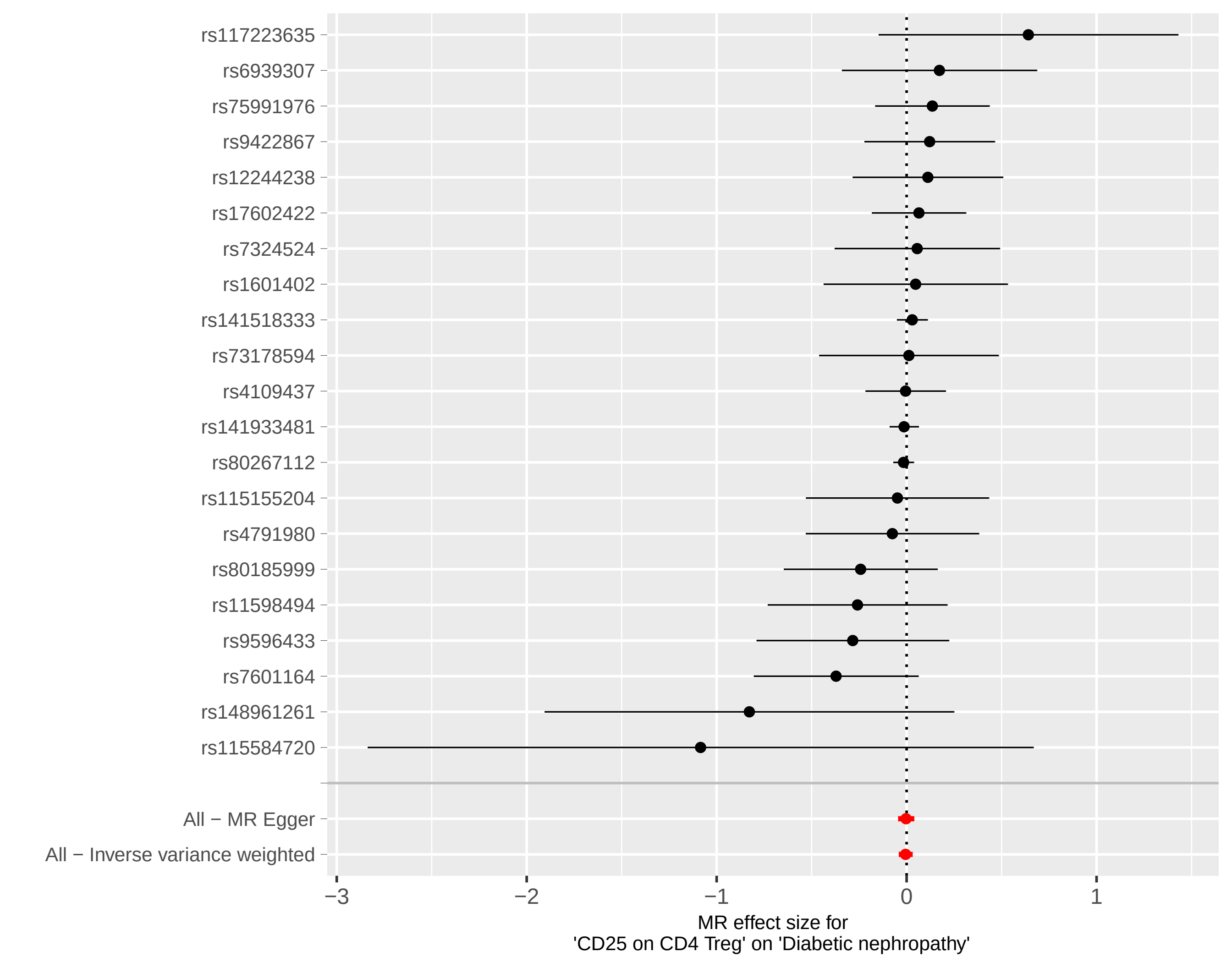

rs117223635
rs6939307
rs75991976
rs9422867
rs12244238
rs17602422
rs7324524
rs1601402
rs141518333
rs73178594
rs4109437
rs141933481
rs80267112
rs115155204
rs4791980
rs80185999
rs11598494
rs9596433
rs7601164
rs148961261
rs115584720
All − MR Egger
All − Inverse variance weighted
−3
−2
−1
0
1
MR effect size for
'CD25 on CD4 Treg' on 'Diabetic nephropathy'

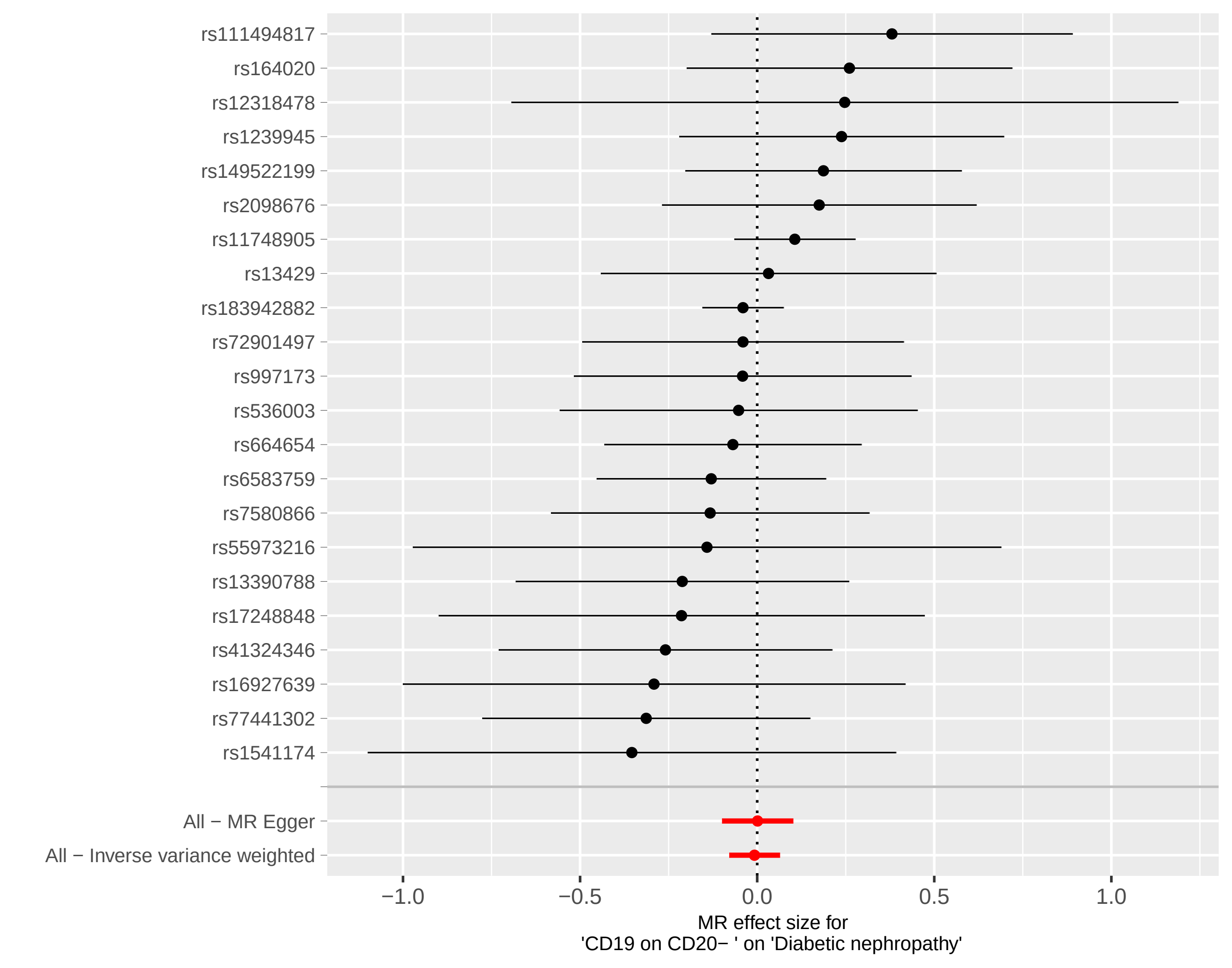
rs111494817
rs164020
rs12318478
rs1239945
rs149522199
rs2098676
rs11748905
rs13429
rs183942882
rs72901497
rs997173
rs536003
rs664654
rs6583759
rs7580866
rs55973216
rs13390788
rs17248848
rs41324346
rs16927639
rs77441302
rs1541174
All − MR Egger
All − Inverse variance weighted
−1.0
−0.5
0.0
0.5
1.0
MR effect size for
'CD19 on CD20− ' on 'Diabetic nephropathy'

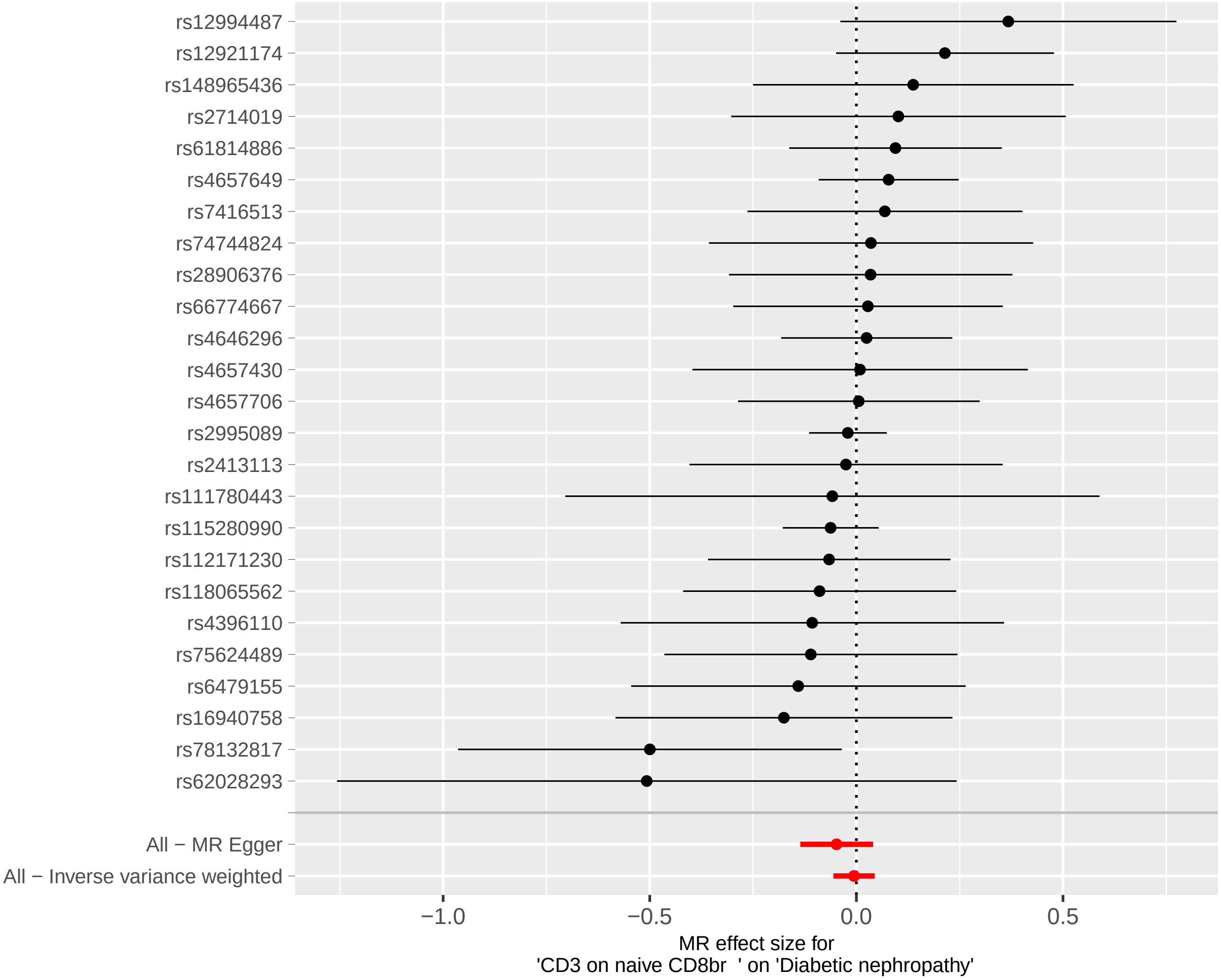
rs12994487
rs12921174
rs148965436
rs2714019
rs61814886
rs4657649
rs7416513
rs74744824
rs28906376
rs66774667
rs4646296
rs4657430
rs4657706
rs2995089
rs2413113
rs111780443
rs115280990
rs112171230
rs118065562
rs4396110
rs75624489
rs6479155
rs16940758
rs78132817
rs62028293
All − MR Egger
All − Inverse variance weighted
−1.0
−0.5
0.0
0.5
MR effect size for
'CD3 on naive CD8br ' on 'Diabetic nephropathy'

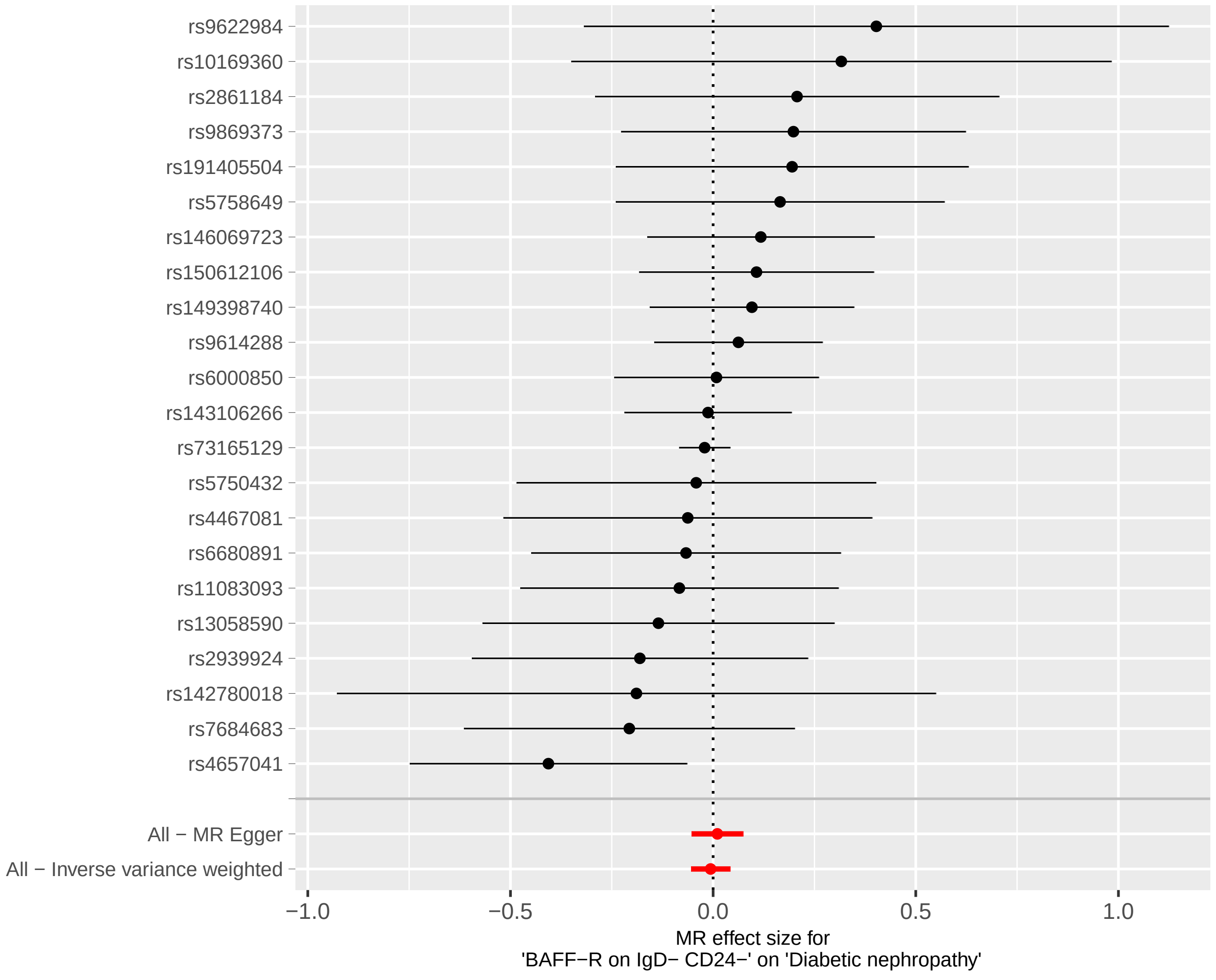

rs9622984
rs10169360
rs2861184
rs9869373
rs191405504
rs5758649
rs146069723
rs150612106
rs149398740
rs9614288
rs6000850
rs143106266
rs73165129
rs5750432
rs4467081
rs6680891
rs11083093
rs13058590
rs2939924
rs142780018
rs7684683
rs4657041
All − MR Egger
All − Inverse variance weighted
−1.0
−0.5
0.0
0.5
1.0
MR effect size for
'BAFF−R on IgD− CD24−' on 'Diabetic nephropathy'

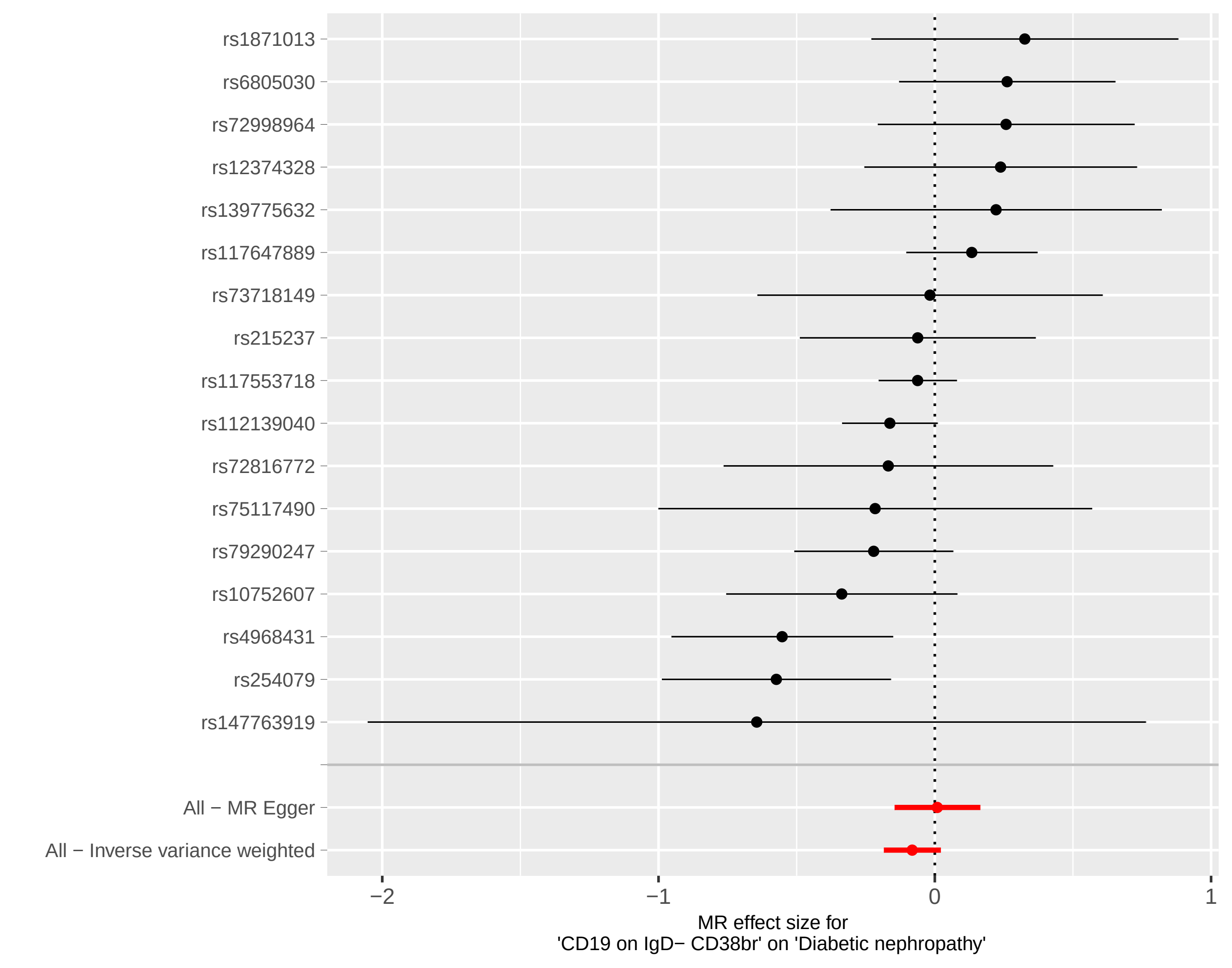
rs1871013
rs6805030
rs72998964
rs12374328
rs139775632
rs117647889
rs73718149
rs215237
rs117553718
rs112139040
rs72816772
rs75117490
rs79290247
rs10752607
rs4968431
rs254079
rs147763919
All − MR Egger
All − Inverse variance weighted
−2
−1
0
1
MR effect size for
'CD19 on IgD− CD38br' on 'Diabetic nephropathy'

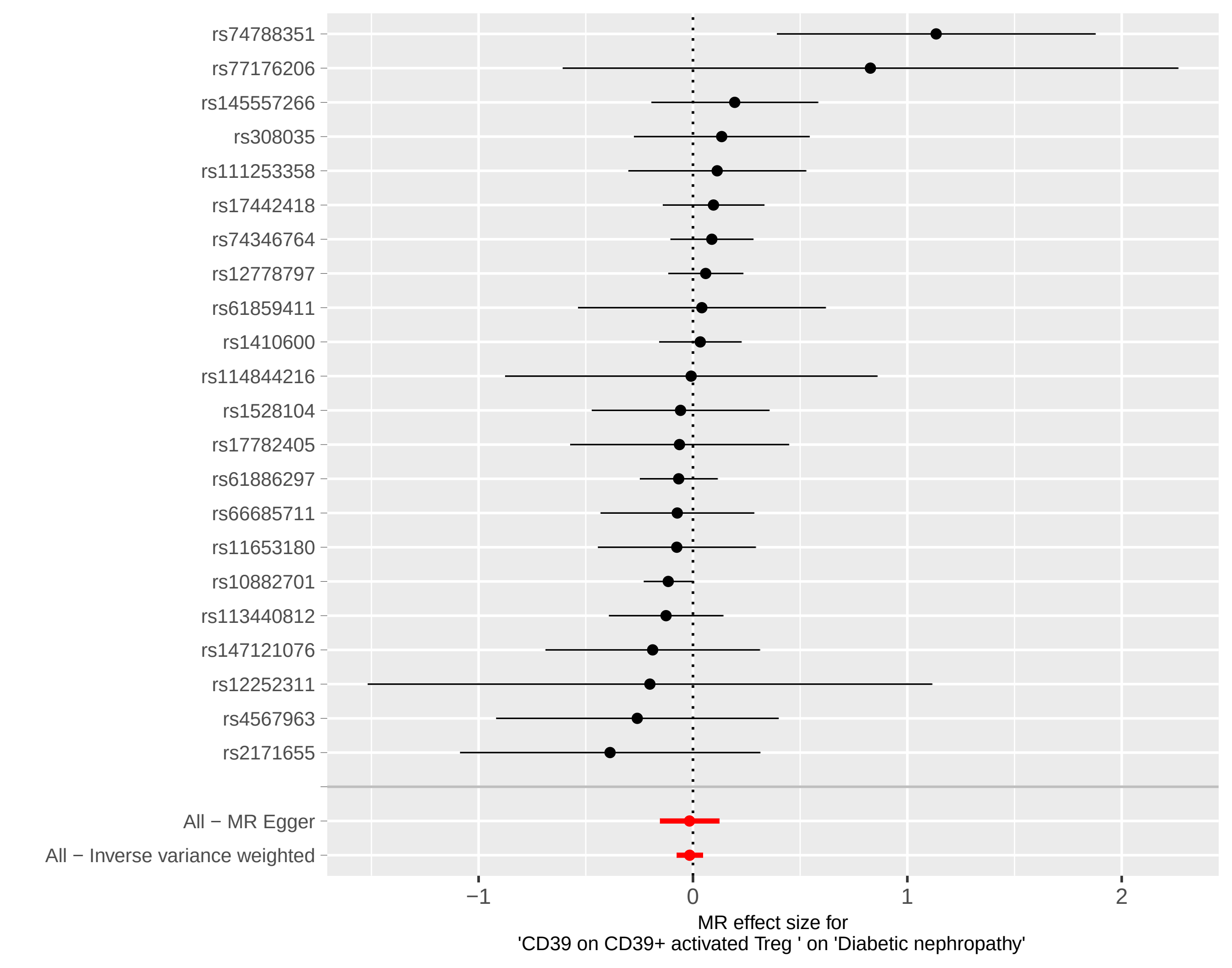
rs74788351
rs77176206
rs145557266
rs308035
rs111253358
rs17442418
rs74346764
rs12778797
rs61859411
rs1410600
rs114844216
rs1528104
rs17782405
rs61886297
rs66685711
rs11653180
rs10882701
rs113440812
rs147121076
rs12252311
rs4567963
rs2171655
All − MR Egger
All − Inverse variance weighted
−1
0
1
2
MR effect size for
'CD39 on CD39+ activated Treg ' on 'Diabetic nephropathy'

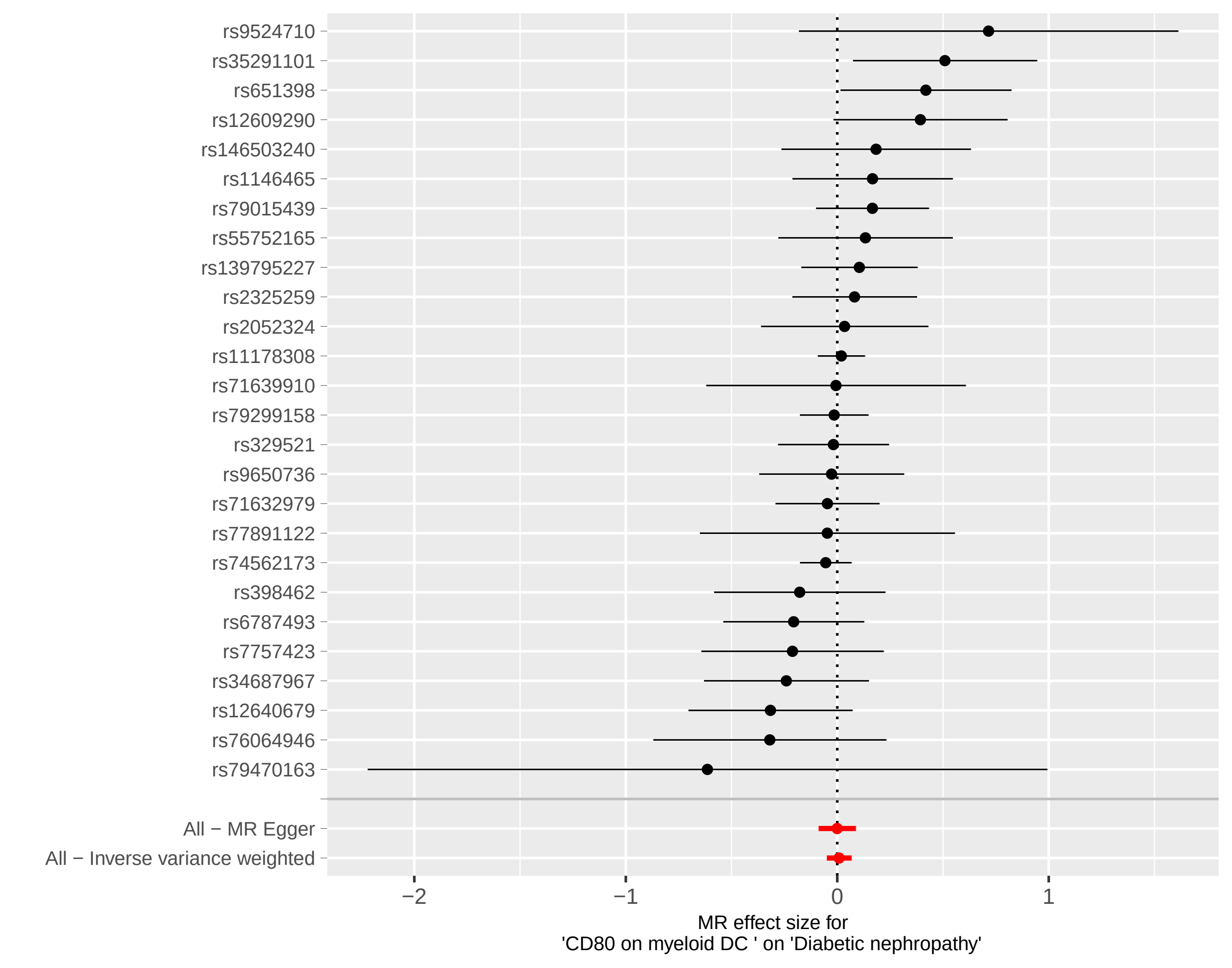
rs9524710
rs35291101
rs651398
rs12609290
rs146503240
rs1146465
rs79015439
rs55752165
rs139795227
rs2325259
rs2052324
rs11178308
rs71639910
rs79299158
rs329521
rs9650736
rs71632979
rs77891122
rs74562173
rs398462
rs6787493
rs7757423
rs34687967
rs12640679
rs76064946
rs79470163
All − MR Egger
All − Inverse variance weighted
−2
−1
0
1
MR effect size for
'CD80 on myeloid DC ' on 'Diabetic nephropathy'

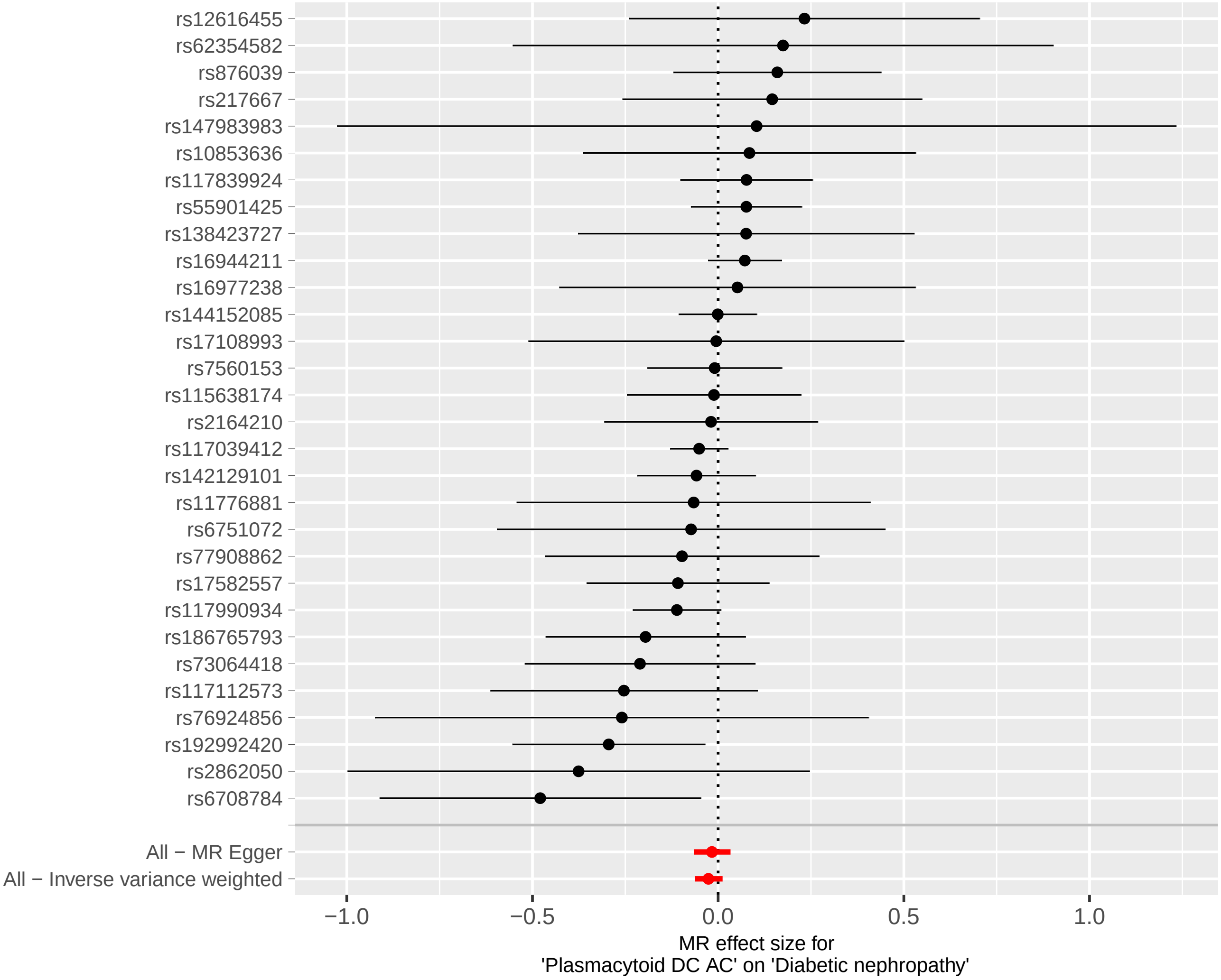

rs12616455
rs62354582
rs876039
rs217667
rs147983983
rs10853636
rs117839924
rs55901425
rs138423727
rs16944211
rs16977238
rs144152085
rs17108993
rs7560153
rs115638174
rs2164210
rs117039412
rs142129101
rs11776881
rs6751072
rs77908862
rs17582557
rs117990934
rs186765793
rs73064418
rs117112573
rs76924856
rs192992420
rs2862050
rs6708784
All − MR Egger
All − Inverse variance weighted
−1.0
−0.5
0.0
0.5
1.0
MR effect size for
'Plasmacytoid DC AC' on 'Diabetic nephropathy'

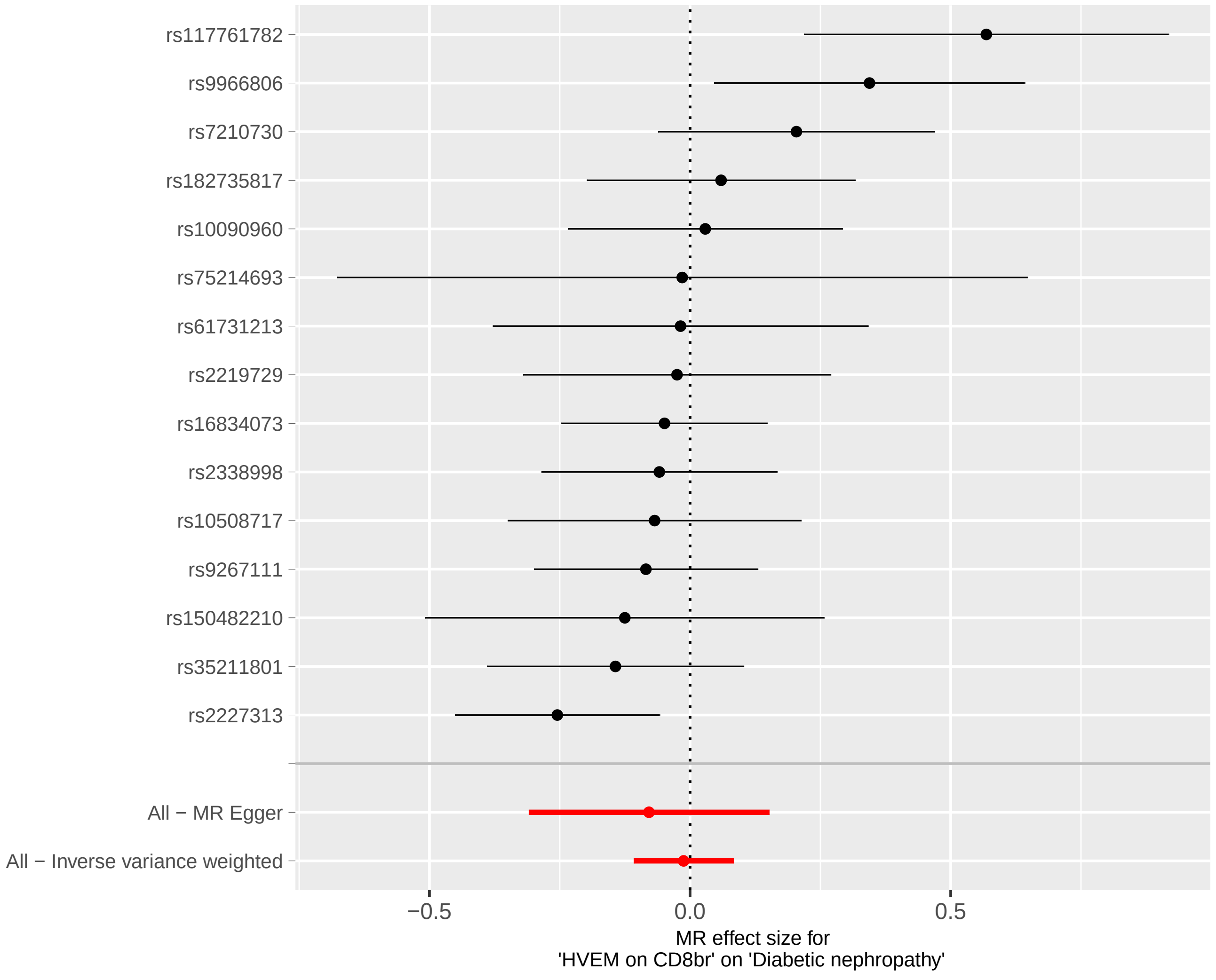
rs117761782
rs9966806
rs7210730
rs182735817
rs10090960
rs75214693
rs61731213
rs2219729
rs16834073
rs2338998
rs10508717
rs9267111
rs150482210
rs35211801
rs2227313
All − MR Egger
All − Inverse variance weighted
−0.5
0.0
0.5
MR effect size for
'HVEM on CD8br' on 'Diabetic nephropathy'

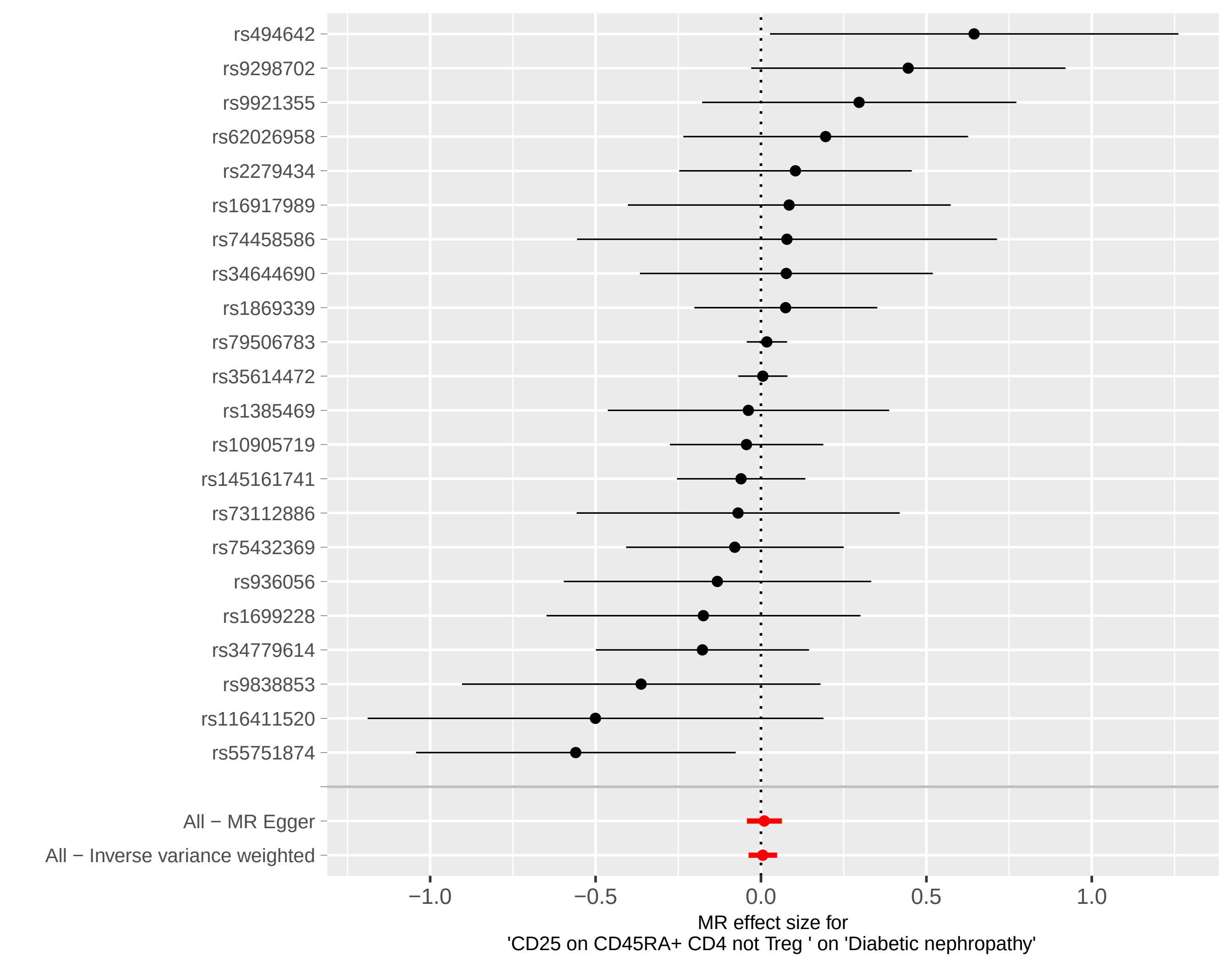
rs494642
rs9298702
rs9921355
rs62026958
rs2279434
rs16917989
rs74458586
rs34644690
rs1869339
rs79506783
rs35614472
rs1385469
rs10905719
rs145161741
rs73112886
rs75432369
rs936056
rs1699228
rs34779614
rs9838853
rs116411520
rs55751874
All − MR Egger
All − Inverse variance weighted
−1.0
−0.5
0.0
0.5
1.0
MR effect size for
'CD25 on CD45RA+ CD4 not Treg ' on 'Diabetic nephropathy'

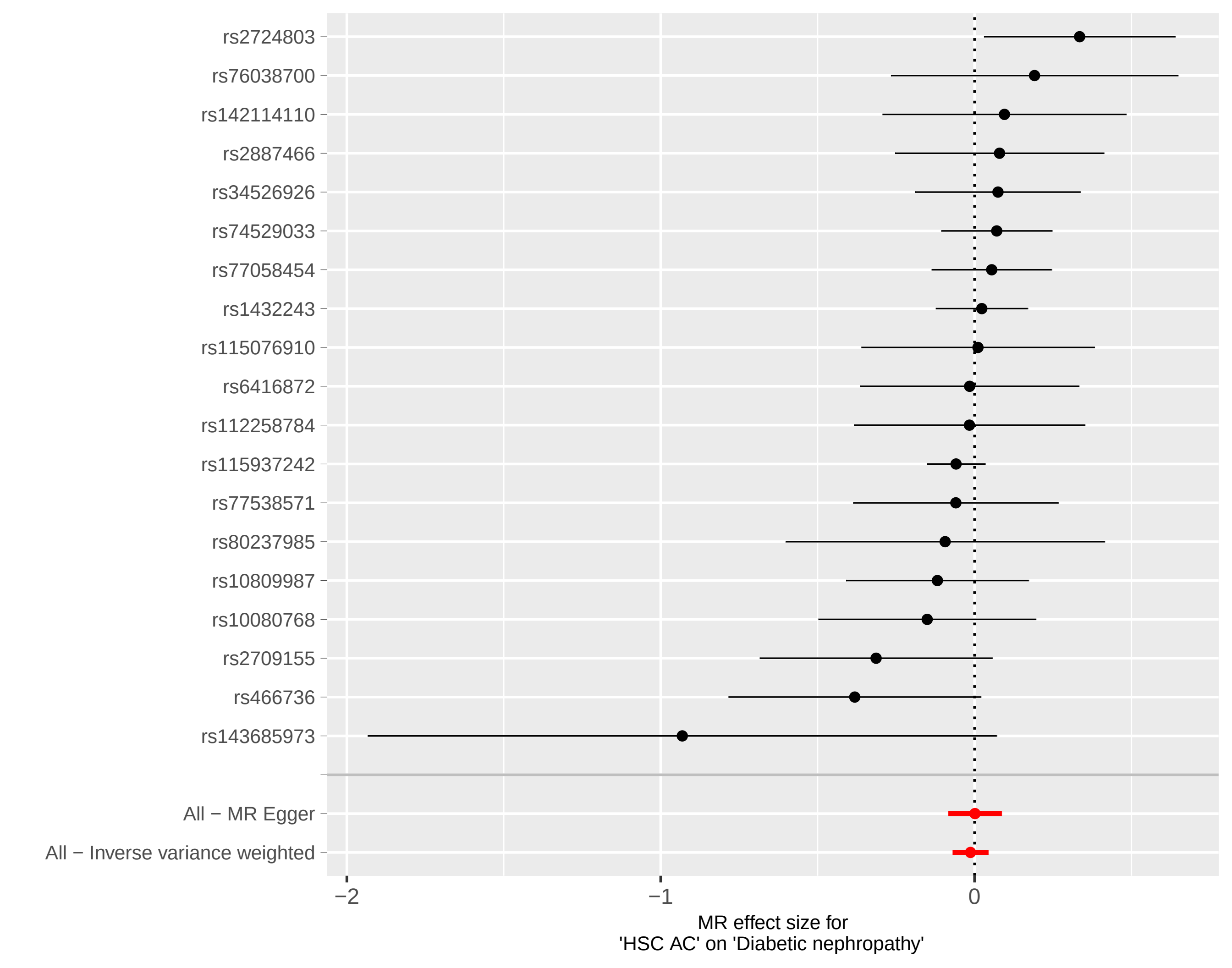
rs2724803
rs76038700
rs142114110
rs2887466
rs34526926
rs74529033
rs77058454
rs1432243
rs115076910
rs6416872
rs112258784
rs115937242
rs77538571
rs80237985
rs10809987
rs10080768
rs2709155
rs466736
rs143685973
All − MR Egger
All − Inverse variance weighted
−2
−1
0
MR effect size for
'HSC AC' on 'Diabetic nephropathy'

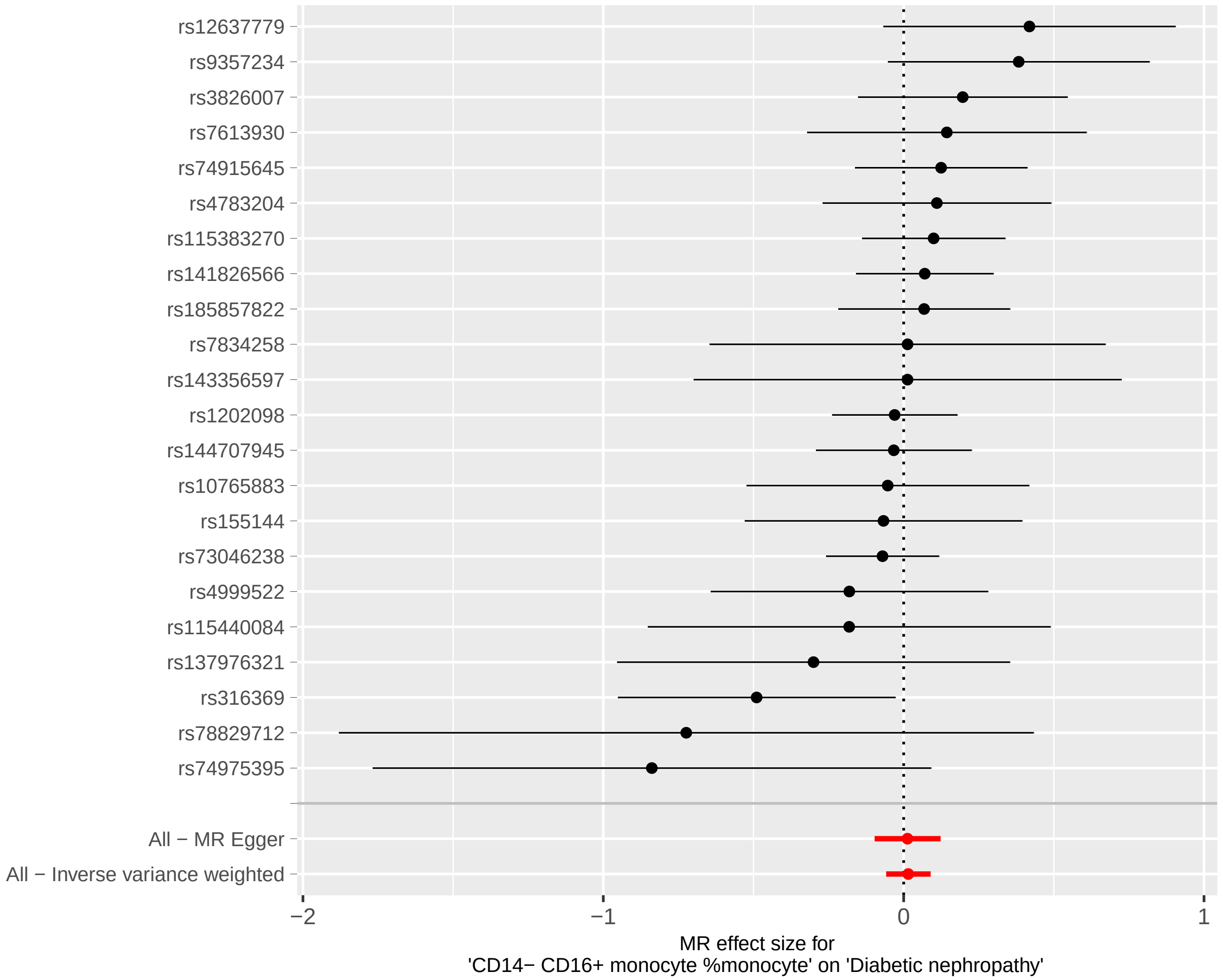
rs12637779
rs9357234
rs3826007
rs7613930
rs74915645
rs4783204
rs115383270
rs141826566
rs185857822
rs7834258
rs143356597
rs1202098
rs144707945
rs10765883
rs155144
rs73046238
rs4999522
rs115440084
rs137976321
rs316369
rs78829712
rs74975395
All − MR Egger
All − Inverse variance weighted
−2
−1
0
1
MR effect size for
'CD14− CD16+ monocyte %monocyte' on 'Diabetic nephropathy'

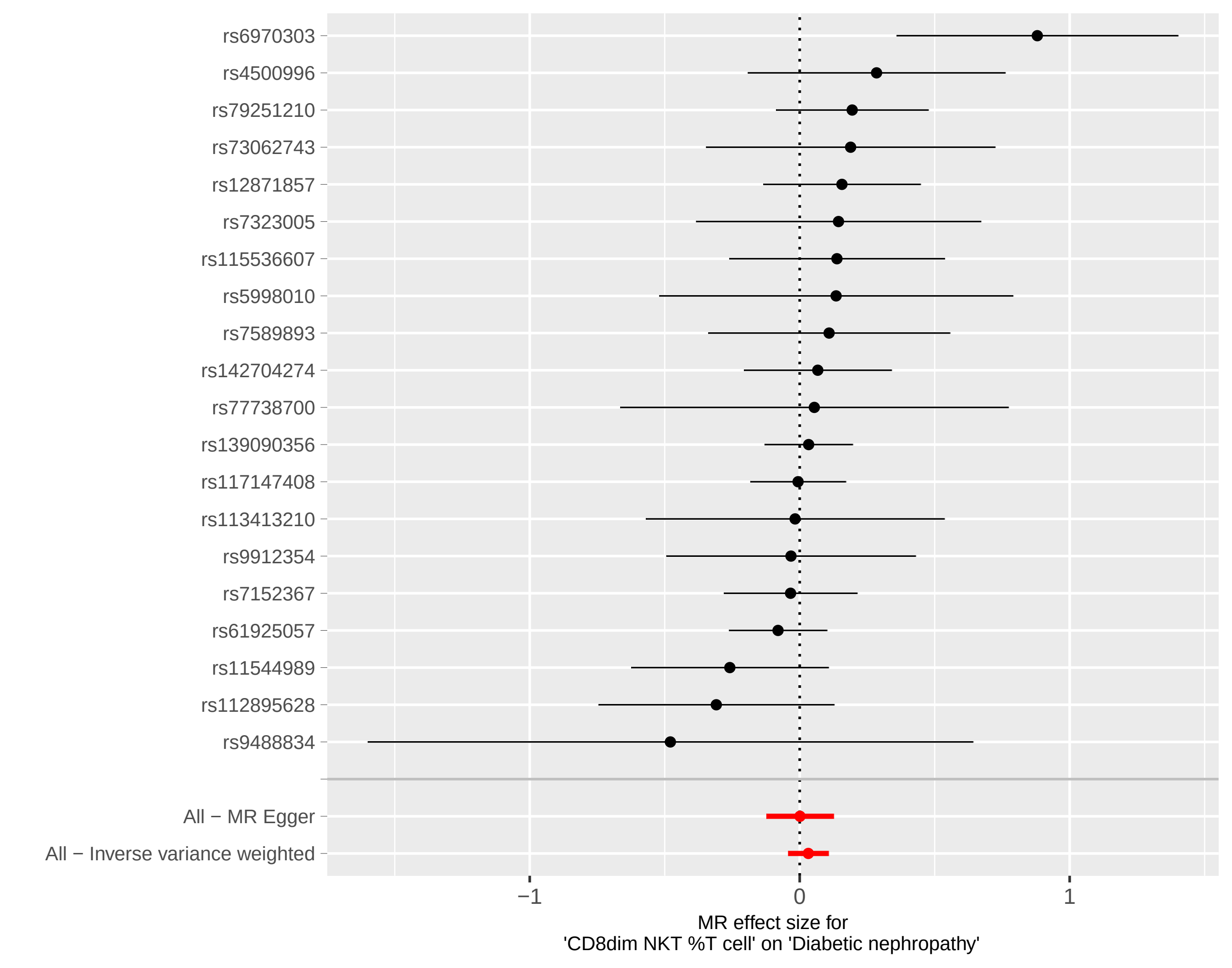
rs6970303
rs4500996
rs79251210
rs73062743
rs12871857
rs7323005
rs115536607
rs5998010
rs7589893
rs142704274
rs77738700
rs139090356
rs117147408
rs113413210
rs9912354
rs7152367
rs61925057
rs11544989
rs112895628
rs9488834
All − MR Egger
All − Inverse variance weighted
−1
0
1
MR effect size for
'CD8dim NKT %T cell' on 'Diabetic nephropathy'

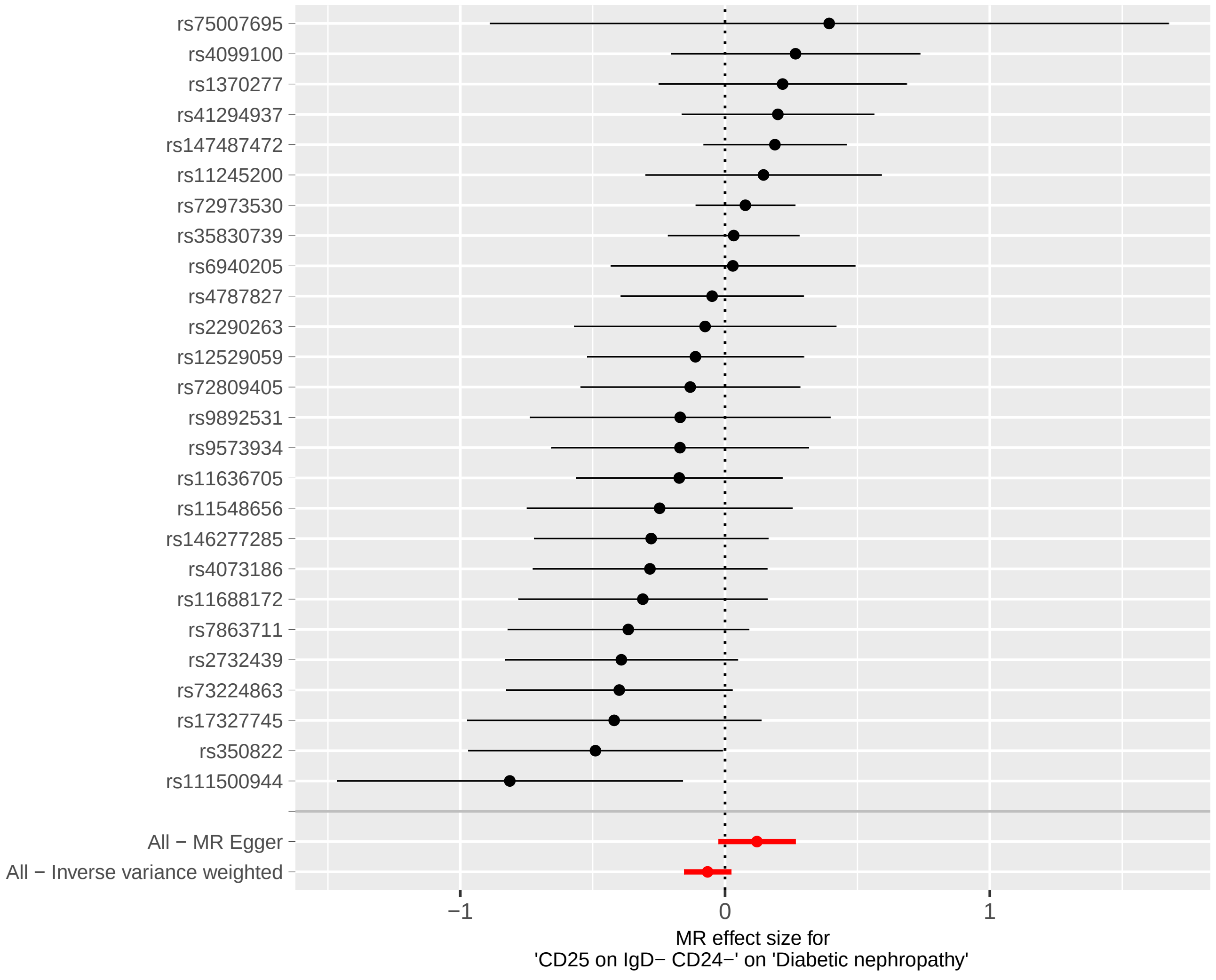

rs75007695
rs4099100
rs1370277
rs41294937
rs147487472
rs11245200
rs72973530
rs35830739
rs6940205
rs4787827
rs2290263
rs12529059
rs72809405
rs9892531
rs9573934
rs11636705
rs11548656
rs146277285
rs4073186
rs11688172
rs7863711
rs2732439
rs73224863
rs17327745
rs350822
rs111500944
All − MR Egger
All − Inverse variance weighted
−1
0
1
MR effect size for
'CD25 on IgD− CD24−' on 'Diabetic nephropathy'

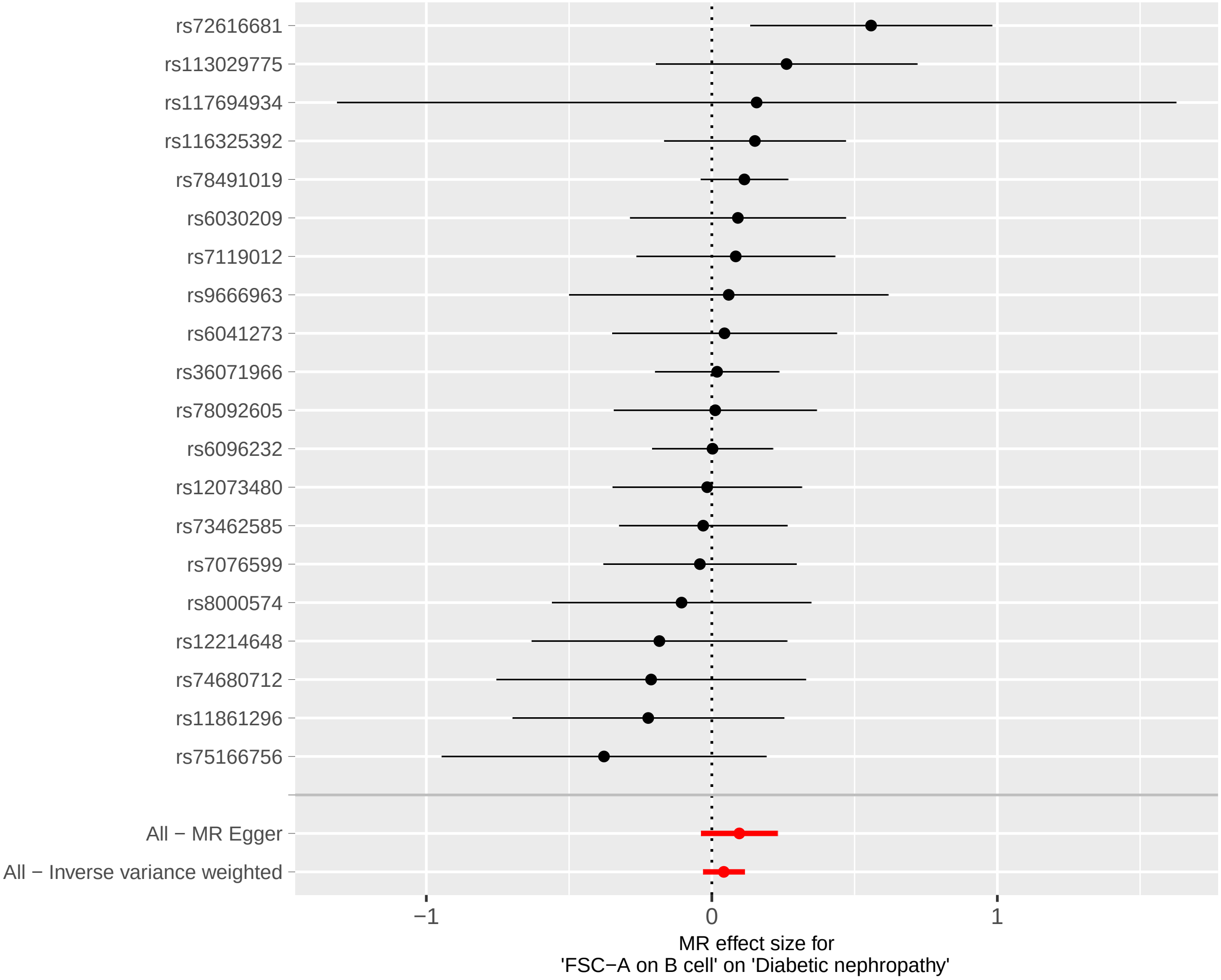
rs72616681
rs113029775
rs117694934
rs116325392
rs78491019
rs6030209
rs7119012
rs9666963
rs6041273
rs36071966
rs78092605
rs6096232
rs12073480
rs73462585
rs7076599
rs8000574
rs12214648
rs74680712
rs11861296
rs75166756
All − MR Egger
All − Inverse variance weighted
−1
0
1
MR effect size for
'FSC−A on B cell' on 'Diabetic nephropathy'

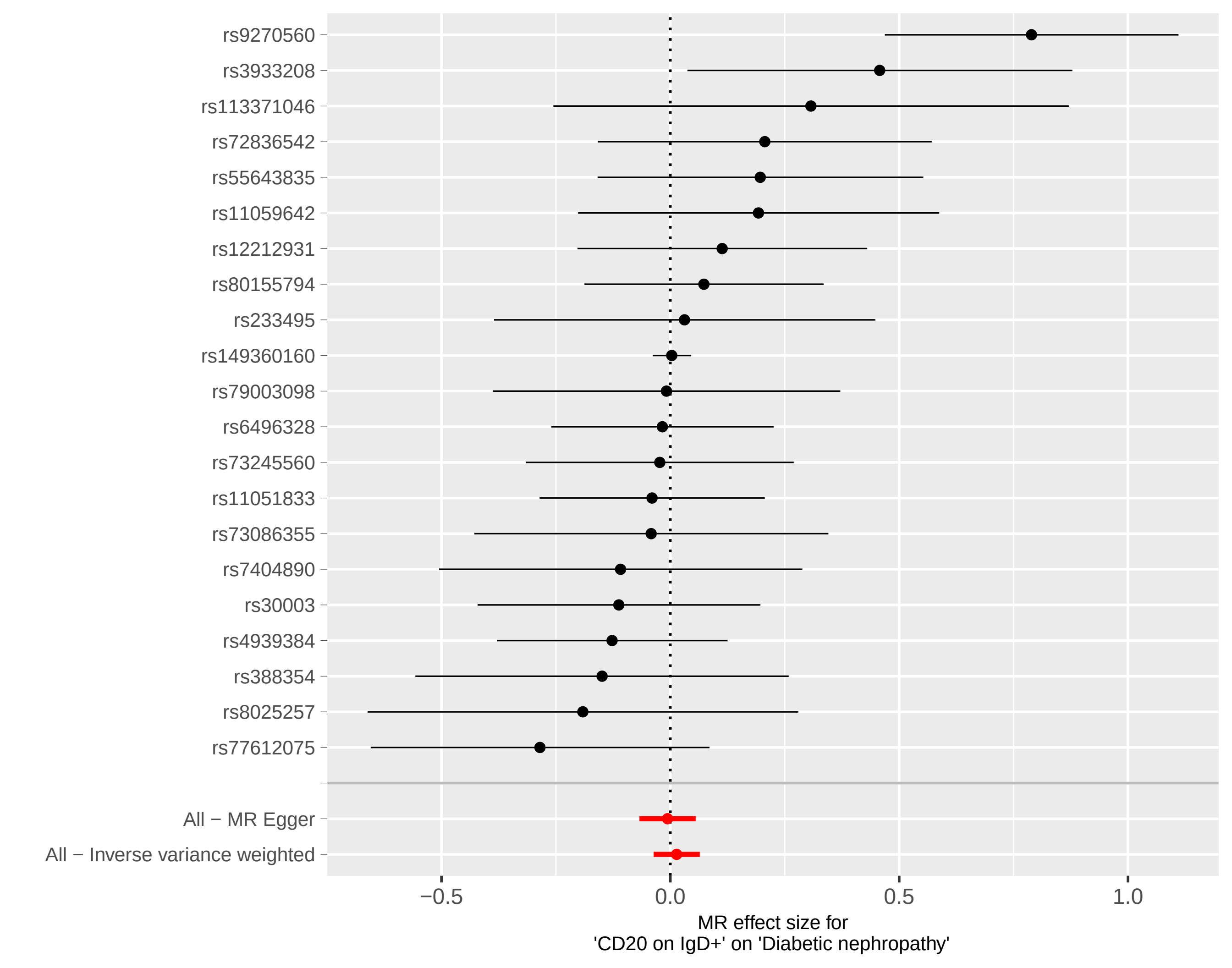

rs9270560
rs3933208
rs113371046
rs72836542
rs55643835
rs11059642
rs12212931
rs80155794
rs233495
rs149360160
rs79003098
rs6496328
rs73245560
rs11051833
rs73086355
rs7404890
rs30003
rs4939384
rs388354
rs8025257
rs77612075
All − MR Egger
All − Inverse variance weighted
−0.5
0.0
0.5
1.0
MR effect size for
'CD20 on IgD+' on 'Diabetic nephropathy'

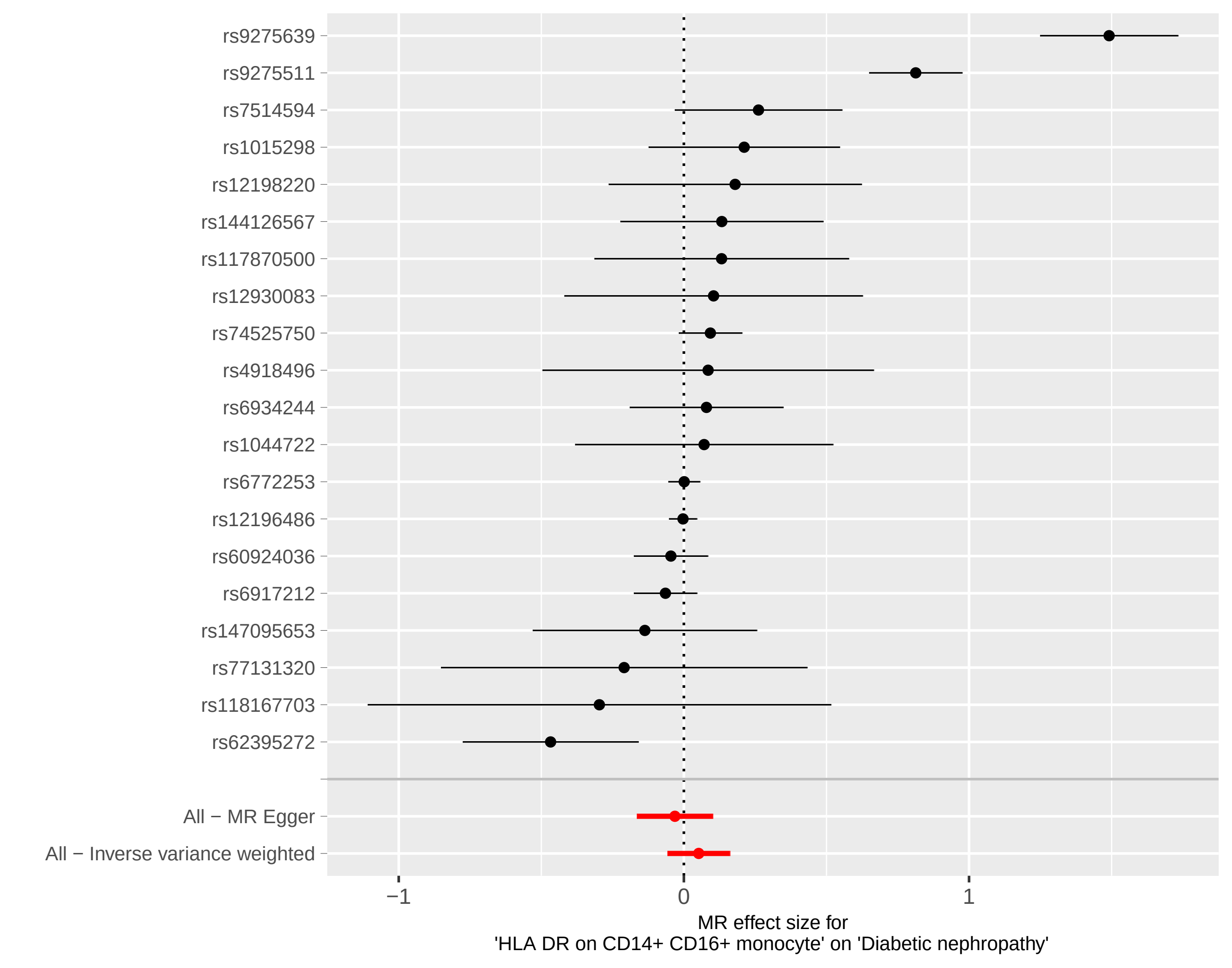
rs9275639
rs9275511
rs7514594
rs1015298
rs12198220
rs144126567
rs117870500
rs12930083
rs74525750
rs4918496
rs6934244
rs1044722
rs6772253
rs12196486
rs60924036
rs6917212
rs147095653
rs77131320
rs118167703
rs62395272
All − MR Egger
All − Inverse variance weighted
−1
0
1
MR effect size for
'HLA DR on CD14+ CD16+ monocyte' on 'Diabetic nephropathy'

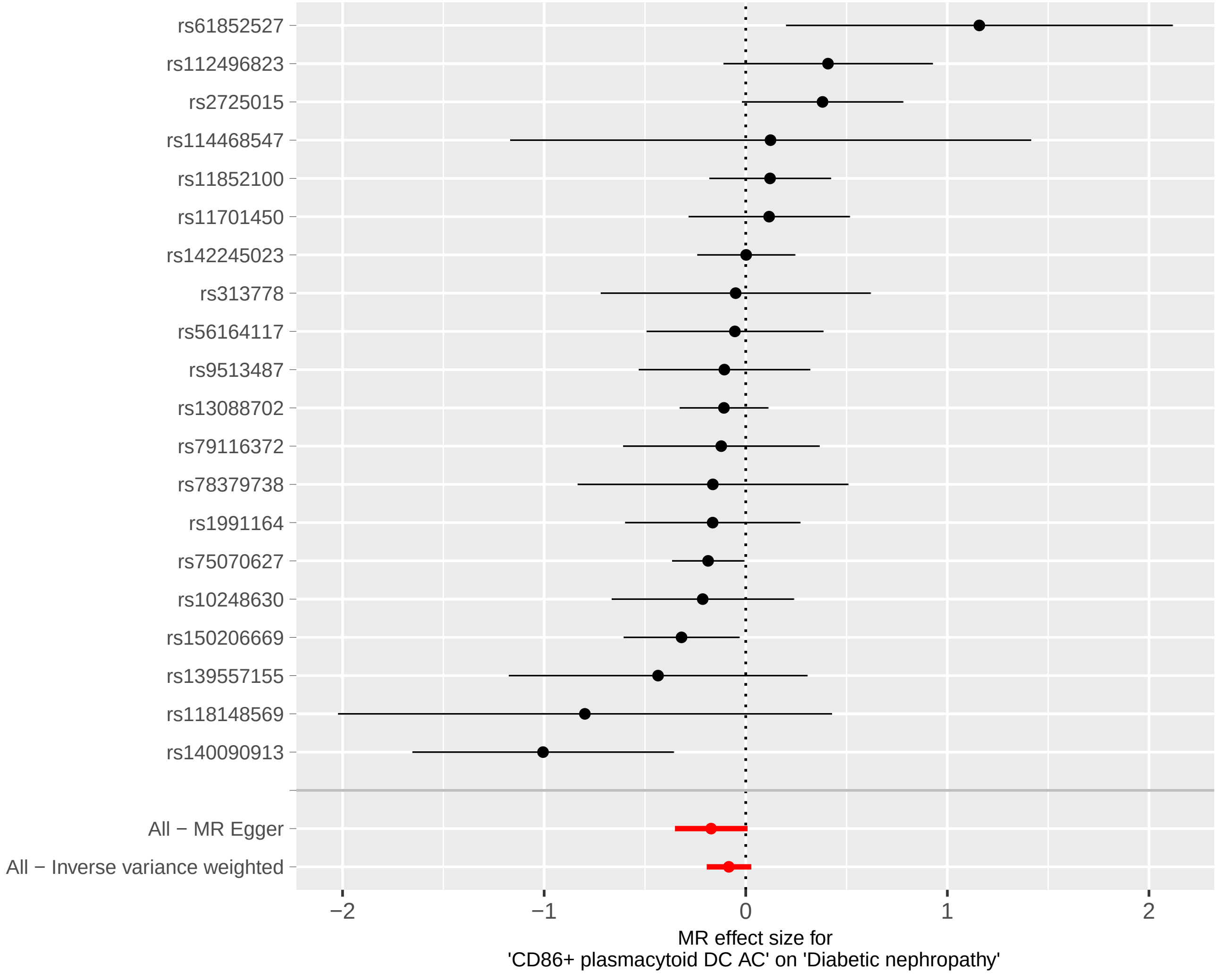
rs61852527
rs112496823
rs2725015
rs114468547
rs11852100
rs11701450
rs142245023
rs313778
rs56164117
rs9513487
rs13088702
rs79116372
rs78379738
rs1991164
rs75070627
rs10248630
rs150206669
rs139557155
rs118148569
rs140090913
All − MR Egger
All − Inverse variance weighted
−2
−1
0
1
2
MR effect size for
'CD86+ plasmacytoid DC AC' on 'Diabetic nephropathy'

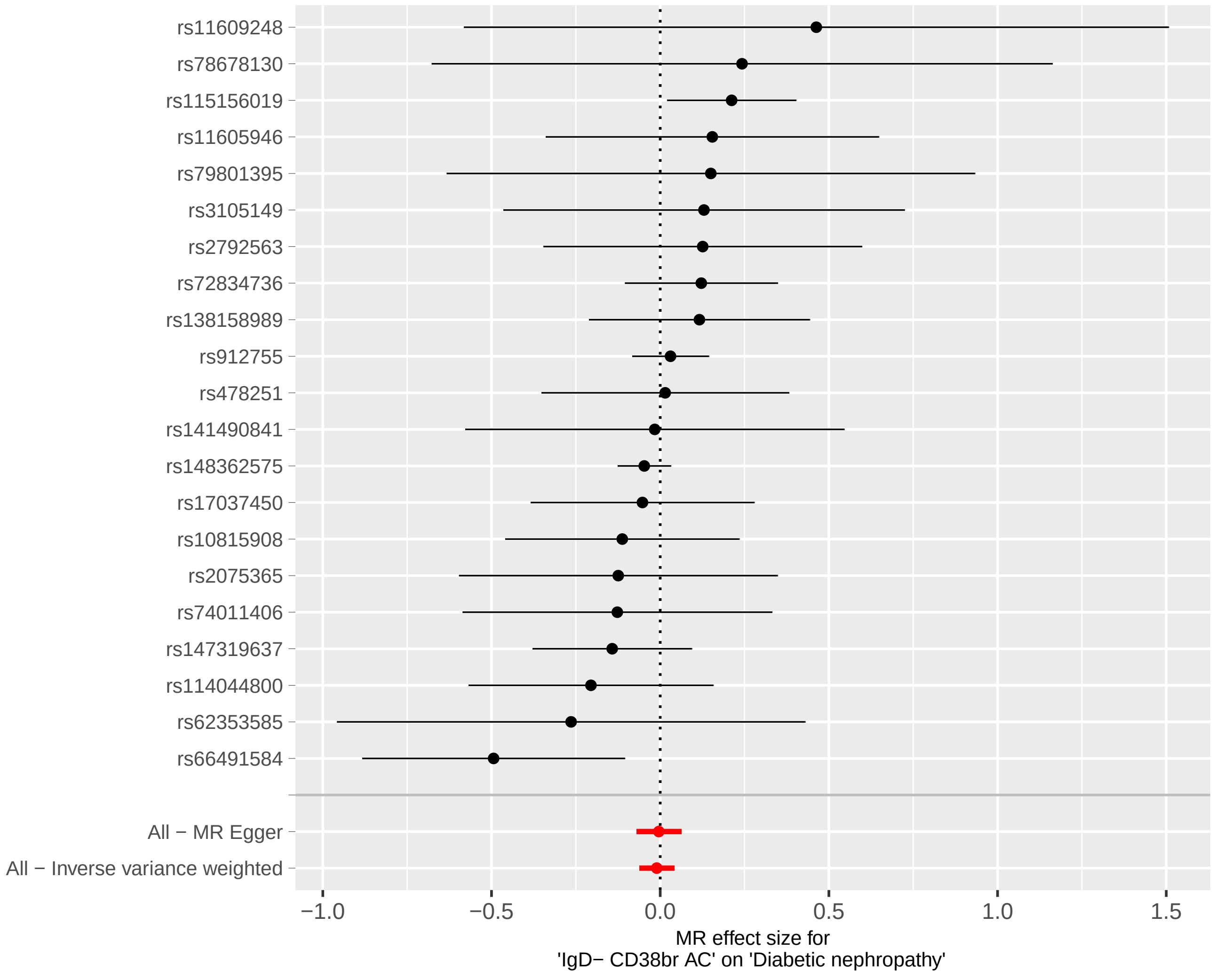

rs11609248
rs78678130
rs115156019
rs11605946
rs79801395
rs3105149
rs2792563
rs72834736
rs138158989
rs912755
rs478251
rs141490841
rs148362575
rs17037450
rs10815908
rs2075365
rs74011406
rs147319637
rs114044800
rs62353585
rs66491584
All − MR Egger
All − Inverse variance weighted
−1.0
−0.5
0.0
0.5
1.0
1.5
MR effect size for
'IgD− CD38br AC' on 'Diabetic nephropathy'

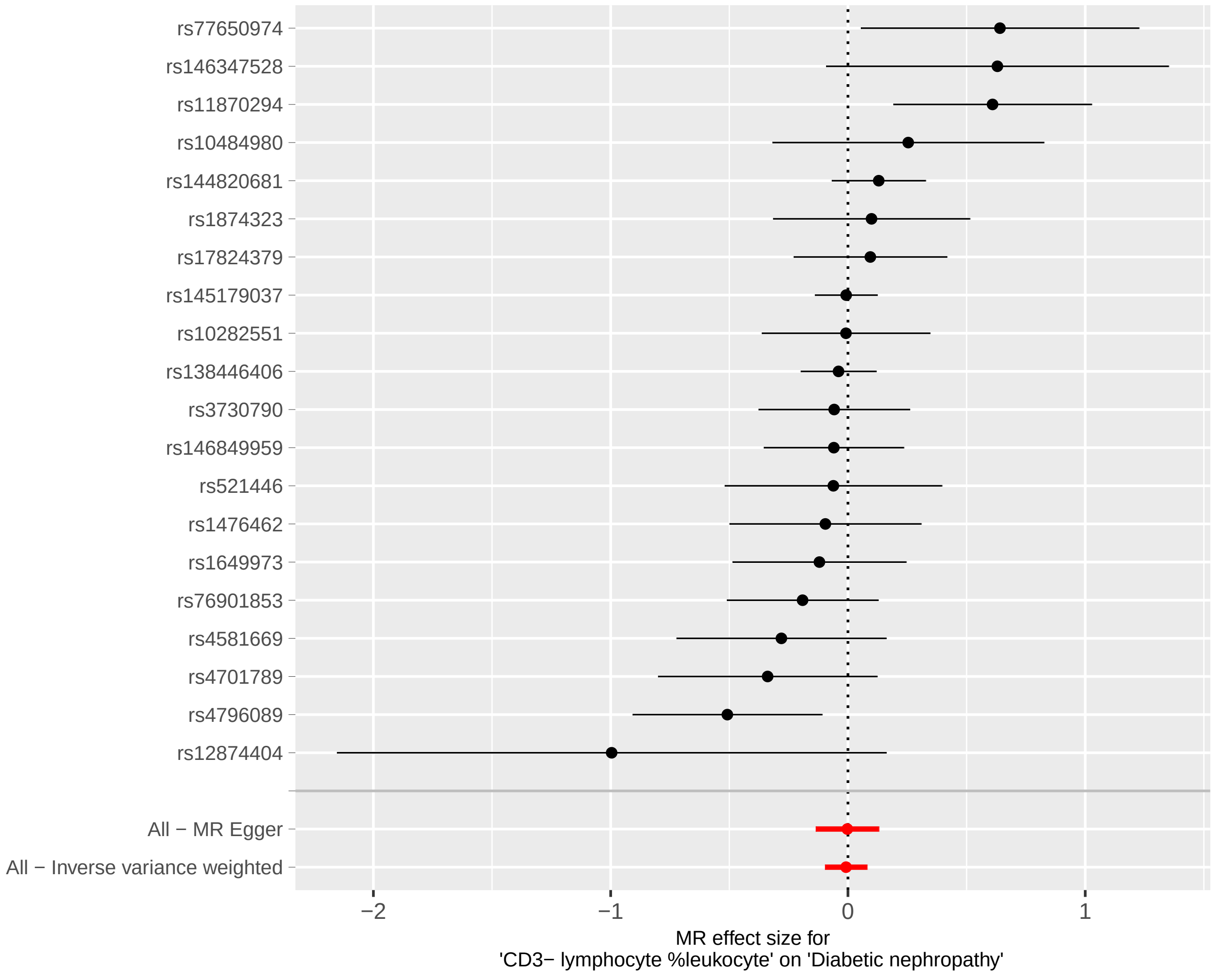

rs77650974
rs146347528
rs11870294
rs10484980
rs144820681
rs1874323
rs17824379
rs145179037
rs10282551
rs138446406
rs3730790
rs146849959
rs521446
rs1476462
rs1649973
rs76901853
rs4581669
rs4701789
rs4796089
rs12874404
All − MR Egger
All − Inverse variance weighted
−2
−1
0
1
MR effect size for
'CD3− lymphocyte %leukocyte' on 'Diabetic nephropathy'

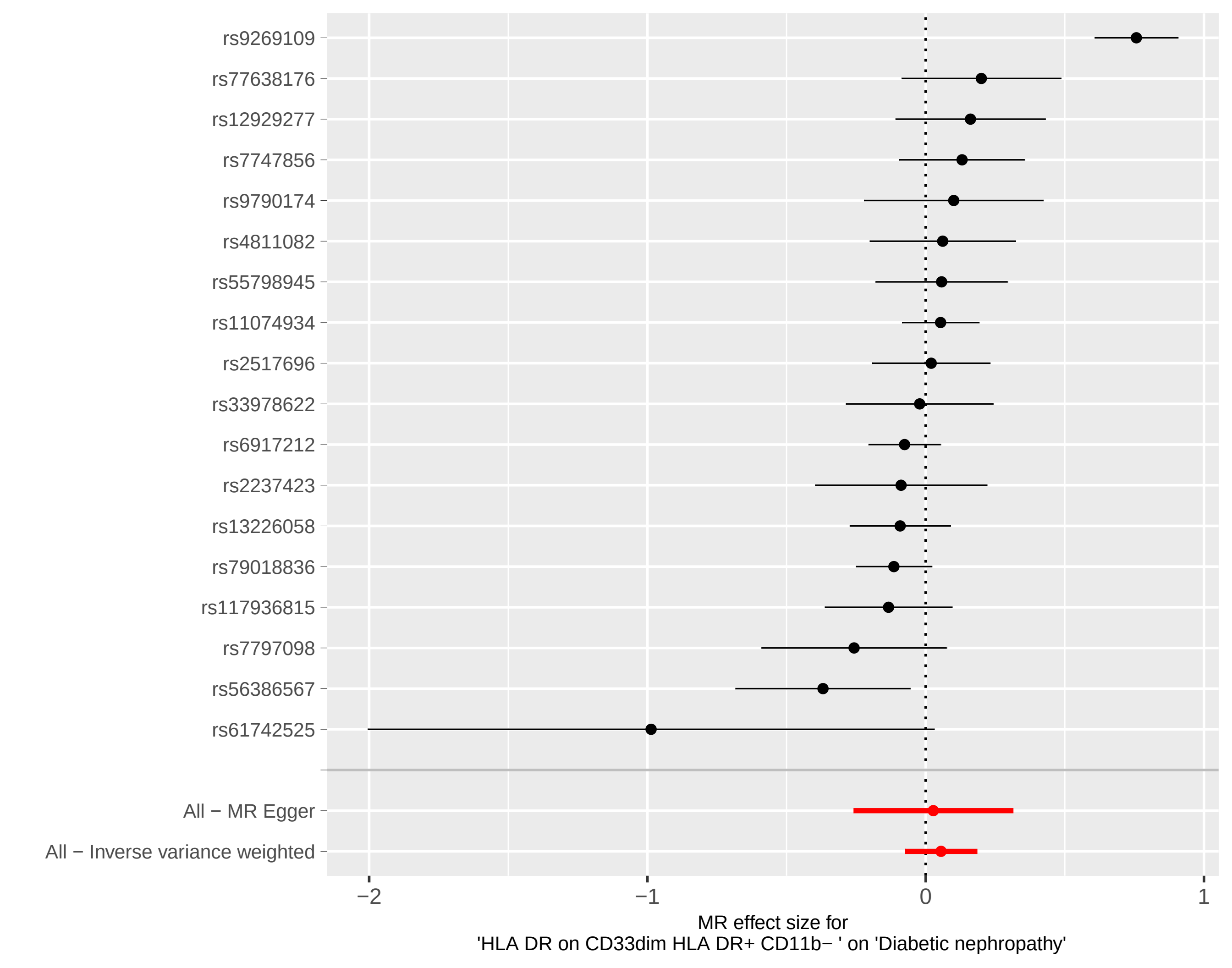
rs9269109
rs77638176
rs12929277
rs7747856
rs9790174
rs4811082
rs55798945
rs11074934
rs2517696
rs33978622
rs6917212
rs2237423
rs13226058
rs79018836
rs117936815
rs7797098
rs56386567
rs61742525
All − MR Egger
All − Inverse variance weighted
−2
−1
0
1
MR effect size for
'HLA DR on CD33dim HLA DR+ CD11b− ' on 'Diabetic nephropathy'

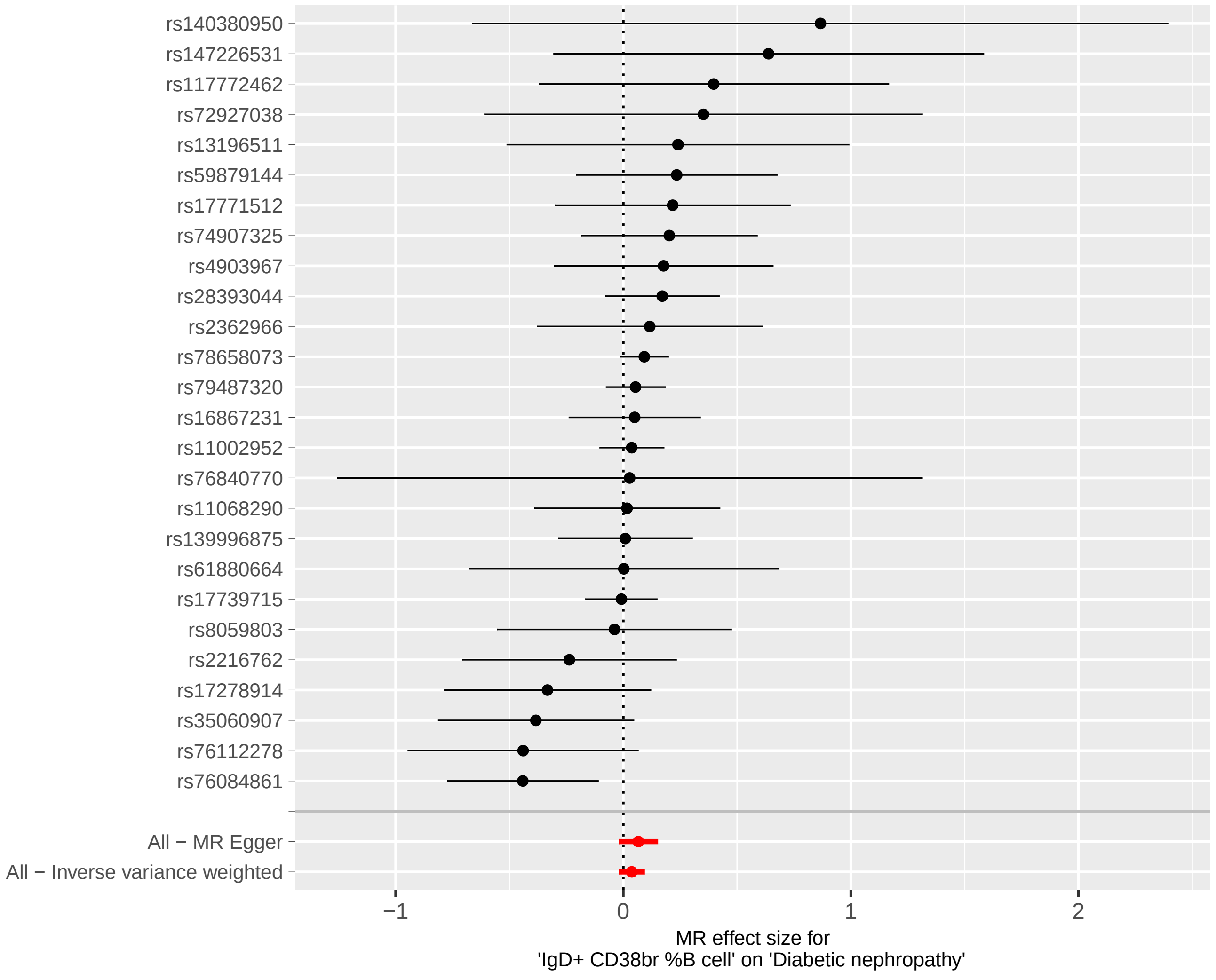
rs140380950
rs147226531
rs117772462
rs72927038
rs13196511
rs59879144
rs17771512
rs74907325
rs4903967
rs28393044
rs2362966
rs78658073
rs79487320
rs16867231
rs11002952
rs76840770
rs11068290
rs139996875
rs61880664
rs17739715
rs8059803
rs2216762
rs17278914
rs35060907
rs76112278
rs76084861
All − MR Egger
All − Inverse variance weighted
−1
0
1
2
MR effect size for
'IgD+ CD38br %B cell' on 'Diabetic nephropathy'

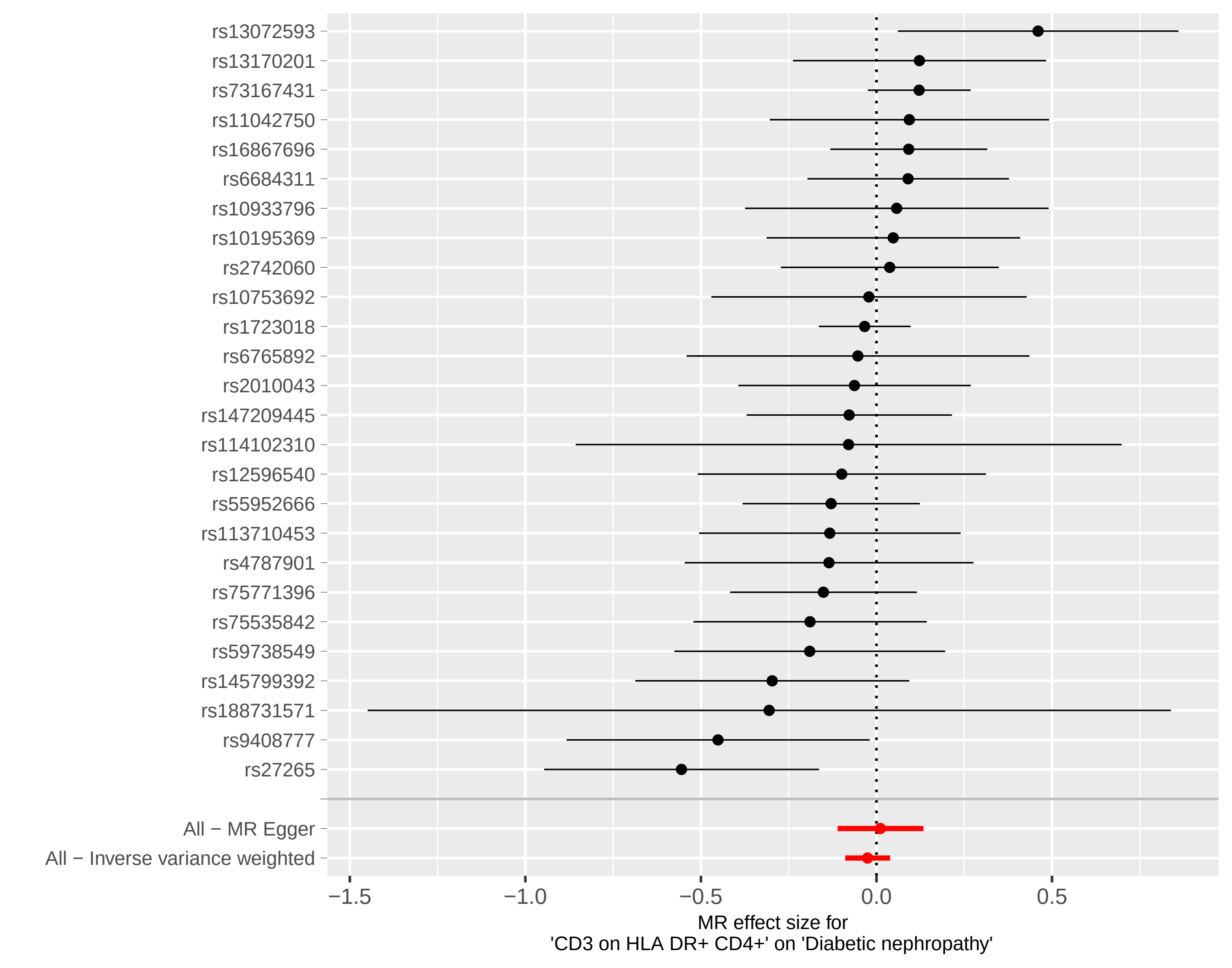

rs13072593
rs13170201
rs73167431
rs11042750
rs16867696
rs6684311
rs10933796
rs10195369
rs2742060
rs10753692
rs1723018
rs6765892
rs2010043
rs147209445
rs114102310
rs12596540
rs55952666
rs113710453
rs4787901
rs75771396
rs75535842
rs59738549
rs145799392
rs188731571
rs9408777
rs27265
All − MR Egger
All − Inverse variance weighted
−1.5
−1.0
−0.5
0.0
0.5
MR effect size for
'CD3 on HLA DR+ CD4+' on 'Diabetic nephropathy'

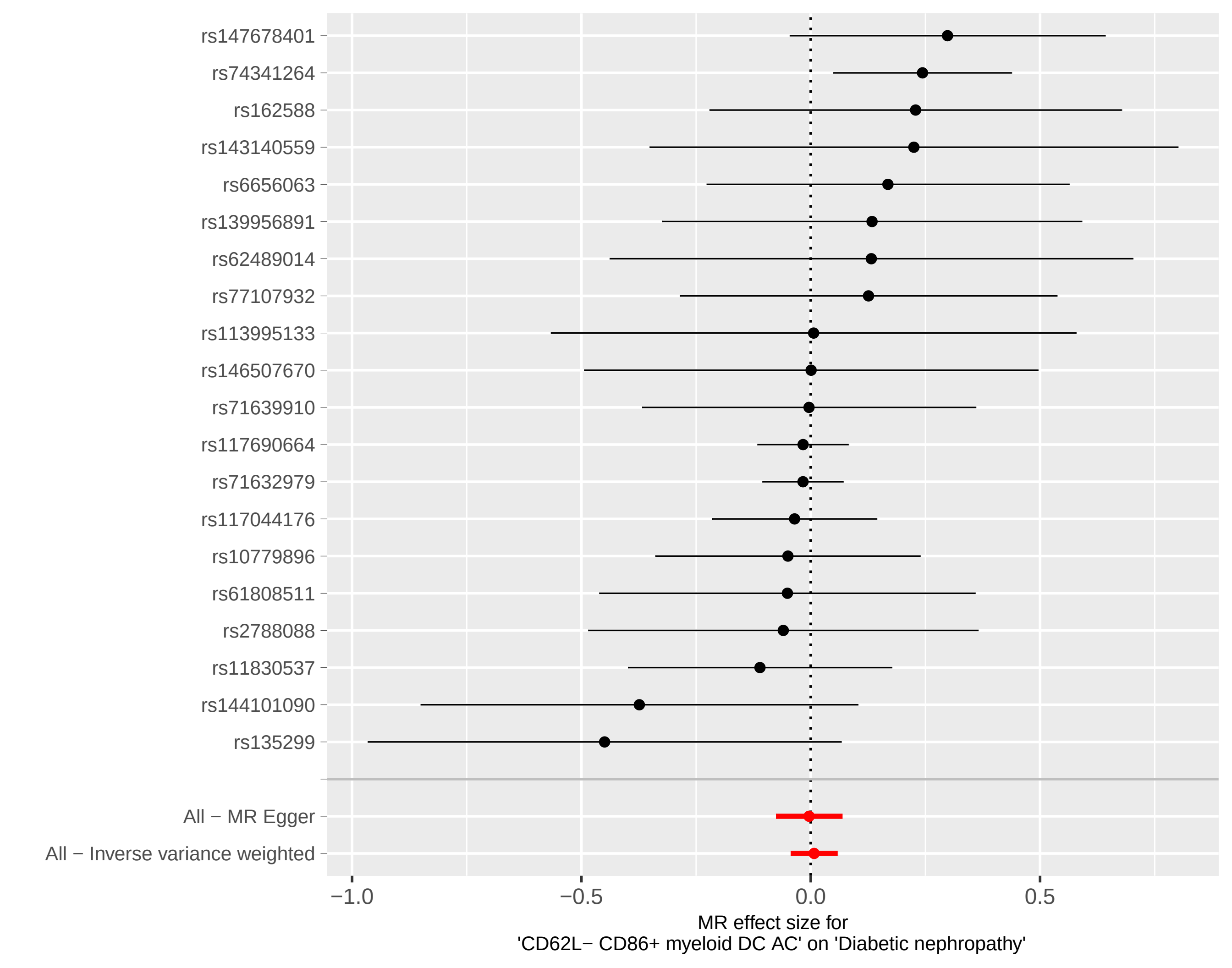
rs147678401
rs74341264
rs162588
rs143140559
rs6656063
rs139956891
rs62489014
rs77107932
rs113995133
rs146507670
rs71639910
rs117690664
rs71632979
rs117044176
rs10779896
rs61808511
rs2788088
rs11830537
rs144101090
rs135299
All − MR Egger
All − Inverse variance weighted
−1.0
−0.5
0.0
0.5
MR effect size for
'CD62L− CD86+ myeloid DC AC' on 'Diabetic nephropathy'

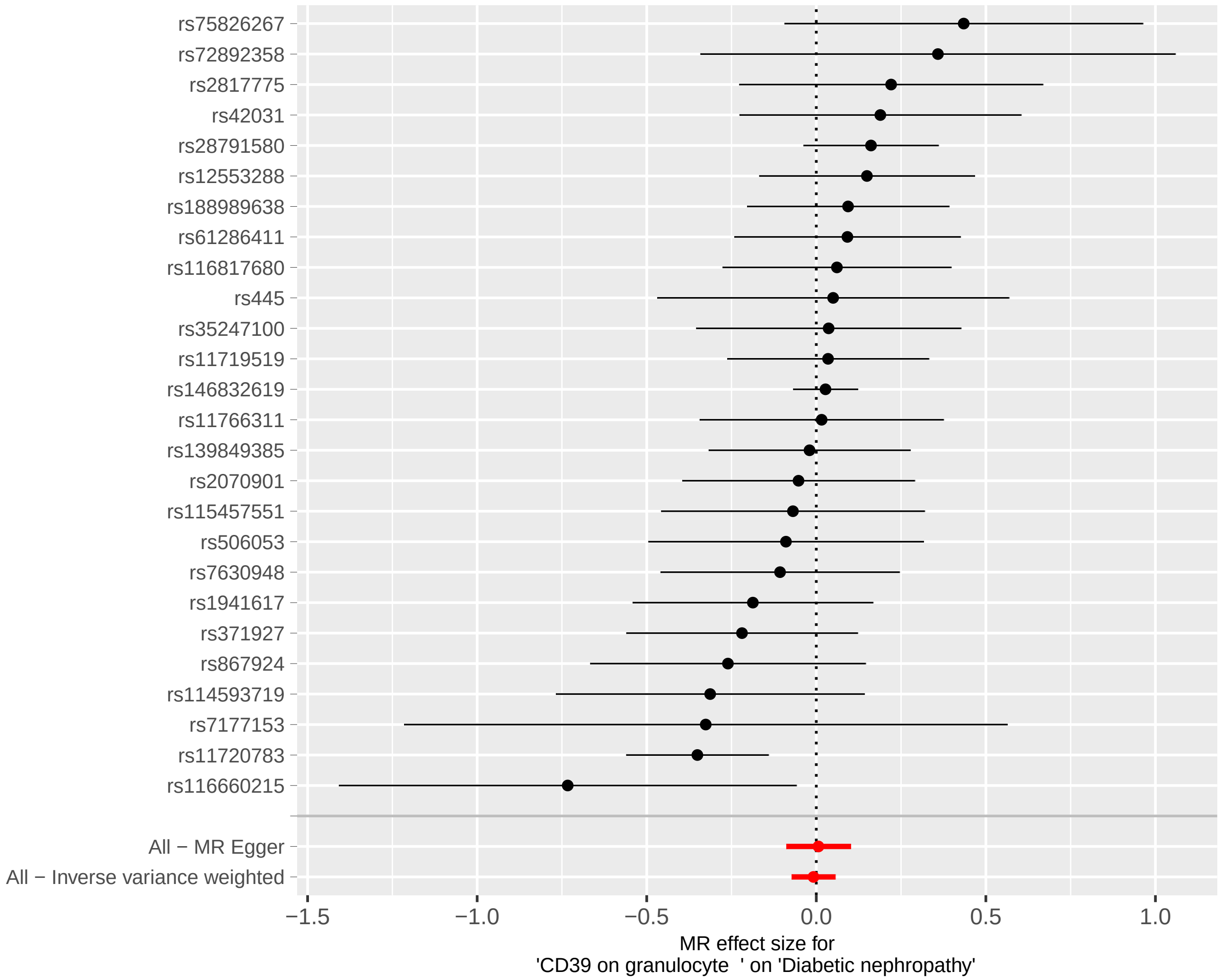
rs75826267
rs72892358
rs2817775
rs42031
rs28791580
rs12553288
rs188989638
rs61286411
rs116817680
rs445
rs35247100
rs11719519
rs146832619
rs11766311
rs139849385
rs2070901
rs115457551
rs506053
rs7630948
rs1941617
rs371927
rs867924
rs114593719
rs7177153
rs11720783
rs116660215
All − MR Egger
All − Inverse variance weighted
−1.5
−1.0
−0.5
0.0
0.5
1.0
MR effect size for
'CD39 on granulocyte ' on 'Diabetic nephropathy'

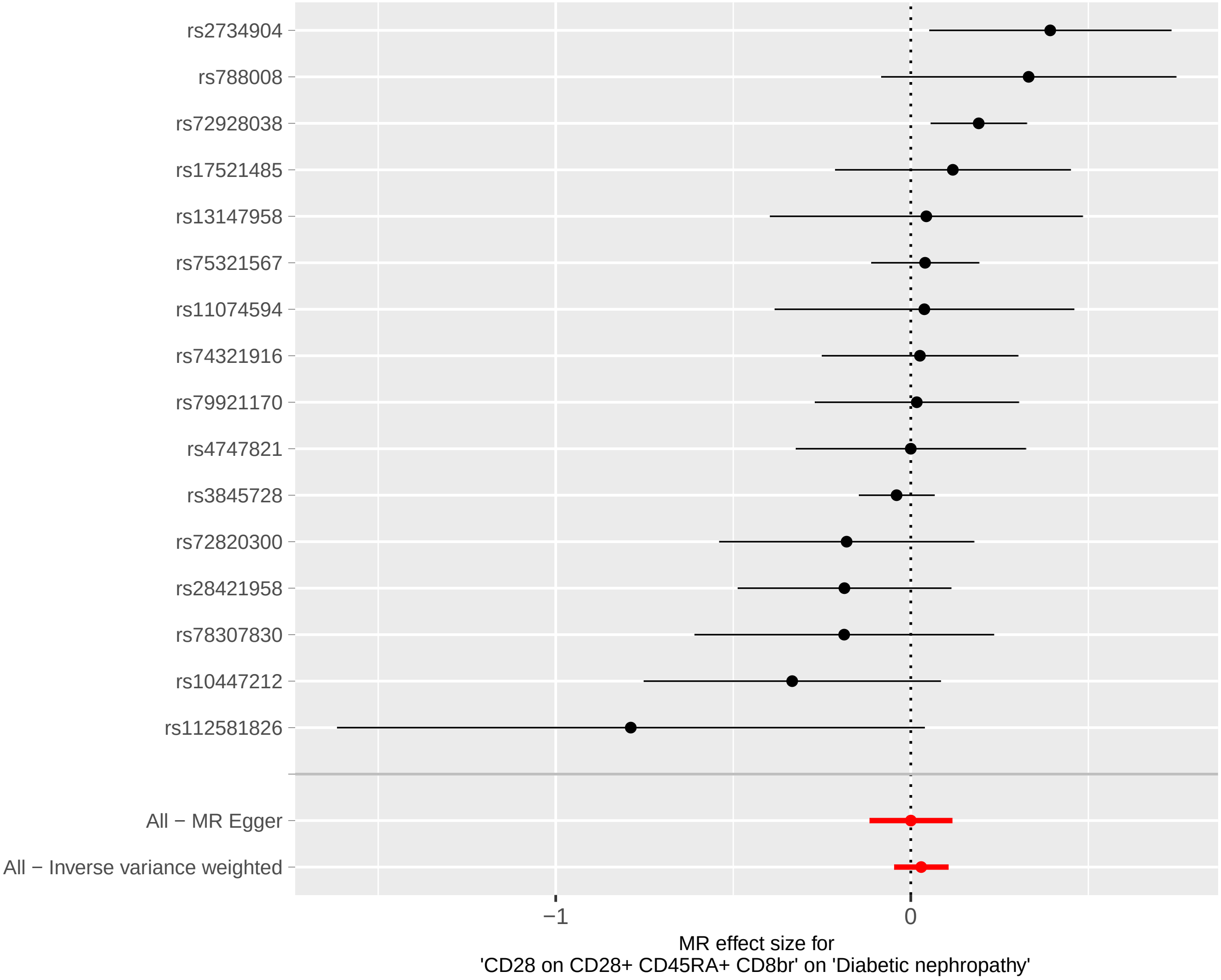
rs2734904
rs788008
rs72928038
rs17521485
rs13147958
rs75321567
rs11074594
rs74321916
rs79921170
rs4747821
rs3845728
rs72820300
rs28421958
rs78307830
rs10447212
rs112581826
All − MR Egger
All − Inverse variance weighted
−1
0
MR effect size for
'CD28 on CD28+ CD45RA+ CD8br' on 'Diabetic nephropathy'

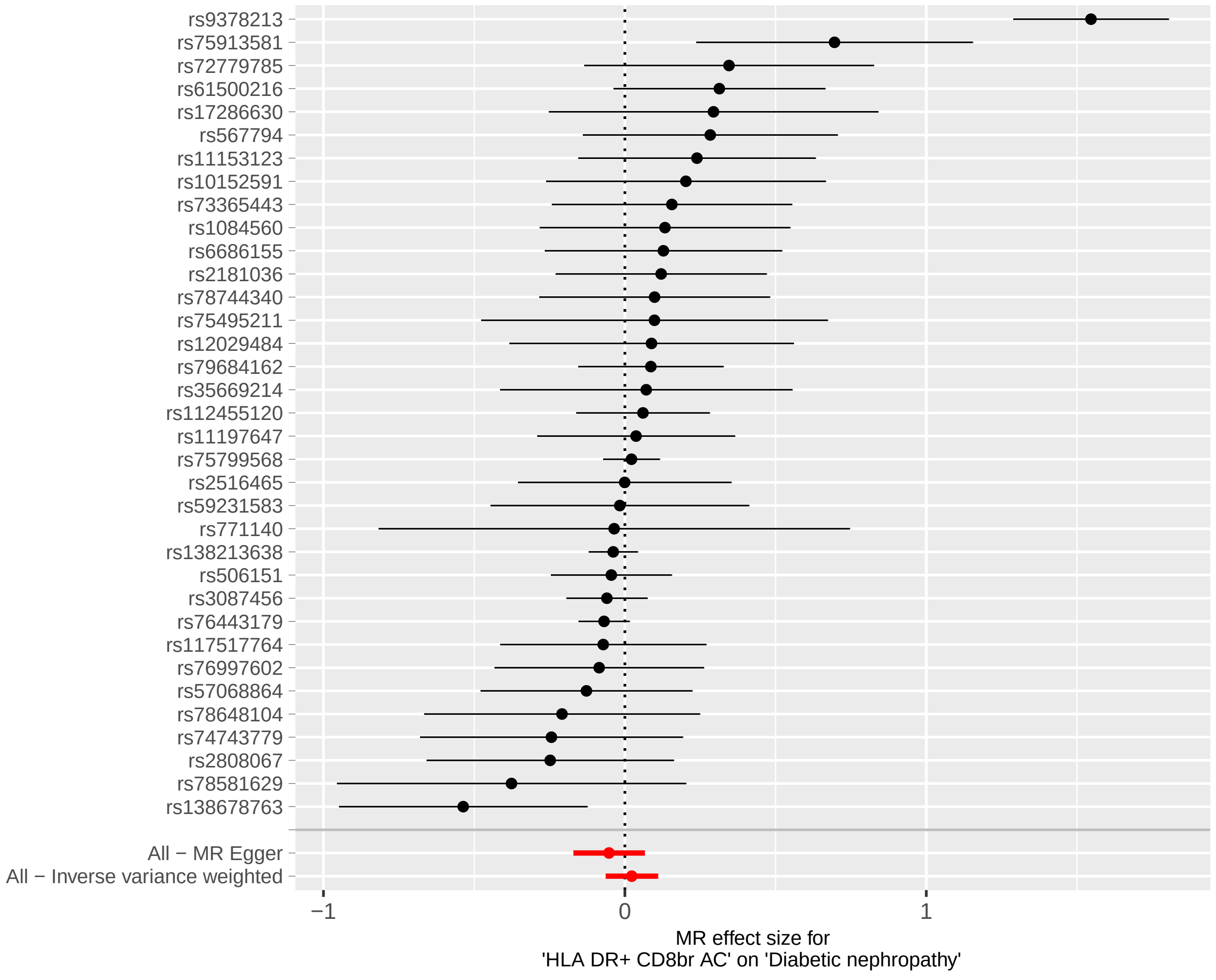

rs9378213
rs75913581
rs72779785
rs61500216
rs17286630
rs567794
rs11153123
rs10152591
rs73365443
rs1084560
rs6686155
rs2181036
rs78744340
rs75495211
rs12029484
rs79684162
rs35669214
rs112455120
rs11197647
rs75799568
rs2516465
rs59231583
rs771140
rs138213638
rs506151
rs3087456
rs76443179
rs117517764
rs76997602
rs57068864
rs78648104
rs74743779
rs2808067
rs78581629
rs138678763
All − MR Egger
All − Inverse variance weighted
−1
0
1
MR effect size for
'HLA DR+ CD8br AC' on 'Diabetic nephropathy'

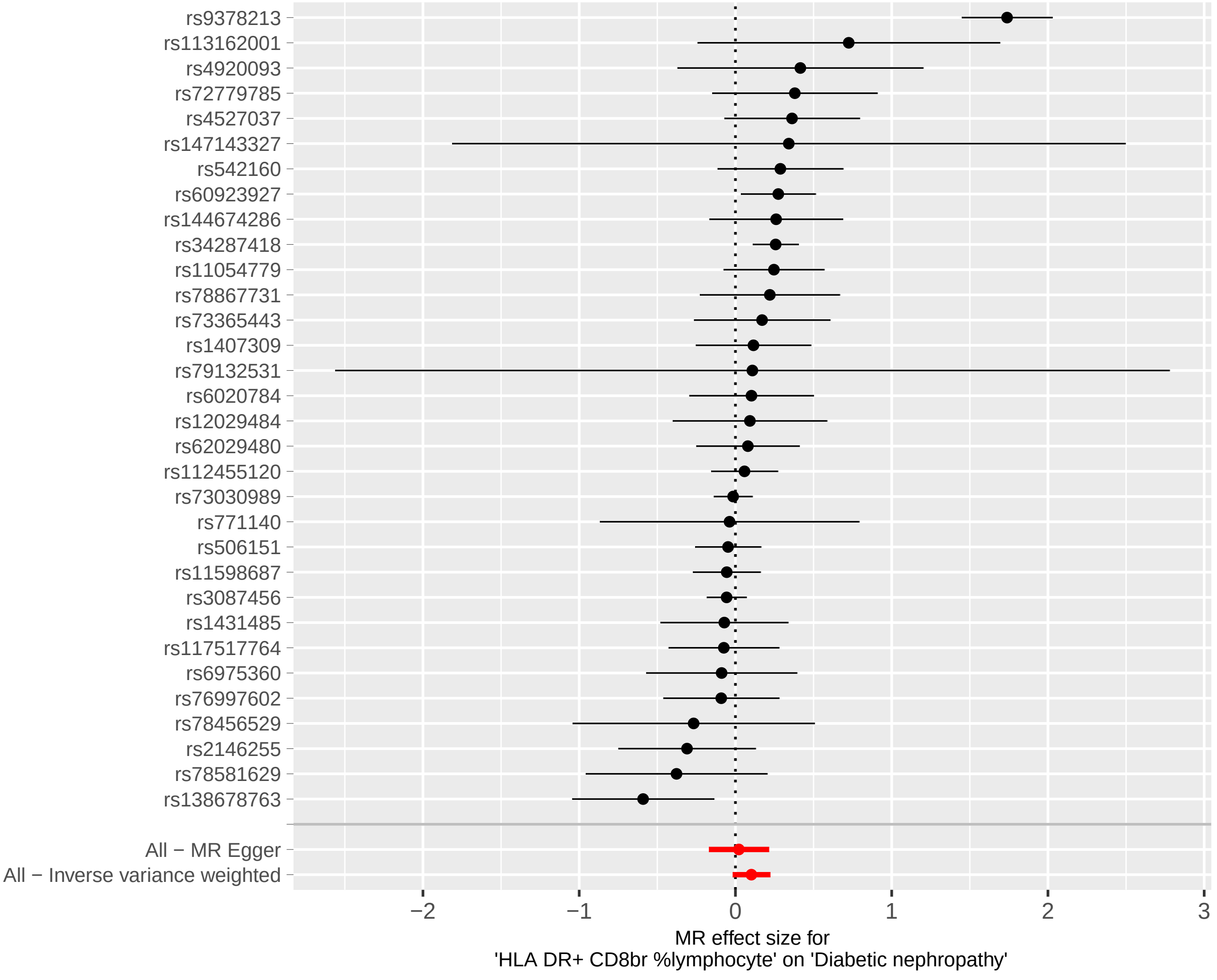
rs9378213
rs113162001
rs4920093
rs72779785
rs4527037
rs147143327
rs542160
rs60923927
rs144674286
rs34287418
rs11054779
rs78867731
rs73365443
rs1407309
rs79132531
rs6020784
rs12029484
rs62029480
rs112455120
rs73030989
rs771140
rs506151
rs11598687
rs3087456
rs1431485
rs117517764
rs6975360
rs76997602
rs78456529
rs2146255
rs78581629
rs138678763
All − MR Egger
All − Inverse variance weighted
−2
−1
0
1
2
3
MR effect size for
'HLA DR+ CD8br %lymphocyte' on 'Diabetic nephropathy'

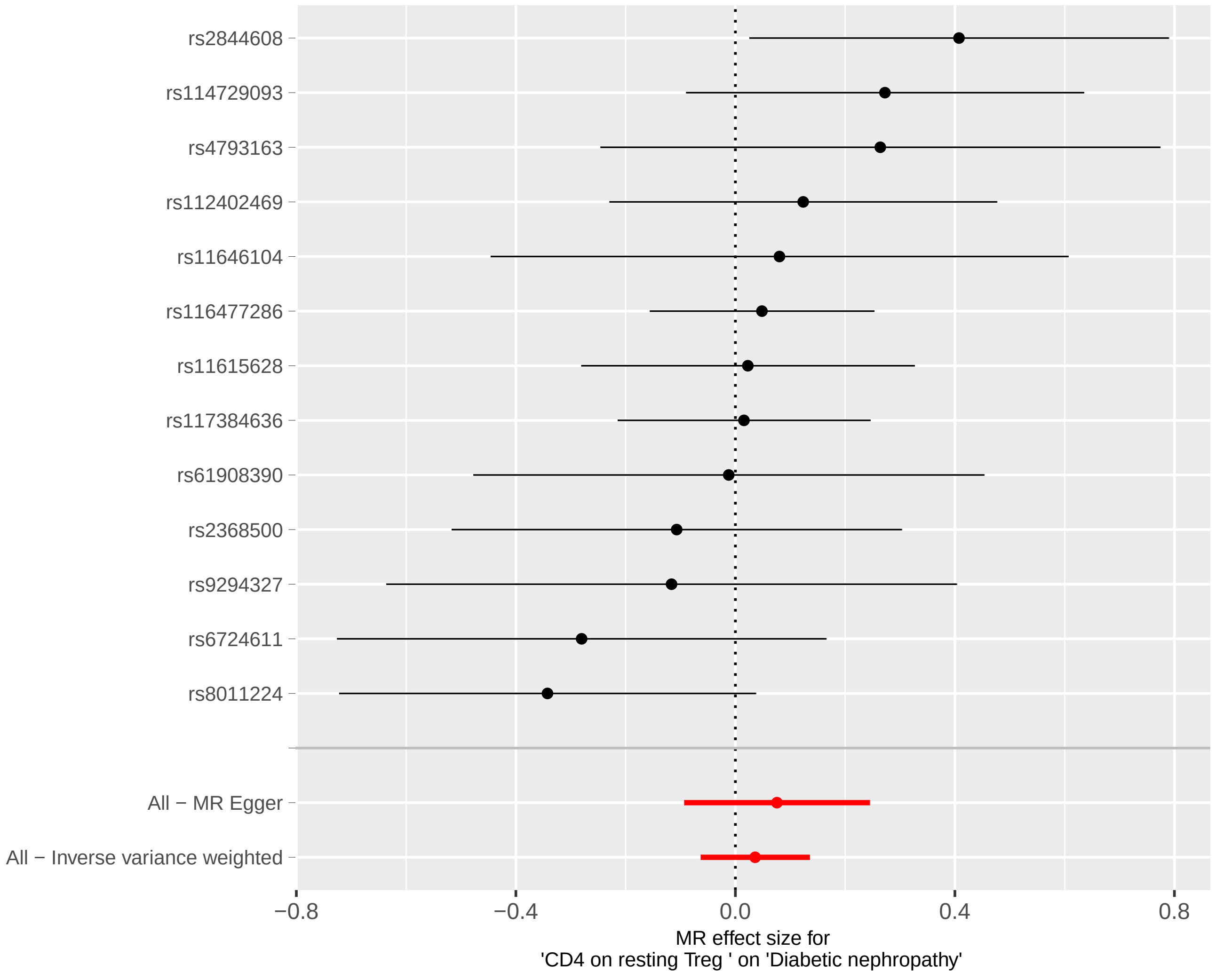
rs2844608
rs114729093
rs4793163
rs112402469
rs11646104
rs116477286
rs11615628
rs117384636
rs61908390
rs2368500
rs9294327
rs6724611
rs8011224
All − MR Egger
All − Inverse variance weighted
−0.8
−0.4
0.0
0.4
0.8
MR effect size for
'CD4 on resting Treg ' on 'Diabetic nephropathy'

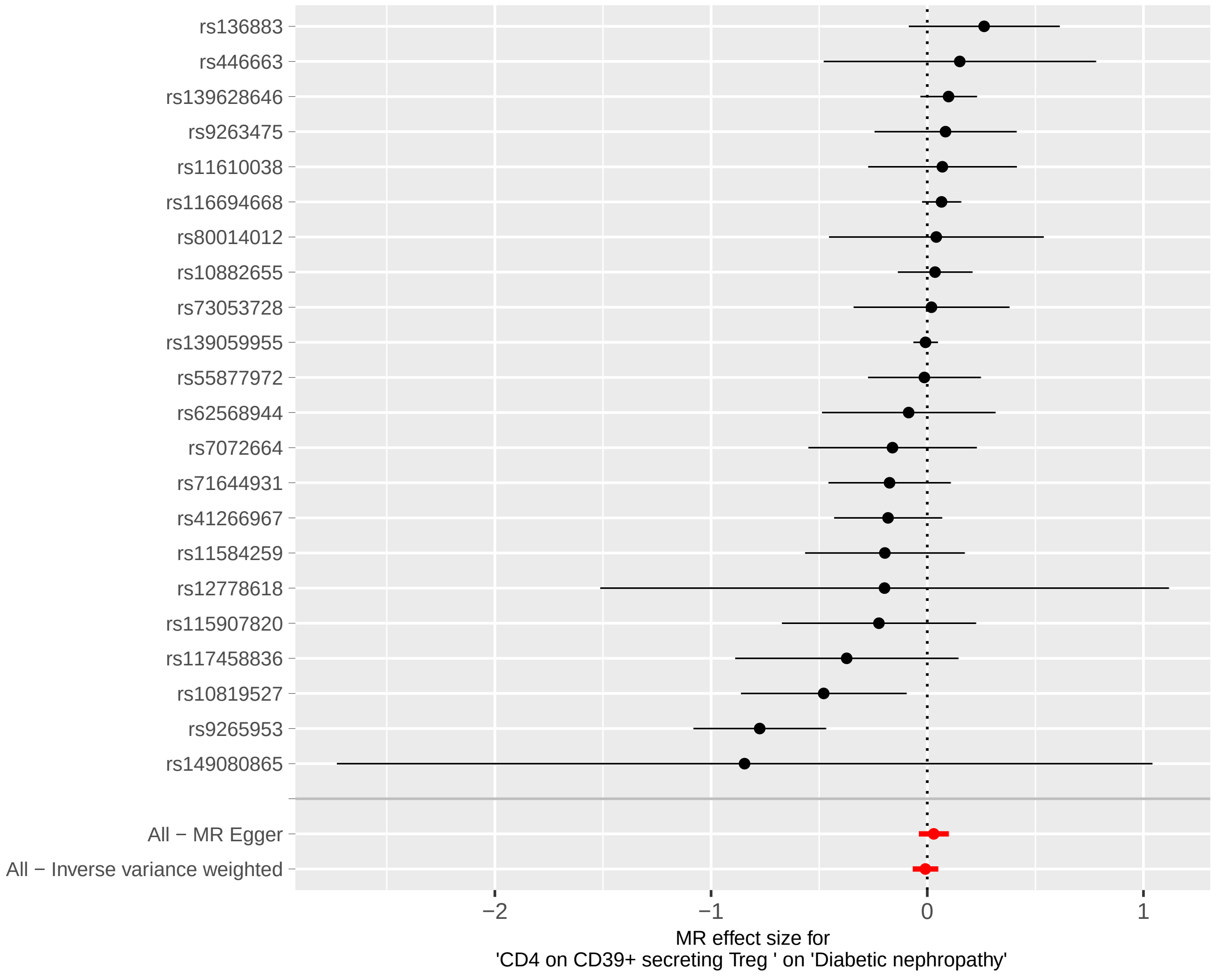
rs136883
rs446663
rs139628646
rs9263475
rs11610038
rs116694668
rs80014012
rs10882655
rs73053728
rs139059955
rs55877972
rs62568944
rs7072664
rs71644931
rs41266967
rs11584259
rs12778618
rs115907820
rs117458836
rs10819527
rs9265953
rs149080865
All − MR Egger
All − Inverse variance weighted
−2
−1
0
1
MR effect size for
'CD4 on CD39+ secreting Treg ' on 'Diabetic nephropathy'

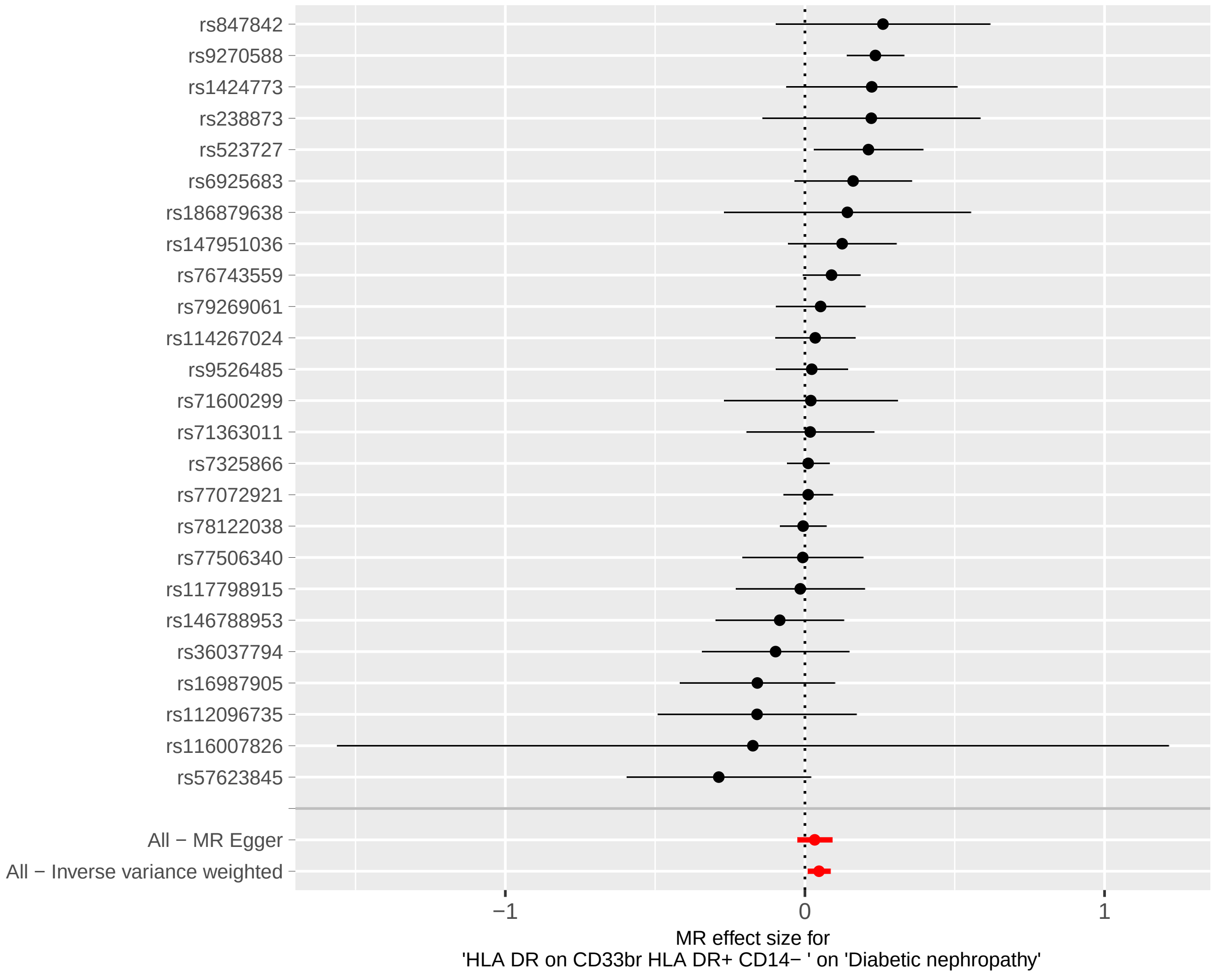
rs847842
rs9270588
rs1424773
rs238873
rs523727
rs6925683
rs186879638
rs147951036
rs76743559
rs79269061
rs114267024
rs9526485
rs71600299
rs71363011
rs7325866
rs77072921
rs78122038
rs77506340
rs117798915
rs146788953
rs36037794
rs16987905
rs112096735
rs116007826
rs57623845
All − MR Egger
All − Inverse variance weighted
−1
0
1
MR effect size for
'HLA DR on CD33br HLA DR+ CD14− ' on 'Diabetic nephropathy'

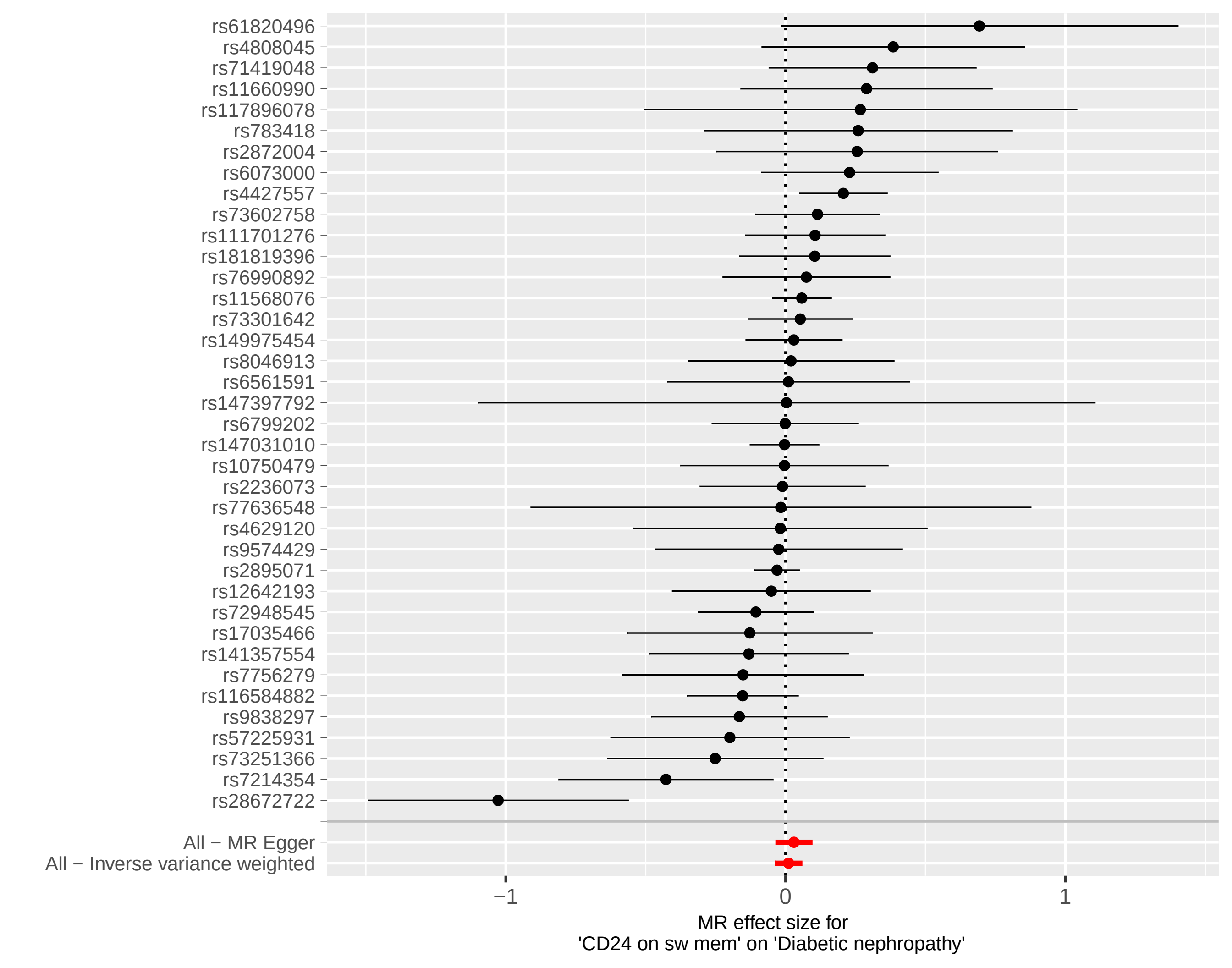
rs61820496
rs4808045
rs71419048
rs11660990
rs117896078
rs783418
rs2872004
rs6073000
rs4427557
rs73602758
rs111701276
rs181819396
rs76990892
rs11568076
rs73301642
rs149975454
rs8046913
rs6561591
rs147397792
rs6799202
rs147031010
rs10750479
rs2236073
rs77636548
rs4629120
rs9574429
rs2895071
rs12642193
rs72948545
rs17035466
rs141357554
rs7756279
rs116584882
rs9838297
rs57225931
rs73251366
rs7214354
rs28672722
All − MR Egger
All − Inverse variance weighted
−1
0
1
MR effect size for
'CD24 on sw mem' on 'Diabetic nephropathy'

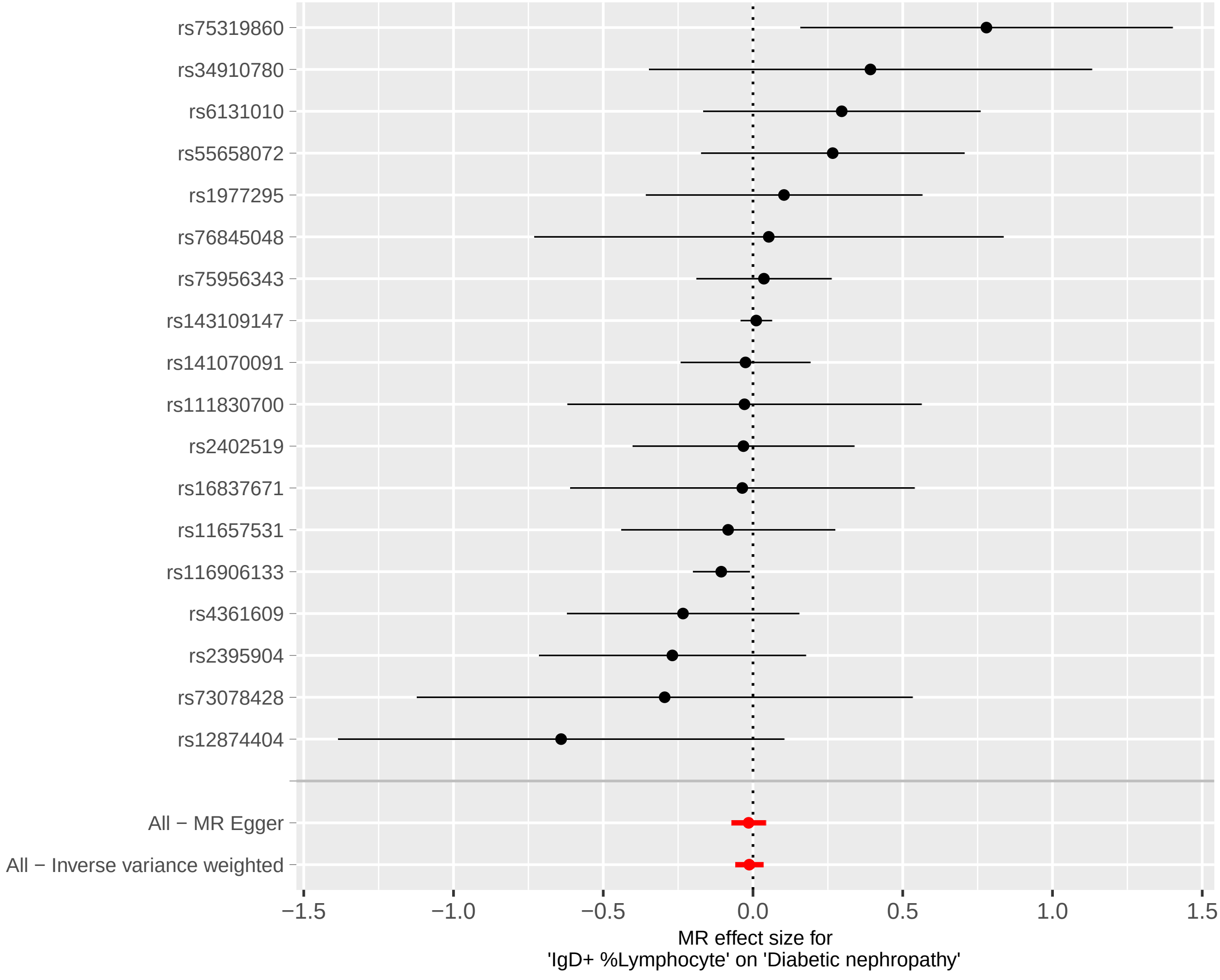
rs75319860
rs34910780
rs6131010
rs55658072
rs1977295
rs76845048
rs75956343
rs143109147
rs141070091
rs111830700
rs2402519
rs16837671
rs11657531
rs116906133
rs4361609
rs2395904
rs73078428
rs12874404
All − MR Egger
All − Inverse variance weighted
−1.5
−1.0
−0.5
0.0
0.5
1.0
1.5
MR effect size for
'IgD+ %Lymphocyte' on 'Diabetic nephropathy'

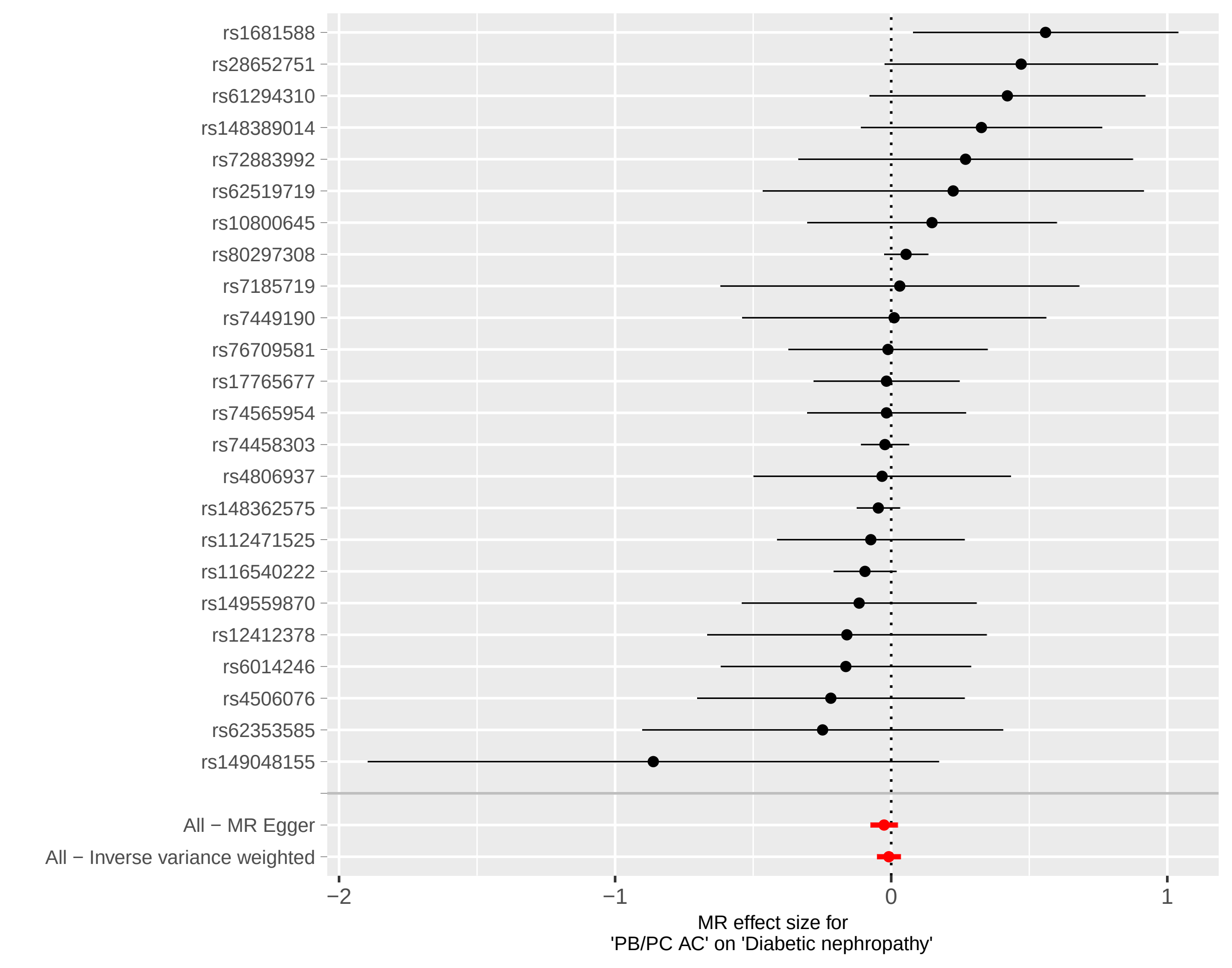
rs1681588
rs28652751
rs61294310
rs148389014
rs72883992
rs62519719
rs10800645
rs80297308
rs7185719
rs7449190
rs76709581
rs17765677
rs74565954
rs74458303
rs4806937
rs148362575
rs112471525
rs116540222
rs149559870
rs12412378
rs6014246
rs4506076
rs62353585
rs149048155
All − MR Egger
All − Inverse variance weighted
−2
−1
0
1
MR effect size for
'PB/PC AC' on 'Diabetic nephropathy'

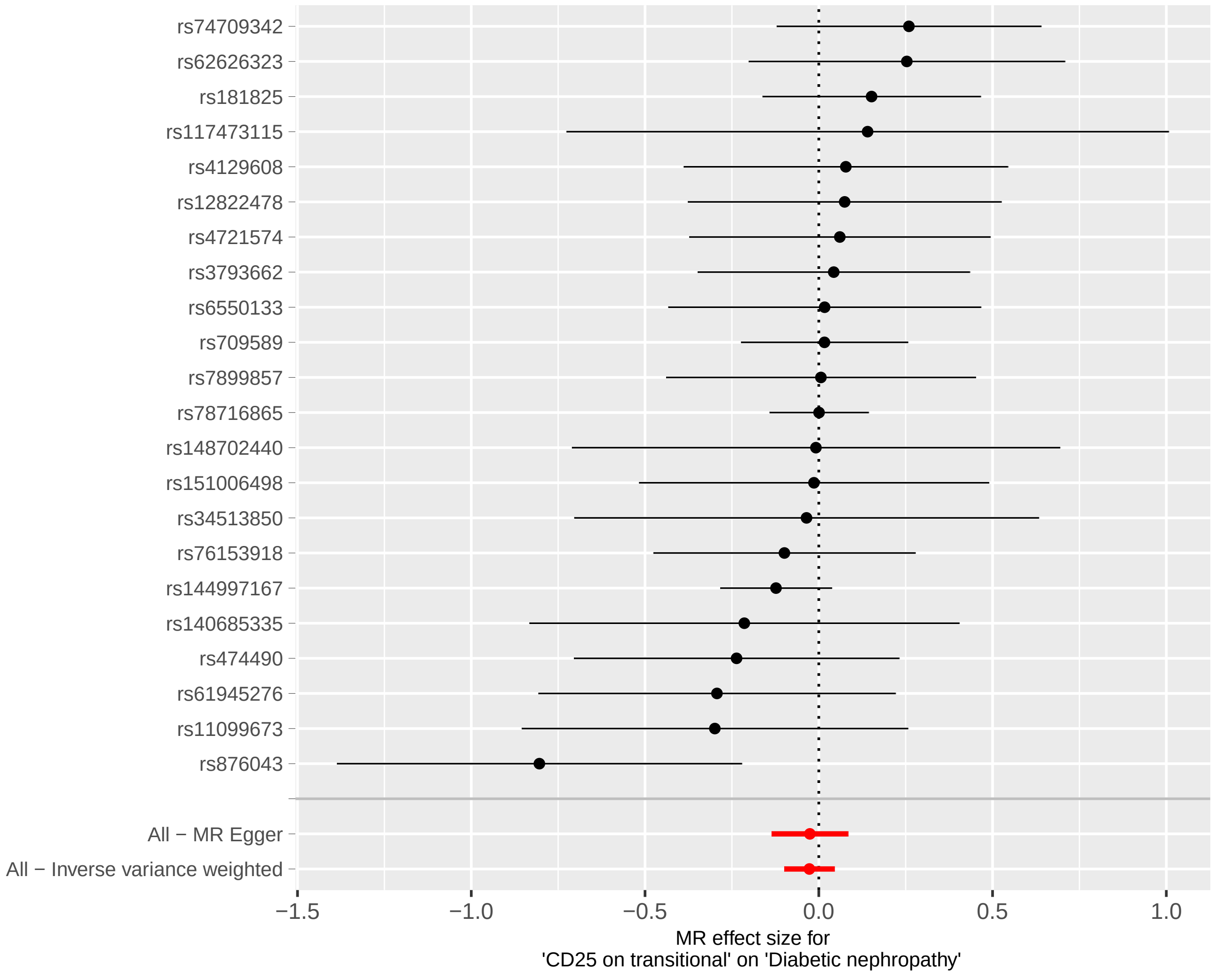
rs74709342
rs62626323
rs181825
rs117473115
rs4129608
rs12822478
rs4721574
rs3793662
rs6550133
rs709589
rs7899857
rs78716865
rs148702440
rs151006498
rs34513850
rs76153918
rs144997167
rs140685335
rs474490
rs61945276
rs11099673
rs876043
All − MR Egger
All − Inverse variance weighted
−1.5
−1.0
−0.5
0.0
0.5
1.0
MR effect size for
'CD25 on transitional' on 'Diabetic nephropathy'

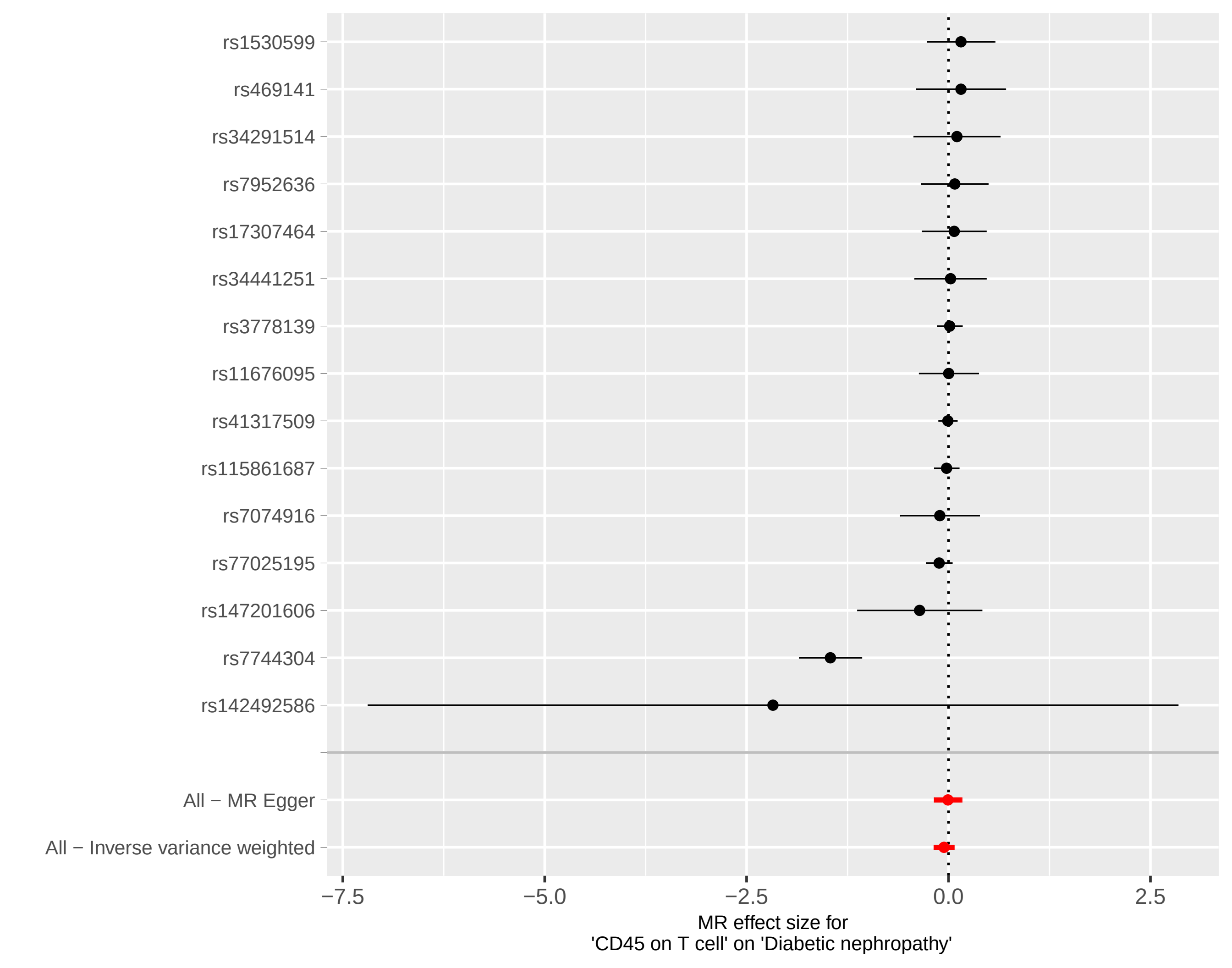
rs1530599
rs469141
rs34291514
rs7952636
rs17307464
rs34441251
rs3778139
rs11676095
rs41317509
rs115861687
rs7074916
rs77025195
rs147201606
rs7744304
rs142492586
All − MR Egger
All − Inverse variance weighted
−7.5
−5.0
−2.5
0.0
2.5
MR effect size for
'CD45 on T cell' on 'Diabetic nephropathy'

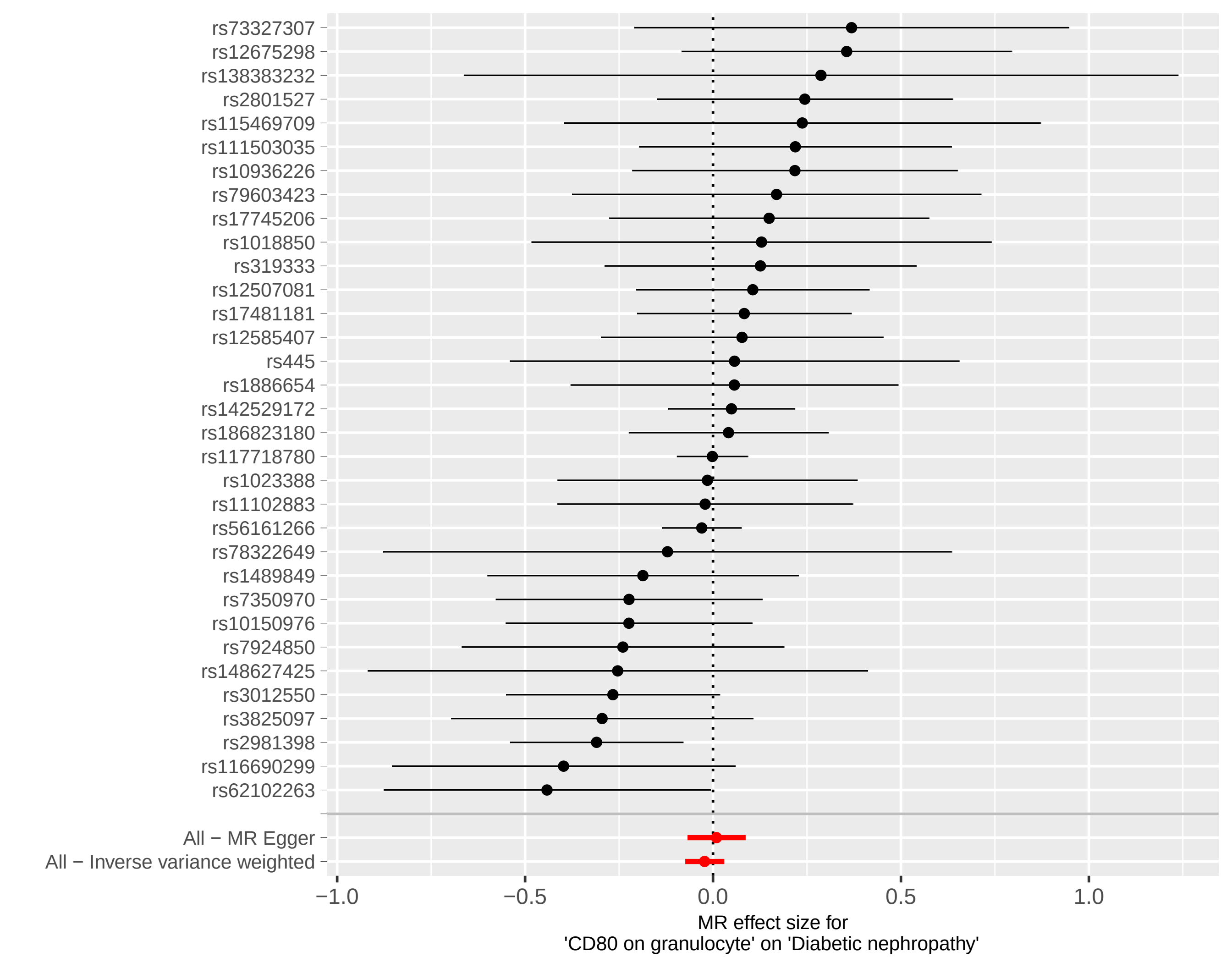
rs73327307
rs12675298
rs138383232
rs2801527
rs115469709
rs111503035
rs10936226
rs79603423
rs17745206
rs1018850
rs319333
rs12507081
rs17481181
rs12585407
rs445
rs1886654
rs142529172
rs186823180
rs117718780
rs1023388
rs11102883
rs56161266
rs78322649
rs1489849
rs7350970
rs10150976
rs7924850
rs148627425
rs3012550
rs3825097
rs2981398
rs116690299
rs62102263
All − MR Egger
All − Inverse variance weighted
−1.0
−0.5
0.0
0.5
1.0
MR effect size for
'CD80 on granulocyte' on 'Diabetic nephropathy'

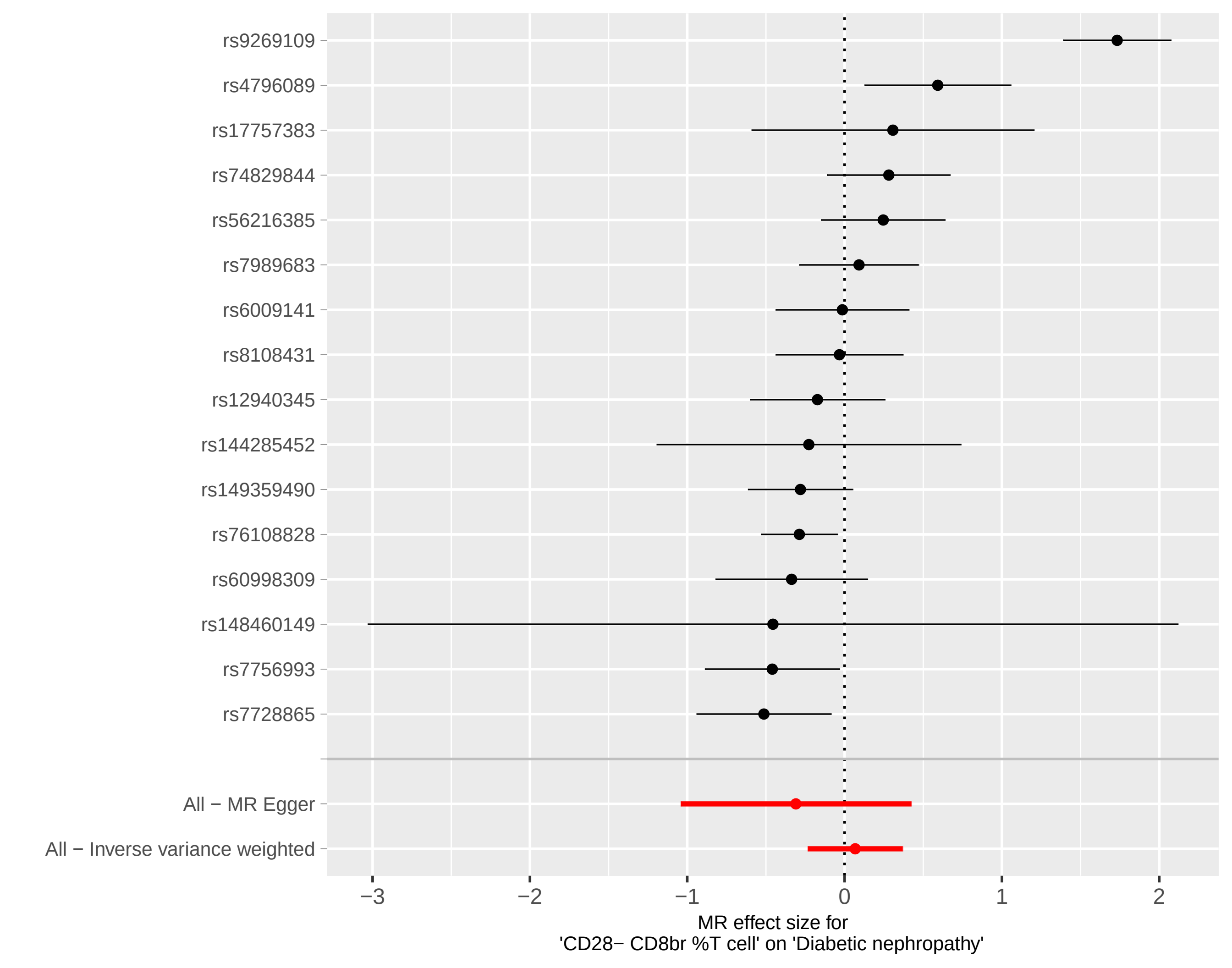
rs9269109
rs4796089
rs17757383
rs74829844
rs56216385
rs7989683
rs6009141
rs8108431
rs12940345
rs144285452
rs149359490
rs76108828
rs60998309
rs148460149
rs7756993
rs7728865
All − MR Egger
All − Inverse variance weighted
−3
−2
−1
0
1
2
MR effect size for
'CD28− CD8br %T cell' on 'Diabetic nephropathy'

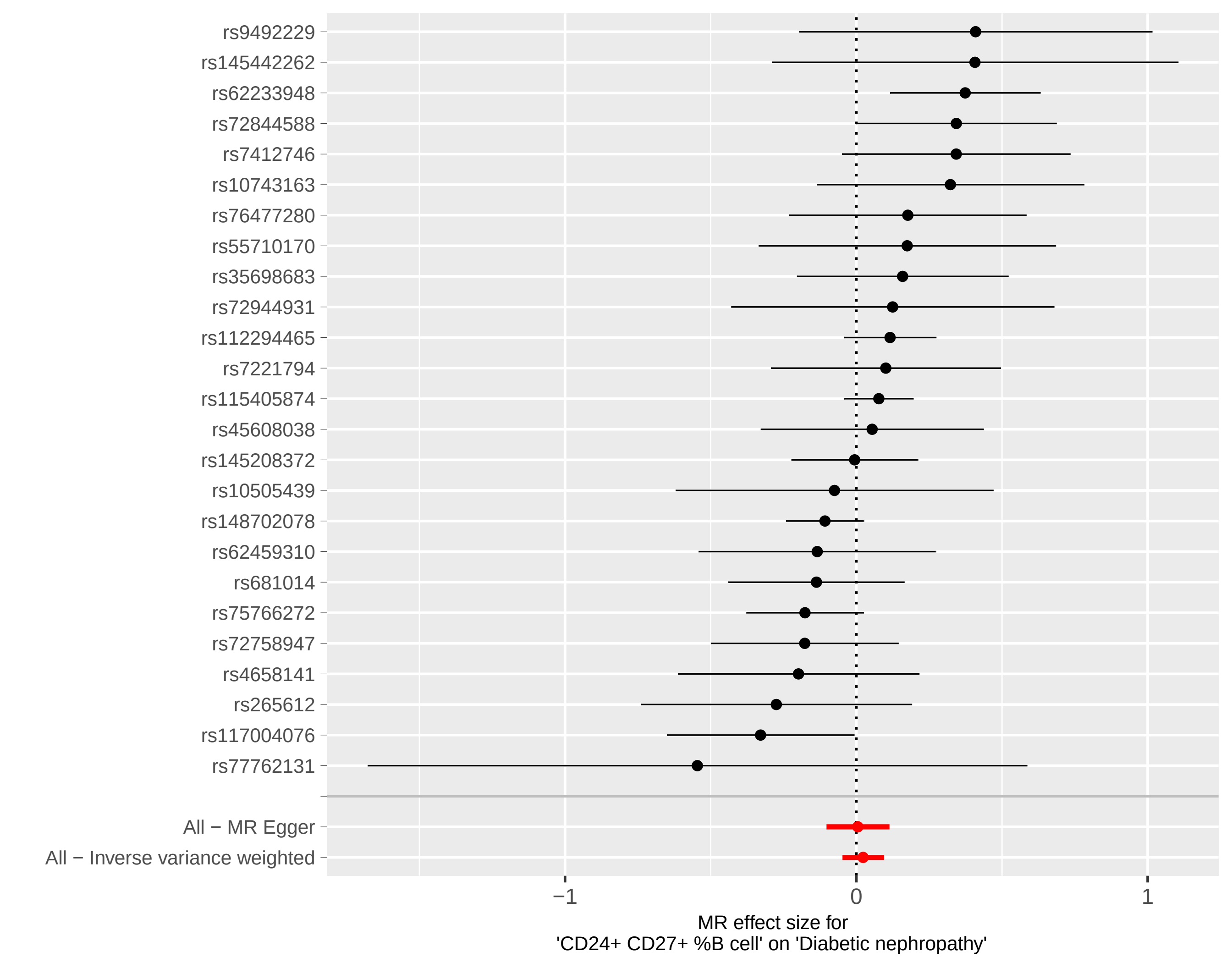
rs9492229
rs145442262
rs62233948
rs72844588
rs7412746
rs10743163
rs76477280
rs55710170
rs35698683
rs72944931
rs112294465
rs7221794
rs115405874
rs45608038
rs145208372
rs10505439
rs148702078
rs62459310
rs681014
rs75766272
rs72758947
rs4658141
rs265612
rs117004076
rs77762131
All − MR Egger
All − Inverse variance weighted
−1
0
1
MR effect size for
'CD24+ CD27+ %B cell' on 'Diabetic nephropathy'

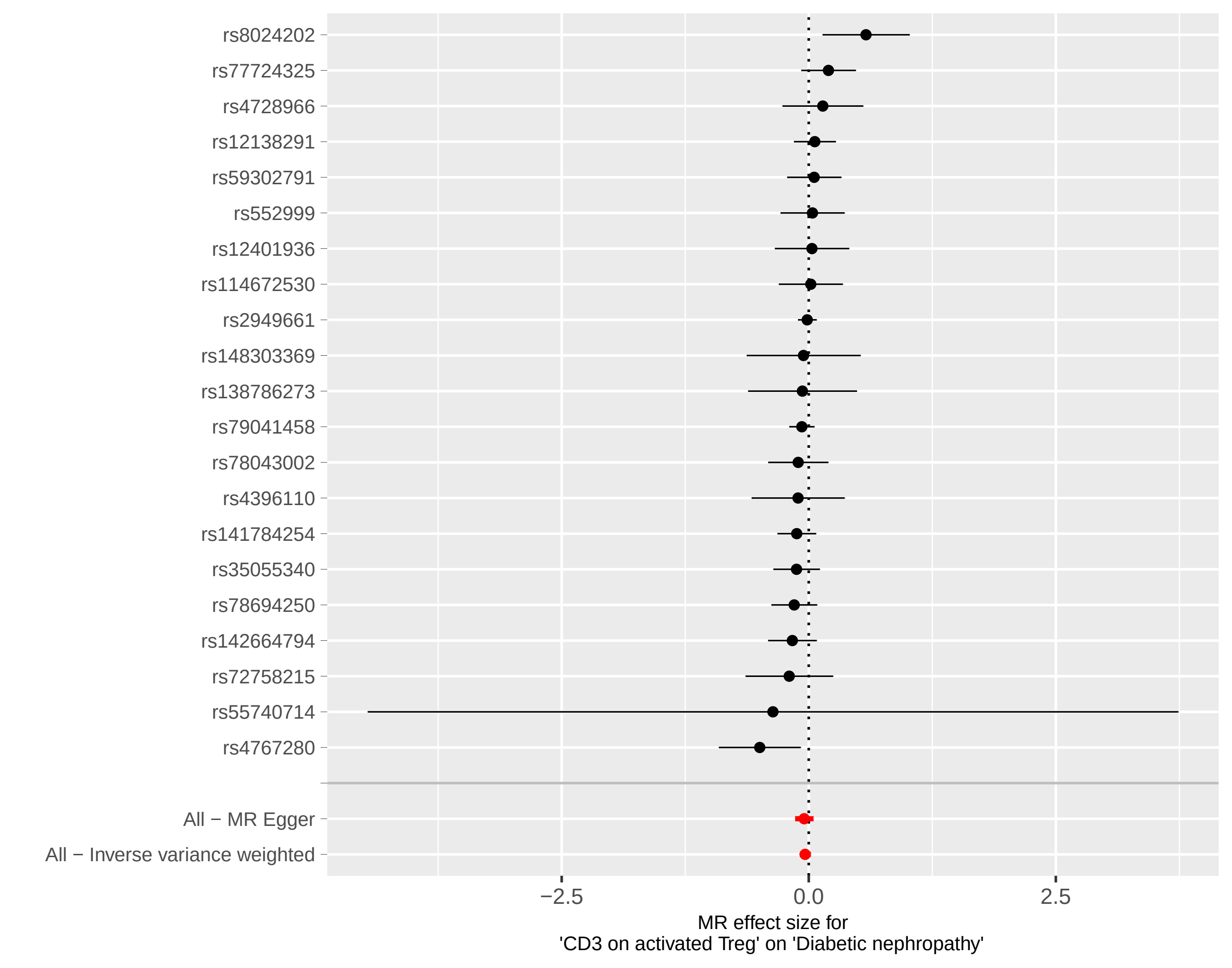

rs8024202
rs77724325
rs4728966
rs12138291
rs59302791
rs552999
rs12401936
rs114672530
rs2949661
rs148303369
rs138786273
rs79041458
rs78043002
rs4396110
rs141784254
rs35055340
rs78694250
rs142664794
rs72758215
rs55740714
rs4767280
All − MR Egger
All − Inverse variance weighted
−2.5
0.0
2.5
MR effect size for
'CD3 on activated Treg' on 'Diabetic nephropathy'

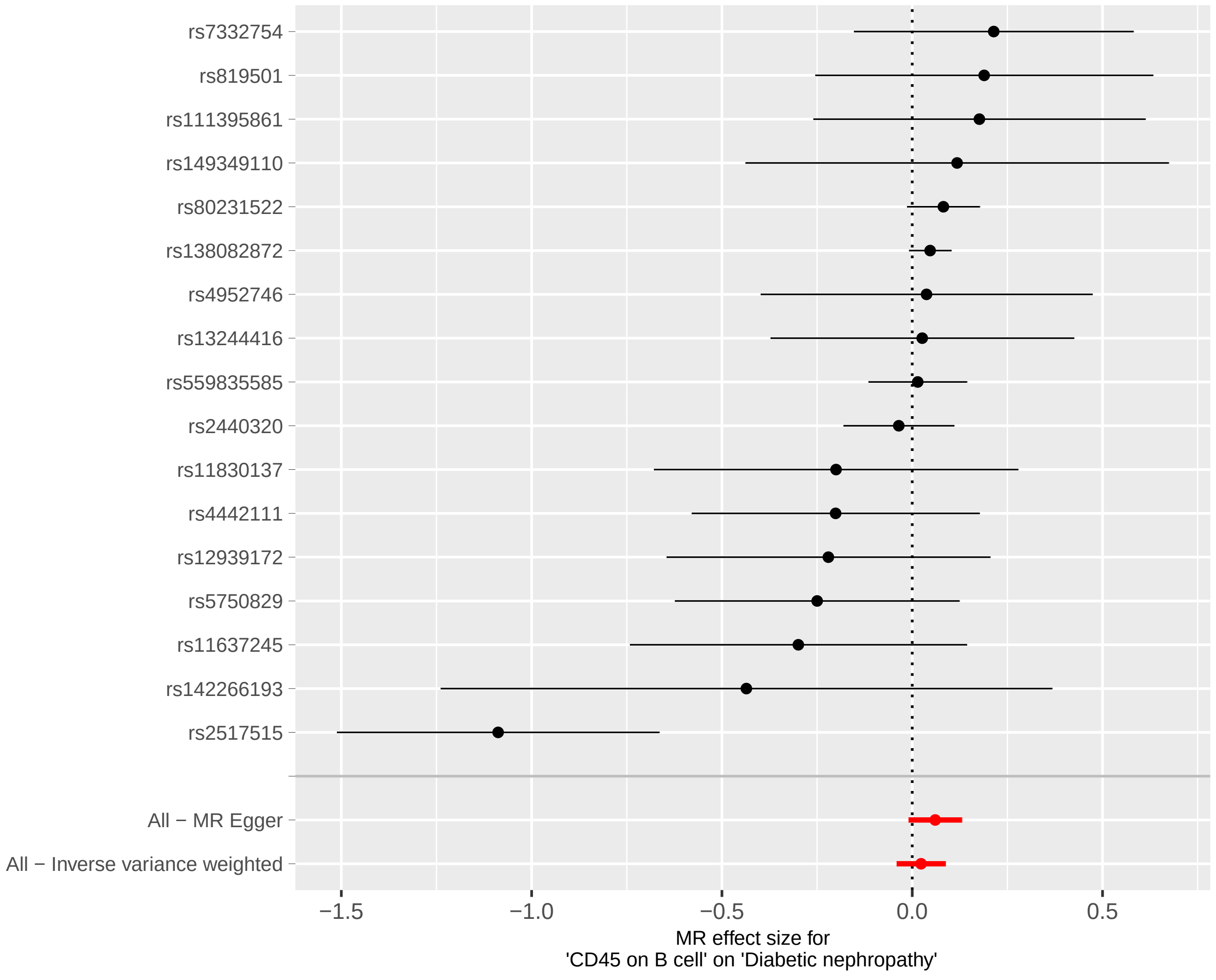
rs7332754
rs819501
rs111395861
rs149349110
rs80231522
rs138082872
rs4952746
rs13244416
rs559835585
rs2440320
rs11830137
rs4442111
rs12939172
rs5750829
rs11637245
rs142266193
rs2517515
All − MR Egger
All − Inverse variance weighted
−1.5
−1.0
−0.5
0.0
0.5
MR effect size for
'CD45 on B cell' on 'Diabetic nephropathy'

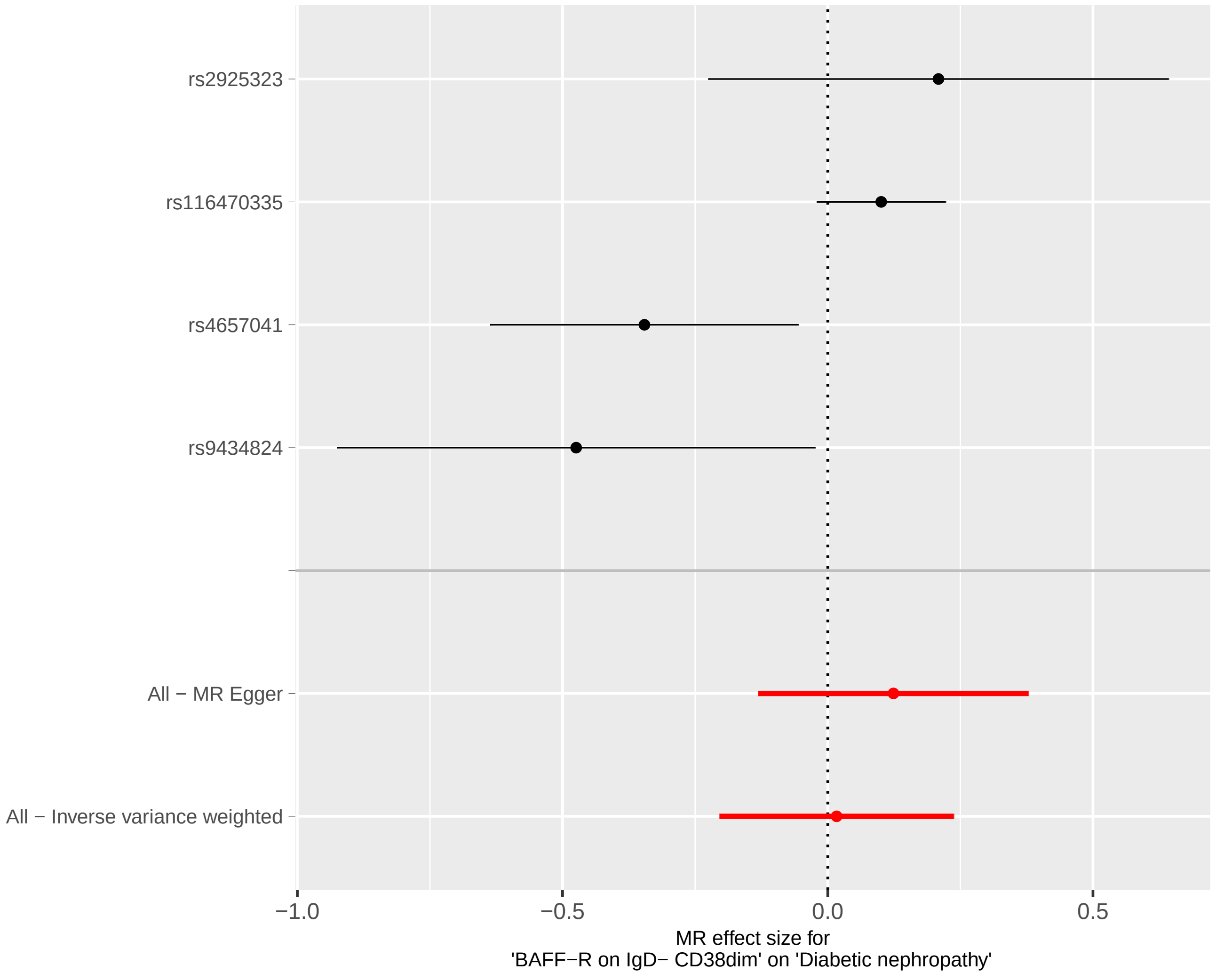

rs2925323
rs116470335
rs4657041
rs9434824
All − MR Egger
All − Inverse variance weighted
−1.0
−0.5
0.0
0.5
MR effect size for
'BAFF−R on IgD− CD38dim' on 'Diabetic nephropathy'

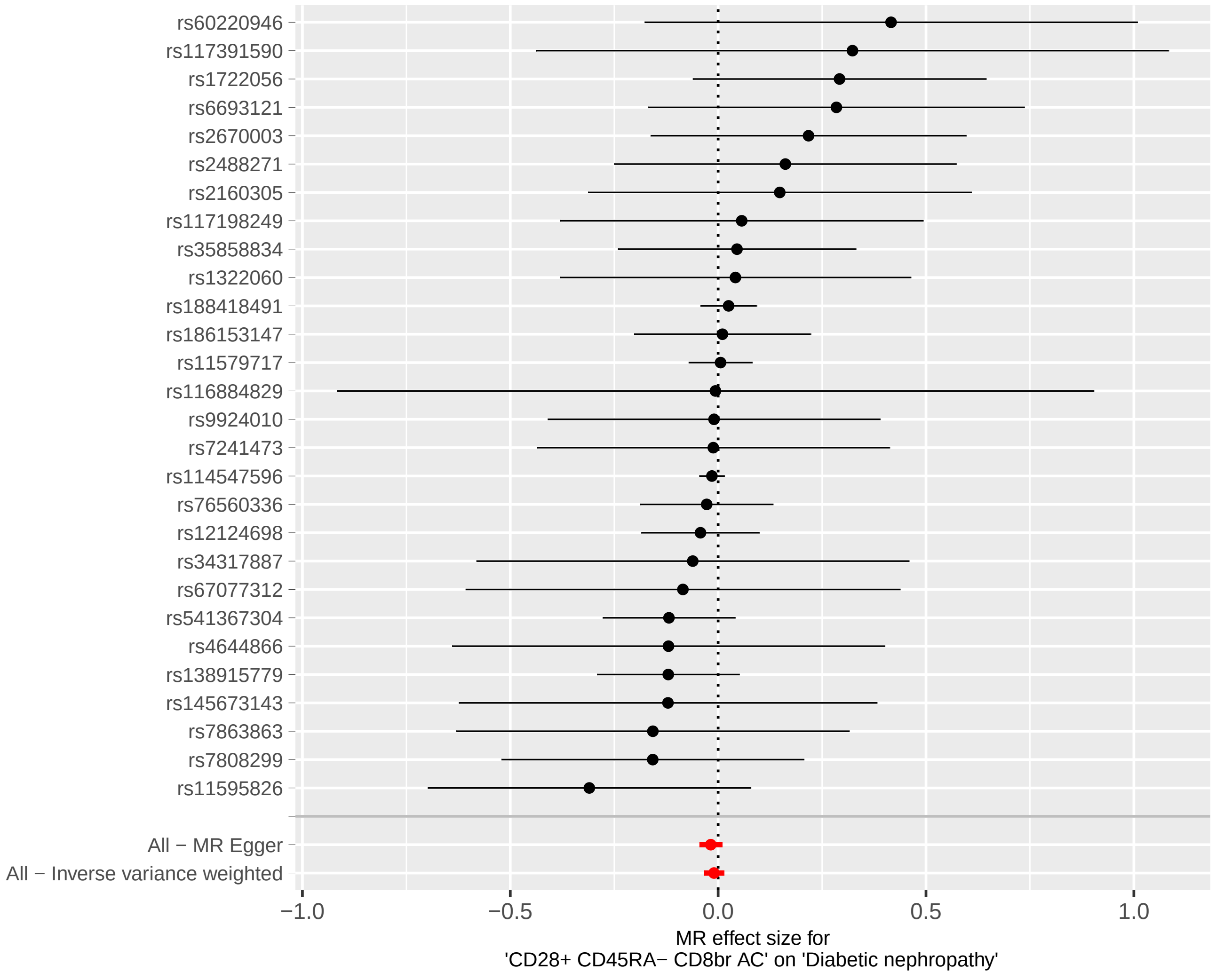

rs60220946
rs117391590
rs1722056
rs6693121
rs2670003
rs2488271
rs2160305
rs117198249
rs35858834
rs1322060
rs188418491
rs186153147
rs11579717
rs116884829
rs9924010
rs7241473
rs114547596
rs76560336
rs12124698
rs34317887
rs67077312
rs541367304
rs4644866
rs138915779
rs145673143
rs7863863
rs7808299
rs11595826
All − MR Egger
All − Inverse variance weighted
−1.0
−0.5
0.0
0.5
1.0
MR effect size for
'CD28+ CD45RA− CD8br AC' on 'Diabetic nephropathy'

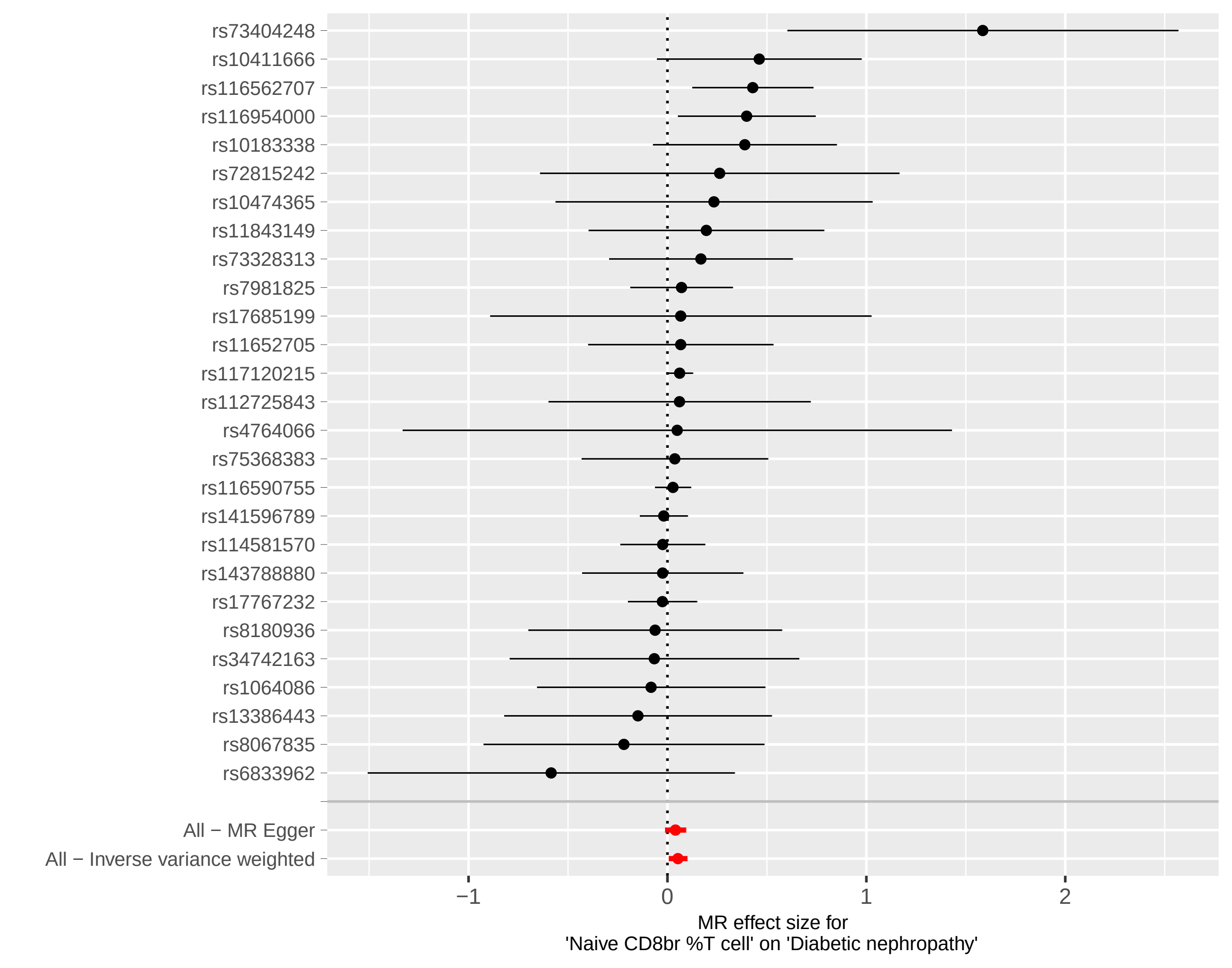
rs73404248
rs10411666
rs116562707
rs116954000
rs10183338
rs72815242
rs10474365
rs11843149
rs73328313
rs7981825
rs17685199
rs11652705
rs117120215
rs112725843
rs4764066
rs75368383
rs116590755
rs141596789
rs114581570
rs143788880
rs17767232
rs8180936
rs34742163
rs1064086
rs13386443
rs8067835
rs6833962
All − MR Egger
All − Inverse variance weighted
−1
0
1
2
MR effect size for
'Naive CD8br %T cell' on 'Diabetic nephropathy'

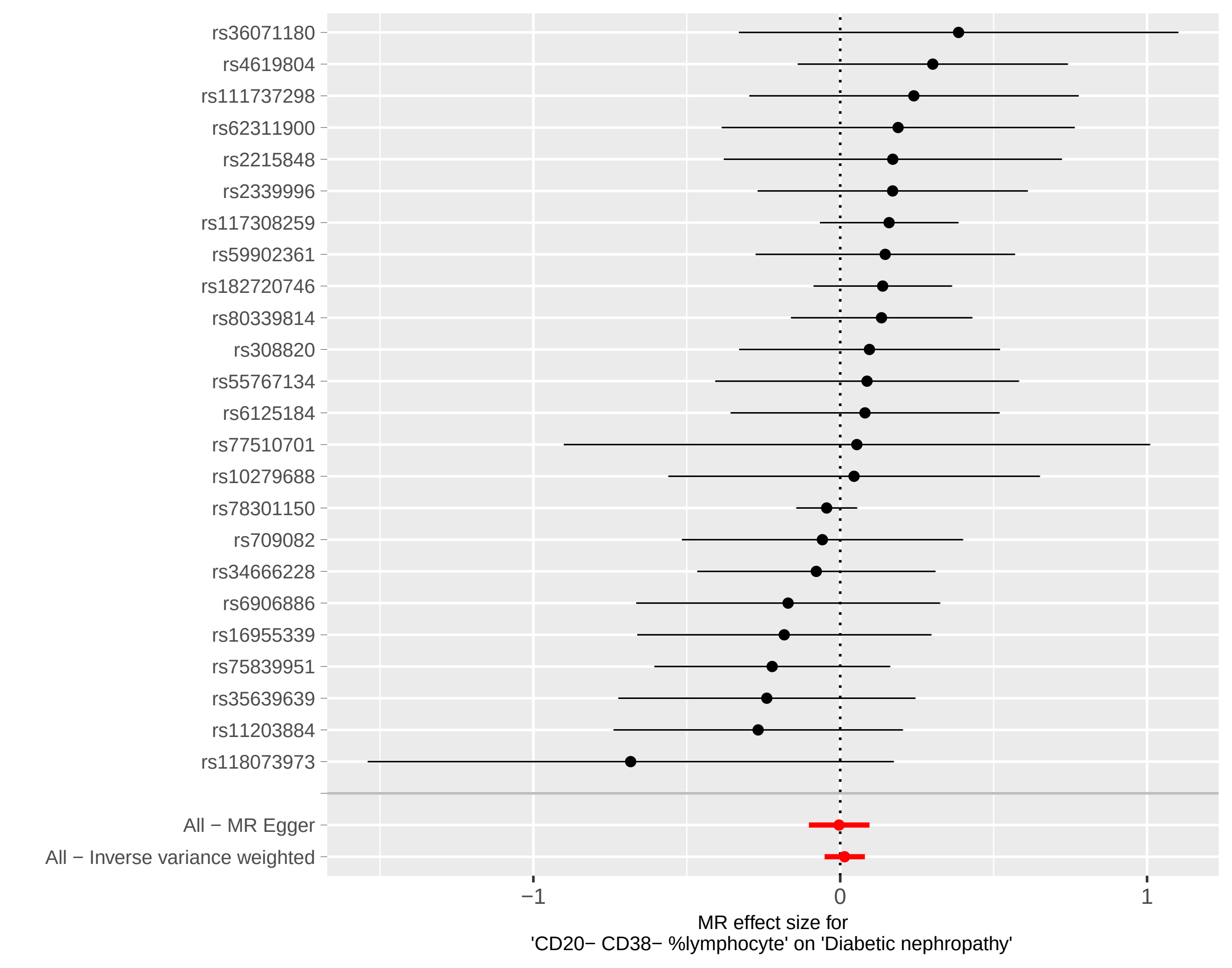
rs36071180
rs4619804
rs111737298
rs62311900
rs2215848
rs2339996
rs117308259
rs59902361
rs182720746
rs80339814
rs308820
rs55767134
rs6125184
rs77510701
rs10279688
rs78301150
rs709082
rs34666228
rs6906886
rs16955339
rs75839951
rs35639639
rs11203884
rs118073973
All − MR Egger
All − Inverse variance weighted
−1
0
1
MR effect size for
'CD20− CD38− %lymphocyte' on 'Diabetic nephropathy'

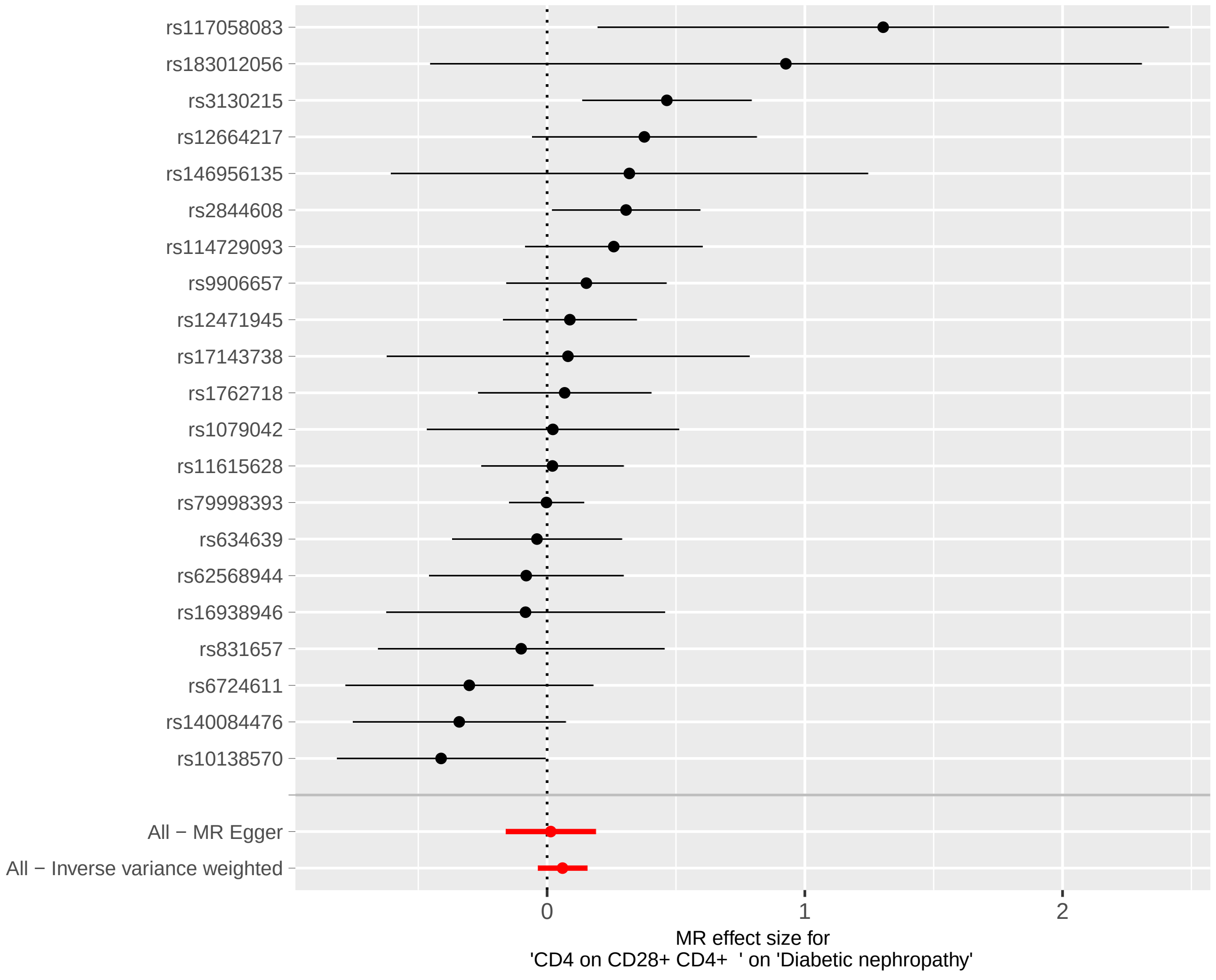
rs117058083
rs183012056
rs3130215
rs12664217
rs146956135
rs2844608
rs114729093
rs9906657
rs12471945
rs17143738
rs1762718
rs1079042
rs11615628
rs79998393
rs634639
rs62568944
rs16938946
rs831657
rs6724611
rs140084476
rs10138570
All − MR Egger
All − Inverse variance weighted
0
1
2
MR effect size for
'CD4 on CD28+ CD4+ ' on 'Diabetic nephropathy'

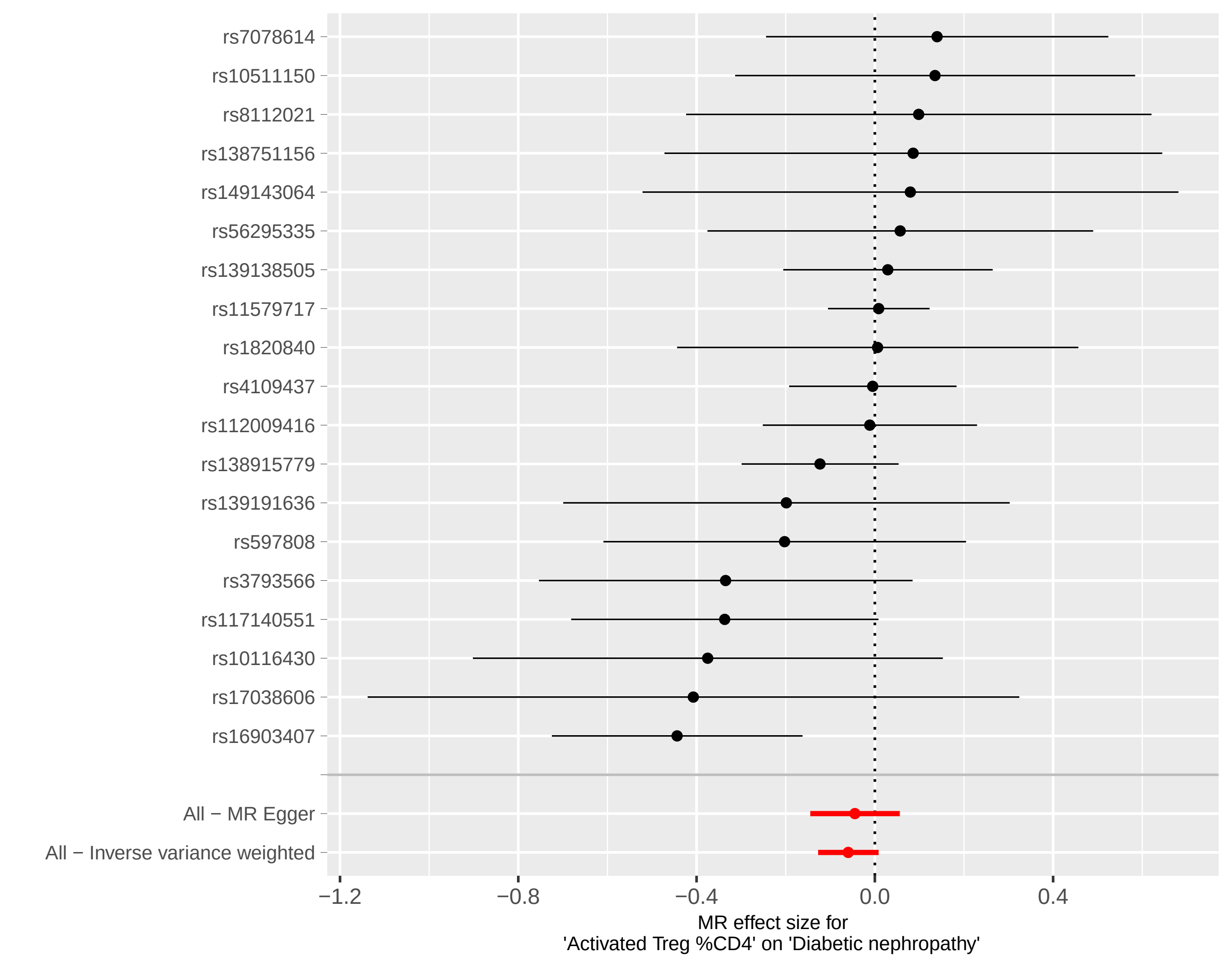
rs7078614
rs10511150
rs8112021
rs138751156
rs149143064
rs56295335
rs139138505
rs11579717
rs1820840
rs4109437
rs112009416
rs138915779
rs139191636
rs597808
rs3793566
rs117140551
rs10116430
rs17038606
rs16903407
All − MR Egger
All − Inverse variance weighted
−1.2
−0.8
−0.4
0.0
0.4
MR effect size for
'Activated Treg %CD4' on 'Diabetic nephropathy'

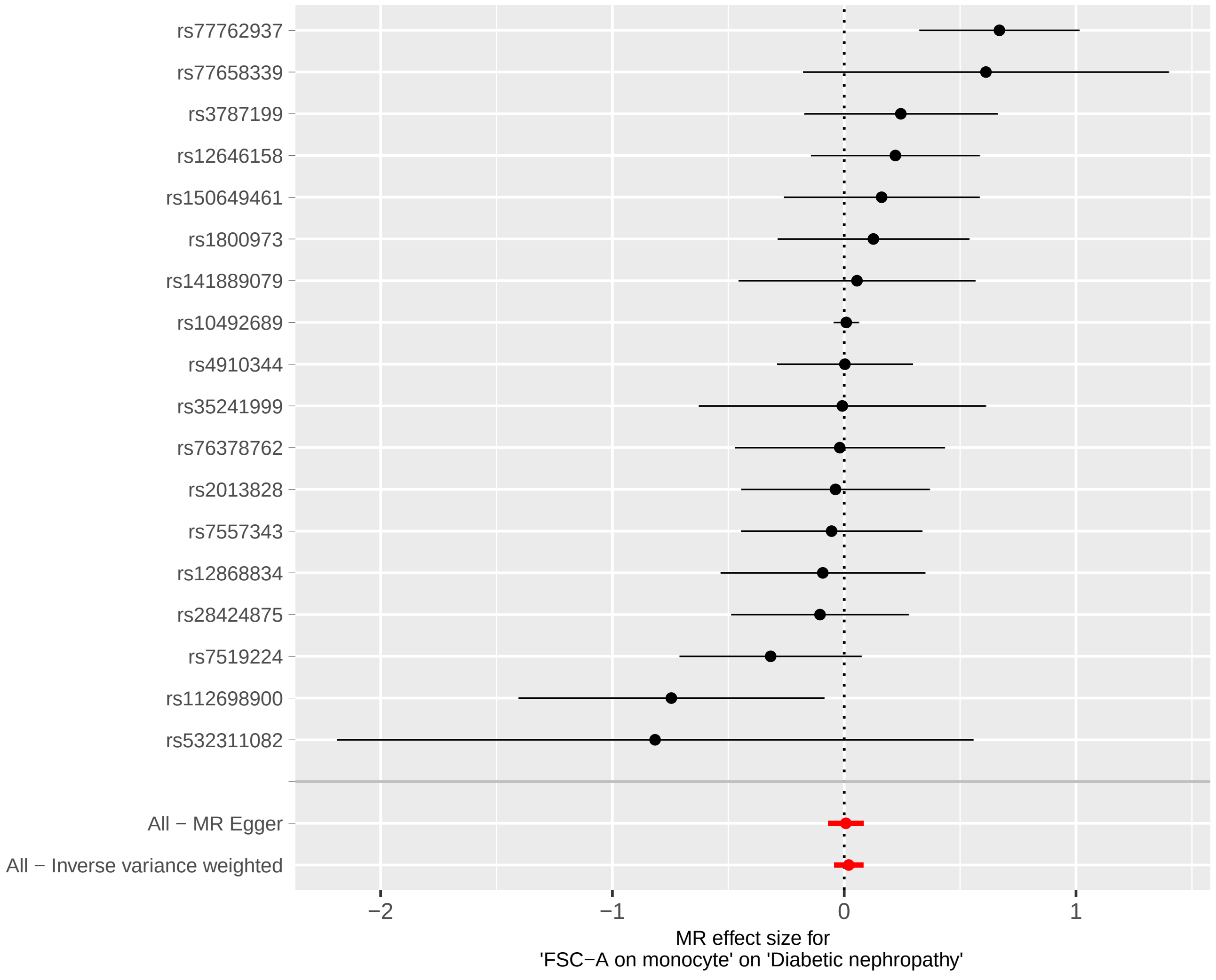

rs77762937
rs77658339
rs3787199
rs12646158
rs150649461
rs1800973
rs141889079
rs10492689
rs4910344
rs35241999
rs76378762
rs2013828
rs7557343
rs12868834
rs28424875
rs7519224
rs112698900
rs532311082
All − MR Egger
All − Inverse variance weighted
−2
−1
0
1
MR effect size for
'FSC−A on monocyte' on 'Diabetic nephropathy'

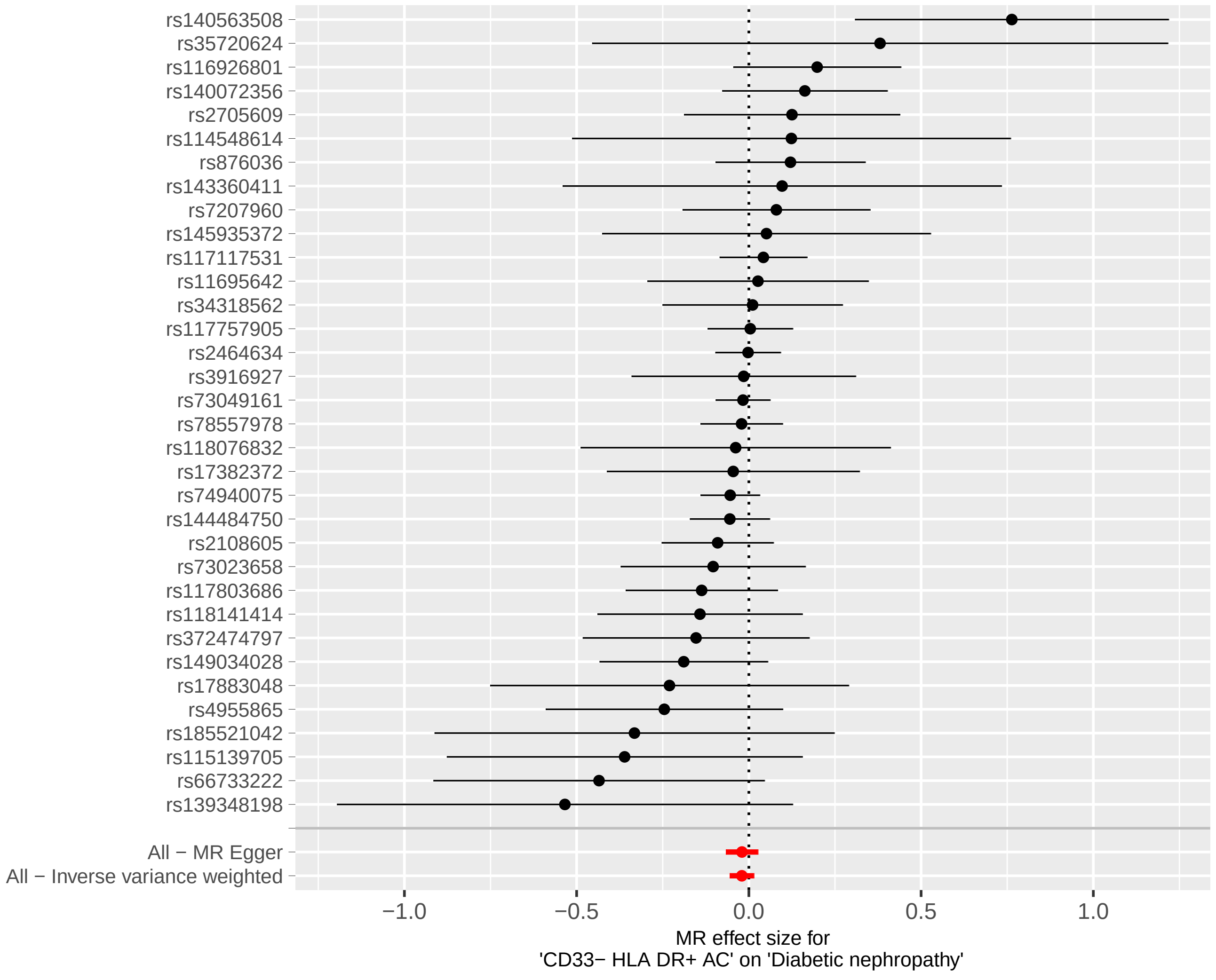
rs140563508
rs35720624
rs116926801
rs140072356
rs2705609
rs114548614
rs876036
rs143360411
rs7207960
rs145935372
rs117117531
rs11695642
rs34318562
rs117757905
rs2464634
rs3916927
rs73049161
rs78557978
rs118076832
rs17382372
rs74940075
rs144484750
rs2108605
rs73023658
rs117803686
rs118141414
rs372474797
rs149034028
rs17883048
rs4955865
rs185521042
rs115139705
rs66733222
rs139348198
All − MR Egger
All − Inverse variance weighted
−1.0
−0.5
0.0
0.5
1.0
MR effect size for
'CD33− HLA DR+ AC' on 'Diabetic nephropathy'

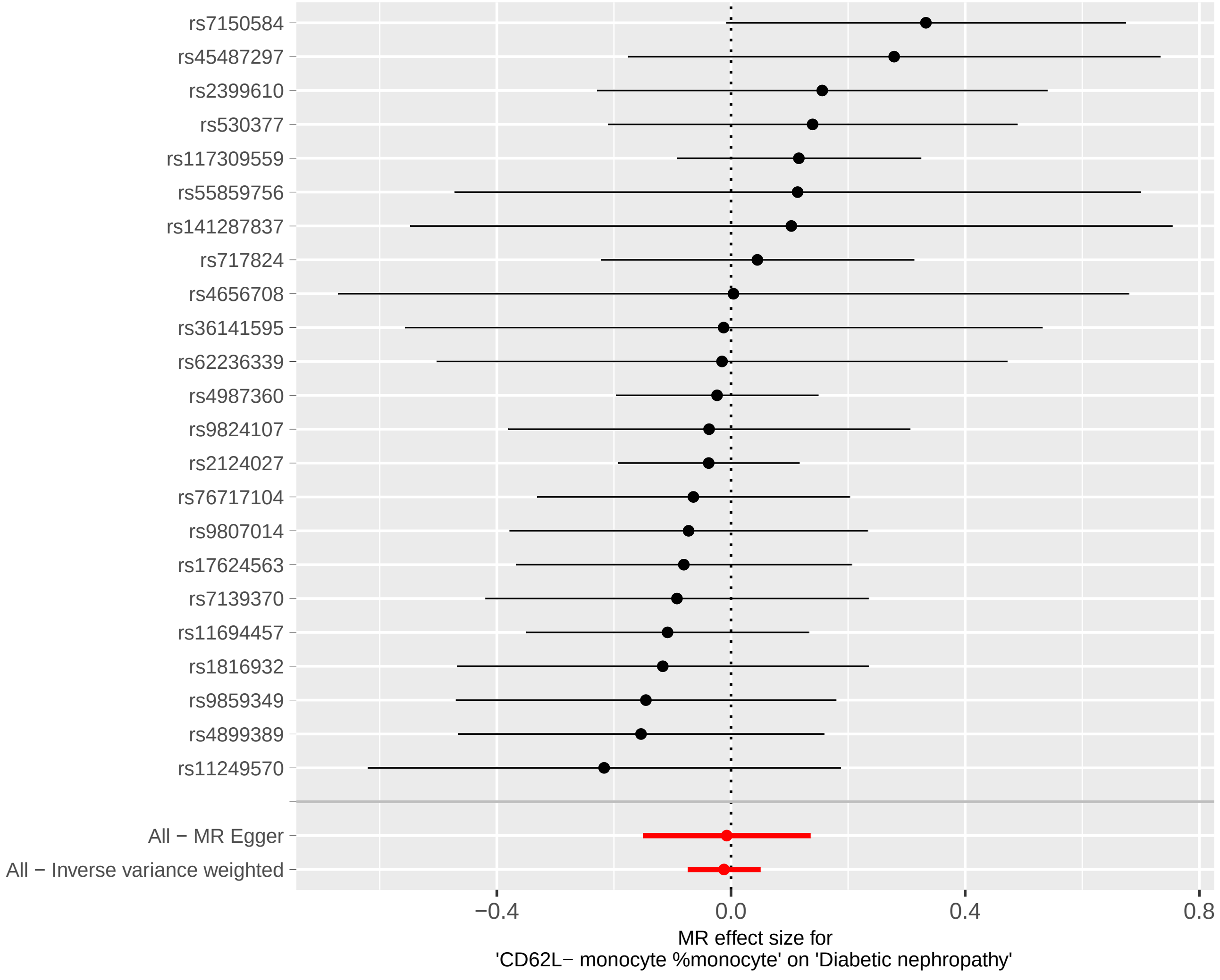

rs7150584
rs45487297
rs2399610
rs530377
rs117309559
rs55859756
rs141287837
rs717824
rs4656708
rs36141595
rs62236339
rs4987360
rs9824107
rs2124027
rs76717104
rs9807014
rs17624563
rs7139370
rs11694457
rs1816932
rs9859349
rs4899389
rs11249570
All − MR Egger
All − Inverse variance weighted
−0.4
0.0
0.4
0.8
MR effect size for
'CD62L− monocyte %monocyte' on 'Diabetic nephropathy'

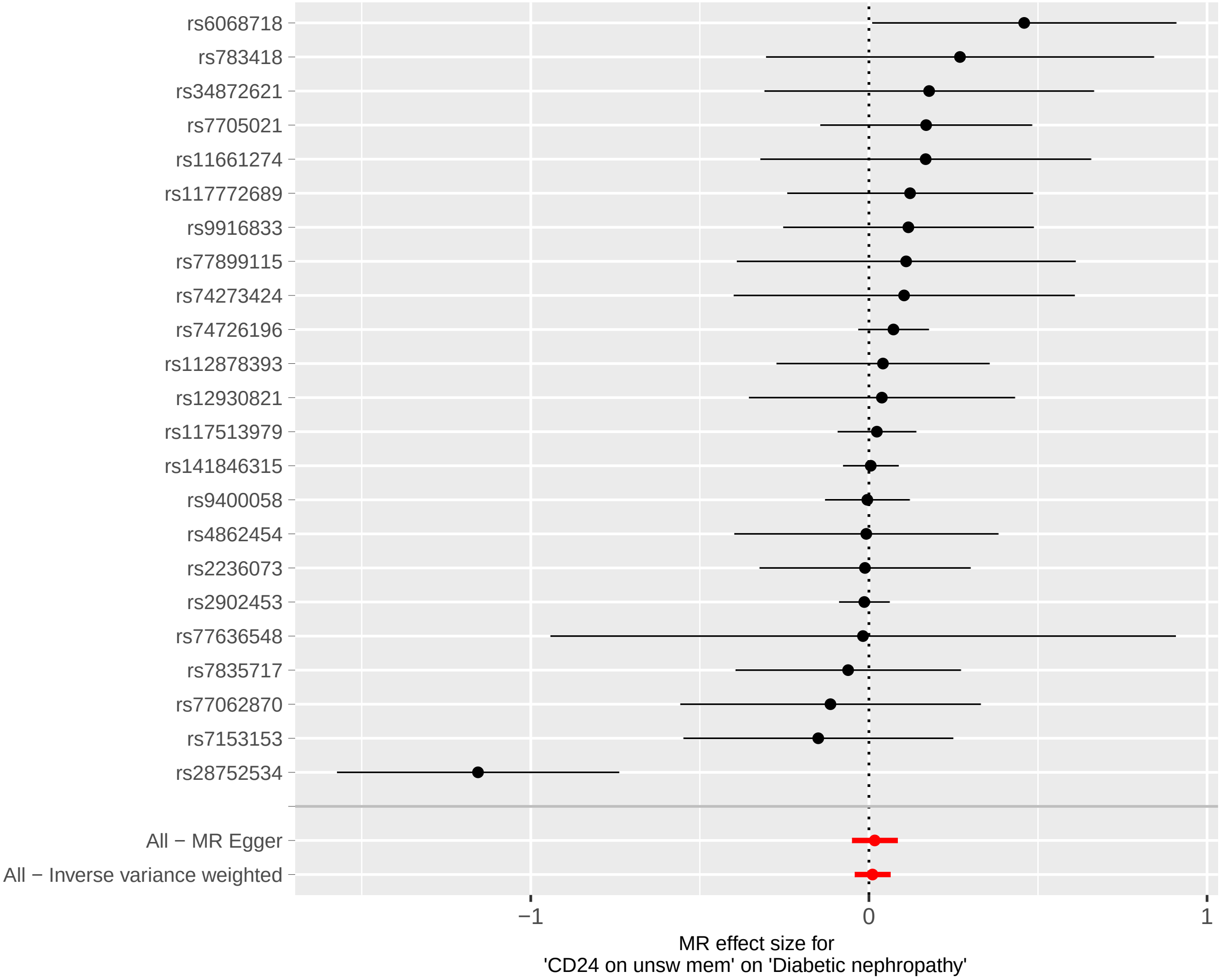

rs6068718
rs783418
rs34872621
rs7705021
rs11661274
rs117772689
rs9916833
rs77899115
rs74273424
rs74726196
rs112878393
rs12930821
rs117513979
rs141846315
rs9400058
rs4862454
rs2236073
rs2902453
rs77636548
rs7835717
rs77062870
rs7153153
rs28752534
All − MR Egger
All − Inverse variance weighted
−1
0
1
MR effect size for
'CD24 on unsw mem' on 'Diabetic nephropathy'

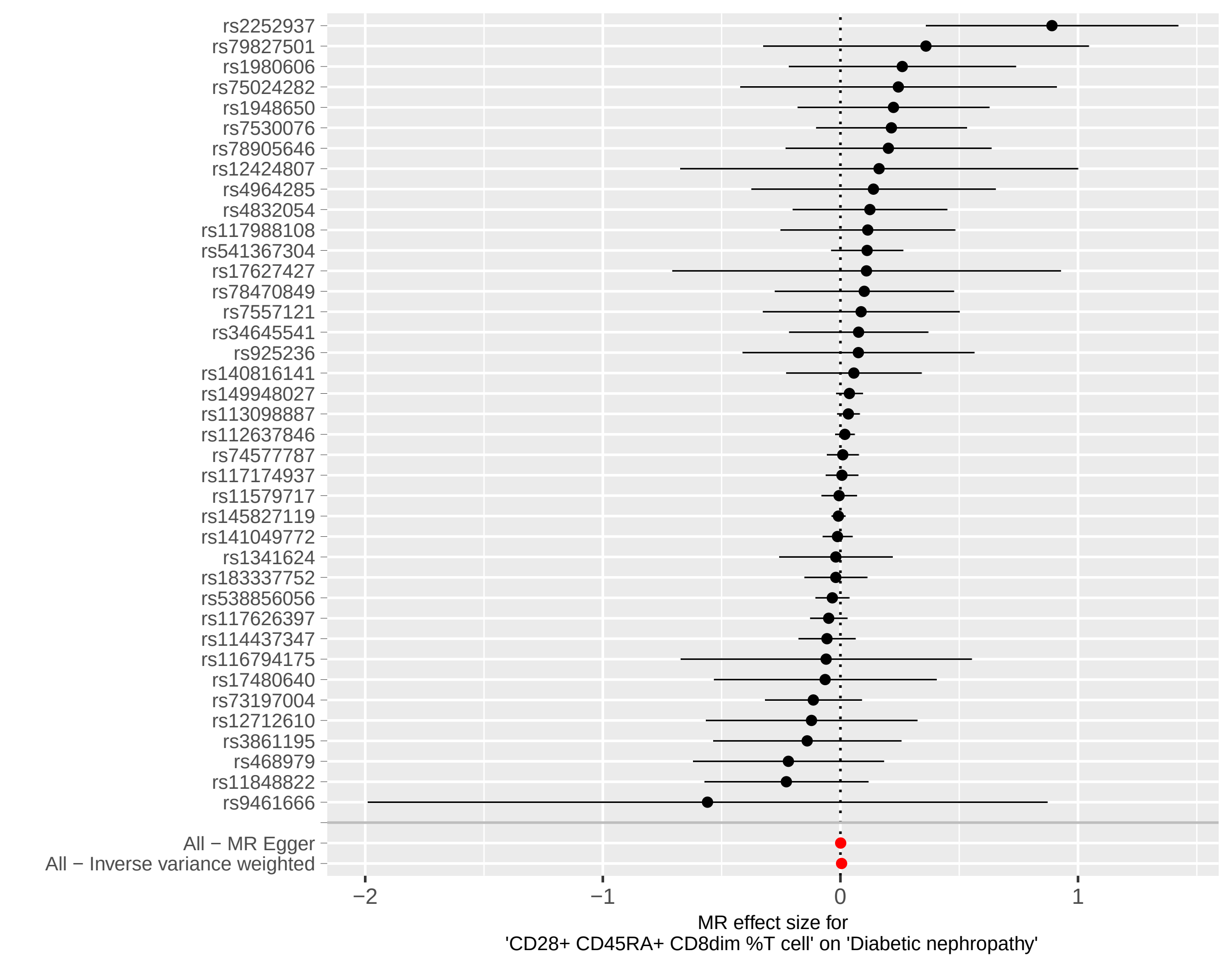
rs2252937
rs79827501
rs1980606
rs75024282
rs1948650
rs7530076
rs78905646
rs12424807
rs4964285
rs4832054
rs117988108
rs541367304
rs17627427
rs78470849
rs7557121
rs34645541
rs925236
rs140816141
rs149948027
rs113098887
rs112637846
rs74577787
rs117174937
rs11579717
rs145827119
rs141049772
rs1341624
rs183337752
rs538856056
rs117626397
rs114437347
rs116794175
rs17480640
rs73197004
rs12712610
rs3861195
rs468979
rs11848822
rs9461666
All − MR Egger
All − Inverse variance weighted
−2
−1
0
1
MR effect size for
'CD28+ CD45RA+ CD8dim %T cell' on 'Diabetic nephropathy'

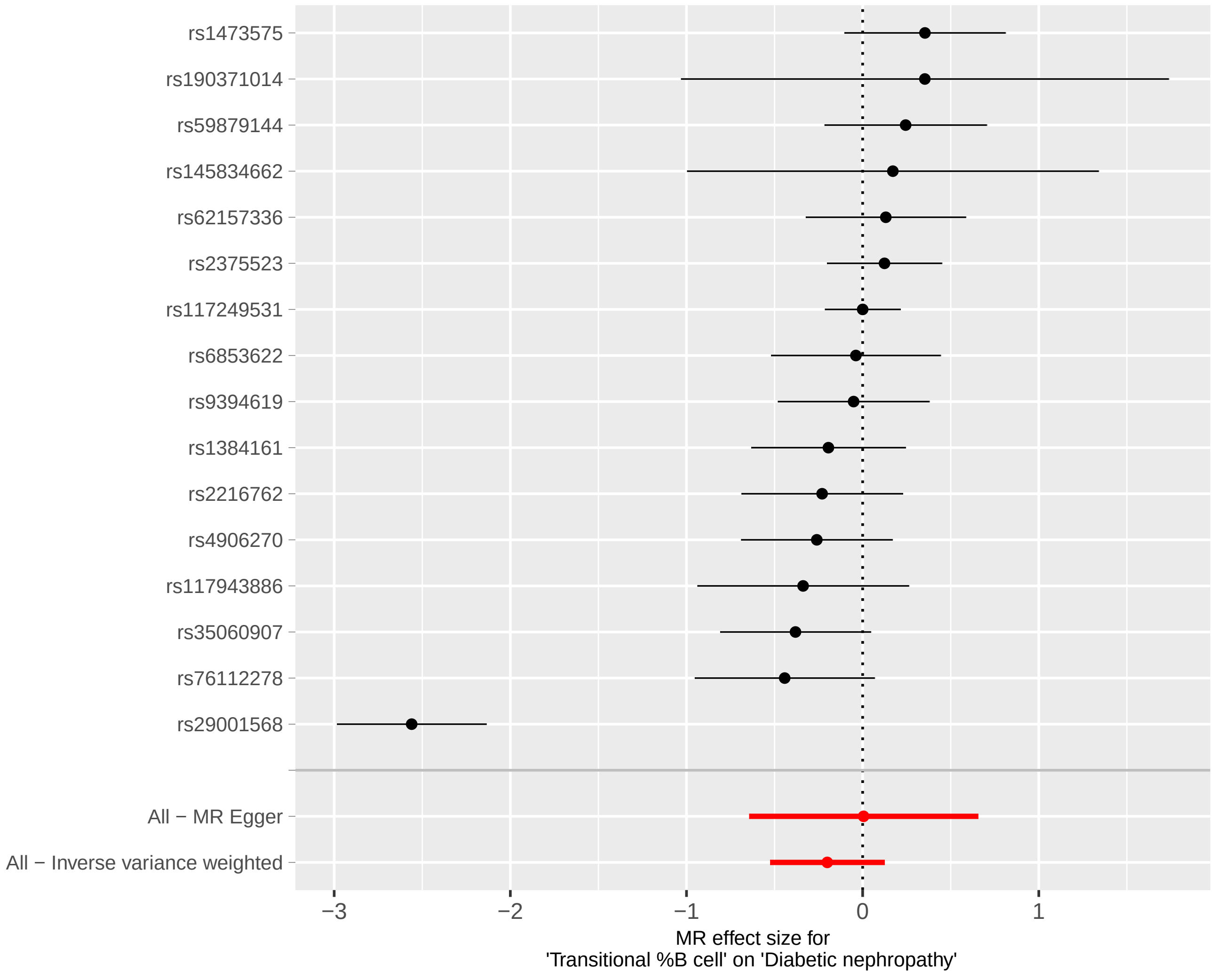

rs1473575
rs190371014
rs59879144
rs145834662
rs62157336
rs2375523
rs117249531
rs6853622
rs9394619
rs1384161
rs2216762
rs4906270
rs117943886
rs35060907
rs76112278
rs29001568
All − MR Egger
All − Inverse variance weighted
−3
−2
−1
0
1
MR effect size for
'Transitional %B cell' on 'Diabetic nephropathy'

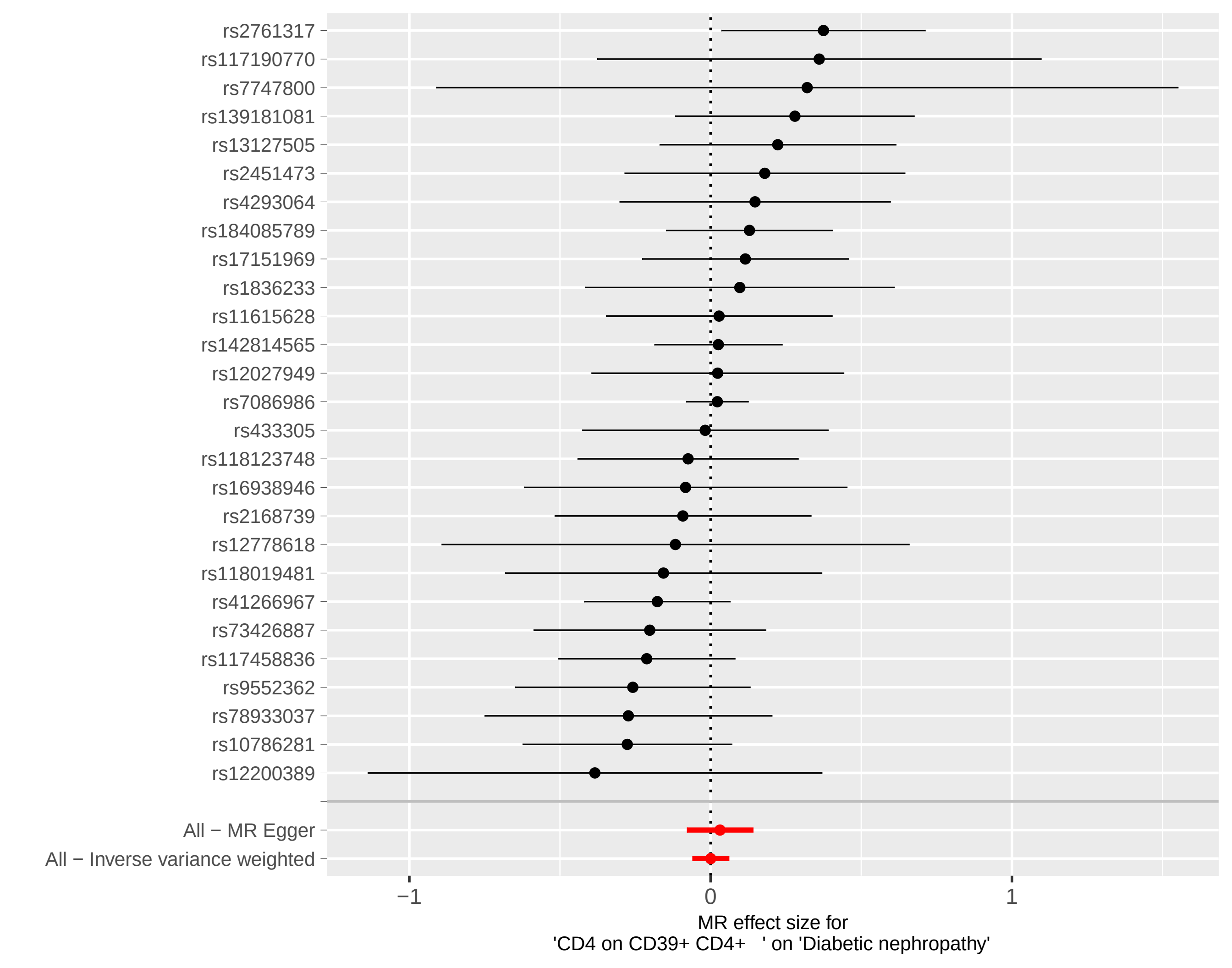
rs2761317
rs117190770
rs7747800
rs139181081
rs13127505
rs2451473
rs4293064
rs184085789
rs17151969
rs1836233
rs11615628
rs142814565
rs12027949
rs7086986
rs433305
rs118123748
rs16938946
rs2168739
rs12778618
rs118019481
rs41266967
rs73426887
rs117458836
rs9552362
rs78933037
rs10786281
rs12200389
All − MR Egger
All − Inverse variance weighted
−1
0
1
MR effect size for
'CD4 on CD39+ CD4+ ' on 'Diabetic nephropathy'

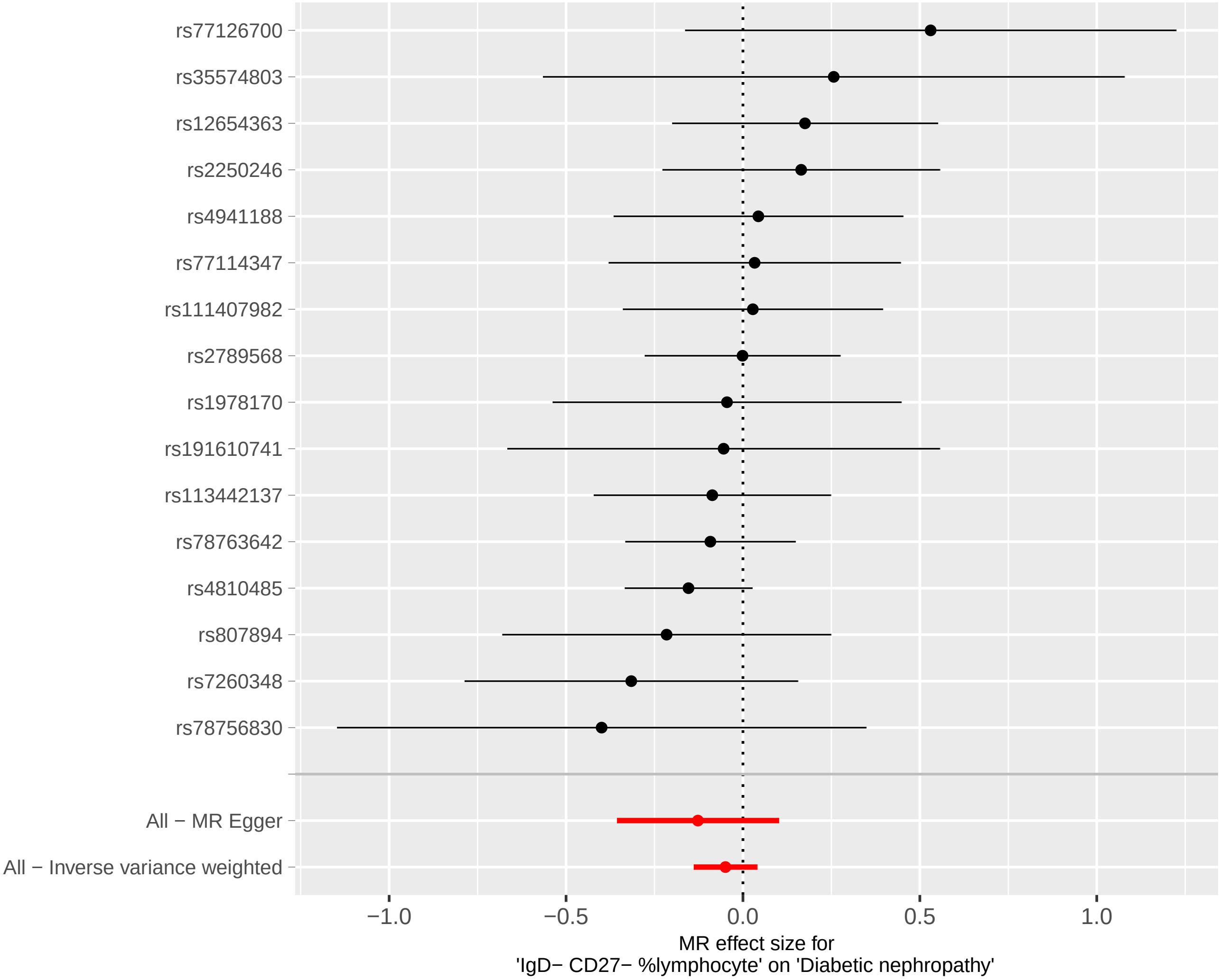
rs77126700
rs35574803
rs12654363
rs2250246
rs4941188
rs77114347
rs111407982
rs2789568
rs1978170
rs191610741
rs113442137
rs78763642
rs4810485
rs807894
rs7260348
rs78756830
All − MR Egger
All − Inverse variance weighted
−1.0
−0.5
0.0
0.5
1.0
MR effect size for
'IgD− CD27− %lymphocyte' on 'Diabetic nephropathy'

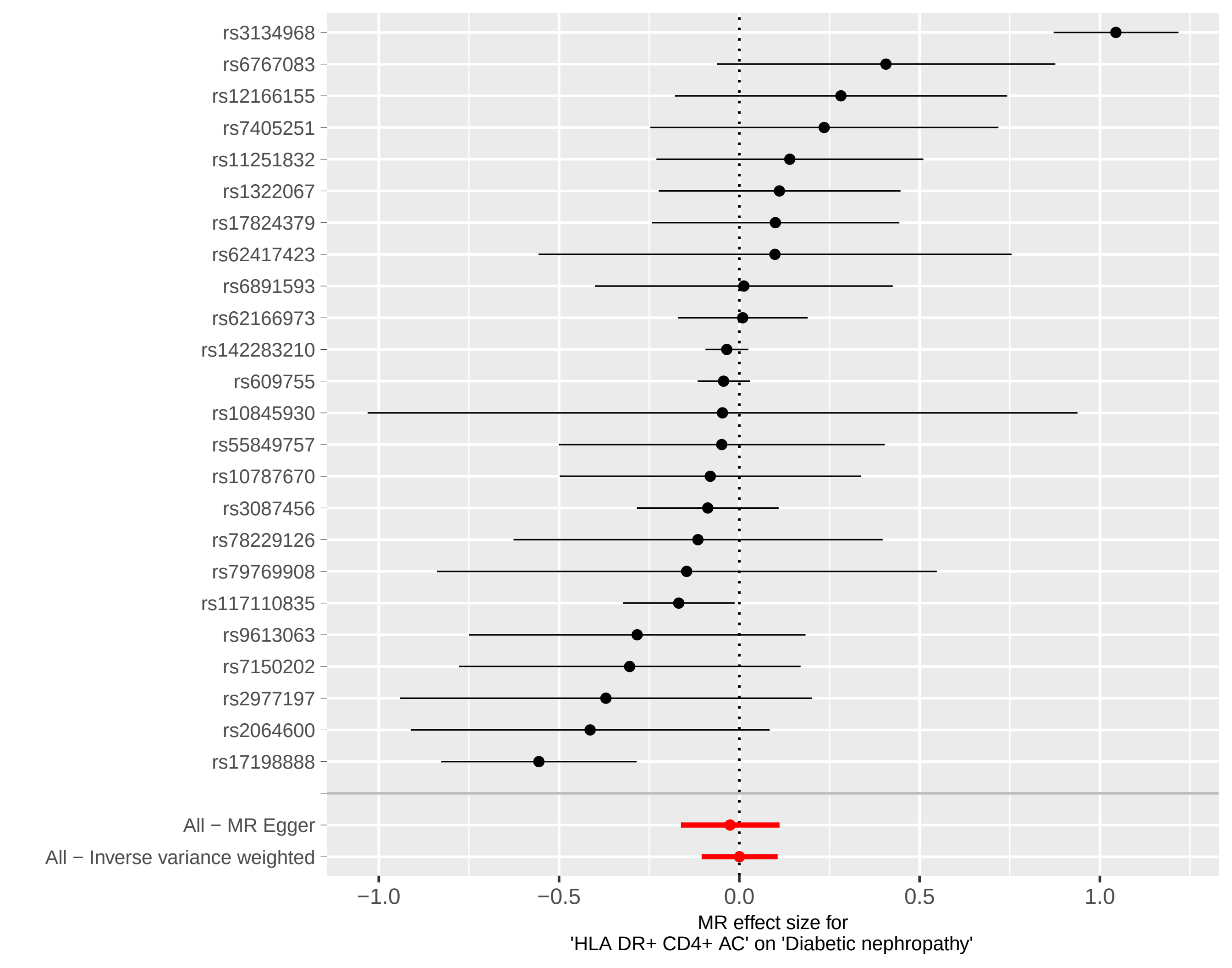

rs3134968
rs6767083
rs12166155
rs7405251
rs11251832
rs1322067
rs17824379
rs62417423
rs6891593
rs62166973
rs142283210
rs609755
rs10845930
rs55849757
rs10787670
rs3087456
rs78229126
rs79769908
rs117110835
rs9613063
rs7150202
rs2977197
rs2064600
rs17198888
All − MR Egger
All − Inverse variance weighted
−1.0
−0.5
0.0
0.5
1.0
MR effect size for
'HLA DR+ CD4+ AC' on 'Diabetic nephropathy'

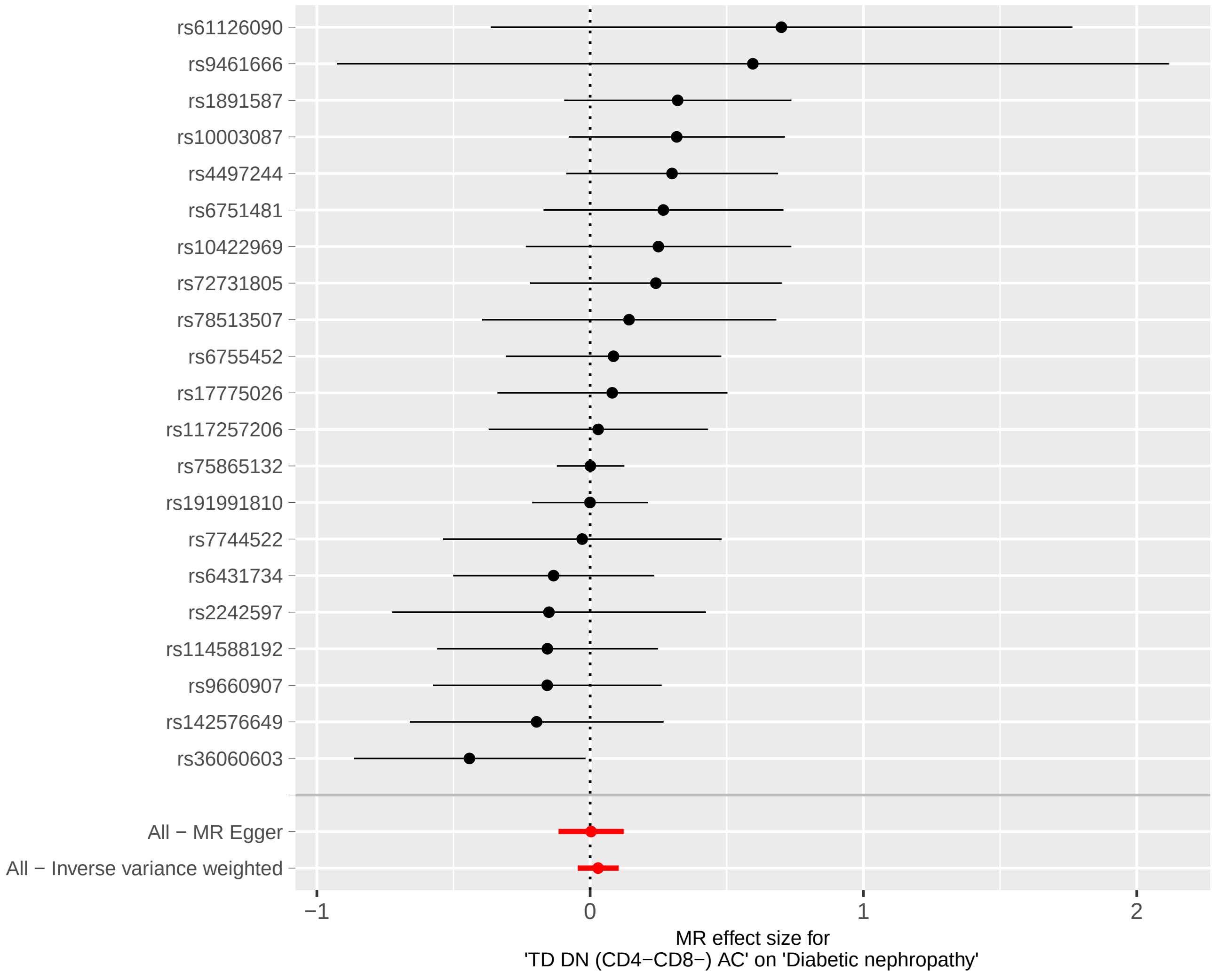
rs61126090
rs9461666
rs1891587
rs10003087
rs4497244
rs6751481
rs10422969
rs72731805
rs78513507
rs6755452
rs17775026
rs117257206
rs75865132
rs191991810
rs7744522
rs6431734
rs2242597
rs114588192
rs9660907
rs142576649
rs36060603
All − MR Egger
All − Inverse variance weighted
−1
0
1
2
MR effect size for
'TD DN (CD4−CD8−) AC' on 'Diabetic nephropathy'

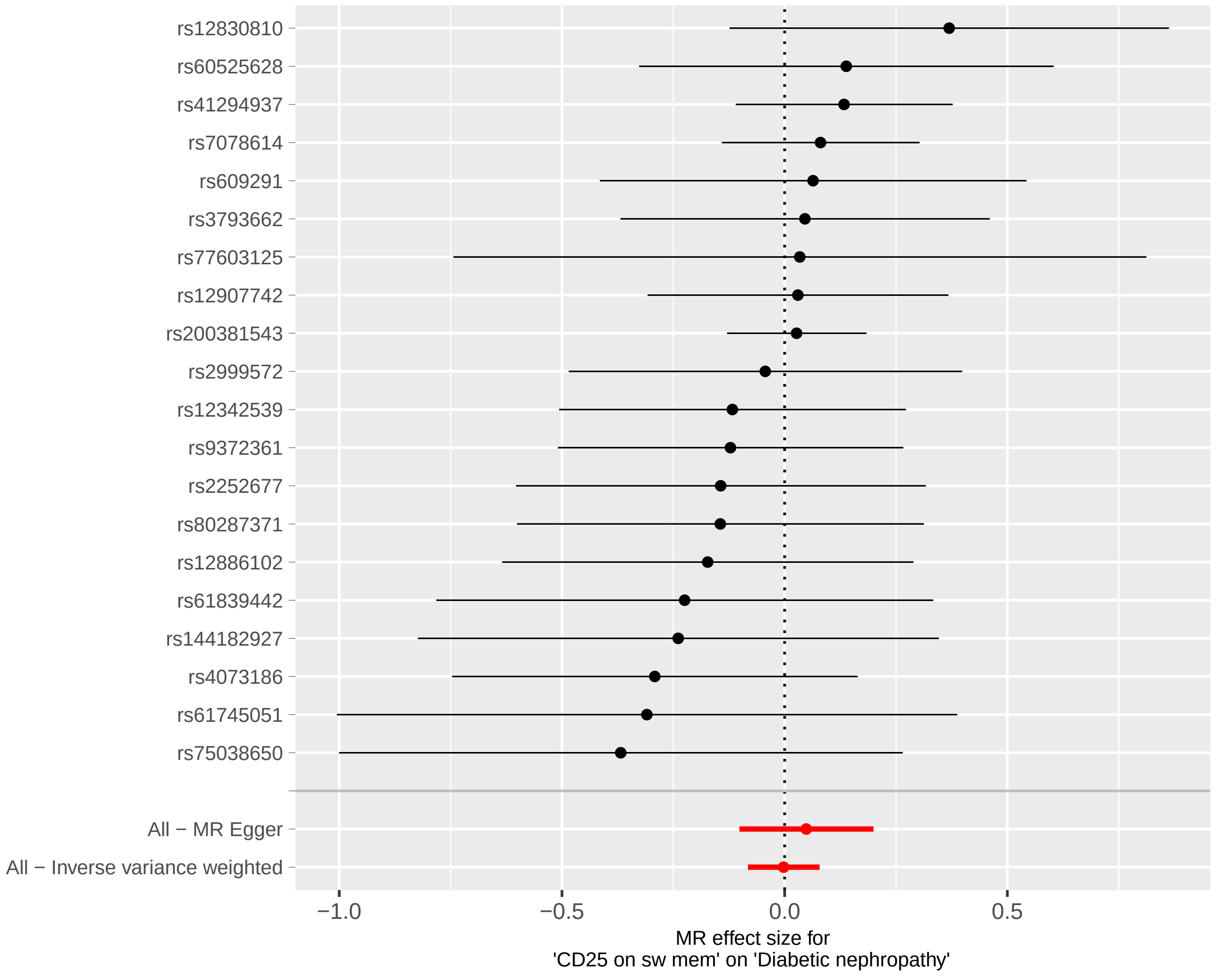
rs12830810
rs60525628
rs41294937
rs7078614
rs609291
rs3793662
rs77603125
rs12907742
rs200381543
rs2999572
rs12342539
rs9372361
rs2252677
rs80287371
rs12886102
rs61839442
rs144182927
rs4073186
rs61745051
rs75038650
All − MR Egger
All − Inverse variance weighted
−1.0
−0.5
0.0
0.5
MR effect size for
'CD25 on sw mem' on 'Diabetic nephropathy'

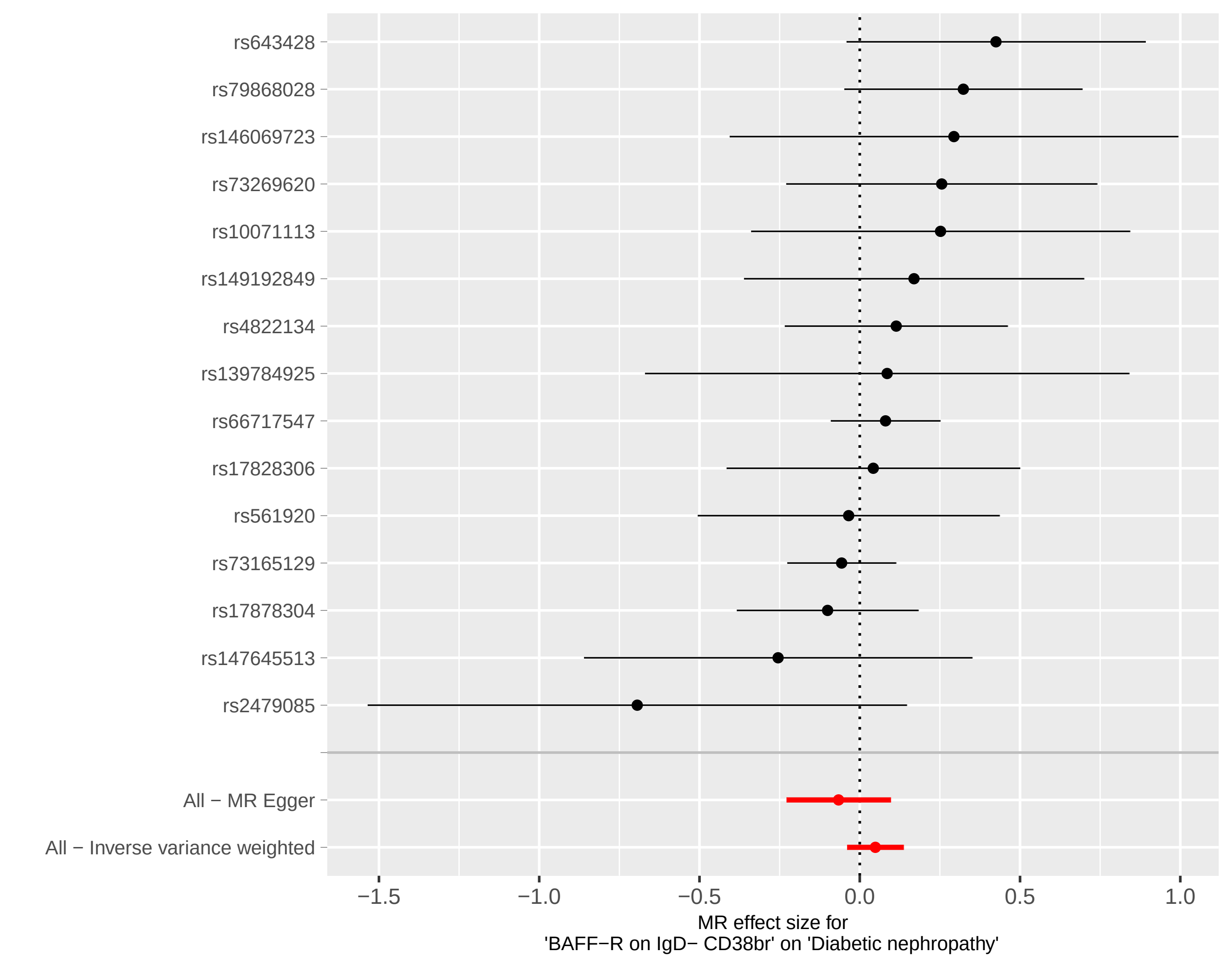
rs643428
rs79868028
rs146069723
rs73269620
rs10071113
rs149192849
rs4822134
rs139784925
rs66717547
rs17828306
rs561920
rs73165129
rs17878304
rs147645513
rs2479085
All − MR Egger
All − Inverse variance weighted
−1.5
−1.0
−0.5
0.0
0.5
1.0
MR effect size for
'BAFF−R on IgD− CD38br' on 'Diabetic nephropathy'

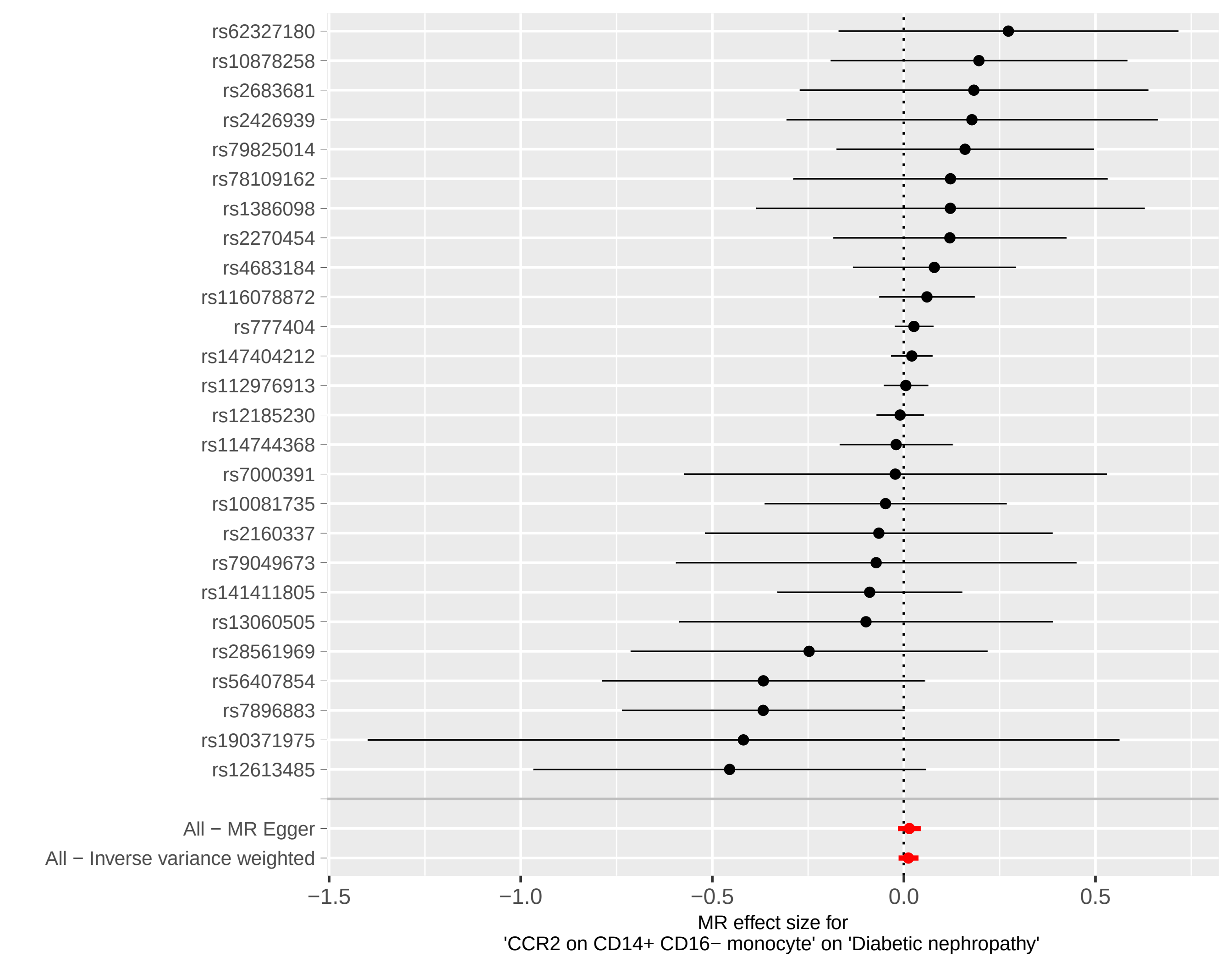
rs62327180
rs10878258
rs2683681
rs2426939
rs79825014
rs78109162
rs1386098
rs2270454
rs4683184
rs116078872
rs777404
rs147404212
rs112976913
rs12185230
rs114744368
rs7000391
rs10081735
rs2160337
rs79049673
rs141411805
rs13060505
rs28561969
rs56407854
rs7896883
rs190371975
rs12613485
All − MR Egger
All − Inverse variance weighted
−1.5
−1.0
−0.5
0.0
0.5
MR effect size for
'CCR2 on CD14+ CD16− monocyte' on 'Diabetic nephropathy'

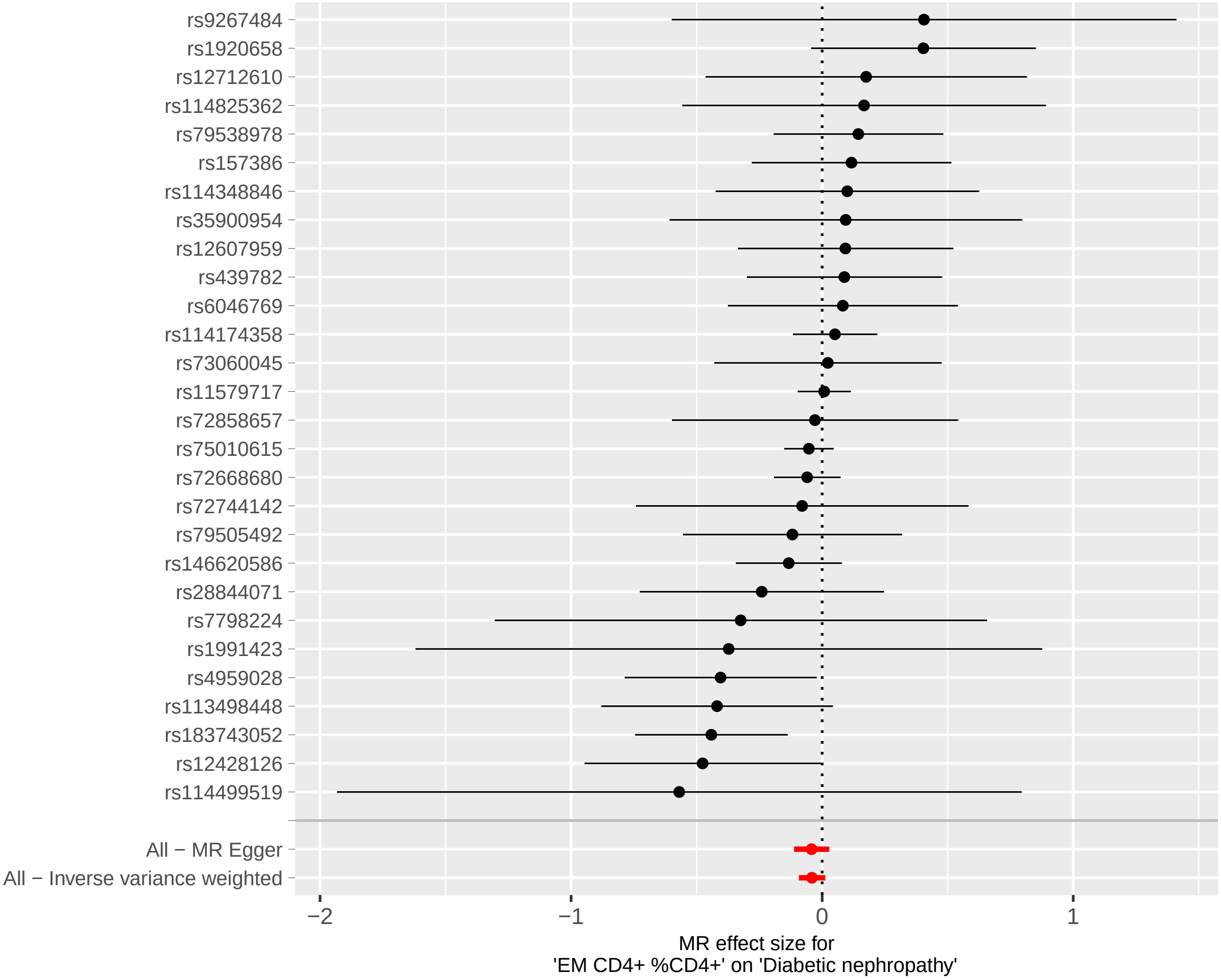
rs9267484
rs1920658
rs12712610
rs114825362
rs79538978
rs157386
rs114348846
rs35900954
rs12607959
rs439782
rs6046769
rs114174358
rs73060045
rs11579717
rs72858657
rs75010615
rs72668680
rs72744142
rs79505492
rs146620586
rs28844071
rs7798224
rs1991423
rs4959028
rs113498448
rs183743052
rs12428126
rs114499519
All − MR Egger
All − Inverse variance weighted
−2
−1
0
1
MR effect size for
'EM CD4+ %CD4+' on 'Diabetic nephropathy'

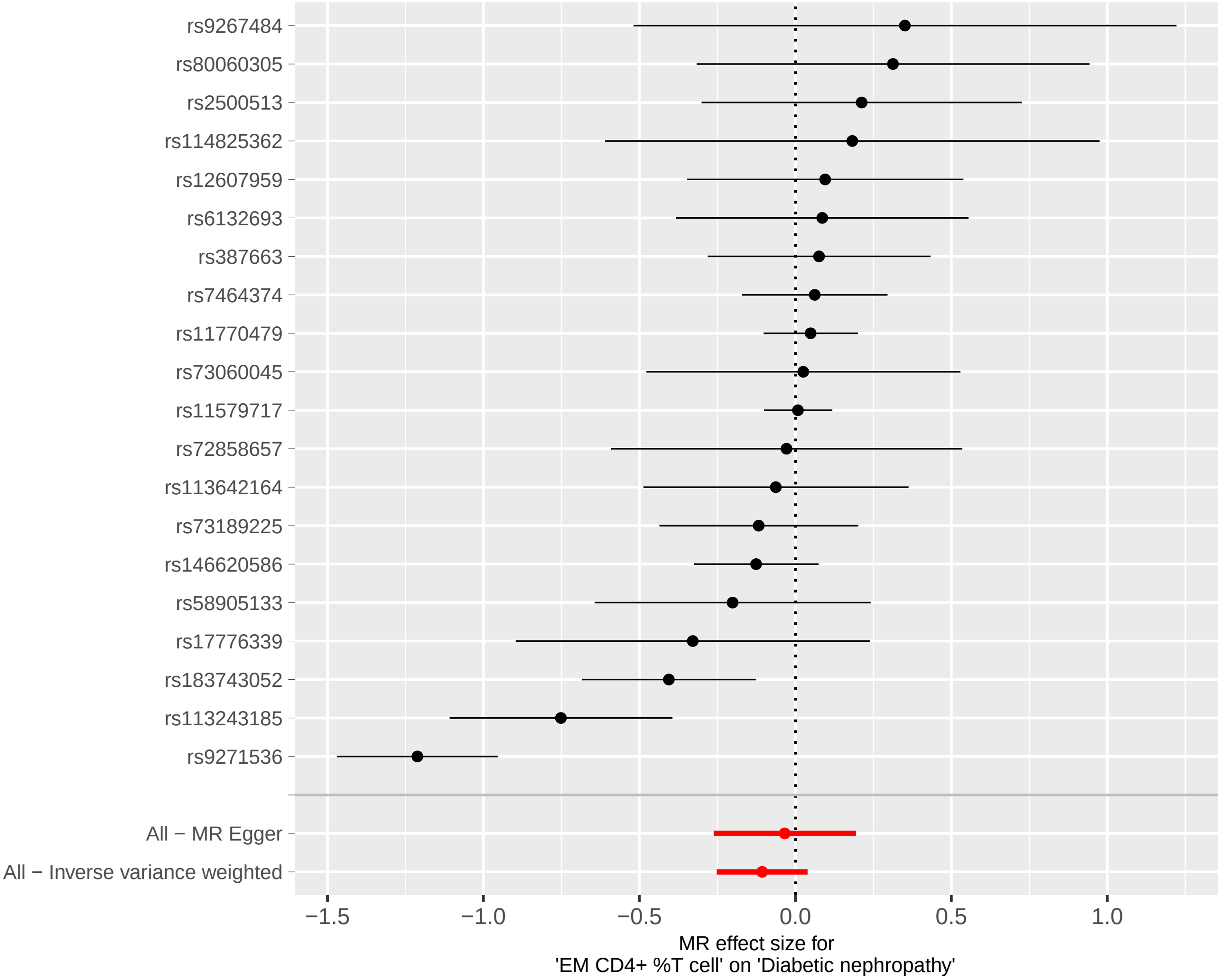

rs9267484
rs80060305
rs2500513
rs114825362
rs12607959
rs6132693
rs387663
rs7464374
rs11770479
rs73060045
rs11579717
rs72858657
rs113642164
rs73189225
rs146620586
rs58905133
rs17776339
rs183743052
rs113243185
rs9271536
All − MR Egger
All − Inverse variance weighted
−1.5
−1.0
−0.5
0.0
0.5
1.0
MR effect size for
'EM CD4+ %T cell' on 'Diabetic nephropathy'

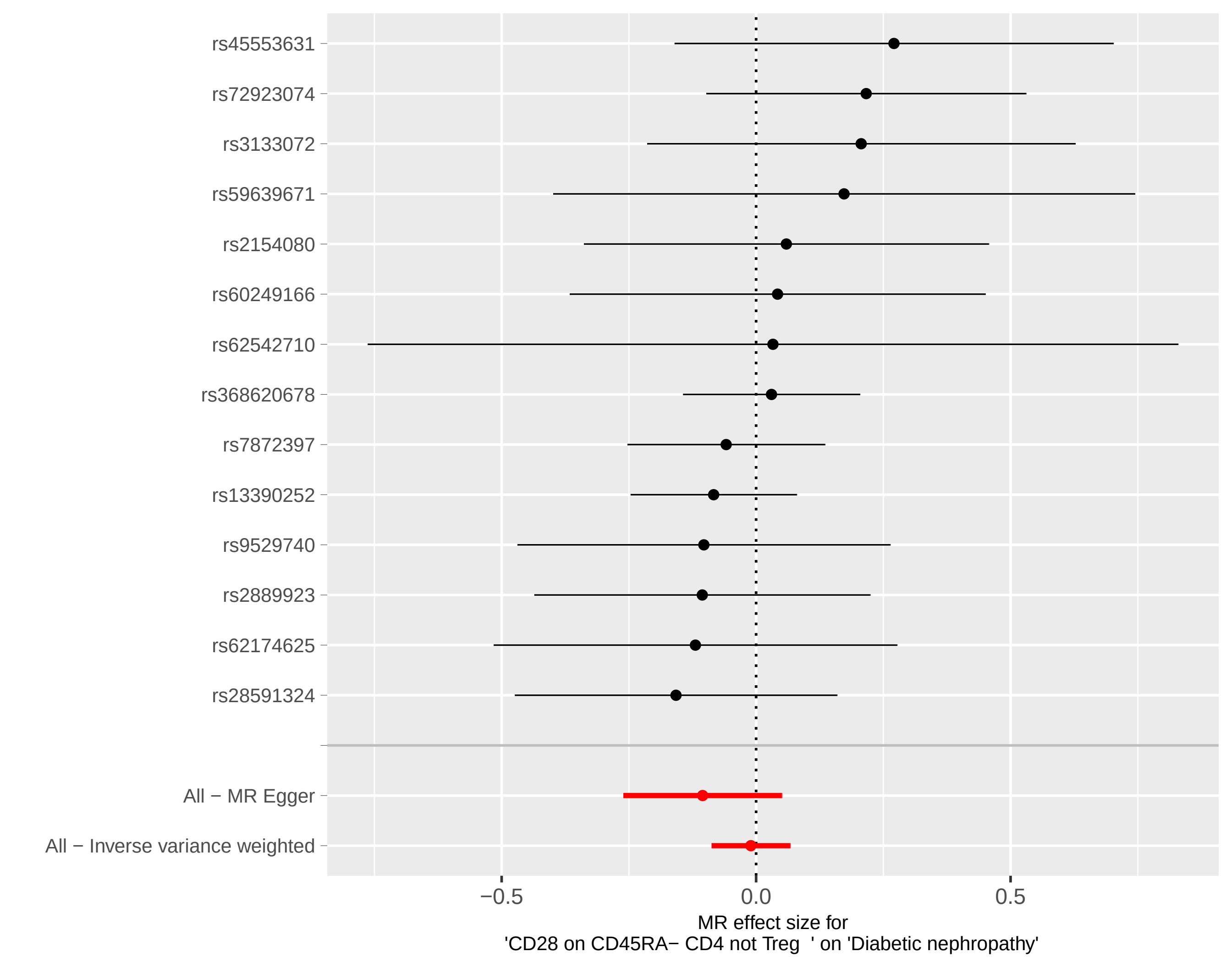
rs45553631
rs72923074
rs3133072
rs59639671
rs2154080
rs60249166
rs62542710
rs368620678
rs7872397
rs13390252
rs9529740
rs2889923
rs62174625
rs28591324
All − MR Egger
All − Inverse variance weighted
−0.5
0.0
0.5
MR effect size for
'CD28 on CD45RA− CD4 not Treg ' on 'Diabetic nephropathy'

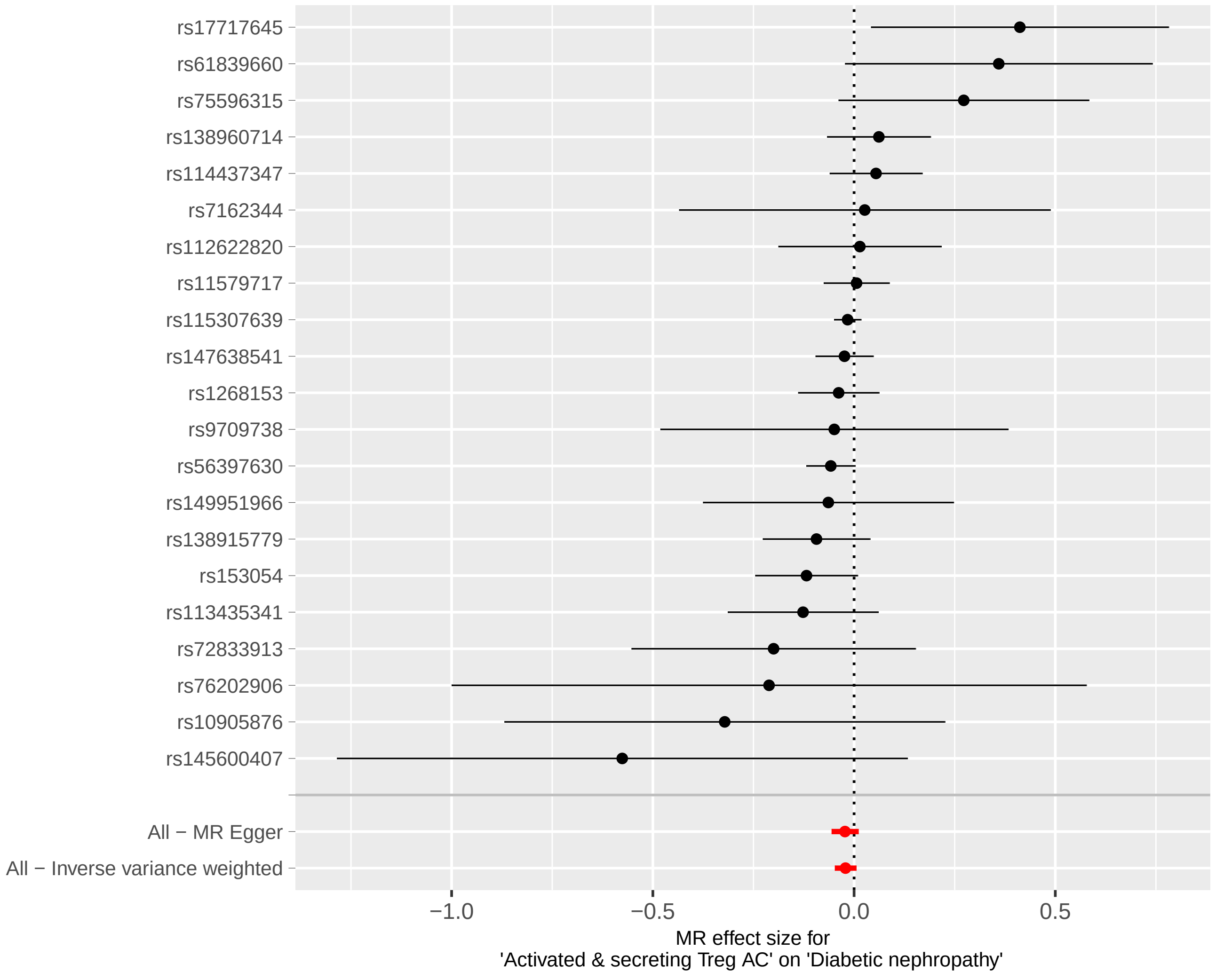
rs17717645
rs61839660
rs75596315
rs138960714
rs114437347
rs7162344
rs112622820
rs11579717
rs115307639
rs147638541
rs1268153
rs9709738
rs56397630
rs149951966
rs138915779
rs153054
rs113435341
rs72833913
rs76202906
rs10905876
rs145600407
All − MR Egger
All − Inverse variance weighted
−1.0
−0.5
0.0
0.5
MR effect size for
'Activated & secreting Treg AC' on 'Diabetic nephropathy'

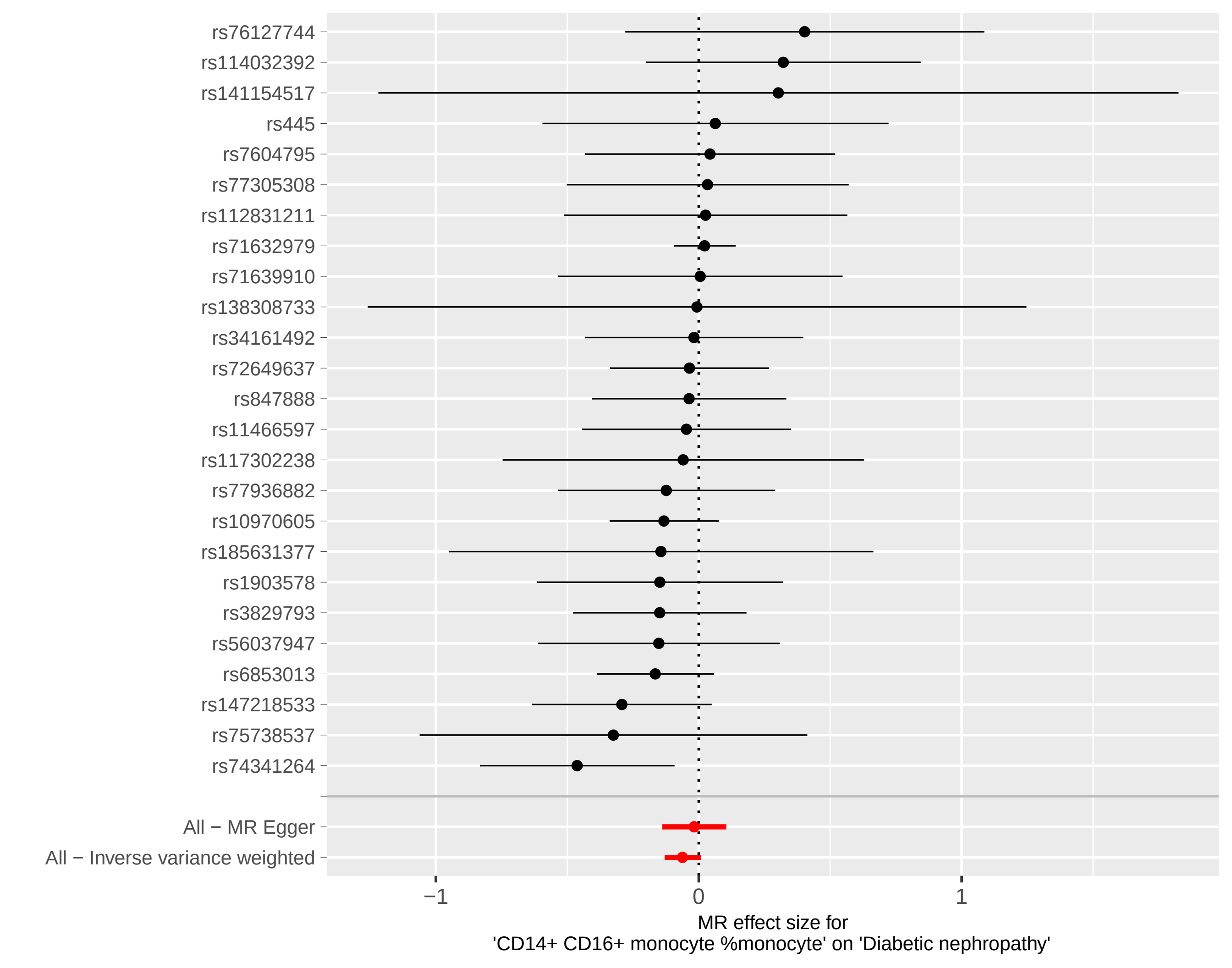

rs76127744
rs114032392
rs141154517
rs445
rs7604795
rs77305308
rs112831211
rs71632979
rs71639910
rs138308733
rs34161492
rs72649637
rs847888
rs11466597
rs117302238
rs77936882
rs10970605
rs185631377
rs1903578
rs3829793
rs56037947
rs6853013
rs147218533
rs75738537
rs74341264
All − MR Egger
All − Inverse variance weighted
−1
0
1
MR effect size for
'CD14+ CD16+ monocyte %monocyte' on 'Diabetic nephropathy'

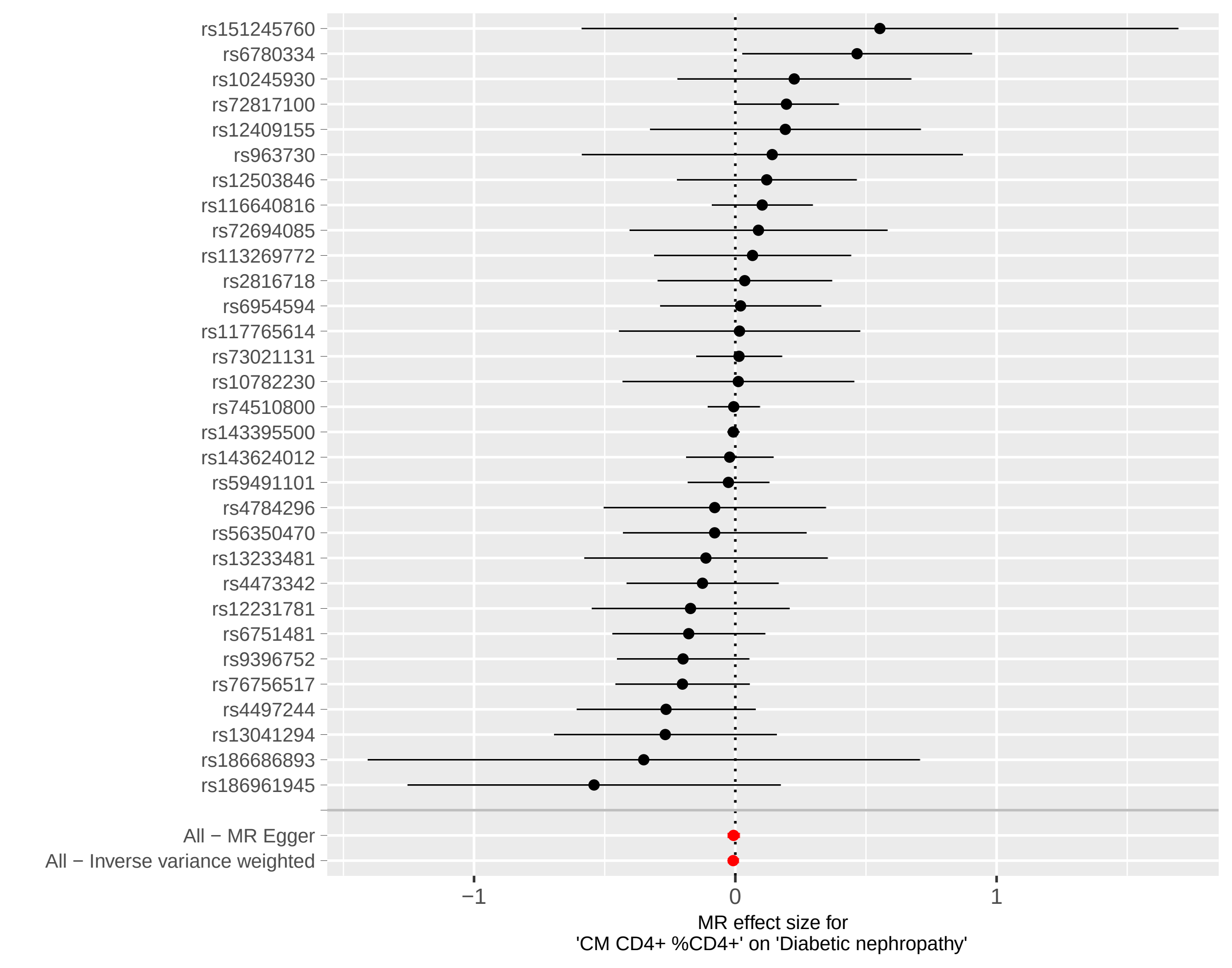
rs151245760
rs6780334
rs10245930
rs72817100
rs12409155
rs963730
rs12503846
rs116640816
rs72694085
rs113269772
rs2816718
rs6954594
rs117765614
rs73021131
rs10782230
rs74510800
rs143395500
rs143624012
rs59491101
rs4784296
rs56350470
rs13233481
rs4473342
rs12231781
rs6751481
rs9396752
rs76756517
rs4497244
rs13041294
rs186686893
rs186961945
All − MR Egger
All − Inverse variance weighted
−1
0
1
MR effect size for
'CM CD4+ %CD4+' on 'Diabetic nephropathy'

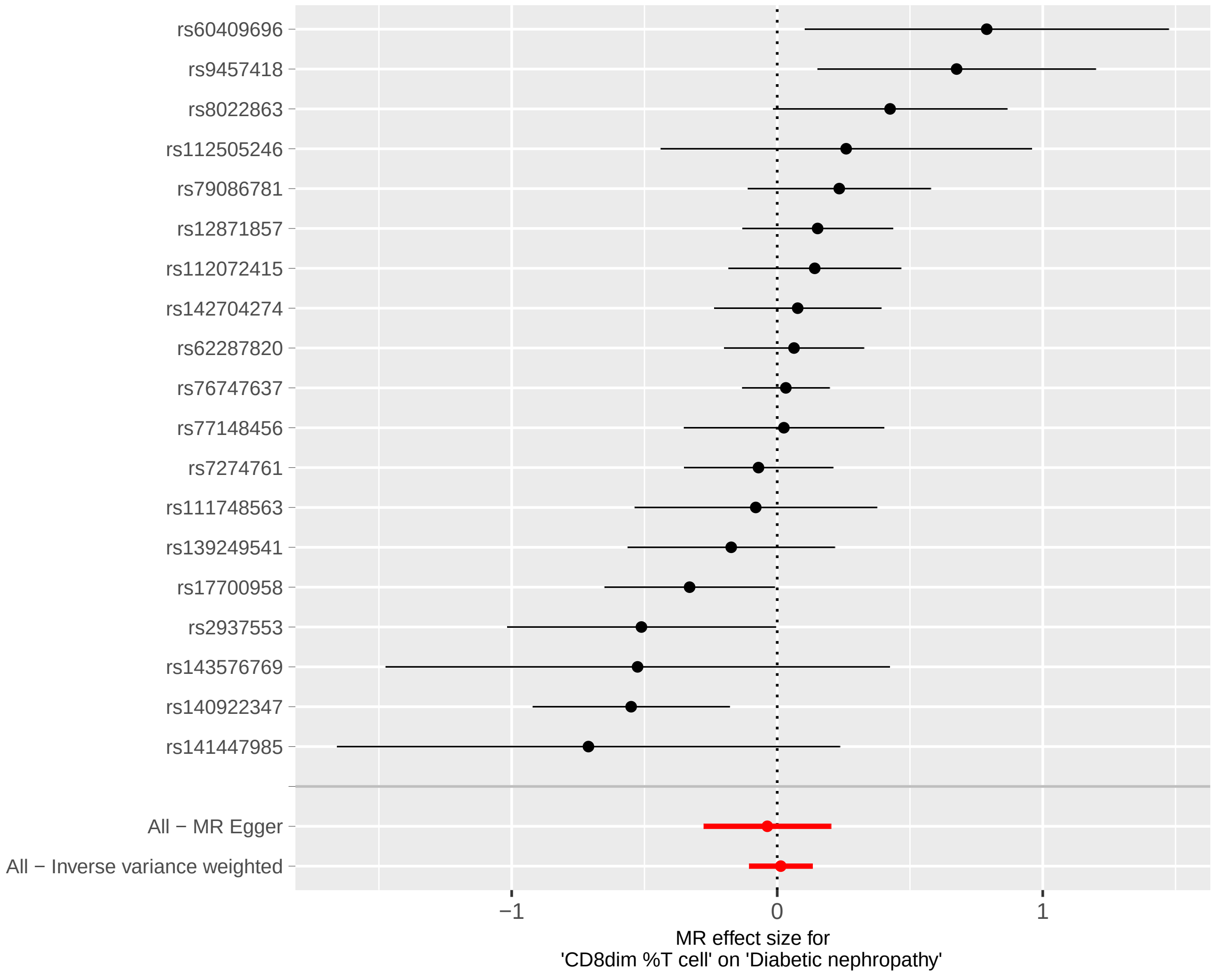
rs60409696
rs9457418
rs8022863
rs112505246
rs79086781
rs12871857
rs112072415
rs142704274
rs62287820
rs76747637
rs77148456
rs7274761
rs111748563
rs139249541
rs17700958
rs2937553
rs143576769
rs140922347
rs141447985
All − MR Egger
All − Inverse variance weighted
−1
0
1
MR effect size for
'CD8dim %T cell' on 'Diabetic nephropathy'

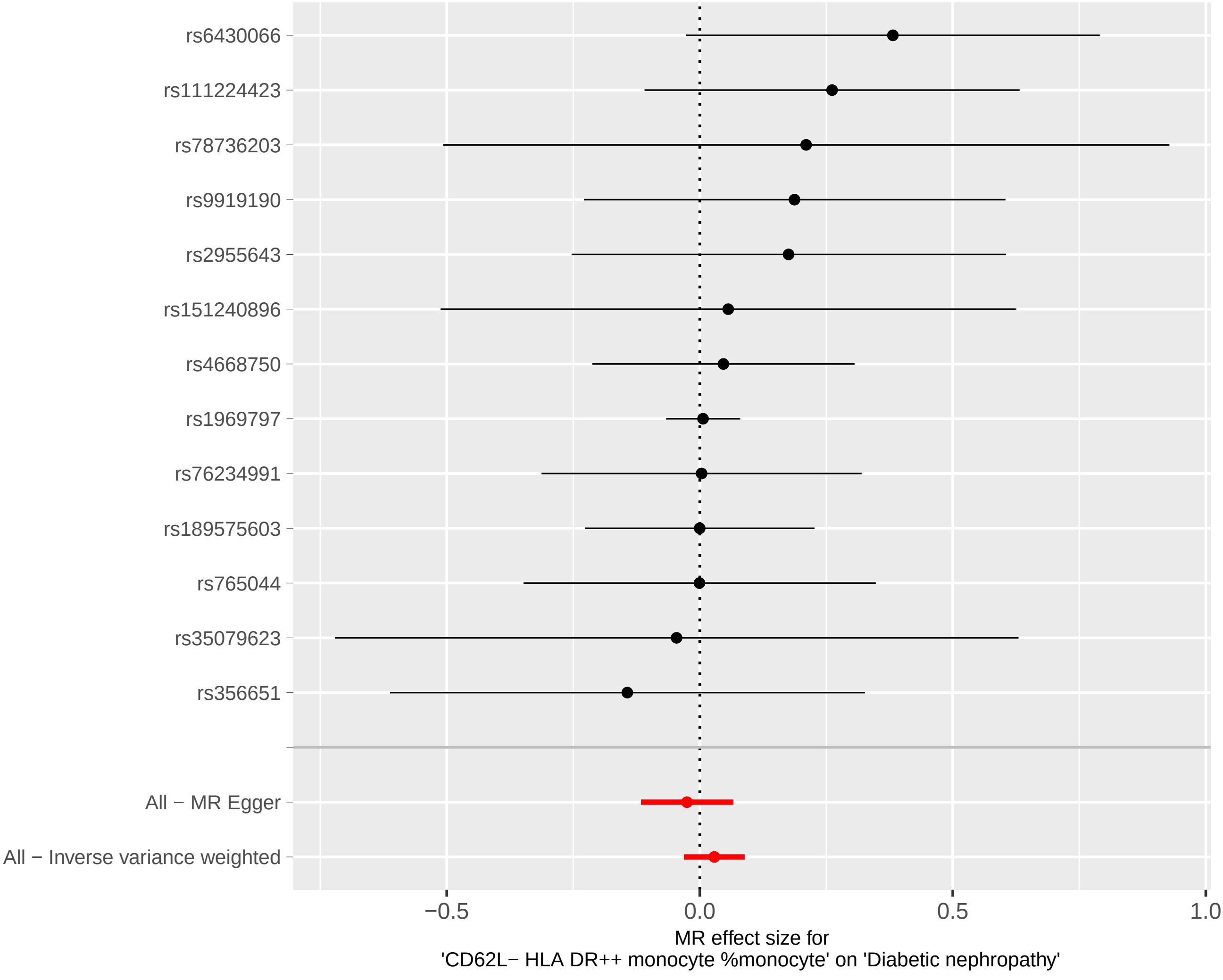
rs6430066
rs111224423
rs78736203
rs9919190
rs2955643
rs151240896
rs4668750
rs1969797
rs76234991
rs189575603
rs765044
rs35079623
rs356651
All − MR Egger
All − Inverse variance weighted
−0.5
0.0
0.5
1.0
MR effect size for
'CD62L− HLA DR++ monocyte %monocyte' on 'Diabetic nephropathy'

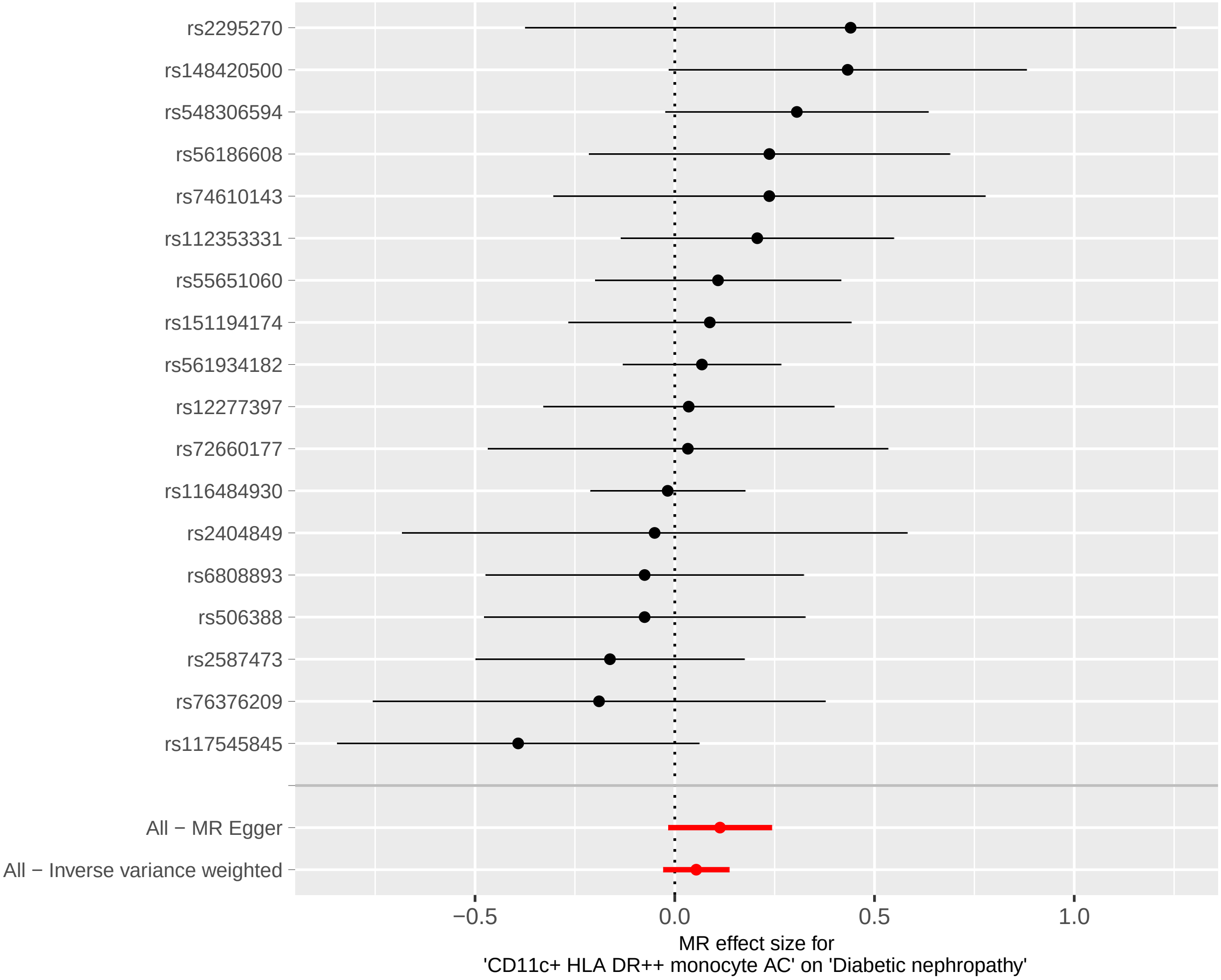
rs2295270
rs148420500
rs548306594
rs56186608
rs74610143
rs112353331
rs55651060
rs151194174
rs561934182
rs12277397
rs72660177
rs116484930
rs2404849
rs6808893
rs506388
rs2587473
rs76376209
rs117545845
All − MR Egger
All − Inverse variance weighted
−0.5
0.0
0.5
1.0
MR effect size for
'CD11c+ HLA DR++ monocyte AC' on 'Diabetic nephropathy'

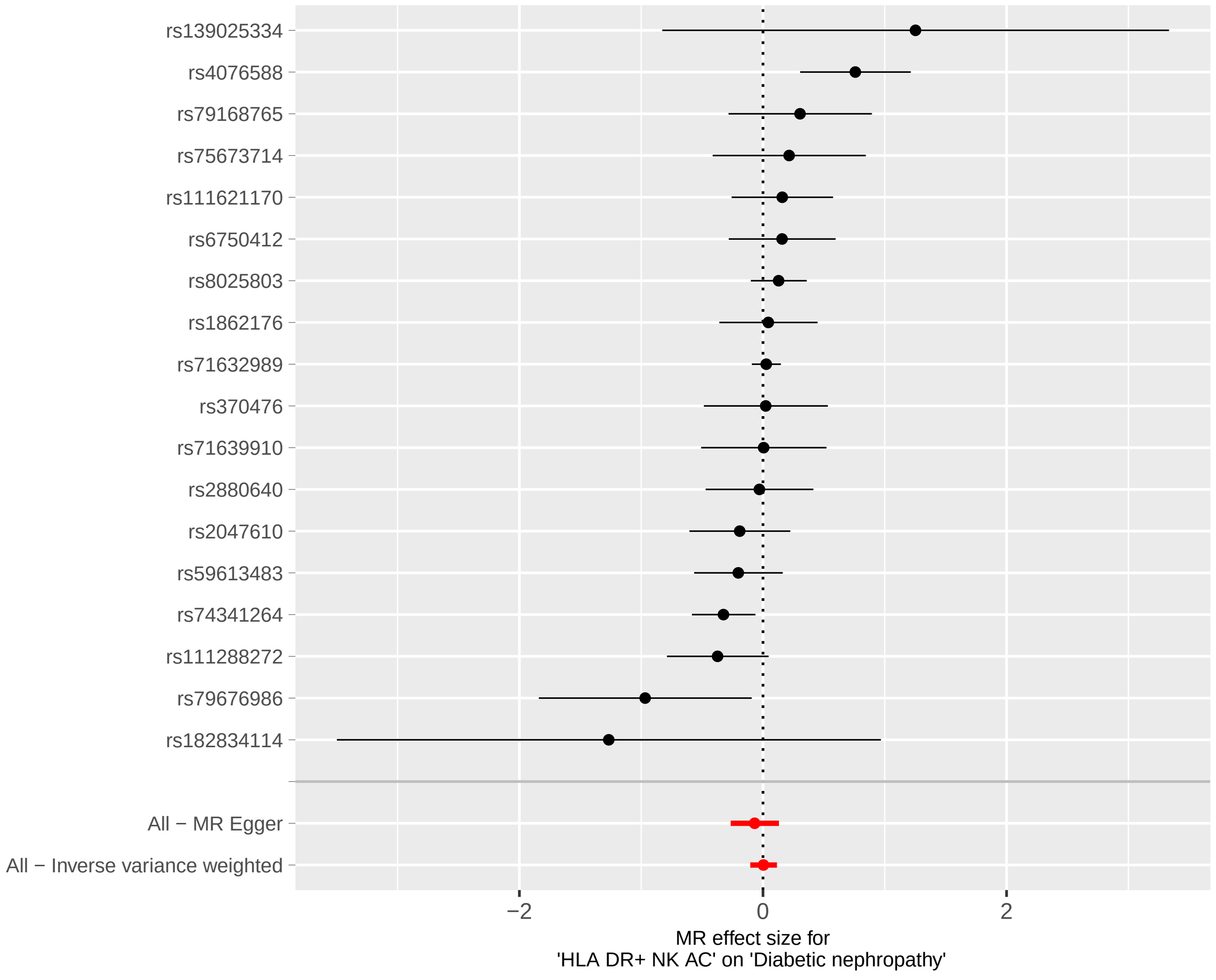
rs139025334
rs4076588
rs79168765
rs75673714
rs111621170
rs6750412
rs8025803
rs1862176
rs71632989
rs370476
rs71639910
rs2880640
rs2047610
rs59613483
rs74341264
rs111288272
rs79676986
rs182834114
All − MR Egger
All − Inverse variance weighted
−2
0
2
MR effect size for
'HLA DR+ NK AC' on 'Diabetic nephropathy'

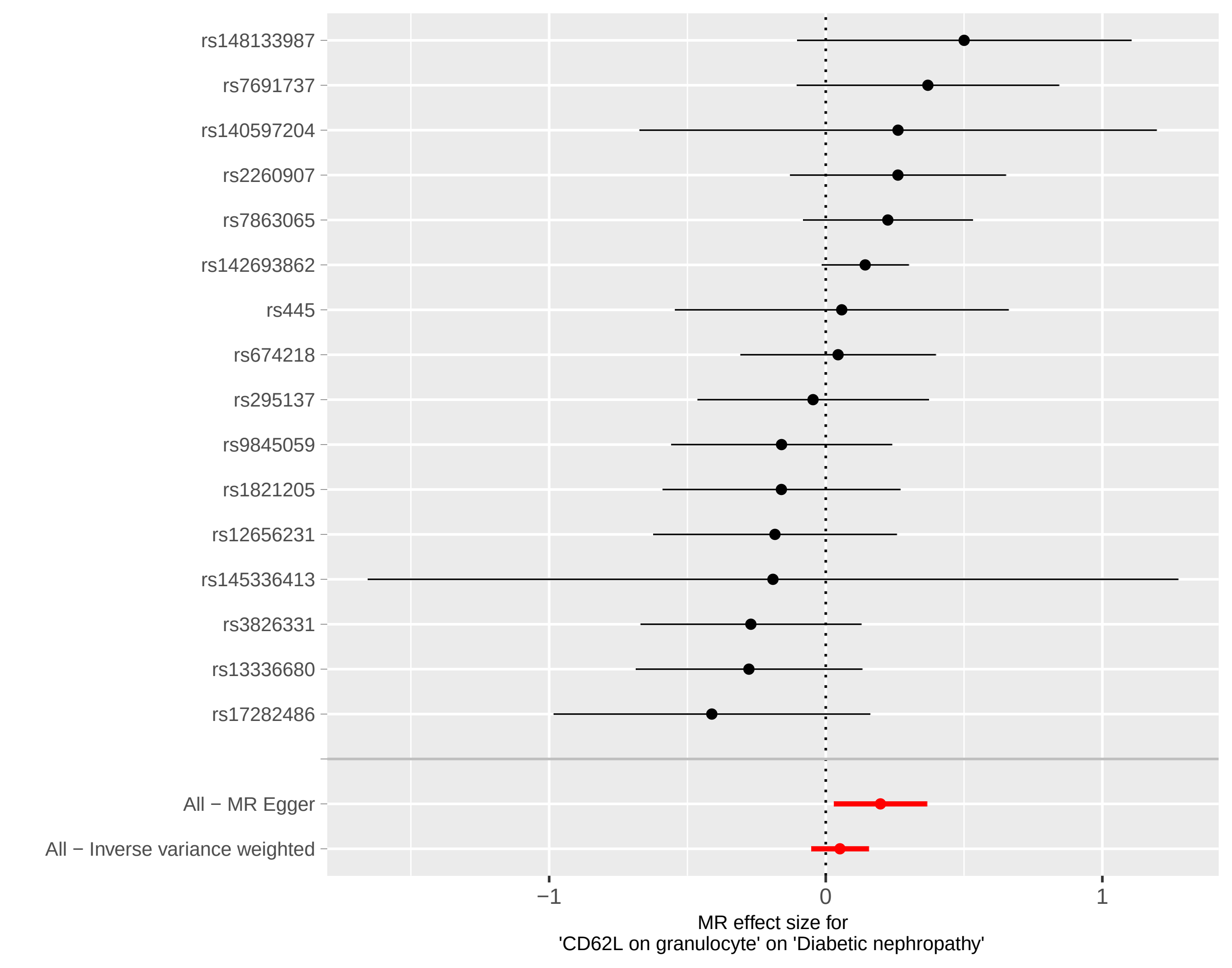

rs148133987
rs7691737
rs140597204
rs2260907
rs7863065
rs142693862
rs445
rs674218
rs295137
rs9845059
rs1821205
rs12656231
rs145336413
rs3826331
rs13336680
rs17282486
All − MR Egger
All − Inverse variance weighted
−1
0
1
MR effect size for
'CD62L on granulocyte' on 'Diabetic nephropathy'

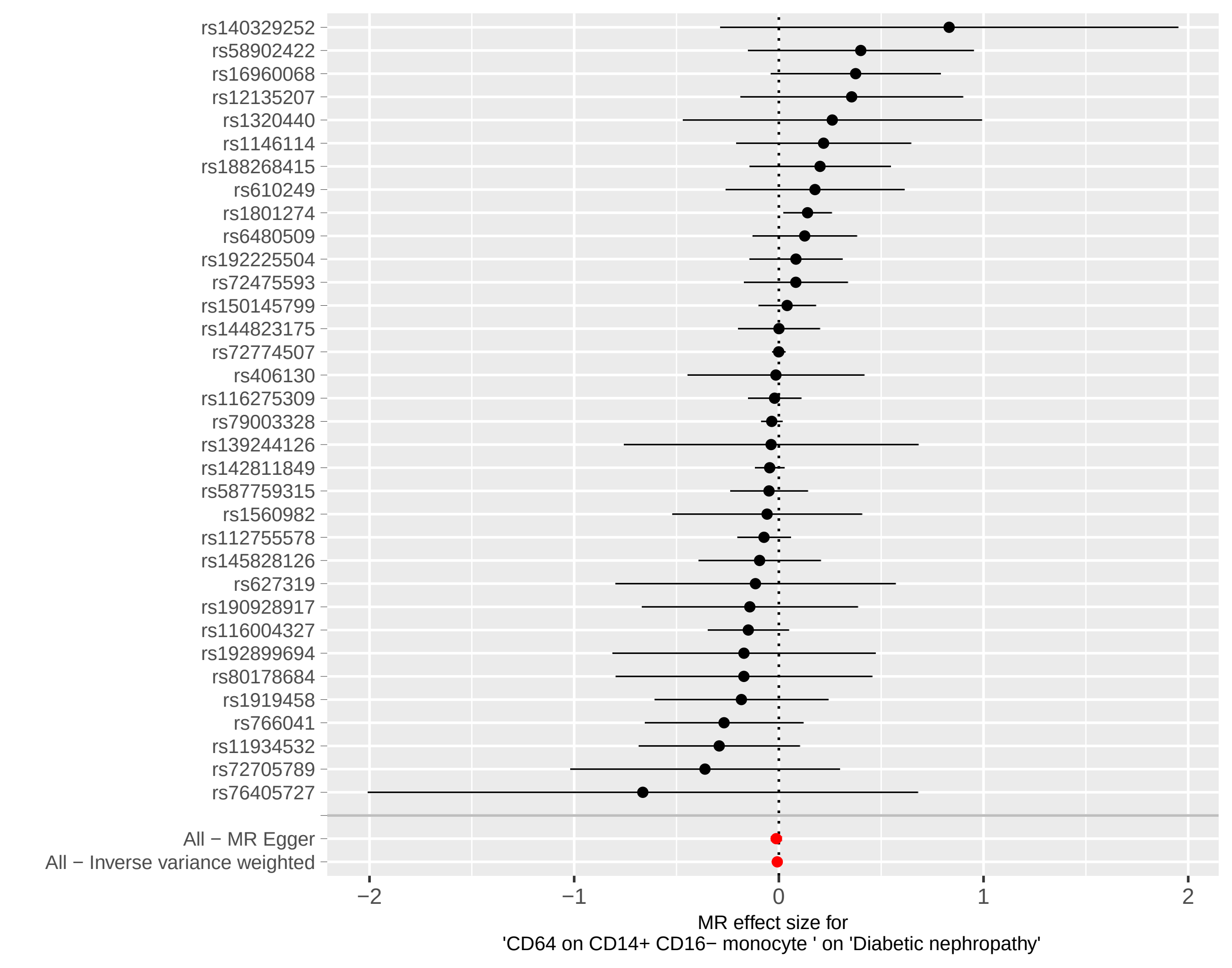

rs140329252
rs58902422
rs16960068
rs12135207
rs1320440
rs1146114
rs188268415
rs610249
rs1801274
rs6480509
rs192225504
rs72475593
rs150145799
rs144823175
rs72774507
rs406130
rs116275309
rs79003328
rs139244126
rs142811849
rs587759315
rs1560982
rs112755578
rs145828126
rs627319
rs190928917
rs116004327
rs192899694
rs80178684
rs1919458
rs766041
rs11934532
rs72705789
rs76405727
All − MR Egger
All − Inverse variance weighted
−2
−1
0
1
2
MR effect size for
'CD64 on CD14+ CD16− monocyte ' on 'Diabetic nephropathy'

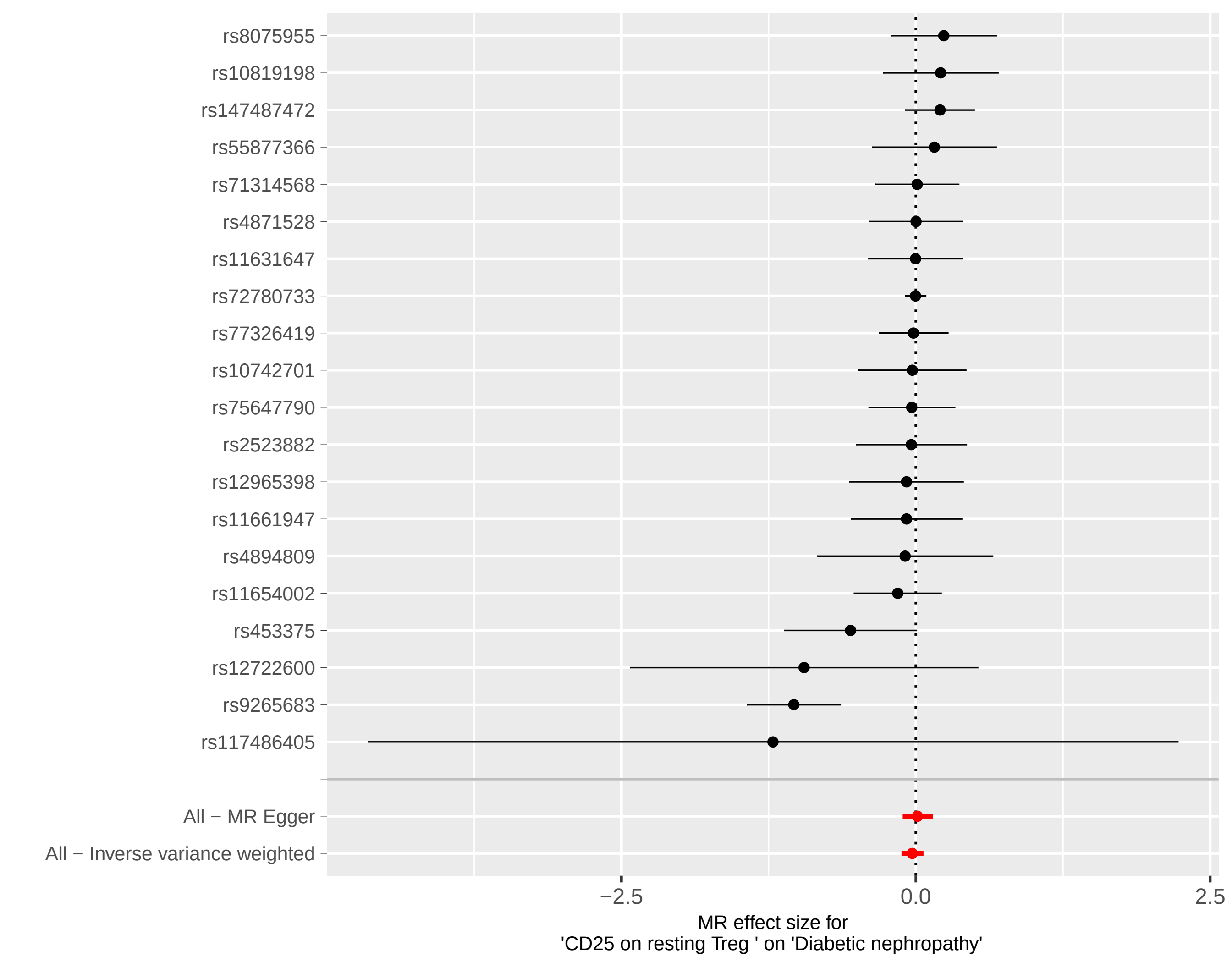
rs8075955
rs10819198
rs147487472
rs55877366
rs71314568
rs4871528
rs11631647
rs72780733
rs77326419
rs10742701
rs75647790
rs2523882
rs12965398
rs11661947
rs4894809
rs11654002
rs453375
rs12722600
rs9265683
rs117486405
All − MR Egger
All − Inverse variance weighted
−2.5
0.0
2.5
MR effect size for
'CD25 on resting Treg ' on 'Diabetic nephropathy'

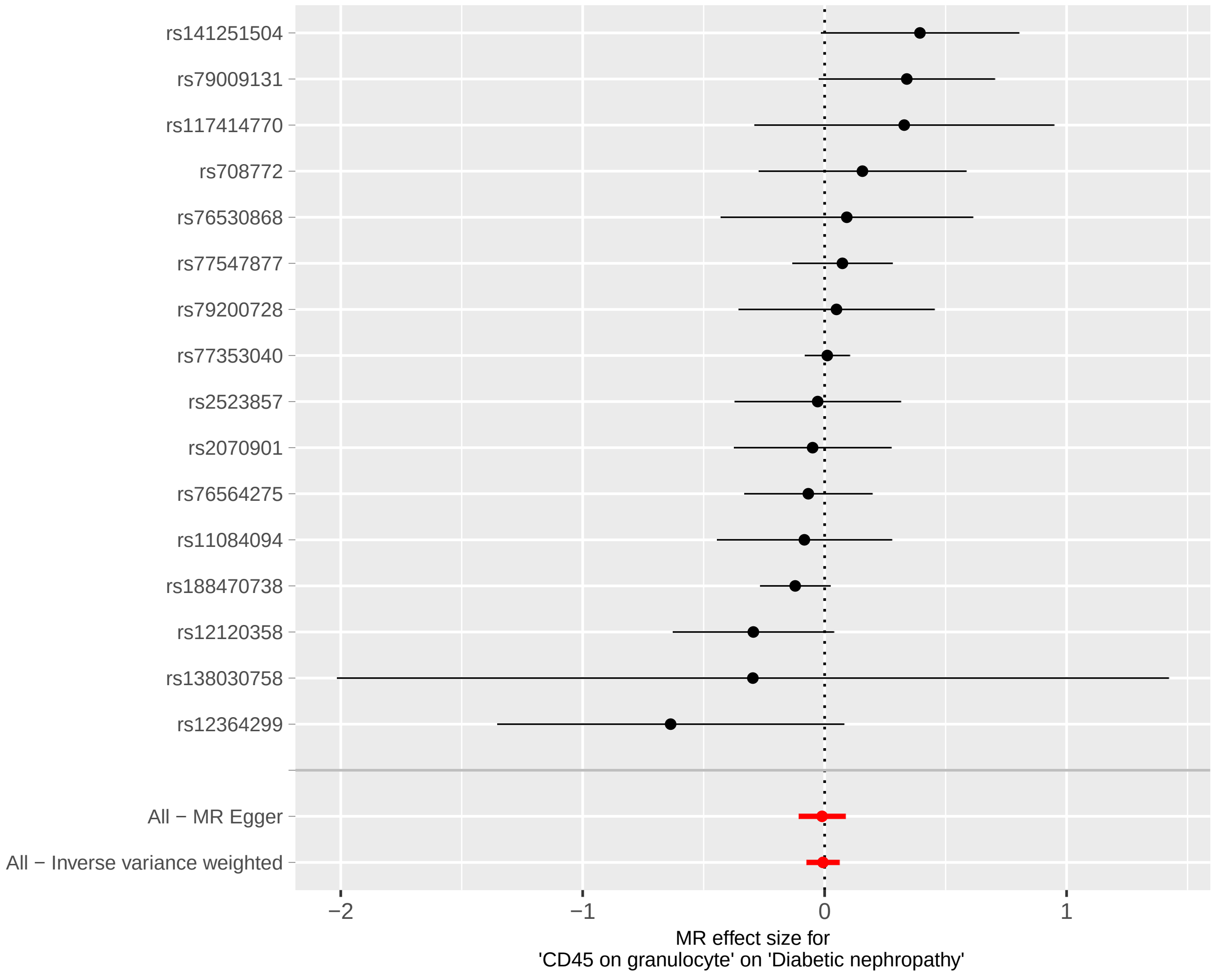
rs141251504
rs79009131
rs117414770
rs708772
rs76530868
rs77547877
rs79200728
rs77353040
rs2523857
rs2070901
rs76564275
rs11084094
rs188470738
rs12120358
rs138030758
rs12364299
All − MR Egger
All − Inverse variance weighted
−2
−1
0
1
MR effect size for
'CD45 on granulocyte' on 'Diabetic nephropathy'

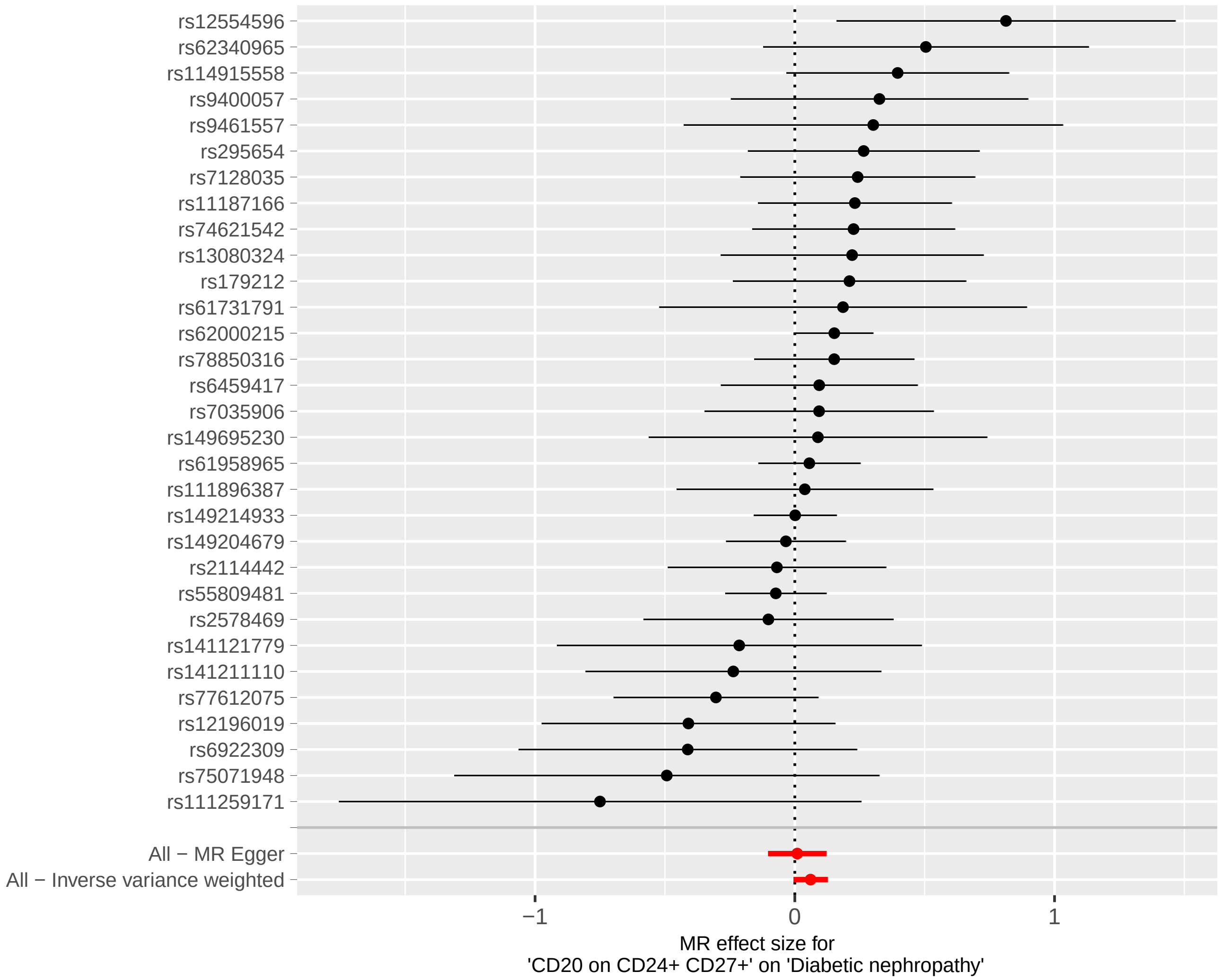
rs12554596
rs62340965
rs114915558
rs9400057
rs9461557
rs295654
rs7128035
rs11187166
rs74621542
rs13080324
rs179212
rs61731791
rs62000215
rs78850316
rs6459417
rs7035906
rs149695230
rs61958965
rs111896387
rs149214933
rs149204679
rs2114442
rs55809481
rs2578469
rs141121779
rs141211110
rs77612075
rs12196019
rs6922309
rs75071948
rs111259171
All − MR Egger
All − Inverse variance weighted
−1
0
1
MR effect size for
'CD20 on CD24+ CD27+' on 'Diabetic nephropathy'

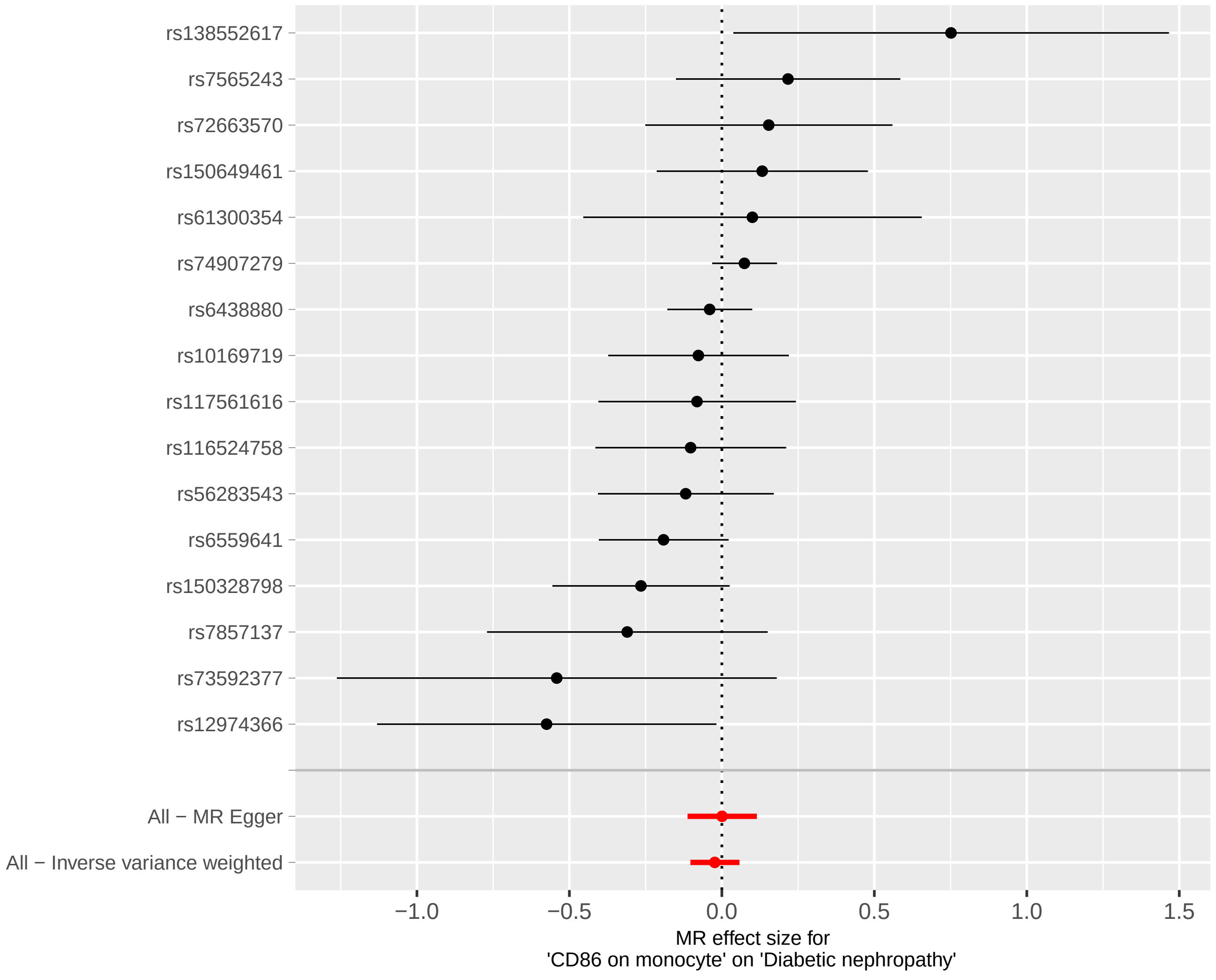
rs138552617
rs7565243
rs72663570
rs150649461
rs61300354
rs74907279
rs6438880
rs10169719
rs117561616
rs116524758
rs56283543
rs6559641
rs150328798
rs7857137
rs73592377
rs12974366
All − MR Egger
All − Inverse variance weighted
−1.0
−0.5
0.0
0.5
1.0
1.5
MR effect size for
'CD86 on monocyte' on 'Diabetic nephropathy'

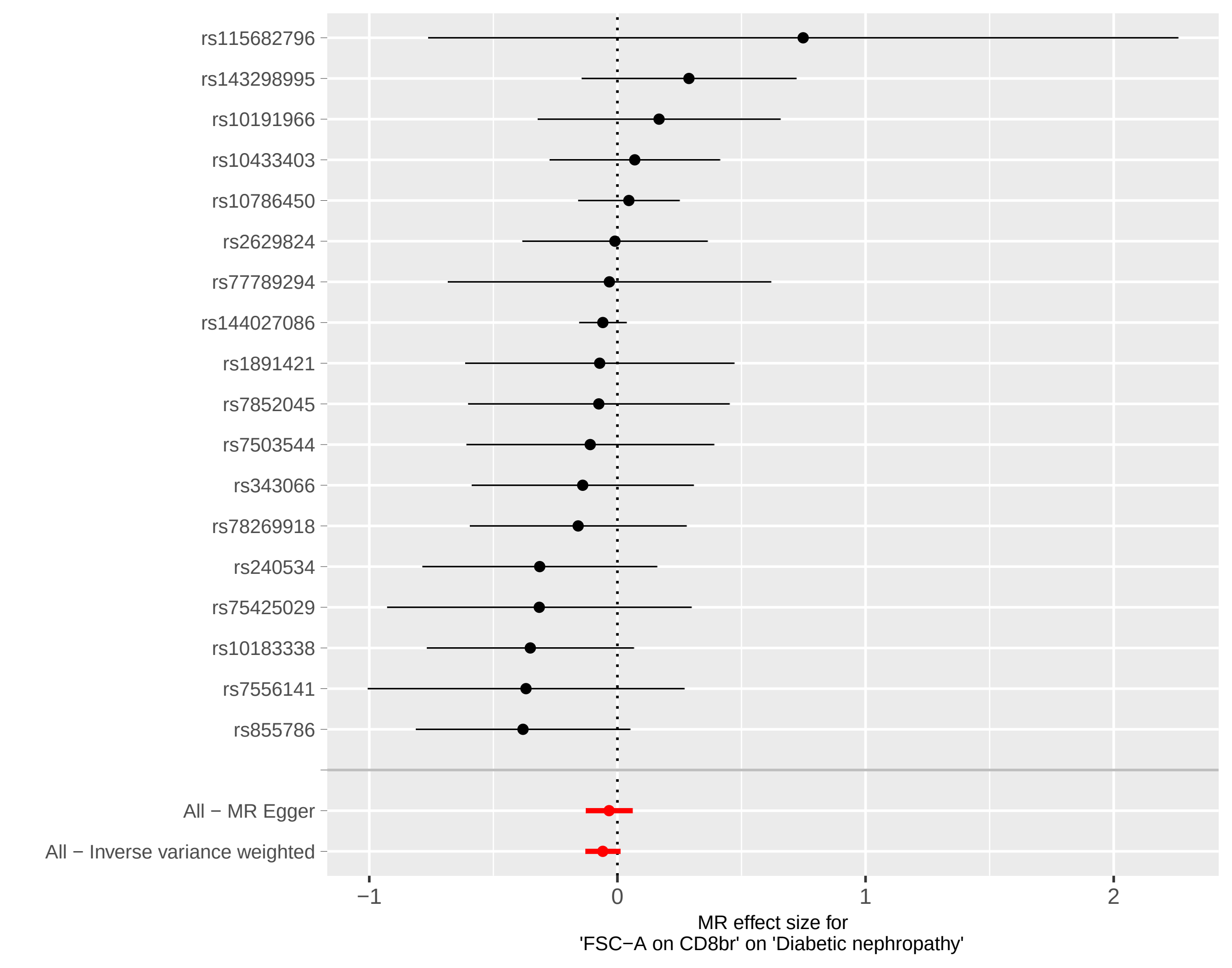
rs115682796
rs143298995
rs10191966
rs10433403
rs10786450
rs2629824
rs77789294
rs144027086
rs1891421
rs7852045
rs7503544
rs343066
rs78269918
rs240534
rs75425029
rs10183338
rs7556141
rs855786
All − MR Egger
All − Inverse variance weighted
−1
0
1
2
MR effect size for
'FSC−A on CD8br' on 'Diabetic nephropathy'

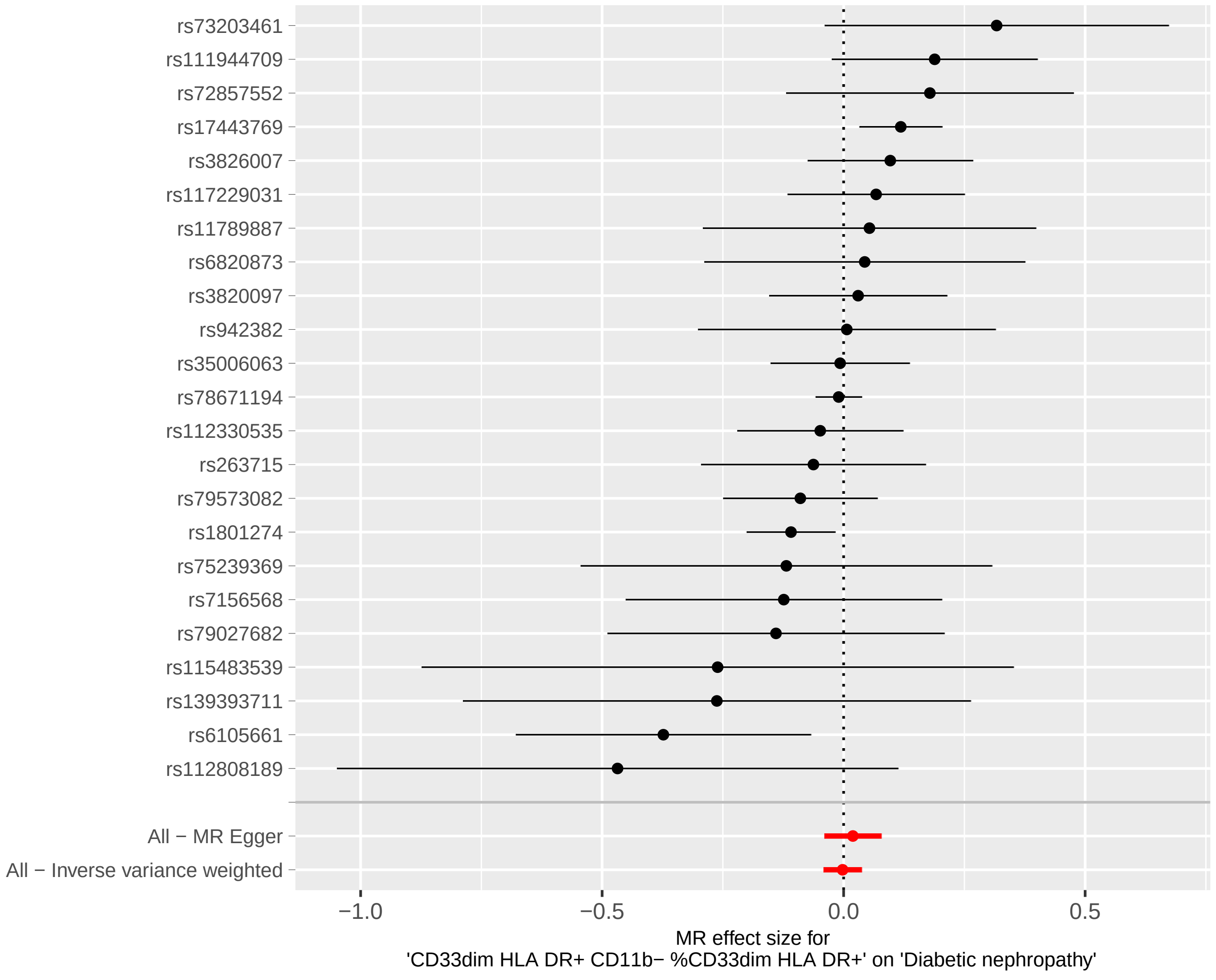

rs73203461
rs111944709
rs72857552
rs17443769
rs3826007
rs117229031
rs11789887
rs6820873
rs3820097
rs942382
rs35006063
rs78671194
rs112330535
rs263715
rs79573082
rs1801274
rs75239369
rs7156568
rs79027682
rs115483539
rs139393711
rs6105661
rs112808189
All − MR Egger
All − Inverse variance weighted
−1.0
−0.5
0.0
0.5
MR effect size for
'CD33dim HLA DR+ CD11b− %CD33dim HLA DR+' on 'Diabetic nephropathy'

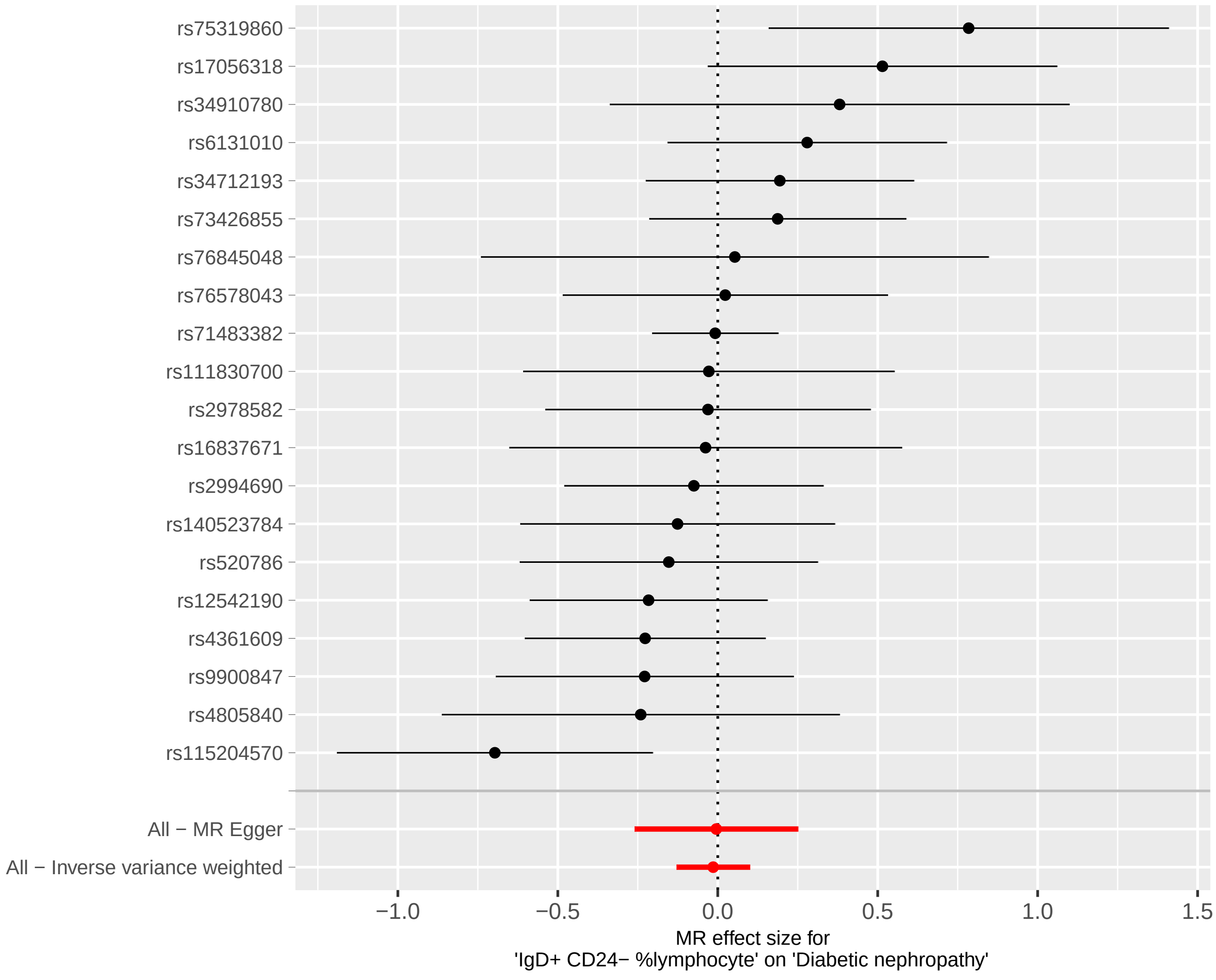
rs75319860
rs17056318
rs34910780
rs6131010
rs34712193
rs73426855
rs76845048
rs76578043
rs71483382
rs111830700
rs2978582
rs16837671
rs2994690
rs140523784
rs520786
rs12542190
rs4361609
rs9900847
rs4805840
rs115204570
All − MR Egger
All − Inverse variance weighted
−1.0
−0.5
0.0
0.5
1.0
1.5
MR effect size for
'IgD+ CD24− %lymphocyte' on 'Diabetic nephropathy'

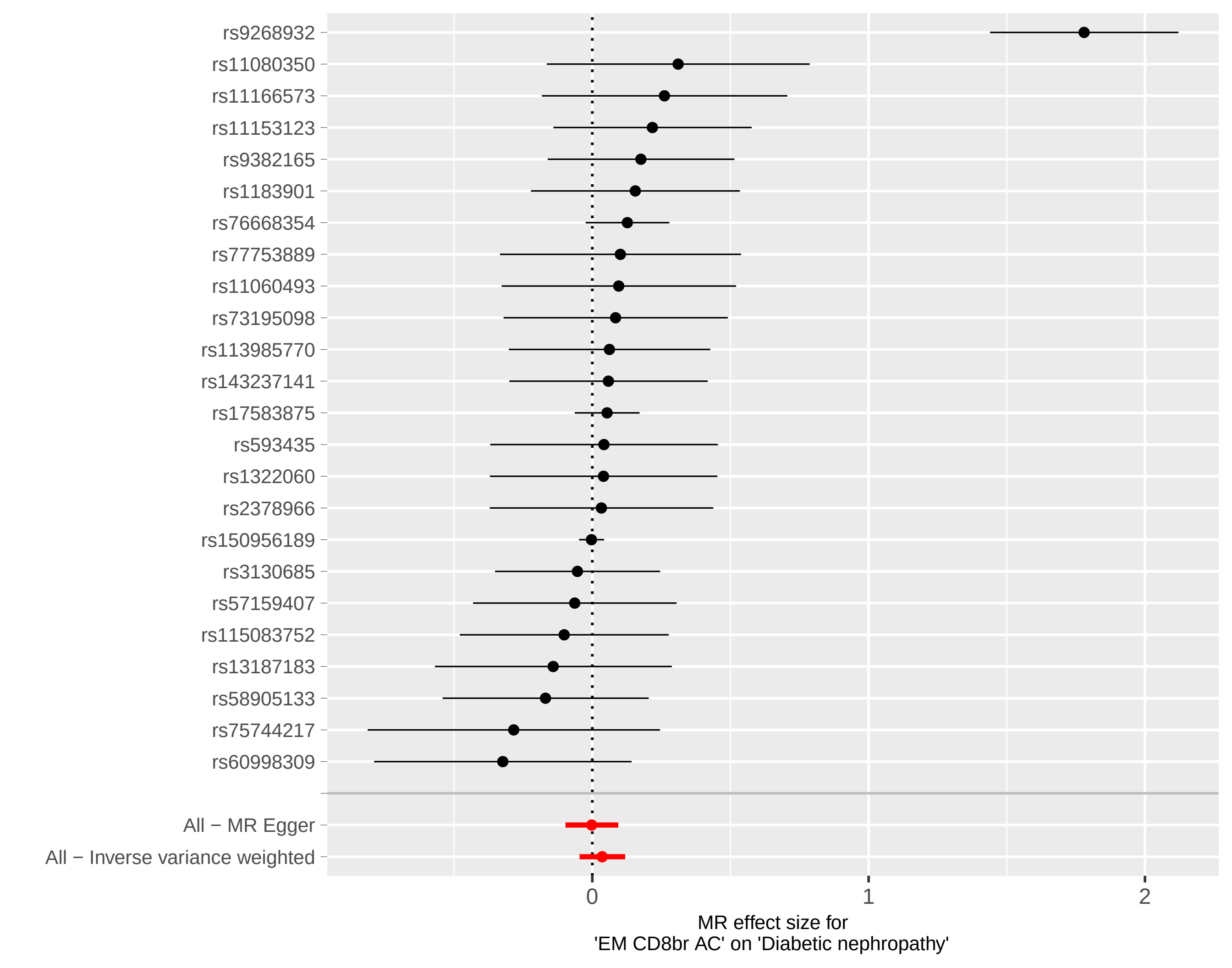

rs9268932
rs11080350
rs11166573
rs11153123
rs9382165
rs1183901
rs76668354
rs77753889
rs11060493
rs73195098
rs113985770
rs143237141
rs17583875
rs593435
rs1322060
rs2378966
rs150956189
rs3130685
rs57159407
rs115083752
rs13187183
rs58905133
rs75744217
rs60998309
All − MR Egger
All − Inverse variance weighted
0
1
2
MR effect size for
'EM CD8br AC' on 'Diabetic nephropathy'

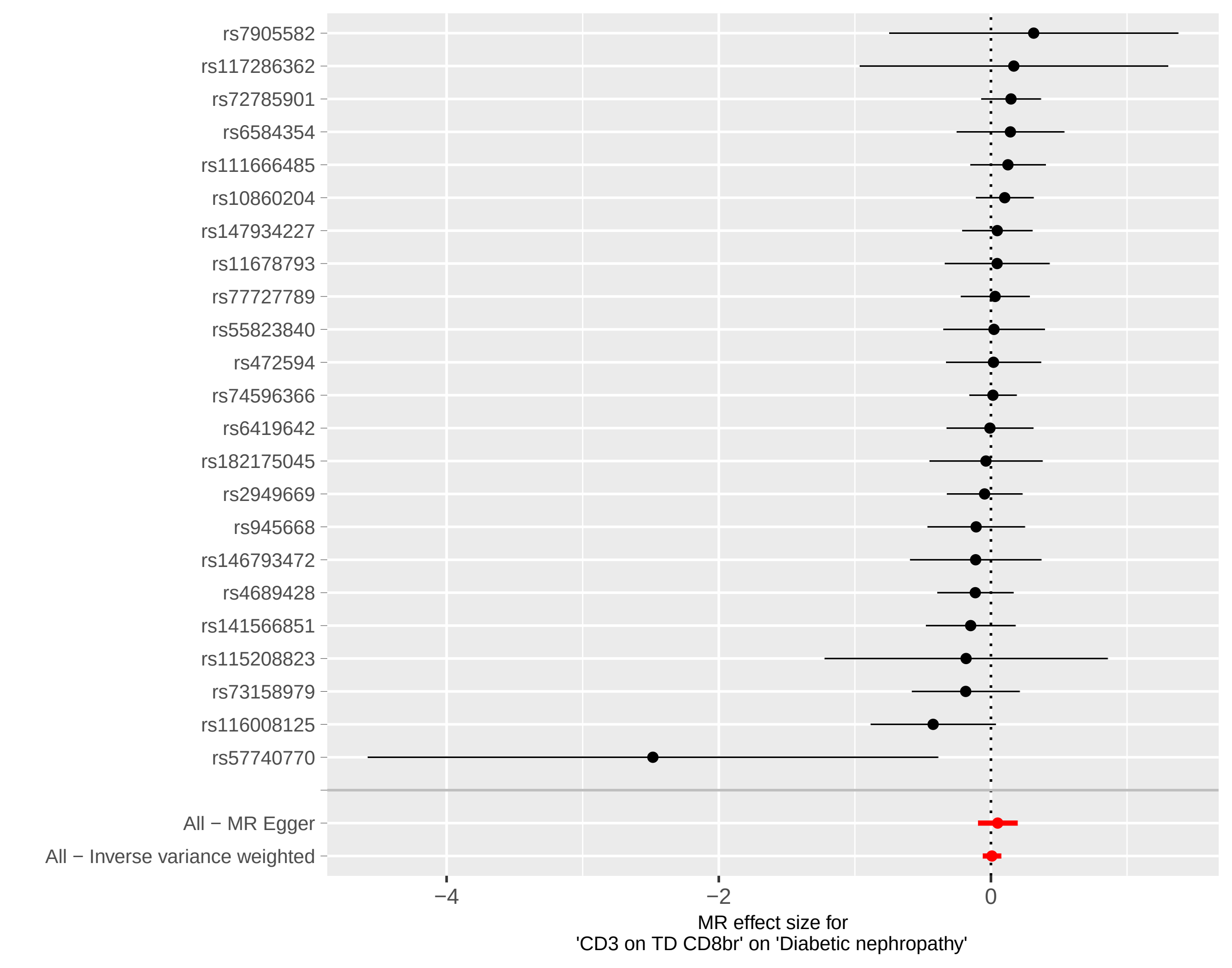

rs7905582
rs117286362
rs72785901
rs6584354
rs111666485
rs10860204
rs147934227
rs11678793
rs77727789
rs55823840
rs472594
rs74596366
rs6419642
rs182175045
rs2949669
rs945668
rs146793472
rs4689428
rs141566851
rs115208823
rs73158979
rs116008125
rs57740770
All − MR Egger
All − Inverse variance weighted
−4
−2
0
MR effect size for
'CD3 on TD CD8br' on 'Diabetic nephropathy'

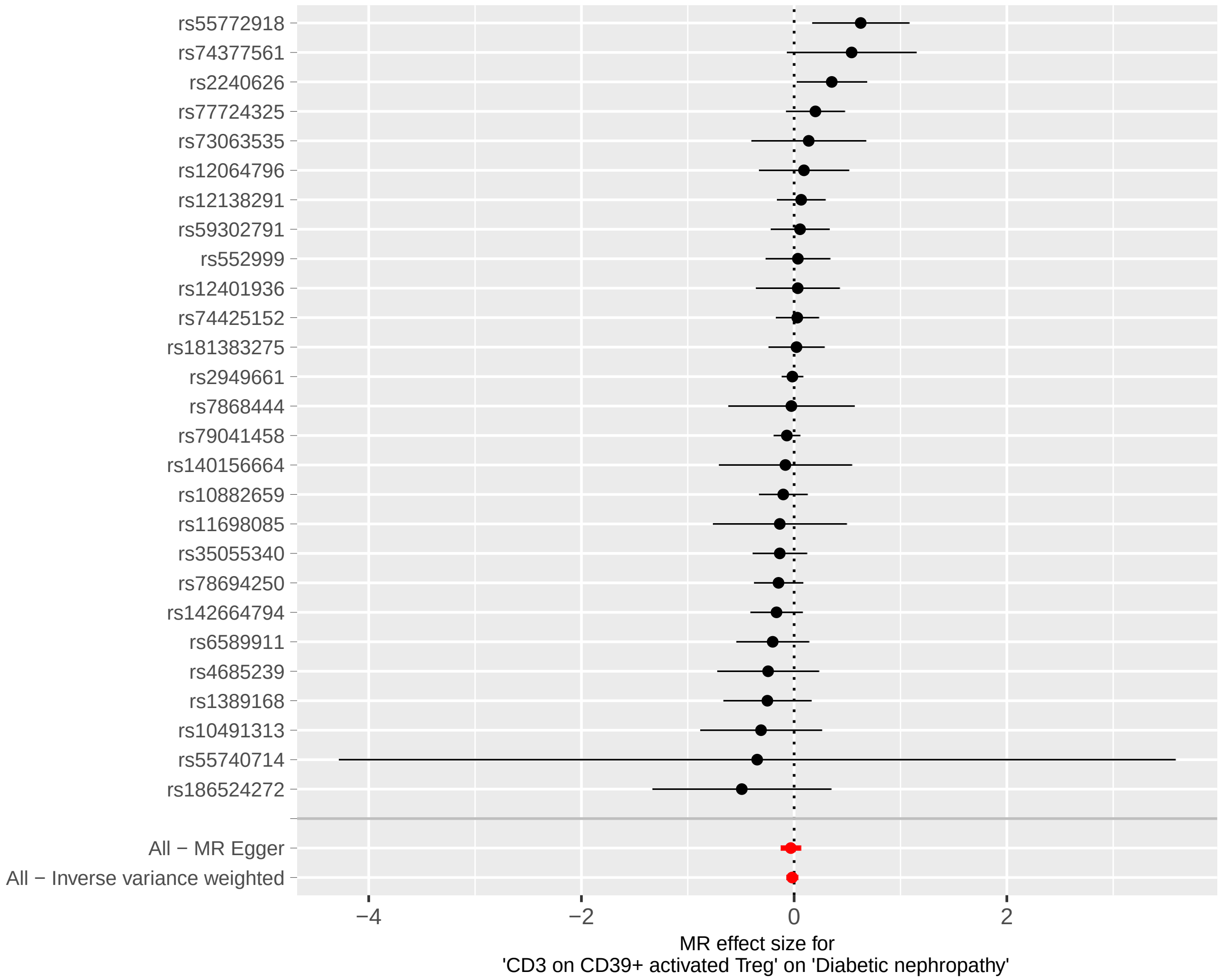
rs55772918
rs74377561
rs2240626
rs77724325
rs73063535
rs12064796
rs12138291
rs59302791
rs552999
rs12401936
rs74425152
rs181383275
rs2949661
rs7868444
rs79041458
rs140156664
rs10882659
rs11698085
rs35055340
rs78694250
rs142664794
rs6589911
rs4685239
rs1389168
rs10491313
rs55740714
rs186524272
All − MR Egger
All − Inverse variance weighted
−4
−2
0
2
MR effect size for
'CD3 on CD39+ activated Treg' on 'Diabetic nephropathy'

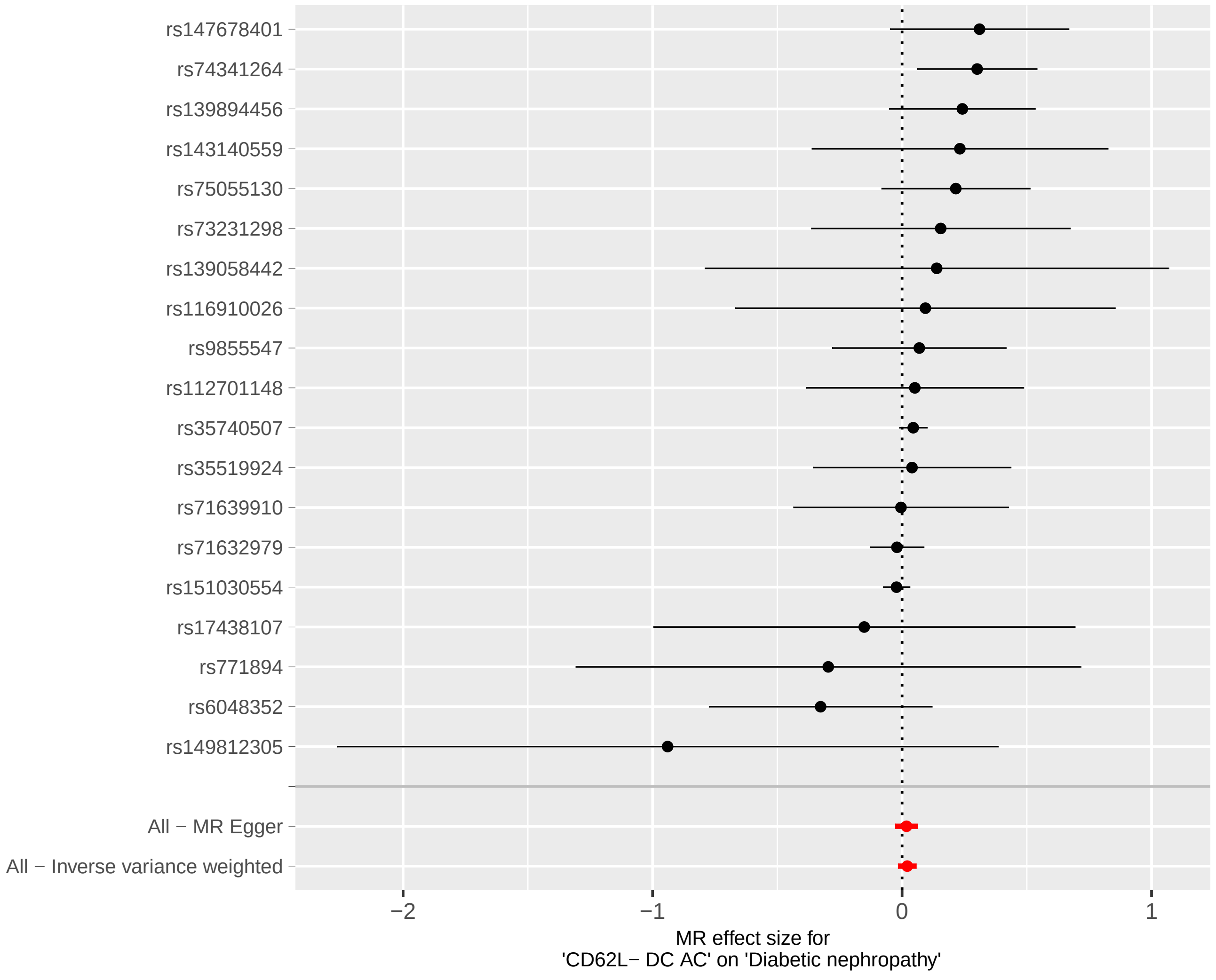
rs147678401
rs74341264
rs139894456
rs143140559
rs75055130
rs73231298
rs139058442
rs116910026
rs9855547
rs112701148
rs35740507
rs35519924
rs71639910
rs71632979
rs151030554
rs17438107
rs771894
rs6048352
rs149812305
All − MR Egger
All − Inverse variance weighted
−2
−1
0
1
MR effect size for
'CD62L− DC AC' on 'Diabetic nephropathy'

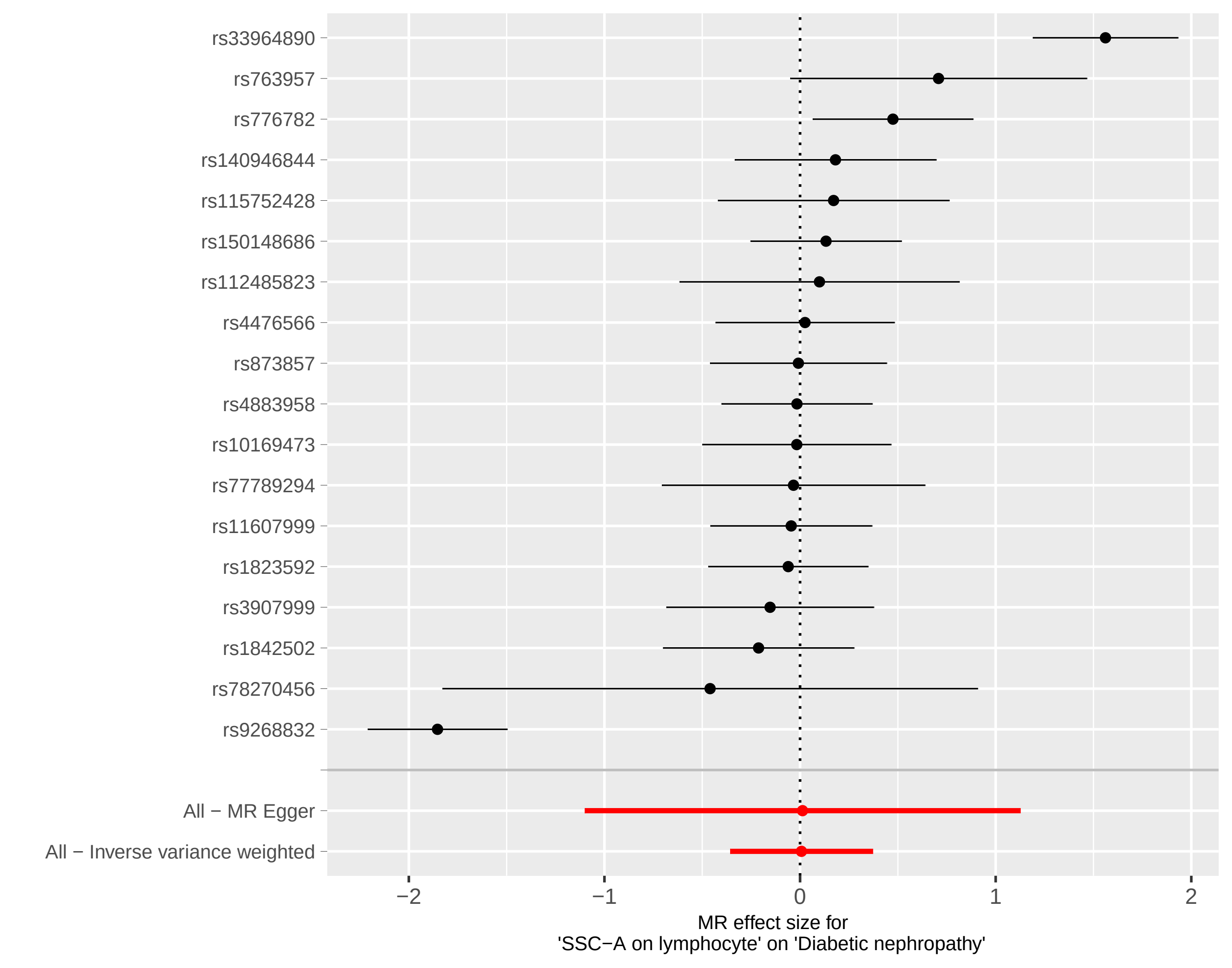

rs33964890
rs763957
rs776782
rs140946844
rs115752428
rs150148686
rs112485823
rs4476566
rs873857
rs4883958
rs10169473
rs77789294
rs11607999
rs1823592
rs3907999
rs1842502
rs78270456
rs9268832
All − MR Egger
All − Inverse variance weighted
−2
−1
0
1
2
MR effect size for
'SSC−A on lymphocyte' on 'Diabetic nephropathy'

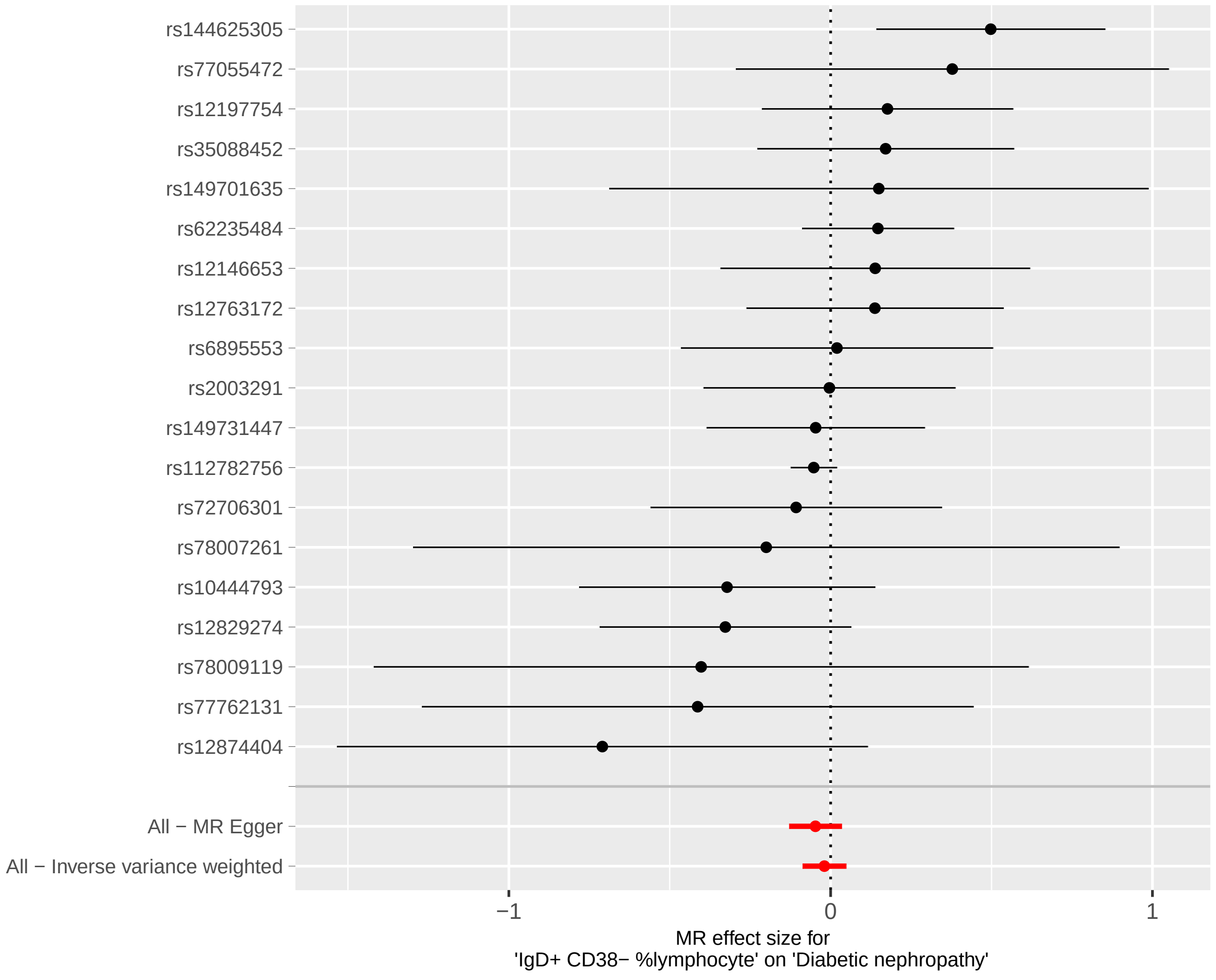
rs144625305
rs77055472
rs12197754
rs35088452
rs149701635
rs62235484
rs12146653
rs12763172
rs6895553
rs2003291
rs149731447
rs112782756
rs72706301
rs78007261
rs10444793
rs12829274
rs78009119
rs77762131
rs12874404
All − MR Egger
All − Inverse variance weighted
−1
0
1
MR effect size for
'IgD+ CD38− %lymphocyte' on 'Diabetic nephropathy'

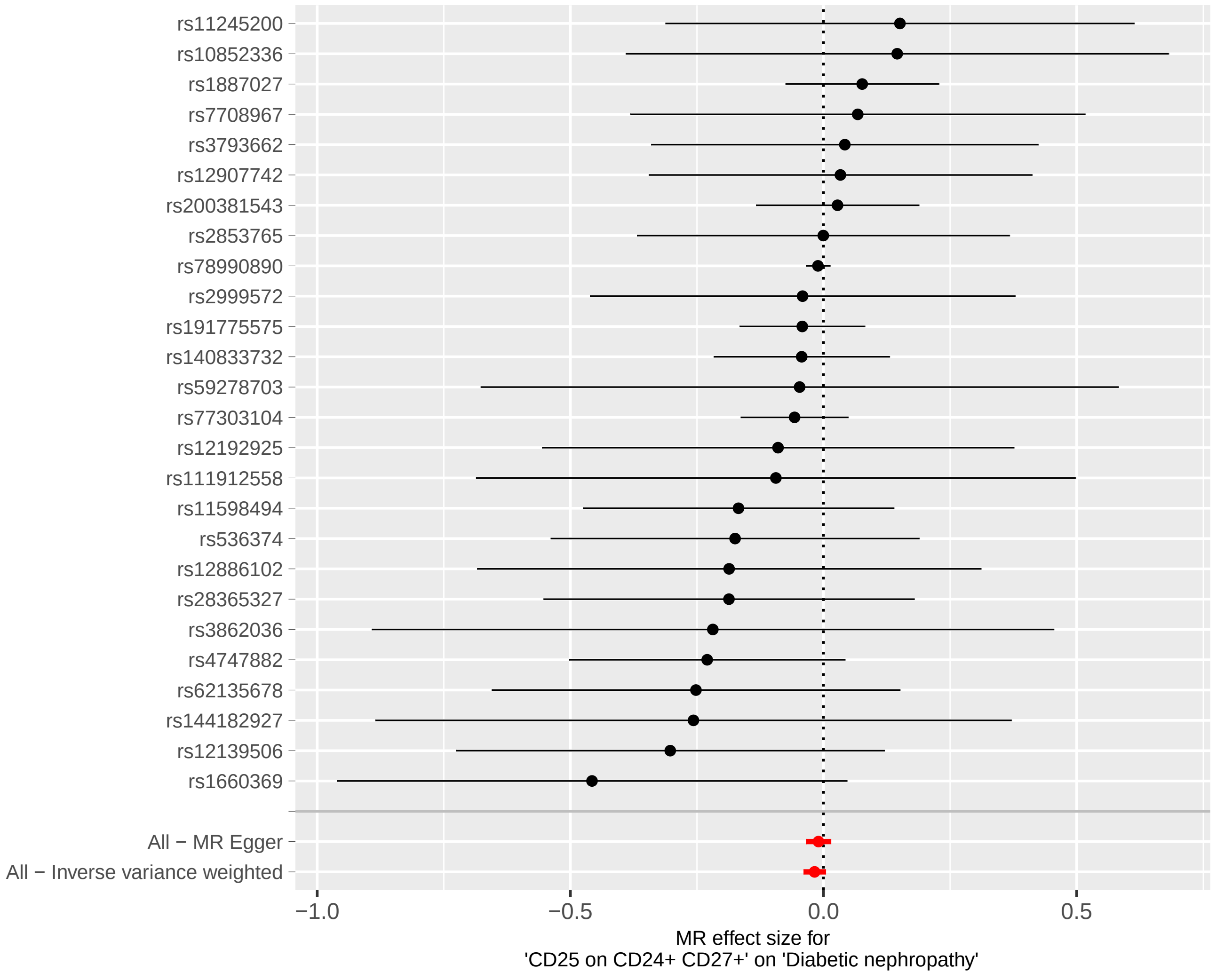
rs11245200
rs10852336
rs1887027
rs7708967
rs3793662
rs12907742
rs200381543
rs2853765
rs78990890
rs2999572
rs191775575
rs140833732
rs59278703
rs77303104
rs12192925
rs111912558
rs11598494
rs536374
rs12886102
rs28365327
rs3862036
rs4747882
rs62135678
rs144182927
rs12139506
rs1660369
All − MR Egger
All − Inverse variance weighted
−1.0
−0.5
0.0
0.5
MR effect size for
'CD25 on CD24+ CD27+' on 'Diabetic nephropathy'

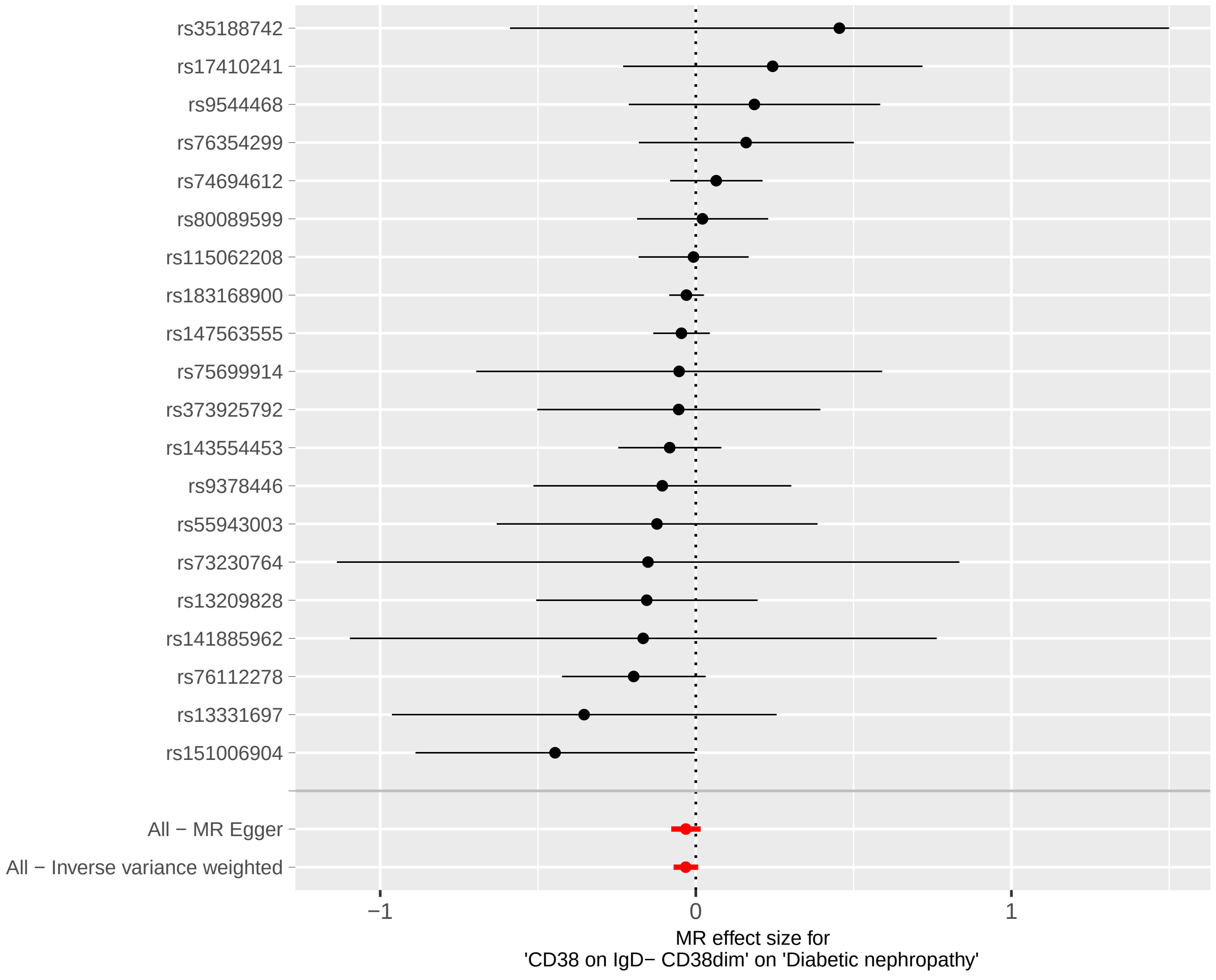
rs35188742
rs17410241
rs9544468
rs76354299
rs74694612
rs80089599
rs115062208
rs183168900
rs147563555
rs75699914
rs373925792
rs143554453
rs9378446
rs55943003
rs73230764
rs13209828
rs141885962
rs76112278
rs13331697
rs151006904
All − MR Egger
All − Inverse variance weighted
−1
0
1
MR effect size for
'CD38 on IgD− CD38dim' on 'Diabetic nephropathy'

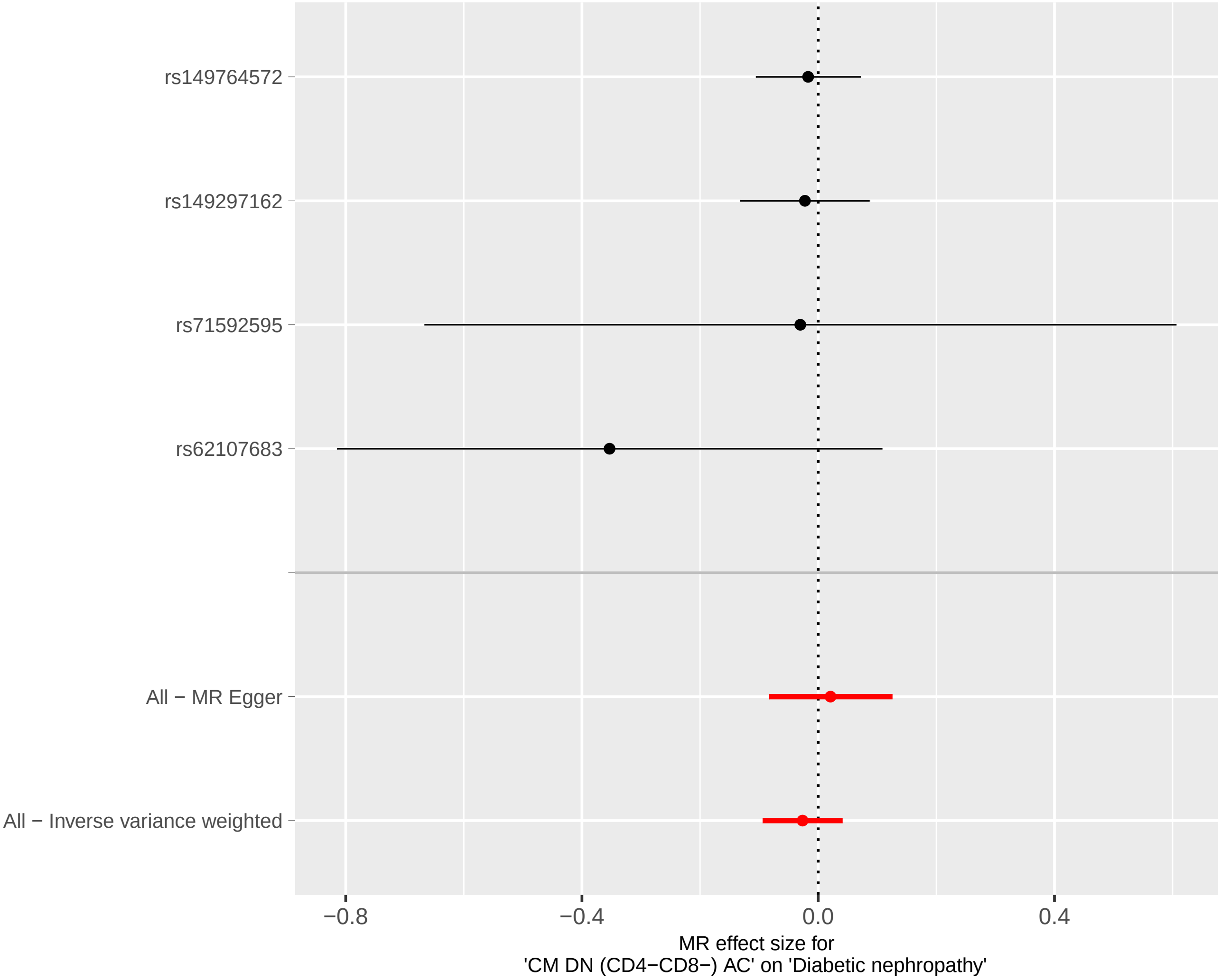
rs149764572
rs149297162
rs71592595
rs62107683
All − MR Egger
All − Inverse variance weighted
−0.8
−0.4
0.0
0.4
MR effect size for
'CM DN (CD4−CD8−) AC' on 'Diabetic nephropathy'

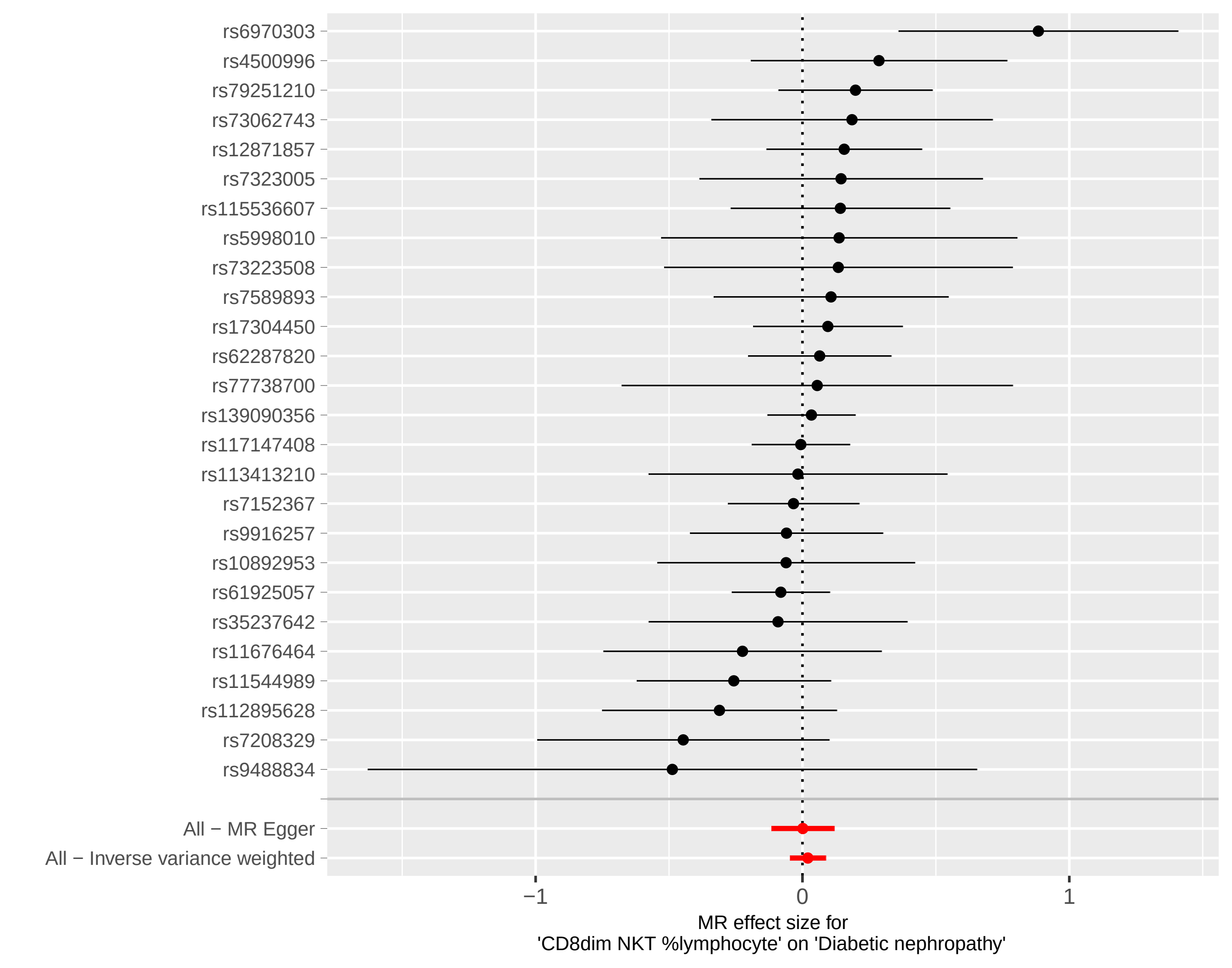
rs6970303
rs4500996
rs79251210
rs73062743
rs12871857
rs7323005
rs115536607
rs5998010
rs73223508
rs7589893
rs17304450
rs62287820
rs77738700
rs139090356
rs117147408
rs113413210
rs7152367
rs9916257
rs10892953
rs61925057
rs35237642
rs11676464
rs11544989
rs112895628
rs7208329
rs9488834
All − MR Egger
All − Inverse variance weighted
−1
0
1
MR effect size for
'CD8dim NKT %lymphocyte' on 'Diabetic nephropathy'

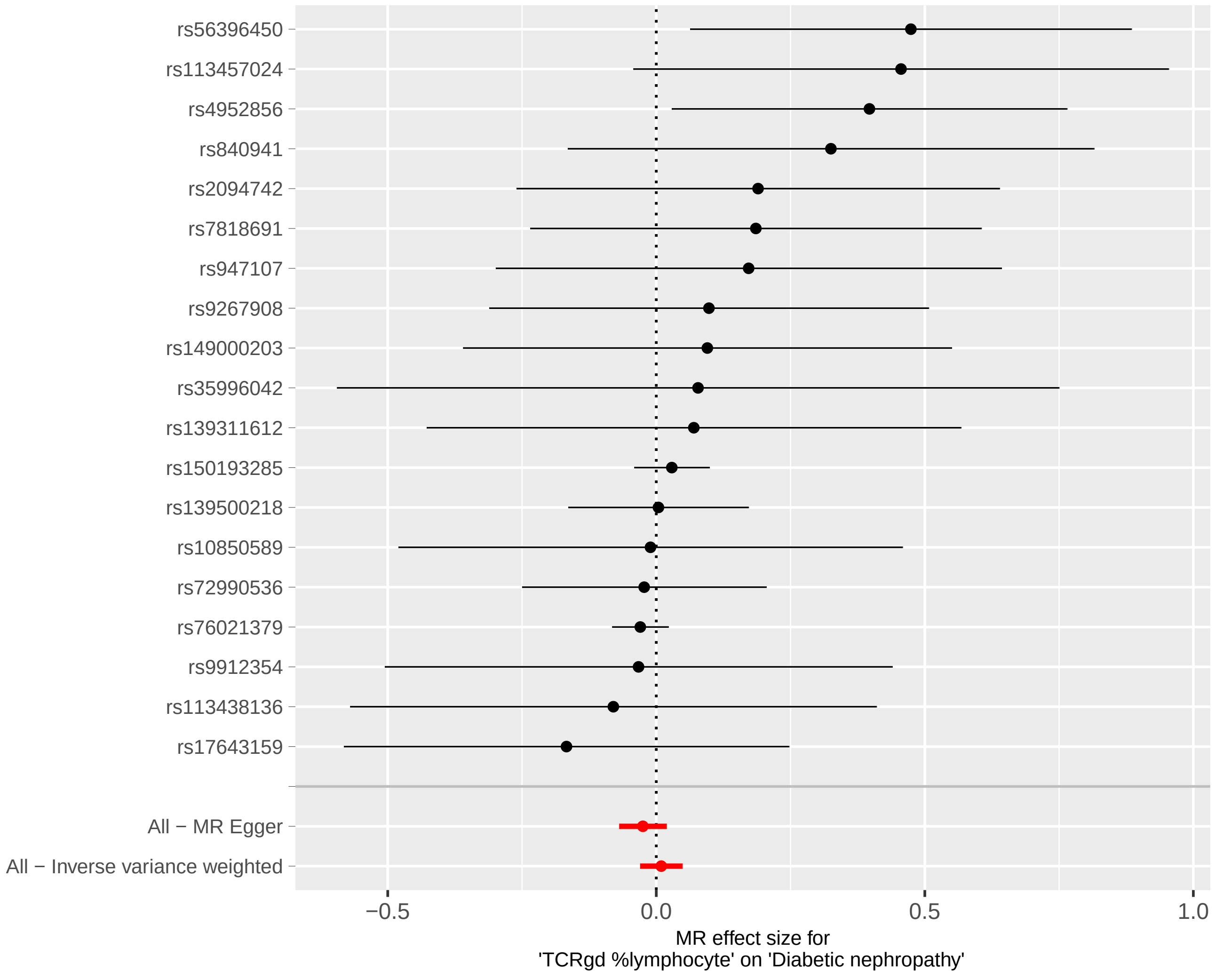
rs56396450
rs113457024
rs4952856
rs840941
rs2094742
rs7818691
rs947107
rs9267908
rs149000203
rs35996042
rs139311612
rs150193285
rs139500218
rs10850589
rs72990536
rs76021379
rs9912354
rs113438136
rs17643159
All − MR Egger
All − Inverse variance weighted
−0.5
0.0
0.5
1.0
MR effect size for
'TCRgd %lymphocyte' on 'Diabetic nephropathy'

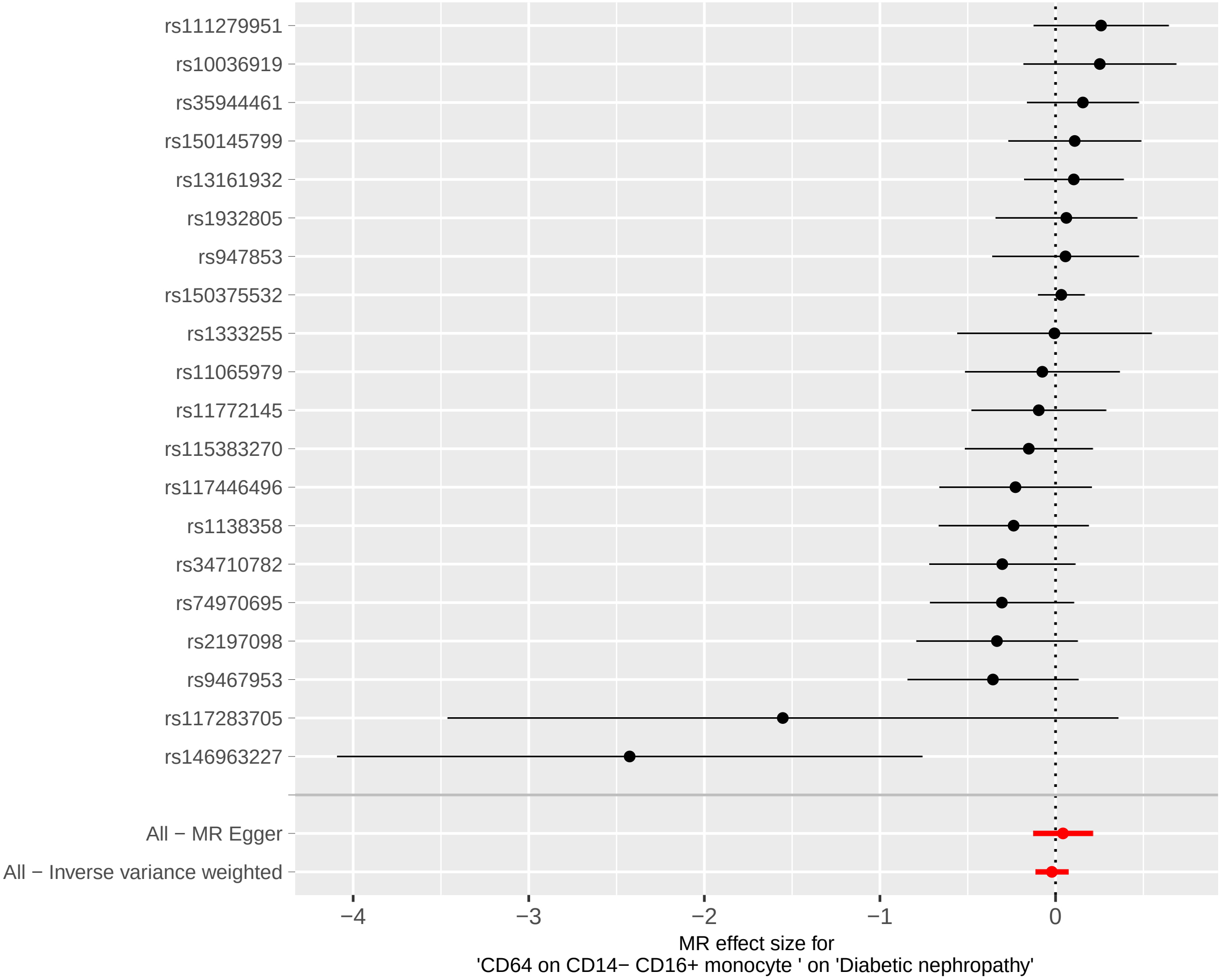
rs111279951
rs10036919
rs35944461
rs150145799
rs13161932
rs1932805
rs947853
rs150375532
rs1333255
rs11065979
rs11772145
rs115383270
rs117446496
rs1138358
rs34710782
rs74970695
rs2197098
rs9467953
rs117283705
rs146963227
All − MR Egger
All − Inverse variance weighted
−4
−3
−2
−1
0
MR effect size for
'CD64 on CD14− CD16+ monocyte ' on 'Diabetic nephropathy'

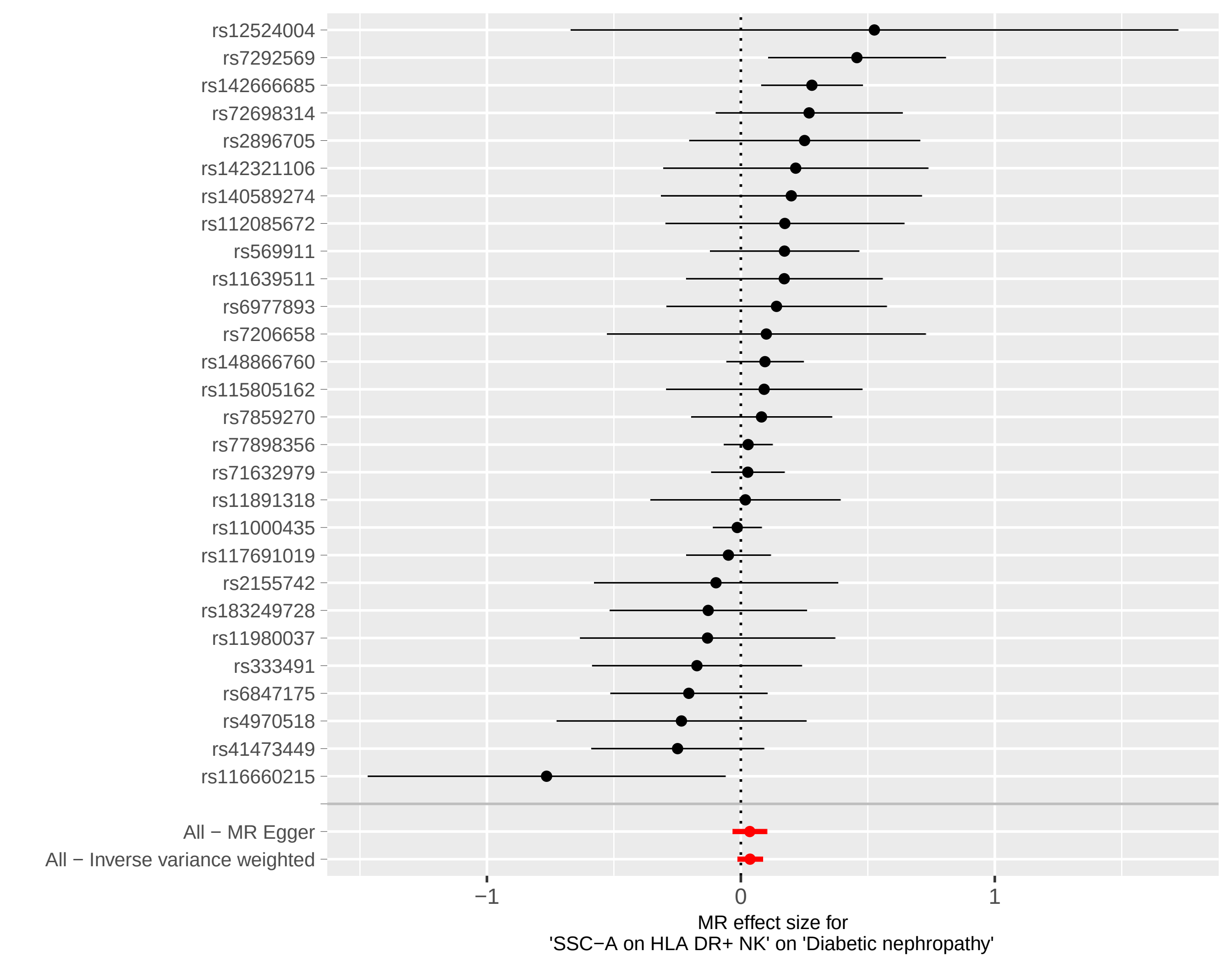
rs12524004
rs7292569
rs142666685
rs72698314
rs2896705
rs142321106
rs140589274
rs112085672
rs569911
rs11639511
rs6977893
rs7206658
rs148866760
rs115805162
rs7859270
rs77898356
rs71632979
rs11891318
rs11000435
rs117691019
rs2155742
rs183249728
rs11980037
rs333491
rs6847175
rs4970518
rs41473449
rs116660215
All − MR Egger
All − Inverse variance weighted
−1
0
1
MR effect size for
'SSC−A on HLA DR+ NK' on 'Diabetic nephropathy'

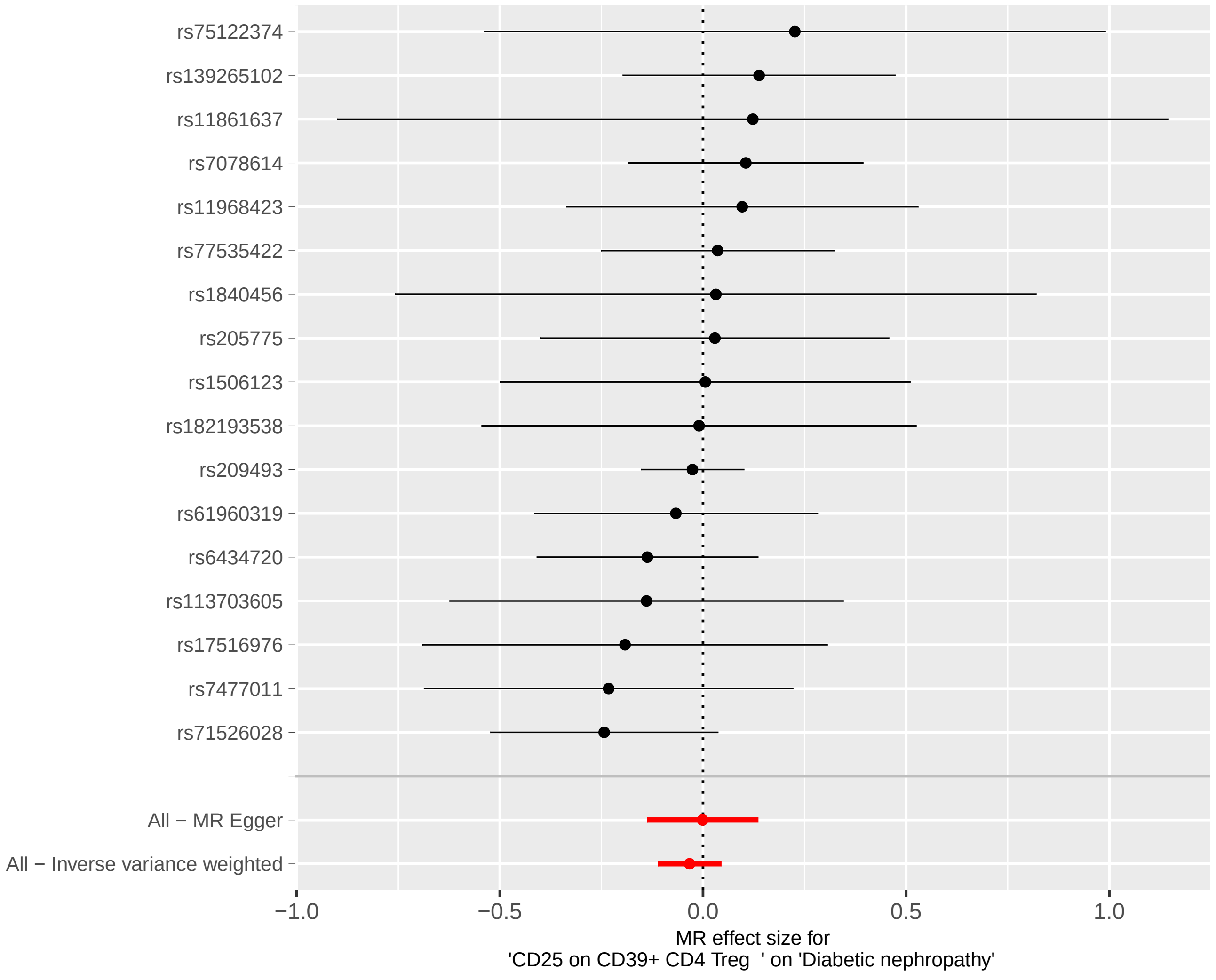

rs75122374
rs139265102
rs11861637
rs7078614
rs11968423
rs77535422
rs1840456
rs205775
rs1506123
rs182193538
rs209493
rs61960319
rs6434720
rs113703605
rs17516976
rs7477011
rs71526028
All − MR Egger
All − Inverse variance weighted
−1.0
−0.5
0.0
0.5
1.0
MR effect size for
'CD25 on CD39+ CD4 Treg ' on 'Diabetic nephropathy'

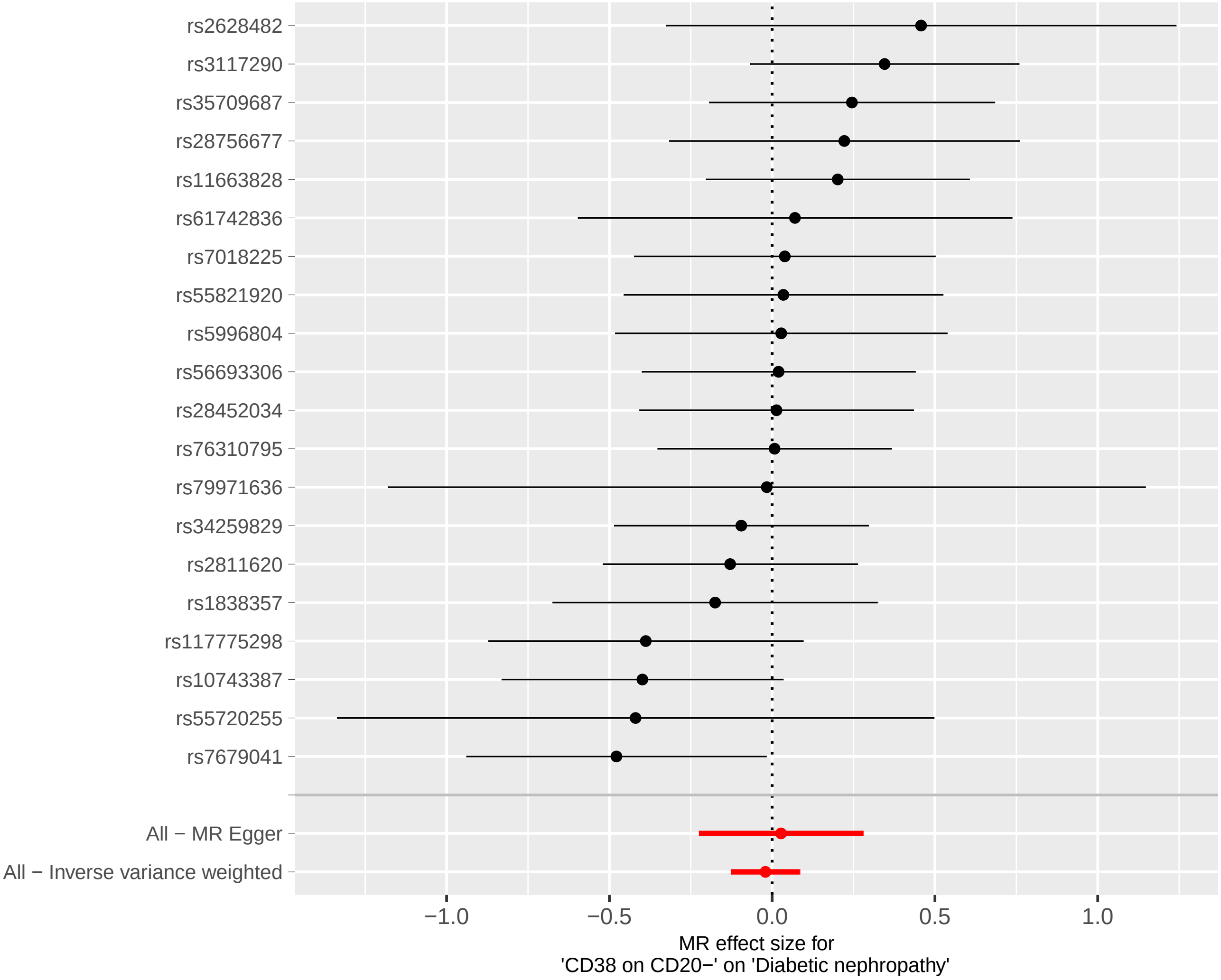
rs2628482
rs3117290
rs35709687
rs28756677
rs11663828
rs61742836
rs7018225
rs55821920
rs5996804
rs56693306
rs28452034
rs76310795
rs79971636
rs34259829
rs2811620
rs1838357
rs117775298
rs10743387
rs55720255
rs7679041
All − MR Egger
All − Inverse variance weighted
−1.0
−0.5
0.0
0.5
1.0
MR effect size for
'CD38 on CD20−' on 'Diabetic nephropathy'

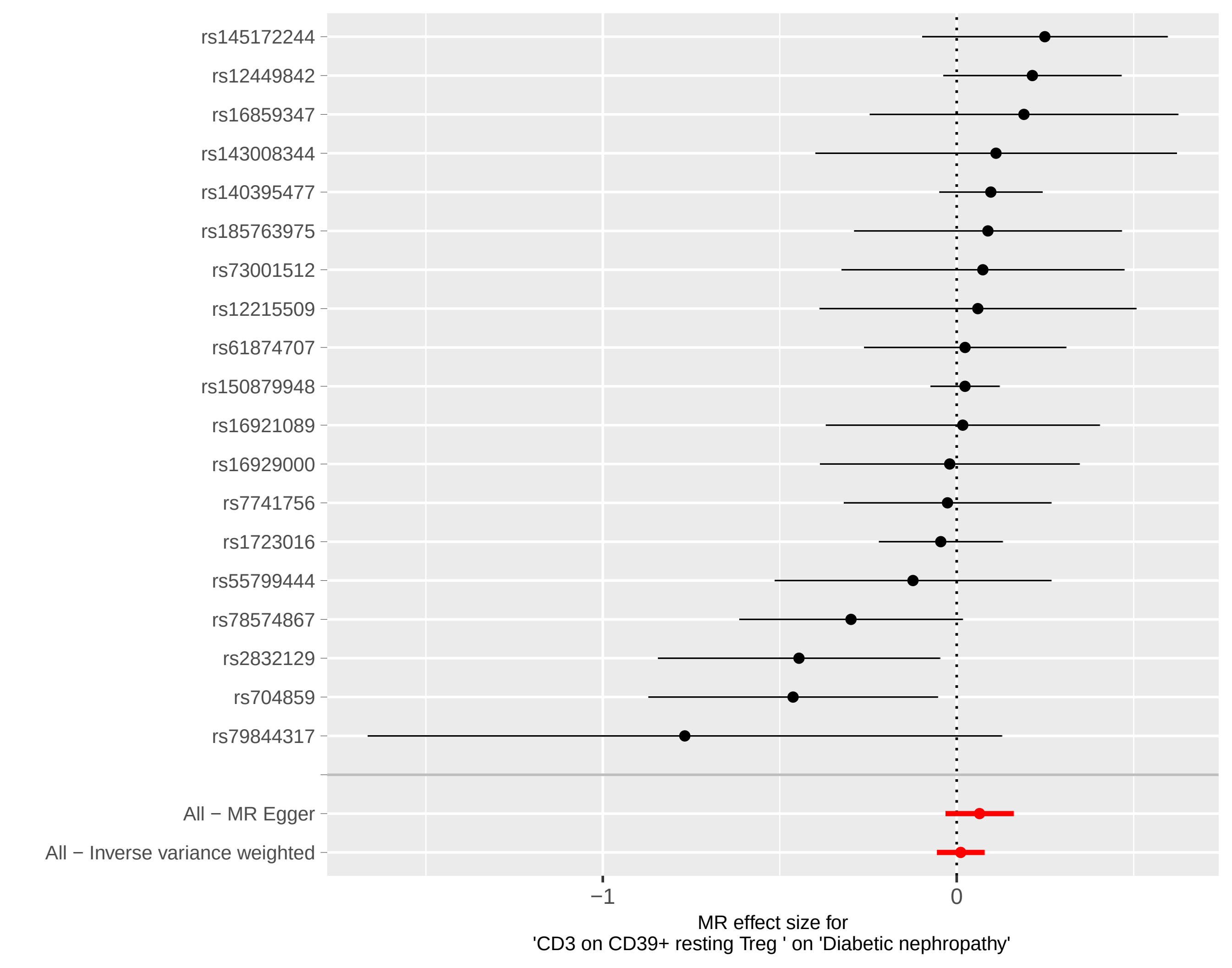

rs145172244
rs12449842
rs16859347
rs143008344
rs140395477
rs185763975
rs73001512
rs12215509
rs61874707
rs150879948
rs16921089
rs16929000
rs7741756
rs1723016
rs55799444
rs78574867
rs2832129
rs704859
rs79844317
All − MR Egger
All − Inverse variance weighted
−1
0
MR effect size for
'CD3 on CD39+ resting Treg ' on 'Diabetic nephropathy'

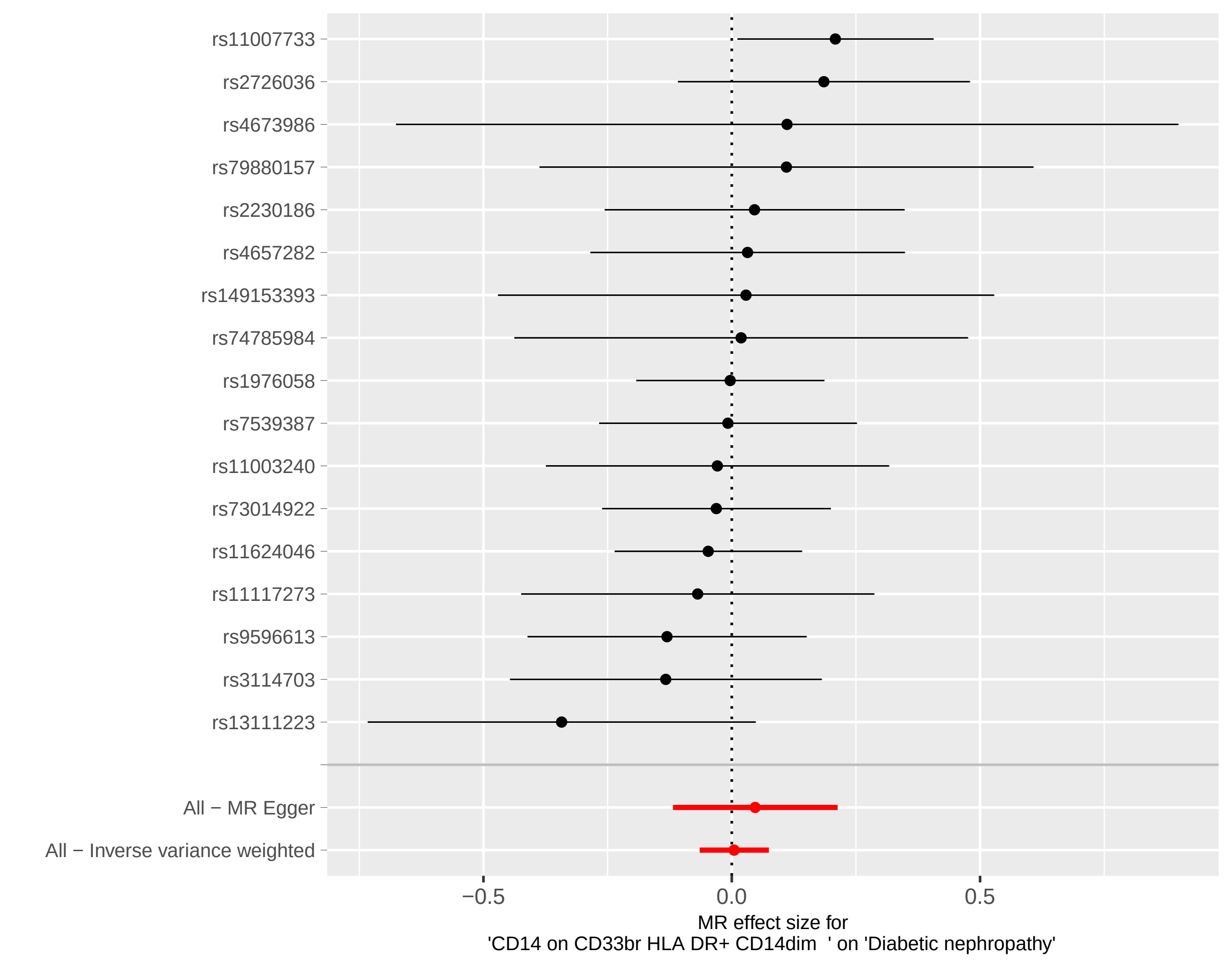
rs11007733
rs2726036
rs4673986
rs79880157
rs2230186
rs4657282
rs149153393
rs74785984
rs1976058
rs7539387
rs11003240
rs73014922
rs11624046
rs11117273
rs9596613
rs3114703
rs13111223
All − MR Egger
All − Inverse variance weighted
−0.5
0.0
0.5
MR effect size for
'CD14 on CD33br HLA DR+ CD14dim ' on 'Diabetic nephropathy'

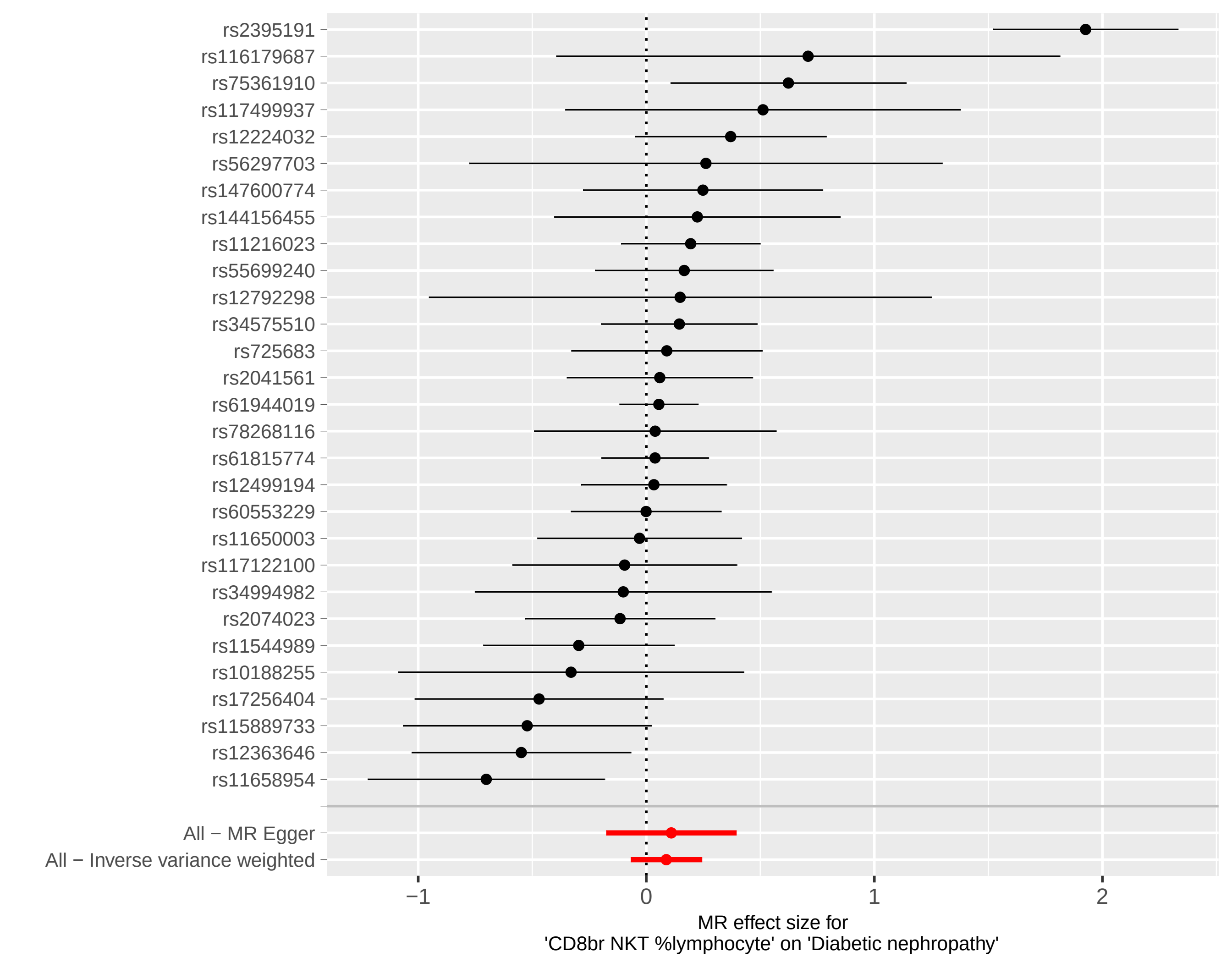

rs2395191
rs116179687
rs75361910
rs117499937
rs12224032
rs56297703
rs147600774
rs144156455
rs11216023
rs55699240
rs12792298
rs34575510
rs725683
rs2041561
rs61944019
rs78268116
rs61815774
rs12499194
rs60553229
rs11650003
rs117122100
rs34994982
rs2074023
rs11544989
rs10188255
rs17256404
rs115889733
rs12363646
rs11658954
All − MR Egger
All − Inverse variance weighted
−1
0
1
2
MR effect size for
'CD8br NKT %lymphocyte' on 'Diabetic nephropathy'

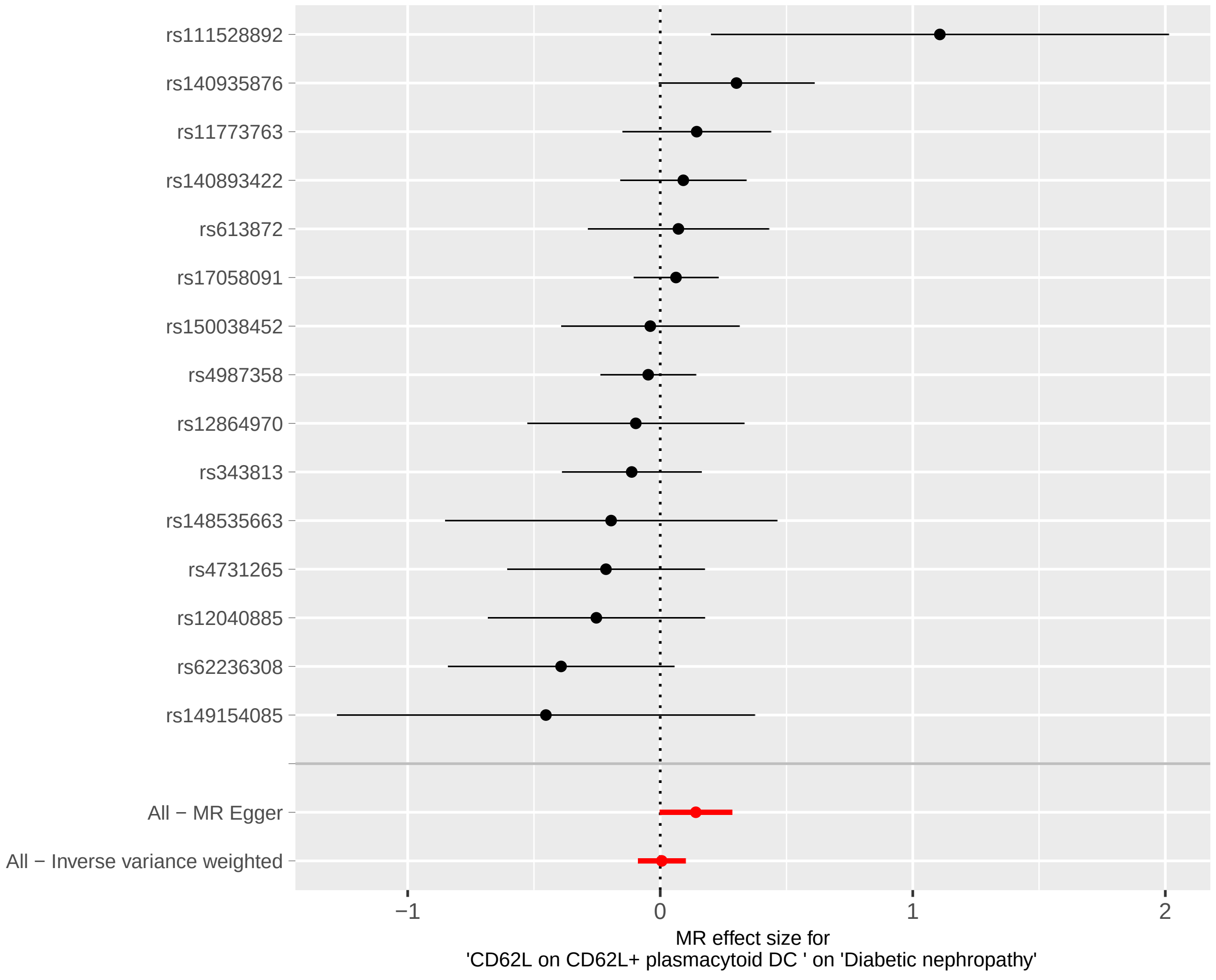
rs111528892
rs140935876
rs11773763
rs140893422
rs613872
rs17058091
rs150038452
rs4987358
rs12864970
rs343813
rs148535663
rs4731265
rs12040885
rs62236308
rs149154085
All − MR Egger
All − Inverse variance weighted
−1
0
1
2
MR effect size for
'CD62L on CD62L+ plasmacytoid DC ' on 'Diabetic nephropathy'

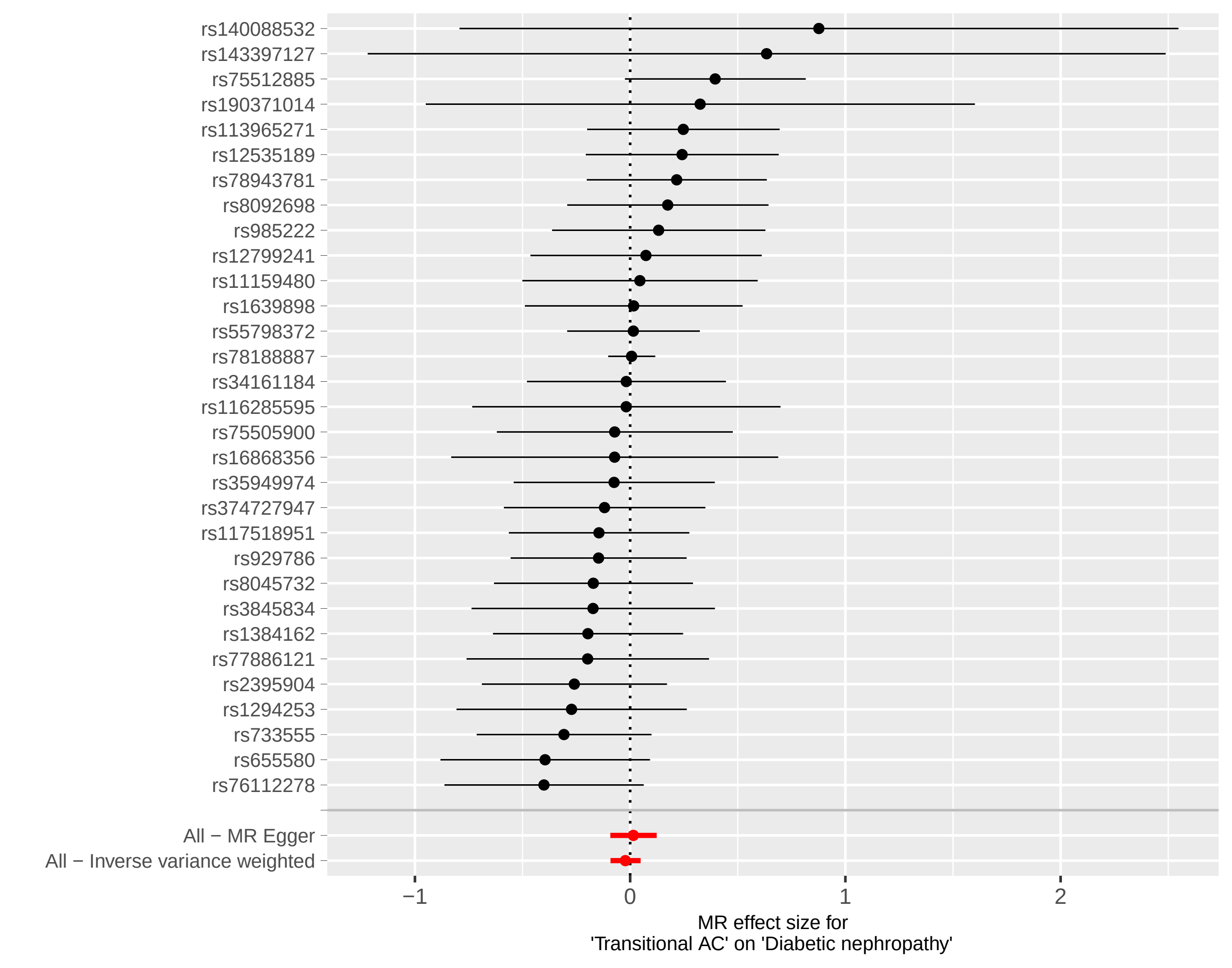
rs140088532
rs143397127
rs75512885
rs190371014
rs113965271
rs12535189
rs78943781
rs8092698
rs985222
rs12799241
rs11159480
rs1639898
rs55798372
rs78188887
rs34161184
rs116285595
rs75505900
rs16868356
rs35949974
rs374727947
rs117518951
rs929786
rs8045732
rs3845834
rs1384162
rs77886121
rs2395904
rs1294253
rs733555
rs655580
rs76112278
All − MR Egger
All − Inverse variance weighted
−1
0
1
2
MR effect size for
'Transitional AC' on 'Diabetic nephropathy'

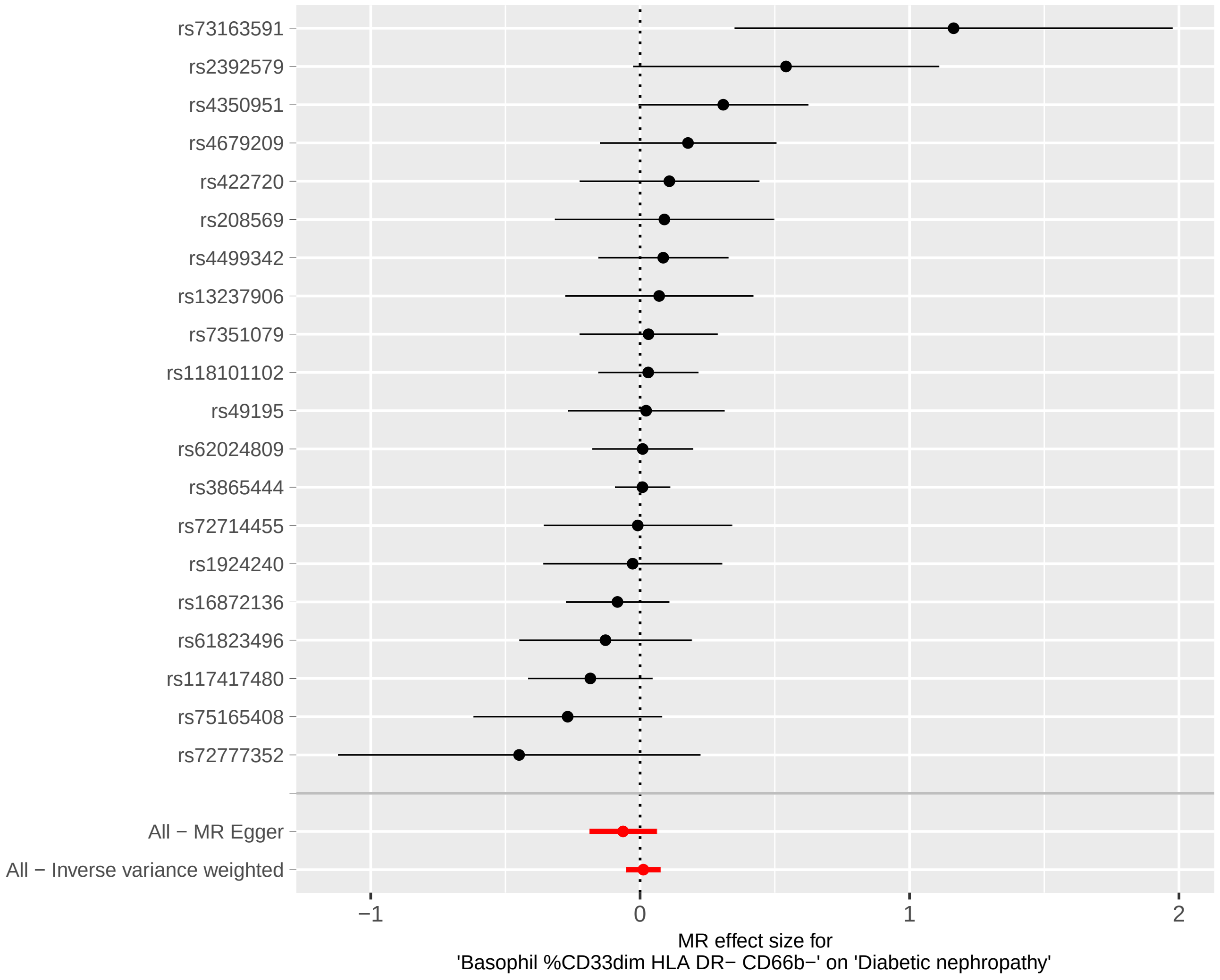
rs73163591
rs2392579
rs4350951
rs4679209
rs422720
rs208569
rs4499342
rs13237906
rs7351079
rs118101102
rs49195
rs62024809
rs3865444
rs72714455
rs1924240
rs16872136
rs61823496
rs117417480
rs75165408
rs72777352
All − MR Egger
All − Inverse variance weighted
−1
0
1
2
MR effect size for
'Basophil %CD33dim HLA DR− CD66b−' on 'Diabetic nephropathy'

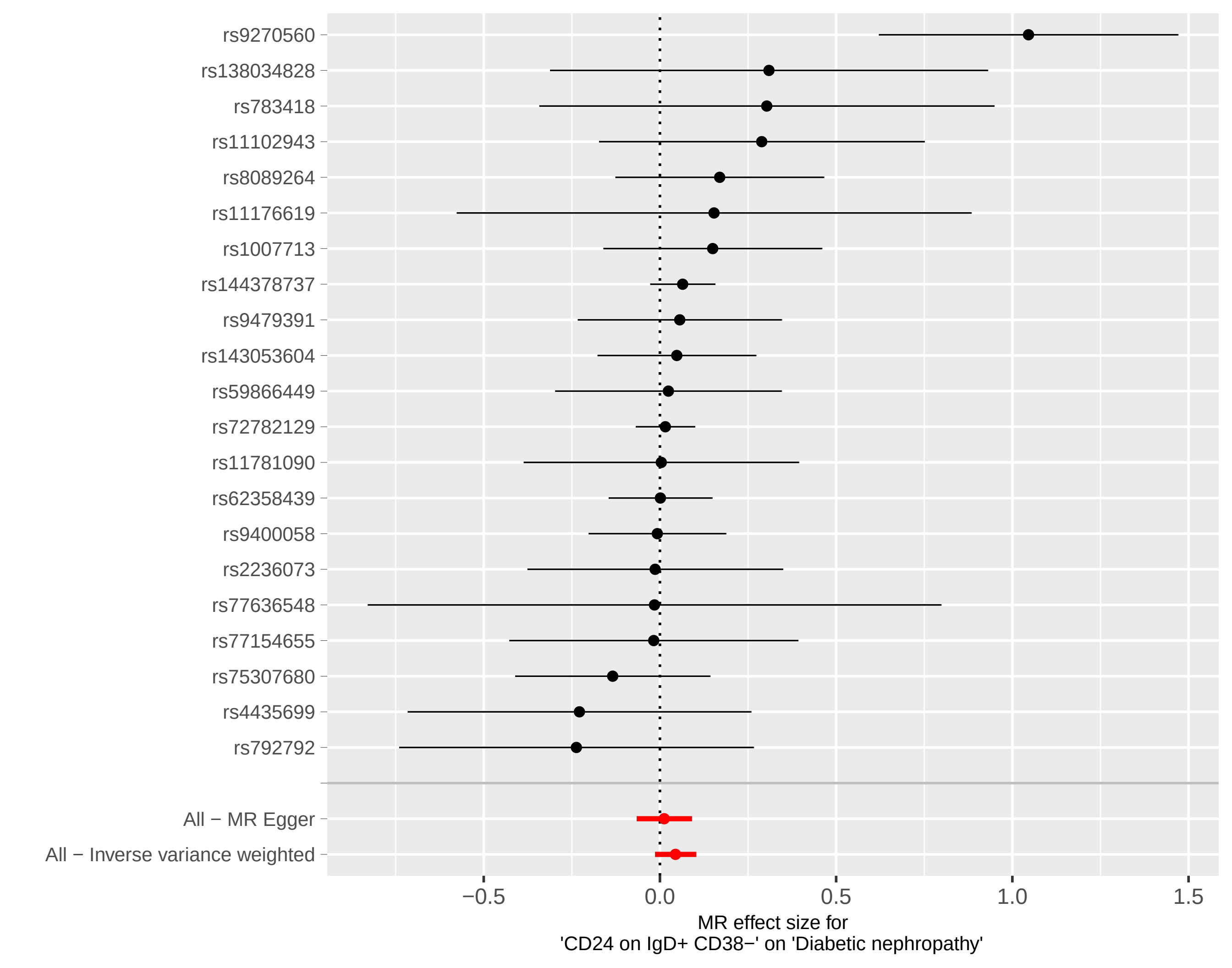
rs9270560
rs138034828
rs783418
rs11102943
rs8089264
rs11176619
rs1007713
rs144378737
rs9479391
rs143053604
rs59866449
rs72782129
rs11781090
rs62358439
rs9400058
rs2236073
rs77636548
rs77154655
rs75307680
rs4435699
rs792792
All − MR Egger
All − Inverse variance weighted
−0.5
0.0
0.5
1.0
1.5
MR effect size for
'CD24 on IgD+ CD38−' on 'Diabetic nephropathy'

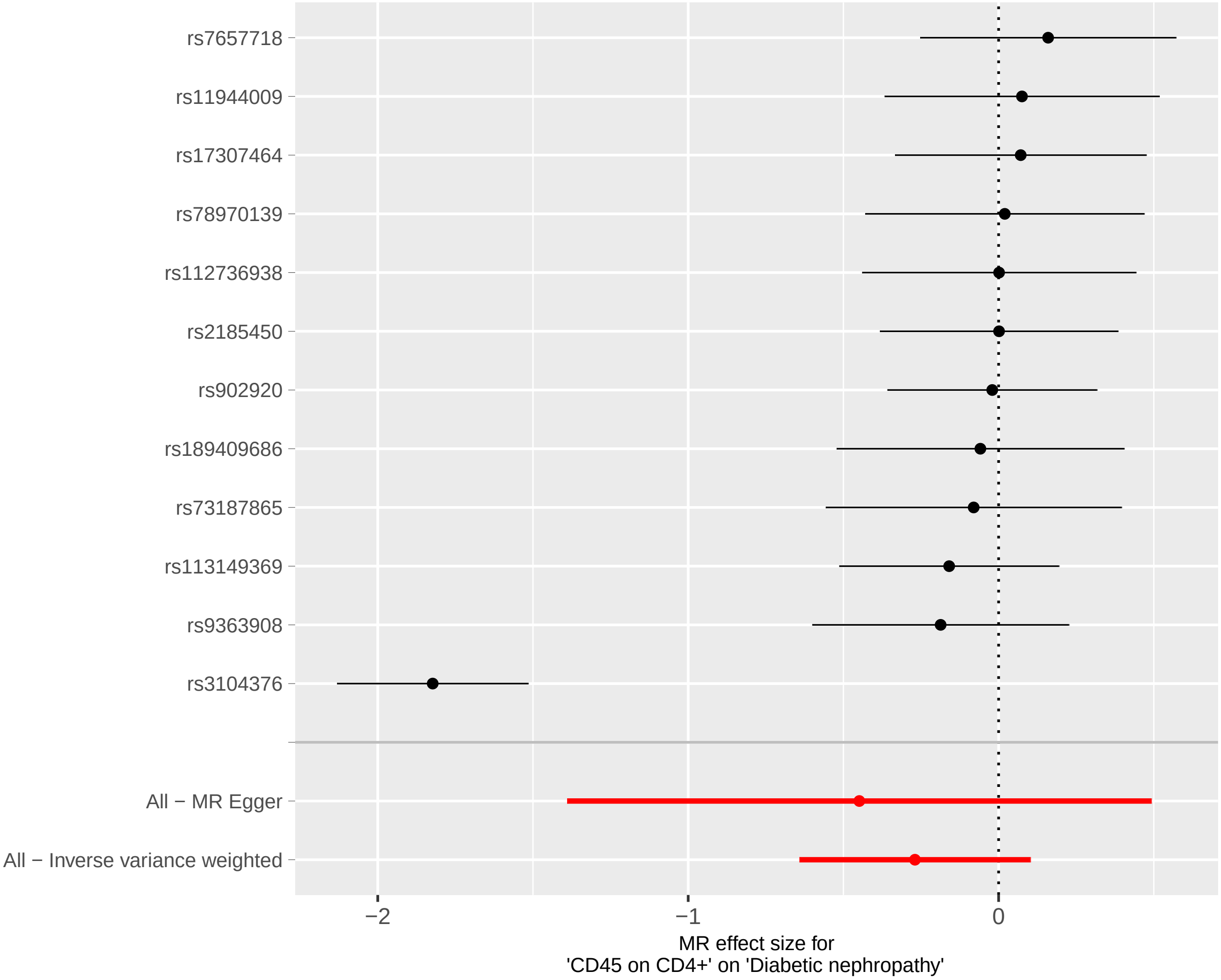

rs7657718
rs11944009
rs17307464
rs78970139
rs112736938
rs2185450
rs902920
rs189409686
rs73187865
rs113149369
rs9363908
rs3104376
All − MR Egger
All − Inverse variance weighted
−2
−1
0
MR effect size for
'CD45 on CD4+' on 'Diabetic nephropathy'

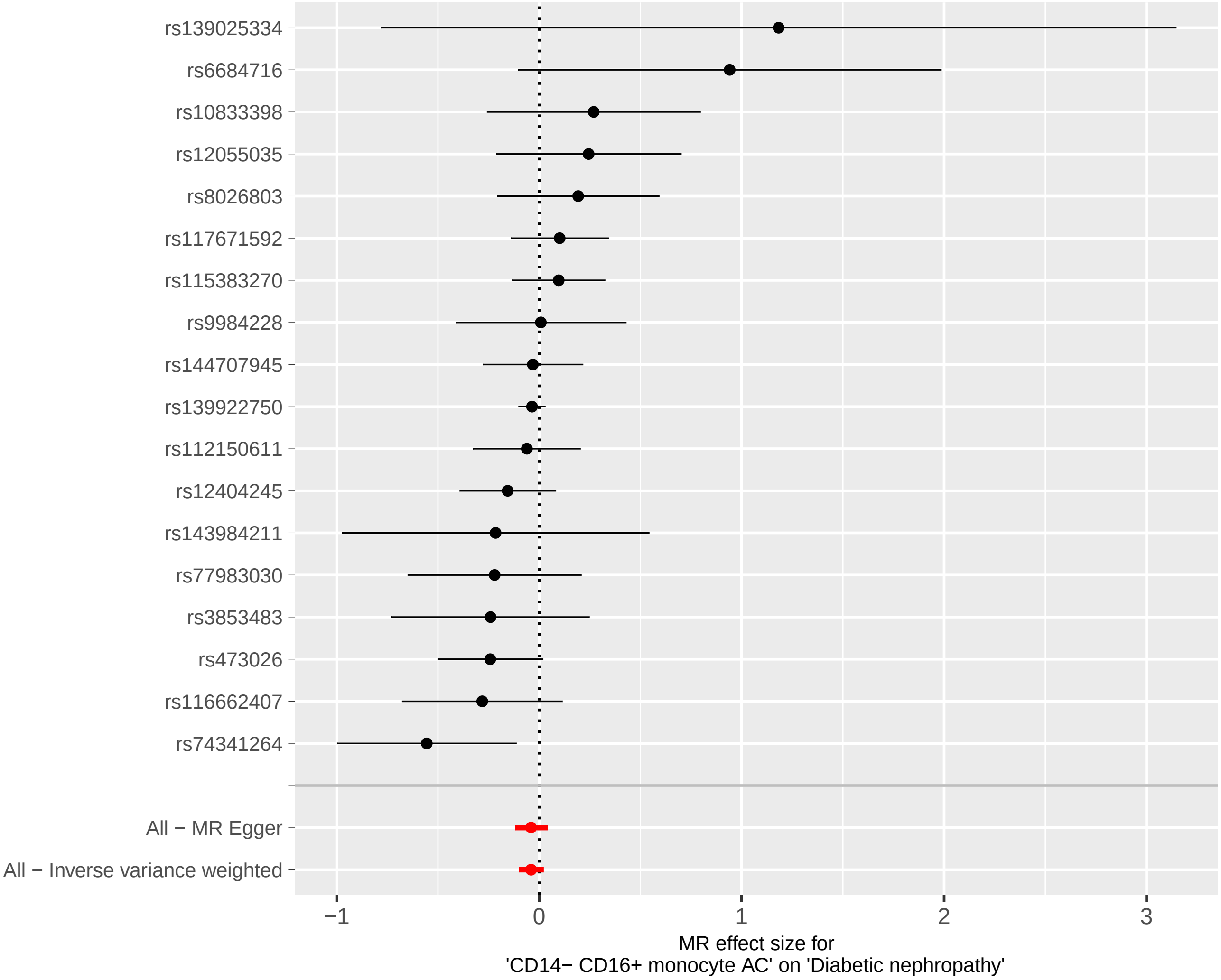
rs139025334
rs6684716
rs10833398
rs12055035
rs8026803
rs117671592
rs115383270
rs9984228
rs144707945
rs139922750
rs112150611
rs12404245
rs143984211
rs77983030
rs3853483
rs473026
rs116662407
rs74341264
All − MR Egger
All − Inverse variance weighted
−1
0
1
2
3
MR effect size for
'CD14− CD16+ monocyte AC' on 'Diabetic nephropathy'

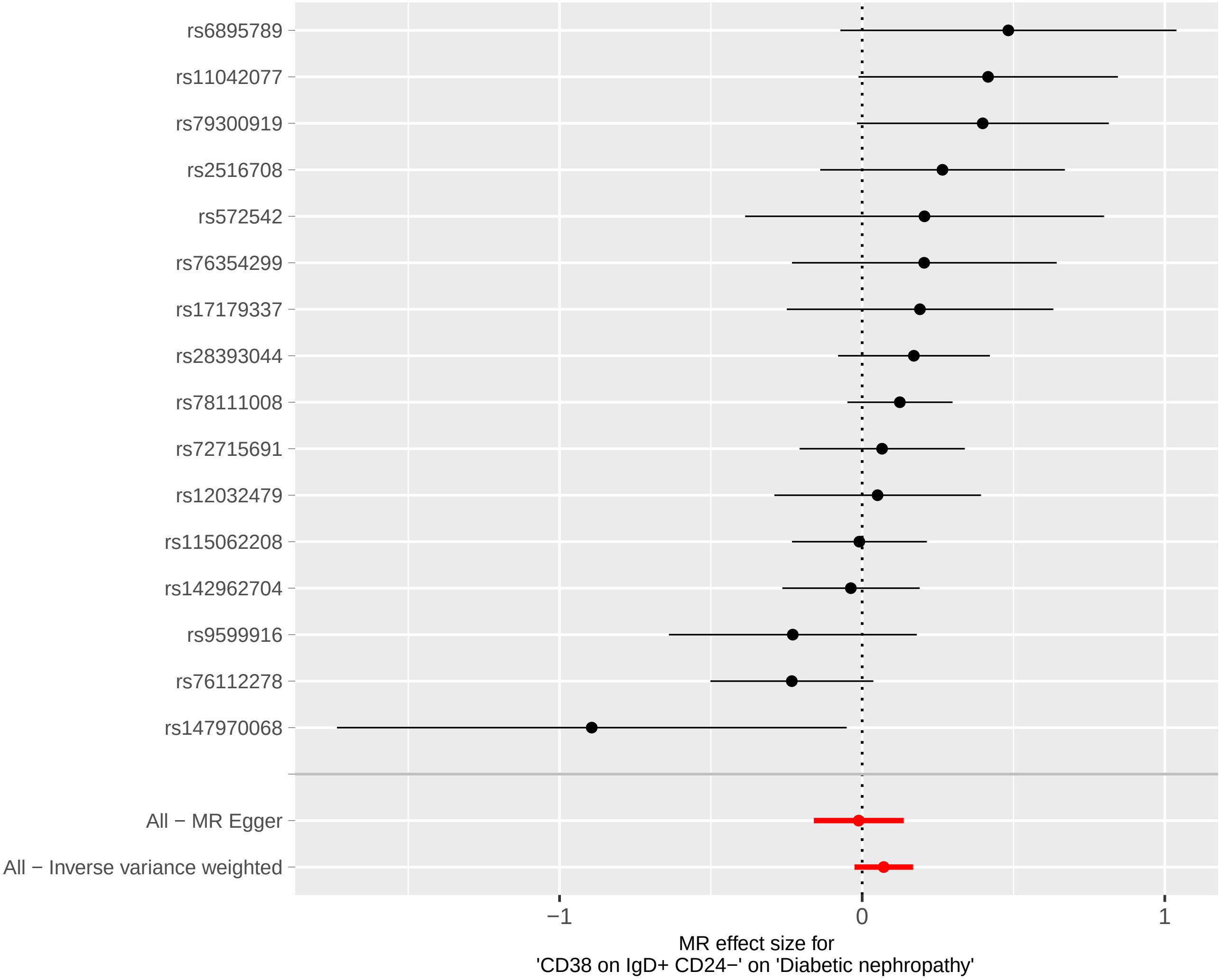
rs6895789
rs11042077
rs79300919
rs2516708
rs572542
rs76354299
rs17179337
rs28393044
rs78111008
rs72715691
rs12032479
rs115062208
rs142962704
rs9599916
rs76112278
rs147970068
All − MR Egger
All − Inverse variance weighted
−1
0
1
MR effect size for
'CD38 on IgD+ CD24−' on 'Diabetic nephropathy'

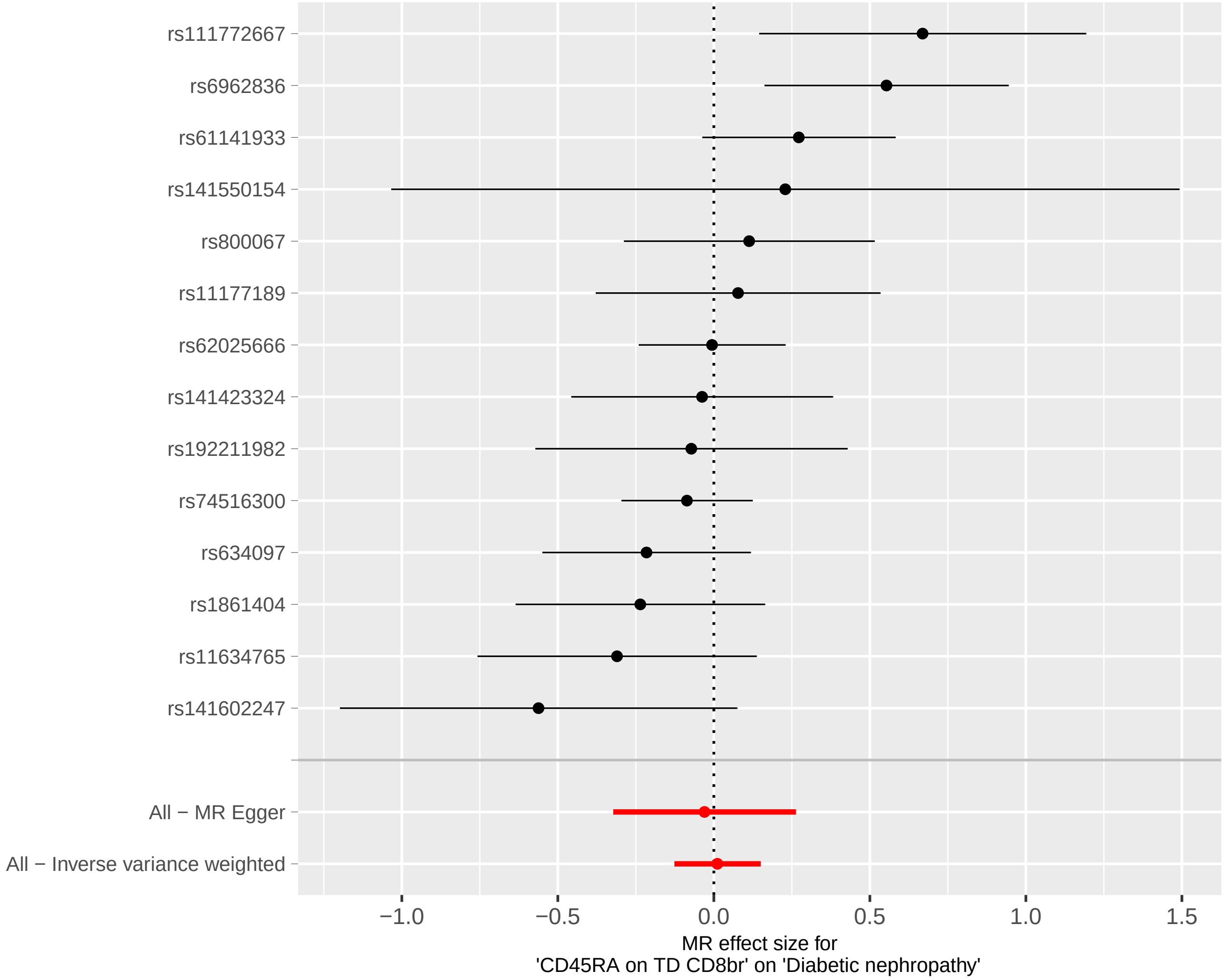

rs111772667
rs6962836
rs61141933
rs141550154
rs800067
rs11177189
rs62025666
rs141423324
rs192211982
rs74516300
rs634097
rs1861404
rs11634765
rs141602247
All − MR Egger
All − Inverse variance weighted
−1.0
−0.5
0.0
0.5
1.0
1.5
MR effect size for
'CD45RA on TD CD8br' on 'Diabetic nephropathy'

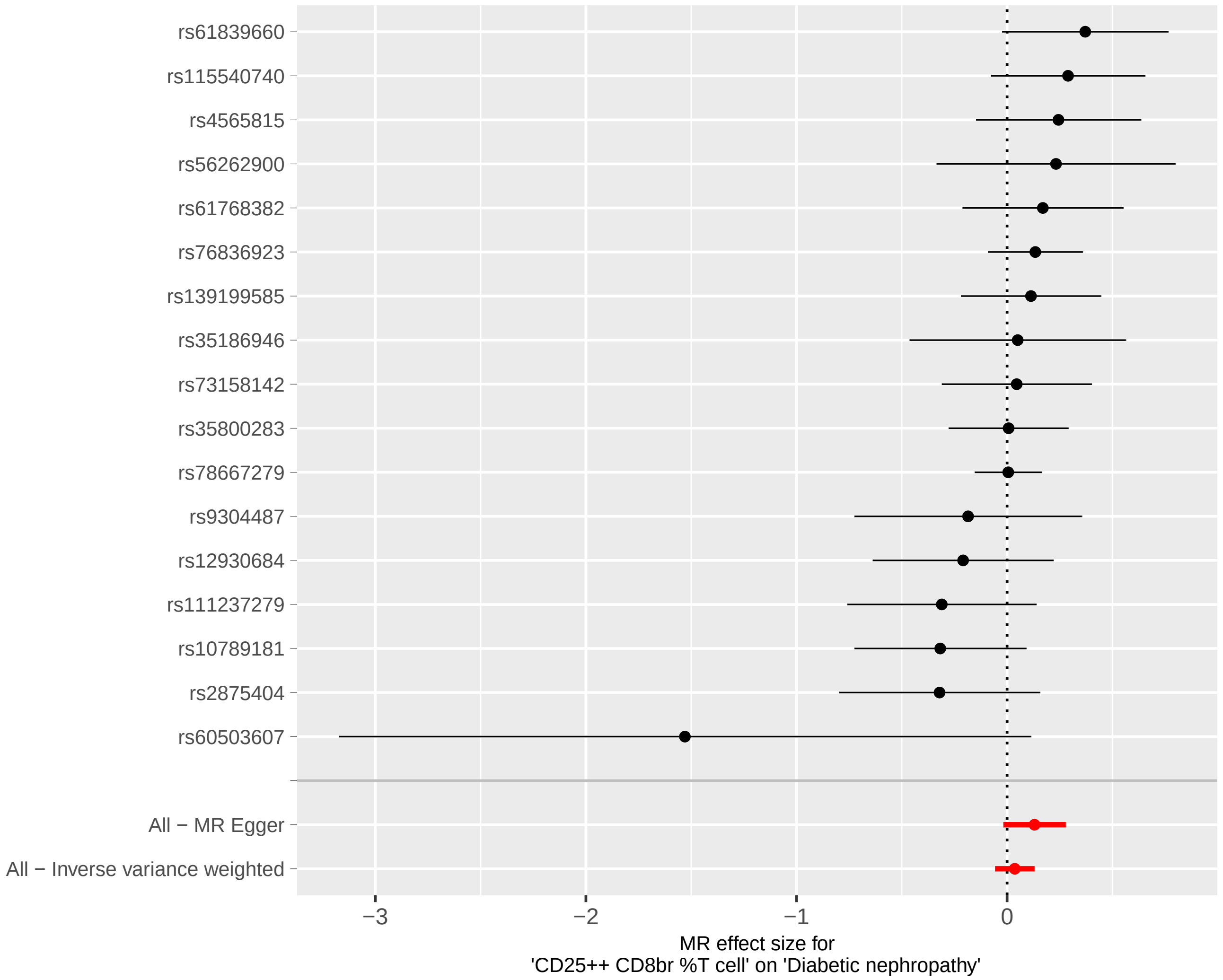
rs61839660
rs115540740
rs4565815
rs56262900
rs61768382
rs76836923
rs139199585
rs35186946
rs73158142
rs35800283
rs78667279
rs9304487
rs12930684
rs111237279
rs10789181
rs2875404
rs60503607
All − MR Egger
All − Inverse variance weighted
−3
−2
−1
0
MR effect size for
'CD25++ CD8br %T cell' on 'Diabetic nephropathy'

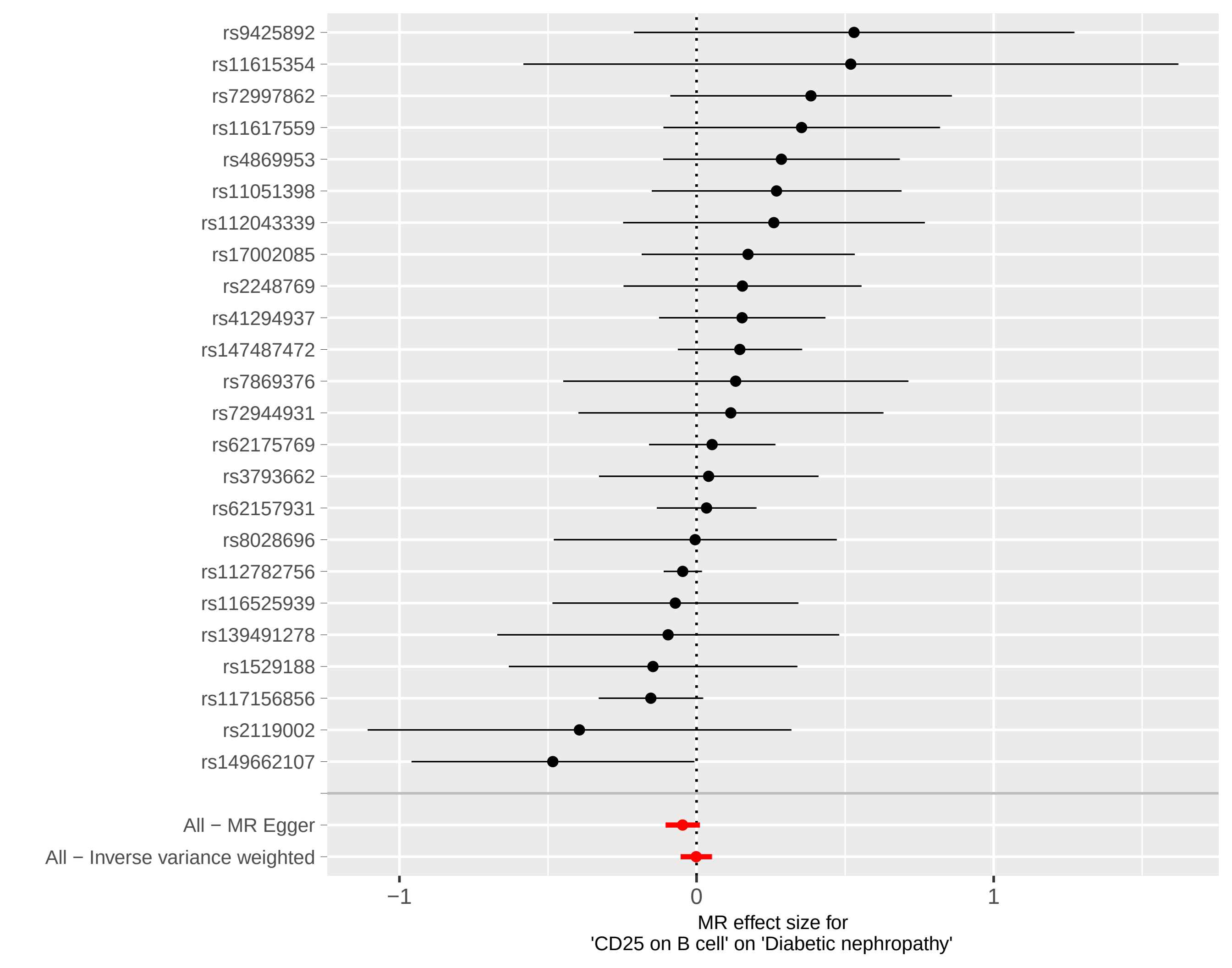
rs9425892
rs11615354
rs72997862
rs11617559
rs4869953
rs11051398
rs112043339
rs17002085
rs2248769
rs41294937
rs147487472
rs7869376
rs72944931
rs62175769
rs3793662
rs62157931
rs8028696
rs112782756
rs116525939
rs139491278
rs1529188
rs117156856
rs2119002
rs149662107
All − MR Egger
All − Inverse variance weighted
−1
0
1
MR effect size for
'CD25 on B cell' on 'Diabetic nephropathy'

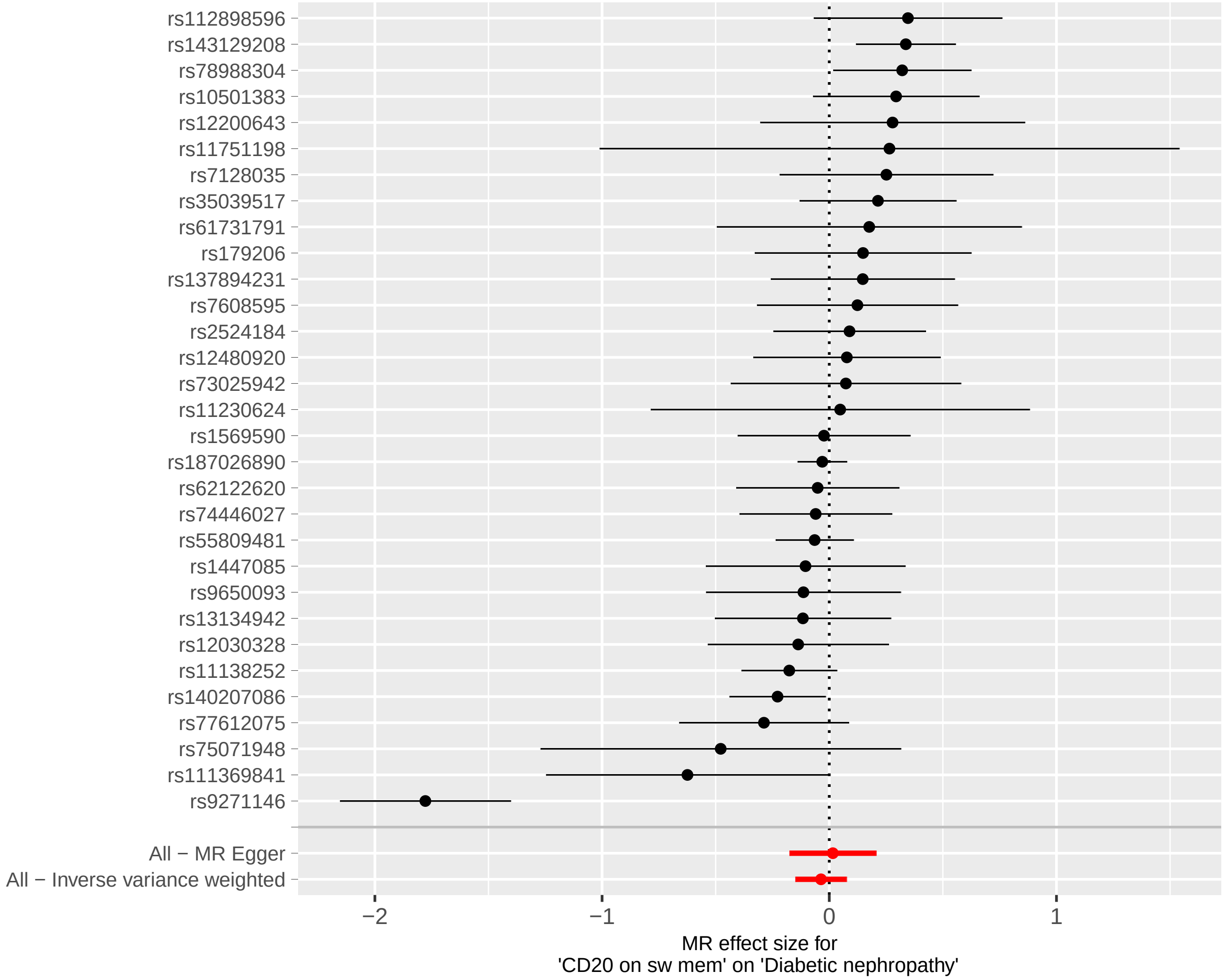
rs112898596
rs143129208
rs78988304
rs10501383
rs12200643
rs11751198
rs7128035
rs35039517
rs61731791
rs179206
rs137894231
rs7608595
rs2524184
rs12480920
rs73025942
rs11230624
rs1569590
rs187026890
rs62122620
rs74446027
rs55809481
rs1447085
rs9650093
rs13134942
rs12030328
rs11138252
rs140207086
rs77612075
rs75071948
rs111369841
rs9271146
All − MR Egger
All − Inverse variance weighted
−2
−1
0
1
MR effect size for
'CD20 on sw mem' on 'Diabetic nephropathy'

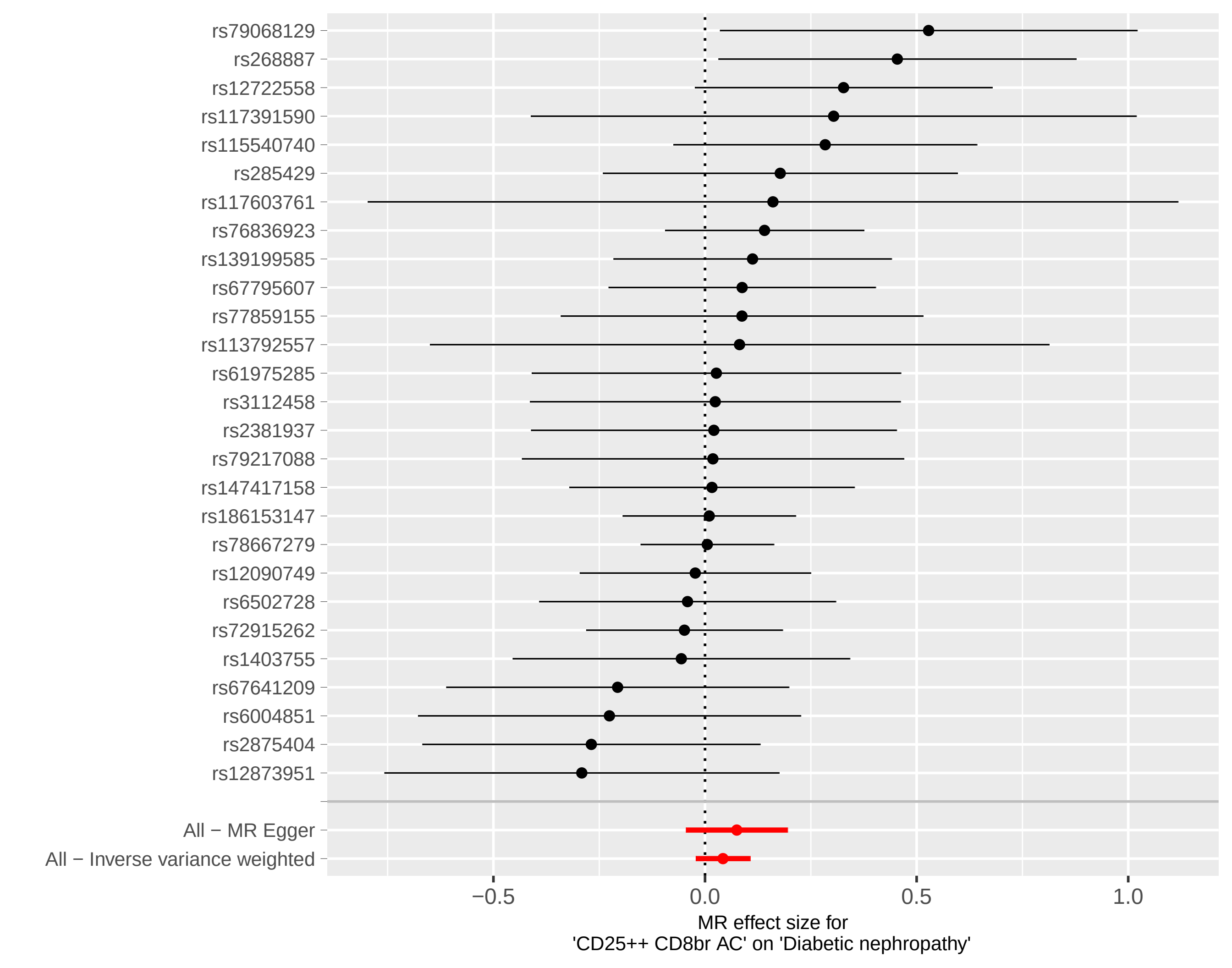

rs79068129
rs268887
rs12722558
rs117391590
rs115540740
rs285429
rs117603761
rs76836923
rs139199585
rs67795607
rs77859155
rs113792557
rs61975285
rs3112458
rs2381937
rs79217088
rs147417158
rs186153147
rs78667279
rs12090749
rs6502728
rs72915262
rs1403755
rs67641209
rs6004851
rs2875404
rs12873951
All − MR Egger
All − Inverse variance weighted
−0.5
0.0
0.5
1.0
MR effect size for
'CD25++ CD8br AC' on 'Diabetic nephropathy'

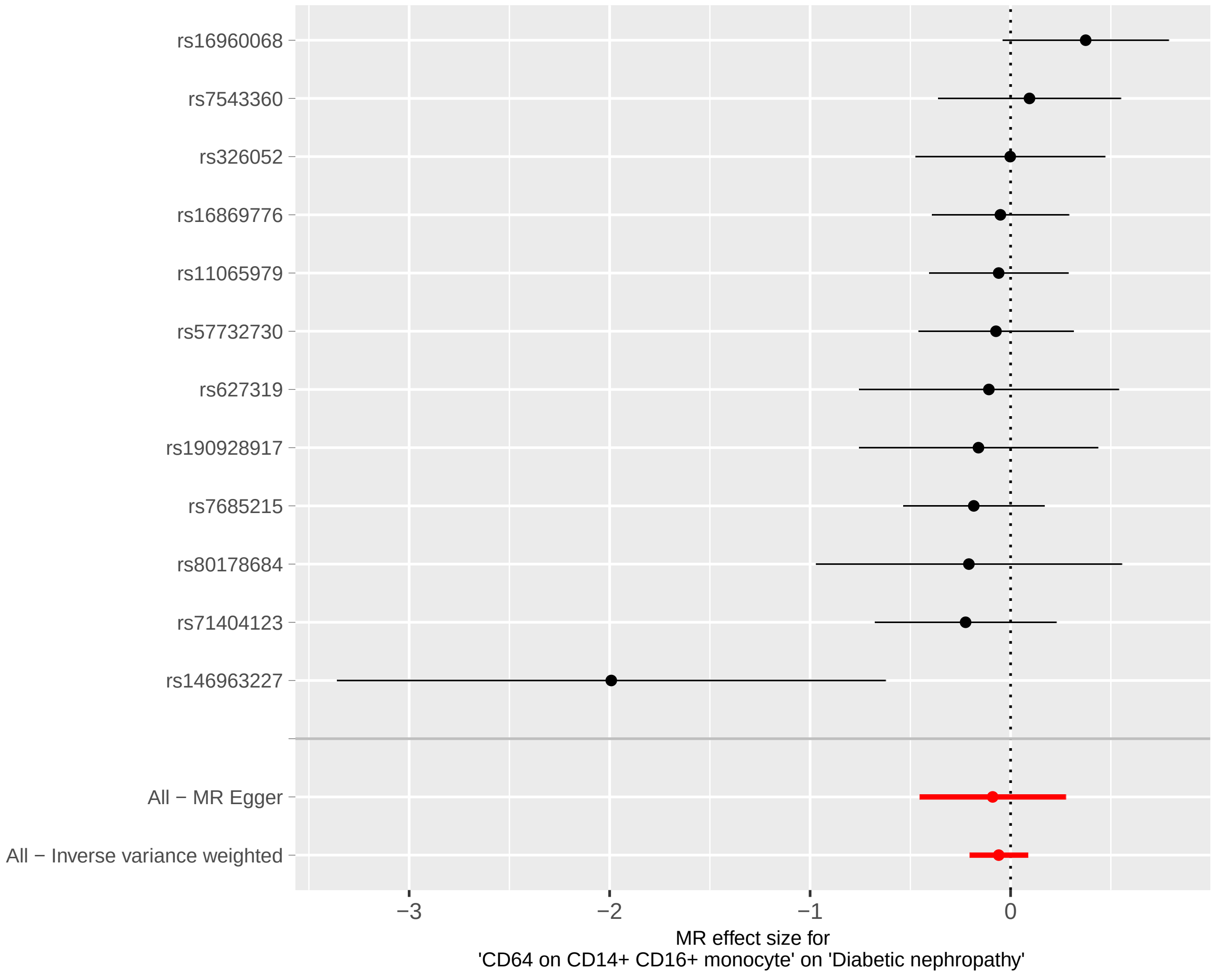

rs16960068
rs7543360
rs326052
rs16869776
rs11065979
rs57732730
rs627319
rs190928917
rs7685215
rs80178684
rs71404123
rs146963227
All − MR Egger
All − Inverse variance weighted
−3
−2
−1
0
MR effect size for
'CD64 on CD14+ CD16+ monocyte' on 'Diabetic nephropathy'

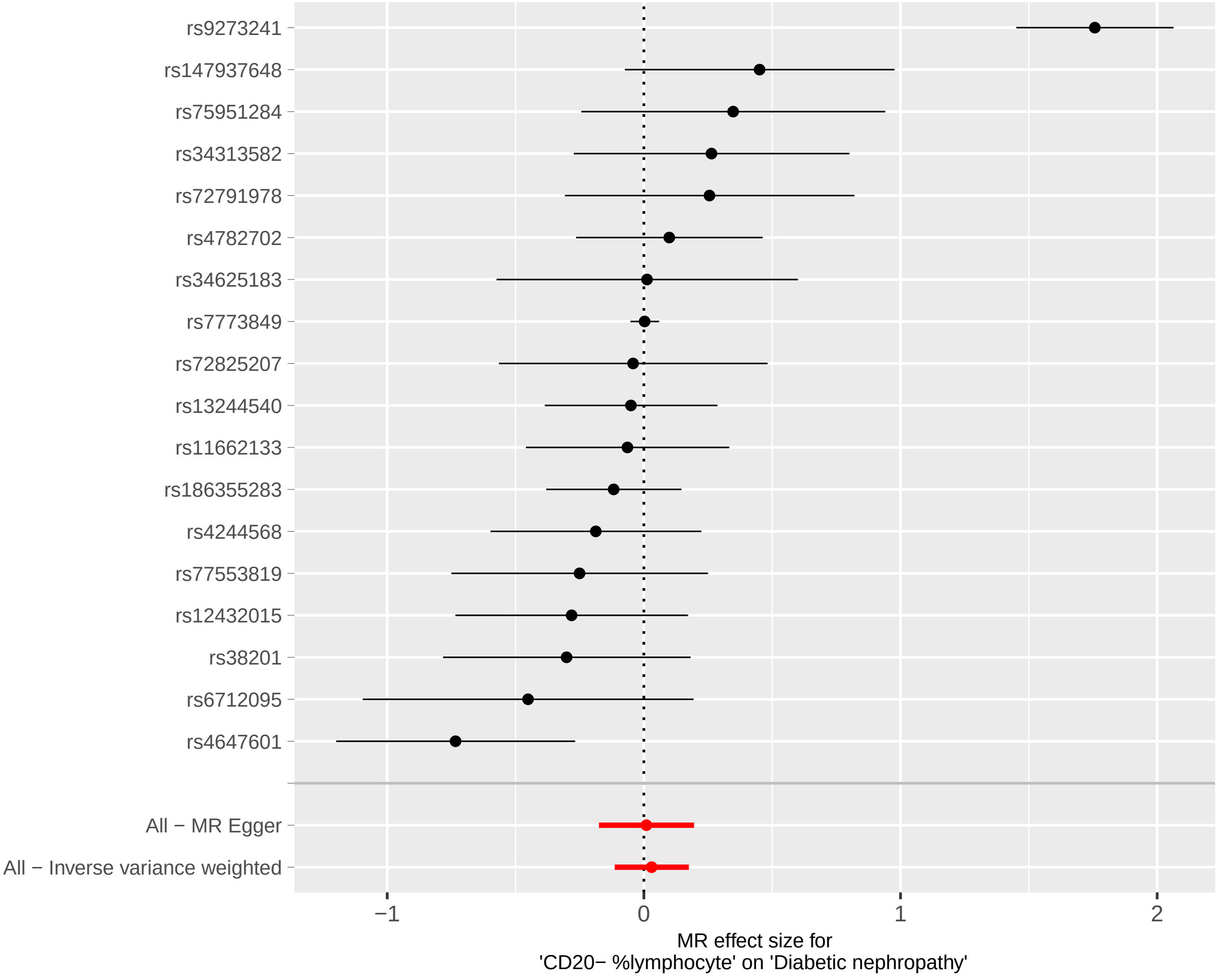
rs9273241
rs147937648
rs75951284
rs34313582
rs72791978
rs4782702
rs34625183
rs7773849
rs72825207
rs13244540
rs11662133
rs186355283
rs4244568
rs77553819
rs12432015
rs38201
rs6712095
rs4647601
All − MR Egger
All − Inverse variance weighted
−1
0
1
2
MR effect size for
'CD20− %lymphocyte' on 'Diabetic nephropathy'

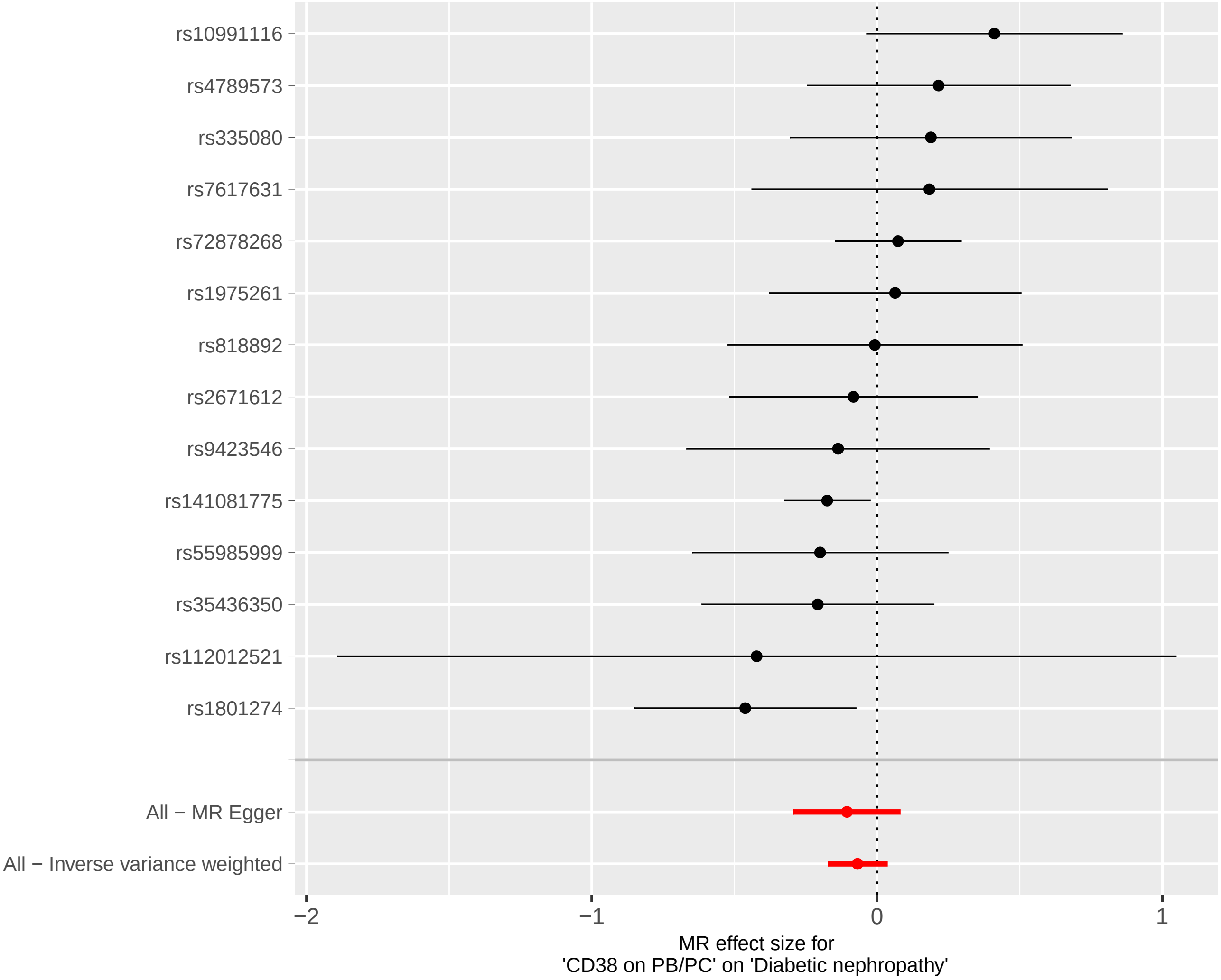
rs10991116
rs4789573
rs335080
rs7617631
rs72878268
rs1975261
rs818892
rs2671612
rs9423546
rs141081775
rs55985999
rs35436350
rs112012521
rs1801274
All − MR Egger
All − Inverse variance weighted
−2
−1
0
1
MR effect size for
'CD38 on PB/PC' on 'Diabetic nephropathy'

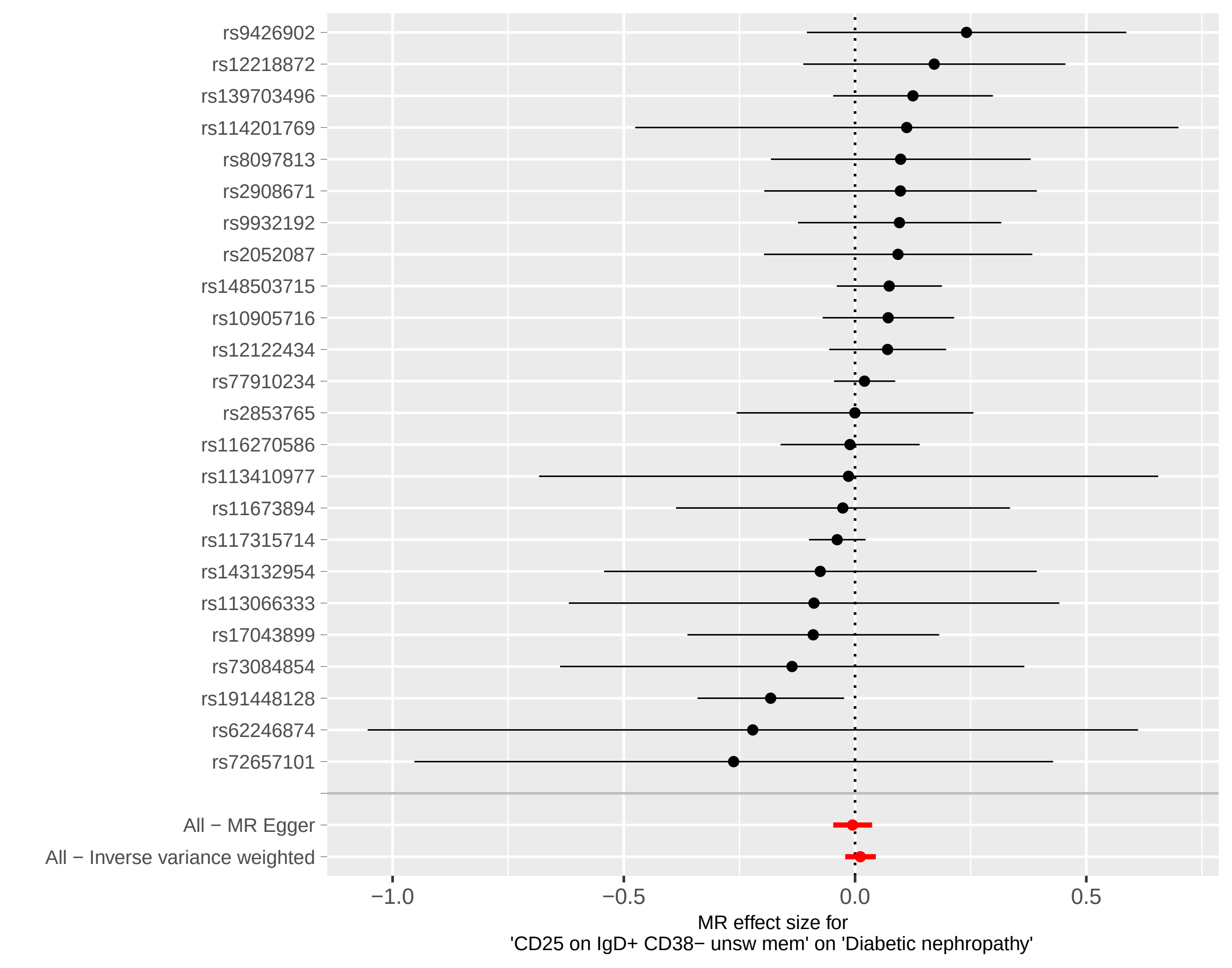
rs9426902
rs12218872
rs139703496
rs114201769
rs8097813
rs2908671
rs9932192
rs2052087
rs148503715
rs10905716
rs12122434
rs77910234
rs2853765
rs116270586
rs113410977
rs11673894
rs117315714
rs143132954
rs113066333
rs17043899
rs73084854
rs191448128
rs62246874
rs72657101
All − MR Egger
All − Inverse variance weighted
−1.0
−0.5
0.0
0.5
MR effect size for
'CD25 on IgD+ CD38− unsw mem' on 'Diabetic nephropathy'

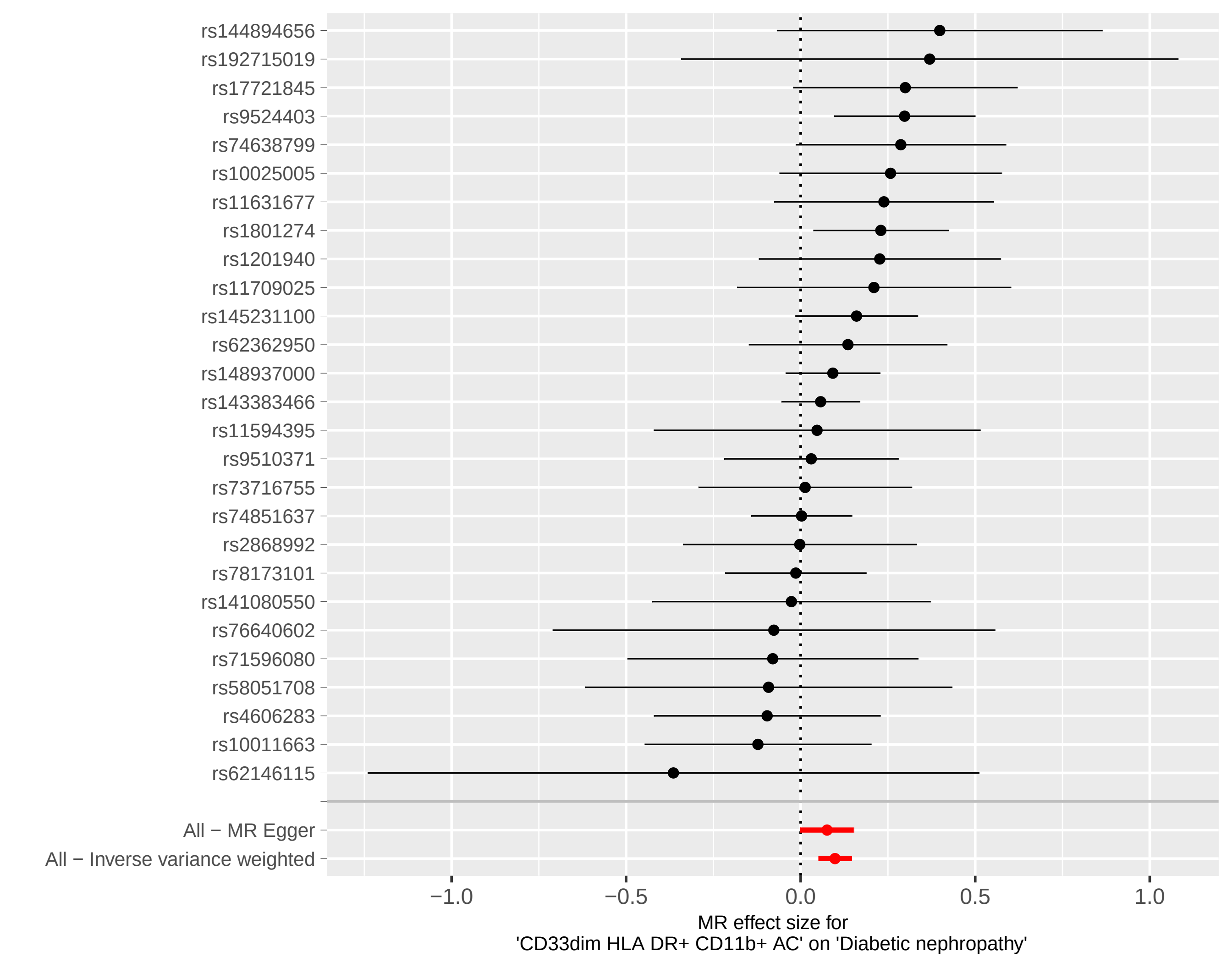
rs144894656
rs192715019
rs17721845
rs9524403
rs74638799
rs10025005
rs11631677
rs1801274
rs1201940
rs11709025
rs145231100
rs62362950
rs148937000
rs143383466
rs11594395
rs9510371
rs73716755
rs74851637
rs2868992
rs78173101
rs141080550
rs76640602
rs71596080
rs58051708
rs4606283
rs10011663
rs62146115
All − MR Egger
All − Inverse variance weighted
−1.0
−0.5
0.0
0.5
1.0
MR effect size for
'CD33dim HLA DR+ CD11b+ AC' on 'Diabetic nephropathy'

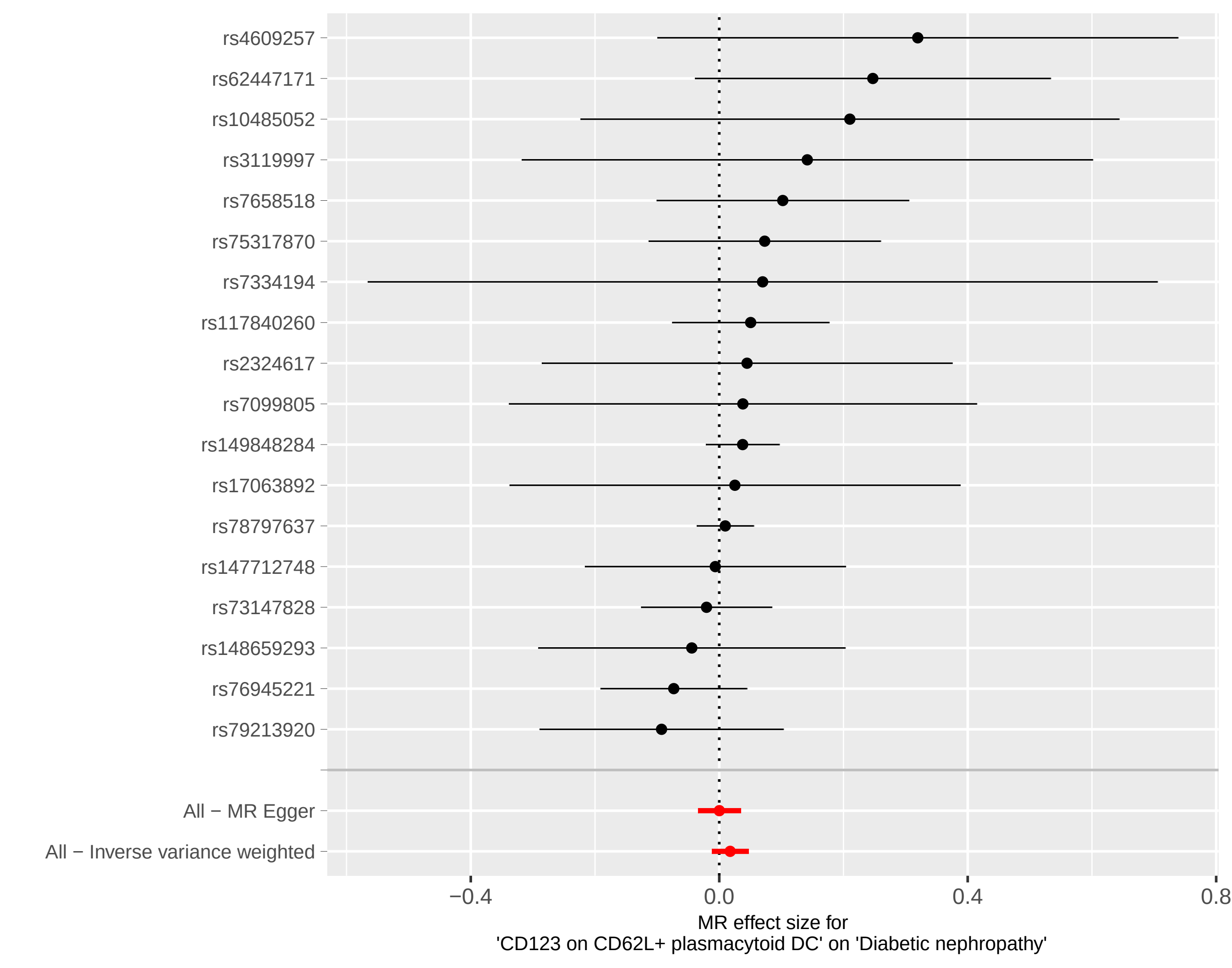
rs4609257
rs62447171
rs10485052
rs3119997
rs7658518
rs75317870
rs7334194
rs117840260
rs2324617
rs7099805
rs149848284
rs17063892
rs78797637
rs147712748
rs73147828
rs148659293
rs76945221
rs79213920
All − MR Egger
All − Inverse variance weighted
−0.4
0.0
0.4
0.8
MR effect size for
'CD123 on CD62L+ plasmacytoid DC' on 'Diabetic nephropathy'

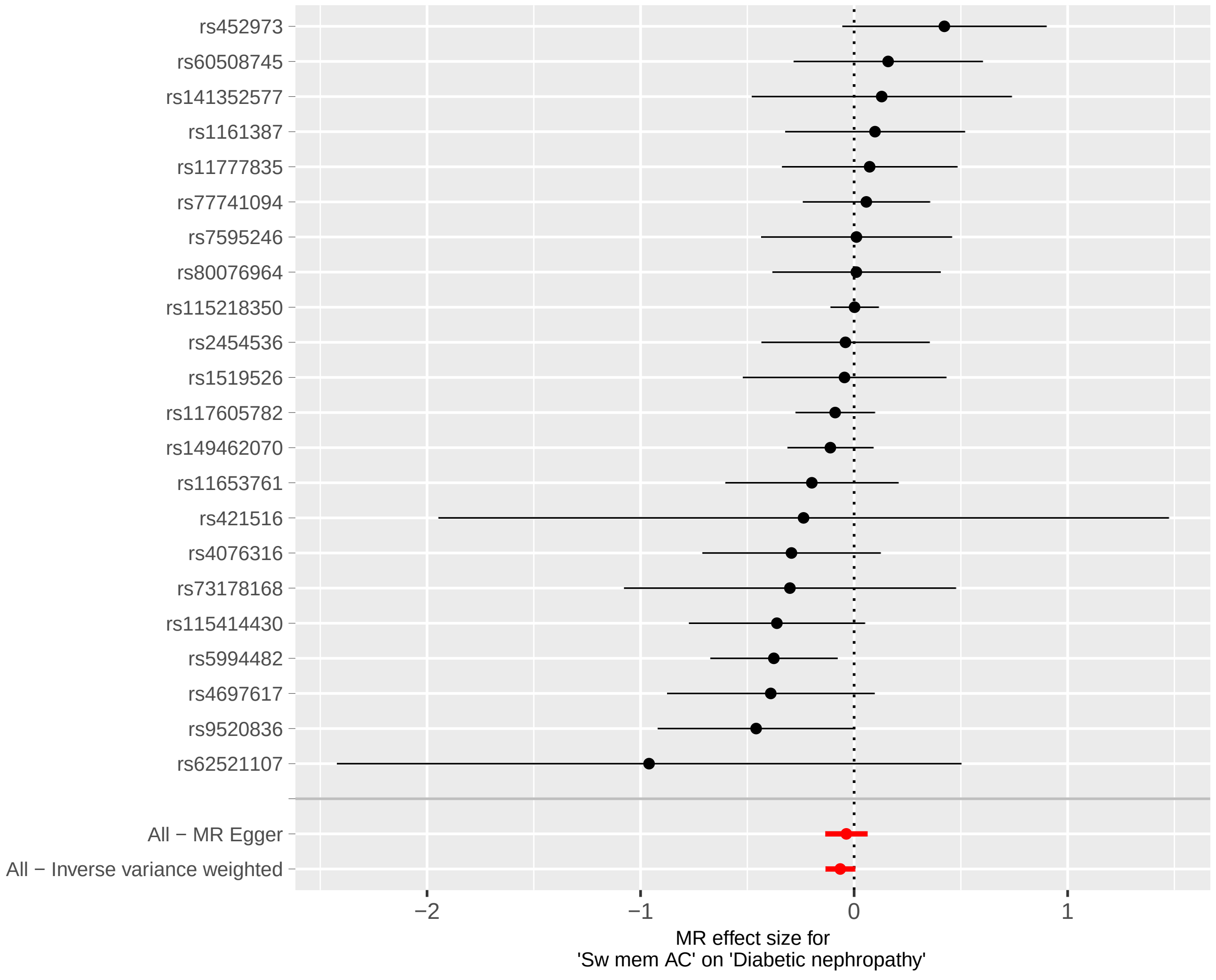
rs452973
rs60508745
rs141352577
rs1161387
rs11777835
rs77741094
rs7595246
rs80076964
rs115218350
rs2454536
rs1519526
rs117605782
rs149462070
rs11653761
rs421516
rs4076316
rs73178168
rs115414430
rs5994482
rs4697617
rs9520836
rs62521107
All − MR Egger
All − Inverse variance weighted
−2
−1
0
1
MR effect size for
'Sw mem AC' on 'Diabetic nephropathy'

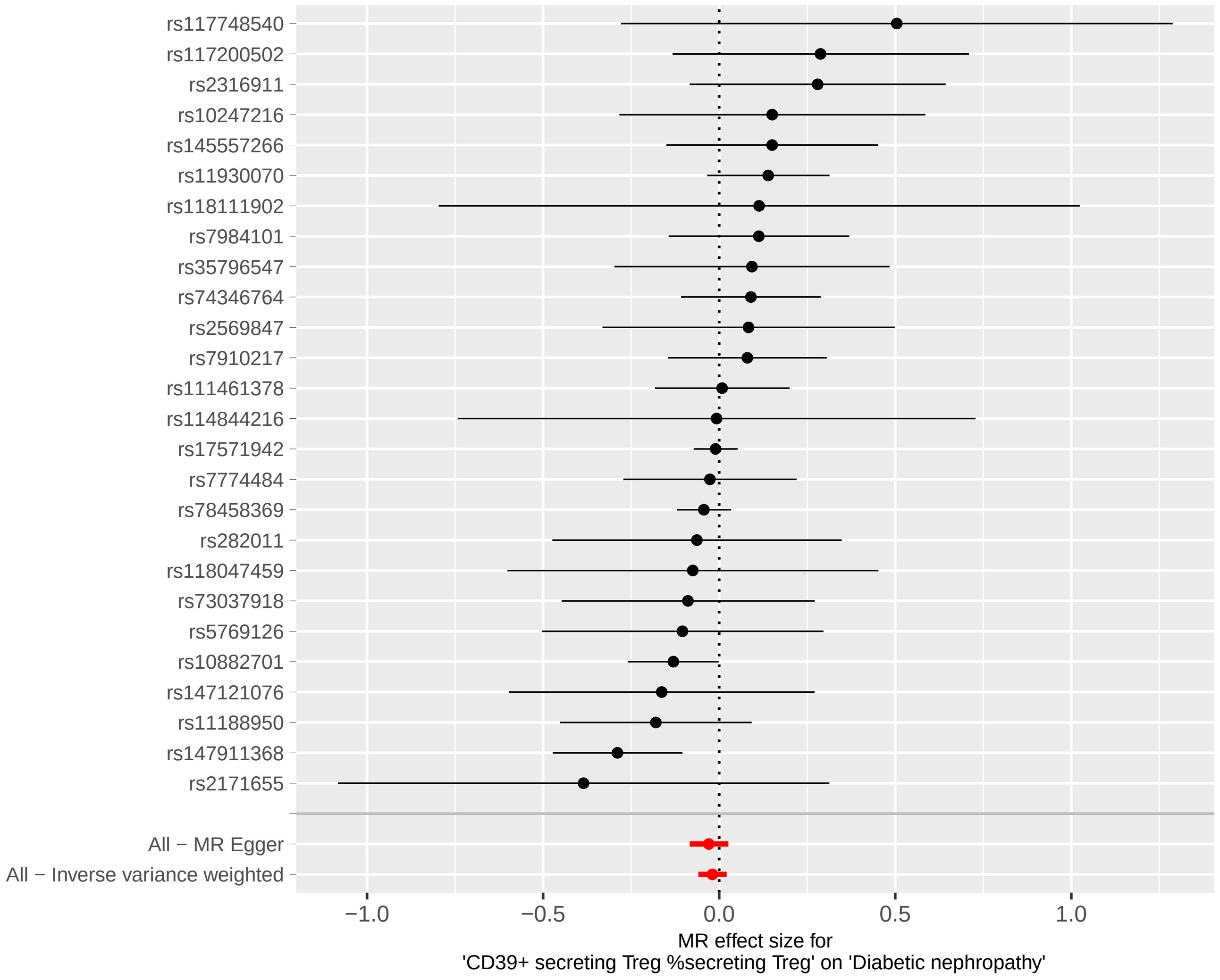

rs117748540
rs117200502
rs2316911
rs10247216
rs145557266
rs11930070
rs118111902
rs7984101
rs35796547
rs74346764
rs2569847
rs7910217
rs111461378
rs114844216
rs17571942
rs7774484
rs78458369
rs282011
rs118047459
rs73037918
rs5769126
rs10882701
rs147121076
rs11188950
rs147911368
rs2171655
All − MR Egger
All − Inverse variance weighted
−1.0
−0.5
0.0
0.5
1.0
MR effect size for
'CD39+ secreting Treg %secreting Treg' on 'Diabetic nephropathy'

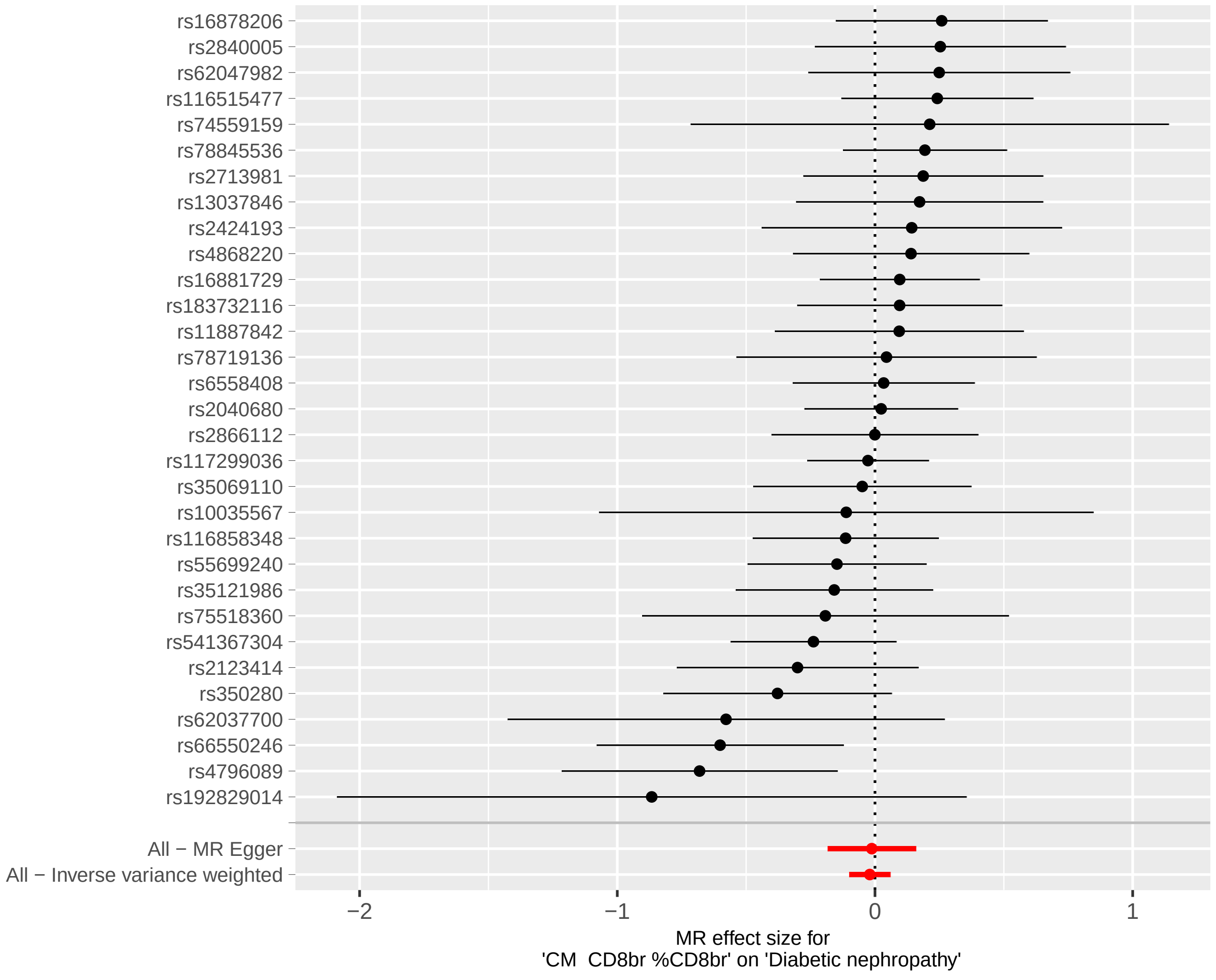
rs16878206
rs2840005
rs62047982
rs116515477
rs74559159
rs78845536
rs2713981
rs13037846
rs2424193
rs4868220
rs16881729
rs183732116
rs11887842
rs78719136
rs6558408
rs2040680
rs2866112
rs117299036
rs35069110
rs10035567
rs116858348
rs55699240
rs35121986
rs75518360
rs541367304
rs2123414
rs350280
rs62037700
rs66550246
rs4796089
rs192829014
All − MR Egger
All − Inverse variance weighted
−2
−1
0
1
MR effect size for
'CM CD8br %CD8br' on 'Diabetic nephropathy'

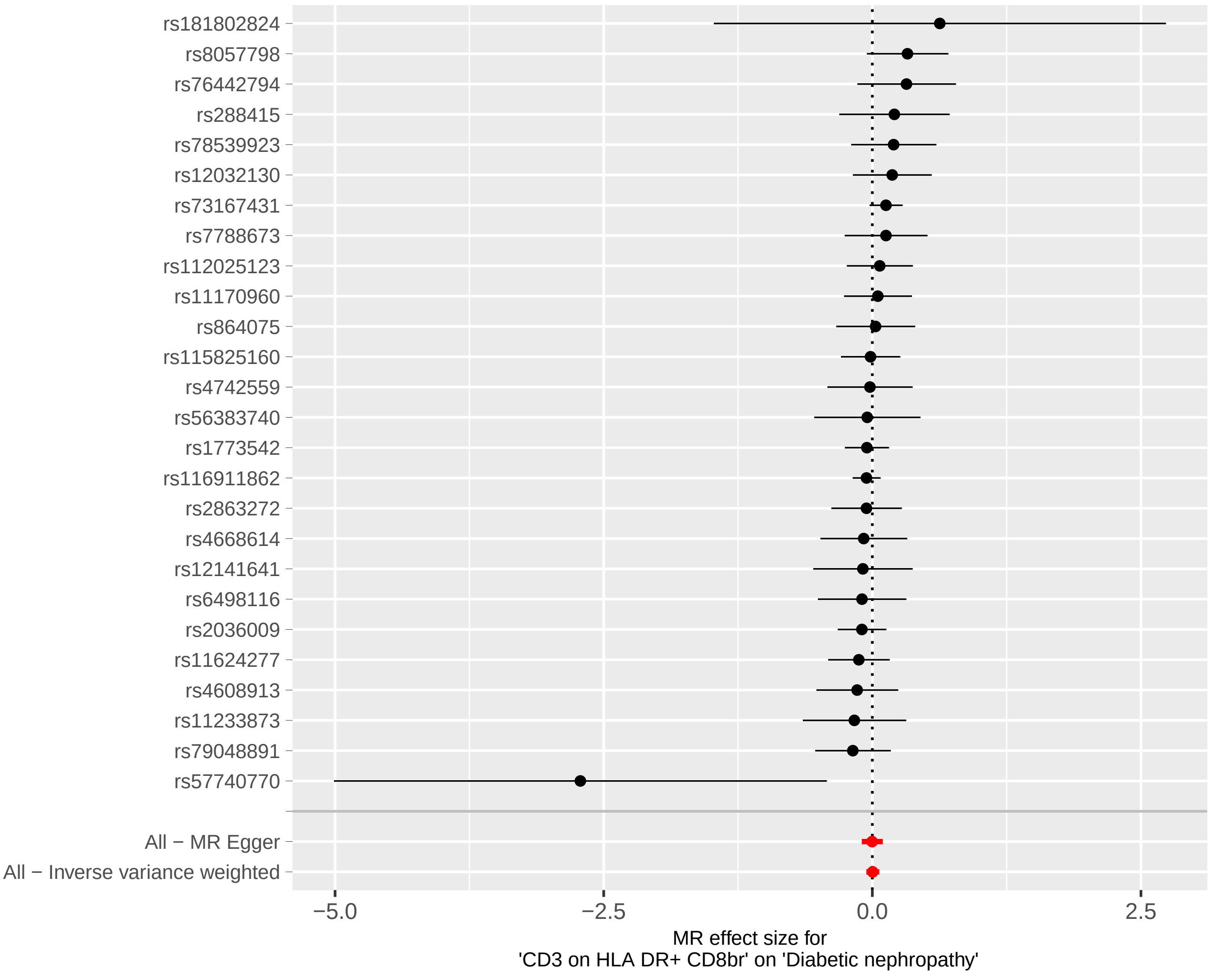

rs181802824
rs8057798
rs76442794
rs288415
rs78539923
rs12032130
rs73167431
rs7788673
rs112025123
rs11170960
rs864075
rs115825160
rs4742559
rs56383740
rs1773542
rs116911862
rs2863272
rs4668614
rs12141641
rs6498116
rs2036009
rs11624277
rs4608913
rs11233873
rs79048891
rs57740770
All − MR Egger
All − Inverse variance weighted
−5.0
−2.5
0.0
2.5
MR effect size for
'CD3 on HLA DR+ CD8br' on 'Diabetic nephropathy'

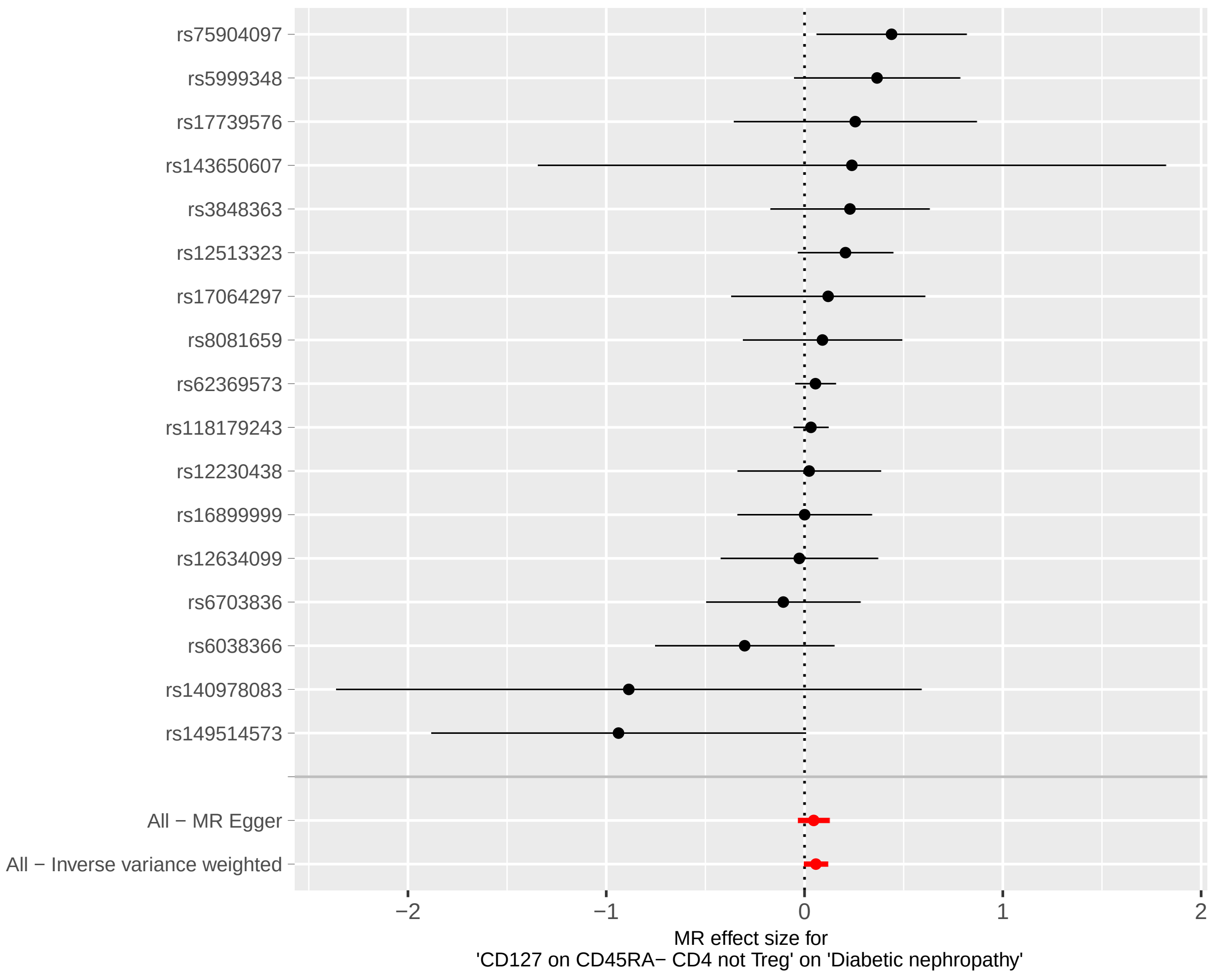
rs75904097
rs5999348
rs17739576
rs143650607
rs3848363
rs12513323
rs17064297
rs8081659
rs62369573
rs118179243
rs12230438
rs16899999
rs12634099
rs6703836
rs6038366
rs140978083
rs149514573
All − MR Egger
All − Inverse variance weighted
−2
−1
0
1
2
MR effect size for
'CD127 on CD45RA− CD4 not Treg' on 'Diabetic nephropathy'

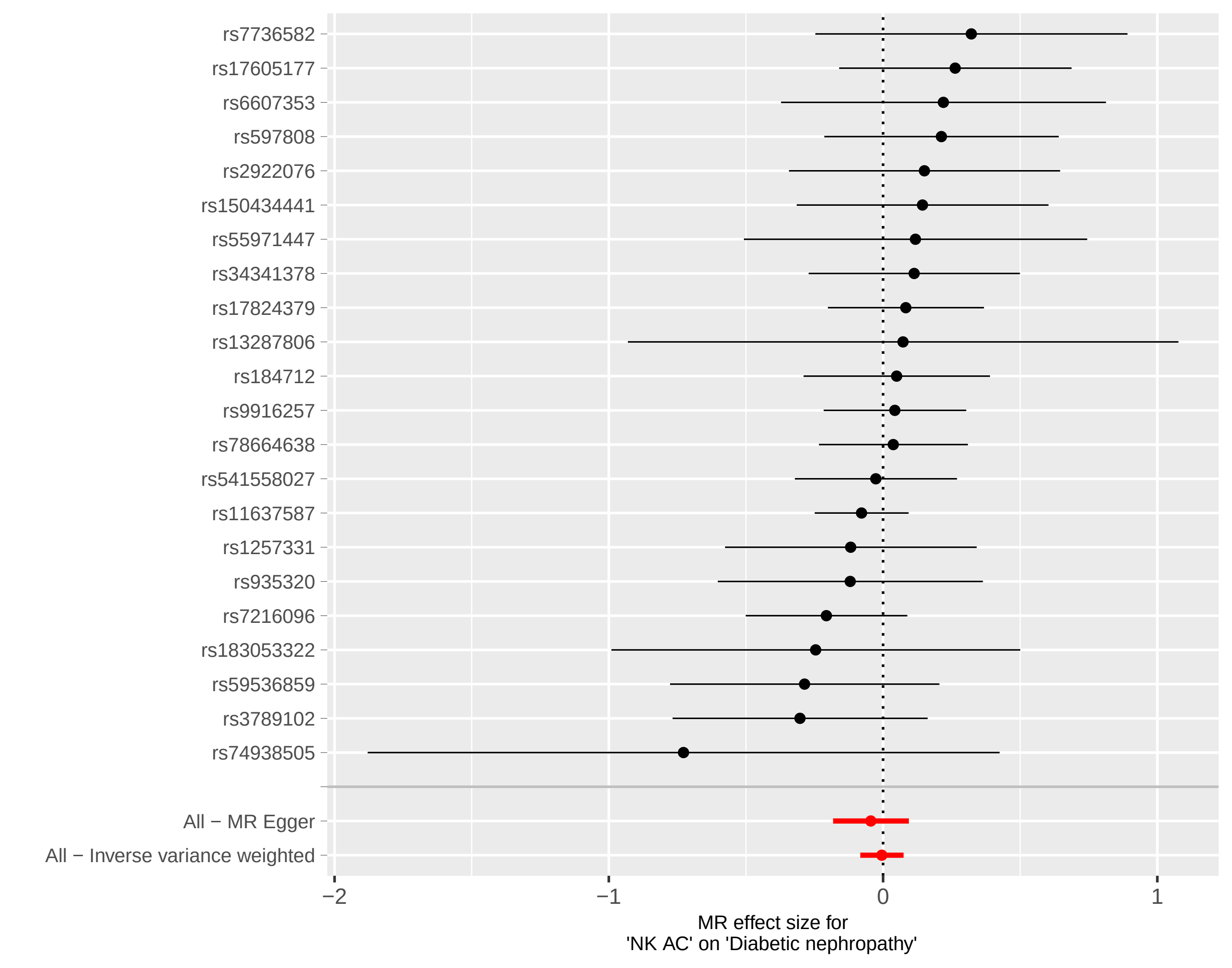
rs7736582
rs17605177
rs6607353
rs597808
rs2922076
rs150434441
rs55971447
rs34341378
rs17824379
rs13287806
rs184712
rs9916257
rs78664638
rs541558027
rs11637587
rs1257331
rs935320
rs7216096
rs183053322
rs59536859
rs3789102
rs74938505
All − MR Egger
All − Inverse variance weighted
−2
−1
0
1
MR effect size for
'NK AC' on 'Diabetic nephropathy'

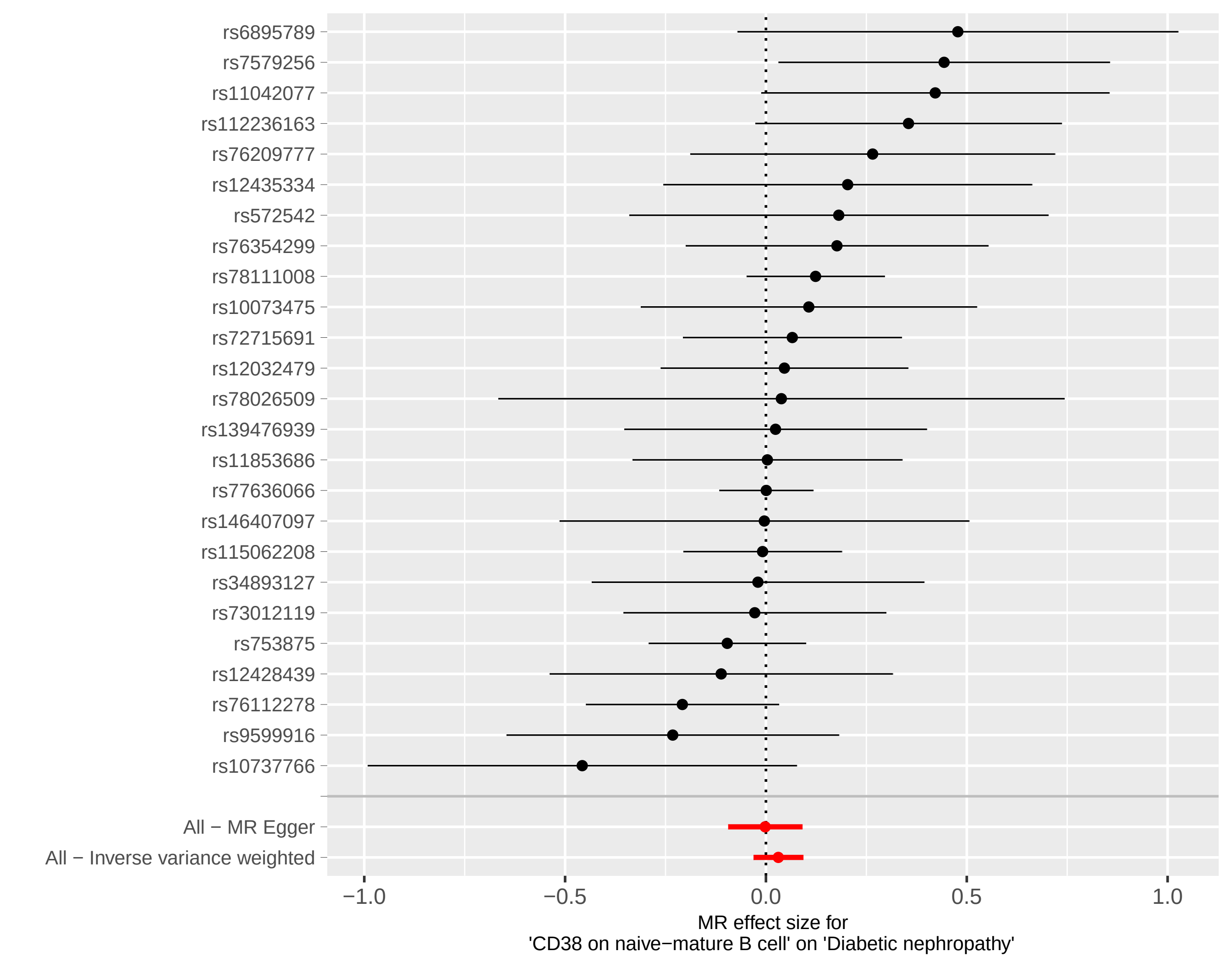

rs6895789
rs7579256
rs11042077
rs112236163
rs76209777
rs12435334
rs572542
rs76354299
rs78111008
rs10073475
rs72715691
rs12032479
rs78026509
rs139476939
rs11853686
rs77636066
rs146407097
rs115062208
rs34893127
rs73012119
rs753875
rs12428439
rs76112278
rs9599916
rs10737766
All − MR Egger
All − Inverse variance weighted
−1.0
−0.5
0.0
0.5
1.0
MR effect size for
'CD38 on naive−mature B cell' on 'Diabetic nephropathy'

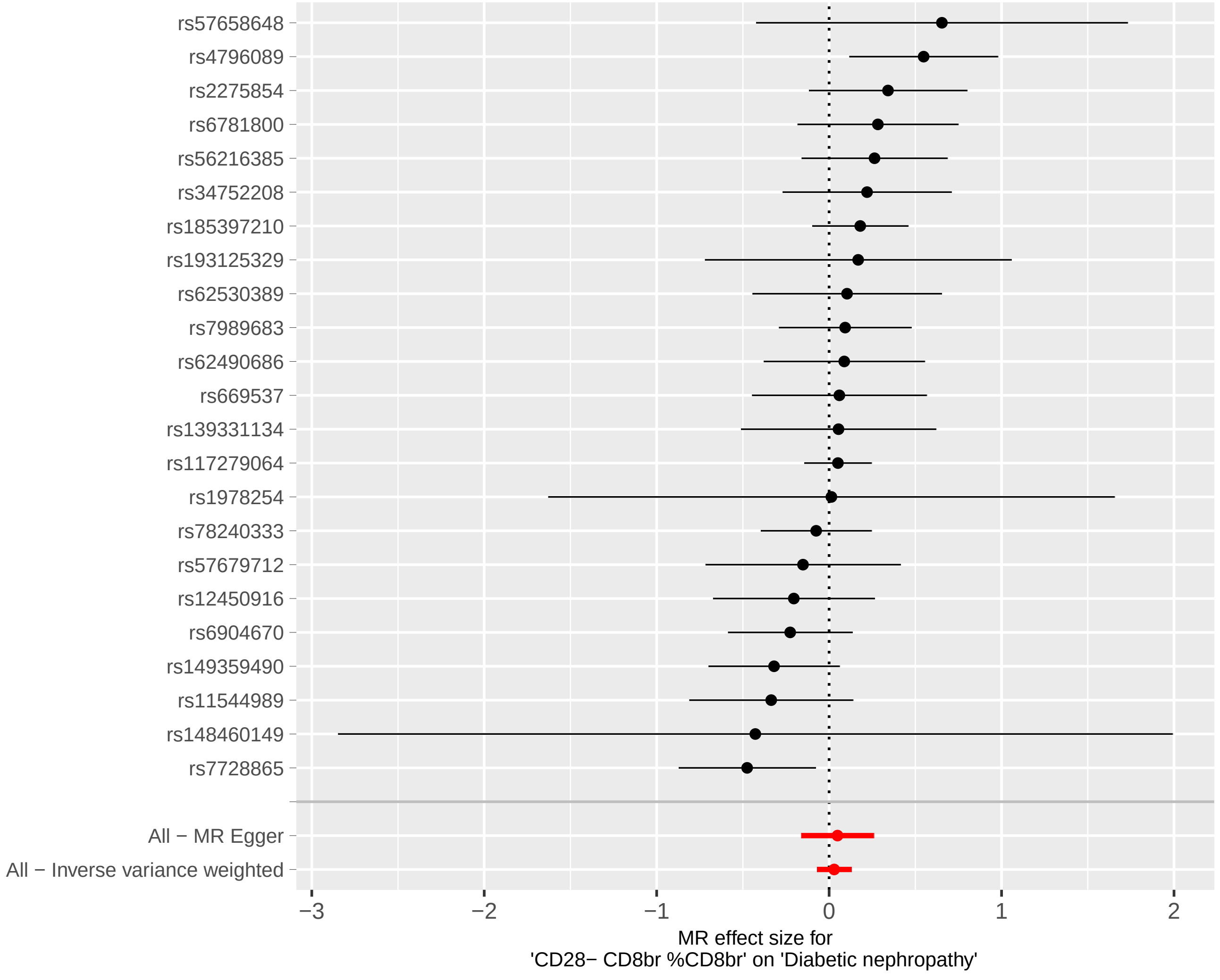
rs57658648
rs4796089
rs2275854
rs6781800
rs56216385
rs34752208
rs185397210
rs193125329
rs62530389
rs7989683
rs62490686
rs669537
rs139331134
rs117279064
rs1978254
rs78240333
rs57679712
rs12450916
rs6904670
rs149359490
rs11544989
rs148460149
rs7728865
All − MR Egger
All − Inverse variance weighted
−3
−2
−1
0
1
2
MR effect size for
'CD28− CD8br %CD8br' on 'Diabetic nephropathy'

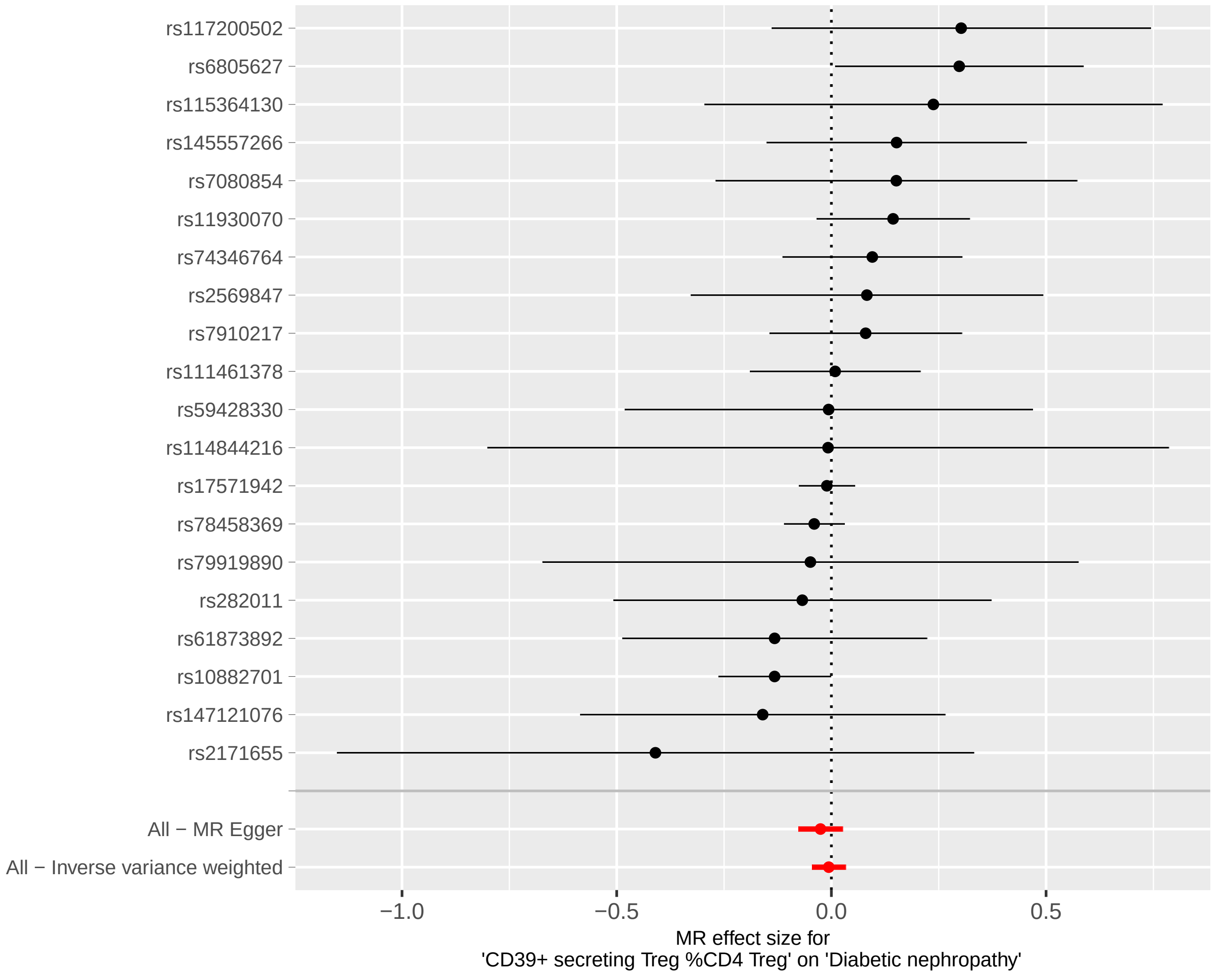
rs117200502
rs6805627
rs115364130
rs145557266
rs7080854
rs11930070
rs74346764
rs2569847
rs7910217
rs111461378
rs59428330
rs114844216
rs17571942
rs78458369
rs79919890
rs282011
rs61873892
rs10882701
rs147121076
rs2171655
All − MR Egger
All − Inverse variance weighted
−1.0
−0.5
0.0
0.5
MR effect size for
'CD39+ secreting Treg %CD4 Treg' on 'Diabetic nephropathy'

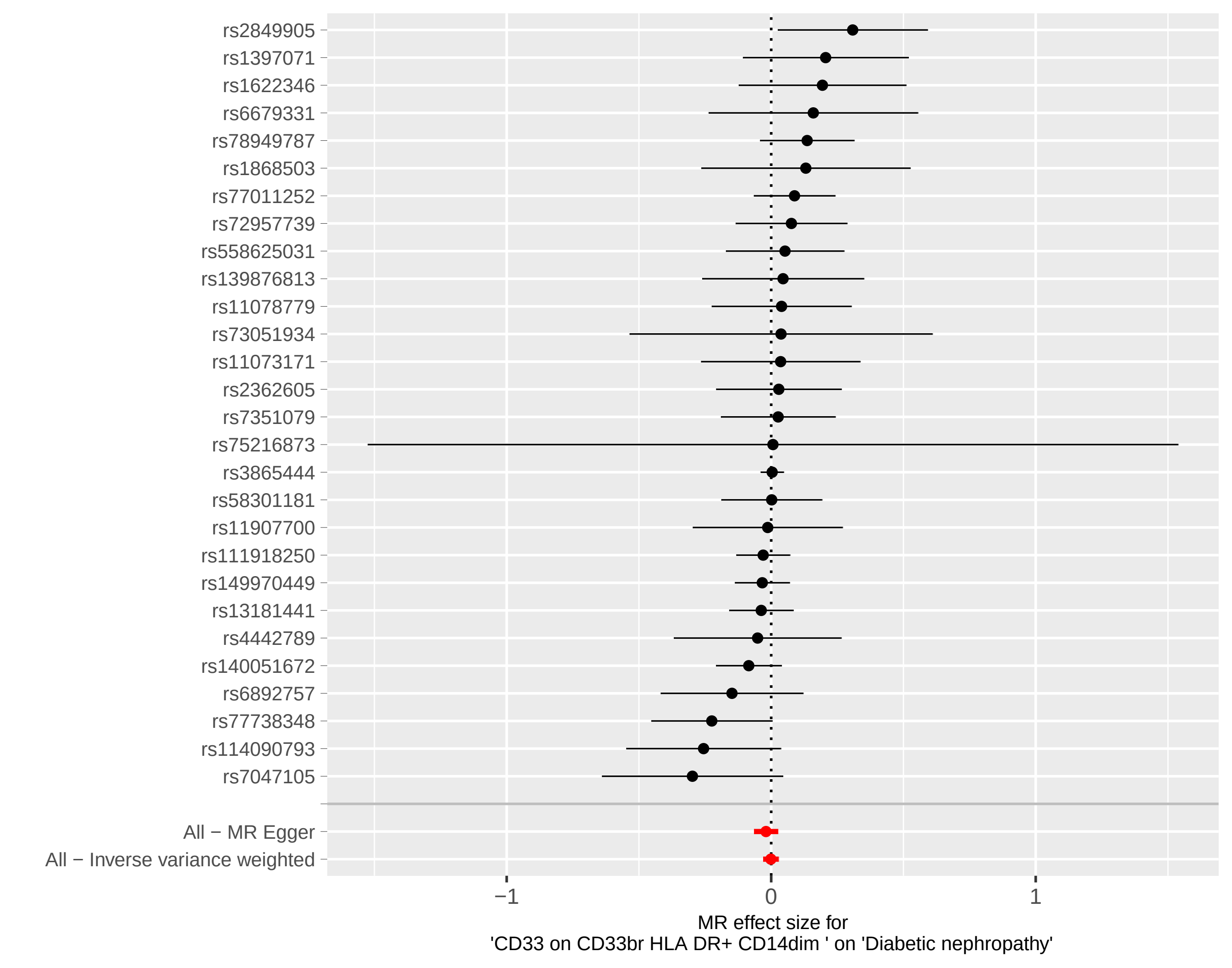

rs2849905
rs1397071
rs1622346
rs6679331
rs78949787
rs1868503
rs77011252
rs72957739
rs558625031
rs139876813
rs11078779
rs73051934
rs11073171
rs2362605
rs7351079
rs75216873
rs3865444
rs58301181
rs11907700
rs111918250
rs149970449
rs13181441
rs4442789
rs140051672
rs6892757
rs77738348
rs114090793
rs7047105
All − MR Egger
All − Inverse variance weighted
−1
0
1
MR effect size for
'CD33 on CD33br HLA DR+ CD14dim ' on 'Diabetic nephropathy'

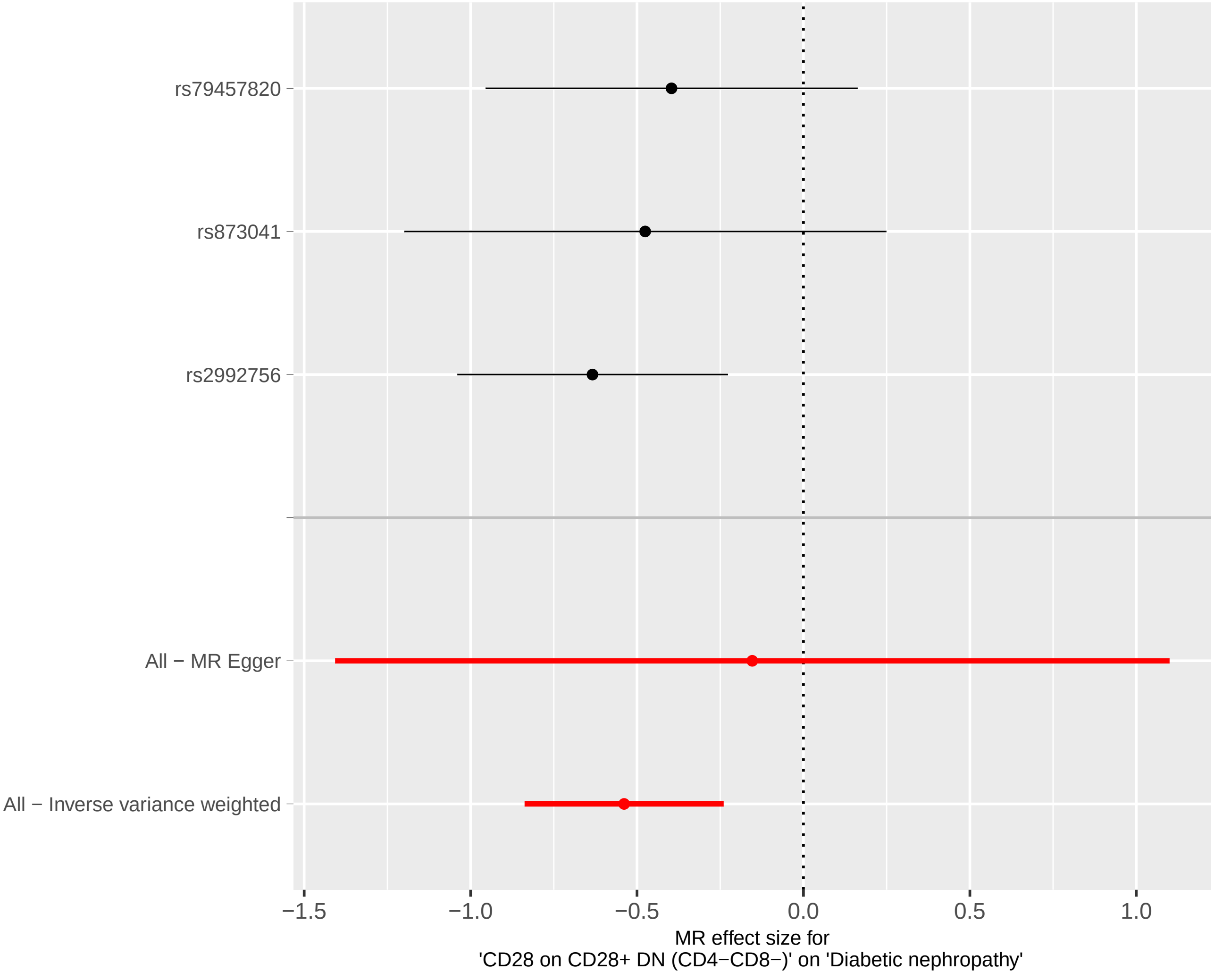

rs79457820
rs873041
rs2992756
All − MR Egger
All − Inverse variance weighted
−1.5
−1.0
−0.5
0.0
0.5
1.0
MR effect size for
'CD28 on CD28+ DN (CD4−CD8−)' on 'Diabetic nephropathy'

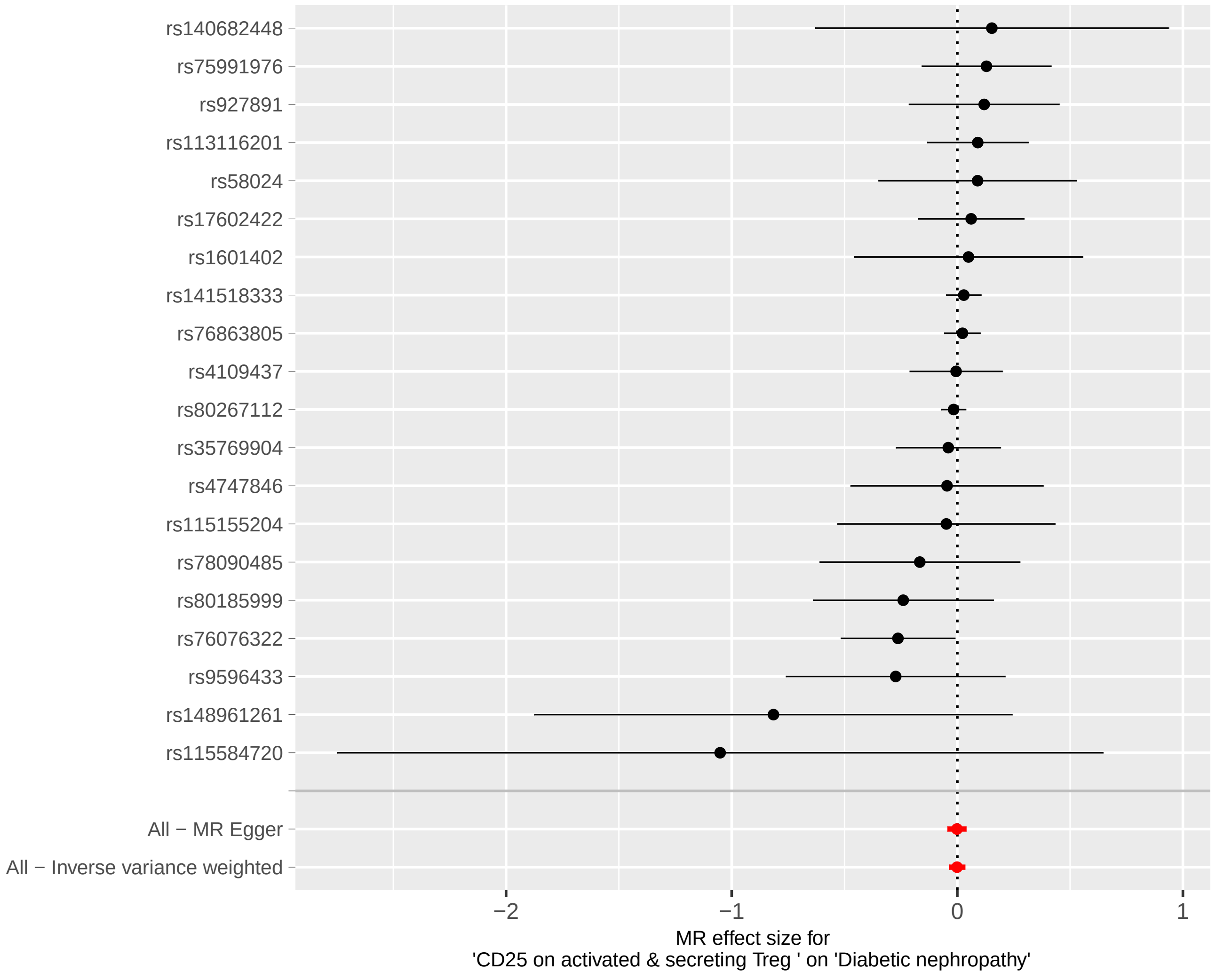
rs140682448
rs75991976
rs927891
rs113116201
rs58024
rs17602422
rs1601402
rs141518333
rs76863805
rs4109437
rs80267112
rs35769904
rs4747846
rs115155204
rs78090485
rs80185999
rs76076322
rs9596433
rs148961261
rs115584720
All − MR Egger
All − Inverse variance weighted
−2
−1
0
1
MR effect size for
'CD25 on activated & secreting Treg ' on 'Diabetic nephropathy'

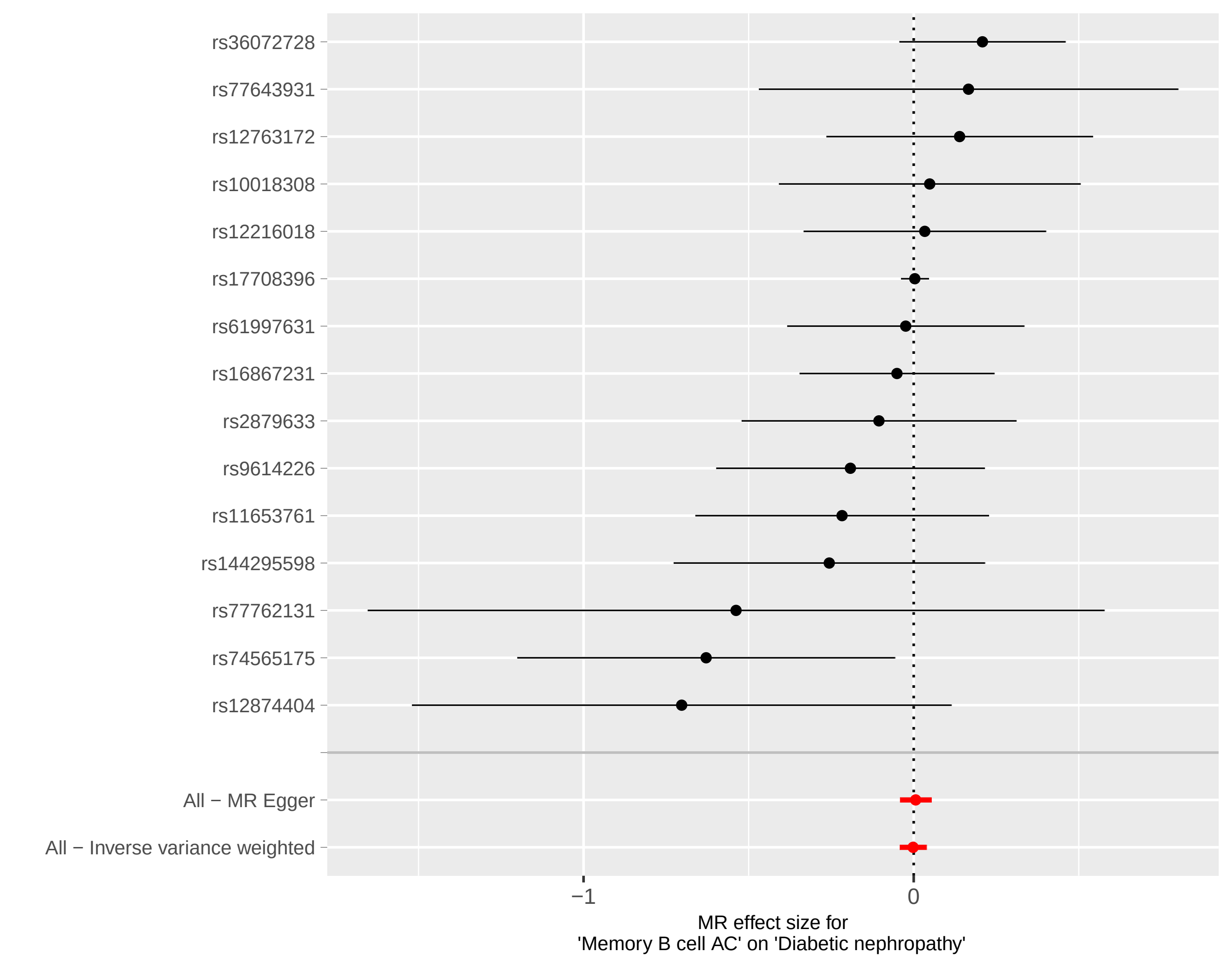
rs36072728
rs77643931
rs12763172
rs10018308
rs12216018
rs17708396
rs61997631
rs16867231
rs2879633
rs9614226
rs11653761
rs144295598
rs77762131
rs74565175
rs12874404
All − MR Egger
All − Inverse variance weighted
−1
0
MR effect size for
'Memory B cell AC' on 'Diabetic nephropathy'

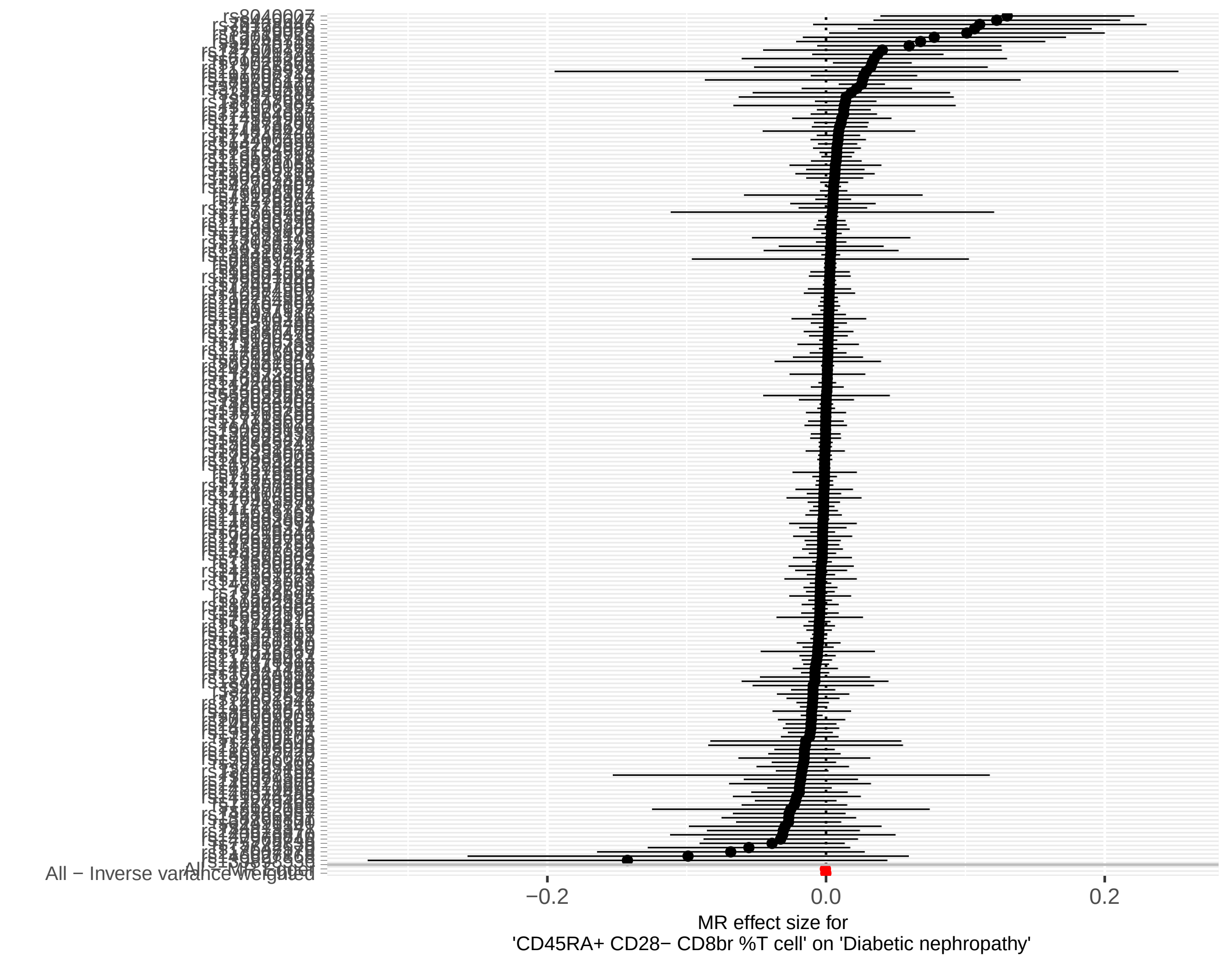
rs8040007
All − MR Egger
All − Inverse variance weighted
−0.2
0.0
0.2
MR effect size for
'CD45RA+ CD28− CD8br %T cell' on 'Diabetic nephropathy'

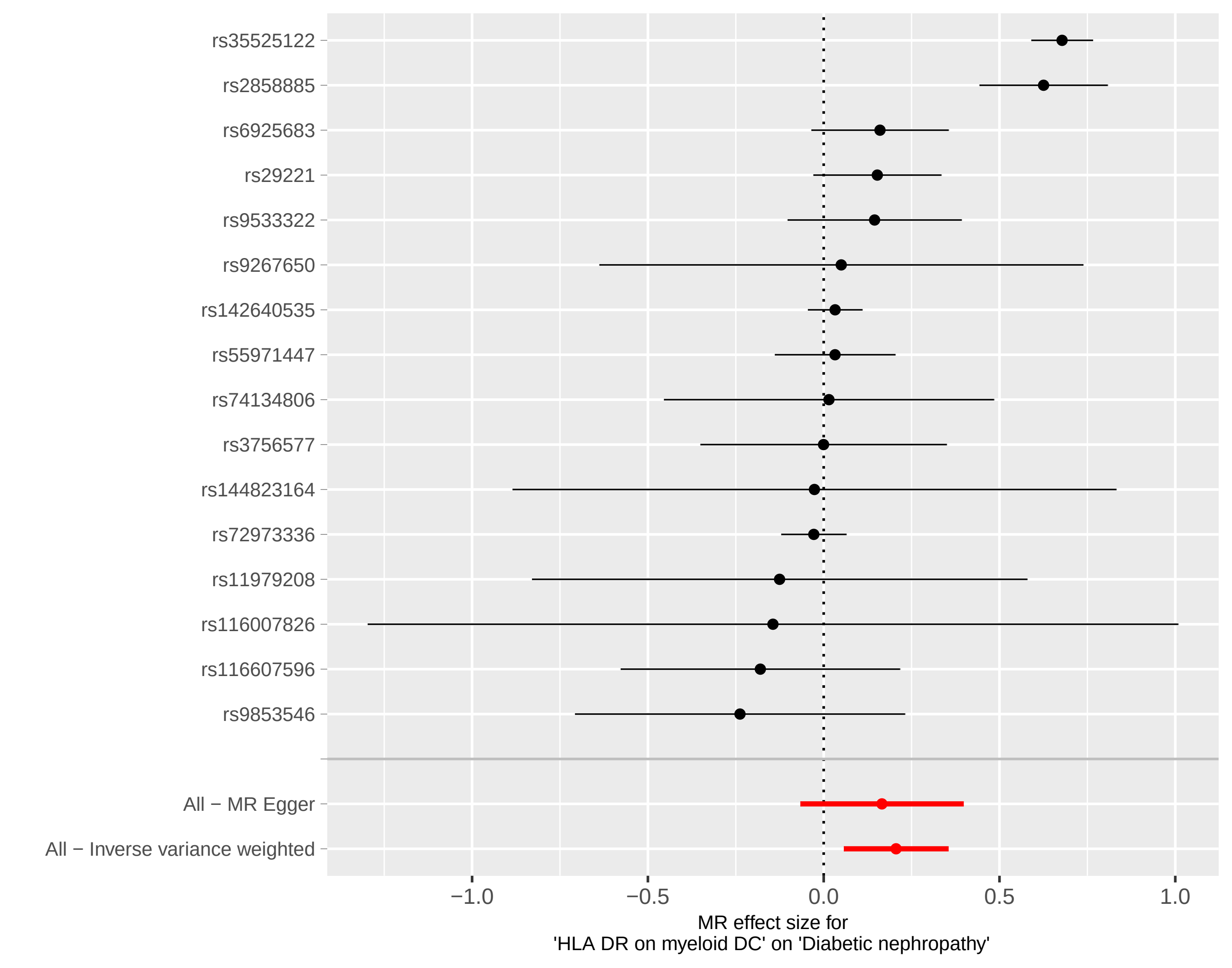
rs35525122
rs2858885
rs6925683
rs29221
rs9533322
rs9267650
rs142640535
rs55971447
rs74134806
rs3756577
rs144823164
rs72973336
rs11979208
rs116007826
rs116607596
rs9853546
All − MR Egger
All − Inverse variance weighted
−1.0
−0.5
0.0
0.5
1.0
MR effect size for
'HLA DR on myeloid DC' on 'Diabetic nephropathy'

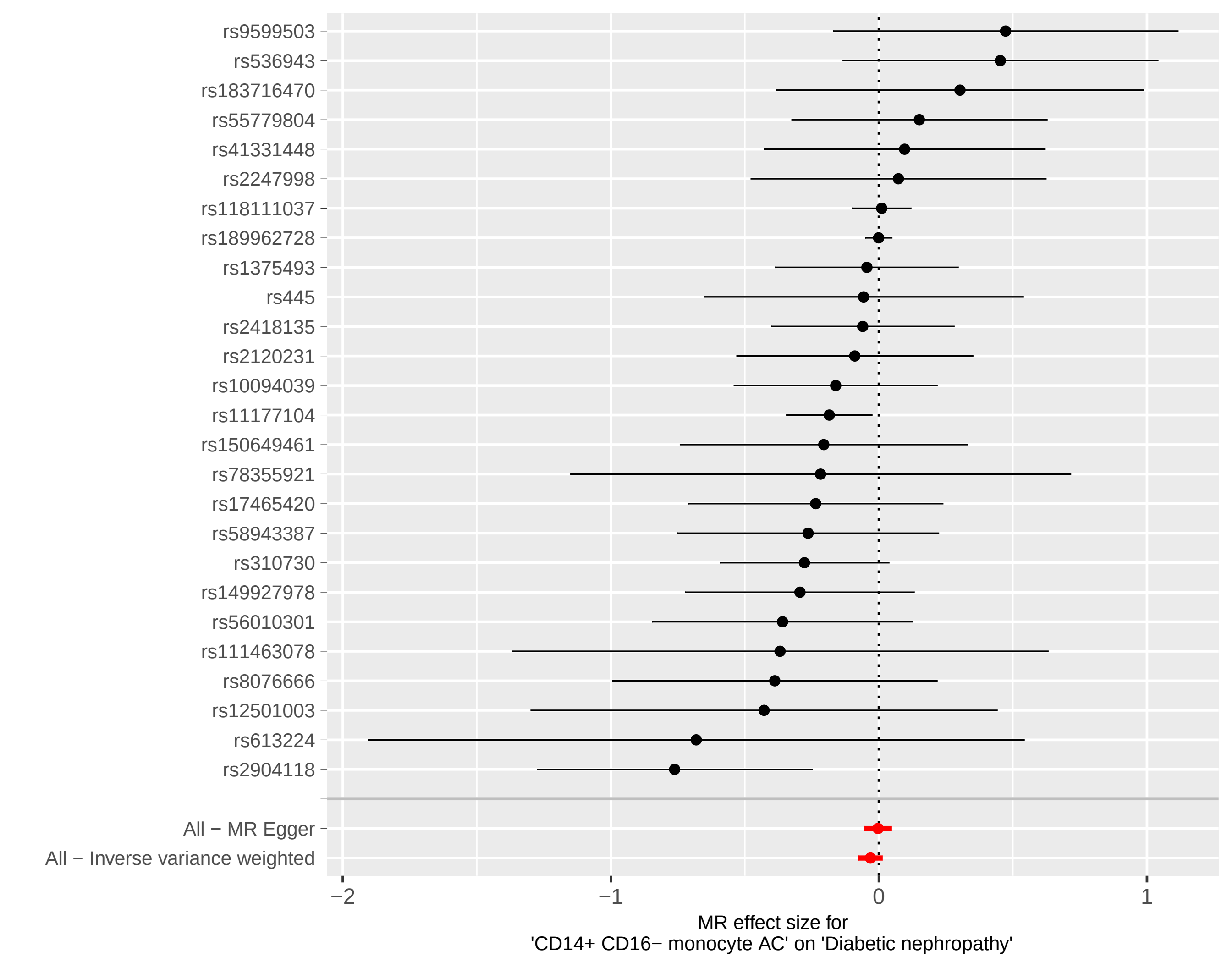

rs9599503
rs536943
rs183716470
rs55779804
rs41331448
rs2247998
rs118111037
rs189962728
rs1375493
rs445
rs2418135
rs2120231
rs10094039
rs11177104
rs150649461
rs78355921
rs17465420
rs58943387
rs310730
rs149927978
rs56010301
rs111463078
rs8076666
rs12501003
rs613224
rs2904118
All − MR Egger
All − Inverse variance weighted
−2
−1
0
1
MR effect size for
'CD14+ CD16− monocyte AC' on 'Diabetic nephropathy'

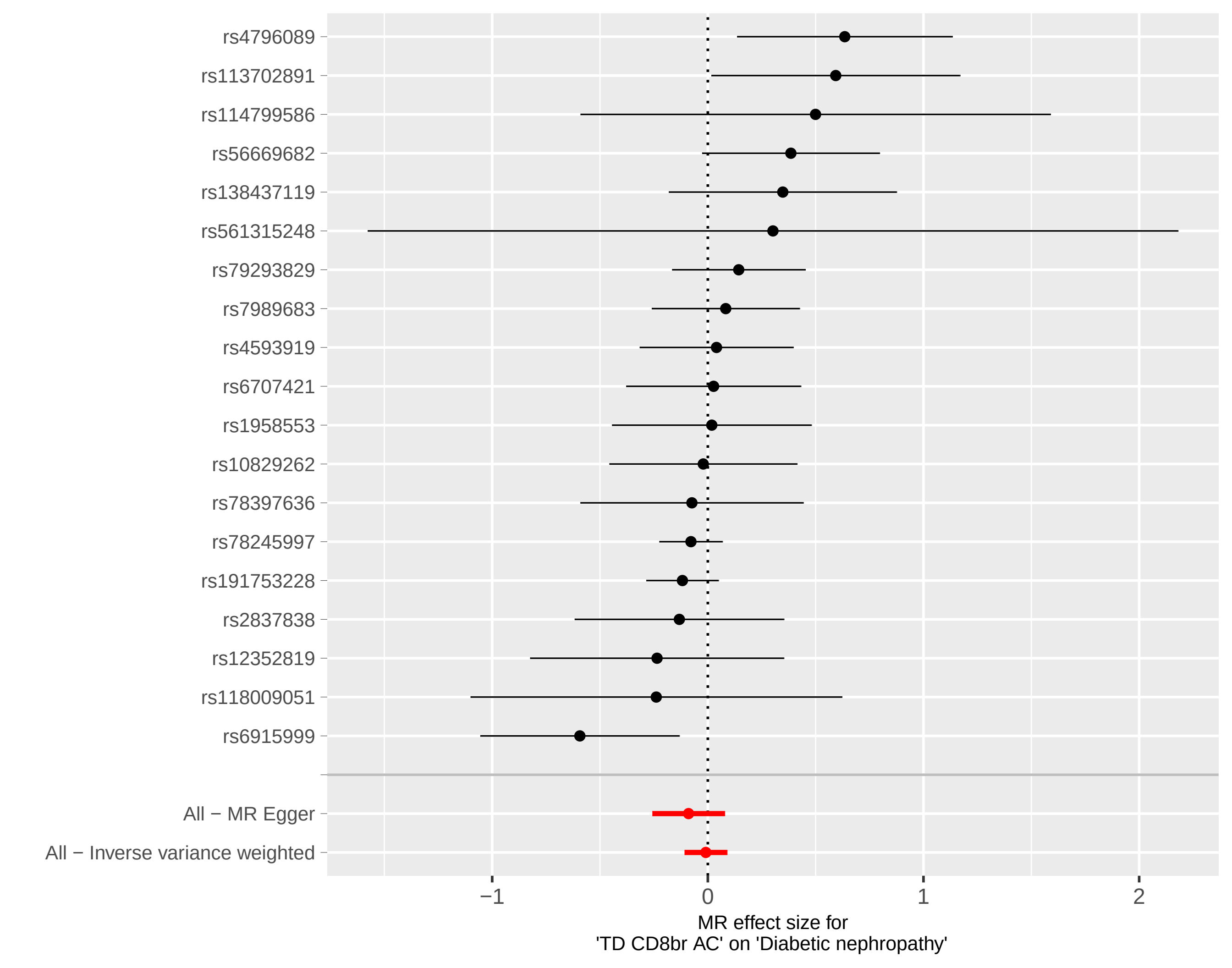
rs4796089
rs113702891
rs114799586
rs56669682
rs138437119
rs561315248
rs79293829
rs7989683
rs4593919
rs6707421
rs1958553
rs10829262
rs78397636
rs78245997
rs191753228
rs2837838
rs12352819
rs118009051
rs6915999
All − MR Egger
All − Inverse variance weighted
−1
0
1
2
MR effect size for
'TD CD8br AC' on 'Diabetic nephropathy'

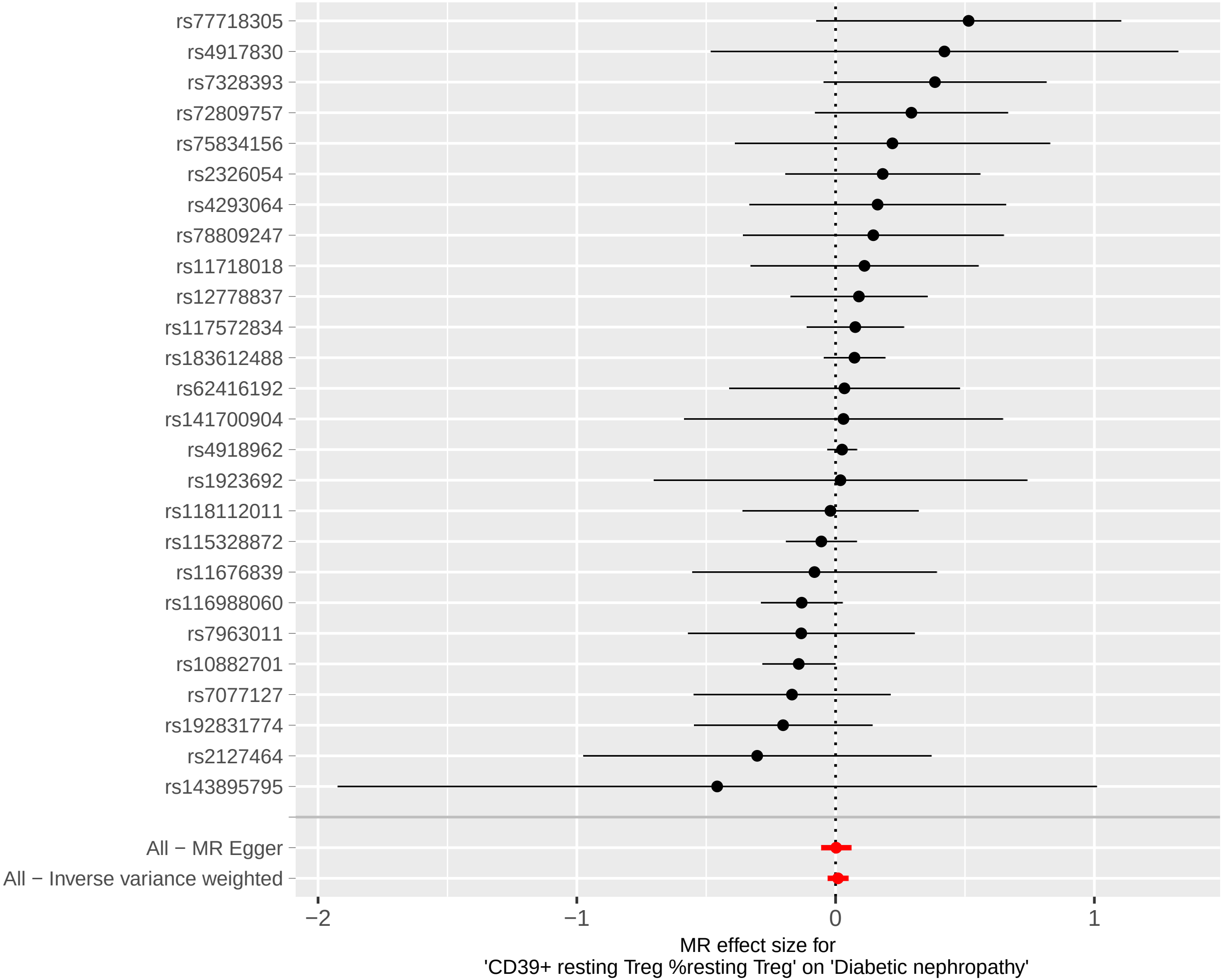

rs77718305
rs4917830
rs7328393
rs72809757
rs75834156
rs2326054
rs4293064
rs78809247
rs11718018
rs12778837
rs117572834
rs183612488
rs62416192
rs141700904
rs4918962
rs1923692
rs118112011
rs115328872
rs11676839
rs116988060
rs7963011
rs10882701
rs7077127
rs192831774
rs2127464
rs143895795
All − MR Egger
All − Inverse variance weighted
−2
−1
0
1
MR effect size for
'CD39+ resting Treg %resting Treg' on 'Diabetic nephropathy'

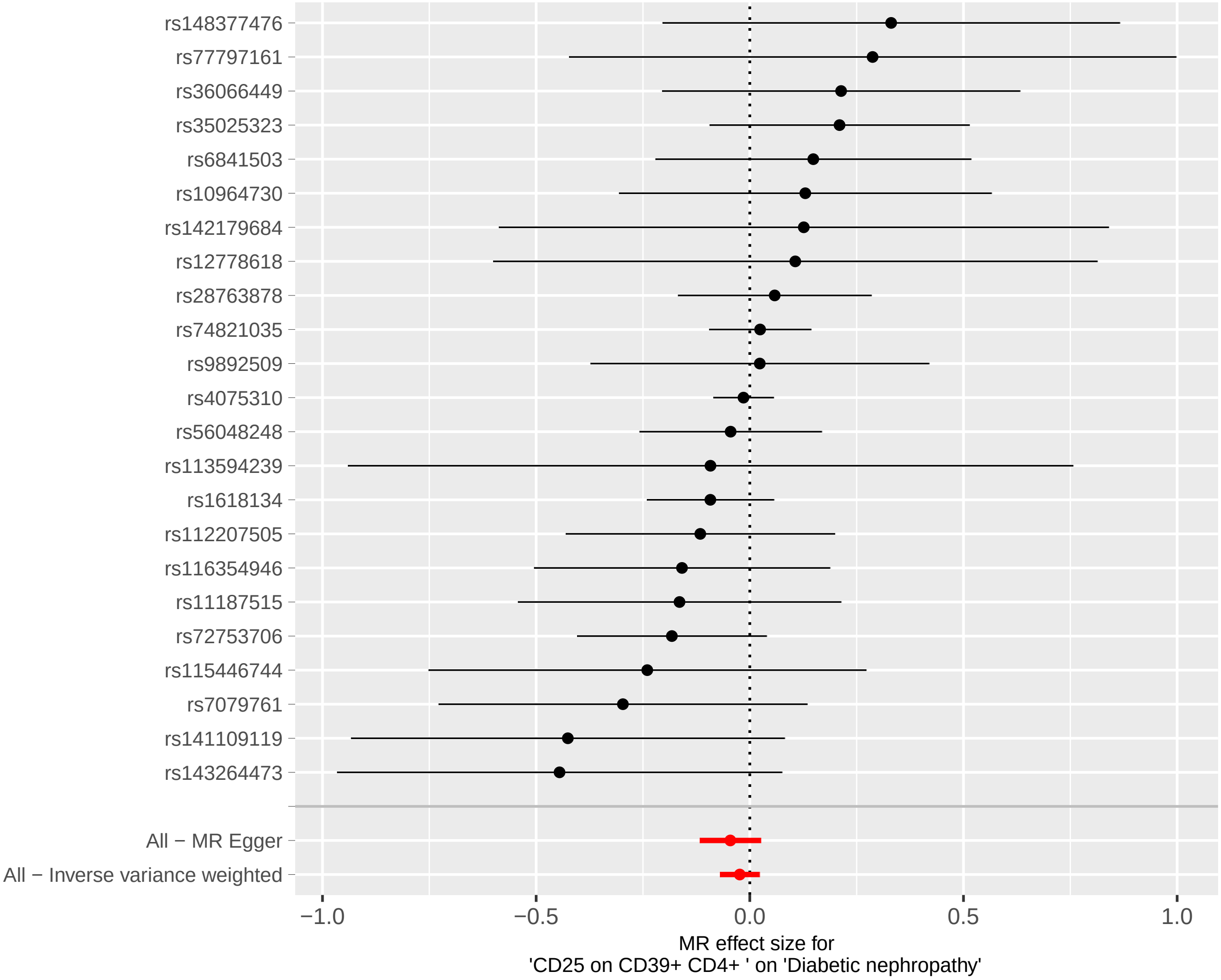

rs148377476
rs77797161
rs36066449
rs35025323
rs6841503
rs10964730
rs142179684
rs12778618
rs28763878
rs74821035
rs9892509
rs4075310
rs56048248
rs113594239
rs1618134
rs112207505
rs116354946
rs11187515
rs72753706
rs115446744
rs7079761
rs141109119
rs143264473
All − MR Egger
All − Inverse variance weighted
−1.0
−0.5
0.0
0.5
1.0
MR effect size for
'CD25 on CD39+ CD4+ ' on 'Diabetic nephropathy'

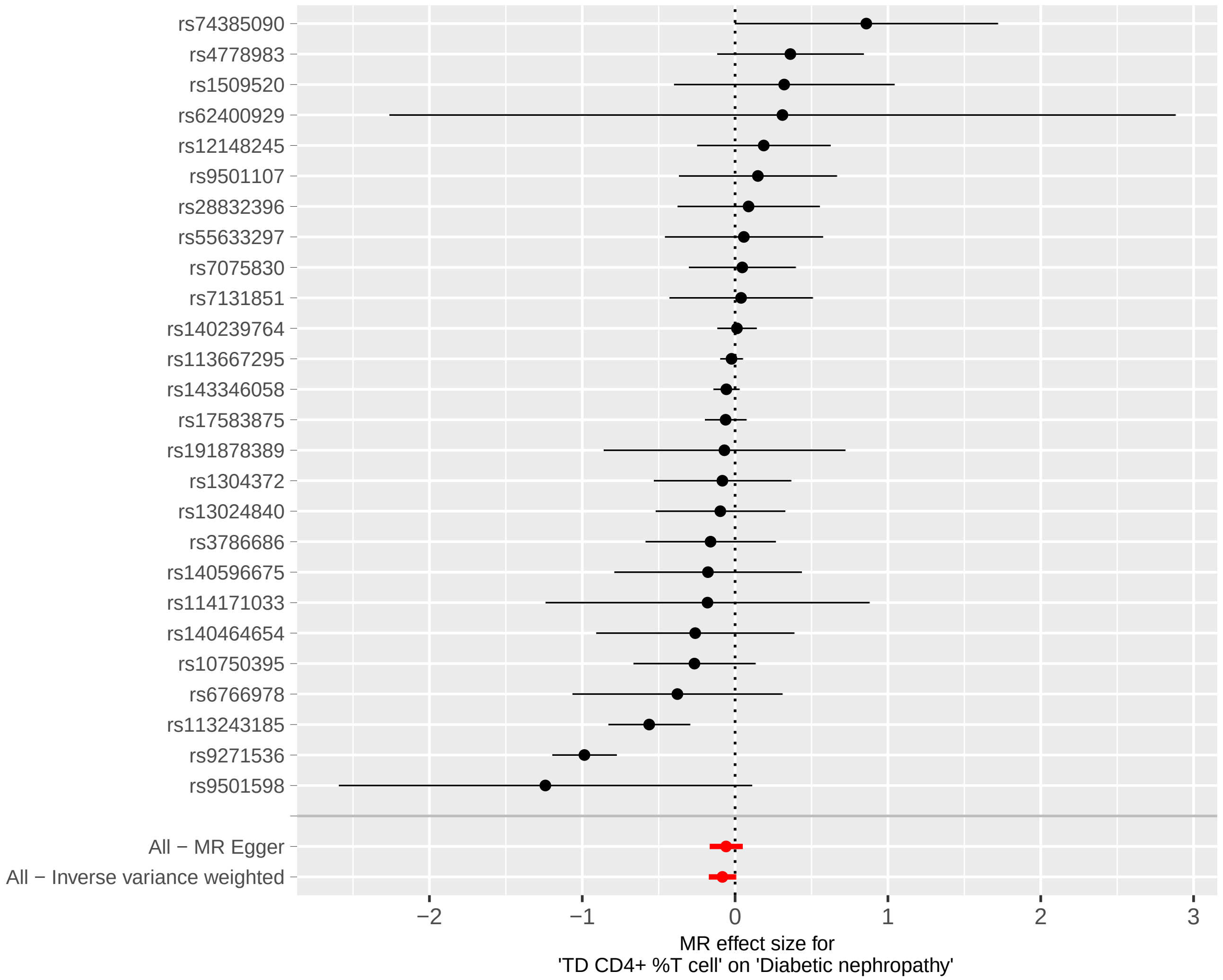
rs74385090
rs4778983
rs1509520
rs62400929
rs12148245
rs9501107
rs28832396
rs55633297
rs7075830
rs7131851
rs140239764
rs113667295
rs143346058
rs17583875
rs191878389
rs1304372
rs13024840
rs3786686
rs140596675
rs114171033
rs140464654
rs10750395
rs6766978
rs113243185
rs9271536
rs9501598
All − MR Egger
All − Inverse variance weighted
−2
−1
0
1
2
3
MR effect size for
'TD CD4+ %T cell' on 'Diabetic nephropathy'

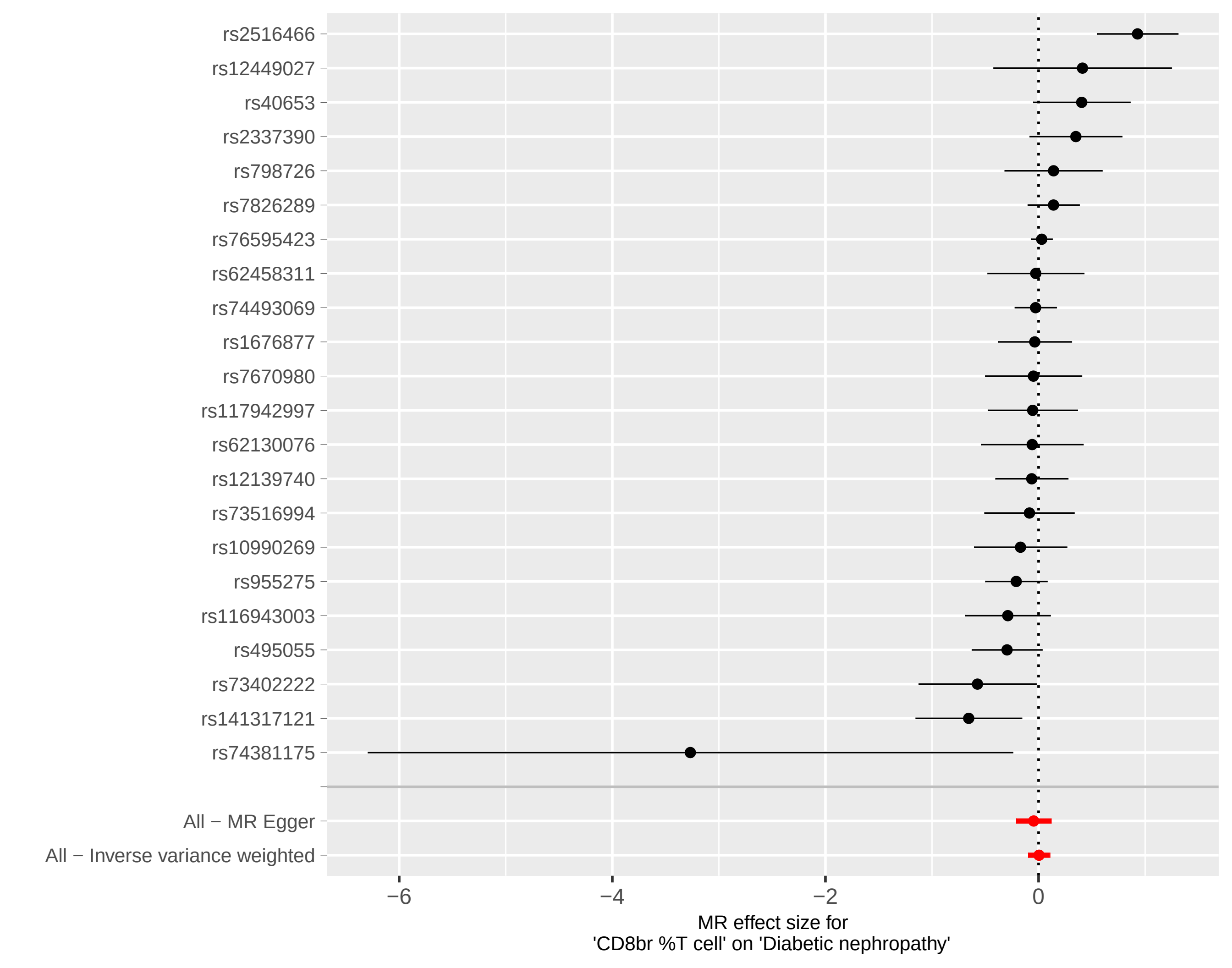
rs2516466
rs12449027
rs40653
rs2337390
rs798726
rs7826289
rs76595423
rs62458311
rs74493069
rs1676877
rs7670980
rs117942997
rs62130076
rs12139740
rs73516994
rs10990269
rs955275
rs116943003
rs495055
rs73402222
rs141317121
rs74381175
All − MR Egger
All − Inverse variance weighted
−6
−4
−2
0
MR effect size for
'CD8br %T cell' on 'Diabetic nephropathy'

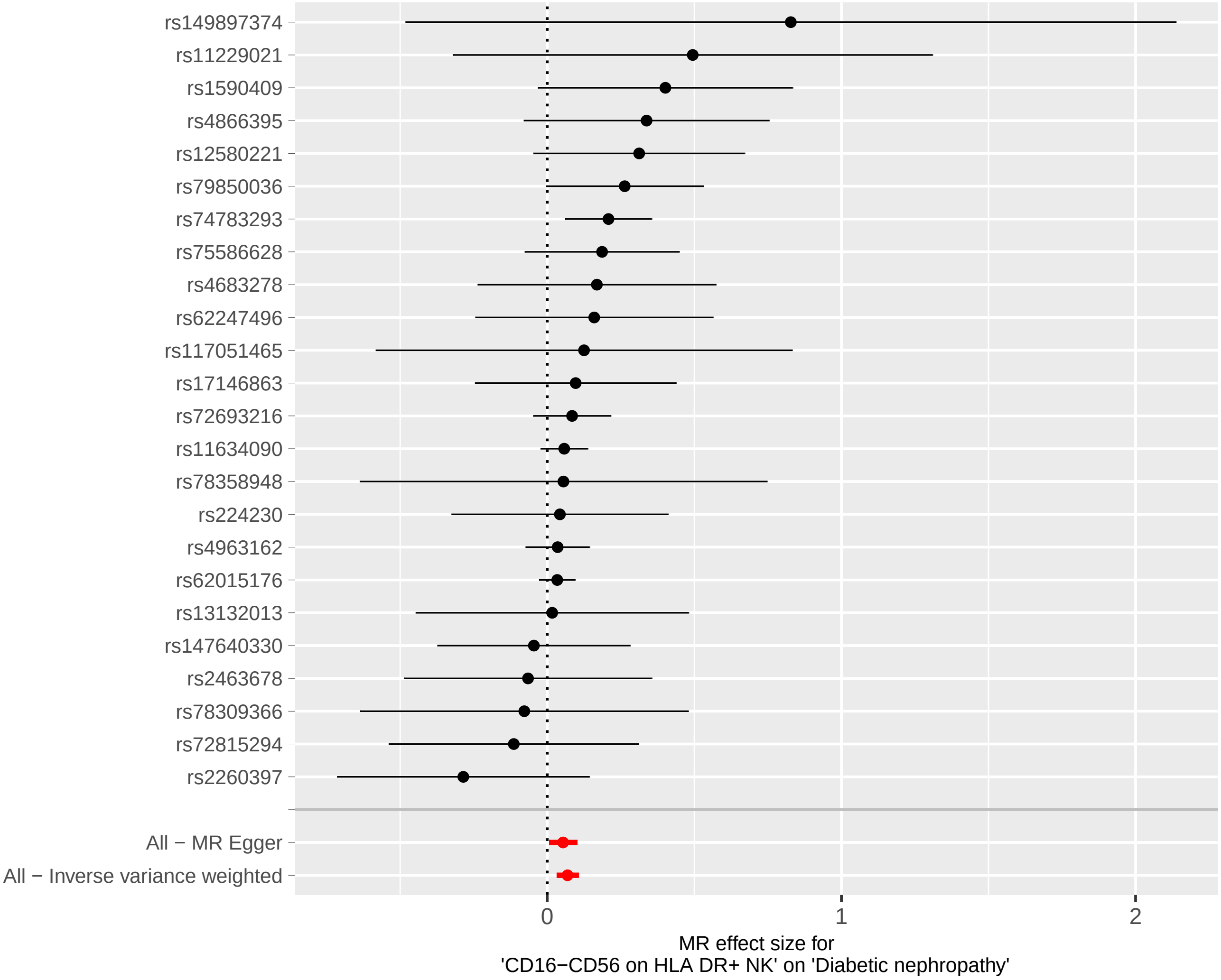

rs149897374
rs11229021
rs1590409
rs4866395
rs12580221
rs79850036
rs74783293
rs75586628
rs4683278
rs62247496
rs117051465
rs17146863
rs72693216
rs11634090
rs78358948
rs224230
rs4963162
rs62015176
rs13132013
rs147640330
rs2463678
rs78309366
rs72815294
rs2260397
All − MR Egger
All − Inverse variance weighted
0
1
2
MR effect size for
'CD16−CD56 on HLA DR+ NK' on 'Diabetic nephropathy'

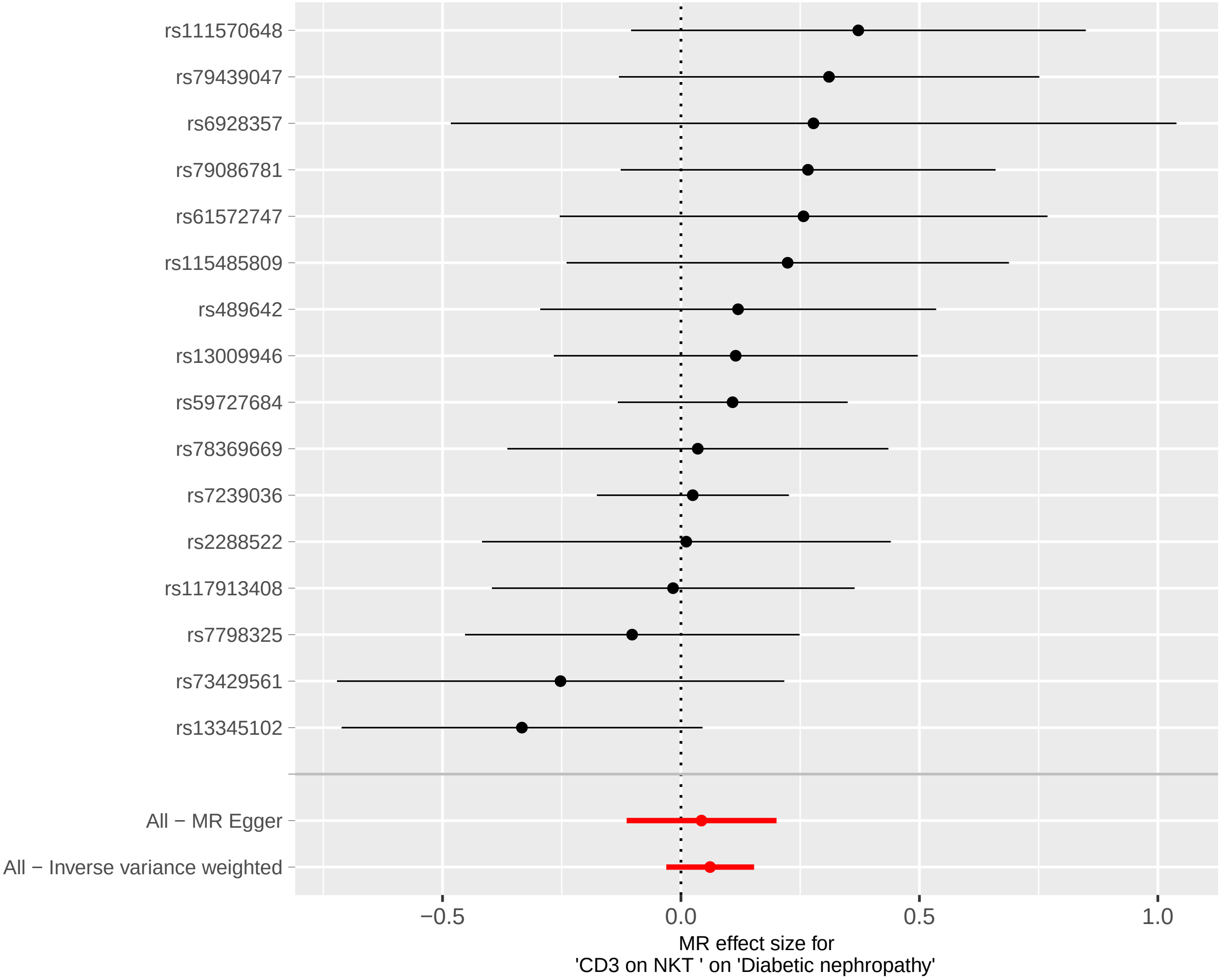

rs111570648
rs79439047
rs6928357
rs79086781
rs61572747
rs115485809
rs489642
rs13009946
rs59727684
rs78369669
rs7239036
rs2288522
rs117913408
rs7798325
rs73429561
rs13345102
All − MR Egger
All − Inverse variance weighted
−0.5
0.0
0.5
1.0
MR effect size for
'CD3 on NKT ' on 'Diabetic nephropathy'

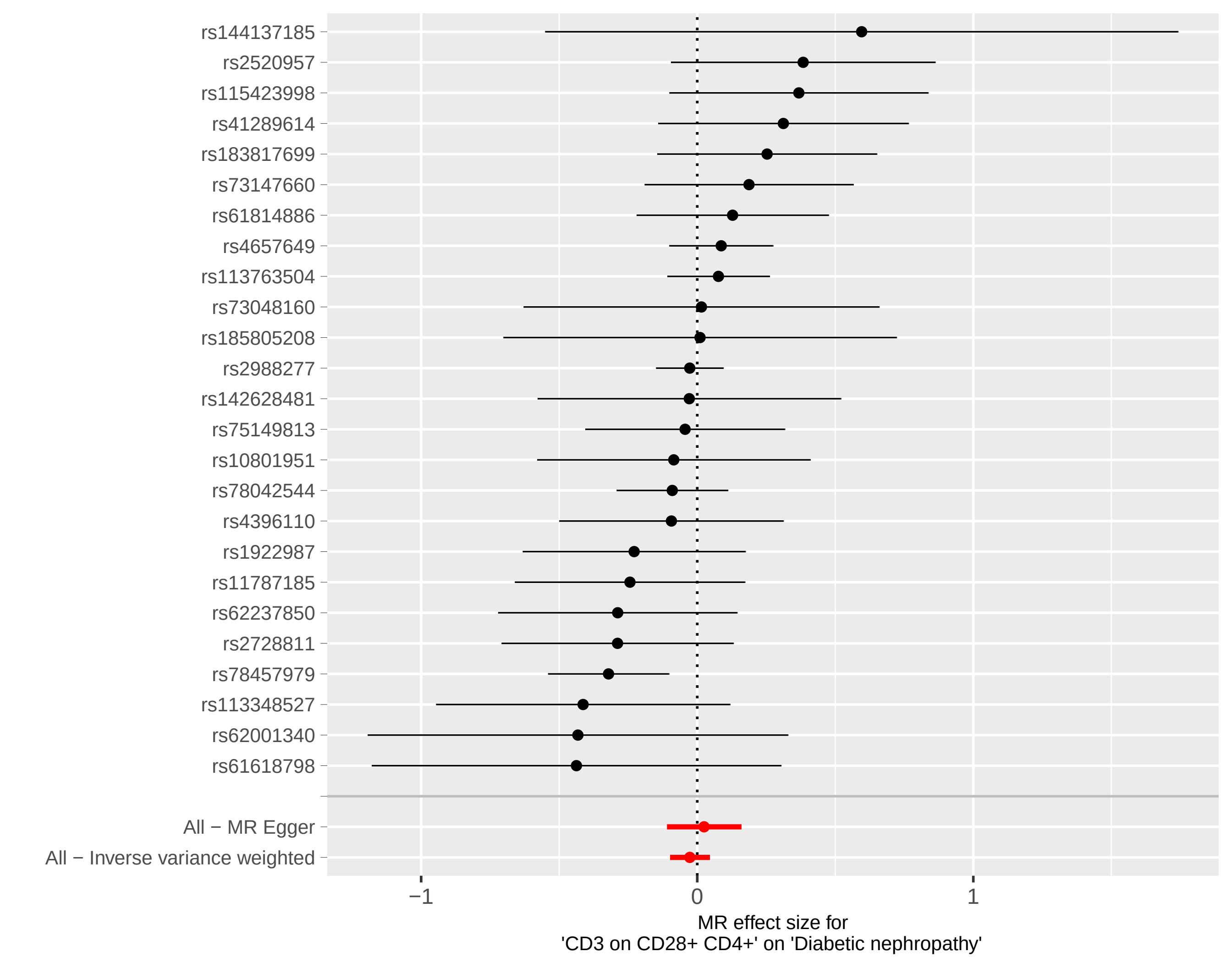
rs144137185
rs2520957
rs115423998
rs41289614
rs183817699
rs73147660
rs61814886
rs4657649
rs113763504
rs73048160
rs185805208
rs2988277
rs142628481
rs75149813
rs10801951
rs78042544
rs4396110
rs1922987
rs11787185
rs62237850
rs2728811
rs78457979
rs113348527
rs62001340
rs61618798
All − MR Egger
All − Inverse variance weighted
−1
0
1
MR effect size for
'CD3 on CD28+ CD4+' on 'Diabetic nephropathy'

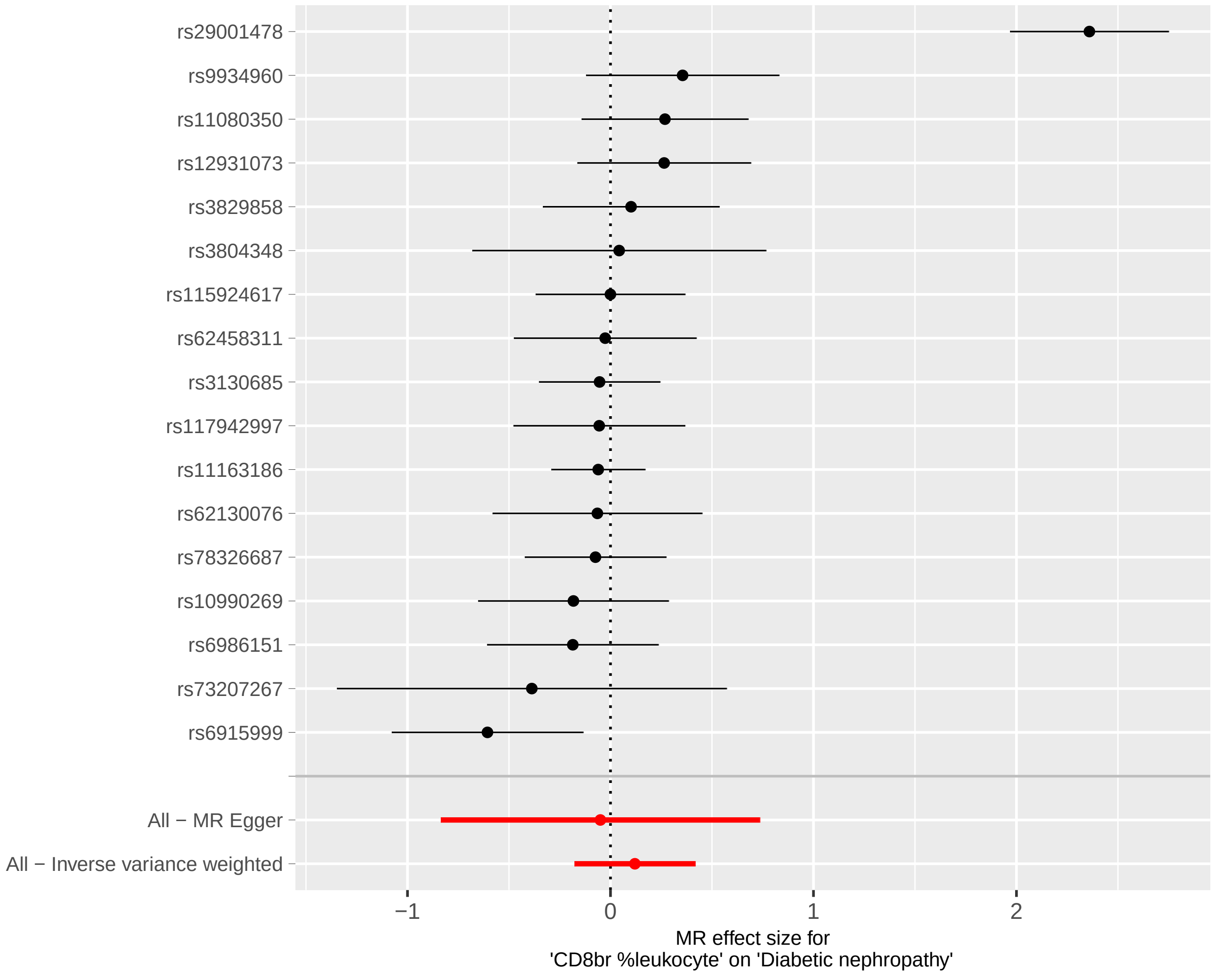

rs29001478
rs9934960
rs11080350
rs12931073
rs3829858
rs3804348
rs115924617
rs62458311
rs3130685
rs117942997
rs11163186
rs62130076
rs78326687
rs10990269
rs6986151
rs73207267
rs6915999
All − MR Egger
All − Inverse variance weighted
−1
0
1
2
MR effect size for
'CD8br %leukocyte' on 'Diabetic nephropathy'

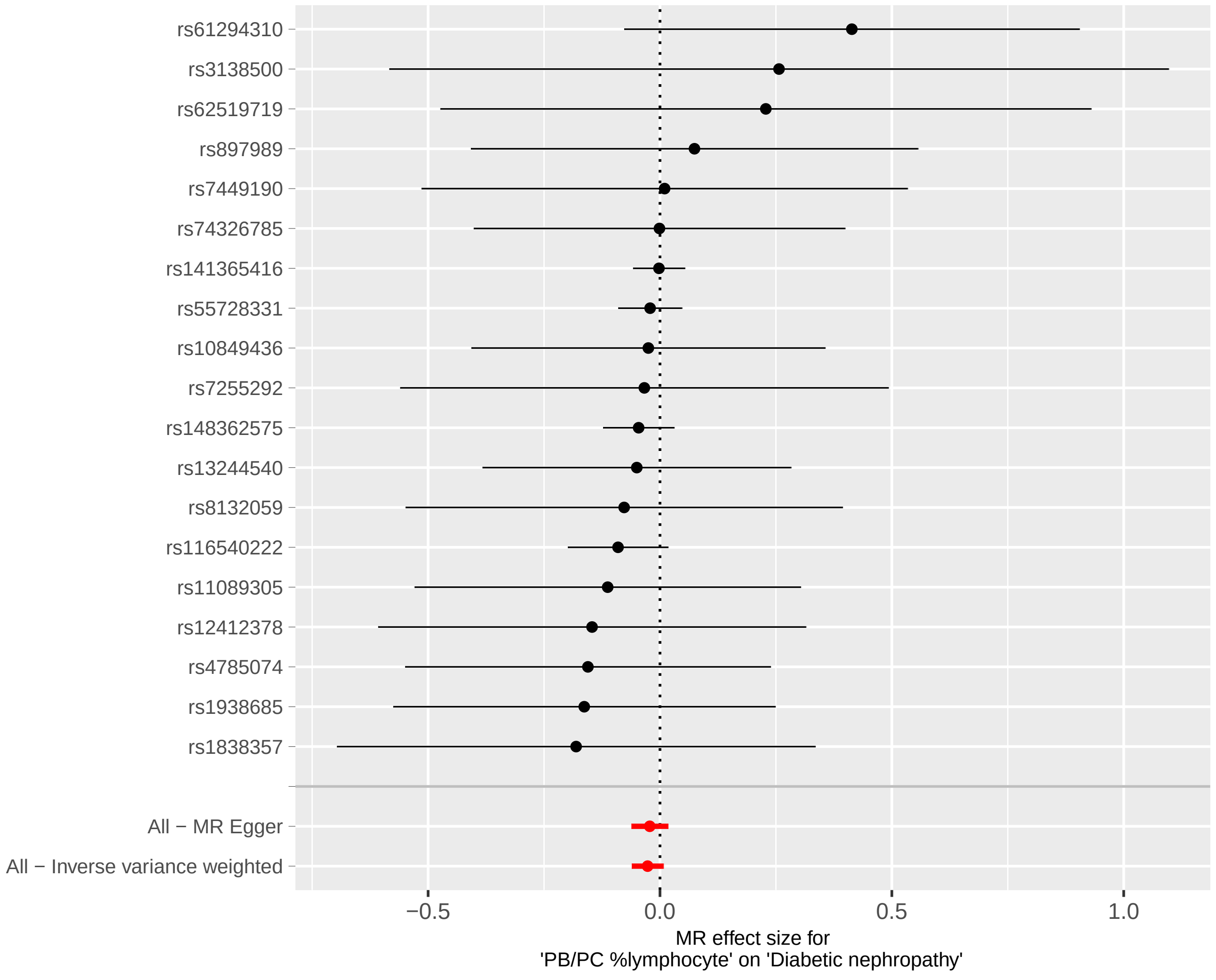
rs61294310
rs3138500
rs62519719
rs897989
rs7449190
rs74326785
rs141365416
rs55728331
rs10849436
rs7255292
rs148362575
rs13244540
rs8132059
rs116540222
rs11089305
rs12412378
rs4785074
rs1938685
rs1838357
All − MR Egger
All − Inverse variance weighted
−0.5
0.0
0.5
1.0
MR effect size for
'PB/PC %lymphocyte' on 'Diabetic nephropathy'

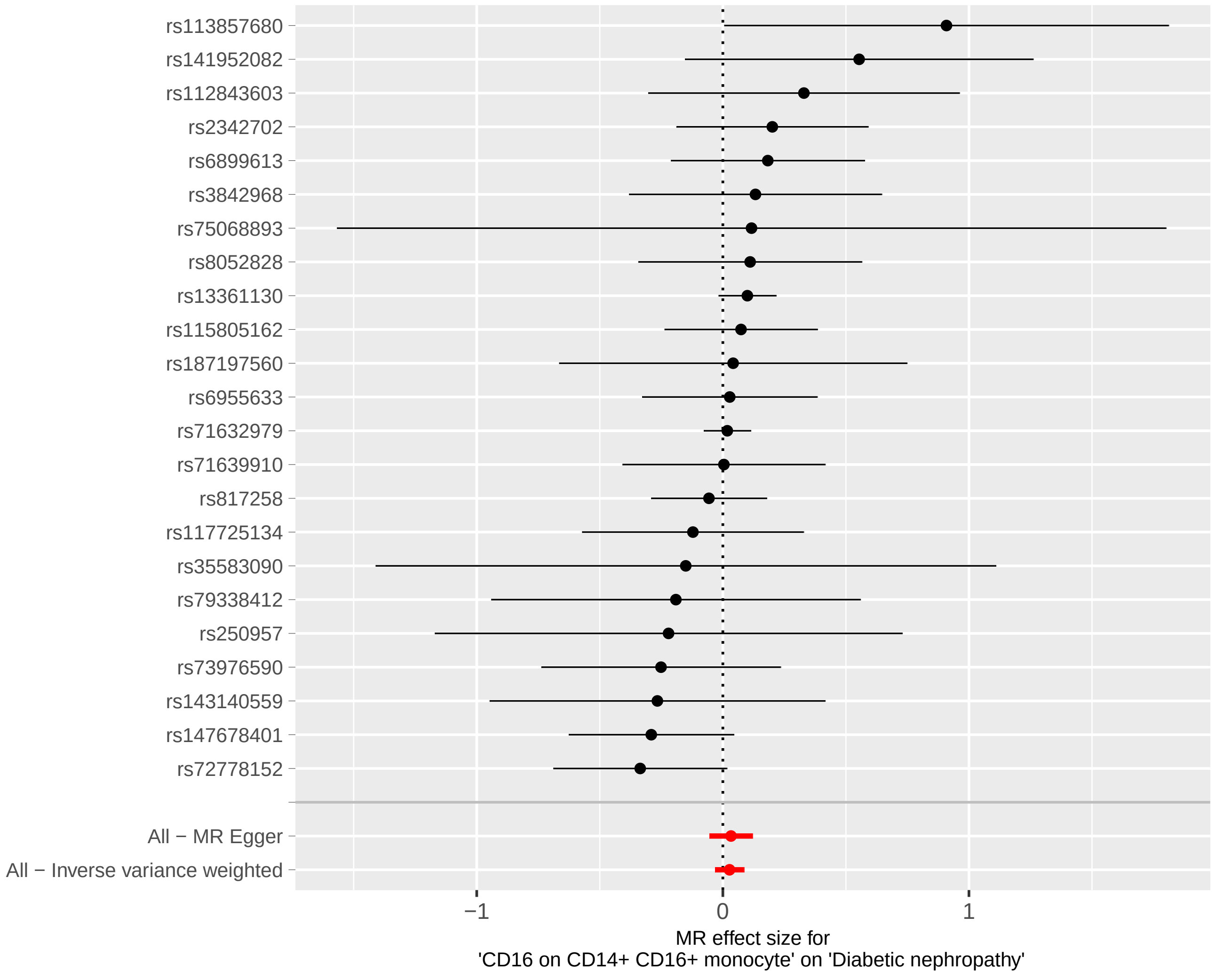

rs113857680
rs141952082
rs112843603
rs2342702
rs6899613
rs3842968
rs75068893
rs8052828
rs13361130
rs115805162
rs187197560
rs6955633
rs71632979
rs71639910
rs817258
rs117725134
rs35583090
rs79338412
rs250957
rs73976590
rs143140559
rs147678401
rs72778152
All − MR Egger
All − Inverse variance weighted
−1
0
1
MR effect size for
'CD16 on CD14+ CD16+ monocyte' on 'Diabetic nephropathy'

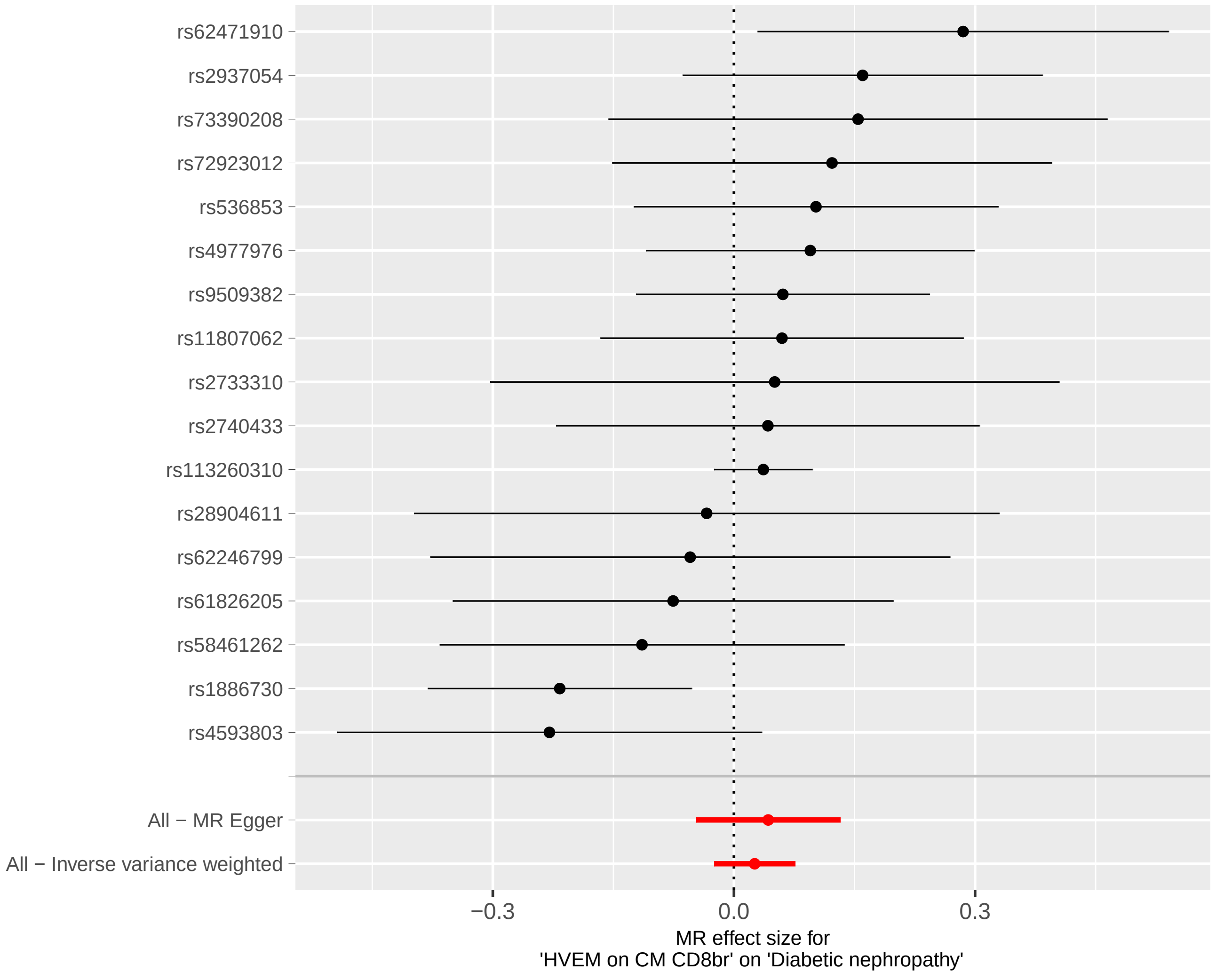

rs62471910
rs2937054
rs73390208
rs72923012
rs536853
rs4977976
rs9509382
rs11807062
rs2733310
rs2740433
rs113260310
rs28904611
rs62246799
rs61826205
rs58461262
rs1886730
rs4593803
All − MR Egger
All − Inverse variance weighted
−0.3
0.0
0.3
MR effect size for
'HVEM on CM CD8br' on 'Diabetic nephropathy'

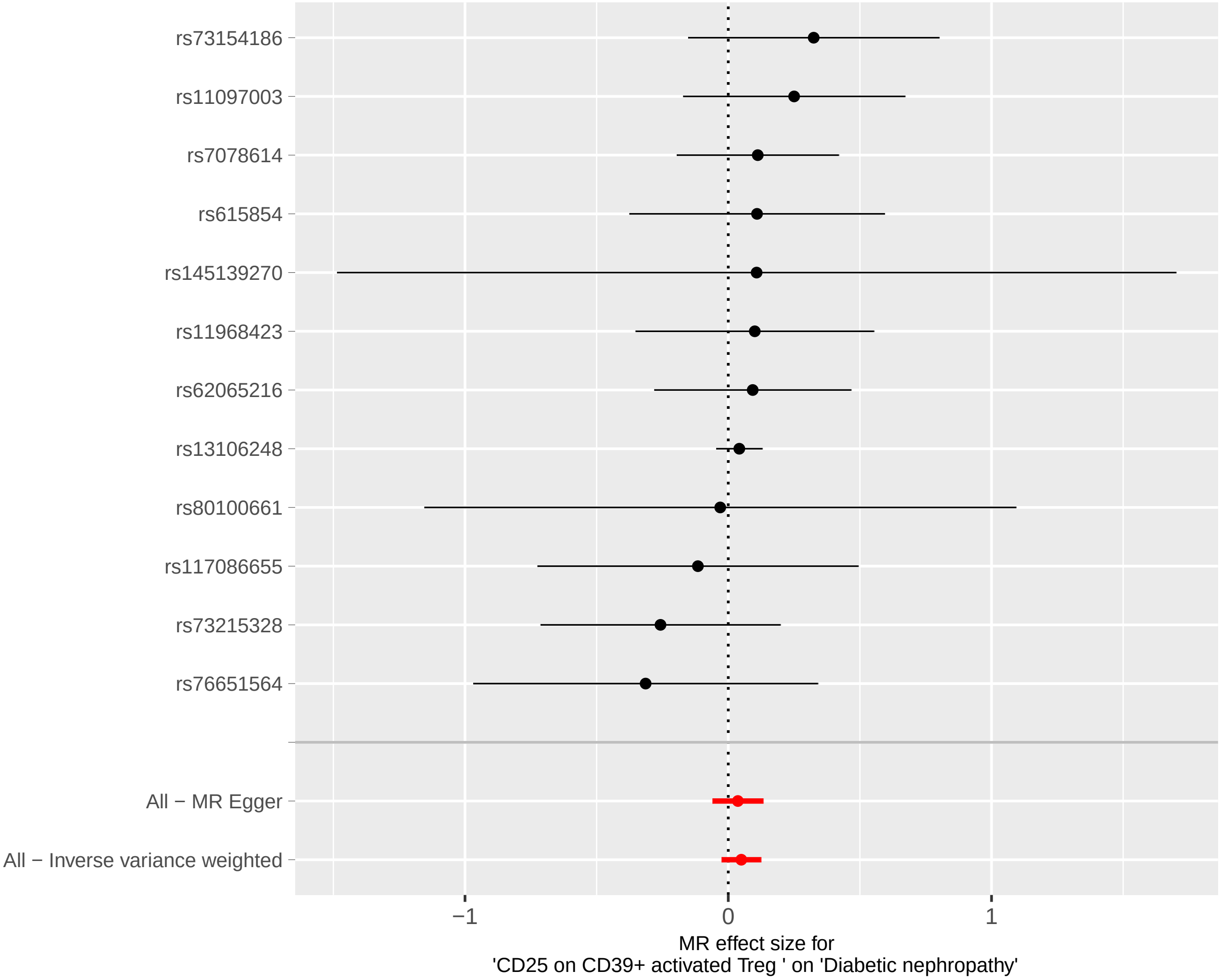
rs73154186
rs11097003
rs7078614
rs615854
rs145139270
rs11968423
rs62065216
rs13106248
rs80100661
rs117086655
rs73215328
rs76651564
All − MR Egger
All − Inverse variance weighted
−1
0
1
MR effect size for
'CD25 on CD39+ activated Treg ' on 'Diabetic nephropathy'

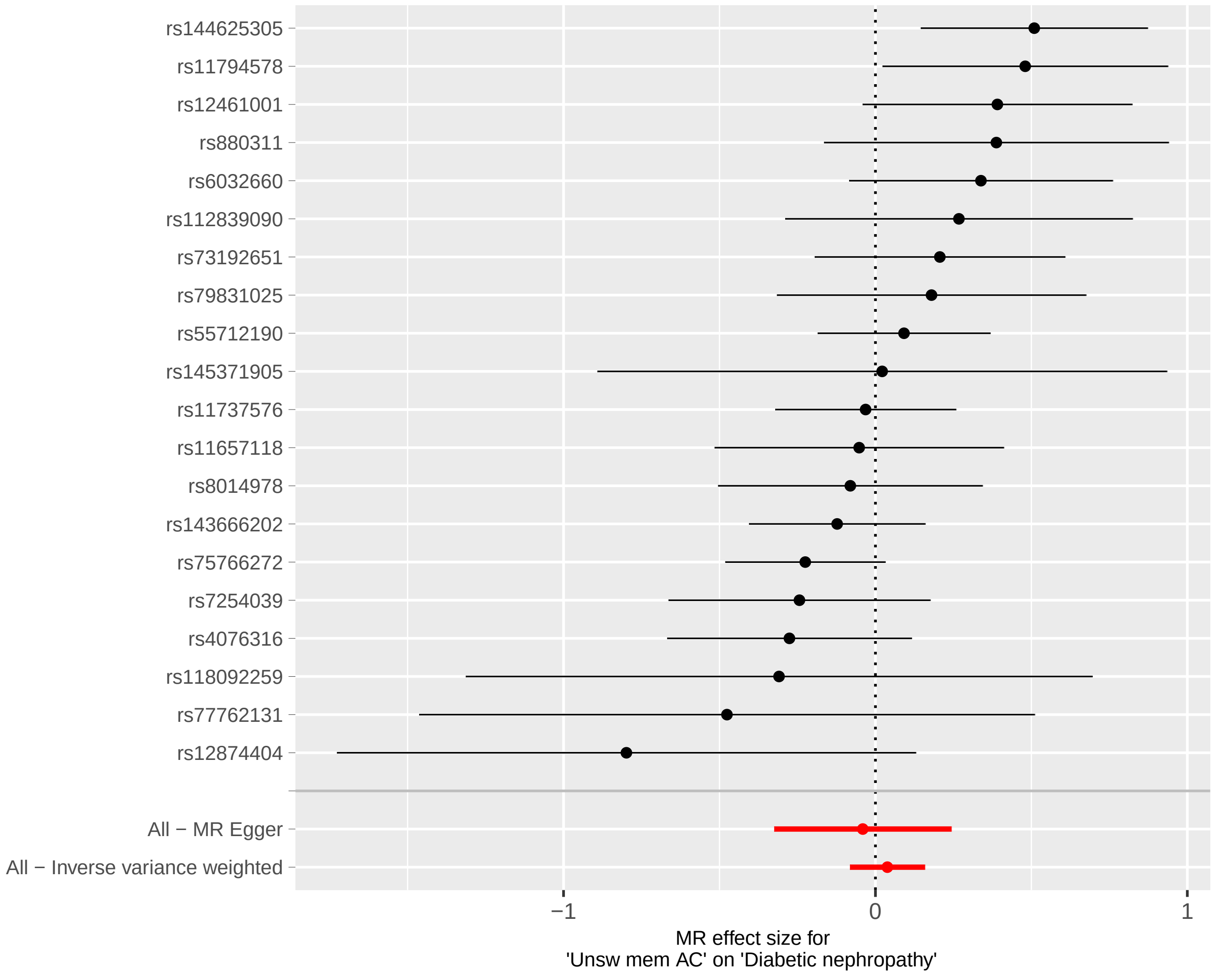
rs144625305
rs11794578
rs12461001
rs880311
rs6032660
rs112839090
rs73192651
rs79831025
rs55712190
rs145371905
rs11737576
rs11657118
rs8014978
rs143666202
rs75766272
rs7254039
rs4076316
rs118092259
rs77762131
rs12874404
All − MR Egger
All − Inverse variance weighted
−1
0
1
MR effect size for
'Unsw mem AC' on 'Diabetic nephropathy'

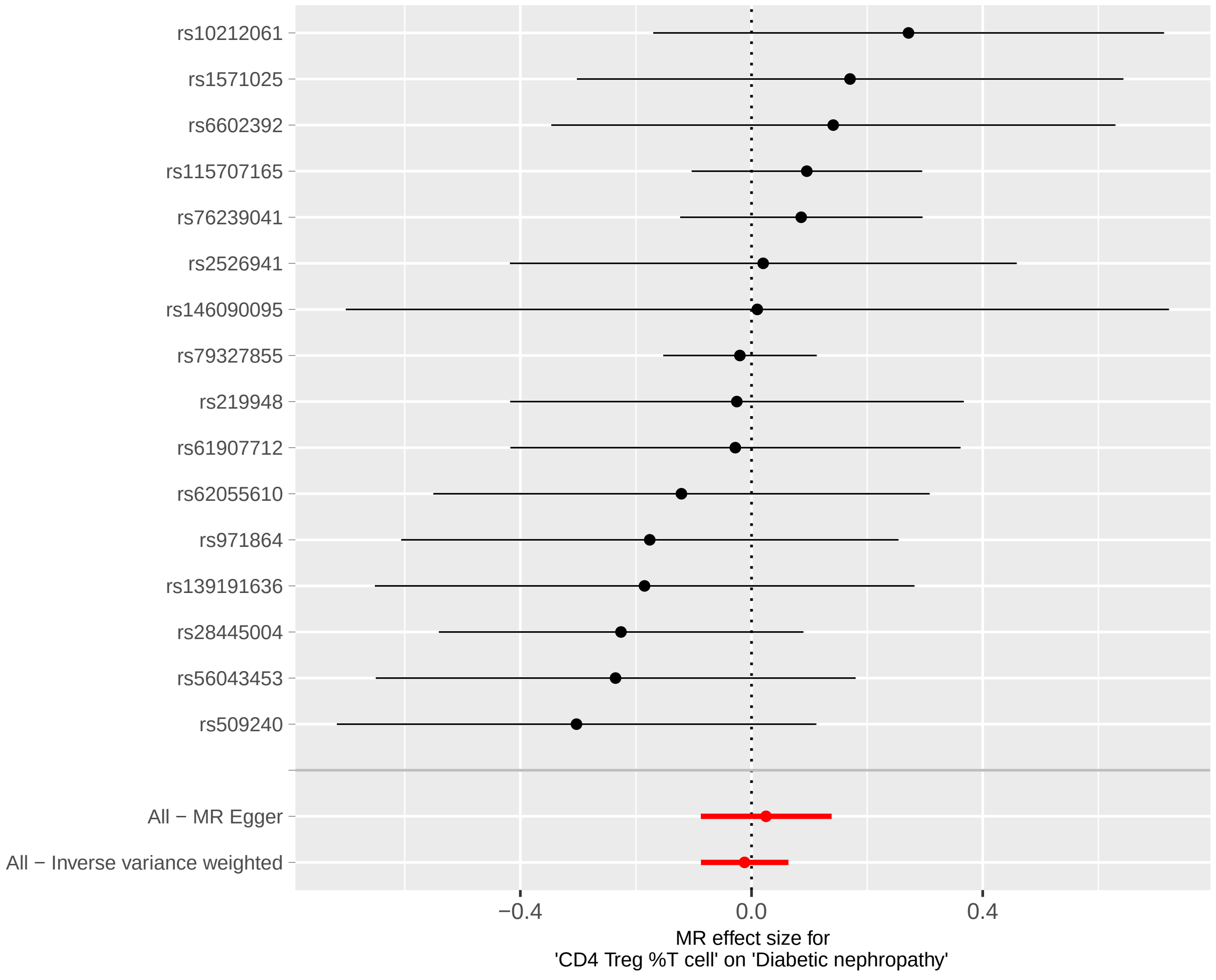
rs10212061
rs1571025
rs6602392
rs115707165
rs76239041
rs2526941
rs146090095
rs79327855
rs219948
rs61907712
rs62055610
rs971864
rs139191636
rs28445004
rs56043453
rs509240
All − MR Egger
All − Inverse variance weighted
−0.4
0.0
0.4
MR effect size for
'CD4 Treg %T cell' on 'Diabetic nephropathy'

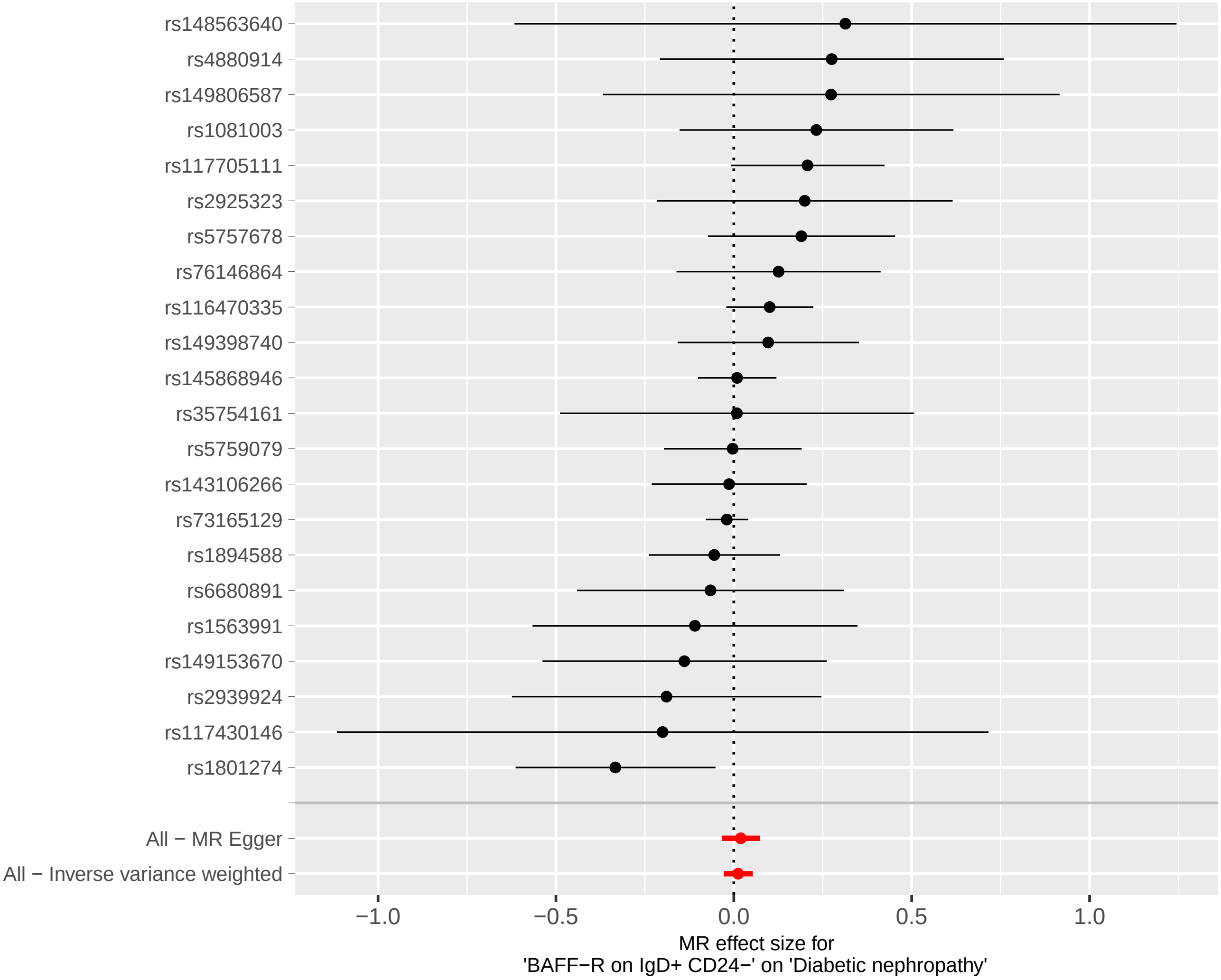
rs148563640
rs4880914
rs149806587
rs1081003
rs117705111
rs2925323
rs5757678
rs76146864
rs116470335
rs149398740
rs145868946
rs35754161
rs5759079
rs143106266
rs73165129
rs1894588
rs6680891
rs1563991
rs149153670
rs2939924
rs117430146
rs1801274
All − MR Egger
All − Inverse variance weighted
−1.0
−0.5
0.0
0.5
1.0
MR effect size for
'BAFF−R on IgD+ CD24−' on 'Diabetic nephropathy'

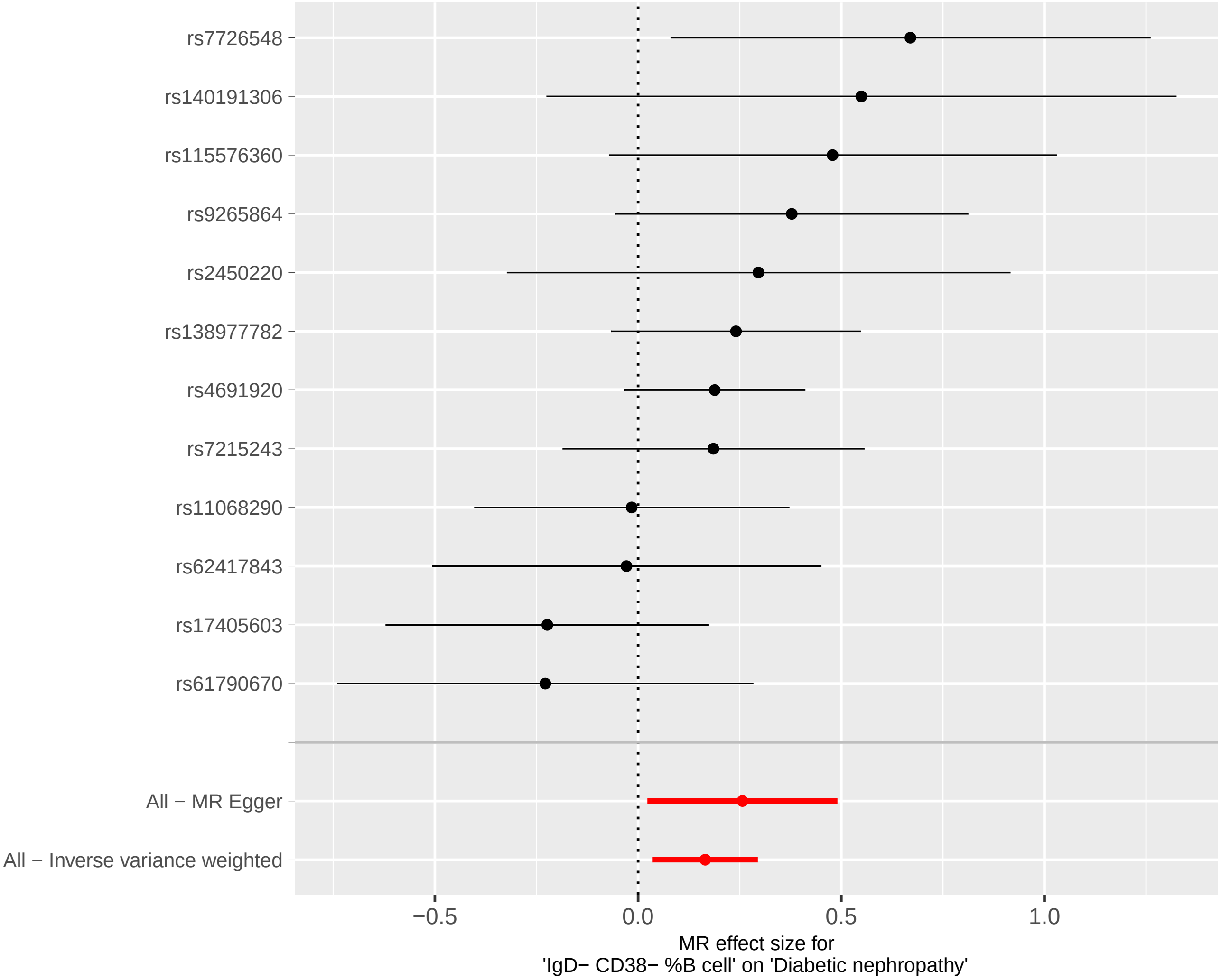

rs7726548
rs140191306
rs115576360
rs9265864
rs2450220
rs138977782
rs4691920
rs7215243
rs11068290
rs62417843
rs17405603
rs61790670
All − MR Egger
All − Inverse variance weighted
−0.5
0.0
0.5
1.0
MR effect size for
'IgD− CD38− %B cell' on 'Diabetic nephropathy'

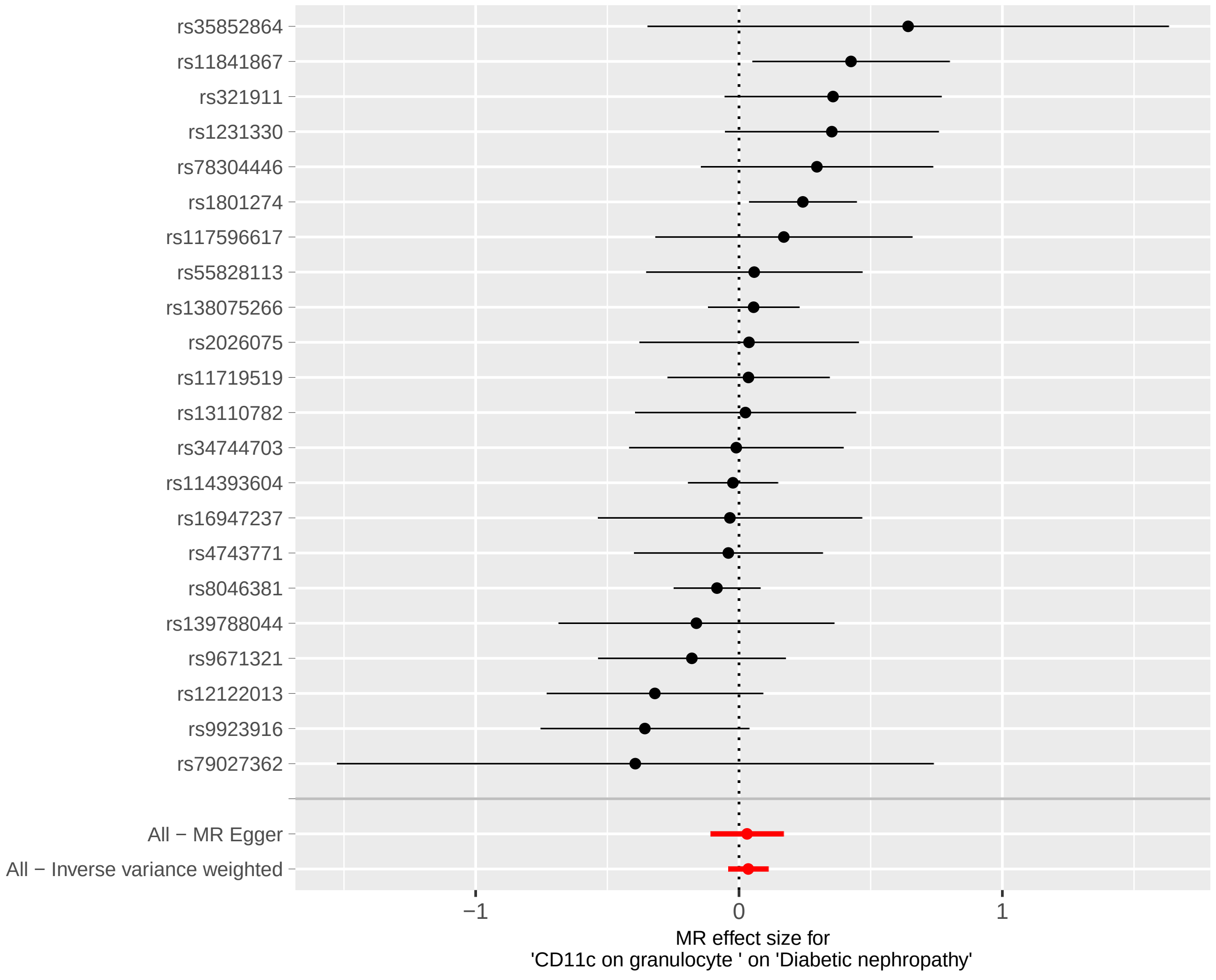

rs35852864
rs11841867
rs321911
rs1231330
rs78304446
rs1801274
rs117596617
rs55828113
rs138075266
rs2026075
rs11719519
rs13110782
rs34744703
rs114393604
rs16947237
rs4743771
rs8046381
rs139788044
rs9671321
rs12122013
rs9923916
rs79027362
All − MR Egger
All − Inverse variance weighted
−1
0
1
MR effect size for
'CD11c on granulocyte ' on 'Diabetic nephropathy'

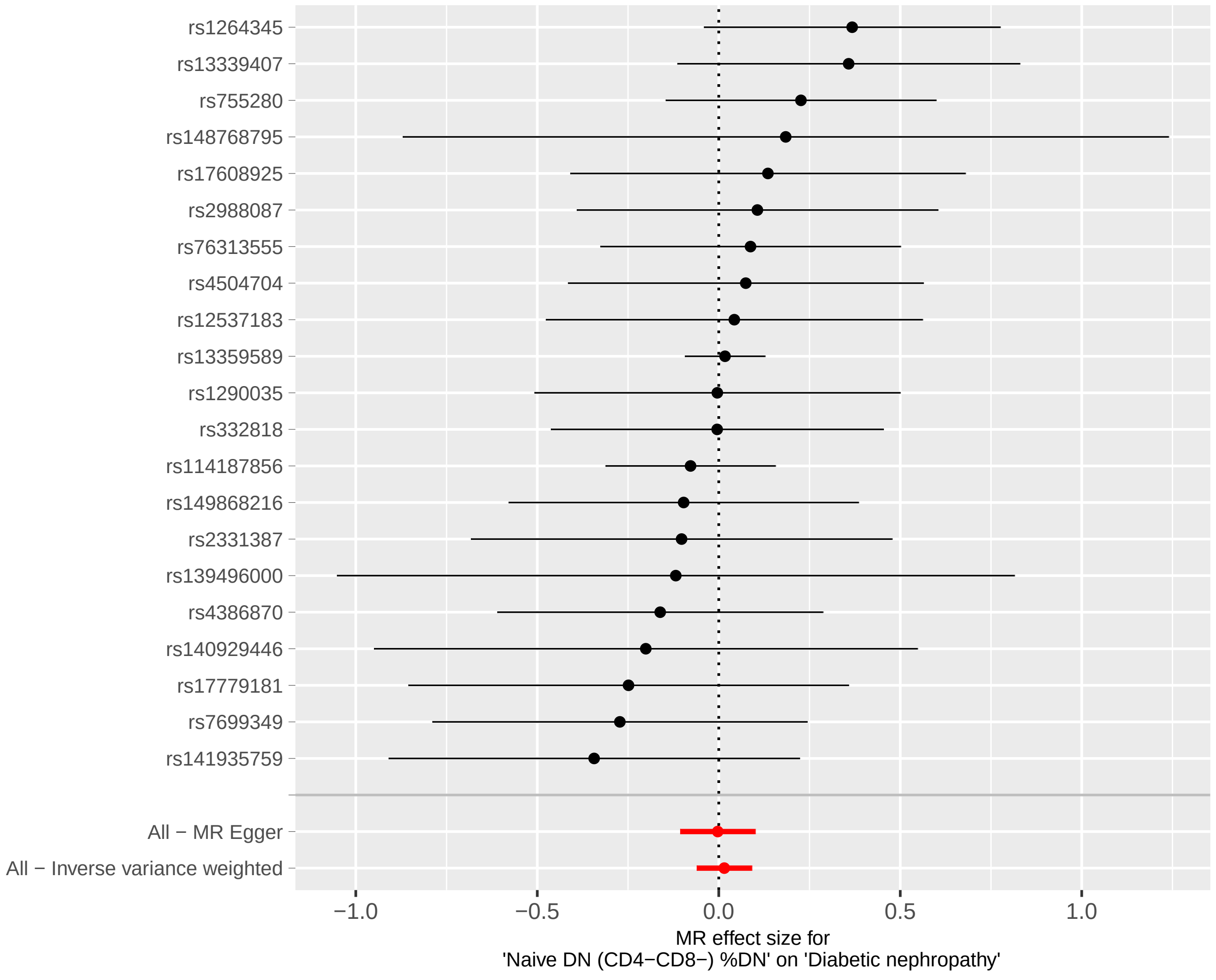

rs1264345
rs13339407
rs755280
rs148768795
rs17608925
rs2988087
rs76313555
rs4504704
rs12537183
rs13359589
rs1290035
rs332818
rs114187856
rs149868216
rs2331387
rs139496000
rs4386870
rs140929446
rs17779181
rs7699349
rs141935759
All − MR Egger
All − Inverse variance weighted
−1.0
−0.5
0.0
0.5
1.0
MR effect size for
'Naive DN (CD4−CD8−) %DN' on 'Diabetic nephropathy'

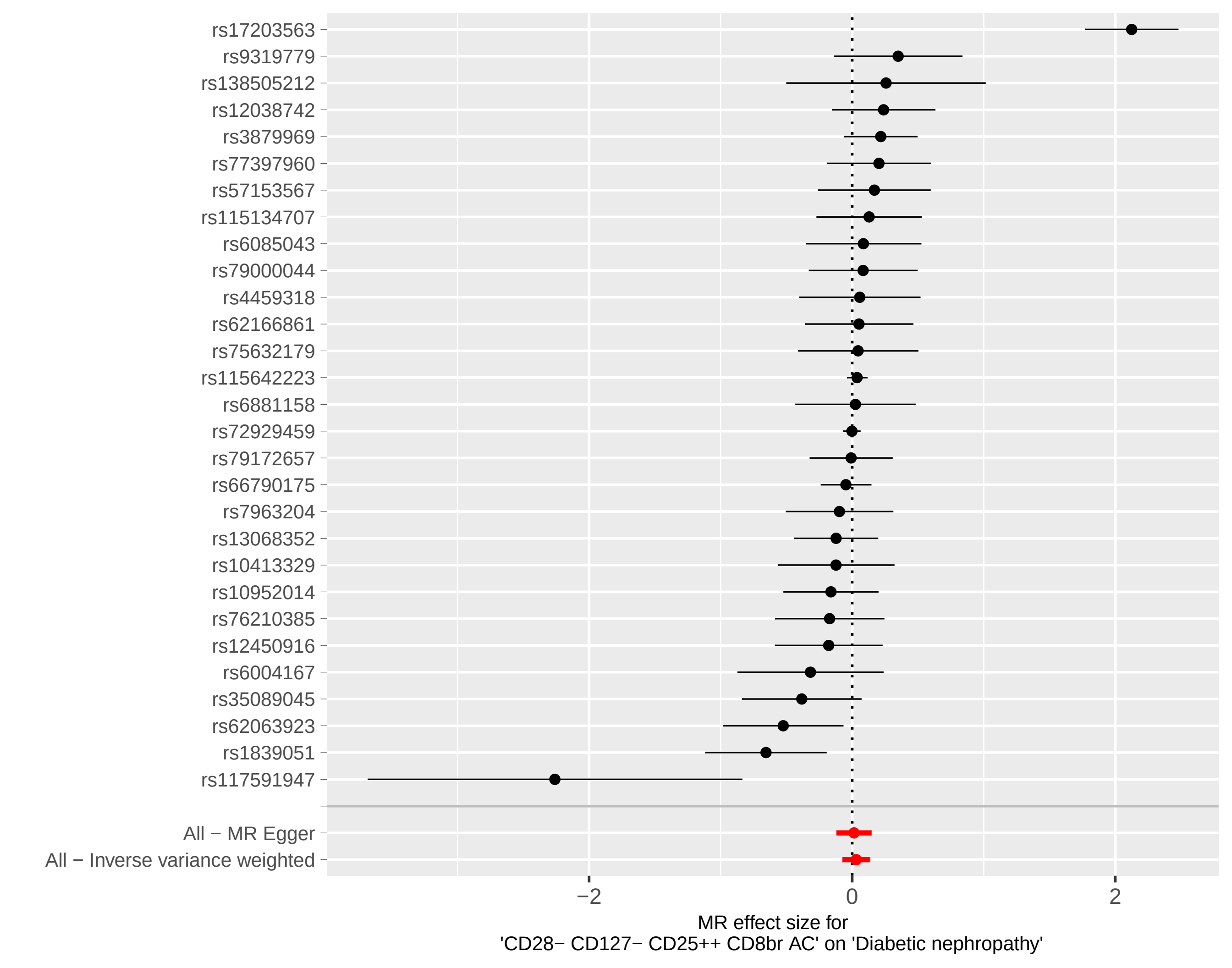

rs17203563
rs9319779
rs138505212
rs12038742
rs3879969
rs77397960
rs57153567
rs115134707
rs6085043
rs79000044
rs4459318
rs62166861
rs75632179
rs115642223
rs6881158
rs72929459
rs79172657
rs66790175
rs7963204
rs13068352
rs10413329
rs10952014
rs76210385
rs12450916
rs6004167
rs35089045
rs62063923
rs1839051
rs117591947
All − MR Egger
All − Inverse variance weighted
−2
0
2
MR effect size for
'CD28− CD127− CD25++ CD8br AC' on 'Diabetic nephropathy'

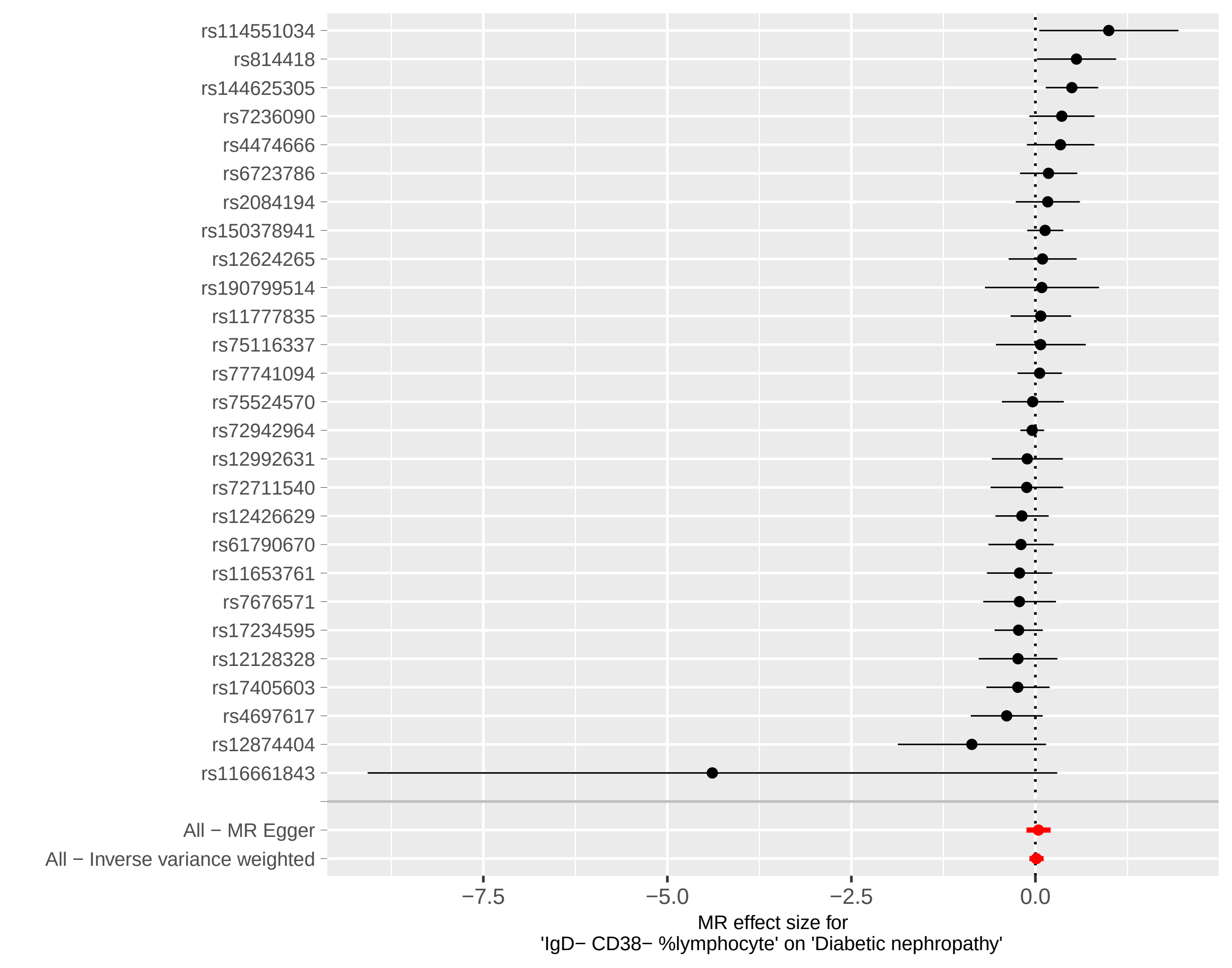
rs114551034
rs814418
rs144625305
rs7236090
rs4474666
rs6723786
rs2084194
rs150378941
rs12624265
rs190799514
rs11777835
rs75116337
rs77741094
rs75524570
rs72942964
rs12992631
rs72711540
rs12426629
rs61790670
rs11653761
rs7676571
rs17234595
rs12128328
rs17405603
rs4697617
rs12874404
rs116661843
All − MR Egger
All − Inverse variance weighted
−7.5
−5.0
−2.5
0.0
MR effect size for
'IgD− CD38− %lymphocyte' on 'Diabetic nephropathy'

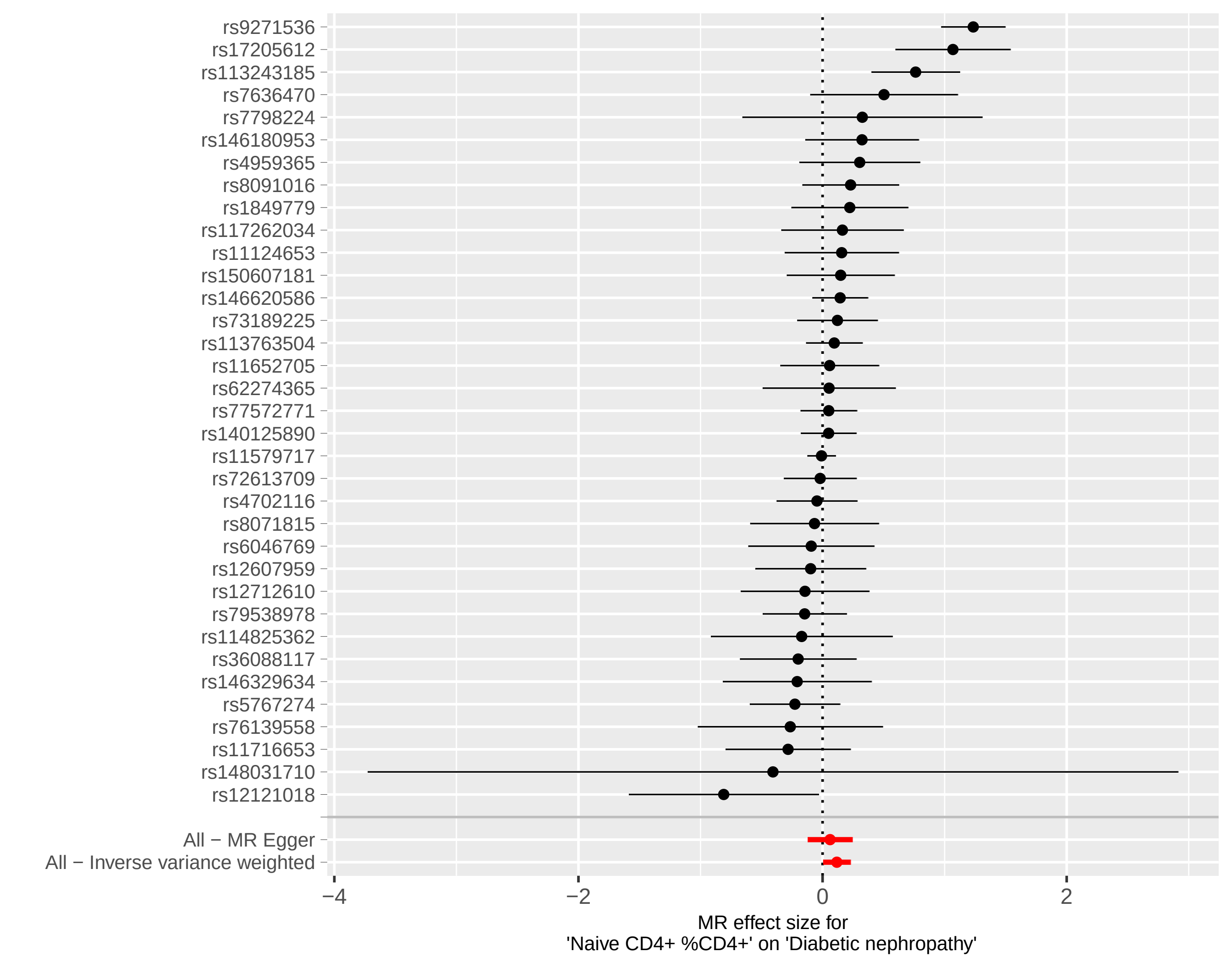

rs9271536
rs17205612
rs113243185
rs7636470
rs7798224
rs146180953
rs4959365
rs8091016
rs1849779
rs117262034
rs11124653
rs150607181
rs146620586
rs73189225
rs113763504
rs11652705
rs62274365
rs77572771
rs140125890
rs11579717
rs72613709
rs4702116
rs8071815
rs6046769
rs12607959
rs12712610
rs79538978
rs114825362
rs36088117
rs146329634
rs5767274
rs76139558
rs11716653
rs148031710
rs12121018
All − MR Egger
All − Inverse variance weighted
−4
−2
0
2
MR effect size for
'Naive CD4+ %CD4+' on 'Diabetic nephropathy'

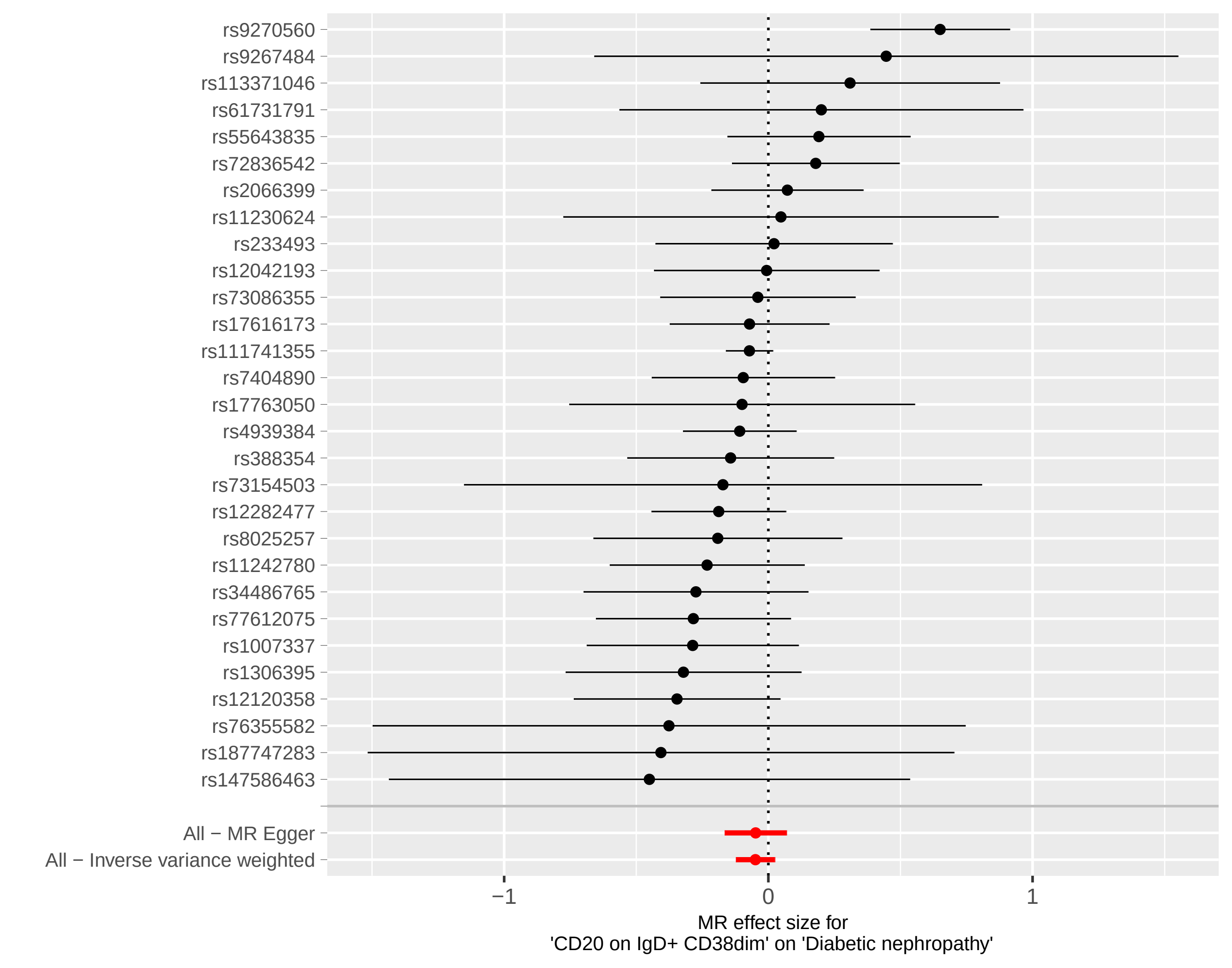

rs9270560
rs9267484
rs113371046
rs61731791
rs55643835
rs72836542
rs2066399
rs11230624
rs233493
rs12042193
rs73086355
rs17616173
rs111741355
rs7404890
rs17763050
rs4939384
rs388354
rs73154503
rs12282477
rs8025257
rs11242780
rs34486765
rs77612075
rs1007337
rs1306395
rs12120358
rs76355582
rs187747283
rs147586463
All − MR Egger
All − Inverse variance weighted
−1
0
1
MR effect size for
'CD20 on IgD+ CD38dim' on 'Diabetic nephropathy'

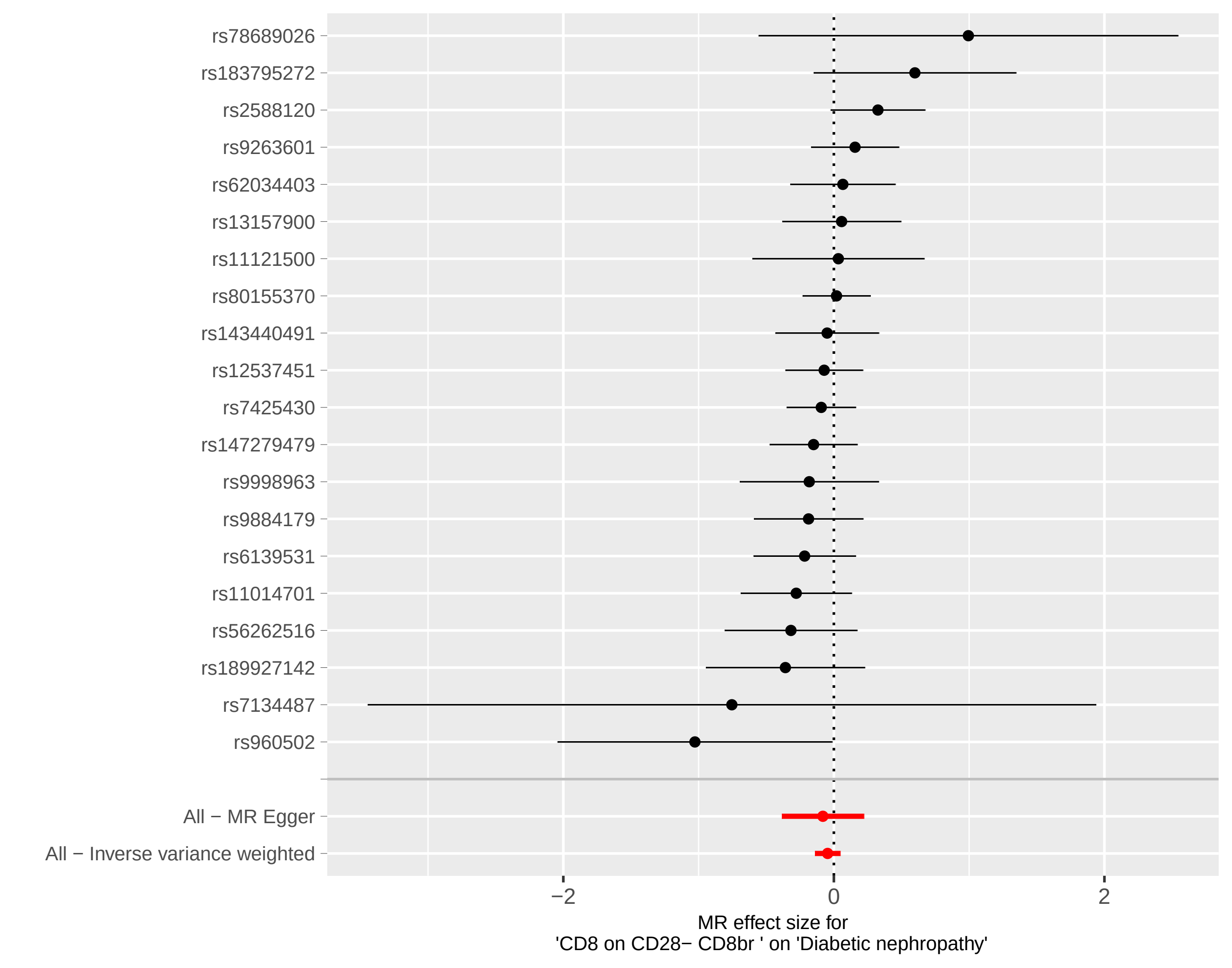
rs78689026
rs183795272
rs2588120
rs9263601
rs62034403
rs13157900
rs11121500
rs80155370
rs143440491
rs12537451
rs7425430
rs147279479
rs9998963
rs9884179
rs6139531
rs11014701
rs56262516
rs189927142
rs7134487
rs960502
All − MR Egger
All − Inverse variance weighted
−2
0
2
MR effect size for
'CD8 on CD28− CD8br ' on 'Diabetic nephropathy'

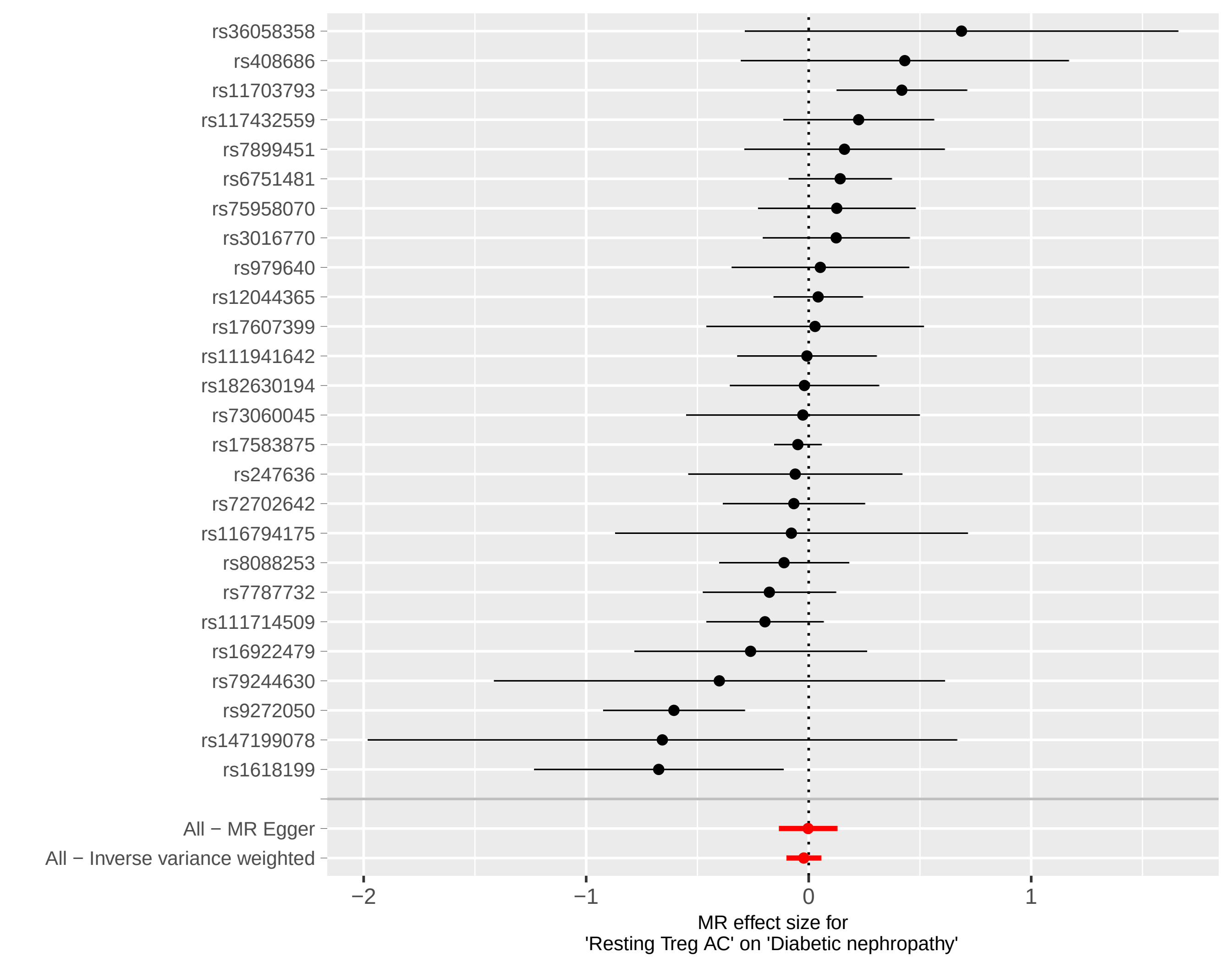
rs36058358
rs408686
rs11703793
rs117432559
rs7899451
rs6751481
rs75958070
rs3016770
rs979640
rs12044365
rs17607399
rs111941642
rs182630194
rs73060045
rs17583875
rs247636
rs72702642
rs116794175
rs8088253
rs7787732
rs111714509
rs16922479
rs79244630
rs9272050
rs147199078
rs1618199
All − MR Egger
All − Inverse variance weighted
−2
−1
0
1
MR effect size for
'Resting Treg AC' on 'Diabetic nephropathy'

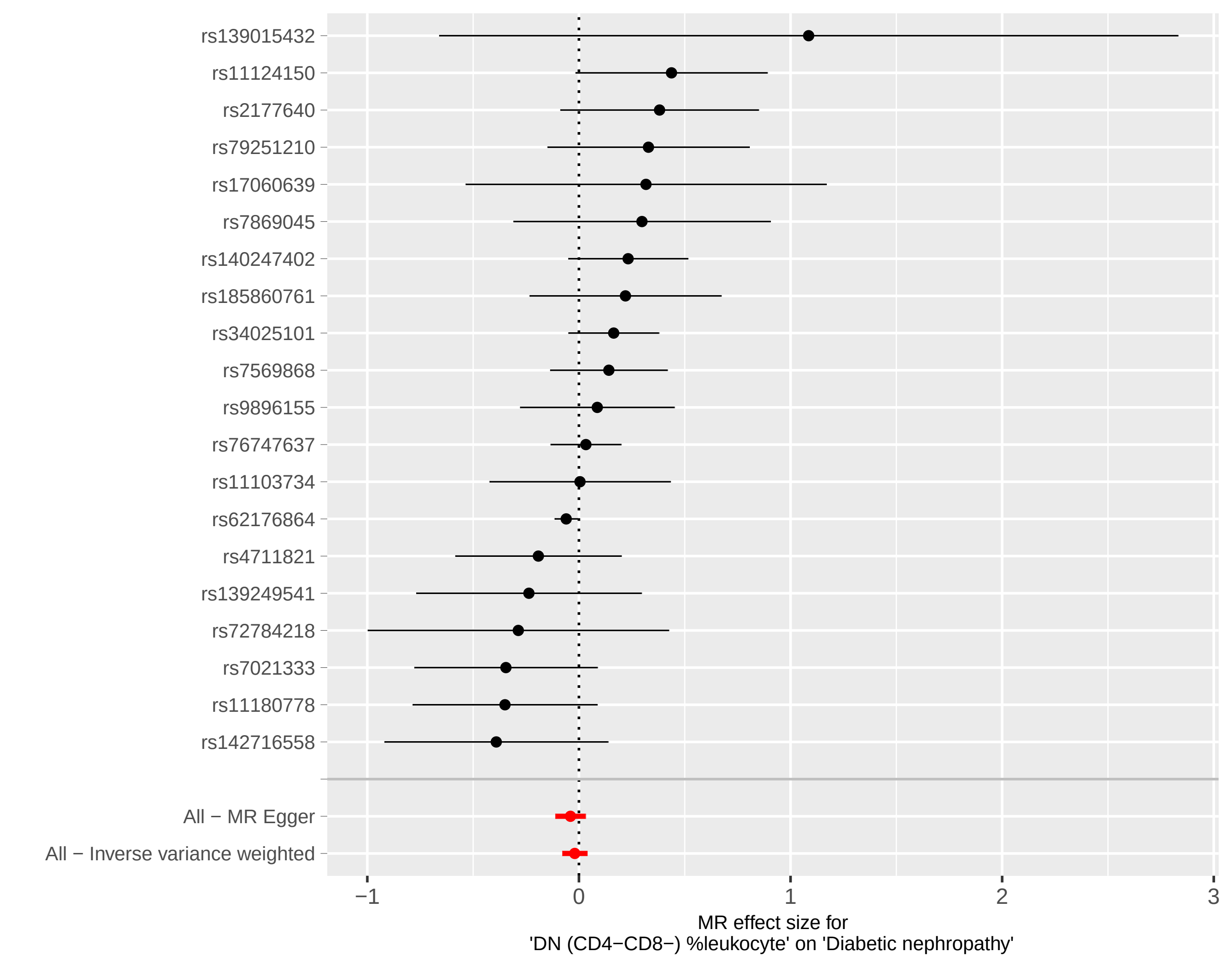
rs139015432
rs11124150
rs2177640
rs79251210
rs17060639
rs7869045
rs140247402
rs185860761
rs34025101
rs7569868
rs9896155
rs76747637
rs11103734
rs62176864
rs4711821
rs139249541
rs72784218
rs7021333
rs11180778
rs142716558
All − MR Egger
All − Inverse variance weighted
−1
0
1
2
3
MR effect size for
'DN (CD4−CD8−) %leukocyte' on 'Diabetic nephropathy'

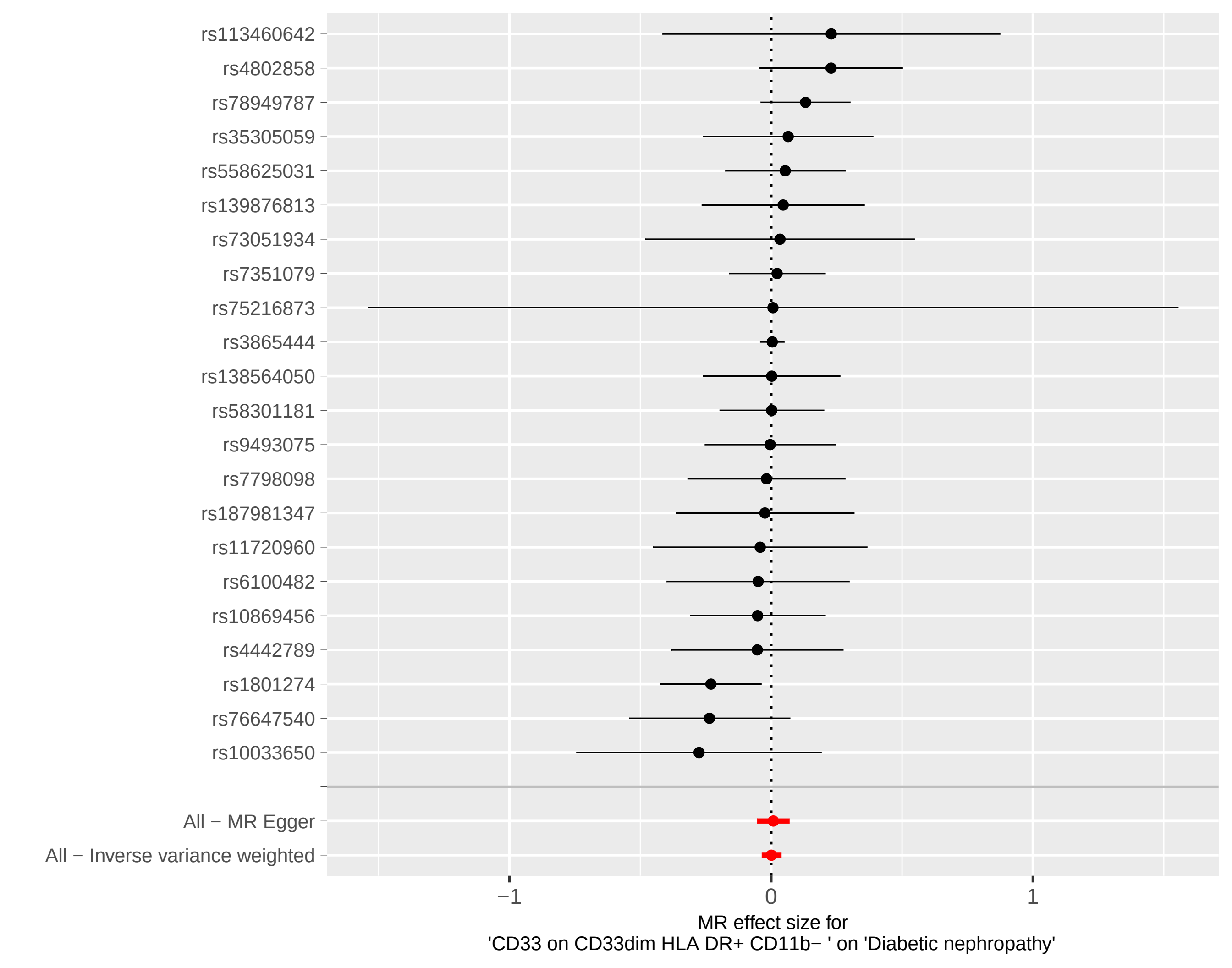
rs113460642
rs4802858
rs78949787
rs35305059
rs558625031
rs139876813
rs73051934
rs7351079
rs75216873
rs3865444
rs138564050
rs58301181
rs9493075
rs7798098
rs187981347
rs11720960
rs6100482
rs10869456
rs4442789
rs1801274
rs76647540
rs10033650
All − MR Egger
All − Inverse variance weighted
−1
0
1
MR effect size for
'CD33 on CD33dim HLA DR+ CD11b− ' on 'Diabetic nephropathy'

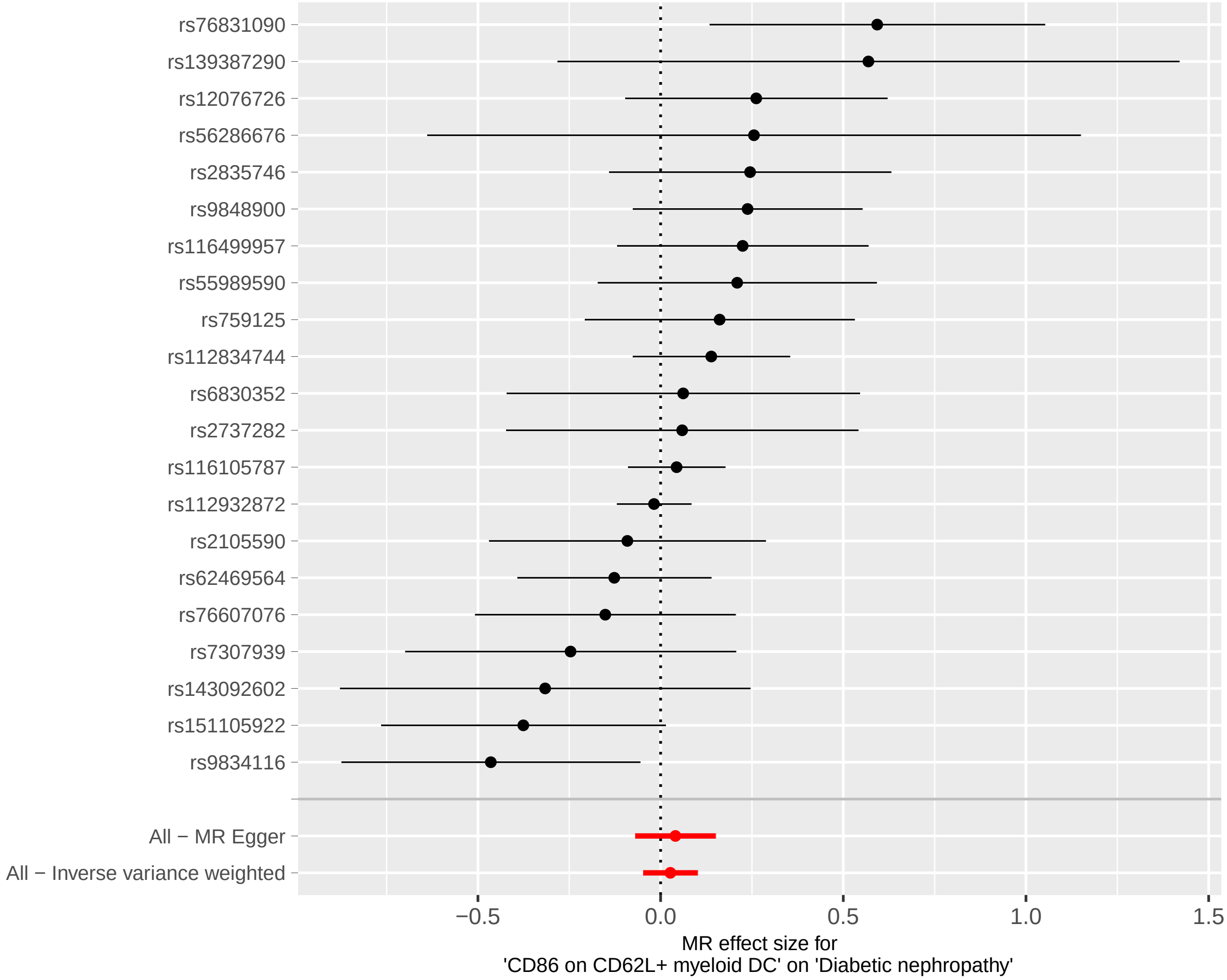

rs76831090
rs139387290
rs12076726
rs56286676
rs2835746
rs9848900
rs116499957
rs55989590
rs759125
rs112834744
rs6830352
rs2737282
rs116105787
rs112932872
rs2105590
rs62469564
rs76607076
rs7307939
rs143092602
rs151105922
rs9834116
All − MR Egger
All − Inverse variance weighted
−0.5
0.0
0.5
1.0
1.5
MR effect size for
'CD86 on CD62L+ myeloid DC' on 'Diabetic nephropathy'

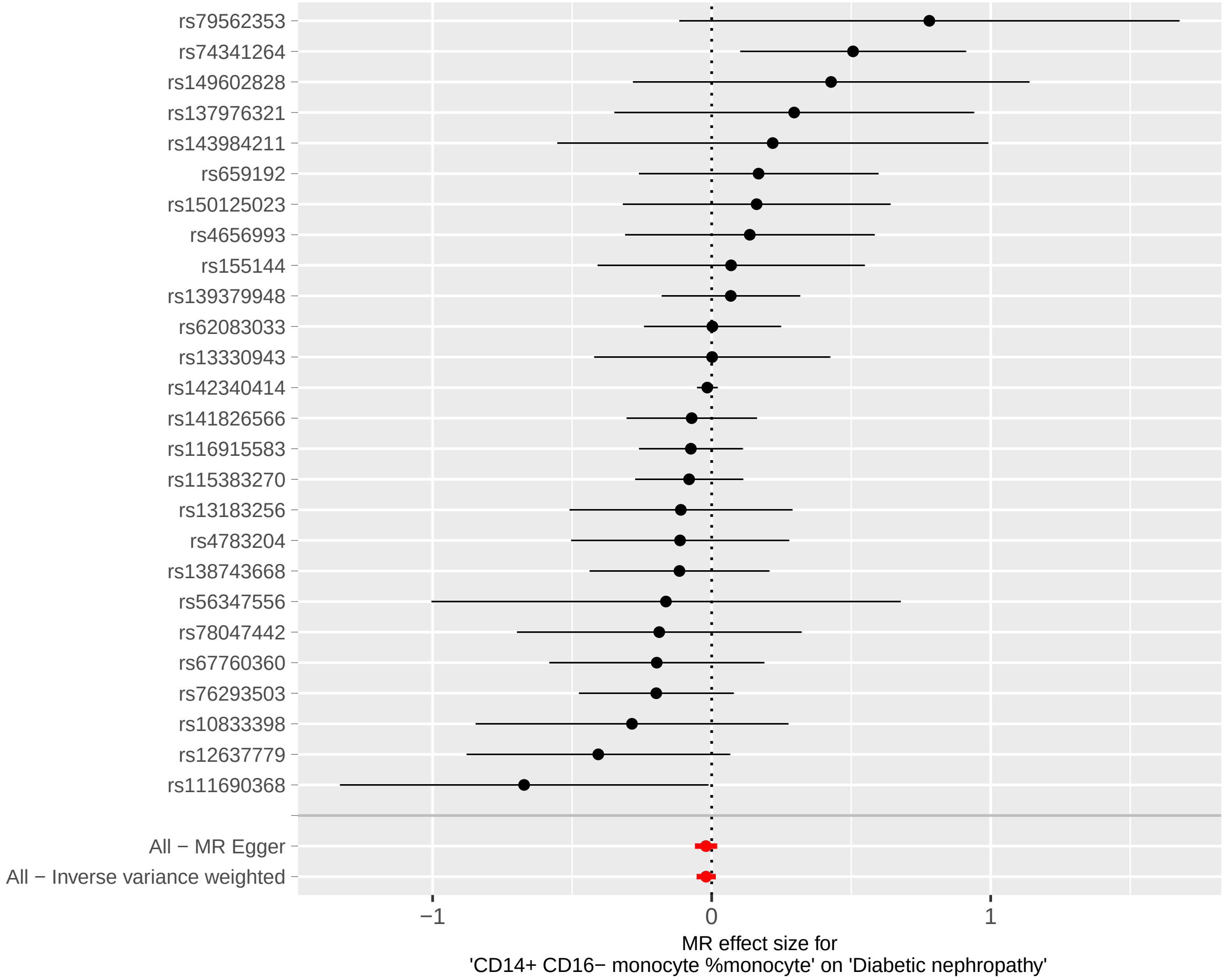
rs79562353
rs74341264
rs149602828
rs137976321
rs143984211
rs659192
rs150125023
rs4656993
rs155144
rs139379948
rs62083033
rs13330943
rs142340414
rs141826566
rs116915583
rs115383270
rs13183256
rs4783204
rs138743668
rs56347556
rs78047442
rs67760360
rs76293503
rs10833398
rs12637779
rs111690368
All − MR Egger
All − Inverse variance weighted
−1
0
1
MR effect size for
'CD14+ CD16− monocyte %monocyte' on 'Diabetic nephropathy'

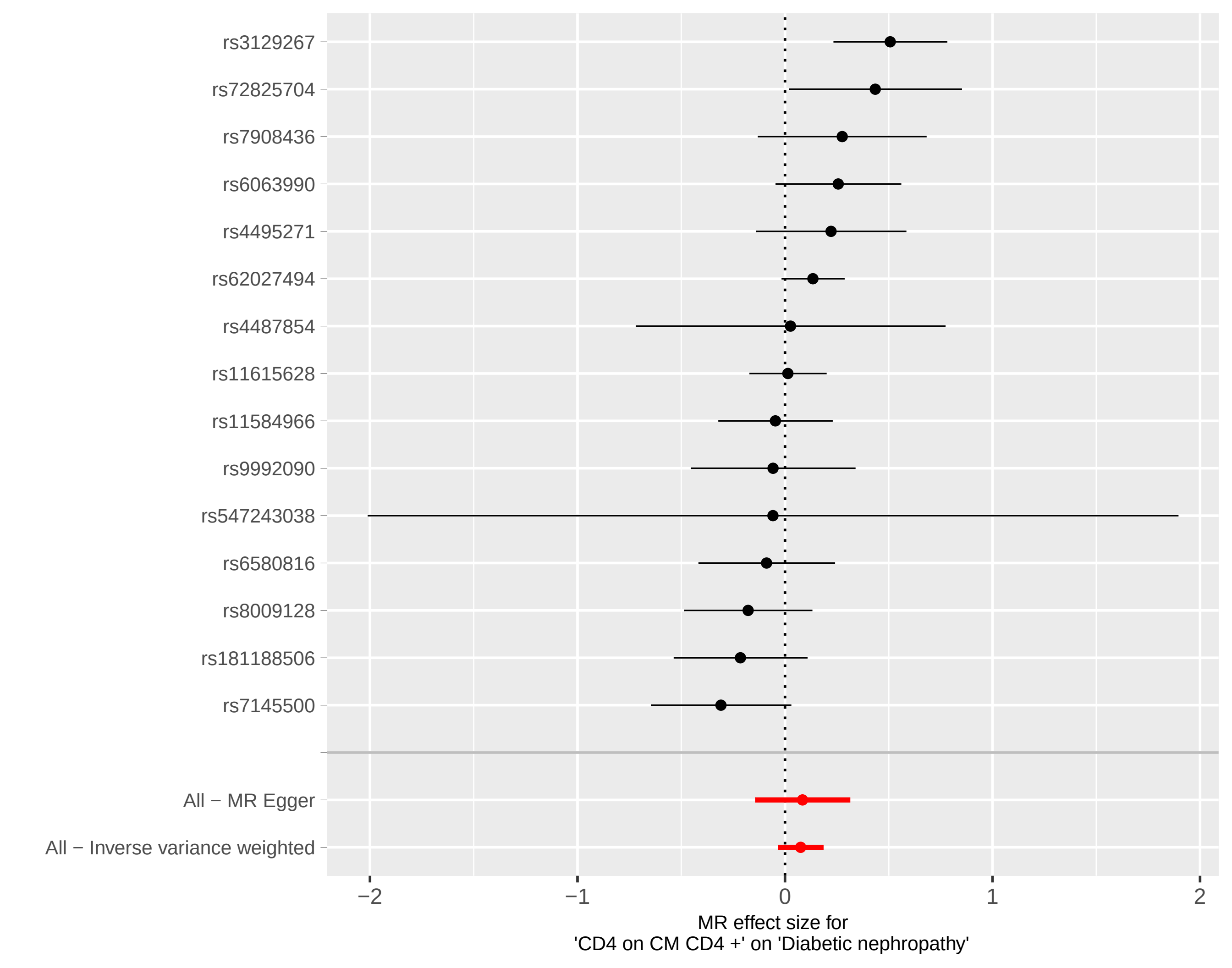
rs3129267
rs72825704
rs7908436
rs6063990
rs4495271
rs62027494
rs4487854
rs11615628
rs11584966
rs9992090
rs547243038
rs6580816
rs8009128
rs181188506
rs7145500
All − MR Egger
All − Inverse variance weighted
−2
−1
0
1
2
MR effect size for
'CD4 on CM CD4 +' on 'Diabetic nephropathy'

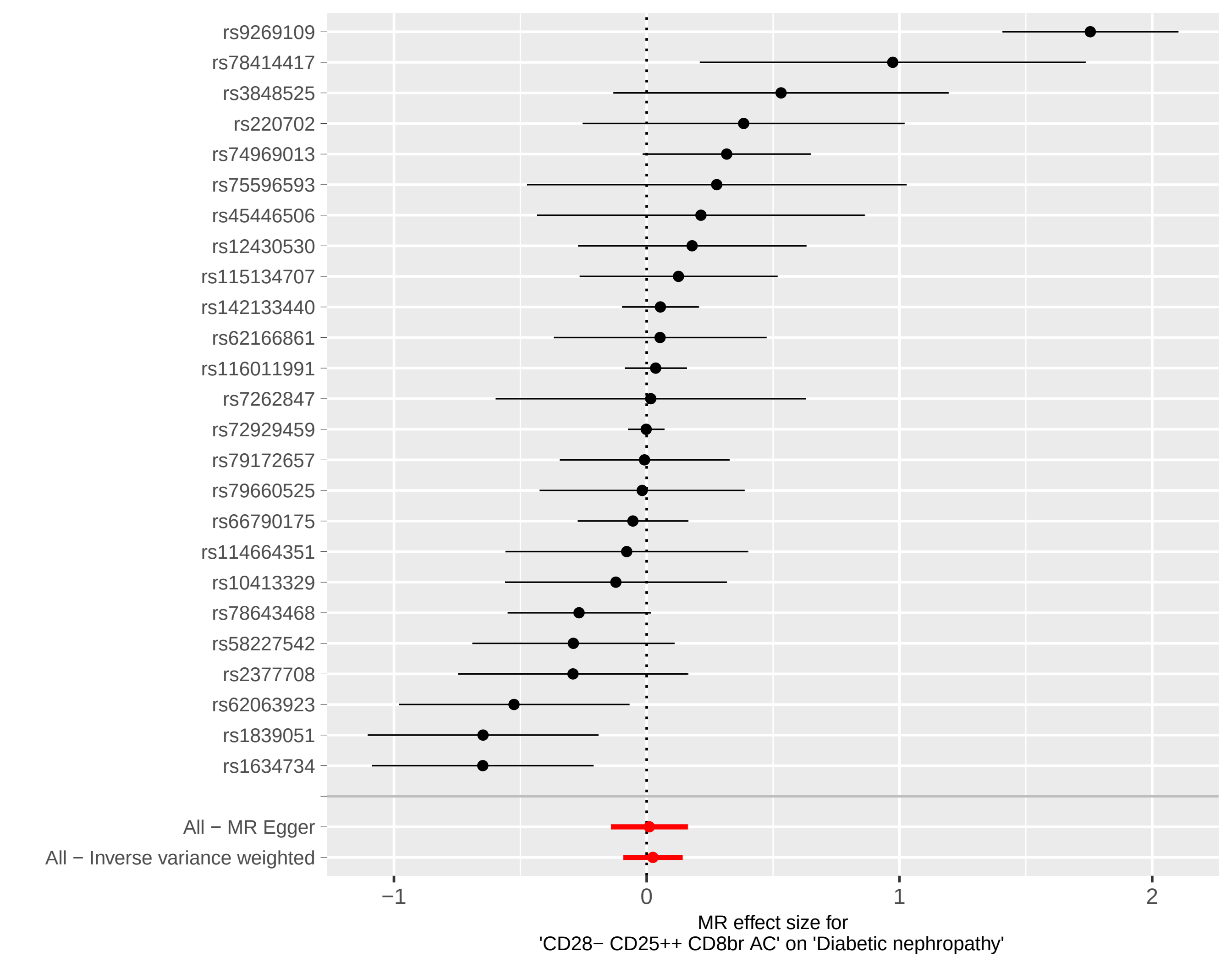
rs9269109
rs78414417
rs3848525
rs220702
rs74969013
rs75596593
rs45446506
rs12430530
rs115134707
rs142133440
rs62166861
rs116011991
rs7262847
rs72929459
rs79172657
rs79660525
rs66790175
rs114664351
rs10413329
rs78643468
rs58227542
rs2377708
rs62063923
rs1839051
rs1634734
All − MR Egger
All − Inverse variance weighted
−1
0
1
2
MR effect size for
'CD28− CD25++ CD8br AC' on 'Diabetic nephropathy'

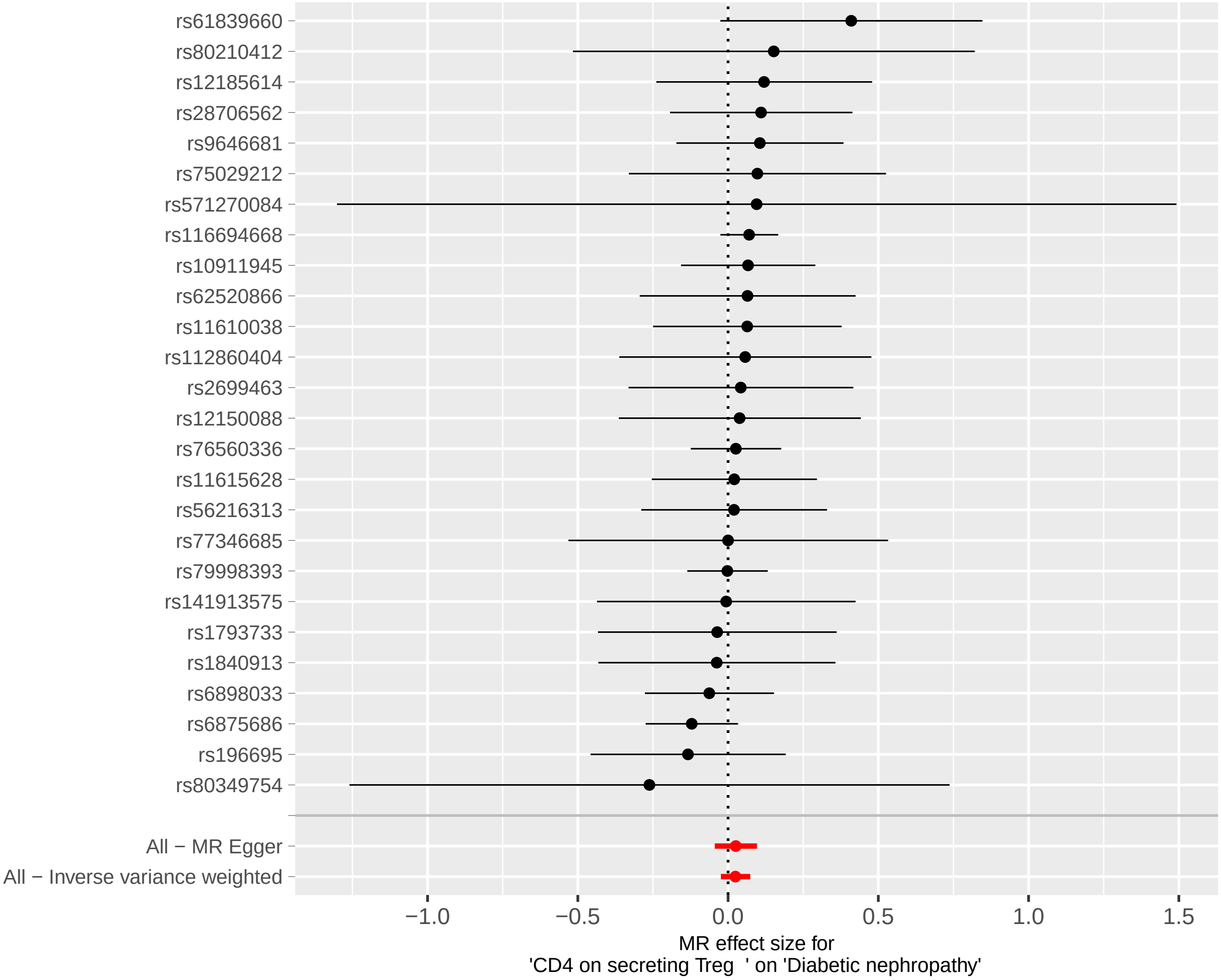
rs61839660
rs80210412
rs12185614
rs28706562
rs9646681
rs75029212
rs571270084
rs116694668
rs10911945
rs62520866
rs11610038
rs112860404
rs2699463
rs12150088
rs76560336
rs11615628
rs56216313
rs77346685
rs79998393
rs141913575
rs1793733
rs1840913
rs6898033
rs6875686
rs196695
rs80349754
All − MR Egger
All − Inverse variance weighted
−1.0
−0.5
0.0
0.5
1.0
1.5
MR effect size for
'CD4 on secreting Treg ' on 'Diabetic nephropathy'

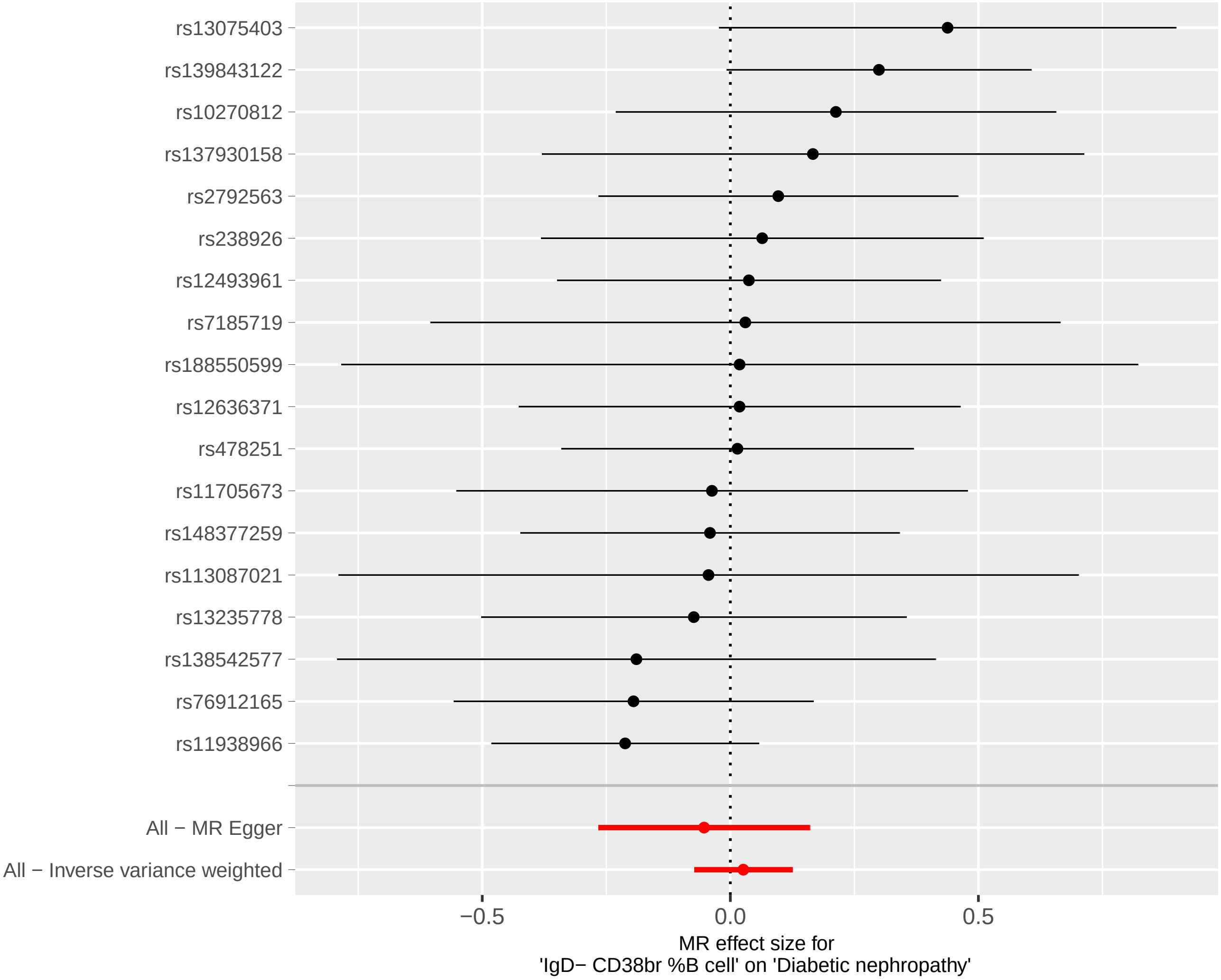
rs13075403
rs139843122
rs10270812
rs137930158
rs2792563
rs238926
rs12493961
rs7185719
rs188550599
rs12636371
rs478251
rs11705673
rs148377259
rs113087021
rs13235778
rs138542577
rs76912165
rs11938966
All − MR Egger
All − Inverse variance weighted
−0.5
0.0
0.5
MR effect size for
'IgD− CD38br %B cell' on 'Diabetic nephropathy'

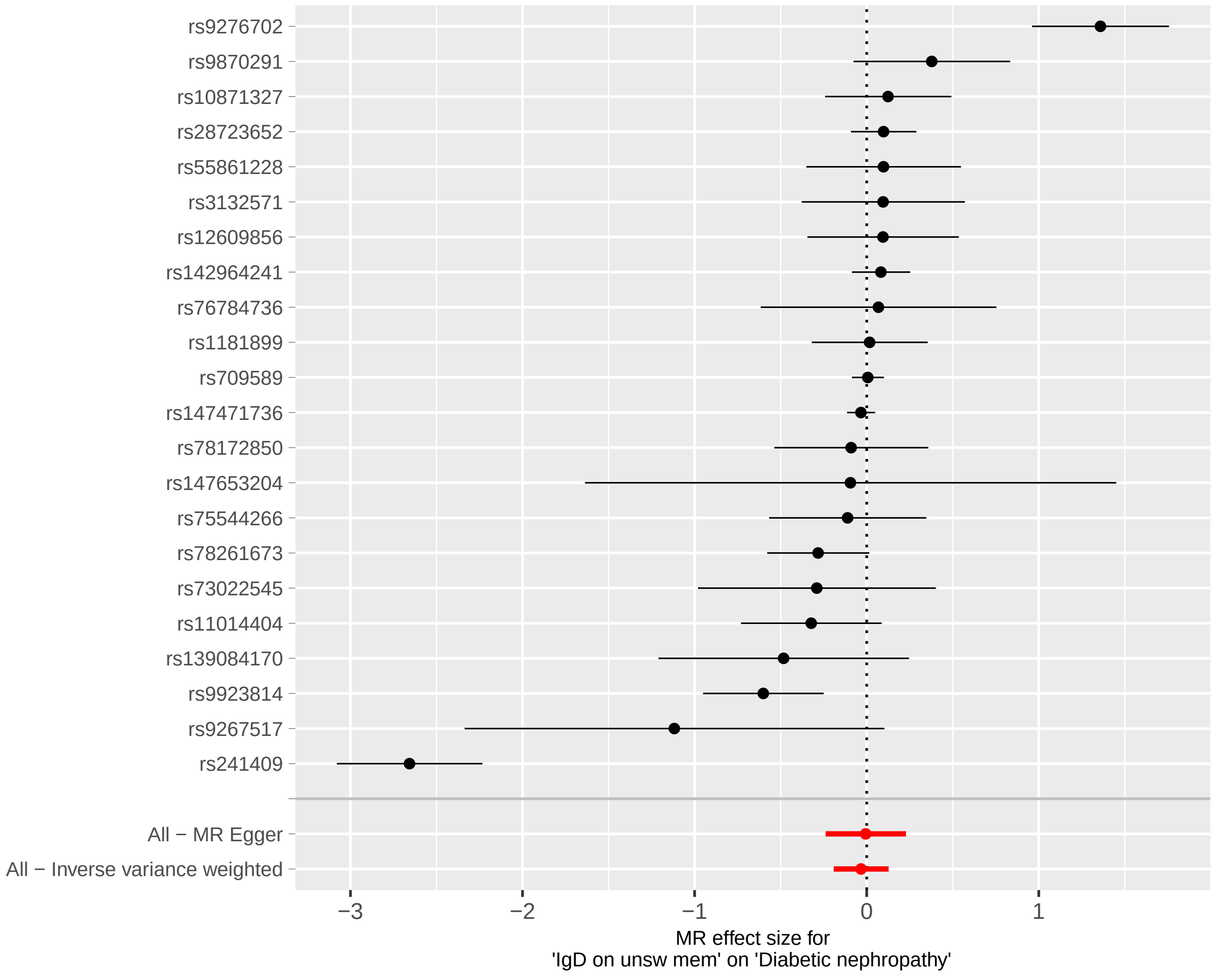

rs9276702
rs9870291
rs10871327
rs28723652
rs55861228
rs3132571
rs12609856
rs142964241
rs76784736
rs1181899
rs709589
rs147471736
rs78172850
rs147653204
rs75544266
rs78261673
rs73022545
rs11014404
rs139084170
rs9923814
rs9267517
rs241409
All − MR Egger
All − Inverse variance weighted
−3
−2
−1
0
1
MR effect size for
'IgD on unsw mem' on 'Diabetic nephropathy'

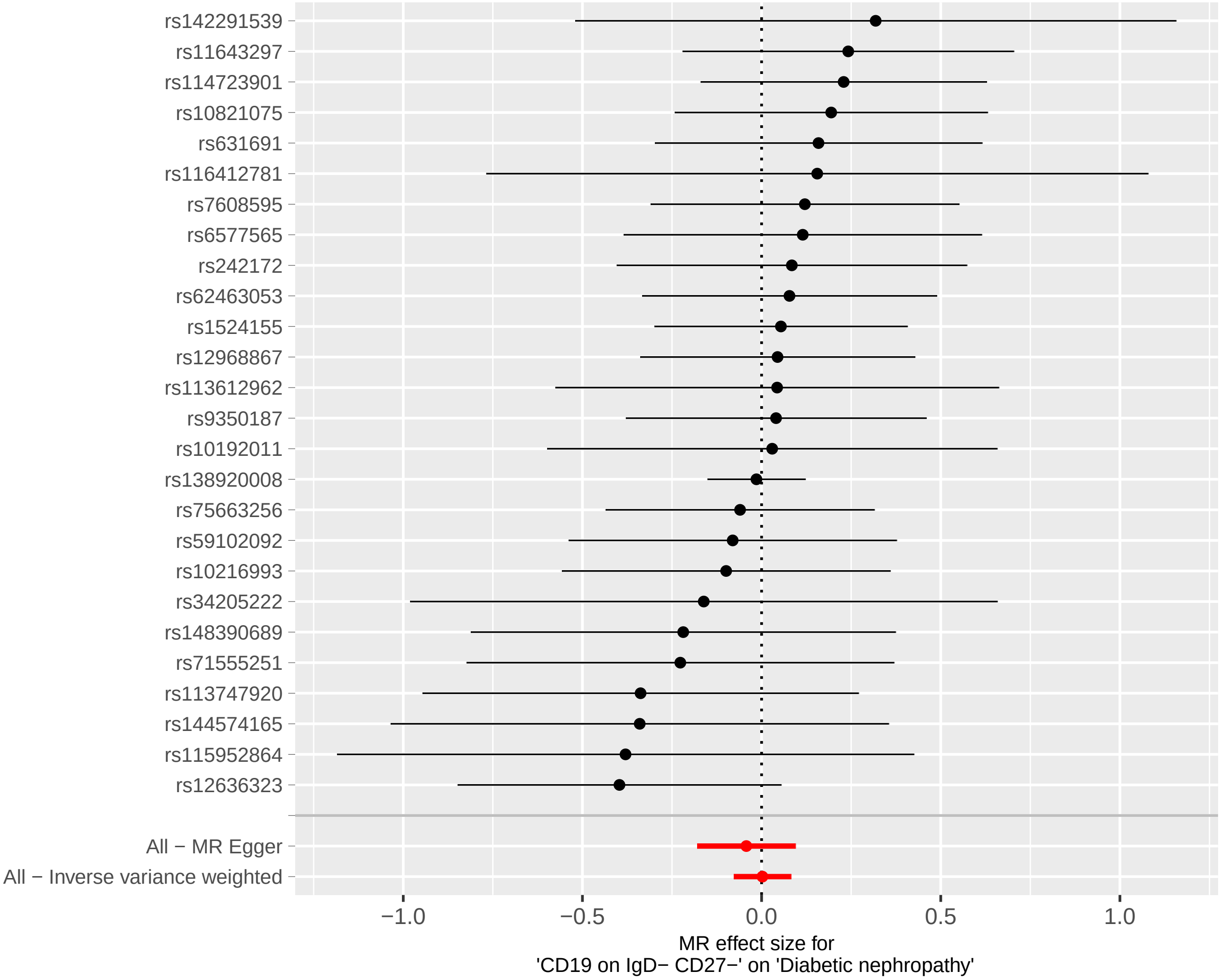
rs142291539
rs11643297
rs114723901
rs10821075
rs631691
rs116412781
rs7608595
rs6577565
rs242172
rs62463053
rs1524155
rs12968867
rs113612962
rs9350187
rs10192011
rs138920008
rs75663256
rs59102092
rs10216993
rs34205222
rs148390689
rs71555251
rs113747920
rs144574165
rs115952864
rs12636323
All − MR Egger
All − Inverse variance weighted
−1.0
−0.5
0.0
0.5
1.0
MR effect size for
'CD19 on IgD− CD27−' on 'Diabetic nephropathy'

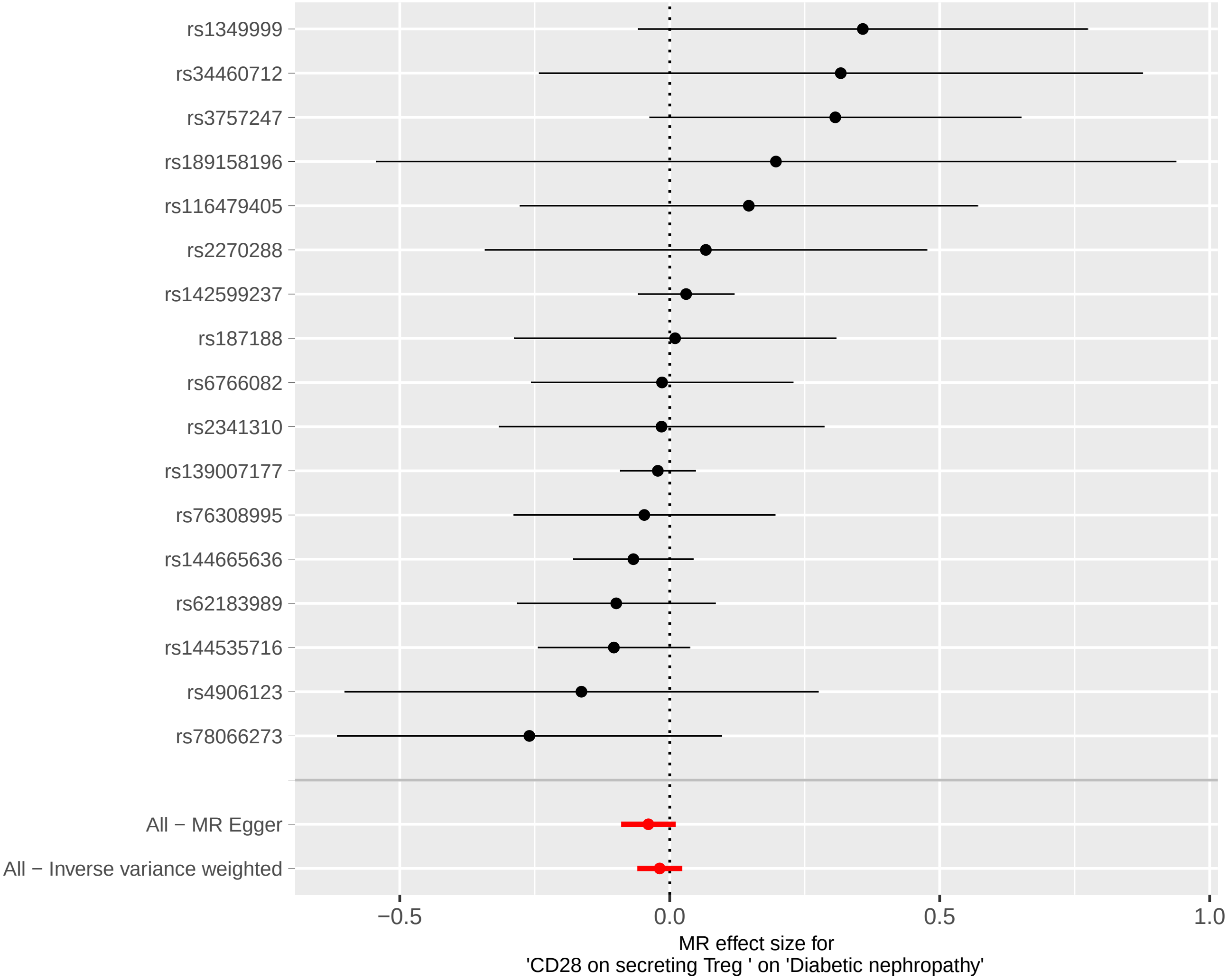

rs1349999
rs34460712
rs3757247
rs189158196
rs116479405
rs2270288
rs142599237
rs187188
rs6766082
rs2341310
rs139007177
rs76308995
rs144665636
rs62183989
rs144535716
rs4906123
rs78066273
All − MR Egger
All − Inverse variance weighted
−0.5
0.0
0.5
1.0
MR effect size for
'CD28 on secreting Treg ' on 'Diabetic nephropathy'

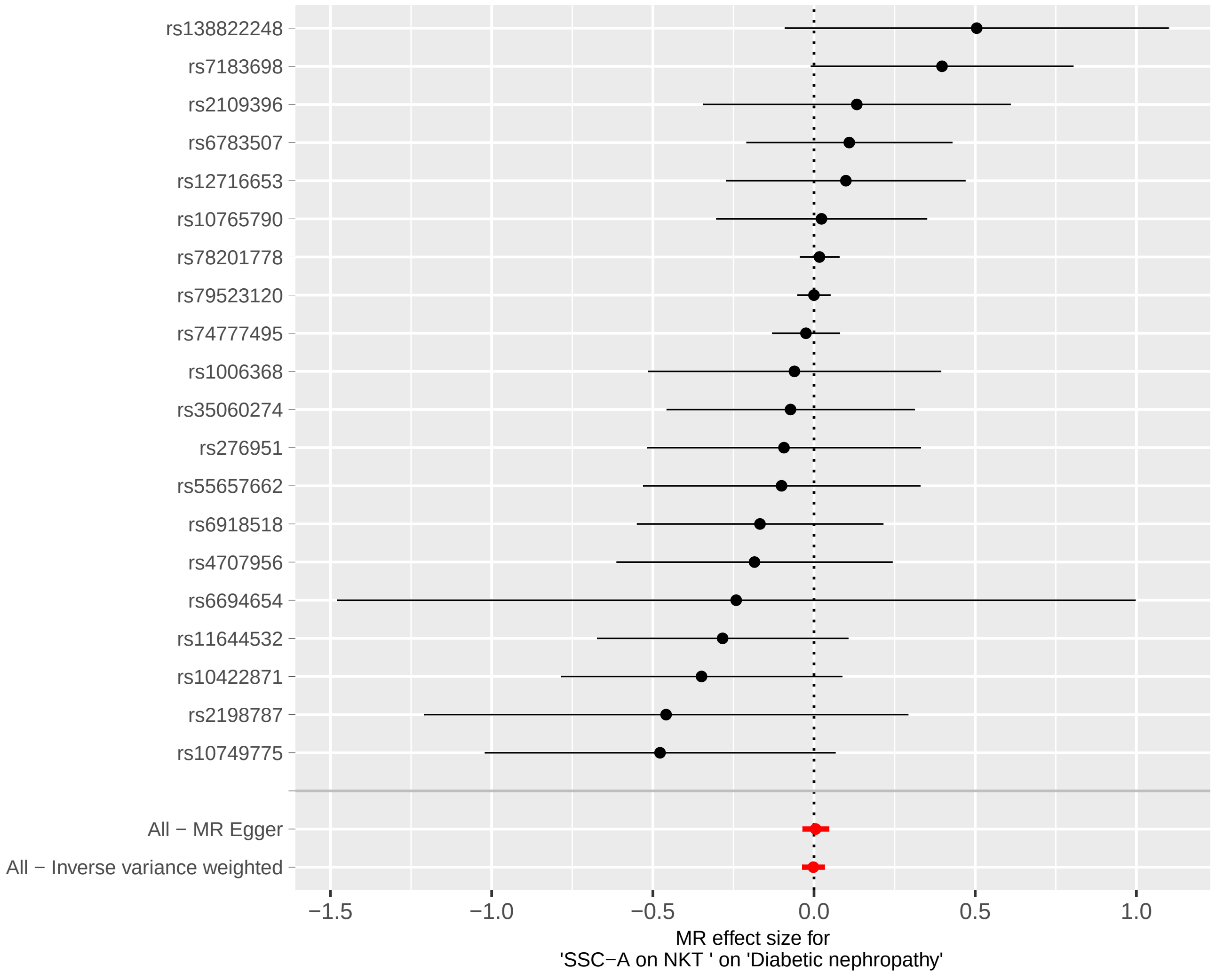
rs138822248
rs7183698
rs2109396
rs6783507
rs12716653
rs10765790
rs78201778
rs79523120
rs74777495
rs1006368
rs35060274
rs276951
rs55657662
rs6918518
rs4707956
rs6694654
rs11644532
rs10422871
rs2198787
rs10749775
All − MR Egger
All − Inverse variance weighted
−1.5
−1.0
−0.5
0.0
0.5
1.0
MR effect size for
'SSC−A on NKT ' on 'Diabetic nephropathy'

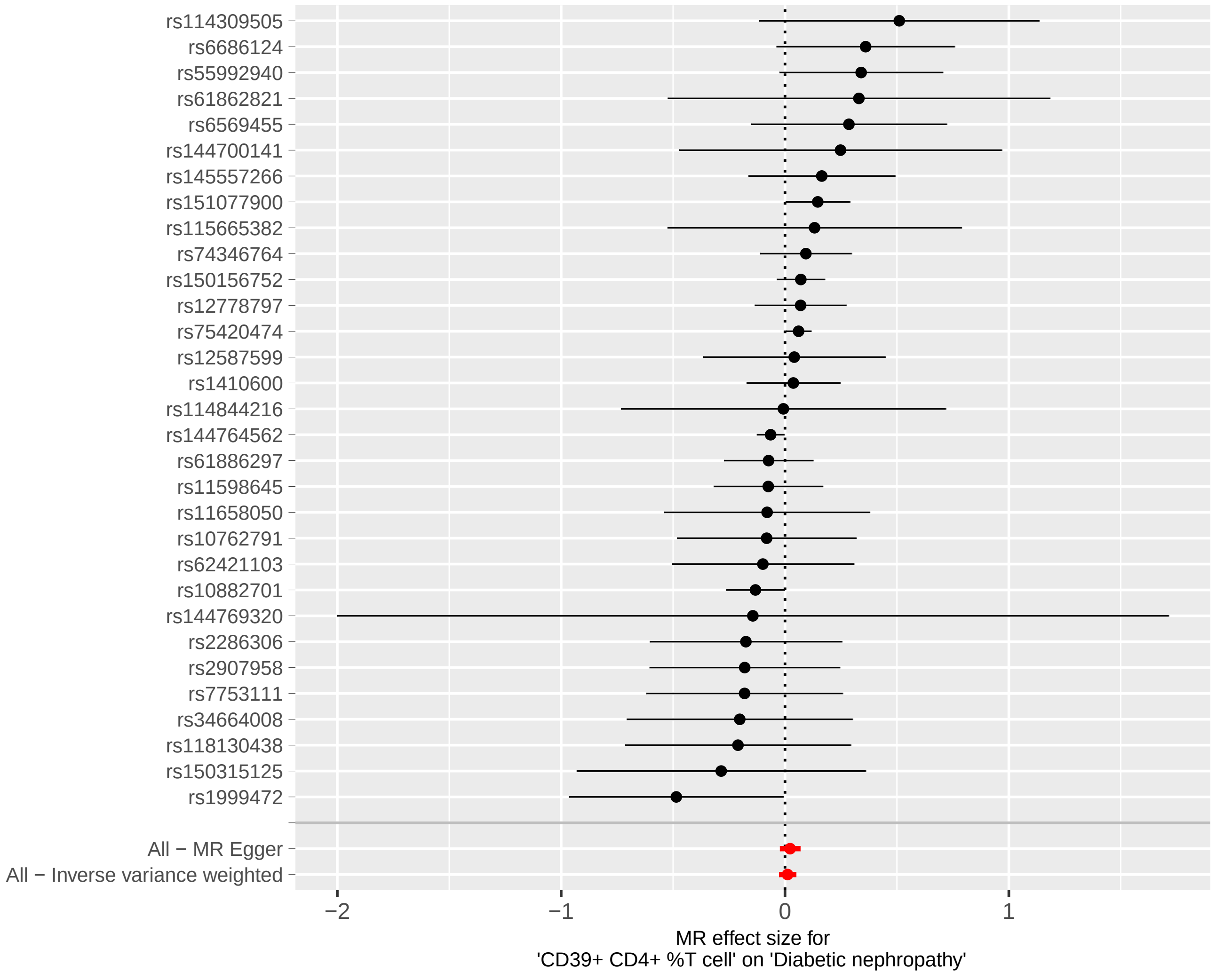
rs114309505
rs6686124
rs55992940
rs61862821
rs6569455
rs144700141
rs145557266
rs151077900
rs115665382
rs74346764
rs150156752
rs12778797
rs75420474
rs12587599
rs1410600
rs114844216
rs144764562
rs61886297
rs11598645
rs11658050
rs10762791
rs62421103
rs10882701
rs144769320
rs2286306
rs2907958
rs7753111
rs34664008
rs118130438
rs150315125
rs1999472
All − MR Egger
All − Inverse variance weighted
−2
−1
0
1
MR effect size for
'CD39+ CD4+ %T cell' on 'Diabetic nephropathy'

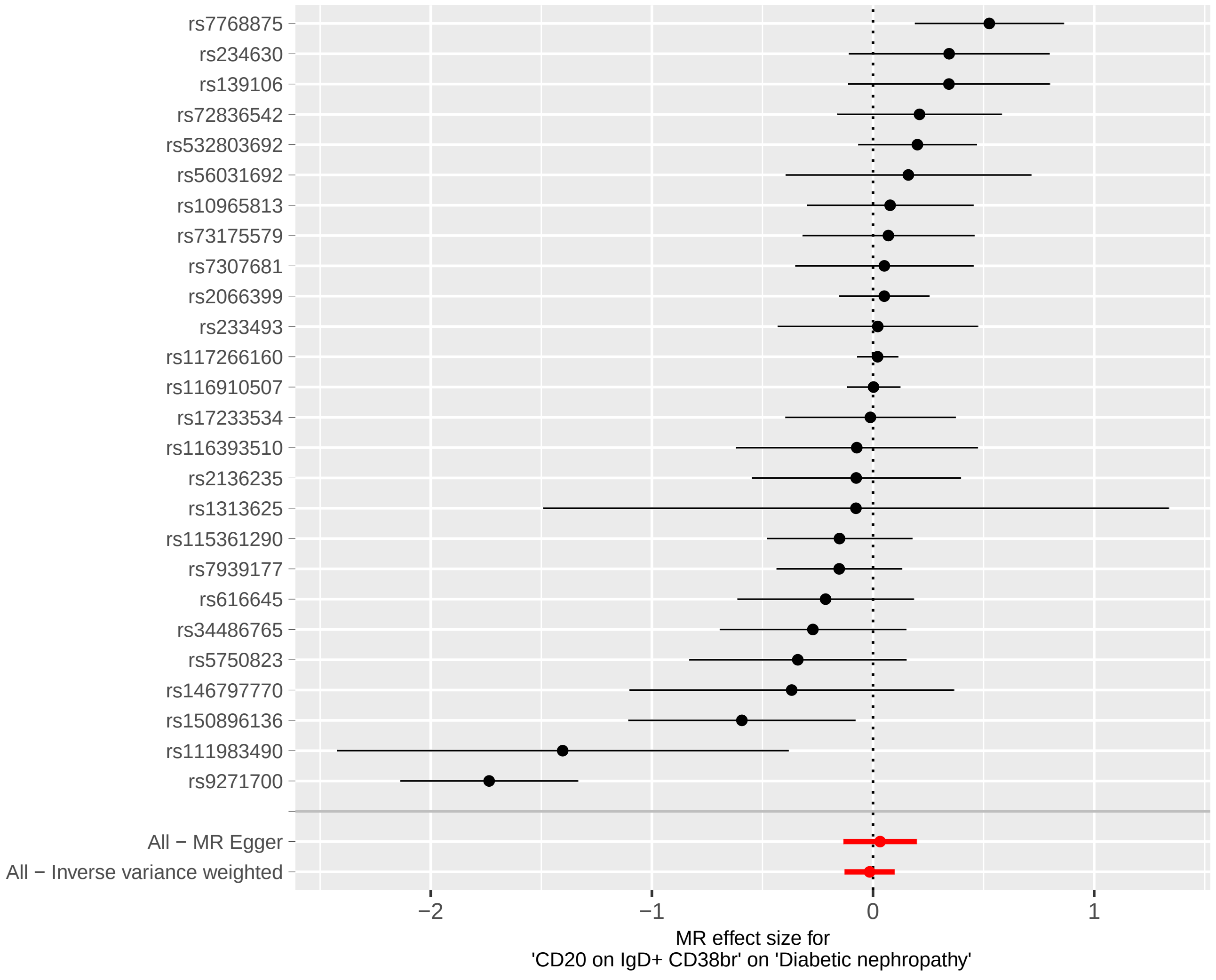
rs7768875
rs234630
rs139106
rs72836542
rs532803692
rs56031692
rs10965813
rs73175579
rs7307681
rs2066399
rs233493
rs117266160
rs116910507
rs17233534
rs116393510
rs2136235
rs1313625
rs115361290
rs7939177
rs616645
rs34486765
rs5750823
rs146797770
rs150896136
rs111983490
rs9271700
All − MR Egger
All − Inverse variance weighted
−2
−1
0
1
MR effect size for
'CD20 on IgD+ CD38br' on 'Diabetic nephropathy'

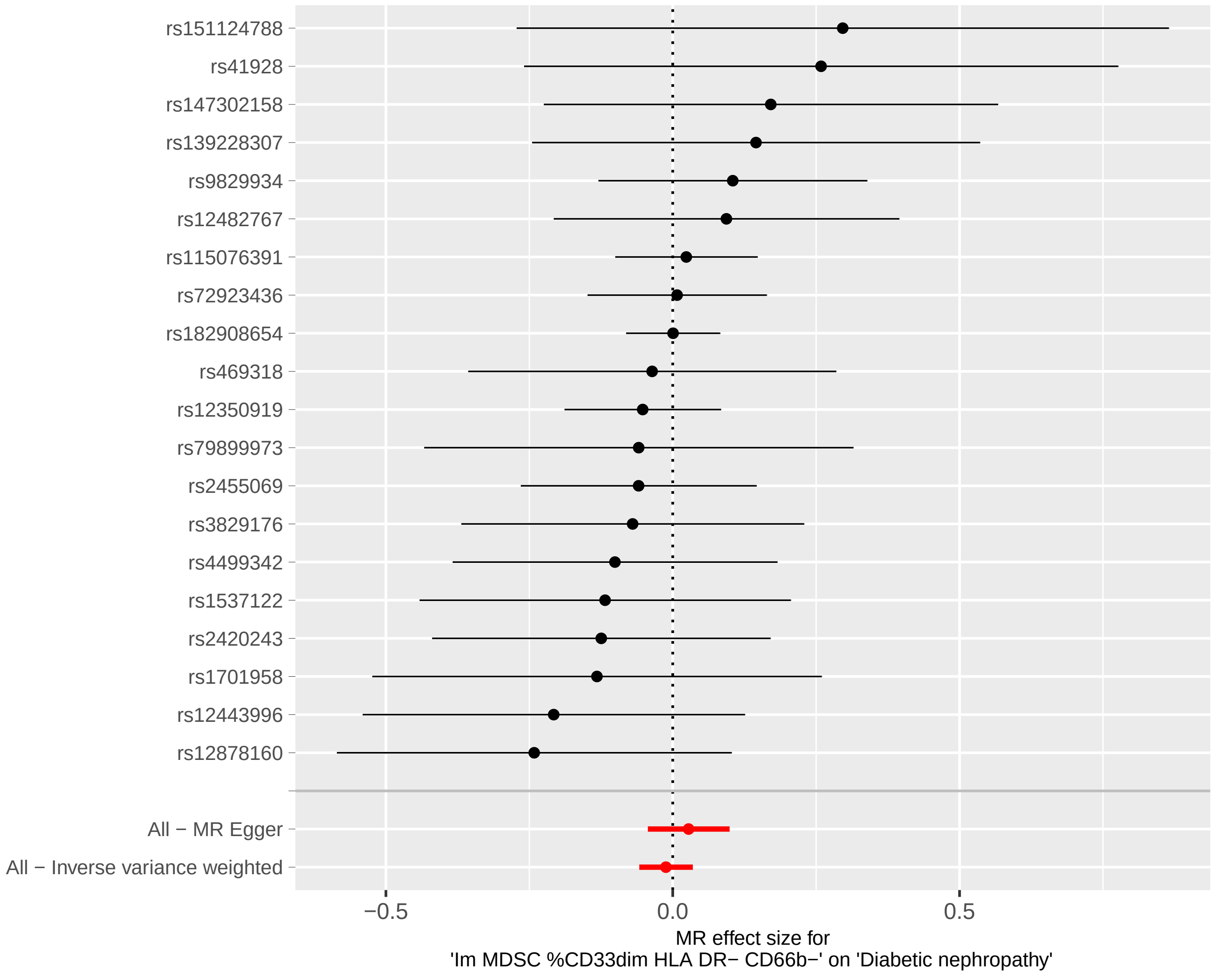
rs151124788
rs41928
rs147302158
rs139228307
rs9829934
rs12482767
rs115076391
rs72923436
rs182908654
rs469318
rs12350919
rs79899973
rs2455069
rs3829176
rs4499342
rs1537122
rs2420243
rs1701958
rs12443996
rs12878160
All − MR Egger
All − Inverse variance weighted
−0.5
0.0
0.5
MR effect size for
'Im MDSC %CD33dim HLA DR− CD66b−' on 'Diabetic nephropathy'

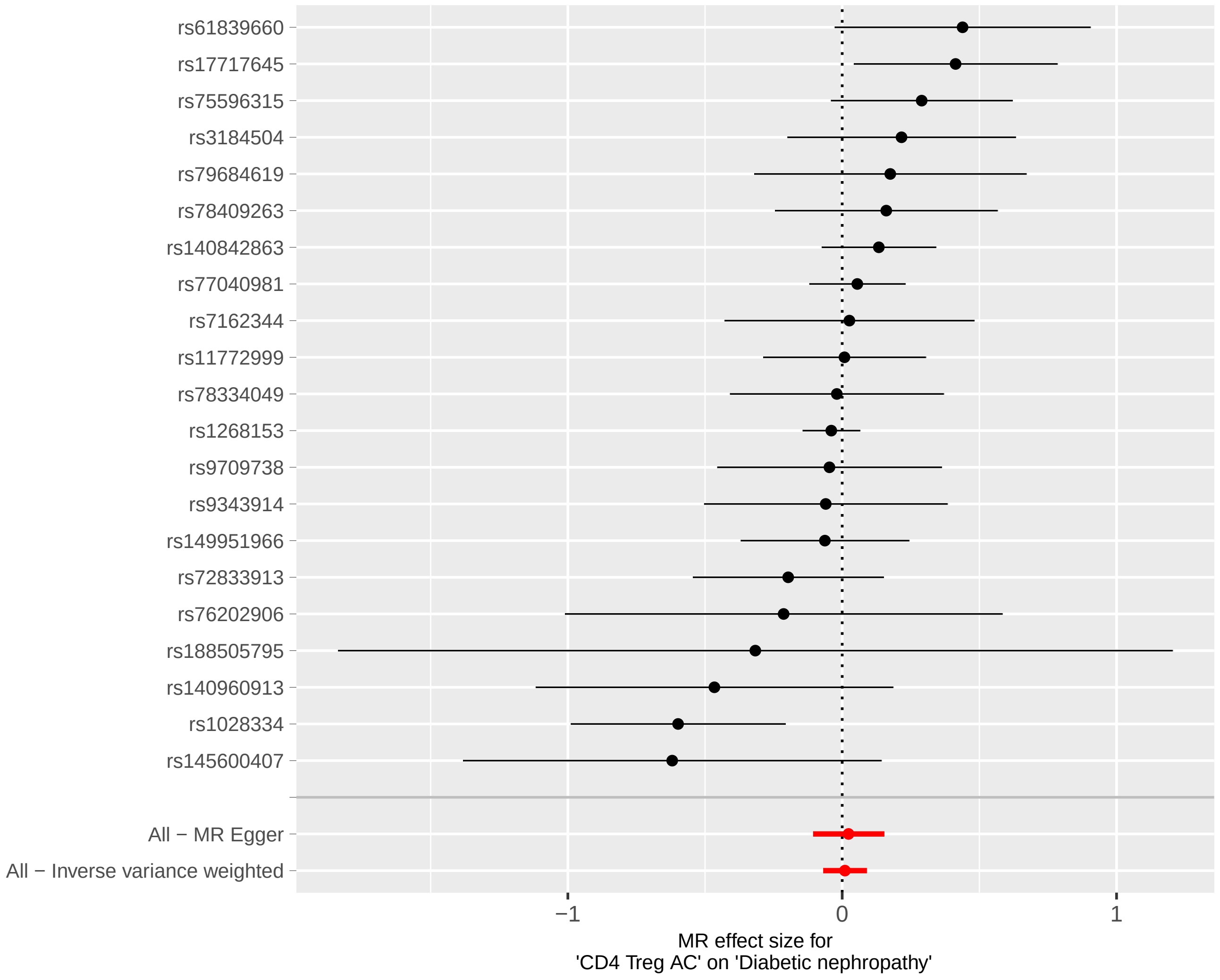
rs61839660
rs17717645
rs75596315
rs3184504
rs79684619
rs78409263
rs140842863
rs77040981
rs7162344
rs11772999
rs78334049
rs1268153
rs9709738
rs9343914
rs149951966
rs72833913
rs76202906
rs188505795
rs140960913
rs1028334
rs145600407
All − MR Egger
All − Inverse variance weighted
−1
0
1
MR effect size for
'CD4 Treg AC' on 'Diabetic nephropathy'

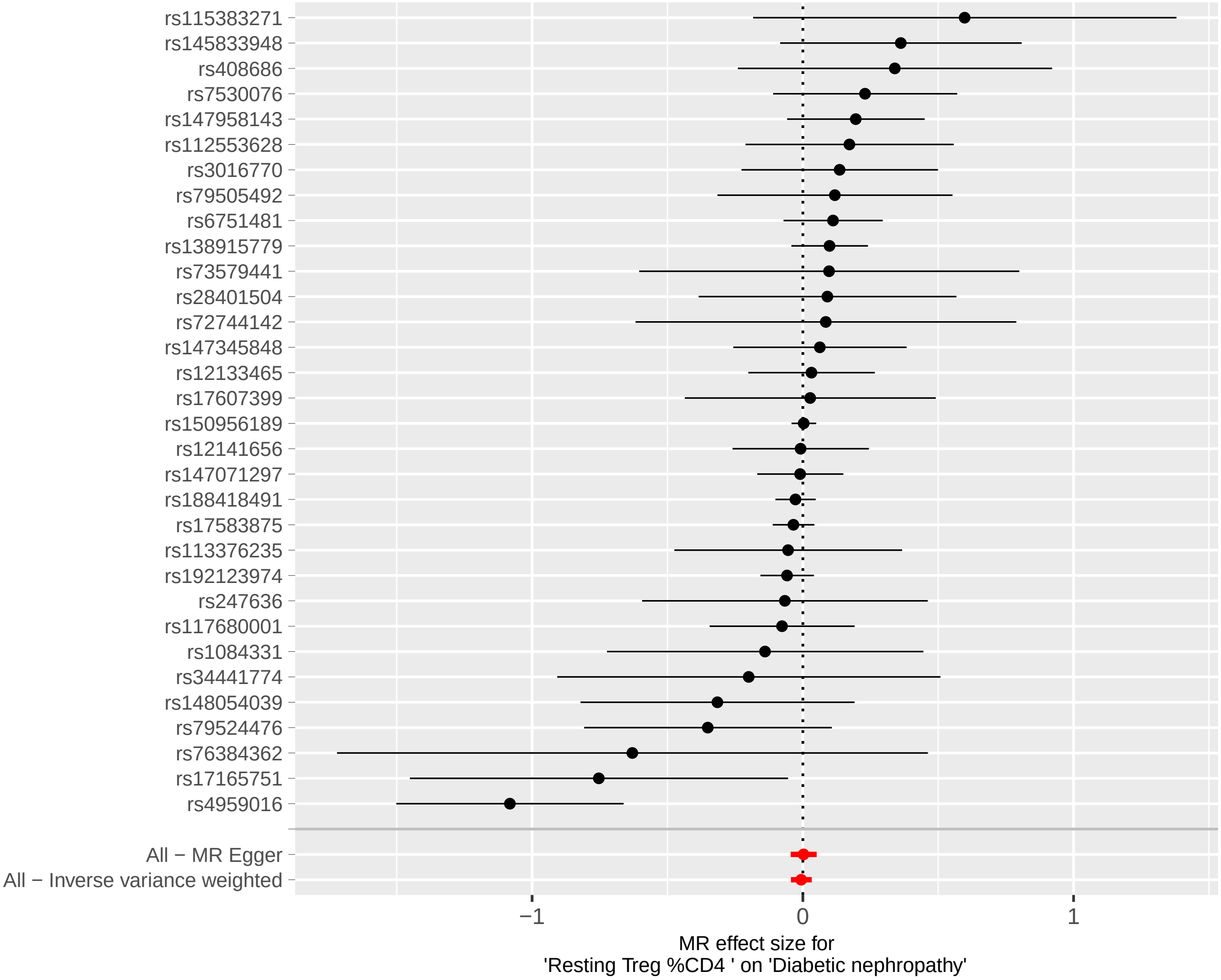
rs115383271
rs145833948
rs408686
rs7530076
rs147958143
rs112553628
rs3016770
rs79505492
rs6751481
rs138915779
rs73579441
rs28401504
rs72744142
rs147345848
rs12133465
rs17607399
rs150956189
rs12141656
rs147071297
rs188418491
rs17583875
rs113376235
rs192123974
rs247636
rs117680001
rs1084331
rs34441774
rs148054039
rs79524476
rs76384362
rs17165751
rs4959016
All − MR Egger
All − Inverse variance weighted
−1
0
1
MR effect size for
'Resting Treg %CD4 ' on 'Diabetic nephropathy'

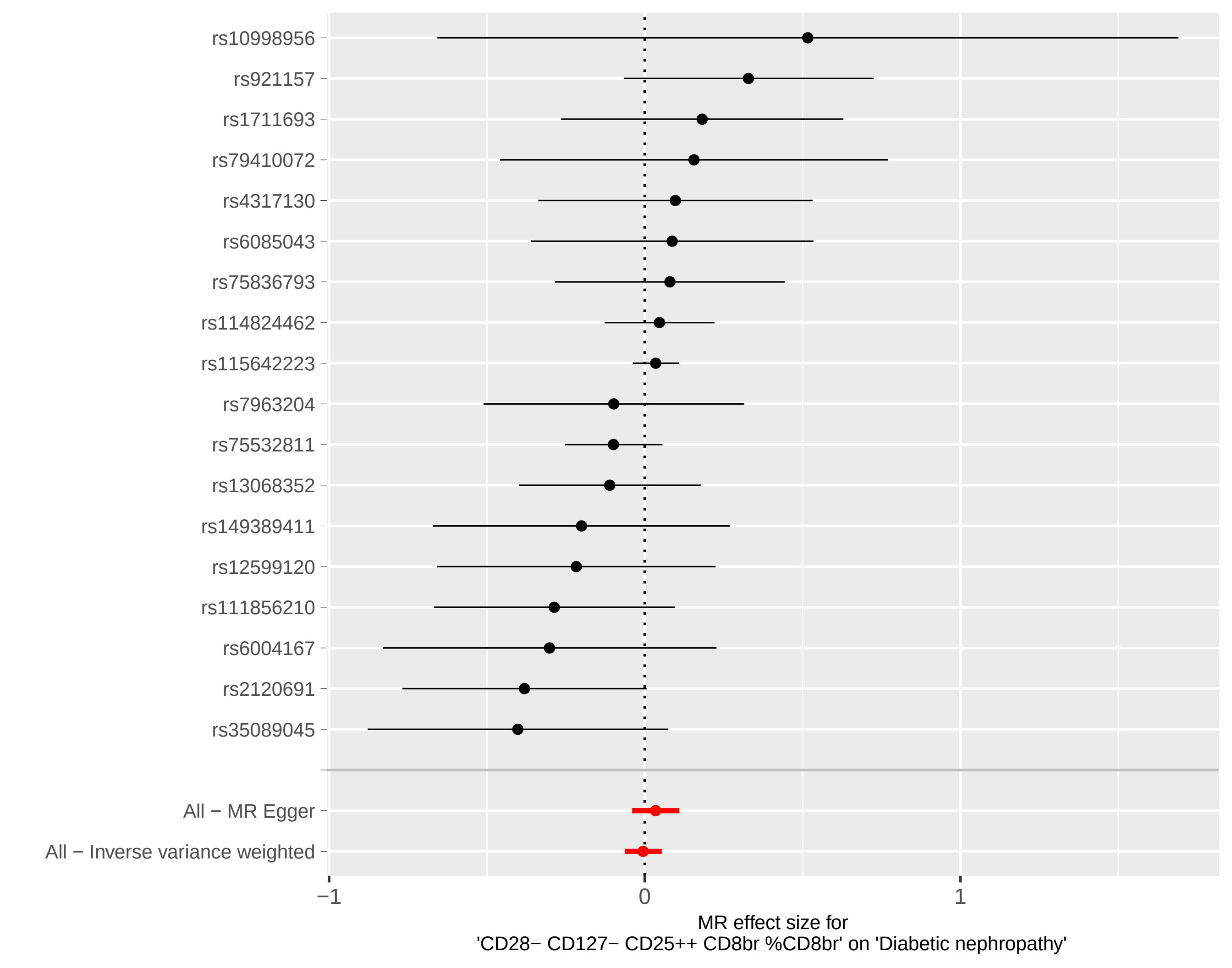
rs10998956
rs921157
rs1711693
rs79410072
rs4317130
rs6085043
rs75836793
rs114824462
rs115642223
rs7963204
rs75532811
rs13068352
rs149389411
rs12599120
rs111856210
rs6004167
rs2120691
rs35089045
All − MR Egger
All − Inverse variance weighted
−1
0
1
MR effect size for
'CD28− CD127− CD25++ CD8br %CD8br' on 'Diabetic nephropathy'

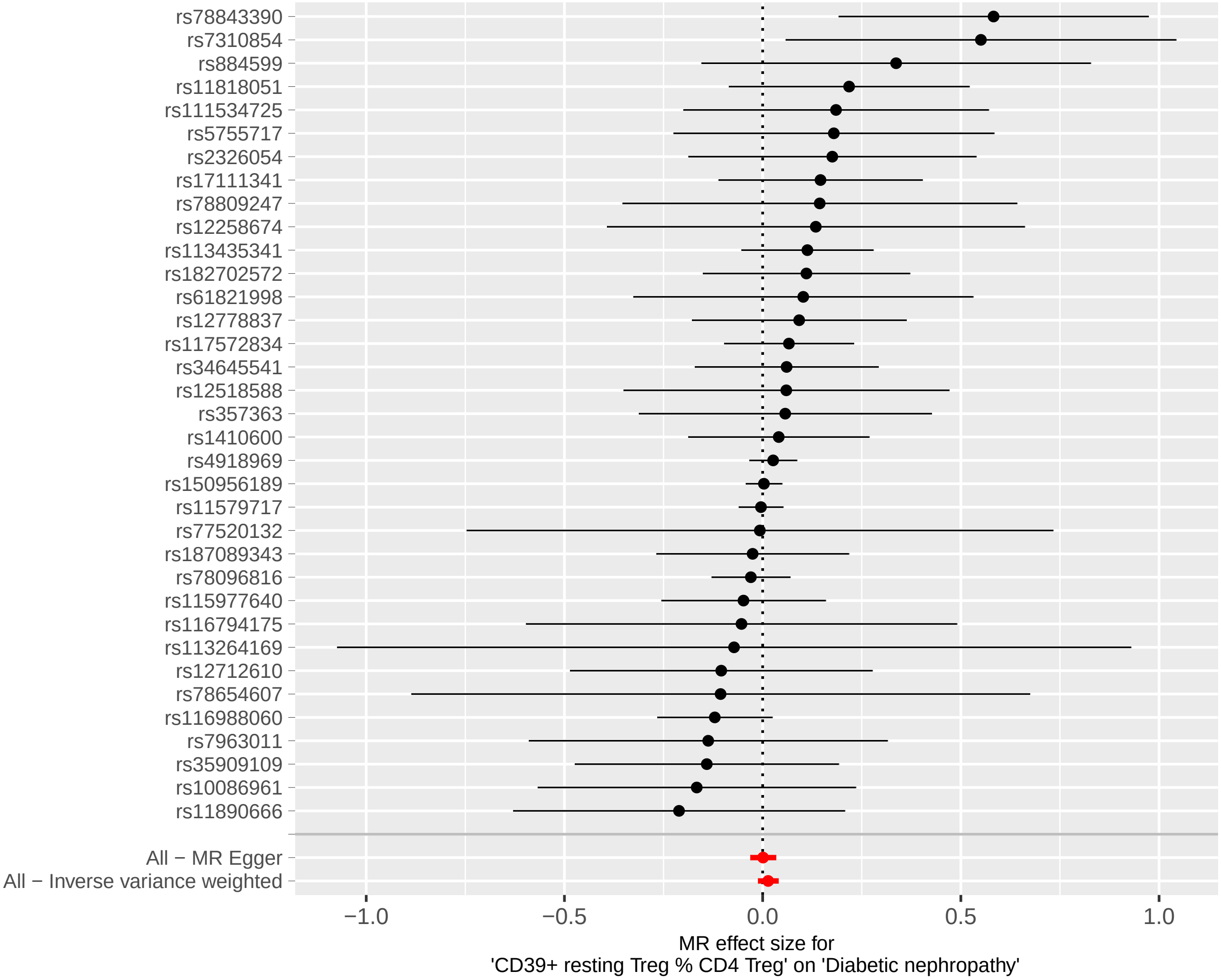

rs78843390
rs7310854
rs884599
rs11818051
rs111534725
rs5755717
rs2326054
rs17111341
rs78809247
rs12258674
rs113435341
rs182702572
rs61821998
rs12778837
rs117572834
rs34645541
rs12518588
rs357363
rs1410600
rs4918969
rs150956189
rs11579717
rs77520132
rs187089343
rs78096816
rs115977640
rs116794175
rs113264169
rs12712610
rs78654607
rs116988060
rs7963011
rs35909109
rs10086961
rs11890666
All − MR Egger
All − Inverse variance weighted
−1.0
−0.5
0.0
0.5
1.0
MR effect size for
'CD39+ resting Treg % CD4 Treg' on 'Diabetic nephropathy'

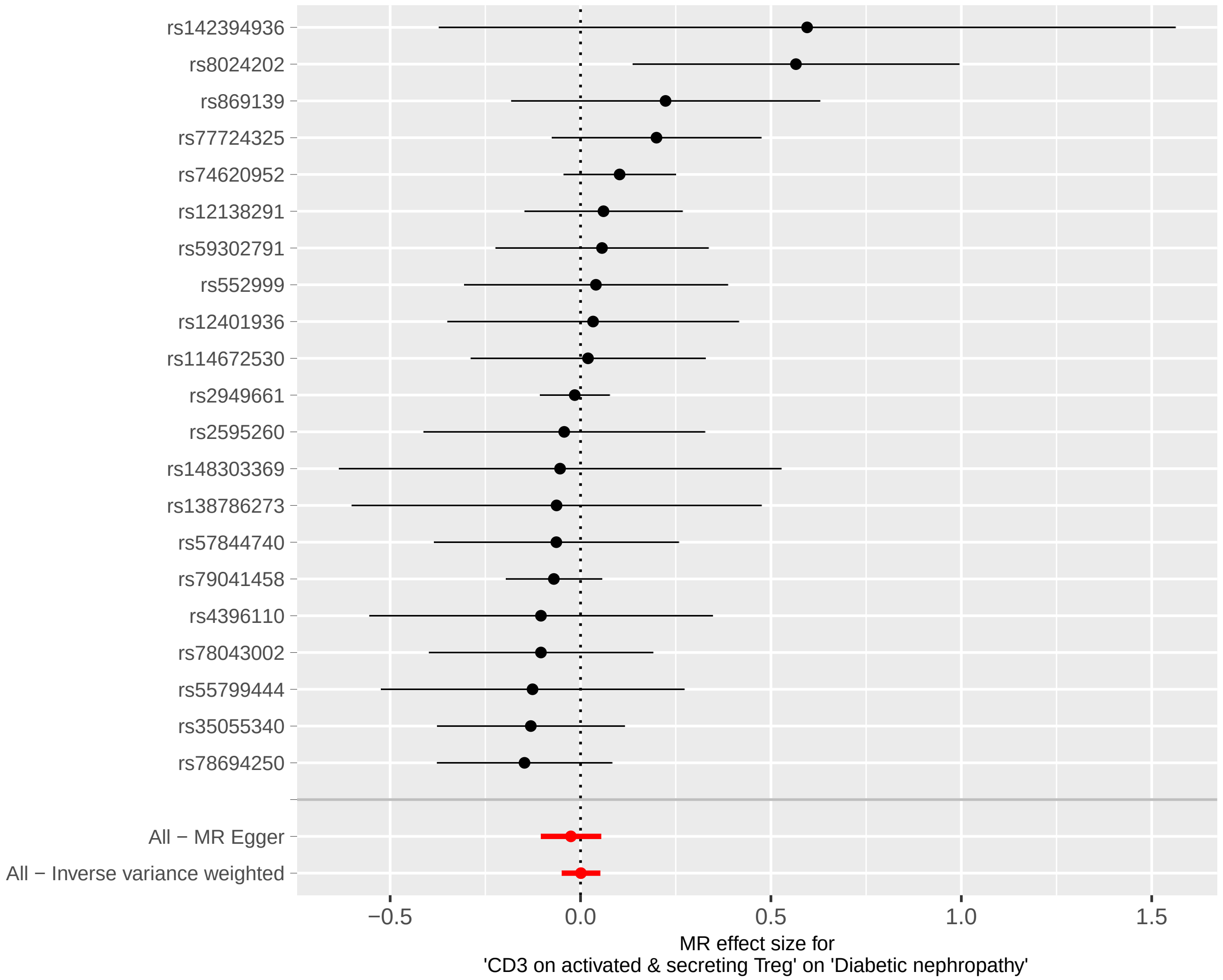
rs142394936
rs8024202
rs869139
rs77724325
rs74620952
rs12138291
rs59302791
rs552999
rs12401936
rs114672530
rs2949661
rs2595260
rs148303369
rs138786273
rs57844740
rs79041458
rs4396110
rs78043002
rs55799444
rs35055340
rs78694250
All − MR Egger
All − Inverse variance weighted
−0.5
0.0
0.5
1.0
1.5
MR effect size for
'CD3 on activated & secreting Treg' on 'Diabetic nephropathy'

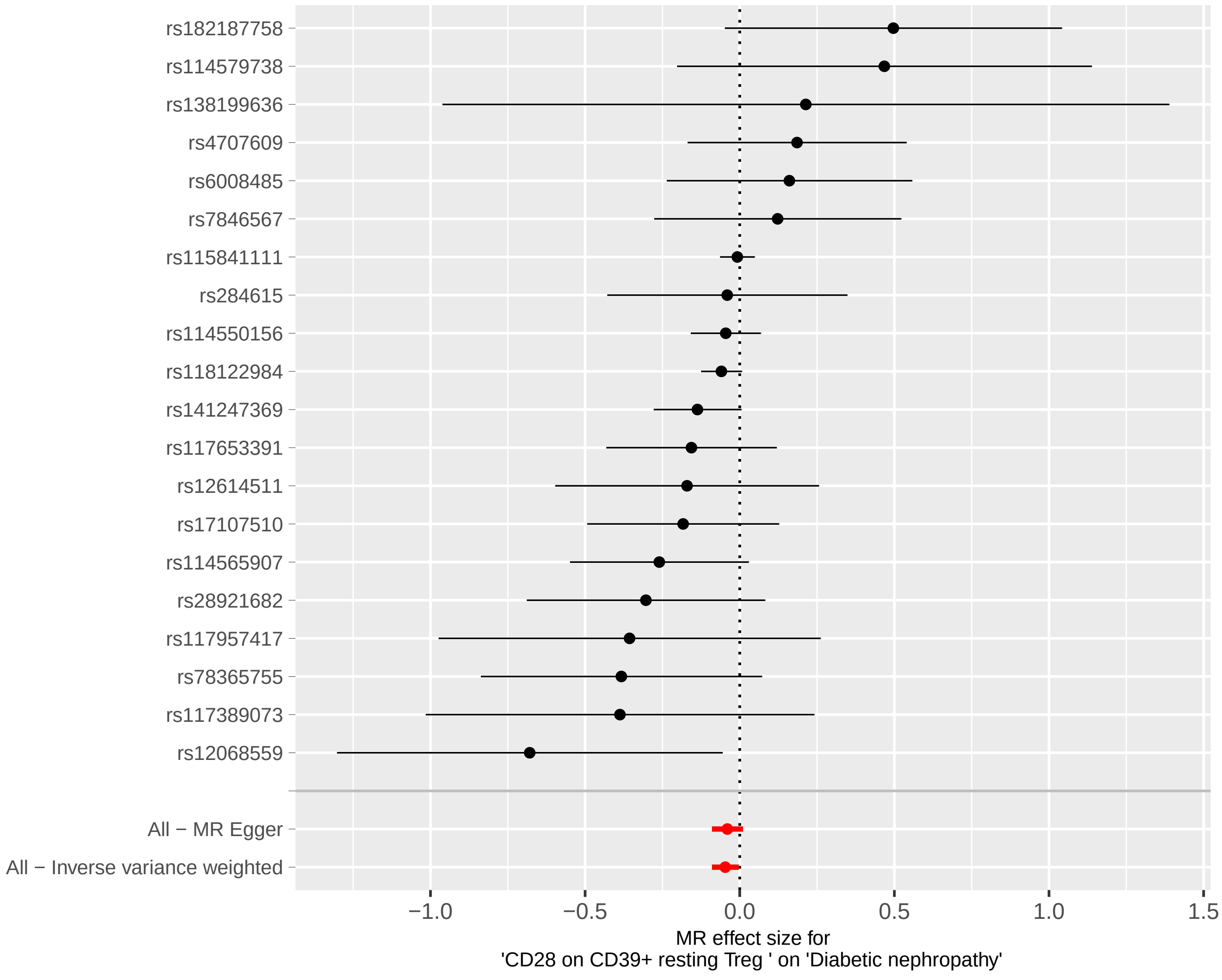

rs182187758
rs114579738
rs138199636
rs4707609
rs6008485
rs7846567
rs115841111
rs284615
rs114550156
rs118122984
rs141247369
rs117653391
rs12614511
rs17107510
rs114565907
rs28921682
rs117957417
rs78365755
rs117389073
rs12068559
All − MR Egger
All − Inverse variance weighted
−1.0
−0.5
0.0
0.5
1.0
1.5
MR effect size for
'CD28 on CD39+ resting Treg ' on 'Diabetic nephropathy'

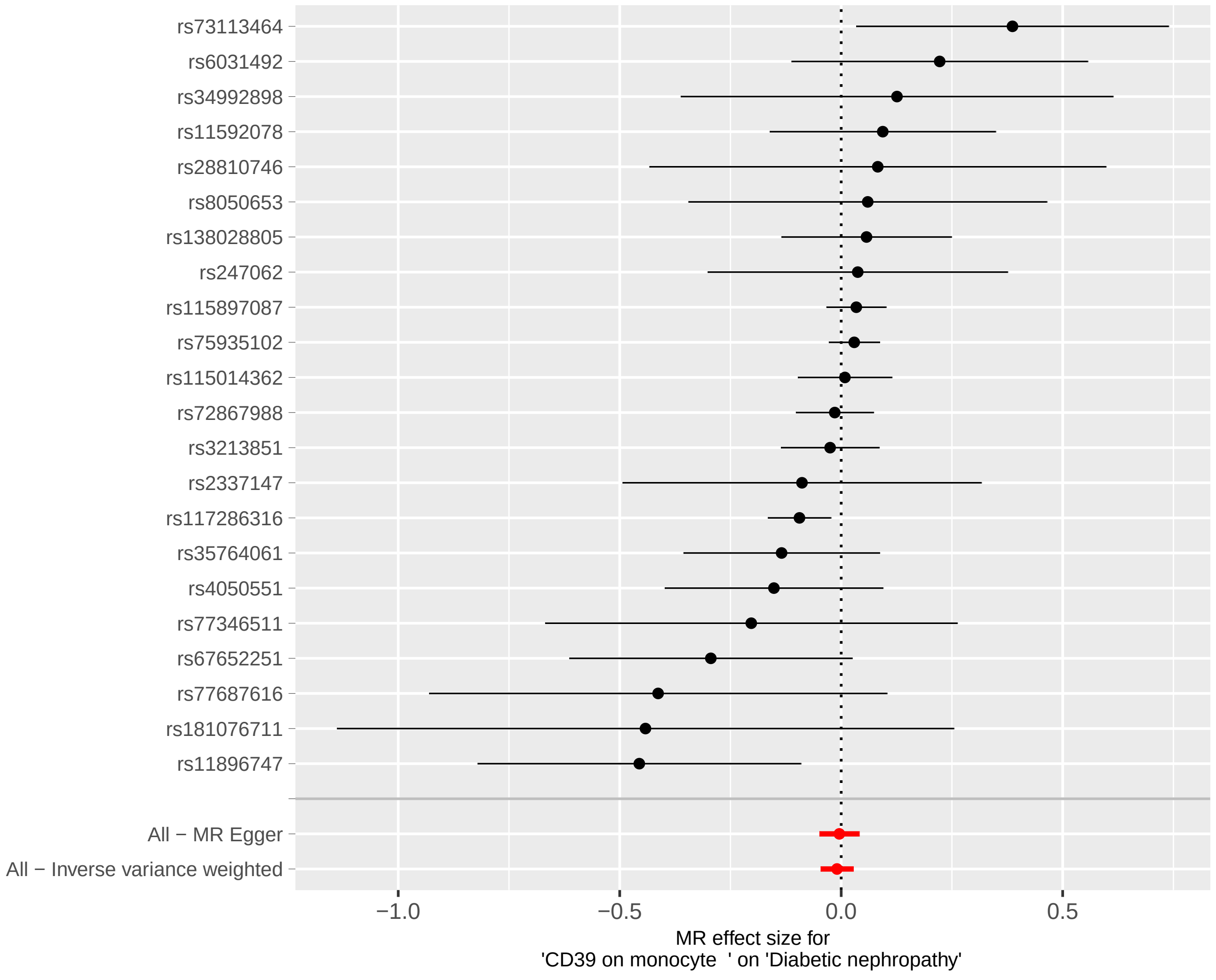
rs73113464
rs6031492
rs34992898
rs11592078
rs28810746
rs8050653
rs138028805
rs247062
rs115897087
rs75935102
rs115014362
rs72867988
rs3213851
rs2337147
rs117286316
rs35764061
rs4050551
rs77346511
rs67652251
rs77687616
rs181076711
rs11896747
All − MR Egger
All − Inverse variance weighted
−1.0
−0.5
0.0
0.5
MR effect size for
'CD39 on monocyte ' on 'Diabetic nephropathy'

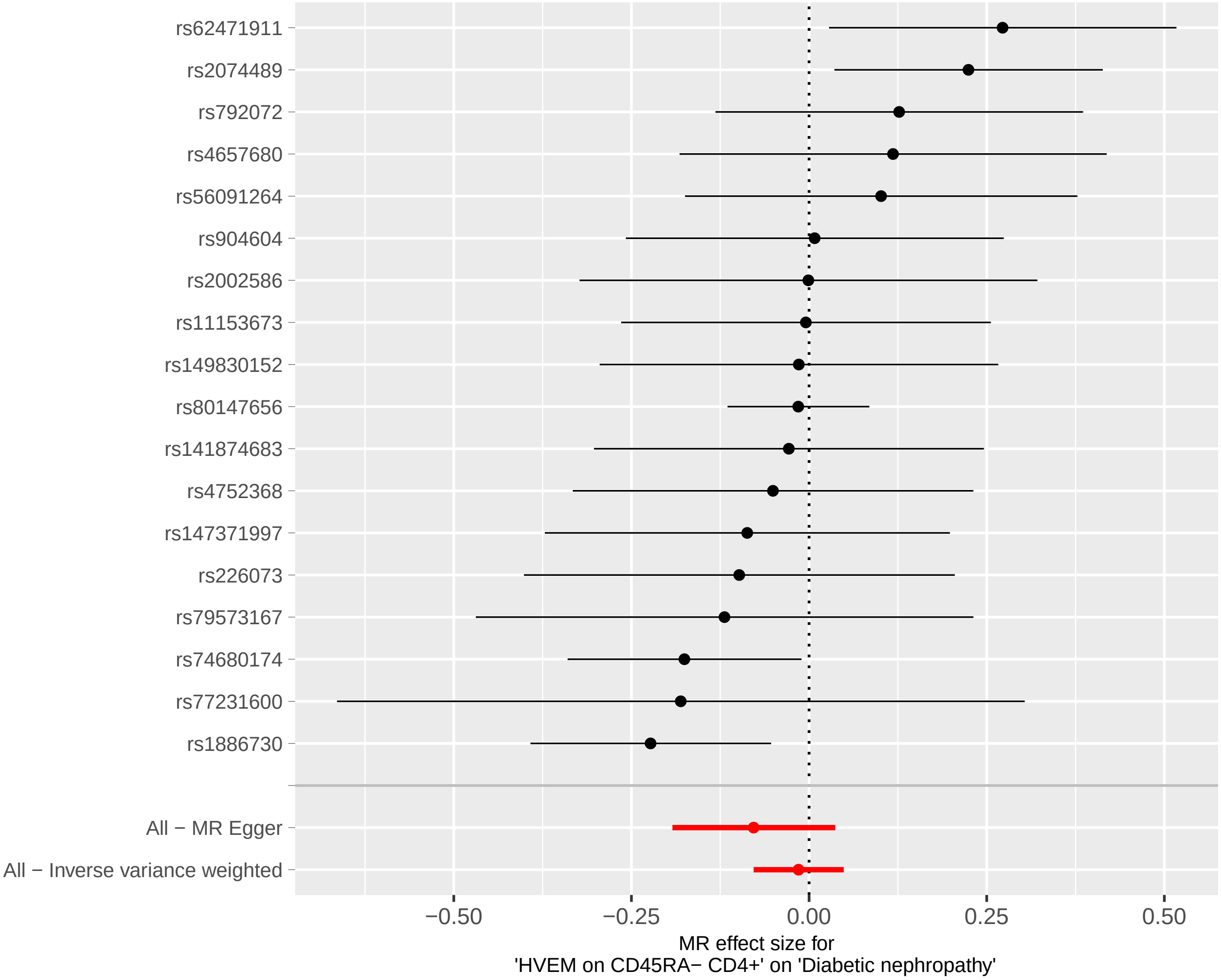
rs62471911
rs2074489
rs792072
rs4657680
rs56091264
rs904604
rs2002586
rs11153673
rs149830152
rs80147656
rs141874683
rs4752368
rs147371997
rs226073
rs79573167
rs74680174
rs77231600
rs1886730
All − MR Egger
All − Inverse variance weighted
−0.50
−0.25
0.00
0.25
0.50
MR effect size for
'HVEM on CD45RA− CD4+' on 'Diabetic nephropathy'

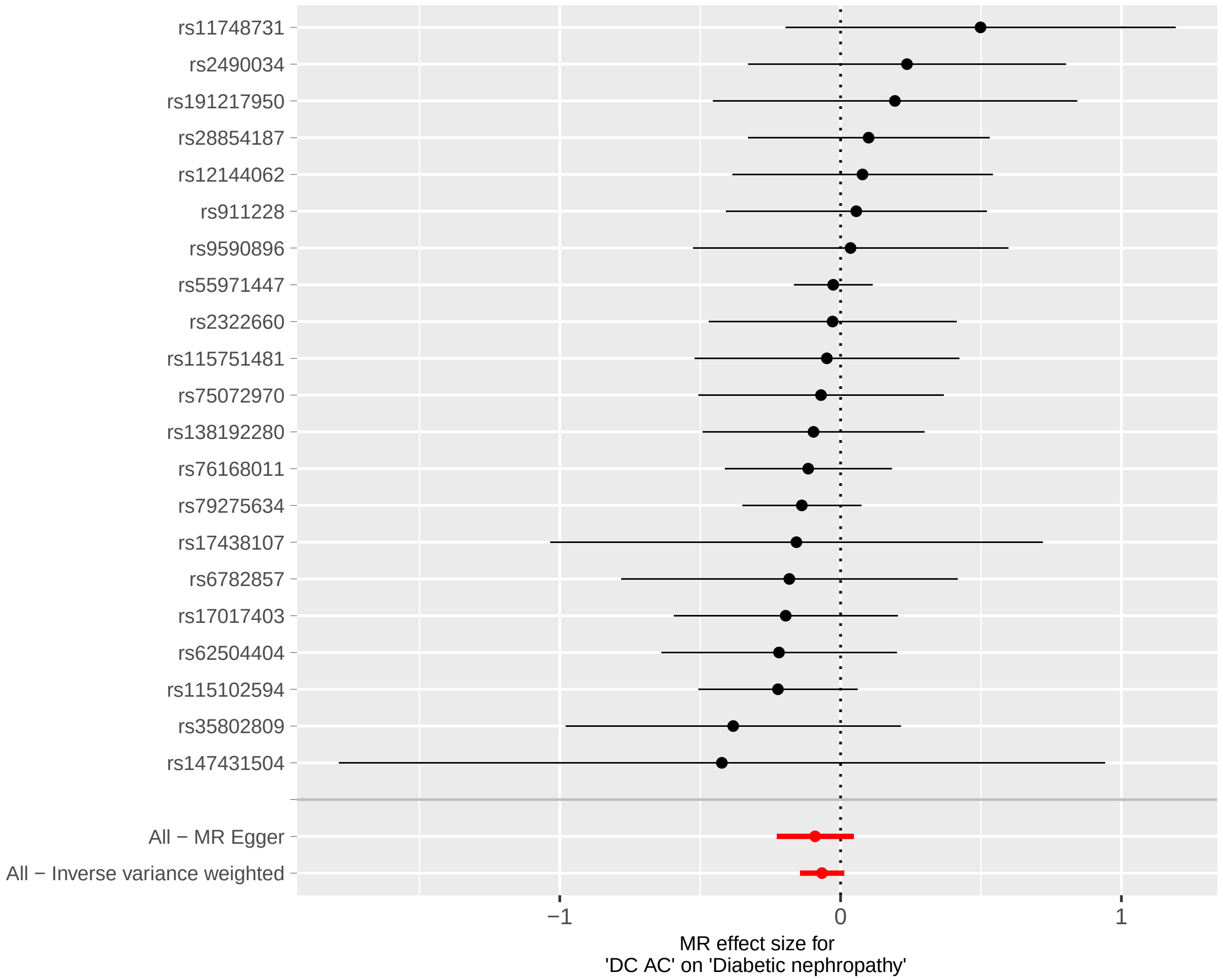
rs11748731
rs2490034
rs191217950
rs28854187
rs12144062
rs911228
rs9590896
rs55971447
rs2322660
rs115751481
rs75072970
rs138192280
rs76168011
rs79275634
rs17438107
rs6782857
rs17017403
rs62504404
rs115102594
rs35802809
rs147431504
All − MR Egger
All − Inverse variance weighted
−1
0
1
MR effect size for
'DC AC' on 'Diabetic nephropathy'

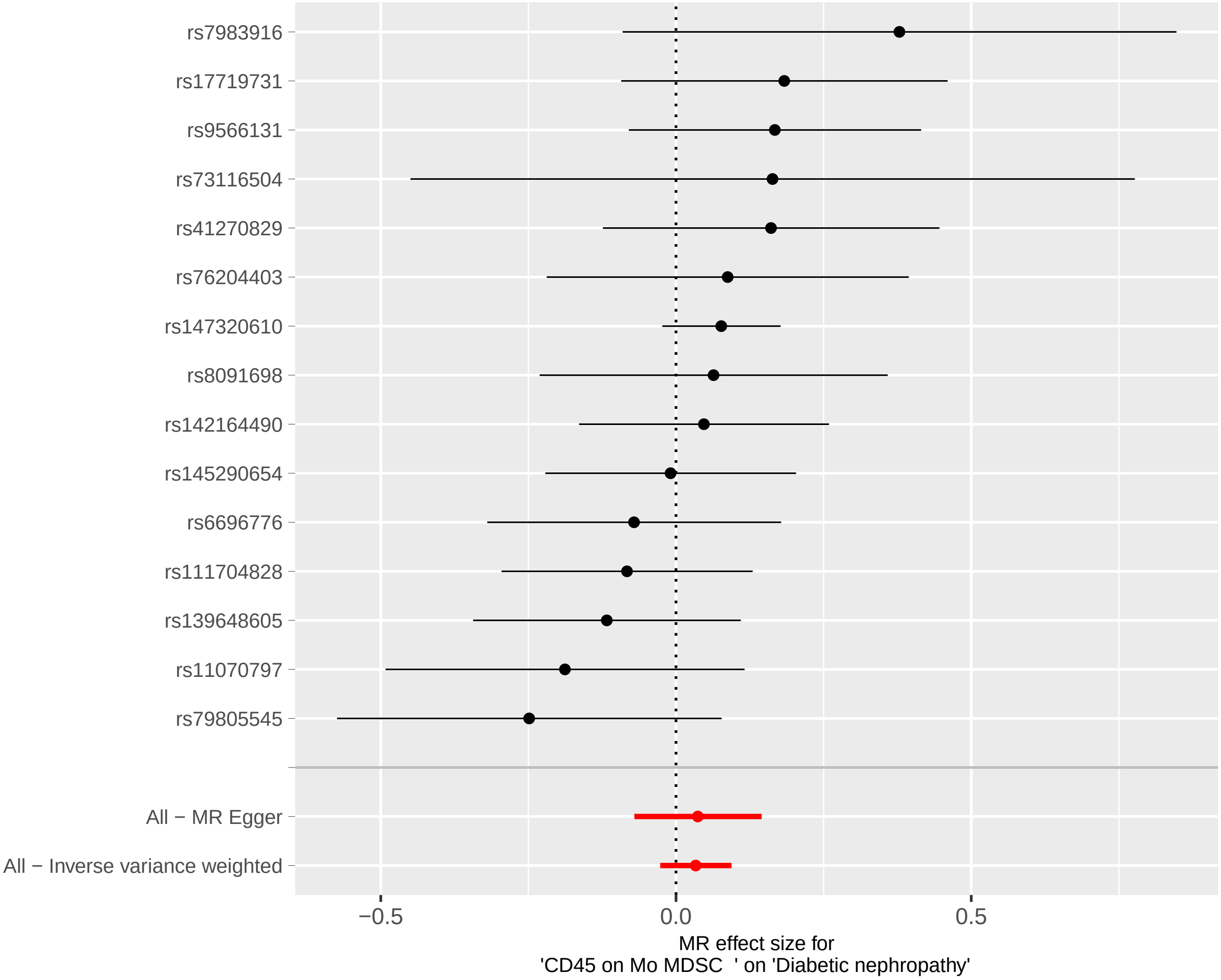

rs7983916
rs17719731
rs9566131
rs73116504
rs41270829
rs76204403
rs147320610
rs8091698
rs142164490
rs145290654
rs6696776
rs111704828
rs139648605
rs11070797
rs79805545
All − MR Egger
All − Inverse variance weighted
−0.5
0.0
0.5
MR effect size for
'CD45 on Mo MDSC ' on 'Diabetic nephropathy'

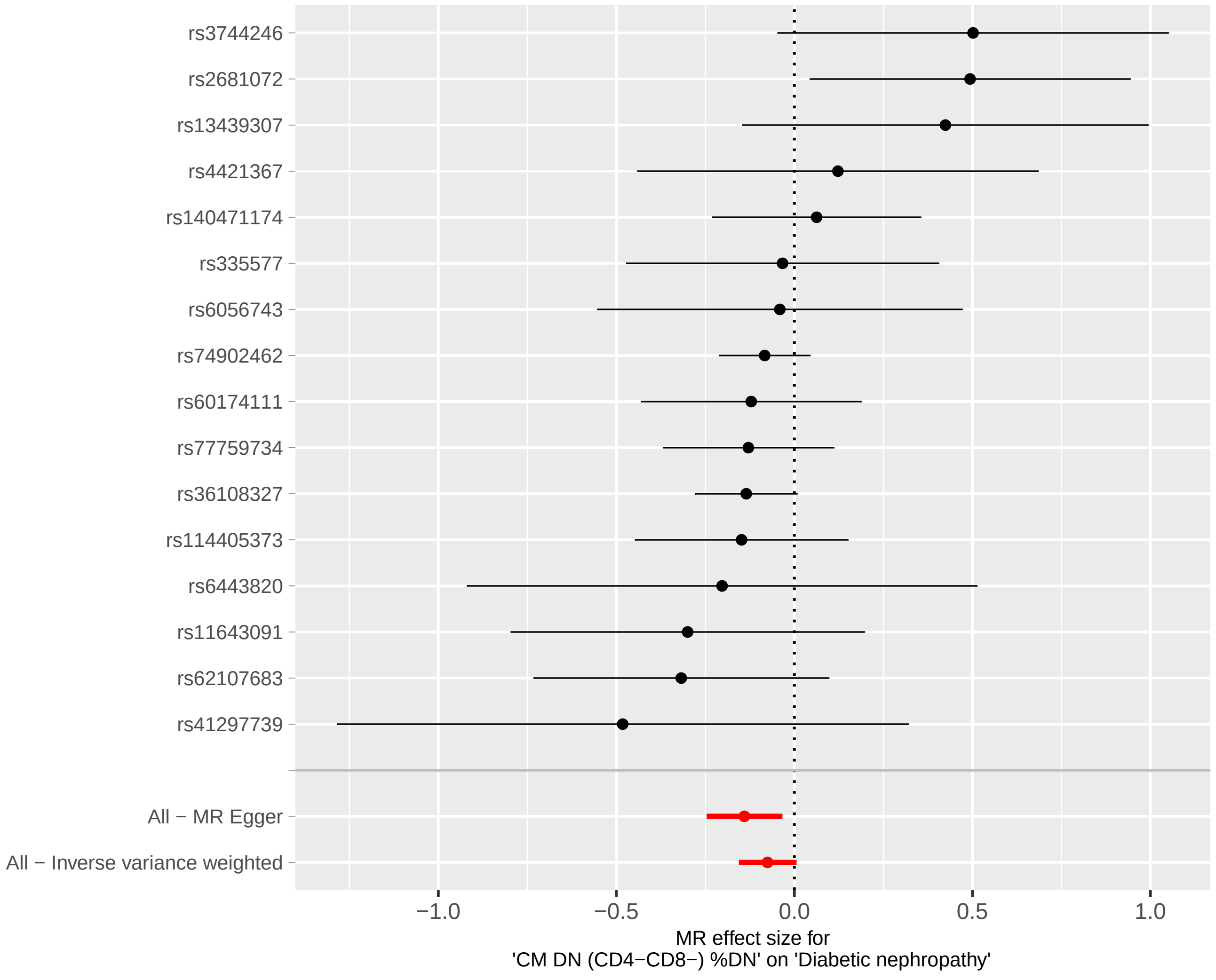
rs3744246
rs2681072
rs13439307
rs4421367
rs140471174
rs335577
rs6056743
rs74902462
rs60174111
rs77759734
rs36108327
rs114405373
rs6443820
rs11643091
rs62107683
rs41297739
All − MR Egger
All − Inverse variance weighted
−1.0
−0.5
0.0
0.5
1.0
MR effect size for
'CM DN (CD4−CD8−) %DN' on 'Diabetic nephropathy'

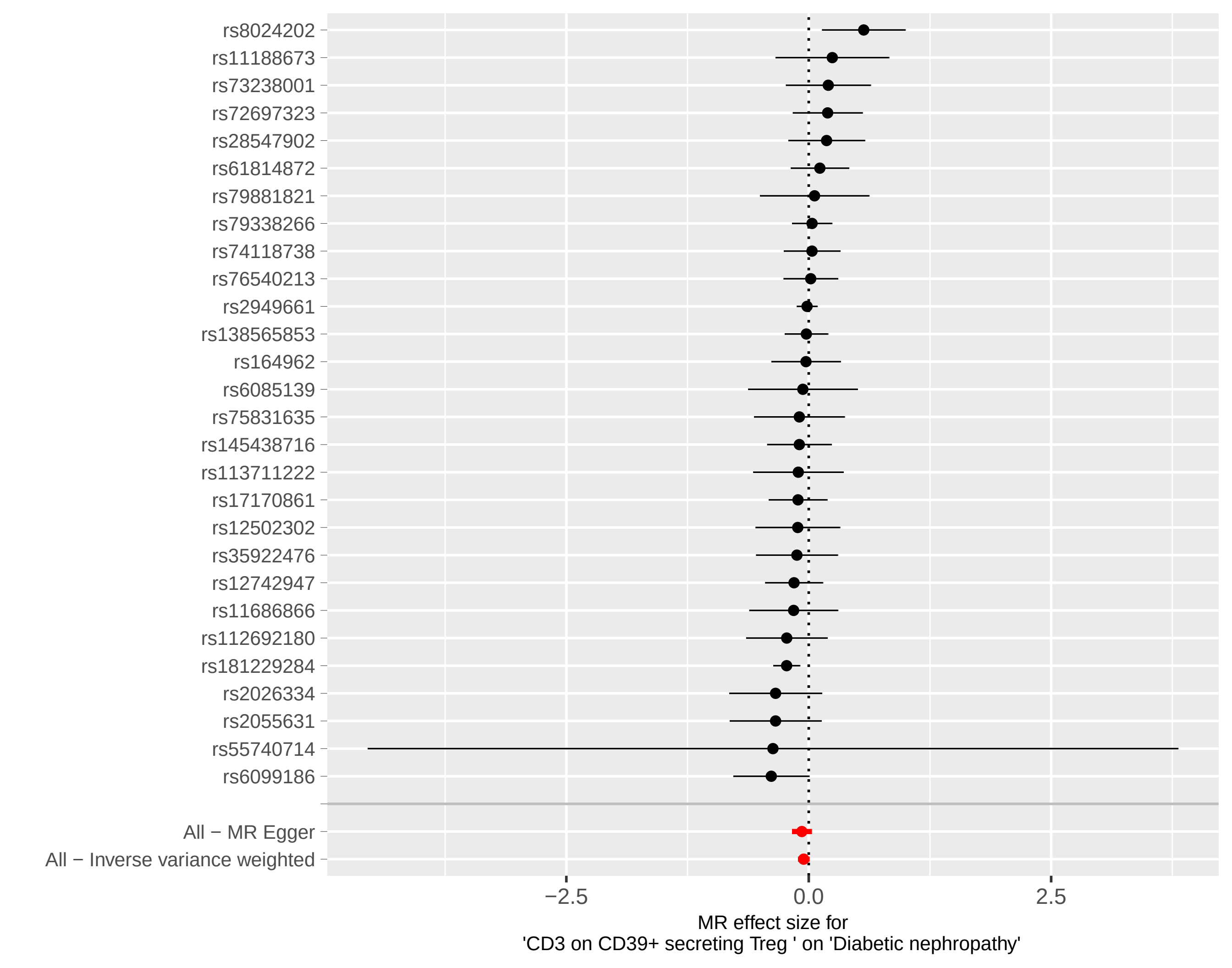

rs8024202
rs11188673
rs73238001
rs72697323
rs28547902
rs61814872
rs79881821
rs79338266
rs74118738
rs76540213
rs2949661
rs138565853
rs164962
rs6085139
rs75831635
rs145438716
rs113711222
rs17170861
rs12502302
rs35922476
rs12742947
rs11686866
rs112692180
rs181229284
rs2026334
rs2055631
rs55740714
rs6099186
All − MR Egger
All − Inverse variance weighted
−2.5
0.0
2.5
MR effect size for
'CD3 on CD39+ secreting Treg ' on 'Diabetic nephropathy'

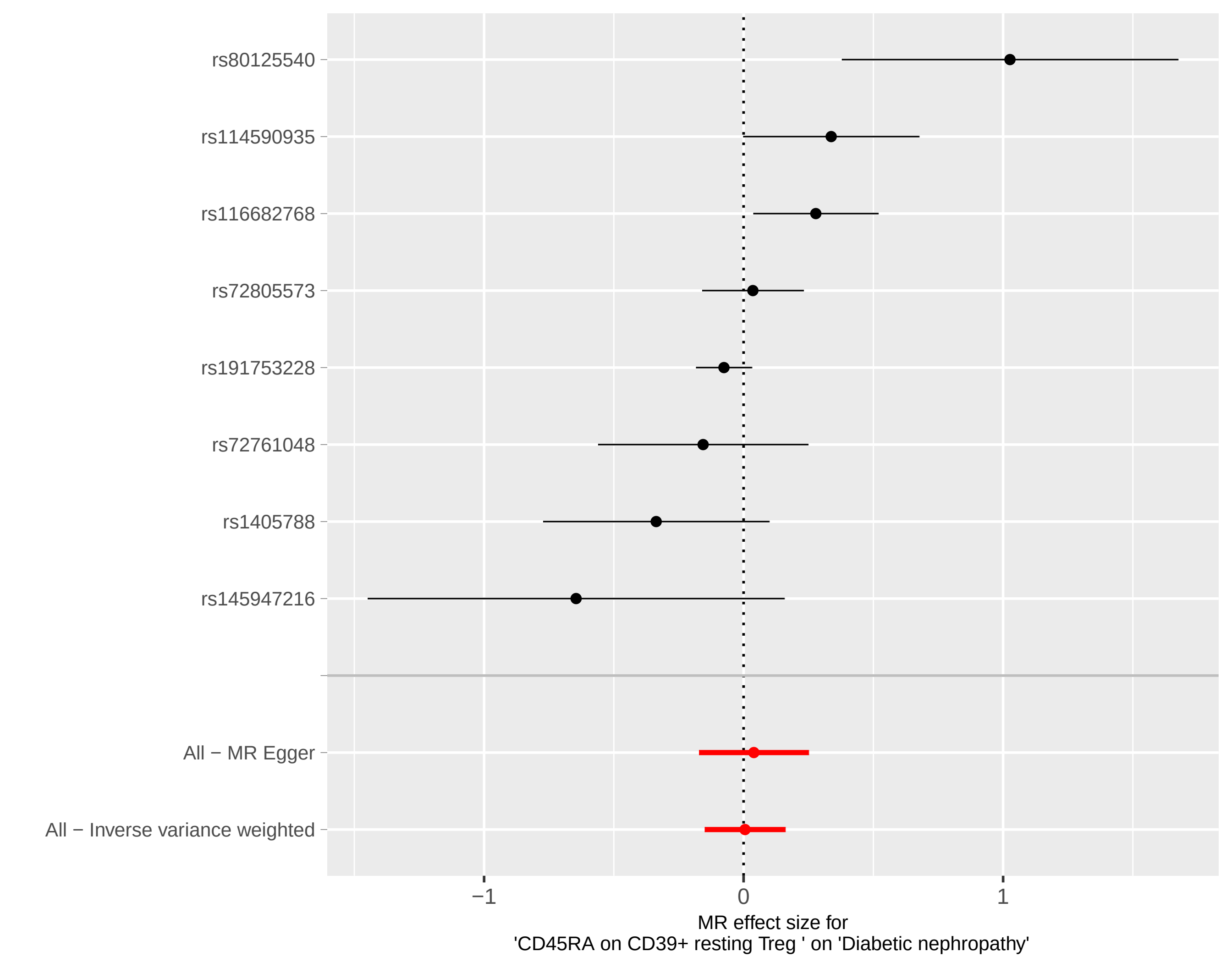
rs80125540
rs114590935
rs116682768
rs72805573
rs191753228
rs72761048
rs1405788
rs145947216
All − MR Egger
All − Inverse variance weighted
−1
0
1
MR effect size for
'CD45RA on CD39+ resting Treg ' on 'Diabetic nephropathy'

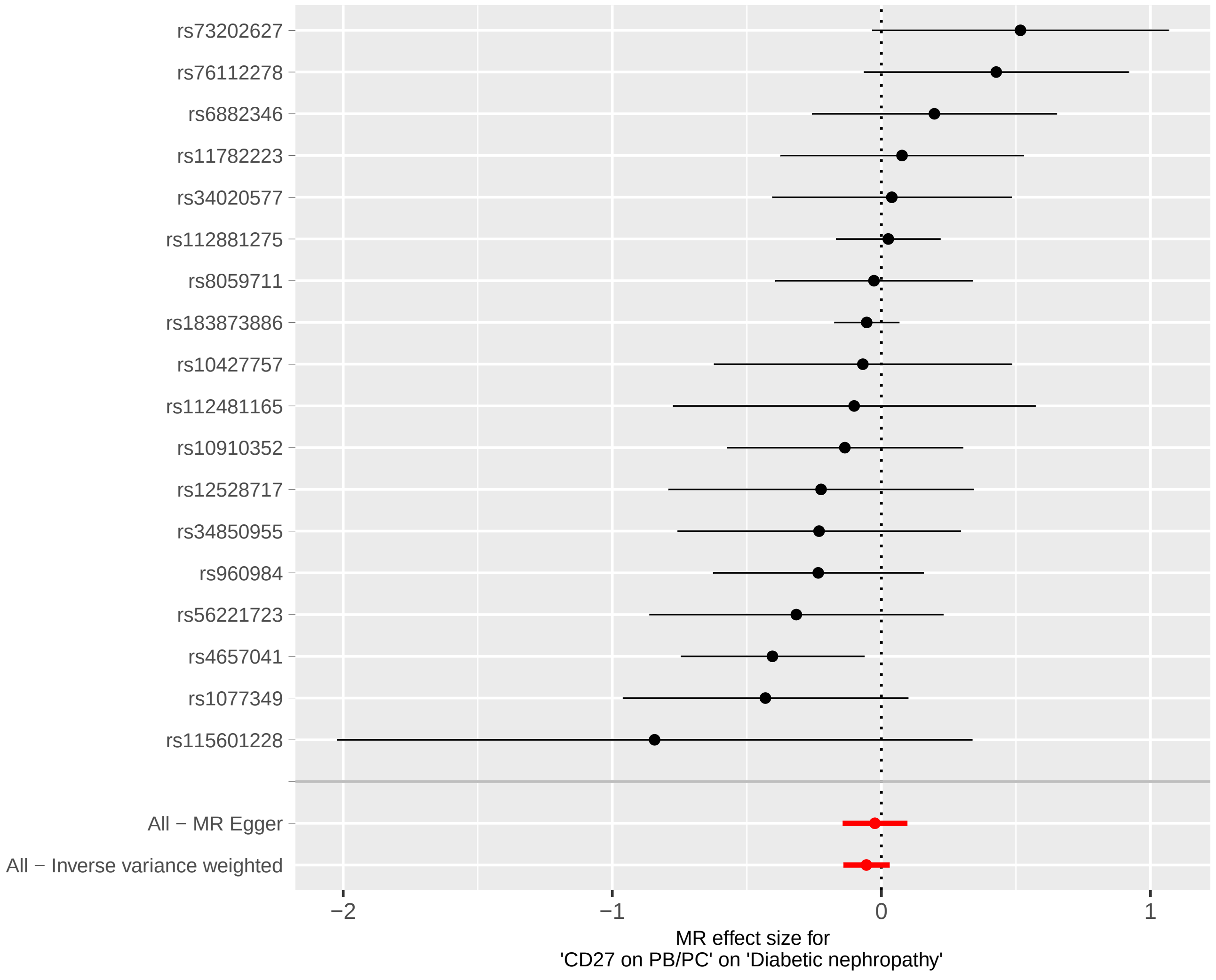
rs73202627
rs76112278
rs6882346
rs11782223
rs34020577
rs112881275
rs8059711
rs183873886
rs10427757
rs112481165
rs10910352
rs12528717
rs34850955
rs960984
rs56221723
rs4657041
rs1077349
rs115601228
All − MR Egger
All − Inverse variance weighted
−2
−1
0
1
MR effect size for
'CD27 on PB/PC' on 'Diabetic nephropathy'

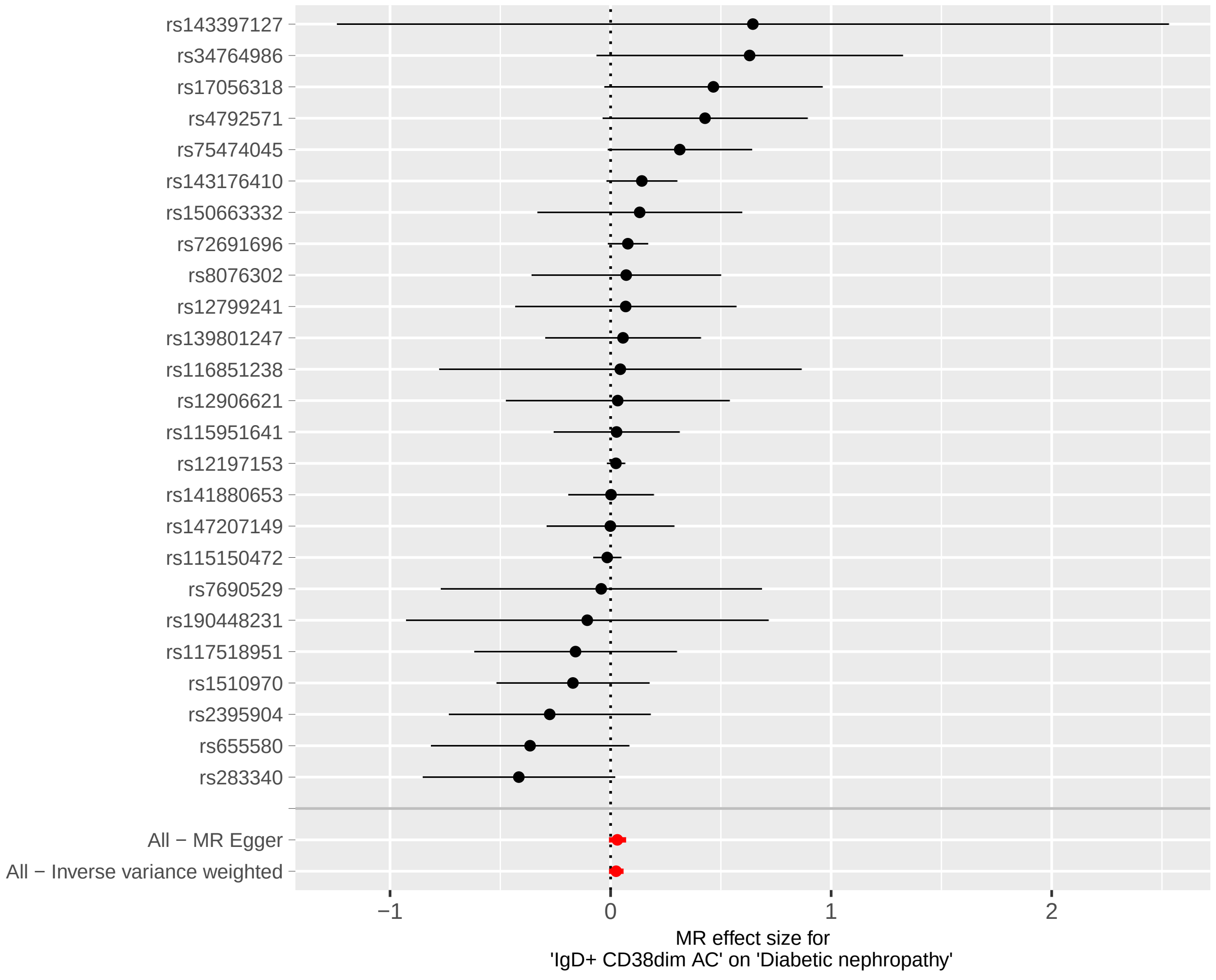
rs143397127
rs34764986
rs17056318
rs4792571
rs75474045
rs143176410
rs150663332
rs72691696
rs8076302
rs12799241
rs139801247
rs116851238
rs12906621
rs115951641
rs12197153
rs141880653
rs147207149
rs115150472
rs7690529
rs190448231
rs117518951
rs1510970
rs2395904
rs655580
rs283340
All − MR Egger
All − Inverse variance weighted
−1
0
1
2
MR effect size for
'IgD+ CD38dim AC' on 'Diabetic nephropathy'

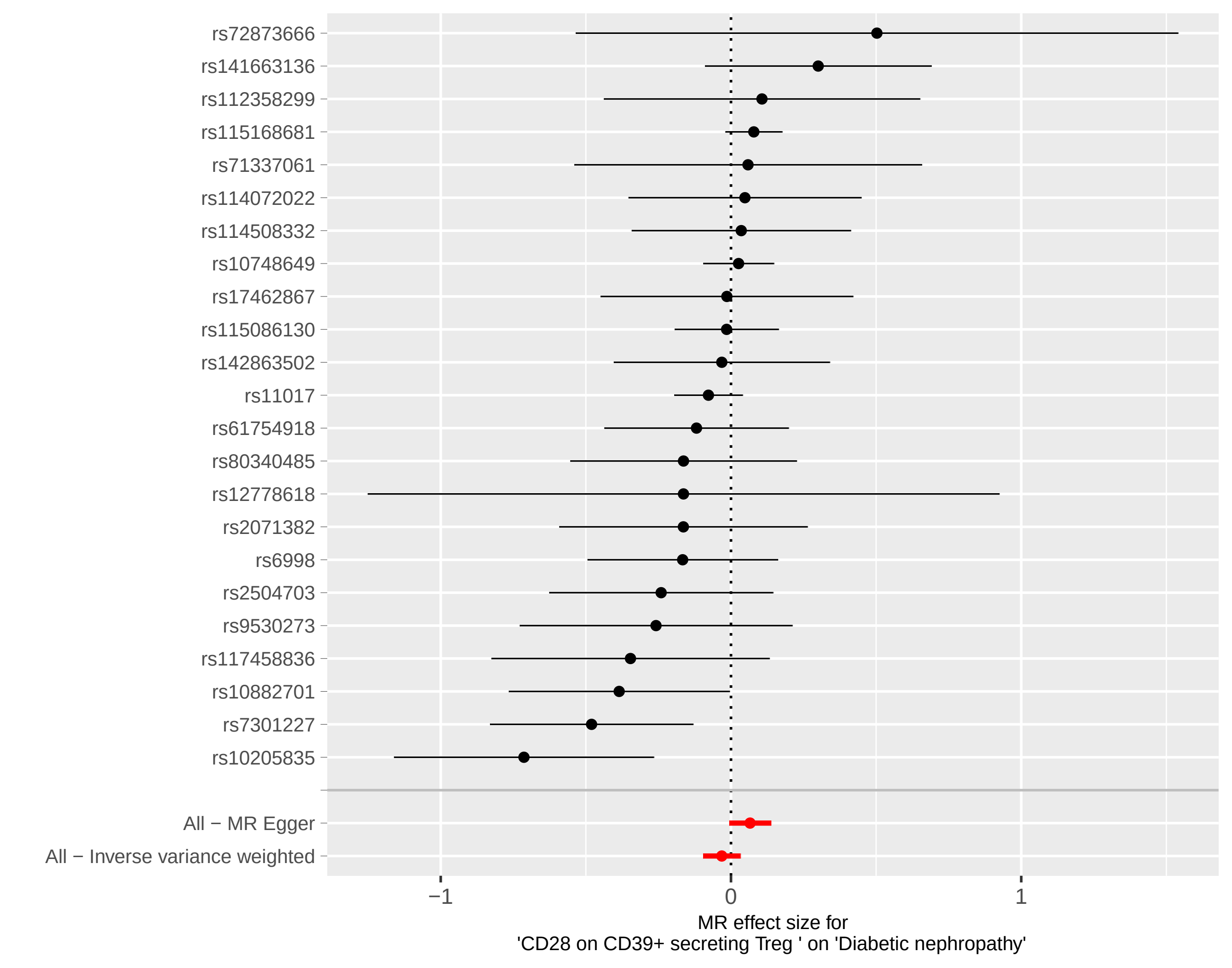
rs72873666
rs141663136
rs112358299
rs115168681
rs71337061
rs114072022
rs114508332
rs10748649
rs17462867
rs115086130
rs142863502
rs11017
rs61754918
rs80340485
rs12778618
rs2071382
rs6998
rs2504703
rs9530273
rs117458836
rs10882701
rs7301227
rs10205835
All − MR Egger
All − Inverse variance weighted
−1
0
1
MR effect size for
'CD28 on CD39+ secreting Treg ' on 'Diabetic nephropathy'

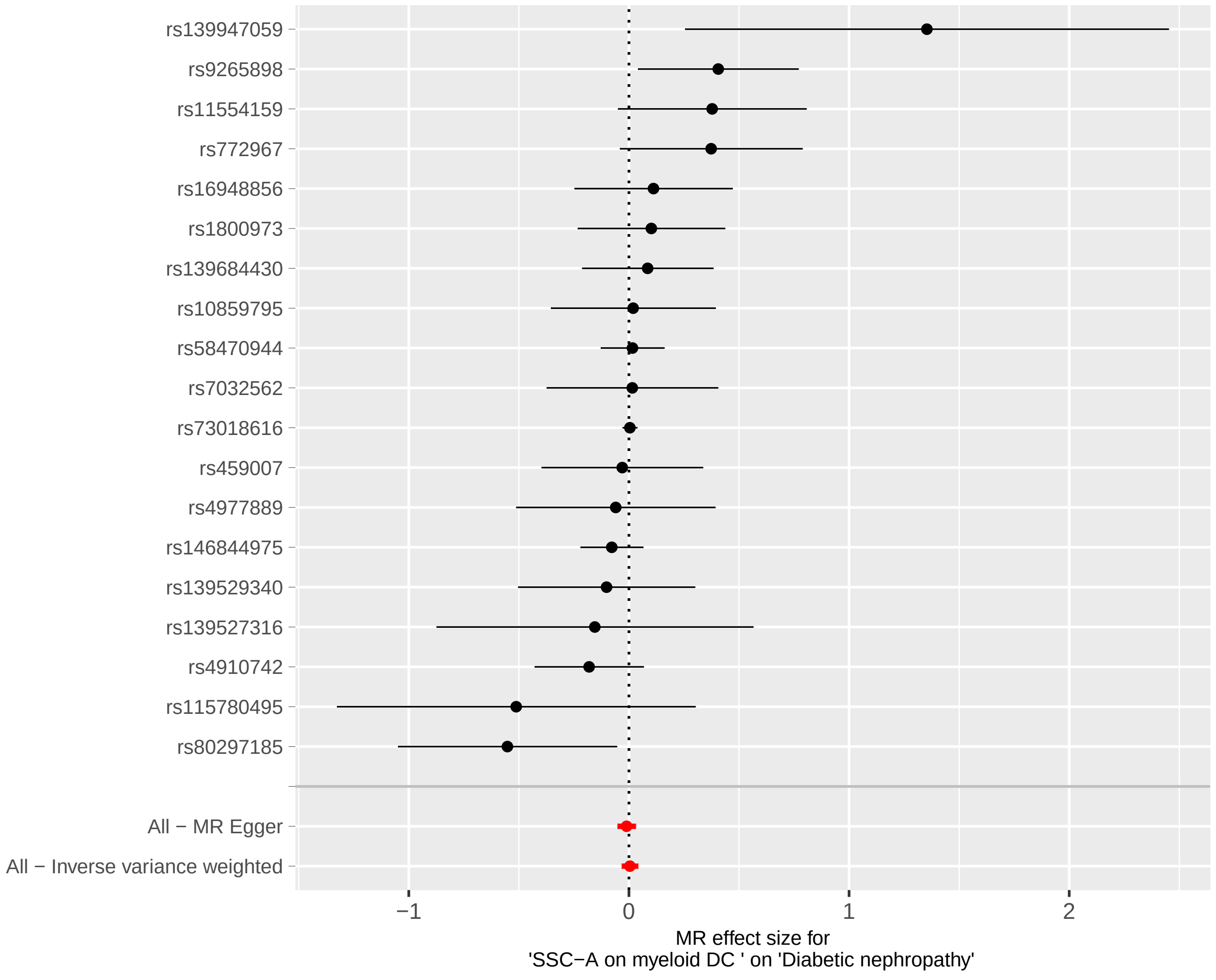

rs139947059
rs9265898
rs11554159
rs772967
rs16948856
rs1800973
rs139684430
rs10859795
rs58470944
rs7032562
rs73018616
rs459007
rs4977889
rs146844975
rs139529340
rs139527316
rs4910742
rs115780495
rs80297185
All − MR Egger
All − Inverse variance weighted
−1
0
1
2
MR effect size for
'SSC−A on myeloid DC ' on 'Diabetic nephropathy'

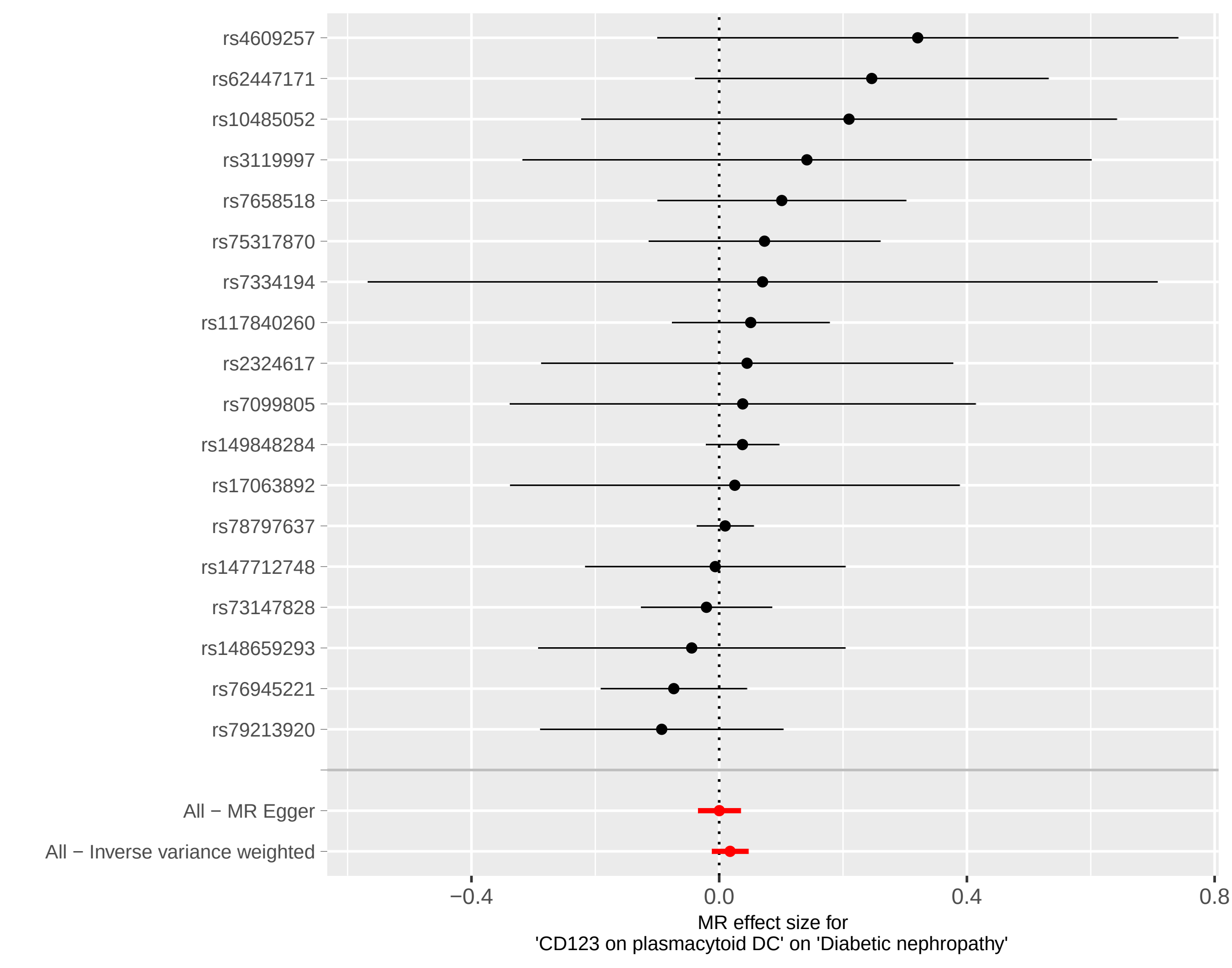
rs4609257
rs62447171
rs10485052
rs3119997
rs7658518
rs75317870
rs7334194
rs117840260
rs2324617
rs7099805
rs149848284
rs17063892
rs78797637
rs147712748
rs73147828
rs148659293
rs76945221
rs79213920
All − MR Egger
All − Inverse variance weighted
−0.4
0.0
0.4
0.8
MR effect size for
'CD123 on plasmacytoid DC' on 'Diabetic nephropathy'

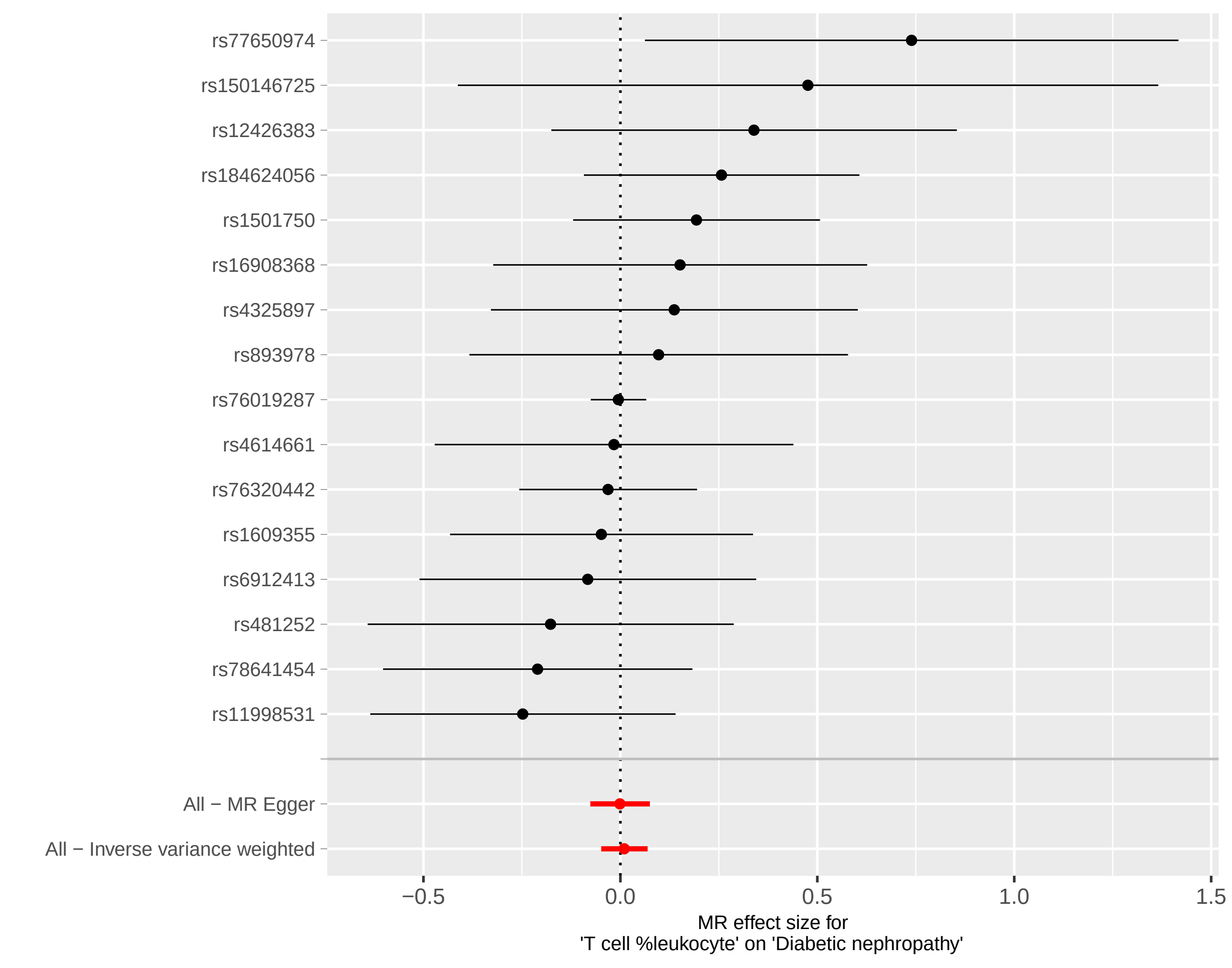

rs77650974
rs150146725
rs12426383
rs184624056
rs1501750
rs16908368
rs4325897
rs893978
rs76019287
rs4614661
rs76320442
rs1609355
rs6912413
rs481252
rs78641454
rs11998531
All − MR Egger
All − Inverse variance weighted
−0.5
0.0
0.5
1.0
1.5
MR effect size for
'T cell %leukocyte' on 'Diabetic nephropathy'

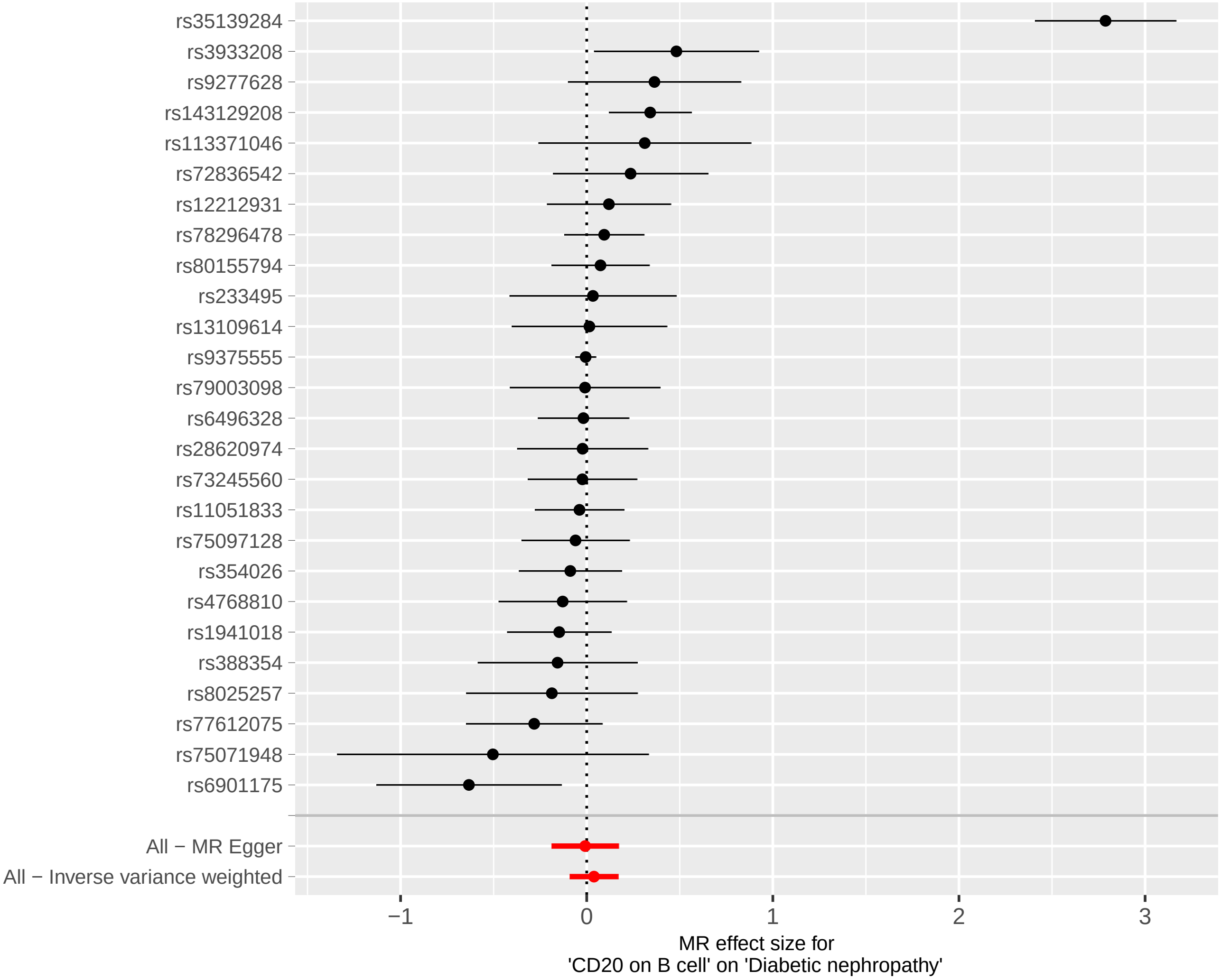
rs35139284
rs3933208
rs9277628
rs143129208
rs113371046
rs72836542
rs12212931
rs78296478
rs80155794
rs233495
rs13109614
rs9375555
rs79003098
rs6496328
rs28620974
rs73245560
rs11051833
rs75097128
rs354026
rs4768810
rs1941018
rs388354
rs8025257
rs77612075
rs75071948
rs6901175
All − MR Egger
All − Inverse variance weighted
−1
0
1
2
3
MR effect size for
'CD20 on B cell' on 'Diabetic nephropathy'

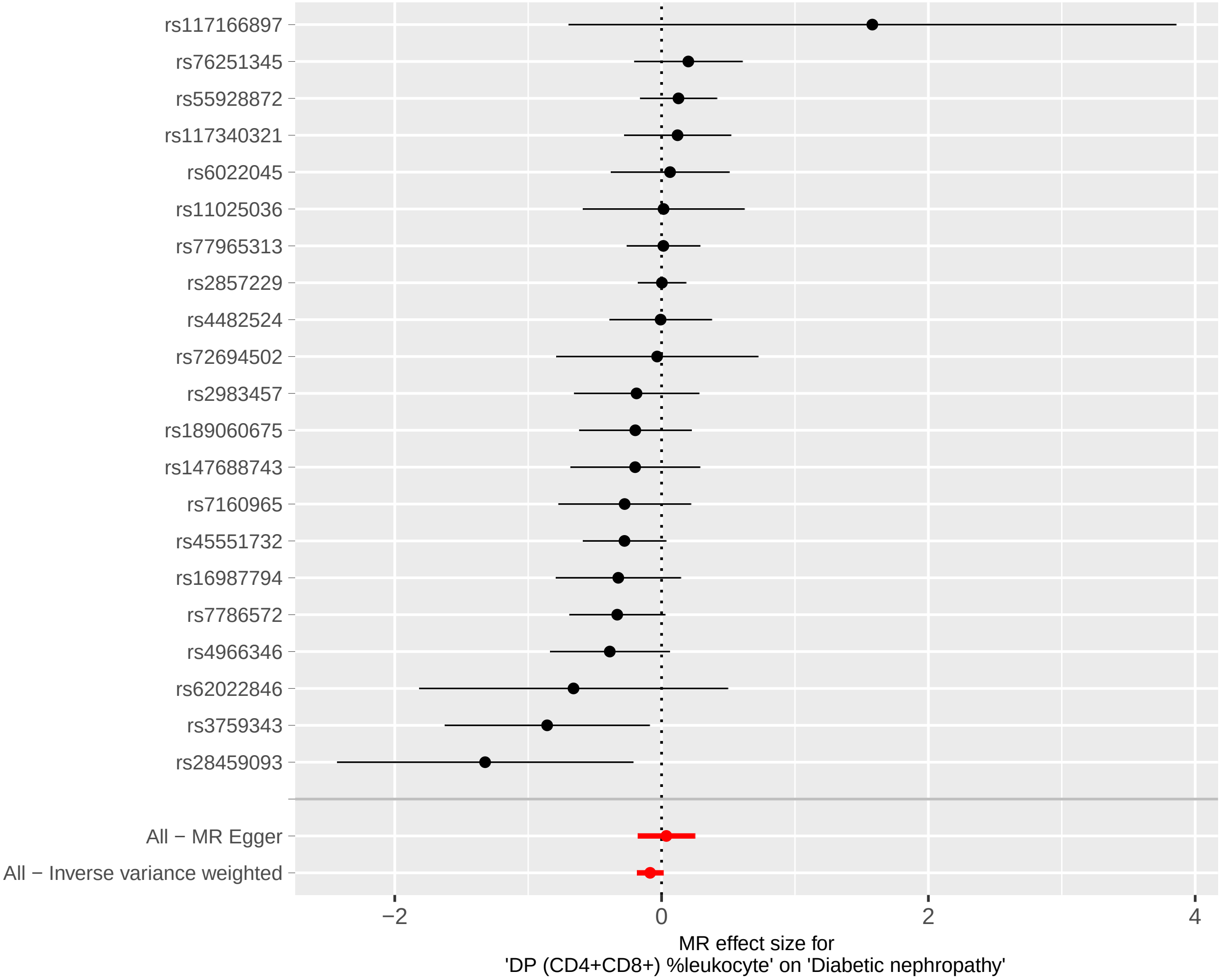
rs117166897
rs76251345
rs55928872
rs117340321
rs6022045
rs11025036
rs77965313
rs2857229
rs4482524
rs72694502
rs2983457
rs189060675
rs147688743
rs7160965
rs45551732
rs16987794
rs7786572
rs4966346
rs62022846
rs3759343
rs28459093
All − MR Egger
All − Inverse variance weighted
−2
0
2
4
MR effect size for
'DP (CD4+CD8+) %leukocyte' on 'Diabetic nephropathy'

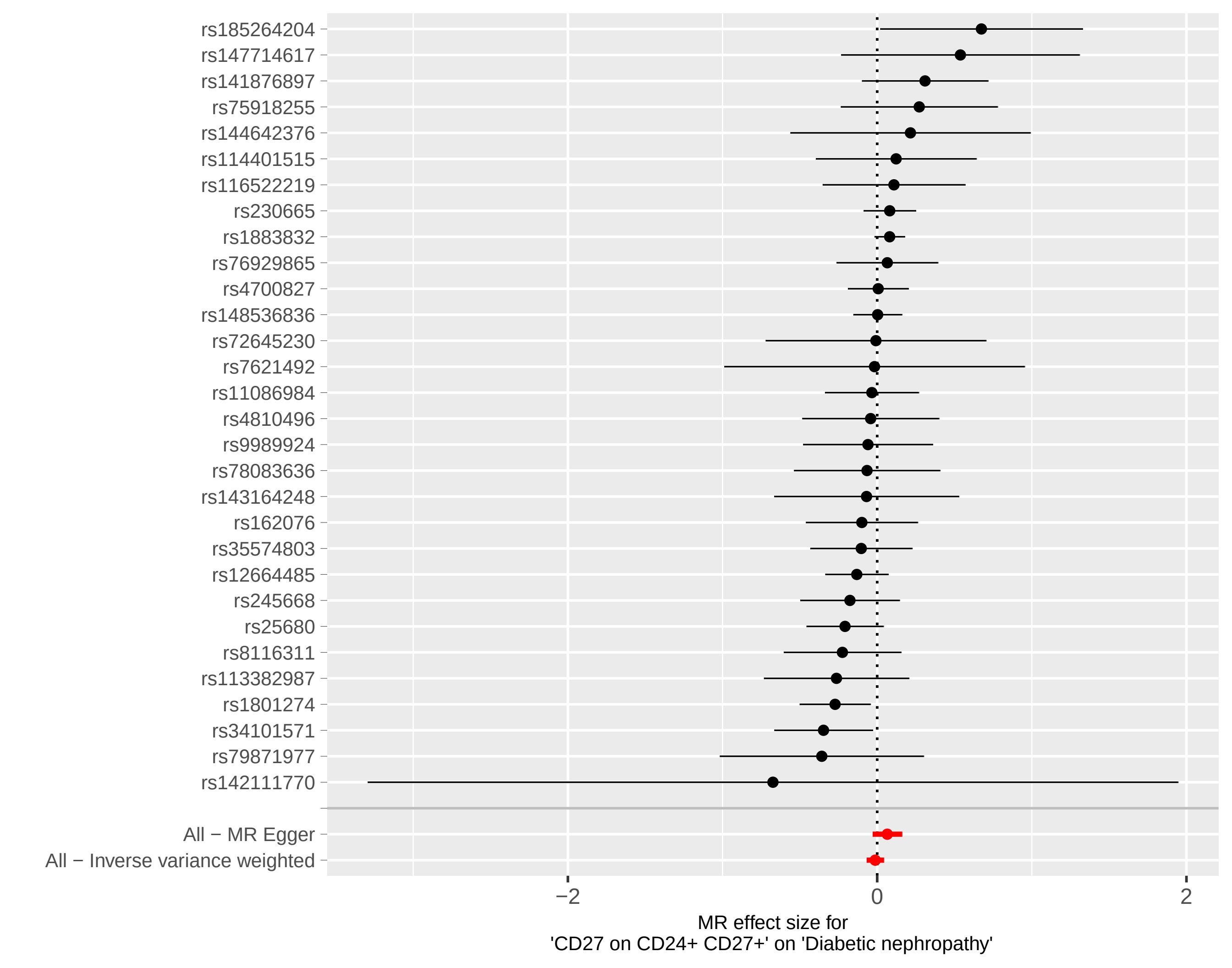
rs185264204
rs147714617
rs141876897
rs75918255
rs144642376
rs114401515
rs116522219
rs230665
rs1883832
rs76929865
rs4700827
rs148536836
rs72645230
rs7621492
rs11086984
rs4810496
rs9989924
rs78083636
rs143164248
rs162076
rs35574803
rs12664485
rs245668
rs25680
rs8116311
rs113382987
rs1801274
rs34101571
rs79871977
rs142111770
All − MR Egger
All − Inverse variance weighted
−2
0
2
MR effect size for
'CD27 on CD24+ CD27+' on 'Diabetic nephropathy'

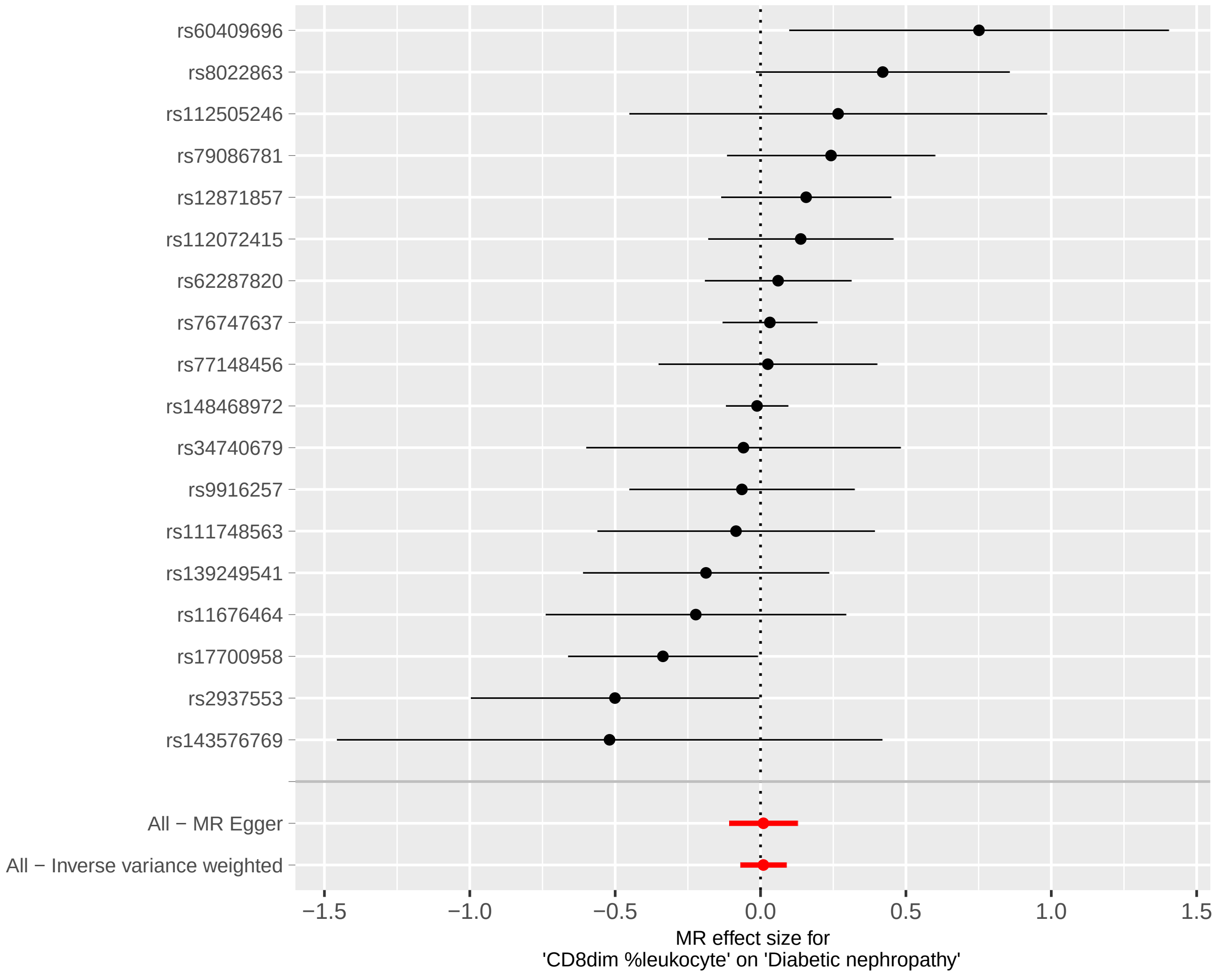

rs60409696
rs8022863
rs112505246
rs79086781
rs12871857
rs112072415
rs62287820
rs76747637
rs77148456
rs148468972
rs34740679
rs9916257
rs111748563
rs139249541
rs11676464
rs17700958
rs2937553
rs143576769
All − MR Egger
All − Inverse variance weighted
−1.5
−1.0
−0.5
0.0
0.5
1.0
1.5
MR effect size for
'CD8dim %leukocyte' on 'Diabetic nephropathy'

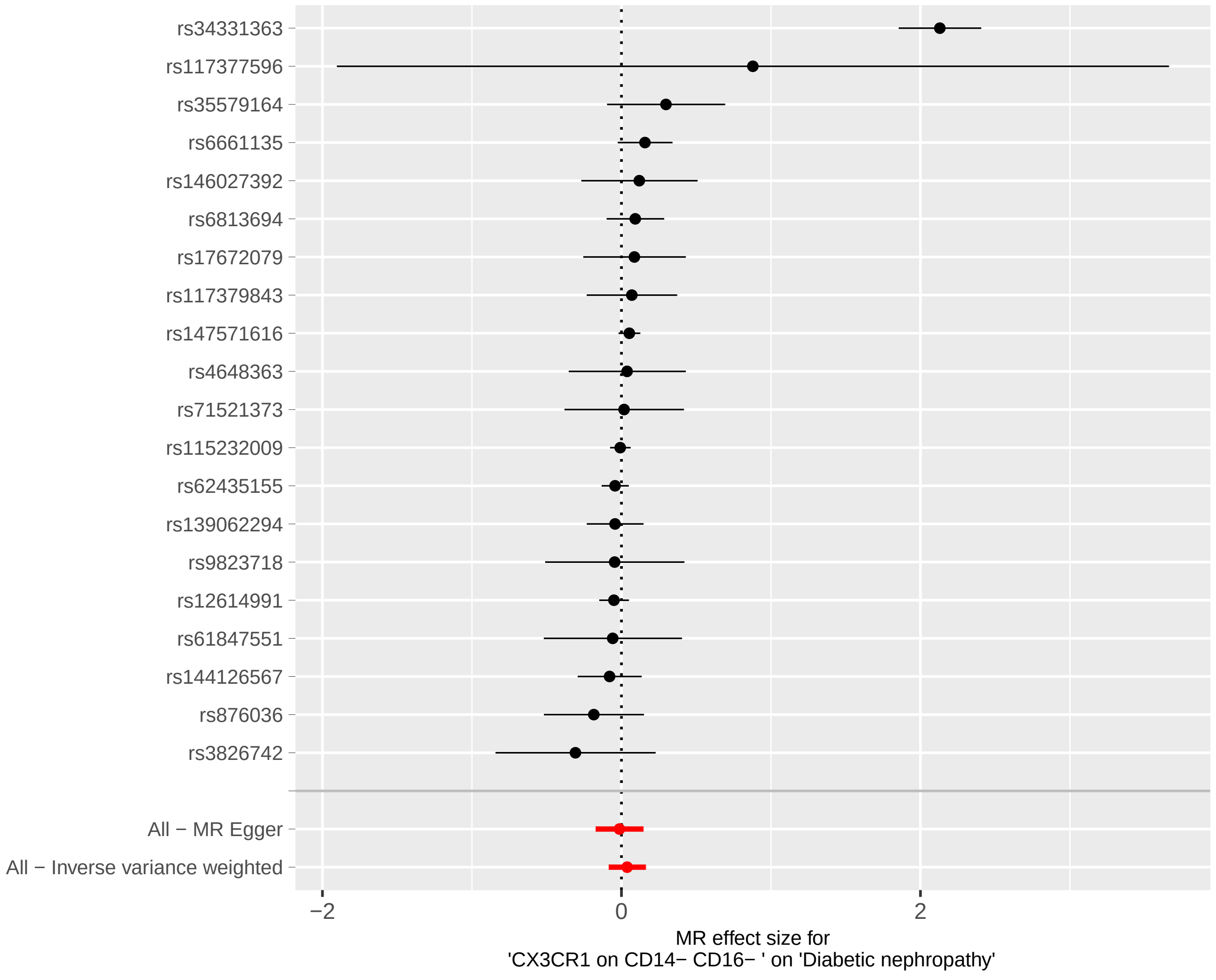
rs34331363
rs117377596
rs35579164
rs6661135
rs146027392
rs6813694
rs17672079
rs117379843
rs147571616
rs4648363
rs71521373
rs115232009
rs62435155
rs139062294
rs9823718
rs12614991
rs61847551
rs144126567
rs876036
rs3826742
All − MR Egger
All − Inverse variance weighted
−2
0
2
MR effect size for
'CX3CR1 on CD14− CD16− ' on 'Diabetic nephropathy'

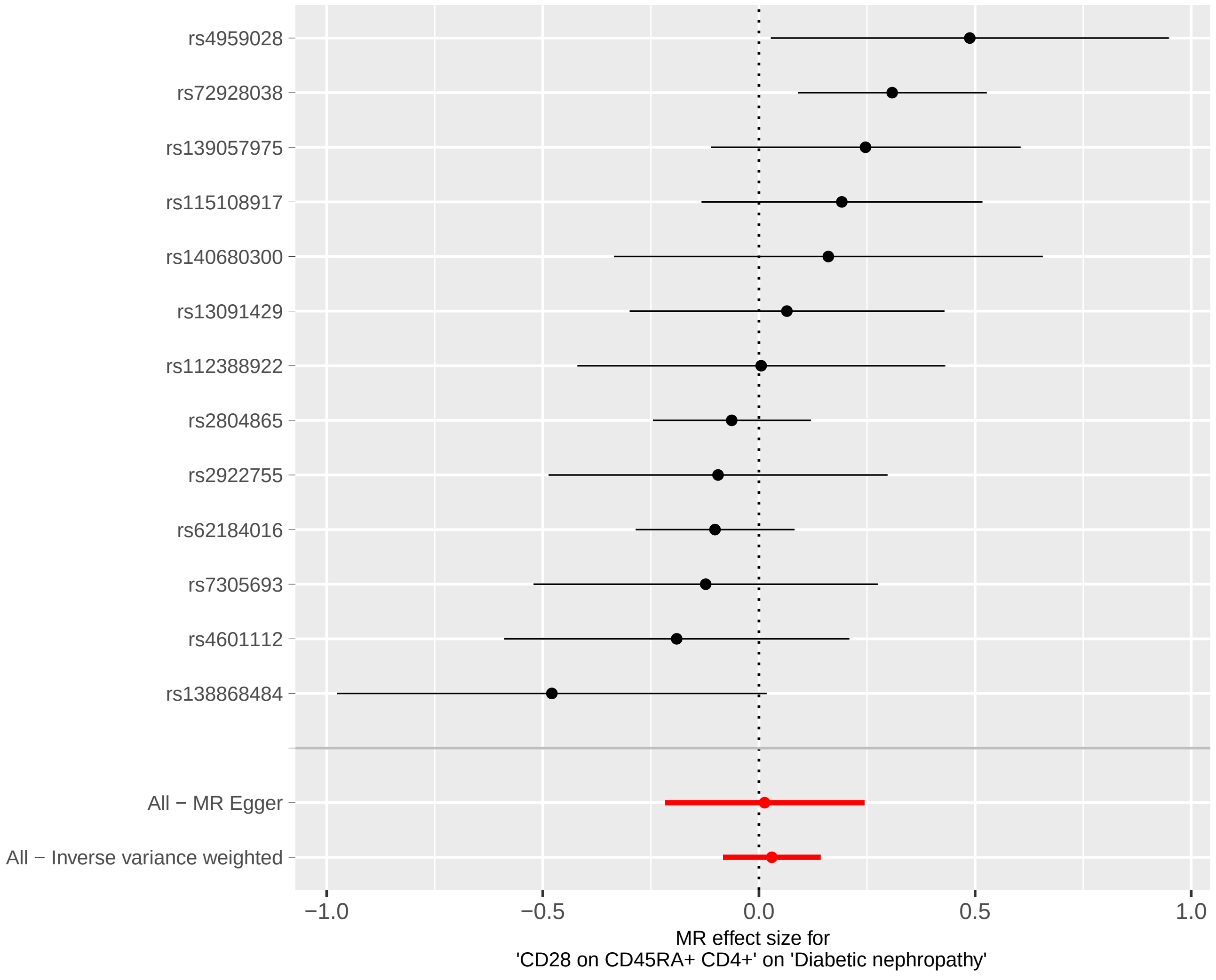
rs4959028
rs72928038
rs139057975
rs115108917
rs140680300
rs13091429
rs112388922
rs2804865
rs2922755
rs62184016
rs7305693
rs4601112
rs138868484
All − MR Egger
All − Inverse variance weighted
−1.0
−0.5
0.0
0.5
1.0
MR effect size for
'CD28 on CD45RA+ CD4+' on 'Diabetic nephropathy'

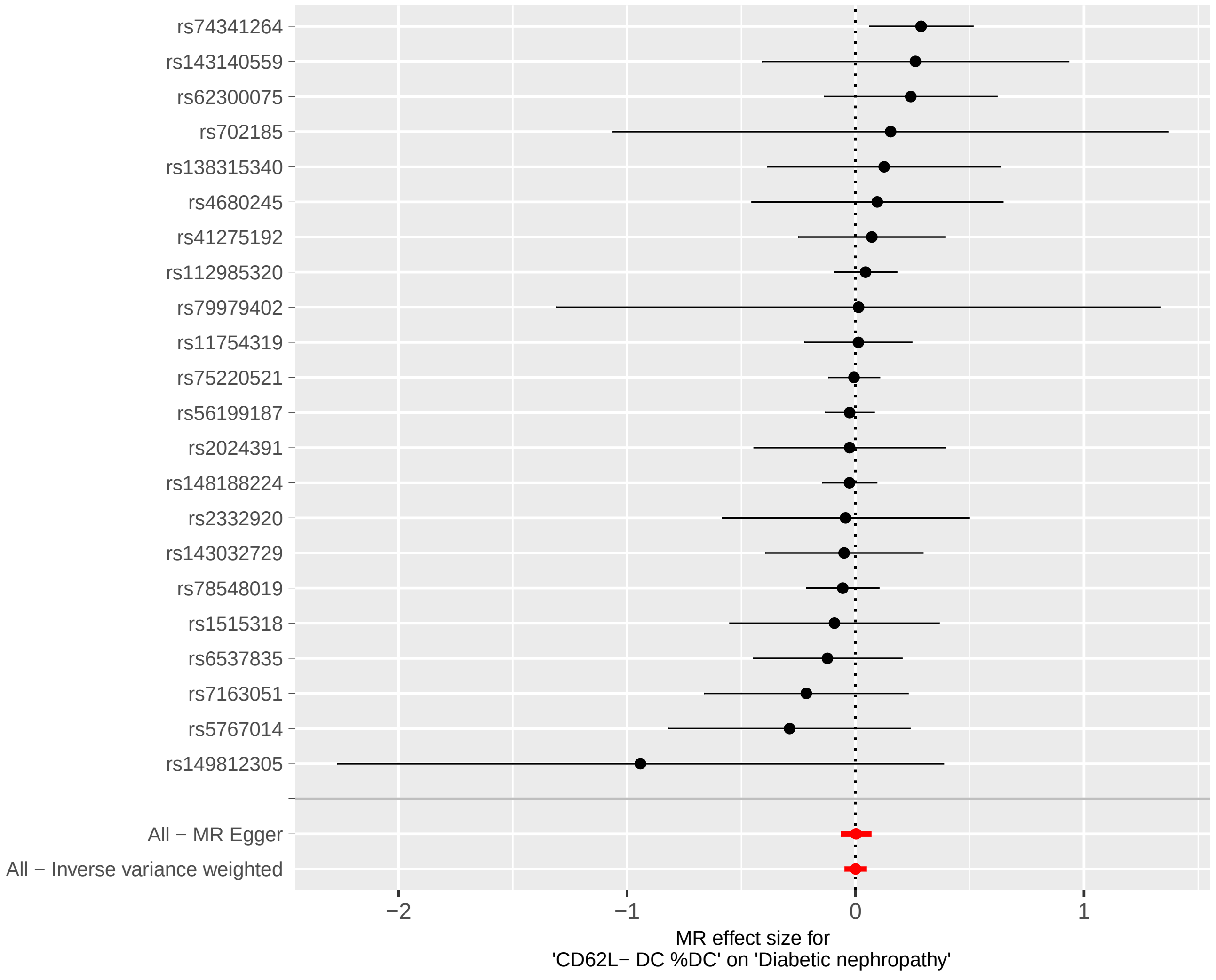
rs74341264
rs143140559
rs62300075
rs702185
rs138315340
rs4680245
rs41275192
rs112985320
rs79979402
rs11754319
rs75220521
rs56199187
rs2024391
rs148188224
rs2332920
rs143032729
rs78548019
rs1515318
rs6537835
rs7163051
rs5767014
rs149812305
All − MR Egger
All − Inverse variance weighted
−2
−1
0
1
MR effect size for
'CD62L− DC %DC' on 'Diabetic nephropathy'

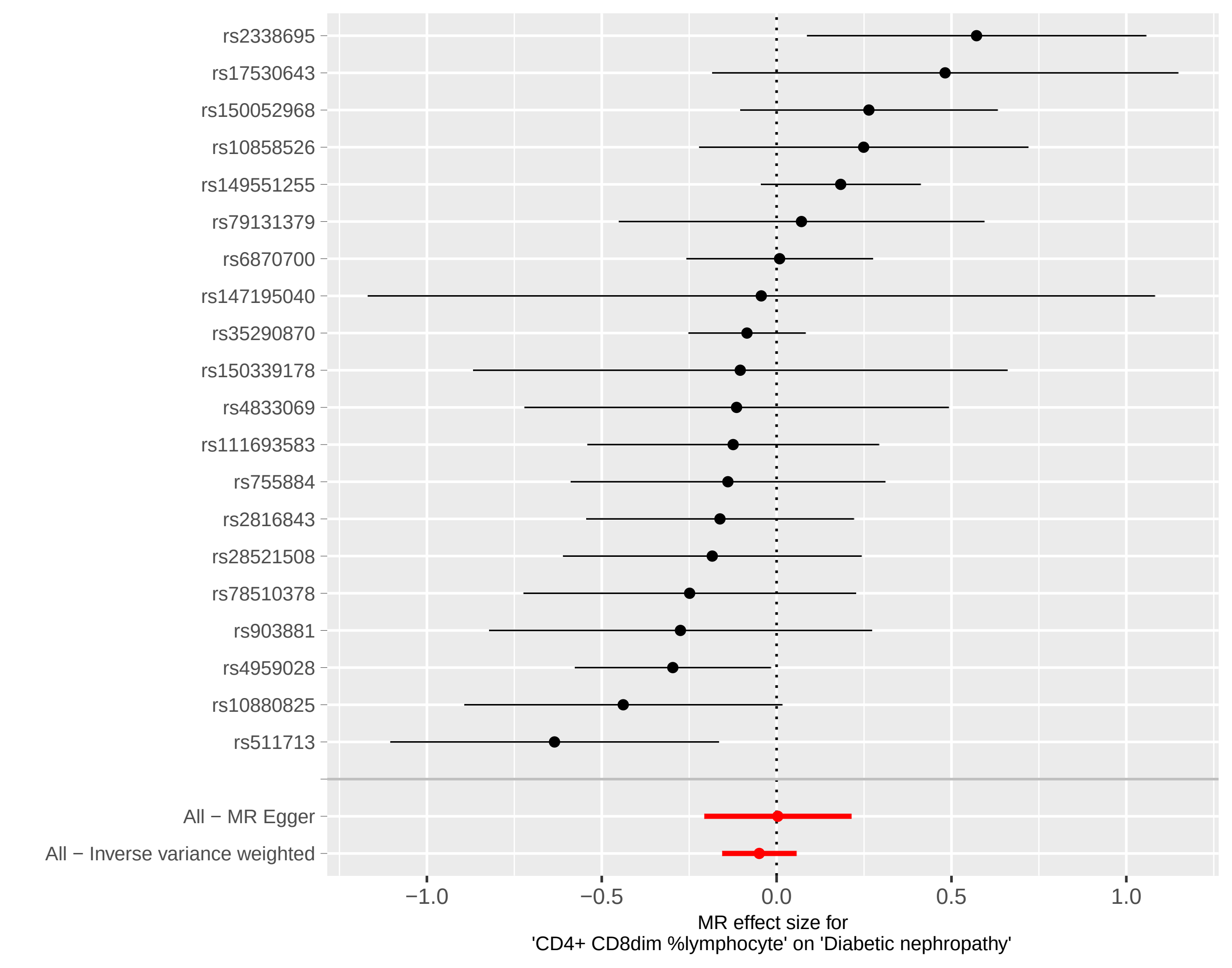
rs2338695
rs17530643
rs150052968
rs10858526
rs149551255
rs79131379
rs6870700
rs147195040
rs35290870
rs150339178
rs4833069
rs111693583
rs755884
rs2816843
rs28521508
rs78510378
rs903881
rs4959028
rs10880825
rs511713
All − MR Egger
All − Inverse variance weighted
−1.0
−0.5
0.0
0.5
1.0
MR effect size for
'CD4+ CD8dim %lymphocyte' on 'Diabetic nephropathy'

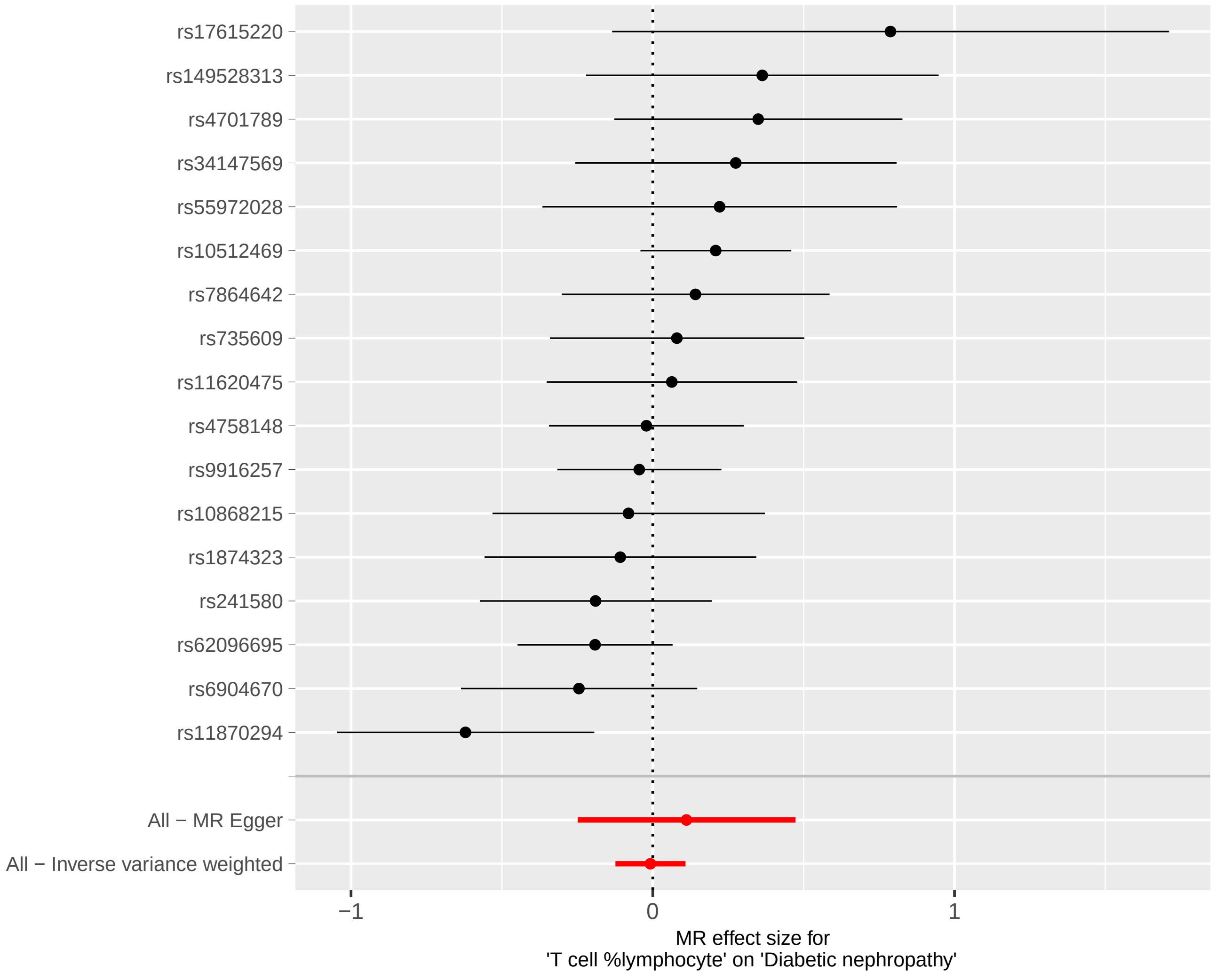

rs17615220
rs149528313
rs4701789
rs34147569
rs55972028
rs10512469
rs7864642
rs735609
rs11620475
rs4758148
rs9916257
rs10868215
rs1874323
rs241580
rs62096695
rs6904670
rs11870294
All − MR Egger
All − Inverse variance weighted
−1
0
1
MR effect size for
'T cell %lymphocyte' on 'Diabetic nephropathy'

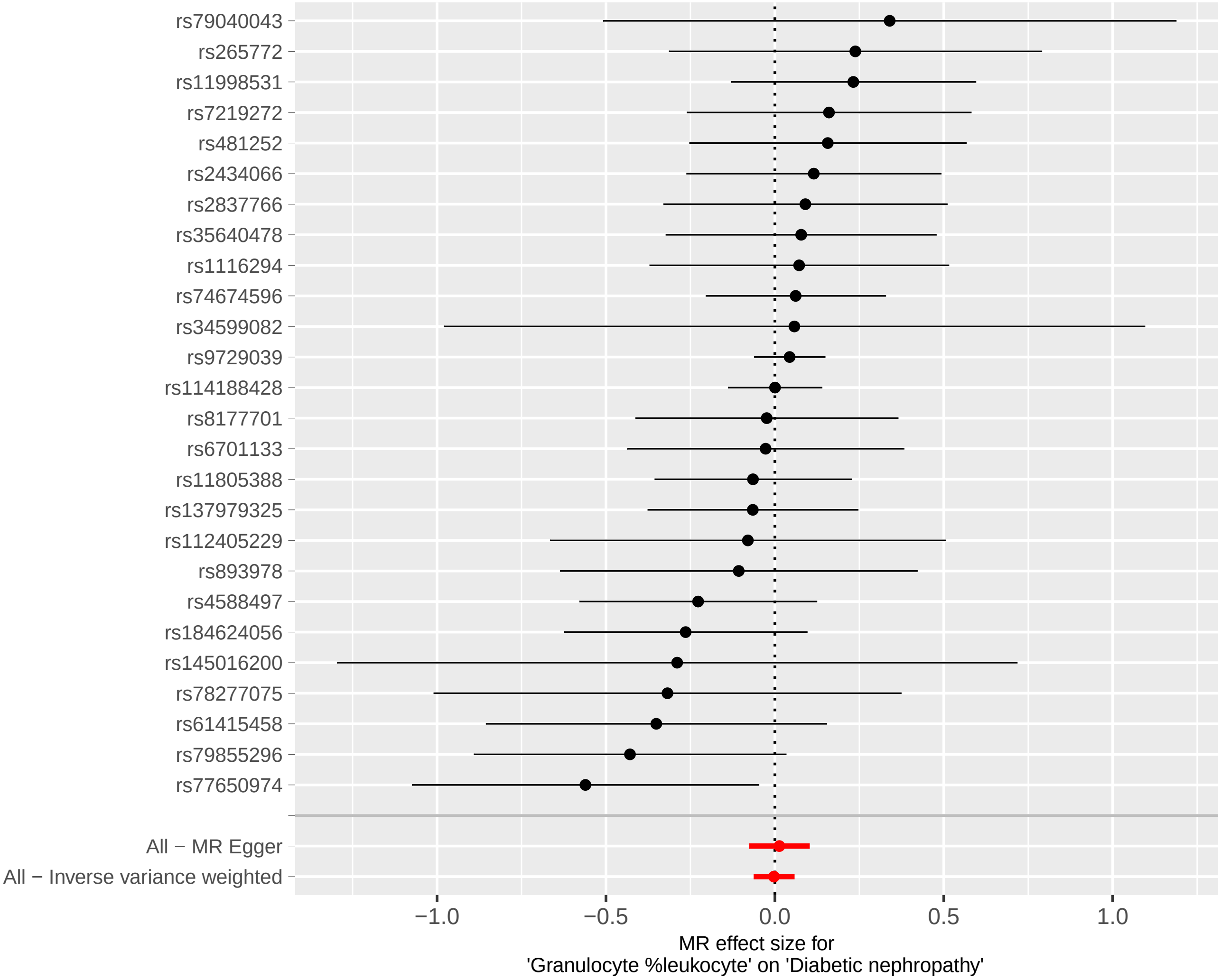
rs79040043
rs265772
rs11998531
rs7219272
rs481252
rs2434066
rs2837766
rs35640478
rs1116294
rs74674596
rs34599082
rs9729039
rs114188428
rs8177701
rs6701133
rs11805388
rs137979325
rs112405229
rs893978
rs4588497
rs184624056
rs145016200
rs78277075
rs61415458
rs79855296
rs77650974
All − MR Egger
All − Inverse variance weighted
−1.0
−0.5
0.0
0.5
1.0
MR effect size for
'Granulocyte %leukocyte' on 'Diabetic nephropathy'

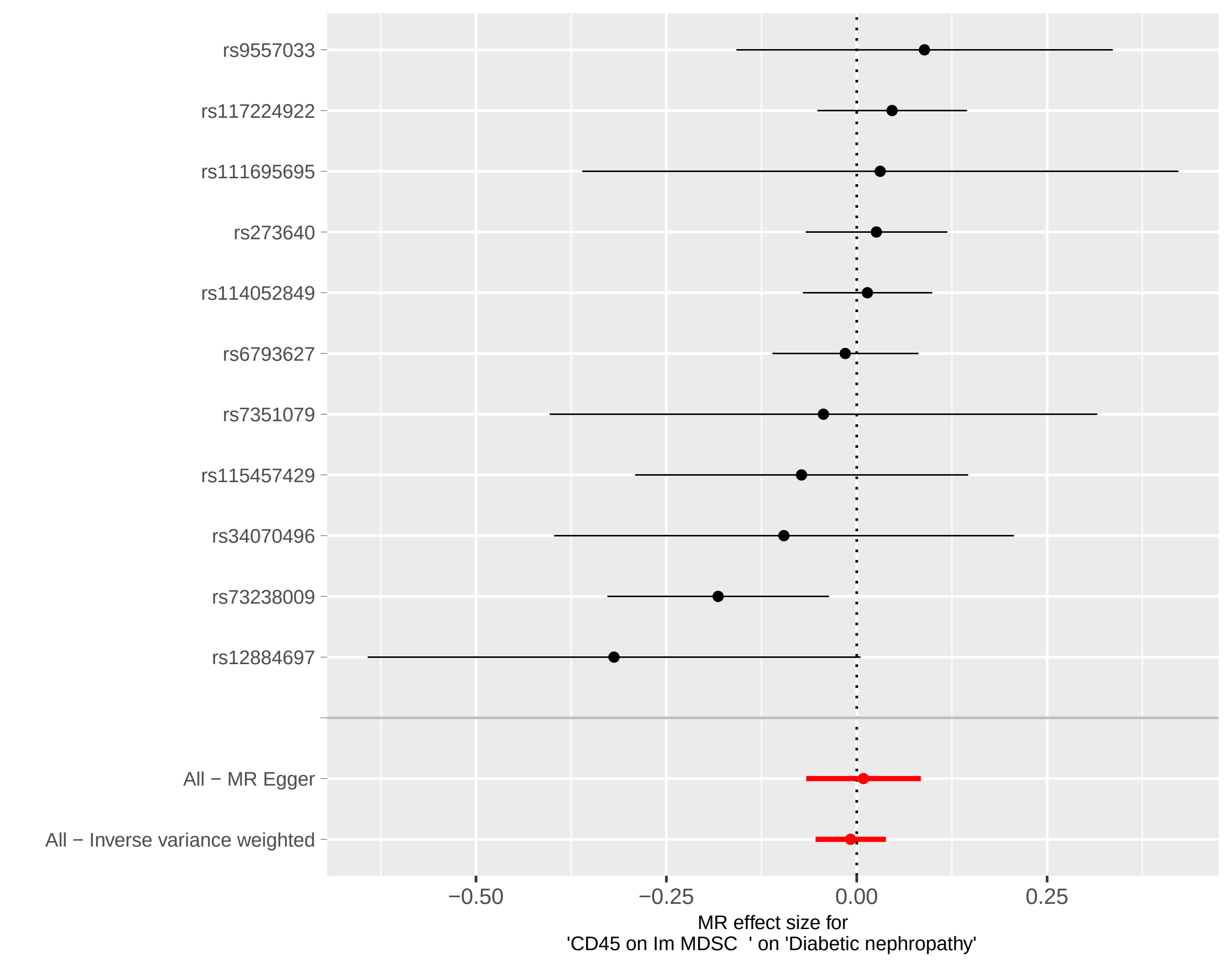

rs9557033
rs117224922
rs111695695
rs273640
rs114052849
rs6793627
rs7351079
rs115457429
rs34070496
rs73238009
rs12884697
All − MR Egger
All − Inverse variance weighted
−0.50
−0.25
0.00
0.25
MR effect size for
'CD45 on Im MDSC ' on 'Diabetic nephropathy'

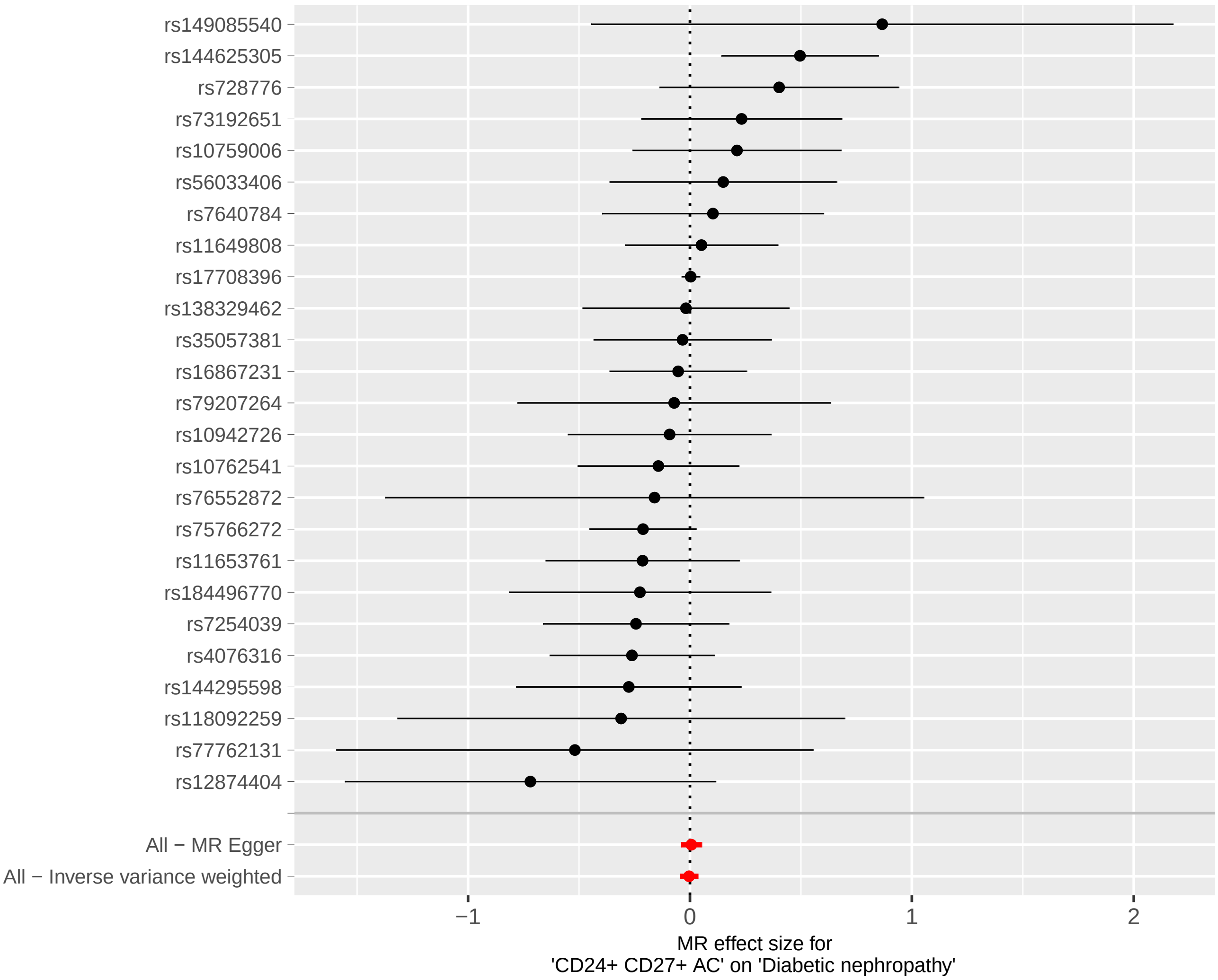
rs149085540
rs144625305
rs728776
rs73192651
rs10759006
rs56033406
rs7640784
rs11649808
rs17708396
rs138329462
rs35057381
rs16867231
rs79207264
rs10942726
rs10762541
rs76552872
rs75766272
rs11653761
rs184496770
rs7254039
rs4076316
rs144295598
rs118092259
rs77762131
rs12874404
All − MR Egger
All − Inverse variance weighted
−1
0
1
2
MR effect size for
'CD24+ CD27+ AC' on 'Diabetic nephropathy'

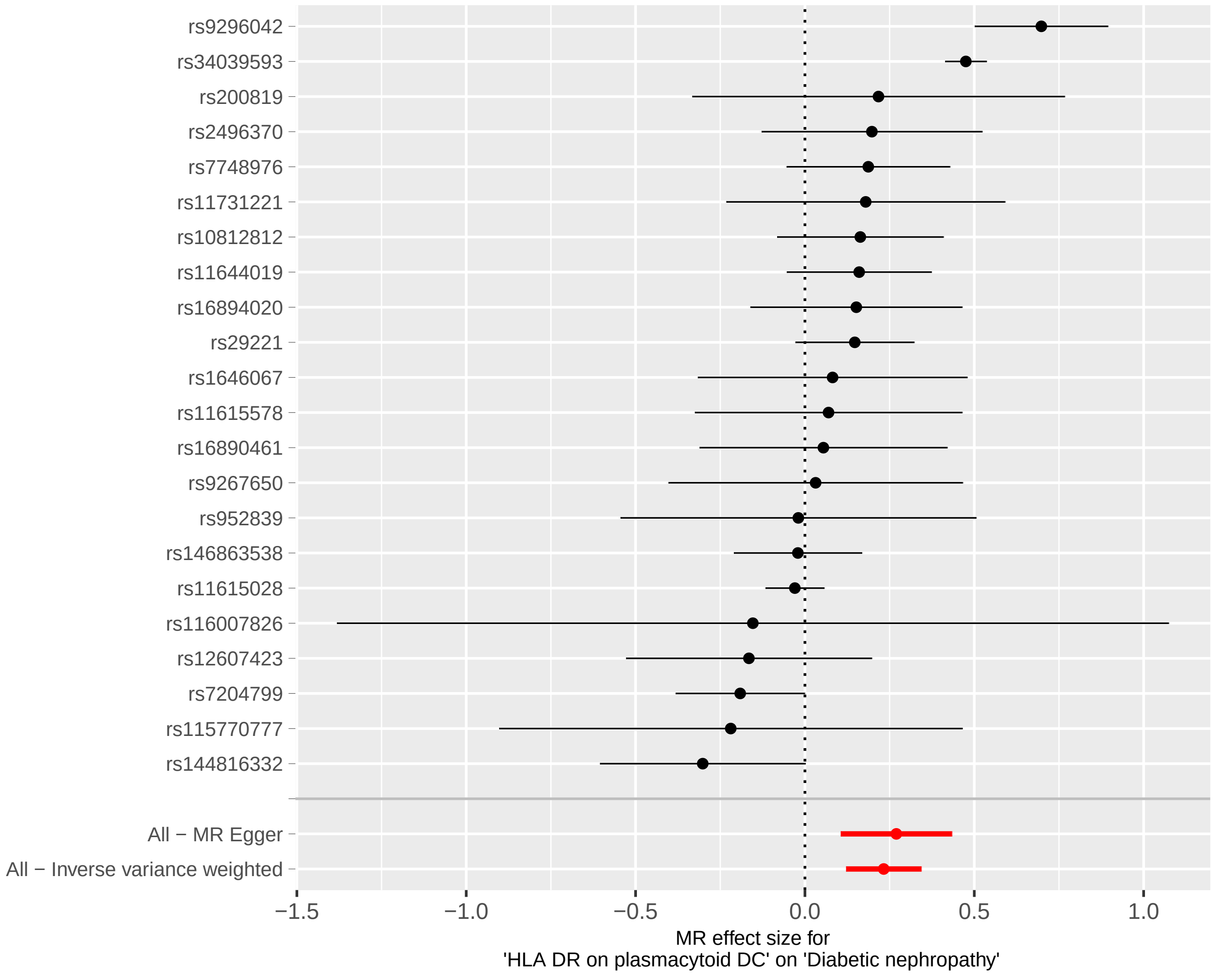

rs9296042
rs34039593
rs200819
rs2496370
rs7748976
rs11731221
rs10812812
rs11644019
rs16894020
rs29221
rs1646067
rs11615578
rs16890461
rs9267650
rs952839
rs146863538
rs11615028
rs116007826
rs12607423
rs7204799
rs115770777
rs144816332
All − MR Egger
All − Inverse variance weighted
−1.5
−1.0
−0.5
0.0
0.5
1.0
MR effect size for
'HLA DR on plasmacytoid DC' on 'Diabetic nephropathy'

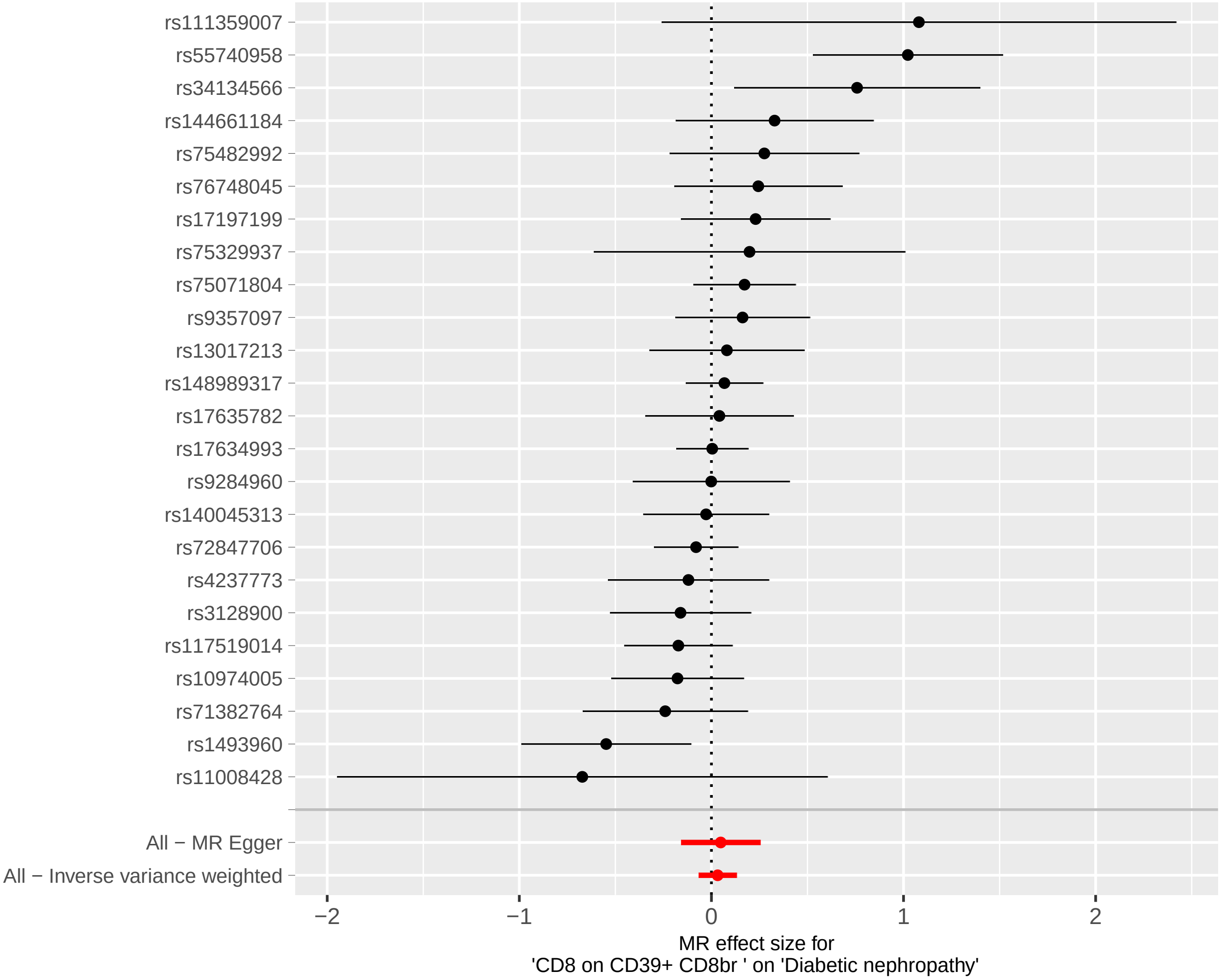

rs111359007
rs55740958
rs34134566
rs144661184
rs75482992
rs76748045
rs17197199
rs75329937
rs75071804
rs9357097
rs13017213
rs148989317
rs17635782
rs17634993
rs9284960
rs140045313
rs72847706
rs4237773
rs3128900
rs117519014
rs10974005
rs71382764
rs1493960
rs11008428
All − MR Egger
All − Inverse variance weighted
−2
−1
0
1
2
MR effect size for
'CD8 on CD39+ CD8br ' on 'Diabetic nephropathy'

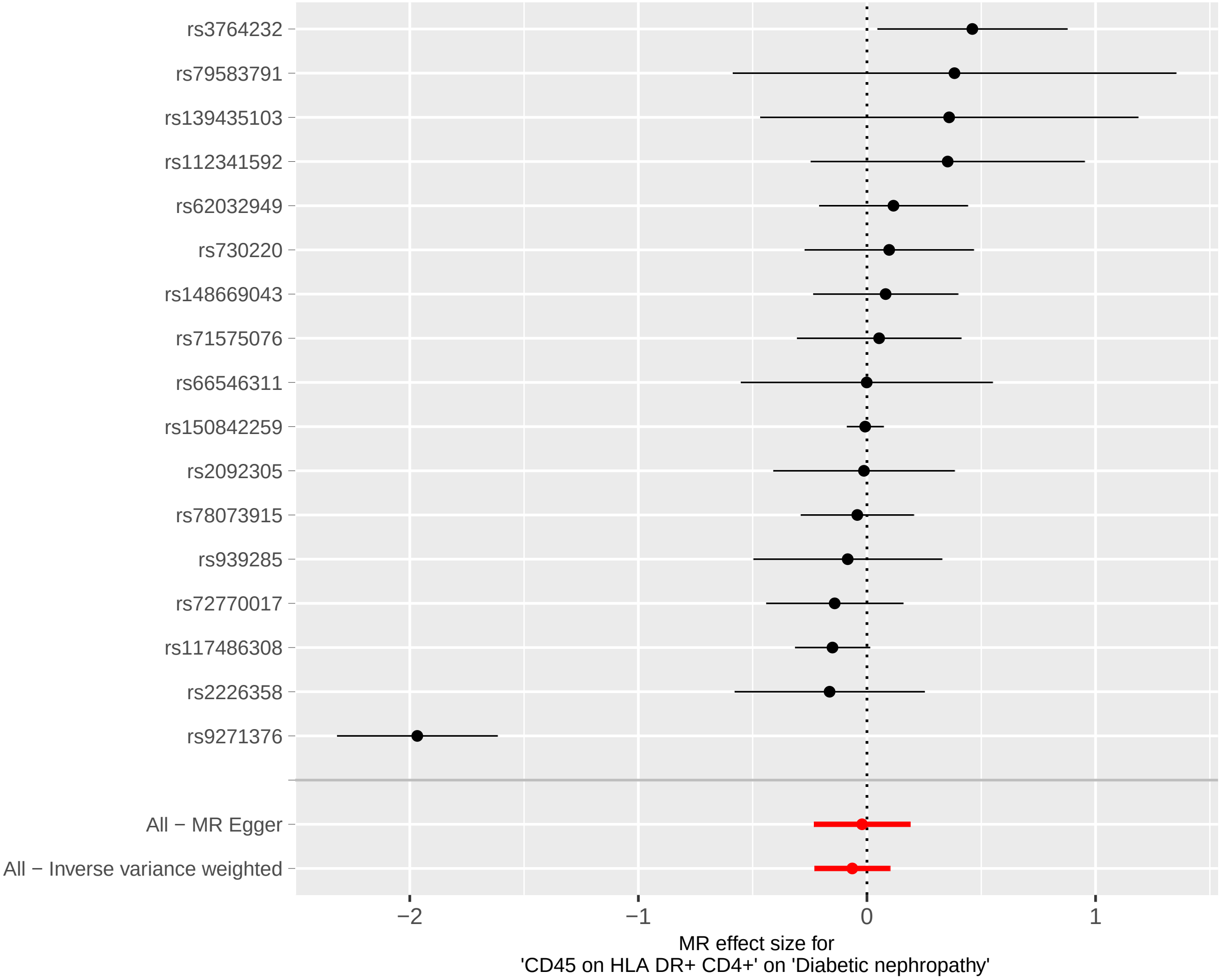
rs3764232
rs79583791
rs139435103
rs112341592
rs62032949
rs730220
rs148669043
rs71575076
rs66546311
rs150842259
rs2092305
rs78073915
rs939285
rs72770017
rs117486308
rs2226358
rs9271376
All − MR Egger
All − Inverse variance weighted
−2
−1
0
1
MR effect size for
'CD45 on HLA DR+ CD4+' on 'Diabetic nephropathy'

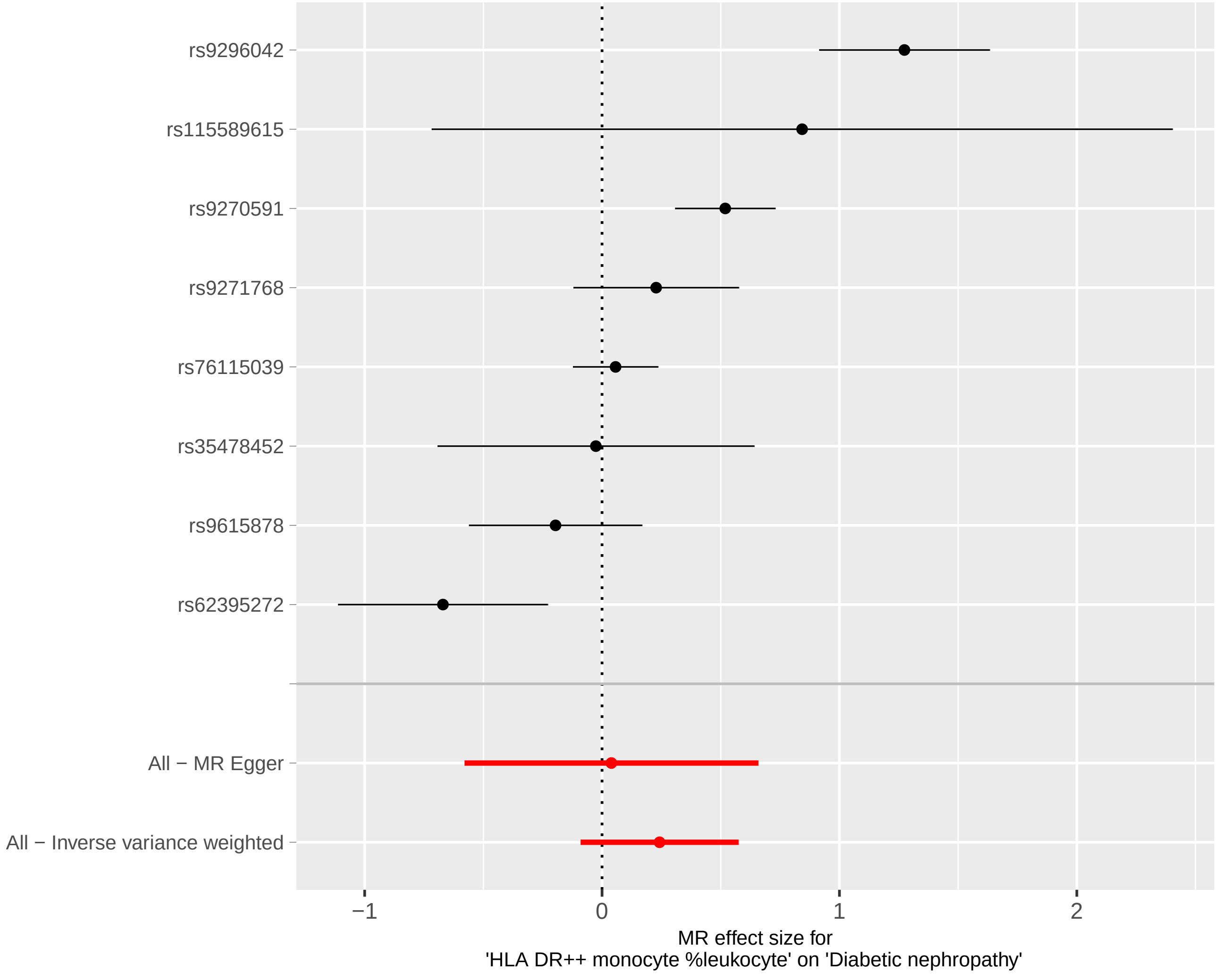

rs9296042
rs115589615
rs9270591
rs9271768
rs76115039
rs35478452
rs9615878
rs62395272
All − MR Egger
All − Inverse variance weighted
−1
0
1
2
MR effect size for
'HLA DR++ monocyte %leukocyte' on 'Diabetic nephropathy'

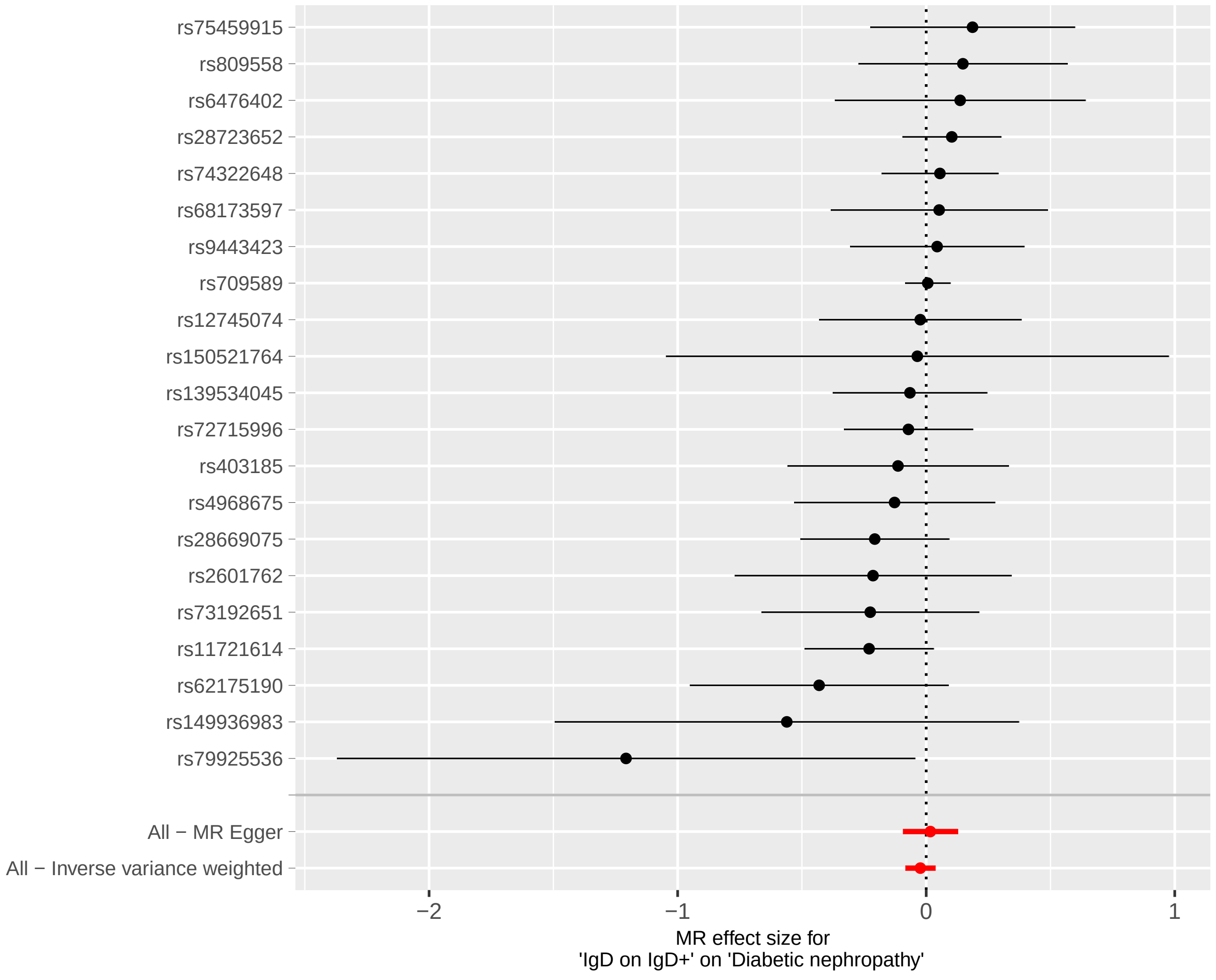
rs75459915
rs809558
rs6476402
rs28723652
rs74322648
rs68173597
rs9443423
rs709589
rs12745074
rs150521764
rs139534045
rs72715996
rs403185
rs4968675
rs28669075
rs2601762
rs73192651
rs11721614
rs62175190
rs149936983
rs79925536
All − MR Egger
All − Inverse variance weighted
−2
−1
0
1
MR effect size for
'IgD on IgD+' on 'Diabetic nephropathy'

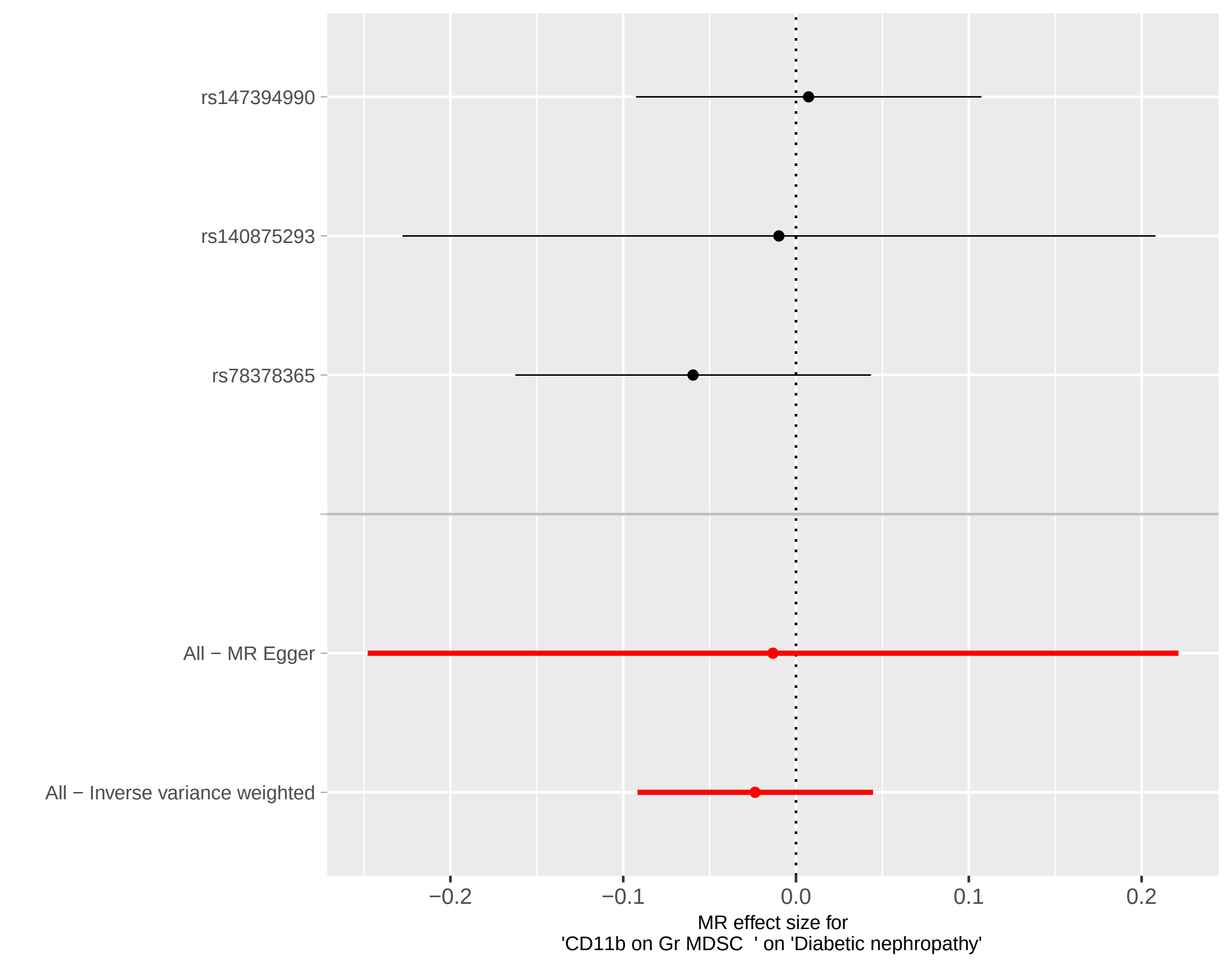

rs147394990
rs140875293
rs78378365
All − MR Egger
All − Inverse variance weighted
−0.2
−0.1
0.0
0.1
0.2
MR effect size for
'CD11b on Gr MDSC ' on 'Diabetic nephropathy'

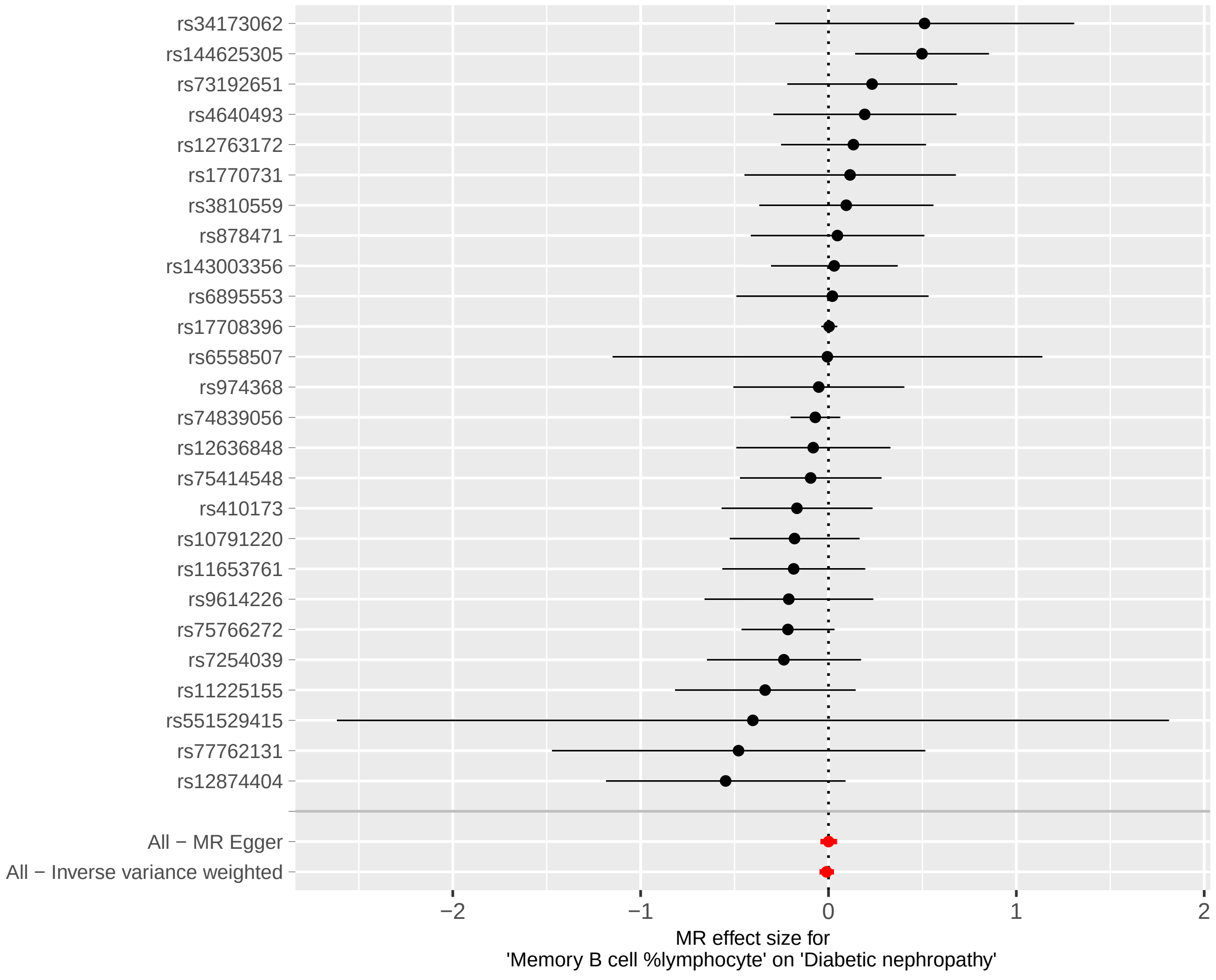
rs34173062
rs144625305
rs73192651
rs4640493
rs12763172
rs1770731
rs3810559
rs878471
rs143003356
rs6895553
rs17708396
rs6558507
rs974368
rs74839056
rs12636848
rs75414548
rs410173
rs10791220
rs11653761
rs9614226
rs75766272
rs7254039
rs11225155
rs551529415
rs77762131
rs12874404
All − MR Egger
All − Inverse variance weighted
−2
−1
0
1
2
MR effect size for
'Memory B cell %lymphocyte' on 'Diabetic nephropathy'

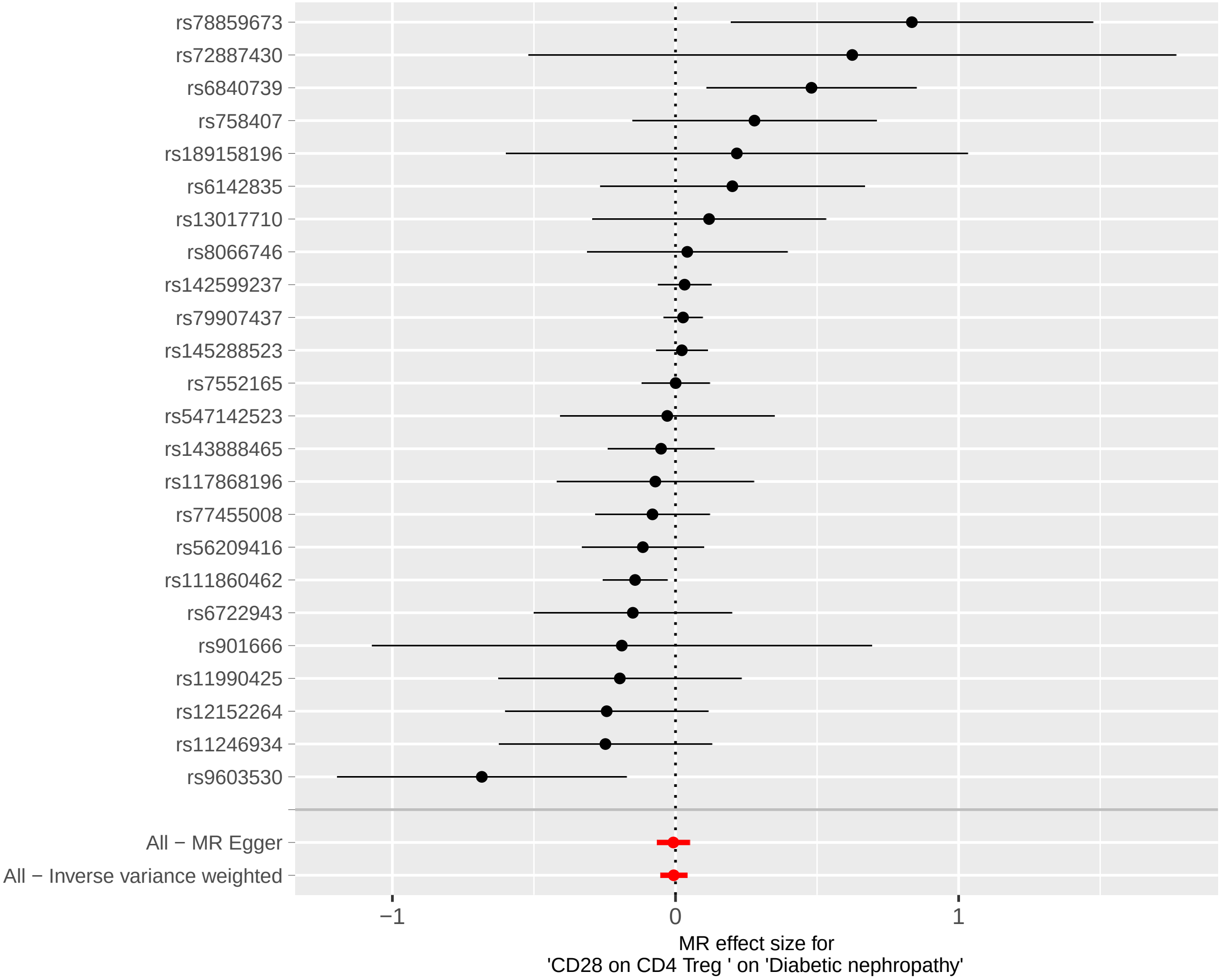
rs78859673
rs72887430
rs6840739
rs758407
rs189158196
rs6142835
rs13017710
rs8066746
rs142599237
rs79907437
rs145288523
rs7552165
rs547142523
rs143888465
rs117868196
rs77455008
rs56209416
rs111860462
rs6722943
rs901666
rs11990425
rs12152264
rs11246934
rs9603530
All − MR Egger
All − Inverse variance weighted
−1
0
1
MR effect size for
'CD28 on CD4 Treg ' on 'Diabetic nephropathy'

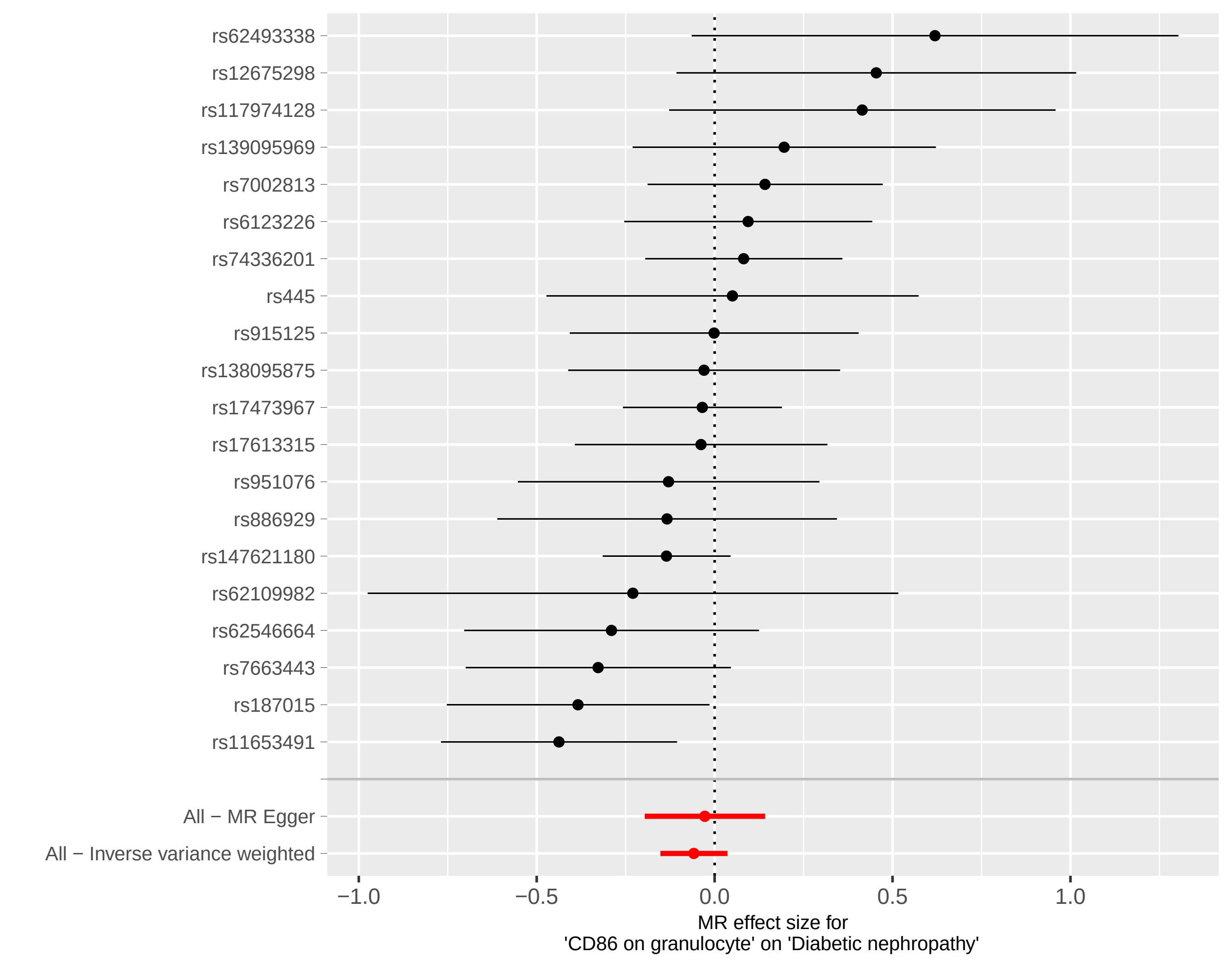
rs62493338
rs12675298
rs117974128
rs139095969
rs7002813
rs6123226
rs74336201
rs445
rs915125
rs138095875
rs17473967
rs17613315
rs951076
rs886929
rs147621180
rs62109982
rs62546664
rs7663443
rs187015
rs11653491
All − MR Egger
All − Inverse variance weighted
−1.0
−0.5
0.0
0.5
1.0
MR effect size for
'CD86 on granulocyte' on 'Diabetic nephropathy'

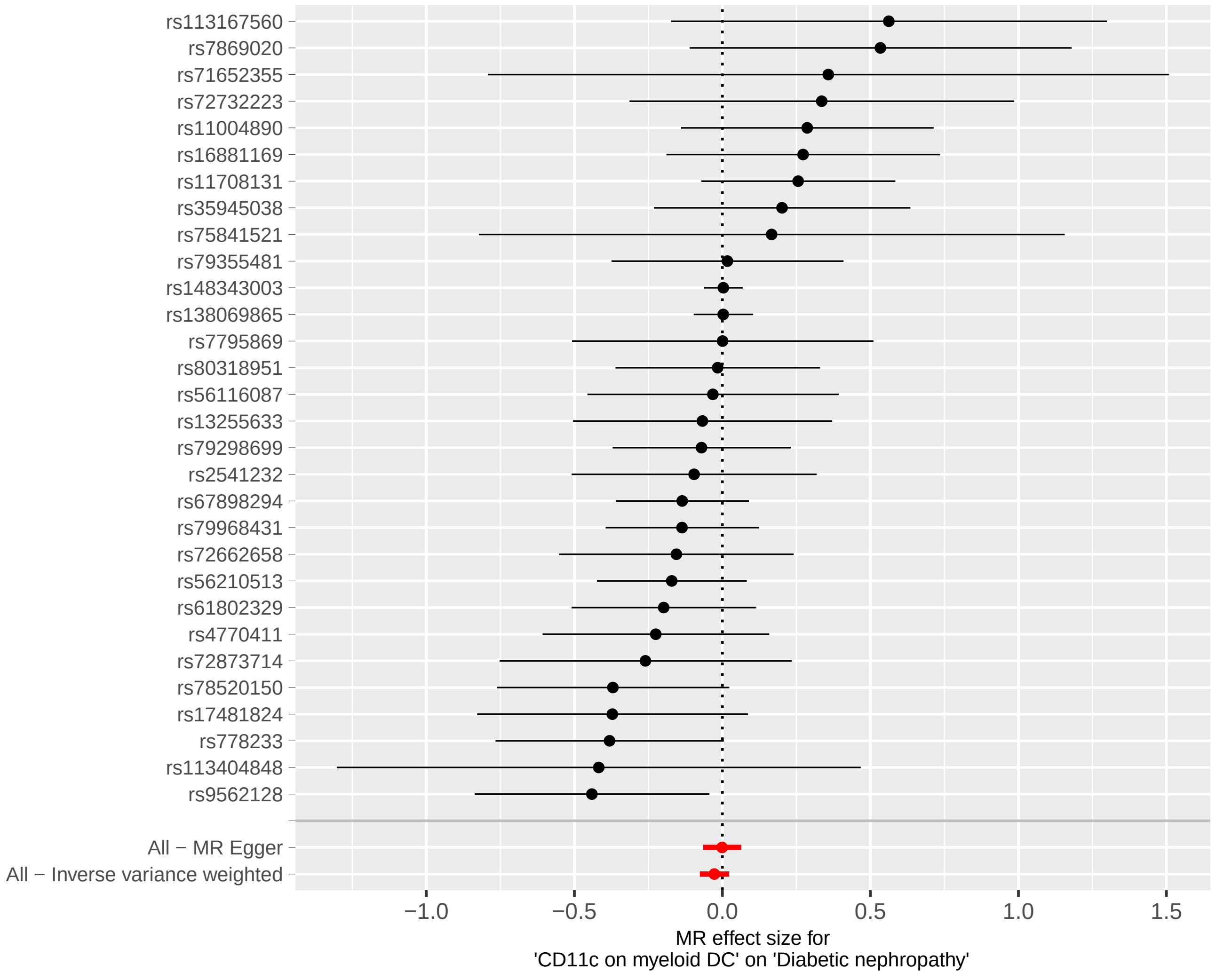
rs113167560
rs7869020
rs71652355
rs72732223
rs11004890
rs16881169
rs11708131
rs35945038
rs75841521
rs79355481
rs148343003
rs138069865
rs7795869
rs80318951
rs56116087
rs13255633
rs79298699
rs2541232
rs67898294
rs79968431
rs72662658
rs56210513
rs61802329
rs4770411
rs72873714
rs78520150
rs17481824
rs778233
rs113404848
rs9562128
All − MR Egger
All − Inverse variance weighted
−1.0
−0.5
0.0
0.5
1.0
1.5
MR effect size for
'CD11c on myeloid DC' on 'Diabetic nephropathy'

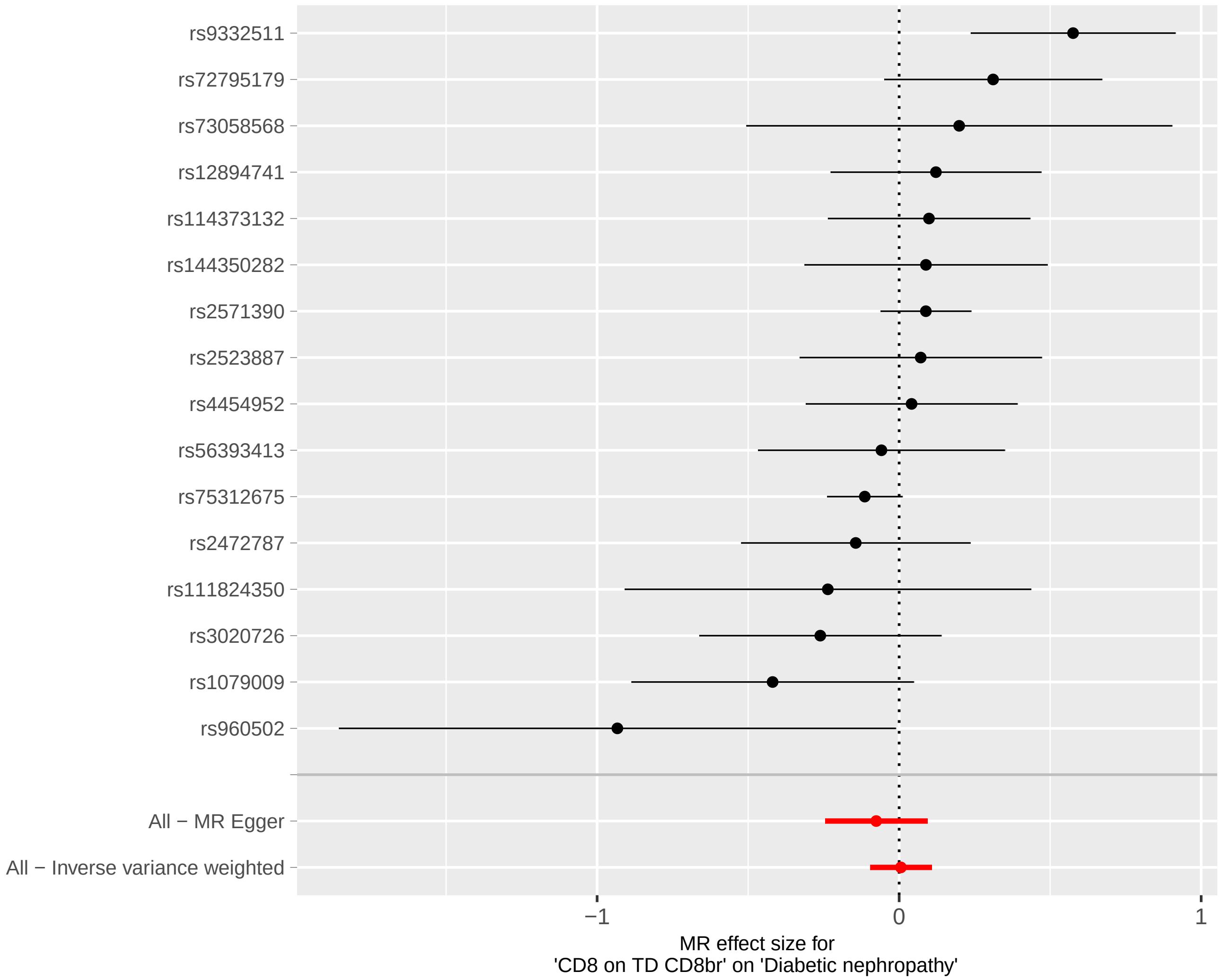
rs9332511
rs72795179
rs73058568
rs12894741
rs114373132
rs144350282
rs2571390
rs2523887
rs4454952
rs56393413
rs75312675
rs2472787
rs111824350
rs3020726
rs1079009
rs960502
All − MR Egger
All − Inverse variance weighted
−1
0
1
MR effect size for
'CD8 on TD CD8br' on 'Diabetic nephropathy'

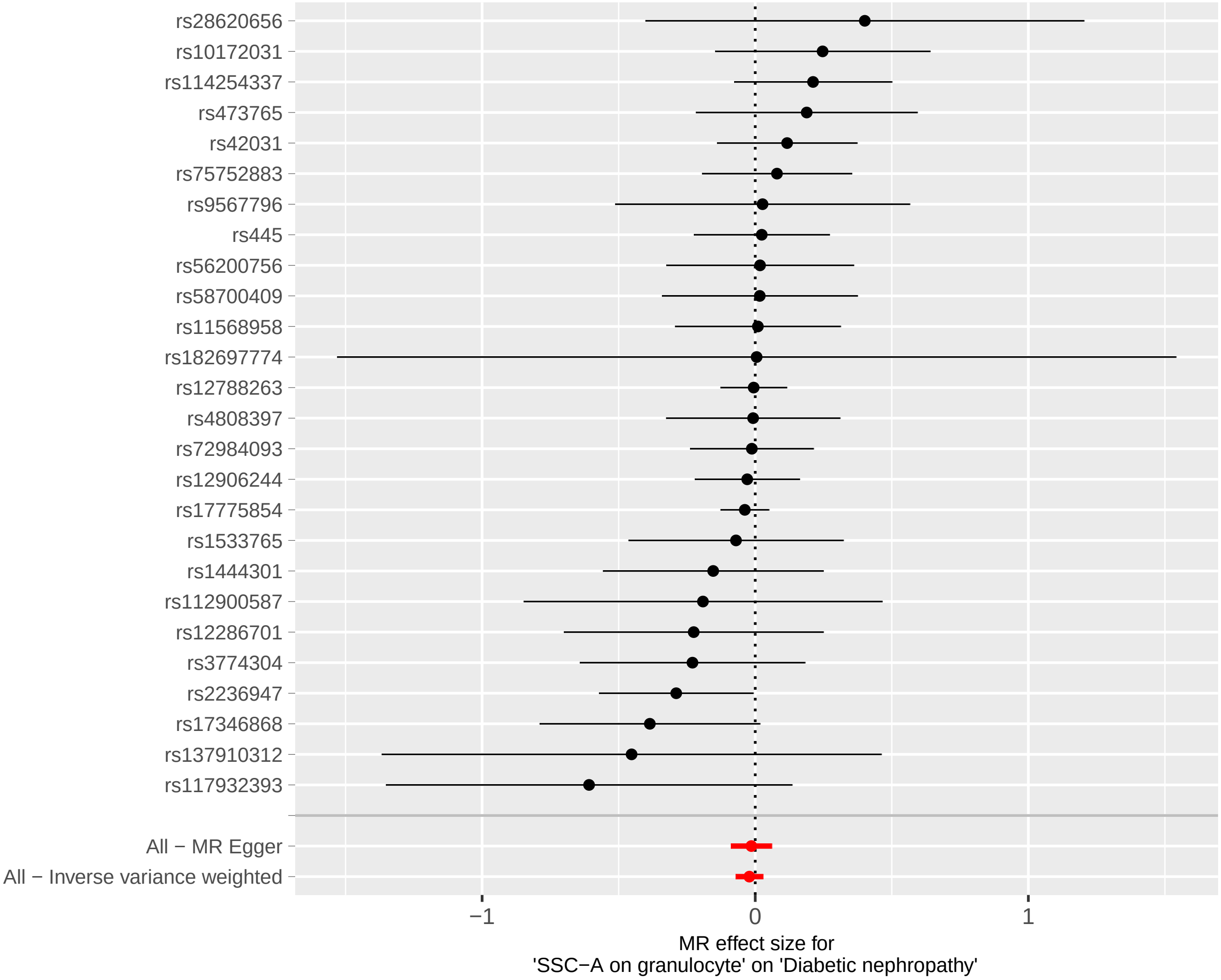

rs28620656
rs10172031
rs114254337
rs473765
rs42031
rs75752883
rs9567796
rs445
rs56200756
rs58700409
rs11568958
rs182697774
rs12788263
rs4808397
rs72984093
rs12906244
rs17775854
rs1533765
rs1444301
rs112900587
rs12286701
rs3774304
rs2236947
rs17346868
rs137910312
rs117932393
All − MR Egger
All − Inverse variance weighted
−1
0
1
MR effect size for
'SSC−A on granulocyte' on 'Diabetic nephropathy'

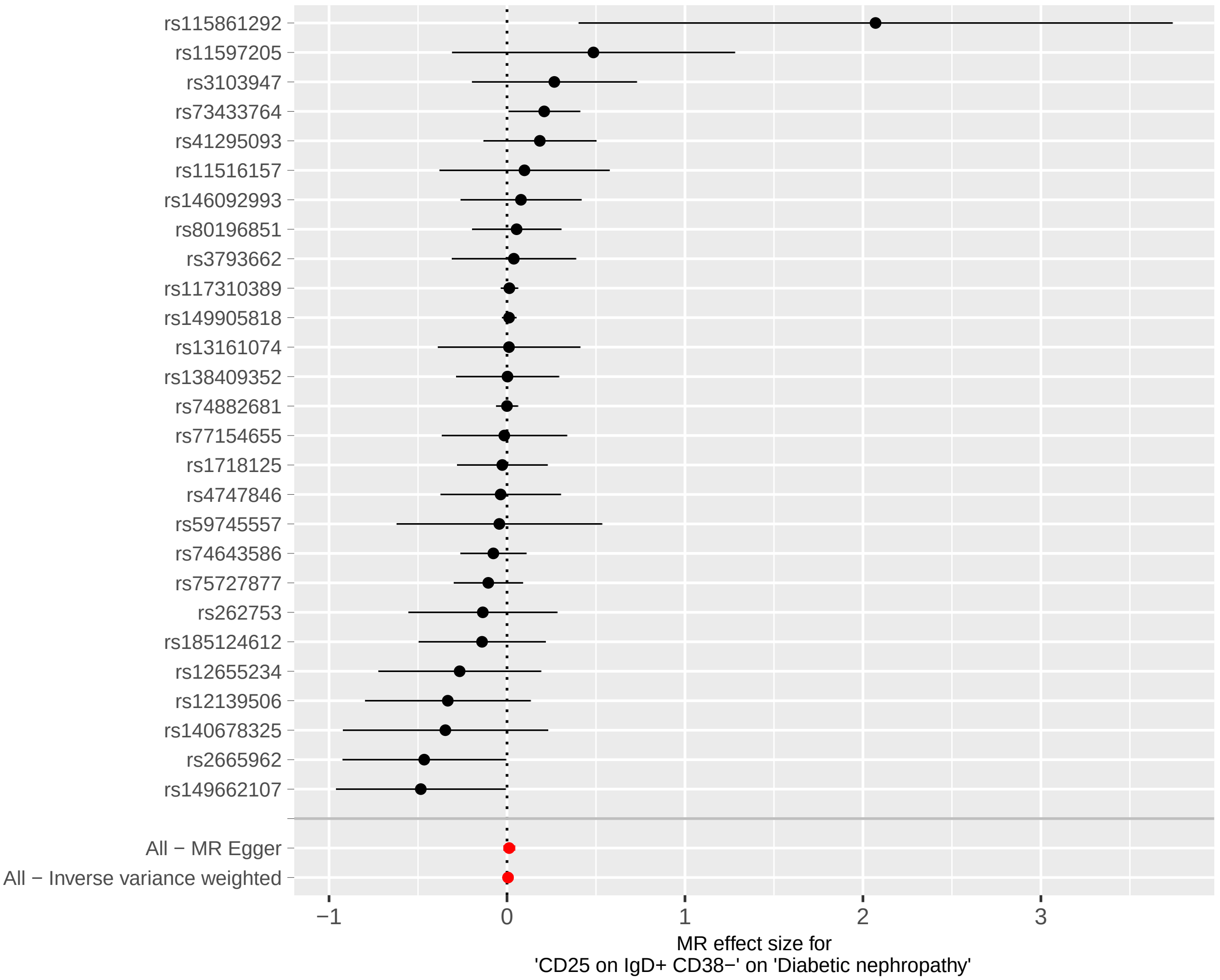
rs115861292
rs11597205
rs3103947
rs73433764
rs41295093
rs11516157
rs146092993
rs80196851
rs3793662
rs117310389
rs149905818
rs13161074
rs138409352
rs74882681
rs77154655
rs1718125
rs4747846
rs59745557
rs74643586
rs75727877
rs262753
rs185124612
rs12655234
rs12139506
rs140678325
rs2665962
rs149662107
All − MR Egger
All − Inverse variance weighted
−1
0
1
2
3
MR effect size for
'CD25 on IgD+ CD38−' on 'Diabetic nephropathy'

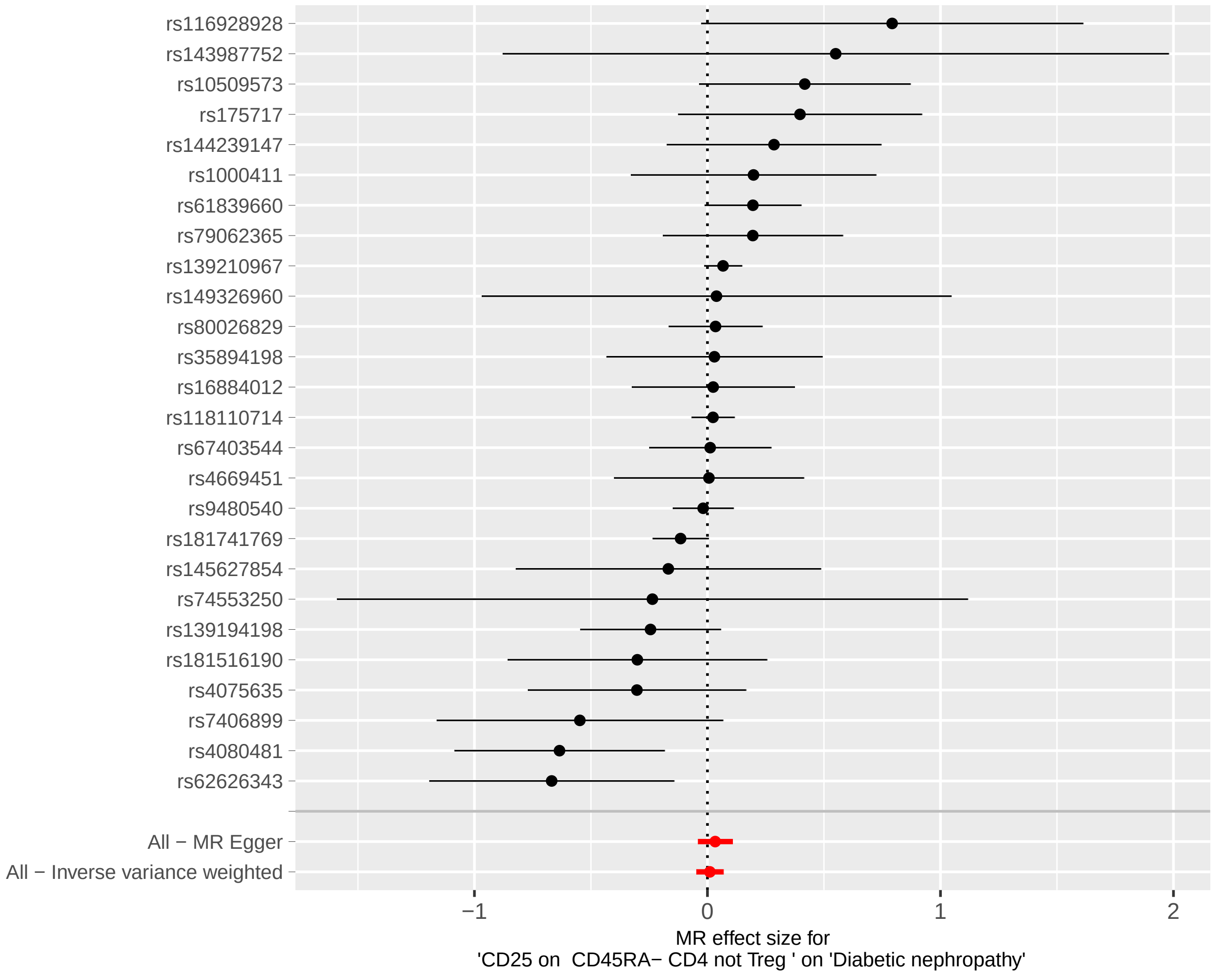
rs116928928
rs143987752
rs10509573
rs175717
rs144239147
rs1000411
rs61839660
rs79062365
rs139210967
rs149326960
rs80026829
rs35894198
rs16884012
rs118110714
rs67403544
rs4669451
rs9480540
rs181741769
rs145627854
rs74553250
rs139194198
rs181516190
rs4075635
rs7406899
rs4080481
rs62626343
All − MR Egger
All − Inverse variance weighted
−1
0
1
2
MR effect size for
'CD25 on CD45RA− CD4 not Treg ' on 'Diabetic nephropathy'
